# Characterization of Spanish Olive Virome by High Throughput Sequencing Opens New Insights and Uncertainties

**DOI:** 10.3390/v13112233

**Published:** 2021-11-06

**Authors:** Ana Belén Ruiz-García, Celia Canales, Félix Morán, Manuel Ruiz-Torres, Magdalena Herrera-Mármol, Antonio Olmos

**Affiliations:** 1 Centro de Protección Vegetal y Biotecnología, Instituto Valenciano de Investigaciones Agrarias (IVIA), Ctra, Moncada-Náquera Km 4.5, 46113 Moncada, Spain; ana.belen.ruiz@uv.es (A.B.R.-G.); canales_cel@externos.gva.es (C.C.); moran_fel@gva.es (F.M.); 2 Laboratorio de Producción y Sanidad Vegetal de Jaén, Junta de Andalucía, Sierra Morena, 12b, 23620 Mengíbar, Spain; manuelj.ruiz.torres@juntadeandalucia.es (M.R.-T.); magdalena.herrera@juntadeandalucia.es (M.H.-M.)

**Keywords:** olive, OLYaV, OEGV, OLV-3, HTS

## Abstract

The use of high throughput sequencing (HTS) for the analysis of Spanish olive trees showing leaf yellowing discoloration, defoliation, and/or decline has provided new insights into the olive viruses present in Spain and has opened discussions about the pros and cons of these technologies for diagnostic purposes. In this study, we report for the first time in Spanish orchards the presence of olive leaf yellowing-associated virus (OLYaV), for which the second full coding sequence has been determined. This virus has also been detected in a putative vector, the psyllid *Euphyllura olivina*. In addition, the presence in Spain of Olea europaea geminivirus (OEGV), recently reported in Italy, has been confirmed, and the full-length sequence of two isolates was obtained by HTS and Sanger sequencing. These results, as well as the detection of other viral sequences related to olive latent virus 3 (OLV-3) and olive viral satellite RNA, raises questions on the biological significance of the findings, about the requirement of standardization on the interpretation of HTS results, and the necessity of additional tests to confirm the relevance of the HTS detection of viral sequences.

## 1. Introduction

Olive (*Olea europaea*) represents a strategic crop in Spain, with more than 309 million productive trees cultivated in the country [1]. According to the latest data available in the FAO database (2019), Spain is the world’s largest producer, with 5,965,080 tons representing 30.6% of the world’s production, and 2,601,900 ha. of growing area [2]. In Spain, this crop is threatened by different pathogens, especially fungi and bacteria, for which measures for containment and control are continuously taken. However, scarce information is related to the viruses that infect olive crop, due to the fact that most olive viruses seem to induce latent infections. To date, two viral olive syndromes have been described, “bumpy fruits”, caused by strawberry latent ringspot virus, and “leaf yellowing complex”, associated to three different filamentous viruses: olive vein yellowing-associated virus, olive yellow mottling-and-decline-associated virus, and olive leaf yellowing-associated virus (OLYaV) [3]. Bumpy fruits disease produces puckered fruits, narrow and twisted leaves, and bushy growth, whereas leaf yellowing syndrome produces bright yellow discolorations of the foliage, poor fruit set, mottling, necrosis, extensive defoliation, and dieback [3]. Among the 17 viruses described infecting olive [4,5,6,7], only cucumber mosaic virus, cherry leafroll virus, and strawberry latent ringspot virus had been occasionally described infecting olive symptomless trees in Spain [8]. However, during the summer of 2020, in a regular inspection of the sanitary status of olive trees in the main olive-producing area in Spain, Jaén (Andalucía, South of Spain), an increasing number of trees showing yellow leaf discoloration and extensive defoliation was observed. A similar symptomatology was detected in another Spanish olive-producing area, Castellón (Valencian Community, Easter region of the country). Two trees showing this symptomatology were selected, one from Jaén and another one from Castellón, representing different climate, soil conditions, and geographical location, for high throughput sequencing (HTS) analysis, with the aim of identifying the etiology of this disorder that alerted growers and plant health services. HTS offers an advantage over traditional methods because it does not require previous knowledge of the virus species/isolates present in the sample and is potentially able to detect any viral sequence present in the plant. The results revealed the presence of olive leaf yellowing-associated virus (OLYaV), Olea europaea geminivirus (OEGV), and olive latent virus 3 (OLV-3), which would represent the first report of these viral species in Spain. In addition, 94 samples and 14 insect samples of OLYaV candidate vectors were also analyzed. OLYaV has been officially reported in California (USA) [9], Italy [10], Tunisia [11], Lebanon [12], Syria [13], Greece [14], Albania [15], and Brazil [16]. The presence of OLYaV in the USDA National Clonal Germplasm Repository (NCGR) at the University of California from imported plant material suggested its possible presence in Spain, France, Cyprus, Chile, Israel, and Australia [9]. However, to the best of our knowledge, the presence of this virus in Spanish orchards has never been reported. OLYaV has remained for a long time as an unassigned member of the family *Closteroviridae*; however, its full-length genome has recently been determined and the analysis of its five broadly conserved closteroviral proteins has shown that OLYaV, Actidinia virus 1, and persimmon virus B systematically cluster together, supporting the proposal of a new genus inside the *Closteroviridae* family [16]. The geminivirus OEGV has recently been identified infecting olive trees in Italy [7], and its bipartite genome consists of a DNA-A with a genome organization similar to the New World begomoviruses and a DNA-B containing a movement protein in the virion sense and a protein with unknown function on the complementary sense. OLV-3 is a latent olive virus that belongs to the genus *Marafivirus* and has been reported in Italy, Syria, Malta, Tunisia, Portugal, Turkey, Lebanon, and Greece [17]. In this study, the analysis of olive trees by HTS offers new insights into the olive viruses present in Spain and the arising uncertainties about the biological significance of the findings opens the discussion on the pros and cons of the application of HTS on plant virus diagnosis.

## 2. Materials and Methods

### 2.1. Plant Material and Sample Preparation

A total of 94 samples from both symptomatic and asymptomatic olive plants (cultivars Picual and Serrana) were collected in 2020 and 2021 during the summer season in the main olive-producing area in the South of Spain (Jaén) and in a small olive-producing area in the eastern region of the country (Castellón).

Leaf tissue from all olive trees analyzed was individually extracted in plastic bags (Bioreba, Reinach, Switzerland). Extraction buffer (PBS containing 0.2% of diethyldithiocarbamate and 2% of PVP-10) was added in a 1:5 ratio (*w:v*). Samples were grinded using Homex 6 homogenizer (Bioreba, Reinach, Switzerland). The resulting plant extract was kept on ice until subsequent processing.

Total RNA was purified from 200 µL of plant extract using the Plant/Fungi RNA isolation kit (Norgen Biotek Corporation, Thorold, ON, Canada) following the manufacturer’s instructions. Total DNA was purified from 200 µL of plant extract using the Plant/Fungi DNA isolation kit (Norgen Biotek Corporation, Thorold, ON, Canada) following the manufacturer’s instructions. After determining its concentration by spectrophotometry (DeNovix DS-11 spectrophotometer, DeNovix Inc., Wilmington, DE, USA), RNA and DNA samples were stored at −80 °C for subsequent analysis.

For HTS analysis, total RNA samples were treated with DNase using RNase-Free DNase I kit (Norgen Biotek Corporation, Thorold, ON, Canada) following the manufacturer’s instructions.

### 2.2. Insect Sample Preparation

A total of 14 samples of different insects were collected from olive trees in Jaén in summer 2021: 7 samples containing 5 different undetermined Cicadellidae (Hemiptera) species; 2 samples containing 1 undetermined Aphididae (Hemiptera) species; 2 samples of *Agalmatium flavescens*; and 3 samples of *Euphyllura olivina*. Total RNA was extracted from insects using the Plant/Fungi Total RNA Purification Kit (Norgen Biotek Corporation, Thorold, ON, Canada) with slight modifications. Individual insects were placed in Eppendorf tubes. A volume of 100 μL of lysis buffer and 212–300 μm glass beads (Sigma-Aldrich, Misuri, TX, USA) were added. After vortexing for 2′ the suspension was centrifuged for 5′ at 10,000× *g*, the supernatant was transferred to a fresh tube and the extraction completed following the kit manufacturer’s instructions.

### 2.3. Analysis of Symptomatic Olive Samples by HTS

Total RNA quality control, generation of libraries, and sequencing were performed at Macrogen Inc. (Seoul, Republic of Korea). Library construction was carried out using the TruSeq Stranded Total RNA Ribo-Zero Plant Kit (Illumina, San Diego, CA, USA) and the library protocol TruSeq Stranded Total RNA Sample Prep Guide, Part #15031048 Rev. E. Sequencing of libraries (2 × 150 bp paired-end reads) was performed in a NovaSeq 6000 platform (Illumina, San Diego, CA, USA).

HTS raw data were subjected to bioinformatic analyses using CLC Genomics Workbench v.10.1.1 (Qiagen Bioinformatics, Hilden, Germany) and Geneious Prime v2020.2.5 (Biomatters Ltd., Auckland, New Zealand). The initial steps in the analysis were completed by CLC Genomics Workbench software: trimming of adapters, read quality control, and *de novo* assembly. BLAST analysis (BLASTN/X) against local and online virus, viroids, and nt/nr databases with a cut-off e-value of 10^−4^ allowed the annotation of contigs longer than 200 nt. Extension of the virus-related contigs and complete viral genome recovery were performed by mapping the reads against the contigs using Geneious Prime software. A similar analysis of the HTS data was conducted, introducing a host genome subtraction step prior to the *de novo* assembly, performed in CLC Genomics Workbench by mapping the reads against *Olea europaea* genome Oe6 scaffolds [18] and *Olea europaea* subsp. europaea plastid complete genome (NC_015401).

### 2.4. Detection of Olive Viruses by RT-PCR

OLYaV, OEGV, and OLV-3 were tested for detection by RT-PCR using newly designed primers or primers previously reported in the literature, modified based on the HTS sequences recovered in this study (Table 1).

All RT-PCR reactions were carried out using AgPath One-Step RT-PCR kit (Applied biosystems, Foster City, CA, USA) following the manufacturer’s instructions. The reaction mixture contained 0.5 µM concentration of each primer and 100 ng of total RNA in a final volume of 25 µL. Amplification protocol consisted of: for OLYaV and OEGV detection, one step at 45 °C for 45 min, one step at 95 °C for 10 min, and 35 cycles of amplification (95 °C for 30 s, 50 °C for 30 s, and 60 °C for 30 s), with a final step at 60 °C for 5 min; and, for OLV-3 detection, one step at 45 °C for 45 min, one step at 95 °C for 10 min, and 35 cycles of amplification (95 °C for 30 s, 52 °C for 30 s, and 60 °C for 1 min), with a final step at 60 °C for 5 min. Amplicons were Sanger-sequenced in both directions after purification using mi-PCR Purification Kit (Metabion International AG, Martinsried, Germany) following the manufacturer’s instructions.

### 2.5. Amplification of OEGV Complete Genome by PCR

The complete genome of OEGV, both DNA-A and DNA-B segments, were amplified by PCR using newly designed primers based on the HTS-recovered full-length genome (Table 2). All PCR reactions were carried out using AgPath One-Step RT-PCR kit (Applied biosystems, Foster City, CA, USA) following the manufacturer’s instructions. The reaction mixture contained 0.5 µM concentration of each primer and 50 ng of total DNA in a final volume of 25 µL. Amplification protocol consisted of: one step at 95 °C for 10 min and 35 cycles of amplification (95 °C for 30 s, 50 °C for 30 s, and 60 °C for 1 min), with a final step at 60 °C for 5 min. Amplicons were Sanger-sequenced in both directions after purification using mi-PCR Purification Kit (Metabion International AG, Martinsried, Germany) following the manufacturer’s instructions.

## 3. Results

### 3.1. Olive Plant Symptoms

**Figure 1:**
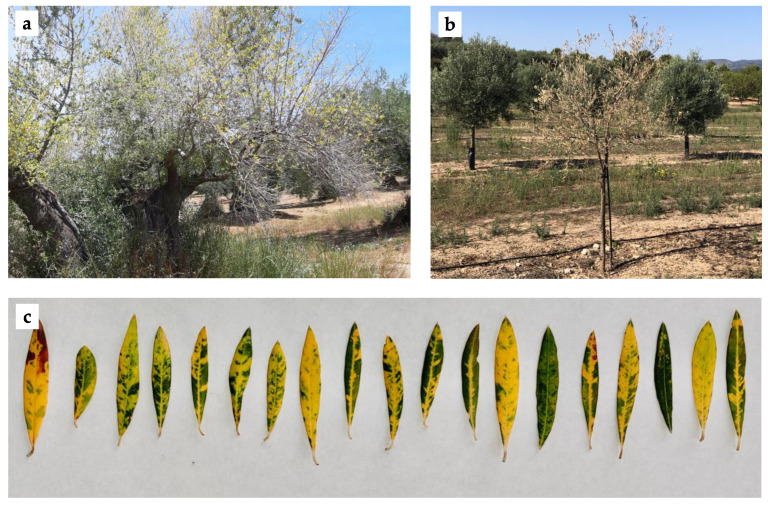
Virus-like symptoms observed in olive trees. (**a**) Defoliation; (**b**) tree decline; (**c**) yellow leaf discolorations.

Different virus-like symptoms, such as yellow leaf discoloration, defoliation, and tree decline (Figure 1), were observed in 24 out of the 94 olive plants analyzed in this study (25.5%). Two plants were selected for HTS analysis: sample 64.1 showing yellow leaf discoloration, defoliation, and tree decline, cultivar Serrana collected from Castellón; and sample 66.5 showing yellow leaf discoloration and extensive defoliation, cultivar Picual collected from Jaén.

### 3.2. HTS Analysis of Olive Symptomatic Plants

HTS raw data obtained from sample 64.1 were subjected to adapter trimming and read quality control in CLC Genomics Workbench, yielding a total of 37,891,771 quality reads. A host genome subtraction step was performed, resulting in 2,489,383 olive-unrelated reads. *De novo* assembly produced a total of 7762 contigs longer than 200 nt. BLASTN/X analysis of the contigs revealed the presence of three contigs related to plant viruses: two contigs related to OLYaV (10,452 nt and 5917 nt) and one contig related to OEGV DNA-A segment (1034 nt).

Contig extension by mapping the reads against the contigs by Geneious Prime software allowed the recovery of a near full-length OLYaV genome of 16,700 nt (average coverage 149×) that was deposited in the databases (isolate V64, accession number MW056495). These results indicate the presence of OLYaV in the sample. To the best of our knowledge, this is the first OLYaV complete genomic sequence of a Spanish isolate and the second full-length available genome for this viral species.

A similar analysis was performed to recover a near complete OEGV DNA-A genomic segment of 2557 nt (average coverage 323×, genomic position 14-2570) representing the complete coding sequence. It is important to note that no sequences corresponding to the DNA-B genomic segment of the virus were found in this analysis. However, the HTS results indicate the presence of OEGV in sample 64.1. To our knowledge, this is the first report of OEGV in olive in Spain.

With the aim of detecting OEGV DNA-B sequences and recovering the full-length genome, the total 37,891,771 quality reads (avoiding a host genome subtraction step) were mapped against a complete OEGV genome available in the databases (MW316657 and MW316658) using CLC Genomics Workbench. This analysis allowed the recovery of the OEGV full-length genome of 2775 nt (DNA-A, average coverage 386.9×) and 2763 nt (DNA-B, average coverage 156.3×), named isolate OEGV-V64.1 (deposited in the databases, accession numbers OK475023 and OK475024).

HTS analysis of sample 66.5 yielded a total of 43,970,177 reads after trimming of adapters and read quality control steps. A subsequent genome subtraction phase resulted in 2,599,785 olive-unrelated reads that were used to assemble 28,917 *de novo* contigs longer than 200 nt. Among them, BLAST analysis revealed the presence of 29 contigs related to viral sequences: 13 contigs related to OLYaV (between 290 and 2948 nt in size); 14 contigs related to OLV-3 (between 206 and 477 nt in size); and 2 contigs related to olive viral satellite RNA (1059 and 1085 nt in size). Attempts to extend these contigs for the recovery of complete genomes of OLYaV and OLV-3 were not successful. However, these results support the occurrence of OLYaV in Spanish olive orchards and suggest the presence of OLV-3 and the olive viral satellite RNA in olive in Spain.

### 3.3. Molecular Characterization of OLYaV Spanish Isolates

**Figure 2:**
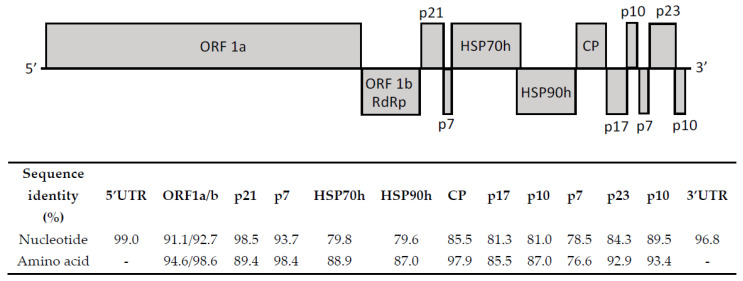
Genomic structure of OLYaV and ORFs is indicated. Percentages of sequence similarity at nucleotide and amino acid levels for each ORF, as well as pairwise identity for 5′ and 3′ UTRs between the Spanish OLYaV isolate V64 and the Brazilian isolate CS1 are shown.

OLYaV Spanish isolate V64 showed 87.8% nucleotide identity with the Brazilian isolate CS1, the only complete sequence reported to date. Nucleotide and amino acid sequence similarity between both full-length isolates was evaluated for all ORFs, as well as 5′ and 3′ UTRs (Figure 2). The results of this analysis showed the highest percentages of identity at the nucleotide level at 5′UTR and p21 (thaumatin-like protein) genomic regions. In the case of the protein sequences, the highest sequence similarity was found at ORF1b (RdRp) and p7 (putative transmembrane protein).

In order to evaluate the spreading of OLYaV in Spanish orchards, 92 additional symptomatic and symptomless samples from both of the geographical origins surveyed (Castellón and Jaén) were tested by RT-PCR using one set of primers designed on the HTS-recovered sequence (Table 1) targeting a 425-nt genomic fragment corresponding to the 3′ end region of the ORF1b gene (RdRp) and the 5′ end region of ORF2 (p21, thaumatin-like protein). A total of 40 out of 92 samples (43.5%) tested positive for OLYaV, 32 out of 69 samples (46.4%) from Castellón and 8 out of 23 samples (34.8%) from Jaén. It is important to note that these percentages do not correspond to the real incidence of the virus, as the survey was not randomly performed, but influenced by the symptomatology observed. A detailed list of olive samples that tested positive for at least one olive virus is shown in Supplementary Table S1. A sequence analysis comparison on 10 of the 40 OLYaV-positive plants showed a nucleotide identity ranging from 90.2 to 99.5% with respect to isolate V64. Although many of the samples that tested positive for OLYaV showed some virus-like symptoms, no symptomatology could be clearly associated to OLYaV infection, with some of the positive samples being symptomless and several asymptomatic samples being negative.

Along with some of the olive samples surveyed in Jaén, 14 samples of different insects were collected from symptomatic trees, classified, and analyzed by RT-PCR. Among them, a sample corresponding to the psyllid *Euphyllura olivina* tested positive for OLYaV, thus indicating the ability of this insect to acquire the virus.

### 3.4. First Detection and Molecular Characterization of OEGV in Olive in Spain

In order to confirm the presence of OEGV in sample 64.1, RT-PCR analysis using two sets of primers designed on the HTS-recovered sequences (listed in Table 1), one targeting a 480 nt region of the AV1 (CP) gene (DNA-A) and another one targeting a genomic region of 320 nt in the BV1 gene (DNA-B), was carried out. Successful amplification and Sanger sequencing of the amplicon confirmed 100% of the genomic sequences recovered by HTS.

RT-PCR using the OEGV-specific primers described above was conducted on the 92 samples surveyed in this study. In this analysis, 60 out of 92 samples (65.2%) tested positive for OEGV, 59 out of 69 samples (85.5%) from Castellón and 1 out of 23 samples (4.3%) from Jaén. As for OLYaV, these data do not represent the real incidence of this viral species in Spanish orchards because of a nonrandom survey. This also accounts for the symptomatology observed in the analyzed plants that could not be associated to the presence of OEGV, nor to the simultaneous infection by both OLYaV and OEGV. A detailed list of olive samples that tested positive for at least one olive virus is shown in Supplementary Table S1.

**Figure 3:**
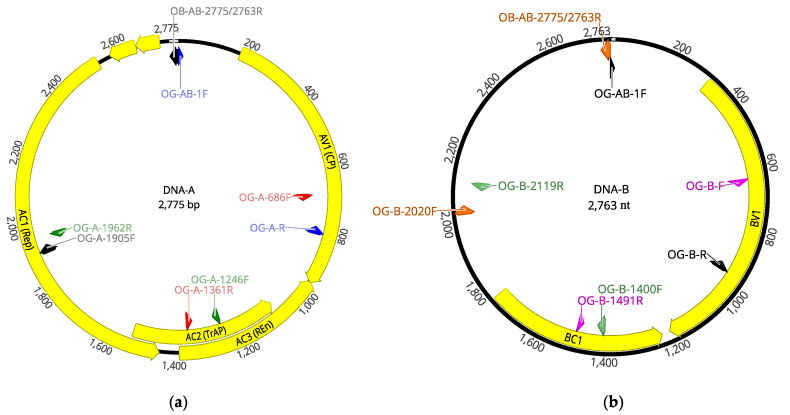
Genomic structure of OEGV isolate V64.2 segments DNA-A (**a**) and DNA-B (**b**). Position of specific primers used to amplify the complete genome is indicated. Each pair of primers used to amplify a specific region of the genome is indicated with the same color. Yellow arrows indicate ORFs.

Intriguingly, when the OEGV-specific amplification products were sequenced and compared, they turned out to be very conserved sequences, with a nucleotide identity higher than 99% and many of the amplicons sequenced being 100% identical to the isolate V64.1. Taking into account this very low genetic diversity that had been evaluated only in two small genomic regions, a positive OEGV sample (64.2) was selected for complete genomic sequencing. Overlapping PCR fragments covering the complete sequence of both genomic segments, DNA-A and DNA-B, were amplified by PCR using specific primers designed on the HTS-recovered full-length genome (listed in Table 2). Amplicons were Sanger sequenced and used to recover a complete OEGV genome (Figure 3), isolate V64.2 (accession numbers OK475021 and OK475022).

Sequence comparison between the two complete OEGV genomes determined in this study, V64.1 and V64.2, showed 99.96% nucleotide identity in the DNA-A genomic segment, as both sequences only differed in one nucleotide at the genomic position 1474. The percentage of sequence similarity between both isolates at the DNA-B genomic segment was 100%.

### 3.5. Other Viral Sequences Detected by HTS

The HTS analysis of olive plants performed in this study also detected the presence of sequences related to OLV-3. A total of 14 contigs (290 nt to 510 nt in size) showing sequence homology with OLV-3 (75.43% to 87.13% nucleotide identity) were found. The biggest contig, located on the CP region of OLV-3 genome, was extended up to 510 nt (average coverage 15.6×) by mapping the reads against the contigs. This contig (accession number OK475025) shared an 84.7% nucleotide identity with the CP region of OLV-3 isolate CN1/1 (FJ444852). These HTS data indicate the presence of OLV-3 infecting olive in Spain. However, not only did the bioinformatic analysis of the HTS data fail to recover a complete OLV-3 genome, but, also, the presence of OLV-3 in the sample could not be confirmed by RT-PCR. Moreover, any of the 94 olive samples analyzed in this study tested positive for OLV-3, questioning the relevance of the HTS results.

In addition, the BLAST analysis performed after *de novo* assembly revealed the presence of two contigs (1059 nt and 1085 nt) related to olive viral satellite RNA. The longest contig (1085 nt), named OVsatRNA-J66 (OK484515), showed 83.1% nucleotide identity with the olive viral satellite RNA isolate Caltabellotta1 (KC133073) and comprised an ORF encoding a putative 17 kDa protein containing a potexvirus coat protein domain (superfamily member pfam06184) at nucleotide position 460 to 858.

## 4. Discussion

HTS is a powerful technology currently being applied in plant virology more and more because of its enormous potential for virus discovery, viral characterization, and pathogen diagnosis. However, as with any other technique, HTS shows important limitations that need to be addressed by other experimental approaches. In this study, HTS has been applied to the virome analysis of olive trees. The presence of three viral species and one olive viral satellite RNA has been detected. These data offer new insights into the olive viruses present in Spain, but also raise questions and uncertainties about the biological significance of the findings.

The presence of OLYaV, a *Closteroviridae* member believed to be associated to the leaf yellowing complex disease, in Spanish olive orchards has been confirmed for the first time in this study, and the complete coding sequence of one Spanish isolate (V64) determined, the second isolate of this species to be completely sequenced. A comparison with the other complete available genome, the Brazilian isolate CS1, shows that, for detection purposes, the best region of the genome for primer/probe designing is the 5′ UTR (99% nucleotide identity). The high level of conservation of this region between the two isolates is in agreement with its possible involvement in virus replication [16]. Interestingly, the traditional region of the genome used for detection to date (HSP70) only shows 79.8% nucleotide homology between these two isolates, suggesting that the diversity in this region is too high for a broad detection of this viral species. Other regions of the genome, such as p21, harboring the thaumatin-like protein, the 3′UTR, or the RdRp show high percentages of homology at the nucleotide level between these two isolates (98.5, 96.8%, and 92.7%, respectively), which makes these regions candidates for designing high inclusivity PCR methods. Thus, Hsp70 and Hsp90 could be more appropriately used for studies of genetic variability instead of detection. In this study, a new RT-PCR based on primers designed in the 3′ end region of the ORF1b gene (RdRp) and the 5′ end region of ORF2 (p21, thaumatin-like protein) demonstrated the presence of the virus in symptomatic and symptomless trees and showed a sequence variability of up to 9.8% between isolates. These results, as well as the scarce number of full-length sequences currently available, highlight the need for a broader genetic diversity study on this virus, which should be performed in order to develop more accurate detection methods. To this end, HTS is a perfect tool able to provide valuable genetic diversity data supporting the development of accurate detection methods. In addition, and taking into account that the symptomatology observed has not been clearly associated to OLYaV infection, the existence of mild and aggressive isolates of this viral species, which could improve the control of the disease, should be investigated. This is the case for other closteroviruses, such as citrus tristeza virus, characterized by the presence of mild isolates that cause tristeza disease, as well as aggressive non-European isolates that induce stem-pitting syndrome or seedling yellows syndrome [19] and little cherry virus 1 isolate V2356, which has been associated to Shirofugen stunt disease [20]. OLYaV has been detected in many olive-producing countries and, in this study, a high level of incidence of the virus has been observed (43.5%), although this percentage does not correspond to the real prevalence of the virus because the survey was not randomly performed but influenced by the symptomatology observed, as previously mentioned. Nevertheless, the indications of the high prevalence of the virus found, not only in Spain, but also in many producing areas around the world, suggests the possible role of a vector in viral transmission. Related to this issue in this study, the detection of OLYaV in the psyllid *Euphyllura olivina* confirms that the virus can be acquired by this insect species, as previously reported [21]. Further studies will be needed to clarify the putative role of *Euphyllura olivina* on the transmission of this viral species.

In conclusion, HTS has allowed the recovery of one full-length Spanish OLYaV genome and the presence of this virus in Spanish olive orchards has been confirmed. The molecular characterization of the genome provides valuable information for the designing of future diagnostic methods. OLYaV has been detected in both symptomatic and symptomless trees, as well as in a putative transmission vector, raising questions on the epidemiology of this viral species. Another virus identified by HTS in Spanish olive trees in this study is the recently reported OEGV, representing the first report of this viral species in Spain. In the case of the detection of this olive geminivirus in Spain, some uncertainties arise from the analysis of the sequences. Interestingly, identical or almost identical sequences were recovered among all Spanish isolates, and also between them and the sequences of the Italian isolate DNA-A and DNA-B (MW316657 and MW316658), as well as with the DNA-B sequence available in Genbank from USA (MW814511). These findings suggest that the virus could be integrated in the olive genome due to a high level of adaptation of this geminivirus to the host. The high prevalence of the geminivirus with the same sequences between different trees and countries supports virus integration in the olive genome and raises the question of the putative coexistence between integrated sequences and episomal forms. This extremely great conservation of the sequence is against a general feature of the *Geminiviridae* family, characterized by its high diversity and variability of sequences intra-species. The biological significance of the presence of this geminivirus in olive remains to be studied because of several reasons. OEGV does not seem to produce symptoms in olive, as it has been detected with a high degree of sequence conservation in both symptomless trees and trees showing leaf yellowing discolorations. Interestingly, although the genome organization of the DNA-A is similar to New World begomoviruses, it lacks a functional C4 protein, a powerful viral effector that contributes to both geminivirus diversification and host jumping [22]. A putative ancestral C4 gene could be present from 2722–2654 nt, which could be followed by a stop–start element from 2652–2575, although no similarity with other C4 proteins have been found. However, more probably, the putative translation of a C4 protein could be based on the presence of a “slippery” heptanucleotide sequence where the shift in reading frame takes place and a 3′-adjacent stimulatory element, which, in general, comprises an RNA stem-loop or pseudoknot structure separated by a spacer region of 5–9 nt [23]. The consensus motif for the slippery heptanucleotide is X XXY YYZ, XX X being any three identical nucleotides, YYY representing AAA or UUU, Z representing A, C, or U, and spaces codons [24]. Thus, into AC1 (Rep) two potential slippery heptanucleotides (2427nt-T TTA AAA-2422nt) and (2262 nt-G GGT TTA-2256 nt) suggest the possible ribosomal frameshift harbored into AC1 that would yield C4 protein. The presence of another small ORF from 2023–1928nt also harbored into AC1(Rep) would complete candidate partial sequences for the C4 protein.

Open questions are also related to DNA-B segment, which contains two open reading frames. The results of the analyses with Interproscan implemented in Geneious Prime reveal that Pfam and InterPro analysis show in BV1 protein a gemini BL_1 domain (MP) from 26–289 nt (264 aa), pfam (Id: PF00845) and IntePro (Id: IPR00021), whilst, in the analysis of BC1, the ORF located in the typical position of the MP in begomovirus’ genome, no known domains have been found. Determination of the function of BC1 in OEGV remains to be studied. However, a potential nuclear localization signal can be found in BC1 at position 708–809 nt that results in a fragment of 34 aa (RIKGRLKLCASKRVEEIEFRSPRINILSKRHEAK) with consecutive basic amino acids or stretches of basic amino acids [25], which could be compatible for an NSP function of this protein.

In conclusion, the presence of OEGV in olive in Spain has been found for the first time by HTS and Sanger sequencing. The high incidence and sequence conservation among OEGV isolates is in agreement with an integration of this virus into the olive genome and raises questions about the biological significance of the presence of this virus in olive that will have to be addressed by means of other techniques.

HTS results presented in this study also show the presence of OLV-3, and these results highlight the importance of establishing harmonized thresholds for HTS detection. This virus species was detected only by HTS, and different attempts to detect OLV-3 by RT-PCR were unsuccessful. Despite the advances in the field of diagnostics based on HTS technology, there are no established thresholds for the number of reads necessary to identify the presence of a virus species in a sample, or to identify possible contaminations between samples analyzed simultaneously or the presence of artifacts produced during HTS reactions that could map against a viral sequence by chance. As is the case of OLV-3, the detection of a low number of reads from a viral genome could indicate the presence of a virus in a low titer. However, no confirmation by RT-PCR opens clearly the question of the interpretation and validation of the results to consider if the sample is negative or if it should be considered a positive sample due to the high sensitivity of the technique. Therefore, some open questions are, if a low number of reads distributed along the genome can be considered a positive result, how many reads, in how many regions, and which coverage level are required to consider the presence of a virus in a sample, as well as if additional molecular/serological techniques are still necessary to confirm such findings.

Consequently, HTS analysis seems to indicate the presence of OLV-3 in olive in Spain. However, due to the lack of confirmation by RT-PCR, the real significance of this finding needs to be further evaluated. In this sense, the establishment of HTS parameters providing a clear threshold, leading to a reliable detection of plant viruses by this technique, has to be addressed.

The biological significance of HTS results is also questioned in this study, where the presence of a contig related to olive viral satellite RNA containing a potexvirus coat protein domain has been found. This result opens the discussion of the effect of this type of satellite in olive plants and if one of the detected viruses acts as a helper virus or an unknown potexvirus is present. Further studies on samples where the olive viral satellite RNA is present will be required in order to answer these questions.

In conclusion, HTS has been demonstrated to be a powerful tool for detection, diagnosis, and virus discovery; however, as with other methods, it has its advantages and limitations. Plant virologists need to address how to manage the identification of new species of viruses that could affect commercial trade between countries, and highlight the urgent necessity to obtain biological data as soon as possible after their identification in order to better assess their relevance.

## Figures and Tables

**Table 1:** Primers used for RT-PCR detection of OLYaV, OEGV, and OLV-3.

Virus	Primer	Sequence (5′-3′)	Size (bp)	Reference
OLYaV	9527F	GAGAGGTTTATCCCGATGTGG	425	this study
	9952R	TAAAAGCACAGATTAGGTAAAACGC		
OEGV	OG-A-F	TGTCAGAGTTAATAATATTGTGGACGT	480	this study
	OG-A-R	CCACTAAGTTTCTTCCTCACAGA		
	OG-B-F	GTCTGATGAGATGCCTTGGAA	320	this study
	OG-B-R	CTGGATATCTCACTGTAACACATTC		
OLV-3	CN7up-M	CCAATCGAACAAGTTGTCTACCAGA	854	[17] modified
	i15R-M	CCGAAGTAGTCGGTCTCGTC		
	CN10F-M	AATTCTACCGGCCAACACCT	659	[17] modified
	OLV3-SR	GAGGGCCGGAATCTGAGT		

**Table 2:** Primers used for the complete amplification of OEGV genome by RT-PCR.

Primer	Sequence (5′-3′)
OG-A-R	CCACTAAGTTTCTTCCTCACAGA
OG-B-F	GTCTGATGAGATGCCTTGGAA
OG-B-R	CTGGATATCTCACTGTAACACATTC
OG-AB-1F	ACTGGCTTGCCCGCG
OG-A-686F	AAACGATACGTTCTAGAAGGTC
OG-A-1361R	TCACATGGCAGCAACTACAAG
OG-A-1246F	CGTGAAGATGTGATGGAACGGT
OG-A-1962R	ATTCGTTGTTCCACAAGAGTTAC
OG-A-1905F	CTCAGGTGAGAATATATTATGGGTC
OG-AB-2775/2763R	AATATTATATTGGCTTGCCCCACGGTC
OG-B-1491R	GATCAGCATTCCCAACAGTG
OG-B-1400 F	TGCATTCCAGTTCTTCGTAATAGTT
OG-B-2119R	GATGATATCGGATCTTTCATTATTCA
OG-B-2020F	TGATCTAAGTCAGCCACTTACATATAC

## Data Availability

All data supporting the results are contained within the article or Supplementary Material.

## Supplementary Materials

The following are available online at https://www.mdpi.com/article/10.3390/v13112233/s1, Table S1: RT-PCR detection of OLYaV, OEGV and OLV-3 in the olive samples analysed in this study. Samples testing positive for one or more viruses are listed, indicating the positive (+) or negative (-) result for each virus tested., FASTAQ files containing the reads covering the HTS-assembled OLYaV, OEGV DNA-A, and OEGV DNA-B.
