# Supplementary material for: Characterization of Spanish Olive Virome by High Throughput Sequencing Opens New Insights and Uncertainties
Source: Viruses. 2021 Nov 6;13(11):2233. doi: 10.3390/v13112233 (PMC8622421; doi:10.3390/v13112233)
Supplement: Supplementary file 1 [file viruses-13-02233-s001.zip › Supplementary_table s1.pdf]

Table S1. RT-PCR detection of OLYaV, OEGV and OLV-3 in the olive samples analysed in this study. Samples testing positive for one or more viruses are listed, indicating the positive (+) or negative (-) result for each virus tested.

Sample	Variety	Symptomatology	OLYaV	OEGV	OLV-3
64.1	Serrana	Yellow leaf discoloration, defoliation and tree decline	+	+	-
64.2	Serrana	Yellow leaf discoloration	+	+	-
64.3	Serrana	Yellow leaf discoloration	+	+	-
64.4	Serrana	Yellow leaf discoloration	+	+	-
64.5	Serrana	Yellow leaf discoloration	+	+	-
64.6	Serrana	Yellow leaf discoloration	+	+	-
64.7	Serrana	Yellow leaf discoloration	+	-	-
64.8	Serrana	Yellow leaf discoloration	+	-	-
64.9	Serrana	Yellow leaf discoloration	-	+	-
64.10	Serrana	Yellow leaf discoloration	+	-	-
66.3	Picual	Yellow leaf discoloration and defoliation	+	-	-
66.4	Picual	Yellow leaf discoloration and defoliation	-	+	-
66.5	Picual	Yellow leaf discoloration and defoliation	+	-	-
66.7	Picual	Yellow leaf discoloration and defoliation	+	-	-
67.1	Serrana	Symptomless	+	-	-
67.2	Serrana	Symptomless	+	+	-
67.3	Serrana	Symptomless	-	+	-
67.4	Serrana	Symptomless	+	+	-
67.5	Serrana	Yellow leaf discoloration	-	+	-
67.6	Serrana	Symptomless	-	+	-
67.7	Serrana	Symptomless	+	+	-
67.9	Serrana	Symptomless	-	+	-
67.10	Serrana	Symptomless	-	+	-
67.11	Serrana	Symptomless	-	+	-
67.12	Serrana	Symptomless	-	+	-
67.14	Serrana	Yellow leaf discoloration	-	+	-
67.15	Serrana	Yellow leaf discoloration	-	+	-
67.19	Serrana	Symptomless	-	+	-
67.20	Serrana	Symptomless	-	+	-
67.21	Serrana	Symptomless	+	+	-
67.22	Serrana	Symptomless	-	+	-
67.23	Serrana	Symptomless	-	+	-
67.25	Serrana	Symptomless	+	+	-
67.26	Serrana	Symptomless	-	+	-
67.27	Serrana	Symptomless	+	+	-
67.28	Serrana	Symptomless	-	+	-
67.29	Serrana	Symptomless	+	+	-
67.30	Serrana	Symptomless	+	+	-
67.31	Serrana	Symptomless	+	+	-
67.32	Serrana	Symptomless	+	+	-
67.33	Serrana	Symptomless	+	+	-
67.34	Serrana	Tree decline	+	+	-
67.35	Serrana	Tree decline	+	+	-
67.36	Serrana	Tree decline	+	+	-
67.37	Serrana	Symptomless	+	+	-

67.38	Serrana	Symptomless	+	+	-
67.39	Serrana	Symptomless	+	+	-
67.40	Serrana	Symptomless	-	+	-
67.41	Serrana	Symptomless	+	+	-
67.42	Serrana	Yellow leaf discoloration	+	+	-
67.43	Serrana	Symptomless	+	+	-
67.44	Serrana	Symptomless	-	+	-
67.45	Serrana	Symptomless	+	+	-
67.46	Serrana	Symptomless	-	+	-
67.47	Serrana	Symptomless	-	+	-
67.48	Serrana	Symptomless	-	+	-
67.49	Serrana	Symptomless	+	+	-
67.50	Serrana	Symptomless	+	+	-
67.51	Serrana	Symptomless	-	+	-
67.52	Serrana	Symptomless	-	+	-
67.53	Serrana	Yellow leaf discoloration	-	+	-
67.54	Serrana	Symptomless	-	+	-
67.55	Serrana	Symptomless	-	+	-
67.56	Serrana	Symptomless	-	+	-
67.57	Serrana	Symptomless	-	+	-
67.58	Serrana	Symptomless	-	+	-
67.59	Serrana	Symptomless	-	+	-
67.60	Serrana	Symptomless	-	+	-
101.2	Picual	Yellow leaf discoloration	+	-	-
101.3	Picual	Yellow leaf discoloration	+	-	-
101.5	Picual	Yellow leaf discoloration	+	-	-
101.6	Picual	Yellow leaf discoloration	+	-	-
101.11	Picual	Defoliation	+	-	-
101.13	Picual	Defoliation	+	-	-
