# Supplementary material for: Characterization of Spanish Olive Virome by High Throughput Sequencing Opens New Insights and Uncertainties
Source: Viruses. 2021 Nov 6;13(11):2233. doi: 10.3390/v13112233 (PMC8622421; doi:10.3390/v13112233)
Supplement: Supplementary file 1 [file DNA-A reads.pdf › DNA-A reads.pdf]

```
@A00721:190:HGJW7DSXY:4:1123:28791:14653 2:N:0:TCTGCAAG+GCTGCACT
AATAAATATAAATTTTGAATTTCAAAATTGACCGTCGATTCTACTGCGTCACTATAGAATTCATTTTT
TAGATGGTTATGATCGTGACGCGTGGTCTAATGACAAATCGACACGTAAGCGTACGGAGGTGTCCCCG
GCGCGGGCAAGCCAG
+
FFFFFFFFFFFFFFFFFFFFFFFFFFFFFFFFFFFFFFFFFFFFF:FFFFFF:FFFF:FFFFFFFFFFFF
FFFFFFFFFFFFF:FFFFF,:FFFFFFFFFFFF:FFFFFFFFFFFFFFFFFFFFFFFFFFFFFFFFFFFF
FFFFFFFFFF:FFFF
@A00721:190:HGJW7DSXY:4:1308:4553:21715 2:N:0:TCTGCAAG+GCTGCACT
AATAAATATAAATTTTGAATTTCAAAATTGACCGTCGATTCTACTGCGTCACTATAGAATTCATTTTT
TAGATGGTTATGATCGTGACGCGTGGTCTAATGACAAATCGACACGTAAGCGTACGGAGGTGTCCCCG
GCGCGGGCAAGCCAG
+
FFF:FFFFFFFFFFFFFFFFFF:FFFFFFFFFFFFFFFFFFFFFFFFFFFF:FF,:FFFF,FFFFFFFFF
FFF:FFFFFFFF:FFFFFFFFFF:FF:FFFFFFFFFFFFFFFFFFFFFF:FFFFF:FFF,FF,FFFFFFF
FFFFFFFF:FFFF:F
@A00721:190:HGJW7DSXY:4:1367:31656:30514 2:N:0:TCTGCAAG+GCTGCACT
AATAAATATAAATTTTGAATTTCAAAATTGACCGTCGATTCTACTGCGTCACTATAGAATTCATTTTT
TAGATGGTTATGATCGTGACGCGTGGTCTAATGACAAATCGACACGTAAGCGTACGGAGGTGTCCCCG
GCGCGGGCAAGCCAG
+
FFFFF:FFFFFFFFFFFFFFFFFFFFFFFFFFFFFFFFFF,FFFFFFFFF:F:FF,::F::F::FFFFF,
:FF:FFFFFFFFFFF:FFF:FFFFFFFFFFFF:FF::FFFF,FFF:FFFFFFF,F:FFFFFF,:FFFFFF
FFFFFFFF,,::FFF
@A00721:190:HGJW7DSXY:4:1464:24804:36276 2:N:0:TCTGCAAG+GCTGCACT
AATAAATATAAATTTTGAATTTCAAAATTGACCGTCGATTCTACTGCGTCACTATAGAATTCATTTTT
TAGATGGTTATGATCGTGACGCGTGGTCTAATGACAAATCGACACGTAAGCGTACGGAGGTGTCCCCG
GCGCGGGCAAGCCAG
+
FFFFFFFFFFFFFFFFFFFFFFFFFFFFFFFFFFFFFFFFFFFFFFFFFFFFFFFFFFFFFFFFFFFFFF
F:FF:FFFFFFFFFFFFFFFFFFF:FFFFFFFFFFFFFFFFFFFFFFFFFFFFFFFFFFFFFFFFFFFFF
FFFFFFFFFFF:F,F
@A00721:190:HGJW7DSXY:4:1470:32072:7811 2:N:0:TCTGCAAG+GCTGCACT
AATAAATATAAATTTTGAATTTCAAAATTGACCGTCGATTCTACTGCGTCACTATAGAATTCATTTTT
TAGATGGTTATGATCGTGACGCGTGGTCTAATGACAAATCGACACGTAAGCGTACGGAGGTGTCCCCG
GCGCGGGCAAGCCAG
+
FFFFFFFFFFFFFFFFF,FF:FFFF,FFFFFFFFFFFFFFFFFFFFFFFFFFFF::::::FFF,FFFFF:
FFFF:F:FFFFFFFFFFFFFF,FFFFFFF:FFFFFFFFFFFFF:FFFFFFF:FFFFFFF:FFFFFFF:FF
FFFFFFFFFFFFF,F
@A00721:190:HGJW7DSXY:4:1566:24053:9893 2:N:0:TCTGCAAG+GCTGCACT
AATAAATATAAATTTTGAATTTCAAAATTGACCGTCGATTCTACTGCGTCACTATAGAATTCATTTTT
TAGATGGTTATGATCGTGACGCGTGGTCTAATGACAAATCGACACGTAAGCGTACGGAGGTGTCCCCG
GCGCGGGCAAGCCAG
+
FFFF:FFFFFFFFFFFFFFFFFFFFFFFFFFFFFFFFFFF:FFFFFFFFF::FFF:F,,:FFFFFFFFFF
FFFFFFFFFFFFFFFFFFF:FFFFFFFFFFFFFFFFFFFFFFFFFFFFFFFFFFFFFFFFFFFFFFF:FF
FFFFFFFFFFFFFF:
@A00721:190:HGJW7DSXY:4:2611:17237:2315 2:N:0:TCTGCAAG+GCTGCACT
AATAAATATAAATTTTGAATTTCAAAATTGACCGTCGATTCTACTGCGTCACTATAGAATTCATTTTT
TAGATGGTTATGATCGTGACGCGTGGTCTAATGACAAATCGACACGTAAGCGTACGGAGGTGTCCCCG
GCGCGGGCAAGCCAG
+
FFFFFFFFFFFFFFFFFFFFFFFFFFFFFFFFFFFFFFFFFFFFFFFFFFFFF,FF:,FF:FFFFFFFFF
```

FFFFFFFFFFFFFFFFFFFFFFFFFFFFFFFFFFFFFFFFFF:FFFFFFFFFFFFFF::FFFFFFFFF
FFFFFFFF:FFFF,F
@A00721:190:HGJW7DSXY:4:2612:5168:33207 2:N:0:TCTGCAAG+GCTGCACT
AATAAATATAAATTTTGAATTTCAAAATTGACCGTCGATTCTACTGCGTCACTATAGAATTCATTTTT
TAGATGGTTATGATCGTGACGCGTGGTCTAATGACAAATCGACACGTAAGCGTACGGAGGTGTCCCCG
GCGCGGGCAAGCCAG
+
F:FFFFFFFFFFFFFFFFFFFFFFFFFFFFFFFFFFFFFFFFFFFFFFFFFFFFF,:FFFFFFFFFF:
FFFF:FFFFFFFFFFFFFFFFFF,FFFFFF:FF,FFFFF:FFFFFFFFFFFFFFFFFFFF:FFFFFFF
FFFFFFFF:,FFF:F
@A00721:190:HGJW7DSXY:4:1402:4146:21793 2:N:0:TCTGCAAG+GCTGCACT
AATAAATATAAATTTTGAATTTCAAAATTGACCGTCGATTCTACTGCGTCACTATAGAATTCATTTTT
TAGATGGTTATGATCGTGACGCGTGGTCTAATGACAAATCGACACGTAAGCGTACGGAGGTGTCCCCG
GCGCGGGCAAGCCCG
+
FFFFFF:FFFFFF:FFFFFFFFFFFFFFFFFFFFFFFFF::FFFFFFFFFF:F,F:FFF:FFFFFFFFF
F:FF:FFFFF:::FFFFFFFFFFFFFFFFFFFF:FFFFFFFFFFF::F::FFF:FFFFFFFFFFFFFF
FFFFFFFF::FFF,F
@A00721:190:HGJW7DSXY:4:1215:27733:12352 2:N:0:TCTGCAAG+GCTGCACT
ACACAATAAATATAAATTTTGAATTTCAAAATTGACCGTCGATTCTACTGCGTCACTATAGAATTCAT
TTTTTAGATGGTTATGATCGTGACGCGTGGTCTAATGACAAATCGACACGTAAGCGTACGGAGGTGTC
CCCGGCGCGGGCAAG
+
FFFFFFFFFFFFFFFFFFFFFFFFFFFFFFFFFFFFFFFFFF,FFFFFFFFF,FFF:FFFFFF:FFFF
FFFFFFFFFFFFFFFFFFFFFFFFFFF,FFFFFFFFFFFFFFF:FF:FFFFF,FFFFFFFFFFFFFFF
FFFFFFFFFFFFFFF
@A00721:190:HGJW7DSXY:4:1331:21504:6731 2:N:0:TCTGCAAG+GCTGCACT
ACACAATAAATATAAATTTTGAATTTCAAAATTGACCGTCGATTCTACTGCGTCACTATAGAATTCAT
TTTTTAGATGGTTATGATCGTGACGCGTGGTCTAATGACAAATCGACACGTAAGAGTACGGAGGTGTC
CCCGGCGCGGGCAAG
+
F:F:FFFFFF,F:FFFFFFFFFFFFFFFFFFFFFFFF:FFFF:FFF::F:FF:,F:FF,,:F,FFFFF
FFF,FFFF,:FF:FFFF:::F:FFFFFFFFFFFFFFFF,FFF,FFFFFFFFFF:,::FF:FF,F,:FF
:FF,FFF:F::FFF:
@A00721:190:HGJW7DSXY:4:1512:4869:5541 2:N:0:TCTGCAAG+GCTGCACT
ACACAATAAATATAAATTTTGAATTTCAAAATTGACCGTCGATTCTACTGCGTCACTATAGAATTCAT
TTTTTAGATGGTTATGATCGTGACGCGTGGTCTAATGACAAATCGACACGTAAGCGTACGGAGGTGTC
CCCGGCGCGGGCAAG
+
FFFFFFFFFFFFFFFFFFFFFFFFFFFFF:FFFFFFFFFFFF:F,FFFF:FFFFFF:F::F:FFF:FF
FFFFFFF,FFFFFFF::F:FFFFFFFFFFFFFFFFFFFFFFFF,FFFFFFFFFFFFFFFFF,FFFFF,
FFFFFFFFFFFFFFF
@A00721:190:HGJW7DSXY:4:1650:3866:34616 2:N:0:TCTGCAAG+GCTGCACT
ACACAATAAATATAAATTTTGAATTTCAAAATTGACCGTCGATTCTACTGCGTCACTATAGAATTCAT
TTTTTAGATGGTTATGATCGTGACGCGTGGTCTAATGACAAATCGACACGTAAGCGTACGGAGGTGTC
CCCGGCGCGGGCAAG
+
FFFFFFFFFFFFFFFFFFFFFFFFFFFFFFFFFFFFFFFFFFFFFFFFFFFFFFFFFFFFFFFFFFFF
FFFFFFFFFFFFFFFFFFFFFFFFFFF:FFFFFFFFFFFFFFFFFFFFFFFFFFFFFFFFFFFFFFFF
FFFFFFFFFFFFFFF
@A00721:190:HGJW7DSXY:4:2205:8612:13839 2:N:0:TCTGCAAG+GCTGCACT
ACACAATAAATATAAATTTTGAATTTCAAAATTGACCGTCGATTCTACTGCGTCACTATAGAATTCAT
TTTTTAGATGGTTATGATCGTGACGCGTGGTCTAATGACAAATCGACACGTAAGCGTACGGAGGTGTC
CCCGGCGCGGGCAAG

```
+
FFFFFFFFFFFFFFFFFFFFFFFFFFFFFFFFFFFFFFFFFFFFFFFFFFFFFFFFFF,FFFFFFFFF
FFFFFFF:FFFFFFFF,F:FFFFFFFFFFFFFFFFFFFFFFFFFFFFFFFF:FFFFFFFFF:FFFFFF
FFFFFFFFFFFFF,F
@A00721:190:HGJW7DSXY:4:2447:6641:25989 2:N:0:TCTGCAAG+GCTGCACT
ACACAATAAATATAAATTTTGAATTTCAAAATTGACCGTCGATTCTACTGCGTCACTATAGAATTCAT
TTTTTAGATGGTTATGATCGTGACGCGTGGTCTAATGACAAATCGACACGTAAGCGTACGGAGGTGTC
CCCGGCGCGGGCAAG
+
FFFFFFFFFFFFFFFFFFFFFFFFFFFFFFFFFFFFFFFFFFFFFFFFFFFFFFFFFFFFFFFFFFFF
FFFFFFFFFFFFFFFFFFFFFFFFFFFFFFFFFFFFFFFFFFFFFFFFFFFFFFFFFFFFFFFFFFFF
FFFFFFFFFFFFF,F
@A00721:190:HGJW7DSXY:4:2450:20003:1376 2:N:0:TCTGCAAG+GCTGCACT
ACACAATAAATATAAATTTTGAATTTCAAAATTGACCGTCGATTCTACTGCGTCACTATAGAATTCAT
TTTTTAGATGGTTATGATCGTGACGCGTGGTCTAATGACAAATCGACACGTAAGCGTACGGAGGTGTC
CCCGGCGCGGGCAAG
+
FFFFF,FFFFFFFFFFFFFFFFFFFFFFFFFFFFFFFFFFFFFFFFFFFFFFFFFFF:F,F:FFFFFF
FF:FFFF:FFFFFFFFFFFFFF:FFFFFFFFFFFFF:FFFF:FF:FFFFFFFFFFFFF:FFFFFFFFF
FFFFFFFFFFFFF:F
@A00721:190:HGJW7DSXY:4:2502:26829:2738 2:N:0:TCTGCAAG+GCTGCACT
ACACAATAAATATAAATTTTGAATTTCAAAATTGACCGTCGATTCTACTGCGTCACTATAGAATTCAT
TTTTTAGATGGTTATGATCGTGACGCGTGGTCTAATGACAAATCGACACGTAAGCGTACGGAGGTGTC
CCCGGCGCGGGCAAG
+
FFFFFFFFFFFFFFFFFFFFFFFFFFFFFFFFFF:FFFFFFF:FFFF:FFFF:F,:FFFFFFFFFFFF
FFFFFFFFFFFFFFFFFFFFFFFFFFF:FFFFFFFFF:FFFFFFFF:FFFFFF,FFFFFF,FFFF:F:
FFFFFFFF::FF:FF
@A00721:190:HGJW7DSXY:4:2319:23854:34569 2:N:0:TCTGCAAG+GCTGCACT
CAACACAATAAATATAAATTTTGAATTTCAAAATTGACCGTCGATTCTACTGCGTCACTATAGAATTC
ATTTTTTAGATGGTTATGATCGTGACGCGTGGTCTAATGACAAATCGACACGTAAGCGTACGGAGGTG
TCCCCGGCGCGGGCA
+
FFFFFFFFFFFFFFFFFFFFFFFFFFFFFFFFFFFFFFFFFFFF:FFFFFFFFFFFFFFF:FFFFFFF
FFFFFFFFFFFFFFFFFFFFFFFFFFFFFFFFFFFFFFF:FFFFFFFFFFFFFFFFFFFFFFFFFFFF
FFFFFFFFFFFFFFF
@A00721:190:HGJW7DSXY:4:1159:3676:24142 2:N:0:TCTGCAAG+GCTGCACT
TTTTAACAGTCTATTGGTTATCAAAAAGACGATGATATAACATATCATCTGCCGTCGATTTTACTGCG
TCACTATAGAATTCATTTTTTAGATGGTCATGATCGTGACGCGTGGTCTAATGACAAATCGACACGTA
AGCGTACGGAGGTGT
+
FFFFFFFFFFFFF,FFFFFFFFFFFFFFFFFFFFFFFFFFFFFFFFFF:FF:FFFFFFFFFFFF:FFF
FFFFFFFFFFFFFFFFFFFFFF:FFFFFFFFFFFFFFFFFFFFFFFFF:FFFFFFFF:F:FFFFFFFF
FFFFFFFFFFFFF:F
@A00721:190:HGJW7DSXY:4:1163:25925:9533 2:N:0:TCTGCAAG+GCTGCACT
TTTTAACAGTCTATTGGTTATCAAAAAGACGATGATATAACATATCATCTGCCGTCGATTTTACTGCG
TCACTATAGAATTCATTTTTTAGATGGTCATGATCGTGACGCGTGGTCTAATGACAAATCGACACGTA
AGCGTACGGAGGTGT
+
FFFFFFFFFFFFFFFFFFFFFFFFFFFFFFFFFFFFFFFFFFFFFFFFFFFFFFFFFFFFFFFFFFFF
FFFFF:FFFFFFFFFFFFFFFFFFFFFFFFFFFFFFFFFFFFFFFFFFFFFFFFF:FFFFFFFF
FFFFFFFFFFFFFFF
@A00721:190:HGJW7DSXY:4:2138:10447:24565 2:N:0:TCTGCAAG+GCTGCACT
TTTTAACAGTCTATTGGTTATCAAAAAGACGATGATATAACATATCATCTGCCGTCGATTTTACTGCG
```

TCACTATAGAATTCATTTTTTAGATGGTCATGATCGTGACGCGTGGTCTAATGACAAATCGACACGTA
AGCGTACGGAGGTGT
+
FFFFFFFFFFFFFFFFFFF:FFFFFFFFFFFFFFFFFFFFFFFFFFF:FFFF:FFFFFF:FFFFFFF:
FFFFFFFFFFFF,FF:FFFFFFFFF:FFFFF,FF:FFFFFFF:FFFFFFFFF:F,FFFF:F:FFFFFF
FF,FFF::FFFFFFF
@A00721:190:HGJW7DSXY:4:2249:4824:21840 2:N:0:TCTGCAAG+GCTGCACT
TTTTAACAGTCTATTGGTTATCAAAAAGACGATGATATAACATATCATCTGCCGTCGATTTTACTGCG
TCACTATAGAATTCATTTTTTAGATGGTCATGATCGTGACGCGTGGTCTAATGACAAATCGACACGTA
AGCGTACGGAGGTGT
+
FFFFFFFFFFFFFFFFFFFFFFFFFFFFFFFFFFFFFFFFFFFFFFFFFFFF:FFFFFFFFFFFFFFF
FFFFFFFFFFFFFFFFFFFFFFFFFFFFFFFFFFFFFFFFFFFFFFFFFFFFFFFFFFF:FFFFFFFF
FF:FFF:FFFFFFFF
@A00721:190:HGJW7DSXY:4:2258:9932:28025 2:N:0:TCTGCAAG+GCTGCACT
TTTTAACAGTCTATTGGTTATCAAAAAGACGATGATATAACATATCATCTGCCGTCGATTTTACTGCG
TCACTATAGAATTCATTTTTTAGATGGTCATGATCGTGACGCGTGGTCTAATGACAAATCGACACGTA
AGCGTACGGAGGTGT
+
FFFFFFFFFFFFFFFFFFFFFFFFFFFFFFFFFFFFFFFFFFFFFFFFFFFFFFFFFFFFFFFFFFFF
:FFFF:FFFFFFFFFFFFFFFFFFFFFFFFFFFFFFFFFFFFFFFFFFFFFFFFFFFFFFFFFFFFFF
FFFFFFFFFFFFFFF
@A00721:190:HGJW7DSXY:4:2543:12491:31735 2:N:0:TCTGCAAG+GCTGCACT
TTTTAACAGTCTATTGGTTATCAAAAAGACGATGATATAACATATCATCTGCCGTCGATTTTACTGCG
TCACTATAGAATTCATTTTTTAGATGGTCATGATCGTGACGCGTGGTCTAATGACAAATCGACACGTA
AGCGTACGGAGGTGT
+
F:FFFFFFFFFFFFFFFFFFFFFFFFFFFF:FFFFFFFFFFFFFFFFFFFFF:FFFFFFFFFFFFFFF
FFFFFF:FFF:FFFFFFFFFFFFFFF:FFFFFFFFFFFFFFFFFF:FFFFFFFFFFFFFFFFFF:FFF
F,FFFFFFFF:FFFF
@A00721:190:HGJW7DSXY:4:1104:5593:22357 2:N:0:TCTGCAAG+GCTGCACT
TGCTAAAGCAGTCATCAACACAATAAATATAAATTTTGAATTTCAAAATTGACCGTCGATTCTACTGC
GTCACTATAGAATTCATTTTTTAGATGGTTATGATCGTGACGCGTGGTCTAATGACAAATCGACACGT
AAGCGTACGGAGGTG
+
FFFFFFFFFFFFFFFF:FFFFFFFFFFFFFFFFFFFFFFFFFFFFFFFFFFFFFFFFFFFFFFFFFFF
FFFFFF,F:F:FFFFFFFFFFFFF,FFF:FFF:FFFFF:,FFFFFFF:FFFFFFF,FFFFFFFFFFFF
FFFFFFFFFFFFFFF
@A00721:190:HGJW7DSXY:4:1209:13440:29277 2:N:0:TCTGCAAG+GCTGCACT
CTTTTAACAGTCTATTGGTTATCAAAAAGACGATGATATAACATATCATCTGCCGTCGATTTTACTGC
GTCACTATAGAATTCATTTTTTAGATGGTCATGATCGTGACGCGTGGTCTAATGACAAATCGACACGT
AAGCGTACGGAGGTG
+
FF:FFFFFFFFFFFFFFFFFFFFFFFFFFFFF:FFFFFFFFFFFFFFFFFFFFFFFFFFFFFFFFFFF
FFFFFFFFF::FFFFFFFFFFFFFFFFFFFFF::FFF:FFFFFFFFFFFFFFFFF:FFFFFFFFFFFF
FF,FFFFFFFFF:FF
@A00721:190:HGJW7DSXY:4:1262:31051:32565 2:N:0:TCTGCAAG+GCTGCACT
CTTTTAACAGTCTATTGGTTATCAAAAAGACGATGATATAACATATCATCTGCCGTCGATTTTACTGC
GTCACTATAGAATTCATTTTTTAGATGGTCATGATCGTGACGCGTGGTCTAATGACAAATCGACACGT
AAGCGTACGGAGGTG
+
FFFFFFFFFFFFFFFFFFFFFFFFFFFFFFFFFFFFFFFFFFFFFFFFFFFFFFFFFFFFFFFFFFFF
FFFFFFFFFFFFFFFFFFFFFFFFFFFFF:FFFFFFFFFFFFFFFFFFFFFFFFFFFFFFFFFFFFFF
FFFFFFFFFFFFFFF

```
@A00721:190:HGJW7DSXY:4:1262:31575:33661 2:N:0:TCTGCAAG+GCTGCACT
CTTTTAACAGTCTATTGGTTATCAAAAAGACGATGATATAACATATCATCTGCCGTCGATTTTACTGC
GTCACTATAGAATTCATTTTTTAGATGGTCATGATCGTGACGCGTGGTCTAATGACAAATCGACACGT
AAGCGTACGGAGGTG
+
FFFFFFFFFFFF:FFFFFFFFFFFFFFFFFFFFFFFFFFFFFFFFFFFFFFFF:FFFFFFFFFFFFFF
FFFFFFFFFFFFFFFFFFFFFFFFFFFFFFFFFFFFFFFFFFFFFFFFFFFFFFFFFFFFFF:F:F:F
FFFFF:FFFFFFFFF
@A00721:190:HGJW7DSXY:4:1339:12255:29951 2:N:0:TCTGCAAG+GCTGCACT
TGCTAAAGCAGTCATCAACACAATAAATATAAATTTTGAATTTCAAAATTGACCGTCGATTCTACTGC
GTCACTATAGAATTCATTTTTTAGATGGTTATGATCGTGACGCGTGGTCTAATGACAAATCGACACGT
AAGCGTACGGAGGTG
+
FFF:FFFFFFFFFFFFFFFFFFFFFFFFFFFFFFFFFFFFFFFFFFFFFFFFFFFFFFFFFFFFFFFF
FFFFFFFF:FFFFFFFFFF::FFFF:FFFF:FFFFFFFFFFFFF,FFFFFFFF,FFFFFFFFFFFFFF
F:FFFFFFFFFFF::
@A00721:190:HGJW7DSXY:4:1522:2410:36793 2:N:0:TCTGCAAG+GCTGCACT
CTTTTAACAGTCTATTGGTTATCAAAAAGACGATGATATAACATATCATCTGCCGTCGATTTTACTGC
GTCACTATAGAATTCATTTTTTAGATGGTCATGATCGTGACGCGTGGTCTAATGACAAATCGACACGT
AAGCGTACGGAGGTG
+
FFFFFFFFFFFFFFFFFFFFFFFFFFFFFFFFFFFFFFFFFFFFFFFFFFFFFFFFFFFFFFFFFFFF
FFFFFFFFFFFFFFFFFFFFFFFFFFFFFFFFFFFFFFFFFFFFFFFFFFFFFFFFF:FF:FFFFFFF
F:FFFFFFFFFFFFF
@A00721:190:HGJW7DSXY:4:1544:8648:17347 2:N:0:TCTGCAAG+GCTGCACT
CTTTTAACAGTCTATTGGTTATCAAAAAGACGATGATATAACATATCATCTGCCGTCGATTTTACTGC
GTCACTATAGAATTCATTTTTTAGATGGTCATGATCGTGACGCGTGGTCTAATGACAAATCGACACGT
AAGCGTACGGAGGTG
+
FFFFFFFFFFFFFFFFFFFFFFFFFFF:F:FFFFFFFFFFFFFFFFFFFFFFFFFFFFFFFFFFFFF:
FFFFFFF:FFFFFFFF:FFFFFFFFF:FFFFFFFFFFFFFFFFFFFFFFFFFFFFF:FFFFFFFFFFF
F,,FFFFFFFFFFFF
@A00721:190:HGJW7DSXY:4:1602:7129:24893 2:N:0:TCTGCAAG+GCTGCACT
CTTTTAACAGTCTATTGGTTATCAAAAAGACGATGATATAACATATCATCTGCCGTCGATTTTACTGC
GTCACTATAGAATTCATTTTTTAGATGGTCATGATCGTGACGCGTGGTCTAATGACAAATCGACACGT
AAGCGTACGGAGGTG
+
FFFFFFFFFFFFFFFFFFFFFFFFFFFFFFFFFFFFFFFFFFFFFFFFFFFFFFFFFFFFFFFFFFFF
FFFFFFFFFFFFFF,FFFFFFFFFFFFFFF:F:FFFFF:FFFFFFFFFFFFFFFFFFFFFFFFFFFFF
FFFFFFFFFFFF:FF
@A00721:190:HGJW7DSXY:4:1645:5873:25347 2:N:0:TCTGCAAG+GCTGCACT
TGCTAAAGCAGTCATCAACACAATAAATATAAATTTTGAATTTCAAAATTGACCGTCGATTCTACTGC
GTCACTATAGAATTCATTTTTTAGATGGTTATGATCGTGACGCGTGGTCTAATGACAAATCGACACGT
AAGCGTACGGAGGTG
+
FFFFFFFFFFFFFFFFFFFFFFFFFFFFFFFFFFFFFFFFFFFFFFFFFFFFFFFFFFFFFFFFFFFF
FFFFFFFF:FFFFFFF:FFFFFFF:FFFFFFFFFFF:F:FFFFFFFFFFFF:FFFFFFFFFFFFFFFF
FFFFFFFFFF:FFFF
@A00721:190:HGJW7DSXY:4:1667:12301:20948 2:N:0:TCTGCAAG+GCTGCACT
CTTTTAACAGTCTATTGGTTATCAAAAAGACGATGATATAACATATCATCTGCCGTCGATTTTACTGC
GTCACTATAGAATTCATTTTTTAGATGGTCATGATCGTGACGCGTGGACTAATGACAAATCGACACGT
AAGCGTACGGAGGTG
+
:F:FFFFFFFFFFF:FFFFFFFFFFFFFFFFFF:FFFFFFFFFFF::F:FFFF:F,,:FF::FFFFFF
```

,FFFFFFF:F::FFFF,:FFFFF,F:::FFF::FFFF,FFFFFF,F:,FFFFF,FFFFFFFFF,FFFF
FF:FF:FFFFF:,F:
@A00721:190:HGJW7DSXY:4:2123:21151:29168 2:N:0:TCTGCAAG+GCTGCACT
CTTTTAACAGTCTATTGGTTATCAAAAAGACGATGATATAACATATCATCTGCCGTCGATTTTACTGC
GTCACTATAGAATTCATTTTTTAGATGGTCATGATCGTGACGCGTGGTCTAATGACAAATCGACACGT
AAGCGTACGGAGGTG
+
:FFFFFFFFFFFFFFFFFFFFFFFFFFFFFFFFFFFFFFFFFFFFFFFFFFFF:FFFFFFFFFFFFFF
FFFFFFFFFF:FFFFFFFFF:FFFFFF,FFFFFFFFFFFFFFFFFFFFFFFFFFFFFFFF,FF:FF:F
:F:FFFFF,FF::F:
@A00721:190:HGJW7DSXY:4:2175:12057:9502 2:N:0:TCTGCAAG+GCTGCACT
TGCTAAAGCAGTCATCAACACAATAAATATAAATTTTGAATTTCAAAATTGACCGTCGATTCTACTGC
GTCACTATAGAATTCATTTTTTAGATGGTTATGATCGTGACGCGTGGTCTAATGACAAATCGACACGT
AAGCGTACGGAGGTG
+
FFFFFFFFFFFFFFFFFFFFFFFFFFFFF:FFFFFFFFFFFFFFFFFFFFFFFFFFFFFFFFFFFFFF
FFFFFFFFFFFFFFFFFFFFFFFFFFFFFFFFF,FFFFFF:FFFFFFFFFFFFFFFFFFFFF:FFFFFF
FFFFFFFF:FFFFFF
@A00721:190:HGJW7DSXY:4:2340:16504:6840 2:N:0:TCTGCAAG+GCTGCACT
CTTTTAACAGTCTATTGGTTATCAAAAAGACGATGATATAACATATCATCTGCCGTCGATTTTACTGC
GTCACTATAGAATTCATTTTTTAGATGGTCATGATCGTGACGCGTGGTCTAATGACAAATCGACACGT
AAGCGTACGGAGGTG
+
FFFFFFFFFFFFFFFFFFFFFFF:FFFFFF:FFFFFFFF:FFFFFFFFFFFFFFFFFFFFFFFFFFFF
FFFFFFFFFFFFFFFFFFFFFFFFFFFFFFFFFFFFFFFFFFFFF:FFFFFFFFFF:FFFFFFFFFFFF
FF,F:FFFFFFFFFF
@A00721:190:HGJW7DSXY:4:2462:10303:26287 2:N:0:TCTGCAAG+GCTGCACT
CTTTTAACAGTCTATTGGTTATCAAAAAGACGATGATATAACATATCATCTGCCGTCGATTTTACTGC
GTCACTATAGAATTCATTTTTTAGATGGTCATGATCGTGACGCGTGGTCTAATGACAAATCGACACGT
AAGCGTACGGAGGTG
+
FFFFFFFFFFFFFFFFFFFFFFFFFFFFFFFFFFFFF:FFFFFFFFFFFFFFFFFFFFFFFFFFFFFF
FFFFFFFFF:FFFFFFFFFFFFFFFFFFFFFFFFFFF:FFFFFFFFFFFFFFF:FFFFFFF:FFFFFFF
FFFFFFFFFFFFFFF
@A00721:190:HGJW7DSXY:4:2553:13602:11929 2:N:0:TCTGCAAG+GCTGCACT
CTTTTAACAGTCTATTGGTTATCAAAAAGACGATGATATAACATATCATCTGCCGTCGATTTTACTGC
GTCACTATAGAATTCATTTTTTAGATGGTCATGATCGTGACGCGTGGTCTAATGACAAATCGACACGT
AAGCGTACGGAGGTG
+
FFFFFFFFFFFFFFF,FFFFFFFFFFFFFFFFFFFFFFFFFFFFFFFFFFFFFFFFFFF:FFFFFFFF
FFFFFFFFFFFFFFFFFFFFFFFFFFFFFFFFFFFFFFFFF:FFFFFFFFFF:FFFFFFFFF:FFFFFFF
FFFFFFFFFFFFFF:
@A00721:190:HGJW7DSXY:4:2608:5484:6887 2:N:0:TCTGCAAG+GCTGCACT
CTTTTAACAGTCTATTGGTTATCAAAAAGACGATGATATAACATATCATCTGCCGTCGATTTTACTGC
GTCACTATAGAATTCATTTTTTAGATGGTCATGATCGTGACGCGTGGTCTAATGACAAATCGACACGT
AAGCGTACGGAGGTG
+
FFFFFFFFFF:FFF:FFFFFFFFFFFFFF:FFFFFF:FFFFFFFFFFFFF:FFFFFFFFFFFFFFFF:
FF:FFF,FFFFFFFFFFFFFFFFFFF:FFF,FF:FFFF,:FFFFFFFFFFFFFFFFFFFFF:,FFFFFFF
FFFF:FFF,FF,FFF
@A00721:190:HGJW7DSXY:4:1427:1642:12853 2:N:0:TCTGCAAG+GCTGCACT
AATCAACTGCTAAAGCAGTCATCAACACAATAAATATAAATTTTGAATTTCAAAATTGACCGTCGATT
CTACTGCGTCACTATAGAATTCATTTTTTAGATGGTTATGATCGTGACGCGTGGTCTAATGACAAATC
GACACGTAAGCGTAC

```
+
FFFFFFFFFFFFFFFFFFFFFFFFFFFFFFFFFFFFFFFFFFFFFFFFFFFFFFFFFFFFFFFFFFFF
FFFFFFFFFFFFFFF:FFFFFFFFFFFFFFFFFFFFFFFFFFFFFFFFFFFFFFFFFFFFFFFFFFFF
FFF:FFFF,FFFFFF
@A00721:190:HGJW7DSXY:4:1512:16477:34381 2:N:0:TCTGCAAG+GCTGCACT
AATCAACTGCTAAAGCAGTCATCAACACAATAAATATAAATTTTGAATTTCAAAATTGACCGTCGATT
CTACTGCGTCACTATAGAATTCATTTTTTAGATGGTTATGATCGTGACGCGTGGTCTAATGACAAATC
GACACGTAAGCGTAC
+
FFFFFFFFFFFFFFFFFFFFFFFFFFFFFFFFFFFFFFFFFFFFFFFFFFFFFFFFFFFFFFFFFFFF
FFFFFFFFFFFFFFF:F:FFFFFFFFFF:FFF:FFFFFFF:FFFFFF:FFFFFFFFFFFF,FFFFFFF
FFFFFFFFFFFFFFF
@A00721:190:HGJW7DSXY:4:1512:18584:35117 2:N:0:TCTGCAAG+GCTGCACT
AATCAACTGCTAAAGCAGTCATCAACACAATAAATATAAATTTTGAATTTCAAAATTGACCGTCGATT
CTACTGCGTCACTATAGAATTCATTTTTTAGATGGTTATGATCGTGACGCGTGGTCTAATGACAAATC
GACACGTAAGCGTAC
+
FFFFFFFFFFFFFFFFFFFFFFFFFFFFFFFFFFFFFFFFFFFFFFFFFFFFFFFFFFFFFFFFFFFF
FFFFFFFFFFFFF:::FFFFFFFFFFFFFFFFFFFFFFFFFFFFFFFFFFFF:FFFFFFFFFFFFFFF
FFFFF:FFFFFFFFF
@A00721:190:HGJW7DSXY:4:2445:12762:23688 2:N:0:TCTGCAAG+GCTGCACT
AATCAACTGCTAAAGCAGTCATCAACACAATAAATATAAATTTTGAATTTCAAAATTGACCGTCGATT
CTACTGCGTCACTATAGAATTCATTTTTTAGATGGTTATGATCGTGACGCGTGGTCTAATGACAAATC
GACACGTAAGCGTAC
+
FFFFFFFFFFFFFFFFFFFFFFFFFFFFFFFFFFFFFFFFFFFFFFFFFFFFFFFFFFFFFFFFFFFF
FFFFFFFFFFFFFFFFFFFFFFFFF:FFFFFFFFFFFFFFFFFFFFFFFFFFFFFFFFFFFFFFFFFF
FFFFFFFFFFFFFFF
@A00721:190:HGJW7DSXY:4:2468:5367:7623 2:N:0:TCTGCAAG+GCTGCACT
AATCAACTGCTAAAGCAGTCATCAACACAATAAATATAAATTTTGAATTTCAAAATTGACCGTCGATT
CTACTGCGTCACTATAGAATTCATTTTTTAGATGGTTATGATCGTGACGCGTGGTCTAATGACAAATC
GACACGTAAGCGTAC
+
FFFFFFFFFFFFFFFFFFFFFFFFFFFFFFFFFFFFFFFFFFFFFFFFFFFFFFFFFFFFFFFFFFFF
FFFFFFFFFFFFFFFFFFFFFFFFFFFFF:FFFFFFFFFFFFFFFFFFFFFFFFFFFFFFFFFFFFFF
:FFFFFFFFFFFFFF
@A00721:190:HGJW7DSXY:4:2545:15881:17096 2:N:0:TCTGCAAG+GCTGCACT
AATCAACTGCTAAAGCAGTCATCAACACAATAAATATAAATTTTGAATTTCAAAATTGACCGTCGATT
CTACTGCGTCACTATAGAATTCATTTTTTAGATGGTTATGATCGTGACGCGTGGTCTAATGACAAATC
GACACGTAAGCGTAC
+
FFFFFFFFFFFFFFFFFFFFFFFFFFFFFFFFFFFFFFFFFFFFFFFFFFFFFFFFFFFFFFFFFF,F
FFFFFFFFFFFFF:FFF:FFFFFFFFFFFFF,FFFFFFFFFFFFFFFFFFFFFFFFFF:FFFFF:FFF
FFFFFFFFFFFFFFF
@A00721:190:HGJW7DSXY:4:1240:24876:2769 2:N:0:TCTGCAAG+GCTGCACT
ATTAGGTCTAGCCGTGCAGCCTCTTTGCTACCCACAATATTTATAAATTTTGAATTTCAAAATTGACC
GTCGATTCTACTGCGTCACTATAGAATTCATTTTTTAGATGGTTATGATCGTGACGCGTGGTCTAATG
ACAAATCGACACGTA
+
FFFFFFFFFFFFFFFFFFFFFFFF,FFFFFFFFFFF:FFFFFFFFFFFFFFFFFFFFFFFFFFFF:FF
FFFFFFFFFFFFFFF:FFFF:FFFFFFFFFFFFFFFFFFFFFFFFFFFFFFFFFFFFFFFFFFF,FFFFF:,
FFFFFFFFFFFF:FF
@A00721:190:HGJW7DSXY:4:1341:18484:7294 2:N:0:TCTGCAAG+GCTGCACT
ATTAGGTCTAGCCGTGCAGCCTCTTTGCTACCCACAATATTTATAAATTTTGAATTTCAAAATTGACC
```

```
GTCGATTCTACTGCGTCACTATAGAATTCATTTTTTAGATGGTTATGATCGTGACGCGTGGTCTAATG
ACAAATCGACACGTA
+
:FFFFFFFFFFFFFFFFFFFFFFFFFFFFFFFFFFFFFFFFFFFFFFFFFFFFFFFFFFFFFFFFFFF
FFFFFFFFFFFFFFFFFFFFFFFFFFFFFFFFFFF,:FFFFFFFFFFFFFFFFFFFFFFFFFFFF:FF
FFFFFFFFFFFFFFF
@A00721:190:HGJW7DSXY:4:1532:10737:6715 2:N:0:TCTGCAAG+GCTGCACT
ATTAGGTCTAGCCGTGCAGCCTCTTTGCTACCCACAATATTTATAAATTTTGAATTTCAAAATTGACC
GTCGATTCTACTGCGTCACTATAGAATTCATTTTTTAGATGGTTATGATCGTGACGCGTGGTCTAATG
ACAAATCGACACGTA
+
F:FFFFFFFFFFFFFFFFFFFFFFFFFFFFFFFFFFFF:FFFFFFFFFFFF:FFFFFFFFFFFFFFFF
FFFFFFFFFFFFFFFFFFFFFFF:FFFFFFFFFFFFFFFFF:FFFFFFFFFFF:FFFFFFFFFFFFFF
FFFFFFFFFFFFFFF
@A00721:190:HGJW7DSXY:4:1671:13946:2973 2:N:0:TCTGCAAG+GCTGCACT
ATTAGGTCTAGCCGTGCAGCCTCTTTGCTACCCACAATATTTATAAATTTTGAATTTCAAAATTGACC
GTCGATTCTACTGCGTCACTATAGAATTCATTTTTTAGATGGTTATGATCGTGACGCGTGGTCTAATG
ACAAATCGACACGTA
+
FFF:FFFFFF:FFFFFFFFFF:FFFFFFFFFFFFFFF:FFFFFFFFFFFFFFFF:FFFFFFFFFFFFF
FFFF:FFFFFFFFFFFFFFFFFF:FFFFFFFFFFFFFFFFFFFFFFFFFFFFF:FFFFFFFFFFFFFF
::FFFFFFFFFFFFF
@A00721:190:HGJW7DSXY:4:2131:7319:21746 2:N:0:TCTGCAAG+GCTGCACT
ATTAGGTCTAGCCGTGCAGCCTCTTTGCTACCCACAATATTTATAAATTTTGAATTTCAAAATTGACC
GTCGATTCTACTGCGTCACTATAGAATTCATTTTTTAGATGGTTATGATCGTGTCGCGTGGTCTAATG
ACAAATCGACACGTA
+
FFFFFFFFFFFFFFFFFFFFFFFFFFFF:FFFFF:,F:FFFFFFFFFFFFFFFFFFF:FF,FFFFFF:
FFFFFFFFFFFFFFFFFFFF:FF:F,F::FF:F:FFFFFFF:FFFF,FF:FFF,F::FFFFFFFF,F:
,FFF:FF,FFFFFFF
@A00721:190:HGJW7DSXY:4:2163:26856:11647 2:N:0:TCTGCAAG+GCTGCACT
ATTAGGTCTAGCCGTGCAGCCTCTTTGCTACCCACAATATTTATAAATTTTGAATTTCAAAATTGACC
GTCGATTCTACTGCGTCACTATAGAATTCATTTTTTAGATGGTTATGATCGTGACGCGTGGTCTAATG
ACAAATCGACACGTA
+
FFFFFFFFFFFFFFFFFFFFFFFFFFFFFFFFFFFFFFFFFFFFFFFFFFFFFFFFFFFFFFFFFFFF
FFFFFFFFFFFFFFFFFFFFFF:FFFFFFFFFFFFFFFFFFFFFFFFFFFFFF,FFFFFF:FFFFFFF
FFFFF:FFFFFFFFF
@A00721:190:HGJW7DSXY:4:2210:15917:11240 2:N:0:TCTGCAAG+GCTGCACT
ATTAGGTCTAGCCGTGCAGCCTCTTTGCTACCCACAATATTTATAAATTTTGAATTTCAAAATTGACC
GTCGATTCTACTGCGTCACTATAGAATTCATTTTTTAGATGGTTATGATCGTGACGCGTGGTCTAATG
ACAAATCGACACGTA
+
FFFFFFFFFFFFFFFFFFFFFFFFFFFFFFFFFF:,FFFF,FFFFFFFFFFFFFFFFFFFFFFF::FF
FFFF:FFFFFFFFFF:FF:FFFFF::FF:FFF:FFF:FFFFF,FFFFFFFFFF:FFFFFFF:FF,FF,
FFFFFFFF:FFFFFF
@A00721:190:HGJW7DSXY:4:2362:15917:7670 2:N:0:TCTGCAAG+GCTGCACT
ATTAGGTCTAGCCGTGCAGCCTCTTTGCTACCCACAATATTTATAAATTTTGAATTTCAAAATTGACC
GTCGATTCTACTGCGTCACTATAGAATTCATTTTTTAGATGGTTATGATCGTGACGCGTGGTCTAATG
ACAAATCGACACGTA
+
FFFFFFFFFFFFFFFFFFFFFFFFFFFFFFFFFFFFFFFFFFFFFFFFFFFFFFFF::FFFFFFFFFF
FFFFFFFFFFFFFFFFFFFFFF,F::FFFFF:FFFF,F:FFFFFFFFFFFFFF:FFFFFFFFFFFFFF
FFFF:FFFFFFFFF:
```

```
@A00721:190:HGJW7DSXY:4:2445:4291:14591 2:N:0:TCTGCAAG+GCTGCACT
ATTAGGTCTAGCCGTGCAGCCTCTTTGCTACCCACAATATTTATAAATTTTGAATTTCAAAATTGACC
GTCGATTCTACTGCGTCACTATAGAATTCATTTTTTAGATGGTTATGATCGTGACGCGTGGTCTAATG
ACAAATCGACACGTA
+
FFFFFFFFFFFFFFFFFFFFFFFFFFFFFFFFFFFFFFFF:FFFFFFFFFFFFFFFF:FFFFFFFFFF
FFFFFFFFFFFFFFFFFFF,FFFFFF,FFF:FF:FFFFFFFFFFFFFFF:F,FF:FFF:FFFFF::F:
FFFFFFF,FFF,FFF
@A00721:190:HGJW7DSXY:4:2445:4734:17362 2:N:0:TCTGCAAG+GCTGCACT
ATTAGGTCTAGCCGTGCAGCCTCTTTGCTACCCACAATATTTATAAATTTTGAATTTCAAAATTGACC
GTCGATTCTACTGCGTCACTATAGAATTCATTTTTTAGATGGTTATGATCGTGACGCGTGGTCTAATG
ACAAATCGACACGTA
+
FFFFFFFFFFFFFFFFFFFFFFF:FFFFFFFFFFFFFFFFFFFFFF:FFFFFFFFFFFFFF:FF,FFF
FFF:FF:FFFFFFFF::FFFFFFFFFFFFF:FFFFFFFFFFFFFFFFFFF:FFFFFFFFFFFFFFFFF
FFFFFFF,FFFFFFF
@A00721:190:HGJW7DSXY:4:2525:27525:32659 2:N:0:TCTGCAAG+GCTGCACT
TTAGGTCTAGCCGTGCAGCCTCTTTGCTACCCACAATATTTATAAATTTTGAATTTCAAAATTGACCG
TCGATTCTACTGCGTCACTATAGAATTCATTTTTTAGATGGTTATGATCGTGACGCGTGGTCTAATGA
CAAATCGACACGTA
+
FFFF:FFFFFFFFFFFFFFFFFFFFFFFFFFFFF::FFFFFFFFFFF:FFFFFFF:FFFFFFFFFFFF
FFFFFFFFFFFF:FFFFFFFF:FFF:F:FFFF:FFFFF:FFFFFFFF:FFFFFF,FFFFFFFFFFFF:
FF:FF,FFFFFF,F
@A00721:190:HGJW7DSXY:4:2577:16333:29058 2:N:0:TCTGCAAG+GCTGCACT
ATTAGGTCTAGCCGTGCAGCCTCTTTGCTACCCACAATATTTATAAATTTTGAATTTCAAAATTGACC
GTCGATTCTACTGCGTCACTATAGAATTCATTTTTTAGATGGTTATGATCGTGACGCGTGGTCTAATG
ACAAATCGACACGTA
+
FFFFFFFFFFFFFFFFFFFFFFFFFFFFFFFFFFFFFFFFFFFFFFFFFFFFFFFFFFF:FFFFFFFF
FFFFFFFFFFFFFFFFFFFFFFFF:FFFFFFFFFFFFFFFFF:FFFFFFF:FFFFFFFFFF:FFFFF,
FFFFFF:FFFFFFFF
@A00721:190:HGJW7DSXY:4:1103:9561:34867 2:N:0:TCTGCAAG+GCTGCACT
CTTCTTTGTAATGTATTAGGTCTAGCCGTGCAGCCTCTTTGCTACCCACAATATTTATAAATTTTGAA
TTTCAAAATTGACCGTCGATTCTACTGCGTCACTATAGAATTCATTTTTTAGATGGTTATGATCGTGA
CGCGTGGTCTAATGA
+
FFFFFFFFFFFFFFFFFFFFFFFFFFFFFFFFFFFFFFFFFFFFFFFFFF:FFFFFFFFFFFFFFFFF
FFFFFFFFFFFFFFFFFF,FFFFFF:F:FFFFFFFF:FFFFFFF:FFFFFFFFFFFFFFFFFFFFFFF
FFFFFFFFFFFFFFF
@A00721:190:HGJW7DSXY:4:1121:22996:18646 2:N:0:TCTGCAAG+GCTGCACT
CTTCTTTGTAATGTATTAGGTCTAGCCGTGCAGCCTCTTTGCTACCCACAATATTTATAAATTTTGAA
TTTCAAAATTGACCGTCGATTCTACTGCGTCACTATAGAATTCATTTTTTAGATGGTTATGATCGTGA
CGCGTGGTCTAATGA
+
FFFFFFFFFFFFF:FFFFFFFFFFFFFFFFFFFFFFFFFFFFFFFFFFFFFFFFFFFFFFFFFFFFFF
FFFFFFFFFFFFFFFFFFF:FFFFFF:FFFFFFFFFFF:FFFFFFFFFFFFFFFFFFFFFFFFFFFFF
FFFFFFFFFFFFFFF
@A00721:190:HGJW7DSXY:4:1210:2862:33442 2:N:0:TCTGCAAG+GCTGCACT
CTTCTTTGTAATGTATTAGGTCTAGCCGTGCAGCCTCTTTGCTACCCACAATATTTATAAATTTTGAA
TTTCAAAATTGACCGTCGATTCTACTGCGTCACTATAGAATTCATTTTTTAGATGGTTATGATCGTGA
CGCGTGGTCTAATGA
+
FFFFF:FFFFFFF:FFFFF:FFFFFF:FFFFFFFFFFFFFFFFFFFFFFFFFFFFFFFFFFFFFFFFF
```

FFFFFFFFFFFFFFFFFFFFFFFFFFFFFFFFFF:F,:FFFFFFFFFFFFFF:FFFFFFFFFFFFFFF:
FFFF,FFFFFF,F:F
@A00721:190:HGJW7DSXY:4:1223:10917:35900 2:N:0:TCTGCAAG+GCTGCACT
CTTCTTTGTAATGTATTAGGTCTAGCCGTGCAGCCTCTTTGCTACCCACAATATTTATAAATTTTGAA
TTTCAAAATTGACCGTCGATTCTACTGCGTCACTATAGAATTCATTTTTTAGATGGTTATGATCGTGA
CGCGTGGTCTAATGA
+
FFFFFFFFFFFFFFFFFFFFFFFF,FFFFFFFFF:FFFFFFFFF:F:FFFFFFFFFFFFFFFFFFFFF
FFFFF:FFFFFFFFFFFFFFFFFFFFFFF:FFFF,:FFFFFF:,F,FFFFFF,:F:FFFFFFF:FFF,F
FFFFFFFFFFFFFFF
@A00721:190:HGJW7DSXY:4:1234:28827:29277 2:N:0:TCTGCAAG+GCTGCACT
CTTCTTTGTAATGTATTAGGTCTAGCCGTGCAGCCTCTTTGCTACCCACAATATTTATAAATTTTGAA
TTTCAAAATTGACCGTCGATTCTACTGCGTCACTATAGAATTCATTTTTTAGATGGTTATGATCGTGA
CGCGTGGTCTAATGA
+
FFFFF:FFFFFFFFFFFFFFFFFFFFFFFF:FFFFFFFFF:FFFFFFFFFFFFFFFFFFFFFFFFFF:F
FF:FFF,,FFFFFFFFFFF:FFFFFFFFFFFFF,FFFFFFFF:F:FFFFFFFFFFFFFFFFFFFFFFFF
F,FFFFFFFFFFFFF
@A00721:190:HGJW7DSXY:4:1240:2790:33505 2:N:0:TCTGCAAG+GCTGCACT
CTTCTTTGTAATGTATTAGGTCTAGCCGTGCAGCCTCTTTGCTACCCACAATATTTATAAATTTTGAA
TTTCAAAATTGACCGTCGATTCTACTGCGTCACTATAGAATTCATTTTTTAGATGGTTATGATCGTGA
CGCGTGGTCTAATGA
+
FFFFFFFFF:FFFFFFFFFFFF:FFFFF:FFFFF:FFF:FFFFFFFFFF:FFFFFFFF:FFFFFFFFF
FFFFFFFFFFFFFF:FFFFFFFFFFFFFFFFFF:FFFFFF:FFFFFFFFFF::FFFFFFFFFFF:F,
::FF::FFFFFFFFF
@A00721:190:HGJW7DSXY:4:1246:3739:17049 2:N:0:TCTGCAAG+GCTGCACT
CTTCTTTGTAATGTATTAGGTCTAGCCGTGCAGCCTCTTTGCTACCCACAATATTTATAAATTTTGAA
TTTCAAAATTGACCGTCGATTCTACTGCGTCACTATAGAATTCATTTTTTAGATGGTTATGATCGTGA
CGCGTGGTCTAATGA
+
FFFFFF:FFFFFFFFFFFFFFFFF:FFFFFFFFFFFFFFFFFFFFFF:FFFFFFFFFFFF:FFFFFFF
FFFFFFFFFFFFFFFFFFF:FFFFFFFFF:FFF:FFFFFFFFFF:FFFFFFFFFFFFFFFFFFFFFFF:
FFFFFFFFFFFFFFF
@A00721:190:HGJW7DSXY:4:1530:25048:7670 2:N:0:TCTGCAAG+GCTGCACT
CTTCTTTGTAATGTATTAGGTCTAGCCGTGCAGCCTCTTTGCTACCCACAATATTTATAAATTTTGAA
TTTCAAAATTGACCGTCGATTCTACTGCGTCACGATAGAATTCATTTTTTAGATGGTTATGATCGTGA
CGCGTGGTCTAATGA
+
FFFFFFFFFF:FFFFFFFFFFFFFFFFFFFFFFFFFFFFFFFFFFFFFFFFFFFFFFFFFFF:FFFFFF
FFFFFFFFFFFFFFFFF:F,FF:F:,FFFFFF,,FF:,FFFFFFFFFFFFFF,FFFFFFFFFFFFFF:
FFFFFFFFFFFFF:F
@A00721:190:HGJW7DSXY:4:1634:13801:1783 2:N:0:TCTGCAAG+GCTGCACT
CTTCTTTGTAATGTATTAGGTCTAGCCGTGCAGCCTCTTTGCTACCCACAATATTTATAAATTTTGAA
TTTCAAAATTGACCGTCGATTCTACTGCGTCACTATAGAATTCATTTTTTAGATGGTTATGATCGTGA
CGCGTGGTCTAATGA
+
FFFFFFFFFFFFFFFFFFFFFFFFFFFFFFFFFFFFFFFFFFFFFFFFFFFFFFFFFFFFFFFFFFFF
FFFFFFFFFFFFF,FF:FFFFFFFFFFFFFFFFF,F:FFFFF:FFFFFFFFFFF:FFFFF,FFFFFFF
:FFFFFFFFFFFFFF
@A00721:190:HGJW7DSXY:4:1674:17924:7200 2:N:0:TCTGCAAG+GCTGCACT
CTTCTTTGTAATGTATTAGGTCTAGCCGTGCAGCCTCTTTGCTACCCACAATATTTATAAATTTTGAA
TTTCAAAATTGACCGTCGATTCTACTGCGTCACTATAGAATTCATTTTTTAGATGGTTATGATCGTGA
CGCGTGGTCTAATGA

```
+
FFFFFFFF:FFFFFFFFFFFFFFFFFFFFFFFFFFFFFFFFFFFFFFFFFFFFFFFFFFFFFFFFFFFFF
FFFFFFFFFFFFFFFFFFFFFFFFFFFFFFFFFFF::FFFFFFFFFFFFFFFFFFFFFFFFFFFFFFFFF
FFFFFFFFFFFFFFF
@A00721:190:HGJW7DSXY:4:2124:28809:9298 2:N:0:TCTGCAAG+GCTGCACT
CTTCTTTGTAATGTATTAGGTCTAGCCGTGCAGCCTCTTTGCTACCCACAATATTTATAAATTTTGAA
TTTCAAAATTGACCGTCGATTCTACTGCGTCACTATAGAATTCATTTTTTAGATGGTTATGATCGTGA
CGCGTGGTCTAATGA
+
FFFFFFFFFFFFFFFFFFFFFFFFFFFFFFFFFFFFFFFFFFFFFFFFFFFFFFFFFFFFFFFFFFFFF
FFFFFFFFFFFFF:FFFFF:FFFFFFFFFFF:FFFFFFFFFF,F:FFFFFF,:FF,FFFFFFF:FFF:
FFFFFF:FFFFFF:F
@A00721:190:HGJW7DSXY:4:2210:6081:26584 2:N:0:TCTGCAAG+GCTGCACT
CTTCTTTGTAATGTATTAGGTCTAGCCGTGCAGCCTCTTTGCTACCCACAATATTTATAAATTTTGAA
TTTCAAAATTGACCGTCGATTCTACTGCGTCACTATAGAATTCATTTTTTAGATGGTTATGATCGTGA
CGCGTGGTCTAATGA
+
FFFFFFFFFF::F,FFFFFFFFF:FFFFFFFFFFFFFFFFFFFFFFFFFFF::FFFFFFFFFFFFFFF
FFFFFFFFFFFFFFFFFFFFFFFFFFFF,FFFFFF::FFFFF:,FFFFFFFFFFFFFFFFF,F:FFFFF
:,FF,FFFFFFFF:F
@A00721:190:HGJW7DSXY:4:2238:20311:11616 2:N:0:TCTGCAAG+GCTGCACT
CTTCTTTGTAATGTATTAGGTCTAGCCGTGCAGCCTCTTTGCTACCCACAATATTTATAAATTTTGAA
TTTCAAAATTGACCGTCGATTCTACTGCGTCACTATAGAATTCATTTTTTAGATGGTTATGATCGTGA
CGCGTGGTCTAATGA
+
FFFFFFFFFFFFFFFFFFFFFFFFFF:FFFFFFFFFFFFFFFFFFFFFFFFFFFFFFFFFFFFFFFFFF
FFFFFFFFFFFFFFFFFFFFFFFFFFF:F:FFFFFFF::FFFFFFFFFFFFFFFFFFFFFFFFFFFFFF
:FFFFFFFFFF:FFF
@A00721:190:HGJW7DSXY:4:2257:4589:26005 2:N:0:TCTGCAAG+GCTGCACT
CTTCTTTGTAATGTATTAGGTCTAGCCGTGCAGCCTCTTTGCTACCCACAATATTTATAAATTTTGAA
TTTCAAAATTGACCGTCGATTCTACTGCGTCACTATAGAATTCATTTTTTAGATGGTTATGATCGTGA
CGCGTGGTCTAATGA
+
FFFFFFFFFFF:FFFFFFFFFF:FF:FFFFFFFFFFFFFFFFFFFFFFFFFFFFFFFFFFFFFFFFFFF
FFFFFFFFFFFFF:FFFFFFFFFFF:FFFFFFFFF:FFFFFF:FFFFFFFFF,F:FFFFFFF:,FFFF
FFFFFFFFFFFFFFF
@A00721:190:HGJW7DSXY:4:2257:4833:27179 2:N:0:TCTGCAAG+GCTGCACT
CTTCTTTGTAATGTATTAGGTCTAGCCGTGCAGCCTCTTTGCTACCCACAATATTTATAAATTTTGAA
TTTCAAAATTGACCGTCGATTCTACTGCGTCACTATAGAATTCATTTTTTAGATGGTTATGATCGTGA
CGCGTGGTCTAATGA
+
FFFFFFFFFFFFFFFFFFFFFFFFFFFFFFFFFFFFFFFFFFFFFFFFFFFFFFFFFFFFFFFFFFFFF
FFFFFFFFFFFFFFFFFFFFFFFFFFFFFFFFFFFFFFFFFFFFFFFFFFFFFFFFFFFFFF:FFFFFF
FFFFFF,FFFFFFFF
@A00721:190:HGJW7DSXY:4:2348:22073:16235 2:N:0:TCTGCAAG+GCTGCACT
CTTCTTTGTAATGTATTAGGTCTAGCCGTGCAGCCTCTTTGCTACCCACAATATTTATAAATTTTGAA
TTTCAAAATTGACCGTCGATTCTACTGCGTCACTATAGAATTCATTTTTTAGATGGTTATGATCGTGA
CGCGTGGTCTAATGA
+
FF:FFFFFFFFFFFFFFFFFFFFFFFFFFFFFFFFFFFFFFFFFFFFFFFFFFFFFFF:FFFFFFFFFF
FFFFFFFFFFFFFFFFFFFFFFFFFFFFFFFFF:FFFFFFFFFFFFFFFFFFFFFFFFFFFFFFFFFFF
FF:FFFFFFFFFFFF
@A00721:190:HGJW7DSXY:4:2630:6560:23657 2:N:0:TCTGCAAG+GCTGCACT
CTTCTTTGTAATGTATTAGGTCTAGCCGTGCAGCCTCTTTGCTACCCACAATATTTATAAATTTTGAA
```

TTTCAAAATTGACCGTCGATTCTACTGCGTCACTATAGAATTCATTTTTAGATGGTTATGATCGTGA
CGCGTGGTCTAATGA
+
FFFFFFFFFFF,FFFFFFFFFFFFFFFF:FFFFFFFFFFFFFFFFFFFFFFFFFFFFFFFFFFFFF
FFFFFFFFFFFFFFFFFFFFFFFFFFFFFFFFF:F,FFFFFFF:FFFFFFFFFFFFFFFFFFFFFF
FFFFFFFFFFFFFFF
@A00721:190:HGJW7DSXY:4:1304:11912:1297 2:N:0:TCTGCAAG+GCTGCACT
GACCGCCTGACCACTTCCTCTTCTTGGAAGACGAATCCATCAATCAACTGCTAAAGCAGTCATCAACA
CAATAAATATAAATTTTGAATTTCAAAATTGACCGTCGATTCTACTGCGTCACTATAGAATTCATTTT
TTAGATGGTTATGAT
+
F:FFFFFFFFFFFFFFFFF:FFFFFFFFFFFFFFFFFFFFF:FFFFFFFF:FFFFFFFFFFF:FFF
FFFFFFF:FFFFFFFFFFFFF:FFFFFFFFFFFFFFFFFFFFFFF:FFFFFF:FFF:FFF,FFFFF
FFFFF,FFFFFFFFF
@A00721:190:HGJW7DSXY:4:1622:31665:13056 2:N:0:TCTGCAAG+GCTGCACT
GACCGCCTGACCACTTCCTCTTCTTGGAAGACGAATCCATCAATCAACTGCTAAAGCAGTCATCAACA
CAATAAATATAAATTTTGAATTTCAAAATTGACCGTCGATTCTACTGCGTCACTATAGAATTCATTTT
TTAGATGGTTATGAT
+
FFFFFFFFFFFFFFFFFFFFFFFFFFFFFFFFFFFFFFFFFFFFFFFFFFFFFFFFFFFFFFFFFF
FFFFFFFFFFFFFFFFFFFFFFFFFFFFFFFFFFFFF:FFFFFFFFFFFFFF,:FFFFFFFFFFF:
FF:F:FFFFFFFFFF
@A00721:190:HGJW7DSXY:4:2168:15510:14512 2:N:0:TCTGCAAG+GCTGCACT
GACCGCCTGACCACTTCCTCTTCTTGGAAGACGAATCCATCAATCAACTGCTAAAGCAGTCATCAACA
CAATAAATATAAATTTTGAATTTCAAAATTGACCGTCGATTCTACTGCGTCACTATAGAATTCATTTT
TTAGATGGTTATGAT
+
FFFFFFFFFFFFFFFFFFFFF:FFFFFFFFFFFFFFFFFFFFFFFFFFFF:FFFFFFFFFFFFFFF
FFFFF::FFFFFFFFFFFFFFFFFFFFFFFFF:FFFF:FF:FFFFFFFFFF:FF,F::FFFFFFFF
FF,FFFF:FFFFFFF
@A00721:190:HGJW7DSXY:4:2611:14977:25520 2:N:0:TCTGCAAG+GCTGCACT
GACCGCCTGACCACTTCCTCTTCTTGGAAGACGAATCCATCAATCAACTGCTAAAGCAGTCATCAACA
CAATAAATATAAATTTTGAATTTCAAAATTGACCGTCGATTCTACTGCGTCACTATAGAATTCATTTT
TTAGATGGTTATGAT
+
FFFFFFFFFFFFFFFFFFFFFFFFFFFFFFFFFFFFFFFFFFFFFFFFFFFFFFFFFFFFFFFFFF
FFFFFFFFFFFFFFFFFFFFFFFFFFFFFFFFFFFFFF:FFFFFFFFFFFFFFFFFFFFFFF:FFF
FFFFFF:FFFFFFFF
@A00721:190:HGJW7DSXY:4:1168:13883:7153 2:N:0:TCTGCAAG+GCTGCACT
GACCGCCTGACCACTTCCTCTTCTTGGAAGACGAATCCATCAATCAACTGCTAAAGCAGTCATCAACA
CAATAAATATAAATTTTGAATTTCAAAATTGACCGTCGATTCTACTGCGTCACTATAGAATTCATTTT
TTAGATGGTTATGA
+
:F:F:::FFFF:FFFF:F,FFFF,,F,F:FF::FFFFFFFFFFFF:F,F:,FFFFF:F::FFFFFF
,FF,FFF,F:FFF:F::FFF,,F,FFFF,FFFFF,F:,F,:FFFF,,F,F::F,F,F:FFFF:F:,F,
,FF:FF:,FFF,:F
@A00721:190:HGJW7DSXY:4:1315:15691:1924 2:N:0:TCTGCAAG+GCTGCACT
CGATAACCGGCGGTCCTAAACGGACCGCCTGACCACTTCCTCTTCTTGGAAGACGAATCCATCAATCA
ACTGCTAAAGCAGTCATCAACACAATAAATATAAATTTTGAATTTCAAAATTGACCGTCGATTCTACT
GCGTCACTATAGAAT
+
FFFFFFFFFFFFFFFFFFFFFFFFFFFFFF:FFFFFFFFFFFFFFFFFFFFFFFFFFFFFFFFFFF
FFFFFFFFFFFFF:FFFFFFF:FFFFFFFFFFFFFFFFFF:FFFFFFFFFFFFFFFFFFFFFFFFF
FFFFFFF:,FFFF:F

```
@A00721:190:HGJW7DSXY:4:1350:24749:14544 2:N:0:TCTGCAAG+GCTGCACT
CGATAACCGGCGGTCCTAAACGGACCGCCTGACCACTTCCTCTTCTTGGAAGACGAATCCATCAATCA
ACTGCTAAAGCAGTCATCAACACAATAAATATAAATTTTGAATTTCAAAATTGACCGTCGATTCTACT
GCGTCACTATAGAAT
+
FF:FFFFFFFFFFFFFFFFFFFFFFFFFFFFFFFFFFFFFFFFFFFFFFFFFFFFFFFFFFFFFFFFF
FFFFFFFFFFFFFFFFFFFFFFFFFFFFFFFFFFFFFFFFFFFFFFFFFFFFFFFFFFFFFFFFFFFF
FF:FFFFFFF:FF:F
@A00721:190:HGJW7DSXY:4:1370:28058:17801 2:N:0:TCTGCAAG+GCTGCACT
CGATAACCGGCGGTCCTAAACGGACCGCCTGACCACTTCCTCTTCTTGGAAGACGAATCCATCAATCA
ACTGCTAAAGCAGTCATCAACACAATAAATATAAATTTTGAATTTCAAAATTGACCGTCGAGTCTACT
GCGTCACTATAGAAT
+
FFFFFFFFFFFFFFFFFFF:FFFFFFFFFFFFFFFFFFFFFF:FFFFFFFFFFFFFFFFFF:FFFFFF
FFFFFFFFFFFFFFFFFFFFF:FFFFFFFFFFFFFFFFFFFF:FFF:FF:FFFFFFFFFFF,FFFFFF
FFF:FFFF::FFFFF
@A00721:190:HGJW7DSXY:4:1531:6090:35931 2:N:0:TCTGCAAG+GCTGCACT
CGATAACCGGCGGTCCTAAACGGACCGCCTGACCACTTCCTCTTCTTGGAAGACGAATCCATCAATCA
ACTGCTAAAGCAGTCATCAACACAATAAATATAAATTTTGAATTTCAAAATTGACCGTCGATTCTACT
GCGTCACTATAGAAT
+
FF,FFFFFFFFFFFFFFFFFFFFFFFFFFFFFFFFFFFFFFFFFFFFFFFFFFFFFFFFFFFFFFFFF
FFFFFFFFFFFFFFFFFFFFFFFFFFFFFFFFFFFFFFFFFFFFFFFFFFFFFFFFFFFFFFFFFFF:
FFFFFFF:F::,F:F
@A00721:190:HGJW7DSXY:4:2322:10881:19366 2:N:0:TCTGCAAG+GCTGCACT
CGATAACCGGCGGTCCTAAACGGACCGCCTGACCACTTCCTCTTCTTGGAAGACGAATCCATCAATCA
ACTGCTAAAGCAGTCATCAACACAATAAATATAAATTTTGAATTTCAAAATTGACCGTCGATTCTACT
GCGTCACTATAGAAT
+
FFFFFFFFFFFFFFFFFFFFFFFFFFFFFFFFFFFFFFFFFFFFFFFFFFFFFFFF:FFFFFFFFFFF
FFFFFFFFFFFFFFFFFF:FFFFFFFFFFFFFFFFFFFFFFFFFFFFFFFFFFFFFFFFF:FFFFFFFF
FFFFFF:FFF,:FFF
@A00721:190:HGJW7DSXY:4:2615:25337:20729 2:N:0:TCTGCAAG+GCTGCACT
CGATAACCGGCGGTCCTAAACGGACCGCCTGACCACTTCCTCTTCTTGGAAGACGAATCCATCAATCA
ACTGCTAAAGCAGTCATCAACACAATAAATATAAATTTTGAATTTCAAAATTGACCGTCGATTCTACT
GCGTCACTATAGAAT
+
FFFFF:FFFFFFFFFFFFFFFFFFFFFFFFFFFFFFF:FFFF:FFFFFFFFFFFFFFFFFF:FFF,FF
FFFFFFFFFFFFFFF:FFFFFFFFFFFFFFFFFFFFFFFFFFFFF:FF:FFFFFFFFFFFFFF,:FFF
FFF:,FFFFF,FFFF
@A00721:190:HGJW7DSXY:4:1223:13847:2957 2:N:0:TCTGCAAG+GCTGCACT
CTATAGTGACGCAGTAGAATCGACGGTCAATTTTGAAATTCAAAATTTATATTTATTGTGTTGATGAC
TGCTTTAGCAGTTGATTGATGGATTCGTCTTCCAAGAAGAGGAAGTGGTAAAAGGACCGCCGGTTATC
GTAAAACACGACGAG
+
FFFFFFFFFFFFFFFFFFFFFFFFFFFFFFFFFFFFFFFFFFFF:FFFFFFFFFFFFFFFFFF:FFFF
FFFFFFFF:FFFFFFFFFFFF,FFFFFFFFFF:FFFFF:FFFFFFFFFFFFFFF,FFFFFFFFFFFFF
,FFF:FF:FFFFFFF
@A00721:190:HGJW7DSXY:4:1647:24288:27085 2:N:0:TCTGCAAG+GCTGCACT
CTATAGTGACGCAGTAGAATCGACGGTCAATTTTGAAATTCAAAATTTATATTTATTGTGTTGATGAC
TGCTTTAGCAGTTGATTGATGGATTCGTCTTCCAAGAAGAGGAAGTGGTAAAAGGACCGCCGGTTATC
GTAAAACACGACGAG
+
FFFFFFFFFFFFFFFFFFFFFFFFFFFFFFFFFFFFFFFFFFFFFFFFFFFF:FFF:F:FFFFFFFFF
```

```
FFFFFFFFFFF:FFFFFFFFFFFFFFFFFFF:FFFFFFFFFFFFFFFFFFFFFFFFFFFFFFF,:F:F
FFFFFFFFFFFFFFF
@A00721:190:HGJW7DSXY:4:2102:22697:10363 2:N:0:TCTGCAAG+GCTGCACT
CTATAGTGACGCAGTAGAATCGACGGTCAATTTTGAAATTCAAAATTTATATTTATTGTGTTGATGAC
TGCTTTAGCAGTTGATTGATGGATTCGTCTTCCAAGAAGAGGAAGTGGTAAAAGGACCGCCGGTTATC
GTAAAACACGACGAG
+
FFFFFFFFFFFFFFFF:FFFFFFFFFFFFFFFFFFFFFFFFFFFFFFFFFFFFFF:FFFFFFFFFFFF
FFFFFFFFFFFFFFFFFFFFFFFFFFFFFFFFFFFFFFFFFFFFFFFFFFFFFFFFF:FFF:FFFFFF
FFFFF:FFF:FFFFF
@A00721:190:HGJW7DSXY:4:2154:14832:16376 2:N:0:TCTGCAAG+GCTGCACT
CTATAGTGACGCAGTAGAATCGACGGTCAATTTTGAAATTCAAAATTTATATTTATTGTGTTGATGAC
TGCTTTAGCAGTTGATTGATGGATTCGTCTTCCAAGAAGAGGAAGTGGTAAAAGGACCGCCGGTTATC
GTAAAACACGACGAG
+
FFFFFFFFFFFFFFFFFFFFFFFFFFFFFFFFFFFFFFFFFFFFFFFFFFFFFFF:,FFF:FFFFFFF
FFFFFFFFFFFFFFFFFFFF:FFFFFFFFFFFFFFFFFFFFFFFFFFFFFFFFFF:FFFFFFFFFFFF
FFFF:FFFFFFFFFF
@A00721:190:HGJW7DSXY:4:2231:29089:5682 2:N:0:TCTGCAAG+GCTGCACT
CTATAGTGACGCAGTAGAATCGACGGTCAATTTTGAAATTCAAAATTTATATTTATTGTGTTGATGAC
TGCTTTAGCAGTTGATTGATGGATTCGTCTTCCAAGAAGAGGAAGTGGTAAAAGGACCGCCGGTTATC
GTAAAACACGACGAG
+
FFFFFFFFFFFFFFFFFFFFFFFFFFFFFFFFFFFFFFFFFFFFF:FFFFFF:F:FFF:FFFFF:FFF
FFFF:FFFF:FFFFFFFFFFFFFFFFFFFFFFFFFFFFF:F:FFFFFF:FF,FFFFFFFFFFFF,FFF
FFFF::FFFFFFFFF
@A00721:190:HGJW7DSXY:4:2413:25925:4210 2:N:0:TCTGCAAG+GCTGCACT
CTATAGTGACGCAGTAGAATCGACGGTCAATTTTGAAATTCAAAATTTATATTTATTGTGTTGATGAC
TGCTTTAGCAGTTGATTGATGGATTCGTCTTCCAAGAAGAGGAAGTGGTAAAAGGACCGCCGGTTATC
GTAAAACACGACGAG
+
FFFFFFFFFFFFFFFFFFFFFFFFFFFFFFFFFFFFFFFFFFFF:FFF,FFFFFFFFFFFF,F:FFFF
FFFFFFFFFFFFFFFF:F:F:FFFFFFFFFFFFF:FFFFF:FFFFFFFFFF,:FFFFFFFFFFFFFFF
FFFFFFFFFFFFFFF
@A00721:190:HGJW7DSXY:4:2465:19786:16501 2:N:0:TCTGCAAG+GCTGCACT
CTATAGTGACGCAGTAGAATCGACGGTCAATTTTGAAATTCAAAATTTATATTTATTGTGTTGATGAC
TGCTTTAGCAGTTGATTGATGGATTCGTCTTCCAAGAAGAGGAAGTGGTAAAAGGACCGCCGGTTATC
GTAAAACACGACGAG
+
FFFFFFFFFFFFFFFFFFFFFFFFFF:FFFFFFFFFFFFFFFFFFF:FFFFFFF:FFF:FFFFFFFFF
FFF:FFFFFFFF:FFFFFFFFFFFFFFFFFFFFFFFFFFFFFF:FFFFFFFFFFFFFFFFFFF:FFFF
FFFFFFFFFFFFFFF
@A00721:190:HGJW7DSXY:4:2475:30544:13651 2:N:0:TCTGCAAG+GCTGCACT
CGATAACCGGCGGTCCTAAACGGACCGCCTGACCACTTCCTCTTCTTGGAAGACGAATCCATCAATCA
ACTGCTAAAGCAGTCATCAACACAATAAATATAAATTTTGAATTTCAAAATTGACCGTCGATTCTACT
GCGTCACTATAG
+
FF:F:FFFFFFFFFFFFFFFFFFFFFFFFFFFFFFFFFFFFFFFFFFFFFFFFFFFFFFFFFFFFFFF
FFFFFFFFFFFFFFFFFFFFFFFFFFFFFFFFFFFFFFFFFFFFF:FFFFFFFFFFFFFFFFFFFFFF
FFFFF,FFFFFF
@A00721:190:HGJW7DSXY:4:2518:7129:2347 2:N:0:TCTGCAAG+GCTGCACT
CTATAGTGACGCAGTAGAATCGACGGTCAATTTTGAAATTCAAAATTTATATTTATTGTGTTGATGAC
TGCTTTAGCAGTTGATTGATGGATTCGTCTTCCAAGAAGAGGAAGTGGTAAAAGGACCGCCGGTTATC
GTAAAACACGACGA
```

+
FFFFFFFFF:FFFFFFFFFFFFFFFFFFFFFFFFFFFFFFFFFFFFF,FFF::FF:F:FFFFFF::FF
,FFFFFFF,FFF,FF:FFFF::FFFF,:FF:F,FFFFFFFFFFF:FF:FFFFFF,FFFFFF::FFFF:
F,FFFFFF:,FF:F
@A00721:190:HGJW7DSXY:4:1109:9941:23688 2:N:0:TCTGCAAG+GCTGCACT
TTTTACGATAACCGGCGGTCCTAAACGGACCGCCTGACCACTTCCTCTTCTTGGAAGACGAATCCATC
AATCAACTGCTAAAGCAGTCATCAACACAATAAATATAAATTTTGAATTTCAAAATTGACCGTCGATT
CTACTGCGTCACTAT
+
FFFFFFFFFFFFFFFFFFFFFFFFFFFFFFFFFFFFFFFFFFFFFFF:FFFFFFFFFFFFFFFFFFFF
FFFFFFFFFFFFFFFFFFFFFFFFFFFFF:FFFFFFFFFFFFFFFFFFFFFFFFFFFFFF:FFFFF,F
FFFFFFFFFFFFF,F
@A00721:190:HGJW7DSXY:4:1419:15917:16720 2:N:0:TCTGCAAG+GCTGCACT
TTTTACGATAACCGGCGGTCCTAAACGGACCGCCTGACCACTTCCTCTTCTTGGAAGACGAATCCATC
AATCAACTGCTAAAGCAGTCATCAACACAATAAATATAAATTTTGAATTTCAAAATTGACCGTCGATT
CTACTGCGTCACTAT
+
FFFFFFFFFFFFFFFFFFFFFFFF,FFFFFFFFFFFFFFFFFFFFFFFFF:FFFFFFFFFFFFFFFFF
FFFFFFFFFFFFFFFFFFFFFFFFFFFFFFFFFFFFFFFFFFFFFFFFFFF:FFFFFFFFFFFFFFFF
FFFFF::FFF:::FF
@A00721:190:HGJW7DSXY:4:1525:20573:2362 2:N:0:TCTGCAAG+GCTGCACT
TTTTACGATAACCGGCGGTCCTAAACGGACCGCCTGACCACTTCCTCTTCTTGGAAGACGAATCCATC
AATCAACTGCTAAAGCAGTCATCAACACAATAAATATAAATTTTGAATTTCAAAATTGACCGTCGATT
CTACTGCGTCACTAT
+
FFFFFFFFFFFFFFFFFFFFFFFFFFFFFFFFFFFFFFFFFFFFFFFF:FFFFFFFFFFFFFFFF:FF
FFFFFFFFFFF:FFFFFFFFFFFF:FFFFFFFFFFFFFFFFFFFFFFFFFFFFFFFFFFFFF:FF:FF
FFFFFFFFFFFF,FF
@A00721:190:HGJW7DSXY:4:1138:13376:9345 2:N:0:TCTGCAAG+GCTGCACT
GTTTTACGATAACCGGCGGTCCTAAACGGACCGCCTGACCACTTCCTCTTCTTGGAAGACGAATCCAT
CAATCAACTGCTAAAGCAGTCATCAACACAATAAATATAAATTTTGAATTTCAAAATTGACCGTCGAT
TCTACTGCGTCACTA
+
FFFFFFFFFFFFFFFFFFFFFFFFFFFFFFFFFFFFFFFFFFFFFFFFFFF:FFF:FFFFFFFFFFFF
FFFFFFFFFFFFFFFFFFFFF,FFF:FFFFF:FFFFFFFFFFFFFFFFFFFFFFFF:FFFFFFFFFF,
FFFFFFFFFFF:FFF
@A00721:190:HGJW7DSXY:4:1160:23439:19883 2:N:0:TCTGCAAG+GCTGCACT
GTTTTACGATAACCGGCGGTCCTAAACGGACCGCCTGACCACTTCCTCTTCTTGGAAGACGAATCCAT
CAATCAACTGCTAAAGCAGTCATCAACACAATAAATATAAATTTTGAATTTCAAAATTGACCGTCGAT
TCTACTGCGTCACTA
+
FF:FFFFFFFFFFFFFFFFFFFFFFFFFFFFFFFFFFFFFF:FFFFFFFF:FFFFFFFFFFFFFFFF,
FFFFFFFFFFFFFFFFFFFFF:FFFFFFFFFFFFFFFFFFFFFFFFFFFFFFFFFFFFFFFFFFFFF,
FFFFFFFFFFFFFFF
@A00721:190:HGJW7DSXY:4:1249:23041:12587 2:N:0:TCTGCAAG+GCTGCACT
GTTTTACGATAACCGGCGGTCCTAAACGGACCGCCTGACCACTTCCTCTTCTTGGAAGACGAATCCAT
CAATCAACTGCTAAAGCAGTCATCAACACAATAAATATAAATTTTGAATTTCAAAATTGACCGTCGAT
TCTACTGCGTCACTA
+
FFFFFFFFFFFFFFFFFFFFFFFFF:FFFFFFFFFFFFFFFFFFFFFFFFFFFFFFFFFFFFFFFFFF
FFFFFFFFFFFFFFFFFFFFFFFFFFFFFFFFFFFFFFFFFFFFFFFF:FFFFFFFFFFFFFFFFFFF
FFFFFFFFFFFFF:F
@A00721:190:HGJW7DSXY:4:1253:8983:29042 2:N:0:TCTGCAAG+GCTGCACT
GTTTTACGATAACCGGCGGTCCTAAACGGACCGCCTGACCACTTCCTCTTCTTGGAAGACGAATCCAT

CAATCAACTGCTAAAGCAGTCATCAACACAATAAATATAAATTTTGAATTTCAAAATTGACCGTCGAT
TCTACTGCGTCACTA
+
FFFFFFFFFF:FFFFFFFFFFFFFF:FFFFFFFFFFFFFFFFFFFFFF:FFFFFFFFFFFFFFFFFFF
FFFFFFFFFFFFFFFFFFFFFFFFFFFFFF:FFFFFFFFFFFFFFFF:FFFFFFFF:FFFFFFFFF:F
FFFFFFFFF::FFF:
@A00721:190:HGJW7DSXY:4:1253:8802:29074 2:N:0:TCTGCAAG+GCTGCACT
GTTTTACGATAACCGGCGGTCCTAAACGGACCGCCTGACCACTTCCTCTTCTTGGAAGACGAATCCAT
CAATCAACTGCTAAAGCAGTCATCAACACAATAAATATAAATTTTGAATTTCAAAATTGACCGTCGAT
TCTACTGCGTCACTA
+
FFFFFFFFFFFFFFFFFFFFFFFFFFFFFFFFFFFFFFFFFFFFFFFFF:FFFFFFFFFFFF,FFFFF
FFFFFFFFFFFFFFFFFFFFFFFFFFFFF:FFFFFFFF:FFFFFFFFFFFFFF:FFFFFFFFF,FF:F
F:FFFFF,FFFFFF:
@A00721:190:HGJW7DSXY:4:1430:27127:3192 2:N:0:TCTGCAAG+GCTGCACT
GTTTTACGATAACCGGCGGTCCTAAACGGACCGCCTGACCACTTCCTCTTCTTGGAAGACGAATCCAT
CAATCAACTGCTAAAGCAGTCATCAACACAATAAATATAAATTTTGAATTTCAAAATTGACCGTCGAT
TCTACTGCGTCACTA
+
FFFFFFFF:FFFFFFFFFFFFFFFFFFFFFFFFFFFFFFFFFFFFFFFFFFFFFFF,FFFFFFFFF:F
FFFFFFFFFFFFFFFFFFFFFFFFFFFFFFFFFFFFFFFFFFFFFFFFFFFFFFFFFFFF:FFFFF:F
FFFFFFFFFFFFFFF
@A00721:190:HGJW7DSXY:4:1457:4101:3427 2:N:0:TCTGCAAG+GCTGCACT
GTTTTACGATAACCGGCGGTCCTAAACGGACCGCCTGACCACTTCCTCTTCTTGGAAGACGAATCCAT
CAATCAACTGCTAAAGCAGTCATCAACACAATAAATATAAATTTTGAATTTCAAAATTGACCGTCGAT
TCTACTGCGTCACTA
+
FFFFFFFFFFFFFFFFFFFFFFFFFFFFFFFFFFFFFFFFFFFFFFFFF:FFFFFF:FFFFFFFFFFF
FFFFFFFFFFFFFFFFFFFFFF:FFFFFFF,FFFFFFFFFFFFFFFFF,FFFF,FFFFFFFFFFFFFF
FFFFFFFFF,FFF:F
@A00721:190:HGJW7DSXY:4:1619:11785:6151 2:N:0:TCTGCAAG+GCTGCACT
GTTTTACGATAACCGGCGGTCCTAAACGGACCGCCTGACCACTTCCTCTTCTTGGAAGACGAATCCAT
CAATCAACTGCTAAAGCAGTCATCAACACAATAAATATAAATTTTGAATTTCAAAATTGACCGTCGAT
TCTACTGCGTCACTA
+
FFFFFFFF,FFFFFFFFFFFFFFF:FFFFFFFFFFFFFFFFFFFFFFFFFFFFFFFFFFFFFFFFFFF
FFFFFFFFFFFFFFFFFFFFFFFF:FFFFFFFFFFFFFFFFFFFFFFFFFFFFFFFFFFFFFFFFFFF
FFFFFFFFFFFFFFF
@A00721:190:HGJW7DSXY:4:2132:10755:12164 2:N:0:TCTGCAAG+GCTGCACT
GTTTTACGATAACCGGCGGTCCTAAACGGACCGCCTGACCACTTCCTCTTCTTGGAAGACGAATCCAT
CAATCAACTGCTAAAGCAGTCATCAACACAATAAATATAAATTTTGAATTTCAAAATTGACCGTCGAT
TCTACTGCGTCACTA
+
FFFFFFFFFFFFFFFFFFFFFFFFFFFFFFFFFFFFF:FFFFFFFFFFFFFFFFFFFFFFFFFFFFFF
FFFFFFFFFFFFFFFFFFFFFFFFFFFFFFFFFFFFFFFFFFFFFFFFFFFFFFFFFFFFF:FFFFF:
F:FFFFFFFFFFFF:
@A00721:190:HGJW7DSXY:4:2138:16523:35243 2:N:0:TCTGCAAG+GCTGCACT
GTTTTACGATAACCGGCGGTCCTAAACGGACCGCCTGACCACTTCCTCTTCTTGGAAGACGAATCCAT
CAATCAACTGCTAAAGCAGTCATCAACACAATAAATATAAATTTTGAATTTCAAAATTGACCGTCGAT
TCTACTGCGGCACTA
+
FFFFFFFF:,FFF:FFFFFF:F:FFFFFFFFFFFFFFFFFFFFFFFFFFFFF:F::FF,FFFFFFFFF
FFFF:FFF:FFFFFF,FFFFF,FFF:FFFFFFFFFFFFFFFFFFFFFFFFFFFFFFFFFFFFFFFFF:
FFFFFFFFF,FFFFF

@A00721:190:HGJW7DSXY:4:2162:13865:8750 2:N:0:TCTGCAAG+GCTGCACT
GTTTTACGATAACCGGCGGTCCTAAACGGACCGCCTGACCACTTCCTCTTCTTGGAAGACGAATCCAT
CAATCAACTGCTAAAGCAGTCATCAACACAATAAATATAAATTTTGAATTTCAAAATTGACCGTCGAT
TCTACTGCGTCACTA
+
FFFFFFFFFFFFFFFFFFFFFFFFFFFFFFFFFFFFFFFFFFFFFFFFFFF:FFFFFFFFFFFFFFFF
FFFFFFFFFFFFFFFFFFFFF:FFFFFFFFFFFFFFFFFF:F:FFFFF:FFFFFFF:FFFFFFFFF,,
F:FFFFFFFFFFF::
@A00721:190:HGJW7DSXY:4:2447:10719:17112 2:N:0:TCTGCAAG+GCTGCACT
GTTTTACGATAACCGGCGGTCCTAAACGGACCGCCTGACCACTTCCTCTTCTTGGAAGACGAATCCAT
CAATCAACTGCTAAAGCAGTCATCAACACAATAAATATAAATTTTGAATTTCAAAATTGACCGTCGAT
TCTACTGCGTCACTA
+
F:FF,FFFFFFFFFFFFF:FFFF,FFFFFFFF::FFFFFFFFFFF:FF,FFFFFFFFFFFFF,FFFF,
FFFFFFFFFFFFF,:FF:FFFFFFFFFFF:FFFFF:FFFFFFFFFF:FF:FFFF:F,FFFFFFFF:FF
FFFFFF:FFF,:FFF
@A00721:190:HGJW7DSXY:4:2612:19560:36057 2:N:0:TCTGCAAG+GCTGCACT
GTTTTACGATAACCGGCGGTCCTAAACGGACCGCCTGACCACTTCCTCTTCTTGGAAGACGAATCCAT
CAATCAACTGCTAAAGCAGTCATCAACACAATAAATATAAATTTTGAATTTCAAAATTGACCGTCGAT
TCTACTGCGTCACTA
+
FFFFFFFFFFFFFFFFFFFFFFFFFFFFFFFFFFFFFFFFFFFFFFFFFF:FFFFFFFFFFFFFFFFF
FFFFFFFFFFFFFFFFFFFFFFFFFFFFF:FFFFFFFFFFFF,FFFFFFFFFFF:FFFFFFFFFFFFF
:FFFFFFFFFFFFF:
@A00721:190:HGJW7DSXY:4:2108:15917:19946 2:N:0:TCTGCAAG+GCTGCACT
GTTTTACGATAACCGGCGGTCCTAAACGGACCGCCTGACCACTTCCTCTTCTTGGAAGACGAATCCAT
CAATCAACTGCTAAAGCAGTCATCAACACAATAAATATAAATTTTGAATTTCAAAATTGACCGTCGAG
TCTACTGCGTCACT
+
FFFFFFFFFFFFFFFFFFFFFFFFF,FFFFFFFFFFFFFFFFFFFFFFFFF,FFFFFFFFFFFFFF::
FFF:FFFFFFFFF:FFFFFFFFFFFFF,FFF,FFFFFFFFFF,:FFF,FFFF:,F::FF,FFFFFFF,
:FF,:FF:FF:F,:
@A00721:190:HGJW7DSXY:4:2162:13928:7983 2:N:0:TCTGCAAG+GCTGCACT
GTTTTACGATAACCGGCGGTCCTAAACGGACCGCCTGACCACTTCCTCTTCTTGGAAGACGAATCCAT
CAATCAACTGCTAAAGCAGTCATCAACACAATAAATATAAATTTTGAATTTCAAAATTGACCGTCGAT
TCTACTGCGTCACT
+
FFFFFFFFFFFFFFFFFFFFFFFF:FFFFFFFFFFFFFFFFFFFFFFFFFFFFFFFFFFFFF:FF::F
FFFFFFFFFFFFFFFFFFFFFFFFFFFFFFFFFFFFFFFFFFFFFFFFFFFF:FFFFF::FFFFFF:F
FFFF:FFFFFFF:F
@A00721:190:HGJW7DSXY:4:2505:15067:21167 2:N:0:TCTGCAAG+GCTGCACT
TTTTACGATAACCGGCGGTCCTAAACGGACCGCCTGACCACTTCCTCTTCTTGGAAGACGAATCCATC
AATCAACTGCTAAAGCAGTCATCAACACAATAAATATAAATTTTGAATTTCAAAATTGACCGTCGATT
CTACTGCGTCAC
+
FFFFFFFFFFFFFFFFFFFFFFFFFFFFFFFFFFFFFFFFFFFFFFFFFFFFFF:FFFFFFFFFF:FF
F:FF:FFFFFFFFFFFFFFFFFFFFFFFFF:FFFFFFFFFFFFFFFFF:FFFFF:FFFFF:FFFFF:F
FFF,FF:F::F:
@A00721:190:HGJW7DSXY:4:2354:6153:9737 2:N:0:TCTGCAAG+GCTGCACT
GTTTTACGATAACCGGCGGTCCTAAACGGACCGCCTGACCACTTCCTCTGCTTGGAAGACGAATCCAT
CAATCAACTGCTAAAGCAGTCATCAACACAATAAATATAAATTTTGAATTTCAAAATTGACCGTCGAT
TCTACTGCGTC
+
F:FF:FFF:FFFFFFFFFFFFF:FF:FFFFFFFFFFF,FFFFFFFF:FF,FFFFFFFFFFF::FFFFF

FFFFFFF:FFFFF:FFFFFFFFFF,FFFFFFF,FFFFFFFFFFF:FFF::FFFFFFFFFFFFFFFFFF
FFFFFFFFFFF
@A00721:190:HGJW7DSXY:4:1153:20030:5838 2:N:0:TCTGCAAG+GCTGCACT
GTTGAATTGCAACACTCGTCGTGTTTTACGATAACCGGCGGTCCTAAACGGACCGCCTGACCACTTCC
TCTTCTTGGAAGACGAATCCATCAATCAACTGCTAAAGCAGTCATCAACACAATAAATTTTGAATTTC
AAAATTGACCGTCGA
+
F,FFFFFFFFFFFFFFFFFFFFFFFFFFFFFFFFFFFFFFFFFFFFFFF,FFFFFFFFFFFFFFFFFF
FFFFFFFFFFFFFFFFFFFFFFFFFFFFFFFFFFFFFFFFFFFFFFFFFFFFFFFFFF:FFFFFFFFF
FFFFF:FF:,FFFFF
@A00721:190:HGJW7DSXY:4:1309:18295:35462 2:N:0:TCTGCAAG+GCTGCACT
GTTGAATTGCAACACTCGTCGTGTTTTACGATAACCGGCGGTCCTAAACGGACCGCCTGACCACTTCC
TCTTCTTGGAAGACGAATCCATCAATCAACTGCTAAAGCAGTCATCAACACAATAAATTTTGAATTTC
AAAATTGACCGTCGA
+
FFFFFFFFFFFFFFFFFFFFFFFFFFFFFFFFFFFFFFFFFFFFFFFFFFFFFFFFFFFFFFFFFFFF
FFFFFFFFFFFFFFFFFFFFFFFFFFFFFFFFFFFFFFFFFFFFFFFFFFFFFFFFFFF:FFFFF:FF
F:FFFFFFFFFFFF:
@A00721:190:HGJW7DSXY:4:1331:21667:36417 2:N:0:TCTGCAAG+GCTGCACT
GTTGAATTGCAACACTCGTCGTGTTTTACGATAACCGGCGGTCCTAAACGGACCGCCTGACCACTTCC
TCTTCTTGGAAGACGAATCCATCAATCAACTGCTAAAGCAGTCATCAACACAATAAATTTTGAATTTC
AAAATTGACCGTCGA
+
FFFFFFFFFFFFFFFFFFFFFFFFFFFFFF:FFFFFFFFFFFFFFFF:FFFFFFFFFFFFFFFFFFFF
FFFFFFFFFFFFFFFFFFFFFFFF:FF:FFFFFFFFF,FFFFFFFFFFFFFFFFFFFFFFFFFFFFFF
FFFFF:FFFFFFFFF
@A00721:190:HGJW7DSXY:4:1332:32678:4789 2:N:0:TCTGCAAG+GCTGCACT
GTTGAATTGCAACACTCGTCGTGTTTTACGATAACCGGCGGTCCTAAACGGACCGCCTGACCACTTCC
TCTTCTTGGAAGACGAATCCATCAATCAACTGCTAAAGCAGTCATCAACACAATAAATTTTGAATTTC
AAAATTGACCGTCGA
+
FFFFFF:FFFFFFFFFFFFFFFF:FFFFFF,FFFFFFFFFFF,FFFFFF,FFFFFFF:FFFFFFFFFF
:FFFF:FFF,FFFFFFFFFFFF:FFFFFFFFFFFFFF:FF:,:FF:FF:FFFFFFF:FFF,FFFFFFF
FFFFF,::F,FFF:F
@A00721:190:HGJW7DSXY:4:1345:8513:19022 2:N:0:TCTGCAAG+GCTGCACT
TTGCAACACTCGTCGTGTTTTACGATAACCGGCGGTCCTAAACGGACCGCCTGACCACTTCCTCTTCT
TGGAAGACGAATCCATCAATCAACTGCTAAAGCAGTCATCAACACAATAAATATAAATTTTGAATTTC
AAAATTGACCGTCGA
+
FFFFFFFFFFFFFFFFFFFFFFFFFFFFFFFFFFFFFFFFFFFFFFFFFFFFFFFFFFFFFFFFFFFF
FFFFFFFFFFFFFFFFFFFFFFFFFFFFFFFFFFFFFFFFFFFFFFFFFFFFF:FFFFFFFFFFFFFF
FFFFFFFFFFFFFFF
@A00721:190:HGJW7DSXY:4:1539:18159:22013 2:N:0:TCTGCAAG+GCTGCACT
TTGCAACACTCGTCGTGTTTTACGATAACCGGCGGTCCTAAACGGACCGCCTGACCACTTCCTCTTCT
TGGAAGACGAATCCATCAATCAACTGCTAAAGCAGTCATCAACACAATAAATATAAATTTTGAATTTC
AAAATTGACCGTCGA
+
FFFFFFFFFFFFFFFFFFFFFFFFFFFFFFFFFFFFFFFFFFFFFFFFFFFFFFFFFFFFFFFFFFFF
FFFFFFFFFF:FFFFFFFFFFFFFFFFFFFFFFFFFFFFFFFFFFFFFFF:FFFFFFFFFFFFFFFFF
FFF:FF:FFFFFFFF
@A00721:190:HGJW7DSXY:4:2153:11930:29888 2:N:0:TCTGCAAG+GCTGCACT
TTGCAACACTCGTCGTGTTTTACGATAACCGGCGGTCCTAAACGGACCGCCTGACCACTTCCTCTTCT
TGGAAGACGAATCCATCAATCAACTGCTAAAGCAGTCATCAACACAATAAATATAAATTTTGAATTTC
AAAATTGACCGTCGA

+
FFFFFFFFFFFFFFFFFFFFFFFFFFFFFFFFFFFFFFFFF:FFFFFFFFFFFFFFFFFFFFFFFFFF
FFFFFFFFFFFFFFFFFFFFFFFFFFFFFFFFFFFFFFFFFFFFF:FFFFFFFFFFFFFFFFFF:FFF
FFFFFFFFFFFFFFF
@A00721:190:HGJW7DSXY:4:2153:10728:30311 2:N:0:TCTGCAAG+GCTGCACT
TTGCAACACTCGTCGTGTTTTACGATAACCGGCGGTCCTAAACGGACCGCCTGACCACTTCCTCTTCT
TGGAAGACGAATCCATCAATCAACTGCTAAAGCAGTCATCAACACAATAAATATAAATTTTGAATTTC
AAAATTGACCGTCGA
+
FFFFFFFFFFFFFFFFFFFFFFFFFFFFFFFFFFFFFFFFFFFFFFFFFFFFFFFFFFFFFFFFFFFF
FFFFFFFFFFFFFFFFFFFFFFFFFFFFFFFFFFFFFFFFFFFFFFFFFFFFFFFF:FFFF,FFFFFF
FFFFFFFFF:FFFFF
@A00721:190:HGJW7DSXY:4:2271:13015:4210 2:N:0:TCTGCAAG+GCTGCACT
GTTGAATTGCAACACTCGTCGTGTTTTACGATAACCGGCGGTCCTAAACGGACCGCCTGACCACTTCC
TCTTCTTGGAAGACGAATCCATCAATCAACTGCTAAAGCAGTCATCAACACAATAAATTTTGAATTTC
AAAATTGACCGTCGA
+
FFFFFFFFFFFFFFFFFFFFFFFFFFFFFFFFFFFFFFFFFFFFFFFFFFFFFFFFFFFFFFFFFFFF
FFFFFFFFFFFFFFFFFFFFFFF:FFFFFFFFFFFFFFFFFFFFFFFFFFFFFFFF:FFFFFFFFFFF
FFFFFFFFFF:FFFF
@A00721:190:HGJW7DSXY:4:2563:11207:28291 2:N:0:TCTGCAAG+GCTGCACT
TTGCAACACTCGTCGTGTTTTACGATAACCGGCGGTCCTAAACGGACCGCCTGACCACTTCCTCTTCT
TGGAAGACGAATCCATCAATCAACTGCTAAAGCAGTCATCAACACAATAAATATAAATTTTGAATTTC
AAAATTGACCGTCGA
+
FFFFFFFFF:FFFFFFFFFFFFFFFF:FFFFFFFFFFFFFFFFFFFFFFFFFFFFFFF,FFFFFFFFF
FFFFFFFFFF,FFFF:FFFFFFFFFFFFF:FFFFFFFFFFFFFFFFFFFF:FFFFFFFFFFFFFFFFF
FFFFFFFFF:FFFFF
@A00721:190:HGJW7DSXY:4:2614:18105:11710 2:N:0:TCTGCAAG+GCTGCACT
TGCAACACTCGTCGTGTTTTACGATAACCGGCGGTCCTAAACGGACCGCCTGACCACTTCCTCTTCTT
GGAAGACGAATCCATCAATCAACTGCTAAAGCAGTCATCAACACAATAAATATAAATTTTGAATTTCA
AAATTGACCGTCGA
+
F:FF,FFFFFFFF:FFFFFFFFFF:FFFFF:FFFFFFFFFFF:FFF,FFFFFFFFFFF:FFF,FF,FF
FFFFFF:FFFF:FFFFFFFFFFFFFFFFFFFFFFFF:,FFFFFFFFF:FF:F,FFF,F:F,FFFF:FF
FFFFFF:F:FFF,F
@A00721:190:HGJW7DSXY:4:2642:29559:11475 2:N:0:TCTGCAAG+GCTGCACT
GTTGAATTGCAACACTCGTCGTGTTTTACGATAACCGGCGGTCCTAAACGGACCGCCTGACCACTTCC
TCTTCTTGGAAGACGAATCCATCAATCAACTGCTAAAGCAGTCATCAACACAATAAATTTTGAATTTC
AAAATTGACCGTCGA
+
F:FFFFFFFFFFFFFFFFFFFFFFFFFFFF:FFFFFFFFFFFFFFFFFFFFFFFFFFFFFFFFFFFFF
FFFFFFFFFFFFFFFFFFFFFFFFFFFFFFFFFFFFFFFFFFFFFFFFFFFFFFFFFFFFFFFFFFFF
FFFFFFFFFFFFFFF
@A00721:190:HGJW7DSXY:4:1374:32145:4679 2:N:0:TCTGCAAG+GCTGCACT
GACGGTCAATTTTGAAATTCAAAATTTATATTTATTGTGTTGATGACTGCTTTAGCAGTTGATTGATG
GATTCGTCTTCCAAGAAGAGGAAGTGGTAAAAGGACCGCCGGTTATCGTAAAACACGACGAGTGTTGC
AATTCAACAGGACTC
+
FF:FFFFFFFFFFFFFFFFFFFF:FFF:FFF:FF:FFFFFFF:FFF:FFFFFFFFFFFFFF:,FF:,F
FFFFFFFFFFF:F:F,FF:FFFFFFFFFFF:,FFFFFFF:FFFFFFFFFFF,FFFFFF,FFFFFFFFF
:FFFFFFFFFFFFFF
@A00721:190:HGJW7DSXY:4:1501:32407:31125 2:N:0:TCTGCAAG+GCTGCACT
GACGGTCAATTTTGAAATTCAAAATTTATATTTATTGTGTTGATGACTGCTTTAGCAGTTGATTGATG

```
GATTCGTCTTCCAAGAAGAGGAAGTGGTAAAAGGACCGCCGGTTATCGTAAAACACGACGAGTGTTGC
AATTCAACAGGACTC
+
FFFFFFFFFFFFFFFFFFFFFF:FFFFFF:F:FFFFFFFFFFFFFFFFFFFFFFFFFFFFFFFFFF:FF
FFFFFFFFFFFFFFFF:FFFFF:F:FF,FFFFFFFFFFFFFF:FFFFFFFFFFFFFFFFFFFFFFFFFF
FFFFFF,FFFFFFFF
@A00721:190:HGJW7DSXY:4:1442:18403:17268 2:N:0:TCTGCAAG+GCTGCACT
TGTTGAATTGCAACACTCGTCGTGTTTTACGATAACCGGCGGTCCTAAACGGACCGCCTGACCACTTC
CTCTTCTTGGAAGACGAATCCATCAATCAACTGCTAAAGCAGTCATCAACACAATAAATATAAATTTT
GAATTTCAAAATTGA
+
FFF:,FFFF:FFFFFF,:F:FF,:F::,FFFFFFFFFF:FFF:FFFFFFF:FF:FFF:FFFFFFFF:F
FFF::FF,,FFF,FFFFFFF,FFFFFFFFFFFF:FFFFF:F:FFFFFFFFFFFFF:FFFFFFFFF:,,
:FF,FFFFFFFF,:F
@A00721:190:HGJW7DSXY:4:1448:14796:13996 2:N:0:TCTGCAAG+GCTGCACT
TGTTGAATTGCAACACTCGTCGTGTTTTACGATAACCGGCGGTCCTAAACGGACCGCCTGACCACTTC
CTCTTCTTGGAAGACGAATCCATCAATCAACTGCTAAAGCAGTCATCAACACAATAAATATAAATTTT
GAATTTCAAAATTGA
+
FFFFFFFFFFFFFFFFFFFFFFFFFFFF:FF:F:FFFFFFFFFFFFFFFFFFFFFFFFFFFFFFFFFF
FFFFFFFFFFFFFFFFFFFFFFFFFFFFFFFF:FFFFFFFFFFFFFFFFFFFFFFFFFFFFFFFFFFF
:FFFFFFFFFFFFFF
@A00721:190:HGJW7DSXY:4:2159:14588:17895 2:N:0:TCTGCAAG+GCTGCACT
TGTTGAATTGCAACACTCGTCGTGTTTTACGATAACCGGCGGTCCTAAACGGACCGCCTGACCACTTC
CTCTTCTTGGAAGACGAATCCATCAATCAACTGCTAAAGCAGTCATCAACACAATAAATATAAATTTT
GAATTTCAAAATTGA
+
FFFFF:FFFFFFFFFF:FFFFFFFFFFFFFFFFFFFFFFFFFFFFFFFFFFFFFFFFFFFFFFFFFFF
:FFFFFFF:FFFFFFFF:FFFF:FFFF:FFFFFFFFFFFFFFFFFFFFFFFFFFFFFFFFFFF:FFFF
FFFFFFF:FFFFFFF
@A00721:190:HGJW7DSXY:4:2618:30897:21590 2:N:0:TCTGCAAG+GCTGCACT
TGTTGAATTGCAACACTCGTCGTGTTTTACGATAACCGGCGGTCCTAAACGGACCGCCTGACCACTTC
CTCTTCTTGGAAGACGAATCCATCAATCAACTGCTAAAGCAGTCATCAACACAATAAATATAAATTTT
GAATTTCAAAATTGA
+
FFFFFFFFFFFFFFFFFFFFFFFFFFFFFFFFFF:FFFFFFFFFFFFFF:FFFFFF:FFFFFFFFFFFF
FFFFFFFFFFFFFFFFFFFFFFFFFFFFFFFFFFFFFFFFFFFFFFFF:FFFFFF,FFFFFFFFFF
FFFFFF:FFF:FFFF
@A00721:190:HGJW7DSXY:4:2677:8793:23265 2:N:0:TCTGCAAG+GCTGCACT
TGTTGAATTGCAACACTCGTCGTGTTTTACGATAACCGGCGGTCCTAAACGGACCGCCTGACCACTTC
CTCTTCTTGGAAGACGAATCCATCAATCAACTGCTAAAGCAGTCATCAACACAATAAATATAAATTTT
GAATTTCAAAATTGA
+
FFFFFFFF:FFFFFFFFFFFFFFF:FFFFFFFFFFFFFFFFF:FFFFFFFFFFFFFFFFFFFFFFFFFF
FFFFFFFF:FFFFFFFFFFFFFFFFFFFFFFFF:FFFFFFFFFFF:FFFFFFFF:FFFFFFFF:FF::F
FFFFF:FFFFFFF,F
@A00721:190:HGJW7DSXY:4:2677:8730:24158 2:N:0:TCTGCAAG+GCTGCACT
TGTTGAATTGCAACACTCGTCGTGTTTTACGATAACCGGCGGTCCTAAACGGACCGCCTGACCACTTC
CTCTTCTTGGAAGACGAATCCATCAATCAACTGCTAAAGCAGTCATCAACACAATAAATATAAATTTT
GAATTTCAAAATTGA
+
FFF:FFFFFFFFF:FFFFFFFFFFFFFFFFF:FFFFFFFFFFFFFFFFFF:FFFFFFF,FFFFFFFFF
FFFFFFFFFFFFFFFFFFFFFFF:FFFFFFFFF:FFFFFFF,FFFFFFFFFFFFFFFFFF:F,FFFFF:F
FFF,FF:FFFFFFFF
```

```
@A00721:190:HGJW7DSXY:4:1539:31765:17487 2:N:0:TCTGCAAG+GCTGCACT
TGACCGCTGAGTCCTGTTGAATTGCAACACTCGTCGTGTTTTACGATAACCGGCGGTCCTAAACGGAC
CGCCTGACCACTTCCTCTTCTTGGAAGACGAATCCATCAATCAACTGCTAAAGCAGTCATCAACACAA
TAAATATAAATTTTG
+
FFFFFFFFFFFFFFFFFFFFFFFFFFFFFFFFFFFFFFFFFFFFFFF,FF:FFFFFFFFFFFFFFFFFFF
FFFFFFFFFFFFFFFFFFFFFFFFFFFFFFFFFFFFFFFFFFFFFFFFFFFFFFFFFFFFFFFFFFFFFF
FFFFFFFFFFFFFFF
@A00721:190:HGJW7DSXY:4:1233:6289:28103 2:N:0:TCTGCAAG+GCTGCACT
TTTATATTTATTGTGTTGATGACTGCTTTAGCAGTTGATTGATGGATTCGTCTTCCAAGAAGAGGAAG
TGGTCAGGCGGTCCGTTTAGGACCGCCGGTTATCGTAAAACACGACGAGTGTTGCAATTCAACAGGAC
TCAGCGGTCACAGCT
+
F:FFF:FFFFFFFFFFFFFFFFFFFFFFFFFFFFFFFFFFFFFFFFFFFFFFFFFFFFFFFFFFFF:FFF
FFFFFFF:FFFFFFFFFFFFFFF:FFFFFFFFFFFFFFFF:FFFF,FF:FFFFFFFFFFFFFFFFF:FFF
FFFFFFFFFFFFFFF
@A00721:190:HGJW7DSXY:4:1247:31937:35540 2:N:0:TCTGCAAG+GCTGCACT
TTTATATTTATTGTGTTGATGACTGCTTTAGCAGTTGATTGATGGATTCGTCTTCCAAGAAGAGGAAG
TGGTCAGGCGGTCCGTTTAGGACCGCCGGTTATCGTAAAACACGACGAGTGTTGCAATTCAACAGGAC
TCAGCGGTCACAGCT
+
FFFFFFFFFFFFFFFFFFFFFFFFFFFFFFFFFFFFFFFFFFFFFFFFFFFFFFFFFFFFF:FFFFFFFF
FFFFFFFFFFFFFFFFFFFFFFFFFFFFFFFFF:FFFFFF:FFFFFFFFFFFFFFFFFFFFFFFFF,FFF
F:FFFFFFFF:FFFF
@A00721:190:HGJW7DSXY:4:1258:31304:27179 2:N:0:TCTGCAAG+GCTGCACT
TTTATATTTATTGTGTTGATGACTGCTTTAGCAGTTGATTGATGGATTCGTCTTCCAAGAAGAGGAAG
TGGTCAGGCGGTCCGTTTAGGACCGCCGGTTATCGTAAAACACGACGAGTGTTGCAATTCAACAGGAC
TCAGCGGTCACAGCT
+
FFFFFFFFFFFFFFFFFFFFFFFFFFFFFFFFFFFFFFFFFFFFFFFFFFFFFFFFFFFFFFFFFFFFFF
FFFFFFFFFFFFFFFFFFFFFFFFFFFFFFFFFFFFFFFF,FFFFFFFFFFFFFFFFFFFFFFFFFFFFF
FFFFFFFFFFFFFFF
@A00721:190:HGJW7DSXY:4:1536:31358:2096 2:N:0:TCTGCAAG+GCTGCACT
TTTATATTTATTGTGTTGATGACTGCTTTAGCAGTTGATTGATGGATTCGTCTTCCAAGAAGAGGAAG
TGGTCAGGCGGTCCGTTTAGGACCGCCGGTTATCGTAAAACACGACGAGTGTTGCAATTCAACAGGAC
TCAGCGGTCACAGCT
+
FFFFFFFFFFFFFFFFFFFFFFFFFFFFFFFFFFFFFFFFFFFFFFFFFFF:FFFFFFFFFFFFFFFFFF
FFFFFFFFFFFFFFFFFFFFFFFFFFFFFFFFFFFF:FF::F,FFFFFFFFFFFFFFF:FFFFFFFFFFF
FFFFFFFF:FFFF:F
@A00721:190:HGJW7DSXY:4:1536:31792:2190 2:N:0:TCTGCAAG+GCTGCACT
TTTATATTTATTGTGTTGATGACTGCTTTAGCAGTTGATTGATGGATTCGTCTTCCAAGAAGAGGAAG
TGGTCAGGCGGTCCGTTTAGGACCGCCGGTTATCGTAAAACACGACGAGTGTTGCAATTCAACAGGAC
TCAGCGGTCACAGCT
+
F:FFFFFFFFFFFFFFFF:FFFFFFFFFFFFF,FFFFFFFFFFFFFFFFFFFF,FFFFFFFFFF:FFFFF
FF,FFFFF:FFFFFFFFFFFFFFFFFFFFFFFFFFFFFFFF:FFFF:FF:FFFFFFFFFFFFFFFFFFFF
FFFFF,FFFFFFF:F
@A00721:190:HGJW7DSXY:4:1655:26359:30232 2:N:0:TCTGCAAG+GCTGCACT
TTTATATTTATTGTGTTGATGACTGCTTTAGCAGTTGATTGATGGATTCGTCTTCCAAGAAGAGGAAG
TGGTCAGGCGGTCCGTTTAGGACCGCCGGTTATCGTAAAACACGACGAGTGTTGCAATTCAACAGGAC
TCAGCGGTCACAGCT
+
FF:FF:FFFFFF:FFFFFFFFFFFFFFFFFFFFFFFFFFFFF:FFFFFFFFFFFFFFFFFFFFFFFFFFF
```

FFFFFFFFFFFFFFFFFFFFFFFFFFFFFFFFFFFFFFFFFFFFFFFFFFFFFFFF:FFFFFFFFFFFFF
FFFFF:FFFFFFFFF
@A00721:190:HGJW7DSXY:4:2373:25807:4507 2:N:0:TCTGCAAG+GCTGCACT
TTTATATTTATTGTGTTGATGACTGCTTTAGCAGTTGATTGATGGATTCGTCTTCCAAGAAGAGGAAG
TGGTCAGGCGGTCCGTTTAGGACCGCCGGTTATCGTAAAACACGACGAGTGTTGCAATTCAACAGGAC
TCAGCGGTCACAGCT
+
FF:FFFF:FFFF:FF:FFFFFFFFFFFFFFFFFF:FFFFFFFF:FFFFFFFFFFFFFF:FF:FF:FFF
::FFFFFF,FF::F:F:FF:FFFF,FF,FF::FFF,FFFFFF:FFF:FFFFF:FFFF:FFFFFFF:FF
FFFF,,FFFFFF:FF
@A00721:190:HGJW7DSXY:4:2545:10691:29747 2:N:0:TCTGCAAG+GCTGCACT
TTTATATTTATTGTGTTGATGACTGCTTTAGCAGTTGATTGATGGATTCGTCTTCCAAGAAGAGGAAG
TGGTCAGGCGGTCCGTTTAGGACCGCCGGTTATCGTAAAACACGACGAGTGTTGCAATTCAACAGGAC
TCAGCGGTCACAGCT
+
FFFFFFFFF:FFFFFFFFFFFFFFFFFFFFFFFFFFFFFFFFFFFFFFF:FFFFFFFFFFFFFFFFFF
FFFFFFFFFFFFFFFFFFFFFFFFFFFFFFFFFFFFFFFFFFFFFFFFFFFFFFF:FFFFFFFFFFF
FFFFFFFFFFFFFFF
@A00721:190:HGJW7DSXY:4:2666:25970:31657 2:N:0:TCTGCAAG+GCTGCACT
TTTATATTTATTGTGTTGATGACTGCTTTAGCAGTTGATTGATGGATTCGTCTTCCAAGAAGAGGAAG
TGGTCAGGCGGTCCGTTTAGGACCGCCGGTTATCGTAAAACACGACGAGTGTTGCAATTCAACAGGAC
TCAGCGGTCACAGCT
+
FFFFFFFFFFFFFFFFFFFFFFFFFFFFFFF:FFFFFFFFFFFFFFFFFF:FFF:FFFFFF::FFFFF
FFFFFFFFFFFFFFFFFFF:FFFFFFF:FFFFF:FFF:,:FF:FFFFFFF:F:F:FFFFFFF:FFFFF
F:FFFFFFFFFFFFF
@A00721:190:HGJW7DSXY:4:2160:16396:12915 2:N:0:TCTGCAAG+GCTGCACT
TTATATTTATTGTGTTGATGACTGCTTTAGCAGTTGATTGATGGATTCGTCTTCCAAGAAGAGGAAGT
GGTCAGGCGGTCCGTTTAGGACCGCCGGTTATCGTAAAACACGACGAGTGTTGCAATTCAACAGGACT
CAGCGGTCACAGCT
+
FFFFFF:FFFFFFFFFF:FFFFFFFFFFFFFFFFFFFFFFFFFFFFFFFFFFFFFFFFFFFFFFFFFF
FFFFFFF:FFFFFFFFF:FFFFF::FFF:FFFFFFFFF::F:FFFFFFFFFFFFFFFFFFF:FFF:FF
FFF:FFFFF:FFFF
@A00721:190:HGJW7DSXY:4:1336:31891:11287 2:N:0:TCTGCAAG+GCTGCACT
TGTATATTTATTGTGTTGATGACTGCTTTAGCAGTTGATTGATGGATTCGTCTTCCAAGAAGAGGAAG
TGGTCAGGCGGTCCGTTTAGGACCGCCGGTTATCGTAAAACACGACGAGTGTTGCAATTCAACAGGAC
TCAGCGGTCACAGCT
+
F,FF:FFFFFFFFFFFFFFFFFFFFFFFFFFFFFFFFFFFFFFFFFFFFFFFFFFFFFFFFFFFFFFF
FFFFFFFFFFFFFFF:FFFFFFFFFFF:FFFFFFFFFF:FFFFFFFFFFFFFFFFFFFFFFFFFFFFFF
FFFFFFFFFFFFFFF
@A00721:190:HGJW7DSXY:4:1439:4987:19993 2:N:0:TCTGCAAG+GCTGCACT
TGAACCGGCAGCTGTGACCGCTGAGTCCTGTTGAATTGCAACACTCGTCGTGTTTTACGATAACCGGC
GGTCCTAAACGGACCGCCTGACCACTTCCTCTTCTTGGAAGACGAATCCATCAATCAACTGCTAAAGC
AGTCATCAACACAAT
+
FFFFFFFFFFFFFFFFFFFFFFFFFFFFFFFFFFFFFFFFFFFFFFFFFFFFFFFFF:FFFFFFFFFFF
FFFFFFFF,FFFFFFFFFFFFFFFFFFFFFFFFFF:FFFFFFFFFFFFF:FFFFFFFFFFFFFFFFFF
FFFF:FFFFFFFFFF
@A00721:190:HGJW7DSXY:4:2138:24795:32002 2:N:0:TCTGCAAG+GCTGCACT
TGAACCGGCAGCTGTGACCGCTGAGTCCTGTTGAATTGCAACACTCGTCGTGTTTTACGATAACCGGC
GGTCCTAAACGGACCGCCTGACCACTTCCTCTTCTTGGAAGACGAATCCATCAATCAACTGCTAAAGC
AGTCATCAACACAAT

+
FFFFFFFFFFF:FFF:FFFFFFFFFFFFFF:FFFFFFFFFF,FFFF::FFFFFFFFFFFFF:FFFFFF
FFFFFFFFFFFFF:FFFFFF:FFFFFFFFFFFFFFFF:FFFFF:FFFFFF::FFFFFF:FFFFFFFFF
FFFFF,FF:FFF:F:
@A00721:190:HGJW7DSXY:4:2356:5873:16986 2:N:0:TCTGCAAG+GCTGCACT
TGAACCGGCAGCTGTGACCGCTGAGTCCTGTTGAATTGCAACACTCGTCGTGTTTTACGATAACCGGC
GGTCCTAAACGGACCGCCTGACCACTTCCTCTTCTTGGAAGACGAATCCATCAATCAACTGCTAAAGC
AGTCATCAACACAAT
+
FFFFFFFFFFFFFFFFFFFFFFFFFFFFFFFFFFFFFFFFFFFFFFFFFFFFFFFFFFFF:FFFFFFFF
FFFFFFFFFFFFFFFF:FFFFFFFFFFFFFFFFFFFFFFFFFFFFFFFFFFFFFFFFFFFFFFFFFFF
FFFFFFFFFFFFFF:
@A00721:190:HGJW7DSXY:4:2516:22489:9314 2:N:0:TCTGCAAG+GCTGCACT
TGAACCGGCAGCTGTGACCGCTGAGTCCTGTTGAATTGCAACACTCGTCGTGTTTTACGATAACCGGC
GGTCCTAAACGGACCGCCTGACCACTTCCTCTTCTTGGAAGACGAATCCATCAATCAACTGCTAAAGC
AGTCATCAACACAAT
+
FFFFFFFFFFFFFFFFFFFFFFFFFFFFFFFFFFFFFFFFFFFFFFFFFFFFF:FFFFFFFFFFFFFF
FFFFFFFFFFFFFFFFFFFFFFFFFFFFFFFFFFFFFFFF:FFFFFFFFFFFFFFFFFFFFFFFFFFF
FFFFF:FF,FFFFFF
@A00721:190:HGJW7DSXY:4:1513:27444:5024 2:N:0:TCTGCAAG+GCTGCACT
CACTGAACCGGCAGCTGTGACCGCTGAGTCCTGTTGAATTGCAACACTCGTCGTGTTTTACGATAACC
GGCGGTCCTAAACGGACCGCCTGACCACTTCCTCTTCTTGGAAGACGAATCCATCAATCAACTGCTAA
AGCAGTCATCAACAC
+
FFFFFFFFFFFFFFFFFFFFFFFFFFFFFFFFFFFFFFFFFFFFFFFFFFFFFFFFFFFFFFF:FFFF
FFFFFFFFFFFFFFFFFFFFFFFFFFF:FFFFFFFFFFFFFFFFFFFFFFFFFFFFFFFFFFFFFFFF
FFFFFFFFFFF:FFF
@A00721:190:HGJW7DSXY:4:2207:20193:31391 2:N:0:TCTGCAAG+GCTGCACT
CACTGAACCGGCAGCTGTGACCGCTGAGTCCTGTTGAATTGCAACACTCGTCGTGTTTTACGATAACC
GGCGGTCCTAAACGGACCGCCTGACCACTTCCTCTTCTTGGAAGACGAATCCATCAATCAACTGCTAA
AGCAGTCATCAACAC
+
FFFFFFFFFFFFFFFFFFFFFFFFFFFFFFFF:FFFFFFFFFFFFF,FFFFFFFFFFFFF:F:FFFFF
FFFFFFFFFFFFFFFFFFFFFFFFFFFFFFFFFFF:FFFFF:FFFFFFFFF:FFFFFFFFFFFFFFFF
FFFFFFF:FFF:FFF
@A00721:190:HGJW7DSXY:4:2232:26250:9032 2:N:0:TCTGCAAG+GCTGCACT
CACTGAACCGGCAGCTGTGACCGCTGAGTCCTGTTGAATTGCAACACTCGTCGTGTTTTACGATAACC
GGCGGTCCTAAACGGACCGCCTGACCACTTCCTCTTCTTGGAAGACGAATCCATCAATCAACTGCTAA
AGCAGTCATCAACAC
+
FFFFFFFFFFFFFFFFFFFFFFFFFFFFFFFFFF:FFFFFFFFFFFFFFFFFFFFFFFFFFFFFFFFF
FFFFFFFFFFF:FFFFFFFFFFFFFFFFFFFFFFFFFFFFFFFFFFFFFFFFFFFFFFFFFFFFFFFF
FFFFFFFFFFFFFFF
@A00721:190:HGJW7DSXY:4:2427:24858:14011 2:N:0:TCTGCAAG+GCTGCACT
CACTGAACCGGCAGCTGTGACCGCTGAGTCCTGTTGAATTGCAACACTCGTCGTGTTTTACGATAACC
GGCGGTCCTAAACGGACCGCCTGACCACTTCCTCTTCTTGGAAGACGAATCCATCAATCAACTGCTAA
AGCAGTCATCAACAC
+
FFFFFFFFFFFFFFFFFFFFFFF:FFFFFFFFFFFFFFFFFFFFFFFFFFFFFFFFFFFFFFFFFF:F
FFFFFFFFFFFFFFFFF:FFFFFFFFFFFFFFFFFFFFFFFFFFFFFFFFFFFFFFFFFFFFFFFF:F
FF:FF:FFFFFFFFF
@A00721:190:HGJW7DSXY:4:2348:8052:22420 2:N:0:TCTGCAAG+GCTGCACT
CCACTGAACCGGCAGCTGTGACCGCTGAGTCCTGTTGAATTGCAACACTCGTCGTGTTTTACGATAAC

CGGCGGTCCTAAACGGACCGCCTGACCACTTCCTCTTCTTGGAAGACGAATCCATCAATCAACTGCTA
AAGCAGTCATCAACA
+
FFFFFFFFFFFFFFFFFFFFFFFFFFFFFFFFFFFFFFFFFFFFFFFFFFFFFFFFFFFFFFFFFFFF
FFFFFFFFFFFFFFFFFFFFFFFFFFFFFFFFFFFFFFFFFFFFFFFFFFFFF:FF:FFFFFFFFFFF
FFFFFF:FFFFFFFF
@A00721:190:HGJW7DSXY:4:2217:18322:16094 2:N:0:TCTGCAAG+GCTGCACT
CGACCACTGAACCGGCAGCTGTGACCGCTGAGTCCTGTTGAATTGCAACACTCGTCGTGTTTTACGAT
AACCGGCGGTCCTAAACGGACCGCCTGACCACTTCCTCTTCTTGGAAGACGAATCCATCAATCAACTG
CTAAAGCAGTCATCA
+
:FFFFFFFFFFF:FFFFFFFFFFFFFF:FFFFFFFFF:,FF,FFFFFF:FFFFFFFFF:F:,FFFF:F
F,FFF,,F::FFFFFFFFFFFFF,FFFFFFFFFFFFFFFF:,:FFFFFFFFF,:FFFFFFFFFFFFFF
FFFFFFFFFFFFF:F
@A00721:190:HGJW7DSXY:4:2332:18087:11616 2:N:0:TCTGCAAG+GCTGCACT
CGACCACTGAACCGGCAGCTGTGACCGCTGAGTCCTGTTGAATTGCAACACTCGTCGTGTTTTACGAT
AACCGGCGGTCCTAAACGGACCGCCTGACCACTTCCTCTTCTTGGAAGACGACTCCATCAATCAACTG
CTAAAGCAGTCATCA
+
FFFFFFFFFFFFFFFFFFFFFFFFFFFFFFFFFFFFFFFFFFFFFFFFF:FFFFFFFFFFFFFFFFFF
FFFFF:FFFFFFFFFFFFFFF,FFFFF,FFFFFFFFFFFFFFFFFFFFFFFF,FFFFFFFFFF:,FFF
FFFFFFFFFFFFFF:
@A00721:190:HGJW7DSXY:4:1348:8350:9032 2:N:0:TCTGCAAG+GCTGCACT
CTCAGAGACCTCGACCACTGAACCGGCAGCTGTGACCGCTGAGTCCTGTTGAATTGCAACACTCGTCG
TGTTTTACGATAACCGGCGGTCCTAAACGGACCGCCTGACCACTTCCTCTTCTTGGAAGACGAATCCA
TCAATCAACTGCTAA
+
FFFFFFFFFFFFFFFFFFFFFFFFFFFFFFFFFFFFFFFFFFFFFFFFFFFFFFFFFFFF:FFFFFFF
FFF:FFFFFFFFFFFFFFFFFFFFFFFF,FFFFFFFFFFFFFFFFFFFFFFFFFFFFFFFFFFFFFFF
FFFFFFFFFFFFFFF
@A00721:190:HGJW7DSXY:4:1611:9082:35790 2:N:0:TCTGCAAG+GCTGCACT
CTCAGAGACCTCGACCACTGAACCGGCAGCTGTGACCGCTGAGTCCTGTTGAATTGCAACACTCGTCG
TGTTTTACGATAACCGGCGGTCCTAAACGGACCGCCTGACCACTTCCTCTTCTTGGAAGACGAATCCA
TCAATCAACTGCTAA
+
FFFFFFFFFFFFFFFFFFFFFFFFFFFFFFFFFFFFFFFFFFFFFFFFFFFF:FFFFFFFFFFFFFFF
:FFFFFFFFFFFFFFFFFFFFFFFFFFFFFFFFFFFFFFFFFFFFFFFFFF:FFFFFFFFFFFFFFFF
FFFFFF:FFF:FFFF
@A00721:190:HGJW7DSXY:4:1618:15989:26991 2:N:0:TCTGCAAG+GCTGCACT
CTCAGAGACCTCGACCACTGAACCGGCAGCTGTGACCGCTGAGTCCTGTTGAATTGCAACACTCGTCG
TGTTTTACGATAACCGGCGGTCCTAAACGGACCGCCTGACCACTTCCTCTTCTTGGAAGACGAATCCA
TCAATCAACTGCTAA
+
FFFFFFFFFFFFFFFFFFFF:FFFFFFFFFFFFFFFFFFFFFFF:FFFFFFFFFFFFFFFFFFFFFFF
FFFFFFFFFFFFFFFFFFFF:FFFFF:FFFFFFFFFFFFFFFFFFFFFFFFFFFFFFFF:FFFFFFFF
FFFFFF:FFFFFFFF
@A00721:190:HGJW7DSXY:4:1618:17933:28604 2:N:0:TCTGCAAG+GCTGCACT
CTCAGAGACCTCGACCACTGAACCGGCAGCTGTGACCGCTGAGTCCTGTTGAATTGCAACACTCGTCG
TGTTTTACGATAACCGGCGGTCCTAAACGGACCGCCTGACCACTTCCTCTTCTTGGAAGACGAATCCA
TCAATCAACTGCTAA
+
FFFFFFF:FFFFFFFFFFFFFFFFFFFFFFFFFFFFFFFFFFFFFFFFFFFFFFFFFFFFFFFFFFFF
FFF:FFFFFFFFFFFFFFFFFFFFFFFFFFFFFFFFFFFFFFFFFFFFFFFFFFFFFFFFFFFFFFFF
FFFFFFFFFFFFFFF

```
@A00721:190:HGJW7DSXY:4:1618:17951:28667 2:N:0:TCTGCAAG+GCTGCACT
CTCAGAGACCTCGACCACTGAACCGGCAGCTGTGACCGCTGAGTCCTGTTGAATTGCAACACTCGTCG
TGTTTTACGATAACCGGCGGTCCTAAACGGACCGCCTGACCACTTCCTCTTCTTGGAAGACGAATCCA
TCAATCAACTGCTAA
+
FFFFF:FFFF:FFFFFFFFF:FFFFFFFFFFFFFFFFFFFFFFFFFFFFFFFFFFFFFFFFFFFFFFF
FFFFFFFFFFFFFFFFFFFFFFFFFF:FFFFFFFFFFFFFFFFFFFFFFFFFFFFFFFFFFFFFFFFF
:FFFFFFFFFFFFFF
@A00721:190:HGJW7DSXY:4:1618:31259:33865 2:N:0:TCTGCAAG+GCTGCACT
CTCAGAGACCTCGACCACTGAACCGGCAGCTGTGACCGCTGAGTCCTGTTGAATTGCAACACTCGTCG
TGTTTTACGATAACCGGCGGTCCTAAACGGACCGCCTGACCACTTCCTCTTCTTGGAAGACGAATCCA
TCAATCAACTGCTAA
+
FFFFFFFFFFFFFFFFFFFF:FFFFFFFFFFFFFFFFFFFFF:FFFFFFFFFFFFFFFFFFFFFFFFF
FFFFFFFFFFFFFFFFFFFFFFFFFFFFFFFFFFFFFFFFFFFFFFFFFFFFFFFFFFFFFFF:FFFF
FFFFFFFFFFFFF::
@A00721:190:HGJW7DSXY:4:2245:1913:3302 2:N:0:TCTGCAAG+GCTGCACT
CTCAGAGACCTCGACCACTGAACCGGCAGCTGTGACCGCTGAGTCCTGTTGAATTGCAACACTCGTCG
TGTTTTACGATAACCGGCGGTCCTAAACGGACCGCCTGACCACTTCCTCTTCTTGGAAGACGAATCCA
TCAATCAACTGCTAA
+
FFFFFFFFFFFFFFFFFFFFFFFFFFFFFFFFFFFFFFFFFFFFF:FFFFFFF:FFFFFFFF:FFFFF
:F:FFFFFFFFFFFFFFFFFFFF,FFFFFFFFFFFFFFFFFFFFFFFFFF::FFFFFFFF:FF,FFFF
FF:FFFFFFFFF:FF
@A00721:190:HGJW7DSXY:4:2362:3314:34037 2:N:0:TCTGCAAG+GCTGCACT
CTCAGAGACCTCGACCACTGAACCGGCAGCTGTGACCGCTGAGTCCTGTTGAATTGCAACACTCGTCG
TGTTTTACGATAACCGGCGGTCCTAAACGGACCGCCTGACCACTTCCTCTTCTTGGAAGACGAATCCA
TCAATCAACTGCTAA
+
FFFFFFFFFFFFFFFFFFFFFFFFFFFFFFFFFFFFF,FFFFFFFFFFFFFFFFFFFFFFFFFFFFFF
FFFFFFFFFFF:FFFFFFFFFFFFFFFFFFFFFFFFFFFFFFFFFFFFF,FF:FFFF,F:FFFF:FF,
:FFFFFFFF,FFF,F
@A00721:190:HGJW7DSXY:4:2523:9878:23390 2:N:0:TCTGCAAG+GCTGCACT
CTCAGAGACCTCGACCACTGAACCGGCAGCTGTGACCGCTGAGTCCTGTTGAATTGCAACACTCGTCG
TGTTTTACGATAACCGGCGGTCCTAAACGGACCGCCTGACCACTTCCTCTTCTTGGAAGACGAATCCA
TCAATCAACTGCTAA
+
FFFFFFFFFFFFFFFFFFFFFFFFFFFFFFFFFFFFFFFFFFF:FFFF,FFFFFFFFFFFFFFFFFFF
FFFFFFFFF:FFFFFFFFFFFFFFFFFFFFFFFFFFFFFFFFFFFFFFFFF:::FFFFFFFFF:FFFF
FFFFFFFFFFFFFFF
@A00721:190:HGJW7DSXY:4:1344:22679:12524 2:N:0:TCTGCAAG+GCTGCACT
AGCAGTTGATTGATGGATTCGTCTTCCAAGAAGAGGAAGTGGTCAGGCGGTCCGTTTAGGACCGCCGG
TTATCGTAAAACACGACGAGTGTTGCAATTCAACAGGACTCAGCGGTCACAGCTGCCGGTTCAGTGGT
CGAGGTCT
+
FFFFFFFFFFFFFFFFFFFFFFFFFFFFFFFFFFFFFFFFFFFFFFFFFFFFFFFFFFFFFFFFFFFF
FFFFFFFFFFFFFFFFFFFFFFFFFFFFFFFFFFFFFFFFFFFFFFFFFFFFFFFFFFFFFFFFFFFF
FFFFFFFF
@A00721:190:HGJW7DSXY:4:1411:18602:30201 2:N:0:TCTGCAAG+GCTGCACT
AGCAGTTGATTGATGGATTCGTCTTCCAAGAAGAGGAAGTGGTCAGGCGGTCCGTTTAGGACCGCCGG
TTATCGTAAAACACGACGAGTGTTGCAATTCAACAGGACTCAGCGGTCACAGCTGCCGGTTCAGTGGT
CGAGGTCT
+
FFFFFFFFFFFFFFFFFFFFFFFFFFFFFFFFFFFFFFFFFFFFFFFFFFFFFFFFFFFFFFFFFFFF
```

FFFFFFFFF:FFFFFFFFFFFF:FFFFFFFFFFFFFFFFFFF:FFFFF:FFFFFFFFFFFFFFFFFFF
FFFFFFFF
@A00721:190:HGJW7DSXY:4:2514:9435:22091 2:N:0:TCTGCAAG+GCTGCACT
AGCAGTTGATTGATGGATTCGTCTTCCAAGAAGAGGAAGTGGTCAGGCGGTCCGTTTAGGACCGCCGG
TTATCGTAAAACACGACGAGTGTTGCAATACAACAGGACTCAGCGGTCACAGCTGCCGGTTCAGTGGT
CGAGGTCT
+
FFFFFFFFFFFFFFFFFFFFFFFFFFFFFFFFFFFF:FFFFFFFFFFFFFFFFFF:FFFFFFFFFFFFF
,FFFFFFFFFFFFFFFFFFFFFFFFFFFFF,FFFFFFFFFFFFFFFFFFFFFFFFFFFFFFFFFFFFF
FFFFFFFF
@A00721:190:HGJW7DSXY:4:1155:8350:14450 2:N:0:TCTGCAAG+GCTGCACT
TATCTCAGAGACCTCGACCACTGAACCGGCAGCTGTGACCGCTGAGTCCTGTTGAATTGCAACACTCG
TCGTGTTTTACGATAACCGGCGGTCCTAAACGGACCGCCTGACCACTTCCTCTTCTTGGAAGACGAAT
CCATCAATCAACTGC
+
FFFFFFFFFFFFFFFFFFFFFFFFFFFFFFFFFFFFFFFFFFFFFFFFFFFFFFFFFFFFFFFFFFFF
FFFFFFFFFFFFFFFFFFFFFFFFFFFFFFFFFFFFFFFFFFFFFFFFFFFFFFFFFF:FFF:FFFFF
FFFFFFFFFFFFFFF
@A00721:190:HGJW7DSXY:4:2112:6705:13949 2:N:0:TCTGCAAG+GCTGCACT
CTCAGAGACCTCGACCACTGAACCGGCAGCTGTGACCGCTGAGTCCTGTTGAATTGCAACACTCGTCG
TGTTTTACGATAACCGGCGGTCCTAAACGGACCGCCTGACCACTTCCTCTTCTTGGAAGACGAATCCA
TCAATCAACTGC
+
FFFFFFFFFFFFFFFF:FFFFFF:FFF:FFFFFFFFFFFFFFFF,FFF:FF,FFFFFFFFFFFFF,FF
FF,,FFFFF:FFF:FFFFFFF:FF:FF:FFF:FF::,F::F,F:F:F,F,,F,FFF,:FF:F:FF:::
,F,:F:F:FFFF
@A00721:190:HGJW7DSXY:4:2669:22878:31626 2:N:0:TCTGCAAG+GCTGCACT
TATCTCAGAGACCTCGACCACTGAACCGGCAGCTGTGACCGCTGAGTCCTGTTGAATTGCAACACTCG
TCGTGTTTTACGATAACCGGCGGTCCTAAACGGACCGCCTGACCACTTCCTCTTCTTGGAAGACGAAT
CCATCAATCAACTGC
+
FFFFFFFFFFFFFFFFFFFF:FFFF:FFFFFFFFFFFFFFFFFFFFFFFFFFFFFF:FFFFFFFFFFF:
FFFFFFFFFFFFFFFFFFFFFFFFFFFFF,FFFFFFFFFFFF:FFFFFFFFFFFFFFFFF::FFFFFF
FFF,FFFFFF:FFFF
@A00721:190:HGJW7DSXY:4:1418:13819:21167 2:N:0:TCTGCAAG+GCTGCACT
GTTGATTGATGGATTCGTCTTCCAAGAAGAGGAAGTGGTCAGGCGGTCCGTTTAGGACCGCCGGTTAT
CGTAAAACACGACGAGTGTTGCAATTCAACAGGACTCAGCGGTCACAGCTGCCGGTTCAGTGGTCGAG
GTCTCTGAGATATAA
+
FFFFFFFFFFFFFFFFFFFFFFFFFFFFF,FFFFFFFFFFFFFFFFFFFFFFFFFFFFFFFFFFFFF,F
FFFFFFFFFFFFFFFFFFFFFFFFFFFFFFFFF:FFFFF:FFFFFFFFFF:FFFFFFFFFFFF:FFFFF
FFFFFFFFFFFFFFF
@A00721:190:HGJW7DSXY:4:1617:21920:26804 2:N:0:TCTGCAAG+GCTGCACT
TTGATTGATGGATTCGTCTTCCAAGAAGAGGAAGTGGTCAGGCGGTCCGTTTAGGACCGCCGGTTATC
GTAAAACACGACGAGTGTTGCAATTCAACAGGACTCAGCGGTCACAGCTGCCGGTTCAGTGGTCGAGG
TCTCTGAGATATAA
+
F:FFFFFFFFFF,FFF:FFFFFFFFFFFFFFFFFFFFFFFFFFF:FFFFFFFFFFFFFFFFFFFFF:,F
FFFFFFFFFFFFFFFFFFFFFFFFFF:FFFFFFFFFFFFFFFFFFFFFFFFFFFF,::FFF::F:FFFFFF
FFFFFFFFFFFF:F
@A00721:190:HGJW7DSXY:4:1271:10809:9878 2:N:0:TCTGCAAG+GCTGCACT
CTTCGATTATATCTCAGAGACCTCGACCACTGAACCGGCAGCTGTGACCGCTGAGTCCTGTTGAATTG
CAACACTCGTCGTGTTTTACGATAACCGGCGGTCCTAAACGGACCGCCTGACCACTTCCTCTTCTTGG
AAGACGAATCCATCA

+
FFFFFFFFFFFFFFFFFFFFFFFFFFFFFFFFFFFFFFFFFFFFFFFFFFFFFFFFFFFFFFFFFFFFF
FFFFFFFFFFFFFFFFFFFFFFFFFFFFFFFFFFFFFFFFFFFFFFFFFFFFFFFFFFFFFFFFFFFFF
FFFFFFFFFFFFFFF
@A00721:190:HGJW7DSXY:4:1540:7835:19758 2:N:0:TCTGCAAG+GCTGCACT
CTTCGATTATATCTCAGAGACCTCGACCACTGAACCGGCAGCTGTGACCGCTGAGTCCTGTTGAATTG
CAACACTCGTCGTGTTTTACGATAACCGGCGGTCCTAAACGGACCGCCTGACCACTTCATCTTCTTGG
AAGACGAATCCATCA
+
F:FFFF:FF:FFFFFFF,FFFFFF::F:FF:FF:F:F:,:F,:F,F:FF::F:FFFFF:FFF:FFF,F:
:FF,F:F:FFF,FFFFFFFF:F:FFFF,:FFF,F,:FFF,FF:F:,,,FFFF,,F,:FF,F:FF,:,FF
FFFFF:F:FFFF::F
@A00721:190:HGJW7DSXY:4:1559:12255:16986 2:N:0:TCTGCAAG+GCTGCACT
CTTCGATTATATCTCAGAGACCTCGACCACTGAACCGGCAGCTGTGACCGCTGAGTCCTGTTGAATTG
CAACACTCGTCGTGTTTTACGATAACCGGCGGTCCTAAACGGACCGCCTGACCACTTCCTCTTCTTGG
AAGACGAATCCATCA
+
FFFFFFFFFFFFFFFFFFFFFFFFFFFFFFFFFFFFFFFFFFFFFFFFFFFFFFFFFFFFFFFFFFFFF
FFFFFFFFFFFFFFFFFFFFFFFFFFFFFFFFFFFFFFFFFFFFFFFFFFFFFFFFFFFFFFFFFFFFF
FFFFFFFFFFFFFFF
@A00721:190:HGJW7DSXY:4:1635:28501:35571 2:N:0:TCTGCAAG+GCTGCACT
CTTCGATTATATCTCAGAGACCTCGACCACTGAACCGGCAGCTGTGACCGCTGAGTCCTGTTGAATTG
CAACACTCGTCGTGTTTTACGATAACCGGCGGTCCTAAACGGACCGCCTGACCACTTCCTCTTCTTGG
AAGACGAATCCATCA
+
FFFFFFFFFFFFFFFFFFFFFFFFFFFFFFFFFFFFFFFFFFFFFFFFFFFFFFFFFFFFFFFFFFFFF
FFFFFFFFFFFFFFFFFFFFF:F::FFFFFFFFFFFFF:FFFFFFFFFFFFFFFFFFFFFFFFFFFFFF
F:FF:FFFF:FFFFF
@A00721:190:HGJW7DSXY:4:2106:25961:33708 2:N:0:TCTGCAAG+GCTGCACT
CTTCGATTATATCTCAGAGACCTCGACCACTGAACCGGCAGCTGTGACCGCTGAGTCCTGTTGAATTG
CAACACTCGTCGTGTTTTACGATAACCGGCGGTCCTAAACGGACCGCCTGACCACTTCCTCTTCTTGG
AAGACGAATCCATCA
+
FFFFFFFFFFFFFFFFFFFFFFFFFFFFFFFFFFFFFFFFFFFFFFFFFFFFFFFFFFFFFFFFFFFFF
FFFFFFFFFFFFFFFFFFFFFFFF:FFFFFFFFFFFFF:FFFFFFFFFFFFFFFFFFFFFFFFFFFFFF
FFFFFFFFFFFFFFF
@A00721:190:HGJW7DSXY:4:2212:9182:6245 2:N:0:TCTGCAAG+GCTGCACT
CTTCGATTATATCTCAGAGACCTCGACCACTGAACCGGCAGCTGTGACCGCTGAGTCCTGTTGAATTG
CAACACTCGTCGTGTTTTACGATAACCGGCGGTCCTAAACGGACCGCCTGACCACTTCCTCTTCTTGG
AAGACGAATCCATCA
+
FFFFFFFFFFFFFFFFFFFFFFFFFFFFFFFFFFFFFFFFFFFFFFFFFFFFFFFFFFFFFFFFFFFFF
FFFFFF:FFFFFFFF:FFFFFFFFFFFFFFFFFFFFFFFFFFFFFFFFFFFFFFFFFFFFFFFFFFFFF
F:FFFFF,FFFF:FF
@A00721:190:HGJW7DSXY:4:2261:19343:28886 2:N:0:TCTGCAAG+GCTGCACT
CTTCGATTATATCTCAGAGACCTCGACCACTGAACCGGCAGCTGTGACCGCTGAGTCCTGTTGAATTG
CAACACTCGTCGTGTTTTACGATAACCGGCGGTCCTAAACGGACCGCCTGACCACTTCCTCTTCTTGG
AAGACGAATCCATCA
+
F:FFFFFFFFFFFFFFFFFFFFFFFFFFFFFFFFFFFFFFFFFFFFFFFFFFFFFFFFFFFFFFFFFFF
FFFFFFFFFFFFFFFFFFFFFFFFFFFFFFFFFFFFFFFFFFFFFFFFFFFFFFFFFFFFFFFFFFFFF
F:FFFFFFFFFFFFF
@A00721:190:HGJW7DSXY:4:2618:12635:32769 2:N:0:TCTGCAAG+GCTGCACT
CTTCGATTATATCTCAGAGACCTCGACCACTGAACCGGCAGCTGTGACCGCTGAGTCCTGTTGAATTG

```
CAACACTCGTCGTGTTTTACGATAACCGGCGGTCCTAAACGGACCGCCTGACCACTTCCTCTTCTTGG
AAGACGAATCCATCA
+
FFFFFFFFFFFFFFFFFFFFFFFFFFFFFFFFFFFFFFFFFFFFFFFFFFFFFFFFFFFFFFFFFFFF
FFFFFFFFFFFFFFFFFFFFFFF:FFFFFFFFFFFF:FFFFFFFFFFFFFFFFFFFFFFFFFFFFFFF
FF:FFFFFFFFFFFF
@A00721:190:HGJW7DSXY:4:2618:12626:32784 2:N:0:TCTGCAAG+GCTGCACT
CTTCGATTATATCTCAGAGACCTCGACCACTGAACCGGCAGCTGTGACCGCTGAGTCCTGTTGAATTG
CAACACTCGTCGTGTTTTACGATAACCGGCGGTCCTAAACGGACCGCCTGACCACTTCCTCTTCTTGG
AAGACGAATCCATC
+
F:FFFFFFFFFFFFFFFFFFFF,FFFFFFFFFFFFFFFFFFFFFFFFFFFFFFFFF:FFF:FFFFFFFF
FFFFFF,FFFFFFFFF,FFFF:FFFFFFFFFFFFFFFFF:FFFFFFFFFFFFFFFFFFFFFFFFFFFF
FFFFFFFFF,FF:F
@A00721:190:HGJW7DSXY:4:1101:21432:30405 2:N:0:TCTGCAAG+GCTGCACT
CAAGTCTTCGATTATATCTCAGAGACCTCGACCACTGAACCGGCAGCTGTGACCGCTGAGTCCTGTTG
AATTGCAACACTCGTCGTGTTTTACGATAACCGGCGGTCCTAAACGGACCGCCTGACCACTTCCTCTT
CTTGGAAGACGAATC
+
FFF:FFFFFFF:FFFFFFFFFFFFF:FFFFFFFFFFFFFFFFFFFFFFFFFFFFFFFFFFFFF:FFFFF
FFFFFFFFFFFF:FFFFFFFFFFFF,:F:::F,FFFFF::FFFFF,FF,FFFF,,FF:FF,FFFF:FF
FF,F:FF:FFFFFFF
@A00721:190:HGJW7DSXY:4:2217:25690:7560 2:N:0:TCTGCAAG+GCTGCACT
CAAGTCTTCGATTATATCTCAGAGACCTCGACCACTGAACCGGCAGCTGTGACCGCTGAGTCCTGTTG
AATTGCAACACTCGTCGTGTTTTACGATAACCGGCGGTCCTAAACGGACCGCCTGACCACTTCCTCTT
CTTGGAAGACGAATC
+
FFFFFFFFFFFFFFFFFFFFFFF:FFFFFFFFFFFFFFFFFFFFFFFFFFFFFFFFFFFFFFFFFFFFF
FFFFFFFFFFFFFFFFFFFFFFFF:FFFFFFFFFF:FFFFFFFFFFFFFFFFFFFFFFFFFFFFFFFFF
FFFFFFFFFFFFFFF
@A00721:190:HGJW7DSXY:4:1114:31132:21465 2:N:0:TCTGCAAG+GCTGCACT
ACCAAGTCTTCGATTATATCTCAGAGACCTCGACCACTGAACCGGCAGCTGTGACCGCTGAGTCCTGT
TGAATTGCAACACTCGTCGTGTTTTACGATAACCGGCGGTCCTAAACGGACCGCCTGACCACTTCCTC
TTCTTGGAAGACGAA
+
FFFFFFFFFFFFFFFFFFFFFFFFFF:F:FFFF:FFFFF:FFFFFFFFFFFFFFFFFFFFFFFFFFFF
FFFFFFFFFFFFFFFFFFFFFFFFFF:FF:FFFFFFFFFFFFFFFF:FFFF:FFFFFF,FFFFFFFFFF
FFFFFFF:FF,FFFF
@A00721:190:HGJW7DSXY:4:1552:27751:28855 2:N:0:TCTGCAAG+GCTGCACT
ACCAAGTCTTCGATTATATCTCAGAGACCTCGACCACTGAACCGGCAGCTGTGACCGCTGAGTCCTGT
TGAATTGCAACACTCGTCGTGTTTTACGATAACCGGCGGTCCTAAACGGACCGCCTGACCACTTCCTC
TTCTTGGAAGACGAA
+
FFFFFFFFFFFFFFFFFFFFFFFFFFFFFFFFFFFFFFFFFFFFF:FFFFF:FFFFFFFFFFFFFF,
FFFFFFFFFFFFF:FFFFF:FFFFFFFFFFFFFFFFFFFFFFFFFFFFFFFFFFFFFFFFFFFFFFFF
FFFFFFFFFFFFFFF
@A00721:190:HGJW7DSXY:4:2119:19054:35493 2:N:0:TCTGCAAG+GCTGCACT
CCAAGTCTTCGATTAAATCTCAGAGACCTCGACCACTGAACCGGAAGCTGTTACCGCTGAGTCCTGTA
GAATTGCAACACTCGTCGTGTTTTACGATAACCGGCGGTCCTAAACGGACCGCATGACCACTTACACT
TCTTGGAAGACGAA
+
FFF,:FF,:::F,:F,FF,F:FFF:FFFFF,FF,FFFFFFF,FF,F:FF:::FFF,F,FFFFFFFF,,
F,FF:F,FFFF:F,:FF,FF,FF,F:::F:F::FFF,F,,FFFFF:,:F,:,F,FF:F:F:F:,F,F,
FF,F:F::,:FFFF
```

```
@A00721:190:HGJW7DSXY:4:2234:19135:2190 2:N:0:TCTGCAAG+GCTGCACT
ACCAAGTCTTCGATTATATCTCAGAGACCTCGACCACTGAACCGGCAGCTGTGACCGCTGAGTCCTGT
TGAATTGCAACACTCGTCGTGTTTTACGATAACCGGCGGTCCTAAACGGACCGCCTGACCACTTCCTC
TTCTTGGAAGACGAA
+
FFFFFFFFFFFFF::FFFFFFFFFFF:FFFFFFFFFFFFFFFFFFFFFFFFFF:FFFFFFFFFFFFFFFF
FFFFFFF:FFFFFFFFFFFFFFFFFFFFFFFFFFFFFFFFFFFFFFFFFFFFFFF:FFFFFFFFFF
FFFFFFFF:FFFFFF
@A00721:190:HGJW7DSXY:4:2402:11451:15969 2:N:0:TCTGCAAG+GCTGCACT
ACCAAGTCTTCGATTATATCTCAGAGACCTCGACCACTGAACCGGCAGCTGTGACCGCTGAGTCCTGT
TGAATTGCAACACTCGTCGTGTTTTACGATAACCGGCGGTCCTAAACGGACCGCCTGACCACTTCCTC
TTCTTGGAAGACGAA
+
FFFFFFFFFFFFFFFFFFFF:FFF:FFFFFFFFFFFFFFFFFFF:FFFFFFFFFFFFFFFFFFFFFFFFF
FFFFFFFFFFFFFFFFFFFF:FFFFFFFFFFFFFFFFFFFFFFFFFF:FFFFFFFFFFFFFFFFFFFFFF
FFFFFFFFFFFFFFF
@A00721:190:HGJW7DSXY:4:1136:31991:13463 2:N:0:TCTGCAAG+GCTGCACT
ACACCAAGTCTTCGATTATATCTCAGAGACCTCGACCACTGAACCGGCAGCTGTGACCGCTGAGTCCT
GTTGAATTGCAACACTCGTCGTGTTTTACGATAACCGGCGGTCCTAAACGGACCGCCTGACCACTTCC
TCTTCTTGGAAGACG
+
FFFFFFFFFFFFFFFFFFFFFFFFFFFFF:FFFFFFFFFFFFFFFFFFFFFFFFFFF:FFFFFFFFFFFF
FFFFFFFFFFFFFFFFFFFFFFFFFFFFFFF:FFFFFFFFFFFFFFFFFFFFFFFFFFFF:FFFFFFFFF
FFFFFFFFFF:FFFF
@A00721:190:HGJW7DSXY:4:1228:30761:31876 2:N:0:TCTGCAAG+GCTGCACT
ACACCAAGTCTTCGATTATATCTCAGAGACCTCGACCACTGAACCGGCAGCTGTGACCGCTGAGTCCT
GTTGAATTGCAACACTCGTCGTGTTTTACGATAACCGGCGGTCCTAAACGGACCGCCTGACCACTTCC
TCTTCTTGGAAGACG
+
FFFFFFFFFFFFF:FFFFFFFFFFFFFFFFFFFFFFF:FF:FFFFFFFFFFFFFFFFFFFFFFFFFFFFF
FFFFFFFF:FFFFFFFFFFFFFFFFFFFFFFFFFF:,F:FFF:F:FFFFFF:FFFFFFFFFFFFFFFFFF
FFFFFF:FFFFFFF:
@A00721:190:HGJW7DSXY:4:1412:32705:34272 2:N:0:TCTGCAAG+GCTGCACT
ACACCAAGTCTTCGATTATATCTCAGAGACCTCGACCACTGAACCGGCAGCTGTGACCGCTGAGTCCT
GTTGAATTGCAACACTCGTCGTGTTTTACGATAACCGGCGGTCCTAAACGGACCGCCTGACCACTTCC
TCTTCTTGGAAGACG
+
FFFFFFFFFFFFFFFFFFFFFFFFFFFFFFFFFFFFFFFFFFFFFFFFFFFFFFFFFFFFFFFFFFFFFF
FFFFFFFFFFFFFFFFFFFFFFFFFFFFFFFFFFFFFFFFFFFFFFFF:FFFFFFFFFFFFFFFFFFFFF
FFFFFFFFFFFFFFF
@A00721:190:HGJW7DSXY:4:1465:32588:13777 2:N:0:TCTGCAAG+GCTGCACT
ACACCAAGTCTTCGATTATATCTCAGAGACCTCGACCACTGAACCGGCAGCTGTGACCGCTGAGTCCT
GTTGAATTGCAACACTCGTCGTGTTTTACGATAACCGGCGGTCCTAAACGGACCGCCTGACCACTTCC
TCTTCTTGGAAGACG
+
FFFFFFFFFFFFFFFFFFFFFFFFFFFFFFFFFFFFFFFFFFFFFFFFFFFFFFFFFFFFFFFFFFFFFF
FFFFFFFFFFFFFFFFFFFFFFFFFFFFFFF:FFFFFFFFFFFFFFFFFFFFFFFFFFFFFFFFFFFFFF
FFFFFFFFFFFFFFF
@A00721:190:HGJW7DSXY:4:1465:32570:13902 2:N:0:TCTGCAAG+GCTGCACT
ACACCAAGTCTTCGATTATATCTCAGAGACCTCGACCACTGAACCGGCAGCTGTGACCGCTGAGTCCT
GTTGAATTGCAACACTCGTCGTGTTTTACGATAACCGGCGGTCCTAAACGGACCGCCTGACCACTTCC
TCTTCTTGGAAGACG
+
FFFFFFFFFFFFFFFFFFFFFFFFFFFFFFFFFFFFFFFFFFFFFFFFFFFFFFFFFFFFFFFFFFFF:F
```

```
FFFFFFFFFFFFFFFFFFFFFFFFFFF:FFFFFFFFFFFFFFFFFFFFF:FFFFFFFFFFFFFFFFFFFF
FFFFFFFFFFFFFFF
@A00721:190:HGJW7DSXY:4:1469:26096:13964 2:N:0:TCTGCAAG+GCTGCACT
ACACCAAGTCTTCGATTATATCTCAGAGACCTCGACCACTGAACCGGCAGCTGTGACCGCTGAGTCCT
GTTGAATTGCAACACTCGTCGTGTTTTACGATAACCGGCGGTCCTAAACGGACCGCCTGACCACTTCC
TCTTCTTGGAAGACG
+
FFFFFFFFFFFFFFFFFFFFFFFFFFFFFFFFFFFFFFFFFFFFFFFFFFFFFFFFFFFFFFFFFFFFFF
FFFFFFFFFFFFFFFFFFFFFFFFFFFFFFFFFFFFFFFFFFFFFFFFFFFFFFFFFFFFFFFF:FFFFF
FFFFFFFFFFFFFFF
@A00721:190:HGJW7DSXY:4:1531:18204:21465 2:N:0:TCTGCAAG+GCTGCACT
ACACCAAGTCTTCGATTATATCTCAGAGACCTCGACCACTGAACCGGCAGCTGTGACCGCTGAGTCCT
GTTGAATTGCAACACTCGTCGTGTTTTACGATAACCGGCGGTCCTAAACGGACCGCCTGACCACTTCC
TCTTCTTGGAAGACG
+
FFFFFFFFFFFFFFFFFFFFFFFFFFFFFFFFFFFFFFFFFFFFFFFFFFFFFFFFFFFFFFFFFFFFFF
FFFFFFFFFFFFFFFFFFFFFFFFFFFFFFFFFFFFFFFFFFFFFFFFFFFFFFFFFFFFFFFFFFFFFF
FFFFFFFFFFFFFFF
@A00721:190:HGJW7DSXY:4:1536:15989:34663 2:N:0:TCTGCAAG+GCTGCACT
ACACCAAGTCTTCGATTATATCTCAGAGACCTCGACCACTGAACCGGCAGCTGTGACCGCTGAGTCCT
GTTGAATTGCAACACTCGTCGTGTTTTACGATAACCGGCGGTCCTAAACTGACCGCCTGACCACTTCC
TCTTCTTGGAAGACG
+
FFFFFF:FFFFFFFF::FFFFFFFFFFFFFFFFFFFFFFFFFFFFFFF:FFFFFFFFFFFFF:FFFFFFF
FFFFFFFFFFF:FFFFFFFFF:FFF:FFF:FFFFFF:FFFFFFFFFFFFF,FFFFFFFFFFFFFFFFF:F
FF,FF,FFFF:FFFF
@A00721:190:HGJW7DSXY:4:1658:27914:31610 2:N:0:TCTGCAAG+GCTGCACT
ACACCAAGTCTTCGATTATATCTCAGAGACCTCGACCACTGAACCGGCAGCTGTGACCGCTGAGTCCT
GTTGAATTGCAACACTCGTCGTGTTTTACGATAACCGGCGGTCCTAAACGGACCGCCTGACCACTTCC
TCTTCTTGGAAGACG
+
FFFFFFFFFFFFFFFFFFFFFFFFFFFFFFFFFFFFFF:FFFFFFFFFFFFFFFFFFFFFFFFFFFFFF:
FFFFFFFFFFFFFFFFFF:FFFFFFFFFFFFFF:FFFFFFFFFFFFFFFFFFFFFFFFFFFFFF:FFFFF
FFFFFFFFFFFFFFF
@A00721:190:HGJW7DSXY:4:1659:17336:27038 2:N:0:TCTGCAAG+GCTGCACT
ACACCAAGTCTTCGATTATATCTCAGAGACCTCGACCACTGAACCGGCAGCTGTGACCGCTGAGTCCT
GTTGAATTGCAACACTCGTCGTGTTTTACGATAACCGGCGGTCCTAAACGGACCGCCTGACCACTTCC
TCTTCTTGGAAGACG
+
FFFFFFFFFFFFFFFFFFFFFFFFFFFFFFFFFFFFFFFFFFFFFFFFFFFFFFFFFFFFFFFFFFFFFF
FFFFFFFFFFFFFFFFFFFFFFFFFFFFFFFFFFFFFFFFFFFFFFFFFFFFFFFFFFFFFFFFFFFFFF
FFFFFFFFFFFFFFF
@A00721:190:HGJW7DSXY:4:2505:16984:13714 2:N:0:TCTGCAAG+GCTGCACT
ACACCAAGTCTTCGATTATATCTCAGAGACCTCGACCACTGAACCGGCAGCTGTGACCGCTGAGTCCT
GTTGAATTGCAACACTCGTCGTGTTTTACGATAACCGGCGGTCCTAAACGGACCGCCTGACCACTTCC
TCTTCTTGGAAGACG
+
FFFFFF:FFFFFFFFFFFFFFFFFFFFFFFFFFFFFFFFFFFFFFFFFFFFFFFFFFFFFFFFFFFFFFF
FFFFFFFFFFFFFFFFFF:FF:FFFFFFFFFFFFFFFFFFFFFFFFF,:FFFFF:FFFFFFFFFFFFFFF
FFFFFFFFFFFFFFF
@A00721:190:HGJW7DSXY:4:2554:17345:20885 2:N:0:TCTGCAAG+GCTGCACT
ACACCAAGTCTTCGATTATATCTCAGAGACCTCGACCACTGAACCGGCAGCTGTGACCGCTGAGTCCT
GTTGAATTGCAACACTCGTCGTGTTTTACGATAACCGGCGGTCCTAAACGGACCGCCTGACCACTTCC
TCTTCTTGGAAGACG
```

+
FFFFFFFFFFFFFFFFFFFFFFFFFF:FFFFFFFFFFFFFFFFFFFFFFFF:FFFFFFFFFFFFFFF:
FFFFFFFFFFFFFFFFFF,FFFF:FFFFFF:FFFFFFFFFFFFFFFFFFFFFFFFFFFFFFFFFFFFF
FFFFFFFFFFFFFFF
@A00721:190:HGJW7DSXY:4:1243:8793:26209 2:N:0:TCTGCAAG+GCTGCACT
CACACCAAGTCTTCGATTATATCTCAGAGACCTCGACCACTGAACCGGCAGCTGTGACCGCTGAGTCC
TGTTGAATTGCAACACTCGTCGTGTTTTACGATAACCGGCGGTCCTAAACGGACCGCCTGACCACTTC
CTCTTCTTGGAAGAC
+
FFFFFFFFFFFFFFFFFFFFFFFFFFFFFFFFFFFFFFFFFFFFFFFFFFFFFFFFFFFFFFF:FFFF
FFFFF:F:FFFFFFFFFFFFFFFFFFFFFFFFFFFFFFFFFFFFFFFFFFFFFFFFFFFFFFFFFFFF
FFFFFFFFFF:FFFF
@A00721:190:HGJW7DSXY:4:1561:10059:18255 2:N:0:TCTGCAAG+GCTGCACT
CACACCAAGTCTTCGATTATATCTCAGAGACCTCGACCACTGAACCGGCAGCTGTGACCGCTGAGTCC
TGTTGAATTGCAACACTCGTCGTGTTTTACGATAACCGGCGGTCCTAAACGGACCGCCTGACCACTTC
CTCTTCTTGGAAGAC
+
FFFFFFFFFFFFFFF:FFFFFFFFFFFFFFFFFFFFFFFFFFFFFFFFFFFFFFFFFFFFFFFFFFFF
FF:FFFFFF:FFFFFFFFFFFFFFFFFFFFFFFFFFFFFFFFFFFFFFFFFFFFFFFFFFFFFFFFFF
FFFFFFFFFFF:FFF
@A00721:190:HGJW7DSXY:4:2217:17219:24643 2:N:0:TCTGCAAG+GCTGCACT
ACACCAAGTCTTCGATTATATCTCAGAGACCTCGACCACTGAACCGGCAGCTGTGACCGCTGAGTCCT
GTTGAATTGCAACACTCGTCGTGTTTTACGATAACCGGCGGTCCTAAACGGACCGCCTGACCACTTCC
TCTTCTTGGAAGAC
+
F:FFFFFFFFFFFFFF:FFFFFFFFFFFFFFFFFFFFFFFFFFFFFFFFFFFFFFFFFFFFFFFFFFF
FFFFFF::FFFFFFFF:F:FFFFF:::FFFFFFF:FFFFFFFFFFFFFFFFFF:FFFF:FFFFFFF:F
:FF,FF:F:FFFFF
@A00721:190:HGJW7DSXY:4:2265:21197:29121 2:N:0:TCTGCAAG+GCTGCACT
ACACCAAGTCTTCGATTATATCTCAGAGACCTCGACCACTGAACCGGCAGCTGTGACCGCTGAGTCCT
GTTGAATTGCAACACTCGTCGTGTTTTACGATAACCGGCGGTCCTAAACGGACCGCCTGACCACTTCC
TCTTCTTGGAAGAC
+
FFFFFFFFFFFFFFFFFFFFFFFFFFFFFFFFFFFFFFFFFFFFFFFFFFFFFFFFFFFFFFFFFFF:
FFFFFFFFFFFFFFFFFFFFFFFFFFFFFFFFFFFFFFFFFFFFFFFFFFFFFFFFFFFFFFFFF:FF
FFFFFFFFFFFFFF
@A00721:190:HGJW7DSXY:4:2275:20916:20776 2:N:0:TCTGCAAG+GCTGCACT
ACACCAAGTCTTCGATTATATCTCAGAGACCTCGACCACTGAACCGGCAGCTGTGACCGCTGAGTCCT
GTTGAATTGCAACACACGTCGTGTTTTACGATAACCGGCGGTCCTAAACTGACCGCCAGACCACTTCC
TCTTCTTGGAAGAC
+
FFF:FFFFFF:,F::,,F:F,FFFF,F,F:FFFF::FF:FFFFF,,F,FF,,FFFFFFFFF:FFFF,F
,F,FFFFF,F:FFFF,:FFF,,,FF,,:FF:,:FFF,,F:F:F,FFFFF,F:FFF,F,:F,FFFF:FF
FFF,F,FF:FF,FF
@A00721:190:HGJW7DSXY:4:2524:27489:13463 2:N:0:TCTGCAAG+GCTGCACT
CACACCAAGTCTTCGATTATATCTCAGAGACCTCGACCACTGAACCGGCAGCTGTGACCGCTGAGTCC
TGTTGAATTGCAACACTCGTCGTGTTTTACGATAACCGGCGGTCCTAAACGGACCGCCTGACCACTTC
CTCTTCTTGGAAGAC
+
FFFFFFFFFFFFFFFFFFFFFFFFFFFFFFFFFFFFFFFFFFFFFFFFFFFFFFFFFF:FFFFFFFFF
FFFFFFFFFFFFFFFFFFFFFFFFFFFFFFFFFFFFFFF:FFFFFFFFFFFFFFFFFFFFFFFFFFFF
FFFFFFFFFFF,FFF
@A00721:190:HGJW7DSXY:4:2637:30318:36276 2:N:0:TCTGCAAG+GCTGCACT
CACACCAAGTCTTCGATTATATCTCAGAGACCTCGACCACTGAACCGGCAGCTGTGACCGCTGAGTCC

```
TGTTGAATTGCAACACTCGTCGTGTTTTACGATAACCGGCGGTCCTAAACGGACCGCCTGACCACTTC
CTCTTCTTGGAAGAC
+
FFFFFFFFFFFFFFFFFFFFFFFFFFFFFFFFFFFFFFFFFFFFFFFFFFFFFFFFFFFFFFFFFFFF
FFFFFFFFFFFFFFFFFFFFFFFFFFFFFFFF:FFFFFFFFFFFFFFFFFFFFFF:FFFFFFFFFFFFF
FFFFFFFFFFFF:FF
@A00721:190:HGJW7DSXY:4:2665:32606:32315 2:N:0:TCTGCAAG+GCTGCACT
CACACCAAGTCTTCGATTATATCTCAGAGACCTCGACCACTGAACCGGCAGCTGTGACCGCTGAGTCC
TGTTGAATTGCAACACTCGTCGTGTTTTACGATAACCGGCGGTCCTAAACGGACCGCCTGACCACTTC
CTCTTCTTGGAAGAC
+
FFFFFFFFFFFFFFFFFFFF:FFFFFFFFFFFFFFFFFFFFFFFFFFFFFFFFFFFFFFFFFFFFFFF
FF,FFFFFFFFFFFFFFFFFFFFFFFFFFFFFFFFFFFFFFFFFFF:FFFFFFFFFFFFFFFFF:F
FFFFFFF:FFFFFFF
@A00721:190:HGJW7DSXY:4:1135:17282:30514 2:N:0:TCTGCAAG+GCTGCACT
TGTGAACACACCAAGTCTTCGATTATATCTCAGAGACCTCGACCACTGAACCGGCAGCTGTGACCGCT
GAGTCCTGTTGAATTGCAACACTCGTCGTGTTTTACGATAACCGGCGGTCCTAAACGGACCGCCTGAC
CACTTCCTCTTCTTG
+
FFFFFFFFFFFFFFFFFFFFFFFFFFFFFFFFFFFFFFFFFFFFFFFFFFFFFFFFFFFFFFFFFFFF
FFFFFFFFFFFFFFFFFFFFFFFFFFFFFFFFFFFFFFFF:FFFFFFFFFFFFFFFFFFFFFFFFFFF
FFFFFFFFFFFFFFF
@A00721:190:HGJW7DSXY:4:1156:15998:24878 2:N:0:TCTGCAAG+GCTGCACT
TGTGAACACACCAAGTCTTCGATTATATCTCAGAGACCTCGACCACTGAACCGGCAGCTGTGACCGCT
GAGTCCTGTTGAATTGCAACACTCGTCGTGTTTTACGATAACCGGCGGTCCTAAACGGACCGCCTGAC
CACTTCCTCTTCTTG
+
FFFFFFFFFFFFFFFFFFFFFFFFFFFFFFFFFFFFFFFFFFFFFFFFFFFFFFFFFFFFFFFFFFFF
FFFFFFFFFFFFFFFFFFFFFFFFFFFFFFFFFFFFFFFF,FFFFFFFFFFFFFFFFFFFFFFFFFFF
FFFFFFFFFFFFFFF
@A00721:190:HGJW7DSXY:4:1351:28221:11663 2:N:0:TCTGCAAG+GCTGCACT
TGTGAACACACCAAGTCTTCGATTATATCTCAGAGACCTCGACCACTGAACCGGCAGCTGTGACCGCT
GAGTCCTGTTGAATTGCAACACTCGTCGTGTTTTACGATAACCGGCGGTCCTAAACGGACCGCCTGAC
CACTTCCTCTTCTTG
+
FFFFFFFFFFFFFFFFFFFFFF:F,FFFFFFFFFFFFFFFFFFFFFFFFFFFFFFFFFFFFFFFFFF
FFFFFFFFFFFFFFFFFFFFFFFFFFFFFFFFFFFFFFFFFFFFFFFFFFFFFFFFFFFFFFFFFFFF
FFFFFFFFFFFFFFF
@A00721:190:HGJW7DSXY:4:1448:29161:6590 2:N:0:TCTGCAAG+GCTGCACT
TGTGAACACACCAAGTCTTCGATTATATCTCAGAGACCTCGACCACTGAACCGGCAGCTGTGACCGCT
GAGTCCTGTTGAATTGCAACACTCGTCGTGTTTTACGATAACCGGCGGTCCTAAACGGACCGCCTGAC
CACTTCCTCTTCTTG
+
:FFFFFFFFFFFFFFFFFFFFFFFFFFFFFFFFFFFFFFFFFFFFFFFFFFFFFFFFFFFFFFFFFFF
FFFFFFFFFFFFF:FFFFFFFFFFFFFFFFFFFFFFFFFFFFFFFFFFFFF::FFFFFFFFFFFFFFF
FFFFFFFFFFFFFFF
@A00721:190:HGJW7DSXY:4:1644:17427:28354 2:N:0:TCTGCAAG+GCTGCACT
TGTGAACACACCAAGTCTTCGATTATATCTCAGAGACCTCGACCACTGAACCGGCAGCTGTGACCGCT
GAGTCCTGTTGAATTGCAACACTCGTCGTGTTTTACGATAACCGGCGGTCCTAAACGGACCGCCTGAC
CACTTCCTCTTCTTG
+
FFFFFFFFFFFFFFFFFFFFFF:FFFFFFFFFFFFFFFFFFFFFFFFFFFFFFFFFFFFFFFFFFFFF
FFFFFFFFFFFFFFFFFFFFFFFFFFFFFFFF:FFFFFFFFFFFFFFFFFFFFFFFFFFFFFFFFFFF
FFFFFFFFFFFFFFF
```

@A00721:190:HGJW7DSXY:4:2418:14380:33692 2:N:0:TCTGCAAG+GCTGCACT
TGTGAACACACCAAGTCTTCGATTATATCTCAGAGACCTCGACCACTGAACCGGCAGCTGTGACCGCT
GAGTCCTGTTGAATTGCAACACTCGTCGTGTTTTACGATAACCGGCGGTCCTAAACGGACCGCCTGAC
CACTTCCTCTTCTTG
+
FFFFFFFFFFFFFFFFFFFFFFFFFFFFFFFFFFFFFFFFFFFFFFFFFFFFFFFFFFFFFFFFFFFF
FFFFFF:FFFFFFFFFFFFFFFFFFFFFFFFFFFFFFFFFFFFFFFFFFFFFFFFFFFFFFFFFFFFF
FFFFFFFFFFFFFFF
@A00721:190:HGJW7DSXY:4:2611:16333:6668 2:N:0:TCTGCAAG+GCTGCACT
TGTGAACACACCAAGTCTTCGATTATATCTCAGAGACCTCGACCACTGAACCGGCAGCTGTGACCGCT
GAGTCCTGTTGAATTGCAACACTCGTCGTGTTTTACGATAACCGGCGGTCCTAAACGGACCGCCTGAC
CACTTCCTCTTCTTG
+
FFFFF:F:FFFFFFFFFF:FFF:FFFFFFFFFFFFFFF:F:FFFFFFFFFFFFF:FFFFFFFFFFFFF
FFFFFFFFFFFFFFF::FFFF:FFFFFFFFFFFFFFFFFFFFFFF:FFFFFFFFFF,FF:FFFF:FFF
FF::,FFFFF:FFFF
@A00721:190:HGJW7DSXY:4:1260:24894:29794 2:N:0:TCTGCAAG+GCTGCACT
GAACACACCAAGTCTTCGATTATATCTCAGAGACCTCGACCACTGAACCGGCAGCTGTGACCGCTGAG
TCCTGTTGAATTGCAACACTCGTCGTGTTTTACGATAACCGGCGGTCCTAAACGGACCGCCTGACCAC
TTCCTCTTCTT
+
FFFFFFFFFFFFFFFFFFFFFFFFFFFFFFFFFFFFFFFFFFFFFFFFFFFFFFFFFFFFFFFFFFFF
FFFFFFFFFFFFFFFFFF:FFFFFFFFFFFFFFF:FFFFFFFFFFFFFFFFFFFFFFFFFFFFFFFFF
FFFFFFF:FFF
@A00721:190:HGJW7DSXY:4:1260:23845:29951 2:N:0:TCTGCAAG+GCTGCACT
GAACACACCAAGTCTTCGATTATATCTCAGAGACCTCGACCACTGAACCGGCAGCTGTGACCGCTGAG
TCCTGTTGAATTGCAACACTCGTCGTGTTTTACGATAACCGGCGGTCCTAAACGGACCGCCTGACCAC
TTCCTCTTCTT
+
FFFFFFFFFFFFFFFFFFFFFFFFFFFFFFF:FFFFFFFFFFFFFFFFFFFFFFFFFF:FFFFFFFFF
FFFFFFFFFFFFFFFFFFFFFFFFFF:FFFFFFFFFFFFFFFFFFFFFFFF,FFFFFFF,FFFFFFFF
FFFFFFF,FFF
@A00721:190:HGJW7DSXY:4:1305:29704:20588 2:N:0:TCTGCAAG+GCTGCACT
GAACACACCAAGTCTTCGATTATATCTCAGAGACCTCGACCACTGAACCGGCAGCTGTGACCGCTGAG
TCCTGTTGAATTGCAACACTCGTCGTGTTTTACGATAACCGGCGGTCCTAAACGGACCGCCTGACCAC
TTCCTCTTCTT
+
FFFFFFFFFFFFFFFFFFFFFFFFFFFFF:FFFFFFFFFFFFFFFFFFF:FFFFFFFFFFFFFFFFFF
FFFFFFFF:FFFFFFFFFFFFFFFFFFFFFFFFFFFFFFFFFFFFFFFFFFFFFFFFFFFFFFFFFFF
FFFFFFFFFFF
@A00721:190:HGJW7DSXY:4:2120:12373:15781 2:N:0:TCTGCAAG+GCTGCACT
GAACACACCAAGTCTTCGATTATATCTCAGAGACCTCGACCACTGAACCGGCAGCTGTGACCGCTGAG
TCCTGTTGAATTGCAACACTCGTCGTGTTTTACGATAACCGGCGGTCCTAAACGGACCGCCTGACCAC
TTCCTCTTCTT
+
FFFFFFFFFFFFFFFFFFFFFFFFFFFFFFFFFFFFFFFFFFFFFFFFFFFFFFFFFFFFFFFFFFFF
FFFFFFFFFFFFFFFFFFFFFFFFFFFFFFFFFFFFFFFFFFFFFFFFFFFFFFFFFFFFFFFFFFFF
FFFFFFFFFFF
@A00721:190:HGJW7DSXY:4:2120:11867:17347 2:N:0:TCTGCAAG+GCTGCACT
GAACACACCAAGTCTTCGATTATATCTCAGAGACCTCGACCACTGAACCGGCAGCTGTGACCGCTGAG
TCCTGTTGAATTGCAACACTCGTCGTGTTTTACGATAACCGGCGGTCCTAAACGGACCGCCTGACCAC
TTCCTCTTCTT
+
FFFFFFFFFFFFFFFFFFFFFFFFFFFF:FFFFFFFFFFFFFFFFFFFFFFFFFFFFFFFFFFFFFFF

FFFFFFFFFF:FFFFFFFFFFFFFFFFFFFFFFFFFFFFFFFFFFFFFFF::FFFFFFFFFFFFFFFF
FFFFFFFFFFF
@A00721:190:HGJW7DSXY:4:2219:4716:16673 2:N:0:TCTGCAAG+GCTGCACT
GAACACACCAAGTCTTCGATTATATCTCAGAGACCTCGACCACTGAACCGGCAGCTGTGACCGCTGAG
TCCTGTTGAATTGCAACACTCGTCGTGTTTTACGATAACCGGCGGTCCTAAACGGACCGCCTGACCAC
TTCCTCTTCTT
+
FFFFFFFFFFFFFFFFFFFFFFFFFFFFFFFFFFFFFFFFFFFFFFFFFFFFFFFFFFFFFFFFFFFF
FFFFFFFFFFFFFFFFFFFFFFFFFFFFFFFFFFFFFFFF:FFFFFFFFFFFFFFFFFFFFFFFFFFF
FFFFFFFFFFF
@A00721:190:HGJW7DSXY:4:2529:5791:13808 2:N:0:TCTGCAAG+GCTGCACT
GAACACACCAAGTCTTCGATTATATCTCAGAGACCTCGACCACTGAACCGGCAGCTGTGACCGCTGAG
TCCTGTTGAATTGCAACACTCGTCGTGTTTTACGATAACCGGCGGTCCTAAACGGACCGCCTGACCAC
TTCCTCTTCTT
+
FFFFFFF:FFFFF:FFFFFFFFFFFFFFFFF,FFFFFFFFFFFFFF:FFFFFFFFFFFFFFFFFFFFF
FFFFFFFFFFFFFFFFFFFFFFFFFFFFFFFFFFFFFFFFFFFFFFF::FFFFFFFFFFFFFFFFFFF
FFFFFFFFFFF
@A00721:190:HGJW7DSXY:4:2629:13847:8625 2:N:0:TCTGCAAG+GCTGCACT
GAACACACCAAGTCTTCGATTATATCTCAGAGACCTCGACCACTGAACCGGCAGCTGTGACCGCTGAG
TCCTGTTGAATTGCAACACTCGTCGTGTTTTACGATAACCGGCGGTCCTAAACGGACCGCCTGACCAC
TTCCTCTTCTT
+
FFFFFFFFFFFFFFFFFFFFFFFFFFFFFFFFFFFFFFFFFFFFFFFFFFFFFFFFFFFFFFFFFFFF
FFFFFFFFF:FFFFFFFFFFFFFFFFFFFFFFFF:FFF:F:FFFFFFFFFFFFFFFFFFFFFFFFFFF
F:FFFFFFFFF
@A00721:190:HGJW7DSXY:4:1241:29143:1047 2:N:0:TCTGCAAG+GCTGCACT
GAGGAAGTGGTCAGGCGGTCCGTTTAGGACCGCCGGTTATCGTAAAACACGACGAGTGTTGCAATTCA
ACAGGACTCAGCGGTCACAGCTGCCGGTTCAGTGGTCGAGGTCTCTGAGATATAATCGAAGACTTGGT
GTGTTCACGCCTCCT
+
F:F:FF:FF:FFFFFFF:FFFFFF:F:FFFFFFF:FFFFFFFFFFFFFF:,F:FF:FFFF,FFFFFFF
FFFFFFF:FFFFFFFFF,F:F::F:F:FFFF,F:FFFF:FFFFFFF,FFFFFF:FFFFFFF:F,F:FF
FFFFF:F,:F:FFFF
@A00721:190:HGJW7DSXY:4:1241:29857:2597 2:N:0:TCTGCAAG+GCTGCACT
GAGGAAGTGGTCAGGCGGTCCGTTTAGGACCGCCGGTTATCGTAAAACACGACGAGTGTTGCAATTCA
ACAGGACTCAGCGGTCACAGCTGCCGGTTCAGTGGTCGAGGTCTCTGAGATATAATCGAAGACTTGGT
GTGTTCACGCCTCCT
+
FFFFFFFFFFFFFF:FFF:FFFFFFFFFFFFFFFFFFFFFFFFFFFFFFFFFFFFFFFFFFFFFFFFF
FFFFFFFFFFFFFFFFFFFFFFFFFFFFFFFFF:FFFFFFFFFFFFFFFFFFFFFFFFFFFFFFFFFF
FFFFFFFFFFFFFFF
@A00721:190:HGJW7DSXY:4:1264:10700:21684 2:N:0:TCTGCAAG+GCTGCACT
GAGGAAGTGGTCAGGCGGTCCGTTTAGGACCGCCGGTTATCGTAAAACACGACGAGTGTTGCAATTCA
ACAGGACTCAGCGGTCACAGCTGCCGGTTCAGTGGTCGAGGTCTCTGAGATATAATCGAAGACTTGGT
GTGTTCACGCCTCCT
+
FFFFFFFFFFFFFFFFFFFFFFFFFFFFFFFFFFFFFFFFFFFFFF::FFFFFFFFF:FFFFFFFFFF
FFFFFFFFFFFFFFFFFFFFFFFFFFFFFFFFFFFFFFFFFF,FFF,FFFFFF:FFFF:FFFF:FF:
FFFFFFFF:FFFFFF
@A00721:190:HGJW7DSXY:4:1546:29261:12712 2:N:0:TCTGCAAG+GCTGCACT
GAGGAAGTGGTCAGGCGGTCCGTTTAGGACCGCCGGTTATCGTAAAACACGACGAGTGTTGCAATTCA
ACAGGACTCAGCGGTCACAGCTGCCGGTTCAGTGGTCGAGGTCTCTGAGATATAATCGAAGACTTGGT
GTGTTCACGCCTCCT

```
+
FFFF:FF,F:FFFFFFFFFF:F:FFFFF:,FFFFFFFFFFFF,FFFFFFFFF:FFFFFF:FFF:FFFF
FFFFFFFFFF:,FFFF:FFFFF::FFFFFFF:FFFFFFFFFFFFFFFFFF,,FFF:FFFFF:FFFFFF
F:FFFFFFFFFFFFF
@A00721:190:HGJW7DSXY:4:1634:3604:11960 2:N:0:TCTGCAAG+GCTGCACT
GAGGAAGTGGTCAGGCGGTCCGTTTAGGACCGCCGGTTATCGTAAAACACGACGAGTGTTGCAATTCA
ACAGGACTCAGCGGTCACAGCTGCCGGTTCAGTGGTCGAGGTCTCTGAGATATAATCGAAGACTTGGT
GTGTTCACGCCTCCT
+
FFFFFFF:FFFFFFFFFFFFFF:FFFFFFFFFFFFFFFFFFFFFFFFFFFFFFFFFFFFFFFFFFFFF
FFFFFFFFFFFFFFFFFFFFFFFFFFFFFFFFFFFFFFFFFFFFFFFFFFFFFFFFFFFFFFFFFFFF
F:FFFFFFFFFFFFF
@A00721:190:HGJW7DSXY:4:2329:5728:30890 2:N:0:TCTGCAAG+GCTGCACT
GAGGAAGTGGTCAGGCGGTCCGTTTAGGACCGCCGGTTATCGTAAAACACGACGAGTGTTGCAATTCA
ACAGGACTCAGCGGTCACAGCTGCCGGTTCAGTGGTCGAGGTCTCTGAGATATAATCGAAGACTGGGT
GTGTTCACGCCTCCT
+
FFFFFFFFFFFFFFFFFFFFFFF:FFFFFFFFFFFF:FFFFFFFFFFF:FFFFFFFFFFFFFFFFFF
FFF:FFFFFFFFFFFFFFFFF,FFFFFFFF:FFFFFFFFFFFFFFFFFFFFFF,FFFFFFFFFF,FF,
FFFF:F:FFFFFFFF
@A00721:190:HGJW7DSXY:4:1175:21278:28855 2:N:0:TCTGCAAG+GCTGCACT
GGAAGTGGTCAGGCGGTCCGTTTAGGACCGCCGGTTATCGTAAAACACGACGAGTGTTGCAATTCAAC
AGGACTCAGCGGTCACAGCTGCCGGTTCAGTGGTCGAGGTCTCTGAGATATAATCGAAGACTTGGTGT
GTTCACGCCTCCTAT
+
FFFFFFFFFFFFFFFFFFFFFFFFFFFFFFFFFFFFFFFFFFF:FFFFFF:FFFFFFFFFFFFFFFFF
FFFFFFFFFFFFFFFFFFF:FFFFFFFFFFFFFFFFFFFFFFFFFFFFFFFFFFFFFFFFFFFFFFFF
FFFFFFFFFFFFFFF
@A00721:190:HGJW7DSXY:4:1219:23809:2988 2:N:0:TCTGCAAG+GCTGCACT
GGAAGTGGTCAGGCGGTCCGTTTAGGACCGCCGGTTATCGTAAAACACGACGAGTGTTGCAATTCAAC
AGGACTCAGCGGTCACAGCTGCCGGTTCAGTGGTCGAGGTCTCTGAGATATAATCGAAGACTTGGTGT
GTTCACGCCTCCTAT
+
FFFFFFFFFFFFFFFFFFF:FFFFFFFFFFFFFFFFFFFFFFFFF:FFFFFFFFFFFFFFFFFFFFFF
FFFFFFFFFFFFFFFFFFFFFFFFFFFFFFFFFFFFFFFFFFFFFFFFFFFFFFFFFFFF:FFFFFFF
FFFFFFFFFFFFFFF
@A00721:190:HGJW7DSXY:4:1219:23475:3161 2:N:0:TCTGCAAG+GCTGCACT
GGAAGTGGTCAGGCGGTCCGTTTAGGACCGCCGGTTATCGTAAAACACGACGAGTGTTGCAATTCAAC
AGGACTCAGCGGTCACAGCTGCCGGTTCAGTGGTCGAGGTCTCTGAGATATAATCGAAGACTTGGTGT
GTTCACGCCTCCTAT
+
FFF:FFFFFFFFFFFFFFFFFFFFFFFFFFFFFFFFFFFFFFFFFFFFFFFFFFFFFFFFFFFFFFFF
FFFFFFFFFFFFFFFFFFFFFFFFFFFF:FFFFFFFFFFFFFFFFFFFFFFFFFFFFFFFFFFFFFFF
FFFFFFFFFFFFFFF
@A00721:190:HGJW7DSXY:4:1337:23791:1799 2:N:0:TCTGCAAG+GCTGCACT
GGAAGTGGTCAGGCGGTCCGTTTAGGACCGCCGGTTATCGTAAAACACGACGAGTGTTGCAATTCAAC
AGGACTCAGCGGTCACAGCTGCCGGTTCAGTGGTCGAGGTCTCTGAGATATAATCGAAGACTTGGTGT
GTTCACGCCTCCTAT
+
FFFFFFFFF,FFFFFFFFFFFFFFFFFFFFFFFFFFFFFFFFFFFFFFFFFF:FFFFFFFFFFFFFF
FF,FFFFF:FF:FFFFFFFFFF:FFFFFFFFFFFFFFFFFFFFFFFFFFFFFF,FFFFFF,:FFFFFFF
FFFFFFFFFFF:FFF
@A00721:190:HGJW7DSXY:4:1347:11939:8829 2:N:0:TCTGCAAG+GCTGCACT
GGAAGTGGTCAGGCGGTCCGTTTAGGACCGCCGGTTATCGTAAAACACGACGAGTGTTGCAATTCAAC
```

```
AGGACTCAGCGGTCACAGCTGCCGGTTCAGTGGTCGAGGTCTCTGAGATATAATCGAAGACTTGGTGT
GTTCACGCCTCCTAT
+
FFFFFFFFFFFFFFFFFFFF,,FFFF:FFFFFFFFFFFFFFFF:FFFFFFFFFFFFFFFFFFFFFFFF
FFFFFFFFFFFFFFFFFFFFFFFFFFFFFFFFFFFFFFFF:FFFFFFFFFFFFFFFFFFFFFFFFFFF
FFFFFFFFFFFFFFF
@A00721:190:HGJW7DSXY:4:2111:12084:16783 2:N:0:TCTGCAAG+GCTGCACT
GGAAGTGGTCAGGCGGTCCGTTTAGGACCGCCGGTTATCGTAAAACACGACGAGTGTTGCAATTCAAC
AGGACTCAGCGGTCACAGCTGCCGGTTCAGTGGTCGAGGTCTCTGAGATATAATCGAAGACTTGGTGT
GTTCACGCCTCCTAT
+
FFF:FFFFFFFFFFFFFFFFFFFFFFFFFFFFFFFFFFFFF:FFFFFFFFFFFFFFFFF:FFFFFF
FFFFFFFFFFFFFFFFFFFFFFFFFFFFFFFFFFFFFFFFFFFFFFFFFFF:FFFF:FFFFFFFFFFFF
FFFFFFFFFFFFFFF
@A00721:190:HGJW7DSXY:4:2158:4499:12571 2:N:0:TCTGCAAG+GCTGCACT
GGAAGTTGTCAGGCGGTCCGTTTAGGACCGCCGGATATCGTAAAACACGACGAGTGTTGCAATTCAAC
AGGACTCAGCGGTCACAGCTGACGGTTCAGTGGTCGAGTTCTCTGAGATATAATCGAAGACTTGGTGT
GTTAACGCATCCTAT
+
FF:F::,FFFFF:F:,::,,,,FFFF:F,FF,FF,:FFFFF:FFFFFF:FFFFFFF,::FFFFF,FFF
FF:FFF:FF,F,FFF:FF,::,:F,FFFFFFFFFFFFF,,,:FFFFFF,:FFF,FFFFFF,FF:F:,F
FF:,F,,F,FF,,FF
@A00721:190:HGJW7DSXY:4:2222:22806:18380 2:N:0:TCTGCAAG+GCTGCACT
GGAAGTGGTCAGGCGGTCCGTTTAGGACCGCCGGTTATCGTAAAACACGACGAGTGTTGCAATTCAAC
AGGACTCAGCGGTCACAGCTGCCGGTTCAGTGGTCGAGGTCTCTGAGATATAATCGAAGACTTGGTGT
GTTCACGCCTCCTAT
+
FFFFFFFFFFFFFFFFFFFFFFFFFFFFFFFFFF:FFFFFFFFFFFFFFFFF:FFFFFFF:FFFFFFF
FFFFFFFFFFFFFFFFFFFFFFFFFFFFFFFFFFFF,FFFFF:FFFFFFFFF:FFFFFFFFFFFFFFF
FFFFFFFFFFFFFFF
@A00721:190:HGJW7DSXY:4:2370:18412:3662 2:N:0:TCTGCAAG+GCTGCACT
GGAAGTGGTCAGGCGGTCCGTTTAGGACCGCCGGTTATCGTAAAACACGACGAGTGTTGCAATTCAAC
AGGACTCAGCGGTCACAGCTGCCGGTTCAGTGGTCGAGGTCTCTGAGATATAATCGAAGACTTGGTGT
GTTCACGCCTCCTAT
+
FFFFFFFFFFFFFFFFFFFFFFFFFFFFFFF:FFFFF,FFFF:FFFFFFFFFFFFFFFFFFFFFFFFFF
FFFFFFFFFFFFFFFFFFFFFFFFFFFFFFFFFFFFFFFFFFFFFFFFFFFF:FFFFFF:FF:FFFFFF
FFFFFF:FFFFFFFF
@A00721:190:HGJW7DSXY:4:1129:17056:6073 2:N:0:TCTGCAAG+GCTGCACT
GAAGTGGTCAGGCGGTCCGTTTAGGACCGCCGTTTATCGTAAAACACGACGAGTGTTGCAATTCAACA
GTACACAGCGGTCACAGCTGCCGGTTCAGTGGTCGAGGTCTCTGAGATATAATCGAAGACTTGGTGTG
TTCACTCCTCCAA
+
FFF,FFFF:F,:FF:F:FF::FF,FFFFFFFF,F,FFF:FFFF::FF,FF,FF,::FFF:FF,FFFF:
:,F,,,:FFF,FFFFF,F,F::FFF,,FF,,:FF,::,,,FF,,:FFFF:FFF,,::,F,FF:,,,FF
F,F:F,FF,F:,:
@A00721:190:HGJW7DSXY:4:1412:32181:12884 2:N:0:TCTGCAAG+GCTGCACT
GAAGTGGTCAGGCGGTCCGTTTAGGACCGCCGGTTATCGTAAAACACGACGAGTGTTGCAATTCAAAA
GGAATCAGCGGTCACAGCTGCCTGTTCAGTGGTCGAGGTCTCTGAGATATAATCTAAGACTGGGTGTG
TTCACGCCTCCTAT
+
FFFFFFFFFFFF::FF:::FFFFF:F::::F,FFFF:FFFFF,F,FFFF,:F,FFF:F::F:FFFF,:
F:F,FF::FFFF,FFFFF:FFF,:,FFFF::FF,:FFFF,FFF:FFF:FFFFF:,FFFF:F,:,F:FF
FFFFF:FFFF:F,F
```

```
@A00721:190:HGJW7DSXY:4:2231:28483:22795 2:N:0:TCTGCAAG+GCTGCACT
GCAAAGGAGGTGTGAACACACCAAGTCTTCGATTATATCTCAGAGACCTCGACCACTGAACCGGCAGC
TGTGACCGCTGAGTCCTGTTGACTTGCAACACTGGGCGTGTTTTACGATAACCGGCGGTCCTAAACGG
ACCGCCTGACCACTT
+
FFF,FF,F,F,,FFFFFFFFFFF:FFFFFF:FF,FFF,FFFFFFFFFFFFFFFFFFFFFFFFFFFFFF
FFFFFFFFF,,FFFF,FFFFFF,,FFFFFFFF,F:,FFFFFFFF:FFFF,F:F,,F:,F:FFFFF:,F
FFFFFFFFF,FFFF:
@A00721:190:HGJW7DSXY:4:2660:21504:32409 2:N:0:TCTGCAAG+GCTGCACT
GCATAGGAGGTGTGAACACACCAAGTCTTCGATTATATCTCAGAGACCTCGACCACTGAACCGGCAGC
TGTGACCGCTGAGTCCTGTTGAATTGCAACACTCGTCGTGTTTTACGATAACCGGCGGTCCTAAACGG
ACCGCCTGACCACTT
+
FFFFFFFFFFFFFFFFFFFFFFFFFFFFFFFF:FFFFFFFFFFFFFFFFFFFFFFFFFFFFFFFFFFF
FFFFFFFFFFFFFFF:FF:FFFFFFFFF:FFFFFFFFFFFFFFFFFFFFFFFFFFFFFFFFFFFFFFF
FFFFFFFFFFFFFFF
@A00721:190:HGJW7DSXY:4:2231:29351:22075 2:N:0:TCTGCAAG+GCTGCACT
GCATAGGAGGTGTGAACACACCAAGTCTTCGATTATATCTCAGAGACCTCGACCACTGAACCGGCAGC
TGTGACCGCTGAGTCCTGTTGAATTGCAACACTCGTCGTGTTTTACGATAACCGGCGGTCCTAAACGG
ACCGCCTGACCACT
+
FFFFFFFFFFFFFFFFFFFFFFFFFFFFFFFFFFFFFFFFFFFFFFFFFFFFFFFFFFFFF:FFFFFF
FFFFFFFFFFFFFFFFFFFFFFFFFFFFFFFFFFFFFFFFF,FF::FFFFFFFFFFFFFFFFFFFFFF
FFFFFFFF,FFFFF
@A00721:190:HGJW7DSXY:4:1256:31367:15045 2:N:0:TCTGCAAG+GCTGCACT
CTGCATAGGAGGTGTGAACACACCAAGTCTTCGATTATATCTCAGAGACCTCGACCACTGAACCGGCA
GCTGTGACCGCTGAGTCCTGTTGAATTGCAACACTCGTCGTGTTTTACGATAACCGGCGGTCCTAAAC
GGACCGCCTGACCAC
+
:FFFFFFFFFFFFFFFFFFFFFFFFFFFFFFFFFFFFFFFFFFFFFFFFFFFFFFFFFFFFFFFFFFF
FFFFFFFFFFFFFFFFF:FFFFFFFFFFFFFFFFFFFFFFFFFFFFFFFFFFFFFFFFFFFFFFFFFF
FFFFFFFFFFFFFFF
@A00721:190:HGJW7DSXY:4:1601:22896:32064 2:N:0:TCTGCAAG+GCTGCACT
CTGCATAGGAGGTGTGAACACACCAAGTCTTCGATTATATCTCAGAGACCTCGACCACTGAACCGGCA
GCTGTGACCGCTGAGTCCTGTTGAATTGCAACACTCGTCGTGTTTTACGATAACCGGCGGTCCTAAAC
GGACCGCCTGACCAC
+
FFFFFFFFFFFFFFFFFFFFFFFFFFFFFFFFFFFFFFFFFFFFFFFFFFFFFFFFFFFFFFFFFFFF
FFFFFFFFFFFFFFF:FFFFFFFFFFFFFFFFFFFFFFFFFFFFF:FF:FFFFF:FFFFFFFFFFFFF
FFFFFFFFFFFFFFF
@A00721:190:HGJW7DSXY:4:1672:28275:3396 2:N:0:TCTGCAAG+GCTGCACT
CTGCATAGGAGGTGTGAACACACCAAGTCTTCGATTATATCTCAGAGACCTCGACCACTGAACCGGCA
GCTGTGACCGCTGAGTCCTGTTGAATTGCAACACTCGTCGTGTTTTACGATAACCGGCGGTCCTAAAC
GGACCGCCTGACCAC
+
FFFFFFFFFFFFFFFFFFFFFFFFFFFFFFFFFFFFFFFFFFFFFFFFFFFFFFFFFFFFFFFFFFFF
FFFFFFFFFFFFFFFFFFFF:FFFFFFFFFFFFFFFFFFFFFFFFFFFF:FFFFFFFFFFFFFFFFFF
FFFFFFFFFFFFFFF
@A00721:190:HGJW7DSXY:4:2308:22498:35571 2:N:0:TCTGCAAG+GCTGCACT
CTGCATAGGAGGTGTGAACACACCAAGTCTTCGATTATATCTCAGAGACCTCGACCACTGAACCGGCA
GCTGTGACCGCTGAGTCCTGTTGAATTGCAACACTCGTCGTGTTTTACGATAACCGGCGGTCCTAAAC
GGACCGCCTGACCAC
+
FFFFFFFFFFFFFFFFFFFFFFFFFFFFFFFFFFFFFFFFFFFFFFFFFFFFFFFFFFFFFFFFFFFF
```

```
:FFFFFFF,FFFFFFFF:FFFFFFFFFFFF:FFFFFFFFFFFFFFFFFFFFFF:FFFF:FF,FFFFF
FFFF:FFFF:FFFFF
@A00721:190:HGJW7DSXY:4:2402:12644:5415 2:N:0:TCTGCAAG+GCTGCACT
CTGCATAGGAGGTGTGAACACACCAAGTCTTCGATTATATCTCAGAGACCTCGACCACTGAACCGGCA
GCTGTGACCGCTGAGTCCTGTTGAATTGCAACACTCGTCGTGTTTTACGATAACCGGCGGTCCTAAAC
GGACCGCCTGACCAC
+
FFFF:FFFFFF:FFFFFFFFFFFF:FFFF:FF:FFFFFFFFFFFFFFFFFFFFF,FF,FFF:FFFFF
FFFFFFFFFFFFFFFFFFFF,FFFFFFFFFFFFFFFF:F,FFFFF:FF,FFF:F:,FF:FF:FFFF:
:FF:FF::FFFF:FF
@A00721:190:HGJW7DSXY:4:2574:3667:29293 2:N:0:TCTGCAAG+GCTGCACT
CTGCATAGGAGGTGTGAACACACCAAGTCTTCGATTATATCTCAGAGACCTCGACCACTGAACCGGCA
GCTGTGACCGCTGAGTCCTGTTGAATTGCAACACTCGTCGTGTTTTACGATAACCGGCGGTCCTAAAC
GGACCGCCTGACCAC
+
FFFFFFFFFFFFFFFFFFFFFFFFFFFFFFFFFFFFFFFFFFFFFFFFFFFFFFFFFFFFFFFFFF,
FFFF:FFFFFFFFFFFFFFFFFFFFFFFFFFFFFFF:FFFFFFFFFFFFFF:FFFFFFFFFFFFFFF
FFFFFFFF,FFFFFF
@A00721:190:HGJW7DSXY:4:2637:18331:14888 2:N:0:TCTGCAAG+GCTGCACT
CTGCATAGGAGGTGTGAACACACCAAGTCTTCGATTATATCTCAGAGACCTCGACCACTGAACCGGCA
GCTGTGACCGCTGAGTCCTGTTGAATTGCAACACTCGTCGTGTTTTACGATAACCGGCGGTCCTAAAC
GGACCGCCTGACCAC
+
FFFFFFFFFFFFFFFFFFFFF:FFFFFFFFFFFFFFFFFFFFFFFFFFFFFFFFFFFFFFFFFFFFF
FFFFFFFFFFFFFFFFFFFFFFFFFFFFFFFFFFFFFFFFFFFFFFFF:F:FFFFFFFFFFFFFFFF
FFFFFFFFFFFFFFF
@A00721:190:HGJW7DSXY:4:1130:32009:16908 2:N:0:TCTGCAAG+GCTGCACT
ACTGCATAGGAGGTGTGAACACACCAAGTCTTCGATTATATCTCAGAGACCTCGACCACTGAACCGGC
AGCTGTGACCGCTGAGTCCTGTTGAATTGCAACACTCGTCGTGTTTTACGATAACCGGCGGTCCTAAA
CGGACCGCCTGACCA
+
FFFFFFFFFFFFFFFFFFFFFFFFFFFFFFFFFFFFFFFFFFFFFFFFFFFFFFFFFFFFFFFFFFF
FFFFFFFFFFFFFFFFFFFFFFFFFFFFFFFFFFFFFFFFFFFFFFFFFFFFFF:FFFFFFFFFFFF
FFFFFFFFFFF:FFF
@A00721:190:HGJW7DSXY:4:1647:1732:13135 2:N:0:TCTGCAAG+GCTGCACT
ACTGCATAGGAGGTGTGAACACACCAAGTCTTCGATTATATCACAGAGACCTCGACCACTGAACCGGC
AGCTGTGACCGCTGAGTCCTGTTGAATTGCAACACTCGTCGTGTTTTACGATAACCGGCGGTCCTAAA
CGGACCGCCTGACCA
+
FFFFF:FFFFFFFFFFFFFFFFFFFFFFFFFFFFFFFFFFF:FFFFFFFFFFFFFFFFFFFFFFFFF
FFFFFFFFFFFFFFFFFFFFFF,FFFFFFFF:FFFFFFFFFFFFF:FFFFFFFFFFFFFFFFFFFFF
FFFFFFFFFFFFFF:
@A00721:190:HGJW7DSXY:4:2303:6460:6793 2:N:0:TCTGCAAG+GCTGCACT
ACTGCATAGGAGGTGTGAACACACCAAGTCTTCGATTATATCTCAGAGACCTCGACCACTGAACCGGC
AGCTGTGACCGCTGAGTCCTGTTGAATTGCAACACTCGTCGTGTTTTACGATAACCGGCGGTCCTAAA
CGGACCGCCTGACCA
+
FFFFFFFFFFFFFFFFFFFFFFFFFFFFFFFFFFFFFFFFFFFFFFFFFFFFFFFFFFFFFFFFFFF
:FFFFFFFFFFFFFFFFFF:FFFFFFFFFFFFFFFFFFFFFFFFFFFFFFFFFFFFFFFFFFFFFFF
FFFFFFFFFFFFFFF
@A00721:190:HGJW7DSXY:4:2303:7310:7858 2:N:0:TCTGCAAG+GCTGCACT
ACTGCATAGGAGGTGTGAACACACCAAGTCTTCGATTATATCTCAGAGACCTCGACCACTGAACCGGC
AGCTGTGACCGCTGAGTCCTGTTGAATTGCAACACTCGTCGTGTTTTACGATAACCGGCGGTCCTAAA
CGGACCGCCTGACCA
```

```
+
FF:FFFFFFFFFFFFFFFFFFFFFFFFFFF:FFFFFFFFFFFFFFFFFFFFFFFFFFFFFFFFFFFFFFF
FFFFFFFFF,FFFFFFFFFFFFFFFFFFFFFFFFFFFFFFFFFFFFFFFFF::FFFFF:FFFFFFFFFFF
FFFFF,FFFFFFFFF
@A00721:190:HGJW7DSXY:4:2469:30463:23406 2:N:0:TCTGCAAG+GCTGCACT
ACTGCATAGGAGGTGTGAACACACCAAGTCTTCGATTATATCTCAGAGACCTCGACCACTGAACCGGC
AGCTGTGACCGCTGAGTCCTGTTGAATTGCAACACTCGTCGTGTTTTACGATAACCGGCGGTCCTAAA
CGGACCGCCTGACCA
+
FFFFFFFFFFFFFFFFFFFFFFFFFFFFFFFFFFFFFFFFFFFFFFFFFFFFFFFFFFFFFFFFFFFFFF
FFFFFFFFFFFFFFFFFFFFFFFFFFFFFFFFFFFFFFFFFFFFFFFFF:FFFF:FFFFFFFFFFFFFFF
FFFFFFFFFFFFFFF
@A00721:190:HGJW7DSXY:4:2469:30816:24173 2:N:0:TCTGCAAG+GCTGCACT
ACTGCATAGGAGGTGTGAACACACCAAGTCTTCGATTATATCTCAGAGACCTCGACCACTGAACCGGC
AGCTGTGACCGCTGAGTCCTGTTGAATTGCAACACTCGTCGTGTTTTACGATAACCGGCGGTCCTAAA
CGGACCGCCTGACCA
+
FFFFFFFFFF:FFFFFFFFFFFFFFFFFFFFFFFFFF:FFFFFFFFFF:FFFFFFFFFFFFFFFFFFFFF
FFFFFFFFFFFFFF::FFFFFFFFFFFFFFFFFFFFFFFFFFFFFFFFF:FF:FFFFFFFFFFFFFFFFF
FFFFFFFFFFFFFFF
@A00721:190:HGJW7DSXY:4:2617:24144:15217 2:N:0:TCTGCAAG+GCTGCACT
ACTGCATAGGAGGTGTGAACACACCAAGTCTTCGATTATATCTCAGAGACCTCGACCACTGAACCGGC
AGCTGTGACCGCTGAGTCCTGTTGAATTGCAACACTCGTCGTGTTTTACGATAACCGGCGGTCCTAAA
CGGACCGCCTGACCA
+
FFFFFFFFFFFFFFFFFFFFFFFFFFFFFFFFFFFFFFFFFFFFFFFFFFFFFFFFFFFFFFFFFFFFFF
FFFFFFFFFFFFFFFFFFFFFFFFFFFFFFFFFFFFFFFFFFFFFFFFFFFFFFFFFFFFFFFFFFF,FF
FFFFFFFFFFFFFFF
@A00721:190:HGJW7DSXY:4:1554:13738:4022 2:N:0:TCTGCAAG+GCTGCACT
CTCTGACAAACTCCGTCTTGTACTGCATAGGAGATGTGAACACACCAAGTCTTCGATTATATCTCAGA
GACCTCGACCACTGAACCGGCAGCTGTGACCGCTGAGTCCTGTTGAATTGCAACACTCGTCGTGTTTT
ACGATAACCGGCGGT
+
FFFFFFFFFFFFFFFFFFFFFFFFFFFFFFFFFFFFFFFFFFFFFFFFFFFFFFFFFFFFFFFFFFFFFF
FFFFFFFFFFFFFF:FFFFFFFFFF:FFFFFFFFFFFFFFFFFFFFFFFFFFFFFFFFFFFFFFFFFFFF
F:FFFFF:,F:FFFF
@A00721:190:HGJW7DSXY:4:2158:26187:28369 2:N:0:TCTGCAAG+GCTGCACT
CTCTGACAAACTCCGTCTTGTACTGCATAGGAGATGTGAACACACCAAGTCTTCGATTATATCTCAGA
GACCTCGACCACTGAACCGGCAGCTGTGACCGCTGAGTCCTGTTGAATTGCAACACTCGTCGTGTTTT
ACGATAACCGGCGGT
+
FFFFFFFFFFFFFFFFFFFFFFFFFFFFFFFFFFFFFFFFFFF:FFFFFFFFFFFFFFFFFFFFFFFFFF
FFFFFFFFFFFFFFFFFFFFFFFFFFFFFFFFFFFFFFFFFFFFFFFFFFFFFF:FFFFFFFFFFFFFFF
FFFFFFFFFFFFFFF
@A00721:190:HGJW7DSXY:4:2229:11749:12070 2:N:0:TCTGCAAG+GCTGCACT
CTCTGACAAACTCCGTCTTGTACTGCATAGGAGATGTGAACACACCAAGTCTTCGATTATATCTCAGA
GACCTCGACCACTGAACCGGCAGCTGTGACCGCTGAGTCCTGTTGAATTGCAACACTCGTCGTGTTTT
ACGATAACCGGCGGT
+
FFFFFFF:FFFFFFFFFFFFFFF:FFFFFFFFFFFFFFFFFFFFFFFFFFFFFFFFFFFFFFFFFFFFFF
FFFFFFFFFFFFFFF,FF:FFFFFFFFFFFFFFFFFFFFFFF:FFFF:FFFFFFFFFFFFFFFFFFFFFF
FFFFFFFFFFFFFFF
@A00721:190:HGJW7DSXY:4:2475:21206:6057 2:N:0:TCTGCAAG+GCTGCACT
CTCTGACAAACTCCGTCTTGTACTGCATAGGAGATGTGAACACACCAAGTCTTCGATTATATCTCAGA
```

```
GACCTCGACCACTGAACCGGCAGCTGTGACCGCTGAGTCCTGTTGAATTGCAACACTCGTCGTGTTTT
ACGATAACCGGCGGT
+
FFFFFFFFFFFFFFFFF:FFFFFFFFFFFFFFFFFFFFFFFFFFFFFFFFFFFFFFFFFFFFFFFFFF
FFFFFFFFFFFFFFFFFFFFFFFFFFFFFFFFFFFFFFFFFF,FFFFFFFFFFFFFFFFFFFFFFFFF
FFFFFFFFFFFFFFF
@A00721:190:HGJW7DSXY:4:1102:5909:18771 2:N:0:TCTGCAAG+GCTGCACT
GTCTTCCAAGAAGAGGAAGTGGTCAGCCGGTTATCGTAAAACACGACGAGTGTTGCAATTCAACAGGA
CTCAGCGGTCACAGCTGCCGGTTCAGTGGTCGAGGTCTCTGAGATATAATCGAAGACTTGGTGTGTTC
ACACCTCCTATGCAG
+
FFFFFFFFFFFFFFFFFFFFFFFFFFFFFFFFFFFFFFF:FFFFFFFFFFFFFFFFFFFFFFFFFFFF
FFFFFFFFFFFFFFFFFFFFFFFFFFFFFFFFFFFFFFFFFFFFFFFFFFFFFFFFFFFFFFFFFFFF
FFFFFFFFFFFFFFF
@A00721:190:HGJW7DSXY:4:1151:12255:8061 2:N:0:TCTGCAAG+GCTGCACT
GTCTTCCAAGAAGAGGAAGTGGTCAGCCGGTTATCGTAAAACACGACGAGTGTTGCAATTCAACAGGA
CTCAGCGGTCACAGCTGCCGGTTCAGTGGTCGAGGTCTCTGAGATATAATCGAAGACTTGGTGTGTTC
ACACCTCCTATGCAG
+
FFFFFFFFFFFFFFFFFFFFFFFFFFFFFFFFFFFFFFFFFFFFFFFFFFFFFFFFFFFFFFFFFFFF
FFFFFFFFFFFFFFFFFFFFFFFFFFFFFFFFFFFFFFFFFFFFFFFFFFFFFFFFFFFFFFFFFFFF
FFFFFFFFFFFFFFF
@A00721:190:HGJW7DSXY:4:1232:16306:25347 2:N:0:TCTGCAAG+GCTGCACT
GTCTTCCAAGAAGAGGAAGTGGTCAGCCGGTTATCGTAAAACACGACGAGTGTTGCAATTCAACAGGA
CTCAGCGGTCACAGCTGCCGGTTCAGTGGTCGAGGTCTCTGAGATATAATCGAAGACTTGGTGTGTTC
ACACCTCCTATGCAG
+
FFFFFFFFFFFFFFFFFFFFFFFFFFFFFFFFFFFFFFF:FFFFFFFFFFFFFFFFF:FFFFFFFFFF
FFFFFFFFFFFFFFFFFFFFFFFFFFFFFFFFFFFFFFFFFFFFF:FFFFFFFFFFFFFFFFFFFFFF
FFFFFFFFFFFFFFF
@A00721:190:HGJW7DSXY:4:1435:17445:29418 2:N:0:TCTGCAAG+GCTGCACT
GTCTTCCAAGAAGAGGAAGTGGTCAGCCGGTTATCGTAAAACACGACGAGTGTTGCAATTCAACAGGA
CTCAGCGGTCACAGCTGCCGGTTCAGTGGTCGAGGTCTCTGAGATATAATCGAAGACTTGGTGTGTTC
ACACCTCCTATGCAG
+
FF::FF:FFFFF:F,,F::FFFFFF:FFFFFFFF:,FFF:FFFFFFFFFFF:F,F:FFF:FFF:F:FF
FFFFFF:FFFFFFF:FF:::F:FFFFFFFFFFF::F:FFFFFFFFFFFFF:FFFFFFFFF:FFFFFFF
FFFF:FFFFFFFFFF
@A00721:190:HGJW7DSXY:4:2223:3911:5071 2:N:0:TCTGCAAG+GCTGCACT
GTCTTCCAAGAAGAGGAAGTGGTCAGCCGGTTATCGTAAAACACGACGAGTGTTGCAATTCAACAGGA
CTCAGCGGTCACAGCTGCCGGTTCAGTGGTCGAGGTCTCTGAGATATAATCGAAGACTTGGTGTGTTC
ACACCTCCTATGCAG
+
FFFFFFF:FFFFFFFFFFFFFFFFFFFFFFFFFFFFFFF:F:FFFFFFFFFFFFFFFFFFFFFFFFFF
FFFFFFFFFFFFFFFF:FFFFFFFFFFFFFFFFFFFFFFFFFFFFFFFFFFFFFFFFFFFFFFFFFF
:FFF,FFFF:FFFFF
@A00721:190:HGJW7DSXY:4:2359:16984:29653 2:N:0:TCTGCAAG+GCTGCACT
GTCTTCCAAGAAGAGGAAGTGGTCAGCCGGTTATCGTAAAACACGACGAGTGTTGCAATTCAACAGGA
CTCAGCGGTCACAGCTGCCGGTTCAGTGGTCGAGGTCTCTGAGATATAATCGAAGACTTGGTGTGTTC
ACACCTCCTATGCAG
+
FFFFFFFFFFFFFFFFFFFFFFFFFFFFFFFFFFFFFFFFFFFFFFFFFFFFFFFFFFFFFFFFFFFF
FFFFFFFFFFFFFFFFFFFFFFFFFFFFFFFFFFFFFFFFFFFF:FFFFFFFFFFFFFFFFFFFFFFF
FFFFFFFFFFFFFFF
```

```
@A00721:190:HGJW7DSXY:4:2628:4038:10833 2:N:0:TCTGCAAG+GCTGCACT
GTCTTCCAAGAAGAGGAAGTGGTCAGCCGGTTATCGTAAAACACGACGAGTGTTGCAATTCAACAGGA
CTCAGCGGTCACAGCTGCCGGTTCAGTGGTCGAGGTCTCTGAGATATAATCGAAGACTTGGTGTGTTC
ACACCTCCTATGCAG
+
FFFFFFFFFFFFFFFFFFFFFFFFFFFF:FFFFFFFFFFFFFFFFFFFFFFFFFFFFFFF:FFFFFFFFFF
FFFFFFFFFFFFFFFFFFFFFFFFFFFFFFFFFFFFFFFFFFFFFF,:FFFFFFFFFFFFFFFFFF:FFFF
:FFFFFFF:FFFFF:
@A00721:190:HGJW7DSXY:4:1320:9525:18646 2:N:0:TCTGCAAG+GCTGCACT
ATTAACTCTGACAAACTCCGTCTTGTACTGCATAGGAGGTGTGAACACACCAAGTCTTCGATTATATC
TCAGAGACCTCGACCACTGAACCGGCAGCTGTGACCGCTGAGTCCTGTTGAATTGCAACACTCGTCGT
GTTTTACGATAACCG
+
FFFFFFFFFFFFFFFFFFFFFFFFFFFFFFFFFFFFFFFFFFFFFFFFFFFFFFFFFFFFFFFFFFFFFFF
FFFFFFFFFFFFFFFFFFFFFFFFFFFFFFFFFFFFFFFFFFFFFFFFFFFFFFFFFFFFFFFFFFFFFFF
FFFFFFFFFFFFFFF
@A00721:190:HGJW7DSXY:4:1535:26377:8124 2:N:0:TCTGCAAG+GCTGCACT
ATTAACTCTGACAAACTCCGTCTTGTACTGCATAGGAGGTGTGAACACACCAAGTCTTCGATTATATC
TCAGAGACCTCGACCACTGAACCGGCAGCTGTGACCGCTGAGTCCTGTTGAATTGCAACACTCGTCGT
GTTTTACGATAACCG
+
FFFFFFFFFFFFFFFFFFFFFFFFFFFFFFFFFFFFFFFFFFFFFFFFFFFFFFFFFFFFFFFFFFFFFFF
FFFFFFFFFFFFFFFFFFFFFFFFFFFFFFFFFFFFFFFFFFFFFF:FFFFFFFFFFFFFFFFFFFFFFFF
FFFFFFFFFFFFFFF
@A00721:190:HGJW7DSXY:4:1615:12210:4946 2:N:0:TCTGCAAG+GCTGCACT
TTAACTCTGACAAACTCCGTCTTGTACTGCATAGGAGGTGTGAACACACCAAGTCTTCGATTATATCT
CAGAGACCTCGACCACTGAACCGGCAGCTGTGACCGCCGAGTCCTGTTGAATTGCAACACTCGTCGTG
TTTTACGATAACCG
+
FFFFFFFFFFFFFFFFFFFFFFFFFFFFFFFFFFFFFFFFFFFFFFFFFFFFFFFFFFFFFFFFFFFFFFF
F:FFFFFFFFFFFFFFFFFFFFFFFFFFFFFFFFFFFFF,FFFFFFFFFFFFFFFFFFFFFFFFFFFFFFF
F:FFFFF:FFFFFF
@A00721:190:HGJW7DSXY:4:1615:13747:5822 2:N:0:TCTGCAAG+GCTGCACT
ATTAACTCTGACAAACTCCGTCTTGTACTGCATAGGAGGTGTGAACACACCAAGTCTTCGATTATATC
TCAGAGACCTCGACCACTGAACCGGCAGCTGTGACCGCTGAGTCCTGTTGAATTGCAACACTCGTCGT
GTTTTACGATAACCG
+
FFFFFF:FFFFFF:FFFFFFFFF:FFFFFFFFFFFFFFFFFFFFFFFFFFFFFFFFFFFFFFFFFFFFFFF
FFFFFFFFFFFFFFF:FFFFFFFFFFFFFFFFFFFFFFFFFFFFFFFFFF,:F:FFFFFFFFFFFFFFFFF
F:FFF:FFFFFFFFF
@A00721:190:HGJW7DSXY:4:1630:9082:19601 2:N:0:TCTGCAAG+GCTGCACT
ATTAACTCTGACAAACTCCGTCTTGTACTGCATAGGAGGTGTGAACACACCAAGTCTTCGATTATATC
TCAGAGACCTCGACCACTGAACCGGCAGCTGTGACCGCTGAGTCCTGTTGAATTGCAACACTCGTCGT
GTTTTACGATAACCG
+
FFFFFFFFFF:F,FFFFFFFFFFFFFFFFFFFF:FFFFFFFFFFFFFFFFFFFFFFFFFFFFFFFFFFFFF
FFFFFFFFFFFFFFFFFFFFFFFFFFFFFFFFFFFFFFFFFFFF:FFFFFFFFFFFFFFFFFFFFFFFFF:
FF,FFFFFFFFFFFF
@A00721:190:HGJW7DSXY:4:2339:8820:10347 2:N:0:TCTGCAAG+GCTGCACT
ATTAACTCTGACAAACTCCGTCTTGTACTGCATAGGAGGTGTGAACACACCAAGTCTTCGATTATATC
TCAGAGACCTCGACCACTGAACCGGCAGCTGTGACCGCTGAGTCCTGTTGAATTGCAACACTCGTCGT
GTTTTACGATAACCG
+
FFFFFFFFFFFFFFFFFFFFFFFFFFFFF:FFFFFFFFFFFFFFFFFFFFFFFFFFFFFFFFFFFFFFFFF
```

```
FFFFFFFFFFFFFFFFFFFFFFFFFFFFFFFFFFFFFFFFFFFFFFF:FFFFFFFFFFFFFFFFFFFF
FFFFFFFFFFFFFFF
@A00721:190:HGJW7DSXY:4:2420:17571:14606 2:N:0:TCTGCAAG+GCTGCACT
ATTAACTCTGACAAACTCCGTCTTGTACTGCATAGGAGGTGTGAACACACCAAGTCTTCGATTATATC
TCAGAGACCTCGACCACTGAACCGGCAGCTGTGACCGCTGAGTCCTGTTGAATTGCAACACTCGTCGT
GTTTTACGATAACCG
+
FFFFFFFFFFFFFFFFFFFFFFFFFFFFFFFFFFFFFFFFFFFFFFFFFFFFFFFFFFFFFFFFFFFF
FFFFFFFFFFFFFFFFFFFFFFFFFFFFFFFFFFFFFFFFFFFFFFFFFFFFFFFFFFFFFFFFFFFF
FFFFFFFFFFFFF:F
@A00721:190:HGJW7DSXY:4:2476:7591:1579 2:N:0:TCTGCAAG+GCTGCACT
ATTAACTCTGACAAACTCCGTCTTGTACTGCATAGGAGGTGTGAACACACCAAGTCTTCGATTATATC
TCAGAGACCTCGACCACTGAACCGGCAGCTGTGACCGCTGAGTCCTGTTGAATTGCAACACTCGTCGT
GTTTTACGATAACCG
+
FFFFFFFFFFFF:FFFFFFFFFFFFFFFFFFFFFFFFFFFFFFFFFFFFFFF:FFFFFFFFFFFFFFF
FFFFFFFFFFFFFFFFFFF:FFFFFFFFFFFFFFFFFFFFFFFFFFFFFFF:FFFFFFFFFFFFFFFF
FFFFFFFFFFFFFFF
@A00721:190:HGJW7DSXY:4:2523:28863:25864 2:N:0:TCTGCAAG+GCTGCACT
ATTAACTCTGACAAACTCCGTCTTGTACTGCATAGGAGGTGTGAACACACCAAGTCTTCGATTATATC
TCAGAGACCTCGACCACTGAACCGGCAGCTGTGACCGCTGAGTCCTGTTGAATTGCAACACTCGTCGT
GTTTTACGATAACCG
+
FFFFFFFFFFFFFFFFFFFFFFFFFFFFFFFFFFFFFFFFFFFFFFFFFFFFFFFF:FFFFFFFFFFF
FFFFFFFFFFFFFFFFFFFFFFFF:FFFFFFFFFFFFFFFFFFFFFFF:FFFFFFFFFFFFFFFFFFF
FFFFFFFFFFFFFFF
@A00721:190:HGJW7DSXY:4:2535:28628:11804 2:N:0:TCTGCAAG+GCTGCACT
ATTAACTCTGACAAACTCCGTCTTGTACTGCATAGGAGGTGTGAACACACCAAGTCTTCGATTATATC
TCAGAGACCTCGACCACTGAACCGGCAGCTGTGACCGCTGAGTCCTGTTGAATTGCAACACTCGTCGT
GTTTTACGATAACCG
+
FFFFFFFFFFFFFFFFFFFFFFF:FFFFFFFFFFFFFFFFFFFFFFFFFFF:FFFFFFFFFFFFFFFF
FFFFFFFFFFFFFFFFFFF:FFFFFFFFFFFFFFFFFFFFFFFFF:FFFFFFFFFFFFFFFFFFFFFF
FFFFFFFFFFFFFFF
@A00721:190:HGJW7DSXY:4:2536:23411:33802 2:N:0:TCTGCAAG+GCTGCACT
ATTAACTCTGACAAACTCCGTCTTGTACTGCATAGGAGGTGTGAACACACCAAGTCTTCGATTATATC
TCAGAGACCTCGACCACTGAACCGGCAGCTGTGACCGCTGAGTCCTGTTGAATTGCAACACTCGTCGT
GTTTTACGATAACCG
+
FFFFFFFFFFFFFFFFFFF:FFFFFFFFFFFF:FFFFF:FFFFF:FFFFFFFFFFFFFFFFFFFF:FF
FFFFFFFFFFFFFFFFFFFFFFFFFFFFFFFFFFFFFFFFFFFFFFFFFFFFFFFFFFFFFFFFFFF:
FFFFFFF:FFFFFFF
@A00721:190:HGJW7DSXY:4:2618:30445:3396 2:N:0:TCTGCAAG+GCTGCACT
ATTAACTCTGACAAACTCCGTCTTGTACTGCATAGGAGGTGTGAACACACCAAGTCTTCGATTATATC
TCAGAGACCTCGACCACTGAACCGGCAGCTGTGACCGCTGAGTCCTGTTGAATTGCAACACTCGTCGT
GTTTTACGATAACCG
+
FFFFFFFFFFFFFFFFFFFFFFFFFFFFFF:FFFFFFFFFFFFFFFFFFFFFFFFFFFFFFFFFFFFF
FFFFFFFFFFFFFFFFFFF:FFFFFFFFFFFFFFFFFFFFFFFFFFFFFFFFFFFFFFFFFFFFFFFF
FFFF::FFFFFFFFF
@A00721:190:HGJW7DSXY:4:1105:27326:11960 2:N:0:TCTGCAAG+GCTGCACT
TATTAACTCTGACAAACTCCGTCTTGTACTGCATAGGAGGTGTGAACACACCAAGTCTTCGATTATAT
CTCAGAGACCTCGACCACTGAACCGGCAGCTGTGACCGCTGAGTCCTGTTGAATTGCAACACTCGTCG
TGTTTTACGATAACC
```

```
+
FFFFFFFFFFFFFFFFFFFFFFFFFFFFFFFFFFFFFFFFFFFFFFFFFFFFFFFFFFFFFFFFFFFF
FFFFFFFFFFFFFFFFFFFFFFFFFFFFFFFFFFFFFFFFFFFFFFFFFFFFFFFFFFFFFFFFFFFF
FFFFFFFFFFFFFFF
@A00721:190:HGJW7DSXY:4:1169:5448:31939 2:N:0:TCTGCAAG+GCTGCACT
TATTAACTCTGACAAACTCCGTCTTGTACTGCATAGGAGGTGTGAACACACCAAGTCTTCGATTATAT
CTCAGAGACCTCGACCACTGAACCGGCAGCTGTGACCGCTGAGTCCTGTTGAATTGCAACACTCGTCG
TGTTTTACGATAACC
+
FFFFFFFFFFFFFFFFFFFFFFFFFFFFFFFFFFFFFFFFFFFFFFFFFFFFFFFFFFFFFFFFFFFF
FFFFF:FFFFFFFFFFFFFFFFFFFFFFFFFFFFFFFFFFFFFF:FFFFFFFFFFFFFFFFFFFFFFF
FFFFFFFFF:FFFFF
@A00721:190:HGJW7DSXY:4:1203:17689:8735 2:N:0:TCTGCAAG+GCTGCACT
TATTAACTCTGACAAACTCCGTCTTGTACTGCATAGGAGGTGTGAACACACCAAGTCTTCGATTATAT
CTCAGAGACCTCGACCACTGAACCGGCAGCTGTGACCGCTGAGTCCTGTTGAATTGCAACACTCGTCG
TGTTTTACGATAACC
+
FFFFFFFFFFFFFFFFFFFFFFFFFFFFFFFFFFFFFFFFFFFFFFFFF:FFFFFFFFFFFFFFFFFF
FFFFFFFFFFFFFFFFFFFFFFFFFFFFFFFFFFFFFFFFFFFF:F,FF:FFFFFFFFFFFFFFFFFF
FFFFFFFFFFFFFFF
@A00721:190:HGJW7DSXY:4:1311:11406:23312 2:N:0:TCTGCAAG+GCTGCACT
TATTAACTCTGACAAACTCCGTCTTGTACTGCATAGGAGGTGTGAACACACCAAGTCTTCGATTATAT
CTCAGAGACCTCGACCACTGAACCGGCAGCTGTGACCGCTGAGTCCTGTTGAATTGCAACACTCGTCG
TGTTTTACGATAACC
+
FFFFFFFFFFFFFFFFFFFFFFFFFFFFFFFFFFFFFFFFFFFFFFFFFFFFFFFFF:FFFFFFFFFF
FFFFFFFFFFFFFFFFFFFFFFFFFFFFFFFFFFFFFFFFFFFFFF,FFFFFFFFF:FFFFFFFFFFF
FFFFFF:FFFFFF:F
@A00721:190:HGJW7DSXY:4:1349:28691:32111 2:N:0:TCTGCAAG+GCTGCACT
TATTAACTCTGACAAACTCCGTCTTGTACTGCATAGGAGGTGTGAACACACCAAGTCTTCGATTATAT
CTCAGAGACCTCGACCACTGAACCGGCAGCTGTGACCGCTGAGTCCTGTTGAATTGCAACACTCGTCG
TGTTTTACGATAACC
+
FFFFFFFFFFFFF:FFFFFFFFFFFFFFFFFFFFFFFFFFFFFFFFFFFFFFFFFFFFFFFFFFFFFF
FFFFFFFFFFFFFFFFFFFFFFFFFFFFFFFFFFFFFFFFFFFFFFFFFFFFFFFFFFFFFFFFFFFF
FFFFFFFFFFFFFFF
@A00721:190:HGJW7DSXY:4:1441:8504:20259 2:N:0:TCTGCAAG+GCTGCACT
TATTAACTCTGACAAACTCCGTCTTGTACTGCATAGGAGGTGTGAACACACCAAGTCTTCGATTATAT
CTCAGAGACCTCGACCACTGAACCGGCAGCTGTGACCGCTGAGTCCTGTTGAATTGCAACACTCGTCG
TGTTTTACGATAACC
+
FFFFFFFFFFFFFFFFFFFFFFFFFFFFFFFFFFFFFFFFFFFFFFFFFFFFFFFFFFFFFFFFFFFF
FFFFFFFFFFFFFFFFFFFFFFFFFFFFFFFFFFFFFFFFFFFFF:FFFFF:FFFFFFFFFFFFFFFF
FFFFFFFFFFFF,FF
@A00721:190:HGJW7DSXY:4:2214:18954:2597 2:N:0:TCTGCAAG+GCTGCACT
TATTAACTCTGACAAACTCCGTCTTGTACTGCATAGGAGGTGTGAACACACCAAGTCTTCGATTATAT
CTCAGAGACCTCGACCACTGAACCGGCAGCTGTGACCGCTGAGTCCTGTTGAATTGCAACACTCGTCG
TGTTTTACGATAACC
+
FFFFFFFFFFFFFFFFFFFFFFFFFFFFFFFFFFFFFFFFFFFFFFFFFFFFFFFFFFFFFFFFFFFF
FFFFFFFFFFFFFFFFFFFFFFFFFFFFFFFFFFFFFFFFFFFFFFFFFFFFFFFFFFFFFFFFFFFF
FFFFFFFFFFFFFFF
@A00721:190:HGJW7DSXY:4:2216:14724:27211 2:N:0:TCTGCAAG+GCTGCACT
TATTAACTCTGACAAACTCCGTCTTGTACTGCATAGGAGGTGTGAACACACCAAGTCTTCGATTATAT
```

CTCAGAGACCTCGACCACTGAACCGGCAGCTGTGACCGCTGAGTCCTGTTGAATTGCAACACTCGTCG
TGTTTTACGATAACC
+
:FFFFF,FFFFF,FFFF:::FF,F:FFFFF:FF:F::FFFFFFFFFF:FF:FFFFFF:F:FF:FFFF
FFFF,F,FFF:FFFFFFFFFF:FF,FFF::F:FFFFF,F:,FF:F,:F:F:FF:,,FFF:F:FF,FF:
F:F,FFFFFF,FF,:
@A00721:190:HGJW7DSXY:4:2240:1136:12352 2:N:0:TCTGCAAG+GCTGCACT
TATTAACTCTGACAAACTCCGTCTTGTACTGCATAGGAGGTGTGAACACACCAAGTCTTCGATTATAT
CTCAGAGACCTCGACCACTGAACCGGCAGCTGTGACCGCTGAGTCCTGTTGAATTGCAACACTCGTCG
TGTTTTACGATAACC
+
FFFFFFFFFFFFFFFFFFFFFFFFFFFFFFFFFFFFFFFFFFFFFFFFFFFFFFFFFFFFFFFFFFFF
FFFFFFFFFFFFFFFFFFFFFFFFFFFFFFFFFFFFFFFFFFFFFFFFFFFFFFFFFFFFFFFFFFFF
FFFFFF,FFFFFFFF
@A00721:190:HGJW7DSXY:4:1243:19678:25144 2:N:0:TCTGCAAG+GCTGCACT
ATATTATTAACTCTGACAAACTCCGTCTTGTACTGCATAGGAGGTGTGAACACACCAAGTCTTCGATT
ATATCTCAGAGACCTCGACCACTGAACCGGCAGCTGTGACCGCTGAGTCCTGTTGAATTGCAACACTC
GTCGTGTTTTACGAT
+
FFFF:FFFFFFFFFFFFFFFFFFFFFFFFFFFFFFFFFFFFFFFFFFFFFFFFFFFFFFFFFFFFF:F
FFFFF:FFFFFFFFFFFFFFFFFFFFFFFFF:FFFFFFFFFFFFFFF:FFFFF:FFFFFF:FFFF:FF
FFFFFFFFFFFFFFF
@A00721:190:HGJW7DSXY:4:2303:12924:20807 2:N:0:TCTGCAAG+GCTGCACT
ATATTATTAACTCTGACAAACTCCGTCTTGTACTGCATAGGAGGTGTGAACACACCAAGTCTTCGATT
ATATCTCAGAGACCTCGACCACTGAACCGGCAGCTGTGACCGCTGAGTCCTGTTGAATTGCAACACTC
GTCGTGTTTTACGAT
+
FFF:F:FFFFFFFFFFF:FFFFFFF:FFFFFFFFFFFFFFFFFFFFFFFFFFFFFFFFFF:FFFFFFF
FF:FFFFFFFFFFFFFFFFFFFFFFFFFFFFFFFFFFFFFFFFFFFFFFFFFFFFF:FFF,:FFFFF
FFFFFFFFFFFFF,F
@A00721:190:HGJW7DSXY:4:2476:23999:18255 2:N:0:TCTGCAAG+GCTGCACT
ATATTATTAACTCTGACAAACTCCGTCTTGTACTGCATAGGAGGTGTGAACACACCAAGTCTTCGATT
ATATCTCAGAGACCTCGACCACTGAACCGGCAGCTGTGACCGCTGAGTCCTGTTGAATTGCAACACTC
GTCGTGTTTTACGAT
+
FFFFFFFFFFFFFFFFFFFFFFFFFFFFFFFFFFFFF:FFFFFFFFFFFFFFF:FFF:FFFFFFFFFF
FFFFFFFFFFFFFFFFFFFFFFFFFFFFFFFFFFFFFFFFFFFFFFFFFF:FFFFFFF:FFFFFFFFF
F,FFFFFFFFFFFFF
@A00721:190:HGJW7DSXY:4:1452:20681:19116 2:N:0:TCTGCAAG+GCTGCACT
CCACAATATTATTAACTCTGACAAACTCCGTCTTGTACTGCATAGGAGGTGTGAACACACCAAGTCTT
CGATTATATCTCAGAGACCTCGACCACTGAACCGGCAGCTGTGACCGCTGAGTCCTGTTGAATTGCAA
CACTCGTCGTGTTTT
+
FFFFFFFFFFFFFFFFFFFFFFFF::FFFFFFFFFFFFFFFFF:FFFFFFFFFFFFFFFFFFFFFFFF
FFFF:FFFFFFFFFFFFFFFFFFFFFFFFFFFFFFFFFFFFFFFFFFFFFFFFFF:F:FFFFFFFFF
FFFFFFFFFFFFFFF
@A00721:190:HGJW7DSXY:4:2353:19054:22404 2:N:0:TCTGCAAG+GCTGCACT
CCACAATATTATTAACTCTGACAAACTCCGTCTTGTACTGCATAGGAGGTGTGAACACACCAAGTCTT
CGATTATATCTCAGAGACCTCGACCACTGAACCGGCAGCTGTGACCGCTGAGTCCTGTTGAATTGCAA
CACTCGTCGTGTTTT
+
FFFFFFFFFFFFFF:FFFFFFFFFFFFFFFFFFFFFFFFFFFFFFFFFFFFFFFFFFFFFFFFFFFFF
FFFFFFF:FFFFFFFFFFFFFFFFFFFFFFFFFFFFFFFFFFFFFFFFFFFFFFF:FFFFFFFFFFFF
FFFFFFFFFFFFFFF

@A00721:190:HGJW7DSXY:4:1162:5041:17738 2:N:0:TCTGCAAG+GCTGCACT
CGTCCACAATATTATTAACTCTGACAAACTCCGTCTTGTACTGCATAGGAGGTGTGAACACCAAGTCT
TCGATTATATCTCAGAGACCTCGACCACTGAACCGGCAGCTGTGACCGCTGAGTCCTGTTGAATTGCA
ACACTCGTCGTGTTT
+
FFFFFFF::FFF:FFFF:FFFFFFFFFFFFFFFFFFFFFFFFFFFF:FFF:FFFFFFFFFFFFFFFFFFF
FFFFFFFFFFFFFFFFFFFFFFFFFFFFFFFFF:FFFFFFFFFFFFFFFFFFFFFFFFFFFF:FFFFFFF
FFFFFFFFFFFFFFF
@A00721:190:HGJW7DSXY:4:1162:25681:35196 2:N:0:TCTGCAAG+GCTGCACT
CGTCCACAATATTATTAACTCTGACAAACTCCGTCTTGTACTGCATAGGAGGTGTGAACACCAAGTCT
TCGATTATATCTCAGAGACCTCGACCACTGAACCGGCAGCTGTGACCGCTGAGTCCTGTTGAATTGCA
ACACTCGTCGTGTTT
+
FFFFFFFFFFFFFF:FFFFFFFFFFFFFFFFFFFFFFFFFFFFFFFFFFFFFFFFFFFFFFFFFFFFFFF
FFFFFFFFFFFFFFFFFFFFFFFFFFFFFFFFFFFFFFFFFFFFFFFFFFFFFFFFFFFFFFFFFF:FFF
FFF,FFFFFFFFFFF
@A00721:190:HGJW7DSXY:4:2224:11930:35055 2:N:0:TCTGCAAG+GCTGCACT
CGTCCACAATATTATTAACTCTGACAAACTCCGTCTTGTACTGCATAGGAGGTGTGAACACCAAGTCT
TCGATTATATCTCAGAGACCTCGACCACTGAACCGGCAGCTGTGACCGCTGAGTCCTGTTGAATTGCA
ACACTCGTCGTGTTT
+
FFFFFFFFFFFFFFFFFFFFFFFFFFFFFFFFFFFFFFFFFFFFFFFFFFFFFFFFFFFFFFFFFFFFFF
FF:FFFFFFFFFFFFFFFFFFFFFFFFFFFFFFFFFFFFFFFFFFFF:FFFFFFFFFFFFFFFFFFFFF:
FFFFFFFFFFFFFFF
@A00721:190:HGJW7DSXY:4:2240:5620:31266 2:N:0:TCTGCAAG+GCTGCACT
CGTCCACAATATTATTAACTCTGACAAACTCCGTCTTGTACTGCATAGGAGGTGTGAACACCAAGTCT
TCGATTATATCTCAGAGACCTCGACCACTGAACCGGCAGCTGTGACCGCTGAGTCCTGTTGAATTGCA
ACACTCGTCGTGTTT
+
FFFFFFFFFFFFFFFFFFFFFFFFFFF:FFFFFFFFFFFFFFFFF:FFFFFFFFFFFFFFFFFFFFFFFF
FFFFFFFFFFFFFFFFFFFFFFFFFFFFFFFFFFFFFFFFFFFFFFFFFFFFFFFFFFFFFFFFFFFFFF
FFFFFFFFFFFFFFF
@A00721:190:HGJW7DSXY:4:2248:9444:7889 2:N:0:TCTGCAAG+GCTGCACT
CGTCCACAATATTATTAACTCTGACAAACTCCGTCTTGTACTGCATAGGAGGTGTGAACACCAAGTCT
TCGATTATATCTCAGAGACCTCGACCACTGAACCGGCAGCTGTGACCGCTGAGTCCTGTTGAATTGCA
ACACTCGTCGTGTTT
+
FFFFFFFFFFFFFFFFFFFFFFFFFFFFFFFFFFFFFFFFFFFFFFFFFFFFFFFFFFFFFFFFFFFFFF
FFFFFFFFFFFFFFFFFFFFFFFFFFFFFFFFFFFFFFFFFFFFF:FFFFFFFFFFFFFFFFFFFFFFFF
:FFFFFFFFFFFFFF
@A00721:190:HGJW7DSXY:4:2254:8359:18818 2:N:0:TCTGCAAG+GCTGCACT
CGTCCACAATATTATTAACTCTGACAAACTCCGTCTTGTACTGCATAGGAGGTGTGAACACCAAGTCT
TCGATTATATCTCAGAGACCTCGACCACTGAACCGGCAGCTGTGACCGCTGAGTCCTGTTGAATTGCA
ACACTCGTCGTGTTT
+
FFFFFFFFFFFFFFFFFFFFFFFFFFFFFFFFFFFFFFFFFFFFFFFFFFFFFFFFFFFFF:FFFFFFFF
FFFFFFF:FFFFFFFFFFFFFFFFFFFFFFFFFFFFFFFFFFFFFFFFFF:FF:FFFFFFFF
FFFFFFFFFFFFFFF
@A00721:190:HGJW7DSXY:4:2463:5692:3865 2:N:0:TCTGCAAG+GCTGCACT
CGTCCACAATATTATTAACTCTGACAAACTCCGTCTTGTACTGCATAGGAGGTGTGAACACCAAGTCT
TCGATTATATCTCAGAGACCTCGACCACTGAACCGGCAGCTGTGACCGCTGAGTCCTGTTGAATTGCA
ACACTCGTCGTGTTT
+
FFFFFFFFFFFFFFFFFFFFFFFFFFFFFFFFFFFFFFFFFFFFFFFFFFFFFFFFFFFFFFFFFFFFFF

```
FFFFFFFFFFFFFFFFFFFFFFFFFFFFFFFFFFFFFFFFFFFFFFFFFFFFFFFFFFFFFFFFFFFFFF
FFFFFFFFFFFFFFF
@A00721:190:HGJW7DSXY:4:2532:2456:2519 2:N:0:TCTGCAAG+GCTGCACT
CGTCCACAATATTATTAACTCTGACAAACTCCGTCTTGTACTGCATAGGAGGTGTGAACACCAAGTCT
TCGATTATATCTCAGAGACCTCGACCACTGAACCGGCAGCTGTGACCGCTGAGTCCTGTTGAATTGCA
ACACTCGTCGTGTTT
+
FFFFFFFFFFFFFFFFFFFFFFFFFFFFFFFFFFFFFFFFFFFFFFFFFFFFFFFFFFFFFFFFFFFFFF
FFFFFFFFFFFFFFFFFFFFFFFFFFFFFFFFFFFFFFFFFFFFFFFFFFFFFFFFFFFF:FFFFF:FFF
FFFFFFFFFFFFFFF
@A00721:190:HGJW7DSXY:4:2672:7708:24361 2:N:0:TCTGCAAG+GCTGCACT
CGTCCACAATATTATTAACTCTGACAAACTCCGTCTTGTACTGCATAGGAGGTGTGAACACCAAGTCT
TCGATTATATCTCAGAGACCTCGACCACTGAACCGGCAGCTGTGACCGCTGAGTCCTGTTGAATTGCA
ACACTCGTCGTGTTT
+
FFFFFFFFFFFFFFFFFFFFFFFFFFFFFFFFFFFFFFFFFFFFFFFFFFFFFFFFFFFFFFFFFFFFFF
FFFFFFFFFFFFFFFFFFFFFFFFFFFFFFFFFFFFFFFFFFFFFFFFFFFFFFFFFFFFFFFFFFFFFF
FFFFFF,FFFFFFFF
@A00721:190:HGJW7DSXY:4:1166:14253:23484 2:N:0:TCTGCAAG+GCTGCACT
CTGCCAACGTCCACAATATTATTAACTCTGACAAACTCCGTCTTGTACTGCATAGGAGGTGTGAACAC
ACCAAGTCTTCGATTATATCTCAGAGACCTCGACCACTGAACCGGCAGCTGTGACCGCTGAGTCCTGT
TGAATTGCAACACTC
+
FFFFFFFFFFFFFFFFFFFFFFFFFFFFFFFFFFF:FFFFFFFFFFFFFFFFFFFFFFFFFFFFFFFFFF
FFFFFFFFFFFFFFFFFFFFFFFFFFFFFFFFFFFFFFFFFFFFFF:FFFFFFFFFFFFFFFFFFFFFFF
FF:FFFFFFFFFFFF
@A00721:190:HGJW7DSXY:4:1337:14027:27289 2:N:0:TCTGCAAG+GCTGCACT
CTGCCAACGTCCACAATATTATTAACTCTGACAAACTCCGTCTTGTACTGCATAGGAGGTGTGAACAC
ACCAAGTCTTCGATTATATCTCAGAGACCTCGACCACTGAACCGGCAGCTGTGACCGCTGAGTCCTGT
TGAATTGCAACACTC
+
FFFFFFFFFFFFFFFFFFFFFFFFFFFFFFFFFFFFFFFFFFFFFFFFFFFFFF:FFFFFFFFFFFFFFF
FFFFFFFFFFFFFFFFFFFFFFFFFFFFFFFFFFFFFFFFFFFFFFFFFFFFFFFFFFFFFFFFFFFFFF
FFFFFFFFFFFFFFF
@A00721:190:HGJW7DSXY:4:1501:25753:13557 2:N:0:TCTGCAAG+GCTGCACT
CTGCCAACGTCCACAATATTATTAACTCTGACAAACTCCGTCTTGTACTGCATAGGAGGTGTGAACAC
ACCAAGTCTTCGATTATATCTCAGAGACCTCGACCACTGAACCGGCAGCTGTGACCGCTGAGTCCTGT
TGAATTGCAACACTC
+
FFFFFFFFFFFFFFFFFFFFFFFFFFFFFFFFFFFFFFFFFFFFFFFFFFFFFFFFFFFFFFFFFFFFFF
FFFFFFFFFFFFFFFFFFFFFFFFFFFFFFFFFFFFFFFFFFFFFFFFFFFFFFFFFFFFFFFFFFFFFF
FFFFFFFFF:FFFFF
@A00721:190:HGJW7DSXY:4:1611:15745:21903 2:N:0:TCTGCAAG+GCTGCACT
CTGCCAACGTCCACAATATTATTAACTCTGACAAACTCCGTCTTGTACTGCATAGGAGGTGTGAACAC
ACCAAGTCTTCGATTATATCTCAGAGACCTCGACCACTGAACCGGCAGCTGTGACCGCTGAGTCCTGT
TGAATTGCAACACTC
+
FFFFF:FFFFFFFFF:FFFFF:FFFFFFFFFF,FFFFFFFFFFFFFFFFFFFFFFFFFFFFFFFFFFFFF
FFFFFFFFFFFFF:FFFFFFFFFFFFFFFFFFFFFFFFFFFFFFFFFFFFFFFFFFFFFFFFFFFF:FF:
FFFFFFFFFFF:FFF
@A00721:190:HGJW7DSXY:4:2140:1850:21386 2:N:0:TCTGCAAG+GCTGCACT
CTGCCAACGTCCACAATATTATTAACTCTGACAAACTCCGTCTTGTACTGCATAGGAGGTGTGAACAC
ACCAAGTCTTCGATTATATCTCAGAGACCTCGACCACTGAACCGGCAGCTGTGACCGCTGAGTCCTGT
TGAATTGCAACACTC
```

```
+
FFFFFFFFFFFFFFFFFFFFFFFFFFFFFFFFFFFFFFFFFFFFFFFFFFFFFFFFFFFFFFFFFFFFFF
FFFFFFFF:FFFFFF:FFFFF:FFFFFFFF:FFFFFFFFFFF:FFFFFFF:FFFFFFFFFFFFFFFFF
FFFFFFFFFFFFFFF
@A00721:190:HGJW7DSXY:4:2308:7437:34381 2:N:0:TCTGCAAG+GCTGCACT
CTGCCAACGTCCACAATATTATTAACTCTGACAAACTCCGTCTTGTACTGCATAGGAGGTGTGAACAC
ACCAAGTCTTCGATTATATCTCAGAGACCTCGACCACTGAACCGGCAGCTGTGACCGCTGAGTCCTGT
TGAATTGCAACACTC
+
FFFFFFFFF:FFFFFFFFF,FFFFFFFFFFFFFFFFF:FFFFFFFFFFFFFF:FFFFFFFFFFFFFFF
FFFFFFFFFFFFFFFFFFFFFFFFFFFFFFFFFFFFFFFFFFFFFFFFFFFFFFFFFFFFFFFFFFF:F
FFFFFFF:FFFFFFF
@A00721:190:HGJW7DSXY:4:2369:1163:23954 2:N:0:TCTGCAAG+GCTGCACT
CTGCCAACGTCCACAATATTATTAACTCTGACAAACACCGTCTTGTACTGCATAGGAGGTGTGAACAC
ACCAAGTCTTCGATTATATCTCAGAGACCTCGACCACTGAACCGGCAGCTGTGACCGCTGAGTCCTGT
TGAATTGCAACACTC
+
FFFF:FFFFFFFF:FFFFFFFFFFF:FF,FFFFFF:,FF:F::,F,FFFFFFFFF,FFF:,FFF::FF
FF:FF:FF::FFF:FF:FFFFFFFFFFFFFFFF:FFFFFFF::F:FFFF:FFFF:FF,FFFF:FFFF:
FFFFFFF,:FF:F:F
@A00721:190:HGJW7DSXY:4:1228:20410:7247 2:N:0:TCTGCAAG+GCTGCACT
CCAACGTCCACAATATTATTAACTCTGACAAACTCCGTCTTGTACTGCATAGGAGATGTGAACACACC
AAGTCTTCGATTATATCTCAGAGACCTCGACCACTGAACCGGCAGCTGTGACCGCTGAGTCCTGTTGA
ATTGCAAGATCGGAA
+
FFFFFFFFFFFFFFFFFFFFFFFFFFFFFFFFFFFFFFFFFFFFFFFFFFFFFFFFFFF:FFFFFFFF
FFFFFFFFFFFFFFFFFFFFFF:FFFFFFFFFFFFFFFFFFFFFFFFFFFFFF:FFFFFFFFFFFFFFF
FFFFFFFFFFFFFFF
@A00721:190:HGJW7DSXY:4:1304:9525:26788 2:N:0:TCTGCAAG+GCTGCACT
CCAACGTCCACAATATTATTAACTCTGACAAACTCCGTCTTGTACTGCATAGGAGATGTGAACACACC
AAGTCTTCGATTATATCTCAGAGACCTCGACCACTGAACCGGCAGCTGTGACCGCTGAGTCCTGTTGA
ATTGCAAGATCGGAA
+
FFFFFFFFFFFFFFFFFFFFFFFFFFFFFFFFFFFFFFFFFFFFFFFFFFFFFFFFFFFFFFFFFFFFF
FFFFFFFFFFFFFFFFFFFFFFFFFFFFFFFFFFFFFFFFFFFFFFFFFFFFFFFFFFFFFFF:FFFFF
FFFFFFFFFFFFFFF
@A00721:190:HGJW7DSXY:4:1375:5195:9236 2:N:0:TCTGCAAG+GCTGCACT
CCAACGTCCACAATATTATTAACTCTGACAAACTCCGTCTTGTACTGCATAGGAGATGTGAACACACC
AAGTCTTCGATTATATCTCAGAGACCTCGACCACTGAACCGGCAGCTGTGACCGCTGAGTCCTGTTGA
ATTGCAAGATCGGAA
+
FFFFFFFFFFFF:FFFFFFFF:FFFFFFFFFFFFFFFFFFFFFFFFFFFFFFFFFFFFFFFFFFFFFFF
FFFFFFFFFFFFFFFFFFFFFFFFFFFFFFFFFFFFFFFFFFFFFFFFFFFFFFFFFFFFFFF,FFFFF
,FFFFFFFFFFFFFF
@A00721:190:HGJW7DSXY:4:1441:28754:18693 2:N:0:TCTGCAAG+GCTGCACT
CCAACGTCCACAATATTATTAACTCTGACAAACTCCGTCTTGTACTGCATAGGAGATGTGAACACACC
AAGTCTTCGATTATATCTCAGAGACCTCGACCACTGAACCGGCAGCTGTGACCGCTGAGTCCTGTTGA
ATTGCAAGATCGGAA
+
FFFFFFFFFFFFFFFFFFFFFFFFFFFFFFFFFFFFFFFFFFFFFFFFFFFFFFFFFFFFFFFFFFFFF
F:FFFFFFFF:FFFFFFFFFFFFFFFFFFFFFFFFFFFFFFFFFFFFFFFFFFFFFFFFFFFFFFFFFF
FFFFFFFFFFFFFF:
@A00721:190:HGJW7DSXY:4:1633:14154:7999 2:N:0:TCTGCAAG+GCTGCACT
CCAACGTCCACAATATTATTAACTCTGACAAACTCCGTCTTGTACTGCATAGGAGATGTGAACACACC
```

AAGTCTTCGATTATATCTCAGAGACCTCGACCACTGAACCGGCAGCTGTGACCGCTGATTCCTGTTGA
ATTGCAAGATCGGAA
+
FFFFFFFFFFFFFFFFFFFFFFFFFFFFFFF:FFFFFFFFFFFFFFFF:FFFFFFFFFFFFFFFFFFF
FFFFFF:,FFFFF:FFFFFFFFFFF:FFF,FFFFFFFF:FFFFFFFFFFFFFFFFFFF,,FFFFFFFF
FFFF,FFFFFFFFFF
@A00721:190:HGJW7DSXY:4:2345:30436:26303 2:N:0:TCTGCAAG+GCTGCACT
CCAACGTCCACAATATTATTAACTCTGACAAACTCCGTCTTGTACTGCATAGGAGATGTGAACACACC
AAGTCTTCGATTATATCTCAGAGACCTCGACCACTGAACCGGCAGCTGTGACCGCTGAGTCCTGTTGA
ATTGCAAGATCGGAA
+
FFFFFFFFFFFFFFFFFFFFFFFFFFFFFFFFFFFFFFFFFFFFFFFF:FFFFFFFFFFFFFFFFFFF
FFFFFFFFFFFFFFFFFFFFF:FFFFFFFFFFFFFFFFFFFFFFFFFFFFFFFFFFFFFFF::FFFFF
FFFFFFFFFFFFFF:
@A00721:190:HGJW7DSXY:4:2508:1416:9987 2:N:0:TCTGCAAG+GCTGCACT
CCAACGTCCACAATATTATTAACTCTGACAAACTCCGTCTTGTACTGCATAGGAGATGTGAACACACC
AAGTCTTCGATTATATCTCAGAGACCTCGACCACTGAACCGGCAGCTGTGACCGCTGAGTCCTGTTGA
ATTGCAAGATCGGAA
+
FFFFFFFFFFFFFFFFFFFFFFFFFFFFFFFFFF:FFFFFFFFFFFFFFFFFFFFFFFFFFFFFFFFF
FF:FFFFFFFFFF:FFFFFFFFFFFF:FFFFFFF:FFFFFF:FFFFFFFFFFFFFFFFFFFF,:FFFF
FFFFFFFFF:FFFFF
@A00721:190:HGJW7DSXY:4:1228:20066:7122 2:N:0:TCTGCAAG+GCTGCACT
CCAACGTCCACAATATTATTAACTCTGACAAACTCCGTCTTGTACTGCATAGGAGATGTGAACACACC
AAGTCTTCGATTATATCTCAGAGACCTCGACCACTGAACCGGCAGCTGTGACCGCTGAGTCCTGTTGA
ATTGCAAGATCGGA
+
FFFFFFFFFFFFFFFFFFFFFFFFFFFFFFFFF:FFFFFFFFFFFFFFFFFFFFFFFFFFFFFFFFFF
FFFFFFFFFFFFFFFFFFFFFFFFFFFFFFFFFFFFFFF,FFFFFFFFFFFFFFFFFFFFFFFFFFFF
,FFFFFFFFFFFFF
@A00721:190:HGJW7DSXY:4:1126:32235:25347 2:N:0:TCTGCAAG+GCTGCACT
AACGATACTGCCAACGTCCACAATATTATTAACTCTGACAAACTCCGTCTTGTACTGCATAGGAGGTG
TGAACACACCAAGTCTTCGATTATATCTCAGAGACCTCGACCACTGAACCGGCAGCTGTGACCGCTGA
GTCCTGTTGAATTGC
+
FFFFFFFFFFFFFFFFFFFFFFFFFFFFFFFFFFFFFFFFFFFFFFFFFFFFFFFFFFFFFFFFFFFF
FFFFFFFFFFFFFFFFFFFFFFFFFFFFFFFFFFFFFFFFFFFFFFFFFFFFFFFFFFFFFFFFFFFF
FFF:FF:FFFFFFFF
@A00721:190:HGJW7DSXY:4:1211:32642:23860 2:N:0:TCTGCAAG+GCTGCACT
AACGATACTGCCAACGTCCACAATATTATTAACTCTGACAAACTCCGTCTTGTACTGCATAGGAGGTG
TGAACACACCAAGTCTTCGATTATATCTCAGAGACCTCGACCACTGAACCGGCAGCTGTGACCGCTGA
GTCCTGTTGAATTGC
+
FFFFFFFFFFFFFFFFFFFFFFFFFFF:FFFFFFFFFFFFFFFFFFFFFFFFFFFFFFFFFFFFFFFF
FFFFFFFFFFFFFFFF:FFFF,FFFFFFFFFFFFFF,F,FFFFFFFFFFFFFFFFFFFFFFFFFFFFF
FFF:FF,FF:FFFFF
@A00721:190:HGJW7DSXY:4:1339:6488:2174 2:N:0:TCTGCAAG+GCTGCACT
AACGATACTGCCAACGTCCACAATATTATTAACTCTGACAAACTCCGTCTTGTACTGCATAGGAGGTG
TGAACACACCAAGTCTTCGATTATATCTCAGAGACCTCGACCACTGAACCGGCAGCTGTGACCGCTGA
GTCCTGTTGAATTGC
+
F:FFFFFFFFFFFFFFFFFFFFFFFFFFFFFFFFFFFFFFFFFFFFFFFFFFFFFFFFFFFFFFFFFF
FFFFFFFFFFFFFFFFFFFF:FFFFFFFFFFFFFFFFFFFFFFFFF:FFFFFFFFFFFFFFFFFFFFF
FFFFFFFFFFFFFFF

```
@A00721:190:HGJW7DSXY:4:1464:18014:19914 2:N:0:TCTGCAAG+GCTGCACT
AACGATACTGCCAACGTCCACAATATTATTAACTCTGACAAACTCCGTCTTGTACTGCATAGGAGGTG
TGAACACACCAAGTCTTCGATTATATCTCAGAGACCTCGACCACTGAACCGGCAGCTGTGACCGCTGA
GTCCTGTTGAATTGC
+
FFFFFFFFFFFFFFFFFFFFFFFFFFFFFFFFFFFFFFFFFFFFFFFFFFFFFFFFFFFFFFFFFFFFFF
FFFFFFFFFFFFFFFFFFFFFFFFFFFFFFFFFFFFFFFFFFFFFFF:FFFFFFFFFFFFFFFFFFFFFF
FF,:FFFFFFFFFFF
@A00721:190:HGJW7DSXY:4:2202:14615:10207 2:N:0:TCTGCAAG+GCTGCACT
AACGATACTGCCAACGTCCACAATATTATTAACTCTGACAAACTCCGTCTTGTACTGCATAGGAGGTG
TGAACACACCAAGTCTTCGATTATATCTCAGAGACCTCGACCACTGAACCGGCAGCTGTGACCGCTGA
GTCCTGTTGAATTGC
+
FFFFFFFFF:FFFFFFFFFFFFFFFFF:F:FFF:FFFFFFFFFFFFFFFFF:FFFFFFF::FF:FF:FFF
FFF,FFF,FFFF:FFFFFFFFFFF:FFFFFFF:FFFF:FF,FFFFF:F:FF:F:FFFF:F:FF:FF:F:,
FFF,FFFFFFFFFF:
@A00721:190:HGJW7DSXY:4:2268:19063:26960 2:N:0:TCTGCAAG+GCTGCACT
AACGATACTGCCAACGTCCACAATATTATTAACTCTGACAAACTCCGTCTTGTACTGCATAGGAGGTG
TGAACACACCAAGTCTTCGATTATATCTCAGAGACCTCGACCACTGAACCGGCAGCTGTGACCGCTGA
GTCCTGTTGAATTGC
+
FFFFFFFFFFFFFFFFFFFFFFFFFFFFFFFFFFFFFFFFFFFFFFFFFFFFFFFFFFFFFFFFFFFFFF
FFFFFFFFFFFFFFFFFFFF,FFFFFFFFFFFFFFFFFFF:FFFFFFFFFFFFFFFFFFFFFFFFFFFFF
FF:FFF:FFFFFFFF
@A00721:190:HGJW7DSXY:4:2104:3821:14184 2:N:0:TCTGCAAG+GCTGCACT
ACAAACGATACTGCCAACGTCCACAATATTATTAACTCTGACAAACTCCGTCTTGTACTGCATAGGAG
GTGTGAACACACCAAGTCTTCGATTATATCTCAGAGACCTCGACCACTGAACCGGCAGCTGTGACCGC
TGAGTCCTGTTGAAT
+
FFFFFFFFFFFFFFFFFFFFFFFFFFFFFFFFFFFFFFFFFFFFFFFFFFFFFFFFFFFFFFFF:FFFFF
FFFFFFFFFFFFFFFFFFFFFFFFFF:FFFFFFFFFFFFFFFFFFFFFFFFFFFFFFFFFFFFFFFFFFF
FFFFFF:FFFFFFFF
@A00721:190:HGJW7DSXY:4:2109:25979:23187 2:N:0:TCTGCAAG+GCTGCACT
ACAAACGATACTGCCAACGTCCACAATATTATTAACTCTGACAAACTCCGTCTTGTACTGCATAGGAG
GTGTGAACACACCAAGTCTTCGATTATATCTCAGAGACCTCGACCACTGAACCGGCAGCTGTGACCGC
TGAGTCCTGTTGAAT
+
FFFFFFFFFFFFFFFFFFFFFFFFFFFFFFFFFFFFFFFFFFFFFFFFFFFFFFFFFFFFFFFFFFFFFF
FFFFFFFFFFFFFFFFFFFFFFFF:FFFFFFFFFFFFFFFFFFFFFFFFFFF:FFFFFFFFFFFFFFFFF
FFFFFFFFFFFFFFF
@A00721:190:HGJW7DSXY:4:2348:14199:19163 2:N:0:TCTGCAAG+GCTGCACT
ACAAACGATACTGCCAACGTCCACAATATTATTAACTCTGACAAACTCCGTCTTGTACTGCATAGGAG
GTGTGAACACACCAAGTCTTCGATTATATCTCAGAGACCTCGACCACTGAACCGGCAGCTGTGACCGC
TGAGTCCTGTTGAAT
+
FFFFFFFFFFFFFFFFFFFFFF:FFFFFFFFFFFFFFFFFFFFFFFFFFFFFFFFFFFFFFFF,FFFFFF
FFFFFFFFFFFFFFFFFFFFFFFFFFFFFFFFFFFFFFFFFFFFFFFFFFFFFFFFFFFFF:FFFFFFFF
FFFFFFFFFFFFF:F
@A00721:190:HGJW7DSXY:4:2349:13349:22200 2:N:0:TCTGCAAG+GCTGCACT
ACAAACGATACTGCCAACGTCCACAATATTATTAACTCTGACAAACTCCGTCTTGTACTGCATAGGAG
GTGTGAACACACCAAGTCTTCGATTATATCTCAGAGACCTCGACCACTGAACCGGCAGCTGTGACCGC
TGAGTCCTGTTGAAT
+
FFFFFFFFFFFFFFFFFFFFFFFFFFFF:FFFFFFFFFFFFFFFFFFFFFFFFF:FFFFFFFFFFFFFFF
```

FFFFFFFFFFFFFFFFFFFFFFFFFFFFFFFFFFFFFFFFFFFFFFFFFFFFFFFFFFFFFFFFFFFF
FFFFFFF,FFFFFFF
@A00721:190:HGJW7DSXY:4:2441:2085:8171 2:N:0:TCTGCAAG+GCTGCACT
ACAAACGATACTGCCAACGTCCACAATATTATTAACTCTGACAAACTCCGTCTTGTACTGCATAGGAG
GTGTGAACACACCAAGTCTTCGATTATATCTCAGAGACCTCGACCACTGAACCGGCAGCTGTGACCGC
TGAGTCCTGTTGAAT
+
FFF:FFFFFFFFFFFFFFFFFFFFFFFFF:,FFFFFFF:FFFF:FFF:F:FF:FFFFFFFF:FFFF:FF
FFF,FFFFF:FFFFFFFF:FFFFFFF::F:FFFFFFFFFFFFFF,FFFF::FFFFFFFFFFFFFFFFF
:FFFFF,FFFFFFF:
@A00721:190:HGJW7DSXY:4:2444:19605:23797 2:N:0:TCTGCAAG+GCTGCACT
ACAAACGATACTGCCAACGTCCACAATATTATTAACTCTGACAAACTCCGTCTTGTACTGCATAGGAG
GTGTGAACACACCAAGTCTTCGATTATATCTCAGAGACCTCGACCACTGAACCGGCAGCTGTGACCGC
TGAGTCCTGTTGAAT
+
FFFFFFFFFFFFFFFFFFFFFFFFFFFFFFFFFF:FFFFFFFFFFFFFFFFFFFFFFFFFFFFFFFFFF
FFFFFFFFFFFFFFFFFFFFFFFFFFFFFFFFFFFFFFFFFFFFFFFFFFFFFFFFFFFFFFFFFFFF
FFFFFFFFFFFF:,F
@A00721:190:HGJW7DSXY:4:2461:1877:7592 2:N:0:TCTGCAAG+GCTGCACT
ACAAACGATACTGCCAACGTCCACAATATTATTAACTCTGACAAACTCCGTCTTGTACTGCATAGGAG
GTGTGAACACACCAAGTCTTCGATTATATCTCAGAGACCTCGACCACTGAACCGGCAGCTGTGACCGC
TGAGTCCTGTTGAAT
+
FFFFFFFFFFFFFFFFFFFFFFFFFF::FFFFFFFFFFFFFFFFFFFFFFFFFFF,FFFFFFFFFFFFFF
FFFFFFFFFFFFFFFFFFFFFFFF:FFFFFFFFFFFFFFFFFFFFFF:FFFFFFFFFFFFFFFFFFFFFF
FFFFFFF,F:FFFF:
@A00721:190:HGJW7DSXY:4:2669:19569:18630 2:N:0:TCTGCAAG+GCTGCACT
ACAAACGATACTGCCAACGTCCACAATATTATTAACTCTGACAAACTCCGTCTTGTACTGCATAGGAG
GTGTGAACACACCAAGTCTTCGATTATATCTCAGAGACCTCGACCACTGAACCGGCAGCTGTGACCGC
TGAGTCCTGTTGAAT
+
FFFFFFFFFFFFFFFFFFFFFFFFFFFFFFFFFFFFFFFFFFFFFFFFFFFFFFFFFFFFFFFFFFFF
FFFFFFFFFFFFFFFFFFFFFFFFFFFF:FFFFFFFFFF:FFFFFFFFFFFFFFFFFFFFFFFFFFFFF
:FFFFFFFF,FFFFF
@A00721:190:HGJW7DSXY:4:2203:25500:14434 2:N:0:TCTGCAAG+GCTGCACT
ATTTATACAAACGATACTGCCAACGTCCACAATATTATTAACTCTGACAAACTCCGTCTTGTACTGCA
TAGGAGGTGTGAACACACCAAGTCTTCGATTATATCTCAGAGACCTCGACCACTGAACCGGCAGCTGT
GACCGCTGAGTCCTG
+
FFFFFFFFFFFFFFFFFFFFFFFFFFFFFFFFFFFFFFFFFFFFFFFFFFFFFFFFFFFFFFFFFFFF
FFFFFFFFFFFFFFFFFFFFFFFFFFFFFFFFFFFFFFFFFFFFFFFFFFFFFFFFFFFFFFFFFFFF
FFFFFFFFFFFFFFF
@A00721:190:HGJW7DSXY:4:2346:4110:31219 2:N:0:TCTGCAAG+GCTGCACT
ATTTATACAAACGATACTGCCAACGTCCACAATATTATTAACTCTGACAAACTCCGTCTTGTACTGCA
TAGGAGGTGTGAACACACCAAGTCTTCGATTATATCTCAGAGACCTCGACCACTGAACCGGCAGCTGT
GACCGCTGAGTCCTG
+
FF,FF:FFFFFF,F,F:FFF:FFF:FFFFFFFFFFFFF,FFFFF,FFFFFF:FF,FF:FFFFF:,FFF
FFFFFF,FFF:FFFFFF:,FF,F,FF,FF,:FFF,FFF:FF,F:F:,FFF:FFF:FFFF:FF,FFFFF
FF,F:FFFFFFFFFF
@A00721:190:HGJW7DSXY:4:2346:4137:31234 2:N:0:TCTGCAAG+GCTGCACT
ATTTATACAAACGATACTGCCAACGTCCACAATATTATTAACTCTGACAAACTCCGTCTTGTACTGCA
TAGGAGGTGTGAACACACCAAGTCTTCGATTATATCTCAGAGACCTCGACCACTGAACCGGCAGCTGT
GACCGCTGAGTCCTG

+
FFFFFFFFFFFFFFFFFFFFFFFFFFFFFFFFFFFFFFFFFFFFFFFFFFFFFFFFFFFFFFFF:FFFF:
FFFFFFFFFFFFFFFFFFFFFFFFFFFFFFFFFFFFFFFFFFFFFFFFFFFFFFFFFFFFFFFFFFFFFF
FFFFFFFFFFFFFFF
@A00721:190:HGJW7DSXY:4:2538:5882:14403 2:N:0:TCTGCAAG+GCTGCACT
ATTTATACAAACGATACTGCCAACGTCCACAATATTATTAACTCTGACAAACTCCGTCTTGTACTGCA
TAGGAGGTGTGAACACACCAAGTCTTCGATTATATCTCAGAGACCTCGACCACTGAACCGGCAGCTGT
GACCGCTGAGTCCTG
+
FFFFFFFFFFFFFFFFFFFFFFFFFFFFFFFFFFFFFFFFFFFFFFFFFFFF:FFFFFFFFFFF:FFFFF
FFFFFFFFFFFFFFFFFFFFFFFFFFFFFFFFFFFFFFFFFFFFFF:FFFFFFFFFFFFFFFFFFFFFFF
FFFFFFFF,FFFFFF
@A00721:190:HGJW7DSXY:4:1132:11071:4131 2:N:0:TCTGCAAG+GCTGCACT
ATCATTTATACAAACGATACTGCCAACGTCCACAATATTATTAACTCTGACAAACTCCGTCTTGTACT
GCATAGGAGGTGTGAACACACCAAGTCTTCGATTATATCTCAGAGACCTCGACCACTGAACCGGCAGC
TGTGACCGCTGAGTC
+
FFFFFFFFFFFFF,FFFFFFFFFFFFFFFFFFFFFFFFFFFFFFFFFFFFFFFFFFFFFFFFFFFFFFFF
:FFFFFFFFFFFFFFFFFFFFFFFFFFFFFFFFFFFFFFFFFFFF:FFFFFFFFFFFFFFFFFFFFF:FF
FFFFFFFFFFFFFFF
@A00721:190:HGJW7DSXY:4:2155:17038:31250 2:N:0:TCTGCAAG+GCTGCACT
CAATCATTTATACAAACGATACTGCCAACGTCCACAATATTATTAACTCTGACAAACTCCGTCTTGTA
CTGCATAGGAGGTGTGAACACACCAAGTCTTCGATTATATCTCAGAGACCTCGACCACTGAACCGGCA
GCTGTGACCGCTGAG
+
FFFFFFFFFFFFFFFFFFFFFFFFFFFFFFFFFFFFFFFFFFFFFFFFFFFFFFFFFFFFFFFFFFFFFF
FFFFFFFFFFFFFFFFFFFFFFFFFF:FFFFFFFF:FFFFFFFFFF:FFFFFFFFFFFFFFFFFFFFFFF
FFFFFFFFFFFFFFF
@A00721:190:HGJW7DSXY:4:2352:18792:31814 2:N:0:TCTGCAAG+GCTGCACT
CAATCATTTATACAAACGATACTGCCAACGTCCACAATATTATTAACTCTGACAAACTCCGTCTTGTA
CTGCATAGGAGGTGTGAACACACCAAGTCTTCGATTATATCTCAGAGACCTCGACCACTGAACCGGCA
GCTGTGACCGCTGAG
+
FFFFFFFFFFFFFFF:FFFFFFFFFFFFFFFFFFFFFFFFFFFFFFFFFFFFFFFFFFFFFFFFFFFFFF
FFFFFFFFFFFFFFFFFFFFFFFFFFFFFFFFFFFFFFFFFFFFFFFFFFFFFFFFFFFFFF:FFFFFFF
FFFFFFFFFFFFFFF
@A00721:190:HGJW7DSXY:4:2433:9824:11146 2:N:0:TCTGCAAG+GCTGCACT
CAATCATTTATACAAACGATACTGCCAACGTCCACAATATTATTAACTCTGACAAACTCCGTCTTGTA
CTGCATAGGAGGTGTGAACACACCAAGTCTTCGATTATATCTCAGAGACCTCGACCACTGAACCGGCA
GCTGTGACCGCTGAG
+
FFFFFFFFFF,FFFF:FFFFFFFFFFFFFFFFFFFFF:FFFFF:FFFFFFFFFFFFFFFFFFFFFFFF:F
FFFF,FFFFFFFFFFFFFFFFFFFFFFFFFFFFFFFFFFFFFFFFFFFFFFFFFFFFFFFFFFFFFFFFF
FF,FFFFF:FFFFFF
@A00721:190:HGJW7DSXY:4:2622:9643:14278 2:N:0:TCTGCAAG+GCTGCACT
CAATCATTTATACAAACGATACTGCCAACGTCCACAATATTATTAACTCTGACAAACTCCGTCTTGTA
CTGCATAGGAGGTGTGAACACACCAAGTCTTCGATTATATCTCAGAGACCTCGACCACTGAACCGGCA
GCTGTGACCGCTGAG
+
FFFFFFFFFFFFFFFFFFFFFFFFFFFFFFFFFFFFFFFFFFFFFFFFFFFFFFFFFFFFFFFFFFFFFF
FFFFFFFFFFFFFFFFFFFFFFFFFFFFFFFFFFF,FFFFFFFFFFFFFFFFFFFFFFFFFFFFFFFFFF
FFFFFFFFFFFFFFF
@A00721:190:HGJW7DSXY:4:1272:1805:1986 2:N:0:TCTGCAAG+GCTGCACT
CGAGGTTCCAGTAGGACAATCATTTATACAAACGATACTGCCAACGTCCACAATATTATTAACTCTGA

```
CAAACTCCGTCTTGTACTGCATAGGAGGTGTGAACACACCAAGTCTTCGATTATATCTCAGAGACCTC
GACCACTGAACCGGC
+
FFFFFFFFFFFFFFFFFFFFFFFFFFFFFFFFFFFFFFFFFFFF:FFFFFFFFFFFFFFFFFF:FFFFFF
FFFFFFFFFFFFFFFFFFFFFFFFFFFFFFFFFFFFFFFFFFFFFFFFFFF:FFFFFFFFFFFFFFFFFF
FFFFFFFFFFFFFFF
@A00721:190:HGJW7DSXY:4:1411:7102:1799 2:N:0:TCTGCAAG+GCTGCACT
CGAGGTTCCAGTAGGACAATCATTTATACAAACGATACTGCCAACGTCCACAATATTATTAACTCTGA
CAAACTCCGTCTTGTACTGCATAGGAGGTGTGAACACACCAAGTCTTCGATTATATCTCAGAGACCTC
GACCACTGAACCGGC
+
FFFFFFFFFFFFFFFFFFFFFFFFFFFFFFFF:FFFFFFFFFFFFFFFFFFFFFFFFFFFFFFFFFFFFF
FFFFFFFFFFFFFFFFFFFFFFFFFFFFFFFFFFFFFFFFFFFFFFFFFFFFFFFFFFFFFFF,FFFFFF
FFFFFFFFFFFFFFF
@A00721:190:HGJW7DSXY:4:1435:12355:29559 2:N:0:TCTGCAAG+GCTGCACT
CGAGGTTCCAGTAGGACAATCATTTATACAAACGATACTGCCAACGTCCACAATATTATTAACTCTGA
CAAACTCCGTCTTGTACTGCATAGGAGGTGTGAACACACCAAGTCTTCGATTATATCTCAGAGACCTC
GACCACTGAACCGGC
+
FFFFFFFFFF:FF:FFFFF:FF:FFF,FFFFFFFFFFFF,FFFFF:FFFFFFFFFFFFFFFFFFFFFFFF
FFFF,FFFFF:FFFFFFFF,FFFFFFF:FFFFFFFFFFFFFF:FFFFFFFFFFFFFFFFFFFFFFFFFFF
FFFFFFFFFFFFFFF
@A00721:190:HGJW7DSXY:4:1456:23439:9111 2:N:0:TCTGCAAG+GCTGCACT
CGAGGTTCCAGTAGGACAATCATTTATACAAACGATACTGCCAACGTCCACAATATTATTAACTCTGA
CAAACTCCGTCTTGTACTGCATAGGAGGTGTGAACACACCAAGTCTTCGATTATATCTCAGAGACCTC
GACCACTGAACCGGC
+
FFFFFFFFFFFFFFFFFFFFFFFFFFFFFFFFFFFFFFFFFFF:FFFFFFFFFFFFFFFFFFFFFFFFFF
FFFFFFFFFFFFFFFFFFFFFFFFFFFFFFFFFFFFFFFFFFFFFFFF:FFFFFFFFFFFFFFFFFFFFF
FFFFFFFF:FFFFFF
@A00721:190:HGJW7DSXY:4:1616:17327:9361 2:N:0:TCTGCAAG+GCTGCACT
CGAGGTTCCAGTAGGACAATCATTTATACAAACGATACTGCCAACGTCCACAATATTATTAACTCTGA
CAAACTCCGTCTTGTACTGCATAGGAGGTGTGAACACACCAAGTCTTCGATTATATCTCAGAGACCTC
GACCACTGAACCGGC
+
FFFFFFFF:FFFFFFFFFFFFFFFFFFFFFFFFFFFF:FFFFFFFFFFFFFFFFFF:F::FF:FFFFFFF
FFFFFFFFFFFF:FFFF:FFFF:FFFFFFFFFFFFFFFFFFFFF:FFFFFFF,FFFFFFF,FFFFFFFFF
FFF:FFFFFFFFFFF
@A00721:190:HGJW7DSXY:4:1657:7744:21167 2:N:0:TCTGCAAG+GCTGCACT
CGAGGTTCCAGTAGGACAATCATTTATACAAACGATACTGCCAACGTCCACAATATTATTAACTCTGA
CAAACTCCGTCTTGTACTGCATAGGAGGTGTGAACACACCAAGTCTTCGATTATATCTCAGAGACCTC
GACCACTGAACCGGC
+
FFFFFFFFFFFFFFFFFFFFFFFFFFFFFFF:FFFFFFFFFFFFFFFFFFFFFFFFFFFFFFFFFFFFFF
FFFFFFFFFFFFFFFF:FFFF:FFFFFFFFFFFFFFFFFFFFFFFFFFFFF,FFFFFFFFFFFFFFFFFF
FFFFFFFF:FFFFFF
@A00721:190:HGJW7DSXY:4:1670:19045:18599 2:N:0:TCTGCAAG+GCTGCACT
CGAGGTTCCAGTAGGACAATCATTTATACAAACGATACTGCCAACGTCCACAATATTATTAACTCTGA
CAAACTCCGTCTTGTACTGCATAGGAGGTGTGAACACACCAAGTCTTCGATTATATCTCAGAGACCTC
GACCACTGAACCGGC
+
FFFFFFFFFFFFFFFFFFFFFFFFFFFFFFFFFFFFFFFFFFFFFFFFFFFFFFFFFFFFFFFFFFFFFF
FFFFFFFFFFFFFFFFFFFFFFFFFFFFFFFFFFFFFFFFFFFFFFFFFFFFFFFFFFFFFFFFFFFFFF
FFFFFFFFFFFFFFF
```

```
@A00721:190:HGJW7DSXY:4:2172:28899:8140 2:N:0:TCTGCAAG+GCTGCACT
CGAGGTTCCAGTAGGACAATCATTTATACAAACGATACTGCCAACGTCCACAATATTATTAACTCTGA
CAAACTCCGTCTTGTACTGCATAGGAGGTGTGAACACACCAAGTCTTCGATTATATCTCAGAGACCTC
GACCACTGAACCGGC
+
FFFFFFFFFFFF:FFFFFFFFFFFFFFFFFFFFFFFFFFFFFFFFFFFFFFFFFFFFFFFFFFFFFFFFF
FF:FFFFFFFFFFFFFFFFFFFFFFFFFFFFFFFFFFFFFFFFFFFFFFFFFFFFFFFFFFFFFFFFFFF
FFFFFFFFFFFFFFF
@A00721:190:HGJW7DSXY:4:2172:29152:8328 2:N:0:TCTGCAAG+GCTGCACT
CGAGGTTCCAGTAGGACAATCATTTATACAAACGATACTGCCAACGTCCACAATATTATTAACTCTGA
CAAACTCCGTCTTGTACTGCATAGGAGGTGTGAACACACCAAGTCTTCGATTATATCTCAGAGACCTC
GACCACTGAACCGGC
+
FFFFFFFFFFFFFFFFFFFFFFFFFFFFFFFFFFFFFFFFFFFFFFFFFFFFFFFFFFFFFFFFFFFFFF
FFFFFFFFFFFFFFFFFFFFFFFFFFFFFFFFFFFFFFFFFFFFFFFFFFFFFFFFFFFFFFFFFFFFFF
FFFFFFFFFFFFFFF
@A00721:190:HGJW7DSXY:4:2246:17110:2785 2:N:0:TCTGCAAG+GCTGCACT
CGAGGTTCCAGTAGGACAATCATTTATACAAACGATACTGCCAACGTCCACAATATTATTAACTCTGA
CAAACTCCGTCTTGTACTGCATAGGAGGTGTGAACACACCAAGTCTTCGATTATATCTCAGAGACCTC
GACCACTGAACCGGC
+
FFFFFFFFFFFF,FFFFFFFFFFF,FFFFFFFFFFFFFF:FFFFFFF:FFFFFFFFFFF:FFFFFFFFFF
FF:FFFFFFFFFFFFFFFFFFFFFFFFFFFFFFFFFFFFFFFFFFFFFF:F:FFFFFFFFFFFFFFFFFF
FFFFFFFFFFFFFFF
@A00721:190:HGJW7DSXY:4:2246:26838:11428 2:N:0:TCTGCAAG+GCTGCACT
CGAGGTTCCAGTAGGACAATCATTTATACAAACGATACTGCCAACGTCCACAATATTATTAACTCTGA
CAAACTCCGTCTTGTACTGCATAGGAGGTGTGAACACACCAAGTCTTCGATTATATCTCAGAGACCTC
GACCACTGAACCGGC
+
FFFFFFFFFFFFFFFFFFFFFFFFFFFFFFFFFFFFFFFFFFFFFFFFFFFFFFFFFFFFFFFFFFFFFF
FFFFFFFFFFFFFFFFFFFFFFFFFFFFFFFFFFFFFFFFFFFFFFFFFFFFFFFFFFFFFFFFFFFFFF
FFFFFFFFFFFFFFF
@A00721:190:HGJW7DSXY:4:2251:22535:18818 2:N:0:TCTGCAAG+GCTGCACT
CGAGGTTCCAGTAGGACAATCATTTATACAAACGATACTGCCAACGTCCACAATATTATTAACTCTGA
CAAACTCCGTCTTGTACTGCATAGGAGGTGTGAACACACCAAGTCTTCGATTATATCTCAGAGACCTC
GACCACTGAACCGGC
+
FFFFFFFFFFFFFFFFFFFFFFFFFFFFFFFFFFFFFFFFFFFFFFFFFFFFFFFFFFFFFFFFFFFFFF
FFFFFFFFF:FFFFFFFFFFFFFFFFFFFFFFFFFFFFFFFFFFFFFFFFFFF:FFFFFFFFFFFFFFFF
FFFFFFF:FFFFFFF
@A00721:190:HGJW7DSXY:4:2307:9136:15781 2:N:0:TCTGCAAG+GCTGCACT
CGAGGTTCCAGTAGGACAATCATTTATACAAACGATACTGCCAACGTCCACAATATTATTAACTCTGA
CAAACTCCGTCTTGTACTGCATAGGAGGTGTGAACACACCAAGTCTTCGATTATATCTCAGAGACCTC
GACCACTGAACCGGC
+
:FFFFFFFFFFFFFFFFFFFFFFFFFFFFFFFFFFFFFFFFFFFFFFFFFFFFFFFFFFFFFFFFFFFFF
FFFFFFFFFFFFFFFFFFFFFFFFFFFFFFFFFFFFFFFFFFFFFFFFFFFFFFFFFFFFFFFFFFFFFF
FFFFFFFFFFFFFFF
@A00721:190:HGJW7DSXY:4:2447:2049:26459 2:N:0:TCTGCAAG+GCTGCACT
CGAGGTTCCAGTAGGACAATCATTTATACAAACGATACTGCCAACGTCCACAATATTATTAACTCTGA
CAAACTCCGTCTTGTACTGCATAGGAGGTGTGAACACACCAAGTCTTCGATTATATCTCAGAGACCTC
GACCACTGAACCGGC
+
FFFFFFFFFFFFFFFFFFFFFFFFFFFFFFFFFFFFFFFFFFFFFFFFFFFFF,F:FF:FFFFFFFFFFF
```

FFFFFFFFFFFFFFFFFFFFFFFFFFFFFFFFFFFFFFFFFFFFFFFFFFFF,FFFFFFFFFFFFFFF
FFFFFFFFFFFFFFF
@A00721:190:HGJW7DSXY:4:2463:4001:26804 2:N:0:TCTGCAAG+GCTGCACT
CGAGGTTCCAGTAGGACAATCATTTATACAAACGATACTGCCAACGTCCACAATATTATTAACTCTGA
CAAACTCCGTCTTGTACTGCATAGGAGGTGTGAACACACCAAGTCTTCGATTATATCTCAGAGACCTC
GACCACTGAACCGGC
+
FFFFFFFFFFFFFFFFFFFFFFFFFFFFFFFFFFFFFFFFFFFFFFFFFFFFFFFFFFFFFFFFFFFF
FF:FFFFFFFFFFFFFFFFFFFFFFFFFFFFFFFFFFFFFFFFFFFFFFFFF:FFFFFFFFFFFFFFF
FFF:FFFFFFFFF:F
@A00721:190:HGJW7DSXY:4:2601:29686:21527 2:N:0:TCTGCAAG+GCTGCACT
CGAGGTTCCAGTAGGACAATCATTTATACAAACGATACTGCCAACGTCCACAATATTATTAACTCTGA
CAAACTCCGTCTTGTACTGCATAGGAGGTGTGAACACACCAAGTCTTCGATTATATCTCAGAGACCTC
GACCACTGAACCGGC
+
FFFFFFFFFFFFFFFFFFFFFFFFFFFFFFFFFFFFFFFFFFFFFFFFFFFFF:FFFFFFFFFFFFFF
FFFFFFFFFFFFFFFFFFFFFFFFFFFFFFFFFFFFFFFFFFFFFFFFFFFFFF:FF:FFFFFFFFFF
FFFFFFFFFFFFFFF
@A00721:190:HGJW7DSXY:4:2627:24749:30890 2:N:0:TCTGCAAG+GCTGCACT
CGAGGTTCCAGTAGGACAATCATTTATACAAACGATACTGCCAACGTCCACAATATTATTAACTCTGA
CAAACTCCGTCTTGTACTGCATAGGAGGTGTGAACACACCAAGTCTTCGATTATATCTCAGAGACCTC
GACCACTGAACCGGC
+
FFFFFFFFFFFFFFFFFFFFFFF:FFFFFFFFFFFFFFFFFFFFFFFFFFFFFFFFFFFFFFFFFFFF
FFFFFFFFFFFF:FF,FFFFFF:FFFFFFFFFFFFFFFFF:FFFFFFFFFFFF:FFFFFFFFFFF,FF
FFFFFFFFFFFFFFF
@A00721:190:HGJW7DSXY:4:1420:12933:17315 2:N:0:TCTGCAAG+GCTGCACT
CCGAGGTTCCAGTAGGACAATCATTTATACAAACGATACTGCCAACGTCCACAATATTATTAACTCTG
ACAAACTCCGTCTTGTACTGCATAGGAGGTGTGAACACACCAAGTCTTCGATTATATCTCAGAGACCT
CGACCACTGAACCGG
+
FFFFFFFFFFFFFFFFFFFFFFFFFFFFFFFFFFFFFFFFFFFFFFFFFFFFFFFFFFFFFFFFFFFF
FFFFFFFFFFFFFFFFFFFFFFF,FFFFF:FFFFFFFFFFFFFFFFFFFFFFFFFFFFFFFFFFFFFF
FFFFFFFFFF:FFFF
@A00721:190:HGJW7DSXY:4:1456:32868:28134 2:N:0:TCTGCAAG+GCTGCACT
CCGAGGTTCCAGTAGGACAATCATTTATACAAACGATACTGCCAACGTCCACAATATTATTAACTCTG
ACAAACTCCGTCTTGTACTGCATAGGAGGTGTGAACACACCAAGTCTTCGATTATATCTCAGAGACCT
CGACCACTGAACCGG
+
:FFFFFFFFFFFFFFFFFFFFFFFFFFFFFFFFFFFFFFFFFFFFFFFFFFFFFFFFFFFFFFFFFFF
FFFFFFFFF:FFFFFFFFFF:FFFFFFFFFFF:FFFFFFFFFF:F:FF,FFFFFFFFFFFF,FFF:FF
FFF,FFFF:FFFF:F
@A00721:190:HGJW7DSXY:4:2458:5936:5290 2:N:0:TCTGCAAG+GCTGCACT
CCGAGGTTCCAGTAGGACAATCATTTATACAAACGATACTGCCAACGTCCACAATATTATTAACTCTG
ACAAACTCCGTCTTGTACTGCATAGGAGGTGTGAACACACCAAGTCTTCGATTATATCTCAGAGACCT
CGACCACTGAACCGG
+
FFFFFFFFFFFFFFFFFFFFFFFFF:FFFFFFFFFFFFFFFFFFFFFFFFFFFFFFFFF:FFFFFFFF
FFFFFFFFFFFFFFFFFFFFFFFFFFFFFFFFFFFFFFFFFFFFFFFFFFFFFFFFFFFFFFFFFFFF
FFFFFFFFFFFFFFF
@A00721:190:HGJW7DSXY:4:2609:5285:5228 2:N:0:TCTGCAAG+GCTGCACT
CCGAGGTTCCAGTAGGACAATCATTTATACAAACGATACTGCCAACGTCCACAATATTATTAACTCTG
ACAAACTCCGTCTTGTACTGCATAGGAGGTGTGAACACACCAAGTCTTCGATTATATCTCAGAGACCT
CGACCACTGAACCGG

+
FFFFFFFFFFFFFFFFFFFFFFFFFFFFFFFFFFFFFFFFFFFFFFFFFFFFFFFFFFFFFFFFFFFF
FFFFFFFFFFFFFFFFFFFFFFF:FFFFFFFFFFFFFFFFFFFFFFFFFFFFFFFFFFFFFFFFFFFF
FFFFFFFFFFFFFFF
@A00721:190:HGJW7DSXY:4:2639:4851:1407 2:N:0:TCTGCAAG+GCTGCACT
CCGAGGTTCCAGTAGGACAATCATTTATACAAACGATACTGCCAACGTCCACAATATTATTAACTCTG
ACAAACTCCGTCTTGTACTGCATAGGAGGTGTGAACACACCAAGTCTTCGATTATATCTCAGAGACCT
CGACCACTGAACCGG
+
FFFFFFFFFFFFFFFFFFFF:FFFFFFFFFFFFFFFFFFFFFFFFFFFFFFFFFFFFFFFFFFFFFFF
FFF:FFFFFFFFFFFFFFFFFFFFFFFFFFFFFFFFFFFFFFFFFFFFFFFFFFFFFFFFFFFFFFFF
F:FFFFFFFFFFFFF
@A00721:190:HGJW7DSXY:4:1167:28501:19852 2:N:0:TCTGCAAG+GCTGCACT
TATCGACCGAGGTTCCAGTAGGACAATCATTTATACAAACGATACTGCCAACGTCCACAATATTATTA
ACTCTGACAAACTCCGTCTTGTACTGCATAGGAGGTGTGAACACACCAAGTCTTCGATTATATCTCAG
AGACCTCGACCACTG
+
FFFFFFFFFFFFFFFFFFFFFFFF,FFFFFFFFFFFFFFFFFFFFFFFFFFFFFFFFFFFFFFFFFF
,FFFFFFFFFFFF:FFFFFFFFFFFFFFFFFFFFFFFFFFFFFFFFFFFFFFFFFFFFFF:FFFFFFFFFF
FFFFFFFFFFFFFFF
@A00721:190:HGJW7DSXY:4:1256:1895:6183 2:N:0:TCTGCAAG+GCTGCACT
TATCGACCGAGGTTCCAGTAGGACAATCATTTATACAAACGATACTGCCAACGTCCACAATATTATTA
ACTCTGACAAACTCCGTCTTGTACTGCATAGGAGGTGTGAACACACCAAGTCTTCGATTATATCTCAG
AGACCTCGACCACTG
+
FFFFFFFFFFFFFFFFFFFFFFFFFF:FFFFFFFFFFFFFFFFFFFFFFFFFFFFFFFFFFFFFFFFF
FFFFFFFFFFFFFFFFFFFFFFFFFFFF:FFFFFFFFFFFFFFFFFFFFFFFFF:FFFFFFFFFFFFF
FFFFFFFFFFFFFFF
@A00721:190:HGJW7DSXY:4:1268:4869:5635 2:N:0:TCTGCAAG+GCTGCACT
TATCGACCGAGGTTCCAGTAGGACAATCATTTATACAAACGATACTGCCAACGTCCACAATATTATTA
ACTCTGACAAACTCCGTCTTGTACTGCATAGGAGGTGTGAACACACCAAGTCTTCGATTATATCTCAG
AGACCTCGACCACTG
+
FFFFFFFFFFFFFFFFFFFFFFFFFFFFFFFFFFFFF:FFFFFFFFFFFFFFFFFFFFFFFFFFFFFF
FFFFFFFFF:FFFFFFFFFFFFFFFFFFFFFFFFFFFFFFFFFFFFFFFFFFFFFF:FFFFFFFFFFF
FFFFFFFFFFFFFFF
@A00721:190:HGJW7DSXY:4:1317:10429:32142 2:N:0:TCTGCAAG+GCTGCACT
TATCGACCGAGGTTCCAGTAGGACAATCATTTATACAAACGATACTGCCAACGTCCACAATATTATTA
ACTCTGACAAACTCCGTCTTGTACTGCATAGGAGGTGTGAACACACCAAGTCTTCGATTATATCTCAG
AGACCTCGACCACTG
+
FFFFFFFFFFFFFFFFFFFFFFFFFFFFFFFFFFFFFFFFFFFFFFFFFFFFFFFFFFF:FFFFF:FF
FFFFFFFFFFFFFFFFFFFFFFFFFFFFFFFFFFFFFFFFFFFFFFFFFFFFFFFFFFFFFFFFFFFF
FFFFFFFFFFFFFF:
@A00721:190:HGJW7DSXY:4:1672:27371:21339 2:N:0:TCTGCAAG+GCTGCACT
TATCGACCGAGGTTCCAGTAGGACAATCATTTATACAAACGATACTGCCAACGTCCACAATATTATTA
ACTCTGACAAACTCCGTCTTGTACTGCATAGGAGGTGTGAACACACCAAGTCTTCGATTATATCTCAG
AGACCTCGACCACTG
+
FFFFFFFFFFFFFFFFFFFFFFFFFFFFFFFFFFFFFFFFFFFFFFFFFFFFFFFFFFFFFFFFFFFF
FFFFFFFFFFFFFFFFFFFFFFFFFFFFFFFFFFFFFFFFFFFFFFFFFFFFFFFFFFFFFFFFFFFF
FFFFFFFFFFFFFFF
@A00721:190:HGJW7DSXY:4:1445:9570:1939 2:N:0:TCTGCAAG+GCTGCACT
CTCGTATCGACCGAGGTTCCAGTAGGACAATCATTTATACAAACGATACTGCCAACGTCCACAATATT

ATTAACTCTGACAAACTCCGTCTTGTACTGCATAGGAGGTGTGAACACACCAAGTCTTCGATTATATC
TCAGAGACCTCGACC
+
FFFFFFFFFFFFFFFFFFFFFFFF:FFFFFFFFFFFFFFFFFFFFFFFFFFFFFFFFFFFFFFF:FFFFF
FFFFFFFFFFFFFFFFFFFFF,FF:FF:FFFFF:FFFFFFFFFFFFFFFFFFFFFFFFFFFFF,F:FFFF
FFFF:FFFF,FFFFF
@A00721:190:HGJW7DSXY:4:2240:2672:35963 2:N:0:TCTGCAAG+GCTGCACT
CTCGTATCGACCGAGGTTCCAGTAGGACAATCATTTATACAAACGATACTGCCAACGTCCACAATATT
ATTAACTCTGACAAACTCCGTCTTGTACTGCATAGGAGGTGTGAACACACCAAGTCTTCGATTATATC
TCAGAGACCTCGACC
+
FFFFFFFFFFFFFFFFFFFFFFFFFFFFFFFFFFFFFFFFFFFFFFFFFFFFFFFFFFFFFF:FFFFFFF
FFFFFFFFFFFFFFFFFFFFFFFFFFFFFFF:FFFFFFFFFFFFFFFFFFFFF:FFFFFFFFFFFF:F:F
FFFFFFFFFFFFFFF
@A00721:190:HGJW7DSXY:4:2464:10746:6605 2:N:0:TCTGCAAG+GCTGCACT
CTCGTATCGACCGAGGTTCCAGTAGGACAATCATTTATACAAACGATACTGCCAACGTCCACAATATT
ATTAACTCTGACAAACTCCGTCTTGTACTGCATAGGAGGTGTGAACACACCAAGTCTTCGATTATATC
TCAGAGACCTCGACC
+
FFFFFFFFFFFFFFFFFFFFFFFFFFFFFFFFFFFFFFFFFFFFFFFFFFFFFFFFFFFFFFFFFFFFFF
FFFFFFFFFFFFFFFFFFFFFFFFFFFFFFFFFFFFFFFFFFFFFFFFFFFFFFFFFFFFFFFFFFFFFF
FFFFFFFFFFFFFFF
@A00721:190:HGJW7DSXY:4:2464:6171:37012 2:N:0:TCTGCAAG+GCTGCACT
CTCGTATCGACCGAGGTTCCAGTAGGACAATCATTTATACAAACGATACTGCCAACGTCCACAATATT
ATTAACTCTGACAAACTCCGTCTTGTACTGCATAGGAGGTGTGAACACACCAAGTCTTCGATTATATC
TCAGAGACCTCGACC
+
FFFFFFFFFFFFFFFFFFFFFFFFFFFFFFFFFFFFFFFFFFFFFFFFFFFFFFFFFFFFFFFFFFFFFF
FFFFFFFFFFFFFFFFFFFFFFFFFFFFFFFFFFFFFFFFFFFFFFFFFFFFFFFFFFFFFFFFFFFFFF
FFFFFFFFFFFFFFF
@A00721:190:HGJW7DSXY:4:2623:19388:29340 2:N:0:TCTGCAAG+GCTGCACT
CTCGTATCGACCGAGGTTCCAGTAGGACAATCATTTATACAAACGATACTGCCAACGTCCACAATATT
ATTAACTCTGACAAACTCCGTCTTGTACTGCATAGGAGGTGTGAACACACCAAGTCTTCGATTATATC
TCAGAGACCTCGACC
+
FFFFFFFFFFFFFFFFFFFFFFFFFFFFFFFFFFFFFFFFFFFFFFFFFFFFFFFFFFFFFFFFFFF:FF
FFFFFFFFFFFFFFFFFFFFFFFFFFFFFFFFFFFFFFFFFFFFFFFFFFFFFFFFFFFFFFFFFFFFFF
FFFFFFFFFFFFFFF
@A00721:190:HGJW7DSXY:4:2645:15736:20040 2:N:0:TCTGCAAG+GCTGCACT
CTCGTATCGACCGAGGTTCCAGTAGGACAATCATTTATACAAACGATACTGCCAACGTCCACAATATT
ATTAACTCTGACAAACTCCGTCTTGTACTGCATAGGAGGTGTGAACACACCAAGTCTTCGATTATATC
CCAGAGACCTCGACC
+
FFFFFFFFFFFFFFFFFFFFFFFFFFFFFFFFFFFFFFFFFFFFFFFFFFFFFFFFFFFFFFFFFFFFFF
FFFFFFFFFFFFFFFFFFFFFFFFFFFFFFFFFFFFFFFFFFFFFFFFFFFFFFFFFFFFFFFFF:FFFF
FFFFFFFFFFFFFFF
@A00721:190:HGJW7DSXY:4:2650:13602:7012 2:N:0:TCTGCAAG+GCTGCACT
CTCGTATCGACCGAGGTTCCAGTAGGACAATCATTTATACAAACGATACTGCCAACGTCCACAATATT
ATTAACTCTGACAAACTCCGTCTTGTACTGCATAGGAGGTGTGAACACACCAAGTCTTCGATTATATC
TCAGAGACCTCGACC
+
FFFFF::FFFFFFF:FFFFFFFFFFFFFFFFFFFFFFFFFFFFFFFFFFFFFFFFFFFFFFFFFFFFFFF
FFFFFFFFFFFFFFFFFFFFFFFFFFFF:FF:FFFFFFFFFFFFFFFFFFFFFFFFFFFFFFFFFFFFF:
FFFFFFFFFFFFFFF

@A00721:190:HGJW7DSXY:4:1126:21296:12101 2:N:0:TCTGCAAG+GCTGCACT
GTCGAGGTCTCTGAGATATAATCGAAGACTTGGTGTGTTCACACCTCCTATGCAGTACAAGACGGAGT
TTGTCAGAGTTAATAATATTGTGGACGTTGGCAGTATCGTTTGTATAAATGATTGTCCTACTGGAACC
TCGGTCGATACGAGA
+
FFFFFFFFFFFFFFFFFFFFFFFFFFFFFFFFFFFFFFFFFFFFFFFF:FFFFFFFFFFFFFFFFFFF
FFFFFFFFFFFFFFFF:FFFFFFFFFFFFFFFFFF:FFFFFFFFFFFFFFF:FFFFFFFFFFFFFFFF
FFFFFFFFFFFFFFF
@A00721:190:HGJW7DSXY:4:1509:9824:33004 2:N:0:TCTGCAAG+GCTGCACT
GTCGAGGTCTCTGAGATATAATCGAAGACTTGGTGTGTTCACACCTCCTATGCAGTACAAGACGGAGT
TTGTCAGAGTTAATAATATTGTGGACGTTGGCAGTATCGTTTGTATAAATGATTGTCCTACTGGAACC
TCGGTCGATACGAGA
+
FFFFFFFFFFFFFFFFFFFFFFFFFFFFFFFFFFFFFFFFFFFFFFFFFFFFFFFFFFFFFFFFFFFF
FFFFF:FFFFFFF,FFFFFFFFFFFFFFFFFFFFFFFFFFFFFFFFFFFFFFFFFFFFFFFFFFFFFF
FFFFFFFFFFFFFFF
@A00721:190:HGJW7DSXY:4:2258:22697:31939 2:N:0:TCTGCAAG+GCTGCACT
GTCGAGGTCTCTGAGATATAATCGAAGACTTGGTGTGTTCACGCCTCCTATGCAGTACAAGACGGAGT
TTGTCAGAGTTAATAATATTGTGGACGTTGGCAGTATCGTTTGTATAAATGATTGTCCTACTGGAACC
TCGGTCGATACGAGA
+
FFFFFFFFFFFFFFFFFFFFFFFFFFFFFFFFFFFFFFFFFFFFFFFFFFFFFFFFFFFFFFFFFFFF
FFFFFFFFFFFFFFFFFFFFFFFFFFFFFFFFFFFFFFFFFFFFFFFFFFFFFFFFFFFFFFF:FFFF
FFFFFFFFFFFFFFF
@A00721:190:HGJW7DSXY:4:2455:3947:28244 2:N:0:TCTGCAAG+GCTGCACT
GTCGAGGTCTCTGAGATATAATCGAAGACTTGGTGTGTTCACACCTCCTATGCAGTACAAGACGGAGT
TTGTCAGAGTTAATAATATTGTGGACGTTGGCAGTATCGTTTGTATAAATGATTGTCCTACTGGAACC
TCGGTCGATACGAGA
+
FFFFFFFFFFFFFFFFFFFFFFFFFFFFFFFFFFFFFFFFFFFFFFFFFFFFFFFFFFFFFFFFFFFF
FFFFFFFFFFFFFFFFFFFFFFFFFFF:FFFFFFF,FFFFFFFFFFFFFFFFFFFFF:FFFFFFF,FF
FFFFFFFFFFFFFFF
@A00721:190:HGJW7DSXY:4:2527:16685:21245 2:N:0:TCTGCAAG+GCTGCACT
GTCGAGGTCTCTGAGATATAATCGAAGACTTGGTGTGTTCACACCTCCTATGCAGTACAAGACGGAGT
TTGTCAGAGTTAATAATATTGTGGACGTTGGCAGTATCGTTTGTATAAATGATTGTCCTACTGGAACC
TCGGTCGATACGAGA
+
FFFFFFFFFFFFFFFFFFFFFFFFFFFFFFFFFFFFFFFFFFFFFFFFFFFFFFFFFFFFFFFFFFF:
FFFFFFFFFFFFFFFFFFFFFFFFFFFFFFFFFFFFFFFFFFFFFFFFFFFFFFFFFFFFFFFFF:FF
FFFFFF:FF:FFFFF
@A00721:190:HGJW7DSXY:4:2550:31548:1924 2:N:0:TCTGCAAG+GCTGCACT
GTCGAGGTCTCTGAGATATAATCGAAGACTTGGTGTGTTCACACCTCCTATGCAGTACAAGACGGAGT
TTGTCAGAGTTAATAATATTGTGGACGTTGGCAGTATCGTTTGTATAAATGATTGTCCTACTGGAACC
TCGGTCGATACGAGA
+
FFFFFFFFFFFFFFFFFFFFFFFFFFF:FFFFFFFFFFFFFFFFFFFFFFFFFFFFFFFFFFFFFFFF
FFFFF:FFFFFFFFFFFFFFFFFFFFFFFFFF:FFFFFFFFFFFFFFFFFFFFFFFF:FFFFFFFFFF
FFFFFFFFFFFFFFF
@A00721:190:HGJW7DSXY:4:2601:4526:31501 2:N:0:TCTGCAAG+GCTGCACT
GTCGAGGTCTCTGAGATATAATCGAAGACTTGGTGTGTTCACACCTCCTATGCAGTACAAGACGGAGT
TTGTCAGAGTTAATAATATTGTGGACGTTGGCAGTATCGTTTGTATAAATGATTGTCCTACTGGAACC
TCGGTCGATACGAGA
+
FFFFFFFFFFFFFFFFFFFFFFFFFFFFFFFFFFFFFFFFFFFFFFFFFFFFFFFFFFFFFFFFFFFF

FFFFFFFFFFFFFFFFFFFFFFFFFFFFFFFFFFFFFFFFFFFFFFFFFFFFFFFFFFFFFFFFFFFF
FFFFFFFFFFFFFFF
@A00721:190:HGJW7DSXY:4:2614:8133:35493 2:N:0:TCTGCAAG+GCTGCACT
GTCGAGGTCTCTGAGATATAATCGAAGACTTGGTGTGTTCACGCCTCCTATGCAGTACAAGACGGAGT
TTGTCAGAGTTAATAATATTGTGGACGTTGGCAGTATCGTTTGTATAAATGATTGTCCTACTGGAACC
TCGGTCGATACGAGA
+
FFFFFFFFFFFFFFFFFFFFFFF:FFFFFFFFFFFFFFFFFFFFFFFF:FF:FFFFFFFFFFFFFFF:
FFFF:FFFFFFFFFFF,FFFFFFFFFFFFFFFFFFFF:FFFFFFFFFFFFFFFFFFFFFFFF:FFFFF
FF:FFFFFFFF,FFF
@A00721:190:HGJW7DSXY:4:1156:15121:17534 2:N:0:TCTGCAAG+GCTGCACT
TTCTGGGTATGTCTCGTATCGACCGAGGTTCCAGTAGGACAATCATTTATACAAACGATACTGCCAAC
GTCCACAATATTATTAACTCTGACAAACTCCGTCTTGTACTGCATAGGAGGTGTGAACACACCAAGTC
TTCGATTATATCTCA
+
FFFFFFFFFFFFFFFF:F:FFFFFFFFFF,:FFF,FFF:FFF::FF,:F,F:FFFFFFFFF,FFFFFF
FFF:FFF:,F:FFF:FFFF,F:FFFFFFFF:FFFFFFFFFFFFF,FFFFFF:FFFFFFF:FFFFFF:F
F:FFFFFFFFFFFFF
@A00721:190:HGJW7DSXY:4:1164:1163:17973 2:N:0:TCTGCAAG+GCTGCACT
TTCTGGGTATGTCTCGTATCGACCGAGGTTCCAGTAGGACAATCATTTATACAAACGATACTGCCAAC
GTCCACAATATTATTAACTCTGACAAACTCCGTCTTGTACTGCATAGGAGATGTGAACACACCAAGTC
TTCGATTATATCTCA
+
FFF:FFFFFFFFFFFFFFFFFFFFFFFFFFFFFFFFFFFFFFFFFFFFFFFFFFFFFFFFFFFFFFFF
FFFFFFFFFFFFFFFFFFFFFFFFFFFFFFFFFFFFFFFFF:FFFFFFFFFFFFFFFFFFFFFFFFFF
FFFFFFFFFFFFFFF
@A00721:190:HGJW7DSXY:4:1619:24985:30358 2:N:0:TCTGCAAG+GCTGCACT
TTCTGGGTATGTCTCGTATCGACCGAGGTTCCAGTAGGACAATCATTTATACAAACGATACTGCCAAC
GTCCACAATATTATTAACTCTGACAAACTCCGTCTTGTACTGCATAGGAGATGTGAACACACCAAGTC
TTCGATTATATCTCA
+
FFFFFFFFFFFFFFFFFFFFFFFFFFFFFFFFFFFFFFFFFFFFFFFFFFFFFFFFFFFFFFFFFFFF
FFFFFFFFFFFFFFFFFFFFFFFFFFFFFFFFFFFFFFFFFFFFFFFFFFFFFFFFFFFFFFFFFFFF
FFFFFFFFFFFFFFF
@A00721:190:HGJW7DSXY:4:2134:24379:30530 2:N:0:TCTGCAAG+GCTGCACT
TTCTGGGTATGTCTCGTATCGACCGAGGTTCCAGTAGGACAATCATTTATACAAACGATACTGCCAAC
GTCCACAATATTATTAACTCTGACAAACTCCGTCTTGTACTGCATAGGAGATGTGAACACACCAAGTC
TTCGATTATATCTCA
+
FFFFFFFFFFFFFFFFFFFFFFFFFFFFFFFFFFFFFFFFFFFFFFFFFFFFFFFFFFFFFFFFFFFF
FFFFFFFFFFFFFFFFFFFFFFFFFFFFFFFFFFFFFFFFFFFFFFFFFFFFFFFFFFFFFFFFFFFF
FFFFFFFFFFFFFFF
@A00721:190:HGJW7DSXY:4:2431:9534:14246 2:N:0:TCTGCAAG+GCTGCACT
TTCTGGGTATGTCTCGTATCGACCGAGGTTCCAGTAGGACAATCATTTATACAAACGATACTGCCAAC
GTCCACAATATTATTAACTCTGACAAACTCCGTCTTGTACTGCATAGGAGGTGTGAACACACCAAGTC
TTCGATTATATCTCA
+
FFFFFFFFFFFFFFFFFFFFFFFFFFFFFFFFFFFFFFFFFFFFFFFFFFFFFFFFFFFFFFFFFFFF
FFFFFFFFFFFFFFFFFFFFFFFFFFFFFFFFFFFFFFFFFFFFFFFFFFFFFFFFFFFFFFFFFFFF
FFFFFFFFFFFFFFF
@A00721:190:HGJW7DSXY:4:2458:22480:2190 2:N:0:TCTGCAAG+GCTGCACT
TTCTGGGTATGTCTCGTATCGACCGAGGTTCCAGTAGGACAATCATTTATACAAACGATACTGCCAAC
GTCCACAATATTATTAACTCTGACAAACTCCGTCTTGTACTGCATAGGAGATGTGAACACACCAAGTC
TTCGATTATATCTCA

+
FFFFFFFFFFFFFFFFFFFFFFFFFFFFFFFFFFFFFFFFFFFFFFFFFFFFFFFFFFFFFFFFFFFF
FFFFFFFFFFFFFFFFFFFFFFFFFFFFFFFFFFFFFFFFFFFFFFFFF:FFFFFFFFFFFFFFFFFF
FFFFFFFFFFFFFFF
@A00721:190:HGJW7DSXY:4:2551:24596:33348 2:N:0:TCTGCAAG+GCTGCACT
TTCTGGGTATGTCTCGTATCGACCGAGGTTCCAGTAGGACAATCATTTATACAAACGATACTGCCAAC
GTCCACAATATTATTAACTCTGACAAACTCCGTCTTGTACTGCATAGGAGGTGTGAACACACCAAGTC
TTCGATTATATCTCA
+
FFFFFFFFFFFFFFFFFFFFFFFFFFFFFFFFFFFFFFFFFFFFFFFFFFFFFFFFFFFFFFFFFFFF
FFFFFFFFFFFFFFFFFFFFFFFFFFFFFFFFFFFFFFFFFFFFFFFFFFFFFFFFFFFFFFFFFFFF
FFFFFFFFFFFFFFF
@A00721:190:HGJW7DSXY:4:1136:1398:19883 2:N:0:TCTGCAAG+GCTGCACT
GAGATATAATCGAAGACTTGGTGTGTTCACACCTCCTATGCAGTACAAGACGGAGTTTGTCAGAGTTA
ATAATATTGTGGACGTTGGCAGTATCGTTTGTATAAATGATTGTCCTACTGGAACCTCGGTCGATACG
AGACATACCCAGAAA
+
FFF:FFFFFFFFFFFFFFFFFFFFFFFFFFFFFFFFFFFFFFFFFFFF,FFFFFFF::FFF:FFFFFF
F,:FFFFFFFFFFFFFF,:F::FFFFFF:FFFFFFFFFF::FFFFFFFFFF:FFF,FFFFFFFFF,F,
FFFF:FFFFFFFFFF
@A00721:190:HGJW7DSXY:4:1359:20184:27743 2:N:0:TCTGCAAG+GCTGCACT
GAGATATAATCGAAGACTTGGTGTGTTCACACCTCCTATGCAGTACAAGACGGAGTTTGTCAGAGTTA
ATAATATTGTGGACGTTGGCAGTATCGTTTGTATAAATGATTGTCCTACTGGAACCTCGGTCGATACG
AGACATACCCAGAAA
+
FFFFFFFFFFFFFFFFFFFFFFFFFFFFFFFFFFFFFFFFFFFFFFFFFFFFFFFFFFFFFFFFFFF:
FFFFFFFFFFFFFFFFFFFFFFFFFFFFFFFFFFFFFFFFFFFFFFFFFFFFFFFFFFFFFFFF:FFF
FFFFFFFFFFFFFFF
@A00721:190:HGJW7DSXY:4:1359:19569:28902 2:N:0:TCTGCAAG+GCTGCACT
GAGATATAATCGAAGACTTGGTGTGTTCACACCTCCTATGCAGTACAAGACGGAGTTTGTCAGAGTTA
ATAATATTGTGGACGTTGGCAGTATCGTTTGTATAAATGATTGTCCTACTGGAACCTCGGTCGATACG
AGACATACCCAGAAA
+
FFFFFFFFFFFFFFFFFFFFFFFFFFFFFFFFFFFFFFFFFFFFFFFFFFFFFFFFFFFFFFFFFFFF
FFFFFFFFFFFFFFFFFFFFFFFFFFFFFFFFFFFFFFFFFFFFFFFFFFFFFFFFFFFFFFFFFFFF
FFFFFFFFFFFFFFF
@A00721:190:HGJW7DSXY:4:1574:15203:9502 2:N:0:TCTGCAAG+GCTGCACT
GAGATATAATCGAAGACTTGGTGTGTTCACACCTCCTATGCAGTACAAGACGGAGTTTGTCAGAGTTA
ATAATATTGTGGACGTTGGCAGTATCGTTTGTATAAATGATTGTCCTACTGGAACCTCGGTCGATACG
AGACATACCCAGAAA
+
FFFFFFFFFFFFFFFFFFFFFFFFFFFFFFFFFFFF:FFFFFFFFFFFFFFFFFFFFFFFFFFFFFFF
FFFFFFFFFFFFFFFFFFFFFFFF:FFFFFFFFFFFFFFFFFFFFFFFFFFFFFFFFFFFFFFFFFFF
FFFFFFFFFFFFFFF
@A00721:190:HGJW7DSXY:4:1148:18521:18630 2:N:0:TCTGCAAG+GCTGCACT
AGATATAATCGAAGACTTGGTGTGTTCACACCTCCTATGCAGTACAAGACGGAGTTTGTCAGAGTTAA
TAATATTGTGGACGTTGGCAGTATCGTTTGTATAAATGATTGTCATACTGGAACCTCGGTCGATACTA
GACATACCCAGAAA
+
FFF:F,FFF,FFF,FFFFFF::::FFFFFF:,FF,:FFF:F:FFFFFFF:F,F:FFF:FFFFFFFFF:
F:FFFFF:FF,F:,FFF:,FFFFF:FFFFFFFFFFFF,FFF:FF,,F,:::FF,FF,FFFF:FFFF,F
:F:F,F:,FFFFFF
@A00721:190:HGJW7DSXY:4:1315:10583:24017 2:N:0:TCTGCAAG+GCTGCACT
CACTTTCTGGGTATGTCTCGTATCGACCGAGGTTCCAGTAGGACAATCATTTATACAAACGATACTGC

```
CAACGTCCACAATATTATTAACTCTGACAAACTCCGTCTTGTACTGCATAGGAGGTGTGAACACACCA
AGTCTTCGATTATAT
+
FFFFFFFFFFFFFFFFFFFFFFFFFFFFFFFFFFFFFFFFFFFFFFFFFFFFFFFFFFFFFFFFFFFF
FFFFFFFFFFFFFFF,FFFFFFFFFFFF:FFFFFFFFFFFFFFFFFFF:FFFFFFFFFFFFFFFFF:
FFFFFFFFFFFFFFF
@A00721:190:HGJW7DSXY:4:1460:25654:17362 2:N:0:TCTGCAAG+GCTGCACT
CACTTTCTGGGTATGTCTCGTATCGACCGAGGTTCCAGTAGGACAATCATTTATACAAACGATACTGC
CAACGTCCACAATATTATTAACTCTGACAAACTCCGTCTTGTACTGCATAGGAGGTGTGAACACACCA
AGTCTTCGGTTATAT
+
FFFFFFFFFFFFFFFFFFFFFFFFFFFFFFFFFFFFFFFFFFFFFFFFFFFFFFFFFFFFFFFFFFFF
FFFFFFFFFFFFFFFFF,FFFFFFFFFFFFFFFFFFFFFFFFFFFFFFFFFFFFFFFFFFFFF:FFFFF
FFFFFFFFFFFFFFF
@A00721:190:HGJW7DSXY:4:1463:4752:12978 2:N:0:TCTGCAAG+GCTGCACT
CACTTTCTGGGTATGTCTCGTATCGACCGAGGTTCCAGTAGGACAATCATTTATACAAACGATACTGC
CAACGTCCACAATATTATTAACTCTGACAAACTCCGTCTTGTACTGCATAGGAGGTGTGAACACACCA
AGTCTTCGATTATAT
+
FFFFFFFFFFFFFFFFFFFFFFFFFFFFFFFFFFFFFFFFFFFFFFFFFFFFFFFFFFFFFFFFFFFF
FFFFFFFFFFFFFFFFFFFF,FFFFFFFFFFFFFFFFFFFFFFFFFFFFFFFFFF:FFFFFFFFFFFF
FFFFFFFFFFF:F:F
@A00721:190:HGJW7DSXY:4:2242:17635:29904 2:N:0:TCTGCAAG+GCTGCACT
CACTTTCTGGGTATGTCTCGTATCGACCGAGGTTCCAGTAGGACAATCATTTATACAAACGATACTGC
CAACGTCCACAATATTATTAACTCTGACAAACTCCGTCTTGTACTGCATAGGAGGTGTGAACACACCA
AGTCTTCGATTATAT
+
FFFFFFFFFFFFFFFFFFFFFFFFFFFFFFFFFFFFFFFFFFFFFFFFFFFFFFFFFFFFFFFFFFF:
FF:FFFFFFFFFFFFFFFFFFFFFFFFFFFFFFFFFFFFFFFFFFFFFFFFFFFFFFFFFFFFFFFFFF
FFFFFFF,FFFFFFF
@A00721:190:HGJW7DSXY:4:2247:11505:31939 2:N:0:TCTGCAAG+GCTGCACT
CACTTTCTGGGTATGTCTCGTATCGACCGAGGTTCCAGTAGGACAATCATTTATACAAACGATACTGC
CAACGTCCACAATATTATTAACTCTGACAAACTCCGTCTTGTACTGCATAGGAGGTGTGAACACACCA
AGTCTTCGATTATAT
+
FFFFFFFFFFF:FFFFFFFFFFFFFFFFFFFFFFFFFFFFFFFFFFFFFFF:FFFFFFFFFFFFFFFFF
FFFFFFFFFFFFFFFFFFFFFFFFFFFFFFFFFFFFFFFFF:FFFF,FFFFFFFFFFFF:FFFFFFF
FFFFFFFFF:FFFFF
@A00721:190:HGJW7DSXY:4:2335:19452:18584 2:N:0:TCTGCAAG+GCTGCACT
CACTTTCTGGGTATGTCTCGTATCGACCGAGGTTCCAGTAGGACAATCATTTATACAAACGATACTGC
CAACGTCCACAATATTATTAACTCTGACAAACTCCGTCTTGTACTGCATAGGAGGTGTGAACACACCA
AGTCTTCGATTATAT
+
FFFFFFFFFFFFFFFFFFFFFFFFFFFFFFFFFFFFFFFF:FFFFF:FFFFFFFFFFFFFFFFFFFFFF
FFFFFFFFFFFFFFFFFFFFFFFFFFFFFFFFFFFFFFFFFFFFFFFFFFFFFFFFFFFFFFFFFFFF
FFFFFFFFFFFFFFF
@A00721:190:HGJW7DSXY:4:2335:19895:18818 2:N:0:TCTGCAAG+GCTGCACT
CACTTTCTGGGTATGTCTCGTATCGACCGAGGTTCCAGTAGGACAATCATTTATACAAACGATACTGC
CAACGTCCACAATATTATTAACTCTGACAAACTCCGTCTTGTACTGCATAGGAGGTGTGAACACACCA
AGTCTTCGATTATAT
+
FFFFFF:FFFFFFFFFFFFFFFFFFFFFFFFFFFFFFFFFFFFFFFFFFFFFFFFFFFFFFFFFFFFFF
FFFFFFFFFFFFFFFFFFFFFFFFFFFF,FFFFF,FFFFFFFFFFFFF,FFFFFFFFFFFFFFFFFFF
FFFFFFFFFFFFFFF
```

```
@A00721:190:HGJW7DSXY:4:2413:7726:29089 2:N:0:TCTGCAAG+GCTGCACT
CACTTTCTGGGTATGTCTCGTATCGACCGAGGTTCCAGTAGGACAATCATTTATACAAACGATACTGC
CAACGTCCACAATATTATTAACTCTGACAAACTCCGTCTTGTACTGCATAGGAGGTGTGAACACACCA
AGTCTTCGATTATAT
+
FF:FF::FFF,FFF,FFFFFFFFFFFFFFF,FFFFFFFFFF:FFFFFFFF,FFFF,FFF,FFFFFFFF
FFFFFF,FF,FF::FFFFFFF,,,FF:FFFFF:::,F,F,FFFFF::F:F,FF,,FFFFFFFFFFF:FF
FFFFFFFFFFFFFFF
@A00721:190:HGJW7DSXY:4:1353:19153:9862 2:N:0:TCTGCAAG+GCTGCACT
ATTCCATCTCACTTTCTGGGTATGTCTCGTATCGACCGAGGTTCCAGTAGGACAATCATTTATACAAA
CGATACTGCCAACGTCCACAATATTATTAACTCTGACAAACTCCGTCTTGTACTGCATAGGAGGTGTG
AACACACCAAGTCTT
+
FFFFFFFFFFFFFFFFFFFFFFFFFFFFFFFFFFFFFFFFF:FFFFFFFFFFFFFFFFFFFFFFFFFF
:FFFFFFFFFFFF:FFFFFFFFFFFFFFFFFFFFFFFFFFFFFFFFFFFFFFFFFFFFF,FFFFFFF
FFFFFFFFFFFFFFF
@A00721:190:HGJW7DSXY:4:1357:6424:15781 2:N:0:TCTGCAAG+GCTGCACT
ATTCCATCTCACTTTCTGGGTATGTCTCGTATCGACCGAGGTTCCAGTAGGACAATCATTTATACAAA
CGATACTGCCAACGTCCACAATATTATTAACTCTGACAAACTCCGTCTTGTACTGCATAGGAGGTGTG
AACACACCAAGTCTT
+
FFFFFFFFFFFFFFFFFFFFFFFFFFFFFFFFFFFFFFFFFFFFFFFFFFFFFFFFFFFFFFFFFFFF
FFFFFFFFFFFFFFFFFFFFF:FFFFFFFFFFFFFFFF::FFFFFFFFFFFFFFFFFFFFFFFFFFFF
FFFFF:FF:FFFFFF
@A00721:190:HGJW7DSXY:4:1404:8775:13902 2:N:0:TCTGCAAG+GCTGCACT
ATTCCATCTCACTTTCTGGGTATGTCTCGTATCGACCGAGGTTCCAGTAGGACAATCATTTATACAAA
CGATACTGCCAACGTCCACAATATTATTAACTCTGACAAACTCCGTCTTGTACTGCATAGGAGGTGTG
AACACACCAAGTCTT
+
FFFFFFFFFFFFFFFFFFFFFFFFFFFFFFFFFFFFFFFFFFFFFFFFFFFFFFFFFFFFFFFFFFFF
FFFFFFFFFFFFFFFFFFFFFFFFFFFFFFFFFFFFFFFFFFFFFFFFFFFFFFFFFFFFFFFFFFFF
FFFFFFFFFFFFFFF
@A00721:190:HGJW7DSXY:4:1513:15718:28494 2:N:0:TCTGCAAG+GCTGCACT
ATTCCATCTCACTTTCTGGGTATGTCTCGTATCGACCGAGGTTCCAGTAGGACAATCATTTATACAAA
CGATACTGCCAACGTCCACAATATTATTAACTCTGACAAACTCCGTCTTGTACTGCATAGGAGGTGTG
AACACACCAAGTCTT
+
FFF:FFFFFFFFFFFFFFFFF:FFFF:FFFFFFFFFFFFFFFFFFFF:FFFFFFFFFFFFFFFFFFFF
FFFFFFF:FFFFFFFFFFFFFFFFF,FFFFFFFFF,FFFFFFFFFFFF:FFFFFFFFFFFFFF,FFFF
FFFFFFFFFFFFFF:
@A00721:190:HGJW7DSXY:4:1564:17644:19586 2:N:0:TCTGCAAG+GCTGCACT
ATTCCATCTCACTTTCTGGGTATGTCTCGTATCGACCGAGGTTCCAGTAGGACAATCATTTATACAAA
CGATACTGCCAACGTCCACAATATTATTAACTCTGACAAACTCCGTCTTGTACTGCATAGGAGGTGTG
AACACACCAAGTCTT
+
FFFFFFFFFFFFF:FFFFFFFFFFFFFFFFFFFFFFFFFFFFFFFFFFFFFFFFFFFFFFFFFFFFFF
FFFFFFFFFFFFFFFFFFFFFFFFFFFFFFFFFFFFFFFFFFFFFFFFFFFFFFFFFFFFFFFFFFF:
FFFFFFFFFFFFFFF
@A00721:190:HGJW7DSXY:4:2407:8223:32988 2:N:0:TCTGCAAG+GCTGCACT
ATTCCATCTCACTTTCTGGGTATGTCTCGTATCGACCGAGGTTCCAGTAGGACAATCATTTATACAAA
CGATACTGCCAACGTCCACAATATTATTAACTCTGACAAACTCCGTCTTGTACTGCATAGGAGGTGTG
AACACACCAAGTCTT
+
F:FFFFFFFFFFFFFFFFFFFFFFF:FFFFFFFFFFFFFFFFFFFFFFFFFFFFFFFFFFFFFFFFFF
```

F,FFF:FFFFFFFFFFFFFFFFFFFFFFFFFFFFFFFFFFF:FFFFFFFFFFFFFFFF:FF:FFFF
FFFFFFF:FFFFFFF
@A00721:190:HGJW7DSXY:4:2518:31340:24236 2:N:0:TCTGCAAG+GCTGCACT
ATTCCATCTCACTTTCTGGGTATGTCTCGTATCGACCGAGGTTCCAGTAGGACAATCATTTATACAAA
CGATACTGCCAACGTCCACAATATTATTAACTCTGACAAACTCCGTCTTGTACTGCATAGGAGGTGTG
AACACACCAAGTCTT
+
FFFFFFFFFFFFFFFFFFFFFFFFFFFFFFFFFFFFFFFFFFFFFFFFFFFFFFFFFFFFFFFFFFFF
FFFFFFFF:FFFFFFFFFFFFFFFFFFFFFFFFFF:FFF:FFF,FFFFFFFF:FF:FFFF:FFFFFFF
FFFFFFFFFFFFF:F
@A00721:190:HGJW7DSXY:4:2576:15167:19805 2:N:0:TCTGCAAG+GCTGCACT
ATTCCATCTCACTTTCTGGGTATGTCTCGTATCGACCGAGGTTCCAGTAGGACAATCATTTATACAAA
CGATACTGCCAACGTCCACAATATTATTAACTCTGACAAACTCCGTCTTGTACTGCATAGGAGGTGTG
AACACACCAAGTCT
+
FFFFFFFF,FFFFFFFFFFFFFFFFFFFFFFFFFFFFFFFFFFFFFFFFFFFFFFFFFFFFFFFFFF:
FFFFFFFFFFF:FFFFFFFFFFFFFFFFFFFFFFFFFFFFFFFFFFFFFFFFFFFFFFFFFFFFFFFF
FFFFFFFFFFFFFF
@A00721:190:HGJW7DSXY:4:1160:19443:33974 2:N:0:TCTGCAAG+GCTGCACT
CACAATTCGATTCCATCTCACTTTCTGGGTATGTCTCGTATCGACCGAGGTTCCAGTAGGACAATCAT
TTATACAAACGATACTGCCAACGTCCACAATATTATTAACTCTGACAAACTCCGTCTTGTACTGCATA
GGAGGTGTGAACACA
+
FFFFFFFFFFFFFF:FFFFFFFFFFFFFFFFFFFFFFFFFFFFFFFFFFFFFFFFFFFFFFFFFFFFF
FFFFFFFFFFFFFFFFFFFFFFFFFFFFFFFFFFFFFFFFFFFFFFFFFFFFFFFFFFFFFFFFFFFF
FFFFFFFFFFFFFFF
@A00721:190:HGJW7DSXY:4:1244:12825:8234 2:N:0:TCTGCAAG+GCTGCACT
CACAATTCGATTCCATCTCACTTTCTGGGTATGTCTCGTATCGACCGAGGTTCCAGTAGGACAATCAT
TTATACAAACGATACTGCCAACGTCCACAATATTATTAACTCTGACAAACTCCGTCTTGTACTGCATA
GGAGGTGTGAACACA
+
FFFFFFFFFFFFFFFFFFFFFFFFFFFFFFFFFFFFFFFFFFFFFFFFFFFFFFFFFFFFFFFFFFFF
FFFFFFFFFFFFFFFFFFFFFFFFFFFFFFFFFFFFFFFFFFFFFFFFFFFFFFFFFFFFFFFFFFFF
FFFFFFFFFFFFFFF
@A00721:190:HGJW7DSXY:4:1349:1136:6402 2:N:0:TCTGCAAG+GCTGCACT
CACAATTCGATTCCATCTCACTTTCTGGGTATGTCTCGTATCGACCGAGGTTCCAGTAGGACAATCAT
TTATACAAACGATACTGCCAACGTCCACAATATTATTAACTCTGACAAACTCCGTCTTGTACTGCATA
GGAGGTGTGAACACA
+
FFFF::FF:FFFFFF:F,FFFF:F:,F,:::,::FFF:,FF:FFFFFFFFFFFFFFFFFFFFF:F,FFF
:,FFF,FFF,FF:FFFFF:FFF,,F,FFF::F,FFFFFFFFFFFFFFFFFFFF:F:FFFFFFFF:F,F
FFFF:FFFFFF:FFF
@A00721:190:HGJW7DSXY:4:1417:10574:21746 2:N:0:TCTGCAAG+GCTGCACT
CACAATTCGATTCCATCTCACTTTCTGGGTATGTCTCGTATCGACCGAGGTTCCAGTAGGACAATCAT
TTATACAAACGATACTGCCAACGTCCACAATATTATTAACTCTGACAAACTCCGTCTTGTACTGCATA
GGAGGTGTGAACACA
+
FFFFFFFFFFFFFFFFFFFFFFFFFFFFFFFFFFFFFFFFFFFFFFFFFFFFFFFFFFFFFFFFFFFF
FFFFFFFF:FFFFFFFFFFFFFFFFFFFFFFFFFFFFFFFFFFFFFFF:FFFFFFFFFFFFFFFFFFF
FFFFFFFFFFFF:FF
@A00721:190:HGJW7DSXY:4:1467:15329:25034 2:N:0:TCTGCAAG+GCTGCACT
CACAATTCGATTCCATCTCACTTTCTGGGTATGTCTCGTATCGACCGAGGTTCCAGTAGGACAATCAT
TTATACAAACGATACTGCCAACGTCCACAATATTATTAACTCTGACAAACTCCGTCTTGTACTGCATA
GGAGGTGTGAACACA

+
FFFFFFFFFFFFFFFFFFFFFFFFFFFFFFFFFFFFFFFFFFFFFFFFFFFFFFFFFFFFFFFFFFFFFF
FFFFFFFFFFFFFFFFFFFFFFFFFFFFFFFFFFFFFFFFFFFFFFFFFFFFFFFFFFFFFFFFFFFFFF
FFFFFFFFFFFFFFF
@A00721:190:HGJW7DSXY:4:1621:22381:31078 2:N:0:TCTGCAAG+GCTGCACT
CACAATTCGATTCCATCTCACTTTCTGGGTATGTCTCGTATCGACCGAGGTTCCAGTAGGACAATCAT
TTATACAAACGATACTGCCAACGTCCACAATATTATTAACTCTGACAAACTCCGTCTTGTACTGCATA
GGAGGTGTGAACACA
+
FFFFFFFFFFFFFF,FFFFFFFFFFFFFFFFFFFFFFFFFFFFFFFFFFFFFFFFFFFFFFFFFFFFFFF
FFFFFFFFFFFFFFFFFFFFFFFFFFFFFFFFFFFFFFFFFFFFFFFFFFFFFFFFFFFFFFFFFF:FFF
FFFFFFFFFFFFFFF
@A00721:190:HGJW7DSXY:4:1641:27959:20384 2:N:0:TCTGCAAG+GCTGCACT
CACAATTCGATTCCATCTCACTTTCTGGGTATGTCTCGTATCGACCGAGGTTCCAGTAGGACAATCAT
TTATACAAACGATACTGCCAACGTCCACAATATTATTAACTCTGACAAACTCCGTCTTGTACTGCATA
GGAGGTGTGAACACA
+
FFFFFFFFFFFFFFF:FFFFFFFFFFFFFFFFFFFFFFFFFFFFFFFFFFFFFFFFFFFFFFFFF:FFFF
FFFFFFFFFFFFFFFFF:F,FFFFFFFFFF,FFFFFFFFFFFFFFFFFFFFFFFFFFFFFFF:FFFFFFF
FF:FFFFFFFFFFF:
@A00721:190:HGJW7DSXY:4:1657:17607:33144 2:N:0:TCTGCAAG+GCTGCACT
CACAATTCGATTCCATCTCACTTTCTGGGTATGTCTCGTATCGACCGAGGTTCCAGTAGGACAATCAT
TTATACAAACGATACTGCCAACGTCCACAATATTATTAACTCTGACAAACTCCGTCTTGTACTGCATA
GGAGGTGTGAACACA
+
FFFFFFFFFFFFFFFFFFFFFFF:FFFFFFFFFFFFFFFFFFFFFFFFFFFFFFFFFFFFFFFFFFFFFF
FFFFFFFFFFFFFFFFFFFFFFF:FFFFFFFFFFFFFFFFFFFFFFFFFFFFFFFFFFF:FFFFFFFFF:
FFFFFFFFFFFFFFF
@A00721:190:HGJW7DSXY:4:1674:12843:14747 2:N:0:TCTGCAAG+GCTGCACT
CACAATTCGATTCCATCTCACTTTCTGGGTATGTCTCGTATCGACCGAGGTTCCAGTAGGACAATCAT
TTATACAAACGATACTGCCAACGTCCACAATATTATTAACTCTGACAAACTCCGTCTTGTACTGCATA
GGAGGTGTGAACACA
+
FFFFFFFFFFFFFFFFFFFFFFFFF:FFFFFFFFFFFFFFFFFFFFFFFFFFFFFFFFFFFFFF:FFFFF
FFFFFFFF:FFFFFFFFFFFFFFFFFFFFFFFFFFFFFFF:FFFFFFFFFFFFFFFFFFFFFFFFFFF:F
FFFFFFFFFFF::FF
@A00721:190:HGJW7DSXY:4:2102:22996:10848 2:N:0:TCTGCAAG+GCTGCACT
CACAATTCGATTCCATCTCACTTTCTGGGTATGTCTCGTATCGACCGAGGTTCCAGTAGGACAATCAT
TTATACAAACGATACTGCCAACGTCCACAATATTATTAACTCTGACAAACTCCGTCTTGTACTGCATA
GGAGGTGTGAACACA
+
FFFFFFFFFFFFFFFFFFFFFFFFFFFFFFFFFFFFFFFFFFFFFFFFFFFFFFFFFFFFFFFFFFFFFF
FFFFFFFFFFFFFFFFFFFFFFFFFFFFFFFFFFFFFFFFFFFFFFFFFFFFFFFFFFFFFFFFFFFFFF
FFFFFFFFFFFFFFF
@A00721:190:HGJW7DSXY:4:2139:27227:14606 2:N:0:TCTGCAAG+GCTGCACT
CACAATTCGATTCCATCTCACTTTCTGGGTATGTCTCGTATCGACCGAGGTTCCAGTAGGACAATCAT
TTATACAAACGATACTGCCAACGTCCACAATATTATTAACTCTGACAAACTCCGTCTTGTACTGCATA
GGAGGTGTGAACACA
+
FFFFFFFFFFFFFFFFFFFFFFFFFFFFFFFFFFFFFFFFFFFFFFFFFFFFFFFFFFFFFFFFFFFFFF
FFFFFFFFFFFFFFFFFFFFFFFFFFFFFF:FFFFFFFFFFFFFFFFFFFFFFFFFFFFFFFFFFFFFFF
FFFFFFFFFFFFFFF
@A00721:190:HGJW7DSXY:4:2227:15718:5040 2:N:0:TCTGCAAG+GCTGCACT
CACAATTCGATTCCATCTCACTTTCTGGGTATGTCTCGTATCGACCGAGGTTCCAGTAGGACAATCAT

TTATACAAACGATACTGCCAACGTCCACAATATTATTAACTCTGACAAACTCCGTCTTGTACTGCATA
GGAGGTGTGAACACA
+
FFFFFFFFFFFFFFFFFFFFFFFFFFFFFFFFFFFFFFFFFFFFFFFFFFFFFFFFFFFFFFFFFFFFFF
FFFFFFFF:FFFFFFFFFFFFFFFFFFFFFFFFFFFFFFFFFFFFFFFFFFFFFFFFFFFFFFFFFFFF
,FFFFFFFFFFFFFF
@A00721:190:HGJW7DSXY:4:2503:8088:31939 2:N:0:TCTGCAAG+GCTGCACT
CACAATTCGATTCCATCTCACTTTCTGGGTATGTCTCGTATCGACCGAGGTTCCAGTAGGACAATCAT
TTATACAAACGATACTGCCAACGTCCACAATATTATTAACTCTGACAAACTCCGTCTTGTACTGCATA
GGAGGTGTGAACACA
+
FFFFFFFFFFFFFF:FFFFFFFFFFFF:FFFFFFFFFFFFFFF:FFFFFF:FFFFFFFFFFFFFFFFFFF
FFFFFFFFFFFF:FF:FFFFFFFFFFFFFFFFFFFFFFFFFF:FFFFFFFFFFF,FFFFFF:FFFFFFFF
FFFFFFFFFF:,FFF
@A00721:190:HGJW7DSXY:4:2572:15483:5822 2:N:0:TCTGCAAG+GCTGCACT
CACAATTCGATTCCATCTCACTTTCTGGGTATGTCTCGTATCGACCGAGGTTCCAGTAGGACAATCAT
TTATACAAACGATACTGCCAACGTCCACAATATTATTAACTCTGACAAACTCCGTCTTGTACTGCATA
GGAGGTGTGAACACA
+
FFFFF,::FF:FFF,FFFFFFFF:FFFF:FFFFFFFF,F:FF,FFFFFFFFF,FFFFF,FFFFFFFFFFF
::FFF:FFFF:F,F:FFFFFFFF:FFFFFFFF:FF,FFFFF,FF:,FFFFF:FF::FFF,FFFFF:FFFF
FFFF,F:FFFF,FFF
@A00721:190:HGJW7DSXY:4:1117:10357:30295 2:N:0:TCTGCAAG+GCTGCACT
GCACAATTCGATTCCATCTCACTTTCTGGGTATGTCTCGTATCGACCGAGGTTCCAGTAGGACAATCA
TTTATACAAACGATACTGCCAACGTCCACAATATTATTAACTCTGACAAACTCCGTCTTGTACTGCAT
AGGAGGTGTGAACAC
+
FFFFFFFFFFFFFFFFFFFFFFFFFFFFFFFFFFFFFFFFFFFFFFFFFFFFFFFFFFFFFFFFFFFFFF
FFFFFF:FFFFFFFFFFFFFFFFFFFFFFFFFFFFFFFFFFFFFFFFFFFFFFFFFFFFFFFF,FFFFF
FFFFFFFFFFFFFFF
@A00721:190:HGJW7DSXY:4:1169:24885:34507 2:N:0:TCTGCAAG+GCTGCACT
GCACAATTCGATTCCATCTCACTTTCTGGGTATGTCTCGTATCGACCGAGGTTCCAGTAGGACAATCA
TTTATACAAACGATACTGCCAACGTCCACAATATTATTAACTCTGACAAACTCCGTCTTGTACTGCAT
AGGAGGTGTGAACAC
+
FFFFFFFFFF:FFFFFFFFFFFFFFFFFFFFFFFFFFFFFFFFFFFFFFFFFFFFFFFFFFFFFFF:FFF
FFFFFFFFFFFFFFFFFFFFFFFFFFFFFFFFFFFFFFFFFFFFFFFFF:FFFFFFFFFFFFFFFFFFFF
FFFFFFFFFFFFFFF
@A00721:190:HGJW7DSXY:4:1442:28980:18239 2:N:0:TCTGCAAG+GCTGCACT
GCACAATTCGATTCCATCTCACTTTCTGGGTATGTCTCGTATCGACCGAGGTTCCAGTAGGACAATCA
TTTATACAAACGATACTGCCAACGTCCACAATATTATTAACTCTGACAAACTCCGTCTTGTACTGCAT
AGGAGGTGTGAACAC
+
FFFFFFFFFFFFFFFFFFFFFFFFFFFFFFFFFFFFFFFFFFFFFFFFFFFFFFFFFFFFFFFFFFFFFF
FFF:FFFFFFFFFFFFFFFFFFFFFFFFFFFFFF:FF:FFFFFFFFFFFFF:FFFFFFFFFFFFFFFFFF
FFFFFFFFFFFFF:F
@A00721:190:HGJW7DSXY:4:1620:26386:6480 2:N:0:TCTGCAAG+GCTGCACT
GCACAATTCGATTCCATCTCACTTTCTGGGTATGTCTCGTATCGACCGAGGTTCCAGTAGGACAATCA
TTTATACAAACGATACTGCCAACGTCCACAATATTATTAACTCTGACAAACTCCGTCTTGTACTGCAT
AGGAGGTGTGAACAC
+
F:FFFFFFFFFFFFFFFFF,FFFFFFFFFFFFFFFFFFFFFF:FFFFFFFFFFFFFFFFFFFFFFFFFFF
F:FFFFFFF:FFF,FFFFFFFFFFFFFFFFFFFF,FFFFFFFFFFFFFFFFFFFF:,:FF:F::FF:FFF
FFFF:FFFFFFFFFF

```
@A00721:190:HGJW7DSXY:4:2642:5484:14278 2:N:0:TCTGCAAG+GCTGCACT
GCACAATTCGATTCCATCTCACTTTCTGGGTATGTCTCGTATCGACCGAGGTTCCAGTAGGACAATCA
TTTATACAAACGATACTGCCAACGTCCACAATATTATTAACTCTGACAAACTCCGTCTTGTACTGCAT
AGGAGGTGTGAACAC
+
FFFFFFFFFFFFFFFFFFFFFFFFFFFFFFFFFFFFFFFFFFFFFFFFFFFFFFF:FFFFFFFFFFFF
FFFFFFFFFFFFFFFFFFFFFFFFFFFFFFFFFFFFFFFFFFFFFFFFFFFFF:F,FFFFFFFFFFFFF
FFFFFFFFFFFFFFF
@A00721:190:HGJW7DSXY:4:2677:3992:36589 2:N:0:TCTGCAAG+GCTGCACT
GCACAATTCGATTCCATCTCACTTTCTGGGTATGTCTCGTATCGACCGAGGTTCCAGTAGGACAATCA
TTTATACAAACGATACTGCCAACGTCCACAATATTATTAACTCTGACAAACTCCGTCTTGTACTGCAT
AGGAGGTGTGAACAC
+
FFFFFFFFFFFFFFFFFFFFFFFFFFFFFFFFFFFFFFFFFFFFFFFFFFFFFFFFFFFFFFFFFFFF
FFFFFFFFFFFFFFFFFFFFFFFFFFFFFFFFFFFFFFFFFFFFFFFFFFFFFFFFFFFFFFFFFF:F
FFFFFFFFFFFFFFF
@A00721:190:HGJW7DSXY:4:1304:3423:18255 2:N:0:TCTGCAAG+GCTGCACT
AAGCACAATTCGATTCCATCTCACTTTCTGGGTATGTCTCGTATCGACCGAGGTTCCAGTAGGACAAT
CATTTATACAAACGATACTGCCAACGTCCACAATATTATTAACTCTGACAAACTCCGTCTTGTACTGC
ATAGGAGGTGTGAAC
+
FF:FFFFFFFFFFFFFFFFFFFFFFFFFFFFFFFFFFF,FFFFFFFFFFF:FFFFFFFFFFF:FFFFFF
FFFFFFFFFFFF:FF:F:FFFFFFFFFFFFFFFFFFFFF:FFFFFFFF::FFFF:FF:FFF,FFFFFF
FFF:FF:FFF:FFF:
@A00721:190:HGJW7DSXY:4:1304:2980:18333 2:N:0:TCTGCAAG+GCTGCACT
AAGCACAATTCGATTCCATCTCACTTTCTGGGTATGTCTCGTATCGACCGAGGTTCCAGTAGGACAAT
CATTTATACAAACGATACTGCCAACGTCCACAATATTATTAACTCTGACAAACTCCGTCTTGTACTGC
ATAGGAGGTGTGAAC
+
FFFFFFFFFFFFF:FFFFFFFFFFFFFFFFFFFFFFFFFFFFFFFFFFFFFFFFFFFFFFFFFFFFFF
FFFFFFFFFFFFFFFFFFFFFFFFFFFFFFFFFFFFFFFFFFFFFFFFFFFFFFFFFFFFFFFFFFFF
FFFFFFFFFFFFFFF
@A00721:190:HGJW7DSXY:4:1346:10963:35102 2:N:0:TCTGCAAG+GCTGCACT
AAGCACAATTCGATTCCATCTCACTTTCTGGGTATGTCTCGTATCGACCGAGGTTCCAGTAGGACAAT
CATTTATACAAACGATACTGCCAACGTCCACAATATTATTAACTCTGACAAACTCCGTCTTGTACTGC
ATAGGAGGTGTGAAC
+
FFFFFFFFFFFFFFFFF:FFFFFFFFFFFFFFFFFFFFFFFFFFFFFFFFFFFFFFFFFFFFFFFFFF
FFFFFFFFFFFFFFFFFFFFFFFFFFFFFFFFFFFFFFFFFFFFFFFFFFFFFFFFFFFFFFFFFFFF
FFFFFFFFFFFFFFF
@A00721:190:HGJW7DSXY:4:1467:1814:7764 2:N:0:TCTGCAAG+GCTGCACT
AAGCACAATTCGATTCCATCTCACTTTCTGGGTATGTCTCGTATCGACCGAGGTTCCAGTAGGACAAT
CATTTATACAAACGATACTGCCAACGTCCACAATATTATTAACTCTGACAAACTCCGTCTTGTACTGC
ATAGGAGGTGTGAAC
+
FFFFFFFFFFFFFFFFFFFFFFFFFFFFFFFFFFFFFFFFFFFFFFFFFFFFFFFFFFFFFFFFFFFF
FFFFFFFFFFFFFFFFFFFFFFFFFFFFFFFFFFFFFFFFFFFFFFFFFFFFFFFFFFFFFFFFFFFF
F:FFFFFFFFFFFFF
@A00721:190:HGJW7DSXY:4:1658:25491:33646 2:N:0:TCTGCAAG+GCTGCACT
AAGCACAATTCGATTCCATCTCACTTTCTGGGTATGTCTCGTATCGACCGAGGTTCCAGTAGGACAAT
CATTTATACAAACGATACTGCCAACGTCCACAATATTATTAACTCTGACAAACTCCGTCTTGTACTGC
ATAGGAGGTGTGAAC
+
FFFFFFFFFFFFFFFFFFFFFFF:FFFFFFFFFFFFFFFFFFFFFFFFFFFFFFFFFFFFFF,FFFFF:F
```

```
FFFFFFFFFFF:FFFFFFFF:FFFFFFFFFFFF:F:FFFFF,FFFFFFFFFFFFFFFFFFFFFFFFFFF
FFFFFFFFFFFFFFF
@A00721:190:HGJW7DSXY:4:2402:12807:25363 2:N:0:TCTGCAAG+GCTGCACT
AATCACAATTCGATTCCATCTCACTTTCTGGGTATGTCTCGTATCGACCGAGGTTCCAGTAGGACAAT
CATTTATACAAACGATACTGCCAACGTCCACAATATTATTAACTCTGACAAACTCCGTCTTGTACTGC
ATAGGAGGTGTGAAC
+
FF,:F:FFF,::F,FF:FFF,:F:FFFFFFFF,FFF,F,F:,FFF,F:FFF,,F,FFFF,F:,FFFFF
FF:,F:FF,FFF:FF,F:F,F,FFF:FFFFFFF:F,FF::FFF:FF:F:FFFFFF,:,FFFFFF:FFF
FFFF:FF:F:FFFF:
@A00721:190:HGJW7DSXY:4:2577:15221:4993 2:N:0:TCTGCAAG+GCTGCACT
AAGCACAATTCGATTCCATCTCACTTTCTGGGTATGTCTCGTATCGACCGAGGTTCCAGTAGGACAAT
CATTTATACAAACGATACTGCCAACGTCCACAATATTATTAACTCTGACAAACTCCGTCTTGTACTGC
ATAGGAGGTGTGAAC
+
F:FFFFFFFFFFFFFFFFFFFFFFFFFFFFFFFFFFFFFFFFFFFFFFFFFFFFFFFFFFFFFFFFFF
FFFFFFFFFFFFFFFFFFFFFFFFFFFFFFFFFFFFFFFFFFFFFFFFFFFFFFFFFFFFFFFFFFFF
FFFFFFF:FFFFFFF
@A00721:190:HGJW7DSXY:4:1328:31358:20791 2:N:0:TCTGCAAG+GCTGCACT
GTTAAGCACAATTCGATTCCATCTCACTTTCTGGGTATGTCTCGTATCGACCGAGGTTCCAGTAGGAC
AATCATTTATACAAACGATACTGCCAACGTCCACAATATTATTAACTCTGACAAACTCCGTCTTGTAC
TGCATAGGAGGTGTG
+
FFFFFFFFFFFFFFF:FFFFFFFFFFFFFFFFFFFFFFFFFFFFFFFFFFFFFFFFFFFFFFFFFFFF
F:FFFFFFFF,FFFFFFFFFFFFFFFFFFFFFFFF:F:FFFFFFFFFFFFFFFFFFFFFFFFFF:FFFF
FFFFFFFFFFFFFFF
@A00721:190:HGJW7DSXY:4:1547:4942:23484 2:N:0:TCTGCAAG+GCTGCACT
GTTAAGCACAATTCGATTCCATCTCACTTTCTGGGTATGTCTCGTATCGACCGAGGTTCCAGTAGGAC
AATCATTTATACAAACGATACTGCCAACGTCCACAATATTATTAACTCTGACAAACTCCGTCTTGTAC
TGCATAGGAGGTGTG
+
FFFFFFFFF:F:FFFFFFFFFFFFFFFFF:FFFFFFFFFFFFFFFFFFFFFFFFFFFFFFF:FFFFFFF
FFFFFFFFFFFFFFFFFFFFFFFFFFFFFFFFFFFFF,FFFF:FFFFFFFFFFFFFFFFFFFFFFFFFFF
FFFFFFFFFFFFFFF
@A00721:190:HGJW7DSXY:4:1551:20726:2534 2:N:0:TCTGCAAG+GCTGCACT
CACACCTCCTATGCAGTACAAGACGGAGTTTGTCAGAGTTAATAATATTGTGGACGTTGGCAGTATCG
TTTGTATAAATGATTGTCCTACTGGAACCTCGGTCGATACGAGACATACCCAGAAAGTGAGATGGAAT
CGAATTGTGCTTAAC
+
FFFFFFFFFFFFFFFFFFFFFFFFFFFFF:FFFFFFFFFFFFFFFFF,FFFFFFFFFF:FFFFFFFFFF
FFFFFFFFF:F:FFFFFFFFFFFFFFFFFFFFFFFFFF:FFFFFFFFFFFFFFFFFFFFFFFFFFFFFFF
FFFFFFFFFFFF:FF
@A00721:190:HGJW7DSXY:4:1619:5810:6574 2:N:0:TCTGCAAG+GCTGCACT
CACACCTCCTATGCAGTACAAGACGGAGTTTGTCAGAGTTAATAATATTGTGGACGTTGGCAGTATCG
TTTGTATAAATGATTGTCCTACTGGAACCTCGGTCGATACGAGACATACCCAGAAAGTGAGATGGAAT
CGAATTGTGCTTAAC
+
FFFFFFFFFFFFFFFFFFFFFFFFFFFFFFFFFFFFFFFFFFFFF:FFFFFFFFFFF:FFFFFFFFFF
FFFFFFFFFFFFFFFFFFFFFFFFFFFFFFFFFFFFFF,FFFFFFFFFFFFFFFFFFFFFFFF:FFFFF
FF,F:FFFFFFFFFF
@A00721:190:HGJW7DSXY:4:1643:21269:16157 2:N:0:TCTGCAAG+GCTGCACT
CACACCTCCTATGCAGTACAAGACGGAGTTTGTCAGAGTTAATAATATTGTGGACGTTGGCAGTATCG
TTTGTATAAATGATTGTCCTACTGGAACCTCGGTCGATACGAGACATACCCAGAAAGTGAGATGGAAT
CGAATTGTGCTTAAC
```

```
+
FFFFFFFFFFFFFFFFFFFFFFFFFFFFFFFFFFFFFFFFFFFFFFFFFFFFFFFFFFFFFFFFFFFFFF
FFFFFFFFFFFFFFFFFFFFFFFFFFFFFFFFFFFFFFFFFFFFFFFFFFFFFFFF:FFFFFFFFFFFFF
FFFFFFFFFFFFFF:
@A00721:190:HGJW7DSXY:4:1675:26142:20275 2:N:0:TCTGCAAG+GCTGCACT
GTTAAGCACAATTCGATTCCATCTCACTTTCTGGGTATGTCTCGTATCGACCGAGGTTCCAGTAGGAC
AATCATTTATACAAACGATACTGCCAACGTCCACAATATTATTAACTCTGACAAACTCCGTCTTGTAC
TGCATAGGAGGTGTG
+
FFF:FFFFFFFFFFFFFFFFFFFFFFFFFFFFFFFFFFFFFFFFFFFFFFFFFFFFFFFFFFFFFFFFFF
FFFFFFFFFFFFFFFFFFFFFFFFFFFFFFFFFFF:FFFFFFFFFFFFFFFFFFFFFFFF,FFFFFFFFF
FFFFFFFFFFFFFFF
@A00721:190:HGJW7DSXY:4:2278:7808:13197 2:N:0:TCTGCAAG+GCTGCACT
GTTAAGCACAATTCGATTCCATCTCACTTTCTGGGTATGTCTCGTATCGACCGAGGTTCCAGTAGGAC
AATCATTTATACAAACGATACTGCCAACGTCCACAATATTATTAACTCTGACAAACTCCGTCTTGTAC
TGCATAGGAGGTGTG
+
FFFFFFFFFFFFFFFFFFFFFFFFFFFFFFFFFFFFFFFFFFFFFFFFFFFFFFFFFFFFFFFFFFFFFF
FFFFFF:FFFFFFFFFFFFFFFFFFFFFFFFFFFFFFFFFFFFFFFFFFFFFFFFFFFFFFFFFFFFFFF
FFFFFFFFFFFFFFF
@A00721:190:HGJW7DSXY:4:2451:19931:25676 2:N:0:TCTGCAAG+GCTGCACT
GTTAAGCACAATTCGATTCCATCTCACTTTCTGGGTATGTCTCGTATCGACCGAGGTTCCAGTAGGAC
AATCATTTATACAAACGATACTGCCAACGTCCACAATATTATTAACTCTGACAAACTCCGTCTTGTAC
TGCATAGGAGGTGTG
+
FFFFFFFFFFFFFFFFFFFFFFFFFFFF:FFFFFFFFFFFFFFFFFFFFFFFFFFFFFFFFFFFFFFFFF
,F:FFFFFFFFFFFFFFFFFFFFFFF:FFFFFFFFFFF,FFFFFFFFFFFFFFFFFFFFFFFFFFFF,:
,FF:FFFFFFFFFFF
@A00721:190:HGJW7DSXY:4:2514:4779:32534 2:N:0:TCTGCAAG+GCTGCACT
GTTAAGCACAATTCGATTCCATCTCACTTTCTGGGTATGTCTCGTATCGACCGAGGTTCCAGTAGGAC
AATCATTTATACAAACGATACTGCCAACGTCCACAATATTATTAACTCTGACAAACTCCGTCTTGTAC
TGCATAGGAGGTGTG
+
FFFFFFFFFFFFFFFFFFFFFFFFFFFFFFFFFFFFFFFFFFFFFFFFFFFFFFFFFFFFFFFFFFFFFF
FFFFFFFFFFFFFFFF:FFFFFFFFFFFFFFFFFFFFFFFFFFFFFFFFFFFFFFFFFFFFFFFFFFFF
FFFFFFFFFFFFFFF
@A00721:190:HGJW7DSXY:4:1122:3830:26913 2:N:0:TCTGCAAG+GCTGCACT
TACAAGACGGAGTTTGTCAGAGTTAATAATATTGTGGACGTTGGCAGTATCGTTTGTATAAATGATTG
TCCTACTGGACCCTCGGTCGATACGAGACATACCCAGCAAGTGAGATGGAATCGAATTGTGCTTAACG
GTTCAGTTAAGTTGA
+
FFFFF,FFFFFFFFFFFFFFFFFFFFFFFF:FFFFFFFFFF,,FFFFFF:FFF:,FFFFFFFFFFFFFF
FFFFFFF:FF,FFFFF::FFFFFFFFFFF:FF:FFFF,:FFFFFFF,:FFFFFFFFFFFFFFFFFF:F
FFFF:F:FFFF,FFF
@A00721:190:HGJW7DSXY:4:1161:3215:19460 2:N:0:TCTGCAAG+GCTGCACT
TACAAGACGGAGTTTGTCAGAGTTAATAATATTGTGGACGTTGGCAGTATCGTTTGTATAAATGATTG
TCCTACTGGAACCTCGGTCGATACGAGACATACCCAGAAAGTGAGATGGAATCGAATTGTGCTTAACG
GTTCAGTTAAGTTGA
+
FFFFFFFFFFFFFFFFFFFFFFFFF:FFFFFFFFFFFFFFFFFFFFFFFFFFFFFF,FFFFFF:FFFFFFF
FFFFFFFFFFFFFFFFFFFFFFFFFFFFFFFFFFFFFFFFFFFFFFFF,FFFFFFFFFFFFFFFFFFFFF
FFFFFF:FFFFFFFF
@A00721:190:HGJW7DSXY:4:1223:27905:35853 2:N:0:TCTGCAAG+GCTGCACT
TACAAGACGGAGTTTGTCAGAGTTAATAATATTGTGGACGTTGGCAGTATCGTTTGTATAAATGATTG
```

```
TCCTACTGGAACCTCGGTCGATACGAGACATACCCAGAAAGTGAGATGGAATCGAAGTGTGCTTAACG
GTTCAGTTAAGTTGA
+
FFF:FFFFFF:FFFFFFFFFFFFFFFFFFFFFFFFFFFFFFFFFFFFF,FFFF:FFF:FFFFFFFFFFFF
FFFFFFFFF:FFFFFFFFFFFFFFFFFFFF:FFFFFFFF::FFFFFFFFFF,FFF,F,FFFFFFFFFFFF
F:FFFFF:FFFFFFF
@A00721:190:HGJW7DSXY:4:1662:13449:28197 2:N:0:TCTGCAAG+GCTGCACT
TACAAGACGGAGTTTGTCAGAGTTAATAATATTGTGGACGTTGGCAGTATCGTTTGTATAAATGATTG
TCCTACTGGAACCTCGGTCGATACGAGACATACCCAGAAAGTGAGATGGAATCGAATTGTGCTTAACG
GTTCAGTTAAGTTGA
+
FFFFFFFFFFFFFFFFFFFFFFFFFFFFFFFFFFFFFFFFFFFFFFFFFFFFFFFFFFFFFFFFFFFFFF
FFFFFFFFFFFFFFFFFFFFFFFFFFFFFFFFFFFFFFFFFFFFFFFFFFFFFFFFFFFFFFFFFFFFFF
FFFFFFFFFFFFFFF
@A00721:190:HGJW7DSXY:4:1665:29152:14747 2:N:0:TCTGCAAG+GCTGCACT
TACAAGACGGAGTTTGTCAGAGTTAATAATATTGTGGACGTTGGCAGTATCGTTTGTATAAATGATTG
TCCTACTGGAACCTCGGTCGATACGAGACATACCCAGAAAGTGAGATGGAATCGAATTGTGCTTAACG
GTTCAGTTAAGTTGA
+
FFFFFFFFFFF:FFFFFFFFFFFFFFFFFFFFFFFFFFFFFFFFFFFFFFFFFFFFFFFFFFFFFFFFFF
FFFFFFFFFFFFFFFFFFFFFFFFFFFFFFFFFFFFFFFFFFFFFFFFFFFFFFFFFFFFFFFFFFFFFF
FF:FFFFFFFFFFFF
@A00721:190:HGJW7DSXY:4:2627:14045:29606 2:N:0:TCTGCAAG+GCTGCACT
TACAAGACGGAGTTTGTCAGAGTTAATAATATTGTGGACGTTGGCAGTATCGTTTGTATAAATGATTG
TCCTACTGGAACCTCGGTCGATACGAGACATACCCAGAAAGTGAGATGGAATCGAATTGTGCTTAACG
GTTCAGTTAAGTTGA
+
FFFFFFFFFFFFFFFFFFFFFFFFFFFFFFFFFFFFFFFFFFFFFFFFFFFFFFFFFFFFFFFFFFFFFF
FFFFFFFFFFFFFFFFFFFFFFFFF:FFFFFFFFFFFFFFFFFFFFFFFFFF:FFFFFFFFFFFFFFFFF
FFFFFFFFFFFFFFF
@A00721:190:HGJW7DSXY:4:2675:28980:35493 2:N:0:TCTGCAAG+GCTGCACT
TACAAGACGGAGTTTGTCAGAGTTAATAATATTGTGGACGTTGGCAGTATCGTTTGTATAAATGATTG
TCCTACTGGAACCTCGGTCGATACGAGACATACCCAGAAAGTGAGATTGAATCGAATTGTGCTTAACG
GTTCTGTTAAGTTGA
+
F:FFF::,FFF,,FF:FFF:FF:F:FFFFFF,:,FFFFF:F:F:FFFF:F:FF,FFFFFFFFFFFF:F
,F,F,FF,FFFFFF,F,,F,F,F,FF,FFFFFF:FF:,:FFF:FFFF,FFFFF,,FF:FFF,F,FF,:
,:F,,,F:FF:FFF:
@A00721:190:HGJW7DSXY:4:2678:25265:34037 2:N:0:TCTGCAAG+GCTGCACT
TACAAGACGGAGTTTGTCAGAGTTAATAATATTGTGGACGTTGGCAGTATCGTTTGTATAAATGATTG
TCCTACTGGAACCTCGGTCGATACGAGACATACCCAGAAAGTGAGATGGAATCGAATTGTGCTTAACG
GTTCAGTTAAGTTGA
+
FFFFFF:FFFFFFFFFFFFFFFFFFFFFFFFFFFFFFFFFFFFFFFFFFFFFFFFFFFFFFFFFFFFFFF
FFFFFFFFFFFFFFFFFFFFFFFFFFF:FFFFFFFFFFFFFFFFFFFFFFFF:FFFFFFFFFFFFFFFFF
FFFFFFFFFFFFFFF
@A00721:190:HGJW7DSXY:4:1204:3414:8970 2:N:0:TCTGCAAG+GCTGCACT
ACAATACGGAGTTTGTCAGAGTTAATAATATTGTGGACGTTGGCAGAATCGTTTGTATAAAAGAATGT
CATACAGGAACCTCGGTCGAAAAGAGACATACCCATAAAGTTAGATGGAATCGAATTGTGATAAAAGC
ATCAGTTAAGATGA
+
FFFF,FF,,F,,F,,:FF,FF,FFF,FF,F,,FFFFFF:,F,,F:,,F,F,FF:,FF,FFF,:F,F,,
:,,F:,,,FFF,F,F,FFFF,F,FF,:,FF::F,,,FFF,:,FFF,F:FF:F,FF:FFFF,F,F:F:,
,FFF,F,FFF,FF:
```

```
@A00721:190:HGJW7DSXY:4:1375:31403:12132 2:N:0:TCTGCAAG+GCTGCACT
ACTTAACTGAACCGTTAAGCACAATTCGATTCCATCTCACTTTCTGGGTATGTCTCGTATCGACCGAG
GTTCCAGTAGGACAATCATTTATACAAACGATACTGCCAACGTCCACAATATTATTAACTCTGACAAA
CTCCGTCTTGT
+
FFFFFFFFFFFFFFFFFFFFFFFFFFFFFFFFF,FFFFFFFFFFFFFFFFFFFFFFFFFFFFFFFFFF
FFFFFFFFFFFFFFFFFFFFF:FFFF:FFFFFFFFFFFFFFFFFFFFFFFFFF:FFFFFFFFFFFFFF
FFFFFFFFFFF
@A00721:190:HGJW7DSXY:4:1557:25464:7106 2:N:0:TCTGCAAG+GCTGCACT
ACTTAACTGAACCGTTAAGCACAATTCGATTCCATCTCACTTTCTGGGTATGTCACGTATCGACCGAG
GTTCCAGTAGGACAATCATTTATACAAACGATACTGCCAACGTCCACAATATTATTAACTCTGACAAA
CTCCGTCTTGT
+
FFFFFFFFFFFFFFFFFFFFFFFFFFFFFFFFFFFFFFFFFFFFFFFFFFFFFF,FFFFFFFFFFF:F
FFFFFFFF:FFFF,FFFFFFFFFFFFFFFFFFFFFFFFFFFFFFFFFFFFFFFFFFFFFFFFFFFFFF
FFFFFFFFFFF
@A00721:190:HGJW7DSXY:4:1644:29948:7326 2:N:0:TCTGCAAG+GCTGCACT
ACTTAACTGAACCGTTAAGCACAATTCGATTCCATCTCACTTTCTGGGTATGTCTCGTATCGACCGAG
GTTCCAGTAGGACAATCATTTATACAAACGATACTGCCAACGTCCACAATATTATTAACTCTGACAAA
CTCCGTCTTGT
+
FFFFFFFFFFFFFFFFFFFFFFFFFFFFF,FFFFFFFFFFF:FFFFFFFFFFFFFFFFFFFFFFFFFF
FFFFFFFFFFFFFFFFFFFFFFFFFFF:F,FFFFFFFFFFFFFFFFFFF:FFFFFFFFFFFFFFFFFF
FFFFFFFFFFF
@A00721:190:HGJW7DSXY:4:2118:5629:13996 2:N:0:TCTGCAAG+GCTGCACT
ACTTAACTGAACCGTTAAGCACAATTCGATTCCATCTCACTTTCTGGGTATGTCTCGTATCGACCGAG
GTTCCAGTAGGACAATCATTTATACAAACGATACTGCCAACGTCCACAATATTATTAACTCTGACAAA
CTCCGTCTTGT
+
FFFFFFFFFFFFFFFFFFFFFFF,FFFFFFFFFF,FF:FFFF::FFFFF,FFFFFF,FFFFFFFFFFF
FFFF,FFFF,FFFFFFFF:,FFF:FFFF,FFFFFFF:FFFFFFFFFFF,:FFF,FF:F,FF,F:FFFF
FF:FFFFF,F:
@A00721:190:HGJW7DSXY:4:2121:11496:9815 2:N:0:TCTGCAAG+GCTGCACT
ACTTAACTGAACCGTTAAGCACAATTCGATTCCATCTCACTTTCTGGGTATGTCTCGTATCGACCGAG
GTTCCAGTAGGACAATCATTTATACAAACGATACTGCCAACGTCCACAATATTATTAACTCTGACAAA
CTCCGTCTTGT
+
FFFFFFFFFFFFFF:FFFFFFF:FFFFFFFFFFFFFFFFFFFFFFFFFFFFFFFFFFFFFFFFFFFFF
FFFFFFFF:FFFFFFFFFFFFFFFFFFFFFFFFFFFFFFFFFFFFFFFFFFFFFFFFFFFFFFFFFFF
FFFFFFFFFFF
@A00721:190:HGJW7DSXY:4:2635:16604:25081 2:N:0:TCTGCAAG+GCTGCACT
ACTTAACTGAACCGTTAAGCACAATTCGATTCCATCTCACTTTCTGGGTATGTCTCGTATCGACCGAG
GTTCCAGTAGGACAATCATTTATACAAACGATACTGCCAACGTCCACAATATTATTAACTCTGACAAA
CTCCGTCTTGT
+
FFFFFFFFFFFFFFFF:FFFFFFF,FFFFFFFFFFFFFFFFFFFFFFFF,FFFFFFFFFFFFFFFFFF
FFFFFFFFFFFFFFF:FFFFFFFFFFFFFFFFFFFFFFFFFFFFFFFFFFFFFFFFFFFFFFFFFFFF
FFFFFFFFFFF
@A00721:190:HGJW7DSXY:4:1125:20699:22185 2:N:0:TCTGCAAG+GCTGCACT
ACTTAACTGAACCGTTAAGCACAATTCGATTCCATCTCACTTTCTGGGTATGTCTCGTATCGACCGAG
GTTCCAGTAGGACAATCATTTATACAAACGATACTGCCAACGTCCACAATATTATTAACTCTGACAAA
CTCCGTCAGAT
+
FFFFFFFFFFFFFFFFFFFFFFFFFFFFFFFFFFFFFFFFFFFFFFFFFFFFFFFFFFFFFFFFFFFF
```

```
FFFFFFFFFFFFFFFFFFFFFFFFFFFFFFFFFFFFFFFFFFFFFFFFFF:FFFFFFFFFFFFFFFFFFF
FFFFFFFFFFF
@A00721:190:HGJW7DSXY:4:1126:15474:25160 2:N:0:TCTGCAAG+GCTGCACT
ACTTAACTGAACCGTTAAGCACAATTCGATTCCATCTCACTTTCTGGGTATGTCTCGTATCGACCGAG
GTTCCAGTAGGACAATCATTTATACAAACGATACTGCCAACGTCCACAATATTATTAACTCTGACAAA
CTCCGTCAGAT
+
FFFFFFFFFFFFFFFFFFFFFFFF:FFFF:FFFFFFFFFFFFFFFFFFFFFFFFFFFFFFFFFFFFFFFF
FFFFFFFFFFFFF:FFFFFFFFFFFFFFFFFFFFFFFFFFFF:FFFFFFFFFFFFFFFFFFFFFFFFFFF
FFFFFFFFFF:
@A00721:190:HGJW7DSXY:4:1355:6352:28056 2:N:0:TCTGCAAG+GCTGCACT
ACTTAACTGAACCGTTAAGCACAATTCGATTCCATCTCACTTTCTGGGTATGTCTCGTATCGACCGAG
GTTCCAGTAGGACAATCATTTATACAAACGATACTGCCAACGTCCACAATATTATTAACTCTGACAAA
CTCCGTCAGAT
+
FFFFFFFFFFFFFF:FFFFFFFFFFFFF:FFFFFF:FFFFFFFFFFFFFFFFFFFFFFFFFFFFFFFFFF
FFFFFF:FFFFFFFFFFFFFFFFFFFFFFFFFFFFFFFFFFF:FFFFFFFFFFFFFFFFFFFFFF:FFFF
FFFFFFFFF,F
@A00721:190:HGJW7DSXY:4:1510:12843:36104 2:N:0:TCTGCAAG+GCTGCACT
ACTTAACTGAACCGTTAAGCACAATTCGATTCCATCTCACTTTCTGGGTATGTCTCGTATCGACCGAG
GTTCCAGTAGGACAATCATTTATACAAACGATACTGCCAACGTCCACAATATTATTAACTCTGACAAA
CTCCGTCAGAT
+
FFFFFFFFFFFFFFFFFFFFFFFFFFFFFFFFFFFFFFFF:FFFFFFFFFFFFFFFFFFF:FFFFFFFFF
FFFFFFFFFFFFFFF::FFFFFFFFFFF:FFFFFFFFFFFFFFFFFFFFFFFFFFFFFF,FFFFFFFFFF
FFFFFFFFFFF
@A00721:190:HGJW7DSXY:4:2142:18159:22420 2:N:0:TCTGCAAG+GCTGCACT
ACTTAACTGAACCGTTAAGCACAATTCGATTCCATCTCACTTTCTGGGTATGTCTCGTATCGACCGAG
GTTCCAGTAGGACAATCATTTATACAAACGATACTGCCAACGTCCACAATATTATTAACTCTGACAAA
CTCCGTCAGAT
+
FFFFFFFFFFFFFFFFFFFFFFFFFFFFFFFFFFFFFFFFF,FFFFFFFFFFFFFFFFFFFFFFFFFFFF
FFFFFFFFFFFFFFFFFFFFFFFFFFFFFFFFFFFFFFFFFFFFFFFFFFFFFFFFFF:FFFFFFFFFFF
FFFFFFF:FFF
@A00721:190:HGJW7DSXY:4:2253:29713:5415 2:N:0:TCTGCAAG+GCTGCACT
ACTTAACTGAACCGTTAAGCACAATTCGATTCCATCTCACTTTCTGGGTATGTCTCGTATCGACCGAG
GTTCCAGTAGGACAATCATTTATACAAACGATACTGCCAACGTCCACAATATTATTAACTCTGACAAA
CTCCGTCAGAT
+
FFFFFFFFFFFFFFFFFFFFFFFFFFFFF:FFFFFFFF:FFFFFFFFFFFFFFFFFFFFFFFFFFFFFFF
FFFFFFFFFFFFFFFFFFF,FFFFFFFFFFFFFFFFFFFFFFFFFFFFFFF:F:FFFFFFFFFFF,FFFF
FFFFFFFFFFF
@A00721:190:HGJW7DSXY:4:2477:14877:9032 2:N:0:TCTGCAAG+GCTGCACT
ACTTAACTGAACCGTTAAGCACAATTCGATTCCATCTCACTTTCTGGGTATGTCTCGTATCGACCGAG
GTTCCAGTAGGACAATCATTTATACAAACGATACTGCCAACGTCCACAATATTATTAACTCTGACAAA
CTCCGTCAGAT
+
FFFFFFFFFFFFFFFFFFFFFFFFFFFFFFFFFFFFFFFFFFFFFFFFFFFFFFFFFFFFFFFFFFFFFF
FFFFFFFFFFFFFFFFFFFFFFFFFFFFFFFFFFFFFFFFFFFFF:FFFFFFFFFFFFFFFFFFFFFF:F
FFFFFFFFFFF
@A00721:190:HGJW7DSXY:4:2665:7473:31031 2:N:0:TCTGCAAG+GCTGCACT
ACTTAACTGAACCGTTAAGCACAATTCGATTCCATCTCACTTTCTGGGTATGTCTCGTATCGACCGAG
GTTCCAGTAGGACAATCATTTATACAAACGATACTGCCAACGTCCACAATATTATTAACTCTGACAAA
CTCCGTCAGAT
```

+
FFFFFFFFFFFFFFFFFFFFFFFFFFFFFFFFFFFFFFFFFFFFFFFFFFFFFFFFFFFFFFFFFFFFFF
FFFFFFFFFFFFFFFFFFFFFFFFFFFFFFFFFFFFFFFFFFFFFFFFFFFFFFFFFFFFFFFFFFFFFF
FFFFFFFFFFF
@A00721:190:HGJW7DSXY:4:1140:9688:20619 2:N:0:TCTGCAAG+GCTGCACT
GTTTGTCAGAGTTAATAATATTGTGGACGTTGGCAGTATCGTTTGTATAAATGATTGTCCTACTGGAA
CCTCGGTCGATACGAGACATACCCAGAAAGTGAGATGGAATCGAATTGTGCTTAACGGTTCAGTTAAG
TTGACTAGCGAAGCC
+
FFFFFFFFFFFFFFFFFFFFFFFFFFFFF:FFFFFFF:FFFFFFFFFFFFFFFFFFFFFFFFFFFFFFFF
FFFFFFFFFFFFFFFFFFFFFFFFFFFFFFFFFFFFFFFFFFFFFFFFFFFFFFFFFFFFFFFFFFFFFF
FFF:FFF,FF:FFFF
@A00721:190:HGJW7DSXY:4:1357:27299:21402 2:N:0:TCTGCAAG+GCTGCACT
GTTTGTCAGAGTTAATAATATTGTGGACGTTGGCAGTATCGTTTGTATAAATGATTGTCCTACTGGAA
CCTCGGTCGATACGAGACATACCCAGAAAGTGAGATGGAATCGAATTGTGCTTAACGGTTCAGTTAAG
TTGACTAGCGAAGCC
+
FFFFFFFFFFFFFFFFFFFFFFFFFFFFFFFFFFFFFFFFFFFFFFFFF:FFFFFFFFFFFFFFFFFFFF
FFFFFFFFFFFFFFFFFFFFFFFFFFFFFFFFFFFFFFF:FFFFFFFFFFFFFFFFFFFFFFFFFFFFFF
FFFFFFFFFFFFFFF
@A00721:190:HGJW7DSXY:4:1460:18050:4789 2:N:0:TCTGCAAG+GCTGCACT
GTTTGTCAGAGTTAATAATATTGTGGACGTTGGCAGTATCGTTTGTATAAATGATTGTCCTACTGGAA
CCTCGGTCGATACGAGACATACCCAGAAAGTGAGATGGAATCGAATTGTGCTTAACGGTTCAGTTAAG
TTGACTAGCGAAGCC
+
FFFFFFFFFFFFFFFF::FF:FFFFFFFF:FFFFFFFFFFFFFF:FFFFFFFFFFFFFFFFFFFFFFFFF
FFFFFFFFFFFFFFFF:FFFFFFF:FFFFFFFFFFFFFFFFFFF:FFFFFFFFFFFFFFFFFFFFFFFFF
FFFFFFFFF:FFFFF
@A00721:190:HGJW7DSXY:4:2215:18412:33536 2:N:0:TCTGCAAG+GCTGCACT
GTTTGTCAGAGTTAATAATATTGTGGACGTTGGCAGTATCGTTTGTATAAATGATTGTCCTACTGGAA
CCTCGGTCGATACGAGACATACCCAGAAAGTGAGATGGAATCGAATTGTGCTTAACGGTTCAGTTAAG
TTGACTAGCGAAGCC
+
FFFFFFFFFFFFFFFFFFFFFFFFFFFFFFFFFFFFFFFFFFFFFFFFFFFFFFFFFFFFFFFFFFFFFF
FFFFFFFFFFFFFFFFFFFFFFFFFFFFFFFFFFFFFFFFFFFFFFFFFFFFFFFFFFFFFFFFFFFFFF
FFFFFFF,F,FFFFF
@A00721:190:HGJW7DSXY:4:2249:15447:11459 2:N:0:TCTGCAAG+GCTGCACT
GTTTGTCAGAGTTAATAATATTGTGGACGTTGGCAGTATCGTTTGTATAAATGATTGTCCTACTGGAA
CCTCGGTCGATACGAGACATACCCAGAAAGTGAGATGGAATCGAATTGTGCTTAACGGTTCAGTTAAG
TTGACTAGCGAAGCC
+
F:FFFFFFFFFFFFFFF:FFFFFFFFFFFFFFFFFFFFFFFFFFFFFFFFFFFFFFFFFFFFFFFFFFFF
FFFFFFFFFFFFFFFFFFFFFF:FFFFFFFF:FFFFFFFFFFFFFFFFF,FFFFFFFFFFFFFFFFFFFF
FF:FFFF:FFFF::F
@A00721:190:HGJW7DSXY:4:1137:21775:33348 2:N:0:TCTGCAAG+GCTGCACT
TCAGAGTTAATAATATTGTGGACGTTGGCAGTATCGTTTGTATAAATGATTGTCCTACTGGAACCTCG
GTCGATACGAGACATACCCAGAAAGTGAGATGGAATCGAATTGTGCTTAACGGTTCAGTTAAGTTGAC
TAGCGAAGCCAATGT
+
FFFFFFFFFFFFFFFFFFFFFFFFFFFFFFFFFFFFFFFFFFFFFFFFFFFFFFFFFFFFFFFFFFFFFF
FFFFFFFFFFFFFFFFFFFFFFFFFFFFFFFFFFFFFFFFFFFFFFFFFFFFFFFFFFFFFFFFFFFFFF
FFFFFFFFFFFFFFF
@A00721:190:HGJW7DSXY:4:1413:24451:17503 2:N:0:TCTGCAAG+GCTGCACT
TCAGAGTTAATAATATTGTGGACGTTGGCAGTATCGTTTGTATAAATGATTGTCCTACTGGAACCTCG

```
GTCGATACGAGACATACCCAGAAAGTGAGATGGAATCGAATTGTGCTTAACGGTTCAGTTAAGTTGAC
TAGCGAAGCCAATGT
+
FFFFFFFFFFF:FFFFFFFFFFFFFFFFFFFFFFFFFFFFFFFFFFFFFFFFFFFFFFFFFFFFFFFF
FFFFFFFFFFFFFFFFFFFFFFFFFFFFFFFFFF,FFFFFFFFFFFFFFFFFFFF:FFFFFFFFFF
FFFFFF:FFFFFFFF
@A00721:190:HGJW7DSXY:4:1529:16179:20682 2:N:0:TCTGCAAG+GCTGCACT
TCAGAGTTAATAATATTGTGGACGTTGGCAGTATCGTTTGTATAAATGATTGTCCTACTGGAACCTCG
GTCGATACGAGACATACCCAGAAAGTGAGATGGAATCGAATTGTGCTTAACGGTTCAGTTAAGTTGAC
TAGCGAAGCCAATG
+
F:FFFF:FFF:FFFFF::,FFFFFFF,F:FF:F:,:,FFFFFFFFFF:F:FFF,:FFF:F:FFFFF:F
F,,FFFFFFFFFFFFFFFF:F,F,F:FFF:FFFFFFF,FF,FF:FF:FFF::FFFF,:FFFFFFFFF
FFF:FFF:FFFFFF
@A00721:190:HGJW7DSXY:4:1549:12536:22733 2:N:0:TCTGCAAG+GCTGCACT
ACATTGGCTTCGCTAGTCAACTTAACTGAACCGTTAAGCACAATTCGATTCCATCTCACTTTCTGGGT
ATGTCTCGTATCGACCGAGGTTCCAGTAGGACAATCATTTATACAAACGATACTGCCAACGTCCACAA
TATTATTAACTCTGA
+
FFFFFFFFFFFFFFFFFFFFFFFFFFFFFFFFFFFFFFFFFFFFFFFF:FFFFFFFF:FFFFFFFFFF
FFFFFFFFFFFFFFFFFFFFFFFFFFFFFFFFFFFFFFFFFF:FFFFFFFFFFFFFFFFFFFFFFFFFF
FFFFFFFFFFFFFFF
@A00721:190:HGJW7DSXY:4:2340:32859:18724 2:N:0:TCTGCAAG+GCTGCACT
TCAGAGTTAATAATATTGTGGACGTTGGCAGTATCGTTTGTATAAATGATTGTCCTACTGGAACCTCG
GTCGATACGAGACATACCCAGAAAGTGAGATGGAATCGAATTGTGCTTAACGGTTCAGTTAAGTTGAC
TAGAGAAGCCAATTT
+
:FF,F:FFFFFFFFFFF,F::FFFFFF,FFFFFF:::FF,:FFFFF:,F:::F,FFFF:,FFF:FF:F
FFFF:FFF,:FFFFFF::FF,FFF,FFF:FF,:FFF:,FF:FFF,,FFFF:::FFFF,FFFFFFFFF:
FF,,:FF::FFFF,F
@A00721:190:HGJW7DSXY:4:2403:1353:22623 2:N:0:TCTGCAAG+GCTGCACT
ACATTGGCTTCGCTAGTCAACTTAACTGAACCGTTAAGCACAATTCGATTCCATCTCACTTTCTGGGT
ATGTCTCGTATCGACCGAGGTTCCAGTAGGACAATCATTTATACAAACGATACTGCCAACGTCCACAA
TATTATTAACTCTGA
+
FFFFFFFFFFFFFFFFFFFFFFFFFFFFFFFFFFFFFFFFFFFFFFFFFFFFFFFFFFFFFFFFFFFF
FFFFFFFFFFFFFFFFFFFFFFFFFFFFFFFFFFFFFFFFFFFFFFFFFFFFFFFFFFFFFFFFFFFF
FFFFFFFFFFFFFFF
@A00721:190:HGJW7DSXY:4:2420:26106:29951 2:N:0:TCTGCAAG+GCTGCACT
ACATTGGCTTCGCTAGTCAACTTAACTGAACCGTTAAGCACAATTCGATTCCATCTCACTTTCTGGGT
ATGTCTCGTATCGACCGAGGTTCCAGTAGGACAATCATTTATACAAACGATACTGCCAACGTCCACAA
TATTATTAACTCTGA
+
FFFFFFFFFFFFFFFFFFFFFFFFFFFFFFFFFFFFFFFFFFFFFFFFFFFFFFFFFFFFFFFFFFFF
FFFFFFFFFFFFFFFFFFFFFFFFFFFFFFFFFFFFFFFFFFFFFFFFFFFFFFFFFFFFFFFFFFFF
FFFFFFFFFFFFFFF
@A00721:190:HGJW7DSXY:4:1355:12798:10661 2:N:0:TCTGCAAG+GCTGCACT
TATGAACATTGGCTTCGCTAGTCAACTTAACTGAACCGTTAAGCACAATTCGATTCCATCTCACTTTC
TGGGTATGTCTCGTATCGACCGAGGTTCCAGTAGGACAATCATTTATACAAACGATACTGCCAACGTC
CACAATATTATTAAC
+
FFFFFFFFFFFFFFFFFFFFFFFFFFFFFFFFFFFFFFF:FFFFFFFFFF:FFFFFFFFFFFFFFFFFF
FFFFFFFFFFFFFFF::FFFFFFFFFFFFFFFFFFFFFFFFFFFFFFFFFFF:FF:F:F:FFFFFF:F:F
FFFFFF:,:FFFF,F
```

```
@A00721:190:HGJW7DSXY:4:1514:32606:25676 2:N:0:TCTGCAAG+GCTGCACT
TATGAACATTGGCTTCGCTAGTCAACTTAACTGAACCGTTAAGCACAATTCGATTCCATCTCACTTTC
TGGGTATGTCTCGTATCGACCGAGGTTCCAGTAGGACAATCATTTATACAAACGATACTGCCAACGTC
CACAATATTATTAAC
+
FFFFFFFFFFFFFFFFFFFFFFFFFFFFFFFFFFFFFFFFFFFFFFFFFFFFFFFFFFFFFFFFFFFF
FFFFFFFFFFFFFFFFFFFFFFFFFFFFFF:FFFFFFFFFFFFFFFFFFFFFF,FFFFFFFFFFFFFF
FFF:F:FFFFFFF:F
@A00721:190:HGJW7DSXY:4:1573:1723:30686 2:N:0:TCTGCAAG+GCTGCACT
TATGAACATTGGCTTCGCTAGTCAACTTAACTGAACCGTTAAGCACAATTCGATTCCATCTCACTTTC
TGGGTATGTCTCGTATCGACCGAGGTTCCAGTAGGACAATCATTTATACAAACGATACTGCCAACGTC
CACAATATTATTAAC
+
F:FFFFFFF,FFFFFFFFFFFFFFFFFFFFFFFFFFFFFFFFFFFFFFFFFFFFFFFFFFFFFF,FFF
FFFFF:FFFFFFFFFFFFFFFFFFF:FFFFFFFFFFFFFFFFFFFFFFFFFFFFFFFFF:FFFFFFFF
FFF:,:FFFFFFFFF
@A00721:190:HGJW7DSXY:4:2157:4833:12399 2:N:0:TCTGCAAG+GCTGCACT
TATGAACATTGGCTTCGCTAGTCAACTTAACTGAACCGTTAAGCACAATTCGATTCCATCTCACTTTC
TGGGTATGTCTCGTATCGACCGAGGTTCCAGTAGGACAATCATTTATACAAACGATACTGCCAACGTC
CACAATATTATTAAC
+
FFFFFFFFFFFFFFFFFFFFFFFFFFFFFFFFFFFFFFFFFFFFFFFFFFFFFFFFFFFFFFFFFFFF
FFFFFFFFFFFFFFFFFFFFFFFFFFFFFFFFFFFFFFFFFFFFFFFFFFFFFFFFFFFF:FFFF::F
FFFFFFFFFFFFFFF
@A00721:190:HGJW7DSXY:4:2409:22896:27962 2:N:0:TCTGCAAG+GCTGCACT
TATGAACATTGGCTTCGCTAGTCAACTTAACTGAACCGTTAAGCACAATTCGATTCCATCTCACTTTC
TGGGTATGTCTCGTATCGACCGAGGTTCCAGTAGGACAATCATTTATACAAACGATACTGCCAACGTC
CACAATATTATTAAC
+
F,FFFFFFFFFFFFFFFFFFFFFFFFFFFFFFFFFFFFFFFFFFFFFFFFF:FFFFFFFFFFFFFFFF
FF:FFFFFF:FFFFFFFFFFFFFFFFFFFFFFFFFFFFFFFFF:FFFF:FFFF,FFFFFF:FFF,F:F
:FFFFFFF:FFFFFF
@A00721:190:HGJW7DSXY:4:2430:15185:17801 2:N:0:TCTGCAAG+GCTGCACT
TATGAACATTGGCTTCGCTAGTCAACTTAACTGAACCGTTAAGCACAATTCGATTCCATCTCACTTTC
TGGGTATGTCTCGTATCGACCGAGGTTCCAGTAGGACAATCATTTATACAAACGATACTGCCAACGTC
CACAATATTATTAAC
+
FFFFFFFFFFFFFFFFFFFFFFFFFFFFFFFFFFFFFFFFFFFFFFF:FFFFFFFFFFFFFFFFFFFF
FFFFFFFFFFFFFFFFFFFFFFFFFFFFF:FFFFFFFFFFFFFFFFFFFFFFFFFFFFFFFFFFFF:F
FFFF,FFFFFFFFFF
@A00721:190:HGJW7DSXY:4:2430:14850:18693 2:N:0:TCTGCAAG+GCTGCACT
TATGAACATTGGCTTCGCTAGTCAACTTAACTGAACCGTTAAGCACAATTCGATTCCATCTCACTTTC
TGGGTATGTCTCGTATCGACCGAGGTTCCAGTAGGACAATCATTTATACAAACGATACTGCCAACGTC
CACAATATTATTAAC
+
FFFFFFF:FFFFFFFFFFF:FFFFFFFFFFF:FFFFFFFFFFFFFFFFFFFF::FFFFFFF:FFFFFF
FFFFFF:FFFFFFFFFFFFFFFFFFF:FFFFFFFFFFFFFFFFFFFFFFFFFF:FFFF:FFFFFF:FF
FFFFFFFFF::FFFF
@A00721:190:HGJW7DSXY:4:1447:6189:34413 2:N:0:TCTGCAAG+GCTGCACT
CCACAGCCACTGCTTCATATTATGAACATTGGCTTCGCTAGTCAACTTAACTGAACCGTTAAGCACAA
TTCGATTCCATCTCACTTTCTGGGTATGTCTCGTATCGACCGAGGTTCCAGTAGGACAATCATTTATA
CAAACGATACTGCCA
+
FFFFFFFFFFFFFFFFFFFFFFFFFF:FFFFFFFFFFFFFFFFFFFFFFFFFFFFFFFFFFFFFFF,F
```

```
FFFFFFFFF:FFFFFFFFFFF:FFFFFFFFFF::FFFFFFFFFFFFFFFFFFFFFFFFFFFFFFFFFFF:
FFFFFF:FFF:FFFF
@A00721:190:HGJW7DSXY:4:1519:8983:35368 2:N:0:TCTGCAAG+GCTGCACT
CCACAGCCACTGCTTCATATTATGAACATTGGCTTCGCTAGTCAACTTAACTGAACCGTTAAGCACAA
TTCGATTCCATCTCACTTTCTGGGTATGTCTCGTATCGACCGAGGTTCCAGTAGGACAATCATTTATA
CAAACGATACTGCCA
+
FFFFFFFFFFFFFFFFFFFFFFFFFFFFFFFFFF:FFFFFFFFFFFFFFFFFFFFFFFFFFFFFFFFFFF
FFFFFFFFFFFFFFFFFFFFFFFFFFFFFFFFFFFFFFFFFFFFFFFFFF:FFFFFFFFFFFFFFFFFFF
FF:FFFFFFFFFFFF
@A00721:190:HGJW7DSXY:4:1608:18973:5447 2:N:0:TCTGCAAG+GCTGCACT
CCACAGCCACTGCTTCATATTATGAACATTGGCTTCGCTAGTCAACTTAACTGAACCGTTAAGCACAA
TTCGATTCCATCTCACTTTCTGGGTATGTCTCGTATCGACCGAGGTTCCAGTAGGACAATCATTTATA
CAAACGATACTGCCA
+
FFFFFFFFFFFFFFFFFFFFFFFFFFFFF:FFFFFFFFF::FFFFFFFFFFFFFFFFFFFFFFFFFFFFF
FFFFFFFFFFFFFFFFF::FFFFFFFFFFFFFFFFFFFFFFFFFFFFFFFFFF,FFFFFFFFFFFFFFFF
FFFFFFFFFFFF:F:
@A00721:190:HGJW7DSXY:4:1634:12726:19429 2:N:0:TCTGCAAG+GCTGCACT
CCACAGCCACTGCTTCATATTATGAACATTGGCTTCGCTAGTCAACTTAACTGAACCGTTAAGCACAA
TTCGATTCCATCTCACTTTCTGGGTATGTCTCGTATCGACCGAGGTTCCAGTAGGACAATCATTTATA
CAAACGATACTGCCA
+
FFFFFFFFFFFFFFFFFFFF::FFFFF,FFFFFFFFFFFFFFFFFFFFFFFFFFFFFFFFFFFFFFFF:F
FFFF,FFFF:FFFFFFFFFFFFFFFF,FFFFFFFF,FFFFFFFFFFFFFFFFFFFFFFFFFFFFFFFFFF
FFFFFFFFFFFFFF:
@A00721:190:HGJW7DSXY:4:2152:1172:10911 2:N:0:TCTGCAAG+GCTGCACT
CCACAGCCACTGCTTCATATTATGAACATTGGCTTCGCTAGTCAACTTAACTGAACCGTTAAGCACAA
TTCGATTCCATCTCACTTTCTGGGTATGTCTCGTATCGACCGAGGTTCCAGTAGGACAATCATTTATA
CAAACGATACTGCCA
+
FFFFFFFFFFFFFFFFFFFFFFFFFFFFFFFFFFFFFFF:FFFFFFFFFFFFFFFFFFFFFFFFFFFFFF
:FFFFFFFFFFFFFFFFFFFFFFFFFFFFFFFFFFFFFFFFF:FFF:FFFFFFF:FFFFFFFFFFFFFFF
:FF:FFFFFFFFFFF
@A00721:190:HGJW7DSXY:4:2557:4092:21355 2:N:0:TCTGCAAG+GCTGCACT
CCACAGCCACTGCTTCATATTATGAACATTGGCTTCGCTAGTCAACTTAACTGAACCGTTAAGCACAA
TTCGATTCCATCTCACTTTCTGGGTATGTCTCGTATCGACCGAGGTTCCAGTAGGACAATCATTTATA
CAAACGATACTGCCA
+
FFFFFFFFFFFFFFFFFFFFFFFFFFFFF:FFFFFFFFF:FFFFFFFFFFFFFFFFFFFFFFFFF:FFFF
FFFFFFFFFFFFFFFFFFFFFFFFFFFFFFFFFFFFFFFFFFFFFFFFFFFFFFFFFFFFFFFFFFFFFF
FFFFFFFFFFFFFFF
@A00721:190:HGJW7DSXY:4:2562:25211:33912 2:N:0:TCTGCAAG+GCTGCACT
CCACAGCCACTGCTTCATATTATGAACATTGGCTTCGCTAGTCAACTTAACTGAACCGTTAAGCACAA
TACGATTCCATCTCACTGTCTGGGTATGTCTCGTATCGACCGAGGTTCCAGTAGGACAATCATTTATA
CAAACGATACTGCCA
+
FFFFF:,FF:FFF:FFF,F,FF::FFFFF,,,,FFFFFF,FFFF:FFFFFFFFFFFFFFF,FFF:FFFFF
F,FFF,,FFFFFFFFFFF,,F,:F:F:F,FF:F:::FFFFFFFFFFFFFFFFFFF,FFFFF:FFF,FFFF
FF,F,:FFFFF,F,F
@A00721:190:HGJW7DSXY:4:2548:26368:10802 2:N:0:TCTGCAAG+GCTGCACT
CCACAGCCACTGCTTCATATTATGAACATTGGCTTCGCTAGTCAACTTAACTGAACCGTTAAGCACAA
TTCGATTCCATCTCACTTTCTGGGTATGTCTCGTATCGACCGAGGTTCCAGTAGGACAATCATTTATA
CAAACGATACTGCC
```

+
FFFFFFFFFFFFFFFFFFFFFFFFF,FFFFFFFFFFFFFFFFFFFFFFFFFFFFFFFFFFFFFFFFFF
FFFFFFFFFFFFFFFFFFFFFFFFFFFFFFFFFFFFFFFFFFFFFFFFFFFFFFFFFFFFFFFFFFFF
FFFFFFFFFFFF:F
@A00721:190:HGJW7DSXY:4:1163:32850:2049 2:N:0:TCTGCAAG+GCTGCACT
CACATCCACAGCCACTGCTTCATATTATGAACATTGGCTTCGCTAGTCAACTTAACTGAACCGTTAAG
CACAATTCGATTCCATCTCACTTTCTGGGTATGTCTCGTATCGACCGAGGTTCCAGTAGGACAATCAT
TTATACAAACGATAC
+
FFFFFFFFFFFFFFFFFFFFFFFFFFFFFFFFFFFFFFFFFFFFFFFFFFFFFFFFFFFFFFFFFFFF
FFFFFFFFFFFFFFFFFFFFFFFFFFFFFFFFFFFFFFFFFFFFFFFFFFFFFFFFFFFFFFFFFFFF
FFFFFFFFFFFFFF:
@A00721:190:HGJW7DSXY:4:1337:11650:28964 2:N:0:TCTGCAAG+GCTGCACT
CACATCCACAGCCACTGCTTCATATTATGAACATTGGCTTCGCTAGTCAACTTAACTGAACCGTTAAG
CACAATTCGATTCCATCTCACTTTCTGGGTATGTCTCGTATCGACCGAGGTTCCAGTAGGACAATCAT
TTATACAAACGATAC
+
FFFFFFFFFFFFFFFFFFFFFFFFFFFFFFFFFFFFFFFFFFFFFFFFFFFFFFFFFFFFFFFFFFFF
FFFFFFFFFFFFFFFFFFFFFFFFFFFFFFFFFFFFFFFFFFFFFFFFFFFFFFFFFFFFFFFFFFF:
FFFFFFFFFFFFFFF
@A00721:190:HGJW7DSXY:4:2152:32823:34225 2:N:0:TCTGCAAG+GCTGCACT
CACATCCACAGCCACTGCTTCATATTATGAACATTGGCTTCGCTAGTCAACTTAACTGAACCGTTAAG
CACAATTCGATTCCATCTCACTTTCTGGGTATGTCTCGTATCGACCGAGGTTCCAGTAGGACAATCAT
TTATACAAACGATAC
+
FFFFFFFFFFFFFFFFFFFFFFFFFFFFFFFFFFFFFFFFFFFFFFFFFFFFFFFFFFFFFFFFFFFF
FFFFFFFFFFFFFFFFFFFFFFFFFFFFFFFFFFFFFFFFFFFFFFFFFFFFFFFFFFFFFFFFFFFF
FFFFFFFF:FFFFF:
@A00721:190:HGJW7DSXY:4:2459:5204:17174 2:N:0:TCTGCAAG+GCTGCACT
CAAATCCACAGCCACTGCTTCATATTATGAACATTGGCTTCGCTAGTCAACTTAACTGAACCGTTAAG
CACAATTCGATTCCATCTCACTTTCTGGGTATGTCTCGTATCGACCGAGGTTCCAGTAGGACAATCAT
TTATACAAACGATAC
+
FF,FFFFFFFFFFFFFFFFFFFFFFFFFFFFFFFFFFFFFF,FFFFFFFFFFFFFFFFFFFFF:FFFF
FFFFF:FFFF::FFF:FF,FFFFFF:FFFF,FFFFFFFFFFFFFFFFFFFFFFFFFFFFFFFFF:FFF
:FFFFFFFF,FFFFF
@A00721:190:HGJW7DSXY:4:1357:5448:31219 2:N:0:TCTGCAAG+GCTGCACT
CGAGCCACATCCACAGCCACTGCTTCATATTATGAACATTGGCTTCGCTAGTCAACTTAACTGAACCG
TTAAGCACAATTCGATTCCATCTCACTTTCTGGGTATGTCTCGTATCGACCGAGGTTCCAGTAGGACA
ATCATTTATACAAAC
+
F::FFFFFFFFFFFFFFFFFFFFFFFFFFFFFFF,FF:FFFFFFFFFFFFFFFFFFFFFFFFFFFFFF
FFFFFFFFFFFFFFFFFFFFFFFFFFFFFFFFFFFFFFFFFFF:FFFFFFFFFFFFFFFFFFFFFFFF
FFFFFFFFFFFFFFF
@A00721:190:HGJW7DSXY:4:2109:28980:14575 2:N:0:TCTGCAAG+GCTGCACT
CGAGCCACATCCACAGCCACTGCTTCATATTATGAACATTGGCTTCGCTAGTCAACTTAACTGAACCG
TTAAGCACAATTCGATTCCATCTCACTTTCTGGGTATGTCTCGTATCGACCGAGGTTCCAGTAGGACA
ATCATTTATACAAAC
+
FFFFFFFFFFFFFFFFFFFFFFFFFFFFFFFFFFFFFFFFFFFFFFFFFFFFFFFFFFFFFFFFFFFF
FFFFFFFFFFFFFFFFFFFFFFFFFFFFFFFFFFFFFFFFFFFFFFFFFFFFFFFFFFFFFFFFFFFF
FFFFFFFFFFFFFFF
@A00721:190:HGJW7DSXY:4:2248:11207:34303 2:N:0:TCTGCAAG+GCTGCACT
CGAGCCACATCCACAGCCACTGCTTCATATTATGAACATTGGCTTCGCTAGTCAACTTAACTGAACCG

```
TTAAGCACAATTCGATTCCATCTCACTTTCTGGGTATGTCTCGTATCGACCGAGGTTCCAGTAGGACA
ATCATTTATACAAAC
+
FFFFFFFFFFFFFFFFFFFFFFFFFFFFFFFFFFFFFFFFFFFFFFFFFFFFFFFFFFFFFFFFFFFF
FFFFFFFF,FFFFFFFFFFFFFFFFFF:FFFF:FFFFFFFFFFFFFFFFFF:FFFFF:FFFF:FFFFF
FFFFFFFFFFFFFFF
@A00721:190:HGJW7DSXY:4:2650:11776:11209 2:N:0:TCTGCAAG+GCTGCACT
CGAGCCACATCCACAGCCACTGCTTCATATTATGAACATTGGCTTCGCTAGTCAACTTAACTGAACCG
TTAAGCACAATTCGATTCCATCTCACTTTCTGGGTATGTCTCGTATCGACCGAGGTTCCAGTAGGACA
ATCATTTATACAAAC
+
FFFFFFFFFFFFFFFFFFFFFFFFFFFFFFFFFFFFFF:FFFFFFFFFFFFFFFFFFFFFFFFFFFFF
FFFFFFFFF:FFFFFFFFFFFFFFFFFFFFFFFFFFFFFFFFFFFFFFFFFFFFFFFFFFFFFFFFFF
FFFFFFFFFFFFFFF
@A00721:190:HGJW7DSXY:4:1418:32145:21809 2:N:0:TCTGCAAG+GCTGCACT
TCATACACGAGCCACATCCACAGCCACTGCTTCATATTATGAACATTGGCTTCGCTAGTCAACTTAAC
TGAACCGTTAAGCACAATTCGATTCCATCTCACTTTCTGGGTATGTCTCGTATCGACCGAGGTTCCAG
TAGGACAATCATTTA
+
FFFFFFFFFFFFFFFFFFFFFFFFFFFFFFFFFFFFFFFFFFFFFFFFFFFFFFFFFFFFFFFFFF:F
FFFFFFFFFFFFFFFFFFFFFFFFFFFFFFFFFFF:FF:F:FF:FFFFFFFFFFFFFFFF:FFFF:FFF
FFFFFFFFFF:FFF:
@A00721:190:HGJW7DSXY:4:1505:18014:1877 2:N:0:TCTGCAAG+GCTGCACT
TCATACACGAGCCACATCCACAGCCACTGCTTCATATTATGAACATTGGCTTCGCTAGTCAACTTAAC
TGAACCGTTAAGCACAATTCGATTCCATCTCACTTTCTGGGTATGTCTCGTATCGACCGAGGTTCCAG
TAGGACAATCATTTA
+
FFFFFFFFFFFFFFFFFFFFFFFFFFFFFFFFFFFFFFFFFFFFFFFFFFFFFFFFFFFFFFFFFFFF
FFFFFFFFFFFFFFFFFFFFFFFFFFFFFFFFFFFFFFFFFFFFFFFFFFFFFFFFFFFFFFFFFFFF
FFFFFFFFFFFFFFF
@A00721:190:HGJW7DSXY:4:1571:2184:9345 2:N:0:TCTGCAAG+GCTGCACT
TCATACACGAGCCACATCCACAGCCACTGCTTCATATTATGAACATTGGCTTCGCTAGTCAACTTAAC
TGAACCGTTAAGCACAATTCGATTCCATCTCACTTTCTGGGTATGTCTCGTATCGACCGAGGTTCCAG
TAGGACAATCATTTA
+
F:FFFFFFFFFFFFFFFFFFFFFFFFFFFFFFFFFFFFFFFFFFFFFFFFFFFFFFFFFFFFFFFFFF
FFFFFFFFFFFFFFFFFFFFFFFFFFFFFFFFFFFFFFFFFFFFFFFFFFFFFFFFFFFFFFFFF:FF
FFFFFFFFFFF:FFF
@A00721:190:HGJW7DSXY:4:2432:23149:26897 2:N:0:TCTGCAAG+GCTGCACT
TCATACACGAGCCACATCCACAGCCACTGCTTCATATTATGAACATTGGCTTCGCTAGTCAACTTAAC
TGAACCGTTAAGCACAATTCGATTCCATCTCACTTTCTGGGTATGTCTCGTATCGACCGAGGTTCCAG
TAGGACAATCATTTA
+
FFFFFFFFFFFFFFFFFFFFFFFFFFFFFFFFFFFFFFFFFFFFFFFFFFFFFFFFFFFFFFFFFFFF
FFFFFFFFFFFFFFFFFFFFFFFFFFFFFFFFFFF:FFFFFFFFFFFFFFFFFFFFFFFFFFFFFFFF
FFFFFFF:FFFFFFF
@A00721:190:HGJW7DSXY:4:2447:28366:8375 2:N:0:TCTGCAAG+GCTGCACT
TCATACACGAGCCACATCCACAGCCACTGCTTCATATTATGAACATTGGCTTCGCTAGTCAACTTAAC
TGAACCGTTAAGCACAATTCGATTCCATCTCACTTTCTGGGTATGTCTCGTATCGACCGAGGTTCCAG
TAGGACAATCATTTA
+
FFFFFFFFFFFFFFFFFFFFFFFFFFFFFFFFFFFFFFFFFFFFFFFFFFFFFFFFFFFFFFFFFFFF
FFFFFFFFFFFFFFFFFFFFFFFFFFFFFFFFFFFFFFFFFFFFFFFFFFFFFFFFFFFFFFFFFFFF
FFFFFFFFFFFFFFF
```

```
@A00721:190:HGJW7DSXY:4:1337:4616:2754 2:N:0:TCTGCAAG+GCTGCACT
ATCATACACGAGCCACATCCACAGCCACTGCTTCATATTATGAACATTGGCTTCGCTAGTCAACTTAA
CTGAACCGTTAAGCACAATTCGATTCCATCTCACTTTCTGGGTATGTCTCGTATCGACCGAGGTTCCA
GTAGGACAATCATTT
+
FFFFFFFFFFFFFFFFFFFFFFFFFFFFFFFFFFFFFFFFFFFFFFFFFFFFFFFFFFFFFFFFFFFF
FFFFFFFFFFF:FFFF,FFFFF:FFFFFFFFFFFFFFFFFFFF,FFFFFFFFFFFFFFFFFFFFFFFF
FFFFFFFF,FFFFFF
@A00721:190:HGJW7DSXY:4:1548:21920:27899 2:N:0:TCTGCAAG+GCTGCACT
ATCATACACGAGCCACATCCACAGCCACTGCTTCATATTATGAACATTGGCTTCGCTAGTCAACTTAA
CTGAACCGTTAAGCACAATTCGATTCCATCTCACTTTCTGGGTATGTCTCGTATCGACCGAGGTTCCA
GTAGGACAATCATTT
+
FFFFFFFFFFFFFFFFFFFFFFFFFFFFFFFFFFFFFFFFFFFFFFFFFFFFFFFFFFFFFFFFF:FF
FFFFFFFF:FFFFFFFFFFFFFFFFFFFFFFFFFF:FFFFFFF:FFFFFFFFFFFFFFFFFF:FFFFF
F:FF:FFFFFFFF,F
@A00721:190:HGJW7DSXY:4:1612:7048:14450 2:N:0:TCTGCAAG+GCTGCACT
ATCATACACGAGCCACATCCACAGCCACTGCTTCATATTATGAACATTGGCTTCGCTAGTCAACTTAA
CTGAACCGTTAAGCACAATTCGATTCCATCTCACTTTCTGGGTATGTCTCGTATCGACCGAGGTTCCA
GTAGGACAATCATTT
+
FFFFFFFFFFFFFFFFFFFFFFFFFFFFFFFFFFFFFFFFFFFFFFFFFFFFFFFFFFFFFFFFFFFF
FFFFFFFF:FFFFFFFFFFFFFFFFFFFFFFFFFF,FFFFFFFFFFFFFFFFFFFFFFFF:FFFFFFF
FFFFFFFFFFFFFFF
@A00721:190:HGJW7DSXY:4:2109:32714:25770 2:N:0:TCTGCAAG+GCTGCACT
ATCATACACGAGCCACATCCACAGCCACTGCTTCATATTATGAACATTGGCTTCGCTAGTCAACTTAA
CTGAACCGTTAAGCACAATTCGATTCCATCTCACTTTCTGGGTATGTCTCGTATCGACCGAGGTTCCA
GTAGGACAATCATTT
+
:FFFFF:FFFFFFFFFFFF:FFFFFFFFFFFFFFFFFFFFFFFFFFFFFFFFFFFFFFFFFFFFFFFF
,:F,FFF:FFFF,FF:FFFF:::FFF:FFFF:F,FFF,FFFFFFF:FFF:,FFFFFF::FF:,FF:,F
:FFF::FFFFFF:FF
@A00721:190:HGJW7DSXY:4:2271:4227:34053 2:N:0:TCTGCAAG+GCTGCACT
ATCATACACGAGCCACATCCACAGCCACTGCTTCATATTATGAACATTGGCTTCGCTAGTCAACTTAA
CTGAACCGTTAAGCACAATTCGATTCCATCTCACTTTCTGGGTATGTCTCGTATCGACCGAGGTTCCA
GTAGGACAATCATTT
+
FFFFFFFFFFFFFFFFFFFFFFFFFFFFFFFFFFFFFFFFFFFFFFFFFFFFFFFFFFFFFFFFFFFF
FFFFFFFFFFFFFFFF:FFFFFFFFFFFFFFFFF:FFF:FFFF,FFFFFFF:FFFFFFFFFFFFFFFF
FFFFFFFFFFFF,FF
@A00721:190:HGJW7DSXY:4:2377:14091:20917 2:N:0:TCTGCAAG+GCTGCACT
ATCATACACGAGCCACATCCACAGCCACTGCTTCATATTATGAACATTGGCTTCGCTAGTCAACTTAA
CTGAACCGTTAAGCACAATTCGATTCCATCTCACTTTCTGGGTATGTCTCGTATCGACCGAGGTTCCA
GTAGGACAATCATTT
+
FFFFFFFFFFFFFFFFFFFFFFFFFFFFFFFFFFFFFFFFFFFFFFFFFFFFFFFFFFFFFFFFFFFF
FFFFFFFFFFFFFFFFFFFFFFFFFFFFFFFFFF:FFFFFFFFFFFFFFFFFFFFFFFFFFFFFFFFF
FFFFFFFFFFFFF,F
@A00721:190:HGJW7DSXY:4:2438:27380:6511 2:N:0:TCTGCAAG+GCTGCACT
ATCATACACGAGCCACATCCACAGCCACTGCTTCATATTATGAACATTGGCTTCGCTAGTCAACTTAA
CTGAACCGTTAAGCACAATTCGATTCCATCTCACTTTCTGGGTATGTCTCGTATCGACCGAGGTTCCA
GTAGGACAATCATTT
+
FFFFFFFFFFFFFFFFFFFFFFFFFFFFFFFFFFFFFFFFFFFFFFFFFFFFFFFFF:FFFFFFFFFF
```

FFFFFFFF:FFFFFFFFFFFFFFFFFFFFFFFFFF:FFFFFFFFFFFFFFFFFFFFFFFFFFFFFFFF
FFFFFFFFFFFFFFF
@A00721:190:HGJW7DSXY:4:2614:12979:1141 2:N:0:TCTGCAAG+GCTGCACT
ATCATACACGAGCCACATCCACAGCCACTGCTTCATATTATGAACATTGGCTTCGCTAGTCAACTTAA
CTGAACCGTTAAGCACAATTCGATTCCATCTCACTTTCTGGGTATGTCTCGTATCGACCGAGGTTCCA
GTAGGACAATCATTT
+
FFFFFFFFFFFFFFFFFFFFFFFFFF:FFFFFFFFFFFFFFFFFFFFFFFFFFFFFFFFFFFFFFFFF
FFF:FF:FFFFFFFFFFFFFFFF,FF::FFFF::FFFFFF:FF:FFFFFFFFFFFFFFF:FFFFFFFF
FFFFFFFF,FFFFFF
@A00721:190:HGJW7DSXY:4:1161:25744:3803 2:N:0:TCTGCAAG+GCTGCACT
CTATCATACACGAGCCACATCCACAGCCACTGCTTCATATTATGAACATTGGCTTCGCTAGTCAACTT
AACTGAACCGTTAAGCACAATTCGATTCCATCTCACTTTCTGGGTATGTCTCGTATCGACCGAGGTTC
CAGTAGGACAATCAT
+
F,FFFFFFFFFFFFFFFF:FFFFFFFFF:FFFF::FFFFFFFFFFFFFFFFFF:FFFFFFFFFFFFFF
FFFFFFFFFFFFFFFFFFF:,FFF:FFFFFFFFFFFFFFFFFF:FF:FFFFFFFFF:F:FF:F::FFF
:FFFFFFFFFFFF:F
@A00721:190:HGJW7DSXY:4:1166:17833:19445 2:N:0:TCTGCAAG+GCTGCACT
CTATCATACACGAGCCACATCCACAGCCACTGCTTCATATTATGAACATTGGCTTCGCTAGTCAACTT
AACTGAACCGTTAAGCACAATTCGATTCCATCTCACTTTCTGGGTATGTCTCGTATCGACCGAGGTTC
CAGTAGGACAATCAT
+
FFFFFFFFFFFFFFFFFFFFFFFFFFFFFFFFFFFFFFFFF:FFFFFF:FFFFFFFFFFFFFFFFFFF
FFFFFFFFFFFFFFFFFFFFFFFFFFFFFFFFFFFFFFFFFFFFFFFFFFFFFFFFFFFFFFFFFFFF
FFFFFFFFFFFFFFF
@A00721:190:HGJW7DSXY:4:1241:23583:18223 2:N:0:TCTGCAAG+GCTGCACT
CTATCATACACGAGCCACATCCACAGCCACTGCTTCATATTATGAACATTGGCTTCGCTAGTCAACTT
AACTGAACCGTTAAGCACAATTCGATTCCATCTCACTTTCTGGGTATGTCTCGTATCGACCGAGGTTC
CAGTAGGACAATCAT
+
FFFFFFFFFFFFFFFFFFFFFFFFFFFFFFFFFFFFFFFFFFFFFFFFFFFFFFFFFFFF:FFFFFFFF
FFFFFFFFFFFFFFFFFFFFFFFFFFFFFFFFFFFFFFFFFFFFFFFFFFFFFFFFFFFFFFFFFFFF
FFFF:FFFFFFFFFF
@A00721:190:HGJW7DSXY:4:1514:4589:7467 2:N:0:TCTGCAAG+GCTGCACT
CTATCATACACGAGCCACATCCACAGCCACTGCTTCATATTATGAACATTGGCTTCGCTAGTCAACTT
AACTGAACCGTTAAGCACAATTCGATTCCATCTCACTTTCTGGGTATGTCTCGTATCGACCGAGGTTC
CAGTAGGACAATCAT
+
FFFFFFFFFFFFFFFFFFFFFFFFFFFFFFFFFFFFFFFFFFFFFFFFFFFFFFFFFFFFFFFFFFFF
FFFFFFFFFFFFFFFFFFFFFFFFFFFFFFFFFFFFFFFFFFFFFFFFFFFFFFFFFFFFFFFFFFFF
FFFFFFFFFFFFFFF
@A00721:190:HGJW7DSXY:4:1578:3314:7357 2:N:0:TCTGCAAG+GCTGCACT
CTATCATACACGAGCCACATCCACAGCCACTGCTTCATATTATGAACATTGGCTTCGCTAGTCAACTT
AACTGAACCGTTAAGCACAATTCGATTCCATCTCACTTTCTGGGTATGTCTCGTATCGACCGAGGTTC
CAGTAGGACAATCAT
+
FFFFFFFFFFFFFFFFFFFFFF:F:FFFFFFFF:FFFFFFFFFF:FFFFFFFF:FFFFFFFFFFFFFF
FFFFFFFFFFFFFFFFFFF:FFFFFFFFFFFFFFFF,FFFFFFFFF:FFFFFFFFFFFFFFFFFFFFF
FFF:FFFFFFFFFFF
@A00721:190:HGJW7DSXY:4:1653:31675:5807 2:N:0:TCTGCAAG+GCTGCACT
CTATCATACACGAGCCACATCCACAGCCACTGCTTCATATTATGAACATTGGCTTCGCTAGTCAACTT
AACTGAACCGTTAAGCACAATTCGATTCCATCTCACTTTCTGGGTATGTCTCGTATCGACCGAGGTTC
CAGTAGGACAATCAT

+
FFFFFFFFFFFFFFFFFFFFFFFFFFFFFFFFFFFFFFFFFFFFFFFFFFFFFFFFFFFFFFFFFFFFFF
FFFFFFFFFFFFFFFFFFFFFFFFFFFFFFFFFFFFFFFFFFFFFFFFFFFFFF:FFFFFFFFFFFFFFF
FFFFFFFFFFFFFFF
@A00721:190:HGJW7DSXY:4:1673:29903:23563 2:N:0:TCTGCAAG+GCTGCACT
CTATCATACACGAGCCACATCCACAGCCACTGCTTCATATTATGAACATTGGCTTCGCTAGTCAACTT
AACTGAACCGTTAAGCACAATTCGATTCCATCTCACTTTCTGGGTATGTCTCGTATCGACCGAGGTTC
CAGTAGGACAATCAT
+
FFFFFFFFF:FFFFFFFFFFFFFFFFFFFFFFFFFFFFFFFFFFFFFFFFFFFFFFFFFFFFFFFFFFFF
FFFFFFFFFFFFFFFFFFFFFFFFFF::FFFF:FFFFFFFFFFFFFFFFFFFFFFFFFFFFFFFFFFFFF
FFFFFFFFFFFFFFF
@A00721:190:HGJW7DSXY:4:2204:17824:20369 2:N:0:TCTGCAAG+GCTGCACT
CTATCATACACGAGCCACATCCACAGCCACTGCTTCATATTATGAACATTGGCTTCGCTAGTCAACTT
AACTGAACCGTTAAGCACAATTCGATTCCATCTCACTTTCTGGGTATGTCTCGTATCGACCGAGGTTC
CAGTAGGACAATCAT
+
FFFFFFFFFFFFFFFFFFFFFFFFFFFFFFFFFFFFFFFFFFFFFFFFFFFFFFFFFFFFFFFFFFFFFF
FFFFFFFFFFFFFFFFFFFFFFFFFFFFFFFFFFFFFFFFFFFFFFFFFFFFFFFFFFF:FFFFFFFFFF
F:FF,FFFFF:FFFF
@A00721:190:HGJW7DSXY:4:2368:22553:4914 2:N:0:TCTGCAAG+GCTGCACT
CTATCATACACGAGCCACATCCACAGCCACTGCTTCATATTATGAACATTGGCTTCGCTAGTCAACTT
AACTGAACCGTTAAGCACAATTCGATTCCATCTCACTTTCTGGGTATGTCTCGTATCGACCGAGGTTC
CAGTAGGACAATCAT
+
FFFFFFFFF:FFFFFFFFFFFFFFFFFFFFFFFFFFFFFFFFFFFFFFFFFFFFFFFFFFFFFFFFFFFF
FFFFFFFFFFFFFFFFFFFFFFFFFFFFFFF:FFF:FFFFFFF:FFFFFFFFFF:FFFFFFFFFFFFFF:
FFFFF:FF:FFFFFF
@A00721:190:HGJW7DSXY:4:2378:15320:3098 2:N:0:TCTGCAAG+GCTGCACT
CTATCATACACGAGCCACATCCACAGCCACTGCTTCATATTATGAACATTGGCTTCGCTAGTCAACTT
AACTGAACCGTTAAGCACAATTCGATTCCATCTCACTTTCTGGGTATGTCTCGTATCGACCGAGGTTC
CAGTAGGACAATCAT
+
FFFFFFFFFFFFFFFFFFFFFFFFFFFFFFFFFFFFFFFFFFFFFFFFFFFFFFFFFFFFFFFFFFFFFF
FFFFFFFFFFFFFFFFFFFFFFFFFFFFFFFFFFFFFFFFFFFFFFFFFFFFFFFFFFFFFFFFFFFFFF
FFFFFFFFFFFFFFF
@A00721:190:HGJW7DSXY:4:2407:31955:35196 2:N:0:TCTGCAAG+GCTGCACT
CTATCATACACGAGCCACATCCACAGCCACTGCTTCATATTATGAACATTGGCTTCGCTAGTCAACTT
AACTGAACCGTTAAGCACAATTCGATTCCATCTCACTTTCTGGGTATGTCTCGTATCGACCGAGGTTC
CAGTAGGACAATCAT
+
FFFFFFFFFFFFFFFFFF:FFFF:F:FFFFFFFFFFFFFFFFFFFFFF:FFFFFFFFFFFFFFFFFFFFF
FFFFFF:FFFFFFFFFFFF:FFFFFFFFFF,FFFFFFFFFFFFFFFFFFFFFFFFFFFF,FF:FFFFFF,
FFFFFFFFFF:FFFF
@A00721:190:HGJW7DSXY:4:2509:14371:19053 2:N:0:TCTGCAAG+GCTGCACT
CTATCATACACGAGCCACATCCACAGCCACTGCTTCATATTATGAACATTGGCTTCGCTAGTCAACTT
AACTGAACCGTTAAGCACAATTCGATTCCATCTCACTTTCTGGGTATGTCTCGTATCGACCGAGGTTC
CAGTAGGACAATCAT
+
FFFFFFFFFFFFFFFFFFFFFFFFFFFFFFFFFFFFFF:FFFFFFFFFFFFFFFFFFFFFFFFFFFFFFF
FFFFFFFFFFFFFFFFFFFFFFFFFFFFFFFFFFFFFFFFFFFFFFFFFFFFFFFFFFFFFFFFFFFFFF
FFFFF:FFFF:FFFF
@A00721:190:HGJW7DSXY:4:2569:17996:35227 2:N:0:TCTGCAAG+GCTGCACT
CTATCATACACGAGCCACATCCACAGCCACTGCTTCATATTATGAACATTGGCTTCGCTAGTCAACTT

```
AACTGAACCGTTAAGCACAATTCGATTCCATCTCACTTTCTGGGTATGTCTCGTATCGACCGAGGTTC
CAGTAGGACAATCAT
+
FFFFFFFFFFFFFFFFFFFFFFFFFFFFFFFFFFFFFFFFFFFFFFFFFFFFFFFFFFFFFFFFFFFF
FFFFFFFFFFFFFFFFFFFFFFFFFFFF:FFFFFFFFFFFFFFFFFFFFFFFFFFFF,FFFFFFFFF
FFFFFFFFFFFFFFF
@A00721:190:HGJW7DSXY:4:1106:12897:18223 2:N:0:TCTGCAAG+GCTGCACT
TCTATCATACACGAGCCACATCCACAGCCACTGCTTCATATTATGAACATTGGCTTCGCTAGTCAACT
TAACTGAACCGTTAAGCACAATTCGATTCCATCTCACTTTCTGGGTATGTCTCGTATCGACCGAGGTT
CCAGTAGGACAATCA
+
FFFF:FFFFFFFFFFFFFFFFFFFFFFFFFFFFFFFFFFFFFFFFFFFFFFFFFFFFFFFFFFFFFFF
FFFFFFFFFFFFFFFFFFFFFFFFFFFFFFFFFFFFFFFFFFFFFFFFFFFFFFFFFFFFFFFFFFFF
FFFFFFFFFFFFFFF
@A00721:190:HGJW7DSXY:4:1456:16929:20102 2:N:0:TCTGCAAG+GCTGCACT
ATCATACACGAGCCACATCCACAGCCACTGCTTCATATTATGAACATTGGCTTCGCTAGTCAACTTAA
CTGAACCGTTAAGCACAATTCGATTCCATCTCACTTTCTGGGTATGTCTCGTATCGACCGAGGTTCCA
GTAGGACAATCA
+
FFFFFFFFFFF:FFFFF:FFFFF,FFFFFFFFFFFFFFFFFFFFFFFFFFFFFFFFF:F:FFFFFF:FF
FF,:FFFFFFFFF:F:F:FFFFFFFF:F:FFFFF,:FFFFFFF,FFFFFFFFF:FFFFFFFF:F:,FF
:,F:FFFFFFFF
@A00721:190:HGJW7DSXY:4:1564:21775:10394 2:N:0:TCTGCAAG+GCTGCACT
TCTATCATACACGAGCCACATCCACAGCCACTGCTTCATATTATGAACATTGGCTTCGCTAGTCAACT
TAACTGAACCGTTAAGCACAATTCGATTCCATCTCACTTTCTGGGTATGTCTCGTATCGACCGAGGTT
CCAGTAGGACAATCA
+
FFFFFFFFFFFFFFFFFFFFFFFFFFFFFFFFFFFFFFFFFFFFFFFFFFFFFFFFFFFFFFFFFFFF
FFFFFFFFFFFFFFFFFFFFFFFFFFFFFFFFFFFFFFFFFFFFFFFFFFFFFFFFFFFFFFFFFFFF
FFFFFFFFFFFFFFF
@A00721:190:HGJW7DSXY:4:2217:30572:10128 2:N:0:TCTGCAAG+GCTGCACT
TCTATCATACACGAGCCACATCCACAGCCACTGCTTCATATTATGAACATTGGCTTCGCTAGTCAACT
TAACTGAACCGTTAAGCACAATTCGATTCCATCTCACTTTCTGGGTATGTCTCGTATCGACCGAGGTT
CCAGTAGGACAATCA
+
FFFFFFFFFFFFFFFFFFFFFFF:FFFFFFFFFFFFFFFFFFFFFFFFFFFFFFFFFFFFFFFFFFFF
FFFF:FFFFFFFFFFFFFFFFFFF,FFFFFFFFFFFFFFFFFFFFFFFFFFFFFFFFFF:F:FFFFFFF
FFFFFFFFFFFFFFF
@A00721:190:HGJW7DSXY:4:2243:32334:31845 2:N:0:TCTGCAAG+GCTGCACT
CTATCATACACGAGCCACATCCACAGCCACTGCTTCATATTATGAACATTGGCTTCGCTAGTCAACTT
AACTGAACCGTTAAGCACAATTCGATTCCATCTCACTTTCTGGGTATGTCTCGTATCGACCGAGGTTC
CAGTAGGACAATCA
+
FFFFFFFFFFFFFFFFFFFFFFFFFFFFFFFFFFFFFFFFFFFFFFFFF:FFFFFFFFFFFFFFFFFF
FFFF:FFFFF,FFFFFF:FFF:FF:FFF:FF:F,FFFFF:FFFFF:FFFFFFFFFFFFFFF,FFFFFF
FFFFFFFFFF:FFF
@A00721:190:HGJW7DSXY:4:2301:11921:4852 2:N:0:TCTGCAAG+GCTGCACT
TCTATCATACACGAGCCACATCCACAGCCACTGCTTCATATTATGAACATTGGCTTCGCTAGTCAACT
TAACTGAACCGTTAAGCACAATTCGATTCCATCTCACTTTCTGGGTATGTCTCGTATCGACCGAGGTT
CCAGTAGGACAATCA
+
FFFFFFFFFFFFFFFFFFFFFFFFFFFFFFFFFFFFFFFFFFFFFFFFFFFFFFFFFFFFFFFFFFFF
FFFFFFFFFFFFFFFFFFFFFFFFFFFFFFFFFFFFFFFFFFFFFFFFFFFFFFFFFFFFF:FFFFFF
FFFFFFFFFFFFFFF
```

```
@A00721:190:HGJW7DSXY:4:2325:27299:6308 2:N:0:TCTGCAAG+GCTGCACT
TCTATCATACACGAGCCACATCCACAGCCACTGCTTCATATTATGAACATTGGCTTCGCTAGTCAACT
TAACTGAACCGTTAAGCACAATTCGATTCCATCTCACTTTCTGGGTATGTCTCGTATCGACCGAGGTT
CCAGTAGGACAATCA
+
FFFFFFFFFFFFFFFFFFFFFFFFFFFFFFFFFFFFFFFFFFFFFFFFFFFFFFFFFFFFFFFFFFFF
FFFFFFFFFFFFFFFFFFFFFFFFFFFFFF:FFFFFFFFFFFFFFFFFFFFFFFFFFFFFFFFFFFFF
FFFFFFFFFFF:FFF
@A00721:190:HGJW7DSXY:4:2603:28890:22686 2:N:0:TCTGCAAG+GCTGCACT
TCTATCATACACGAGCCACATCCACAGCCACTGCTTCATATTATGAACATTGGCTTCGCTAGTCAACT
TAACTGAACCGTTAAGCACAATTCGATTCCATCTCACTTTCTGGGTATGTCTCGTATCGACCGAGGTT
CCAGTAGGACAATCA
+
FFFFFFFFFFFFFFFFFFFFFFFFFFFFFFFFFFFFFFFFFFFFFFFFFFFFFFFFFFFFFFFFFFFF
FFFFFFFFFFFFFFFFFFFFFFFFFFFFFFFFFFFFFFFFFFFFFFFFFFFFFFFFFFFFFFFFFFFF
FFFFFFFFFFFFFF:
@A00721:190:HGJW7DSXY:4:1324:4878:27508 2:N:0:TCTGCAAG+GCTGCACT
TATCATACACGAGCCACATCCACAGCCACTGCTTCATATTATGAACATTGGCTTCGCTAGTCAACTTA
ACTGAACCGTTAAGCACAATTCGATTCCATCTCACTTTCTGGGTATGTCTCGTATCGACCGAGGTTCC
AGTAGGACAGATCGG
+
F:FFFFFFFFFFFFFFFFFFFFFFFFFFFFFFFFFFFFFFFFFFFFFFFFFFFFFFFFFFFFFFFFFF
FFFFFFFFFFFFFFFFFFFFFFFFFFFFFFFFFFFFFFFFFFFFFFFFFFFFFFFFFFFFFFFFFFF:
FFFF:FFFFFFFFFF
@A00721:190:HGJW7DSXY:4:1342:13485:29481 2:N:0:TCTGCAAG+GCTGCACT
TATCATACACGAGCCACATCCACAGCCACTGCTTCATATTATGAACATTGGCTTCGCTAGTCAACTTA
ACTGAACCGTTAAGCACAATTCGATTCCATCTCACTTTCTGGGTATGTCTCGTATCGACCGAGGTTCC
AGTAGGACAGATCGG
+
FFFFFFFFFFFFFFFFFFFFFFFFFFFFFFFFFFFFFFFFFFFFFFFFFFFFFFF:FFFFFFFFFFFF
FFFFFFFFFFFFFFFFFFFFFFFFFFFFFFFFFFFFFFFFFFFFFFFFFFFFFFFFFFFFFFFFFFFF
FFFFFFFF:FFFFFF
@A00721:190:HGJW7DSXY:4:1367:28565:27696 2:N:0:TCTGCAAG+GCTGCACT
TATCATACACGAGCCACATCCACAGCCACTGCTTCATATTATGAACATTGGCTTCGCTAGTCAACTTA
ACTGAACCGTTAAGCACAATTCGATTCCATCTCACTTTCTGGGTATGTCTCGTATCGACCGAGGTTCC
AGTAGGACAGATCGG
+
FFFFFFFFFFFFFFFFFFFFFFFFFFFFFFFFFFFFFFFFFFFFFFFFFFFFFFFFFFFFFFFFFFFF
FFFFFFFFFFFFFFFFFFFFFFFFFFFFFFFFFFFFFF:FFFFFFFFFF:FFFFFFFFFFFFFFFF:F
:FFFFFFFFFFFFFF
@A00721:190:HGJW7DSXY:4:1374:27624:8093 2:N:0:TCTGCAAG+GCTGCACT
TATCATACACGAGCCACATCCACAGCCACTGCTTCATATTATGAACATTGGCTTCGCTAGTCAACTTA
ACTGAACCGTTAAGCACAATTCGATTCCATCTCACTTTCTGGGTATGTCTCGTATCGACCGAGGTTCC
AGTAGGACAGATCGG
+
FFFFFFFFFFFFFFFFFFFFFFFFFFFFFFFFF:FFFFFFFFFFFFFFFFFFFFFFFFFFFFF:FFFF
FFFFFFFFFFFFFFF:FFFFFFFFFFFFFFFFFFFFFFFFFFFFFFFFFFFFFFFFFFFFFFFFFFFF
FF:FFFFFFFFFFFF
@A00721:190:HGJW7DSXY:4:1455:10691:34914 2:N:0:TCTGCAAG+GCTGCACT
TATCATACACGAGCCACATCCACAGCCACTGCTTCATATTATGAACATTGGCTTCGCTAGTCAACTTA
ACTGAACCGTTAAGCACAATTCGATTCCATCTCACTTTCTGGGTATGTCTCGTATCGACCGAGGTTCC
AGTAGGACAGATCGG
+
FFFFFFFFFFFFFFFFFFFFFFFFFFFFFFFFFFFFFFFFFFFFFFFFFFFFFFFFFFFFFFFFFFFF
```

FFFFFFFFFFFFFFFFFF:FFFF:FFFFFFFFFFFFFFF:FFFFFFFFFFFFFFFFFFFFFFFFFF:F
FF:FFFFFFFFFFFF
@A00721:190:HGJW7DSXY:4:2208:8648:2440 2:N:0:TCTGCAAG+GCTGCACT
TATCATACACGAGCCACATCCACAGCCACTGCTTCATATTATGAACATTGGCTTCGCTAGTCAACTTA
ACTGAACCGTTAAGCACAATTCGATTCCATCTCACTTTCTGGGTATGTCTCGTATCGACCGAGGTTCC
AGTAGGACAGATCGG
+
FFFFFFFFFFFFFFFFFFFFFFFFFFFFFFFFFFFFFFFFFFFFFFFFFFFFFFFFFFFFFFFFF:FF
FFFFFFFFF:FFFFFFFFFFFFFFFFFFFFFFFFFFFFFFFFFFFFFFFFF:,FFFFFFFFFFFFFFF
FFF:FFFF,FFFF:F
@A00721:190:HGJW7DSXY:4:2247:10420:35759 2:N:0:TCTGCAAG+GCTGCACT
TATCATACACGAGCCACATCCACAGCCACTGCTTCATATTATGAACATTGGCTTCGCTAGTCAACTTA
ACTGAACCGTTAAGCACAATTCGATTCCATCTCACTTTCTGGGTATGTCTCGTATCGACCGAGGTTCC
AGTAGGACAGATCGG
+
FFFFFF:FFFFFFFFFFFFF:FFFFFFFFFFFFF:FFFFFFF:FFFF,FFFFFFFFFFFFFFFFFFFF
FF,FFF:FFFFFF:FFFFFFFF:FFFFFFFFFFFF:FFFFFF,FFFFFFFFFFFF:FFFFFFFFFFFF
FFFFFFFF:FFFFFF
@A00721:190:HGJW7DSXY:4:2557:2184:7811 2:N:0:TCTGCAAG+GCTGCACT
TATCATACACGAGCCACATCCACAGCCACTGCTTCATATTATGAACATTGGCTTCGCTAGTCAACTTA
ACTGAACCGTTAAGCACAATTCGATTCCATCTCACTTTCTGGGTATGTCTCGTATCGACCGAGGTTCC
AGTAGGACAGATCGG
+
FFFFFFFFFFFFFFFFFFFFFFFFFFFFFFFFFFFF:FFFFFFFFFFFFFFFFFFFFFFFFFFFFFFF
FFFFFFFFF:FFFFFFFFFF:FFFFFFFFFFFFFFFFFFFFFFFFFFFFFFFFFFFFFFFFFFFFFFF
FFFFFFFFFFFFFFF
@A00721:190:HGJW7DSXY:4:2372:18304:36542 2:N:0:TCTGCAAG+GCTGCACT
GTCTATCATACACGAGCCACATCCACAGCCACTGCTTCATATTATGAACATTGGCTTCGCTAGTCAAC
TTAACTGAACCGTTAAGCACAATTCGATTCCATCTCACTTTCTGGGTATGTCTCGTATCGACCGAGGT
TCCAGTAGGACAATC
+
:FFFFFFFFFFFFFFFFFFFFFFFFFFFFFFFFFFFFFFFFFFFFFFFFFFFFFFFFFFFFFFFFFFF
FFFFFFFFFFFFFFFFFFFFFFFFFFFFFFFFFFFFFFFFFFFFFFFFFFFFFFFFFFFFFFFFFFFF
FFFFFFFFFFFFFFF
@A00721:190:HGJW7DSXY:4:2372:18222:36558 2:N:0:TCTGCAAG+GCTGCACT
GTCTATCATACACGAGCCACATCCACAGCCACTGCTTCATATTATGAACATTGGCTTCGCTAGTCAAC
TTAACTGAACCGTTAAGCACAATTCGATTCCATCTCACTTTCTGGGTATGTCTCGTATCGACCGAGGT
TCCAGTAGGACAATC
+
FFFFFFFFFFFFFFFFFFFFFFFFFFFFFFFFFFFFFFFFFFFFFFFFFFFFFFFFFFFFFFFFF:FF
FFFFFFFFFFFFFFFFFFFFFFFFFFFFFFFFFFFFFFF:FFFFFFFFFFFFFFFFFFFFFFFFFFFF
FFFFFFFFFFF::FF
@A00721:190:HGJW7DSXY:4:1336:5575:34287 2:N:0:TCTGCAAG+GCTGCACT
ACCGGGACGTCTATCATACACGAGCCACATCCACAGCCACTGCTTCATATTATGAACATTGGCTTCGC
TAGTCAACTTAACTGAACCGTTAAGCACAATTCGATTCCATCTCACTTTCTGGGTATGTCTCGTATCG
ACCGAGGTTCCAGTA
+
FFFFFFFFFFFFFFFFFFFFFFFFFFFFFFFFFFFFFFFFFFFFFFFFFFFFFFFFFFFFFFFFFFFF
FFFFFFFFFFFFFFFFFFFFFFFFFFFFFFFFFFFFFFFFFFFFFFFFFFFFFFFFFFFFFFFFFFFF
FFFFFFFFFFFFFFF
@A00721:190:HGJW7DSXY:4:1625:5213:31344 2:N:0:TCTGCAAG+GCTGCACT
ACCGGGACGTCTATCATACACGAGCCACATCCACAGCCACTGCTTCATATTATGAACATTGGCTTCGC
TAGTCAACTTAACTGAACCGTTAAGCACAATTCGATTCCATCTCACTTTCTGGGTATGTCTCGTATCG
ACCGAGGTTCCAGTA

```
+
FFFFFFFFFFFFFFFFFFFFFFFFFFFFFFFFFFFFFFFFFFFFFFFFFFFFFFFFFFFFFFFFFFFFFFF
FFFFFFFFFFFFFFFFFFFFF:FFFFFFFFFFFFFFFFFFFFFFFFFFFFFFFFFFFFFFFFFFFFFFFFF
FFFFFFFFFFFFFFF
@A00721:190:HGJW7DSXY:4:2102:12888:7467 2:N:0:TCTGCAAG+GCTGCACT
ACCGGGACGTCTATCATACACGAGCCACATCCACAGCCACTGCTTCATATTATGAACATTGGCTTCGC
TAGTCAACTTAACTGAACCGTTAAGCACAATTCGATTCCATCTCACTTTCTGGGTATGTCTCGTATCG
ACCGAGGTTCCAGTA
+
FFFFFFFFFFFFFF:FFFFFFFFFFFFFFFFFFFFFFFFFFFFFFFFFFFFFFFFFFFFFFFFFFFFFFFF
F:FFFFFFFFFFFFFFFFFFFFFFFFFFFFFFFFFFFFFFFFFFFFFFFFFFFFFFFFFFFFFFFFFFFFF
FFFFFFFFFFFFFFF
@A00721:190:HGJW7DSXY:4:2108:10565:9674 2:N:0:TCTGCAAG+GCTGCACT
ACCGGGACGTCTATCATACACGAGCCACATCCACAGCCACTGCTTCATATTATGAACATTGGCTTCGC
TAGTCAACTTAACTGAACCGTTAAGCACAATTCGATTCCATCTCACTTTCTGGGTATGTCTCGTATCG
ACCGAGGTTCCAGTA
+
FFFFFFFFFFFFFFFFFFFFFFFFFFFFFFFFFFFFFFFFFFFFFFFFFFFFFFFFFFFF:FFFFFFFFFF
FFFFFFFFFFFFFFFFFFFFFFFFFFFFFFFFFFFFFFFFFFFFFFFFFFFFFFFFFFFFFFF:FFFFFFF
FFFFFFFFFFFFFFF
@A00721:190:HGJW7DSXY:4:2531:19768:35102 2:N:0:TCTGCAAG+GCTGCACT
ACCGGGACGTCTATCATACACGAGCCACATCCACAGCCACTGCTTCATATTATGAACATTGGCTTCGC
TAGTCAACTTAACTGAACCGTTAAGCACAATTCGATTCCATCTCACTTTCTGGGTATGTCTCGTATCG
ACCGAGGTTCCAGTA
+
FFFFFFFFFFFFFFFFFFFFFFFFFFFFFFFFFFFFFFFFFFFFFFFFFFFFFFFFFFFFFFFFFFFFFFF
FFFFFFFFFFF:FFFFFFFFFFFFFFFFFFFFFFFFFFFFFFFFFFF:FFF::FFFFFFFFFFFFFFFFFF
FFFFFFFFFFFFFF:
@A00721:190:HGJW7DSXY:4:2625:6090:26506 2:N:0:TCTGCAAG+GCTGCACT
ACCGGGACGTCTATCATACACGAGCCACATCCACAGCCACTGCTTCATATTATGAACATTGGCTTCGC
TAGTCAACTTAACTGAACCGTTAAGCACAATTCGATTCCATCTCACTTTCTGGGTATGTCTCGTATCG
ACCGAGGTTCCAGTA
+
FFFFFFFFFFFFFFFFFFFFFFFFFFFFFFFFFFFFFFFFFFFFFFFFFFFFFFFFFFFFFFFFFFFFFFF
FFFFFFFFFFFFFFFFFFFFFFFFFFFFFFFFFFFFFFFFFFFFFFFFFFFFFFFFFFFFFFFFFFFFFFF
FFFFFFFFFFFFFFF
@A00721:190:HGJW7DSXY:4:1273:6361:29356 2:N:0:TCTGCAAG+GCTGCACT
TACCGGGACGTCTATCATACACGAGCCACATCCACAGCCACTGCTTCATATTATGAACATTGGCTTCG
CTAGTCAACTTAACTGAACCGTTAAGCACAATTCGATTCCATCTCACTTTCTGGGTATGTCTCGTATC
GACCGAGGTTCCAGT
+
FFFFFFFFFFFFFFFFFFFFFFFFFFFFFFFFFFFFFFFFFFFFFFFFFFFFFFFFFFFFFFFFFFFFFFF
FFFFFFFFFFFFFFFFFFFFFFFFFFFFFFFFFFFFFFFFFFFFFFFFFFFFFFFFFFFFFFFFFFFFFFF
FFFFFFFFFFFFFFF
@A00721:190:HGJW7DSXY:4:1303:7491:30843 2:N:0:TCTGCAAG+GCTGCACT
AATTACCGGGACGTCTATCATACACGAGCCACATCCACAGCCACTGCTTCATATTATGAACATTGGCT
TCGCTAGTCAACTTAACTGAACCGTTAAGCACAATTCGATTCCATCTCACTTTCTGGGTATGTCTCGT
ATCGACCGAGGTTCC
+
FFFFFFFFFFFFFFFFFFFFFFFFFFFFFFFFFFFFFFFFFFFFFFFFFFFFFFFFFFFFFFFFFFFFFFF
FFFFFFFFFFFFFFFFFFFFFFFFFFFFFFFFFFFFFFFFFFFFFFFFFFFFFFFFFFFFFFFFFFFFFFF
:FFFFFFFFFFFFFF
@A00721:190:HGJW7DSXY:4:1346:23628:3521 2:N:0:TCTGCAAG+GCTGCACT
AATTACCGGGACGTCTATCATACACGAGCCACATCCACAGCCACTGCTTCATATTATGAACATTGGCT
```

```
TCGCTAGTCAACTTAACTGAACCGTTAAGCACAATTCGATTCCATCTCACTTTCTGGGTATGTCTCGT
ATCGACCGAGGTTCC
+
FFFFFFFFFFFFFFFFFFFFFFFFFFFFFFFFFFFFFFFFFFFFFFFFFFFFFFFFFFFFFFFFFFFF
FFFFF:FFFFFFFFFFFFFFFFFFFFFFFFFFFFFFFFFFFFFFFFFFF:FFFFFFFFFFFFFFFF
FFFFFFFF:FFFFFF
@A00721:190:HGJW7DSXY:4:1404:20401:34914 2:N:0:TCTGCAAG+GCTGCACT
AATTACCGGGACGTCTATCATACACGAGCCACATCCACAGCCACTGCTTCATATTATGAACATTGGCT
TCGCTAGTCAACTTAACTGAACCGTTAAGCACAATTCGATTCCATCTCACTTTCTGGGTATGTCTCGT
ATCGACCGAGGTTCC
+
FFFFFFFFFFFFFFFFFFFFFFFFFFFFFFFFFFFFFFFFFFFFFFFFFFFFFFFFFFFFFFFFFFFF
FFFFFFFFF:FFFFFFFF:FFFFFFFFFFFFFFFFFF:FFFF:FFFFFFFFFFFFFFFF,FFFFFFF,
FFFFFFFFFFFFFFF
@A00721:190:HGJW7DSXY:4:1554:19425:13526 2:N:0:TCTGCAAG+GCTGCACT
AATTACCGGGACGTCTATCATACACGAGCCACATCCACAGCCACTGCTTCATATTATGAACATTGGCT
TCGCTAGTCAACTTAACTGAACCGTTAAGCACAATTCGATTCCATCTCACTTTCTGGGTATGTCTCGT
ATCGACCGAGGTTCC
+
FFFFFFFFFFFFFFFFFFFFFFFFFFFFFFFFFFFFFFFFFFFFFFFFFFFFFFFFFFFFFFFFFFFF
FFFFFFFFFFFFFFFFFFFFFFFFFFFFFFFFFFFFFFFFFFFFFFFF:FF,FFFFFFFF,FFFFFF:F
FFFFFFFFFFFFFFF
@A00721:190:HGJW7DSXY:4:2122:13937:36808 2:N:0:TCTGCAAG+GCTGCACT
AATTACCGGGACGTCTATCATACACGAGCCACATCCACAGCCACTGCTTCATATTATGAACATTGGCT
TCGCTAGTCAACTTAACTGAACCGTTAAGCACAATTCGATTCCATCTCACTTTCTGGGTATGTCTCGT
ATCGACCGAGGTTCC
+
FFFFFFFFFFFFFFFFFFFFFFFFFFFFFFFFFFFFFFFFFFFFFFFFFFFFFFFFFFFFFFFFFFFF
FFFFFFFFFFFFFFFFFFFFFFFFFFFFFFFFFFFFFFFFFFF::FFFFFFFFFFFFFFFFFFFFFFFFF
FFFFFFFFFFFFFF:
@A00721:190:HGJW7DSXY:4:2337:14253:29121 2:N:0:TCTGCAAG+GCTGCACT
AATTACCGGGACGTCTATCATACACGAGCCACATCCACAGCCACTGCTTCATATTATGAACATTGGCT
TCGCTAGTCAACTTAACTGAACCGTTAAGCACAATTCGATTCCATCTCACTTTCTGGGTATGTCTCGT
ATCGACCGAGGTTCC
+
FFFFFFFFFFFFFFFFFFFFFFFFFFFFFFFFFFFFFFFFFFFFFFFFFFFFFFFFFFFFFFFFFFFF
FFFFFFFFFFFFF:FFF:FF:FFFFFFFFFFFFFFFFFFFFFFFFFFFFFFFFFFFFF:FFFF:FFFFFF
FFFFFFFFFFFFFFF
@A00721:190:HGJW7DSXY:4:1328:32199:18364 2:N:0:TCTGCAAG+GCTGCACT
GAATTACCGGGACGTCTATCATACACGAGCCACATCCACAGCCACTGCTTCATATTATGAACATTGGC
TTCGCTAGTCAACTTAACTGAACCGTTAAGCACAATTCGATTCCATCTCACTTTCTGGGTATGTCTCG
TATCGACCGAGGTTC
+
FFFFFFFFFFFFFFFFFFFFFFFFFFFFFFFFFFFFFFFFFFFFFFFFFFFFFFFFFFFFFFFFFFFF
FFFFFFFFFFFFFFFF:FFFFFFFFFFFFFFFFFFFFFFFFFFFFFFFFFF:FFFFFFFFFFFFFFFFF
FFFFFFFFFFFFFFF
@A00721:190:HGJW7DSXY:4:1601:14407:7122 2:N:0:TCTGCAAG+GCTGCACT
GAATTACCGGGACGTCTATCATACACGAGCCACATCCACAGCCACTGCTTCATATTATGAACATTGGC
TTCGCTAGTCAACTTAACTGAACCGTTAAGCACAATTCGATTCCATCTCACTTTCTGGGTATGTCTCG
TATCGACCGAGGTTC
+
FFFFFFFFFFFFFFFFFF:FFFFFFFFFFFFFFFFFFFFFFFFFFFFFFFFFFFFFFFFFFFFFFFFFF
FFFFFFFFFFFFFFFFFFFFFFFFFFFFFFFFFFFFFFFFFFFFFFFFFFFFFFFFFFFFFFFFFFFF
FFFFFFFFFFFFFFF
```

```
@A00721:190:HGJW7DSXY:4:1614:27317:18208 2:N:0:TCTGCAAG+GCTGCACT
GAATTACCGGGACGTCTATCATACACGAGCCACATCCACAGCCACTGCTTCATATTATGAACATTGGC
TTCGCTAGTCAACTTAACTGAACCGTTAAGCACAATTCGATTCCATCTCACTTTCTGGGTATGTCTCG
TATCGACCGAGGTTC
+
FF:FFFFFFFFFFFFFFFFFFFFFFFFFFFFFFFFFFFFFFFFFFFFFFFFFFFF:FFFFFFFFFFFF
FFFFFF:FFF:FFFFFFFFFFFFFFFFFFFFFFFFFFFFFFFFFFFFFFFFFFFFFFFFFFFFFFFFF
FFFFFFFFFFFFFFF
@A00721:190:HGJW7DSXY:4:2152:5231:36949 2:N:0:TCTGCAAG+GCTGCACT
GAATTACCGGGACGTCTATCATACACGAGCCACATCCACAGCCACTGCTTCATATTATGAACATTGGC
TTCGCTAGTCAACTTAACTGAACCGTTAAGCACAATTCGATTCCATCTCACTTTCTGGGTATGTCTCG
TATCGACCGAGGTTC
+
FFFFFFFFFFFFFFFFFFFFFFFFFFFFFFFFFFFFFFFFFFFFFFFFFFFFFFFFFFFFFFFFFFFF
FFFFFFFFFFFFFFFFFFFFFFFFFFFFFFFFFFFFFFFFFFFFFFFFFFFFFFFFFFFFFFFFFFFF
FFFFFFFFFFFFFFF
@A00721:190:HGJW7DSXY:4:2417:3956:32612 2:N:0:TCTGCAAG+GCTGCACT
GAATTACCGGGACGTCTATCATACACGAGCCACATCCACAGCCACTGCTTCATATTATGAACATTGGC
TTCGCTAGTCAACTTAACTGAACCGTTAAGCACAATTCGATTCCATCTCACTTTCTGGGTATGTCTCG
TATCGACCGAGGTTC
+
F:FFFFFFFFFFFFFFFFFFFFFFFFFFFFFFFFFFFFFFFFFFFFFFFFFFFFFFFFFFFFFFFFFF
FFFFFFFFFFFFFFFFFFFFFFFFFFFFFFFFFFFFFFFFFFFFFFFFFFFFFFFFFFFFFFFFFFFF
FFFFFFFFFFFFFFF
@A00721:190:HGJW7DSXY:4:2577:12409:26428 2:N:0:TCTGCAAG+GCTGCACT
GAATTACCGGGACGTCTATCATACACGAGCCACATCCACAGCCACTGCTTCATATTATGAACATTGGC
TTCGCTAGTCAACTTAACTGAACCGTTAAGCACAATTCGATTCCATCTCACTTTCTGGGTATGTCTCG
TATCGACCGAGGTTC
+
FFFFFFFFFFFFFFFFFFFFFFFFFFFFFFFFFFFFFFFFFFFFFFFFFFFFFFFFFFFFFFFFFFFF
FFFFFFFFFFFFFFFFFFFFFFFFFFFFFFFFFFFFFFFFFFFFFFFFFFFFFFFFFFFFFFFFFFFF
FFFFFFFFFFFFFFF
@A00721:190:HGJW7DSXY:4:2614:28465:10582 2:N:0:TCTGCAAG+GCTGCACT
GAATTACCGGGACGTCTATCATACACGAGCCACATCCACAGCCACTGCTTCATATTATGAACATTGGC
TTCGCTAGTCAACTTAACTGAACCGTTAAGCACAATTCGATTCCATCTCACTTTCTGGGTCTGTCTCG
TATCGACCGATGTTC
+
FF::FF::F:FFF:FF,FFFFFFF::FF,FFFFFFFFFFFF:FFF:FFFFFFFFFFFFFFF:F:F::F
FFFFF:FFF:FFF:FF,FFFFFFFF:,FFF:FFFF:FF:FF:F::,FF,FF::FF,,FFF,FFFFFF:
F,FF:,FFFF,FFF:
@A00721:190:HGJW7DSXY:4:2670:14326:35822 2:N:0:TCTGCAAG+GCTGCACT
GAATTACCGGGACGTCTATCATACACGAGCCACATCCACAGCCACTGCTTCATATTATGAACATTGGC
TTCGCTAGTCAACTTAACTGAACCGTTAAGCACAATTCGATTCCATCTCACTTTCTGGGTATGTCTCG
TATCGACCGAGGTTC
+
FFFFFFFFFFFFFFFFFFFFFFFFFFFFFFFFFFFFFFFFFFFFFFFFFFFFFFFFFFFFFFFFFFFF
FFFFFFFFFFFFFFFFFFFFFFFFFFFFFFFFFFFFFFFFFFFFFFFFFFFFFFFF:FFFFFFFFFFF
FFFF:FFFFFFFFFF
@A00721:190:HGJW7DSXY:4:1234:3577:22936 2:N:0:TCTGCAAG+GCTGCACT
ATTAGAATTACCGGGACGTCTATCATACACGAGCCACATCCACAGCCACTGCTTCATATTATGAACAT
TGGCTTCGCTAGTCAACTTAACTGAACCGTTAAGCACAATTCGATTCCATCTCACTTTCTGGGTATGT
CTCGTATCGACCGAG
+
FFFFFFFFFFFFFFFFFFFFFFFFFFFFFFFFFFFFFFFFFFFFFFFFFFFFFFFFFFFFFFFFFFFF
```

FFFFFFFFFFFFFFFFFFFFFFFFFFFFFF,FFFFFFFFFFFFFFFFFFFFFFFFFFFFFFFFFFFFFFF
FFFFFFFFFFFFFFF
@A00721:190:HGJW7DSXY:4:1350:24659:17644 2:N:0:TCTGCAAG+GCTGCACT
ATTAGAATTACCGGGACGTCTATCATACACGAGCCACATCCACAGCCACTGCTTCATATTATGAACAT
TGGCTTCGCTAGTCAACTTAACTGAACCGTTAAGCACAATTCGATTCCATCTCACTTTCTGGGTATGT
CTCGTATCGACCGAG
+
FFFFFFFFFFFFFFFFFFFFFFFFFFFFFFFFFFFFFFFFFFFFFFFFFFFFFFFFFFFFFFFFFFFFFF
FFFFFFFFFFFFFFFFFFFFFFFFFFFFFFFFFFFFFFFFFFFFFFFFFFFFFFFFFFFFFFFF:F:FFF
FFFFFFF:FFFFFFF
@A00721:190:HGJW7DSXY:4:1578:15546:17425 2:N:0:TCTGCAAG+GCTGCACT
ATTAGAATTACCGGGACGTCTATCATACACGAGCCACATCCACAGCCACTGCTTCATATTATGAACAT
TGGCTTCGCTAGTCAACTTAACTGAACCGTTAAGCACAATTCGATTCCATCTCACTTTCTGGGTATGT
CTCGTATCGACCGAG
+
FFFFFFFFFFFFFFFFFFFFFFFFFFFFFFFFFFFFFFFFFFFFFFFFFFFFFFFFFFFFFFFFFFFFF:
FFFFFFFFFFFFFFFFFFFF:FFFFFFFFFFFFFFFFFFFFFFFFFFFFFFFFFFFFFFFFFFFFFFFFF
FFFFFFFFFFFFFFF
@A00721:190:HGJW7DSXY:4:2169:4761:3693 2:N:0:TCTGCAAG+GCTGCACT
ATTAGAATTACCGGGACGTCTATCATACACGAGCCACATCCACAGCCACTGCTTCATATTATGAACAT
TGGCTTCGCTAGTCAACTTAACTGAACCGTTAAGCACAATTCGATTCCATCTCACTTTCTGGGTATGT
CTCGTATCGACCGAG
+
FFFFFFFFFFFFFFFFFFFFFFFFFFFFFFFFFFFFFFFFFFFFFFFFFFFFFFFFFFFF:FFFFFFFFF
FFFFFFFFFFFFFFFFFFFFFFFFFFFFFFFFFFFFFFFFFFF:FFFFFFFFFFFFFFFFFFFFFFFFFF
FFFFFFFFFFFFFFF
@A00721:190:HGJW7DSXY:4:2237:5394:16877 2:N:0:TCTGCAAG+GCTGCACT
ATTAGAATTACCGGGACGTCTATCATACACGAGCCACATCCACAGCCACTGCTTCATATTATGAACAT
TGGCTTCGCTAGTCAACTTAACTGAACCGTTAAGCACAATTCGATTCCATCTCACTTTCTGGGTATGT
CTCGTATCGACCGAG
+
FFFFFFFFFFFFFFFFFFFFFFFFFFFFFFFFFFFFFFFFFFFFFFFFFFFFFFFFFFFFFFFFFFFFFF
FFFFFFFFFFFFFFFFFFFFFFFFFFFFFFFFFFFFFFFFFFFFFFFFFFFFFFFFFFFFFFFFFFFFFF
FFFFFFFFFFFFFFF
@A00721:190:HGJW7DSXY:4:2259:13268:3834 2:N:0:TCTGCAAG+GCTGCACT
ATTAGAATTACCGGGACGTCTATCATACACGAGCCACATCCACAGCCACTGCTTCATATTATGAACAT
TGGCTTCGCTAGTCAACTTAACTGAACCGTTAAGCACAATTCGATTCCATCTCACTTTCTGGGTATGT
CTCGTATCGACCGAG
+
FFFFFFFFFFFFFFFFFFFFFFFFFFFFFFFFFFFFFFFFFFFFFFFFFFFFFFFFFFFFFFFFFFFFFF
FFFFFFFFFFFFFFFFFFFFFFFFFFFFFFFFFFFFFFFFFFFFFFFFFFFFFFFFFFF:FFFFFFFF:F
FFFFFFFFFFFF:FF
@A00721:190:HGJW7DSXY:4:2450:31467:20290 2:N:0:TCTGCAAG+GCTGCACT
ATTAGAATTACCGGGACGTCTATCATACACGAGCCACATCCACAGCCACTGCTTCATATTATGAACAT
TGGCTTCGCTAGTCAACTTAACTGAACCGTTAAGCACAATTCGATTCCATCTCACTTTCTGGGTATGT
CTCGTATCGACCGAG
+
FFFFFFFFFFFFFFFFFFFFFFFFFFFF:FFFFFFFFFFFFFFFFFFFFFFFFFFFFFFFFFFFFFFFFF
FFFFFFFFFFFFFFFFFFFFFFFFF:FFFFFFFF:FFFFFF:FFFFFFFFFF:FFF::FFFFF,FFFFFF
FFFFFFFFFFFFFFF
@A00721:190:HGJW7DSXY:4:2508:4643:6151 2:N:0:TCTGCAAG+GCTGCACT
ATTAGAATTACCGGGACGTCTATCATACACGAGCCACATCCACAGCCACTGCTTCATATTATGAACAT
TGGCTTCGCTAGTCAACTTAACTGAACCGTTAAGCACAATTCGATTCCATCTCACTTTCTGGGTATGT
CTCGTATCGACCGAG

+
FFFFFFFFFFFFFFFFFFFFFFFFFFFFFFFFFFFF:FFFFFFFFFFFFFFFFFFFF:FFFFFFFFFFF,
FFFFFFFFFFFFFFFFFFFFFFFFFFFFFFFFFFFFFFF:FFFFFFFFFFFFFFFFFFFFFFFFFFFFFF
FFFFFFFFFFFFFFF
@A00721:190:HGJW7DSXY:4:2543:21007:23437 2:N:0:TCTGCAAG+GCTGCACT
ATTAGAATTACCGGGACGTCTATCATACACGAGCCACATCCACAGCCACTGCTTCATATTATGAACAT
TGGCTTCGCTAGTCAACTTAACTGAACCGTTAAGCACAATTCGATTCCATCTCACTTTCTGGGTATGT
CTCGTATCGACCGAG
+
FFFFFFFFFFFFFFFFFFFFFFFFFFFFFFFFFFFFFFFFFFFFFFFFFFFFFFFFFFFFFFFFFFFFFF
FFFFFFFFFFFFFFFFFFFFFFFFFFFFFFFFFFFFFFFFFFFFFFFFFFFFFFFFFFFFFFFFFFFFFF
FFFFFFFFFFFFFFF
@A00721:190:HGJW7DSXY:4:1104:4960:3944 2:N:0:TCTGCAAG+GCTGCACT
ACGAGACATACCCAGAAAGTGAGATGGAATCGAATTGTGCTTAACGGTTCAGTTAAGTTGACTAGCGA
AGCCAATGTTCATAATATGAAGCAGTGGCTGTGGATGTGGCTCGTGTATGATAGACGTCCCGGTAATT
CTAATCCCAGAGATC
+
FFFFFFFFFFFFF:FFFFFFFFFFFFFFFFFFFFFFFFFFFFFFFFFFFFFFFFFFFFFFFFFFFFFFFFFF
FFFFFFFFFFFFFF:FFFFFFFFFF:FFFFFFFFF,FFFFFFFFFFFFFFFFFFFFFFFFFFFFFF:FFF
FFFFFFFFFFFFFFF
@A00721:190:HGJW7DSXY:4:1104:5005:4022 2:N:0:TCTGCAAG+GCTGCACT
ACGAGACATACCCAGAAAGTGAGATGGAATCGAATTGTGCTTAACGGTTCAGTTAAGTTGACTAGCGA
AGCCAATGTTCATAATATGAAGCAGTGGCTGTGGATGTGGCTCGTGTATGATAGACGTCCCGGTAATT
CTAATCCCAGAGATC
+
FFFFFFFFFFFFFFFFFFFFFFFFFFFFFFFFFFFFFFFFFFFFFFFFFFFFFFFFFFFFFFFFFFFFFF
:FFFFFFFFFFFFFF:FFFFFFF:FFFFFFFFFFF:FFFFFFFFFFFFFFFFFFFFFFFFFFFFFFFFFF
FFFFFFFFFFFFF:F
@A00721:190:HGJW7DSXY:4:1253:2944:30013 2:N:0:TCTGCAAG+GCTGCACT
ACGAGACATACCCAGAAAGTGAGATGGAATCGAATTGTGCTTAACGGTTCAGTTAAGTTGACTAGCGA
AGCCAATGTTCATAATATGAAGCAGTGGCTGTGGATGTGGCTCGTGTATGATAGACGTCCCGGTCATT
CTAATCCCAGAGATC
+
FFFFFFFFFFFFFFFF:FFFFFFFFFFF:FFFFFFFFFFFFFFFFFFFFFFFFFFFFFFFFFFFFFFFFF
FFFFFFFFFFFFFFFFF:FFFFFFFFFFFFFFFFFFFFFFFFFFFFFFFFFFFFFFFFFFFFFFFF,FFF
FFFFFFFFFFFFFFF
@A00721:190:HGJW7DSXY:4:1421:9914:32158 2:N:0:TCTGCAAG+GCTGCACT
ACGAGACATACCCAGAAAGTGAGATGGAATCGAATTGTGCTTAACGGTTCAGTTAAGTTGACTAGCGA
AGCCAATGTTCATAATATGAAGCAGTGGCTGTGGATGTGGCTCGTGTATGATAGACGTCCCGGTAATT
CTAATCCCAGAGATC
+
FFFFFFFFFFFFFFFFFFFFFFFFFFFFFFFFFFFFFFFFFFFFFFFFFFFFFFFFFFFFFFFFFFFFFF
FFFFFFFFFFFFFFFFFFFFFFFFFFFFFFFFFFFFFF:FFFFFFFFFFFFFFFFFFFFFFFFFFFFFFF
FFFFFFFFFFFFFFF
@A00721:190:HGJW7DSXY:4:1421:10520:32831 2:N:0:TCTGCAAG+GCTGCACT
ACGAGACATACCCAGAAAGTGAGATGGAATCGAATTGTGCTTAACGGTTCAGTTAAGTTGACTAGCGA
AGCCAATGTTCATAATATGAAGCAGTGGCTGTGGATGTGGCTCGTGTATGATAGACGTCCCGGTAATT
CTAATCCCAGAGATC
+
FFFFFFFFFFFFFF:FFF,FFFFFFF::F::FFF:FFFF,FFFFFFFF:,FFFFFFFFF::FF:FFFF
FFF,:FFFFFFFFF:F,FF:FFF,FFFF:FFF:FF,FFFF:F,F:F,:F::F,FFFFFFFFFFFF:FF
:FFFFF:F:FFFFFF
@A00721:190:HGJW7DSXY:4:1569:20754:19398 2:N:0:TCTGCAAG+GCTGCACT
ACGAGACATACCCAGAAAGTGAGATGGAATCGAATTGTGCTTAACGGTTCAGTTAAGTTGACTAGCGA

AGCCAATGTTCATAATATGAAGCAGTGGCTGTGGATGTGGCTCGTGTATGATAGACGTCCCGGTAATT
CTAATCCCAGAGATC
+
FFFFFFFFFFFFFFFFFFFFFFFFFFFF:FFFFFFFFFFFFFFFFFFFFFFFFFFFFFFFFFFFFFFF
FFFFFFFFFFFFFFFFFFFFFF:FFFFFFFFFFFFFFFFFFFFFFFFFFFFFFFFFFFFFFFFFFFFF
FFFFFFFFFFFFFFF
@A00721:190:HGJW7DSXY:4:1668:31647:4413 2:N:0:TCTGCAAG+GCTGCACT
ACGAGACATACCCAGAAAGTGAGATGGAATCGAATTGTGCTTAACGGTTCAGTTAAGTTGACTAGCGA
AGCCAATGTTCATAATATGAAGCAGTGGCTGTGGATGTGGCTCGTGTATGATAGACGTCCCGGTAATT
CTAATCCCAGAGATC
+
FFFFFFFFFFFFFFFFFFFFFFFFFFFF,FFFF:FFFFFFFFF,FFFFFFFFFFFFFFFFFFFFFFFF
FFFFFFFFFFFFFFFFF:FFFFFFFFFFFFFFFFF:F:FFFFFFFFFFFFFFFFFFFFFFFFFFFFFF
FFFFFFFFFFFFFFF
@A00721:190:HGJW7DSXY:4:2516:6931:5822 2:N:0:TCTGCAAG+GCTGCACT
ACGAGACATACCCAGAAAGTGAGATGGAATCGAATTGTGCTTAACGGTTCAGTTAAGTTGACTAGCGA
AGCCAATGTTCATAATATGAAGCAGTGGCTGTGGATGTGGCTCGTGTATGATAGACGTCCCGGTAATT
CTAATCCCAGAGATC
+
FFFFFFFFFFFFFFFFFFFFFFFFFFFFFFFFFFFFFFFFFFFFFFF:FFFFFFFFFFFFF:FFFFFF
FFFFFFFFFFFFFFF:FFFFFF:FFFFFF,FFFFF:FFFFF:F:FFFFFFFFFFFFFFFFFFFFFFFF
FFFFFFFFFFFFFFF
@A00721:190:HGJW7DSXY:4:1104:4381:4006 2:N:0:TCTGCAAG+GCTGCACT
AGACATACCCAGAAAGTGAGATGGAATCGAATTGTGCTTAACGGTTCAGTTAAGTTGACTAGCGAAGC
CAATGTTCATAATATGAAGCAGTGGCTGTGGATGTGGCTCGTGTATGATAGACGTCCCGGTAATTCTA
ATCCAAGAGATC
+
F,F:FFFF:FFFFFFFFFFF:F:FF,:,FFF:FF::FF:FFFF:FFFF::,FFF::FFFFFF:,FFFF
FFFFFFFFFFFFFFFFFF,FF:F:FFF,F,FF:F,::FFF:F,FFFFFFFFF,F:F:F,FF:FF:F:F
FFFF,FFFFF:F
@A00721:190:HGJW7DSXY:4:1131:12897:27430 2:N:0:TCTGCAAG+GCTGCACT
CTCATTCACTCTGGGATTAGAATTACCGGGACGTCTATCATACACGAGCCACATCCACAGCCACTGCT
TCATATTATGAACATTGGCTTCGCTAGTCAACTTAACTGAACCGTTAAGCACAATTCGATTCCATCTC
ACTTTCTGGGTATGT
+
FFFFFFFFFFFFFFFFFFFFFFFFFFFFFFFFFFFFFFFFFFFFFFFFFFFFFFFFFFFFFFFFFFFF
FFFFFFFFFFFFFFFFFFFFFFFFFFFFFFFFFFFFFFFFFFFFFFFFFFFFFFFFFFFFFFFFFFFF
FFFFFFFFFFFFFFF
@A00721:190:HGJW7DSXY:4:1248:14561:27398 2:N:0:TCTGCAAG+GCTGCACT
CTCATTCACTCTGGGATTAGAATTACCGGGACGTCTATCATACACGAGCCACATCCACAGCCACTGCT
TCATATTATGAACATTGGCTTCGCTAGTCAACTTAACTGAACCGTTAAGCACAATTCGATTCCATCTC
ACTTTCTGGGTATGT
+
FFFFFFFFFFFFFFFFFFFFFFFFFFFFFFFFFFFFFFFFFFFFFFFFFFFFFFFFFFFFFFFFFFFF
FFFFFFFFFFFFFFFFFFFFFFFFFFFFFFFFFFFFFF:FFFFFFFFFFFFFFFFFF:FFFFFFFFFF
FFFFFFFFFFFFFFF
@A00721:190:HGJW7DSXY:4:1330:30002:24236 2:N:0:TCTGCAAG+GCTGCACT
CTCATTCACTCTGGGATTAGAATTACCGGGACGTCTATCATACACGAGCCACATCCACAGCCACTGCT
TCATATTATGAACATTGGCTTCGCTAGTCAACTTAACTGAACCGTTAAGCACAATTCGATTCCATCTC
ACTTTCTGGGTATGT
+
FFFFFFFFFFFFFFFFFFFFFFFFFFFFFFFFFFFFFFFFFFFFFFFFFFFFFFFFFFFFFFFFFFFF
FFFFFFFFFFFFFFFFFFFFFFFFFFFFFFFFFFFFFFFFFFFFFFFFFFFFF:FFFFFFFFFFFFFF
FFFFFFFFFFFFFFF

```
@A00721:190:HGJW7DSXY:4:1543:8133:35399 2:N:0:TCTGCAAG+GCTGCACT
CTCATTCACTCTGGGATTAGAATTACCGGGACGTCTATCATACACGAGCCACATCCACAGCCACTGCT
TCATATTATGAACATTGGCTTCGCTAGTCAACTTAACTGAACCGTTAAGCACAATTCGATTCCATCTC
ACTTTCTGGGTATGT
+
FFFFFFFFFFFFFFFFFFFFFFFFFFFFFFFFFFFFFFFFFFFFFFFFFFFFFFFFFFFFFFFFFFFF
FFFFFFFFFFFFFFFFFFFFFF:FFFFFF:FFFFFFFFF:FFF:FFFFFFFFFFFFFFFFFFFFFFFF
FFFFFFFFFFFFFFF
@A00721:190:HGJW7DSXY:4:1564:3522:1454 2:N:0:TCTGCAAG+GCTGCACT
CTCATTCACTCTGGGATTAGAATTACCGGGACGTCTATCATACACGAGCCACATCCACAGCCACTGCT
TCATATTATGAACATTGGCTTCGCTAGTCAACTTAACTGAACCGTTAAGCACAATTCGATTCCATCTC
ACTTTCTGGGTATGT
+
FFFFFFFFFFFFFFFFFFFFFFFFFFFFFFFFFFFFFFFFFFFFFFFFFFFFFFFFFFFFFFFFFFFF
FFFFFFFFFFFFFFFFFFFFFFFFFFFFFFFFFFFFFFFFFFFFFFFFFFFFFFFFFFFFFFFFFFFF
FFFFFFFFFFFFFFF
@A00721:190:HGJW7DSXY:4:2169:5258:33614 2:N:0:TCTGCAAG+GCTGCACT
CTCATTCACTCTGGGATTAGAATTACCGGGACGTCTATCATACACGAGCCACATCCACAGCCACTGCT
TCATATTATGAACATTGGCTTCGCTAGTCAACTTAACTGAACCGTTAAGCACAATTCGATTCCATCTC
ACTTTCTGGGTATGT
+
FFFFFFFFFFFFFFFFFFFFFFFFFFFFFFFFFFFF:FFFFFFFFFFFFFFFFFFFFFFFFFFFFFFF
FFFFFFFFFFFFFFFFF:FF:FFFF,FFFFFFFFFFFFFFFFFFFFFFFFFFFFFFFFFFFFFFFFFF
FFFF:FFFFFFFFFF
@A00721:190:HGJW7DSXY:4:2231:21187:30232 2:N:0:TCTGCAAG+GCTGCACT
CTCATTCACTCTGGGATTAGAATTACCGGGACGTCTATCATACACGAGCCACATCCACAGCCACTGCT
TCATATTATGAACATTGGCTTCGCTAGTCAACTTAACTGAACCGTTAAGCACAATTCGATTCCATCTC
ACTTTCTGGGTATGT
+
FFFFFFFFFFFFFFFFFFFFFFFFFFFFFFFFFFFFFFFFFFFFFFFFFFFFFFFFFFFFFFFFFFFF
FFFFFFFFFFFFFFFFFFFFFFFFFFFFFFFFFFFFFFFFFFFFFFFFFFFFF:FFFFFFFFFFFFFF
FFFFFFFFFFFFFFF
@A00721:190:HGJW7DSXY:4:2264:31250:35383 2:N:0:TCTGCAAG+GCTGCACT
CTCATTCACTCTGGGATTAGAATTACCGGGACGTCTATCATACACGAGCCACATCCACAGCCACTGCT
TCATATTATGAACATTGGCTTCGCTAGTCAACTTAACTGAACCGTTAAGCACAATTCGATTCCATCTC
ACTTTCTGGGTATGT
+
FFFFFFFFFFFFFFFFFFFFFFFFFFFFFFFFFFFFFFFFFFFFFFFFFFFFFFFFFFFFFFFFFFFF
FFFFFFFFFFFFFFFFFFFFFFFFFFFFFFFFFFFFFFFFFFFFFFFFFFFFFFFFFFFFFF:FFFFF
FFFFFFFFFFFFFFF
@A00721:190:HGJW7DSXY:4:2264:31376:35916 2:N:0:TCTGCAAG+GCTGCACT
CTCATTCACTCTGGGATTAGAATTACCGGGACGTCTATCATACACGAGCCACATCCACAGCCACTGCT
TCATATTATGAACATTGGCTTCGCTAGTCAACTTAACTGAACCGTTAAGCACAATTCGATTCCATCTC
ACTTTCTGGGTATGT
+
FFFFFFFFFFFFFFFFF:FFFFFFFFFFFFFFFFF:FFFFFFFFFFFFFFFFFFFFFFFFFFFFFFFF
FFFFFFFFFFFFFFFFFFFFFFFFFFFFFFFFF:FFFFFFFFFF:FFFFFFFFF,FFFFFFFFFFFFF
FFFFFFFFF:FFFFF
@A00721:190:HGJW7DSXY:4:2267:8422:12320 2:N:0:TCTGCAAG+GCTGCACT
CTCATTCACTCTGGGATTAGAATTACCGGGACGTCTATCATACACGAGCCACATCCACAGCCACTGCT
TCATATTATGAACATTGGCTTCGCTAGTCAACTTAACTGAACCGTTAAGCACAATTCGATTCCATCTC
ACTTTCTGGGTATGT
+
FFFFFFFFFFFFFFFFFFFFFFFFFFFFFFFFFFFFFFFFFFFFFFFFFFFFFFFFFFFFFFFFFFFF
```

FFFFFFFFFFFFFFFFFFFFFFFFF,FFFFFFFFFFFFF:FFFFFFFFFFFFFFFFFFFFFFFFFFFF
FFF,FFFFFFFFFFF
@A00721:190:HGJW7DSXY:4:2408:8250:6825 2:N:0:TCTGCAAG+GCTGCACT
CTCATTCACTCTGGGATTAGAATTACCGGGACGTCTATCATACACGAGCCACATCCACAGCCACTGCT
TCATATTATGAACATTGGCTTCGCTAGTCAACTTAACTGAACCGTTAAGCACAATTCGATTCCATCTC
ACTTTCTGGGTATGT
+
FFFFFFFFFFFFFFFFFFFFFFFFFFFFFFFFFFFFFFFFFFFFFFFFFFFFFFFFFFFFFFFFFFFF
FFFFFFFFFFFFFFFFFFFFFFFFFFFFFFFFFFFFFFFFFFFFFFFFFFFFF,:FFFFFFFFFFFFF
FFFFFFFFFFFFFFF
@A00721:190:HGJW7DSXY:4:2621:31295:22122 2:N:0:TCTGCAAG+GCTGCACT
CTCATTCACTCTGGGATTAGAATTACCGGGACGTCTATCATACACGAGCCACATCCACAGCCACTGCT
TCATATTATGAACATTGGCTTCGCTAGTCAACTTAACTGAACCGTTAAGCACAATTCGATTCCATCTC
ACTTTCTGGGTATGT
+
FFFFFFFFFFFFFFFFFFFFFFFFFFFFFFFFFFFFFFFFFFFFFFFFFFFFFFFFFFFFFFFFFFFF
FFFFFFFFFFFFFFFFFFFFFFFFFFFFFFFFFFFFFFFFFFFFFFFFFFFFFFFFFFFFFFFFFFFF
FFFFFFFFFFFFFFF
@A00721:190:HGJW7DSXY:4:2666:30011:8061 2:N:0:TCTGCAAG+GCTGCACT
CTCATTCACTCTGGGATTAGAATTACCGGGACGTCTATCATACACGAGCCACATCCACAGCCACTGCT
TCATATTATGAACATTGGCTTCGCTAGTCAACTTAACTGAACCGTTAAGCACAATTCGATTCCATCTC
ACTTTCTGGGTATGT
+
FFFFFFFFFFFFFFFFFFFFFFFFFFFFFFFFFFFFFFFFFFFFFFFFFFFFFFFFFFFFFFFFFFFF
FFFFFFFFFFFFFFFFFFFFFFFFFFFFFFFFFFFFFFFFFFFFFFFFFFFF:FFFFFFFFFFFFFFF
FFFFFFFFFFFFFFF
@A00721:190:HGJW7DSXY:4:1103:32660:8578 2:N:0:TCTGCAAG+GCTGCACT
CATACCCAGAAAGTGAGATGGAATCGAATTGTGCTTAACGGTTCAGTTAAGTTGACTAGCGAAGCCAA
TGTTCATAATATGAAGCAGTGGCTGTGGATGTGGCTCGTGTATGATAGACGTCCCGGTAATTCTAATC
CCAGAGTGAATGAGA
+
FFFFFFFFFFFFFFFFFFFFFFF:FF:FFFFF:FFFF,:FFFFFFFFFF:FFFFFFFFFFFFFFFFFF
FFFF:FF,FFFFFFFFFFFFFFF:FF:FF:FFFFFF:FFFFFFFF:FFFFFFFFFFF::FFFFFFFFF
FFFFFFFFF:FFFFF
@A00721:190:HGJW7DSXY:4:1355:14778:30499 2:N:0:TCTGCAAG+GCTGCACT
CATACCCAGAAAGTGAGATGGAATCGAATTGTGCTTAACGGTTCAGTTAAGTTGACTAGCGAAGCCAA
TGTTCATAATATGAAGCAGTGGCTGTGGATGTGGCTCGTGTATGATAGACGTCCCGGTAATTCTAATC
CCAGAGTGAATGAGA
+
FFFFFFFFFFFFFFFFFFFFFF:FFFFFFFFFFFFFFFFFFFFFFFFFFFFFFFFFFFFFFFFFFFFF
FFFFFFFFFFFFFFFFFFFFFFFFFFFFFFFFFFFFFFFFF:FFFFFF:FFFFFFFFFFFFFFFFFFF
FFFFFFFFFFFFFFF
@A00721:190:HGJW7DSXY:4:1565:23095:11052 2:N:0:TCTGCAAG+GCTGCACT
CATACCCAGAAAGTGAGATGGAATCGAATTGTGCTTAACGGTTCAGTTAAGTTGACTAGCGAAGCCAA
TGTTCATAATATGAAGCAGTGGCTGTGGATGTGGCTCGTGTATGATAGACGTCCCGGTAATTCTAATC
CCAGAGTGAATGAGA
+
FFFFFFFFFFFFFFFFFFFFFF:FFFFFFFFFFFFFFFFFFFFFFFFFFFFFFFFFF:FFFFFFFFFF
FFFFFF:FFFFFFFFFFFFFFFFFFFFFFFFFFFFFFFFFFFFFFFFFFFFFFFFFFFFFFFFFFFFF
FFFFFFFFF:FFFFF
@A00721:190:HGJW7DSXY:4:1658:31322:2879 2:N:0:TCTGCAAG+GCTGCACT
CATACCCAGAAAGTGAGATGGAATCGAATTGTGCTTAACGGTTCAGTTAAGTTGACTAGCGAAGCCAA
TGTTCATAATATGAAGCAGTGGCTGTGGATGTGGCTCGTGTATGATAGACGTCCCGGTAATTCTAATC
CCAGAGTGAATGAGA

```
+
FFFFFFFFFFFFFFFFFFFFFFFFFFFFFFFFFF::FFFFFFFFFFFFFFFFFFFFFFFFFFFFFFFFF
FFFFFFFFFFFFFFFFFFFFFFFFFFFFFFFFFFFFFFFF:FFFF:FFFFFFFFFFFFFFFF,F:F
FFFFFF:FFFFFFFF
@A00721:190:HGJW7DSXY:4:2101:31656:29293 2:N:0:TCTGCAAG+GCTGCACT
CATACCCAGAAAGTGAGATGGAATCGAATTGTGCTTAACGGTTCAGTTAAGTTGACTAGCGAAGCCAA
TGTTCATAATATGAAGCAGTGGCTGTGGATGTGGCTCGTGTATGATAGACGTCCCGGTAATTCTAATC
CCAGAGTGAATGAGA
+
FFFF:FFFFFF,FFFFF:FFFFFFFFFFFFFFFFFFFFFFFFFF:FFFFFFFFFF:FFF:FFFFFFFFF
FFFFFFFFFF:FFFF,:FFFFFFFFFFFFFF:F:FFFFF:FFFFFFF,FFFFFFFFFFFFFFFF,FFF
FFFFFFFFFFFFFF:
@A00721:190:HGJW7DSXY:4:2167:6831:17581 2:N:0:TCTGCAAG+GCTGCACT
CATACCCAGAAAGTGAGATGGAATCGAATTGTGCTTAACGGTTCAGTTAAGTTGACTAGCGAAGCCAA
TGTTCATAATATGAAGCAGTGGCTGTGGATGTGGCTCGTGTATGATAGACGTCCCGGTAATTCTAATC
CCAGAGTGAATGAGA
+
FFFFFFFFFFFFFFFFFFFFFFFFFFFFFFFFFFFFFFFFFFFFFFFFFFFFFFFFFFFFFFFFFFFF
FFFFFFFFFFFFFFFFFFFFFFFFFFFFFFFFFFFFFFFFFFFFFFFFFFFFFFFFFFFFFFFFFFFF
FFFFFFFFFFFFFFF
@A00721:190:HGJW7DSXY:4:1274:16315:24706 2:N:0:TCTGCAAG+GCTGCACT
CATTCACTCTGGGATTAGAATTACCGGGACGTCTATCATACACGAGCCACATCCACAGCCACTGCTTC
ATATTATGAACATTGGCTTCGCTAGTCAACTTAACTGAACCGTTAAGCACAATTCGATTCCATCTCAC
TTTCTGGGTAT
+
FF,FFF,FFF:FFF,FFFFF,:F:FF::F,FFFFFFFFFF:FFFFFFFFFF,,FFFF:FFFFF,:FF:
F,FFFFFFFF:FFF:F:FF:FFFFF,FFFFFF:FFFFFFFFFF,FF:FFFFF,F:FF:FFFFFF:FFF
FF,FF:,FFFF
@A00721:190:HGJW7DSXY:4:1330:30454:11083 2:N:0:TCTGCAAG+GCTGCACT
CATTCACTCTGGGATTAGAATTACCGGGACGTCTATCATACACGAGCCACATCCACAGCCACTGCTTC
ATATTATGAACATTGGCTTCGCTAGTCAACTTAACTGAACCGTTAAGCACAATTCGATTCCATCTCAC
TTTCTGGTTAT
+
FFFFFFFFFFFFFFFFFFFFFFFFFFFFFFFFFFFFFFFFFFFFF:FFFFFFFFFFFFFFFFFFFFFF
F:FFFFF:FFFFFFF,FFFF::FFFFFFFFFFFFFFFFFF:F:FFFFFFFFF:F,FFFFFFFFFFFFF
:FF,F:F,FFF
@A00721:190:HGJW7DSXY:4:1337:13711:8171 2:N:0:TCTGCAAG+GCTGCACT
CATTCACTCTGGGATTAGAATTACCGGGACGTCTATCATACACGAGCCACATCCACAGCCACTGCTTC
ATATTATGAACATTGGCTTCGCTAGTCAACTTAACTGAACCGTTAAGCACAATTCGATTCCATCTCAC
TTTCTGGGTAT
+
FFFFFFFFFFFFFFFFFFFFFFFFFFFFFFFFFFFFFFFFFFFFFFFFFFFFFFFFFFFFFFFFFFFF
FFFFFFFF:FFFFFFFFFFFFFFFFFF,FFFFFFFFFFFFFFFFFFFFFFFFFFFFFFFFFFFFFFFFF
FFFFFFFFFFF
@A00721:190:HGJW7DSXY:4:1376:17056:1626 2:N:0:TCTGCAAG+GCTGCACT
CATTCACTCTGGGATTAGAATTACCGGGACGTCTATCATACACGAGCCACATCCACAGCCACTGCTTC
ATATTATGAACATTGGCTTCGCTAGTCAACTTAACTGAACCGTTAAGCACAATTCGATTCCATCTCAC
TTTCTGGGTAT
+
FFFFFFFFFFFFFFFFFFFFFFFFFFFFFFFFFF:FFFFFFFFFFFFFFFFFFFFFFFFFFFFFFFFF
FFFFFFFFFFFFFFF:FFFFFFFFFFFFFFFFFFFFFFFFFFFFFFFFFFFFFFFFFFFFFFFFFFFF
FFFFFFFFFFF
@A00721:190:HGJW7DSXY:4:1376:16288:2080 2:N:0:TCTGCAAG+GCTGCACT
CATTCACTCTGGGATTAGAATTACCGGGACGTCTATCATACACGAGCCACATCCACAGCCACTGCTTC
```

```
ATATTATGAACATTGGCTTCGCTAGTCAACTTAACTGAACCGTTAAGCACAATTCGATTCCATCTCAC
TTTCTGGGTAT
+
FFFFFFFFFFFFFFFFFFFFFFFFFFFFFFFFFFFFFFFFFFFFFFFFFFFFFFFFFFFFFFFFFFFF
FFFFFFFF:FFFFFFFFFFFFFFFFFFFFFFFFFFFFFFFFFFFFFFFFFFFFFFFFFFFFFFFFFFF
:FFFFF:FFFF
@A00721:190:HGJW7DSXY:4:1502:11026:4554 2:N:0:TCTGCAAG+GCTGCACT
CATTCACTCTGGGATTAGAATTACCGGGACGTCTATCATACACGAGCCACATCCACAGCCACTGCTTC
ATATTATGAACATTGGCTTCGCTAGTCAACTTAACTGAACCGTTAAGCACAATTCGATTCCATCTCAC
TTTCTGGGTAT
+
FFFFFFFFFFFFFFFFFFFFFFFFFFFFFFFFFFFFFFFFFFF:FFFFFFFFFFFFFFFFFF:FFFFF
FFFFFFFFFFF:FFFFFFFFFFFFFFFFFFFFFFF:FFFFFFFFFFFFFF:FFFFFFF,FFFFFFFFF
FFFFFFFFFFF
@A00721:190:HGJW7DSXY:4:1663:19352:11397 2:N:0:TCTGCAAG+GCTGCACT
CATTCACTCTGGGATTAGAATTACCGGGACGTCTATCATACACGAGCCACATCCACAGCCACTGCTTC
ATATTATGAACATTGGCTTCGCTAGTCAACTTAACTGAACCGTTAAGCACAATTCGATTCCATCTCAC
TTTCTGGGTAT
+
FFFFFFFFFFFFFFFFFFFFFFFFFFFFFFFFFFFFFFFFFFFFFFFFFFFFFFFFFFFFFFFFFFFF
FFFFFFFFFFFFFFFFFFFF:FFFFFFFFFFFFFFFFFFFFFFFFFFFFFFFF:FFFFFFFFFFFFF:
FFFFFFFFFFF
@A00721:190:HGJW7DSXY:4:2328:3043:7294 2:N:0:TCTGCAAG+GCTGCACT
CATTCACTCTGGGATTAGAATTACCGGGACGACTATCATACACGAGCCACATCCACAGCCACTGCTTC
ATATTATGAACATAGGCTTCGCTAGTCAACTTAACTGAACCGTTAAGCACAATTCGATTCCATCTCAC
ATTCTGGGTAT
+
:::FFFFFF:FF:FFFFFFF:F:FF:FFFFF,FF:FFFFFFFFFF:FFF:FFFF:FFF,FFFF:FF:F
FFFFFFFFFFFFF,,FFFF:FFFFFFFFFFF,FF:,,:FFFFFFFF,FFFF,:F:,F,F::FFF:FFF
,,,F:FF,FFF
@A00721:190:HGJW7DSXY:4:2507:21558:13589 2:N:0:TCTGCAAG+GCTGCACT
CATTCACTCTGGGATTAGAATTACCGGGACGTCTATCATACACGAGCCACATCCACAGCCACTGCTTC
ATATTATGAACATTGGCTTCGCTAGTCAACTTAACTGAACCGTTAAGCACAATTCGATTCCATCTCAC
TTTCTGGGTAT
+
FFFFFFFFFFFFFFFFFFFFFFFFFFFFFFFFFFFFFFFFFFFFFFFFFFFFFFFFFFFFFFFFFFFF
FFFFFFFFFFFFFFFFFFFFFFFFFFFFFFFFFFFFFFFFFF,FFFFFFFF:FFFFF:F:FFFFFFFF
FFF,FFFFFFF
@A00721:190:HGJW7DSXY:4:2507:31186:15953 2:N:0:TCTGCAAG+GCTGCACT
CATTCACTCTGGGATTAGAATTACCGGGACGTCTATCATACACGAGCCACATCCACAGCCACTGCTTC
ATATTATGAACATTGGCTTCGCTAGTCAACTTAACTGAACCGTTAAGCACAATTCGATTCCATCTCAC
TTTCTGGGTAT
+
FFFFFFFFFFFFFFFFFFFFFFFFFFFFFFFFFFFFFFFFFFFFFFFFFFFFFFFFFFFFFFFFFFFF
FFFFFFFFFFFFFFFFFFFFFFFFFFFFFFFFFFFFFFFFFFFFFFFFFFFFFFFFFFFFFFFFFFFF
FFFFFFFFFFF
@A00721:190:HGJW7DSXY:4:2615:2166:23156 2:N:0:TCTGCAAG+GCTGCACT
CATTCACTCTGGGATTAGAATTACCGGGACGTCTATCATACACGAGCCACATCCACAGCCACTGCTTC
ATATTATGAACATTGGCTTCGCTAGTCAACTTAACTGAACCGTTAAGCACAATTCGATTCCATCTCAC
TTTCTGGGTAT
+
FFFFFFFFFFFFFFFFF:FFFFFFFFFFFFFFFFFF:FFF:FFFFFFFFF:FF:FFFFFFFFFFFFFF
FFFFFFFFFFF:FFFFFFFFFFFFFFF:FFFFFFFFFF:FFFFFFFF:FFF:FF:FFFFFFFFFF,FF
F:FFFFFFF:F
```

```
@A00721:190:HGJW7DSXY:4:2626:9091:31704 2:N:0:TCTGCAAG+GCTGCACT
CATTCACTCTGGGATTAGAATTACCGGGACGTCTATCATACACGAGCCACATCCACAGCCACTGCTTC
ATATTATGAACATTGGCTTCGCTAGTCAACTTAACTGAACCGTTAGGCACAATTCGATTCCATCTCAC
TTTCTGGGTAT
+
FFFFFFFFFFFFFFFFFFFFFFFFFFFFFFFFFFFFFFFFFFFFFFFFFFFFFFFFFFFFFFFFFFFF
FFFFFFFFFFFFFFFFFFFFF:FFFFFFFFFFFF:FFFFFFFFFFF,FFFFFFFFFFFFFFFFFFFFFF
F:FFFFFFFFF
@A00721:190:HGJW7DSXY:4:2635:30626:8719 2:N:0:TCTGCAAG+GCTGCACT
CATTCACTCTGGGATTAGAATTACCGGGACGTCTATCATACACGAGCCACATCCACAGCCACTGCTTC
ATATTATGAACATTGGCTTCGCTAGTCAACTTAACTGAACCGTTAAGCACAATTCGATTCCATCTCAC
TTTCTGGGTAT
+
FFFFFF:FFFFFFFF:FFFFFFFFFFFFFFFFFFFFFFFFFFFFFFFFFFFFFFFFFFFFFFFFFFFF
FFFFFFFFFFFFFFFFFFFFFFFFFFFFFFFFFFFFFFFFFFFFFFFFFFFFFFFFFFFFFFFF:FFFF
FFF:FFFFFFF
@A00721:190:HGJW7DSXY:4:1530:18837:32487 2:N:0:TCTGCAAG+GCTGCACT
TATCTCATTCACTCTGGGATTAGAATTACCGGGACGTCTATCATACACGAGCCACATCCACAGCCACT
GCTTCATATTATGAACATTGGCTTCGCTAGTCAACTTAACTGAACCGTTAAGCACAATTCGATTCCAT
CTCACTTTCTGGGTA
+
:FFFFFFFFF,FFFFFFFF:FFFFFFFFFF,FFFFFFFFFFFFFFFFFFFFF,FF:FFFFFFFFFFFF
FFFFFFFFFFFFFFFFFFFFFFFFFFFFFFFFFFFFFFFFFFFFFFFF:F:FFFFFFF,FFFF::FFFF
FFFF:F:FFFFFFFF
@A00721:190:HGJW7DSXY:4:2242:22815:21402 2:N:0:TCTGCAAG+GCTGCACT
TATCTCATTCACTCTGGGATTAGAATTACCGGGACGTCTATCATACACGAGCCACATCCACAGCCACT
GCTTCATATTATGAACATTGGCTTCGCTAGTCAACTTAACTGAACCGTTAAGCACAATTCGATTCCAT
CTCACTTTCTGGGTA
+
FFFFFFFFFFFFFFFFFFFFFFFFFFFFFFFFFFFFFFFFFFFFFFFFFFFFFFFFFFFFFFFFFFFF
FFFFFFFFFFFFFFFFFFFFFFFFFFFFF:FFFFFFFFFFFFF:FFFFFFFFFFFFFFFFF:FFFFFFF
:FFFFF:FFFFFFFF
@A00721:190:HGJW7DSXY:4:2457:12138:23422 2:N:0:TCTGCAAG+GCTGCACT
TATCTCATTCACTCTGGGATTAGAATTACCGGGACGTCTATCATACACGAGCCACATCCACAGCCACT
GCTTCATATTATGAACATTGGCTTCGCTAGTCAACTTAACTGAACCGTTAAGCACAATTCGATTCCAT
CTCACTTTCTGGGTA
+
FFFFFFFFFF:FF::,F,FF:FFFFFFFFFFFFFFFFFFFFFFFFFFFFFF:FFFFFFFFFFFFFFFF
FFFFFFFFFFFFFFFFFF:FF:FFFFFFFFFFFFFFFFFFFFFFF:FFFFFFFFFFFFFFF:FFFFFFF
FFFFFFFFFFFFF:FF
@A00721:190:HGJW7DSXY:4:2635:32705:9095 2:N:0:TCTGCAAG+GCTGCACT
CATTCACTCTGGGATTAGAATTACCGGGACGTCTATCATACACGAGCCACATCCACAGCCACTGCTTC
ATATTATGAACATTGGCTTCGCTAGTCAACTTAACTGAACCGTTAAGCACAATTCGATTCCATCTCAC
TTTCTGGG
+
FFFFFF,FFFFFFFFFFFFFFFFFFFFFFF:FFFFFFFFFFFFFFFFFFFFFFF:FFFFFFFFFFFFFFF
FFFFFFFFFFFFFFFFFFFFFF:FFFFFFFFF:FFFFFFFF:F:FF:F,FFFF::F:FFF:F:FFFFF,
::F,,FFF
@A00721:190:HGJW7DSXY:4:2278:1705:20572 2:N:0:TCTGCAAG+GCTGCACT
CTCATTCACTCTGGGATTAGAATTACCGGGACGTCTATCATACACGAGCCACATCCACAGCCACTGCT
TCATATTATGAACATTGGCTTCGCTAGTCAACTTAACTGAACCGTTAAGCACAATTCGATTCCATCTC
ACTATCTGAGATCG
+
:FFFFF:F:F:F,,FFF:FFFF::FFFFF,FF:FFFFFFFFFFFF,F,:FFFF,FFFFFFFFFF,FFF
```

```
,FFFFF:FFFFFFF:,:FFF:F:FFFF:FFFFF,FFFFFFFF,:::FFF:FFFF,:F,FFFFFFFFFF
FFF,F::FFFFFF:
@A00721:190:HGJW7DSXY:4:2539:27145:19758 2:N:0:TCTGCAAG+GCTGCACT
TCTCATTCACTCTGGGATTAGAATTACCGGGACGTCTATCATACACGAGCCACATCCACAGCCACTGC
TTCATATTATGAACATTGGCTTCGCTAGTCAACTTAACTGAACCGTTAAGCACAATTCGATTCCATCT
CACTTTCTGAGATCG
+
FFFFFFFFFF,FFFFFFFFFFFFFFFFFFFFFFFFFFFFFFFFFFFFFFFFFFFFFFFFFFFFFFFFF
FFFFFFFFFFFFFFFFFFFFFFFFFFFFFFFFFFFFFFFFF:F:FFFFFFFFF:FFFFFFFFFFFFFF
FFFFFFFFFFFFFFF
@A00721:190:HGJW7DSXY:4:2633:6705:24283 2:N:0:TCTGCAAG+GCTGCACT
TCTCATTCACTCTGGGATTAGAATTACCGGGACGTCTATCATACACGAGCCACATCCACAGCCACTGC
TTCATATTATGAACATTGGCTTCGCTAGTCAACTTAACTGAACCGTTAAGCACAATTCGATTCCATCT
CACTTTCTGAGATCG
+
FFFFFFFFFFFFFFFFFFFFFFFFFFF::::FFFFFFFFFFFFFFFFFFFFFFFFFFFFFFFFFFFFF
FFFFFFFFFFFFFFF:FFFFFFFFFFFFFFFFFFFFFFFFF:FFFFFF:FFFFFFFFFFFFFFFFFFF
FFFF,FFFFFFFFFF
@A00721:190:HGJW7DSXY:4:1271:29080:7733 2:N:0:TCTGCAAG+GCTGCACT
TGAATATCTCATTCACTCTGGGATTAGAATTACCGGGACGTCTATCATACACGAGCCACATCCACAGC
CACTGCTTCATATTATGAACATTGGCTTCGCTAGTCAACTTAACTGAACCGTTAAGCACAATTCGATT
CCATCTCACTTTCTG
+
FFFFFFFFFFFFFFFFFF,FFFFF:FFFFFFFFFFFFFFFFFFFFFFFFFFFFFFFFFFFFFFF:FFFF
FFFFFFFFFFFFFFFFFFFFFFFFFFFFFFFFFFFFFFFFFF:FFFFFFFFFFFFFFFFFFFFFFFFF
FFFFFFFFFFFFFFF
@A00721:190:HGJW7DSXY:4:1372:25355:14967 2:N:0:TCTGCAAG+GCTGCACT
TGAATATCTCATTCACTCTGGGATTAGAATTACCGGGACGTCTATCATACACGAGCCACATCCACAGC
CACTGCTTCATATTATGAACATTGGCTTCGCTAGTCAACTTAACTGAACCGTTAAGCACAATTCGATT
CCATCTCACTTTCTG
+
FFFF,FFFFFFFFFFFFFFFFFFFFFFFFFFFFFFFFFFFFFFFFFFFFFFFFFFFFFFFFFFFFFFF
FFFFFFFFFFFFFFFFFFFFFFFFFFFFFFFFFFFFFFFFFFFFFFFFFFFFFFFFFFFFFFFFFFFF
FFFFFFFFFFFFFFF
@A00721:190:HGJW7DSXY:4:2402:16559:5181 2:N:0:TCTGCAAG+GCTGCACT
TGAATATCTCATTCACTCTGGGATTAGAATTACCGGGACGTCTATCATACACGAGCCACATCCACAGC
CACTGCTTCATATTATGAACATTGGCTTCGCTAGTCAACTTAACTGAACCGTTAAGCACAATTCGATT
CCATCTCACTTTCTG
+
FFFFFFFFFFFFFFFFFFFFFFFFFFFFFFFFFFFFFFFFFFFFFFFFFFFFFFFFFFFFFFFFFFFF
FFFFFFFFFFFFFFFFFFFFFFFFFFFFFFFFFFFFFFFFFFFFFFFFFFFFFFFFFFFFFFFFFFFF
FFFFFFFFFFFFFFF
@A00721:190:HGJW7DSXY:4:1308:7030:20087 2:N:0:TCTGCAAG+GCTGCACT
GATGAATATCTCATTCACTCTGGGATTAGAATTACCGGGACGTCTATCATACACGAGCCACATCCACA
GCCACTGCTTCATATTATGAACATTGGCTTCGCTAGTCAACTTAACTGAACCGTTAAGCACAATTCGA
TTCCATCTCACTTTC
+
FFFFFFFFFFFFFFFFFFFFFFFFFFFFFFFFFFFFFFFFFFFFFFFFFFF,FF:FFFFFFFFFFFFF
FFFFFFFFFFFFFFFFFFFFFFFFFF:FFFFFF:FFFFFFFFFFF:FFFF:FFFF:FFFFFFFFFF,F
FFF:FFFFFFFFFFF
@A00721:190:HGJW7DSXY:4:1308:8106:20854 2:N:0:TCTGCAAG+GCTGCACT
GATGAATATCTCATTCACTCTGGGATTAGAATTACCGGGACGTCTATCATACACGAGCCACATCCACA
GCCACTGCTTCATATTATGAACATTGGCTTCGCTAGTCAACTTAACTGAACCGTTAAGCACAATTCGA
TTCCATCTCACTTTC
```

+
FFFFFFFFFFFFFFFFFFFF:FFFFFFFFFFFFFFFFFFFFFFFFFFFFFFFFFFFFFFFFFFFFFFF
FFFFFFFFFFFFFFFFFFFFFF:FFFFFFFFF:FFFFFFFFFFFFFFFFFFFFFFFFFFFFFFFFFFF
FFFFFFFFFFFFFFF
@A00721:190:HGJW7DSXY:4:1356:24478:34773 2:N:0:TCTGCAAG+GCTGCACT
GATGAATATCTCATTCACTCTGGGATTAGAATTACCGGGACGTCTATCATACACGAGCCACATCCACA
GCCACTGCTTCATATTATGAACATTGGCTTCGCTAGTCAACTTAACTGAACCGTTAAGCACAATTCGA
TTCCATCTCACTTTC
+
FFFFFFFFFF,FF,FFFFFFFFFFFF:FFFFF::FFF:FFFFFFFFFFFFFFF,:FFFFFFFFFFF,F
:,FFF:FF:,FF:FFFFFFFFFFF,:FFFFF:FFFFFFF,F:FFFF:FFFFF::,FF:FFFFFF:F,F
FFFFFFFFFFF,FFF
@A00721:190:HGJW7DSXY:4:1516:6876:35227 2:N:0:TCTGCAAG+GCTGCACT
GATGAATATCTCATTCACTCTGGGATTAGAATTACCGGGACGTCTATCATACACGAGCCACATCCACA
GCCACTGCTTCATATTATGAACATTGGCTTCGCTAGTCAACTTAACTGAACCGTTAAGCACAATTCGA
TTCCATCTCACTTTC
+
FFFFF,FFFFFFFFFFFFFF:FFFFF:FFFFFFFFFFFFFFFFFFFFFFFFFFFFFFFFFFFFFFFFF
FFFFFFFFFFFFFFFFFFFFFFFFF:,FFFFFFFFFFFFFFFF:FFFFFFFFFFFFFFFFFFFFFFFF
FFFFF:FFFFFFFF:
@A00721:190:HGJW7DSXY:4:2420:10945:9173 2:N:0:TCTGCAAG+GCTGCACT
GATGAATATCTCATTCACTCTGGGATTAGAATTACCGGGACGTCTATCATACACGAGCCACATCCACA
GCCACTGCTTCATATTATGAACATTGGCTTCGCTAGTCAACTTAACTGAACCGTTAAGCACAATTCGA
TTCCATCTCACTTTC
+
FFFFFFFFFFFFFFFFFFFFFFFFFFFFFF:FFFFFFFFFFFFFFFFFFFFFFFFFFFFFFFFFFFFF
FFFFFFFFFFFFFFFFFFFF:FFFFFFFFFFFFF:FFFFFFFFFFFFFFFFFFFFFFFFFFFFFFFFF
FFFFFFFFFFFFFFF
@A00721:190:HGJW7DSXY:4:2465:1145:26303 2:N:0:TCTGCAAG+GCTGCACT
GATGAATATCTCATTCACTCTGGGATTAGAATTACCGGGACGTCTATCATACACGAGCCACATCCACA
GCCACTGCTTCATATTATGAACATTGGCTTCGCTAGTCAACTTAACTGAACCGTTAAGCACAATTCGA
TTCCATCTCACTTTC
+
FFFFFFFFFFFFFFFFFFFFFFFFFFFFFFFFFFFFF:FFF:FFFFFFFFFFFFFFFFFFFFFFFFFF
FFFFFF:FFFFFFFFFFFFFFFFFFFFFFFFF:FF:FFFFFFFFFFFFFF:FF:FFFFFFFFFFFFFF
FFFFFFFFFFFFFFF
@A00721:190:HGJW7DSXY:4:1143:28501:9361 2:N:0:TCTGCAAG+GCTGCACT
CGATGAATATCTCATTCACTCTGGGATTAGAATTACCGGGACGTCTATCATACACGAGCCACATCCAC
AGCCACTGCTTCATATTATGAACATTGGCTTCGCTAGTCAACTTAACTGAACCGTTAAGCACAATTCG
ATTCCATCTCACTTT
+
FFFFFFFFFFFFFFFFFFFFFFFFFFFFFFFFFFFFFFFFFFFFFFFFFFFFFFFFFFFFFFFFFFFF
FFFFFFFFFFFFFFFFFFFFFFFFFFFFFFFFFFFFFFFFFFFFFFFFFFFFFFFFFFFFFFFFFFFF
FFFFFFFFFFFFFFF
@A00721:190:HGJW7DSXY:4:1408:13865:27727 2:N:0:TCTGCAAG+GCTGCACT
CGATGAATATCTCATTCACTCTGGGATTAGAATTACCGGGACGTCTATCATACACGAGCCACATCCAC
AGCCACTGCTTCATATTATGAACATTGGCTTCGCTAGTCAACTTAACTGAACCGTTAAGCACAATTCG
ATTCCATCTCACTTT
+
FFFFFFFFFFFFFFFFFFFFFFFFFFFFFFFFFFFFFFFFFFFFFFFFFFFFFFFFFFFFFFFFFFFF
FFFFFFFFFFFFFFFFFFFFFFFFFFFFFFFFFFFFFFFFFFFFFFFFFFFFFFFFFFFFFF:FFF:F
FFFFFFFFFFFFFFF
@A00721:190:HGJW7DSXY:4:2177:16215:21496 2:N:0:TCTGCAAG+GCTGCACT
CGATGAATATCTCATTCACTCTGGGATTAGAATTACCGGGACGTCTATCATACACGAGCCACATCCAC

AGCCACTGCTTCATATTATGAACATTGGCTTCGCTAGTCAACTTAACTGAACCGTTAAGCACAATTCG
ATTCCATCTCACTTT
+
FFFFFFFFFFFFFFFFFFFFFFFFFFFFFFFFFFFFFFFFFFFFFFFFFFFFFFFFFFFFFFFFFFFF
FFFFFFFFFFFFFFFFFFFFFFFFFFFFFFFFFFFFFFFFFFFFFFFFFFFFFFFFFFFFFFFFFFFF
FFFFFFFFFFFFFFF
@A00721:190:HGJW7DSXY:4:2238:16541:31704 2:N:0:TCTGCAAG+GCTGCACT
CGATGAATATCTCATTCACTCTGGGATTAGAATTACCGGGACGTCTATCATACACGAGCCACATCCAC
AGCCACTGCTTCATATTATGAACATTGGCTTCGCTAGTCAACTTAACTGAACCGTTAAGCACAATTCG
ATTCCATCTCACTTT
+
FFFFFFFFFFFFFFFFFFFFFFFFFFFFFFFFFFFFFFFFFFFFFFFFFFFFFFFFFFFFFFFFFFFF
FFFFFFFFFFFFFFFFFFF::FFFFFFFFFFFFFF:FFFFFFFFFFFFFFFFF:FFFF:FFFFFFFFF
FFFFFFF:FFFFFFF
@A00721:190:HGJW7DSXY:4:2307:6623:9424 2:N:0:TCTGCAAG+GCTGCACT
CGATGAATATCTCATTCACTCTGGGATTAGAATTACCGGGACGTCTATCATACACGAGCCACATCCAC
AGCCACTGCTTCATATTATGAACATTGGCTTCGCTAGTCAACTTAACTGAACCGTTAAGCACAATTCG
ATTCCATCTCACTTT
+
FFFFFF,FFFFFFFFFFFFFFFFFFFFFFFFFFFFFFFFFFFFFFFFFFFFFFFFFFFFFFFFFFFFF
FFFFFFFFFFFFFFFFFFFFFFFF:FFFFFFFFFF,FFFFFFFFFFFFFFFFF:FFFFFFFFFFFFFF
FFFFFF:FFFFFF:F
@A00721:190:HGJW7DSXY:4:2677:3549:36104 2:N:0:TCTGCAAG+GCTGCACT
CGATGAATATCTCATTCACTCTGGGATTAGAATTACCGGGACGTCTATCATACACGAGCCACATCCAC
AGCCACTGCTTCATATTATGAACATTGGCTTCGCTAGTCAACTTAACTGAACCGTTAAGCACAATTCG
ATTCCATCTCACTTT
+
FFFFFFFFFFFFFFFFFFFFFFFFFFFFFFFFFFFFFFFFFFFFFFFFFFFFFFFFFFFFFFFFFFFF
FFFFFFFFFFFFFFFFFFFF:FFFFFFFFFFFFF,FFFFFFFFFFFFFFFFFFFFFFFFFFFFFFFFF
FFFFFFFFFFFFF,F
@A00721:190:HGJW7DSXY:4:1102:8847:6261 2:N:0:TCTGCAAG+GCTGCACT
CCGATGAATATCTCATTCACTCTGGGATTAGAATTACCGGGACGTCTATCATACACGAGCCACATCCA
CAGCCACTGCTTCATATTATGAACATTGGCTTCGCTAGTCAACTTAACTGAACCGTTAAGCACAATTC
GATTCCATCTCACTT
+
FFFFFFFF,FFFFFFFFFF,FF,:FFF:FFFFFFFFFFFFFFFFFFFFFFFFFFFF:F:FF:FFFFFF
FFFFFFFFFF:FFFFFFF:FFFFF:FFFFFFFFFFFFFFFFFFFFFFFFFFFFFFF:FFFFFFFFFFF
FFFFF:FFFFFFFF:
@A00721:190:HGJW7DSXY:4:1206:28248:5478 2:N:0:TCTGCAAG+GCTGCACT
CCGATGAATATCTCATTCACTCTGGGATTAGAATTACCGGGACGTCTATCATACACGAGCCACATCCA
CAGCCACTGCTTCATATTATGAACATTGGCTTCGCTAGTCAACTTAACTGAACCGTTAAGCACAATTC
GATTCCATCTCACTT
+
FFFFFFFFFFFFFFFFFFFFFFFFFFFFFFFFFFFFFFFFFFFFFFFFFFFFFFFFFFFFFFFFFFFF
FFFFFFFFFFFFFFFFFFFFFF:FFFFFFFFFFFFFFFFF:FFFFFFFFFFFFFFFFFFFFFFFFF:F
FFFFFFFFFFFFFFF
@A00721:190:HGJW7DSXY:4:1239:16532:3035 2:N:0:TCTGCAAG+GCTGCACT
CGATGAATATCTCATTCACTCTGGGATTAGAATTACCGGGACGTCTATCATACACGAGCCACATCCAC
AGCCACTGCTTCATATTATGAACATTGGCTTCGCTAGTCAACTTAACTGAACCGTTAAGCACAATTCG
ATTCCATCTCACTT
+
FFF:FFFF:FFFFF:FFFFFFFFFFF,FFFF::FFFF:FFFFFFFF::F,FFFFFFFFFFFFFFFFFF
FF::FFFF:FFFFFF:FFFFFFFFF:FFFFFF,FFFF:FFFFFFFFFFFFFFFF:FFFF:FFFF:,FF
,,FFFFFFFFFF:F

```
@A00721:190:HGJW7DSXY:4:1267:18493:6402 2:N:0:TCTGCAAG+GCTGCACT
CCGATGAATATCTCATTCACTCTGGGATTAGAATTACCGGGACGTCTATCATACACGAGCCACATCCA
CAGCCACTGCTTCATATTATGAACATTGGCTTCGCTAGTCAACTTAACTGAACCGTTAAGCACAATTC
GATTCCATCTCACTT
+
FFFFFFFFFFFFFFFFFFFFFFFFFFFFFFFFFFFFFFFFFFFFFFFFFFFFFFFFFFFFFFFFFFFFFF
FFFFFFFFFFFFFFFFFFFFFFFFFFFFFFFFFFFFFFFFFFFFFFFFFFFFFFFFFFFFFFFFFFFFFF
FFFFFFFFFFFFFFF
@A00721:190:HGJW7DSXY:4:2219:24099:10535 2:N:0:TCTGCAAG+GCTGCACT
CCGATGAATATCTCATTCACTCTGGGATTAGAATTACCGGGACGTCTATCATACACGAGCCACATCCA
CAGCCACTGCTTCATATTATGAACATTGGCTTCGCTAGTCAACTTAACTGAACCGTTAAGCACAATTC
GATTCCATCTCACTT
+
FFFFFFFFFFFFFFFFFFF,FFFFFFFFFFFFFFFFFFFFFFFFFFFFFFFFFFFFFFFFFFFFFFFFFF
FFFFFFFFFFFFFFFFFFFFFFFFFFFFFFFFFFFFFFFFFFFFFFFFFFFFFFFFFFFFFFFFFFFFFF
FFFFFFFFFFFFFFF
@A00721:190:HGJW7DSXY:4:2239:21215:34350 2:N:0:TCTGCAAG+GCTGCACT
CCGATGAATATCTCATTCACTCTGGGATTAGAATTACCGGGACGTCTATCATACACGAGCCACATCCA
CAGCCACTGCTTCATATTATGAACATTGGCTTCGCTAGTCAACTTAACTGAACCGTTAAGCACAATTC
GATTCCATCTCACTT
+
FFFFFFF:FFFFFFFFFFFFFFFFFFFFFFFFFFFFFFFFFFFFFFFFFFFFFFFFFFFFFFFFF,FFFF
FFFFFFFFFFFFFFFFFFFFFFFFFF:FFFFFFFFFFFFFFFFFFFFFFFFFFF:FFFFFFFFFFFFFFF
:FFFF,FFFFFFFFF
@A00721:190:HGJW7DSXY:4:2170:2618:18176 2:N:0:TCTGCAAG+GCTGCACT
CCCCGATGAATATCTCATTCACTCTGGGATTAGAATTACCGGGACGTCTATCATACACGAGCCACATC
CACAGCCACTGCTTCATATTATGAACATTGGCTTCGCTAGTCAACTTAACTGAACCGTTAAGCACAAT
TCGATTCCATCTCAC
+
FFFFFF:FFFFFFFFFFFFFFFFFFFFFFFFFFFFFFFFFFFFFFFFFFFFFFFFFFFFFFFFFFFFFFF
FFFFFFFFFFFFFFFFFFFFFFFFF:FF:FFFFFFFFFF:FFFFFFFFFFFFFFFFFFFFFFFFFFFFFF
FFFFFFFFFFFFFFF
@A00721:190:HGJW7DSXY:4:2411:17888:10833 2:N:0:TCTGCAAG+GCTGCACT
CCCCGATGAATATCTCATTCACTCTGGGATTAGAATTACCGGGACGTCTATCATACACGAGCCACATC
CACAGCCACTGCTTCATATTATGAACATTGGCTTCGCTAGTCAACTTAACTGAACCGTTAAGCACAAT
TCGATTCCATCTCAC
+
FFFFFFFFFFF,FFFFFFFFFFFFF,:F,FFFFFFF:FFFFFFFFFFFFFFFFFFFFFFFFFFFFFFFFF
FFFFFFFFFFFFFFFFFFFFFFFFF:FFFFFF:F,FFFFFFFFFFFFFFFFFFFFFFFF:FFFF:FFF:F
FFFFFF:FF:FFFF:
@A00721:190:HGJW7DSXY:4:2525:30653:31876 2:N:0:TCTGCAAG+GCTGCACT
CCCCGATGAATATCTCATTCACTCTGGGATTAGAATTACCGGGACGTCTATCATACACGAGCCACATC
CACAGCCACTGCTTCATATTATGAACATTGGCTTCGCTAGTCAACTTAACTGAACCGTTAAGCACAAT
TCGATTCCATCTCAC
+
FFFFFFFFFFFFFFFFFFFFFFFFFFFFFFFFFFFFFFFFFFFFFFFFFFFFFFFFFFFFFFFFFFFFFF
FFFFFFFFFFFFFFFFFFFFFFF:FFFFFFFFFFFFFFFFFFFFFFFFFFFFFFF:FFFFFFFFFFFFF:
FFFFFFFFFFFFFF:
@A00721:190:HGJW7DSXY:4:1140:31186:26162 2:N:0:TCTGCAAG+GCTGCACT
CTTTCCCCGATGAATATCTCATTCACTCTGGGATTAGAATTACCGGGACGTCTATCATACACGAGCCA
CATCCACAGCCACTGCTTCATATTATGAACATTGGCTTCGCTAGTCAACTTAACTGAACCGTTAAGCA
CAATTCGATTCCATC
+
FFFFFFFFFFFFF:FFFFFFFFF:FFFFFFFFFFFFF:FFFFFFFFFFFFFFFFFFFFFFF:FFFFFFFF
```

```
FFFFFFFFFFFFFFFFFFFFFFFFFFFFFFFFFFFFFFFFFFFFFFFFFFFFFFFF:F:FFFFFFFFF
FFFFFFFFFFFFFFF
@A00721:190:HGJW7DSXY:4:1177:19569:18630 2:N:0:TCTGCAAG+GCTGCACT
CTTTCCCCGATGAATATCTCATTCACTCTGGGATTAGAATTACCGGGACGTCTATCATACACGAGCCA
CATCCACAGCCACTGCTTCATATTATGAACATTGGCTTCGCTAGTCAACTTAACTGAACCGTTAAGCA
CAATTCGATTCCATC
+
FFFFFFFFFFFFFFFFFFFFFFFFFFFFFFFFFFFFFFFFFFFFFFFFFFFFFFFFFFFFFFFFFFFF
FFFFFFFFFFFFFFFFFFFFFFFFFFFFFFFFFFFFFFFFFFFFFFFFFFFFFFFFFFFFFFFFFFFF
FF:FFFFFFFFFFFF
@A00721:190:HGJW7DSXY:4:1209:17861:14606 2:N:0:TCTGCAAG+GCTGCACT
CTTTCCCCGATGAATATCTCATTCACTCTGGGATTAGAATTACCGGGACGTCTATCATACACGAGCCA
CATCCACAGCCACTGCTTCATATTATGAACATTGGCTTCGCTAGTCAACTTAACTGAACCGTTAAGCA
CAATTCGATTCCATC
+
F::FFFFFFFFFFFFFFFFFFFFFFF,FFFFFFF:FFFFFFFFFFFFF,FFFFFF:FF:FFFFFFFFF
FFFFFFFFFFFFFFFFFFFFFFFFFF:FFFFFFFFFFFFFFF:F,FFFFFFFFFF:FFFFFFF:FFFF
FFFFFFFFFFFFFFF
@A00721:190:HGJW7DSXY:4:1221:30337:31109 2:N:0:TCTGCAAG+GCTGCACT
CTTTCCCCGATGAATATCTCATTCACTCTGGGATTAGAATTACCGGGACGTCTATCATACACGAGCCA
CATCCACAGCCACTGCTTCATATTATGAACATTGGCTTCGCTAGTCAACTTAACTGAACCGTTAAGCA
CAATTCGATTCCATC
+
FFFFFFFFFFFFFFFFFFFFFFFFFFFFFFFFFFFFFFFFFFFFFFFFFFFFFFFFFFFFFFFFFFFF
FFFFFFFFFFFFFFFFFFFFFFFFFFFFFFFFFFFFFFFFFFFFFFFFFFFFFFFFFFFFFFFFFFFF
FFFFFFFFFFFFFFF
@A00721:190:HGJW7DSXY:4:1261:19054:26005 2:N:0:TCTGCAAG+GCTGCACT
CTTTCCCCGATGAATATCTCATTCACTCTGGGATTAGAATTACCGGGACGTCTATCATACACGAGCCA
CATCCACAGCCACTGCTTCATATTATGAACATTGGCTTCGCTAGTCAACTTAACTGAACCGTTAAGCA
CAATTCGATTCCATC
+
FFFFFFFFFFFFFFFFFFFFFFFFFFFFFFFFF,FFFFFFFFFFFFFFFFFFFFFFFFFFFFFFFFFF
FFFFFFFFFFFFFFFFFFFFFFFFFFFFFFF:FFFFFFFFFFFFFFFFFFFFFFFFFFFFFFFFFFFF
FFFFFFFFFFFFFFF
@A00721:190:HGJW7DSXY:4:1273:17192:7529 2:N:0:TCTGCAAG+GCTGCACT
CTTTCCCCGATGAATATCTCATTCACTCTGGGATTAGAATTACCGGGACGTCTATCATACACGAGCCA
CATCCACAGCCACTGCTTCATATTATGAACATTGGCTTCGCTAGTCAACTTAACTGAACCGTTAAGCA
CAATTCGATTCCATC
+
FFFFFFFFFFFFFFFFFFFFFFFFFFFFFFFFFFFFFFFFFFFFFFFFFFFFFFFFFFFFFFFFFFFF
FFFFFFFFFFFFFFFF:FFFFFFFFFFFFFFFFFFFFFFFF:FFFFFFFFFFFFFFFFFFFFFFFFFF
FF:F:FFFFFFFFFF
@A00721:190:HGJW7DSXY:4:1313:28872:5901 2:N:0:TCTGCAAG+GCTGCACT
CTTTCCCCGATGAATATCTCATTCACTCTGGGATTAGAATTACCGGGACGTCTATCATACACGAGCCA
CATCCACAGCCACTGCTTCATATTATGAACATTGGCTTCGCTAGTCAACTTAACTGAACCGTTAAGCA
CAATTCGATTCCATC
+
FFFFFFFFFFFFFFFFFFFFFFFF:FFFFFFFFFFFFFFFFFFFFFFFFFFFF:FFFFFFFFFFFFFF
F:FFFFFFFFFFFFFFFFFFFFFFFFFFFFFFFFFFFFFFFFFFFFFFFFFFFFFFFFFFFFFFFFFF
FFFFFFFFFFFFFFF
@A00721:190:HGJW7DSXY:4:1314:31295:7435 2:N:0:TCTGCAAG+GCTGCACT
CTTTCCCCGATGAATATCTCATTCACTCTGGGATTAGAATTACCGGGACGTCTATCATACACGAGCCA
CATCCACAGCCACTGCTTCATATTATGAACATTGGCTTCGCTAGTCAACTTAACTGAACCGTTAAGCA
CAATTCGATTCCATC
```

+
FFFFFFFFFFFFF,FF,FFFFFF:FFFF:FFFFFFFF:FFFFFFFFFFFFFFFFFFFFFFFFFFFFFFF
FFFFFFFFFFFFFFFFFFFFFFFFFFFFF:FFFFFFFFFFFF:FFFFFFFFF:FFFFF:FFFFFFFFF
FFFFFFFFFFFFFFF
@A00721:190:HGJW7DSXY:4:1315:22525:20870 2:N:0:TCTGCAAG+GCTGCACT
CTTTCCCCGATGAATATCTCATTCACTCTGGGATTAGAATTACCGGGACGTCTATCATACACGAGCCA
CATCCACAGCCACTGCTTCATATTATGAACATTGGCTTCGCTAGTCAACTTAACTGAACCGTTAAGCA
CAATTCGATTCCATC
+
FFFFFFFFFFFFFFFFFFFFFFFFFFFFFFFFFFFFFFFFFFFFFFFFFFFFFFFFFFFFFFFFFFFF
FFFFFFFFFFFFFFFFFFFFFFFFFFFFFFFFFFFFFFFFFFFFFFFFFFFFFFFFFFFFFFFFFFFF
FFFFFFFFFFFFFFF
@A00721:190:HGJW7DSXY:4:1444:30029:25410 2:N:0:TCTGCAAG+GCTGCACT
CTTTCCCCGATGAATATCTCATTCACTCTGGGATTAGAATTACCGGGACGTCTATCATACACGAGCCA
CATCCACAGCCACTGCTTCATATTATGAACATTGGCTTCGCTAGTCAACTTAACTGAACCGTTAAGCA
CAATTCGATTCCATC
+
FFFFFFFFFFFFFFFFFFFFFFFFFFFFFFFFFFFFFFFFFFFFFF,FFFFFFFFFFFFFFFFF:FFF
F:FFFFFFFFFFFFFFF:FFFFFFFFFFFFFFFF:FFFFFFF:FFFFFFFFFFFFFFF:FFFFFFFFF
FFF,FFFFFFF:FFF
@A00721:190:HGJW7DSXY:4:2148:8757:34976 2:N:0:TCTGCAAG+GCTGCACT
CTTTCCCCGATGAATATCTCATTCACTCTGGGATTAGAATTACCGGGACGTCTATCATACACGAGCCA
CATCCACAGCCACTGCTTCATATTATGAACATTGGCTTCGCTAGTCAACTTAACTGAACCGTTAAGCA
CAATTCGATTCCATC
+
FFFFFFFFFFFFFFFFFFFFFFFFFFFFFFFFFFF:FFFFFFFFFFFFFFFFFFFFFFFFFFFFFFFF
FFFFFFFFFFFFFFFFFFFFFFFFFFFFFFFFFFFFFFFFFFFFFFFFFFFFFFFFFFFFFFFFFFFF
FFFFFFFFFFFFFFF
@A00721:190:HGJW7DSXY:4:2148:11704:35728 2:N:0:TCTGCAAG+GCTGCACT
CTTTCCCCGATGAATATCTCATTCACTCTGGGATTAGAATTACCGGGACGTCTATCATACACGAGCCA
CATCCACAGCCACTGCTTCATATTATGAACATTGGCTTCGCTAGTCAACTTAACTGAACCGTTAAGCA
CAATTCGATTCCATC
+
FFFFFFFFFFFFFFFFFFFFFFFFFFFFFFFFFFFFFFFFFFFFFFFFFFFFFFFFFFFFFFFFFFFF
FFFFFFFFFFFFFFFFFFFFFFFFFFFFFFFFFFFFFFFFFFFFFFFFFFFFFFFFFFFFFFFFFFFF
FFFFFFFFFFFFFFF
@A00721:190:HGJW7DSXY:4:2204:30165:35258 2:N:0:TCTGCAAG+GCTGCACT
CTTTCCCCGATGAATATCTCATTCACTCTGGGATTAGAATTACCGGGACGTCTATCATACACGAGCCA
CATCCACAGCCACTGCTTCATATTATGAACATTGGCTTCGCTAGTCAACTTAACTGAACCGTTAAGCA
CAATTCGATTCCATC
+
FFFFFFFFFFFF:FFFFFFFFFFFFFFFFFFFFFFFFFFFFFFFFFFFFFFFFFFFFFFFFFFFFFFF
FFFFFFFFFFFFFFFFFFFFFFFFFFFFFFFFFFFFFFFFFFFFFFFFFFFFFFFFFFFFFFFFFFFF
FFFFFFFFFFF:FFF
@A00721:190:HGJW7DSXY:4:2206:1434:31751 2:N:0:TCTGCAAG+GCTGCACT
CTTTCCCCGATGAATATCTCATTCACTCTGGGATTAGAATTACCGGGACGTCTATCATACACGAGCCA
CATCCACAGCCACTGCTTCATATTATGAACATTGGCTTCGCTAGTCAACTTAACTGAACCGTTAAGCA
CAATTCGATTCCATC
+
FFFFFFFFFFFFFFFFFFFFFFFFFFFFFFFFFFFFFFFFFFFFFFFFFFFFFFFFFFFFFFFFFFFF
FFFFFFFFFFFFFFFFFFFFFFFFFFFFFFFFFFFFFFFFFFFFFFFFFFFFFFFFFFFFFFFFFFFF
FFFFFFFFFFFFFFF
@A00721:190:HGJW7DSXY:4:2260:15257:31454 2:N:0:TCTGCAAG+GCTGCACT
CTTTCCCCGATGAATATCTCATTCACTCTGGGATTAGAATTACCGGGACGTCTATCATACACGAGCCA

```
CATCCACAGCCACTGCTTCATATTATGAACATTGGCTTCGCTAGTCAACTTAACTGAACCGTTAAGCA
CAATTCGATTCCATC
+
FFFFFFFFFFFFFFF:FFFFFFFFFF,FFFFFFFFFFFFFFFFFFFFFFFFFFFFFFFFFFFFFFFFFF
FFFFFFFFFFFFFFFFFFFFFFFFFFFFFF:FFFFFFFFFFFFFFFFFFFFFFFFFFFFFFFFFFFFF
FFFFFFFFFFFFFFF
@A00721:190:HGJW7DSXY:4:2349:20139:1830 2:N:0:TCTGCAAG+GCTGCACT
CTTTCCCCGATGAATATCTCATTCACTCGGGGATTAGAATTACCGGGACGTCTATCATACACGAGCCA
CATCCACAGCCACTGCTTCATATTATGAACATTGGCTTCGCTAGTCAACTTAACTGAACCGTTAAGCA
CAATTCGATTCCATC
+
FFFFFFFFFFFF:FFFFFFFFFFFFF,F,FFFFFFFFFFFFFFFFFFFFFFFFFFFFFFFFFFFFFFFF
FFFFFFFFFFFFFFFFFFFF,FFFFFFF:FFFFFFFFFFFFFFFFFFFFFFFFFFF:FFFFF:FFFFF
FFF:FFFFFFFFFFF
@A00721:190:HGJW7DSXY:4:2349:12572:11052 2:N:0:TCTGCAAG+GCTGCACT
CTTTCCCCGATGAATATCTCATTCACTCTGGGATTAGAATTACCGGGACGTCTATCATACACGAGCCA
CATCCACAGCCACTGCTTCATATTATGAACATTGGCTTCGCTAGTCAACTTAACTGAACCGTTAAGCA
CAATTCGATTCCATC
+
F::FFFFFFFFFF:FFFFFF,,FF::FFFFFFF,FFF:FFFFFFFFFFFF,FF:FF:FFFFFF:FFFF
FFFFFFF:FFFFFFFF:,FFFFF:FF:FFFFF:FFFF,FFFFFF,FFFFFFFFFFFFFFF:FFFFFF
FFFFFFF,FFFFFFF
@A00721:190:HGJW7DSXY:4:2357:24641:5149 2:N:0:TCTGCAAG+GCTGCACT
CTTTCCCCGATGAATATCTCATTCACTCTGGGATTAGAATTACCGGGACGTCTATCATACACGAGCCA
CATCCACAGCCACTGCTTCATATTATGAACATTGGCTTCGCTAGTCAACTTAACTGAACCGTTAAGCA
CAATTCGATTCCATC
+
FFFFFFFFFFFFFFFFFFFFFFFFFFFF,F:FFF:FFFFFFFFFFFFFFFFFFFF:FFFFFFFFFFFFF
FFFFFFFFFFFFFFFFFFFFFFFFFFFFFFFFFFFFFFFFFFFFFFFFFFF:FFFFFFFFFFFFFFFF
FF,FFF:FFFFFFFF
@A00721:190:HGJW7DSXY:4:2601:17318:4617 2:N:0:TCTGCAAG+GCTGCACT
CTTTCCCCGATGAATATCTCATTCACTCTGGGATTAGAATTACCGGGACGTCTATCATACACGAGCCA
CATCCACAGCCACTGCTTCATATTATGAACATTGGCTTCGCTAGTCAACTTAACTGAACCGTTAAGCA
CAATTCGATTCCATC
+
FFFFFFFFFFFFFFF:FFFFFFFFFFFFFFFFFFFFFFFFFFFFFFFFFFFFFFFFFFFFFFFFFFFFF
FFFFFFFFFFFFFFFFFFFFFFFFFFFFFFFFFFFFFFFFFFF:FFFFFFFFFFFFFFFFFFFFFFFFF
FFFFFFFFFFFFFFF
@A00721:190:HGJW7DSXY:4:2601:17128:4633 2:N:0:TCTGCAAG+GCTGCACT
CTTTCCCCGATGAATATCTCATTCACTCTGGGATTAGAATTACCGGGACGTCTATCATACACGAGCCA
CATCCACAGCCACTGCTTCATATTATGAACATTGGCTTCGCTAGTCAACTTAACTGAACCGTTAAGCA
CAATTCGATTCCATC
+
FFFFFFFFFFFFFF:FFFFFFFFFFFF:FFFFFFFFFFFFFFFFFFFFFFFFFFFFFFFFFFFFFFFFF
FFFFFFFFFFFFFFFFFFFFFFFFFFFFFFFFFFFFFFFFFFFFFFFFFFFFFFFFFFFFFFFFFFFF
FFFFFFFFFFFFFFF
@A00721:190:HGJW7DSXY:4:2664:18014:21010 2:N:0:TCTGCAAG+GCTGCACT
CTTTCCCCGATGAATATCTCATTCACTCTGGGATTAGAATTACCGGGACGTCTATCATACACGAGCCA
CATCCACAGCCACTGCTTCATATTATGAACATTGGCTTCGCTAGTCAACTTAACTGAACCGTTAAGCA
CAATTCGATTCCATC
+
FFFFFFFFFFFFFFF:FFFFFFFFFFFFFFFFFFFFFFFFFFFFFFF:FFFFFF,FFFFFFFFFFFFF
FFFFFFFFFFFFFFFFFFFFFFFFFFFFFFFFFFFFFFFFFFFFFFFFFFFFFFFFFFFFFFFFFFFF
FFFFFFFFFFFFFFF
```

```
@A00721:190:HGJW7DSXY:4:1316:28646:10645 2:N:0:TCTGCAAG+GCTGCACT
CGACTTTCCCCGATGAATATCTCATTCACTCTGGGATTAGAATTACCGGGACGTCTATCATACACGAG
CCACATCCACAGCCACTGCTTCATATTATGAACATTGGCTTCGCTAGTCAACTTAACTGAACCGTTAA
GCACAATTCGATTCC
+
FFFFFFFF::FFFFFFFF:FFFFF,FFFFFFFFF:F:FFF:FFFF,FFFFFFFFFFFF:FFF,FF:F,
FFFFFFFFFFF:FFFF:FF,F:FFFFFFF:FFFFF:F:FFFFF,:,FFFFFFF:FFFFFFF,FFF:FF
FFFFFFFFFFFF:FF
@A00721:190:HGJW7DSXY:4:1454:26729:33098 2:N:0:TCTGCAAG+GCTGCACT
CGACTTTCCCCGATGAATATCTCATTCACTCTGGGATTAGAATTACCGGGACGTCTATCATACACGAG
CCACATCCACAGCCACTGCTTCATATTATGAACATTGGCTTCGCTAGTCAACTTAACTGAACCGTTAA
GCACAATTCGATTCC
+
FFFFFFFFFFFFFFFF,FFFFFFFFFFFFFFFFFFFFFFFFFFFFFFFFFFFFFFFFFFFFFFFFFFF
FFFFFFFFFFFFFFFFFFFFFFFFFFFFFFFFFFFFFFFFFFFFFFFFFFFFFFFFFFFFFFFFFFFF
FFFFFFFFFFFFFFF
@A00721:190:HGJW7DSXY:4:1512:1678:22060 2:N:0:TCTGCAAG+GCTGCACT
CGACTTTCCCCGATGAATATCTCATTCACTCTGGGATTAGAATTACCGGGACGTCTATCATACACGAG
CCACATCCACAGCCACTGCTTCATATTATGAACATTGGCTTCGCTAGTCAACTTAACTGAACCGTTAA
GCACAATTCGATTCC
+
FFFFFFFFFFFFFFFFFFFFFFFFFFFFF:FFFFFFFFFFFFFFFFFFFFFFFFFFFFFFFFFFFFFF
FFFFFFFFFFFFFFFFFFFFFFFFFFFFFFFFFFFFFFFFFFFFFFFFFFFFFFFFFFFFFFFFFFFF
FFFFFFFFFFFFFFF
@A00721:190:HGJW7DSXY:4:1571:16794:29262 2:N:0:TCTGCAAG+GCTGCACT
CGACTTTCCCCGATGAATATCTCATTCACTCTGGGATTAGAATTACCGGGACGTCTATCATACACGAG
CCACATCCACAGCCACTGCTTCATATTATGAACATTGGCTTCGCTAGTCAACTTAACTGAACCGTTAA
GCACAATTCGATTCC
+
FFFFFFFFFFFFFFFF:FFFFFFFFFFFFFFFFFFFFFFFFFFFFFFFFFFFFFFFFFFFFFFFFFFF
FFFFFFFFFFFFFFFFFFFFFFFFFFFFFFFFFFFFFFFFFFFFFFFFFFFFFFFFFFFFFFFFFFFF
FFFFFFFFFFFFFFF
@A00721:190:HGJW7DSXY:4:2624:12355:24799 2:N:0:TCTGCAAG+GCTGCACT
CGACTTTCCCCGATGAATATCTCATTCACTCTGGGATTAGAATTACCGGGACGTCTATCATACACGAG
CCACATCCACAGCCACTGCTTCATATTATGAACATTGGCTTCGCTAGTCAACTTAACTGAACCGTTAA
GCACAATTCGATTCC
+
FFFFFFFFFFFFFFFFFFFFFFFFFFFFFFFFFFFFFFFFFFFFFFFFFFFFFFFFFFFFFFFFFFFF
FFFFFFFFFFFFFFFFFFFF:FFFF:FFFFFFFFFFFFFFFFFFFFFFFFFFFFFFFFFFFFFFFFFF
FFFFFFFF:FFFFFF
@A00721:190:HGJW7DSXY:4:2638:16812:15358 2:N:0:TCTGCAAG+GCTGCACT
CGACTTTCCCCGATGAATATCTCATTCACTCTGGGATTAGAATTACCGGGACGTCTATCATACACGAG
CCACATCCACAGCCACTGCTTCATATTATGAACATTGGCTTCGCTAGTCAACTTAACTGAACCGTTAA
GCACAATTCGATTCC
+
FFFFFFFFFFFFFFFFFFFFFFFFFFFFFFF:FFFFFFFFFFFFFFFF:F:FFFFFFFFFFFFFFFFF
F:FFFFFFFFFFFFFFFFFFFFFFFFFFF,:FFFFF:FF:FF:FFFF:FFF:FFFFFFFFFFFFFFFF
FFFFFF:FFFFFF:F
@A00721:190:HGJW7DSXY:4:2660:22327:25379 2:N:0:TCTGCAAG+GCTGCACT
CGACTTTCCCCGATGAATATCTCATTCACTCTGGGATTAGAATTACCGGGACGTCTATCATACACGAG
CCACATCCACAGCCACTGCTTCATATTATGAACATTGGCTTCGCTAGTCAACTTAACTGAACCGTTAA
GCACAATTCGATTC
+
F:F:FFF:F,FFF::FFFFFFFFFFF:FFF,FFFFFFFF,FF:FF:::F:FFFFFFFF,FFFFFF:F,
```

```
,:FFFFF:FFFFFFFFF,,FFFFFFF:F:,FF:F::F,FFFF,,FFFFFFFF:FFFFFFFF:,:F,FF
,,FFFFFFF:FFFF
@A00721:190:HGJW7DSXY:4:1145:2157:8891 2:N:0:TCTGCAAG+GCTGCACT
CCCGACTTTCCCCGATGAATATCTCATTCACTCTGGGATTAGAATTACCGGGACGTCTATCATACACG
AGCCACATCCACAGCCACTGCTTCATATTATGAACATTGGCTTCGCTAGTCAACTTAACTGAACCGTT
AAGCACAATTCGATT
+
:FFFFFFFFFFFFFFFFF,FFFFFF:FFF:FFFFFFFF:FFFFFFFFFFF:FFFFFFFFFFF:FFFFF
FFFFFFFFFFFFFFFF:FFFFFFFFFFFFFFFFFFFFF:FFFFFFFFFFFFFFFFFFF:FFFFFFFFF
FFFFFFFF,FFFFFF
@A00721:190:HGJW7DSXY:4:1159:14271:29716 2:N:0:TCTGCAAG+GCTGCACT
CCCGACTTTCCCCGATGAATATCTCATTCACTCTGGGATTAGAATTACCGGGACGTCTATCATACACG
AGCCACATCCACAGCCACTGCTTCATATTATGAACATTGGCTTCGCTAGTCAACTTAACTGAACCGTT
AAGCACAATTCGATT
+
FFFFFFFFFFFFFFFFFFFFFFFFFFFFFFFFF:FFFF:FFFFFFFFFFFFFFFFFFFFFFFFFFFFF
FFFFFFFFFFFFFFFFFFFFFFFFFFFFFFFFFFFFFFFFFFFFFFFFFFFFFFFFFFFFFFFFFFFF
FFFFFFFFFFFFFFF
@A00721:190:HGJW7DSXY:4:1313:12066:26271 2:N:0:TCTGCAAG+GCTGCACT
CCCGACTTTCCCCGATGAATATCTCATTCACTCTGGGATTAGAATTACCGGGACGTCTATCATACACG
AGCCACATCCACAGCCACTGCTTCATATTATGAACATTGGCTTCGCTAGTCAACTTAACTGAACCGTT
AAGCACAATTCGATT
+
FFFFFFFFFFFFFFFFFFFFFFFFFFFFFF:FFFFFFFFFFFFFFFFFFFFFFFFFFFFFFFFFFFFF
FFFFFFFFFFFFFFFFFFFFFFFFFFFFFFFFFFFFFFFFFFFFFFFFFFFFFFFFFFFFFFFFFFFF
FFFFFFF:FFFFFFF
@A00721:190:HGJW7DSXY:4:1635:4083:16329 2:N:0:TCTGCAAG+GCTGCACT
CCCGACTTTCCCCGATGAATATCTCATTCACTCTGGGATTAGAATTACCGGGACGTCTATCATACACG
AGCCACATCCACAGCCACTGCTTCATATTATGAACATTGGCTTCGCTAGTCAACTTAACTGAACCGTT
AAGCACAATTCGATT
+
FFFFFFFFFFFFFFFFFFFFFFFFFFFFFFFFFFFFFFFFFFFFFFFFFFFFFFFFFFFFFFFFFFFF
FFFFFFFFFFFFFFFFFFFFFFFFFFFFFFFFFFFFFFFFFFFFFFFFFFFFFFFFFFFFFFFFFFFF
FFFFFFFFFFFFFFF
@A00721:190:HGJW7DSXY:4:1664:16776:26256 2:N:0:TCTGCAAG+GCTGCACT
CCCGACTTTCCCCGATGAATATCTCATTCACTCTGGGATTAGAATTACCGGGACGTCTATCATACACG
AGCCACATCCACAGCCACTGCTTCATATTATGAACATTGGCTTCGCTAGTCAACTTAACTGAACCGTT
AAGCACAATTCGATT
+
FFFFFFFFFFFFFFFFFFFFFFFFFFFFF:FFFFFFFFFFFFFFFFFFFFFFFFFFFFFFFFFFFFFF
FFFFFFFFFFFFFFFFFFFFFFFFFFFFFFFFFFFFFFFFFFFFFFFFFFFFFFFFFFFFFFFFFFFF
FFFFFFF,FFFFFFF
@A00721:190:HGJW7DSXY:4:1676:14724:13745 2:N:0:TCTGCAAG+GCTGCACT
CCCGACTTTCCCCGATGAATATCTCATTCACTCTGGGATTAGAATTACCGGGACGTCTATCATACACG
AGCCACATCCACAGCCACTGCTTCATATTATGAACATTGGCTTCGCTAGTCAACTTAACTGAACCGTT
AAGCACAATTCGATT
+
FFFFFFFFFFFFFFFFFFFFFFFFFFFFFFFFFFFFFFFFFFFFFFFFFFFFFFFFFFFFFFFFFFFF
FFFFFFFFFFFFFFFFFFFFFFFFFFFFFFFFFFFFFFFFFFFFFFFFFFFFFFF:FFFFF:FFFFFF
FFFFFFFFFFFFFFF
@A00721:190:HGJW7DSXY:4:2117:16812:19930 2:N:0:TCTGCAAG+GCTGCACT
CCCGACTTTCCCCGATGAATATCTCATTCACTCTGGGATTAGAATTACCGGGACGTCTATCATACACG
AGCCACATCCACAGCCACTGCTTCATATTATGAACATTGGCTTCGCTAGTCAACTTAACTGAACCGTT
AAGCACAATTCGATT
```

```
+
FFFFFFFFFF:F:FFFFFFFF:FFFFFFFF:FFFFFFF:FFFF:FFF:FFFFFF:FFFFFFFFFFFF
FFFFFFFFFFFFFFFFFFFFFF:FFFFF:FFFFFF:F:FF:FFFFFFF:FFFFFFFFFFFFFFFF::
FFFFFFFF:FFF,FF
@A00721:190:HGJW7DSXY:4:2137:18765:1110 2:N:0:TCTGCAAG+GCTGCACT
CCCGACTTTCCCCGATGAATATCTCATTCACTCTGGGATTAGAATTACCGGGACGTCTATCATACACG
AGCCACATCCACAGCCACTGCTTCATATTATGAACATTGGCTTCGCTAGTCAACTTAACTGAACCGTT
AAGCACAATTCGATT
+
FFFFFFFFFFFFFFFFFFFFFFFFFFFFFFFFFFFFFFFFFFFFFFFFFFFFFFFFFFFFFFFFFFFF
:FFFFFFFFFFFFFFFFFFFFFFFFFFFFFFFFFFFFFFFFFFFFFFFFFFFFFFFFFFFFFFFFFF:
FFFFFFFF:FFFFFF
@A00721:190:HGJW7DSXY:4:2352:2248:17347 2:N:0:TCTGCAAG+GCTGCACT
CCCGACTTTCCCCGATGAATATCTCATTCACTCTGGGATTAGAATTACCGGGACGTCTATCATACACG
AGCCACATCCACAGCCACTGCTTCATATTATGAACATTGGCTTCGCTAGTCAACTTAACTGAACCGTT
AAGCACAATTCGATT
+
FFFFFFFFFFFFFFF:FFFFFFFFFFFFF:F:FFF:F:F:FFFFF,FFFFFFFF:FFF:FFFFFFFFF
FFFFFFFFFFFFFFFFFFFFFFFFF:FFFFFFFFFFF:FF:F,FFFF:FFFFFFF:FFFFFFFFFF,F
FF:FFF:FFFFFFFF
@A00721:190:HGJW7DSXY:4:2440:21802:29794 2:N:0:TCTGCAAG+GCTGCACT
CCCGACTTTCCCCGATGAATATCTCATTCACTCTGGGATTAGAATTACCGGGACGTCTATCATACACG
AGCCACATCCACAGCCACTGCTTCATATTATGAACATTGGCTTCGCTAGTCAACTTAACTGAACCGTT
AAGCACAATTCGATT
+
FFFFFFFFFFFFFFFFFFFFFFFFFFFFFFFFFFFFFFFFFFFFFFFFFFFFFFFFFFFFFFFFFFFF
F,F:FFFFFFFFFFFFFFFFFFFFFFFFFFFFFFFFFFFFF:FFF:FFF:FFFFFFFFFFFFFFFFFF
:F:FFFF,FF:FFFF
@A00721:190:HGJW7DSXY:4:2652:26576:7435 2:N:0:TCTGCAAG+GCTGCACT
CCCGACTTTCCCCGATGAATATCTCATTCACTCTGGGATTAGAATTACCGGGACGTCTATCATACACG
AGCCACATCCACAGCCACTGCTTCATATTATGAACATTGGCTTCGCTAGTCAACTTAACTGAACCGTT
AAGCACAATTCGATT
+
FFFFFFFFFFFFFFFFFFFFFFFFFFFFFFFFFFFFFFFFFFFFFFFFFFFFFFFFFFFFFFFFFFFF
FFFFFFFFFFFFFFFFFFFFFFFFFFFFFFFFFFFFFFFFFFFF:FFFFFFFFFFFFFFFFFFFFFF:F
,FFFFFFFFFFFFFF
@A00721:190:HGJW7DSXY:4:2656:23773:24158 2:N:0:TCTGCAAG+GCTGCACT
CCCGACTTTCCCCGATGAATATCTCATTCACTCTGGGATTAGAATTACCGGGACGTCTATCATACACG
AGCCACATCCACAGCCACTGCTTCATATTATGAACATTGGCTTCGCTAGTCAACTTAACTGAACCGTT
AAGCACAATTCGATT
+
FFFFFFFFFFFFFFFFFFFFFFFFFFFFFFFFFFFFFFFFFFFFFFFFFFFFFFFFFFFFFFFFFFFF
FFFFFFFFFFFFFFFFFFFFFFFFFFFFFFFFFFFFFFFFFFFFFFFFFFFFFFFFFFFFFFFFFFFF
FFFFFFFFFFFFFF:
@A00721:190:HGJW7DSXY:4:2656:23909:24236 2:N:0:TCTGCAAG+GCTGCACT
CCCGACTTTCCCCGATGAATATCTCATTCACTCTGGGATTAGAATTACCGGGACGTCTATCATACACG
AGCCACATCCACAGCCACTGCTTCATATTATGAACATTGGCTTCGCTAGTCAACTTAACTGAACCGTT
AAGCACAATTCGATT
+
FFFFFFFFFFFFFFFFFFFFFFFFFFFFFFFFFF:FFFFFFFFFFFFFFFFFFFFFFFFFFFFFFFFF
FFFFFFFFFFFFFFFFFFFFFFFFFFFFFFFFFFFFFFFFFFFFFFFFFFFFFFFFFFFFFFFFFF:F
FFFFFFFFFFFFFFF
@A00721:190:HGJW7DSXY:4:1311:23710:28244 2:N:0:TCTGCAAG+GCTGCACT
AAGTATCTGGATCCCGACTTTCCCCGATGAATATCTCATTCACTCTGGGATTAGAATTACCGGGACGT
```

```
CTATCATACACGAGCCACATCCACAGCCACTGCTTCATATTATGAACATTGGCTTCGCTAGTCAACTT
AACTGAACCGTTAAG
+
FFFFFFFFFFFFFFFFFFFFFFFFFFFFFFFFFFFFFFFFFFFF:FFFFFFFFFFFFFFFFFFFFFFF
FFFFFFFFF,FFFFFFFFFFFFFFFFFFFFFFFFFFFFFFFFFFFFFFFFFFFFFFFFF:FFFFFFFF
FFFFFFFFFFFFFFF
@A00721:190:HGJW7DSXY:4:1643:29948:13369 2:N:0:TCTGCAAG+GCTGCACT
AAGTATCTGGATCCCGACTTTCCCCGATGAATATCTCATTCACTCTGGGATTAGAATTACCGGGACGT
CTATCATACACGAGCCACATCCACAGCCACTGCTTCATATTATGAACATTGGCTTCGCTAGTCAACTT
AACTGAACCGTTAAG
+
FFFFFFFFFFFFFFFFFFFFFFFFFFFFFF:FFFFFFFFFFFFFFFFFFFFFFFFFFFFFFFFFFFFF
FFFFFFFFFFFFFFFFFFFFFF:FFFFFFFFFFFFFFFFFFFFFFFFFFFFFFFFFFFFFFFFFFFFF
FFFFFFFFFFFFFFF
@A00721:190:HGJW7DSXY:4:2215:27633:11021 2:N:0:TCTGCAAG+GCTGCACT
AAGTATCTGGATCCCGACTTTCCCCGATGAATATCTCATTCACTCTGGGATTAGAATTACCGGGACGT
CTATCATACACGAGCCACATCCACAGCCACTGCTTCATATTATGAACATTGGCTTCGCTAGTCAACTT
AACTGAACCGTTAAG
+
FFFFFFFFFFFFFFFFFFFF,FFFFFFFFFFFFFFFFFFFFF,FF:FFFFFFF::FF:FFFFFFFFFF
FF,F:FFFFFFFFFFFFFFF,FFFFFFFFF,FFFF:FFFF:FFFFFFFF::,FFFF,FFF:F:F:F:F
FFFF:FFFFF:,FFF
@A00721:190:HGJW7DSXY:4:2413:28311:7968 2:N:0:TCTGCAAG+GCTGCACT
AAGTATCTGGATCCCGACTTTCCCCGATGAATATCTCATTCACTCTGGGATTAGAATTACCGGGACGT
CTATCATACACGAGCCACATCCACAGCCACTGCTTCATATTATGAACATTGGCTTCGCTAGTCAACTT
AACTGAACCGTTAAG
+
FFFFFFFFFFFFFFFFFFFFFFFFFFFFFFFFFFFFFFFFFFFFFFFFFFFFFFFFFFFFFFFFFFFF
FFFFFFFFFFFFFFFFFFFFFFFFFFFFFFFFFFFFFFFFFFFFFFFFFFFFFFFFFFFFFFFFFFFF
FFFFFFFFFFFFFFF
@A00721:190:HGJW7DSXY:4:2426:9444:30311 2:N:0:TCTGCAAG+GCTGCACT
AAGTATCTGGATCCCGACTTTCCCCGATGAATATCTCATTCACTCTGGGATTAGAATTACCGGGACGT
CTATCATACACGAGCCACATCCACAGCCACTGCTTCATATTATGAACATTGGCTTCGCTAGTCAACTT
AACTGAACCGTTAAG
+
FFFFFFFFFFFFFFFFFFFFFFFFFFFFFFFFFFFFFFFFFFFFFFFFFFFFFFF:FFFFFFFFFFF
FFFFFFFFFFFFFFFFFFFFFFFFFF:FFFFFFFFFFFFFFFFFFFFFFFF:FFFFFFFFFFFFFFFF
FFFFFFFFFF:FFFF
@A00721:190:HGJW7DSXY:4:2426:9362:30859 2:N:0:TCTGCAAG+GCTGCACT
AAGTATCTGGATCCCGACTTTCCCCGATGAATATCTCATTCACTCTGGGATTAGAATTACCGGGACGT
CTATCATACACGAGCCACATCCACAGCCACTGCTTCATATTATGAACATTGGCTTCGCTAGTCAACTT
AACTGAACCGTTAAG
+
FFFFFFFFFFFFFFFFFFFFFFFFFFFFFFFFFFFFFFFFFFFFFFFFFF:FFFFFFFFFFFFFFFFF
FFFFFFFFFFFFFFFFFFFFFFFFFFFFFFFFFFFFFFFFFFFFFFFF,FFFFFFFFF:FFFFFFFFF
FFFFFFFFFF,FFFF
@A00721:190:HGJW7DSXY:4:2605:23285:10567 2:N:0:TCTGCAAG+GCTGCACT
AAGTATCTGGATCCCGACTTTCCCCGATGAATATCTCATTCACTCTGGGATTAGAATTACCGGGACGT
CTATCATACACGAGCCACATCCACAGCCACTGCTTCATATTATGAACATTGGCTTCGCTAGTCAACTT
AACTGAACCGTTAAG
+
FFFFFFFFFFFFFFFFFFFFFFFFFFFFFFFFFFFFFFFFFFFFFFFFFFFFFFFFFFFFFFFFFFFF
FFFFFFFFFFFFFFFFFFFFFFFFFFFFFFFFFFFFFFFFFFFFFFFFFFFFFFFFFFFFFFFFFFFF
FFFFFFFFFFFFFFF
```

@A00721:190:HGJW7DSXY:4:1377:20943:29027 2:N:0:TCTGCAAG+GCTGCACT
CAAGTATCTGGATCCCGACTTTCCCCGATGAATATCTCATTCACTCTGGGATTAGAATTACCGGGACG
TCTATCATACACGAGCCACATCCACAGCCACTGCTTCATATTATGAACATTGGCTTCGCTAGTCAACT
TAACTGAACCGTTAA
+
FFFFF:FFFFFFFFFFFFFFFFFFFFFFFFF:FFFFFFFFFFFFFFFFFFFFFFFFFFFFFFFFFFFF
FFFFFFFFFFFFFFFFFFFFFFFFFFFFFFFFFFFFFFFFFFFFFFFFFFFFFFFFFFFFFFFFFFFF
FFFFFFFFFFFFFFF
@A00721:190:HGJW7DSXY:4:1637:13792:16736 2:N:0:TCTGCAAG+GCTGCACT
CAAGTATCTGGATCCCGACTTTCCCCGATGAATATCTCATTCACTCGGGGATTAGAATTACCGGGACG
TCTATCATACACGAGCCACATCCACAGCCACTGCTTCATATTATGAACATTGGCTTCGCTAGTCAACT
TAACTGAACCGTTAA
+
FFFFFFFFFFFFFFFFFFFFFFFFFF:FFF,FFF,FFFFFFFF:,F,FFF:FFFFFFFFF:FFFFFFF
F,FFFF:FFFFFFFFFFFFF:FFFF:FFFFF:FF,FFF:FFFFFF,FFFFFFFFFFFFFFF:FFFF:F
FFFFFFF:FFF:FFF
@A00721:190:HGJW7DSXY:4:2158:2338:21104 2:N:0:TCTGCAAG+GCTGCACT
CAAGTATCTGGATCCCGACTTTCCCCGATGAATATCTCATTCACTCTGGGATTAGAATTACCGGGACG
TCTATCATACACGAGCCACATCCACAGCCACTGCTTCATATTATGAACATTGGCTTCGCTAGTCAACT
TAACTGAACCGTTAA
+
FFFFFFFFFFFFFFFFFFF:FFFFFFFFFFFFFFFFFFFFFFFFFFFFFFF:FFFFFFFFFFF:FFFF
FFF:FFFFFFFFFF:FFFFFFFFFFFFFF:FFFFFFFFFFFFFFFFFF:FF::F,FFFFFFFFFFFFF
FFFFFFFFFFFFFFF
@A00721:190:HGJW7DSXY:4:2212:17833:27868 2:N:0:TCTGCAAG+GCTGCACT
AAGTATCTGGATCCCGACTTTCCCCGATGAATATCTCATTCACTCTGGGATTAGAATTACCGGGACGT
CTATCATACACGAGCCACATCCACAGCCACTGCTTCATATTATGAACATTGGCTTCGCTAGTCAACTT
AACTGAACCGTTAA
+
:F:FFFFF,FF:FFF:FFFFFFFF:FF:FFFFFFFFFFFFFFF:FFFFFF:FFFFFFFFF,FF,F,F:
:F:FFFFFFFFFF,FFFFFF:FFFFF:FFFF,,:,:F,FFFF,FFF:FFF::,:FFFFFFFFFFF,FF
FF:F,FF:,FFFFF
@A00721:190:HGJW7DSXY:4:2470:13621:29340 2:N:0:TCTGCAAG+GCTGCACT
CAAGTATCTGGATCCCGACTTTCCCCGATGAATATCTCATTCACTCTGGGATTAGAATTACCGGGACG
TCTATCATACACGAGCCACATCCACAGCCACTGCTTCATATTATGAACATTGGCTTCGCTAGTCAACT
TAACTGAACCGTTAA
+
FFFFFFFFFFFFFFFFFFFFFFFFFFFFFFFFFFFFFFFFFFFFFFFFFFFFFFFFFFFFFFFFFFFF
FFFFFFFFFFFFFFFFFFFFFFFFFFFFFFFFFFFFFFFFFFFFFFFFFFFFFFFFFFFFFFFFFFFF
FFFFFFFFFFFFFFF
@A00721:190:HGJW7DSXY:4:1126:7744:7075 2:N:0:TCTGCAAG+GCTGCACT
CCAAGTATCTGGATCCCGACTTTCCCCGATGAATATCTCATTCACTCTGGGATTAGAATTACCGGGAC
GTCTATCATACACGAGCCACATCCACAGCCACTGCTTCATATTATGAACATTGGCTTCGCTAGTCAAC
TTAACTGAACCGTTA
+
FFFFFFFFFFF:FFFFFF::FFFFFFFF,F:FF,FFFFF,FFF::F:FFFFFFFFFFFFFF:FFFFFF
FFFFFFFFFFFFF:FFFFFFFFFFF:FFFFFFFFFFFFFFFFFFFF,FFFFFFFFFFF:FFFF:FFFF
FFF:FFFFFFFFFFF
@A00721:190:HGJW7DSXY:4:1362:22073:28260 2:N:0:TCTGCAAG+GCTGCACT
CCAAGTATCTGGATCCCGACTTTCCCCGATGAATATCTCATTCACTCTGGGATTAGAATTACCGGGAC
GTCTATCATACACGAGCCACATCCACAGCCACTGCTTCATATTATGAACATTGGCTTCGCTAGTCAAC
TTAACTGAACCGTTA
+
FFFFFFFFFFFFFFFFFFFFFFFFFFFFFFFFFFFFFFFFFFFFFFFFFFFFF:FFFFFFFFFFFFFF

FFFFFFFFFFFFFFFFFFFFFFFFFFFFFFFFFFFFFFFFFFFFFFFFFFFFFFFFFFFFFFFFFFFFFF
FFFFFFFFFFFFFFF
@A00721:190:HGJW7DSXY:4:1411:32280:29183 2:N:0:TCTGCAAG+GCTGCACT
CCAAGTATCTGGATCCCGACTTTCCCCGATGAATATCTCATTCACTCTGGGATTAGAATTACCGGGAC
GTCTATCATACACGAGCCACATCCACAGCCACTGCTTCATATTATGAACATTGGCTTCGCTAGTCAAC
TTAACTGAACCGTTA
+
FFFFFFFFFFFFFFFFFFFFFFFFFFFFFFFF:FFFFFFF:FFFF:FF,FFFFFFFFFFFFFFFFFFFFF
FFFFFFFFFFFFFFFFFFFFFFFFFFFFFFFFFFFFFFFFFFFFFFFF:FFFFFFFFFFFFFFFFFFFFF
FFFFFFFFFFFFFFF
@A00721:190:HGJW7DSXY:4:1441:20048:8437 2:N:0:TCTGCAAG+GCTGCACT
CCAAGTATCTGGATCCCGACTTTCCCCGATGAATATCTCATTCACTCTGGGATTAGAATTACCGGGAC
GTCTATCATACACGAGCCACATCCACAGCCACTGCTTCATATTATGAACATTGGCTTCGCTAGTCAAC
TTAACTGAACCGTTA
+
FFFFFFFFFFFFFFFFFFFFFFFFFFFFFFFFFFFFFFFFFFFFFFFFFFFFFFFFFFFFFFFFFFFFFF
FFFFFFFFFFFFFFFFFFFFFFFFFFFFFFFFFFFFFFFFFFFFFFFFFFFFFFFFFFFFFFFFFFFFFF
FFFFFFF:FFFFFFF
@A00721:190:HGJW7DSXY:4:1474:11867:36229 2:N:0:TCTGCAAG+GCTGCACT
CCAAGTATCTGGATCCCGACTTTCCCCGATGAATATCTCATTCACTCTGGGATTAGAATTACCGGGAC
GTCTATCATACACGAGCCACATCCACAGCCACTGCTTCATATTATGAACATTGGCTTCGCTAGTCAAC
TTAACTGAACCGTTA
+
FFFFFFFFFFFFFFFFFFFFFFFFFFFFFFFFFFFFFFFFFFFFFFFFFFFFFFFFFFF:FFFFFFFFFF
FFFFFFFFFFF:FFFFFFFFFFFFFFFFFFFFFFFFFFFFFFFFFFFFFFFFFFFFFFFFFFFFFFFFFF
FFFFFFFFFFFFFFF
@A00721:190:HGJW7DSXY:4:1543:19886:23750 2:N:0:TCTGCAAG+GCTGCACT
CCAAGTATCTGGATCCCGACTTTCCCCGATGAATATCTCATTCACTCTGGGATTAGAATTACCGGGAC
GTCTATCATACACGAGCCACATCCACAGCCACTGCTTCATATTATGAACATTGGCTTCGCTAGTCAAC
TTAACTGAACCGTTA
+
FFFFFFF:FFFFFFFFFFFFF:FFFFFFFFFFF:FFFFFFFFFFFF:F,FFFFFFFFFFFFFFFFFFFFF
FFFFF:FFFFFFFFFFFFFFFFFFFFFFFFF,FFFFFFFF:FFFFFFFFFFFFFFFFFFFFFFFFFFFFF
FFFFFFFFFFFFF:F
@A00721:190:HGJW7DSXY:4:2265:18349:30170 2:N:0:TCTGCAAG+GCTGCACT
CCAAGTATCTGGATCCCGACTTTCCCCGATGAATATCTCATTCACTCTGGGATTAGAATTACCGGGAC
GTCTATCATACACGAGCCACATCCACAGCCACTGCTTCATATTATGAACATTGGCTTCGCTAGTCAAC
TTAACTGAACCGTTA
+
FFFFFFFFFFFFFFFFFFFFFFFFFFFFFFFFF,FFFFFFFFFFFFFF:FFFFFFFFFFFFFFFFFFFFF
FFFFFFFFFFFFFFFFFFFFFFFFFFFFFFFFFFFFFFFFFFFF,FFFFFFFFFFFFFFFFFFF:FFFFF
FFFFFFFFFFF:F::
@A00721:190:HGJW7DSXY:4:2507:11966:28823 2:N:0:TCTGCAAG+GCTGCACT
CCAAGTATCTGGATCCCGACTTTCCCCGATGAATATCTCATTCACTCTGGGATTAGAATTACCGGGAC
GTCTATCATACACGAGCCACATCCACAGCCACTGCTTCATATTATGAACATTGGCTTCGCTAGTCAAC
TTAACTGAACCGTTA
+
FFFFFFFFFFFFFFFFFFFFFFFFFFFFFFFFFFFFFFFFFFFFFFFFFFFFFFFFFFFFFFFFFFFFFF
FFFFFFFFFFFFFFFFFFFFFFFFFFFFFFFFFFFFFFFFFFFFFFFFFFFFFFFFFFFFFFFFFFFFFF
FFFFFFFFFFFFFFF
@A00721:190:HGJW7DSXY:4:2658:18258:26318 2:N:0:TCTGCAAG+GCTGCACT
CCAAGTATCTGGATCCCGACTTTCCCCGATGAATATCTCATTCACTCTGGGATTAGAATTACCGGGAC
GTCTATCATACACGAGCCACATCCACAGCCACTGCTTCATATTATGAACATTGGCTTCGCTAGTCAAC
TTAACTGAACCGTTA

```
+
FFFFFFFFFFFFFFFFFFFFFFFFFFFFFFFFFFFFFFFFFFFFFFFFFFFFFFFFFFFFFFFFFFFF
FFFFFFFFFFFFFFFFFFFFFFFFFFFFFFFFFFFFFFFFFFFFFFFFFFFFFFFFFFFFFF:FFFFF
FFFFFFFFFFFFFFF
@A00721:190:HGJW7DSXY:4:1578:18530:7341 2:N:0:TCTGCAAG+GCTGCACT
CATCCAAGTATCTGGATCCCGACTTTCCCCGATGAATATCTCATTCACTCTGGGATTAGAATTACCGG
GACGTCTATCATACACGAGCCACATCCACAGCCACTGCTTCATATTATGAACATTGGCTTCGCTAGTC
AACTTAACTGAACCG
+
FFFFFFFFFFFFFFFFFFFFF:FFFFFFFFFFFFFFFFFFFFFFFFF:FFFFFFFFFFFFFFFFFFFF
FFFFFFFFFFFFFFFFFFFFFFFFFFFFFFFFFFFFFFFFFFFFFFFFFFFFFFFFFFFFFFFFFFFF
FFFFFFFFFFFFFFF
@A00721:190:HGJW7DSXY:4:1610:19768:23140 2:N:0:TCTGCAAG+GCTGCACT
CATCCAAGTATCTGGATCCCGACTTTCCCCGATGAATATCTCATTCACTCTGGGATTAGAATTACCGG
GACGTCTATCATACACGAGCCACATCCACAGCCACTGCTTCATATTATGAACATTGGCTTCGCTAGTC
AACTTAACTGAACCG
+
FFFFFFFFFFFFFFFFFFFFFFFFFFFFFFFFFFF,FFFFFFFFFFFFFFFFF:FFFFFFFFFFFFFF
FFFFFFFFFFFFFFFFFFFFFFFFFFFFFFFFFFFFFFFFFFFFFFFFFFFFFFFFFFFFFFFFFFFF
FFFFFFFFFFFFFFF
@A00721:190:HGJW7DSXY:4:2660:11605:2300 2:N:0:TCTGCAAG+GCTGCACT
CATCCAAGTATCTGGATCCCGACTTTCCCCGATGAATATCTCATTCACTCTGGGATTAGAATTACCGG
GACGTCTATCATACACGAGCCACATCCACAGCCACTGCTTCATATTATGAACATTGGCTTCGCTAGTC
AACTTAACTGAACCG
+
FFFFFF:FF:FFFFFFFFFFFFFFFFFFFFFFFFFFFFFFFFFFFFFFFFFFFFFFFFFFFFFFFF:F
FFFFFFF:FFFFFFFFFFFFFFFFFFFFFF:FFFFFFFFFFFFFFFFFFFFFFFFFFFFFFFFF:FFF
FFFFFFFFFFFFFFF
@A00721:190:HGJW7DSXY:4:1244:15130:31735 2:N:0:TCTGCAAG+GCTGCACT
GTTCAGTTAAGTTGACTAGCGAAGCCAATGTTCATAATATGAAGCAGTGGCTGTGGATGTGGCTCGTG
TATGATAGACGTCCCGGTAATTCTAATCCCAGAGTGAATGAGATATTCATCGGGGAAAGTCGGGATCC
AGATACTTGGATGAT
+
FFFFFFFFF:FFFFFFFFFFFFFFFFFFFFFFFFFFFF:FFFFFFFFFFFFFFFFFFFFFFFFFFFFF
FFFFFFFFFFFFFFFFFFFFFFFFFFFFFFFFFFFFF,FFFFF:FFFFFFFFFFFFFFFFFFFFFFFF
FFFFFFFFFFFFFFF
@A00721:190:HGJW7DSXY:4:1312:29568:13088 2:N:0:TCTGCAAG+GCTGCACT
GTTCAGTTAAGTTGACTAGCGAAGCCAATGTTCATAATATGAAGCAGTGGCTGTGGATGTGGCTCGTG
TATGATAGACGTCCCGGTAATTCTAATCCCAGAGTGAATGAGATATTCATCGGGGAAAGTCGGGATCC
AGATACTTGGATGAT
+
FFFFFFFFFFFFFFFFFFFFFFFFFFFFFFFFFFFFFFFFFFFFFFFFFFFFFFFFFFFFFFFFFFFF
FFFFFFFFFFFFFFFFFFFFFFFFFFFFFFFFFFFFFFFFFFFFFFFFFFFFF,FFFFFFF:FFFFFF
FFF::,FFFFFFF:F
@A00721:190:HGJW7DSXY:4:1444:13503:16454 2:N:0:TCTGCAAG+GCTGCACT
GTTCAGTTAAGTTGACTAGCGAAGCCAATGTTCATAATATGAAGCAGTGGCTGTGGATGTGGCTCGTG
TATGATAGACGTCCCGGTAATTCTAATCCCAGAGTGAATGAGATATTCATCGGGGAAAGTCGGGATCC
AGATACTTGGATGAT
+
:FFFFFFFFFFFFFFFFFFFFFFFFFFF:FFFFFFFFFFFFFFFFFFFFFFFFFFFFFFFFFFFFFFF
FFFFFF:FFFFFFFFFFFFFFFFFFFFFFFFFFFFFFFFFFFFF:FFFFFFFFFFF:FFFFFFFFFF:
FFFFFFFFFFFF:FF
@A00721:190:HGJW7DSXY:4:2104:18466:29684 2:N:0:TCTGCAAG+GCTGCACT
GTTCAGTTAAGTTGACTAGCGAAGCCAATGTTCATAATATGAAGCAGTGTCTGTGGATGTGGCTCGTG
```

TATGATAGACGTCCCGGTAATTCTAATCCCAGAGTGAATGAGATATTCATCGGGGAAAGTCGGGATCC
AGATACTTGGATGAT
+
F,FFF:FF:F,F:FFFFFFF:FF:FFFF:,FFFF,FF,:,FFF,FFFFF,FFF,F:F:,,,FFFFFFF
F,FFFFF,F,FF,:FFFF:::,FFFFFFFFFFF:FFFF:FFFF,FFFFFF,,FFFF,FFFFF:F,FFF
FFF,F,FFFFFFFFF
@A00721:190:HGJW7DSXY:4:2228:5321:17660 2:N:0:TCTGCAAG+GCTGCACT
GTTCAGTTAAGTTGACTAGCGAAGCCAATGTTCATAATATGAAGCAGTGGCTGTGGATGTGGCTCGTG
TATGATAGACGTCCCGGTAATTCTAATCCCAGAGTGAATGAGATATTCATCGGGGAAAGTCGGGATCC
AGATACTTGGATGAT
+
FFFFFFFFFFFFFFFFFFFFFFFFFFFFFFFFFFFFFFFFFFFF:FFFFFFFFFFFFFFFFFFFFFFF
:FFFFFFFFFFFFFFFFFFFFFFFFFFFFF:FFFFFFFFFFFFFFFFFFFFFFFFFFFFFFFFFFFF:
FFFFFFFFFFFFFFF
@A00721:190:HGJW7DSXY:4:2265:15293:30358 2:N:0:TCTGCAAG+GCTGCACT
GTTCAGTTAAGTTGACTAGCGAAGCCAATGTTCATAATATGAAGCAGTGGCTGTGGATGTGGCTCGTG
TATGATAGACGTCCCGGTAATTCTAATCCCAGAGTGAATGAGATATTCATCGGGGAAAGTCGGGATCC
AGATACTTGGATGAT
+
FFFFFFFFFFFFFFFFFFFFFFFFFFFFFFFFFFFFFFFFFFFFFFFFFFFFFFFFFFFFFFFFFFFF
FFFFFFFFFFFFFFFFFFFFFFFFFFFFFFFFFFFFFFFFFFFFFFFFFFFF:FFFFFFFFFFFFFFF
FFFFFFF:FFFFFFF
@A00721:190:HGJW7DSXY:4:2571:14687:18756 2:N:0:TCTGCAAG+GCTGCACT
GTTCAGTTAAGTTGACTAGCGAAGCCAATGTTCATAATATGAAGCAGTGGCTGTGGATGTGGCTCGTG
TATGATAGACGTCCCGGTAATTCTAATCCCAGAGTGAATGAGATATTCATCGGGGAAAGTCGGGATCC
AGATACTTGGATGAT
+
FFFFFFFFFFFFFFFFFFFFFFFFFFFFFFFFFFFFFFFFFFFF:FFFFFFFFFFFFFFFFFFFFFFF
FFFFFFFFFFFFFFFFFFFFFFFFFFFFFFFFFFFFFFFFFFFFFFFFFFFFFFFFFFFFFFFFFFFF
FFFFFFFFFFFFFFF
@A00721:190:HGJW7DSXY:4:1306:13612:22561 2:N:0:TCTGCAAG+GCTGCACT
TGATCATCCAAGTATCTGGATCCCGACTTTCCCCGATGAATATCTCATTCACTCTGGGATTAGAATTA
CCGGGACGTCTATCATACACGAGCCACATCCACAGCCACTGCTTCATATTATGAACATTGGCTTCGCT
AGTCAACTTAACTGA
+
FFFFFFFFFFFFFFFFFFFFFFFFFFFFFFFFFFFFFFFFFFFFFFFFFFFFFFF:FFFFFFFFFFFF
FFFFFFFFFFFFFFFFFFFFFFFFFFFFFFFFFFFFFFFFFFFFFFFFFFFFFFF:FFFF:FFFFFFF
:FFFFF:F:FFFFFF
@A00721:190:HGJW7DSXY:4:2119:11559:25708 2:N:0:TCTGCAAG+GCTGCACT
TGATCATCCAAGTATCTGGATCCCGACTTTCCCCGATGAATATCTCATTCACTCTGGGATTAGAATTA
CCGGGACGTCTATCATACACGAGCCACATCCACAGCCACTGCTTCATATTATGAACATTGGCTTCGCT
AGTCAACTTAACTGA
+
FFFFFFFFFFFFF,FFFFFFFFFFFFFFFFFFFFFFFFFFF,FFFFFFFFF:F:FFFFFFFFFFFFFF
FFFFFFFFFFFFFFFFFFFFFFFFF,FFFFFFFFF:FFFFFF::FFFFFFFFFFFFFFFFFFFFFF::
FFFFFFFFFFFFFFF
@A00721:190:HGJW7DSXY:4:2333:17363:25207 2:N:0:TCTGCAAG+GCTGCACT
TGATCATCCAAGTATCTGGATCCCGACTTTCCCCGATGAATATCTCATTCATTCTGGGATTAGAATTA
CCGGGACGTCTATCATACACGAGCCACATCCACAGCCACTGCTTCATATTATGAACATTGGCTTCGCT
AGTCAACTTAACTGA
+
FFFFFFFFFFFFFFFFFFFFFFFFFFFFFFFFFFFFFFFFFFFFFFFFFFFFFFFFFFFFFFFFFFFF
FFFFFFFFFFFFFFFFFFFFFFFFFFFFFFFFFFFFFFFFFFFFFFFFFFFFFFFFFFFFFFFFFFFF
FFFFFFFFFFFFFFF

```
@A00721:190:HGJW7DSXY:4:2464:5764:26256 2:N:0:TCTGCAAG+GCTGCACT
TGATCATCCAAGTATCTGGATCCCGACTTTCCCCGATGAATATCTCATTCACTCTGGGATTAGAATTA
CCGGGACGTCTATCATACACGAGCCACATCCACAGCCACTGCTTCATATTATGAACATTGGCTTCGCT
AGACAACTTAACTGA
+
FFFFFFF:F,FF:FFF:F:F:F,:FFF:FF,,FF,F:,FFFF:FFFF:F:F,FFF:FFFFFF,FFFFF
F:FFFFF,,,:FFFFFFFF,FF,F:FFFF,,F:FFF:,FFFFF,:FFFFFFFFFF:F:F,:,,,F,F,
F:,FFF:FF:F,FF:
@A00721:190:HGJW7DSXY:4:1101:25979:11381 2:N:0:TCTGCAAG+GCTGCACT
TGTTGATCATCCAAGTATCTGGATCCCGACTTTCCCCGATGAATATCTCATTCACTCTGGGATTAGAA
TTACCGGGACGTCTATCATACACGAGCCACATCCACAGCCACTGCTTCATATTATGAACATTGGCTTC
GCTAGTCAACTTAAC
+
FFFFFFFFFFFFFFFFFFFFFFFFFFFFFFFFFFFFFFFFFFFFFFFFFFFFFFFFF,FFFF,FFFF:
FFFFFFFFFFFFFFFFFFFFFFFFFFFFFFFFFFFFFFFFFFFFFFFFFFFFFFFFFFFFFFFFFFFF
FFFFFFFFFFFFFFF
@A00721:190:HGJW7DSXY:4:2212:27679:10254 2:N:0:TCTGCAAG+GCTGCACT
TGTTGATCATCCAAGTATCTGGATCCCGACTTTCCCCGATGAATATCTCATTCACTCTGGGATTAGAA
TTACCGGGACGTCTATCATACACGAGCCACATCCACAGCCACTGCTTCATATTATGAACATTGGCTTC
GCTAGTCAACTTAAC
+
FFFFFFFFFFFFFFFFFFFFFFFFFFFFFFFFFFFFFFFFFFFFFFFFFFFFFFFFFFFFFFFFFFFF
FFFFFFFFFFFFFFFFFFFFFFFFFFFFFFFFFFFFFFFFFFFFFFFFFFFFFFFFFFFFFFFFFFFF
FFF:FFFFFFFFFFF
@A00721:190:HGJW7DSXY:4:1268:19840:21668 2:N:0:TCTGCAAG+GCTGCACT
TCCATGTTGATCATCCAAGTATCTGGATCCCGACTTTCCCCGATGAATATCTCATTCACTCTGGGATT
AGAATTACCGGGACGTCTATCATACACGAGCCACATCCACAGCCACTGCTTCATATTATGAACATTGG
CTTCGCTAGTCAACT
+
FFFFFFF,FFFFFFFFFFFFFFFFFFFFF:FFF:F,FFFFFFFF:FFFFFFFFFFFFFFFFFFFFFFF
FFFFFFFFFFF:FFFFFFFFFFFFFFF,FFFFFFFFF,FF:FFFFFFFFFFFF:FFFFF:FFFFFFFF
FFFFFFFFFFFFFFF
@A00721:190:HGJW7DSXY:4:1336:19678:31782 2:N:0:TCTGCAAG+GCTGCACT
TCCATGTTGATCATCCAAGTATCTGGATCCCGACTTTCCCCGATGAATATCTCATTCACTCTGGGATT
AGAATTACCGGGACGTCTATCATACACGAGCCACATCCACAGCCACTGCTTCATATTATGAACATTGG
CTTCGCTAGTCAACT
+
FFFFFFFFFFFFFFFFFFFFFFFFFFFFFFFFFFFFFFFFFFFFFFFFFFFFFFFFFFFFFFFFFFFF
FFFFFFFFFFFFFFFFFFFFFFFFFFFFFFFFFFFFFFFFFFFFFFFFFFFFFFFFFFFFFFFFFFFF
FFFF:FFFFFFFFFF
@A00721:190:HGJW7DSXY:4:1377:11984:33614 2:N:0:TCTGCAAG+GCTGCACT
TCCATGTTGATCATCCAAGTATCTGGATCCCGACTTTCCCCGATGAATATCTCATTCACTCTGGGATT
AGAATTACCGGGACGTCTATCATACACGAGCCACATCCACAGCCACTGCTTCATATTATGAACATTGG
CTTCGCTAGTCAACT
+
FFFFFFFFFFFFFFFFFFFFFFFFFFFFFFFFFFFFFFFFFFFFFFFFFFFFFFFFFFFFFFFFFFFF
FFFFFFFFFFFFFFFFFFFFFFFFFFFFFFFFFFFFFFFFFFFFFFFFFFFFFFFFFFFFFFFFFF:F
FFFFFFFFFFFFFFF
@A00721:190:HGJW7DSXY:4:1522:3830:24627 2:N:0:TCTGCAAG+GCTGCACT
TCCATGTTGATCATCCAAGTATCTGGATCCCGACTTTCCCCGATGAATATCTCATTCACTCTGGGATT
AGAATTACCGGGACGTCTATCATACACGAGCCACATCCACAGCCACTGCTTCATATTATGAACATTGG
CTTCGCTAGTCAACT
+
FFFFFFFFFFFFFFFFFFFFFFFFFFFFFFFFFFFFFFFFFFFFFFFFFFFFFFFFFFFFFFFFFFF:
```

```
FFFFFFFFFFFFFFFFFFFFFFFFFFFFFFFFFFFFFFFFFFF:FFFFFFF:FFFFFFFFFFFFFFFFFF
FFFFFFFFFFFF:FF
@A00721:190:HGJW7DSXY:4:1530:3884:18270 2:N:0:TCTGCAAG+GCTGCACT
TCCATGTTGATCATCCAAGTATCTGGATCCCGACTTTCCCCGATGAATATCTCATTCACTCTGGGATT
AGAATTACCGGGACGTCTATCATACACGAGCCACATCCACAGCCACTGCTTCATATTATGAACATTGG
CTTCGCTAGTCAACT
+
FFFFF:FFFFFFFFFFFFFFFFFFFF:FFFFFFFFFFFFFFFFF,FFFFFFFFFFFFFFFFF:FFFFFFF
FFFFFFF:FF:FFFFFFFFFFFFFFFFFFFFFFFFFFFFFFFFFFFF:FFFFFF:FFFFFFFFFFF:FF
FFFFF::FFFFFFFF
@A00721:190:HGJW7DSXY:4:1530:5746:20087 2:N:0:TCTGCAAG+GCTGCACT
TCCATGTTGATCATCCAAGTATCTGGATCCCGACTTTCCCCGATGAATATCTCATTCACTCTGGGATT
AGAATTACCGGGACGTCTATCATACACGAGCCACATCCACAGCCACTGCTTCATATTATGAACATTGG
CTTCGCTAGTCAACT
+
FFFFFFFFFFFFFFFFFFFFFFFF:FFFFFFFFFFFFFFFFFFFFFFFFF:FFFFFFFFFFFFFFFFF:
FFFFFFFFFFFFFFFFFFFFFFFFFFF:FFFFFFFFFFFFFFFFFFFFFFFFFFFFFFFFFF,FFFFF,
FFFFFFFFFFFFFFF
@A00721:190:HGJW7DSXY:4:1577:10285:24158 2:N:0:TCTGCAAG+GCTGCACT
TCCATGTTGATCATCCAAGTATCTGGATCCCGACTTTCCCCGATGAATATCTCATTCACTCTGGGATT
AGAATTACCGGGACGTCTATCATACACGAGCCACATCCACAGCCACTGCTTCATATTATGAACATTGG
CTTCGCTAGTCAACT
+
FFFFFFF:FFFF:FFFFFF:FFFFFFFFFFFFFFFFFFFFFFFFFFF:F:FFFFFFFFFFFFFFFFFF:
FFFFFFFFFFFFFFFFFFFFFFFFFFFFFFFFFFFFFFFFFFFFFFFFFFFFFFFFFFFFFFFFFFFFFF
FF:FFFF,FFFFFFF
@A00721:190:HGJW7DSXY:4:1627:26051:6496 2:N:0:TCTGCAAG+GCTGCACT
TCCATGTTGATCATCCAAGTATCTGGATCCCGACTTTCCCCGATGAATATCTCATTCACTCTGGGATT
AGAATTACCGGGACGTCTATCATACACGAGCCACATCCACAGCCACTGCTTCATATTATGAACATTGG
CTTCGCTAGTCAACT
+
FF:FFFF,FFFFFFFFFFFFFFF:FFF::FF:FF:FFFF:FFFFFFFFF,FFF:FFFFF:F:FF:FFF
FFFFFFFFFFFFFFFFF:F,FF,FFF::FFFFFF:FFFFFFFFFFFFFF:FFFFFFFF:FFFFF,F:F
FFFFFFFFF:F::FF
@A00721:190:HGJW7DSXY:4:1627:25319:7545 2:N:0:TCTGCAAG+GCTGCACT
TCCATGTTGATCATCCAAGTATCTGGATCCCGACTTTCCCCGATGAATATCTCATTCACTCTGGGATT
AGAATTACCGGGACGTCTATCATACACGAGCCACATCCACAGCCACTGCTTCATATTATGAACATTGG
CTTCGCTAGTCAACT
+
FFFFFFFFFFFFFFFFFFFFFFFFFFFFFFFFFFFFFFFFFFFFFF:FFF:FFFFFFFFF:FFFFFFF
FFFFFFFFFFFFFFFFFFFFFFFFFFFFFFFFFFFFFFFFFFFFFFFFFFFFFFF:FFFFFFFFFF
FFFFFFFFFFFFFFF
@A00721:190:HGJW7DSXY:4:2207:5457:11412 2:N:0:TCTGCAAG+GCTGCACT
TCCATGTTGATCATCCAAGTATCTGGATCCCGACTTTCCCCGATGAATATCTCATTCACTCTGGGATT
AGAATTACCGGGACGTCTATCATACACGAGCCACATCCACAGCCACTGCTTCATATTATGAACATTGG
CTTCGCTAGTCAACT
+
FFFFFFFFFFFFFFFFFFFFFF,FFFFF:FFFFFFF::FFFFFFFFFFFFFFFFFFFFFFFFF,:FFFF
FF:FFFFFFFFF,FFFFFFFFFFFFF:FFFFFFFFF:FFFFFFFFFFFFFFFFFFFFFFFFFFFFFFFF
:FFFFFFF:FFFFFF
@A00721:190:HGJW7DSXY:4:2216:7102:1548 2:N:0:TCTGCAAG+GCTGCACT
TCCATGTTGATCATCCAAGTATCTGGATCCCGACTTTCCCCGATGAATATCTCATTCACCCTGGGATT
AGAATTACCGGGACGTCTATCATACACGAGCCACATCCACAGCCACTGCTTCATATTATGAACATTGG
CTTCGCTAGTCAACT
```

+
FFFFFFF::FFFF:F:FF,FF:FF:FF,FFF,FFFFFFFFFFF,FFFFFFFFFFFF:F,,:F,,FF,:
F,FF::FFFF::FFF,FFFF:F:F,FFFFF:FFFF:F:FFFF::FFFF,::FFFFF,F,:FFFFF,:,
FFFF:FFF,FFFFFF
@A00721:190:HGJW7DSXY:4:2274:31394:14434 2:N:0:TCTGCAAG+GCTGCACT
TCCATGTTGATCATCCAAGTATCTGGATCCCGACTTTCCCCGATGAATATCTCATTCACTCTGGGATT
AGAATTACCGGGACGTCTATCATACACGAGCCACATCCACAGCCACTGCTTCATATTATGAACATTGG
CTTCGCTAGTCAACT
+
FFFFFFFFFFFFFFFF:FFFFFFFFFFFFFFFFFFF:FFFFFFFFF,FFFFFFFFFFFF:FFFFF,F
FFFFFFFFFFFFFFF:FFFFFFFFFFFF:FFFFFFFFFFFFF:FFFFFFFFF:FFF:FFFF:FF:FFF
F,F,FFFFFF,FFFF
@A00721:190:HGJW7DSXY:4:2325:9091:12289 2:N:0:TCTGCAAG+GCTGCACT
TCCATGTTGATCATCCAAGTATCTGGATCCCGACTTTCCCCGATGAATATCTCATTCACTCTGGGATT
AGAATTACCGGGACGTCTATCATACACGAGCCACATCCACAGCCACTGCTTCATATTATGAACATTGG
CTTCGCTAGTCAACT
+
FFFFFFFFFFFFFFFFFFFFFFFFFFFFFFFFFFFFFFFFFFFFFFFFFFFFFFFFFFFFFFFFFFFF
FFFFFFFFFF:FFFFFFFFFFFFFFFFFFFFFFFFFFFFFFFFFFFFFFFFFFFFFFF:FFFFFFF:
FFFFFFFFF:FFFFF
@A00721:190:HGJW7DSXY:4:2372:5095:24377 2:N:0:TCTGCAAG+GCTGCACT
TCCATGTTGATCATCCAAGTATCTGGATCCCGACTTTCCCCGATGAATATCTCATTCACTCTGGGATT
AGAATTACCGGGACGTCTATCATACACGAGCCACATCCACAGCCACTGCTTCATATTATGAACATTGG
CTTCGCTAGTCAACT
+
FFFFFFFFFFFFFFFFFFFFFFFFFFFFFFFFFFFFFFFFFFFFFFFFFFFFFFFFFFFFFFFFFFFF
FFFFFFFFFFFFFFFFFFFFFFFFFFFFFFFFFFFFFFFFFFFFFFFFFFFFFFFFF:FFFFFFFFFF
FFFF,FF:FFFFFFF
@A00721:190:HGJW7DSXY:4:2466:17635:23985 2:N:0:TCTGCAAG+GCTGCACT
TCCATGTTGATCATCCAAGTATCTGGATCCCGACTTTCCCCGATGAATATCTCATTCACTCTGGGATT
AGAATTACCGGGACGTCTATCATACACGAGCCACATCCACAGCCACTGCTTCATATTATGAACATTGG
CTTCGCTAGTCAACT
+
FFFFFFFFFFFFFFFFFFFFFFFFFFFFFFFFFFFFFFFFFFFFFFFFFFFFFFFFFFFFFFFFFFFF
FFFFFFFFFFFFFFFFFFFFFFFFFFFFFFFFFFFFFFFFFFFFFFFFFFFFFFFFFFFFFFFFFFF:
FFFFFFFFFFFFFFF
@A00721:190:HGJW7DSXY:4:2503:5764:24283 2:N:0:TCTGCAAG+GCTGCACT
TCCATGTTGATCATCCAAGTATCTGGATCCCGACTTTCCCCGATGAATATCTCATTCACTCTGGGATT
AGAATTACCGGGACGTCTATCATACACGAGCCACATCCACAGCCACTGCTTCATATTATGAACATTGG
CTTCGCTAGTCAACT
+
FFFFFF:FFFFFFFFFFFFFFFFFFFFFFFFFFFFFFFFFFFFFFFFFFFFFFFFFFFFFFFFFF:FF
FFFFFFFFFFFFFFFFFFFFFFFFFFFFFFFFFFFFFFFFFFFFFFFFFFFFFFFFFFFFFFFF:F:F
FFFFFFF:FFFFFFF
@A00721:190:HGJW7DSXY:4:2576:7482:32080 2:N:0:TCTGCAAG+GCTGCACT
TCCATGTTGATCATCCAAGTATCTGGATCCCGACTTTCCCCGATGAATATCTCATTCACTCTGGGATT
AGAATTACCGGGACGTCTATCATACACGAGCCACATCCACAGCCACTGCTTCATATTATGAACATTGG
CTTCGCTAGTCAACT
+
FFFFFFFFFFFFFFFFFFFFFFFFFFFFFFFFFFFFFFFFFFFFFFFFFFFFFFFF:FFFFFFF:F
FFFFFFFFFFFFFFFFFFFFFFFFFFFFFFFFFFFFFFF:FFFFFFFFFFFFFFFFFFFFFFFFFF::F
FFFFFFF:FFFFFFF
@A00721:190:HGJW7DSXY:4:2634:1859:26631 2:N:0:TCTGCAAG+GCTGCACT
TCCATGTTGATCATCCAAGTATCTGGATCCCGACTTTCCCCGATGAATATCTCATTCACTCTGGGATT

```
AGAATTACCGGGACGTCTATCATACACGAGCCACATCCACAGCCACTGCTTCATATTATGAACATTGG
CTTCGCTAGTCAACT
+
FFFFFFFFFFFF:FFFFFFFFFF:FFFFFFFFFFFFFF:F,FFFFFFFFFF,FFFFFFFFF:FFFFFF
FFFFFFFFFFFFFFFFFFFF:F,FFFFFFFFFFFF:FFFFFFFFFFFFFFFFF:FFFFFF:FFFFFF:
FFFFFFFFFFFFFFF
@A00721:190:HGJW7DSXY:4:1148:10366:3317 2:N:0:TCTGCAAG+GCTGCACT
CATTATCCATGTTGATCATCCAAGTATCTGGATCCCGACTTTCCCCGATGAATATCTCATTCACTCTG
GGATTAGAATTACCGGGACGTCTATCATACACGAGCCACATCCACAGCCACTGCTTCATATTATGAAC
ATTGGCTTCGCTAGT
+
FFFFFFFFFFFFFFFFFFFFFFFFF:FFFFFFFFFFFFFFFFFFFFFFFFFFFFF:FFFFFFFFFFFF
FFFFFFFFFFFFFFFFFFFFFFF:FFFFFFFFFFFFFFFFFFFFFFFFFFF:FFFFFFFFFFFFFFFF
FFFFFFFFFFFF:F:
@A00721:190:HGJW7DSXY:4:1444:25536:23923 2:N:0:TCTGCAAG+GCTGCACT
CATTATCCATGTTGATCATCCAAGTATCTGGATCCCGACTTTCCCCGATGAATATCTCATTCACTCTG
GGATTAGAATTACCGGGACGTCTATCATACACGAGCCACATCCACAGCCACTGCTTCATATTATGAAC
ATTGGCTTCGCTAGT
+
FFFFFFFFFFFFFF:FFFFFFFFFFFFFFFFFFFFFFFFFFFFFFFFFFFFFF:FFFFFFFFFFF::F
FFFF,FFFFFFF,F,FFFFFFFFFFFFFFFFFFFFFFFFFFFFFFFFFFFFFFFFFFF:FFFFFFF:
FFFFFFFF:FFF:FF
@A00721:190:HGJW7DSXY:4:1472:29161:27821 2:N:0:TCTGCAAG+GCTGCACT
CATTATCCATGTTGATCATCCAAGTATCTGGATCCCGACTTTCCCCGATGAATATCTCATTCACTCTG
GGATTAGAATTACCGGGACGTCTATCATACACGAGCCACATCCACAGCCACTGCTTCATATTATGAAC
ATTGGCTTCGCTAGT
+
FFFFFFFFFFFFFFFFFFFFFFFFFFFFFFFFFFFFFFFFFFFFFFFFFFFFFFFFFFFFFFFFFFFF
FFFFFFFFFFFFFFFFFFFFFFFFFFFFFFFFFFFFFFFFFFFFFFFFFFFFFFFFFFFFFFFFFFFF
FFFFFFFFFFF:FFF
@A00721:190:HGJW7DSXY:4:1512:17906:22889 2:N:0:TCTGCAAG+GCTGCACT
CATTATCCATGTTGATCATCCAAGTATCTGGATCCCGACTTTCCCCGATGAATATCTCATTCACTCTG
GGATTAGAATTACCGGGACGTCTATCATACACGAGCCACATCCACAGCCACTGCTTCATATTATGAAC
ATTGGCTTCGCTAGT
+
FFFFF:FFFFFFFFFFFFFFFFFFFFFFFFFFFFFFFFFFFFFFFFFFFFFFFFFFFFFFFFFFFFFF
FFFF,FFFFFFFFFFFFFFFFFFFFFFFFFFFFFFFFFFFFFFFFFFFFFFFFFFFFFFFFFFFFFFF
FFF::FFFFF:FFFF
@A00721:190:HGJW7DSXY:4:1516:28610:20635 2:N:0:TCTGCAAG+GCTGCACT
CATTATCCATGTTGATCATCCAAGTATCTGGATCCCGACTTTCCCCGATGAATATCTCATTCACTCTG
GGATTAGAATTACCGGGACGTCTATCATACACGAGCCACATCCACAGCCACTGCTTCATATTATGAAC
ATTGGCTTCGCTAGT
+
FFFFFFFFFFFFFFFFFFFFFFFFFFFFFFFFFFFFFFFFFFFFFFFFFFFFFFFFFFFFFFFFFFFF
FFFFFFFFFFFFFFFFFFFFFFFFFFFFFFFFFFFFFFFFFFFFFFFFFFFFFFFFFFFFFFFFF:FF
FFFF,FFFF:FFFFF
@A00721:190:HGJW7DSXY:4:1601:27814:36323 2:N:0:TCTGCAAG+GCTGCACT
CATTATCCATGTTGATCATCCAAGTATCTGGATCCCGACTTTCCCCGATGAATATCTCATTCACTCTG
GGATTAGAATTACCGGGACGTCTATCATACACGAGCCACATCCACAGCCACTGCTTCATATTATGAAC
ATTGGCTTCGCTAGT
+
FFFFF:FFFFFFFFFFFFFFFF:FFFFFFFFFFFFFFFFFFFFFFFFFFFFFFFFFFFFFFFF:FFFF
FFF:FFFFFFFFFFFFFFFFFFFFFFFFFFFFFFFFFFF:FFFFFFFFFFFFFFFFFFFFFFFFFFFF
FFF:FFFFF::FF:F
```

```
@A00721:190:HGJW7DSXY:4:2131:14705:33505 2:N:0:TCTGCAAG+GCTGCACT
CATTATCCATGTTGATCATCCAAGTATCTGGATCCCGACTTTCCCCGATGAATATCTCATTCACTCTG
GGATTAGAATTACCGGGACGTCTATCATACACGAGCCACATCCACAGCCACTGCTTCATATTATGAAC
ATTGGCTTCGCTAGT
+
FFFFFFFFFFFFFFFFFFFFFFFF:FFFFFFFFFFFFFFFFFFFFFFFFFFFFFFFFFFFFFFFFFFFF:
FFFFFFFFFFFFFFFFFFFFFFFFFFFFFFFFFFFFFFFFFFFFFFFFFFFFFFFFFFFFFFFFFFFFFF
FFFFFFFFF,FFF:F
@A00721:190:HGJW7DSXY:4:2225:12102:27461 2:N:0:TCTGCAAG+GCTGCACT
CATTATCCATGTTGATCATCCAAGTATCTGGATCCCGACTTTCCCCGATGAATATCTCATTCACTCTG
GGATTAGAATTACCGGGACGTCTATCATACACGAGCCACATCCACAGCCACTGCTTCATATTATGAAC
ATTGGCTTCGCTAGT
+
FFFFFFF:FFFFFFFFFFFFFFFFFFFFFFFFFFFFFFFFFF:FFF:FFFFFF:FFFFFFFFFFFF:FFF
:FFF:FFFFF:FFF,FFFFFFFFFFF:FFFFFFF:FF:FFFFFFFFFFFFFFFFFFFFF:F:FFFFFFFF
F:F,FFFFF,FFFF:
@A00721:190:HGJW7DSXY:4:2244:13901:9095 2:N:0:TCTGCAAG+GCTGCACT
CATTATCCATGTTGATCATCCAAGTATCTGGATCCCGACTTTCCCCGATGAATATCTCATTCACTCTG
GGATTAGAATTACCGGGACGTCTATCATACACGAGCCACATCCACAGCCACTGCTTCATATTATGAAC
ATTGGCTTCGCTAGT
+
FFFF:FFFF:FFFFFFFFFFFFFFFFFFFFFFFFFFFFFFFFFFFFFFFFF:FFFF:FFFFFFFF:FFFF
FFFFFFFFFFFF:F:F:F:FFFFFFFFFFFFFFFF,FF:FFF:FFFFFFFFFFF,FFFFFFFFFFFFFFF
FFFF:FFFFFFFF:FF
@A00721:190:HGJW7DSXY:4:2265:6108:36119 2:N:0:TCTGCAAG+GCTGCACT
CATTATCCATGTTGATCATCCAAGTATCTGGATCCCGACTTTCCCCGATGAATATCTCATTCACTCTG
GGATTAGAATTACCGGGACGTCTATCATACACGAGCCACATCCACAGCCACTGCTTCATATTATGAAC
ATTGGCTTCGCTAGT
+
FFFFFFFFFFFFFFFFFFFFFFFFFFFFFFFFFFFFFFFFFFFFFFFFFFFFFFFFFFFFFFFFFFFFFF
FFFFFFFFFFFFFFFFFFFFFFFFFFFFFFFFFFFFFFFFFFFFFFFFFFFFFFFFFFFFFFFFFFFFFF
:FFFFFFFFFFFFFF
@A00721:190:HGJW7DSXY:4:2265:6551:36323 2:N:0:TCTGCAAG+GCTGCACT
CATTATCCATGTTGATCATCCAAGTATCTGGATCCCGACTTTCCCCGATGAATATCTCATTCACTCTG
GGATTAGAATTACCGGGACGTCTATCATACACGAGCCACATCCACAGCCACTGCTTCATATTATGAAC
ATTGGCTTCGCTAGT
+
FFFFFFFFFFFFFFFFFFFFFFFFFFFFFFFFFFFFFFFFFFFFFFFFFFFFFFFFFFFFFFFFFFFFFF
FFFFFFFFFFFFFFFFFFFFFFFFFFFFFFFFFFFFFFFFFFFFFFFFFFFFFFFFFFFFFFFFFFFFFF
F,FFFFFFFFFFFFF
@A00721:190:HGJW7DSXY:4:2431:9498:35289 2:N:0:TCTGCAAG+GCTGCACT
CATTATCCATGTTGATCATCCAAGTATCTGGATCCCGACTTTCCCCGATGAATATCTCATTCACTCTG
GGATTAGAATTACCGGGACGTCTATCATACACGAGCCACATCCACAGCCACTGCTTCATATTATGAAC
ATTGGCTTCGCTAGT
+
FFFFFFFFFFFFFFFFFFFFFFFFFFFFFFFFFFFFFFFFFFFFFFFFFFFFFFFFFFFFFFFFFFFFFF
FFFFFFFFFFFFFFFFFFFFFFFFFFFFFFFFFFFF:FFFFFFFFFFFFFFFFFFFFFFF:FFFFFFFFF
FFFFFFF:FFFFFFF
@A00721:190:HGJW7DSXY:4:2467:1434:20603 2:N:0:TCTGCAAG+GCTGCACT
CATTATCCATGTTGATCATCCAAGTATCTGGATCCCGACTTTCCCCGATGAATATCTCATTCACTCTG
GGATTAGAATTACCTGGACGTCTATCATACACGAGCCACATCCACAGCCACTGCTTCATATTATGAAC
ATTGGCTTCGCTAGT
+
F:,F:,F,FF:::FFFFF,F:FFFFFFFFFFFFFFF::FFFF:FFF:::FF:FFFFFFFFF:FFF:FF,F
```

FFFFFFFFFFFFF,,:FFF:FFFFF,FFFFFFFF:F,FFFFFFFFFFFF,:FF:FFFFFFF::FF:FFFF
FFFFF:F::FF:,F:
@A00721:190:HGJW7DSXY:4:2649:10402:35634 2:N:0:TCTGCAAG+GCTGCACT
CATTATCCATGTTGATCATCCAAGTATCTGGATCCCGACTTTCCCCGATGAATATCTCATTCACTCTG
GGATTAGAATTACCGGGACGTCTATCATACACGAGCCACATCCACAGCCACTGCTTCATATTATGAAC
ATTGGCTTCGCTAGT
+
FFFFFFFFFFFFFFFFFFFFFFFFFFFFFFFFFFFFFFFFFFFFFFFFFFFFFFFFFFFFFFFFFFFFFF
FFFFFFFFFFFFFFFFFFFFFFFFFFFFFFFFFFFFFFFFFFFFFFFFFFFFFFFFFFFFFFFFFFFFFF
FFFFFFFFFFFFFFF
@A00721:190:HGJW7DSXY:4:1153:28836:3239 2:N:0:TCTGCAAG+GCTGCACT
CAACATTATCCATGTTGATCATCCAAGTATCTGGATCCCGACTTTCCCCGATGAATATCTCATTCACT
CTGGGATTAGAATTACCGGGACGTCTATCATACACGAGCCACATCCACAGCCACTGCTTCATATTATG
AACATTGGCTTCGCT
+
FFFFFFFFFFFFFFFFFFFFFFFFFFFFFFFFFFFFFFFFFFFFFFFFFFFFFFFFFFFFFFFFFFFFFF
FFFFFFFFFFFFFFFFFFFFFFFFFFFFFFFFFFFFF:FFFFFFFFFFFFFFFFFFFFFFFFFFFFFFFF
FFFFFFFFFFFFFFF
@A00721:190:HGJW7DSXY:4:1222:18566:6684 2:N:0:TCTGCAAG+GCTGCACT
CAACATTATCCATGTTGATCATCCAAGTATCTGGATCCCGACTTTCCCCGATGAATATCTCATTCACT
CTGGGATTAGAATTACCGGGACGTCTATCATACACGAGCCACATCCACAGCCACTGCTTCATATTATG
AACATTGGCTTCGCT
+
FFFFFFFFFFFFFFF:FFFFFFFFFFFFFFFFFFFFFFFFFFFFFFFFFFFFFFFFFFFFFFFFFFFFFF
:FF:FFFFFFFFFFFF:FFFFFFFFFFFFFFFFFFFFFFFFFFFFFFFFFFFFFFFFFFFFFFFFFFFF:
FFFFFFFFFFFFFFF
@A00721:190:HGJW7DSXY:4:1266:2193:33536 2:N:0:TCTGCAAG+GCTGCACT
CAACATTATCCATGTTGATCATCCAAGTATCTGGATCCCGACTTTCCCCGATGAATATCTCATTCACT
CTGGGATTAGAATTACCGGGACGTCTATCATACACGAGCCACATCCACAGCCACTGCTTCATATTATG
AACATTGGCTTCGCT
+
FFFFFFFFFFFFFFFFFFFFFFFFFFFF:FFFFFFFFFFFFFFFFFFFFFFFFFFFFFFFFFFFFFF:FF
FFFFFFFFFFFFFFFFFFFFFFFFFFFFFFFFFFFFFFFFFFFFFFFFFFFFFFFFFFFFFFFFFFFFFF
FFFFFFFFFF:FFFF
@A00721:190:HGJW7DSXY:4:1421:18349:36151 2:N:0:TCTGCAAG+GCTGCACT
CAACATTATCCATGTTGATCATCCAAGTATCTGGATCCCGACTTTCCCCGATGAATATCTCATTCACT
CTGGGATTAGAATTACCGGGACGTCTATCATACACGAGCCACATCCACAGCCACTGCTTCATATTATG
AACATTGGCTTCGCT
+
FFFFFFFFFFFFFFFFFFFFFFFFFFFFFFFFFFFFFFFFFFFFFFFFFFFFFFFFFFFFFFFFFFF:FF
F:FFFF:FFFFFFFFFFFFFFFFFFFFFFFFFFF:FFFFFFFFFFFFFFFFFFFFFFFFFFFFFFFFFFF
FFFFFFFFF:FFFFF
@A00721:190:HGJW7DSXY:4:1426:16993:28761 2:N:0:TCTGCAAG+GCTGCACT
CAACATTATCCATGTTGATCATCCAAGTATCTGGATCCCGACTTTCCCCGATGAATATCTCATTCACT
CTGGGATTAGAATTACCGGGACGTCTATCATACACGAGCCACATCCACAGCCACTGCTTCATATTATG
AACATTGGCTTCGCT
+
FFF,F,FF,F:FFFF,:F,FFFF,FF,FF:FFF:FF,FFFFFFFFF,FF:FFFFFFFFFF:FFFFF:F
FFFFFF,FFFF:FFF,F:FFFF,FFFFF,FFF,F,FF::F:FFFFFFFFFF:FFFF:FFFF:FFFFFF
FFFFFFF:FFF:F:F
@A00721:190:HGJW7DSXY:4:1429:30427:26725 2:N:0:TCTGCAAG+GCTGCACT
CAACATTATCCATGTTGATCATCCAAGTATCTGGATCCCGACTTTCCCCGATGAATATCTCATTCACT
CTGGGATTAGAATTACCGGGACGTCTATCATACACGAGCCACATCCACAGCCACTGCTTCATATTATG
AACATTGGCTTCGCT

```
+
FFFFFFFFFFFFFFFFFFFFFFFFFFFFFFFFFFFFFFFFFFFFFFFFFFFFFFFFFFFFFFFFFFFFFF
FFFFFFFFFFFFFFFFFFFFFFFFFFFFFFFFFFFFFFFFFFFFFFFFFFF:FFFFFFFFFFFFFFFFFF
FFFFFFFFFFFFFFF
@A00721:190:HGJW7DSXY:4:1515:23882:12070 2:N:0:TCTGCAAG+GCTGCACT
CAACATTATCCATGTTGATCATCCAAGTATCTGGATCCCGACTTTCCCCGATGAATATCTCATTCACT
CTGGGATTAGAATTACCGGGACGTCTATCATACACGAGCCACATCCACAGCCACTGCTTCATATTATG
AACATTGGCTTCGCT
+
FF:FF,:FFFFFFFFFFFFFFFFFFFFFFFFFFFFFFFFFFFFFFFFFFFFFFFFFFFFFFFFFFFFFFF
FFFFFFFFFFFF:FFFFF:,FFFFFF:FFFFFFFFF:FFFFFFFFF:FFFFFFFFF:FFFF:FFFFFFF,
FF,FFF:FFFFFFFF
@A00721:190:HGJW7DSXY:4:1653:21983:30639 2:N:0:TCTGCAAG+GCTGCACT
CAACATTATCCATGTTGATCATCCAAGTATCTGGATCCCGACTTTCCCCGATGAATATCTCATTCACT
CTGGGATTAGAATTACCGGGACGTCTATCATACACGAGCCACATCCACAGCCACTGCTTCATATTATG
AACATTGGCTTCGCT
+
FFFFFFFFFFFFFFFFFFFFFFFFFFFFFFFFFFFFFFFFFFFFFFFFFFFFFFFFFFFFFFFFFFFFFF
FFFFFFFFFFFFFFFFFFFFFFFFFFFFFFFFFFFFFFFFFFFFFFF:FFFFFFFFFFFFFFFFFFFFFF
F:FFFFFFFFFFFFF
@A00721:190:HGJW7DSXY:4:2253:2085:21887 2:N:0:TCTGCAAG+GCTGCACT
CAACATTATCCATGTTGATCATCCAAGTATCTGGATCCCGACTTTCCCCGATGAATATCTCATTCACT
CTGGGATTAGAATTACCGGGACGTCTATCATACACGAGCCACATCCACAGCCACTGCTTCATATTATG
AACATTGGCTTCGCT
+
FFFFFFFFFFFFFFFFFFFFFFFFFFFF:F:FFFFFFFFFFFFFFFFFF:FFFFFF:FFFFFFFFFFFF:
FFFFFFFFFFFFFFFFFFFFFFFFFFFFFFFFFFFFFFFFFFFFFFFFFFFFFFFFFFFF,FFFF:FFFF
FFFFFFFFFFFFFFF
@A00721:190:HGJW7DSXY:4:2317:12400:11099 2:N:0:TCTGCAAG+GCTGCACT
CAACATTATCCATGTTGATCATCCAAGTATCTGGATCCCGACTTTCCCCGATGAATATCTCATTCACT
CTGGGATTAGAATTACCGGGACGTCTATCATACACGAGCCACATCCACAGCCACTGCTTCATATTATG
AACATTGGCTTCGCT
+
FFFFFFFFFFFFFFFFFFFFFFFFFFFFFFFFFFFFFFFFFFFFFFFFFFFFFF:FFFFFFFFFFFFFFF
FFFFFFFFFFFFFFFFFFFFFFFFFFFFFFFFFFFFFFFFFFFFFFFFFFFFFFFFFFFF:FFFFF:FFF
FFFFF,FFFFFFFFF
@A00721:190:HGJW7DSXY:4:2336:9706:8312 2:N:0:TCTGCAAG+GCTGCACT
CAACATTATCCATGTTGATCATCCAAGTATCTGGATCCCGACTTTCCCCGATGAATATCTCATTCACT
CTGGGATTAGAATTACCGGGACGTCTATCATACACGAGCCACATCCACAGCCACTGCTTCATATTATG
AACATTGGCTTCGCT
+
FFFFFFFFFFFFFFFFFFFFFFFFFFFFFFFFFFFFFFFFFFFFFFFFFFFFFFFFFFFFFFFFFFFFFF
FFFFFFFFFFFFFFFFFFFFFFFFFFFFFFFFFFFFFF:FFFFFFFFFFFFFFFFFFFFFFFFFFFFFFF
FFFFFFFFFFFFFFF
@A00721:190:HGJW7DSXY:4:2338:12816:2926 2:N:0:TCTGCAAG+GCTGCACT
CAACATTATCCATGTTGATCATCCAAGTATCTGGATCCCGACTTTCCCCGATGAATATCTCATTCACT
CTGGGATTAGAATTACCGGGACGTCTATCATACACGAGCCACATCCACAGCCACTGCTTCATATTATG
AACATTGGCTTCGCT
+
FFFFFFFFFFFFFFFFFFFFFFFFFFFFFFFFFFFFFFFFFFFFFFFFFFFFFFFFFFFFFFFFFFFFFF
FFFFFFFFFFF:FFF:FFFFFFFFFFFFFFFFFFFFFFFFFFFFFFFFFFFFFFFFFFFFFFFFFFFFFF
FFFFFFFFFFFF,FF
@A00721:190:HGJW7DSXY:4:2338:12807:2942 2:N:0:TCTGCAAG+GCTGCACT
CAACATTATCCATGTTGATCATCCAAGTATCTGGATCCCGACTTTCCCCGATGAATATCTCATTCACT
```

CTGGGATTAGAATTACCGGGACGTCTATCATACACGAGCCACATCCACAGCCACTGCTTCATATTATG
AACATTGGCTTCGCT
+
FFFFFFFFFFFFFFFFFFFFFFFFFFFFFFFFFFFFFFFFFFFFFFFFFFFFFFFFFFFFFFFFFFFF
FFFFFFFFFFFFFFFFFFFFFFFFFFFFFFFFFFFFFFFFFFFFFFFFFFFFFFFFFFFFFFFFFFFF
FFFFFFFFFFFF:FF
@A00721:190:HGJW7DSXY:4:1142:23818:9518 2:N:0:TCTGCAAG+GCTGCACT
CTATCAACATTATCCATGTTGATCATCCAAGTATCTGGATCCCGACTTTCCCCGATGAATATCTCATT
CACTCTGGGATTAGAATTACCGGGACGTCTATCATACACGAGCCACATCCACAGCCACTGCTTCATAT
TATGAACATTGGCTT
+
FFFFFFFFFFFFFFFFFFFFFFFFFFFFFFFFFFFFFFFFFFFFFFFFFFFFFFFFFFFFFFFFFFFF
FFFFFFFFFF:FFFFFFFFFFFFFFFFFFFFFFFFFFFFFFFFFFFFFFFFFFF:FFFFFFFFFFFFF
FFFFFFFFF:FFFFF
@A00721:190:HGJW7DSXY:4:1349:18475:10504 2:N:0:TCTGCAAG+GCTGCACT
CTATCAACATTATCCATGTTGATCATCCAAGTATCTGGATCCCGACTTTCCCCGATGAATATCTCATT
CACTCTGGGATTAGAATTACCGGGACGTCTATCATACACGAGCCACATCCACAGCCACTGCTTCATAT
TATGAACATTGGCTT
+
FFFFFFFFFFFFFFFFFFFFFFFFFFF:FFFFFFFFFFFFFFFFFFFFFFFFFFFF:FFFFFFFFFFF
FFFFFFFFFFFFFFFFFFFFFFFFFFFFFFFFFFFFFFFFFFFFFFFFFFFFFFFF:FFFFFFFFFFF
FFFFFFFFFFFFFFF
@A00721:190:HGJW7DSXY:4:1566:7319:21778 2:N:0:TCTGCAAG+GCTGCACT
CTATCAACATTATCCATGTTGATCATCCAAGTATCTGGATCCCGACTTTCCCCGATGAATATCTCATT
CACTCTGGGATTAGAATTACCGGGACGTCTATCATACACGAGCCACATCCACAGCCACTGCTTCATAT
TATGAACATTGGCTT
+
FFFFFFFFFFFFFFFFFFFFFFFFFFFFFFFFFFFFFFFFFFFFFFFFFFFFFFFFFFFFFFFFFFFF
FFFFFFFFFFFFFFFFFFFFFFFF:FFFFFFFFFFFFFFFFFFFFFFFFFFFFFFFFFFFFFFFFFF
FFFFFFFFFFFFFFF
@A00721:190:HGJW7DSXY:4:1604:6460:19633 2:N:0:TCTGCAAG+GCTGCACT
CTATCAACATTATCCATGTTGATCATCCAAGTATCTGGATCCCGACTTTCCCCGATGAATATCTCATT
CACTCTGGGATTAGAATTACCGGGACGTCTATCATACACGAGCCACATCCACAGCCACTGCTTCATAT
TATGAACATTGGCTT
+
FFF,FFFFFFFF,FFFFFFFFFFFF:FFFFFFF,FF,:FFF:FFFFFFFFFFF:FFFF,F:FFFFFF:
FFFFFF,FFFFFFFFFFFFFFFFFFFF:FFFFFFFFFF:FFFFFFFFFFFFFFFFFFF:,FFF:F,FF
FFFFFFF,,FFF:::
@A00721:190:HGJW7DSXY:4:2137:27471:24298 2:N:0:TCTGCAAG+GCTGCACT
CTATCAACATTATCCATGTTGATCATCCAAGTATCTGGATCCCGACTTTCCCCGATGAATATCTCATT
CACTCTGGGATTAGAATTACCGGGACGTCTATCATACACGAGCCACATCCACAGCCACTGCTTCATAT
TATGAACATTGGCTT
+
FFFFFFFFFFF:FFFFFFFFFFFFFFFFFFFFF:FFFFFFFFFFFFFFFFFFFFFFFFFFFFFFFFFF
FFFFFFFFFFFFFFFFFFFFFFFFFFFFFFFFFFFFFFF:FFFF:FFFFFFFFFFFFFFFFFFFFFFF
FFFFFFFFF,FFFFF
@A00721:190:HGJW7DSXY:4:2142:15564:10347 2:N:0:TCTGCAAG+GCTGCACT
CTATCAACATTATCCATGTTGATCATCCAAGTATCTGGATCCCGACTTTCCCCGATGAATATCTCATT
CACTCTGGGATTAGAATTACCGGGACGTCTATCATACACGAGCCACATCCACAGCCACTGCTTCATAT
TATGAACATTGGCTT
+
FFFFFFFFFFFFFFFFFFFFFFFFFFFFFFFFFFFFFFFFFFFFFFFFFFFFFFFFFFFFFFFFFFFF
FFFFFFFFFFFFFFFFFFFFFFFFFFFFFFFFFFFFFFFFFFFFFFFFFFFFFFFFFFFFFFFFFFFF
FFFFFFFF:FFFFFF

```
@A00721:190:HGJW7DSXY:4:2352:1226:9408 2:N:0:TCTGCAAG+GCTGCACT
CTATCAACATTATCCATGTTGATCATCCAAGTATCTGGATCCCGACTTTCCCCGATGAATATCTCATT
CACTCTGGGATTAGAATTACCGGGACGTCTATCATACACGAGCCACATCCACAGCCACTGCTTCATAT
TATGAACATTGGCTT
+
FFFFFFFFFFFFFFFFFFFFFFFFFFFFFFFFFFFFFFFFFFFFFFFFFFFFFFFFFFFFFFFFFFFFFF
FFFFFFFFFFFFFFFFFFFFFFFFFFFFFFFFFFFFFFFFFFFFFFFFFFFFFFFFFFFFFFFFFFFFFF
FFFFFFFFFFFFFFF
@A00721:190:HGJW7DSXY:4:2628:14642:36307 2:N:0:TCTGCAAG+GCTGCACT
CTATCAACATTATCCATGTTGATCATCCAAGTATCTGGATCCCGACTTTCCCCGATGAATATCTCATT
CACTCTGGGATTAGAATTACCGGGACGTCTATCATACACGAGCCACATCCACAGCCACTGCTTCATAT
TATGAACATTGGCTT
+
FFFFFFFFFFFFFFFFFFFFFFFFFFFFFFFFFFFFFFFFFFFFFFFFFFFFFFFFFFFFFFFFFFFFFF
FFFFFFFFFFFFFFFFFFFFFFFFFFFFFFFFFFFFFFFFFFFFFFFFFFFFFFFFFFFFFFFFFFFFFF
FFFF:F:FFFFFFFF
@A00721:190:HGJW7DSXY:4:1144:5963:1673 2:N:0:TCTGCAAG+GCTGCACT
CCTATCAACATTATCCATGTTGATCATCCAAGTATCTGGATCCCGACTTTCCCCGATGAATATCTCAT
TCACTCTGGGATTAGAATTACCGGGACGTCTATCATACACGAGCCACATCCACAGCCACTGCTTCATA
TTATGAACATTGGCT
+
FFFFFFFFFFFF:FFFFFF:FFFFFFFFFFFFFFFFFFFFFFFFFFFFFFFFFFFFFFFFFFFFFFFFFF
FFFFFFFFFFFFFFFFFFFFFFFFFFFFFFFFFFFFFFFFFFFFFFFFFFFFFFFFFFFFFFFFFFFFFF
FFFFFFF:FFF:FFF
@A00721:190:HGJW7DSXY:4:1152:4679:15984 2:N:0:TCTGCAAG+GCTGCACT
CCTATCAACATTATCCATGTTGATCATCCAAGTATCTGGATCCCGACTTTCCCCGATGAATATCTCAT
TCACTCTGGGATTAGAATTACCGGGACGTCTATCATACACGAGCCACATCCACAGCCACTGCTTCATA
TTATGAACATTGGCT
+
FFFFFFFFFFFFFFFFFFFFFFFFFFFFFFFFFF,FFFFFFFFFFFFFFFFFFFFFFFFFFF:FFFFFFF
FFFFFFFFFFFFFFFFFFFFFFFFFFFFFFFFFFFFFFFFFFFFFFFFFFFFFFFFF:FFFFFFFFFF:F
FFFFFFFFFFFFFFF
@A00721:190:HGJW7DSXY:4:1447:22354:9204 2:N:0:TCTGCAAG+GCTGCACT
CCTATCAACATTATCCATGTTGATCATCCAAGTATCTGGATCCCGACTTTCCCCGATGAATATCTCAT
TCACTCTGGGATTAGAATTACCGGGACGTCTATCATACACGAGCCACATCCACAGCCACTGCTTCATA
TTATGAACATTGGCT
+
FFF:FFFFFFFFFFFFFFFFFFFFFFFFFFFFFFFFFFFFFFFFFFFFFFFFFFFFFFFFFFFFFFFFFF
FFFFFFFFFFFFFFFFFFFFFFFFFFFFFFFFFFFFFFFFFFFFFFFFFFFFFFFFFFFFFFFFFFFFFF
FFFFFFFFFFFFFFF
@A00721:190:HGJW7DSXY:4:1652:17155:1548 2:N:0:TCTGCAAG+GCTGCACT
CCTATCAACATTATCCATGTTGATCATCCAAGTATCTGGATCCCGACTTTCCCCGATGAATATCTCAT
TCACTCTGGGATTAGAATTACCGGGACGTCTATCATACACGAGCCACATCCACAGCCACTGCTTCATA
TTATGAACATTGGCT
+
FFFFFFFFFFFFFFFFFFFFFFFFFFFFFFFF:FFFFFFFFFFFFFFFFFFFFF:FFFFFFFFFFFFFFF
FFFFFFFFFFFFFFFFFFFFFFFFFFFFFFFFFFFFFFFFFFFFFFFFFFFFFFFFFFFFFFFFFFFF,F
FFFFFFFFF:FFFFF
@A00721:190:HGJW7DSXY:4:1652:17381:2002 2:N:0:TCTGCAAG+GCTGCACT
CCTATCAACATTATCCATGTTGATCATCCAAGTATCTGGATCCCGACTTTCCCCGATGAATATCTCAT
TCACTCTGGGATTAGAATTACCGGGACGTCTATCATACACGAGCCACATCCACAGCCACTGCTTCATA
TTATGAACATTGGCT
+
FFFFFFFFFFFFFFFFFFFFFFFFFFFFFF:FFFFFFFFFFFFFFFFFFFFFFFFFFFFFFFFFFFFFFF
```

FFFFFFFFFFFFFFFFFFFFFFFFFFFFFFFFFFFFFFFFFFFFFFFFFFFFFFFFFFFFFFFFFFFFFF
FFFFFFFFFFFFFFF
@A00721:190:HGJW7DSXY:4:2172:8205:26475 2:N:0:TCTGCAAG+GCTGCACT
CCTATCAACATTATCCATGTTGATCATCCAAGTATCTGGATCCCGACTTTCCCCGATGAATATCTCAT
TCACTCTGGGATTAGAATTACCGGGACGTCTATCATACACGAGCCACATCCACAGCCACTGCTTCATA
TTATGAACATTGGCT
+
FFFFFFFFFFFFFFFFFFFFFFFFFFFFFFFFFFFFFFFFFFFFFFFFFFFFFFFFFFFFFFFFFFFFFF
FFFFFFFFFFFFFFFFFFFFFFFFFFFFFFFFFFFFFFFFFFFFFFFFFFFFFFFFFFFFFFFFFFFF:F
FFFFFFFFFF:FFFF
@A00721:190:HGJW7DSXY:4:2301:18177:13463 2:N:0:TCTGCAAG+GCTGCACT
CTATCAACATTATCCATGTTGATCATCCAAGTATCTGGATCCCGACTTTCCCCGATGAATATCTCATT
CACTCTGGGATTAGAATTACCGGGACGTCTATCATACACGAGCCACATCCACAGCCACTGCTTCATAT
TATGAACATTGGCT
+
FFFFFFFFFFFFF:FF:FFFFFFFFFFFFFF,F:F,F,F:FF::FFF:FF:F:FF:FFFFFF:FFFF,
:FF,F,,FF:FF::FFFFFF:FFFFF,,FF::FFFFFFFFFF,FFFFF::FFF:FFFF::::,FFF::
:FF,FFFFF,,,:F
@A00721:190:HGJW7DSXY:4:2303:31593:23422 2:N:0:TCTGCAAG+GCTGCACT
CCTATCAACATTATCCATGTTGATCATCCAAGTATCTGGATCCCGACTTTCCCCGATGAATATCTCAT
TCACTCTGGGATTAGAATTACCGGGACGTCTATCATACACGAGCCACATCCACAGCCACTGCTTCATA
TTATGAACATTGGCT
+
FFFFFFFFFFFFFFFFFFFFFFFFFFFFFFFFFFFFFFFFFFFFFFFFFFFFFFFFFFFFFFFFFFFFFF
FFFFFFFFFFFFFFFFFFFFFFFFFFFFFFFFFFFFFFFFFFFFFFFFFFFFFFFFFFFFFFFFFFFFFF
FFFFFFFFFFFFFFF
@A00721:190:HGJW7DSXY:4:2314:6686:26224 2:N:0:TCTGCAAG+GCTGCACT
CCTATCAACATTATCCATGTTGATCATCCAAGTATCTGGATCCCGACTTTCCCCGATGAATATCTCAT
TCACTCTGGGATTAGAATTACCGGGACGTCTATCATACACGAGCCACATCCACAGCCACTGCTTCATA
TTATGAACATTGGCT
+
FFFFFFFFFFFFFFFFFFFFFFFFFFFFFFFFFFFFFFFFFFFFFFFFFFFFFFFFFFFFFFFFFFFFFF
FFFFFFFFFFFFFFFFFFFFFFFFFFFFFFFFFFFFFFFFFFFFFFFFFFFFFFFFFFFFFFFFFFFFFF
FFFFFFFFFFFFFFF
@A00721:190:HGJW7DSXY:4:2340:22733:34851 2:N:0:TCTGCAAG+GCTGCACT
CCTATCAACATTATCCATGTTGATCATCCAAGTATCTGGATCCCGACTTTCCCCGATGAATATCTCAT
TCACTCTGGGATTAGAATTACCGGGACGTCTATCATACACGAGCCACATCCACAGCCACTGCTTCATA
TTATGAACATTGGCT
+
FFFFFFFFFFFFFFFFFFFFFFFFFFFFFFFFFFFFFFFFFFFFFFFFFFFFFFFFFFFFFFFFFFFFFF
FFFFFFFFFFFFFFFFFFFFFFFFFFFFFFFFFFFFFFFFFFFFFFFFFFFFFFFFFFFFFFFFFFFFFF
FFFFF:FFFFFFFFF
@A00721:190:HGJW7DSXY:4:2372:29080:36104 2:N:0:TCTGCAAG+GCTGCACT
CCTATCAACATTATCCATGTTGATCATCCAAGTATCTGGATCCCGACTTTCCCCGATGAATATCTCAT
TCACTCTGGGATTAGAATTACCGGGACGTCTATCATACACGAGCCACATCCACAGCCACTGCTTCATA
TTATGAACATTGGCT
+
FFFFFFFFFFFFFFFFFFFFFFFFFFFFFFFFFFFFFFFFFFFFFFFFFFFFFFFFFFFFFFFFFFFFFF
FFFFFFFFFFFFFFFFFFFFFFFFFFFFFFFFFFFFFFFFFFFFFFFFFFFFFFFFFFFFFFFFFFFFF:
FFFFFFFFFFFFFFF
@A00721:190:HGJW7DSXY:4:2374:24361:26428 2:N:0:TCTGCAAG+GCTGCACT
CCTATCAACATTATCCATGTTGATCATCCAAGTATCTGGATCCCGACTTTCCCCGATGAATATCTCAT
TCACTCTGGGATTAGAATTACCGGGACGTCTATCATACACGAGCCACATCCACAGCCACTGCTTCATA
TTATGAACATTGGCT

```
+
FFFFFFFFFFFFFFFFFFFFFFFFFFFFFF:FFFFFFFFFFFFFFFFFFFFFFFFFFFFFFFFFFFFFF
FFFFFFFFFFFFFFFFFFFFFFFFFFFFFFFFFFFFFFFFFFFFFFFFFFFFFFFFFFFFFFFFFFFFF
:F:FF::FFFFFFFF
@A00721:190:HGJW7DSXY:4:2278:32108:21840 2:N:0:TCTGCAAG+GCTGCACT
CCTATCAACATTATCCATGTTGATCATCCAAGTATCTGGATCCCGACTTTCCCCGATGAATATCTCAT
TCACTCTGGGATTAGAATTACCGGGACGTCTATCATACACGAGCCACATCCACAGCCACTGCTTCATA
TTATGAACATTG
+
FFFFFFFFFFFFFFFFFFFFFFFF:FFFFFFFFFFFFFFFFFFFFFFFFFFFF:FFFFFF:FFFFFFF
FFFFFFFFFFFFFFF:FFFF::FFFFFFFFFF,FFFFFFFFF:FFF:FF,F:FF,,:FFFFFFFFFFF
FFFF,FF:FFFF
@A00721:190:HGJW7DSXY:4:1176:1072:8234 2:N:0:TCTGCAAG+GCTGCACT
TAAACCTATCAACATTATCCATGTTGATCATCCAAGTATCTGGATCCCGACTTTCCCCGATGAATATC
TCATTCACTCTGGGATTAGAATTACCGGGACGTCTATCATACACGAGCCACATCCACAGCCACTGCTT
CATATTATGAACATT
+
FFF,FFFFFFFFFFFFFFFFFFFFFFFFFFFFFFFFFFFFFFFFFFFFFFFFFFFFFFFFFFFFFFFF
FF:FFFF:FFFFFFFFFFFFFFFFFFFFFFFFFFFFFFFFF:FFFFFFFFFFFFFFFFFFFFFFFFFF
FFFFFFFFF:FFFFF
@A00721:190:HGJW7DSXY:4:1202:14660:18615 2:N:0:TCTGCAAG+GCTGCACT
TAAACCTATCAACATTATCCATGTTGATCATCCAAGTATCTGGATCCCGACTTTCCCCGATGAATATC
TCATTCACTCTGGGATTAGAATTACCGGGACGTCTATCATACACGAGCCACATCCACAGCCACTGCTT
CATATTATGAACATT
+
FFFFFFFFFFF:FFFFFFFFFFF:FFFFFFFFFFFF,FFFFFFFFFFFFFFFFFFFFFFFFFFFFFFF
FFFFFFFF,FFFFFFFFFFF,FFFFFFFF:FFFFFFFFFFFFFFFFFFFFFFFFFFFFFFFFFFFFFF
FFFFF:FFFFFFF:F
@A00721:190:HGJW7DSXY:4:1634:8757:23422 2:N:0:TCTGCAAG+GCTGCACT
TAAACCTATCAACATTATCCATGTTGATCATCCAAGTATCTGGATCCCGACTTTCCCCGATGAATATC
TCATTCACTCTGGGATTAGAATTACCGGGACGTCTATCATACACGAGCCACATCCACAGCCACTGCTT
CATATTATGAACATT
+
FFFFFFFFFFFFFF,FFFFFFFFFFFFFFFFFFFFFF:F::FF:FFF:,FF:FFFFFFFFFFF:FFFFF
FFF:FFFFFFFF:FFFFF:FFFFFFFFFFFFFFFF:FFFFFF:FFFFFFFFFFFFFFFFFFFFFFFFF
FFFFFF:FFFFFFFF
@A00721:190:HGJW7DSXY:4:2134:4372:26443 2:N:0:TCTGCAAG+GCTGCACT
TAAACCTATCAACATTATCCATGTTGATCATCCAAGTATCTGGATCCCGACTTTCCCCGATGAATATC
TCATTCACTCTGGGATTAGAATTACCGGGACGTCTATCATACACGAGCCACATCCACAGCCACTGCTT
CATATTATGAACATT
+
FFFFFFFFFFFFFFFFFFFFFFFFFFFFFFFFFFFFFFFFFFFFFFFFFFFFFFFFFFFFFFFFFFFFF
FFFFFFFFFFFFFFFFFFFFFFFFFFFFFFFFFFFFFFFFFFFFFFFFFFFFFFFFFFFFFFFFFFFFF
FFFFFFFFFFFFFFF
@A00721:190:HGJW7DSXY:4:2134:1452:27461 2:N:0:TCTGCAAG+GCTGCACT
TAAACCTATCAACATTATCCATGTTGATCATCCAAGTATCTGGATCCCGACTTTCCCCGATGAATATC
TCATTCACTCTGGGATTAGAATTACCGGGACGTCTATCATACACGAGCCACATCCACAGCCACTGCTT
CATATTATGAACATT
+
FFFFFFFFFFFFFFFFFFFFFFFFFFFF:FFFFF:FFFFFFFFFFFFFFFFFFFFFFFFFFFFFFFFFFF
FFFF:FFFFFFFFFFFFFFFFFFFFFFFFFFFFFFFFFFFFFFFF:FFFFFFFFFFFFFFFFFFFF:F:
FFFFFFFFFFFFFF:
@A00721:190:HGJW7DSXY:4:2348:27805:17926 2:N:0:TCTGCAAG+GCTGCACT
TAAACCTATCAACATTATCCATGTTGATCATCCAAGTATCTGGATCCCGACTTTCCCCGATGAATATC
```

TCATTCACTCTGGGATTAGAATTACCGGGACGTCTATCATACACGAGCCACATCCACAGCCACTGCTT
CATATTATGAACATT
+
FFFFFFFFFFFFFFFFF:FFFFF:FFFFFFFFFFFFFFFFFF:FFFFFFFFFFFFFFF:FFF:,FFFF
FFFFFF,FFFFFFFF:FFFFFFFFFFFF:FFFFFFFFFFFFF:FFFFFFFFFFFFFFFFFFFFFFFFF
FFF:FFFFFFFFFFF
@A00721:190:HGJW7DSXY:4:2416:10294:25582 2:N:0:TCTGCAAG+GCTGCACT
TAAACCTATCAACATTATCCATGTTGATCATCCAAGTATCTGGATCCCGACTTTCCCCGATGAATATC
TCATTCACTCTGGGATTAGAATTACCGGGACGTCTATCATACACGAGCCACATCCACAGCCACTGCTT
CATATTATGAACATT
+
FFFFFFFFFFFFFFFFFFFFFFFFFFFFFFFFFFFFFFFFFFFFFFFFFFFFFFFFFFFFFFFFFFFF
FFFFFFFFFFFFFFFFFFFFFFFFFFFFFFFFFFFFFFFFFFFFFFFFFFFFFFFFFFFFFFFFFFFF
FFFFFFFFFFFFFFF
@A00721:190:HGJW7DSXY:4:2673:29197:9502 2:N:0:TCTGCAAG+GCTGCACT
TAAACCTATCAACATTATCCATGTTGATCATCCAAGTATCTGGATCCCGACTTTCCCCGATGAATATC
TCATTCACTCTGGGATTAGAATTACCGGGACGTCTATCATACACGAGCCACATCCACAGCCACTGCTT
CATATTATGAACATT
+
FFFFFFFFFFFFFFFFFFFFFFFFFFFFFFFFFFFFFFFFFFFFFFFFFFFFFFFFFFFFFFFFFFFF
FFFFFFFFFFFFFFFFF:FFFFFFFFFFF:FFFFFF:FFFFFFFFFFFFFF:FFFFFFFFFFFFFFFF
FF,FFFFFFFF:FFF
@A00721:190:HGJW7DSXY:4:1118:31114:28698 2:N:0:TCTGCAAG+GCTGCACT
CTAAACCTATCAACATTATCCATGTTGATCATCCAAGTATCTGGATCCCGACTTTCCCCGATGAATAT
CTCATTCACTCTGGGATTAGAATTACCGGGACGTCTATCATACACGAGCCACATCCACAGCCACTGCT
TCATATTATGAACAT
+
FFFFFFFFFFFFFFF:F:FFFFFFFFFFFFFFF,FFFFFFFFFFFFFFFFFFFFFFFFFFFFF::FFF
FFFFFFFFF,:FFFFF:FFFFFFFF:FFFFFFFFFFFFFFFF:FFFFFFFFFFFFFFFFFFFFFFFFF
FFFFF:FFFFFFFFF
@A00721:190:HGJW7DSXY:4:1161:12066:9298 2:N:0:TCTGCAAG+GCTGCACT
CTAAACCTATCAACATTATCCATGTTGATCATCCAAGTATCTGGATCCCGACTTTCCCCGATGAATAT
CTCATTCACTCTGGGATTAGAATTACCGGGACGTCTATCATACACGAGCCACATCCACAGCCACTGCT
TCATATTATGAACAT
+
FFFFFFFFFFFFFFFFFFFFFFFFFFFFFFFFFFFFFFF:FFFFFFFFFFFFFFFFFFFFFFFFFFFF
FFFFFFFFFFFFFFFFFFFFFFFFF:FFFFFFFFFFFFFFFFFFFFFFFFFFFFFFFFFFFFFFFFFF
FFFFFFFFFFFFFFF
@A00721:190:HGJW7DSXY:4:1365:18864:34507 2:N:0:TCTGCAAG+GCTGCACT
CTAAACCTATCAACATTATCCATGTTGATCATCCAAGTATCTGGATCCCGACTTTCCCCGATGAATAT
CTCATTCACTCTGGGATTAGAATTACCGGGACGTCTATCATACACGAGCCACATCCACAGCCACTGCT
TCATATTATGAACAT
+
FFFF:FFFFFFFFFFFFF:FFFFFFFFFFFFFFFFFFFFFFFF:FFFFFFFFFFFFFFFFFFFFFFFF
FFFFFFFFFFFFFFFFFFFFFFFFFFFFFFFFFFFFFFFFFFFFFFFFFFFFFFFFFFFFFF:FFFFF
FFFFFFFFFFFFFFF
@A00721:190:HGJW7DSXY:4:1638:28546:20181 2:N:0:TCTGCAAG+GCTGCACT
CTAAACCTATCAACATTATCCATGTTGATCATCCAAGTATCTGGATCCCGACTTTCCCCGATGAATAT
CTCATTCACTCTGGGATTAGAATTACCGGGACGTCTATCATACACGAGCCACATCCACAGCCACTGCT
TCATATTATGAACAT
+
FFFFFFFFFFFFFFFFFFFFFFFFFFFFFFFFFFFFF:FFFFFFFFFFFFFFFFFFFFFFFFFFFFFF
FFFFFFFFFFFFFFFFFFFF:FFFFFFFFFFFFFFFFFFFFFFFFFFFFFFFF:FFFFFFFFFFFFFF
:FFFFFFFFF:FFFF

```
@A00721:190:HGJW7DSXY:4:1646:9498:23672 2:N:0:TCTGCAAG+GCTGCACT
CTAAACCTATCAACATTATCCATGTTGATCATCCAAGTATCTGGATCCCGACTTTCCCCGATGAATAT
CTCATTCACTCTGGGATTAGAATTACCGGGACGTCTATCATACACGAGCCACATCCACAGCCACTGCT
TCATATTATGAACAT
+
FFFFFFFFFFFFFFFFFFFFFFFFFFFFFFFFFFFFFFFFFFFFFFFFFFFFFFFFFFFFFFFFFFFFFF
FFFFFFFFFFF:FFFFFFFFFFFFFFFFFFFFFFFFFFFFFFFF:FFFFFFFFFFFFFFFFFFFFFF:FF
FFFFFFFFFFFFFFF
@A00721:190:HGJW7DSXY:4:2139:4544:6762 2:N:0:TCTGCAAG+GCTGCACT
CTAAACCTATCAACATTATCCATGTTGATCATCCAAGTATCTGGATCCCGACTTTCCCCGATGAATAT
CTCATTCACTCTGGGATTAGAATTACCGGGACGTCTATCATACACGAGCCACATCCACAGCCACTGCT
TCATATTATGAACAT
+
FFFFFFFFFFFFFFFFFFFFFFFFFFFFFFFFFF:FFFFFF:FFF:FFFFFFFFFFFFFFFFFFFFFFFF
FFFFFFFFFFFFFFFFF,FFFFFFFFFFFFFFFFFFFFFFFFFFFFFFFFFFFFFFFFFFFFFFFFFFFF
FFFFF:FFFFF:FFF
@A00721:190:HGJW7DSXY:4:2621:16215:3646 2:N:0:TCTGCAAG+GCTGCACT
CTAAACCTATCAACATTATCCATGTTGATCATCCAAGTATCTGGATCCCGACTTTCCCCGATGAATAT
CTCATTCACTCTGGGATTAGAATTACCGGGACGTCTATCATACACGAGCCACATCCACAGCCACTGCT
TCATATTATGAACAT
+
FFFFFFFFFFFFFFFFFFFFFFFFFFFFFFFFFFFFFFFFFFFFFFFFFFFFFFFFFFFF:FFFFFFFFF
FFFFFFFFFFFFFFFFFFFFFFFFFFFFFFFFFFFFFFFFFFFFFFFFFFFFFFFFFFFFFFFFFFFFFF
FFFFFFFFFFFFFFF
@A00721:190:HGJW7DSXY:4:2645:14561:12211 2:N:0:TCTGCAAG+GCTGCACT
CTAAACCTATCAACATTATCCATGTTGATCATCCAAGTATCTGGATCCCGACTTTCCCCGATGAATAT
CTCATTCACTCTGGGATTAGAATTACCGGGACGTCTATCATACACGAGCCACATCCACAGCCACTGCT
TCATATTATGAACAT
+
FFFFFFFFFFFFFFFFFFFFFFFFFFFFFFFFFFFFFFFFFFFFFFFFFFFFFFFFFFFFFFFFFFFFFF
FFFFFFFFFFFFFFFFFFFFFFFFFF:FFFFFFFFFFFFFFFFFFFFFFFFFFFFFFFFFFFFFFF:FFF
FFFFFFFFFFFFFFF
@A00721:190:HGJW7DSXY:4:2645:14235:12461 2:N:0:TCTGCAAG+GCTGCACT
CTAAACCTATCAACATTATCCATGTTGATCATCCAAGTATCTGGATCCCGACTTTCCCCGATGAATAT
CTCATTCACTCTGGGATTAGAATTACCGGGACGTCTATCATACACGAGCCACATCCACAGCCACTGCT
TCATATTATGAACAT
+
FFFFFFFFFFFFFFFFFFFFFFFFFFFFFFFFFFFFFFFFFFFFFFFFFFFFFFFFFFFFFFFFFF:FFF
FFFFFFFFFFFFFFFFFFFFFFFFFFF:FFFFFFFFFFFFFFFFFFFFFFFFFFFFFFFFFFFFFFFFFF
FFFFFFFFF:FFFFF
@A00721:190:HGJW7DSXY:4:1165:21233:6981 2:N:0:TCTGCAAG+GCTGCACT
CACTAAACCTATCAACATTATCCATGTTGATCATCCAAGTATCTGGATCCCGACTTTCCCCGATGAAT
ATCTCATTCACTCTGGGATTAGAATTACCGGGACGTCTATCATACACGAGCCACATCCACAGCCACTG
CTTCATATTATGAAC
+
FFFFFFFFFFFFFFFFF:FFFFFFFFFFFFFFFFFFFFFFFFFFFFFFFFFFFFFFFFFFFFFFFFFFFF
FFFFFFFFFFFFF,FFFFFFFFFFFFFFFFFFFFFFFFFFFFFFFFFFFFFFFFFFFFFFFFFFFFFFFF
FFFFFFFFFFFFFFF
@A00721:190:HGJW7DSXY:4:1545:4408:17268 2:N:0:TCTGCAAG+GCTGCACT
CACTAAACCTATCAACATTATCCATGTTGATCATCCAAGTATCTGGATCCCGACTTTCCCCGATGAAT
ATCTCATTCACTCTGGGATTAGAATTACCGGGACGTCTATCATACACGAGCCACATCCACAGCCACTG
CTTCATATTATGAAC
+
FFFFFFFFFFFFFFFFFFFFFFFFFFFFFFFFFFFFFFFFFFFFFFFFFFFFFFFFFFFFFFFFFFF:FF
```

FFFFFFFFFFFFF,FFFFFFFFFFFFFFFFFFFFFFFFFFFFFFFFFFFFFFFFFFFFFFFFFFFFF
FFFFFFFFFFFFFFF
@A00721:190:HGJW7DSXY:4:1638:17381:4382 2:N:0:TCTGCAAG+GCTGCACT
CACTAAACCTATCAACATTATCCATGTTGATCATCCAAGTATCTGGATCCCGACTTTCCCCGATGAAT
ATCTCATTCACTCTGGGATTAGAATTACCGGGACGTCTATCATACACGAGCCACATCCACAGCCACTG
CTTCATATTATGAAC
+
FFFFFFFF:FF:FFFFFFFFFFFFFFFFF:FFFFFF:FFFFFFFFFFF:FFF:FFFFFFFFFFFFFFF
F,,FFFF,,F,FFFFFFF:FFFFFFFFF:FFFFFFFFFFFFFFFFFFFF,FFFFFFFFFFFFFFFFFF
FF:FFFFFFFFFFFF
@A00721:190:HGJW7DSXY:4:2169:14362:6324 2:N:0:TCTGCAAG+GCTGCACT
CACTAAACCTATCAACATTATCCATGTTGATCATCCAAGTATCTGGATCCCGACTTTCCCCGATGAAT
ATCTCATTCACTCTGGGATTAGAATTACCGGGACGTCTATCATACACGAGCCACATCCACAGCCACTG
CTTCATATTATGAAC
+
FFFFFFFFFFFFFFFFFFFFFFFFFFFFFFFFFFFFFFFFFFFFFFFFFFFFFFFFFFFFFFFFFFFF
FFFFFFFFFFFFFFFFFFFFFFFFFFFFFFFFFFFFFFFFFFFFFF:FFFFFFFFFFFFFFFFFFFFF
FFFFFFFFFFFF:FF
@A00721:190:HGJW7DSXY:4:2358:22083:34851 2:N:0:TCTGCAAG+GCTGCACT
CACTAAACCTATCAACATTATCCATGTTGATCATCCAAGTATCTGGATCCCGACTTTCCCCGATGAAT
ATCTCATTCACTCTGGGATTAGAATTACCGGGACGTCTATCATACACGAGCCACATCCACAGCCACTG
CTTCATATTATGAAC
+
FFFF:FFFFFFFFFFFFFFFFFFFFFFFFFFFFFFFFFFFFFFFFFFFFFFFFFFFFFFFFFFFFFFF
:FFFFF:FFFFFFFFFFFFFFFFFFFFFFFFFFFFFFFFFFFFFFFFFFFFFFFFFFFFFFF:F:FFF
FFFFF:FFFFFFFFF
@A00721:190:HGJW7DSXY:4:2543:5448:7451 2:N:0:TCTGCAAG+GCTGCACT
CACTAAACCTATCAACATTATCCATGTTGATCATCCAAGTATCTGGATCCCGACTTTCCCCGATGAAT
ATCTCATTCACTCTGGGATTAGAATTACCGGGACGTCTATCATACACGAGCCACATCCACAGCCACTG
CTTCATATTATGAAC
+
FFFFFFFFFFFFFFFFFFFFFFFFFFFFFFFFFFFFFFFFFFFFFFFFFFFFFFFFFFFFFFFFFFFF
FFFFFFFFFFFFFFFFFFFFFFFFFFFFFFFFFFFFFFFFFFFFFFFFFFFFFFFFFFFFFFFFFFFF
FF:FFFFFFFFFFFF
@A00721:190:HGJW7DSXY:4:2628:18747:17080 2:N:0:TCTGCAAG+GCTGCACT
CACTAAACCTATCAACATTATCCATGTTGATCATCCAAGTATCTGGATCCCGACTTTCCCCGATGAAT
ATCTCATTCACTCTGGGATTAGAATTACCGGGACGTCTATCATACACGAGCCACATCCACAGCCACTG
CTTCATATTATGAAC
+
FFFFFFFFFFFFFFFFFFF,FFFFFF:FFFFFFFFFFFFFFFFFFFFFFFFFFFFFFFFFFFFFFFFF
FFFFFFFFF:FFFFFFFFFFF:FFFFF:,FFFFFFFFFFFFFFFFFFFFF:FFFFFFFFFFFFF::FF
FFFFFFFFFFFFFFF
@A00721:190:HGJW7DSXY:4:1375:24930:18646 2:N:0:TCTGCAAG+GCTGCACT
CACTAAACCTATCAACATTATCCATGTTGATCATCCAAGTATCTGGATCCCGACTTTCCCCGATGAAT
ATCTCATTCACTCTGGGATTAGAATTACCGGGACGTCTATCATACACGAGCCACATCCACAGCCACTG
CTTCATATTATGAA
+
FFFFFFFFFFFFFF:FFFFFFFFFFFFFFFFFFFFFFFFFFFFFFFFFFFFFFFFFFF:FFFFFFFFF,F
FFFFFFFF:FFFFFFFFF,FFFFF:FFFF,FFFFFFFFFFFFFFF:FFFFFFFFF,FFFFF,FFFFFF
:F,,FFF:FFFF::
@A00721:190:HGJW7DSXY:4:1368:8892:28635 2:N:0:TCTGCAAG+GCTGCACT
CGACACTAAACCTATCAACATTATCCATGTTGATCATCCAAGTATCTGGATCCCGACTTTCCCCGATG
AATATCTCATTCACTCTGGGATTAGAATTACCGGGACGTCTATCATACACGAGCCACATCCACAGCCA
CTGCTTCAGATCGGA

```
+
F,FFFFFF:FFFFFFFFFFFFFFFFFF::FFFFFFFFFFFFFFFFFFFFFFFFFFFFFFFFFFF:FFF
FFFFFFFFFF,FF:FF:F:FFFFFFFFFFF:FFFFFF:FFFFFFF:F:FFFF,FFFFFFFFFFFFFF,
FFFFFFFFFFFFFFF
@A00721:190:HGJW7DSXY:4:2402:18719:26835 2:N:0:TCTGCAAG+GCTGCACT
CGACACTAAACCTATCAACATTATCCATGTTGATCATCCAAGTATCTGGATCCCGACTTTCCCCGATG
AATATCTCATTCACTCTGGGATTAGAATTACCGGGACGTCTATCATACACGAGCCACATCCACAGCCA
CTGCTTCAGATCGGA
+
FFFFFFFFFFFFFFFFFFFFFFFFFFFFFFFFFFFFFFFFFFFFFFFFFFFFFFFFFFFFFFFFFFFF
FFF:FFFFFFFFFFFFFFFFFFFFFFFFFFFFFFFFFFFFFFFFFFFFFFFFFFFFFFFFFFFFFFFF
FFFFFFFFFFFFFFF
@A00721:190:HGJW7DSXY:4:2454:30129:5415 2:N:0:TCTGCAAG+GCTGCACT
CGACACTAAACCTATCAACATTATCCATGTTGATCATCCAAGTATCTGGATCCCGACTTTCCCCGATG
AATATCTCATTCACTCTGGGATTAGAATTACCGGGACGTCTATCATACACGAGCCACATCCACAGCCA
CTGCTTCAGATCGGA
+
FFFFFFFFFFFFFFFFFFFFFFFFFFFFFFFFFFFFFFFFFFF:FFFFFFFFFFFFFFFFFFFFFFFF
FFFFFFFF:FFFFFFFFFFFFFFFFFFFFFFFFFFFFFFFFFFFFFFFFFFFFFFFFFFFFFFFFFF
FFFFFFFFFFFFFFF
@A00721:190:HGJW7DSXY:4:2628:5005:9251 2:N:0:TCTGCAAG+GCTGCACT
CGACACTAAACCTATCAACATTATCCATGTTGATCATCCAAGTATCTGGATCCCGACTTTCCCCGATG
AATATCTCATTCACTCTGGGATTAGAATTACCGGGACGTCTATCATACACGAGCCACATCCACAGCCA
CTGCTTCAGATCGGA
+
FFFFFFFFFFFFFFFFFFFF,FFFFFFFFFFFFFFFFFFFFFFFFFFFFFFFFFFFFFFFFFFFFFFF
FFFFFFFFFFFFFF,FFFFFF:FFFFFFFFFFFFFFFFFFFFFFFFFFFFFFFFFFFFFFFFFFFFFF
FFFFF,F:FFFFFFF
@A00721:190:HGJW7DSXY:4:1174:8422:16517 2:N:0:TCTGCAAG+GCTGCACT
GTTTTTCACAAACGACACTAAACCTATCAACATTATCCATGTTGATCATCCAAGTATCTGGATCCCGA
CTTTCCCCGATGAATATCTCATTCACTCTGGGATTAGAATTACCGGGACGTCTATCATACACGAGCCA
CATCCACAGCCACTG
+
FFFFFFFFFFFFFFFFFF:FFFFFFFFFFFFFFFFFFFFFFFFFFFFFFFFFFFFFFFFFFFFFFFFF
FFFFFFFFFFFFFFFFFF:FFFFFFFFFFFFFFFFFFFFFFFFFFFFFFFFFFFFFFFFFFFFFFFFF
FFFFFFFFFFFFFFF
@A00721:190:HGJW7DSXY:4:1251:19162:13291 2:N:0:TCTGCAAG+GCTGCACT
GTTTTTCACAAACGACACTAAACCTATCAACATTATCCATGTTGATCATCCAAGTATCTGGATCCCGA
CTTTCCCCGATGAATATCTCATTCACTCTGGGATTAGAATTACCGGGACGTCTATCATACACGAGCCA
CATCCACAGCCACTG
+
FFFFFFFFFFFFFFFFFFFF:FFFFFFFFFFFFFFFFFFFFFFFFFFFFFFFF:FF:FFFFFFFFFFF
FFFFFFFFFFFFFFFFFFFFFFFFFFFFFFFFFFFFFFFFFFFFFFFFFFFFF:FFFFFFFFFFFFFF
FFFFFFFFFFFFFFF
@A00721:190:HGJW7DSXY:4:1472:4797:20760 2:N:0:TCTGCAAG+GCTGCACT
GTTTTTCACAAACGACACTAAACCTATCAACATTATCCATGTTGATCATCCAAGTATCTGGATCCCGA
CTTTCCCCGATGAATATCTCATTCACTCTGGGATTAGAATTACCGGGACGTCTATCATACACGAGCCA
CATCCACAGCCACTG
+
FFFFFFFFFFFFFFFFFFFFFFFFFFFFFFFFFFFFFFFFFFFFFFFFFFFFFFFFFFFFFFFFFFFF
FFFFFFFFFFFFFFFFFFFFFFFFFFFFFFFFF:FFFFFFFFFFFFFFFFFFFFFF:FFFFFFFFFFF
FFFFFFFFFFFFFFF
@A00721:190:HGJW7DSXY:4:1472:5864:21449 2:N:0:TCTGCAAG+GCTGCACT
GTTTTTCACAAACGACACTAAACCTATCAACATTATCCATGTTGATCATCCAAGTATCTGGATCCCGA
```

CTTTCCCCGATGAATATCTCATTCACTCTGGGATTAGAATTACCGGGACGTCTATCATACACGAGCCA
CATCCACAGCCACTG
+
FFFFFFFFFFFFFFFFFFFFFFFFFFFFFFFFFFFFFFFFFFFFFFFFFFFFFFFFFFFFFF:FFFFFFF
FFFF:FFFFF:,FFFFF:FFFFF:FFFFFFFFFFFFFFFFFFFF,FFF,FF:FFFFFFFFFF:F:FF:FF
FFFFFFFFFFFFFFF
@A00721:190:HGJW7DSXY:4:1564:26341:4554 2:N:0:TCTGCAAG+GCTGCACT
GTTTTTCACAAACGACACTAAACCTATCAACATTATCCATGTTGATCATCCAAGTATCTGGATCCCGA
CTTTCCCCGATGAATATCTCATTCACTCTGGGATTAGAATTACCGGGACGTCTATCATACACGAGCCA
CATCCACAGCCACTG
+
FFFFFFFFFFFFFFFFFFFFF:FFFFFFFFFFFFFFFFFFFFFFFFFFFFFFF:FFFFFFFFFFFFFFFF
FFFFFFFFFFFFFFFFFFFFFFFFFF:FFFFFFFFFFFFFFF:FFFFFFFFFFFFFFFFFFFFFF:FFFF
FFFFFFFFFFFFFFF
@A00721:190:HGJW7DSXY:4:1618:17409:21183 2:N:0:TCTGCAAG+GCTGCACT
GTTTTTCACAAACGACACTAAACCTATCAACATTATCCATGTTGATCATCCAAGTATCTGGATCCCGA
CTTTCCCCGATGAATATCTCATTCACTCTGGGATTAGAATTACCGGGACGTCTATCATACACGAGCCA
CATCCACAGCCACTG
+
FFFFFFFFFFFFFFFFFFFFFFFFFFFFFFFFFFFFFFFF:FFFFFFFFFFFFFFFF:FFFFFFFFFFFF
F:FFFFFFFFFFFFFFFFFFFFFFFFFFFFFFF:FFFFF:FFFFFFFFFFFFFFFFFFFFFFFFFFFFFF
FFFFFFFFFFFFFFF
@A00721:190:HGJW7DSXY:4:2213:18349:22811 2:N:0:TCTGCAAG+GCTGCACT
GTTTTTCACAAACGACACTAAACCTATCAACATTATCCATGTTGATCATCCAAGTATCTGGATCCCGA
CTTTCCCCGATGAATATCTCATTCACTCTGGGATTAGAATTACCGGGACGTCTATCATACACGAGCCA
CATCCACAGCCACTG
+
FFFFFFFFFFFFFFFFFFFFFFFFFFFFFFFFFFFFFFFFFFFFFFFFFFFFFFFFFFFFFFFFFFFFFF
FFFFFFFFFFFFFFFFFFFFFFFFFFFFFFFFFFFFFFFFFFFF:FFFFFFFFFFFFFFFFFFFFFFFFF
FFFFFFFFFFFFFFF
@A00721:190:HGJW7DSXY:4:2271:4182:29465 2:N:0:TCTGCAAG+GCTGCACT
GTTTTTCACAAACGACACTAAACCTATCAACATTATCCATGTTGATCATCCAAGTATCTGGATCCCGA
CTTTCCCCGATGAATATCTCATTCACTCTGGGATTAGAATTACCGGGACGTCTATCATACACGAGCCA
CATCCACAGCCACTG
+
FFFFFFFFFFF:FFFFFFFFFFFFFFFFFFFFFFFFFFFFFFFFFFFFFFFFFFFFFFFFFFFFFFFFFF
FFFFFFF:FFFFFFFFFFFFFFFFFFFFFFFFFFFFFFFFFFFFFFFFFFFFFFFFFFFFFFFFFFFFFF
FFFFFFFFFFFFFF:
@A00721:190:HGJW7DSXY:4:2341:2745:19648 2:N:0:TCTGCAAG+GCTGCACT
GTTTTTCACAAACGACACTAAACCTATCAACATTATCCATGTTGATCATCCAAGTATCTGGATCCCGA
CTTTCCCCGATGAATATCTCATTCACTCTGGGATTAGAATTACCGGGACGTCTATCATACACGAGCCA
CATCCACAGCCACTG
+
FFFFFFFFFFFFFFFFFF:FFFFFFFFFFFFFFFFFFFFFFFFFFFFFFFFFFFFFFFFFFFFFFFFFFF
FFFFFFF:FFFFFFFFFFFFFFFFFFFFFFFFF:FFFFF:FFFFFFFFFFFFFFFFFFFF:FFFFFFFFF
FFFFFFFFFFFFFFF
@A00721:190:HGJW7DSXY:4:1225:19786:20572 2:N:0:TCTGCAAG+GCTGCACT
GTTTTTCACAAACGACACTAAACCTATCAACATTATCCATGTTGATCATCCAAGTATCTGGATCCCGA
CTTTCCCCGATGAATATCTCATTCACTCTGGGATTAGAATTACCGGGACGTCTATCATACACGAGCCA
CATCCACAGCCACT
+
FF:FFFFFFFFFFFFFFFFFFFFFFFFF:FFFFFFFFFFFFFFFFFFFFF,FFFFFFFFFFFFFFFFFFF
FFFF:FFFFFFFFFFFFFFFFFFFFFFF:FF:FFFFFFFFFFFFFFFFF,FFFFFFFFFFFFFFFFFF,F
FFFFFFFFFFFFFF

@A00721:190:HGJW7DSXY:4:1220:18927:34585 2:N:0:TCTGCAAG+GCTGCACT
TCGTTTTTCACAAACGACACTAAACCTATCAACATTATCCATGTTGATCATCCAAGTATCTGGATCCC
GACTTTCCCCGATGAATATCTCATTCACTCTGGGATTAGAATTACCGGGACGTCTATCATACACGAGC
CACATCCACAGCCAC
+
FF::FFFFFF:FFFFFFFFFFF:FFFFFFFFF:FFFFFF,F:FFFFFFFFF,:FFFFFFFFFFFF,F
:FFFFF:FF,:FFFFFFFFF,FFF:FFFFFFFFFFFFFFFFFFF,,FF,FF::FFFFFFFFFFF:F,F
,FFFF:FFFF:FFFF
@A00721:190:HGJW7DSXY:4:1234:23981:32471 2:N:0:TCTGCAAG+GCTGCACT
TCGTTTTTCACAAACGACACTAAACCTATCAACATTATCCATGTTGATCATCCAAGTATCTGGATCCC
GACTTTCCCCGATGAATATCTCATTCACTCTGGGATTAGAATTACCGGGACGTCTATCATACACGAGC
CACATCCACAGCCAC
+
FFFFF:FFFFFFF,FFFFFFFFFFFFFFFFFFFFFFFFFFFFFFFFFFFFFFFFFFFF:FFFFFFFFFF
FFF:FFFFFFFFFFF::FFFFFFFFFFFFF,FFFF:,FFFFFFFFFFFFFFFFFFFFFFFFF:FFFFFF
FFFFFFF:FFFFFFF
@A00721:190:HGJW7DSXY:4:1501:29722:19711 2:N:0:TCTGCAAG+GCTGCACT
TCGTTTTTCACAAACGACACTAAACCTATCAACATTATCCATGTTGATCATCCAAGTATCTGGATCCC
GACTTTCCCCGATGAATATCTCATTCACTCTGGGATTAGAATTACCGGGACGTCTATCATACACGAGC
CACATCCACAGCCAC
+
FFFFFFFFFFFFFFFFFFFFFFFFFFFFFFFFFFFFFFFFFFFFFFFFFFFFFFFFFFFFFFFFFFFFF
FFFFFFFFFFFFFFFF:FFFFFFFFFFFFFFFFFF:FFFFFFFFFFFFFFFFFFF:FFFFFFFFFFFF
FFFFFFFFFFFFFFF
@A00721:190:HGJW7DSXY:4:1626:5981:6590 2:N:0:TCTGCAAG+GCTGCACT
TCGTTTTTCACAAACGACACTAAACCTATCAACATTATCCATGTTGATCATCCAAGTATCTGGATCCC
GACTTTCCCCGATGAATATCTCATTCACTCTGGGATTAGAATTACCGGGACGTCTATCATACACGAGC
CACATCCACAGCCAC
+
FFFFFFFFFFFFFFFFFFFFFFFFFFFFFFFFFFFFFFFFFFFFFFFFFFFFFFFFFF:FFFFFFFFFF
FFFF:FFFFFFFFFFF:FFFFFF::FFFFFFFFFF,FFFF:FFFFFFFFFFFFFFFFFFFFFFFFFFFF
FFFFFFFFFF:FFFF
@A00721:190:HGJW7DSXY:4:2128:24876:15358 2:N:0:TCTGCAAG+GCTGCACT
TCGTTTTTCACAAACGACACTAAACCTATCAACATTATCCATGTTGATCATCCAAGTATCTGGATCCC
GACTTTCCCCGATGAATATCTCATTGACTCTGGGATTAGAATTAACGGGACGTCTATAATACACGAGC
CACATCCACAGCCAC
+
FFF:FFFFFFFFFFFFFFFFFF::FFFFFFFFFFFFFFFFFFFFFF:FFFFFFFFFF:FFFFFFFFFF
FFFFFFFFFF,FF,:F,F:,,FF:F,FF:,,FFF:FFF,,FFF,,F:FF,:FFF,::,F,,,F,::,F
FF:F,F:FFF,FFFF
@A00721:190:HGJW7DSXY:4:2213:7708:26334 2:N:0:TCTGCAAG+GCTGCACT
TCGTTTTTCACAAACGACACTAAACCTATCAACATTATCCATGTTGATCATCCAAGTATCTGGATCCC
GACTTTCCCCGATGAATATCTCATTCACTCTGGGATTAGAATTACCGGGACGTCTATCATACACGAGC
CACATCCACAGCCAC
+
FFFFFFFFFFFFFFFFFFFFFFFF:FFFFFFFFFFFFFFFFFFFFFFFFFFFFFFFF:F,FFFFFFFFFF
FFFFFFFFFFFFFFFFFFFFFFFFFFFFFFFFFFFFFFFFFFFFFFFFFFFFFFFFFFFFFFFFFFFF:F
FFFFFFFFFFFFFFF
@A00721:190:HGJW7DSXY:4:2227:12608:4852 2:N:0:TCTGCAAG+GCTGCACT
TCGTTTTTCACAAACGACACTAAACCTATCAACATTATCCATGTTGATCATCCAAGTATCTGGATCCC
GACTTTCCCCGATGAATATCTCATTCACTCTGGGATTAGAATTACCGGGACGTCTATCATACACGAGC
CACATCCACAGCCAC
+
FFFFFFFFFFFFFFFFFFFFFFFFFFFFFFFFFFFFFFFFFFFFFFFFFFFFFFFFFFFFFFFFFFFFF

FFFFFFFFFFFFFFFFFFFFFFFFFFFFFFFFFFFFFFFFFFFFFFFFFFFFFFFFFFFFFFFFFFFFFF
FFFFFFFFFFFFFFF
@A00721:190:HGJW7DSXY:4:2543:12753:33254 2:N:0:TCTGCAAG+GCTGCACT
TCGTTTTTCACAAACGACACTAAACCTATCAACATTATCCATGTTGATCATCCAAGTATCTGGATCCC
GACTTTCCCCGATGAATATCTCATTCACTCTGGGATTAGAATTACCGGGACGTCTATCATACACGAGC
CACATCCACAGCCAC
+
FFFFFFFFFFFFFFFFFFFFFFFFFFFFFFFFFFFFFFFFFFFFFFFFF:FFFFFFFFFFFFFFFFFFFF
FFFFFFFFFFFFFFFFFFFFFFFFFFFFFFFFFFFFFFFFFFFFFFFFFFFFFFFFFFFFFFFFFFFFFF
FFFFFFFFFFFFFFF
@A00721:190:HGJW7DSXY:4:1204:22435:29011 2:N:0:TCTGCAAG+GCTGCACT
ATCGTTTTTCACAAACGACACTAAACCTATCAACATTATCCATGTTGATCATCCAAGTATCTGGATCC
CGACTATCCCCGATGAATATCTCATTCACTCTGGGATTAGAATTACCGGGACGTCTATCATACACGAG
CCACATCCACAGCCA
+
FFFFFFFFFFFFFFFFFFFFFFFFFFFFFFFFFFFFFFFFFFFFFFFFFFFFFFFFFFFFFFFFFFFFFF
FFFFF,FFFFFFFFFFFF:FFFFFFFFFFFFFF:FFFFFFFFFFFFFFFFFFFFFFFFFFFFFFFFFFFF
FFFFFFFFFFFFFFF
@A00721:190:HGJW7DSXY:4:1556:31801:17738 2:N:0:TCTGCAAG+GCTGCACT
ATCGTTTTTCACAAACGACACTAAACCTATCAACATTATCCATGTTGATCATCCAAGTATCTGGATCC
CGACTTTCCCCGATGAATATCTCATTCACTCTGGGATTAGAATTACCGGGACGTCTATCATACACGAG
CCACATCCACAGCCA
+
FFFFFFFFFFFFFFFFFFFFFFFF:,F:FFFFFFFFFFFFFFFFFFFFF:FFFFFFFFFFFFFFFFFFFF
FFFFFFFFFFFFFFFFFFFFFFFFFFFF:FFFFFFFFFFFFFFFFFFFFFFFFFFFFFFFFFFFFFFFFF
FFFFFFFFFFFFFFF
@A00721:190:HGJW7DSXY:4:2160:11153:7059 2:N:0:TCTGCAAG+GCTGCACT
ATCGTTTTTCACAAACGACACTAAACCTATCAACATTATCCATGTTGATCATCCAAGTATCTGGATCC
CGACTTTCCCCGATGAATATCTCATTCACTCTGGGATTAGAATTACCGGGACGTCTATCATACACGAG
CCACATCCACAGCCA
+
FF::FFF:F,FFFF:,:FFFFFFFF,FFFF,FFFFFFFFFFF:FF,FF:F:FFFF,FF:FFF:F,FFFF:
,FFFFF:F,:FFFF:FFFF,FFFFFFFFFFF:F:,FFFF:FFFFF:,,,FFF:F,FFFFFFFF,FFF,
,FFFFFF:FFF:,FF
@A00721:190:HGJW7DSXY:4:2308:19126:24439 2:N:0:TCTGCAAG+GCTGCACT
ATCGTTTTTCACAAACGACACTAAACCTATCAACATTATCCATGTTGATCATCCAAGTATCTGGATCC
CGACTTTCCCCGATGAATATCTCATTCACTCTGGGATTAGAATTACCGGGACGTCTATCATACACGAG
CCACATCCACAGCCA
+
:FFFFFFFFFFFFFFFFFFFFFFFFFFFFFFFFFFFFFFFFFFFFFFFFFFFFFFFFFFFFFFFFFFFFF
FFFFFFFFFFFFFFFFFFFFFFFFFFFFFFFFFFFFFFFFFFFFFFFFFFFFFFFFFFFFFFFFFFFFFF
FFFFFFFFFFFFFFF
@A00721:190:HGJW7DSXY:4:2368:31458:13072 2:N:0:TCTGCAAG+GCTGCACT
ATCGTTTTTCACAAACGACACTAAACCTATCAACATTATCCATGTTGATCATCCAAGTATCTGGATCC
CGACTTTCCCCGATGAATATCTCATTCACTCTGGGATTAGAATTACCGGGACGTCTATCATACACGAG
CCACATCCACAGCCA
+
:FFFFFFFFFFFFFFFFFFFFFFFFFFFFFFFFFFFFFFFFFFFFFFFFFFFFFFFFFFFFFFFFFFFFF
FFFFFFFFF:F:FFFFFFFFFFFFFFFFF:F:F::FFFFFFFFFFFF::FFFFFFFFFFFFFFFFFFF:FF
:FF:FFF:FFF::::
@A00721:190:HGJW7DSXY:4:2527:4553:5149 2:N:0:TCTGCAAG+GCTGCACT
TCGTTTTTCACAAACGACACTAAACCTATCAACATTATCCATGTTGATCATCCAAGTATCTGGATCCC
GACTTTCCCCCGATGAATATCTCATTCACTCTGGGATTAGAATTACCGGGACGTCTATCATACACGAG
CCACATCCACAGCCA

+
FFFFFFFFFFFFFFFFFFFFFFFFFFFFFFFFFFFFFFFFFFFFFFFFFFFFFFF:FFFFFFFFFFF
FFFFFFFFFFFFFFFFFFFFFFFFFFFFFFFFFFFF:FFFFFFFFFFFFFFFFFFFFFF:FFFFFFF
FFFFFFFF,FFFFFF
@A00721:190:HGJW7DSXY:4:1107:21459:4867 2:N:0:TCTGCAAG+GCTGCACT
ATCGTTTTCACAAACGACACTAAACCTATCAACATTATCCATGTTGATCATCCAAGTATCTGGATCC
CGACTTTCCCCGATGAATATCTCATTCACTCTGGGATTAGAATTACCGGGACGTCTATCATACACGAG
CCACATCCACAGCC
+
FFFFFFFFFFFFFF,FFFFFFF:F:FFFFFFFFFFFFFFFFFFFFFFFFFFFFFFFFFFFFFFFFFF
FFFFFFFFFFF:FFF:F:FFFFFFFFFFFFFFFFFFFFFFFF:F,FFFFFFFFFFFFFFFFFFFFF,
FFFFFFFFFFFFFF
@A00721:190:HGJW7DSXY:4:2544:6479:34131 2:N:0:TCTGCAAG+GCTGCACT
TTTTCACAAACGACACTAAACCTATCAACATTATCCATGTTGATCATCCAAGTATCTGGATCCCGACT
TTCCCCGATGAATATCTCATTCACTCTGGGATTAGAATTACCGGGACGTCTATCATACACGAGCCACA
TCCACAGAGATCGGA
+
FFFFFFFFFFFFFFFFFFFFFFFFFFFFFFFFFFFFFFFFFFFFFFFFFFFFFFFFFFFFFFFFFFF
FFF:FFFFFFFFFFFFFFFFFFF:FFFFFFFFFFFFFFFFFFFFFFFFFFFFFFFFFFFFFFFFFFF
FFFFFFFFFFFFFFF
@A00721:190:HGJW7DSXY:4:2556:31675:4805 2:N:0:TCTGCAAG+GCTGCACT
TTTTCACAAACGACACTAAACCTATCAACATTATCCATGTTGATCATCCAAGTATCTGGATCCCGACT
TTCCCCGATGAATATCTCATTCACTCTGGGATTAGAATTACCGGGACGTCTATCATACACGAGCCACA
TCCACAGAGATCGGA
+
FFFFFFFFFFFFFFFFFFFFFFFFFFFFFFFFFFFFFFFFFFFFFFFFFFFFFFFFFFFFFFFFFFF
FFFFFFFFFF:FFFFFFFFFFFF:FFFFFFFFFFFFFFFFF:FFFFFFFFFFFFFFF:FFFFFFFFF
FFFFFFFFFFFFFFF
@A00721:190:HGJW7DSXY:4:2656:7536:20932 2:N:0:TCTGCAAG+GCTGCACT
TTTTCACAAACGACACTAAACCTATCAACATTATCCATGTTGATCATCCAAGTATCTGGATCCCGACT
TTCCCCGATGAATATCTCATTCACTCTGGGATTAGAATTACCGGGACGTCTATCATACACGAGCCACA
TCCACAGAGATCGGA
+
FFFFFFFFFFFFFFFFFFFFFFFFFFFFFFFFFFFFFFFFFFFFFFFFFFFFFFFFFFFFFFFFFFF
FFFFFFFFFFF:FFFFFFFFFFFFFFFFFFFFFFFFFFFFFFFFFFFFFFFFFFFFFFFFFFFFFFF
FFFFFFFFFFFFFFF
@A00721:190:HGJW7DSXY:4:2178:17635:23328 2:N:0:TCTGCAAG+GCTGCACT
GACCTTCTAGAACGTATCGTTTTTCACAAACGACACTAAACCTATCAACATTATCCATGTTGATCATC
CAAGTATCTGGATCCCGACTTTCCCCGATGAATATCTCATTCACTCTGGGATTAGAATTACCGGGACG
TCTATCATACACGAG
+
FFFFFFFFFFFFFFFFFFFFFFFFFFFFFFFFFFFFFFFFFFFFFFFFFFFFFFFFFFFFFFFFFFF
FFFFFFFFFFFFFFFFFFFFFFFFF:FFFFFFFFFFFFFFFFFFFFFFFFFFF,FFFFFFFFFFFFF
FFFFFFFFFF:FFFF
@A00721:190:HGJW7DSXY:4:2434:1316:4147 2:N:0:TCTGCAAG+GCTGCACT
GACCTTCTAGAACGTATCGTTTTTCACAAACGACACTAAACCTATCAACATTATCCATGTTGATCATC
CAAGTATCTGGATCCCGACTTTCCCCGATGAATATCTCATTCACTCTGGGATTAGAATTACCGGGACG
TCTATCATACACGAG
+
FFF,FFF:FFFF:FFFFFFFFFF:FFFFFFFFFFFFFFFFFFFF:FFFFFFFFF,FFFF:FFFFFFF
,FFFFFFFFFFFFFFFFFFFFFFFF,FFFFFFFFFFFFF:FF:F,F:FFFFFFFFFFFF:FFFFFF:
FFFFFFF:F,FFFF:
@A00721:190:HGJW7DSXY:4:2509:11306:9173 2:N:0:TCTGCAAG+GCTGCACT
GACCTTCTAGAACGTATCGTTTTTCACAAACGACACTAAACCTATCAACATTATCCATGTTGATCATC

```
CAAGTATCTGGATCCCGACTTTCCCCGATGAATATCTCATTCACTCTGGGATTAGAATTACCGGGACG
TCTATCATACACGAG
+
FFFFFFFFFFFFFFFFFFFFFFFFFFFFFFFFFFFFFFFFFFFFFFFFFFFFFFFFFFFFFFFFFFFFFF
FFFFF:FFFFFFFFFFFFFFFFFFFFFFFFFFFFFFFFFFFFFFFFFFFFFFFFFFFFFFFFFFFFFFF:
FFFFFFFFFFFF,FF
@A00721:190:HGJW7DSXY:4:2528:15420:18333 2:N:0:TCTGCAAG+GCTGCACT
GACCTTCTAGAACGTATCGTTTTTCACAAACGACACTAAACCTATCAACATTATCCATGTTGATCATC
CAAGTATCTGGATCCCGACTTTCCCCGATGAATATCTCATTCACTCTGGGATTAGAATTACCGGGACG
TCTATCATACACGAG
+
FFFFFFFFF:FFFFFFFFFFFFFFFFFFFFFFFFFFFFFFFFFFFFFFFFFFFFFFFFFFFFFFFFFFFF
FFFFFFFFFFFFFFFFFFFFFFFFFFFFFFFFFFFFFFFFFFFFFFFFFFFFFFFFFFFFFF:FFFFFFF
FFFFFFFFFFFFFFF
@A00721:190:HGJW7DSXY:4:2610:15971:16376 2:N:0:TCTGCAAG+GCTGCACT
GACCTTCTAGAACGTATCGTTTTTCACAAACGACACTAAACCTATCAACATTATCCATGTTGATCATC
CAAGTATCTGGATCCCGACTTTCCCCGATGAATATCTCATTCACTCTGGGATTAGAATTACCGGGACG
TCTATCATACACGAG
+
FFFFFFFFFFFFFFF:FFFFFFFF:FFFFFFFFFFFFFFFFFFFFFFFFFFFFFFFFFFFFFFFFFFFFF
FFFF:FFFFFFFFFFFFFFFFFFFFFFFFFFFFFFFFFFFFFFFFFFFFFFFF:F:FFFFFFFFFFFFFF
FFFFFFFFFFFFFFF
@A00721:190:HGJW7DSXY:4:1150:22589:32315 2:N:0:TCTGCAAG+GCTGCACT
CGACCTTCTAGAACGTATCGTTTTTCACAAACGACACTAAACCTATCAACATTATCCATGTTGATCAT
CCAAGTATCTGGATCCCGACTTTCCCCGATGAATATCTCATTCACTCTGGGATTAGAATTACCGGGAC
GTCTATCATACACGA
+
FFFFFFFFFFFFFFFFFFFFFFFFFFFFFFFFFFFFFFFFFFFFFFFFFFFFFFF:FFFFFFFFFFFFFF
FFF:FF:FFFFFFFFFFFFFFFFFFFFFFFFFFFFFFFFFFFFFFFFFFFFFFFF:FFFFFFFFFFFFFF
F:FFFFFFFFFFFFF
@A00721:190:HGJW7DSXY:4:1263:21802:34115 2:N:0:TCTGCAAG+GCTGCACT
CGACCTTCTAGAACGTATCGTTTTTCACAAACGACACTAAACCTATCAACATTATCCATGTTGATCAT
CCAAGTATCTGGATCCCGACTTTCCCCGATGAATATCTCATTCACTCTGGGATTAGAATTACCGGGAC
GTCTATCATACACGA
+
FFFFFFFFFFFFFFFFFFFFFFFFFFFFFFFFFFFFFFFFFFFFFFFFFFFFFFFFFFFFFFFFFFFFFF
FFFFFFFFFFFFFFFFFFFFFFFFFFFFFFFFFFFFFFFFFFFFFFFFFFFFFF,FFFFFFFFFFFFFFF
FFFFFFFFFFFFFFF
@A00721:190:HGJW7DSXY:4:1329:14669:33348 2:N:0:TCTGCAAG+GCTGCACT
CGACCTTCTAGAACGTATCGTTTTTCACAAACGACACTAAACCTATCAACATTATCCATGTTGATCAT
CCAAGTATCTGGATCCCGACTTTCCCCGATGAATATCTCATTCACTCTGGGATTAGAATTACCGGGAC
GTCTATCATACACGA
+
FFF:FFFF:FFFFFFFFFFFFFFFFFFFFFFFFFFFFFFFFFFFFFFFFFFFFFFFFFFFFFFFFFFFFF
FFFF:FFFF,F:FF:F:FFFFFF:FF:FFFFF::FFFFFFFF:FFFFFFFF:FFF:F:FFFFFF:FFFFF
FFFFFFFF:FFFFFF
@A00721:190:HGJW7DSXY:4:1540:14904:7921 2:N:0:TCTGCAAG+GCTGCACT
CGACCTTCTAGAACGTATCGTTTTTCACAAACGACACTAAACCTATCAACATTATCCATGTTGATCAT
CCAAGTATCTGGATCCCGACTTTCCCCGATGAATATCTCATTCACTCTGGGATTAGAATTACCGGGAC
GTCTATCATACACGA
+
FFFFFFFFFFFFFFFFFFFFFFFFFFFFFFFFFFFFFF:FF:FFFFFFFFFFFFFFFF:FFFFFFFFFFF
FFFF:FFFFFFFFFFFFFFFFFFFFFF:FFFFFFFF:FFFFFFF:FFFFFFFFFFFFFFFFFFF,:FFFF
FFFFFFFFFFFFFFF
```

```
@A00721:190:HGJW7DSXY:4:1609:24587:30921 2:N:0:TCTGCAAG+GCTGCACT
CGACCTTCTAGAACGTATCGTTTTTCACAAACGACACTAAACCTATCAACATTATCCATGTTGATCAT
CCAAGTATCTGGATCCCGACTTTCCCCGATGAATATCTCATTCACTCTGGGATTAGAATTACCGGGAC
GTCTATCATACACGA
+
FFFFFFFFFFFFFFFFFFFFFFFFFFFFFFFFFFFFFFFFFFFFFFFFFFFFFFFFFFFFFFFFFFFF
FFFFFFFFFFFFFFFFFFFFFFFFFFFFFFFFFFFFFFFFFFFFFFFFFFFFFFFFFFFFF:FFFFFF
FFFFFFFFFFFFFFF
@A00721:190:HGJW7DSXY:4:2102:28772:18129 2:N:0:TCTGCAAG+GCTGCACT
CGACCTTCTAGAACGTATCGTTTTTCACAAACGACACTAAACCTATCAACATTATCCATGTTGATCAT
CCAAGTATCTGGATCCCGACTTTCCCCGATGAATATCTCATTCACTCTGGGATTAGAATTACCGGGAC
GTCTATCATACACGA
+
FFFFF:,FFFFFFFFFFFFFFFFFFFFFFFFFFFFFFFFFFFFFFFFFFFFFFFF:FFFFFFF:FF:
FFFFFFFFFFF:FFFFF:FFFFFF,FF::FFFFF,FFFFF,FFFFFFFF,FFFFFFFF:FFFF:FFFF
:FFFFFFFFFF:F:F
@A00721:190:HGJW7DSXY:4:2115:25093:25817 2:N:0:TCTGCAAG+GCTGCACT
CGACCTTCTAGAACGTATCGTTTTTCACAAACGACACTAAACCTATCAACATTATCCATGTTGATCAT
CCAAGTATCTGGATCCCGACTTTCCCCGATGAATATCTCATTCACTCTGGGATTAGAATTACCGGGAC
GTCTATCATACACGA
+
FFFFFFFFFFFFFFFFFFFFFFFFFFFFFFFFFFFFFFFFFFFFFFFFFFFFFFFFFFFFFFFFFFFF
FFFFFFFFFFFFFFFFFFFFFFFFFFFFFFFFFFFFFFFFFFFFFFFFFFFFFFFFFFFFFFFFFFFF
FFFFFFFFFFFFFFF
@A00721:190:HGJW7DSXY:4:2133:23240:18035 2:N:0:TCTGCAAG+GCTGCACT
CGACCTTCTAGAACGTATCGTTTTTCACAAACGACACTAAACCTATCAACATTATCCATGTTGATCAT
CCAAGTATCTGGATCCCGACTTTCCCCGATGAATATCTCATTCACTCTGGGATTAGAATTACCGGGAC
GTCTATCATACACGA
+
FFFFFFFFFFFFFFFFFFFFFFFFFFFFFFFFFFFFFFFFFFFFFFFFFFFFFFFFFFFFFFFFFFFF
FFFFFFFFFFFFFFFFFFFFFFFFFFFFFFFFFFFFFFFFFFFFFFFFFFFFFFFFFFFFFFFFFFFF
FFFFFFFFFFFFFFF
@A00721:190:HGJW7DSXY:4:2420:28926:18646 2:N:0:TCTGCAAG+GCTGCACT
CGACCTTCTAGAACGTATCGTTTTTCACAAACGACACTAAACCTATCAACATTATCCATGTTGATCAT
CCAAGTATCTGGATCCCGACTTTCCCCGATGAATATCTCATTCACTCTGGGATTAGAATTACCGGGAC
GTCTATCATACACGA
+
FFFFFFFFFFFFFFFFFFFFFFFFFFFFFFFFFFFFFFFFFFFFFFFFFFFFFFFFFFFFFFFFFFFF
FFFFFFFFFFFFFFFFFFFFFFFFFFFFFFFFFFFFFFFFFFFFFFFFFFFF:FFFFFFFF:,FFFFF
FFFFFFFFFFFFFFF
@A00721:190:HGJW7DSXY:4:2512:30572:36839 2:N:0:TCTGCAAG+GCTGCACT
CGACCTTCTAGAACGTATCGTTTTTCACAAACGACACTAAACCTATCAACATTATCCATGTTGATCAT
CCAAGTATCTGGATCCCGACTTTCCCCGATGAATATCTCATTCACTCTGGGATTAGAATTACCGGGAC
GTCTATCATACACGA
+
FF:FFFFFFFFFFFFFFFFFFFFFFFFFFF:FFFFFFFFFFFFFFFFFFFFFFFFFFFFFFFFFFFFF
FF:FFFFFFFFFFFFFFFFFFFFFFFFFFFFFFFFFFFFFFFFFFFFFFFFFFFFFFFFFFFFFFFFF
FFFFFFFFFFF:FFF
@A00721:190:HGJW7DSXY:4:1217:3631:30170 2:N:0:TCTGCAAG+GCTGCACT
ACGACCTTCTAGAACGTATCGTTTTTCACAAACGACACTAAACCTATCAACATTATCCATGTTGATCA
TCCAAGTATCTGGATCCCGACTTTCCCCGATGAATATCTCATTCACTCTGGGATTAGAATTACCGGGA
CGTCTATCATACACG
+
FFFFFFFFFFFFFFFFFFFFFFFFFFFFFFFFFFFFFFFFFFFFFFFFFFFFFFFF:FFFFFFFFFFF
```

```
FFFFFFFFF:FFFFF,FFFFFFFFF,FFFFFFFFFFFFFFFFFFFFF:FF::FFFFFFFFFFFFFF::
:FFFFFFFFFFFFFF
@A00721:190:HGJW7DSXY:4:2278:15429:25238 2:N:0:TCTGCAAG+GCTGCACT
ACGACCTTCTAGAACGTATCGTTTTTCACAAACGACACTAAACCTATCAACATTATCCATGTTGATCA
TCCAAGTATCTGGATCCCGACTTTCCCCGATGAATATCTCATTCACTCTGGGATTAGAATTACCGGGA
CGTCTATCATACACG
+
FFFFFFFFFFFFFFFFFFFFFFFFFFFFFFFFFFFFFFFFFFFFFFFFFFFFFFFFFFFFFFFFFFFF
FFFFFFFFFFFFFFFFFFFFFFFFFFFFFFFFFFFFFFFFFFFFFFFFFFFFFFFFFFFFFFFFFFFF
FFFFFFFFFFFFFFF
@A00721:190:HGJW7DSXY:4:1222:9390:22263 2:N:0:TCTGCAAG+GCTGCACT
CCACGACCTTCTAGAACGTATCGTTTTTCACAAACGACACTAAACCTATCAACATTATCCATGTTGAT
CATCCAAGTATCTGGATCCCGACTTTCCCCGATGAATATCTCATTCACTCTGGGATTAGAATTACCGG
GACGTCTATCATACA
+
FFFFFFFF:FFFF:FFFFFFFF:FFFFFFFFFFFFFFFFFFFFFFFF:FFFFFF,FFFFFFFFFFFFF
FF:FF,FFF:FFFFFFFFFFFFFF,FFFFFFFFFF:FFFFFFFFFFF:FFFFFFFFFFFFFFFFFFFF
FFFFFFFFFFF:FFF
@A00721:190:HGJW7DSXY:4:1250:30563:5415 2:N:0:TCTGCAAG+GCTGCACT
CCACGACCTTCTAGAACGTATCGTTTTTCACAAACGACACTAAACCTATCAACATTATCCATGTTGAT
CATCCAAGTATCTGGATCCCGACTTTCCCCGATGAATATCTCATTCACTCTGGGATTAGAATTACCGG
GACGTCTATCATACA
+
FFFFFFFFFFFFFFFFFFFFFFFFFFFFFFFFFFFFFFFFFFFFFFFFFFFFFFFFFFFFFFFFFFFF
FFFFFFFFFFFFFFFFFFFFFFFFFFFFFF:FFFFF:FFFFFFFFFFFFFFFFFFFFFFFFFF:FFFFFF
FFFFFFFFFFF:FFF
@A00721:190:HGJW7DSXY:4:1268:11162:20666 2:N:0:TCTGCAAG+GCTGCACT
CCACGACCTTCTAGAACGTATCGTTTTTCACAAACGACACTAAACCTATCAACATTATCCATGTTGAT
CATCCAAGTATCTGGATCCCGACTTTCCCCGATGAATATCTCATTCACTCTGGGATTAGAATTACCGG
GACGTCTATCATACA
+
FFFFFFFFFFFFFFF:FFFFFFFFFFFFFFFFFFFFFFFFFFFFF:FFFFFFFFFFFFFFFFFFFFFFFF
FFFFFFFFFFFFFFFFFFFFFFFFFFFFFFFFFFFFFFFFFFFFFFFFFFFFFFFFFFFFFFFFFFFF
FFFFFFFFFFFFFF:
@A00721:190:HGJW7DSXY:4:1345:2157:5071 2:N:0:TCTGCAAG+GCTGCACT
CCACGACCTTCTAGAACGTATCGTTTTTCACAAACGACACTAAACCTATCAACATTATCCATGTTGAT
CATCCAAGTATCTGGATCCCGACTTTCCCCGATGAATATCTCATTCACTCTGGGATTAGAATTACCGG
GACGTCTATCATACA
+
FFFFFFFFFFFFFFFFFFF:FFFFFFFFFFFF:FFFFFFFF:FFFFFFFFFFFFFFFFFFFFFFFFFFF
FFFFFFFFFFFFFFFFFFFFFFFFFFFFFF:FFFF,FFFFFFFFFFFFFFFFFFFFFFFFFFFFFFFF
FFFFFFFFFFFFFFF
@A00721:190:HGJW7DSXY:4:1363:16984:21480 2:N:0:TCTGCAAG+GCTGCACT
CCACGACCTTCTAGAACGTATCGTTTTTCACAAACGACACTAAACCTATCAACATTATCCATGTTGAT
CATCCAAGTATCTGGATCCCGACTTTCCCCGATGAATATCTCATTCACTCTGGGATTAGAATTACCGG
GACGTCTATCATACA
+
F,FF:F::FFF,F,F:F::F,F:FFFFFFF:FFFFFFFFFFFFFF,,FF,FFFF,:F:F:F:,,FFFF
:FFF:FFF,F,FF:FF,F,F:FF:F,FFF:,FFFFFFFFFFFF:FFFF,FFF:FFFFF,FFFFFF,,F
FFFFFF:FFFFFFFF
@A00721:190:HGJW7DSXY:4:1621:12057:13730 2:N:0:TCTGCAAG+GCTGCACT
CCACGACCTTCTAGAACGTATCGTTTTTCACAAACGACACTAAACCTATCAACATTATCCATGTTGAT
CATCCAAGTATCTGGATCCCGACTTTCCCCGATGAATATCTCATTCACTCTGGGATTAGAATTACCGG
GACGTCTATCATACA
```

+
FFFFFFFFFFFFFFFFFFFFFFFFFFFFFFFFFFFFFFFFFFFFFFFFFFFFFFFFFFFFFFFFFFFF
FFFFFFFFFFFFFFFFFFFFFFFFFFFFFFFFFFF:FFFFFFFFFFFFFFFFFFFFFFFFFFFFFFFF
FFFFFFFFFFFFFFF
@A00721:190:HGJW7DSXY:4:1668:19253:9721 2:N:0:TCTGCAAG+GCTGCACT
CCACGACCTTCTAGAACGTATCGTTTTTCACAAACGACACTAAACCTATCAACATTATCCATGTTGAT
CATCCAAGTATCTGGATCCCGACTTTCCCCGATGAATATCTCATTCACTCTGGGATTAGAATTACCGG
GACGTCTATCATACA
+
FFFFFFFFFFFFFFFF:FFFFFFFFFFFFFFFF,FFFFFFFFFFFF,FFFFFFF:FFFFFFFFFFFFF
FFFFFFFFFFFFFFFF:FFFFFF:FFFFFFFFF:FFFFFFFFFFFFFFFFFFFFFFFFFFFFFFFFFF
FFFFFFFFFFFFFFF
@A00721:190:HGJW7DSXY:4:1668:18900:9799 2:N:0:TCTGCAAG+GCTGCACT
CCACGACCTTCTAGAACGTATCGTTTTTCACAAACGACACTAAACCTATCAACATTATCCATGTTGAT
CATCCAAGTATCTGGATCCCGACTTTCCCCGATGAATATCTCATTCACTCTGGGATTAGAATTACCGG
GACGTCTATCATACA
+
FFFFFFFFFFFFFFFFFFFFFFFFFFFFFFFFFF:FFFFFFFFFFFFFFFFFFFFFFFFFFFF:FFFF
FFFFFFFFFFFF:F:FFF,FFFFFFFFFFFFFFFFFFFFFFFFF::FFFFF:FFFFFFFFFFFFFFFF
FFF::FFFF,FFFFF
@A00721:190:HGJW7DSXY:4:2220:22914:10708 2:N:0:TCTGCAAG+GCTGCACT
CCACGACCTTCTAGAACGTATCGTTTTTCACAAACGACACTAAACCTATCAACATTATCCATGTTGAT
CATCCAAGTATCTGGATCCCGACTTTCCCCGATGAATATCTCATTCACTCTGGGATTAGAATTACCGG
GACGTCTATCATACA
+
FFFFFFF:FFFFFF:FFFFFFFFFFFFFFFF,F:FFFFFFFFFFFFFFFFF,F,FFF,FFFFFFF:F,
:FFFFF,FF::FFFFF,F,FFFFFF,FFFF:F,F::::FFFFFFFFFF,FFFFFFF:FFFFFFF:F,,
:FFFFFFFFFF,FFF
@A00721:190:HGJW7DSXY:4:2249:31286:3599 2:N:0:TCTGCAAG+GCTGCACT
CCACGACCTTCTAGAACGTATCGTTTTTCACAAACGACACTAAACCTATCAACATTATCCATGTTGAT
CATCCAAGTATCTGGATCCCGACTTTCCCCGATGAATATCTCATTCACTCTGGGATTAGAATTACCGG
GACGTCTATCATACA
+
FFFFFFFFFFFFFFFFFFFFFFFFFFFFFFFFFFFFFFFFFFF:FFFFFFFFFFFFFFFFFFFFFFFF
FFFFFFFFFFFFFFFFFFFFFFFFFFFFFFFFFFFFFFFFFFFFFF:FFFFFFFFFFFFFFFFF:FFF
FFFFFFFFFFFFFFF
@A00721:190:HGJW7DSXY:4:2301:5873:25911 2:N:0:TCTGCAAG+GCTGCACT
CCACGACCTTCTAGAACGTATCGTTTTTCACAAACGACACTAAACCTATCAACATTATCCATGTTGAT
CATCCAAGTATCTGGATCCCGACTTTCCCCGATGAATATCTCATTCACTCTGGGATTAGAATTACCGG
GACGTCTATCATACA
+
FFFFFFFFFFFFFFFFFFFFFFFFFFFFFFFFFFFFFFFFFF:FFFFFFFFFFFFFFFFFFFF:FFFF
FFFFF:FFFFFFFFFFFF,FFFFF:FFF:FFFFFFFFFFFFFFFFFF,FFFFFFFFFFFF,FFFFFFF
FFFFFFFFFFFFFF:
@A00721:190:HGJW7DSXY:4:2348:9010:35446 2:N:0:TCTGCAAG+GCTGCACT
CCACGACCTTCTAGAACGTATCGTTTTTCACAAACGACACTAAACCTATCAACATTATCCATGTTGAT
CATCCAAGTATCTGGATCCCGACTTTCCCCGATGAATATCTCATTCACTCTGGGATTAGAATTACCGG
GACGTCTATCATACA
+
FFFFFFFF:FFFFFFFFFFFFFFFFFFFFFFFFFFFFFFFFFFFFFFFFFFFFFFFFFFFFFFFFFFF
FFFFFFFFFFFFFFFFFFFFFFFFFFFFFFFFFFFFFFFFFFFFFFFFFFFFFFFFFFFFFFFFFFFF
FFFFFFFFFFFFFFF
@A00721:190:HGJW7DSXY:4:2375:12120:6480 2:N:0:TCTGCAAG+GCTGCACT
CCACGACCTTCTAGAACGTATCGTTTTTCACAAACGACACTAAACCTATCAACATTATCCATGTTGAT

CATCCAAGTATCTGGATCCCGACTTTCCCCGATGAATATCTCATTCACTCTGGGATTAGAATTACCGG
GACGTCTATCATACA
+
FFFFFFFFFFFFFFFFFFFFFFFFFFFFFFFFFFFFFFFFFFFFFFFFFFFFFFFFFFFFFFFFFFFFFF
FFFFFFFFFFFFFFFFFFFFFFFFF:FFF:FFFFFFFFFFFFFFFFFFFFFFFFFFFFFFFFFFFFFFFF
FFFFFFFFFFFFFFF
@A00721:190:HGJW7DSXY:4:2453:28944:14199 2:N:0:TCTGCAAG+GCTGCACT
CCACGACCTTCTAGAACGTATCGTTTTTCACAAACGACACTAAACCTATCAACATTATCCATGTTGAT
CATCCAAGTATCTGGATCCCGACTTTCCCCGATGAATATCTCATTCACTCTGGGATTAGAATTACCGG
GACGTCTATCATACA
+
FFFFFFFFFFFFF:FFFFFFF:FFFFFFFFFFFFF:FFFFFFFFFFFF:FF:FFFFFFFF:FF::FFFFF
FFFFFFFFFFF:FFFFFF,FFFFFFFFFFFFFFFF:FF,FFFFFFF:FFFFFF:FFFFFFFFFFFFFFF:
,FFFFFFFFFF:F:F
@A00721:190:HGJW7DSXY:4:2533:1154:28040 2:N:0:TCTGCAAG+GCTGCACT
CCACGACCTTCTAGAACGTATCGTTTTTCACAAACGACACTAAACCTATCAACATTATCCATGTTGAT
CATCCAAGTATCTGGATCCCGACTTTCCCCGATGAATATCTCATTCACTCTGGGATTAGAATTACCGG
GACGTCTATCATACA
+
FFFF,F:FFFFFFFFFFF,F:,:FFFFFFFFFF:FFFFF:FFFFFFFF:FFFFFFFF,FFFFFFFFF::F
FFFF:F,FFFFF:FFFFFFF:FFFFFFF:F::F:FFFF,:FFFFFFFF:FFFF,FFFFFFFFFF:FF,F,
FFF,,F::FF:FFFF
@A00721:190:HGJW7DSXY:4:2570:25093:21151 2:N:0:TCTGCAAG+GCTGCACT
CCACGACCTTCTAGAACGTATCGTTTTTCACAAACGACACTAAACCTATCAACATTATCCATGTTGAT
CATCCAAGTATCTGGATCCCGACTTTCCCCGATGAATATCTCATTCACTCTGGGATTAGAATTACCGG
GACGTCTATCATACA
+
FFFFFFFF:FFFFFFFFFFFFFFFFFF:FFFFFFFFFFFFFFFFFFFFFF:FFFFFFFFFFFFFFFFFFF
FFFFFFFFFFFFFFFFFFFFFFF:FFFFFFFFFFFFFFFFFFFFFFFFFFF:::F:FFFFFFFFFFF:F:
:FFFFFFFFFFFFFF
@A00721:190:HGJW7DSXY:4:2655:25482:12399 2:N:0:TCTGCAAG+GCTGCACT
CCACGACCTTCTAGAACGTATCGTTTTTCACAAACGACACTAAACCTATCAACATTATCCATGTTGAT
CATCCAAGTATCTGGATCCCGACTTTCCCCGATGAATATCTCATTCACTCTGGGATTAGAATTACCGG
GACGTCTATCATACA
+
FFFFFFFFFFFFFFFFFFFFFFFFFFFFFFFFFFFFFFFFFFFFFFFFFFFFF:FFFFFFFFFFFFFFFF
FFFFF,FFFFFFFFFFFFFFFFFFFFFFFFFFFFFFFFFFFFFFFFFFFFFFF:FFFFFFFFFFFF
FFFFFFFFFFFFFFF
@A00721:190:HGJW7DSXY:4:1167:17589:12571 2:N:0:TCTGCAAG+GCTGCACT
CAACGTAACCACGACCTTCTAGAACGTATCGTTTTTCACAAACGACACTAAACCTATCAACATTATCC
ATGTTGATCATCCAAGTATCTGGATCCCGACTTTCCCCGATGAATATCTCATTCACTCTGGGATTAGA
ATTACCGGGACGTCT
+
FFFFFFFFFFFFFFFFFFFFFFFFFFFFFFFFFFFFFFFFFFFFFFFFFFFFFFFFFFFFFFFFFFFFFF
FFFFFFFFFFFFFFFFFF,FFFFFFFFFFFFFFFFFFFFFFFFFFFFFFFFFFFFFFFFFFFFFFFFFF
FFFFFFFFFFFFFFF
@A00721:190:HGJW7DSXY:4:1273:13584:29590 2:N:0:TCTGCAAG+GCTGCACT
CAACGTAACCACGACCTTCTAGAACGTATCGTTTTTCACAAACGACACTAAACCTATCAACATTATCC
ATGTTGATCATCCAAGTATCTGGATCCCGACTTTCCCCGATGAATATCTCATTCACTCTGGGATTAGA
ATTACCGGGACGTCT
+
FFFFFFFFFFFFFFFFFFFFFFFFFFFFFFFFFFFFFFFFFFFFFFFFFFFFF:FFFFFFFFFFFFFFFF
FFFFFFFFFFFFFFFFFFFFFFFFFFFFFFFFFFFFFFFFFFFFFFFFFFFFFFFFFFFFFFFFFFFFFF
FFFFF:FFFFFFFFF

```
@A00721:190:HGJW7DSXY:4:1326:3640:18443 2:N:0:TCTGCAAG+GCTGCACT
CAACGTAACCACGACCTTCTAGAACGTATCGTTTTTCACAAACGACACTAAACCTATCAACATTATCC
ATGTTGATCATCCAAGTATCTGGATCCCGACTTTCCCCGATGAATATCTCATTCACTCTGGGATTAGA
ATTACCGGGACGTCT
+
FFFFFFFFFFFFFFFFFFFFFFFFFFFFFFFFFFFFFFFFFFFFFFFFFFFFFFFFFFFFFFFFFFFFFF
,FFFFFFFFFFFFFFFFFFFFFFFFFFFFFFFFFFFFFFFFFFFFFFFFFFFFFFFFFFFFFFF:FFFF
FFFFFFFFFFFFFFF
@A00721:190:HGJW7DSXY:4:2271:25364:26537 2:N:0:TCTGCAAG+GCTGCACT
CAACGTAACCACGACCTTCTAGAACGTATCGTTTTTCACAAACGACACTAAACCTATCAACATTATCC
ATGTTGATCATCCAAGTATCTGGATCCCGACTTTCCCCGATGAATATCTCATTCACTCTGGGATTAGA
ATTACCGGGACGTCT
+
FFFFFFFFFFFFFFFF:FFFFFFFFFFFFFFFFFFFFFFFFFFFFFFFFFFFFFFFFFFFFFFFFFFFFF
FFFFFFFFFFFFFFFFFFFFFFFFFFFFFFF:FFFFFFFFFF:FFFFFFFFFFFFFFFFFFFFFFFFFFF
FFFFFFFFFFFFFFF
@A00721:190:HGJW7DSXY:4:2315:13630:8249 2:N:0:TCTGCAAG+GCTGCACT
CAACGTAACCACGACCTTCTAGAACGTATCGTTTTTCACAAACGACACTAAACCTATCAACATTATCC
ATGTTGATCATCCAAGTATCTGGATCCCGACTTTCCCCGATGAATATCTCATTCACTCTGGGATTAGA
ATTACCGGGACGTCT
+
FFFFFFFFFFFFFFFFFFFF:FFFFFFFFFFFFFFFFFFFFFFFFFFFFFFFFFFFFFFFFFFFFFFFFF
FFFFFF,FFFF::FFFFFFFFFFF:FFFFFFFFFFFFFFFFFFFFF::FFFFFFFF:FFFFFFFFFFFFF
FFFFF,FFFFFF:FF
@A00721:190:HGJW7DSXY:4:2433:22019:6840 2:N:0:TCTGCAAG+GCTGCACT
CAACGTAACCACGACCTTCTAGAACGTATCGTTTTTCACAAACGACACTAAACCTATCAACATTATCC
ATGTTGATCATCCAAGTATCTGGATCCCGACTTTCCCCGATGAATATCTCATTCACTCTGGGATTAGA
ATTACCGGGACGTCT
+
FFFFFFFFFFFFFFFFFFFFFFFFFFFFFFFFFFFFFFFFFFFFFFFFF:FFFFFFFFFFFFFFFFFFFF
FFFFFFFFFFFFFFFFFFFFFFFFFFFFFFFFFFFFFFFFFFFFFFFFFFFFFFFFFFFFFFFFFFFFFF
FFFFF,FFFFFFFFF
@A00721:190:HGJW7DSXY:4:2449:10050:10128 2:N:0:TCTGCAAG+GCTGCACT
CAACGTAACCACGACCTTCTAGAACGTATCGTTTTTCACAAACGACACTAAACCTATCAACATTATCC
ATGTTGATCATCCAAGTATCTGGATCCCGACTTTCCCCGATGAATATCTCATTCACTCTGGGATTAGA
ATTACCGGGACGTCT
+
FFFFFFFFFFFFFFFFFFFFFFFFFFFFFFFFFFFFFFFFFFFFFFFFFFFFFFFFFFFFFFFFFFFFFF
FFFFFFFFFFFFFFFFFFFFFFFFFFFFFFFFFFFFFFF:FFFFF:F:FFFFFFFFFFFFFFFFFFFFFF
:FFFFFFFFFFFFFF
@A00721:190:HGJW7DSXY:4:2462:14235:6981 2:N:0:TCTGCAAG+GCTGCACT
CAACGTAACCACGACCTTCTAGAACGTATCGTTTTTCACAAACGACACTAAACCTATCAACATTATCC
ATGTTGATCATCCAAGTATCTGGATCCCGACTTTCCCCGATGAATATCTCATTCACTCTGGGATTAGA
ATTACCGGGACGTCT
+
FFFFFFFFFFFFFFFFFFFFFFFFFFFFFFFFFFFFFFFFFFFFFFFFFFFFFFFFFFFFFFFFFFFFFF
FFFFFFFFFFFFFFFFFFFFFFFFFFFFFFFFFFFFFFFFFFFFFFFFFFFFFFFFFFFFFFFFFFFFFF
FFFFFFFFFFFFFF:
@A00721:190:HGJW7DSXY:4:2513:30201:15186 2:N:0:TCTGCAAG+GCTGCACT
CAACGTAACCACGACCTTCTAGAACGTATCGTTTTTCACAAACGACACTAAACCTATCAACATTATCC
ATGTTGATCATCCAAGTATCTGGATCCCGACTTTCCCCGATGAATATCTCATTCACTCTGGGATTAGA
ATTACCGGGACGTCT
+
FFFFFFFFFFFFFFFFFFFFFFFFFFFFFFFFFFFFFFFFFFFFFFFFFFFFFF,FFFFFFFFFFFFFFF
```

FFFFFFFFFFFFF:FFFFFFFFFFFFFFFFFFFF:FFFFFFFFFFFFFFFFFFFFFFFFFFFFF:FFFF
FFFFF:FF:FFFFFF
@A00721:190:HGJW7DSXY:4:2549:11903:30029 2:N:0:TCTGCAAG+GCTGCACT
CAACGTAACCACGACCTTCTAGAACGTATCGTTTTTCACAAACGACACTAAACCTATCAACATTATCC
ATGTTGATCATCCAAGTATCTGGATCCCGACTTTCCCCGATGAATATCTCATTCACTCTGGGATTAGA
ATTACCGGGACGTCT
+
FFFFFFFFFFFFFFFFFFFFFFFFFFFFFFFFFFFFFFFFFFFFFFFFF:FFFFFFFFFFFFFFFFFFF
FFFFFFFFFFFFFFFFFFFFFFFFFFFFFFFFFFFF::FFFFFFFFFFFFFFFFFFFFFFFFFFFFFFF
FFFFFFFFFFFFFFF
@A00721:190:HGJW7DSXY:4:2636:4960:20979 2:N:0:TCTGCAAG+GCTGCACT
CAACGTAACCACGACCTTCTAGAACGTATCGTTTTTCACAAACGACACTAAACCTATCAACATTATCC
ATGTTGATCATCCAAGTATCTGGATCCCGACTTTCCCCGATGAATATCTCATTCACTCTGGGATTAGA
ATTACCGGGACGTCT
+
FFFFFFFFFFFFFFFFFFFFFFFFFFFFFFFFFFFFFFFFFFFFFFFFFFFFFFFFFFFFFFF:FFFFF
FFFFFFFFFFFFFFFFFFFFFFFFFFFFFFFFFFFFFFFFFFFFFFFFFFFFFFFFFFF:FFFF,FFFF
FFFFFFFFFF:,FFF
@A00721:190:HGJW7DSXY:4:1163:29903:11318 2:N:0:TCTGCAAG+GCTGCACT
GATGAATCAACGTAACCACGACCTTCTAGAACGTATCGTTTTTCACAAACGACACTAAACCTATCAAC
ATTATCCATGTTGATCATCCAAGTATCTGGATCCCGACTTTCCCCGATGAATATCTCATTCACTCTGG
GATTAGAATTACCGG
+
FFFFFFFFFFFFFFFFFFFFFFF:FFFFFFFFFFFFFFFFFFFFF:FFF:FFFFFFFFFFFFFFFFFFF
FFFFFFFFFFFFFFFFF:FFFFFF:FFFFFFFFF:FFFFFFFFFFFFFFFFFFFFFFFFFFFFFFFFFF
FFFFFFFFFFFFFFF
@A00721:190:HGJW7DSXY:4:1353:18005:7780 2:N:0:TCTGCAAG+GCTGCACT
GATGAATCAACGTAACCACGACCTTCTAGAACGTATCGTTTTTCACAAACGACACTAAACCTATCAAC
ATTATCCATGTTGATCATCCAAGTATCTGGATCCCGACTTTCCCCGATGAATATCTCATTCACTCTGG
GATTAGAATTACCGG
+
FFFFF:FFFFFFFFFFFFFFFFFFFFFFFFFFFFFFFFFFFFFFFFFFFFFFFFFFFFFFFFFFFFFFF
FFFFFFFFFFFFFFFFFFF:FFFFFFFFFFFFFFFFFFFFFFFFFFFFFFFFFFFFFFFFFFFFFFFFF
FFFFFFF:FFFFFFF
@A00721:190:HGJW7DSXY:4:2446:10872:32597 2:N:0:TCTGCAAG+GCTGCACT
GATGAATCAACGTAACCACGACCTTCTAGAACGTATCGTTTTTCACAAACGACACTAAACCTATCAAC
ATTATCCATGTTGATCATCCAAGTATCTGGATCCCGACTTTCCCCGATGAATATCTCATTCACTCTGG
GATTAGAATTACCGG
+
FFFFFFFFFFFFFFFFFFFFFFFFFFFFFFFFFFFFFFFFFFF:FFFFF:FFFFFFFFFFFFFFFFFFF
F:FFFFFFFFFFFFFFFFFFFFFFFFFFFFFFFFFFFFFFFFFFFFFFFFF:FFFFFFFFFFFFFFFFF
FFFFFFF:FFFFFFF
@A00721:190:HGJW7DSXY:4:2643:32334:27899 2:N:0:TCTGCAAG+GCTGCACT
GATGAATCAACGTAACCACGACCTTCTAGAACGTATCGTTTTTCACAAACGACACTAAACCTATCAAC
ATTATCCATGTTGATCATCCAAGTATCTGGATCCCGACTTTCCCCGATGAATATCTCATTCACTCTGG
GATTAGAATTACCGG
+
FFFFFFFFFFFF:FFFFFFFFFFFFFFFFFFFFFFFFFFFFFFFFFFFF,F:FFFFFFF,FFFFFFFFF
F:FFFFF,FFFFFFFFFFFFFFFFFFFF:FFFFFFFFFFFFFFFFFFFFF:FFFFFFFFFFFFFFF,FF
F:FFFFFFFFFFF,F
@A00721:190:HGJW7DSXY:4:1121:9100:22232 2:N:0:TCTGCAAG+GCTGCACT
GGATGAATCAACGTAACCACGACCTTCTAGAACGTATCGTTTTTCACAAACGACACTAAACCTATCAA
CATTATCCATGTTGATCATCCAAGTATCTGGATCCCGACTTTCCCCGATGAATATCTCATTCACTCTG
GGATTAGAATTACCG

+
FFF,FFFFFFFFF:F:FFFFFF:FFFFFFFFF,FFFFFFFFFFFFFFFF,FFFFFFFFFFFFF:FFFF
FFFFFFFFFFFFFFF:FFFF:FFFF:FFFFFFFFFFFFFF:FFFFFFFFFFF,FFFFFFFFFFFFFFF
:F:,FF:F:FFFFF:
@A00721:190:HGJW7DSXY:4:1361:31801:3865 2:N:0:TCTGCAAG+GCTGCACT
GGATGAATCAACGTAACCACGACCTTCTAGAACGTATCGTTTTTCACAAACGACACTAAACCTATCAA
CATTATCCATGTTGATCATCCAAGTATCTGGATCCCGACTTTCCCCGATGAATATCTCATTCACTCTG
GGATTAGAATTACCG
+
FFFFFFFFFFFFFFF:FF:FFFFFFFFFFFFFFFFFFFFFFFFFFFFFFFFFFFFFFFF:FFFFFFFF
FFFFFFFFFFFFFFFFFFFFFFFFFF:FFFFF:FFFFFF,FFFFFFFFFFFFFFFFFFFFFFF,F:FF
FFFFFFFFFFFFFFF
@A00721:190:HGJW7DSXY:4:1361:4381:5791 2:N:0:TCTGCAAG+GCTGCACT
GGATGAATCAACGTAACCACGACCTTCTAGAACGTATCGTTTTTCACAAACGACACTAAACCTATCAA
CATTATCCATGTTGATCATCCAAGTATCTGGATCCCGACTTTCCCCGATGAATATCTCATTCACTCTG
GGATTAGAATTACCG
+
FFFFFFFFFFFFFFFFFFFF:FFFFFFFFFFFF,FFFF:FFFFFFFFFFFFFFFFFFFFFFFFFFFFF
FFFFFFFFFFFFFFFFFFF:FFFFFFFFFFFFFFFFFFFFFFFFFFFFFFFFFFFFFFFFFFFFFFFF
FFFFFFFFFFFF,FF
@A00721:190:HGJW7DSXY:4:1466:28592:12587 2:N:0:TCTGCAAG+GCTGCACT
GGATGAATCAACGTAACCACGACCTTCTAGAACGTATCGTTTTTCACAAACGACACTAAACCTATCAA
CATTATCCATGTTGATCATCCAAGTATCTGGATCCCGACTTTCCCCGATGAATATCTCATTCACTCTG
GGATTAGAATTACCG
+
FF:FFFFFFFFFFFFFFFFFFFFFFFFFFFFFFFFFFFFFFFFFFFFFFFFFFFFFFFFFFFFFFFFF
FFFFFFFFFFFFFFFFFFFFFFFFFFFFFFFFFFFFFFFFFFFFFFFFFFFFFFFFFFFFFFFFFFFF
FFFFFFFFFFFFFFF
@A00721:190:HGJW7DSXY:4:2116:19199:21527 2:N:0:TCTGCAAG+GCTGCACT
GGATGAATCAACGTAACCACGACCTTCTAGAACGTATCGTTTTTCACAAACGACACTAAACCTATCAA
CATTATCCATGTTGATCATCCAAGTATCTGGATCCCGACTTTCCCCGATGAATATCTCATTCACTCTG
GGATTAGAATTACCG
+
FFFFFFFFFFFFFFFFFFFFFFFFFFFFFFFFFFFFFFFFFFFFFFFFFFFFFFFFFFFFFFFFFFFF
FFFFFFFFFFFFFFFFFFFF:FFFFFFFFFFFFFFFFFFFFFFFFFFFFFFFFFFFFFFFFFFFFFF
FFFFFFFFFFFFFFF
@A00721:190:HGJW7DSXY:4:2116:19298:22451 2:N:0:TCTGCAAG+GCTGCACT
GGATGAATCAACGTAACCACGACCTTCTAGAACGTATCGTTTTTCACAAACGACACTAAACCTATCAA
CATTATCCATGTTGATCATCCAAGTATCTGGATCCCGACTTTCCCCGATGAATATCTCATTCACTCTG
GGATTAGAATTACCG
+
FFFFFFFFFFFFFFFFFFFFFFFFFFFFFFFFFFFFFFFFFFFFFFFFF:FFFFFFFFFFFFFFFFFF
FF:FFFFFFFFFFFFFFFFFFFFFFFFFFFFFFFFFFFFFFFFFFFFFFFFFFFFFFFFFFFF:FFFF
FFFFFFFFFFF:FFF
@A00721:190:HGJW7DSXY:4:2233:25201:17863 2:N:0:TCTGCAAG+GCTGCACT
GGATGAATCAACGTAACCACGACCTTCTAGAACGTATCGTTTTTCACAAACGACACTAAACCTATCAA
CATTATCCATGTTGATCATCCAAGTATCTGGATCCCGACTTTCCCCGATGAATATCTCATTCACTCTG
GGATTAGAATTACCG
+
FFFFFFFFFFFFFFFFFFFFFFFFFFFFFF:FFFFFFFFFFFFFFFFFFFFFFFFFFFFFFFFFFFFF
FFFFFFFFFFFFFFFF:FFFFFFFFFFFFFFFFFFFFFFFF:FFF,FFFFFFFFFFFFFFF:F:FFFF
FFFFFFF:FFFF:,F
@A00721:190:HGJW7DSXY:4:2234:5376:19727 2:N:0:TCTGCAAG+GCTGCACT
GGATGAATCAACGTAACCACGACCTTCTAGAACGTATCGTTTTTCACAAACGACACTAAACCTATCAA

```
CATTATCCATGTTGATCATCCAAGTATCTGGATCCCGACTTTCCCCGATGAATATCTCATTCACTCTG
GGATTAGAATTACCG
+
FFFFFFFFFFFFFFFFFFFFFFFFFFFFFFFFFFFFFFFFFFFFFFFFFFFFFFFFFF:FFFFFFFFFFF
FFFF:FFFF:FFFFFFFFFFFF,:FFF:::F:FFFFFF:::FFFFFF::FF:F,FF:FFFFF,:::::F
FFF::,FF:F::F::
@A00721:190:HGJW7DSXY:4:2238:10303:29199 2:N:0:TCTGCAAG+GCTGCACT
GGATGAATCAACGTAACCACGACCTTCTAGAACGTATCGTTTTTCACAAACGACACTAAACCTATCAA
CATTATCCATGTTGATCATCCAAGTATCTGGATCCCGACTTTCCCCGATGAATATCTCATTCACTCTG
GGATTAGAATTACCG
+
FFFFFFFFFFFFFFFFFFFFFFFFFFFFFFFFFFFFFFFFFFFFFFFFFFFFFFFFFFFFFFFFFFFFFF
FFFFFFFFFFFFFFFFFFFFFFFFFFFFFFFFFFFF:FFFFFFFFFFFFFFFFFFFFFFFFFFFFFFFFF
FF:FFFFFFFFFF:F
@A00721:190:HGJW7DSXY:4:2271:14082:19836 2:N:0:TCTGCAAG+GCTGCACT
GGATGAATCAACGTAACCACGACCTTCTAGAACGTATCGTTTTTCACAAACGACACTAAACCTATCAA
CATTATCCATGTTGATCATCCAAGTATCTGGATCCCGACTTTCCCCGATGAATATCTCATTCACTCTG
GGATTAGAATTACCG
+
FFFFFFFFFFFFF:FFFFFFFF:FFFFF:FFFFFFFFFF:,:FFFFFFFFFFFFFF,FFFFFFFFFFF
FF,FFFFFFFF,FF:FFFFFFFFFFFFFFF:FFFFFFFFFF:FFFF:F:FFFFFFFFFF,:FFFFFFF
FFFFFFFF:FFFFFF
@A00721:190:HGJW7DSXY:4:2574:10764:24799 2:N:0:TCTGCAAG+GCTGCACT
GGATGAATCAACGTAACCACGACCTTCTAGAACGTATCGTTTTTCACAAACGACACTAAACCTATCAA
CATTATCCATGTTGATCATCCAAGTATCTGGATCCCGACTTTCCCCGATGAATATCTCATTCACTCTG
GGATTAGAATTACCG
+
FFFFFFFFFFFFFFFFFFFFFFFFFFFFFFFFFFFFFFFFFFFFFFFFFFFFFFFFFFFFFFFFFFFFFF
FFFFFFFFFFFFFFFF:FFFFFFF:FFFFFFFFFFFFFFFFFFFFFFFFFFFFFFFFFFFFFFFFFFFF
FFFFFFFFFFFFFFF
@A00721:190:HGJW7DSXY:4:1120:15899:32252 2:N:0:TCTGCAAG+GCTGCACT
CATGGATGAATCAACGTAACCACGACCTTCTAGAACGTATCGTTTTTCACAAACGACACTAAACCTAT
CAACATTATCCATGTTGATCATCCAAGTATCTGGATCCCGACTTTCCCCGATGAATATCTCATTCACT
CTGGGATTAGAATTA
+
FF,FFFFFFFFFFFFFFFFFFFFFFFFF:FFFFFFFFFFFFFF:FFFFFFFFFFFFFFFFFFFFFFFFF
FFFFFFFFFFFFFFFFFFFFFFFFFFF:FFFFFFFFFFFFFFFFFFF:FFFFFFFFFFFFFFFFFFFFF
FFFFFFFFFFFFF:F
@A00721:190:HGJW7DSXY:4:1145:23710:2347 2:N:0:TCTGCAAG+GCTGCACT
CATGGATGAATCAACGTAACCACGACCTTCTAGAACGTATCGTTTTTCACAAACGACACTAAACCTAT
CAACATTATCCATGTTGATCATCCAAGTATCTGGATCCCGACTTTCCCCGATGAATATCTCATTCACT
CTGGGATTAGAATTA
+
FFFFFFFFFFFFFFFFFFFFFFFFFFFFFFFFFFFFFFFFFFFFFFFFFFFFFFFFFFFFFFFFFFFF,F
FFFFFFFFFFFFFFFFFFFFFFFFFFFFFFFFFFFFFFFFFFFFFFFFFFFFFFFFFFFFFFFF:FFFF
FFFFFFFFFFFFFFF
@A00721:190:HGJW7DSXY:4:1250:30065:23688 2:N:0:TCTGCAAG+GCTGCACT
CATGGATGAATCAACGTAACCACGACCTTCTAGAACGTATCGTTTTTCACAAACGACACTAAACCTAT
CAACATTATCCATGTTGATCATCCAAGTATCTGGATCCCGACTTTCCCCGATGAATATCTCATTCACT
CTGGGATTAGAATTA
+
FFFFF::FFF:FFFFFFFFFF:FFFFFFFFF:FFFFFFFFF:FFFF:FFFFFFFFFFFF:FF:FFFFF
FFFFF,F:FF,FF::FFFFFFFFF:F:FFFF,FF:FFFFFFFFFFFFFFFF,FFFFFFFFFFFFFF,F
F:FF:FFFFFFFFFF
```

@A00721:190:HGJW7DSXY:4:1259:25907:1517 2:N:0:TCTGCAAG+GCTGCACT
CATGGATGAATCAACGTAACCACGACCTTCTAGAACGTATCGTTTTTCACAAACGACACTAAACCTAT
CAACATTATCCATGTTGATCATCCAAGTATCTGGATCCCGACTTTCCCCGATGAATATCTCATTCACT
CTGGGATTAGAATTA
+
FFFFFFFFFFFFFFFFFFFFFFFFFFFFFFFFFFFFFFFFFFFFFFFFFFFFFFFFFFFFFFFFFFFF
FFFFFFFFFFFFFFFFFFFFFFFFFFFFFFFFFFFFFFFFFFFFFFFF:FFFFF:FFF:FFFFFFFFF
FFFFFF:FFFFFFFF
@A00721:190:HGJW7DSXY:4:1446:16098:29152 2:N:0:TCTGCAAG+GCTGCACT
CATGGATGAATCAACGTAACCACGACCTTCTAGAACGTATCGTTTTTCACAAACGACACTAAACCTAT
CAACATTATCCATGTTGATCATCCAAGTATCTGGATCCCGACTTTCCCCGATGAATATCTCATTCACT
CTGGGATTAGAATTA
+
FFFFFFFFFFFFFFFFFFFFFFFFFFFFFFFFFFFFFFFFFFFFFFFFFFFFFFFFFFFFFFFFFF:F
FFFFFFFFFFFFFFFFFFFFFFFFFFFF:FFFFFFFFFFFFFFFFFFFFFFFFFFFFFFFFFFFFFFFF
FFFFFFFF,FFFFFF
@A00721:190:HGJW7DSXY:4:2414:31521:16877 2:N:0:TCTGCAAG+GCTGCACT
CATGGATGAATCAACGTAACCACGACCTTCTAGAACGTATCGTTTTTCACAAACGACACTAAACCTAT
CAACATTATCCATGTTGATCATCCAAGTATCTGGATCCCGACTTTCCCCGATGAATATCTCATTCACT
CTGGGATTAGAATTA
+
FFFFFFFFFFFFFFFFFFFFFFFFFFFFFFFFF:FFFFFFFFFFFFFF:FFFFFFFFFFFFF,FFFFF
FFFFFFFFFFFFFFFFFFFFFFFFFFFFFFFFFFFFFFFFFFFFFF:FFFFFFFFFFFFFFFFFFFFF
FF:FFFFFF::FFFF
@A00721:190:HGJW7DSXY:4:2522:27190:32988 2:N:0:TCTGCAAG+GCTGCACT
CATGGATGAATCAACGTAACCACGACCTTCTAGAACGTATCGTTTTTCACAAACGACACTAAACCTAT
CAACATTATCCATGTTGATCATCCAAGTATCTGGATCCCGACTTTCCCCGATGAATATCTCATTCACT
CTGGGATTAGAATTA
+
FFFFFFFFFFFFFFFFFFFFFFFFFFFFFFFFFFFFFFFFFFFFFFFFFFFFFFFFFFFFFFFFFFFF
FFFFFFFFFFFFFFFFFFFFFFFFF,FFFFFFFFFFFFFFFFFFFFFFFFFFF:F:FFFFFFFFF:FF
FFFFFF:,FFFFFF:
@A00721:190:HGJW7DSXY:4:2647:4101:28855 2:N:0:TCTGCAAG+GCTGCACT
CATGGATGAATCAACGTAACCACGACCTTCTAGAACGTATCGTTTTTCACAAACGACACTAAACCTAT
CAACATTATCCATGTTGATCATCCAAGTATCTGGATCCCGACTTTCCCCGATGAATATCTCATTCACT
CTGGGATTAGAATTA
+
FFFFFFFFFFFFFFFFFFFFFFFFFFFFFFFFFFFFFFFFFFFFFFFFFFFFFFFFFFFFFFFFFFFF
FFFFFFFFFFFFFFFFFFFFFFFFFFFFFFFFFFFFFFFFFFFFFFFFFFFFFFFFFFFFFFFFFFFF
FFFFFFF,FFFFFFF
@A00721:190:HGJW7DSXY:4:2662:5204:21997 2:N:0:TCTGCAAG+GCTGCACT
CATGGATGAATCAACGTAACCACGACCTTCTAGAACGTATCGTTTTTCACAAACGACACTAAACCTAT
CAACATTATCCATGTTGATCATCCAAGTATCTGGATCCCGACTTTCCCCGATGAATATCTCATTCACT
CTGGGATTAGAATTA
+
FFFFFFFFFFFFFFFFFFFFFFFFFFFFFFFFFFFFFFFFFFFFFFFFFFFFFFFFFFFFFFFFFFFF
FFFFFFFFFFFFFFFFFFFFFFFFFFFFFFFFFFFFFFFFFFFFFFFFFFFFFFFFFFFFFFFFFFFF
FFFFFFFFFFFFFFF
@A00721:190:HGJW7DSXY:4:1157:30535:35493 2:N:0:TCTGCAAG+GCTGCACT
TCTTAACGTCGTCGTCCGCATCCATGGATGAATCAACGTAACCACGACCTTCTAGAACGTATCGTTTT
TCACAAACGACACTAAACCTATCAACATTATCCATGTTGATCATCCAAGTATCTGGATCCCGACTTTC
CCCGATGAATATCTC
+
FFFFF:FFFFFFFFFFFFFFFFFFFFFFFFFFFFFFFFFFFFFFFFFFFFFFFFFFFFFFFFFFFFFF

FFFFFF:FFFFFFFFFFFFFFFFFFFFFFFFF:FFFFFFFFFFFF:FFFF:FFFFFFFFFFFFFFFF:
FFFFFFFFFFFFFFF
@A00721:190:HGJW7DSXY:4:1560:6967:29528 2:N:0:TCTGCAAG+GCTGCACT
TCTTAACGTCGTCGTCCGCATCCATGGATGAATCAACGTAACCACGACCTTCTAGAACGTATCGTTTT
TCACAAACGACACTAAACCTATCAACATTATCCATGTTGATCATCCAAGTATCTGGATCCCGACTTTC
CCCGATGAATATCTC
+
FFFFFFFFFFFFFFFFFFFFFFFFFFFFFFFFFFFFFFFFFFFFFFFFFFFFFFFFFFFFFFFFFFFF
FFFFFF:FFFFFFFFF:FFFFFFFFFFFFFFFFFFFFFFFFFFFFFFFFFFFFFFFFFFFFFF:FFF
FFF:FFFFFFFFFFF
@A00721:190:HGJW7DSXY:4:2341:28800:2988 2:N:0:TCTGCAAG+GCTGCACT
TCTTAACGTCGTCGTCCGCATCCATGGATGAATCAACGTAACCACGACCTTCTAGAACGTATCGTTTT
TCACAAACGACACTAAACCTATCAACATTATCCATGTTGATCATCCAAGTATCTGGATCCCGACTTTC
CCCGATGAATATCTC
+
FFFFFFFFFFFFFFFFFFFFFFFFFFFFFFFFFFFFFFFFFFFFFFFFFFFFFFFFFFFFFFFFFFFF
FF:FFFFFFFFFFFFF,FFFFFFF:FFFFFFFFFFFFFFFFFFFFFFFFFFFFFFFFFFFFFFFFFFF
FFFFFFFF:FFFFFF
@A00721:190:HGJW7DSXY:4:2351:30409:30671 2:N:0:TCTGCAAG+GCTGCACT
TCTTAACGTCGTCGTCCGCATCCATGGATGAATCAACGTAACCACGACCTTCTAGAACGTATCGTTTT
TCACAAACGACACTAAACCTATCAACATTATCCATGTTGATCATCCAAGTATCTGGATCCCGACTTTC
CCCGATGAATATCTC
+
FFFFFFFFFFFFFFFFFFFFFFFFFFFFFFFFFFFFFFFFFFFFFFFFFFFFFFF:FFFFFFFFFFFF
FFFFFFFFFFFFFFFF:FFFFF,FFFFFFFFF:FFFFFFFFFFFFFFFFFFFFFF:FFFFFFFFFFFF
F:FFFFFFFFFFFFF
@A00721:190:HGJW7DSXY:4:2366:29930:32409 2:N:0:TCTGCAAG+GCTGCACT
TCTTAACGTCGTCGTCCGCATCCATGGATGAATCAACGTAACCACGACCTTCTAGAACGTATCGTTTT
TCACAAACGACACTAAACCTATCAACATTATCCATGTTGATCATCCAAGTATCTGGATCCCGACTTTC
CCCGATGAATATCTC
+
FFFFFFFFFFFFFFFFFFFFFFFFFFFFFFFFFFFFFFFFFFFFFFFFFFFFFFFFFFFFFFFFFFFF
FFFFFFFFFFFFFFFF:FFFFFFFFFFFFFFFFFFFFFFFFFFFFFFFFFFFFFFFFFFFFFFFFFF
FFFFFFFFFFFFFFF
@A00721:190:HGJW7DSXY:4:2534:32868:2206 2:N:0:TCTGCAAG+GCTGCACT
TCTTAACGTCGTCGTCCGCATCCATGGATGAATCAACGTAACCACGACCTTCTAGAACGTATCGTTTT
TCACAAACGACACTAAACCTATCAACATTATCCATGTTGATCATCCAAGTATCTGGATCCCGACTTTC
CCCGATGAATATCTC
+
FFFFFFFFFFFFFFFFFFFFFFFFFFFFFFFFFFFFFFFFFFFFFFF:FFFFFFFFFF:FFFFFFFFF
FFFFFF:FFFFFFFFFFFFFFFFFFFFFFFFFFFFFFFFFFFFFF,FFFF:FFFFFFF:FFFFFFFFF
FFFFFFFFFFFFFFF
@A00721:190:HGJW7DSXY:4:1102:30544:36072 2:N:0:TCTGCAAG+GCTGCACT
AAGCGTTCTTAACGTCGTCGTCCGCATCCATGGATGAATCAACGTAACCACGACCTTCTAGAACGTAT
CGTTTTTCACAAACGACACTAAACCTATCAACATTATCCATGTTGATCATCCAAGTATCTGGATCCCG
ACTTTCCCCGATGAA
+
FFFFFFFFFFFFFFFFFFFFFFFFFFFFFFFFFFFFFFFFFFFFFFFFFFFFFFFFFFFFFFFFFFFF
FFFFFFFFFFFFFFFFFFFFFF:FFFFFFFFFFFFFFFFFFFFFFFFFFFFFFFFFFFFFFFFFFFFF
FFFFFFFFFFFFFFF
@A00721:190:HGJW7DSXY:4:1163:31060:2832 2:N:0:TCTGCAAG+GCTGCACT
AAGCGTTCTTAACGTCGTCGTCCGCATCCATGGATGAATCAACGTAACCACGACCTTCTAGAACGTAT
CGTTTTTCACAAACGACACTAAACCTATCAACATTATCCATGTTGATCATCCAAGTATCTGGATCCCG
ACTTTCCCCGATGAA

+
FFFFFFFFFFFFFFFFFFFFFFFFFFFFFFFFFFFFFFFFFFFFFFFFFFFFFFFFFFFFFFFFFFFF
FFFFFFFFFFFFFFFFFFFFFFFFFFFFFFFFFFFFFFFFFFFFFFFFFFFFF:FFFFFFFFFFFFFF
FFFFFFFFFFFFFFF
@A00721:190:HGJW7DSXY:4:2556:8070:2065 2:N:0:TCTGCAAG+GCTGCACT
AAGCGTTCTTAACGTCGTCGTCCGCATCCATGGATGAATCAACGTAACCACGACCTTCTAGAACGTAT
CGTTTTTCACAAACGACACTAAACCTATCAACATTATCCATGTTGATCATCCAAGTATCTGGATCCCG
ACTTTCCCCGATGAA
+
FFFFFFFFFFFFFFFFFFFFFFFFFFFFFFFFFFFFFFFFFFFFFFFFFFFFFFFFFFFFFFFFFFFF
FFFFFFFFFFFFFFFFFFFFFFFFFFFFFFFFFFFFFFFFFFFFFFFFFFFFFFFFFFFFFFFFFFFF
FFFFFFFFFFFFFF:
@A00721:190:HGJW7DSXY:4:2607:32877:10113 2:N:0:TCTGCAAG+GCTGCACT
AAGCGTTCTTAACGTCGTCGTCCGCATCCATGGATGAATCAACGTAACCACGACCTTCTAGAACGTAT
CGTTTTTCACAAACGACACTAAACCTATCAACATTATCCATGTTGATCATCCAAGTATCTGGATCCCG
ACTTTCCCCGATGA
+
FFFFFFF:FFFFFFFFFFFFFFFFFFFFFFFFFFFFFFFFFFFFFFFFFFFFFFFFF:FFFFFFFFFF
FFFFFFFFF:FFF,,FFFFFF:FFFFFFFFFFFFFFFF,FFFFF,FF,FF:FF:FFFF:FF:FFF,F:
FFFF::FFFFFFFF
@A00721:190:HGJW7DSXY:4:1224:16360:5901 2:N:0:TCTGCAAG+GCTGCACT
CTCATATAACAGAAGTAAGCGTTCTTAACGTCGTCGTCCGCATCCATGGATGAATCAACGTAACCACG
ACCTTCTAGAACGTATCGTTTTTCACAAACGACACTAAACCTATCAACATTATCCATGTTGATCATCC
AAGTATCTGGATCCC
+
FFFFFFFFFFFFFFFFFFFFFFFFFFFFFFFFFFFFFFFFFFFFFFFFFFFFFFFFFFFFFFFFFFFF
FFFFFFFFFFFFFFFFFFF:FFFFFFFFFFFFFFFFFFFFFFFFFFFFFFFFFFFFFFFFFFFFFFFF
FFFFFFFFFFFFFFF
@A00721:190:HGJW7DSXY:4:1245:5430:36104 2:N:0:TCTGCAAG+GCTGCACT
CTCATATAACAGAAGTAAGCGTTCTTAACGTCGTCGTCCGCATCCATGGATGAATCAACGTAACCACG
ACCTTCTAGAACGTATCGTTTTTCACAAACGACACTAAACCTATCAACATTATCCATGTTGATCATCC
AAGTATCTGGATCCC
+
FFFFFFFF:FFFFFFFFFFFFFFFFFFFFFFFFFFFFFFFFFFFF:FFFFFFFFFFFFFFFFFFFFFF
FFFFFFFFFFFFFFFFFFFFFFFFFFFFFFFFFFFFFFFFFFFFFFFFFFFFFFFF:FFFFFFFFFFF
FFFFF:FFFFFFFFF
@A00721:190:HGJW7DSXY:4:1301:6876:6104 2:N:0:TCTGCAAG+GCTGCACT
CTCATATAACAGAAGTAAGCGTTCTTAACGTCGTCGTCCGCATCCATGGATGAATCAACGTAACCACG
ACCTTCTAGAACGTATCGTTTTTCACAAACGACACTAAACCTATCAACATTATCCATGTTGATCATCC
AAGTATCTGGATCCC
+
FFFFFFFFFFFFFFFFFFF:FFFFFFFFFF:FFFFF:FFFFFFFFFFFFFFFFFFFFFFFFFFFFFFF
FFFF:FFFFFFFFFFFFFFFFFFFFFFF,FFFFFFFFFFFFFFFFF:FFFFFFFFF,FFFFFFFFFFF
FFFFF:FFFFFFFFF
@A00721:190:HGJW7DSXY:4:1301:6180:6277 2:N:0:TCTGCAAG+GCTGCACT
CTCATATAACAGAAGTAAGCGTTCTTAACGTCGTCGTCCGCATCCATGGATGAATCAACGTAACCACG
ACCTTCTAGAACGTATCGTTTTTCACAAACGACACTAAACCTATCAACATTATCCATGTTGATCATCC
AAGTATCTGGATCCC
+
:FFFFFFFFFFFFFFFFFFFFFFFFFFFFFFFFFFFFFFFFFFFFFFFFF:FFFFFFFFFFFFFFFFF
FFFFFFFFFFFFFFFFFFFFFFFFFFFF:FFFFFFFFFFFFFFFFFFFFFFFFFFFFFFFFFFFFFFF
FFFFFFFFFFFFFFF
@A00721:190:HGJW7DSXY:4:1334:9679:21668 2:N:0:TCTGCAAG+GCTGCACT
CTCATATAACAGAAGTAAGCGTTCTTAACGTCGTCGTCCGCATCCATGGATGAATCAACGTAACCACG

```
ACCTTCTAGAACGTATCGTTTTTCACAAACGACACTAAACCTATCAACATTATCCATGTTGATCATCC
AAGTATCTGGATCCC
+
FFFFFFFFFFFFFFFFFFFFFFFFFFF:FFFFFFFFFFFFFFFFFFFFFFFFFFFFFFFFFFFFFFFFFF
FFF,FFFFFFFFFFFFFFFFFFFF:FFFFFFFFFFFFF:FFF:FFF:FFFFF,FFFFFFFFFFFFFFFFF
FFFFF:FFF:FFFFF
@A00721:190:HGJW7DSXY:4:1448:29197:17331 2:N:0:TCTGCAAG+GCTGCACT
CTCATATAACAGAAGTAAGCGTTCTTAACGTCGTCGTCCGCATCCATGGATGAATCAACGTAACCACG
ACCTTCTAGAACGTATCGTTTTTCACAAACGACACTAAACCTATCAACATTATCCATGTTGATCATCC
AAGTATCTGGATCCC
+
FFFFFFFFFFFFFFFFFFFFFFFFFFFFFFFFFFFFFFFFFFFFFFFFFFFFFFFFFFFFFFFFFFFFFF
FFFFFFFFFFFFFFFFFFFFFFFFFFFFFFFFFFFFFFFFFFFFFFFFFFFFFFFFFFFFFFFFFFFFFF
FFFFFFFFFFFFFFF
@A00721:190:HGJW7DSXY:4:1467:23990:21026 2:N:0:TCTGCAAG+GCTGCACT
CTCATATAACAGAAGTAAGCGTTCTTAACGTCGTCGTCCGCATCCATGGATGAATCAACGTAACCACG
ACCTTCTAGAACGTATCGTTTTTCACAAACGACACTAAACCTATCAACATTATCCATGTTGATCATCC
AAGTATCTGGATCCC
+
FFFFFFFFFFFFFFFFFFFFFFFFFFFFFFFFFFFFFFFFFFFFFFFFFFFFFFFFFFFFFFFFFFFFFF
FFFFFFFF:FFFFFFFFFFFFFFFFFFFFFFFFFFFFFFFFFFFFFFFFFFFFFFFFFFFFFFFFFFFF
FFFFFFFFFFFFFFF
@A00721:190:HGJW7DSXY:4:1524:9706:16047 2:N:0:TCTGCAAG+GCTGCACT
CTCATATAACAGAAGTAAGCGTTCTTAACGTCGTCGTCCGCATCCATGGATGAATCAACGTAACCACG
ACCTTCTAGAACGTATCGTTTTTCACAAACGACACTAAACCTATCAACATTATCCATGTTGATCATCC
AAGTATCTGGATCCC
+
FFFFFFFFFFFFFFFFFFFFFFFFFFFFFFFFFFFFFFFF:FFFFFFFFFFFFFFFFFFFFFF:FFFFFF
FFFFFFFFFFFFFFFFFFFFFFFF:FFFFFFFFFFFFFFFFFFFFFFFFFFFFFFFFFFFFFFFFFFFF
FFFFFFFFF:FFFFF
@A00721:190:HGJW7DSXY:4:1537:6081:21668 2:N:0:TCTGCAAG+GCTGCACT
CTCATATAACAGAAGTAAGCGTTCTTAACGTCGTCGTCCGCATCCATGGATGAATCAACGTAACCACG
ACCTTCTAGAACGTATCGTTTTTCACAAACGACACTAAACCTATCAACATTATCCATGTTGATCATCC
AAGTATCTGGATCCC
+
FFFFFFFFFFFFFFFFFFF:FF:FFFFFFFFFFFFFFFFFFFFFFFFFFFFFFFFFFFFFFFFFFFFFFF
FFF:FFFFFFFFFFFFFFFFF:FFFFF:FFFFFFFFF:FFFFFFFFFFFFFFFFFFFFFFFFFFF:FFFF
FFFFFFFFFFFFFFF
@A00721:190:HGJW7DSXY:4:1612:21151:16078 2:N:0:TCTGCAAG+GCTGCACT
CTCATATAACAGAAGTAAGCGTTCTTAACGTCGTCGTCCGCATCCATGGATGAATCAACGTAACCACG
ACCTTCTAGAACGTATCGTTTTTCACAAACGACACTAAACCTATCAACATTATCCATGTTGATCATCC
AAGTATCTGGATCCC
+
FFFFFFFFFFFFFFFFFFFFFFFFFFFFFFFFFFFFFFFFFFFFFFFFFFFFFFFFFFFFFFFFFFFFFF
FFFFFFFFFFFFFFFFFFFFFFFFFFFFFFFFFFFFFFFFFFFFFFFFFFFFFFFFFFFFFFFFFFFFFF
FFFFFFFFF:FFFFF
@A00721:190:HGJW7DSXY:4:1630:24668:33692 2:N:0:TCTGCAAG+GCTGCACT
CTCATATAACAGAAGTAAGCGTTCTTAACGTCGTCGTCCGCATCCATGGATGAATCAACGTAACCACG
ACCTTCTAGAACGTATCGTTTTTCACAAACGACACTAAACCTATCAACATTATCCATGTTGATCATCC
AAGTATCTGGATCCC
+
FFFFFFFFFFFFFFFFFFFFFFFFFFFFFFFFFFFFFFFFFFFFFFFFFFFFFFFFFFFFFFFFFFFFFF
FFFFFFFF:FFF:FFFFFFFFFFFFFFFFFFFFFFFFFFFFFFFFFFFFFFFFFFFFFFFFFFFFFFFF
FFFFFFFFFFFFFFF
```

```
@A00721:190:HGJW7DSXY:4:2119:16251:8531 2:N:0:TCTGCAAG+GCTGCACT
CTCATATAACAGAAGTAAGCGTTCTTAACGTCGTCGTCCGCATCCATGGATGAATCAACGTAACCACG
ACCTTCTAGAACGTATCGTTTTTCACAAACGACACTAAACCTATCAACATTATCCATGTTGATCATCC
AAGTATCTGGATCCC
+
FFFFFFFFFFFFFFFFFFFF:FFFFFFFFFFFFFFFFFFFFFFFFFFFFFFFFFFFFFFFFFFFFFFFFF
FFFFFFFFFF:FFFFFFFFF:FFFFFFFFFFFFFFFFFFFFFF:FFFFF:::FFFFFFFF:FFFF:FFFF
FFFF:FFFFF::FFF
@A00721:190:HGJW7DSXY:4:2136:31882:11490 2:N:0:TCTGCAAG+GCTGCACT
CTCATATAACAGAAGTAAGCGTTCTTAACGTCGTCGTCCGCATCCATGGATGAATCAACGTAACCACG
ACCTTCTAGAACGTATCGTTTTTCACAAACGACACTAAACCTATCAACATTATCAATGTTGATCATCC
AAGTATCTGGATCCC
+
FFFFFFFFFFFF:FFFFF:FFFFFFFFFFFFFFFFFFFFFFFFFFFFFFFFFFFFFFFFFF,FFFFFFFF
FFFFFFFF:FF:FFFFFFFFFFFF:FFFFFFFFFFFFFF:FFFFFFFFF,FFFFF,,F:F::,FFFFFF,
FF:FF::FF,FFF,F
@A00721:190:HGJW7DSXY:4:2222:16758:7592 2:N:0:TCTGCAAG+GCTGCACT
CTCATATAACAGAAGTAAGCGTTCTTAACGTCGTCGTCCGCATCCATGGATGAATCAACGTAACCACG
ACCTTCTAGAACGTATCGTTTTTCACAAACGACACTAAACCTATCAACATTATCCATGTTGATCATCC
AAGTATCTGGATCCC
+
FFFFFFFFFFFFFFFFFFFFFFFFFFFFFFFFFFFFFFFFFFFFFFFFFFFFFFFFFFFFFFFFFFFFFF
FFFFFFFFFFFFFFFFFFFFFFFFFFFFFFFFFFFFFFFFFFFFFFFFFFFFFFFFFFFFFFFFF:FFFF
FFFFFFFFFFFFFFF
@A00721:190:HGJW7DSXY:4:2608:29053:25848 2:N:0:TCTGCAAG+GCTGCACT
CTCATATAACAGAAGTAAGCGTTCTTAACGTCGTCGTCCGCATCCATGGATGAATCAACGTAACCACG
ACCTTCTAGAACGTATCGTTTTTCACAAACGACACTAAACCTATCAACATTATCCATGTTGATCATCC
AAGTATCTGGATCCC
+
FFFFFFFFFFFFFFFFFFFFFFFFFFFFFFFFFFFFFFFFFFFFFFFFFFFFFFFFFFFFFFFFFF:FFF
FFFFFFFFFFFFFFFFFFFFFFFFFFFFFFFFFFF::FFFFFFFFFFFFFFFFFFFFFFFFFFFFFFFFF
F:FFFFFFFFFFFFF
@A00721:190:HGJW7DSXY:4:2654:30228:30326 2:N:0:TCTGCAAG+GCTGCACT
CTCATATAACAGAAGTAAGCGTTCTTAACGTCGTCGTCCGCAACCATGGATGAATCAACGTAACCACG
ACCTTCTAGAACGTATCGTTTTTCACAAACGACACTAAACCTATCAACATTATCAAAGATTATCATCC
AAGTATCTGGATCC
+
F::FFFFFF:FFF:FFFF,,F,,,FFFFFF,,FF,FFF,,,:F,FFF,F,FFF:FFFFFFFFF:F,FF:F
FFFFFF:FFF:FFF,FF,F:F,FF:F,FFF:FFFFFFFF,FF,FFFFFF:F:FF:,:,F,:,FFFFFFFF
FFFFFFFF,,:F,F
@A00721:190:HGJW7DSXY:4:1104:4200:29058 2:N:0:TCTGCAAG+GCTGCACT
TCTAGACCTCATATAACAGAAGTAAGCGTTCTTAACGTCGTCGTCCGCATCCATGGATGAATCAACGT
AACCACGACCTTCTAGAACGTATCGTTTTTCACAAACGACACTAAACCTATCAACATTATCCATGTTG
ATCATCCAAGTATCT
+
FFFFFFFFFFFFFFFFFFFFFFFFFFFFFFFFFFFFFFFFFFFFFFFFFFFFFFFFFFFFFFFFFFFFFF
FFFFFFFFFFFFFFFFFFFFFFFFFFFFFFFFFFFFFFFFFFFFFFFFFFFFFFFFFFFFFFFFFFFFFF
FFFFFFFFFFFFFFF
@A00721:190:HGJW7DSXY:4:1104:4255:29371 2:N:0:TCTGCAAG+GCTGCACT
TCTAGACCTCATATAACAGAAGTAAGCGTTCTTAACGTCGTCGTCCGCATCCATGGATGAATCAACGT
AACCACGACCTTCTAGAACGTATCGTTTTTCACAAACGACACTAAACCTATCAACATTATCCATGTTG
ATCATCCAAGTATCT
+
FFFFFFFFFFFFFFFFFFFFFFFFFFFFFFFFFFFFFFFFFFFFFFFFFFFFFFFFFFFFFFFFFF:FFF
```

FFFFFFFFFFFFFFFFFFFFFFFFFFFFFFFFFFFFFFFFFFFFFFFFFFFFFFFFFFFFFFFFFFFFFF
FFFFFFFFFFFFFFF
@A00721:190:HGJW7DSXY:4:1134:23854:20384 2:N:0:TCTGCAAG+GCTGCACT
TCTAGACCTCATATAACAGAAGTAAGCGTTCTTAACGTCGTCGTCCGCATCCATGGATGAATCAACGT
AACCACGACCTTCTAGAACGTATCGTTTTTCACAAACGACACTAAACCTATCAACATTATCCATGTTG
ATCATCCAAGTATCT
+
FFFFFFFFFFFFFFFFFFFFFFFFFFFFFFFFFFFFFFFFFFFFFFFFFFFFFFFFFFFFFFF:FFFFFF
FFFFFFFFFFFFFFFFFFFFFFFFFFFFFFFFFFFFFFFFFFFFFFFF:FFFFFFFFFFFFFFFFFFFFF
FFFFFFFFFFFFFFF
@A00721:190:HGJW7DSXY:4:1157:24948:14199 2:N:0:TCTGCAAG+GCTGCACT
AGATACTTGGATGATCAACATGGATAATGTTGATAGGTTTAGTGTCGTTTGTGAAAAACGATACGTTC
TAGAAGGTCGTGGTTACGTTGATTCATCCATGGATGCGGACGACGACGTTAAGAACGCTTACTTCTGT
TATATGAGGTCTAGA
+
F:FFF:FF:FFF:FFFFFFFFFFFFFFFFFFFFFFFFFFFF:FFF,FFFFFFFFFFF::::FF:,FF:
FF,FF,::,:F:FFFF:F:FFFFF:FF:FFFFFFF,FFFF:,F,:FF:FFFF:FF,:FFFFFFFFF:F
FFFF::FFFFFF::F
@A00721:190:HGJW7DSXY:4:1421:6253:5619 2:N:0:TCTGCAAG+GCTGCACT
AGATACTTGGATGATCAACATGGATAATGTTGATAGGTTTAGTGTCGTTTGTGAAAAACGATACGTTC
TAGAAGGTCGTGGTTACGTTGATTCATCCATTGATGCGGACGACGACCTTAAGAACGCATACAGCTGT
TATATGAGGTCTAGA
+
F:FFFFFFF,F,FF,:FFFF,F:FFFFF:F:FFFFFFFF:FF,FF,FFF,FF,FFFFF::::F:,,F,
:F,FF,:F:,,FFF:F,:F::F:F,F,FFFF:,F,,FF::F:FF,,F,:,F:,FF,,F,,FF,,,:FF
FF,FFFF,FF::F::
@A00721:190:HGJW7DSXY:4:1421:29188:6981 2:N:0:TCTGCAAG+GCTGCACT
TCTAGACCTCATATAACAGAAGTAAGCGTTCTTAACGTCGTCGTCCGCATCCATGGATGAATCAACGT
AACCACGACCTTCTAGAACGTATCGTTTTTCACAAACGACACTAAACCTATCAACATTATCCATGTTG
ATCATCCAAGTATCT
+
FFFFFFFFFFFFFFFFFFFFFFFFFFFFFFFFFFFFFFFFFFFFFFFFFFFFFFFFFFFFFFFFFFFFFF
FFFFFFFFFFFFFFFFFFFFFFFFFFFFFFFFFFFFFFFFFFFFFFFFFFFFFFFFFFFFFFFFFFFFFF
FFFFFFFFFFFFFFF
@A00721:190:HGJW7DSXY:4:1629:26847:4460 2:N:0:TCTGCAAG+GCTGCACT
TCTAGACCTCATATAACAGAAGTAAGCGTTCTTAACGTCGTCGTCCGCATCCATGGATGAATCAACGT
AACCACGACCTTCTAGAACGTATCGTTTTTCACAAACGACACTAAACCTATCAACATTATCCATGTTG
ATCATCCAAGTATCT
+
FFFFFFFFFFFFFFFFFFFFFFFFFFFFFFFFFFFFFFFFFFFFFFFFFFFFFFFFFFFFFFFFFFFFFF
FFFFFFFFFFFFFFFFFFFFFFFFFFFFFFFFFFFFFFFFFFFFFFFFFFFFFFFFFFFFFFFFFFFFFF
FFFFFFFFFFFFFFF
@A00721:190:HGJW7DSXY:4:1642:30852:17503 2:N:0:TCTGCAAG+GCTGCACT
TCTAGACCTCATATAACAGAAGTAAGCGTTCTTAACGTCGTCGTCCGCATCCATGGATGAATCAACGT
AACCACGACCTTCTAGAACGTATCGTTTTTCACAAACGACACTAAACCTATCAACATTATCCATGTTG
ATCATCCAAGTATCT
+
FFFFFFFFFFFFFFFFF:FFFF:FFFFFFFFFFFFFFFFFFFFFFFFFFFFFFFFFFFFFFFFFFFFFFF
FFFFFFFFFFFFFFFFFFFFFFFFFFFFFF:FFFFFFFFFFFFFFFFFFFF:FFFFFFFFFFFFFFFFFF
FFFFFFFFFFFFFFF
@A00721:190:HGJW7DSXY:4:2473:4643:29168 2:N:0:TCTGCAAG+GCTGCACT
TCTAGACCTCATATAACAGAAGTAAGCGTTCTTAACGTCGTCGTCCGCATCCATGGATGAATCAACGT
AACCACGACCTTCTAGAACGTATCGTTTTTCACAAACGACACTAAACCTATCAACATTATCCATGTTG
ATCATCCAAGTATCT

```
+
FFFFFFFFFFFFFFFFFFFFFFFFFFFFFFFFFFFFFFFFFFFFFFFFFFFFFFFFFFFFFFFFFFFFFF
FFFFFFFFFFFFFFFFFFFFFFFFFFFFFFFFFFFFFFFFFFFFFFFFFFFFFFFFFFFFFFFFFFFFFF
FFFFFFFFFFFFFF:
@A00721:190:HGJW7DSXY:4:2554:24551:16861 2:N:0:TCTGCAAG+GCTGCACT
TCTAGACCTCATATAACAGAAGTAAGCGTTCTTAACGTCGTCGTCCGCATCCATGGATGAATCAACGT
AACCACGACCTTCTAGAACGTATCGTTTTTCACAAACGACACTAAACCTATCAACATTATCCATGTTG
ATCATCCAAGTATCT
+
FFFFFFFFFFFFFFFFFFFFFFFFFFFFFFFFFFFFFFFFFFFFFFFFFFFFFFFFFFFFFFFFFFFFFF
FFFFFFFFFFFFFFFFFFFFFFFFFFFFFFFFFFFFFFFFFFFFFFFFFFFFFFFFFFFFFFFFFFFFFF
FFFFFFFFFFFFFFF
@A00721:190:HGJW7DSXY:4:2658:27995:36323 2:N:0:TCTGCAAG+GCTGCACT
TCTAGACCTCATATAACAGAAGTAAGCGTTCTTAACGTCGTCGTCCGCATCCATGGATGAATCAACGT
AACCACGACCTTCTAGAACGTATCGTTTTTCACAAACGACACTAAACCTATCAACATTATCCATGTTG
ATCATCCAAGTATCT
+
FFFFFFFFFFFFFFFFFFFFFFFFFFFFFFFFFFFFFFFFFFFFFFFFFFFFFFFFFFFFFFFFFFFFFF
FFFFFFFFFFFFFFFFFFFFFFFFFFFFFFFFFFFFFFFFFFFFFFFFFFFFFFFFFFFFFFFFFFFFFF
FFFFFFFFFFFFFFF
@A00721:190:HGJW7DSXY:4:1262:4065:5462 2:N:0:TCTGCAAG+GCTGCACT
GATGATCAACATGGATAATGTTGATAGGTTTAGTGTCGTTTGTGAAAAACGATACGTTCTAGAAGGTC
GTGGTTACGTTGATTCATCCATGGATGCGGACGACGACGTTAAGAACGCTTACTTCTGTTATATGAGG
TCTAGACCGAT
+
FFFFFFFFFFFFFFFFFFFFFFFFFFFFFFFFFFFFFFFFFFFFFFFFFFFFFFFFFFF::F,FF,:FF
FF:F,FFFF:F:FFFFFFFFFFFFFFFFFFFFFFF:FFFF:FFFFFFFFFFFFF:F:F:FFFFFFFFFF
F:FF:F:F:FF
@A00721:190:HGJW7DSXY:4:1605:5584:12477 2:N:0:TCTGCAAG+GCTGCACT
GATGATCAACATGGATAATGTTGATAGGTTTAGTGTCGTTTGTGAAAAACGATACGTTCTATAAGGTC
GTGGTTACGTTGATTCATCCATGGATGCGGACGACGACGTTAAGAACGCTTACTTCTGTTATATGAGG
TCTAGACCGAT
+
FFFFFFFFFFFFFFFFFFFFFFFFFFFFFFFFFFFFFFFF::FFFFFF:FFFF:FFFFFF,:F,FF,
F:FF:FFF::F,F:F,FFFFFF:F:FF:FFFFFFFF:FFFFFF:FF,:::FFF:F:,,FFFFFF,FFF
F::F:F,FFFF
@A00721:190:HGJW7DSXY:4:2228:23213:12226 2:N:0:TCTGCAAG+GCTGCACT
GATGATCAACATGGATAATGTTGATAGGTTTAGTGTCGTTTGTGAAAAAAGATACGTTCTAGAAGGTC
GTGGTTACGTTGATTCATCCATGGATGCGGACGACGACGTTAAGAACGCTTACTTCTGTTATATGAGG
TCTAGACCGAT
+
FFFFFFFFFFFFFF:FFFFFFFFFFFFFFFFFFFFFFFFF:FFFF:FFFF,FFF:FF,FF,F:FF,FF:
FFFF:FFFF,FF,FFFFF:F,:F:F:FF:FFFFFF::FF,FFF,,F:FF,FFF,F,FF,FFF,F,:,F
,F:F:F:FFFF
@A00721:190:HGJW7DSXY:4:2250:5466:6386 2:N:0:TCTGCAAG+GCTGCACT
GATGATCAACATGGATAATGTTGATAGGTTTAGTGTCGTTTGTGAAAAACGATACGTTCTAGAAGGTC
GTGGTTACGTTGATTCATCCATGGATGCGGACGACGACGTTAAGAACGCTTACTTCTGTTATATGAGG
TCTAGACCGAT
+
FFFFFFFFFFFFFFFFFFFFFFFFF:FFFFFFFFFFFFFFFF:FFFFFFF:FFFFFFFFFFFFFFFFFF
FF:FFFFFFFFFFFFFFFF,F:FFFFFFFFFFFF:FFFFFFFFF,FF,FFFF,,FF,:FFFFFF:F,:
F:FFFF:FFFF
@A00721:190:HGJW7DSXY:4:2425:1904:2347 2:N:0:TCTGCAAG+GCTGCACT
GATGATCAACATGGATAATGTTGATAGGTTTAGTGTCGTTTGTGAAAAACGATACGTTCTAGAAGGTC
```

GTGGTTACGTTGATTCATCCATGGATGCGGACGACGACGTTAAGAACGCTTACTTCTGTTATATGAGG
TCTAGACCGAT
+
FFFFFFFFFFFFFFFFFFFFFFFFFFFFFFFFFFFFFFFFFFFFFFFFF,FFFFF:FFFFF,FF,FFF
F:FFFFF,:FFFFFFFF::FF,:FFF:FFFF:FFF:F::FFFF,FF::FFFFF,FF,FFFFFF:,FF:
FFFF,FF:FFF
@A00721:190:HGJW7DSXY:4:2569:22055:29199 2:N:0:TCTGCAAG+GCTGCACT
GATGATCAACATGGATAATGTTGATAGGTTTAGTGTCGTTTGTGAAAAACGATACGTTCTAGAAGGTC
GTGGTTACGTTGATTCATCCATGGATGCGGACGACGACGTTAAGAACGCTTACTTCTGTTATATGAGG
TCTAGACCGAT
+
FFFFFFFFFFFFFFFFFFFFFFFFFFFFFFFFFFFFFFF,FFFFFFFFFFFFFFF:FFFFFF:FFFFF
FFFFFFF:FFF:FFFFFFFFFFFFFFFFFFFFFFFFFFFFFFFFFF,FF:FF:FFFFFFFFFFFFFFF
FFFF,FFFFFF
@A00721:190:HGJW7DSXY:4:1369:8395:4977 2:N:0:TCTGCAAG+GCTGCACT
CAGAAATCGGTCTAGACCTCATATAACAGAAGTAAGCGTTCTTAACGTCGTCGTCCGCATCCATGGAT
GAATCAACGTAACCACGACCTTCTAGAACGTATCGTTTTTCACAAACGACACTAAACCTATCAACATT
ATCCATGTTGATCAT
+
FFFFFFFFFFFFFFFFFFFFFFFFFFFFFFFFFFFFFFFFFFFFFFFFFFFF:FFFFFFFFFF:FFFF
FFFFFFFFFFFFFFFFFFFFFFFFFFFFFFFFFFFFFF:FFFFFF:FFFFFFFFF:FFFFFFF:FF:F
FFFFFFFFFFFFFFF
@A00721:190:HGJW7DSXY:4:1375:19560:9784 2:N:0:TCTGCAAG+GCTGCACT
CAGAAATCGGTCTAGACCTCATATAACAGAAGTAAGCGTTCTTAACGTCGTCGTCCGCATCCATGGAT
GAATCAACGTAACCACGACCTTCTAGAACGTATCGTTTTTCACAAACGACACTAAACCTATCAACATT
ATCCATGTTGATCAT
+
FFFFFFFFFFFFFFFFFFFFFFFFFFFFFFFFFFFFFFFFFFFF:FFFFFFFFFFFFFFFFFFFFFFF
FFFFFFFFFFFFFFFFFFF:FFFFFFFFFFFFFFFFFFFFF:FFF:FFFFFFFFFFFFFFFFFFFFFF
FFFFFFFFFFFFFFF
@A00721:190:HGJW7DSXY:4:2326:19325:32142 2:N:0:TCTGCAAG+GCTGCACT
CAGAAATCGGTCTAGACCTCATATAACAGAAGTAAGCGTTCTTAACGTCGTCGTCCGCATCCATGGAT
GAATCAACGTAACCACGACCTTCTAGAACGTATCGTTTTTCACAAACGACACTAAACCTATCAACATT
ATCCATGTTGATCAT
+
FFFFFFFFFFFFFFFFFFFFFFFFFFFFFFFFFFFFFFFFFFFFFFFFFFFFFFFFFFFFFFFFFFFF
FFFFFFFFFFFFFFFFFFFFFFFFFFFFFFFFFFFFFFFFF:FF:FFFFFFFFFFFFFFFFFFFFFFF
FFFFFFFFFFFFFFF
@A00721:190:HGJW7DSXY:4:2339:2871:6590 2:N:0:TCTGCAAG+GCTGCACT
CAGAAATCGGTCTAGACCTCATATAACAGAAGTAAGCGTTCTTAGCGTCGTCGTCCGCATCCATGGAT
GAATCAACGTAACCACGACCTTCTAGAACGTATCGTTTTTCACAAACGACACTAAACCTATCAACATT
ATCCATGTTGATCAT
+
FFFFFFFFFFFFFFFFFFFFFFFFF:FFFFFFFFFFFFFFFFFF,FFFFFFFFFFFFFFFFFFFFFFF
FFFFFFFFFFF:FFFFFFFF:FFFFFFFFFFFFFFFFFFFFFFFFFFFFFFFFFF:FFFFFFFFFFFF
FF:FFFFFFFFF:FF
@A00721:190:HGJW7DSXY:4:2416:14886:8609 2:N:0:TCTGCAAG+GCTGCACT
CAGAAATCGGTCTAGACCTCATATAACAGAAGTAAGCGTTCTTAACGTCGTCGTCCGCATCCATGGAT
GAATCAACGTAACCACGACCTTCTAGAACGTATCGTTTTTCACAAACGACACTAAACCTATCAACATT
ATCCATGTTGATCAT
+
FFFFFFFF:FFFFFFFFFFFFFFFFFFFFFFFFFFFFFFFFFFFFFFFFFFFFFFFFFFFFFFFFFFF
FFFFFFFFFFFFFFFFFFFFFFFFFFFFFFFFFFFFFFFFFFFFFFFFFFFFFFFFFFFFFFFFFFF:
FFFFFFFFFFFFFFF

@A00721:190:HGJW7DSXY:4:2436:6271:25974 2:N:0:TCTGCAAG+GCTGCACT
CAGAAATCGGTCTAGACCTCATATAACAGAAGTAAGCGTTCTTAACGTCGTCGTCCGCATCCATGGAT
GAATCAACGTAACCACGACCTTCTAGAACGTATCGTTTTTCACAAACGACACTAAACCTATCAACATT
ATCCATGTTGATCAT
+
FFFFFF:FFF,FFFFFFFFFFFFFFFFFFFFF:FFFFFFFFFFFFFFFFFFFFFFFFF:FFFF,FFFF
FFFFFFFFFFFFFFFFFFFFFFFF:FFFFFFFFFFFF:FFFFFFFF,FFFFFFFFF:FFFFFFFFFF:F
FF:FFFFFFFFFFFF
@A00721:190:HGJW7DSXY:4:2449:31620:17456 2:N:0:TCTGCAAG+GCTGCACT
CAGAAATCGGTCTAGACCTCATATAACAGAAGTAAGCGTTCTTAACGTCGTCGTCCGCATCCATGGAT
GAATCAACGTAACCACGACCTTCTAGAACGTATCGTTTTTCACAAACGACACTAAACCTATCAACATT
ATCCATGTTGATCAT
+
FFFFFFFFFFFFFFFFFFFFFFFFFFFFFFFFFF:FFFFFFFFFFFFFFFFFFFFFFFFFFFFFFFFFF
FFFFFFFFFFFFFFFFFFFFFFFFFFF:FFFFFFFFFFFFFFFFFFFFFFFFFFFFF,FFFFFFFFFF:F
FFFFFFFFFFFFFFF
@A00721:190:HGJW7DSXY:4:2644:22679:2221 2:N:0:TCTGCAAG+GCTGCACT
CAGAAATCGGTCTAGACCTCATATAACAGAAGTAAGCGTTCTTAACGTCGTCGTCCGCATCCATGGAT
GAATCAACGTAACCACGACCTTCTAGAACGTATCGTTTTTCACAAACGACACTAAACCTATCAACATT
ATCCATGTTGATCAT
+
FFFFFFFFFFFFFFFFFFFFFFFFFFFFFFFFFFFFFFFFFFFFFFFFFFFFFFFFFFFFFFFFFFFFF
FFFFFFFFFFFFFFFFFFFFFFFFFFFFFFFFFFFFFFFFFFFFFFFFF:FFFFFFFFFFFFFFFFFFF
FFFF:FFFFFFFFFF
@A00721:190:HGJW7DSXY:4:1237:32949:14622 2:N:0:TCTGCAAG+GCTGCACT
CTCACAGAAATCGGTCTAGACCTCATATAACAGAAGTAAGCGTTCTTAACGTCGTCGTCCGCATCCAT
GGATGAATCAACGTAACCACGACCTTCTAGAACGTATCGTTTTTCACAAACGACACTAAACCTATCAA
CATTATCCATGTTGA
+
FFFFFFFFFFFFFFFFFFFFFFFFFFFFFFFFFFFFFFFFFFFFFFFFFFFFFFFFFFFFFFFFFFFFF
FFFFFFFFFFFFFFFFFFFFFFFFFFFFFFF:FFFFFFFFFFFFFFFFFFFFFFFFFFF:FFFFFFFFFF
FFFFFFFFFFF:FFF
@A00721:190:HGJW7DSXY:4:1358:8693:6558 2:N:0:TCTGCAAG+GCTGCACT
CTCACAGAAATCGGTCTAGACCTCATATAACAGAAGTAAGCGTTCTTAACGTCGTCGTCCGCATCCAT
GGATGAATCAACGTAACCACGACCTTCTAGAACGTATCGTTTTTCACAAACGACACTAAACCTATCAA
CATTATCCATGTTGA
+
FFFFFFFFFFFFFFFFFFFFFFFFFFFFFFFFFF:FFFF:FFFFFFFFFFFFFFFFFFFFFFFFFFFFF
FFFFFFFFFFFFFFFFFFFFFFFFFFF:F:FFFFFFFF:FFFFFF:FFF,FFFFFFFFFFFFFFFFF:
FF,FFFFFFFF:FFF
@A00721:190:HGJW7DSXY:4:2449:20419:4100 2:N:0:TCTGCAAG+GCTGCACT
CTCACAGAAATCGGTCTAGACCTCATATAACAGAAGTAAGCGTTCTTAACGTCGTCGTCCGCATCCAT
GGATGAATCAACGTAACCACGACCTTCTAGAACGTATCGTTTTTCACAAACGACACTAAACCTATCAA
CATTATCCATGTTGA
+
FFFFFFF:FFFFFFFF,FFFF:FFFFFFFFFFFFFFFFFFFFFFFFFFF:F:FFFFFFFFFFFF:F:FF
FF:FFFFFFFFFFFFFFFFFFFFFFFFFFFFFFFFFFF:FFF,FFFF:FFFFFFFFFFFFFFFFFFFFFF
FFFFFFFFFFFFFFF
@A00721:190:HGJW7DSXY:4:1366:3775:8249 2:N:0:TCTGCAAG+GCTGCACT
GTTTCTTCCTCACAGAAATCGGTCTAGACCTCATATAACAGAAGTAAGCGTTCTTAACGTCGTCGTCC
GCATCCATGGATGAATCAACGTAACCACGACCTTCTAGAACGTATCGTTTTTCACAAACGACACTAAA
CCTATCAACATTATC
+
FFFFFFFFFFFFFFFFFFFFFFFFFFFFFFFFFFFFFFFFFFFFFFFFFFFFFFFFFFFFFFFFFFFFF

FFFFFFFFFFFFFFFFFFFFFFFFFFFFFFFFFFFFFFFFFFFFFFFFFFFFFFFFF:FFFFFFF:F:
FFF:FFFFFFFFFFF
@A00721:190:HGJW7DSXY:4:1537:30129:9204 2:N:0:TCTGCAAG+GCTGCACT
GTTTCTTCCTCACAGAAATCGGTCTAGACCTCATATAACAGAAGTAAGCGTTCTTAACGTCGTCGTCC
GCATCCATGGATGAATCAACGTAACCACGACCTTCTAGAACGTATCGTTTTTCACAAACGACACTAAA
CCTATCAACATTATC
+
FFFFFFFFFFFFFFFFFFFFFFFFFFFFFFFFFFFFFFFFFFFFFF,FFFFFFFFFFFFFFFFFFFFF
FF,FFFFFFFFFFFFFFFFFFFFFFF:FFFFFFFFFFFFFFFFFFFFFFFFFFFFFFFFFFFFFFFFF
FFFFFFFFF:FFFFF
@A00721:190:HGJW7DSXY:4:1567:3432:1110 2:N:0:TCTGCAAG+GCTGCACT
GTTTCTTCCTCACAGAAATCGGTCTAGACCTCATATAACAGAAGTAAGCGTTCTTAACGTCGTCGTCC
GCATCCATGGATGAATCAACGTAACCACGACCTTCTAGAACGTATCGTTTTTCACAAACGACACTAAA
CCTATCAACATTATC
+
FFFFFFFFFFFFFFFFFFFFFFFFFFFFFFFFFFFFFFFFFFFFFFF:FFFFFFFFFFFFFFFFFFFF
FFFFFFFFFFFFFFFFFF:FFFFFFFFFFFFFFFFFF,FFF,FFFFFFFFF:FFFFFF:FFFF,FFFF
F:FFFFFFFFFFFFF
@A00721:190:HGJW7DSXY:4:1601:30942:19695 2:N:0:TCTGCAAG+GCTGCACT
GTTTCTTCCTCACAGAAATCGGTCTAGACCTCATATAACAGAAGTAAGCGTTCTTAACGTCGTCGTCC
GCATCCATGGATGAATCAACGTAACCACGACCTTCTAGAACGTATCGTTTTTCACAAACGACACTAAA
CCTATCAACATTATC
+
FFFFFFFFFFFFFFFFFFFFFFFFFFFFFFFFFFFFFFFFF,FFFFFFFFFFFFFFFFFFFFFFFFFF
FFFFFFFFFFFFFF,FFFFFFFFFFFFFFFFFFFFFFFFFFFFFFFFFFFFFFFFFFFF:FFFFFFF:
FFFFFFFFFFFFFFF
@A00721:190:HGJW7DSXY:4:2154:20546:18255 2:N:0:TCTGCAAG+GCTGCACT
GTTTCTTCCTCACAGAAATCGGTCTAGACCTCATATAACAGAAGTAAGCGTTCTTAACGTCGTCGTCC
GCATCCATGGATGAATCAACGTAACCACGACCTTCTAGAACGTATCGTTTTTCACAAACGACACTAAA
CCTATCAACATTATC
+
F:FFFFFFFFFFFFFFFFFFFFFFFFFFFFFFFFFFFFFFFFFFFF,FFFFFFFFFFFFFFFFFFFFF
FFFFFFFFFFFFFFFFFFFFFFFFFF:FFFF:FFFFFFFFFFFFFFFF:FFFFFFFFFFFFFFFF:F:
FF:,FFFFFFFFFFF
@A00721:190:HGJW7DSXY:4:2531:13566:22326 2:N:0:TCTGCAAG+GCTGCACT
GTTTCTTCCTCACAGAAATCGGTCTAGACCTCATATAACAGAAGTAAGCGTTCTTAACGTCGTCGTCC
GCATCCATGGATGAATCAACGTAACCACGACCTTCTAGAACGTATCGTTTTTCACAAACGACACTAAA
CCTATCAACATTATC
+
FFFFFFFFFFFFFFFFFFFFFFFFFFFFFFFFFFFFFFFFFFFFFFFFFFFFFFFFFFFFFFFFFFFF
FFFFFFFFFFFFFFFFFFFFFFFFFFFFFFFFFFFFFFFFFFFFFFFFFFFFFFFFFFFFFFFFFF:F
FFFFFFFFFFFFFFF
@A00721:190:HGJW7DSXY:4:2676:9326:17581 2:N:0:TCTGCAAG+GCTGCACT
GTTTCTTCCTCACAGAAATCGGTCTAGACCTCATATAACAGAAGTAAGCGTTCTTAACGTCGTCGTCC
GCATCCATGGATGAATCAACGTAACCACGACCTTCTAGAACGTATCGTTTTTCACAAACGACACTAAA
CCTATCAACATTATC
+
FFFFFFFFFFFFFFFFFFFFFFFFFF:FFFFFFFFFFFFFFFFFFFFFFFFFFFFFFFFFFFFFFFFF
FFFFFFFFFFFFFFFFFFFFFFFFFFFFFFFFFFFFFFFFFFFFFFFFFFFFFFFFFFFFFFFF:FF:
FFFFFFFFFFFFFFF
@A00721:190:HGJW7DSXY:4:1168:12183:18427 2:N:0:TCTGCAAG+GCTGCACT
AAGTTTCTTCCTCACAGAAATCGGTCTAGACCTCATATAACAGAAGTAAGCGTTCTTAACGTCGTCGT
CCGCATCCATGGATGAATCAACGTAACCACGACCTTCTAGAACGTATCGTTTTTCACAAACGACACTA
AACCTATCAACATTA

+
FFFFFFFFFFFFFFFFFFFFFFFFFFFFFFFFFFFFFFFFFFFFFFFFFFFFFFFFFFFFFFFFFFFFFF
FFFFFFFFFFFF:FFFFFFFFFFFFFFFFFFFFFFFFFFFFF:FFFFFFFFFFFFFFFFFFFFFFFFFFF
FFFFFFFFFFFFFFF
@A00721:190:HGJW7DSXY:4:1403:5819:18584 2:N:0:TCTGCAAG+GCTGCACT
AAGTTTCTTCCTCACAGAAATCGGTCTAGACCTCATATAACAGAAGTAAGCGTTCTTAACGTCGTCGT
CCGCATCCATGGATGAATCAACGTAACCACGACCTTCTAGAACGTATCGTTTTTCACAAACGACACTA
AACCTATCAACATTA
+
FFFFFFFFFFFFFFFFFFFFFFFFFFFFFFFFFFFFFFFFFFFFFFFFFFFFFFFFFFFFFFFFFFFFFF
FFFFFFFFFFFFFFFFFFFFFFFFFFFFFFFFFFFFFFFFFFFFFFFFFFFFFFFFFFFFFFFFFFFFFF
FFFFFFFFFFFFFFF
@A00721:190:HGJW7DSXY:4:2257:19443:32064 2:N:0:TCTGCAAG+GCTGCACT
AAGTTTCTTCCTCACAGAAATCGGTCTAGACCTCATATAACAGAAGTAAGCGTTCTTAACGTCGTCGT
CCGCATCCATGGATGAATCAACGTAACCACGACCTTCTAGAACGTATCGTTTTTCACAAACGACACTA
AACCTATCAACATTA
+
FFFFFFFFFFFFFFFFFFFFFFFFFFFFFFFFFFFF:FFFFFFFFFFFFFFFFFFFFFFFFFFFFFFFFF
FFFFFFFFFFFFFFFFFFFFFFFFFFFFFFFFF:FFFFFFFFFFFFFFFFFFFFFFFF:FFFFFFFFFFFF
FFFFFFFFFFFFFFF
@A00721:190:HGJW7DSXY:4:2505:8757:19789 2:N:0:TCTGCAAG+GCTGCACT
AAGTTTCTTCCTCACAGAAATCGGTCTAGACCTCATATAACAGAAGTAAGCGTTCTTAACGTCGTCGT
CCGCATCCATGGATGAATCAACGTAACCACGACCTTCTAGAACGTATCGTTTTTCACAAACGACACTA
AACCTATCAACATTA
+
FFFFFFFFFFFFFFFFFFFFFFFFFFFFFFFFFFFFFFFFFFFFFFFFFFFFFFFFFFFFFFFFFF,FFF
FFFFFFFFFFFFFFFFFFFFFFFFFFFFFFFFFFFFFFFFFFFFFFFFFFFFFFFFFFFFFF:FFFFFFF,
FFFFF:FFFFFF:FF
@A00721:190:HGJW7DSXY:4:1201:25563:27070 2:N:0:TCTGCAAG+GCTGCACT
CTAAGTTTCTTCCTCACAGAAATCGGTCTAGACCTCATATAACAGAAGTAAGCGTTCTTAACGTCGTC
GTCCGCATCCATGGATGAATCAACGTAACCACGACCTTCTAGAACGTATCGTTTTTCACAAACGACAC
TAAACCTATCAACAT
+
FFFFFFFFFFFFFFFFFFFFFFFFFFFFFFFFFFFFFFFFFFFFFFFFFFFFFFFFFFFFFFFFFFFFFF
FFFFFFFFFFFFFFFFFFFFFFFFFFFFFFFFFFFFFFFFFFFFFFFFFFFFFFFFFFFFFFFFFFFFFF
FFFFF:FFFFFFFFF
@A00721:190:HGJW7DSXY:4:1534:11650:28808 2:N:0:TCTGCAAG+GCTGCACT
CTAAGTTTCTTCCTCACAGAAATCGGTCTAGACCTCATATAACAGAAGTAAGCGTTCTTAACGTCGTC
GTCCGCATCCATGGATGAATCAACGTAACCACGACCTTCTAGAACGTATCGTTTTTCACAAACGACAC
TAAACCTATCAACAT
+
FFFFFFFFFFFFFFFFFFFFFFFFFFFFFFFFFFFFFFFFFFFFFFFFFFFFFFFFFFFFFFFFFFFFFF
FFFFFFFFFFFFF:FFFFFFFFFFFFFFFFFFFFFFFFFFFFFFFFFFFFFF:FFFFFFFFFFFFFFFFF
FFFFF,:FFFFFFFF
@A00721:190:HGJW7DSXY:4:1633:8739:26835 2:N:0:TCTGCAAG+GCTGCACT
CTAAGTTTCTTCCTCACAGAAATCGGTCTAGACCTCATATAACAGAAGTAAGCGTTCTTAACGTCGTC
GTCCGCATCCATGGATGAATCAACGTAACCACGACCTTCTAGAACGTATCGTTTTTCACAAACGACAC
TAAACCTATCAACAT
+
FFFFFFFFFFFFFFFFFFFFFFFFFFFFFFFFFFFFFFFFFFFFFFFFFFFFFFFFFFFFFFFFFFFFFF
FFFFFFF:FFFFFFFFFFFFFFFFFFFFFFFFFFFFFFF:FF:FFFFFFFFFFFFFFFFFFFFFFFFFFF
FFFFFFFFFFFFFFF
@A00721:190:HGJW7DSXY:4:2166:32913:14465 2:N:0:TCTGCAAG+GCTGCACT
CTAAGTTTCTTCCTCACAGAAATCGGTCTAGACCTCATATAACAGAAGTAAGCGTTCTTAACGTCGTC

GTCCGCATCCATGGATGAATCAACGTAACCACGACCTTCTAGAACGTATCGTTTTTCACAAACGACAC
TAAACCTATCAACAT
+
FFFFFFFFFFFFFFFFFFFFFFFFFFFFFFF:FFF:FFFFFFFFFFFFFFFFFFF:FFFFFFFFFFFFFF
FFFFFFFFFFFFFFFFFFFFFFFFFFFFFFFFFFFF:FFFFFFFFFFFFFFFFFFFFFFFFFFFF:FFFF
FFFFFFFFFFFFFFF
@A00721:190:HGJW7DSXY:4:2312:15492:6997 2:N:0:TCTGCAAG+GCTGCACT
CTAAGTTTCTTCCTCACAGAAATCGGTCTAGACCTCATATAACAGAAGTAAGCGTTCTTAACGTCGTC
GTCCGCATCCATGGATGAATCAACGTAACCACGACCTTCTAGAACGTATCGTTTTTCACAAACGACAC
TAAACCTATCAACAT
+
FFFFFFFFFFFFFFFFFFFFFFFFFFFFFFFFFFFFFFFFFFFFFFFFFFFFFFFFFFFFFFFFFFFFFF
FFFFFFFFFFFFFFFFFF,FFFFFFFFFFFFFFFFFFFFFFFFFFFFFFFFFFFFFFFFFFFFFFFFFF:,FFFFFF
FFFFFFFFFFFFFFF
@A00721:190:HGJW7DSXY:4:2322:18548:21245 2:N:0:TCTGCAAG+GCTGCACT
CTAAGTTTCTTCCTCACAGAAATCGGTCTAGACCTCATATAACAGAAGTAAGCGTTCTTAACGTCGTC
GTCCGCATCCATGGATGAATCAACGTAACCACGACCTTCTAGAACGTATCGTTTTTCACAAACGACAC
TAAACCTATCAACAT
+
FFFFFFFFFFFFFFFFFFFFFFFFFFFFFFFFFFFFFFFFFFFFFFFFFFFFFFFFFFFFFFFFFFFFFF
FFFFFFFFFFFFFFFFFFFFFFFFFFFFFFFFFFFFFFFFFFFFFFFFFFFFFFFFFFFFFFFF:FFFFF
FFF:F:FFFFFFFFF
@A00721:190:HGJW7DSXY:4:2441:28854:26162 2:N:0:TCTGCAAG+GCTGCACT
CTAAGTTTCTTCCTCACAGAAATCGGTCTAGACCTCATATAACAGAAGTAAGCGTTCTTAACGTCGTC
GTCCGCATCCATGGATGAATCAACGTAACCACGACCTTCTAGAACGTATCGTTTTTCACAAACGACAC
TAAACCTATCAACAT
+
FFFFFFFFFFFFFFFFFFFFFFFFFFFFFFFFFFFFFFFFFFFFFFFFFFFFFFFFFFFFFFFFFFFFFF
FFFFFFFFFFFFFFFFFFFFFFFFFFFFFFFFFFFFFFFFFFFFFFFFFFFFFFFFFFFFFFFFFFFFFF
FFFFFFFFFFFFFFF
@A00721:190:HGJW7DSXY:4:1511:11324:20509 2:N:0:TCTGCAAG+GCTGCACT
TGTTGATAGGTTTAGTGTCGTTTGTGAAAAACGATACGTTCTAGAAGGTCGTGGTTACGTTGATTCAT
CCATGGATGCGGACGACGACGTTAAGAACGCTTACTTATGTTATATTAGGTCTAGACCGATTTCTGTG
AGGAAGAAACTTAGT
+
FFFFFFF:FFFFFFF:FFFF::F:FFFFFFFFFFFF,:FF:FF:FF,FF:F:F:FFF::,FF,,F,F,
:::F,FFF,FFFF,,FF,FFF:FFF::F,,:,FFF:F,,FF:FFF:,F:FF,:F,F:FFFFFF,F:FF
F:FFF::FF,:FF:F
@A00721:190:HGJW7DSXY:4:1533:28393:13495 2:N:0:TCTGCAAG+GCTGCACT
TGTTGATAGGTTTAGTGTCGTTTGTGAAAAACGATACGTTCTAGAAGGTCGTGGTTACGTTGATTCAT
CCATGGATGCGGACGACGACGTTAAGAACGCTTACTTCTGTTATATGAGGTCTAGACCGATTTCTGTG
AGGAAGAAACTTAGT
+
FFFFFFFFFFFFFFFFFFFFF:FFFFFFFF:FFFFFFFFF,,F:FFFFFFFFFF:FFFFFF:FFFFFF
FFF,FFFFFFFFFFFFFFFFFFFFFF:FF,F:FF:,FFFFF::FFFFF,FFFF::F:FF:FFF,F:FF
FFFFF::FF:,FFFF
@A00721:190:HGJW7DSXY:4:1637:21495:26475 2:N:0:TCTGCAAG+GCTGCACT
TGTTGATAGGTTTAGTGTCGTTTGTGAAAAACGATACGTTCTAGAAGGTCGTGGTTACGTTGATTCAT
CCATGGATGCGGACGACGACGTTAAGAACGCTTACTTCTGTTATATGAGGTCTAGACCGATTTCTGTG
AGGAAGAAACTTAGT
+
FFFFFFFFFFFFFFFFFF:FF,F:FFFFFFFFF,FF:FFFFFFFFFFF::FFFFFFFFFFFFF,FF:F
FFFFFFFF:FFFFFFFF:F::FF,FFFFFFFFFFFF:,:FFFFFFF,FF,FF:FF,F,FFFFFF:FFFF
FFFFFFFFF,FFFFF

```
@A00721:190:HGJW7DSXY:4:2227:27507:34976 2:N:0:TCTGCAAG+GCTGCACT
TGTTGATAGGTTTAGTGTCGTTTGTGAAAAACGATACGTTCTAGAAGGTCGTGGTTACGTTGATTCAT
CCATGGATGCGGACGACGACGTTAAGAACGCTTACTTCTGTTATATGAGGTCTAGACCGATTTCTGTG
AGGAAGAAACATAGT
+
FFFFFFFFFFFFFFFFFFFFFFFFFFFFFFFF:F:F::,F,,F:FF,F,F:,FF::FF:,FFFFF:F,
,F:,,F:F,F:FFF:FF:FFFFFFF:FF:FF,FFF,F:,::FFFFFFF,FF:FFFFFFFF:FFFF:FF
FFFFF:FFFF:FF,F
@A00721:190:HGJW7DSXY:4:2474:29053:26944 2:N:0:TCTGCAAG+GCTGCACT
TGTTGATAGGTTTAGTGTCGTTTGTGAAAAACGATACGTTCTAGAAGGTCGTGGTTACGTTGATTCAT
CCATGGATGCGGACGACGACGTTAAGAACGCTTACTTCTGTTATATGAGGTCTAGACCGATTTCTGTG
AGGAAGAAACTTAGT
+
FFFFFFFFFFFFFFFFFFFFFFFFFFFFFFFFFFFFFFFFFFFFFF,FFFF,FFFFFFFFFFFFFFFF
FFFFFFFFFFFFFFFFFFFFF:FFFFFFFFFFFFFFF:FFF:FFFF:FFFFFFFFFFFFFFFFFFFFF
F:FFFFFFFFFFFFF
@A00721:190:HGJW7DSXY:4:2634:19994:33364 2:N:0:TCTGCAAG+GCTGCACT
AAGTTTCTTCCTCACAGAAATCGGTCTAGACCTCATATAACAGAAGTAAGCGTTCTTAACGTCGTCGT
CCGCATCCATGGATGAATCAACGAAACCACGACCTTCTAGAACGTATCGTTTTTCACAAACGACACTA
AAACTAAAAACA
+
F:FFFF:FF::,:FF:FFF,F,,F:,:F:FF,FFF,FFFFFFFFF,,FF,,FF,F,,FF,F,FF:,,F
:,,F::,FF,FFFF,FFFFFFF:,FFFFFFFFF,FFF:F:FFFFFFF,:F,,FFF:FFFFF,F,FF,F
,F,,,F,,FFFF
@A00721:190:HGJW7DSXY:4:1375:25807:1438 2:N:0:TCTGCAAG+GCTGCACT
TCCACTAAGTTTCTTCCTCACAGAAATCGGTCTAGACCTCATATAACAGAAGTAAGCGTTCTTAACGT
CGTCGTCCGCATCCATGGATGAATCAACGTAACCACGACCTTCTAGAACGTATCGTTTTTCACAAACG
ACACTAAACCTATCA
+
FFFFFFFFFFFFFFFFFFFFFFFFFFFFFFFFFF:FFFFF:FFFFFFFFFFFFFFFFFFFFFFFFFFF
FFFFFFFFFFFFFFFFFFF:FFFFFFFFFFFFFFFFFFFFFFFFFFFFFFFFFFFFFFFFFFFFFFFF
:FFFFFFFFFFFFFF
@A00721:190:HGJW7DSXY:4:1504:17824:21652 2:N:0:TCTGCAAG+GCTGCACT
TCCACTAAGTTTCTTCCTCACAGAAATCGGTCTAGACCTCATATAACAGAAGTAAGCGTTCTTAACGT
CGTCGTCCGCATCCATGGATGAATCAACGTAACCACGACCTTCTAGAACGTATCGTTTTTCACAAACG
ACACTAAACCTATCA
+
FFFFFFFFFFFFFFFFFFFFFFFFFFFFFFFFFFFFFFFFFFFFFFFFFFFFFFFFFFFFFFFFFFFF
FFFFF:FFFFFFFFFFFFFFFFFFFFFFFFFFFFFFFFFFFFFFFFFFFFFFFFFFFFFFFFFFFFFF
FFFFFFFFFFF:FFF
@A00721:190:HGJW7DSXY:4:2128:11767:13228 2:N:0:TCTGCAAG+GCTGCACT
TCCACTAAGTTTCTTCCTCACAGAAATCGGTCTAGACCTCATATAACAGAAGTAAGCGTTCTTAACGT
CGTCGTCCGCATCCATGGATGAATCAACGTAACCACGACCTTCTAGAACGTATCGTTTTTCACAAACG
ACACTAAACCTATCA
+
FFFFFFFFFFFFFFFFFFFFFFFFFFFFFFFFFFFFFFFFFFFFFFFFFFFFFFFFFFFFFFFFFFFF
FFFFFFFFFFFFFFFFFFFFFFFFFFFFFFFFFFFFFFFFFFFFF:FFFFFFFFFFFFFF:FFFFF:F
FFFFFFF:FFFF:FF
@A00721:190:HGJW7DSXY:4:2132:32723:8406 2:N:0:TCTGCAAG+GCTGCACT
TCCACTAAGTTTCTTCCTCACAGAAATCGGTCTAGACCTCATATAACAGAAGTAAGCGTTCTTAACGT
CGTCGTCCGCATCCATGGATGAATCAACGTAACCACGACCTTCTAGAACGTATCGTTTTTCACAAACG
ACACTAAACCTATCA
+
FFFFFFFFFFFFFFFFFFFFFFFFFFFFFFFFFFFFFFFFFFFFFFFFF:FFFFFFFFFFFFFFFFFF
```

```
FFFFFFFFFFFFFFFFFFFFFFFFFFFFFFFFFFFFFFFFFFFFFFFFFF:FFFFFFFFFFFFFFFF
FFFFFFFFFFFFFFF
@A00721:190:HGJW7DSXY:4:2151:2446:4883 2:N:0:TCTGCAAG+GCTGCACT
TCCACTAAGTTTCTTCCTCACAGAAATCGGTCTAGACCTCATATAACAGAAGTAAGCGTTCTTAACGT
CGTCGTCCGCATCCATGGATGAATCAACGTAACCACGACCTTCTAGAACGTATCGTTTTTCACAAACG
ACACTAAACCTATCA
+
FFFFFFFFFFFFFFFFFFFFFFFFFFFFFFFFFFFFFFFF:FFFFFFFF,FFFFFFFFFFFFFFFFF
FFFFFFFFFFFFFFFFFFF:FFF:FFFFFFFFF:FFFFFFFFFFF:FFFF:F:FFFFFFFFFFFFFFF
FFFF:FFFFFFFF:F
@A00721:190:HGJW7DSXY:4:2269:8919:31469 2:N:0:TCTGCAAG+GCTGCACT
TCCACTAAGTTTCTTCCTCACAGAAATCGGTCTAGACCTCATATAACAGAAGTAAGCGTTCTTAACGT
CGTCGTCCGCATCCATGGATGAATCAACGTAACCACGACCTTCTAGAACGTATCGTTTTTCACAAACG
ACACTAAACCTATCA
+
FFFFFFFFFFFFFFFFFFFFFFFFFFFFFFFFFFFFFFFFFFFFFFFFFFFFFFFFFFFFFFFFFFFF
FFFFFFFFFFFFFFFFFFFFFFFFFFFFFFFFFFFFFFFFFFFFFFFFFFFFFFFFFFFFFFFFFFFF
FFFF:FFFFFFFFFF
@A00721:190:HGJW7DSXY:4:2627:4887:13902 2:N:0:TCTGCAAG+GCTGCACT
TCCACTAAGTTTCTTCCTCACAGAAATCGGTCTAGACCTCATATAACAGAAGTAAGCGTTCTTAACGT
CGTCGTCCGCATCCATGGATGAATCAACGTAACCACGACCTTCTAGAACGTATCGTTTTTCACAAACG
ACACTAAACCTATCA
+
FFFFFFFFFFFFFFFFFFFFFFFFFFFFFFFFFFFFFFFFFFFFFFFFFFFFFFFFFFFFFFFFFFFF
FFFFFFFFFF:FFFFFFFFFFFFFFFFFFFFFFFFFFFFFFFFFFFFFFFFFFFFFFFFFFFFFFFFF
FFFFFF:FFFFFFFF
@A00721:190:HGJW7DSXY:4:1239:4209:8625 2:N:0:TCTGCAAG+GCTGCACT
GATAGGTTTAGTGTCGTTTGTGAAAAACGATACGTTCTAGAAGGTCGTGGTTACGTTGATTCATCCAT
GGATGCGGACGACGACGTTAAGAACGCTTACTTCTGTTATATGAGGTCTAGACCGATTTCTGTGAGGA
AGAAACTTAGTGGAC
+
FFFFFFFFFFFFFFFFFFFFFFFFFFF::FFFFF,F,FFFFFFF:FFFFF,FFFFFFFFFF:FFFFF,
FFFFFFFFFF:F:,FFFFFFF:FF:F::FFFF::,F:FFFF::FFFFFFFFFF:FF:FFFFFF::,FF
FFFFF:FFFFFFFFF
@A00721:190:HGJW7DSXY:4:1254:21784:36902 2:N:0:TCTGCAAG+GCTGCACT
GATAGGTTTAGTGTCGTTTGTGAAAAACGATACGTTCTAGAAGGTCGTGGTTACGTTGATTCATCCAT
GGATGCGGACGACGACGTTAAGAACGCTTACTTCTGTTATATGAGGTCTAGACCGATTTCTGTGAGGA
AGAAACTTAGTGGAC
+
FFFFFFFFFFFFFFFFFFFFFFFFFF:FF:FF:FFF,FFFFF:FF:FFFFFFFFFFFFFFF:FFFFFF
FFFFFFFFFFFFFFFFF:FFFFFF:FF:FFF:F,FFFFFFFFFF,FFFFF:FFFFFFFFFFFFF:FFF
FFFFFFFFF::FFFF
@A00721:190:HGJW7DSXY:4:1340:18511:14606 2:N:0:TCTGCAAG+GCTGCACT
GATAGGTTTAGTGTCGTTTGTGAAAAACGATACGTTCTAGAAGGTCGTGGTTACGTTGATTCATCCAT
GGATGCGGACGACGACGTTAAGAAAGCTTACTTCTGTTATATGAGGTCTAGACCGATTTCTGTGAGGA
AGAAACTTAGTGGAC
+
FFFFFFFFFFFFFFFFFFFFF,:FFFFFFFFFFFFF,FF,:F,FF:F::FFFF,FFF,FFFFFFFFFF
:FFFFFFFFFFF:F:FFFFF:,F,FFFFF,FF,F,FFFFFF:FF:F,FF:F,FFFFFFF:FFFFFFF
FF::F,,FFFFFFFF
@A00721:190:HGJW7DSXY:4:1417:31132:3803 2:N:0:TCTGCAAG+GCTGCACT
GATAGGTTTAGTGTCGTTTGTGAAAAACGATACGTTCTAGAAGGTCGTGGTTACGTTGATTCATCCAT
GGATGCGGACGACGACGTTAAGAACGCTTACTTCTGTTATATGAGGTCTAGACCGATTTCTGTGAGGA
AGAAACTTAGTGGAC
```

+
FFFFFF:FFFFFFFFFF,F:FFFFFFF:F:FF,,FF:FFFFFFF:F::FFFF,FFFFF,FFFFFFF:,
FFF:FF:FFFFF,FFF:FFFF,:FF::,F,F,F,,:FFFF:F,FFFF:FFF:FFFFFFF,:,::FFFF
F::FFF:FF::FF,F
@A00721:190:HGJW7DSXY:4:1440:4074:6104 2:N:0:TCTGCAAG+GCTGCACT
GATAGGTTTAGTGTCGTTTTTGAAAAACGATACGTTCTAGAAGGTAGTGGTTACGTTGTTTCAACCAT
GTATGCGGACGAAGACGTTAAGAACGCTAACTTCTGTTATATGATGTCTAGAACGATTTCTGTGAGGA
AGAAACTTAGTGTA
+
FFFFFF,FFFF::FF,,,F,,,FFFFFF,FFFFFF,,FF,F:F,,,:,,FFFFF::F,,FFFF,,,,:
F,F,,:,,F:,F,,F:,,:F:,FF,,,F,FF:FF,F:,F,FF,F,F:,:F,F,,,:,:::F,F:,F:F
F,FFF:F,FFF,FF
@A00721:190:HGJW7DSXY:4:1524:15374:16188 2:N:0:TCTGCAAG+GCTGCACT
GATAGGTTTAGTGTCGTTTGTGAAAAACGATACGTTCTAGAAGGTCGTGGTTACGTTGATTCATCCAT
GGATGCGGACGACGACGTTAAGAACGCTTACTTCTGTTATATGAGGTCTAGACCGATTTCTGTGAGGA
AGAAACTTAGTGGAC
+
FFFFFFFFFFFFFFFFF,FFFFFFFFFF,FFF:::FF:F:FF,:F:F,:FFFFFF,FFFFFFF::F::
:FFFFFFFFF:FFF:FFFFFF:FF:FF:FFF,F:,F:FFFF:,FFFFFFF,F::FFFFF:F:FFF,FF
F:FFF::FF,F:FFF
@A00721:190:HGJW7DSXY:4:1632:29143:7059 2:N:0:TCTGCAAG+GCTGCACT
GATAGGTTTAGTGTCGTTTGTGAAAAACGATACGTTCTAGAAGGTCGTGGTTACGTTGATTCATCCAT
GGATGCGGACGACGACGTTAAGAACGCTTACTTCTGTTATATGAGGTCTAGACCGATTTCTGTGAGGA
AGAAACTTAGTGGAC
+
FFFFFFFFFFFFFFFFF:FFFFFFFFF:FFFF,FFF:FFFFF:F:,F:FFFFFFFFFFFFF,FF:F:F
FFFFFFFFFFFFFFFFF:FFF,F,FFFFFF:F:::F,:FFFFFF,FFFFFFFFFFF:FF,FFFFFFFF
FFFFFF,FF,FF,FF
@A00721:190:HGJW7DSXY:4:2231:20763:32346 2:N:0:TCTGCAAG+GCTGCACT
GATAGGTTTAGTGTCGTTTGTGAAAAACGATACGTTCTAGAAGGTCGTGGTTACGTTGATTCATCCAT
GGATGCGGACGACGACGTTAAGAACGCTTACTTCTGTTATATGAGGTCTAGACCGATTTCTGTGAGGA
AGAAACTTAGTGGA
+
::F:FFFFFFFFFFFFFFF:FFFFFFF:FFFFF:FFFFF:FF,FFFFFFFFFFFFFF:FFFFFF,FF,
FFF:FFFFFFFFFFFF:FFFF,FF:FF:FFF:FF:FFFFFFF:F:FFFFF:F,F:F:FF:,FFF::FF
FFFFF,:FF:FFFF
@A00721:190:HGJW7DSXY:4:2270:27832:31344 2:N:0:TCTGCAAG+GCTGCACT
GATAGGTTTAGTGTCGTTTGTGAAAAACGATACGTTCTAGAAGGTCGTGGTTACGTTGATTCATCCAT
GGATGCGGACGACGACGTTAAGAACGCTTACTTCTGTTATATGAGGTCTAGACCGATTTCTGTGAGGA
AGAAACTTAGTGGAC
+
FFFFFFFFFFFFFFFFFFFFFFFFFFFFFFFF::F:FFFFF,FFFFFFFFFF:FFFFFF:FFFFFFFFF
FF,FFFFFFFFFFF:FFFFFFFFFFF:FFF::FF:F,FFFFFFFFFFFFF,F:FF:FFFFFFFF:FFF
:FFFFFFFFFFFFF:
@A00721:190:HGJW7DSXY:4:2321:1163:1282 2:N:0:TCTGCAAG+GCTGCACT
GATAGGTTTAGTGTCGTTTGTGAAAAACGATACGTTCTATAAGGTCGTGGTTACGTTGATTAATCCAT
GGATGCGGACGACGACGTTAATAACGCTTACTTCTGTTATATGAGGTCTAGACCGATCTCTGTGAGGA
AGAAACTTATTGGA
+
FF:FFFFFFFFFFF:FF,F,FF:FF:F,FF:,F:FF,FF,FFFFF,,:FFFFF,::F:FFF,F:F:FF
,FF:FFFFF,,F:F,,::FFF,FF,FFFFF,,:,,FFF:FF:,F:,:,FFF::,::FFFFFFF:F,F,
FFFFF::FF,F:::
@A00721:190:HGJW7DSXY:4:2324:23547:30342 2:N:0:TCTGCAAG+GCTGCACT
GATAGGTTTAGTGTCGTTTGTGAAAAACGATACGTTCTAGAAGGTCGTGGTTACGTTGATTCATCCAT

```
GGATGCGGACGACGACGTTAAGAACGCTTACTTCTGTTATATGAGGTCTAGACCGATTTCTGTGAGGA
AGAAACTTAGTGGAC
+
::FFFFFFFFFFFFFFFFFFFFFFFFFFFFFF::FF:FF:FF:FFFFFFFFFF:FFFFFFFFF,:F:,
:FFF:FFFF:FFFFFF,FFFF,FFF:FFFFFFF:FFFFFFF:,F,FF,FF,F:F:FFFF::,F,F,FF
F,FFF,:FF:F,FFF
@A00721:190:HGJW7DSXY:4:2350:25229:35039 2:N:0:TCTGCAAG+GCTGCACT
GATAGGTTTAGTGTCGTTTGTGAAAAACGATACGTTCTAGAAGGTCGTGGTTACGTTGATTCATCCAT
GGATGCGGACGACGACGTTAAGAACGCTTACTTCTGTTATATGAGGTCTAGACCGATTTCTGTGAGGA
AGAAACTTAGTGGAC
+
FFFFFFFFFFFFFFFFFFFFFFFFFFFFFFFF:FFF:FFFFFFFFFFFFFFFFF:FFFFFFFFF:FFF
,FFFFFFFFFFFFFF:FFFFF:FF,FFFFFFFF:FFFFFFFFFF,FF,FFFFFFFF:FF:F,F:FFF:
F,FFF,:FF,F,FFF
@A00721:190:HGJW7DSXY:4:2502:6903:26256 2:N:0:TCTGCAAG+GCTGCACT
GATAGGTTTAGTGTCGTTTGTGAAAAACGATAAGTTCTAGAAGGTCGTGGTTACGGTGATTCATCCAT
GGATGCGGACGACGACGTTAAGAACGCTTACTTCTGTTATATGAGGTCTAGAACGATTTATGTGAGGA
AGAAACATAGTGGA
+
FFFFFFFFFFFFFFFFFFF:F,FFFFF,,:,F,,,F,,F,:F,FFFF:FF:F:,:,FF,FF,F::F:,
,FFF:FFFFF,FF,::FFFFF,FF,,F,FF:,F,:,FFF:,FFFFF:,FF,F,F:FFFF,::F:,F::
F,FFF,,FF,F:FF
@A00721:190:HGJW7DSXY:4:2550:32768:9392 2:N:0:TCTGCAAG+GCTGCACT
GATAGGTTTAGTGTCGTTTGTGAAAAACGATACGTTCTAGAAGGTCGTGGTTACGTTGATTCATCCAT
GGATGCGGACGACGACGTTAAGAACGCTTACTTCTGTTATATGAGGTCTAGACCGATTTCTGTGAGGA
AGAAACTTAGTGGAC
+
FFFFFFFFFFFFFFFFF:FFFFFFFFF,F:FFFFFF:FF::FFFF:FFFFFFFFFFFF:FF:FFFF:F
FF:FFFFFFFF,FF:FF,F:F,FF,F:,F,F:F:FF,FFF:F::FFFFF:,FFFFFFFFFF:,F,FFF
FFFFFF:FF:F,F,:
@A00721:190:HGJW7DSXY:4:2557:10140:4398 2:N:0:TCTGCAAG+GCTGCACT
GATAGGTTTAGTGTCGTTTGTGAAAAACGATACGTTCTAGAAGGTCGTGGTTACGTTGATTCATCCAT
GGATGCGGACGACGACGTTAAGAACGCTTACTTCTGTTATATGAGGTCTAGACCGATTTCTGTGAGGA
AGAAACTTAGTGGAC
+
F:FFFFFFFFFFFFFFFFFF::FFFFFFFFFFFFFFFF:FF,F:FF,F:FFFFF:FFFFFFFFF:FF:
:FFF:F:FFFFF:FFF,:FFFFFFF:FFFFFFF:,FFFFFFF,F:FF:FF,FF:FFFFF,FFF:FFFF
F,FFFF:FF,FFFFF
@A00721:190:HGJW7DSXY:4:1114:29586:34068 2:N:0:TCTGCAAG+GCTGCACT
CTTAAAAACTGTGAGTCCACTAAGTTTCTTCCTCACAGAAATCGGTCTAGACCTCATATAACAGAAGT
AAGCGTTCTTAACGTCGTCGTCCGCATCCATGGATGAATCAACGTAACCACGACCTTCTAGAACGTAT
CGTTTTTCACAAACG
+
FFFFFFFFFFFFFFFFFFFFFFFFFFFFFFFFFFFFFFFFFFFFFFFFFFF:FFFFFFFFFFFFFFFF
FFFFFFFF:FFFFFFFFFFFFFFFFFFFFF,FFFFFFFFFFFFFFFFFFFFFFFFFFFFFFFFFFFFF
FFFFFFFFFFFFFFF
@A00721:190:HGJW7DSXY:4:1114:29577:34366 2:N:0:TCTGCAAG+GCTGCACT
CTTAAAAACTGTGAGTCCACTAAGTTTCTTCCTCACAGAAATCGGTCTAGACCTCATATAACAGAAGT
AAGCGTTCTTAACGTCGTCGTCCGCATCCATGGATGAATCAACGTAACCACGACCTTCTAGAACGTAT
CGTTTTTCACAAACG
+
FFFFFFFFFFFFFFFFFFFFFFFFFFFFFFFFFF:FFFFFFFFFFFFFFFFFFFFFFFFFFFFFFFFF
FFFFFFFFFFFFFFFFFFFFFFFFFFFFFFFFFFFFFF:FFFFFFFFF:FFFFFFFFFFFFFFFFFFF
FFFFFFFFFFFFFFF
```

@A00721:190:HGJW7DSXY:4:1266:29939:23500 2:N:0:TCTGCAAG+GCTGCACT
CTTAAAAACTGTGAGTCCACTAAGTTTCTTCCTCACAGAAATCGGTCTAGACCTCATATAACAGAAGT
AAGCGTTCTTAACGTCGTCGTCCGCATCCATGGATGAATCAACGTAACCACGACCTTCTAGAACGTAT
CGTTTTTCACAAACG
+
FFFFFFFFFFFFFFFFFFFFFFFFFFFFFFFFFFFFFFFFFFFFFFFFFFFFFFFFFFFFFFFFFFFFF
FFFFFFFFFFFFFFFFFFFFFFFFFFFFFFFFFFFFFF:FFFFFFFFFFFFFFFFF:FFFFFFFFFFFF
FFFFFFFFFFFF,:F
@A00721:190:HGJW7DSXY:4:1271:25681:32503 2:N:0:TCTGCAAG+GCTGCACT
CGTTTGTGAAAAACGATACGTTCTAGAAGGTCGTGGTTACGTTGATTCATCCATGGATGCGGACGACG
ACGTTAAGAACGCTTACTTCTGTTATATGAGGTCTAGACCGATTTCTGTGAGGAAGAAACTTAGTGGA
CTCACAGTTTTTAAG
+
FF:FFFFFFFFFFFFFFFFFFFFFFFFFFFFFFFFFFFFFFFFFFFFFFFFFFFFF:FF:FFFFFF:FF
FFFFFFFFFFF:FFFFFFFFFFFFFFFFFFFFFFFFFFFFF:FFFFFFFFFFFFF:FFFFFFFFFFFFF
:FF:FFFFFFFFF:F
@A00721:190:HGJW7DSXY:4:1349:32280:10207 2:N:0:TCTGCAAG+GCTGCACT
CGTTTGTGAAAAACGATACGTTCTAGAAGGTCGTGGTTACGTTGATTCATCCATGGATGCGGACGACG
ACGTTAAGAACGCTTACTTCTGTTATATGAGGTCTAGACCGATTTCTGTGAGGAAGAAACTTAGTGGA
CTCACAGTTTTTAAG
+
FFFFFFFFFFFFFFF:FFFFFFFFFFFFFFFF:FFFF:F::FFFFFFFFFFFFFFFFF:FFFFF,FFF
FFFFFFFF:FFFFFFF:FFFFFFFFFF:FFFFFFFFFFFFFF:FFFFFFFFFFFFFF,FFFFFFFFF:F
:FF:F:FFFFFFFFF
@A00721:190:HGJW7DSXY:4:1364:26395:20588 2:N:0:TCTGCAAG+GCTGCACT
CGTTTGTGAAAAACGATACGTTCTAGAAGGTCGTGGTTACGTTGATTCATCCATGGATGCGGACGACG
ACGTTAAGAACGCTTACTTCTGTTATATGAGGTCTAGACCGATTTCTGTGAGGAAGAAACTTAGTGGA
CTCACAGTTTTTAAG
+
FFFFFFFFFFFFFFFFFFFFFFFFFFFFFFFFFFFFFF:FFFFFFF:FFFFFFFFFFFFFFFFFFFFFF
FFFFFFFF:FFFFFFFFFFFFF:F:FFFFFFFFFFFFFFFFFFFFFFFFFFF:FFFFFFFFFFFFFFFF
FFFFF:FFFFFFF::
@A00721:190:HGJW7DSXY:4:1371:30454:33567 2:N:0:TCTGCAAG+GCTGCACT
CTTAAAAACTGTGAGTCCACTAAGTTTCTTCCTCACAGAAATCGGTCTAGACCTCATATAACAGAAGT
AAGCGTTCTTAACGTCGTCGTCCGCATCCATGGATGAATCAACGTAACCACGACCTTCTAGAACGTAT
CGTTTTTCACAAACG
+
FFFFFFFFFFFFFFFFFF,FFFFFFFFFFFFFFFFFFFFFFFFFFFFFFFFFFFFFFFFFFFFFFFFFF
FFFFFFFFFFFFFFFFF:FFFFFFFFFFFFFFFFFFFFFFFFFFFFFFFFFFF:FFFFFFFFFFFFFFF
FFFFFFFFFFFFFFF
@A00721:190:HGJW7DSXY:4:1517:1696:16767 2:N:0:TCTGCAAG+GCTGCACT
CTTAAAAACTGTGAGTCCACTAAGTTTCTTCCTCACAGAAATCGGTCTAGACCTCATATAACAGAAGT
AAGCGTTCTTAACGTCGTCGTCCGCATCCATGGATGAATCAACGTAACCACGACCTTCTAGAACGTAT
CGTTTTTCACAAACG
+
FFFFFFFFFFFFFFFFFFFFFFFFFFFFF:FFFFFFFFFFFFFFFFFFFFFFFFFFFFFFFFFFFFFFF
FFFFFFFFFFFFFFFFFFFFFFFFFFFFFFFFFFFFF:FFFFFFFFFFFFFFFFFFF,FFFFFFFFFFF
FFFFFFF:FFFFFFF
@A00721:190:HGJW7DSXY:4:1603:7139:33990 2:N:0:TCTGCAAG+GCTGCACT
CTTAAAAACTGTGAGTCCACTAAGTTTCTTCCTCACAGAAATCGGTCTAGACCTCATATAACAGAAGT
AAGCGTTCTTAACGTCGTCGTCCGCATCCATGGATGAATCAACGTAACCACGACCTTCTAGAACGTAT
CGTTTTTCACAAACG
+
FF:FFFFFFFFFFFFFFF:FFFFFFFFFFFFFFFFFFFFFFFFFFFFFFFFFFFFFFFFFFFFFFF:FF

```
F:FFFFFFFFFFFFFFFFFFFFFFFFFFF:FFFFFFFFFFFFFFFFFFFFFFFFFFFFFFFFFFF:FF
FFFFFFFFFFFFFFF
@A00721:190:HGJW7DSXY:4:1652:29695:2722 2:N:0:TCTGCAAG+GCTGCACT
CTTAAAAACTGTGAGTCCACTAAGTTTCTTCCTCACAGAAATCGGTCTAGACCTCATATAACAGAAGT
AAGCGTTCTTAACGTCGTCGTCCGCATCCATGGATGAATCAACGTAACCACGACCTTCTAGAACGTAT
CGTTTTTCACAAACG
+
FFFFFFFFFFFFFFFFFFFFFFFFFFFFFFFFFFFFFFFFFFFFFFFFF:FFFFFFFFFFFFF:FFFF
FFFFFFFFFFFFFFFFFFFFFFFFFFFFF,FFFFFFFFFFFFFFFF:FFFFFFFFFFFFFFFFFFFFF
FFFFFFFFFFFFFFF
@A00721:190:HGJW7DSXY:4:1662:11017:13213 2:N:0:TCTGCAAG+GCTGCACT
CTTAAAAACTGTGAGTCCACTAAGTTTCTTCCTCACAGAAATCGGTCTAGACCTCATATAACAGAAGT
AAGCGTTCTTAACGTCGTCGTCCGCATCCATGGATGAATCAACGTAACCACGACCTTCTAGAACGTAT
CGTTTTTCACAAACG
+
FFFFFFFFFFFFFFFFFFFFFFFFFFFFFFFFFFFFFFFFFFF:FFFFFFFFFFFFFFFFFFFFFFFF
F,FFFFFFFFFFFFFFF:FFFFFFFFFFFFFFFFFFFFFFFFFFFFFFFFFFFFFFFFFFFFFFFFFF
FFFFFFFFFFFFFFF
@A00721:190:HGJW7DSXY:4:2137:1976:31814 2:N:0:TCTGCAAG+GCTGCACT
CGTTTGTGAAAAACGATACGTTCTAGAAGGTCGTGGTTACGTTGATTCATCCATGGATGCGGACGACG
ACGTTAAGAACGCTTACTTCTGTTATATGAGGTCTAGACCGATTTCTGTGAGGAAGAAACTTAGTGGA
CTCACAGTTTTTAAG
+
FFFFFFFFFFFFFFFFFFFFFFFFFFFFFFFFFFFFFFFFFFFFFFFFFF:FFFFFFFFFFFFFFFFF
FFFFFFFFFF:FFFFFFF:FFFFFFFFFFFFFFFFFFFFFFFFFFFFFFFFFFFFFFFFFFFFFFFFF
FFFFFFFFFFFFFFF
@A00721:190:HGJW7DSXY:4:2243:20103:2769 2:N:0:TCTGCAAG+GCTGCACT
CTTAAAAACTGTGAGTCCACTAAGTTTCTTCCTCACAGAAATCGGTCTAGACCTCATATAACAGAAGT
AAGCGTTCTTAACGTCGTCGTCCGCATCCATGGATGAATCAACGTAACCACGACCTTCTAGAACGTAT
CGTTTTTCACAAACG
+
FF::FFFFFFFFFFFFFFFFFFFFFFFFFFFFFFFFFFFFFFFFFFFF:FFFFFFFFFFFFFFFFFFF
F,FFFFFFFFFFFFFF::FFFFFFFFFFFF:FF::F::FFFFFFF:FFFFFFFFFFFFFFFFFFFFFF
FFF:FF:FFFFFFFF
@A00721:190:HGJW7DSXY:4:2250:5367:17926 2:N:0:TCTGCAAG+GCTGCACT
CGTTTGTGAAAAACGATACGTTCTAGAAGGTCGTGGTTACGTTGATTCATCCATGGATGCGGACGACG
ACGTTAAGAACGCTTACTTCTGTTATATGAGGTCTAGACCGATTTCTGTGAGGAAGAAACTTAGTGGA
CTCACAGTTTTTAAG
+
FF:FFFFFF:FFFFFFFFFF:FFFFFFFFFFFFFFFFFFFFFFFFFFFFFFFFFFFFFFFFFFFFFFF
FFFFFFFFFFFFFFFFF:FFFFF:F:FFFFFFFFFFF:FFFFFFFFFF,FFFFFFFFFFFFFFFFF,F
FFFFFFFFFFF,F,F
@A00721:190:HGJW7DSXY:4:2350:22498:29309 2:N:0:TCTGCAAG+GCTGCACT
CTTAAAAACTGTGAGTCCACTAAGTTTCTTCCTCACAGAAATCGGTCTAGACCTCATATAACAGAAGT
AAGCGTTCTTAACGTCGTCGTCCGCATCCATGGATGAATCAACGTAACCACGACCTTCTAGAACGTAT
CGTTTTTCACAAACG
+
FFFFFFFFFFFFFFFFFFFFF:FFFFFFFFFFFFFFFFFFFFFFFFFFFFFFFFFFFFFFFFFFFFFF
FFFFFFFFFFFFFFFFFFFFFFFFFFFFFFFFFFFFFFFFFFFFFFFFFFFFFFFFFFFF:FFFFFFF
FFFFFFFFFFFFFFF
@A00721:190:HGJW7DSXY:4:2374:8874:26193 2:N:0:TCTGCAAG+GCTGCACT
CTTAAAAACTGTGAGTCCACTAAGTTTCTTCCTCACAGAAATCGGTCTAGACCTCATATAACAGAAGT
AAGCGTTCTTAACGTCGTCGTCCGCATCCATGGATGAATCAACGTAACCACGACCTTCTAGAACGTAT
CGTTTTTCACAAACG
```

```
+
FFFFFFF:FFFFFFFFFFFFFFFFFFFFFFFFFFFFFFFFFFFF:FFF:FFFFFFFFFF:FF:FFFFFF
FFFFFFFFFFFFFFFFFFFFFFFFFFFFFFFFFFFFFFFFFFFFFFFFFFFFFFFFFFFFFFFFFF:FF
FFFFFFFFFFFFFFF
@A00721:190:HGJW7DSXY:4:2433:13865:33458 2:N:0:TCTGCAAG+GCTGCACT
CTTAAAAACTGTGAGTCCACTAAGTTTCTTCCTCACAGAAATCGGTCTAGACCTCATATAACAGAAGT
AAGCGTTCTTAACGTCGTCGTCCGCATCCATGGATGAATCAACGTAACCACGACCTTCTAGAACGTAT
CGTTTTTCACAAACG
+
FFFFFFFFFFFFFFFFFFFFFFFFFFFFFFFFFFFFFFFFFFFFFFFFFFFFFFFFFFFFFFFFFFFFF
FFFFFFFFFFFFFFFFFFFFFFFFFFFFFFFFFFF:FFFFFFFFFFFFFFFFFFFFFFFFFFFFFFFFF
FFFFFFFFFFFFFF:
@A00721:190:HGJW7DSXY:4:2541:18295:32049 2:N:0:TCTGCAAG+GCTGCACT
CTTAAAAACTGTGAGTCCACTAAGTTTCTTCCTCACAGAAATCGGTCTAGACCTCATATAACAGAAGT
AAGCGTTCTTAACGTCGTCGTCCGCATCCATGGATGAATCAACGTAACCACGACCTTCTAGAACGTAT
CGTTTTTCACAAACG
+
FFFFFFFFFFFFFFFFFFFFFFFFFFFFFFFFFFFFFFFFFFFFFFFFFFFFFFFFFFFFFFFFFFFFF
FFFFFFFFFFFFFFFFFFFFFFFFFFFFFFFFFFFFFFFFFFFFFFFFF:FFFFFFFFFFFFFFFFFFF
FFFFFFFFFFFF:F:
@A00721:190:HGJW7DSXY:4:2611:1913:22842 2:N:0:TCTGCAAG+GCTGCACT
CTTAAAAACTGTGAGTCCACTAAGTTTCTTCCTCACAGAAATCGGTCTAGACCTCATATAACAGAAGT
AAGCGTTCTTAACGTCGTCGTCCGCATCCATGGATGAATCAACGTAACCACGACCTTCTAGAACGTAT
CGTTTTTCACAAACG
+
FFFFFFFF:FFFFFFFFFFFFFFFFFFFFFFFFF:FFFFFFFFFF:FFF:FFFFFFFFFFFFFFFFFF
FFF:FFFFFFFFFFFFFFFFFFFFFFFFFFFFFFFFFFFFFFFFFFFFFFFFFFFF:FF,FFFFFFFFF
FFFFFFFFFFFFFFF
@A00721:190:HGJW7DSXY:4:2615:23104:3709 2:N:0:TCTGCAAG+GCTGCACT
CGTTTGTGAAAAACGATACGTTCTAGAAGGTCGTGGTTACGTTGATTCATCCATGGATGCGGACGACG
ACGTTAAGAACGCTTACTTCTGTTATATGAGGTCTAGACCGATTTCTGTGAGGAAGAAACTTATTGGA
CTCACAGTTTTTAAG
+
FFF:F:FFFFFFF:FFFFFFFFFFFFFF:F:,F,F:FFF::FFFFF:FFF::FF:FFFFFF:FFFFF:
FFFFFFF::F:F:FFF:F,,FFFFF:FFFFF:FFFF,F,F:FFFFFFFFFF:,F:F:FFFFFF,FFFF
FFF:FFFFFFFFF,:
@A00721:190:HGJW7DSXY:4:2674:15429:34444 2:N:0:TCTGCAAG+GCTGCACT
CTTAAAAACTGTGAGTCCACTAAGTTTCTTCCTCACAGAAATCGGTCTAGACCTCATATAACAGAAGT
AAGCGTTCTTAACGTCGTCGTCCGCATCCATGGATGAATCAACGTAACCACGACCTTCTAGAACGTAT
CGTTTTTCACAAACG
+
FFFFFFFFFFFFFFFFFFFF:FFFFFFFFFFFFFFFFFFFFFFFFFFF:FFFFFFFFFFFFFFFFFFFF
FFFFFFFFFFFFFFFFFFFFFFFFFFFFFFFFFFFFFFFFFFFFFFFFFFFFFFFFFFFFFFFFFFFFF
FFFFFFFFFFFFFFF
@A00721:190:HGJW7DSXY:4:1367:32452:31015 2:N:0:TCTGCAAG+GCTGCACT
GTTTGTGAAAAACGATACGTTCTAGAAGGTCGTGGTTACGTTGATTCATCCATGGATGCGGACGACGA
CGTTAAGAACGCTTACTTCTGTTATATGAGGTCTAGACCGATTTCTGTGAGGAAGAAACTTAGTGGAC
TCACAGTTTTTAAG
+
FFFFFFFFFFFFFFFFFFFFFFFFFFFFFFFFFFFFFFFFFFFFFFFFFFFFFFFFFFFFFFFFFFFFF
FFFFFFFFFFF:FFFFFFFFFFFFFFFFFFFFFFFFFFFFFFFFFFFFFFFFFFFFFFFFFFFFFFFFF
FFFFFFFFFFFFFF
@A00721:190:HGJW7DSXY:4:2561:5394:27774 2:N:0:TCTGCAAG+GCTGCACT
CTTAAAAACTGTGAGTCCACTAAGTTTCTTCCTCACAGAAATCGGTCTAGACCTCATATAACAGAAGT
```

```
AAGCGTTCTTAACGTCGTCGTCCGCATCCATGGATGAATCAACGTAACCACGACCTTCTAGAACGTAT
CGTTTTTCACAAAC
+
FFFFFFFFFFFFFFFFFFFFFFFFFFFFFFFFFFFFFFFFFFFFFFFFFFFFFFFFFFFFFFFFFFFF
FFFFFFFFFFFFFFFFFFFFFFFFFFFFFFFFFFFFFFFFFFFFFFFFFFFFFFFFFFFFFFFFFFFF
FFFFFFFFFFFF:F
@A00721:190:HGJW7DSXY:4:2160:20952:21371 2:N:0:TCTGCAAG+GCTGCACT
TGTCCTTAAAAACTGTGAGTCCACTAAGTTTCTTCCTCACAGAAATCGGTCTAGACCTCATATAACAG
AAGTAAGCGTTCTTAACGTCGTCGTCCGCATCCATGGATGAATCAACGTAACCACGACCTTCTAGAAC
GTATCGTTTTTCACA
+
FFFFFF:FFFF:FFFFFFFFFFFFFFFFF:FFFFFFFFFF:FFFFFFFFFFFFFFFFF:FFFFFFFFF
:FFF:FFFFFFFFFFFFFFFFFFFFFFFFF,FFFFF:FFFFFFFFFFFFFFFFFFFFFFFF:FFFFFF
F:FFFFFF,FFF:FF
@A00721:190:HGJW7DSXY:4:2328:4182:5979 2:N:0:TCTGCAAG+GCTGCACT
TGTGAAAAACGAAACGTTCTAGAAGGTCGTGGTAACGTTGATTCATCCATGGATGCCGACGACGACGT
TAAGAACGCTTACATCTGTTATATGAGGTCTAGACCGATTTCTGTGAGGAAGAAACTAAGAGCACTCA
CAGTTTTTAAG
+
:,FF:FFFF:FFFFF,F,:FF:FFFFFF,FF,:,FFF::FFFFFF::FFF::FFFF,:::FF:FFFFF
FFF,F,,:FF:F,,,FFF,:FF:FFF,F,FFFFFF,F:,,:F:FF,F::FF,FFFFF,FF,:,FF,:F
FF::FFFFFFF
@A00721:190:HGJW7DSXY:4:2328:3721:6339 2:N:0:TCTGCAAG+GCTGCACT
GAAAAACAATACGTACTAGAAGGTCGTGCATACGTAGAATCAACCATCGATGCTGACGACGAAGATAA
GAACGCTTACAACTGTTATATGAGGTCTAGACCGATTTCAGAGAGGAATAAAATTAGAGGACTAAAAG
ATATAAA
+
::F:FF,,,FFF,F,:FFF:FFFF,,F:,,:FFF,:FFFF,F,,:FF::FF,F:FF:FF:FF,:,FF:
F:FF,,,,F:F,:,,,F::F,,:F:F:FF,F:,,F,F:::FF:::FF,F::,F,F,FF,F::FF,F,
F,,F:FF
@A00721:190:HGJW7DSXY:4:1331:22046:6355 2:N:0:TCTGCAAG+GCTGCACT
CTCTACACTGCCACCAGTGGTGTCCTTAAAAACTGTGAGTCCACTAAGTTTCTTCCTCACAGAAATCG
GTCTAGACCTCATATAACAGAAGTAAGCGTTCTTAACGTCGTCGTCCGCATCCATGGATGAATCAACG
TAACCACGACCTTCT
+
FFFFFFFFFFFFFFFFFFFFFFFFFFFFFFFFFFFFFFFFFFFFFFFFFFFFFFFFFFFFFFFFFF:F
FFFFFFFFFFFFFFFFFFFFFFFFFFFFFFFFFFFFFFFFFFFFFFFFFFFFFFFFFFFFFFFFFFFF
FFFFFFFFFFFFFFF
@A00721:190:HGJW7DSXY:4:1369:20012:14137 2:N:0:TCTGCAAG+GCTGCACT
CTCTACACTGCCACCAGTGGTGTCCTTAAAAACTGTGAGTCCACTAAGTTTCTTCCTCACAGAAATCG
GTCTAGACCTCATATAACAGAAGTAAGCGTTCTTAACGTCGTCGTCCGCATCCATGGATGAATCAACG
TAACCACGACCTTCT
+
FFFFFFFFFFFFFFFFFFFFFFFFFFFFFFFFFFFFFFFFFFFFFFFFFFFFFFFFFFFFFFFFFFFF
FFFFFFFFFFFFFFFFFFFFFFFFFFFFFFFFFFF,FFFFFFFFFFFFFFFFFFFFFFFFFF,FFFFF
FFFFFFFFFFFFFFF
@A00721:190:HGJW7DSXY:4:1408:13548:19132 2:N:0:TCTGCAAG+GCTGCACT
CTCTACACTGCCACCAGTGGTGTCCTTAAAAACTGTGAGTCCACTAAGTTTCTGCCTCACAGAAATCG
GTCTAGACCTCATATAACAGAAGTAAGCGTTCTTAACGTCGTCGTCCGCATCCATGGATGAATCAACG
TAACCACGACCTTCT
+
FFFFFFFFFFFFFFFFFFFFFFFFFFFFFFFFFFFFFFFFFFFFFFFFFFFFF,FFFFFFFFFFFFFF
FFFFFFFFFFFFFFFFFFFFFFFFFFFFFFFFFFFFFFFFFFFFFFFFFFFFFFFFFFFFFFFFFFFF
FFFFFFFFFFFFFFF
```

```
@A00721:190:HGJW7DSXY:4:1451:5231:21449 2:N:0:TCTGCAAG+GCTGCACT
CTCTACACTGCCACCAGTGGTGTCCTTAAAAACTGTGAGTCCACTAAGTTTCTTCCTCACAGAAATCG
GTCTAGACCTCATATAACAGAAGTAAGCGTTCTTAACGTCGTCGTCCGCATCCATGGATGAATCAACG
TAACCACGACCTTCT
+
FFFFFFFFFFFFFFFFFFFFFF:FFFFFFFFFFFFFFFFFFFFFFFFFFFFFFFFFFFFFFFFFFFFF
FFF:FFFFFFFFFFFFFFFFFFFFFFFFFFFFFFFFFFFFFFFFFFFFFFFFFFFFFFFFFFFFFFFF
FFFFFFFFFFF::FF
@A00721:190:HGJW7DSXY:4:2302:24297:16329 2:N:0:TCTGCAAG+GCTGCACT
CTCTACACTGCCACCAGTGGTGTCCTTAAAAACTGTGAGTCCACTAAGTTTCTTCCTCACAGAAATCG
GTCTAGACCTCATATAACAGAAGTAAGCGTTCTTAACGTCGTCGTCCGCATCCATGGATGAATCAACG
TAACCACGACCTTCT
+
FFFFFFFFFFFFFFFFFFFFFFFFFFFFFFFFFFFFFFFFFFFFFFFFFFFFFFFFFFFFFFFFFFFF
FFFFFFFFFFFFFFFFFFFFFFFFFFFFFFFFFFFFFFFFFFFFFFFFFFFFFFFFFFFFFFFFFFFF
FFFFFFFFFFFFFFF
@A00721:190:HGJW7DSXY:4:2324:15609:2127 2:N:0:TCTGCAAG+GCTGCACT
CTCTACACTGCCACCAGTGGTGTCCTTAAAAACTGTGAGTCCACTAAGTTTCTTCCTCACAGAAATCG
GTCTAGACCTCATATAACAGAAGTAAGCGTTCTTAACGTCGTCGTCCGCATCCATGGATGAATCAACG
TAACCACGACCTTCT
+
FFFFFFFFFFFFFFFFFFFFFFFFFFFFFFFFFFFFFFFFFFFFFFFFFFFFFFFFFFFFFFFFFFFF
FFFFFFFFFFFFFFFFFFFFFFFFFFFFFFFFFFFFFFFFFFFFFFFFFFFFFFFFFF:FFFFFFFFF
FFFFFFFFFFFF:FF
@A00721:190:HGJW7DSXY:4:2468:14769:5243 2:N:0:TCTGCAAG+GCTGCACT
CTCTACACTGCCACCAGTGGTGTCCTTAAAAACTGTGAGTCCACTAAGTTTCTTCCTCACAGAAATCG
GTCTAGACCTCATATAACAGAAGTAAGCGTTCTTAACGTCGTCGTCCGCATCCATGGATGAATCAACG
TAACCACGACCTTCT
+
FFFFFFFFFFFFFFFFFFFFFFFFFFFFFFFFFFFFFFFFFFFFFFFFFFFFFFFFFF:FFFFFFFFF
FFFFFFFFFFFF:FFFFFFFFFFFFFFFFFFFFFFFFFFFFFFFFFFFFFFFFFFFFFFFFFFFFFF
FFFFFFFFFFFFFFF
@A00721:190:HGJW7DSXY:4:2666:24686:5635 2:N:0:TCTGCAAG+GCTGCACT
CTCTACACTGCCACCAGTGGTGTCCTTAAAAACTGTGAGTCCACTAAGTTTCTTCCTCACAGAAATCG
GTCTAGACCTCATATAACAGAAGTAAGCGTTCTTAACGTCGTCGTCCGCATCCATGGATGAATCAACG
TAACCACGACCTTCT
+
FFFFFFFFFFFFFFFFFFFFFFFFFFFFFFFFFFFFFFFFFFFFFFFFFFFFFFFFFFFFFFFFFFFF
FFFFFFFFFFFFFFFFFFFFFFFFFFFFFFFFFFFFFFFFFFFFFFFFFFFFFFFFFFFFFFFFFFFF
FFFFFFFFFFFFFFF
@A00721:190:HGJW7DSXY:4:1119:25174:21919 2:N:0:TCTGCAAG+GCTGCACT
CCTCTACACTGCCACCAGTGGTGTCCTTAAAAACTGTGAGTCCACTAAGTTTCTTCCTCACAGAAATC
GGTCTAGACCTCATATAACAGAAGTAAGCGTTCTTAACGTCGTCGTCCGCATCCATGGATGAATCAAC
GTAACCACGACCTTC
+
FFFFFFFFFFFFFFFFFFFFFFFFFFF:FFFFFFFFFFFFFFFFFFFFFFFFFFFFFFFFFFFFFFFF
FFFFFFFF:FFFF:FFFFFFFFFFFFFFFF:FFFF:FFFFFFFFFFFFFFFFFFFFFFF:FFFFFFFF
FFFFFFFFFFFFFFF
@A00721:190:HGJW7DSXY:4:1413:4354:11663 2:N:0:TCTGCAAG+GCTGCACT
CCTCTACACTGCCACCAGTGGTGTCCTTAAAAACTGTGAGTCCACTAAGTTTCTTCCTCACAGAAATC
GGTCTAGACCTCATATAACAGAAGTAAGCGTTCTTAACGTCGTCGTCCGCATCCATGGATGAATCAAC
GTAACCACGACCTTC
+
FFFFFFFFFFFFFFFFFFFFFFFFFFFFFFFFFFFFFFFFFFFFFFFFFFFFFFFFFFFFFFFFFFFF
```

FFFFFFFFFF,FFFFFFFFFFFFFFF:FFFFFFFFFFFFFFFFFFFFFFFFFFFFFFFFFFFFFFFFF
FFFFFFFFFFFFFFF
@A00721:190:HGJW7DSXY:4:2112:25771:6261 2:N:0:TCTGCAAG+GCTGCACT
CCTCTACACTGCCACCAGTGGTGTCCTTAAAAACTGTGAGTCCACTAAGTTTCTTCCTCACAGAAATC
GGTCTAGACCTCATATAACAGAAGTAAGCGTTCTTAACGTCGTCGTCCGCATCCATGGATGAATCAAC
GTAACCACGACCTTC
+
FFFFFFFFFFFFFFFFFFFFFFFFFFFFFFFFFFFFFFFF:FFFFFFFFFFFFFFFFFFFFFFFFFFF
FFFFFFFFFFFFFFFFFFFFFFFFFFFFFFFFFFFFFFFFFFFFFFFFFFFFFFFFFFFFFFFFFFFF
FFFFFFFFFFFFFFF
@A00721:190:HGJW7DSXY:4:2452:20500:18302 2:N:0:TCTGCAAG+GCTGCACT
CCTCTACACTGCCACCAGTGGTGTCCTTAAAAACTGTGAGTCCACTAAGTTTCTTCCTCACAGAAATC
GGTCTAGACCTCATATAACAGAAGTAAGCGTTCTTAACGTCGTCGTCCGCATCCATGGATGAATCAAC
GTAACCACGACCTTC
+
FFFFFFFFFFFFFFFFFFFFFFFFFFFFFFFFFFFFFFFFFFFFFFFFF:FFFFFFFFF:FFFFFFFF
FFFFFF:FFFFFFFFFFFFFFFFFF:FFFFFFFFFFFFFFFF,FFFFFFFFFFFFFFFFFFFFFFFFF
FFFFFFFFFFFFFFF
@A00721:190:HGJW7DSXY:4:2572:30942:8422 2:N:0:TCTGCAAG+GCTGCACT
CCTCTACACTGCCACCAGTGGTGTCCTTAAAAACTGTGAGTCCACTAAGTTTCTTCCTCACAGAAATC
GGTCTAGACCTCATATAACAGAAGTAAGCGTTCTTAACGTCGTCGTCCGCATCCATGGATGAATCAAC
GTAACCACGACCTTC
+
FFFFFFFFFFFFFFFFFFFFFFFFFFF:FFFFFFFFFFFFFFFFFFFFFFFFFFFFFFFFFFFFFFFF
FFFFFFFFFFFFFFFFFFFFFFFFFFFFFFFFFFFFFFFFFFFFFFFFFFFFFFFFFFFFFFFFF::F
FFFFFFFFFFFFFFF
@A00721:190:HGJW7DSXY:4:2606:11957:15718 2:N:0:TCTGCAAG+GCTGCACT
CCTCTACACTGCCACCAGTGGTGTCCTTAAAAACTGTGAGTCCACTAAGTTTCTTCCTCACAGAAATC
GGTCTAGACCTCATATAACAGAAGTAAGCGTTCTTAACGTCGTCGTCCGCATCCATGGATGAATCAAC
GTAACCACGACCTTC
+
FFFFFFFFFFFFFFFFFFFFFFFFFFFFFFFFFFFFFFFFFFFFFF,FFFFFFFFFFFFFFFFFFFFF
FFFFFFFFFFFFFFFFFFFFFFFFFFFFFFFFFFFFFFFFFFFFFFFFFFFFFFFFFFFFFFFFFFFF
FFFFFFFFFFFFFFF
@A00721:190:HGJW7DSXY:4:2632:7654:8829 2:N:0:TCTGCAAG+GCTGCACT
CCTCTACACTGCCACCAGTGGTGTCCTTAAAAACTGTGAGTCCACTAAGTTTCTTCCTCACAGAAATC
GGTCTAGACCTCATATAACAGAAGTAAGCGTTCTTAACGTCGTCGTCCGCATCCATGGATGAATCAAC
GTAACCACGACCTTC
+
FFFFFFFFFFFFFFFFFFFFFFFFFFFFFFFFFFFFFFFFFFFFFFFFFFFFFFFFFFFFFFFFFFFF
FFFFFFFFFFFFFFFFFFFFFFFFFFFF:FFFFFFFFFFFFFFFFFFFFFFFFFFFFFFFFFFFFFFF
FFFF:FFFFFFFFFF
@A00721:190:HGJW7DSXY:4:1417:31647:32440 2:N:0:TCTGCAAG+GCTGCACT
GTCCTCTACACTGCCACCAGTGGTGTCCTTAAAAACTGTGAGTCCACTAAGTTTCTTCCTCACAGAAA
TCGGTCTAGACCTCATATAACAGAAGTAAGCGTTCTTAACGTCGTCGTCCGCATCCATGGATGAATCA
ACGTAACCACGACCT
+
FFFFFFFFFFFFFFFFFFFFFFFFFFFFFFFFFFFFFFFFFFFFFFFFFFFFFFFFFFFFFFFFFFFF
FFFFFFFFFFFFFFFFFFFFFFFFFFFFFFFFFFFFFFFFFFFFFFFFFFFFFFFFF:FFFFFFF:FF
FFFFFFFFFFF:FFF
@A00721:190:HGJW7DSXY:4:1475:32045:31876 2:N:0:TCTGCAAG+GCTGCACT
GTCCTCTACACTGCCACCAGTGGTGTCCTTAAAAACTGTGAGTCCACTAAGTTTCTTCCTCACAGAAA
TCGGTCTAGACCTCATATAACAGAAGTAAGCGTTCTTAACGTCGTCGTCCGCATCCATGGATGAATCA
ACGTAACCACGACCT

```
+
FFFFFFFFFFFFFFFFFFFFFFFFFFFFFFFFFFFFFFFFFFFFFFFFFFFFFFFFFFFFFFFFFFFFFF
FFFFFFFFFFFFFFFFFFFFFFFFFFFFFFFFFFFFFFF,FFFFFFFFFFFFFFFFFFFFFFFFFF:FFF
FFFFFFFF,FFFFFF
@A00721:190:HGJW7DSXY:4:1571:22345:3709 2:N:0:TCTGCAAG+GCTGCACT
GTCCTCTACACTGCCACCAGTGGTGTCCTTAAAAACTGTGAGTCCACTAAGTTTCTTCCTCACAGAAA
TCGGTCTAGACCTCATATAACAGAAGTAAGCGTTCTTAACGTCGTCGTCCGCATCCATGGATGAATCA
ACGTAACCACGACCT
+
FFFFFFFFFFFFFFFFFFFFFFFFFFFFFFFFFFFFFFFFFFFFFFFFFFFFFFFFFFFFFFFFFFFFFF
FFFFFFFFFFFFFFFFFFFFFFFFFFFFFFFFFFFFFFFFFFFFFFFFFFFFFFFFFFF:FFFFFF:FFF
FFFFFFFFFFFFFFF
@A00721:190:HGJW7DSXY:4:2301:11207:5682 2:N:0:TCTGCAAG+GCTGCACT
GTCCTCTACACTGCCACCAGTGGTGTCCTTAAAAACTGTGAGTCCACTAAGTTTCTTCCTCACAGAAA
TCGGTCTAGACCTCATATAACAGAAGTAAGCGTTCTTAACGTCGTCGTCCGCATCCATGGATGAATCA
ACGTAACCACGACCT
+
FFFFFFFFFFFFFFFFFFFFFFFFFFFFFFFFFFFFFFFFFFFFFFFF:FFFFFFFFFFFFFFFFFFFFF
FFFFFFFFFFFFFFFFFFFFFFFFFFFFFFFFFFFFFFFFFFFFFFFFFFFFFFFFFFFFFFFFFFFFFF
FFFFFFFFFFFFFFF
@A00721:190:HGJW7DSXY:4:2443:28275:34867 2:N:0:TCTGCAAG+GCTGCACT
GTCCTCTACACTGCCACCAGTGGTGTCCTTAAAAACTGTGAGTCCACTAAGTTTCTTCCTCACAGAAA
TCGGTCTAGACCTCATATAACAGAAGTAAGCGTTCTTAACGTCGTCGTCCGCATCCATGGATGAATCA
ACGTAACCACGACCT
+
FFFFFFFFFFFFFFFFFFFFFFFFFFFFFFFFFFFFFFFFFFFFFFFFFFFFFFFFFFFFFFFFFFFFFF
FFFFFFFFFFFFFFFFFFFF:FFFFFFFFFFFFFFFFFFFFF:FFFFFFFFFFFFFFFFFFF:FFFFFFF
FF:FFFFFFFFFFFF
@A00721:190:HGJW7DSXY:4:2448:29785:1814 2:N:0:TCTGCAAG+GCTGCACT
GTCCTCTACACTGCCACCAGTGGTGTCCTTAAAAACTGTGAGTCCACTAAGTTTCTTCCTCACAGAAA
TCGGTCTAGACCTCATATAACAGAAGTAAGCGTTCTTAACGTCGTCGTCCGCATCCATGGATGAATCA
ACGTAACCACGACCT
+
:FFFFFFFFFFFFFFFFFFFFFFFFFFFFFFFFFFFFFFFFFFFFFFFFFFFFFFFFFFFFFFFFFFFFF
FFFFFFFFFFFFFFFFFFFFFFFFFFFFFFFFFFFFFFFFFFFFFFFFFFF:FFFFFFFFFFFFFFFFFF
FFFFFFFFFFFFFFF
@A00721:190:HGJW7DSXY:4:2578:6099:8046 2:N:0:TCTGCAAG+GCTGCACT
GTCCTCTACACTGCCACCAGTGGTGTCCTTAAAAACTGTGAGTCCACTAAGTTTCTTCCTCACAGAAA
TCGGTCTAGACCTCATATAACAGAAGTAAGCGTTCTTAACGTCGTCGTCCGCATCCATGGATGAATCA
ACGTAACCACGACCT
+
FFFFFFFFFFFFFFFFFFFFFFFFFFFFFFFFFFFFFFFF:FFFFFFFF,FFFFFFFFFF:FFFFFFFFF
FFFFFF,FFFFFFFFFFFFFF:FF:FF:FFFFFFFFFFF,FFFFFFFFFFFFFFFFFFFFFFFFFFFFFF
FFFFFFFFFFFFFFF
@A00721:190:HGJW7DSXY:4:2641:18249:16125 2:N:0:TCTGCAAG+GCTGCACT
GTCCTCTACACTGCCACCAGTGGTGTCCTTAAAAACTGTGAGTCCACTAAGTTTCTTCCTCACAGAAA
TCGGTCTAGACCTCATATAACAGAAGTAAGCGTTCTTAACGTCGTCGTCCGCATCCATGGATGAATCA
ACGTAACCACGACCT
+
FFFFFFFFFFFFFFFFFFFFFFFFFFFFFF,FFFFFFFFFFFFFFFFFFFFFFFFFFFFFFFFFFFFFFF
FFFFFFFFFFFF:FFFFFFFFFFFFFF:FFFFFFFFFFFFFFFFFFFFFFFFFFFFFFFFFFF:FF,FFF
FFFFFFFFFFFFFFF
@A00721:190:HGJW7DSXY:4:2675:19018:32080 2:N:0:TCTGCAAG+GCTGCACT
GTCCTCTACACTGCCACCAGTGGTGTCCTTAAAAACTGTGAGTCCACTAAGTTTCTTCCTCACAGAAA
```

```
TCGGTCTAGACCTCATATAACAGAAGTAAGCGTTCTTAACGTCGTCGTCCGCATCCATGGATGAATCA
ACGTAACCACGACCT
+
FFFFFFFFFFFFFFFFFFFFFFFFFFFFFFFFFFFFFFFFFFFFFFFFFFFFFFFFFFFFFFFFFF:FFF
FFFFFFFFFFFFFFFFFFFFFFFFFFFFFFFFFFFFFFFFFFFFFFFFFFFFFFFFFFFFFFFFFFFFFF
FFFFFFFFFFFFFFF
@A00721:190:HGJW7DSXY:4:1139:9561:35837 2:N:0:TCTGCAAG+GCTGCACT
TACGTTGATTCATCCATGGATGCGGACGACGACGTTAAGAACGCTTACTTCTGTTATATGAGGTCTAG
ACCGATTTCTGTGAGGAAGAAACTTAGTGGACTCACAGTTTTTAAGGACACCACTGGTGGCAGTGTAG
AGGACATGATGAAAG
+
FFFFFFFFFFFFF::FFFFFFFFFFFFFFFFFFFFFFFFFFFFFFFFF:FFFFF:FF:FFFFFFFFFFFF
FFFFFFFFFFFFFFFFFFFFFFFFFFFFFFFFFFFFFFFFFFFFFFFFFFFFFFFFFFFFFFFFFFFFFF
FFFFFFFFFFFFFFF
@A00721:190:HGJW7DSXY:4:1276:11478:23563 2:N:0:TCTGCAAG+GCTGCACT
TACGTTGATTCATCCATGGATGCGGACGACGACGTTAAGAACGCTTACTTCTGTTATATGAGGTCTAG
ACCGATTTCTGTGAGGAAGAAACTTAGTGGACTCACAGTTTTTAAGGACACCACTGGTGGCAGTGTAG
AGGACATGATGAAAG
+
FFFFFFFFFFFFF:FFFFFFFFFFFFFFFFFFFFFFFFFFFFFFFFFFFFFFFFFFFFFFFFFFFFFFFF
FFFFFFFFFFFFFFFFFFFFFFFFFFFFFFFFFFFFFFFFFFFFFFFFFF,FFFFFFFFFFFFFFFFFFF
FFFFFFFFFFFF:FF
@A00721:190:HGJW7DSXY:4:1318:3893:6637 2:N:0:TCTGCAAG+GCTGCACT
TACGTTGATTCATCCATGGATGCGGACGACGACGTTAAGAACGCTTACTTCTGTTATATGAGGTCTAG
ACCGATTTCTGTGAGGAAGAAACTTAGTGGACTCACAGTTTTTAAGGACACCACTGGTGGCAGTGTAG
AGGACATGATGAAAG
+
FFFFFFFFFFFFF:FFFFFFFFFFFFFFFFF:FFFFFFFFFFFFFFFFFFFF:FFFFFFFFFFFFFFFFF
FFFF:FFFFFFFFFFFFFFFFFFFFFFFFFFFFFFFFFFFFFFF:FFFFFFFFFFFFFFF:FFF:FFFFF
FFFFFFFFFFFFFFF
@A00721:190:HGJW7DSXY:4:1462:18195:26553 2:N:0:TCTGCAAG+GCTGCACT
TACGTTGATTCATCCATGGATGCGGACGACGACGTTAAGAACGCTTACTTCTGTTATATGAGGTCTAG
ACCGATTTCTGTGAGGAAGAAACTTAGTGGACTCACAGTTTTTAAGGACACCACTGGTGGCAGTGTAG
AGGACATGATGAAAG
+
FFFF:FFFFFFFFFFFFFFFFFFFFFFFFFFFFFFFFFFF:FFFFFFFFFFF,FF:FFFFFFFFFFFFFF
FFFFFFFFFFFFFFFFFFFFFFFFFFFFFFF,FFFFFFFF,:,FFFFFFFFFFFFFFFFFFFFFF:F,:F
FFFFFFFFFFFFFFF
@A00721:190:HGJW7DSXY:4:1524:19560:19241 2:N:0:TCTGCAAG+GCTGCACT
TACGTTGATTCATCCATGGATGCGGACGACGACGTTAAGAACGCTTACTTCTGTTATATGAGGTCTAG
ACCGATTTCTGTGAGGAAGAAACTTAGTGGACTCACAGTTTTTAAGGACACCACTGGTGGCAGTGTAG
AGGACATGATGAAAG
+
FFFFFFFFFFFFF:FFFFFFFFFFFFFFFFFFFFFFFFFFFFFFFFFFFFFFFFFFFFFFFFFFFFFFFF
FFFFFFFFFFFFFFFFFFFFFFFFFFFFFFFFFFFFFFFFFFFFFFFFFFFFFFFFFFFFF:FFFFFFFF
FFFFFFFFFFFFFFF
@A00721:190:HGJW7DSXY:4:1624:7654:33599 2:N:0:TCTGCAAG+GCTGCACT
TACGTTGATTCATCCATGGATGCGGACGACGACGTTAAGAACGCTTACTTCTGTTATATGAGGTCTAG
ACCGATTTCTGTGAGGAAGAAACTTAGTGGACTCACAGTTTTTAAGGACACCACTGGTGGCAGTGTAG
AGGACATGATGAAAG
+
FFFFFFFFFFFFFF:FFFFFFFFFFFFFFFFFFFFFFFFFFFFFFF:FFFFFFFFFFFFFFFFFFFFFFF
FFFFF:FFFFFFFFFFFFFFFFFFFFFFFFFFFFFFFFFFFFFF:FFFFFFFFFFFFFFFFFFFFFFFFF
FFFFFFFFFFFFFFF
```

@A00721:190:HGJW7DSXY:4:1656:13114:16376 2:N:0:TCTGCAAG+GCTGCACT
TACGTTGATTCATCCATGGATGCGGACGACGACGTTAAGAACGCTTACTTCTGTTATATGAGGTCTAG
ACCGATTTCTGTGAGGAAGAAACTTAGTGGACTCACAGTTTTTAAGGACACCACTGGTGGCAGTGTAG
AGGACATGATGAAAG
+
FFFFFFFFFF:FFFFFFFFFFFFFFFFFFFFFFFFFFFFFFFFFFFFFFFFFFFFFFFFFFFFFFFFF
FFFFFFFFFFFFFFFFFFF:FFFFFFFFFFFFFFFFFFFFFFFFFFFFFFFFFFFFFFFFFFFFFFFF
FFFFFFFFFFFFFFF
@A00721:190:HGJW7DSXY:4:1549:14100:9972 2:N:0:TCTGCAAG+GCTGCACT
ACGTTGATTCATCCATGGATGCGGACGACGACGTTAAGAACGCTTACTTCTGTTATATGAGGTCTAGA
CCGATTTCTGTGAGGAAGAAACTTAGTGGACTCACAGTTTTTAAGGACACCACTGGTGGCAGTGTAGA
GGACATGATGAAAG
+
FF:F:FFFFFFFF:FFFFFFFFFFFFFFFFFFF:FFFFFFF:,F:FF,FFFFFFFFFFF:FFFF,FFF
FFFF:FFFFFFFFFFFFFF:FFFFF:FF:FFFFFFFFFFF:FFFFFFFFFFFFFFFFFFFFF:FFFF:
FFFFF,FFFFFFFF
@A00721:190:HGJW7DSXY:4:1103:4056:16094 2:N:0:TCTGCAAG+GCTGCACT
GCTCCTTTCATCATGTCCTCTACACTGCCACCAGTGGTGTCCTTAAAAACTGTGAGTCCACTAAGTTT
CTTCCTCACAGAAATCGGTCTAGACCTCATATAACAGAAGTAAGCGTTCTTAACGTCGTCGTCCGCAT
CCATGGATGAATCAA
+
FFFFFFFFFFFFFFFFFFFFFFFFFFFFFFFFFFFFFFFFFFFFFFFFFFFFFFFFFFFFFFFFFFFF
FFFFFFFFFFFFFFFFFFFFFFFFFF:FFFFFFFF:FFFFFFFFFFFFFFFFFFFFFFFFFFFFFFFF
FFF:FFFFFFFFFFF
@A00721:190:HGJW7DSXY:4:1250:21043:1830 2:N:0:TCTGCAAG+GCTGCACT
CCATGGATGCGGACGACGACGTTAAGAACGCTTACTTCTGTTATATGAGGTCTAGACCGATTTCTGTG
AGGAAGAAACTTAGTGGACTCACAGTTTTTAAGGACACCACTGGTGGCAGTGTAGAGGACATGATGAA
AGGAGCTTATTATTT
+
FFFFFFFFFFFFFFFFFFFFFFFFFFF:FFF:FF:,FF:F:,FFF:FFFFFFFFF:FFFFFFFFFF,F
FFFFFFFFFFFFFFFFFFFFFFFFFFFFFFFFFFFFFFFFF:FFFFFFFFFFF:FFFFFFFFF:,FF:
FFFFFFFFFFFFFFF
@A00721:190:HGJW7DSXY:4:1251:9209:35477 2:N:0:TCTGCAAG+GCTGCACT
CCATGGATGCGGACGACGACGTTAAGAACGCTTACTTCTGTTATATGAGGTCTCGACCGATTTCTGTG
AGGAAGAAACTTAGTGGACTCACAGTTTTTAAGGACACCACTGGTGGCAGTGTAGAGGACATGATGAA
AGGAGCTTATTATTT
+
FF:FFFFFFFFFFFFFFFFFFFFFFFFFFFFFFFF:FF,FF:FFF:FFFFFFF,:FFFFFFFFFFFFF
FFFFFFFFFFFFFFF:FFFFFFFFFFF:FFFFFFFF:FFFF,FFFFFFFF,FF,FFFFFFFFFFFFFF
:FFFFF:FFFF,FFF
@A00721:190:HGJW7DSXY:4:1262:3784:30436 2:N:0:TCTGCAAG+GCTGCACT
CCATGGATGCGGACGACGACGTTAAGAACGCTTACTTCTGTTATATGAGGTCTAGACCGATTTCTGTG
AGGAAGAAACTTAGTGGACTCACAGTTTTTAAGGACACCACTGGTGGCAGTGTAGAGGACATGATGAA
AGGAGCTTATTATTT
+
FFFFFFFFFFFFFFFFFFFFFFFFFFFFFFFFFFFFFFFFFFFF:FFFFFFFFFFFFFFFFFFFFFFF
FFFFFFFFFFFFFFFFFFFFFFFF:FFFFFFFFFFFFFFFFFFFFF:FFFFFFFFFFFFFFFFFFFFF
FFFFFFFFFFFFFFF
@A00721:190:HGJW7DSXY:4:1347:16640:19288 2:N:0:TCTGCAAG+GCTGCACT
CCATGGATGCGGACGACGACGTTAAGAACGCTTACTTCTGTTATATGAGGTCTAGACCGATTTCTGTG
AGGAAGAAACTTAGTGGACTCACAGTTTTTAAGGACACCACTGGTGGCAGTGTAGAGGACATGATGAA
AGGAGCTTATTATTT
+
FFFFFFFFFFFFFFFFFFFFFFFFFFFFFFF:FF,:FFFFFFFFF:FFFFFFFFFFFFFFFFFFFFFF

```
FFFFFFFFFFFFFFFFFFFFFFFFFFFFFFFFFFFFF:FFFFFFFFFFFFFFFFFFFFFFFFFFFF:,
FFFFFFFFFFF:F:F
@A00721:190:HGJW7DSXY:4:2307:19958:8343 2:N:0:TCTGCAAG+GCTGCACT
CCATGGATGCGGACGACGACGTTAAGAACGCTTACTTCTGTTATATGAGGTCTAGACCGATTTCTGTG
AGGAAGAAACTTAGTGGACTCACAGTTTTTAAGGACACCACTGGTGGCAGTGTAGAGGACATGATGAA
AGGAGCTTATTATTT
+
FF:FFFF:FFFFFFF:FFFFFFF,FFFFFFFF,:,FFFFF:FFFFFFF:FF:,FFF,FFFFFFFFFFF
:FFFFF,:FFFFFF:FFFFFFFFFFFF:FF:FFFFFFF,FF,FFFFFFFFFFFFFFFFFFFFFFFFFF
FFFFFFF:FFFFF,F
@A00721:190:HGJW7DSXY:4:2326:19045:34601 2:N:0:TCTGCAAG+GCTGCACT
CCATGGATGCGGACGACGACGTTAAGAACGCTTACTTCTGTTATATGAGGTCTAGACCGATTTCTGTG
AGGAAGAAACTTAGTGGACTCACAGTTTTTAAGGACACCACTGGTGGCAGTGTAGAGGACATGATGAA
AGGAGCTTATTATTT
+
FFFFFFFFFFFFFFFFFFFFFFFFFFFFFFFFFFF:FFFFFFFFFFFFFFFFFFFFFFFFFFFFFFFF
FFFFFFFFFFFFFFFFFFFFFFFFFFFFFFFFFFFFFFFFFFFFFFFFFFFFFFFFFFFFFFFFFFFF
FFFFFFFFFFFFFFF
@A00721:190:HGJW7DSXY:4:2361:22968:14967 2:N:0:TCTGCAAG+GCTGCACT
CCATGGATGCGGACGACGACGTTAAGAACGCTTACTTCTGTTATATGAGGTCTAGACCGATTTCTGTG
AGGAAGAAACTTAGTGGACTCACAGTTTTTAAGGACACCACTGGTGGCAGTGTAGAGGACATGATGAA
AGGAGCTTATTATTT
+
FFFFFFFFFFFFFFFFFFFFFFFFFFFFFFFFFFF::FFFFFFFFFFFFFFFFFFFFFFFFFFFFFFF
FFFFFFFFFFFFFFFFFFFFFFFFFFFFFFFFFFFFFFFFFFFFFFFFFFFFF,:FFFFFFFFFFFFF
FFFFFFFFFFFFFFF
@A00721:190:HGJW7DSXY:4:1611:27254:11334 2:N:0:TCTGCAAG+GCTGCACT
CAAAAACTAAATAATAAGCTCCTTTCATCATGTCCTCTACACTGCCACCAGTGGTGTCCTTAAAAACT
GTGAGTCCACTAAGTTTCTTCCTCACAGAAATCGGTCTAGACCTCATATAACAGAAGTAAGCGTTCTT
AACGTCGTCGTCCGC
+
FFFFF:FFFFFFFFFFFFFFFFFFFFFFFFFFFFFFFFFFFFFFFFFFFFF:FFFFFFFFFFFFFFFF
FFFFFFFFFFFFFFF:FFFFFFFFFFFFFFFFFFFFFFFFFFFFFFF:FFFFFFFFFF,:FFFF:FFF
FFFFFFFFFF:FFFF
@A00721:190:HGJW7DSXY:4:1612:29333:7200 2:N:0:TCTGCAAG+GCTGCACT
CAAAAACTAAATAATAAGCTCCTTTCATCATGTCCTCTACACTGCCACCAGTGGTGTCCTTAAAAACT
GTGAGTCCACTAAGTTTCTTCCTCACAGAAATCGGTCTAGACCTCATATAACAGAAGTAAGCGTTCTT
AACGTCGTCGTCCGC
+
FFFFFFFFFFFFF:FFFFFFFFFFFFFFFFFFFFFFFFFFFFFFFFFFFFFFFFFFFFFFFFFFFFFF
FFFFFFFFFFFFFFFFFFFFFFFFFFFFFFFFFFFFFFFFFFFFFFFFFFFFFFFFFFF,FFFFFF:F
FFFFFFFFFFFFFFF
@A00721:190:HGJW7DSXY:4:2202:18918:5697 2:N:0:TCTGCAAG+GCTGCACT
CAAAAACTAAATAATAAGCTCCTTTCATCATGTCCTCTACACTGCCACCAGTGGTGTCCTTAAAAACT
GTGAGTCCACTAAGTTTCTTCCTCACAGAAATCGGTCTAGACCTCATATAACAGAAGTAAGCGTTCTT
AACGTCGTCGTCCGC
+
FFFFFFFFFFFFFFFFFFFFFFFFFFFFFFFFFFFFFFFFFFFFFFFFFFFFFFFFFFFFFFFFFFFF
FFFFFFFFFFF:FFFFFFFFFFFFFFFFFFFF,FFFFFFFFFFFFFFFFFFFFFFFFFFFFFF:FFFF
F:FFFFFFFFFFFFF
@A00721:190:HGJW7DSXY:4:2230:22905:29324 2:N:0:TCTGCAAG+GCTGCACT
CAAAAACTAAATAATAAGCTCCTTTCATCATGTCCTCTACACTGCCACCAGTGGTGTCCTTAAAAACT
GTGAGTCCACTAAGTTTCTTCCTCACAGAAATCGGTCTAGACCTCATATAACAGAAGTAAGCGTTCTT
AACGTCGTCGTCCGC
```

```
+
FFFF:FFFFFFFFFFFFFFFF:FFFFFFFFFFFFFFFFFFF:F:FFFFFFFFF:FFFFFFFFFFFFFF
:F:FFFF:FFFFFF,FFFF:,FFFFFF:FFFFFFFF,FF,F,FFFFFFFFFFF,FFFF:F,FFFFFFF
FFF:FFFFFFFFFF:
@A00721:190:HGJW7DSXY:4:2249:6515:18944 2:N:0:TCTGCAAG+GCTGCACT
CAAAAACTAAATAATAAGCTCCTTTCATCATGTCCTCTACACTGCCACCAGTGGTGTCCTTAAAAACT
GTGAGTCCACTAAGTTTCTTCCTCACAGAAATCGGTCTAGACCTCATATAACAGAAGTAAGCGTTCTT
AACGTCGTCGTCCGC
+
FFFFFFFFFFFFFFFFFFFFFFFFFFFFFFFFFFFFFFFFFFFFFFFFFFFFFFFFFFFFFFFFFFFF
FFFFFFFFFFFFFFFFFFFFFFFFFFFFFFFFFFFFFFFFFFFFFFFFFFFFFFFFFFF:FFFFFFFF
FFFFFFFFFFFFFFF
@A00721:190:HGJW7DSXY:4:2301:14633:11898 2:N:0:TCTGCAAG+GCTGCACT
CAAAAACTAAATAATAAGCTCCTTTCATCATGTCCTCTACACTGCCACCAGTGGTGTCCTTAAAAACT
GTGAGTCCACTAAGTTTCTTCCTCACAGAAATCGGTCTAGACCTCATATAACAGAAGTAAGCGTTCTT
AACGTCGTCGTCCGC
+
FFFFFFFFFFFFFFFFFFFFFFFFFFFFFFFFFFFFFFFFFFFFF:FFFFF,FFFFFFFFFFFFFFFFFF
FFF:FFFFFF:FFFFFFFFFFFFFFFFFFFFFF:FFFFFFFFFF:FF:FFFF:FFFFF:FFFFFFFFFF
FFFFFFFFFFFFFFF
@A00721:190:HGJW7DSXY:4:2520:4743:1752 2:N:0:TCTGCAAG+GCTGCACT
CAAAAACTAAATAATAAGCTCCTTTCATCATGTCCTCTACACTGCCACCAGTGGTGTCCATAAAAACT
GTGAGTCCACTAAGTTTCTACCTCACAGAAATCGGTCTAGACCTCATCTAACAGAAGTAAGCGTTCTT
AACGTCGTCGTCCGC
+
FFF,FFF,F:FFF,FFFFF:F,FFFFF:FFF:FF:FF,FF::::FF:F:F,FF,F,FFF,,FF:FFF:
,:,F,FFFF:F,FFF:F:F,F::FFFFFF,FFFFFFF:FFFFFF:F,,:FF,::FFF,FF,FF:FFF,
,FFFFFF,F:F:FFF
@A00721:190:HGJW7DSXY:4:1653:32524:11506 2:N:0:TCTGCAAG+GCTGCACT
GTCGCAAAAACTAAATAATAAGCTCCTTTCATCATGTCCTCTACACTGCCACCAGTGGTGTCCTTAAA
AACTGTGAGTCCACTAAGTTTCTTCCTCACAGAAATCGGTCTAGACCTCATATAACAGAAGTAAGCGT
TCTTAACGTCGTCGT
+
FFFFFFFFFFFFFFFFFFFFFFFFFFFFFFFFFFFFFFFFFFFFFFFFFFFFFFFFFFFFFFFFFFFF
F:FFFFFFFFF:FFFFFFFFFFFFFFFFFFFFFFFFFFFFFFFFFFFFFFFFFFF:FF:FFFFFFFF
FFFFF:FFFFFFFFF
@A00721:190:HGJW7DSXY:4:1661:16278:30436 2:N:0:TCTGCAAG+GCTGCACT
GTCGCAAAAACTAAATAATAAGCTCCTTTCATCATGTCCTCTACACTGCCACCAGTGGTGTCCTTAAA
AACTGTGAGTCCACTAAGTTTCTTCCTCACAGAAATCGGTCTAGACCTCATATAACAGAAGTAAGCGT
TCTTAACGTCGTCGT
+
FFFFFFFFFFFFFFFFFFFFFFFFFF:FFFFFFFFFFFFFFFFFFFFFFFFFFFFFFFFFFFFFFFFF:FFF
F:FFFFFFFFFFFFFFFFFFFFFFFFFFFFFFFFFFFFF:FFFFFFFFFFFFFFFFFFFFFFFFFFFFFF
FFFFFFFFFFFFFFF
@A00721:190:HGJW7DSXY:4:2312:19271:21527 2:N:0:TCTGCAAG+GCTGCACT
GTCGCAAAAACTAAATAATAAGCTCCTTTCATCATGTCCTCTACACTGCCACCAGTGGTGTCCTTAAA
AACTGTGAGTCCACTAAGTTTCTTCCTCACAGAAATCGGTCTAGACCTCATATAACAGAAGTAAGCGT
TCTTAACGTCGTCGT
+
FFFFFFFFFFFFFFFFFFFFFFFFFFFFFFFFFFFFFFFFFFFFFFFFFF:FFFFFFFFFFFFFFFFF
FFFFFFFFFFFFFFFFFFFFFFFFFFFFFFFFFFFFFFFFFFFFFFFFFFFFFFFFFFFFFFFFFF:FF
FFFFFFFFFF:FFFF
@A00721:190:HGJW7DSXY:4:1533:26205:15468 2:N:0:TCTGCAAG+GCTGCACT
CGTTAAGAACGCTTACTTCTGTTATATGAGGTCTAGACCGATTTCTGTGAGGAAGAAACTTAGTGGAC
```

TCACAGTTTTTAAGGACACCACTGGTGGCAGTGTAGAGGACATGATGAAAGGAGCTTATTATTTAGTT
TTTGCGAGATCGGAA
+
FFFFFFFFFFFFFFFF::FFFFFFFFFFFFFFFFFFFFFFFFFFFFFFFFFFFFFFFFFFFFFFFFFFFF
FFFFFFFFFFFFFFFFFFFFFFFFFFFFFFFFFFFFFFFFFFFFFFFFFFFFFFFFFFFF,FF,FFFFFF
FFFFFFFFFFFFFFF
@A00721:190:HGJW7DSXY:4:1553:11803:5932 2:N:0:TCTGCAAG+GCTGCACT
CGTTAAGAACGCTTACTTCTGTTATATGAGGTCTAGACCGATTTCTGTGAGGAAGAAACTTAGTGGAC
TCACAGTTTTTAAGGACACCACTGGTGGCAGTGTAGAGGACATGATGAAAGGAGCTTATTATTTAGTT
TTTGCGAGATCGGAA
+
FFFFFFFFFFFF:FFFFFFFFFF:FFFFFFFFFFFFFFFFFF:FFFFFFFFFFFFFFFFFFFFFFFFFFF
FFFFFFFFFFFFFFFFFFFFFFFFFFFFFFFFFF,FFFFFFFFFFFFFFFFFFFFFFFF:F,FFFFFFFF
FFFFFFFFFFFFFFF
@A00721:190:HGJW7DSXY:4:2424:21242:9721 2:N:0:TCTGCAAG+GCTGCACT
CGTTAAGAACGCTTACTTCTGATATATGAGGTCTAGACCGATTTCTGTTAGGAAGAAACTTAGTGGAC
TCACAGTATTTAAGGACACCACTGGTGGCAGTGTAGAGGACATGATGAAAGGAGCTTATTATTTAGTT
TTTGCGAGATCGGA
+
F::,FF,FFF,F,,FF,F,,:,,:FF,,FF,FFFF:FFF:FF,,F::F,F,,F,,FFFF,,FFF,,:F
F,F:F:,,F,FFFF:FFFF:FFF,,F,,F:,:,FF:FF::FFFFF::FF:,,::F::FFFF,,FFF,F
FFFF,FFFFF,FFF
@A00721:190:HGJW7DSXY:4:2555:25699:14246 2:N:0:TCTGCAAG+GCTGCACT
CGTTAAGAACGCTTACTTCTGTTATATGAGGTCTAGACCGATTTCTGTGAGGAAGAAACTTAGTGGAC
TCACAGTTTTTAAGGACACCACTGGTGGCAGTGTAGAGGACATGATGAAAGGAGCTTATTATTTAGTT
TTTGCGAGATCGGAA
+
FFFFFFFFFFFFFFFFFFFFFFFFFFFFFFFFFFFFFFFFFFFFFFFFFFFFFFFFFFFFFFFFFFFFFF
FFFFFFFFFFFFFFFFFFFFFFFFFFFFFFFFFFFFFFFFFFFFFFFFFFFFFFFFFFFFFFFFFFFFFF
FFFFFFFFFFFFFFF
@A00721:190:HGJW7DSXY:4:1265:11623:36495 2:N:0:TCTGCAAG+GCTGCACT
GATGAAATGTCGCAAAAACTAAATAATAAGCTCCTTTCATCATGTCCTCTACACTGCCACCAGTGGTG
TCCTTAAAAACTGTGAGTCCACTAAGTTTCTTCCTCACAGAAATCGGTCTAGACCTCATATAACAGAA
GTAAGCGTTCTTAAC
+
FFFFFFFFFFFFFFFFFFFFFFFFFFFFFFFFFFFFFFFFFFFFFFFFFF:FFFFFFFF:FFFFFFFF:F
FFFFFFFFFFFFFFFFFFFFFFFFFF:FFFFFFFFFFFFFFFFFFFFFFFFFFFFFFFFFFFFFFFFFFF
FFFFFFFFFFFFFFF
@A00721:190:HGJW7DSXY:4:1448:25961:10473 2:N:0:TCTGCAAG+GCTGCACT
GATGAAATGTCGCAAAAACTAAATAATAAGCTCCTTTCATCATGTCCTCTACACTGCCACCAGTGGTG
TCCTTAAAAACTGTGAGTCCACTAAGTTTCTTCCTCACAGAAATCGGTCTAGACCTCATATAACAGAA
GTAAGCGTTCTTAAC
+
FFFFFFFFFFFFFFFF:FFFFFFFFFFFF:FFFFFFFFFFFFFFFFFFFFFFFFFFFFFFFFFFFFFFFF
FFFFFFFFFFFFFFFFFFFFFF::FF:FFFFFFFFFFFFFFFFFF:FFFFFFFFFF:FFFFFF:FFFFF
FFFFFFF:FFFFFFF
@A00721:190:HGJW7DSXY:4:1461:10827:9189 2:N:0:TCTGCAAG+GCTGCACT
GATGAAATGTCGCAAAAACTAAATAATAAGCTCCTTTCATCATGTCCTCTACACTGCCACCAGTGGTG
TCCTTAAAAACTGTGAGTCCACTAAGTTTCTTCCTCACAGAAATCGGTCTAGACCTCATATAACAGAA
GTAAGCGTTCTTAAC
+
FFFFFFFFFFFFFFFFFFFFFFFFFFFFFFFFFFFFFFFFFFFFFFFFFFFFFFFFFFFFFFFFFFFFFF
FFFF:FFFFFFFFFFFFFFFFFFFFFFFFFFFFFFFFFFFFFFFFFFFFFF:FFFFFFFFFFFFFF
FFFF:FFFFFFFFFF

```
@A00721:190:HGJW7DSXY:4:1475:18756:36041 2:N:0:TCTGCAAG+GCTGCACT
GATGAAATGTCGCAAAAACTAAATAATAAGCTCCTTTCATCATGTCCTCTACACTGCCACCAGTGGTG
TCCTTAAAAACTGTGAGTCCACTAAGTTTCTTCCTCACAGAAATCGGTCTAGACCTCATATAACAGAA
GTAAGCGTTCTTAAC
+
FFFFFFFFFFFFFFFFFFFFFFFFFFFFFFFFFFFFFFFFFFFFFFFFFFFFFFFFFFFFFFFFFFFFFF
FFFFFFFFFFFFFFFFFFFFFFFFFFFFFFFFFFFFFFFFFFFFFFFFFFFFFFFFFFFF,FFFFFFF:F
FFFFFF:FFFFFFFF
@A00721:190:HGJW7DSXY:4:1558:16288:18865 2:N:0:TCTGCAAG+GCTGCACT
GATGAAATGTCGCAAAAACTAAATAATAAGCTCCTTTCATCATGTCCTCTACACTGCCACCAGTGGTG
TCCTTAAAAACTGTGAGTCCACTAAGTTTCTTCCTCACAGAAATCGGTCTAGACCTCATATAACAGAA
GTAAGCGTTCTTAAC
+
FFFFFFFFFFFFFFFFFFFFFFFFFFFFFFFFFFFFFFFFFFFFFFFFFFFFFFFFFFFFFFFFFFFFFF
FF::FFFFFFFF,FFFFFF,FFFFF:FFFFFF:,FFFFFFFFFFF,F::FF:FF:FFFFF:F:FFFFF
:FFFFFFFFFFFFFF
@A00721:190:HGJW7DSXY:4:2104:22263:13620 2:N:0:TCTGCAAG+GCTGCACT
GATGAAATGTCGCAAAAACTAAATAATAAGCTCCTTTCATCATGTCCTCTACACTGCCACCAGTGGTG
TCCTTAAAAACTGTGAGTCCACTAAGTTTCTTCCTCACAGAAATCGGTCTAGACCTCATATAACAGAA
GTAAGCGTTCTTAAC
+
FFFFFFFFFFFFFFFFFFFFFFFFFFFFFFFFFFFFFFFFFFFFFFFFFFFFFFFFFFFFFFFFFFFFFF
FFFFF:FFFFFFFFFFFFFFFFFFFFFFFFFFFFFFFFFFFFFFFFFFFFFFFFFFFFFFFFFFFFFFFF
FFFF,FFFFFFFFF:
@A00721:190:HGJW7DSXY:4:2157:18041:25598 2:N:0:TCTGCAAG+GCTGCACT
GATGAAATGTCGCAAAAACTAAATAATAAGCTCCTTTCATCATGTCCTCTACACTGCCACCAGTGGTG
TCCTTAAAAACTGTGAGTCCACTAAGTTTCTTCCTCACAGAAATCGGTCTAGACCTCATATAACAGAA
GTAAGCGTTCTTAAC
+
FFFFFFFFFFFFFF:FFFFFFFFFFFFFFFFFFFFFFFFFFFFFFFFFFFFFFFFFFFFFFFFFFFFFF
FFFFFFFFFFFFFFFFFFFF:FFFFFFFFFFFFFFFFF:FFFF:F:FFFFFFFFFFFFFFFFFFFFFFF
FFFF:F:FFFFFFFF
@A00721:190:HGJW7DSXY:4:2268:28411:14372 2:N:0:TCTGCAAG+GCTGCACT
GATGAAATGTCGCAAAAACTAAATAATAAGCTCCTTTCATCATGTCCTCTACACTGCCACCAGTGGTG
TCCTTAAAAACTGTGAGTCCACTAAGTTTCTTCCTCACAGAAATCGGTCTAGACCTCATATAACAGAA
GTAAGCGTTCTTAAC
+
FFFFFFFFFFFFFFFFFFFFFFFFFFFFFFFFFFFFFFFFFFFFFFFFFFFFFFFFFFFFFFFFFFFFFF
FFFFFFFFFFFFFFFFFFFFFFFFFFFFFFFFFFFFFFFFFFFFFFFFFFFFFFFFFFFFFFFFFFFFFF
FFF:FFFFFFFFFFF
@A00721:190:HGJW7DSXY:4:2420:19651:33771 2:N:0:TCTGCAAG+GCTGCACT
GATGAAATGTCGCAAAAACTAAATAATAAGCTCCTTTCATCATGTCCTCTACACTGCCACCAGTGGTG
TCCTTAAAAACTGTGAGTCCACTAAGTTTCTTCCTCACAGAAATCGGTCTAGACCTCATATAACAGAA
GTAAGCGTTCTTAAC
+
FFFFFFFFFFFFFFFFFFFFFFFFFFFFFFFFFFFFFFFFFFFFFFFFFFFFFFFFFFFFFFFFFFFFFF
FFFFFFFFFFFFFFFFFFFFFFFFFFFFFFFFFFFFFFFFFFFFFFFFFFFFFFFFFFFFFFFFFFFFFF
FFFFFFFFFFFFFF:
@A00721:190:HGJW7DSXY:4:2575:30183:36385 2:N:0:TCTGCAAG+GCTGCACT
GATGAAATGTCGCAAAAACTAAATAATAAGCTCCTTTCATCATGTCCTCTACACTGCCACCAGTGGTG
TCCTTAAAAACTGTGAGTCCACTAAGTTTCTTCCTCACAGAAATCGGTCTAGACCTCATATAACAGAA
GTAAGCGTTCTTAAC
+
:FFFFFFFFFFFFFFF:FFFFFFFFFFFFFFFFFFFFFFFFFFFFFFFFFFFFFFFFFFFFFFFFFFFF
```

```
FFFFFFF:FFFFFFFFFFFFFFFFFFFFFFFFFFFFFFFFFFFFFFFFFFFFFFFFFFFFFFFFFFFFFF
FFFF:FFFFFFFF,F
@A00721:190:HGJW7DSXY:4:2656:27724:5760 2:N:0:TCTGCAAG+GCTGCACT
GATGAAATGTCGCAAAAACTAAATAATAAGCTCCTTTCATCATGTCCTCTACACTGCCACCAGTGGTG
TCCTTAAAAACTGTGAGTCCACTAAGTTTCTTCCTCACAGAAATCGGTCTAGACCTCATATAACAGAA
GTAAGCGTTCTTAAC
+
FFFFFFFFFFFFFFFFFFFFFFFFFFFFFFFFFFFFFFFFFFFFFFFFFFFFFFFFFFFFFFF:FFFFFF
FFFFFFFFFFFFFFFFFFFFFFFFFFFFFFFFFFFFFFFFFFFFFFFFFFFFFFFFFFFFFFFFFFFFFF
FFFFFFFFFFFFFFF
@A00721:190:HGJW7DSXY:4:2565:6280:15311 2:N:0:TCTGCAAG+GCTGCACT
ATGAAATGTCGCAAAAACTAAATAATAAGCTCCTTTCATCATGTCCTCTACACTGCCACCAGTGGTGT
CCTTAAAAACTGTGAGTCCACTAAGTTTCTTCCTCACAGAAATCGGTCTAGACCTCATATAACAGAAG
TAAGCGTTCTTAA
+
:::FFFFFFFFFFFFFFFFFFFFFFFFFFFFFFF:F:FFF:FFFFFFFFFFFFFF:F:FF:FFFFFFFFF
FFFFFFFFFF:FFFFFF:F:FFFFF:FFFFFFF:FFFFFFFFFF:F,FFFFF:FFFFFFFFFFF:FFFF,
FFFFFFFFFFFFF
@A00721:190:HGJW7DSXY:4:1270:29432:33708 2:N:0:TCTGCAAG+GCTGCACT
CGCATATTGATGAAATGTCGCAAAAACTAAATAATAAGCTCCTTTCATCATGTCCTCTACACTGCCAC
CAGTGGTGTCCTTAAAAACTGTGAGTCCACTAAGTTTCTTCCTCACAGAAATCGGTCTAGACCTCATA
TAACAGAAGTAAGCG
+
FFFFFFFFFFFFFFFFFFFFFFF:FFFFFFFFFFFFFFFFFFFFFFFFFFFFFFFFFFFFFFFFFFFFFF
FFFFFFFFFFFFFFFFFFFFFFFFFFFFFFFF:FFFFFFFFFF,FFFFFFFFFFFFFFFFFFFFFFF:FF
FFFFFFFFF:F,FFF
@A00721:190:HGJW7DSXY:4:1450:20808:8437 2:N:0:TCTGCAAG+GCTGCACT
CGCATATTGATGAAATGTCGCAAAAACTAAATAATAAGCTCCTTTCATCATGTCCTCTACACTGCCAC
CAGTGGTGTCCTTAAAAACTGTGAGTCCACTAAGTTTCTTCCTCACAGAAATCGGTCTAGACCTCATA
TAACAGAAGTAAGCG
+
FFFFFFFFFFFFFFFFFFFFFFFFFFFFFFFFFFFFF:F:FFFFFFFFFFFFFFFFFFFFFFFF:FFFFF
FFFFFFFFFFFFFFFFFFFFFFFFFFFFFFFFFFFFFFFFFFFFFFFFFFFFFFFFFFFFFFF:FFFFFF
FFFFFFFFFFFFFF:
@A00721:190:HGJW7DSXY:4:1675:31304:34914 2:N:0:TCTGCAAG+GCTGCACT
CGCATATTGATGAAATGTCGCAAAAACTAAATAATAAGCTCCTTTCATCATGTCCTCTACACTGCCAC
CAGTGGTGTCCTTAAAAACTGTGAGTCCACTAAGTTTCTTCCTCACAGAAATCGGTCTAGACCTCATA
TAACAGAAGTAAGCG
+
FFFFFFFFFFFFFFFFFFFFFFFFFFFFFFFFFFFFFFFFFFFFFFFFFFF:FFF,FFFFFFFF:FFFFF
FFFF:FFFFF,FFFFFF,FFFFF,FFF:FFFFFFFFFFFFFFF,FFFFF,FFF:F,FF::FFFF,FFFFF
FFFFFF:FFFFF:FF
@A00721:190:HGJW7DSXY:4:2138:30553:24815 2:N:0:TCTGCAAG+GCTGCACT
CGCATATTGATGAAATGTCGCAAAAACTAAATAATAAGCTCCTTTCATCATGTCCTCTACACTGCCAC
CAGTGGTGTCCTTAAAAACTGTGAGTCCACTAAGTTTCTTCCTCACAGAAATCGGTCTAGACCTCATA
TAACAGAAGTAAGCG
+
FFFFFF:FFFFF:FFFFFFFFFFF:F:FFFFFFFFFFFFFFFFFFFFFFFFFFF:FFFFF:FFF,FFFFF
FF:FFFFFFFFFFFFFFFFFFFFFFFFFFFF:FFFFFFFFFF:FFFFF:F:FFFFFFFFFFFFFFFFFFF
FFFFFFFFFFFF,FF
@A00721:190:HGJW7DSXY:4:2314:20003:20572 2:N:0:TCTGCAAG+GCTGCACT
CGCATATTGATGAAATGTCGCAAAAACTAAATAATAAGCTCCTTTCATCATGTCCTCTACACTGCCAC
CAGTGGTGTCCTTAAAAACTGTGAGTCCACTAAGTTTCTTCCTCACAGAAATCGGTCTAGACCTCATA
TAACAGAAGTAAGCG
```

```
+
:FFFFFFFFF:FFFFFFFFFFFFFFFFFFFFFFFFFFFFFFFFF:FFFFFFFFFFFFFFFFFFFFFFFF
FFFFFFFFFFFFFFFFFFFFFFFFFFFFFFFFFFFFFFFFFF:FFFFFFFFFFFFFFFFFFF::FF:F:
FFFFFFFFFFFFFFF
@A00721:190:HGJW7DSXY:4:2666:12626:35258 2:N:0:TCTGCAAG+GCTGCACT
CGCATATTGATGAAATGTCGCAAAAACTAAATAATAAGCTCCTTTCATCATGTCCTCTACACTGCCAC
CAGTGGTGTCCTTAAAAACTGTGAGTCCACTAAGTTTCTTCCTCACAGAAATCGGTCTAGACCTCATA
TAACAGAAGTAAGCG
+
FFFFFFFF:FFFFFFFFFFFFFFF:F:FFFFFFFFFFFFFFFF:F,FFFFFF:F,FF:FFFF:FFF
,,:F:F:F,F,:FFFFFFFF:FFFFF:FFFFFF::,FFFFF,FFFFF:FFFF:::FFFF,F::FFFF,
FFF:FFFFF:FFFF:
@A00721:190:HGJW7DSXY:4:1566:12635:7874 2:N:0:TCTGCAAG+GCTGCACT
CGCATATTGATGAAATGTCGCAAAAACTAAATAATAAGCTCCTTTCATCATGTCCTCTACACTGCCAC
CAGTGGTGTCCTTAAAAACTGTGAGTCCACTAAGTTTCTTCCTCACAGAAATCGGTCTAGACCTCATA
TAACAGAAGTAAGC
+
FFFFFFFFFFFFFFFFFFFFF:,FFFFFFFFF:FFFFFFF:FFFFFFFFFFFFFFFFFFFFFFFFFFF
FFFFFFFFFFFF::FFFFFFFFF:FFFFFFFFFFF:FFFFFFFFFFFFFFFFFFFFFFF:FFFFFFFF
FFFFFFFFFFFFFF
@A00721:190:HGJW7DSXY:4:1501:9046:2910 2:N:0:TCTGCAAG+GCTGCACT
GCAACTTCCATCGCATATTGATGAAATGTCGCAAAAACTAAATAATAAGCTCCTTTCATCATGTCCTC
TACACTGCCACCAGTGGTGTCCTTAAAAACTGTGAGTCCACTAAGTTTCTTCCTCACAGAAATCGGTC
TAGACCTCATATAAC
+
FFFFFFFFFFFFFFFFFFFFFFFFFFFFFFFFF:FFFFFFFFFFFFFFFFFFFFFFFFFFFFFFFFFF
FFFFFFFFFFFFFFFFFFFFFFFFFFFFFFFFFFFFFFFFFFFFFFF:FFFFFFFFFFFFFF:FFFFFFF
FFFFFFFFFFFFFFF
@A00721:190:HGJW7DSXY:4:1564:8106:7388 2:N:0:TCTGCAAG+GCTGCACT
GCAACTTCCATCGCATATTGATGAAATGTCGCAAAAACTAAATAATAAGCTCCTTTCATCATGTCCTC
TACACTGCCACCAGTGGTGTCCTTAAAAACTGTGAGTCCACTAAGTTTCTTCCTCACAGAAATCGGTC
TAGACCTCATATAAC
+
F:FFFFFFFFFFFFFFFFFFFFFFFFFFFFFFFFFF:FFFFFFFFFFFFFFFFFFFFFFFFFFFFFFF
FFFFFFFFFFFFFFFFFFFFFFFFFFFFFFFFFFFFFFFFFFFFFFFFFFFFFFFFFFFFFFFFFFFF
FFFFFFFFFFFFF:F
@A00721:190:HGJW7DSXY:4:2635:6072:29230 2:N:0:TCTGCAAG+GCTGCACT
AACTTCCATCGCATATTGATGAAATGTCGCAAAAACTAAATAATAAGCTCCTTTCATCATGTCCTCTA
CACTGCCACCAGTGGTGTCCTTAAAAACTGTGAGTCCACTAAGTTTCTTCCTCACAGAAATCGGTCTA
GACCTCATATAAC
+
:FFFFFFFFFFFFFFFFFFFFFFFFFFFFFFFFFFFFFFFFFFFFFFFFFFFFFFFFFFFFFFFFFFF
FFFFFFFFFFFFFFFFFFFFFFFFFFFFFFFFFFFFFFFFFFFFFFFFFFFFFFFFFFFFF:FFFFFF
FFFFFFFFFFFF:
@A00721:190:HGJW7DSXY:4:1140:4652:2660 2:N:0:TCTGCAAG+GCTGCACT
ATTTAATGCAACTTCCATCGCATATTGATGAAATGTCGCAAAAACTAAATAATAAGCTCCTTTCATCA
TGTCCTCTACACTGCCACCAGTGGTGTCCTTAAAAACTGTGAGTCCACTAAGTTTCTTCCTCACAGAA
ATCGGTCTAGACCTC
+
FFFFFFFFFFFFFFFFFFFFFFFFFFFFFFFFF:FFFFFFFFF:FFFFFFFFFFFFFFFFFFFFFFFFF
FFFFFFFFFFFFFFFFF:FFFFFFFFFFFFFFFFFFFFFFFFFFFFFFFFFFFFFFFFFFFFFFFFFF
,FFFFFFFFFFFFFF
@A00721:190:HGJW7DSXY:4:2365:32859:35665 2:N:0:TCTGCAAG+GCTGCACT
ATTTAATGCAACTTCCATCGCATATTGATGAAATGTCGCAAAAACTAAATAATAAGCTCCTTTCATCA
```

TGTCCTCTACACTGCCACCAGTGGTGTCCTTAAAAACTGTGAGTCCACTAAGTTTCTTCCTCACAGAA
ATCGGTCTAGACCTC
+
FFFFFFFFFFFFFFFFFFFFFFFF:FFFFFFFFFFFFFFFF:F:FFFFFFFFFFFFFFFFF:FFFFFF
FFFFFFFFFFFF:FFFFFFFFFFFFFFFFFFFFFFFFFFFFFFFFFFFFFFFFFFFFFFFFFFFFFFF
FFFFFFFFFFF,:FF
@A00721:190:HGJW7DSXY:4:2408:11008:15953 2:N:0:TCTGCAAG+GCTGCACT
ATTTAATGCAACTTCCATCGCATATTGATGAAATGTCGCAAAAACTAAATAATAAGCTCCTTTCATCA
TGTCCTCTACACTGCCACCAGTGGTGTCCTTAAAAACTGTGAGTCCACTAAGTTTCTTCCTCACAGAA
ATCGGTCTAGACCTC
+
FFFFFFFFFFFFFFFFFFFFFFFFFFFFFFFFFFFFFFFFFFFFFFFFFFFFFFFFFFFFFFFFFFFF
FFFFFFFFFFFFFFFFFFFFFFFFFFFFFFFFFFFFFFFFFFFFFFFFFFFFFFFFFFFFFFFFFFFF
FFFFFFFFFFFFFFF
@A00721:190:HGJW7DSXY:4:2574:1479:26976 2:N:0:TCTGCAAG+GCTGCACT
ATTTAATGCAACTTCCATCGCATATTGATGAAATGTCGCAAAAACTAAATAATAAGCTCCTTTCATCA
TGTCCTCTACACTGCCACCAGTGGTGTCCTTAAAAACTGTGAGTCCACTAAGTTTCTTCCTCACAGAA
ATCGGTCTAGACCTC
+
FFFFFFFFFFFFFFFFFFFFFFFFFFFFFFFFFFFFFFFF:F:FFF,FFFFFFFFFFFFFFFFFFFFF
FFFFFFFFFFFFFFFFFFFFFFFFFFFFFFFFFFF:FFFFFFFFFFFFFFFFFFFFFFFFFFFFFFFF
FFFFFFFFFFFFFFF
@A00721:190:HGJW7DSXY:4:1220:1714:9439 2:N:0:TCTGCAAG+GCTGCACT
TACATTTAATGCAACTTCCATCGCATATTGATGAAATGTCGCAAAAACTAAATAATAAGCTCCTTTCA
TCATGTCCTCTACACTGCCACCAGTGGTGTCCTTAAAAACTGTGAGTCCACTAAGTTTCTTCCTCACA
GAAATCGGTCTAGAC
+
FFFFFFFFFFFFFFFFFFFFFFFFFFFFFFFFFFFFFFFFFFFFF::FFFFFFFFFFFFFFFFFFFFF
FFFFFFFFFFF,FFFFFFFFFFFFFFFFFFFFFFFFFFFFFFFFFFFFFFFFFFFFFFFFFFFFFFFF
F:FFFFFFFFFFFFF
@A00721:190:HGJW7DSXY:4:1220:1452:9455 2:N:0:TCTGCAAG+GCTGCACT
TACATTTAATGCAACTTCCATCGCATATTGATGAAATGTCGCAAAAACTAATTAATAAGCTCCTTTCA
TCATGTCCTCTACACTGCCACCAGTGGTGTCCTTAAAAACTGTGAGTCCACTAAGTTTCTTACTCACA
GAAATCGGTCTAGAC
+
FFFFF:,FFFF:FFFFFFFFFF:FFFFF:FFF:FFFFFF:FFF,FFFFFFF,FFFFF:FF,:FFFFF:
FFFFFFFFFFFFFFFFF:FFFFF,FFFFFFFF,FFFFFFFFFF:FFFFFFFFFFFFFF:::,:FF,FF
,FF:FFFF,FF,FFF
@A00721:190:HGJW7DSXY:4:1412:4227:16767 2:N:0:TCTGCAAG+GCTGCACT
TACATTTAATGCAACTTCCATCGCATATTGATGAAATGTCGCAAAAACTAAATAATAAGCTCCTTTCA
TCATGTCCTCTACACTGCCACCAGTGGTGTCCTTAAAAACTGTGAGTCCACTAAGTTTCTTCCTCACA
GAAATCGGTCTAGAC
+
FFFFFFFFFFFFFFFFFFFFFFFFFF:FFFFFFFFF:FFFFFFFFFFFFF:FFFFFFFFFFFFFFFFF
FFFFFFFFFFFFFFFFFFFFFFFFFFFFFFFFFFF:FFFFFFFFFFFFFFFFF:FFFFFFFFFFFFFF
FF:FFFFFFFFFFFF
@A00721:190:HGJW7DSXY:4:1412:28293:17394 2:N:0:TCTGCAAG+GCTGCACT
TACATTTAATGCAACTTCCATCGCATATTGATGAAATGTCGCAAAAACTAAATAATAAGCTCCTTTCA
TCATGTCCTCTACACTGCCACCAGTGGTGTCCTTAAAAACTGTGAGTCCACTAAGTTTCTTCCTCACA
GAAATCGGTCTAGAC
+
FFFFFFFFFFFFFFFFFFFFFFFFFF,FFFFFFFFFFFFFFFFFFFFFFFFFFFFFFFFFFFFFFFFF
FFFFFFFFFFFFFFFFFFFFFFFFFFFFFFFFFFFFFFFFF:FFFFFFFFFFFFFFFFFFFFFFF:FFFFFF
:FFFFFFFFFFFFFF

@A00721:190:HGJW7DSXY:4:1477:10755:16579 2:N:0:TCTGCAAG+GCTGCACT
TACATTTAATGCAACTTCCATCGCATATTGATGAAATGTCGCAAAAACTAAATAATAAGCTCCTTTCA
TCATGTCCTCTACACTGCCACCAGTGGTGTCCTTAAAAACTGTGAGTCCACTAAGTTTCTTCCTCACA
GAAATCGGTCTAGAC
+
FFFFFFFFFFFF:FFFFFFFFFFFFFFFFFFFFFFFFFFF:FFFFFFFFFFFFFFFFFFFFFFFFFFFFF
FFFFF:FFFFFFFFFFFFFFFFFFFFFFFFFFFF,F:FFFFFFFFFFFFFFFFFFFFF:FFF:FFFFFF
FFFFFFFFFFFF,FF
@A00721:190:HGJW7DSXY:4:1542:9652:7686 2:N:0:TCTGCAAG+GCTGCACT
TACATTTAATGCAACTTCCATCGCATATTGATGAAATGTCGCAAAAACTAAATAATAAGCTCCTTTCA
TCATGTCCTCTACACTGCCACCAGTGGTGTCCTTAAAAACTGTGAGTCCACTAAGTTTCTTCCTCACA
GAAATCGGTCTAGAC
+
FFFFFFFFFFFFFFFFFFFFFFFFFFFFFFFFFFFFFFFFFFFFFFFFFFFFFFFFFFFFFFFFFFFFFF
FFFFFFFFFFFFFFFFFFFFFFFFFFFFFFFFFFFFFFFFFFFFFFFFFFFFFFFFFFFFFFF,FFFFF
FFFFFFFFFFFFFFF
@A00721:190:HGJW7DSXY:4:2504:24216:34507 2:N:0:TCTGCAAG+GCTGCACT
TACATTTAATGCAACTTCCATCGCATATTGATGAAATGTCGCAAAAACTAAATAATAAGCTCCTTTCA
TCATGTCCTCTACACTGCCACCAGTGGTGTCCTTAAAAACTGTGAGTCCACTAAGTTTCTTCCTCACA
GAAATCGGTCTAGAC
+
:FFFFFFFFFFFF:FFFFFFF:FFFFFFFFFFFFFFFFFFFFFF,FFFFFFFFFFFFFFFFFFFFFFFFF
FFFFFFF,FFFFFFF:FFFF:FFFFFFFFFF:FFFFFFFF,:FFFFFFFFFFFFFFFFFFFFFFFFFFF
FFFFFFFFFFFFFFF
@A00721:190:HGJW7DSXY:4:2613:4887:16094 2:N:0:TCTGCAAG+GCTGCACT
TACATTTAATGCAACTTCCATCGCATATTGATGAAATGTCGCAAAAACTAAATAATAAGCTCCTTTCA
TCATGTCCTCTACACTGCCACCAGTGGTGTCCTTAAAAACTGTGAGTCCACTAAGTTTCTTCCTCACA
GAAATCGGTCTAGAC
+
FFFFFFFFFFFFFFFFFFFFFFFFFFFFFFFFFFFFFFFFFFFFFFFFFFFFFFFFFFFFFFFFFFFFFF
FFFFFFFFFFFFFFFFFFFFFFFFFFFFFFFFFFFFFFFFFFFFFFFFFF:FFFFFFFFFFFFFFFFFFF
FFFFFFFFFFFFFFF
@A00721:190:HGJW7DSXY:4:1235:2293:14763 2:N:0:TCTGCAAG+GCTGCACT
GTACATTTAATGCAACTTCCATCGCATATTGATGAAATGTCGCAAAAACTAAATAATAAGCTCCTTTC
ATCATGTCCTCTACACTGCCACCAGTGGTGTCCTTAAAAACTGTGAGTCCACTAAGTTTCTTCCTCAC
AGAAATCGGTCTAGA
+
FFFFFF:FFFFFFFFFFFFFF:F:FFFFFFFFFFFFFFFFFFF:F,FFFF:FFF,FFFFFFFF,FFF
FF:FFFFFFFFFFFFFFFFF:FFFFFFFFF::FFFFFFFFFFFF:FF:FF:,FFFFFFFFFF,FFFF
FFFF:FFFFFFFFFF
@A00721:190:HGJW7DSXY:4:1577:28763:31767 2:N:0:TCTGCAAG+GCTGCACT
GTACATTTAATGCAACTTCCATCGCATATTGATGAAATGTCGCAAAAACTAAATAATAAGCTCCTTTC
ATCATGTCCTCTACACTGCCACCAGTGGTGTCCTTAAAAACTGTGAGTCCACTAAGTTTCTTCCTCAC
AGAAATCGGTCTAGA
+
FFFFFFFFFFFFFFFFFFFFFFFFF:FFFFFFFFFFFFFFFFFFFFFF:FFFF:FFFFFFFFFFFFFFF
FFFFFFFFFFFFFFFFFFFFFFFFFFFFFFF:FFFFFFFFFFFFFFFFFFFFFFFFFFFFFFFFFFFF:F
FF,FFFFFFFFFFFF
@A00721:190:HGJW7DSXY:4:1609:7500:32142 2:N:0:TCTGCAAG+GCTGCACT
GTACATTTAATGCAACTTCCATCGCATATTGATGAAATGTCGCAAAAACTAAATAATAAGCTCCTTTC
ATCATGTCCTCTACACTGCCACCAGTGGTGTCCTTAAAAACTGTGAGTCCACTAAGTTTCTTCCTCAC
AGAAATCGGTCTAGA
+
:FFFFFFFFFFFFFFFFFFFFFFFFFFFFFFFFFFFFFFFFFFFFFF:FFFFFF:FFF:FFFFFFFFFF

FFFFFFFFFFFFFFFFF:FFFFFFF:FFFFFFF:::FFFFFFFFFFFFFFFF,:FF:FFFFFF::FFF
FFFFFFFFFFFFFFF
@A00721:190:HGJW7DSXY:4:2121:7374:13260 2:N:0:TCTGCAAG+GCTGCACT
GTACATTTAATGCAACTTCCATCGCATATTGATGAAATGTCGCAAAAACTAAATAATAAGCTCCTTTC
ATCATGTCCTCTACACTGCCACCAGTGGTGTCCTTAAAAACTGTGAGTCCACTAAGTTTCTTCCTCAC
AGAAATCGGTCTAGA
+
:FFFFFFFFFFFFFFFFFF:FFFFF,FFFFFFFF:FFFFFFFFFFFFFFFFFFFFFFFFF,FFFFFFF
FFFFFFFF:FFFFFF,FF,FFF,FFFFFFFF::F,FFFFFFFFFFFFFFFFFFFFFFFF,FF:,FF,F
F:FFFFFFFFFF:FF
@A00721:190:HGJW7DSXY:4:2248:1127:23046 2:N:0:TCTGCAAG+GCTGCACT
GTACATTTAATGCAACTTCCATCGCATATTGATGAAATGTCGCAAAAACTAAATAATAAGCTCCTTTC
ATCATGTCCTCTACACTGCCACCAGTGGTGTCCTTAAAAACTGTGAGTCCACTAAGTTTCTTCCTCAC
AGAAATCGGTCTAGA
+
FFFFFFFFFFFFFFFFFFFFFFFFFFFFFFFFFFFFFFFFFFFFFFFFFFFFFFFFFFFFFFFFFFFF
FFFFFFFFFFFFFFFFFFFFFFFFFFFFFFFFFFFFFFFFFFFFFFFFFFFFFFFFFFFFFFFFFFFF
FFFFFFFFFFFFFFF
@A00721:190:HGJW7DSXY:4:2248:1307:23077 2:N:0:TCTGCAAG+GCTGCACT
GTACATTTAATGCAACTTCCATCGCATATTGATGAAATGTCGCAAAAACTAAATAATAAGCTCCTTTC
ATCATGTCCTCTACACTGCCACCAGTGGTGTCCTTAAAAACTGTGAGTCCACTAAGTTTCTTCCTCAC
AGAAATCGGTCTAGA
+
FFFFFFFFFFFFFFFFFFFFFFFFFFFFFFFFFFFFFFFFFFFFFFFFFFFFFFFFFFFFFFFFFFFF
FFFFFFFFFFFFFFFFFFFFFFF,FFFFFFFFFFFFFFFFFFFFFFFFFFFFFFFFFFFFFFFFFFFF
FFFFFFFFFFFFFFF
@A00721:190:HGJW7DSXY:4:2365:12093:17206 2:N:0:TCTGCAAG+GCTGCACT
GTACATTTAATGCAACTTCCATCGCATATTGATGAAATGTCGCAAAAACTAAATAATAAGCTCCTTTC
ATCATGTCCTCTACACTGCCACCAGTGGTGTCCTTAAAAACTGTGAGTCCACTAAGTTTCTTCCTCAC
AGAAATCGGTCTAGA
+
FFFFFFFFFFFFFFFFFFFFFFFFFFFFFFFFFFFFFFFFFFFFFFFFFFFFFFFFFFFFFFFFFFFF
FFFFFFFFFFFFFFFFFFFFFFFFFFFFFFFFFF:FFFFFFFFFFFFFFFFFFFFFFFFFFFFFFFFF
FFF:FFFFFFFF:FF
@A00721:190:HGJW7DSXY:4:2454:32940:21840 2:N:0:TCTGCAAG+GCTGCACT
GTACATTTAATGCAACTTCCATCGCATATTGATGAAATGTCGCAAAAACTAAATAATAAGCTCCTTTC
ATCATGTCCTCTACACTGCCACCAGTGGTGTCCTTAAAAACTGTGAGTCCACTAAGTTTCTTCCTCAC
AGAAATCGGTCTAGA
+
FFFFFFFFFFFFFFFFFFFFFFFFFFFFFFFFFFFFFFFFFFFFFFFFFFFFFFFFFFFFFFFFFFFF
FFFFFFFFFFFFFFFFFFFFFFFFFFFFFFFFFFFFFFFFFFFFFFFFF,:FFF:FFFFFFFF:FFFF
FFFFFFFFFFFF:FF
@A00721:190:HGJW7DSXY:4:2514:10411:7842 2:N:0:TCTGCAAG+GCTGCACT
GTACATTTAATGCAACTTCCATCGCATATTGATGAAATGTCGCAAAAACTAAATAATAAGCTCCTTTC
ATCATGTCCTCTACACTGCCACCAGTGGTGTCCTTAAAAACTGTGAGTCCACTAAGTTTCTTCCTCAC
AGAAATCGGTCTAGA
+
FF,FFFFFFFFFFFFFFFFFFFFFFFFFFFFFFFFFFFFFFFF:FFFFFFFFFFFFF,FFFFFFFFFF
FFFF:FFFFFFFFFFFFFFFFFFFFFFF:FFFFF:FFFFFFFFFFFFFFF:F,FFFFFFFFFFFFFFF
FFFF,FFFFFFFFFF
@A00721:190:HGJW7DSXY:4:2542:9941:26537 2:N:0:TCTGCAAG+GCTGCACT
GTACATTTAATGCAACTTCCATCGCATATTGATGAAATGTCGCAAAAACTAAATAATAAGCTCCTTTC
ATCATGTCCTCTACACTGCCACCAGTGGTGTCCTTAAAAACTGTGAGTCCACTAAGTTTCTTCCTCAC
AGAAATCGGTCTAGA

```
+
F:FFFFFFFFFFFFFFFFFFFFFFFFFFFFFFFFFFFFFFFFFFFFFFFFFFFFFFFFFFFFFFFFFFFF
FFFFFFFFFFFFFFFFFFFFFFFFFFFFFFFFFFFFFFFFFFFFFFFFFFFFFFFFFFFFFFFFFFFFFF
FFFFFF::FFFFFFF
@A00721:190:HGJW7DSXY:4:1214:4435:11334 2:N:0:TCTGCAAG+GCTGCACT
CGTACATTTAATGCAACTTCCATCGCATATTGATGAAATGTCGCAAAAACTAAATAATAAGCTCCTTT
CATCATGTCCTCTACACTGCCACCAGTGGTGTCCTTAAAAACTGTGAGTCCACTAAGTTTCTTCCTCA
CAGAAATCGGTCTAG
+
FFFFFFFFFFFFFFFFFFFFFFFFFFFFFFFFFFFFFFFFFFFFFFFFFFFFFFFFFFFFFFFFFFFFFF
FFFFFFFFFFFFFFFFFFFFFFFFFFFFFFFFFFFFFFFFFFFFFFFFFFFFFFFFFFFFFFFFFFFFFF
FFFFFFF:FFFFFF:
@A00721:190:HGJW7DSXY:4:1275:21748:22780 2:N:0:TCTGCAAG+GCTGCACT
CGTACATTTAATGCAACTTCCATCGCATATTGATGAAATGTCGCAAAAACTAAATAATAAGCTCCTTT
CATCATGTCCTCTACACTGCCACCAGTGGTGTCCTTAAAAACTGTGAGTCCACTAAGTTTCTTCCTCA
CAGAAATCGGTCTAG
+
FFFFFFFFFFFFFFFFFFFFFFFFFFFFFFFFFFFFFFFFFFFFFFFFFFFFFFFFFFFFFFFFFFFFFF
FFFFFFFFFFFFFFFFFFFFFFF:FFFFFFFF:FFFF:FFF:FFFFFFFFF:FFF:FFFFFFFFF:FFFF
FFFFFFFFFFFFF:F
@A00721:190:HGJW7DSXY:4:1446:21206:7028 2:N:0:TCTGCAAG+GCTGCACT
CGTACATTTAATGCAACTTCCATCGCATATTGATGAAATGTCGCAAAAACTAAATAATAAGCTCCTTT
CATCATGTCCTCTACACTGCCACCAGTGGTGTCCTTAAAAACTGTGAGTCCACTAAGTTTCTTCCTCA
CAGAAATCGGTCTAG
+
FFFFFFF:FFFFFFFFFFFFFFFFFFFFFFFFFFFFFFFFFFFFFF,FFFFFFFFFFFFFFFFFFFFFFF
FFFFFFFFFFFFFFFFFFFFFFFFF:FFFFFFFFFFFFFFFFFFFFFFFFFFFFFFFFFFFFFFFFFFFF
FFFFFFFFFFFFFFF
@A00721:190:HGJW7DSXY:4:1462:3378:17331 2:N:0:TCTGCAAG+GCTGCACT
CGTACATTTAATGCAACTTCCATCGCATATTGATGAAATGTCGCAAAAACTAAATAATAAGCTCCTTT
CATCATGTCCTCTACACTGCCACCAGTGGTGTCCTTAAAAACTGTGAGTCCACTAAGTTTCTTCCTCA
CAGAAATCGGTCTAG
+
FFFFFFFFFFFFFFFFFFFFFFFFFFFFFFFFFFFFFFFFFFFF:FFFFFFFF:FFFFFFFFFFFFFFFF
FFFFFFFFFFFFFFFFFFFFFFFFFFFFFFFF:FFFFFFFFFFFFFFFFFFFFFFFFFFFFFFFFFFFFF
FFFFFFFFFFFFFFF
@A00721:190:HGJW7DSXY:4:1504:22978:20713 2:N:0:TCTGCAAG+GCTGCACT
CGTACATTTAATGCAACTTCCATCGCATATTGATGAAATGTCGCAAAAACTAAATAATAAGCTCCTTT
CATCATGTCCTCTACACTGCCACCAGTGGTGTCCTTAAAAACTGTGAGTCCACTAAGTTTCTTCCTCA
CAGAAATCGGTCTAG
+
FFFFFFFFFFFFFFF:FFFFFFFFFFFFFFFFFFFFFFFFFFFFF:FFF:FFFFFFFFFFFFFFFFFFFF
FFFFF:FFFFFFFFFFFFFFFFFFFFFFFFFFFFF:FFFFFFFFFFFFFFFFFFFFFFFFFFFFFFFFF,
FFFFF,FFFFFFFFF
@A00721:190:HGJW7DSXY:4:1536:11216:36135 2:N:0:TCTGCAAG+GCTGCACT
CGTACATTTAATGCAACTTCCATCGCATATTGATGAAATGTCGCAAAAACTAAATAATAAGCTCCTTT
CATCATGTCCTCTACACTGCCACCAGTGGTGTCCTTAAAAACTGTGAGTCCACTAAGTTTCTTCCTCA
CAGAAATCGGTCTAG
+
FFFFFFFFFFFFFFFFFFFFFFF:FFFFFFFFFFFFFFFFFFFFFFFFF:FFFFFFFFFFFFFFFFFFFF
F:FFFFFFFFFFFFFFFFFFFFFFFFFFFFFF:FFFFF:FFFFFFFFFFFFFFFFFF:FFFFFFFFFFFF
FFFFFFFFFFFFFFF
@A00721:190:HGJW7DSXY:4:2133:24153:5525 2:N:0:TCTGCAAG+GCTGCACT
CGTACATTTAATGCAACTTCCATCGCATATTGATGAAATGTCGCAAAAACTAAATAATAAGCTCCTTT
```

CATCATGTCCTCTACACTGCCACCAGTGGTGTCCTTAAAAACTGTGAGTCCACTAAGTTTCTTCCTCA
CAGAAATCGGTCTAG
+
FFFFFFFFFFFFFFFFFFFFFFFFFFFFFFFFFFFFFFFFFFFFFFFFFFFFFFFFFFFFFFFFFFFF
FFFFFFFFFFFFFFFFFFFFFFFFFFFFFFFFFFFFFFFFFFFFFFFFFFFFFFFFFFFFFFFF:FFF
FFFFFFFF:FFFFFF
@A00721:190:HGJW7DSXY:4:2178:5095:13260 2:N:0:TCTGCAAG+GCTGCACT
CGTACATTTAATGCAACTTCCATCGCATATTGATGAAATGTCGCAAAAACTAAATAATAAGCTCCTTT
CATCATGTCCTCTACACTGCCACCAGTGGTGTCCTTAAAAACTGTGAGTCCACTAAGTTTCTTCCTCA
CAGAAATCGGTCTAG
+
FFFFFFFFFFFFFFFFFFFFFFFFFFFFFFFFFFFFFFFFFFFFFFFFFFFFFFFFFFFFFFFFFFFF
FFFFFFFFFFFFFFFFFFFFFFFFFFFFFFFFFFFFFFFFFFFFFFFFFFFFFFFFFFFFFFFFFFFF
FFFFFFFFFFFFFFF
@A00721:190:HGJW7DSXY:4:2178:4490:14591 2:N:0:TCTGCAAG+GCTGCACT
CGTACATTTAATGCAACTTCCATCGCATATTGATGAAATGTCGCAAAAACTAAATAATAAGCTCCTTT
CATCATGTCCTCTACACTGCCACCAGTGGTGTCCTTAAAAACTGTGAGTCCACTAAGTTTCTTCCTCA
CAGAAATCGGTCTAG
+
FFFFFFFFFFFFFFF:FFFFFFFFFFFFFFFFFFFFFFFFFFFFFFFFFFFFFFFFFFFFFFFFFFFF
FFFFFFFFFFFFFFFFFFFFFFFFFFFFFFFFFFFFFFFFFFFFFFFFFFFFFFFFFFFFFFFFFFFF
FFFFFFFFFFFFFFF
@A00721:190:HGJW7DSXY:4:2346:1479:24158 2:N:0:TCTGCAAG+GCTGCACT
CGTACATTTAATGCAACTTCCATCGCATATTGATGAAATGTCGCAAAAACTAAATAATAAGCTCCTTT
CATCATGTCCTCTACACTGCCACCAGTGGTGTCCTTAAAAACTGTGAGTCCACTAAGTTTCTTCCTCA
CAGAAATCGGTCTAG
+
FFFFFFFFFFFFFFFFFFFFFFFFFFFFFFFFFFFFFFFFFFFFFFFFFFFFFFFFFFFFFFFFFFFF
FFFFFFFFFFFFFFFFFFFFFFFFFFFFFFFFFFFFFFFFFFFFFFFFFFFFFFFFFFFFFFFFFFFF
FFFFFFFFFFFFFFF
@A00721:190:HGJW7DSXY:4:2354:23882:36996 2:N:0:TCTGCAAG+GCTGCACT
CGTACATTTAATGCAACTTCCATCGCATATTGATGAAATGTCGCAAAAACTAAATAATAAGCTCCTTT
CATCATGTCCTCTACACTGCCACCAGTGGTGTCCTTAAAAACTGTGAGTCCACTAAGTTTCTTCCTCA
CAGAAATCGGTCTAG
+
FFFFFFFFFFFFFFFFFFFFFFFFFFFFFFFFFFFFFFFFFFFFFFFFFFFFFFFFFFFFFFFFFFFF
FFFFFFFFFFFFFFFFFFFFFFFFFFFFFFFFFFFFFFFFFFFFFFFFFFFFFFFFFFFFFFFFFFFF
FFFFFFFFFFFFFF:
@A00721:190:HGJW7DSXY:4:1144:14859:6308 2:N:0:TCTGCAAG+GCTGCACT
ATTCGTACATTTAATGCAACTTCCATCGCATATTGATGAAATGTCGCAAAAACTAAATAATAAGCTCC
TTTCATCATGTCCTCTACACTGCCACCAGTGGTGTCCTTAAAAACTGTGAGTCCACTAAGTTTCTTCC
TCACAGAAATCGGTC
+
F,FFFFF,F,:FFFF:FFFFFFFFF:FFFFFFFFFFFFFFFF:FFFFFFFFFFFFFFFFFFFF:F,FF
FFFFFFFFF:FFFFF:FFF,FFFFFF:FFFFFFFFFFF:FFFF::::FFFFFFFFFFFFFFFFFFFF:
F:FFFFFFFFFFFFF
@A00721:190:HGJW7DSXY:4:1144:15790:6355 2:N:0:TCTGCAAG+GCTGCACT
ATTCGTACATTTAATGCAACTTCCATCGCATATTGATGAAATGTCGCAAAAACTAAATAATAAGCTCC
TTTCATCATGTCCTCTACACTGCCACCAGTGGTGTCCTTAAAAACTGTGAGTCCACTAAGTTTCTTCC
TCACAGAAATCGGTC
+
FFFFFFFFFFFFFFFFFFFFFFFFFFFFFFFFFFFFFFFFFFFFFFFFFFFFFFFFFFFFFFFFFFFF
FFFFFFFFFFFFFFFFFFFFFFFFFFFFFFFFFFFFFFFFFFFFFFFFFFFFFFFFFFFFFFFFFFFF
FFFFFFFFFFFFFFF

```
@A00721:190:HGJW7DSXY:4:1323:12617:11569 2:N:0:TCTGCAAG+GCTGCACT
ATTCGTACATTTAATGCAACTTCCATCGCATATTGATGAAATGTCGCAAAAACTAAATAATAAGCTCC
TTTCATCATGTCCTCTACACTGCCACCAGTGGTGTCCTTAAAAACTGTGAGTCCACTAAGTTTCTTCC
TCACAGAAATCGGTC
+
FFF:FFFFFFFFFFFFFFFFFFFFFFFFFFFFFFFFFFFFFFFFFFFFFF,:FFFFFFFFFFFFFFFF
FFFFFFFFFFFFFFFFFFFFFFFFFFFFFFFFFFFFFFFFFFFFFFFFF:FFFFFFFFFFFFFFFFF:
FFFFFFFFFFFFFFF
@A00721:190:HGJW7DSXY:4:1403:32307:8281 2:N:0:TCTGCAAG+GCTGCACT
ATTCGTACATTTAATGCAACTTCCATCGCATATTGATGAAATGTCGCAAAAACTAAATAATAAGCTCC
TTTCATCATGTCCTCTACACTGCCACCAGTGGTGTCCTTAAAAACTGTGAGTCCACTAAGTTTCTTCC
TCACAGAAATCGGTC
+
FFFFFFFFFFFFFFFFFFFFFFFFFFFFFFFFFFFFFFFFFFFFFFFFFFFFFFFFFFFFFFF:FFFF
FFFFFFFFFFFFFFFFFFFFFFFFFFFFFFFFFFFFFFFFFFFFFFFFFFFFFFFFFFFFFFFFFFFF
FFFFFFFFFFFF:FF
@A00721:190:HGJW7DSXY:4:1566:9227:8265 2:N:0:TCTGCAAG+GCTGCACT
ATTCGTACATTTAATGCAACTTCCATCGCATATTGATGAAATGTCGCAAAAACTAAATAATAAGCTCC
TTTCATCATGTCCTCTACACTGCCACCAGTGGTGTCCTTAAAAACTGTGAGTCCACTAAGTTTCTTCC
TCACAGAAATCGGTC
+
FFFFFFFFFFFFFFFFFF:FFFFFFFF:FFFFFFFFFFFFFFFFFFFFFFFFFFFFFFFFFFFFFFFF
FFFFFFFFFFFFFFFFFFFFFFFFFFFFFFFFFFFFFFFFFFFFFFFFFFFFFFFFFFFFFFFFFFFF
FFFFF:FFFFFFFFF
@A00721:190:HGJW7DSXY:4:2571:26313:11334 2:N:0:TCTGCAAG+GCTGCACT
ATTCGTACATTTAATGCAACTTCCATCGCATATTGATGAAATGTCGCAAAAACTAAATAATAAGCTCC
TTTCATCATGTCCTCTACACTGCCACCAGTGGTGTCCTTAAAAACTGTGAGTCCACTAAGTTTCTTCC
TCACAGAAATCGGTC
+
FFFFFFFFFFFFFFFFFFFFFFFFFFFFFFFFFFFFFFFFFFFFFFFFFFFFFFFFFFFFFFFFFFFF
FFFFFFFFFFFFFFFFFFFFFF:FFFFFFFFFFFFFFFFFFFFFFFFFFFFFFFFFFFFFFFFFFFFF
FFFFF:FFFFFFFFF
@A00721:190:HGJW7DSXY:4:1457:28619:13542 2:N:0:TCTGCAAG+GCTGCACT
CACATTCGTACATTTAATGCAACTTCCATCGCATATTGATGAAATGTCGCAAAAACTAAATAATAAGC
TCCTTTCATCATGTCCTCTACACTGCCACCAGTGGTGTCCTTAAAAACTGTGAGTCCACTAAGTTTCT
TCCTCACAGAAATCG
+
FFFFFFFFFFFFFF:FFFFFFFFFFFF:FFFFFFFFFFFFFFFFFFFFFFFFFFFFFFFFFFFFFF,FF
FF:FFFFFFFFFFFFFFFFFFFFFF:FFFFFF:FFFFFFFFF,:FFFFFFFFFFF,FFF:FFFFFFFF
F,FFFFFFFFF:,FF
@A00721:190:HGJW7DSXY:4:1576:25084:30029 2:N:0:TCTGCAAG+GCTGCACT
CACATTCGTACATTTAATGCAACTTCCATCGCATATTGATGAAATGTCGCAAAAACTAAATAATAAGC
TCCTTTCATCATGTCCTCTACACTGCCACCAGTGGTGTCCTTAAAAACTGTGAGTCCACTAAGTTTCT
TCCTCACAGAAATCG
+
FFFFFFFFFFFFFFFFFFFFFFFFFFFFFFFFFFFFFFFFFFFFF:FFFFFFFFFF:FFFFFFFFFF:FF
FFFFFFFFFFFFFFFFFFFFFFFFFFFFFFFFFFFFFFFFFFFF,,:FFFFFFFFFFFF:FFFFFFFFF
FFFFFFFFFFFFFFF
@A00721:190:HGJW7DSXY:4:2117:14769:10285 2:N:0:TCTGCAAG+GCTGCACT
ACATTCGTACATTTAATGCAACTTCCATCGCATATTGATGAAATGTCGCAAAAACTAAATAATAAGCT
CCTTTCATCATGTCCTCTAAACTGCCACCAGTGGTGTCATTAAAAACTGTGAGTCCACTAAGTTTCTT
CCTCACAGAAATCG
+
FF:,:FFFFFFFFFFFFFFFFFFFF,FFFF:FFFF:FFFFFFFF:F,FFF:FFFFFFFF:FFFFFFFF
```

:::FFFFFFF,:FF,F:FF,FFFFFFFFFFFFFFFFFF,FFFFFFFF:FFFF::F,FFF,FFF:FF:F
F,F:F:FF,FFF:F
@A00721:190:HGJW7DSXY:4:2165:7419:29496 2:N:0:TCTGCAAG+GCTGCACT
CACATTCGTACATTTAATGCAACTTCCATCGCATATTGATGAAATGTCGCAAAAACTAAATAATAAGC
TCCTTTCATCATGTCCTCTACACTGCCACCAGTGGTGTCCTTAAAAACTGTGAGTCCACTAAGTTTCT
TCCTCACAGAAATCG
+
FFFFFFFFFFFFFFFFFFFFFFFFFFFFFFFFFFFFFFFFFFFFFFFFFFFFF:FFFFFFFFFFFFFF
FFFFFFFFFFFFFFFFFFFFFFFFFFFFFFFF:FFFFFFFFFFFFFF:FFFFFFFFFFFFFFFF:FFF
FFFFFFFFFF,FFFF
@A00721:190:HGJW7DSXY:4:2471:9824:28651 2:N:0:TCTGCAAG+GCTGCACT
CACATTCGTACATTTAATGCAACTTCCATCGCATATTGATGAAATGTCGCAAAAACTAAATAATAAGC
TCCTTTCATCATGTCCTCTACACTGCCACCAGTGGTGTCCTTAAAAACTGTGAGTCCACTAAGTTTCT
TCCTCACAGAAATCG
+
FFFFFFFFFFFFFFFFFFFFFFFFFFFFFFFFFFFFFFFFFFFFFFFFFFFF:FFFFFFFFFFFFFFF
FFFF:FFFFFFFFFFFFFFFFFFFFFFFFFFFFFFFFFFFF,FFFFFFFFFFFFFFFFFFFFFFFFFF
FFFFFFFF,FFF,FF
@A00721:190:HGJW7DSXY:4:2535:23981:15781 2:N:0:TCTGCAAG+GCTGCACT
CACATTCGTACATTTAATGCAACTTCCATCGCATATTGATGAAATGTCGCAAAAACTAAATAATAAGC
TCCTTTCATCATGTCCTCTACACTGCCACCAGTGGTGTCCTTAAAAACTGTGAGTCCACTAAGTTTCT
TCCTCACAGAAATCG
+
FFFFFFFFFFFFFFFFFFFFFFFFFFFFFFFF:FFFFFFFFFFFFFFFF:FFFFFFFFFFFFFFFF:F
FFFFFFFFFFF:FFFFFFFFFFFFFF:FFFFFFFFFFFFFFFFFFFFFFFFFFFFFF:FF,FFF:FFF
FF,FFFFFFFFFFFF
@A00721:190:HGJW7DSXY:4:1102:12545:14418 2:N:0:TCTGCAAG+GCTGCACT
CCACATTCGTACATTTAATGCAACTTCCATCGCATATTGATGAAATGTCGCAAAAACTAAATAATAAG
CTCCTTTCATCATGTCCTCTACACTGCCACCAGTGGTGTCCTTAAAAACTGTGAGTCCACTAAGTTTC
TTCCTCACAGAAATC
+
FFFFFFFFFFFFFFFFFFFFFFFFFFFFFFFFFFFFFFFFFFFFFFFFFFFFFFF:FFFFFFFFFFFF
FFFFFFFFFFFFFFFFFFFFFFFFFFFFFFFFFFFFFFFFFFFFFFFFFFFFFFFFFFFFFFFFFFFF
FFFFFFFFFFFF,FF
@A00721:190:HGJW7DSXY:4:1104:10601:22639 2:N:0:TCTGCAAG+GCTGCACT
CCACATTCGTACATTTAATGCAACTTCCATCGCATATTGATGAAATGTCGCAAAAACTAAATAATAAG
CTCCTTTCATCATGTCCTCTACACTGCCACCAGTGGTGTCCTTAAAAACTGTGAGTCCACTAAGTTTC
TTCCTCACAGAAATC
+
FFFFFFFFFFFFFFFFFFFFFFFFFFFFFFFFFFFFFFFFFFFFFFFFFFFFFFFFFFFFFFFFFFFF
FFFFFFFFFFFFFFFFFFFFFFFFFFFFFFFFFFFFFFFFFFFFFFFFFFFFFFFFFFFFFFFFFFFF
FFFFFFFFFFFFFFF
@A00721:190:HGJW7DSXY:4:1127:14543:20791 2:N:0:TCTGCAAG+GCTGCACT
CCACATTCGTACATTTAATGCAACTTCCATCGCATATTGATGAAATGTCGCAAAAACTAAATAATAAG
CTCCTTTCATCATGTCCTCTACACTGCCACCAGTGGTGTCCTTAAAAACTGTGAGTCCACTAAGTTTC
TTCCTCACAGAAATC
+
FFFFFFFFFFFFFFFFFFFFFFFFFFFFFFFFFFFFFFFFFFFFFFFFFFFFFFF:FFFFFFFFFFFF
FFF:FFFFFFFFFFFFFFFFFFFFFFFFFFFFFFFFFFFFFFFFFFFFFFFFFFFFFFFFFFFFFFFF
FFF:FFFFFFFF:FF
@A00721:190:HGJW7DSXY:4:1150:2239:13730 2:N:0:TCTGCAAG+GCTGCACT
GCACATTCGTACATTTAATGCAACTTCCATCGCATATTGATGAAATGTCGCAAAAACTAAATAATAAG
CTCCTTTCATCATGTCCTCTACACTGCCACCAGTGGTGTCCTTAAAAACTGTGAGTCCACTAAGTTTC
TTCCTCACAGAAATC

+
FFFFFFFFFFFFFFFFFFFFFFFFFFFFFFFFFFFFFFFFFFFFFFFFFFFFFFFFFFFFFFFFFFFFF
FFFFFFFFFFFFFF:FFFFFFFFFFFFFFFFFFFFFFFFFFFFFFFFFFFFFFFFFFFFFFF:FFFFFF
FF:FFFFFFFFFFFF
@A00721:190:HGJW7DSXY:4:1169:21829:25770 2:N:0:TCTGCAAG+GCTGCACT
CCACATTCGTACATTTAATGCAACTTCCATCGCATATTGATGAAATGTCGCAAAAACTAAATAATAAG
CTCCTTTCATCATGTCCTCTACACTGCCACCAGTGGTGTCCTTAAAAACTGTGAGTCCACTAAGTTTC
TTCCTCACAGAAATC
+
FFFFFFFFFFFFFFFFFFFFFFFFFFFFFFFFFFFFFFFFFFFFFFFFFFFFFFFFFFFFFFFFFFFFF
FFFFFFFFFFFFFFFFFFFFFFFFFFFF:FFFFFFFFFFFFFFFFFFFFFFFFFFFFFFFFFFFFFFFF
FFFFFFFFFF:FFFF
@A00721:190:HGJW7DSXY:4:1232:3721:19774 2:N:0:TCTGCAAG+GCTGCACT
CCACATTCGTACATTTAATGCAACTTCCATCGCATATTGATGAAATGTCGCAAAAACTAAATAATAAG
CTCCTTTCATCATGTCCTCTACACTGCCACCAGTGGTGTCCTTAAAAACTGTGAGTCCACTAAGTTTC
TTCCTCACAGAAATC
+
FFFF:FFFFFFFFFFFFFFFFFFFFFFFFFFFFFFFFFFFFFFFFFFFFFFFFFFFFFFFFFFFFFFFF
FFFFFFFFFFFFFFFFFFFFFFFFFFFFFFFFFFFFFFFFFFFFFFFFFFFFFFFFFFFFFFFFFFFFF
FFFFFFFFFFFFFFF
@A00721:190:HGJW7DSXY:4:1237:5321:24267 2:N:0:TCTGCAAG+GCTGCACT
CCACATTCGTACATTTAATGCAACTTCCATCGCATATTGATGAAATGTCGCAAAAACTAAATAATAAG
CTCCTTTCATCATGTCCTCTACACTGCCACCAGTGGTGTCCTTAAAAACTGTGAGTCCACTAAGTTTC
TTCCTCACAGAAATC
+
FFFFFFFFFFFFFFFFFFFFFFFFFFFFFFFFFFFFFFFFFFFFFFFFFFFFFFFFFFFFFFFFFFFFF
FFF:FFFFFFFFFFFFFFFFFFFFFFFFFFFFFFF:FFFFFFFFFFFFFFFFFFFFFFFFFFFFFFFFF
FFFFFFFFFFFFFFF
@A00721:190:HGJW7DSXY:4:1243:21486:16814 2:N:0:TCTGCAAG+GCTGCACT
CCACATTCGTACATTTAATGCAACTTCCATCGCATATTGATGAAATGTCGCAAAAACTAAATAATAAG
CTCCTTTCATCATGTCCTCTACACTGCCACCAGTGGTGTCCTTAAAAACTGTGAGTCCACTAAGTTTC
TTCCTCACAGAAATC
+
FFFFFFFFFFFFFFFFFFFFFFFFFFFFFFFFFFFFFFFFFFFFFFFFFFFFFFFFFFFFFFFFFFFFF
FFFFFFFFFFFFFFFFFFFFFFFFFFFFFFFFFFFFFFFFFFFFFFFFFFFFFFFFFFFFFFFFFFFFF
FFFFFFFFFFFFFFF
@A00721:190:HGJW7DSXY:4:1266:4264:23657 2:N:0:TCTGCAAG+GCTGCACT
GCACATTCGTACATTTAATGCAACTTCCATCGCATATTGATGAAATGTCGCAAAAACTAAATAATAAG
CTCCTTTCATCATGTCCTCTACACTGCCACCAGTGGTGTCCTTAAAAACTGTGAGTCCACTAAGTTTC
TTCCTCACAGAAATC
+
FFFF:FFFFFFFFFFFFFFFFFFFFFFFFFFFFFFFFFFFFFF:FFFFFFFFFFFFF:FFFFFFFFFF
FFFF,FFFFFFFFFFFFFFFFFFFFFFFFFFFFFFFFFFFFFFFFFFFFFFFFFFFFFFFFFFFFFFFF
FFFFFFFFFFFFFFF
@A00721:190:HGJW7DSXY:4:1269:24234:31845 2:N:0:TCTGCAAG+GCTGCACT
CCACATTCGTACATTTAATGCAACTTCCATCGCATATTGATGAAATGTCGCAAAAACTAAATAATAAG
CTCCTTTCATCATGTCCTCTACACTGCCACCAGTGGTGTCCTTAAAAACTGTGAGTCCACTAAGTTTC
TTCCTCACAGAAATC
+
F:FFFFFFFFFFFFFFFFFFFFFFFFFFFFFFFFFFFFFFFFFFFFFFFFFFFFF:FFFFFFFFFFFFF
FFFFFFFFFFFFFFFFFFFFFFFFFFFFFFFFFFFFFFFFFFFFFFFFFFFFFFFFFFFFFFFFFFFFF
FFFFFFFFFFFFFFF
@A00721:190:HGJW7DSXY:4:1275:10013:33238 2:N:0:TCTGCAAG+GCTGCACT
CCACATTCGTACATTTAATGCAACTTCCATCGCATATTGATGAAATGTCGCAAAAACTAAATAATAAG

CTCCTTTCATCATGTCCTCTACACTGCCACCAGTGGTGTCCTTAAAAACTGTGAGTCCACTAAGTTTC
TTCCTCACAGAAATC
+
FFFFFFFFFFFFFFFFFFFFFFFFFFFFFFFFFFFFFFF:FFFFFFFFFFFFFFFFFFFFFFFFFFFF
FFFFFFFFFFFFFFFFFFFFFF:FFFFFFFFFFFFFFFFF:FFFFFFFFFFFFFFFFFFFFFFFFFFF
FFFFF:FF:FFFFFF
@A00721:190:HGJW7DSXY:4:1307:20220:15280 2:N:0:TCTGCAAG+GCTGCACT
CCACATTCGTACATTTAATGCAACTTCCATCGCATATTGATGAAATGTCGCAAAAACTAAATAATAAG
CTCCTTTCATCATGTCCTCTACACTGCCACCAGTGGTGTCCTTAAAAACTGTGAGTCCACTAAGTTTC
TTCCTCACAGAAATC
+
F:FFFFFFFFFFFFFFFFFFFFFFFFFFFFFFFF,FFFFFFFFFFFFFFFFFFFF:FFFF:FFFFFFF
FFFFFFF:FFFFFFFFFFFFFFF,F:F:FFFFF:FFFFFF:FFFFFFFFFFF::FFFFFFFFF,FFFF
FFFF:FFFF:F,F:F
@A00721:190:HGJW7DSXY:4:1307:19596:15389 2:N:0:TCTGCAAG+GCTGCACT
CCACATTCGTACATTTAATGCAACTTCCATCGCATATTGATGAAATGTCGCAAAAACTAAATAATAAG
CTCCTTTCATCATGTCCTCTACACTGCCACCAGTGGTGTCCTTAAAAACTGTGAGTCCACTAAGTTTC
TTCCTCACAGAAATC
+
FFFFFFFFFFFFFFFFFFFFFFFFFFFFFFFFFFFFFFFFFFFFFFFFFFFFFFFFFFFFFFFFFFFF
FFFFFFFFFFFFFFFFFFFFFFFFFFFFFFFFFFFFFFFFFFFFFFFFFFFFFFFFFFF:FFFFFFFF
FFFFFFFFFFFFF,F
@A00721:190:HGJW7DSXY:4:1312:30273:32252 2:N:0:TCTGCAAG+GCTGCACT
CCACATTCGTACATTTAATGCAACTTCCATCGCATATTGATGAAATGTCGCAAAAACTAAATAATAAG
CTCCTTTCATCATGTCCTCTACACTGCCACCAGTGGTGTCCTTAAAAACTGTGAGTCCACTAAGTTTC
TTCCTCACAGAAATC
+
:FFFFFFFFFFFFFFFFFFFFFFFFFFFFFFFFFFFFFFFFFFFFFFFFFFFFFF:FFFFFFFFFFFF
FFFFFFFFFFFFFFFFFFFFFFFFFFFFFFFFFFFFFFFFFFFFFFFFFFFFFFFFFFFFFFFFFFFF
FFFFFFFFFFFFFFF
@A00721:190:HGJW7DSXY:4:1323:1741:29778 2:N:0:TCTGCAAG+GCTGCACT
CCACATTCGTACATTTAATGCAACTTCCATCGCATATTGATGAAATGTCGCAAAAACTAAATAATAAG
CTCCTTTCATCATGTCCTCTACACTGCCACCAGTGGTGTCCTTAAAAACTGTGAGTCCACTAAGTTTC
TTCCTCACAGAAATC
+
FFFFFFFFFFFFFFFFFFFFFFFFFFFFFFFFFFFFFFFFFFFF:FFFFFFF,FFFFFFFFFFFFFFF
FFFFFFFFFFFFFFFF:FFFFFFFFFFFFFFFFFFFFFFFFFFFFFFFFFFFFFFFFFFFFFFFFFF
FFFFFFFFFFFFFFF
@A00721:190:HGJW7DSXY:4:1324:13015:34021 2:N:0:TCTGCAAG+GCTGCACT
CCACATTCGTACATTTAATGCAACTTCCATCGCATATTGATGAAATGTCGCAAAAACTAAATAATAAG
CTCCTTTCATCATGTCCTCTACACTGCCACCAGTGGTGTCCTTAAAAACTGTGAGTCCACTAAGTTTC
TTCCTCACAGAAATC
+
FFFFFFFFFFFFFFFFFFFFFFFFFFFFFFFFFFFFFFFFFFFFFFFFFFFFFFFFFFFFFFFFFFFF
FFFFFFFFFFFFFFFFFFFFFFFFFFFFFFFFFFFFFFFFFFFFFFFFFFFFFFFFFFFFFFFFFFFF
FFFFFFFFFFFFFFF
@A00721:190:HGJW7DSXY:4:1360:15989:10081 2:N:0:TCTGCAAG+GCTGCACT
CCACATTCGTACATTTAATGCAACTTCCATCGCATATTGATGAAATGTCGCAAAAACTAAATAATAAG
CTCCTTTCATCATGTCCTCTACACTGCCACCAGTGGTGTCCTTAAAAACTGTGAGTCCACTAAGTTTC
TTCCTCACAGAAATC
+
FFFFFFFFFFFFFFFFFFFFFFFFFFFFFFFFFFFFFFFFFFFFFFFFFFFFFFFFFFFFFFFFFFFF
FFFFFFFFFFFFFFFFFFFFFFFFFFFFFFFFFFFFFFFFFF,FFFFFF:FFFFFFFFFFFFFFFFFF
FFFFFFFFFFFFFFF

```
@A00721:190:HGJW7DSXY:4:1411:29568:29465 2:N:0:TCTGCAAG+GCTGCACT
CCACATTCGTACATTTAATGCAACTTCCATCGCATATTGATGAAATGTCGCAAAAACTAAATAATAAG
CTCCTTTCATCATGTCCTCTACACTGCCACCAGTGGTGTCCTTAAAAACTGTGAGTCCACTAAGTTTC
TTCCTCACAGAAATC
+
F,FFFFFFFFFFFFFFFFFFFFFFFFFFFFF:FFFFFFFFFFFFFFFFFFF,F::F,FFFFFFFFFF:
:FFFFFF:FFFFFFF::FFFFFF:FFFFFFFF:FFFFFFF,FFFFFFFFF,:,FFFF,F,::F:FFF,
FF:FFFF,FFFFFF:
@A00721:190:HGJW7DSXY:4:1414:28917:10614 2:N:0:TCTGCAAG+GCTGCACT
CCACATTCGTACATTTAATGCAACTTCCATCGCATATTGATGAAATGTCGCAAAAACTAAATAATAAG
CTCCTTTCATCATGTCCTCTACACTGCCACCAGTGGTGTCCTTAAAAACTGTGAGTCCACTAAGTTTC
TTCCTCACAGAAATC
+
:FFFF::F:FFFFFF,FFFF:FF:FF:FFFFF,FFFFFFFFFFFFF,FF,FFFFF,F:FF:F:FFFFF
,FFFFFFFFFFFFFFFFFFFFFFFF:FFFFFF:F::FFFFFFF:F::FF,FFF,F:F,,FFFF,:FF,F
FFFFFFFFF::F:FF
@A00721:190:HGJW7DSXY:4:1472:13919:20400 2:N:0:TCTGCAAG+GCTGCACT
GCACATTCGTACATTTAATGCAACTTCCATCGCATATTGATGAAATGTCGCAAAAACTAAATAATAAG
CTCCTTTCATCATGTCCTCTACACTGCCACCAGTGGTGTCCTTAAAAACTGTGAGTCCACTAAGTTTC
TTCCTCACAGAAATC
+
FFFFFFFFFFFFF,FFFFFFFFFFFFFFFFFFFFFFFFFFFF:FFFFFFFFFFFFFFFFFF::FFFFF
FFFFFF:FF:FFFFF:FFFFF,:FFF:FFFFFFFFF:FFFFF:FFFFFFFFFFFFF:,FF,FF:FFFF
FFF,FFFFFFFFFFF
@A00721:190:HGJW7DSXY:4:1501:12780:15358 2:N:0:TCTGCAAG+GCTGCACT
GCACATTCGTACATTTAATGCAACTTCCATCGCATATTGATGAAATGTCGCAAAAACTAAATAATAAG
CTCCTTTCATCATGTCCTCTACACTGCCACCAGTGGTGTCCTTAAAAACTGTGAGTCCACTAAGTTTC
TTCCTCACAGAAATC
+
FFFFFFFFFFFFFFFFFFFFFFFFFFFFFFFFFFFFFFFFFFFFFFFFFF:FFFFF:FFFFFFFFFFF
FF:FFFFFFFFFFFFFFFFFFFFFFFFFFFFFFFFFFFFFFFFFFFFFFFFFFFFFFFFFFFF:FFFF
FFFFFFFFFFFFFFF
@A00721:190:HGJW7DSXY:4:1508:12066:23891 2:N:0:TCTGCAAG+GCTGCACT
CCACATTCGTACATTTAATGCAACTTCCATCGCATATTGATGAAATGTCGCAAAAACTAAATAATAAG
CTCCTTTCATCATGTCCTCTACACTGCCACCAGTGGTGTCCTTAAAAACTGTGAGTCCACTAAGTTTC
TTCCTCACAGAAATC
+
FFFFFFFFFFFFFFFFFFFFFFFFFFFFFFFFFFFFFFFFFFFFFFFFFFFFFFFFFFFFFFFFFFFF
FFFFFFFFFFFFFFFFFFFFFFFFFFFFFFFFFFFFFFFFFFFFFFFFFFFFFFF:FFFFFFFFFFFF
FFFFFFFFFFFFFFF
@A00721:190:HGJW7DSXY:4:1509:29668:7874 2:N:0:TCTGCAAG+GCTGCACT
CCACATTCGTACATTTAATGCAACTTCCATCGCATATTGATGAAATGTCGCAAAAACTAAATAATAAG
CTCCTTTCATCATGTCCTCTACACTGCCACCAGTGGTGTCCTTAAAAACTGTGAGTCCACTAAGTTTC
TTCCTCACAGAAATC
+
FFFFFFFFFFFFFFFFFFFFFFFFFFFFFFFFFFFFFFFFFFFFFFFFFFFFFFFFFFFFFFFFFFFF
FFFFFFFFFFFFFFFFFFFFFFFFFFFFFFFFFFFFFFFFFFFFFFFFFFFFFFFFFFFFFFFFFFFF
FFFFFFFFFFFFFFF
@A00721:190:HGJW7DSXY:4:1522:23873:4319 2:N:0:TCTGCAAG+GCTGCACT
CCACATTCGTACATTTAATGCAACTTCCATCGCATATTGATGAAATGTCGCAAAAACTAAATAATAAG
CTCCTTTCATCATGTCCTCTACACTGCCACCAGTGGTGTCCTTAAAAACTGTGAGTCCACTAAGTTTC
TTCCTCACAGAAATC
+
FFFFFF:FFFFFFFFFFFFFFFFFFFFFFFFFFFFFFFFFFFFFFFFFFFF:FFFFFFFFFFFFFFFF
```

FFFFFFFFFFFFFFFFFFFFFFFFFFFFFFFFFFFFFFFFFFFFFFFFFFFFFFFFFFFFFFFFFFFF
FFFFFFFFFFFFFFF
@A00721:190:HGJW7DSXY:4:1565:25880:21543 2:N:0:TCTGCAAG+GCTGCACT
CCACATTCGTACATTTAATGCAACTTCCATCGCATATTGATGAAATGTCGCAAAAACTAAATAATAAG
CTCCTTTCATCATGTCCTCTACACTGCCACCAGTGGTGTCCTTAAAAACTGTGAGTCCACTAAGTTTC
TTCCTCACAGAAATC
+
FFFFFFFFFFFFFFFFFFFFFFFFFFFFFFFFFFFFFFFFFFFFFFFFFFFFFFFFFFFFFFFFFFFF
FFFF:FFFFFFFFFFFFFFFFFFFFFFFFFFFFFFFFFFFFFFFFFFFFFFFFFFFFFFFFFFFFFFF
FFFFFFFFFFFFFFF
@A00721:190:HGJW7DSXY:4:1565:24948:21840 2:N:0:TCTGCAAG+GCTGCACT
CCACATTCGTACATTTAATGCAACTTCCATCGCATATTGATGAAATGTCGCAAAAACTAAATAATAAG
CTCCTTTCATCATGTCCTCTACACTGCCACCAGTGGTGTCCTTAAAAACTGTGAGTCCACTAAGTTTC
TTCCTCACAGAAATC
+
FFFFFFFFFFFFFFFFFF:FFFFFFFFFFFFFFFFFFFFFFFFFFFFFFFF:FFFF:FFF:FFFFFFF
FFF,FFFFFFFFFFF:FFFFFFFFFFFFFFFFFFFFFFFF,FFFFFFF:FFFFFFFF:FFFFFFF:F:
FFFFFFFFFFFFFFF
@A00721:190:HGJW7DSXY:4:1610:23502:25723 2:N:0:TCTGCAAG+GCTGCACT
CCACATTCGTACATTTAATGCAACTTCCATCGCATATTGATGAAATGTCGCAAAAACTAAATAATAAG
CTCCTTTCATCATGTCCTCTACACTGCCACCAGTGGTGTCCTTAAAAACTGTGAGTCCACTAAGTTTC
TTCCTCACAGAAATC
+
FFFFFFFFFFFFFFFFFFFFFF:FF:FFFFFFFFFFFFFFFFFFFFFFFFFFFFFFFFFFFFFFFFFF
FFFFF:FFFFF:FFFFFFF:FFF:FFFFFFFFF:FFFFFF:FFFFFFFFFFFFFFFFFFFFFF:FFFF
FFFFFFFFFFFFFFF
@A00721:190:HGJW7DSXY:4:1654:25211:35759 2:N:0:TCTGCAAG+GCTGCACT
CCACATTCGTACATTTAATGCAACTTCCATCGCATATTGATGAAATGTCGCAAAAACTAAATAATAAG
CTCCTTTCATCATGTCCTCTACACTGCCACCAGTGGTGTCCTTAAAAACTGTGAGTCCACTAAGTTTC
TTCCTCACAGAAATC
+
FFFFFFFFFFFFFFFFFFFFFFFFFFFF:FFFFFFFFFFFFFFFFFFFFFFFFFFFFFFFFFFFFFFF
FFFFFFFFFFFFFFFFFFFFFFFFFFFFFFFFFFF:FFFFFFFFFFFFFFFFFFFFFFFFFFFFFFFF
FFF:FFFFFFFFFFF
@A00721:190:HGJW7DSXY:4:2105:25663:23547 2:N:0:TCTGCAAG+GCTGCACT
CCACATTCGTACATTTAATGCAACTTCCATCGCATATTGATGAAATGTCGCAAAAACTAAATAATAAG
CTCCTTTCATCATGTCCTCTACACTGCCACCAGTGGTGTCCTTAAAAACTGTGAGTCCACTAAGTTTC
TTCCTCACAGAAATC
+
FFFFFFFFFFFFFFFFFFFFFFFFFFFFFFFFFFFFFFFFFFFFFFFFFFFFFFFFFFFFFFFFFFFF
FFFFFFFFFFFFFFFFFFFFFFFFFFFFFFFFFFFFFFFFFFFFFFFFFFFFFFFFFFFFFFFFFFFF
FFFFFFFFFFFFFFF
@A00721:190:HGJW7DSXY:4:2126:3676:32409 2:N:0:TCTGCAAG+GCTGCACT
CCACATTCGTACATTTAATGCAACTTCCATCGCATATTGATGAAATGTCGCAAAAACTAAATAATAAG
CTCCTTTCATCATGTCCTCTACACTGCCACCAGTGGTGTCCTTAAAAACTGTGAGTCCACTAAGTTTC
TTCCTCACAGAAATC
+
FFFFFFFFFFFFFFFFFFFFFFFFFFFFFFFFFFFFFFFFFFFFFFFFFFFFFFFFFFFFFFFFFFFF
FFFFFFFFFFFFFFFFFFFFFFFFFFFFFFF:FFFFFFFFFF,FFFFFFFFFFFFFFFFFFFFFFFFF
FFFFFFFFFFFFFFF
@A00721:190:HGJW7DSXY:4:2212:16143:34334 2:N:0:TCTGCAAG+GCTGCACT
CCACATTCGTACATTTAATGCAACTTCCATCGCATATTGATGAAATGTCGCAAAAACTAAATAATAAG
CTCCTTTCATCATGTCCTCTACACTGCCACCAGTGGTGTCCTTAAAAACTGTGAGTCCACTAAGTTTC
TTCCTCACAGAAATC

```
+
FFFFFFFFFFFFFFFFFFFFFFFFFFFFFFFFFFFFFFFFFFFFFFFFFFFFFFFFFFFFFFFFFFFF
FFFFFFFFFFFFFFFFFFFFFFFFFFFFFFFFFFFFFFFFFFFFFFFFFFFFFFFFFFFFFFFFFFFF
FFFFFFFFFFFFFFF
@A00721:190:HGJW7DSXY:4:2266:5728:1204 2:N:0:TCTGCAAG+GCTGCACT
CCACATTCGTACATTTAATGCAACTTCCATCGCATATTGATGAAATGTCGCAAAAACTAAATAATAAG
CTCCTTTCATCATGTCCTCTACACTGCCACCAGTGGTGTCCTTAAAAACTGTGAGTCCACTAAGTTTC
TTCCTCACAGAAATC
+
FFFFFFFFFFFFFFFFFFFFFFFFFFFFFFFFFFFFFFFFFFFFFFFFFFFFFFFFFFFFFFFFFFFF
FFFFFFFFFFFFFFFFFFFFFFFFFFFFFFFFFFFFFFFFFFFFFFFFFFFFFFFFFFFFFFFFFFFF
FFFFFFFFFFFFFFF
@A00721:190:HGJW7DSXY:4:2302:2781:25254 2:N:0:TCTGCAAG+GCTGCACT
CCACATTCGTACATTTAATGCAACTTCCATCGCATATTGATGAAATGTCGCAAAAACTAAATAATAAG
CTCCTTTCATCATGTCCTCTACACTGCCACCAGTGGTGTCCTTAAAAACTGTGAGTCCACTAAGTTTC
TTCCTCACAGAAATC
+
FFFF:FFFFFFFFFFFFFFFFFFFFFFFFFFFFFFFFFFFFFFFFFFFFFFFFFFFFFFFFFFFFFFF
FFFFFFFFFFFFFFFFFFFFFFFFFFFFFFFFFFFF:FFFFFFFFFFF,FFFFFFFFFFFFFFFFFFF
FFFFFFFFFFFFFFF
@A00721:190:HGJW7DSXY:4:2334:26648:20369 2:N:0:TCTGCAAG+GCTGCACT
CCACATTCGTACATTTAATGCAACTTCCATCGCATATTGATGAAATGTCGCAAAAACTAAATAATAAG
CTCCTTTCATCATGTCCTCTACACTGCCACCAGTGGTGTCCTTAAAAACTGTGAGTCCACTAAGTTTC
TTCCTCACAGAAATC
+
FFFF:FFFFFFFFFFFFFFFFF,FFFFFFFFFFFFFFFFFFFFFFFFFFFFFFFFFFFFFFFFFFF:F
FFFFFFFFFFFFFFFFFFFFFFFFFFFFFFFFFFFFFFFFFFFFFFFFFFFFFF:FFFFFFF:FFFFF
FFFFFFFFFFFF,FF
@A00721:190:HGJW7DSXY:4:2355:28004:4398 2:N:0:TCTGCAAG+GCTGCACT
CCACATTCGTACATTTAATGCAACTTCCATCGCATATTGATGAAATGTCGCAACAACTAAATAATAAG
CTCCTTTCATCATGTCCTCTACACTGCCACCAGTGGTGTCCTTAAAAACTGTGAGTCCACTAAGTTTC
TTCCTCACAGAAATC
+
FFFFFFFFFFFFFFFFFFFFF,FFFFFFFFFFFFFFFFFFFFFFFFFFFFFFF,F,FFFFF:FFFFFF
FFFFFFFFF:FF:FFFFFFFFFFFFFFFFFFFFFFFFFFFFF:FFFFFFFF,FFFFFF:FF,:FFFFF
FFFFFFFFFF,F::F
@A00721:190:HGJW7DSXY:4:2362:28854:30295 2:N:0:TCTGCAAG+GCTGCACT
CCACATTCGTACATTTAATGCAACTTCCATCGCATATTGATGAAATGTCGCAAAAACTAAATAATAAG
CTCCTTTCATCATGTCCTCTACACTGCCACCAGTGGTGTCCTTAAAAACTGTGAGTCCACTAAGTCTC
TTCCTCACAGAAATC
+
FFFFFFFFFFFFFFFFFFFFFFFFFFFFFFFFFFFFFFFFFFFFFFFFFFFFFFFFFFFFFFFFFFFF
FFFFFFFFFFFFFFFFFFFFFFFFFFFFFFFFFFFFFFFFFFFFFFFFFFFFFFFFFFFFFFFFFFFF
FFFFFFFFFFFFFFF
@A00721:190:HGJW7DSXY:4:2434:12952:3756 2:N:0:TCTGCAAG+GCTGCACT
CCACATTCGTACATTTAATGCAACTTCCATCGCATATTGATGAAATGTCGCAAAAACTAAATAATAAG
CTCCTTTCATCATGTCCTCTACACTGCCACCAGTGGTGTCCTTAAAAACTGTGAGTCCACTAATTTTC
TTCCTCACAGAAATC
+
:FFFFF:,FFFFFFFFFFFFFFFFFFFFFFF,,FFF:,:FFFFF:,FF:::FFFFFFFFF:FFFFFFF
FFFF:FFFFF:FF,FFF:FFFFFFF:,FFFFFFF::F::FFFFFFFFFFFFFF,FF:F:FF,F,F:FF
FFFFF,:,F,FFFFF
@A00721:190:HGJW7DSXY:4:2442:29695:26991 2:N:0:TCTGCAAG+GCTGCACT
GCACATTCGTACATTTAATGCAACTTCCATCGCATATTGATGAAATGTCGCAAAAACTAAATAATAAG
```

CTCCTTTCATCATGTCCTCTACACTGCCACCAGTGGTGTCCTTAAAAACTGTGAGTCCACTAAGTTTC
TTCCTCACAGAAATC
+
FFFFFFFFFFFFFFFFFFFFFFFFFFFFFFFFFFFFFFFFFFFFFFFFFFFFF:FFFFFFFFFFFFFF
FFFFFFFFFFFFFFFFFFFFFFFFFFFFFFFFFFFFFFFFFFFFFFFFFFFFFFFFFFFF:FFFFFFF
FFF:FFFFFFF:FFF
@A00721:190:HGJW7DSXY:4:2443:23601:4695 2:N:0:TCTGCAAG+GCTGCACT
GCACATTCGTACATTTAATGCAACTTCCATCGCATATTGATGAAATGTCGCAAAAACTAAATAATAAG
CTCCTTTCATCATGTCCTCTACACTGCCACCAGTGGTGTCCTTAAAAACTGTGAGTCCACTAAGTTTC
TTCCTCACAGAAATC
+
F,FFFFFFFFFFFFFFFFFFFFFFFFFFFFFFFFFFFFFFFFFFFFFFFFFFFFFFFFFFFFFFFFFF
FFFFFFFFFFFFFFFFFFFFFFFFFFFFFFFFFFFFFFFFFFFFFFFFFFFFFFFFFFFFFFFFFFFF
FFFFFFFFFFFFFFF
@A00721:190:HGJW7DSXY:4:2456:5963:35274 2:N:0:TCTGCAAG+GCTGCACT
CCACATTCGTACATTTAATGCAACTTCCATCGCATATTGATGAAATGTCGCAAAAACTAAATAATAAG
CTCCTTTCATCATGTCCTCTACACTGCCACCAGTGGTGTCCTTAAAAACTGTGAGTCCACTAAGTTTC
TTCCTCACAGAAATC
+
FFFFFFFFFFFFFFFFFFFFFFFFFFFFFFFFFFFFFFFFFFFFFFFFFFFFFFFFFFFFFFFFFFFF
FFFFFFFFFFFFFFFFFFFFFFFFFFFFFFFFFFFFFFFFFFFFFFFFFFF,FFFFFFFFFF:FFFFF
FFFFFFFFFFFFFFF
@A00721:190:HGJW7DSXY:4:2478:18041:35712 2:N:0:TCTGCAAG+GCTGCACT
CCACATTCGTACATTTAATGCAACTTCCATCGCATATTGATGAAATGTCGCAAAAACTAAATAATAAG
CTCCTTTCATCATGTCCTCTACACTGCCACCAGTGGTGTCCTTAAAAACTGTGAGTCCACTAAGTTTC
TTCCTCACAGAAATC
+
FFFFFFFFFFFFFFFFFFFFFFFFFFFFFFFFFFFFFFFFFFFFFFFFFFFFFFFFFFFFFFFFFFFF
FFFFFFFFFFFFFFFFFFFFFFFFFFFFFFFFFFFFFFFFFFFFF,F:FFFFFFFFFFFFFFFFFFFF
FFFFFF,FFFFFFFF
@A00721:190:HGJW7DSXY:4:2478:16731:36041 2:N:0:TCTGCAAG+GCTGCACT
CCACATTCGTACATTTAATGCAACTTCCATCGCATATTGATGAAATGTCGCAAAAACTAAATAATAAG
CTCCTTTCATCATGTCCTCTACACTGCCACCAGTGGTGTCCTTAAAAACTGTGAGTCCACTAAGTTTC
TTCCTCACAGAAATC
+
FFFFFFFFFFFFFFFFFFFFFFFFFFFFFFFFFFFFFFFFFFFFFFFFFFFFF:FFFFFFFFFFFFFF
FFFFFFFFFFFFFFFFFFFFFFFFFFFFFFFFFFFFFFFFFFFFFFFFFFFFFFFFFFFFFFFFFFFF
FFFFFFFFFFFFFFF
@A00721:190:HGJW7DSXY:4:2523:11116:16673 2:N:0:TCTGCAAG+GCTGCACT
CCACATTCGTACATTTAATGCAACTTCCATCGCATATTGATGAAATGTCGCAAAAACTAAATAATAAG
CTCCTTTCATCATGTCCTCTACACTGCCACCAGTGGTGTCCTTAAAAACTGTGAGTCCACTAAGTTTC
TTCCTCACAGAAATC
+
FFFFFFFF::FFF:FFFF::,FFFFFFF:FF::FFFFFFFFFFFF:FFFFFFFF:FFFFFFFFFFFFF
FFFF:FFFFFFFFFFFFFFFFFFF:FFFFF,F::FFFFFFFF:FFFFFFFFFFF,,FFFFFF:F:FFF
F::FFFFFFFF::FF
@A00721:190:HGJW7DSXY:4:2546:21920:21042 2:N:0:TCTGCAAG+GCTGCACT
CCACATTCGTACATTTAATGCAACTTCCATCGCATATTGATGAAATGTCGCAAAAACTAAATAATAAG
CTCCTTTCATCATGTCCTCTACACTGCCACCAGTGGTGTCCTTAAAAACTGTGAGTCCACTAAGTTTC
TTCCTCACAGAAATC
+
FFFFFFFFFFFFFFFFFFFFFFFFFFFFFFFFFFFFF:FFFFFFFFFFFFFFFFF,FFFFFFFFFFFF
FFFFFFFFFFFFF:FFFFFFFFFFFFFFFFFFFFFFFFFFFFF:F:FFFFFFFFFFFFFFFFFFFFFF
FFFFFFFFFFFFFFF

```
@A00721:190:HGJW7DSXY:4:2601:5882:29089 2:N:0:TCTGCAAG+GCTGCACT
CCACATTCGTACATTTAATGCAACTTCCATCGCATATTGATGAAATGTCGCAAAAACTAAATAATAAG
CTCCTTTCATCATGTCCTCTACACTGCCACCAGTGGTGTCCTTAAAAACTGTGAGTCCACTAAGTTTC
TTCCTCACAGAAATC
+
FFFFFFFFFFFFFFFFFFFFFFFFFFFFFFFFFFFFFFFFFFFFFFFFFFFFFFFFFFFFFFFFFFFF
FFFFFFFFFFFFFFFFFFFFFFFFFFFFFFFFFFFFFFFFFFFFFFFFFFFFFFFFFFFFFFFFFFFF
FFFFFFFFFFFFFFF
@A00721:190:HGJW7DSXY:4:2603:13232:15076 2:N:0:TCTGCAAG+GCTGCACT
CCACATTCGTACATTTAATGCAACTTCCATCGCATATTGATGAAATGTCGCAAAAACTAAATAATAAG
CTCCTTTCATCATGTCCTCTACACTGCCACCAGTGGTGTCCTTAAAAACTGTGAGTCCACTAAGTTTC
TTCCTCACAGAAATC
+
FFFFFFFFFFFFFFFFFFFFFFFFFFFFFFFFFFFFFFFFFFFFFFFFFFFFFFFFFFFFFFFFFFFF
FFFFFFFFFFFFFFFFFFFFFFFFFFFFFFFFFFFFFFFFFFFFFFFFFFFFFFFFFFFFFFFFFFFF
FFFFFFFFFFFFFFF
@A00721:190:HGJW7DSXY:4:2611:6533:4601 2:N:0:TCTGCAAG+GCTGCACT
CCACATTCGTACATTTAATGCAACTTCCATCGCATATTGATGAAATGTCGCAAAAACTAAATAATAAG
CTCCTTTCATCATGTCCTCTACACTGCCACCAGTGGTGTCCTTAAAAACTGTGAGTCCACTAAGTTTC
TTCCTCACAGAAATC
+
FFFFFFFFFFFFFFFFFFFFFFFFFFFFFFFFFFFFFFFFFFFFFFFFFFFFFFFFFFFFFFFFFFFF
FFFFFFFFFFF:FFFFFFFFFFFFFFFFFFFFFFFFFFFFFFFFFFFFFFFFFFFFFFFFFFFFFFFF
FFFFFFFFFFFFFFF
@A00721:190:HGJW7DSXY:4:2630:20980:24987 2:N:0:TCTGCAAG+GCTGCACT
CACATTCGTACATTTAATGCAACTTCCATCGCATATTGATGAAATGTCGCAAAAACTAAATAATAAGC
TCCTTTCATCATGTCCTCTACACTGCCACCAGTGGTGTCCTTAAAAACTGTGAGTCCACTAAGTTTCT
TCCTCACAGAAATC
+
FFFFFFFFFFFFFFFFFFFFFFFFFFFFFFFFFFFFFFFFFFFFFFFFF:FFFFFFFFFFFFFFFFFF
FFFFF:FFFFFFFFFFFFFFFFFFFFFFFFFFFFFFFFFFFFFFFFFFFFFFFFFFF:FFFF::FFFF
FFFFFFFFFFFFFF
@A00721:190:HGJW7DSXY:4:2634:22616:27477 2:N:0:TCTGCAAG+GCTGCACT
CCACATTCGTACATTTAATGCAACTTCCATCGCATATTGATGAAATGTCGCAAAAACTAAATAATAAG
CTCCTTTCATCATGTCCTCTACACTGCCACCAGTGGTGTCCTTAAAAACTGTGAGTCCACTAAGTTTC
TTCCTCACAGAAATC
+
FFFFFFFFFFFFFFFFFFFFFFFFFFFFFFFFFF:FFFFFFFFFFFFFFFFFFFFFFFFFFFFFFFFF
FFFFFFFFFFFFFFFFFFFFFFFFFFFFFFFFFFFFFFFFFFFFFFFFFFFFFFFFFFFFFFFFFFFF
FFFFFFFFFFFFFFF
@A00721:190:HGJW7DSXY:4:2639:14742:4100 2:N:0:TCTGCAAG+GCTGCACT
CCACATTCGTACATTTAATGCAACTTCCATCGCATATTGATGAAATGTCGCAAAAACTAAATAATAAG
CTCCTTTCATCATGTCCTCTACACTGCCACCAGTGGTGTCCTTAAAAACTGTGAGTCCACTAAGTTTC
TTCCTCACAGAAATC
+
FFFFFF:FFFFFFFFFFFFFFFFFFFFFFFFFFFFFFFFFFFFFFFFFFFFFFFFFFFFFFFFFFFFF
FFFFFFFFFFFFFFFFFFFFFFFFFFFFFFFFFFFFFFFFFFFFFFFFFFFFFFFFFFFFFFFFFFF:
FFFFF:FFFFFFFFF
@A00721:190:HGJW7DSXY:4:2666:25961:5682 2:N:0:TCTGCAAG+GCTGCACT
CCACATTCGTACATTTAATGCAACTTCCATCGCATATTGATGAAATGTCGCAAAAACTAAATAATAAG
CTCCTTTCATCATGTCCTCTACACTGCCACCAGTGGTGTCCTTAAAAACTGTGAGTCCACTAAGTTTC
TTCCTCACAGAAATC
+
FFFFFFFFFFFFFFFFFFFFFFFFFFFFFFFFFFFFFFFFFFFFFFFFFFFFFFFFF:FFFFFFFFFFFF
```

```
FFFFFFFFFFFFFFFFFFFFFFFFFFFFFFFFFFFFFFFFFFFFFFFFFFFFFFFFFFFFFFFFFFFFFFFF
FFFFFFFFFFFFFFF
@A00721:190:HGJW7DSXY:4:2352:32832:11631 2:N:0:TCTGCAAG+GCTGCACT
GCACATTCGTACATTTAATGCAACTTCCATCGCATATTGATGAAATGTCGCAAAAACTAAATAATAAG
CTCCTTTCATCATGTCCTCTACACTGCCACCAGTGGTGTCCTTAAAAACTGTGAGTCCACTAAGTTTC
TTCCTCACAGAAAT
+
F,FFFFFF:FFFFFFFFF:FFFFFFFFFFFFFFFFFFFF:FFFFFF:F:FFFFFF:FFFFFFFFFFFF
FFFFFFFFFFF:FFFFFFFFFFFFFFF:FFFF,FFFFFFF,FFF:FFF,FFFFFFF:FF,FFFFFFFF
FF::F,F:FFF:FF
@A00721:190:HGJW7DSXY:4:1339:31051:2284 2:N:0:TCTGCAAG+GCTGCACT
CTGTGAGGAAGAAACTTAGTGGACTCACAGTTTTTAAGGACACCACTGGTGGCAGTGTAGAGGACATG
ATGAAAGGAGCTTATTATTTAGTTTTTGCGACATTTCATCAATATGCGATGGAAGTTGCATTAAATGT
ACGAATGT
+
FFFFFFFFFFFFFFFFFFFFFFFFFFFFFFFF:FFFFFFFFFFFFFFFFFFFFFFFFFFFFFFFFFFF
FFFFF:FFFFF::FFFFFFFFFFFFFFFFFFFFFFFFFFFFFFFFFFFFFFFF,FFFFFFFFF:FFFF
FFFF:FFF
@A00721:190:HGJW7DSXY:4:1511:2401:25848 2:N:0:TCTGCAAG+GCTGCACT
CTGTGAGGAAGAAACTTAGTGGACTCACAGTTTTTAAGGACACCACTGGTGGCAGTGTAGAGGACATG
ATGAAAGGAGCTTATTATTTAGTTTTTGCGACATTTCATCAATATGCGATGGAAGTTGCATTAAATGT
ACGAATGT
+
FFFFFFFFFFFFFFFFFFFFFFFFFFFFFFFFFFFFFFFFFFFFFFFFFFFFFFFFFFFFFFFFFFFF
FFFFFFFFFFF:FF:FF:FFFFFFFFFFFFFFFFFFFFFFFFFF:FFFFFFFFFFFFFFFFFFFFFFFF
FFFFFFFF
@A00721:190:HGJW7DSXY:4:2309:24460:29763 2:N:0:TCTGCAAG+GCTGCACT
CTGTGAGGAAGAAACTTAGTGGACTCACAGTTTTTAAGGACACCACTGGTGGCAGTGTAGAGGACATG
ATGAAAGGAGCTTATTATTTAGTTTTTGCGACATTTCATCAATATGCGATGGAAGTTGCATTAAATGT
ACGAATGT
+
FFFFFFF:FFF,FFFFFFFFFFFFF:FFFFFFFFFF::FFFFFFFFFFFFFFFFFFFF,F::FFF:FF
FFFFFFFFFFFFFFFFFFFFFFFFFFFFF:FFFFFFFFFFFFF:FF,,FFFFFF:FFFFFFFF:FFFF
FFFFFF,F
@A00721:190:HGJW7DSXY:4:1454:24460:14105 2:N:0:TCTGCAAG+GCTGCACT
TACATTTAATGCAACTTCCATCGCATATTGATGAAATGTCGCAAAAACTAAATAATAAGCTCCTTTCA
TCATGTCCTCTACACTGCCACCAGTGGTGTCCTTAAAAACTGTGAGTCCACTAAGTTTCTTCCT
+
FFFFFFFFFFFFFFFFFFFFFFFFFFFFFFFFFFFFFFF:FFFFFF:FFFFFFFFFFFFFFFFFFFFF
FFFFFFFFFFFFFFFFFFFFFFFFFFFFFFFFFFFFFFFFFFFFFFFFFFFFFFFFFFFFFFFF
@A00721:190:HGJW7DSXY:4:2308:19407:11365 2:N:0:TCTGCAAG+GCTGCACT
TACATTTAATGCAACTTCCATCGCATATTGATGAAATGTCGCAAAAACTAAATAATAAGCTCCTTTCA
TCATGTCCTCTACACTGCCACCAGTGGTGTCCTTAAAAACTGTGAGTCCACTAAGTTTCTTCCT
+
FFFFFFFFFFFFFFFFFFFFFFFFFFFFFFFFFFFFFFFFFF:FF,FFFFFFFF:FFFFFFFFFFFF
FFFFFFFFFF:FFFFFFFFFFFFF:FFFF:FFFFFFFFFF:F,FFFFFFFFF,FFFFFFFFFFF
@A00721:190:HGJW7DSXY:4:2528:28592:36667 2:N:0:TCTGCAAG+GCTGCACT
TACATTTAATGCAACTTCCATCGCATATTGATGAAATGTCGCAAAAACTAAATAATAAGCTCCTTTCA
TCATGTCCTCTACACTGCCACCAGTGGTGTCCTTAAAAACTGTGAGTCCACTAAGTTTCTTCCT
+
FFFFFFFFFFFFFFFFFFFFFFFFFFFFFFFFFFFFFFFFFFFFFFFFFFFFFFFFFFFFFFFFFFFF
FFFFFFFFFFFFFFFFFFFFFFFFFFFFFFFF:FFFFFFFFFFFFFFFFFFFFFFFFFFFFFFF
@A00721:190:HGJW7DSXY:4:2532:29993:22780 2:N:0:TCTGCAAG+GCTGCACT
TACATTTAATGCAACTTCCATCGCATATTGATGAAATGTCGCAAAAACTAAATAATAAGCTCCTTTCA
```

```
TCATGTCCTCTACACTGCCACCAGTGGTGTCCTTAAAAACTGTGAGTCCACTAAGTTTCTTCCT
+
FFFFFFFFFFFFFFFFFFFFFFFFFFFFFFFFFFFFFFFFFFFFFFFFFFFFFFFFFFFFFFFFFFFFFF
FFFFFFFFFFFFFFFFFFFFFFFFFFFFFFFFFFFFFFFFFFF:FFFFFFFFFFFFFFFFFFFFF
@A00721:190:HGJW7DSXY:4:2635:10601:18662 2:N:0:TCTGCAAG+GCTGCACT
TACATTTAATGCAACTTCCATCGCATATTGATGAAATGTCGCAAAAACTAAATAATAAGCTCCTTTCA
TCATGTCCTCTACACTGCCACCAGTGGTGTCCTTAAAAACTGTGAGTCCACTAAGTTTCTTCCT
+
FFFFFFFFFFFFFFFFFFFFFFFFFFFFFFFFFFFFFFFFFFFFFFFFFFFFFFFFFFFFFFFFFFFFFF
FFFFFFFFFFFFFFFFFFFFFFFFFFFFFFFFFFFFFFFFFFF:FFFFFFFFFFFFFFFFFFF,F
@A00721:190:HGJW7DSXY:4:1408:15212:35446 2:N:0:TCTGCAAG+GCTGCACT
TACATTTAATGCAACTTCCATCGCATATTGATGAAATGTCGCAAAAACTAAATAATAAGCTCCTTTCA
TCATGTCCTCTACACTGCCACCAGTGGTGTCCTTAAAAACTGTGAGTCCACTAAGTTTCTTCC
+
,FFFFFFFFFFFFFFFFFFFFFFFFFFFFFFFFFFFF,,,FFF:FFF:FFFFFFFFFFF,:FFF,,::
FFFF,:F,::FF,F,::F,FF:FFFF:F:FFF,FFFFF:F:FF:FF:,FFF,FFF:FF,:F::
@A00721:190:HGJW7DSXY:4:1143:24297:27633 2:N:0:TCTGCAAG+GCTGCACT
TATATATCATTTTATTAAACACTTCTGAACCACATTCGTACATTTAATACAACTTCCATCGCATATTG
ATGAAATGTCGCAAAAACTAAATAATAAGCTCCTTTCATCATGTCCTCTACACTGCCACCAGTGGTGT
CCTTAAAAACTGTGA
+
FFFFFFFFFFFFFFFFFFFFFFFFFFFFFFFFFFFFFFFFFFFFFFFFFFFFFFFFFFFFFFFFFFFFFF
FFFF:FFFFFFF:FFF:FFFFFFFFFFFF:FFFFFFFFFFFFFFFFFFFFFFFFFFFFFFFFFFFFFFF
FFFFFFFFF:FFFF:
@A00721:190:HGJW7DSXY:4:1315:8404:27258 2:N:0:TCTGCAAG+GCTGCACT
TATATATCATTTTATTAAACACTTCTGAACCACATTCGTACATTTAATACAACTTCCATCGCATATTG
ATGAAATGTCGCAAAAACTAAATAATAAGCTCCTTTCATCATGTCCTCTACACTGCCACCAGTGGTGT
CCTTAAAAACTGTGA
+
FFFFFFFFFFFFFFFFFFFFFFFFFFFFFFFFFFFFFFFFFFFFFFFFFFFFFFFFFFFFFFFFFFFFFF
FFFFFFFFFFFFFFFFFFFFFFFFFFFFFFFFFFFFFFFFFFFFFFFFFFFFFFFFFFFFFFFFFFFFFF
FFFFFFFFFFFFFFF
@A00721:190:HGJW7DSXY:4:1359:12635:13416 2:N:0:TCTGCAAG+GCTGCACT
TATATATCATTTTATTAAACACTTCTGAACCACATTCGTACATTTAATACAACTTCCATCGCATATTG
ATGAAATGTCGCAAAAACTAAATAATAAGCTCCTTTCATCATGTCCTCTACACTGCCACCAGTGGTGT
CCTTAAAAACTGTGA
+
F:FFFFFFFFFFFFFFFFFFFFFFFFFFFFFFFFFFFFFFFFFFFFFFFFFFFFFFFFFFFFFFFFFFFF
FFFFFFFFFFFFFFFFFFFFFFFFFFFFFFFFFFFFFFFFFFFFFFFFFFFFFFFFFFFFFFFFFFFFFF
FFFFFFFFFFFFFFF
@A00721:190:HGJW7DSXY:4:2143:27787:31548 2:N:0:TCTGCAAG+GCTGCACT
TATATATCATTTTATTAAACACTTCTGAACCACATTCGTACATTTAATACAACTTCCATCGCATATTG
ATGAAATGTCGCAAAAACTAAATAATAAGCTCCTTTCATCATGTCCTCTACACTGCCACCAGTGGTGT
CCTTAAAAACTGTGA
+
FFFFFFFFFFFFFFFFFFFFFFFFFFFFFFFFFFFFFFFFFFFFFFFFFFFFFFFFFFFFFFFFFFFFFF
FFFFFFFFFFFFFFFFFFFFFFFFFFFFFFFFFFFFFFFFFFFFFFFFFFFFFFFFFFF:FFFFFFFFF
F:FFF,FFFFFFFFF
@A00721:190:HGJW7DSXY:4:2265:21802:2080 2:N:0:TCTGCAAG+GCTGCACT
TATATATCATTTTATTAAACACTTCTGAACCACATTCGTACATTTAATACAACTTCCATCGCATATTG
ATGAAATGTCGCAAAAACTAAATAATAAGCTCCTTTCATCATGTCCTCTACACTGCCACCAGTGGTGT
CCTTAAAAACTGTGA
+
F:FFFFFFFFFFFFFFFFFFFFFFFFFFFFFF:FFFFFFFFFFFFFFFFFFFF:FFFFFFFFFFFFFFF
```

```
FFF:FFFFFFFFFFFFFFFFFFFFFFF,FFFFFFFFFFFFFFFFFFFFFFFFFFFFFFFFFFFFFFFFFF
FFFFFFFFFFFFFFF
@A00721:190:HGJW7DSXY:4:2405:29740:24471 2:N:0:TCTGCAAG+GCTGCACT
TATATATCATTTTATTAAACACTTCTGAACCACATTCGTACATTTAATACAACTTCCATCGCATATTG
ATGAAATGTCGCAAAAACTAAATAATAAGCTCCTTTCATCATGTCCTCTACACTGCCACCAGTGGTGT
CCTTAAAAACTGTGA
+
F:FFFFFFFFFFFFFFFFFFFFFFFFFFFFFFFFFFFFFFFFFFFFFFFFFFFFFFFFFFFFFFFFFF
FFFF:FFFFFFFF:FFFFFFFFFFFFFFFFFFFFFFFFFFFFFFFFFFFFFFFFFFFFFFFFFFFFF
FFFFFFFFFFFFFFF
@A00721:190:HGJW7DSXY:4:2456:6153:2597 2:N:0:TCTGCAAG+GCTGCACT
TATATATCATTTTATTAAACACTTCTGAACCACATTCGTACATTTAATACAACTTCCATCGCATATTG
ATGAAATGTCGCAAAAACTAAATAATAAGCTCCTTTCATCATGTCCTCTACACTGCCACCAGTGGTGT
CCTTAAAAACTGTGA
+
FFFFFFFFFFFFFFFFFF:FFFFFFFFFFFFFFF:FFFFFF:F:FFFFFFF,F,FFFFFFFFF:FFFF
FFFFFFFFFFFFFF,FFFFFFFFFFFFFFFFFF,FFFFFFFFFFFFFF:FFFFFFF:FFFFFFFFFF
F,F,FFFFFFFFFFF
@A00721:190:HGJW7DSXY:4:2641:22634:5744 2:N:0:TCTGCAAG+GCTGCACT
TATATATCATTTTATTAAACACTTCTGAACCACATTCGTACATTTAATACAACTTCCATCGCATATTG
ATGAAATGTCGCAAAAACTAAATAATAAGCTCCTTTCATCATGTCCTCTACACTGCCACCAGTGGTGT
CCTTAAAAACTGTGA
+
FFFFFFFFFFFFFFFFFFFFFFFFFFFFFFFFFFFF:FFFFFFFFFFFFFFFFFFFFFFF:FFFFFFFF
FFFFFFFFFFFFFFFFF:FFFFFFFFFF,:F:FFFF,F:FFF:FFFFF:FFFFFFF:FFFFF:F:,:F
FFFFFFFFFFFFFFF
@A00721:190:HGJW7DSXY:4:2678:14823:4335 2:N:0:TCTGCAAG+GCTGCACT
TATATATCATTTTATTAAACACTTCTGAACCACATTCGTACATTTAATACAACTTCCATCGCATATTG
ATGAAATGTCGCAAAAACTAAATAATAAGCTCCTTTCATCATGTCCTCTACACTGCCACCAGTGGTGT
CCTGAAAAACTGTGA
+
FFFFFFFFFFFFFFFFFFFFFFFFFFFFFF:FFFF:FFFFFFFFFFFFFFF:FFFFFFF,:FFFFFFF:
FFFFFFFFFFFFFFFFFFFFFFFFFFFFFFF:FFFFFFF:FFFFFFF:FFFFFFFFFFFFFFFFFF:FF
FFF,FFFFFF:FFFF
@A00721:190:HGJW7DSXY:4:2134:11514:3302 2:N:0:TCTGCAAG+GCTGCACT
TATATATCATTTTATTAAACACTTCTGAACCACATTCGTACATTTAATACAACTTCCATCGCATATTG
ATGAAATGTCGCAAAAACTAAATAATAAGCTCCTTTCATCATGTCCTCTACACTGCCACCAGTGGTGT
CCTTAAAAACTGTGG
+
FFFFFFFFFFFFFFFFFFFFFFFFFFFFFFFFFFF:FFFFFFFFFFFFFFFFFFFFFFFFFFFFFFFFF
FFFFFFFFFFFFFFFF:FFFFFFFFF,FFFFFFFFFFFFFFFFFF:FFFFFFFFFFFFFFFFFFFFFFF
FFFFFFFFFFFFFF:
@A00721:190:HGJW7DSXY:4:1554:32280:29747 2:N:0:TCTGCAAG+GCTGCACT
TGGAAAATGAATATATATCATTTTATTAAACACTTCTGAACCACATTCGTACATTTAATACAACTTCC
ATCGCATATTGATGAAATGTCGCAAAAACTAAATAATAAGCTCCTTTCATCATGTCCTCTACACTGCC
ACCAGTGGTGTCCTT
+
FFFFFFFFFFFFFFFFFFFFFFFFFFFFFFFFFFFFFFFFFFFFFFFFFFFFFFFFFFFFFFFFFFFF
FFFFFFFFFFFFFFF:FFFFFFFFFFFF:FFFFFFFFFFFFFFFFFFFFFFFFFFFFFFFFFFFFFFFF
FFFFFFFFFFF::FF
@A00721:190:HGJW7DSXY:4:2223:18059:28166 2:N:0:TCTGCAAG+GCTGCACT
TGGAAAATGAATATATATCATTTTATTAAACACTTCTGAACCACATTCGTACATTTAATACAACTTCC
ATCGCATATTGATGAAATGTCGCAAAAACTAAATAATAAGCTCCTTTCATCATGTCCTCTACACTGCC
ACCAGTGGTGTCCTT
```

```
+
FFFFFF,FFFF,FF:FFFFFFFFFFFFFFFFFFFFFFFFFFFFFF:FFFFFFFFFFFFFFFFFFFFFFF
FFFFFFFFFFFFFFF:FFFFFF:FFF:FF:FFFFFF:FF,FFFFFFFF,FFFFFFF:FFFFFFFFFFF
FFFFFFFFFFFFFFF
@A00721:190:HGJW7DSXY:4:2223:16794:29199 2:N:0:TCTGCAAG+GCTGCACT
TGGAAAATGAATATATATCATTTTATTAAACACTTCTGAACCACATTCGTACATTTAATACAACTTCC
ATCGCATATTGATGAAATGTCGCAAAAACTAAATAATAAGCTCCTTTCATCATGTCCTCTACACTGCC
ACCAGTGGTGTCCTT
+
FFFFFFFFFFFFFFFFFFFFFFFFFFFFFFFFFFFFFFFFFFFFFFFFFFFFFFFFFFFFFF:FFFFFF
FFFFFFFFFFFFFFFF:FFFFFFFFFFFFFFFFFFFFFFFFFFFFFFFFFFFFF:FFFFFFFF:FFFFF
FFF:F:FFFFFFFFF
@A00721:190:HGJW7DSXY:4:2472:12970:26897 2:N:0:TCTGCAAG+GCTGCACT
TGGAAAATGAATATATATCATTTTATTAAACACTTCTGAACCACATTCGTACATTTAATACAACTTCC
ATCGCATATTGATGAAATGTCGCAAAAACTAAATAATAAGCTCCTTTCATCATGTCCTCTACACTGCC
ACCAGTGGTGTCCTT
+
FFFFFFFFFFFFFFFFFFFFFFFFFFFFFFFFFFFFFFFFFFFFFFFFFFFFFFFFFFFFFFFFFFFF
FFFFFFFFFFFFFFFFFFFFFFFFFFFFFFFFFFFFFFFFFFFFFFFFFFFFFFFFFFFFFFFFFFFF
FFFFF:FFFFFFFFF
@A00721:190:HGJW7DSXY:4:1320:9353:15499 2:N:0:TCTGCAAG+GCTGCACT
ATTATCGAATGGAAAATGAATATATATCATTTTATTAAACACTTCTGAACCACATTCGTACATTTAAT
GCAACTTCCATCGCATATTGATGAAATGTCGCAAAAACTAAATAATAAGCTCCTTTCATCATGTCCTC
TACACTGCCACCAGT
+
FFFFFFFFFFFFFFFFFFFFFFFFFFFFFFFFFFFFFFFFFFFFFFFFFFFFFFFFFFFFFFFFFFFF
FFFFFFFFFFFFFFFFFFFFFFFFFFFFFFFFFFFFFFFFFFFFFFFFFFFFFFFFFFFFFFFFFFFF
FFFFFFFFFFFFFFF
@A00721:190:HGJW7DSXY:4:1406:13358:35336 2:N:0:TCTGCAAG+GCTGCACT
ATTATCGAATGGAAAATGAATATATATCATTTTATTAAACACTTCTGAACCACATTCGTACATTTAAT
GCAACTTCCATCGCATATTGATGAAATGTCGCAAAAACTAAATAATAAGCTCCTTTCATCATGTCCTC
TACACTGCCACCAGT
+
FFFFFFFFFFFFFFFFFFFFFFFFFFFFFFFFFFFFFFFFFFFFFFFFFFFFFFFFFFFFFFFFFFFF
FFFFFFFFFFFFFFFFFFFFFFFFFFFFFFFFFFFFFFFFFFFFFFFFFFFFFFFFFFFFFFFFFFFF
FFFFFFFFFFFFFFF
@A00721:190:HGJW7DSXY:4:1448:1118:20744 2:N:0:TCTGCAAG+GCTGCACT
ATTATCGAATGGAAAATGAATATATATCATTTTATTAAACACTTCTGAACCACATTCGTACATTTAAT
GCAACTTCCATCGCATATTGATGAAATGTCGCAAAAACTAAATAATAAGCTCCTTTCATCATGTCCTC
TACACTGCCACCAGT
+
FFFFFFFFFFFFFFFFFFFFFFFFFFFFFFFFFFFFFFFFFFFFFFFFFFFFFFFFFFFFFFFFFFFF
FFFFFFFFFFFFFFFFFFFFFFFFFFFFFFFFFFFFFFFFFFFFFFFFFFFFFFFFFFFFFFFFFFFF
FFFFFFFFFFFFFFF
@A00721:190:HGJW7DSXY:4:2354:20555:12477 2:N:0:TCTGCAAG+GCTGCACT
ATTATCGAATGGAAAATGAATATATATCATTTTATTAAACACTTCTGAACCACATTCGTACATTTAAT
GCAACTTCCATCGCATATTGATGAAATGTCGCAAAAACTAAATAATAAGCTCCTTTCATCATGTCCTC
TACACTGCCACCAGT
+
FFFFFFFFFFFFFFFFFFFFFFFF:FFFFFFFFFFFFFFFFFFFFFFFFFFFFFFFFFFFFFFFFFFF
FFFFFFFFFFFFFFFFFFFFFFFFFFFFFFFFFFFFFFFFFFFFFFFFFFFFFFFFFFFFF:FFFFFFF
FFFFFFFFFFFFFFF
@A00721:190:HGJW7DSXY:4:2468:23574:32550 2:N:0:TCTGCAAG+GCTGCACT
ATTATCGAATGGAAAATGAATATATATCATTTTATTAAACACTTCTGAACCACATTCGTACATTTAAT
```

GCAACTTCCATCGCATATTGATGAAATGTCGCAAAAACTAAATAATAAGCTCCTTTCATCATGTCCTC
TACACTGCCACCAGT
+
FFFFFFFFFFFFFFFFFFF:FFFFF,FFFFFFFFFFF,:FFFF:FFFFFFFFFFFFFFFFFFFFFFFFF
FFFFFFFFFFFFFFFFFFFFFFFFFFFFFFFFFFFFFFFFFFFFFFFFFFFFFFFFFFFFFFFFFF:FF
FFFFFFFFFFFFFFF
@A00721:190:HGJW7DSXY:4:2561:14235:10207 2:N:0:TCTGCAAG+GCTGCACT
ATTATCGAATGGAAAATGAATATATATCATTTTATTAAACACTTCTGAACCACATTCGTACATTTAAT
GCAACTTCCATCGCATATTGATGAAATGTCGCAAAAACTAAATAATAAGCTCCTTTCATCATGTCCTC
TACACTGCCACCAGT
+
FFFFFFFFFFFFFFFFFFFFFFFFFFFFFFFFFFFFFFFFFFFFFFFFFFFFFFFFFFFFFFFFFFFFF
FFFFFFFFFFFFFFFFFFFFFFFFFFFFFFFFFFFFFFFFFFFFFFFFFFFFFFFFFFFFFFFFFFFFF
FFFFFFFFFFFFFFF
@A00721:190:HGJW7DSXY:4:2544:23601:21167 2:N:0:TCTGCAAG+GCTGCACT
ATTATCGAATGGAAAATGAATATATATCATTTTATTAAACACTTCTGAACCACATTCGTACATTTAAT
GCAACTTCCATCGCATATTGATGAAATGTCGCAAAAACTAAATAATAAGCTCCTTTCATCATGTCCTC
TACACTGCCACCAG
+
FFFFFFFFF::FFFFF,FFF:FF:FFFFFF,,FFFFFFFFFFFFFFFFFFFFFF,FF:FFFFF,:FF:
:FFFFFFF,FF,F:F:FFF,FFFFFFF,F,,FFFFFF:FF:FFFFFFF::,F,FFFFFFFF,F::F,F
F:FF:F,,FFFFF:
@A00721:190:HGJW7DSXY:4:1315:9399:22936 2:N:0:TCTGCAAG+GCTGCACT
AGATTATCGAATGGAAAATGAATATATATCATTTTATTAAACACTTCTGAACCACATTCGTACATTTA
ATGCAACTTCCATCGCATATTGATGAAATGTCGCAAAAACTAAATAATAAGCTCCTTTCATCATGTCC
TCTACACTGCCACCA
+
FFF,FFFF:FFFFFFFFFFFFFFFFFFFFFFFFFFFFFFFFFFFFFFFFFFFFFFFFFFFFFFFFFFFF
FFFFFFFFFFFFFF:FFFFFFFFFFFFFFFFFFFFFFFFFFFFFFFFFFFFFFFFFFFFFFFFFF:FFF
FFFFFFFFFFFFFFF
@A00721:190:HGJW7DSXY:4:1510:17743:36292 2:N:0:TCTGCAAG+GCTGCACT
AGATTATCGAATGGAAAATGAATATATATCATTTTATTAAACACTTCTGAACCACATTCGTACATTTA
ATGCAACTTCCATCGCATATTGATGAAATGTCGCAAAAACTAAATAATAAGCTCCTTTCATCATGTCC
TCTACACTGCCACCA
+
FFFFFFFFFFFFFFFFFFFFFFFFFFF:FFFFFFFFFFFFFFFFFFFFFFFFFFFFFFFFFFFFFFFFF
FFFFF:FFFFFFFFFFFFFFFFFFFFFFFFFFFFFFFFFFFFFFFFFFFFFFFFFFFFFFFFFFFFFFF
FFFFFFFFFFFFFFF
@A00721:190:HGJW7DSXY:4:2333:17942:29872 2:N:0:TCTGCAAG+GCTGCACT
AGATTATCGAATGGAAAATGAATATATATCATTTTATTAAACACTTCTGAACCACATTCGTACATTTA
ATGCAACTTCCATCGCATATTGATGAAATGTCGCAAAAACTAAATAATAAGCTCCTTTCATCATGTCC
TCTACACTGCCACCA
+
FFFFFFFFFFFFFFFFFFFFFFFFFFFFFFFFFFFFFFFFFFFFFFFFFFFFFFFFFFFFFFFFFFFFF
FFFFFFFFFFFFFFFFFFFFFFFFFFFFFFFFFFF:FFFFFFFFFFFFFFFFFFF:FFFFFFFFFFFFF
FFFFFFFFFFFFFFF
@A00721:190:HGJW7DSXY:4:1328:7554:7028 2:N:0:TCTGCAAG+GCTGCACT
CAAAGATTATCGAATGGAAAATGAATATATATCATTTTATTAAACACTTCTGAACCACATTCGTACAT
TTAATGCAACTTCCATCGCATATTGATGAAATGTCGCAAAAACTAAATAATAAGCTCCTTTCATCATG
TCCTCTACACTGCCA
+
FFFFF:FFFFFFFFFFFFFFFFFFFFFFFFFFFFFFFFFFFFFFFFFFFFFFFFFFFFFFFFFFFFFFF
FFFFFFFFFFFFFFFFF:FFFFFFFFFFFFFFFFFFFF,FFF:FFFFFFFFFFFFFFFFFFFFFFFFFF
FF:FFFFFFFFF:FF

```
@A00721:190:HGJW7DSXY:4:1653:4029:25473 2:N:0:TCTGCAAG+GCTGCACT
CAAAGATTATCGAATGGAAAATGAATATATATCATTTTATTAAACACTTCTGAACCACATTCGTACAT
TTAATGCAACTTCCATCGCATATTGATGAAATGTCGCAAAAACTAAATAATAAGCTCCTTTCATCATG
TCCTCTACACTGCCA
+
FFFFFFFFFFFFFFFFFFFFFFFFFFFF:FFFFFFFFFFFFFFFFFFFFFFFFFFFFFFF:FFFFFFF
FFFFFFFFFFFFFFFFFFFFFFFFFFFFFFFFFFFFFFFFF,FFFFFFFFFFFFFFFFFFFFFFFFFF
FFFFFFFFFFFFF:F
@A00721:190:HGJW7DSXY:4:2216:26838:20666 2:N:0:TCTGCAAG+GCTGCACT
CAAAGATTATCGAATGGAAAATGAATATATATCATTTTATTAAACACTTCTGAACCACATTCGTACAT
TTAATGCAACTTCCATCGCATATTGATGAAATGTCGCAAAAACTAAATAATAAGCTCCTTTCATCATG
TCCTCTACACTGCCA
+
FFFFFFFFFFFFFFFFFFFFFFFFFFFFFFFFFFFFFFFFFFFFFFFFFFFFFFFFFFF:FFFFFFFF
FFFFFFFFFFFFFFFFFFFFFFF:FFFFFFFFFFFFFFFFF:FFFFFFFFFFF:FFFFFFFFFFFFFF
FFFFFFFFFFFFFFF
@A00721:190:HGJW7DSXY:4:1223:26069:34178 2:N:0:TCTGCAAG+GCTGCACT
TGTAATCAAAGATTATCGAATGGAAAATGAATATATATCATTTTATTAAACACTTCTGAACCACATTC
GTACATTTAATACAACTTCCATCGCATATTGATGAAATGTCGCAAAAACTAAATAATAAGCTCCTTTC
ATCATGTCCTCTACA
+
FFFFFFFFF:FFFF:FFFFFFFFFFFFFFFFFFFFFFFFFFFFFFFFFFFFFFFFFFFFFFFFFFFFF
FFFFFFFFFFFFFFFFFFFFFFFFFFFFFFFFFFFFFFFFFFF:FFF:FFFFFFFFFFF:FFF:FFFF
FFFFFFFFFFFFFFF
@A00721:190:HGJW7DSXY:4:1304:22426:7451 2:N:0:TCTGCAAG+GCTGCACT
TGTAATCAAAGATTATCGAATGGAAAATGAATATATATCATTTTATTAAACACTTCTGAACCACATTC
GTACATTTAATACAACTTCCATCGCATATTGATGAAATGTCGCAAAAACTAAATAATAAGCTCCTTTC
ATCATGTCCTCTACA
+
FFFFFFFFFFFFFFFFFFFFFFFFFFFFFFFFFFFFFFFFFFFFFFFFFFFFFFFFFFFFFFFFFFFF
FFFF:FFFFFFFFFFFFFFFFFFFFFFFFFFFFFFFFFFF:FFFFFFFFFFFFFFFF:FFFFFFFFF
FFFFFFFFFFFFFFF
@A00721:190:HGJW7DSXY:4:1467:30861:20181 2:N:0:TCTGCAAG+GCTGCACT
TGTAATCAAAGATTATCGAATGGAAAATGAATATATATCATTTTATTAAACACTTCTGAACCACATTC
GTACATTTAATACAACTTCCATCGCATATTGATGAAATGTCGCAAAAACTAAATAATAAGCTCCTTTC
ATCATGTCCTCTACA
+
FFFFFFFFFFFFFFFFFFFFFFFFFFFFFFFFFFFFFFFFFFFFFFFF::FFFFFFFFFFF:FFFFFF
FFFFFFFFFFFFFFFFFFFFFFFFFFFFFFFFFF,FFFFFFFFFF:FFFF:FFFFFFFFFFFFFFFFF
FFFFFFFFFFFFFFF
@A00721:190:HGJW7DSXY:4:1519:22354:18443 2:N:0:TCTGCAAG+GCTGCACT
TGTAATCAAAGATTATCGAATGGAAAATGAATATATATCATTTTATTAAACACTTCTGAACCACATTC
GTACATTTAATACAACTTCCATCGCATATTGATGAAATGTCGCAAAAACTAAATAATAAGCTCCTTTC
ATCATGTCCTCTACA
+
FFFFFFFFFFFFFFFFFFFFFFFF:FFFFFFFFFFFFFFFFFFFFFFFFFFFFFFFFFFFFFFFFFFF
FFFFFFFFFFFFFFFFFFFFFFFFFFFFFFFFFFFFFFFFFFFFFFFFFFFFFFFFFFFFFFFFFFFF
FFFFFFFFFFFFFFF
@A00721:190:HGJW7DSXY:4:1526:15709:9659 2:N:0:TCTGCAAG+GCTGCACT
TGTAATCAAAGATTATCGAATGGAAAATGAATATATATCATTTTATTAAACACTTCTGAACCACATTC
GTACATTTAATACAACTTCCATCGCATATTGATGAAATGTCGCAAAAACTAAATAATAAGCTCCTTTC
ATCATGTCCTCTACA
+
FFFFFFFFFFFFFFFFFFFFFFFFFFFFFFFFFFFFFFFFFFFFFFFFFFFFFFFFFFFFFFFFFFFF
```

FFFFFFFFFFFFFFFFFFFFFFFFFFFFFFFFFFFFFFFFFFFFFFFFFFFFFFF:FFF:FFFFFFF
FFFFFFFFFFFFFFF
@A00721:190:HGJW7DSXY:4:2347:15845:17284 2:N:0:TCTGCAAG+GCTGCACT
TGTAATCAAAGATTATCGAATGGAAAATGAATATATATCATTTTATTAAACACTTCTGAACCACATTC
GTACATTTAATACAACTTCCATCGCATATTGATGAAATGTCGCAAAAACTAAATAATAAGCTCCTTTC
ATCATGTCCTCTACA
+
FFFFFFFFFFFFFFFFFFFFFFFFFFFFFFFFFFFFFF:FFFFFFFFFFFFFFFFFFFFFFFFFFF:FF
FFFFFFFFFF:FFFFFFFFFFFF:FFFFFFFFFFFFFFFFFFF:F:FFF:FFFFFFFFFFFFFFFFFF
:FFFFF:FFFFFFFF
@A00721:190:HGJW7DSXY:4:2517:13196:17550 2:N:0:TCTGCAAG+GCTGCACT
TGTAATCAAAGATTATCGAATGGAAAATGAATATATATCATTTTATTAAACACTTCTGAACCACATTC
GTACATTTAATACAACTTCCATCGCATATTGATGAAATGTCGCAAAAACTAAATAATAAGCTCCTTTC
ATCATGTCCTCTACA
+
FFFF,FFFFFFFFFFFFFFFFFFFFFFFFFFFFF,FFFFFFFFFFFFFFFFFFFFFFFFFFFFFFFFFF
FFFFFFFFFFFFFFFFFFFFFFFFFFFFFFFFFFFFFFFFFFF::FFFFFFFFFFFFFFFFFFFFFFFF
FFFFFF::FFFFFFF
@A00721:190:HGJW7DSXY:4:2663:23999:32346 2:N:0:TCTGCAAG+GCTGCACT
TGTAATCAAAGATTATCGAATGGAAAATGAATATATATCATTTTATTAAACACTTCTGAACCACATTC
GTACATTTAATACAACTTCCATCGCATATTGATGAAATGTCGCAAAAACTAAATAATAAGCTCCTTTC
ATCATGTCCTCTACA
+
FFFFFFFFFFFFFFFFFFFFFFFFFFFFFFFFFFFFFFFFFFFFFFFFFFFFFFFFFFFFFFFFFFFF
FFFFFFFFFFFFFFFFFFFFFFFFFFFFFFFFFFFFFFFFF,FFFFFFFFFFFFFFFFFFFFFFFFFF
FFFFFFFFFFFFFFF
@A00721:190:HGJW7DSXY:4:1375:23204:33442 2:N:0:TCTGCAAG+GCTGCACT
AAGCAATGGAAAATCTAGATCATGTAATCAAAGATTATCGAATGGAAAATGAATATATATCATTTTAT
TAAACACTTCTGAACCACATTCGTACATTTAATGCAACTTCCATCGCATATTGATGAAATGTCGCAAA
AACTAAATAATAAGC
+
FFFFFFFFFFFFFFFFFF,FFFFF:F:FFFFFFFFFFFFFFFFFFFFFFFFFFFFFFFFFFFFFFFFFF
FFFFFFFFFFFFFFFFFFFFFFFFFFFFFFFFFFFFFFFFFFF:FFFFFFFFFFFFFFFFFFFFFF,FF
FFFFFFFFFFFFFF:
@A00721:190:HGJW7DSXY:4:2566:29577:33113 2:N:0:TCTGCAAG+GCTGCACT
AAGCAATGGAAAATCTAGATCTTGTAATCAAAGATTATCGAATGGAAAATGAATATATATCATTTTAT
TAAACACTTCTGAACCACATTCGTACATTTAATGCAACTTCCATCGCATATTGATGAAATGTCGCAAA
AACTAAATAATAAGC
+
FFFFFFFFFFFFFFFFFF:FF,FFFFFFFFFFFFFFFFFFFFFFFFFF:FFFFFFFFFFFFFFFFFFFF
FFFFFFFFFFFFFFFFFFFFFFFFFFFFFFFFFFFFFFFFFFF:FFFFFFFFFFFFFFFFFFFFFFFFF
FFFFFFFFFFFFFF:
@A00721:190:HGJW7DSXY:4:2660:20329:24956 2:N:0:TCTGCAAG+GCTGCACT
AAGCAATGGAAAATCTAGATCATGTAATCAAAGATTATCGAATGGAAAATGAATATATATCATTTTAT
TAAACACTTCTGAACCACATTCGTACATTTAATGCAACTTCCATCGCATATTGATGAAATGTCGCAAA
AACTAAATAATAAGC
+
FFFFFFFFFFFFFFFFFF:FFFFFFFFFFFF,FF,FFFFFFFFFFFFFFFFFFFFFFFFFFFFFFFFFF
FFFFFF:FFFFFFFFFFFF::FF:FFFFFFFFFFFFFFFFFFFFFFFFFFFFF:FFFFFFFFFFFFFF:
F,FFFFF:FFFFFFF
@A00721:190:HGJW7DSXY:4:1104:7934:30545 2:N:0:TCTGCAAG+GCTGCACT
AAGCAATGGAAAATCTAGATCATGTAATCAAAGATTATCGAATGGAAAATGAATATATATCATTTTAT
TAAACACTTCTGAACCACATTCGTACATTTAATGCAACTTCCATCGCATATTGATGAAATGTCGCAAA
AACTAAATAATAAG

+
FFFFFFFFFFFFFFFFFFFFFFFFFFFFFFFF:FFFFFFFFFFFFFFFFFFFFF:FFFFFFFFFFFFFFF
FFFFFFFFFFFFFFFFFFFF:FFFFFFFFFFFFFFFFFFFFFFFFFFFFFFFFFFFFFFFFFFFFF,FF
FFFFFFFFFFFFFF
@A00721:190:HGJW7DSXY:4:2604:4408:8281 2:N:0:TCTGCAAG+GCTGCACT
AAGAAATGGAAAATCTAGATCATGTAATCAAAGATTATCGAATGGAAAATGAATATATATCATTTTAT
TAAACACTTCTGAACCACATTCGTACATTTAATTCAACTTCCATCGAATATTGATGAAATGTCGCAAA
AACTAAATAATAAG
+
FF,,FFFF,F:FF,FFFF,FFF::FFFFFFFFFFFFFF::,FFFF:FFFF:FFFFF:F,F,FFFFFFFF
:FFF,F,,FFF:FFF,FFF:FFFFFFFF:FFFF,,FFFFF,,FF,,,,:FF,F,FF,FFF:,FF:FFFF
F,:,F:F:FF,:FF
@A00721:190:HGJW7DSXY:4:1115:26341:8844 2:N:0:TCTGCAAG+GCTGCACT
TTGTAATTTAGGGGTAATTTCAATTTATAATTTTTGTTATGCGTGGCATAAAGCAATGGAAAATCTAG
ATCATGTAATCAAAGATTATCGAATGGAAAATGAATATATATCATTTTATTAAACACTTCTGAACCAC
ATTCGTACATTTAAT
+
FFFFFFFFFFFFFFFFFFFFFFFFFFFFFFFFFFFFFFFFFFFFFFFFFFFFFFFFFFFFFFFFFFFFFF
:FFFFF:FFFFFFFFFFFFFFF:FFFFFFFFFFFFFFFFFFFFFFFFFFFFFFFFFFFFFFFFFFFFFF
FFFF,FFFFFFFFFF
@A00721:190:HGJW7DSXY:4:1454:15465:13182 2:N:0:TCTGCAAG+GCTGCACT
TTGTAATTTAGGGGTAATTTCAATTTATAATTTTTGTTATGCGTGGCATAAAGCAATGGAAAATCTAG
ATCATGTAATCAAAGATTATCGAATGGAAAATGAATATATATCATTTTATTAAACACTTCTGAACCAC
ATTCGTACATTTAAT
+
FFFFFFFFFFFFFFFFFFFFFFFFFFFFFFFFFFFFFFFFFFFFFFF:FFFFFFFFFFFFFFFFFFFF:F
:FFFFFFFFFFFFFFFFFFFFFFFFFF,FFFFFF:FFFFFFFFF:FFFFFFFFFFFF,FFFFFFF:FFF,:F
FFF:FFFFFFFFFFF
@A00721:190:HGJW7DSXY:4:2262:6361:14826 2:N:0:TCTGCAAG+GCTGCACT
TTGTAATTTAGGGGTAATTTCAATTTATAATTTTTGTTATGCGTGGCATAAAGCAATGGAAAATCTAG
ATCATGTAATCAAAGATTATCGAATGGAAAATGAATATATATCATTTTATTAAACACTTCTGAACCAC
ATTCGTACATTTAAT
+
FFFFFFFFFFFFFFFFFFFFFFFFFFFFFFFFFFFFFFFFFFFFFFFFFFFFF:FFFFFFFFFFFFFFF
:FFFFFFFFFFFFFFFFFFFFFFFFFFFFFFFFFFFFFFFFFFFFFFFFFFFFFFFFF:FFFFFFFFF:
FFF:FFFFFFFFFFF
@A00721:190:HGJW7DSXY:4:2554:4842:28823 2:N:0:TCTGCAAG+GCTGCACT
TTGTAATTTAGGGGTAATTTCAATTTATAATTTTTGTTATGCGTGGCATAAAGCAATGGAAAATCTAG
ATCATGTAATCAAAGATTATCGAATGGAAAATGAATATATATCATTTTATTAAACACTTCTGAACCAC
ATTCGTACATTTAAT
+
FFFFFFF:FFF:FFFFFFFFFFFFFFFFFFFFFFFFFFFFFFFFFFFFFFFFF:FFFFFFFFFFFFFFFF
:FFFFFFFFFFFFF,,FFFFF:FFFFFFFFFFF:FFFF,FFFFF:F:FF:FFFFFF:FFFFFFFFFFFFF:
FFFFFFFFFFF:FFF
@A00721:190:HGJW7DSXY:4:1157:27887:31438 2:N:0:TCTGCAAG+GCTGCACT
AAAATCTTTGTAATTTAGGGGTAATTTCAATTTATAATTTTTGTTATGCGTGGCATAAAGCAATGGAA
AATCTAGATCATGTAATCAAAGATTATCGAATGGAAAATGAATATATATCATTTTATTAAACACTTCT
GAACCACATTCGTAC
+
FFFFFFFFFFFFFF,FFFFFFFFF:FFFFFFFFFFFFFFF:FFFFFFFFFFFFFFFFFFFFFFFFFFFFFF
FFFFFFFFFFFFFFFFFFFFF:FFFFFFFFFFFFFFF,FFFFF:FFFFFFFFFFF,FFFFFFFFFFFFFFFF
FFFFFFFFFFF::F:F
@A00721:190:HGJW7DSXY:4:1219:2781:13698 2:N:0:TCTGCAAG+GCTGCACT
AAAATCTTTGTAATTTAGGGGTAATTTCAATTTATAATTTTTGTTATGCGTGGCATAAAGCAATGGAA

AATCTAGATCATGTAATCAAAGATTATCGAATGGAAAATGAATATATATCATTTTATTAAACACTTCT
GAACCACATTCGTAC
+
FFFFFFFFFFFFFFFFFFFFFF,FFFFFFFFFFFFFFFFFFFFFFFFFF:FFFFFFFFFFFFFFFFFF
FFFFFFF:FFFFFFFFFFFFFFFFFFFF:FFFFFFFFFFFFFF:FFFFFFFFFFFFF:FFFFFFFFFF
FFFFFFFFFFFFFFF
@A00721:190:HGJW7DSXY:4:1361:7346:15092 2:N:0:TCTGCAAG+GCTGCACT
AAAATCTTTGTAATTTAGGGGTAATTTCAATTTATAATTTTTGTTATGCGTGGCATAAAGCAATGGAA
AATCTAGATCATGTAATCAAAGATTATCGAATGGAAAATGAATATATATCATTTTATTAAACACTTCT
GAACCACATTCGTAC
+
FFFFFFFFFFFFFFFFFFFFFFFFFFFFFF:FFFFFFFFFFFFFFFFFFFFFFFFFFFFFFFFFFFFF
FFFFFFFFFFFFF:FFFFFF:FFFFFFFFFFFFFFFFFFFFFFFFFFFFFFFFFFFFFFFFFFFF:FF
:FFF:FFFFFFFFF:
@A00721:190:HGJW7DSXY:4:1368:9173:13839 2:N:0:TCTGCAAG+GCTGCACT
AAAATCTTTGTAATTTAGGGGTAATGTCAATTTATAATTTTTGTTATGCGTGGCATAAAGCAATGGAA
AATCTAGATCATGTAATCAAAGATTATCGAATGGAAAATGAATATATATCATTTTATTAAACACTTCT
GAACCACATTCGTAC
+
FFFFF,FFFFFFF,,,F:FFFFF::,,F,FFFFFFFFFF:,FFF:FFF,F:FFF,F,FFFFFF,:FFF
FF,FFF:FFFFFF:F,::FFF::F:F,FFFFF:,FFF,,FFFF:::F,FFFFFF,FF,,FFFFFFFF,
FFFF,,FF,FFFFFF
@A00721:190:HGJW7DSXY:4:1427:27796:26303 2:N:0:TCTGCAAG+GCTGCACT
AAAATCTTTGTAATTTAGGGGTAATTTCAATTTATAATTTTTGTTATGCGTGGCATAAAGCAATGGAA
AATCTAGATCATGTAATCAAAGATTATCGAATGGAAAATGAATATATATCATTTTATTAAACACTTCT
GAACCACATTCGTAC
+
FFFFFFFFFFFFFFFFFFFFFFF:FFFFFFFFFFFFFFFFFFFFFFFFFFFFFFFFFFFFFFFFF:FF
FFFFFFFFFFFFFFFFF:FFFFF:FFFFFF:FF,FF:FFFFFFFFFF:FFF:FFFFFFFFFFFFFFF:
FFFFFFF:F:FFFFF
@A00721:190:HGJW7DSXY:4:2247:10276:36855 2:N:0:TCTGCAAG+GCTGCACT
AAAATCTTTGTAATTTAGGGGTAATTTCAATTTATAATTTTTGTTATGCGTGGCATAAAGCAATGGAA
AATCTAGATCATGTAATCAAAGATTATCGAATGGAAAATGAATATATATCATTTTATTAAACACTTCT
GAACCACATTCGTAC
+
FFFFFFFFFFFFFFFFFFFFFFFFF:FFFFFFF,FFF:F:FFFFFFFFFFFFFFFF:FF:FFFFFFFF
FFFFFFF:FFFFFFFFFFF::FFFFFFFFFFFFFF:FFFFFFFFF:F,FFFFFFFFF,FFFFF:FFFF
FFFF,:FF:FFFFFF
@A00721:190:HGJW7DSXY:4:2338:7310:12743 2:N:0:TCTGCAAG+GCTGCACT
AAAATCTTTGTAATTTAGGGGTAATTTCAATTTATAATTTTTGTTATGCGTGGCATAAAGCAATGGAA
AATCTAGATCATGTAATCAAAGATTATCGAATGGAAAATGAATATATATCATTTTATTAAACACTTCT
GAACCACATTCGTAC
+
FFFFFFFFFFFFFFFFFFFFFF,FFFFFFFFFFFFFFFFFFFFFFFFFFFFFFFFFFFFFFFFFFFFF
FFFFFFFFFFFFFFFFFFFFFFFFFFFF:FFFFFF:FFFFFF:FFFFFFFFFF:FFFFFFFFFFFFFF
:FFF,FFFF:FFFF:
@A00721:190:HGJW7DSXY:4:2345:11704:37012 2:N:0:TCTGCAAG+GCTGCACT
AAAATCTTTGTAATTTAGGGGTAATTTCAATTTATAATTTTTGTTATGCGTGGCATAAAGCAATGGAA
AATCTAGATCATGTAATCAAAGATTATCGAATGGAAAATGAATATATATCATTTTATTAAACACTTCT
GAACCACATTCGTAC
+
FFFFFFFFFFFFFFFFFFFFFFFFFFFFFFFFFFFFFFFFFFFFFFFFFFFFFFFFFFFFFFFFFFFF
FFFFFFF:FFFFFF:FFFFFFFFFFFFFFFFFFFFFFFFFFFFFFFFFFFFFFFFFFFFFFFF:FFFF
F,:FFFFFFFFFFFF

```
@A00721:190:HGJW7DSXY:4:2464:22851:33270 2:N:0:TCTGCAAG+GCTGCACT
AAAATCTTTGTAATTTAGGGGTAATTTCAATTTATAATTTTTGTTATGCGTGGCATAAAGCAATGGAA
AATCTAGATCATGTAATCAAAGATTATCGAATGGAAAATGAATATATATCATTTTATTAAACACTTCT
GAACCACATTCGTAC
+
FFFFFFFFFFF:FFFFFFFFFFFFFFFFFFFFFFFFFFFFFFFFFFFFFFFFFFFFFFFFFFFFFFFF
FFFFFFF:FFFFFFFFFFFFFFFFFFFFFFFFFFFFFFFFFFFFFFFFFFFFFFFFFFFF:F:FFFFF
FFFFFFFFFFFFFFF
@A00721:190:HGJW7DSXY:4:1201:16297:23484 2:N:0:TCTGCAAG+GCTGCACT
GGAAGACGATGATTTCTGGGACTTTATCCCGACGGCTTTATTTTTATATCCATAAAAATCTTTGTAAT
TTAGGGGTAATTTCAATTTATAATTTTTGTTATGCGTGGCATAAAGCAATGGAAAATCTAGATCATGT
AATCAAAGATTATCG
+
FFFFFFFFFFFFFFFFFFFFFFFFFFFFFFFFFFFFFFFFFFFFFFFFFFFFFFFFFFFFFFFFFFF:
FF:FFFFFFFFFFFFFFFFFFFFFFFFFFFFFFFFF:FFFFFF:FFFFFFFFFFFFFFFFFFFFFFFFFF
FFFFFFF:FFFFFFF
@A00721:190:HGJW7DSXY:4:1674:25265:1376 2:N:0:TCTGCAAG+GCTGCACT
GGAAGACGATGATTTCTGGGACTTTATCCCGACGGCTTTATTTTTATATCCATAAAAATCTTTGTAAT
TTAGGGGTAATTTCAATTTATAATTTTTGTTATGCGTGGCATAAAGCAATGGAAAATCTAGATCATGT
AATCAAAGATTATCG
+
FFFFFFFFFFFFFFFFFFFFFFFFFFFFFFFFFFFFFFFFFFFFFFFFFFFFFFFF:FFFFFFFFFFF
F:FFFFFFFFFFFFFFF:FFFFFFFFFFFFFFFFFFFFF:FFFFFFFFFFFFFFFFFFFFFF:FFFFFFF
FFFFFFFFF:FFFFF
@A00721:190:HGJW7DSXY:4:2220:1741:36605 2:N:0:TCTGCAAG+GCTGCACT
GGAAGACGATGATTTCTGGGACTTTATCCCGACGGCTTTATTTTTATATCCATAAAAATCTTTGTAAT
TTAGGGGTAATTTCAATTTATAATTTTTGTTATGCGTGGCATAAAGCAATGGAAAATCTAGATCATGT
AATCAAAGATTATCG
+
FFFFFFFFFFFFFFFFFFFFFFFFFFFFFFFFFFFFFFFFFFFFFFFFFFFFFFFFFFFFFFFFFFFF
FFFFFFFFFFFFFFFFFFFFFFFFFFFFFFFFFFFFFFFFFFFFFFFFFFFFFFFFFFFFF:FFFF:F
FFFFFFFFFFFFFFF
@A00721:190:HGJW7DSXY:4:2231:29776:35556 2:N:0:TCTGCAAG+GCTGCACT
GGAAGACGATGATTTCTGGGACTTTATCCCGACGGCTTTATTTTTATATCCATAAAAATCTTTGTCAT
TTAGGGGTAATTTCAATTTATAATTTTTGTTATGCGTGGCATAAAGCAATGGAAAATCTAGATCATGT
AATCAAAGAGTATCG
+
FFFFFFFFFFF::FFF:F:FFF,FFFFFFFFFFF:FF:FFFF:FFF,FFFFF:FFFF:FFFF,FF,::
F,,:FFFFF::FFF:FFFFFFF:FFFF,,FFFFFFF,FFFF,FF:,:,:F::FF,:F:FFF:FFFF,F
:FFFFFFFF,FFFFF
@A00721:190:HGJW7DSXY:4:1202:22516:15624 2:N:0:TCTGCAAG+GCTGCACT
GGAAGACGATGATTTCTGGGACTTTATCCCGACGGCTTTATTTTTATATCCATAAAAATCTTTGTAAT
TTAGGGGTAATTTCAATTTATAATTTTTGTTATGCGTGGCATAAAGCAATGGAAAATCTAGATCATGT
AATCAAAGATTATC
+
FFF:FFFFFFFFFFFFFFFFFFFFFFFFFFFFFFFFFFFFFFFFFFFFFFFFFFFFF:FFFFFFFFFF
FFFFFF:FF:FFFFFFFFFFFFFFFFFFFFFFFFFF:F:FFFFFFFFFFFFFFFFFFFFFFFFFFFF,F
FFFFFF:FFFFFFF
@A00721:190:HGJW7DSXY:4:1503:25554:15405 2:N:0:TCTGCAAG+GCTGCACT
GGAAGACGATGATTTCTGGGACTTTATCCCGACGGCTTTATTTTTATATCCATAAAAATCTTTGTAAT
TTAGGGGTAATTTCAATTTATAATTTTTGTTATGCGTGGCATAAAGCAATGGAAAATCTAGATCATGT
AATCAAAGATTATC
+
FFF:FFFF:FFFFFFFFFFFFFFFFFFFFFFFFFFFFFFFFFFFFFFFFFFFFFFFFFFFFFFFFFFF
```

FFFFFFFF:,F:FFF:FF:FFFFFFFFFFFFFFFFFFFFFFFFFF,:FFFFFFFF:FFFFFFF:FFFF:F
FFFF:FF,FFFFFF
@A00721:190:HGJW7DSXY:4:1626:1750:28792 2:N:0:TCTGCAAG+GCTGCACT
GGAAGACGATGATTTCTGGGACTTTATCCCGACGGCTTTATTTTTATATCCATAAAAATCTTTGTAAG
TTAGGGGTAATTTCAATTTATAATTTTTGTTATGCGTGGCATAAAGCAATGGAAAATCTAGATCATGT
AATCAAAGATTATC
+
FFF,FFFFFFFFFFFFFFFFFFFFFFFFFFFFFFFFFFFFFFFFFF:F,FFFFFFFFFFFF:F:FFFFF,
FFFFF:FFFFFFFFFFFFFFFFFFFFFFF,FFFFFFFFF:FFFFF,FFFFFFFFFFFFFFFFF,FFFF,F
FFFF,F,FFFFFFF
@A00721:190:HGJW7DSXY:4:1650:20383:8547 2:N:0:TCTGCAAG+GCTGCACT
GGAAGACGATGATTTCTGGGACTTTATCCCGACGGCTTTATTTTTATATCCATAAAAATCTTTGTAAT
TTAGGGGTAATTTCAATTTATAATTTTTGTTATGCGTGGCATAAAGCAATGGAAAATCTAGATCATGT
AATCAAAGATTATC
+
FFFFFFFFFFFFFFFFFFFFFFFFFFFFFFFFFFFFFFFFFFFFFFFFFFFFFFFFFFFFFFFFFFFFFF
FFFFFFFFF:F,FFFFF:FFFFFFFFFF:FFFFFFFFFFFFFFFF:,FFFFF,FFFF,FFFFFFF:FFFF
FFFFFFFFFFFFFF
@A00721:190:HGJW7DSXY:4:1306:6677:12587 2:N:0:TCTGCAAG+GCTGCACT
ATTACATGATCTAGATTTTCCATTGCTTTATGCCACGCATAACAAAAATTATAAATTGAAATTACCCC
TAAATTACAAAGATTTTTATGGATATAAAAATAAAGCCGTCGGGATAAAGTCCCAGAAATCATCGTCT
TCCATGACGGGTTCT
+
FFFFFFFFFFFFFFFFFFFFFFFFFFFFFFFFFFFFFFFFFFFFFFFFFFFFFFFFFFFFFFFFFFFFFF
FFFFFFFFFFFFFFFFFFFFFFFFFFFFFFFFFFFF:FFFFFFFFFFFFFFFFF:FFFFFFFFFFFF:FF
FFFFFFFFFFFFFFF
@A00721:190:HGJW7DSXY:4:1513:28076:15608 2:N:0:TCTGCAAG+GCTGCACT
ATTACATGATCTAGATTTTCCATTGCTTTATGCCACGCATAACAAAAATTATAAATTGAAATTACCCC
TAAATTACAAAGATTTTTATGGATATAAAAATAAAGCCGTCGGGATAAAGTCCCAGAAATCATCGTCT
TCCATGACGGGTTCT
+
FFFFFFFFFFFFFFFFFFFFFFFFFFFFFFFFFFFFFFFFFFFFFFFFFFFFFFFFFFFFFFFFFFFFFF
FFFFFFFFFFFFFFFFFFFFFFFFFFFFFFFFF:FFFFFFFFFFFFFFFFFFFF:F:FFFFFFFFFFFFF
FFFFFFFFFFFFFFF
@A00721:190:HGJW7DSXY:4:1523:21124:26647 2:N:0:TCTGCAAG+GCTGCACT
ATTACATGATCTAGATTTTCCATTGCTTTATGCCACGCATAACAAAAATTATAAATTGAAATTACCCC
TAAATTACAAAGATTTTTATGGATATAAAAATAAAGCCGTCGGGATAAAGTCCCAGAAATCATCGTCT
TCCATGACGGGTTCT
+
FFF:FFFFFFFFFFFFFFFFFFFFFFFFFFFFFFFFF:FFFFFFFFFFFFFFFFFFFFFFFFFFFFFFFF
F:FF:FFFFFFFFFFFFFFFFFFFFFFFFFFFFFFFFFFF:FFFFFFFFF:FFFFFFFFFFFFFFFFFFF
FFFF,FFF:FFFFFF
@A00721:190:HGJW7DSXY:4:2104:16007:14340 2:N:0:TCTGCAAG+GCTGCACT
ATTACATGATCTAGATTTTCCATTGCTTTATGCCACGCATAACAAAAATTATAAATTGAAATTACCCC
TAAATTACAAAGATTTTTATGGATATAAAAATAAAGCCGTCGGGATAAAGTCCCAGAAATCATCGTCT
TCCATGACGGGTTCT
+
FF,FF:FFFFFFFF:FFFFFFFFF,FFFFFFFFFFF,FFFFFFFFFFFFFFFFFFFFFFFFF:FFFFFF:
FFFFFFFFFFF,FFFFFFFFFFFFFFFFFFFFFFFF,FFFFF::FFFFFF:FFFFF:FFFFFFFFFFFFF
FFFFFF,F,F:FFFF
@A00721:190:HGJW7DSXY:4:1265:21866:7670 2:N:0:TCTGCAAG+GCTGCACT
TGCAGAACCCGTCATGGAAGACGATGATTTCTGGGACTTTATCCCGACGGCTTTATTTTTATATCCAT
AAAAATCTTTGTAATTTAGGGGTAATTTCAATTTATAATTTTTGTTATGCGTGGCATAAAGCAATGGA
AAATCTAGATCATGT

```
+
FFFFFFFFF:FFFFFFFFFFFFFFFFFFFFFFFFFFFFFFFFFFFFFFFFFFFFFFFFFFFFFF
FFFFFFFFFFFFFFF:FFFFFFFFFFFFFFFF:FFFFFFFFFFFFFFFFFFFFFFFFFFFFFFF
FFFFFFFF:FFFFFF
@A00721:190:HGJW7DSXY:4:1311:24939:11678 2:N:0:TCTGCAAG+GCTGCACT
ACATGATCTAGATTTTCCATTGCTTTATTCAACGCATAACAAAAATTATAAATAGAAAATACCCCTAA
ATAACAAAGATTTTTATGGATATAAAAATAAATCAGTCGGGATAAAGTCCCAGAAATCATCGTCTTCC
ATGACGGGTTCT
+
FFF:,,:F,FFFFF:FF,FF:FFF,FFF,F,F,,F,,::,FFFF,,FFFF:F,,FFFF,FF,FF,FF:
F,:FFFFF,FF:,FFFF,FFFFFF,FFFFFFF,F,:F,,,,F,FFF:,,FFF:FFF,FFFFFF,,:,,
FF,FF,F,:FFF
@A00721:190:HGJW7DSXY:4:1320:16740:26349 2:N:0:TCTGCAAG+GCTGCACT
TGCAGAACCCGTCATGGAAGACGATGATTTCTGGGACTTTATCCCGACGGCTTTATTTTTATATCCAT
AAAAATCTTTGTAATTTAGGGGTAATTTCAATTTATAATTTTTGTTATGCGTGGCATAAAGCAATGGA
AAATCTAGATCATGT
+
FFFFFFFFFFFFFFFFFFFFFFFFFFFFFFFFFFFFFFFFFFFFFFFFFFFFFFFFFFFFFFFFFFFF
FFFFFFFFFFFFF::F,FFFFFFFFFFFFFFFFFFFFFFFFFFFFFFFFFFFFFFFFFFFFFFFFFFF
FF:FFFFF:FFFFFF
@A00721:190:HGJW7DSXY:4:1667:8124:13839 2:N:0:TCTGCAAG+GCTGCACT
TGCAGAACCCGTCATGGAAGACGATGATTTCTGGGACTTTATCCCGACGGCTTTATTTTTATATCCAT
AAAAATCTTTGTAATTTAGGGGTAATTTCAATTTATAATTTTTGTTATGCGTGGCATAAAGCAATGGA
AAATCTAGATCATGT
+
FFFFFF,FFFFFFFFFFFFFFFFFFFFFFFFFFFFFFFFFFFFFFFFFFFFFFFFFFFFFFFFFFFFF
FFFFFFFFFFFFFFFFFFFFFFFFFFFFFFF,FFFFFFFFFFFFFFFFFF:FFFFFFFFFFFFFFFFF
FF,FFFF:,FFFFFF
@A00721:190:HGJW7DSXY:4:2167:31937:19758 2:N:0:TCTGCAAG+GCTGCACT
TGCAGAACCCGTCATGGAAGACGATGATTTCTGGGACTTTATCCCGACGGCTTTATTTTTATATCCAT
AAAAATCTTTGTAATTTAGGGGTAATTTCAATTTATAATTTTTGTTATGCGTGGCATAAAGCAATGGA
AAATCTAGATCATGT
+
FFFFFFFFFFFFF:FFFFFFFFFFFFFFFFFFFFFFFFFFFFFFFFFFFFFFFFFFFFFFFFFFFFFF
FF:FFFFFFFFFF::F:FFFFFFFFFFFFF:F,FFFFFFFFFFFFFFFFFFF:FF,F:,:FF:FFF::
FF,F:FFF:FFFFF:
@A00721:190:HGJW7DSXY:4:2259:9760:25786 2:N:0:TCTGCAAG+GCTGCACT
TGCAGAACCCGTCATGGAAGACGATGATTTCTGGGACTTTATCCCGACGGCTTTATTTTTATATCCAT
AAAAATCTTTGTAATTTAGGGGTAATTTCAATTTATAATTTTTGTTATGCGTGGCATAAAGCAATGGA
AAATCTAGATCATGT
+
F::FFFFFFFFFFFFFFFFFF:FFFFFFFFF,,::FFFFFF:FFF,FFF:FF:FFFFFFFFFFFFFFF
FFFFFFF,FFF:FFFFFFFFFFF:FFF,FF,,FFFFFFF,:FFF:,FFF::FFF,FFFFFFFFFFFFF
FFFF:FF:FFFFFF:
@A00721:190:HGJW7DSXY:4:2427:19678:22388 2:N:0:TCTGCAAG+GCTGCACT
TGCAGAACCCGTCATGGAAGACGATGATTTCTGGGACTTTATCCCGACGGCTTTATTTTTATATCCAT
AAAAATCTTTGTAATTTAGGGGTAATTTCAATTTATAATTTTTGTTATGCGTGGCATAAAGCAATGGA
AAATCTAGATCATGT
+
FFFFFFFFFFFFF:FFFFFFFFF,FFFFFFFFFFFFFFFFFFFFFFFFFFFFFFFFFFFFFFFFFFFF
FFFF:FFFFFFFFF::F,FFFFFF,FFFFFFF:F:FFFFFFFFFFFFFFF:FFFFF:FF,FFFFFFFF
FFFFFFFFFFFFFFF
@A00721:190:HGJW7DSXY:4:2559:15085:3662 2:N:0:TCTGCAAG+GCTGCACT
TGCAGAACCCGTCATGGAAGACGATGATTTCTGGGACTTTATCCCGACGGCTTTATTTTTATATCCAT
```

```
AAAAATCTTTGTAATTTAGGGGTAATTTCAATTTATAATTTTTGTTATGCGTGGCATAAAGCAATGGA
AAATCTAGATCATGT
+
FFFFFFFFFFFFFFFFFFFFFFFFFFF:FFFFFFFFFFFFFFFFFFFFFFFFFFFFFFFFFFFFFFFF
FFFFFFFFFFFFFFFF:FFFFFFFFFFFFFFFFFFFFFFFFFFFFFFFFFFFFFFFFFFFFFF:FFFF
FFFFFFFFFFFFF:F
@A00721:190:HGJW7DSXY:4:2620:7500:29919 2:N:0:TCTGCAAG+GCTGCACT
TGCAGAACCCGTCATGGAAGACGATGATTTCTGGGACTTTATCCCGACGGCTTTATTTTTATATCCAT
AAAAATCTTTGTAATTTAGGGGTAATTTCAATTTATAATTTTTGTTATGCGTGGCATAAAGCAATGGA
AAATCTAGATCATGT
+
FFFFFFFFFFFFFFFFFFFFFFFFFFFFFFFFFFFFFFFFFFFFFFFFFFFFFFFFFFFFFFFFFFFF
FFFFFFFFFFFFFFFFF:FFFF:FFFFFFF:FFFF:FFFFFFFFFFFF:FFFFFFF:FFFF:FFFFFF
FFFFFFFFFFFFF:FF
@A00721:190:HGJW7DSXY:4:1246:23421:1846 2:N:0:TCTGCAAG+GCTGCACT
GCAGAACCCGTCATGGAAGACGATGATTTCTGGGACTTTATCTCGACGGCTTTATTTTTATATCCATA
AAAATCTTTGTAATTTAGGGGTAATTTCAATTTATAATTTTTGTTATGCGTGGCATAAAGCAATGGAA
AATCTAGAGATCGGA
+
FFFFFFFFFFFFFFFFFFFFFFFFFFFFFFFFFFFFFFFFFFFFFFFFFFFFFFFFFFFFFFFFFFFF
FFFFFFFFFFFFFF:FFFFFFFFFFFFFFFFFFFFFFFFFFFFFFFFFFFFFFFF:FFFFFFFFFFFFF
FFFFFFFFFFFFFFF
@A00721:190:HGJW7DSXY:4:1246:7084:3176 2:N:0:TCTGCAAG+GCTGCACT
GCAGAACCCGTCATGGAAGACGATGATTTCTGGGACTTTATCTCGACGGCTTTATTTTTATATCCATA
AAAATCTTTGTAATTTAGGGGTAATTTCAATTTATAATTTTTGTTATGCGTGGCATAAAGCAATGGAA
AATCTAGAGATCGGA
+
FFFFFFFFFFFFFFFFFFFFFFFFFF:FF:FFFFFFFFFFFFFFFFFFFFFFFFFFFFFFFFFFFFFF
FFFFFFFFFFF:FFFFFFFFFFFFFFFFFFFFFFFFFFFFFFFFFFFFFFFFFFFFFFFFFFFFFFF:
FFFFFFFFFFFFFFF
@A00721:190:HGJW7DSXY:4:1459:16676:22983 2:N:0:TCTGCAAG+GCTGCACT
GCAGAACCCGTCATGGAAGACGATGATTTCTGGGACTTTATCTCGACGGCTTTATTTTTATATCCATA
AAAATCTTTGTAATTTAGGGGTAATTTCAATTTATAATTTTTGTTATGCGTGGCATAAAGCAATGGAA
AATCTAGAGATCGGA
+
FFFFFFFFFFFFFFFFFFFFFFFFFFFFFFFFFFFFFFFFFFFFFFFFFFFFFFFFFFFFFFFFFFFF
FFFFFFFFFFFFFFFFFFFFFFFFFFFFFFFFFFFFFFFFFFFFFFFFFFFFFFFFFFFFFF:FFFFF
FFFFFFFFFFFFF:FF
@A00721:190:HGJW7DSXY:4:1513:8214:31219 2:N:0:TCTGCAAG+GCTGCACT
GCAGAACCCGTCATGGAAGACGATGATTTCTGGGACTTTATCTCGACGGCTTTATTTTTATATCCATA
AAAATCTTTGTAATTTAGGGGTAATTTCAATTTATAATTTTTGTTATGCGTGGCATAAAGCAATGGAA
AATCTAGAGATCGGA
+
F:FFFFFFFFFFFFFFFFFFFFFFFFFFFFFFFFFFFFFFFFFFFFFFFFFFFFFFFFFFFFFFFFFF
FFFFFFFFFFFFFFFFFFFFFFFFFFFFFFFFFFFFFFFFFFFFFFFFFFFFFFFFFFFFFFFFF:FF
FFFFFFFFFFFFFFF
@A00721:190:HGJW7DSXY:4:2167:9634:30827 2:N:0:TCTGCAAG+GCTGCACT
GCAGAACCCGTCATGGAAGACGATGATTTCTGGGACTTTATCTCGACGGCTTTATTTTTATATCCATA
AAAATCTTTGTAATTTAGGGGTAATTTCAATTTATAATTTTTGTTATGCGTGGCATAAAGCAATGGAA
AATCTAGAGATCGGA
+
FFFFFFFFFFFFFFFFFFFFFFFFFFFFFFFFFFFFFFFFFFFFFFFFFFFFFFFFFFFFFFFFFFFF
FFFFFFFFFFFFFFFFFFFFFFFFFFFFFFFFFFFFFFFFFFFFFFFF:FFFFFFFFFFFFFFFFFFF
FFFFFFFFFFFFFFF
```

@A00721:190:HGJW7DSXY:4:2216:16884:8375 2:N:0:TCTGCAAG+GCTGCACT
GCAGAACCCGTCATGGAAGACGATGATTTCTGGGACTTTATCTCGACGGCTTTATTTTTATATCCATA
AAAATCTTTGTAATTTAGGGGTAATTTCAATTTATAATTTTTGTTATGCGTGGCATAAAGCAATGGAA
AATCTAGAGATCGGA
+
FFFFFFFFFFFFFFFFFFFFFFFFFF:FFFFFFFFFFFFFFFFFFFFFFFFFFFFFF:FFFFFFFFFFFF
F:FFFFFFFFF,FFFFFFFFFFFFFFFFFFFFFFFFFFFFFFFFFFFFFFFFFFFFFFFFFFFFFFFFFF
FFFFFF:FFFFFF:F
@A00721:190:HGJW7DSXY:4:2304:11008:33332 2:N:0:TCTGCAAG+GCTGCACT
GCAGAACCCGTCATGGAAGACGATGATTTCTGGGACTTTATCTCGACGGCTTTATTTTTATATCCATA
AAAATCTTTGTAATTTAGGGGTAATTTCAATTTATAATTTTTGTTATGCGTGGCATAAAGCAATGGAA
AATCTAGAGATCGGA
+
::FFFFFFFFFFFFFFFFFFFFFFFFFFFFFFFFFFFFFFFFFFFFFFFFFFFFFFFFFFFFFFFFFFFF
FFFFFFFFFFFFFFFFFFFFFFFFFFFFFFFFFFFFFFFFFFFFFFFF::FFFFFFFFFF::FFFFFFFF
FFFFFF:FFFFFFFF
@A00721:190:HGJW7DSXY:4:2558:26820:12743 2:N:0:TCTGCAAG+GCTGCACT
GCAGAACCCGTCATGGAAGACGATGATTTCTGGGACTTTATCTCGACGGCTTTATTTTTATATCCATA
AAAATCTTTGTAATTTAGGGGTAATTTCAATTTATAATTTTTGTTATGCGTGGCATAAAGCAATGGAA
AATCTAGAGATCGGA
+
FFFFFFFFFFFFFFFFFFFFFFFFFFFFFFFFFFFFFFFFFFFFFFFFFFFFFFFFFFFFFFFFFFFFFF
FFFFFFFFFFFFFF,FFFFFFFFFFFFFFFFFFFFFFFFFFFFFFFFFFFFFFFFFFFFFFFFFFFFFFF
FFFF:F:FFFFFFFF
@A00721:190:HGJW7DSXY:4:1459:17445:23531 2:N:0:TCTGCAAG+GCTGCACT
GCAGAACCCGTCATGGAAGACGATGATTTCTGGGACTTTATCTCGACGGCTTTATTTTTATATCCATA
AAAATCTTTGTAATTTAGGGGTAATTTCAATTTATAATTTTTGTTATGCGTGGCATAAAGCAATGGAA
AATCTAGAGATC
+
FF,:FFF:FFFFFFFFFFFF:FF:FFFF:FF,,:F,FFFFFF,:,F,F:,FFFFFFFFFFFFF:FFFF
FFFFF,:,F:FFFFF,FFFF:FFFFFF:F:FFFFFFFFFF:F:FFFF,,FF:FFFFFFFFFF,F:FFF
FFF,FF:FFFFF
@A00721:190:HGJW7DSXY:4:1106:20057:33943 2:N:0:TCTGCAAG+GCTGCACT
GAATTACTGCAGAACCCGTCATGGAAGACGATGATTTCTGGGACTTTATCCCGACGGCTTTATTTTTA
TATCCATAAAAATCTTTGTAATTTAGGGGTAATTTCAATTTATAATTTTTGTTATGCGTGGCATAAAG
CAATGGAAAATCTAG
+
FFFFFFFFFFFFFFFF:FFFFFFFFFFFFFFFFFFFFFFFFFFFFFFFFFFFFFFFFFFFFFFFFFFFF
FFFFFFFFFFFFFFFFFFFFF:FFFFFFFFFFFFFFFFF:FFFFFFFFFFFFFFFFFFFFFFFFFFFFFF
FFFFFFFFFFFFFFF
@A00721:190:HGJW7DSXY:4:1302:11433:34037 2:N:0:TCTGCAAG+GCTGCACT
GAATTACTGCAGAACCCGTCATGGAAGACGATGATTTCTGGGACTTTATCCCGACGGCTTTATTTTTA
TATCCATAAAAATCTTTGTAATTTAGGGGTAATTTCAATTTATAATTTTTGTTATGCGTGGCATAAAG
CAATGGAAAATCTAG
+
:FFFFFFFFFFFFFFFFF:FFFFFFFFFFFFFFFFFFFFFFFFFFFFFFFFFFF:FFFFFFFF:FFF:FFF
FFFFFFFFFFFFFFF,FFFFFFFFFFFFFFFFFFFFFFFFFFFFFFFFFF:FFFFFFFFFF:FFFFFFFF
FFF:F:FFFF:FFFF
@A00721:190:HGJW7DSXY:4:1425:29559:20494 2:N:0:TCTGCAAG+GCTGCACT
GAATTACTGCAGAACCCGTCATGGAAGACGATGATTTCTGGGACTTTATCCCGACGGCTTTATTTTTA
TATCCATAAAAATCTTTGTAATTTAGGGGTAATTTCAATTTATAATTTTTGTTATGCGTGGCATAAAG
CAATGGAAAATCTAG
+
FFFFFFFFFFFFFFFFFFFFFFFFFFFFFFFFFFFFFFFFFFFFFFFFFFFFFFFFFFFFFFFFFFFFFF

FFFFFFFFFFFFFFFFFFFFFFFF:FFFFFF:FFFFFFFFFFFFFFFFFFFFFFFFFFFFF:FFFFF
FFFFFFFFF:FFFF:
@A00721:190:HGJW7DSXY:4:1450:19352:22075 2:N:0:TCTGCAAG+GCTGCACT
GAATTACTGCAGAACCCGTCATGGAAGACGATGATTTCTGGGACTTTATCCCGACGGCTTTATTTTTA
TATCCATAAAAATCTTTGTAATTTAGGGGTAATTTCAATTTATAATTTTTGTTATGCGTGGCATAAAG
CAATGGAAAATCTAG
+
FFFF:FFFFFFFFFFFFFFFFFFFF,FFFFFFFFFFFFFFFFFFFFFFFF:FFFF:FFFFFFFF,:FF
FFFFFFFFFFF:FFF:FFFFFF::FFFFFFFFFFF,F:FFFFFFFFFFFFFFFFFFF:FF,FF,FFFF
FFFFFFFFFFF,FF:
@A00721:190:HGJW7DSXY:4:1521:5204:8750 2:N:0:TCTGCAAG+GCTGCACT
GAATTACTGCAGAACCCGTCATGGAAGACGATGATTTCTGGGACTTTATCCCGACGGCTTTATTTTTA
TATCCATAAAAATCTTTGTAATTTAGGGGTAATTTCAATTTATAATTTTTGTTATGCGTGGCATAAAG
CAATGGAAAATCTAG
+
FFFFFFFFFFFFFFFFFFFFFFFFFFF:FFFFFFFFFFFFFFFFFFFFFFFFFFFFFFFFFFFFFFFF
FFFFFFFFFFFFFFFF,FFF:FFFF:FFFFF,FFFF:FFFFFFFFFFFFFFFFFFFFFFFFFFFFFFF
FF,FFFFFFFFFFFF
@A00721:190:HGJW7DSXY:4:2531:19578:2300 2:N:0:TCTGCAAG+GCTGCACT
GAATTACTGCAGAACCCGTCATGGAAGACGATGATTTCTGGGACTTTATCCCGACGGCTTTATTTTTA
TATCCATAAAAATCTTTGTAATTTAGGGGTAATTTCAATTTATAATTTTTGTTATGCGTGGCATAAAG
CAATGGAAAATCTAG
+
FFFFFFFFFFFFFFFFFFFFFFFFFFFFFFFFFFFFFFFFFFFFFFFFFFFFFFFFFFFFFFFFFFFF
FFFFFFFFFFFFFFFFFFFFFFFFFFFFFFFFF,FFFFFFF:FFFFFFFFFFFFFFFFFFFFFF:FFFF
FFFFFFFFFFFFFFF
@A00721:190:HGJW7DSXY:4:2533:29568:33849 2:N:0:TCTGCAAG+GCTGCACT
GAATTACTGCAGAACCCGTCATGGAAGACGATGATTTCTGGGACTTTATCCCGACGGCTTTATTTTTA
TATCCATAAAAATCTTTGTAATTTAGGGGTAATTTCAATTTATAATTTTTGTTATGCGTGGCATAAAG
CAATGGAAAATCTAG
+
FFFFFFFFFFFFFFFFFFFFFFFFFFFFFFFFFFFFFFFFFFFFFFFFFFFFFFFFFFFFFFFFFFFF
FFFFFFFFFFFFFFFFFFFFFFFF,FFF:FFFF:FFFFFFFFFFFFFFFFFFFFFFFFFFFFF:FFFF
F:FFFFF:FFFFFFF
@A00721:190:HGJW7DSXY:4:2538:12075:23750 2:N:0:TCTGCAAG+GCTGCACT
GAATTACTGCAGAACCCGTCATGGAAGACGATGATTTCTGGGACTTTATCCCGACGGCTTTATTTTTA
TATCCATAAAAATCTTTGTAATTTAGGGGTAATTTCAATTTATAATTTTTGTTATGCGTGGCATAAAG
CAATGGAAAATCTAG
+
FFFFFFFFFFFFFFFFFFFFFFFFFFFFFFFFFFFFFFFFFFFFFFFFFFFFFFFFFFFFFFFFFFFF
FFFFFFFFFFFFFFFFFFFFFFFFFFFFFFFFFFFFFF:FFFFFFFFFFFFFFFFF:FFFFFFFFFFF
:FFFFFFFFFFFFF:
@A00721:190:HGJW7DSXY:4:2178:24433:18599 2:N:0:TCTGCAAG+GCTGCACT
GAATTACTGCAGAACCCGTCATGGAAGACGATGATTTCTGGGACTTTATCCCGACGGCTTTATTTTTA
TATCCATAAAAATCTTTGTAATTTAGGGGTAATTTCAATTTATAATTTTTGTTATGCGTGGCATAAAG
CAATGGAAAATCTA
+
FFFFFFFFFFFFFFFFFFFFFFFFFFFFFFFFFFFFFFFFFFFFFFFFFFFFFFFFFFFFFFFFFFFF
FFFFFFFFFFFFFFFFFFFFF,FFFFFFFFFFFFFFFFFFFFFFFFFFFFFFFFFFFFFFFFFFF:FF
FFFFFFFFFFFFFF
@A00721:190:HGJW7DSXY:4:2678:24921:25426 2:N:0:TCTGCAAG+GCTGCACT
CAACCAGTCCCAGAATTACTGCAGAACCCGTCATGGAAGACGATGATTTCTGGGACTTTATCCCGACG
GCTTTATTTTTATATCCATAAAAATCTTTGTAATTTAGGGGTAATTTCAATTTATAATTTTTGTTATG
CGTGGCATAAAGCAA

```
+
FFFFFFFFFFFFFFFFFFFFFFFFFFFFFFFFFFFFFFFFFFFFFFFFFFFFFFFFFFFFFFFFFFFFFFF
FFFFFFFFFFFFFFFFFFFFFFFFFFFFFFFFFFFFFFFFFFFFFFFFFFFFFFFFFFFFFFFFFFFFFFF
FFFF:FFFF:FFFFF
@A00721:190:HGJW7DSXY:4:1246:21811:10614 2:N:0:TCTGCAAG+GCTGCACT
ATTACAACCAGTCCCAGAATTACTGCAGAACCCGTCATGGAAGACGATGATTTCTGGGACTTTATCCC
GACGGCTTTATTTTTATATCCATAAAAATCTTTGTAATTTAGGGGTAATTTCAATTTATAATTTTTGT
TATGCGTGGCATAAA
+
FFFFFFFFFFFFFFFFFFFFFFFFFFFFFFFFFFFFFFFFFFFFFFFFFFFFFFFFFFFFF:FFFFFFFF
FFFFFFFFFFFFFFFFFFFFFFFFFFFFFFFFFFFF,FFFFFFFFFFFFFFF:FFFFFFFFFFFFFF
FFF,FFFFFFFFFF:
@A00721:190:HGJW7DSXY:4:2248:23936:33583 2:N:0:TCTGCAAG+GCTGCACT
ATTACAACCAGTCCCAGAATTACTGCAGAACCCGTCATGGAAGACGATGATTTCTGGGACTTTATCCC
GACGGCTTTATTTTTATATCCATAAAAATCTTTGTAATTTAGGGGTAATTTCAATTTATAATTTTTGT
TATGCGTGGCATAAA
+
FFFFFFFFFFFFFFFFFFFFFFFFFFFFFF:FFFFFFFFFFF:FFFFFFFFF,FFFFFFFFFFFFFFFF
FFFFFFFFFFFFFFFFFFFFFFFFFFFFFFFFFFFFFFFFFFFFFFFFFFFFFFF:FFFFFFFFFFFF
FFFF:FFFFFF:FFF
@A00721:190:HGJW7DSXY:4:2264:21893:30984 2:N:0:TCTGCAAG+GCTGCACT
ATTACAACCAGTCCCAGAATTACTGCAGAACCCGTCATGGAAGACGATGATTTCTGGGACTTTATCCC
GACGGCTTTATTTTTATATCCATAAAAATCTTTGTAATTTAGGGGTAATTTCAATTTATAATTTTTGT
TATGCGTGGCATAAA
+
FFFFFFFFFFFFFFF:FFFFFFFFFFFFFFFFFFFFFFFFFFFF:FFFFFFFFFFFFFFFFFFFFFFFFF
FFFFFFFFFFFFFFFFFFFFF:FFFFFFFFFFFFFFFFFF,FFFFFFFFFFFFF:FFFFFFFFFFFFFF
FFF:F:FF:FFFF:F
@A00721:190:HGJW7DSXY:4:2270:9625:22263 2:N:0:TCTGCAAG+GCTGCACT
ATTACAACCAGTCCCAGAATTACTGCAGAACCCGTCATGGAAGACGATGATTTCTGGGACTTTATCCC
GACGGCTTTATTTTTATATCCATAAAAATCTTTGTAATTTAGGGGTAATTTCAATTTATAATTTTTGT
TATGCGTGGCATAAA
+
FFFFFFFFFFFFFFFFFFFFFFFFFFFFFFFFFFFFFFFFFFFFFFFFFFFFFFFFFFFFFFFFFFFFFFF
FFFFFFFFFFFFFFFFFFFF:FFFFFFFFFFFFFFFFF,FFFFFFFFFFFFFFFFFFFFFFFFFFFFFF
FFF:FFFFFFFFFFF
@A00721:190:HGJW7DSXY:4:1505:24198:24017 2:N:0:TCTGCAAG+GCTGCACT
ATTACAACCAGTCCCAGAATTACTGCAGAACCCGTCATGGAAGACGATGATTTCTGGGACTTTATCCC
GACGGCTTTATTTTTATATCCATAAAAATCTTTGTAATTTAGGGGTAATTTCAATTTATAATTTTTGT
TATGCGTGGCATAA
+
FFFFFFFFFFFFFFFFFFFFFFFFFFFFFFFFFFFFFFFFFFFFFFFFFFFFFFFFFFFFFFFFFFFFFFF
FFFFFFFFFFFFFFFFFFFFFFFFFFFFFFFFFFFF,FFFFFFFFFFFFFFF:FFFFFFFFFFFFFF
FFFFFFFFFFFFFF
@A00721:190:HGJW7DSXY:4:1162:24903:32784 2:N:0:TCTGCAAG+GCTGCACT
GGACGACATTACAACCAGTCCCAGAATTACTGCAGAACCCGTCATGGAAGACGATGATTTCTGGGACT
TTATCCCGACGGCTTTATTTTTATATCCATAAAAATCTTTGTAATTTAGGGGTAATTTCAATTTATAA
TTTTTTTTATGCGTG
+
FFFFFFFFFFFFFFFFFFFF:FFFFFFFFFFFFFFFFF:FFFFFFFFFFFFFFFFFFF,FFFF:FFFF
FFFFFFF:FF,F:FFFFFFFFFFFFFFFFFFFFFFFF,FFFFFFF:FFFFFF:FFFFFF,FFFFFFFFF
FFFF:,FFFFFFFF:
@A00721:190:HGJW7DSXY:4:1234:29640:34162 2:N:0:TCTGCAAG+GCTGCACT
GGACGACATTACAACCAGTCCCAGAATTACTGCAGAACCCGTCATGGAAGACGATGATTTCTGGGACT
```

```
TTATCCCGACGGCTTTATTTTTATATCCATAAAAATCTTTGTAATTTAGGGGTAATTTCAATTTATAA
TTTTTGTTATGCGTG
+
FFF:FFFFFFFFFFFFFFFFFFFFFFFFFFFFFFFFFFFFFFFFFFF:FFFFFFFFFFFFFFFFFFFFFF
FFFFFFFFFFFFFFF:FFFFFFFFFFFFFFFFFFFFFFFFFFFFFFFFFFFFFFFFFFFFFFFFFFFFFF
FFFFFFFFFFFFFFF
@A00721:190:HGJW7DSXY:4:1252:24768:14951 2:N:0:TCTGCAAG+GCTGCACT
GGACGACATTACAACCAGTCCCAGAATTACTGCAGAACCCGTCATGGAAGACGATGATTTCTGGGACT
TTATCCCGACGGCTTTATTTTTATATCCATAAAAATCTTTGTAATTTAGGGGTAATTTCAATTTATAA
TTTTTGTTATGCGTG
+
FFFFFFFFFFFFFFFFFFFFFFF:FFFFFFFFFFFFFFFFFFFFFFFFFFFFFFFFFFFFFFFFFFFFFF
FFFFFFFFFFFFFFFFFFFFFFFFFFFFFF:FFFFFFFFFFFFFFFFFFFFFFFFFFFFFFFFFFFFFFF
FFFFFFFFFFF:FFF
@A00721:190:HGJW7DSXY:4:1252:24686:14967 2:N:0:TCTGCAAG+GCTGCACT
GACGACATTACAACCAGTCCCAGAATTACTGCAGAACCCGTCATGGAAGACGATGATTTCTGGGACTT
TATCCCGACGGCTTTATTTTTATATCCATAAAAATCTTTGAAATTTAGGGTTAATTTCAATTTATAAT
TTTTGTTATGCGTG
+
FFFFFFF::FFFFFF::FF:FFFFFF,FF,FFFFFFFF:,F:FFF:F:FFFFFFF:,F:FF,::,FF,,:
FF,FFFFF:FFFFFFFF,F:FFFFFF::F,FFFFFF,,F:FF,FFF,FF:,:,FFFFF::FF:FFFFFFF
F:FFFF:FF:,,FF
@A00721:190:HGJW7DSXY:4:1254:24017:32659 2:N:0:TCTGCAAG+GCTGCACT
GGACGACATTACAACCAGTCCCTGAATTACTGCAGAACCCGTCATGGAAGACGATGATTTCTGGGACT
TTATCCCGACGGCTTTATTTTTATATCCATAAAAATCTTTGTAATTTAGGGGTAATTTCAATTTATAA
TTTTTGTTATGCGTG
+
F:F:,:FFFFFFFF:FFFFFFF,FFFFFFFFF,FFFFF:FF,FFFFFFFF:FFFFFFFFFFFFFFFFF:F
FFFFF,FFF,F:FFFFFFFFFFFFFF:FFFFFFFFF,FFFFFFFFF:FF:FF:FFFFFFFFFF:FFFFFF
FFFFFFFFFF:,FFF
@A00721:190:HGJW7DSXY:4:1303:30626:30201 2:N:0:TCTGCAAG+GCTGCACT
GGACGACATTACAACCAGTCCCAGAATTACTGCAGAACCCGTCATGGAAGACGATGATTTCTGGGACT
TTATCCCGACGGCTTTATTTTTATATCCATAAAAATCTTTGTAATTTAGGGGTAATTTCAATTTATAA
TTTTTGTTATGCGTG
+
FFFFFFFFFFFFF:FFFFFFFFFFFFFFFFFFFFFFFFFFFFFFFFFFFFF:FFFFFFFFFFFFFFFFFF
FFFFFFFF:FFFFFFFFFF:FFFFFFFFFFFFFFFFFFFFFFFFF:FFFFFFFFFFFFFFFF,F:FFFFF
FFFFFFFFFFFFFFF
@A00721:190:HGJW7DSXY:4:1416:12635:29293 2:N:0:TCTGCAAG+GCTGCACT
GGACGACATTACAACCAGTCCCAGAATTACTGCAGAACCCGTCATGGAAGACGATGATTTCTGGGACT
TTATCCCGACGGCTTTATTTTTATATCCATAAAAATCTTTGTAATTTAGGGGTAATTTCAATTTATAA
TTTTTGTTATGCGTG
+
FFFFFFFFFFFFFFFFFFFFFFFFFFFFFFFFFFFFFFFF:FFFFFFFFFFFFFFFFFFFFFFFFFFFFF
FFFFFFFFFFFFFFFFFFFFFFFFFFF:FFFFFFFFFFFFFFFFFFFFFFFFFFFFFFFFFFFFFFFFFF
FFFFFFF:FFFFFFF
@A00721:190:HGJW7DSXY:4:1465:1316:13385 2:N:0:TCTGCAAG+GCTGCACT
GGACGACATTACAACCAGTCCCAGAATTACTGCAGAACCCGTCATGGAAGACGATGATTTCTGGGACT
TTATCCCGACGGCTTTATTTTTATATCCATAAAAATCTTTGTAATTTAGGGGTAATTTCAATTTATAA
TTTTTGTTATGCGTG
+
FFFFFFFFFFFFFFFFFFFFFFFFFFFFFFFFFFFFFFFFFFFFFFFFFF,FFFFFFFF:FFFFFFFFF:
FFFFFFFFFFFFFFFFFFFFFFFFFFFFFFFFFFF:FFFFFFFF:FFFFFFFFFFFFFFFFFFFFFFFFF
FFFFFFFFFFFFFFF
```

@A00721:190:HGJW7DSXY:4:1501:5873:34021 2:N:0:TCTGCAAG+GCTGCACT
CACGCATAACAAAAATTATAAATTGAAATTACCCCTAAATTACAAAGATTTTTATGGATATAAAAATA
AAGCCGTCGGGATAAAGTCCCAGAAATCATCGTCTTCCATGACGGGTTCTGCAGTAATTCTGGGACTG
GTTGTAATGTCGTCC
+
FFFFFFFFFFFFFFFFFFFFFFFFFFFFFFFFFFFFFFFFFFFFFFFFFFFFFFFFFFFFFFFFFFFF
FFFFFFFFFFFFFFFFFFFF:FFFFFFFFFFFFFFFFFFFFFFFFFFFFFFFFFFFFF:FFFFFFFFFFF
FFFFFFFFFFFFFFF
@A00721:190:HGJW7DSXY:4:1529:32163:4586 2:N:0:TCTGCAAG+GCTGCACT
ACGACATTACAACCAGTCCCAGAATTACTGCAGAACCCGTCATGTAAGACGATGATTTCTTGTACTTT
ATCACGACGTCTTTATTTTTATATCCATAAAAATATTTGTAATTTAGGTTTAATTTAAATTTATAATT
TTTGTTATGAGTG
+
FFFFFFFFFFFFFFFFF::F:FFFF:FF::FFFFF:FFF,:FF,,FFFFFF:F,F,FFFF,F,F::FF
FF,,FFFFF,FFFFFFFFF:FFFFFFFFFFFF,::FFFF::FFFFFFF,,:FFF:F,F:FFFFF,FFF
FFFFFFFFF,FFF
@A00721:190:HGJW7DSXY:4:1550:18105:29465 2:N:0:TCTGCAAG+GCTGCACT
GGACGACAGTACAACCAGTCCCAGAATTACTGCAGAACCCGTCATGGAAGACGATGATTTCTGGGACT
TTATCCCGACGGCTTTATTTTTATATCCATAAAAATCTTTGTAAGTTAGGGGTAATGTCAATTTATAA
TTTTTGTTATGCGTG
+
FFFFFFF:,FFFFFFFFFFFFFFFF:F:FFFFFFFFFFFFFFF:FFF:FFFFFFFFFFFFFFFFFF:F
FFFFFFFFFFFFFFFFF:FFF,FFFFFFFFFFFFFFFFFFFFFFFF,FFFFFFFFF,F,FFFFFFFFFFF
FFFFFFFFFFFFFFF
@A00721:190:HGJW7DSXY:4:1551:29333:14779 2:N:0:TCTGCAAG+GCTGCACT
GGACGACATTACAACCAGTCCCAGAATTACTGCAGAACCCGTCATGGAAGACGATGATTTCTGGGACT
TTATCCCGACGGCTTTATTTTTATATCCATAAAAATCTTTGTAATTTAGGGGTAATTTCAATTTATAA
TTTTTGTTATGCGTG
+
::F:FFFFFFFFFF:FFFFFFFFFFFFFF:FF:F:FFFFF::FFFFFFF:FFFFFFFFF:FFF::FFF
FFFFFFFFFFF,FFFFFFF,FFFFFF:FFFFF:FFFFFFFFFFFFFFF,:::FFFF:FFFFFFFFFFF
FFFFF:FFFF,F:F:
@A00721:190:HGJW7DSXY:4:1578:23104:1736 2:N:0:TCTGCAAG+GCTGCACT
GGACGACATTACAACCAGTCCCAGAATTACTGCAGAACCCGTCATGGAAGACGATGATTTCTGGGACT
TTATCCCGACGGCTTTATTTTTATATCCATAAAAATCTTTGTAATTTAGGGGTAATTTCAATTTATAA
TTTTTGTTATGCGTG
+
FFFFFFFFFFFFFFFFFFFFFFFFFF:FFFFFFFFFFFFFFFFFFFFFFFFFFFFFFF,FFFFFFFFFF
FFFFFFFFFFFFFFFFFFFFFFFFFFFFFFFFFFFFFFFFFFFFFFFFFFFFFFF:FFFFFFFFFFFFF
F,FFFFFFF:FFFFF
@A00721:190:HGJW7DSXY:4:2304:9634:1673 2:N:0:TCTGCAAG+GCTGCACT
CACGCAAAACAAAAATTATAAATTGAAATTACCCCTAAATTACAAACATTAATATGGATATAAAAATA
AAGCCGTCGGGATAAATTCCCAGAAATCAAAGACTTCCATGACGGGTTCTGCAGTAATTCTGGGACTG
GTTGTAATGTCGTAC
+
FFF,FF,F:,FFFFFFFFF:FFF:FF:F,,FF:F,FFF:FFFFF:F,F,,,,F:,F,F::F,FF:F,F
F,FFFF,FFFFFFFFF,F,,,F,FFFF,F,,,,FF::,:FFF,,F::,FF:FFF,FFFFFF,F,:F:,
:F,F,FF:,FFFF,:
@A00721:190:HGJW7DSXY:4:2450:4417:23766 2:N:0:TCTGCAAG+GCTGCACT
GGACGACATTACAACCAGTCCCAGAATTACTGCAGAACCCGTCATGGAAGACGATGATTTCTGGGACT
TTATCCCGACGGCTTTATTTTTATATCCATAAAAATCTTTGTAATTTAGGGGTAATTTCAATTTATAA
TTTTTGTTATGCGTG
+
FFFFFFFFFFFFFFFFFFFFFFFFFFFFFFFFFFFFFFFFFFFFFFFFFFFFFFFFFFFFFFFFFFFF

```
FFFFFFFFFFFFFFFFFFFFFFFFFFFFFFFFFFFFFFFFFFFFFFFFFFFFFFFF:FFFF,FFFFFF
FFFFFFFFFFFFFFF
@A00721:190:HGJW7DSXY:4:2576:14262:7122 2:N:0:TCTGCAAG+GCTGCACT
GGACGACATTACAACCAGTCCCAGAATTACTGCAGAACCCGTCATGGAAGACGATGATTTCTGGGACT
TTATCCCGACGGCTTTATTTTTATATCCATAAAAATCTTTGTAATTTAGGGGTAATTTCAATTTATAA
TTTTTGTTATGCGTG
+
FFFFFFFFFFFFFFFFFFFFFFFFFFFFFFFFFFFFFFFFFFFFFFFFFFFFFFFFFFFFFFFFFFFF
FFFFFFFFFFFFFFFFFFFFFFFFFFFFFFFFFFFFFFFFFFFFFFFFF:FFFFFFFFFFFFFFFFFF
FFFFFFFFFFFFFFF
@A00721:190:HGJW7DSXY:4:2647:12997:10066 2:N:0:TCTGCAAG+GCTGCACT
GGACGACATTACAACCAGTCCCAGAATTACTGCAGAACCCGTCATGGAAGACGATGATTTCTGGGACT
TTATCCCGACGGCTTTATTTTTATATCCATAAAAATCTTTGTAATTTAGGGGTAATTTCAATTTATAA
TTTTTGTTATGCGTG
+
FFFFFFFFFFFFFFFFFFFFFFFFFFFFFFFFFFFFFFFFFFFFFFFFFFFFFFFFFFFFFFFFFFFF
FFFFFFFFFFFFFFF:FFFFFFFFFFFFFFFFFFF:FFFFFF:FFFFFFFFFFFFFFFFFFFFFFFFF
FFFFF,FFFFFF:FF
@A00721:190:HGJW7DSXY:4:2662:23936:7435 2:N:0:TCTGCAAG+GCTGCACT
GGACGACATTACAACCAGTCCCAGAATTACTGCAGAACCCGTCATGGAAGACGATGATTTCTGGGACT
TTATCCCGACGGCTTTATTTTTATATCCATAAAAATCTTTGTAATTTAGGGGTAATTTCAATTTATAA
TTTTTGTTATGCGTG
+
FFFFFFF:FFFFFFFFFFFFFFFFFFFFFFFFFFFFFFFFFFFFFFFFFFFFFFFFFFFF:FFFFFFF
FFFFFFFFFFFFFFFFFFFFFFFFFFFFFFFFFF:FFFFFFFFFFFFFFFFFFFFFFFFFF,FFFF:F
:FFFFFFFFFF,FFF
@A00721:190:HGJW7DSXY:4:2678:27127:7889 2:N:0:TCTGCAAG+GCTGCACT
GGACGACATTACAACCAGTCCCAGAATTACTGCAGAACCCGTCATGGAAGACGATGATTTCTGGGACT
TTATCCCGACGGCTTTATTTTTATATCCATAAAAATCTTTGTAATTTAGGGGTAATTTCAATTTATAA
TTTTTGTTATGCGTG
+
FFFFFFF:FFFFFFFFFFFFFFFFFFFFFFFFFFFFFFFFFFFFFFFFFFFFFFFFFFFFFFFFFFFF
FFFFFFFFFFFFFFFFFFFFFFFFFFFFFFFFFFFFFFFFFFFFFFFFF:FFFFFFFFFFFFFFFFFF
FFFFFFFFFFFF:FF
@A00721:190:HGJW7DSXY:4:1344:3143:9721 2:N:0:TCTGCAAG+GCTGCACT
GGACGACATTACAACCAGTCCCAGAATTACTGCAGAACCCGTCATGGAAGACGATGATTTCTGGGACT
TTATCCCGACGGCTTTATTTTTATATCCATAAAAATCTTTGTAATTTAGGGGTAATTTCAATTTATAA
TTTTTG
+
FFFFFFFFFFFFFFFFFFFFFFFFFFFFFFFFFFFFFFFFFFFFFFFFFFFFFFFFFFFFFFFFFFFF
FFFFFFFFFFFFFFFFFFFFFFFFFFFFFFFF::FFFFFFFFFF,F:FFFFFF,FFFFF:FFFFFFFF
FFFFFF
@A00721:190:HGJW7DSXY:4:1567:22761:12696 2:N:0:TCTGCAAG+GCTGCACT
GGACGACATTACAACCAGTCCCAGAATTACTGCAGAACCCGTCATGGAAGACGATGATTTCTGGGACT
TTATCCCGACGGCTTTATTTTTATATCCATAAAAATCTTTGTAATTTAGGGGTAATTTCAATTTATAA
TTTTTG
+
FFFFFFFFFFFFFFFFFFFFFFFFFFFFFFFFFFFFFFFFFFFFFFFFFFFFFFFFFFFFFFFFFFFF
FFFFFFFFFFFFFFFFFFFFFFFFFFFFFFFFFFFFFFFFFFFFFFFFFFFFF:FFFFFFFFFFFFFF
FFFFFF
@A00721:190:HGJW7DSXY:4:2342:27308:7106 2:N:0:TCTGCAAG+GCTGCACT
GGACGACATTACAACCAGTCCCAGAATTACTGCAGAACCCGTCATGGAAGACGATGATTTCTGGGACT
TTATCCCGACGGCTTTATTTTTATATCCATAAAAATCTTTGTAAGTTAGGGGTAATTTCAATTTATAA
TTTTTG
```

+
FFFFFFFF:FFFFFFFFFFFFFFFFF:FFFFFFFFFFFFFFFFFF,FFF,FFFFFFFFFFFFFFFFFFFF
:FFFFFFFFFFFFFFFFFFFFFFFFF:FFFFFF:F:FFFFFFFFF,,F,FF:FFFF:F:FFFFFFFFFFF
F::FFF
@A00721:190:HGJW7DSXY:4:2357:31611:22357 2:N:0:TCTGCAAG+GCTGCACT
GGACGACATTACAACCAGTCCCAGAATTACTGCAGAACCCGTCATGGAAGACGATGATTTCTGGGACT
TTATCCCGACGGCTTTATTTTTATATCCATAAAAATCTTTGTAATTTAGGGGTAATTTCAATTTATAA
TTTTTG
+
FFFFFFFFFFFFFFFFFFFFFFFFFFFFFFFFFF:F:FFFFFFFFFFFFFFFFFFFFFFFFFFFFFFFFF
FFFFFFFFFFFFFFFFFFFFFFFFFFFFFFFFFFFFFFFFFFFFFFFFFFF:FFFFFFFFFF,FFFFFFF
FF:FF:
@A00721:190:HGJW7DSXY:4:2648:21594:16658 2:N:0:TCTGCAAG+GCTGCACT
GGACGACATTACAACCAGTCCCAGAATTACTGCAGAACCCGTCATGGAAGACGATGATTTCTGGGACT
TTATCCCGACGGCTTTATTTTTATATCCATAAAAATCTTTGTAATTTAGGGGTAATTTCAATTTATAA
TTTTTG
+
FFFFFFFF:FFFFFFFFFFFFFFFFFFFFFFFFFFFFFFFFFFFFFFFFFFFFFFFFFFFFFFFFFFFFF
FFFFFFFFFFFFFF:FFFFFFFFFFFFFFFFFFFFFFF::FFFFFFFFFFFFFF:FFFF:F:FFFFFFFF
FFFFF:
@A00721:190:HGJW7DSXY:4:1118:31548:6934 2:N:0:TCTGCAAG+GCTGCACT
GGACGACATTACAACCAGTCCCAGAATTACTGCAGAACCCGTCATGGAAGACGATGATTTCTGGGACT
TTATCCCGACGGCTTTATTTTTATATCCATAAAAATCTTTGTAATTTAGGGGTAATTTCAATTTATAA
TTTT
+
F:FFFFFF,FFFFFFFFFFFFFFFFFF:FFFFFFFFFFFF,F:FFFFFFFFFFF:FF::,FFF:FF:F,,
FFFFF:::FFFF:F::FF:,FFFFFFFFFF:FFFFFFFF,FFF:FFFFFF:FFFFFFFFFFFFFFF:FFF
:F:F
@A00721:190:HGJW7DSXY:4:1213:20546:10551 2:N:0:TCTGCAAG+GCTGCACT
GGACGACATTACAACCAGTCCCAGAATTACTGCAGAACCCGTCATGGAAGACGATGATTTCTGGGACT
TTATCCCGACGGCTTTATTTTTATATCCATAAAAATCTTTGTAATTTAGGGGTAATTTCAATTTATAA
TTT
+
FFFFFFFFFFFFFFFFFFFFFFFFFFFFFFFFFFFFFFFFFFFFFFFFFFFFFFFFFFFFFFFFFFFFFF
FFFFFFFFFFFFFFFFFFFFFFFFFFFFFFFFFFFFFFFFFFFF:F,FFFFFFFFFFFFFFFFFFFFFF:
:FF
@A00721:190:HGJW7DSXY:4:1236:26060:1094 2:N:0:TCTGCAAG+GCTGCACT
GGACGACATTACAACCAGTCCCAGAATTACTGCAGAACCCGTCATGGAAGACGATGATTTCTGGGACT
TTATCCCGACGGCTTTATTTTTATATCCATAAAAATCTTTGTAATTTAGGGGTAATTTCAATTTATAA
TTT
+
FFFFFFFFFFFFFFFFFFFFFFFFFFFFFFFFFFFFFFFFFFFFFFFFFFFFFFFFFFFFFFFFFFF:F,
FF:FFFFFFFFFFFFFFFFFFFFFFFFFFFF:FFFFFFFFFFFF,F,FFFFFFFFF,:FFFFFF,FFFFF
FFF
@A00721:190:HGJW7DSXY:4:1434:10230:33332 2:N:0:TCTGCAAG+GCTGCACT
GGACGACATTACAACCAGTCCCAGAATTACTGCAGAACCCGTCATGGAAGACGATGATTTCTGGGACT
TTATCCCGACGGCTTTATTTTTATATCCATAAAAATCTTTGTAATTTAGGGGTAATTTCAATTTATAA
TTT
+
FFFFFFFFFFFFFFFFFFFFFFFFFFFFFFFFFFFFFFFFFFFFFFFFFFFFFFFFFFFFFFFFFFFFFF
FFFFFFFFFFFFFFFFFFFFFFFFFFFFFFFFFFF,FFFFFFFFFFFFFFFFFFFFFFFFFFFFFFFFFF
FFF
@A00721:190:HGJW7DSXY:4:1437:3938:20932 2:N:0:TCTGCAAG+GCTGCACT
GGACGACATTACAACCAGTCCCAGAATTACTGCAGAACCCGTCATGGAAGACGATGATTTCTGGGACT

```
TTATCCCGACGGCTTTATTTTTATATCCATAAAAATCTTTGTAATGTAGGGGTAATTTCAATTTATAA
TTT
+
FFFFFFFFFFFFFFFFFFFFFFFFFFFFFFFFFFFFFFFFFFFFFFFFFFFFFFFFFFFFFFFFFFFF,
FFFFFFFFFFFFF:FFFFFFFFFFFFFFFFFFFFFFFFFFFFFFFF,FFF,FFFFFFFFFFFFFFFFF
FFF
@A00721:190:HGJW7DSXY:4:1617:4047:11882 2:N:0:TCTGCAAG+GCTGCACT
GGACGACATTACAACCAGTCCCAGAATTACTGCAGAACCCGTCATGGAAGACGATGATTTCTGGGACT
TTATCCCGACGGCTTTATTTTTATATCCATAAAAATCTTTGTAATTTAGGGGTAATTTCAATTTATAA
TTT
+
FFFFFFFFFFFFFFFFFFFFFFFFFFFFFFFFFFFFFFFFFFFFFFFFFFFFFFFFFFFFFFFFFFFFF
FFFFF:FFFFFFFFFFFFFFFFFFFFFFFFFFFFFFFFFF:FF:FF:FF:FFFFFFFFFFFF,FF:FFF
FFF
@A00721:190:HGJW7DSXY:4:2132:11514:10629 2:N:0:TCTGCAAG+GCTGCACT
GGACGACATTACAACCAGTCCCAGAATTACTGCAGAACCCGTCATGGAAGACGATGATTTCTGGGACT
TTATCCCGACGGCTTTATTTTTATATCCATAAAAATCTTTGTAATTTAGGGGTAATTTCAATTTATAA
TTT
+
FFFFFFFFFFFFFFFFFFFFFFFFFFFFFFFFFFFFFFFF:FFFFFFFFFFFFFFFFFFFFFFFFFFFF
FFFFFFFFFFFFFFFFFFFFFFFFFFFFFFFFFFFF:FFFFFFFFFFFFF,FFFF:FFFFFFF,FFFFFF
FFF
@A00721:190:HGJW7DSXY:4:2642:23213:18552 2:N:0:TCTGCAAG+GCTGCACT
GGACGACATTACAACCAGTCCCAGAATTACTGCAGAACCCGTCATGGAAGACGATGATTTCTGGGACT
TTATCCCGACGGCTTTATTTTTATATCCATAAAAATCTTTGTAATTTAGGGGTAATTTCAATTTATAA
TTT
+
FFFFFFFFFFFFFFFFFFFFFFFFFFFFFFFFFFFFFFFFFFFFFFFFFFFFFFFFFFFFFFFFFFFFF
FFFFFFFFFFFFFFFFFFFFFFFFFFFFFFFFFFFFFFFFFFFFF:FFFFFFFFFFFFFFFFFFFFFFFF
FFF
@A00721:190:HGJW7DSXY:4:1441:19144:22404 2:N:0:TCTGCAAG+GCTGCACT
GGACTATCCGCATCTGGACGACATTACAACCAGTCCCAGAATTACTGCAGAACCCGTCATGGAAGACG
ATGATTTCTGGGACTTTATCCCGACGGCTTTATTTTTATATCCATAAAAATCTTTGTAATTTAGGGGT
AATTTCAATTTATAA
+
FFFFFFFFFFFFFFFFFFFFFFFFFFFFFFFFFFFFFFFFFFFFFFFFFFFFFF:FFFFFFFFFFFFF
FFFFFFFFFFFFFFFFFFFFFFFFFFFFFFFFFFFFFFFFF:FF:FFFFFFFFFFFFFFFFFFFFFFFFFFF
,FFFFFFFFFFFFFF
@A00721:190:HGJW7DSXY:4:1623:32271:15139 2:N:0:TCTGCAAG+GCTGCACT
GGACTATCCGCATCTGGACGACATTACAACCAGTCCCAGAATTACTGCAGAACCCGTCATGGAAGACG
ATGATTTCTGGGACTTTATCCCGACGGCTTTATTTTTATATCCATAAAAATCTTTGTAATTTAGGGGT
AATTTCAATTTATAA
+
FFFFFFFFFFFFFFFFFFFFFFFF:FFFFFFFFFFFFFFFFFFFFFFFFFFFFFFFFFFFF,FFFFFFFFF
FFF:FFFFFF,F:FF:FFFFFF::FFF:FFFFFFFFFFFFFFFFFFFFFFFFFFFFFFFF,,FFFFFFFF
FFF:FFFF:F:FF:F
@A00721:190:HGJW7DSXY:4:1642:15582:24189 2:N:0:TCTGCAAG+GCTGCACT
GGACTATCCGCATCTGGACGACATTACAACCAGTCCCAGAATTACTGCAGAACCCGTCATGGAAGACG
ATGATTTCTGGGACTTTATCCCGACGGCTTTATTTTTATATCCATAAAAATCTTTGTAATTTAGGGGT
AATTTCAATTTATAA
+
FFFFFFFFFFFFFFFFFFFFFFFFFFFFFFFFFFFFFFFFFFFFFFFFFFFFFFFFFFFFFFFFFFFFF
FFFFFFFF:FFFFFFFFFFFFFFFFFFFFFFFFFFFFFFFFFFFFFFFF:FFFFFFFFFF:FFFFFFFFF
FFF:FFFFFFFFFFF
```

@A00721:190:HGJW7DSXY:4:1645:5611:23703 2:N:0:TCTGCAAG+GCTGCACT
GGACTATCCGCATCTGGACGACATTACAACCAGTCCCAGAATTACTGCAGAACCCGTCATGGAAGACG
ATGATTTCTGGGACTTTATCCCGACGGCTTTATTTTTATATCCATAAAAATCTTTGTAATTTAGGGGT
AATTTCAATTTATAA
+
FFFFFFFFFFFFFFFFFFFFFFFF:FFFFFFFFFFFFFFFFFFFFFFFFFFFFFFFFFFFFFFFFFF,FF
FFFFFFFFFFFFFFFFFFFFFFFFFFFFFFFFFFFFFFFFF:FFFFFFFFFFFFFF:FFFFFFFFFFFFF
FFFFFFFFF:FFFFF
@A00721:190:HGJW7DSXY:4:2343:3974:9032 2:N:0:TCTGCAAG+GCTGCACT
GGACTATCCGCATCTGGACGACATTACAACCAGTCCCAGAATTACTGCAGAACCCGTCATGGAAGACG
ATGATTTCTGGGACTTTATCCCGACGGCATTATTTTTATATCCATAAAAATCTTTGTAATTTAGGGGT
AATTTCAATTTATAA
+
FF:FFFFFFFFFFFFFFFFFFFFFFFFFFFFFFFFFFFFFFFFFFFFFFFFFFFFFFFFFFFFFFFFFFF
FFFFFFFFFFFFFFFFFFFFFFFFFFFFF,FFFFFFFFFFFFFFFFFFFFFFFFFFF:FFFFFFFFF:FF
FFFFFFFFFFFFFFF
@A00721:190:HGJW7DSXY:4:2358:21748:2049 2:N:0:TCTGCAAG+GCTGCACT
GGACTATCCGCATCTGGACGACATTACAACCAGTCCCAGAATTACTGCAGAACCCGTCATGGAAGACG
ATGATTTCTGGGACTTTATCCCGACGGCTTTATTTTTATATCCATAAAAATCTTTGTAATTTAGGGGT
AATTTCAATTTATAA
+
FFFFFFFFFFFFFFFFFFFFFFFFFFFFFFFFFFFFFF:FFFFFFFFFFFFFFFFFFFFFFFFFFFFFFF
FFFF:FFFFFFFFFFFFFFFFFFFFFFFFFFFFFFFFFFFFFFFFFFFFFFFFFFFFFFFFFFF:FFFF
F:FFFFF::FFFFFF
@A00721:190:HGJW7DSXY:4:2554:18891:28385 2:N:0:TCTGCAAG+GCTGCACT
GGACTATCCGCATCTGGACGACATTACAACCAGTCCCAGAATTACTGCAGAACCCGTCATGGAAGACG
ATGATTTCTGGGACTTTATCCCGACGGCTTTATTTTTATATCCATAAAAATCTTTGTAATTTAGGGGT
AATTTCAATTTATAA
+
FFFFFFFFFFFFFFFFFFFFFFFFFFFFFFFFFFFFFF:FFFFFFFFFFFFFFFFFFFFFFFFFF:FFFF
FFFFFFFFFFFFFFFFFFFFFFFFFFFFFFFFFFFFFFFFFFFFFFFFFFFFFFFFFFFFFFF,FFFFFF
FFFFFFFFFFFFFFF
@A00721:190:HGJW7DSXY:4:1111:3097:21512 2:N:0:TCTGCAAG+GCTGCACT
TGAAATTACCCCTAAATTACAAAGATTTTTATGGATATAAAAATAAAGCCGTCGGGATAAAGTCCCAG
AAATCATCGTCTTCCATGACGGGTTCTGCAGTAATTCTGGGACTGGTTGTAATGTCGTCCAGATGCGG
ATAGTCCACCATGCT
+
FFFFFFFFFFFFFFFFFFFFFFFFFFFFFFFFFFFFFFFFFFFFFFF:FFFFFFFFFFFFFFFFFFFFFF
FFFFFFFFFFFFFFFFFFFFFFFFFFFFFFFFFFFFFFFFFFFFFFF:FFFFFFFFFFFFFFFFFFFFFF
FFFFFFFFFFFFFFF
@A00721:190:HGJW7DSXY:4:1223:9381:5650 2:N:0:TCTGCAAG+GCTGCACT
TGAAATTACCCCTAAATTACAAAGATTTTTATGGATATAAAAATAAAGCCGTCGGGATAAAGTCCCAG
AAATCATCGTCTTCCATGACGGGTTCTGCAGTAATTCTGGGACTGGTTGTAATGTCGTCCAGATGCGG
ATAGTCCACCATGCT
+
FFFFFFFFFFFFFFFFFFFFFFFFFFFFFFFFFFFFF,FFFFFFF:F:FFFFFFFFFFFFFFFFFFFFFF
F:FFFFFFFFFFFFFFFFFFFFFFFFFFFFFFF:FFFFFFFFFFFFFFFFFFFFFFFFFFFFFFFFFFFF
FFFFFFFFFFFFFFF
@A00721:190:HGJW7DSXY:4:1444:15537:33880 2:N:0:TCTGCAAG+GCTGCACT
TGAAATTACCCCTAAATTACAAAGATTTTTATGGATATAAAAATAAAGCCGTCGGGATAAAGTCCCAG
AAATCATCGTCTTCCATGACGGGTTCTGCAGTAATTCTGGGACTGGTTGTAATGTCGTCCAGATGCGG
ATAGTCCACCATGCT
+
FFFFFFFFFFFFFFFFFFFFFFFFFFFFFFFFFFFFFFFFFFFFFFF:FFFFFFFFF::FFFFFFFFFFF

FFFFFFFFFFFFFFFFFFFFFFFFFFFFFFFFFFFFFFFFFFFF:FFFF:FF:FFFFFFFFFFFFFFFFF
:FFFFFFFFFFFFFF
@A00721:190:HGJW7DSXY:4:2311:3730:24298 2:N:0:TCTGCAAG+GCTGCACT
TGAAATTACCCCTAAATTACAAAGATTTTTATGGATATAAAAATAAAGCCGTCGGGATAAAGTCCCAG
AAATCATCGTCTTCCATGACGGGTTCTGCAGTAATTCTGGGACTGGTTGTAATGTCGTCCAGATGCGG
ATAGTCCACCATGCT
+
FFFFFFFFFFFFFFFFFFFFFFFFFFFFFFFFFFFFFFFFFFFFFFFFF:F:FFF:FFFFFFFF:FFFFF
FFFFFFFFF:FFFFFFFFFFFFFFFFFFFFFFFFFFFFFF::FFF:F:FFFFFFFFFF:FF,FFFFFFFF
FFFFFF:FFFFFFFF
@A00721:190:HGJW7DSXY:4:2640:2456:31266 2:N:0:TCTGCAAG+GCTGCACT
TGAAATTACCCCTAAATTACAAAGATTTTTATGGATATAAAAATAAAGCCGTCGGGATAAAGTCCCAG
AAATCATCGTCTTCCATGACGGGTTCTGCAGTAATTCTGGGACTGGTTGTAATGTCGTCCAGATGCGG
ATAGTCCACCATGCT
+
FFFFFFFFFFFFFFFFFFFFFFFFFFFFFFF:FFFF:FFFFFFFFFFFFFFFFFFFFFFFFFFFFFFFFF
FFFFFFFFFFFFFFFFFFFFFFFFFFFFFFFFFFFFFFFFFFF::FFFFFFFFFFFFFFFFFFFFFFFFF
FFFFFFFFFFF:FFF
@A00721:190:HGJW7DSXY:4:1264:26539:7780 2:N:0:TCTGCAAG+GCTGCACT
CGAGCATGGTGGACTATCCGCATCTGGACGACATTACAACCAGTCCCAGAATTACTGCAGAACCCGTC
ATGGAAGACGATGATTTCTGGGACTTTATCCCGACGGCTTTATTTTTATATCCATAAAAATCTTTGTA
ATTTAGGGGTAATTT
+
FFFFFFFFFFFFFFFFFFFFFFFFFFFFFFFFFFFFFFFFFFFFFFFFFFFFFFFFFFFFFFFFFFFFFF
FFFFFFFFFFFFFFFFFFFFFFFFFFFFFFFFFFFFFFFFFFFFFFFFFFFFF:FFFFFFF:FFFFFFFF
FFFFFFFFFFFFFFF
@A00721:190:HGJW7DSXY:4:1264:26250:7968 2:N:0:TCTGCAAG+GCTGCACT
CGAGCATGGTGGACTATCCGCATCTGGACGACATTACAACCAGTCCCAGAATTACTGCAGAACCCGTC
ATGGAAGACGATGATTTCTGGGACTTTATCCCGACGGCTTTATTTTTATATCCATAAAAATCTTTGTA
ATTTAGGGGTAATTT
+
FFFFFFFFFFFFFFFFFFFFFFFFFFFFFFFFFFFFFFF:FFFFFFFFFFFFFFFFFFFFF:FFFFFFFF
FFFFFFFFFF:FFFFFFFFFFFFFFFFFFFFFFFFFFFFFFFFFFFFFFFFFFFFFFFFFFFFFFFFFFF
FFFFFFFFFFFF:FF
@A00721:190:HGJW7DSXY:4:1329:32633:6903 2:N:0:TCTGCAAG+GCTGCACT
CGAGCATGGTGGACTATCCGCATCTGGACGACATTACAACCAGTCCCAGAATTACTGCAGAACCCGTC
ATGGAAGACGATGATTTCTGGGACTTTATCCCGACGGCTTTATTTTTATATCCATAAAAATCTTTGTA
ATTTAGGGGTAATTT
+
FFFFFFFFFFFFFFFFFFFFFFF:FFFFFFFFFFFFFFFFFFFFFFFFFFFFFFFFFFFFFFFFFFFFFF
FFFFFFF:FFFFFFFFFFFFFFFFFFFFFFFFFFFFFFFFF:FFFFFFFFFFFFFFFFFFF,FFFFFFFF
FFFFFFFFFF,FFFF
@A00721:190:HGJW7DSXY:4:1466:16740:24220 2:N:0:TCTGCAAG+GCTGCACT
CGAGCATGGTGGACTATCCGCATCTGGACGACATTACAACCAGTCCCAGAATTACTGCAGAACCCGTC
ATGGAAGACGATGATTTCTGGGACTTTATCCCGACGGCTTTATTTTTATATCCATAAAAATCTTTGTA
ATTTAGGGGTAATTT
+
FFFFFFFFFFFFFFFFFFFFFFFFFFFFFFFFFFFFFFFFFFFFFFFFFFFFFFFFFFFFFFFFFFF:FF
FFFFFFFFFFFFFFFFFFFFFFFFFFFFFFFFFFFFFFFFFFFFFFFFFFFFFFFFFFFFF,FFFFFFFF
FFFFFF:FFFFFFF:
@A00721:190:HGJW7DSXY:4:2508:13286:33959 2:N:0:TCTGCAAG+GCTGCACT
CGAGCATGGTGGACTATCCGCATCTGGACGACATTACAACCAGTCCCAGAATTACTGCAGAACCCGTC
ATGGAAGACGATGATTTCTGGGACTTTATCCCGACGGCTTTATTTTTATATCCATAAAAATCTTTGTA
ATTTAGGGGTAATTT

+
FFFFFFFFFFFFFFFFFFFFFFFFFFFFFFFFFFFFFFFFFFFFFFFFFFFFFFFFFFFFFFFFFFFF
FFFFFFFFFFFFFFFFFFFFFFFFFFFFFFFFFFFFFFFFFFFFFFFFFFFFFFFFFFFFFFFFFFFF
FFFFFFFF:FFFFFF
@A00721:190:HGJW7DSXY:4:1343:30436:36260 2:N:0:TCTGCAAG+GCTGCACT
AATTACCCCTAAATTACAAAGATTTTTATGGATATAAAAATAAAGCCGTCGGGATAAAGTCCCAGAAA
TCATCGTCTTCCATGACGGGTTCTGCAGTAATTCTGGGACTGGTTGTAATGTCGTCCAGATGCGGATA
GTCCACCATGCTCGC
+
FFFFFFFFFFFFFFFFFFFFFFFFFFFFFFF:FFFFFFFFFFFFFF:FFFFFFFF:FFFFF:FFF:FF
FFFFFFFFFFFFFFF:F:FFFFFFF,FFFFFFFFFFFFFFFFFFFFFFFFFFFFFFFFF:FFFFFFF,
FFFF:F,::FFFFFF
@A00721:190:HGJW7DSXY:4:1344:27633:1094 2:N:0:TCTGCAAG+GCTGCACT
AATTACCCCTAAATTACAAAGATTTTTATGGATATAAAAATAAAGCCGTCGGGATAAAGTCCCAGAAA
TCATCGTCTTCCATGACGGGTTCTGCAGTAATTCTGGGACTGGTTGTAATGTCGTCCAGATGCGGATA
GTCCACCATGCTCGC
+
F,FFFFFFFFFFFFFFFFF:FF:FFFFFFFFFF:FF:FFFFFFFFFFFFFFFFFFFFFFFFFFFFFFF
FFFFFFFFFFFF:FFFFFFFFFFFFFFFF,FFFFFFFF,FFFFFFF:FFFF:FFFFFFFFFFFFFFF:
FFFFFFFFFFFFFFF
@A00721:190:HGJW7DSXY:4:1656:3332:8954 2:N:0:TCTGCAAG+GCTGCACT
AATTACCCCTAAATTACAAAGATTTTTATGGATATAAAAATAAAGCCGTCGGGATAAAGTCCCAGAAA
TCATCGTCTTCCATGACGGGTTCTGCAGTAATTCTGGGACTGGTTGTAATGTCGTCCAGATGCGGATA
GTCCACCATGCTCGC
+
FFFFFFFFFFFFFFFFFFF:FFFFFFFFFFFFFF:FFFFFFFFFFFFFFFFFFF:FFFFFFFF:FFFF
,FFFFFFFFFFFFFFFFFFFFFFFFF:FF:FFFFFFFFFF:FFF,FFFFFFFFFFFFFFFFFFFFF,F
FFFFFFFFFFFFFFF
@A00721:190:HGJW7DSXY:4:2305:21341:8547 2:N:0:TCTGCAAG+GCTGCACT
AATTACCCCTAAATTACAAAGATTTTTATGGATATAAAAATAAAGCCGTCGGGATAAAGTCCCAGAAA
TCATCGTCTTCCATGACGGGTTCTGCAGTAATTCTGGGACTGGTTGTAATGTCGTCCAGATGCGGATA
GTCCACCATGCTCGC
+
F:FFFFFFFFFFFFFFFFFF:FFFFF:FFFFFFFF:FFFFFFFFFFFFFFFFFFFFFFFFF,FFFFFF
FFFFFFFFFFF:FFFFFFFFFFFFFFFFFFFFF,FFFFFFFFF:FFFF,FFFFFFFFFFFFFFFFFFF
F:F:FFFFFFFFFFF
@A00721:190:HGJW7DSXY:4:2448:2031:11083 2:N:0:TCTGCAAG+GCTGCACT
AATTACCCCTAAATTACAAAGATTTTTATGGATATAAAAATAAAGCCGTCGGGATAAAGTCCCAGAAA
TCATCGTCTTCCATGACGGGTTCTGCAGTAATTCTGGGACTGGTTGTAATGTCGTCCAGATGCGGATA
GTCCACCATGCTCGC
+
FFFFFFFFFFFFFFFFFFFFFFFFFFFFFFFFFF:FFFFFF,F:FFFFFFFFFFFFFFFFFFFFFFFF
FFFFFFFFFFFFFFFFFFFFFFFFFFFFFFFFFFFFFFFFFFFFFFFFFFFFFFFFFFFFFFFFFFFF
FFFFFFFF:FFFFFF
@A00721:190:HGJW7DSXY:4:1511:4264:21057 2:N:0:TCTGCAAG+GCTGCACT
TTTAAAGATTTGGACAGACTATCCGCATCTGGTCGACATTACAACCAGTCCCAGAATTACTGCAGAAC
CCGTCATGGAAGACGAGGATTTCTGGGACTTTATCCCGACGGCTTTATTTTTATATCCATAAAAATCT
TTGTAATTTAGGGGT
+
FFFFFFFFFFFFFFFFFFFFFFFFFFFFFFFF,FFFFFFFFFFFFFFFFFFFFFFFFFFFFFFFFFFF
FFFFFFFFFFF:FFFF,FFFFFFFFFFFFFFFFFFFFFFFFFFFFFFFFFFFF:FFFFFFFF,FFFFF
FFFFFF,FFFFFFFF
@A00721:190:HGJW7DSXY:4:1217:29550:5854 2:N:0:TCTGCAAG+GCTGCACT
GCGGATAAAGCGAGCATGGTGGACTATCCGCATCTGGACGACATTACAACCAGTCCCAGAATTACTGC

AGAACCCGTCATGGAAGACGATGATTTCTGGGACTTTATCCCGACGGCTTTATTTTTATATCCATAAA
AATCTTTGTAATTTA
+
FFFFFFFFFFFFFFFFFFFFFFFFFFFFFFFFFFFFFFFFFFFFFFFFFFFFFFFFFFF:FFFFFFFFFF
FFFFFFFFFF:FFFFFFFFFFFFF:FFFFFFFFFFFFFFFFFFFFFFFFFFFFFFFFFFFFFFFFFFFFF
F:FFFFFFFFFFFFF
@A00721:190:HGJW7DSXY:4:1308:25012:21073 2:N:0:TCTGCAAG+GCTGCACT
GCGGATAAAGCGAGCATGGTGGACTATCCGCATCTGGACGACATTACAACCAGTCCCAGAATTACTGC
AGAACCCGTCATGGAAGACGATGATTTCTGGGACTTTATCCCGACGGCTTTATTTTTATATCCATAAA
AATCTTTGTAATTTA
+
FFF:FFFF:FFFFFFFF:FFFFF:FFFFFFFFFF:FFFFFFFFFFFFFFFFFFFFFFFFF:FFF:FFF,F
FFFFFFF:FFFF:FFFFFFFF:FFFF:FF:F:F:FFFFFF,F:FF,FFFFFFF:FF:F:FFFFFFFFFFF
FFFFFFFFFFFF,F:
@A00721:190:HGJW7DSXY:4:1504:4345:31187 2:N:0:TCTGCAAG+GCTGCACT
GCGGATAAAGCGAGCATGGTGGACTATCCGCATCTGGACGACATTACAACCAGTCCCAGAATTACTGC
AGAACCCGTCATGGAAGACGATGATTTCTGGGACTTTATCCCGACGGCTTTATTTTTATATCCATAAA
AATCTTTGTAATTTA
+
F::FFFFFFFFFFFFFFFFFFFFFFFFFFFFFFFFFFFFFFFFFFFFFFFFFFFFFFFFFFFFFFFFFFF
FFFFFFFFFFFFFFFFFFFFFFFF:FFFFFFFFFFFFFFFFFFFFFFFFFFFFFFFFFFFFFFFFFFFFF
FFFFFFFFFFFFFFF
@A00721:190:HGJW7DSXY:4:2440:28763:33990 2:N:0:TCTGCAAG+GCTGCACT
GCGGATAAAGCGAGCATGGTGGACTATCCGCATCTGGACGACATTACAACCAGTCCCAGAATTACTGC
AGAACCCGTCATGGAAGACGATGATTTCTGGGACTTTATCCCGACGGCTTTATTTTTATATCCATAAA
AATCTTTGTAATTTA
+
FFFFFFFFFFFFFFFFFFF:FFFFFFFFFFFFFFFFFFFFFFFFFFFFFFFFFFFFFFF:FFFFFFFFFF
FFFFFFFFFFFFFFFFFFFFFFFFFFFFFFFFFFFFFFFFFFFFFFFFFFFFFFFFFFFFFFFFFFFFFF
FFFFFFFFFFFFFFF
@A00721:190:HGJW7DSXY:4:1109:32253:26005 2:N:0:TCTGCAAG+GCTGCACT
TGCGGATAAAGCGAGCATGGTGGACTATCCGCATCTGGACGACATTACAACCAGTCCCAGAATTACTG
CAGAACCCGTCATGGAAGACGATGATTTCTGGGACTTTATCCCGACGGCTTTATTTTTATATCCATAA
AAATCTTTGTAATTT
+
FFFFFFFFFFFFFFFFFFFFFFFFFFFFFFFFFFFFFFFFFFFFFFFFFFFFFFFFFFFFFFFFFFFFFF
FFFFFFFFFFFFFFFFFFFFFFFFFFFFFFFFFFFFFFFFF:FFFFFFFFFFFFFFFFFFFFFFFFFFFF
FF:FFFFFFFFFFFF
@A00721:190:HGJW7DSXY:4:1176:25066:7420 2:N:0:TCTGCAAG+GCTGCACT
TGCGGATAAAGCGAGCATGGTGGACTATCCGCATCTGGACGACATTACAACCAGTCCCAGAATTACTG
CAGAACCCGTCATGGAAGACGATGATTTCTGGGACTTTATCCCGACGGCTTTATTTTTATATCCATAA
AAATCTTTGTAATTT
+
FFFFFFFFFFFFFFFFFFFFFFFFFFFFFFFFFFFFFFFFFFFFFFFFFFFFFFFFFFFFFFFFFFFFFF
FFFFFFFFFFFFFFFFFFFFFFFFFFFFFFFFFFFFFFFFFFFFFFFFFFFFFFFFFFFFFFFFFFFFFF
:FFFFFFFFFFFFFF
@A00721:190:HGJW7DSXY:4:1245:15908:1078 2:N:0:TCTGCAAG+GCTGCACT
TGCGGATAAAGCGAGCATGGTGGACTATCCGCATCTGGACGACATTACAACCAGTCCCAGAATTACTG
CAGAACCCGTCATGGAAGACGATGATTTCTGGGACTTTATCCCGACGGCTTTATTTTTATATCCATAA
AAATCTTTGTAATTT
+
FFFFFFFFFFFFFFFFFFFFFFFFFFFFFFFFFFFFFFFFFFFF,FFFF:FFFFFFFFFFFFFFFFFFFF
FFFFFFF:FFFFFFFFFFFFFFFFFFFFFFFF:FFFFFFFFFFFFFFFF:FFFFFFFFFFFFFFFFFFFF
FFFFFFFFFFFFFFF

@A00721:190:HGJW7DSXY:4:1438:9923:9846 2:N:0:TCTGCAAG+GCTGCACT
TGCGGATAAAGCGAGCATGGTGGACTATCCGCATCTGGACGACATTACAACCAGTCCCAGAATTACTG
CAGAACCCGTCATGGAAGACGATGATTTCTGGGACTTTATCCCGACGGCTTTATTTTTATATCCATAA
AAATCTTTGTAATTT
+
FFFFFFFFFFFFFFFFFFFFFFFFFFFFFFFFFFFFFFFFFFFFFFFFFFFFFFFFFFFFFFFFFFFFFF
FFFFFFFFFFFFFFFFFFFFFFFFFFFFFFFFFFFFFFFFFFFFFFFFFFFFFFFFFFFFFFFFFFFFFF
FFFFFFFFFFFF:FF
@A00721:190:HGJW7DSXY:4:2225:3685:22748 2:N:0:TCTGCAAG+GCTGCACT
TGCGGATAAAGCGAGCATGGTGGACTATCCGCATCTGGACGACATTACAACCAGTCCCAGAATTACTG
CAGAACCCGTCATGGAAGACGATGATTTCTGGGACTTTATCCCGACGGCTTTATTTTTATATCCATAA
AAATCTTTGTAATTT
+
FFFFFFFFFFFFFFFFFFFFFFFFFFFFFFFFFFFFFFFFFFFFFFFFFFFFFFFFFFFFFFFFFFFFFF
FFFFFFF:FFFFFFFFFFFFFFFFFFFFFFFFFFFFFFFFFFFFFFFFFFFFF:FFFFFFFFFFF:F:FF
FFFFFFFFF:FFF:F
@A00721:190:HGJW7DSXY:4:2243:7943:28526 2:N:0:TCTGCAAG+GCTGCACT
TGCGGATAAAGCGAGCATGGTGGACTATCCGCATCTGGACGACATTACAACCAGTCCCAGAATTACTG
CAGAACCCGTCATGGAAGACGATGATTTCTGGGACTTTATCCCGACGGCTTTATTTTTATATCCATAA
AAATCTTTGTAATTT
+
FFFFFFFFFFFFFFFFFFFFFFFFFFFFFFFFFFFFFFFFFFFFFFFFFFFFFFFFFFFFFFFFFFFFFF
FFFFFFF:FFFFFFFFFFFFFFFFF:FFFFFFFFFFFFFFFFFFFFFF:FFFFFFFFFFFFFFFFFFFFF
FFFFFFFFFFFFFFF
@A00721:190:HGJW7DSXY:4:2342:9570:19977 2:N:0:TCTGCAAG+GCTGCACT
TGCGGATAAAGCGAGCATGGTGGACTATCCGCATCTGGACGACATTACAACCAGTCCCAGAATTACTG
CAGAACCCGTCATGGAAGACGATGATTTCTGGGACTTTATCCCGACGGCTTTATTTTTATATCCATAA
AAATCTTTGTAATTT
+
FFFFFFFFFFFFFFFFFFFFFFFFFFFFFFFFFFFFFFFFFFFFFFFFFFFFFFFFFFFFFFFFFFFFFF
FFFFFFFFFFFF:FFFFFFFFF:FFFFFFFFFFFFFFFFFFFFFFFFFFFFFFFFFFFFFFFFFFFFFFF
FFFFFFFFFFFFFFF
@A00721:190:HGJW7DSXY:4:2358:19524:33708 2:N:0:TCTGCAAG+GCTGCACT
TGCGGATAAAGCGAGCATGGTGGACTATCCGCATCTGGACGACATTACAACCAGTCCCAGAATTACTG
CAGAACCCGTCATGGAAGACGATGATTTCTGGGACTTTATCCCGACGGCTTTATTTTTATATCCATAA
AAATCTTTGTAATTT
+
FFFFFFFFFFFFFFFFFFFFFFFFFFFFFFFFFFFFFFFFFFFFFFFFFFFFFFFFFFFFFFFFFFFFFF
FFFFFFFFFFFFFFFF:FFFFFFFFFFFFFFFFFFFFFFFFFFFFFFFFFFFFFFFFFFFFFFFFFFFFF
FFFFFFFFFFFFFFF
@A00721:190:HGJW7DSXY:4:2416:12834:32800 2:N:0:TCTGCAAG+GCTGCACT
TGCGGATAAAGCGAGCATGGTGGACTATCCGCATCTGGACGACATTACAACCAGTCCCAGAATTACTG
CAGAACCCGTCATGGAAGACGATGATTTCTGGGACTTTATCCCGACGGCTTTATTTTTATATCCATAA
AAATCTTTGTAATTT
+
FFFFFFFFFFFFFFFFF:FFFFFFFFFFFFFFFFFFFFFFFFFFFFFFFFFFFFFFFFFFFFF,FFFFFF
FFFFFFFFFFFFFFFFFFFFFFFFFFFFFFFFFFFFFFFFFFF:FFFFFFFFFFFFFFFFFFFFFFFFFF
FFFFFFFFFFFFFFF
@A00721:190:HGJW7DSXY:4:2471:7148:18192 2:N:0:TCTGCAAG+GCTGCACT
TGCGGATAAAGCGAGCATGGTGGACTATCCGCATCTGGACGACATTACAACCAGTCCCAGAATTACTG
CAGAACCCGTCATGGAAGACGATGATTTCTGGGACTTTATCCCGACGGCTTTATTTTTATATCCATAA
AAATCTTTGTAATTT
+
F:FFFFFFFF:,FF::FFFFFFFFF:FFFF,FF::F:FFFFF:FFFFFFFFFFFFFFFFFF:FFFFFFF:

```
:FFFFFFFFFFFFFFFF,F:FFFFF:FFFFF::FFF:FFF:F::F::FF:FFFFFFFFFFFFFFF:FF
FFFFFF:FFFFF:F:
@A00721:190:HGJW7DSXY:4:2554:14362:7858 2:N:0:TCTGCAAG+GCTGCACT
TGCGGATAAAGCGAGCATGGTGGACTATCCGCATCTGGACGACATTACAACCAGTCCCAGAATTACTG
CAGAACCCGTCATGGAAGACGATGATTTCTGGGACTTTATCCCGACGGCTTTATTTTTATATCCATAA
AAATCTTTGTAATTT
+
FFFFFFFFFFFFFFFFFFFFFFFFFFFFFFFFFFFFFFFFFFFFFFFFFFFFFFFFFFFFFFFFFFFF
FFFFFFFFFFFFFFFFFFFFFFFFFFFFFFFFFFFFFFFFFF:FFFFFFFF:FFFFFFFFFFFFFFFF
FFFFFFFFF:FF,FF
@A00721:190:HGJW7DSXY:4:2570:19859:12399 2:N:0:TCTGCAAG+GCTGCACT
TGCGGATAAAGCGAGCATGGTGGACTATCCGCATCTGGACGACATTACAACCAGTCCCAGAATTACTG
CAGAACCCGTCATGGAAGACGATGATTTCTGGGACTTTATCCCGACGGCTTTATTTTTATATCCATAA
AAATCTTTGTAATTT
+
FFFFFFFFFFFFFFFFFFFFFFFFFFFFFFFFFFFFFFFFFFFFFFFFFFFFFFFFFFFFFFFFFFFF
FFFFFFFFFFFF:FFFFFFFFFFFF,FFFFFFFFFFFFFFFFFFFFFFFFFFFFFFFFFF,FFFFFFF
FFFFFFFFF:FFFFF
@A00721:190:HGJW7DSXY:4:2570:20717:12602 2:N:0:TCTGCAAG+GCTGCACT
TGCGGATAAAGCGAGCATGGTGGACTATCCGCATCTGGACGACATTACAACCAGTCCCAGAATTACTG
CAGAACCCGTCATGGAAGACGATGATTTCTGGGACTTTATCCCGACGGCTTTATTTTTATATCCATAA
AAATCTTTGTAATTT
+
FFFFFFFFFFFFFFFFFFFFFFFFFFFFFFFFFFFFFFFFFFFFFFFFFFFFFFFFFFFFFFFFFFFF
FFFFFFFFFFFFFFFFFFFFFFFFFFFFFFFFFFFFFFFFFFFFFFFFFFFFFFFFFFFFFFFFFFFF
FFFFFFFFFFFFFFF
@A00721:190:HGJW7DSXY:4:2665:8232:9486 2:N:0:TCTGCAAG+GCTGCACT
TGCGGATAAAGCGAGCATGGTGGACTATCCGCATCTGGACGACATTACAACCAGTCCCAGAATTACTG
CAGAACCCGTCATGGAAGACGATGATTTCTGGGACTTTATCCCGACGGCTTTATTTTTATATCCATAA
AAATCTTTGTAATTT
+
FFFFFFFFFFFFFFFFFFFFFFFFFFFFFFFFFFFFFFFFFFFFFFFFFFFFFFFFFFFFFFFFFFFF
FFFFFFFFFFFFFFFFFFFFFFFFFFFFFFFFFFFFFFFFFFFFFFFFFFFFFFFFFFFFFFFFFFFF
:FFFFFFFFFF:FFF
@A00721:190:HGJW7DSXY:4:1176:8612:33004 2:N:0:TCTGCAAG+GCTGCACT
GTTGCGGATAAAGCGAGCATGGTGGACTATCCGCATCTGGACGACATTACAACCAGTCCCAGAATTAC
TGCAGAACCCGTCATGGAAGACGATGATTTCTGGGACTTTATCCCGACGGCTTTATTTTTATATCCAT
AAAAATCTTTGTAAT
+
FFFFFFFFFFFFFFFFFFFFFFFFFFFFFFFFFFFFFFFFFFFFFFFFFFFFFFFFFFFFFFFFFFFF
FFFFFFFFFFFFFFFFFFFF:FFFFFFFFFFFFFFFFFFFFFFFFFF:FFFFFFFFFFFFFFFFFFFF
FFFFFFFFFFFFFFF
@A00721:190:HGJW7DSXY:4:1177:23204:10426 2:N:0:TCTGCAAG+GCTGCACT
GTTGCGGATAAAGCGAGCATGGTGGACTATCCGCATCTGGACGACATTACAACCAGTCCCAGAATTAC
TGCAGAACCCGTCATGGAAGACGATGATTTCTGGGACTTTATCCCGACGGCTTTATTTTTATATCCAT
AAAAATCTTTGTAAT
+
FFFFFFFFFFFFFFFFFFFFFFFFFFFFFFFFFFFFFFFFFFFFFFFFFFFFFFFFFFFFFFFFFFFF
FFFFFFFFFFFFFFFFFFFFFFFFFFFF:FFFFFFFFFFFFFFFFFFFFFFFFFFFFFFFFFFFFFFF
FFFFFFFFFFFFF:F
@A00721:190:HGJW7DSXY:4:1223:29785:8797 2:N:0:TCTGCAAG+GCTGCACT
GTTGCGGATAAAGCGAGCATGGTGGACTATCCGCATCTGGACGACATTACAACCAGTCCCAGAATTAC
TGCAGAACCCGTCATGGAAGACGATGATTTCTGGGACTTTATCTCGACGGCTTTATTTTTATATCCAT
AAAAATCTTTGTAAT
```

+
FFFFFFFFFFFFFFFFFFFFFFFFFFFFFFFFFFFFFFFFFFFFFFFFFFFFFFFFFFFFFFFFFFFFFF
FFFFFFFFF:FFFFFFFFFFFFFFFFFFFFFFFFFFFFFFFFFFFFFFFFFFFFFFFFFF:FFFFFF:
FFFFFFFFFF:FFFF
@A00721:190:HGJW7DSXY:4:1269:10068:35368 2:N:0:TCTGCAAG+GCTGCACT
GTTGCGGATAAAGCGAGCATGGTGGACTATCCGCATCTGGACGACATTACAACCAGTCCCAGAATTAC
TGCAGAACCCGTCATGGAAGACGATGATTTCTGGGACTTTATCCCGACGGCTTTATTTTTATATCCAT
AAAAATCTTTGTAAT
+
FFFFFFFFFFFFFFFFFFFFFFFFFFFFFFFFFFFFFFFFFFFFFFFFFFFFFFFFFFFFFFFFFFFFFF
FFFFFFFFFFFFFF,FFFFFFFFFFFFFFFFFFFFFFFFFFFFFFFFFFFFFFFFFFFFFFFFFFFFFF
FFFFFFFF:FFFFFF
@A00721:190:HGJW7DSXY:4:1308:24795:23453 2:N:0:TCTGCAAG+GCTGCACT
AATTCATCTTTAAAGATTTGGACAGACTATCCGCATCTGGACGACATTACAACCAGTCCCAGAATTAC
TGCAGAACCCGTCATGGAAGACGATGATTTCTGGGACTTTATCCCGACGGCTTTATTTTTATATCCAT
AAAAATCTTTGTAAT
+
FFFFFFFFFFFFFFFFFFFFFFFFFFFFFFFFFFFFFFFFFFFFFFFFFFFFFFFFFFFFFFFFFFFFFF
FFFFFFFFFFFFFFFFFFFFFFFFFFFFFFFFFFFFFFFFFFFFFFFFFFFFFFFFFFFFFFFFFFFFFF
FFFF:FFFFFFFFFF
@A00721:190:HGJW7DSXY:4:1367:11153:1924 2:N:0:TCTGCAAG+GCTGCACT
GTTGCGGATAAAGCGAGCATGGTGGACTATCCGCATCTGGACGACATTACAACCAGTCCCAGAATTAC
TGCAGAACCCGTCATGGAAGACGATGATTTCTGGGACTTTATATCTACGGCTTTATTTTTATATCAAT
AAAAATCTTTGTAAT
+
:,FFF:,FF:F,FF,F:FFFFFFF,FFFFFF:F,FFF:,:FF,FFFF,FFFFF,FFFFFFF,:F:,F:
F,FF:FFFF:,:FFF,FFFFFFFF,:,FFFFFFF,FFFFF:F,FF,FFF,F:FFFFFFFFFFFFF,F,
:FFF:,FF,FFF:,F
@A00721:190:HGJW7DSXY:4:1367:10682:2237 2:N:0:TCTGCAAG+GCTGCACT
GTTGCGGATAAAGCGAGCATGGTGGACTATCCGCATCTGGACGACATTACAACCAGTCCCAGAATTAC
TGCAGAACCCGTCATGGAAGACGATGATTTCTGGGACTTTATCTCGACGGCTTTATTTTTATATCCAT
AAAAATCTTTGTAAT
+
FFFFFFFFFFFFFFFFFFFFFFFFFFFFFFFFFFFFFFFFFFFFFFFFFFFFFFFFFFFFFFFFFFFFFF
FFFFFFFFF:FFFFFFFFFFFFFFFFFFFFFFFFFFFFFFFFFFFFFFFFFFFFFFFFFFFFFFFFFFFF
FFFFFFFFFFFFFFF
@A00721:190:HGJW7DSXY:4:1375:24831:32377 2:N:0:TCTGCAAG+GCTGCACT
GTTGCGGATAAAGCGAGCATGGTGGACTATCCGCATCTGGACGACATTACAACCAGTCCCAGAATTAC
TGCAGAACCCGTCATGGAAGACGATGATTTCTGGGACTTTATCTCGACGGCTTTATTTTTATATCCAT
AAAAATCTTTGTAAT
+
FFFFFF:FFFFFFFFFFFFFFFFFFFFFFFFFFFFFFFFFFFFFFFFFFFFFFFFFFFFFFFFFFFFFFF
F:FF:FFFFFFFFFFFFFFFFFFFFFFF:FFFFFFFFFFFFFFFFFF:FFFFFFFFFFFFFFFFFF:FF
FF:FFF,FFFFFFFF
@A00721:190:HGJW7DSXY:4:1557:5023:16454 2:N:0:TCTGCAAG+GCTGCACT
GTTGCGGATAAAGCGAGCATGGTGGACTATCCGCATCTGGACGACATTACAACCAGTCCCAGAATTAC
TGCAGAACCCGTCATGGAAGACGATGATTTCTGGGACTTTATCCCGACGGCTTTATTTTTATATCCAT
AAAAATCTTTGTAAT
+
FFFFFFFFFFFFFFFFFFFFFFFFFFFFFFFFFFFFFFFFFFFFFF:FFFFFFFFFFFFFFFFFFFFFFF
FFFFFFFFFF:FFFFFFF,FFFFFFFFFFFFFFFFFFFFFFFFFFFFFFFFFFFFFFF:FFFFFFFFFF
FFFFFFFFFFFFFFF
@A00721:190:HGJW7DSXY:4:1618:3296:12774 2:N:0:TCTGCAAG+GCTGCACT
GTTGCGGATAAAGCGAGCATGGTGGACTATCCGCATCTGGACGACATTACAACCAGTCCCAGAATTAC

```
TGCAGAACCCGTCATGGAAGACGATGATTTCTGGGACTTTATCCCGACGGCTTTATTTTTATATCCAT
AAAAATCTTTGTAAT
+
FFFFFFFFFFFFFFFFFFFFFFFFFFFFFFFFFFFFFFFFFFFFFFFFFFFFFFFFFFFFFFFFFFFF
FFFFFFFFFFFFFFFFFFFFFFFFFFFFFFFFFFFFFFFFFFFFFFFFFFFFFFFFFFFFFFFFFFFF
FFFFFFFFFFFFFFF
@A00721:190:HGJW7DSXY:4:1671:5999:35274 2:N:0:TCTGCAAG+GCTGCACT
GTTGCGGATAAAGCGAGCATGGTGGACTATCCGCATCTGGACGACATTACAACCAGTCCCAGAATTAC
TGCAGAACCCGTCATGGAAGACGATGATTTCTGGGACTTTATCTCGACGGCTTTATTTTTATATCCAT
AAAAATCTTTGTAAT
+
FF,FFFFF:FFFFF,FFFFFFF,FFFFFF,,F:FFFFFFFF:FFFFFFFFF:FFF,FFFFFFF:F:FF
FF,F,:FF,FF:FFFF:FF:FF,FF:FF:,,FF::F,F,FFF:FFFFFFFFFFFFFFF:F:FFFF,F,
FF:FFFFFFF::FFF
@A00721:190:HGJW7DSXY:4:2108:3070:18333 2:N:0:TCTGCAAG+GCTGCACT
AATTCATCTTTAAAGATTTGGACAGACTATCCGCATCTGGACGACATTACAACCAGTCCCAGAATTAC
TGCAGAACCCGTCATGGAAGACGATGATTTCTGGGACTTTATCCCGACGGCTTTATTTTTATATCCAT
AAAAATCTTTGTAAT
+
F,FFFFFFFFFFFFFFFFFFFFFFFFFFF::FFFFFFFFFFFFFFFFFFFFFFFFFFFFF:FFFFFFFF
FFFFFFFF:FFFFFFFFFFFFFFFFFFFFFFFFFFFFFFFFFFFFFFFFFFFFFFFF:FF,FFFF,FFF
FFFF:FFF:F,FF,F
@A00721:190:HGJW7DSXY:4:2165:5665:32503 2:N:0:TCTGCAAG+GCTGCACT
GTTGCGGATAAAGCGAGCATGGTGGACTATCCGCATCTGGACGACATTACAACCAGTCCCAGAATTAC
TGCAGAACCCGTCATGGAAGACGATGATTTCTGGGACTTTATCCCGACGGCTTTATTTTTATATCCAT
AAAAATCTTTGTAAT
+
F:FFFFFFFFFFFFFFFFFFFFFFFFFFFFFFFFFFFFFFFFFFFFFFFFFFFFFFFFFFFFFFFFFF
FFFFFFFFFFFFFFFFFFFFFFFFFFFFFFFFFFFFFFFFFFFFFFFFFFFFFFFFFFFFFFFFFFFF
FFFFFFFFFFFFFFF
@A00721:190:HGJW7DSXY:4:2177:24053:12837 2:N:0:TCTGCAAG+GCTGCACT
GTTGCGGATAAAGCGAGCATGGTGGACTATCCGCATCTGGACGACATTACAACCAGTCCCAGAATTAC
TGCAGAACCCGTCATGGAAGACGATGATTTCTGGGACTTTATCCCGACGGCTTTATTTTTATATCCAT
AAAAATCTTTGTAAT
+
FF:FFFFF:FFFFFFFFFFF:FFFFFFFFFFFFFFFFFFFFFFFFFFFFFFFFFFFF,FFFFFFFFFF:
FFFF:FF:FFFFFF:FFF:FFFFFFFFFFFFFFFFFFF,FFFFFF:FFFFFF,FFFFFFFFFF:FFF,
FFFFF:FF,F:FFFF
@A00721:190:HGJW7DSXY:4:2209:22209:14497 2:N:0:TCTGCAAG+GCTGCACT
GTTGCGGATAAAGCGAGCATGGTGGACTATCCGCATCTGGACGACATTACAACCAGTCCCAGAATTAC
TGCAGAACCCGTCATGGAAGACGATGATTTCTGGGACTTTATCCCGACGGCTTTATTTTTATATCCAT
AAAAATCTTTGTAAT
+
FFFF:FFFFFFFFFF:FFFFFFFFFFFFFFFF:FFFFFF,FFFFFFFFFFFFFFFFFFFFFFFFFFFF
FFFFFFFFFFFFFFFFFF,FFFFFFFFFFFFFFFFFFFFFFF:F:FFF:FFFFFFFFFFFFFFFFFFFF
FF:FFF:FFFFFF:F
@A00721:190:HGJW7DSXY:4:2222:6605:6637 2:N:0:TCTGCAAG+GCTGCACT
GTTGCGGATAAAGCGAGCATGGTGGACTATCCGCATCTGGACGACATTACAACCAGTCCCAGAATTAC
TGCAGAACCCGTCATGGAAGACGATGATTTCTGGGACTTTATCCCGACGGCTTTATTTTTATATCCAT
AAAAATCTTTGTAAT
+
FFF:FF:FFF,FFFFFFFFFFFFFFFFFFFFFFFFFFFFFFFF:FF:FFFFFFFFFFFFFFFF::FFFF
FFFFFFFFF:FFFFFFFFFFFFF::FF:FFFFFFFFFF:FFFFFF:FFFFFFFFFFFF,FFFF:FFFF
FFFFFFFFF::FF:F
```

```
@A00721:190:HGJW7DSXY:4:2226:2555:20071 2:N:0:TCTGCAAG+GCTGCACT
GTTGCGGATAAAGCGAGCATGGTGGACTATCCGCATCTGGACGACATTACAACCAGTCCCAGAATTAC
TGCAGAACCCGTCATGGAAGACGATGATTTCTGGGACTTTATCCCGACGGCTTTATTTTTATATCCAT
AAAAATCTTTGTAAT
+
FFFFFFFFFFFFFFFFFFFFFFFFFFFFFFFFFFFFFFFFFFFFFFFFFFFFFFFFFFFFFFFFFFFFFF
FFFFFFFFF:FFFFFFFFFFF:FFFFFFFFFFFFFFFFFFFF:F:FFFFFFFFF:FFFFFFFFFFF:FF
FFFFFFFFFFFFFFF
@A00721:190:HGJW7DSXY:4:2252:27127:17315 2:N:0:TCTGCAAG+GCTGCACT
GTTGCGGATAAAGCGAGCATGGTGGACTATCCGCATCTGGACGACATTACAACCAGTCCCAGAATTAC
TGCAGAACCCGTCATGGAAGACGATGATTTCTGGGACTTTATCCCGACGGCTTTATTTTTATATCCAT
AAAAATCTTTGTAAT
+
FFFFFFFFFFFFFFFFFFFFFFFFFFFFF:FFFFFFFFFFFFFFFFFFFFFFFFFFFFFFFFFFFFFFFF
FFFFFFFFFFFFFFFFFF:FFFFFFFF:FFFFFFFFFFFFFFFFFFFFFFFFFFFFFFFFFFFFFFFFFF
FFFFFFFFFFFFFFF
@A00721:190:HGJW7DSXY:4:2327:24605:9377 2:N:0:TCTGCAAG+GCTGCACT
GTTGCGGATAAAGCGAGCATGGTGGACTATCCGCATCTGGACGACATTACAACCAGTCCCAGAATTAC
TGCAGAACCCGTCATGGAAGACGATGATTTCTGGGACTTTATCTCGACGGCTTTATTTTTATATCCAT
AAAAATCTTTGTAAT
+
FFFFFFFFFFF,FFFFFFFFFF:FFFFFFFFFFFFFFFFFFFFFFFFFFFFFFFFFFFFFFFFFFFFFFF
FFFFFFFF:FFFFFFFFFFFFFFFFFFF:FFFFFFFF:FFFFFFF:FFFFFFFFFFFFFFFFFFFFFFF
FFFFFFFF:FFFFFF
@A00721:190:HGJW7DSXY:4:2408:8458:6997 2:N:0:TCTGCAAG+GCTGCACT
GTTGCGGATAAAGCGAGCATGGTGGACTATCCGCATCTGGACGACATTACAACCAGTCCCAGAATTAC
TGCAGAACCCGTCATGGAAGACGATGATTTCTGGGACTTTATCTCGACGGCTTTATTTTTATATCCAT
AAAAATCTTTGTAAT
+
FFFFFFFFFFFFFFFFFFFFFFFFFFFFFFFFFFFFFFFFFFFFFFFFFFFFFFFFFFFFFFFFFFFFFF
FFFFFFFFFFFFFFFFFFFFFFFFFFFFFFFFFFFFFFFFFFFFFF:FFFFFFFFFFFFFFFFFFFFFFF
FFFFFFFFFFFFFFF
@A00721:190:HGJW7DSXY:4:2603:4227:10441 2:N:0:TCTGCAAG+GCTGCACT
GTTGCGGATAAAGCGAGCATGGTGGACTATCCGCATCTGGACGACATTACAACCAGTCCCAGAATTAC
TGCAGAACCCGTCATGGAAGACGATGATTTCTGGGACTTTATCTCGACGGCTTTATTTTTATATCCAT
AAAAATCTTTGTAAT
+
F,FFFFFFFFF:FFFFFF::FFFFFFF::FFFFFF:FFFFFFFFFFFFFFFFFFFFFFFFFFFFFFFF:F
FFFFFFFFFFFFFFF:FFFFFFFFFFF:FFFFFFFFFFFFFFFFFFFFFFFFFFFFFFFFFFFFFFFF,F
:FFFFFFFFFFFFFF
@A00721:190:HGJW7DSXY:4:2611:6262:9580 2:N:0:TCTGCAAG+GCTGCACT
GTTGCGGATAAAGCGAGCATGGTGGACTATCCGCATCTGGACGACATTACAACCAGTCCCAGAATTAC
TGCAGAACCCGTCATGGAAGACGATGATTTCTGGGACTTTATCTCGACGGCTTTATTTTTATATCCAT
AAAAATCTTTGTAAT
+
FFFFFFFFFFFFFFFFFFFFFF:FFFFFFFFFFFFFFFFFFFFFFFFFFFFFFFFFFFF,FFFFFF:FF
FFFFFFFFFFF:FFFFFFFFFFFFFFF:FFFFFFFFFFFFFFFFFFFFFFFFFFFFFFFFFFFFFFFFF,
FFFFFFFF,FFFFFF
@A00721:190:HGJW7DSXY:4:2616:29143:2832 2:N:0:TCTGCAAG+GCTGCACT
AATTCATCTTTAAAGATTTGGACAGACTATCCGCATCTGGACGACATTACAACCAGTCCCAGAATTAC
TGCAGAACCCGTCATGGAAGACGATGATTTCTGGGACTTTATCCCGACGGCTTTATTTTTATATCCAT
AAAAATCTTTGTAAT
+
FFFFFFFFFFFFF:FFFFFFFFFFFFFFFFFFFFFFFFFFFFFFFFFFFFFFFFFFFFFFFFFFFFFFFF
```

```
FFFFFFFFFFFFFFFFFFFFFFFFFFFF:FFFFFFFFF:FFFFFFFFFFFFFFFFFFFFFFFFFFFFFFF
FFFF:FFFFFFFFFF
@A00721:190:HGJW7DSXY:4:2632:22824:17347 2:N:0:TCTGCAAG+GCTGCACT
AATTCATCTTTAAAGATTTGGACAGACTATCCGCATCTGGACGACATTACAACCAGTCCCAGAATTAC
TGCAGAACCCGTCATGGAAGACGATGATTTCTGGGACTTTATCCCGACGGCTTTATTTTTATATCCAT
AAAAATCTTTGTAAT
+
FFFFFFFFFFFFFFFFFFFFFFFFFFFFFFFFFFFFFFFFFFFFFFFFFFFFFFFFFFFFFFFFFFFFFF
FFFFFFFFFFFFFFFFFFFFFFFFFFFFFFFFFFFFFFFFFFFFFFFFFFFFFFFFFFFFFFFFFFF:FF
FFFFFFFFFFFFFFF
@A00721:190:HGJW7DSXY:4:1659:27823:3396 2:N:0:TCTGCAAG+GCTGCACT
AATTCATCTTTAAAGATTTGGACAGACTATCCGCATCTGGACGACATTACAACCAGTCCCAGAATTAC
TGCAGAACCCGTCATGGAAGACGATGATTTCTGGGACTTTATCCCGACGGCTTTATTTTTATATCCAT
AAAAATCTTTGTAA
+
FFFFFFFFFFFFFFFFFF:FFFFFFFFFFFFFFFFFFFFFFFFFFFFFFFFFFFFF:FFFFFFFF:FFFF
FF,FFFFFF:FFFFFFFFFFFFFFFFFFFFFF:FFFFFFFFFFFFFFFFFFFFFFFFFFFFFFFFFFF:F
FFFFFFFFFF:FFF
@A00721:190:HGJW7DSXY:4:2308:25012:30217 2:N:0:TCTGCAAG+GCTGCACT
AATTCATCTTTAAAGATTTGGACAGACTATCAGCATCTGGACGACATTACAACCAGTCCCAGAATTAC
TGCAGAACCCGTCAAGGAAGACGATGATTTCTGGTACATTATCCCGACGGCTTTATTTTTATATCCAT
AAAAATCTTAGTAA
+
FFFFFF,:FF,F:F,,F,F:::F:,F:,FF:,,:F:,,F,:FFFFFFFF:FF:FFF,FFF:F,FFFFFF
F:F:F,,:F,FFFF,F,F:,FFFF,,FFFFF:,:,FF,F,FFFF,:F,,,FF,F:FFFFFFFFFF:,F:
F:FF:,,FF,,FFF
@A00721:190:HGJW7DSXY:4:1132:29894:14904 2:N:0:TCTGCAAG+GCTGCACT
AAAGATTTTTATGGATATAAAAATAAATCCGTCGGGATAAAGTCCCAGAAATCATCGTCTTCCATTAC
GTGTTCTGCAGTAATTCTGTGACTGGTTGTAATGTCGTCCAGATGCGGATAGTCCACAATGCTCGCTT
TATCCGCAACTGCAT
+
FFFFF:FFFFFFF,FF:FFFFFFFFFFF:,,F:F:,F:FFFFF:FFF,F:F:FF:FF,F:FFF,FFF,F:
F,FFFFF,:FFFF,FF:F,,F:::FF:FFFFFFFFFFF:F:F:F:FFFFFFFFFF:F:,FF,FFFFFFF
FFFF,F:F:FFF,FF
@A00721:190:HGJW7DSXY:4:1152:5538:6511 2:N:0:TCTGCAAG+GCTGCACT
AAAGATTTTTATGGATATAAAAATAAAGCCGTCGGGATAAAGTCCCAGAAATCATCGTCTTCCATGAC
GGGTTCTGCAGTAATTCTGGGACTGGTTGTAATGTCGTCCAGATGCGGATAGTCCACCATGCTCGCTT
TATCCGCAACTGCAT
+
FFFFFFFFFFFFFFFFFFFFFFFFFFFFFFFFFFFFFFFFFFFFFFFFFFFFFFFFFFFFFFFFFFFFFF
FFFFFFFFFFFFFFFFFFFFFFFFFFFFFFFFFFFFFFFFFFFFFFFFFFFFFFFFFFFFFFFFFFFFFF
FFFFFFFFFFFFFFF
@A00721:190:HGJW7DSXY:4:1276:4137:9752 2:N:0:TCTGCAAG+GCTGCACT
AAAGATTTTTATGGATATAAAAATAAAGCCGTCGGGATAAAGTCCCAGAAATCATCGTCTTCCATGAC
GGGTTCTGCAGTAATTCTGGGACTGGTTGTAATGTCGTCCAGATGCGGATAGTCCACCATGCTCGCTT
TATCCGCAACTGCAT
+
FFFFFFFFFFFFFFFFF:FFFFFFFFFFFFFFFFFFFFFFFFFF:FFFFFFFFFFFFFFFFFFFFFFFFF
FFFFFFFFFFFFFFFFFFFFFFFFFFFFFFFFFFFFFFFFFFFFFFFFFFFFFFFFFFFFFFFFFFFFFF
FFFFFFFFFFFFFFF
@A00721:190:HGJW7DSXY:4:1302:5312:5400 2:N:0:TCTGCAAG+GCTGCACT
AAAGATTTTTATGGATATAAAAATAAAGCCGTCGGGATAAAGTCCCAGAAATCATCGTCTTCCATGAC
GGGTTCTGCAGTAATTCTGGGACTGGTTGTAATGTCGTCCAGATGCGGATAGTCCACCATGCTCGCTT
TATCCGCAACTGCAT
```

```
+
FFFFFFFFFFF:FFFFF,FFFFF,FFFFFFFFFFFFFFFFFFFFFFFFF,FFFFFFFFF:FFFFFFFF
FFFFFFFFFFFF:,FFFFF,FFF,FF,FFFF:FFFFFFFFFFFFFFFFFF,FFFFFFFFFFFFFFF,,
FFFFFFFFFF:FFFF
@A00721:190:HGJW7DSXY:4:1462:25437:7467 2:N:0:TCTGCAAG+GCTGCACT
ATGCCAATTCATCTTTAAAGATTTGGACAGACTATCCGCATCTGGACGACATTACAACCAGTCCCAGA
ATTACTGCAGAACCCGTCATGGAAGACGATGATTTCTGGGACTTTATCCCGACGGCTTTATTTTTATA
TCCATAAAAATCTTT
+
FFFFFFF:FFFFFFFFFFFFFFFFFFFFFFFFFFFFFFFFFFFFFFFFFFFFFFFFFFFFFFFFFFFF
FFFFFFFFFFFFFFFFFFFFFFFFFFFFFFFFF:FFFFFFFFFFFFFFFFFFFFFFFFFFFFFFFFFF
FFF:FFFFFFFFF,F
@A00721:190:HGJW7DSXY:4:2104:15600:5932 2:N:0:TCTGCAAG+GCTGCACT
ATGCCAATTCATCTTTAAAGATTTGGACAGACTATCCGCATCTGGACGACATTACAACCAGTCCCAGA
ATTACTGCAGAACCCGTCATGGAAGACGATGATTTCTGGGACTTTATCCCGACGGCTTTATTTTTACA
TCCATAAAAATCTTT
+
FFFFFFFFFFFFFFFFFFFFFFFFFFFFFF:FFFFFFFFFFFFFFFFFFFFFFFFFFFFFFFFFFF:FF
FFFFFFFFFFFFFFFFFFFFFFFFFFFFFFFFFFFFFF:FFFFFFFFFFFFFFFFFFFFFFFFFF:F,F
FFFFFFFFFFFFFFF
@A00721:190:HGJW7DSXY:4:2114:16658:22545 2:N:0:TCTGCAAG+GCTGCACT
ATGCCAATTCATCTTTAAAGATTTGGACAGACTATCCGCATCTGGACGACATTACAACCAGTCCCAGA
ATTACTGCAGAACCCGTCATGGAAGACGATGATTTCTGGGACTTTATCCCGACGGCTTTATTTTTATA
TCCATAAAAATCTTT
+
FFFFFFFFFFFFFFFFFFFFFFFFFFFFFFFFFFFFFFFFFFFFFFFFFFFFFFFFFFFFFFFFFFFF
FFFFFFFFFFFFFFFFFFFFFFFFFFFFFFFFFFFFFFFFFFFFFFFFFF:FFFFFFFFFFFFFFFFF
F:FFFFFFF:FFFFF
@A00721:190:HGJW7DSXY:4:2119:4842:24314 2:N:0:TCTGCAAG+GCTGCACT
AAAGATTTTTATGGATATAAAAATAAAGCCGTCGGGATAAAGTCCCAGAAATCATCGTCTTCCATGAC
GGGTTCTGCAGTAATTCTGGGACTGGTTGTAATGTCGTCCAGATGCGGATAGTCCACCATGCTCGCTT
TATCCGCAACTGCAT
+
FFFFFFFFFFFFFFFFFFFFFFFFFFFFFFFFFFFFFFFFFFFFFFFFFFFFFFFFFFFFFFFFFFFF
FFFFFFFFFFFFFFFFFFFFFFFFFFFFFFFFFFFFFFFFFFFFFFFFFFFFFFFFFFFFFFFFFFFF
FFFFFFFF:FFFFFF
@A00721:190:HGJW7DSXY:4:2503:12174:30217 2:N:0:TCTGCAAG+GCTGCACT
ATGCCAATTCATCTTTAAAGATTTGGACAGACTATCCGCATCTGGACGACATTACAACCAGTCCCAGA
ATTACTGCAGAACCCGTCATGGAAGACGATGATTTCTGGGACTTTATCCCGACGGCTTTATTTTTATA
TCCATAAAAATCTTT
+
FFFFFFF,FFFFFFFFFFFFFFFF:FFFFFFFFFFFFFFFFFFFFFFFF:FFFFFFFFFFFFFFFFFFF
FFFFFFFFFFFFFFFFFFFFFFFFFFFFFFFFFFFFF:FFFFFFFFFFFFFFFFFFFFFFFFFFFF:FFF
FF,FFFFFFFFFFFF
@A00721:190:HGJW7DSXY:4:2623:20564:31250 2:N:0:TCTGCAAG+GCTGCACT
ATGCCAATTCATCTTTAAAGATTTGGACAGACTATCCGCATCTGGACGACATTACAACCAGTCCCAGA
ATTACTGCAGAACCCGTCATGGAAGACGATGATTTCTGGGACTTTATCCCGACGGCTTTATTTTTATA
TCCATAAAAATCTTT
+
FFFFFFF:FFFFFFFFFF,FFFFFFFFFFFFFFFFFFFFFFFFFFFFFFFFFFFFFFFFFFFFFFFFF
FFFFFFFFFFFFFF:FFFFFFFFFFFFFFFFFFFFFFFFFFFFFFFFFFFFFFFFFFFFFFFFFFFFF
FFFFFFFFFFFFFFF
@A00721:190:HGJW7DSXY:4:1173:6433:2644 2:N:0:TCTGCAAG+GCTGCACT
AAGATTTTTATGGATATAAAAATAAAGCCGTCGGGATAAAGTCCCAGAAATCATCGTCTTCCATGACG
```

GGTTCTGCAGTAATTCTGGGACTGGTTGTAATGTCGTCCAGATGCGGATAGTCCACCATGCTCGCTTT
ATCCGCAACTGCATT
+
FFFFFFFFFFFFFFFFFFFFFFFFFFFFFFFFFFFFFFFFFFFFFFFFFFFFFFFFFFFFFFFFFFFF
FFFFFFFFFFFFFFFFFFFFFFFFFFFFFFFFFFFFFFFFFFFFFFFFFFFFFFFFFFFFFFFFFFFF
FFFFFFFFFFFFFFF
@A00721:190:HGJW7DSXY:4:1204:8169:33739 2:N:0:TCTGCAAG+GCTGCACT
AAGATTTTTATGGATATAAAAATAAAGCCGTCGGGATAAAGTCCCAGAAATCATCGTCTTCCATGACG
GGTTCTGCAGTAATTCTGGGACTGGTTGTAATGTCGTCCAGATGCGGATAGTCCACCATGCTCGCTTT
ATCCGCAACTGCATT
+
FFFFFFF,FFFFFFFFFFFFFFFFFFFFFFFFFFFFFFFFFFFFFFFFFFFFFFFFFFFFFFFFFFFF
FFFFFFFFFFFFFFFFFFFFFFFFFFFFFFFFFFFFFFFFFFFFFFFFFFFFF:FFFFFFFFFFFFFF
FFFFFFFFFFFFFFF
@A00721:190:HGJW7DSXY:4:1224:21522:12367 2:N:0:TCTGCAAG+GCTGCACT
AAGATTTTTATGGATATAAAAATAAAGCCGTCGGGATAAAGTCCCAGAAATCATCGTCTTCCATGACG
GGTTCTGCAGTAATTCTGGGACTGGTTGTAATGTCGTCCAGATGCGGATAGTCCACCATGCTCGCTTT
ATCCGCAACTGCAT
+
FFFFFFFFF:FFFFFFFFFFFFFFFFFFFFFFFFFFFFFFFFFFFFFFFFFFFFFFFFFFFFFFFF:F
FFFFFFFFFFFFFFF:FFFFFFFFFFFFFFFFFFFFFFFFFFFFFFFFFFFFFFFFFFFFFFFFFFFF
FFFFFFFFFFFFFF
@A00721:190:HGJW7DSXY:4:1362:11180:22608 2:N:0:TCTGCAAG+GCTGCACT
AATGCAGTTGCGGATAAAGCGAGCATGGTGGACTATCCGCATCTGGACGACATTACAACCACTCCCAG
AATTACTGCAGAACCCGTCATGGAAGACGATGATTTCTGGGACTTTATCCCGACGGCTTTATTTTTAT
ATCCATAAAAATCTT
+
FFFFFFFFFFFFFFFFFFFFFFFFFFFFFFFFFFFFFFFFFFFFFFFFFFFFFFFFFFFFFFFFFFFF
FFFFFFFFFFFFFFFFFFFFFFFFFFFFFFFFFFFFFFFFFFFFFFFFFFFFFFFFFFFFFFFFFFFF
FFFFFFFFFFFFFFF
@A00721:190:HGJW7DSXY:4:1533:6379:4523 2:N:0:TCTGCAAG+GCTGCACT
AATGCAGTTGCGGATAAAGCGAGCATGGTGGACTATCCGCATCTGGACGACATTACAACCACTCCCAG
AATTACTGCAGAACCCGTCATGGAAGACGATGATTTCTGGGACTTTATCCCGACGGCTTTATTTTTAT
ATCCATAAAAATCTT
+
FFFFFFFFFFFFFFFFFFFFFFFFFFFFFFFFFFFFFFFFFFFFFFFFFFFFFFFFFFFFFFFFFFFF
FFFFFFFFFFFFFFFFFFFFFFFFFFFFFFFFF:FFFFFFFFFFFFFFFFFFFFFFFFFFFFFFFFFF
FFFFFFFFFFFFFFF
@A00721:190:HGJW7DSXY:4:2246:12807:37012 2:N:0:TCTGCAAG+GCTGCACT
AAGATTTTTATGGATATAAAAATAAAGCCGTCGGGATAAAGTCCCAGAAATCATCGTCTTCCATGACG
GGTTCTGCAGTAATTCTGGGACTGGTTGTAATGTCGTCCAGATGCGGATAGTCCACCATGCTCGCGTT
ATCCGCAACTGCATT
+
FFFFF:FFFFFFFFF:FFFFFFFFF:FFFFFFFFFFFFFFFFFFFFFFFFFFFFFFFFFF:FFFFFFF
FFFFFFFF:FFFFFFFFFFFFFFFFFFFFFFFFFFFFF:FFFFFFFFFFFFFFFFFFFFFFFFFF,FF
FFFFFFFFFFFFF:F
@A00721:190:HGJW7DSXY:4:2303:26802:19132 2:N:0:TCTGCAAG+GCTGCACT
AATGCAGTTGCGGATAAAGCGAGCATGGTGGACTATCCGCATCTGGACGACATTACAACCACTCCCAG
AATTACTGCAGAACCCGTCATGGAAGACGATGATTTCTGGGACTTTATCCCGACGGCTTTATTTTTAT
ATCCATAAAAATCTT
+
FFFFFFFFFFFFFFFFFFFFFFFFFFFFFFFFFFFFFFFFFFFFFFFFFFFFFFFFFFFFFFFFFFFF
FFFFFFFFFFFFFFFFFFFFFFFF:FFFFFFFFFFFFFFFFFFFFFFFFF:FFFFFFFFFFFFFFFFF
FFFFFFFFFFFFFFF

```
@A00721:190:HGJW7DSXY:4:2445:32606:23202 2:N:0:TCTGCAAG+GCTGCACT
AATGCAGTTGCGGATAAAGCGAGCATGGTGGACTATCCGCATCTGGACGACATTACAACCACTCCCAG
AATTACTGCAGAACCCGTCATGGAAGACGATGATTTCTGGGACTTTATCCCGACGGCTTTATTTTTAT
ATCCATAAAAATCTT
+
FFFFFFFFFFFFFFFFFFFFFFFFFFFFFFFFFFFFFFFFFFFFFFFFFFFFFFFFFFFFFFFFFFFFFF
FFFFFFFFFFFFFFFFFFFFFFFFFFFFFFFFFFFFFFFFFFFFFFFFFFFFFFFFFFFFFFFFFFFFFF
FFFFFFFF,FFFFFF
@A00721:190:HGJW7DSXY:4:2467:8160:5259 2:N:0:TCTGCAAG+GCTGCACT
AAGATTTTTATGGATATAAAAATAAAGCCGTCGGGATAAAGTCCCAGAAATCATCGTCTTCCATGACG
GGTTCTGCAGTAATTCTGGGACTGGTTGTAATGTCGTCCAGATGCGGATAGTCCACCATGCTCGCTTT
ATCCGCAACTGCATT
+
FFFFFFFFFFFFFFFFFFFFFFFFFFFFFFFFFFFFFFFFFFFFFFFFFFFFFFFFFFFFFFFFFFFFFF
FFFFFFFFFFFFFFFFFFFFFFFFFFFFFFFFFFFFFFFFFFFFFFFFFFFFFFFFFFFFFFFFFFFFFF
FFFFFFFFFFFFFFF
@A00721:190:HGJW7DSXY:4:2537:18728:15640 2:N:0:TCTGCAAG+GCTGCACT
AAGATTTTTATGGATATAAAAATAAAGCCGTCGGGATAAAGTCCCAGAAATCATCGTCTTCCATGACG
GGTTCTGCAGTAATTCTGGGACTGGTTGTAATGTCGTCCAGATGCGGATAGTCCACCATGCTCGCTTT
ATCCGCAACTGCATT
+
FFFFFFFFFF:FFFFFFFFFFFFFFFFFFFFFFFFFFFFFFFFFFFFFFFFFFFFFFFFFFFFFFFFFFF
FFFFFFFFFFFF:FFFFFFFFFFFFFFFFFF,FFFFFFFFFFFFFFFFFFFFFFFFFFFFFFFFFFFFFF
FFFFFFF,FFFFFFF
@A00721:190:HGJW7DSXY:4:2562:15103:7858 2:N:0:TCTGCAAG+GCTGCACT
AAGATTTTTATGGATATAAAAATAAAGCCGTCGGGATAAAGTCCCAGAAATCATCGTCTTCCATGACG
GGTTCTGCAGTAATTCTGGGACTGGTTGTAATGTCGTCCAGATGCGGATAGTCCACCATGCTCGCTTT
ATCCGCAACTGCATT
+
FFFFFFFFFFFFFFFFFFFFFF:FFFFFFFFFFFFFFFFFFFFFFFFFFFFFFFFFFFFFFFFFFFFFFF
FFFFFFFFFFFFFFFFFFFFFFFFFFFFFFFFFFFFFFFFFFFFFFFFFFFFFFFFFFFFFFFFFFFFFF
FFFFFFFFFFFFFFF
@A00721:190:HGJW7DSXY:4:2669:6316:20791 2:N:0:TCTGCAAG+GCTGCACT
AAGATTTTTATGGATATAAAAATAAAGCCGTCGGGATAAAGTCCCAGAAATCATCGTCTTCCATGACG
GGTTCTGCAGTAATTCTGGGACTGGTTGTAATGTCGTCCAGATGCGGATAGTCCACCATGCTCGCTTT
ATCCGCAACTGCATT
+
FFFFFFFFFFFFFFFFFFFFFFFFFFFFFFFFFFFFFFFFFFFFFFFFFFFFFFFFFFFFFFFFFFFFFF
FFFFFFFFFFFFFFFFFFFFFFFFFFFFFFFFFFFFFFFFFFFFFFFFFFFFFFFFFFFFFFFFFFFFFF
FFFFFFFFFFFFFFF
@A00721:190:HGJW7DSXY:4:1108:17255:23672 2:N:0:TCTGCAAG+GCTGCACT
AGATTTTTATGGATATAAAAATAAAGCCGTCGGGATAAAGTCCCAGAAATCATCGTCTTCCATGACGG
GTTCTGCAGTAATTCTGGGACTGGTTGTAATGTCGTCCAGATGCGGATAGTCCACCATGCTCGCTTTA
TCCGCAACTGCATTC
+
FFFFFFFF::FF:F,FFFFFFFFFFFFF:F::FFFF:FFFFFFFFFFFFFFFFFFFFFFFFFFFFFFFFF
F:FFFFFFFFFFFFFFFFFFFFFFFFFFFFFFFFFFFFFFFFFFFFF:FFFFFF:FFFFFFFFFFFFFFF
FF,FFFFFFFFFFFF
@A00721:190:HGJW7DSXY:4:1552:3070:20776 2:N:0:TCTGCAAG+GCTGCACT
AGATTTTTATGGATATAAAAATAAAGCCGTCGGGATAAAGTCCCAGAAATCATCGTCTTCCATGACGG
GTTCTGCAGTAATTCTGGGACTGGTTGTAATGTCGTCCAGATGCGGATAGTCCACCATGCTCGCTTTA
TCCGCAACTGCATTC
+
FFFFFFFFFFFFFF:FFFFFFFFFFFFFFF:FFFFFFFFFFFFFFFFFFFFFFFFFFF:FFFFFFFFFFF
```

```
FFFFFFFFF:FFFFFFFFFF,FFFFFFFFFFFFF,FFFFFFFFFFFFFFFFFFFFFFFFFFFFFFFFF
FFFFFFFFFFFFFFF
@A00721:190:HGJW7DSXY:4:1622:15483:34820 2:N:0:TCTGCAAG+GCTGCACT
AGATTTTTATGGATATAAAAATAAAGCCGTCGGGATAAAGTCCCAGAAATCATCGTCTTCCATGACGG
GTTCTGCAGTAATTCTGGGACTGGTTGTAATGTCGTCCAGATGCGGATAGTCCACCATGCTCGCTTTA
TCCGCACCTGCATTC
+
FFFFFFFFF:FFFFFFFFFFFFFFFFFFF:FFFFFFFFFFFFFFFFFFFFFFFFFFFFFFFFFFFFFF
FFFFFFFFFFFFFFFFFFFFFFFFFFFFFFF:FFFFFFFFFFFFFFFFFFFFFFFFFFFFFFFFFFFF
,FFFFF,FFFFFF,:
@A00721:190:HGJW7DSXY:4:1667:4237:20384 2:N:0:TCTGCAAG+GCTGCACT
AGATTTTTATGGATATAAAAATAAAGCCGTCGGGATAAAGTCCCAGAAATCATCGTCTTCCATGACGG
GTTCTGCAGTAATTCTGGGACTGGTTGTAATGTCGTCCAGATGCGGATAGTCCACCATGCTCGCTTTA
TCCGCAACTGCATTC
+
FFFFFFFFF,FFFFFFFFFFFFFFFFFFFFFFFFFFFFFFFFFFFFFFFFFFFFFFFFFFFFFFFFFF
FFF:FFFFF:FFFFFFFFFFF:FFF:FFFFFFFFFFFFFFFFFFFFFF:FFFFFFFF:FFFFFFFFFF
FFFFFF,FFFFFFFF
@A00721:190:HGJW7DSXY:4:2104:2040:10661 2:N:0:TCTGCAAG+GCTGCACT
AGATTTTTATGGATATAAAAATAAAGCCGTCGGGATAAAGTCCCAGAAATCATCGTCTTCCATGACGG
GTTCTGCAGTAATTCTGGGACTGGTTGTAATGTCGTCCAGATGCGGATAGTCCACCATGCTCGCTTTA
TCCGCAACTGCATTC
+
FFFFFFFFFFFFFFFFFFFFFFFFFFFFFFFFFFFFFFFFFFFFFFFFFFFFFFFFFFFFFFFFFFFF
FFFFFFFFFFFFFFFFFFF::FFFFFFFFFF:FFFFFFFFFFFFFFFFFFFFFFFFFFFFFFFFFFF:
FFFFFF:FFFFF:FF
@A00721:190:HGJW7DSXY:4:2364:30472:15781 2:N:0:TCTGCAAG+GCTGCACT
AGATTTTTATGGATATAAAAATAAAGCCGTCGGGATAAAGTCCCAGAAATCATCGTCTTCCATGACGG
GTTCTGCAGTAATTCTGGGACTGGTTGTAATGTCGTCCAGATGCGGATAGTCCACCATGCTCGCTTTA
TCCGCAACTGCATT
+
FFFFFFFF,FFF,F:,FFFFFFFFF,F,:FFFFF:FFFFFFF:FFFFFF:FFFFFFFFFFFFF:F,F:
,FF:FF,FFFFFFFFFFFFF,FFF,FFFFFFFF,:F::F,:FF::FFFFFFFFF:FFFFFFF:,,FFF
FF,:FFFFFFFFFF
@A00721:190:HGJW7DSXY:4:2368:19551:24017 2:N:0:TCTGCAAG+GCTGCACT
AGATTTTTATGGATATAAAAATAAAGCCGTCGGGATAAAGTCCCAGAAATCATCGTCTTCCATGACGG
GTTCTGCAGTAATTCTGGGACTGGTTGTAATGTCGTCCAGATGCGGATAGTCCACCATGCTCGCTTTA
TCCGCAACTGCATTC
+
FFFFFFFFFFFFFFFFFFFFFFF,F,FFFFFFFFFFFFFFFFFFFFFFFFFFFFFFFFFFFFFFFFFF
FFFFFFFFFFFFFFFFFFFFFF::FFFFFFFFFFFFFFFFFFFF:FFFFFFFFFFFFFFFFFFFFFFFF
FFFFFFFFFFFFFFF
@A00721:190:HGJW7DSXY:4:2506:7355:34147 2:N:0:TCTGCAAG+GCTGCACT
AGATTTTTATGGATATAAAAATAAAGCCGTCGGGATAAAGTCCCAGAAATCATCGTCTTCCATGACGG
GTTCTGCAGTAATTCTGGGACTGGTTGTAATGTCGTCCAGATGCGGATAGTCCACCATGCTCGCTTTA
TCCGCAACTGCATTC
+
FFFF::FFFFFFFFF::FFF:FFFF:FFFFFFFFFF,FFFFFFFFFFFFFFFFFFFFFF:FFFFF:FF
:FFFFFFFFFFFFFFFFFFF:FF:FFFFFFFFFFFFFFFFFFF:FFFFFFFFFFFFFFFFFFFFFFFF
FFFFFFFFFF,F:FF
@A00721:190:HGJW7DSXY:4:1230:1362:22263 2:N:0:TCTGCAAG+GCTGCACT
CCTGGACAAGGATGCCAATTCATCTTTAAAGATTTGGACAGACTATCCGCATCTGGACGACATTACAA
CCAGTCCCAGAATTACTGCAGAACCCGTCATGGAAGACGATGATTTCTGGGACTTTATCCCGACGGCT
TTATTTTTATATCCA
```

```
+
FFFFFFFFFFFFFFFFFF,FFFFFFFFFF:FFFFFFFFFFFFFFFFFFFFFFFFFFFFFFFFFFFFF,FF
FF:FFFFFFFFFFFFFFFFFFFFFFFFFFFFFFFFFFFFFFFFFFF:FFFFFF,FFFFFFFFFFFFFFFF
FFFFFFFFFFFFFFF
@A00721:190:HGJW7DSXY:4:2101:10664:36338 2:N:0:TCTGCAAG+GCTGCACT
CCTGGACAAGGATGCCAATTCATCTTTAAAGATTTGGACAGACTATCCGCATCTGGACGACATTACAA
CCAGTCCCAGAATTACTGCAGAACCCGTCATGGAAGACGATGATTTCTGGGACTTTATCCCGACGGCT
TTATTTTTATATCCA
+
FFFFFFFFFFFFFFFFFFFFFFFFFFFFFFFFFFFFFFFFFFFFFFFFFFFFFFFFFFFFFFFFFFFFFF
FFFFFFFFFFFFFFFFFFFFFFFFFF:FFFFFFFFFFFFFFFFFFFFFFFFFFFFFFFFFFFFFFF:FFF
F:FFFFFFFFFFFF:
@A00721:190:HGJW7DSXY:4:2151:7654:4726 2:N:0:TCTGCAAG+GCTGCACT
CCTGGACAAGGATGCCAATTCATCTTTAAAGATTTGGACAGACTATCCGCATCTGGACGACATTACAA
CCAGTCCCAGAATTACTGCAGAACCCGTCATGGAAGACGATGATTTCTGGGACTTTATCCCGACGGCT
TTATTTTTATATCCA
+
FFFFFFFFFFFFFFFFFFFFFFFFFFFFFFFF:F:FFFFFFFFFFFFFFFFFFFFFFFFFFFFFFFFFFF
FFFFFFFFFFFFFFFFFFFFFFFFFFFFFFFFFFFFFF:FFFFFFF:FFFFFFFFFFFFFFF:FFFFFFF
FFFFF:FFFFFFFFF
@A00721:190:HGJW7DSXY:4:2310:7654:31407 2:N:0:TCTGCAAG+GCTGCACT
CCTGGACAAGGATGCCAATTCATCTTTAAAGATTTGGACAGACTATCCGCATCTGGACGACATTACAA
CCAGTCCCAGAATTACTGCAGAACCCGTCATGGAAGACGATGATTTCTGGGACTTTATCCCGACGGCT
TTATTTTTATATCCA
+
FFFFFFFFFFFFFFFFFF,FFFFFFFFFFFFFFFFFFFFFFFFFFFFFFFFFFFFFFFFFFFFFFFFFFF
FFFFFFFFFFFFFFFFFFFFFFFFFFFFFFFFFFFFFFFFFFFFFFFFFFFFFFFFFFFFFFFFFFFFFF
FFFFFFFFFFFFFFF
@A00721:190:HGJW7DSXY:4:1273:9435:23876 2:N:0:TCTGCAAG+GCTGCACT
CTACAATCTCAGAATGCAGTTGCGGATAAAGCGAGCATGGTGGACTATCCGCATCTGGACGACATTAC
AACCAGTCCCAGAATTACTGCAGAACCCGTCATGGAAGACGATGATTTCTGGGACTTTATCCCGACGG
CTTTATTTTTATATC
+
FFFFFFFFFFFFFFFFFFFFFFFFFFFFFFFFFFFFFFFFFFFFFFFFFFFFFFFFFFFFFFFFFFFFFF
FFFFFFFFFFFFFFFFFFFFFF:FFFFFFFFFFFFFFFFFFFFFFF::FFFFFFFFFFFFFFFFFFFFFF
FFFFFFFFFFFFFFF
@A00721:190:HGJW7DSXY:4:2231:26178:29794 2:N:0:TCTGCAAG+GCTGCACT
CTACAATCTCAGAATGCAGTTGCGGATAAAGCGAGCATGGTGGACTATCCGCATCTGGACGACATTAC
AACCAGTCCCAGAATTACTGCAGAACCCGTCATGGAAGACGATGATTTCTGGGACTTTATCCCGACGG
CTTTATTTTTATATC
+
FFFFFFFFFFFFFFFFFFFFFFFFFFFFFFFFFFFFFFFFFFFFFFFFFFFFFFFFFFFFFFFFFFFFFF
FFFFFFFFFFFFFFFFFFFFFFFFFFFFFFFFFFFFFFFFFFFFFFFFFFFFFFFFFFFFFFFFFFFFFF
FFFFFFFFFFFFFFF
@A00721:190:HGJW7DSXY:4:2259:25066:3944 2:N:0:TCTGCAAG+GCTGCACT
CTACAATCTCAGAATGCAGTTGCGGATAAAGCGAGCATGGTGGACTATCCGCATCTGGACGACATTAC
AACCAGTCCCAGAATTACTGCAGAACCCGTCATGGAAGACGATGATTTCTGGGACTTTATCCCGACGG
CTTTATTTTTATATC
+
FFFFFFFFFFFFFFFFFFFFFFFFFFFF:FFFFFFFFFFFFFFFFFFFFFFFFFFFFFFFFFFFFFFFFF
FFFFFFFFFFFFFFFFFFFFFFFFFFFFFFFFF:FFFF,FFFFFFFFFFFFFF,FFFFFFFFFFFFFFFF
FFFFFFFFFFFFFFF
@A00721:190:HGJW7DSXY:4:2570:16188:24643 2:N:0:TCTGCAAG+GCTGCACT
CTACAATCTCAGAATGCAGTTGCGGATAAAGCGAGCATGGTGGACTATCCGCATCTGGACGACATTAC
```

```
AACCAGTCCCAGAATTACTGCAGAACCCGTCATGGAAGACGATGATTTCTGGGACTTTATCCCGACGG
CTTTATTTTTATATC
+
FFFFFFFFFFFFFFFFFFFFFFFFFFFFFFFFFFFFFFFFFFFFFFFFFFFFFFFFFFFFFFFFFFFF
FFFFFFFFFFFFFFFFFFFFFFFFFFF:FFFFFFFFFFFFFFFF:FFFFFFFFFFFFFFFFF:FFFFF
FFFFFFFFFFFFFFF
@A00721:190:HGJW7DSXY:4:2651:31675:13792 2:N:0:TCTGCAAG+GCTGCACT
CTACAATCTCAGAATGCAGTTGCGGATAAAGCGAGCATGGTGGACTATCCGCATCTGGACGACATTAC
AACCAGTCCCAGAATTACTGCAGAACCCGTCATGGAAGACGATGATTTCTGGGACTTTATCCCGACGG
CTTTATTTTTATATC
+
FFF:FFFFFFFFFFFFFFFFFFFFFFFFFFFFFFFFFFF:FFFFFFFFFFFFFFFFFFFFFFFFFFFF
FFFFFFFFFFFFFFFFFFFFFFFFFFFFF:FFFFFFFFFFFFFFFFFFFFFFFFFFFFFFFFFFFFFF
FFFFFFFFFFFFFFF
@A00721:190:HGJW7DSXY:4:1133:7491:20290 2:N:0:TCTGCAAG+GCTGCACT
AAAAATAAAGCCGTCGGGATAAAGTCCCAGAAATCATCGTCTTCCATGACGGGTTCTGCAGTAATTCT
GGGACTGGTTGTAATGTCGTCCAGATGCGGATAGTCCACCATGCTCGCTTTATCCGCAACTGCATTCT
GAGATTGTAGTTGAA
+
FFFFFFFFFFFFFFFFFFFFFFFFFFFFFFFFFFFFFFFFFFFFFFFFFFFFFFF:FFFFFF,FFFF
FFFFFFFFFFFFFFFFFFFFFFFFFFFFFFFFF:FFFFFFFFFFFFFFFFFFFFFFFFFFFFFFFFFF
FFFF:FFFFF:FFFF
@A00721:190:HGJW7DSXY:4:1239:23637:17848 2:N:0:TCTGCAAG+GCTGCACT
AAAAATAAAGCCGTCGGGATAAAGTCCCAGAAATCATCGTCTTCCATGACGGGTTCTGCAGTAATTCT
GGGACTGGTTGTAATGTCGTCCAGATGCGGATAGTCCACCATGCTCGCTTTATCCGCAACTGCATTCT
GAGATTGTAGTTGAA
+
FFFFFFFFFFFFFFFFFFFFFFFFFFFFFFFFFFFFFFFFFFFFFFFFFFFFFFFFFFFFFFFFFFFF
FFFFFFFFFFFFFFFFFFFFFFFFFFFFFFFFFF:FFFFFFFFFFFFFFFFFFFFFFFFFFFFFFFFF
FFFF:FFFFFFFFFF
@A00721:190:HGJW7DSXY:4:1523:22752:4038 2:N:0:TCTGCAAG+GCTGCACT
AAAAATAAAGCCGTCGGGATAAAGTCCCAGAAATCATCGTCTTCCATGACGGGTTCTGCAGTAATTCT
GGGACTGGTTGTAATGTCGTCCAGATGCGGATAGTCCACCATGCTCGCTTTATCCGCAACTGCATTCT
GAGATTGTAGTTGAA
+
FFFFFFFFFFFFFFFFFFFFFFFFFFFFFFFFFFFFFFFFFFFFFFFFFFFFFFFFFFFFFFFFFFFF
FFFFFFFFFFFFFFFFFFFFFFFFFFFFFFFFFFFFFFFFFFFFFFFFFFFFFFFFFFFFFFFFFFFF
FFFFFFFF:FFFFFF
@A00721:190:HGJW7DSXY:4:1532:26467:7373 2:N:0:TCTGCAAG+GCTGCACT
AAAAATAAAGCCGTCGGGATAAAGTCCCAGAAATCATCGTCTTCCATGACGGGTTCTGCAGTAATTCT
GGGACTGGTTGTAATGTCGTCCAGATGCGGATAGTCCACCATGCTCGCTTTATCCGCAACTGCATTCT
GAGATTGTAGTTGAA
+
FFFFFFFF:FFFFFFFFFFFFFFFFFFFFFFFFFFFFFFFFFFFFFFFFFFFFFFFFFFFFFFFFFFF
FFFFFFFFFFFFFFFFFFFFFFFFFFFFFFFFFFFFFFFFFFFFFFF:FFFFFFFFFFFFFFFFF,FFF
FFFF,FFFF:FFFFF
@A00721:190:HGJW7DSXY:4:1603:18358:5635 2:N:0:TCTGCAAG+GCTGCACT
ATTCAACTACAATCTCAGAATGCAGTTGCGGATAAAGCGAGCATGGTGGACTATCCGCATCTGGACGA
CATTACAACCAGTCCCAGAATTACTGCAGAACCCGTCATGGAAGACGATGATTTCTGGGACTTTATCC
CGACGGCTTTATTTT
+
FFFFFFFFFFFFFFFFFF:FFFFFFFFFFFFFFFFFFFFFFFFFFFFFFFFFFFFFFFFFFFFFFFFF
FFFFFFFFFFFFFFFFFFFFFFFFFFFFFFFFFFFFFFFFFFFFFFFFFFFFFFFFFFFFFFF:FFFFF
FFFFF:FFFFFFFFF
```

```
@A00721:190:HGJW7DSXY:4:2139:31901:32910 2:N:0:TCTGCAAG+GCTGCACT
ATTCAACTACAATCTCAGAATGCAGTTGCGGATAAAGCGAGCATGGTGGACTATCCGCATCTGGACGA
CATTACAACCAGTCCCAGAATTACTGCAGAACCCGTCATGGAAGACGATGATTTCTGGGACTTTATCC
CGACGGCTTTATTTT
+
FFF,FFFFFFFFFFF::FFFFFFFFFFFFFFFFFFFFF:FFFFFFFFFFFFFFFFFFFFFFFFFFFFFFF
FFFFFFFFFFFFFF:FFFFFFFFFFFFFFFFF,F::F:FFFFFFFFFFFF:FFFFFFFFFFFFFFFFFFF
:FFFF,FFFFFFFFF
@A00721:190:HGJW7DSXY:4:2260:14253:8296 2:N:0:TCTGCAAG+GCTGCACT
ATTCAACTACAATCTCAGAATGCAGTTGCGGATAAAGCGAGCATGGTGGACTATCCGCATCTGGACGA
CATTACAACCAGTCCCAGAATTACTGCAGAACCCGTCATGGAAGACGATGATTTCTGGGACTTTATCC
CGACGGCTTTATTTT
+
FFFFFFFFFFFFFFFFFFFFFFFFFFFFFFFFFFFFFFFFFFFFFFFFFFFFFFFFFFFFFFFFFFFFFF
FFFFFFFFFFFFFFFFFFFFFFFFFFFFFFFFFFFFFFFFFFFFFFFFFFFFFFFFFFFFFFFFFFFFFF
:FFFFFFFFFFFFFF
@A00721:190:HGJW7DSXY:4:2318:23484:29418 2:N:0:TCTGCAAG+GCTGCACT
ATTCAACTACAATCTCAGAATGCAGTTGCGGATAAAGCGAGCATGGTGGACTATCCGCATCTGGACGA
CATTACAACCAGTCCCAGAATTACTGCAGAACCCGTCATGGAAGACGATGATTTCTGGGACTTTATCC
CGACGGCTTTATTTT
+
FFFFFFFFFFFFFFFFFFFFFFFFFFFFFFFFFFFFFFFFFFFFFFFFFFFFFFFFFFFFFFFFFFFFFF
FFFFFFFFFFFFFFFFFFFFFFFFFFFFFFFFFFFFFFFFFFFFFFFFFFFFFFFFFFFFFFFFFFFFFF
FFFFFFFFFFFFFFF
@A00721:190:HGJW7DSXY:4:2328:10474:19820 2:N:0:TCTGCAAG+GCTGCACT
ATTCAACTACAATCTCAGAATGCAGTTGCGGATAAAGCGAGCATGGTGGACTATCCGCATCTGGACGA
CATTACAACCAGTCCCAGAATTACTGCAGAACCCGTCATGGAAGACGATGATTTCTGGGACTTTATCC
CGACGGCTTTATTTT
+
FFFFFFFFFFFF:FFFFFFFFFFFF:FFFFFFFFFFFFFFFFFFFFFFFFFFFFFFFFFFFFFFFFFFF,
FFFFFFFFFFFFFFFFFFFFFFFFFFFFFFFFFFFFFFFFFFFFF:,FFFFFFFFFFFFFF::FFFFFFF
FFFFFFFFFFFF:FF
@A00721:190:HGJW7DSXY:4:2349:5113:3020 2:N:0:TCTGCAAG+GCTGCACT
ATTCAACTACAATCTCAGAATGCAGTTGCGGATAAAGCGAGCATGGTGGACTATCCGCATCTGGACGA
CATTACAACCAGTCCCAGAATTACTGCAGAACCCGTCAGGGAAGACGAGGATTTCTGGGACTTTATCC
CGACGGCTTTATTTT
+
F:FFFFFFFF:FFFFFFFFFFFFFFFFFFFFF:,FFFFFFFFFFFFFFFFFFF:FFFFFFFF:FFFFFFF
F,FFFFFFFFFFFFFFFFFFFFFFF:FFFFFFFFFFFFFF,FFFFFFFFF,FFF:FFFF:FFF,:FFFFF
FFF:FFFFFFF,:FF
@A00721:190:HGJW7DSXY:4:2469:29957:36245 2:N:0:TCTGCAAG+GCTGCACT
ATTCAACTACAATCTCAGAATGCAGTTGCGGATAAAGCGAGCATGGTGGACTATCCGCATCTGGACGA
CATTACAACCAGTCCCAGAATTACTGCAGAACCCGTCATGGAAGACGATGATTTCTGGGACTTTATCC
CGACGGCTTTATTTT
+
FFFFFFFFFFFFFFFFFFFFFFFFFFFFFFFFFFFFFFFFFFFFFFFFFFFFFFFFFFFFFFFFFFFFFF
FFFFFFFFFFFFFFFFFFFFFFFFFFFFFFFFFFFFFFFFFFFFFFFFFFFFFFFFFFFFFFFFFFFF:F
:FFFFFFFF:FFFFF
@A00721:190:HGJW7DSXY:4:2635:18738:30592 2:N:0:TCTGCAAG+GCTGCACT
ATTCAACTACAATCTCAGAATGCAGTTGCGGATAAAGCGAGCATGGTGGACTATCCGCATCTGGACGA
CATTACAACCAGTCCCAGAATTACTGCAGAACCCGTCATGGAAGACGATGATTTCTGGGACTTTATCC
CGACGGCTTTATTTT
+
FFFFFFFFFFFFFFFFFFFFFFFFFFFFFFFFFFFFFFFFFFFFFFFFFFFFFFFFFFFFFFFFFFFFFF
```

FFFFFFFFFFFFFFFFFFFFFFFFFFFFFFFFFFFFFFF,FFFFFFFFFFFFFFFFFFFFFFFFFFFF:F
:FFFFFFFFF,FFFF
@A00721:190:HGJW7DSXY:4:2678:27597:13589 2:N:0:TCTGCAAG+GCTGCACT
ATTCAACTACAATCTCAGAATGCAGTTGCGGATAAAGCGAGCATGGTGGACTATCCGCATCTGGACGA
CATTACAACCAGTCCCAGAATTACTGCAGAACCCGTCATGGAAGACGATGATTTCTGGGACTTTATCC
CGACGGCTTTATTTT
+
FFFFFFFFFFFFFFFFFFFFFFFFF:FFFFFFFFFFFFFFFFFFFFFFFFFFFFFFFFFFFFFFFFFFFF
FFFFFFFFFFFFFFFFFFF,FFFFFFFFFFFFFFFFFFFFFFFF,FFFFFFFFFFFFFFFFFF:FFFFFF
FFFFFFFFFFFFFFF
@A00721:190:HGJW7DSXY:4:1345:30789:9659 2:N:0:TCTGCAAG+GCTGCACT
GATTCAACTACAATCTCAGAATGCAGTTGCGGATAAAGCGAGCATGGTGGACTATCCGCATCTGGACG
ACATTACAACCAGTCCCAGAATTACTGCAGAACCCGTCATGGAAGACGATGATTTCTGGGACTTTATC
CCGACGGCTTTATTT
+
FFFFFFFFFFFFFFFFFFFFFFFFFFFFFFFFFFFFFFFFFFFFFFFFFFFFFFFFFFFFFFFFFFFFFF
FFFFFFFFFFFFFFFFFFFFFFFFFFFFFFFFFFFFFFFFFFFFFFFFFFFFFFFFFFFFFFF,FFFFFF
FFFFFFFFFFFFFFF
@A00721:190:HGJW7DSXY:4:1529:23737:22748 2:N:0:TCTGCAAG+GCTGCACT
GATTCAACTACAATCTCAGAATGCAGTTGCGGATAAAGCGAGCATGGTGGACTATCCGCATCTGGACG
ACATTACAACCAGTCCCAGAATTACTGCAGAACCCGTCATGGAAGACGATGATTTCTGGGACTTTATC
CCGACGGCTTTATTT
+
FFFFFFFFFFFFFFFFFFFFFFFFFFFFFFFFFFFFFFFFFFFFF:FFFFFFFFFFFFFFFFFFFFFFFF
FFFFFFFFFFFFFFFFFFFFFFFFFFFFFFFFFFFFFFFFFFFFFFFFFFFFFFFFFFFFFFFFFFFFF:
FFFFFFFFFFFFFFF
@A00721:190:HGJW7DSXY:4:1553:5113:30326 2:N:0:TCTGCAAG+GCTGCACT
GATTCAACTACAATCTCAGAATGCAGTTGCGGATAAAGCGAGCATGGTGGACTATCCGCATCTGGACG
ACATTACAACCAGTCCCAGAATTACTGCAGAACCCGTCATGGAAGACGATGATTTCTGGGACTTTATC
CCGACGGCTTTATTT
+
FFFFFFFFFFFFFFFFFFFFFFFFFFFFFFFFFFFFFFFFFFFFFFFFFFFFFFFFFFFFFFFFFFFFFF
FFFFFFFFFF:FFFFFFFFFFFFFFFFFFFFFFFF:FFFFFFFFFFFFFFFFFFFFFFFF:FFFFFFFFF
FFFFFFFFFFFFFFF
@A00721:190:HGJW7DSXY:4:1578:10945:30530 2:N:0:TCTGCAAG+GCTGCACT
GATTCAACTACAATCTCAGAATGCAGTTGCGGATAAAGCGAGCATGGTGGACTATCCGCATCTGGACG
ACATTACAACCAGTCCCAGAATTACTGCAGAACCCGTCATGGAAGACGATGATTTCTGGGACTTTATC
CCGACGGCTTTATTT
+
FFFFFFFFFFFFFFFFFFFFFFFFFFFFFFFFFF:FFFFFFFFFFFFFFFFFFFFFFFFFFFFFFFFFFF
FFFFFFFFFFFFFFFFFFFFFFFFFFFFFFF:FFFFFFFFFFFFFFFFFFFFFFFFFFFFFFFFFFFFFF
FFFFF:FFFFFFFFF
@A00721:190:HGJW7DSXY:4:2262:20166:19100 2:N:0:TCTGCAAG+GCTGCACT
GATTCAACTACAATCTCAGAATGCAGTTGCGGATAAAGCGAGCATGGTGGACTATCCGCATCTGGACG
ACATTACAACCAGTCCCAGAATTACTGCAGAACCCGTCATGGAAGACGATGATTTCTGGGACTTTATC
CCGACGGCTTTATTT
+
FFFFFFFFFFFFFFFFFFFFFFFFFFFFFFFFFFFFFFFFFFFFFFFFFFFFFFFFFFFFFFFFFFFFFF
FFFFFFFFFFFFFFFFFFFFF,FFFFFFFFFFFFFFFFFFFFFF:FFFFFFFFF:FFFFFFFFFFFFFFF
FFFFFFFFFFFFFFF
@A00721:190:HGJW7DSXY:4:2553:3992:36432 2:N:0:TCTGCAAG+GCTGCACT
GATTCAACTACAATCTCAGAATGCAGTTGCGGATAAAGCGAGCATGGTGGACTATCCGCATCTGGACG
ACATTACAACCAGTCCCAGAATTACTGCAGAACCCGTCATGGAAGACGATGATTTCTGGGACTTTATC
CCGACGGCTTTATTT

+
FFFFFFFFFFFFFFFFFFFFFFFFFFFFFFFFFFFFFFFFFFFFFFFFFFFFFFFFFFFFFFFFFFFF
FFFFFFFFFFFFFFFFFFFFFFFFFFFFFFFFFFFFFFFFFFFFFFFFFFFFFFFFFFFFFFFFFFFF
FFFFFFFFFFFFFFF
@A00721:190:HGJW7DSXY:4:2567:2483:16501 2:N:0:TCTGCAAG+GCTGCACT
GATTCAACTACAATCTCAGAATGCAGTTGCGGATAAAGCGAGCATGGTGGACTATCCGCATCTGGACG
ACATTACAACCAGTCCCAGAATTACTGCAGAACCCGTCATGGAAGACGATGATTTCTGGGACTTTATC
CCGACGGCTTTATTT
+
FFFFFFFFFFFFFFFFFFFFFFFFFFFFFFFFFFFFFFFFFFFFFFFFFFFFFFFFFFFFFFFFF:FF
FFFFFFFFFFFFFFFFFFFFFFFFFFFFFFFFFFFFFFFFFFFFFFFFFFFF:FFFFFFFFFFFFFFF
FFFFFFFFFFFFFFF
@A00721:190:HGJW7DSXY:4:2607:25843:31469 2:N:0:TCTGCAAG+GCTGCACT
GATTCAACTACAATCTCAGAATGCAGTTGCGGATAAAGCGAGCATGGTGGACTATCCGCATCTGGACG
ACATTACAACCAGTCCCAGAATTACTGCAGAACCCGTCATGGAAGACGATGATTTCTGGGACTTTATC
CCGACGGCTTTATTT
+
FFFFFFFFF:FFFFFFFFFFFFFFFFFFFFFFFFFFFFFFFFFFFFFFFFFFFFFFFFFFFFFFFFFF
FFFFFFFFFFFFFFFFF:FFFFFFFFFFFFFFFFFFFFFFFFFFFFFFFFFFFFFFFFFFFFFFFFFF
FFFFFFFFFFFFFF:
@A00721:190:HGJW7DSXY:4:2404:21314:35524 2:N:0:TCTGCAAG+GCTGCACT
ATTCAACTACAATCTCAGAATGCAGTTGCGGATAAAGCGAGCATGGTGTACTATCCGCATCAGGACGA
CATTACAACCAGTCCCAGAATTACTGCAGAACCCGTCATGGAAGACTATGATTTCTGGGACTTTATCC
CGACGGCTTTATTAT
+
:FF,FFF,FFFFF:FFFFFFF:,FF,:FF::FFFFFFF:FF:FF::,,,FF:FFF,F::FF,:,F,FF
F:FFFFFFFF:,,FF:F,FFF:F,,,FFFFF,,::,FF,,,FFF:,,FF,FFF,F,,,,FFF::FF,,
:FFF:FFF,FFF:,:
@A00721:190:HGJW7DSXY:4:1130:29550:12994 2:N:0:TCTGCAAG+GCTGCACT
AAGATTCAACTACAATCTCAGAATGCAGTTGCGGATAAAGCGAGCATGGTGGACTATCCGCATCTGGA
CGACATTACAACCAGTCCCAGAATTACTGCAGAACCCGTCATGGAAGACGATGATTTCTGGGACTTTA
TCCCGACGGCTTTAT
+
F:FFFFFFFFFFFFFFFFFFFFFFFFFFFFFFFFFFFFFFFFFFF:FFFFFFFFFFFFFFFFFFFFFF
FFFFFFFFFFFFFFFFFFFFFFFFFFFFFFFFFFFFFFFFF:FFFFFFFFFFFFFFFFFFFFFF:FFF
FFFFFFFFFFFFFFF
@A00721:190:HGJW7DSXY:4:1465:10438:31125 2:N:0:TCTGCAAG+GCTGCACT
AAGATTCAACTACAATCTCAGAATGCAGTTGCGGATAAAGCGAGCATGGTGGACTATCCGCATCTGGA
CGACATTACAACCAGTCCCAGAATTACTGCAGAACCCGTCATGGAAGACGATGATTTCTGGGACTTTA
TCCCGACGGCTTTAT
+
FFFFFFFFFFFFFFFFFFFFFFFFFFFFFFFFFFFFFFFFFFFFFFFFFFFFFFFFFFFFFFFFFFFF
FFFFFFFFFFFFFFFFFFFFFFFFFFFFFFFFFFFFFFFFFFFFFFFFFFF:FF:FFFFFFFFFFFFF
FFFFFFFFFFFFFFF
@A00721:190:HGJW7DSXY:4:1530:27091:26741 2:N:0:TCTGCAAG+GCTGCACT
AAGATTCAACTACAATCTCAGAATGCAGTTGCGGATAAAGCGAGCATGGTGGACTATCCGCATCTGGA
CGACATTACAACCAGTCCCAGAATTACTGCAGAACCCGTCATGGAAGACGATGATTTCTGGGACTTTA
TCCCGACGGCTTTAT
+
FFFFFFFFFFFFFFFFFFFFFFFFFFFFFFFFFFFFFFFFFFFFFFFFFFFFFFFFFFFFFFFFFFFF
FFFFFFFFFFFFF:FFFFFFFFFFFFFFFFFFFFFFFFFFFFFFFFFFFFF:FF,FFFFFFFFFFFFF
FFFFFFFFFFFFFFF
@A00721:190:HGJW7DSXY:4:1544:20961:31093 2:N:0:TCTGCAAG+GCTGCACT
AAGATTCAACTACAATCTCAGAATGCAGTTGCGGATAAAGCGAGCATGGTGGACTATCCGCATCTGGA

CGACATTACAACCAGTCCCAGAATTACTGCAGAACCCGTCATGGAAGACGATGATTTCTGGGACTTTA
TCCCGACGGCTTTAT
+
FFFFFFFFFFFFFFFFFFFFFFFFFFFFFFFFFFFFFFFFFFFFFFFFFFFFFFFFFFFFFFFFFFFF
FFFFFFF:FFFFFFFFFFFFFFFFFFFFFFFFFFFFFFFFFFFFF:FFFFFFFFFFFFFFFFFFFFFF
FF:FFFFFFFFFFFF
@A00721:190:HGJW7DSXY:4:1578:28845:29590 2:N:0:TCTGCAAG+GCTGCACT
AAGATTCAACTACAATCTCAGAATGCAGTTGCGGATAAAGCGAGCATGGTGGACTATCCGCATCTGGA
CGACATTACAACCAGTCCCAGAATTACTGCAGAACCCGTCATGGAAGACGATGATTTCTGGGACTTTA
TCCCGACGGCTTTAT
+
FFFFFF:FFFFFFFFFFFFFFFFFFFFFFF,::FFFFFFFF:FFFFFFFFFFFFFFFFFFFF:FFF:F
FFFFFFFFFFFF,F:FFFFF:FFFFF:F:FFFFF:FF,FFFFFFFFFFFFFFFFFFFFFF,FFFFFFF
FFF:FFFFFFFFFFF
@A00721:190:HGJW7DSXY:4:1660:4634:6731 2:N:0:TCTGCAAG+GCTGCACT
AAGATTCAACTACAATCTCAGAATGCAGTTGCGGATAAAGCGAGCATGGTGGACTATCCGCATCTGGA
CGACATTACAACCAGTCCCAGAATTACTGCAGAACCCGTCATGGAAGACGATGATTTCTGGGACTTTA
TCCCGACGGCTTTAT
+
FFFFF,,FFFFF:F:FF,,FFFFF,::,FF,:,,FFFFF:FFFF:FF,F::,FFFFFF:,F:F:FF,F
:FFFFFFF,FFFFFFF:FF:,FF:F:,F:FF:FFFF::FFFF,FFFFF,FFFFFF,F::F,:FFF,,F
F,F:FF:,,,:F:F:
@A00721:190:HGJW7DSXY:4:1676:6903:6746 2:N:0:TCTGCAAG+GCTGCACT
AAGATTCAACTACAATCTCAGAATGCAGTTGCGGATAAAGCGAGCATGGTGGACTATCCGCATCTGGA
CGACATTACAACCAGTCCCAGAATTACTGCAGAACCCGTCATGGAAGACGATGATTTCTGGGACTTTA
TCCCGACGGCTTTAT
+
FFFFFFFF,FFFFFFFFFFFFFFFFFFFFFFFFFFFFFFFFFFFFFFFFFFFFFFFFFFFFFFFFFFF
FFFFFFFFFFFFF:FFFFF,FFFFFFFFFFFFFFFFFFFFFFFFFFFFFFFFFFFFFFFFFFFF,FFF
FFFFFFFFFFFFFFF
@A00721:190:HGJW7DSXY:4:2120:9959:18771 2:N:0:TCTGCAAG+GCTGCACT
AAGATTCAACTACAATCTCAGAATGCAGTTGCGGATAAAGCGAGCATGGTGGACTATCCGCATCTGGA
CGACATTACAACCAGTCCCAGAATTACTGCAGAACCCGTCATGGAAGACGATGATTTCTGGGACTTTA
TCCCGACGGCTTTAT
+
FFFFFFFFFFFFFFFFFFFFFFFFFFFFFFFFFFFFFFFFFFFFFFFFFFFFFFFFFFFFFFFFFFFF
FFFFFFFFFFFFFFFFF:FFFFFFFFFFFFFFFFFFFFFFF:FFFFFFFFFFFFFF:FFFFFFFFFFF
FFFFFFFFFFFFFFF
@A00721:190:HGJW7DSXY:4:2356:26558:16579 2:N:0:TCTGCAAG+GCTGCACT
AAGATTCAACTACAATCTCAGAATGCAGTTGCGGATAAAGCGAGCATGGTGGACTATCCGCATCTGGA
CGACATTACAACCAGTCCCAGAATTACTGCAGAACCCGTCATGGAAGACGATGATTTCTGGGACTTTA
TCCCGACGGCTTTAT
+
FFFFFFFFFFFFFFFFFFFFFFFFFFFFFFFFFFFFFFFFFFFFFFFFFFFFFFFFFFFFFFFFFFFF
FFFFFFFFFFFFFFFFFFFFFFFFFFFFFFFFFFFFFFFFFFFFFFFFFFFFFFFFFFFFFFFFFFFF
FFFFFFFFFFFFFFF
@A00721:190:HGJW7DSXY:4:2357:17788:31829 2:N:0:TCTGCAAG+GCTGCACT
AAGATTCAACTACAATCTCAGAATGCAGTTGCGGATAAAGCGAGCATGGTGGACTATCCGCATCTGGA
CGACATTACAACCAGTCCCAGAATTACTGCAGAACCCGTCATGGAAGACGATGATTTCTGGGACTTTA
TCCCGACGGCTTTAT
+
F:FFFFFFFFFFFFFFFFFFFFFFFFFFFFFFFFFFFFFFFFFFFFFFFFFFFFFFFFFFFFFFFFFF
FFFFFFFFFFFFFFFFFFF:FFFFFFFFFFFFFFFFFFFFFFFF:FFFFFFFF:FFFFFF:FFFFFFF
FFFFFFFFFFFFFFF

```
@A00721:190:HGJW7DSXY:4:2447:30807:5337 2:N:0:TCTGCAAG+GCTGCACT
AAGATTCAACTACAATCTCAGAATGCAGTTGCGGATAAAGCGAGCATGGTGGACTATCCGCATCTGGA
CGACATTACAACCAGTCCCAGAATTACTGCAGAACCCGTCATGGAAGACGATGATTTCTGGGACTTTA
TCCCGACGGCTTTAT
+
FFFFFFFFFFFFFFFFFFFFFFFFFFFFFFFFFFFFFFFFFFFFFFFFFFFFFFFFFFFFFFFFFFFF
FFFFFFFFFFFFFFFFFFFFFFFFFFFFFFFFFFFFFFFFFFFFFFFFFFFFFFFFFFFFFFFFFFFF
FFF:FFF:FFFFFFF
@A00721:190:HGJW7DSXY:4:2458:24189:7373 2:N:0:TCTGCAAG+GCTGCACT
AAGATTCAACTACAATCTCAGAATGCAGTTGCGGATAAAGCGAGCATGGTGGACTATCCGCATCTGGA
CGACATTACAACCAGTCCCAGAATTACTGCAGAACCCGTCATGGAAGACGATGATTTCTGGGACTTTA
TCCCGACGGCTTTAT
+
FFFFFFFFFFFFFFFFFFFFFFFFFFFFFFFFFFFFFFFFFFFFFFFFFFFFFFFFFFFFFFFFFFFF
FFFFFFFFFFFFFFFFFFFFFFFFFFFFFFFFFFFFFFFFFFFFFFFFFFFFFFFFFFFFFFFFFFFF
FFFFFFFFFFFFFFF
@A00721:190:HGJW7DSXY:4:1108:14850:35070 2:N:0:TCTGCAAG+GCTGCACT
CTAAGATTCAACTACAATCTCAGAATGCAGTTGCGGATAAAGCGAGCATGGTGGACTATCCGCATCTG
GACGACATTACAACCAGTCCCAGAATTACTGCAGAACCCGTCATGGAAGACGATGATTTCTGGGACTT
TATCCCGACGGCTTT
+
FFFFFFFFFFFFFFFFFFFFFFFFFFFFFFFFFFFFFFFFFFFFFFFFFFFFFFFF:FFFFFFFFFFF
FFFFFFFFFFFFFFFFFFFFFFFFFFFFFFFFFFFFFFFFFFFFFF:FFFFFFFFFFFFFFFFFFFFF
FFFFFFFFFFFFFFF
@A00721:190:HGJW7DSXY:4:1202:8269:27461 2:N:0:TCTGCAAG+GCTGCACT
CTAAGATTCAACTACAATCTCAGAATGCAGTTGCGGATAAAGCGAGCATGGTGGACTATCCGCATCTG
GACGACATTACAACCAGTCCCAGAATTACTGCAGAACCCGTCATGGAAGACGATGATTTCTGGGACTT
TATCCCGACGGCTTT
+
FFFFFFFFFFFFFFFFFFFFFFFFFFFFFFFFFFFFFFFFFFFFFFFFFFFFFFFFFFFF:FFFFFFF
FFFFFFFFFFFFFFFFFFFFFFFFFFFFFFFFFFFFFFFFFFFFFFFFFFF:FFFFFFFFFFFFFFFF
FFFFFFFFFFFFF:F
@A00721:190:HGJW7DSXY:4:1215:17255:19758 2:N:0:TCTGCAAG+GCTGCACT
CTAAGATTCAACTACAATCTCAGAATGCAGTTGCGGATAAAGCGAGCATGGTGGACTATCCGCATCTG
GACGACATTACAACCAGTCCCAGAATTACTGCAGAACCCGTCATGGAAGACGATGATTTCTGGGACTT
TATCTCGACGGCTTT
+
FFFFFFFFFFFFFFFFFFFFFFFFFFFFFFFFFFFFFFFFFFFFFFFFFFFFFFFFFFFFFFFFFFFF
FFFFFFFFFFFFF:FFFFFFFFFFFFFFFFFFFFFFFFFFFFFFFFFFFFFFFFF:FFFFFFFFFFFF
FFFFFFFFFFFFFF:
@A00721:190:HGJW7DSXY:4:1215:17454:20228 2:N:0:TCTGCAAG+GCTGCACT
CTAAGATTCAACTACAATCTCAGAATGCAGTTGCGGATAAAGCGAGCATGGTGGACTATCCGCATCTG
GACGACATTACAACCAGTCCCAGAATTACTGCAGAACCCGTCATGGAAGACGATGATTTCTGGGACTT
TATCTCGACGGCTTT
+
FFFFFFFFFFFFFFFFFFFFFFFFFFFFFF:FFFFFFFFFFFFFFFF:FFFFFFFFFFFFFFFFFFFF
FFFFFFFFFFFFFFFFFFFFFFFFFFFFFFFFFFFFFFFFFFFFFFFFFFFFFFFF:FFFFFFFFF:F
FFFFFFFFF:FFFFF
@A00721:190:HGJW7DSXY:4:1225:18810:7294 2:N:0:TCTGCAAG+GCTGCACT
CTAAGATTCAACTACAATCTCATAATGCAGTTGCGGATAAAGCGAGCATGGTGGACTATCCGCATCTG
GACGACATTACAACCAGTCCCAGAATTACTGCAGAACCCGTCATGGAAGACGATGATTTCTGGGACTT
TATCCCGACGGCTTT
+
FFFFFFFFFFFFFFFFFFFFFFFFFFFFF:FFFFFFFF:FFFFFFFFFF:FFFFF:FFFFFFFFFFFF
```

```
FFFFFFFF:FFFFFFFFFFFFFFFFFFFFFFFFFFFFFFFFFFFFFFF:FFFFFFFF:FFFFFFFFFF
FFFFF:FFFFFFFF:
@A00721:190:HGJW7DSXY:4:1257:21784:6715 2:N:0:TCTGCAAG+GCTGCACT
CTAAGATTCAACTACAATCTCAGAATGCAGTTGCGGATAAAGCGAGCATGGTGGACTATCCGCATCTG
GACGACATTACAACCAGTCCCAGAATTACTGCAGAACCCGTCATGGAAGACGATGATTTCTGGGACTT
TATCCCGACGGCTTT
+
FFFFFFFFFFFFFFF:FFFFFFFFFFFFFFFFFFFFF:FFFFFFFFF,FFF:FFFFFFFFFFFFFFFF
FFFFFFFFFFF:FFF,FFFFF,FF,FFF::FFFFF:FFFFFF,FFF,:F,FF:FF,,:FFFFFF,,F,
F:FFFFF:,FF,FFF
@A00721:190:HGJW7DSXY:4:1335:23583:2503 2:N:0:TCTGCAAG+GCTGCACT
CTAAGATTCAACTACAATCTCAGAATGCAGTTGCGGATAAAGCGAGCATGGTGGACTATCCGCATCTG
GACGACATTACAACCAGTCCCAGAATTACTGCAGAACCCGTCATGGAAGACGATGATTTCTGGGACTT
TATCCCGACGGCTTT
+
FFFFFFFFFFFFFFFFFFFFFFFFFFFFFFFFFFFFFFFFF:FFFFFFFFFFFFFFFFFFFFFFFFFF
FFFFFFFFFFFFFFFFFFFFFFFFFFFFFFFFFFFFFFFFFFFFFFFFFFFFFF,FFFFFFFFFFFFF
FF:FFFFFFFFFFFF
@A00721:190:HGJW7DSXY:4:1340:20799:33755 2:N:0:TCTGCAAG+GCTGCACT
CTAAGATTCAACTACAATCTCATAATGCAGTTGCGGATAAAGCGAGCATGGTGGACTATCCGCATCTG
GACGACATTACAACCAGTCCCAGAATTACTGCAGAACCCGTCATGGAAGACGATGATTTCTGGGACTT
TATCCCGACGGCTTT
+
FFFFFFFFFFFFFFFFF:FFF:FFFFFFFF:FFFFFFFFFFFFFFFF:F:FFFFFFFFFFFFFFFFFF
FFFFFFFFFFFFFFFFFFFFFFFF:FFFFFFFFFFFFFFFFFFFFFFFFFFFFFF:FF:FFFFFFFFFF
FF:FFFFFFFFFFFF
@A00721:190:HGJW7DSXY:4:1340:21061:33927 2:N:0:TCTGCAAG+GCTGCACT
CTAAGATTCAACTACAATCTCATAATGCAGTTGCGGATAAAGCGAGCATGGTGGACTATCCGCATCTG
GACGACATTACAACCAGTCCCAGAATTACTGCAGAACCCGTCATGGAAGACGATGATTTCTGGGACTT
TATCCCGACGGCTTT
+
FFFFFFFFFFFFFFFFFFFFFFFFFFFFFFFFFFFFFFFFFFFFFFFFFFFFFFFFFFFFFFFFFFFF
FFFFFFFFFFFFFFFFFFFFFFFFFFFFFFFFFFFFFFFFFFFFFFFFFFFFFFFFFFFFFFFFFFFF
FFFFF:FFF:FFFFF
@A00721:190:HGJW7DSXY:4:1347:11071:15624 2:N:0:TCTGCAAG+GCTGCACT
CTAAGATTCAACTACAATCTCATAATGCAGTTGCGGATAAAGCGAGCATGGTGGACTATCCGCATCTG
GACGACATTACAACCAGTCCCAGAATTACTGCAGAACCCGTCATGGAAGACGATGATTTCTGGGACTT
TATCCCGACGGCTTT
+
FFFFFFFFFFFFFFFFFFFFFFFFFFFFFFFFFFFFFFFFFFFFFFFFFFFFFFFFFFFFFFFFFFFF
FFFFFFFFFFFFFFFFFFFFFFFFFFFFFFFFFFFFFFFFFFFFFFFFFFFFFFFFFFFFFFFFFFFF
FFFFFFFFFFFFFFF
@A00721:190:HGJW7DSXY:4:1431:5141:7607 2:N:0:TCTGCAAG+GCTGCACT
CTAAGATTCAACTACAATCTCAGAATGCAGTTGCGGATAAAGCGAGCATGGTGGACTATCCGCATCTG
GACGACAGTACAACCAGTCCCAGAATTACTGCAGAACCCGTCATGGAAGACGATGATTTCTGGGACTT
TATCCCGACGGCTTT
+
FFFFFFFFFFFFFFFFF,FFFFFFFFFFFFFFFFFFFFFFFFFFFFFFFFFFFFF:FFF:FFFF,,FFF
F:FFFFF,FFFFFFF:FFFFFFFFFFFFFFFFFFFFFFFFFFFFFFFF:FFFFF:FF::FFFFFFFF:F
FFFFFFFFFFFFFFF
@A00721:190:HGJW7DSXY:4:1465:19316:22169 2:N:0:TCTGCAAG+GCTGCACT
CTAAGATTCAACTACAATCTCAGAATGCAGTTGCGGATAAAGCGAGCATGGTGGACTATCCGCATCTG
GACGACATTACAACCAGTCCCAGAATTACTGCAGAACCCGTCATGGAAGACGATGATTTCTGGGACTT
TATCTCGACGGCTTT
```

```
+
FFFFFFFFFFFFFFFFFFFFFFFFFFFFFFFFFFFFFFFFFFFFFFFFFFFFFFFFFFFFFFFFFFFFFF
FFFFFFFFFFFFFFFFFFFFFF:FFFFFFFFFFFFFFFFFFFFFFFFFFFFFFFFFFFFFFFFFFFFFFF
FFFFFFFFFFFFF:F
@A00721:190:HGJW7DSXY:4:1508:16288:34992 2:N:0:TCTGCAAG+GCTGCACT
CTAAGATTCAACTACAATCTCAGAATGCAGTTGCGGATAAAGCGAGCATGGTGGACTATCCGCATCTG
GACGACATTACAACCAGTCCCAGAATTACTGCAGAACCCGTCATGGAAGACGATGATTTCTGGGACTT
TATCCCGACGGCTTT
+
FFFFFFFFFFFFFFFFFFFFFFFFFFFFFFFFFFFFFFFFFFFFFFFFFFFFFFFFFFFFFFFFFFFFFF
FFFFFFFFFFFFFFFFFFFFFFFFFFFFFFFF:FFFFFFFFFFFFFFFFFFFFFFFFFFFFFF:FFFFFF
FFFFFFFFFFFFFF:
@A00721:190:HGJW7DSXY:4:1552:8712:15984 2:N:0:TCTGCAAG+GCTGCACT
CTAAGATTCAACTACAATCTCAGAATGCAGTTGCGGATAAAGCGAGCATGGTGGACTATCCGCATCTG
GACGACATTACAACCAGTCCCAGAATTACTGCAGAACCCGTCATGGAAGACGATGATTTCTGGGACTT
TATCCCGACGGCTTT
+
FFFFFFFFFFFFFFFFFFF:FFFFFFFFFFFFFFFFFFFFFFFF:FFFFFFFFFFFFFFFFFFFFFFFFF
FFFFFFFFFFFFFFFFF:FFFFFFFFFFFFFFFFFFFFFFFFFFFFFF,FFFFFFFFFFFFFFFFFFFFF
FFFFFFFFFFFFFFF
@A00721:190:HGJW7DSXY:4:1568:27055:21355 2:N:0:TCTGCAAG+GCTGCACT
CTAAGATTCAACTACAATCTCAGAATGCAGTTGCGGATAAAGCGAGCATGGTGGACTATCCGCATCTG
GACGACATTACAACCAGTCCCAGAATTACTGCAGAACCCGTCATGGAAGACGATGATTTCTGGGACTT
TATCCCGACGGCTTT
+
FFFFFFFFFFFFFFFFFFFFFFFFFFFFFFFFFFFFFFFFFFFFFFFFFFFFFFFFFFFFFFFFFFFFFF
FFFFFFFFFFFFFFFFFFFFFFFFFFFFFFFFFFFFFFFFFFFFFFFFFFFFFFFFFFFFFFFFFFFFFF
FFFF:FFFFFFFFFF
@A00721:190:HGJW7DSXY:4:1602:10511:19507 2:N:0:TCTGCAAG+GCTGCACT
CTAAGATTCAACTACAATCTCAGAATGCAGTTGCGGATAAAGCGAGCATGGTGGACTATCCGCATCTG
GACGACATTACAACCAGTCCCAGAATTACTGCAGAACCCGTCATGGAAGACGATGATTTCTGGGACTT
TATCCCGACGGCTTT
+
FFFFFFFFFFFFFFFFF:FFFFFFF:FFFFFFFFFFFFFFFFFFFFFFFFFFFFFFFFFFFFFFFFFFFF
FFFFFFFFFFFFFFFFFFFFFFFFFFFFFFFFFFFFFFFFFFFFFFFFFFFFFFFFFFFFFFFFFFFFFF
FFFFFFFFFFFFFF:
@A00721:190:HGJW7DSXY:4:1602:10438:19852 2:N:0:TCTGCAAG+GCTGCACT
CTAAGATTCAACTACAATCTCAGAATGCAGTTGCGGATAAAGCGAGCATGGTGGACTATCCGCATCTG
GACGACATTACAACCAGTCCCAGAATTACTGCAGAACCCGTCATGGAAGACGATGATTTCTGGGACTT
TATCCCGACGGCTTT
+
FFFFFF:,FFFFFFFFFFFFFFFFFFFFFFFFFFFFFFFFFFFFFFFFFFFFFFFF:FFFFFF:FF,FFF
FFFFFFFFFFFFFFFFFFFFFFFFFFFFFFFFFFFFFFFFFFFFFFFFF:FFFFFFFF,,FFFF:FFFF:
FFF:FF,FFFFFFFF
@A00721:190:HGJW7DSXY:4:1602:12834:21308 2:N:0:TCTGCAAG+GCTGCACT
CTAAGATTCAACTACAATCTCAGAATGCAGTTGCGGATAAAGCGAGCATGGTGGACTATCCGCATCTG
GACGACATTACAACCAGTCCCAGAATTACTGCAGAACCCGTCATGGAAGACGATGATTTCTGGGACTT
TATCCCGACGGCTTT
+
FFFFFFFFFF:FFFFFFFFFFFFFFFFFFFFFFFFFFFFFFFFFFFFFFFFFFFFFFFF:FFFFFFFFFF
FFFFFFFFFFFFFFFFFFFFF:FFFFFFFFFFFFFFFFFFFFFFFFFFFFFFFFFFFFFFFFFFFFFFFF
FFFFFFFFFFFFFFF
@A00721:190:HGJW7DSXY:4:1620:17824:21966 2:N:0:TCTGCAAG+GCTGCACT
CTAAGATTCAACTACAATCTCAGAATGCAGTTGCGGATAAAGCGAGCATGGTGGACTATCCGCATCTG
```

```
GACGACATTACAACCAGTCCCAGAATTACTGCAGAACCCGTCATGGAAGACGATGATTTCTGGGACTT
TATCCCGACGGCTTT
+
FFFFFFFFFFFFFFFFFFFFFFFFFFFFFFFFFFFFFFFFFFFFFFFFFFFFFFFFFFFFFFFFFFFFFF
FFFFFFFFFFFFFFFFFFFFFFFFFFFFFFFFFFFFFFFFFFFFFFFFFFFFFFFFFF,FFFFFFFFFFF
FFFF:FFFFFFFFFF
@A00721:190:HGJW7DSXY:4:1630:28122:21793 2:N:0:TCTGCAAG+GCTGCACT
CTAAGATTCAACTACAATCTCAGAATGCAGTTGCGGATAAAGCGAGCATGGTGGACTATCCGCATCTG
GACGACATTACAACCAGTCCCAGAATTACTGCAGAACCCGTCATGGAAGACGATGATTTCTGGGACTT
TATCCCGACGGCTTT
+
FFFFFFFFFFFFFFFFFFFFFFFFF,FFFFFFFFFFFFFFFFFFFFFFFFFFFFFFFFFFFFFFFFFFFF
FFFFFFFFFFFFFFFFFFFFFFFFFFFFFFFFFFFFFFFFFFFFFFFFFFFFFFFFFFF,FFFFFFFFFF
FFFFFFFFFFFFFFF
@A00721:190:HGJW7DSXY:4:1634:28474:31548 2:N:0:TCTGCAAG+GCTGCACT
CTAAGATTCAACTACAATCTCAGAATGCAGTTGCGGATAAAGCGAGCATGGTGGACTATCCGCATCTG
GACGACATTACAACCAGTCCCAGAATTACTGCAGAACCCGTCATGGAAGACGATGATTTCTGGGACTT
TATCCCGACGGCTTT
+
FFFFFFFFFFFFFFFFFFFFFF:FFFFFFFFFFFFFFFFFFFFFFFFFFFFFFFFFFFFFFFFFFFFFFF
FFFFFFFFFFFFFFFFF:FFFFFFFFFFFFFFFFFFFFFFFFFFFFFFFFFFFFFFFFFFFFFFFFFFFF
FFFFFFFFFFFFFFF
@A00721:190:HGJW7DSXY:4:1649:15718:16658 2:N:0:TCTGCAAG+GCTGCACT
CTAAGATTCAACTACAATCTCAGAATGCAGTTGCGGATAAAGCGAGCATGGTGGACTATCCGCATCTG
GACGACATTACAACCAGTCCCAGAATTACTGCAGAACCCGTCATGGAAGACGATGATTTCTGGGACTT
TATCCCGACGGCTTT
+
FFFFFFFFFFFFFFFFFFFFFFFFFFFFFFFFFFFFFFFFFFFFFFFFFFFFFFFFFFFFFFFFFFFFFF
FFFFFFFFFFFFFFFFFFFFFFFFFFFFFFFFFFFFFFFFFFFFFFFFFFFFFFFFFFFFFFFFFFFFFF
FFFFFFFFFFFFFFF
@A00721:190:HGJW7DSXY:4:1659:31530:24815 2:N:0:TCTGCAAG+GCTGCACT
CTAAGATTCAACTACAATCTCAGAATGCAGTTGCGGATAAAGCGAGCATGGTGGACTATCCGCATCTG
GACGACATTACAACCAGTCCCAGAATTACTGCAGAACCCGTCATGGAAGACGATGATTTCTGGGACTT
TATCTCGACGGCTTT
+
FFFFF:FFFFFFFFFFFFFFFFFFFFFFFFFFFFFFFFFFFFFFFFFFFFFFFFFFFFFFFFFFFFFFFF
FFFFFFFFFFFFFFFFFFFFFFFFFFFFFFFFFFFFFFFFFFFFFFFFFFFFFFFFFFFFFFFFFFFFFF
FFFFFF,FFFFFFFF
@A00721:190:HGJW7DSXY:4:1661:26160:6778 2:N:0:TCTGCAAG+GCTGCACT
CTAAGATTCAACTACAATCTCAGAATGCAGTTGCGGATAAAGCGAGCATGGTGGACTATCCGCATCTG
GACGACATTACAACCAGTCCCAGAATTACTGCAGAACCCGTCATGGAAGACGATGATTTCTGGGACTT
TATCCCGACGGCTTT
+
FFFFFFFFFFFFFFFFFFFFFFFFFFFFFFFFFFFFFFFFFFFFFFFFFFFFFFFFFFFFFFFFFFFFFF
FFFFFFFFFFFFFFFFFFFFF:FFFFFFFFFFFFFFFFFFFFFFFFFFFFFFFFFFFFFFFFFFFFFFFF
FFFFFFFFFFFFFFF
@A00721:190:HGJW7DSXY:4:1662:21124:13620 2:N:0:TCTGCAAG+GCTGCACT
CTAAGATTCAACTACAATCTCAGAATGCAGTTGCGGATAAAGCGAGCATGGTGGACTATCCGCATCTG
GACGACATTACAACCAGTCCCAGAATTACTGCAGAACCCGTCATGGAAGACGATGATTTCTGGGACTT
TATCTCGACGGCTTT
+
FFFFFFFFFFFFFFFFFFFFFFFFFFFFFFFFFFFFFFFFFFFFFFFFFFFFFFFFFFFFFFFFFFFFFF
F:FFFFFFFFFFFFFFFFFFFFFFFFFFFFFFFFFFFFFFFFFFFFFFFFFFFFFFFFFFFFFFFFFFFF
FFFFFF:FFFFFFFF
```

```
@A00721:190:HGJW7DSXY:4:1664:2284:35383 2:N:0:TCTGCAAG+GCTGCACT
CTAAGATTCAACTACAATCTCATAATGCAGTTGCGGATAAAGCGAGCATGGTGGACTATCCGCATCTG
GACGACATTACAACCAGTCCCAGAATTACTGCAGAACCCGTCATGGAAGACGATGATTTCTGGGACTT
TATCCCGACGGCTTT
+
FFFFFFFFFFFFFFFFFF:FFFFFFFFFFFFFFFFFFFFFFFFFFFFFFFFFFFFFFFFFFFFFFFFFFF
FFFFFFFFFFFFFFFFFFFFFFFFFF:FFFFFFFFFFFFFFFFF:FFFFFFFFFFFFFFFFFFFFFFFFF
FFFFFFF:FFFFFFF
@A00721:190:HGJW7DSXY:4:1670:8332:12915 2:N:0:TCTGCAAG+GCTGCACT
CTAAGATTCAACTACAATCTCAGAATGCAGTTGCGGATAAAGCGAGCATGGTGGACTATCCGCATCTG
GACGACATTACAACCAGTCCCAGAATTACTGCAGAACCCGTCATGGAAGACGATGATTTCTGGGACTT
TATCCCGACGGCTTT
+
FFFFFFFFFFFFFFFFFFFFFFFFFFFFFFFFFFFFFFFFFFFFFFFF,FFFFFFFFFFFFFFFFFFFFF
FFFFFFFFFFFFFFFFFFFFFFFFFFFFFFFFFF:FFFFFFFFFFFFFFFFFFFFFFFFFFFFFFFFFFF
FFFFFFFFFFFFFFF
@A00721:190:HGJW7DSXY:4:2122:19831:4304 2:N:0:TCTGCAAG+GCTGCACT
CTAAGATTCAACTACAATCTCAGAATGCAGTTGCGGATAAAGCGAGCATGGTGGACTATCCGCATCTG
GACGACATTACAACCAGTCCCAGAATTACTGCAGAACCCGTCATGGAAGACGATGATTTCTGGGACTT
TATCCCGACGGCTTT
+
FFFFFFFFFFFFFFFFFFFFFFFFFFFFFFFFFFFFFFFFFFFFFFFFFFFFFFFFFFFFFFFFFFFFFF
FFFFFFFFFFFFFFFFFFFFFFFFFFFFFFFFFFFFFFFFFFFFFFFFFFFFFFFFFFFFFFFFFFFFFF
FFFFFFFFFFFFFFF
@A00721:190:HGJW7DSXY:4:2157:4056:11021 2:N:0:TCTGCAAG+GCTGCACT
CTAAGATTCAACTACAATCTCATAATGCAGTTGCGGATAAAGCGAGCATGGTGGACTATCCGCATCTG
GACGACATTACAACCAGTCCCAGAATTACTGCAGAACCCGTCATGGAAGACGATGATTTCTGGGACTT
TATCCCGACGGCTTT
+
FFFFFFFFFFFFFFFFFFFFFFFFFFFFFFFFFFFFFF,FFFFFFFFFFFFFFFFFFFFFFFFFFFFFFF
FFFFFFFFFFFFFFFFFFFFFFFFF,FFFFFFFFFFFFFFFFFFFF:F:FFFFFFFFFFFFFFFFFFFFF
FFFFF:FFFFFFFFF
@A00721:190:HGJW7DSXY:4:2167:26521:21715 2:N:0:TCTGCAAG+GCTGCACT
CTAAGATTCAACTACAATCTCAGAATGCAGTTGCGGATAAAGCGAGCATGGTGGACTATCCGCATCTG
GACGACATTACAACCAGTCCCAGAATTACTGCAGAACCCGTCATGGAAGACGATGATTTCTGGGACTT
TATCCCGACGGCTTT
+
FFFFFFFFFFFFFFFFFFFFFFFFFFFFFFFFFFFFFFFFFFFFFFFFFFFFFFFFFFFFFFFFFFFFFF
FFFFFFFFFFFFFFFFFFFFFFFFFFFFFFFFFFFFFFFFFFFFFFFFFFFFFFF:FFFFFFFFFFFFFF
FFFFFFFFFFFFFFF
@A00721:190:HGJW7DSXY:4:2207:25319:4601 2:N:0:TCTGCAAG+GCTGCACT
CTAAGATTCAACTACAATCTCAGAATGCAGTTGCGGATAAAGCGAGCATGGTGGACTATCCGCATCTG
GACGACATTACAACCAGTCCCAGAATTACTGCAGAACCCGTCATGGAAGACGATGATTTCTGGGACTT
TATCTCGACGGCTTT
+
FFFFFFFFFFFFFFFFFFFFFFFFFFFFFFF:FFFFFFFFFFFFFFFFFFFFFFFFFFFFFFFFFFFFFF
FFFFFFFFFFFFFFFFFFFFFFFFF:FFFFFFFFFFFFFFFFFFFFFFFFFFFFFFFFF:FFFFFFFFFF
FFFFFFFFFFFFFFF
@A00721:190:HGJW7DSXY:4:2217:3305:18145 2:N:0:TCTGCAAG+GCTGCACT
CTAAGATTCAACTACAATCTCATAATGCAGTTGCGGATAAAGCGAGCATGGTGGACTATCCGCATCTG
GACGACATTACAACCAGTCCCAGAATTACTGCAGAACCCGTCATGGAAGACGATGATTTCTGGGACTT
TATCCCGACGGCTTT
+
FFFFFFFFFFFFFFFFFFFFFFFFFFFFFFFFFFFFFFFFFFFFFFFFFFFFFFFFFFFF:FFFFFFFF:
```

```
FFFFFF:FFFFFFFFFFFFFFFFFFFFFFFFFFFFFFFF:FFFFFFF:FFFFFFFFFFFFFFFFFF:F
FFFFFFFFFFFFFFF
@A00721:190:HGJW7DSXY:4:2251:1814:36699 2:N:0:TCTGCAAG+GCTGCACT
CTAAGATTCAACTACAATCTCAGAATGCAGTTGCGGATAAAGCGAGCATGGTGGACTATCCGCATCTG
GACGACATTACAACCAGTCCCAGAATTACTGCAGAACCCGTCATGGAAGACGATGATTTCTGGGACTT
TATCTCGACGGCTTT
+
FFFFFFFFFFFFFFFFFFFFFFFFFFFFFFFFFFFFFFFFFFFFFFFFFFFFFFFFFFFFFFFFFFFF
FFFFFFFFFFFFFFF:FFFFFFFFFFFFFFFFFFFFFFFFFFFFFF:FFFFFFFFFFFFFFFFFFFFF
FFFFF:FFF:FFFF:
@A00721:190:HGJW7DSXY:4:2310:31213:17378 2:N:0:TCTGCAAG+GCTGCACT
CTAAGATTCAACTACAATCTCATAATGCAGTTGCGGATAAAGCGAGCATGGTGGACTATCCGCATCTG
GACGACATTACAACCAGTCCCAGAATTACTGCAGAACCCGTCATGGAAGACGATGATTTCTGGGACTT
TATCCCGACGGCTTT
+
FFFFFFF:FF:FFFFFFFFFFFFFFF:FF,FFFFF:FF,FFFFFFFFF,:FFFFFFFFFFFFFFFFF:
FFFFFFFF:FFFFF:FFFF,FFFFFF,F:F:FFFFFFFFFFFFFFFFFFFFFFFFFF,FFFFF:FFF:
FFFFFFFFF:FFFFF
@A00721:190:HGJW7DSXY:4:2317:29884:17738 2:N:0:TCTGCAAG+GCTGCACT
CTAAGATTCAACTACAATCTCAGAATGCAGTTGCGGATAAAGCGAGCATGGTGGACTATCCGCATCTG
GACGACATTACAACCAGTCCCAGAATTACTGCAGAACCCGTCATGGAAGACGATGATTTCTGGGACTT
TATCCCGACGGCTTT
+
F,:FFFFFFFFFFFFFFFFFFFFFFFFFFFFFFFFFF:FFF:FFFFFFFFFFFFFFFFFFFFFF:FFF
FFFFFFFFFFFFFFFFFFFFFFFFFFFFFFFFFFF,FFFFFFFFFFFFFFFFFFFFF:FFFFFFFFFF
FFFFFFFFFFFFFFF
@A00721:190:HGJW7DSXY:4:2320:28492:21684 2:N:0:TCTGCAAG+GCTGCACT
CTAAGATTCAACTACAATCTCAGAATGCAGTTGCGGATAAAGCGAGCATGGTGGACTATCCGCATCTG
GACGACATTACAACCAGTCCCAGAATTACTGCAGAACCCGTCATGGAAGACGATGATTTCTGGGACTT
TATCCCGACGGCTTT
+
FFFFF:FFFFFFFFF::FFFFFFFFFFFFFFFFFFFFFFFFFFFFFFFFFFFFFFFFFFFFFFFFFFF
FFFFFFFFFFFFFFFFFFFFFFFFFFFFFFFFFFFFFFFFFFFFFFF:FFFFFFFFFFFFFFFFFFFF
FFFFFFFFFFFF:FF
@A00721:190:HGJW7DSXY:4:2326:9833:31986 2:N:0:TCTGCAAG+GCTGCACT
CTAAGATTCAACTACAATCTCATAATGCAGTTGCGGATAAAGCGAGCATGGTGGACTATCCGCATCTG
GACGACATTACAACCAGTCCCAGAATTACTGCAGAACCCGTCATGGAAGACGATGATTTCTGGGACTT
TATCCCGACGGCTTT
+
FFFFFFFFFFFFFFFFF:FFFFFFFFFFFFFFFFFFFFFFFFFFFFFFFFFFFFFFFFFFFFFFFFFF
FFFFFFFFFFFFFFFFFFFFFFFFFFFFFFFFFFFFFFFFFFFFFFFFFFFFFFFFFFFFFFFFFFFF
FFFFFFFFFFFFFFF
@A00721:190:HGJW7DSXY:4:2359:18837:19492 2:N:0:TCTGCAAG+GCTGCACT
CTAAGATTCAACTACAATCTCAGAATGCAGTTGCGGATAAAGCGAGCATGGTGGACTATCCGCATCTG
GACGACATTACAACCAGTCCCAGAATTACTGCAGAACCCGTCATGGAAGACGATGATTTCTGGGACTT
TATCCCGACGGCTTT
+
FFFFFFFFFFFFFFFFFFFFFFFFFFFFFFFFFFFFFFFFFFFFFFFFFFFFFFFFFFFFFFFFFFFF
FFFFFFFFFFFFFFFFFFFFFFFFFFFFFFFFFFFFFFFFFFFFFFFFFFFFFFFFFFFFFFFFFFFF
FFFFFFFFFFFFFF:
@A00721:190:HGJW7DSXY:4:2362:5348:26068 2:N:0:TCTGCAAG+GCTGCACT
CTAAGATTCAACTACAATCTCAGAATGCAGTTGCGGATAAAGCGAGCATGGTGGACTATCCGCATCTG
GACGACATTACAACCAGTCCCAGAATTACTGCAGAACCCGTCATGGAAGACGATGATTTCTGGGACTT
TATCCCGACGGCTTT
```

```
+
FFFFFFFFFFFFFFFFFFFFFFFFFFFFFFFFFFFFFFFFFFFFFFFFF:FFFFFFFFFFFFFFFFFFF
FFFFFFFFFFFFFFFFFFFFFFFFF:FFFFFFFFFFFFFFFFFFFFFFFFFFFFFFFFFFFFFFFFFFF
FFFFFFFFFFFFFFF
@A00721:190:HGJW7DSXY:4:2406:30581:5854 2:N:0:TCTGCAAG+GCTGCACT
CTAAGATTCAACTACAATCTCAGAATGCAGTTGCGGATAAAGCGAGCATGGTGGACTATCCGCATCTG
GACGACATTACAACCAGTCCCAGAATTACTGCAGAACCCGTCATGGAAGACGATGATTTCTGGGACTT
TATCTCGACGGCTTT
+
FFFFFFFFFFFFFFFFFFFFFFFFFFFFFFFFFFFFFFFFFFFFFFFFFFFFFFFFFFFFFFFFFFFFF
FFFFFFFFFFFFFFFFFFFFF:FFFFFFFFFFFFFFFFFFFFFFFFFFFFFFFFFFFFFFFFFFFFFFF
FFFFFFFFFFFFFFF
@A00721:190:HGJW7DSXY:4:2453:30897:9940 2:N:0:TCTGCAAG+GCTGCACT
CTAAGATTCAACTACAATCTCAGAATGCAGTTGCGGATAAAGCGAGCATGGTGGACTATCCGCATCTG
GACGACATTACAACCAGTCCCAGAATTACTGCAGAACCCGTCATGGAAGACGATGATTTCTGGGACTT
TATCCCGACGGCTTT
+
FFFFFFFFFFFFFFFFFFFFFFFFFFFFFFFFFFFFFFFFFFFFFFFFFFFFFFFFFFFFFFFFFFFFF
FFFFFFFFFFFFFFFFFFFFFFFFFFFFFFFFFFFFFFFFFFFFFFFFFFFFF::FFFFF,FF:FF
FFFF:FFFFFFFFFF
@A00721:190:HGJW7DSXY:4:2477:10728:31093 2:N:0:TCTGCAAG+GCTGCACT
CTAAGATTCAACTACAATCTCAGAATGCAGTTGCGGATAAAGCGAGCATGGTGGACTATCCGCATCTG
GACGACATTACAACCAGTCCCAGAATTACTGCAGAACCCGTCATGGAAGACGATGATTTCTGGGACTT
TATCCCGACGGCTTT
+
FFFFFFFFFFFFFFFFFFFFFFFFFF:FFFFFFFFFFFFFFFFFFFFFFFFFFFFFFFFFFFFFFFFFF
FFFFFFFFFFFFFFFFFFFFFF:FFFFFFFFFFFFFFFFFFFFFFFFFFFFFFFFF:FFFFFFFFF:FFFF
FFFFF:F,F:FFFFF
@A00721:190:HGJW7DSXY:4:2505:14823:21746 2:N:0:TCTGCAAG+GCTGCACT
CTAAGATTCAACTACAATCTCAGAATGCAGTTGCGGATAAAGCGAGCATGGTGGACTATCCGCATCTG
GACGACATTACAACCAGTCCCAGAATTACTGCAGAACCCGTCATGGAAGACGATGATTTCTGGGACTT
TATCTCGACGGCTTT
+
FFFFFFFFFFFFFFFFFFFFFFFFF:FFFFFFFFF,FFFFFFFFFFFF:FFFFFFFFFFFF:FFFFFF
FFFFFFFFFFFFFF:FF:F:FFFF:FFFFFFFFFFFFFFFFFFFFFFFFFFF:FFFFF,FFFF:FFFFF
FFFF:FFFFFFFFFF
@A00721:190:HGJW7DSXY:4:2540:9408:23516 2:N:0:TCTGCAAG+GCTGCACT
CTAAGATTCAACTACAATCTCAGAATGCAGTTGCGGATAAAGCGAGCATGGTGGACTATCCGCATCTG
GACGACATTACAACCAGTCCCAGAATTACTGCAGAACCCGTCATGGAAGACGATGATTTCTGGGACTT
TATCCCGACGGCTTT
+
FFFFF:FFFFFFFFFFFFFFFFFFFFFFFFFFFFFFFFFFFFFFFFFFFFFFFFFFFFFFFFFFFFFF
FFFFFFFFFFFFFFFFF:FFFFFFFFFFF:FFFFFFFF:FFFFFFFFFFFFFFFFFFFFFFFFFFFFFF
FFFFF:FFFFFFFF:
@A00721:190:HGJW7DSXY:4:2563:10095:34413 2:N:0:TCTGCAAG+GCTGCACT
CTAAGATTCAACTACAATCTCAGAATGCAGTTGCGGATAAAGCGAGCATGGTGGACTATCCGCATCTG
GACGACATTACAACCAGTCCCAGAATTACTGCAGAACCCGTCATGGAAGACGATGATTTCTGGGACTT
TATCCCGACGGCTTT
+
FFFFFFFFFFFFFFFFFFFF:FFFFFFFFFFFFFFFFFFFFFFFFFFFFFFFFFFFFFFFFFFFFFFFFF
FFFFFFFFFFFFFFFFFFFFFFFFFFFFFFFFFFFFFFFFFFFFFFFFFFFFFFFFFFFFFFF:F:FFF
FFFFFFFFF:FFFFF
@A00721:190:HGJW7DSXY:4:2601:9227:1783 2:N:0:TCTGCAAG+GCTGCACT
CTAAGATTCAACTACAATCTCAGAATGCAGTTGCGGATAAAGCGAGCATGGTGGACTATCCGCATCTG
```

GACGACATTACAACCAGTCCCAGAATTACTGCAGAACCCGTCATGGAAGACGATGATTTCTGGGACTT
TATCCCGACGGCTTT
+
FFFFFFFFFFFFFFFFFFFFFFFFFFFFFFFFFFFFFFFFFFFFFFFFFFFFFFFFFFFFFFFFFFFFFF
FFFFFFFFFFFFFFFFFFFFFFFFFFFFFFFFFFFFFFFFFFFFFFFFFFFFFFFFFF:FFFFFFFFFFF
FFFFFFFFFFFFFFF
@A00721:190:HGJW7DSXY:4:2621:26341:16924 2:N:0:TCTGCAAG+GCTGCACT
CTAAGATTCAACTACAATCTCAGAATGCAGTTGCGGATAAAGCGAGCATGGTGGACTATCCGCATCTG
GACGACATTACAACCAGTCCCAGAATTACTGCAGAACCCGTCATGGAAGACGATGATTTCTGGGACTT
TATCCCGACGGCTTT
+
::FFFFFFFFFFFFFFFFFFFFFFFFFFFFFFFFFFFFFFFFFFFFFFFFFFFFFFFFFFFFFFF:FFFF
FFFFFFFFFFFFFFFFFFFFFF:FFFFFFFFFFFF:FFFFFFF:FFFFFFFFFFFFFFFFFFFFFFFFFF
FFFFFFFFFFFFFFF
@A00721:190:HGJW7DSXY:4:2623:7337:19241 2:N:0:TCTGCAAG+GCTGCACT
CTAAGATTCAACTACAATCTCAGAATGCAGTTGCGGATAAAGCGAGCATGGTGGACTATCCGCATCTG
GACGACATTACAACCAGTCCCAGAATTACTGCAGAACCCGTCATGGAAGACGATGATTTCTGGGACTT
TATCTCGACGGCTTT
+
FFFFFFFFFFFFFFFFFFFFFFFFFFFFFFFFFFFFFFFFFFFFFFFFFFFFFFFFFFFFFFFFFFFFFF
FFFFFFFFFFFFFFFFFFFFFFFFFFFFFFFFFFFFFFFFFFFFFFFFFFFFFF,FFFFFFFFFFFFFFF
FFFFFFFFFFFFFFF
@A00721:190:HGJW7DSXY:4:2624:15609:4069 2:N:0:TCTGCAAG+GCTGCACT
CTAAGATTCAACTACAATCTCAGAATGCAGTTGCGGATAAAGCGAGCATGGTGGACTATCCGCATCTG
GACGACATTACAACCAGTCCCAGAATTACTGCAGAACCCGTCATGGAAGACGATGATTTCTGGGACTT
TATCCCGACGGCTTT
+
FFFFFF,FFFFFFFFFFFFFFFFFFFFFFFFFFFFFFFFFFFFFFFFFFFFFFFFFFFFFFFF:FFFFFF
FFFFFFFFFFFFFFFFFFFFFFFFFFFFFFFFFFFF:F:F:FFF:FFFFFFFFFFFFFFFFFFFFFFFFF
FFFFFF:FFFFFFFF
@A00721:190:HGJW7DSXY:4:2635:15835:28855 2:N:0:TCTGCAAG+GCTGCACT
CTAAGATTCAACTACAATCTCATAATGCAGTTGCGGATAAAGCGAGCATGGTGGACTATCCGCATCTG
GACGACATTACAACCAGTCCCAGAATTACTGCAGAACCCGTCATGGAAGACGATGATTTCTGGGACTT
TATCCCGACGGCTTT
+
FFFFFFFFFFFFFFFFFFFFFFFFFFFFFF:FFFFFFFFFFFFFFFFFFFFFFFFFFFFFFFFFFFFFFF
FFFFFFFFFFFFFFFFFFFFFFFFFFF:FFFFFFFFFF:FFFFFFFFFFFFFFFFFFFFF:FFFFFFFF:
FFFF:FFFFFFFFFF
@A00721:190:HGJW7DSXY:4:2643:6795:30201 2:N:0:TCTGCAAG+GCTGCACT
CTAAGATTCAACTACAATCTCAGAATGCAGTTGCGGATAAAGCGAGCATGGTGGACTATCCGCATCTG
GACGACATTACAACCAGTCCCAGAATTACTGCAGAACCCGTCATGGAAGACGATGATTTCTGGGACTT
TATCTCGACGGCTTT
+
FFFFFFFFFFFFFFFFFFFFFFFFFFFFFFFFFFFFFFFFFFFFFFFFFFFFFFFFFFFFFFFFFFFFFF
FFFFFFFFFFFFFFF:FFFFFFFFFFFFFFFFFFFFFF:FFFFFFFFFFFFFFFFFFFFFFFFFFFFFFF
FFFFFFFFFFFFFFF
@A00721:190:HGJW7DSXY:4:2678:31503:30655 2:N:0:TCTGCAAG+GCTGCACT
CTAAGATTCAACTACAATCTCATAATGCAGTTGCGGATAAAGCGAGCATGGTGGACTATCCGCATCTG
GACGACATTACAACCAGTCCCAGAATTACTGCAGAACCCGTCATGGAAGACGATGATTTCTGGGACTT
TATCCCGACGGCTTT
+
FFFFF,FFFFFFFFFFFFFFFFFFFFFFFFFFFFFFFFFFFFFFFFFFFFFFF:FFFFFFFFFFFFFFFF
FFFFFFFFFFFFFFFFFFFFFFFFF:FFFFFFFFFFFFFFFFFFFFFF:FFFFFFFFFFFFFFFFFFFFF
FFFFFFFFFFFFFFF

@A00721:190:HGJW7DSXY:4:1413:6180:6778 2:N:0:TCTGCAAG+GCTGCACT
ATTCTTATAAGACGTTCCTGGACAAGGATGCCAATTCATCTTTAAAGATTTGGACAGACTATCCGCAT
CTGGACGACATTACAACCAGTCCCAGAATTACTGCAGAACCCGTCATGGAAGACGATGATTTCTGGGA
CTTTATCCCGACGGC
+
FFFFFFFFFFFFFFFFFFFFFFFFFFFFFFFFFFFFFFFFFFFFFFFFFFFFFFFFFFFFFFFFFFFF
FFFFFFFFFFFFFFFFFFFFFFFFFFFF:FFFFFFFFFFFFFFFFFFFFF:FFFFFFFFFFFFFFFFF
FFFFFFFFFFFF:FF
@A00721:190:HGJW7DSXY:4:2515:20853:5822 2:N:0:TCTGCAAG+GCTGCACT
ATTCTTATAAGACGTTCCTGGACAAGGATGCCAATTCATCTTTAAAGATTTGGACAGACTATCCGCAT
CTGGACGACATTACAACCAGTCCCAGAATTACTGCAGAACCCGTCATGGAAGACGATGATTTCTGGGA
CTTTATCCCGACGGC
+
FFFFFFFFFFFFFFFFFFFFFFFFFFFFFFFFFFFFFFFFFFFFFFFFFFFFFF:FFFFFFFFFFFFF
FFFFFFFFFFFFFFFFFFFFFFFFFFFFFFFFFFFFFFFFFFFFFFFFFFFFFFFFFFFFFFFFFFFF
FFFFFFFF:FFFFFF
@A00721:190:HGJW7DSXY:4:2648:18059:31078 2:N:0:TCTGCAAG+GCTGCACT
ATTCTTATAAGACGTTCCTGGACAAGGATGCCAATTCATCTTTAAAGATTTGGACAGACTATCCGCAT
CTGGACGACATTACAACCAGTCCCAGAATTACTGCAGAACCCGTCATGGAAGACGATGATTTCTGGGA
CTTTATCCCGACGGC
+
FFFFFFFFFFFFFFFFFFFFFFFFFFFFFFFFFFFFFFFFFFFFFFFFFFFFFFFFFFFFFFFFFFFF
FFFFFFFFFFFFFFFFFF,FFFFFFFFFFFFFFFFFFFFFFFFFFFFFFFFFFFFFFFFFFFFFFFFF
FFFFFFFFFFFFFFF
@A00721:190:HGJW7DSXY:4:1261:27308:18035 2:N:0:TCTGCAAG+GCTGCACT
GAAGAATATTCTTATAAGACGTTCCTGGACAAGGATGCCAATTCATCTTTAAAGATTTGGACAGACTA
TCCGCATCTGGACGACATTACAACCAGTCCCAGAATTACTGCAGAACCCGTCATGGAAGACGATGATT
TCTGGGACTTTATCC
+
FFFFFFFFFFFFFFFFFFFFFFFFFFFFFFFFFFFFFFFFFFFFFFFFFFFFFFFFFFFFFFFFFFFF
FFFFFFFFFFFFFFFFFFFFFFFFFFFFFFFFFFFFFFFFFFFFFFFFFFFFFFFFFFFFFFFFFFFF
FFFFFFFFFFFFFFF
@A00721:190:HGJW7DSXY:4:1306:22833:14544 2:N:0:TCTGCAAG+GCTGCACT
GAAGAATATTCTTATAAGACGTTCCTGGACAAGGATGCCAATTCATCTTTAAAGATTTGGACAGACTA
TCCGCATCTGGACGACATTACAACCAGTCCCAGAATTACTGCAGAACCCGTCATGGAAGACGATGATT
TCTGGGACTTTATCC
+
FFFFFFFFFFFFFFFFFFFFFFFFFFFFFFFFFFFFFFFFFFFFFFFFFFFFFFFFFFFFFFFFFFFF
FFFFFFFFFF:FFFFFFFFFFFFFFFFFFFFFFFFFFFFFFFFFFFFFFFFFFFFFFFFFF:FFFFFF
FFFFFFFFFFFFFFF
@A00721:190:HGJW7DSXY:4:2262:2049:20290 2:N:0:TCTGCAAG+GCTGCACT
GAAGAATATTCTTATAAGACGTTCCTGGACAAGGATGCCAATTCATCTTTAAAGATTTGGACAGACTA
TCCGCATCTGGACGACATTACAACCAGTCCCAGAATTACTGCAGAACCCGTCATGGAAGACGATGATT
TCTGGGACTTTATCC
+
FFFFFFFFFFFFFFFFFFFFFFFFFFFFFFFFFFFFFFFFFFFFFFFFFFFFFFFFFFFFFFFFFFFF
:FFFFFFFFFFFFFFFFFFFFFFFF:FFFFFFFF:FFFFFFFFFFFFF:FFFFFFFFFFFFFF:FFFF
FFFFFFFFFFFFFFF
@A00721:190:HGJW7DSXY:4:2454:5882:5228 2:N:0:TCTGCAAG+GCTGCACT
GAAGAATATTCTTATAAGACGTTCCTGGACAAGGATGCCAATTCATCTTTAAAGATTTGGACAGACTA
TCCGCATCTGGACGACATTACAACCAGTCCCAGAATTACTGCAGAACCCGTCATGGAAGACGATGATT
TCTGGGACTTTATCC
+
FFFFFF:FFFFFFFFFFFFFFFFFFFFFFFFFFFFFFFFFFFFFFFFFFFFFFFFFFFFFFFFFFFFF

```
FFFFFFFFFFFFFFFFFFFFFFFFFFFFFFF,FFFFFFFFFFFFFFFFFFFFFFFFFFFFFFFFFF:F
FFFFFFFFFFFFF:F
@A00721:190:HGJW7DSXY:4:2478:3260:14998 2:N:0:TCTGCAAG+GCTGCACT
GAAGAATATTCTTATAAGACGTTCCTGGACAAGGATGCCAATTCATCTTTAAAGATTTGGACAGACTA
TCCGCATCTGGACGACATTACAACCAGTCCCAGAATTACTGCAGAACCCGTCATGGAAGACGATGATT
TCTGGGACTTTATCC
+
F:FFFFFFFFFFFFFFFFFFFFFFFFFFFFFFFFFFFFFFFFFFFFFFFFFFFFFFFFFFFFFFFFFF
FFFFFFFFFFFFFFFFFFFFFFFFFFFFFFF:FF,FFFFFFFFFFFFFF,FF:FFF:FFFFFFFFFF
FFFFFFFFFFFFFFF
@A00721:190:HGJW7DSXY:4:1642:22191:32972 2:N:0:TCTGCAAG+GCTGCACT
CACCGTTCCATCACATCTTCACGGTGGAACTAAGATTCAACTACAATCTCAGAATGCAGTTGCGGATA
AAGCGAGCATGGTGGACTATCCGCATCTGGACGACATTACAACCAGTCCCAGAATTACTGCAGAACCC
GTCATGGAAGACGAT
+
FFFFFFFFFFFFFFFFFFFFFFFFFFFFFFFFFFFFFFFFFFFFFFFFFFFFFFFFFFFFFFFFFFFF
FFFFFFFFFFFFFFFFFFFFFFFFFFFFFFFFFFFFFFFFFFFFFFFFFFFFFFFFFFFFFFFFFFFF
FFFFFFFFFFFFFFF
@A00721:190:HGJW7DSXY:4:2159:8992:21136 2:N:0:TCTGCAAG+GCTGCACT
CACCGTTCCATCACATCTTCACGGTGGAACTAAGATTCAACTACAATCTCAGAATGCAGTTGCGGATA
AAGCGAGCATGGTGGACTATCCGCATCTGGACGACATTACAACCAGTCCCAGAATTACTGCAGAACCC
GTCATGGAAGACGAT
+
FFFFFFFFFFFFFFFFFFFFFFFFFFFFFFFFFFFFFFFFFFFFFFFFFFF:F:FFFFFFFFFFFFFFF
FFFFFFFF:FFFFFFFFFFFFFFFFFFFFFFFFFFFFFFFFFFFFFFFFF:FFFFFFFFFFFFFFFFF
FFFFFFFFF:FFFFF
@A00721:190:HGJW7DSXY:4:2207:16369:3662 2:N:0:TCTGCAAG+GCTGCACT
CACCGTTCCATCACATCTTCACGGTGGAACTAAGATTCAACTACAATCTCAGAATGCAGTTGCGGATA
AAGCGAGCATGGTGGACTATCCGCATCTGGACGACATTACAACCAGTCCCAGAATTACTGCAGAACCC
GTCATGGAAGACGAT
+
FFFFFFFFFFFFFFFFFFFFFFFFFFFFFFFFFFFFFFFFFFFFFFFFFFFFFFFFFFFFFFFFFFFF
FFFFFFFFFFFFFFFFFFFFFFFFFFFFFFFFFFFFFFFFFFFFFFFFFFFFFFFFFFFFFFF,FFFF
FFFFFFFFFFFFFFF
@A00721:190:HGJW7DSXY:4:2444:1425:23218 2:N:0:TCTGCAAG+GCTGCACT
CACCGTTCCATCACATCTTCACGGTGGAACTAAGATTCAACTACAATCTCAGAATGCAGTTGCGGATA
AAGCGAGCATGGTGGACTATCCGCATCTGGACGACATTACAACCAGTCCCAGAATTACTGCAGAACCC
GTCATGGAAGACGAT
+
FFFFFFFFFFFFFFFFFFFFFFFFFFFFFFFFFFFFFFFFFFFFFFFFFFFFFFFFFFFFFFFFFFFF
FFFFFFFFFFFFFFFFFFFFFFFFFFFFFFFFFFFFFFFFFFFFFFFFFFFFFFFFFFFFFFFFFFFF
FFFFFFFFFFFFFFF
@A00721:190:HGJW7DSXY:4:2528:26386:3881 2:N:0:TCTGCAAG+GCTGCACT
CACCGTTCCATCACATCTTCACGGTGGAACTAAGATTCAACTACAATCTCAGAATGCAGTTGCGGATA
AAGCGAGCATGGTGGACTATCCGCATCTGGACGACATTACAACCAGTCCCAGAATTACTGCAGAACCC
GTCATGGAAGACGAT
+
FFFFFFFFFFFFFFFFFFFFFFFFFFFF::FFFFFFFFFFFFFFFFFFFFFFFFFFFF,FF:F,FFFFFF
FFFFFFFFFFFFFFFFFFFFFFFFFFFFFFFFFFFFFFFFFFFFFFFFF:FFFFFFFFFFFFFFFFFF:
FFFFFFFFFFFFFF:
@A00721:190:HGJW7DSXY:4:2678:9028:28275 2:N:0:TCTGCAAG+GCTGCACT
CACCGTTCCATCACATCTTCACGGTGGAACTAAGATTCAACTACAATCTCAGAATGCAGTTGCGGATA
AAGCGAGCATGGTGGACTATCCGCATCTGGACGACATTACAACCAGTCCCAGAATTACTGCAGAACCC
GTCATGGAAGACGAT
```

+
FFFFFFFFFFFFFFFFFFFFFFFFFFFFFFFFFFFFFFFFFFFFFFFFFFFFFFFFFFFFFFFFFFFFFF
FFFFFFFFFFFFFFFFFFFFFFFFFFFFFFFFFFFFFFFFFFFFFFFFFFFFFFFFFFFFFFFFFFFFFF
FFFFFFFFFFFFFFF
@A00721:190:HGJW7DSXY:4:2678:9317:31187 2:N:0:TCTGCAAG+GCTGCACT
CACCGTTCCATCACATCTTCACGGTGGAACTAAGATTCAACTACAATCTCAGAATGCAGTTGCGGATA
AAGCGAGCATGGTGGACTATCCGCATCTGGACGACATTACAACCAGTCCCAGAATTACTGCAGAACCC
GTCATGGAAGACGAT
+
FFFFFFFFFFFFFFFFFFFFFFFFFFFF:FFFFFFFFFFFFFFFFFFFFFFFFFFFFFFF:FFFFFFFF
FFFFFFFFFFFFFFFFFFFFFFFFFFFFFFFFFFFFFFFFF:FFFFFFFFFFFFFFFFFFFFFFFFFFF
FFFFFFFFFFFFFFF
@A00721:190:HGJW7DSXY:4:1139:11523:1188 2:N:0:TCTGCAAG+GCTGCACT
CACCGTTCCATCACATCTTCACGGTGGAACTAAGATTCAACTACAATCTCAGAATGCAGTTGCGGATA
AAGCGAGCATGGTGGACTATCCGCATCTGGACGACATTACAACCAGTCCCAGAATTACTGCAGAACCC
GTCATGGAAGACGA
+
FFF,:,,FFFFFFFF,FF,F:FF::F:FF:FFF:FFFFFFFFFFFF:FFFFFFFF:FFFFFFFFFF,F
FF:FFFFF:,F:FFFFFFFFFFFFFFFFF::FFFFF,FFF,FFFFF,FFFFFF:,FFF,FFFF:FFFF
,FF:FF,FFFFF:F
@A00721:190:HGJW7DSXY:4:1207:3332:13777 2:N:0:TCTGCAAG+GCTGCACT
GACGAGTCCACCGTTCCATCACATCTTCACGGTGGAACTAAGATTCAACTACAATCTCAGAATGCAGT
TGCGGATAAAGCGAGCATGGTGGACTATCCGCATCTGGACGACATTACAACCAGTCCCAGAATTACTG
CAGAACCCGTCATGG
+
FFFFFFFFFFFFFFFFFFFFFFFFFFFFFFFFFFFFFFFFFF,FFFFFF:FFFFFFFFFFFFFF,FF
FFFFFFFFFFFFFFFFFFFFFFFFFFFFFFFFFFFFFFFFFFFFFFFFFFFFFFFFFF::,FFFFFF
FF:FFFFFFFFFFFF
@A00721:190:HGJW7DSXY:4:2531:17020:31876 2:N:0:TCTGCAAG+GCTGCACT
GACGAGTCCACCGTTCCATCACATCTTCACGGTGGAACTAAGATTCAACTACAATCTCAGAATGCAGT
TGCGGATAAAGCGAGCATGGTGGACTATCCGCATCTGGACGACATTACAACCAGTCCCAGAATTACTG
CAGAACCCGTCATGG
+
FFFFFFFFFFFFFFFFFFFFFFFFFFFFFFFFFFFFFFFFFFFFFFFFFFFFFFFFFFFFFFFFFFFFFF
FFFFFFFFFFFFFFFFFFFFFFFFFFFFFFFFFFFFFFFFFFFFFFFFFFFFFFFFFFFFFFFFFFFFFF
FFFFFFFFFFFFFFF
@A00721:190:HGJW7DSXY:4:2617:5439:21684 2:N:0:TCTGCAAG+GCTGCACT
GACGAGTCCACCGTTCCATCACATCTTCACGGTGGAACTAAGATTCAACTACAATCTCAGAATGCAGT
TGCGGATAAAGCGAGCATGGTGGACTATCCGCATCTGGACGACATTACAACCAGTCCCAGAATTACTG
CAGAACCCGTCATGG
+
FFFFFFFFFFFFF:FFFFFFFFFFFFFFFFFFFFFFFF:FFFFFFFFFFFFFFFFFF:FFFFFFFFFFFFF
FFFFFFFFFFFFFFFFFFFFFFFFFFFFFFFFFFFFFFFFFFFFFFFFFFFFFFFFFFFFFFFFFFFFFF
FFFFFFFFFFFFFFF
@A00721:190:HGJW7DSXY:4:1102:21902:22013 2:N:0:TCTGCAAG+GCTGCACT
CGACGAGTCCACCGTTCCATCACATCTTCACGGTGGAACTAAGATTCAACTACAATCTCAGAATGCAG
TTGCGGATAAAGCGAGCATGGTGGACTATCCGCATCTGGACGACATTACAACCAGTCCCAGAATTACT
GCAGAACCCGTCATG
+
FFFFFFFFFFFFFFFFFFFFFFFFFFFFFFFFFFFFFFFFFFFFFFFFFFFFF,FFFFFFFFFFFFFF
FFFFFFFFFFFFFFFFFFFFFFFFFFFFFFFFFFFFFFFFFFFFFFFFFF:FFFFFFFFFFF:F:FFFF
FFFFFFFFFFFFFFF
@A00721:190:HGJW7DSXY:4:1107:2953:22921 2:N:0:TCTGCAAG+GCTGCACT
CGACGAGTCCACCGTTCCATCACATCTTCACGGTGGAACTAAGATTCAACTACAATCTCAGAATGCAG

TTGCGGATAAAGCGAGCATGGTGGACTATCCGCATCTGGACGACATTACAACCAGTCCCAGAATTACT
GCAGAACCCGTCATG
+
FFFFFFFFFFFFFFFFFFFFFFFFFFFFFFFFFFFFFFFFFFFFFFFFFFFFFFFFFFFFFFFFFFFFFF
FFFFFFFFFFFFFFFFFFFFFFFFFFFFFFFFFFFFFFFFFFFFFFFFFFFFFFFFFFFFFFFFFFFFFF
FFFFF:FFFFFFFFF
@A00721:190:HGJW7DSXY:4:1267:7111:33098 2:N:0:TCTGCAAG+GCTGCACT
CGACGAGTCCACCGTTCCATCACATCTTCACGGTGGAACTAAGATTCAACTACAATCTCAGAATGCAG
TTGCGGATAAAGCGAGCATGGTGGACTATCCGCATCTGGACGACATTACAACCAGTCCCAGAATTACT
GCAGAACCCGTCATG
+
FFFFFFFFFFFFFFFFFFFFFFFFFFFFFFFFFFFFFFFFFFFFFFFFFFFFFFFFFFFFFFFFFFFFFF
FFFFFFFFFFFFFFFFFFFFFFFFFFFFFFFFFFFFFFFFFFFFFFFFFFFFFFFFFFFFFFFFFFFFFF
FFFFFFFFFFFFFFF
@A00721:190:HGJW7DSXY:4:1350:3712:18912 2:N:0:TCTGCAAG+GCTGCACT
CGACGAGTCCACCGTTCCATCACATCTTCACGGTGGAACTAAGATTCAACTACAATCTCAGAATGCAG
TTGCGGATAAAGCGAGCATGGTGGACTATCCGCATCTGGACGACATTACAACCAGTCCCAGAATTACT
GCAGAACCCGTCATG
+
FFFFFFFFFFFFFFFFFFFFFFFFFFFFFFFFFFFFFFFFFFFFFF:FFFFFFFFFFFFFFFFFFFFFFF
FFFFFFF,FFFFFFFFFFFFFFFFFFFFFFFFFFFFFFFFFFFFFFFFF:FFFFFFFFFFFF
FFFFFF:FF:FFFFF
@A00721:190:HGJW7DSXY:4:1510:29875:27868 2:N:0:TCTGCAAG+GCTGCACT
CGACGAGTCCACCGTTCCATCACATCTTCACGGTGGAACTAAGATTCAACTACAATCTCAGAATGCAG
TTGCGGATAAAGCGAGCATGGTGGACTATCCGCATCTGGACGACATTACAACCAGTCCCAGAATTACT
GCAGAACCCGTCATG
+
FFFFFFFFFFFFFFFFFFFFFFFFFFFFFFFFFFFFFFFFFFFFFFFFFFFFFFFFFFFFFFFFFFFFFF
FFFFFFFFFFFFFFFFFFFFFFFFFFFFFFFFFFFFFFFFFFFFFFFFFFFFFFFFFFFFFFFFFFFFFF
FFFFF,FFFFFF:FF
@A00721:190:HGJW7DSXY:4:1550:28637:16799 2:N:0:TCTGCAAG+GCTGCACT
CGACGAGTCCACCGTTCCATCACATCTTCACGGTGGAACTAAGATTCAACTACAATCTCAGAATGCAG
TTGCGGATAAAGCGAGCATGGTGGACTATCCGCATCTGGACGACATTACAACCAGTCCCAGAATTACT
GCAGAACCCGTCATG
+
FFFFFFFFFFFFFFFFFFFFFFFFFFFFFFFFFFFFFFFFFFFFFFFFFFFFFFFFFFFFFFFFFFFFFF
FFFFFFFFFFFFFF:FFFFFFFFFFFFFFFFFFFFF,FFFFFFFFF:FFFFFFFFFFFFFFFFF:FFFFF
FFFFFFFFFFFFFFF
@A00721:190:HGJW7DSXY:4:1571:29631:25817 2:N:0:TCTGCAAG+GCTGCACT
CGACGAGTCCACCGTTCCATCACATCTTCACGGTGGAACTAAGATTCAACTACAATCTCAGAATGCAG
TTGCGGATAAAGCGAGCATGGTGGACTATCCGCATCTGGACGACATTACAACCAGTCCCAGAATTACT
GCAGAACCCGTCATG
+
FFFFFFFFFFFFFFFFFFFFFFFFFFFFFFFFFFFFFF:FFFFFFFFFFFFFF:FFFFFFFFFFFFFFFF
FFFFFFFFFFFFFFFFFFFFFFFFFFFFFFFFFFFFFFFFFFFFFF:FFFFFFFF,FFFFFFFFF,FFFF
FFFFFFFFFFFFFFF
@A00721:190:HGJW7DSXY:4:2130:24966:4366 2:N:0:TCTGCAAG+GCTGCACT
CGACGAGTCCACCGTTCCATCACATCTTCACGGTGGAACTAAGATTCAACTACAATCTCAGAATGCAG
TTGCGGATAAAGCGAGCATGGTGGACTATCCGCATCTGGACGACATTACAACCAGTCCCAGAATTACT
GCAGAACCCGTCATG
+
FFFFFFFFFFFFFFFFFFFFFFFFFFFFFFFFFFFFFFF:FFFFFFFFFFFFFFFFFFFFFFFFFFFFFF
FFFFFFFFFFFFFFFFFFFFFFFFFFFFFFFFFFFFFFFFFFFFFFFFFFFFFFFFFFFFFFFFFFFFFF
FFFFFFFFFFFFFFF

```
@A00721:190:HGJW7DSXY:4:2147:29640:8015 2:N:0:TCTGCAAG+GCTGCACT
CGACGAGTCCACCGTTCCATCACATCTTCACGGTGGAACTAAGATTCAACTACAATCTCAGAATGCAG
TTGCGGATAAAGCGAGCATGGTGGACTATCCGCATCTGGACGACATTACAACCAGTCCCAGAATTACT
GCAGAACCCGTCATG
+
FFFFFFFFFFFFFFFFFFFFFFFFFFFFFFFFFFFFFFFFFFFFFFFFFFF:FFFFFFFFFFFFFFFF
FFFFFFFFFFFFFF:F:FFFFFFFFFFFFFFFFFFFFFFFFFFFF,FFFFFFF:FFFFFFFFFFFFFF
FFFFF,FFFFFFF:F
@A00721:190:HGJW7DSXY:4:2549:19108:17049 2:N:0:TCTGCAAG+GCTGCACT
CGACGAGTCCACCGTTCCATCACATCTTCACGGTGGAACTAAGATTCAACTACAATCTCAGAATGCAG
TTGCGGATAAAGCGAGCATGGTGGACTATCCGCATCTGGACGACATTACAACCAGTCCCAGAATTACT
GCAGAACCCGTCATG
+
FFFFFFFFFFFFFFFFFFFFFFFFFFFFFFFFFFFFFFFFFFFFFFFFFFFFFFFFFFFFFF:FFFFF
F:FFFFFFFFFFFFFFFFFFFFFFFFFFFFFFFFFFFFFFFFFFFFFFFFFFFFFFFFFFFFFFFFFF
FFFFFFF:FFFFFFF
@A00721:190:HGJW7DSXY:4:2663:18747:3458 2:N:0:TCTGCAAG+GCTGCACT
CGACGAGTCCACCGTTCCATCACATCTTCACGGTGGAACTAAGATTCAACTACAATCTCAGAATGCAG
TTGCGGATAAAGCGAGCATGGTGGACTATCCGCATCTGGACGACATTACAACCAGTCCCAGAATTACT
GCAGAACCCGTCAGG
+
FFFFFFFFFFFFFFF:FFFFFFFF:FFFFFFFFFFFFFFFFFFFFFFFFFFFF,F:FFFFFFFFFFFF
FFFFFFFFF:FFFFFFFFFFFFFF:,F,FFFFFFFFF:FFFF:FF,F:FFFFFFFF:FFFFFFFFF:,
FF:FFFFFFF:F:,F
@A00721:190:HGJW7DSXY:4:1113:18159:17190 2:N:0:TCTGCAAG+GCTGCACT
CAGACGACGAGTCCACCGTTCCATCACATCTTCACGGTGGAACTAAGATTCAACTACAATCTCAGAAT
GCAGTTGCGGATAAAGCGAGCATGGTGGACTATCCGCATCTGGACGACATTACAACCAGTCCCAGAAT
TACTGCAGAACCCGT
+
FFFFFFFFFFFFFFFFFFFFFFFFFFFFFFFFFFFFFFFFFFFFFFFFFFFFF:FFFFFFFFFFFFFF
FFFFFFFFFFFFFFFFFFFFFFFFFFFFFFFFFF:FFFFFFFFFFFFFFFFFFFFFFFFFFFFFFF:F
FFFFFFFFFFFFFFF
@A00721:190:HGJW7DSXY:4:1457:8440:24502 2:N:0:TCTGCAAG+GCTGCACT
CAGACGACGAGTCCACCGTTCCATCACATCTTCACGGTGGAACTAAGATTCAACTACAATCTCAGAAT
GCAGTTGCGGATAAAGCGAGCATGGTGGACTATCCGCATCTGGACGACATTACAACCAGTCCCAGAAT
TACTGCAGAACCCGT
+
FFFFFFFFFF:FFFFFFFFFFFF:FFFFF,FFFFF:FFFFFFFFFFFFFFF:FFFFFFFFFFFFF:FF
:FFFFFFFFFF,FFFFFFFFFF:FFFFF:FFFFFFFFFFFFFFFF:FFFFFFFFFFFF:FFFFFFFF:
FFFFFFFFFFFFFFF
@A00721:190:HGJW7DSXY:4:1562:10827:4523 2:N:0:TCTGCAAG+GCTGCACT
CAGACGACGAGTCCACCGTTCCATCACATCTTCACGGTGGAACTAAGATTCAACTACAATCTCAGAAT
GCAGTTGCGGATAAAGCGAGCATGGTGGACTATCCGCATCTGGACGACATTACAACCAGTCCCAGAAT
TACTGCAGAACCCGT
+
FFFFFFFFFFFFFFFFFFFFFFFFFFFFFFFFFFFFFFFFFFFFFFFFFFFFFFFFFF:FFFFFFFFF
FFFF,FFFFFFF:FFFFFFFFF:FFFFFFFFFFFFFFFFFFFFFFFFFFFFFFFFFFFFFFFFFFFFF
FFFFFFFFFFFFFFF
@A00721:190:HGJW7DSXY:4:1621:17770:35462 2:N:0:TCTGCAAG+GCTGCACT
CAGACGACGAGTCCACCGTTCCATCACATCTTCACGGTGGAACTAAGATTCAACTACAATCTCAGAAT
GCAGGTGCGGATAAAGCGAGCATGGTGGACTATCCGCATCTGGACGACATTACAACCAGTCCCAGAAT
TACTGCAGAACCCGT
+
FFFFFFFFFFFFFFFFFFFFFFFFFFFFFFFFFFFFFFFFFFFFFFFFFFFFFFFFFFFFFFFFFFFF
```

FFFF,FFFFFFFFFFFFFFFFFFFFFFFFFFFFFFFFFFFFFFFFFFFFFFFFFFFFFFFFFFFFFFF
FFFFFFFFF:FFFF:
@A00721:190:HGJW7DSXY:4:1671:28284:12273 2:N:0:TCTGCAAG+GCTGCACT
CAGACGACGAGTCCACCGTTCCATCACATCTTCACGGTGGAACTAAGATTCAACTACAATCTCAGAAT
GCAGTTGCGGATAAAGCGAGCATGGTGGACTATCCGCATCTGGACGACATTACAACCAGTCCCAGAAT
TACTGCAGAACCCGT
+
FF:FFFFFFFFFFFFFFFF:FFFFFFFFFFFFFFFFFFFFF:FFFFFFFFFFFFFFFFFFFFFFFFFF
FFFFFFFFFF:FFFFFFFFFFFFF:FFFFFFFFFFFFFFFFFFFFFFFFFFFFFFFFFFFFFF,FFFF
FFFFFFFFF:FF:FF
@A00721:190:HGJW7DSXY:4:2165:10502:15796 2:N:0:TCTGCAAG+GCTGCACT
CAGACGACGAGTCCACCGTTCCATCACATCTTCACGGTGGAACTAAGATTCAACTACAATCTCAGAAT
GCAGTTGCGGATAAAGCGAGCATGGTGGACTATCCGCATCTGGACGACATTACAACCAGTCCCAGAAT
TACTGCAGAACCCGT
+
FFFFFFFFFFFFFFFFFFFFFFFFFFFFFFFFFFFFFFFFF,FFFFFFFFFFFFFFFFF:FFFFFFFF
FFFFFFFFFFFFFFFFFFFFFFFFFFFFFFFFFFFFFFFFFFFFFFFFFFF:FFFFFFFFFFFFFFFF
FFFFFFFFFFFFFFF
@A00721:190:HGJW7DSXY:4:2228:15121:33536 2:N:0:TCTGCAAG+GCTGCACT
CAGACGACGAGTCCACCGTTCCATCACATCTTCACGGTGGAACTAAGATTCAACTACAATCTCAGAAT
GCAGTTGCGGATAAAGCGAGCATGGTGGACTATCCGCATCTGGACGACATTACAACCAGTCCCAGAAT
TACTGCAGAACCCGT
+
FFFFFFFFFFFFFFFFFFFFFFFFFFFFFFFFFFFFFFFFFFFFF:FFFFFFFFFFFFFFFFF:F:FF
FFFFFFFFFFFFFF:FFFFFF:FFFFFFFFFFFFFFFFFFFFFFFFFFF,FFFFFFFFFFFFFFFFFF
FFFFFFFFFFFFFFF
@A00721:190:HGJW7DSXY:4:2473:30038:7420 2:N:0:TCTGCAAG+GCTGCACT
CAGACGACGAGTCCACCGTTCCATCACATCTTCACGGTGGAACTAAGATTCAACTACAATCTCAGAAT
GCAGTTGCGGATAAAGCGAGCATGGTGGACTATCCGCATCTGGACGACATTACAACCAGTCCCAGAAT
TACTGCAGAACCCGT
+
FFFFFFFFFFFFFFFFFFFFFFFFFFFFFFFFFFFFFFFFFFFFFFFFFFFFFFFFFF:FFFFFFFFF
FFFFFFFFFFFFFFFFFFFFFF:FFFFFFFFFFFFFFF:FFFFFFFFFFFFFFFFFFFFFFFF,FFFF
FFFFFF:FFFFFFFF
@A00721:190:HGJW7DSXY:4:2503:14778:1689 2:N:0:TCTGCAAG+GCTGCACT
CAGACGACGAGTCCACCGTTCCATCACATCTTCACGGTGGAACTAAGATTCAACTACAATCTCAGAAT
GCAGTTGCGGATAAAGCGAGCATGGTGGACTATCCGCATCTGGACGACATTACAACCAGTCCCAGAAT
TACTGCAGAACCCGT
+
FFFFFF,FFFFFFFFFFFFFFFFFFFFFFFFFFFFFFFFFFFFFFFFFFFFFFFFFFFFFFFFFFFFF
FFFFFFFF:FFFFFFFFFFFFFFFFFFFFFFFFFFFFFFFFFFFFFFFFFFFFF,FFFFFFFF:FFFF
FFFFFFFFFFFFFFF
@A00721:190:HGJW7DSXY:4:2503:17734:14231 2:N:0:TCTGCAAG+GCTGCACT
CAGACGACGAGTCCACCGTTCCATCACATCTTCACGGTGGAACTAAGATTCAACTACAATCTCAGAAT
GCAGTTGCGGATAAAGCGAGCATGGTGGACTATCCGCATCTGGACGACATTACAACCAGTCCCAGAAT
TACTGCAGAACCCGT
+
FFFFFFFFFFFFFFFFFFFFFFFFFFFFFFFFFFFFFFFFFFFFFFFFFFFFFFFFFFFFFFFFFFFF
FFFFF:FFFFFFFFFFFFFFFFFFFFFFFFFFFFFFFFFFFFFFFFFFFFFFFFFFFFFFFFFFFFFF
F:FFFFFFFFFFFFF
@A00721:190:HGJW7DSXY:4:2575:20518:21840 2:N:0:TCTGCAAG+GCTGCACT
CAGACGACGAGTCCACCGTTCCATCACATCTTCACGGTGGAACTAAGATTCAACTACAATCTCAGAAT
GCAGTTGCGGATAAAGCGAGCATGGTGGACTATCCGCATCTGGACGACATTACAACCAGTCCCAGAAT
TACTGCAGAACCCGT

+
FF:FFFFFFFFFFFFFFFFFFFFFFFFFFFFFFFFFFFFFFFFFFFFFFFFFFFFFFFFFFFFFFFF:FFF
FFFFFF:FFFFFFFFFFFFFFFFFFFFFFFFFFFFFFFFFFFFFFFFFFFFF:FFFFFF:FFFFFFFF:F
FFFFFFFFFFFF:F:
@A00721:190:HGJW7DSXY:4:1254:15826:28776 2:N:0:TCTGCAAG+GCTGCACT
TGGACAGACGACGAGTCCACCGTTCCATCACATCTTCACGGTGGAACTAAGATTCAACTACAATCTCA
GAATGCAGTTGCGGATAAAGCGAGCATGGTGGACTATCCGCATCTGGACGACATTACAACCAGTCCCA
GAATTACTGCAGAAC
+
FFFFFFFFFFFFFFFFFFFFFFFFFFFFFFFFFFFFFFFFFFFFFFFFF:FFFFFFFFF,F,FFFF
FFFFFFFFFFFFFFFFFFFFFFFFFF:FFFFFFFFFFFFFFFFFFFFFFFFFFFFFFFFFFFFFFFFF
FFFFFFFFFFFFFFF
@A00721:190:HGJW7DSXY:4:1254:15148:29074 2:N:0:TCTGCAAG+GCTGCACT
TGGACAGACGACGAGTCCACCGTTCCATCACATCTTCACGGTGGAACTAAGATTCAACTACAATCTCA
GAATGCAGTTGCGGATAAAGCGAGCATGGTGGACTATCCGCATCTGGACGACATTACAACCAGTCCCA
GAATTACTGCAGAAC
+
FFFFFFFFFF:FFFFFFFFFFFFFFFFFFFFFFFFFFFFFFFFFFF:FF:FF:FFFFFFFFFFFF:FF:
FFFFF:FFFFFF:FFFFFFFFFFFFFFFFFFFFFFFFFFFFFFFFFFFFFFFFFFFFFFFFFFFFFFFF
FF,FFFFFFFFFFFF
@A00721:190:HGJW7DSXY:4:1304:30978:2816 2:N:0:TCTGCAAG+GCTGCACT
TGGACAGACGACGAGTCCACCGTTCCATCACATCTTCACGGTGGAACTAAGATTCAACTACAATCTCA
GAATGCAGTTGCGGATAAAGCGAGCATGGTGGACTATCCGCATCTGGACGACATTACAACCAGTCCCA
GAATTACTGCAGAAC
+
FFFFFFFFFFFFFFFFFFFFFFFFFFFFFFFFFFFFFFFFFFFFFF,F:FFFFFFFFFFFFFFFFFFFF
FFFFFFFFFF:FFFFFFFFFFFFFFFFFFFFFFFFFFFFFFFFFFFFFFFFFFFFFFFFFFFFFFFFFF
FFFFFFFFFFFFFFF
@A00721:190:HGJW7DSXY:4:1304:29776:3020 2:N:0:TCTGCAAG+GCTGCACT
TGGACAGACGACGAGTCCACCGTTCCATCACATCTTCACGGTGGAACTAAGATTCAACTACAATCTCA
GAATGCAGTTGCGGATAAAGCGAGCATGGTGGACTATCCGCATCTGGACGACATTACAACCAGTCCCA
GAATTACTGCAGAAC
+
FFFFFFFFFFFFFFFFFFFFFFFFFFFFFFFFFFFFFFFFFFFFFFFFFFFFFFFFFFFFFFFFFFFFF
FFFFFFFFFFFFFFFFFFFFFFFFFF:FFFFFFFFFFFFFFFFFFFFFFFFFFFFFFFFFFFFFFFFFF
FFFFFFFFFFFFFFF
@A00721:190:HGJW7DSXY:4:1313:9019:2237 2:N:0:TCTGCAAG+GCTGCACT
TGGACAGACGACGAGTCCACCGTTCCATCACATCTTCACGGTGGAACTAAGATTCAACTACAATCTCA
GAATGCAGTTGCGGATAAAGCGAGCATGGTGGACTATCCGCATCTGGACGACATTACAACCAGTCCCA
GAATTACTGCAGAAC
+
FFFFFFFFFFFFFFFFFFFFFFFFFFFFFFFFFFFFFFFFFFFFFFFFFFFFFFFFFFFFFFFFFFFFF
FFFFFFFFFFFFFFFFFFFFFFFFFFFFFFFFFFFFFFFFFFFFFFFFFFFFFFFFFFFFFFFFFFFFF
FFFFFFFFFF:FFFF
@A00721:190:HGJW7DSXY:4:1345:27868:5228 2:N:0:TCTGCAAG+GCTGCACT
TGGACAGACGACGAGTCCACCGTTCCATCACATCTTCACGGTGGAACTAAGATTCAACTACAATCTCA
GAATGCAGTTGCGGATAAAGCGAGCATGGTGGACTATCCGCATCTGGACGACATTACAACCAGTCCCA
GAATTACTGCAGAAC
+
FFFFFFFFFFFFFFFFFFFFFFFFFFFFFFFFFFFFFFFFFFFFFFFFFFFFFFF:FFFFFFFFFFFF
FFFFFFFFFFFFFFFFFFFFFFFFFFFFFFFFFFFFFFFFFFFFFFFFFFFFFFFFFFF:FF:FFFFFF
FFFFFFFFFFFFFFF
@A00721:190:HGJW7DSXY:4:1345:27579:5854 2:N:0:TCTGCAAG+GCTGCACT
TGGACAGACGACGAGTCCACCGTTCCATCACATCTTCACGGTGGAACTAAGATTCAACTACAATCTCA

GAATGCAGTTGCGGATAAAGCGAGCATGGTGGACTATCCGCATCTGGACGACATTACAACCAGTCCCA
GAATTACTGCAGAAC
+
FFFFFFFFFFFFFFFFFFFFFFFFFFFFFFFFFFFFFFFFFFFFF,FFFFFFFFFFFFFFFFFFFFFF
FFF:FFFFFFFFFFF::FFF:FFFFFFFFFFFFFFFFFFFFF:FFFFFFFFFF,FFFFFFF:FFFFFF
FF,FF:FFFFFFFFF
@A00721:190:HGJW7DSXY:4:1515:20862:29825 2:N:0:TCTGCAAG+GCTGCACT
TGGACAGACGACGAGTCCACCGTTCCATCACATCTTCACGGTGGAACTAAGATTCAACTACAATCTCA
GAATGCAGTTGCGGATAAAGCGAGCATGGTGGACTATCCGCATCTGGACGACATTACAACCAGTCCCA
GAATTACTGCAGAAC
+
FFFFFFFFFFFFFFFFFFFFFFFFFFFFFFFFFFFFFFFFFFFFFFFFFFFFFFFFFFFFFFFFFFFF
FFFFFFFFFFFFFFFFFFFFFFFFFFFFFFFFFFFFFFFFFFFFFFFFFFFFFFFFFFFFFFFFF:FF
FFFFFFFFFFFFFFF
@A00721:190:HGJW7DSXY:4:1563:21178:17127 2:N:0:TCTGCAAG+GCTGCACT
TGGACAGACGACGAGTCCACCGTTCCATCACATCTTCACGGTGGAACTAAGATTCAACTACAATCTCA
GAATGCAGTTGCGGATAAAGCGAGCATGGTGGACTATCCGCATCTGGACGACATTACAACCAGTCCCA
GAATTACTGCAGAAC
+
FFFFFFFFFFFFFFFFFFFFFFFFFFFFFFFFFFFFFFFFFFFFF:FFFFFFFFFFFFFFFFFFFFFF
F:FFFFFFFFFFFFFFFFFFFFFFFFFFFFFFFFFFFFFFFFFFFFFFFFFFFFFFFFFFFFFFFFFF
FFFFFFFFFFFFFFF
@A00721:190:HGJW7DSXY:4:1603:8278:22623 2:N:0:TCTGCAAG+GCTGCACT
TGGACAGACGACGAGTCCACCGTTCCATCACATCTTCACGGTGGAACTAAGATTCAACTACAATCTCA
GAATGCAGTTGCGGATAAAGCGAGCATGGTGGACTATCCGCATCTGGACGACATTACAACCAGTCCCA
GAATTACTGCAGAAC
+
FFFFFFFFFFFFFFFFFFFFFFFFFFFFFFFFFFFFFFFFFFFFFFFFFFFFFFFFFFFFFFFFFFFF
FFFFFFFFFFFFFFFFFFFFFFFFFFFFFFFFFFFFFFFFFFFFFFFFFFFFFFFFFFFFFFFFFFFF
FFFFFFFFFFFFFFF
@A00721:190:HGJW7DSXY:4:2130:16993:19241 2:N:0:TCTGCAAG+GCTGCACT
TGGACAGACGACGAGTCCACCGTTCCATCACATCTTCACGGTGGAACTAAGATTCAACTACAATCTCA
GAATGCAGTTGCGGATAAAGCGAGCATGGTGGACTATCCGCATCTGGACGACATTACAACCAGTCCCA
GAATTACTGCAGAAC
+
FFFFFFFFFFFFFFFFFFFFFFFFFFFFFFFFFFFFFFFFFFFFFFFFFFFFFFFFFFFFFFFFFFFF
F:FFFFFFFFFFFFFFFFFFFFFFFFFFFFFFFFFFFFFFFFFFFFFFFFFFFFFFFFFFF:FFFFFF
FFFFFFFFFFFFFFF
@A00721:190:HGJW7DSXY:4:2275:11207:6339 2:N:0:TCTGCAAG+GCTGCACT
TGGACAGACGACGAGTCCACCGTTCCATCACATCTTCACGGTGGAACTAAGATTCAACTACAATCTCA
GAATGCAGTTGCGGATAAAGCGAGCATGGTGGACTATCCGCATCTGGACGACATTACAACCAGTCCCA
GAATTACTGCAGAAC
+
FFFFFFFFFF:FFFFFFFFFFFFFFFFFFFFFFFFFFFFFFFFFFFFFFFFFFFFFFFFFFFFFFFFF
FFFFFFFFFFFFFFFFFFFFFFFFFFFFFFFFFFF:FFFFFFFFFFFFFFFFFFFFFFFFFFFFFFFF
FFFFFFFFFFFFFFF
@A00721:190:HGJW7DSXY:4:2275:10447:6558 2:N:0:TCTGCAAG+GCTGCACT
TGGACAGACGACGAGTCCACCGTTCCATCACATCTTCACGGTGGAACTAAGATTCAACTACAATCTCA
GAATGCAGTTGCGGATAAAGCGAGCATGGTGGACTATCCGCATCTGGACGACATTACAACCAGTCCCA
GAATTACTGCAGAAC
+
FFFFFFFFFFFFFFFFFFFFFFFFFFFFFFFFFFFFFFFFFFFFFFFFFFFFFFFFFFFFFFFFFFFF
FFFFFFFFFFFFFFFFFFFFFFFFFFFFFFFFFFFFFFFFFFFFFFFFFFFFFFFFFFFFFFFFFFFF
FFFFFFFFFFFFFFF

```
@A00721:190:HGJW7DSXY:4:2532:4435:10332 2:N:0:TCTGCAAG+GCTGCACT
TGGACAGACGACGAGTCCACCGTTCCATCACATCTTCACGGTGGAACTAAGATTCAACTACAATCTCA
GAATGCAGTTGCGGATAAAGCGAGCATGGTGGACTATCCGCATCTGGACGACATTACAACCAGTCCCA
GAATTACTGCAGAAC
+
FFFFFFFFFFFFFFFFFFFFFFFFFFFFFFFFFFFFFFFFFFFFFFFFFFFFFFFFFFFFFF:FFFFFFF
FFFFFFFFFFFFFFFFFFFFFFFFFFF,FFFFFFFFFFFFFFFFFFFFFFFFF:FFFFFFFFFFFFFFFF
FFFFFFFFFFFFFFF
@A00721:190:HGJW7DSXY:4:2671:32389:23234 2:N:0:TCTGCAAG+GCTGCACT
TGGACAGACGACGAGTCCACCGTTCCATCACATCTTCACGGTGGAACTAAGATTCAACTACAATCTCA
GAATGCAGTTGCGGATAAAGCGAGCATGGTGGACTATCCGCATCTGGACGACATTACAACCAGTCCCA
GAATTACTGCAGAAC
+
FFFFFFF:FFFFFFFFFFFFFFFFFFFFFFFFFFFFFFFFFFFFFFFFFFFFFFFFFFFFFFFFFFFFFF
FFFFFFFFFFFFFFFFFFFFFFFFFFFFFFFFFFFFFFFFFFFFFFFFFFFFFFFFFFFFFFFFFFF:FF
FFFFFFFFFFFFFFF
@A00721:190:HGJW7DSXY:4:1306:26232:29481 2:N:0:TCTGCAAG+GCTGCACT
CTCTGGACAGACGACGAGTCCACCGTTCCATCACATCTTCACGGTGGAACTAAGATTCAACTACAATC
TCAGAATGCAGTTGCGGATAAAGCGAGCATGGTGGACTATCCGCATCTGGACGACATTACAACCAGTC
CCAGAATTACTGCAG
+
FFFFFFFFFFFFFFFFFFFFFFFFFFFFFFFFFF,FFFFFFFFFFFFFFFFFFFFFFFFFFFFFF:FFF:
FFFF:FF:FFFFFF:FFFFFFFFF:FFFFFFFFFFFFFFFFFFFFFFFFF::FFF::FFFFFFFF:FFFF
,::FFFFFFFFFFFF
@A00721:190:HGJW7DSXY:4:1411:8241:10692 2:N:0:TCTGCAAG+GCTGCACT
CTCTGGACAGACGACGAGTCCACCGTTCCATCACATCTTCACGGTGGAACTAAGATTCAACTACAATC
TCAGAATGCAGTTGCGGATAAAGCGAGCATGGTGGACTATCCGCATCTGGACGACATTACAACCAGTC
CCAGAATTACTGCAG
+
FFFFFFFFFFFFFFFFFFFFFFFFFFFFFFFFFFFFFFFFFFFFFFFFFFFFFFFFFFFFFFFFFFFFFF
FFFFFFFFFFFFFFFFFFFFFFFFFFFFFFFFFFFFFFFFFFFFFFFFFFFFFFFFFFFFFFFFFFFFFF
FFFFFFFFFFFFFFF
@A00721:190:HGJW7DSXY:4:1659:8648:4539 2:N:0:TCTGCAAG+GCTGCACT
CTCTGGACAGACGACGAGTCCACCGTTCCATCACATCTTCACGGTGGAACTAAGATTCAACTACAATC
TCAGAATGCAGTTGCGGATAAAGCGAGCATGGTGGACTATCCGCATCTGGACGACATTACAACCAGTC
CCAGAATTACTGCAG
+
FFFFFFFFFFFFFFFFFFFFFFFFFFFFFF:FFFFFFFFFFFFFFFFFF:FFFFFFFFFFFFF:FFFFFF
FFFFFFFFFFFFFFFFFFFFFFFFFFFFFFFFFFFFFFFFFFFFFFFFFFFFFFFFFFFFFFFFFFFFFF
FFFFFFFFFFFFFFF
@A00721:190:HGJW7DSXY:4:2215:4056:22733 2:N:0:TCTGCAAG+GCTGCACT
CTCTGGACAGACGACGAGTCCACCGTTCCATCACATCTTCACGGTGGAACTAAGATTCAACTACAATC
TCAGAATGCAGTTGCGGATAAAGCGAGCATGGTGGACTATCCGCATCTGGACGACATTACAACCAGTC
CCAGAATTACTGCAG
+
FFFFFFFFFFFFFFFFFFFFFFFFFFFFFFFFFFFFFFFFFFFFFFFFFFFFFFFFFFFFFFFFFFFFFF
FFFFFFFFFFFFFFFFFFFFFFFFFFFFFFFFFFFFFFFFFFFFFFFFFFFFFFFFFFFFFFFFFFFFFF
FFFFFFFFFFFFFFF
@A00721:190:HGJW7DSXY:4:2372:4535:31454 2:N:0:TCTGCAAG+GCTGCACT
CTCTGGACAGACGACGAGTCCACCGTTCCATCACATCTTCACGGTGGAACTAAGATTCAACTACAATC
TCAGAATGCAGTTGCGGATAAAGCGAGCATGGTGGACTATCCGCATCTGGACGACATTACAACCAGTC
CCAGAATTACTGCAG
+
FFFFFFFFFFFFFFFFFFFFFFFFFFFFFFFFFFFFFFFFFFFFFFFFFFFFFFFFFFFFFFFFFFFFFF
```

```
FFFFFFFFFFFFFFFFFFFFFFFFFFFFFFFFFFFFFFFFFFFFFFFFFFFFFFFFFFFFFFFFFFFF
FFFFF,FFFFFFFFF
@A00721:190:HGJW7DSXY:4:2471:27507:19539 2:N:0:TCTGCAAG+GCTGCACT
CTCTGGACAGACGACGAGTCCACCGTTCCATCACATCTTCACGGTGGAACTAAGATTCAACTACAATC
TCAGAATGCAGTTGCGGATAAAGCGAGCATGGTGGACTATCCGCATCTGGACGACATTACAACCAGTC
CCAGAATTACTGCAG
+
FFFFFFFFFFFFFFFFFFFFFFFFFFFFFFFFFFFFFFFFFFFFFFFF,F:FFFFFFFFFF:FFFFFF
FFFFFFFFFFFFFFFFF:FFFFFFFFFF:FFFFFFFFFFFFFFFFFFFFFFFFFFF:F:FFFFFFFFF
FFFFFFFFFFFFFFF
@A00721:190:HGJW7DSXY:4:2471:27706:19758 2:N:0:TCTGCAAG+GCTGCACT
CTCTGGACAGACGACGAGTCCACCGTTCCATCACATCTTCACGGTGGAACTAAGATTCAACTACAATC
TCAGAATGCAGTTGCGGATAAAGCGAGCATGGTGGACTATCCGCATCTGGACGACATTACAACCAGTC
CCAGAATTACTGCAG
+
FFFFFFFFFFFFFFFFFFFFFFFFFFFFFFFFFFFFFFFFFFFFFFFFFFFFFFFFFFFFFFFFFFFF
FFFFFFFFFFFFFFFFFFFFFFFFFFFF:FFFFFFFFFFFFFFFFFFFFFFFFFFFFFFFFFFFFFFF
FFFFFFFFFFFFFF:
@A00721:190:HGJW7DSXY:4:1173:16803:32409 2:N:0:TCTGCAAG+GCTGCACT
CCAACACTCTGGACAGACGACGAGTCCACCGTTCCATCACATCTTCACGGTGGAACTAAGATTCAACT
ACAATCTCAGAATGCAGTTGCGGATAAAGCGAGCATGGTGGACTATCCGCATCTGGACGACATTACAA
CCAGTCCCAGAATTA
+
FFFFFFFFFFFFFFFFFFF:FFFFFFFFFFFFFFFFFFFFFFFFFFFFFFFFFFFFFFFFFFFFFFFF
FFFFFFFFFFFFFFFFFFFFFFFFF:FFFFFFFFFFFFF:FFFF,FFFFFFFFFFFFFFFFFFFFFFF
FF:FFFFFFFFFFFF
@A00721:190:HGJW7DSXY:4:1319:6777:4460 2:N:0:TCTGCAAG+GCTGCACT
CCAACACTCTGGACAGACGACGAGTCCACCGTTCCATCACATCTTCACGGTGGAACTAAGATTCAACT
ACAATCTCAGAATGCAGTTGCGGATAAAGCGAGCATGGTGGACTATCCGCATCTGGACGACATTACAA
CCAGTCCCAGAATTA
+
FFFFFFFFFFFFFFFFFFFFFFFFFFFFFFFFFFFFFFFFFFFFFFFFFFFFFFFF,FFFFFFFFFFF
FFFFFFFFFFFFFFFFFFFFFFFFFFFFFFFFFFFFFFFFFFFFFFFFFFFFFFFFFFFFFFFFFFFF
FFFFFFFFFFFFFF:
@A00721:190:HGJW7DSXY:4:2131:23773:27915 2:N:0:TCTGCAAG+GCTGCACT
CCAACACTCTGGACAGACGACGAGTCCACCGTTCCATCACATCTTCACGGTGGAACTAAGATTCAACT
ACAATCTCAGAATGCAGTTGCGGATAAAGCGAGCATGGTGGACTATCCGCATCTGGACGACATTACAA
CCAGTCCCAGAATTA
+
FFFFFFFFFFFFFFFFFFFFFFFFFFFFFFFFFFFFFFFFFFFFFFFFFFF:FFFFFFFFFFFFFFFF
FFFFFFFFFFFFFFFFFFFFFFFFF:FFFFFFFFFFFFFFFFFFFFFFFFFFFFFFFFFFFFFFFFFF
FFFFFFFFFFFFFFF
@A00721:190:HGJW7DSXY:4:2221:25798:27633 2:N:0:TCTGCAAG+GCTGCACT
CCAACACTCTGGACAGACGACGAGTCCACCGTTCCATCACATCTTCACGGTGGAACTAAGATTCAACT
ACAATCTCAGAATGCAGTTGCGGATAAAGCGAGCATGGTGGACTATCCGCATCTGGACGACATTACAA
CCAGTCCCAGAATTA
+
FFFFFFFFFFFFFFFFFFFFFFFFFFFFFFFFFFFFFFFFFFFFFFFFFFFFFFFFFFFF:FFFFFFF
FFFFFFFFFFFFFFFFFFFFFFFFFFFFFFFFFFFFFFFFFFFFFFFFFFFFFFFFFFFFFFFFFFFF
FFFFFFFFFFFFFFF
@A00721:190:HGJW7DSXY:4:2529:22896:9267 2:N:0:TCTGCAAG+GCTGCACT
CCAACACTCTGGACAGACGACGAGTCCACCGTTCCATCACATCTTCACGGTGGAACTAAGATTCAACT
ACAATCTCAGAATGCAGTTGCGGATAAAGCGAGCATGGTGGACTATCCGCATCTGGACGACATTACAA
CCAGTCCCAGAATTA
```

+
FFF:FFFFFFFFFFFFFFFFFFFFFF:FFFFFFFFFFFFFF:FFFFFFFFFFFFFFFFFFFFF:FFFF
FFFFFFFFFFFFFFFFFFFFFFFFFFFFFFFFFFF:FFFFFFFFFFFFFFFFFFFFFFFFFF:FFF:F
FFFFFFFFFFFFF,:
@A00721:190:HGJW7DSXY:4:2529:22968:9267 2:N:0:TCTGCAAG+GCTGCACT
CCAACACTCTGGACAGACGACGAGTCCACCGTTCCATCACATCTTCACGGTGGAACTAAGATTCAACT
ACAATCTCAGAATGCAGTTGCGGATAAAGCGAGCATGGTGGACTATCCGCATCTGGACGACATTACAA
CCAGTCCCAGAATTA
+
FFFFFFFFFFFFFFFFFFFFFFFFFFFFFFFFFFFFFFFFFFFFFFFFFFFFFFFFFFFFFFFFFFFF
FFFF::FFFFFFFFFFFFFFFFFFFFFFFFFFFFFFFFFFFFFFFFFFF:FFFFF:FFFFFFFFFFFF
FFFFFFFFFFFFFFF
@A00721:190:HGJW7DSXY:4:2633:7319:6965 2:N:0:TCTGCAAG+GCTGCACT
CCAACACTCTGGACAGACGACGAGTCCACCGTTCCATCACATCTTCACGGTGGAACTAAGATTCAACT
ACAATCTCAGAATGCAGTTGCGGATAAAGCGAGCATGGTGGACTATCCGCATCTGGACGACATTACAA
CCAGTCCCAGAATTA
+
FFFFF:FFFFFFFFFFFFFFFFFFFFFFFFFFFFFFFFFFFFFFFFFFFFFFFFFFFFFFFFFFF:FF
FFFFFFFFFFFFFFFFFFFFFFFFFFFFFFFFFFFFFFFFFFFFFF:FFFFFF:FFFFFFFFF:FFFF
FFFFFFFFFFFFFFF
@A00721:190:HGJW7DSXY:4:1563:20546:32440 2:N:0:TCTGCAAG+GCTGCACT
CCAACACTCTGGACAGACGACGAGTCCACCGTTCCATCACATCTTCACGGTGGAACTAAGATTCAACT
ACAATCTCAGAATGCAGTTGCGGATAAAGCGAGCATGGTGGACTATCCGCATCTGGACGACATTACAA
CCAGTCCCAGAATT
+
FFFFFFFFFFFFFFFFFFFFFFFFFFFFF:FFFFFFFFFFFFFFFFFFFFFFFFFF:FFF:FFFFFFF
FFFFFFFFFFFFFFFFFFFFFFFFFFFFFFFFFFFFFFFFFFFFFFFFFFFFFFFFFFFFFFFFFFFF
FFFFFF:FFFFFFF
@A00721:190:HGJW7DSXY:4:1103:5330:4022 2:N:0:TCTGCAAG+GCTGCACT
TACGAGACCAACACTCTGGACAGACGACGAGTCCACCGTTCCATCACATCTTCACGGTGGAACTAAGA
TTCAACTACAATCTCAGAATGCAGTTGCGGATAAAGCGAGCATGGTGGACTATCCGCATCTGGACGAC
ATTACAACCAGTCCC
+
FFFFFF,FFFFFFFFFFFFFFFFFFFFFFFFFFFFFFFFFFFFFFFFFFFFFFFFFFFFFF:FFFFFF
:FFFFFFFFFFFFFFFFFFFFFFFFFFFFFFFFFFFFFFFFFFFFFFFFFFFFFFFFFFFFFFFFFFF
FFFFFF:FF:FFFFF
@A00721:190:HGJW7DSXY:4:1239:31828:23891 2:N:0:TCTGCAAG+GCTGCACT
TACGAGTCCAACACTCTGGACAGACGACGAGTCCACCGTTCCATCACATCTTCACGGTGGAACTAAGA
TTCAACTACAATCTCAGAATGCAGTTGCGGATAAAGCGAGCATGGTGGACTATCCGCATCTGGACGAC
ATTACAACCAGTCCC
+
FFFFFFFFFFF:FFFFFFFFFFFFFFFFFFFFFFFFFFFFFFFFFFFFFFFFFFFFFFFFF,FF,:FF
FFFFFFFFFFFFFFFFFFFFFFFFFFFFFFFFFFFFFFFFF:FFFFFFFFFFFFFFFFFFFFFFFFFF
FFFFFFFFF:FFFFF
@A00721:190:HGJW7DSXY:4:1344:23402:27899 2:N:0:TCTGCAAG+GCTGCACT
TACGAGTCCAACACTCTGGACAGACGACGAGTCCACCGTTCCATCACATCTTCACGGTGGAACTAAGA
TTCAACTACAATCTCAGAATGCAGTTGCGGATAAAGCGAGCATGGTGGACTATCCGCATCTGGACGAC
ATTACAACCAGTCCC
+
FFFFFFFFFFFFFFFFFFFFFFFFFFFFFFFFFFFFFFFFFFFFFFFFFFFFFFFFFFFFFFFFFFFF
FFFFFFFFFFFFFFFFFFFFFF:FFFFFFFFFFFFFFFFFFFFFFFFFFFFFFFFFFFFFFFFFFFFF
FFFFFFFFF,FFFFF
@A00721:190:HGJW7DSXY:4:1415:31901:33160 2:N:0:TCTGCAAG+GCTGCACT
TACGAGTCCAACACTCTGGACAGACGACGAGTCCACCGTTCCATCACATCTTCACGGTGGAACTAAGA

```
TTCAACTACAATCTCAGAATGCAGTTGCGGATAAAGCGAGCATGGTGGACTATCCGCATCTGGACGAC
ATTACAACCAGTCCC
+
FFFFFFFFFFFFFFFFFFFFFFFFFFFFFFFFFFFFFFFFFFFFFFFFFFFFFFFFFFFFFFFFFFFF
FFFFFFFFFFFFFFFFFFFFFFFF:FFFFFFFFF:FFFFFFFFFFFFFFFFFFFFFFFFFFFFFFFFF
F:FFFFFFFFFFFFF
@A00721:190:HGJW7DSXY:4:1415:30770:33739 2:N:0:TCTGCAAG+GCTGCACT
TACGAGTCCAACACTCTGGACAGACGACGAGTCCACCGTTCCATCACATCTTCACGGTGGAACTAAGA
TTCAACTACAATCTCAGAATGCAGTTGCGGATAAAGCGAGCATGGTGGACTATCCGCATCTGGACGAC
ATTACAACCAGTCCC
+
FFF:FFFFFF:FFFFFFFFFFFFFFFFFFFFFFF:FFFFFFFFFFFFFFFFFFFFF:F:FFF:FFFFF
FFFFFFFF,FFF,FFFFFFFFFFFFF,FFFFF::F:FFFFFFFFFFFFFFFFFFFFFFFFFFFFF,FF
:FFFFFF:FF::F,F
@A00721:190:HGJW7DSXY:4:1547:4770:15295 2:N:0:TCTGCAAG+GCTGCACT
TACGAGTCCAACACTCTGGACAGACGACGAGTCCACCGTTCCATCACATCTTCACGGTGGAACTAAGA
TTCAACTACAATCTCAGAATGCAGTTGCGGATAAAGCGAGCATGGTGGACTATCCGCATCTGGACGAC
ATTACAACCAGTCCC
+
FFFFF:FFFF:FFFFFFFFFFFFFFFFFFFFFFFFFFFFFFFFFFFFFFF:FFFF:FFF:FFFF,FFF
FFFFFFFFFFFFFFFFFFFFFFFFFFF:FFFFFFFFFFFFFFFFFFFF:FF,FFFFF:,FFF:FFFFF
F:F,:FFFFFFFFFF
@A00721:190:HGJW7DSXY:4:2129:3314:13401 2:N:0:TCTGCAAG+GCTGCACT
TACGAGTCCAACACTCTGGACAGACGACGAGTCCACCGTTCCATCACATCTTCACGGTGGAACTAAGA
TTCAACTACAATCTCAGAATGCAGTTGCGGATAAAGCGAGCATGGTGGACTATCCGCATCTGGACGAC
ATTACAACCAGTCCC
+
FFFFFFFFFFFFFFFFFFFFFFFFFFFFFFFFFFFFFF:FFFFFFFFFFFFFFFFFFFFFF:FFFFFF
FFFFFFFFFFFFFFFFFFFF:FFFFF:FFFFFFFFFFFFFFFFFFFFFFFFFFFFFFFFFFFFFFFFF
FFFFFFF:FFFFF,F
@A00721:190:HGJW7DSXY:4:2139:29225:35446 2:N:0:TCTGCAAG+GCTGCACT
TACGAGTCCAACACTCTGGACAGACGACGAGTCCACCGTTCCATCACATCTTCACGGTGGAACTAAGA
TTCAACTACAATCTCAGAATGCAGTTGCGGATAAAGCGAGCATGGTGGACTATCCGCATCTGGACGAC
ATTACAACCAGTCCC
+
FFFFFFFFFFFFFFFFFFFFFFFFFFFFFFFFFFFFFFFFFFFFFFFFFFFFFFFFFFFFFFFFFFFF
FFFFFFFFFFFFFFFFFFFFFFFFFFFFFFFFFFFFFFFFFFFFFFFFFFFFFFFFFFFFFFFFFFFF
FFFFFFFFFFFFFFF
@A00721:190:HGJW7DSXY:4:2326:4779:25645 2:N:0:TCTGCAAG+GCTGCACT
TACGAGTCCAACACTCTGGACAGACGACGAGTCCACCGTTCCATCACATCTTCACGGTGGAACTAAGA
TTCAACTACAATCTCAGAATGCAGTTGCGGATAAAGCGAGCATGGTGGACTATCCGCATCTGGACGAC
ATTACAACCAGTCCC
+
F:FFFFFFFFFFFFFFFFFFFFFFFFFFFFFFFFFFFFFFFFFFFFFFFFFFFFFFFFFFF,FFFFFF
FFFFFFFFFFFFFFFFFFFFFFFFFFFFFFFFFFFFFFFFFFFFFFFFFFFFFFFFFFFFFFFFFFFF
FFFFFFFFF:FFFFF
@A00721:190:HGJW7DSXY:4:2333:6234:26537 2:N:0:TCTGCAAG+GCTGCACT
TACGAGTCCAACACTCTGGACAGACGACGAGTCCACCGTTCCATCACATCTTCACGGTGGAACTAAGA
TTCAACTACAATCTCAGAATGCAGTTGCGGATAAAGCGAGCATGGTGGACTATCCGCATCTGGACGAC
ATTACAACCAGTCCC
+
FFFFFFFFFFFFFF:FFFFFFFF:FFFFFFFFFFFFFFFFFFFFFFFFFFFFFFFF:FFFFFFFF:FF
FFFFFFF:FFFFFFFFFFFFFFFFFFFFFFFFFFFFFFFFF,FFFFFFFFF:FFFFFFFFFFFFFFFF
FFFFFFFFFFFFF,F
```

@A00721:190:HGJW7DSXY:4:2372:19524:9690 2:N:0:TCTGCAAG+GCTGCACT
TACGAGTCCAACACTCTGGACAGACGACGAGTCCACCGTTCCATCACATCTTCACGGTGGAACTAAGA
TTCAACTACAATCTCAGAATGCAGTTGCGGATAAAGCGAGCATGGTGGACTATCCGCATCTGGACGAC
ATTACAACCAGTCCC
+
FFFFFFFFFFFFFFFFFFFFFFFFFFFFFFFFFFFFFFFFFFFFFFFFFFFFFFFFFFFFFFFFFFFFFF
FFFFFFFFFFFFFFFFFFFFFFFFFFFFFF:FFFFFFFFFFFFFFFFFFFFFFFFFFFFFFFFFFFFFFF
FFFFFFFFFFFFFFF
@A00721:190:HGJW7DSXY:4:2460:24361:28307 2:N:0:TCTGCAAG+GCTGCACT
TACGAGTCCAACACTCTGGACAGACGACGAGTCCACCGTTCCATCACATCTTCACGGTGGAACTAAGA
TTCAACTACAATCTCAGAATGCAGTTGCGGATAAAGCGAGCATGGTGGACTATCCGCATCTGGACGAC
ATTACAACCAGTCCC
+
FFFFFFFFFFFFFFFFFFFFFFFFFFFFFFFFFFFFFFFFFFFFFFFFFFFFFFFFFFFFFFFFFFFFFF
FFFFFFFFFFFFFFFFFFFFFFFFF:FFFFFFFFFFFFFFFFFFFFFFFFFFFFFFFFFFFFFFFFFFFF
FFFFFFFFFFFFFFF
@A00721:190:HGJW7DSXY:4:2652:25807:20134 2:N:0:TCTGCAAG+GCTGCACT
TACGAGTCCAACACTCTGGACAGACGACGAGTCCACCGTTCCATCACATCTTCACGGTGGAACTAAGA
TTCAACTACAATCTCAGAATGCAGTTGCGGATAAAGCGAGCATGGTGGACTATCCGCATCTGGACGAC
ATTACAACCAGTCCC
+
FFFFFFFFFFFFFFFFFFFFFFFFFFFFFFFFFFFFFFFFFFFFFFFFFFFFFFFFFFFFFFFFFFFFFF
FFFFFFFFFFFFFFFFFFFFFFFFFFFFFFFFFFFFFFFFFFFFFFFFFFFFFFFFFFF,FFFFFFFFFF
FFFFFFFFFFFFFFF
@A00721:190:HGJW7DSXY:4:1543:3197:4460 2:N:0:TCTGCAAG+GCTGCACT
AACAATCCAATATGGATACGAGTCCAACACTCTGGACAGACGACGAGTCCACCGTTCCATCACATCTT
CACGGTGGAACTAAGATTCAACTACAATCTCAGAATGCAGTTGCGGATAAAGCGAGCATGGTGGACTA
TCCGCATCTGGACGA
+
FFFFFFFFFFFFFFFFFFFFFFFFFFFFFFFFFFFFFFFFFFFFFFFFFFFFFFFFFFFFFFFFFFFFFF
FFFFFFFFFFFFFFFFF:FFFFFFFFFFFFFFFFFFFFFFFFFFFFFFFFFFFFFFFFFFFFFFFFFFFF
FFFFFFFFFFFFFFF
@A00721:190:HGJW7DSXY:4:1605:18982:27884 2:N:0:TCTGCAAG+GCTGCACT
AACAATCCAATATGGATACGAGTCCAACACTCTGGACAGACGACGAGTCCACCGTTCCATCACATCTT
CACGGTGGAACTAAGATTCAACTACAATCTCAGAATGCAGTTGCGGATAAAGCGAGCATGGTGGACTA
TCCGCATCTGGACGA
+
FFFFFFFFFFFFFFFFFFFFFFFFFFF:FFFFFFFFFFFFFFFFFFFFFFFFFFFFFFFFFFFFFFFFFF
FFFFFFFFFFFFFFFFFFFFFFFF:FFFFFFFFFFFFFFFFFFFFFFFFFFFFFFFFFFF,FFFFFFF:F
FFFFFFFFFFFFFFF
@A00721:190:HGJW7DSXY:4:2125:6795:17456 2:N:0:TCTGCAAG+GCTGCACT
AACAATCCAATATGGATACGAGTCCAACACTCTGGACAGACGACGAGTCCACCGTTCCATCACATCTT
CACGGTGGAACTAAGATTCAACTACAATCTCAGAATGCAGTTGCGGATAAAGCGAGCATGGTGGACTA
TCCGCATCTGGACGA
+
FFFFFFFFFFFFFFFFFFFFFFFFFFFFFFFFFFFFFFFFFFFFFFFFFFFFFFFFFFFFFFFFFFFFFF
FFFFFFFFFFFFFFFFFFFFFFFFFFFFFFFFFFFFFFFFFFFFFFFFFFFFFFFFFFF:FFFFFFFFFF
FFFFFFFFFFFFFFF
@A00721:190:HGJW7DSXY:4:2231:29215:15577 2:N:0:TCTGCAAG+GCTGCACT
AACAATCCAATATGGATACGAGTCCAACACTCTGGACAGACGACGAGTCCACCGTTCCATCACATCTT
CACGGTGGAACTAAGATTCAACTACAATCTCAGAATGCAGTTGCGGATAAAGCGAGCATGGTGGACTA
TCCGCATCTGGACGA
+
FFFFFFFFFFFFFFFFFFFFFFFFFFF:F,FFFFFFFFFFFFF:FFFFFFFFFFFFFFFFFFFFFF,FFF

F:FFFFFFFFFF:FFFFFFF:FFFFF:FFFFFFFFFFFFFFFFFFFFF,FFFFFFFF:FFFFFF,FFF
FFFFFFFFFFFFFFF
@A00721:190:HGJW7DSXY:4:2312:1253:25958 2:N:0:TCTGCAAG+GCTGCACT
AACAATCCAATATGGATACGAGTCCAACACTCTGGACAGACGACGAGTCCACCGTTCCATCACATCTT
CACGGTGGAACTAAGATTCAACTACAATCTCAGAATGCAGTTGCGGATAAAGCGAGCATGGTGGACTA
TCCGCATCTGGACGA
+
FFFFFFFFFFFFFFFFFFFFFFFFFFFFFFFFFFFFFFFFFFFFFFFFFFFFFFFFFFFFFFFFFFFF
FFFFFFFFFFFFFFFFFFFFFFFFFFFFFFFFFFFFFFFFFFFFFFFFFFFFFFFFFF:FFFFFFFFF
FFFFFFFFFFFFFFF
@A00721:190:HGJW7DSXY:4:2448:31232:32095 2:N:0:TCTGCAAG+GCTGCACT
AACAATCCAATATGGATACGAGTCCAACACTCTGGACAGACGACGAGTCCACCGTTCCATCACATCTT
CACGGTGGAACTAAGATTCAACTACAATCTCAGAATGCAGTTGCGGATAAAGCGAGCATGGTGGACTA
TCCGCATCTGGACGA
+
FFFFFFFFFFFFFFFFFFFFFFFFFF:FFFFFFFFFFFFFFFFFFFFFFFFFFFFFFFFFFFFFFFFF
FFFFFFFF:FFF:FFFFFF:FFFFFFFFFFFFFFFFFFFFFFFFFFFFFFFFFFFFFFFFFFFFFFFF
FFFFFF:FFFFFFFF
@A00721:190:HGJW7DSXY:4:1350:25825:7921 2:N:0:TCTGCAAG+GCTGCACT
AAACAATCCAATATGGATACGAGTCCAACACTCTGGACAGACGACGAGTCCACCGTTCCATCACATCT
TCACGGTGGAACTAAGATTCAACTACAATCTCAGAATGCAGTTGCGGATAAAGCGAGCATGGTGGACT
ATCCGCATCTGGACG
+
FFFFFFFFFFFFFFFFFFFFFFFFFFFFFFFFFFFFFFFFFFFFFFFFFFFFFFFFFFFFFFFFFFFF
FFFFFFFFFFFFFFFFFFFFFFFFFFFFFFFFF:FFFFFFFFFFFFFFFFFFFFFFFFFFFFFFFFFF
FFFFFFFFFFFFFFF
@A00721:190:HGJW7DSXY:4:1415:17499:15953 2:N:0:TCTGCAAG+GCTGCACT
AAACAATCCAATATGGATACGAGTCCAACACTCTGGACAGACGACGAGTCCACCGTTCCATCACATCT
TCACGGTGGAACTAAGATTCAACTACAATCTCAGAATGCAGTTGCGGATAAAGCGAGCATGGTGGACT
ATCCGCATCTGGACG
+
FFFFFFFFFFFFFFFFFFFFFFFFFFFFFFFFFFFFFFFFFFFFFFFFFFFFFFFFFFFFFFFFFFFF
FFFFFF:FFF:FFFFFFFFFFFFFFFFFFFFFFFFF::FFFF:FFFFFFFFFFFFFFFFF:FFFFFFFF
FFFFFFFFFFFFFFF
@A00721:190:HGJW7DSXY:4:2232:4869:13369 2:N:0:TCTGCAAG+GCTGCACT
AAACAATCCAATATGGATACGAGTCCAACACTCTGGACAGACGACGAGTCCACCGTTCCATCACATCT
TCACGGTGGAACTAAGATTCAACTACAATCTCAGAATGCAGTTGCGGATAAAGCGAGCATGGTGGACT
ATCCGCATCTGGACG
+
FFFFFFFFFFFFFFFFFFFFFFFFFFFFFFFFFFFFFFFFFFFFFFFFFFFFFFFFFFFFFFFFFFFF
FFFFFFFFFFFFFFFFFF:FFFFFFFFFFFFFFFFFFFFFFFFFFFFFFFFFFFFFFFFFFFFFFFFF
FFFFFFFFFFFFFF:
@A00721:190:HGJW7DSXY:4:2246:28565:20337 2:N:0:TCTGCAAG+GCTGCACT
AAACAATCCAATATGGATACGAGTCCAACACTCTGGACAGACGACGAGTCCACCGTTCCATCACATCT
TCACGGTGGAACTAAGATTCAACTACAATCTCAGAATGCAGTTGCGGATAAAGCGAGCATGGTGGACT
ATCCGCATCTGGACG
+
FFFFFFFFFFFFFFFFFFFFFFFFFFFFFFFFFFFFFFFFFFFFFFFFFFFFFFFFFFFFFFFFFFFF
FFFFFFFFFFFFFFFFFFFFFFFFFFFFFFFFFFFFFFFFFFFFF:FFFFFFFFFFFFFFFFFFFFFFFF
FFFFFFFFFFFFFFF
@A00721:190:HGJW7DSXY:4:2678:2645:10363 2:N:0:TCTGCAAG+GCTGCACT
AAACAATCCAATATGGATACGAGTCCAACACTCTGGACAGACGACGAGTCCACCGTTCCATCACATCT
TCACGGTGGAACTAAGATTCAACTACAATCTCAGAATGCAGTTGCGGATAAAGCGAGCATGGTGGACT
ATCCGCATCTGGACG

```
+
FFFFFFFFFFFFFFFFFFFFFFFFFFFFFFFFFFFFFFFFFFFFFFFFFFFFFFFFFFFFFFFFFFFFFF
FFFFFFFFFFFFFFFFFFFFFFFFFFFFFFFFFFFFFFFFFFFFFFFFFFFFFFFFFFFFFFFFFFFFFF
FFFFFFFFFFFFFFF
@A00721:190:HGJW7DSXY:4:1309:14633:10457 2:N:0:TCTGCAAG+GCTGCACT
AAACAATCCAATATGGATACGAGTCCAACACTCTGGACAGACGACGAGTCCACCGTTCCATCACATCT
TCACGGTGGAACTAAGATTCAACTACAATCTCAGAATGCAGTTGCGGATAAAGCGAGCATGGTGGACT
ATCCGCATCTGGACA
+
FFFFFFFFFFFFFFFFFFFFFFFFFFFFFFFFFFFFFFFFF:FFFFFFFFFFFFFFF,FFFFFFFFFFFF
FFFFFFFFFFFFFFFFFFFFFFFFFFFFFFFFFFFFFFFFFFFFFFFFFFFFFFFFFFFFFFFFFFFFFF
FFFFFFFFFFFFFFF
@A00721:190:HGJW7DSXY:4:1309:14678:10504 2:N:0:TCTGCAAG+GCTGCACT
AAACAATCCAATATGGATACGAGTCCAACACTCTGGACAGACGACGAGTCCACCGTTCCATCACATCT
TCACGGTGGAACTAAGATTCAACTACAATCTCAGAATGCAGTTGCGGATAAAGCGAGCATGGTGGACT
ATCCGCATCTGGACA
+
FFFFFFFFFFFFFFFFFFFFFFFFFFFFFFFFFFFFFFFFFFFFFFFF:FFFFFFFFFFFFFFFFFFFFF
FFFFFFFFFFFFFFFFFFFF:FFFFFFFFFF:FFFFFFFFFFFFFFFFFFFFFFFFFFFFF:FFFFFFFF
FFFFFFF,FFFFFFF
@A00721:190:HGJW7DSXY:4:2643:29423:15812 2:N:0:TCTGCAAG+GCTGCACT
AAACAATCCAATATGGATACGAGTCCAACACTCTGGACAGACGACGAGTCCACCGTTCCATCACATCT
TCACGGTGGAACTAAGATTCAACTACAATCTCAGAATGCAGTTGCGGATAAAGCGAGCATGGTGGACT
ATCCGCATCTGGACA
+
FFFFFFFFFFFFFFFFFFFFFFFFFFFFFFFFFFFFFFFFFFFFFFFFFFFFFFFFFFFFFFFFFFFFFF
FFFFFFFFFFFFFFFFFFFFFFFFFFFFFFFFFFFFFFFFFFFFFFFFFFFFFFFFFFFFFFFFFFFFFF
FFFFFFF:FFFFFFF
@A00721:190:HGJW7DSXY:4:1617:17788:21089 2:N:0:TCTGCAAG+GCTGCACT
AAACAATCCAATATGGATACGAGTCCAACACTCTGGACAGACGACGAGTCCACCGTTCCATCACATCT
TCACGGTGGAACTAAGATTCAACTACAATCTCAGAATGCAGTTGCGGATAAAGCGAGCATGGTGGACT
ATCCGCATCTGGAC
+
FFFFFFFFFFFF:FFFFFF:FFFFFFFFFFFFFFFFFFFFFFFFFFFFF:FFFFFFFFFFFF,FFFF,F:
FFFFFFFFFFF:FFFFFFFFFF:FF:F,FFFFF:FFFFFFF:FFFFFFFFFFFFFFFFFFF,FFF:,FFF
FFFFFFFFFFFFFF
@A00721:190:HGJW7DSXY:4:1235:27010:19523 2:N:0:TCTGCAAG+GCTGCACT
AAACAATCCAATATGGATACGAGTCCAACACTCTGGACAGACGACGAGTCCACCGTTCCATCACATCT
TCACGGTGGAACTAAGATTCAACTACAATCTCAGAATGCAGTTGCGGATAAAGCGAGCATGGTGGACT
ATCCGCATCCGGAGA
+
FFFFFFFFFFFFFFFFFFFFFFFFFFFFFFFF:FFF:FFFFFFFFFFFFFFFFFFFFFFFFFFFFFFFFF
FF,FFFFFFFFFFFFFFFFFFFFFFF:FFFFFFFFFFFFFFF:FFFFFFFFFFFFFFFFFF,FFFFFFF,
FFFFFF,FFFFFFFF
@A00721:190:HGJW7DSXY:4:1269:2139:6637 2:N:0:TCTGCAAG+GCTGCACT
AAACAATCCAATATGGATACGAGTCCAACACTCTGGACAGACGACGAGTCCACCGTTCCATCACATCT
TCACGGTGGAACTAAGATTCAACTACAATCTCAGAATGCAGTTGCGGATAAAGCGAGCATGGTGGACT
ATCCGCATCCGGAGA
+
FFFFFFFFFFFFFFFFFFFFFFFFFFFFFFFFFFFFFFFFFFFFFFFFFFFFFFFFFFFFFFFFFFFFFF
FFFFFFFFFF,F:FFFFFFFFFFFFFFFFFFFFFFFFFFFFFFFFFFFFFFFFFFFFFFFFFFFFFFFFF
FFFFFFFFFFFFFFF
@A00721:190:HGJW7DSXY:4:1302:28402:3490 2:N:0:TCTGCAAG+GCTGCACT
AAACAATCCAATATGGATACGAGTCCAACACTCTGGACAGACGACGAGTCCACCGTTCCATCACATCT
```

```
TCACGGTGGAACTAAGATTCAACTACAATCTCAGAATGCAGTTGCGGATAAAGCGAGCATGGTGGACT
ATCCGCATCCGGAGA
+
FFFFFFFFFFF:FFFFFFFFFFFFFFFFFFFFFFFFFFFFFFFFFFFFFFFFFFFFFFFFFFFFFFFFFF
FFFFFFFFFFFFF,FFFFFFFFFFFFFFFFFFF:FFFFFFFFFFFFFFFFF:FFFFF:FFF,FFFFFFFF
FFFFFFFFFFFFFFF
@A00721:190:HGJW7DSXY:4:1513:17056:31156 2:N:0:TCTGCAAG+GCTGCACT
AAACAATCCAATATGGATACGAGTCCAACACTCTGGACAGACGACGAGTCCACCGTTCCATCACATCT
TCACGGTGGAACTAAGATTCAACTACAATCTCAGAATGCAGTTGCGGATAAAGCGAGCATGGTGGACT
ATCCGCATCCGGAGA
+
FFFFFFFFFFFFFFFFFFFFFFFFFFFFFFFFFFFFFFFFFFFFFFFFFFFFFFFFFFFFFFFFFFFFFF
FFFFFFFFFFFFFFFFFFFFFFFFFFFFFFFFFFFFFFFFFFFFFFFFFFFFFFFFFFFFFFFFFFFFFF
FFFFFFFFFFFFFFF
@A00721:190:HGJW7DSXY:4:1602:13141:1799 2:N:0:TCTGCAAG+GCTGCACT
AAACAATCCAATATGGATACGAGTCCAACACTCTGGACAGACGACGAGTCCACCGTTCCATCACATCT
TCACGGTGGAACTAAGATTCAACTACAATCTCAGAATGCAGTTGCGGATAAAGCGAGCATGGTGGACT
ATCCGCATCCGGAGA
+
FFFFFFFFFFFFFFFFFFFFFFFFFFFFFFFFFFFFFFFFFFFFFFFFFFFFFFFFFFFFFFFFFFFFFF
FFFFFFFFFFFFFFFFFFFFFFFFFFFFFFFFFFFFFFFFFFFFFFFFFFFFFFFFFFFFFFFFFFFFFF
,FFFFFFFFFFFFFF
@A00721:190:HGJW7DSXY:4:1662:3269:20462 2:N:0:TCTGCAAG+GCTGCACT
AAACAATCCAATATGGATACGAGTCCAACACTCTGGACAGACGACGAGTCCACCGTTCCATCACATCT
TCACGGTGGAACTAAGATTCAACTACAATCTCAGAATGCAGTTGCGGATAAAGCGAGCATGGTGGACT
ATCCGCATCCGGAGA
+
FFFFFFFFFFFFFFFFFFFFFFFFFFFFFFFFFFFFFFFFFFFFFFFFFFFFFFFFFFFFFFFFFFFFFF
FFFFFFFFFF,FFFFFFFFFFFFFFFFFFFFFFFFFFFFFFFFFFFFFFFFF:FFFF:FFFFFF:FFFFF
FFFFFFFFFFFFFFF
@A00721:190:HGJW7DSXY:4:1667:16369:6981 2:N:0:TCTGCAAG+GCTGCACT
AAACAATCCAATATGGATACGAGTCCAACACTCTGGACAGACGACGAGTCCACCGTTCCATCACATCT
TCACGGTGGAACTAAGATTCAACTACAATCTCAGAATGCAGTTGCGGATAAAGCGAGCATGGTGGACT
ATCCGCATCCGGAGA
+
FFFFFFFFFFFFFFFFFFFFFFFFFFFFFFFFFFFFFFFFFFFFFFFFFFFFFFFFFFFFFFFFFFFFFF
FFFFFFFFFF:FF,FFFFFFFFFFFFFFFFFFFFFFFFFFFFFFFFFFFFF,FFFFFFFFF,FFFFFFFF
FFFFFFFFFFFFFFF
@A00721:190:HGJW7DSXY:4:2210:2627:12273 2:N:0:TCTGCAAG+GCTGCACT
AAACAATCCAATATGGATACGAGTCCAACACTCTGGACAGACGACGAGTCCACCGTTCCATCACATCT
TCACGGTGGAACTAAGATTCAACTACAATCTCAGAATGCAGTTGCGGATAAAGCGAGCATGGTGGACT
ATCCGCATCCGGAGA
+
FFFFFFFFFFFFFFFFFFFFFFFFFFFFFFFFFFFFFFFFFFFFFFFFFFFFFFFFFFFFFFFFFFFFFF
FFFFFFFFFFFFFFFFFFFFFFFFFFFFFFFFFFFFFFFFFFFFFFFFF:FFFFFFFFFFFFFFFFFFFF
FFFFFFFFFFFFFFF
@A00721:190:HGJW7DSXY:4:2307:29894:26616 2:N:0:TCTGCAAG+GCTGCACT
AAACAATCCAATATGGATACGAGTCCAACACTCTGGACAGACGACGAGTCCACCGTTCCATCACATCT
TCACGGTGGAACTAAGATTCAACTACAATCTCAGAATGCAGTTGCGGATAAAGCGAGCATGGTGGACT
ATCCGCATCCGGAGA
+
FFFFFFFFFFFFFFFFFFFFFFFFFFFFFFFFFFFFFFFFFFFFFFFFFFFFFFFFFFFFFFFFFFFFFF
FFFFFFFFFF:FFFFFFFFFFFFFFFFFFFFFFFFFFFFFFFFFFFFFFFFFFFFFFFFFFFFFFFFFFF
FFFFFFFFFFFFFFF
```

```
@A00721:190:HGJW7DSXY:4:2307:29785:26772 2:N:0:TCTGCAAG+GCTGCACT
AAACAATCCAATATGGATACGAGTCCAACACTCTGGACAGACGACGAGTCCACCGTTCCATCACATCT
TCACGGTGGAACTAAGATTCAACTACAATCTCAGAATGCAGTTGCGGATAAAGCGAGCATGGTGGACT
ATCCGCATCCGGAGA
+
FFFFFFFFFFFFFFFFFFFFFFFFFFFFFF:FFFFFFFFFFFFFFFFFFFFFFFFFFFFFFFFFFFFFF
FFFFFFFFFFFFFFFFFFFFFFFFFFFFFFFFFFFFFFFFFFFFFFFFFFFFFFFFFFF::FFFFFFFF
:FFFFFFFFFFFFFF
@A00721:190:HGJW7DSXY:4:2421:30101:35493 2:N:0:TCTGCAAG+GCTGCACT
AAACAATCCAATATGGATACGAGTCCAACACTCTGGACAGACGACGAGTCCACCGTTCCATCACATCT
TCACGGTGGAACTAAGATTCAACTACAATCTCAGAATGCAGTTGCGGATAAAGCGAGCATGGTGGACT
ATCCGCATCCGGAGA
+
FFFFFFFFFFFFFFFFFFFFFFFFFFFFFFFFFFFFFFFFFFFFFFFFFFFFFFFFFFFFFFFFFFFFF
FFFFFFFFFF:FFFFFFFFFFFFFFFFFFFFFFFFFFFFFFF:FFFFFFFFF:FFFFFFFFFFFFFFFF
FFFFFFFFFFFFFFF
@A00721:190:HGJW7DSXY:4:2177:9091:36714 2:N:0:TCTGCAAG+GCTGCACT
CCTAAACAATCCAATATGGATACGAGTCCAACACTCTGGACAGACGACGAGTCCACCGTTCCATCACA
TCTTCACGGTGGAACTAAGATTCAACTACAATCTCAGAATGCAGTTGCGGATAAAGCGAGCATGGTGG
ACTATCCGCATCTGG
+
FFFFFFFFFFFFFFFFF:FFFFFFFFFFFFFFFFFFFFFFFFFFFFFFFFFFFFFFFFFFFFFFFFFFF
FFFFFFF:FFFFFFFFFFF:FFFFFFFFFFFFFFFFFFFFFFF,FFFFFFFFFF:,FFFFFFFFFFFFF
FFFFFFFFFFFFFFF
@A00721:190:HGJW7DSXY:4:2543:4282:9314 2:N:0:TCTGCAAG+GCTGCACT
CCTAAACAATCCAATATGGATACGAGTCCAACACTCTGGACAGACGACGAGTCCACCGTTCCATCACA
TCTTCACGGTGGAACTAAGATTCAACTACAATCTCAGAATGCAGTTGCGGATAAAGCGAGCATGGTGG
ACTATCCGCATCTGG
+
FFFFFFFFFFFFFFFFFFFFF:FFFFFFFFFFFFFFFFFFFFFFFFFFFFFFFFFFFFFFFFFFFFFFF
FFFFFFFFFFFFFFFFFFFFFFFFFFFFFFFFFFFFFFFFFFFFFFFFFFFFFFFFFFFFFFFFFFFFF
FFFFFFFFFFFFFFF
@A00721:190:HGJW7DSXY:4:2543:4942:9831 2:N:0:TCTGCAAG+GCTGCACT
CCTAAACAATCCAATATGGATACGAGTCCAACACTCTGGACAGACGACGAGTCCACCGTTCCATCACA
TCTTCACGGTGGAACTAAGATTCAACTACAATCTCAGAATGCAGTTGCGGATAAAGCGAGCATGGTGG
ACTATCCGCATCTGG
+
FFFFFFFFFFFFFFFFFFFFFFFFFFFFFFFFFFFFFFFFFFFFFFFFFFFFFFFFFFFFFFFFFFFFF
FFFFFFFFFFFFFFFFFFFFFFFF:FFFFFFFFFFFFFFFFFFFFFFFFFFFFFFFFFFFFFFFFFFFF
FFF:FFFFFFFFFFF
@A00721:190:HGJW7DSXY:4:2601:13494:21668 2:N:0:TCTGCAAG+GCTGCACT
CCTAAACAATCCAATATGGATACGAGTCCAACACTCTGGACAGACGACGAGTCCACCGTTCCATCACA
TCTTCACGGTGGAACTAAGATTCAACTACAATCTCAGAATGCAGTTGCGGATAAAGCGAGCATGGTGG
ACTATCCGCATCTGG
+
FFFFFFFFFFFFFFFFFFFFFFFFFFFFFFFFFFFFFFFFFFFFFFFFFFFFFFFFFFFFFFFFFFFFF
FFFFFFFFFFFFF:FFFFFFFFFFFFFFFFFFFFFFFFFFFFFFFFFFFFFFFFFFFFF,FFFFFFFFF
FFF:FFFFFFFFFFF
@A00721:190:HGJW7DSXY:4:1636:10140:1172 2:N:0:TCTGCAAG+GCTGCACT
CTGGAACCTAAACAATCCAATATGGATACGAGTCCAACACTCTGGACAGACGACGAGTCCACCGTTCC
ATCACATCTTCACGGTGGAACTAAGATTCAACTACAATCTCAGAATGCAGTTGCGGATAAAGCGAGCA
TGGTGGACTATCCGC
+
FFFFFFFFFFFFFFFFFFFFFFFFFFFFFFFFFFFFFFFFFFFFFFFFFFFFFFFFFFFFFFFFFFFFF
```

```
FFFFFFFFFFFFFFFFFFFFFFFFFFFFFFFFFFFFFFFFFFFFFFFFFFFFFFFFFFFFFFFFFFFF
FFFFFFFFFFFFFFF
@A00721:190:HGJW7DSXY:4:1640:4860:13041 2:N:0:TCTGCAAG+GCTGCACT
CTGGAACCTAAACAATCCAATATGGATACGAGTCCAACACTCTGGACAGACGACGAGTCCACCGTTCC
ATCACATCTTCACGGTGGAACTAAGATTCAACTACAATCTCAGAATGCAGTTGCGGATAAAGCGAGCA
TGGTGGACTATCCGC
+
FFFFFFFFFFFFFFFFFFFFFFFFFFFFFFFFFFFFFFFFFFFFFFFFFFFFFFFFFFFFFFFFFFFF
FFFFFFFFFFFFFFFFFFFFFFFFFFFFFFFFFFFFFFFFFFFFFFFFFFFF:FFFFF:FFFFFFFFFFF
,FFFFFFFFFFFFFF
@A00721:190:HGJW7DSXY:4:2355:4435:23328 2:N:0:TCTGCAAG+GCTGCACT
CTGGAACCTAAACAATCCAATATGGATACGAGTCCAACACTCTGGACAGACGACGAGTCCACCGTTCC
ATCACATCTTCACGGTGGAACTAAGATTCAACTACAATCTCAGAATGCAGTTGCGGATAAAGCGAGCA
TGGTGGACTATCCGC
+
FFFFFFFFFFFFFFFFFFFFFFFFFFFFFFFFFFFFFFFF:FFFFFFFFFFFFFFFFFFFFFFFF:FFF
FFFFFFFFFFFFFF:FFFFFFFFFFFF:FFFF,FFFFFFFFFFFFF:FFFFFFFFFFFFFF:FFFFF,
FFFFFFFFFFFFFFF
@A00721:190:HGJW7DSXY:4:2607:15347:23657 2:N:0:TCTGCAAG+GCTGCACT
CTGGAACCTAAACAATCCAATATGGATACGAGTCCAACACTCTGGACAGACGACGAGTCCACCGTTCC
ATCACATCTTCACGGTGGAACTAAGATTCAACTACAATCTCAGAATGCAGTTGCGGATAAAGCGAGCA
TGGTGGACTATCCGC
+
FFFFF::FF:F,FFFFFFFFF:FFFFFFFFFFFFFFFFFFF:FFFFFFFFFFFF:FFFFFFFFFFFFFF
FFFFFFFFFFF:FFFFFFF:F:FF:F:FFFFFFFFFFFFFFFFF:FFF,FFFFFFFFF:FFFFFFFF,
FFFFFFFFFFFFFFF
@A00721:190:HGJW7DSXY:4:1127:19596:2675 2:N:0:TCTGCAAG+GCTGCACT
CTGGAACCTAAACAATCCAATATGGATACGAGTCCAACACTCTGGACAGAAGACGAGTCCACCGTTCC
ATCACAGCTTCACGGTGGAACTAAGATTCAACTACAATCTCAGAATGCAGTTGCGGATAAAGCGAGCA
TGGTGGACTATCCG
+
FFFFF:FF::FFFFF:FFFFF:FFFFFF:FF:F:FF:FFFFFFFFFFFFF,FFFFF:FF,FFFFFFFF
FFFFF,,FFFFFFFFFF:FFFFFF:FFF:FFFF,FFFFFF:FF:FFFFFFFFFFFFF:FFF,FFFF,,
,:FFFFFF:FFFFF
@A00721:190:HGJW7DSXY:4:1501:31611:23578 2:N:0:TCTGCAAG+GCTGCACT
ACTGGAACCTAAACAATCCAATATGGATACGAGTCCAACACTCTGGACAGACGACGAGTCCACCGTTC
CATCACATCTTCACGGTGGAACTAAGATTCAACTACAATCTCAGAATGCAGTTGCGGATAAAGCGAGC
ATGGTGGACTATCCG
+
FFFFFFFFFFFFFFFFFFFFFFFFFFF:FFFFFFFFFFFFFFFFFFFFFFFFFFF:FFFFFFFFFFFFFF
FFFFFFFFFFFFFFFFFFFFFFF:FFFFFFFFFFFFFFFFFFFFFFFFFFFFFFFFFFFFFFFFFFFFF
FFFFFFFFFFFFFFF
@A00721:190:HGJW7DSXY:4:1502:24831:24142 2:N:0:TCTGCAAG+GCTGCACT
ACTGGAACCTAAACAATCCAATATGGATACGAGTCCAACACTCTGGACAGACGACGAGTCCACCGTTC
CATCACATCTTCACGGTGGAACTAAGATTCAACTACAATCTCAGAATGCAGTTGCGGATAAAGCGAGC
ATGGTGGACTATCCG
+
FFFFFFFFF:FFFFFFFFFFFFFFFFFFFFFFFFFFFFF:FFFFFFFFFFFFFFFFFFFF:FFFFFFF,,
FFFFFFFFFFFFFFFFFFFFFFFFFFFFFFFFFFFFFFFFFFFFFFFF:FFFFFFFFFF:FFFFFFFF
F,FFFFFFFFFFFFF
@A00721:190:HGJW7DSXY:4:1523:19506:28917 2:N:0:TCTGCAAG+GCTGCACT
ACTGGAACCTAAACAATCCAATATGGATACGAGTCCAACACTCTGGACAGACGACGAGTCCACCGTTC
CATCACATCTTCACGGTGGAACTAAGATTCAACTACAATCTCAGAATGCAGTTGCGGATAAAGCGAGC
ATGGTGGACTATCCG
```

```
+
FFFFFFFFFFFFFFFFFFFFFFFFFFFFFFFFFFFFFFFFFFFFF:FFFFFFFFFFFFFFFFFFFFFFFF
FFFFFFFFFFFFFFFFFFFFFFFFFFFFFFFFFFFFFFFFFFFFFFFFFFFFFFFFFFFFFFFFFFFFFF
FFFFFFFFFFFFFFF
@A00721:190:HGJW7DSXY:4:1546:1696:19711 2:N:0:TCTGCAAG+GCTGCACT
ACTGGAACCTAAACAATCCAATATGGATACGAGTCCAACACTCTGGACAGACGACGAGTCCACCGTTC
CATCACATCTTCACGGTGGAACTAAGATTCAACTACAATCTCAGAATGCAGTTGCGGATAAAGCGAGC
ATGGTGGACTATCCG
+
:FFFFF:FFFFF:FFFFFFFFFFFFFFFFFFFFFFFFFFFFFFFFFFFFFFFFFFFFFFFFFFFFFF:FF
FFFFFFF,FFFFFFFFFFFF:FFFFFFFFFFFFFFFFFFFFFFFFF:FFFFFFF:FFFFFFFFFFF,FFF
,FFFFFFFF:FFFFF
@A00721:190:HGJW7DSXY:4:1555:11632:34695 2:N:0:TCTGCAAG+GCTGCACT
ACTGGAACCTAAACAATCCAATATGGATACGAGTCCAACACTCTGGACAGACGACGAGTCCACCGTTC
CATCACATCTTCACGGTGGAACTAAGATTCAACTACAATCTCAGAATGCAGTTGCGGATAAAGCGAGC
ATGGTGGACTATCCG
+
FFFFFFFFFFFFFFFFFFFFFFFFFFFFFFFFFFFFFFFFFFFFFFFFFFFFFFFFFFFFFFFFFFFFFF
FFFFFFFFFFFFFFFFFFFFFFFFFFFFFFFFFFFFFFFFFFFFFFFFFFFFFFFFFFFFFFFFFFFFFF
FFFFFFF:FFFFFFF
@A00721:190:HGJW7DSXY:4:1565:22679:25426 2:N:0:TCTGCAAG+GCTGCACT
ACTGGAACCTAAACAATCCAATATGGATACGAGTCCAACACTCTGGACAGACGACGAGTCCACCGTTC
CATCACATCTTCACGGTGGAACTAAGATTCAACTACAATCTCAGAATGCAGTTGCGGATAAAGCGAGC
ATGGTGGACTATCCG
+
FFFFFFFFFFFFFFFFFFFFFFFFFFFFFFFFFFFFFFFFFFFFFFFFFFFFFFFFFFFFFFFFFFFFFF
FFFFFFFFFFFFFFFFFFFFFFFFFFFFF:FFFFFFFFFFF:FFFF:FFFFFFFFFFFFFFFFFFFFFFF
:FFFFFFFFFFFFFF
@A00721:190:HGJW7DSXY:4:1573:22815:3552 2:N:0:TCTGCAAG+GCTGCACT
ACTGGAACCTAAACAATCCAATATGGATACGAGTCCAACACTCTGGACAGACGACGAGTCCACCGTTC
CATCACATCTTCACGGTGGAACTAAGATTCAACTACAATCTCAGAATGCAGTTGCGGATAAAGCGAGC
ATGGTGGACTATCCG
+
FFFFFFFFFFFF:FFFFFFFFFFFFFFFFFFFFFFFFF:FFFFFFFFFFFFFFFFFFFFFFFFFFFFFFF
FFFFFFFFFFFFFFFFFFFF:FFFFFFFFFFFFFFFFFFFFFFFFFFFFFFFFFFFFFFFFFFFFFFFFF
FFFFFFFFFFFFFFF
@A00721:190:HGJW7DSXY:4:1658:13340:22373 2:N:0:TCTGCAAG+GCTGCACT
ACTGGAACCTAAACAATCCAATATGGATACGAGTCCAACACTCTGGACAGACGACGAGTCCACCGTTC
CATCACATCTTCACGGTGGAACTAAGATTCAACTACAATCTCAGAATGCAGTTGCGGATAAAGCGAGC
ATGGTGGACTATCCG
+
FFFFFFFFFFFFFFFFFFFFFFFFFFFFFFFFFFFFFFFFFFFFFFFFFFFFFFFFFFFFFFFFFFFFFF
FFFFFFFFFFFFFFFFFFFFFFFFFFFFFFFFFFFFFFFFFFFFFFFFFFFFFFFFFFFFFFFFFFFFFF
:FFFFFFFFFFFFFF
@A00721:190:HGJW7DSXY:4:2148:5430:25958 2:N:0:TCTGCAAG+GCTGCACT
ACTGGAACCTAAACAATCCAATATGGATACGAGTCCAACACTCTGGACAGACGACGAGTCCACCGTTC
CATCACATCTTCACGGTGGAACTAAGATTCAACTACAATCTCAGAATGCAGTTGCGGATAAAGCGAGC
ATGGTGGACTATCCG
+
FFFFFFFFFFFFFFFFFFFFFFFFFFFFFFFFFFFFFFFFFFFFFFFFFFFFFFFFFFFFFFFFFFFFFF
FFFFFFFFFFFFFFFFFFFFFFFFFFFFFFFFFFFFFFFFFFFFFFFFFFFFFFFFFFFFFFFFFFFFFF
FFFFFFFFFFFFFFF
@A00721:190:HGJW7DSXY:4:2148:5475:26130 2:N:0:TCTGCAAG+GCTGCACT
ACTGGAACCTAAACAATCCAATATGGATACGAGTCCAACACTCTGGACAGACGACGAGTCCACCGTTC
```

CATCACATCTTCACGGTGGAACTAAGATTCAACTACAATCTCAGAATGCAGTTGCGGATAAAGCGAGC
ATGGTGGACTATCCG
+
FFFFFF,FFFFFFFFFFFF:FFFFFFFF:FFFFFFFFFFFFFFFFFFFFFFFFFFFFFFFFFFFFFFF
FFFFFFFFFFFFFFFFFFFF:FFFFFFFFFFFFFFFFFFFFFFFFFFFFFFFFFFFFFFFFFFFFFFF
FFFFFFFFFFFFFFF
@A00721:190:HGJW7DSXY:4:2446:2736:34882 2:N:0:TCTGCAAG+GCTGCACT
ACTGGAACCTAAACAATCCAATATGGATACGAGTCCAACACTCTGGACAGACGACGAGTCCACCGTTC
CATCACATCTTCACGGTGGAACTAAGATTCAACTACAATCTCAGAATGCAGTTGCGGATAAAGCGAGC
ATGGTGGACTATCCG
+
FFFFFFFFFFFFFFFFFFFFFFFFFFFFFFFFFFFFFFFFFFFFFF:FFFFFFFFFFFFFFFFFFFFF
FFFFFFFFFFFFFFFFFFFFFFF,FFFFFFFFFFFFFF:FFFFFFFFFFFFFFFFFFFFFFFFFFFFF
FFFFFFFFFF:FFFF
@A00721:190:HGJW7DSXY:4:2478:2275:10128 2:N:0:TCTGCAAG+GCTGCACT
ACTGGAACCTAAACAATCCAATATGGATACGAGTCCAACACTCTGGACAGACGACGAGTCCACCGTTC
CATCACATCTTCACGGTGGAACTAAGATTCAACTACAATCTCAGAATGCAGTTGCGGATAAAGCGAGC
ATGGTGGACTATCCG
+
FFFFFFFFFFFFFFFFFFFFF:FFFFFFFFFFFFFFFFFFFFFFFFFFFFFFFFFFFFFFFFFFFFFF
FFFFFFF:FFFFFFFFFFFFFFFFFFFFFFFFFFFFFFFFFFFFFFFFFFFFFFFFF::FFFFFFFFF
,FFFFFFFFFFFFF:
@A00721:190:HGJW7DSXY:4:2620:5764:12633 2:N:0:TCTGCAAG+GCTGCACT
ACTGGAACCTAAACAATCCAATATGGATACGAGTCCAACACTCTGGACAGACGACGAGTCCACCGTTC
CATCACATCTTCACGGTGGAACTAAGATTCAACTACAATCTCAGAATGCAGTTGCGGATAAAGCGAGC
ATGGTGGACTATCCG
+
FFFFFF:FFFFFFFFFFFFFFFFFFFFFFFFFFFFFFFFFFFFFFFFFFFFFFFFFFFFFFFFFFFFF
FFFFFFFFFFFFFFFFFFFFFFFFFFFFFFFFFFFFFFFFFFFFFFFFFFFFFFFFFFFFFFFFFFFF
,FFFFFFFFFFFFFF
@A00721:190:HGJW7DSXY:4:1251:28718:17848 2:N:0:TCTGCAAG+GCTGCACT
CTGGAACCTAAACAATCCAATATGGATACGAGTCCAACACTCTGGACAGACGACGAGTCCACCGTTCC
ATCACATCTTCACGGTGGAACTAAGATTCAACTACAATCTCAGAATGCAGTTGCGGATAAAGCGAGCA
TGGTGGACTATCC
+
FF,FFF:,FFFFFFFF,:FFFFF::FFF::F,FF,FFFF,F,FFFFFF:F,:F:FFF:FFF,:F,F:F
FF:F,FFFF,FF:FFFF:FF:FFFFFFFFFFF:FFFFFFFFFFFF,FFFFFF,F:,FFFFFF,FFF:F
F:FF,FFFFFFF:
@A00721:190:HGJW7DSXY:4:1109:25048:17440 2:N:0:TCTGCAAG+GCTGCACT
ACTACTGGAACCTAAACAATCCAATATGGATACGAGTCCAACACTCTGGACAGACGACGAGTCCACCG
TTCCATCACATCTTCACGGTGGAACTAAGATTCAACTACAATCTCAGAATGCAGTTGCGGATAAAGCG
AGCATGGTGGACTAT
+
FFFFFFFFFFFFFFFFFFFFFFFFFFFFFFFFFFFFFFFFFFFFFFFFFFFFFFFFFFFFFFFFFFFF
FFFFFFFFFFFFFFFFFFFFFFFFFFFFFF,FFFFFFFF:FFFFFFFFFFFFFFFFFFFFFFFFFFFF
FFFFFFFFFFFFFFF
@A00721:190:HGJW7DSXY:4:1109:24994:17472 2:N:0:TCTGCAAG+GCTGCACT
ACTACTGGAACCTAAACAATCCAATATGGATACGAGTCCAACACTCTGGACAGACGACGAGTCCACCG
TTCCATCACATCTTCACGGTGGAACTAAGATTCAACTACAATCTCAGAATGCAGTTGCGGATAAAGCG
AGCATGGTGGACTAT
+
FFFFFFFFFFFFFFFFFFFFFFFFFFFFFFFFFFFFFFFFFFFFFFFFFFFFFFFFFFFFFFFFFFFF
FFFFFFFFFFFFFFFFFFFFFFF,FFFFFFFFFFFFFFFFFFFFFFFFFFFFFFFFFFFFFFF:FFFF
FFF:FFFFFFFFF:F

```
@A00721:190:HGJW7DSXY:4:1554:24768:20212 2:N:0:TCTGCAAG+GCTGCACT
ACTACTGGAACCTAAACAATCCAATATGGATACGAGTCCAACACTCTGGACAGACGACGATTCCACCG
TTCCATCACATCTTCACGGTGGAACTAAGATTCAACTACACTCGCAGAATGCAGTTGCGGATAAAGCG
AGCATGGTGGACTAT
+
FF,FF,F:FF,FFFFF:FFFFFFFFFFFFFF:FFFFFFFFFFFFFFFFFF:FFFF:FF,F,F:,:FFF
F,FFFFFFF:::,:FFF,FF:FFFFFFFF:F,FFFFFF::,FF,FFFF:,FFF,FFFF:FFFFF:FFF
F,,,,F:,FF:FF:F
@A00721:190:HGJW7DSXY:4:1572:6388:15655 2:N:0:TCTGCAAG+GCTGCACT
ACTACTGGAACCTAAACAATCCAATATGGATACGAGTCCAACACTCTGGACAGACGACGAGTCCACCG
TTCCATCACATCTTCACGGTGGAACTAAGATTCAACTACAATCTCAGAATGCAGTTGCGGATAAAGCG
AGCATGGTGGACTAT
+
FFFFFFFFFFFFFFFF:FFFFFFFFFFFFFFFFFFFFFFFFFFFFFFFFFFFFFFFFFFFFFFFFFFF
FFFFFFFFFFFFFFFFFFFFFFFFFFFFFFFFFFFFFFFFFFFFFFFFFFFFFFFFFFFFFFFFFFFF
FFFFFFFFFFFFFFF
@A00721:190:HGJW7DSXY:4:2524:15700:18662 2:N:0:TCTGCAAG+GCTGCACT
ACTACTGGAACCTAAACAATCCAATATGGATACGAGTCCAACACTCTGGACAGACGACGAGTCCACCG
TTCCATCACATCTTCACGGTGGAACTAAGATTCAACTACAATCTCAGACTGCAGTTGCGGATAAAGCG
AGCATGGTGGACTAT
+
FFFFFFFFF:FFFFFFFFFFFFFFFFFFFFFFFFFFFFFFFFFFFFFFFFFFFFFFFFFFFFFFFFFF
FFFFFFFFFFFFFFFFFFFFFFF,FFFFFFFFFFFFFFFFFFFFFFFF,FFFFFFFFFFFF:FFFFF:
FFFFFFFFFFFF::F
@A00721:190:HGJW7DSXY:4:2540:17445:4053 2:N:0:TCTGCAAG+GCTGCACT
ACTACTGGAACCTAAACAATCCAATATGGATACGAGTCCAACACTCTGGACAGACGACGAGTCCACCG
TTCCATCACATCTTCACGGTGGAACTAAGATTCAACTACAATCTCAGAATGCAGTTGCGGATAAAGCG
AGCATGGTGGACTAT
+
FFFFFFFFFFFFFFFFFFFFFFFFFFFFFFFFFFFFFFFFFFFFFFFFFFFFFFFFFFFFFFFFFFFF
FFFFFFFFFFFFFFFFFFFFFFFFFFFFFFFFFFFFFFFFFFFFFFFFFFFFFFFFFFFFFFFFFFFF
FFFFFFFFFFFFFFF
@A00721:190:HGJW7DSXY:4:1240:24605:32831 2:N:0:TCTGCAAG+GCTGCACT
AGTCCACCATGCTCGCTTTATCCGCAACTGCATTCTGAGATTGTAGTTGAATCTTAGTTCCACCGTGA
AGATGTGATGGAACGGTGGACTCGTCGTCTGTCCAGAGTGTTGGACTCGTATCCATATTGGATTGTTT
AGGTTCCAGTAGTAC
+
FFFFFFFFFFFFFFFFFFFFFFFFFFFFFFFFFFFFFFFFFFFFFFFFFFFFFFFFFFFFFFFFFFFF
FFFFFFFFFFFFFFFFFFFFFFFFFFFFFFFFFFFFFFFFFFFFFFFFFFFFFFFFFFF:FFFFFFFF
F:FFFFFFFFFFFFF
@A00721:190:HGJW7DSXY:4:1278:31702:22764 2:N:0:TCTGCAAG+GCTGCACT
AGTCCACCATGCTCGCTTTATCCGCAACTGCATTCTGAGATTGTAGTTGAATCTTAGTTCCACCGTGA
AGATGTGATGGAACGGTGGACTCGTCGTCTGTCCAGAGTGTTGGACTCGTATCCATATTGGATTGTTT
AGGTTCCAGTAGTAC
+
FFFFFFFFFFFFFFFFFFFFFFFFFFFF:FFFFFFFFFFFFFFFFFFFFFFFFFFFFFFFFFFFFFFF
FFF:FFFFFFFFFFFFFFFFF:FFFFF:FFFFFFFFFFFFFFFFFFFFFFFFFF,,FFFFFF,,FF:F
F:FFFFFFFFFFF::
@A00721:190:HGJW7DSXY:4:1508:7961:1094 2:N:0:TCTGCAAG+GCTGCACT
AGTCCACCATGCTCGCTTTATCCGCAACTGCATTCTGAGATTGTAGTTGAATCTTAGTTCCACCGTGA
AGATGTGATGGAACGGTGGACTCGTCGTCTGTCCAGAGTGTTGGACTCGTATCCATATTGGATTGTTT
AGGTTCCAGTAGTAC
+
FFFFFFFFFFFFFFFFFFFFFFFFFFFFFFFFFFFFFFFF:FFFFFFFFFFFFFFFFFFFFFFFF:FF
```

```
::FFFFFFFFFFFFFFFFFFFFFFFFFFFFFFFFFFFFFFFFFFFFFFFFFFFF:F,FFFFF,FFFFF
FFFFFFFFFFFFFFF
@A00721:190:HGJW7DSXY:4:2162:4264:5243 2:N:0:TCTGCAAG+GCTGCACT
AGTCCACCATGCTCGCTTTATCCGCAACTGCATTCTGAGATTGTAGTTGAATCTTAGTTCCACCGTGA
AGATGTGATGGAACGGTGGACTCGTCGTCTGTCCAGAGTGTTGGACTCGTATCCATATTGGATTGTTT
AGGTTCCAGTAGTAC
+
FFFFFFFFFFFFFFFF:FFFFFFFFFFFFFFFFFFFFFFF:FFFFFFFFFFFFFFFFFFFFFFFFFFF
FFFFFFFFFFFFFFFFFFFFFFFFFFFFFFF:FFFFFFFFFFFFFFFFFFFFFFFFFFFFFFFFFFFF
FFFFFFFFFFFFFFF
@A00721:190:HGJW7DSXY:4:2314:3568:3317 2:N:0:TCTGCAAG+GCTGCACT
AGTCCACCATGCTCGCTTTATCCGCAACTGCATTCTGAGATTGTAGTTGAATCTTAGTTCCACCGTGA
AGATGTGATGGAACGGTGGACTCGTCGTCTGTCCAGAGTGTTGGACTCGTATCCATATTGGATTGTTT
AGGTTCCAGTAGTAC
+
FFFFFFFFF:FFFFFFFFFFFF:FFF:FFFFFFF:FFFFFFFFFFF,:FFFF:FFFF,FFFFFF:FFF
F:FFFFFFFFFF:FFF:FFF:FF,:F:FFFF:FF:FFFFFFF,FFFFFFFFFFF,,:FFF:FFF:FFF
FFFFFF,FFFFFFF:
@A00721:190:HGJW7DSXY:4:2419:5421:27602 2:N:0:TCTGCAAG+GCTGCACT
AGTCCACCATGCTCGCTTTATCCGCAACTGCATTCTGAGATTGTAGTTGAATCTTAGTTCCACCGTGA
AGATGTGATGGAACGGTGGACTCGTCGTCTGTCCAGAGTGTTGGACTCGTATCCATATTGGATTGTTT
AGGTTCCAGTAGTAC
+
FFFFFFFFFFFFFFFFFFFFFF:FFF:FFFFFFFFFFFFFFFF:FFFFFFFFFFFFFFFF:FFFFFFF
FFFFFFFFFFFFFFFFFFFFFFFFFFFFFFFFFFFFFFFFFFFFFFFFFFFFFFFFFFFFFF,FF:FF
FF:FFFFFFFFFFFF
@A00721:190:HGJW7DSXY:4:2520:9950:19758 2:N:0:TCTGCAAG+GCTGCACT
AGTCCACCATGCTCGCTTTATCCGCAACTGCATTCTGAGATTGTAGTTGAATCTTAGTTCCACCGTGA
AGATGTGATGGAACGGTGGACTCGTCGTCTGTCCAGAGTGTTGGACTCGTATCCATATTGGATTGTTT
AGGTTCCAGTAGTAC
+
FFFFFFFFFFFFFFFFFFFFFFFFFFFFFFFFFFFFFFFFFFFFFFFFFFFFFFFFFFFFFFFFFFFF
FFFFFFFFFFFF:FFFFFFFFFFFFFFFFFF:FFFFFFFFFFFFFFFFFFFFFFFFFFFFFFFFFFFF
FFFFFFFFFFFFFFF
@A00721:190:HGJW7DSXY:4:1238:16721:26318 2:N:0:TCTGCAAG+GCTGCACT
GTCCACCATGCTCGCTTTATCCGCAACTGCATTCTGAGATTGTAGTTGAATCTTAGTTCCACCGTGAA
GATGTGATGGAACGGTGGACTCGTCGTCTGTCCAGAGTGTTGGACTCGTATCCATATTGGATTGTTTA
GGTTCCAGTAGTACT
+
FFFFFFFFFFFFFFFFFFFFFFFFFFFFFFFFFFFFFFFFFFFFFFFFFFFFFFFFFFFFFFFFFFFF
FFFFFFFFFFF:FFFFFFFFFFFFFFFFFFFFFFFFFFFFFFFFFFFFF:FFFFF::FFFFFFFF:FF
FFFFFFFFFFFFFFF
@A00721:190:HGJW7DSXY:4:1431:17553:10191 2:N:0:TCTGCAAG+GCTGCACT
GTCCACCATGCTCGCTTTATCCGCAACTGCATTCTGAGATTGTAGTTGAATCTTAGTTCCACCGTGAA
GATGTGATGGAACGGTGGACTCGTCGTCTGTCCAGAGTGTTGGACTCGTATCCATATTGGATTGTTTA
GGTTCCAGTAGTACT
+
FFFFFFFFFFFFFFFFFFFFFFFFF:FFFFFFFFFFFFFF:FFFFFFFFFFFFFFFFFFFFFFFFFFF
FFFFFFFFFFFFFFFFFFFFFFFFFFFFFFFFFFFFFFFFFFFFFFFFFFFFFFFFFFFF::FFFFFF
FF:FFFFFFFFFFFF
@A00721:190:HGJW7DSXY:4:1619:23312:7420 2:N:0:TCTGCAAG+GCTGCACT
GTCCACCATGCTCGCTTTATCCGCAACTGCATTCTGAGATTGTAGTTGAATCTTAGTTCCACCGTGAA
GATGTGATGGAACGGTGGACTCGTCGTCTGTCCAGAGTGTTGGACTCGTATCCATATTGGATTGTTTA
GGTTCCAGTAGTACT
```

```
+
FFFFFFFF:FFFFFFFFFFFFFFFFFFFFFFFFFFFFFFFFFFFFFFFFFFFFFFFFFFFFFFFFFFF
FF:FFFFFFFF,FFFFFFFFFFFFFFFFFFFFFFFFFFFFFFFFFFFFFFFF:F:FFFFFF,FFFFFF
FFFFFFFFFFFFFFF
@A00721:190:HGJW7DSXY:4:1619:25464:8108 2:N:0:TCTGCAAG+GCTGCACT
GTCCACCATGCTCGCTTTATCCGCAACTGCATTCTGAGATTGTAGTTGAATCTTAGTTCCACCGTGAA
GATGTGATGGAACGGTGGACTCGTCGTCTGTCCAGAGTGTTGGACTCGTATCCATATTGGATTGTTTA
GGTTCCAGTAGTACT
+
FFF:FFFFFFF:F,FFFFFFFF:FFFF::FFFF:FFFFFFF:F,:::FFFFFFFFF:FFFF:F:FFFF
FFFFFFFFF:FFFFFF:FF:FF,FF:FF:FFFFF,F,F::F::FFF,,FFFF,,FFFF,FFFFF:FFF
::FFF,FFFFF,FFF
@A00721:190:HGJW7DSXY:4:2120:11234:29590 2:N:0:TCTGCAAG+GCTGCACT
GTCCACCATGCTCGCTTTATCCGCAACTGCATTCTGAGATTGTAGTTGAATCTTAGTTCCACCGTGAA
GATGTGATGGAACGGTGGACTCGTCGTCTGTCCAGAGTGTTGGACTCGTATCCATATTGGATTGTTTA
GGTTCCAGTAGTACT
+
FFFFFFFFFFFFFFFFFFFFFFFFFFFFFFFFFFFFFFFFFFFFFFFFFFFFFFFFFFFFFFFFFFF:
FFFFFFFFFFFFFFFFFFFFFFFFFFFFFFFFFFFFFFFFFFFFFFFFFFFFFFFF:FFFF,FFFFFF
FFFFFFFFFFFFFFF
@A00721:190:HGJW7DSXY:4:1133:23339:3114 2:N:0:TCTGCAAG+GCTGCACT
GGGAGTACTACTGGAACCTAAACAATCCAATATGGATACGAGTCCAACACTCTGGACAGACGACGAGT
CCACCGTTCCATCACATCTTCACGGTGGAACTAAGATTCAACTACAATCTCAGAATGCAGTTGCGGAT
AAAGCGAGCATGGTG
+
FFFFFFFFFFFFFFFFFFFFFFFFFFFFFFFFFFFFFFF:FFFFFFFFFFFFFFFFFFFFFFFFF:FF
FFFFFFFFFFFFFFFFFFFFFFFFF:FFFFFFFFFFFFFFFFFFFFFFFFFFFFFFFFFFFFFFF:FFF
FFFFFFFFFFFF,FF
@A00721:190:HGJW7DSXY:4:1136:22498:3568 2:N:0:TCTGCAAG+GCTGCACT
GGGAGTACTACTGGAACCTAAACAATCCAATATGGATACGAGTCCAACACTCTGGACAGACGACGAGT
CCACCGTTCCATCACATCTTCACGGTGGAACTAAGATTCAACTACAATCTCAGAATGCAGTTGCGGAT
AAAGCGAGCATGGTG
+
FFFFFFFFFFFFFFFFFFFFFFFFFFFFFFFFFFFFFFFFFFFFFFFFFFFFFFFFFFFFFFFFFFFF
FFFFFFFFFFFFFFFFFFFFFFFFFFFFFFFFFFFFF:FFFFFFFFFFFFFFFFFFFFFFFFFFFFFF
FFFFF:FFFFFFFFF
@A00721:190:HGJW7DSXY:4:1534:26594:2957 2:N:0:TCTGCAAG+GCTGCACT
GGGAGTACTACTGGAACCTAAACAATCCAATATGGATACGAGTCCAACACTCTGGACAGACGACGAGT
CCACCGTTCCATCACATCTTCACGGTGGAACTAAGATTCAACTACAATCTCAGAATGCAGTTGCGGAT
AAAGCGAGCATGGTG
+
FFFFFFFFFFFFFFFFFFFFFFFFFFFFFFFFFFFFFFFFFFFFFFFFFFFFFFFFFFFFFFFFFFFF
FFFFFFFFFFFFFFFFFFFFFFFFFFFFF,FFFFFFFFFFFFFFFFFFFFFFFFFFFFFFFFFFFFFF
FFFFFFFFFFFFFFF
@A00721:190:HGJW7DSXY:4:1535:30101:1548 2:N:0:TCTGCAAG+GCTGCACT
GGGAGTACTACTGGAACCTAAACAATCCAATATGGATACGAGTCCAACACTCTGGACAGACGACGAGT
CCACCGTTCCATCACATCTTCACGGTGGAACTAAGATTCAACTACAATCTCAGAATGCAGTTGCGGAT
AAAGCGAGCATGGTG
+
FFFFFFFFFFFFFFF,FFFFFFFFFFFFFFFFFFFFFFFFFFFFFFFFFFFFFFFFFFFFFFFFFFFF
FFFFFFFFFFFFFFFFFFFFFFFFFFFFFFFFFFFFFFFFFFFFFFFFFFFFFFFFFFFFFFFFFFFF
FFFFFFFFF:FFFFF
@A00721:190:HGJW7DSXY:4:1542:7374:21903 2:N:0:TCTGCAAG+GCTGCACT
GGGAGTACTACTGGAACCTAAACAATCCAATATGGATACGAGTCCAACACTCTGGACAGACGACGAGT
```

```
CCACCGTTCCATCACATCTTCACGGTGGAACTAAGATTCAACTACAATCTCAGAATGCAGTTGCGGAT
AAAGCGAGCATGGTG
+
FFFFFFFFFFFFFFFFFF:FFFFFFFFFFFFFFFFFFFFFFFFFFFFFFFFFFFFFFFFFFFFFFFFF
FFFFFFFFFFFFFFFFFFFFFFFFFFFFFFFFF:FFFFFFFFFFFFFFFFFFFFFFFFFFFFFFFFFF
FFFFFFFFFFFFFFF
@A00721:190:HGJW7DSXY:4:1638:19750:16313 2:N:0:TCTGCAAG+GCTGCACT
GGGAGTACTACTGGAACCTAAACAATCCAATATGGATACGAGTCCAACACTCTGGACAGACGACGAGT
CCACCGTTCCATCACATCTTCACGGTGGAACTAAGATTCAACTACAATCTCAGAATGCAGTTGCGGAT
AAAGCGAGCATGGTG
+
FFFFFFFFFFFFFFFFFFFFFFFFFFFFFFFFFFFFFFFFFFFFFFFFFFFFFFFFFFFFFFFFFFFF
FFFFFFFFFFFFFFFFFFFFFFFFFFFFF,FFFFFFFFFFFFFFFFFFFFFFFF,FFFFFFFFFFFFF
FFFFFFFF:FFFFFF
@A00721:190:HGJW7DSXY:4:1664:18900:20478 2:N:0:TCTGCAAG+GCTGCACT
GGGAGTACTACTGGAACCTAAACAATCCAATATGGATACGAGTCCAACACTCTGGACAGACGACGAGT
CCACCGTTCCATCACATCTTCACGGTGGAACTAAGATTCAACTACAATCTCAGAATGCAGTTGCGGAT
AAAGCGAGCATGGTG
+
FFFFFFFFFFFFFFFFFF:FFFFF:FFFFFFFFFFFFFFFFFFFFFFFFFFFFFFFFFFFFF:FFFFF
FFFFFFFFFFFFFFFFFFFFFFFFFFFFF:FFFFFFFFFFFFF:FFFFFFFFFFFFFFFFFFFFFFFF
FFFFFFFFFFFFFFF
@A00721:190:HGJW7DSXY:4:2120:18602:9659 2:N:0:TCTGCAAG+GCTGCACT
GGGAGTACTACTGGAACCTAAACAATCCAATATGGATACGAGTCCAACACTCTGGACAGACGACGAGT
CCACCGTTCCATCACATCTTCACGGTGGAACTAAGATTCAACTACAATCTCAGAATGCAGTTGCGGAT
AAAGCGAGCATGGTG
+
FFF:FFFFFFFFFFF,FFFFFFFFFFFFFFFFFFFFFFFFFFFFFFFFFFFFFFFFFFFFFFFFFFFF
FFFFFFFFFFFFFFFFFFFFFFFFFFFFFFFFFFFFFFFFFFFFFFFFFFFFF:FFFFFFFFFFFFF:
,FFFFFFFFFFFFFF
@A00721:190:HGJW7DSXY:4:1208:18484:24017 2:N:0:TCTGCAAG+GCTGCACT
CTACAAGTAGGGGAGTACTACTGGAACCTAAACAATCCAATATGGATACGAGTCCAACACTCTGGACA
GACGACGAGTCCACCGTTCCATCACATCTTCACGGTGGAACTAAGATTCAACTACAATCTCAGAATGC
AGTTGCGGATAAAGC
+
FFFFFFFFFFFFFFFFFFFFFFFFFFFFFFF:FFFF:FFFFFFFFFFFFFFFFFFFFFFFFFFFFFFF
FFFFFFFFFFFF:FFFFFFFFFFFFFFF:FFFFF:FFFFFFFFFFFFFFF:FFFFFFFFFFFFFFF:F
FFFFFFFFFFFFFFF
@A00721:190:HGJW7DSXY:4:1470:7880:4554 2:N:0:TCTGCAAG+GCTGCACT
CTACAAGTAGGGGAGTACTACTGGAACCTAAACAATCCAATATGGATACGAGTCCAACACTCTGGACA
GACGACGAGTCCACCGTTCCATCACATCTTCACGGTGGAACTAAGATTCAACTACAATCTCAGAATGC
AGTTGCGGATAAAGC
+
FFFFFFF,FFFFFFFFFFFFFFFFFFFFFFFFFFFFFFFFFFFFFF:FFFFFFFFFFFFFFFFFFFFF
FFFFFFFFFFFFFFFFF:FFFFFFFFFFFFFFFFFFFFFFFFFFFFFFFFFFFFFF:FFFFFFFFFFF
FFFFFFFFFFFFFFF
@A00721:190:HGJW7DSXY:4:1470:7102:6214 2:N:0:TCTGCAAG+GCTGCACT
CTACAAGTAGGGGAGTACTACTGGAACCTAAACAATCCAATATGGATACGAGTCCAACACTCTGGACA
GACGACGAGTCCACCGTTCCATCACATCTTCACGGTGGAACTAAGATTCAACTACAATCTCAGAATGC
AGTTGCGGATAAAGC
+
FFFFFFFFFFFFFFFFFFFFFFFFFFFFFFFFFFFFFFFFFFFFFFFFFFFFFFFFFFFFFFFFFFFF
FFFFFFFFFFFFFFFFFFFFFFFFFFFFFFFFFFFFFFFFFFFFFFFFFFFFFFFFFFFFFFFFFFFF
FFFFFFFFFFFFFFF
```

```
@A00721:190:HGJW7DSXY:4:2353:28185:4272 2:N:0:TCTGCAAG+GCTGCACT
CTACAAGTAGGGGAGTACTACTGGAACCTAAACAATCCAATATGGATACGAGTCCAACACTCTGGACA
GACGACGAGTCCACCGTTCCATCACATCTTCACGGTGGAACTAAGATTCAACTACAATCTCAGAATGC
AGTTGCGGATAAAGC
+
FFFFFFFFFFFFFFFFFFFFFFFFFFFFFFFFFFFFFFFFFFFFFFFFFFFFFFFFFFFFFFFFFFFF
FFFFFFFFFFFFFFFFFFFFFFFFFFFFFFFFFFFFFFFFFFFFFFFFFFFFF:FFFFFFFFFF,FFF
FFFFFFFFFFFFFFF
@A00721:190:HGJW7DSXY:4:2629:30047:6308 2:N:0:TCTGCAAG+GCTGCACT
CTACAAGTAGGGGAGTACTACTGGAACCTAAACAATCCAATATGGATACGAGTCCAACACTCTGGACA
GACGACGAGTCCACCGTTCCATCACATCTTCACGGTGGAACTAAGATTCAACTACAATCTCAGAATGC
AGTTGCGGATAAAGC
+
FFFFFFFFFFFFFFFFFFFFFFFFFFFFFF:FFFFFFFFFFFFFFFFFFFFFFFFFFFFFFFFFFFFF
FFFFFFFFFFFFFFFFFFFFFFFFFFFFFFFFFFFFFFFFFFFFFFFFFFFFFF:FFFFFFFFFFFFF
FFFFFFFFFFFFFFF
@A00721:190:HGJW7DSXY:4:1574:7111:20854 2:N:0:TCTGCAAG+GCTGCACT
ACTACAAGTAGGGGAGTACTACTGGAACCTAAACAATCCAATATGGATACGAGTCCAACACTCTGGAC
AGACGACGAGTCCACCGTTCCATCACATCTTCACGGTGGAACTAAGATTCAACTACAATCTCAGAATG
CAGTTGCGGATAAAG
+
FFFFFFFFFFFFFFFFFFFFFFFFFFFFFFFFFFFFFFFFFFFFFFFFFFFFFFFFFFFFFFFFFFFF
FFFFFFFFFFFFFFFFFFFFFFFFFFFFFFFFFFFFFFFFFFFFFFFFFFFFFFFFFFFFFFFFFFFF
FFFFFFFFFFFFFFF
@A00721:190:HGJW7DSXY:4:2329:32660:18881 2:N:0:TCTGCAAG+GCTGCACT
CTACAAGTAGGGGAGTACTACTGGAACCTAAACAATCCAATATGGATACGAGTCCAACACTCTGGACA
GACGACGAGTCCACCGTTCCATCACATCTTCACGGTGGAACTAAGATTCAACTACAATCTCAGAATGC
AGTTGCGGATAAA
+
:FFFFF,FFFFF:FFFFFFFFF:FFFF::FFF:FFFFFFF:FFFFF,FF,F,FFFFF,F::FF:FF:F
FF,,F:FF,,F,FF,FF:::F:FF:FF:,F:F:,,,F,FFF,FF:F:FFFF:,FFFF,::,F:FFF,,
F,:::,F:F:FFF
@A00721:190:HGJW7DSXY:4:1116:26087:2018 2:N:0:TCTGCAAG+GCTGCACT
GCAACTACAAGTAGGGGAGTACTACTGGAACCTAAACAATCCAATATGGATACGAGTCCAACACTCTG
GACAGACGACGAGTCCACCGTTCCATCACATCTTCACGGTGGAACTAAGATTCAACTACAATCTCAGA
ATGCAGTTGCGGATA
+
F:FFFFFFFFFFFFFFFFFFFFFFFFFFFFFFFFFFFFFFFFFFFFFFFFFFFFFFFFFFFFFFFFFF
FFFFFFFFFFFFFFFFFFFFFFFFFFFFFFFFFFFFFFFFFFFFFFFFFFFFFFFFFFFFFFFFFFFF
FFFFFFFFFFFFFFF
@A00721:190:HGJW7DSXY:4:1171:24361:36824 2:N:0:TCTGCAAG+GCTGCACT
GCAACTACAAGTAGGGGAGTACTACTGGAACCTAAACAATCCAATATGGATACGAGTCCAACACTCTG
GACAGACGACGAGTCCACCGATCCATCACATATTCACGGTGGAACTAAGATTCAACAACAATCTCAGA
ATGCAGATGCGGATA
+
F,FF:,FFFF:FFFF,:FF:FF,FF,FFFF,F:FFFFFFF,FFFFFFF:FFF,,F,:::F:,FFF,F,
,F:FF:::F:,FFF,:F,,:,:F:FF:F:F,,F::F,,,:F,FF,FFF:FF::F::,FFFFFF:,F,F
F:,FFF,,:,F:FFF
@A00721:190:HGJW7DSXY:4:1416:22354:25739 2:N:0:TCTGCAAG+GCTGCACT
GCAACTACAAGTAGGGGAGTACTACTGGAACCTAAACAATCCAATATGGATACGAGTCCAACACTCTG
GACAGACGACGAGTCCACCGTTCCATCACATCTTCACGGTGGAACTAAGATTCAACTACAATCTCAGA
ATGCAGTTGCGGATA
+
FFFFFFFF:FFFFFFFFFFFFFFFFFFFFFFFFFFFFFFFFFFFFFFFFFFFFFFFFFFFFFFFFFFF
```

```
FFFFFFFFFFFFFFFFFFFFFFFFFFFFFFFFFFFFFFFFFFFF:F,FFFFFFFFFFFFFFFFFFF,F
FFFFFF::FFFFFFF
@A00721:190:HGJW7DSXY:4:1416:22797:26569 2:N:0:TCTGCAAG+GCTGCACT
GCAACTACAAGTAGGGGAGTACTACTGGAACCTAAACAATCCAATATGGATACGAGTCCAACACTCTG
GACAGACGACGAGTCCACCGTTCCATCACATCTTCACGGTGGAACTAAGATTCAACTACAATCTCAGA
ATGCAGTTGCGGATA
+
FFFFFFFFFFFFFFFFFFFFFFFFFFFFFFFFFFFFFFFFFFFFFFFFFFFFFFFFFFFFFFFFFFFF
FFFFFFFFFFFFFFFFFFFFFFFFFFFFFFFFFFFFFFFFFFFFFFFFFFFFFFFFFFFFFFFFFFFF
F:FFFFFFFFFFF:F
@A00721:190:HGJW7DSXY:4:1426:17210:22874 2:N:0:TCTGCAAG+GCTGCACT
GCAACTACAAGTAGGGGAGTACTACTGGAACCTAAACAATCCAATATGGATACGAGTCCAACACTCTG
GACAGACGACGAGTCCACCGTTCCATCACATCTTCACGGTGGAACTAAGATTCAACTACAATCTCAGA
ATGCAGTTGCGGATA
+
FFF:FFFFFFFFFFFFFFFFFFFFFFFFFFFFFFFFFFFFFFFFFFFFFFFFFFFFFFFF:FFFFFFF
F:FFFFFFFFFFFFFFFFFFFFFFFFFFFFFFFFFFFFFFFFFFFFFFFFFF:FFFFFFFFFFFFFFF
FFFFFFFFFFFFFFF
@A00721:190:HGJW7DSXY:4:1532:7012:11162 2:N:0:TCTGCAAG+GCTGCACT
GCAACTACAAGTAGGGGAGTACTACTGGAACCTAAACAATCCAATATGGATACGAGTCCAACACTCTG
GACAGACGACGAGTCCACCGTTCCATCACATCTTCACGGTGGAACTAAGATTCAACTACAATCTCAGA
ATGCAGTTGCGGATA
+
FFFFFFFFFFFFFFFFFFFFFFFFFFFFFFFFFFFFFFFFFFFFFFFFFFFFFFFFFFFFFFFFFFFF
FFFFFFFFFFFFFFFFFFFFFFFFFFFFFFFFFFFFFFFFFFF:FFFFFFFFFFFFFFFFFFFFFF,F
FFFFFFFFFFFFF:F
@A00721:190:HGJW7DSXY:4:2150:3468:4085 2:N:0:TCTGCAAG+GCTGCACT
GCAACTACAAGTAGGGGAGTACTACTGGAACCTAAACAATCCAATATGGATACGAGTCCAACACTCTG
GACAGACGACGAGTCCACCGTTCCATCACATCTTCACGGTGGAACTAAGATTCAACTACAATCTCAGA
ATGCAGTTGCGGATA
+
FFFFFFFFFFFFF:FFFFF:FFFFFFF:FFFFFF:FFFFFF,FFFFFFFFFFFFF,FFFFFFFFFFFF
,FFFFFF:F,FFFFFFFFF::FFFF,FF:FF:FFFFFFFFFFFFFFFFFFFF,FFFFFFFFF::FFFF
FFF,FFFF,F::FFF
@A00721:190:HGJW7DSXY:4:2163:2971:15217 2:N:0:TCTGCAAG+GCTGCACT
GCAACTACAAGTAGGGGAGTACTACTGGAACCTAAACAATCCAATATGGATACGAGTCCAACACTCTG
GACAGACGACGAGTCCACCGTTCCATCACATCTTCACGGTGGAACTAAGATTCAACTACAATCTCAGA
ATGCAGTTGCGGATA
+
FFFFFFFFFFFFFFFFFFFFFFFFFFFFFFFFFFFFFFFFFFFFFFFFFFFFFF:FFFFFFFFFFFFF
FFFFFFFFFFFFFFFFFFFF:FFFF:FFFFFFFFFFFFFFFFF:FFFFFFFFFFFFFFFFFFFFFFFF
FFFFFFFFFFFFFF:
@A00721:190:HGJW7DSXY:4:2261:5457:4460 2:N:0:TCTGCAAG+GCTGCACT
GCAACTACAAGTAGGGGAGTACTACTGGAACCTAAACAATCCAATATGGATACGAGTCCAACACTCTG
GACAGACGACGAGTCCACCGTTCCATCACATCTTCACGGTGGAACTAAGATTCAACTACAATCTCAGA
ATGCAGTTGCGGATA
+
FFFFFFFFFFFFFFFFFFFFFFFFFFFFFFFFFFFFFFFFFFFFFFFFFFFFFFFFFFFFFFFFFFFF
FFFF:FFFFFFFFFFFFFFFFFFFFFFFFFFFFFFFFF:FFFFFFFFFFFFFFFFFFFFFFFFFFFFF
:FFFFFFFFFFFFFF
@A00721:190:HGJW7DSXY:4:2465:18141:36104 2:N:0:TCTGCAAG+GCTGCACT
GCAACTACAAGTAGGGGAGTACTACTGGAACCTAAACAATCCAATATGGATACGAGTCCAACACTCTG
GACAGACGACGAGTCCACCGTTCCATCACATCTTCACGGTGGAACTAAGATTCAACTACAATCTCAGA
ATGCAGTTGCGGATA
```

+
FFFFFFFFFFF:FFFFFFFFFFFFFFFFFFFFFFFF:FFFFFFFFFFFFFFFFFFF:FFFFFFFFFFFF
FFFFFFFFFFFFFFFFFFF:FFFFFFFFFFFFFFFFFFFFFFFFFFFFFF:FFFFFFFFFFFFFFFFFFFFFF
FFFFFFFFFFFFFFF
@A00721:190:HGJW7DSXY:4:2549:17716:36276 2:N:0:TCTGCAAG+GCTGCACT
GCAACTACAAGTAGGGGAGTACTACTGGAACCTAAACAATCCAATATGGATACGAGTCCAACACTCTG
GACAGACGACGAGTCCACCGTTCCATCACATCTTCACGGTGGAACTAAGATTCAACTACAATCTCAGA
ATGCAGTTGCGGATA
+
FFFFFFFFFFFFFFFFFFFFFFFFFFFFFFFFF,FF:FFFFFFFFFFFFFFFFFFFFFFFFFFFFFFFFF
FFFFFFFFFFFFFFFFFFFFFFFFFFFFF:FFFFFFFFFFFFF,F:FFFFFFFFFFFFFFFFFFFFFFF
F:FFFFFFFFFFFFF
@A00721:190:HGJW7DSXY:4:1244:22381:24972 2:N:0:TCTGCAAG+GCTGCACT
AGCAACTACAAGTAGGGGAGTACTACTGGAACCTAAACAATCCAATATGGATACGAGTCCAACACTCT
GGACAGACGACGAGTCCACCGTTCCATCACATCTTCACGGTGGAACTAAGATTCAACTACAATCTCAG
AATGCAAGATCGGA
+
FFFFFFFFFFFFFFFFFFFFFFF:F:FF:FFFFFFFFFFF:FFFFFFFFFFFFF:F:FFFFFFFFF:F
::FFFFFF:FF:FFFFFF:F,FFFFFFFFFFFFFFFFFFFF::FF:FFFFF:FFFF,FF,FFF,:FFF
FF:,FFF:FF,::F
@A00721:190:HGJW7DSXY:4:1417:5963:16642 2:N:0:TCTGCAAG+GCTGCACT
CAGCAACTACAAGTAGGGGAGTACTACTGGAACCTAAACAATCCAATATGGATACGAGTCCAACACTC
TGGACAGACGACGAGTCCACCGTTCCATCACATCTTCACGGTGGAACTAAGATTCAACTACAATCTCA
GAATGCAAGATCGGA
+
FFFFFFFFFFFFFFFFFFFFFFFFFFFFFFFFFFFFFFFFFFFFFFFFFFFFFFFFFFFFFFFFFFFF
FFFFFFF:FFFFFFFFFFFFFFFFFFFFFFFFFFFFFFFFFFFFFFF,FFFFFFFFFFFFFFFFFFFFFF
FFFFFFFFFFFFFFF
@A00721:190:HGJW7DSXY:4:1204:21115:7435 2:N:0:TCTGCAAG+GCTGCACT
GCATTCTGAGATTGTAGTTGAATCTTAGTTCCACCGTGAAGATGTGATGGAACGGTGGACTCGTCGTC
TGTCCAGAGTGTTGGACTCGTATCCATATTGGATTGTTTAGGTTCCAGTAGTACTCCCCTACTTGTAG
TTGCTGCCATGTGAG
+
FFFFFFFFFFFFFFFFFFFFFFFFFFFFFFFFFFFFFFFFFFFFFFFFFFFF,FFFFFFFFFFFFFFFF
FFFFFFFFFFFFFF:FFFFFFFFFF,FFFFFFFFFFF:FFFFFFF:FF:FFFFFFFFFFFFFFFFF:F
FFFFFFFFFFFFFFF
@A00721:190:HGJW7DSXY:4:1211:16396:25379 2:N:0:TCTGCAAG+GCTGCACT
CTCACATGGCAGCAACTACAAGTAGGGGAGTACTACTGGAACCTAAACAATCCAATATGGATACGAGT
CCAACACTCTGGACAGACGACGAGTCCACCGTTCCATCACATCTTCACGGTGGAACTAAGATTCAACT
ACAATCTCAGAATGC
+
F,FFF:FFFFFFFFFFFFFFFF,FFFFFFFF:FF:FFFFFFFF,F:FF::FFF,FFFFFFFF,FFFFF
F:FFFFFFFFFFFFFFFFFFFFFFFFFFFFFFFFFFFFFFF:FFFFFFFF:FFFFFFFF:FFFFF:FFFF
,:FFFFF:F,FFFFF
@A00721:190:HGJW7DSXY:4:1226:31991:29434 2:N:0:TCTGCAAG+GCTGCACT
CTCACATGGCAGCAACTACAAGTAGGGGAGTACTACTGGAACCTAAACAATCCAATATGGATACGAGT
CCAACACTCTGGACAGACGACGAGTCCACCGTTCCATCACATCTTCACGGTGGAACTAAGATTCAACT
ACAATCTCAGAATGC
+
FFFFFFFFFFFFFFFFFFFFFFFFFFFFFFFFFFFFFFFFFFFFFFFFFFFFFFFFFFFFFFFFFFFF
FFFFFFFFFFFFFFFFFFFFFFFFFFFFFFFFF:FFFFFFFFFFFFFFFFFFFFF:FFFFFFFFFFFF
FFFFFFFFFFFFFFF
@A00721:190:HGJW7DSXY:4:1348:16532:7983 2:N:0:TCTGCAAG+GCTGCACT
CTCACATGGCAGCAACTACAAGTAGGGGAGTACTACTGGAACCTAAACAATCCAATATGGATACGAGT

```
CCAACACTCTGGACAGACGACGAGTCCACCGTTCCATCACATCTTCACGGTGGAACTAAGATTCAACT
ACAATCTCAGAATGC
+
FFFFFFFFFF:FFFFFFFFFFFFFFFFFFFFFFFFFFFFFFFFFFFFFFFFFFFFFFFFFFFFF:FFFFF
FFFFFFFFFFFFFFFFFFFFFFFFFFFFFFFFFFFFFFFFFFFFFFFFFFFFFFFFFFFFFFFFFFFF:F
FFFFFFFFFFFFFFF
@A00721:190:HGJW7DSXY:4:1404:10357:1799 2:N:0:TCTGCAAG+GCTGCACT
CTCACATGGCAGCAACTACAAGTAGGGGAGTACTACTGGAACCTAAACAATCCAATATGGATACGAGT
CCAACACTCTGGACAGACGACGAGTCCACCGTTCCATCACATCTTCACGGTGGAACTAAGATTCAACT
ACAATCTCAGAATGC
+
FFFFF:FFFFFFFFFFFFFFFFFFFFFFFFFFFFFFFFFFFFFFFFFFFFFFFFFFFFFFFF,FFFFF
FFFFFFFFFFFFFFFFFFFFFFFFFFFFFFFFFFFFFFFFFFFFFFFFFFFFFFFFFFFFFFFFFFFFFF
FFFFFFFFFFF:FFF
@A00721:190:HGJW7DSXY:4:1419:6108:36182 2:N:0:TCTGCAAG+GCTGCACT
CTCACATGGCAGCAACTACAAGTAGGGGAGTACTACTGGAACCTAAACAATCCAATATGGATACGAGT
CCAACACTCTGGACAGACGACGAGTCCACCGTTCCATCACATCTTCACGGTGGACCTAAGATTCAACT
ACAATCTCAGAATGC
+
FFFFFF:FFF:FFFFFF:FFFFF,FFFF:FFFFFFFFFFFFFFFFFFFFF,FFFFFFFFFFFFFFFFFFF
FFFFFFFFFFFFFFFFFFFFFFFFFFFFFFFFFFFFFFFFFFFFFFFFFFFFFFFF:FF:FFFFFFFFFF
FFFFFFFFFFFFFFF
@A00721:190:HGJW7DSXY:4:1441:5611:33536 2:N:0:TCTGCAAG+GCTGCACT
CTCACATGGCAGCAACTACAAGTAGGGGAGTACTACTGGAACCTAAACAATCCAATATGGATACGAGT
CCAACACTCTGGACAGACGACGAGTCCACCGTTCCATCACATCTTCACGGTGGAACTAAGATTCAACT
ACAATCTCAGAATGC
+
FFFFFFFFFFFFFFFFFFFFFFFFFFFFFFFFFFFFFFFFFFFFFFFF:FFFFFFFF:FFFFFFFFF:FF
FFFFFFFFFFFFFFFFFFFFFFFFFFFFFFFFFFFFFFFFFFFFFFFFFFFFFFF:F:F,FFFFFFFFFF
FFFFF:FFFFFFF,F
@A00721:190:HGJW7DSXY:4:1468:30400:16783 2:N:0:TCTGCAAG+GCTGCACT
CTCACATGGCAGCAACTACAAGTAGGGGAGTACTACTGGAACCTAAACAATCCAATATGGATACGAGT
CCAACACTCTGGACAGACGACGAGTCCACCGTTCCATCACATCTTCACGGTGGAACTAAGATTCAACT
ACAATCTCAGAATGC
+
FFFFFFFFFFFFFFFFFFFFFFFFFFFFFFFFFFFFFFFFFFFFFFFFFFFFFFFFFFFFFFFFFFFFFF
FFFFFFFFFFFFFFFFFFFFFFFFFFFFFFFFFFFFFFFFFFFFFFFFFFFFFFFF:FFFFFFFFFFFFF
FFFFFFFFFFFFFFF
@A00721:190:HGJW7DSXY:4:1509:3866:8531 2:N:0:TCTGCAAG+GCTGCACT
CTCACATGGCAGCAACTACAAGTAGGGGAGTACTACTGGAACCTAAACAATCCAATATGGATACGAGT
CCAACACTCTGGACAGACGACGAGTCCACCGTTCCATCACATCTTCACGGTGGAACTAAGATTCAACT
ACAATCTCAGAATGC
+
FFFFFFFFFFFFFFFFFFFFFFFFFFFFFFFFFFFFFFFFFFFFFFFFFFFFFFFFFFFFFFFFFFFFFF
FFFFFFFFFFFFFFFFFFFFFFFFFFFFFFFFFFFFFFFFFFFFFFFFFFFFFFFFFFFFFFFFFFFFFF
FFFFFFFFFFFFFFF
@A00721:190:HGJW7DSXY:4:1619:11858:30326 2:N:0:TCTGCAAG+GCTGCACT
CTCACATGGCAGCAACTACAAGTAGGGGAGTACTACTGGAACCTAAACAATCCAATATGGATACGAGT
CCAACACTCTGGACAGACGACGAGTCCACCGTTCCATCACATCTTCACGGTGGAACTAAGATTCAACT
ACAATCTCAGAATGC
+
FFFFFFFFFFFFFFFFFFFFFFFFFFFFFFFFFFFFFFFFFFFFFFFFFFFFFFFFFFFFFFFFFFFFFF
FFFFFFFFFFFFFFFFFFFFFFFFFFF:FFFFFFFFFFFFFFFFFFFFFFFFFFFFFFFFFFFFFFFFFF
FFFFFFFFFFFFFFF
```

```
@A00721:190:HGJW7DSXY:4:1621:30264:36965 2:N:0:TCTGCAAG+GCTGCACT
CTCACATGGCAGCAACTACAAGTAGGGGAGTACTACTGGAACCTAAACAATCCAATATGGATACGAGT
CCAACACTCTGGACAGACGACGAGTCCACCGTTCCATCACATCTTCACGGTGGAACTAAGATTCAACT
ACAATCTCAGAATGC
+
FFFFFFFFFFFFFFFFFFFFFFFFFFFFFFFFFFFFFFFFFFFFFFFFFFFFFFFFFFFFFFFFFFFFFF
FFFFFFFFFFFFFFFFFFFFFFFFFFFFFFFFFFFFFFFFFFFFFFFFFFFFFFFFFFFFFFFFFFFFFF
FFFFFFFFFFFFFFF
@A00721:190:HGJW7DSXY:4:1635:16224:24674 2:N:0:TCTGCAAG+GCTGCACT
CTCACATGGCAGCAACTACAAGTAGGGGAGTACTACTGGAACCTAAACAATCCAATATGGATACGAGT
CCAACACTCTGGACAGACGACGAGTCCACCGTTCCATCACATCTTCACGGTGGAACTAAGATTCAACT
ACAATCTCAGAATGC
+
FFFFFFFFFFFFFFFFFFFFFFFFFFFFFFFFFFFFFFFFFFFFFFFFFFFFFFFFFFFFFFFFFFFFFF
FFFFFFFFFFFF:FFFFFFFFFFFFFFFFFFFFFFFFFFF:FFFFFFFFFFFFFFF,F,FFFFFFFFFFF
FFF:FFFFFFFFFFF
@A00721:190:HGJW7DSXY:4:2103:9670:5181 2:N:0:TCTGCAAG+GCTGCACT
CTCACATGGCAGCAACTACAAGTAGGGGAGTACTACTGGAACCTAAACAATCCAATATGGATACGAGT
CCAACACTCTGGACAGACGACGAGTCCACCGTTCCATCACATCTTCACGGTGGAACTAAGATTCAACT
ACAATCTCAGAATGC
+
FFFFFFFFFFFFFFFFFFFFFFFFFFFFFFFFFFFFFFFFFFFFFFFFFFFFFFFFFFFFFFFFFFFFFF
FFFFFFFFFFFFFFFFFFFFFFFFFFFFFFFFFFFFFFFFFFFFFFFFFFFFFFFFFFFFFFFFFFFFFF
FFFFFFFFFFFFFFF
@A00721:190:HGJW7DSXY:4:2110:29767:8015 2:N:0:TCTGCAAG+GCTGCACT
CTCACATGGCAGCAACTACAAGTAGGGGAGTACTACTGGAACCTAAACAATCCAATATGGATACGAGT
CCAACACTCTGGACAGACGACGAGTCCACCGTTCCATCACATCTTCACGGTGGAACTAAGATTCAACT
ACAATCTCAGAATGC
+
FFFFFFFFFFF:FFFFF,FFF:FFF:FFFFF,FFFFFFFFFFFFFFFFFFFF:FFFFFF:FFFFF:FFFF
F:F:FFFFFFFFFFFFFFFFFFF:,FFFFF:FFF:FFFFFFFF,FFFFFF:F,FFF,F,FFFFFFFFFFF
FFFFFFFFFFFFFFF
@A00721:190:HGJW7DSXY:4:2145:4634:26240 2:N:0:TCTGCAAG+GCTGCACT
CTCACATGGCAGCAACTACAAGTAGGGGAGTACTACTGGAACCTAAACAATCCAATATGGATACGAGT
CCAACACTCTGGACAGACGACGAGTCCACCGTTCCATCACATCTTCACGGTGGAACTAAGATTCAACT
ACAATCTCAGAATGC
+
FFFFFFFFFFFFFFFFFFFFFFFFFFFFFFFFFFFFFFFFFFFFFFFFFFFFFFFFFF:FFFFFFFFFFF
FFFFFFFFFFFFFFFFFFFFFFFFFFFFFFFFFFFFFFFFFFFFFFFFFFFFFFF:FFFFFFFFFFFFFF
FFFF:FFFFFFFFFF
@A00721:190:HGJW7DSXY:4:2168:29586:24142 2:N:0:TCTGCAAG+GCTGCACT
GCATTCTGAGATTGTAGTTGAATCTTAGTTCCACCGTGAAGATGTGATGGAACGGTGGACTCGTCGTC
TGTCCAGAGTGTTGGACTCGTATCCATATTGGATTGTTTAGGTTCCAGTAGTACTCCCCTACTTGTAG
TTGCTGCCATGTGAG
+
FFFFFFFFFFFFFFFFFFFFFFFFFFFFFFFFFFFFFFFFFF:FFFFFFFFFF:FFFFFFFFFFFFFFFF
FF:FFFFFFFFFFFFFFFFFFFFFFFFFFFFFFFFFFFFFFFFFFFFFFFFFFFFFFFFFFFFFFFFFFF
FFFFFFFFFFFFFFF
@A00721:190:HGJW7DSXY:4:2302:26901:3270 2:N:0:TCTGCAAG+GCTGCACT
CTCACATGGCAGCAACTACAAGTAGGGGAGTACTACTGGAACCTAAACAATCCAATATGGATACGAGT
CCAACACTCTGGACAGACGACGAGTCCACCGTTCCATCACATCTTCACGGTGGAACTAAGATTCAACT
ACAATCTCAGAATGC
+
FFFFF:FFFFFFFFFFFFFFFFFFFFFFF:FFFFFFFFFFFFFFFFFFFFFFFFFFFFF:FFFFFFFFFF
```

```
FFFFFFFFFFFFFFFFFFFFFFFFFFFFFFFFFFFFFFFFFFFFFFFFFF:FFFFFFFFFFFFFFFFFFF
FFFFFFFFFFFFFFF
@A00721:190:HGJW7DSXY:4:2350:8422:23938 2:N:0:TCTGCAAG+GCTGCACT
GCATTCTGAGATTGTAGTTGAATCTTAGTTCCACCGTGAAGATGTGATGGAACGGTGGACTCGTCGTC
TGTCCAGAGTGTTGGACTCGTATCCATATTGGATTGTTTAGGTTCCAGTAGTACTCCCCTACTTGTAG
TTGCTGCCATGTGAG
+
FFFFFFFFFFFFFFFFFFFFFFFFFFFFFFFFFFFFFFFFFFFFFFFFFFFFFFFFFFFFFFFFFFFFFF
FFFFFFFFFFFFFFFFFFFFFFFFFFF::FFFFFFFFFFFFFFFFFFFFFFFFFFFFFFFFFFF::FFFF
FFFFFFFFFFFFFFF
@A00721:190:HGJW7DSXY:4:2356:20889:14027 2:N:0:TCTGCAAG+GCTGCACT
GCATTCTGAGATTGTAGTTGAATCTTAGTTCCACCGTGAAGATGTGATGGAACGGTGGACTCGTCGTC
TGTCCAGAGTGTTGGACTCGTATCCATATTGGATTGTTTAGGTTCCAGTAGTACTCCCCTACTTGTAG
TTGCTGCCATGTGAG
+
FFFFFFFFFFFFFFFFFFFFFFFFFFFFFFFFFFFFFFFFFFF:FFFFFFFFFFFFFFF:FFFFFFFFFF
FFFFFFFFFFFFFFFFFFFFFFFFFFFF:FFF:F:FFFFFFFFFFFFFFFF:FFFF:FFFFFFFFFFFFF
FFFFFFFFFFFFFFF
@A00721:190:HGJW7DSXY:4:2356:21278:14137 2:N:0:TCTGCAAG+GCTGCACT
GCATTCTGAGATTGTAGTTGAATCTTAGTTCCACCGTGAAGATGTGATGGAACGGTGGACTCGTCGTC
TGTCCAGAGTGTTGGACTCGTATCCATATTGGATTGTTTAGGTTCCAGTAGTACTCCCCTACTTGTAG
TTGCTGCCATGTGAG
+
F,FFFFFFF:F:FFFFFFFFFFFFFFFFFFFFFFFFFFFFFFFFFFFFFFFFFFFFFFFFFFFFFFFFFF
FF:FFFFFFFFFFFFFFFFFFFFF:FFFFF:FFF:,FFFFFFFFFFFFFFF:FFFFFFFFFFFF:FFFFF
::FFFF,F:FFFFFF
@A00721:190:HGJW7DSXY:4:2371:7997:30499 2:N:0:TCTGCAAG+GCTGCACT
GCATTCTGAGATTGTAGTTGAATCTTAGTTCCACCGTGAAGATGTGATGGAACGGTGGACTCGTCGTC
TGTCCAGAGTGTTGGACTCGTATCCATATTGGATTGTTTAGGTTCCAGTAGTACTCCCCTACTTGTAG
TTGCTGCCATGTGAG
+
FFFFFFFFFFFFFFFFFFFFFFFFFFFFFFFFFFFFFFFFFFFFFFFFFFFFFFFFFFFFFFFFFFFFFF
FFFFFFFFFFFFFFFFFFFFFFFFFFFFF:FFFFFFFFFFFFFFFFFFFFFFFFFFFFFFFFFFFFFFF:
FFFFFFFFFFFFFFF
@A00721:190:HGJW7DSXY:4:2373:1316:31078 2:N:0:TCTGCAAG+GCTGCACT
CTCACATGGCAGCAACTACAAGTAGGGGAGTACTACTGGAACCTAAACAATCCAATATGGATACGAGT
CCAACACTCTGGACAGACGACGAGTCCACCGTTCCATCACATCTTCACGGTGGAACTAAGATTCAACT
ACAATCTCAGAATGC
+
FFFFFFFFFFFFFFFFFFFFFFFFFFFFFFFFFFFFFFFFFF:FFFFFFFFFFFFFFFFFFFFFFFFFFF
FFFFFFFFFFFFFFFFFFFFFFFFFFFFFFFFFFFFFFFFFFFFF:FFFFFFFFFF::FFFF:FFFFFFF
FFFFFFFFFFFFF:F
@A00721:190:HGJW7DSXY:4:2414:17038:2942 2:N:0:TCTGCAAG+GCTGCACT
CTCACATGGCAGCAACTACAAGTAGGGGAGTACTACTGGAACCTAAACAATCCAATATGGATACGAGT
CCAACACTCTGGACAGACGACGAGTCCACCGTTCCATCACATCTTCACGGTGGAACTAAGATTCAACT
ACAATCTCAGAATGC
+
FFFFFFFFFFFFFFFFFFFFFFFFFFFFFFFFFFFFFFFFFFFFFFFFFFFFFFFFFFFFFFFFFFFFFF
FFFFFFFFFFFFFFFFFFFFFFFFFF:FFFFFFFFFFFFFFFFFFFFFFFFFFFF,FFFFFFFFFFFFFF
,F:FFFFFFFFFFF:
@A00721:190:HGJW7DSXY:4:2418:27082:36714 2:N:0:TCTGCAAG+GCTGCACT
GCATTCTGAGATTGTAGTTGAATCTTAGTTCCACCGTGAAGATGTGATGGAACGGTGGACTCGTCGTC
TGTCCAGAGTGTTGGACTCGTATCCATATTGGATTGTTTAGGTTCCAGTAGTACTCCCCTACTTGTAG
TTGCTGCCATGTGA
```

```
+
FFFFFFFFFFFFFFFFFFFFFFFFFFFFFFF:FFFFFFFFFFFFFFFFFFFFFFFFFFFFFFFFFFFF
FF:FFFFFFFFFFFFFFF:FFFFFFFFFFFFF:FFFFF:FFFFFFF:FF:FF:FFFFFFF:FF,FFFF
FFFFFFFFFFFFFF
@A00721:190:HGJW7DSXY:4:2419:7563:27993 2:N:0:TCTGCAAG+GCTGCACT
GCATTCTGAGATTGTAGTTGAATCTTAGTTCCACCGTGAAGATGTGATGGAACGGTGGACTCGTCGTC
TGTCCAGAGTGTTGGACTCGTATCCATATTGGATTGTTTAGGTTCCAGTAGTACTCCCCTACTTGTAG
TTGCTGCCATGTGAG
+
FF:FFFFFFFFFFFFFFFFFFFFFFFFFFFFFFFFFFFFFFFFFFFFFFFFFFFFFFFFFFFFFFFFF
FF:FFFFFFFFFFFFFFFFFFFFFFFFFFFFFF:FFFFFFFFFFFFFFFFFFFFFFFFFF:FFFFFFF
FFFFF:FFF:FFFF:
@A00721:190:HGJW7DSXY:4:2457:27028:28291 2:N:0:TCTGCAAG+GCTGCACT
CTCACATGGCAGCAACTACAAGTAGGGGAGTACTACTGGAACCTAAACAATCCAATATGGATACGAGT
CCAACACTCTGGACAGACGACGAGTCCACCGTTCCATCACATCTTCACGGTGGAACTAAGATTCAACT
ACAATCTCAGAATGC
+
FFFFFFFFFFFFFFFFFFFFFFFFFFFFFFFFFFFFFFFFFFFFFFFFFFFFFFFFFFFFFF:FFFFF
FFFFFFFFFFFFFFFFFFFFFFFFFFFFFFFFFFF:FFFFFFFFFFFFFFFFFF,FFFFFFFFFFFFF
FF,FFFFFFFF:FFF
@A00721:190:HGJW7DSXY:4:2530:28646:25457 2:N:0:TCTGCAAG+GCTGCACT
CTCACATGGCAGCAACTACAAGTAGGGGAGTACTACTGGAACCTAAACAATCCAATATGGATACGAGT
CCAACACTCTGGACAGACGACGAGTCCACCGTTCCATCACATCTTCACGGTGGAACTAAGATTCAACT
ACAATCTCAGAATGC
+
FFFFFFFFFFFFFFFFFFFFFFFFFFFF:FFFFFFFFFFFFFFFFF:FFFFFFFFFFFFFFFFFFFFF
FFFFFFFFFFFFFFFFFFFFFFFFFFFFFFFFFFFFFFFFFFFFFFFFFFFFFF,FFFFFFFFFFFFF
FFFFFFFFFFFFFFF
@A00721:190:HGJW7DSXY:4:2637:8196:11303 2:N:0:TCTGCAAG+GCTGCACT
GCATTCTGAGATTGTAGTTGAATCTTAGTTCCACCGTGAAGATGTGATGGAACGGTGGACTCGTCGTC
TGTCCAGAGTGTTGGACTCGTATCCATATTGGATTGTTTAGGTTCCAGTAGTACTCCCCTACTTGTAG
TTGCTGCCATGTGAG
+
FFFFFFFFFFFFFFFFFFFFFFFFFFFFFFFFFFFFFFFFFFFFFFFFFFFFFFFFFFFFFFFFFFFF
FF:FFFFFFFFFFFFFFFFFFFFFFFFFFFFFFF:FFFFFF:FFFFFFFFFFFFFF:FFFF:FFFFFF
FFFFFFFFFFFFFFF
@A00721:190:HGJW7DSXY:4:2675:16486:24659 2:N:0:TCTGCAAG+GCTGCACT
GCATTCTGAGATTGTAGTTGAATCTTAGTTCCACCGTGAAGATGTGATGGAACGGTGGACTCGTCGTC
TGTCCAGAGTGTTGGACTCGTATCCATATTGGATTGTTTAGGTTCCAGTAGTACTCCCCTACTTGTAG
TTGCTGCCATGTGAG
+
FFFFFFFFFFFFFFFFFFFFFFFFFFFFFFFFFFFFFFFFFFFFFFFFFFFFFFFFFFFFFFFFFFFF
FFFFFFFFFFFFFFFFFFFFFFFFFFFFFFFFFFFFFFFFFFFFFFFFFFFFFFFFFFFFFFFFFFFF
FFF:FFFFFFFFFFF
@A00721:190:HGJW7DSXY:4:2219:25482:9173 2:N:0:TCTGCAAG+GCTGCACT
CTCACATGGCAGCAACTACAAGTAGGGGAGTACTACTGGAACCTAAACAATCCAATATGGATACGAGT
CCAACACTCTGGACAGACGACGAGTCCACCGTTCCATCACATCTTCACGGTGGAACTAAGATTCAACT
ACAATCTCAGAATG
+
FFFFFFFFFFFFFFFFFFFFFFFFFFF:FFFFFFFFFFFFFFFFFFF:FFFFFFF,FFFFFF:FFFFF
FFFFFFFFFFF:FFFFFF:FFFFFFFFFFFFFFFFFFFFFFFFF:FFFFFFFFF,FFFFFFFFFFFFF
FFFFFFFFFFFFFF
@A00721:190:HGJW7DSXY:4:2304:28592:34726 2:N:0:TCTGCAAG+GCTGCACT
CTCACATGGCAGCAACTACAAGTAGGGGAGTACTACTGGAACCTAAACAATCCAATATGGATACGAGT
```

```
CCAACACTCTGGACAGACGACGAGTCCACCGTTCCATCACATCTTCACGGTGGAACTAAGATTCAACT
ACAATCTCAGAATG
+
FFFFFFFF:FFFFFFFFFFFF:FFFFFFFFFFFFFFFF:FFFFFFFFFFFFFFFFFFFFFFFFFFFFFFF
FFFFFFFFFFF:FFFFFFFFF:FFFFFFFFFFFFFFFFFFFFFF:FFFFFFFFFFFFFFFFFFF:FFFF:
F:FFFF:FFFFFFF
@A00721:190:HGJW7DSXY:4:1263:19985:19038 2:N:0:TCTGCAAG+GCTGCACT
CACTCACATGGCAGCAACTACAAGTAGGGGAGTACTACTGGAACCTAAACAATCCAATATGGATACGA
GTCCAACACTCTGGACAGACGACGAGTCCACCGTTCCATCACATCTTCACGGTGGAACTAAGATTCAA
CTACAATCTCAGAGA
+
FFFFFFF:FFFFFFFFFFFFFFFFFFFFFFFFFFFFFFFFFFFFFFFFFFFFFFFFFFFFFFFFFF:FFF
FFFFFFFFFFFFFFFFFFFFFFFFFFFFFFFFFFFFFFFFFFFFFFFFFFFFFFFFFF,FFFFFFFFFFF
FFFFF:FFFFFFFFF
@A00721:190:HGJW7DSXY:4:1455:31575:14372 2:N:0:TCTGCAAG+GCTGCACT
CACTCACATGGCAGCAACTACAAGTAGGGGAGTACTACTGGAACCTAAACAATCCAATATGGATACGA
GTCCAACACTCTGGACAGACGACGAGTCCACCGTTCCATCACATCTTCACGGTGGAACTAAGATTCAA
CTACAATCTCAGAGA
+
FFFFFFFFFFFFFFFFFFFFFFFFFF:FFFFFFFFFFFFFFFFFFFFF:FFFFFFFFFFFFFFFFFFFFF
FFFFFFFFFFFFFFFFFFFFFF:FFFFFFFFFFFFFFFFFFFFFFFFFFFFFFFFFFF,FFFFFF:FF:F
FFFFFFFFFFFFFF:
@A00721:190:HGJW7DSXY:4:1458:6099:30686 2:N:0:TCTGCAAG+GCTGCACT
CACTCACATGGCAGCAACTACAAGTAGGGGAGTACTACTGGAACCTAAACAATCCAATATGGATACGA
GTCCAACACTCTGGACAGACGACGAGTCCACCGTTCCATCACATCTTCACGGTGGAACTAAGATTCAA
CTACAATCTCAGAGA
+
FFFFFFFFFFFF:FFFFFFFFFFFFFFFFFFFFFFFFFFFFFFFFFFFFFFFFFFFFFFFFFFFFFFFFF
FFFFFFFFFFFFFFFFFFFFFFFFFFFFFFFFFFFFFFFFFFFFFFFFFFFFFFFFFFFFFFFFFFFFFF
FFFFFFFFFFFFFFF
@A00721:190:HGJW7DSXY:4:1540:5095:7968 2:N:0:TCTGCAAG+GCTGCACT
CACTCACATGGCAGCAACTACAAGTAGGGGAGTACTACTGGAACCTAAACAATCCAATATGGATACGA
GTCCAACACTCTGGACAGACGACGAGTCCACCGTTCCATCACATCTTCACGGTGGAACTAAGATTCAA
CTACAATCTCAGAGA
+
FFFFFFFFFFFFFFFFFFFFFFFFFFFFFFFFFFFFFFFFFFFFFF:FFFFFFFFFFFFFFFFFFFFFFF
FFFFF:FFFFFFFFFFFFFFFFFFFFFFFFFFFFFFFFFFFFFFFFFFFFFFFFFFFFFFF:FFFFFFFF
FFFFFFFFFFFFFFF
@A00721:190:HGJW7DSXY:4:1540:5321:8140 2:N:0:TCTGCAAG+GCTGCACT
CACTCACATGGCAGCAACTACAAGTAGGGGAGTACTACTGGAACCTAAACAATCCAATATGGATACGA
GTCCAACACTCTGGACAGACGACGAGTCCACCGTTCCATCACATCTTCACGGTGGAACTAAGATTCAA
CTACAATCTCAGAGA
+
FFFFFFFFFFFFFFFFFFFFFFFFFFFFFFFFFFFFFFFFFFFF:FFFFFFFFFFFFFFFFFFFFFFFFF
FFFFFFFFFFFFFFFFFFFFFFFFFFFFFFFFFFFFFFFFFFFFFFFFFFFFFFFFFFFFFFFFFFFFFF
FFFFFFFFFFFFFFF
@A00721:190:HGJW7DSXY:4:1621:32407:23891 2:N:0:TCTGCAAG+GCTGCACT
CACTCACATGGCAGCAACTACAAGTAGGGGAGTACTACTGGAACCTAAACAATCCAATATGGATACGA
GTCCAACACTCTGGACAGACGACGAGTCCACCGTTCCATCACATCTTCACGGTGGAACTAAGATTCAA
CTACAATCTCAGAGA
+
FFFFFFFFFFFFFFFFFFF::FFFFFFFFFFFFFFFFFFFFFFFFFFF:FF:FFFF::FFFFFFFFFFFF
FFFF:FFFFFFFFFFFFFFFFFFFFFFFFFFFFFFFFFFFFFFFFFFFF,FFFFFF:FFFF:FFFFFFF
FFFFFFFFFFFFF,F
```

```
@A00721:190:HGJW7DSXY:4:2123:10077:1094 2:N:0:TCTGCAAG+GCTGCACT
CACTCACATGGCAGCAACTACAAGTAGGGGAGTACTACTGGAACCTAAACAATCCAATATGGATACGA
GTCCAACACTCTGGACAGACGACGAGTCCACCGTTCCATCACATCTTCACGGTGGAACTAAGATTCAA
CTACAATCTCAGAGA
+
FFFFFFFFFFFFFFFFFFFFFFFFFFFFFFFFFFFFFFFFFFFFFFF,FFFFFFFFFFFFFFFFFFFF
FFFFFFFFFFFFFFFFFFFFFFFFFFFFFFFFFFFFFFFF:FFFFFFFFFFFFFFFFFFFFFFFFF:F
FFFFFFFFFFFFFFF
@A00721:190:HGJW7DSXY:4:2166:22263:34006 2:N:0:TCTGCAAG+GCTGCACT
CACTCACATGGCAGCAACTACAAGTAGGGGAGTACTACTGGAACCTAAACAATCCAATATGGATACGA
GTCCAACACTCTGGACAGACGACGAGTCCACCGTTCCATCACATCTTCACGGTGGAACTAAGATTCAA
CTACAATCTCAGAGA
+
FFFFFFFFFFFFFFFFFFFFFFFFFFFFFFFFFFFFFFFFFFFFFFFFFFFFFFFFFFFFFFFFFFFF
FFFFFFFFFFFFFFFFFFFFFFFFFFFFFFFFFFFFFFFFFFFFFFFFFFFFFFFF:FFFFFFFFFFF
FFFFFFFFFFFFFFF
@A00721:190:HGJW7DSXY:4:2258:15239:12665 2:N:0:TCTGCAAG+GCTGCACT
CACTCACATGGCAGCAACTACAAGTAGGGGAGTACTACTGGAACCTAAACAATCCAATATGGATACGA
GTCCAACACTCTGGACAGACGACGAGTCCACCGTTCCATCACATCTTCACGGTGGAACTAAGATTCAA
CTACAATCTCAGAGA
+
FFFFFFFFFFFFFFFFFFFFFFFFFFFFFFFFFFFFFFFFFFFFFFFFFFFFFFFFFFFFFFFFFFFF
FFFFFFFFFFFFFFFFFFFFFFFFFFFFFFFFFFFFFFFFFFFFFFFFFFFFFFFFFFFFFFFFFFFF
FFFFFFFFFFFFFFF
@A00721:190:HGJW7DSXY:4:2366:18611:35289 2:N:0:TCTGCAAG+GCTGCACT
CACTCACATGGCAGCAACTACAAGTAGGGGAGTACTACTGGAACCTAAACAATCCAATATGGATACGA
GTCCAACACTCTGGACAGACGACGAGTCCACCGTTCCATCACATCTTCACGGTGGAACTAAGATTCAA
CTACAATCTCAGAGA
+
FFFFFFFFFFFFFFFFFFFFF:FFFFFFFFFFFFFFFFFFFFFFFFFF:FFFFFFFFFFFFFFFFFFF
FFFFFFFFFFFFFFFFFFFFFFFFFFFFFFFFFFFFFFFF:FFFFFFFFFFFFFFF,FFFFFFFFFFF
FFFFFFFFFFFFFFF
@A00721:190:HGJW7DSXY:4:2538:31367:28604 2:N:0:TCTGCAAG+GCTGCACT
CACTCACATGGCAGCAACTACAAGTAGGGGAGTACTACTGGAACCTAAACAATCCAATATGGATACGA
GTCCAACACTCTGGACAGACGACGAGTCCACCGTTCCATCACATCTTCACGGTGGAACTAAGATTCAA
CTACAATCTCAGAGA
+
FFFFFFFFFFFFFFFFFFFFFFFFFFFFFFFFFFFFFFFFFFFFFFFFFFFFFFFFFFFFFFFFFFFF
FFFFFFFFFFFFFFFFFFFFFFFFFFFFFFFFFFFFFFFFFFFFFFFFFFFFFFFFFFFFFFFFFFFF
FFFFFFFFFFFFFFF
@A00721:190:HGJW7DSXY:4:1573:28565:24721 2:N:0:TCTGCAAG+GCTGCACT
CTGAGATTGTAGTTGAATCTTAGTTCCACCGTGAAGATGTGATGGAACGGTGGACTCGTCGTCTGTCC
AGAGTGTTGGACTCGTATCCATATTGGATTGTTTAGGTTCCAGTAGTACTCCCCTACTTGTAGTTGCT
GCCATGTGAGTGGCA
+
FFFF:FFFFFFFFFFFFFFFFFFFFFFFFFFFFFFFFFFFFFFFFFFFFFFFFFFFFFFFFFFFFFFF
FFFFFFFFFFFFFFFFFFFFFF:FFFFFFFFFFFFFFFFFFFFFFFFFFFFFFFFFFFFFFFFFFFFF
FFFFFFFFFFFFFFF
@A00721:190:HGJW7DSXY:4:2204:27823:2957 2:N:0:TCTGCAAG+GCTGCACT
CTGAGATTGTAGTTGAATCTTAGTTCCACCGTGAAGATGTGATGGAACGGTGGACTCGTCGTCTGTCC
AGAGTGTTGGACTCGTATCCATATTGGATTGTTTAGGTTCCAGTAGTACTCCCCTACTTGTAGTTGCT
GCCATGTGAGTGGCA
+
FFFFFFFFFFFFFFFFFFFFFFFFFF:FFFFFFFFFFFFFFFFFFFFFFFFFFFFFFFFFFFFFF:FF
```

FFFFFFFFFFFFFFFFFFFFFFFFFFFFFFFFFFFFFFFFFFFFFFFFFFFFF:FFFFFFFFFFFF:FFF
FFFFFFFFFFFF::F
@A00721:190:HGJW7DSXY:4:1235:16378:2895 2:N:0:TCTGCAAG+GCTGCACT
GGATTCGTCCACAAGGGTGCCACTCACATGGCAGCAACTACAAGTAGGGGAGTACTACTGGAACCTAA
ACAATCCAATATGGATACGAGTCCAACACTCTGGACAGACGACGAGTCCACCGTTCCATCACATCTTC
ACGGTGGAACTAAGA
+
FFFFFFFFFFFFFFFFFFFFFFFFFFFFFFFFFFFFFFFFFFFFFFFFFFFFFFFFFFFFFFFFFFFFFF
FFFFFFFFFFFFFFFFFFFFFFFFFFFFFFFFFFFFFFFFFFFFFFFFFFFFF:FFFFFFFFFFFFFFFF
FFFFFF:FFFFFFFF
@A00721:190:HGJW7DSXY:4:1236:21884:34413 2:N:0:TCTGCAAG+GCTGCACT
TCTTAGTTCCACCGTGAAGATGTGATGGAACGGTGGACTCGTCGTCTGTCCAGAGTGTTGGACTCGTA
TCCATATTGGATTGTTTAGGTTCCAGTAGTACTCCCCTACTTGTAGTTGCTGCCATGTGAGTGGCACC
CTTGCGGACAGATCG
+
FFFFFFFFFFFFFFFFFFFFFFFFFFFFFFFFFFFFFFFFFFFFFFFFFFFFFFFFFFFFFFFFFFFFFF
FFFFFFFFFFFFFFFFFFFFFFFFFFFFFFFFFFFFFFFFFFFFFFFFFFFFFFFFFFFFFFFFFFFFFF
FFFFFFFFFFFFFFF
@A00721:190:HGJW7DSXY:4:1238:17707:25770 2:N:0:TCTGCAAG+GCTGCACT
TCTTAGTTCCACCGTGAAGATGTGATGGAACGGTGGACTCGTCGTCTGTCCAGAGTGTTGGACTCGTA
TCCATATTGGATTGTTTAGGTTCCAGTAGTACTCCCCTACTTGTAGTTGCTGCCATGTGAGTGGCACC
CTTGCGGACAGATCG
+
FFFFFFFFFFFFFFFFFFFFFFFFFFFFFFFFFFFFFFFFFFFFFFFFFFFFFFFFFFFFFFFFFFFFFF
FFFFFFFFFFF:FFFFFFFFFFFFFFF,FFFFFFFFFF,FFFFFFFFFFFFFFFFFFFFFFFFFFFFFFF
FFFFFFFFFFFFFFF
@A00721:190:HGJW7DSXY:4:1457:24334:25848 2:N:0:TCTGCAAG+GCTGCACT
GGATTCGTCCACAAGGGTGCCACTCACATGGCAGCAACTACAAGTAGGGGAGTACTACTGGAACCTAA
ACAATCCAATATGGATACGAGTCCAACACTCTGGACAGACGACGAGTCCACCGTTCCATCACATCTTC
ACGGTGGAACTAAGA
+
FFFFFFFFFFFFFFFFFFFFFFFFFFFFFFFFFFFFFFFFFFFFFFFFFFFFFFFFFFFFFFFFFFFFFF
FFFFFFFFFFFFFFFFFFFFFFFFFFFFFFFFFFFFFFFFFFFFFFFFFFFFFFFFFFFFFFFFFFFFFF
FFFFFFFFFFFFFFF
@A00721:190:HGJW7DSXY:4:2117:8504:30561 2:N:0:TCTGCAAG+GCTGCACT
TCTTAGTTCCACCGTGAAGATGTGATGGAACGGTGGACTCGTCGTCTGTCCAGAGTGTTGGACTCGTA
TCCATATTGGATTGTTTAGGTTCCAGTAGTACTCCCCTACTTGTAGTTGCTGCCATGTGAGTGGCACC
CTTGCGGACAGATCG
+
FFFFFFFFFFFFFFFFFFFFFFFFFFFFFFFFFFFFFFFFFFFFFFFFFFFFFFFFFFFFFFFFFFFFFF
FFFFFFFFFFFFFFFFFFFFFFFFFFFFFFFFFFFFFFFFFFFFFFFFFFFFFFFFFFFFFFFFFFFFFF
FFFFFFFFFFFFFFF
@A00721:190:HGJW7DSXY:4:2117:10393:31955 2:N:0:TCTGCAAG+GCTGCACT
TCTTAGTTCCACCGTGAAGATGTGATGGAACGGTGGACTCGTCGTCTGTCCAGAGTGTTGGACTCGTA
TCCATATTGGATTGTTTAGGTTCCAGTAGTACTCCCCTACTTGTAGTTGCTGCCATGTGAGTGGCACC
CTTGCGGACAGATCG
+
FFFFFFFFFFFFFFFFFFFFFFFFFFFFFF:FFFFFFFFFFFFFFFFFFF:FFFFFFFFFFFFFFFFFFF
FFF:FFFFFFF:FFFFFFFFFFFFFFFFFFFFFFFFFFFFF:FFFFFFFFFFFFFFFFFFFFFFFFFFFF
FF:FFFFFFFFFF:F
@A00721:190:HGJW7DSXY:4:2243:22435:31579 2:N:0:TCTGCAAG+GCTGCACT
GGATTCGTCCACAAGGGTGCCACTCACATGGCAGCAACTACAAGTAGGGGAGTACTACTGGAACCTAA
ACAATCCAATATGGATACGAGTCCAACACTCTGGACAGACGACGAGTCCACCGTTCCATCACATCTTC
ACGGTGGAACTAAGA

```
+
FFFFFFFFFFFFFFFFFFFFFFFFFFFFFFFFFFFFFFFFFFFFFFFFFFFFFFFFFFFFFFFFFFFF
FFFFFFFFFFFFFFFFFFFFFFFFFFFFFFFFFFFFFFFFFFFFFFFFFFFFFFFFFFFFFFFFFFFF
FFFFFFFFFFFFFFF
@A00721:190:HGJW7DSXY:4:2345:4598:4820 2:N:0:TCTGCAAG+GCTGCACT
GGATTCGTCCACAAGGGTGCCACTCACATGGCAGCAACTACAAGTAGGGGAGTACTACTGGAACCTAA
ACAATCCAATATGGATACGAGTCCAACACTCTGGACAGACGACGAGTCCACCGTTCCATCACATCTTC
ACGGTGGAACTAAGA
+
FFFFFFFFFFFFFFFFFFFFFFFFFFFFFFFFFFFFFFFFFFFFFFFFFF:FFFFFFFFFFFFFFFFF
FFFFFFFFFFFFFFFFFFFFFFFFFFFFFFFFFFFFFFFFFFFFFFFFFFFFFFFFFFFFFFFFFFFF
FFFFFFFF:FF,FF:
@A00721:190:HGJW7DSXY:4:2556:20148:33755 2:N:0:TCTGCAAG+GCTGCACT
GGATTCGTCCACAAGGGTGCCACTCACATGGCAGCAACTACAAGTAGGGGAGTACTACTGGAACCTAA
ACAATCCAATATGGATACGAGTCCAACACTCTGGACAGACGACGAGTCCACCGTTCCATCACATCTTC
ACGGTGGAACTAAGA
+
FF:FFFFFFFFFFFFFFFFFFFFFFFFFFFFFFFFFFFFFFFFFFFFFFFFFFFFFFFFFFFFFFFFF
FFFFFFFFFFFFFFFFFFFFFFFFFFFFFFFFFFFFFFFFFFFFFFFFFFFFFFFFFFFFFFFFFFFF
FFFFFFFFFFFFFFF
@A00721:190:HGJW7DSXY:4:2573:29830:29543 2:N:0:TCTGCAAG+GCTGCACT
TCTTAGTTCCACCGTGAAGATGTGATGGAACGGTGGACTCGTCGTCTGTCCAGAGTGTTGGACTCGTA
TCCATATTGGATTGTTTAGGTTCCAGTAGTACTCCCCTACTTGTAGTTGCTGCCATGTGAGTGGCACC
CTTGCGGACAGATCG
+
FFFFFFFFFFFFFFFFFFFFFFFFFFFFF:FFFFFFFFFFF:FFFFFFFFFFFFFFFFFFFFFFFFFF
FFFFFFFFFFFFFFFFFFFFFFFFFFFFFFFFFFFFFFFFFFFFFFFFFFFFFFFFFFFFFFFFFFFF
F:FFFFFFFFFFFFF
@A00721:190:HGJW7DSXY:4:2626:3839:25739 2:N:0:TCTGCAAG+GCTGCACT
GGATTCGTCCACAAGGGTGCCACTCACATGGCAGCAACTACAAGTAGGGGAGTACTACTGGAACCTAA
ACAATCCAATATGGATACGAGTCCAACACTCTGGACAGACGACGAGTCCACCGTTCCATCACATCTTC
ACGGTGGAACTAAGA
+
FFFFFFFFFFFFFFFFFFFFFFFFFFFFFFFFFFFFFFFFFFFFFFFFFFFFFFFFFFFFFFFFFFFF
FFFFFFFFFFFFFFFFFFFFFFFFFFFFFFFFFFFFFFFFFFFFFFFFFFFFFFFFFFFFFFFFFFFF
FFFFFF:F:FF,FFF
@A00721:190:HGJW7DSXY:4:2667:25816:35900 2:N:0:TCTGCAAG+GCTGCACT
GGATTCGTCCACAAGGGTGCCACTCACATGGCAGCAACTACAAGTAGGGGAGTACTACTGGAACCTAA
ACAATCCAATATGGATACGAGTCCAACACTCTGGACAGACGACGAGTCCACCGTTCCATCACATCTTC
ACGGTGGAACTAAGA
+
FFFFFFFFFFFFFFFFFFFFFFFFFFFFFFFFFFFFFFFFFFFFFFFFFFFFFFFFFFFFFFFFFFFF
FFFFFFFFFFFFFFFFFFFFFFFFFFFFFFFFFFFFFFFFFFFFFFFF:FFFFFFFFFFFFFFFFFFF
FFFFFFFFFFFFFFF
@A00721:190:HGJW7DSXY:4:2668:27877:16016 2:N:0:TCTGCAAG+GCTGCACT
GGATTCGTCCACAAGGGTGCCACTCACATGGCAGCAACTACAAGTAGGGGAGTACTACTGGAACCTAA
ACAATCCAATATGGATACGAGTCCAACACTCTGGACAGACGACGAGTCCACCGTTCCATCACATCTTC
ACGGTGGAACTAAGA
+
FFFFFFFFFFFFFFFFFFFFFFFFFFFFFFFFFFFFFFFFFFFFFFFFFFFFFFFFFFFFFFFFFFFF
:FFFFFFFFFFFFFFFFFFFFFFFFFFFFFFFFF:FFFFFFFFFFFFFFFFFFFFFFFFFFFFFFFFF
FFFFFFF:F:FFFFF
@A00721:190:HGJW7DSXY:4:1107:5629:29778 2:N:0:TCTGCAAG+GCTGCACT
AATGGATTCGTCCACAAGGGTGCCACTCACATGGCAGCAACTACAAGTAGGGGAGTACTACTGGAACC
```

```
TAAACAATCCAATATGGATACGAGTCCAACACTCTGGACAGACGACGAGTCCACCGTTCCATCACATC
TTCACGGTGGAACTA
+
FFFFFFFFFFFFFFFFFFFFFFFFFFFFFFFFFFFFFFFFFFFFFFFFFFFFFFFFFFFFFFFFFFFF
FFFFFFFFFFFFFFFFFFFFFFFFFFFFFFFFFFFFFFFFFFFFFFFF:FFFFFFFFFFFFFFFFFFF
FFFFFFFFFFFFFFF
@A00721:190:HGJW7DSXY:4:1126:12183:21903 2:N:0:TCTGCAAG+GCTGCACT
AATGGATTCGTCCACAAGGGTGCCACTCACATGGCAGCAACTACAAGTAGGGGAGTACTACTGGAACC
TAAACAATCCAATATGGATACGAGTCCAACACTCTGGACAGACGACGAGTCCACCGTTCCATCACATC
TTCACGGTGGAACTA
+
FFFFFFFFFFFFFFFFFFFFFFFFFFFFFFFFFFFFFFF:FF:FFFFFFFFFFFFFFFFFFFFFFFFF
:FFFFFFFFFFFFFFFFFFFFFFFFFFFFFFFFFFFFFFFFFFFFFFFFFFFFFFFFFFFFFFFF:F
FFFFFFFFFFFFFFF
@A00721:190:HGJW7DSXY:4:1134:14579:28870 2:N:0:TCTGCAAG+GCTGCACT
AATGGATTCGTCCACAAGGGTGCCACTCACATGGCAGCAACTACAAGTAGGGGAGTACTACTGGAACC
TAAACAATCCAATATGGATACGAGTCCAACACTCTGGACAGACGACGAGTCCACCGTTCCATCACATC
TTCACGGTGGAACTA
+
FFFFFFFFFFFFFFFFFF:FFFFFFFFFFFFFFFFFFFFFFFFFFFFFFFFFFFFFFFFFFFFFFFFF
FFFFFFFFFFFFFFFFFFFFFFFFFFFFFFFFFFFFFFFFFFFFFFFFFFFFFFFFFFFFFFFFFFFF
FFFFFFFFFFF:FFF
@A00721:190:HGJW7DSXY:4:1446:15673:10942 2:N:0:TCTGCAAG+GCTGCACT
AATGGATTCGTCCACAAGGGTGCCACTCACATGGCAGCAACTACAAGTAGGGGAGTACTACTGGAACC
TAAACAATCCAATATGGATACGAGTCCAACACTCTGGACAGACGACGAGTCCACCGTTCCATCACATC
TTCACGGTGGAACTA
+
FFFFFFFFFFFFFFFFFFFFFFFFFFFFFFFFFFFFFFFFFFFFFFFFFFFFFFFFFFFFFFFFFFFF
FFFFFFFFFFFFFFFFFFFFFFFFFFFFFFFFFFFFFFFFFFFFFFFFFFFFFFFFFFFFFFFFFFFF
FFFFFFFFFFFFFFF
@A00721:190:HGJW7DSXY:4:1649:17589:24878 2:N:0:TCTGCAAG+GCTGCACT
AATGGATTCGTCCACAAGGGTGCCACTCACATGGCAGCAACTACAAGTAGGGGAGTACTACTGGAACC
TAAACAATCCAATATGGATACGAGTCCAACACTCTGGACAGACGACGAGTCCACCGTTCCATCACATC
TTCACGGTGGAACTA
+
FFFFFFFFFFFFFFFFFFFF:FFF:FFFFFFFFFFFFFFFFFFFFFFFFFFFFFFFFFFFFFFFF:FF
FFFFFFF:FFFFFFFFFFFFFFFFFF:FFFFF:FFFFFFFFFFFFFFFFFFFFFFF:FFFF:FFFFFF
FFFFF,FFFFFFFFF
@A00721:190:HGJW7DSXY:4:2322:14009:7686 2:N:0:TCTGCAAG+GCTGCACT
AATGGATTCGTCCACAAGGGTGCCACTCACATGGCAGCAACTACAAGTAGGGGAGTACTACTGGAACC
TAAACAATCCAATATGGATACGAGTCCAACACTCTGGACAGACGACGAGTCCACCGTTCCATCACATC
TTCACGGTGGAACTA
+
FFFFFFFFF:FFFFFF,:FFFFFFFFFFFFFFF:FFFFFFFFFFFF:FFFFFFFFFFFFFFFFFFFFF
FFFFFFFFFFFFFFF:FFF:FFFFFFFFFFFFFFFFFFFFFFFFFFFFFFFFFFFFFFFF::FFFFFF
FFFFFFFFFFFFFFF
@A00721:190:HGJW7DSXY:4:2433:5746:13009 2:N:0:TCTGCAAG+GCTGCACT
AATGGATTCGTCCACAAGGGTGCCACTCACATGGCAGCAACTACAAGTAGGGGAGTACTACTGGAACC
TAAACAATCCAATATGGATACGAGTCCAACACTCTGGACAGACGACGAGTCCACCGTTCCATCACATC
TTCACGGTGGAACTA
+
FFFFFFFFFFFFFFF:FFFFFFFFFFFFFFFFFFFFFFFFFF:FFFFFFFFFFFFFFFFFFFFFF:FF
FFFFFF:FFFFF:FFFFFFFFFFFFFFFFFFFFFFFFFFFFFFFFFFFFFFFFFFFFFFFFFFFFFFF
FFFFFFFFFFFFFFF
```

@A00721:190:HGJW7DSXY:4:2542:8621:7717 2:N:0:TCTGCAAG+GCTGCACT
AATGGATTCGTCCACAAGGGTGCCACTCACATGGCAGCAACTACAAGTAGGGGAGTACTACTGGAACC
TAAACAATCCAATATGGATACGAGTCCAACACTCTGGACAGACGACGAGTCCACCGTTCCATCACATC
TTCACGGTGGAACTA
+
FFFFFF:FFFFFFFFFFFFFFFFFFFFFFFFFFFFFFFFFFFFFFFFF,FFFFFFFFFFFFFFFFFF:FF
FFFFFFFFFFFFFFFFFFFFFFFFFFFFFFFFFFFFFFFFFFFFFFFFFFFFFFFFFFFFFFFFFFFFFF
FFFFFFFFFFFFFFF
@A00721:190:HGJW7DSXY:4:1110:25663:17691 2:N:0:TCTGCAAG+GCTGCACT
AAACAATGGATTCGTCCACAAGGGTGCCACTCACATGGCAGCAACTACAAGTAGGGGAGTACTACTGG
AACCTAAACAATCCAATATGGATACGAGTCCAACACTCTGGACAGACGACGAGTCCACCGTTCCATCA
CATCTTCACGGTGGA
+
FFFFFFFFFFFFFFFFFFFFFFFFFFFFFFFFFFFFFFFFFFFFFFFFFFFFFFFFFFFFFFFFFFFFFF
FFFFFFFFFF:FFFFFFFFFFFF:FFFFFFFFFFFFFFFFFFFFFFFFFFFFFF,FFFFFFFFFFFFFFF
FFFFFFFFFFFFFFF
@A00721:190:HGJW7DSXY:4:1209:23285:9596 2:N:0:TCTGCAAG+GCTGCACT
AAACAATGGATTCGTCCACAAGGGTGCCACTCACATGGCAGCAACTACAAGTAGGGGAGTACTACTGG
AACCTAAACAATCCAATATGGATACGAGTCCAACACTCTGGACAGACGACGAGTCCACCGTTCCATCA
CATCTTCACGGTGGA
+
FFFFFFFFFFFFFFFFFFFFFFFFFFFFFFFFFFFFFFFFFFFFFF,FFFFFF,FFFFFFFFFFF:FFFF
F:FFFFFFFFFFFFFFFFFFFFFFFFFFFFFFFFFFFFFFFFFFFFFFFFFFFFFFFFFFFFFFFF:FFF
FFFFF,FFFFFFFFF
@A00721:190:HGJW7DSXY:4:2611:6994:10347 2:N:0:TCTGCAAG+GCTGCACT
AAACAATGGATTCGTCCACAAGGGTGCCACTCACATGGCCGCAACTACAAGTAGGGGAGTACTACTGG
AACCTAAACAATCCAATATGGATACGAGTCCAACACTCTGGACAGACGACGAGTCCACCGTTCCATCA
CATCTTCACGGTGGA
+
FFFFFFFFF,FFFFFFF,FF:FFFFFFFFFFFFFFFFFFFF,FFF,FF:FFFFFFFFFF:FFFFFFFFFF
FFFFFFFFFFFFFFFFFFFFFFFFFFFFFFFFFFFFFFF:FFFFFFFFFFFFFFFFFFFFFFFFFFFFFF
FFFFF:FFFFFFFFF
@A00721:190:HGJW7DSXY:4:1455:31060:8876 2:N:0:TCTGCAAG+GCTGCACT
TGCAGAAACAATGGATTCGTCCACAAGGGTGCCACTCACATGGCAGCAACTACAAGTAGGGGAGTACT
ACTGGAACCTAAACAATCCAATATGGATACGAGTCCAACACTCTGGACAGACGACGAGTCCACCGTTC
CATCACATCTTCACG
+
FFFFFFFFFFFFFF:FFFFFFFFF,FFFFFFFFFFFFFFFFFFFFFFFF:FFFFFFFFFFFFFFFFFFFF
FFFFFFFFFFF:FFFFFFFFFFFFFFFFFFFFFFFFFFFFFFFFFFFFFFFFFFFFFFFFFFFFF:FFFF
FFFFFFFFFFFFFFF
@A00721:190:HGJW7DSXY:4:1535:6723:3145 2:N:0:TCTGCAAG+GCTGCACT
TGCAGAAACAATGGATTCGTCCACAAGGGTGCCACTCACATGGCAGCAACTACAAGTAGGGGAGTACT
ACTGGAACCTAAACAATCCAATATGGATACGAGTCCAACACTCTGGACAGACGACGAGTCCACCGTTC
CATCACATCTTCACG
+
FFFFFFFFFFFFFFFFFFFFFFFFFFFFFFFFFFFFFFFFFFFFFFFFFFFFFFFFFFFFFFFFFFFFFF
FFFFFFFFFFFFFFFFFFFFFFFFFFFFFFFFFFFFFFFFFFFFFFFFFFFFFFFFFFFFFFFFFFFFFF
FFFFFFFFFFFFFFF
@A00721:190:HGJW7DSXY:4:1655:31286:15937 2:N:0:TCTGCAAG+GCTGCACT
TGCAGAAACAATGGATTCGTCCACAAGGGTGCCACTCACATGGCAGCAACTACAAGTAGGGGAGTACT
ACTGGAACCTAAACAATCCAATATGGATACGAGTCCAACACTCTGGACAGACGACGAGTCCACCGTTC
CATCACATCTTCACG
+
FFFFFFFFFFFFFFFFFFFFFFFFFFFFFFFFFFFFFFFFFFFFFFFFFFFFFFFFFFFFFFFFFFFFFF

```
FFFFFFFFFFFFFFFFFFFFFFFFFFFFFFFF:FFFFFFFFFFFFFFFFFFFFFFFFFFFFFFFFFFF
FFFFFFFFFFFFFFF
@A00721:190:HGJW7DSXY:4:1655:31412:17127 2:N:0:TCTGCAAG+GCTGCACT
TGCAGAAACAATGGATTCGTCCACAAGGGTGCCACTCACATGGCAGCAACTACAAGTAGGGGAGTACT
ACTGGAACCTAAACAATCCAATATGGATACGAGTCCAACACTCTGGACAGACGACGAGTCCACCGTTC
CATCACATCTTCACG
+
FFFFFFFFFFFFFFFFFFFFFFFFFFFFFFFFFFFFFFFFFFFFFFFFFFFFFFFF:FFFFFFFFFFF
FFFFFFFFFFFFFFFFFFFFFFFFFFFFFFFFFFFFFFFFFFFFFFFFFFFFF:FFFFFFFFFFFFFF
FFFFFFFFFFFFFFF
@A00721:190:HGJW7DSXY:4:2150:29098:31501 2:N:0:TCTGCAAG+GCTGCACT
TGCAGAAACAATGGATTCGTCCACAAGGGTGCCACTCACATGGCAGCAACTACAAGTAGGGGAGTACT
ACTGGAACCTAAACAATCCAATATGGATACGAGTCCAACACTCTGGACAGACGACGAGTCCACCGTTC
CATCACATCTTCACG
+
FFFFFFFFFFFFFFFFFFFFFFFFFFFFFFFFFFFFFFF:FFFF:FFF,FF:FFFFFFFFFFFFFFF:
FFFFFFFFFFFFFFFFFFFFFFFFFFFF:FFFFFFFFFFFFFFFFF,FFFFFFFFFFFFFFFFFFFFF
FFFFFFFFFFFFFFF
@A00721:190:HGJW7DSXY:4:2233:9127:28416 2:N:0:TCTGCAAG+GCTGCACT
TGCAGAAACAATGGATTCGTCCACAAGGGTGCCACTCACATGGCAGCAACTACAAGTAGGGGAGTACT
ACTGGAACCTAAACAATCCAATATGGATACGAGTCCAACACTCTGGACAGACGACGAGTCCACCGTTC
CATCACATCTTCACG
+
FFFFFFFFFFFFFFFFFFFFFFFFFFFFFFFFFFFFFFFFFFFFFFFFFFFFFFFFFFFFFFFFFFFF
FFFFFFFFFFFFFFFFFFFFFFFFFFFFFFFFFFFFFFFFFFFFFFFFFFFFFFFFFFFFFFFFFFFF
,FFFFFFFFFFFFFF
@A00721:190:HGJW7DSXY:4:2262:12156:3098 2:N:0:TCTGCAAG+GCTGCACT
TGCAGAAACAATGGATTCGTCCACAAGGGTGCCACTCACATGGCAGCAACTACAAGTAGGGGAGTACT
ACTGGAACCTAAACAATCCAATATGGATACGAGTCCAACACTCTGGACAGACGACGAGTCCACCGTTC
CATCACATCTTCACG
+
FFFFFFFFFFFFFFFFFFFFFFFFFFFFFFFFFFFFFFFFFFFFFFFFFFFFFFFFFFFFFFFFFFFF
FFFFFFFFFFFFFFFFFFFFFF:FFF:FFFFFFFFFFFFFFFFFFFFFFFFFFFFFFFFFFFFFFFFF
FFFFFFFFFFFFFFF
@A00721:190:HGJW7DSXY:4:2407:17879:9784 2:N:0:TCTGCAAG+GCTGCACT
TGCAGAAACAATGGATTCGTCCACAAGGGTGCCACTCACATGGCAGCAACTACAAGTAGGGGAGTACT
ACTGGAACCTAAACAATCCAATATGGATACGAGTCCAACACTCTGGACAGACGACGAGTCCACCGTTC
CATCACATCTTCACG
+
FFFFF:FFFFFFFFFFFFFFFF:F:FFFFFFFF:FFFFFFFFFFFFFF:FF,FFFF:FFFFF:FFF:F
:FFFFF,FFFFF:FF:FFFFFFFFFFFFFFFFFFFFFFFFFFFFFFFFFFFFFFFFFFFFFFFFFFFF
FFFFFFFFFFFFFFF
@A00721:190:HGJW7DSXY:4:2407:18448:9799 2:N:0:TCTGCAAG+GCTGCACT
TGCAGAAACAATGGATTCGTCCACAAGGGTGCCACTCACATGGCAGCAACTACAAGTAGGGGAGTACT
ACTGGAACCTAAACAATCCAATATGGATACGAGTCCAACACTCTGGACAGACGACGAGTCCACCGTTC
CATCACATCTTCACG
+
FFFFFFFFFFFFFFFFFFFFFFFFFFFFFFFFFFFFFFFFFFFFFFFFFFFFFFFFFFFFFFFFFFFF
FFFFFF:FFFFFFFFFFFFFFFFFFFFFFFFFFFFFFFFFFFFFFFFFFFFFFFFFFFFFFFFFFFFF
FFFFFFFFFFFFFFF
@A00721:190:HGJW7DSXY:4:2434:12364:4241 2:N:0:TCTGCAAG+GCTGCACT
TGCAGAAACAATGGATTCGTCCACAAGGGTGCCACTCACATGGCAGCAACTACAAGTAGGGGAGTACT
ACTGGAACCTAAACAATCCAATATGGATACGAGTCCAACACTCTGGACAGACGACGAGTCCACCGTTC
CATCACATCTTCACG
```

+
FFFFFFFFFFFFFFFFFFFFFFFFFFFFFFFFFFFFFFFFFFFFFFFFFFFFFFFFFFFFFFFFFFFFFF
FFFFFFFFFFFFFFFFFFFFFFFFFFFFFFFFFFFFFFFFFFFFFFFFFFFFFFFFFFFFFFFFFFFFFF
FFFFFFFFFFFFFFF
@A00721:190:HGJW7DSXY:4:2444:2609:7420 2:N:0:TCTGCAAG+GCTGCACT
TGCAGAAACAATGGATTCGTCCACAAGGGTGCCACTCACATGGCAGCAACTACAAGTAGGGGAGTACT
ACTGGAACCTAAACAATCCAATATGGATACGAGTCCAACACTCTGGACAGACGACGAGTCCACCGTTC
CATCACATCTTCACG
+
FFFFFFFFFFFFFFFFFFFFFFFFFFF:FFFFFFFFFFFFFFFFFFFFFFFFFFFFFFFFFFFF:FFFFF
FFFFFFFFFFFFFFFFFFFFFFFFFF:FFFFFFFFFFFFFFFFFFFFFFFFFFFFFFFFFFFFFFFFFFF
FFFFFF:FFFFFFFF
@A00721:190:HGJW7DSXY:4:2450:22625:28244 2:N:0:TCTGCAAG+GCTGCACT
TGCAGAAACAATGGATTCGTCCACAAGGGTGCCACTCACATGGCAGCAACTACAAGTAGGGGAGTACT
ACTGGAACCTAAACAATCCAATATGGATACGAGTCCAACACTCTGGACAGACGACGAGTCCACCGTTC
CATCACATCTTCACG
+
FFFFFFFFFFFFFFFFFFFFFFFFFFFFFFFFFFFFFFFFFFFFFFFFFFFFFFFFFF:FFFFFFFFFFF
FFFFFFFFFFFFFFFFFFFFFFFFFFFFFFFFFFFFFFFFFFFFFFFFFFFFFFFFFFFFFFFFFFF:FF
FFFFFFFFFFFFFFF
@A00721:190:HGJW7DSXY:4:2562:25563:25911 2:N:0:TCTGCAAG+GCTGCACT
TGCAGAAACAATGGATTCGTCCACAAGGGTGCCACTCACATGGCAGCAACTACAAGTAGGGGAGTACT
ACTGGAACCTAAACAATCCAATATGGATACGAGTCCAACACTCTGGACAGACGACGAGTCCACCGTTC
CATCACAACTTCACG
+
:FFFFFFF,FFFFFFFFFFFFF,FFFFFFFFFFFFFFFFFFFFFFFFFFFFF,FFFFF:FF:FFFFFFF:
FFF:FF,FFFFFFFFFFFFFFFFFF,FFFFFFF:FFF:FFF,FFF,FFFFFFFFFFFFFFFFFFF,:FFF
FFFFFFF,FFFFF,F
@A00721:190:HGJW7DSXY:4:2131:7157:34867 2:N:0:TCTGCAAG+GCTGCACT
AGACTATAGATTGCAGAAACAATGGATTCGTCCACAAGGGTGCCACTCACATGGCAGCAACTACAAGT
AGGGGAGTACTACTGGAACCTAAACAATCCAATATGGATACGAGTCCAACACTCTGGACAGACGACGA
GTCCACCGTTCCATC
+
FFFFFFFFFFFFFFFFFFFFFFFFFFFFFFFFFFFFFFFFFFFFFFFFFFFFFFFFFFFFFFFFFFFFFF
:FFFFFFFFFFFFFFFFFFFFFFFFFFFFFFFFFFFFFFFFFFFFFFFFFFFFFFFFFFFFFFFFFFFFF
FFFFFFFFFFFFFFF
@A00721:190:HGJW7DSXY:4:2433:27208:8437 2:N:0:TCTGCAAG+GCTGCACT
AGACTATAGATTGCAGAAACAATGGATTCGTCCACAAGGGTGCCACTCACATGGCAGCAACTACAAGT
AGGGGAGTACTACTGGAACCTAAACAATCCAATATGGATACGAGTCCAACACTCTGGACAGACGACGA
GTCCACCGTTCCATC
+
FFFFFFFFFFFFFFFFFFFFFFFFFFFFFFFFFFFFF:FFFFFFFFFFFFFFFFFFF,FFFFFFFFF:FF
FFFFFFFFFFFFFFFFFFFFFFFFFFF,FFFFFFFFFFFFFFFFFFFFFFF,FFFFFFFFFFFFFFFFFF
FFFFFFFFFFFFFFF
@A00721:190:HGJW7DSXY:4:2433:27290:9580 2:N:0:TCTGCAAG+GCTGCACT
AGACTATAGATTGCAGAAACAATGGATTCGTCCACAAGGGTGCCACTCACATGGCAGCAACTACAAGT
AGGGGAGTACTACTGGAACCTAAACAATCCAATATGGATACGAGTCCAACACTCTGGACAGACGACGA
GTCCACCGTTCCATC
+
FFFFFFFFFFFFFFFFFFFFFFFFFFFFFFFFFFFFFFFFFFFFF,FFFFFFFFFFFFFFFFFFFFFFFF
FFFFFFFFFFFFFFFFFF:FFFFFFFF:FFFFFFFFFFFFFFFFFFFFFFFFFFFFFFFFFFFFFFFFFF
FFFFFFFFFFFFFFF
@A00721:190:HGJW7DSXY:4:2464:26630:29825 2:N:0:TCTGCAAG+GCTGCACT
AGACTATAGATTGCAGAAACAATGGATTCGTCCACAAGGGTGCCACTCACATGGCAGCAACTACAAGT

```
AGGGGAGTACTACTGGAACCTAAACAATCCAATATGGATACGAGTCCAACACTCTGGACAGACGACGA
GTCCACCGTTCCATC
+
FFFFFFFFFFFFFFFFFFFFFFFFFFFFFFFFFFFFFFFFFFFFFFFFFFFFFFFFFFFFFFFFFFFF,
:FFFFFFFFFFFFFFFFFFF:FFFFFFFFFFFFFFFF:FFFFFFFFFFFFFFFFFFFF:FFFFFFFF
FFFFFFFFFFFFFFF
@A00721:190:HGJW7DSXY:4:1166:18439:35180 2:N:0:TCTGCAAG+GCTGCACT
GACTATAGATTGCAGAAACAATGGATTCGTCCACAAGGGTGCCACTCACATGGCAGCAACTACAAGTA
GGGGAGTACTACTGGAACCTAAACAATCCAATATGGATACGAGTCCAACACTCTGGACAGACGACGAG
TCCACCGTTCCAGAT
+
FFFFFFFFFFFFFFFFFFFFFFFFFFFFFFFFFFFFFFFFFFFFFFFFFFFFFFFFFFFFF,FFFFFF
FFFFFFFFFFFFFFFFFFFFFFFFFFFFFFFFFFFFFFFFFFFFFFFFFFFFFFFFFF:FFFF:FFFF
FFF,FFFFFFFFF:F
@A00721:190:HGJW7DSXY:4:1308:22815:29450 2:N:0:TCTGCAAG+GCTGCACT
GACTATAGATTGCAGAAACAATGGATTCGTCCACAAGGGTGCCACTCACATGGCAGCAACTACAAGTA
GGGGAGTACTACTGGAACCTAAACAATCCAATATGGATACGAGTCCAACACTCTGGACAGACGACGAG
TCCACCGTTCCAGAT
+
FFFFFFF:FFFFFFFFFFFFFFFFFFFFFFFFFFFF:FFFFFFFFFFFF:FFFFFFFFFFFFFF:FFF
FFFFFFFFFFFFFFFFF:FFFFFFFFFF:FFFFFFFFF:FFFFFFFFFFFFFFFFFFFFFFFF:FFFF
FF,FFFFFFF:FFFF
@A00721:190:HGJW7DSXY:4:1321:30273:30874 2:N:0:TCTGCAAG+GCTGCACT
GACTATAGATTGCAGAAACAATGGATTCGTCCACAAGGGTGCCACTCACATGGCAGCAACTACAAGTA
GGGGAGTACTACTGGAACCTAAACAATCCAATATGGATACGAGTCCAACACTCTGGACAGACGACGAG
TCCACCGTTCCAGAT
+
FFFFFFFFFFFFFFFFFFFFFFFFFFFFFFFFFFFFFFFFFFFFFFFFFFFFFFFFFF:FFFFFFFFF
FFFFFFFFFFFFFFFFFFFFFFFFFFFFFFFFFFFFFFFFFFFFFFFFFFFFFFFFFFFFFFFFFFFF
FFFFFFFFFFFFFFF
@A00721:190:HGJW7DSXY:4:1333:31177:3881 2:N:0:TCTGCAAG+GCTGCACT
GACTATAGATTGCAGAAACAATGGATTCGTCCACAAGGGTGCCACTCACATGGCAGCAACTACAAGTA
GGGGAGTACTACTGGAACCTAAACAATCCAATATGGATACGAGTCCAACACTCTGGACAGACGACGAG
TCCACCGTTCCAGAT
+
F:FFFFFFFFFFFFFFFFFFFFFFFFFFFFFFFFFFFFFFFFFFFFFFFFFFFFFFFFFFFFFFFFFF
FFFFFFFFFFFFFFFFFFFFFFFFFFFFFFFFFFFFFFFFFFFFFFFFFFFFFFFFFFFFFFFFFFFF
FFFFFFFFFFFFFFF
@A00721:190:HGJW7DSXY:4:1416:16975:23437 2:N:0:TCTGCAAG+GCTGCACT
GACTATAGATTGCAGAAACAATGGATTCGTCCACAAGGGTGCCACTCACATGGCAGCAACTACAAGTA
GGGGAGTACTACTGGAACCTAAACAATCCAATATGGATACGAGTCCAACACTCTGGACAGACGACGAG
TCCACCGTTCCAGAT
+
FFFFFFFFFFFFFFFFFFFFFFFFFFFFFFFFFFFFFFFFFFFFFFFFF:FFFFFFFF,FFFFFFF:F
FFFF:FFFFFFFFFFFFFFFFFFFFFFFFFFFFFFFFFFFFFFFFFFFFFFFFFFFFFFFFFFFFFFF
FFFFFFFFFFFFFFF
@A00721:190:HGJW7DSXY:4:1423:5168:10191 2:N:0:TCTGCAAG+GCTGCACT
GACTATAGATTGCAGAAACAATGGATTCGTCCACAAGGGTGCCACTCACATGGCAGCAACTACAAGTA
GGGGAGTACTACTGGAACCTAAACAATCCAATATGGATACGAGTCCAACACTCTGGACAGACGACGAG
TCCACCGTTCCAGAT
+
FFFFFFFFFFFF:F:FFFFFFFFFF:FFFFFFFFFFFFFFFFF:FFFF::FFFFFFFFFFFFFFFFFF
FFFFFFFFFF:FFF:FFF,FFFFFFFFFFFFF::FFFFFF,FFFFFFFFFF,FFF:F:F:FF,:FFFF
FFFFFFF,,FFFFF:
```

```
@A00721:190:HGJW7DSXY:4:2104:31159:27054 2:N:0:TCTGCAAG+GCTGCACT
GACTATAGATTGCAGAAACAATGGATTCGTCCACAAGGGTGCCACTCACATGGCAGCAACTACAAGTA
GGGGAGTACTACTGGAACCTAAACAATCCAATATGGATACGAGTCCAACACTCTGGACAGACGACGAG
TCCACCGTTCCAGAT
+
F:FFFFFF:FFFFFFFFFFFFFFFFFFFFFFFFFFFFFFFFFFFFFFFFFFFFFFFFFFFFFFFFFFF
FFFFFFFFFFFFFFFFFFFFFFFFFFFFFFFFFF:FFFFFFFFFFFFFFFFF::FFFFFFFFFFF,FF
FFFFFFF,FFFFFF:
@A00721:190:HGJW7DSXY:4:2104:28293:29136 2:N:0:TCTGCAAG+GCTGCACT
GACTATAGATTGCAGAAACAATGGATTCGTCCACAAGGGTGCCACTCACATGGCAGCAACTACAAGTA
GGGGAGTACTACTGGAACCTAAACAATCCAATATGGATACGAGTCCAACACTCTGGACAGACGACGAG
TCCACCGTTCCAGAT
+
FFFFFFFFFFFFFFFFFFFFFFFFFFFFFFFFFFFFFFFFFFFFFFFFFFFFFFFFFFFFF:FFFFFF
FFFFFFFFFFFFFFFFFFFFFFFFFFFFFFFFFFFFFFFFFFFFFFFFFFFFFFFFFFFFFFFFFFFF
FFFFFFFFFFFFFFF
@A00721:190:HGJW7DSXY:4:2378:18530:33364 2:N:0:TCTGCAAG+GCTGCACT
GACTATAGATTGCAGAAACAATGGATTCGTCCACAAGGGTGCCACTCACATGGCAGCAACTACAAGTA
GGGGAGTACTACTGGAACCTAAACAATCCAATATGGATACGAGTCCAACACTCTGGACAGACGACGAG
TCCACCGTTCCAGAT
+
FFFFFFFFFFFFFFFFFFFFFFFFFFFFFFFFFFFFFFFFFFFFFFFFFFFFFFFFFFFFFFFFFFFF
FFFFFFFFFFFFFFFFFFFFFF:FFFFFFFFFFFFFFFFFFFFFFFFFFFFFFFFFFFFFFFFFFFFF
FFFFFFFFFFFFFFF
@A00721:190:HGJW7DSXY:4:2378:19642:34757 2:N:0:TCTGCAAG+GCTGCACT
GACTATAGATTGCAGAAACAATGGATTCGTCCACAAGGGTGCCACTCACATGGCAGCAACTACAAGTA
GGGGAGTACTACTGGAACCTAAACAATCCAATATGGATACGAGTCCAACACTCTGGACAGACGACGAG
TCCACCGTTCCAGAT
+
FFFFFFFFFFFFFFFFFFFFFFFFFFFFFFFFFFFFFFFFFFFFFFFFFFFFFFFFFFFFFFFFFFFF
FFFFFFFFFFFFFFFFFFFFFFFFFFFFFFFFFFFFFFFFFFFFFFFFFFFFFFFFFFFFFFFFFFFF
FFFFFFFFFFFFFFF
@A00721:190:HGJW7DSXY:4:1559:29821:4445 2:N:0:TCTGCAAG+GCTGCACT
GCATCATTTATCAGACTATAGATTGCAGAAACAATGGATTCGTCCACAAGGGTGCCACTCACATGGCA
GCAACTACAAGTAGGGGAGTACTACTGGAACCTAAACAATCCAATATGGATACGAGTCCAACACTCTG
GACAGACGACGAGTC
+
FFFFFFFFFF:FFFFFFFFFFFFFFF:FFFFFFFFFFFFFFFFFFFFFFFFFFFFFFFFFFFFFFFFF
FFFFFFFF,FF:FF,FFFFFFFF:FFFFFFFFFFFFFFF:FFFFFFF:FFFFFFFFFFFFFFFFFFFF
:FFFFFFFFFFFFFF
@A00721:190:HGJW7DSXY:4:2230:20528:2347 2:N:0:TCTGCAAG+GCTGCACT
GCATCATTTATCAGACTATAGATTGCAGAAACAATGGATTCGTCCACAAGGGTGCCACTCACATGGCA
GCAACTACAAGTAGGGGAGTACTACTGGAACCTAAACAATCCAATATGGATACGAGTCCAACACTCTG
GACAGACGACGAGTC
+
FFFFFFFFFFFFFFFFFFFFFFFFFFFFFFFFFFFFFFFFFFFFFFFF:FFFFFFFFFFFFFFFFFFF
FFFFFFFFFFFFFFFFFFFFFFFFFFFFFFFFFFFFFFFFFFFFF:FFFFFFFFFFFFFFFFFFFFFF
FFFFFFFFFFFFFFF
@A00721:190:HGJW7DSXY:4:1104:31024:17206 2:N:0:TCTGCAAG+GCTGCACT
CTGCATCATTTATCAGACTATAGATTGCAGAAACAATGGATTCGTCCACAAGGGTGCCACTCACATGG
CAGCAACTACAAGTAGGGGAGTACTACTGGAACCTAAACAATCCAATATGGATACGAGTCCAACACTC
TGGACAGACGACGAG
+
FFFFFFFFFFFFFFFFFFFFFFFFFFFFFF:FFFFFFFFFFFFFFFFFFFFFFFFFFFFFFFFFFFFF
```

FFFFFFFFFFFFFFFFFFFFFFFFFFFFFFFFFFFFFFFFFFF:FFFFF:FFFFFFFFFFFFFFFFFFFF
FFFFFFFFFFFFFFF
@A00721:190:HGJW7DSXY:4:1113:14642:11068 2:N:0:TCTGCAAG+GCTGCACT
CTGCATCATTTATCAGACTATAGATTGCAGAAACAATGGATTCGTCCACAAGGGTGCCACTCACATGG
CAGCAACTACAAGTAGGGGAGTACTACTGGAACCTAAACAATCCAATATGGATACGAGTCCAACACTC
TGGACAGACGACGAG
+
FFFFFFF:FFFFFFFFFFFFFFFFFFFFFFFFFFFFFFFFFFFFFFFFFFFFFFFFFFFFFFFFFFFFFF
FFFFF,FFFFFFFFFFFFFFFFFFF:F,FFFFFFFFFFFFFFFFFFFFF:FFFFFFFFFFFFFFFFFFFF
FFFFFFFFF:FFFFF
@A00721:190:HGJW7DSXY:4:1202:2745:26506 2:N:0:TCTGCAAG+GCTGCACT
CTGCATCATTTATCAGACTATAGATTGCAGAAACAATGGATTCGTCCACAAGGGTGCCACTCACATGG
CAGCAACTACAAGTAGGGGAGTACTACTGGAACCTAAACAATCCAATATGGATACGAGTCCAACACTC
TGGACAGACGACGAG
+
FFFFFFFF::FFFFFFFFFFFFFFFFFFFFFFFFFFFFFF::FFFFFFFFFFFFFFFFFFFFFFFFFFFF
FFFFFFFFFFFFFFFFFFFFFFFFFFFFFFFFFFFFFFFFFFFFFFFFFFFFFFFFFFFFFFFFFFFFFF
FFFFFFFFFFFFFFF
@A00721:190:HGJW7DSXY:4:1367:22073:6089 2:N:0:TCTGCAAG+GCTGCACT
CTGCATCATTTATCAGACTATAGATTGCAGAAACAATGGATTCGTCCACAAGGGTGCCACTCACATGG
CAGCAACTACAAGTAGGGGAGTACTACTGGAACCTAAACAATCCAATATGGATACGAGTCCAACACTC
TGGACAGACGACGAG
+
FFFFFFFFFFFFFFFFFFF,FFFFFFFFFFFFFFFFFFFFFFFFFFFFFFFFFFFFFFFFFFFFFFFFFF
FFFFFFFFFFFFFFFFFFFFFFFFFFFFFFF:FFFFFFFFFFFFFFFFFFFFFF:FFFFFFFFFFFFFFF
FFFFFFFFFFFFFFF
@A00721:190:HGJW7DSXY:4:1368:8938:20509 2:N:0:TCTGCAAG+GCTGCACT
CTGCATCATTTATCAGACTATAGATTGCAGAAACAATGGATTCGTCCACAAGGGTGCCACTCACATGG
CAGCAACTACAAGTAGGGGAGTACTACTGGAACCTAAACAATCCAATATGGATACGAGTCCAACACTC
TGGACAGACGACGAG
+
FFFFFFFFFFFFFFFFFFFFFFFFFFFFFFFFFFFFFFFFFFFFFFFFFFFFFFFFFFFFFFFFFFFFFF
FFFFFFFFFFFFFFFFFFFFFFFFFFFFFFF:FFF:FFFFFFFFFFFFFFFFFFFFFFFFFFFFFFFFFF
FFFFFFFFFFFFFFF
@A00721:190:HGJW7DSXY:4:1436:12653:14888 2:N:0:TCTGCAAG+GCTGCACT
CTGCATCATTTATCAGACTATAGATTGCAGAAACAATGGATTCGTCCACAAGGGTGCCACTCACATGG
CAGCAACTACAAGTAGGGGAGTACTACTGGAACCTAAACAATCCAATATGGATACGAGTCCAACACTC
TGGACAGACGACGAG
+
FFFFFFFFFFFFFFFFFFFFFFFFFFFFFFFFFFFFFFFFFFFFFFFFFFFFFFFFFFFFFFFFFFFFFF
FFFFFFFFFFFFFFFFFFFFFFFFFFFFFFFFFFFFFFFFFFFFFFFFFFFFFFFFFFFFFFFFFFFFFF
FFFFFFFFFFFFFFF
@A00721:190:HGJW7DSXY:4:2118:25852:3302 2:N:0:TCTGCAAG+GCTGCACT
CTGCATCATTTATCAGACTATAGATTGCAGAAACAATGGATTCGTCCACAAGGGTGCCACTCACATGG
CAGCAACTACAAGTAGGGGAGTACTACTGGAACCTAAACAATCCAATATGGATACGAGTCCAACACTC
TGGACAGACGACGAG
+
FFFFFFFFFFFFFFFFFFFFFFFFFF:FFFFFFFFFFFFFFFFFFFFFFFFFFFFFFFFFFFFFFFFFFF
FFFFFFFFFFFFFFFFFFFFFFFFFFFFFFFFFFFFFFFFFFFFFFFFFFFFFFFFFFFFFFFFFFFFFF
FFFFFFFFFFFFFF:
@A00721:190:HGJW7DSXY:4:2430:12915:9455 2:N:0:TCTGCAAG+GCTGCACT
CTGCATCATTTATCAGACTATAGATTGCAGAAACAATGGATTCGTCCACAAGGGTGCCACTCACATGG
CAGCAACTACAAGTAGGGGAGTACTACTGGAACCTAAACAATCCAATATGGATACGAGTCCAACACTC
TGGACAGACGACGAG

```
+
FFFFFFFFFFFFFFFFFFFFFFFFFFFFFFFFFF:FFFFFFFFFFFFFFFFFFFFFFFFFFFFFFFFFFF
F:FFFFFFFFFF:FFFFFFFFFFFFFFFFFFFFFFFFFFFFFFFFFFFFFFFFFFFFFFFF:FFFFFFFF
FFFFFFFFFFFFFFF
@A00721:190:HGJW7DSXY:4:2536:28103:10520 2:N:0:TCTGCAAG+GCTGCACT
CTGCATCATTTATCAGACTATAGATTGCAGAAACAATGGATTCGTCCACAAGGGTGCCACTCACATGG
CAGCAACTACAAGTAGGGGAGTACTACTGGAACCTAAACAATCCAATATGGATACGAGTCCAACACTC
TGGACAGACGACGAG
+
FFFFFFFF,FFFFFFFFFFFFFFFFFFFFFFFFFFFFFFFFFFFFFFFFFFFFFFFFFFFFFFFFFFFF
FFFFFFFFFFFFFFFFFFFFFFFFFFFFFFFFFFFFFFFFFFFFFFFFFFF:FFFFFFFFFFFFFFFFFF
FFFFFFFFFFFFFFF
@A00721:190:HGJW7DSXY:4:2611:10276:11209 2:N:0:TCTGCAAG+GCTGCACT
CTGCATCATTTATCAGACTATAGATTGCAGAAACAATGGATTCGTCCACAAGGGTGCCACTCACATGG
CAGCAACTACAAGTAGGGGAGTACTACTGGAACCTAAACAATCCAATATGGATACGAGTCCAACACTC
TGGACAGACGACGAG
+
FFFFFFFFFFFFFFFFFFFFFFFFFFFFFFFFFFFFFFFFFFFFFFFFFFFFFFFFFFFFFFFFFFFFFF
FFFFFFFFFFFFFFFFFFFFFFFFFFFFFFFFFFFFFFFFFFFFFFFFFFFFFFFFF:FFFFFFFFFFFF
FFFFFFFFFFFFFFF
@A00721:190:HGJW7DSXY:4:2111:30129:10582 2:N:0:TCTGCAAG+GCTGCACT
CTGCATCATTTATCAGACTATAGATTGCAGAAACAATGGATTCGTCCACAAGGGTGCCACTCACATGG
CAGCAACTACAAGTAGGGGAGTACTACTGGAACCTAAACAATCCAATATGGATACGAGTCCAACACTC
TGGACAGACGACTA
+
FFF,FFFFFFFFFFF:FFFFF:FFFFFFFFFFFFFFFFFFFFFFFFFFFFFFFFFFFFFFFFFFFFFFFF
FFFFFFFFFFFFFF:FFF:FFFFFFFFFFFF,FFFFFFF:FF,FFFFFFFFFFFF:FFFFFF:FFFFFFF
F:FFFF:FF,FF,F
@A00721:190:HGJW7DSXY:4:1156:28239:9032 2:N:0:TCTGCAAG+GCTGCACT
CTTACACTACCGTGCGGCTGCATCATTTATCAGACTATAGATTGCAGAAACAATGGATTCGTCCACAA
GGGTGCCACTCACATGGCAGCAACTACAAGTAGGGGAGTACTACTGGAACCTAAACAATCCAATATGG
ATACGAGTCCAACAC
+
FFFFFFFFFFFFFFFFFFFFFFFFFF:FFFFFFFFFFFFFFFF:FFFFFFFFFFFFFFF:FFFFFFFF,F
FFFFFFFFFFFFFFFFFFFFFFFFFFFFFFFFFFFFFFFFFFFFFFFFFF:FF:FFFFFF:FFFFFFFFF
FFFFFFFFFFFFFFF
@A00721:190:HGJW7DSXY:4:2149:2582:26506 2:N:0:TCTGCAAG+GCTGCACT
CTTACACTACCGTGCGGCTGCATCATTTATCAGACTATAGATTGCAGAAACAATGGATTCGTCCACAA
GGGTGCCACTCACATGGCAGCAACTACAAGTAGGGGAGTACTACTGGAACCTAAACAATCCAATATGG
ATACGAGTCCAACAC
+
:FFFFFFFFFFFFFFFFFFFFFFFFFFFFFFFFFFFFFFFFFFFFFFFFFFFFFFFFFFFFFFFFFFFFF
FFFFFFFFFFFFFFFFFFFFFFFFFFFFFFFFFFFFFFFFFFFFFFFFFFFFFFFFFFFFFFFFFFFFFF
FFFFFFFFFFFFFFF
@A00721:190:HGJW7DSXY:4:2643:13259:20134 2:N:0:TCTGCAAG+GCTGCACT
CTTACACTACCGTGCGGCTGCATCATTTATCAGACTATAGATTGCAGAAACAATGGATTCGTCCACAA
GGGTGCCACTCACATGGCAGCAACTACAAGTAGGGGAGTACTACTGGAACCTAAACAATCCAATATGG
ATACGAGTCCAACAC
+
FFF:FFFFFFFFFFFFFFFFFFFFFFF:FFFFFFFFFFFF:FFFFFFFFFFFFFFFFFFFFFFFFFFFFF
FFFFFFFFFFFFFFFFFFFFFFFFFFFFFFFFFFFFFFFFFFFF:FFFFFFFF,FFFFFFFFFFFFFFFF
FFFFF:FFFFFFFFF
@A00721:190:HGJW7DSXY:4:1526:10890:12273 2:N:0:TCTGCAAG+GCTGCACT
GTCTTACACTACCGTGCGGCTGCATCATTTATCAGACTATAGATTGCAGAAACAATGGATTCGTCCAC
```

AAGGGTGCCACTCACATGGCAGCAACTACAAGTAGGGGAGTACTACTGGAACCTAAACAATCCAATAT
GGATACGAGTCCAAC
+
FFFFFFFFFFFFFFFFFFFFFFFFFFFFFFFFFFFFFFFFFFFFFFFFFFFFFFFFFFFFFFFFFFFFF
FFFFFFFFFFFFFFFFFFFFFFFFFFFFFFFFFFFFFFFFFFFFFFFFFFFFFFFFFFFFFFFFFF:FF
FFFF,FFFFFFFFFF
@A00721:190:HGJW7DSXY:4:1616:3197:29982 2:N:0:TCTGCAAG+GCTGCACT
GTCTTACACTACCGTGCGGCTGCATCATTTATCAGACTATAGATTGCAGAAACAATGGATTCGTCCAC
AAGGGTGCCACTCACATGGCAGCAACTACAAGTAGGGGAGTACTACTGGAACCTAAACAATCCAATAT
GGATACGAGTCCAAC
+
FFFFFFFFFFFFFFFFFFFFFFFFFFFFFFFFFFFFFFFFFFFFFFFFFFFFFFFFFFFFFFFFFFFFF
FF:FFFFFFFFFFFFFFFFFFFFFFFFFFFFFFFFFFFFFFFFFFFFFFFFFFFFFFFFFFFFFFFFFF
F:FFFFFFFFFFFFF
@A00721:190:HGJW7DSXY:4:1636:16089:1642 2:N:0:TCTGCAAG+GCTGCACT
GTCTTACACTACCGTGCGGCTGCATCATTTATCAGACTATAGATTGCAGAAACAATGGATTCGTCCAC
AAGGGTGCCACTCACATGGCAGCAACTACAAGTAGGGGAGTACTACTGGAACCTAAACAATCCAATAT
GGATACGAGTCCAAC
+
FFFFFFFFFFFFFFFFFFFFFFFFFFFFFFFFFFFFFFFFFF:FFFFFFFFFFFFFFFFFFFFFFFFFF
FFFFFFFFFFFFFFFFFFFFFFFFF,FFFFFFFFFFFFFFFFFFFFFFFFFFFFFFFFFFFFFFFFFFF
FFFFFFFFFFFF:FF
@A00721:190:HGJW7DSXY:4:2114:15772:6136 2:N:0:TCTGCAAG+GCTGCACT
GTCTTACACTACCGTGCGGCTGCATCATTTATCAGACTATAGATTGCAGAAACAATGGATTCGTCCAC
AAGGGTGCCACTCACATGGCAGCAACTACAAGTAGGGGAGTACTACTGGAACCTAAACAATCCAATAT
GGATACGAGTCCAAC
+
FFFFFFFFFFFFFFFFFFFFFFFFFF:FFFFFFFFFFFFFFF,FFFF:FF:FFFFFF:FFF::FFFFFF
FFFFFFFFFFFFFFFFFFF,FFFFFFFF,,FFFFFFFFFFF:F:FFFFFFFFFF,FF,FFFFFFFFF:F
F:FFFFFFFFFFFFF
@A00721:190:HGJW7DSXY:4:2128:30056:9142 2:N:0:TCTGCAAG+GCTGCACT
GTCTTACACTACCGTGCGGCTGCATCATTTATCAGACTATAGATTGCAGAAACAATGGATTCGTCCAC
AAGGGTGCCACTCACATGGCAGCAACTACAAGTAGGGGAGTACTACTGGAACCTAAACAATCCAATAT
GGATACGAGTCCAAC
+
FFFFFFFFFFFFFFFFFFFFFFFFFFFFFFFFFFFFFFFFFFFFFFFFFFFFFFFFFFFFFFFFFFFFF
FFFFFFFFFFFFFFFFFFFFFFFFFFFFFFFFFFFFFFFFFFFFFFFFFFFFFFFFF:FFFFFFFFFF
FFFFFFFFFFFFFFF
@A00721:190:HGJW7DSXY:4:2222:31901:5134 2:N:0:TCTGCAAG+GCTGCACT
GTCTTACACTACCGTGCGGCTGCATCATTTATCAGACTATAGATTGCAGAAACAATGGATTCGTCCAC
AAGGGTGCCACTCACATGGCAGCAACTACAAGTAGGGGAGTACTACTGGAACCTAAACAATCCAATAT
GGATACGAGTCCAAC
+
FFFFFFFFFFFFFFFFFFFFFFFFFFFFFFFFFF:FFFFFFFFFFFFFFFFFFFFFFFFFFFFFFFFFF
FFFFFF:F:FFFFF,:FFFFFFFFFFFFFFFFFFFFFFFFFFFFFFFFF:FFFFFFFFF:FFFFFFFFFF
FFFFFFFFF:FF,FF
@A00721:190:HGJW7DSXY:4:2353:30409:18991 2:N:0:TCTGCAAG+GCTGCACT
GTCTTACACTACCGTGCGGCTGCATCATTTATCAGACTATAGATTGCAGAAACAATGGATTCGTCCAC
AAGGGTGCCACTCACATGGCAGCAACTACAAGTAGGGGAGTACTACTGGAACCTAAACAATCCAATAT
GGATACGAGTCCAAC
+
FFFFFFFFFFFFFFFFFFFFFFFFFFFFFFFFFFFFFFFFFFFFFFFFFFFFFFFFFFFFFFFFFFFFF
FFFFFFFFFFFFFFFFFFFFFFFFFFFFFFFFFFFFFFFFFFFFFFFFFFFFFFFFFFFFFFFFFFFFF
FFFFFFFFFFFFFFF

```
@A00721:190:HGJW7DSXY:4:2603:2184:10097 2:N:0:TCTGCAAG+GCTGCACT
GTCTTACACTACCGTGCGGCTGCATCATTTATCAGACTATAGATTGCAGAAACAATGGATTCGTCCAC
AAGGGTGCCACTCACATGGCAGCAACTACAAGTAGGGGAGTACTACTGGAACCTAAACAATCCAATAT
GGATACGAGTCCAAC
+
FFF:FFFFFFFFFF,FFFFFFFFFFFF:FFFFFFFFFFFFFFFFFFFFFFFFFFFFFFFFFFFFFFFF
FF:FFFFFFFFFFFFFFFFF:FF:FFFFFFFF::FFFFFFFFFFFFFFFF,FFFFFFFF:FFFFF,FF
FFFF,FFFFFFFFFF
@A00721:190:HGJW7DSXY:4:1178:20148:28682 2:N:0:TCTGCAAG+GCTGCACT
CTCGTATCCATATTGGATTGTTTAGGTTCCAGTAGTACTCCCCTACTTGTAGTTGCTGCCATGTGAGT
GGCACCCTTGTGGACGAATCCATTGTTTCTGCAATCTATAGTCTGATAAATGATGCAGCCGCACGGTA
GTGTAAGACGTCTAG
+
FFFFFFFFFFFFFFFFFFFFFFFFFFFFFFFFFFFFFFFFFFFFFFFFFFFFFFFFFFFFFFFFFFFF
FFFFFFFFFFFFFFFFFFFFFFFFFFFFFFFFFFFFFFFFFFFFFFFFFFF:FFFFFFFFFFFFFFFF
FFFFFFFFFFFFFFF
@A00721:190:HGJW7DSXY:4:1268:6108:2394 2:N:0:TCTGCAAG+GCTGCACT
CTCGTATCCATATTGGATTGTTTAGGTTCCAGTAGTACTCCCCTACTTGTAGTTGCTGCCATGTGAGT
GGCACCCTTGTGGACGAATCCATTGTTTCTGCAATCTATAGTCTGATAAATGATGCAGCCGCACGGTA
GTGTAAGACGTCTAG
+
FFFFFFFFFFFFFFFFFFFFFFFFFFFFFFFFFFFFFFFFFFFFFFFFFFFFFFFFFFFFFFFFFFFF
FFFFFFFFFFFFFFFFFFFFFFFFFFFFFFFFFFFFFFFFFF:FFFFFFFFFFFFFFFFFFFFFFFFF
FFFFFFFFFFFFFFF
@A00721:190:HGJW7DSXY:4:1435:24849:29183 2:N:0:TCTGCAAG+GCTGCACT
CTCGTATCCATATTGGATTGTTTAGGTTCCAGTAGTACTCCCCTACTTGTAGTTGCTGCCATGTGAGT
GGCACCCTTGTGGACGAATCCATTGTTTCTGCAATCTATAGTCTGATAAATGATGCAGCCGCACGGTA
GTGTAAGACGTCTAG
+
FFFFFFFFFFFFFFFF:FFFFFFFFFFFFFFFFFFFFFFFFFFFF:::FFFFFFFFFFFFFFFFFFFFF
FFFFFFFFFFF:FFFFFFFFFFFFFFFFFFFFFFFFFFFFFFFFFFFFFFFFFFFFFFFFFFFFFFFF
FFFFFFFFFFFFFFF
@A00721:190:HGJW7DSXY:4:2162:25391:24549 2:N:0:TCTGCAAG+GCTGCACT
CTCGTATCCATATTGGATTGTTTAGGTTCCAGTAGTACTCCCCTACTTGTAGTTGCTGCCATGTGAGT
GGCACCCTTGTGGACGAATCCATTGTTTCTGCAATCTATAGTCTGATAAATGATGCAGCCGCACGGTA
GTGTAAGACGTCTAG
+
FFFFFFFFFFFFFFFFFFFFFFFFFF::FFFFFFFFFFFFFFFFFFFFFFFFFFFFFFFFFFFFFFFF
FFFFFFFFFFFFFFFFFFFFFFFFFFFFFFFFFFFFFFFFF,FFF,FF:FFF:FFFFFFFFFFFFFFFF,
FFFFFF:FFFFFFFF
@A00721:190:HGJW7DSXY:4:2357:27715:33520 2:N:0:TCTGCAAG+GCTGCACT
CTCGTATCCATATTGGATTGTTTAGGTTCCAGTAGTACTCCCCTACTTGTAGTTGCTGCCATGTGAGT
GGCACCCTTGTGGACGAATCCATTGTTTCTGCAATCTATAGTCTGATAAATGATGCAGCCGCACGGTA
GTGTAAGACGTCTAG
+
FFFFFFFFFFFFFFFF,F:FFFFFFFFFFFFFFFFFFFFFFFFFFFFFFFFFFFFFFFFFFFFFFFFF
FFFFFFFFFFFFFFFFFFFFFF:FFFFFFFFFFFFFFFFFFFFFFFFFFFFFFFFFFF,FFFFFFFFF
FFFFFFFFFFFFFFF
@A00721:190:HGJW7DSXY:4:2417:27733:8406 2:N:0:TCTGCAAG+GCTGCACT
CTCGTATCCATATTGGATTGTTTAGGTTCCAGTAGTACTCCCCTACTTGTAGTTGCTGCCATGTGAGT
GGCACCCTTGTGGACGAATCCATTGTTTCTGCAATCTATAGTCTGATAAATGATGCAGCCGCACGGTA
GTGTAAGACGTCTAG
+
FFFFFFFFFFFFFFFFFFFFFFFFFFFFFFFFFFFFFFFFFFFFFFFFFFF:FFFFFFFFFFFFFFFF
```

FFFFFFFFFFFFFFFFFFFFFFFFFFFFFFFFFFFFFFFFFFFF:FFFFFFFFFFFFFFFFFFFFFFF
FFFFFFFFFF:FFFF
@A00721:190:HGJW7DSXY:4:2418:12798:12038 2:N:0:TCTGCAAG+GCTGCACT
CTCGTATCCATATTGGATTGTTTAGGTTCCAGTAGTACTCCCCTACTTGTAGTTGCTGCCATGTGAGT
GGCACCCTTGTGGACGAATCCATTGTTTCTGCAATCTATAGTCTGATAAATGATGCAGCCGCACGGTA
GTGTAAGACGTCTAG
+
FFFFFFFFFFFFFFFFFFFFFFFFFFFFFFFFFFFFFFFFFFFFFFFFFFFFFFFFFFFFFFFFFFFF
FFFFFFF,FFFFFFFFFFFFFFFFFFFFFFFFFFFFFFFFFFFFFFFFFFFFF:FFFFFFFFFFFFFF
FFFFF:FFFFFFFFF
@A00721:190:HGJW7DSXY:4:2543:2528:2268 2:N:0:TCTGCAAG+GCTGCACT
CTCGTATCCATATTGGATTGTTTAGGTTCCAGTAGTACTCCCCTACTTGTAGTTGCTGCCATGTGAGT
GGCACCCTTGTGGACGAATCCATTGTTTCTGCAATCTATAGTCTGATAAATGATGCAGCCGCACGGTA
GTGTAAGACGTCTAG
+
FFFFFFFFFFFFFFFFF:FFFFFFFFFFFFFFFFFFFFFFFFFFFFFFFFFFFFFFFFFFFFFFFFFF
FFFFFFFF:FFFFFFFFFFFFFFFFFFFFFFFFFFFFFFFFFFFFFFFFFF:FFFFFFFFFFFFFF:F
FFFFFFFFFFFFFFF
@A00721:190:HGJW7DSXY:4:2569:11044:3082 2:N:0:TCTGCAAG+GCTGCACT
CTCGTATCCATATTGGATTGTTTAGGTTCCAGTAGTACTCCCCTACTTGTAGTTGCTGCCATGTGAGT
GGCACCCTTGTGGACGAATCCATTGTTTCTGCAATCTATAGTCTGATAAATGATGCAGCCGCACGGTA
GTGTAAGACGTCTAG
+
FFFFFFFFFFFFFFFFFFFFFFFFFFFFFFFFFFFFFFFFFFFFFFFFFFFFFFFFFFFFFFFFFFFF
FFFFFFFFFFFFFFFFFFFFFF:FFFFFFFFFFFFFF:FFFFFFFFFFFFFFFFFFFFFFFFFFFFFF
FFFFFFF:FFFFFFF
@A00721:190:HGJW7DSXY:4:2621:32488:5431 2:N:0:TCTGCAAG+GCTGCACT
CTCGTATCCATATTGGATTGTTTAGGTTCCAGTAGTACTCCCCTACTTGTAGTTGCTGCCATGTGAGT
GGCACCCTTGTGGACGAATCCATTGTTTCTGCAATCTATAGTCTGATAAATGATGCAGCCGCACGGTA
GTGTAAGACGTCTAG
+
FFFF:FFFFFFFFFFFFFFF:FFFFFF:FFFFFFFFFFFFFFFFFF:FFFFFFFFFFFFFFFF:FFFF
FFFFFFFFFFFFFFFFFFFFFFFFFFFFFFFFFFFFFFFFFFFFFFFFFFFFFFFFF:FFFFFFFF::
FFFFFFF:FFFFFFF
@A00721:190:HGJW7DSXY:4:1225:2202:1266 2:N:0:TCTGCAAG+GCTGCACT
CAAGCCTAGACGTCTTACACTACCGTGCGGCTGCATCATTTATCAGACTATAGATTGCAGAAACAATG
GATTCGTCCACAAGGGTGCCACTCACATGGCAGCAACTACAAGTAGGGGAGTACTACTGGAACCTAAA
CAATCCAATATGGAT
+
FFFFFFFFFFFFFFFFFFFFFFFFFFFFFFFFFFFFFFFFFFFFFFFFFFFFFFFFFFFFFFFFFFFF
FFFFFFFFFFFFFFFFFFFFFFFFFFFFFFFFFFFFFFFFFFFFFFFFFFFFFFFFFFFFFFFFFFFF
FFFFFFFFFFFFFFF
@A00721:190:HGJW7DSXY:4:1240:20347:24549 2:N:0:TCTGCAAG+GCTGCACT
CAAGCCTAGACGTCTTACACTACCGTGCGGCTGCATCATTTATCAGACTATAGATTGCAGAAACAATG
GATTCGTCCACAAGGGTGCCACTCACATGGCAGCAACTACAAGTAGGGGAGTACTACTGGAACCTAAA
CAATCCAATATGGAT
+
FFFFFFFFFFFFFFFFFFFFFFFFF:FFFFFFFFFFFFFFFFFFFFFFFFFFFFFFFFFFFFFFFFFF
FFFFFFFFFFFF:FFFFFFFFFFFFFFFFFFFFFF:FFFFF:FFFFFFFFFFFFFFFFFFFFFFFFF:
FFFFFFF:FFFFFFF
@A00721:190:HGJW7DSXY:4:1246:24071:16313 2:N:0:TCTGCAAG+GCTGCACT
CAAGCCTAGACGTCTTACACTACCGTGCGGCTGCATCATTTATCAGACTATAGATTGCAGAAACAATG
GATTCGTCCACAAGGGTGCCACTCACATGGCAGCAACTACAAGTAGGGGAGTACTACTGGAACCTAAA
CAATCCAATATGGAT

```
+
FFFFFF:FFFFFFFFFFFFFFFFFFFFFFFFFFFFFFFFFFFFFFFFFFFFFFFFFFFFFFFFFFFFFFF
FFFFFFFFFFFFF:FFFFFFFFFFFFFFFFFFFFFF:FFFFFFFFFFFFFFFFFFFFFFFFFFFF:FFFF
FFFFFFFFFFFFFFF
@A00721:190:HGJW7DSXY:4:1313:8341:6167 2:N:0:TCTGCAAG+GCTGCACT
CAAGCCTAGACGTCTTACACTACCGTGCGGCTGCATCATTTATCAGACTATAGATTGCAGAAACAATG
GATTCGTCCACAAGGGTGCCACTCACATGGCAGCAACTACAAGTAGGGGAGTACTACTGGAACCTAAA
CAATCCAATATGGAT
+
FFFFFFF:FFFFFFFFFFFFFFFFFFFFFFFFFFFFFFFFFFFFFFFFFFFFFFFFFFFFFFFFF:FFFF
FFFFF:FFFFFF:FFFFFFFFFFFFFFFFFFFFFFFFFFFFFFFFFFFFFFFFFFFFFFFFFFFFFF:F
FFFFFFFFFFFFFFF
@A00721:190:HGJW7DSXY:4:1557:17707:21919 2:N:0:TCTGCAAG+GCTGCACT
CAAGCCTAGACGTCTTACACTACCGTGCGGCTGCATCATTTATCAGACTATAGATTGCAGAAACAATG
GATTCGTCCACAAGGGTGCCACTCACATGGCAGCAACTACAAGTAGGGGAGTACTACTGGAACCTAAA
CAATCCAATATGGAT
+
FFFFFFFFFFFFFFFFFFFFFFFFFFFFFFFFFFFFFFFFFFFFFFFFFFFFFFFFFFFFFFFFFFFFFF
FFFFFFFFFFFFFFFFFFFFFFFFFFFFFFFFFFFFFFF:FFFFF:FFFFFFFFFFFFFFFFFFFFFFFF
FFFFFFFFFFFFFFF
@A00721:190:HGJW7DSXY:4:1623:3794:33646 2:N:0:TCTGCAAG+GCTGCACT
CAAGCCTAGACGTCTTACACTACCGTGCGGCTGCATCATTTATCAGACTATAGATTGCAGAAACAATG
GATTCGTCCACAAGGGTGCCACTCACATGGCAGCAACTACAAGTAGGGGAGTACTACTGGAACCTAAA
CAATCCAATATGGAT
+
FFFFFFFFFFFFFFFFFFFFFFFFFFFFFFFFFFFFFFFFFFFFFFFFFFFFFFFFFF:FFFFFFFFFFF
FFFFFFFFFFFFFFFFFFFFFFFFFFFFFFFFFFFFFFFFFFFFFFFFFFFFFFFFFFFFFFFFFFFFFF
FFFFFFFFFFFFFFF
@A00721:190:HGJW7DSXY:4:2272:8721:32628 2:N:0:TCTGCAAG+GCTGCACT
CAAGCCTAGACGTCTTACACTACCGTGCGGCTGCATCATTTATCAGACTATAGATTGCAGAAACAATG
GATTCGTCCACAAGGGTGCCACTCACATGGCAGCAACTACAAGTAGGGGAGTACTACTGGAACCTAAA
CAATCCAATATGGAT
+
FFFFFFFFFFFFFFFFFFFFFFFFFFFFFFFFFFFFFFFFFFFFFFFFFFFFFFFFFFFFFFFFFFFFFF
FFFFFFFFFFFFFFFFFFFFFFFFFFFFFFFFFFFFFFFFFFFFFFFFFFFFFFFFFFFF:FFFFFFFFF
FFFFFFFFFF,FFFF
@A00721:190:HGJW7DSXY:4:2358:32886:19867 2:N:0:TCTGCAAG+GCTGCACT
CAAGCCTAGACGTCTTACACTACCGTGCGGCTGCATCATTTATCAGACTATAGATTGCAGAAACAATG
GATTCGTCCACAAGGGTGCCACTCACATGGCAGCAACTACAAGTAGGGGAGTACTACTGGAACCTAAA
CAATCCAATATGGAT
+
FFFFFFFFFFFFFFFFFFFFFFFFFFFFFFFFFFFFFFFFFFFFFFFFFFFFFFFFFFFFFFFFFFFFFF
FFFFFFFFFFFFFFFFFFFFFFFFFFFFFFFFFFFFFFFFFFFFFFFFFFFFFFFFFFFFFFFFFFFF:F
FFFFFFFFFFFFFFF
@A00721:190:HGJW7DSXY:4:2665:11704:9893 2:N:0:TCTGCAAG+GCTGCACT
CAAGCCTAGACGTCTTACACTACCGTGCGGCTGCATCATTTATCAGACTATAGATTGCAGAAACAATG
GATTCGTCCACAAGGGTGCCACTCACATGGCAGCAACTACAAGTAGGGGAGTACTACTGGAACCTAAA
CAATCCAATATGGAT
+
FFFFFFFFFFFFFFFFFFFFFFFFFFFFFFFFFFFFFFFFFFFFFFFFFFFFFFFFFFFFFFFFFFFFFF
FFFFFFFFFFFFFFFFFFFFFFFFFFFFFFFFFFFFFFF:FFFFFFFFFFFFFFFFFFFFFFFFFFF,FF
FFFFFFFFFFFFFFF
@A00721:190:HGJW7DSXY:4:1407:17933:24314 2:N:0:TCTGCAAG+GCTGCACT
CGTCTAGCGATCAAGCCTAGACGTCTTACACTACCGTGCGGCTGCATCATTTATCAGACTATAGATTG
```

```
CAGAAACAATGGATTCGTCCACAAGGGTGCCACTCACATGGCAGCAACTACAAGTAGGGGAGTACTAC
TGGAACCTAAACAAT
+
FFFFFFFFFFFFFFFFFFFFFFFFFFFFFFFFFFFFFFFFFFFFFFFFFFFFFFFFFFFFFFFFFFFF
FFFFFFFFFFFFFFFFFFFFFFFFFFFFFFFFFFFFFFFFFFFFFFFFFFFFFFFFFFFFFFFFFFFF
FFFFFFFFFFFFFFF
@A00721:190:HGJW7DSXY:4:1515:19777:24408 2:N:0:TCTGCAAG+GCTGCACT
CGTCTAGCGATCAAGCCTAGACGTCTTACACTACCGTGCGGCTGCATCATTTATCAGACTATAGATTG
CAGAAACAATGGATTCGTCCACAAGGGTGCCACTCACATGGCAGCAACTACAAGTAGGGGAGTACTAC
TGGAACCTAAACAAT
+
FFFFFFFFFFFFFFFFFFFFFFFFFFFFFFFFFFFFFFFFFFFFFFFFF:FFFFFFFFFFFFFFFFFF
FFFFFFFFFFFFFFFFFFFFFFFFFFFFFFFFFFFFFFFFFFFFFFF:FFFF:FFFFFFFFFFFFFFF
FFFFFFFFFF:FFFF
@A00721:190:HGJW7DSXY:4:1554:30924:33285 2:N:0:TCTGCAAG+GCTGCACT
CGTCTAGCGATCAAGCCTAGACGTCTTACACTACCGTGCGGCTGCATCATTTATCAGACTATAGATTG
CAGAAACAATGGATTCGTCCACAAGGGTGCCACTCACATGGCAGCAACTACAAGTAGGGGAGTACTAC
TGGAACCTAAACAAT
+
FFFFFFFFFFFFFFFFFFFFFFFFFFFFFFFFFFFFFFFFFFFFFFFFFFFFFFFFFFFFFFFFFFFF
FFFFFFFFFFFFFFFFFFFFFFFFFFFFFFFFFFFFFFFFFFFFFFFFFFFFFFFFFFFF:FFFFFFF
FFFFFFFFFF:FFFF
@A00721:190:HGJW7DSXY:4:2103:1334:23062 2:N:0:TCTGCAAG+GCTGCACT
CGTCTAGCGATCAAGCCTAGACGTCTTACACTACCGTGCGGCTGCATCATTTATCAGACTATAGATTG
CAGAAACAATGGATTCGTCCACAAGGGTGTCACTCACATGGCAGCAACTACAAGTAGGGGAGTACTAC
TGGAACCTAAACAAT
+
FFFFFFFFFFFFFFFFFFFFFFFFFFFFFFFFFFFFFFFFFFFFFFFFFFFFFFFFFFFFFFFFFFFF
FFFFFFFFFFFFFFFFFFFFFFFFFFFFFFFFFFFFFFFFFFFFFFFFFFFFFFFFFFFFFFFFFFFF
FFFFFFFFFFFFFFF
@A00721:190:HGJW7DSXY:4:2268:31774:11052 2:N:0:TCTGCAAG+GCTGCACT
CGTCTAGCGATCAAGCCTAGACGTCTTACACTACCGTGCGGCTGCATCATTTATCAGACTATAGATTG
CAGAAACAATGGATTCGTCCACAAGGGTGTCACTCACATGGCAGCAACTACAAGTAGGGGAGTACTAC
TGGAACCTAAACAAT
+
FFFFFFFFFFFFFFFFFFF:FFFFFFFFFFFFFFFFFFFFFFFFFFFFFFFFFFFFFFFFFFF:FFFF
FFFFFFFFFFFFFFFFFFFFFFFF,FFFFFFFFFFFFFFFFFFFFF:FFFFFF:FFFFFFFFFFFFFF
FFFFF:FFF:FFFFF
@A00721:190:HGJW7DSXY:4:2371:17725:4914 2:N:0:TCTGCAAG+GCTGCACT
CGTCTAGCGATCAAGCCTAGACGTCTTACACTACCGTGCGGCTGCATCATTTATCAGACTATAGATTG
CAGAAACAATGGATTCGTCCACAAGGCTGTCACTCACATGGCAGCAACTACAAGTAGGGGAGTACTAC
TGGAACCTAAACAAT
+
FF:FFFFFFFFFFFFFFFFFF:FFFFFFFFFFFFFFFFFFFFFFFFFFFFFFFFFF:FFFFFFFFFFF
FFFFFFFFFF:FFFFFFFFFFFFFFFF,FFFFFFFFFFF::FFFFFFFFFFFFFFFFFFFFFFFFFFF
FFFFFFFFFF:FFFF
@A00721:190:HGJW7DSXY:4:2469:18882:1814 2:N:0:TCTGCAAG+GCTGCACT
CGTCTAGCGATCAAGCCTAGACGTCTTACACTACCGTGCGGCTGCATCATTTATCAGACTATAGATTG
CAGAAACAATGGATTCGTCCACAAGGGTGCCACTCACATGGCAGCAACTACAAGTAGGGGAGTACTAC
TGGAACCTAAACAAT
+
FFFFFFFFFFFFFFFFFFFFFFFFFFFFFFFF:FFFFFFFFFFFFFFFFFF:FFFFFFFFFFFFFFFF
:FFFFFFFFFF:FFFFFF:FFFFF,FFFFFFFFFFFFF:FFFFFFF,FFFFFFFFFFFFFFFFFFFFF
FFFFF:FFFFFFF:F
```

```
@A00721:190:HGJW7DSXY:4:2650:26793:8469 2:N:0:TCTGCAAG+GCTGCACT
CGTCTAGCGATCAAGCCTAGACGTCTTACACTACCGTGCGGCTGCATCATTTATCAGACTATAGATTG
CAGAAACAATGGATTCGTCCACAAGGGTGCCACTCACATGGCAGCAACTACAAGTAGGGGAGTACTAC
TGGAACCTAAACAAT
+
FFFFFF:FFFFFFFFFFFFFFFFFFFFFFFFFFFFFFFFFFFFFFFFFFFFFFFFFFFFFFFFFFFFF
FFFFFF:FFFFFFFFFFFFFFFF:FFFFFFFFFF:FFFFFFFFFFFFFFFFFFFF:FFFF:FFFFFFF
FFFFFFFFFFFFFFF
@A00721:190:HGJW7DSXY:4:2650:27950:9064 2:N:0:TCTGCAAG+GCTGCACT
CGTCTAGCGATCAAGCCTAGACGTCTTACACTACCGTGCGGCTGCATCATTTATCAGACTATAGATTG
CAGAAACAATGGATTCGTCCACAAGGGTGCCACTCACATGGCAGCAACTACAAGTAGGGGAGTACTAC
TGGAACCTAAACAAT
+
FFFFFFFFFFFFFFFFFFFFFFFFFFFFFFFFFFFFFFFFFFFFFFFFFFFFFFFFFFFFFFFFFFFF
FFFFFFFFFFFFFFFFFFFFFFFFFFFFFFFFFFFFFFFFFFFFFFFFFF:FFFFFFFFFFFFFFFF:F
FFFFFFFFF:FFFF:
@A00721:190:HGJW7DSXY:4:1146:25861:4100 2:N:0:TCTGCAAG+GCTGCACT
GTTTAGGTTCCAGTAGTACTCCCCTACTTGTAGTTGCTGCCATGTGAGTGGCACCCTTGTGGACGAAT
CCATTGTTTCTGCAATCTATAGTCTGATAAATGATGCAGCCGCACGGTAGTGTAAGACGTCTAGGCTT
GATCGCTAGACGTCT
+
FFFFFFFFFFFFFFF:FFFFFFFFFFFF:F:FFFFFFFFFFFFFFFFFFFFFFFFFF,FFFFFF:FFF
FFFFFFFFFFFFFFFFFFFFFFFFFFFFFFFFFFFFFFFFFFFFFFFFFFFFFFFFFFFFFFFFFFFF
FFFFFFF:FFFFFFF
@A00721:190:HGJW7DSXY:4:1206:2564:31798 2:N:0:TCTGCAAG+GCTGCACT
GTTTAGGTTCCAGTAGTACTCCCCTACTTGTAGTTGCTGCCATGTGAGTGGCACCCTTGTGGACGAAT
CCATTGTTTCTGCAATCTATAGTCTGATAAATGATGCAGCCGCACGGTAGTGTAAGACGTCTAGGCTT
GATCGCTAGACGTCT
+
FFFFFFFFFFFFF:FFFFFFFFFFFFFFFFFFFFFFFFFFFFFFFFFFFFFFFFFFFFFFFFFFFFFF
FFFFFFFFFFFFFFFFFF:FFFFFFFFFFF:FFFFFFFFFFFFFFFFF,FFFFFFFFFFFFFFFFFFF
FFFFFFFFFFFFFFF
@A00721:190:HGJW7DSXY:4:1362:22480:25582 2:N:0:TCTGCAAG+GCTGCACT
GTTTAGGTTCCAGTAGTACTCCCCTACTTGTAGTTGCTGCCATGTGAGTGGCACCCTTGTGGACGAAT
CCATTGTTTCTGCAATCTATAGTCTGATAAATGATGCAGCCGCACGGTAGTGTAAGACGTCTAGGCTT
GATCGCTAGACGTCT
+
FFFFFFFFFFFFFFFFFFFFFFFFFFFFFFFFFFFFFFFFFFFFFFFFFFFFFFFFFFFFFFFFFFFF
FFFFFFFFFFFFFFFFFFFFFFFFFFFFFFFFFFFFFFFFFFFFFFFFF::FFFFFFFFFFFFFFFFFF
FFFFFFFFFFFFFFF
@A00721:190:HGJW7DSXY:4:1362:23204:27179 2:N:0:TCTGCAAG+GCTGCACT
GTTTAGGTTCCAGTAGTACTCCCCTACTTGTAGTTGCTGCCATGTGAGTGGCACCCTTGTGGACGAAT
CCATTGTTTCTGCAATCTATAGTCTGATAAATGATGCAGCCGCACGGTAGTGTAAGACGTCTAGGCTT
GATCGCTAGACGTCT
+
FFFFFFFFFFFFFFFFFFFFFFFFFFFFFFFFFFFFFFFFFFFFFFF:FFFFFFFFFFFFFFFFFFFF
FFF,:FFFFFFFF,FFFFFF,FFFFFFFFFFFFFFFFFFFFFFFFFFFFFFFFFFF:FFFFFFFFFFF
FFFFFF:FF:FFFFF
@A00721:190:HGJW7DSXY:4:2414:18511:33802 2:N:0:TCTGCAAG+GCTGCACT
GTTTAGGTTCCAGTAGTACTCCCCTACTTGTAGTTGCTGCCATGTGAGTGGCACCCTTGTGGACGAAT
CCATTGTTTCTGCAATCTATAGTCTGATAAATGATGCAGCCGCACGGTAGTGTAAGACGTCTAGGCTT
GATCGCTAGACGTCT
+
FFFFFFFFFFFFFFFFFFFFFFFFFFFFFFFFFFFFFFFFFFFFFFFFFFFFFFFFF:FFFFFFFFFF
```

```
FFFFFFFFFFFFFFFFFF:FFFFFFFFFFFFFFFFFFFFFFFFFFFFFFFFFFFFFFF:FFFFF:FFF
FFFFFFF:FFFFFFF
@A00721:190:HGJW7DSXY:4:2357:27868:33724 2:N:0:TCTGCAAG+GCTGCACT
TTTAGGTTCCAGTAGTACTCCCCTACTTGTAGTTGCTGCCATGTGAGTGTCACACTTGTGGACGAATC
CATTGTTTCTGCAATCTATAGTCTGATAAATGATTCAGCCGCACGGTAGTGTAAGACTTATA
+
:FFF,F,FFFF:,FF,F:FF,F:FF:FF,:F,F,F,,,F,FF:F,FFFF,FFF,,:FFF,,F:FFF:,
,F,,,FF,,::FFF:F,FFFFF,:,FFFFFFFF:,FF:F,FFF,,FFF,F,,:F,:,,:,FF
@A00721:190:HGJW7DSXY:4:1241:22770:9298 2:N:0:TCTGCAAG+GCTGCACT
GAAGAGACGTCTAGCGATCAAGCCTAGACGTCTTACACTACCGTGCGGCTGCATCATTTATCAGACTA
TAGATTGCAGAAACAATGGATTCGTCCACAAGGGTGCCACTCACATGGCAGCAACTACAAGTAGGGGA
GTACTACTGGAACCT
+
FFFFFFFFFFFFFFFFFFFFFFFFFFFFFFFFFFFFFFFFFFFFFFFFFFFFFFFF:FFFFFFFFFFF
FFFFFFFFFFFFFFFFFFFF:FFFFFFFFFFFFFFFFFFFFFFFFF:FFFFFFF:FFFFF,:FFFFFFF
FFFFFFFFFFFFFFF
@A00721:190:HGJW7DSXY:4:1242:26512:9925 2:N:0:TCTGCAAG+GCTGCACT
GAAGAGACGTCTAGCGATCAAGCCTAGACGTCTTACACTACCGTGCGGCTGCATCATTTATCAGACTA
TAGATTGCAGAAACAATGGATTCGTCCACAAGGGTGCCACTCACATGGCAGCAACTACAAGTAGGGGA
GTACTACTGGAACCT
+
FFFFFFFFFFFFFFFFFFFFFFFFFFFFFFFFFFFFFFFFFFFFFFFFFFFFFFFFFFFFFFFFFFFF
FFFFFFFFFFFFFFFF:FFFFFFFFFFFFFFFFFFFFFFFFFFFFFFFFFFFF:FFFFFFFFFFFFFF
FFFFFF:FFF:FFFF
@A00721:190:HGJW7DSXY:4:1602:24903:34381 2:N:0:TCTGCAAG+GCTGCACT
GAAGAGACGTCTAGCGATCAAGCCTAGACGTCTTACACTACCGTGCGGCTGCATCATTTATCAGACTA
TAGATTGCAGAAACAATGGATTCGTCCACAAGGGTGCCACTCACATGGCAGCAACTACAAGTAGGGGA
GTACTACTGGAACCT
+
FFFFFFFFFFFFFFFFFFFFFFFFFFFFFFFFFFFFFFFFFFFFFFFFFFFFFFFFFFFFFFFFFFFF
FFFFFFFFFFFFFFFFFFFFFFFFFFFFFFFFFFFFFFFFFFFFFFFFFFFFFFFFFFFFFFFFFFFF
FFFFF:FFFFFFFFF
@A00721:190:HGJW7DSXY:4:2163:24370:6684 2:N:0:TCTGCAAG+GCTGCACT
GAAGAGACGTCTAGCGATCAAGCCTAGACGTCTTACACTACCGTGCGGCTGCATCATTTATCAGACTA
TAGATTGCAGAAACAATGGATTCGTCCACAAGGGTGCCACTCACATGGCAGCAACTACAAGTAGGGGA
GTACTACTGGAACCT
+
FFF,FFFFF:F:F,:FF:FFF:FFFFFFFFFFFFFFFFFFF:FFFFF:FFFFFFFFFF:FFFFFF:FF
FF:FFF:FF,FFFFFFF:FFFFF:FF:FFFF,F:FFFFF:FFFFFFFFFF::FFFFFFFF,FF,F,FF
:FF,FFF:FFF:::F
@A00721:190:HGJW7DSXY:4:2303:25509:23907 2:N:0:TCTGCAAG+GCTGCACT
GAAGAGACGTCTAGCGATCAAGCCTAGACGTCTTACACTACCGTGCGGCTGCATCATTTATCAGACTA
TAGATTGCAGAAACAATGGATTCGTCCACAAGGGTGCCACTCACATGGCAGCAACTACAAGTAGGGGA
GTACTACTGGAACCT
+
FFFFFFFFFFFFFFFFFFFFFFFFFFFFFFFFFFFFFFFFFFFFFFFFFFFFFFFF:FFFFFFFFFFFF
FFFFFFFFFFFFFFFFFFFFFFFFFFFFFFFFFFFFFFFFFFFFFFFFFFFFF::FFFFFFFFFFFFF
FFFFF,FFFF,FFFF
@A00721:190:HGJW7DSXY:4:2339:16089:36902 2:N:0:TCTGCAAG+GCTGCACT
GAAGAGACGTCTAGCGATCAAGCCTAGACGTCTTACACTACCGTGCGGCTGCATCATTTATCAGACTA
TAGATTGCAGAAACAATGGATTCGTCCACAAGGGTGCCACTCACATGGCAGCAACTACAAGTAGGGGA
GTACTACTGGAACCT
+
FFFFFFFFFFFFFFFFFFFFFFFFFFFFFFFFFFFFFFFFF:FFFFFFFFFFFFFFFFFFFFFFFFFFF
```

FFFFFFFFFFFFFFFFFFFFFFFFFFFFFFFFFFFFFFFFFFFFF:F:FFFFFFFFFFFFFFFFFFFFF
FF:FFFFFFFF,FFF
@A00721:190:HGJW7DSXY:4:1121:1362:30499 2:N:0:TCTGCAAG+GCTGCACT
CGGAAGAGACGTCTAGCGATCAAGCCTAGACGTCTTACACTACCGTGCGGCTGCATCATTTATCAGAC
TATAGATTGCAGAAACAATGGATTCGTCCACAAGGGTGCCACTCACATGGCAGCAACTACAAGTAGGG
GAGTACTACTGGAAC
+
FFFFFFFFFFFFFFFFFFFFFFFFFFFFFFFFFFFFFFFFFFFFFFFFFFFFFFFFFFFFFFFFFFFF
FFFFFFFFFFFFFFFFFFFFFFF,FFFFFFFFFFFFFFFFFFFFFFFFFFFFFFFFFFFFFFF,FFFF
FFFFFFFFFFFFF,F
@A00721:190:HGJW7DSXY:4:2125:8269:34851 2:N:0:TCTGCAAG+GCTGCACT
CGGAAGAGACGTCTAGCGATCAAGCCTAGACGTCTTACACTACCGTGCGGCTGCATCATTTATCAGAC
TATAGATTGCAGAAACAATGGATTCGTCCACAAGGGTGCCACTCACATGGCAGCAACTACAAGGAGGG
GAGTACTACTGGAAC
+
FFFFFFFFFFFFFFFFFFFFFFFFFFFFFFFFFFFFF:FFFFFFFFFFFFFFF::FF,:FFFFF,FF
FFFFFF:FFFFFFFFF:FFFFFFFFFFFFFFFFFFFFFFFFFFFFFFFFFFFFFF,FFF:FFFF,FFFF
FFFFFFFFFFFF:FF
@A00721:190:HGJW7DSXY:4:2416:28646:22200 2:N:0:TCTGCAAG+GCTGCACT
CGGAAGAGACGTCTAGCGATCAAGCCTAGACGTCTTACACTACCGTGCGGCTGCATCATTTATCAGAC
TATAGATTGCAGAAACAATGGATTCGTCCACAAGGGTGCCACTCACATGGCAGCAACTACAAGTAGGG
GAGTACTACTGGAAC
+
FFFFFFFFFFFFFFFFFFFFFFFFFFFFFFFFFFFFFFFFFFFFFFFFFFFFFFFFFFFFFFFFFFFF
FFFFFFFFFFFFFFFFFFFFFFFFFFFFFFFFFFFFFFFFFFFFFFFFFFFFFFFFFFFFFFFFFFFF
FFFFFFFFFFFFFFF
@A00721:190:HGJW7DSXY:4:1267:19587:26741 2:N:0:TCTGCAAG+GCTGCACT
TAGTACTCCCCTACTTGTAGTTGCTGCCATGTGAGTGGCACCCTTGTGGACGAATCCATTGTTTCTGC
AATCTATGTCTGATAAATGATGCAGCCGCACGGTAGTGTAAGACGTCTAGGCTTGATCGCTAGACGTC
TCTTCCGTTCGCATT
+
FFFFFFFFFFFFFFFFFFFFFFFFFFFFFFFFFFFFFFFFFFFFFFFFFFFFFFFFFFF,FFFFFFFFF
FFFFFFFFFF:FFFFFFFFFFFFFFFFFFFFFFFFFFFFFFFFFFFFFFFFFFFFFFFFFF:FFFFF
FFFFFFFFFFFFFFF
@A00721:190:HGJW7DSXY:4:1272:11767:5588 2:N:0:TCTGCAAG+GCTGCACT
TAGTACTCCCCTACTTGTAGTTGCTGCCATGTGAGTGGCACCCTTGTGGACGAATCCATTGTTTCTGC
AATCTATGTCTGATAAATGATGCAGCCGCACGGTAGTGTAAGACGTCTAGGCTTGATCGCTAGACGTC
TCTTCCGTTCGCATT
+
FFFFFFFFFFFFFFFFFFFFFFFFFFFFFFFFFFFFFFFFFFFFFFFFFFFFFFFFFFFFFFFFFFFF
F:FFFFFFFFFFFFFFFFFFFFFFFFFFFFF:FFFFFFFFFFFF:FFFFF:FFFFFFFFFFFFFF:FF
FFFFFFFFFFFFFFF
@A00721:190:HGJW7DSXY:4:1373:7491:3693 2:N:0:TCTGCAAG+GCTGCACT
TAGTACTCCCCTACTTGTAGTTGCTGCCATGTGAGTGGCACCCTTGTGGACGAATCCATTGTTTCTGC
AATCTATGTCTGATAAATGATGCAGCCGCACGGTAGTGTAAGACGTCTAGGCTTGATCGCTAGACGTC
TCTTCCGTTCGCATT
+
FFFFFFFFFFFFFF,FFFFFFFFFFFFFFFFFFFFFFFFFFFFFFFFFFFFFFF:FFFFFFFFFFFFF
FFFFFF:FFFFFFFFFFFF:FFFFFFFFFFFFF:FFFFFFFF,FFFFFFFFFFFFFFFFFFFFFFFFFF
FFFFFFFFFFFF:FF
@A00721:190:HGJW7DSXY:4:1373:8034:5697 2:N:0:TCTGCAAG+GCTGCACT
TAGTACTCCCCTACTTGTAGTTGCTGCCATGTGAGTGGCACCCTTGTGGACGAATCCATTGTTTCTGC
AATCTATGTCTGATAAATGATGCAGCCGCACGGTAGTGTAAGACGTCTAGGCTTGATCGCTAGACGTC
TCTTCCGTTCGCATT

+
FFFFFFFFFFFFFFFFFFFFFFFFFFFFFFFFFFFFFFFFFFFFFFFFFFFFFFFFFFFFFFFFFFFFFF
FFFFFFFFFFFFFFFFFFFFFFFFFFFFFFFFFFFFFFFFFFFFFFFFFFFFFFFFFFFFFFFFFFFFFF
FFFFFFFFFFFFFFF
@A00721:190:HGJW7DSXY:4:1510:13304:21465 2:N:0:TCTGCAAG+GCTGCACT
TAGTACTCCCCTACTTGTAGTTGCTGCCATGTGAGTGGCACCCTTGTGGACGAATCCATTGTTTCTGC
AATCTATGTCTGATAAATGATGCAGCCGCACGGTAGTGTAAGACGTCTAGGCTTGATCGCTAGACGTC
TCTTCCGTTCGCATT
+
FFFFFFFFFFFFFFF:FFFFFFFFFFFFFFFFFFFFFFFFFFFFFFFFFFFFFFFFFFFF:FFFFFFFFF
FFFFF:FFFFFFFFFFFFFFFFFFFFFFFFFFFFFFFFFFFFFFFFFFFFFFFFFFFFFFFFFFFF
FFFFFFFFFFFFF,F
@A00721:190:HGJW7DSXY:4:1605:11939:9674 2:N:0:TCTGCAAG+GCTGCACT
TAGTACTCCCCTACTTGTAGTTGCTGCCATGTGAGTGGCACCCTTGTGGACGAATCCATTGTTTCTGC
AATCTATGTCTGATAAATGATGCAGCCGCACGGTAGTGTAAGACGTCTAGGCTTGATCGCTAGACGTC
TCTTCCGTTCGCATT
+
FFFFFFFFFFFFFFFFFFFFFFFFFFFFFFFFFFFFFFFFFFFFFFFFFFFFFFFFFFFFFFFFFFFFFF
FFFFFFFFFFFFFFFFFFF:FFFFFFFFFFFFFFFFFFFFFFFFFFFFFFFFFFFFFFFFFFFFFFFFFF
FFFFFFFFFFFFFFF
@A00721:190:HGJW7DSXY:4:2313:21974:22952 2:N:0:TCTGCAAG+GCTGCACT
TAGTACTCCCCTACTTGTAGTTGCTGCCATGTGAGTGGCACCCTTGTGGACGAATCCATTGTTTCTGC
AATCTATGTCTGATAAATGATGCAGCCGCACGGTAGTGTAAGACGTCTAGGCTTGATCGCTAGACGTC
TCTTCCGTTCGCATT
+
FFFFFFFFFFFFFFFFFFFFFFFFFFFFFFFFFFFFFFFFFFFFFFFFFFFFFFFFFFFFFFFFFFFFFF
FFFFFFFFFFFFFFFFFFFFFFFFFFFFFFFFFFFFFFFFFFFFFFFFFFFFFFFFFFFFFFFFFFFFFF
FFFFFFFFFFFFFFF
@A00721:190:HGJW7DSXY:4:2508:17119:2644 2:N:0:TCTGCAAG+GCTGCACT
TAGTACTCCCCTACTTGTAGTTGCTGCCATGTGAGTGGCACCCTTGTGGACGAATCCATTGTTTCTGC
AATCTATGTCTGATAAATGATGCAGCCGCACGGTAGTGTAAGACGTCTAGGCTTGATCGCTAGACGTC
TCTTCCGTTCGCATT
+
FFFFFFFFFFFFFFFFFFFFFFFFFFFFFFFFFFFFFFFFFFFFFFFFFFFFFFFFFFFFFFFFFFFFFF
FFFFF,FFFFFFFFFFFFFFFFFFFFFFFFFFFFFFFFFFFFFFFFFFFFFFFFFFFFF:F:FFFF
FFFFFFFFFFFFFFF
@A00721:190:HGJW7DSXY:4:2517:16025:20478 2:N:0:TCTGCAAG+GCTGCACT
TAGTACTCCCCTACTTGTAGTTGCTGCCATGTGAGTGGCACCCTTGTGGACGAATCCATTGTTTCTGC
AATCTATGTCTGATAAATGATGCAGCCGCACGGTAGTGTAAGACGTCTAGGCTTGATCGCTAGACGTC
TCTTCCGTTCGCATT
+
FFFFFFFFFFFFFFF:FF:FFFFFFFFFFFFFFFFFFFFFFFFF:FFF:FFFFFFFFFFFFFFFFFFFFF
FFFFFFFFFFFFFFFFFFFFFFFFFFFFFFFFFFFFFFFFFFFFFF:FFFF:FFFFFFFFFFFF:FFFFF
FFFFFFFFFFFFFFF
@A00721:190:HGJW7DSXY:4:2623:20672:23234 2:N:0:TCTGCAAG+GCTGCACT
TAGTACTCCCCTACTTGTAGTTGCTGCCATGTGAGTGGCACCCTTGTGGACGAATCCATTGTTTCTGC
AATCTATGTCTGATAAATGATGCAGCCGCACGGTAGTGTAAGACGTCTAGGCTTGATCGCTAGACGTC
TCTTCCGTTCGCATT
+
FFFFFFFFFFFFFFFFFFFFFFFF:FFFFFFFFFFFFFFFFFFFFFFFFFFFFFFFFFFFFFFFFFFFF
FFFFFFFFFFFFFFFFFFFFFFFFFF:FFFFFFFFFFFFFFFFFFFFFFFFFFFFFFFFFFFFFFFFFF
FFFFFFFFFFFFFFF
@A00721:190:HGJW7DSXY:4:2637:9733:33786 2:N:0:TCTGCAAG+GCTGCACT
TAGTACTCCCCTACTTGTAGTTGCTGCCATGTGAGTGGCACCCTTGTGGACGAATCCATTGTTTCTGC

```
AATCTATGTCTGATAAATGATGCAGCCGCACGGTAGTGTAAGACGTCTAGGCTTGATCGCTAGACGTC
TCTTCCGTTCGCATT
+
FFFFFFFFFFFFFF:FFFFFFFFFFFFFFFFFFFFFFFFFFFFFFFFFFFFFFFFFFFFFFFFFFFFF
FFFFFFFFFFFFFFFFFFFFFFFFFFFFFFFFFFFFFFFFFFF:FFFFFFFFFFFFFFFFFFFFFFFF
FFFFFFFFFFFFFFF
@A00721:190:HGJW7DSXY:4:1108:16242:20760 2:N:0:TCTGCAAG+GCTGCACT
GTACTCCCCTACTTGTAGTTGCTGCCATGTGAGTGGCACCCTTGTGGACGAATCCATTGTTTCTGCAA
TCTATAGTCTGATAAATGATGCAGCCGCACGGTAGTGTAAGACGTCTAGGCTTGATCGCTAGACGTCT
CTTCCGTTCGCATTG
+
FFFFFFFFFFFFFFFFFFFFFFFFFFFFFFFFFFFFFFFFFFFFFFFFFFFFFFFFF:FFFFFFFFFF
FFFFFFFFFFFFFFFFFFFFFFFFFFFFF:FFFFFFFFFFFFFF,FFFFFFFFFFFFFFFFFFFFFFFF
FFFFFFFFFFFFFFF
@A00721:190:HGJW7DSXY:4:1350:26847:32925 2:N:0:TCTGCAAG+GCTGCACT
GTACTCCCCTACTTGTAGTTGCTGCCATGTGAGTGGCACCCTTGTGGACGAATCCATTGTTTCTGCAA
TCTATAGTCTGATAAATGATGCAGCCGCACGGTAGTGTAAGACGTCTAGGCTTGATCGCTAGACGTCT
CTTCCGTTCGCATTG
+
:FFFFFFFFFFFFFFFFFFFFFFFFFFFFFFFFFFFFFFFFFFFFFFFFFFFFFFFFFFFFFFFF:FF
F:FFFFFFFFFFFFFFFFFFFFFFFFFFFFFFFFFF:FFFFFFFFFFFFFFFFFFFFFFFFFFFFFFF
FFFFFFFFFFFFFFF
@A00721:190:HGJW7DSXY:4:1476:15411:25207 2:N:0:TCTGCAAG+GCTGCACT
GTACTCCCCTACTTGTAGTTGCTGCCATGTGAGTGGCACCCTTGTGGACGAATCCATTGTTTCTGCAA
TCTATAGTCTGATAAATGATGCAGCCGCACGGTAGTGTAAGACGTCTAGGCTTGATCGCTAGACGTCT
CTTCCGTTCGCATTG
+
FFFFFFFFFF,FFFFFFFFFFFFFFFFFFFFFFFFFFFFFFFFFFFFFFFFFFFFFFFFFFFFFFFFF
FFFFFFF,FFFFFFF:FFFFFFFFFFFFFFFF:FFFFFFFFFFFFFFFFFFF:FFFFFFFFF:FFFFF
FFFFFFFFFFFFFFF
@A00721:190:HGJW7DSXY:4:2177:10610:17519 2:N:0:TCTGCAAG+GCTGCACT
GTACTCCCCTACTTGTAGTTGCTGCCATGTGAGTGGCACCCTTGTGGACGAATCCATTGTTTCTGCAA
TCTATAGTCTGATAAATGATGCAGCCGCACGGTAGTGTAAGACGTCTAGGCTTGATCGCTAGACGTCT
CTTCCGTTCGCATTG
+
FFFFFFFFFFFFFFFFFFFFFFFFFFFFFFFFFFFFFFFFFFFFFFFFFFF:FFFFFFFFFFFFFFFF
FFFFFFFFFFFFFFFFFFFFFFFFFFFFFFFFFFFFFFFFFFFFFFFFFFFFFFFFF:FFFFFFFFFF
FFFFFFFFFFFFFFF
@A00721:190:HGJW7DSXY:4:2535:26720:16924 2:N:0:TCTGCAAG+GCTGCACT
GTACTCCCCTACTTGTAGTTGCTGCCATGTGAGTGGCACCCTTGTGGACGAATCCATTGTTTCTGCAA
TCTATAGTCTGATAAATGATGCAGCCGCACGGTAGTGTAAGACGTCTAGGCTTGATCGCTAGACGTCT
CTTCCGTTCGCATTG
+
FFFFFFFFFFFFFFFFFFFFFFFFFFFFFFFFFFFFFFFFFFFFFFFFFFFFFFF:FFFFFFFFFFFF
FFFFFFFFFFFFFFFFFFFFFFFFFFFFFFFFFFFFFFFFFFFFFFFFFFFFFFFFFFFFFFFFFFFF
FFFFFFFFFFFFFFF
@A00721:190:HGJW7DSXY:4:2538:12798:9377 2:N:0:TCTGCAAG+GCTGCACT
GTACTCCCCTACTTGTAGTTGCTGCCATGTGAGTGGCACCCTTGTGGACGAATCCATTGTTTCTGCAA
TCTATAGTCTGATAAATGATGCAGCCGCACGGTAGTGTAAGACGTCTAGGCTTGATCGCTAGACGTCT
CTTCCGTTCGCATTG
+
FFFFFFFFFFFFF:FFFFFFFFFFFFFFFFFFFFFFFFFFFFFFFFFFFFFFFFFF::FFFFFFFFFF
FFFFFFFFFFFFFFFFFFFFFFFFFFFFFFFFFFFFFFFFF:FFF:FFFFFFFFFF:FFFFFFF:FFFFF
FFFFFFFF:FFFF:F
```

```
@A00721:190:HGJW7DSXY:4:2569:28691:5838 2:N:0:TCTGCAAG+GCTGCACT
GTACTCCCCTACTTGTAGTTGCTGCCATGTGAGTGGCACCCTTGTGGACGAATCCATTGTTTCTGCAA
TCTATAGTCTGATAAATGATGCAGCCGCACGGTAGTGTAAGACGTCTAGGCTTGATCGCTAGACGTCT
CTTCCGTTCGCATTG
+
FFFFFFFFFFFFF:FFFFFFFFFFFFFFFFFFFFFFFFFFFFFFFFFFFFFFFFFFFFFFFFFFFFFFF
FFFFFFFFFFFFFFFFFFFFFFFFFFFFFFFFFFFFFFFFFFFFFFFFFFFFFFFFFFFFFFFFFFFFF
FFFFFFFFF:FFF:F
@A00721:190:HGJW7DSXY:4:2641:14488:4382 2:N:0:TCTGCAAG+GCTGCACT
GTACTCCCCTACTTGTAGTTGCTGCCATGTGAGTGGCACCCTTGTGGACGAATCCATTGTTTCTGCAA
TCTATAGTCTGATAAATGATGCAGCCGCACGGTAGTGTAAGACGTCTAGGCTTGATCGCTAGACGTCT
CTTCCGTTCGCATTG
+
FFFFFFFFFFFFFFFFFFFFFFFF:FFFFFFFFFFFFFFFFFFFFFFFFFFFFFFFFFFFFFFFFFFFF
FFF:FFFFFFFFFFFFFFFFFFFF,FFFFF:FFFFFFFFFFFFFFFFFF:FFFFFFFFFFFFFFFFFF
FF::FFFFFF:FF:F
@A00721:190:HGJW7DSXY:4:1144:26422:24205 2:N:0:TCTGCAAG+GCTGCACT
ATGCAAACGGAAGAGACGTCTAGCGATCAAGCCTAGACGTCTTACACTACCGTGCGGCTGCATCATTT
ATCAGACTATAGATTGCAGAAACAATGGATTCGTCCACAAGGGTGCCACTCACATGGCAGCAACTACA
AGTAGGGGAGTAGAT
+
FFFFFFFFFFFFFFFFFF:FFFFFFFFFFFFFFFFFFFFFFFFFFFFF,FFFFFFFFFFFFFFFFF:FF
FFFFFFFF::FFFFFFFFFFFF:FFF:FFFFFFFFFFF:FFFFFFFFFF:FFFFFFFFFFFFFFFFFF
FFFFFFF:FFFFFFF
@A00721:190:HGJW7DSXY:4:1419:14253:6856 2:N:0:TCTGCAAG+GCTGCACT
ATGCAAACGGAAGAGACGTCTAGCGATCAAGCCTAGACGTCTTACACTACCGTGCGGCTGCATCATTT
ATCAGACTATAGATTGCAGAAACAATGGATTCGTCCACAAGGGTGCCACTCACATGGCAGCAACTACA
AGTAGGGGAGTAGAT
+
FFFFFFFFFFFFFFFFFFFFFFFFF,:FFFFFFFFFFFFFFFFFFFFFFFFFFFFFFFFFF:FFFFFF,F
FFFFFFFFFFF,FFFFFFFFFFFFFFFF:FFFFFF:F:F,FF:FFFF:FFFFFFFFFF,FFFFFF:FF
F::,:F:FFFFF,F:
@A00721:190:HGJW7DSXY:4:1435:10664:24659 2:N:0:TCTGCAAG+GCTGCACT
ATGCAAACGGAAGAGACGTCTAGCGATCAAGCCTAGACGTCTTACACTACCGTGCGGCTGCATCATTT
ATCAGACTATAGATTGCAGAAACAATGGATTCGTCCACAAGGGTGCCACTCACATGGCAGCAACTACA
AGTAGGGGAGTAGAT
+
FFFFFF:FFFFFFFFFFFFFFFFFFFFFFFFFFFFFFFFFFFFFFFFFFFFFFFFFFFFFFFFFFFFFF
FFFFF,F:FFFFFFFFFFFFFFFFFFFFFFFFFFFFFFFFFFFFFFFFFFFFFFFFFFFFFFFFFFF,
FFFFFFFFFFFFFFF
@A00721:190:HGJW7DSXY:4:1460:19578:26224 2:N:0:TCTGCAAG+GCTGCACT
ATGCAAACGGAAGAGACGTCTAGCGATCAAGCCTAGACGTCTTACACTACCGTGCGGCTGCATCATTT
ATCAGACTATAGATTGCAGAAACAATGGATTCGTCCACAAGGGTGCCACTCACATGGCAGCAACTACA
AGTAGGGGAGTAGAT
+
FFFFFFFFFFFFFFFFFFFFFFFFFFFFFFFFFFFFFFFFFFFFFFFFFFFFFFFFFFFFFFFFFFFFF
FFFFFFFFFFFFFFFFFFFFFF:FFFFFFFFFFFFFFFF:FFFFFFFFFFFFFFFFFFFFFFFFFFFFF
FFFFFFFFFFFFFFF
@A00721:190:HGJW7DSXY:4:1468:30472:27586 2:N:0:TCTGCAAG+GCTGCACT
ATGCAAACGGAAGAGACGTCTAGCGATCAAGCCTAGACGTCTTACACTACCGTGCGGCTGCATCATTT
ATCAGACTATAGATTGCAGAAACAATGGATTCGTCCACAAGGGTGCCACTCACATGGCAGCAACTACA
AGTAGGGGAGTAGAT
+
FFFFFFFFFFF:FFFFFFFFFFFFFFFFFFFFFFFFFFFFFFFFFFFFFFFFFFFFFFFFFFFFFFFFF
```

FFFFFFFF:FFFFFFFFFFFFFFFF:FFFFFFFFFFFFFFFFFFFFFFFFFFFFFFFFFFFFFF:FFF::
FF:FFFFFFFF:FFF
@A00721:190:HGJW7DSXY:4:2210:6189:6073 2:N:0:TCTGCAAG+GCTGCACT
ATGCAAACGGAAGAGACGTCTAGCGATCAAGCCTAGACGTCTTACACTACCGTGCGGCTGCATCATTT
ATCAGACTATAGATTGCAGAAACAATGGATTCGTCCACAAGGGTGCCACTCACATGGCAGCAACTACA
AGTAGGGGAGTAGAT
+
FFFFFFFFFFF:FFFFFFFFFFFFFFFFFFFFFFFFFFFFFFFFFFFFFFFFFFFFFFFFFFFFFFFFFF
:FFFFFFFFFFFFFFFFFFFFFFFFFFFFFFFFFFFFFF:FFFFFFFFFFFFFFFFFFFFFFFFFFFFFF
FF:FFFFFFFFFFFF
@A00721:190:HGJW7DSXY:4:2266:10059:25113 2:N:0:TCTGCAAG+GCTGCACT
ATGCAAACGGAAGAGACGTCTAGCGATCAAGCCTAGACGTCTTACACTACCGTGCGGCTGCATCATTT
ATCAGACTATAGATTGCAGAAACAATGGATTCGTCCACAAGGGTGCCACTCACATGGCAGCAACTACA
AGTAGGGGAGTAGAT
+
FFFFFFFFFFFFFFFFFFFFFFFFFFFFFFFFFFFFFFFFFFFFFFFFFFFFFFFFFFFFFFFFFFFFFF
FFFFFFF:FFFFFFFFFFFFFFFFFFFFFFFFFFFFFFFFFFFFFFFF:FFFFFFFFFFFFFFFFFFFFF
FF:,FFFFFFFFFFF
@A00721:190:HGJW7DSXY:4:2476:23845:11318 2:N:0:TCTGCAAG+GCTGCACT
ATGCAAACGGAAGAGACGTCTAGCGATCAAGCCTAGACGTCTTACACTACCGTGCGGCTGCATCATTT
ATCAGACTATAGATTGCAGAAACAATGGATTCGTCCACAAGGGTGCCACTCACATGGCAGCAACTACA
AGTAGGGGAGTAGAT
+
FFFFFFF:FFFFFFFFFFFFFFFFFFFFFFFFFFFFFFFFFFFFFFFFFFFFFFFFFFFFFFFFFFFFFF
FFFFFFFFFFFFFFFFFFFFFFFFFFFFFFFFFFFFFFFFFFFFFFFFFFFFFFFFFFFFFFFFFFFFFF
FFFF:FFFFFFFFFF
@A00721:190:HGJW7DSXY:4:2615:15049:25175 2:N:0:TCTGCAAG+GCTGCACT
ATGCAAACGGAAGAGACGTCTAGCGATCAAGCCTAGACGTCTTACACTACCGTGCGGCTGCATCATTT
ATCAGACTATAGATTGCAGAAACAATGGATTCGTCCACAAGGGTGCCACTCACATGGCAGCAACTACA
AGTAGGGGAGTAGAT
+
FFFFFFFFFFFFFFFFFFFFFFFFFFFFFFFFFFFFFFFFFFFFFFFFFFFFFFFFFFFFFFFFFFFFFF
FFFFFFFF:FFFFFFFFFFFFFFFFFFFFFFFFFFFFFFFFFFFFFFFFFFFFFFFFFFFFFF:FFFFF
FFFFFFFFFFFFFFF
@A00721:190:HGJW7DSXY:4:2668:11351:28886 2:N:0:TCTGCAAG+GCTGCACT
ATGCAAACGGAAGAGACGTCTAGCGATCAAGCCTAGACGTCTTACACTACCGTGCGGCTGCATCATTT
ATCAGACTATAGATTGCAGAAACAATGGATTCGTCCACAAGGGTGCCACTCACATGGCAGCAACTACA
AGTAGGGGAGTAGAT
+
FFFFFFFFFFFFFFFFFFFFFFFFFFFFFFFFFFFFFFFFFFFFFFFFFFFFFFFFFFFFFFFFFFFFFF
FFFFFFFFFFFFFFFFFFFFFFFFF:FFFFF:FFFFFFFFFFF:FFFFFFFFFFFF:FFFFFFFFFF:FF
FFFF:FFFFFFFFFF
@A00721:190:HGJW7DSXY:4:1160:20283:4460 2:N:0:TCTGCAAG+GCTGCACT
CTCCCCTACTTGTAGTTGCTGCCATGTGAGTGGCACCCTTGTGGACGAATCCATTGTTTCTGCAATCT
ATAGTCTGATAAATGATGCAGCCGCACGGTAGTGTAAGACGTCTAGGCTTGATCGCTAGACGTCTCTT
CCGTTCGCATTGCTG
+
FFFFFFFFFFFFFFFFFFFFFFFFFFFFFFFFFFFFFF:FFFFF:FFFFFFFFFFFFFFFFFFFFFFFFF
FFFFFFFFFFFFFFFFFFFFFFFFFFFF:,F:FFFFFFFFFFFFFFFFFFFFFFFFFFFFFFFFFFFF:F
FFFFFFFFFFF:FFF
@A00721:190:HGJW7DSXY:4:1229:2682:27430 2:N:0:TCTGCAAG+GCTGCACT
CTCCCCTCCTTGTAGTTGCTGCCATGTGAGTGGCACCCTTGTGGACGAATCCATGGTTTCTGCAATCT
ATAGTCTGATAAATGATGCAGCCGCACGGTAGTGTAAGACGTCTAGGCTTGATCGCTAGACGTCTCTT
CCGTTCGCATTGCTG

```
+
FFFFFFF,F,FFFFFFFFFFFFFFFFFFFFFFFFFFF:FFFFFFFFFFF:FFF::,FFFFFFFFFFFF,
F:FF:FFFFFFFFFFFFFF,FFFFF:FFF:,FFF:FF:FFFFFFFFFF:FF:FFFFFFFFFF:FFFFF
FFFF:FFFFFFFF,F
@A00721:190:HGJW7DSXY:4:1435:8214:19727 2:N:0:TCTGCAAG+GCTGCACT
CTCCCCTACTTGTAGTTGCTGCCATGTGAGTGGCACCCTTGTGGACGAATCCATTGTTTCTGCAATCT
ATAGTCTGATAAATGATGCAGCCGCACGGTAGTGTAAGACGTCTAGGCTTGATCGCTAGACGTCTCTT
CCGTTCGCATTGCTG
+
FFFFFFFFFFFFFFFFFFFFFFFFFFFFFFFFFFFFFFFFFF:FFFFFFFFF,FFFFFFFFFFF,F
FFFFFFFFFFFFFFFFFFFFFFFFFFFFFFF,FFFFFFF:FFFFFFFF:,FFFFFFFFF,FFFFFFFF,
FFFFFFFF:FFFFF:
@A00721:190:HGJW7DSXY:4:2120:10710:8641 2:N:0:TCTGCAAG+GCTGCACT
CTCCCCTACTTGTAGTTGCTGCCATGTGAGTGGCACCCTTGTGGACGAATCCATTGTTTCTGCAATCT
ATAGTCTGATAAATGATGCAGCCGCACGGTAGTGTAAGACGTCTAGGCTTGATCGCTAGACGTCTCTT
CCGTTCGCATTGCTG
+
FFFFFFFFFFFFFFFFFFFFFFFFFFFFFFFFFFFFFFFFFFFFFFFFFFFFFFFFFFFFFFFFFFFF
F,FFFFFFFFFFFFFFFFFFFFFFFFFFFFFFF:FFFFFFFFFFFFFFFFFFFF,FF::FFFFF:FFF
FFFFFF,FF:FFFFF
@A00721:190:HGJW7DSXY:4:2120:11243:9032 2:N:0:TCTGCAAG+GCTGCACT
CTCCCCTACTTGTAGTTGCTGCCATGTGAGTGGCACCCTTGTGGACGAATCCATTGTTTCTGCAATCT
ATAGTCTGATAAATGATGCAGCCGCACGGTAGTGTAAGACGTCTAGGCTTGATCGCTAGACGTCTCTT
CCGTTCGCATTGCTG
+
FFFFFFFFFFFFFFFFFFFFFFFFFFFFFFFFFFFFFFFFFFFFFFFFFFFFFFFFFFFFFFFFFFFF
FFFFFFFFFFFFFFFFFFFFFFFFFFFFFFFFFFFFFFFFFFFFFFFFFFFFFFFFFFF:FFFFFFFF
FFFFFF:FFFFFFFF
@A00721:190:HGJW7DSXY:4:2209:4065:27164 2:N:0:TCTGCAAG+GCTGCACT
CTCCCCTACTTGTAGTTGCTGCCATGTGAGTGGCACCCTTGTGGACGAATCCATTGTTTCTGCAATCT
ATAGTCTGATAAATGATGCAGCCGCACGGTAGTGTAAGACGTCTAGGCTTGATCGCTAGACGTCTCTT
CCGTTCGCATTGCTG
+
FFFFFFFFFF:FFFFFFFFFFFFFFFFFFFFFFFFFFFFFFFFFFFFFFFFFFFFFFFFFFFFFFFFF
FFFFFFFFFFFFFFFFFFFFFFFFFFFFFFFFFFFFFFFFFFFFFFFFFFFFFFFFF:FFFFFFFFFF
FFFFFFFFFFFFFFF
@A00721:190:HGJW7DSXY:4:2330:3206:19132 2:N:0:TCTGCAAG+GCTGCACT
CTCCCCTACTTGTAGTTGCTGCCATGTGAGTGGCACCCTTGTGGACGAATCCATTGTTTCTGCAATCT
ATAGTCTGATAAATGATGCAGCCGCACGGTAGTGTAAGACGTCTAGGCTTGATCGCTAGACGTCTCTT
CCGTTCGCATTGCTG
+
FFFFFFFFFFFFFFFFFFFFFFFFFFFFFFFFFFFFFFFFFFFFFFFFFFFFFFFFFFFFFFFFFFFF
FFFFFFFFF:FFFFFFFFFFFFFFFFFFFFFFFFFFFFFFFFFFFFFFFFFFFFFFFFFFFFFFFFFF
FFFFFFFFFFFFFFF
@A00721:190:HGJW7DSXY:4:2438:11143:11804 2:N:0:TCTGCAAG+GCTGCACT
CTCCCCTACTTGTAGTTGCTGCCATGTGAGTGGCACCCTTGTGGACGAATCCATTGTTTCTGCAATCT
ATAGTCTGATAAATGATGCAGCCGCACGGTAGTGTAAGACGTCTAGGCTTGATCGCTAGACGTCTCTT
CCGTTCGCATTGCTG
+
FFFFFFFF:FFFFFFFFFFFFFFFFFFFFFFFFFFFFFF:FFFFFFFFFFFFFFF:FFFFFFFFFFFF
FFFFFFFFFFFFFFF:FFFFFFFFFFFFFF,:FFFFFFF:FFFFFFFFFF:FFFFFFFFFFFFFFFF
FFFFFFFFFFFFFFF
@A00721:190:HGJW7DSXY:4:2648:13639:9236 2:N:0:TCTGCAAG+GCTGCACT
CTCCCCTACTTGTAGTTGCTGCCATGTGAGTGGCACCCTTGTGGACGAATCCATTGTTTCTGCAATCT
```

```
ATAGTCTGATAAATGATGCAGCCGCACGGTAGTGTAAGACGTCTAGGCTTGATCGCTAGACGTCTCTT
CCGTTCGCATTGCTG
+
FFFFFFFFFFFFFFFFFFF:FFFFFFFFFFFFFFFFFFF:FFFFFFFFFFFFFFFFFFFFFFFFFFFFF
FFFFFFFFFFFFFFFFFFFFFFFFFFFFFFFF:FFFFFFFFFFFFFFFF:FFFF:FF:F:FFFFFFFF
FFFFFFFFFFFFFF:
@A00721:190:HGJW7DSXY:4:1267:8269:21950 2:N:0:TCTGCAAG+GCTGCACT
GCAATGCAAACGGAAGAGACGTCTAGCGATCAAGCCTAGACGTCTTACACTACCGTGCGGCTGCATCA
TTTATCAGACTATAGATTGCAGAAACAATGGATTCGTCCACAAGGGTGCCACTCACATGGCAGCAACT
ACAAGTAGGGAGATC
+
FFFFFFFFF:FFFFFFFFFFFFFFFFFFFFFFFFFFFFFFFFFFFFFFFFFFFFFFFFFFFFFFFFFFF
FFFFFFFFFFF:FFFFFFFFFFFFFFFFFFFFFFFFFFFFFFFFFFFFFFFFFFFFFFFFFFFFFFFF
FFFFFFFFF:FFFFF
@A00721:190:HGJW7DSXY:4:1673:10447:25128 2:N:0:TCTGCAAG+GCTGCACT
GCAATGCAAACGGAAGAGACGTCTAGCGATCAAGCCTAGACGTCTTACACTACCGTGCGGCTGCATCA
TTTATCAGACTATAGATTGCAGAAACAATGGATTCGTCCACAAGGGTGCCACTCACATGGCAGCAACT
ACAAGTAGGGAGATC
+
FFFFFFFFFFFFFFFFFFFFFFFFFFFFFFFFFFFFFFFFFFFFFFFFFFFFFFFFFFFFFFFFFFFF
FFFFFFFFFFFFFFFFFFFFFFFFFFFFFFFFFFFFFFFFFFFFFFFFFFFFFFFFFFFFFFFFFFFF
FF,FFFFFFFFFFFF
@A00721:190:HGJW7DSXY:4:2166:16071:13949 2:N:0:TCTGCAAG+GCTGCACT
GCAATGCAAACGGAAGAGACGTCTAGCGATCAAGCCTAGACGTCTTACACTACCGTGCGGCTGCATCA
TTTATCAGACTATAGATTGCAGAAACAATGGATTCGTCCACAAGGGTGCCACTCACATGGCAGCAACT
ACAAGTAGGGAGATC
+
:FFFFFFFF:FFFFFFFFFFFFFFFFFFFFFFFFFFFFFFFFFFFFFFFFFFFFFFFFFFFFFFFFFFF
:FFFFFFFFFF:FFFFFFFFFFFFFFFFFFFFFFFFFFFFFFFF,F,FFFFFFFFFFFFFFFFFFFFFFF
FFF:FFFFFFFFFFF
@A00721:190:HGJW7DSXY:4:2364:1750:9690 2:N:0:TCTGCAAG+GCTGCACT
GCAATGCAAACGGAAGAGACGTCTAGCGATCAAGCCTAGACGTCTTACACTACCGTGCGGCTGCATCA
TTTATCAGACTATAGATTGCAGAAACAATGGATTCGTCCACAAGGGTGCCACTCACATGGCAGCAACT
ACAAGTAGGGAGATC
+
FFF:FFFFFFFFFFFFFFFFFFFFFFFFFFFFFFFFFFFFFFFFFFFFFFFFFFFFFFFFFFFFFFFF
F:FFFFFFFF,:FFFFFFFFFFFFFFFFFFFFFFFFFFFFFFFFFFFFFFFFFFFFFFFFFFFFFFFF
F:FFFF,FFF,FFFF
@A00721:190:HGJW7DSXY:4:1506:1931:22905 2:N:0:TCTGCAAG+GCTGCACT
AGCGAAGCATCAGCAATGCAAACGGAAGAGACGTCTAGCGATCAAGCCTAGACGTCTTACACTACCGT
GCGGCTGCATCATTTATCAGACTATAGATTGCAGAAACAATGGATTCGTCCACAAGGGTGCCACTCAC
ATGGCAGCAACTACA
+
FFFFFFFFFFFFFFFFFFFFFFFFFFFFFFFFFFFFFFFFFFFFFFFFF:FFFFFFFFFFFFFFFFFF
FFFFFFFFFFFFFFFFFFFFFFFFFFFFFFFFFFFFFFFFFFFFF:F:FFFFFFFFFFFFFFFFFFFFF
FFFFFFFFFFFFFFF
@A00721:190:HGJW7DSXY:4:2112:2139:31845 2:N:0:TCTGCAAG+GCTGCACT
AGCGAAGCATCAGCAATGCAAACGGAAGAGACGTCTAGCGATCAAGCCTAGACGTCTTACACTACCGT
GCGGCTGCATCATTTATCAGACTATAGATTGCAGAAACAATGGATTCGTCCACAAGGGTGCCACTCAC
ATGGCAGCAACTACA
+
FFFFFFFFFFFFFFFFFFFFFFFFFFFFFFFFFFFFFFFFFFFFFFFFF:FFFFF:FFFFFFFFFFFF:
FFFFFFFFFFFF,FFFFF:FFF,F,FFFF:FFFFFFFFFFFFFFFFFFFFFFFFFFFFFFFFFF:FFFFF
F,FFFFFFF:FF,FF
```

```
@A00721:190:HGJW7DSXY:4:2116:13621:9330 2:N:0:TCTGCAAG+GCTGCACT
AGCGAAGCATCAGCAATGCAAACGGAAGAGACGTCTAGCGATCAAGCCTAGACGTCTTACACTACCGT
GCGGCTGCATCATTTATCAGACTATAGATTGCAGAAACAATGGATTCGTCCACAAGGGTGCCACTCAC
ATGGCAGCAACTACA
+
FFFFFFFFFFFFFFFFFFFFFFFFFFFFFFFFFFFFFFFFFFFFFFFFFFFFFFFFFFFFFFFFF:FFFF
FFFFFFFFFFFFFFFFFFFFFFFFFFFFFFFFFFFFFFFFFFFFFFFFFFFFFFFFFFFFFFFFFFFFFF
FFFFFFFFFFFF,FF
@A00721:190:HGJW7DSXY:4:2169:26684:28072 2:N:0:TCTGCAAG+GCTGCACT
AGCGAAGCATCAGCAATGCAAACGGAAGAGACGTCTAGCGATCAAGCCTAGACGTCTTACACTACCGT
GCGGCTGCATCATTTATCAGACTATAGATTGCAGAAACAATGGATTCGTCCACAAGGGTGCCACTCAC
ATGGCAGCAACTACA
+
FFFFFFFFFFFFFFFFFFFFFFFFFFFFFFFFFFFFFFFFFF:FFFFFFFFFFFFFFFFFFFFFFFFFFF
FFFFFFFFFFFF:FFFFFFFFFFFFFFFFFFFFFFFFFFFFFFFFFFFFFFFFFFFFFFFFFFFFFFFFF
FFFFFFFFFFFFFFF
@A00721:190:HGJW7DSXY:4:2272:16405:31062 2:N:0:TCTGCAAG+GCTGCACT
AGCGAAGCATCAGCAATGCAAACGGAAGAGACGTCTAGCGATCAAGCCTAGACGTCTTACACTACCGT
GCGGCTGCATCATTTATCAGACTATAGATTGCAGAAACAATGGATTCGTCCACAAGGGTGCCACTCAC
ATGGCAGCAACTACA
+
FFFFFFFFFFFFFFFFFFFFFFFFF:FFFFFFFFFFFFFFFF:FFFFFFFFFF:FFFFFFFFFFFFFFFF
FFFFFFFF:FFF:FFFFFFF:F:FFFFF:FFFFFFFFFFFFFFFFFFFFFFFFFFFFFFFFFFFFFFFFF
FFFFFFFFFFFFFFF
@A00721:190:HGJW7DSXY:4:2341:28402:9345 2:N:0:TCTGCAAG+GCTGCACT
AGCGAAGCATCAGCAATGCAAACGGAAGAGACGTCTAGCGATCAAGCCTAGACGTCTTACACTACCGT
GCGGCTGCATCATTTATCAGACTATAGATTGCAGAAACAATGGATTCGTCCACAAGGGTGCCACTCAC
ATGGCAGCAACTACA
+
FFFFFFFFFFFFFFFFFFFFFFFFFFFFFFFFFFFFFFFFFFFFFFFFFFFFFFFFFFFFFFFFFFFFFF
FFFFFFFFFFFFFFFFFFFFFFFFFFFFFFFFFFFFFFFFFFFFFFFFFFFFFFFFFFFFFFFF,FFFFF
FFFFFFFFF:FF,FF
@A00721:190:HGJW7DSXY:4:2428:20627:26162 2:N:0:TCTGCAAG+GCTGCACT
AGCGAAGCATCAGCAATGCAAACGGAAGAGACGTCTAGCGATCAAGCCTAGACGTCTTACACTACCGT
GCGGCTGCATCATTTATCAGACTATAGATTGCAGAAACAATGGATTCGTCCACAAGGGTGCCACTCAC
ATGGCAGCAACTACA
+
FFFFFFFFFFFFFFFFFFFFFFFFFFFFFFFFFFFFFFFFFFFFFFFFFFFFFFFFFFFFFFFFFFFFFF
FFFFFFFFFFFFFFFFFFFFFF:FFFFFFFFFFFFFFFFFF:FFFFFFFFFFFFFFFFFFFFFFFFFFFF
FFFFFFFFFFFFFFF
@A00721:190:HGJW7DSXY:4:2539:30101:6464 2:N:0:TCTGCAAG+GCTGCACT
AGCGAAGCATCAGCAATGCAAACGGAAGAGACGTCTAGCGATCAAGCCTAGACGTCTTACACTACCGT
GCGGCTGCATCATTTATCAGACTATAGATTGCAGAAACAATGGATTCGTCCACAAGGGTGCCACTCAC
ATGGCAGCAACTACA
+
FFFFFFFFFFFFFFFFFFFFFFFFFFFFFFFFFFFFFFFFFFFFFFFFFFFFFFFFFFFFFFFFFFFFFF
FFFFFFFFFFFFFFFFFFFFFFFFFFFFFFFFFFFFFFFFFFFFFFFFFFFFFFFFFFFFFFFFFFFFFF
FFFFFFFFFFFFFFF
@A00721:190:HGJW7DSXY:4:2611:3703:13886 2:N:0:TCTGCAAG+GCTGCACT
AGCGAAGCATCAGCAATGCAAACGGAAGAGACGTCTAGCGATCAAGCCTAGACGTCTTACACTACCGT
GCGGCTGCATCATTTATCAGACTATAGATTGCAGAAACAATGGATTCGTCCACAAGGGTGCCACTCAC
ATGGCAGCAACTACA
+
FFFFFFFFFFFFFFFFFFFFFFFFFFFFFFFFFFFFFFFFFFFFFFFFFFFFFFFFFFFFFFFFFFFFFF
```

```
FFFFFFFFFFFFFFF:FFFFFFFFFFFFFFFFFFFFFFFFFFFFFFFFFFFFF:FFFFFFFFFFFFFF
FFFFFFFFFFFFFFF
@A00721:190:HGJW7DSXY:4:2652:29894:2253 2:N:0:TCTGCAAG+GCTGCACT
AGCGAAGCATCAGCAATGCAAACGGAAGAGACGTCTAGCGATCAAGCCTAGACGTCTTACACTACCGT
GCGGCTGCATCATTTATCAGACTATAGATTGCAGAAACAATGGATTCGTCCACAAGGGTGCCACTCAC
ATGGCAGCAACTACA
+
F:FFFFFFFFFFFFFFFFFFFFFFFFFF:FF,FFFFFFFFFFFFFF:FFFFFFFFFFFFFFFFFFFFF
FFFFFFFFFFFFFFFFFFFFFFF:FFFFFFFFFFFFFFFFFFFFFFFFFFFFFFFFFFFFFF:FFFFF
FFFFFFFFF:FFFFF
@A00721:190:HGJW7DSXY:4:2658:24668:27085 2:N:0:TCTGCAAG+GCTGCACT
AGCGAAGCATCAGCAATGCAAACGGAAGAGACGTCTAGCGATCAAGCCTAGACGTCTTACACTACCGT
GCGGCTGCATCATTTATCAGACTATAGATTGCAGAAACAATGGATTCGTCCACAAGGGTGCCACTCAC
ATGGCAGCAACTACA
+
FFFFFFFFFFFFFFFFFFFFFFFFFFFFFFFFFFFFFFFFF:FFFFFFFFFFFFFFFFFFFFFFFFFF
FFFFFFFFFFFFFFFFFFFF:FF:FFFF:FFFFFFFFFFFFFFFFFFFFFFFFFFFFFFFFFFFFFFF
FFFFFFFFFFFFFFF
@A00721:190:HGJW7DSXY:4:1134:3170:1063 2:N:0:TCTGCAAG+GCTGCACT
GCCTTCTATAGAAGCGAAGCATCAGCAATGCAAACGGAAGAGACGTCTAGCGATCAAGCCTAGACGTC
TTACACTACCGTGCGGCTGCATCATTTATCAGACTATAGATTGCAGAAACAATGGATTCGTCCACAAG
GGTGCCACTCACATG
+
FFFFFFFFFFFFFFFFFFFFFFFFFFFFFFFFFFFFFFFFFFFFFFFFFFFFFFFFFFFFFFFFFFFF
FFFFFFFFFFFFFFFFFFFFFFFFFFFFFFFFFFFF:FFFFFFFFF:FFFF:FFFFFFFFFFF:FFFF
FFFFFFFFFFFFFFF
@A00721:190:HGJW7DSXY:4:1633:23746:15123 2:N:0:TCTGCAAG+GCTGCACT
GCCTTCTATAGAAGCGAAGCATCAGCAATGCAAACGGAAGAGACGTCTAGCGATCAAGCCTAGACGTC
TTACACTACCGTGCGGCTGCATCATTTATCAGACTATAGATTGCAGAAACAATGGATTCGTCCACAAG
GGTGCCACTCACATG
+
FFFFFFFFFFFFFFFFFFFFFFFFFFFFFFFFFFFFFFFFFFFFFFFFFFFFFFFFFF:FFFFFFFFF
FFFFFFFFFFFFFFFFFFFFFFFFFFFFFFFFFFFFF:FFFFFFFFFFFFFFF:FFF:FFFFFFFFFF
FFFF:FFFFFFFFFF
@A00721:190:HGJW7DSXY:4:2207:11315:28667 2:N:0:TCTGCAAG+GCTGCACT
GCCTTCTATAGAAGCGAAGCATCAGCAATGCAAACGGAAGAGACGTCTAGCGATCAAGCCTAGACGTC
TTACACTACCGTGCGGCTGCATCATTTATCAGACTATAGATTGCAGAAACAATGGATTCGTCCACAAG
GGTGCCACTCACATG
+
FFFFFFFFFFFFFFFFFFFFFFFFFFFFFFFFFFFFFFFFFFFFFFFFF:FFFFFFFFFFFFFFFF,F
FFFFFFFFFFFFFFFFFFFFFFFF:FFFFFFF:FF:FFFFFFFFFFFFFFFFFFFFFFFFFFFFFFFF
FFFFFFFFFFFFFFF
@A00721:190:HGJW7DSXY:4:2562:20338:3615 2:N:0:TCTGCAAG+GCTGCACT
GCCTTCTATAGAAGCGAAGCATCAGCAATGCAAACGGAAGAGACGTCTAGCGATCAAGCCTAGACGTC
TTACACTACCGTGCGGCTGCATCATTTATCAGACTATAGATTGCAGAAACAATGGATTCGTCCACAAG
GGTGCCACTCACATG
+
F::,FFFFFFFFF::FFFFFF,:F,F:F,:FF:FFF,FFFF:FFF:F,F:F:FFFFF,F,FFFFFFFF
F:FFFFF,FF,,FFFFFF:FFF:F,,,FFF:FF::FFF,FF::FFFFFFFFFFFFFFFF:FFFFFFFF
FFFFFFFFFF:FF,F
@A00721:190:HGJW7DSXY:4:2562:20283:3771 2:N:0:TCTGCAAG+GCTGCACT
GCCTTCTATAGAAGCGAAGCATCAGCAATGCAAACGGAAGAGACGTCTAGCGATCAAGCCTAGACGTC
TTACACTACCGTGCGGCTGCATCATTTATCAGACTATAGATTGCAGAAACAATGGATTCGTCCACAAG
GGTGCCACTCACATG
```

+
FFFFFFFFFFFFFFFFFFFFFFFFFFFFFFFFFFFFFFFFFFFFFFFF:FFFFFFFFFFFFFFFFFFF
FFFFFFFFFFFFFFFFFFFFFFFFFFFFFFFFFFFFFFFF:FFFFFFFFFFFFFFFFFFFFFFFFFFF
FFFFFFFFFFFFFFF
@A00721:190:HGJW7DSXY:4:2616:5050:35978 2:N:0:TCTGCAAG+GCTGCACT
GCCTTCTATAGAAGCGAAGCATCAGCAATGCAAACGGAAGAGACGTCTAGCGATCAAGCCTAGACGTC
TTACACTACCGTGCGGCTGCATCATTTATCAGACTATAGATTGCAGAAACAATGGATTCGTCCACAAG
GGTGCCACTCACATG
+
FFFFFFFFFFFFFFFFFFFFFFFFFFF:FFFFFFFFFFFFFFFFFFFFFFFFFFFFFFFFFFFFFFFF
FFFFFFFFFFFFFFFFFFFFFFFFF:FFFFFFFFFF,FFFFFFFFFFFFFFFFFFFFFFFFFFFFF:F
FFFFFFFFFFFFFFF
@A00721:190:HGJW7DSXY:4:2619:15185:25410 2:N:0:TCTGCAAG+GCTGCACT
GCCTTCTATAGAAGCGAAGCATCAGCAATGCAAACGGAAGAGACGTCTAGCGATCAAGCCTAGACGTC
TTACACTACCGTGCGGCTGCATCATTTATCAGACTATAGATTGCAGAAACAATGGATTCGTCCACAAG
GGTGCCACTCACATG
+
FFFFFFFFFFFFFFFFFFFFFFFFFFFFFFFFFFFFFFFFFFFFFFFFFFFFFFFFFFFFFFFFFFFF
FFFFFFFFFFFFFFFFFFFFFFFFFFFFFFFFFFF:FFFFFFFFFFFFFFFFFFFFFFFFFFFFFFFF
FFFFFFFFFFFFFFF
@A00721:190:HGJW7DSXY:4:2632:26937:22999 2:N:0:TCTGCAAG+GCTGCACT
GCCTTCTATAGAAGCGAAGCATCAGCAATGCAAACGGAAGAGACGTCTAGCGATCAAGCCTAGACGTC
TTACACTACCGTGCGGCTGCATCATTTATCAGACTATAGATTGCAGAAACAATGGATTCGTCCACAAG
GGTGCCACTCACATG
+
FFFFFFFFFFFFFFFFFFFFFFFFFFFFFFFFFFFFFFFFFFFFFFFFFFFFFFFFFFFFFFFFFFFF
FFFFFFFFFFFFFFFFFFFFFFFFFFFFFFFFFFF,FFFFFFFFFFFFFFFFFFFFFFFFFFFFFFFF
FFFFFFFFFFFF:FF
@A00721:190:HGJW7DSXY:4:2634:14877:15608 2:N:0:TCTGCAAG+GCTGCACT
GCCTTCTATAGAAGCGAAGCATCAGCAATGCAAACGGAAGAGACGTCTAGCGATCAAGCCTAGACGTC
TTACACTACCGTGCGGCTGCATCATTTATCAGACTATAGATTGCAGAAACAATGGATTCGTCCACAAG
GGTGCCACTCACATG
+
FFFFFFFFFFFFFF:FFFFFFFFFFFFFFFFFFFFFFFFFFFFFFFFFFFFFFFFFFFFFFFFFFFFF
FFFFFFFFFFFFFFF:FFFFFFFFFFFFFFFFFFFFFF:FFFFFFFFFFFFFFFFFFFFFFFFFFFFF
FFFFFF,F:FFFFFF
@A00721:190:HGJW7DSXY:4:1158:9968:25457 2:N:0:TCTGCAAG+GCTGCACT
CGCCTTCTATAGAAGCGAAGCATCAGCAATGCAAACGGAAGAGACGTCTAGCGATCAAGCCTAGACGT
CTTACACTACCGTGCGGCTGCATCATTTATCAGACTATAGATTGCAGAAACAATGGATTCGTCCACAA
GGGTGCCACTCACAT
+
FFFFFFFFFFFFFFFFFFFFFFFFFFFFFFFFFFFFFFFFFFFFFFFFFFFFFFFFFFFFFF:FFFFF
FFFFF:FFFFFF:FFF:FFFFFFFFF:FFFFFFFF:FFFFFFFFFFFFFFFFFFFFFFFFFFFFFFFF
FFFFFFFFFFFFFFF
@A00721:190:HGJW7DSXY:4:1162:1208:15013 2:N:0:TCTGCAAG+GCTGCACT
CGCCTTCTATAGAAGCGAAGCATCAGCAATGCAAACGGAAGAGACGTCTAGCGATCAAGCCTAGACGT
CTTACACTACCGTGCGGCTGCATCATTTATCAGACTATAGATTGCAGAAACAATGGATTCGTCCACAA
GGGTGCCACTCACAT
+
FFFFFFFFFFFFFFFFFFFFFFFFFFFFFFFFFFFFFFFFFFFFFFFFFFFFFFFFFFFFF:FFFFFF
FFFFFFFFFFFF:FFFFFFFFFFFFFFFFFFFFFFFFFFFFFFFFFFFFFFFFFFFF::FFFFFFFFF
FFFFFFFFFFFFFFF
@A00721:190:HGJW7DSXY:4:1274:17445:23594 2:N:0:TCTGCAAG+GCTGCACT
CGCCTTCTATAGAAGCGAAGCATCAGCAATGCAAACGGAAGAGACGTCTAGCGATCAAGCCTAGACGT

CTTACACTACCGTGCGGCTGCATCATTTATCAGACTATAGATTGCAGAAACAATGGATTCGTCCACAC
GGGTGCCACTCACAT
+
FFFFFFFFFFFFFFFFFFFFFFFFFFFFFFFFFFFFFFFFFFFFFFFFFFFFFFFFFFFFFFFFF:FFF,
FFFFFFFFFFFFFFFFFFFFFFFFFFFFFFFFFFFFFFFFFFFFFFFFFFFFFFFFFFFFFFFFFF,FF,
FFFFFFF,FFFFFFF
@A00721:190:HGJW7DSXY:4:1471:13187:8046 2:N:0:TCTGCAAG+GCTGCACT
CGCCTTCTATAGAAGCGAAGCATCAGCAATGCAAACGGAAGAGACGTCTAGCGATCAAGCCTAGACGT
CTTACACTACCGTGCGGCTGCATCATTTATCAGACTATAGATTGCAGAAACAATGGATTCGTCCACAA
GGGTGCCACTCACAT
+
FFFFFFFFFFFFFFFFFFFFFFFFFFFFFFFFFFFFFFFFF:FFFFFF:FFFFFFFFFFFFFFFFFFF
FFFFFFFFFFFFFFFFFFFFFFFFFFFFFFFFF:FFFFFF:FFFF:FFFFFFFFFFFFFFFFFFFFFFF
FFFFFFFFFFFFFFF
@A00721:190:HGJW7DSXY:4:1123:1723:2409 2:N:0:TCTGCAAG+GCTGCACT
CCGCCTTCTATAGAAGCGAAGCATCAGCAATGCAAACGGAAGAGACGTCTAGCGATCAAGCCTAGACG
TCTTACACTACCGTGCGGCTGCATCATTTATCAGACTATAGATTGCAGAAACAATGGATTCGTCCACA
AGGGTGCCACTCACA
+
FFFFFFFFFFFFFFFFFFFFFFFFFFFF,FFFFF:FFFFFFFFFFFFFFFFFFFF:FFFFFFFFFFFF
FFFFFFFFFFFFFFFFFFFFFFFFFFFFFFFFF:FFFFFFFF:,FFFFFFFFFFFFFFFFFFFFFFFFF
FFFFFFFF:FFFFFF
@A00721:190:HGJW7DSXY:4:1160:24668:15562 2:N:0:TCTGCAAG+GCTGCACT
CCGCCTTCTATAGAAGCGAAGCATCAGCAATGCAAACGGAAGAGACGTCTAGCGATCAAGCCTAGACG
TCTTACACTACCGTGCGGCTGCATCATTTATCAGACTATAGATTGCAGAAACAATGGATTCGTCCACA
AGGGTGCCACTCACA
+
FFFFFFFFFFFFFFFFFFFFFFFFFFFFFFFFFFFFFFFFFFFFFFFFFFFFFFFFFFFFFFFFFFFF
FFFFFFFFF,:FFFFFFFFFFFFFFFFFFFFFFFFFFFFFFFFFFFFFFFFFFFFFFFFF:FFFFFF
FFFFFFFFFFFFFFF
@A00721:190:HGJW7DSXY:4:1571:1732:15358 2:N:0:TCTGCAAG+GCTGCACT
CCGCCTTCTATAGAAGCGAAGCATCAGCAATGCAAACGGAAGAGACGTCTAGCGATCAAGCCTAGACG
TCTTACACTACCGTGCGGCTGCATCATTTATCAGACTATAGATTGCAGAAACAATGGATTCGTCCACA
AGGGTGCCACTCACA
+
FFFFFFFFFFFFFFFFFFFFFFFF:FFFFFFFFFFFFFFFFFFFFFFFFFFFFFFFFFFFFFFFFFFF
FFFFFFFFF:FFFFFFFFFFFFFFFFFFFFFFFFFFFFFFFFFFFFFFFFF:FF:FFFFFFFFFFFFF
FFFFFFFFFFFFFFF
@A00721:190:HGJW7DSXY:4:1676:1931:26944 2:N:0:TCTGCAAG+GCTGCACT
CCGCCTTCTATAGAAGCGAAGCATCAGCAATGCAAACGGAAGAGACGTCTAGCGATCAAGCCTAGACG
TCTTACACTACCGTGCGGCTGCATCATTTATCAGACTATAGATTGCAGAAACAATGGATTCGTCCACA
AGGGTGCCACTCACA
+
FFFFFFFFFFFFFFFFFFFFFFFFFFFFFFFFFFFFFFFFFFFFFFFF:FFFFFFFFFFFFFFFFFFFF
FFFFFFFFFFFFFFFFFFFFFFFFFFF:FFFFFF:FFFFFFFFFFFFFFFFF:FFFFFFFFFFFFFFFF:
FFFFFFFFFFFFFFF
@A00721:190:HGJW7DSXY:4:2537:30101:3396 2:N:0:TCTGCAAG+GCTGCACT
CCGCCTTCTATAGAAGCGAAGCATCAGCAATGCAAACGGAAGAGACGTCTAGCGATCAAGCCTAGACG
TCTTACACTACCGTGCGGCTGCATCATTTATCAGACTATAGATTGCAGAAACAATGGATTCGTCCACA
AGGGTGCCACTCACA
+
FFFFFFFFFFFFFFFFFFFFFFFFFFFFFFFFFFFFFFFFFFFFFFFFFFFFFFFFFFFFFFFFFFFF
FFFFFFFFFFFFFFFFFFFFFFFFFFF:FFFFFFFFFFF:FFF:FFFFFFFFFFFFFFFFFFFFFFFFFF
FFFFFFFFFFFFFFF

```
@A00721:190:HGJW7DSXY:4:1136:23809:9815 2:N:0:TCTGCAAG+GCTGCACT
CGCCGAATCCGCCTTCTATAGAAGCGAAGCATCAGCAATGCAAACGGAAGAGACGTCTAGCGATCAAG
CCTAGACGTCTTACACTACCGTGCGGCTGCATCATTTATCAGACTATAGATTGCAGAAACAATGGATT
CGTCCACAAGGGTGC
+
FFFFFFFFFFFFFFFFFFFFFFFFFFFFFFFFFFFFFFFFFFFFFFFFFFFFFFFFFFFFFFFFFFFF
FFFFFFFFFFFFFFFFFFFFFFFFFFFFFFFFFFFFFFFFFFFFFFFFFFFFFFFFFFFFFFFFFFFF
FFFFFFFFFFFFFFF
@A00721:190:HGJW7DSXY:4:1226:15872:18740 2:N:0:TCTGCAAG+GCTGCACT
CGCCGAATCCGCCTTCTATAGAAGCGAAGCATCAGCAATGCAAACGGAAGAGACGTCTAGCGATCAAG
CCTAGACGTCTTACACTACCGTGCGGCTGCATCATTTATCAGACTATAGATTGCAGAAACAATGGATT
CGTCCACAAGGGTGC
+
FFFFFFFFFFFFFFFFFFFFFFFFFFFFFFFFFFFFFFFFFFFFFFFFFFFFFFFFFFFFFFFFFFFF
FFFF:FFFFFFFFFFFFFFFFFFFFFFFFF:FFFFF:FFFFFFFFFFFFFFFFFFFFFFFFFF,FFFF
F,FFFFFFFFFFFFF
@A00721:190:HGJW7DSXY:4:1262:28447:22576 2:N:0:TCTGCAAG+GCTGCACT
CGCCGAATCCGCCTTCTATAGAAGCGAAGCATCAGCAATGCAAACGGAAGAGACGTCTAGCGATCAAG
CCTAGACGTCTTACACTACCGTGCGGCTGCATCATTTATCAGACTATAGATTGCAGAAACAATGGATT
CGTCCACAAGGGTGC
+
FFFFFFFFFFFFFFFFFFFFFFFFFFFFFFFFFFFFFFFFFFFFFFFFFFFFFFFFFFFFFFFFFFFF
FFFFFFFFFFFFFFFFFFFFFFFFFFFFFFFFFF::FFFFFFFFFFFFFFFFFFFF,FFFFFFFFFFF
FFFFFFFFFFFFFFF
@A00721:190:HGJW7DSXY:4:2460:23737:22216 2:N:0:TCTGCAAG+GCTGCACT
CGCCGAATCCGCCTTCTATAGAAGCGAAGCATCAGCAATGCAAACGGAAGAGACGTCTAGCGATCAAG
CCTAGACGTCTTACACTACCGTGCGGCTGCATCATTTATCAGACTATAGATTGCAGAAACAATGGATT
CGTCCACAAGGGTGC
+
FFFFFFFFFFFFFFFFFFFFFFFFFFFFFFFFFFFFFFFFFFF:FFF:FFFFFFFFFFFFFFFFFFFF
FFFFFFFFFF:FFFFFFFFFFFFFFFFFFFFFFFFFFFFFFFFFFFFFF:FFFFFFFFFFFFFFFFFF
FFFFFFFFFFFFFFF
@A00721:190:HGJW7DSXY:4:2649:12608:13025 2:N:0:TCTGCAAG+GCTGCACT
CGCCGAATCCGCCTTCTATAGAAGCGAAGCATCAGCAATGCAAACGGAAGAGACGTCTAGCGATCAAG
CCTAGACGTCTTACACTACCGTGCGGCTGCATCATTTATCAGACTATAGATTGCAGAAACAATGGATT
CGTCCACAAGGGTGC
+
FFFFFFFFFFFFFFFFFFF,FFFFFFFFFFFFFFFFFFFFFFFFFFFFFFFFFFFFFF:FFFFFFFFF
FFFFFFFFFFFFFFFFFFFFFFFFFFFFFF:FFFFFFFFFFFFFFFFFFFFFFFFFFFFFFFFFFFFF
FFFFFFFFFFFFFFF
@A00721:190:HGJW7DSXY:4:1227:4553:19586 2:N:0:TCTGCAAG+GCTGCACT
GCAGAATTCTTCTTCGCCGAATCCGCCTTCTATAGAAGCGAAGCATCAGCAATGCAAACGGAAGAGAC
GTCTAGCGATCAAGCCTAGACGTCTTACACTACCGTGCGGCTGCATCATTTATCAGACTATAGATTGC
AGAAACAATGGATTC
+
FFFFFFFFFFFFFFFFFFFFFFFFFFF:FFFFFFFFFFFFF:FFFFFFFFFFFFFFFFFFFFFFFFFF
FFFFFFFFFFFFF:FFFFFFFFFF:FFFFFFFFFFFFFFFFFFFFFFF:FFFFFFFFFFFFFFFFFFF
FFFFFFF:F:FFFFF
@A00721:190:HGJW7DSXY:4:1468:23927:32252 2:N:0:TCTGCAAG+GCTGCACT
GCAGAATTCTTCTTCGCCGAATCCGCCTTCTATAGAAGCGAAGCATCAGCAATGCAAACGGAAGAGAC
GTCTAGCGATCAAGCCTAGACGTCTTACACTACCGTGCGGCTGCATCATTTATCAGACTATAGATTGC
AGAAACAATGGATTC
+
FFFFFFFFFFFFFFFFFFFFFFFFFFFFFFFFFFFFFFFFFFFFFFFFFFFFFFFF:FFFFFFFFFFF
```

FFFF::FFFFFFFFFFFFFFFFFFFFFFFFFFFFFFFFFFFFFFFF,FF,FF:FFF:FFFFFFFFF
FFFFFFFFFFFFFFF
@A00721:190:HGJW7DSXY:4:1508:19768:10645 2:N:0:TCTGCAAG+GCTGCACT
GCAGAATTCTTCTTCGCCGAATCCGCCTTCTATAGAAGCGAAGCATCAGCAATGCAAACGGAAGAGAC
GTCTAGCGATCAAGCCTAGACGTCTTACACTACCGTGCGGCTGCATCATTTATCAGACTATAGATTGC
AGAAACAATGGATTC
+
:FFFFFFFFFFFFFFFFFFFFFFFFFFFFFFFFFFFFFFFFFFFFFFFFFFFFFFFFFFFFFFFFFFF
FFFFF:FFFFFFFFFFFF:FFFFFFFFFFFFFFFFFFFFFFFFFFFF:FFFFFFFFFFF,FF,FFFFF
FFFFFFFFFFFFFFF
@A00721:190:HGJW7DSXY:4:1508:19425:12305 2:N:0:TCTGCAAG+GCTGCACT
GCAGAATTCTTCTTCGCCGAATCCGCCTTCTATAGAAGCGAAGCATCAGCAATGCAAACGGAAGAGAC
GTCTAGCGATCAAGCCTAGACGTCTTACACTACCGTGCGGCTGCATCATTTATCAGACTATAGATTGC
AGAAACAATGGATTC
+
FFFFFFFFFFFFFFFFFFFFFFFFFFFFFFFFFFFF,FFFFFFFFFFFFFFFFFFFFFFFFFFFFFFF
FFFFFFFFFFFFFFFFFFFFFFFFFFFFFFFFFFFFFFFFFFFFFFFFFFFFFFFFFFF:FFFFFFFFF
FFFFFFFFFFFFF:F
@A00721:190:HGJW7DSXY:4:2137:22706:29512 2:N:0:TCTGCAAG+GCTGCACT
GCAGAATTCTTCTTCGCCGAATCCGCCTTCTATAGAAGCGAAGCATCAGCAATGCAAACGGAAGAGAC
GTCTAGCGATCAAGCCTAGACGTCTTACACTACCGTGCGGCTGCATCATTTATCAGACTATAGATTGC
AGAAACAATGGATTC
+
FFFFFFFFFFFFFFFFFFFFFFFFFFFFFFFFFFF:FFFFF:FFFFFFFF:FFFFFF,FFFFFFFFFF
FFFFFFFFFFFFFFFFFFFFFFFFF:FFFFFFFFFFFFFFFFFFFFFFFFFFFFFFFFF:FFFFFFFFF
FFFFFFF:FFFF:FF
@A00721:190:HGJW7DSXY:4:2653:17454:8171 2:N:0:TCTGCAAG+GCTGCACT
GCAGAATTCTTCTTCGCCGAATCCGCCTTCTATAGAAGCGAAGCATCAGCAATGCAAACGGAAGAGAC
GTCTAGCGATCAAGCCTAGACGTCTTACACTACCGTGCGGCTGCATCATTTATCAGACTATAGATTGC
AGAAACAATGGATTC
+
FFFFFFFFFFFFFFFFFFFFFFFF:FFFFFFFFFFFFFFFFFFFFFFFFFFFFFFFFF:FFFFFFFFFF
FFFFFFFFFFFFFFFFFFFFFFFFFFFFFFFFFFFFFFFFFFFFFFFFFFFFFF:FFF:,FFFFFFFF
FFFFFFFFFFFFFFF
@A00721:190:HGJW7DSXY:4:2359:22625:7545 2:N:0:TCTGCAAG+GCTGCACT
TGCAGAATTCTTCTTCGCCGAATCCGCCTTCTATAGAAGCGAAGCATCAGCAATGCAAACGGAAGAGA
CGTCTAGCGATCAAGCCTAGACGTCTTACACTACCGTGCGGCTGCATCATTTATCAGACTATAGATTG
CAGAAACAATGGATT
+
FFFFFFFFFFFFFFFFFFFFFF:FFFFFFFFFFFFFFFFFFFFFFFFFFFFFFFFFFFFFFFFFFFFFF
FFFFFFFFFFFFFFFFFFFFFFFFFFFFFFFFFFFFFFFFFFFFFFFFFFFFFFFFFFFFFF:FFFFFF
FFFFFFFFFFFFFFF
@A00721:190:HGJW7DSXY:4:2601:26304:29982 2:N:0:TCTGCAAG+GCTGCACT
TGCAGAATTCTTCTTCGCCGAATCCGCCTTCTATAGAAGCGAAGCATCAGCAATGCAAACGGAAGAGA
CGTCTAGCGATCAAGCCTAGACGTCTTACACTACCGTGCGGCTGCATCATTTATCAGACTATAGATTG
CAGAAACAATGGATT
+
FFFFFFFFFFFFFFFFFFFFFFFFFFFFFFFFFFFFFFFFFFFFFFFFFFFFFFFFFFFFFFFFFFFFF
FFFFFFFFFFFFFFFFFFFFFFFFFFFFFFFFFFFFFFFFFFFFFFFFFFFFFFFFFFFFFFFFFFFFF
FFFFFFFFFFFFFFF
@A00721:190:HGJW7DSXY:4:2638:16360:33990 2:N:0:TCTGCAAG+GCTGCACT
TGCAGAATTCTTCTTCGCCGAATCCGCCTTCTATAGAAGCGAAGCATCAGCAATGCAAACGGAAGAGA
CGTCTAGCGATCAAGCCTAGACGTCTTACACTACCGTGCGGCTGCATCATTTATCAGACTATAGATTG
CAGAAACAATGGATT

+
FFFFFFFFFFFFFFFFFFFFFFFFFFFFFFFFFFFFFFFFFFFFFFFFFFFFFFFFFFFFFFFFFFFF
FFFFFFFFFFFFFFFFFFFFFFFFFFFFFFFFFFFFFFFFFFFFFFFFFFFFFFFFFFFFFFFFFFFF
FFFFFFFFFFFFFF:
@A00721:190:HGJW7DSXY:4:1129:26521:16986 2:N:0:TCTGCAAG+GCTGCACT
CAGAGACAAATGCAGAATTCTTCTTCGCCGAATCCGCCTTCTATAGAAGCGAAGCATCAGCAATGCAA
ACGGAAGAGACGTCTAGCGATCAAGCCTAGACGTCTTACACTACCGTGCGGCTGCATCATTTATCAGA
CTATAGATTGCAGAA
+
FFFFFFFFFFFFFF:FFFFFFFFFFFFFFFFFFFFFFFFFFFFFFFFFFFFFFFFFFFFFFFFFFFFF
FFFFFFFFFFFFFFFFFFFFFFFFFFFFF,FFFFFFFFFFFFFFFFFFFFFFFFFFFFFFFFFFFFFF
FFFFFFFFFFFFFFF
@A00721:190:HGJW7DSXY:4:1161:21956:32033 2:N:0:TCTGCAAG+GCTGCACT
CAGAGACAAATGCAGAATTCTTCTTCGCCGAATCCGCCTTCTATAGAAGCGAAGCATCAGCAATGCAA
ACGGAAGAGACGTCTAGCGATCAAGCCTAGACGTCTTACACTACCGTGCGGCTGCATCATTTATCAGA
CTATAGATTGCAGAA
+
FFFFFFFFFFFFFFFFFFFFFFFFFFFFFFFFFFFFFFFFFFFFFFF:FFFFFFFFFFFFFFFFFFFF
FFFFFFFFFFFFFFFFFFFFFFFFFFFFFFFFFFFFFFFFFFFFFFFFFFFFFFFFFFFFFFFFFFFF
FFFFFFFFFFFFFFF
@A00721:190:HGJW7DSXY:4:1206:2889:29512 2:N:0:TCTGCAAG+GCTGCACT
CAGAGACAAATGCAGAATTCTTCTTCGCCGAATCCGCCTTCTATAGAAGCGAAGCATCAGCAATGCAA
ACGGAAGAGACGTCTAGCGATCAAGCCTAGACGTCTTACACTACCGTGCGGCTGCATCATTTATCAGA
CTATAGATTGCAGAA
+
FFFFFFFFFFFFFFFFFF:FFFFFFFFFFFFFFFFFFF:FFFFFFFF,FFFFFFFFFFFFFFFFFFFF
FFFFFFFFFFFFFFFFFFFFFFFFFFFFFFFFFFFFFFFFFFFFFFFFFFFFFFFFFFFFFFFFFFFF
FFFFFFFFFFFFFFF
@A00721:190:HGJW7DSXY:4:1427:20971:31234 2:N:0:TCTGCAAG+GCTGCACT
CAGAGACAAATGCAGAATTCTTCTTCGCCGAATCCGCCTTCTATAGAAGCGAAGCATCAGCAATGCAA
ACGGAAGAGACGTCTAGCGATCAAGCCTAGACGTCTTACACTACCGTGCGGCTGCATCATTTATCAGA
CTATAGATTGCAGAA
+
FFFFFFFF:FFFFFFFFFFFFFFFFFFFFFFFFFFFFFFFFFFFFFFFFFFFFFFFFFFFFFFFFFFF
FFFFFFFFFFFFFFFFFFFFFFFFFFFFFF:F,FFFFFFFFFFFFFFFFFFFFFFF:FFFFFFFFFFF
FFFFFFFFF,FFFFF
@A00721:190:HGJW7DSXY:4:1509:8612:10895 2:N:0:TCTGCAAG+GCTGCACT
CAGAGACAAATGCAGAATTCTTCTTCGCCGAATCCGCCTTCTATAGAAGCGAAGCATCAGCAATGCAA
ACGGAAGAGACGTCTAGCGATCAAGCCTAGACGTCTTACACTACCGTGCGGCTGCATCATTTATCAGA
CTATAGATTGCAGAA
+
FFFFFFFFFFFFFFFFFFFFFFFFFFFFFFFFFFFFFFFFF:FFFFFFFFFFFFFFFFFFFFFFFFFF
FFFFFFFFFFFFFFFFFFFFFFFFFFFFFFFFFFFFFFFFFFFFFFFFFFFFFFFFFFFFFFFFFFFF
FFFFFFFFFFFFFFF
@A00721:190:HGJW7DSXY:4:1568:3712:31657 2:N:0:TCTGCAAG+GCTGCACT
CAGAGACAAATGCAGAATTCTTCTTCGCCGAATCCGCCTTCTATAGAAGCGAAGCATCAGCAATGCAA
ACGGAAGAGACGTCTAGCGATCAAGCCTAGACGTCTTACACTACCGTGCGGCTGCATCATTTATCAGA
CTATAGATTGCAGAA
+
FFFFFFFFFFFFF:FFFFFFFFFFFFFFFFFFFFFFFFFFFFFF:FFFFFFFFFFFFFFFFFFFFFFF
FFFFFFFFFFFFFFFFFFFFFFFFFFFFFFFFFFFFFFFFFFFFFFFFFFFFFFFFFFFFFFFFFFFF
FFFFFFFFFFFFF:F
@A00721:190:HGJW7DSXY:4:2260:6696:4570 2:N:0:TCTGCAAG+GCTGCACT
CAGAGACAAATGCAGAATTCTTCTTCGCCGAATCCGCCTTCTATAGAAGCGAAGCATCAGCAATGCAA

```
ACGGAAGAGACGTCTAGCGATCAAGCCTAGACGTCTTACACTACCGTGCGGCTGCATCATTTATCAGA
CTATAGATTGCAGAA
+
FFFFFFFFFFFFFFFFFFFFFFFFFFFFFFFFFFFFFFFF:FFFFFFFFFFFFFFFFFFFFFFFFFFFFF
FFFFFFFFFFFFFF:FFFFFFFFFFFFFFFFFFFFFFFFFFFFFFFFFFFFFFFFFFFFFFFFFFFFFFF
FFF:FFF:FFFFFFF
@A00721:190:HGJW7DSXY:4:2463:28221:18364 2:N:0:TCTGCAAG+GCTGCACT
CAGAGACAAATGCAGAATTCTTCTTCGCCGAATCCGCCTTCTATAGAAGCGAAGCATCAGCAATGCAA
ACGGAAGAGACGTCTAGCGATCAAGCCTAGACGTCTTACACTACCGTGCGGCTGCATCATTTATCAGA
CTATAGATTGCAGAA
+
FFFFFFFFFFFFFFFFFFFFFFFFFFFFFFFFFFFFFFFFFFFFFFFFFFFFFFFFFFFFFFFFFFFFFF
FFFFFFFFFFFFFFFFFFFFFFFFFFFFFFFFFFFFFFFFFFFFFFFFFFFFFFFFFFFFFFFFFFFFFF
FFFFFFFFFFFFFFF
@A00721:190:HGJW7DSXY:4:1114:28167:24972 2:N:0:TCTGCAAG+GCTGCACT
ATTTGGACAGAGACAAATGCAGAATTCTTCTTCGCCGAATCCGCCTTCTATAGAAGCGAAGCATCAGC
AATGCAAACGGAAGAGACGTCTAGCGATCAAGCCTAGACGTCTTACACTACCGTGCGGCTGCATCATT
TATCAGACTATAGAT
+
FFFFFFFFFFFFFFFFFFFFFFFFFFFFFFFFFFFFFFFFFFFFFFFF:F:FFFFFFFFFF:FFFFFFFF
FFFFFF:FFFFFFFFFFFFFFFFFFFFFFFFFFFFF:FFFFFFFFFFFFF:FFFFFFFFFFFF:FFFFFF
FFFFFFFFF:F:FFF
@A00721:190:HGJW7DSXY:4:1442:15754:23046 2:N:0:TCTGCAAG+GCTGCACT
ATTTGGACAGAGACAAATGCAGAATTCTTCTTCGCCGAATCCGCCTTCTATAGAAGCGAAGCATCAGC
AATGCAAACGGAAGAGACGTCTAGCGATCAAGCCTAGACGTCTTACACTACCGTGCGGCTGCATCATT
TATCAGACTATAGAT
+
FFFFFFFFFFFFFFFFFFFFFFFFFFFFFFFFFFFFFFFFFFFFFFFFFFFFFFFFFFFFFFFFFFFFFF
FFFFFFFFFFFFFFFFFFFFFFFFFFFFFFFFFFFFFFFFFFFFFFFFFFFFFFFFFFFFFFFFFFF:FF
FFFFFFFFFF:FFFF
@A00721:190:HGJW7DSXY:4:1507:17454:13714 2:N:0:TCTGCAAG+GCTGCACT
ATTTGGACAGAGACAAATGCAGAATTCTTCTTCGCCGAATCCGCCTTCTATAGAAGCGAAGCATCAGC
AATGCAAACGGAAGAGACGACTAGCGATCAAGCCTAGACGTCTTACACTACCGTGCGGCTGCATCATT
TATCAGACTATAGAT
+
F:FFF,FFFFFFFFFFF:FFFFFFFFFFFFFFFFFFFFFFF::,F,FFF::FFFFFF,:FFFFFF,FFF:
FFF:FFFF,F,:FFFFFF,,FF:FFFFFFFFFFFFFFF:F:FFFFFF:FFFFFFFFFFF,FF:FF,:FF,
:FFFFFFFFFFFFF:
@A00721:190:HGJW7DSXY:4:1540:4435:24424 2:N:0:TCTGCAAG+GCTGCACT
ATTTGGACAGAGACAAATGCAGAATTCTTCTTCGCCGAATCCGCCTTCTATAGAAGCGAAGCATCAGC
AATGCAAACGGAAGAGACGTCTAGCGATCAAGCCTAGACGTCTTACACTACCGTGCGGCTGCATCATT
TATCAGACTATAGAT
+
FFFFFFFFFFFFFFFF:FFFFFFFFFFFFFFFFFFFFFFFFFFFFFFFFFFFFFFFF:FFFFFFFFFFFF
FFFFFFFFFFFFFFFFFFFFFFFFFFFFFFFFFFFFFFFFFFFFFFFFFFFFFFFFFFFFFFFFFFFFFF
:FFFFFFFFFFFFFF
@A00721:190:HGJW7DSXY:4:2205:13521:26537 2:N:0:TCTGCAAG+GCTGCACT
ATTTGGACAGAGACAAATGCAGAATTCTTCTTCGCCGAATCCGCCTTCTATAGAAGCGAAGCATCAGC
AATGCAAACGGAAGAGACGTCTAGCGATCAAGCCTAGACGTCTTACACTACCGTGCGGCTGCATCATT
TATCAGACTATAGAT
+
FFFFFFFFFFFFFFFFFFFFFFFFFFFFFFFFFFFFFFFFFFFFFFFFFFFFFFFFFFFFFFFFFFFFFF
F:FFFFFFFFFFFFFFFFFFFFFFFFFFFFFFFFFFFFFFFFFFFFFFFFFFFFFFFFFFFFFFFFFFFF
FFFFFFFFFFFFFFF
```

```
@A00721:190:HGJW7DSXY:4:2628:24325:8609 2:N:0:TCTGCAAG+GCTGCACT
ATTTGGACAGAGACAAATGCAGAATTCTTCTTCGCCGAATCCGCCTTCTATAGAAGCGAAGCATCAGC
AATGCAAACGGAAGAGACGTCTAGCGATCAAGCCTAGACGTCTTACACTACCGTGCGGCTGCATCATT
TATCAGACTATAGAT
+
FFFFFFFFFFFFFFFFFFFFFFFFFFFFFFFFFFFFFFFFFFFFFFFFFFFFFFFF,FFFFFFFFFFFFF
FFFFFFFFFFFFFFFFFFFFFFFFFFFFFFFFFFFF,FFFFFFFFFFFFFFFFFFFFFFFFFFFFFFFFF
FFF:F:FFF:FFFFF
@A00721:190:HGJW7DSXY:4:2653:20437:15812 2:N:0:TCTGCAAG+GCTGCACT
ATTTGGACAGAGACAAATGCAGAATTCTTCTTCGCCGAATCCGCCTTCTATAGAAGCGAAGCATCAGC
AATGCAAACGGAAGAGACGTCTAGCGATCAAGCCTAGACGTCTTACACTACCGTGCGGCTGCATCATT
TATCAGACTATAGAT
+
F:FF:FFFFFFFFFFFFFFFFF,F,FFFFFFFFFFFFFFFFFFFFFFFFFFFFFFFFF:FFFFFF:FFFF
FFFFFFFFFFFFF:FFFF:FFFFFFFFFFFFF,F,FFF:FF,FF,FFFFFFFFFF,FFFFFFFFFFFFFF
:FFFFFFF:FFFFFF
@A00721:190:HGJW7DSXY:4:1167:4544:27492 2:N:0:TCTGCAAG+GCTGCACT
AAAGATTTGGACAGAGACAAATGCAGAATTCTTCTTCGCCGAATCCGCCTTCTATAGAAGCGAAGCAT
CAGCAATGCAAACGGAAGAGACGTCTAGCGATCAAGCCTAGACGTCTTACACTACCGTGCGGCTGCAT
CATTTATCAGACTAT
+
FFFFFFFFFFFFFFFFFFF:FFFFFFFFFFFFFFFFF:FFFFFFFFFFFFF:FFFFFFFFFFFFFFFFFF
FFFFFFF:FFFFFFFFFF,FFFFFFFFFFFFFFFFFFFFFFF:FFFFFFFFFFF,F,FFFFFFFFFFFFF
FFF:FFFFFFFFFFF
@A00721:190:HGJW7DSXY:4:1304:1814:31062 2:N:0:TCTGCAAG+GCTGCACT
AAAGATTTGGACAGAGACAAATGCAGAATTCTTCTTCGCCGAATCCGCCTTCTATAGAAGCGAAGCAT
CAGCAATGCAAACGGAAGAGACGTCTAGCGATCAAGCCTAGACGTCTTACACTACCGTGCGGCTGCAT
CATTTATCAGACTAT
+
FFFFFFFFFFFFFFFFFFFFFFFFFFFFFFFFFFFFFFFFFFFFFFFFFFFFFFFFFFFFFFFFFFFFFF
FFFFFF:FFFFFFFFFFFFFFFFFFFFF:FFFFFFFFFFFFFFFFFFFFFFFFFFFFFF:FFFFFFFFFF
FFF:FFFFFFFFFFF
@A00721:190:HGJW7DSXY:4:1342:10077:35383 2:N:0:TCTGCAAG+GCTGCACT
AAAGATTTGGACAGAGACAAATGCAGAATTCTTCTTCGCCGAATCCGCCTTCTATAGAAGCGAAGCAT
CAGCAATGCAAACGGAAGAGACGTCTAGCGATCAAGCCTAGACGTCTTACACTACCGTGCGGCTGCAT
CATTTATCAGACTAT
+
FFFFFFFFFFFFFFFFFFFFFFFFFFFF:FFFFFF:FFFFFFFFFFFFFFFFFFFFFFFFFFFFF,FFFF
FFFFFFFFFFFFFFFFFFFFFFFFFFF:FFFFFFFFFFFFFFFFFFFFFFFFFFFFFFFFFFFFFFFFFF
F:FFFFFFFFFFFFF
@A00721:190:HGJW7DSXY:4:1362:32479:26083 2:N:0:TCTGCAAG+GCTGCACT
AAAGATTTGGACAGAGACAAATGCAGAATTCTTCTTCGCCGAATCCGCCTTCTATAGAAGCGAAGCAT
CAGCAATGCAAACGGAAGAGACGTCTAGCGATCAAGCCTAGACGTCTTACACTACCGTGCGGCTGCAT
CATTTATCAGACTAT
+
FFFFFF:FFFFFFFFFFFFFFFFFFFFFFFFFFFFFFFFFFFFFFFFFFFFFFFFFFFFFFFF:FFFFF
FFFFFFFFFFFFFFFFFFFFFFFFFFFFFFF:FFFFFFFFFFFFFFFFFFFFFFFFFFFFFFFFFFFFFF
FFFFFFFFFFFFFFF
@A00721:190:HGJW7DSXY:4:1423:12246:30686 2:N:0:TCTGCAAG+GCTGCACT
AAAGATTTGGACAGAGACAAATGCAGAATTCTTCTTCGCCGAATCCGCCTTCTATAGAAGCGAAGCAT
CAGCAATGCAAACGGAAGAGACGTCTAGCGATCAAGCCTAGACGTCTTACACTACCGTGCGGCTGCAT
CATTTATCAGACTAT
+
FFFFFFFFFFFFFFFFFFFFFFFFFFFFFFFFFFFFFFFFFFFFFFFFFFFFFF:FFFFFFFFFFFFFFF
```

FFFFFFFFFFFFFFFFFFFFFFFFFFFFFFFFFFFFFFFFFFFFFFFFFFFFFFFFFFFFFFFFFFFF
FFFFFFFFFFFF:FF
@A00721:190:HGJW7DSXY:4:1471:28284:24612 2:N:0:TCTGCAAG+GCTGCACT
AAAGATTTGGACAGAGACAAATGCAGAATTCTTCTTCGCCGAATCCGCCTTCTATAGAAGCGAAGCAT
CAGCAATGCAAACGGAAGAGACGTCTAGCGATCAAGCCTAGACGTCTTACACTACCGTGCGGCTGCAT
CATTTATCAGACTAT
+
F:FFFFFFFFFFFFFFFFFFFFFFFFFFFFFFFFFFFFFFFFFFFFFFFFFF:FFFFFFFFFFFFFFF
FFFFFFFFFFFFFFFFFFFF:FFFFFFFFFFFFFFFFFFFFFFFFFFFFFFFFFFFFFFFFFFFFFF
FFFFFFFFFFFFFFF
@A00721:190:HGJW7DSXY:4:1471:28275:24659 2:N:0:TCTGCAAG+GCTGCACT
AAAGATTTGGACAGAGACAAATGCAGAATTCTTCTTCGCCGAATCCGCCTTCTATAGAAGCGAAGCAT
CAGCAATGCAAACGGAAGAGACGTCTAGCGATCAAGCCTAGACGTCTTACACTACCGTGCGGCTGCAT
CATTTATCAGACTAT
+
FFFFFFFFFFFFFFFFFFFFFFFFFFFFFFFFFFFFFFFFFF:FFFFFFFFFFFFFF:FFFFFFFFFF
FFFFFFFFFFFFFFFFFFFFFFFFFFFFFFFFFFFFFFFFFFFFFFFFFFFFFFFFFFFFFFFFFFFF
FFFFFFFFFFFFFFF
@A00721:190:HGJW7DSXY:4:1667:22426:28213 2:N:0:TCTGCAAG+GCTGCACT
AAAGATTTGGACAGAGACAAATGCAGAATTCTTCTTCGCCGAATCCGCCTTCTATAGAAGCGAAGCAT
CAGCAATGCAAACGGAAGAGACGTCTAGCGATCAAGCCTAGACGTCTTACACTACCGTGCGGCTGCAT
CATTTATCAGACTAT
+
FFFFFFFFFFFFFFFFFFFFFFFFFFFFFFFFFFFFFFFFFFFFFFFFFFFFFFFFFFFFFFFFFFFF
FFFFFFFFFFFFFFFFFFFFFFF:FFFFFFFFF:FFFFFF:FFF:FF,FFFFFFFF:FFFFFFFFFFF
FFFFFFFFFFFFF:F
@A00721:190:HGJW7DSXY:4:2361:16532:18035 2:N:0:TCTGCAAG+GCTGCACT
AAAGATTTGGACAGAGACAAATGCAGAATTCTTCTTCGCCGAATCCGCCTTCTATAGAAGCGAAGCAT
CAGCAATGCAAACGGAAGAGACGTCTAGCGATCAAGCCTAGACGTCTTACACTACCGTGCGGCTGCAT
CATTTATCAGACTAT
+
FFFFFFFFFFFF,FFFFFFF:FFFFFFFFFFFFFFFFFFFFFFFFFFFF::FFFFFFFFFFFFFFFFF
FFFFF:FFFFF:FFFFFFFFFFFFFFFFFFFFFFFFFFFFFFFFFFFFFFFFFFFFFFFF
FFFFFFFFFFFF:,F
@A00721:190:HGJW7DSXY:4:2515:7048:16892 2:N:0:TCTGCAAG+GCTGCACT
AAAGATTTGGACAGAGACAAATGCAGAATTCTTCTTCGCCGAATCCGCCTTCTATAGAAGCGAAGCAT
CAGCAATGCAAACGGAAGAGACGTCTAGCGATCAAGCCTAGACGTCTTACACTACCGTGCGGCTGCAT
CATTTATCAGACTAT
+
FFFFFFFFFFFFFFFFFFFFFFFF:FFF:FFFFFFFFFFFFFFFFFFFFF,FFFFFFFFFFFFFFFFF
FFFFFFFFFFFFFFFFFFFFFFFFFFFFFFFFFFFFFFFFF:FFFFFFFFFFFFFFFFFFFFFFFFFF
FFFFFFFFFFFFFFF
@A00721:190:HGJW7DSXY:4:2524:17002:4006 2:N:0:TCTGCAAG+GCTGCACT
AAAGATTTGGACAGAGACAAATGCAGAATTCTTCTTCGCCGAATCCGCCTTCTATAGAAGCGAAGCAT
CAGCAATGCAAACGGAAGAGACGTCTAGCGATCAAGCCTAGACGTCTTACACTACCGTGCGGCTGCAT
CATTTATCAGACTAT
+
FFFFFFFFFF:FFFFFFFFFFFFFFFFFFFFFFFFFFFFFFFF:FFFFFFFFFF:FFFFFFFFFFFFF
FFFFFFFFFFF:FFFF,FFFFFFFFF,FFF:FFFFFFFFFFFFFFFFF:FFFFFFFFFFFFFFFFFFF
FF,FFFFFFFFFFFF
@A00721:190:HGJW7DSXY:4:2553:23249:7185 2:N:0:TCTGCAAG+GCTGCACT
AAAGATTTGGACAGAGACAAATGCAGAATTCTTCTTCGCCGAATCCGCCTTCTATAGAAGCGAAGCAT
CAGCAATGCAAACGGAAGAGACGTCTAGCGATCAAGCCTAGACGTCTTACACTACCGTGCGGCTGCAT
CATTTATCAGACTAT

```
+
FFFFFFFFFFFFFFFFFFFFFFFFFFFFFFFFFFFFFFFFFFFFFFFFFFFFFFFFFFFFFFFFFFFF
FFFFFFFFFFFFFFFFFFFFFFFFFFFFFFFFFFFFFFFFFFFFFFFFFFFFFFFFFFFFFFFFFFFF
FFFFFFFFFFFFFFF
@A00721:190:HGJW7DSXY:4:2604:18855:28197 2:N:0:TCTGCAAG+GCTGCACT
AAAGATTTGGACAGAGACAAATGCAGAATTCTTCTTCGCCGAATCCGCCTTCTATAGAAGCGAAGCAT
CAGCAATGCAAACGGAAGAGACGTCTAGCGATCAAGCCTAGACGTCTTACACTACCGTGCGGCTGCAT
CATTTATCAGACTAT
+
FFFFF:FFFFFFFFFFFFFFFFFFFFFF,FFF:FFFFFFFFFFFFFFFFFFFFFFFFFFFFFFFFFFF
FFFFFFFFFFFFFFFFFFFFFFFFFFFF:FFFFFFF:FFFFFF:FFFFFFFFFFFFFFFFFFFFFFFFF
FF:FFF:FFFFFF:F
@A00721:190:HGJW7DSXY:4:2618:31910:27602 2:N:0:TCTGCAAG+GCTGCACT
AAAGATTTGGACAGAGACAAATGCAGAATTCTTCTTCGCCGAATCCGCCTTCTATAGAAGCGAAGCAT
CAGCAATGCAAACGGAAGAGACGTCTAGCGATCAAGCCTAGACGTCTTACACTACCGTGCGGCTGCAT
CATTTATCAGACTAT
+
FFFFFFFFFFFF:FFFFFFFFFFFFF:FFFFFFFFFFFFFFFFFFFFFFFFFFFFFFFFFFFFFFFFF
FFFF,FFFFFF:FFFFFFFFFFF:FFFFFFFFFFFFFFFFFFFFFFFFFFFFF,FFFFFFFFFFFFF:F
FF:FFFFFFFFFF,F
@A00721:190:HGJW7DSXY:4:2645:29866:1329 2:N:0:TCTGCAAG+GCTGCACT
AAAGATTTGGACAGAGACAAATGCAGAATTCTTCTTCGCCGAATCCGCCTTCTATAGAAGCGAAGCAT
CAGCAATGCAAACGGAAGAGACGTCTAGCGATCAAGCCTAGACGTCTTACACTACCGTGCGGCTGCAT
CATTTATCAGACTAT
+
FFFFFFFFFFFFFFFFFFFFFFFFFFFFFFFFFFFFFFFFFFFFFFFFFFFFFFF:FFFFFFFFFFF
FFFFFFFFFFFFF:FFFFFFFFFFFFFFFFFFFFFFFFFFFFFFFFFFFFFFFFFFFFFFFFFFFFFF
FFFFFFF:FFFFFF:
@A00721:190:HGJW7DSXY:4:1206:18728:14669 2:N:0:TCTGCAAG+GCTGCACT
TAAAGATTTGGACAGAGACAAATGCAGAATTCTTCTTCGCCGAATCCGCCTTCTATAGAAGCGAAGCA
TCAGCAATGCAAACGGAAGAGACGTCTAGCGATCAAGCCTAGACGTCTTACACTACCGTGCGGCTGCA
TCATTTATCAGACTA
+
FFFFFF:FFFFFFFFFFFFFFFFFFFFFFFFFFFFF:FFFFFFFFFFFFFFFFFFF::FFFFFFFFFF
FFFFFFFF,FFFFFFFFFFFFFFFFFFFFFFFFFFFFFFFFFFFF:FFFFFFFFFFFFFFF:FFFFFF
FFFFFFFFFFFFF:F
@A00721:190:HGJW7DSXY:4:2371:10863:35712 2:N:0:TCTGCAAG+GCTGCACT
TAAAGATTTGGACAGAGACAAATGCAGAATTCTTCTTCGCCGAATCCGCCTTCTATAGAAGCGAAGCA
TCAGCAATGCAAACGGAAGAGACGTCTAGCGATCAAGCCTAGACGTCTTACACTACCGTGCGGCTGCA
TCATTTATCAGACTA
+
FFFFFFFFFFFFFFFFFFFFFFFFFFFFFFFFFFFFFFFFFFFFFFFFFF:FFFFFFFFFFFFFFFFF
FFFFFFFFFFFFFFFFFFFFFFFFFFFFFFFFFFFFFFFFFFFFF:FFFFFFFFFFFFFFFFFFFFFFF
FFFFFFFFFFFFF:F
@A00721:190:HGJW7DSXY:4:2133:5593:12587 2:N:0:TCTGCAAG+GCTGCACT
TTCATCTTTAAAGATTTGGACAGAGACAAATGCAGAATTCTTCTTCGCCGAATCCGCCTTCTATAGAA
GCGAAGCATCAGCAATGCAAACGGAAGAGACGTCTAGCGATCAAGCCTAGACGTCTTACACTACCGTG
CGGCTGCATCATTTA
+
FFFFFFFFFFFFFFFFFFFFFFFFFFFFFFFFFFFFFFFFFFFFFFFFFFFFFFFFFFFFF:FFFFFF
FFFFFFFFFFFFFFFFFFFFFFFFFFFFFFFFFFFFFFFFF:FFFFFFFFFFFFFFFFFFFF:FFFFFF
FFFFFFFFFFFFFFF
@A00721:190:HGJW7DSXY:4:2141:25211:23265 2:N:0:TCTGCAAG+GCTGCACT
TTCATCTTTAAAGATTTGGACAGAGACAAATGCAGAATTCTTCTTCGCCGAATCCGCCTTCTATAGAA
```

```
GCGAAGCATCAGCAATGCAAACGGAAGAGACGTCTAGCGATCAAGCCTAGACGTCTTACACTACCGTG
CGGCTGCATCATTTA
+
FFFFFFFFFFFFFFFFFFFFFF:FFFFFFFFFFF:FFFFF:FFFF:FFF:FF:FFF,:FFFFFFFFFF
,FFFFF,FFFFFFFFFFFFFF,FFFFFFFFFFF:FF,,FFF,FFFFFFFFFFFFFFFFFF:FFFFFFF
FF:FFFFFFFFFFFF
@A00721:190:HGJW7DSXY:4:2153:11098:11600 2:N:0:TCTGCAAG+GCTGCACT
TTCATCTTTAAAGATTTGGACAGAGACAAATGCAGAATTCTTCTTCGCCGAATCCGCCTTCTATAGAA
GCGAAGCATCAGCAATGCAAACGGAAGAGACGTCTAGCGATCAAGCCTAGACGTCTTACACTACCGTG
CGGCTGCATCATTTA
+
FFFFFFFFFFFFFFFFFFFFFFFFFFFFFFFFFFFFFFFFFFFFFFFFFFFFFFFFFFFFFFFFFFFF
FFFFFFFFFFFFFFFFFFFFFFFFFFFFFFFFFFFFFFFFFFF:FFFFFFFFFFFFFFFFFFFFFFFF
FFFFFFFFFFFFFFF
@A00721:190:HGJW7DSXY:4:2255:6262:28056 2:N:0:TCTGCAAG+GCTGCACT
TTCATCTTTAAAGATTTGGACAGAGACAAATGCAGAATTCTTCTTCGCCGAATCCGCCTTCTATAGAA
GCGAAGCATCAGCAATGCAAACGGAAGAGACGTCTAGCGATCAAGCCTAGACGTCTTACACTACCGTG
CGGCTGCATCATTTA
+
FFFFFFFFFFFFFFFFFFFFFFFFFFFFFFFFFFFFFFFFFFFFFFFFFFFFFF:FFFFFFFFFFFFF
FFFFFFFFFFFFFFFFFFFFFFFFFFFFFFFFFFFFFFFFFFFFFFFFFFFFFFFFFFFFFFFFFFFF
FFFFFFFFFFFFFFF
@A00721:190:HGJW7DSXY:4:2319:6949:8484 2:N:0:TCTGCAAG+GCTGCACT
TTCATCTTTAAAGATTTGGACAGAGACAAATGCAGAATTCTTCTTCGCCGAATCCGCCTTCTATAGAA
GCGAAGCATCAGCAATGCAAACGGAAGAGACGTCTAGCGATCAAGCCTAGACGTCTTACACTACCGTG
CGGCTGCATCATTTA
+
F:FFFFFFFFFFFFF:FFFFFFFFFFFFFFFFFFFF:,FFFFF:FFFFFFFFFFFFFF,FFFFFFFFF
FFFFFFFFFFFFFFFFFFFFFFFFFFFFFFFFFFF,FFFFFFFFFFFFFFFFFFFFFFF,F:FFFFFF
FFFFFFF:FFF:F:F
@A00721:190:HGJW7DSXY:4:2334:19678:2284 2:N:0:TCTGCAAG+GCTGCACT
TTCATCTTTAAAGATTTGGACAGAGACAAATGCAGAATTCTTCTTCGCCGAATCCGCCTTCTATAGAA
GCGAAGCATCAGCAATGCAAACGGAAGAGACGTCTAGCGATCAAGCCTAGACGTCTTACACTACCGTG
CGGCTGCATCATTTA
+
FFFFFFFFFFF:FFFFFFFFFFFFFFFFFFFFFFFFF,FFFFFFFFFFFFFFFF:FFFFFFFFFFFFF
FFFFFFFFFFFFFFF:FFFFFFFFFFFFFFFFFFFFFFFFFFFFFFFFFFFFFFFFFFFFF:FFFFFF
FFFFFFFFFFFFFFF
@A00721:190:HGJW7DSXY:4:2430:26214:7216 2:N:0:TCTGCAAG+GCTGCACT
TTCATCTTTAAAGATTTGGACAGAGACAAATGCAGAATTCTTCTTCGCCGAATCCGCCTTCTATAGAA
GCGAAGCATCAGCAATGCAAACGGAAGAGACGTCTAGCGATCAAGCCTAGACGTCTTACACTACCGTG
CGGCTGCATCATTTA
+
FFFFFFFFFFFFFFFFFFFFFFFFFFFFFFFFFFFFFFFFFFFFFFFFFFFFFFFFFFFFFFFFF:FF
,FFFFFFFFFFFFFFFFFFFFFFFFFFFFFFFFFF:FFFFFFFFFFFFFFFFFFFFFFFFFFFFFFFF
FFFFFFFFFFF:FFF
@A00721:190:HGJW7DSXY:4:2551:27444:25034 2:N:0:TCTGCAAG+GCTGCACT
TTCATCTTTAAAGATTTGGACAGAGACAAATGCAGAATTCTTCTTCGCCGAATCCGCCTTCTATAGAA
GCGAAGCATCAGCAATGCAAACGGAAGAGACGTCTAGCGATCAAGCCTAGACGTCTTACACTACCGTG
CGGCTGCATCATTTA
+
FFFFFFFFFFFFFFFFFFFFFFFFFFFF:FFFFFFF,FFFFFFFFFFFFFFFFFFFFFFFFF:FFFF:
FFFF:FFFFFFFFFFFFFFFFFFFFFFFFFFFFFFFFFF,FFFFFFFFFFFF:FFFFFFFFFFFFFFF
FFFFFFFFFFFFFFF
```

```
@A00721:190:HGJW7DSXY:4:1224:27308:25520 2:N:0:TCTGCAAG+GCTGCACT
AATTCATCTTTAAAGATTTGGACAGAGACAAATGCAGAATTCTTCTTCGCCGAATCCGCCTTCTATAG
AAGCGAAGCATCAGCAATGCAAACGGAAGAGACGTCTAGCGATCAAGCCTAGACGTCTTACACTACCG
TGCGGCTGCATCATT
+
FFFFFFFFFFFFFFFFFFFFFFFFFFFFFFFFFFFFFFFFFFFFFFFFFFFFFFFFFFFFFFFFFFFF
FFFFFF:FFFFFFFFFFFFFFFFFFFFFFFFFFFFFFFFFFFFFFFFFFFFFFFFFFFFFFFFF:FFF
FF,FFFFFFFFFFFF
@A00721:190:HGJW7DSXY:4:2236:7003:2253 2:N:0:TCTGCAAG+GCTGCACT
AATTCATCTTTAAAGATTTGGACAGAGACAAATGCAGAATTCTTCTTCGCCGAATCCGCCTTCTATAG
AAGCGAAGCATCAGCAATGCAAACGGAAGAGACGTCTAGCGATCAAGCCTAGACGTCTTACACTACCG
TGCGGCTGCATCATT
+
FFFFFFFFFFFFFFFFFFFFFFFFFFFFFFFFFFFFFFFFFFFFFFFFFFFFFFFFFFFFFFFFFFFF
FF:FFFFFFFFFFFFFFFFFFFFFFFFFFFFFFFFFFFFFFFFFFFFFFFFFFFFFFFFFFFFFFFFF
FFFFFFFFFFFFFFF
@A00721:190:HGJW7DSXY:4:2411:29586:15749 2:N:0:TCTGCAAG+GCTGCACT
AATTCATCTTTAAAGATTTGGACAGAGACAAATGCAGAATTCTTCTTCGCCGAATCCGCCTTCTATAG
AAGCGAAGCATCAGCAATGCAAACGGAAGAGACGTCTAGCGATCAAGCCTAGACGTCTTACACTACCG
TGCGGCTGCATCATT
+
FFFFFFFFFFFFFFFFFFFFFFFFFFFFFFFFFFFFFFFFFFFFFFFFFFFFFFFFFFFFFFFFFFFF
FFFFFFFFFFFFFFFFFFFFF:FFFFFFFFFFFFFFFFFFFFFFFFFFFFFFFFFFFFFFFFFFFFFF
FFFFFFFFFFFFFF:
@A00721:190:HGJW7DSXY:4:1176:4887:28964 2:N:0:TCTGCAAG+GCTGCACT
CAATTCATCTTTAAAGATTTGGACAGAGACAAATGCAGAATTCTTCTTCGCCGAATCCGCCTTCTATA
GAAGCGAAGCATCAGCAATGCAAACGGAAGAGACGTCTAGCGATCAAGCCTAGACGTCTTACACTACC
GTGCGGCTGCATCAA
+
FFFFFFFFFFFFFFFFFFFFFFFFFFFFFFFFFFFFFFFFFFFFFFFFFFFFFF,FFFFFFFFFFFFF
FFFFFFFFFFFFFFFFFFFFFFFFFFFFFFFFFFFFFFFFFFFFFFFFFFFFF:FFFFFFFFFFF:FF
FFFFFFFFFFFFFFF
@A00721:190:HGJW7DSXY:4:1475:14136:3208 2:N:0:TCTGCAAG+GCTGCACT
CAATTCATCTTTAAAGATTTGGACAGAGACAAATGCAGAATTCTTCTTCGCCGAATCCGCCTTCTATA
GAAGCGAAGCATCAGCAATGCAAACGGAAGAGACGTCTAGCGATCAAGCCTAGACGTCTTACACTACC
GTGCGGCTGCATCAA
+
FFF:FFFFFFFFFFFFFFFFFFFFFFFFFFFFFFFFFFFFFFFFFFFFFFFFFFFFFFFFFFFFFFFF
FFFFFFFFFFFFFFFFFFFFFFFFFFFFFFFFFFFFFFFF:FFFFFFFFFFFFFFFFFFF:FFFFFFF
FFFFFFFFFFFFFF:
@A00721:190:HGJW7DSXY:4:1508:8088:2096 2:N:0:TCTGCAAG+GCTGCACT
CAATTCATCTTTAAAGATTTGGACAGAGACAAATGCAGAATTCTTCTTCGCCGAATCCGCCTTCTATA
GAAGCGAAGCATCAGCAATGCAAACGGAAGAGACGTCTAGCGATCAAGCCTAGACGTCTTACACTACC
GTGCGGCTGCATCAA
+
FFFFFFFFFFFFFFFFFFFFFFFFFFFFFFFFFFFFFFFFFFFFFFFFFFFFFFFFFFFFFFFFFFFF
FFFFFFFFFFFFFFFFFFFFFFFFF:FFF:FFFFFFFFFFFFFFFFFFFFFFFFFFFFFFFFFF:FFF
FFFFFFFFFFFFFFF
@A00721:190:HGJW7DSXY:4:2308:4716:22623 2:N:0:TCTGCAAG+GCTGCACT
CAATTCATCTTTAAAGATTTGGACAGAGACAAATGCAGAATTCTTCTTCGCCGAATCCGCCTTCTATA
GAAGCGAAGCATCAGCAATGCAAACGGAAGAGACGTCTAGCGATCAAGCCTAGACGTCTTACACTACC
GTGCGGCTGCATCAA
+
:FFFFFFFFFFFF:FFFF,FFFFFFFFFFF:FFFFFFFFFFFFFFFFFFFFFFFFFF:FFF,:F,,FF
```

```
FF::FFFF:FFFFFFFF,FFFF,FFFFF,FFFFFFFFFFFFFFFFFFFFFFFFFF:FFFFFFFFFFFF
FFFFFFFFFFFFFFF
@A00721:190:HGJW7DSXY:4:2477:23267:36808 2:N:0:TCTGCAAG+GCTGCACT
CAATTCATCTTTAAAGATTTGGACAGAGACAAATGCAGAATTCTTCTTCGCCGAATCCGCCTTCTATA
GAAGCGAAGCATCAGCAATGCAAACGGAAGAGACGTCTAGCGATCAATCCTAGACGTCTTACACAACC
GTGCGGCTGCATCAA
+
FFFFFFF:FFFFFF,FFFFFFFFFFFFFFFFFFFFFF,FFFFFFFFF:FF::FF:FF,FFFFFFFFFF
,FF,FFF:,FFFFFFFFF::FFFFFF:FFFFFF:FFFF,FFFFFFFF,FFFFFF,,FFFFF:FF,FFF
F,FFFFFF:FFFFFF
@A00721:190:HGJW7DSXY:4:1127:29206:13683 2:N:0:TCTGCAAG+GCTGCACT
AGGATGCCAATTCATCTTTAAAGATTTGGACAGAGACAAATGCAGAATTCTTCTTCGCCGAATCCGCC
TTCTATAGAAGCGAAGCATCAGCAATGCAAACGGAAGAGACGTCTAGCGATCAAGCCTAGACGTCTTA
CACTACCGTGCGGCT
+
FFFFFFFFFFFFFFFFFFFFFFFFFFFFFFFFFFFFFFFFFFFFFFFFFFFFFFFFFFFFFFFFFFFF
FFFFFFFFFFFFFFFFFFFFFFFFFFFFFFF:FFFFFFFFFFFFFFFFFFFFF:FFFFF:FFFFFFF
FFFFFFFFFFFFFFF
@A00721:190:HGJW7DSXY:4:1127:29261:13777 2:N:0:TCTGCAAG+GCTGCACT
AGGATGCCAATTCATCTTTAAAGATTTGGACAGAGACAAATGCAGAATTCTTCTTCGCCGAATCCGCC
TTCTATAGAAGCGAAGCATCAGCAATGCAAACGGAAGAGACGTCTAGCGATCAAGCCTAGACGTCTTA
CACTACCGTGCGGCT
+
FFFFFFFFFFFFFFF:FFFFF:FFFFFFFFFFFFFFFFF:FFFFFFF,FFFFFFFFFFFFFFFFFFFF
FFFFFFFFFFFFFFFFFFFFFFFFFFFFFF:FFFFFFFF:FFF:F,FFFFFFFFFFF:F:FFFF:FF
FFFFFFFFFFFFFFF
@A00721:190:HGJW7DSXY:4:1417:19343:31579 2:N:0:TCTGCAAG+GCTGCACT
AGGATGCCAATTCATCTTTAAAGATTTGGACAGAGACAAATGCAGAATTCTTCTTCGCCGAATCCGCC
TTCTATAGAAGCGAAGCATCAGCAATGCAAACGGAAGAGACGTCTAGCGATCAAGCCTAGACGTCTTA
AACTACCGTGCGGCT
+
FFFFFFFFFFF:F:FFFFFFFFFFFFFFFFFFFFFFFFFFFF:FFFF,FFFFFFFFF:F:FFFFFFF,F
FFFFF:FFFF,FFFFFFFFFFFFFFF:FFFFF,FFFFFFF:FFF:F:FFFFFFFF:FFFFFFFFFFFF
,FFFFF:FF,FFFFF
@A00721:190:HGJW7DSXY:4:1563:18394:19445 2:N:0:TCTGCAAG+GCTGCACT
AGGATGCCAATTCATCTTTAAAGATTTGGACAGAGACAAATGCAGAATTCTTCTTCGCCGAATCCGCC
TTCTATAGAAGCGAAGCATCAGCAATGCAAACGGAAGAGACGTCTAGCGATCAAGCCTAGACGTCTTA
CACTACCGTGCGGCT
+
FFFFFFFFFFFFFFFFFFFFFFFFFFFFFFFFFFFFFFFFFFFFFFFFFFFFFFFFFFFFFFFFFFFF
FFFFFFFFFFFFFFFFFFFF:FFFFFFFFFFFFFFFFFFFFFFFFFFFFFFFFFFFFFFFFFFFFFFF
FFFFFFFFFFFFFFF
@A00721:190:HGJW7DSXY:4:1563:18566:19492 2:N:0:TCTGCAAG+GCTGCACT
AGGATGCCAATTCATCTTTAAAGATTTGGACAGAGACAAATGCAGAATTCTTCTTCGCCGAATCCGCC
TTCTATAGAAGCGAAGCATCAGCAATGCAAACGGAAGAGACGTCTAGCGATCAAGCCTAGACGTCTTA
CACTACCGTGCGGCT
+
FFFFFFFFFFFFFFFFFFFFFFFFFFFFFFFFFFFFFFFFFFFFFFFFFFFFFFFFFFFFFFFFFFFF
FFFFFFFFFFFFFFFFFFFFFFFFFFFFFF:FFFFFFFFFF:FFFFFFFFFFFFFFFFFFFFFFFFFF
FFFFFFFF,FFFFFF
@A00721:190:HGJW7DSXY:4:1563:18204:19617 2:N:0:TCTGCAAG+GCTGCACT
AGGATGCCAATTCATCTTTAAAGATTTGGACAGAGACAAATGCAGAATTCTTCTTCGCCGAATCCGCC
TTCTATAGAAGCGAAGCATCAGCAATGCAAACGGAAGAGACGTCTAGCGATCAAGCCTAGACGTCTTA
CACTACCGTGCGGCT
```

+
FFFFFFFFFFFFFFFFFFFFFFFFFFFFFFFFFFFFFFFFFFFFFFFFFFFFFFFFFFFFFFFFFFFFFF
F:FFFFFF:FFFFFFFFFFFFFFFFFFFFFFFFFFFFFFFFFFFFFFFFFFFFFFFFFFFFFFFFFFFFF
FFFFFFFFFFFFFFF
@A00721:190:HGJW7DSXY:4:2118:1136:30702 2:N:0:TCTGCAAG+GCTGCACT
AGGATGCCAATTCATCTTTAAAGATTTGGACAGAGACAAATGCAGAATTCTTCTTCGCCGAATCCGCC
TTCTATAGAAGCGAAGCATCAGCAATGCAAACGGAAGAGACGTCTAGCGATCAAGCCTAGACGTCTTA
CACTACCGTGCGGCT
+
FFFFFF:FFFFFFFFFFFFFFFFFFFFFFFFFFFFFFFFFFFFFFFFFFFFFFFFFFFFFFFFFFFFFFF
F,FFFFFFFF::FFFFFFFFFFFFFFFFFFFFFFF::F:FFF:FFFFFFFFFFFFFFFFFFFFFFFFFFF
FFFFFF,FFFFFFFF
@A00721:190:HGJW7DSXY:4:2128:1768:27727 2:N:0:TCTGCAAG+GCTGCACT
AGGATGCCAATTCATCTTTAAAGATTTGGACAGAGACAAATGCAGAATTCTTCTTCGCCGAATCCGCC
TTCTATAGAAGCGAAGCATCAGCAATGCAAACGGAAGAGACGTCTAGCGATCAAGCCTAGACGTCTTA
CACTACCGTGCGGCT
+
FFFF:FFFFFFFFFFFFFFFFF,FFFFFFFFFFF:FFFFFF:F,FFFFFFFFFFFFFFFFFFFFFFFFFF
FFFFF:FFF::FFFFF:FFFFF,FFFFFFFFF:FFF:FF,FFFFFFFFFFFFFF,FFFFFFFFFFFFFFF
FFFFFFFF,F,FFF:
@A00721:190:HGJW7DSXY:4:2128:1877:28510 2:N:0:TCTGCAAG+GCTGCACT
AGGATGCCAATTCATCTTTAAAGATTTGGACAGAGACAAATGCAGAATTCTTCTTCGCCGAATCCGCC
TTCTATAGAAGCGAAGCATCAGCAATGCAAACGGAAGAGACGTCTAGCGATCAAGCCTAGACGTCTTA
CACTACCGTGCGGCT
+
FFFFFFFFFFFFFFFFFFFFFFFFFFFFFFFFFFFFFFFFFFFF:FFFFFFFFFFFFFFFFFFFFFFFFF
FFFFFFFFFFFFFFFFFFFFFFFFFFFFFFFFFFFFFFFFFFFFFFFFFFFFFFFFFFFFFFFFFFFFFF
FFFF::FFFFFFFFF
@A00721:190:HGJW7DSXY:4:2142:19226:2691 2:N:0:TCTGCAAG+GCTGCACT
AGGATGCCAATTCATCTTTAAAGATTTGGACAGAGACAAATGCAGAATTCTTCTTCGCCGAATCCGCC
TTCTATAGAAGCGAAGCATCAGCAATGCAAACGGAAGAGACGTCTAGCGATCAAGCCTAGACGTCTTA
CACTACCGTGCGGCT
+
FFFFFFFFFFFFFFFFFFFFFFFFFFFFFFFFFFFFFFFFFFFFFFFFFFFFFFFFFFFFFFFFFFFFFF
FFFFFFFFFFFFFFFFFFFFFFFFFFFFFFFFFFFFFFFFFFFFFFFFFFFFFFFFFFFFF:FFFFFFFF
FFF:FFFFFF,FFFF
@A00721:190:HGJW7DSXY:4:2221:14371:34961 2:N:0:TCTGCAAG+GCTGCACT
AGGATGCCAATTCATCTTTAAAGATTTGGACAGAGACAAATGCAGAATTCTTCTTCGCCGAATCCGCC
TTCTATAGAAGCGAAGCATCAGCAATGCAAACGGAAGAGACGTCTAGCGATCAAGCCTAGACGTCTTA
CACTACCGTGCGGCT
+
FFFFFFFFFFFFFFFFFFFFFFFFFFFFFFFFFFFFFFFFFFFFFFFFFFFFFFFFFFFFFFFFFFFF:F
FFFFFFFFFFFFFFFFFFFFFFFFFFFFFFFFFFFFFFF:FFFFFFFFFFFFFFFFFFFFFFFFFFFFFF
FFFFFFFF:FFFFFF
@A00721:190:HGJW7DSXY:4:2267:22544:4961 2:N:0:TCTGCAAG+GCTGCACT
AGGATGCCAATTCATCTTTAAAGATTTGGACAGAGACAAATGCAGAATTCTTCTTCGCCGAATCCGCC
TTCTATAGAAGCGAAGCATCAGCAATGCAAACGGAAGAGACGTCTAGCGATCAAGCCTAGACGTCTTA
CACTACCGTGCGGCT
+
FFFFFFFFFFFFFFFFFFFFFFFFFFFFFFFFFFFFFFFFFFFFFFFFFFFFFFFFFFFFFFFFFFFFFF
FFFFFFFFFF,FFFFFFFFFFFFFFFFFFF:FFFFFFFFFFFFFFF,FFFFFFFFFFFFFFFFFFFFFFF
FFFFFFFFFFFFFFF
@A00721:190:HGJW7DSXY:4:2512:31629:6355 2:N:0:TCTGCAAG+GCTGCACT
AGGATGCCAATTCATCTTTAAAGATTTGGACAGAGACAAATGCAGAATTCTTCTTCGCCGAATCCGCC

```
TTCTATAGAAGCGAAGCATCAGCAATGCAAACGGAAGAGACGTCTAGCGATCAAGCCTAGACGTCTTA
CACTACCGTGCGGCT
+
FFFFFFF:FFFFFFFFFFFFFFFFFFFFFF:FFFFFFF:FFFFFFFFFFFFFFFFFFFFFFFFFFFFFFF
F:FFFFFF:FFFFFFFFFFFFFFFFFFFF::FFFFFFFFFFFFFFFFFFFFFFFFFFFFFFFFFFFFFFF
FFFFF:FF,FFFFFF
@A00721:190:HGJW7DSXY:4:1623:22978:9408 2:N:0:TCTGCAAG+GCTGCACT
ACAAGGATGCCAATTCATCTTTAAAGATTTGGACAGAGACAAATGCAGAATTCTTCTTCGCCGAATCC
GCCTTCTATAGAAGCGAAGCATCAGCAATGCAAACGGAAGAGACGTCTAGCGATCAAGCCTAGACGTC
TTACACTACCGTGCG
+
FFFFFFFFFFFFFFFFFFFFFFFFFFFFFFFFFFFFFFFFFFFF:FFFFFFFFFFFFFFFFFFFFFFFFF
FFFF:FFFFFFFFFFFFFFFFFFFFFFFFFFFFFFFFFFFFFFFFFFFF:FFFFFFFFFFFFFF:FFFFF
FFFFFFFFFFFFFFF
@A00721:190:HGJW7DSXY:4:2116:20491:14810 2:N:0:TCTGCAAG+GCTGCACT
ACAAGGATGCCAATTCATCTTTAAAGATTTGGACAGAGACAAATGCAGAATTCTTCTTCGCCGAATCC
GCCTTCTATAGAAGCGAAGCATCAGCAATGCAAACGGAAGAGACGTCTAGCGATCAAGCCTAGACGTC
TTACACTACCGTGCG
+
FFFFFFFFFFFFFFFFFFFFFFFF:FFFFFFFF:FFFFFFFFFFFFFFFFFFFFFFFFFFFFFFFFFFFF
FFFFFFFFFFFFFFFFFFFFFFFFFFF:FFFF:FFFFFFFFFFFFFFFFFFFFFFFFFFFFFFFFFFFFF
FFFFFFFFFFFFFFF
@A00721:190:HGJW7DSXY:4:2145:11758:23484 2:N:0:TCTGCAAG+GCTGCACT
ACAAGGATGCCAATTCATCTTTAAAGATTTGGACAGAGACAAATGCAGAATTCTTCTTCGCCGAATCC
GCCTTCTATAGAAGCGAAGCATCAGCAATGCAAACGGAAGAGACGTCTAGCGATCAAGCCTAGACGTC
TTACACTACCGTGCG
+
FFFFFFFFFFFFFFFFFFFFFFFFFFFFFFFFFFFFFFFFFFFFFFFFFFFFFFFFFFFFFFFFFFFFFF
FFFFFFFFFFFFFFFFFFFFFFFFFF:FFFFFFFFFFFFFFFFFFFFFFFFFFFFFFFFFFFFFFFFFFF
FFFFFFFFFFFFFFF
@A00721:190:HGJW7DSXY:4:2145:12048:23860 2:N:0:TCTGCAAG+GCTGCACT
ACAAGGATGCCAATTCATCTTTAAAGATTTGGACAGAGACAAATGCAGAATTCTTCTTCGCCGAATCC
GCCTTCTATAGAAGCGAAGCATCAGCAATGCAAACGGAAGAGACGTCTAGCGATCAAGCCTAGACGTC
TTACACTACCGTGCG
+
FFFFFFFFFFF:FFFFFFFFFFFF:FFFFFFFFFFF:FFFFFFFFFFFFFFFFFFFFFFFFF:FFFFFFF
FFFFFFF:FFFFFF:FFFFFFFFFFFFFF:FFFFFFFFFFFFFFFFFFFFFFFFFFFFFFFFFFFFFFFF
FFFFFFFF,FF:F,F
@A00721:190:HGJW7DSXY:4:2562:29351:14935 2:N:0:TCTGCAAG+GCTGCACT
ACAAGGATGCCAATTCATCTTTAAAGATTTGGACAGAGACAAATGCAGAATTCTTCTTCGCCGAATCC
GCCTTCTATAGAAGCGAAGCATCAGCAATGCAAACGGAAGAGACGTCTAGCGATCAAGCCTAGACGTC
TTACACTACCGTGCG
+
FFFFFFFFFFFFFFFFFFFFFFFFFFFFFFFFFFFFFFFFFFFFFFFFFFFFFFFFFFFFFFFFFFFFFF
FFFFFFFFFFFFFFFFFF:FFFFFFFFFFFFFFFFFFFFFFFFFFFFFFFFFFFFFFFFFFFFFFFFFFF
FFFFFFFFFFFFFFF
@A00721:190:HGJW7DSXY:4:2576:30020:30592 2:N:0:TCTGCAAG+GCTGCACT
ACAAGGATGCCAATTCATCTTTAAAGATTTGGACAGAGACAAATGCAGAATTCTTCTTCGCCGAATCC
GCCTTCTATAGACGCGAAGCATCAGCAATGCAAACGGAAGAGACGTCTAGCGATCAAGCCTAGACGTC
TTACACTACCGTGCG
+
FFFFFF:FFFFFFFFFFFFFFFF:FFFFFFFFFFFFFFFF,FFFF:FFFFFFF:FF:FFFFFFF:FFFFF
FFFF,FF,FFFF,FF:FFFFFFFFFFF,FFFFFFFFFFFFFFFFFFF:FFF::FFFF:FFFFF:FFFFFF
FFFFFFFFFFF,FF:
```

```
@A00721:190:HGJW7DSXY:4:1177:17427:15483 2:N:0:TCTGCAAG+GCTGCACT
GGACAAGGATGCCAATTCATCTTTAAAGATTTGGACAGAGACAAATGCAGAATTCTTCTTCGCCGAAT
CCGCCTTCTATAGAAGCGAAGCATCAGCAATGCAAACGGAAGAGACGTCTAGCGATCAAGCCTAGACG
TCTTACACTACCGTG
+
FFFFFFFFFFFFFFF:FFFFFFFFFFFF:FFFFFFFFFFFFFFFFFFF:FFFF:FFFFFFFFFFFFFFFF
FFFFFFFFFFFFFFFFFFFFFFFFFFFFFFFFFFFFFFFFFFFFFFFFFFFFFFFFFFFFFFFFFFFFFF
FFFFFFFFFFFFFFF
@A00721:190:HGJW7DSXY:4:1333:6795:9627 2:N:0:TCTGCAAG+GCTGCACT
GGACAAGGATGCCAATTCATCTTTAAAGATTTGGACAGAGACAAATGCAGAATTCTTCTTCGCCGAAT
CCGCCTTCTATAGAAGCGAAGCATCAGCAATGCAAACGGAAGAGACGTCTAGCGATCAAGCCTAGACG
TCTTACACTACCGTG
+
FFFFFFF:FFFFF:FFFFFFFFFFFFFFFFFFFFFFFFFFFFFFFFFFFFFFFFFFFFFFFFFFF:FFFF
FFFFFFFFFFFFFFFFFFFFFFFF:FFFF:FFFFF,FFFFFFFFFFF:FFFFFFFFFFFF,FFFFFFFF,
FFFFFFFFF:FFFFF
@A00721:190:HGJW7DSXY:4:2333:5565:24784 2:N:0:TCTGCAAG+GCTGCACT
GGACAAGGATGCCAATTCATCTTTAAAGATTTGGACAGAGACAAATGCAGAATTCTTCTTCGCCGAAT
CCGCCTTCTATAGAAGCGAAGCATCAGCAATGCAAACGGAAGAGACGTCTAGCGATCAAGCCTAGACG
TCTTACACTACCGTG
+
FFFFFFFFFFFFFFFFFFFFFFFFFFFFFFFFFFFFFFFFFFFFFFFFFFFFFFFFFFFFFFFFFFFFFF
FFFFFFFFFFFFFFFFFFFFFFFFF:FFFFFFFFFFFFFFFFFFFFFFFFFFFFFFFFFFFFFF:FFFFF
FFFFFFFFFFFFFFF
@A00721:190:HGJW7DSXY:4:2445:26395:15107 2:N:0:TCTGCAAG+GCTGCACT
GGACAAGGATGCCAATTCATCTTTAAAGATTTGGACAGAGACAAATGCAGAATTCTTCTTCGCCGAAT
CCGCCTTCTATAGAAGCGAAGCATCAGCAATGCAAACGGAAGAGACGTCTAGCGATCAAGCCTAGACG
TCTTACACTACCGTG
+
:FFF,F:FFFFFF:FFFF::FFFFFFFFFFFFFFFFFFFFFFFFFFFFFFFFFFFFFFFFFFFFFFFFFF
FFFFFFFFFFFF:FFFFFFF,FFF:FFFFFF,F,FFFFFF:FFFFFFFFF,:FFF,F:FFFFFFFFFF:F
FFFFFFFFFFFFFFF
@A00721:190:HGJW7DSXY:4:2526:31448:29340 2:N:0:TCTGCAAG+GCTGCACT
GGACAAGGATGCCAATTCATCTTTAAAGATTTGGACAGAGACAAATGCAGAATTCTTCTTCGCCGAAT
CCGCCTTCTATAGAAGCGAAGCATCAGCAATGCAAACGGAAGAGACGTCTAGCGATCAAGCCTAGACG
TCTTACACTACCGTG
+
FFFF:FFFFFFFFFFFFFFFFFFFFFFFFFFFFFFFFFFFFFFF,FFFFFFFFFFFFFFFFFFFFFFFFF
FFFFFFFFFFFFFFFFFFFFFFFFFFFFFFFFFFFF,FFFFFFFFFFFFFFFFFFFFF:FFFFFFFFFFF
:FFFFFFFFFFFFF:
@A00721:190:HGJW7DSXY:4:1105:9932:12806 2:N:0:TCTGCAAG+GCTGCACT
ATAAGACGTTCCTGGACAAGGATGCCAATTCATCTTTAAAGATTTGGACAGAGACAAATGCAGAATTC
TTCTTCGCCGAATCCGCCTTCTATAGAAGCGAAGCATCAGCAATGCAAACGGAAGAGACGTCTAGCGA
TCAAGCCTAGACGTC
+
FFFFFFFFFFFFFFFFFFFFFFF:FFFF,FF::FFFFFF:FFFFFFFFFFFFFFFFFFFFFFFFFFF:FF
FFFFFFFFFFFFFFFFFF:FFFFF:FF,FF:FFFFFFFFFFFFFFFFFFFFFFFFFFFFFF:FFFFFFFF
FFFFFFFFF:FFFFF
@A00721:190:HGJW7DSXY:4:1130:32606:30718 2:N:0:TCTGCAAG+GCTGCACT
ATAAGACGTTCCTGGACAAGGATGCCAATTCATCTTTAAAGATTTGGACAGAGACAAATGCAGAATTC
TTCTTCGCCGAATCCGCCTTCTATAGAAGCGAAGCATCAGCAATGCAAACGGAAGAGACGTCTAGCGA
TCAAGCCTAGACGTC
+
FF:FFFFFFFFFFFFF:FFFFFFFFFFFFFFFFFFFFFF:F,FFFFFFFFFFFFFFFFF:,,FF:FFFFF
```

FFFFFFFFFFFFFFFFFFFFF,:,FFFFFFFFFFFFFFFFFF:FFFFFFFFFF,FFFFFFF:F,,F:F
FFFF:FFFFFFFFFF
@A00721:190:HGJW7DSXY:4:1173:4978:27555 2:N:0:TCTGCAAG+GCTGCACT
ATAAGACGTTCCTGGACAAGGATGCCAATTCATCTTTAAAGATTTGGACAGAGACAAATGCAGAATTC
TTCTTCGCCGAATCCGCCTTCTATAGAAGCGAAGCATCAGCAATGCAAACGGAAGAGACGTCTAGCGA
TCAAGCCTAGACGTC
+
FFFFFFFFFFFFFFFFFF:FFFFFFFFFFFFFFFFFFFFFFFFFFFFFFFFFFFFFFFFFFFFFFFFF
FFFFFFFFFFFFFFFFFF:FFFFF:FFFFFFFFFFFFFFFFFFFFFFFFFFFFFFFFFFFFFFFFFF:
FFFFFFFFFFFFFFF
@A00721:190:HGJW7DSXY:4:1240:11243:33238 2:N:0:TCTGCAAG+GCTGCACT
ATAAGACGTTCCTGGACAAGGATGCCAATTCATCTTTAAAGATTTGGACAGAGACAAATGCAGAATTC
TTCTTCGCCGAATCCGCCTTCTATAGAAGCGAAGCATCAGCAATGCAAACGGAAGAGACGTCTAGCGA
TCAAGCCTAGACGTC
+
FFFFFFFFFFFFFFFFFFFFFFFFFFFFFFFFFF::FFFFFFFFFFFFFFFFFFF,F,FFFFFFFFFF
FFFFFFFFFFFFFFFFFFFFFFFFFFFFFFFFFFFFFFFFFFFFFFFFFFFFFFFFFFFFFFFFFF:F
FFF:FFFFF:FFFFF
@A00721:190:HGJW7DSXY:4:1539:24560:12806 2:N:0:TCTGCAAG+GCTGCACT
ATAAGACGTTCCTGGACAAGGATGCCAATTCATCTTTAAAGATTTGGACAGAGACAAATGCAGAATTC
TTCTTCGCCGAATCCGCCTTCTATAGAAGCGAAGCATCAGCAATGCAAACGGAAGAGACGTCTAGCGA
TCAAGCCTAGACGTC
+
FFFFFFFFFFFFFFFFFFFFFFFFFFFFFFFFFFFFFFFFFFFFFFFFFFFFFFFFFFFFFFFFFFFF
FFFFFFFFFFFFFFFFFFFFFFFFFFFFFFFFFFFFFFFFFFFFFFFFFFFFFFFFFFFFFFFFFFFF
FFFFFFFFFFFFFFF
@A00721:190:HGJW7DSXY:4:1547:9118:18630 2:N:0:TCTGCAAG+GCTGCACT
ATAAGACGTTCCTGGACAAGGATGCCAATTCATCTTTAAAGATTTGGACAGAGACAAATGCAGAATTC
TTCTTCGCCGAATCCGCCTTCTATAGAAGCGAAGCATCAGCAATGCAAACGGAAGAGACGTCTAGCGA
TCAAGCCTAGACGTC
+
FFFFFFFFFFFFFFFFFFFFFFFFFFFF:FFFFFFFFFFFFFFFFFFFFFFFFFFFFFFFFFFFFFFF
FFFFFFFFFFFFFFFFFFFFFFFFFFFFFFFFFFFFFFFFFFFFFFFFFFFFFFFFFFFFFFF:FF:F
FFFF:FFFFFFFFFF
@A00721:190:HGJW7DSXY:4:1637:3622:23547 2:N:0:TCTGCAAG+GCTGCACT
ATAAGACGTTCCTGGACAAGGATGCCAATTCATCTTTAAAGATTTGGACAGAGACAAATGCAGAATTC
TTCTTCGCCGAATCCGCCTTCTATAGAAGCGAAGCATCAGCAATGCAAACGGAAGAGACGTCTAGCGA
TCAAGCCTAGACGTC
+
FFFFFFFFFFFFFFFFF:FFFFFFFFF:FFFFFFFFFFFFFFFFFFFFFFFFFFFFFFFFFFFFFFFF
,FFFFFFFFFFFFFFFFFFFFFFFFFFFFFFFFFFFFFFFFFFFFFFFFFFFFFFFFFFFFFFF:FFF
FFFFFFFFFFFFFFF
@A00721:190:HGJW7DSXY:4:2149:16604:2190 2:N:0:TCTGCAAG+GCTGCACT
ATAAGACGTTCCTGGACAAGGATGCCAATTCATCTTTAAAGATTTGGACAGAGACAAATGCAGAATTC
TTCTTCGCCGAATCCGCCTTCTATAGAAGCGAAGCATCAGCAATGCAAACGGAAGAGACGTCTAGCGA
TCAAGCCTAGACGTC
+
FFFFFFFFFFFFFFFFFFFFFFFFFF,FFFFFFFFFFFFFFF:FFFFFFFFFFFFFFFFFFFFFFFFF
FFFFFFFFFFFFFFFFFFFFFFFFFFFFFFFFFFFFFFFFFFFFFFFFFFFFFFFFFFFFFFFFFFFF
FFFFFFFFFFFFFFF
@A00721:190:HGJW7DSXY:4:2158:16369:30968 2:N:0:TCTGCAAG+GCTGCACT
ATAAGACGTTCCTGGACAAGGATGCCAATTCATCTTTAAAGATTTGGACAGAGACAAATGCAGAATTC
TTCTTCGCCGAATCCGCCTTCTATAGAAGCGAAGCATCAGCAATGCAAACGGAAGAGACGTCTAGCGA
TCAAGCCTAGACGTC

```
+
FFFFFFFFFFFFFFFFFFFFFFFFFFFFFFFFFFFF:FFFFFFFFFFFFFFFFFFFFFFFFFFFFFFFFF
FFFFFFFFFFFFFFFFFFFFFFFFFFFFFFFFFFFFFFFFFFFFFFFFFFFFFFFFFFFFFFFFF:FFFF
FFFFFFFFFFFFFFF
@A00721:190:HGJW7DSXY:4:2253:4878:14982 2:N:0:TCTGCAAG+GCTGCACT
ATAAGACGTTCCTGGACAAGGATGCCAATTCATCTTTAAAGATTTGGACAGAGACAAATGCAGAATTC
TTCTTCGCCGAATCCGCCTTCTATAGAAGCGAAGCATCAGCAATGCAAACGGAAGAGACGTCTAGCGA
TCAAGCCTAGACGTC
+
FFFFFFFFFFFFFFFFF:FFFFFFFFF:,FFFFFFFFFFFFFFFF:FFFFFFFFFFFFFFFFFFFFFFFF
FFFFFF:FFFFFFFFFFFFFFFFF:FFFFFFFFFFFFFFFFFFFFFFFFFFFFFFFFFFFFFFFFFFFFF
FFF:FFFFFFFFFFF
@A00721:190:HGJW7DSXY:4:2317:25030:9799 2:N:0:TCTGCAAG+GCTGCACT
ATAAGACGTTCCTGGACAAGGATGCCAATTCATCTTTAAAGATTTGGACAGAGACAAATGCAGAATTC
TTCTTCGCCGAATCCGCCTTCTATAGAAGCGAAGCATCAGCAATGCAAACGGAAGAGACGTCTAGCGA
TCAAGCCTAGACGTC
+
FFFFFFFFFFFFFFFFFFFFFFFFFFFFFFFFFFFFFFFFFFFFFFFFFFFFFFFFFFFFFFFFFFFFFF
FFFFFFFFFFFFFFFFFFFFFF,FF:FFFFFFFFFFFFFFFFFFFFFFFFFFFFFFFFFFFFFFFFFFFF
FFFFFFFFFFFFFFF
@A00721:190:HGJW7DSXY:4:2405:10764:16595 2:N:0:TCTGCAAG+GCTGCACT
ATAAGACGTTCCTGGACAAGGATGCCAATTCATCTTTAAAGATTTGGACAGAGACAAATGCAGAATTC
TTCTTCGCCGAATCCGCCTTCTATAGAAGCGAAGCATCAGCAATGCAAACGGAAGAGACGTCTAGCGA
TCAAGCCTAGACGTC
+
FFFFFFFFFFFFFFFFFF:FFFFFFFFFFFFFFFFFFFFFFFF:FFFFFFFFFFFFFFF,FFFFFF::FF
FFFFFFFFFFFFFFFFFFFFFF:FFFFFFFFFFFFFFFFFFFFF:FFF::FFFFFFFFFFFFFFFFFFFF
FFFF:FFFFFFFFFF
@A00721:190:HGJW7DSXY:4:2419:12102:11146 2:N:0:TCTGCAAG+GCTGCACT
ATAAGACGTTCCTGGACAAGGATGCCAATTCATCTTTAAAGATTTGGACAGAGACAAATGCAGAATTC
TTCTTCGCCGAATCCGCCTTCTATAGAAGCGAAGCATCAGCAATGCAAACGGAAGAGACGTCTAGCGA
TCAAGCCTAGACGTC
+
FFFFFFFFFFFFFFFFF:F:FFFFFFFF:FF:FFFFFFFFFFFFFFFFFFFFFFFFFFFFFFFFFFFFFF
FFFFFFFFFFFFFFFFFFFFFFFFFFFF,FFFFFFFFFFFFFFFFFFFFFFFFFFFFFFFFFFFFFFFFF
FFFFFFFFFFFFFFF
@A00721:190:HGJW7DSXY:4:2455:21829:21480 2:N:0:TCTGCAAG+GCTGCACT
ATAAGACGTTCCTGGACAAGGATGCCAATTCATCTTTAAAGATTTGGACAGAGACAAATGCAGAATTC
TTCTTCGCCGAATCCGCCTTCTATAGAAGCGAAGCATCAGCAATGCAAACGGAAGAGACGTCTAGCGA
TCAAGCCTAGACGTC
+
FFFFFFFFF:FFFFFFFFFFFFFFFFFFFFFFFFFFFFFFFFFFFFFFFFFFFFFFFFFFFFFFFFFFFF
FFFFFFFFFFFFFFFFFFFFFFFFFF:FFFFFFFFFFFFFFFFFFFFFFFF:FFFFFFFFFFFFFFFFFF
FFFFFFFFFFFFFFF
@A00721:190:HGJW7DSXY:4:2461:21658:6965 2:N:0:TCTGCAAG+GCTGCACT
ATAAGACGTTCCTGGACAAGGATGCCAATTCATCTTTAAAGATTTGGACAGAGACAAATGCAGAATTC
TTCTTCGCCGAATCCGCCTTCTATAGAAGCGAAGCATCAGCAATGCAAACGGAAGAGACGTCTAGCGA
TCAAGCCTAGACGTC
+
FFFFFFFFFFFFFFFFFFFFFFFFFFFFFFFFFFFFFFFFFFFFFFFFFFFFFFFFFFFFFFFFFFFFFF
FFFFFFFFFFFFFFFFFFFFFFFFFFFFFFFFFFFFFFFFFFFFFFFFF:FFFFFFFFFFFFFFFFFFFF
FFFFFFFFFFFFFFF
@A00721:190:HGJW7DSXY:4:2527:4227:17550 2:N:0:TCTGCAAG+GCTGCACT
ATAAGACGTTCCTGGACAAGGATGCCAATTCATCTTTAAAGATTTGGACAGAGACAAATGCAGAATTC
```

TTCTTCGCCGAATCCGCCTTCTATAGAAGCGAAGCATCAGCAATGCAAACGGAAGAGACGTCTAGCGA
TCAAGCCTAGACGTC
+
FFFFFFFFFFFFFFFFFFFFFFFFFFFFFFFFFFFFFFFFFFFFFFFFFFFFFFFFFFFFFFFFFFFFFF
FFFFFFFFFFFFFFFFFFFFF:FFFFFFFFFFFFFFFFFFFFFFFFFFFFFFFFFFFFFFFFFFFFFFFF
FFFFFFFFFFFFFFF
@A00721:190:HGJW7DSXY:4:1306:17210:27383 2:N:0:TCTGCAAG+GCTGCACT
CTTATAAGACGTTCCTGGACAAGGATGCCAATTCATCTTTAAAGATTTGGACAGAGACAAATGCAGAA
TTCTTCTTCGCCGAATCCGCCTTCTATAGAAGCGAAGCATCAGCAATGCAAACGGAAGAGACGTCTAG
CGATCAAGCCTAGAC
+
FFFFFFFFFFFFFFFFFFFFFFFFFFFFFFFFFFFFFFFFFFFFFFFFFFFFFFFFFFFFF:FFFFFFFF
FFFFFFFFFFFFFFFFFFFFFFFF:FFFFFFFFFFFFFFFFFFFFFFFFFFFFFFFFFFFFFFFFFFFF:
FFFFFFF:FFFFFF:
@A00721:190:HGJW7DSXY:4:1344:6705:19053 2:N:0:TCTGCAAG+GCTGCACT
CTTATAAGACGTTCCTGGACAAGGATGCCAATTCATCTTTAAAGATTTGGACAGAGACAAATGCAGAA
TTCTTCTTCGCCGAATCCGCCTTCTATAGAAGCGAAGCATCAGCAATGCAAACGGAAGAGACGTCTAG
CGATCAAGCCTAGAC
+
FFFFFFFFFFFFFFFFFFFFFFFFFFFFFFFFFFFFFFFFFFFFFFFFFFFFFFFFFF,F:FFFFFFFFF
FFFFFFFFFFFFFFFFFFFFFFFFFFFFFFF:FFFFFFFFFFFFFFFFFFFFFFFFFFFFFFFFFFFFF:
FFFFFFFFFFFFFFF
@A00721:190:HGJW7DSXY:4:1452:13367:22106 2:N:0:TCTGCAAG+GCTGCACT
CTTATAAGACGTTCCTGGACAAGGATGCCAATTCATCTTTAAAGATTTGGACAGAGACAAATGCAGAA
TTCTTCTTCGCCGAATCCGCCTTCTATAGAAGCGAAGCATCAGCAATGCAAACGGAAGAGACGTCTAG
CGATCAAGCCTAGAC
+
FFFFFFFFFFFFFFFFFFFFFFFFFFFFFFFFFFFFFFFFFFFFFFFFFFFFFFFFFFFFFFFFFFFFFF
FFFFFFFFFFFFFFFFFFFFFFFFFFFFFFFFFFFFFFFFFFFFFFFFFFFFFFFFFFFFFFFFFFFFFF
FFFFFFFFFFFFFFF
@A00721:190:HGJW7DSXY:4:1655:24722:13808 2:N:0:TCTGCAAG+GCTGCACT
CTTATAAGACGTTCCTGGACAAGGATGCCAATTCATCTTTAAAGATTTGGACAGAGACAAATGCAGAA
TTCTTCTTCGCCGAATCCGCCTTCTATAGAAGCGAAGCATCAGCAATGCAAACGGAAGAGACGTCTAG
CGATCAAGCCTAGAC
+
FFFFFFFFFFFFFFFFFFFFFFFFFFFFFFFFFFFFFFFFFFFFFFFFFFFFFFFFFFFFFFFFFFFFFF
FFFFFFFFFFFFFFFFFFFFFFFF,FFFFFFFFFFFFFFFFFFFFFFFFFFFFFFFFFFFFFFFFFFFFF
FFFFFFF:FFFFFFF
@A00721:190:HGJW7DSXY:4:2531:29342:1673 2:N:0:TCTGCAAG+GCTGCACT
CTTATAAGACGTTCCTGGACAAGGATGCCAATTCATCTTTAAAGATTTGGACAGAGACAAATGCAGAA
TTCTTCTTCGCCGAATCCGCCTTCTATAGAAGCGAAGCATCAGCAATGCAAACGGAAGAGACGTCTAG
CGATCAAGCCTAGAC
+
FFFFFFFFFFFFFFFFFFFFFFFFFFFFFFFFFFFFFFFFFFFFFFFFFFFFFFFFFFFFFFFFFFFFFF
FFFFFFFFFFFFFFFFFFFFFFFFFFFFFFF:FFF:FFFFFFFFFFFFFFFF,FFFFFFFFFFFFFFFFF
FFFFFFFFFFFFFFF
@A00721:190:HGJW7DSXY:4:2563:9281:22013 2:N:0:TCTGCAAG+GCTGCACT
CTTATAAGACGTTCCTGGACAAGGATGCCAATTCATCTTTAAAGATTTGGACAGAGACAAATGCAGAA
TTCTTCTTCGCCGAATCCGCCTTCTATAGAAGCGAAGCATCAGCAATGCAAACGGAAGAGACGTCTAG
CGATCAAGCCTAGAC
+
FFFFFFFFFFFFFFFFFFFFFFFFFFFFFFFFFFFFFF:FFF:FF:FFFFFFFFFFFFFFFFFFFFF:FF
FFFFFFFFFFFFFFFFFFFFFFFFFFFFFF:FFFFFFFFFFFFFFFF:FFFFFFFFFFFFFFFFFFFFFF
FFFFFFFFFFFFFFF

```
@A00721:190:HGJW7DSXY:4:2648:23737:14951 2:N:0:TCTGCAAG+GCTGCACT
CTTATAAGACGTTCCTGGACAAGGATGCCAATTCATCTTTAAAGATTTGGACAGAGACAAATGCAGAA
TTCTTCTTCGCCGAATCCGCCTTCTATAGAAGCGAAGCATCAGCAATGCAAACGGAAGAGACGTCTAG
CGATCAAGCCTAGAC
+
FFFFFFFFFFFFFFFFFFFFFFFFFFFFFF,FFFFFFFFFF:FFFFFFFFFFFFFFFFFFFFFFFFFF
FFFFFFFFFFFFFFFFFFFFFFFFFFFFFFF:FFFFFFFFFFFFFFFFFFFFFFFFFFFFFFFFFFFF
FFFFFFFFFFFFFFF
@A00721:190:HGJW7DSXY:4:2241:14624:29230 2:N:0:TCTGCAAG+GCTGCACT
TCTTATAAGACGTTCCTGGACAAGGATGCCAATTCATCTTTAAAGATTTGGACAGAGACAAATGCAGA
ATTCTTCTTCGCCGAATCCGCCTTCTATAGAAGCGAAGCATCAGCAATGCAAACGGAAGAGACGTCTA
GCGATCAAGCCTAGA
+
FF:FFFFFFFFFFFFFFFFFFFFFFFFFFFFFFFFFFFFFFF:FFFFFFFFFFFFFFFFFFFFFFFFF
F:FFFFFFFFFFFFFFFFFFFFFFFFFFFFFFFFFFFFFFFFFFFFF:FFFFFFFF:FFFFFFFFFFF
FFFFFFFFFFFFFFF
@A00721:190:HGJW7DSXY:4:2442:16568:14152 2:N:0:TCTGCAAG+GCTGCACT
TCTTATAAGACGTTCCTGGACAAGGATGCCAATTCATCTTTAAAGATTTGGACAGAGACAAATGCAGA
ATTCTTCTTCGCCGAATCCGCCTTCTATAGAAGCGAAGCATCAGCAATGCAAACGGAAGAGACGTCTA
GCGATCAAGCCTAGA
+
FFFFFFFFFFFFFFFFFFFFFFFFFFFFFFFFFFFFFFFFFFFFFFFFFFFFFFFFFFFFFFFFFFFF
FFFFFFFFFFFF:FFFFFFFFFFFF,FFFFFF:FFF,F:FFFFFFFFFFFFFFFF:FFFFFFFFFFFF
FFFFFFF:FFFFFFF
@A00721:190:HGJW7DSXY:4:2663:30391:9251 2:N:0:TCTGCAAG+GCTGCACT
TCTTATAAGACGTTCCTGGACAAGGATGCCAATTCATCTTTAAAGATTTGGACAGAGACAAATGCAGA
ATTCTTCTTCGCCGAATCCGCCTTCTATAGAAGCGAAGCATCAGCAATGCAAACGGAAGAGACGTCTA
GCGATCAAGCCTAGA
+
FFFFFFFFFFFFFFFFFFFF:F:FFFFFFF,FFFFFFFFFFFFFFFFFFFFFFFFFFFFFFFFFFFFF
FFFFFFFFFFFFFFFFFFFFFFFFFFFFFFFFFFFFFFFFFFFFFFFFFFFF,FFFFFFFFFFFFFFF
FFFFFFFFFFFF:FF
@A00721:190:HGJW7DSXY:4:1310:27525:22326 2:N:0:TCTGCAAG+GCTGCACT
GAATATTCTTATAAGACGTTCCTGGACAAGGATGCCAATTCATCTTTAAAGATTTGGACAGAGACAAA
TGCAGAATTCTTCTTCGCCGAATCCGCCTTCTATAGAAGCGAAGCATCAGCAATGCAAACGGAAGAGA
CGTCTAGCGATCAAG
+
FFFFFFFFFFFFF:FFFFFFFFFFFFF:FFFFFFFFFFFFF:FFF,FFF:FF,FFFFFFFFFFFFF:F
FFFFFFFFFFFFF:FF,FFFF:FF:FFFFFFFF:FFFFFFFFFFFFFFFFFFFFFFF,FFFFFFFFFF
FFFF::FFFFFFFFF
@A00721:190:HGJW7DSXY:4:1408:10185:1031 2:N:0:TCTGCAAG+GCTGCACT
GAATATTCTTATAAGACGTTCCTGGACAAGGATGCCAATTCATCTTTAAAGATTTGGACAGAGACAAA
TGCAGAATTCTTCTTCGCCGAATCCGCCTTCTATAGAAGCGAAGCATCAGCAATGCAAACGGAAGAGA
CGTCTAGCGATCAAG
+
FFFFFFFFFFFFFFFFFFFFFF:FFFFFFFFFFFFFFFFFFFFFFFFF:FFFFFFFFFFFFFFFFFFF
FFFFFFFFFFFFFFFFFFFFFFFFFFFF,FFFFFFFF::FFFFFFFFFFFFF:FFFFFFFFFFFFFFF
FFFFF:FFFFFFFFF
@A00721:190:HGJW7DSXY:4:1446:30698:22122 2:N:0:TCTGCAAG+GCTGCACT
GAATATTCTTATAAGACGTTCCTGGACAAGGATGCCAATTCATCTTTAAAGATTTGGACAGAGACAAA
TGCAGAATTCTTCTTCGCCGAATCCGCCTTCTATAGAAGCGAAGCATCAGCAATGCAAACGGAAGAGA
CGTCTAGCGATCAAG
+
FFFFFFFFFFFFFFFFFFFFFFFFFFFF,FF:FFFFFFFFFFFFFFFF:FFFFFFFFFFFFFFFFFFF
```

FFFFFF:FFFFFFFFFFFFFFFFFFFFFFFFFFFFFFFFFFFFFFFFFFFFFFFFFFF:FFFFFFFFF
FFFFFFFFFFFFFFF
@A00721:190:HGJW7DSXY:4:1456:12048:25770 2:N:0:TCTGCAAG+GCTGCACT
GAATATTCTTATAAGACGTTCCTGGACAAGGATGCCAATTCATCTTTAAAGATTTGGACAGAGACAAA
TGCAGAATTCTTCTTCGCCGAATCCGCCTTCTATAGAAGCGAAGCATCAGCAATGCAAACGGAAGAGA
CGTCTAGCGATCAAG
+
F:FFFFFFFFFFFFFFFFFFFFFFFFFFFFFFFFFFFFFFFFFFFFFFFFFFFFFFFFFFFFFFFFFF
FFFFFFFFFFFFFFFFFFFFFFFFFFFFFFFF:FFFFFFFFFFFFFFFFFFFFFFFFFFFFFFFFFFF
FFFFFFFFFFFFFFF
@A00721:190:HGJW7DSXY:4:1458:21187:1799 2:N:0:TCTGCAAG+GCTGCACT
GAATATTCTTATAAGACGTTCCTGGACAAGGATGCCAATTCATCTTTAAAGATTTGGACAGAGACAAA
TGCAGAATTCTTCTTCGCCGAATCCGCCTTCTATAGAAGCGAAGCATCAGCAATGCAAACGGAAGAGA
CGTCTAGCGATCAAG
+
FFFFFFFFFFFFFFFFFFFFFFFFFFFFFFFFFFFFFFFFFFFFFFFFFFFFFFFFFFFFFFFFFFFF
FFFFFFFFFFFFFFFFFFFFFFFFFFFFFFFFFFFFFFFFFFFFFFFFFF:FFFFFFFFFFFFFFFFF
F:FFFFFFFFFFFFF
@A00721:190:HGJW7DSXY:4:1601:16450:29105 2:N:0:TCTGCAAG+GCTGCACT
GAATATTCTTATAAGACGTTCCTGGACAAGGATGCCAATTCATCTTTAAAGATTTGGACAGAGACAAA
TGCAGAATTCTTCTTCGCCGAATCCGCCTTCTATAGAAGCGAAGCATCAGCAATGCAAACGGAAGAGA
CGTCTAGCGATCAAG
+
FFFFFFFFFFFFFFFFFFFFFFFFFFFFFFFFFFFFFFFFFFFFFFFF:F:FFFFFFFFFFFFFFFFF
FFFFFFFFFFFFFFFFFFFFFFFFFFFFFFFFFFF:FFFFFFFFFFFFFFFFFFFFF::F:FFFFFFF
FFFFFFFFFFFFFFF
@A00721:190:HGJW7DSXY:4:1613:22941:17957 2:N:0:TCTGCAAG+GCTGCACT
GAATATTCTTATAAGACGTTCCTGGACAAGGATGCCAATTCATCTTTAAAGATTTGGACAGAGACAAA
TGCAGAAGTCTTCTTCGCCGAATCCGCCTTCTATAGAAGCGAAGCATCAGCAATGCAAACGGAAGAGA
CGTCTAGCGATCAAG
+
FFF,FF,FF:FFFFFFFFFF,FFF:FFFFF,F,F,FFFF,FFFFF,,FFF,:FFFF,FFFF:FFF,FF
:FFFFFF,FFFFFFFF:FFFF:FFFF,F,FF:FFF,F::F,FF:,:FFFFFFFFFFFFFF,FF:,FF:
FF,FF,FFFFFFFFF
@A00721:190:HGJW7DSXY:4:2142:14552:10034 2:N:0:TCTGCAAG+GCTGCACT
GAATATTCTTATAAGACGTTCCTGGACAAGGATGCCAATTCATCTTTAAAGATTTGGACAGAGACAAA
TGCAGAATTCTTCTTCGCCGAATCCGCCTTCTATAGAAGCGAAGCATCAGCAATGCAAACGGAAGAGA
CGTCTAGCGATCAAG
+
FFFFFFFFFFFFFFFFFFFFFFFFFFFFFFFFFFFFFFFFFFFFFFFFFFFFFFFFFFFFFFFFFFFF
FFFFFFFFFFFFFFFFFFFFFFFFFFFFFFFFFFFFFFFFFF:FFFFFFFFFF:FFFFF:FFFFFFFF
FFFFF,:FFFFF:FF
@A00721:190:HGJW7DSXY:4:2336:18783:5087 2:N:0:TCTGCAAG+GCTGCACT
GAATATTCTTATAAGACGTTCCTGGACAAGGATGCCAATTCATCTTTAAAGATTTGGACAGAGACAAA
TGCAGAATTCTTCTTCGCCGAATCCGCCTTCTATAGAAGCGAAGCATCAGCAATGCAAACGGAAGAGA
CGTCTAGCGATCAAG
+
FFFFFFFFFFFFFFFFFFFFFFFFFFFFFFFFFFFFFF:FFFFFFFFFFFFFFFFFFFFFFFFFFFFF
FFFFFFFFFFFFFFFFFF:FFFFFFFFFFFFFFFF:FFFFFFFFFFF:FFFFFFFFF:FFFFFFFFFF
F,:FFFFFFFFFF,F
@A00721:190:HGJW7DSXY:4:2402:30210:21684 2:N:0:TCTGCAAG+GCTGCACT
GAATATTCTTATAAGACGTTCCTGGACAAGGATGCCAATTCATCTTTAAAGATTTGGACAGAGACAAA
TGCAGAATTCTTCTTCGCCGAATCCGCCTTCTATAGAAGCGAAGCATCAGCAATGCAAACGGAAGAGA
CGTCTAGCGATCAAG

+
FFFFFFFF,FFFFFF,FFFFFFFFFFFFFFFFFFFFFFFFFFFFFFFFF,FFFFFFFFFFFFFFF:FF
FFFFFFFFFFFFFFFFFFFFFFFFFFFFFFFF:FFFFFFFFFFFFFFFFFFFFFFFFFF::FFFF:FFF
FFFFFFFFFFFFFFF
@A00721:190:HGJW7DSXY:4:2432:9851:14826 2:N:0:TCTGCAAG+GCTGCACT
GAATATTCTTATAAGACGTTCCTGGACAAGGATGCCAATTCATCTTTAAAGATTTGGACAGAGACAAA
TGCAGAATTCTTCTTCGCCGAATCCGCCTTCTATAGAAGCGAAGCATCAGCAATGCAAACGGAAGAGA
CGTCTAGCGATCAAG
+
:F:FFFFFFFFFFFFFFFFFFFFFFFFFFFFFFFFFFFFFFFFFFFFFFFFFFFF:FFFFFFFFFFFF
FFFFFFFFFFFFFFFFFFFFFFFFFFFFFFFFFFFFFFFFFFFFFFFFFFFFFFFFFF:FFFF:FFFF
FFFFFFFFFFFFFFF
@A00721:190:HGJW7DSXY:4:2438:12807:23171 2:N:0:TCTGCAAG+GCTGCACT
GAATATTCTTATAAGACGTTCCTGGACAAGGATGCCAATTCATCTTTAAAGATTTGGACAGAGACAAA
TGCAGAATTCTTCTTCGCCGAATCCGCCTTCTATAGAAGCGAAGCATCAGCAATGCAAACGGAAGAGA
CGTCTAGCGATCAAG
+
FF:FFFFF:FFFFFFFFF:FFFFFFFF:FFFFFFFFFFFFFFFFFFFF:FFFFFFFFFFFFFFFFFF
FFFFFFFFFFFFFFFFFFFFFFFFFFFFF:FFFFFFFFFFFFFFFFFF:FFFFFFFFFFF:FFFFFFF
FFFFFFF::F:FFFF
@A00721:190:HGJW7DSXY:4:2519:2275:29951 2:N:0:TCTGCAAG+GCTGCACT
GAATATTCTTATAAGACGTTCCTGGACAAGGATGCCAATTCATCTTTAAAGATTTGGACAGAGACAAA
TGCAGAATTCTTCTTCGCCGAATCCGCCTTCTATAGAAGCGAAGCATCAGCAATGCAAACGGAAGAGA
CGTCTAGCGATCAAG
+
FFFFFFFFFFFFFFFFFFFFFFFFFFFFFFFFFFFFFFFFFFFFFFFFFFFFFFFFFFFFFFFFFFFF
FFFFFFFFFFFFFFFFFFFFFFFFFFFFFFF:FFFFF:FFFF:FFFFFFFFFFFFFFFFFFFFFFFFF
FFFFFFFFFFFFFFF
@A00721:190:HGJW7DSXY:4:2529:13548:12868 2:N:0:TCTGCAAG+GCTGCACT
GAATATTCTTATAAGACGTTCCTGGACAAGGATGCCAATTCATCTTTAAAGATTTGGACAGAGACAAA
TGCAGAATTCTTCTTCGCCGAATCCGCCTTCTATAGAAGCGAAGCATCAGCAATGCAAACGGAAGAGA
CGTCTAGCGATCAAG
+
FFFFFF:FFFFFFFFFFFFFFFFFFFFFFFFFFFFFF:FFFFFFFFFFFFF::FFFFFF:FFFFFFF
FFFFFFFFFFFFFFFFFFFFFFFFFFFFFFF,FFFFF:FFFF:FFFFFFFFFFFFFF:FFFFFFFFFF
FFFFF,FFFF:FFFF
@A00721:190:HGJW7DSXY:4:2541:31204:22748 2:N:0:TCTGCAAG+GCTGCACT
GAATATTCTTATAAGACGTTCCTGGACAAGGATGCCAATTCATCTTTAAAGATTTGGACAGAGACAAA
TGCAGAATTCTTCTTCGCCGAATCCGCCTTCTATAGAAGCGAAGCATCAGCAATGCAAACGGAAGAGA
CGTCTAGCGATCAAG
+
FFFFFFFFFFFFFFFFFFFFFFFFFFFFFFFFFFFFFFFFFFFFFFFFFFFFFFFFFFFFFFF:FFFF
FFFFFFFFFFFFFFFFFFFFFFFFFFFFFFFFFFFFFFFFFFFFFFFFFFFFFFFFFFFFFFFFFFFF
F:FFF,FF:FFFFFF
@A00721:190:HGJW7DSXY:4:2646:21974:29622 2:N:0:TCTGCAAG+GCTGCACT
GAATATTCTTATAAGACGTTCCTGGACAAGGATGCCAATTCATCTTTAAAGATTTGGACAGAGACAAA
TGCAGAATTCTTCTTCGCCGAATCCGCCTTCTATAGAAGCGAAGCATCAGCAATGCAAACGGAAGAGA
CGTCTAGCGATCAAG
+
FFFFFFFFFFFFFFFFFFFFFFFFFFFFFFFFFFFFFFFFFFFFFFFFFFFFFFFFFFFFFFFFFFFF
FFFFFFFFFFFFFFFF:FFFFFFFFFFFFFFFFFFFFFFFFFFFFFFFFFFFFFFFFFF:FFFFFFFFF
FFFFFFFFFFFFFFF
@A00721:190:HGJW7DSXY:4:1248:15393:15468 2:N:0:TCTGCAAG+GCTGCACT
AAGAATATTCTTATAAGACGTTCCTGGACAAGGATGCCAATTCATCTTTAAAGATTTGGACAGAGACA

```
AATGCAGAATTCTTCTTCGCCGAATCCGCCTTCTATAGAAGCGAAGCATCAGCAATGCAAACGGAAGA
GACGTCTAGCGATCA
+
FFFFFF:FFFFFFFFFFFFFFFFFFFFFFFFFFFFFFFFFFFFFFFFFFFF:FFFFFFFFFFFFFFFF
FFFFFFFFFFFFFFFFFFFFFFFFFFFFFFFFFFFFF:FFFFFFFFFFFFFFFFFFFFFFFFFFFFFF
FFFFFFFFFFFFFFF
@A00721:190:HGJW7DSXY:4:2301:9896:31720 2:N:0:TCTGCAAG+GCTGCACT
AAGAATATTCTTATAAGACGTTCCTGGACAAGGATGCCAATTCATCTTTAAAGATTTGGACAGAGACA
AATGCAGAATTCTTCTTCGCCGAATCCGCCTTCTATAGAAGCGAAGCATCAGCAATGCAAACGGAAGA
GACGTCTAGCGATCA
+
F:FFFFFFFFFFFFFFFFFFFFFFFFFFFFFFFFFFFF,FFFFFFFFFFFFFFFF:FFFFF:FFFFFF
FFFFFFFFFFFFFFFFFFFFFFFFFFFFFFFFFFFFFFFFFFFFFFFFFFFFFFFFFFFFFFFFFFFF
FFFFFFFFFFFFFFF
@A00721:190:HGJW7DSXY:4:1123:21441:22811 2:N:0:TCTGCAAG+GCTGCACT
GAAGAATATTCTTATAAGACGTTCCTGGACAAGGATGCCAATTCATCTTTAAAGATTTGGACAGAGAC
AAATGCAGAATTCTTCTTCGCCGAATCCGCCTTCTATAGAAGCGAAGCATCAGCAATGCAAACGGAAG
AGACGTCTAGCGATC
+
FF:F:FFFFFFFFFFFFFFFFFFFFFFFFFFFFFFFFFFFFFFFFFFFFFFFFFFFFFFFFFFFFFFF
FFFFFFFFFFFFFFFFFFFFFFFFFFFFFFFFFFFFFFFFFFFFF:FFFFFFFFFFFFFFFFFFFFFF
FFFFFFFFFFFFFFF
@A00721:190:HGJW7DSXY:4:1273:25373:22326 2:N:0:TCTGCAAG+GCTGCACT
GAAGAATATTCTTATAAGACGTTCCTGGACAAGGATGCCAATTCATCTTTAAAGATTTGGACAGAGAC
AAATGCAGAATTCTTCTTCGCCGAATCCGCCTTCTATAGAAGCGAAGCATCAGCAATGCAAACGGAAG
AGACGTCTAGCGATC
+
FFFFFFFFFFFFFFFFFFFFFFFFFFFFFFFFFFFFFFFFFFFFFFFFFFF:FFFFFFFFFFFFFFFF
FFFFFFFFFFFFFFFFFFFFFFFFFFFFFFFFFFFFFFFFFFFFFFFFFFFFFFFF:FFF,FFFFFFF
FFFFFFFFFF:FFFF
@A00721:190:HGJW7DSXY:4:1526:12165:19742 2:N:0:TCTGCAAG+GCTGCACT
GAAGAATATTCTTATAAGACGTTCCTGGACAAGGATGCCAATTCATCTTTAAAGATTTGGACAGAGAC
AAATGCAGAATTCTTCTTCGCCGAATCCGCCTTCTATAGAAGCGAAGCATCAGCAATGCAAACGGAAG
AGACGTCTAGCGATC
+
FFFFFFFFFFFFFFFFFFFFFFFFFFFFFFFFFFFFFFFFFFFFFFFFFFFFFFFFFFFFFFFFFFFF
FFFFFFFFFFFFFFFFFFFFFFFFFFFFFFFFFFFFFFFFFFFFFFFFFFFFFFFFFFFFFFFFFFFF
FFFFFFFFFFFFFFF
@A00721:190:HGJW7DSXY:4:1615:8639:9095 2:N:0:TCTGCAAG+GCTGCACT
GAAGAATATTCTTATAAGACGTTCCTGGACAAGGATGCCAATTCATCTTTAAAGATTTGGACAGAGAC
AAATGCAGAATTCTTCTTCGCCGAATCCGCCTTCTATAGAAGCGAAGCATCAGCAATGCAAACGGAAG
AGACGTCTAGCGATC
+
FFFFFFFFFFFFFFFFFFFFFFFFFFFFFFFF:FFFFFFFFFFFFFFFFFFFFFFFFFFFFFFFFFFF
FF,FFFFFFFFFFFFFFFFFFFFFFFFFFFFFFFFFFFFFFFFFFFFFFFFFFFFFFFFFFFFFFFFF
FFFFFFFFFFFFFFF
@A00721:190:HGJW7DSXY:4:2123:21459:35211 2:N:0:TCTGCAAG+GCTGCACT
GAAGAATATTCTTATAAGACGTTCCTGGACAAGGATGCCAATTCATCTTTAAAGATTTGGACAGAGAC
AAATGCAGAATTCTTCTTCGCCGAATCCGCCTTCTATAGAAGCGAAGCATCAGCAATGCAAACGGAAG
AGACGTCTAGCGATC
+
:FF:FFFFFFFFFFFFFF:FF:FFFFFFFFFFFFF:FFFFF:FFFFFFFFFFFFFFFFF,FFFFFFFF
FFFF:FFFFFFFFFFFFFFF,FFFFF,FFF:F:FFFFF,FF:,FFF:FFF:FFFFFF,FFFF,,:FFF
FFFF:FFFF,FFF:F
```

```
@A00721:190:HGJW7DSXY:4:2272:6207:23453 2:N:0:TCTGCAAG+GCTGCACT
GAAGAATATTCTTATAAGACGTTCCTGGACAAGGATGCCAATTCATCTTTAAAGATTTGGACAGAGAC
AAATGCAGAATTCTTCTTCGCCGAATCCGCCTTCTATAGAAGCGAAGCATCAGCAATGCAAACGGAAG
AGACGTCTAGCGATC
+
FFFFFFFFFFFFFFFFFFFFFFFFFFFFFFFFFFFFFFFFFFFFFFFFFFFFFF:FFFFFFFFFFFFFFF
FFFFFFFFFFFFFFFFFFFFFFFFFFFFFFFFFFF:FFFFFFFFFF,FFFFFFFFFFFFFFFFFFFFFFF
FFFFFFFFFFFFFFF
@A00721:190:HGJW7DSXY:4:2615:21965:36652 2:N:0:TCTGCAAG+GCTGCACT
GAAGAATATTCTTATAAGACGTTCCTGGACAAGGATGCCAATTCATCTTTAAAGATTTGGACAGAGAC
AAATGCAGAATTCTTCTTCGCCGAATCCGCCTTCTATAGAAGCGAAGCATCAGCAATGCAAACGGAAG
AGACGTCTAGCGATC
+
FFFFFFFFFFFFFFFFFFFFFFFFFFFFF:FFFFFFFFFFFFFFFFFFF,FFFFFFFFFFFFFFFFFFFF
FFFFFFFFFFFFFFFFFFFFFFFFFFFFFFFFFFFFFFFFFFFFFFFFFFFFFFFFF,FFFF:FFFFFFF
FFFFFFFFFFFFFFF
@A00721:190:HGJW7DSXY:4:2619:16712:10989 2:N:0:TCTGCAAG+GCTGCACT
GAAGAATATTCTTATAAGACGTTCCTGGACAAGGATGCCAATTCATCTTTAAAGATTTGGACAGAGAC
AAATGCAGAATTCTTCTTCGCCGAATCCGCCTTCTATAGAAGCGAAGCATCAGCAATGCAAACGGAAG
AGACGTCTAGCGATC
+
FFFFFFFFFFFFFFFFFFFFFFFFFFFFFFFFFFFFFFFFF:FFFFFFFFFFFFFFFFFFFFFFFFFFFF
FF:FFFFFFFFFFFFFFFFFFFFFFFFFFFFFFFFFFFFFFFFFFFFFFFFFFFFFFFFFFFFFFFFFFF
FFFF,FFFFFFFFFF
@A00721:190:HGJW7DSXY:4:1121:3748:25144 2:N:0:TCTGCAAG+GCTGCACT
CTCAGAAGAATATTCTTATAAGACGTTCCTGGACAAGGATGCCAATTCATCTTTAAAGATTTGGACAG
AGACAAATGCAGAATTCTTCTTCGCCGAATCCGCCTTCTATAGAAGCGAAGCATCAGCAATGCAAACG
GAAGAGACGTCTAGC
+
FFFFFFFFFFFFFFFFFFFFFFFFFFFFFFFFFFF:FFFFFFFF,FFFFFFFFFFFFFFFF:FFFFFFFF
FFFFFFFFFFFFFFFFFFFFFFFFFFFFFFFFFFFFFFFFFFFFFFFFFFFFFFFFFFFFFFFFFFFFFF
FFFFFFFFFFFFFFF
@A00721:190:HGJW7DSXY:4:1134:20030:30295 2:N:0:TCTGCAAG+GCTGCACT
CTCAGAAGAATATTCTTATAAGACGTTCCTGGACAAGGATGCCAATTCATCTTTAAAGATTTGGACAG
AGACAAATGCAGAATTCTTCTTCGCCGAATCCGCCTTCTATAGAAGCGAAGCATCAGCAATGCAAACG
GAAGAGACGTCTAGC
+
FFFFF:FFFFFFFFFFFFFFF:FFFFFFFFFFFFFFFFFFFFF:F:FFFFFFFFFFFFFFFFFFFFFFFF
FFFFFFF:FFFFFFFFFFFFFFFFFFFFFFFFFFFFFFFFFFF:FF,FFF:FFFFFFFFFFFFFFFFFFF
FFFFFFFFFFFFFFF
@A00721:190:HGJW7DSXY:4:1163:23692:20478 2:N:0:TCTGCAAG+GCTGCACT
CTCAGAAGAATATTCTTATAAGACGTTCCTGGACAAGGATGCCAATTCATCTTTAAAGATTTGGACAG
AGACAAATGCAGAATTCTTCTTCGCCGAATCCGCCTTCTATAGAAGCGAAGCATCAGCAATGCAAACG
GAAGAGACGTCTAGC
+
F,FFFFFFFFFFFFFFFFFFFFFFFFFFFFFFFFFFFFFFFFFFFFFFFFFFFFFFFFFFFFFFFFFFFF
:FFFFF:FFFFFF,FFFFFFFFFFFFFFFFFFFFFFFFFFFF:FFFFFFF:FFFFFFFFF:FFFFFFFFF
FFFFFFFFFFFFF:F
@A00721:190:HGJW7DSXY:4:1308:22815:28260 2:N:0:TCTGCAAG+GCTGCACT
CTCAGAAGAATATTCTTATAAGACGTTCCTGGACAAGGATGCCAATTCATCTTTAAAGATTTGGACAG
AGACAAATGCAGAATTCTTCTTCGCCGAATCCGCCTTCTATAGAAGCGAAGCATCAGCAATGCAAACG
GAAGAGACGTCTAGC
+
FFFFFFFFFFFFFF:FFFFFFFFFF:FF:FFFFFFFFFFFFF:FFFF::FFF:FFFFF,FFFFFF,FFFF
```

FFFFFFFFFFFFF:FFFFFFFFFFFF:FFF:F,F:FFFFFFFFFF,:FFFFFFF:FFFFFFFFFFFFF
FF:FFFFF:FFFFF:
@A00721:190:HGJW7DSXY:4:1338:20491:16564 2:N:0:TCTGCAAG+GCTGCACT
CTCAGAAGAATATTCTTATAAGACGTTCCTGGACAAGGATGCCAATTCATCTTTAAAGATTTGGACAG
AGACAAATGCAGAATTCTTCTTCGCCGAATCCGCCTTCTATAGAAGCGAAGCATCAGCAATGCAAACG
GAAGAGACGTCTAGC
+
FFFFFFFFFFFFFFFFFFFFFFFFFFFFFFFFFFFFFFFFFFFFFFFFFFFFFFFF:FFFFFFFFFFF
FFFFFFFFFFF:FFFFFFFFFFFFFFFFFFFFFFFFFFFFFFFFFFFFFFFFFFFFFFFFFFFFFFFF
FFFFF:FFFFFF::F
@A00721:190:HGJW7DSXY:4:1365:13910:5635 2:N:0:TCTGCAAG+GCTGCACT
CTCAGAAGAATATTCTTATAAGACGTTCCTGGACAAGGATGCCAATTCATCTTTAAAGATTTGGACAG
AGACAAATGCAGAATTCTTCTTCGCCGAATCCGCCTTCTATAGAAGCGAAGCATCAGCAATGCAAACG
GAAGAGACGTCTAGC
+
FFFFFFFFFFFFFFFFFFFFFFFFFFFFFFFFFFFFFFFFFFFFFFFFFFFFFFFFFFFFFFFFFFFF
FFFFFFFFFFFFFFFFFFFFFFFFFFFFFFFFFFFFFFFFFFF:FFFFFFFFFFFFFFFFFFFFFFFF
FFFFFFFFFFFF::F
@A00721:190:HGJW7DSXY:4:1524:3414:15358 2:N:0:TCTGCAAG+GCTGCACT
CTCAGAAGAATATTCTTATAAGACGTTCCTGGACAAGGATGCCAATTCATCTTTAAAGATTTGGACAG
GGACAAATGCAGAATTCTTCTTCGCCGAATCCGCCTTCTATAGAAGCGAAGCATCAGCAATGCAAACG
GAAGAGACGTCTAGC
+
FFFFFF,FFFFFFFFFFFFFFFFFFFFFFFFFFFFFFFFFFFFFFFFFFFFFFFFFFFFFFFFFFFFF
FFFFFFFFFFFFFFFFFFFFFFFFFFFFFFFFFFFFFFFFFFFFFFFFF:FFFFFFFFFFFFFFFFFF
FFFFFFFFFFFFFFF
@A00721:190:HGJW7DSXY:4:1641:3016:10755 2:N:0:TCTGCAAG+GCTGCACT
CTCAGAAGAATATTCTTATAAGACGTTCCTGGACAAGGATGCCAATTCATCTTTAAAGATTTGGACAG
AGACAAATGCAGAATTCTTCTTCGCCGAATCCGCCTTCTATAGAAGCGAAGCATCAGCAATGCAAACG
GAAGAGACGTCTAGC
+
FFFFFFFFFFFF:FFFFFFFFFFFFFFFFFFFFFFFFFFFFFF:FFFFFFFFFFFFFFFFFFF,FFFF
FFFFFFFFFFFFFFFFFFFFFFFFFFFFFFFFFFFFFFFFFFFFFFFFFFFFFFFFFFFFFFFF,FFF
FFFFFFFFFFFFFFF
@A00721:190:HGJW7DSXY:4:2112:32136:7670 2:N:0:TCTGCAAG+GCTGCACT
CTCAGAAGAATATTCTTATAAGACGTTCCTGGACAAGGATGCCAATTCATCTTTAAAGATTTGGACAG
AGACAAATGCAGAATTCTTCTTCGCCGAATCCGCCTTCTATAGAAGCGAAGCATCAGCAATGCAAACG
GAAGAGACGTCTAGC
+
FFFFFFFFFFFFFFFFFFFFFFFFFFFFFFFFFFFFFFFFFFFFFFFFFFFFFFFF:FFFFFFFFFFF
FFFFFFFFFFFFFFFFFFFFFFFFFFFFFFFFFFFFFFFFFFFFFFFFFFFFFFFFFFFFFFFFFFFF
FFFF:FFFFFFFFFF
@A00721:190:HGJW7DSXY:4:2114:29487:28040 2:N:0:TCTGCAAG+GCTGCACT
CTCAGAAGAATATTCTTATAAGACGTTCCTGGACAAGGATGCCAATTCATCTTTAAAGATTTGGACAG
AGACAAATGCAGAATTCTTCTTCGCCGAATCCGCCTTCTATAGAAGCGAAGCATCAGCAATGCAAACG
GAAGAGACGTCTAGC
+
FFFFFFFFFFFFFFFFFFFFFFFFFFFFFFFFFFFFFFFFFFFFFFFFFFFFFFFFFFFFFFFFFFFF
FFFFFFFFFFFFFFFFFFFFFFFFFFFFFFFFFFFFFFFFFFFFFFFFFFFFFFFFFFFFFFFFFFFF
FFFFFFFFFFFFFFF
@A00721:190:HGJW7DSXY:4:2148:9679:27993 2:N:0:TCTGCAAG+GCTGCACT
CTCAGAAGAATATTCTTATAAGACGTTCCTGGACAAGGATGCCAATTCATCTTTAAAGATTTGGACAG
AGACAAATGCAGAATTCTTCTTCGCCGAATCCGCCTTCTATAGAAGCGAAGCATCAGCAATGCAAACG
GAAGAGACGTCTAGC

+
FFFFFFFFFFFFFFFFFFFFF:FFFFFFFFFFFFFFFFFFFFFFFFFFFFFFFFFFFFF:FFFFFFFFFFF
FFFFFFFFFF:FFFFFFFFFF,FFFF:FFFFFFFFF,FFFFFFFFFFFFFFFFFFFFFFFFFFF:FFFFF
FFFFFFFFFFFFF:F
@A00721:190:HGJW7DSXY:4:2223:31747:30264 2:N:0:TCTGCAAG+GCTGCACT
CTCAGAAGAATATTCTTATAAGACGTTCCTGGACAAGGATGCCAATTCATCTTTAAAGATTTGGACAG
AGACAAATGCAGAATTCTTCTTCGCCGAATCCGCCTTCTATAGAAGCGAAGCATCAGCAATGCAAACG
GAAGAGACGTCTAGC
+
FFFFF,FFFFFFFF:FFFFFFFFFFFFFFFFFF:FFFFFFFFFFFFFFFFFFFFFFFFF:FFFFFFFFF
FFFFFFFFFFFFFFFFFFFFFFFFFFFFFFFFFFFFFFFFFFFFFFFF:FFFFFFFFFFFFFFFF,FF
F::F,F:FFFFF,FF
@A00721:190:HGJW7DSXY:4:2332:28700:8954 2:N:0:TCTGCAAG+GCTGCACT
CTCAGAAGAATATTCTTATAAGACGTTCCTGGACAAGGATGCCAATTCATCTTTAAAGATTTGGACAG
AGACAAATGCAGAATTCTTCTTCGCCGAATCCGCCTTCTATAGAAGCGAAGCATCAGCAATGCAAACG
GAAGAGACGTCTAGC
+
FFFFFFFFFFFFFFFFFFFFFFFFFFFFFFFFFFFFFFFFFFFFFFFFFFFFFFFFFFFFFFFFFFFF
FFFFFFFFFFFFFFFFFFFFFFFFFFFFFFFFFFFFFFFFFFFFFFFFFF:FFFFFFFFFFFFFFFFFF
FFFF:FFFFFFFFFF
@A00721:190:HGJW7DSXY:4:2338:7509:36010 2:N:0:TCTGCAAG+GCTGCACT
CTCAGAAGAATATTCTTATAAGACGTTCCTGGACAAGGATGCCAATTCATCTTTAAAGATTTGGACAG
AGACAAATGCAGAATTCTTCTTCGCCGAATCCGCCTTCTATAGAAGCGAAGCATCAGCAATGCAAACG
GAAGAGACGTCTAGC
+
FFFFFFFFFFFFFFFFFFFFFFFFF::FF:FFFFFFFFFFFFFFFFFFFFFFFFFFFFFFFFFF:FFFF
FFFFFFFFFFFFFFFFFFF:FFFFFFFFFFFFFFFFFFFFFFF:FFF:FFFFFFFFFFFF:FFFFFFFF
FFFFFFFFFFFFFFF
@A00721:190:HGJW7DSXY:4:2346:19090:9752 2:N:0:TCTGCAAG+GCTGCACT
CTCAGAAGAATATTCTTATAAGACGTTCCTGGACAAGGATGCCAATTCATCTTTAAAGATTTGGACAG
AGACAAATGCAGAATTCTTCTTCGCCGAATCCGCCTTCTATAGAAGCGAAGCATCAGCAATGCAAACG
GAAGAGACGTCTAGC
+
FFFFFFFFFFFFFFFFFFFFFF:FFFFFFFFFFFFFFFFFFFFFFFFFFFFFFFFFF:,FFFFFFFFFFF
FFFFFFFFFFFFFFFFFFFFFFFFFFFFFFF:FFFFFFFFFFFFFFF:FFFFFFFFFFFFFFFFFFFFF
FFFFFFFFFFFFFFF
@A00721:190:HGJW7DSXY:4:2354:15356:31250 2:N:0:TCTGCAAG+GCTGCACT
CTCAGAAGAATATTCTTATAAGACGTTCCTGGACAAGGATGCCAATTCATCTTTAAAGATTTGGACAG
AGACAAATGCAGAATTCTTCTTCGCCGAATCCGCCTTCTATAGAAGCGAAGCATCAGCAATGCAAACG
GAAGAGACGTCTAGC
+
FFFFFFFFFFFFFFFFFFFFFFFFFFFFFFFFFFFFFFFFFFF:FFFFFFFFFFFFFFF:FFFFFFFF
FFFFFFFFFFFFFFFFFFFFFFFFFFFFFFFFFFFFFFFFF:FFFFFF:FFFFFFFFFFFFFFFF:FFFF
FFFFFFFFFFFFFFF
@A00721:190:HGJW7DSXY:4:2449:25572:7451 2:N:0:TCTGCAAG+GCTGCACT
CTCAGAAGAATATTCTTATAAGACGTTCCTGGACAAGGATGCCAATTCATCTTTAAAGATTTGGACAG
AGACAAATGCAGAATTCTTCTTCGCCGAATCCGCCTTCTATAGAAGCGAAGCATCAGCAATGCAAACG
GAAGAGACGTCTAGC
+
FFFFFFFFFFFFFFFFFFFFFFFFFFFFFFFFFFFFFFFFFFFFFFFFFFFFFFFFFFFFFFFFFFFF
FFFFFFFFFFFFFFFFFFFFFFFFFFFFFFFFFFFFFFFFFFFFFFFFFFFFFFFFFFFFFFFFFFFF
FFFFFFFFFFFFFFF
@A00721:190:HGJW7DSXY:4:2521:20989:11068 2:N:0:TCTGCAAG+GCTGCACT
CTCAGAAGAATATTCTTATAAGACGTTCCTGGACAAGGATGCCAATTCATCTTTAAAGATTTGGACAG

```
AGACAAATGCAGAATTCTTCTTCGCCGAATCCGCCTTCTATAGAAGCGAAGCATCAGCAATGCAAACG
GAAGAGACGTCTAGC
+
FFFFFFFFFFFFFFFFFFFFFFFFFFFFFFFFFFFFFFFFFFFFFFFFFFFFFFFFFFFFFFFFFFFF
FFFFFFFFFFFFFFFFFFFFFFFFFFFFFFFFFFFFFFFFFFFFFFFFFFFFFFFFFFFFFFFFFFFF
FFFFFFFFFFFFFFF
@A00721:190:HGJW7DSXY:4:1335:9155:36417 2:N:0:TCTGCAAG+GCTGCACT
GCTCAGAAGAATATTCTTATAAGACGTTCCTGGACAAGGATGCCAATTCATCTTTAAAGATTTGGACA
GAGACAAATGCAGAATTCTTCTTCGCCGAATCCGCCTTCTATAGAAGCGAAGCATCAGCAATGCAAAC
GGAAGAGACGTCTAG
+
FFFFFFFFFFFFFFFFFFFFFFFFFFFFFFFFFFFFFFFFFFFFFFFFFFFFFFFFFFFFFFFFFFFF
FFFFFFFFFFFFFFFFFFFFFFFFFFFFFFFFFFFFFFFFFFFFFFFFFFF:FFFFFFFFFFFFFFFF
FFFFF:FFFFFFFFF
@A00721:190:HGJW7DSXY:4:2239:30933:36276 2:N:0:TCTGCAAG+GCTGCACT
GCTCAGAAGAATATTCTTATAAGACGTTCCTGGACAAGGATGCCAATTCATCTTTAAAGATTTGGACA
GAGACAAATGCAGAATTCTTCTTCGCCGAATCCGCCTTCTATAGAAGCGAAGCATCAGCAATGCAAAC
GGAAGAGACGTCTAG
+
FFFFFFFFFFFFFFFFFFFFFFFFFFFFFFFFFFFFFFFFFFFFFFFFFFFFFFFFFF:FFFFFFFFF
FFFFFFF:FFFFFFFFFFFFFFFFFFFFFFFFFFFFFFFFFFFFF:FFFFFFF:FFFFFFF,FFFFF:F
:FFFFF:FFFFFFFF
@A00721:190:HGJW7DSXY:4:2440:15917:5729 2:N:0:TCTGCAAG+GCTGCACT
GCTCAGAAGAATATTCTTATAAGACGTTCCTGGACAAGGATGCCAATTCATCTTTAAAGATTTGGACA
GAGACAAATGCAGAATTCTTCTTCGCCGAATCCGCCTTCTATAGAAGCGAAGCATCAGCAATGCGAAC
GGAAGAGACGTCTAG
+
F:FFFFFF:FFFFFFFFFFFFFFFFF:FFFF:FFFFFFFFFFFFFFFFFFFFFFFFFF:FFFFFFFFF
:FFFFFFFFFFF,FFF:FFFFFFF:F:FFFFF:FFF:FFFFFFFF::FFFFFFF:FFFFFFFFF,FFF
FFFFFFFFFFFFFF:
@A00721:190:HGJW7DSXY:4:1646:14724:36636 2:N:0:TCTGCAAG+GCTGCACT
CGTCAATCGTGCTTTGCAATCCAGGCTCAGAAGAATATTCTTATAAGACGTTCCTGGACAAGGATGCC
AATTCATCTTTAAAGATTTGGACAGAGACAAATGCAGAATTCTTCTTCGCCGAATCCGCCTTCTATAG
AAGCGAAGCATCAGC
+
FFFFFFFFFFFFFFFFFFFFFFFFFFFFFFFFFFFFFFFFFFFFFFFFFFFFFFFFFFFFFFFFFFFF
FFFFFFFFF:FFFFFFFFFFFFFFFFFFFFFFFFFFFFFFFFFFFFFFFFFFFFFFFFFFFFFFFFF,
FFFFFFFFFFFFFFF
@A00721:190:HGJW7DSXY:4:1653:1380:23578 2:N:0:TCTGCAAG+GCTGCACT
CGTCAATCGTGCTTTGCAATCCAGGCTCAGAAGAATATTCTTATAAGACGTTCCTGGACAAGGATGCC
AATTCATCTTTAAAGATTTGGACAGAGACAAATGCAGAATTCTTCTTCGCCGAATCCGCCTTCTATAG
AAGCGAAGCATCAGC
+
FFFFFF:FFFFFFFFFFFFFFFFFFFFFFFFFFFFFFFFFFFFFFFFFFFFFFFFFFFFFFFFFFFFF
FFFFFFFFFF,FFFFFFFFFFFF:FFFFFFFFFFFFFFFFFFFFFFFFFFFFFFFFFF:FFFFFFFFF:
FFFFFF:FF:FFFFF
@A00721:190:HGJW7DSXY:4:1673:28185:20619 2:N:0:TCTGCAAG+GCTGCACT
CGTCAATCGTGCTTTGCAATCCAGGCTCAGAAGAATATTCTTATAAGACGTTCCTGGACAAGGATGCC
AATTCATCTTTAAAGATTTGGACAGAGACAAATGCAGAATTCTTCTTCGCCGAATCCGCCTTCTATAG
AAGCGAAGCATCAGC
+
FFFFFFFFFFFFFFFFFFFFFFFFFFFFFFFFFFFFFFFFFFFFFFFFFFFFFFFFFFFFFFFFFFFF
FFFFFFFFFFFFFFFFFFFFFFFFFFFFFFFFFFFFFFFFFFFFFFFFFFFFFFFFFFFFFF:FFFFFF
FFFFFFFFFFFFFF:
```

```
@A00721:190:HGJW7DSXY:4:1154:26332:22608 2:N:0:TCTGCAAG+GCTGCACT
CCGTCAATCGTGCTTTGCAATCCAGGCTCAGAAGAATATTCTTATAAGACGTTCCTGGACAAGGATGC
CAATTCATCTTTAAAGATTTGGACAGAGACAAATGCAGAATTCTTCTTCGCCGAATCCGCCTTCTATA
GAAGCGAAGCATCAG
+
FFFFFFFFFFFFFFFFFFFFFFFFFFFFFFFFFFFFFFFFFFFFFFFFFFFFFFF:FFFFFFFFFFFF
FFFFFFFFFFFFFFFFFFFFFFFFFFFFFFFFFFFFFFFFFFFFFFFFFFFFFFFFF:FFFFFFFFFF
FFF,FFF,FF:FFFF
@A00721:190:HGJW7DSXY:4:1655:20980:12305 2:N:0:TCTGCAAG+GCTGCACT
CCGTCAATCGTGCTTTGCAATCCAGGCTCAGAAGAATATTCTTATAAGACGTTCCTGGACAAGGATGC
CAATTCATCTTTAAAGATTTGGACAGAGACAAATGCAGAATTCTTCTTCGCCGAATCCGCCTTCTATA
GAAGCGAAGCATCAG
+
FFFFFFFFFFFFFFFFFFFFFFFFFFFFFFFFFFFFFFFFFFFFFFFFFFFFFFFFFFFFFFFFFFFF
FFFFFFFFFFFFFF:FFFFFFFFFFFFFFFFFFFFFFFFFFFFFFFFFFFFFFFFFFFFFFFFFFFFF
FFFFFFFFFFFFFFF
@A00721:190:HGJW7DSXY:4:2104:3097:25300 2:N:0:TCTGCAAG+GCTGCACT
CGTCAATCGTGCTTTGCAATCCAGGCTCAGAAGAATATTCTTATAAGACGTTCCTGGACAAGGATGCC
AATTCATCTTTAAAGATTTGGACAGAGACAAATGCAGAATTCTTCTTCGCCGAATCCGCCTTCTATAG
AAGCGAAGCATCAG
+
F:FFFFFFFFFFFFFFFFFFFFFFFFFFF:F:FFFFF,FFFFFFFFFFFFFFFFF:FF:FFFFFFF:F
FFFFFFFF:,F,FFFF:,FFFFF,FFFFFFFF:FFFFFFF:FFFFFFFFFFFFF,FFFFF:FF,FFFF
FFFFFFFF:F:FFF
@A00721:190:HGJW7DSXY:4:2347:6171:30561 2:N:0:TCTGCAAG+GCTGCACT
CCGTCAATCGTGCTTTGCAATCCAGGCTCAGAAGAATATTCTTATAAGACGTTCCTGGACAAGGATGC
CAATTCATCTTTAAAGATTTGGACAGAGACAAATGCAGAATTCTTCTTCGCCGAATCCGCCTTCTATA
GAAGCGAAGCATCAG
+
FFFFFFFFFFFFFFFFFFFFFFFFFFFFFFFFFFFFFF:FFFFFFF,FFFFFFFFFFFFFFFFFFFFF
FFFFFFFFFFFFFFFFFFFFFFFFFFFFFFFFFFFFFFFF:FFFFFFFFFFFFFFFFFFFFFFFFFFF
:FFFFFFFFFFFFFF
@A00721:190:HGJW7DSXY:4:1272:13358:33990 2:N:0:TCTGCAAG+GCTGCACT
ATCGTGCTTTGCAATCCAGGCTCAGAAGAATATTCTTATAAGACGTTCCTGGACAAGGATGCCAATTC
ATCTTTAAAGATTTGGACAGAGACAAATGCAGAATTCTTCTTCGCCGAATCCGCCTTCTATAGAAGCG
AAGCATC
+
FFFFFFFFFFFFFFFFFFFFFFFFFFFFFFFFFFFFFFFFFFFFFFFFFFFFFFFFFFFFFFFFFFFF
FFFFFFFF:F:FFFFFFFFFFFFFFFFFFFF:FFFFFFFFFFFFFFFFFFFFFFFFFFFFFF,FF:FF
FFFFFFF
@A00721:190:HGJW7DSXY:4:1331:15139:28557 2:N:0:TCTGCAAG+GCTGCACT
ATCGTGCTTTGCAATCCAGGCTCAGAAGAATATTCTTATAAGACGTTCCTGGACAAGGATGCCAATTC
ATCTTTAAAGATTTGGACAGAGACAAATGCAGAATTCTTCTTCGCCGAATCCGCCTTCTATAGAAGCG
AAGCATC
+
FFFFFFFF:FFFFFFFFFFFFFFFF::FFFFFFFFFFFFFFFFFFFFFFFFFFFF:FFFFFF:FFFFF
FFFFFFF:,F:FFFFFFFFFFFFFFFFFFF:FFFFFFFFFFFFFFFFFFFFFFFFFFFFFFF:FF:FF
F,:FFFF
@A00721:190:HGJW7DSXY:4:1509:27606:30702 2:N:0:TCTGCAAG+GCTGCACT
ATCGTGCTTTGCAATCCAGGCTCAGAAGAATATTCTTATAAGACGTTCCTGGACAAGGATGCCAATTC
ATCTTTAAAGATTTGGACAGAGACAAATGCAGAATTCTTCTTCGCCGAATCCGCCTTCTATAGACGCG
AAGCATC
+
FFFFFFFFFFFFFFFFFFFFFFFFFF,FFFFF:FFFFFFFFFFFFFFFFFFFFFFFFFFFFFFF:,FF
```

```
FFFFFFFFFFF::FFFFF,FFFFFFFFFFFFFF:FFFFFF:FFFFFF,FFFFFFFF,FF,FF:F,FFF
FF:FFFF
@A00721:190:HGJW7DSXY:4:1607:3323:21183 2:N:0:TCTGCAAG+GCTGCACT
ATCGTGCTTTGCAATCCAGGCTCAGAAGAATATTCTTATAAGACGTTCCTGGACAAGGATGCCAATTC
ATCTTTAAAGATTTGGACAGAGACAAATGCAGAATTCTTCTTCGCCGAATCCGCCTTCTATAGAAGCG
AAGCATC
+
FFFFFFFFFFFFFFFFFFFFFFFFFFFFFFFFFFFFFFFFFFFFFFFFFFFFFFFFFFFFFFFFFFFF
FFFFFFFFFFFFFFFFFFFFFF:FFFFFFFFFFFFFFFFFFFFFFFFF:FFFFFFFFFFFF:FFFF:F
F,FFFFF
@A00721:190:HGJW7DSXY:4:2272:8404:27164 2:N:0:TCTGCAAG+GCTGCACT
ATCGTGCTTTGCAATCCAGGCTCAGAAGAATATTCTTATAAGACGTTCCTGGACAAGGATGCCAATTC
ATCTTTAAAGATTTGGACAGAGACAAATGCAGAATTCTTCTTCGCCGAATCCGCCTTCTATAGAAGCG
AAGCATC
+
FFFFFFFFFFFFFFFFFFFFFFFFFFFFFFFFFFFFFFFFFFFFFFFFFFFFFFFFFFFFFFFFFFFF
FFFFFFFFFFFFFFFFFFFFFFFFFFFFFFFFFFFFFFFFFFFFFFFFFFFFFFFFFFFFFFFFFFFF
FFFFFFF
@A00721:190:HGJW7DSXY:4:2276:26549:14465 2:N:0:TCTGCAAG+GCTGCACT
ATCGTGCTTTGCAATCCAGGCTCAGAAGAATATTCTTATAAGACGTTCCTGGACAAGGATGCCAATTC
ATCTTTAAAGATTTGGACAGAGACAAATGCAGAATTCTTCTTCGCCGAATCCGCCTTCTATAGAAGCG
AAGCATC
+
FFFFFFFFFFFFFFFFFFFFFFFFFFFFFFFFF:FFFFFFFFFFFFFFFFFFFFFFFFFFFFFFFFFF
FFFFFFFFFFFFFFFFFFFFFFFFFFFFFFFFFFFFFFFFFFFFFFFFFFFFFFFFFFFFFFFF::F:
FFFFFFF
@A00721:190:HGJW7DSXY:4:2347:4318:12727 2:N:0:TCTGCAAG+GCTGCACT
ATCGTGCTTTGCAATCCAGGCTCAGAAGAATATTCTTATAAGACGTTCCTGGACAAGGATGCCAATTC
ATCTTTAAAGATTTGGACAGAGACAAATGCAGAATTCTTCTTCGCCGAATCCGCCTTCTATAGAAGCG
AAGCATC
+
FFFFFFFFFFFFFFFFFFFFFFFFFFFFFFFFFFFFFFFFFFFFFFFFFFFF:FFFFFFFFF,FFFF
FFFFFFFFFFFFFFFFFF,FFFFFFFFFFFFFFFFFFFFFFFFFFFFFFFFFFFFFF:F:F:FFFFFFF
FFFFFFF
@A00721:190:HGJW7DSXY:4:2563:12364:2049 2:N:0:TCTGCAAG+GCTGCACT
ATCGTGCTTTGCAATCCAGGCTCAGAAGAATATTCTTATAAGACGTTCCTGGACAAGGATGCCAATTC
ATCTTTAAAGATTTGGACAGAGACAAATGCAGAATTCTTCTTCGCCGAATCCGCCTTCTATAGAAGCG
AAGCATC
+
FFFFFFFFFFFFFFFFFFFFFFFFFFF:FFFFFFFFFFFFFFFFFFFFFFFFFFF:FFFFFFFFFFFFF
FFFFFFF,FFFFFFFFFFFFFFFFFFFFFFFFFFFFFFFFFFFFFFFFFFFFFFFFFFFFFFFFFFFF
FFFF:FF
@A00721:190:HGJW7DSXY:4:1107:27272:3255 2:N:0:TCTGCAAG+GCTGCACT
AACCAATTCAAATTAAAGGTGGTATCCCGTCAATCGTGCTTTGCAATCCAGGCTCAGAAGAATATTCT
TATAAGACGTTCCTGGACAAGGATGCCAATTCATCTTTAAAGATTTGGACAGAGACAAATGCAGAATT
CTTCTTCGCCGAATC
+
FFFFFFFFFFFFFFFFFFFFFFFFFFFFFFFFFFFFFFFFFFFFFFFFFFFFFFFFFFFFFFFFFFFF
FFFFFFFFFFFFFFFFFFFFFFFFFFFFFFFFFFFFFFFF:FF:FFFFFFFFFFFFFFFFFFFFFFFF
FFFFFFFFFFFFFFF
@A00721:190:HGJW7DSXY:4:1133:10158:32424 2:N:0:TCTGCAAG+GCTGCACT
AACCAATTCAAATTAAAGGTGGTATCCCGTCAATCGTGCTTTGCAATCCAGGCTCAGAAGAATATTCT
TATAAGACGTTCCTGGACAAGGATGCCAATTCATCTTTAAAGATTTGGACAGAGACAAATGCAGAATT
CTTCTTCGCCGAATC
```

+
FFFFFFFFFFFFFFFFFFFFFFFFFFFFFFFFFFFFFFFFFFFFFFFFFFFFFFFFFFFFFFFFFFFF
FFFFFFFFFFFFFF:FFFFFFFFFFFFFFFFFFFFFFFFFFFFFFFFFFFFFFFFFFFFFF:F,FFFF
FFFFFFFFFFFFFFF
@A00721:190:HGJW7DSXY:4:1165:11704:2660 2:N:0:TCTGCAAG+GCTGCACT
AACCAATTCAAATTAAAGGTGGTATCCCGTCAATCGTGCTTTGCAATCCAGGCTCAGAAGAATATTCT
TATAAGACGTTCCTGGACAAGGATGCCAATTCATCTTTAAAGATTTGGACAGAGACAAATGCAGAATT
CTTCTTCGCCGAATC
+
FFFFFFFFFFFFFFFFFFFFFFFFFFFFFFFFFFFFFFFFFFFFFFFFFFFFFFFFFFFFFFFFFFFF
FFFFFFFFFFFFFFFFFFFFFFFFFFFFFFFFFFFFFFFFFFFFFFF::FFF:FFFFFFFFFFFFFFF
FFFFFFFFFFFFFFF
@A00721:190:HGJW7DSXY:4:1350:1163:34976 2:N:0:TCTGCAAG+GCTGCACT
AACCAATTCAAATTAAAGGTGGTATCCCGTCAATCGTGCTTTGCAATCCAGGCTCAGAAGAATATTCT
TATAAGACGTTCCTGGACAAGGATGCCAATTCATCTTTAAAGATTTGGACAGAGACAAATGCAGAATT
CTTCTTCGCCGAATC
+
FFFFFFFFFFFFFFFFFFFFFFFFFFFFFFFFFFFFFFFFFFFFFFFFFFFFFFFFFFFFFFFFFFFF
FFFFFFFFFFFFFFFFFFFFFFFFFFFFFFFFFFFFFFFF:FFFFFFFFFFFFFFFFFFFFFFFFFFF
FFFFFFFFFFFFFFF
@A00721:190:HGJW7DSXY:4:1448:5222:28197 2:N:0:TCTGCAAG+GCTGCACT
AACCAATTCAAATTAAAGGTGGTATCCCGTCAATCGTGCTTTGCAATCCAGGCTCAGAAGAATATTCT
TATAAGACGTTCCTGGACAAGGATGCCAATTCATCTTTAAAGATTTGGACAGAGACAAATGCAGAATT
CTTCTTCGCCGAATC
+
FFFFFFFFFFFFFF:FFFFF:FFFFFFFFFFFFFFFFFFFFFFFF:FFFFFFFFFFFF:FFFFF:FFF
FFFFFFFFF:FFFFFFFFFFFFFFFFFFFFFFFFFFFFFFFFFFFFFFFFFFFFFFFFF:F:FFFFFF
FFF:FFF,FF,F,FF
@A00721:190:HGJW7DSXY:4:1521:18991:27461 2:N:0:TCTGCAAG+GCTGCACT
AACCAATTCAAATTAAAGGTGGTATCCCGTCAATCGTGCTTTGCAATCCAGGCTCAGAAGAATATTCT
TATAAGACGTTCCTGGACAAGGATGCCAATTCATCTTTAAAGATTTGGACAGAGACAAATGCAGAATT
CTTCTTCGCCGAATC
+
F:FFFFFFFFFFFFFFFFFFFFFFFFFFFFFFFFFFFFFFFFFFFFFFFFFFFFFFFFFFFFFFFFFF
:FFFFFFFFFFFFFFFFFFFFFFFFFFFFFFFFFFFFFFF:FFFFFFFFFFFFFFFFFFFFFFFFFFF
FFFFFFFFFFFFFFF
@A00721:190:HGJW7DSXY:4:1618:3161:30953 2:N:0:TCTGCAAG+GCTGCACT
AACCAATTCAAATTAAAGGTGGTATCCCGTCAATCGTGCTTTGCAATCCAGGCTCAGAAGAATATTCT
TATAAGACGTTCCTGGACAAGGATGCCAATTCATCTTTAAAGATTTGGACAGAGACAAATGCAGAATT
CTTCTTCGCCGAATC
+
FFFFFFFFFFFFFFFFFFFFFFFFFFFFFFFFFFFF:FFFFFFFF:FFFFFFFFFFFFFFFFFFFFFF
FFFFFFFFFFFFFFFFFFF,FFFFFFFFFFFFFFFFF:FFFFFFFFFFFFFFFFFFF:FFFFF,FFFF
FFFFFF::F:FFF:F
@A00721:190:HGJW7DSXY:4:1619:27290:25645 2:N:0:TCTGCAAG+GCTGCACT
AACCAATTCAAATTAAAGGTGGTATCCCGTCAATCGTGCTTTGCAATCCAGGCTCAGAAGAATATTCT
TATAAGACGTTCCTGGACAAGGATGCCAATTCATCTTTAAAGATTTGGACAGAGACAAATGCAGAATT
CTTCTTCGCCGAATC
+
FFFFFFFFFFF:FFFFFFFFFFFFFFFFF:FFFFFFFF:FFFFFFFFFFFFF:FFFFFFFFFFFFFF:
FFF:FFFF:FFFFFFFFFFFFFFFFFF:F:FFFFFF:FFFFFFFFFFFFFFFFFFFFFFFFFF,FFF,
F:FFFFF:FFFFFFF
@A00721:190:HGJW7DSXY:4:2218:2184:5588 2:N:0:TCTGCAAG+GCTGCACT
AACCAATTCAAATTAAAGGTGGTATCCCGTCAATCGTGCTTTGCAATCCAGGCTCAGAAGAATATTCT

TATAAGACGTTCCTGGACAAGGATGCCAATTCATCTTTAAAGATTTGGACAGAGACAAATGCAGAATT
CTTCTTCGCCGAATC
+
FFFFFFFFFFFFFFFFFFFFFFFFFFFFFFFFFFFFFFFFFFFFFFFFFFFFFFFFFFFFFFFFFFFF
FFF:FFFFFFFFFFFFFFFFFFFFFFFFFFFFFFFFFFFFFFF:FFFFFFFFFFFFFFFFFFFFFFFF
FFFFFFFFFFFFFFF
@A00721:190:HGJW7DSXY:4:2333:24288:25488 2:N:0:TCTGCAAG+GCTGCACT
AACCAATTCAAATTAAAGGTGGTATCCCGTCAATCGTGCTTTGCAATCCAGGCTCAGAAGAATATTCT
TATAAGACGTTCCTGGACAAGGATGCCAATTCATCTTTAAAGATTTGGACAGAGACAAATGCAGAATT
CTTCTTCGCCGAATC
+
FFFFFFFFFFFFFFFFFFFFFFFFFFFFFFFFFFFFFFFFFFFFFFF:FFFFFFFFFFFFFFFF:FFF
FFFFFFFFFFFFFFF:FFFFFFFFFFFFFFFFF:FFFFFFFFFFFF:FFFFFFFFFFFFFF:FFFFFF
F:FFFFF:FFFFFFF
@A00721:190:HGJW7DSXY:4:2374:8323:25520 2:N:0:TCTGCAAG+GCTGCACT
AACCAATTCAAATTAAAGGTGGTATCCCGTCAATCGTGCTTTGCAATCCAGGCTCAGAAGAATATTCT
TATAAGACGTTCCTGGACAAGGATGCCAATTCATCTTTAAAGATTTGGACAGAGACAAATGCAGAATT
CTTCTTCGCCGAATC
+
FFFFFFFFFFFF:FFFFFFFFFF::FFFFFFFFFFFFFFFFFFFFFFFFFFFFFFFFFFFFFFFFFFF
FFFFFFFFF:FFF:FFFFFFFFFFFFFFFFFF:FFFFFFFFFFFFFFFFFFFF:FFFFFFFFFF:FFF
FFFFFFFFFFFFFFF
@A00721:190:HGJW7DSXY:4:2545:21902:17597 2:N:0:TCTGCAAG+GCTGCACT
AACCAATTCAAATTAAAGGTGGTATCCCGTCAATCGTGCTTTGCAATCCAGGCTCAGAAGAATATTCT
TATAAGACGTTCCTGGACAAGGATGCCAATTCATCTTTAAAGATTTGGACAGAGACAAATGCAGAATT
CTTCTTCGCCGAATC
+
FFFFFFFFFFFF:FFFFFFFFFFFFFFFFFFFFFFFFFFFFFFFFFFFFFFFFFFFFFFFFFFF:FFFF
FFFFFF:FFFFFFFFFFFFFFFFF:FFFFFFFFFFFFFFFFFF:FFFFFFFFFFFFFFFFFFFFFFFF
FFFFFFFFFFFFFFF
@A00721:190:HGJW7DSXY:4:1126:20808:19961 2:N:0:TCTGCAAG+GCTGCACT
CGGCGAAGAAGAATTCTGCATTTGTCTCTGTCCAAATCTTTAAAGATGAATTGGCATCCTTGTCCAGG
AACGTCTTATAAGAATATTCTTCTGAGCCTGGATTGCAAAGCACGATTGACGGGATACCACCTTTAAT
TTGAATTGGTTTGCC
+
FFFFFFFFFFFFFFFFFFFFFFFFFFFF:FFFFFFFFFFFFFFFFFFF:FFFFFFFFFFFFFFFF:FF
FFFFFFFFFFFFFFFFF:FFFFFFFFFFFFFFFFFFFFFFFFFF:FFFFFFFFF:FFFFFFFFFFFFF
:FFFFFFFFFFFFFF
@A00721:190:HGJW7DSXY:4:1319:19542:11475 2:N:0:TCTGCAAG+GCTGCACT
CGGCGAAGAAGAATTCTGCATTTGTCTCTGTCCAAATCTTTAAAGATGAATTGGCATCCTTGTCCAGG
AACGTCTTATAAGAATATTCTTCTGAGCCTGGATTGCAAAGCACGATTGACGGGATACCACCTTTAAT
TTGAATTGGTTTGCC
+
FFFFFFFFFFFFFFFFFFFFF:FFFFFFFFFFFFFFFFFFFFFFFFFFFFFFFFFFFFFF,FFFFFFF
FFFFFFFF:FFF:FFFFFFFFFFFFFFFFFFFFFFFFFF,FF:FFFFFFFFFFFFFFFFFFFF,FF:F
FFFFFFFFF:FFFFF
@A00721:190:HGJW7DSXY:4:1675:30490:2065 2:N:0:TCTGCAAG+GCTGCACT
CGGCGAAGAAGAATTCTGCATTTGTCTCTGTCCAAATCTTTAAAGATGAATTGGCATCCTTGTCCAGG
AACGTCTTATAAGAATATTCTTCTGAGCCTGGATTGCAAAGCACGATTGACGGGATACCACCTTTAAT
TTGAATTGGTTTGCC
+
FFFFFFFF:FFFFFFFFFFFFFFFFFFFFFFFFFFFFFFFFFFFF:FFFFFFFFFFFFFFFFFFFFFF
FFFFFFFFFFFFFFFFF:FFFFFFFFFFFFFFFFFFFFFFFFFFFFFFFFFFFFFFFFFFFFFFFF:F
FFFFFFFFFFFFFFF

```
@A00721:190:HGJW7DSXY:4:1678:29288:16172 2:N:0:TCTGCAAG+GCTGCACT
CGGCGAAGAAGAATTCTGCATTTGTCTCTGTCCAAATCTTTAAAGATGAATTGGCATCCTTGTCCAGG
AACGTCTTATAAGAATATTCTTCTGAGCCTGGATTGCAAAGCACGATTGACGGGATACCACCTTTAAT
TTGAATTGGTTTGCC
+
FFFFFFFFFFFFFFFFFFFFFFFFFFFFFFFFFFFFFFFFFFFFFFFFFFFFFFFFFFFFFFFFFFFFFF
FFFFFFFFFFFFFFF,FFFFFF:FFFFFFFFFFFFFFFFFFFFFFFFFFFFFFFFFFFFFFFFFF,FF,F
:FFFFFFFFFFFFFF
@A00721:190:HGJW7DSXY:4:2176:24252:20008 2:N:0:TCTGCAAG+GCTGCACT
CGGCGAAGAAGAATTCTGCATTTGTCTCTGTCCAAATCTTTAAAGATGAATTGGCATCCTTGTCCAGG
AACGTCTTATAAGAATATTCTTCTGAGCCTGGATTGCAAAGCACGATTGACGGGATACCACCTTTAAT
TTGAATTGGTTTGCC
+
FFFFFFFFFFFFFFFFFFFFFFFFFFFFFFFFFFFFFFFFFFFFFFFFFFFFFFFFFFFFFFFFFFF:FF
FFFFFFFFFFFFFFFFFFFFFFFFFFFFFFFFFFFFFFFFFFFFFFFFFFFFFFF:FFFFFFFFFFFFFF
FFFFFF:FFFFFFFF
@A00721:190:HGJW7DSXY:4:2247:29857:3693 2:N:0:TCTGCAAG+GCTGCACT
CGGCGAAGAAGAATTCTGCATTTGTCTCTGTCCAAATCTTTAAAGATGAATTGGCATCCTTGTCCAGG
AACGTCTTATAAGAATATTCTTCTGAGCCTGGATTGCAAAGCACGATTGACGGGATACCCCCTTTAAT
TTGAATTGGTTTGC
+
F,::FFFFFFFFF,FFF::F::F,FFFFFFFFFFFFFF,F:FFFFFFFFFFFFFF:FFFFFFFFFF,FFF
FFFFFF:F,F,FFF,,FFF,,FF:FFF,F:FFFFFFFFFF::FFFFF::FFFFFFF:F:F:,FFF,FFFF
,FFF,,F:FFFFFF
@A00721:190:HGJW7DSXY:4:2343:7654:27618 2:N:0:TCTGCAAG+GCTGCACT
CGGCGAAGAAGAATTCTGCATTTGTCTCTGTCCAAATCTTTAAAGATGAATTGGCATCCTTGTCCAGG
AACGTCTTATAAGAATATTCTTCTGAGCCTGGATTGCAAAGCACGATTGACGGGATACCACCTTTAAT
TTGAATTGGTTTGCC
+
:FFFFFFFFFFFFFFFFFFFFFFFFFFFFFFFFFFFFFFFFFFFF:FFFFFFFFFFFFFFFFFFFF::FF
FF:FFF:FFFFFF::FFFF,FFFFFFFFF,FFFFFFFFFFFFFFFFF,F:F:,FFFFF,FFFFFF,FF:F
,FFFF,FFFFFFFFF
@A00721:190:HGJW7DSXY:4:2414:4860:30733 2:N:0:TCTGCAAG+GCTGCACT
CGGCGAAGAAGAATTCTGCATTTGTCTCTGTCCAAATCTTTAAAGATGAATTGGCATCCTTGTCCAGG
AACGTCTTATAAGAATATTCTTCTGAGCCTGGATTGCAAAGCACGATTGACGGGATACCACCTTTAAT
TTGAATTGGTTTGCC
+
FFFFFF:FFFFFFFFFFFFFFFFFFFFFFFFFFFFFFFFFFFF:FFFFFFFFFFFFFFFFFFFFFFFFFF
FFFFFF:FFFF:F,FFFFFFF:FFFFFFFFFFFFFFFFFFFFFFFFFFFFFFFF:FFFFFFFFFF,FFFF
FFFFFFFFFFFFFFF
@A00721:190:HGJW7DSXY:4:2627:1479:36839 2:N:0:TCTGCAAG+GCTGCACT
CGGCGAAGAAGAATTCTGCATTTGTCTCTGTCCAAATCTTTAAAGATGAATTGGCATCCTTGTCCAGG
AACGTCTTATAAGAATATTCTTCTGAGCCTGGATTGCAAAGCACGATTGACGGGATACCACCTTTAAT
TTGAATTGGTTTGCC
+
FFFFFFFFFFFFFFFFFFFFF:FF:FFFFFFFFFFFFFFFFFFFFFFFFFFFFFFFFFFFFF,FFFF:FF
FFFFF:FFFFFFFFFFF:FFFFFFF,FFFFFFFFFFFFFFFFFFFFFFFFFFFFFFFFFFFFFFFFFFFF
FFFFFFFFFFFFFFF
@A00721:190:HGJW7DSXY:4:2673:15139:5196 2:N:0:TCTGCAAG+GCTGCACT
CGGCGAAGAAGAATTCTGCATTTGTCTCTGTCCAAATCTTTAAAGATGAATTGGCATCCTTGTCCAGG
AACGTCTTATAAGAATATTCTTCTGAGCCTGGATTGCAAAGCACGATTGACGGGATACCACCTTTAAT
TTGAATTGGTTTGCC
+
FFFFFFFF,FFFFFFFFFFFFFFFFFFFFFFFFFFFFFFFFFFFFFFFFFFFFFFFFFFFFFFFFFFFFF
```

FFFFFFFFFFFFFFFFFFFFFFFFFFFFFFFFFFFFFFFFFFFFFFFFFFFFFFFFFFFFFFFFFFFF
FFFFFFFFFFFFFFF
@A00721:190:HGJW7DSXY:4:1528:9453:6715 2:N:0:TCTGCAAG+GCTGCACT
GTACGGCAAACCAATTCAAATTAAAGGTGGTATCCCGTCAATCGTGCTTTGCAATCCAGGCTCAGAAG
AATATTCTTATAAGACGTTCCTGGACAAGGATGCCAATTCATCTTTAAAGATTTGGACAGAGACAAAT
GCAGAATTCTTCTTC
+
FFFFFFFFFFFFFFFFFFFFFFFFFFFFFFFFFFFFFFFFFFFFFFFFFFFFFFFFFFFFFFFFFFFF
FFFFFFFFFFFFFFFFFFFFFFFF,FFFFFFFFFFFFFFFFFFFFFFFFFFFFFFFFFFFFFFFFFFF
FFFFFFFFFFFFFFF
@A00721:190:HGJW7DSXY:4:1564:17183:7357 2:N:0:TCTGCAAG+GCTGCACT
GTACGGCAAACCAATTCAAATTAAAGGTGGTATCCCGTCAATCGTGCTTTGCAATCCAGGCTCAGAAG
AATATTCTTATAAGACGTTCCTGGACAAGGATGCCAATTCATCTTTAAAGATTTGGACAGAGACAAAT
GCAGAATTCTTCTTC
+
FFFFFFFFFFFFFFFFFFFFFFFF:FFFFFFFFFFFFFFFFFFFFFFFFFFFFFFFFFFFFFFFFFFF
FFFFFFFFFFFFFFFFFFF:FFFFFFFFFFFFFFFFFFFFFFFFFFF:,:FFFF,FFFFFF:FFFFFF
FFFFFFFFFFFFFFF
@A00721:190:HGJW7DSXY:4:1676:15157:23484 2:N:0:TCTGCAAG+GCTGCACT
GTACGGCAAACCAATTCAAATTAAAGGTGGTATCCCGTCAATCGTGCTTTGCAATCCAGGCTCAGAAG
AATATTCTTATAAGACGTTCCTGGACAAGGATGCCAATTCATCTTTAAAGATTTGGACAGAGACAAAT
GCAGAATTCTTCTTC
+
F:FFFFFFFFFFFFFFFFF:FFF,FFFF,FFFFFFFF:FFFFF:,FFFFF:FFFFFFF:FFFFFF:FF
FFFFF::FFFFFFFFFFFFFFFFFFFF::FFFFF:FFFFFFFFFFF:FFFFFFFFFFFFFFFFFFF:F
FFFF:FFFFFFF:FF
@A00721:190:HGJW7DSXY:4:1213:27597:5447 2:N:0:TCTGCAAG+GCTGCACT
AGAAGAATTCTGCATTTGTCTCTGTCCAAATCTTTAAAGATGAATTGGCATCCTTGTCCAGGAACGTC
TTATAAGAATATTCTTCTGAGCCTGGATTGCAAAGCACGATTGACGGGATACCACCCTTAATTTGAAT
TGGTTTGCCGTACTT
+
FFFFFFFFFFFFFF:FFFFFFFFFFFFFFFFFFFFFFFFFFFFFFFFFFFFFFFFFFFFFFFFFFFFF
FFFF,FFFFFFFFFFFFFFFFFFFFFFFFFFFFFF:FFFFFFFFFFFF:FFFFFFF,FFFF:FFFFFF
FFFFFFFFFFFFFFF
@A00721:190:HGJW7DSXY:4:1228:19813:22561 2:N:0:TCTGCAAG+GCTGCACT
AGAAGAATTCTGCATTTGTCTCTGTCCAAATCTTTAAAGATGAATTGGCATCCTTGTCCAGGAACGTC
TTATAAGAATATTCTTCTGAGCCTGGATTGCAAAGCACGATTGACGGGATACCACCTTTAATTTGAAT
TGGTTTGCCGTACTT
+
FFFFFF:FFFFFFFFFFFFFFFFFFFFFFFFF:FFFFFFFFFFFFFFFFFFFFFFFF:FFFFFFFFFF
FFFFFF:FFFFFFFFFFFFFFFFFFFFFFFFFFFFFFFFFFFFFFFFFFFFFFFFFFFFFFFF:FF:F
FFFFFFFFFFFFFF:
@A00721:190:HGJW7DSXY:4:2134:28203:13542 2:N:0:TCTGCAAG+GCTGCACT
AGAAGAATTCTGCATTTGTCTCTGTCCAAATCTTTAAAGATGAATTGGCATCCTTGTCCAGGAACGTC
TTATAAGAATATTCTTCTGAGCCTGGATTGCAAAGCACGATTGACGGGATACCACCTTTAATTTGAAT
TGGTTTGCCGTACTT
+
FFFFFFF::FFFF:FFF,FFFFFFFFFFFFFFF,F:FFFFFFFFFFFFFFFFF::FFF:FFFFFFF:F
FFFFF,:FF,FFF:FFFFFFFF:,FFFFFF,FFFFFFFFFFFFFFFFF:FFFFFFFF,FFFFFFFFFF
FF,FFF,FFFFFFF:
@A00721:190:HGJW7DSXY:4:2272:32579:21840 2:N:0:TCTGCAAG+GCTGCACT
AGAAGAATTCTGCATTTGTCTCTGTCCAAATCTTTAAAGATGAATTGGCATCCTTGTCCAGGAACGTC
TTATAAGAATATTCTTCTGAGCCTGGATTGCAAAGCACGATTGACGGGATACCACCTTTAATTTGAAT
TGGTTTGCCGTACTT

```
+
FFFFFFFFFFFFFFFFFFFFFFFFFFFFFFFFFFFFF:FFFFFFFFFFFFFFFFFFFFFFFFFFFFFF
FFFFFFFF,F:FFFFFFFF:FF:FFFFF:FFFFF:FFFFFFFFFFFFFFFFFFF:FFFFF:FFFFFFF
F:FFFFFFFFFFFF:
@A00721:190:HGJW7DSXY:4:2616:3025:7012 2:N:0:TCTGCAAG+GCTGCACT
AGAAGAATTCTGCATTTGTCTCTGTCCAAATCTTTAAAGATGAATTGGCATCCTTGTCCAGGAACGTC
TTATAAGAATATTCTTCTGAGCCTGGATTGCAAAGCACGATTGACGGGATACCACCTTTAATTTGAAT
TGGTTTGCCGTACTT
+
FFFFFFFFFFFFFFFFFFFFFFFFFFFFFFFFFFFFFFFFFFFFFFFFFFFFFFFFFFFFFFFFFFFF
FFFFFFFFFFFFFFFFFFFFFFF:FFFFFFFFFFFFFFFFFFFFFFFFFFFFFFFFFFFFFFFFFFFF
FFF:FFFFFFFFFFF
@A00721:190:HGJW7DSXY:4:1227:25997:28510 2:N:0:TCTGCAAG+GCTGCACT
GGTATCCAATGTGAAGTACGGCAAACCAATTCAAATTAAAGGTGGTATCCCGTCAATCGTGCTTTGCA
ATCCAGGCTCAGAAGAATATTCTTATAAGACGTTCCTGGACAAGGATGCCAATTCATCTTTAAAGATT
TGGACAGAGACAAAT
+
FFFFFFFFF,FFFFFFFFFFFFFFFFF:FFFFFFFFFFFFFFFF:FFFFFFFFFFFFFFFFFFFFFFF
:FFFFFFFFFFFFFFFFFFFFFFFFFFFFFFF,FFFFFFFFFFFFFFFF:FFFF:FFFFFFFFF:FFFF
FFFFFFFFFFFFFFF
@A00721:190:HGJW7DSXY:4:2317:9362:1892 2:N:0:TCTGCAAG+GCTGCACT
GGTATCCAATGTGAAGTACGGCAAACCAATTCAAATTAAAGGTGGTATCCCGTCAATCGTGCTTTGCA
ATCCAGGCTCAGAAGAATATTCTTATAAGACGTTCCTGGACAAGGATGCCAATTCATCTTTAAAGATT
TGGACAGAGACAAAT
+
FFFFFFFFFFFFFFFFFFFFFFFFFFF::FFFFF:,FFF:FF:FFFFFFFFFFFFFFFFFFFFFFFFF
:FFFFFFFFFFFFFFFFFFFFFFFFFFFFF:FFFFFFFFFFFFFFFFFFFFFF::FFFFFFFFFFFFFF
FFFFFFFFFFFFFFF
@A00721:190:HGJW7DSXY:4:1453:25536:6762 2:N:0:TCTGCAAG+GCTGCACT
GCTTTGCAATCCAGGCTCAGAAGAATATTCTTATAAGACGTTCCTGGACAAGGATGCCAATTCATCTT
TAAAGATTTGGACAGACTATCCGCATCTGGACGACATTACAACCAGTCCCAGAATTACTGCAGAACCC
GTCATGGAAGACGAT
+
FFFFFFFFFFFFFFFFFFFFFFFFFFFFFFFFFFFFFFFFFFFFFFFFFFFFFFFFFFFF:FFFFFFF
FFFFFFFFFF:FFFFFFFFFFFFFFFFFFFFFFFFFFFFFFFFFFFFFFFFFFFFFFFFFFFFFFFFF
FFFFFFFFFFFFFFF
@A00721:190:HGJW7DSXY:4:1219:25861:36072 2:N:0:TCTGCAAG+GCTGCACT
ATCCCGTCAATCGTGCTTTGCAATCCAGGCTCAGAAGAATATTCTTATAAGACGTTCCTGGACAAGGA
TGCCAATTCATCTTTAAAGATTTGGACAGACTATCCGCATCTGGACGACATTACAACCAGTCCCAGAA
TTACTGCAGAACCCG
+
FFFFFFFFFFFFFFFFFFFFFFFFFFFFFFFFFFF:FFFFFFFFFFFFFFFFFFFFFFFFFFFFFFFF
FFFF:FFFFFFFFFFFFFFFFFFFFFFFFFFFFFFFFFFFFFFFFFFFFFFFFFFFFFFFFFFFFFFF
FFFFFFFFFFFFFFF
@A00721:190:HGJW7DSXY:4:1447:32009:23766 2:N:0:TCTGCAAG+GCTGCACT
ATCCCGTCAATCGTGCTTTGCAATCCAGGCTCAGAAGAATATTCTTATAAGACGTTCCTGGACAAGGA
TGCCAATTCATCTTTAAAGATTTGGACAGACTATCCGCATCTGGACGACATTACAACCAGTCCCAGAA
TTACTGCAGAACCCG
+
F:FFFFFFFF:FFFF::FFFFF:FFFFFFFFFFFFFFF,FFFFFFFFFFFFFFF:FFF:FFFFFFFFF
F:FFFFFFFFFFFFFFFFFFFFFFFFFFFFFFFFFFFFFFFFFFFFFFFF::FFFFFF:F::FFFFFFF
FFFFFFFFFF:FFFF
@A00721:190:HGJW7DSXY:4:1536:28971:25676 2:N:0:TCTGCAAG+GCTGCACT
ATCCCGTCAATCGTGCTTTGCAATCCAGGCTCAGAAGAATATTCTTATAAGACGTTCCTGGACAAGGA
```

```
TGCCAATTCATCTTTAAAGATTTGGACAGACTATCCGCATCTGGACGACATTACAACCAGTCCCAGAA
TTACTGCAGAACCCG
+
FFFFFFFFFFFFFFFFFFFFFFFFFFFFF:FFFFFFFFFFFFFFFFFFFFFFFFFFFFFFFFFFFFFFF
FFFFFFFFFFFFFFFF:F:FFFFF:FFFFFFFFFFFFFFFFFF,FFFFFFFFF,FFFFFFFFFFFFF
F:FFFFFFF:FFFFF
@A00721:190:HGJW7DSXY:4:1570:23619:7513 2:N:0:TCTGCAAG+GCTGCACT
ATCCCGTCAATCGTGCTTTGCAATCCAGGCTCAGAAGAATATTCTTATAAGACGTTCCTGGACAAGGA
TGCCAATTCATCTTTAAAGATTTGGACAGACTATCCGCATCTGGACGACATTACAACCAGTCCCAGAA
TTACTGCAGAACCCG
+
FFFFFFFFFFFFFFFFFFFFFFFFFFFFFFFFFFFFFFFFFFFFFFFFFFFFFFFFFF:FFFFFFFFF
FFFFFFFFFFFFFFFFF:FFFFFF:FFFFFFFFFFFFFFFFFFFFF:FFFFFFFFF:FFFFFFFFFF
FFF:FFFFFFFFF:F
@A00721:190:HGJW7DSXY:4:2343:7527:22451 2:N:0:TCTGCAAG+GCTGCACT
ATCCCGTCAATCGTGCTTTGCAATCCAGGCTCAGAAGAATATTCTTATAAGACGTTCCTGGACAAGGA
TGCCAATTCATCTTTAAAGATTTGGACAGACTATCCGCATCTGGACGACATTACAACCAGTCCCAGAA
TTACTGCAGAACCCG
+
FFFFFFFFFFFFFFFFFFFFFFFFFFFFFFFFFFFFFFFFFFFFFFFFFFFFFFFFFF:FFFFFFFFF
FFFFFFFFFFFFFFFFFFFFFFFFFFFFFFFFFFFFFFFFFFFFFFFFFFFFFFFFFFFFFFFFFFFF
FFFFFFFFFFFFFFF
@A00721:190:HGJW7DSXY:4:2614:20717:25254 2:N:0:TCTGCAAG+GCTGCACT
ATCCCGTCAATCGTGCTTTGCAATCCAGGCTCAGAAGAATATTCTTATAAGACGTTCCTGGACAAGGA
TGCCAATTCATCTTTAAAGATTTGGACAGACTATCCGCATCTGGACGACATTACAACCAGTCCCAGAA
TTACTGCAGAACCCG
+
FFFFFFFFFFFF:FFFFFFFFFFFFFFFFFFFFFFFFFFFFFFFFFFFFFFFFF:FFFFFFFFFFFF:F
FFFFFFFFFFFFFFFFFFFFFFFFFFFFFFFFFFFF:FFFFFFFFFFFFFFFFFFFFFFFFFFF:FFFFF
F:FFFF:FFFFFF:F
@A00721:190:HGJW7DSXY:4:1421:7961:20415 2:N:0:TCTGCAAG+GCTGCACT
CGGCAAACCAATTCAAATTAAAGGTGGTATCCCGTCAATCGTGCTTTGCAATCCAGGCTCAGAAGAAT
ATTCTTATAAGACGTTCCTGGACAAGGATGCCAATTCATCTTTAAAGATTTGGACAGACTATCCGCAT
CTGGACGACATTACA
+
FFFFFFFFFFFFFFFFFFFFFFFFFFFFFFFFFFFFFFFFFFFFFFFFFFFFFFFFFFFFFFFFFFFF
FFFFFFFFFFFFFFFFFFFFFFFFFFFFFFFFFFFFFFFFFFFFFFFFFFFFFFFFFFFFFFFFFFFF
FFFFFFFFFFFFF:F
@A00721:190:HGJW7DSXY:4:1519:28366:17268 2:N:0:TCTGCAAG+GCTGCACT
CGGCAAACCAATTCAAATTAAAGGTGGTATCCCGTCAATCGTGCTTTGCAATCCAGGCTCAGAAGAAT
ATTCTTATAAGACGTTCCTGGACAAGGATGCCAATTCATCTTTAAAGATTTGGACAGACTATCCGCAT
CTGGACGACATTACA
+
FFFFFFFFFFFFFFFFFFFFFFFFFFFFFFFFFFFFFFFFFFFFFFFFFFFFFFFFFFFFFFFFFFFF
FFFFFFFFFFFFFFFFFFFFFFFFFFFFFFFFFFFFFFFFFFFFF:FFFFFFFFFFFFFFFFFFFFFF
FFFFFFFFFFFFFFF
@A00721:190:HGJW7DSXY:4:1560:2193:26678 2:N:0:TCTGCAAG+GCTGCACT
CGGCAAACCAATTCAAATTAAAGGTGGTATCCCGTCAATCGTGCTTTGCAATCCAGGCTCAGAAGAAT
ATTCTTATAAGACGTTCCTGGACAAGGATGCCAATTCATCTTTAAAGATTTGGACAGACTATCCGCAT
CTGGACGACATTACA
+
FFFFFFFFFFFFFFFF:FF:FFFFFFFFFFFFFFFFFFFFFFFFFFFFFFFFFFFFFFFFFFF:FFFFF
FFFFFFFFFFFFFFFFFFFFFFFFFFFFFFFFFFFFFFFFFFFFFFFFFFFFFFFFFFFF:FFFFFFF
FFFFFFFFFFFFFFF
```

```
@A00721:190:HGJW7DSXY:4:2612:27380:33724 2:N:0:TCTGCAAG+GCTGCACT
CGGCAAACCAATTCAAATTAAAGGTGGTATCCCGTCAATCGTGCTTTGCAATCCAGGCTCAGAAGAAT
ATTCTTATAAGACGTTCCTGGACAAGGATGCCAATTCATCTTTAAAGATTTGGACAGACTATCCGCAT
CTGGACGACATTACA
+
FFFFFF:FF:FFFFFFFFFFFFF:F:FFFFFFFFFFFFFFFFFFFFFFFFFFFFFFFFFFFFFF:F:FFF
FFFFFFF:FFFFFFFFFFFFFFFFFFFFFFFFFFFFFFFFFFFFFFFFFFFFFFFFF:FFFF:FFFFFFF
FFFFFFFFFFFFFFF
@A00721:190:HGJW7DSXY:4:2640:19633:22905 2:N:0:TCTGCAAG+GCTGCACT
CGGCAAACCAATTCAAATTAAAGGTGGTATCCCGTCAATCGTGCTTTGCAATCCAGGCTCAGAAGAAT
ATTCTTATAAGACGTTCCTGGACAAGGATGCCAATTCATCTTTAAAGATTTGGACAGACTATCCGCAT
CTGGACGACATTACA
+
FFFFF:FFFFFFFFFFFFFFF:FFFFFF:FFFFFFFFFFFFFFFFFFFFFFF:FFFFFFFFFFFFFFFFF
FFFFFFFF,FFFFFFFFFFF:FFFF:FFFFFFFFFFFFFFFFFFFFF:FFFFFFFFFFFFFF:FFFFFF,
FFFFFF:FFFFFFFF
@A00721:190:HGJW7DSXY:4:1117:6696:13933 2:N:0:TCTGCAAG+GCTGCACT
GCACAATTGGGTATCCAATGTGAAGTACGGCAAACCAATTCAAATTAAAGGTGGTATCCCGTCAATCG
TGCTTTGCAATCCAGGCTCAGAAGAATATTCTTATAAGACGTTCCTGGACAAGGATGCCAATTCATCT
TTAAAGATTTGGACA
+
FFFFFFFFFFFFFFFFFF:FFFFFFFFFFFFFFFFFFFFFFFFFFFFFFFFFFFFFFFFFFFFFFFFFFF
FFFFFFFFFFFFFFFFFFFFFF:FFFFFFFFFFFFFFFFFFFFFFFFFFFFFFFFFFFFFFF:FFFFFFF
FFFFFFFFFFFFFFF
@A00721:190:HGJW7DSXY:4:1147:14714:28322 2:N:0:TCTGCAAG+GCTGCACT
GCACAATTGGGTATCCAATGTGAAGTACGGCAAACCAATTCAAATTAAAGGTGGTATCCCGTCAATCG
TGCTTTGCAATCCAGGCTCAGAAGAATATTCTTATAAGACGTTCCTGGACAAGGATGCCAATTCATCT
TTAAAGATTTGGACA
+
FFFFFFFFFFFFFFFFFFFFFFFFFFFFFFFFFFFFFFFFFFFFFFFFFFFFFFFFFFFFFFFFFFFFFF
FFFFFFFFFFFFFFFFFFFFFF:FFFFFFFFFFFFFFFFFFFFFFFFFFFFFFFF:FFFFFFFFFFFFFF
FFFF:FFFFFFFFFF
@A00721:190:HGJW7DSXY:4:1160:30987:11130 2:N:0:TCTGCAAG+GCTGCACT
GCACAATTGGGTATCCAATGTGAAGTACGGCAAACCAATTCAAATTAAAGGTGGTATCCCGTCAATCG
TGCTTTGCAATCCAGGCTCAGAAGAATATTCTTATAAGACGTTCCTGGACAAGGATGCCAATTCATCT
TTAAAGATTTGGACA
+
FFFFFFFFFFFFFFFFFFFFFFFFFFFFFFFFFFFFFFFFFFFFFFFFF,FFFFFFFFFFFFFFFFFFFF
FFFFFFFFFFFFFFFFFFFFFFFFFFFFFFFFFFFFFFF:FFFFFFFFFFFFFFFFFFFF:FFFFFFFFF
FFFF:FFFFFFFFFF
@A00721:190:HGJW7DSXY:4:1208:6198:18803 2:N:0:TCTGCAAG+GCTGCACT
GCACAATTGGGTATCCAATGTGAAGTACGGCAAACCAATTCAAATTAAAGGTGGTATCCCGTCAATCG
TGCTTTGCAATCCAGGCTCAGAAGAATATTCTTATAAGACGTTCCTGGACAAGGATGCCAATTCATCT
TTAAAGATTTGGACA
+
FFFFF:FFFFFFFFFFFFFFFFFFFFFFFFFFFFFFFFFFFFFFFFFFFFFFFFFFF,FFFFFFFFFFFF
FFFFFFFFFFFFFFFFFFFFFFFFFFFFFFFFFFFFFFFFFFFFFFFFFFFFFFFFFFFFFFFFFFFFFF
FFFF:FFFFF:FFFF
@A00721:190:HGJW7DSXY:4:1258:31910:34835 2:N:0:TCTGCAAG+GCTGCACT
GCACAATTGGGTATCCAATGTGAAGTACGGCAAACCAATTCAAATTAAAGGTGGTATCCCGTCAATCG
TGCTTTGCAATCCAGGCTCAGAAGAATATTCTTATAAGACGTTCCTGGACAAGGATGCCAATTCATCT
TTAAAGATTTGGACA
+
FFFFFF:FFFFFFFFFFFFFFFFFFFFFFFFFFFFFFFFFFFFFFFFFFFFFFFFFFFFFFFFFFFFFFF
```

```
FFFFFFFFFFFFFFFFFFFFFFFFFFFFFFF:FFFFFFFF:FFFFFFFFFFFFFFFFFFFF:FFFFFF
,FF::FF:FF:FFF:
@A00721:190:HGJW7DSXY:4:1276:5141:27211 2:N:0:TCTGCAAG+GCTGCACT
GCACAATTGGGTATCCAATGTGAAGTACGGCAAACCAATTCAAATTAAAGGTGGTATCCCGTCAATCG
TGCTTTGCAATCCAGGCTCAGAAGAATATTCTTATAAGACGTTCCTGGACAAGGATGCCAATTCATCT
TTAAAGATTTGGACA
+
FFFFFFFFFFF:FFFFFFFFFFFFFFFFFFFFFFFFFFFFFFFFFFFF,FFFFFF:,FFFFFFF:FFF
FFFFFFFFFFFFFFFFFFFFFF,FFFFF:FFFFFFFF:FFFFFFFFFFFFFFFFFFFFFFFFFFFFFF
FFF:FFF:FFFFFFF
@A00721:190:HGJW7DSXY:4:1430:25644:20415 2:N:0:TCTGCAAG+GCTGCACT
GCACAATTGGGTATCCAATGTGAAGTACGGCAAACCAATTCAAATTAAAGGTGGTATCCCGTCAATCG
TGCTTTGCAATCCAGGCTCAGAAGAATATTCTTATAAGACGTTCCTGGACAAGGATGCCAATTCATCT
TTAAAGATTTGGACA
+
FFFFFFFFFFFFFFFFFFFFFFFFFFFFFFFFFFFFFF:FFFFFFFFFFFFFFFFFFFFFFFFFFFFF
FFFFFFFFFFFFFFFFFFFFFFFFFFFFFFFFFFFFF:FFFFFFF:FFFFFFFFFFFFFF:FFFFFFF
FFFFFFFFFFFFFFF
@A00721:190:HGJW7DSXY:4:1467:30291:31250 2:N:0:TCTGCAAG+GCTGCACT
GCACAATTGGGTATCCAATGTGAAGTACGGCAAACCAATTCAAATTAAAGGTGGTATCCCGTCAATCG
TGCTTTGCAATCCAGGCTCAGAAGAATATTCTTATAAGACGTTCCTGGACAAGGATGCCAATTCATCT
TTAAAGATTTGGACA
+
FF,FFFFFFFFFFFFFFFFFFFFFFFFFFFFFFFFFFFFFFFFFFFFFFFFFFFFFFFFFFFFFFFFF
FFF:FFFFFFFFFFFFFFFFFFFFFFFFFFF:F:FFFFFFFFFFFFF:FFFFFFFFFFFFFFFFFFFF
FF:::FFFFFFFF:F
@A00721:190:HGJW7DSXY:4:1477:20582:7889 2:N:0:TCTGCAAG+GCTGCACT
GCACAATTGGGTATCCAATGTGAAGTACGGCAAACCAATTCAAATTAAAGGTGGTATCCCGTCAATCG
TGCTTTGCAATCCAGGCTCAGAAGAATATTCTTATAAGACGTTCCTGGACAAGGATGCCAATTCATCT
TTAAAGATTTGGACA
+
FFFFFFFFFFFFFFFFFFFFFFFFFFFFFFFFFFFFFFFFFFFFFFFFFFFFFFFFFFFFFFFFFFFF
FFFFFFFFFFFFFFFFFFFFFF:FFFFFFFFFFFFFFFFFFFFFFFFFFF::FFFFFFFFFFFFFFFF
FF:F,FF,FFFFFFF
@A00721:190:HGJW7DSXY:4:2120:8929:12665 2:N:0:TCTGCAAG+GCTGCACT
GCACAATTGGGTATCCAATGTGAAGTACGGCAAACCAATTCAAATTAAAGGTGGTATCCCGTCAATCG
TGCTTTGCAATCCAGGCTCAGAAGAATATTCTTATAAGACGTTCCTGGACAAGGATGCCAATTCATCT
TTAAAGATTTGGACA
+
F,::FFF:FFFFFFFFFFFFFFFFFFFFFFFFFFFFFFFFFFFFFFFFFFFFFFFFFFF:FFFFFFFF
FF:FFFF:FFFFFFFFFFFFFFFFFFFFF:FFFFFFFFFFFFF:F:FFFFFFF:F:,F:FF:FFFFFF
FFFFFFFFF:F:FFF
@A00721:190:HGJW7DSXY:4:2158:22661:18067 2:N:0:TCTGCAAG+GCTGCACT
GCACAATTGGGTATCCAATGTGAAGTACGGCAAACCAATTCAAATTAAAGGTGGTATCCCGTCAATCG
TGCTTTGCAATCCAGGCTCAGAAGAATATTCTTATAAGACGTTCCTGGACAAGGATGCCAATTCATCT
TTAAAGATTTGGACA
+
FFFFFFFFFFFFFFFFFFFFFFFFFFFFFFFFFFFFFFFFFFFFFFFFFFFFFFFFFFFFFFFFFFFF
FFFF,FFFFFFFFFFFFFFFFFFFFFFFFFFFFFFFFFFFFFFFFFFFFFFFFFFFFF:F:FFFFFF
FFFF:FFFFFFFFF:
@A00721:190:HGJW7DSXY:4:2308:6262:12461 2:N:0:TCTGCAAG+GCTGCACT
GCACAATTGGGTATCCAATGTGAAGTACGGCAAACCAATTCAAATTAAAGGTGGTATCCCGTCAATCG
TGCTTTGCAATCCAGGCTCAGAAGAATATTCTTATAAGACGTTCCTGGACAAGGATGCCAATTCATCT
TTAAAGATTTGGACA
```

+
FFFFFFFFFFFFFFFFFFFFFFFFFFFFFFFFFFFFFFFFFFFFFFFFFFFFFFFFFFFFFFFFFFFF
FFFFFFFFFFFFFFFFFFFFFF:FF:FFFFFFFFFFFFFFFFFFFFFFFFFFFFFFFFFFFFFFFFFF
FFFFFFFFFFFFFFF
@A00721:190:HGJW7DSXY:4:2541:2935:16094 2:N:0:TCTGCAAG+GCTGCACT
GCACAATTGGGTATCCAATGTGAAGTACGGCAAACCAATTCAAATTAAAGGTGGTATCCCGTCAATCG
TGCTTTGCAATCCAGGCTCAGAAGAATATTCTTATAAGACGTTCCTGGACAAGGATGCCAATTCATCT
TTAAAGATTTGGACA
+
F:FFFFFFFFFFFFFFFFFFFFFFFFFFFFFFFFFFFFFFFFFFFFFFFFFFFFFFFFFFFFFFFFFF
FFFFFFFFF:FFFFFFFFFFFFFFFFFFFFFFFFFFFFFFFFFFFFFFFFFFFFFFFFFFFFFFFFFF
FFFFFFFFFFFFFFF
@A00721:190:HGJW7DSXY:4:2603:12319:34851 2:N:0:TCTGCAAG+GCTGCACT
GCACAATTGGGTATCCAATGTGAAGTACGGCAAACCAATTCAAATTAAAGGTGGTATCCCGTCAATCG
TGCTTTGCAATCCAGGCTCAGAAGAATATTCTTATAAGACGTTCCTGGACAAGGATGCCAATTCATCT
TTAAAGATTTGGACA
+
FFFFFFFFFFFFFFFFF:FFFFFFFFFFFFFFFFFFFFF:FFF:FFFFFFFFFFFFFFF:FFFF:FF:
FFFFFFFFFFFFF:FFFFF:FFFFFFFFFF:FFF:FFF:FFFFF:FFFFFFFFFFFFF:F,FFFFFFF
:FF:FFFFFFFFFFF
@A00721:190:HGJW7DSXY:4:2659:29215:1892 2:N:0:TCTGCAAG+GCTGCACT
GCACAATTGGGTATCCAATGTGAAGTACGGCAAACCAATTCAAATTAAAGGTGGTATCCCGTCAATCG
TGCTTTGCAATCCAGGCTCAGAAGAATATTCTTATAAGACGTTCCTGGACAAGGATGCCAATTCATCT
TTAAAGATTTGGACA
+
FFFFFFFFFFFFFFFFFFFFFFFFFFFFFFFFFFFFFFFFFFFFFFFFFFFFFFFFFFFFFFFFFFFF
FFFFFFFFFFFFFFFFFFFFFFFFFFFFFFFFFFFFFFFFFFFFF:FFFFFFFFFFFFF:FFFFFFFF
FFF::FFFFF:FFFF
@A00721:190:HGJW7DSXY:4:1418:32244:35196 2:N:0:TCTGCAAG+GCTGCACT
GCACAATTGGGTATCCAATGTGAAGTACGGCAAACCAATTCAAATTAAAGGTGGTATCCCGTCAATCG
TGCTTTGCAATCCAGGCTCAGAAGAATATTCTTATAAGACGTTCCTGGACAAGGATGCCAATTCATCT
TTAAAGATTTGGAC
+
FFFFFFFFFFFFFFFFFFFFFFFFFFFFFFFFFFFFFFFFFFFFFFFFFF,FFFFFFFFFFFFFFFFF
FFFF:FFFFFFFFFFFFFFFFFFFFFFFFFFFFFFFFFFFFFFFFFFFFF:FFFFFFFFFFFFFFFFF
FFFFFFFFFFF:FF
@A00721:190:HGJW7DSXY:4:1132:3676:14434 2:N:0:TCTGCAAG+GCTGCACT
ACAATTGGGAATCCAATGTGAAGTACGGCAAACCAATTCAAATTAAAGGTGGTATCCCGTCAATCGTG
CTTTGCAATCCAGGCTCAGAATAATAATCTTATAAAACGTTCCTGGACAAGTATGCCAATACATAATT
AAATATTTCTACA
+
:,:::FFF,,:,:::::F,,::F::FF:::::::::,:F::::,:::,:,::,:,,,:::::::,:,,
:F:F,,::::::::,::::::,:::,,,,:,,,::,:,,,,,,,,,:,:::,,:,F,::,,,:,,,F:
:::,:,FF,::,:
@A00721:190:HGJW7DSXY:4:1146:22164:7560 2:N:0:TCTGCAAG+GCTGCACT
ATTGGGGTCCAGCACAATTGGGTATCCAATGTGAAGTACGGCAAACCAATTCAAATTAAAGGTGGTAT
CCCGTCAATCGTGCTTTGCAATCCAGGCTCAGAAGAATATTCTTATAAGACGTTCCTGGACAAGGATG
CCAATTCATCTTTAA
+
FFFFFFFFFFFFFFFFFFFFFFFFFFFFFFFFFFFFFFFFFFFFFFFFFFFFFFFFFFFFFFFFFFFF
FFFFFFFFFFFFFFFFFFFFFFFFFFFFFFFFFFFFFFFFFFFFFFFFFFFFFFFFFFFFFFFFFFFF
FFFFFFFFFFFFFF:
@A00721:190:HGJW7DSXY:4:1272:22435:2895 2:N:0:TCTGCAAG+GCTGCACT
ATTGGGGTCCAGCACAATTGGGTATCCAATGTGAAGTACGGCAAACCAATTCAAATTAAAGGTGGTAT

```
CCCGTCAATCGTGCTTTGCAATCCAGGCTCAGAAGAATATTCTTATAAGACGTTCCTGGACAAGGATG
CCAATTCATCTTTAA
+
FFFFFFFFFFFFFFFFFFFFFFFFFFFFFFFFFFFFFFFFFFFFFFFFFFFFF:FFFFFFFFFFFFFF
FFFFFFFFFFFFFFFFFFFFFFFFFFFFFFFFFFFFFFFFFFFFFFFF:FFFFFFFFF:FFFFFFFFF
FFFFFFFFFFFFFFF
@A00721:190:HGJW7DSXY:4:1331:17662:36464 2:N:0:TCTGCAAG+GCTGCACT
ATTGGGGTCCAGCACAATTGGGTATCCAATGTGAAGTACGGCAAACCAATTCAAATTAAAGGTGGTAT
CCCGTCAATCGTGCTTTGCAATCCAGGCTCAGAAGAATATTCTTATAAGACGTTCCTGGACAAGGATG
CCAATTCATCTTTAA
+
FFFFFFFFFFFFFFFFFFFFFFFFFFF:FFFFFFFFFFFFFFFFFFF:F:FFFFFFF:F,FFFFFFFF
FFFFFFFFFFFFFFFFFFFFFFFFFFFFFFFFFFFFFFFFFFFFFFFFFFFFFFFFFFFFFFFFFFFF
FF,FF:FFFFF:FFF
@A00721:190:HGJW7DSXY:4:1332:15709:1454 2:N:0:TCTGCAAG+GCTGCACT
ATTGGGGTCCAGCACAATTGGGTATCCAATGTGAAGTACGGCAAACCAATTCAAATTAAAGGTGGTAT
CCCGTCAATCGTGCTTTGCAATCCAGGCTCAGAAGAATATTCTTATAAGACGTTCCTGGACAAGGATG
CCAATTCATCTTTAA
+
FFFFFFFFFFFF:FFFFFFFFFFFFFFFFFFFFFFFFFFFFFFFFFFF,FFFFFFFFFFFFFFFFFFF
FFFFFFFFFFFFFFFFFFFFFFFFFFFFFFFFF:FFFFFFFFFFFFFFFFF:FFFFFFFFFFFFFFFF
:FFFFFFFFFF:FFF
@A00721:190:HGJW7DSXY:4:1335:22290:32111 2:N:0:TCTGCAAG+GCTGCACT
ATTGGGGTCCAGCACAATTGGGTATCCAATGTGAAGTACGGCAAACCAATTCAAATTAAAGGTGGTAT
CCCGTCAATCGTGCTTTGCAATCCAGGCTCAGAAGAATATTCTTATAAGACGTTCCTGGACAAGGATG
CCAATTCATCTTTAA
+
FFFFFFFFFFFFFFFFFFFFFFFFFFFFFFFFFFFFFFFFFFFFFFFFFFFFF,FFFFFFFFFFFFFF
FFFFFFFFFFFFFFFFFFFFFFFFFFFFFFFFF:FFFFFFFFFFFFFFFFFFFFFFFFFFFFFFFFFF
FFFFFFFFFFFFFFF
@A00721:190:HGJW7DSXY:4:1544:4562:3912 2:N:0:TCTGCAAG+GCTGCACT
ATTGGGGTCCAGCACAATTGGGTATCCAATGTGAAGTACGGCAAACCAATTCAAATTAAAGGTGGTAT
CCCGTCAATCGTGCTTTGCAATCCAGGCTCAGAAGAATATTCTTATAAGACGTTCCTGGACAAGGATG
CCAATTCATCTTTAA
+
FFFFFFFFFFFF:FFFFFFFFFFFFFFFFFFFFFFFFFF:FFFFFFFFFFFFFFFF:FFFFFFFFFFF
FFFF:FFFFFFFFFFFFFFFFFFFFFFFFF:FFFFFFFFFFFFFFFFF:FFF:FFFFFFFFFFFFFFF
FF,FFFFFFFFFF:F
@A00721:190:HGJW7DSXY:4:2410:8305:2942 2:N:0:TCTGCAAG+GCTGCACT
ATTGGGGTCCAGCACAATTGGGTATCCAATGTGAAGTACGGCAAACCAATTCAAATTAAAGGTGGTAT
CCCGTCAATCGTGCTTTGCAATCCAGGCTCAGAAGAATATTCTTATAAGACGTTCCTGGACAAGGATG
CCAATTCATCTTTAA
+
FFFFFFFFFFFFFFFFFFFFFFFFFFFFFFFFFFFFFFFFFFFFFFFFF:FFFFFFFFF:FFFFFFFF
FFFFFFFFFFFFFFFFFFFFFFFFFFFFFFFFF,FFFFFFFFFFFFFFFFFFFFFFFFFFFFFFFFFF
FFFFFFFFFFFFFFF
@A00721:190:HGJW7DSXY:4:1462:32669:12414 2:N:0:TCTGCAAG+GCTGCACT
CTTATTGGGGTCCAGCACAATTGGGTATCCAATGTGAAGTACGGCAAACCAATTCAAATTAAAGGTGG
TATCCCGTCAATCGTGCTTTGCAATCCAGGCTCAGAAGAATATTCTTATAAGACGTTCCTGGACAAGG
ATGCCAATTCATCTT
+
FFFFFFFFFFFFFFFFFFFFFFFFFFFFFFFFFFFFFFFFFFFFFFFFFFFFFFFFFFFFFFFFFF,FFFFF
FFFFFFFFFFFFFFFFFFFFFFFFFFFFFFFFFFFFFFFFFFFFFFFFFFFFFFFFFFFFFFFFFFFF
FFF:F:FFFFFFFFF
```

```
@A00721:190:HGJW7DSXY:4:1525:15148:2362 2:N:0:TCTGCAAG+GCTGCACT
CTTATTGGGGTCCAGCACAATTGGGTATCCAATGTGAAGTACGGCAAACCAATTCAAATTAAAGGTGG
TATCCCGTCAATCGTGCTTTGCAATCCAGGCTCAGAAGAATATTCTTATAAGACGTTCCTGGACAAGG
ATGCCAATTCATCTT
+
FFFFFFFFFFFFFFFFFFFFFFFFFFFFFFFFFFFFFFFFFFFFFFFFFFFFFFFFFFFFFFFFFFFF
FFFFFFFFFFFFFFFFFFFFFFFFFFFFFFFFFFFFFFFFFFFFFFFFFFFFFFFFFFFFFFFFFFFF
FFFFFFFFFFFFFFF
@A00721:190:HGJW7DSXY:4:2378:21459:13949 2:N:0:TCTGCAAG+GCTGCACT
CTTATTGGGGTCCAGCACAATTGGGTATCCAATGTGAAGTACGGCAAACCAATTCAAATTAAAGGTGG
TATCCCGTCAATCGTGCTTTGCAATCCAGGCTCAGAAGAATATTCTTATAAGACGTTCCTGGACAAGG
ATGCCAATTCATCTT
+
:FFFFFFFFFFFFFFFFFFFFFFFFFFFFFFFFFFFFFFFFFFFFFFFFFF:FFFFFFFFFFFF:FF
FFFFFFFFFFFFFFFFFFFFFFFFFFFFFFFFFFFF:FFFFFFFFFFFFFFFFFFFFFF:FFFF:,FF
FFF:FFF:FFFFFFF
@A00721:190:HGJW7DSXY:4:1164:12210:18630 2:N:0:TCTGCAAG+GCTGCACT
AGCTTATTGGGGTCCAGCACAATTGGGTATCCAATGTGAAGTACGGCAAACCAATTCAAATTAAAGGT
GGTATCCCGTCAATCGTGCTTTGCAATCCAGGCTCAGAAGAATATTCTTATAAGACGTTCCTGGACAA
GGATGCCAATTCATC
+
FFF:FF:FFFFFFF:FFFFFFFFFFFFFFFFFFFFFFFFFFFFFFFFFFFFFFFFFFFFFFFFFFFFF
FFFFFFFF:FFFFF:FFFFFFFFFFFFFFFFFFFFFFFFFFFFFFFFFFFFFFFFFFF:FFF:FFFFF:F
FFFFFFFFFFFFFFF
@A00721:190:HGJW7DSXY:4:1472:13729:8390 2:N:0:TCTGCAAG+GCTGCACT
AGCTTATTGGGGTCCAGCACAATTGGGTATCCAATGTGAAGTACGGCAAACCAATTCAAATTAAAGGT
GGTATCCCGTCAATCGTGCTTTGCAATCCAGGCTCAGAAGAATATTCTTATAAGACGTTCCTGGACAA
GGATGCCAATTCATC
+
FFFFFFFFFFFFFFFFFFFFFFFFFFFFFFFFFFFFFFFFFFFFFFFFFFFFFFFFFFFFFFFFFFFF
FFFFFFFFFFFFFFFFFFFFFFFFFFFFFFFFFFFFFFF:FFFFFFFFFFFFFFFFFFFFFFFFFFFF
FFFFFFFFFFFFFFF
@A00721:190:HGJW7DSXY:4:2442:18801:7216 2:N:0:TCTGCAAG+GCTGCACT
AGCTTATTGGGGTCCAGCACAATTGGGTATCCAATGTGAAGTACGGCAAACCAATTCAAATTAAAGGT
GGTATCCCGTCAATCGTGCTTTGCAATCCAGGCTCAGAAGAATATTCTTATAAGACGTTCCTGGACAA
GGATGCCAATTCATC
+
FFFFFFFFFFFFFFFFFFFFFFFFFFFFFFFFFFFFFFFFF:FFFFFFFFF:F:FFFF:FF:FFFFF
FFFFFFFFFFFFFFFFFFFFFFFFFF:FFFFFFFFFFFFFFFFFFF:FFFFFFFFFFFFFFFFFFFFFFFF
FFFFFFFFFFFFFFF
@A00721:190:HGJW7DSXY:4:2578:7708:11992 2:N:0:TCTGCAAG+GCTGCACT
AGCTTATTGGGGTCCAGCACAATTGGGTATCCAATGTGAAGTACGGCAAACCAATTCAAATTAAAGGT
GGTATCCCGTCAATCGTGCTTTGCAATCCAGGCTCAGAAGAATATTCTTATAAGACGTTCCTGGACAA
GGATGCCAATTCATC
+
FFFFFFFFFFFFFFFFFFFFFFFFFFFFFFFFFFFFFFFFFFFFFFFFFFFFFFFFFFFFFFFFFFFF
FFFFFFFFFFFFFFFFFFFFFFFFFFFFFFFFFFFFFFFFFFFFFFFFFFFFFFFFFFFFFFFFFFFF
FFFFFFFFFFFFFFF
@A00721:190:HGJW7DSXY:4:2641:19623:16438 2:N:0:TCTGCAAG+GCTGCACT
AGCTTATTGGGGTCCAGCACAATTGGGTATCCAATGTGAAGTACGGCAAACCAATTCAAATTAAAGGT
GGTATCCCGTCAATCGTGCTTTGCAATCCAGGCTCAGAAGAATATTCTTATAAGACGTTCCTGGACAA
GGATGCCAATTCATC
+
FF,FF:,,F:FFF::F,FFFFFFFFF,FFFF,FF,FF,:F::FFFFFFFF:,FFFF:FFFF:FFF,F:
```

```
,,FFFF:,:FFFFFF:F:,FF:,:FF,FFFF::F:FFFFFFFFFF:FF:FFFF,FFFFF,FFF,FFFF
,FFF,F:F:,F,FF:
@A00721:190:HGJW7DSXY:4:1336:19434:7467 2:N:0:TCTGCAAG+GCTGCACT
GGAGCTTATTGGGGTCCAGCACAATTGGGTATCCAATGTGAAGTACGGCAAACCAATTCAAATTAAAG
GTGGTATCCCGTCAATCGTGCTTTGCAATCCAGGCTCAGAAGAATATTCTTATAAGACGTTCCTGGAC
AAGGATGCCAATTCA
+
FFFFFFF:F:FFFFFFFFFFFFF:FFFFFF:FFFFFFFFFFFFFFFFFFFF:FFFFFFFFFF:FFFFF
FFFFFFFF:FFFFFFFFFFFFFFFFF:FFFFFFFFFFFFFFFF:FFFFFFFFFFFFFFFFFFFFFFFF
FFFFFFFFFFFFFFF
@A00721:190:HGJW7DSXY:4:1434:20971:14951 2:N:0:TCTGCAAG+GCTGCACT
GGAGCTTATTGGGGTCCAGCACAATTGGGTATCCAATGTGAAGTACGGCAAACCAATTCAAATTAAAG
GTGGTATCCCGTCAATCGTGCTTTGCAATCCAGGCTCAGAAGAATATTCTTATAAGACGTTCCTGGAC
AAGGATGCCAATTCA
+
FFFFF:FFFFFFFFFFFFFFFFFFFFFFF:FFFFFFFFFFFFFFFFFFFFFFFFFFFFFFFFFFFFFF
FFFFFFFFFFFFFFFFFFFFFFFFFFFFFFFFFFFFFFFFFFFFFFFFFFFFFFFFFFFFFFFFFFFF
FFFFFFFFFFFFFFF
@A00721:190:HGJW7DSXY:4:1453:4553:21214 2:N:0:TCTGCAAG+GCTGCACT
GGAGCTTATTGGGGTCCAGCACAATTGGGTATCCAATGTGAAGTACGGCAAACCAATTCAAATTAAAG
GTGGTATCCCGTCAATCGTGCTTTGCAATCCAGGCTCAGAAGAATATTCTTATAAGACGTTCCTGGAC
AAGGATGCCAATTCA
+
FFFFFFFFFFFFFFFFFFFFFFFFFFFFFFFFFFFFFFFFFFFFFFFFFFFFFFFFFFFFFFFF:FFF
FFFFFFFFFFFFFFFFFFFFFFF,FFFFFFFFFFFFFFFFF,FFFFFFFFFFFFFFFFFFFFFFFFFFF
FFFFFFFFFFFFFF:
@A00721:190:HGJW7DSXY:4:2310:25545:26506 2:N:0:TCTGCAAG+GCTGCACT
GGAGCTTATTGGGGTCCAGCACAATTGGGTATCCAATGTGAAGTACGGCAAACCAATTCAAATTAAAG
GTGGTATCCCGTCAATCGTGCTTTGCAATCCAGGCTCAGAAGAATATTCTTATAAGACGTTCCTGGAC
AAGGATGCCAATTCA
+
:FFFFFFFFFFFFFFFFFFFFFFF,FFFFFFFF,FFFFFFFFFFFFF,FF:F::FFFFFFFFFFFF
FFFFFFFFFFFFFFFFFFFFFFF:FFFFFFFFFFFFFFFFF:FFFFFFFFFFFFFFFFFFFFFFFFFFF
F:FFFFFFF,FF,FF
@A00721:190:HGJW7DSXY:4:2602:18620:3302 2:N:0:TCTGCAAG+GCTGCACT
GGAGCTTATTGGGGTCCAGCACAATTGGGTATCCAATGTGAAGTACGGCAAACCAATTCAAATTAAAG
GTGGTATCCCGTCAATCGTGCTTTGCAATCCAGGCTCAGAAGAATATTCTTATAAGACGTTCCTGGAC
AAGGATGCCAATTCA
+
FFFFFFFFFFFFFFFFFFFFFFFFFFFFFFFFFFFFFFFFFFFFFFFFFFFFFFFFFFFFFFFFFFFF
FFFFFFFFFFFFFFFFFFFFFFFFFFFFFFFFFFFFFFFFFFFFFFFFFFFFFFFFFFFFFFFFFFFF
FFFFFFFFFFFFFFF
@A00721:190:HGJW7DSXY:4:1163:19470:18208 2:N:0:TCTGCAAG+GCTGCACT
GGAGCTTATTGGGGTCCAGCACAATTGGGTATCCAATGTGAAGTACGGCAAACCAATTCAAATTAAAG
GTGGTATCCCGTCAATCGTGCTTTGCAATCCAGGCTCAGAAGAATATTCTTATAAGACGTTCCTGGAC
AAGGATGCCAATTC
+
FFFFFFFFFFFFFFFFFFFFFFFFFFFFFFFFFFFFF:FFFFFFFFFFFFFFFFFFFFFFFFFFFFFF
FFFFFFFFFFFFFFFFFFFFFFFFFFFFFFFFFFFFFFFFFFFFFFFFFFFFFFFFFFFFFFFFFFFF
FFFFFFFFFFF,:F
@A00721:190:HGJW7DSXY:4:1150:10936:20807 2:N:0:TCTGCAAG+GCTGCACT
AAGGAGCTTATTGGGGTCCAGCACAATTGGGTATCCAATGTGAAGTACGGCAAACCAATTCAAATTAA
AGGTGGTATCCCGTCAATCGTGCTTTGCAATCCAGGCTCAGAAGAATATTCTTATAAGACGTTCCTGG
ACAAGGATGCCAATT
```

```
+
F:FFFFFFFFFFFFFFFFFFFFFFFFFFFFFFFFFFFFFFFFFFFFFFFFFFFFFFFFFFFFFFFFFFFF
FFFFFFFFFFFFFFFFFFFFFFFFFFFFFFFFFFFFFFFFFFF,FFFFFFFFFFFFFFFFFFFFFFFFFF
FFFFFFFFFFFFFFF
@A00721:190:HGJW7DSXY:4:1242:24361:25895 2:N:0:TCTGCAAG+GCTGCACT
AAGGAGCTTATTGGGGTCCAGCACAATTGGGTATCCAATGTGAAGTACGGCAAACCAATTCAAATTAA
AGGTGGTATCCCGTCAATCGTGCTTTGCAATCCAGGCTCAGAAGAATATTCTTATAAGACGTTCCTGG
ACAAGGATGCCAATT
+
FFFFFFFFFFFFFFFFFFFFFFFFFFFFFFFFFFFFFFFFFFFFFFFFFFFFF:FFFFFFFFFFFFFF
FFFFFFFFFFFFFFFFFFFFFFFFFFFFFFFFFFFFFFFFFFFFFFFFFFFFFFFFFFFFFFFFFFFF
FFFFFFFFFFFFFFF
@A00721:190:HGJW7DSXY:4:1312:31222:25817 2:N:0:TCTGCAAG+GCTGCACT
AAGGAGCTTATTGGGGTCCATCACAATTGGGTATCCAATGTGAAGTACGGCAAACCAATTCAAATTAA
AGGTGTTATCCCGTCAATCGTGCTTTGCAATCCAGGCTCAGAAGAATATTCTTATAAGACGTTCCTGG
ACAAGGATGCCAATT
+
FF,F:F:,:FFFFFFF:F:F,FFFFFFFFF:,F:,FFF,,FFFF:FFF:,FFF:,FFFFFFFF,F:FF
FF,F,,FFFFFF,FFFFFF,F,:FFF,FFFFF:F:,F:F:FF:F:FFFFF:FFFFFF,FFFFFFFF,:
FFFF,FFF::,F:FF
@A00721:190:HGJW7DSXY:4:1317:32127:8375 2:N:0:TCTGCAAG+GCTGCACT
AAGGAGCTTATTGGGGTCCAGCACAATTGGGTATCCAATGTGAAGTACGGCAAACCAATTCAAATTAA
AGGTGGTATCCCGTCAATCGTGCTTTGCAATCCAGGCTCAGAAGAATATTCTTATAAGACGTTCCTGG
ACAAGGATGCCAATT
+
FFFFFFFFFFFFFFFFFFFFFFFFFFFFFFFFFFFFFFFFFFFFFFFFFFFFFFFFFFFFFFFFFFFF
FFFFFFFFFFFFFFFFFFFFFFFFFFFFFFFFFFFFFFFFFFFFFFFFFFF:FFFFFFFFFFFFFFFF
FFFFF:FFFFFFFFF
@A00721:190:HGJW7DSXY:4:1330:28348:15671 2:N:0:TCTGCAAG+GCTGCACT
AAGGAGCTTATTGGGGTCCAGCACAATTGGGTATCCAATGTGAAGTACGGCAAACCAATTCAAATTAA
AGGTGGTATCCCGTCAATCGTGCTTTGCAATCCAGGCTCAGAAGAATATTCTTATAAGACGTTCCTGG
ACAAGGATGCCACTT
+
FFFFFFFF:FFFFFFFFF:FFFFFFFFFFFFFFFFFFF,FFFFFFFFFFFFFFFF:FFFFFFFFFFFF
:FFFFFFFFFFFFFFFFFFF:FFFFFFFFFFFFFFFFFFFFFFFFFFF,FFFFFFFFFFFFFFFFFFFF
FF:FFFFFFFFF,FF
@A00721:190:HGJW7DSXY:4:1359:16007:4351 2:N:0:TCTGCAAG+GCTGCACT
AAGGAGCTTATTGGGGTCCAGCACAATTGGGTATCCAATGTGAAGTACGGCAAACCAATTCAAATTAA
AGGTGGTATCCCGTCAATCGTGCTTTGCAATCCAGGCTCAGAAGAATATTCTTATAAGACGTTCCTGG
ACAAGGATGCCAATT
+
FFFFFFF,FFFFFFFFFFFFFFFFFFFFF:FF:FFFFFFFFFF:FFFFFFFFFFFFFFFFFFFFFFFFF
FFFFFFFFFFFFFFFFFFFFFFFFFFFFFFF:FFFFFFFFFFFFFFF:FFFFF:FFFFFFFF,FFFFFF
FFFFFFFFFFFF:FF
@A00721:190:HGJW7DSXY:4:1469:6533:19758 2:N:0:TCTGCAAG+GCTGCACT
AAGGAGCTTATTGGGGTCCAGCACAATTGGGTATCCAATGTGAAGTACGGCAAACCAATTCAAATTAA
AGGTGGTATCCCGTCAATCGTGCTTTGCAATCCAGGCTCAGAAGAATATTCTTATAAGACGTTCCTGG
ACAAGGATGCCAATT
+
FFFFFFFFFFFFFFFFFFFFFFFFFFFFFFFFFFFFFFFFFFFFFFFFFFFFFFFFFFFFFFFFFFFF
FFFFFFFFFFFFFFFFFFFFFFFFFFFFFFFFFFFFFFFFFFFFFFFFFFFFFFFFFFF:FFFFFFFFF
FFFFFFFFFFF,FFF
@A00721:190:HGJW7DSXY:4:1569:19108:14951 2:N:0:TCTGCAAG+GCTGCACT
AAGGAGCTTATTGGGGTCCAGCACAATTGGGTATCCAATGTGAAGTACGGCAAACCAATTCAAATTAA
```

AGGTGGTATCCCGTCAATCGTGCTTTGCAATCCAGGCTCAGAAGAATATTCTTATAAGACGTTCCTGG
ACAAGGATGCCAATT
+
FFFFFFF,F:FFFFFFFFFFFFFFFFFFFFFFFFFFFFFFFFFFFFFFFFFFFFFFFF:FFFFFFFFF
:FFFFFFFFFFFFFFFFFFFFFFFFFFFFFFFFFFFFFFFFFFFFFFFFFFFFFFFFFFFFFFFFFFF
FFFFFFFFFFFFFF:
@A00721:190:HGJW7DSXY:4:1647:13720:11162 2:N:0:TCTGCAAG+GCTGCACT
AAGGAGCTTATTGGGGTCCAGCACAATTGGGTATCCAATGTGAAGTACGGCAAACCAATTCAAATTAA
AGGTGGTATCCCGTCAATCGTGCTTTGCAATCCAGGCTCAGAAGAATATTCTTATAAGACGTTCCTGG
ACAAGGATGCCAATT
+
FFFFFFFFFFFFFFFFFFFFFFFFFFFFFFFFFFFFFFFFFFFFFFFFFFFFFFFFFFFFFFFF:FFF
FFFFFFFFFFFFFFFFFFFFFFFFFFFFFFFFFFFFFFFFFFFFFFFFFFFFFFFFFFFFFFFFFFFF
FFFFFFFFFFFFFFF
@A00721:190:HGJW7DSXY:4:1668:19461:26647 2:N:0:TCTGCAAG+GCTGCACT
AAGGAGCTTATTGGGGTCCAGCACAATTGGGTATCCAATGTGAAGTACGGCAAACCAATTCAAATTAA
AGGTGGTATCCCGTCAATCGTGCTTTGCAATCCAGGCTCAGAAGAATATTCTTATAAGACGTTCCTGG
ACAAGGATGCCAATT
+
FFFFFFFFFFFFFFFFFFFFFFFFFFFFFFFFFFFFFFFFFFFFFFFFFFFFFFFFFFFFFFFFFFFF
FFFFFFFFFFFFFFFFFFFFFFFFFFFFFFFFFFFFFFFFFFFFFFFFFFFFFFFFFFFFFFFFFFFF
FFFFFFFFFFFFFFF
@A00721:190:HGJW7DSXY:4:2315:14018:24486 2:N:0:TCTGCAAG+GCTGCACT
AAGGAGCTTATTGGGGTCCAGCACAATTGGGTATCCAATGTGAAGTACGGCAAACCAATTCAAATTAA
AGGTGGTATCCCGTCAATCGTGCTTTGCAATCCAGGCTCAGAAGAATATTCTTATAAGACGTTCCTGG
ACAAGGATGCCAATT
+
FFFFFFF:F:FFFFFFFFFFFFFFFFFFFFFFFFFFFFFFFFFFFFFFFFFFFFFFFFFFFFFFFFFF
FFFFFFFFFFFFFFFFFFFFFFFFFFFFFFFFFFFFFFFFFF:FFFFFFFFFFFFFFFFFFFFFFFFF
FFFFFFFFFFFFFFF
@A00721:190:HGJW7DSXY:4:2317:25699:31626 2:N:0:TCTGCAAG+GCTGCACT
AAGGAGCTTATTGGGGTCCAGCACAATTGGGTATCCAATGTGAAGTACGGCAAACCAATTCAAATTAA
AGGTGGTATCCCGTCAATCGTGCTTTGCAATCCAGGCTCAGAAGAATATTCTTATAAGACGTTCCTGG
ACAAGGATGCCAATT
+
FFFFFFFFFFFFFFFFFFFFFFFFFFFFFFFFFFFFFFFFFFFFFFFFFFFFFFFFFFFFFFFFFFFF
FFFFFFFFFFFFFFFFFFFFFFFFFFFFFFFFFFFFFFFFFFFFFFFFFFFFFFFFFFFFFFFFF:FF
FFFFFFFFFFFFFFF
@A00721:190:HGJW7DSXY:4:2438:29170:17534 2:N:0:TCTGCAAG+GCTGCACT
AAGGAGCTTATTGGGGTCCAGCACAATTGGGTATCCAATGTGAAGTACGGCAAACCAATTCAAATTAA
AGGTGGTATCCCGTCAATCGTGCTTTGCAATCCAGGCTCAGAAGAATATTCTTATAAGACGTTCCTGG
ACAAGGATGCCAATT
+
FFFFFFFFFFFFFFFFFFFFFFFFFFFFFFFFFFFFFFFFFF:FFFFFFFFFFFFFFFFFFFFFFFFF
FFFFFFF:FFFFFFFFFFFFFFFFFFFFFFFFFFFFFFFFFF:FF,FFFFFFFFFFFFFFFFFFFFFF
FFFFFFFFFFF,FF:
@A00721:190:HGJW7DSXY:4:2619:21802:28604 2:N:0:TCTGCAAG+GCTGCACT
AAGGAGCTTATTGGGGTCCAGCACAATTGGGTATCCAATGTGAAGTACGGCAAACCAATTCAAATTAA
AGGTGGTATCCCGTCAATCGTGCTTTGCAATCCAGGCTCAGAAGAATATTCTTATAAGACGTTCCTGG
ACAAGGATGCCAATT
+
FFFFFFFFFFFFFFFFFFFFFFFFFFFFFFFFFFFFFFFFFFFFFFFFFFFFFFFFFFFFFFFFFFFF
FFFFFFFFFFFFFFFFFFFFFFFFFFFFFFFFFFFFFFFFFFFFFFFFFFFFFFFFFFFFFFFFFFFF
FFFFFFFFFFF:FFF

```
@A00721:190:HGJW7DSXY:4:1370:23149:14904 2:N:0:TCTGCAAG+GCTGCACT
GAAGGAGCTTATTGGGGTCCAGCACAATTGGGTATCCAATGTGAAGTACGGCAAACCAATTCAAATTA
AAGGTGGTATCCCGTCAATCGTGCTTTGCAATCCAGGCTCAGAAGAATATTCTTATAAGACGTTCCTG
GACAAGGATGCCAAT
+
FFFFFFFFFFFFFFFFFFFFFFFFFFFFFFFFFFFFFFFFFFFFFFFFFFFFFFFFFFFFFFFFFFFF
FFFFFFFFFFFFFFFFFFFFFFFFFFFFFFFFFFFFFFFFFFFFFFFFFFFFFFFFFFFFFFFFFFFF
FFFFFFFFFFFFFFF
@A00721:190:HGJW7DSXY:4:1406:1452:16438 2:N:0:TCTGCAAG+GCTGCACT
GAAGGAGCTTATTGGGGTCCAGCACAATTGGGTATCCAATGTGAAGTACGGCAAACCAATTCAAATTA
AAGGTGGTATCCCGTCAATCGTGCTTTGCAATCCAGGCTCAGAAGAATATTCTTATAAGACGTTCCTG
GACAAGGATGCCAAT
+
F:FFFF:FFFFFFFFFFFFFFFFFFFFFFFFFFFFFFFFFFFFFFFFFFFFFFFFFFFFFFFFFFFFF
FFFFFFFFFFFFFF:FFFFFFFFFFFFFFFFFFFFFFFFF:FFFF,FFFFFFFFFFFFFFFFFFFFFF
FFFFF:FFF:FFFF:
@A00721:190:HGJW7DSXY:4:1463:6668:7028 2:N:0:TCTGCAAG+GCTGCACT
GAAGGAGCTTATTGGGGTCCAGCACAATTGGGTATCCAATGTGAAGTACGGCAAACCAATTCAAATTA
AAGGTGGTATCCCGTCAATCGTGCTTTGCAATCCAGGCTCAGAAGAATATTCTTATAAGACGTTCCTG
GACAAGGATGCCAAT
+
FFFFFFFFFFFFFFFFFFFFFFFFFFFFFFFFFFFFFFFFFFFFFFFFFFFFFFFFFFFFFFFFFFFF
FFFFFFFFFFFFFFFFFFFFFFFFFFFFFFFFFFFFFFFFFFFFFFFFFFFFFFFFFFFFFFFFFFFF
FFFFFFFFFFFFFFF
@A00721:190:HGJW7DSXY:4:1514:15121:4163 2:N:0:TCTGCAAG+GCTGCACT
GAAGGAGCTTATTGGGGTCCAGCACAATTGGGTATCCAATGTGAAGTACGGCAAACCAATTCAAATTA
AAGGTGGTATCCCGTCAATCGTGCTTTGCAATCCAGGCTCAGAAGAATATTCTTATAAGACGTTCCTG
GACAAGGATGCCAAT
+
FFFFFFFFFFFFFFFFFFFFFFFFFFFFFFFFFFFFFFFFFFFFFFFFFFFFFFFFFFFFFFFF::FF
:FFFFFFFFF:FFFFFFFF:FFFFFFFFFFFFFFFFFFFFFFFFFFFFFFFFFFFFFFFFFFFFFFFF
FFFFFF,FFFFFFFF
@A00721:190:HGJW7DSXY:4:2176:1407:19366 2:N:0:TCTGCAAG+GCTGCACT
GAAGGAGCTTATTGGGGTCCAGCACAATTGGGTATCCAATGTGAAGTACGGCAAACCAATTCAAATTA
AAGGTGGTATCCCGTCAATCGTGCTTTGCAATCCAGGCTCAGAAGAATATTCTTATAAGACGTTCCTG
GACAAGGATGCCAAT
+
FFFFFFFFFFFFFFFFFFFFFFFFFFFFFFFFFFFFFFFFFFFFFFFFFFFFFFFFFFF,FFFFFFFF
FFFFFFFFFFFFFFFFFFFFFFFFFFFFFFFFFFFFFFFFFF:FFF:FFFFFFFFFFFFFFFFFFFFF
FFFFFFFFFFFFFFF
@A00721:190:HGJW7DSXY:4:2177:32099:18161 2:N:0:TCTGCAAG+GCTGCACT
AAGGAGCTTATTGGGGTCCAGCACAATTGGGTATCCAATGTGAAGTACGGCAAACCAATTCAAATTAA
AGGTGGTATCCCGTCAATCGTGCTTTGCAATCCAGGCTCAGAATAATATTCTTATAAGACTTTCATGT
ACAAGGATGCCAAT
+
:F::F,FFFFF,:,FF,FFFFFF:FFFF,F,FF,:,FF:FF,FF,FFF,,FFFF:,FFFFFFFF,FF:
:,,F,,:FF,F,,FFFFFF:,:,FF,,FFF,,:F:::F:F,FF,FF,F:,,F,FFF,,FF,:F,,,,,
FFFF,,FF:,FFFF
@A00721:190:HGJW7DSXY:4:2277:28167:34147 2:N:0:TCTGCAAG+GCTGCACT
GAAGGAGCTTATTGGGGTCCAGCACAATTGGGTATCCAATGTGAAGTACGGCAAACCAATTCAAATTA
AAGGTGGTATCCCGTCAATCGTGCTTTGCAATCCAGGCTCAGAAGAATATTCTTATAAGACGTTCCTG
GACAAGGATGCCAAT
+
F,F:FFFFFF::FFFFFFFFFFFFFFFFFFFFFFFFFFFFFFFFFFFFFFFFFFFFFFFFFFF,FFFFF
```

```
F:FF:FFFFFFFF:FFFFFFFFFFFFFFFFFFFFFFFFFFFFF:FF:FFFFFFFFFFFFFFFFFFFFF
FFFFFFFFF:FFFFF
@A00721:190:HGJW7DSXY:4:2277:28872:36777 2:N:0:TCTGCAAG+GCTGCACT
GAAGGAGCTTATTGGGGTCCAGCACAATTGGGTATCCAATGTGAAGTACGGCAAACCAATTCAAATTA
AAGGTGGTATCCCGTCAATCGTGCTTTGCAATCCAGGCTCAGAAGAATATTCTTATAAGACGTTCCTG
GACAAGGATGCCAAT
+
FFFFFFFFFFF,FFFFFFFFFFFFFFF:FFFFFFFFFFFFFFFFFFFFFFFFF,FFFFFFFFFFFFFFF
F,FFFFFFFFF:FFFFF:FFFFFFFFFFFFFFFFFFFFFFFFFFFFFFFFFFF:FFFFFFFF:FFFFF
FFFFF:FFFFFFFFF
@A00721:190:HGJW7DSXY:4:2320:29161:19241 2:N:0:TCTGCAAG+GCTGCACT
GAAGGAGCTTATTGGGGTCCAGCACAATTGGGTATCCAATGTGAAGTACGGCAAACCAATTCAAATTA
AAGGTGGTATCCCGTCAATCGTGCTTTGCAATCCAGGCTCAGAAGAATATTCTTATAAGACGTTCCTG
GACAAGGATGCCAAT
+
FFFFFFFFFFFFFFFFFFFFFFFFFFFFFFFFFFFFFFFFFFFFFFFFFFFFFFFFFFFFFFFFF:FF
FFFFFFFF:FFFFFFFFFFFFFFFFFFFFF:FFFFFFFFFFFFFFFFFFFFFFFFFFFFFFFFFFFFF
FFFFFFFFFFFFFFF
@A00721:190:HGJW7DSXY:4:2320:29423:19445 2:N:0:TCTGCAAG+GCTGCACT
GAAGGAGCTTATTGGGGTCCAGCACAATTGGGTATCCAATGTGAAGTACGGCAAACCAATTCAAATTA
AAGGTGGTATCCCGTCAATCGTGCTTTGCAATCCAGGCTCAGAAGAATATTCTTATAATACGTTCCTG
GACAAGGATGCCAAT
+
:F:FFF::FF:F,,FFFFFFFFFFFF:FFFFFFFFF:FF:FF:FF::FFFFFFFF:FFFFFFFFFFF
FF,FFFF:FFFFFFFF:FF:FFFFFFF:FFFF:FFF,FFFFFFFFFFFFFFFFFFFFF,F,::F::F:
FFFFF,FFF,F:FFF
@A00721:190:HGJW7DSXY:4:2641:11695:30796 2:N:0:TCTGCAAG+GCTGCACT
GAAGGAGCTTATTGGGGTCCAGCACAATTGGGTATCCAATGTGAAGTACGGCAAACCAATTCAAATTA
AAGGTGGTATCCCGTCAATCGTGCTTTGCAATCCAGGCTCAGAAGAATATTCTTATAAGACGTTCCTG
GACAAGGATGCCAAT
+
FFFFFFFFFFF::FFFFFFFFF:FFFFFFFFFF,FFFFFFFFFFFFFFFFFFFFFFF,:FFFF:FFFF
FFFFFFFFFFFFFFFFFFFFFFFFFFFFFFFFFFFFFFF:FF,FFFFFFFFFFFFFFFFFFFFF,FF
FFFFFFFFFFFF:FF
@A00721:190:HGJW7DSXY:4:1121:14561:14340 2:N:0:TCTGCAAG+GCTGCACT
ATTGGAAGGAGCTTATTGGGGTCCAGCACAATTGGGTATCCAATGTGAAGTACGGCAAACCAATTCAA
ATTAAAGGTGGTATCCCGTCAATCGTGCTTTGCAATCCAGGCTCAGAAGAATATTCTTATAAGACGTT
CCTGGACAAGGATGC
+
F:FFFFFFFFFFFFFFFFFFFFFFFFFFFFFFFFFFFFFFFFFFFFFFFFFFFFFFFFFFFFFFFFFF
FFFFFFFFFFFFFFFFFFFFFFFFFFFFFF:FFFFFFFF:FFFFFFFFFFFFFFFFFFFFFF,FFFFF
FF:FFFFFFFFFFFF
@A00721:190:HGJW7DSXY:4:1204:10773:32925 2:N:0:TCTGCAAG+GCTGCACT
ATTGGAAGGAGCTTATTGGGGTCCAGCACAATTGGGTATCCAATGTGAAGTACGGCAAACCAAGTCAA
ATTAAAGGTGGTATCCCGTCAATCGTGCTTTGCAATCCAGGCTCAGAAGAATATTCTTATAAGACGTT
CCTGGACAAGGATGC
+
FFF:FFFFFFFFF:FFFFFFFFFFFFFFFFFFFFFFF:FFFFFFFFFFFFFF,FFFFFFFFF,F,FFFF
FF:FFFFFFFF:FFFFFFFFFFFF:FFFFFF:FF,FFFFFFFFFFFF:,FFFFFFFFFFFFFFFFFFF
FF,FFFFFFFFF:FF
@A00721:190:HGJW7DSXY:4:1403:10854:16125 2:N:0:TCTGCAAG+GCTGCACT
ATTGGAAGGAGCTTATTGGGGTCCAGCACAATTGGGTATCCAATGTGAAGTACGGCAAACCAATTCAA
ATTAAAGGTGGTATCCCGTCAATCGTGCTTTGCAATCCAGGCTCAGAAGAATATTCTTATAAGACGTT
CCTGGACAAGGATGC
```

+
FFFFFFFFFFFFFFFFFFFFFFFFFFFFFFFFFFFFFFFFFFFFFFFFFFFFFFFFFFFFFFFFFFFFFF
FFFFFFFFFFFFFFF:FFFFFFFFFFFFFFF:FFFFFFFFFFFFFFFFFFFFFFFFFFFFFFFFFFFFFF
FFFFFFFFFFFFFFF
@A00721:190:HGJW7DSXY:4:1403:11659:17268 2:N:0:TCTGCAAG+GCTGCACT
ATTGGAAGGAGCTTATTGGGGTCCAGCACAATTGGGTATCCAATGTGAAGTACGGCAAACCAATTCAA
ATTAAAGGTGGTATCCCGTCAATCGTGCTTTGCAATCCAGGCTCAGAAGAATATTCTTATAAGACGTT
CCTGGACAAGGATGC
+
F:FFFFFFFFFFFFFFFFFFFFFFFFFFFFFFFFFFFFFFFFFFFFFFFFFFFFFFFF:FFFFFFFFFF
FFFFFFFFFFFFFFFFFFFFFFFFFFFFFFFFFFFFF:FFFFFFFFFFFFFFFFFFFFFFFFFFFFF:FF
FFFFFFFFFFFFFFF
@A00721:190:HGJW7DSXY:4:1669:3450:36714 2:N:0:TCTGCAAG+GCTGCACT
ATTGGAAGGAGCTTATTGGGGTCCAGCACAATTGGGTATCCAATGTGAAGTACGGCAAACCAATTCAA
ATTAAAGGTGGTATCCCGTCAATCGTGCTTTGCAATCCAGGCTCAGAAGAATATTCTTATAAGACGTT
CCTGGACAAGGATGC
+
FFFFFFFFFF:FFFFFFF:FFFFF,FFFFFFFFFFFFFFF,FFFFF:FFFFFFFF:,FFFFFFFFFFF
FFF:FFF,FFFFFFFFFFFFFFF:F,FFFF:FFF:FFFFFF:FFFFFFFFFFFFFFFFFFFFFFFFF:F
FF:FFFFFFFFFFFF
@A00721:190:HGJW7DSXY:4:2143:28772:21261 2:N:0:TCTGCAAG+GCTGCACT
TTGGAAGGAGCTTATTGGGGTCCAGCACAATTGGGTATCCAATGTGAAGTACGGCAAACCAATTCAAA
TTAAAGGTGGTATCCCGTCAATCGTGCTTTGCAATCCAGGCTCAGAAGAATATTCTTATAAGACGTTC
CTGGACAAGGATGC
+
FF:FFFFFFFFFFFFFFFFFFFFFFFFFFFFFFFFFFF:FFFFFFFFFFFFFFFFFFFFFFFFFFFFFF
FFFFFFFFFFF:FFFFFFFFFFFFFFFFFFFFFFFFFFFFFFFFFFFFFFFFFFFFFFFFFFFFFFFF
FFFFFFFFFFFFFF
@A00721:190:HGJW7DSXY:4:2157:16523:11788 2:N:0:TCTGCAAG+GCTGCACT
ATTGGAAGGAGCTTATTGGGGTCCAGCACAATTGGGTATCCAATGTGAAGTACGGCAAACCAATTCAA
ATTAAAGGTGGTATCCCGTCAATCGTGCTTTGCAATCCAGGCTCAGAAGAATATTCTTATAAGACGTT
CCTGGACAAGGATGC
+
FFFFFF::FF::FFF:FFFF:FFFFFFFFF:F::F:F:FF:FFFF:FFFFFF:F:FF:FFFFF::FFF
FFFFFFFFFFFF:FF,,FFFF:F:F,:FF:,F:FF:F:FFF:FFFFFF,FFFFF:FFFFFFFFF:FFF
:F:FFFF:FFFFFFF
@A00721:190:HGJW7DSXY:4:1625:9914:18537 2:N:0:TCTGCAAG+GCTGCACT
CATCCTTGTCCAGGAACGTCTTATAAGAATATTCTTCTGAGCCTGGATTGCAAAGCACGATTGACGGG
ATACCACCTTTAATTTGAATTGGTTTGCCGTACTTCACATTGGATACCCAATTGTGCTGGACCCCAAT
AAGCTCCTTCCAATG
+
FFFFFF:FFFFFFFFFFFFFFFFFFFFFFFFFFFFFFFFFFFFFFFFFFFFFFFFFFFFFFFFFFFFF
FFFFFFFFFFFFFFFFFFFFFFFFFFFFFFFFFFFFFFFFFFFFFFFFFFFFFFFFFFFFFFFFFFFFF
FFFFFF:FFFFFF,F
@A00721:190:HGJW7DSXY:4:2633:30029:2237 2:N:0:TCTGCAAG+GCTGCACT
CATCCTTGTCCAGGAACGTCTTATAAGAATATTCTTCTGAGCCTGGATTGCAAAGCACGATTGACGGG
ATACCACCTTTAATTTGAATTGGTTTGCCGTACTTCACATTGGATACCCAATTGTGCTGGACCCCAAT
AAGCTCCTTCCAA
+
FFFFFFFFFFFFFFFFF:FFFFFFFFFFF:FFF,:FFF:FFFFFFFFFFFFFFFFFFFFFFFFFFF,FF
FFFFFFFFFFFFFFFFFFFFFFF,FFFFFF:FFFFFFFFFFF:,FFFFFFFF:F,F:,FF:FFFF:FFF
FF:,F:FFF,FFF
@A00721:190:HGJW7DSXY:4:2635:21124:30530 2:N:0:TCTGCAAG+GCTGCACT
CATCCTTGTCCAGGAACGTCTTATAAGAATATTCTTCTGAGCCTGGATTGCAAAGCACGATTGACGGG

ATACCACCTTTAATTTGAATTGGTTTGCCGTACTTCACATTGGATACCCAATTGTGCTGGACCCCAAT
AAGCTCCTTCCAATG
+
FFFFFFFFFFFFFFFFFFFFFFFFFFFFFFFFFFFFFFFFFFFFFFFFFFFFFFFFFFFFFFFFFFFF
FFFFFFFFFFFFFFFFFFFFFFFFFFFFFFFFFFFFFFFFFFFFFFFFFFFFFFFFFFFFFFFFFFFF
FFFFFF:FFFFFFFF
@A00721:190:HGJW7DSXY:4:2652:19533:11804 2:N:0:TCTGCAAG+GCTGCACT
GGAGCTTATTGGGGTCCAGCACAATTGGGTATCCAATGTGAAGTACGGCAAACCAATTCAAATTAAAG
GTGGTATCCCGTCAATCGTGCTTTGCAATCCAGGCTCAGAAGAATATTCTTATAAGACGTTCCTGGAC
AAGGATG
+
FFFF:FFFFFFFFFFFFFFFFFFFFFFFFFFFFFFFFFFFFFFFFFFFFFFFFFFFFFFFFFFFF,FF
FFFFFFFFFFFFF:FFFFFFFFFFFFFFFFFFFFFFFFFF:F,FF:FFFFF:F:FF:FFFFFFFFFF:
,,FF:,F
@A00721:190:HGJW7DSXY:4:1314:27190:1172 2:N:0:TCTGCAAG+GCTGCACT
AAACATTGGAAGGAGCTTATTGGGGTCCAGCACAATTGGGTATCCAATGTGAAGTACGGCAAACCAAT
TCAAATTAAAGGTGGTATCCCGTCAATCGTGCTTTGCAATCCAGGCTCAGAAGAATATTCTTATAAGA
CGTTCCTGGACAAGG
+
FFFFFFFFFFFFFFFFFFFFFFFFFFFFFFFFFFFFFFFFFFFFFFFFFFFFFFFFFFFFFFFFFFFF
FFFFFFFFFFFFFFFFFFFFFFFFFFFFFFFFFFFFFFFFFFFFFFFFFFF,F:FFFFFFFFFFFFFF
FFFFFFFFFFFFFFF
@A00721:190:HGJW7DSXY:4:1268:29044:27993 2:N:0:TCTGCAAG+GCTGCACT
CTTCAAAAGAAACATTGGAAGGAGCTTATTGGGGTCCAGCACAATTGGGTATCCAATGTGAAGTACGG
CAAACCAATTCAAATTAAAGGTGGTATCCCGTCAATCGTGCTTTGCAATCCAGGCTCAGAAGAATATT
CTTATAAGACGTTCC
+
FFFFFFFFFFFFFF:FFFFFFFFFFFFFFFFFFFFFFFFFFFFFFFFFFFFFFFFFFFFFFFFFFFFF
FFFFFFFFFFFFFFFFFFFFFFFFFFFFFFF:FFFFFFFFFFFFFFFFFFFFFFFFFFFFFFFFFFFF
FFFFFFFFFFFFFFF
@A00721:190:HGJW7DSXY:4:1276:19054:16016 2:N:0:TCTGCAAG+GCTGCACT
CTTCAAAAGAAACATTGGAAGGAGCTTATTGGGGTCCAGCACAATTGGGTATCCAATGTGAAGTACGG
CAAACCAATTCAAATTAAAGGTGGTATCCCGTCAATCGTGCTTTGCAATCCAGGCTCAGAAGAATATT
CTTATAAGACGTTCC
+
FFFFFFFFFFFFFFFFFFFFFFFFFFFFFFFFFFFFFFFFFFFFFFFFFFFFFFFFFFFFFFFFFFFF
FFFFFFFFFFFFFFFFFFFFFFFFF,FFFFFFFFFFFFFFF:FFFFFFFFFFFFFFFFF:::FFFFFF
FFFFFFFF:FFFFFF
@A00721:190:HGJW7DSXY:4:1503:23836:17409 2:N:0:TCTGCAAG+GCTGCACT
CTTCAAAAGAAACATTGGAAGGAGCTTATTGGGGTCCAGCACAATTGGGTATCCAATGTGAAGTACGG
CAAACCAATTCAAATTAAAGGTGGTATCCCGTCAATCGTGCTTTGCAATCCAGGCTCAGAAGAATATT
CTTATAAGACGTTCC
+
F,FFFFFFFFFFFFFFFFFFFFFF:FFFFFF,F:FFFFFFFFFFFFFFFFFF::F,F:FFFF:FFF:F
F:FFF:FFF:FFFFF:F:FF:FF:FFFFFFFFFFFF:F,:FFF,:FFFFFFFFFFFFFFFFFFFFF,F
FFFFFFFFFF::FFF
@A00721:190:HGJW7DSXY:4:1550:26096:34569 2:N:0:TCTGCAAG+GCTGCACT
TTCAAAAGAAACATTGGAAGGAGCTTATTGGGGTCCAGCACAATTGGGTATCCAATGTGAAGTACGGC
AAACCAATTCAAATTAAAGGTGGTATCCCGTCAATCGTGCTTTGCAATCCAGGCTCAGAAGAATATTC
TTATAAGACGTTCC
+
FFFFFFF,FF:FFFFFFFFFFFFFFFFFFFFFFFFFFFFF,FFFFF:F,FFFFFFF:FFFFFFF,:FF
FF:F,FFFFFFFFFF:FFF:FFFFFFFF,FFFFFF:FFFFFFF,FFFFFF:F,FFFFFFFFFFFFFFF
F:FFFF:,F:FF:F

```
@A00721:190:HGJW7DSXY:4:1636:8757:16344 2:N:0:TCTGCAAG+GCTGCACT
CTTCAAAAGAAACATTGGAAGGAGCTTATTGGGGTCCAGCACAATTGGGTATCCAATGTGAAGTACGG
CAAACCAATTCAAATTAAAGGTGGTATCCCGTCAATCGTGCTTTGCAATCCAGGCTCAGAAGAATATT
CTTATAAGACGTTCC
+
FFFFFFF:FFFFFF:FFFFFFFFFFFFFFFFFFFFFFFFFFFFFFFFFFFFFFFFFFFFFFFFFFFFF
FFFFFFFFFFFFFFFFFFFFFFFFFFFFFFFFFFFFFFFFFFFFFFFFFFFFFFFFFFFFFFFFFFFF
FFFFFFFFFFFFFFF
@A00721:190:HGJW7DSXY:4:2451:7744:17378 2:N:0:TCTGCAAG+GCTGCACT
CTTCAAAAGAAACATTGGAAGGAGCTTATTGGGGTCCAGCACAATTGGGTATCCAATGTGAAGTACGG
CAAACCAATTCAAATTAAAGGTGGTATCCCGTCAATCGTGCTTTGCAATCCAGGCTCAGAAGAATATT
CTTATAAGACGTTCC
+
FFFFFFFFFFFFFF:FFFFFFFFFFFFFFF:FFFFFFFFFFFFFFFFFFFFFFFFFF,FFFFFFFFFF
FFFFFFFFFFFFFFFFFFFFFFFFFFFFFFFFFFFFFFFFFFFFFFFFFFFFFFFFFFFFFFFFFFFF
FFFFFFFFFFFFFFF
@A00721:190:HGJW7DSXY:4:2609:6343:12164 2:N:0:TCTGCAAG+GCTGCACT
CTTCAAAAGAAACATTGGAAGGAGCTTATTGGGGTCCAGCACAATTGGGTATCCAATGTGAAGTACGG
CAAACCAATTCAAATTAAAGGTGGTATCCCGTCAATCGTGCTTTGCAATCCAGGCTCAGAAGAATATT
CTTATAAGACGTTCC
+
FFFFFFFFFFFFFFFFFFFFFFFFFFFFFFFFFFFFFFFFFFFFFFFFFFFFFFFFFFFFFFFFFFFF
FFFFFFFFFFFFFFFFFFFFFFFFFFFFFFFFFFFFFFFFFFFFFFFFFFFFFFFFFFFFFFFFFFFF
FFFFFFFFFFFFFFF
@A00721:190:HGJW7DSXY:4:1532:19542:14920 2:N:0:TCTGCAAG+GCTGCACT
GAACGTCTTATAAGAATATTCTTCTGAGCCTGGATTGCAAAGCACGATTGACGGGATACCACCTTTAA
TTTGAATTGGTTTGCCGTACTTCACATTGGATACCCAATTGTGCTGGACCCCAATAAGCTCCTTCCAA
TGTTTCTTTTGAAGA
+
FFFFFFFFFFFFFFFFFFFFFFFF:FFFFFFFFFFFFFFFFFFFFFFFFFFFFFFFFFFFFFFFFFFF
FFFFFFFFFFFFFFFFFFFFFFFFFFFFFFFFFFFFF:FFFFFFFFFFFFFFFFFFFFFFFFFFFF:FF
:FFFFFFFFFF:FFF
@A00721:190:HGJW7DSXY:4:1544:7184:36605 2:N:0:TCTGCAAG+GCTGCACT
GAACGTCTTATAAGAATATTCTTCTGAGCCTGGATTGCAAAGCACGATTGACGGGATACCACCTTTAA
TTTGAATTGGTTTGCCGTACTTCACATTGGATACCCAATTGTGCTGGACCCCAATAAGCTCCTTCCAA
TGTTTCTTTTGAAGA
+
FFFFF:FFFFFFFFFFFFFFFFFFFFFFFFFFFFFFFFFFFFFFFFFFFFFFFFFFFFFFFFFFFFFF
FFFFF:FFFFFFF:FFFFFFFFFFF:FFFFFFFFFFFFFFFFFFFFFFFFFFFFFFFFFFFFFFFFFF
FFFFFFFFFFFFFFF
@A00721:190:HGJW7DSXY:4:1566:3016:32643 2:N:0:TCTGCAAG+GCTGCACT
GAACGTCTTATAAGAATATTCTTCTGAGCCTGGATTGCAAAGCACGATTGACGGGATACCACCTTTAA
TTTGAATTGGTTTGCCGTACTTCACATTGGATACCCAATTGTGCTGGACCCCAATAAGCTCCTTCCAA
TGTTTCTTTTGAAGA
+
FFFFFFFFFFFFFFFFF:FFFFFFFFFFFFFFFFFFFFFFFFFFFFFFFFFFFFFFFFFF:FFFFFFFF
FFFFFFFFFFFFFFFFFFFFFFFFFFFFFFFFFFFFFFFFFFFFFFFFFFFFFFFFFFFFFF:FFFFF
:FFFFFFFFF,FFFF
@A00721:190:HGJW7DSXY:4:2357:5023:20337 2:N:0:TCTGCAAG+GCTGCACT
GAACGTCTTATAAGAATATTCTTCTGAGCCTGGATTGCAAAGCACTATTGACGGGATACCACCTTTAA
TTTGAATTGGTTTGCCGTACTTCACATTGGATACCCAATTGTGCTGGACCCCAATAAGCTCCTTCCAA
TGTTTCTTTTGAAGA
+
FFFFFFFFFF:FFFFFFF:FFF,FFFFFFFFFFFF:FFFFF:FFF,,FF,FFFFFFFFF:FF:FFFFF
```

```
F::FFFFF::::FFFFFFFFF:FFFF,FFFFFF,FFF,FFFF:FFFFFF:F:FF:FFFFFFFFFF,FF
FFFFFFF::F:FFF:
@A00721:190:HGJW7DSXY:4:2471:17327:34162 2:N:0:TCTGCAAG+GCTGCACT
GAACGTCTTATAAGAATATTCTTCTGAGCCTGGATTGCAAAGCACGATTGACGGGATACCACCTTTAA
TTTGAATTGGTTTGCCGTACTTCACATTGGATACCCAATTGTGCTGGACCCCAATAAGCTCCTTCCAA
TGTTTCTTTTGAAGA
+
:FFFFFFFFFFFFFFFFFFFFFFFFFFFFFFFFFFFFFFFFFFFFFFFFFFF:FFFFFFFFFFFFFFF
F:FFFFFFFFFFFFFFFFFFFFFFFFFFFFFFFFFFFFFFFFFFFF:FF:FFFFFFFFFFFFFFFFFF
FFFFFFFFFFFFFFF
@A00721:190:HGJW7DSXY:4:2145:8332:22842 2:N:0:TCTGCAAG+GCTGCACT
AGAATATTCTTCTGAGCCTGGATTGCAAAGCACGATTGACGGGATACCACCTTTAATTTGAATTGGTT
TGCCGTACTTCACATTGGATACCCAATTGTGCTGGACCCCAATAAGCTCCTTCCAATGTTTCTTTTGA
AGATATGAAGGGCTT
+
:FFFFFFFFFFFFFFFFFFFFFFFFFFFFFFFFFFFFFFFFFFFFFFFFFFFFFFFFFFFFFFFFFFF
FFFFFFFFFFFFFFFFFFFF:FFFFFFFFFFFFFFFFFFFFFFFFFFFFFFFFFFFFFFF:FFFFFFFFFFFF
FFFFFFFFFF:FFFF
@A00721:190:HGJW7DSXY:4:1265:21251:27492 2:N:0:TCTGCAAG+GCTGCACT
GTAAGCCCTTCATATCTTCAAAAGAAACATTGGAAGGAGCTTATTGGGGTCCAGCACAATTGGGTATC
CAATGTGAAGTACGGCAAACCAATTCAAATTAAAGGTGGTATCCCGTCAATCGTGCTTTGCAATCCAG
GCTCAGAAGAATATT
+
FFFFFFFFFFFFFFFFFFFFFFFFF:FFFFFFFFFFFFFFFFFFFFFFFFFFFFFFFFFFFFFFFFFF
FFFFFFFFFFFFFFFFFFFFFFFFFF,FFF:FFFFFFFFFFFFFFFFFFFFFFFFFFFFFFFFFFFFF
FFFF:FFFFFFFFFF
@A00721:190:HGJW7DSXY:4:1550:32181:29857 2:N:0:TCTGCAAG+GCTGCACT
GTAAGCCCTTCATATCTTCAAAAGAAACATTGGAAGGAGCTTATTGGGGTCCAGCACAATTGGGTATC
CAATGTGAAGTACGGCAAACCAATTCAAATTAAAGGTGGTATCCCGTCAATCGTGCTTTGCAATCCAG
GCTCAGAAGAATATT
+
FFFFFFFFFFFFFFFFFFFFFFFFFFFFFFFFFFFFFFFFFFFFFFFFFFFFFFFFFFFFFFFFFFFF
FFFFFFF:FFFFFFFFFFFFFFF:FFFFFFFF,FFFFFFF:FF:FFFFFFFFFFFFFFFFFFFFFFFFF
FFFFFFFFFF:FFFF
@A00721:190:HGJW7DSXY:4:2326:6424:8516 2:N:0:TCTGCAAG+GCTGCACT
GTAAGCCCTTCATATCTTCAAAAGAAACATTGGAAGGAGCTTATTGGGGTCCAGCACAATTGGGTATC
CAATGTGAAGTACGGCAAACCAATTCAAATTAAAGGTGGTATCCCGTCAATCGTGCTTTGCAATCCAG
GCTCAGAAGAATATT
+
FFFFFFFFFFFFFFFFFFFFFFFFFFFFFFFFFFFFFFFFFFFFFFFFFFFFFFFFFFFFFFFFFF:F
FFFFFFFFFFFFFFFFFFFFFFFF,FFFFFFFFFFFFFFFFFFFFFFFFF:FFFF:FFFFFFFFFFF::F
FFFFFFF:FFFFFFF
@A00721:190:HGJW7DSXY:4:2403:22661:17315 2:N:0:TCTGCAAG+GCTGCACT
GTAAGCCCTTCATATCTTCAAAAGAAACATTGGAAGGAGCTTATTGGGGTCCAGCACAATTGGGTATC
CAATGTGAAGTACGGCAAACCAATTCAAATTAAAGGTGGTATCCCGTCAATCGTGCTTTGCAATCCAG
GCTCAGAAGAATATT
+
FFFFFFFFFFFFFFFFFFFFF:FFFFFF:FFFFFFFFFF:FFF:FFFFFFFFFFFFFFF:FFFFFFFF
FFFFFFFFFFFFFFFFFFFFFFFF:FFFFFFFFFF:FFFFFFFFFFFFFFFFFFFFFFFFFFFF:FFF:F
,FFFFFFFFFFFFFF
@A00721:190:HGJW7DSXY:4:2514:25753:33912 2:N:0:TCTGCAAG+GCTGCACT
GTAAGCCCTTCATATCTTCAAAAGAAACATTGGAAGGAGCTTATTGGGGTCCAGCACAATTGGGTATC
CAATGTGAAGTACGGCAAACCAATTCAAATTAAAGGTGGTATCCCGTCAATCGTGCTTTGCAATCCAG
GCTCAGAAGAATATT
```

+
FFFFFFFFFFFFFFFFFFFFF:FFFFFFFFFFFFFFFFFF:FF:FFFFFFFFFFFFFFFFFFFFFFFFFF
FFFFFFFFFFFFFFFFFFFFFFFFFFFF:FFFF:FFFFFFFFFFFFFF:FFFFFFFFFFFFFFFFFFF:F
FFFFFFFFFF:FFFF
@A00721:190:HGJW7DSXY:4:2564:10791:24345 2:N:0:TCTGCAAG+GCTGCACT
GTAAGCCCTTCATATCTTCAAAAGAAACATTGGAAGGAGCTTATTGGGGTCCAGCACAATTGGGTATC
CAATGTGAAGTACGGCAAACCAATTCAAATTAAAGGTGGTATCCCGTCAATCGTGCTTTGCAATCCAG
GCTCAGAAGAATATT
+
FFFFFFFFFFFFFFFFFFFFFFFFFFFFFFFFFFFFFFFFFFFFFFFFFFFFFFFFFFFFFFFFFFFFFF
FFFFFFFFFFFFFFFFFFFFFFFFFFFFFFFFFFFFFFFFFFFFFFFFFFFFFFFFFFFFFFFFFFFFFF
FFFFFFFFFFFFFFF
@A00721:190:HGJW7DSXY:4:1201:8793:14340 2:N:0:TCTGCAAG+GCTGCACT
CGTAAGCCCTTCATATCTTCAAAAGAAACATTGGAAGGAGCTTATTGGGGTCCAGCACAATTGGGTAT
CCAATGTGAAGTACGGCAAACCAATTCAAATTAAAGGTGGTATCCCGTCAATCGTGCTTTGCAATCCA
GGCTCAGAAGAATAT
+
FFF:FFFFFF:FFFFFFFFFFFFFFFFFFFFF:FFFFFFFFFF:FFFFFFFFFFFFFFFFFFFFFFFFFF
FFFFFFFFFFFFFFFFFFFFFFFFFFFFFFF,F:FFFFFFFF,FFFFFFFFFFFFFFF:FFFFFFFFFFF
FFFFFFFF,FFF:FF
@A00721:190:HGJW7DSXY:4:1363:18385:13698 2:N:0:TCTGCAAG+GCTGCACT
CGTAAGCCCTTCATATCTTCAAAAGAAACATTGGAAGGAGCTTATTGGGGTCCAGCACAATTGGGTAT
CCAATGTGAAGTACGGCAAACCAATTCAAATTAAAGGTGGTATCCCGTCAATCGTGCTTTGCAATCCA
GGCTCAGAAGAATAT
+
FFFFFFFFFFFFFFFFFFFFFFFFFFFFFFFFFFFFFFFFFFFFFFFFFFFFFFFFFFFFFFFFFFFFFF
FFFFFFFFFFFFFFFFFFFFFFFFFFFFFFFFFFFFFFFFFFFFFFFFFFFFFFFFFFFFFFFFFFFFFF
FFFFFFFFFFFFFFF
@A00721:190:HGJW7DSXY:4:2278:17336:14638 2:N:0:TCTGCAAG+GCTGCACT
CGTAAGCCCTTCATATCTTCAAAAGAAACATTGGAAGGAGCTTATTGGGGTCCAGCACAATTGGGTAT
CCAATGTGAAGTACGGCAAACCAATTCAAATTAAAGGTGGTATCCCGTCAATCGTGCTTTGCAATCCA
GGCTCAGACGAATAT
+
FFFFFFFFFFFFFFFFFFFFFFFFFFFFFFFFFFFFFFFFFFFFFFFFFFFFFFFFFFFFFFFFFFFFFF
FFFFFFFFFFFFFFFFFFFFFFFFFFFFFFFFFFFFFFFFFFFFFFFFFFFFFFFFFFFFFFFFFFFFFF
FFFFFFFF,FFFFFF
@A00721:190:HGJW7DSXY:4:2315:2112:12947 2:N:0:TCTGCAAG+GCTGCACT
CGTAAGCCCTTCATATCTTCAAAAGAAACATTGGAAGGAGCTTATTGGGGTCCAGCACAATTGGGTAT
CCAATGTGAAGTACGGCAAACCAATTCAAATTAAAGGTGGTATCCCGTCAATCGTGCTTTGCAATCCA
GGCTCAGAAGAATAT
+
FFFFFFFFFFFFFFFFFFFFFFFFFFFFFFFFFFFFFFFFFFFFF,FFFFFFFFFFFFFFFFFFFFFFFF
FFFFFFFFFFFFFFFFFFF:FFFFFFFFFFFFFFFFFFFFF:FFF:FFFFFFFFFFFFFFFFFFFFFFFF
FFFFFFFFFFFFFFF
@A00721:190:HGJW7DSXY:4:2315:2546:13260 2:N:0:TCTGCAAG+GCTGCACT
CGTAAGCCCTTCATATCTTCAAAAGAAACATTGGAAGGAGCTTATTGGGGTCCAGCACAATTGGGTAT
CCAATGTGAAGTACGGCAAACCAATTCAAATTAAAGGTGGTATCCCGTCAATCGTGCTTTGCAATCCA
GGCTCAGAAGAATAT
+
FFFFFFFFFFFFFFFFFFFFFFFFFFFFFF:FFFFFFFFFFF,FFFFFFFFFFFFFFFFFFFFFFFFFFF
FFFFFFFFFFFFFFFFFFFFFFFFFFFFFFFFFFFFFFFFFFFFFFFFFFFFFFFFFFFFFFFFFFFFFF
FFFFFFFF,FFFF:F
@A00721:190:HGJW7DSXY:4:2326:5981:8531 2:N:0:TCTGCAAG+GCTGCACT
GTAAGCCCTTCATATCTTCAAAAGAAACATTGGAAGGAGCTTATTGGGGTCCAGCACAATTGGGTATC

CAATGTGAAGTACGGCAAACCAATTCAAATTAAAGGTGGTATCCCGTCAATAGTGCTTTGCAATCCAG
GCTCAGAAGAATAT
+
:FFF:F::,,:F,FFF,FFFFFF:FFF:F:,:,FFF::,FFF:FF,,F,FF,F,FFFF::FFF,::F,
FF:,FFFFFFFF:F,F:FFF:FFF:F:FF,FFFFF,FF:FFFFFFF,F,F,,FFFFF,,:FFFFF:FF
FFFFF,FFFFFFF:
@A00721:190:HGJW7DSXY:4:1371:12301:1971 2:N:0:TCTGCAAG+GCTGCACT
CGTAAGCCCTTCATATCTTCAAAAGAAACATTGGAAGGAGCTTATTGGGGTCCAGCACAATTGGGTAT
CCAATGTGAAGTACGGCAAACCAATTCAAATTAAAGGTGGTATCCCGTCAATCGTGCTTTGCAATCCA
GGCTCAGAAGAATA
+
FFFFFFFFFFFFFFFFFFFFFFF:FFFFFFFFFFFFFFFFFFFFFFFFFFFFFFFFFFFFFFFFFFFFFF
FFFF:FFFFFFFFFFFFFFFFFFFFFFFFF,FFFFFFFFFFFFFFFFFFFFFF:FFFFFFFFFFF:FFFF
FFFFFFFFFFFFFF
@A00721:190:HGJW7DSXY:4:1101:12952:34757 2:N:0:TCTGCAAG+GCTGCACT
ATGACGTAAGCCCTTCATATCTTCAAAAGAAACATTGGAAGGAGCTTATTGGGGTCCAGCACAATTGG
GTATCCAATGTGAAGTACGGCAAACCAATTCAAATTAAAGGTGGTATCCCGTCAATCGTGCTTTGCAA
TCCAGGCTCAGAAGA
+
FFFFFFFFFFFFFFFFFFFFFFFFFF:FFFFFFFFFFFFFFFFFFFFF:FFFFFFFFFFFFFFFFFFFFF
FFFFFFFFFFFFFFFFFFFFFFFF:FFFF:FFFFF::FFFFFFFFFFFFFFFF:FFFFFFFFFFFFF:FF
FFFFFFFFFFFFFFF
@A00721:190:HGJW7DSXY:4:1141:4661:15264 2:N:0:TCTGCAAG+GCTGCACT
ATGACGTAAGCCCTTCATATCTTCAAAAGAAACATTGGAAGGAGCTTATTGGGGTCCAGCACAATTGG
GTATCCAATGTGAAGTACGGCAAACCAATTCAAATTAAAGGTGGTATCCCGTCAATCGTGCTTTGCAA
TCCAGGCTCAGAAGA
+
FFFFFFFFFFFFFFFFFFFFFFFFFFFFFFFFFFFFFFFF:FFFFFFFFFFFFFFFFFFFFFFFFFFFFF
FFFF:FFFFFFFFFFFFFFFFFFFFFFFFFFFFFF:FFF:FFFFFFFFFFFFFFFFFFFFFFFFFFFFF:
FFFFFFFFFFFFFFF
@A00721:190:HGJW7DSXY:4:1328:6795:32424 2:N:0:TCTGCAAG+GCTGCACT
ATGACGTAAGCCCTTCATATCTTCAAAAGAAACATTGGAAGGAGCTTATTGGGGTCCAGCACAATTGG
GTATCCAATGTGAAGTACGGCAAACCAATTCAAATTAAAGGTGGTATCCCGTCAATCGTGCTTTGCAA
TCCAGGCTCAGAAGA
+
FFFFFFFFFFFFFFFFFFFFFFFFFFFFFFFFFFFFFFFFFFFFFFFFFFFFFFFFFFFFFFFFFFFFFF
FFFFFFFF:FFFFFFFFFFFFFFFFFFFFF,F:FFFFFF:FFFFFFFFFFFFFFFFFFFFFFFFFFFFFF
FFFFFFFFFFFFFFF
@A00721:190:HGJW7DSXY:4:1417:4797:7169 2:N:0:TCTGCAAG+GCTGCACT
ATGACGTAAGCCCTTCATATCTTCAAAAGAAACATTGGAAGGAGCTTATTGGGGTCCAGCACAATTGG
GTATCCAATGTGAAGTACGGCAAACCAATTCAAATTAAAGGTGGTATCCCGTCAATCGTGCTTTGCAA
TCCAGGCTCAGAAGA
+
FFFFFFFFFFFFFFFFFFFFFFFFFFFFFFFFFFFFFFFF:FFFFFFFFFFFFFFFFFFFFFFFFFFFFF
FFFFFFFFFFFFFFFFFFFFFFFFFFFFFFFFFFFFFFFFFFFFFFFFFFFFFFFFFFFFFFF:FFFFFF
FFFFFFFFFFFFFFF
@A00721:190:HGJW7DSXY:4:1504:11189:23531 2:N:0:TCTGCAAG+GCTGCACT
ATGACGTAAGCCCTTCATATCTTCAAAAGAAACATTGGAAGGAGCTTATTGGGGTCCAGCACAATTGG
GTATCCAATGTGAAGTACGGCAAACCAATTCAAATTAAAGGTGGTATCCCGTCAATCGTGCTTTGCAA
TCCAGGCTCAGAAGA
+
FFFFFFFFFFFFFFFFFFFFFFFFFFFFFFFF,FFFFFFFFFFFFFFFFFFFFFFFFFFFFFFFFFFFFF
F:FFFFFFF:FFFFFFFFFFFFFFFF:FFFFFFFFFF,FFFFFFFFFFFFFFFFFFFFFFFFFFFFFFFF
FFFFFFFFFFFFF:F

```
@A00721:190:HGJW7DSXY:4:1508:15790:30906 2:N:0:TCTGCAAG+GCTGCACT
ATGACGTAAGCCCTTCATATCTTCAAAAGAAACATTGGAAGGAGCTTATTGGGGTCCAGCACAATTGG
GTATCCAATGTGAAGTACGGCAAACCAATTCAAATTAAAGGTGGTATCCCGTCAATCGTGCTTTGCAA
TCCAGGCTCAGAAGA
+
FFFFFFFFFFFFFFFFFFFFFFFFFFFFFFFFFFFFFFFFFFFFFFFFFFFFFFFFFFFFFFFF,FFF
FF:FFFFF:FFFFFFFFFFFFFFFFFFFFFFFF:FFFFFFFFFFFFFFFFFFFFFFFFFFFFFFFFF:
FFFFFFFFFFFFFFF
@A00721:190:HGJW7DSXY:4:2159:17409:12947 2:N:0:TCTGCAAG+GCTGCACT
ATGACGTAAGCCCTTCATATCTTCAAAAGAAACATTGGAAGGAGCTTATTGGGGTCCAGCACAATTGG
GTATCCAATGTGAAGTACGGCAAACCAATTCAAATTAAAGGTGGTATCCCGTCAATCGTGCTTTGCAA
TCCAGGCTCAGAAGA
+
FFFFFFFFFFFFFFFFFFFFFFFFFFFFFFFFFFFFFFFFFFFFF,FFFFFFFFFFFFFFFFFFFFFF
FFFFFFFFFFFFFFFFFFFFFFFFFFFFFFFFFF:FFFFFFFFFFFFFFFFFFFFFFFFFFFFFFFF:
FFFFFFFFFFFF:F:
@A00721:190:HGJW7DSXY:4:2240:26377:4085 2:N:0:TCTGCAAG+GCTGCACT
ATGACGTAAGCCCTTCATATCTTCAAAAGAAACATTGGAAGGAGCTTATTGGGGTCCAGCACAATTGG
GTATCCAATGTGAAGTACGGCAAACCAATTCAAATTAAAGGTGGTATCCCGTCAATCGTGCTTTGCAA
TCCAGGCTCAGAAGA
+
FFF:FFFFFFFFFFFFFFFFFFFFFFFFFFFFFF,FFFFFFFFFFFFFFFFFFFFFFFFFFFFFFFFF
FFFFFFFF,FFFFFFFF,FFFFFFFFFFFFFFFFFFFFFFFFFFFFFFFFFFFFFFFFFFFF:FFFF:
F:FFFFFFFFFFFFF
@A00721:190:HGJW7DSXY:4:2505:29315:29403 2:N:0:TCTGCAAG+GCTGCACT
ATGACGTAAGCCCTTCATATCTTCAAAAGAAACATTGGAAGGAGCTTATTGGGGTCCAGCACAATTGG
GTATCCAATGTGAAGTACGGCAAACCAATTCAAATTAAAGGTGGTATCCCGTCAATCGTGCTTTGCAA
TCCAGGCTCAGAAGA
+
FFFFFFFFFFFFFFFFFFFFFFFFFFFFFFFFFFFFFFFFFFFFFFFFFFFFFFFFFFFFFFFFFFFF
FFFFFFFFFFFFFFFFFFFFFFFFFFFFFFFFFFFFFFFFFFFFFFFFFFFFFFFFFFFFFFFFFFFF
FFFFFFFFFFFFFFF
@A00721:190:HGJW7DSXY:4:2548:25337:29684 2:N:0:TCTGCAAG+GCTGCACT
ATGACGTAAGCCCTTCATATCTTCAAAAGAAACATTGGAAGGAGCTTATTGGGGTCCAGCACAATTGG
GTATCCAATGTGAAGTACGGCAAACCAATTCAAATTAAAGGTGGTATCCCGTCAATCGTGCTTTGCAA
TCCAGGCTCAGAAGA
+
FFFFFFFFFFFFFFFFFFFFFFFFFFFFFFFFFFFFFFFFFFFFFFFFFFFFFFFFFFFFFFFFFFFF
FFFFFFFFFFFFFFFFFFFFFFFFFFFFFFFFFFFFFFFFFFFFFFFFFFFFFFFFFFFFFFFFFFFF
FFFFFFFFFFFFFFF
@A00721:190:HGJW7DSXY:4:2621:3531:28119 2:N:0:TCTGCAAG+GCTGCACT
ATGACGTAAGCCCTTCATATCTTCAAAAGAAACATTGGAAGGAGCTTATTGGGGTCCAGCACAATTGG
GTATCCAAGGTGAAGTACGGCAAACCAATTCAAATTAAAGGTGGTATCCCGTCAATCGTGCTTTGCAA
TCCAGGCTCAGAAGA
+
F,:F,F,FF,:,,:FFF:FFFF:,F:FFFFFF,FFF,FFF,:FFFFFFFFF,FFF:FFFFFFFFFF,,
,,F:F,FF,:F::FF,FFF,FFFFF,FF:F:FF,,FF,FFFFF::FF:::,,FFFF,,F:FF,FFFFF
FFFFF:FF:F,FF:F
@A00721:190:HGJW7DSXY:4:1221:27019:20290 2:N:0:TCTGCAAG+GCTGCACT
AGATGACGTAAGCCCTTCATATCTTCAAAAGAAACATTGGAAGGAGCTTATTGGGGTCCAGCACAATT
GGGTATCCAATGTGAAGTACGGCAAACCAATTCAAATTAAAGGTGGTATCCCGTCAATCGTGCTTTGC
AATCCAGGCTCAGAA
+
FFFFFFFFFFFFFFFFFFFFFFFFFFFFFFFFFFFFFFFFFFFFFFFFFFFFFFFFFFFFFFFFFFFF
```

FFFFFFFFFFFFFFFFFFFFFFFFFFFFFFFFFFFFFFF:FFFFFFFFFFFFFFFFFFFFFFFFFFFFF
FFFFFFFFFFFFFFF
@A00721:190:HGJW7DSXY:4:1221:25762:22811 2:N:0:TCTGCAAG+GCTGCACT
AGATGACGTAAGCCCTTCATATCTTCAAAAGAAACATTGGAAGGAGCTTATTGGGGTCCAGCACAATT
GGGTATCCAATGTGAAGTACGGCAAACCAATTCAAATTAAAGGTGGTATCCCGTCAATCGTGCTTTGC
AATCCAGGCTCAGAA
+
F:FFFFFFFFFFFFF:FFFFFFFFFF:FFFFFFFFFFFFFFFF:FFFFFFFFFFFFFFFFFFFFFFFFF
FFFF:FFFF:FFFFFFFFFFFFFFFFFFFFFFFFFFFF:FFFFFFFFFFFF:FFFFFFFFFFFFFFFFF
FFFFFFFFFFFFFFF
@A00721:190:HGJW7DSXY:4:1271:11279:24158 2:N:0:TCTGCAAG+GCTGCACT
AGATGACGTAAGCCCTTCATATCTTCAAAAGAAACATTGGAAGGAGCTTATTGGGGTCCAGCACAATT
GGGTATCCAATGTGAAGTACGGCAAACCAATTCAAATTAAAGGTGGTATCCCGTCAATCGTGCTTTGC
AATCCAGGCTCAGAA
+
FFFFFFFFFFFFFFFFFFFFFFFFFFFFFFFFFFFFFFFFFFFFFFFFFFFFFFFFFFFFFFFFFFFF
FFFFFFFFFFFFFFFFFFFFFFFFFFFFFFFFFFFFFFFFFFFF:FFFFFFFFFFFFFFFFFFFFFF:F
FFFFFFFFFFFFFFF
@A00721:190:HGJW7DSXY:4:1306:25952:8453 2:N:0:TCTGCAAG+GCTGCACT
AGATGACGTAAGCCCTTCATATCTTCAAAAGAAACATTGGAAGGAGCTTATTGGGGTCCAGCACAATT
GGGTATCCAATGTGAAGTACGGCAAACCAATTCAAATTAAAGGTGGTATCCCGTCAATCGTGCTTTGC
AATCCAGGCTCAGAA
+
FFFFFFFFFFFFFFFFFFFFFFFFFFFFFFFFFFFFFFFFFFFFFFFFF:FFFFFFFFFFFFFFFFFFF
FFFFFFFFFFFFFFFFFFFFFFFFFFFFFFFFFFFFFFFFFFFFFFFFFFFFFFFFFFFFFFFFFFFF
FFFFFFFFFFFFFFF
@A00721:190:HGJW7DSXY:4:1475:21459:15295 2:N:0:TCTGCAAG+GCTGCACT
AGATGACGTAAGCCCTTCATATCTTCAAAAGAAACATTGGAAGGAGCTTATTGGGGTCCAGCACAATT
GGGTATCCAATGTGAAGTACGGCAAACCAATTCAAATTAAAGGTGGTATCCCGTCAATCGTGCTTTGC
AATCCAGGCTCAGAA
+
FFFFFFFFFFFFFFFFFFFFFFFFFFFFFFFFFFFFF:FFFFFFFFFFFFFFFFFFFFFFFFFFFFFFF
FFFFFFFFFFFFFFFFFFFFFFFFF:,FFFFFFFFFFFFFF:FFFFFFFFFFFFFFFFFFFFFFFFFFF
FFFFFFFFFFFFFFF
@A00721:190:HGJW7DSXY:4:2142:7075:19789 2:N:0:TCTGCAAG+GCTGCACT
AGATGACGTAAGCCCTTCATATCTTCAAAAGAAACATTGGAAGGAGCTTATTGGGGTCCAGCACAATT
GGGTATCCAATGTGAAGTACGGCAAACCAATTCAAATTAAAGGTGGTATCCCGTCAATCGTGCTTTGC
AATCCAGGCTCAGAA
+
:FFFFFFFFFFFFFFFFFFFFFFFFFFFFFFFFFFFFFFFFFFFFFFFFFFFFFFFFFFFFFFFFFFF
FFFFFFFFFF:FFFFFFFFFFFFFFFFF:F,FFFFFFFFFFFFFFFFFFFFFFFFFFFFFFFFFFFFFF
FFFFFFFFFFFFFF:
@A00721:190:HGJW7DSXY:4:2509:9498:17190 2:N:0:TCTGCAAG+GCTGCACT
AGATGACGTAAGCCCTTCATATCTTCAAAAGAAACATTGGAAGGAGCTTATTGGGGTCCAGCACAATT
GGGTATCCAATGTGAAGTACGGCAAACCAATTCAAATTAAAGGTGGTATCCCGTCAATCGTGCTTTGC
AATCCAGGCTCAGAA
+
FFFFFFFFFFFFFFFFFFFFFFFFFFFFFFFFFFFFFF:FFFFFFFFFFFFFFFFFFFFFFFFFFFF:F
FFFFFFFFFFFF:FFFFFFFFFFFFFFFFFFFFFFFFFFF:FFFFFFFFFFFFFFFFF:FFFFFFFFFF
F:FFFFFFFFFFFFF
@A00721:190:HGJW7DSXY:4:1125:25807:12806 2:N:0:TCTGCAAG+GCTGCACT
CTGAGCCTGGATTGCAAAGCACGATTGACGGGATACCACCTTTAATTTGAATTGGTTTGCCGTACTTC
ACATTGGATACCCAATTGTGCTGGACCCCAATAAGCTCCTTCCAATGTTTCTTTTGAAGATATGAAGG
GCTTGCGTCATCTAT

```
+
FFFFFFFFFFFFFFFFFFFFFFFF:FFFFFFFFFFFFFFFFFFFFFFFFFFFFFFFFFFFFFFFFFFFF
FFF,FFFFF:FFFFFFFFFFFFFFFFFFFFFFFFFFFFFFFFFFFFFFFFFF:,FFFFFFFFFFFFFF
FFFFFFFFFF:FFF:
@A00721:190:HGJW7DSXY:4:2205:17409:21621 2:N:0:TCTGCAAG+GCTGCACT
CTGAGCCTGGATTGCAAAGCACGATTGACGGGATACCACCTTTAATTTGAATTGGTTTGCCGTACTTC
ACATTGGATACCCAATTGTGCTGGACCCCAATAAGCTCCTTCCAATGTTTCTTTTGAAGATATGAAGG
GCTTGCGTCATCTAT
+
FFFFFFFFFFFFFFFFFFFFFFFF:FFFFFFFFFFFFFFFFFFFFFFFFFFFFFFFFFFFFFFFFFFFF
FFFFFFFFFFFFFFFFFFFFFFFFFFFFFFFFFFFFFFFFFFFFFFFFFFFFFFFFFFF:FFFFFFFF
FFFFFFFFFFFFFFF
@A00721:190:HGJW7DSXY:4:2511:25093:26193 2:N:0:TCTGCAAG+GCTGCACT
CTGAGCCTGGATTGCAAAGCACGATTGACGGGATACCACCTTTAATTTGAATTGGTTTGCCGTACTTC
ACATTGGATACCCAATTGTGCTGGACCCCAATAAGCTCCTTCCAATGTTTCTTTTGAAGATATGAAGG
GCTTGCGTCATCTAT
+
FFFFFFFFFFFFFFFFFFFFFFFFFFFFFFFFFFFFFFFFFFFFFFFFFFFFFFFFFFFFFFFFFFFF
FFFFFFFFFFFFFFF,FFFFFFFFFFFFFFFFFFFFFFFFFFFFFFFFFFFFFFFFFF:FFFFFFFFF
FFFFFFFFFFFFFFF
@A00721:190:HGJW7DSXY:4:1242:19524:9095 2:N:0:TCTGCAAG+GCTGCACT
ATCATAGATGACGTAAGCCCTTCATATCTTCAAAAGAAACATTGGAAGGAGCTTATTGGGGTCCAGCA
CAATTGGGTATCCAATGTGAAGTACGGCAAACCAATTCAAATTAAAGGTGGTATCCCGTCAATCGTGC
TTTGCAATCCAGGCT
+
FFFFFFFFFFFFFFFFFFFFFFFFFFFFFFFFFFFFFFFFFFFFFFFFFFFFFFFFFFFFFFFFFFFF
FFFFFFFFFFFFFFFFFFFFFFFFFFFFFFFFFFFFFFFFFFFFFFFFFFFF:FFFFFFFFFFFFFFF
FFFFFFFFFFFFFFF
@A00721:190:HGJW7DSXY:4:1258:4291:1689 2:N:0:TCTGCAAG+GCTGCACT
ATCATAGATGACGTAAGCCCTTCATATCTTCAAAAGAAACATTGGAAGGAGCTTATTGGGGTCCAGCA
CAATTGGGTATCCAATGTGAAGTACGGCAAACCAATTCAAATTAAAGGTGGTATCCCGTCAATCGTGC
TTTGCAATCCAGGCT
+
:FFFFFFFFFFFFFFFFFFFFFFFFFFFFFFFFFFFFFFFFFFFFFFFFFFFFFFFFFFFFFFFFFFF
FFFFFFFFFFFFF:FFFFFFFFFFFFFFFFFFFFFFFFFFF:FFFFFFFFFFFFFFFFFFFFFFFFFF
FFFFFFF:FFFFFFF
@A00721:190:HGJW7DSXY:4:1362:7952:27101 2:N:0:TCTGCAAG+GCTGCACT
ATCATAGATGACGTAAGCCCTTCATATCTTCAAAAGAAACATTGGAAGGAGCTTATTGGGGTCCAGCA
CAATTGGGTATCCAATGTGAAGTACGGCAAACCAATTCAAATTAAAGGTGGTATCCCGTCAATCGTGC
TTTGCAATCCAGGCT
+
FFFFFFFFFFFFFFFFFFFFFFFFFFFFFFFFFFFFFFFF,FFFFFFFFFFFFFFFFFFFFFFFFFFF
FFFFFFFF,FFFFFFFFFFFFFFFFFFFFFFFFFFFFFFFFFFFFFFFFFFFFFFFFFFFFFFFFFFF
FFFFFF:FFFFFFF:
@A00721:190:HGJW7DSXY:4:1374:21902:28902 2:N:0:TCTGCAAG+GCTGCACT
ATCATAGATGACGTAAGCCCTTCATATCTTCAAAAGAAACATTGGAAGGAGCTTATTGGGGTCCAGCA
CAATTGGGTATCCAATGTGAAGTACGGCAAACCAATTCAAATTAAAGGTGGTATCCCGTCAATCGTGC
TTTGCAATCCAGGCT
+
FFFFFFFFFFFFFFFFFFFFFFFFFFFFFFFFFFFFFFFFFFFFFFFFFFFFFFFFFFFFFFFFFFFF
FFFFFFFFFFFFFFFFFFFFFFFFFFFFFFFFFFFFFFFFFFFFFFFFFFFFFFFFFFFFFFFFFFFF
FFFFFFFFFFFFFFF
@A00721:190:HGJW7DSXY:4:1430:31358:22670 2:N:0:TCTGCAAG+GCTGCACT
ATCATAGATGACGTAAGCCCTTCATATCTTCAAAAGAAACATTGGAAGGAGCTTATTGGGGTCCAGCA
```

CAATTGGGTATCCAATGTGAAGTACGGCAAACCAATTCAAATTAAAGGTGGTATCCCGTCAATCGTGC
TTTGCAATCCAGGCT
+
FFFFFFFFFFFFFFFFFFFFFFFFFFFFFFFFFFFFFFFFFFFFFFFFFFFFFFFFFFFFFFFFFFFF
FFFFFFFFFFFFFFFFFFFFFFFFFFFFFFFFFFFFFFFF,FFFF,:FF:FFFF:FFFFFF:F,,FFF
FFFF:FFFFFFFFFF
@A00721:190:HGJW7DSXY:4:1472:8043:36996 2:N:0:TCTGCAAG+GCTGCACT
ATCATAGATGACGTAAGCCCTTCATATCTTCAAAAGAAACATTGGAAGGAGCTTATTGGGGTCCAGCA
CAATTGGGTATCCAATGTGAAGTACGGCAAACCAATTCAAATTAAAGGTGGTATCCCGTCAATCGTGC
TTTGCAATCCAGGCT
+
FFFFFFFFFF:FFFFF,FFFF:FFFFFFFFFFFFFFFFFF,FFFFFFFFFFFFFFFFFFFFFFFFFFF
FFF:FFFFFFFFF:FFFFFFFFFFF:::FFFFFFFFFFFFFFFFF:FFFFFFFFFFFFFFFFFFFFF:
FFFFFFFFFFFFFFF
@A00721:190:HGJW7DSXY:4:1638:11523:36104 2:N:0:TCTGCAAG+GCTGCACT
ATCATAGATGACGTAAGCCCTTCATATCTTCAAAAGAAACATTGGAAGGAGCTTATTGGGGTCCAGCA
CAATTGGGTATCCAATGTGAAGTACGGCAAACCAATTCAAATTAAAGGTGGTATCCCGTCAATCGTGC
TTTGCAATCCAGGCT
+
FFFFFFFFFFFFFFFFFFFFFFFFFFFFFFFFFFFFFFFFFFFFFF,FFFFFFFFFFFFFFFFFFFFF
FFFFFFFFF:FFFFFFFFFFFFFFFFFFFFFFFFF:FFFFF:FFFFFFFFFFFFFFFFFF,FFFFFFF
FFFFFFFFFFFFFFF
@A00721:190:HGJW7DSXY:4:2122:1850:9267 2:N:0:TCTGCAAG+GCTGCACT
ATCATAGATGACGTAAGCCCTTCATATCTTCAAAAGAAACATTGGAAGGAGCTTATTGGGGTCCAGCA
CAATTGGGTATCCAATGTGAAGTACGGCAAACCAATTCAAATTAAAGGTGGTATCCCGTCAATCGTGC
TTTGCAATCCAGGCT
+
FFFF:F:FFFFFFFFFFFFF:FFFFFFF:FFFFFFFFFFFFFFFFFFFFFFF:FFFFFFFFFFFFFFF
FFF,FFFFFFFFFFFFFFFFF:FFFFFFFFFFFFF:FFF:F:FFFFFFFFFFFFFFFFFFFFFFFFFF
FFFFFFFFFFFFFFF
@A00721:190:HGJW7DSXY:4:2219:32145:34272 2:N:0:TCTGCAAG+GCTGCACT
ATCATAGATGACGTAAGCCCTTCATATCTTCAAAAGAAACATTGGAAGGAGCTTATTGGGGTCCAGCA
CAATTGGGTATCCAATGTGAAGTACGGCAAACCAATTCAAATTAAAGGTGGTATCCCGTCAATCGTGC
TTTGCAATCCAGGCT
+
FFFFFFFFFF:FFFFFFFFFFFFFFFFFFFFF:FFF:FFFFFFFF:F:FFF:FFFFFFFFFFFFFFFF
FFFFFFFFFFFFFFFFFFFFFFFFFF:FFF:FFFFFFFFFFFFF:,:FFF:FFF,FFFFFFFF::FFF
FFFF,FFFF:F:FFF
@A00721:190:HGJW7DSXY:4:1150:5403:24940 2:N:0:TCTGCAAG+GCTGCACT
TATAACATCATAGATGACGTAAGCCCTTCATATCTTCAAAAGAAACATTGGAAGGAGCTTATTGGGGT
CCAGCACAATTGGGTATCCAATGTGAAGTACGGCAAACCAATTCAAATTAAAGGTGGTATCCCGTCAA
TCGTGCTTTGCAATC
+
FFFFFFFFFFFFFFFFFFFFFFFFFFFFFFFFFFFFFFFFFFFFFFFFFFFFFFFFFFFFFFFFFFFF
FFFFFFFFFFFFFFF:FFFFFFFFFFFFF:FFFFFFFFFFFFFFFFFFFFF:FFFFFFFFFFFFFF,F
FFFFFFFFFFFF,FF
@A00721:190:HGJW7DSXY:4:1204:4164:20729 2:N:0:TCTGCAAG+GCTGCACT
TATAACATCATAGATGACGTAAGCCCTTCATATCTTCAAAAGAAACATTGGAAGGAGCTTATTGGGGT
CCAGCACAATTGGGTATCCAATGTGAAGTACGGCAAACCAATTCAAATTAAAGGTGGTATCCCGTCAA
TCGTGCTTTGCAATC
+
::FFFFFFFFFFFFFFFFFFFFFFFF,FFFFFFFFFFFFFF::FF:FFFFFF:F,FFFFFFFF:FFFF
FFFFFFFFFFFFF:FFFFFFF:FFFFFFFFFFFFFFF:FFF,,FFF,FFF,FF:FF,FFF,FFFFFFF
FFFFF,:,FF:FFFF

```
@A00721:190:HGJW7DSXY:4:1356:4227:5932 2:N:0:TCTGCAAG+GCTGCACT
TATAACATCATAGATGACGTAAGCCCTTCATATCTTCAAAAGAAACATTGGAAGGAGCTTATTGGGGT
CCAGCACAATTGGGTATCCAATGTGAAGTACGGCAAACCAATTCAAATTAAAGGTGGTATCCCGTCAA
TCGTGCTTTGCAATC
+
FFFFFFFFFFFFFFFFFFFFFFFFFFFFFFFFF:FFFFFFFFFFFFFFFFFFFFFFFFFFFF,FFFFFFF
FFFFFFFFFFFFFFFFFFFFFFFFFFFFFFFFFFFF::FFF:F:FFFFFFF:F:FFFFFFFFFFFFFFFF
FFFFFFFFFFFF,FF
@A00721:190:HGJW7DSXY:4:1462:14543:32158 2:N:0:TCTGCAAG+GCTGCACT
TATAACATCATAGATGACGTAAGCCCTTCATATCTTCAAAAGAAACATTGGAAGGAGCTTATTGGGGT
CCAGCACAATTGGGTATCCAATGTGAAGTACGGCAAACCAATTCAAATTAAAGGTGGTATCCCGTCAA
TCGTGCTTTGCAATC
+
FFFFFFFFFFFFFFFFFFF:FFFFFFFFFFFFFFFFFFFFFFFFFFFF:,FF:::F:F:FF:FFFFFFFF
FFF:FFFFFFFFFFFFFFFFF:FFFFFFFFFFFFFFFFF,FFFFFFFFFF:FF:F,F:FFFFF:FFFFFF
FFFFFFFFFFFFFFF
@A00721:190:HGJW7DSXY:4:1514:5113:8688 2:N:0:TCTGCAAG+GCTGCACT
TATAACATCATAGATGACGTAAGCCCTTCATATCTTCAAAAGAAACATTGGAAGGAGCTTATTGGGGT
CCAGCACAATTGGGTATCCAATGTGAAGTACGGCAAACCAATTCAAATTAAAGGTGGTATCCCGTCAA
TCGTGCTTTGCAATC
+
FFFFFFFFFFFFFFFFFFFFFFFFFFFFFFFFFFFFFFFFFFFFFFFFFFFFFFFFFFFFFFFFFFFFFF
FFFFFFFFFFFFFFFFFFFFFFFFFFFFFFFFFFFFFFFFFFFFFF:FFFFFFFFFFFFFFFFFFFFFFF
FFFFFFFFFFFFFFF
@A00721:190:HGJW7DSXY:4:1612:7916:28855 2:N:0:TCTGCAAG+GCTGCACT
ATAACATCATAGATGACGTAAGCCCTTCATATCTTCAAAAGAAACATTGGAAGGAGCTTATTGGGGTC
CAGCACAATTGGGTATCCAATGTGAAGTACGGCAAACCAATTCAAATTAAAGGTGGTATCCCGTCAAT
CGTGCTTTGCAATC
+
:FFFFFFFFFFFFFFFFFFFFFFFFFFFFFFFFFFFFFFFFFFFFF:F,FFFFFFFFFFFFFFFFFFFFF
FFFFFFFFFFFFFFFFFFFFFFFFFFFFFFFFFFFF,FFFFFFFFFFFFFFFFFFFFFFFFFFFFFFFFF
FFFFFFFFFFFFFF
@A00721:190:HGJW7DSXY:4:1612:8775:29371 2:N:0:TCTGCAAG+GCTGCACT
TATAACATCATAGATGACGTAAGCCCTTCATATCTTCAAAAGAAACATTGGAAGGAGCTTATTGGGGT
CCAGCACAATTGGGTATCCAATGTGAAGTACGGCAAACCAATTCAAATTAAAGGTGGTATCCCGTCAA
TCGTGCTTTGCAATC
+
FFFFFFFFFFFFFFFF:FFFFFFFFF:FFFFFFFFFFFFFFFFFFFF:FFFFFFFFFFFFFFFFFFFFFF
FFFFFFFFFFFFFFFFFFFFFFFFFFFFFFFFFFFFFFFFFFFFFFFFFFFF,FFFFFFFFFFFFFFFFF
FFFFFFFFFFFFFFF
@A00721:190:HGJW7DSXY:4:1619:4209:9283 2:N:0:TCTGCAAG+GCTGCACT
TATAACATCATAGATGACGTAAGCCCTTCATATCTTCAAAAGAAACATTGGAAGGAGCTTATTGGGGT
CCAGCACAATTGGGTATCCAATGTGAAGTACGGCAAACCAATTCAAATTAAAGGTGGTATCCCGTCAA
TCGTGCTTTGCAATC
+
F:FFFFFFFFFFFFFFFFFFFFFFFFFFFFFFFFFFFFFFFFFFFFF,FFFFFFFFFFFFFFFFFFFFFF
FFFFFFFFFFFFFFFFFFFFF:FFFFFFFFFFFFFFFFFFF:FFFFFFFF:FFFFFFFFFFFFFFFFFFF
FFFFFFFFFFFFFFF
@A00721:190:HGJW7DSXY:4:1631:25635:11694 2:N:0:TCTGCAAG+GCTGCACT
TATAACATCATAGATGACGTAAGCCCTTCATATCTTCAAAAGAAACATTGGAAGGAGCTTATTGGGGT
CCAGCACAATTGGGTATCCAATGTGAAGTACGGCAAACCAATTCAAATTAAAGGTGGTATCCCGTCAA
TCGTGCTTTGCAATC
+
FFFFFFFFFFFFFFFFFFFFFFFFFFFFFFFFFFFFFFFFFFFFFFFFFFFFFFFFFFFFF:FFFFFFFF
```

FFFFFFFFFFFFFFFFFFFFFFFFFFFFFFFFFFFF,FFFF:FFFFFFFFF:FFFFFFFFFFFFFFFF
FFFFFFFFFFFFFFF
@A00721:190:HGJW7DSXY:4:1634:11957:7482 2:N:0:TCTGCAAG+GCTGCACT
TATAACATCATAGATGACGTAAGCCCTTCATATCTTCAAAAGAAACATTGGAAGGAGCTTATTGGGGT
CCAGCACAATTGGGTATCCAATGTGAAGTACGGCAAACCAATTCAAATTAAAGGTGGTATCCCGTCAA
TCGTGCTTTGCAATC
+
FFFFFFFFFFFFFFFFFFFFFFFFFFFFFFFFFFFFFFFFFFFFFFFFFFFFFFFFFFFFFFFFFFFF
FFF:FFFFFFFFFFFFFFFFFFFFFFFFFFFFFFFFFFFFFFFFFFFFFFFFFF:FFFFFFFFFFF
FFFFFFFFFFFFFFF
@A00721:190:HGJW7DSXY:4:1642:8558:28964 2:N:0:TCTGCAAG+GCTGCACT
TATAACATCATAGATGACGTAAGCCCTTCATATCTTCAAAAGAAACATTGGAAGGAGCTTATTGGGGT
CCAGCACAATTGGGTATCCAATGTGAAGTACGGCAAACCAATTCAAATTAAAGGTGGTATCCCGTCAA
TCGTGCTTTGCAATC
+
FFFFFFF:FFFF:FFFFFFFFFF:FF,FFFFF,F,,FFFFFFFFFFFFF,FFFFFFFFFFFF:FF,FF
,FFFFF,FFFFFFFFFF:FF:,FFFFFFFFF:FFFFF::FFFF:FFF,:FFFF:FFFFFFFFFF:FFF
FFFFFF:FFFFFFFF
@A00721:190:HGJW7DSXY:4:2419:1099:16078 2:N:0:TCTGCAAG+GCTGCACT
TATAACATCATAGATGACGTAAGCCCTTCATATCTTCAAAAGAAACATTGGAAGGAGCTTATTGGGGT
CCAGCACAATTGGGTATCCAATGTGAAGTACGGCAAACCAATTCAAATTAAAGGTGGTATCCCGTCAA
TCGTGCTTTGCAATC
+
:FFFFFFFF:FFFFFFFFFFFFFFFFFFFFFFFFFFFFFFFFFFFFFFFFFFFFFFFFFFFFFFFF:F
FFFFFFFFF:FFFFF,FFFFF,:FFFFFFFFFFFF:FF:FF:FFFFFFFFFF:FFFFFFFF:FFFFFF
FFFFFFF:FF:FFFF
@A00721:190:HGJW7DSXY:4:2437:27715:29387 2:N:0:TCTGCAAG+GCTGCACT
TATAACATCATAGATGACGTAAGCCCTTCATATCTTCAAAAGAAACATTGGAAGGAGCTTATTGGGGT
CCAGCACAATTGGGTATCCAATGTGAAGTACGGCAAACCAATTCAAATTAAAGGTGGTATCCCGTCAA
TCGTGCTTTGCAATC
+
FFFFFFFFFFFFFFFFFFFFFFFFFFFFFFFFFFFFFFFFFFFF:FFFFFFF:FFFFFFF,FFFFFFF
FFFFFFFFFFFFFFFFFFFF,FFFFFFFFFFFFFFFF:FFFFFFFFFFFFFFFFFFFFFFFFFFFFFF,
FFFFFFFFFFFFFFF
@A00721:190:HGJW7DSXY:4:2535:1976:23547 2:N:0:TCTGCAAG+GCTGCACT
TATAACATCATAGATGACGTAAGCCCTTCATATCTTCAAAAGAAACATTGGAAGGAGCTTATTGGGGT
CCAGCACAATTGGGTATCCAATGTGAAGTACGGCAAACCAATTCAAATTAAAGGTGGTATCCCGTCAA
TCGTGCTTTGCAATC
+
FFFFFFFFFFFFFFFFFFFFFFFFFFFFFFFFFFFFFFFFFFFFFFFFFFFFFFFFFFFFFF:FFFFFF
FFFFFFFFFFFFFFFFFFFFFFFFFFFFFFFFFFFFFFFFFFFFF:FFFFFFFFFFFFF:FFFFFFFFF
FFFFFFFFFFFFFFF
@A00721:190:HGJW7DSXY:4:2566:30960:1501 2:N:0:TCTGCAAG+GCTGCACT
TATAACATCATAGATGACGTAAGCCCTTCATATCTTCAAAAGAAACATTGGAAGGAGCTTATTGGGGT
CCAGCACAATTGGGTATCCAATGTGAAGTACGGCAAACCAATTCAAATTAAAGGTGGTATCCCGTCAA
TCGTGCTTTGCAATC
+
FFFFFFFFFFFFFFFFFFFFFFFFFFFFFFFFFFFFFFFFFFFFFFFFFFFFFFFFFFFFFFFFFFFF
FFFFFFFFF,FFFFFFFFFFFFFFFFFFFFFF:FFFFFFFFFFFFFFFFFFF:FF:FFFFFFFFFFFFF
FFFFFFFFFFFFFFF
@A00721:190:HGJW7DSXY:4:2608:18041:8406 2:N:0:TCTGCAAG+GCTGCACT
TATAACATCATAGATGACGTAAGCCCTTCATATCTTCAAAAGAAACATTGGAAGGAGCTTATTGGGGT
CCAGCACAATTGGGTATCCAATGTGAAGTACGGCAAACCAATTCAAATTAAAGGTGGTATCCCGTCAA
TCGTGCTTTGCAATC

```
+
FFFFFFFFFFFFFFFFFFFFFFFFFFFFFFFFFFFFFFFFFFFFFFFFFFFFFFFFFFFFFFFFFFFFFF
FFFFFFFFFFFFFFFFFFFFFFFFFFFFFFF:FFFFFFFFFFFFFFFFFFFF:FFFFF:FFFFFFFFFFF
FFFFFFFFFFFFFFF
@A00721:190:HGJW7DSXY:4:1526:10068:33739 2:N:0:TCTGCAAG+GCTGCACT
GGATAACATCATAGATGACTTAAGCCCTTCATATCTTCAAAAGAAACATTGGAAGGAGCTTATTGGGG
TCCAGCACAATAGGGTATCCAATGTGAAGTACGGCAAACCAATTCAAATTAAAGGTGGTATCACGTCA
ATCGTGCTTTGCAAT
+
:,::FFFFF,FF,FFFFF,,,:,FFFFF:,FFFFFF,FFFF:FFFFFF:,:,FFF,F:F,FFFF,:,F
F,,F,,:FFFF,,::,FF,FFFF,,::F,,FFF,:FFF:FFFFFFFFF:FFF:F,:,FFFFF:,,F:F
F,:,:FFFF:F,F:F
@A00721:190:HGJW7DSXY:4:2465:16459:13495 2:N:0:TCTGCAAG+GCTGCACT
GTATAACATCATAGATGACGTAAGCCCTTCATATCTTCAAAAGAAACATTGGAAGGAGCTTATTGGGG
TCCAGCACAATTGGGTATCCAATGTGAAGTACGGCAAACCAATTCAAATTAAAGGTGGTATCCCGTCA
ATCGTGCTTTGCAAT
+
FFF:FFFFFFFFFFFFFFFFFFFFFFFFFFFFFFFFFFFFFFFFFFFFFFFFFF:FFFFF:FFFFFFFFF
FFFFFFFFFFFFFFFFFFFFFFFFFFFFFFFFFFFFFF,FFFFFFFFFFFFFFFFFFFFFFFFFFFFFFF
FFFFFFFFFFFFFFF
@A00721:190:HGJW7DSXY:4:1155:8124:14184 2:N:0:TCTGCAAG+GCTGCACT
CGCATGGTATAACATCATAGATGACGTAAGCCCTTCATATCTTCAAAAGAAACATTGGAAGGAGCTTA
TTGGGGTCCAGCACAATTGGGTATCCAATGTGAAGTACGGCAAACCAATTCAAATTAAAGGTGGTATC
CCGTCAATCGTGCTT
+
FFFFFFFFF:FFFFFFFFF:FFFFFFFFFFFFFFFFFFFFFFFFFFFFFFFFFFFFFFFFF:FFFF:FFF
FF,FFFFFFFFFFFFFFF:FFFFFFF:F:FF,FFFFFFF:FFFFFFFF,FFFF:FFF:,FFF,FFFFF
:FF,FFFFFFFF,FF
@A00721:190:HGJW7DSXY:4:1155:7464:16047 2:N:0:TCTGCAAG+GCTGCACT
CGCATGGTATAACATCATAGATGACGTAAGCCCTTCATATCTTCAAAAGAAACATTGGAAGGAGCTTA
TTGGGGTCCAGCACAATTGGGTATCCAATGTGAAGTACGGCAAACCAATTCAAATTAAAGGTGGTATC
CCGTCAATCGTGCTT
+
:FFFFFFFFFFFFFFFFFFFFFFFFFFFFFFFFFFFFFFFFFFFFFFFFFFFFFFFFFFFFFFFFFFFFF
FFFFFFFFFFFFFFFFFFFFFFFFFFFFFFFFFFFFFFFFFFFFFFFFFFFFFFFFFFFFFFFFFFFFFF
FFFFFFFFFFFFFFF
@A00721:190:HGJW7DSXY:4:1207:17155:1611 2:N:0:TCTGCAAG+GCTGCACT
CGCATGGTATAACATCATAGATGACGTAAGCCCTTCATATCTTCAAAAGAAACATTGGAAGGAGCTTA
TTGGGGTCCAGCACAATTGGGTATCCAATGTGAAGTACGGCAAACCAATTCAAATTAAAGGTGGTATC
CCGTCAATCGTGCTT
+
FFFFFFFFFFFFFFFFFFFFFFFFFFFFFFFFFFFFFFFFFFFFFFFFFFFFFFFFFFFFFFFFFFF,FF
FFFFFFFFFFFFFFFFFFFFFFFFFFFFFFFFFFFFFFFFFFFF,FFFFFFFFFFFFFFF:FFFFFF:FF
FFFFFFFFFFFFFFF
@A00721:190:HGJW7DSXY:4:1310:28926:29700 2:N:0:TCTGCAAG+GCTGCACT
CGCATGGTATAACATCATAGATGACGTAAGCCCTTCATATCTTCAAAAGAAACATTGGAAGGAGCTTA
TTGGGGTCCAGCACAATTGGGTATCCAATGTGAAGTACGGCAAACCAATTCAAATTAAAGGTGGTATC
CCGTCAATCGTGCTT
+
FFFFFFFFFFFFFFFFFFFFFFFFFFFFFFFFFFFFFFFFFFFFFFFF:FFFFFF:FFFFFFFFFFFFFF
FFFFFFFFFFFFFFFFFFFFFFFFFFFFFFFFFFFFFFFFFFFFFFFFFFFFFFFFFFFFFF:FFFFFFF
FFFFFFFFFFFFFFF
@A00721:190:HGJW7DSXY:4:1410:10438:33912 2:N:0:TCTGCAAG+GCTGCACT
CGCATGGTATAACATCATAGATGACGTAAGCCCTTCATATCTTCAAAAGAAACATTGGAAGGAGCTTA
```

```
TTGGGGTCCAGCACAATTGGGTATCCAATGTGAAGTACGGCAAACCAATTCAAATTAAAGGTGGTATC
CCGTCAATCGTGCTT
+
FFFFFFFFFFFFFFFF:FFFFFFFFFFFFFFFF,FFFFFFFFFFFFFFFFFFFFFFFFFFF:FFF:FF
FFFFFFFFFFFFFFFFFFFFFFFFFFFFFFFFFFFFFFFFFFFFFFFF,FFFF:FFFFFFFFFFFFFF
FFFFFFFFFFFFFFF
@A00721:190:HGJW7DSXY:4:2101:25834:15076 2:N:0:TCTGCAAG+GCTGCACT
CGCATGGTATAACATCATAGATGACGTAAGCCCTTCATATCTTCAAAAGAAACATTGGAAGGAGCTTA
TTGGGGTCCAGCACAATTGGGTATCCAATGTGAAGTACGGCAAACCAATTCAAATTAAAGGTGGTATC
CCGTCAATCGTGCTT
+
FFF:FFFFFFFFFFFFFFFFFFFFFFFFFFFFFFFFFFFFFFFFFFFFF:FFFFFFFFFFFFFFFFFF
FFFFFFFFFFFFFFFFFFFFFFFFFFFFFFFFFFFFFFFFFFF:FFFFFFFFF:FFFF,FFFF:FFFF
FFFFFFFFFFFFFFF
@A00721:190:HGJW7DSXY:4:2248:23484:9627 2:N:0:TCTGCAAG+GCTGCACT
CGCATGGTATAACATCATAGATGACGTAAGCCCTTCATATCTTCAAAAGAAACATTGGAAGGAGCTTA
TTGGGGTCCAGCACAATTGGGTATCCAATGTGAAGTACGGCAAACCAATTCAAATTAAAGGTGGTATC
CCGTCAATCGTGCTT
+
FFFFFFFFFFFFFFFFFFFFFFFFFFFFFFFFFFFFFFFFFFFFFFFFFFFFFFFFFFFFFFFFFFFF
FFFFFFFFFFFFFFFFFFFFFFFFFFFFFFFFFFFFFFFFFFFFFFFFFFFFFFFFFFFFFFFFFFFF
:FFFFFFFFFFFFFF
@A00721:190:HGJW7DSXY:4:2303:7663:19554 2:N:0:TCTGCAAG+GCTGCACT
CGCATGGTATAACATCATAGATGACGTAAGCCCTTCATATCTTCAAAAGAAACATTGGAAGGAGCTTA
TTGGGGTCCAGCACAATTGGGTATCCAATGTGAAGTACGGCAAACCAATTCAAATTAAAGGTGGTATC
CCGTCAATCGTGCTT
+
FFFFFFFF:FFFFFFFFFFFFFFFFFFFFFFFFFFFFFFFFFFFFFFFFFFFFFFFFFFFFFFFFFF:
FFFFFFFFFFFFFFFFFFFFFF:FFFFFFFFFFFF:FF:FFFFFFFFFFFFFFFFFFFFF:FFFF:FF
FFFFFFFFFFFF:F:
@A00721:190:HGJW7DSXY:4:2565:13386:28995 2:N:0:TCTGCAAG+GCTGCACT
CGCATGGTATAACATCATAGATGACGTAAGCCCTTCATATCTTCAAAAGAAACATTGGAAGGAGCTTA
TTGGGGTCCAGCACAATTGGGTATCCAATGTGAAGTACGGCAAACCAATTCAAATTAAAGGTGGTATC
CCGTCAATCGTGCTT
+
FFFFFFFFFFFFFFFFFFFFFFFFFFFFFFFFFFFFFFFFFFFFFFFFFFFFFFFFFFFFFFFFFFFF
:FFFFFFFFFFFFFFFFFFFFFFFFFFFFFFFFFFFFFFFFFFFFFFFFFFFFFFFFFFFFFFFFFFF
FFFFFFFFFFFFFFF
@A00721:190:HGJW7DSXY:4:2565:25681:34413 2:N:0:TCTGCAAG+GCTGCACT
CGCATGGTATAACATCATAGATGACGTAAGCCCTTCATATCTTCAAAAGAAACATTGGAAGGAGCTTA
TTGGGGTCCAGCACAATTGGGTATCCAATGTGAAGTACGGCAAACCAATTCAAATTAAAGGTGGTATC
CCGTCAATCGTGCTT
+
F,FFF:FFFFFFFFFFFFFFFFF:FF:FFFFF::FFFFFFFFFFFFF:FFFFFFFFFFFFFFFFFF:F
F:FFFFFF:FF:FFFFFFF,FFFF,F:FFFFFFFFFF:::FFFFFFFF:FFFFFFFFFFF:FFFFFFF
F:FFFFFF,F:F:FF
@A00721:190:HGJW7DSXY:4:2573:1687:31000 2:N:0:TCTGCAAG+GCTGCACT
CGCATGGTATAACATCATAGATGACGTAAGCCCTTCATATCTTCAAAAGAAACATTGGAAGGAGCTTA
TTGGGGTCCAGCACAATTGGGTATCCAATGTGAAGTACGGCAAACCAATTCAAATTAAAGGTGGTATC
CCGTCAATCGTGCTT
+
FFFFFFFFFFFFFFFFFFFFFFFFFFFFFFFFFFFFFFFFFFFFFFFFFFFFFFFFFFFFFFFFFFFF
FFFFFFFFFFFFFFFFFFFFFFFFFFFFFFFFFFFFFFFFFFFFFFFFFFFFFFFFFFFFFFFFFFFF
FFFFFFFFFFFFFFF
```

```
@A00721:190:HGJW7DSXY:4:2621:11279:14074 2:N:0:TCTGCAAG+GCTGCACT
CGCATGGTATAACATCATAGATGACGTAAGCCCTTCATATCTTCAAAAGAAACATTGGAAGGAGCTTA
TTGGGGTCCAGCACAATTGGGTATCCAATGTGAAGTACGGCAAACCAATTCAAATTAAAGGTGGTATC
CCGTCAATCGTGCTT
+
FFF::FFFF:FFFFFFFFFFFFFFFFFFFFFFF:FFF:FFFFFF:F:FFFFFF:F:FFF:FFFFF,FF
FFFFFFFFFFFFFFF:FFFFFF,FFFFF:FFFFFFFFFFFFFFF:FFFFFFFFFFFFFFFFFFFFFFF
FFFFF:FFFFFFFFF
@A00721:190:HGJW7DSXY:4:2611:5755:11992 2:N:0:TCTGCAAG+GCTGCACT
CGCATGGTATAACATCATAGATGACGTAAGCCCTTCATATCTTCAAAAGAAACATTGGAAGGAGCTTA
TTGGGGTCCAGCACAATTGGGTATCCAATGTGAAGTACGGCAAACCAATTCAAATTAAAGGTGGTATC
CCGTCAATCGTGCT
+
FFFFFFF:F:FFFFFFFFFFF:FFFFFFFFFFFFFFFFFFFFFFFFFFFFFFFFFFFFFFFFF:F:F,
F::FFFFFFFFFFFFFFFFFFFFFFFFFF:FFFFFFFFFFFFFFFF:FFFFFFFFFFFFF,::FFFFF
,FFFFFFFFF:FFF
@A00721:190:HGJW7DSXY:4:2361:7464:19648 2:N:0:TCTGCAAG+GCTGCACT
AACGCATGGTATAACATCATAGATGACGTAAGCCCTTCATATCTTCAAAAGAAACATTGGAAGGAGCT
TATTGGGGTCCAGCACAATTGGGTATCCAATGTGAAGTACGGCAAACCAATTCAAATTAAAGGTGGTA
TCCCGTCAATCGTGC
+
F:FFFFFFFFFFFFFFFFFFFFFFFFFFFFFFFFFFFFFFFFFFFFFFFFFFFFFFFFFFFFFFFFF:
,F:FFFFFFFFFFFFFFFFFFFFFFFFFF:FFFFFF,FFF:FFF:FFFFFF,FFFF,F,:FFF,FFFF
FFFFF:FFFFFFFFF
@A00721:190:HGJW7DSXY:4:2536:17879:25473 2:N:0:TCTGCAAG+GCTGCACT
AACGCATGGTATAACATCATAGATGACGTAAGCCCTTCATATCTTCAAAAGAAACATTGGAAGGAGCT
TATTGGGGTCCAGCACAATTGGGTATCCAATGTGAAGTACGGCAAACCAATTCAAATTAAAGGTGGTA
TCCCGTCAATCGTGC
+
F:FFFF:FFFFF:FFFFFFFFFFFFFFFFFFFFFFFFFFFFFFFFFFFFFFFFFFFFFFFFFFFFFFF
FF,FFFFFFFFFFFFFFFFFFFFF:FFFFFFFFFFFFFFFFFFFFFFFFFFFFFFFFFFF:FFFFFFF
FFFFFFFFFFFFFFF
@A00721:190:HGJW7DSXY:4:2670:6063:15186 2:N:0:TCTGCAAG+GCTGCACT
AACGCATGGTATAACATCATAGATGACGTAAGCCCTTCATATCTTCAAAAGAAACATTGGAAGGAGCT
TATTGGGGTCCAGCACAATTGGGTATCCAATGTGAAGTACGGCAAACCAATTCAAATTAAAGGTGGTA
TCCCGTCAATCGTGC
+
FFFFFFFFFFFFFFFFFFFFFFFFFFFFFFFFFFFFFFFFFFFFFFFFFFFFFFFFFFFFFFFFFFFF
FFFFFFFFFFFFFFFFFFFFFFFFFFFFFF:FFFFFFFFFFFFFF:FFFF:FFFFFFFFFFFFFFFFF
FFFFFFFFFFFFFF:
@A00721:190:HGJW7DSXY:4:1264:31756:14904 2:N:0:TCTGCAAG+GCTGCACT
CGAAACTATATTCGAATAACGCATGGTATAACATCATAGATGACGTAAGCCCTTCATATCTTCAAAAG
AAACATTGGAAGGAGCTTATTGGGGTCCAGCACAATTGGGTATCCAATGTGAAGTACGGCAAACCAAT
TCAAATTAAAGGTGG
+
FFFFFFFFFFFFFFFFFFFFFFFFFFFFFFFFFFFFFFFFFFFFFFFFFFFFFFFFFFFFFFFFFFFF
FFF:FFFFFFFFFFFF:FFFFFFFFFFFFFFFFFFFFFFFFFFFFFFFFFF:FFFFFFFFFFFFFFFF
:FFFFFFFFFFFFFF
@A00721:190:HGJW7DSXY:4:1470:2645:12148 2:N:0:TCTGCAAG+GCTGCACT
CGAAACTATATTCGAATAACGCATGGTATAACATCATAGATGACGTAAGCCCTTCATATCTTCAAAAG
AAACATTGGAAGGAGCTTATTGGGGTCCAGCACAATTGGGTATCCAATGTGAAGTACGGCAAACCAAT
TCAAATTAAAGGTGG
+
FFFFFFFFFFFFFFFFFFFFFFFFFFFFFFFFFFFFFFFFFFFFFFFFFFFFFFFFFFFFFFFFFFFF
```

FFFFFFFFFFFFFFFFFFFFFFFFFFFFFFFFFFFFFFFFFFFFFFFFFFFFFFFFFFFFFFFFFFFF
FFFFFFFFFFFFFF:
@A00721:190:HGJW7DSXY:4:1643:14154:11569 2:N:0:TCTGCAAG+GCTGCACT
CGAAACTATATTCGAATAACGCATGGTATAACATCATAGATGACGTAAGCCCTTCATATCTTCAAAAG
AAACATTGGAAGGAGCTTATTGGGGTCCAGCACAATTGGGTATCCAATGTGAAGTACGGCAAACCAAT
TCAAATTAAAGGTGG
+
FFF:FFF,FFFFFFFFFFFFFFFFFFFFFFFFFFFF:FFFFF:FFFFFFFFFFFFFFFFFFFF:FFFF
:FFFFFFFFFFFFFFFFFFFFFFFFFFFFFFFFFFFFFFFFFFFFFFFFFFFFFFFFFFF:FFFFFF
FFFFFFFFF:F:FFF
@A00721:190:HGJW7DSXY:4:2369:32750:32753 2:N:0:TCTGCAAG+GCTGCACT
CGAAACTATATTCGAATAACGCATGGTATAACATCATAGATGACGTAAGCCCTTCATATCTTCAAAAG
AAACATTGGAAGGAGCTTATTGGGGTCCAGCACAATTGGGTATCCAATGTGAAGTACGGCAAACCAAT
TCAAATTAAAGGTGG
+
FFFFFFFFFFFFFFFFFFFFFFFFFFFFFFFFFFFFFFFFFFFFFFFFFFFFFFFFFFFFFFFFFFFF
FFFFFFFFFFFFFFFFFFFFFFFFFFFFFFFFFFF:FFF:FFFFFFFFFFFFFFFFF:FFFFFFFFFF
FFFFFFFF:FF:FFF
@A00721:190:HGJW7DSXY:4:1113:16866:23281 2:N:0:TCTGCAAG+GCTGCACT
CACCTTTAATTTGAATTGGTTTGCCGTACTTCACATTGGATACCCAATTGTGCTGGACCCCAATAAGC
TCCTTCCAATGTTTCTTTTGAAGATATGAAGGGCTTGCGTCATCTATGATGTTATACCATGCGTTATT
CGAATATAGTTTCGA
+
FFFF:FFFFFFFFFFF:FFFFFFFFFFFFFFFFFFFFFFFFFFFFFFFFFF:FFF::FFF:FFFFFFFF
FFFFFFFFFFFFFFFFFFFFFF,FFFFFFFFFFFFFFFFFFFF:FFFFFFF:FFFFFFFFFF:FFFFF
F,FFFFFFFFFFFFF
@A00721:190:HGJW7DSXY:4:1402:22064:33317 2:N:0:TCTGCAAG+GCTGCACT
CACCTTTAATTTGAATTGGTTTGCCGTACTTCACATTGGATACCCAATTGTGCTGGACCCCAATAAGC
TCCTTCCAATGTTTCTTTTGAAGATATGAAGGGCTTGCGTCATCTATGATGTTATACCATGCGTTATT
CGAATATAGTTTCGA
+
FFFFFFFFFFFFFFFFFFFFFFFFFFFFFFFFFFFFFFFFFFFFFFFFFFFFFFFFFFFFFFFFFFFF
FFFFFFFFFFFFF:FFFFFFFFFFFFFFFFFFFFFFFFFFFFFFFFFFFFFFFFFFFFFFFFFFFFFF
FFFFFFFFFFFFFFF
@A00721:190:HGJW7DSXY:4:1450:5746:23062 2:N:0:TCTGCAAG+GCTGCACT
CACCTTTAATTTGAATTGGTTTGCCGTACTTCACATTGGATACCCAATTGTGCTGGACCCCAATAAGC
TCCTTCCAATGTTTCTTTTGAAGATATGAAGGGCTTGCGTCATCTATGATGTTATACCATGCGTTATT
CGAATATAGTTTCGA
+
FFFFFFFFFFFFFFFFFFFFFFFFFFFFFFFFFFFFFFFFFFFFFFFFFFFFFFFFFFFFFFFFFFFF
FFFFFFFFFFFFFFFFFFFFFFFFFFFFFFFFFFFFFFFFFFFFFFFFFFFFFFFFFFFFFFFFFFFF
FFFFFFFFFFFFFFF
@A00721:190:HGJW7DSXY:4:1468:24957:24361 2:N:0:TCTGCAAG+GCTGCACT
CACCTTTAATTTGAATTGGTTTGCCGTACTTCACATTGGATACCCAATTGTGCTGGACCCCAATAAGC
TCCTTCCAATGTTTCTTTTGAAGATATGAAGGGCTTGCGTCATCTATGATGTTATACCATGCGTTATT
CGAATATAGTTTCGA
+
FFFFFFFFFFFFFFFFFFFFFFFFFFFFFFFFFFFFFFFFFFFFFFFFFFFFFFFFFFFFFFFFFFFF
FFFFFFFFFFFFFFFFFFFFFFFFFFFFFFFFFFFFFFFFFFFFFFFFFFFFFFFFFFFFFFFFFFFF
FFFFFFFFFFFFFFF
@A00721:190:HGJW7DSXY:4:1509:14217:14716 2:N:0:TCTGCAAG+GCTGCACT
CACCTTTAATTTGAATTGGTTTGCCGTACTTCACATTGGATACCCAATTGTGCTGGACCCCAATAAGC
TCCTTCCAATGTTTCTTTTGAAGATATGAAGGGCTTGCGTCATCTATGATGTTATACCATGCGTTATT
CGAATATAGTTTCGA

```
+
FFFFFFFFFFFFFFFFFFFFFFFFFFFFFFFFFFFFFFFFFFFFFFFFFFFFFFFFFFFFFFFFFFFFFF
FFFFFFF:FFFFFFFFFFFFFFFFFFFFFFFFFFFFFFFFFFFFFF:FFFFFFFFFFFFFFFFFFFFFFF
FFFFFFFFFFFFF:F
@A00721:190:HGJW7DSXY:4:2139:8386:21840 2:N:0:TCTGCAAG+GCTGCACT
CGAAACTATATTCGAATAACGCATGGTATAACATCATAGATGACGTAAGCCCTTCATATCTTCAAAAG
AAACATTGGAAGGAGCTTATTGGGGTCCAGCACAATTGGGTATCCAATGTGAAGTACGGCAAACCAAT
TCAAATTAAAGGTG
+
FFFFFFFFF:FFFFFFFFFFFFFFFFFFFFFFFFFFFFFFFFFFFFFFFFFFFFFFFFFFFFFFFFFFFF
FFFFFFFFFFFFFFFF:FFFFFFFFFFFFFFFFFFFFFFFFFFFFFFFFFFFFFFFFFFFFFFFFFFFFF
FFFFFFFFFFFFFF
@A00721:190:HGJW7DSXY:4:1171:26512:20979 2:N:0:TCTGCAAG+GCTGCACT
CTTTAATTCGAAACTATATTCGAATAACGCATGGTATAACATCATAGATGACGTAAGCCCTTCATATC
TTCAAAAGAAACATTGGAAGGAGCTTATTGGGGTCCAGCACAATTGGGTATCCAATGTGAAGTACGGC
AAACCAATTCAAATT
+
FFFFFFFFFFFFFF:FFFFFFFFFFFFFFFFFFFFFFFFFFFFFFFFFFFFFFFFFF,FFFFFFFFFFF
FFFF:FFFFFFFFFFFFFFFFFFF,FFFFFFFFFFFFFFFFFFFFFFFFFFFFFFFFFFFFFFFFFFFFF
FFFFFFFFFFFFFF:
@A00721:190:HGJW7DSXY:4:1201:6741:32299 2:N:0:TCTGCAAG+GCTGCACT
CTTTAATTCGAAACTATATTCGAATAACGCATGGTATAACATCATAGATGACGTAAGCCCTTCATATC
TTCAAAAGAAACATTGGAAGGAGCTTATTGGGGTCCAGCACAATTGGGTATCCAATGTGAAGTACGGC
AAACCAATTCAAATT
+
FFFFFFFFFFFFFFF:FFFFFFFFFFFFFFFFFFFFFFFFFFFFFFFFFFFFFFFFFFFFFFFFFFFFFF
FFFFFFFFFFFFFFFFFFFFFFFFFFFFFFFFFFFFFFFFFFFFFFFFFFFFFFFFFFFFFFFFFFFFFF
FFFFFFFFFFFFF:F
@A00721:190:HGJW7DSXY:4:1209:4065:11412 2:N:0:TCTGCAAG+GCTGCACT
CTTTAATTCGAAACTATATTCGAATAACGCATGGTATAACATCATAGATGACGTAAGCCCTTCATATC
TTCAAAAGAAACATTGGAAGGAGCTTATTGGGGTCCAGCACAATTGGGTATCCAATGTGAAGTACGGC
AAACCAATTCAAATT
+
FFFFFFFFFFFFFFF,FFFFFFFFFFFFFFFFFFFFFFFFFFFFFFFFFFF:FFFFFFFFFFFFFFFFFF
FFFFFFFFFFFFFFFFFFFFFFFFFFFFF:FFFFFFFFFFFF:FFFFFFFFFFFFFFFFFFFFFFF,FF
FFFFFFFFFFFFFFF
@A00721:190:HGJW7DSXY:4:1527:21838:23375 2:N:0:TCTGCAAG+GCTGCACT
CTTTAATTCGAAACTATATTCGAATAACGCATGGTATAACATCATAGATGACGTAAGCCCTTCATATC
TTCAAAAGAAACATTGGAAGGAGCTTATTGGGGTCCAGCACAATTGGGTATCCAATGTGAAGTACGGC
AAACCAATTCAAATT
+
FFFFFFFFFFFFFF:FFFFFFFFFFFFFFFFFFFFFFFFFFFFFFFFFFFFFFFFFFFFFFFFFFFFFFFF
FFFFFFFFFFFFFFFFFFFFFFFFFFFFFFFFFFFFFFFFFFFFFFFFFFFFFFFFFFFFFFFFFFFFFF
F:FFFFFFFFFFFFF
@A00721:190:HGJW7DSXY:4:1625:17363:14121 2:N:0:TCTGCAAG+GCTGCACT
CTTTAATTCGAAACTATATTCGAATAACGCATGGTATAACATCATAGATGACGTAAGCCCTTCATATC
TTCAAAAGAAACATTGGAAGGAGCTTATTGGGGTCCAGCACAATTGGGTATCCAATGTGAAGTACGGC
AAACCAATTCAAATT
+
FFFFFFFFFFFFFFFFFFFFFFFFFFF:FFFFFFFFFFFFFFFFFFFFF:FFFFFFFF:F:FFFFFFFFF
FFFFF:FFFFFFFFFFFFFFFFFFFFFFFFFFFFFFFFFFFF:FFFFFFFFFFFFF:FFFFFFFFF:F
FFFFFFF:FFFFFFF
@A00721:190:HGJW7DSXY:4:2242:13304:28855 2:N:0:TCTGCAAG+GCTGCACT
CTTTAATTCGAAACTATATTCGAATAACGCATGGTATAACATCATAGATGACGTAAGCCCTTCATATC
```

TTCAAAAGAAACATTGGAAGGAGCTTATTGGGGTCCAGCACAATTGGGTATCCAATGTGAAGTACGGC
AAACCAATTCAAATT
+
FFFFFFFFFFFFFFFFFFFFFFFFFFFFFFFFFFFFFFFFFFFFF:FFFFFFF:FFFFFFFFFFFFFF
FFFFF:FFFFFFFFFFFFFFFFFFFFFFFFFFFFFFFFFFFFFFFFFFFFFF:FFFFFFFFFFFFFFF
FFFFFFFFFFFFFFF
@A00721:190:HGJW7DSXY:4:2353:10773:27508 2:N:0:TCTGCAAG+GCTGCACT
CTTTAATTCGAAACTATATTCGAATAACGCATGGTATAACATCATAGATGACGTAAGCCCTTCATATC
TTCAAAAGAAACATTGGAAGGAGCTTATTGGGGTCCAGCACAATTGGGTATCCAATGTGAAGTACGGC
AAACCAATTCAAATT
+
FFFFFFFFFFFFFFFFFFFFFFFFFFFFFFFFFFFFFFFFFFFFFFFFFFFFFFFFFFFFFFFFFFFF
FFFFFFFFFFFFFFFFFFFFFFFFFFFFFFFFFFFFFFFFFFFFFFFFFFFFFFFFFFFFFFFFFFFF
FFFFFFFFFFFFFFF
@A00721:190:HGJW7DSXY:4:2557:17616:18818 2:N:0:TCTGCAAG+GCTGCACT
CTTTAATTCGAAACTATATTCGAATAACGCATGGTATAACATCATAGATGACGTAAGCCCTTCATATC
TTCAAAAGAAACATTGGAAGGAGCTTATTGGGGTCCAGCACAATTGGGTATCCAATGTGAAGTACGGC
AAACCAATTCAAATT
+
FFFFFFFFFFFFFFF:F:FFFFFFFFFFFFFFFFFFFFFFFFFFFFFFFF:FFFFFFFFFFFFFFFFF
FFFFFFFFFFFFF:FFFFFFFFFFFFFFFFFFFFFFFFFFFFFFFFFFFFFFFFF:FFFFFFFFFFFF
FFFFFFFFFFFFFFF
@A00721:190:HGJW7DSXY:4:2668:30382:25238 2:N:0:TCTGCAAG+GCTGCACT
CTTTAATTCGAAACTATATTCGAATAACGCATGGTATAACATCATAGATGACGTAAGCCCTTCATATC
TTCAAATGAAACATTGGAAGGAGCTTATTGGGGTCCAGCACAATTGGGTATCCAATGTGAAGTACGGC
AAACCAATTCAAATT
+
FFFFFFFFFFFFFFFFFFFFFFFFFFFFFFFFFFFFFFFFFFFFFFFFFFFFFFFFFFFFFFFFFFFF
FFFFFF,FFFFFFFFFFFFFFFFFFFFFFFFFFFFFFFFFFFFFFFFFFFFFFFFFFFFFFFFFFFFF
FFFFFFF:FFFF,FF
@A00721:190:HGJW7DSXY:4:1207:32669:25066 2:N:0:TCTGCAAG+GCTGCACT
CTAGACTTTAATTCGAAACTATATTCGAATAACGCATGGTATAACATCATAGATGACGTAAGCCCTTC
ATATCTTCAAAAGAAACATTGGAAGGAGCTTATTGGGGTCCAGCACAATTGGGTATCCAATGTGAAGT
ACGGCAAACCAATTC
+
FFFF:FFFFFFFFFFFF:FFFF:FFFFFFFFFFFFFFFF:FFFFFFFFFFFFFFFFFFFFFFFFFFFF
FFFFFFFF:FFFFFFFFFFFF:FFF::FFFFFFFFFFFFFFFFFFFFFFFFFFFFFFFFFFFF:FFFF
FFFFFFFFFFF,FFF
@A00721:190:HGJW7DSXY:4:1227:8395:2159 2:N:0:TCTGCAAG+GCTGCACT
CTAGACTTTAATTCGAAACTATATTCGAATAACGCATGGTATAACATCATAGATGACGTAAGCCCTTC
ATATCTTCAAAAGAAACATTGGAAGGAGCTTATTGGGGTCCAGCACAATTGGGTATCCAATGTGAAGT
ACGGCAAACCAATTC
+
FFFFFFFFFFFFFFFFFFFFFFFFFFFF:FFFFFFFFFFFFFFFFFFFFFFFFFFFFFFFFFFF,FFF
FFFFFFFFFF,FFFFFFF:FFFFFFFFFFFFFFFFFFFFFFFFFFFFFFFFFFF:FFFFFFFFFFFFF
FFFFFFFFFFFFFFF
@A00721:190:HGJW7DSXY:4:1311:14434:31250 2:N:0:TCTGCAAG+GCTGCACT
CTAGACTTTAATTCGAAACTATATTCGAATAACGCATGGTATAACATCATAGATGACGTAAGCCCTTC
ATATCTTCAAAAGAAACATTGGAAGGAGCTTATTGGGGTCCAGCACAATTGGGTATCCAATGTGAAGT
ACGGCAAACCAATTC
+
FFFFFFFFFFFFFFFFFFFFFF:FFFFFFFFFFFFFFFFFFFFFFFFFFFFFFFFFFFFFFFFFFFFF
FFFFFFFFFFFFFFFFFFFFFFFFFFFFFFFFFF:FFFFFFFFFFFFFFFFFFFFFFFFFFFFFFFFF
FFFFFFFFF:FFF::

@A00721:190:HGJW7DSXY:4:1326:12599:2174 2:N:0:TCTGCAAG+GCTGCACT
CTAGACTTTAATTCGAAACTATATTCGAATAACGCATGGTATAACATCATAGATGACGTAAGCCCTTC
ATATCTTCAAAAGAAACATTGGAAGGAGCTTATTGGGGTCCAGCACAATTGGGTATCCAATGTGAAGT
ACGGCAAACCAATTC
+
FFFFFFFFFFFFFFFFFFFFFFFFFFFFFFFFFFFFFFFFFFFFFFFFFFFFFFFFFFFFFFFFFFFFFF
FFFFFFFFFFFFFFFFFFFFFFFFFFFFFFFFFFFFFFFFFFFFFFFFFFFFFFFFFFFFFFFFFFFF:F
FFFFFFFFFFFFFFF
@A00721:190:HGJW7DSXY:4:1343:6659:24205 2:N:0:TCTGCAAG+GCTGCACT
CTAGACTTTAATTCGAAACTATATTCGAATAACGCATGGTATAACATCATAGATGACGTAAGCCCTTC
ATATCTTCAAAAGAAACATTGGAAGGAGCTTATTGGGGTCCAGCACAATTGGGTATCCAATGTGAAGT
ACGGCAAACCAATTC
+
FFFFFFFF:,FFFFF:F:FF,FFFFFFFFFFFFFFFFFFFFFFFFFFF:FFFFFF:FFFFFFFFFFFF:F
FF,FFFFFF:FFFFFFFFFFFFFFFFFF:FFF:FFFFF:FFFFFFFFFF,FFFFFFFFFF:FFFFFFF:F
FFF:FFFFFFFFFFF
@A00721:190:HGJW7DSXY:4:1559:17969:26256 2:N:0:TCTGCAAG+GCTGCACT
CTAGACTTTAATTCGAAACTATATTCGAATAACGCATGGTATAACATCATAGATGACGTAAGCCCTTC
ATATCTTCAAAAGAAACATTGGAAGGAGCTTATTGGGGTCCAGCACAATTGGGTATCCAATGTGAAGT
ACGGCAAACCAATTC
+
FFFFFFFFFFFFFFFFFFFF,FF,FFFFFFFFFFFFF:FFFFFFFFFFFFFFFFFFFFFFFFFFFFFFFF
FFFFFFFFFFFFFFFFFFFFFFFFFFFFFFFFFFFFFFF:FFFFFFFFFFFFFFFFFFFFFFFFFFFFFF
:FFFFFFFFFFFFFF
@A00721:190:HGJW7DSXY:4:2273:3757:2174 2:N:0:TCTGCAAG+GCTGCACT
CTAGACTTTAATTCGAAACTATATTCGAATAACGCATGGTATAACATCATAGATGACGTAAGCCCTTC
ATATCTTCAAAAGAAACATTGGAAGGAGCTTATTGGGGTCCAGCACAATTGGGTATCCAATGTGAAGT
ACGGCAAACCAATTC
+
FFFFFFFFFFFFFFFFFFFFFFFFFFFFFFFFFFFFFFFFFFFFFFFFFFFFFFFFFFFFFFFFFFFFFF
FFFFFFFFFFFFFFFFFFFFFFFFFFFFFFFFFFFFFFFFFFFFFFFFFFFFFFFFFFFFFFFFFFFFFF
FFFFFFFFFFFFFFF
@A00721:190:HGJW7DSXY:4:2345:11912:36245 2:N:0:TCTGCAAG+GCTGCACT
CTAGACTTTAATTCGAAACTATATTCGAATAACGCATGGTATAACATCATAGATGACGTAAGCCCTTC
ATATCTTCAAAAGAAACATTGGAAGGAGCTTATTGGGGTCCAGCACAATTGGGTATCCAATGTGAAGT
ACGGCAAACCAATTC
+
FFFFFFFFFFFFFFFFFFF::FFFFFFFFFFFFFFFFFFFFFFFFFFFFFFFFFFFFFFFFFFFFFFFFF
FFFFFFFF:FFFFFFFFF,FFFFFFFFFFFF,FFFFFFFFFFFFFFFFFF:FFFFFFFFFFFFFFFFF:F
FFFFFFFFFF,FFFF
@A00721:190:HGJW7DSXY:4:2431:3224:2879 2:N:0:TCTGCAAG+GCTGCACT
CTAGACTTTAATTCGAAACTATATTCGAATAACGCATGGTATAACATCATAGATGACGTAAGCCCTTC
ATATCTTCAAAAGAAACATTGGAAGGAGCTTATTGGGGTCCAGCACAATTGGGTATCCAATGTGAAGT
ACGGCAAACCAATTC
+
FFFFFFFFFFFFFFFF:FFF:FFFFFFFFFFFFFFFFFFFF:FFFFFFFFFFFFFFFFFFFFFFFFFFFF
FFFFFFFFFFFFFFFFFFFFFFFFFFFFFFFFF:FFFFFFFFFFFFFFFFFFFFFFFFFFFFFFFFFFFF
FFFFFFFFFF:FFFF
@A00721:190:HGJW7DSXY:4:2577:13738:25128 2:N:0:TCTGCAAG+GCTGCACT
CTAGACTTTAATTCGAAACTATATTCGAATAACGCATGGTATAACATCATAGATGACGTAAGCCCTTC
ATATCTTCAAAAGAAACATTGGAAGGAGCTTATTGGGGTCCAGCACAATTGGGTATCCAATGTGAAGT
ACGGCAAACCAATTC
+
FFFFFFFFFFFFFFFFFFFFFFFFFFFFFFFFFFFFFFFF,FFFFFFFFFFFFFFFFFFFFFFFFFFFFF

FFFFFFFF:FFFFFFFFF:FFFFFFFFFFFFFFFFFFFFFFFFFFFFFFFFFFFF:FFFF:FFFFFFF
FFFFFFFFFFFFFFF
@A00721:190:HGJW7DSXY:4:2675:15510:15890 2:N:0:TCTGCAAG+GCTGCACT
CTAGACTTTAATTCGAAACTATATTCGAATAACGCATGGTATAACATCATAGATGACGTAAGCCCTTC
ATATCTTCAAAAGAAACATTGGAAGGAGCTTATTGGGGTCCAGCACAATTGGGTATCCAATGTGAAGT
ACGGCAAACCAATTC
+
FFFFFFFFFFFFFFFFFFFFFFFFFFFFFFFFFFFFFFFFFFFFFFFFFFFFFFFFFFFFFFFFFFFF
FFFFFFFFFFFFFFFFFFFFFFFFFFFFFFFFFFFFFFFFFFFFFFFF:FFFFFFFFFFFFFFFFFFF
FFFFF,FFFFFFFFF
@A00721:190:HGJW7DSXY:4:2263:12274:1986 2:N:0:TCTGCAAG+GCTGCACT
CTAGACTTTAATTCGAAACTATATTCGAATAACGCATGGTATAACATCATAGATGACGTAAGCCCTTC
ATATCTTCAAAAGAAACATTGGAAGGAGCTTATTGGGGTCCAGCACAATTGGGTATCCAATGTGAAGT
ACGGCAAACCAATT
+
:FFFFFFFFFFFFFFFFFFFFFF:FFFFFFFFFF:FFFFFF:FFFFFFFFF::F:FFFFFF:FFFFFF
FFFF:FFFFFFFFFFFFFFFF,FFFFFFFFFFFFFFFFFFFFFFFFFFFFFF:F:FFFFF:FFFFFFF
FFFFFFFFFFF,FF
@A00721:190:HGJW7DSXY:4:1326:3125:21527 2:N:0:TCTGCAAG+GCTGCACT
AGACTTTAATTCGAAACTATATTCGAATAACGCATGGTATAACATCATAGATGACGTAAGCCCTTCAT
ATCTTCAAAAGAAACATTGGAAGGAGCTTATTGGGGTCCAGCACAATTGGGTATCCAATGTGAAGTAC
GGCAAACCAAT
+
FFFFFFFFFFFFFFFFFFFF:FFFFFFFFFFFFFFFF:FFFFFFFFFFFFFFFFFFFFFFFFFFFFFF
FFFFFFFFFFFFFFFFFFFFFFFFFFFFFFFFFFFFFFFFFFFFFFFFFFFFFFFFFFFFFFFFFFFF
FFFFFFFFFFF
@A00721:190:HGJW7DSXY:4:1336:16324:17487 2:N:0:TCTGCAAG+GCTGCACT
AGACTTTAATTCGAAACTATATTCGAATAACGCATGGTATAACATCATAGATGACGTAAGCCCTTCAT
ATCTTCAAAAGAAACATTGGAAGGAGCTTATTGGGGTCCAGCACAATTGGGTATCCAATGTGAAGTAC
GGCAAACCAAT
+
FFFFFFFFFFFFFFFFFFFFFFFFFFFFFFFFFFFFFF:FFFFFFFFFFFFFFFFFFFFFFFFFFFFF
FFFFFFFFFFFFFFFFFFFFFFFFFFFFFFFFFFFFFFFFFFFFFFFFFFFF:FFFFFFFFFFFFFFF
FFFFFFFFF:F
@A00721:190:HGJW7DSXY:4:2124:16080:8829 2:N:0:TCTGCAAG+GCTGCACT
AGACTTTAATTCGAAACTATATTCGAATAACGCATGGTATAACATCATAGATGACGTAAGCCCTTCAT
ATCTTCAAAAGAAACATTGGAAGGAGCTTATTGGGGTCCAGCACAATTGGGTATCCAATGTGAAGTAC
GGCAAACCAAT
+
FFFFFFFFFFFFFFFFF,:FFFFFFFFFFFFFFFFFFFFFFFFFFFFFFFFFFFFFFFFFFFFFFFFF
FFFFFFFF:,F,FFFFFFFF:FFFFFFFFF:FFFFFFFFFFFFFFFFFFFF:FFFFFFFFFFFFFFFF
FFFFFFFFFFF
@A00721:190:HGJW7DSXY:4:2247:2573:29215 2:N:0:TCTGCAAG+GCTGCACT
AGACTTTAATTCGAAACTATATTCGAATAACGCATGGTATAACATCATAGATGACGTAAGCCCTTCAT
ATCTTCAAAAGAAACATTGGAAGGAGCTTATTGGGGTCCAGCACAATTGGGTATCCAATGTGAAGTAC
GGCAAACCAAT
+
FFFFFFFFFFFFFFFFF:FFFFFFFFFFFFFFFFFFFFFFFFFFFFFFFFFFFFFFFFFFFFFFFFFF
FFF:FFFFFFFFFFFFFFFFFFFFFFFFFFFFFFFFFFFFFFFFFFFFFFFFFFFF,FFFFFFFFF
FFFFFFFFF:F
@A00721:190:HGJW7DSXY:4:2264:19732:7138 2:N:0:TCTGCAAG+GCTGCACT
AGACTTTAATTCGAAACTATATTCGAATAACGCATGGTATAACATCATAGATGACGTAAGCCCTTCAT
ATCTTCAAAAGAAACATTGGAAGGAGCTTATTGGGGTCCAGCACAATTGGGTATCCAATGTGAAGTAC
GGCAAACCAAT

```
+
FFFFFFFFFFFFFFFFFF:FFFFFFFFFFFFFFFFFFFFFFFFFFFFFFFFFFFFFFFFFFFFFFFFFF
FFFFFFFFFFFFFFFFFFFFFFFFFFFFFFFFFFFFFFFFFFFFFFFFFFFFFFFFFFFFFFFFFFFF
FFFF::FFFFF
@A00721:190:HGJW7DSXY:4:2314:28999:5713 2:N:0:TCTGCAAG+GCTGCACT
AGACTTTAATTCGAAACTATATTCGAATAACGCATGGTATAACATCATAGATGACGTAAGCCCTTCAT
ATCTTCAAAAGAAACATTGGAAGGAGCTTATTGGGGTCCAGCACAATTGGGTATCCAATGTGAAGTAC
GGCAAACCAAT
+
FFFFFFFFFFFFFFF,FF:FFFFFFFFFFFFFFFFFFF:FFFFFFFFFFFFFFFFFFFFFFFFFFFFF
FFFFFFFFFFFFFFFF,FFFFFFFFFF:FF:FFFFFFFFFFFFFFFFFFFFFFFFFFFFFFFFFF:FFF
FFFF:FFFFFF
@A00721:190:HGJW7DSXY:4:2350:25174:34914 2:N:0:TCTGCAAG+GCTGCACT
AGACTTTAATTCGAAACTATATTCGAATAACGCATGGTATAACATCATAGATGACGTAAGCCCTTCAT
ATCTTCAAAAGAAACATTGGAAGGAGCTTATTGGGGTCCAGCACAATTGGGTATCCAATGTGAAGTAC
GGCAAACCAAT
+
FFFFFFFFFFFFF:FFFF:FFFFFFFFFFFFFF,FFF:FFFFFFFFFFFFFFFFFFFFFF,:,FFFFFF
FFFFFFFFFFF:FFFFFF:FFFF:FF,FFFFFFFFFFFFFFFFFFFFFF:FFFF::FFFFFFFFFFFF
FFFFF:FFFFF
@A00721:190:HGJW7DSXY:4:2457:16514:9424 2:N:0:TCTGCAAG+GCTGCACT
AGACTTTAATTCGAAACTATATTCGAATAACGCATGGTATAACATCATAGATGTCGTAAGCCCTTCAT
ATCTTCAAAAGAAACATTGGAAGGAGCTTATTGGGGTCCAGCACAATTGGGTATCCAATGTGAAGTAC
GGCAAACCACT
+
FFFFF:FFFFFFFFFFFF:FF:FFF:FFFFFFF,,FF,:FFFFF,FFFFFF:,,FF,FFFFFFFFFFF
FF:FF:FFFF,FFF:FF,F:FFFFFF,,,FFFFFFF:FFFFFFFFFFF:FFFFFFF,FFFFFFFFFFF
FFFF:F,FF,F
@A00721:190:HGJW7DSXY:4:1132:11650:32597 2:N:0:TCTGCAAG+GCTGCACT
ATATATAGACTTTAATTCGAAACTATATTCGAATAACGCATGGTATAACATCATAGATGACGTAAGCC
AATCATATCTACAAAAGAAACATTTGAAGGAGCTTATTGGTTTCCAGCACAATTGGGTATCCAATGTG
AAGTACGGCAAACCA
+
FFF,,F:FFF,:FF:FF,FFF,F:FF,F,,F,,F:,,FF:F:FF:F:FFFFFF,FFFFFF:FFF:FFF
,,FFFFF:FF,FF:,::FFFFF:F,FFF,:F,F:FFF,FF,F,:,FF,FFF,FFFF,F,FFFFFFF,F
:FFF,:,FFF:FFFF
@A00721:190:HGJW7DSXY:4:1434:31078:8782 2:N:0:TCTGCAAG+GCTGCACT
ATATCTAGACTTTAATTCGAAACTATATTCGAATAACGCATGGTATAACATCATAGATGACGTAAGCC
CTTCATATCTTCAAAAGAAACATTGGAAGGAGCTTATTGGGGTCCAGCACAATTGGGTATCCAATGTG
AAGTACGGCAAACCA
+
FFFFFF:FFFFFFFFFFFFFFFFFFF:FFFFFFFFFFFFFFFFFFFFFFFFFFFFFFFFFFFFFFFFF
FFFFFF:FFFFFFFF,FFFFFFFFFF:FFFFFFFFFFFFF,FFFFFFFFFFFFFFFFFFFFFFFFFFF
FFFFFFFFFF:,FFF
@A00721:190:HGJW7DSXY:4:1519:21911:25191 2:N:0:TCTGCAAG+GCTGCACT
ATATCTAGACTTTAATTCGAAACTATATTCGAATAACGCATGGTATAACATCATAGATGACGTAAGCC
CTTCATATCTTCAAAAGAAACATTGGAAGGAGCTTATTGGGGTCCAGCACAATTGGGTATCCAATGTG
AAGTACGGCAAACCA
+
FFFFFF:FFFFFFFFFFFFFFFFFFFFFFFFFFFFFFFFFFFFFFFFFFFFFFFFFFFFFFFFFFFFF
FFFFFFFFFFFFFFFF:FFFFFFFFFFFFFFFFFFFFFFFFFFFFFFFFFFF:FFFFFFFFFFFFFFF
FFFFFFFFFFFFFFF
@A00721:190:HGJW7DSXY:4:1545:7437:18912 2:N:0:TCTGCAAG+GCTGCACT
ATATCTAGACTTTAATTCGAAACTATATTCGAATAACGCATGGTATAACATCATAGATGACGTAAGCC
```

CTTCATATCTTCAAAAGAAACATTGGAAGGAGCTTATTGGGGTCCAGCACAATTGGGTATCCAATGTG
AAGTACGGCAAACCA
+
FFFFFFFFFFFFFFFFFFFFFFFFFFFFFFFFFFFFFFFFFFFFFFFFFFFFFFFFFFFFFFFFFFFFFF
FFFFFFFFFFFFFFFFFFFFFFF:FFFFFFFFFFFFFFF,FFFFFFFFFFFFFFFFFFFF,FFFFFFFFF
FFFFFFFFFFFFFFF
@A00721:190:HGJW7DSXY:4:1545:7491:19257 2:N:0:TCTGCAAG+GCTGCACT
ATATCTAGACTTTAATTCGAAACTATATTCGAATAACGCATGGTATAACATCATAGATGACGTAAGCC
CTTCATATCTTCAAAAGAAACATTGGAAGGAGCTTATTGGGGTCCAGCACAATTGGGTATCCAATGTG
AAGTACGGCAAACCA
+
FFFFFFFFFFFFFFFFFFFFFFFF:FFFFFFFFFFFFFFFFFFFFFFFFFFFFFFFFFFFFFFFFFFFFF
FFFFFFFFFFFFFFFFFFFFFFFFFFFFFFFFFFFFFFFFFFFFFFFFFFFFFFFFFFFFFFFFFFFFFF
FFFFFFFFFFFFFFF
@A00721:190:HGJW7DSXY:4:1545:7934:19398 2:N:0:TCTGCAAG+GCTGCACT
ATATCTAGACTTTAATTCGAAACTATATTCGAATAACGCATGGTATAACATCATAGATGACGTAAGCC
CTTCATATCTTCAAAAGAAACATTGGAAGGAGCTTATTGGGGTCCAGCACAATTGGGTATCCAATGTG
AAGTACGGCAAACCA
+
FFFFFFFFFFFFFFFFFFFFFFF:FFFFFFFFFFFFFFFFFFFFF:FFFFFFFFFFFFFFFFFF:FFFFF
FFFFFFFFFFFFFFFF:FFFFFFFFFFFFFFFFFFFFFFFFFFFFFFFFFFFFFFFFFFFFFFFFFFFFF
FFFFFFFFFFF:FFF
@A00721:190:HGJW7DSXY:4:2435:12545:12477 2:N:0:TCTGCAAG+GCTGCACT
ATATCTAGACTTTAATTCGAAACTATATTCGAATAACGCATGGTATAACATCATAGATGACGTAAGCC
CTTCATATCTTCAAAAGAAACATTGGAAGGAGCTTATTGGGGTCCAGCACAATTGGGTATCCAATGTG
AAGTACGGCAAACCA
+
FFFFFFFFFFFFFFFFFFFFFFFFFFFFFFFFFFFFFFFFFFFFFFFFFFFFFFFFFFFFFFFFFFFFFF
FFFFFFFFFFFFFFFFFFFFFF,FFFFFFFFFFFFFFFFFFFFFFFFFFFFFFFFFFFFFFFFFFFFFFF
FFFFFFFFFFFFFFF
@A00721:190:HGJW7DSXY:4:2470:3251:22780 2:N:0:TCTGCAAG+GCTGCACT
ATATCTAGACTTTAATTCGAAACTATATTCGAATAACGCATGGTATAACATCATAGATGACGTAAGCC
CTTCATATCTTCAAAAGAAACATTGGAAGGAGCTTATTGGGGTCCAGCACAATTGGGTATCCAATGTG
AAGTACGGCAAACCA
+
FFFFFFFFFFFFFFFFFFFFFFFFFFFFFFFFFFFFFFFFFFFFFFFFFFFFFFFFFFFFFFFFFFFFFF
FFFFFFFFFFFFFFFFFFFFFFFFFFFFFFFFFFFFFFFFFFFFFFFFFFFFFFFFFFFF:FFFFF:FFF
FFFFFFFFFFFFFFF
@A00721:190:HGJW7DSXY:4:2669:5339:7075 2:N:0:TCTGCAAG+GCTGCACT
ATATCTAGACTTTAATTCGAAACTATATTCGAATAACGCATGGTATAACATCATAGATGACGTAAGCC
CTTCATATCTTCAAAAGAAACATTGGAAGGAGCTTATTGGGGTCCAGCACAATTGGGTATCCAATGTG
AAGTACGGCAAACCA
+
FFFFFFFFFFFFFFFFFFFFFFFFFFFFFFFFFFFFFFFFFFFFFFFFFFFFFFFFFFFFFFFFFFFFFF
FFFFFFFFFFFFFFFFFFFFFFFFFFFFFFFFFFFFFFFFFFFFFFFFFFFFFFFFFFFFFFFFFFFFFF
FFFFFFFFFFFFFFF
@A00721:190:HGJW7DSXY:4:1219:17553:33364 2:N:0:TCTGCAAG+GCTGCACT
GTTCGGATATCTAGACTTTAATTCGAAACTCTATTCGAATAACGCATGGTATAACATCATAGATGACG
TAAGCCCTTCATATCTTCAAAAGAAACATTGGAAGGAGCTTATTGGGGTCCAGCACAATTGGGTATCC
AATGTGAAGTACGGC
+
FFFFFFFFFFFFFFFFFFFFFFFFFFFFFFF,F:FFFFFFFFFFFFFFFFFFFFFFFFFFFFFFFFFFFF
FFFFFFFFFFFFFFFFFFFFFFFFFFFFFFFFFFFFFFFFFFFFFFFFFFFFFFFFFFF:FFFFFFFFFF
FFFFFFFFFFFFFFF

@A00721:190:HGJW7DSXY:4:1247:10601:9236 2:N:0:TCTGCAAG+GCTGCACT
GTTCGGATATCTAGACTTTAATTCGAAACTATATTCGAATAACGCATGGTATAACATCATAGATGACG
TAAGCCCTTCATATCTTCAAAAGAAACATTGGAAGGAGCTTATTGGGGTCCAGCACAATTGGGTATCC
AATGTGAAGTACGGC
+
FFFFFFFFFFFFFFFFFFFFFFFFFFFFFFFFFFFFFFFFFFFFFFFFFFFFFFFFFFFFFFFFFFFF
FFFFFFFFFFFFFFFFFFFFFFFFFFFFFFFFFFFFFFFFFFFFFFFFFFFFFFFFFFFFFFFFFFFF
FFFFFFFFFFFFFFF
@A00721:190:HGJW7DSXY:4:1412:7988:18427 2:N:0:TCTGCAAG+GCTGCACT
GTTCGGATATCTAGACTTTAATTCGAAACTATATTCGAATAACGCATGGTATAACATCATAGATGACG
TAAGCCCTTCATATCTTCAAAAGAAACATTGGAAGGAGCTTATTGGGGTCCAGCACAATTGGGTATCC
AATGTGAAGTACGGC
+
FFFFFFFFFFFFFFFFFFFFFFFFFFFFFFFFFFFFFFFFFFFFFFFFFFFFFFFFFFFFFFFFF:FF
FFFFFFF:FFFFFFFFFFFFFFFFFFFFFFFFFFFFFFFFFFFFFFFFFFFFFFFFFF:FFFFFFFFF
FFFFFFFFFFFFFFF
@A00721:190:HGJW7DSXY:4:1509:19379:14105 2:N:0:TCTGCAAG+GCTGCACT
GTTCGGATATCTAGACTTTAATTCGAAACTATATTCGAATAACGCATGGTATAACATCATAGATGACG
TAAGCCCTTCATATCTTCAAAAGAAACATTGGAAGGAGCTTATTGGGGTCCAGCACAATTGGGTATCC
AATGTGAAGTACGGC
+
FFFFFFFFFFFFFFFFFFFFFFFFFFFFFFFFFFFFFFFFFFFFFFFFFFFFFFFFFFFFFFFFFFFF
FFFFFFFFFFFFFFFFFFFFFFFFFFFFFFFFFFFFFFFFFFFFFFFFFFFFFFFFFFFFFFFFFFFF
FFFFFFFFFFFF:FF
@A00721:190:HGJW7DSXY:4:2346:10710:5102 2:N:0:TCTGCAAG+GCTGCACT
GTTCGGATATCTAGACTTTAATTCGAAACTATATTCGAATAACGCATGGTATAACATCATAGATGACG
TAAGCCCTTCATATCTTCAAAAGAAACATTGGAAGGAGCTTATTGGGGTCCAGCACAATTGGGTATCC
AATGTGAAGTACGGC
+
FFFFFFFFFFFFFFFFFFFFFFFFFF:FFFFF:,FFFFFFFFFFFFFFF:FFFFF:FFF:FFFFFFFF
FF:FFFFFFFFFFFFFFFFFFFFFFFFFFFFFF,FFFFFFFFFFFFFFFFFFFFFFFFFFFFFFFFFF
FFFFFFFFFFFFFFF
@A00721:190:HGJW7DSXY:4:2659:4589:15327 2:N:0:TCTGCAAG+GCTGCACT
GTTCGGATATCTAGACTTTAATTCGAAACTATATTCGAATAACGCATGGTATAACATCATAGATGACG
TAAGCCCTTCATATCTTCAAAAGAAACATTGGAAGGAGCTTATTGGGGTCCAGCACAATTGGGTATCC
AATGTGAAGTACGGC
+
F:FF:FFFFFFFFFFFFFFFFFF:FFFFFFFFFFFFFFFFFFFFFFFFFF:FFFFFFFFFFFFFFFFF
FFFFFFFFFFFFFFFFFFFFFFFFFFFFFFFFFFFFFFF:FF:FFFFFFFFFFFFFFF:FFFFFFFFF
FFFFFFF,FFFFFFF
@A00721:190:HGJW7DSXY:4:1158:6696:3662 2:N:0:TCTGCAAG+GCTGCACT
ATTACATGTTCGGATATCTAGACTTTAATTCGAAACTATATTCGAATAACGCATGGTATAACATCATA
GATGACGTAAGCCCTTCATATCTTCAAAAGAAACATTGGAAGGAGCTTATTGGGGTCCAGCACAATTG
GGTATCCAATGTGAA
+
FF,FFFF:F,FF:FFFF::F,FFFFFFFFFFFFFFF:F:FFFFFFFFFF,::FFFFFF:FF:F:FFFF
FFFFFF:FFFFF,FF:FF:F:FF:FFFFFFFFFFFFFFFFFFFFFFFFFFF,FFFFFFFFFFFFF,FF
FFFFFFFFFFFFFFF
@A00721:190:HGJW7DSXY:4:1170:14841:30577 2:N:0:TCTGCAAG+GCTGCACT
ATTACATGTTCGGATATCTAGACTTTAATTCGAAACTATATTCGAATAACGCATGGTATAACATCATA
GATGACGTAAGCCCTTCATATCTTCAAAAGAAACATTGGAAGGAGCTTATTGGGGTCCAGCACAATTG
GGTATCCAATGTGAA
+
FFFFFFFFFFFFFFFFFFFFFFFFFFFFFFFFFFFFFFFFFFFFFFFFFFFFF:FFFFFFFFFFFFFF

```
FFFFFFFFFFFFFFFFFFFFFFFFFFFF:FFFFFFFFFFFFFFFF:FFFFFFF:FFFF:FFFFFFFFF
FFFFFFF:FFFFFFF
@A00721:190:HGJW7DSXY:4:1213:3405:31876 2:N:0:TCTGCAAG+GCTGCACT
ATTACATGTTCGGATATCTAGACTTTAATTCGAAACTATATTCGAATAACGCATGGTATAACATCATA
GATGACGTAAGCCCTTCATATCTTCAAAAGAAACATTGGAAGGAGCTTATTGGGGTCCAGCACAATTG
GGTATCCAATGTGAA
+
FFFFFFFFFFFFFFFFFFFFFFFFFFFFFFFFFFFFF,FFFFFFFFFFFFFFFFFFFFFFFFFFFF:F
FFFFFFFFFFFFFFFFFFFFFFFFFFFFFFFFFFFFFFFF:FFFFFF:FFFFFFFFFFFFFFFF,FFF
FFF:FFFFFFFFFFF
@A00721:190:HGJW7DSXY:4:1232:1506:28808 2:N:0:TCTGCAAG+GCTGCACT
ATTACATGTTCGGATATCTAGACTTTAATTCGAAACTATATTCGAATAACGCATGGTATAACATCATA
GATGACGTAAGCCCTTCATATCTTCAAAAGAAACATTGGAAGGAGCTTATTGGGGTCCAGCACAATTG
GGTATCCAATGTGAA
+
FFFFFFFFFFFFFFFFFFFFFFFFFFFFFFFFFFFF:FFFFFFFF:FFFFFFF:FFFFFFFFFFFFFF
FFFFFFFFFFFFFFFFFFFFFFFFFFFFFFFFFFFFFFFFFFFFFFFF,FFFFFFFFFFFFFFF:FFF
FFFFFFFFFFFFFF:
@A00721:190:HGJW7DSXY:4:1340:5330:19805 2:N:0:TCTGCAAG+GCTGCACT
ATTACATGTTCGGATATCTAGACTTTAATTCGAAACTATATTCGAATAACGCATGGTATAACATCATA
GATGACGTAAGCCCTTCATATCTTCAAAAGAAACATTGGAAGGAGCTTATTGGGGTCCAGCACAATTG
GGTATCCAATGTGAA
+
FFFFFFFFFFFFFFFFFFFFFFFFFFFFFFFFFFFFFFFFFFFFFFFFFFFFFFFFFFFFFFFFFFFF
FFFFFFFFFFFFFFFFFFFFFFFFFFFFFFFFFFFFFFFFFFFFFFFFFFFF:FFFFFFFFFFFFFFF
FFFFFFFF::FFFFF
@A00721:190:HGJW7DSXY:4:1562:14498:10848 2:N:0:TCTGCAAG+GCTGCACT
ATTACATGTTCGGATATCTAGACTTTAATTCGAAACTATATTCGAATAACGCATGGTATAACATCATA
GATGACGTAAGCCCTTCATATCTTCAAAAGAAACATTGGAAGGAGCTTATTGGGGTCCAGCACAATTG
GGTATCCAATGTGAA
+
FFFFFFFFFFFFFFFFFFFFFFFFFFFFFFFFFFFFFFFFFFFFFFFFFFFF:FFFFFFFFFFFFFFF
FFFFFFFFFFFFFFFFFFFFFFFFFFFFFFFFFFFFFFFFF:FF:FFFFFFFFFFFFFFF:FFFFFFF
FFFFFFFFFFFFFFF
@A00721:190:HGJW7DSXY:4:1562:14434:11021 2:N:0:TCTGCAAG+GCTGCACT
ATTACATGTTCGGATATCTAGACTTTAATTCGAAACTATATTCGAATAACGCATGGTATAACATCATA
GATGACGTAAGCCCTTCATATCTTCAAAAGAAACATTGGAAGGAGCTTATTGGGGTCCAGCACAATTG
GGTATCCAATGTGAA
+
FFFFFFFFFFFFFFFFFFFFFFFFFFFFFFFFFFFFF:FFFFFFFFFFFFFFFFFFFFFFFFFFFFFF
FFFFFFFFFFFFFFFFFFFFFFFFFFFFF:FFFFFFFFFFFFFFF:FFFFFFFFFFFFFFFFFFFFFF
,FFFFFFFFFFFFFF
@A00721:190:HGJW7DSXY:4:2111:18891:20243 2:N:0:TCTGCAAG+GCTGCACT
ATTACATGTTCGGATATCTAGACTTTAATTCGAAACTATATTCGAATAACGCATGGTATAACATCATA
GATGACGTAAGCCCTTCATATCTTCAAAAGAAACATTGGAAGGAGCTTATTGGGGTCCAGCACAATTG
GGTATCCAATGTGAA
+
FFFFFFFF:FF,FF,FFF:FFFF:FF:F:FF:FFFF,FFFFFFFF:FFFFFFFF,FFFFFF:FFFFFF
FFF:FFF:FFFFFFF,:F:FFF,FFFFFFFFFFFFFF:FFF,:F,F,F,F,FFF:FFFFF:FFF,FFF
,F:FF:FFF:F:FFF
@A00721:190:HGJW7DSXY:4:2261:4915:24283 2:N:0:TCTGCAAG+GCTGCACT
ATTACATGTTCGGATATCTAGACTTTAATTCGAAACTATATTCGAATAACGCATGGTATAACATCATA
GATGACGTAAGCCCTTCATATCTTCAAAAGAAACATTGGAAGGAGCTTATTGGGGTCCAGCACAATTG
GGTATCCAATGTGAA
```

+
FFFFFFFFFFFFFFFFFFFFFFFFFFFFFFFFFFFFFFFFFF:FFFFFFFFFFFFFFFFFFFFFFFFFFF
FFFFFFFFFFFFFF:FFFFFFFFFFFF:FFFFFFFFFFFFFFFFFFFF,FFFFFFFFFFFFFFF:FFF
FFFF:FFFFFFFFFF
@A00721:190:HGJW7DSXY:4:2277:32090:16485 2:N:0:TCTGCAAG+GCTGCACT
ATTACATGTTCGGATATCTAGACTTTAATTCGAAACTATATTCGAATAACGCATGGTATAACATCATA
GATGACGTAAGCCCTTCATATCTTCAAAAGAAACATTGGAAGGAGCTTATTGGGGTCCAGCACAATTG
GGTATCCAATGTGAA
+
FFFFFFFFFFFFFFFFFFFFFFFFFFFFFFFFFFFFFFFFFFFFFFFFFFFFFFFFFFFFFFFFFFFF
FF:FFFFFFFFFFFFFFFFFFFFFFF:FFFFFFFFFFFFFFFFFFFFFFFFFFFFFFFFFFFFFF:FFF
FFFFFFFFFFFFFFF
@A00721:190:HGJW7DSXY:4:2508:21350:16673 2:N:0:TCTGCAAG+GCTGCACT
ATTACATGTTCGGATATCTAGACTTTAATTCGAAACTATATTCGAATAACGCATGGTATAACATCATA
GATGACGTAAGCCCTTCATATCTTCAAAAGAAACATTGGAAGGAGCTTATTGGGGTCCAGCACAATTG
GGTATCCAATGTGAA
+
FFFFFFFFFFFFFFFFFFFFFFFFFFFFFFFFFFFFFFFF:FFFFFFFFFFFFFFFFFFFFFFFFFFFF
FFFFFFFFFFFFFFFFFFFFFFFFFFFFFFFFFFFFFFFFFFFFFFFF:FFFFFFFFFFFFFFFFFFF
FFFFFFFFFFFFFFF
@A00721:190:HGJW7DSXY:4:2551:7844:4961 2:N:0:TCTGCAAG+GCTGCACT
ATTACATGTTCGGATATCTAGACTTTAATTCGAAACTATATTCGAATAACGCATGGTATAACATCATA
GATGACGTAAGCCCTTCATATCTTCAAAAGAAACATTGGAAGGAGCTTATTGGGGTCCAGCACAATTG
GGTATCCAATGTGAA
+
FFF:FFFFFFFFFFFFFFFFFFFFFFFFFFFFFFFFF:FFFF:FF:FFFFFFFFFFFFFFFFFFFFFFF
FFFFFFFFFFFFFFFFFFFFFFFFFFFF:FFFFFFFFFFFFFFFFFFFFF:FF:FFFFFFFFFFFFFF
F:FFFFFFFFFFFFF
@A00721:190:HGJW7DSXY:4:2551:1931:14418 2:N:0:TCTGCAAG+GCTGCACT
ATTACATGTTCGGATATCTAGACTTTAATTCGAAACTATATTCGAATAACGCATGGTATAACATCATA
GATGACGTAAGCCCTTCATATCTTCAAAAGAAACATTGGAAGGAGCTTATTGGGGTCCAGCACAATTG
GGTATCCAATGTGAA
+
FFFFFFFFFFFFFFFFFFFFFFFFFFFFFFFFFFFFFFFFFFFFF:FFFFFFFFFFFFFFFFFFFFFFF
FFFFFFFFFFFFFFFFFF:FFFFFFFFFFFFFFFFFFFFFFFFFFFFFFFFFFFFFFFFFFFFFFFFF
FFFFFFFFFFFFFF:
@A00721:190:HGJW7DSXY:4:2568:20383:16063 2:N:0:TCTGCAAG+GCTGCACT
ATTACATGTTCGGATATCTAGACTTTAATTCGAAACTATATTCGAATAACGCATGGTATAACATCATA
GATGACGTAAGCCCTTCATATCTTCAAAAGAAACATTGGAAGGAGCTTATTGGGGTCCAGCACAATTG
GGTATCCAATGTGAA
+
FFFFFFFFFFFFFFFFFFFFFFFFFFFFFFFFFFFFFFFFFFFFFFFFFFFFFFFFFFFFFFFFFFFF
FFFFFFFFFFFFFFFFFFFFFFFFFFFFFFFFFFFFFFFFFFFFFFFFFFFFFFFFFFFFFFFFFFFF
FFFFFFFFFFFFFFF
@A00721:190:HGJW7DSXY:4:2571:12057:17143 2:N:0:TCTGCAAG+GCTGCACT
ATTACATGTTCGGATATCTAGACTTTAATTCGAAACTATATTCGAATAACGCATGGTATAACATCATA
GATGACGTAAGCCCTTCATATCTTCAAAAGAAACATTGGAAGGAGCTTATTGGGGTCCAGCACAATTG
GGTATCCAATGTGAA
+
FFFFFFFFFFFFFFFFFFFFFFFFFFFFFFFFFFFFFFFFFFFFFFFFFFFFFFFFFFFFFFFFFFFF
FFFFFFFFFFFFFFFFFFFFFFFFFFFFFFFFFFFFFFFFFFFFFFFFFFFFFFFFFFFFFFFFFFFF
:FFFFFFFFFFFFFF
@A00721:190:HGJW7DSXY:4:2616:16224:7357 2:N:0:TCTGCAAG+GCTGCACT
ATTACATGTTCGGATATCTAGACTTTAATTCGAAACTATATTCGAATAACGCATGGTATAACATCATA

```
GATGACGTAAGCCCTTCATATCTTCAAAAGAAACATTGGAAGGAGCGTATTGGGGTCCAGCACAATTG
GGTATCCAATGTGAA
+
FFFFFFFFFFFFFFFFFFFFFFF:FFFFFFFFFFFF:FFFFFF:FFFFF::FFFFFFFFFFFFFFF:F
FFFFFFFFFFFFFFFFFFFFFFF,:FFFFFFFFFFFFFFFFFFFFF,FF:FFFFFFFFFFFFFFFFFF
FFFF:FFFFFFFFFF
@A00721:190:HGJW7DSXY:4:2635:8305:9518 2:N:0:TCTGCAAG+GCTGCACT
ATTACATGTTCGGATATCTAGACTTTAATTCGAAACTATATTCGAATAACGCATGGTATAACATCATA
GATGACGTAAGCCCTTCATATCTTCAAAAGAAACATTGGAAGGAGCTTATTGGGGTCCAGCACAATTG
GGTATCCAATGTGAA
+
FFFFFFFFFFFFFFFFFFFFFFFFFFFFFFFFFFFFF:FFFFFFFFFFFFFFFFFFFFFFFFFFFFFF
FFFFFFF:FFF:FFFFFFFFFF:FFFFFFFFFFFF:FFFFFFFFFFFFFFFFFFFFFFFFFFFFFFFF
FFFFFFFFFFFFFFF
@A00721:190:HGJW7DSXY:4:2645:31132:7717 2:N:0:TCTGCAAG+GCTGCACT
ATTACATGTTCGGATATCTAGACTTTAATTCGAAACTATATTCGAATAACGCATGGTATAACATCATA
GATGACGTAAGCCCTTCATATCTTCAAAAGAAACATTGGAAGGAGCTTATTGGGGTCCAGCACAATTG
GGTATCCAATGTGAA
+
FFFFFFFFFFFFFFFFFFFFFFFFFFFFFFFFFFFFFFFFFFFFFFFFFFFFFFFF:FFFFFFFFFFF
F:FFFF:FFFFFFFFFFFFFFFFFFFFFFFFFFFFFF:FFFFFFFFFFF:FFF:FFF:FFFFFFF,F:
FFF:FFFFFFFFFFF
@A00721:190:HGJW7DSXY:4:1118:18041:6120 2:N:0:TCTGCAAG+GCTGCACT
CATAATTACATGTTCGGATATCTAGACTTTAATTCGAAACTATATTCGAATAACGCATGGTATAACAT
CATAGATGACGTAAGCCCTTCATATCTTCAAAAGAAACATTGGAAGGAGCTTATTGGGGTCCAGCACA
ATTGGGTATCCAATG
+
FFFFFFFFFFFFFFFFFFFFFFFFFFFFFFFFFFFFFFFFFFFFFFFFFFFFFFFFFFFFFFFFFFFF
FFFF:FFFFFFFFFFFFFFFFFF:FFFFFFFFFFFFFFFFFFFFFFFFFFFFFFF,FFFFFFFFFFFF
FF:F:FFFFFFFFFF
@A00721:190:HGJW7DSXY:4:1434:17797:15562 2:N:0:TCTGCAAG+GCTGCACT
CATAATTACATGTTCGGATATCTAGACTTTAATTCGAAACTATATTCGAATAACGCATGGTATAACAT
CATAGATGACGTAAGCCCTTCATATCTTCAAAAGAAACATTGGAAGGAGCTTATTGGGGTCCAGCACA
ATTGGGTATCCAATG
+
FFFFFFFFFFFFFFFFFFFFFFFFFFFFFFFFFFFFFFFFFFFFFFFFFFFFFFFFFFFFFFFFFFFF
FFFFFFFFFFFFFFFFFFFFFFFFFFFFFFFFFFFFFFFFFFFFFFFFFFFFFFFFFFFFFFFFFFFF
FFFFFFFFFFFFFFF
@A00721:190:HGJW7DSXY:4:1557:5095:11068 2:N:0:TCTGCAAG+GCTGCACT
CATAATTACATGTTCGGATATCTAGACTTTAATTCGAAACTATATTCGAATAACGCATGGTATAACAT
CATAGATGACGTAAGCCCTTCATATCTTCAAAAGAAACATTGGAAGGAGCTTATTGGGGTCCAGCACA
ATTGGGTATCCAATG
+
FFFFFFFFFFFFFFFFFFFFFFFFFFFFFFFFFFFFF:FFFFFFFFFFFFFFFFFFFFFFFFFFFFFF
FF:FFFFFFFFFFFFFFFFFFFFFFFFFFFFFFFFFFFFFFFFFFFFFFFFF:FFFFFFFFFFFFFFF
FFFFFFFFFFFFFFF
@A00721:190:HGJW7DSXY:4:1557:5638:11224 2:N:0:TCTGCAAG+GCTGCACT
CATAATTACATGTTCGGATATCTAGACTTTAATTCGAAACTATATTCGAATAACGCATGGTATAACAT
CATAGATGACGTAAGCCCTTCATATCTTCAAAAGAAACATTGGAAGGAGCTTATTGGGGTCCAGCACA
ATTGGGTATCCAATG
+
FFFFFFFFFFFFFFFFFFFFFFFFFFFFFFFFFFFFFFFFFFFFFFFFFFFFFFFFFFFF,FFFFFFF
FFFFFFFFFFFFFFFFFFFFFFFFFFFFFFFFFFFFFFFFFFFFFFFFFFFFFFFFFFFFFFFFFFFF
FFFFFFFFFFFFFFF
```

```
@A00721:190:HGJW7DSXY:4:1557:3830:11475 2:N:0:TCTGCAAG+GCTGCACT
CATAATTACATGTTCGGATATCTAGACTTTAATTCGAAACTATATTCGAATAACGCATGGTATAACAT
CATAGATGACGTAAGCCCTTCATATCTTCAAAAGAAACATTGGAAGGAGCTTATTGGGGTCCAGCACA
ATTGGGTATCCAATG
+
FF:FFFF,FFFFFFFFFFFFFFFFFFFFFFFFFFFFFFFFF:FF,FFFFFFFFFFFFFFFFFFFFFFF
F:FFFFFFFFFFFFFFFFFFFFFFFFFFFFFFFFFFFFFFF:FFFFFFF:FFFFFF:F:FFFFF:FFF
FFFFFFFFFFFFFFF
@A00721:190:HGJW7DSXY:4:1558:22345:5619 2:N:0:TCTGCAAG+GCTGCACT
CATAATTACATGTTCGGATATCTAGACTTTAATTCGAAACTATATTCGAATAACGCATGGTATAACAT
CATAGATGACGTAAGCCCTTCATATCTTCAAAAGAAACATTGGAAGGAGCTTATTGGGGTCCAGCACA
ATTGGGTATCCAATG
+
FFFFFFFFFFFFFFFFFFFFFFFFFFFFFFFFFFFFFFFFFFFFFFFFFFFFFFFFF,FFFFFFFFFF
FFFFFFFFFFFFFF:FFFFFFFFFFFFFFFFFF:FFFFFFFFFFFFFFFFFFFFFFFFFFFFFFFFFF
FFFFFFFFFFFFFF:
@A00721:190:HGJW7DSXY:4:1672:13630:28886 2:N:0:TCTGCAAG+GCTGCACT
CATAATTACATGTTCGGATATCTAGACTTTAATTCGAAACTATATTCGAATAACGCATGGTATAACAT
CATAGATGACGTAAGCCCTTCATATCTTCAAAAGAAACATTGGAAGGAGCTTATTGGGGTCCAGCACA
ATTGGGTATCCAATG
+
FFFFFFFFFFFFFFFFFFFFFFFFFFFFFFFFFFFFFFFFFFF:FFFFFFFFFFFFFFFFFFF:FFFFFF
FFFFFFFFFFFFFFFFFFFFFFFFFFFFFFFFFFFFFFFFFFFFFFFFFFFFFFFFFFFFFFFFFFFF
FFFFFFFFFFFFFF:
@A00721:190:HGJW7DSXY:4:2115:1985:18427 2:N:0:TCTGCAAG+GCTGCACT
CATAATTACATGTTCGGATATCTAGACTTTAATTCGAAACTATATTCGAATAACGCATGGTATAACAT
CAGAGATGACGTAAGCCCTTCATATCTTCAAAAGAAACATTGGAAGGAGCTTATTGGGGTCCAGCACA
ATTGGGTATCCAATG
+
FFFFFFFFFFFFF,FFFFFFFFFFFFFFFFFFFFFFFFF:FF,FFFFFFFFFFFFFFFFFFFF,FFFFF
FF,FFFFFFFFFFFFFFFF:FFFFFFFFFFF,F:FFFFF,:FFFFF:,FFFF:,FFFFFF,FFFF,FF
,FFFFFF,FFFFFFF
@A00721:190:HGJW7DSXY:4:2138:19633:13730 2:N:0:TCTGCAAG+GCTGCACT
CATAATTACATGTTCGGATATCTAGACTTTAATTCGAAACTATATTCGAATAACGCATGGTATAACAT
CATAGATGACGTAAGCCCTTCATATCTTCAAAAGAAACATTGGAAGGAGCTTATTGGGGTCCAGCACA
ATTGGGTATCCAATG
+
FFFFFFFFFFFFFFFFFFFFFFFFFFFFFFFFFFFFFFFFFFF:FFFFFFFFFFFFFFFFFFFFFFFFFF
FFFFFFFFFFFFFFFFFFFFFFFFFFFFFFFFFFFFFFFFFFFFF:FFFFFFFFFFFFFFFFFFFFFFF
FFFFFFF,FFFFFFF
@A00721:190:HGJW7DSXY:4:2377:16776:36182 2:N:0:TCTGCAAG+GCTGCACT
CATAATTACATGTTCGGATATCTAGACTTTAATTCGAAACTATATTCGAATAACGCATGGTATAACAT
CATAGATGACGTAAGCCCTTCATATCTTCAAAAGAAACATTGGAAGGAGCTTATTGGGGTCCAGCACA
ATTGGGTATCCAATG
+
FFFFFFFFFFFFFFFFFFFFFFFFF:FFFFFFFFFFFFFFFFFFFF:FFFFFFFFFFFFFFFFFFFFFFFF
FFFF:FFFFFFFFFFFFFFFFFF:FFFFFFFFFFFFFFFFFFFFFFFFFFFFF:FFFFFFFFFFFFF:FFF
FFFFFFFFFFFFFFF
@A00721:190:HGJW7DSXY:4:2378:16830:1423 2:N:0:TCTGCAAG+GCTGCACT
CATAATTACATGTTCGGATATCTAGACTTTAATTCGAAACTATATTCGAATAACGCATGGTATAACAT
CATAGATGACGTAAGCCCTTCATATCTTCAAAAGAAACATTGGAAGGAGCTTATTGGGGTCCAGCACA
ATTGGGTATCCAATG
+
FFFFFFFFFFFFFFFFFFFFFFFFFFFFFFFFFFFFFFFFFFFFFFFFFFFFFFFFFFFFFF:FFFFFF
```

```
FFFFFFFFFFFFFFFFFFFFFFFFFFFFFFF:FFFFFFFFF:FFFFFFFFFFF:FFFFFFFFFFFFFF
FFFFFFFFFFFFFFF
@A00721:190:HGJW7DSXY:4:2528:6225:27211 2:N:0:TCTGCAAG+GCTGCACT
CATAATTACATGTTCGGATATCTAGACTTTAATTCGAAACTATATTCGAATAACGCATGGTATAACAT
CATAGATGACGTAAGCCCTTCATATCTTCAAAAGAAACATTGGAAGGAGCTTATTGGGGTCCAGCACA
ATTGGGTATCCAATG
+
FFFFFFFFFFFFFFFFFFFFFFFFFFFFFFFFFFFFFFFFF:FFFFFFFFFFFFFFFFFFFFFFFFFF
FFFFFFFFFFFFFFFFFFFFFFFFFFFFFFFF:FF:FFFFFFFFFFFFFFFFFFFFFFFFFFFFFFFF
FFFFFFF:FFFFFFF
@A00721:190:HGJW7DSXY:4:2557:7202:17065 2:N:0:TCTGCAAG+GCTGCACT
CATAATTACATGTTCGGATATCTAGACTTTAATTCGAAACTATATTCGAATAACGCATGGTATAACAT
CATAGATGACGTAAGCCCTTCATATCTTCAAAAGAAACATTGGAAGGAGCTTATTGGGGTCCAGCACA
ATTGGGTATCCAATG
+
FFFFFFFFFFFFFFFFFFFFFFFFFFFFFFFFFFFFFFFFFFFFFFFFFFFFFFFFFFFFFFFFFFFF
FFFFFFFFFFFFFFFFFFFFFFFFFFFFF:FFFFFFFFFFFFFFFFFFFFFFFFFFFFF:FFFFFFF:
FFFFFFFF:FFFFFF
@A00721:190:HGJW7DSXY:4:2559:8449:25175 2:N:0:TCTGCAAG+GCTGCACT
CATAATTACATGTTCGGATATCTAGACTTTAATTCGAAACTATATTCGAATAACGCATGGTATAACAT
CATAGATGACGTAAGCCCTTCATATCTTCAAAAGAAACATTGGAAGGAGCTTATTGGGGTCCAGCACA
ATTGGGTATCCAATG
+
FFFFFFFFFFFFFFFFFFFFFFFFFFFFFFFFFFFFFFFFFFFFFFFFFFFFFFFFFFFFFFFFFFFF
FFFFFFFFFFFFFFFFFFFFFFFFFFF:FFFFFFFFFFFFFFFFFFFFFFFFFFFFFFFFFFFFFFFF
:FFFFFF:FFFFFFF
@A00721:190:HGJW7DSXY:4:2647:15673:16861 2:N:0:TCTGCAAG+GCTGCACT
CATAATTACATGTTCGGATATCTAGACTTTAATTCGAAACTATATTCGAATAACGCATGGTATAACAT
CATAGATGACGTAAGCCCTTCATATCTTCAAAAGAAACATTGGAAGGAGCTTATTGGGGTCCAGCACA
ATTGGGTATCCAATG
+
FFF,FFFFFFFFFFFFFFFFFFFFF::FFFFFFFFFFFFFF:FFFFFFFFFFF,FFFFFFFFFFFFFF
FFFFFFFFFFFFFFFFFFFFFFFFFFFFFFFFFF:FFFFFFF:FFFFFFFFFFFFF,FFFFFFFFFF:
F,FFFFFFFFFFFFF
@A00721:190:HGJW7DSXY:4:1130:13422:1157 2:N:0:TCTGCAAG+GCTGCACT
CATAATTACATGTTCGGATATCTAGACTTTAATTCGAAACTATATTCGAATAACGCATGGTATAACAT
CATAGATGACGTAAGCCCTTCATATCTTCAAAAGAAACATTGGAAGGAGCTTATTGGGGTCCAGCACA
ATTGGGTATCCAAT
+
FF,FFFFFFFF,FFF::F:F::FF:FFFF,FF:FF,FFF,FF,FFF::FFFFF:F:FF,,FFFFFFFF
FF:F,FF:FF,FFFF::,F:FF,FFFF,FFFFF:FFF,F:,F,FFF:F,FF,FFF::,F:F,F,FF:F
FF:,F,FF,:,FFF
@A00721:190:HGJW7DSXY:4:1507:25491:17456 2:N:0:TCTGCAAG+GCTGCACT
ACATAATTACATGTTCGGATATCTAGACTTTAATTCGAAACTATATTCGAATAACGCATGGTATAACA
TCATAGATGACGTAAGCCCTTCATATCTTCAAAAGAAACATTGGAAGGAGCTTATTGGGGTCCAGCAC
AATTGGGTATCCAAT
+
FFFFFFFFFF:FFFFFFFFFFFFFFFFFFFFFFFFFFFFFFFFFFFFFFFFFFFF,FFFFFFFFFFFF
FFFFFFFFFFFFFFFFFFFFFFFFFFFFFFFFFFFFFFFFFFFFFFFFFFFFFFFFFFFFFFFFFFFF
F:FFFFFFFFFFFFF
@A00721:190:HGJW7DSXY:4:2419:18240:30138 2:N:0:TCTGCAAG+GCTGCACT
ACATAATTACATGTTCGGATATCTAGACTTTAATTCGAAACTATATTCGAATAACGCATGGTATAACA
TCATAGATGACGTAAGCCCTTCATATCTTCAAAAGAAACATTGGAAGGAGCTTATTGGGGTCCAGCAC
AATTGGGTATCCAAT
```

+
FFFFFFFFFFFFFFFFFFFFFFFFFFFFFFFFFFFFFFFFFFFFFFFFFFFFFFFFFFFFFFFFFFFFFFF
FFFFFFFFFFFF:FFF:FFFFFFFFFFFFFFFFFF:FFFFFFFFFFFF:FFFFFFFFFFFFFFFFFFF
FFFFFFFF,FFFFFF
@A00721:190:HGJW7DSXY:4:1626:22001:30358 2:N:0:TCTGCAAG+GCTGCACT
CCCAATTGTGCTGGACCCCAATAAGCTCCTTCCAATGTTTCTTTTGAAGATATGAAGGGCTTGCGTCA
TCTATGATGTTATACCATGCGTTATTCGAATATAGTTTCGAATTAAAGTCTAGATATCCGAACATGTA
ATTATGTGGGCCTAG
+
FFFFFFFFFFFFFFFFFFFFFFFFFFFFFF,FFFFFFFFFFFFFFFFFFFFFF:FFFFFFFFFFFFFF
FFFF::FFFFFFFFFFFF:FFFFFFFFFFFFFFFFFFFFFFFFFFFFFFFFFFFFFFFFFF:FFF:F
FFFFFFFFFF:FFFF
@A00721:190:HGJW7DSXY:4:1656:20754:15734 2:N:0:TCTGCAAG+GCTGCACT
CTAGGCCCACATAATTACATGTTCGGATATCTAGACTTTAATTCGAAACTATATTCGAATAACGCATG
GTATAACATCATAGATGACGTAAGCCCTTCATATCTTCAAAAGAAACATTGGAAGGAGCTTATTGGGG
TCCAGCACAATTGGG
+
FFFFFFFFFFFFFFFFFFF:FFFFFFFFFFFFFFFFFFFFFFFFFFFFFFF:FFFFFFFFFFFFFFF,F
FFFFFFFFFFFFFFFFFFFFFFFFFFFFFFFFFFFFFFFFF,FFFFFFFFFFFFFFFFFFF,FFFFFFFF
FFFFFFFFFFFFFFF
@A00721:190:HGJW7DSXY:4:1659:2474:30013 2:N:0:TCTGCAAG+GCTGCACT
CCCAATTGTGCTGGACCCCAATAAGCTCCTTCCAATGTTTCTTTTGAAGATATGAAGGGCTTGCGTCA
TCTATGATGTTATACCATGCGTTATTCGAATATAGTTTCGAATTAAAGTCTAGATATCCGAACATGTA
ATTATGTGGGCCTAG
+
FFFFFFFFFFFFFFFFFFFFFFFFFFFFFFFFFFFFFFFFFFFFFFFFFFFFFFFFFFFFFFFFFFFFFF
FFFFFFFFFFFFFFFFFFFFFFFFFFF:FFFFFFFFFFFFFFFFFFFFFFFFFFFFFFFFFFFFFFFFFF
FFFFFFFFFFFFFFF
@A00721:190:HGJW7DSXY:4:1666:13593:25755 2:N:0:TCTGCAAG+GCTGCACT
CTAGGCCCACATAATTACATGTTCGGATATCTAGACTTTAATTCGAAACTATATTCGAATAACGCATG
GTATAACATCATAGATGACGTAAGCCCTTCATATCTTCAAAAGAAACATTGGAAGGAGCTTATTGGGG
TCCAGCACAATTGGG
+
FFFFFFFFFFFFFFFFFFFFFFFFFFFFFFFFFFFFFFFFFFFFFFFFFFFFFFFFFFFFFFFFFFFFFF
FFFFFFFFFFFFFFFFFFFFFFFFFFFFFFFFFFFFFFFFFFFFFFFFFFFFFFFFFFFFFFFFFFFFFF
FFFFFFFFFFFFFFF
@A00721:190:HGJW7DSXY:4:2327:15465:14591 2:N:0:TCTGCAAG+GCTGCACT
CTAGGCCCACATAATTACATGTTCGGATATCTAGACTTTAATTCGAAACTATATTCGAATAACGCATG
GTATAACATCATAGATGACGTAAGCCCTTCATATCTTCAAAAGAAACATTGGAAGGAGCTTATTGGGG
TCCAGCACAATTGGG
+
FFFFFFFFFFFFFFFFFFFFFFFFFFFFFFFFFFFF:FFFFFFFFFFFFFFFFFFFFFFFFFFFFFFFFF
FFFFFF:FFFFFFFFFFFFFFFFFFFFFFFFFFFFFFFFFFFFFFFFFFFFFFFFFFFF:FFFFFFFF
FFFFFFFFFFFFFFF
@A00721:190:HGJW7DSXY:4:2347:11189:8719 2:N:0:TCTGCAAG+GCTGCACT
CTAGGCCCACATAATTACATGTTCGGATATCTAGACTTTAATTCGAAACTATATTCGAATAACGCATG
GTATAACATCATAGATGACGTAAGCCCTTCATATCTTCAAAAGAAACATTGGAAGGAGCTTATTGGGG
TCCAGCACAATTGGG
+
FFFFFFFFFFFFFFFFFFFFFFFFFFFFFFFFFFFFFFFFFFFFFFFFFFFFFFFFFFFFFFFFFFFFFF
FFFFFFFFFFFFFFFFFFFFFFFFFFFFFFFFFFFFFFFFFFFFFFFFFFFFFFFFFFFFFFFFFFFFFF
FFFFFFFFFFFFFFF
@A00721:190:HGJW7DSXY:4:2503:21260:26694 2:N:0:TCTGCAAG+GCTGCACT
CCCAATTGTGCTGGACCCCAATAAGCTCCTTCCAATGTTTCTTTTGAAGATATGAAGGGCTTGCGTCA

```
TCTATGATGTTATACCATGCGTTATTCGAATATAGTTTCGAATTAAAGTCTAGATATCCGAACATGTA
ATTATGTGGGCCTAG
+
FFFFFFFFFFFFFFFFFFFFFFFFFFFFFFFFFFFFFFFFFFFF:FFFF:FFFFFFFFFFFFFFFFFFF
FFF:FFFFFFFFFFFFF:FFFFFFFFFFFFFFFFFFFFFF:FFFF,,FFF:FFFFFFFFFFFFF:FFF
FFFFFFFFFFFFFFF
@A00721:190:HGJW7DSXY:4:2503:21206:27289 2:N:0:TCTGCAAG+GCTGCACT
CCCAATTGTGCTGGACCCCAATAAGCTCCTTCCAATGTTTCTTTTGAAGATATGAAGGGCTTGCGTCA
TCTATGATGTTATACCATGCGTTATTCGAATATAGTTTCGAATTAAAGTCTAGATATCCGAACATGTA
ATTATGTGGGCCTA
+
FFFFFFFFFFFFFFFFFFFFFFFFFFFFFFFFFFF:FFFFFFFFFFFFFFFFFFFFFFFFFFFFFFFF
FFFFFFFFFFFFFFFFFFFFFFFFFFFFFFFFFFFFFFFFFFFFFFFFFFF,:FFFFFFFF,FFFFFF
FFFFFFFFFFFFFF
@A00721:190:HGJW7DSXY:4:2624:25970:2378 2:N:0:TCTGCAAG+GCTGCACT
CTAGGCCCACATAATTACATGTTCGGATATCTAGACTTTAATTCGAAACTATATTCGAATAACGCATG
GTATAACATCATAGATGACGTAAGCCCTTCATATCTTCAAAAGAAACATTGGAAGGAGCTTATTGGGG
TCCAGCACAATTGGG
+
FFFFFFFFFFFFFFFFFFFFFFFFFFFFFFFFFFFFFFFFFFFFFFFFFFFFFFFFFFFFFFFFFFFF
FFFFFFFFFFFFFFFFFFFFFFFFFFFFFFFFFFFFFFFF:FFFFFFFFFFFFFFFFFFFFFFFFFFF
FFFFFFFFFFFFF,F
@A00721:190:HGJW7DSXY:4:2657:5791:5321 2:N:0:TCTGCAAG+GCTGCACT
CCCAATTGTGCTGGACCCCAATAAGCTCCTTCCAATGTTTCTTTTGAAGATATGAAGGGCTTGCGTCA
TCTATGATGTTATACCATGCGTTATTCGAATATAGTTTCGAATTAAAGTCTAGATATCCGAACATGTA
ATTATGTGGGCCTAG
+
FFFFFFFFFFFFFFFFFFFFF:FFFFFFFFFFF:F,FFFFFF:FFFFFFFFFFFFFFFFFFFFFFFFF
FFFFFFFFFFFFFFFFFFFFFFFFFFFFFFFFFFFFFFFFFFFFFFFFFFFFFFFFFFFFFFFFFFFF
:FFFFFFF:FFFFFF
@A00721:190:HGJW7DSXY:4:2678:26295:23077 2:N:0:TCTGCAAG+GCTGCACT
CCCAATTGTGCTGGACCCCAATAAGCTCCTTCCAATGTTTCTTTTGAAGATATGAAGGGCTTGCGTCA
TCTATGATGTTATACCATGCGTTATTCGAATATAGTTTCGAATTAAAGTCTAGATATCCGAACATGTA
ATTATGTGGGCCTAG
+
FFFFFFFFFFFFFFFFFFFFF:FFFFF:FFFFFFFFFFFFFFFFFFFFFFFFFFFFF:FFFFFFFFFF
::FFFF:FFFFFFFFFFFFFFFFFFFFFFFFFFFFF,:FFFFFFFFFFF:FFFFFFFFFFFFFFFFF::F
F:FFFFF:FFFFFFF
@A00721:190:HGJW7DSXY:4:1155:2826:35227 2:N:0:TCTGCAAG+GCTGCACT
CTCTAGGCCCACATAATTACATGTTCGGATATCTAGACTTTAATTCGAAACTATATTCGAATAACGCA
TGGTATAACATCATAGATGACGTAAGCCCTTCATATCTTCAAAAGAAACATTGGAAGGAGCTTATTGG
GGTCCAGCACAATTG
+
F:FFFF:FFFFF:FFFFFFFFFFF:FFF:FFFFF::FFFF:FF,FFFFFFFF,,FFFFF:FFFFFFFF
FF:FFFFFFFFFFFF:FFFFFFFFFF,FF:FFFFFFFF::F:FFFFFFF:FFFFFFFFFFF,,FFFFF
FFFFF:FFFFFFFF:
@A00721:190:HGJW7DSXY:4:2148:30282:30170 2:N:0:TCTGCAAG+GCTGCACT
CTCTAGGCCCACATAATTACATGTTCGGATATCTAGACTTTAATTCGAAACTATATTCGAATAACGCA
TGGTATAACATCATAGATGACGTAAGCCCTTCATATCTTCAAAAGAAACATTGGAAGGAGCTTATTGG
GGTCCAGCACAATTG
+
FFFFFFFFFFFFFFFFFFFFFFFF:FFFFFFFFFFFFFFFFFF:F:FFFFFF:FFFFFFFFFFFFFFF
FFFFFFFFFFFFFFFFFFFFFFFFFFFFF:FFFFF:FFF:FFF:FFFFFF:F:F::,:F,FFFFFFFF
FFFFF:F,FFFFF:F
```

```
@A00721:190:HGJW7DSXY:4:2222:26313:9017 2:N:0:TCTGCAAG+GCTGCACT
CTCTAGGCCCACATAATTACATGTTCGGATATCTAGACTTTAATTCGAAACTATATTCGAATAACGCA
TGGTATAACATCATAGATGACGTAAGCCCTTCATATCTTCAAAAGAAACATTGGAAGGAGCTTATTGG
GGTCCAGCACAATTG
+
FFFFFFFFFFFFFFFFFFFFFFFFFFFFFFFFFFFFFFFFFFFFFFFFFFFFFFFFFFFFFFFFFFFFFF
FFFFFFFFFFFFFFFFFFFFFFFFFFFFFFFFFFFFFFFFFFFFFFFFFFFFFFFFFFFFFFFFFFFFFF
FFFFFFFFFFFFFFF
@A00721:190:HGJW7DSXY:4:2307:2275:36151 2:N:0:TCTGCAAG+GCTGCACT
CTCTAGGCCCACATAATTACATGTTCGGATATCTAGACTTTAATTCGAAACTATATTCGAATAACGCA
TGGTATAACATCATAGATGACGTAAGCCCTTCATATCTTCAAAAGAAACATTGGAAGGAGCTTATTGG
GGTCCAGCACAATTG
+
FFFFFFFFFFFFF:FFFFFFFFFF:FFFFFFFFFFFFFFFFFFFFFFFFFFFFFFFFFFFFFFFFFFFFF
FFFFF,FFFFFFFFFFFFFFFFFFFFFFFFFFFFFFFFFFFFFFFFF:FFFFFFFF:FFFFFFFFFFFFF
FFFFFFFFFFFFF:F
@A00721:190:HGJW7DSXY:4:2315:22155:7576 2:N:0:TCTGCAAG+GCTGCACT
CTCTAGGCCCACATAATTACATGTTCGGATATCTAGACTTTAATTCGAAACTATATTCGAATAACGCA
TGGTATAACATCATAGATGACGTAAGCCCTTCATATCTTCAAAAGAAACATTGGAAGGAGCTTATTGG
GGTCCAGCACAATTG
+
FFFFFFFFFFFFFFFFFFFFFFFFFFFFFFFFFFFFFFFFFFFFFFFFFFFFFFFFFFFFFFFFFFFFFF
FFFFFFFFFFFFFFFFFFFFFFFFFFFFFFFFFFFFFFFFFFFFFFFFFFFFFFFFFFFFFFFFFFFFFF
FFFFFFFFFFFFFFF
@A00721:190:HGJW7DSXY:4:2433:3640:25864 2:N:0:TCTGCAAG+GCTGCACT
CTCTAGGCCCACATAATTACATGTTCGGATATCTAGACTTTAATTCGAAACTATATTCGAATAACGCA
TGGTATAACATCATAGATGACGTAAGCCCTTCATATCTTCAAAAGAAACATTGGAAGGAGCTTATTGG
GGTCCAGCACAATTG
+
FFFFFFFFFFFFF:FFFFFFFFFFFFFFFFFFFFFFFFFFFFFFFFFFF:FFF,:FFFFFFFFFFFFFFF
FFFFF,FFFFFFF:FFFFFFFFFFFFFFF,FFFF:FFFFFFFFFFFFFFFFFFFFFFFFFFFF,FFFFFF
FFFFFFFFFFF:FFF
@A00721:190:HGJW7DSXY:4:1302:21070:13745 2:N:0:TCTGCAAG+GCTGCACT
CTAGGCCCACATAATTACATGTTCGGATATCTAGACTTTAATTCGAAACTATATTCGAATAACGCATG
GTATAACATCATAGATGACGTAAGCCCTTCATATCTTCAAAAGAAACATTGGAAGGAGCTTATTGGGG
TCCAGCACAA
+
FFFFFFFFFFFFFFFFFFFFFFFFFFFFFFFFFFFFFFFFFFFFFFFFFFFFFFFFFFFFFFFFFFFFFF
FFFFFFFFFFFFFFFFFFFFFFFFFFFFFFFFFFFFFFFFF:FFFFFFFFFFFFFFFFFFFFFFFFFFFF
FFFFFFFFFF
@A00721:190:HGJW7DSXY:4:1313:32117:26459 2:N:0:TCTGCAAG+GCTGCACT
CTAGGCCCACATAATTACATGTTCGGATATCTAGACTTTAATTCGAAACTATATTCGAATAACGCATG
GTATAACATCATAGATGACGTAAGCCCTTCATATCTTCAAAAGAAACATTGGAAGGAGCTTATTGGGG
TCCAGCACAA
+
FF:FFFFFFFFFFFFFF:FFFFFF::FFFFFFFFF:FFFFFFF:FFFFFFFFF:FFFFFFFFF:FFFF
FFFFFFFFF:FFFFFFF:FFF:FFF::FFFFFFFFFF,FFFFF::FFF:,FFFFFFFFFFFFFFFFFF
:FFF::F:FF
@A00721:190:HGJW7DSXY:4:1322:27371:33677 2:N:0:TCTGCAAG+GCTGCACT
CTAGGCCCACATAATTACATGTTCGGATATCTAGACTTTAATTCGAAACTATATTCGAATAACGCATG
GTATAACATCATAGATGACGTAAGCCCTTCATATCTTCAAAAGAAACATTGGAAGGAGCTTATTGGGG
TCCAGCACAA
+
FFFFFFFFFFFFFFFFFFFFFFFFFFFFFFFFFFFFFFFFFFFFFFFFFF:FFFFFFFFFFFFFFFF:FF
```

F:FFFFFFFFF,FFFFFFFFFFFFFFFFFFFFFFFFFFFFF:FFFFFFFFFFFFFFFFFFFFFFFFF:FF
FFFFF,FFFF
@A00721:190:HGJW7DSXY:4:1358:4074:3129 2:N:0:TCTGCAAG+GCTGCACT
CTAGGCCCACATAATTACATGTTCGGATATCTAGACTTTAATTCGAAACTATATTCGAATAACGCATG
GTATAACATCATAGATGACGTAAGCCCTTCATATCTTCAAAAGAAACATTGGAAGGAGCTTATTGGGG
TCCAGCACAA
+
FFFFFFFFFFFFFFFFFFFFFFFFFFFFFFFFFFFFFFFFFFFFFFFFFFFFFF:FFFFFFFFFFFFFFF
FFFFFFFFFFFFFFFFFFFFFFFFFFFFFFFFFFFFFFFFFFFFFFFFFFFFF:FFFFFFF:FFFFFFFF
FFFFFFFFFF
@A00721:190:HGJW7DSXY:4:1519:17707:24392 2:N:0:TCTGCAAG+GCTGCACT
CTAGGCCCACATAATTACATGTTCGGATATCTAGACTTTAATTCGAAACTATATTCGAATAACGCATG
GTATAACATCATAGATGACGTAAGCCCTTCATATCTTCAAAAGAAACATTGGAAGGAGCTTATTGGGG
TCCAGCACAA
+
FFFFFFFFFFFFFFFFFFFFFFFFFFFFFFFFFFFFFFFFFFFFFFFFFFFF:FFFFFFFFFFFFFFFFF
FFFFFFFFFFFFFFFFF:FFFFFFFFFFFFFFFFFFFFFFFFFFFFFFF:FF:FFFFFF:FFFFFFFFFF
FFFFFFFFFF
@A00721:190:HGJW7DSXY:4:1549:6949:18317 2:N:0:TCTGCAAG+GCTGCACT
CTAGGCCCACATAATTACATGTTCGGATATCTAGACTTTAATTCGAAACTATATTCGAATAACGCATG
GTATAACATCATAGATGACGTAAGCCCTTCATATCTTCAAAAGAAACATTGGAAGGAGCTTATTGGGG
TCCAGCACAA
+
F:FF,:FFFFFFFFFFF:FFFFFF:FFF:F:FFFFFFFFFF:F,FFFFFFFFFFFFFFFFFFFFF:FF,F
,FFFFFFFF:FFFFFFFFFFFFFFFFFFFFF:FFFFFFFFFFFFFFFFFFFFF:FFF:FFFFF::FFFF:
FFFFF:FFFF
@A00721:190:HGJW7DSXY:4:1576:1145:4257 2:N:0:TCTGCAAG+GCTGCACT
CTAGGCCCACATAATTACATGTTCGGATATCTAGACTTTAATTCGAAACTATATTCGAATAACGCATG
GTATAACATCATAGATGACGTAAGCCCTTCATATCTTCATAAGAAACATTGGAAGGAGCTTATTGGGG
TCCAGCACAA
+
FFFFFFFFFFFFFFFFFFFFFFFFFFFFFFFFFFFFFFFFFFFFFFFF:FFFFFFFFFFFFFFFFFFFFF
FFFFFFFFFFF:FFFFFFFFFFFFFFFF:FFFFFFFFFFF,,FFFFFF:FFFFFFFF::,FF:F:FFFFF
,FF:FFFFFF
@A00721:190:HGJW7DSXY:4:1622:11722:27868 2:N:0:TCTGCAAG+GCTGCACT
CTAGGCCCACATAATTACATGTTCGGATATCTAGACTTTAATTCGAAACTATATTCGAATAACGCATG
GTATAACATCATAGATGACGTAAGCCCTTCATATCTTCAAAAGAAACATTGGAAGGAGCTTATTGGGG
TCCAGCACAA
+
FFFFFFFFFFFFFFFFFFFFFFFFFFFFFFFFFFFFFF:FFF:FF:FFFFFFFFFFFFFFFFFFFFFFFF
FFFFFFFFFFFFFFFFFF:FFFFF:FFFFFFFFFFFFFFFFFF:FFFFFFFFFFFFFFFF:FFFFFFF:F
FFFFFFFFFF
@A00721:190:HGJW7DSXY:4:1660:32714:32972 2:N:0:TCTGCAAG+GCTGCACT
CTAGGCCCACATAATTACATGTTCGGATATCTAGACTTTAATTCGAAACTATATTCGAATAACGCATG
GTATAACATCATAGATGACGTAAGCCCTTCATATCTTCAAAAGAAACATTGGAAGGAGCTTATTGGGG
TCCAGCACAA
+
FFFFFFFFFFFFFFFFFFFFFFFFFFFFFFFFFFFFFFFFFFFFFFFFFFFFFFFFFFFFFFFFFFFFFF
FFFFFFFFFFFFFFFF,FFFFFFFFFFFFFFFFFF:FFFFFFF,FFF:FFFF,FFFFFFFFFFFFF::FF
FF:FFFFFFF
@A00721:190:HGJW7DSXY:4:2101:2519:27618 2:N:0:TCTGCAAG+GCTGCACT
CTAGGCCCACATAATTACATGTTCGGATATCTAGACTTTAATTCGAAACTATATTCGAATAACGCATG
GTATAACATCATAGATGACGTAAGCCCTTCATATCTTCAAAAGAAACATTGGAAGGAGCTTATTGGGG
TCCAGCACAA

```
+
FFFFFFFFFFFFFFFFFFFFFFFFFFFFFFFFFFFFFFFFFFFFFFFFFFFFF:FFFFFFFFFFFFFF
F:FFFFFFFFFFFFFFFFFFF:FFFFFF:FFF:FFF,FFFFFFFF:FFF:FFFFFF:FFFFF:FFFFF
:FFFF,FFF:
@A00721:190:HGJW7DSXY:4:2341:29921:16579 2:N:0:TCTGCAAG+GCTGCACT
CTAGGCCCACATAATTACATGTTCGGATATCTAGACTTTAATTCGAAACTATATTCGAATAACGCATG
GTATAACATCATAGATGACGTAAGCCCTTCATATCTTCAAAAGAAACATTGGAAGGAGCTTATTGGGG
TCCAGCACAA
+
FFFFFFFFFFFFFFFFFFFFFFFFFFFFFFFFFFFFFFFFFFFFFFFFFFFFFFFFFFFFFFFFFFFF
FFFFFFFFFFFFFFFFFFFFFFFFFFFFFFFFFFFFFFFFFFFFFFFFFFFFFFFFFF:FFFFFFFFF
FFFFFFFFFF
@A00721:190:HGJW7DSXY:4:2453:9842:25363 2:N:0:TCTGCAAG+GCTGCACT
CTAGGCCCACATAATTACATGTTCGGATATCTAGACTTTAATTCGAAACTATATTCGAATAACGCATG
GTATAACATCATAGATGACGTAAGCCCTTCATATCTTCAAAAGAAACATTGGAAGGAGCTTATTGGGG
TCCAGCACAA
+
FFFFFFFFFFFFFFFFFFFFFFFFFFFFFFFFFFFFFFFFFFFFFFFFFFFF,FFFFFFFFFFFFFFF
FFFFFFFFFFFFFFFFFFFFFFFFFFFFFFFFFFFFFFFFFFFFFFFFFFFFFFFFFFF:F:FFFFFF
FFFFFFFFFF
@A00721:190:HGJW7DSXY:4:2453:10276:25520 2:N:0:TCTGCAAG+GCTGCACT
CTAGGCCCACATAATTACATGTTCGGATATCTAGACTTTAATTCGAAACTATATTCGAATAACGCATG
GTATAACATCATAGATGACGTAAGCCCTTCATATCTTCAAAAGAAACATTGGAAGGAGCTTATTGGGG
TCCAGCACAA
+
FFFF:FFFFFFFFFFFFFFFFFFFFFFFFFFFFFFFFFFFFFFFFFFFFFFFFFFFFFFFFFFFFFFF
FFFFFFFFFFFFFFFF:FFFFFFFF:FF:FFFFFFFFFFFFF:FFF:FFFF,FFFFFFFFFFFF,F,F
FF,F,FFFFF
@A00721:190:HGJW7DSXY:4:2465:28863:12461 2:N:0:TCTGCAAG+GCTGCACT
CTAGGCCCACATAATTACATGTTCGGATATCTAGACTTTAATTCGAAACTATATTCGAATAACGCATG
GTATAACATCATAGATGACGTAAGCCCTTCATATCTTCAAAAGAAACATTGGAAGGAGCTTATTGGGG
TCCAGCACAA
+
FFF:FFFFFFFFFFFFFFFFFFFFFFFFFFFFFFFFFFFFFFFFFFFFFFFFFFFFFFFFFFFFFFFF
FFFFFFFFFFFFFFFFFFFFFFFFFFFFFFFFFFFFFFFF:FF:FFFFFFFFFFFFFFFFFFFFFFFF
FFFFFFFFFF
@A00721:190:HGJW7DSXY:4:1153:18150:13322 2:N:0:TCTGCAAG+GCTGCACT
CTAGGCCCACATAATTACATGTTCGGATATCTAGACTTTAATTCGAAACTATATTCGAATAACGCATG
GTATAACATCATAGATGACGTAAGCCCTTCATATCTTCAAAAGAAACATTGGAAGGAGCTTATTGGGG
TCCAGCACA
+
FFFFFFFFFFFFFFFFFFFFFFFFFFFF:FFFFFFFFFFFFFFFF:FFFFFFFFFFFFFFFFFFFFFF
FFFFFFFFFF:FFFFFFFFFFF:,FFFFFFFFFFFFFFFF::FFFFFFF,:FFFFFFFFFF:FFFFFF
FFFFFFFFF
@A00721:190:HGJW7DSXY:4:2530:27615:14340 2:N:0:TCTGCAAG+GCTGCACT
CTAGGCCCACATAATTACATGTTCGGATATCTAGACTTTAATTCGAAACTATATTCGAATAACGCATG
GTATAACATCATAGATGACGTAAGCCCTTCATATCTTCAAAAGAAACATTGGAAGGAGCTTATTGGGG
TCCAGCACA
+
FFFFFFFFFFFFFFFFFFFFFFFFFFFFFFFFFFFFFFFFFFFFFFFFFFFFF:FFFFFFFFFFFF:F
FFFFFFFFFFFFFFFFFFF:FFFFFFFFFFFFFFFFFFFFFFFFFFFFFFFFFFFFFFFFFFFFFFFF
FFFFFFFFF
@A00721:190:HGJW7DSXY:4:1173:28999:30044 2:N:0:TCTGCAAG+GCTGCACT
CTAGATCTCTAGGCCCACATAATTACATGTTCGGATATCTAGACTTTAATTCGAAACTATATTCGAAT
```

AACGCATGGTATAACATCATAGATGACGTAAGCCCTTCATATCTTCAAAAGAAACATTGGAAGGAGCT
TATTGGGGTCCAGCA
+
FFFFFFFFFFFFFFFFFFFFFFFFFFFFFFFFFFFFFFFFFFFFFFFFFFFFFFFFFFFFFFFFFFFF
FFFFFFFFFFFFFFFFFFFFFFFFFFFFFFFFFFFFFFFFFFFFFFFFFFF:FFFFFFFFFFFFFFFF
FFFFFFFFFFFFFFF
@A00721:190:HGJW7DSXY:4:1507:10972:36245 2:N:0:TCTGCAAG+GCTGCACT
CTAGATCTCTAGGCCCACATAATTACATGTTCGGATATCTAGACTTTAATTCGAAACTATATTCGAAT
AACGCATGGTATAACATCATAGATGACGTAAGCCCTTCATATCTTCAAAAGAAACATTGGAAGGAGCT
TATTGTGGTCCAGCA
+
FFF:FFFFFFFFFFFFF:FFFFFFFFFFFFFFFFFFFFFFF:FFFFFFFFFFFFFFFFFFFFF:FFFF
FFFFFFFF,FFFFF:FFFFFFFFFF:FF:FF,FFFFFFFFFFFFFFFFF:FFFFFFFFF,FFF:F:FF
FFFFF,:FFFFFF:F
@A00721:190:HGJW7DSXY:4:1510:9218:1141 2:N:0:TCTGCAAG+GCTGCACT
CTAGATCTCTAGGCCCACATAATTACATGTTCGGATATCTAGACTTTAATTCGAAACTATATTCGAAT
AACGCATGGTATAACATCATAGATGACGTAAGCCCTTCATATCTTCAAAAGAAACATTGGAAGGAGCT
TATTGGGGTCCAGCA
+
FFFFFFFFFFFFFFFFFFFFFFFFF:,FFFFFFFFFFFFFF:FFFF:FFFFFFFFFFF:FFFFFFFFF
FF:FFFFFFFFFFFFFFFFFFFFF:FFF:FFFFFFFFFFFFFFFFFF:,,FFFFFF:FFFFFFFFF:F
FFFFFF:FFFFFFFF
@A00721:190:HGJW7DSXY:4:1556:21097:7592 2:N:0:TCTGCAAG+GCTGCACT
CTAGATCTCTAGGCCCACATAATTACATGTTCGGATATCTAGACTTTAATTCGAAACTATATTCGAAT
AACGCATGGTATAACATCATAGATGACGTAAGCCCTTCATATCTTCAAAAGAAACATTGGAAGGAGCT
TATTGGGGTCCAGCA
+
FFFFFFFFFFFFFFFFFFFFFFFFFFFFF,FFFFFFFFFFFFFFFFFFFFFFFFFFFF:F:FFFFFFF
FFFFFFFFFFFFFFFFFFFFFFFFFFFFFFFFFFFFFFFFFFFFFFFFFFFFFFFFFFFFFFFFFFFF
FFFFFFFFFFFFFFF
@A00721:190:HGJW7DSXY:4:1654:4589:27445 2:N:0:TCTGCAAG+GCTGCACT
CTAGATCTCTAGGCCCACATAATTACATGTTCGGATATCTAGACTTTAATTCGAAACTATATTCGAAT
AACGCATGGTATAACATCATAGATGACGTAAGCCCTTCATATCTTCAAAAGAAACATTGGAAGGAGCT
TATTGGGGTCCAGCA
+
FFFFFFFFFFFFFFFFFFFFFFFFFFFFFFFFFFFFFFFFFFFFFFFFFFFFFFFFFF:FFFFFFFFF
FFFFFFFFFFFFFFFFFFFFFFFFFFFFFFFFFFFFFFFFFFFFFFFFFFFFFFFFFFFFF:FFFFFF
FFFFFFFFFFFFFFF
@A00721:190:HGJW7DSXY:4:2124:12463:30154 2:N:0:TCTGCAAG+GCTGCACT
CTAGATCTCTAGGCCCACATAATTACATGTTCGGATATCTAGACTTTAATTCGAAACTATATTCGAAT
AACGCATGGTATAACATCATAGATGACGTAAGCCCTTCATATCTTCAAAAGAAACATTGGAAGGAGCT
TATTGGGGTCCAGCA
+
FFFFFFFFFFFFFFFFFFFFFFFFFF:FFFFFFFFFFFFFFFFFFFFFFFFFFFFFFFFFFFFFFFFF
FFFFFFFFFFFFFFFFFFFFFFFFFFFFFFFFFFFFFFFFFFFFFFFFF:FFFFFFFFFFFFFFFFFF
:FFFFFFFFFFFFFF
@A00721:190:HGJW7DSXY:4:2164:2157:36166 2:N:0:TCTGCAAG+GCTGCACT
CTAGATCTCTAGGCCCACATAATTACATGTTCGGATATCTAGACTTTAATTCGAAACTATATTCGAAT
AACGCATGGTATAACATCATAGATGACGTAAGCCCTTCATATCTTCAAAAGAAACATTGGAAGGAGCT
GATTGGGGTCCAGCA
+
FFFFFFFFFFFFFFFFFFFFFFFFFFFFFFFFFFFFFFFFFFFFFFFFFFFF:FFFF:FFFFFFF,FF
FFFFFFFFFFFFFFFFFFF:FFFFFFFFFFFFFFFFFFFFFFFFFFFFFFFF:FF:FFFF,FFFFFF
,F:FFFFFFFFFFFF

```
@A00721:190:HGJW7DSXY:4:2240:19741:30264 2:N:0:TCTGCAAG+GCTGCACT
CTAGATCTCTAGGCCCACATAATTACATGTTCGGATATCTAGACTTTAATTCGAAACTATATTCGAAT
AACGCATGGTATAACATCATAGATGACGTAAGCCCTTCATATCTTCAAAAGAAACATTGGAAGGAGCT
TATTGGGGTCCAGCA
+
FFFFFFFFFFFFFFFFFFFFFFFFFFFFFFFFFFFFFFFFFFFFFFFFFFFFFFFFFFFFFFFFFFFF
FFFFFFFFFF:FFFFFFFFFFFFFFFFFFFFFFFFFFFFFFFFFFFFFFFFFFFFFFFFFF,FFFFF:
FF,FFFFFFFFFFFF
@A00721:190:HGJW7DSXY:4:2428:16152:16282 2:N:0:TCTGCAAG+GCTGCACT
CTAGATCTCTAGGCCCACATAATTACATGTTCGGATATCTAGACTTTAATTCGAAACTATATTCGAAT
AACGCATGGTATAACATCATAGATGACGTAAGCCCTTCATATCTTCAAAAGAAACATTGGAAGGAGCT
TATTGGGGTCCAGCA
+
FFFFFFFFFFFFFFFFFFFFFFFFFFFFFFFFFFFFFFFFFFFFFFFFFFFFFFFFFFFFFFFFFFFF
FFFFF,FFFFFFFFFFFFFFFFFFFFFFFFFFFFFFFFFFFFFFFFFFFFFFFFF:FFFFFFFF
FFFF,FFFFFFFFFF
@A00721:190:HGJW7DSXY:4:2578:28203:26850 2:N:0:TCTGCAAG+GCTGCACT
CTAGATCTCTAGGCCCACATAATTACATGTTCGGATATCTAGACTTTAATTCGAAACTATATTCGAAT
AACGCATGGTATAACATCATAGATGACGTAAGCCCTTCATATCTTCAAAAGAAACATTGGAAGGAGCT
TATTGGGGTCCAGCA
+
FFFFFFFFFFFFFFFFFFFFFFFFFFFFFFFFFFFFFFFFFFFFFFFFFFFFFFFFFF,FFFFFFFFF
FFFFFF:FFF:FFFFFFFFFFF:FFFFF:FFFFFFFFFFFFFFFFFFFFFFFFFFF:FFFFFFFFFFF
:FFFFFFFFFFFFFF
@A00721:190:HGJW7DSXY:4:2613:27769:10723 2:N:0:TCTGCAAG+GCTGCACT
CTAGATCTCTAGGCCCACATAATTACATGTTCGGATATCTAGACTTTAATTCGAAACTATATTCGAAT
AACGCATGGTATAACATCATAGATGACGTAAGCCCTTCATATCTTCAAAAGAAACATTGGAAGGAGCT
TATTGGGGTCCAGCA
+
FFFFFFFFFFFFFFFFFFFFFFFFFFFFFFFFFFFFFFFFFFFFFFFFFFFFFFFFFF:FFFFFFFFF
FFFFFFFFFFFFFFFFFFFFFFFFFFFFFFFFFFFFFFFFFFFFFFFFFFFFFFFFFF:FFFFFFFFF
FFFFFFFFFFFFFFF
@A00721:190:HGJW7DSXY:4:2613:5394:21073 2:N:0:TCTGCAAG+GCTGCACT
CTAGATCTCTAGGCCCACATAATTACATGTTCGGATATCTAGACTTTAATTCGAAACTATATTCGAAT
AACGCATGGTATAACATCATAGATGACGTAAGCCCTTCATATCTTCAAAAGAAACATTGGAAGGAGCT
TATTGGGGTCCAGCA
+
FF:FFFFFFF:FFFFFFFFFFFFFFFFFFFFFFFFFFFFFFFFFFFFFFFFFFFFFFFFFFFFFFFFF
FFFFFF,FFFF:FFFFFFF:FFFFFFFF:FFFFFFFFFF:FFFFFFFFFFF:FFFFFFFFFF,FFF:F
FFFF:FFFFFFFFFF
@A00721:190:HGJW7DSXY:4:1339:13774:5243 2:N:0:TCTGCAAG+GCTGCACT
GGAAAAACTCTGTGGGCTAGATCTCTAGGCCCACATAATTACATGTTCGGATATCTAGACTTTAATTC
GAAACTATATTCGAATAACGCATGGTATAACATCATAGATGACGTAAGCCCTTCATATCTTCAAAAGA
AACATTGGAAGGAGC
+
FFFFFFFFFFFFFFFFFFFF,FFFFFFFFFFFFF:FFFFF:FFFFFFFFFFF::FFFFFFFFFFFF:F
FFFFFFFF,:FFFFFFFFFFFFFFF:FFFFFFFFF:FFFFFFFFFFFFFFFFFFFFFFFFFFFFFFFF
FFFFFFFFFFFFFFF
@A00721:190:HGJW7DSXY:4:1340:19605:20071 2:N:0:TCTGCAAG+GCTGCACT
GGAAAAACTCTGTGGGCTAGATCTCTAGGCCCACATAATTACATGTTCGGATATCTAGACTTTAATTC
GAAACTATATTCGAATAACGCATGGTATAACATCATAGATGACGTAAGCCCTTCATATCTTCAAAAGA
AACATTGGAAGGAGC
+
FFFFFFFFFFFFFFFFFFFFFFFFFFFFFFFFFFFFFFFFFFFFFFFFFFFFFFFFFFFFFFFFFFFF
```

FFFFFF,FFFFFFFFFF:FFFFFFFF,:FFFFFFF:FF:FFFFFFFFFFFFFFFFFFFFFFFFFF,F,
FFFFFFFFFF,FFFF
@A00721:190:HGJW7DSXY:4:1355:7238:7075 2:N:0:TCTGCAAG+GCTGCACT
GGAAAAACTCTGTGGGCTAGATCTCTAGGCCCACATAATTACATGTTCGGATATCTAGACTTTAATTC
GAAACTATATTCGAATAACGCATGGTATAACATCATAGATGACGTAAGCCCTTCATATCTTCAAAAGA
AACATTGGAAGGAGC
+
FFFFFFFFFFFFFFFFFFFFFFFFFFFFFFFFFFF:FFFFFFFFF:FFFFFFFFFFFFFFFFFFFFFF
FFFFF,FFFFFFFFFFFFFFFFFFFFFFFFFFFFFFFFFFFFFFFFFFFFFFFFFFFFFFFFF:FF
FFFFFFFFFFFFFFF
@A00721:190:HGJW7DSXY:4:2121:19732:16188 2:N:0:TCTGCAAG+GCTGCACT
GGAAAAACTCTGTGGGCTAGATCTCTAGGCCCACATAATTACATGTTCGGATATCTAGACTTTAATTC
GAAACTATATTCGAATAACGCATGGTATAACATCATAGATGACGTAAGCCCTTCATATCTTCAAAAGA
AACATTGGAAGGAGC
+
FFFFFF:FFFFFFFFFFF,FFFFFFFFFFFFFFF,FFFFFFFFFFFFFFFFFFFFFF:FFFFFFFFFF
FFFFFFFF,FFFFFFFFFF:FF,FFFFFFFFFFFFFFFFFFFFFFFFFFFFFFFFFFFFFFFFFFFFF
FFFF:FF:F:FFFFF
@A00721:190:HGJW7DSXY:4:2121:19804:16313 2:N:0:TCTGCAAG+GCTGCACT
GGAAAAACTCTGTGGGCTAGATCTCTAGGCCCACATAATTACATGTTCGGATATCTAGACTTTAATTC
GAAACTATATTCGAATAACGCATGGTATAACATCATAGATGACGTAAGCCCTTCATATCTTCAAAAGA
AACATTGGAAGGAGC
+
FFFFFFFFFFFFFFFFFFFFFFFFFFFFFFFFFFFFFFFFFFFFFFFFFFFFFFFFFFFFFFFFFFFF
FFFFF,FFFFFFFFFF:FFFFFFFFFFFFFFFFFFFFFFFFFFFFFFFFFF:FFFFFFFFFFF:FFFF
FFFFFFFFFF:FFF:
@A00721:190:HGJW7DSXY:4:2312:4273:14246 2:N:0:TCTGCAAG+GCTGCACT
GGAAAAACTCTGTGGGCTAGATCTCTAGGCCCACATAATTACATGTTCGGATATCTAGACTTTAATTC
GAAACTATATTCGAATAACGCATGGTATAACATCATAGATGACGTAAGCCCTTCATATCTTCAAAAGA
AACATTGGAAGGAGC
+
FFFFFFFFFFFFFFFFFFFF:FFFFFFFFFFFFFFFFFFFFFFFFFFFFFFFFFFFFFFFFFFFFFFF
FFF:FFFF:FFFFFFFFFFFFFFFFF:FFFFFFFF:FF,FFFFFFFFFFFFFFFFFFFFFFFFFFFFF
FFFF,FFFFFFFFFF
@A00721:190:HGJW7DSXY:4:2435:2157:5415 2:N:0:TCTGCAAG+GCTGCACT
GGAAAAACTCTGTGGGCTAGATCTCTAGGCCCACATAATTACATGTTCGGATATCTAGACTTTAATTC
GAAACTATATTCGAATAACGCATGGTATAACATCATAGATGACGTAAGCCCTTCATATCTTCAAAAGA
AACATTGGAAGGAGC
+
FFFFFFFFFFFFFFFFFFFFFFFFFFFFFFFFFFFFFFFFFFFFFFFFFFFFFFFFFFFFFFFFFFFF
FFFFFFFFFFFFFFFFF:FFFFFFFFFFFFFFFFF:FFFFFFFFFFFFFFFFF:FFFFFFFFFFFFFF
FFFFF,FFFFFFFFF
@A00721:190:HGJW7DSXY:4:2476:23122:3709 2:N:0:TCTGCAAG+GCTGCACT
GGAAAAACTCTGTGGGCTAGATCTCTAGGCCCACATAATTACATGTTCGGATATCTAGACTTTAATTC
GAAACTATATTCGAATAACGCATGGTATAACATCATAGATGACGTAAGCCCTTCATATCTTCAAAAGA
AACATTGGAAGGAGC
+
FFFFF:FFFFFFFFFFFFFFFFFFFFFFFFFFFFFFFFFFFFFFFFFFFFFFFFFFFFFFFFFF
FFFFFFFFFFFFFFFFFFFFFFFFFFFFFFFFFFFFF,FFF,FFFFFFFFFFFF:FFFFFFFF,FFFF
FFFFFFFFFFFFFFF
@A00721:190:HGJW7DSXY:4:2573:32000:35837 2:N:0:TCTGCAAG+GCTGCACT
GGAAAAACTCTGTGGGCTAGATCTCTAGGCCCACATAATTACATGTTCGGATATCTAGACTTTAATTC
GAAACTATATTCGAATAACGCATGGTATAACATCATAGATGACGTAAGCCCTTCATATCTTCAAAAGA
AACATTGGAAGGAGC

```
+
FFFFF:,FFFFFFFFFFFFFFFFFFFFFFFFFFFFFFFFFFFFFFFFFFFFFFFFFFFFFFFFFFF:FFF
FFFFFFFF::FFFFFFF:FFFF,FFFFFFFF,FFFF,FFFF::FFFFFFFF,FF,F:FFFF:FF,:FF
FFFFF:FFFFFFFFF
@A00721:190:HGJW7DSXY:4:1304:20690:13025 2:N:0:TCTGCAAG+GCTGCACT
TGGAAAAACTCTGTGGGCTAGATCTCTAGGCCCACATAATTACATGTTCGGATATCTAGACTTTAATT
CGAAACTATATTCGAATAACGCATGGTATAACATCATAGATGACGTAAGCCCTTCATATCTTCAAAAG
AAACATTGGAAGGAG
+
FFFFFFFFFFFFFFFFFFFFFFFFFFFFFFFFFFFFF:FFFFFFFFFFFFFFFFFFFFFFFFFFFFFF
FFFFFFFFFFFFFFFFFFFFFFFFFFFFFFFFFFFFFFFFFFFFFFFF:FFFFFFFFFFFFFFFFFFF
FFFFFFFFFFFFFFF
@A00721:190:HGJW7DSXY:4:1676:25934:9956 2:N:0:TCTGCAAG+GCTGCACT
TGGAAAAACTCTGTGGGCTAGATCTCTAGGCCCACATAATTACATGTTCGGATATCTAGACTTTAATT
CGAAACTATATTCGAATAACGCATGGTATAACATCATAGATGACGTAAGCCCTTCATATCTTCAAAAG
AAACATTGGAAGGAG
+
FFFFFF:FFFFFFFFFFFFFFFFFFFFFFFFFFFFFFFFFFFFFFFFFFFFFFFFFFFFFFFFFFFFF
FFFFFFFFFFFFFFFFFFFFFFFFFFFFFFFFFFFFFFFFFFFFFFFFFFFFFFFFFFFFFFFFFFFF
:FFFFFFFFFFFFFF
@A00721:190:HGJW7DSXY:4:2363:9625:13025 2:N:0:TCTGCAAG+GCTGCACT
TGGAAAAACTCTGTGGGCTAGATCTCTAGGCCCACATAATTACATGTTCGGATATCTAGACTTTAATT
CGAAACTATATTCGAATAACGCATGGTATAACATCATAGATGACGTAAGCCCTTCATATCTTCAAAAG
AAACATTGGAAGGAG
+
FFFFFFF:FFFFFFFFFFFFFFFFFFFFFFFFFFFFFFFFFFFFFFFFFFFFFFFFFFFFFFFFFFFF
FFFFFFFFFFF,FFFFFFFFFFFFFFFFFFFFFFFFFFFFFFFFFFFFFFFFFFFFFFFFFFFFFFFF
FFFFFFFFFFFFFFF
@A00721:190:HGJW7DSXY:4:1204:5367:19586 2:N:0:TCTGCAAG+GCTGCACT
TAGAAGTGGAAAAACTCTGTGGGCTAGATCTCTAGGCCCACATAATTACATGTTCGGATATCTAGACT
TTAATTCGAAACTATATTCGAATAACGCATGGTATAACATCATAGATGACGTAAGCCCTTCATATCTT
CAAAAGAAACATTGG
+
FFFFFFFFFFFFFFFFFFFFFFFFFFFFFFFFFFFFFFFFFFFFFFFFFFFFFFFFFFFFFFFFFFFF
FFFFFFFFFFFF:FFFFFFFFFFFFFFFF,FFFFFFFFFFFFFFFFFFFFFFFFFFFFFFFFFFFFFF
FFFFFFFFFFFFFFF
@A00721:190:HGJW7DSXY:4:1227:18738:30091 2:N:0:TCTGCAAG+GCTGCACT
TAGAAGTGGAAAAACTCTGTGGGCTAGATCTCTAGGCCCACATAATTACATGTTCGGATATCTAGACT
TTAATTCGAAACTATATTCGAATAACGCATGGTATAACATCATAGATGACGTAAGCCCTTCATATCTT
CAAAAGAAACATTGG
+
FFFFFFFFFFFFFFFFFFFFFFFFFFFFFFFFFFFFFFFFFFFFFFFFFFFFFFFFFFFFFFFFFFFF
FFFFFFFFFFFFFFFFFFFFFFFFFFFFFFFFFF:FFFFFFFFFFFFFFFFFFFFFFFFFFFFFFFFF
FFFFFFFFFFFF:F:
@A00721:190:HGJW7DSXY:4:1375:27914:35055 2:N:0:TCTGCAAG+GCTGCACT
TAGAAGTGGAAAAACTCTGTGGGCTAGATCTCTAGGCCCACATAATTACATGTTCGGATATCTAGACT
TTAATTCGAAACTATATTCGAATAACGCATGGTATAACATCATAGATGACGTAAGCCCTTCATATCTT
CAAAAGAAACATTGG
+
F:FFFF:FFF:FFFFFFFFFFFFFFFFFFFFFFFFFFFFFFF:FFFFFFFFFF,FFFFFFFFFFFFFF
FFFFFFFFFFFFFFF:FFFFFFFFFFFFFFFF::FFFFFFFFFFFFFFFFFFFFFFFFFFFFF,FFF:
FFFFFFFFFFFFFFF
@A00721:190:HGJW7DSXY:4:1541:25635:34992 2:N:0:TCTGCAAG+GCTGCACT
TAGAAGTGGAAAAACTCTGTGGGCTAGATCTCTAGGCCCACATAATTACATGTTCGGATATCTAGACT
```

TTAATTCGAAACTATATTCGAATAACGCATGGTATAACATCATAGATGACGTAAGCCCTTCATATCTT
CAAAAGAAACATTGG
+
FFFFFFFFFFFF:FFFFFFFFFFFFFFFFFFFFFFFFFFFFFFFFFFFFFFFFFFFFFFFFFFFFFF
FFFFFFFFFFFFFFFFFFFFFFFFFFFFFFFFFFFFFFFFFFFFFFFFFFFFFFFFFFFFFFFFFFFF
FFFFFFFFFFFFFFF
@A00721:190:HGJW7DSXY:4:1541:25979:36182 2:N:0:TCTGCAAG+GCTGCACT
TAGAAGTGGAAAAACTCTGTGGGCTAGATCTCTAGGCCCACATAATTACATGTTCGGATATCTAGACT
TTAATTCGAAACTATATTCGAATAACGCATGGTATAACATCATAGATGACGTAAGCCCTTCATATCTT
CAAAAGAAACATTGG
+
FFFFFFFFFFFFFFFFFFFFFFFFF:FFFFFFFFFFFFFFFFFFFFFFF:FFFFFFFFFFFFFFFFFF
FFFFFFFFFFFF:FFFFFFFFFFFFFFFFFFFFFFFFFFFFFFFFFFFF:FFFFFFFFFFFFFFFFFF
FFFFFFFFFFF:F:F
@A00721:190:HGJW7DSXY:4:2230:29197:13886 2:N:0:TCTGCAAG+GCTGCACT
TAGAAGTGGAAAAACTCTGTGGGCTAGATCTCTAGGCCCACATAATTACATGTTCGGATATCTAGACT
TTAATTCGAAACTATATTCGAATAACGCATGGTATAACATCATAGATGACGTAAGCCCTTCATATCTT
CAAAAGAAACATTGG
+
FFFFFFFFFFFFFFFFFFFFFFFFFFFFFFFFFFFFFFFFFFFFFFFFFFFFFFFFFFFFFFFFFFFF
FFFFFFFFFFFFFFFFFFFFFFFFFFFFFFFFFFFFFFFFFFFFFFFFFFFFFFFFFFFFFFFFFFFF
FFFFFFFFFFFFFFF
@A00721:190:HGJW7DSXY:4:2265:7392:1830 2:N:0:TCTGCAAG+GCTGCACT
TAGAAGTGGAAAAACTCTGTGGGCTAGATCTCTAGGCCCACATAATTACATGTTCGGATATCTAGACT
TTAATTCGAAACTATATTCGAATAACGCATGGTATAACATCATAGATGACGTAAGCCCTTCATATCTT
CAAAAGAAACATTGG
+
FFFFFFFFFFFFFFFFFFFFFFFFFFFFFFFFFFFFFFFFFFFFFFFFFFFFFFFFFFF:FFFFFFFF
FFFFFFFFFFFFF:FFFFFFFFFFF:FFFFFFF:FFFFFFFFFFFFFFFFFFFFFFFFFFFFFFFFF:
FFFFFF:FFFFFFF:
@A00721:190:HGJW7DSXY:4:2542:24270:3004 2:N:0:TCTGCAAG+GCTGCACT
TAGAAGTGGAAAAACTCTGTGGGCTAGATCTCTAGGCCCACATAATTACATGTTCGGATATCTAGACT
TTAATTCGAAACTATATTCGAATAACGCATGGTATAACATCATAGATGACGTAAGCCCTTCATATCTT
CAAAAGAAACATTGG
+
FFFFFFFFFFFFFFFFFFFFFFFFFFFFFFFFFFFFFFFFFFFFFFFFFFFFFFFFFFFFFFFFFFFF
FFFFFFFFFFFFFFF,FFFFFFFFFFFFF,FFFFFFFFFFFFFFFFFFFFFFFFFFFFFFFFFFFFFF
F,FFFFFFFFF,FFF
@A00721:190:HGJW7DSXY:4:2622:28944:7717 2:N:0:TCTGCAAG+GCTGCACT
TAGAAGTGGAAAAACTCTGTGGGCTAGATCTCTAGGCCCACATAATTACATGTTCGGATATCTAGACT
TTAATTCGAAACTATATTCGAATAACGCATGGTATAACATCATAGATGACGTAAGCCCTTCATATCTT
CAAAAGAAACATTGG
+
FFFFFFFFFFFFFFFFFFFFFFFFFFFFFFFFF:FFFFFFFFFFFFFFFFFFFFFFFFFFFFFFFFFF
FFFFFFFFFFFFFFFFFFF:FFFFFFFFFFFFFFFFFFFFFFFFFFFFFFFFFFFFFFFFFFFFFFFF
FFFFFFFFFF,FFF:
@A00721:190:HGJW7DSXY:4:1460:24758:30968 2:N:0:TCTGCAAG+GCTGCACT
TAGAAGTGGAAAAACTCTGTGGGCTAGATCTCTAGGCCCACATAATTACATGTTCGGATATCTAGACT
TTAATTCGAAACTATATTCGAATAACGCATGGTATAACATCATAGATGACGTAAGCCCTTCATATCTT
CAAAAGAAACATTG
+
FFFFFFFFFFFFFFFFFFFFFFFFFFFFFFFFFF:FFFFFFFFFFFFFFFFFFFFFFFFFFFFFFFFF
FFFFFF:FFFFFFFFFFFFFFFFFFFFFFFFFFFFFFFFFFFFFFFFFFFFFFFFFFFFFFFFFFFFF
FFFFFFFFFFFFFF

```
@A00721:190:HGJW7DSXY:4:1122:10755:29700 2:N:0:TCTGCAAG+GCTGCACT
GTCTGATACTCGAAGGTCCATCTAGAAGTGGAAAAACTCTGTGGGCTAGATCTCTAGGCCCACATAAT
TACATGTTCGGATATCTAGACTTTAATTCGAAACTATATTCGAATAACGCATGGTATAACATCATAGA
TGACGTAAGCCCTTC
+
FFFFFFFFFFFFFFFFFFFFFFFFFFFFFFFFFFFFFFFFFFFFFFFFFFFFFFFFFFFFFFFFFFFF
FFFFFFFFFFFFFFFFFFF:FFFFFFFFFFFFFFFFFFFFFFFFFFFFFFFFFFFFF:FFFFF:FFFF
FFFFFFFFFFFFFFF
@A00721:190:HGJW7DSXY:4:1304:13196:22780 2:N:0:TCTGCAAG+GCTGCACT
GTCTGATACTCGAAGGTCCATCTAGAAGTGGAAAAACTCTGTGGGCTAGATCTCTAGGCCCACATAAT
TACATGTTCGGATATCTAGACTTTAATTCGAAACTATATTCGAATAACGCATGGTATAACATCATAGA
TGACGTAAGCCCTTC
+
FFFFFFFFFFFFFFFFFFFFFFFFFFFF:FFFFFFFFFFFFFFFFFFFFFFFFFFFFFFFFFFF:FFF
FFFFFFF:FFFFFFFFFFFFFFFFFFFFFFFFFFF:FFFFFFFFFFFFFFFFFFFFFFFFFFFFFFFF
FFFFFFFFFFFFFFF
@A00721:190:HGJW7DSXY:4:1431:29857:13307 2:N:0:TCTGCAAG+GCTGCACT
GTCTGATACTCGAAGGTCCATCTAGAAGTGGAAAAACTCTGTGGGCTAGATCTCTAGGCCCACATAAT
TACATGTTCGGATATCTAGACTTTAATTCGAAACTATATTCGAATAACGCATGGTATAACATCATAGA
TGACGTAAGCCCTTC
+
FFFFFFFFFFFFFFFFFFFFFFFFFF:FFFFFFFFFFFFFFFFFFFFFFFFFFFFFFFFFFFFFFFFF
FFFFFFFFFFFFFFFFFFFFFFFFFFFFFFFFFF:FF,FFFFFFFFFFFFFFFFFFFFFFFFFFFFFF
FFFFFFFFFFFFFFF
@A00721:190:HGJW7DSXY:4:2367:15203:6746 2:N:0:TCTGCAAG+GCTGCACT
GTCTGATACTCGAAGGTCCATCTAGAAGTGGAAAAACTCTGTGGGCTAGATCTCTAGGCCCACATAAT
TACATGTTCGGATATCTAGACTTTAATTCGAAACTATATTCGAATAACGCATGGTATAACATCATAGA
TGACGTAAGCCCTTC
+
FFFFFFF:FFFFFFFFFFFFFFFFFFFFFFFFFF,:FFFFFFFFFFFFFFFFFFFFFFFFFFFFFFFF
FFFFFFFFFFFFF:FFFFFFFFFFFFFFFFFFFFFFFFFFFFFFFFFFFFFFFFFFFFFFFFFFFFF:
FFFFFFFFFFFFFFF
@A00721:190:HGJW7DSXY:4:2432:9344:13009 2:N:0:TCTGCAAG+GCTGCACT
GTCTGATACTCGAAGGTCCATCTAGAAGTGGAAAAACTCTGTGGGCTAGATCTCTAGGCCCACATAAT
TACATGTTCGGATATCTAGACTTTAATTCGAAACTATATTCGAATAACGCATGGTATAACATCATAGA
TGACGTAAGCCCTTC
+
FFFFFFF:FFFFFFFFFFFFFFFFFFFFFFFFFFFFFFFFFFFFFFFFFFFFFFFFFFFFFFFFFFFF
FFFFFFFFFFFFFFFFFFFFFFFFFFFFFFF,FFFFFFFFFFFFFFFFFFF:FFFFFFFFFFFFFFFF
FFFFFFFFFFFFFFF
@A00721:190:HGJW7DSXY:4:2531:8657:36025 2:N:0:TCTGCAAG+GCTGCACT
GTCTGATACTCGAAGGTCCATCTAGAAGTGGAAAAACTCTGTGGGCTAGATCTCTAGGCCCACATAAT
TACATGTTCGGATATCTAGACTTTAATTCGAAACTATATTCGAATAACGCATGGTATAACATCATAGA
TGACGTAAGCCCTTC
+
F:FFFFFFFFFFFFFFFFFFFFFFFFFFFFFFFFFFFFFFFFFFFFFFFFFFFFFFFFFFFFFFFFFF
FFFFFFFFFFFFFFFFFFFFFFFFFFFFFFFFFFFFFFFF:FFFFFFFFFFFFFFFFFFFFFFFFFFF
FFFFFFFFFFFFFFF
@A00721:190:HGJW7DSXY:4:2177:27814:6417 2:N:0:TCTGCAAG+GCTGCACT
GTCTGATACTCGAAGGTCCATCTAGAAGTGGAAAAACTCTGTGGGCTAGATCTCTAGGCCCACATAAT
TACATGTTCGGATATCTAGACTTTAATTCGAAACTATATTCGAATAACGCATGGTATAACATCATAGA
TGACGTAAGCCCTT
+
FFFFFFF::FF:F,:FFFFFF:::F:FFFFFFFF,FFFFFFFF:FFF:F,F:FFFFFFF:F:FFFFFF
```

```
FFFFFFFFFFFFFFF::F,FFFFFF:F:,FFFF:FFFF:F:FFFFFF,F,FFFFF:FFFFFF:FFFFF
F,FFFFFF,FF,:F
@A00721:190:HGJW7DSXY:4:2346:32922:13479 2:N:0:TCTGCAAG+GCTGCACT
CTGATACTCGAAGGTCCATCTAGAAGTGGAAAAACTCTGTGGGCTAGATCTCTAGGCCCACATAATTA
CATGTTCGGATATCTAGACTTTAATTCGAAACTATATTCGAATAACGCATGGTATAACATCATAGATG
ACGTAAGCCCTT
+
:,FFFFFFFFF:FFFFFFFFFFFFF:FFFFFFF,F:F:FFF:FFFF:FFFFFFFFFFFFFFF:FFF,F
FFFFFF:F:FFFFFFF,FFF:FFFFFF,FFFFFFFFFFF,FFFFF:,FFFF::FFFFFFFFFFFFF,,
F,,:FF:F,FFF
@A00721:190:HGJW7DSXY:4:2611:30346:30342 2:N:0:TCTGCAAG+GCTGCACT
GTCTGATACTCGAAGGTCCATCTAGAAGTGGAAAAACTCTGTGGGCTAGATCTCTAGGCCCACATAAT
TACATGTTCGGATATCTAGACTTTAATTCGAAACTATATTCGAATAACGCATGGTATAACATCATAGA
TGACGTAAGCCCT
+
FFFFFFF:FFF:FFF:FFFFFFF,:FFFFFFFFFFFFFFFFFFFFFFFFFF:FFFFFFFFFFFFFFFF
FF:FFFFFFF:FFFFFFF:F:F,FFFFFFFF:FFF:FFF,FFFFF::F,F:F,FFFFFF,FFFFFF:,
FFFF,FFFFF::F
@A00721:190:HGJW7DSXY:4:1118:30770:19742 2:N:0:TCTGCAAG+GCTGCACT
CCAAGAGTCTGATACTCGAAGGTCCATCTAGAAGTGGAAAAACTCTGTGGGCTAGATCTCTAGGCCCA
CATAATTACATGTTCGGATATCTAGACTTTAATTCGAAACTATATTCGAATAACGCATGGTATAACAT
CATAGATGACGTAAG
+
FFFFFFFFFFFFFFFFFFFFFFFFFFFFFFFFFFFFFFFFFFFFFFFFFFFFFFFFFFFFFFFFFFFF
FFFFFFFFFF:FFFFFFFFFFFFFFFFFFFF:FFFFF,FFFFFFFFFFFFF:FFFFFFFFFFFFFFFF
FFFF:FFFFFFFFFF
@A00721:190:HGJW7DSXY:4:1425:12671:23688 2:N:0:TCTGCAAG+GCTGCACT
CCAAGAGTCTGATACTCGAAGGTCCATCTAGAAGTGGAAAAACTCTGTGGGCTAGATCTCTAGGCCCA
CATAATTACATGTTCGGATATCTAGACTTTAATTCGAAACTATATTCGAATAACGCATGGTATAACAT
CATAGATGACGTAAG
+
FFFFFFFFFFFFFFFFF:FFFFFFFFFFFFFFFFFFFFFFFFFFFFFFFFFFFFFFFFFFFFFFFFFF
FFFFFFFFFFFFFFFFFF:,FFFFFFF:FFFFF:FFFFFFFFFFFFFFFFFFFF:FFFFFFFFF,FFF
FFFFFFFF:FFFFF:
@A00721:190:HGJW7DSXY:4:1442:6280:19100 2:N:0:TCTGCAAG+GCTGCACT
CCAAGAGTCTGATACTCGAAGGTCCATCTAGAAGTGGAAAAACTCTGTGGGCTAGATCTCTAGGCCCA
CATAATTACATGTTCGGATATCTAGACTTTAATTCGAAACTATATTCGAATAACGCATGGTATAACAT
CATAGATGACGTAAG
+
FFFFFFFFFFFFFFFFFFFFFFFFFFFFFFFFFFFFFFFFFFFFFFFFFFFFFFFFFFFFFFFFFFFF
FFFFFFFFF:FFFFFFFFF:FFFFFFFFFFFFFFFFFFFFF,F,FFFFFFFFFFFFFFFFFFFFFFFF
FFFFFFFFFFFFFFF
@A00721:190:HGJW7DSXY:4:1443:12707:8688 2:N:0:TCTGCAAG+GCTGCACT
CTTACGTCATCTATGATGTTATACCATGCGTTATTCGAATATAGTTTCGAATTAAAGTCTAGATATCC
GAACATGTAATTATGTGGGCCTAGAGATCTAGCCCACAGAGTTTTTCCACTTCTAGATGGACCTTCGA
GTATCAGACTCTTGG
+
FFFFFFFFF:FFFFFFFF,FF:FFFFFFFFFFF:FF:FFFFFFFF:FFFFFFFFFFFFFFFFFFFFFF
FFFFF,F:FFFFFFFFFFFFFFFFFFFFFFFFFFFFFFFFF:FF::FFFFFFFF:FFFFF,FFFFFF:F
,FFFFF:FFFF:FFF
@A00721:190:HGJW7DSXY:4:1508:15085:5760 2:N:0:TCTGCAAG+GCTGCACT
CTTACGTCATCTATGATGTTATACCATGCGTTATTCGAATATAGTTTCGAATTAAAGTCTAGATATCC
GAACATGTAATTATGTGGGCCTAGAGATCTAGCCCACAGAGTTTTTCCACTTCTAGATGGACCTTCGA
GTATCAGACTCTTGG
```

```
+
FFFFFFFFFFFFFFFFFFFFFFFFFFFFFFFFFFFFFFFFFFFFFFFFFFFFFFFFFFFFFFFFFFFF
FFFFFFFFFFFFFFFFFF:FFFFFFFFFFFFFFFFFFFFFFFFFFFFFFFFFFFFFFFFFFFFFFFFF
FFFFFF:FFFFFFF:
@A00721:190:HGJW7DSXY:4:1611:21468:1595 2:N:0:TCTGCAAG+GCTGCACT
CTTACGTCATCTATGATGTTATACCATGCGTTATTCGAATATAGTTTCGAATTAAAGTCTAGATATCC
GAACATGTAATTATGTGGGCCTAGAGATCTAGCCCACAGAGTTTTTCCACTTCTAGATGGACCTTCGA
GTATCAGACTCTTGG
+
FFFFFFFFFFFFFFFFFFFFFFFFFFFFFFFFFFFFFFFFFFFFFFFFFFFFFFFFFFFFFFFFFFFF
FFFFFFFFFFFFFFFFFFFFFFFFFFFFFFFFFFFFFFFFFFFFFFFFFFFFF:FFFFFFFFF,FFFF
FFFFFFFFFFFFFFF
@A00721:190:HGJW7DSXY:4:1621:31412:10332 2:N:0:TCTGCAAG+GCTGCACT
CCAAGAGTCTGATACTCGAAGGTCCATCTAGAAGTGGAAAAACTCTGTGGGCTAGATCTCTAGGCCCA
CATAATTACATGTTCGGATATCTAGACTTTAATTCGAAACTATATTCGAATAACGCATGGTATAACAT
CATAGATGACGTAAG
+
FFFF:FFFFFFFFFFFFFFFFFFFFFFFFFF:FFFFFFFFF:FFFFFFFFFFFFFFFFFFFFF:FFFF
FFFFFFFFFFFFFFFFFFFFFFFFFFFFFFFFFFFFFFFFFFFFFFFFFFFFFFFFFFFFFFFFFFFF
F,FFFFFFFFFFFFF
@A00721:190:HGJW7DSXY:4:2111:5086:36730 2:N:0:TCTGCAAG+GCTGCACT
CCAAGAGTCTGATACTCGAAGGTCCATCTAGAAGTGGAAAAACTCTGTGGGCTAGATCTCTAGGCCCA
CATAATTACATGTTCGGATATCTAGACTTTAATTCGAAACTATATTCGAATAACGCATGGTCTAACAT
CATAGATGACGTAAG
+
FFFFFFFFFFFFFFFFFFFFFFFFFFFFF:FFFFFFFFFFFFFFFFFFFFFFFFFFFFFFF:FFFFFF
FFFFFFFFFFFF::FFF,FFFFFFFFFFFFFF:FF:FF:FFFF::FFFFFFFFFFFFFFFF,FFFFFF
F:FFFFFFF:FFFFF
@A00721:190:HGJW7DSXY:4:2172:27055:5979 2:N:0:TCTGCAAG+GCTGCACT
CCAAGAGTCTGATACTCGAAGGTCCATCTAGAAGTGGAAAAACTCTGTGGGCTAGATCTCTAGGCCCA
CATAATTACATGTTCGGATATCTAGACTTTAATTCGAAACTATATTCGAATAACGCATGGTATAACAT
CATAGATGACGTAAG
+
FFFFFFFFFFFFFFFFFFFFFFFFFFFFFFFFF:FFFFFFFFFFFFFFFFFFFFFFFFFFFFF:FFFF
FFFFFFFFFF,FFFFFFFFFFFFF:FFFFF:F:FFFFFFFFFF:F:FFFFFFFF:FFFFFFFFFFFFF
FFFFF:F:FFF,FFF
@A00721:190:HGJW7DSXY:4:2458:8874:36088 2:N:0:TCTGCAAG+GCTGCACT
CTTACGTCATCTATGATGTTATACCATGCGTTATTCGAATATAGTTTCGAATTAAAGTCTAGATATCC
GAACATGTAATTATGTGGGCCTAGAGATCTAGCCCACAGAGTTTTTCCACTTCTAGATGGACCTTCGA
GTATCAGACTCTTGG
+
FFFFFFFFFFFFFFFFFFFFFFFFFFFFFFFFFFFFFFFFFFFFFFFFFFFFFFFFFFFFFFFFFFFF
FFFFFFFFFFFFFFFFFF:FFFFFFFFFFFFFFFFFFFF:FFFFFFFFFFFFFFFFFFFFFFFFFFFF
FFFFFFFFFFFFFFF
@A00721:190:HGJW7DSXY:4:2473:15854:17456 2:N:0:TCTGCAAG+GCTGCACT
CTTACGTCATCTATGATGTTATACCATGCGTTATTCGAATATAGTTTCGAATTAAAGTCTAGATATCC
GAACATGTAATTATGTGGGCCTAGAGATCTAGCCCACAGAGTTTTTCCACTTCTAGATGGACCTTCGA
GTATCAGACTCTTGG
+
FFFFFFFFFFFFFFFFFFFFFFFFFFFFFFFFFFFFFFFFFFFFFFFFFFFFFFFFFFFFFFFFFFFF
FFFFFFF:FFFFFFFFFFFFFFFFFFFFFFFFFFFFFFFFFFFFFFFF:FFFFFFFFFFFFFFFFFFF
FFFFFFFFFFFFFFF
@A00721:190:HGJW7DSXY:4:2474:6379:6464 2:N:0:TCTGCAAG+GCTGCACT
CCAAGAGTCTGATACTCGAAGGTCCATCTAGAAGTGGAAAAACTCTGTGGGCTAGATCTCTAGGCCCA
```

CATAATTACATGTTCGGATATCTAGACTTTAATTCGAAACTATATTCGAATAACGCATGGTATAACAT
CATAGATGACGTAAG
+
FFFFFFFFFFFFFFFFFFFFFFFFFFFFFFFFFFFFFF:FF:FFFFFFFFFFFFFFFFFFFFFFFFFF
FF:FFFFFFF:FFFFFFFFFFFFFFFFFFFFFFFFFFFFFFFFFFFFFFFFFFFFFFFFFF:FFFFFF
FFFFFFFFFFFFFFF
@A00721:190:HGJW7DSXY:4:2548:28266:10269 2:N:0:TCTGCAAG+GCTGCACT
CTTACGTCATCTATGATGTTATACCATGCGTTATTCGAATATAGTTTCGAATTAAAGTCTAGATATCC
GAACATGTAATTATGTGGGCCTAGAGATCTAGCCCACAGAGTTTTTCCACTTCTAGATGGACCTTCGA
GTATCAGACTCTTGG
+
FFFFFFFFFFFFFFFFFFFFFFFFFFFFFFFFFFFFFFFFFFFFFFFFFFFFFFFFFFFFFFFFFFFF
FFFFFFFFFFFFFFFFFFFFFFFFFFFFFFFFFFFFFFFFFFFFFFFFFFFFFFFFFFFFFFFFFFFF
FFFFFFFFFFFFFFF
@A00721:190:HGJW7DSXY:4:2574:15826:12743 2:N:0:TCTGCAAG+GCTGCACT
CCAAGAGTCTGATACTCGAAGGTCCATCTAGAAGTGGAAAAACTCTGTGGGCTAGATCTCTAGGCCCA
CATAATTACATGTTCGGATATCTAGACTTTAATTCGAAACTATATTCGAATAACGCATGGTATAACAT
CATAGATGACGTAAG
+
FFFFFFFFFFFFFFFFFFFFFFFFFFFFFFFFF:FFFFFFFFFFFFFFFFFFFFFFFFFFFFFFFFFF
FFFFFFFFFFFFFFFFFFF:FFFFFF:FFFFFFFFFFFFFF:FF:FFFFFFFFF,FF,F:FFFF:FFF
FFFFFFFFFFF:FFF
@A00721:190:HGJW7DSXY:4:2609:30318:34397 2:N:0:TCTGCAAG+GCTGCACT
CCAAGAGTCTGATACTCGAAGGTCCATCTAGAAGTGGAAAAACTCTGTGGGCTAGATCTCTAGGCCCA
CATAATTACATGTTCGGATATCTAGACTTTAATTCGAAACTATATTCGAATAACGCATGGTATAACAT
CATAGATGACGTAAG
+
FFF:FFFFFFFFFFFFFFFFFFFFFFFFFFFFFFFFFFFF,FFFF:FFFFFFFFFFFFFFFFFFF:FF
FFFFFFFFFFFFFFFFFFFFFFFFFFFFFFFFFFFFFFFFFFFF:FFFFFFFFFFFFFFFFFFFFFFF
FFFFFFFF:FFFFFF
@A00721:190:HGJW7DSXY:4:2659:15429:8359 2:N:0:TCTGCAAG+GCTGCACT
CCAAGAGTCTGATACTCGAAGGTCCATCTAGAAGTGGAAAAACTCTGTGGGCTAGATCTCTAGGCCCA
CATAATTACATGTTCGGATATCTAGACTTTAATTCGAAACTATATTCGAATAACGCATGGTATAACAT
CATAGATGACGTAAG
+
FFFFFFFFFFFFFFFFFFFFFFFFFFFFFFFFFFFFFFFFFFFFFFFFFFFFFFFFFFFFFFFFFFFF
FFFFFFFFFFFFFFFFFFFFFFFFFFFFFFFFFFFFFFFFFFFFFFFFFFFFFFFFFFFFFFFFFFFF
F:FFFFFFFF:FFFF
@A00721:190:HGJW7DSXY:4:1110:3025:16094 2:N:0:TCTGCAAG+GCTGCACT
CATCTATGATGTTATACCATGCGTTATTCGAATATAGTTTCGAATTAAAGTCTAGATATCCGAACATG
TAATTATGTGGGCCTAGAGATCTAGCCCACAGAGTTTTTCCACTTCTAGATGGACCTTCGAGTATCAG
ACTCTTGGTCCTGTA
+
FFFFFFFFFFFFFFFFFFFFFFFFFFFFFFFFFFFFFFFFFFFFFFFFFFFFFFFFFFFFFFFFFFFF
FFFFFFFFFFFFFFFFFFFFFFFFFFFFFFFFFFFFFFFFFFFFFFFFF:FFFFFFFFFFFFF:FFFF
FFFFFFF:FF:FFFF
@A00721:190:HGJW7DSXY:4:1127:31792:15687 2:N:0:TCTGCAAG+GCTGCACT
CATCTATGATGTTATACCATGCGTTATTCGAATATAGTTTCGAATTAAAGTCTAGATATCCGAACATG
TAATTATGTGGGCCTAGAGATCTAGCCCACAGAGTTTTTCCACTTCTAGATGGACCTTCGAGTATCAG
ACTCTTGGTCCTGTA
+
FFFFFFFFFFFFFFFFFFFFFFFFFFFFFFFFFFFFFFFFFFFFFFFFFFFFFFFFFFFFFFFFFFFF
FFF:FFFFFFFFFFFFFFFFFFFFFFFFFFFFFFFFFFFFFFFFFFFFFFFFFFFFFFFFFFFFFFFF
FFFFFFFFFFFFFFF

```
@A00721:190:HGJW7DSXY:4:1251:32235:1705 2:N:0:TCTGCAAG+GCTGCACT
CATCTATGATGTTATACCATGCGTTATTCGAATATAGTTTCGAATTAAAGTCTAGATATCCGAACATG
TAATTATGTGGGCCTAGAGATCTAGCCCACAGAGTTTTTCCACTTCTAGATGGACCTTCGAGTATCAG
ACTCTTGGTCCTGTA
+
FFFFFFFFFFFFFF:FFFFFFFFFFFFFFFFFFFFFFFFFFFFFFFFF,FFFFFFFFFFFFFFFFFFF
FFFFFFFFFFFFFFFFFFFFFFFFFF:FFFFFFFFFFFFFFFFFFFFFFFFFFFFFFFFFFFFFFFF:
FFFFFFFFF:FFFFF
@A00721:190:HGJW7DSXY:4:1403:1542:7169 2:N:0:TCTGCAAG+GCTGCACT
CATCTATGATGTTATACCATGCGTTATTCGAATATAGTTTCGAATTAAAGTCTAGATATCCGAACATG
TAATTATGTGGGCCTAGAGATCTAGCCCACAGAGTTTTTCCACTTCTAGATGGACCTTCGAGTATCAG
ACTCTTGGTCCTGTA
+
FFFFFFFFFFFFFFFFFF:,FFFFFFFFFFFFFF:FFFFFFFFFFFFFFFFFFFFFFFFFFF:FFFFF
FFFFFFFFFFFFFFFFFFFFFFFFFFFFFFFFFFFFFFFFFFFFFFFFFFFFFFFFFFFFFF:FFFFF
FFFFFFFFFFFFFF:
@A00721:190:HGJW7DSXY:4:1615:19117:18380 2:N:0:TCTGCAAG+GCTGCACT
CATCTATGATGTTATACCATGCGTTATTCGAATATAGTTTCGAATTAAAGTCTAGATATCCGAACATG
TAATTATGTGGGCCTAGAGATCTAGCCCACAGAGTTTTTCCACTTCTAGATGGACCTTCGAGTATCAG
ACTCTTGGTCCTGTA
+
FFFFFFFFFFFFFFFFFFFFFFFFFFFFFFFFFFFFFFFFFFFF:FFFFFFFFFFFFFFFFFF:FFFF
FFFFFFFFFFFFFFFFFFFFFFFFFFFFFFFFFFFFFFFFFFFFFFFFFFFFFFFFFFFFFFFFFFFF
FFFFFFFFFFF:FFF
@A00721:190:HGJW7DSXY:4:2513:18141:13025 2:N:0:TCTGCAAG+GCTGCACT
CATCTATGATGTTATACCATGCGTTATTCGAATATAGTTTCGAATTAAAGTCTAGATATCCGAACATG
TAATTATGTGGGCCTAGAGATCTAGCCCACAGAGTTTTTCCACTTCTAGATGGACCTTCGAGTATCAG
ACTCTTGGTCCTGTA
+
FFFFFFFFFFFFFFFFFFF,FFFFFFFFFFFFFFFFFFFFFFFFFFFFFFFFFFFFFFFFFFFFFFFF
F,FFFFFFFFFFFFFFFFFFFFFFFFFFFFFFFFFFFFFFFFFFFFFFFFFFFFFFFFF:FFFFFFFF
FFFFFFFFFF:FFFF
@A00721:190:HGJW7DSXY:4:2562:18403:32894 2:N:0:TCTGCAAG+GCTGCACT
CATCTATGATGTTATACCATGCGTTATTCGAATATAGTTTCGAATTAAAGTCTAGATATCCGAACATG
TAATTATGTGGGCCTAGAGATCTAGCCCACAGAGTTTTTCCACTTCTAGATGGACCTTCGAGTATCAG
ACTCTTGGTCCTGTA
+
FFF:FFFFFFFFFFFFFF:FFFFFFFFFFFFFFFFFFFFFFFFFFFFFFFFFFFFF:FFFFFFFFFFF
,FFFFFFFFFFFFFFFFFFFFFFFFF:FFFFFFFFFFFFF:FF:FFFF:FFFFFFFFFF:FFFFFFFF
FFFFFFFFF:FFFFF
@A00721:190:HGJW7DSXY:4:1114:32886:3991 2:N:0:TCTGCAAG+GCTGCACT
CTTACAGGACCAAGAGTCTGATACTCGAAGGTCCATCTAGAAGTGGAAAAACTCTGTGGGCTAGATCT
CTAGGCCCACATAATTACATGTTCGGATATCTAGACTTTAATTCGAAACTATATTCGAATAACGCATG
GTATAACATCATAGA
+
FFFFFFFFFFFF,FFFFFFFFFFFFFFFFFFFFFFFFFFFFFFFFFFFFFFFFFFFFFFFFFF::FF
F:FFFFFFFF:FFFFFFFFFFFFFFFFFF,FFFFFFFFFFF:FFFF,FFFFF,FFFFFFFFFF:FF,F
FFFFFFFFFFF:FF:
@A00721:190:HGJW7DSXY:4:1348:15619:3521 2:N:0:TCTGCAAG+GCTGCACT
CTTACAGGACCAAGAGTCTGATACTCGAAGGTCCATCTAGAAGTGGAAAAACTCTGTGGGCTAGATCT
CTAGGCCCACATAATTACATGTTCGGATATCTAGACTTTAATTCGAAACTATATTCGAATAACGCATG
GTATAACATCATAGA
+
FFFFFFFFFFFFFFFFFFFFFFFFFFFFFFFFFFFFFFFFFFFFFFFFF:FFFFFFFFFFFFFFFFFF
```

FFFFFFFFFFFFFFFFFFFFFFFFFFFFFFFFFFFFFFFFFFFFFFFFF:F,FF:FFFFFFFFFFFF
FFFFFFFFFFFFFFF
@A00721:190:HGJW7DSXY:4:1464:6949:28087 2:N:0:TCTGCAAG+GCTGCACT
CTTACAGGACCAAGAGTCTGATACCCGAAGGTCCATCTAGAAGTGGAAAAACTCTGTGGGCTAGATCT
CTAGGCCCACATAATTACATGTTCGGATATCTAGACTTTAATTCGAAACTATATTCGAATAACGCATG
GTATAACATCATAGA
+
FFFFFFFFFFFFFFFFFFFFFFFFF:FFFFFFFFFFFFFFFFFFFFFFFFFFFFFFFFFFFFFFFFFF
FFFFFFFFFFFFFFFFFFFFFFFFFFFFFFFFFFFFFFFFFFFFFFFFFFFFFFFFFFFFFFF:FFFF
FFFFFFFFFFF:FFF
@A00721:190:HGJW7DSXY:4:1469:10628:10567 2:N:0:TCTGCAAG+GCTGCACT
CTTACAGGACCAAGAGTCTGATACTCGAAGGTCCATCTAGAAGTGGAAAAACTCTGTGGGCTAGATCT
CTAGGCCCACATAATTACATGTTCGGATATCTAGACTTTAATTCGAAACTATATTCGAATAACGCATG
GTATAACATCATAGA
+
FFFFFF:,FFFFF:F,,F::F,FFFFFFF:F,:FFFFF,FFF:::FFFFFFFFF,:,F,:F,:FFFFF
FFFFF:FFFFFFFFFFFFFF,,FFF:FFFFFFFFFF::FFFFF:,FFF:F,FFF,F,FFFFF:F:F:F
FFF,FFFFFFFFF:F
@A00721:190:HGJW7DSXY:4:1557:1072:34538 2:N:0:TCTGCAAG+GCTGCACT
CTTACAGGACCAAGAGTCTGATACTCGAAGGTCCATCTAGAAGTGGAAAAACTCTGTGGGCTAGATCT
CTAGGCCCACATAATTACATGTTCGGATATCTAGACTTTAATTCGAAACTATATTCGAATAACGCATG
GTATAACATCATAGA
+
FFFFFFFFFFFFFFFFFFFFFFFFFFFF:FFFFFFFFFFFFFFFFFFFFF:FFFFFFFFFFFFFFFFF
FFFFFFFFFFFFFFFFFFFFFFFFFFFFFFFFFFFFFFFFFFFFFFFFFF:FFF:FFFFFFF:FFF,F
FFFFFFFFFFFFFF:
@A00721:190:HGJW7DSXY:4:2130:19081:32064 2:N:0:TCTGCAAG+GCTGCACT
CTTACAGGACCAAGAGTCTGATACTCGAAGGTCCATCTAGAAGTGGAAAAACTCTGTGGGCTAGATCT
CTAGGCCCACATAATTACATGTTCGGATATCTAGACTTTAATTCGAAACTATATTCGAATAACGCATG
GTATAACATCATAGA
+
FFFFFFFFFFFFFFFFFFFFFFFFFFFFFFFFFFFFFFFFFFFFFFFFFFFFFFFFFFFFFFFFFFFF
FFFFFFFFFFFFFFFFFFFFFFFFFFFFFFFFFFFFFFFFFFFFF:FFFFFFFFFFFFFFFFFFFFFF
FFFFFFFFFFFFFF:
@A00721:190:HGJW7DSXY:4:2403:10429:30483 2:N:0:TCTGCAAG+GCTGCACT
CTTACAGGACCAAGAGTCTGATACTCGAAGGTCCATCTAGAAGTGGAAAAACTCTGTGGGCTAGATCT
CTAGGCCCACATAATTACATGTTCGGATATCTAGACTTTAATTCGAAACTCTATTCGAATAACGCATG
GTATAACATCATAGA
+
FFFFFFFFFFFFFFFFFFFFFFFFFFFFFFFFFFFFFFFFFFFFFFFFFFFFFFF:FFFFFFFFFFF
FFFFFFFFFFFFFFFFFFFFFFFFFFFFF,FFFF:FFFFFFFFFFFFFFF:,F:FFFFFFFFFFFFF,F
FF:FFFFFFFFFFF:
@A00721:190:HGJW7DSXY:4:1154:30309:34225 2:N:0:TCTGCAAG+GCTGCACT
AGACCTTACAGGACCAAGAGTCTGATACTCGAAGGTCCATCTAGAAGTGGAAAAACTCTGTGGGCTAG
ATCTCTAGGCCCACATAATTACATGTTCGGATATCTAGACTTTAATTCGAAACTATATTCGAATAACG
CATGCTATAACATCA
+
FFFFFFFFFFFFFFFFFFFFFFFFFFFFFFFFFFFFFFFFFF:FFF:FFFFFFF:FFFFFFFFFFFFFF
FFFFFFFFFFFFFFFFFFFFFFFFFFFFFFFFFFFF:FFFFFFFFFFFFFFFFFFFFF:FFFFFFFFFF
FFFFFFFFFFFFFFF
@A00721:190:HGJW7DSXY:4:1154:30770:36495 2:N:0:TCTGCAAG+GCTGCACT
AGACCTTACAGGACCAAGAGTCTGATACTCGAAGGTCCATCTAGAAGTGGAAAAACTCTGTGGGCTAG
ATCTCTAGGCCCACATAATTACATGTTCGGATATCTAGACTTTAATTCGAAACTATATTCGAATAACG
CATGCTATAACATCA

```
+
FFFFFFFFFFFFFFFFFFFFFFFFFFFFFFFFFFFFFFFFFFFFFFFFFFFFFFFFFFFFFFFFFFFF
FFFFFFFFFFFFFFFFFFFFFFFFFFFFFFFFFFFFFFFFFFFFFFFFFFFFFF:FFFFFFFFF:FFF
FFFFFFFF:FFFFFF
@A00721:190:HGJW7DSXY:4:1174:10963:24893 2:N:0:TCTGCAAG+GCTGCACT
AGACCTTACAGGACCAAGAGTCTGATACTCGAAGGTCCATCTAGAAGTGGAAAAACTCTGTGGGCTAG
ATCTCTAGGCCCACATAATTACATGTTCGGATATCTAGACTTTAATTCGAAACTATATTCGAATAACG
CATGCTATAACATCA
+
FFFFFFFFFFFFFFFFFFFFFFFFFFFFFFFFFFFFFFFFFFFFFFFFFFFFFFFFFFFFFFFFFFFF
FFFFFFFFFFFFFFFFFFFFFFFFFFFFFFFFFFFFF:FFFFFFFFFFFFFFFFFFFFFFFFFFFFFF
FFFFFF,FFFFFF:F
@A00721:190:HGJW7DSXY:4:1204:27127:34131 2:N:0:TCTGCAAG+GCTGCACT
AGACCTTACAGGACCAAGAGTCTGATACTCGAAGGTCCATCTAGAAGTGGAAAAACTCTGTGGGCTAG
ATCTCTAGGCCCACATAATTACATGTTCGGATATCTAGACATTAATTCGAAACTATATTCGAATAACG
CATGGTATAACATCA
+
FFFFFFFFFFFFFFFFFFFFFFFFFFFFFFFFFFFFFFFFFFFFFFFFFFFFFFFFFFFFFFFFFFFF
FFFFFFFFFFFFFFFFFFFFFFFFFFFFFFFFFFFFFFFF,FFFFFFFFFFFF:FFFFF:FFFFFFFF
FFFFFFFFFFFFFFF
@A00721:190:HGJW7DSXY:4:1434:7310:27586 2:N:0:TCTGCAAG+GCTGCACT
AGACCTTACAGGACCAAGAGTCTGATACTCGAAGGTCCATCTAGAAGTGGAAAAACTCTGTGGGCTAG
ATCTCTAGGCCCACATAATTACATGTTCGGATATCTAGACTTTAATTCGAAACTATATTCGAATAACG
CATGGTATAACATCA
+
F:FFFFFFFFFFFFFFFFFFFFFFFFFFFFFFFFFFFFFFFFFFFFFFFFFFFFFFFFFFFFFFFFFF
FFFFFFFFFFFFFFFFFFFFFFFFFFFFFFFF:FFFFFFFFFFFFFFFFFFFFF:FFFFF:FFFFFFF
FFFFFFFFFFFFFFF
@A00721:190:HGJW7DSXY:4:2143:4065:24283 2:N:0:TCTGCAAG+GCTGCACT
AGACCTTACAGGACCAAGAGTCTGATACTCGAAGGTCCATCTAGAAGTGGAAAAACTCTGTGGGCTAG
ATCTCTAGGCCCACATAATTACATGTTCGGATATCTAGACTTTAATTCGAAACTATATTCGAATAACG
CATGCTATAACATCA
+
FFFFFFFFFFFFFFFFFFFFFFFFFFFFFFFFFFFFFFFFFFFFFFFFFFFFFFFFFFFFFFFFFFFF
FFFFFFFFFFFFFFFFFFFFFFFFFFFFFFFFFFFFFFFFFFFFFFFFFFFFFFFFFFFFFFFFFFFF
FFFFFFFFFFFFFFF
@A00721:190:HGJW7DSXY:4:2163:8006:30170 2:N:0:TCTGCAAG+GCTGCACT
AGACCTTACAGGACCAAGAGTCTGATACTCGAAGGTCCATCTAGAAGTGGAAAAACTCTGTGGGCTAG
ATCTCTAGGCCCACATAATTACATGTTCGGATATCTAGACTTTAATTCGAAACTATATTCGAATAACG
CATGCTATAACATCA
+
FFFFFFFFFFFFFFFFFFFFFFFFFFFFFFFFFFFFFFFFFFFFFFFFFFFFFFFFFFFFFFFFFFFF
FFFFFFFFFFFFFFFFFFFFFFFFFFFFFFFFFFFFFFFFFFFFFFFFFFF:F:FF:FFFFFFFFFF:
FFFFFF:FFFFFFFF
@A00721:190:HGJW7DSXY:4:2228:28908:11945 2:N:0:TCTGCAAG+GCTGCACT
AGACCTTACAGGACCAAGAGTCTGATACTCGAAGGTCCATCTAGAAGTGGAAAAACTCTGTGGGCTAG
ATCTCTAGGCCCACATAATTACATGTTCGGATATCTAGACTTTAATTCGAAACTATATTCGAATAACG
CATGCTATAACATCA
+
FFFFFFFFFFFFFFFFFFFFFFFFFFFFFFFFFFFFFFFFFFFFFFFFFFFFFFFFFFFFFFFFFFFF
FFFFFFFFFFFFFFFFFFFFFFFFFFFFFFFFFFFFFFFFFFFFFFFFFFFFFF:,FFFFFFFFFFFF
FFFFFFFF:FFFFFF
@A00721:190:HGJW7DSXY:4:2403:17933:8469 2:N:0:TCTGCAAG+GCTGCACT
AGACCTTACAGGACCAAGAGTCTGATACTCGAAGGTCCATCTAGAAGTGGAAAAACTCTGTGGGCTAG
```

```
ATCTCTAGGCCCACATAATTACATGTTCGGATATCTAGACTTTAATTCGAAACTATATTCGAATAACG
CATGCTATAACATCA
+
FFFFFFFFF:FFFFFF:FFFFFFFFFFFFFFFFFFFFFFFFFFFFFFFFFFFFFFFFFFFFFFFFFFF
FFFFFFFFFFFFFFFFFFFFFFFFFFFFFFFF,FFFFFFFFFFFFFFFFF:FFF,FFFFFFFFF:FFF
FFFFF::FFFFFFFF
@A00721:190:HGJW7DSXY:4:2403:17770:8563 2:N:0:TCTGCAAG+GCTGCACT
AGACCTTACAGGACCAAGAGTCTGATACTCGAAGGTCCATCTAGAAGTGGAAAAACTCTGTGGGCTAG
ATCTCTAGGCCCACATAATTACATGTTCGGATATCTAGACTTTAATTCGAAACTATATTCGAATAACG
CATGCTATAACATCA
+
FFFFFFFFFFFFFFFFFFFFFFFFFFFFFFFFFFFFFFFFFFFFFFFFFFFFFFFFFFFFFFFFFFFF
F:FFFFFFFFFFFFFFFFF,FF:FFFF:F:FF,FFFFFFFFF:F:FFF:FFFFFFFF:F:FFFFFFFF
FF,FFFFFFF:FF:F
@A00721:190:HGJW7DSXY:4:2431:10890:30060 2:N:0:TCTGCAAG+GCTGCACT
AGACCTTACAGGACCAAGAGTCTGATACTCGAAGGTCCATCTAGAAGTGGAAAAACTCTGTGGGCTAG
ATCTCTAGGCCCACATAATTACATGTTCGGATATCTAGACTTTAATTCGAACCTCTATTCGAATAACG
CATGCTATAACATCA
+
FFFFFFF:FFFFFFFFFFFFFFFFFFFFFFFFFFFFFF:FFFFFFFFFFFFFFFFFFFFFFFFFFFFF
,FFFFFFFFFFFFFFFFFFFFFFFFFFFFFFFFFFF,FFFFFFFFFFFFFF,FF,FFFFFFFFFFFFF
FFFFFFF:FFFFFFF
@A00721:190:HGJW7DSXY:4:2540:20980:35603 2:N:0:TCTGCAAG+GCTGCACT
AGACCTTACAGGACCAAGAGTCTGATACTCGAAGGTCCATCTAGAAGTGGAAAAACTCTGTGGGCTAG
ATCTCTAGGCCCACATAATTACATGTTCGGATATCTAGACTTTAATTCGAAACTCTATTCGAATAACG
CATGGTATAACATCA
+
FFFFFFFFFFFFFFFFFFFFFFFFFFFFFFFFFFFFFFFFFF:FFFFF:FFFF:FFFFFFFFFFFFFF
FFFFFFFFFFFFFFFF:FFFFFFF,FFFFFFFFFFFFFFFFFFFFFFFFFFFFF,FF:FFFFFFFFFF
FFFFFFFFFFFFF:F
@A00721:190:HGJW7DSXY:4:2561:30101:7498 2:N:0:TCTGCAAG+GCTGCACT
AGACCTTACAGGACCAAGAGTCTGATACTCGAAGGTCCATCTAGAAGTGGAAAAACTCTGTGGGCTAG
ATCTCTAGGCCCACATAATTACATGTTCGGATATCTAGACTTTAATTCGAAACTATATTCGAATAACG
CATGGTATAACATCA
+
FFFFFFFFFFFFFFFFFFFFFFFFFFFFFFFFFFFFFFFFFFFFFFFFFFFFFFFFFFFFFFFFFFFF
FFFFFFFFFFFFFFFFFFFFFFFFFFFFFFFFFFFFFFFFFFFFFFFFFFFFFF:FFFFFFFFFFFFF
FFFFFFFFFFFFFFF
@A00721:190:HGJW7DSXY:4:2607:22191:10551 2:N:0:TCTGCAAG+GCTGCACT
AGACCTTACAGGACCAAGAGTCTGATACTCGAAGGTCCATCTAGAAGTGGAAAAACTCTGTGGGCTAG
ATCTCTAGGCCCACATAATTACATGTTCGGATATCTAGACTTTAATTCGAAACTATATTCGAATAACG
CATGGTATAACATCA
+
FFFFFFFFFFFFFFFFFFFFFFFFFFFFFFFFF,FFFFFFFFFFFFFFF,FFFFFFFFFF:,::FFFF
FFFFFFFFFFFFFFFF:FFFFFFFFFFFFFFFFFFFFFFFF:FFFFF,FFF:FFFFFFFFFFFFFF:F
FF:FFFFFFFFFFFF
@A00721:190:HGJW7DSXY:4:1567:30870:8797 2:N:0:TCTGCAAG+GCTGCACT
TCTCACCTGAGTCCAGACCTTACAGGACCAAGAGTCTGATACTCGAAGGTCCATCTAGAAGTGGAAAA
ACTCTGTGGGCTAGATCTCTAGGCCCACATAATTACATGTTCGGATATCTAGACTTTAATTCGAAACT
ATATTCGAATAACGC
+
FFFFFFFFFFFFFFFFFFFFFFFFFFFFFFFFFFFFFFFFFFFFFFFFFFFFFFFFFFFFFFF:FFF,
FFFFFFFFFFFFFFFFFFFFFFFFFFFFFF:FFFFFFFFFFFFFFFFFFFFFFFFFFFFFFF,FFFFF
FFF:FFFFFFFFFFF
```

```
@A00721:190:HGJW7DSXY:4:1547:23665:25473 2:N:0:TCTGCAAG+GCTGCACT
TCTCACCTGAGTCCAGACCTTACAGGACCAAGAGTCTGATACTCGAAGGTCCATCTAGAAGTGGAAAA
ACTCTGTGGGCTAGATCTCTAGGCCCACATAATTACATGTTCGGATATCTATACTTTAATTAGAAACT
ATATTCGAATAAAG
+
FFFFFFFFFFFFFFFFFFFFFFFFFFFFFFFFFFFFFFFF:FFFFFFFFFFFFFFFFFF:FFFFFFF
F,F,F:FFFFFFFFFF,F:FF::FFFFFFFFF,FF:F:,FF,F:FFFF,FF,F,FFFFF:F,,FFF::
F:FF:,,FFFFF,:
@A00721:190:HGJW7DSXY:4:1616:23529:4476 2:N:0:TCTGCAAG+GCTGCACT
TATATTCTCACCTGAGTCCAGACCTTACAGGACCAAGAGTCTGATACTCGAAGTTCCATCTAGAAGTG
GAAAAACTCTGTGGGCTAGATCTCTAGGCCCACATAATTACATGTTCGGATATCTAGACTTTAATTCG
AAACTATATTCGAAT
+
FFFFFFFFFFFFFFFFFFFFFFFFFFFFFFFFFFFFFFFFFFFFFFFFFFFFF,FFFFFFFFFFFFFF
FFFFFFFFFFFFFFFFFFFFFFFFFFFFFFFFFFFFFFFFFFFFFFFFFFFFFFFFFFFFFFFFFFFF
FFFFF:FFFFFFFFF
@A00721:190:HGJW7DSXY:4:1629:16396:17581 2:N:0:TCTGCAAG+GCTGCACT
TATATTCTCACCTGAGTCCAGACCTTACAGGACCAAGAGTCTGATACTCGAAGGTCCATCTAGAAGTG
GAAAAACTCTGTGGGCTAGATCTCTAGGCCCACATAATTACATGTTCGGATATCTAGACTTTAATTCG
AAACTCTATTCGAAT
+
FFFFFFFFFFFFFFFFFFFFFFFFFF:FFFFFFFFFFFFFFFFFFFFFFFFFFFFFFFFFF,FF,:FFF
FFFFF,FFFFFFFFFFFFFFFFFFFFFFFFFFF:FFFFFFFFFFFFFFFFFFFFFFFFFFFFFFFFFF
F,FFF,F,:,FFFFF
@A00721:190:HGJW7DSXY:4:1644:6135:1720 2:N:0:TCTGCAAG+GCTGCACT
TATATTCTCACCTGAGTCCAGACCTTACAGGACCAAGAGTCTGATACTCGAAGGTCCATCTAGAAGTG
GAAAAACTCTGTGGGCTAGATCTCTAGGCCCACATAATTACATGTTCGGATATCTAGACTTTAATTCG
AAACTATATTCGAAT
+
FFFFFFFFFFFFFFFFFFFFFFFFFFFFFFFFFFFFFFFFFFFFF:FFFFFFFFFFFFFFFFFFFFFF
FFFF::FFFFFFFFFFFFFFFFFFF:FFFFFFFFFFFFFFFFFFFFFFFFFFFFFFF:FF:FFFFFFFF
FFFFF:F,FFFFFFF
@A00721:190:HGJW7DSXY:4:1667:17101:9659 2:N:0:TCTGCAAG+GCTGCACT
TATATTCTCACCTGAGTCCAGACCTTACAGGACCAAGAGTCTGATACTCGAAGGTCCATCTAGAAGTG
GAAAAACTCTGTGGGCTAGATCTCTAGGCCCACATAATTACATGTTCGGATATCTAGACTTTAATTCG
AAACTATATTCGAAT
+
FFFFFFFFFFFFFFFFFFFFFFFFFFFFFFFFFFFFFFFFFFFFFFFFFFFFFFFFFFF:FFFFFFFF
FFFF:FFFFFFFFFFFFFFFFFFFFFFFFFFF:FFFFF:FFFFFFFFFFFFFFFFFFFFFFFFFFFFFF
FFFFFFFFFFFFFFF
@A00721:190:HGJW7DSXY:4:2121:29523:32737 2:N:0:TCTGCAAG+GCTGCACT
TATATTCTCACCTGAGTCCAGACCTTACAGGACCAAGAGTCTGATACTCGAAGGTCCATCTAGAAGTG
GAAAAACTCTGTGGGCTAGATCTCTAGGCCCACATAATTACATGTTCGGATATCTAGACTTTAATTCG
AAACTATATTCGAAT
+
FFF:FFFFFFFFFFFFFFFFFFFFFFFFFFFFFFFFFFFFFFFFFFFFFFFFFFFFFFFFFFFFFFFF
FFFFFFFFFFFFFFFFFFFFFFFFFFFFFFFFFFFFFFFFFFFFFFFFFFFFFFFFFFFFFFFFFFFF
FFFF::FF,FFFFFF
@A00721:190:HGJW7DSXY:4:2474:19633:36777 2:N:0:TCTGCAAG+GCTGCACT
TATATTCTCACCTGAGTCCAGACCTTACAGGACCAAGAGTCTGATACTCGAAGGTCCATCTAGAAGTG
GAAAAACTCTGTGGGCTAGATCTCTAGGCCCACATAATTACATGTTCGGATATCTAGACTTTAATTCG
AAACTATATTCGAAT
+
FFFFFFFFFFFFFFFFFFFFFFFFFFFFFFFFFFFFFFFFFFFFFFFFFFFFF:FFFFFFFFFFFFF:F
```

```
FFFFFFFFFFFFFFFFFFFFFFFFFFFFFFFFFFFFFFFFF::FFFFFFFFFFFFFFFFFFFFFFFFF
FFFFFFFF:FFFFFF
@A00721:190:HGJW7DSXY:4:2577:2826:11428 2:N:0:TCTGCAAG+GCTGCACT
TATATTCTCACCTGAGTCCAGACCTTACAGGACCAAGAGTCTGATACTCGAAGGTCCATCTAGAAGTG
GAAAAACTCTGTGGGCTAGATCTCTAGGCCCACATAATTACATGTTCGGATATCTAGACTTTAATTCG
AAACTATATTCGAAT
+
F,FFFFFFFFFFFFFFFFFFFFFF:FFFFFFFFFFFFFFFFFFFFFFFFFFFFFFFFFFFFFFFFFFF
F:FFFFFFFFFFFFFFFFFFFFFFFFF:FFFFFFF,FFFFFFF:FFFFFFF:FFFFFFFFFFFFFFFF:
FFFF,:FFF,FFFFF
@A00721:190:HGJW7DSXY:4:1462:18313:10410 2:N:0:TCTGCAAG+GCTGCACT
ATATATTCTCACCTGAGTCCAGACCTTACAGGACCAAGAGTCTGATACTCGAAGGTCCATCTAGAAGT
GGAAAAACTCTGTGGGCTAGATCTCTAGGCCCACATAATTACATGTTCGGATATCTAGACTTTAATTC
GAAACTATATTCGAA
+
FFFFFFFFFFFFFFFFFFFFFFFFFFFFFFFFFFFFFFFFFFFFFFFFFFFFFFFFFFFFFFFFFFF:
FFFFF:FFFFFFFFFFFFFFFFFFFFFFFFFFFFFFFFFFFFFFFFFFFFFFFFFFFFFFFFFFFFFF
FFF:FF:F:FFFFFF
@A00721:190:HGJW7DSXY:4:1668:20302:6433 2:N:0:TCTGCAAG+GCTGCACT
ATATATTCTCACCTGAGTCCAGACCTTACAGGACCAAGAGTCTGATACTCGAAGGTCCATCTAGAAGT
GGAAAAACTCTGTGGGCTAGATCTCTAGGCCCACATAATTACATGTTCGGATATCTAGACTTTAATTC
GAAACTATATTCGAA
+
FFFFFFFFFFFFFFFFFFFFF:FFFFFFFFFFFFFFFFFFFFFFFFFFFFFFFFF:FFF:FF:FFFFFF
FFFFFFFFFFFFFF:FFFF:FFFFFFFFFFFF:FFFFFFFFFF:FFFFFFFFFFFFFFFFFF,F:FF:
FFFFFF:FFFFFF:F
@A00721:190:HGJW7DSXY:4:2175:30364:11459 2:N:0:TCTGCAAG+GCTGCACT
ATATATTCTCACCTGAGTCCAGACCTTACAGGACCAAGAGTCTGATACTCGAAGGTCCATCTAGAAGT
GGAAAAACTCTGTGGGCTAGATCTCTAGGCCCACATAATTACATGTTCGGATATCTAGACTTTAATTC
TAAACTATATTCGAA
+
FFFFFFFFFFFFFFFFFFFFFFFFFFFFFFFFFFFFFFF:FFFFFFFFFFFFFFFFFFFFFFFFFFFFF
FFFFF,,FFFFFFFFFFFFFFFFFFFFFFFFFFFFFFFFFFFFFFFF,FFFFF:FFFFFFFF:FFFF
,FFFFFFF:FFF,F:
@A00721:190:HGJW7DSXY:4:1509:29866:28103 2:N:0:TCTGCAAG+GCTGCACT
AATATATTCTCACCTGAGTCCAGACCTTACAGGACCAAGAGTCTGATACTCGAAGGTCCATCTAGAAG
TGGAAAAACTCTGTGGGCTAGATCTCTAGGCCCACATAATTACATGTTCGGATATCTAGACTTTAATT
CGAAACTATATTCGA
+
FFFFFFFFFFFF:FFFFFFFFFFFFFFFF:FFFFFFFFFFFFFFFFFFFFFFFFFFFFFFFFFFFFFFF
,FFFFFFFFFFFFFFFFFFFFFFFFFFFFFFFFFFF:FFFFFFFFFFFFFFFFFFFFFFFFFFFFFFFF
FFFFF:F,F:FFFFF
@A00721:190:HGJW7DSXY:4:1608:29894:35102 2:N:0:TCTGCAAG+GCTGCACT
AATATATTCTCACCTGAGTCCAGACCTTACAGGACCAAGAGTCTGATACTCGAAGGTCCATCTAGAAG
TGGAAAAACTCTGTGGGCTAGATCTCTAGGCCCACATAATTACATGTTCGGATATCTAGACTTTAATT
CGAAACTATATTCGA
+
FFFFFFFFFFFFFFFFFFFFFFFFFFFFFFFFFFFFFFF,FFFFFFFFFFFFFFFFFFFFF:FFFFF:FF
FFFFFFFFFFFFFFFFFFFFFFFFFFFFFFFFFFFFFFF,:FFF:FFFFFFFFFFFFFFFFFFFFFFFFF
FFFFFFFFFFFFFFF
@A00721:190:HGJW7DSXY:4:2378:15546:30138 2:N:0:TCTGCAAG+GCTGCACT
AATATATTCTCACCTGAGTCCAGACCTTACAGGACCAAGAGTCTGATACTCGAAGGTCCATCTAGAAG
TGGAAAAACTCTGTGGGCTAGATCTCTAGGCCCACATAATTACATGTTCGGATATCTAGACTTTAATT
CGAAACTATATTCGA
```

```
+
FFFFFFFFF:FFFFFFFFFFFFFFFFFFFFFFFFFFFFFFFFFFFFFFFFFFFFFFFFFFFFFFFFF
F:FFFFFFFFFFFFFF:FFFFFFFFF:FFFF,F:FFFFFFFFF:FFFFF,FFFFFFFF,FFFFFFFFF
F::FF:FFFFFF:FF
@A00721:190:HGJW7DSXY:4:2504:10999:36761 2:N:0:TCTGCAAG+GCTGCACT
AATATATTCTCACCTGAGTCCAGACCTTACAGGACCAAGAGTCTGATACTCGAAGGTCCATCTAGAAG
TGGAAAAACTCTGTGGGCTAGATCTCTAGGCCCACATAATTACATGTTCGGATATCTAGACTTTAATT
CGAAACTATATTCGA
+
FFFFFFFFFFFF:FFFFFFFFFFFFFFFFFFFFFFFFFFFFFFFFFFF:FFFFFFFFFFFFFFFFFFF
FFFFFFFFFFFFFFFFFFF:FFFFFFFFFFFFFFFFFF:FFFFFFFFFFFFFF,FF:FF:FFFFFFFF
FFFFFFFF,FFFFFF
@A00721:190:HGJW7DSXY:4:2275:14027:27853 2:N:0:TCTGCAAG+GCTGCACT
ATAATATATTCTCACCTGAGTCCAGACCTTACAGGACCAAGAGTCTGATACTCGAAGGTCCATCTAGA
AGTGGAAAAACTCTGTGGGCTAGATCTCTAGGCCCACATAATTACATGTTCGGATATCTAGACTTTAA
TTCGAAACTATATTC
+
FFFFFFFFFFFFFFFFFFFFFFFFFFFFFFFFFFFFFFFFFFFFFFFFFF:FFFFFFFFFFFFFFFFF
FFFFFFFFFFFFFFFFFFFFFFFFFFFFFFFFFFFF:FFFFFFFFF,FFFFFFFFFFFFFFFFFFFFFF
FFFFFFFFF:FFFFF
@A00721:190:HGJW7DSXY:4:1113:27570:4304 2:N:0:TCTGCAAG+GCTGCACT
CCATAATATATTCTCACCTGAGTCCAGACCTTACAGGACCAAGAGTCTGATACTCGAAGGTCCATCTA
GAAGTGGAAAAACTCTGTGGGCTAGATCTCTAGGCCCACATAATTACATGTTCGGATATCTAGACTTT
AATTCGAAACTATAT
+
FF,:FFFFFFFFFFFFFFFFFFFFFFFFFFFFFFFFFF:FFFFFFFFFFFFFFFFFFFFFFFFFFFF:F
FFFFFFFFFF:FFFFFF:FFFFFFFF:FFFFFFFFFFFFF:FFFFFFFFFFFFFFFFFFFFFFFFFFFF
FFFFFFF,FFFFF,F
@A00721:190:HGJW7DSXY:4:1134:4164:35383 2:N:0:TCTGCAAG+GCTGCACT
CCATAATATATTCTCACCTGAGTCCAGACCTTACAGGACCAAGAGTCTGATACTCGAAGGTCCATCTA
GAAGTGGAAAAACTCTGTGGGCTAGATCTCTAGGCCCACATAATTACATGTTCGGATATCTAGACTTT
AATTCGAAACTATAT
+
FFFFFFFFFF:FFFFFFFFFFFFFFFFFFFFFFFFFFFF:FFFFFFFFFFFFFFFFFFFFFFFFFF:FFFF
FFFFFFFFFFFFFFFF:FFFFFFFFFFFFFFFFFFFFFFFFFFFFFFFFF:F::FFFFF,FFFFFFFF:F
FFFFFFFFFF:FFFF
@A00721:190:HGJW7DSXY:4:1178:31756:17534 2:N:0:TCTGCAAG+GCTGCACT
CCATAATATATTCTCACCTGAGTCCAGACCTTACAGGACCAAGAGTCTGATACTCGAAGGTCCATCTA
GAAGTGGAAATACTCTGTGGGCTAGATCTCTAGGCCCACATAATTACATGTTCGGATATCTAGACTTT
AATTCGAAACTATAT
+
FFFFFFFFFFFFFFFF:FFFFFFFFFFFFFFFFFFFFFFFFFFFFFFFFFFFFFFFFFFFFFFFFFFF
FFFFFFFF:F,FFFFFFFFFFFFFFFFFFFFFFFFFFFFFFFFFFFFFFFFFFFFFFFFFFFF:FFFF
FFFFFFF:FFFFFFF
@A00721:190:HGJW7DSXY:4:1348:28809:34882 2:N:0:TCTGCAAG+GCTGCACT
CCATAATATATTCTCACCTGAGTCCAGACCTTACAGGACCAAGAGTCTGATACTCGAAGGTCCATCTA
GAAGTGGAAAAACTCTGTGGGCTAGATCTCTAGGCCCACATAATTACATGTTCGGATATCTAGACTTT
AATTCGAAACTATAT
+
FFFFFFFFFFFFFFFFFFFFFFFFFFFFFFFFFFFFFFFFFFFF:FFFFFFFFFFFFFFFFFFFFFFFF
FFFFFFFFFF:FFFFFFFFFFFFFFFFFFFFFFFFFFFFFFFFFFFFFFFFFFFFFFFFFFFFFFFFFF
FFFFFFFFFFF:FFF
@A00721:190:HGJW7DSXY:4:2121:28619:3333 2:N:0:TCTGCAAG+GCTGCACT
CCATAATATATTCTCACCTGAGTCCAGACCTTACAGGACCAAGAGTCTGATACTCGAAGGTCCATCTA
```

GAAGTGGAAAAACTCTGTGGGCTAGATCTCTAGGCCCACATAATTACATGTTCGGATATCTAGACTTT
AATTCGAAACTATAT
+
FFFFFFFFFFFFFFFFFFFFFFFFFFFFFFFFFFFFFFFFFFFFFFFFFFFFFFFFFFFFFFFFFFFFFFF
FFFFFFFFFFFFFFFFFFFFFFFFFFFFFFFFFFFFFFFFFFFFFFFFFFFFFFFFFFFFFFFFFFFFFFF
FFFF,FFFFFF:F,F
@A00721:190:HGJW7DSXY:4:2273:11433:4100 2:N:0:TCTGCAAG+GCTGCACT
CCATAATATATTCTCACCTGAGTCCAGACCTTACAGGACCAAGAGTCTGATACTCGAAGGTCCATCTA
GAAGTGGAAAAACTCTGTGGGCTAGATCTCTAGGCCCACATAATTACATGTTCGGATATCTAGACTTT
AATTCGAAACTATAT
+
FFFFFFFFFFFFFFFFFFFFFFFFFFFFFFFFFF:FFFFFFFFFFFFFFFFFFFFFFFFFFFFFFFFFFFF
FFFFFFFFFFF:FFFFFFFFFFFFFFFFFFF:FFFFFFF:FFFFFFFFFFFFFFFFFFFFFFFFFFFFFF
:FFF:FFFFFFFFFF
@A00721:190:HGJW7DSXY:4:2547:19244:22576 2:N:0:TCTGCAAG+GCTGCACT
CCATAATATATTCTCACCTGAGTCCAGACCTTACAGGACCAAGAGTCTGATACTCGAAGGTCCATCTA
GAAGTGGAAAAACTCTGTGGGCTAGATCTCTAGGCCCACATAATTACATGTTCGGATATCTAGACTTT
AATTCGAAACTATAT
+
FFFFFFFF:F:FFFFFFFFFFFFFFFFFFFFFFFFFFFFFFFFFFFFFFFFF,FFFFFFFFFFFFFFFFF
F:FFFFFFFFFFFFFFFFFFF:FFFFFFFFFFFFFFF:FFFFFFFFFFFF,FFFFFFFFFFFFFFFFF
FFFFFFFF:FF,FFF
@A00721:190:HGJW7DSXY:4:2631:8070:11240 2:N:0:TCTGCAAG+GCTGCACT
CCATAATATATTCTCACCTGAGTCCAGACCTTACAGGACCAAGAGTCTGATACTCGAAGGTCCATCTA
GAAGTGGAAAAACTCTGTGGGCTAGATCTCTAGGCCCACATAATTACATGTTCGGATATCTAGACTTT
AATTCGAAACTATAT
+
FFF:FFFFFFFFFFFFFFFFFFFFFFFFFFFFFFFFFFFFFFFFFFFFFFFFFFFFFFFFFFF,FFFF:
FF:FFFFFFFFFFFFFFFFFFFFF:FFFFFFFFFFFFFFFFFFFFFFFFFFFFF:FFFFFFFF:FFFFFFF
FFFFF,FFFFF,FFF
@A00721:190:HGJW7DSXY:4:2674:16215:16141 2:N:0:TCTGCAAG+GCTGCACT
CCATAATATATTCTCACCTGAGTCCAGACCTTACAGGACCAAGAGTCTGATACTCGAAGGTCCATCTA
GAAGTGGAAAAACTCTGTGGGCTAGATCTCTAGGCCCACATAATTACATGTTCGGATATCTAGACTTT
AATTCGAAACTATAT
+
FFFFFFFFFFFFFFFFFFFFFFFFFFFFFFFFFFFFFFFFFFFFFFFFFFFFFFFFFFFFFFFFFFF::F
FFFF:FFFFFFFFFFFFFFFFFFFFFFFFFFFFFFFFFFFF:FFFFFFFFF:FFFFFFFFFFFFFFF:F
FFFFFF:FFFF,FF:
@A00721:190:HGJW7DSXY:4:1252:22254:1454 2:N:0:TCTGCAAG+GCTGCACT
GGGTGACCCATAATATATTCTCACCTGAGTCCAGACCTTACAGGACCAAGAGTCTGATACTCGAAGGT
CCATCTAGAAGTGGAAAAACTCTGTGGGCTAGATCTCTAGGCCCACATAATTACATGTTCGGATATCT
AGACTTTAATTCGAA
+
FFFFFFF:FFFFFF:FFFFFFFFFFFFFFFFFFFFFFFFFFFFFFFFFFFFFFFFFFFFFFFFFFFFFFF
FFFFFFFF,FFFFFFFFFFFFFFFFFFFFFFFFFFFFFFFFFFFFFFFFFFFFFFF,FFFF:FFF,FFF
,:FFFFFFFFFF:FF
@A00721:190:HGJW7DSXY:4:1332:25590:4006 2:N:0:TCTGCAAG+GCTGCACT
GGGTGACCCATAATATATTCTCACCTGAGTCCAGACCTTACAGGACCAAGAGTCTGATACTCGAAGGT
CCATCTAGAAGTGGAAAAACTCTGTGGGCTAGATCTCTAGGCCCACATAATTACATGTTCGGATATCT
AGACTTTAATTCGAA
+
FFFFFFF:FFFFFFFFFFFFFFFFFFFFFFFFFFFFFFFFFFFFFFFFFFFFFFFFF:FFFFFFFFFFF
FFFFFFFFFFFFFFFFF:FFFFFFFFFFFFFFFFFFFFFFF:FFFFFFFFFFFFFFFFFFFFFFFFFFFFF
FFFFFFFFFFFFFFF

```
@A00721:190:HGJW7DSXY:4:1647:28745:34491 2:N:0:TCTGCAAG+GCTGCACT
GGGTGACCCATAATATATTCTCACCTGAGTCCAGACCTTACAGGACCAAGAGTCTGATACTCGAAGCT
CCATCTAGAAGTGGAAAAACTCTGTGGGCTAGATCTCTAGGCCCACATAATTACATGTTCGGATATCT
AGACTTTAATTCGAA
+
FFFFFFFFFFFFFFFFFFFFFFFFFFFFFFFFFFFFFFFFFFFFFFFFFFFFFFFFFFFFFFFFFF,F
FFFFFFFFFFFFFFFFFFFFFFFFFFFFFFFFFFFFFFFFFFFFFFFFF:FFFFFFFFFFFFFFFFFF
FFFFFFFFFFFF,F:
@A00721:190:HGJW7DSXY:4:2152:6045:1313 2:N:0:TCTGCAAG+GCTGCACT
GGGTGACCCATAATATATTCTCACCTGAGTCCAGACCTTACAGGACCAAGAGTCTGATACTCGAAGGT
CCATCTAGAAGTGGAAAAACTCTGTGGGCTAGATCTCTAGGCCCACATAATTACATGTTCGGATATCT
AGACTTTAATTCGAA
+
FFFFFFF,FFFFF:FFFFFFFFFFFFFFFFFFFFFFFFFFFFFFFFFFFFFFFFFFFFFFFF:FFFFF
F:FFF:FFFFFFFFFFFFFFFFFFFF:FFFFFFFFFFFFFFFFFFFFFF:FFFFFFFFFFFFFFFFFF
F:FFFF:FFFFFFFF
@A00721:190:HGJW7DSXY:4:2152:5177:2660 2:N:0:TCTGCAAG+GCTGCACT
GGGTGACCCATAATATATTCTCACCTGAGTCCAGACCTTACAGGACCAAGAGTCTGATACTCGAAGGT
CCATCTAGAAGTGGAAAAACTCTGTGGGCTAGATCTCTAGGCCCACATAATTACATGTTCGGATATCT
AGACTTTAATTCGAA
+
FFFFFFF,FFFFFFF:F:FFFFFFFFFFFFFFFFFFFFFFFFFFFFFFFFFFFFFFFFFFFFF:FFFFF
FFFFF:FFFFFFFFFFFFFFFFFF:FFFFFFFFFFFFFFF:FFFFFFFF,:FFFFFFFFFFFFFFFFF
F,F:F:FFFFFFFFF
@A00721:190:HGJW7DSXY:4:1140:14651:30655 2:N:0:TCTGCAAG+GCTGCACT
TGGGTGACCCATAATATATTCTCACCTGAGTCCAGACCTTACAGGACCAAGAGTCTGATACTCGAAGG
TCCATCTAGAAGTGGAAAAACTCTGTGGGCTAGATCTCTAGGCCCACATAATTACATGTTCGGATATC
TAGACTTTAATTCGA
+
FFFFFFFF:FFFFFFFF:FFFFFFFFFFFFFFFFF:FFFFFFFFFFFF,FFFFFFFFFFF:FFFFFFF
FFF:FFFFFF,FFFFFFF:FFFFFFFFFFFFFFFF:FFFFFFFFFFFFFFFFFFFFFFFFFFFFFFFF
FFFFFFFFFFF:FFF
@A00721:190:HGJW7DSXY:4:1241:9688:28510 2:N:0:TCTGCAAG+GCTGCACT
TGGGTGACCCATAATATATTCTCACCTGAGTCCAGACCTTACAGGACCAAGAGTCTGATACTCGAAGG
TCCATCTAGAAGTGGAAAAACTCTGTGGGCTAGATCTCTAGGCCCACATAATTACATGTTCGGATATC
TAGACTTTAATTCGA
+
FFFFFFFF:FFFFFFFFFFFFFFFFFFFFFFFFFFFFFFFFFFFFFFFFFFFFFFFFFFFFFF:FFFF
FFFFFFFFF,FFFFFFFFFFFFFFFFFFFFFFFFFFFFFFFFFFFFFFFFFFFFFFF:FFFFFFFF:
FFFFFFFFFFFFFFF
@A00721:190:HGJW7DSXY:4:1249:27841:27759 2:N:0:TCTGCAAG+GCTGCACT
TGGGTGACCCATAATATATTCTCACCTGAGTCCAGACCTTACAGGACCAAGAGTCTGATACTCGAAGG
TCCATCTAGAAGTGGAAAAACTCTGTGGGCTAGATCTCTAGGCCCACATAATTACATGTTCGGATATC
TAGACTTTAATTCGA
+
FFFFFFFF:FFFFFFFFFFFFFFFFFFFFFFFFFFFFFFFFFFFFFFFFFFFFFFFFFFFFFFFFFFF
FFFFFFFFFF:FFFFFFFFFFFFFFFFFFFFFFFFFFFF:FFFFFFF,FFFFFFFF,FFFFFFFFFFF
FFFFFFFFFFFFFFF
@A00721:190:HGJW7DSXY:4:1271:27796:31375 2:N:0:TCTGCAAG+GCTGCACT
TGGGTGACCCATAATATATTCTCACCTGAGTCCAGACCTTACAGGACCAAGAGTCTGATACTCGAAGG
TCCATCTAGAAGTGGAAAAACTCTGTGGGCTAGATCTCTAGGCCCACATAATTACATGTTCGGATATC
TAGACTTTAATTCGA
+
FFFFFFFF:FF:F:FFFFFFFFFF:FFFFFFFFFFFFFFFFFFFFFFFFFFFFFFFFFFFFFFFFFFF
```

FFFFFF:FFFFFFFFFFFFFFFFFFFFFFFFFFFFFFFFFFFFFFFFFFF:FFFFF:FFFFFFFFFFF
FF:FFFFF:FFFFFF
@A00721:190:HGJW7DSXY:4:1341:26341:17237 2:N:0:TCTGCAAG+GCTGCACT
TGGGTGACCCATAATATATTCTCACCTGAGTCCAGACCTTACAGGACCAAGAGTCTGATACTCGAAGG
TCCATCTAGAAGTGGAAAAACTCTGTGGGCTAGATCTCTAGGCCCACATAATTACATGTTCGGATATC
TAGACTTTAATTCGA
+
FFFFFF:FFFFFFFFFFF:F:FFFFFF:FFFFFFFFF:FFFFFFFFFFFFF:FFFFFFFFFFFFFFFF
FFFFFF:FFFFF,FFFFFF:FFFFFFFF:FFFFFFFFFFFF:FFFF,FFFFFFFFFFF,:FF:F:FF,
FFFFFF:FFFFFFFF
@A00721:190:HGJW7DSXY:4:2209:7889:33567 2:N:0:TCTGCAAG+GCTGCACT
TGGGTGACCCATAATATATTCTCACCTGAGTCCAGACCTTACAGGACCAAGAGTCTGATACTCGAAGG
TCCATCTAGAAGTGGAAAAACTCTGTGGGCTAGATCTCTAGGCCCACATAATTACATGTTCGGATATC
TAGACTTTAATTCGA
+
FFFFFFFF:FFFFFFFF,FFFFFFFFFFFFFFFFFFFFFFFFFFFFFF:FFFFFFFFFFF:FFFFFFF
FFFFFFFFFFFFFFFFFFFFFFFFFFFF:FFFFFFFFFFFFFFFFF:FFFFFFFFFFFF,FFFFFFFFF
FFF:FFFFFFFFF:F
@A00721:190:HGJW7DSXY:4:2418:23791:28166 2:N:0:TCTGCAAG+GCTGCACT
TGGGTGACCCATAATATATTCTCACCTGAGTCCAGACCTTACAGGACCAAGAGTCTGATACTCGAAGG
TCCATCTAGAAGTGGAAAAACTCTGTGGGCTAGATCTCTAGGCCCACATAATTACATGTTCGGATATC
TAGACTTTAATTCGA
+
FFFFFFFFFFFFFFFFFF,FFFFFFFFFFFFFFFFFFFFFFFFFFFFFFFFFFFFFFFFFFFFFFFFF
FFFFFFFFFFFFFFFFFFFFFFFFFFFF:FFFFFFFFFFFFFFFFFFFFFFFFFFFFFFFFFFFFF:FF
FF:FFF:FFFFFF:F
@A00721:190:HGJW7DSXY:4:2552:7862:5337 2:N:0:TCTGCAAG+GCTGCACT
TGGGTGACCCATAATATATTCTCACCTGAGTCCAGACCTTACAGGACCAAGAGTCTGATACTCGAAGG
TCCATCTAGAAGTGGAAAAACTCTGTGGGCTAGATCTCTAGGCCCACATAATTACATGTTCGGATATC
TAGACTTTAATTCGA
+
FFFFFFFF:FFFFFFF:F:FFFFFFFFFFFFFFFFFFFFFFFFFFFFFFFFFFFFFFFFFFFFFFFFF
FFFFFFFFFFFFFFFFFFFFFF:FFFFFFFFFFFFFFFFFFFFFFFFFFFFFFFFFFFFFFFFFFFFFF
FFFFFFFFFFFF:FF
@A00721:190:HGJW7DSXY:4:2633:28131:14512 2:N:0:TCTGCAAG+GCTGCACT
TGGGTGACCCATAATATATTCTCACCTGAGTCCAGACCTTACAGGACCAAGAGTCTGATACTCGAAGG
TCCATCTAGAAGTGGAAAAACTCTGTGGGCTAGATCTCTAGGCCCACATAATTACATGTTCGGATATC
TAGACTTTAATTCGA
+
:FFFFFFF:FFFFFFFFFFFFFFFFFFFFFF:FFFFFFFFFFFFFFFFFFFFFFFFFFFFFFFFFF:FF
FFFFFFFFFFFFFFFFFFFFFFFFFFFFFFFFFFFFFFFFFFFF:FFFFFFFFFFFFFFFFFFFFFFF
:,FFFFFFFFFFFFF
@A00721:190:HGJW7DSXY:4:1226:28266:19069 2:N:0:TCTGCAAG+GCTGCACT
ATTGGGTGACCCATAATATATTCTCACCTGAGTCCAGACCTTACAGGACCAAGAGTCTGATACTCGAA
GGTCCATCTAGAAGTGGAAAAACTCTGTGGGCTAGATCTCTAGGCCCACATAATTACATGTTCGGATA
TCTAGACTTTAATTC
+
FFFFF:FF:F:FFFFFFFFFFFFFFF:F::FFFFFFFFFFFFFFFFFFFF::FFF,FFFFFFFFFFFF
FFFFFF,FFFFFFFFFFFFFFFFFF:FFFFFFFFFFFFFFFF:FFFFFFFFF,F,,FFF:F::FFFF:F
FFFFFFFFFFFFFFF
@A00721:190:HGJW7DSXY:4:1323:25527:33270 2:N:0:TCTGCAAG+GCTGCACT
ATTGGGTGACCCATAATATATTCTCACCTGAGTCCAGACCTTACAGGACCAAGAGTCTGATACTCGAA
GGTCCATCTAGAAGTGGAAAAACTCTGTGGGCTAGATCTCTAGGCCCACATAATTACATGTTCGGATA
TCTAGACTTTAATTC

```
+
FFFFFFFFFFFFFFFFFFFF:FFFFFFFFFFFFFFFFFFFFF:FFFFFFFFFFFFFFFFFFFFFFFFF
FFFFFFFF:FFFFFFFFFFFFFFFFFFFFFFFFFFFFFFF:FFFFFFFFF,FFFFFFFFF:FFFF:FF
FFFFFFFFFFFFFFF
@A00721:190:HGJW7DSXY:4:1511:23592:11882 2:N:0:TCTGCAAG+GCTGCACT
ATTGGGTGACCCATAATATATTCTCACCTGAGTCCAGACCTTACAGGACCAAGAGTCTGATACTCGAA
GGTCCATCTAGAAGTGGAAAAACTCTGTGGGCTAGATCTCTAGGCCCACATAATTACATGTTCGGATA
TCTAGACTTTAATTC
+
FFFFFFFFFF:FFFFFFFFFFFFFFFFFFFFFFFFFFFFFFFFFFFFFFFFFFFFFFFFFFFFFFFFF
FFFFFFFFFFFFFFFFFFFFFFFFFFFFFFFFFFFFFFFFFFFFFFFFFFFFFFFFFFFFFFFFFFFF
FFFFFFFFFFFFFFF
@A00721:190:HGJW7DSXY:4:1636:1579:6198 2:N:0:TCTGCAAG+GCTGCACT
ATTGGGTGACCCATAATATATTCTCACCTGAGTCCAGACCTTACAGGACCAAGAGTCTGATACTCGAA
GGTCCATCTAGAAGTGGAAAAACTCTGTGGGCTAGATCTCTAGGCCCACATAATTACATGTTCGGATA
TCTAGACTTTAATTC
+
FFFFFFFFFFFFFFFFFFFFFFFFFFFFFFFFFFFFFFFFFFFFFFFFFFFFFFFFFFFFFFFFFFFF
FFFFFFFFF:FFFFFFFFFFFFFFFFFFFFFFF:FFFFFFFFFFFFFFFFFFFFFFFF,F:FFFFFFF
FFFFFFF:FFFFFFF
@A00721:190:HGJW7DSXY:4:2126:16080:15311 2:N:0:TCTGCAAG+GCTGCACT
ATTGGGTGACCCATAATATATTCTCACCTGAGTCCAGACCTTACAGGACCAAGAGTCTGATACTCGAA
GGTCCATCTAGAAGTGGAAAAACTCTGTGGGCTAGATCTCTAGGCCCACATAATTACATGTTCGGATA
TCTAGACTTTAATTC
+
FFFFFFFFFF:FFFFFFFFFFFFFFFFFFFFFFFFFFFFFFFFFFFFFFFFFFFFFFFFFFFFFFFFF
FFFFFFFFFFFFFFFFFFFFFFFFFFFFFFFFFFFFFFFFFFFFFFFFFF:FFF:FFFFFFFFFFFFF
FFFFFFFFFFFFFFF
@A00721:190:HGJW7DSXY:4:2207:17499:4836 2:N:0:TCTGCAAG+GCTGCACT
ATTGGGTGACCCATAATATATTCTCACCTGAGTCCAGACCTTACAGGACCAAGAGTCTGATACTCGAA
GGTCCATCTAGAAGTGGAAAAACTCTGTGGGCTAGATCTCTAGGCCCACATAATTACATGTTCGGATA
TCTAGACTTTAATTC
+
FFFFFF:FFFFFFF:FFFFFFFFFFFFFFFFFFFFFFFFF:FFFFFFFFFFFFFFFFFFFFFFFFFFF
FFFFFFFFFFFFFFFFFFFF,:FFFFFFFFFFFFFFFFFFFF:FFFFFFFFFFFFFFFFFF,F:FFFF
FFFFFFFFFFFFFFF
@A00721:190:HGJW7DSXY:4:2253:31693:16391 2:N:0:TCTGCAAG+GCTGCACT
ATTGGGTGACCCATAATATATTCTCACCTGAGTCCAGACCTTACAGGACCAAGAGTCTGATACTCGAA
GGTCCATCTAGAAGTGGAAAAACTCTGTGGGCTAGATCTCTAGGCCCACATAATTACATGTTCGGATA
TCTAGACTTTAATTC
+
FFFFFFFFFFFFFFFFFFFFFFFFFFFFFFFFFFFFFFFFFFFFFFFFFFFFFFFFFFFFFF:FFFF:
FFFFFFFFFFFF:FFFFFFFFFFFFFFFF:FFFFFFFFFFFFFFFFFFFFFFFFFF,FFFFFFFFFFF
FFFFF:FFFFFFFFF
@A00721:190:HGJW7DSXY:4:2324:6967:22420 2:N:0:TCTGCAAG+GCTGCACT
ATTGGGTGACCCATAATATATTCTCACCTGAGTCCAGACCTTACAGGACCAAGAGTCTGATACTCGAA
GGTCCATCTAGAAGTGGAAAAACTCTGTGGGCTAGATCTCTAGGCCCACATAATTACATGTTCGGATA
TCTAGACTTTAATTC
+
FFFFFFFFFFFF:FFFFFFFFFFFFFFFFFFFFFFFFFFFFFFFFFFFFFFFFFFFFFFFFFFFFFF
FFFFFFFFF::FFFFFFFFFFFFFFFFFFFFFFFFFFFFFFFFFFFF:FFFFFFFFFFFFFFFFFFFF
FF:F:FF:FFFFFF:
@A00721:190:HGJW7DSXY:4:2453:12753:9173 2:N:0:TCTGCAAG+GCTGCACT
TGGGTGACCCATAATATATTCTCACCTGAGTCCAGACCTTACAGGACCAAGAGTCTGATACTCGAAGG
```

TCCATCTAGAAGTGGAAAAACTCTGTGGGCTAGATCTCTAGGCCCACATAATTACATGTTCGGATATC
TAGACTTTAAT
+
FF,:,:FF,FFFFFFFFFFF:FFFFF::FFFFFFFFFFFFFFFFFF::FF:FF:FFFFFFFF:FFF:,
FF:F:F,FFFF:FFFFFFFFF::F,:,::,FFFFF,F::FF:FFFFFFFFFFFFFFFF,,F::FFF:F
FF,FFFFFFFF
@A00721:190:HGJW7DSXY:4:2459:9462:33786 2:N:0:TCTGCAAG+GCTGCACT
GTATTGGGTGACCCATAATATATTCTCACCTGAGTCCAGACCTTACAGGACCAAGAGTCTGATACTCG
AAGGTCCATCTAGAAGTGGAAAAACTCTGTGGGCTAGATCTCTAGGCCCACATAATTACATGTTCGGA
TATCTAGACTTTAAT
+
FFFFFFFFFFFFFFFFFFFFFFFFFFFFFFFFFFFFFFFFFFFFFFFFFFFFFFFFFFFFFFFFFFFF
FFFFFFFFFFFFFFFFFFFFFFFFFFFFFFFFFFF:FFFFFFFFFFFFFFFFFFFFFFFFFFFFFFFF
F:FFFFFFFFFFFFF
@A00721:190:HGJW7DSXY:4:1411:24270:23422 2:N:0:TCTGCAAG+GCTGCACT
TCTAGATATCCGAACATGTAATTATGTGGGCCTAGAGATCTAGCCCACAGAGTTTTTCCACTTCTAGA
TGGACCTTCGAGTATCAGACTCTTGGTCCTGTAAGGTCTGGACTCAGGTGAGAATATATTATGGGTCA
CCCAATACTGTAACT
+
FFFFFFFFFFFFFFFFFFFFFFFFFFFFFFFFFFFFFFFFFFFFFFFFFFFFFFFFFFFFFFFFFFFF
FFFFFFFFFFFFFFFFFFFFFFFFFFFFFFFFFFFFFFFFFFFFFFFFFFFFFFFFFFFFFF:FFFFF
FFFFFFFFFFFFFFF
@A00721:190:HGJW7DSXY:4:1307:16749:17879 2:N:0:TCTGCAAG+GCTGCACT
GAGTTACAGTATTGGGTGACCCATAATATATTCTCACCTGAGTCCAGACCTTACAGGACCAAGAGTCT
GATACTCGAAGGTCCATCTAGAAGTGGAAAAACTCTGTGGGCTAGATCTCTAGGCCCACATAATTACA
TGTTCGGATATCTAG
+
FFFFFFFFFFFFFFFFFFFF,FFFFFFFFFFFFFFFFFFFFFFFFF,FFFFFFFF::FFFFF:FFFFF
FFFFFFF:F:FF:FFFFFFFFFF:FFFFFF,FFFFFF,FFFF:F:FFFFFFF,FFFFFFFFF:FFF,,
FFFFF:FFFFFF:FF
@A00721:190:HGJW7DSXY:4:1441:24641:30733 2:N:0:TCTGCAAG+GCTGCACT
GAGTTACAGTATTGGGTGACCCATAATATATTCTCACCTGAGTCCAGACCTTACAGGACCAAGAGTCT
GATACTCGAAGGTCCATCTAGAAGTGGAAAAACTCTGTGGGCTAGATCTCTAGGCCCACATAATTACA
TGTTCGGATATCTAG
+
FFFFFFFFFFFFFFFFFFFF:FFFFFFFFFFFFFFF:FFFFFFFFFFFFFFFFFFFFFFFFFFFFFFF
FFFFFFFFFF:FFFFFFFF:FFFFFFFFFFFFFFF:FFF:FFFFFFFFFFFFFFFFFFFFFFFFFFFF
FFFFFFFFFFFFF:F
@A00721:190:HGJW7DSXY:4:1462:29098:19507 2:N:0:TCTGCAAG+GCTGCACT
GAGTTACAGTATTGGGTGACCCATAATATATTCTCACCTGAGTCCAGACCTTACAGGACCAAGAGTCT
GATACTCGAAGGTCCATCTAGAAGTGGAAAAACTCTGTGGGCTAGATCTCTAGGCCCACATAATTACA
TGTTCGGATATCTAG
+
FFFFFFFFFFFFFFFFFFFF:FFFFFF:FF:FFFFFFFFFFFFFFFFFFFFFFFFFFFFFF:FFFFFF
FFFFFFFFFFFFFFFFFFFFFFFFFFFFFFF:FFFFFFFFFFFFFFFFFFFFFFFFFFFFFFFFFFFF
FFFFFFFFFFFFFFF
@A00721:190:HGJW7DSXY:4:1511:16975:18490 2:N:0:TCTGCAAG+GCTGCACT
GAGTTACAGTATTGGGTGACCCATAATATATTCTCACCTGAGTCCAGACCTTACAGGACCAAGAGTCT
GATACTCGAAGGTCCATCTAGAAGTGGAAAAACTCTGTGGGCTAGATCTCTAGGCCCACATAATTACA
TGTTCGGATATCTAG
+
F:FFFFFFF:FFFFFFFFFF,FFFFFFFFFFFFFFF:FFFFFFFFFFF:FFFFFFFFFFFFFFFFFFF
FFFFFFFFFFFFFFFFF:FFFFFFFFFFFFFFFFFFFFFFFFFFFFFFFFFFFFFFFFFFFFFFFFFF
FFFFFFFFFFFFFFF

@A00721:190:HGJW7DSXY:4:1625:18213:31939 2:N:0:TCTGCAAG+GCTGCACT
GAGTTACAGTATTGGGTGACCCATAATATATTCTCACCTGAGTCCAGACCTTACAGGACCAAGAGTCT
GATACTCGAAGGTCCATCTAGAAGTGGAAAAACTCTGTGGGCTAGATCTCTAGGCCCACATAATTACA
TGTTCGGATATCTAG
+
FFFFFFFFFFFFFFFFFFFF,FFFFFFFFFFFFFFF:FFFFFFFFFFFFFFFFFFFFFFFFFFFFFFF
FFFFFFFFFFFFFFFFFFFFFFFFFFFFFFF,FFFFFFFFFFFFFFFFFFFFFFFFFFFFFFFFFFFF
FFFFFFFFF,FFFFF
@A00721:190:HGJW7DSXY:4:1673:8214:8703 2:N:0:TCTGCAAG+GCTGCACT
GAGTTACAGTATTGGGTGACCCATAATATATTCTCACCTGAGTCCAGACCTTACAGGACCAAGAGTCT
GATACTCGAAGGTCCATCTAGAAGTGGAAAAACTCTGTGGGCTAGATCTCTAGGCCCACATAATTACA
TGTTCGGATATCTAG
+
FFF,FFF:FFFFFFFFFFFF:FFFFFFFFFFFFFFFFFFFFFFFFFFFFFFFFFFFFFFFFFFFFFFF
FFFFFFFFFFFFFFFFFFFFFFFFFFFFFFFFFFFFFFFFFFFFFFFFFFFFFFFFFFFFFFFFFFFF
FFFFFFFFFFFFFFF
@A00721:190:HGJW7DSXY:4:2139:32470:1517 2:N:0:TCTGCAAG+GCTGCACT
GAGTTACAGTATTGGGTGACCCATAATATATTCTCACCTGAGTCCAGACCTTACAGGACCAAGAGTCT
GATACTCGAAGGTCCATCTAGAAGTGGAAAAACTCTGTGGGCTAGATCTCTAGGCCCACATAATTACA
TGTTCGGATATCTAG
+
FFFFFFFFFFFFFFFFFFFF:FFFFFFFFFFFFFFFFFFFFFFFFFFFFFFFFFFFFFFFFFFFFFFF
FFFFFFFFFF,FFFFFFFFFFFFFFFFFFFF,FFFFFFFFFFFFFFFFFFFFFF,FFFFF:FFFFFFF
FFFFFFFFFFFFFFF
@A00721:190:HGJW7DSXY:4:2410:14389:4492 2:N:0:TCTGCAAG+GCTGCACT
GAGTTACAGTATTGGGTGACCCATAATATATTCTCACCTGAGTCCAGACCTTACAGGACCAAGAGTCT
GATACTCGAAGGTCCATCTAGAAGTGGAAAAACTCTGTGGGCTAGATCTCTAGGCCCACATAATTACA
TGTTCGGATATCTAG
+
:FFFF:FFFFFFFFFFFFFF,FFFFFF:FFFFF:FF:FFFFFFFFFFFFF,FFFFFFFFFFFFFFFFF
FFFFFFFFFFFFFFFFFFFFFFFFFFFFFFF,FFFFFFFFFFFFFFFFFFFF:FFF,FFFFFFFFFF,
FFFFFFFFF:FFFFF
@A00721:190:HGJW7DSXY:4:2450:15673:3646 2:N:0:TCTGCAAG+GCTGCACT
GAGTTACAGTATTGGGTGACCCATAATATATTCTCACCTGAGTCCAGACCTTACAGGACCAAGAGTCT
GATACTCGAAGGTCCATCTAGAAGTGGAAAAACTCTGTGGGCTAGATCTCTAGGCCCACATAATTACA
TGTTCGGATATCTAG
+
FFFFFFFFFFFFFFFFFFFFFFFFFFFFFFFFFFFFFFFFFFFFFFFFFFFFFFFFFFFFFFFFFFFF
FFFFFFFFFFFFFFFFFFFFFF:FFFFFFFF:FFFFFFFFFFFFFFFFFFFFFFFFFFFFFFFFFFFF
FFFFFFFFFFFFFFF
@A00721:190:HGJW7DSXY:4:2450:16396:3740 2:N:0:TCTGCAAG+GCTGCACT
GAGTTACAGTATTGGGTGACCCATAATATATTCTCACCTGAGTCCAGACCTTACAGGACCAAGAGTCT
GATACTCGAAGGTCCATCTAGAAGTGGAAAAACTCTGTGGGCTAGATCTCTAGGCCCACATAATTACA
TGTTCGGATATCTAG
+
FFFFFFFFFFFFFFFFFFFF:FFFFFFFFFFFFFFFFFFFFFFFFFFFFFFFFFFFFFFFFFFFFFFF
FFFFFFFFFFFFFFFFFFFFFFFFFFFFFFFFFFFFFFFFFFFFFFFFFFF:FFFFFFFFFFFFFFFF
FFFFFFFFFFFFFFF
@A00721:190:HGJW7DSXY:4:2450:25111:21872 2:N:0:TCTGCAAG+GCTGCACT
GAGTTACAGTATTGGGTGACCCATAATATATTCTCACCTGAGTCCAGACCTTACAGGACCAAGAGTCT
GATACTCGAAGGTCCATCTAGAAGTGGAAAAACTCTGTGGGCTAGATCTCTAGGCCCACATAATTACA
TGTTCGGATATCTAG
+
FFFFFFFFFFF:FFFFFFFF,FFFFFFFFFFFFFFF:FFFFFFFFFFFFFFFFFFFFFFFFFFFFFFF

FFFFFFFFFFFFFFFFFFFFFFFFFFFFFFFFFFFFFFFFFFFFFFFFFFFFF:FF:FFFFFFFFFFF
FFFFFFFFF,FFFFF
@A00721:190:HGJW7DSXY:4:2649:12952:8954 2:N:0:TCTGCAAG+GCTGCACT
GAGTTACAGTATTGGGTGACCCATAATATATTCTCACCTGAGTCCAGACCTTACAGGACCAAGAGTCT
GATACTCGAAGGTCCATCTAGAAGTGGAAAAACTCTGTGGGCTAGATCTCTAGGCCCACATAATTACA
TGTTCGGATATCTAG
+
F:FFFFFFFFFFFFFFFFFF:FFFFFFFFFFFFFFFFFFFFFFFFFFFFFFFFFFFFFFFFFFFFFFF
FFFFFFFFFFFFFFFFFFFFFFFFFFFFFFFFFFFF:FFFFFFFFFFFFFFFFFFFFFFFFFFFFFFF
F,FFFFFFFFFFFFF
@A00721:190:HGJW7DSXY:4:1577:2031:24737 2:N:0:TCTGCAAG+GCTGCACT
GAGTTACAGTATTGGGTGACCCATAATATATTCTCACCTGAGTCCAGACCTTACAGGACCAAGAGTCT
GATACTCGAAGGTCCATCTAGAAGTGGAAAAACTCTGTGGGCTAGATCTCTAGGCCCACATAATTACA
TGTTCGGATATCTA
+
FFFFFFFFFFFFFFFFFFFF:FFFFFFFFFFFFFFFFFFFFFFFFFFFFFFFFFFFFFFFFFFFFFFF
FFFFFFFFFFFFFFFFFFFFFFFFFFFFFFFFFFFFFFFFFFFFFFFFFFFFFFFFFFFFFFFFFFFF
FFFFFFFFFFFFFF
@A00721:190:HGJW7DSXY:4:1368:3387:19445 2:N:0:TCTGCAAG+GCTGCACT
TTACAGTATTGGGTGACCCATAATATATTCTCACCTGAGTCCAGACCTTACAGGACCAAGAGTCTGAT
ACTCGAAGGTCCATCTAGAAGTGGAAAAACTCTGTGGGCTAGATCTCTAGGCCCACATAATTACATGT
TCGGATAGATCGGAA
+
FFFFFFFFFFFFFFFFFFFFFFFFFFFFFFFFFFFFFFFFFFFFFFFFFFFFFFFFFFFFFFFFFFFF
FFFFFFFFFFFFFFFFFFFFFFFFFFFFFFFFFFFFFFFFFFFFFFFFFFFFFFFFFFFFFFFFFFFF
FFFFFFFFFFFFFFF
@A00721:190:HGJW7DSXY:4:1374:28601:23093 2:N:0:TCTGCAAG+GCTGCACT
TTACAGTATTGGGTGACCCATAATATATTCTCACCTGAGTCCAGACCTTACAGGACCAAGAGTCTGAT
ACTCGAAGGTCCATCTAGAAGTGGAAAAACTCTGTGGGCTAGATCTCTAGGCCCACATAATTACATGT
TCGGATAGATCGGAA
+
FFFFFFFFFFFFFFFFFFFFFFFFFFFFFFFFFFFFFFFFFFFFFFFFFFFFFFFFFFFFFFFFF:FF
FFFFFFFFFFFFFFFFFFFFFFFFFFFFFFFFFFFF:FFFFFFFFFFFFFFFFFFFFFFFFFFFFFFF
FF,FFFFFFFFFFFF
@A00721:190:HGJW7DSXY:4:1167:8639:9721 2:N:0:TCTGCAAG+GCTGCACT
CACAAGAGTTACAGTATTGGGTGACCCATAATATATTCTCACCTGAGTCCAGACCTTACAGGACCAAG
AGTCTGATACTCGAAGGTCCATCTAGAAGTGGAAAAACTCTGTGGGCTAGATCTCTAGGCCCACATAA
TTACATGTTCGGATA
+
FFFFFFFFFFFFFFFFFFFFFFFFFFFFFFFFFFFFFFFFFFFFFFFFFFFFFFFFFFFFFFFFFFFF
FFFFFFFFFFFFFFFFFFFFFFFFFFFFFFFFFFFFFFFFFFFFFFFF:FFFFFFFFFFFFFFFFFFF
FFFFFFFFFFFFFFF
@A00721:190:HGJW7DSXY:4:1405:28718:22576 2:N:0:TCTGCAAG+GCTGCACT
CACAAGAGTTACAGTATTGGGTGACCCATAATATATTCTCACCTGAGTCCAGACCTTACAGGACCAAG
AGTCTGATACTCGAAGGTCCATCTAGAAGTGGAAAAACTCTGTGGGCTAGATCTCTAGGCCCACATAA
TTACATGTTCGGATA
+
FFFFFFFFFFFFFFFFFFFFFFFFFFFFFFFFFFFFFFFFFFFFFFFFFFFFFFFFFFFFFFFFFFFF
FFFFFFFFFFFFFFFFFFFFFFFFFFFFFFFFFFFF:FFFFFFFFFFFFFFFFFFFFFFFFFFFFFFF
FFFFFFFFFFFFFFF
@A00721:190:HGJW7DSXY:4:1536:23647:13354 2:N:0:TCTGCAAG+GCTGCACT
CACAAGAGTTACAGTATTGGGTGACCCATAATATATTCTCACCTGAGTCCAGACCTTACAGGACCAAG
AGTCTGATACTCGAAGGTCCATCTAGAAGTGGAAAAACTCTGTGGGCTAGATCTCTAGGCCCACATAA
TTACATGTTCGGATA

```
+
FFFFFFFFFFFFFFFFFFFFFFFFFFFFFFFFFFFFFFFFFFFFFFFFFFFFFFFFFFFFFFFFFFFF
FFFFFFFFFFFFFFFFFFFFFFFFFFFFFFFFFFFF:FFFFFFFFFFFFFFFFFFFFFFFFFFFFFFF
FFFFFFFFFFFFFFF
@A00721:190:HGJW7DSXY:4:1108:2184:30295 2:N:0:TCTGCAAG+GCTGCACT
GTTGTTCCACAAGAGTTACAGTATTGGGTGACCCATAATATATTCTCACCTGAGTCCAGACCTTACAG
GACCAAGAGTCTGATACTCGAAGGTCCATCTAGAAGTGGAAAAACTCTGTGGGCTAGATCTCTAGGCC
CACATAATTACATGT
+
FFFFFFFFFFFFFFFFFFF:FFFFFFFFFFFFFFFFFFFFFFFFFFFF:FFFFFFFFF:FFFFFFFFF
FFFFFFFFFFFFFFFFFFFFFFFFFFFFFFFFFFFF:FFFF:FFFFFFFFFFFFFF:FFFFFFF:FF:
FFFFFFFFF:FFFFF
@A00721:190:HGJW7DSXY:4:1155:4942:28087 2:N:0:TCTGCAAG+GCTGCACT
GTTGTTCCACAAGAGTTACAGTATTGGGTGACCCATAATATATTCTCACCTGAGTCCAGACCTTACAG
GACCAAGAGTCTGATACTCGAAGGTCCATCTAGAAGTGGAAAAACTCTGTGGGCTAGATCTCTAGGCC
CACATAATTACATGT
+
FFFFFFFFFFFFFFFFFFFFFFFFFFFFFFFFFFFFFFFFFFFFFFFFFFFFFFFFFFFFFFFFFFFF
FFFFFFFFFFFFFFFFFFFFFFFFFFF:FFFFFFFFFFFFFFF,FFFFFFFFFFFFFFFFFFFFFFFF
FFFFFFFFFFF,FFF
@A00721:190:HGJW7DSXY:4:1155:5367:28385 2:N:0:TCTGCAAG+GCTGCACT
GTTGTTCCACAAGAGTTACAGTATTGGGTGACCCATAATATATTCTCACCTGAGTCCAGACCTTACAG
GACCAAGAGTCTGATACTCGAAGGTCCATCTAGAAGTGGAAAAACTCTGTGGGCTAGATCTCTAGGCC
CACATAATTACATGT
+
FFFFFFFFFFFFFFFFFFFFFFFFFFFFFFFFFFFFFFFFFFFFFFFFFFFFFFFFFFFFFFFFFFFF
FFFFFFFFFFFFFFFFFFFFFFFFFFFFFFFFFFFFFFFFFFFFFFFFFFFFFFFFFFFFFFFFFFFF
FFFFFFFFFFFFFFF
@A00721:190:HGJW7DSXY:4:1165:23167:34851 2:N:0:TCTGCAAG+GCTGCACT
GTTGTTCCACAAGAGTTACAGTATTGGGTGACCCATAATATATTCTCACCTGAGTCCAGACCTTACAG
GACCAAGAGTCTGATACTCGAAGGTCCATCTAGAAGTGGAAAAACTCTGTGGGCTAGATCTCTAGGCC
CACATAATTACATGT
+
FFFFFFFFFFFFFFFFFFFFFFFFFFFFFFFFFFFFFFFFFFFFFFFFFFFFFFFFFFFFFFFFFFFF
FFFFFFFFFFFFFFFFFFFFFFFFFFFFFFF:FFFFFFFFFFFFF:FFFFFFFFFFFFFFFFFFFFFF
FFFF,F:FFFFF:FF
@A00721:190:HGJW7DSXY:4:1242:11867:7889 2:N:0:TCTGCAAG+GCTGCACT
GTTGTTCCACAAGAGTTACAGTATTGGGTGACCCATAATATATTCTCACCTGAGTCCAGACCTTACAG
GACCAAGAGTCTGATACTCGAAGGTCCATCTAGAAGTGGAAAAACTCTGTGGGCTAGATCTCTAGGCC
CACATAATTACATGT
+
FFF:FFFFFFFFFFFFFFFFFFFFFFFFFFFFFFFFFFFFFFFFFFFFFFFFFFFFFFFFFFFFFFFF
FFFFFFFFFFFFFFFFFFFFFFFFFFF:FFFFFFFFFFFFFFF,FFFFFFFFFFFFFFFFFFFFFFFF
FFFFFFFFFFFFFFF
@A00721:190:HGJW7DSXY:4:1307:27407:35336 2:N:0:TCTGCAAG+GCTGCACT
GTTGTTCCACAAGAGTTACAGTATTGGGTGACCCATAATATATTCTCACCTGAGTCCAGACCTTACAG
GACCAAGAGTCTGATACTCGAAGGTCCATCTAGAAGTGGAAAAACTCTGTGGGCTAGATCTCTAGGCC
CACATAATTACATGT
+
F:FFFFFFFFFFFFFFFFFFFFFFFFFFFFFFFFFFFFFFFFFFFFFFFFFFFFFFFFFFFFFFFFFF
FFFFFFFFFFFFFFFFFFFFFFFFFFFFFFFFFFFFFFFFFFFFFFFFFFFFFFFFFFFFFFFFFFFF
FFFFFFFFFFFFFFF
@A00721:190:HGJW7DSXY:4:1328:30337:31015 2:N:0:TCTGCAAG+GCTGCACT
GTTGTTCCACAAGAGTTACAGTATTGGGTGACCCATAATATATTCTCACCTGAGTCCAGACCTTACAG
```

```
GACCAAGAGTCTGATACTCGAAGGTCCATCTAGAAGTGGAAAAACTCTGTGGGCTAGATCTCTAGGCC
CACATAATTACATGT
+
FFFFFFFFFFFFFFFFFFFFFFFFFFFFFFFFFFFFFFFFFFFFFFFFF:FFFFFFFFFFFFFFFFFFF
F:FFFFFFFFFFFFFFFFFFFFFFFFFFFFFFFFFFFFFFFFFFFFFFFFF:FFFFFFFFFFFFFFFF
FFFFFFFFFFFFFFF
@A00721:190:HGJW7DSXY:4:1506:22182:26443 2:N:0:TCTGCAAG+GCTGCACT
GTTGTTCCACAAGAGTTACAGTATTGGGTGACCCATAATATATTCTCACCTGAGTCCAGACCTTACAG
GACCAAGAGTCTGATACTCGAAGGTCCATCTAGAAGTGGAAAAACTCTGTGGGCTAGATCTCTAGGCC
CACATAATTACATGT
+
FFFFFFFFFFFFFFFFFFFFFFF:FFFFFFFFFFF::FFFFFFFFFFFF:FFFFFFFFFFFFFFFFFFF
FFFFFFFFFFFFFFFFFFFFFFFFFFFFFFFFFFFFFFFFFFFFFFFFFFFFFFF,FFFFFFFFFFFF
FFFF:F:FFFFFFFF
@A00721:190:HGJW7DSXY:4:2302:8856:22341 2:N:0:TCTGCAAG+GCTGCACT
GTTGTTCCACAAGAGTTACAGTATTGGGTGACCCATAATATATTCTCACCTGAGTCCAGACCTTACAG
GACCAAGAGTCTGATACTCGAAGGTCCATCTAGAAGTGGAAAAACTCTGTGGGCTAGATCTCTAGGCC
CACATAATTACATGT
+
FFFFFFFFFFFFFFFF:FFFFFFFFFFFFFFFF:F,FFFFFFFFFFFFFFFFFFFFFFFFFFFFFFFFF
FFFFFFFFFFFFFFFFFFFFFFFFFFFFFFFFFFFFFFFFFFFFFFFFFFFFFFFFFFFFFFFFF:FFF
FFFFFFFFFFFFFFF
@A00721:190:HGJW7DSXY:4:2337:19090:31861 2:N:0:TCTGCAAG+GCTGCACT
GTTGTTCCACAAGAGTTACAGTATTGGGTGACCCATAATATATTCTCACCTGAGTCCAGACCTTACAG
GACCAAGAGTCTGATACTCGAAGGTCCATCTAGAAGTGGAAAAACTCTGTGGGCTAGATCTCTAGGCC
CACATAATTACATGT
+
FFFFFFFFFFFFFFFFFFFFFFFFFFFFFFFFFFFFFFFFFFFFFFFFF:FFFFFFFFFFFFFFFFFFF
FFFFF:FFFFFFFFFFFFFFFFFFFFFFFFFFFFFFFFFFFFFFF:FFFFFFFFFFFFFFFFFFFFFFFF
FFFFFFFFFFFFFFF
@A00721:190:HGJW7DSXY:4:2624:23439:31093 2:N:0:TCTGCAAG+GCTGCACT
GTTGTTCCACAAGAGTTACAGTATTGGGTGACCCATAATATATTCTCACCTGAGTCCAGACCTTACAG
GACCAAGAGTCTGATACTCGAAGGTCCATCTAGAAGTGGAAAAACTCTGTGGGCTAGATCTCTAGGCC
CACATAATTACATGT
+
FFFFFFFFFFFFFFF:FFFFFFFFFFFFFFFFFFFFFF:FFFFFFFFFF:FFFFFFFFFFFFFFFFFFF
FFFFFFFFFFFFFFFFFFFFFFFFFFFFFFFF:FFFFFFFFFF,FFFFFFFFFFFFFFFFFFFFFFFFF
FFFFFFFFFFFFFFF
@A00721:190:HGJW7DSXY:4:2302:8802:22341 2:N:0:TCTGCAAG+GCTGCACT
GTTGTTCCACAAGAGTTACAGTATTGGGTGACCCATAATATATTCTCACCTGAGTCCAGACCTTACAG
GACCAAGAGTCTGATACTCGAAGGTCCATCTAGAAGTGGAAAAACTCTGTGGGCTAGATCTCTAGGCC
CACATAATTACATG
+
FFFFFFFFFFFFFFF,FFFFFFFFFFFFFFFFFFFFFFFFFFFFFFFFF:FF:FFFFFFF:FFFFFFFF
FFFFFFFFFFFFFFFFFFFFFFFFFFF,FFF,FFFFFFF:F:FFFFFFFFFFFFFFFFFF:FFFFFF:
FF::F,::FF,FFF
@A00721:190:HGJW7DSXY:4:1213:14877:14450 2:N:0:TCTGCAAG+GCTGCACT
ATTCGTTGTTCCACAAGAGTTACAGTATTGGGTGACCCATAATATATTCTCACCTGAGTCCAGACCTT
ACAGGACCAAGAGTCTGATACTCGAAGGTCCATCTAGAAGTGGAAAAACTCTGTGGGCTAGATCTCTA
GGCCCACATAATTAC
+
FFFFFFFFFFFFFFFFFFFFFFFFFFFFFFFFFFFF:FFFFFFFFFFFFFFFFFFFFFFFFFFFFFFFF
FFFFFFFFFFFFFFFFFFFFFFFFFFFF:FFFFFFFFFFFFFFFFFFFFFFFFFFFFFFFFFFFFFFFF
FFFFFFFFFFFF,FF
```

@A00721:190:HGJW7DSXY:4:1439:1316:19680 2:N:0:TCTGCAAG+GCTGCACT
GTAATTATGTGGGCCTAGAGATCTAGCCCACAGAGTTTTTCCACTTCTAGATGGACCTTCGAGTATCA
GACTCTTGGTCCTGTAAGGTCTGGACTCAGGTGAGAATATATTATGGGTCACCCAATACTGTAACTCT
TGTGGAACAACGAAT
+
F,FFFFFFFF:FFFFFFFFF:FFFFFFFF:FFFFFFF:FFFF:FFFFFFFFFFFFFF:FF:FFFFFFF
,FFF:FFFF,F,F:FF:FFFFFF,F,FFFFF:FFFF:FFFFFFFF,FFFFFFFFF:FF,,FFFFFFFF
FFFF:FFFFFFFF:F
@A00721:190:HGJW7DSXY:4:1459:30888:16689 2:N:0:TCTGCAAG+GCTGCACT
ATTCGTTGTTCCACAAGAGTTACAGTATTGGGTGACCCATAATATATTCTCACCTGAGTCCAGACCTT
ACAGGACCAAGAGTCTGATACTCGAAGGTCCATCTAGAAGTGGAAAAACTCTGTGGGCTAGATCTCTA
GGCCCACATAATTAC
+
FFFFFFFFFFFFFFFFFFFFFFFFFF,FFFFFFFFF:FFFFFFFFFFFFFFFFFFFFFFFFFF:FFFF
FFFF:FFFFF:FFFFFFFFFFFFFFFFFFFFFFFFFFFFFFF:FFF:,FFFFFFFFFFFFFFFFFFFF
FFFFF:FFFFFFFFF
@A00721:190:HGJW7DSXY:4:1518:3983:8672 2:N:0:TCTGCAAG+GCTGCACT
GTAATTATGTGGGCCTAGAGATCTAGCCCACAGAGTTTTTCCACTTCTAGATGGACCTTCGAGTATCA
GACTCTTGGTCCTGTAAGGTCTGGACTCAGGTGAGAATATATTATGGGTCACCCAATACTGTAACTCT
TGTGGAACAACGAAT
+
FFFFFFFFFFFFFFFFFFFFFFFFFFFFFFFFFFFFFFFFFFF:FFFFFFFFFFFFFFFFFFFFFFFF
FFFFFFFFFFFFFFFFFF:FFFFFFFFFFFFFFFFFFFFFFFFFFFFFFFFFFFFFFFFFFFFFFFFF
FFFFFFFFFFFFF:F
@A00721:190:HGJW7DSXY:4:1542:27172:27884 2:N:0:TCTGCAAG+GCTGCACT
ATTCGTTGTTCCACAAGAGTTACAGTATTGGGTGACCCATAATATATTCTCACCTGAGTCCAGACCTT
ACAGGACCAAGAGTCTGATACTCGAAGGTCCATCTAGAAGTGGAAAAACTCTGTGGGCTAGATCTCTA
GGCCCACATAATTAC
+
FFFFFFFFFFFFFFFFFFFFFFFFFFFFFFFFFFFF:FFFFFFFFFFFFFFFFFFFFFFFFFFFFFFF
FFFFFFFFFFFFFFFFFFFFFFFFFFFFFFFFFFFFFFFFFFFFFFFFFFFFFFF:FFFFFFFFFFFF
FF:FFFFFF:F:FFF
@A00721:190:HGJW7DSXY:4:1611:2844:36041 2:N:0:TCTGCAAG+GCTGCACT
ATTCGTTGTTCCACAAGAGTTACAGTATTGGGTGACCCATAATATATTCTCACCTGAGTCCAGACCTT
ACAGGACCAAGAGTCTGATACTCGAAGGTCCATCTAGAAGTGGAAAAACTCTGTGGGCTAGATCTCTA
GGCCCACATAATTAC
+
FFFFFFFFFFFFFFFFFFFFFFFFFFFFFFFFFFFF:FFFFFFFFFF:FFFF:FFFFFFFFFFFFFFF
FFFF:FFFFFFFFFFFFFFFFFFFFFFFFFFFFFFF,FFFFFFFFFFFFFFFFFFFFFFFFFFFFFFF
FFFFFFFFFFFFFFF
@A00721:190:HGJW7DSXY:4:2205:6723:17581 2:N:0:TCTGCAAG+GCTGCACT
ATTCGTTGTTCCACAAGAGTTACAGTATTGGGTGACCCATAATATATTCTCACCTGAGTCCAGACCTT
ACAGGACCAAGAGTCTGATACTCGAAGGTCCATCTAGAAGTGGAAAAACTCTGTGGGCTAGATCTCTA
GGCCCACATAATTAC
+
FFFFFFFFFFFFFFFFFFFFFFFFFFFFFFFFFFFFFFFFFFFFFFFFFFFFFFFFFFFFFFFFFFFF
FFFFFFFFFFFFFFFFFFFFFFFFFFFFFFFFFFFFFFFFFFFFFFFFFFFFFFFFFFFFFFFFFFFF
FFFFFFFFFFFFFF:
@A00721:190:HGJW7DSXY:4:2219:7536:19429 2:N:0:TCTGCAAG+GCTGCACT
ATTCGTTGTTCCACAAGAGTTACAGTATTGGGTGACCCATAATATATTCTCACCTGAGTCCAGACCTT
ACAGGACCAAGAGTCTGATACTCGAAGGTCCATCTAGAAGTGGAAAAACTCTGTGGGCTAGATCTCTA
GGCCCACATAATTAC
+
F:FFFFFFFFFFFFF:FFFFF:FFFF:FFFFFFFFF:FFFFFFFFFFFFFFFFFFFFFFFFFFFFFFF

FFFFFFFFFFFFFFFFFFFFFFFFFFFFFFFFFFFF:FFFFFFFFFFFFFFFFFFFFFFFF::FFFFFFF
FFFFFFF,FFFFFFF
@A00721:190:HGJW7DSXY:4:2219:7383:19539 2:N:0:TCTGCAAG+GCTGCACT
ATTCGTTGTTCCACAAGAGTTACAGTATTGGGTGACCCATAATATATTCTCACCTGAGTCCAGACCTT
ACAGGACCAAGAGTCTGATACTCGAAGGTCCATCTAGAAGTGGAAAAACTCTGTGGGCTAGATCTCTA
GGCCCACATAATTAC
+
FFFFFFFFFFFFFFFFFFFFFFFFFFFFFFFFFFFFFFFFFFFFFFFFFF:FFFF:F:FFFFFFFF
FFFFFFFFFFFFFFFFFFFFFFFFFFFFFFFFFFFFFFFFFFFFFFFFFFFFFFFFFFFFFFFFFFFF
FFFFFFFFF:FFFFF
@A00721:190:HGJW7DSXY:4:2309:10583:34225 2:N:0:TCTGCAAG+GCTGCACT
GTAATTATGTGGGCCTAGAGATCTAGCCCACAGAGTTTTTCCACTTCTAGATGGACCTTCGAGTATCA
GACTCTTGGTCCTGTAAGGTCTGGACTCAGGTGAGAATATATTATGGGTCACCCAATACTGTAACTCT
TGTGGAACAACGAAT
+
F,FFFFFFFFFFFFFFFFFFF:FFFFFFFFFFFFFFFFFFFFFFF:,F::FFFFFFFFFFFFFFFFFFF
FFFFFF:FFFFFFFF,:FFFFFFFFFFFFFFFF:F,F::FFFFFFFFF:FFFFFFFF,FFFFFFFFFF
FFFFFFFFFFFFFFF
@A00721:190:HGJW7DSXY:4:2471:5502:26991 2:N:0:TCTGCAAG+GCTGCACT
ATTCGTTGTTCCACAAGAGTTACAGTATTGGGTGACCCATAATATATTCTCACCTGAGTCCAGACCTT
ACAGGACCAAGAGTCTGATACTCGAAGGTCCATCTAGAAGTGGAAAAACTCTGTGGGCTAGATCTCTA
GGCCCACATAATTAC
+
FFFFFFFFFFFFFFFFFFFFFFFFFFF:FFFFFFFFF:FFFFFFFFFFFFFFFFFFFFFFFFFFFFF,:
FFFFFFFFFFFFFFFFFFFFFFFFFFFFFFFFFFFFFFFFFFFFFFFFFFFFFFFFFFFFFFFFFFFF
:FFFFFFF,FFFFFF
@A00721:190:HGJW7DSXY:4:2544:16550:15781 2:N:0:TCTGCAAG+GCTGCACT
ATTCGTTGTTCCACAAGAGTTACAGTATTGGGTGACCCATAATATATTCTCACCTGAGTCCAGACCTT
ACAGGACCAAGAGTCTGATACTCGAAGGTCCATCTAGAAGTGGAAAAACTCTGTGGGCTAGATCTCTA
GGCCCACATAATTAC
+
FFFFFFFFFFFFFFFFFFFFFFFFFFFFFFFFFFFF:FFFFFFFFFFFFFFFFFFFFFFFFFFFFFFF
FFFFFFFFFFFFFFFFFFFFFFFFFFF:FFFFFFFFFFFFFFFFFFFFFFFFFFFFFFFFFFF:FFFFF
FFFFFFFFFFFFFFF
@A00721:190:HGJW7DSXY:4:1420:21558:36542 2:N:0:TCTGCAAG+GCTGCACT
ATTCGTTGTTCCACAAGAGTTACAGTATTGGGTGACCCATAATATATTCTCACCTGAGTCCAGACCTT
ACAGGACCAAGAGTCTGATACTCGAAGGTCCATCTAGAAGTGGAAAAACTCTGTGGGCTAGATCTCTA
GGCCCACATAA
+
FFFFF:,,:F:::F,F,FFF,FFFF:F:FF,,F:FF,FFFFF:FFFFFFFFFFFFFFFFF,F:FFFFF
F:FF,FF,FF,FFFF,FFFF:,:,FF:FFF:FFFFF,FF:,FFFF:FF::F,,FF::,FFFFFFFFFF
,,FFFFFF:F:
@A00721:190:HGJW7DSXY:4:1104:18891:31266 2:N:0:TCTGCAAG+GCTGCACT
CACAATCATTCGTTGTTCCACAAGAGTTACAGTATTGGGTGACCCATAATATATTCTCACCTGAGTCC
AGACCTTACAGGACCAAGAGTCTGATACTCGAAGGTCCATCTAGAAGTGGAAAAACTCTGTGGGCTAG
ATCTCTAGGCCCACA
+
FFFFFF,,FFFFFFFFFFFFFFFFFF:FFFFFFF:FF:FFFFF:F:FFFFFFFFFFFFFFFFFFFFFF
FFFFFFFFFFFFFFFFFFFFFFFFFFFFFFFFFFFFFFFFF:FFFFFFFFFFF:FFFFFFFFFFFFFF
FFFFFFFFFFFFFFF
@A00721:190:HGJW7DSXY:4:1108:3432:23093 2:N:0:TCTGCAAG+GCTGCACT
CACAATCATTCGTTGTTCCACAAGAGTTACAGTATTGGGTGACCCATAATATATTCTCACCTGAGTCC
AGACCTTACAGGACCAAGAGTCTGATACTCGAAGGTCCATCTAGAAGTGGAAAAACTCTGTGGGCTAG
ATCTCTAGGCCCACA

+
FFFFFF,FFFFFFFFFFFFFFFFFFFFFFFF:FFFFFFFFFFFF,FFFFFFFFFFFFFFFFFFFFFFFF
FFFFFFFFFFFFFFFFFFFFFFFFFFFFFFFFFFFFFFFFFFFFFFFFFFFFFFFFFFF:FFFFFFFF
FFFFFFFFFFFFFFF
@A00721:190:HGJW7DSXY:4:1109:4264:19241 2:N:0:TCTGCAAG+GCTGCACT
CACAATCATTCGTTGTTCCACAAGAGTTACAGTATTGGGTGACCCATAATATATTCTCACCTGAGTCC
AGACCTTACAGGACCAAGAGTCTGATACTCGAAGGTCCATCTAGAAGTGGAAAAACTCTGTGGGCTAG
ATCTCTAGGCCCACA
+
FFFFFF,F:FFFFFF:FFFFFFFFFFF:FFFFFFFFFFFFFFF,FFFFFFFFFFFFFFFFFFFFFFFF
FFFFFFFFFFFFFFFFFFFFFFFFFFFFFFFFFFFFFFFFFFFFFFFFFFFFFFFFFFFFFFFFFFFF
F:FFFFFFFFFFFFF
@A00721:190:HGJW7DSXY:4:1350:14742:36260 2:N:0:TCTGCAAG+GCTGCACT
CACAATCATTCGTTGTTCCACAAGAGTTACAGTATTGGGTGACCCATAATATATTCTCACCTGAGTCC
AGACCTTACAGGACCAAGAGTCTGATACTCGAAGGTCCATCTAGAAGTGGAAAAACTCTGTGGGCTAG
ATCTCTAGGCCCACA
+
FFFFF:,FFFFF:FFFFFF:FF:FFFFFFFFFFFFFFFFFFFF:FFFF:FFFFFFFFFFFF:FF:FFF
FFFFFFFFFFFFFFFFFF:FFFFFFFFFFFFFFFFFFFFFFFFFFFFFFFFFFFFFFFFFFFFFFFFF
FFFFFFFFFFFF,FF
@A00721:190:HGJW7DSXY:4:1443:7048:34240 2:N:0:TCTGCAAG+GCTGCACT
CACAATCATTCGTTGTTCCACAAGAGTTACAGTATTGGGTGACCCATAATATATTCTCACCTGAGTCC
AGACCTTACAGGACCAAGAGTCTGATACTCGAAGGTCCATCTAGAAGTGGAAAAACTCTGTGGGCTAG
ATCTCTAGGCCCACA
+
FFFFFF:F:FFFFFFFFFFFFFFFFFF:FFFFFFFFFFFFFFFFF,FFFFFFFFFFFFFFFFFFFFFF
FFFFFFFFFFFFFFFFFFFFFFFFFFFFFFFFFFFFFFFFFF::FFFFFFFFFFFFFFFFFFFFFFFF
FFFFFF:FFFFFFFF
@A00721:190:HGJW7DSXY:4:1505:26883:13510 2:N:0:TCTGCAAG+GCTGCACT
CACAATCATTCGTTGTTCCACAAGAGTTACAGTATTGGGTGACCCATAATATATTCTCACCTGAGTCC
AGACCTTACAGGACCAAGAGTCTGATACTCGAAGGTCCATCTAGAAGTGGAAAAACTCTGTGGGCTAG
ATCTCTAGGCCCACA
+
FFFFFF,F,:,FFF:FFF:F,FF:FF:FFFF:FFF:F,FFFFF::F:FFFFFFFFFFFFFF::FFFFF
F:F:F::FFFF:FF:F::FFFFF:FFFFFF:FFFFF,FFFFF:FFFF,FFFFFF:::FF:FFFFFFFF
FF,FFFFFFF:FF:F
@A00721:190:HGJW7DSXY:4:1628:25039:30765 2:N:0:TCTGCAAG+GCTGCACT
CACAATCATTCGTTGTTCCACAAGAGTTACAGTATTGGGTGACCCATAATATATTCTCACCTGAGTCC
AGACCTTACAGGACCAAGAGTCTGATACTCGAAGGTCCATCTAGAAGTGGAAAAACTCTGTGGGCTAG
ATCTCTAGGCCCACA
+
FFFFFF,FF:::FFFFFF:FFF,FFF,:FFFF:F:FFFFFFFFFFF:FFFF:,FFFFFF,FFFF:FFF
F,FFFF,FFFF,FFFFFFFFFFFFFFFFFFFFFFFFF:FFFFFFFF::::FFFF:FFFFFFFF::FFF:
F:FFFFFF:FFFFFF
@A00721:190:HGJW7DSXY:4:1673:28257:10473 2:N:0:TCTGCAAG+GCTGCACT
CACAATCATTCGTTGTTCCACAAGAGTTACAGTATTGGGTGACCCATAATATATTCTCACCTGAGTCC
AGACCTTACAGGACCAAGAGTCTGATACTCGAAGGTCCATCTAGAAGTGGAAAAACTCTGTGGGCTAG
ATCTCTAGGCCCACA
+
FFFFFF,FFFFFFFFFFFFFFFFFFFFF:FFFFF:FFFFF:FF:FFFFFFFFFFFFFFFFF:FFFFFF
FFFFF,FFFFFFFFFFFFFFFFFFFFFFFF,FFF:FFFFFFFFFFFFFFFFFFFFFFFFFFFF:FFFFFF
FFFFFFFFFFFFFFF
@A00721:190:HGJW7DSXY:4:2169:3622:4288 2:N:0:TCTGCAAG+GCTGCACT
CACAATCATTCGTTGTTCCACAAGAGTTACAGTATTGGGTGACCCATAATATATTCTCACCTGAGTCC

AGACCTTACAGGACCAAGAGTCTGATACTCGAAGGTCCATCTAGAAGTGGAAAAACTCTGTGGGCTAG
ATCTCTAGGCCCACA
+
FFFFFF:FFFFFFFFFFFFFFFFFFFFFFFF:FFFFFFFFFFFF:FFFFFFFFFFFFFFFFFFFFFFFFF
FFFFFFFFFFFFFFFFFFFFFFFFFFFFFFFFFFFFFFFFFFFFFFFFFFFFFF:FFFFFFFFFFFFF,F
FFFFFF:FFFFFFFF
@A00721:190:HGJW7DSXY:4:2172:6659:36292 2:N:0:TCTGCAAG+GCTGCACT
CACAATCATTCGTTGTTCCACAAGAGTTACAGTATTGGGTGACCCATAATATATTCTCACCTGAGTCC
AGACCTTACAGGACCAAGAGTCTGATACTCGAAGGTCCATCTAGAAGTGGAAAAACTCTGTGGGCTAG
ATCTCTAGGCCCACA
+
FFFFFF,FFFFFFFFFFFFFFFFFFFFFFFFFFFFFFFFFFFFF:FFFFFFFFFFFFFFFFFFFFFFFFF
FFFFFFFFFFFFFFFFFFFFFFFFFFFFFFFFFFFFFFFFFFF:FFFFFFFFFF,FFFFFFFFFFFFFFF
FFFFFFFFFFFFFFF
@A00721:190:HGJW7DSXY:4:2335:6650:32487 2:N:0:TCTGCAAG+GCTGCACT
CACAATCATTCGTTGTTCCACAAGAGTTACAGTATTGGGTGACCCATAATATATTCTCACCTGAGTCC
AGACCTTACAGGACCAAGAGTCTGATACTCGAAGGTCCATCTAGAAGTGGAAAAACTCTGTGGGCTAG
ATCTCTAGGCCCACA
+
FFFFFFFFFFFFFFFFFFFFFFFFFFFFFFFFFFFFFFFFFFFFFFFFFFFFFFFFFFFFFFFFFFFFFF
FFFFFFFFFFFFFFFFFFFFFFFFFFFFF:FFFFFFFFFFFFFFFFFFFFFFFFFFFFFFFFFF:FFFFF
FF:FFFFFFFFFFFF
@A00721:190:HGJW7DSXY:4:2337:30563:13526 2:N:0:TCTGCAAG+GCTGCACT
CACAATCATTCGTTGTTCCACAAGAGTTACAGTATTGGGTGACCCATAATATATTCTCACCTGAGTCC
AGACCTTACAGGACCAAGAGTCTGATACTCGAAGGTCCATCTAGAAGTGGAAAAACTCTGTGGGCTAG
ATCTCTAGGCCCACA
+
FFFFFFFFFFFFFFFFFFFFFFFFFFFFFFFFFFFFFFFFFFFFFFFFFFFFFFFFFFFFFFFFFFFFFF
FFFFFFFFFFFFFFFFFFFFFFFFFFFFFFFFFFFFFFFFFFFFFFFFFFFFFFFFFFFFFFFFFF:FFF
FFFFFFFFFF:FFFF
@A00721:190:HGJW7DSXY:4:2407:14109:23234 2:N:0:TCTGCAAG+GCTGCACT
CACAATCATTCGTTGTTCCACAAGAGTTACAGTATTGGGTGACCCATAATATATTCTCACCTGAGTCC
AGACCTTACAGGACCAAGAGTCTGATACTCGAAGGTCCATCTAGAAGTGGAAAAACTCTGTGGGCTAG
ATCTCTAGGCCCACA
+
FFFFFF,F:FFFFFFFFFFFFFFFFFFF:FFFFF,FFFFFFFFF:FFFFFFFFFFFFFFFFFFFFFFFFF
FFFFFFFFFFFFFFFF:FFFFFFFFFFFFFFFFFFFFFFFFFFFFFFFFFFFFFFFFFFFFFFFFFFFFF
FFFFFFFFFFFFFFF
@A00721:190:HGJW7DSXY:4:2416:25220:5619 2:N:0:TCTGCAAG+GCTGCACT
CACAATCATTCGTTGTTCCACAAGAGTTACAGTATTGGGTGACCCATAATATATTCTCACCTGAGTCC
AGACCTTACAGGACCAAGAGTCTGATACTCGAAGGTCCATCTAGAAGTGGAAAAACTCTGTGGGCTAG
ATCTCTAGGCCCACA
+
FFFFFF,F:FFFFFFFFFF:FFFFFF:FFFFFFFFFFFFFFFFF:FFFFFFFFFFFFFFFFFFFFFFFFF
FFFF:FFFFFF:F,FFFFFFFFFFFFF:FFFFFF:FFFFFFFFFFFFFFFFFF:FFFFFFFFF:FFFFFF
FFFFFFFFFFFFFFF
@A00721:190:HGJW7DSXY:4:2420:6189:1564 2:N:0:TCTGCAAG+GCTGCACT
CACAATCATTCGTTGTTCCACAAGAGTTACAGTATTGGGTGACCCATAATATATTCTCACCTGAGTCC
AGACCTTACAGGACCAAGAGTCTGATACTCGAAGGTCCATCTAGAAGTGGAAAAACTCTGTGGGCTAG
ATCTCTAGGCCCACA
+
FFFFFF,,FFFFFFFFFFFFFF:FFFFF:FFFFFFFFFFFFFFF:FFFFFF:FFFFFFFFFFFFFFFFFF
FFFFFFFFFFFFFFFFFFFFFFFFFFFFFFFFFFFFFFFFFF:FFFFFFFFFFFFFFF:FFF:FFFFFFF
:FFFFFFFFFFFFFF

```
@A00721:190:HGJW7DSXY:4:2432:25798:20400 2:N:0:TCTGCAAG+GCTGCACT
CACAATCATTCGTTGTTCCACAAGAGTTACAGTATTGGGTGACCCATAATATATTCTCACCTGAGTCC
AGACCTTACAGGACCAAGAGTCTGATACTCGAAGGTCCATCTAGAAGTGGAAAAACTCTGTGGGCTAG
ATCTCTAGGCCCACA
+
FFFFFF,FFFFFFFFFFFFFFFFFFFFFFFFFFFFFFFFFFFFFFFF:FFFFFFFFFFFFF:FFFFFFFF
FFFFFFFFFFFFFFFFFFFFFFFFFFFFFFFFFFFFFFFFFFFFFFFFFFFFFFF:FFFFFFFFFFFFFF
FFFFFFFFFF:FFFF
@A00721:190:HGJW7DSXY:4:2443:28664:35289 2:N:0:TCTGCAAG+GCTGCACT
CACAATCATTCGTTGTTCCACAAGAGTTACAGTATTGGGTGACCCATAATATATTCTCACCTGAGTCC
AGACCTTACAGGACCAAGAGTCTGATACTCGAAGGTCCATCTAGAAGTGGAAAAACTCTGTGGGCTAG
ATCTCTAGGCCCACA
+
FFFF:F,,FFFF,FFF:FF,F:FFFFFFFFFFFFFFFFFFFFFFFFFFFFF,FFFFFFFFFFFFF:FFFF
F::FF:FFFFFFFFFFFFFF,FFFFFF:FFF:FFFFFFFF:FF,,FFF,FFFFFFFF,FFFF:FFFFFFF
,FFFF:,FFFF:FFF
@A00721:190:HGJW7DSXY:4:2444:23249:12258 2:N:0:TCTGCAAG+GCTGCACT
CACAATCATTCGTTGTTCCACAAGAGTTACAGTATTGGGTGACCCATAATATATTCTCACCTGAGTCC
AGACCTTACAGGACCAAGAGTCTGATACTCGAAGGTCCATCTAGAAGTGGAAAAACTCTGTGGGCTAG
ATCTCTAGGCCCACA
+
FFFFFF,F,FFFFFFFFFFFFFFFFFFFFFFFFFFFFFFFFFFFF:FF:FFF,FFFFFFFFFFFFFFFFF
FFFF:FFFFFFFFFFFFFFFFFFFFFFFFF:FFF,FFFFFFFFFFFFFFFFFFFFFF:FFFFFFFFFFFF
FFFFFFFFFFFFFFF
@A00721:190:HGJW7DSXY:4:2513:1877:31266 2:N:0:TCTGCAAG+GCTGCACT
CACAATCATTCGTTGTTCCACAAGAGTTACAGTATTGGGTGACCCATAATATATTCTCACCTGAGTCC
AGACCTTACAGGACCAAGAGTCTGATACTCGAAGGTCCATCTAGAAGTGGAAAAACTCTGTGGGCTAG
ATCTCTAGGCCCACA
+
FFFFFFFFFFFFFFFFFFFFFFFFFFFFFFFFFFFFFFFFFFFFFFFFFFFFFFFFFFFFFFFFFFFFFF
FFFFFFFFFFFFFFFFFFFFFFFFFFFFFFFFFFFFFFFFFFFFFFFFFFFFFFFFFFFFFFFFFFFFFF
FFFFFFFFFFFFFFF
@A00721:190:HGJW7DSXY:4:2528:2338:31407 2:N:0:TCTGCAAG+GCTGCACT
CACAATCATTCGTTGTTCCACAAGAGTTACAGTATTGGGTGACCCATAATATATTCTCACCTGAGTCC
AGACCTTACAGGACCAAGAGTCTGATACTCGAAGGTCCATCTAGAAGTGGAAAAACTCTGTGGGCTAG
ATCTCTAGGCCCACA
+
FFFF:F,:F,FFFFFFFFFFFFFFFFFF:FFFFFF:FFFFFFF:FFFFF,F:FFFFFFFFFFFFFFFFFF
,F::F:FFFFFFFFF:FFFFFFFFFFFFFFFFFFFFFFFFFFFF:FF,F,FFFFFFFFFFFFFFFFFFFF
,FFFFF:FFFFFFF:
@A00721:190:HGJW7DSXY:4:2650:20636:13996 2:N:0:TCTGCAAG+GCTGCACT
CACAATCATTCGTTGTTCCACAAGAGTTACAGTATTGGGTGACCCATAATATATTCTCACCTGAGTCC
AGACCTTACAGGACCAAGAGTCTGATACTCGAAGGTCCATCTAGAAGTGGAAAAACTCTGTGGGCTAG
ATCTCTAGGCCCACA
+
FFFFFF,FFFFFFFFFFFFFFFFFFFFFFFFFFFFFFFFFFFFFF:FFFFFFFFFFFFFFFFFFFFFFFF
FFFFFFFFFFFFFFFFFFFFFFFFFFFFFFFFFFFFFFFFFFFF:FFFFFFFFFFF:FFFFFFFFFFFFF
FFFFFFFFFFFFFFF
@A00721:190:HGJW7DSXY:4:2673:19660:23985 2:N:0:TCTGCAAG+GCTGCACT
CACAATCATTCGTTGTTCCACAAGAGTTACAGTATTGGGTGACCCATAATATATTCTCACCTGAGTCC
AGACCTTACAGGACCAAGAGTCTGATACTCGAAGGTCCATCTAGAAGTGGAAAAACTCTGTGGGCTAG
ATCTCTAGGCCCACA
+
FFFFFF,:FFFFFFFFFFFFFFFFFFFFFFFFFF:FFFFFFFFF:FFFFFFFFFFFFFF:FFFFFFFFFF
```

FFFFFFFFFFFFFFFFFFFFFFFFFFFFFFFFFFFFFFFFFFFFFFFFFFFFFFFFFFFFFFFFFF,F
FFFFFFFFFF:FFF:
@A00721:190:HGJW7DSXY:4:1256:15736:8265 2:N:0:TCTGCAAG+GCTGCACT
CCACAATCATTCGTTGTTCCACAAGAGTTACAGTATTGGGTGACCCATAATATATTCTCACCTGAGTC
CAGACCTTACAGGACCAAGAGTCTGATACTCGAAGGTCCATCTAGAAGTGGAAAAACTCTGTGGGCTA
GATCTCTAGGCCCAC
+
FFFFFFF,FFFFFFFFFFFFFFFFFFFFFFFFFFFFFFFFFFFFFFFFFFFFFFFFFFFFFFFFFFFF
FFFFFFFFFFFFFFFFFFF,FFFFFFFFFFFFFFFFFF:FFFFFFFFFFF:FFFFFFFFFFFFFFFF
FFFFFFFFFFFFF:F
@A00721:190:HGJW7DSXY:4:1401:15637:31422 2:N:0:TCTGCAAG+GCTGCACT
CCACAATCATTCGTTGTTCCACAAGAGTTACAGTATTGGGTGACCCATAATATATTCTCACCTGAGTC
CAGACCTTACAGGACCAAGAGTCTGATACTCGAAGGTCCATCTAGAAGTGGAAAAACTCTGTGGGCTA
GATCTCTAGGCCCAC
+
F:FFFFF,FF::F:FF:FFFFFFFF:FFFFFFF,F:FF:FFFFFFFFFFFFFFF:FFFF:FFFFF,F
FFFFF,:FFF:F:FFF:FFFF:FFFFFFFFF:FFFFFF:FFFF:FFF:FF:FF:FFFFF:F,:F:FFF
FFFFFFFFFFFFFFF
@A00721:190:HGJW7DSXY:4:1403:13440:30123 2:N:0:TCTGCAAG+GCTGCACT
CCACAATCATTCGTTGTTCCACAAGAGTTACAGTATTGGGTGACCCATAATATATTCTCACCTGAGTC
CAGACCTTACAGGACCAAGAGTCTGATACTCGAAGGTCCATCTAGAAGTGGAAAAACTCTGTGGGCTA
GATCTCTAGGCCCAC
+
FFFFFFF:FFFFFFFF,FFFFFFFFFFFFFFFF,FFFFFFFFFFFFFFFFFFFFFFFFFF:FFFFFF:
FFFFFFFFFFFFFFFFFFFFFFFFFFFFFFFFFFFFFFFFFFFFFFFFFFF,FFFFFFFFFF:FFFF
FFFFFFFFF:FFFFF
@A00721:190:HGJW7DSXY:4:1470:30192:14137 2:N:0:TCTGCAAG+GCTGCACT
CCACAATCATTCGTTGTTCCACAAGAGTTACAGTATTGGGTGACCCATAATATATTCTCACCTGAGTC
CAGACCTTACAGGACCAAGAGTCTGATACTCGAAGGTCCATCTAGAAGTGGAAAAACTCTGTGGGCTA
GATCTCTAGGCCCAC
+
FFFFFFF:FFFFFFFFFFFFFFFFFFFFFF,FFFFFFFFFFFFFFFFFFFFF:FFFFFFFFFFFFFFF
FFFFFFFFFFFFFFFFFFFFFFFFFFFFFFFFFFFFFFFFFFF:FF:FFFFFFFFF,FFFFFFFFFFFF
FFFFFFFFFFFFFFF
@A00721:190:HGJW7DSXY:4:2111:30183:23328 2:N:0:TCTGCAAG+GCTGCACT
CCACAATCAGTCGTTGTTCCACAAGAGTTACAGTATTGGGTGACCCATAATATATTCTCACCTGAGTC
CAGACCTTACAGGACCAAGAGTCTGATACTCGAAGGTCCATCTAGAAGTGGAAAAACTCTGTGGGCTA
GATCTCTAGGCCCAC
+
FFFFFFF,F:FFF:F:FFFFFFFFF:FF::F:FFF:F::F:FFFFFFFF:F,F:FF,FFF:FFFFFFF
FFFFFFF:FFFFFFFFFFFF:FFFFFFFFFFFFFF,,:F::F:,FFF:FFFFFF:FF:F:FF:FFFF:
FFFFFFF:F:FFF:F
@A00721:190:HGJW7DSXY:4:2347:1705:21136 2:N:0:TCTGCAAG+GCTGCACT
CACAATCATTCGTTGTTCCACAAGAGTTACAGTATTGGGTGACCCATAATATATTCTCACCTGAGTCC
AGACCTTACAGGACCAAGAGTCTGATACTCGAAGGTCCATCTAGAAGTGGAAAAACTCTGTGGGCTAG
ATCTCTAGGCCCAC
+
FFFFFF,FFFFFFFFFFFFFFFFF:FFFFFFFFF,FFFFFFFFFFFFFFFFFFFFFFFFFFFFFFFFFF
FFFFFFFFFFFFFFFFFFFFFFFFFF:F:FF:FFFFFFFFF,FF,FF:FFFF,::FFFFFFFFFFFFF
FF:FF:FFFFFFFF
@A00721:190:HGJW7DSXY:4:2403:29984:10081 2:N:0:TCTGCAAG+GCTGCACT
CCACAATCATTCGTTGTTCCACAAGAGTTACAGTATTGGGTGACCCATAATATATTCTCACCTGAGTC
CAGACCTTACAGGACCAAGAGTCTGATACTCGAAGGTCCATCTAGAAGTGGAAAAACTCTGTGGGCTA
GATCTCTAGGCCCAC

```
+
F,FFFFF,F:FFFFFFFFFFFFFFFFFFFFFFFFFFFFFFFFFF:FFFFFFFFFFFFF:F:FFFFFFF
FFFF:FFFFFFFFFFFFFFFFFFFFFFFFFFFFFFFFFFFFF::FFFFFFFFFFFFFFFFFFF:FFF,
:FFFFFFFF:FF:FF
@A00721:190:HGJW7DSXY:4:2454:21866:3881 2:N:0:TCTGCAAG+GCTGCACT
CCACAATCATTCGTTGTTCCACAAGAGTTACAGTATTGGGTGACCCATAATATATTCTCACCTGAGTC
CAGACCTTACAGGACCAAGAGTCTGATACTCGAAGGTCCATCTAGAAGTGGAAAAACTCTGTGGGCTA
GATCTCTAGGCCCAC
+
:FFFFFF:FFFFFFF:FFF:FFFFFF:FFFFFF,FFFFFFFFFFFFFF:F,FFFFFFFFFFFFFFFFF
:FFFFF:FF:FF,FFFFFFF,F:FFFFFFF:FFF:F:FFFFFFF:FF::F:FFFF:FFFF:FFFFFFF
:F:FFF:,,:FFFF:
@A00721:190:HGJW7DSXY:4:2551:12572:7952 2:N:0:TCTGCAAG+GCTGCACT
CCACAATCATTCGTTGTTCCACAAGAGTTACAGTATTGGGTGACCCATAATATATTCTCACCTGAGTC
CAGACCTTACAGGACCAAGAGTCTGATACTCGAAGGTCCATCTAGAAGTGGAAAAACTCTGTGGGCTA
GATCTCTAGGCCCAC
+
FFFFFFF,FFFFFFFFFFFFFF:FFFFFFFFFFFFFFFFFFFFFFFF:F:FFFFFFFFFFFFFFFFFFF
FFFFFFFFFFFFFF:FFFF:FFFFFFFFFFFFFFFFFFFFFFFFFFF:F:FFFFFFFFFFFFFFFFFF:
FFFFFFFFFFFFF,F
@A00721:190:HGJW7DSXY:4:2562:28492:20556 2:N:0:TCTGCAAG+GCTGCACT
CCACAATCATTCGTTGTTCCACAAGAGTTACAGTATTGGGTGACCCATAATATATTCTCACCTGAGTC
CAGACCTTACAGGACCAAGAGTCTGATACTCGAAGGTCCATCTAGAAGTGGAAAAACTCTGTGGGCTA
GATCTCTAGGCCCAC
+
FFFFFFF,F:FFFFFFFFFFFFFFFFFFFFFFFFF:FFFFFFFFFFFFFFFFFFFFFFFFFFFFFFFFF
FFFFFFFFFFFFFFFFFFFFFFFFFFFFFFFFFFFFFFFFFFFFFFFF:FFFFFFF,FFFFFFFFFFFFF
FFFFFFFFFFFFFFF
@A00721:190:HGJW7DSXY:4:2628:9299:9173 2:N:0:TCTGCAAG+GCTGCACT
CCACAATCATTCGTTGTTCCACAAGAGTTACAGTATTGGGTGACCCATAATATATTCTCACCTGAGTC
CAGACCTTACAGGACCAAGAGTCTGATACTCGAAGGTCCATCTAGAAGTGGAAAAACTCTGTGGGCTA
GATCTCTAGGCCCAC
+
FFFFFFF,F:FFF:FFFFFFFFFFFFFF:FF:FFFFFFFFFFFFFF:F:FFFFFFF:FFFF:FFFFFF
F:FFFFFFFFFFFFFFFFFFFFFFFFFFFFFFFFFFFFFFF,FFF,FF:F,FFFFFFFFFFFFFFF:FF:
FFFFFFFFFFFFFFF
@A00721:190:HGJW7DSXY:4:2647:25735:17691 2:N:0:TCTGCAAG+GCTGCACT
CCACAATCATTCGTTGTTCCACAAGAGTTACAGTATTGGGTGACCCATAATATATTCTCACCTGAGTC
CAGACCTTACAGGACCAAGAGTCTGATACTCGAAGGTCCATCTAGAAGTGGAAAAACTCTGTGGGCTA
GATCTCTAGGCCCAC
+
FFFFFFF:FFFFFFFFFFFFFFFFFFFFFFFFFFFFFFFFFFFFFFFFFFFFFFFFFFFFFFFFFFFFF
FFFFFFFFFFFFFFFFFFFFFFFFFFFFFFFFFFFFFFFFFFFFFFFFFFFFFFFFFFFFFFFFFFFFF
FFFFFFFFFFFFFFF
@A00721:190:HGJW7DSXY:4:2663:12210:21198 2:N:0:TCTGCAAG+GCTGCACT
CCACAATCATTCGTTGTTCCACAAGAGTTACAGTATTGGGTGACCCATAATATATTCTCACCTGAGTC
CAGACCTTACAGGACCAAGAGTCTGATACTCGAAGGTCCATCTAGAAGTGGAAAAACTCTGTGGGCTA
GATCTCTAGGCCCAC
+
FFFFFFFFFFFFFFFFFFFFFFFFFFFFFFFFFFFFFFFFFFFFFFFFFFFFFFFFFFFFFFFFFFFFF
FFFFFFFFFFFFFFFFFFFFFFFFFFFFFFFFFFFFFFFFFFFFFFFFFFFFFFFFFFFFFFFFFFFF:
FFFFFFFFFFFFFFF
@A00721:190:HGJW7DSXY:4:1244:3043:29403 2:N:0:TCTGCAAG+GCTGCACT
ATCCACAATCATTCGTTGTTCCACAAGAGTTACAGTATTGGGTGACCCATAATATATTCTCACCTGAG
```

```
TCCAGACCTTACAGGACCAAGAGTCTGATACTCGAAGGTCCATCTAGAAGTGGAAAAACTCTGTGGGC
TAGATCTCTAGGCCC
+
FFFFFFFFF:FFFFFFFFFFFFFFFFFFFFFFFFFFFFFFFFFFFFFFFFFFFFFFFFFFFFFFFFFF
FFFFFFFFFFFFFFFFFFFFFFFFFFFFFFFFFFFFFFFFFFF:FFFFFFFFFFFF:FFFFFFFFFFF
FFFFFFFFFFFFFFF
@A00721:190:HGJW7DSXY:4:2534:17436:4382 2:N:0:TCTGCAAG+GCTGCACT
ATCCACAATCATTCGTTGTTCCACAAGAGTTACAGTATTGGGTGACCCATAATATATTCTCACCTGAG
TCCAGACCTTACAGGACCAAGAGTCTGATACTCGAAGGTCCATCTAGAAGTGGAAAAACTCTGTGGGC
TAGATCTCTAGGCCC
+
FFFFFFFFF,FFFFFFFFFFFFFF,FFFF:FFFFFFFFFFFFFFFFFF:FFFFFFFFFFFFFFFFFFF
FFFFFFFFFFFFFFFFFFFFFFFFFFFFFFFFFFFFFFFFFFFFFFFF:FFFFFFFFFFF:FFFFFFF
FFFF:FFFFF:FF:F
@A00721:190:HGJW7DSXY:4:2567:25816:3396 2:N:0:TCTGCAAG+GCTGCACT
ATCCACAATCATTCGTTGTTCCACAAGAGTTACAGTATTGGGTGACCCATAATATATTCTCACCTGAG
TCCAGACCTTACAGGACCAAGAGTCTGATACTCGAAGGTCCATCTAGAAGTGGAAAAACTCTGTGGGC
TAGATCTCTAGGCCC
+
FFFFFFFFF,FFFFFFFFFFFFFF:FFFFFFFFFFFFFFFFFFFFFFF:F:FFFFFFFFFFFFFFFFF
FFFFFFFFFFFFFFFFFFFFFFFFFFFFFFFFFF:FFFFFFF:FFFFF,FFFFFFFF:FFFFFFFFFF
FFFFFFFFFFFFFFF
@A00721:190:HGJW7DSXY:4:2644:32244:8829 2:N:0:TCTGCAAG+GCTGCACT
ATCCACAATCATTCGTTGTTCCACAAGAGTTACAGTATTGGGTGACCCATAATATATTCTCACCTGAG
TCCAGACCTTACAGGACCAAGAGTCTGATACTCGAAGGTCCATCTAGAAGTGGAAAAACTCTGTGGGC
TAGATCTCTAGGCCC
+
FFFFFFFFF,FFFFFFFFFFFFFFF:FFFFFFFFFFFFFFFFFFFFFFFFFFFFFFFFFFFFFFFFFF
FFFFFFFFFFFFFFFFFFFFFFFFFFFFF:FFFFF:FFFFFFFFF:FFFFFFFFFFFFFFFFFFFFF:
FFFFFFFFFFFFFFF
@A00721:190:HGJW7DSXY:4:1332:27525:20384 2:N:0:TCTGCAAG+GCTGCACT
GTTCGATCCACAATCATTCGTTGTTCCACAAGAGTTACAGTATTGGGTGACCCATAATATATTCTCAC
CTGAGTCCAGACCTTACAGGACCAAGAGTCTGATACTCGAAGGTCCATCTAGAAGTGGAAAAACTCTG
TGGGCTAGATCTCTA
+
FFFFFFFFFFFFFF,FFFFF:FFFFFFFFFFFFF:FFFFFFFFFFFFFFFFFF,FFF:FFFFFFFFFF
FFFFFFFFFFFFFFFFFFFFFFFF,FFFFFFFFFFFFFFFFFFFFF:FFFFFFFF:F:FFFFFFFFFF
FFFFFF:FFFFFFFF
@A00721:190:HGJW7DSXY:4:2307:19470:4272 2:N:0:TCTGCAAG+GCTGCACT
GTTCGATCCACAATCATTCGTTGTTCCACAAGAGTTACAGTATTGGGTGACCCATAATATATTCTCAC
CTGAGTCCAGACCTTACAGGACCAAGAGTCTGATACTCGAAGGTCCATCTAGAAGTGGAAAAACTCTG
TGGGCTAGATCTCTA
+
FFFFFFFFFFFFFF,FFFFFFFFFFFFFFFFFFFFFFFFFFFFFFFFFFFFFFFF,FFFFFFFFFFFF
FFFFFFFFFFFFFFFFFFFFFFFFFFFFFFFFFFFFFFFFFFFFFFFFFFFFFFFFFFFFFFFFFFFF
FFFFFFFFFFFFFFF
@A00721:190:HGJW7DSXY:4:2123:14714:27445 2:N:0:TCTGCAAG+GCTGCACT
TATCGCCGTTCGATCCACAATCATTCGTTGTTCCACAAGAGTTACAGTATTGGGTGACCCATAATATA
TTCTCACCTGAGTCCAGACCTTACAGGACCAAGAGTCTGATACTCGAAGGTCCATCTAGAAGTGGAAA
AACTCTGTGGGCTAG
+
FFFFFFFFFFFFFFFFFFFFF,FFFFFFFFFFFFFFFFFFFFFFFFFFFFFFFFFFFF:FFFFFFFFF
FFFFFFFF:FFFFFFFFFFFFFFFFFFFFFFFFFFFFFFFFFFFFFFFFFFFFFFFFFFFFFFFFFFF
F,FFFFFFFFFFFFF
```

```
@A00721:190:HGJW7DSXY:4:2223:24144:33536 2:N:0:TCTGCAAG+GCTGCACT
TATCGCCGTTCGATCCACAATCATTCGTTGTTCCACAAGAGTTACAGTATTGGGTGACCCATAATATA
TTCTCACCTGAGTCCAGACCTTACAGGACCAAGAGTCTGATACTCGAAGGTCCATCTAGAAGTGGAAA
AACTCTGTGGGCTAG
+
FFFFFFFFFFFFFFFFFFFFFFFFFFFFFFFFFFFFFFFFF:FFFFFFFFFFFFFFFFFFFFFFFFFF
FFFFFFFFFFFFFFFFFFFFFFFFFFFFFFFFFFFFFFFFFFFFFFFFFFFFFFFFFFFFFFFFFFFF
FFFFFFFFFFFFFFF
@A00721:190:HGJW7DSXY:4:2319:12536:11459 2:N:0:TCTGCAAG+GCTGCACT
TATCGCCGTTCGATCCACAATCATTCGTTGTTCCACAAGAGTTACAGTATTGGGTGACCCATAATATA
TTCTCACCTGAGTCCAGACCTTACAGGACCAAGAGTCTGATACTCGAAGGTCCATCTAGAAGTGGAAA
AACTCTGTGGGCTAG
+
:FFFFFFFFFFFFFFFF:F:F,FFFFFFFFFFFFFFFFFFFF:FFFFFFFFFFFF:FF,FF:FFFFFF
FFFFFF:FFFFFFFFFFFFFFFFFFFFFFFF:FFFFFFF::FFFF,::FFFF:FFFFFFFFFF,FFFFF
FFF:FFFF,FFFFFF
@A00721:190:HGJW7DSXY:4:2319:12843:11522 2:N:0:TCTGCAAG+GCTGCACT
TATCGCCGTTCGATCCACAATCATTCGTTGTTCCACAAGAGTTACAGTATTGGGTGACCCATAATATA
TTCTCACCTGAGTCCAGACCTTACAGGACCAAGAGTCTGATACTCGAAGGTCCATCTAGAAGTGGAAA
AACTCTGTGGGCTAG
+
FFFFFFFFFFFFFFFFFFFFFFFFFFFFFFFFFFFFFFFFFFFFFFFFFFFFFFFFFFFFFFFFFFFF
FFFFFFFFFFFFFFFFFFFFFFFFFFFFFFFFFFFFFFFFFFFFFFFFF:FFFFFFFFFFF::FFFFFF
FFFFFF:FFF,FFFF
@A00721:190:HGJW7DSXY:4:2319:12988:11772 2:N:0:TCTGCAAG+GCTGCACT
TATCGCCGTTCGATCCACAATCATTCGTTGTTCCACAAGAGTTACAGTATTGGGTGACCCATAATATA
TTCTCACCTGAGTCCAGACCTTACAGGACCAAGAGTCTGATACTCGAAGGTCCATCTAGAAGTGGAAA
AACTCTGTGGGCTAG
+
FF:FFFFF:FFFFFFFFFFFF,FFFFFFFFFFFFFFFFFFFFFFFFF:FFFFFFFFFF:FFFFFFFFF
FFFFFFFFFFFFFFFFFFFFFFF:FFFFFFFFFFFF:FFFFFFFFFFFFFFFFFFFFF,FFFF,FFFFF
FFFFFFFFFFFFFFF
@A00721:190:HGJW7DSXY:4:2643:26368:17096 2:N:0:TCTGCAAG+GCTGCACT
TATCGCCGTTCGATCCACAATCATTCGTTGTTCCACAAGAGTTACAGTATTGGGTGACCCATAATATA
TTCTCACCTGAGTCCAGACCTTACAGGACCAAGAGTCTGATACTCGAAGGTCCATCTAGAAGTGGAAA
AACTCTGTGGGCTAG
+
FFFFFFFFFFFFFFFFFFFFF,FFFFFFFFFFFFFFFFFFFFFFFFFFF,FFFFFFFFFFFFFFFFFFF
FFFFFFFFFFFFFFFFFFFFFFFFFFFFFFFFFF:FFFFFFFFFFFFFFFFFFFFFFFFFFFFFFFFFF
FFFFFFFFFFFFFFF
@A00721:190:HGJW7DSXY:4:2332:31222:16986 2:N:0:TCTGCAAG+GCTGCACT
TATCGCCGTTCGATCCACAATCATTCGTTGTTCCACAAGAGTTACAGTATTGGGTGACCCATAATATA
TTCTCACCTGAGTCCAGACCTTACAGGACCAAGAGTCTGATACTCGAAGGTCCATCTAGAAGTGGAAA
AACTCTGTGGGCTA
+
FFFFFFFFFFFFFFFFFFFFF,FFFFFFFFFFFFFF:FFFFFFFFFF:FFFFFFFF:FFFFFFFFFFF
FFFFFFFFFFFFFFFFFFFFFFF:FFFFFFFFFFFFFFFFFFFFFFFFF:FFFFFFFFFFFFFFFFFFFF
,:FFFFFFFFFFF:
@A00721:190:HGJW7DSXY:4:2354:30038:23829 2:N:0:TCTGCAAG+GCTGCACT
TATCGCCGTTCGATCCACAATCATTCGTTGTTCCACAAGAGTTACAGTATTGGGTGACCCATAATATA
TTCTCACCTGAGTCCAGACCTTACAGGACCAAGAGTCTGATACTCGAAGGTCCATCTAGAAGTGGAAA
AACTCTGTGGGCTA
+
FFFFFFF:FFFFFFFFFFFFF,FFFFFF:FFFFFFFFFFF:FFFF:FFFFFFFFFFFF:FFFFF:FFF
```

F:FF:FFFFFFFFFFFFFFFFFFFFFFFFFF:FFF:FFFFFFFFFFFFFFFFFFFFFFFFFFFFFF:FFF
FFFFF::FFFFFFF
@A00721:190:HGJW7DSXY:4:1209:7726:9956 2:N:0:TCTGCAAG+GCTGCACT
AGTTTTTCCACTTCTAGATGGACCTTCGAGTATCAGACTCTTGGTCCTGTAAGGTCTGGACTCAGGTG
AGAATATATTATGGGTCACCCAATACTGTAACTCGTGTGGAACAACGAATGATTGTGGATCGAACGGC
GATAGATATTCTTGT
+
FFFFFFFFFFFFFFFFFFFFFFFFFFFFFFFFFFFFFFFFFFFFFFFFFFFFFFFFFFFFFFFFFFFFFF
FFFFFFFFFFFFFFFFFFFFFFFFFFFFFFF:FFFFFFFFFFFFFFFFFFFFFFFFFFFFFFF:FFFFFF
FFFFFFFFFFFFFFF
@A00721:190:HGJW7DSXY:4:1638:18945:13354 2:N:0:TCTGCAAG+GCTGCACT
AGTTTTTCCACTTCTAGATGGACCTTCGAGTATCAGACTCTTGGTCCTGTAAGGTCTGGACTCAGGTG
AGAATATATTATGGGTCACCCAATACTGTAACTCGTGTGGAACAACGAATGATTGTGGATCGAACGGC
GATAGATATTCTTGT
+
FFFFFFFFFFFFFFFFFFFFFFFFFFFFFFFF,FFFFFFFFFFFFFFFFFFFFFFFFFFFFFF:FFFFFF
FFFFFFFFF:FFFFFFFFFFFFFFFFFFFFFFFFFFFFFFFFFFFFFFFFFFFFFFFFFFFFF:FFFFFF
:FFFFFFFFFFFFFF
@A00721:190:HGJW7DSXY:4:1648:27218:21418 2:N:0:TCTGCAAG+GCTGCACT
AGTTTTTCCACTTCTAGATGGACCTTCGAGTATCAGACTCTTGGTCCTGTAAGGTCTGGACTCAGGTG
AGAATATATTATGGGTCACCCAATACTGTAACTCGTGTGGAACAACGAATGATTGTGGATCGAACGGC
GATAGATATTCTTGT
+
FFFFFFFFFFF,FFFF:FFFFFFFFFFFFFFFFFFFFFFFFFFFFFFFFFFFFFFFFFFFFFFFFFF:FF
FF:FFFFFFFFFFFFFFFFFF,FFFFFFFFFFFFFFFFFFFFFFF,F:FFFFFFFFFFFFFFFFFFFFFF
FF:FFF::FFFFF:F
@A00721:190:HGJW7DSXY:4:1649:12500:13745 2:N:0:TCTGCAAG+GCTGCACT
AGTTTTTCCACTTCTAGATGGACCTTCGAGTATCAGACTCTTGGTCCTGTAAGGTCTGGACTCAGGTG
AGAATATATTATGGGTCACCCAATACTGTAACTCGTGTGGAACAACGAATGATTGTGGATCGAACGGC
GATAGATATTCTTGT
+
FFFFFFFFFFFFFFFFFFFFFFFFFFFFFFFFFFFFFFFFFFFFFFFFFFFFFFF:FFFFFFFFFFFFFF
FFFFFFFFFFFFF,FFFFFFFFFFFFFFFFFFFFFFFFFFFFFFFFFFFFFFFFFFFFFFFFFFFFFFFF
FFFFFF:FFFFFFFF
@A00721:190:HGJW7DSXY:4:2202:31313:36746 2:N:0:TCTGCAAG+GCTGCACT
AGTTTTTCCACTTCTAGATGGACCTTCGAGTATCAGACTCTTGGTCCTGTAAGGTCTGGACTCAGGTG
AGAATATATTATGGGTCACCCAATACTGTAACTCGTGTGGAACAACGAATGATTGTGGATCGAACGGC
GATAGATATTCTTGT
+
FFFFFFFFFFFFFFFFFFFFFFFFFFFFFFFFFFFFFFFFFFFFFFFFFFFFFFFFFFFFFFFFFFFFFF
FFFFFFFFFFFFFFFFFFFFFF:FFFFFFFFFFFFFFFFFFFFFFFFFF:FFFF:FFFFFFFFFFFFFFF
FFFFFFFFFFFFFFF
@A00721:190:HGJW7DSXY:4:2207:22652:11600 2:N:0:TCTGCAAG+GCTGCACT
AGTTTTTCCACTTCTAGATGGACCTTCGAGTATCAGACTCTTGGTCCTGTAAGGTCTGGACTCAGGTG
AGAATATATTATGGGTCACCCAATACTGTAACTCGTGTGGAACAACGAATGATTGTGGATCGAACGGC
GATAGATATTCTTGT
+
F:FF::FFFFF,:FFFFFF:FFFFFF,:FFFFF,F,FFF:FFFF:,FF,F,FF:FFFF,FFFFFF:FFF
FFFFFFFFFFFFF:F:FF,FFFF,FF:,,FFF,F:,FF:F:FFFFFFFFFFFF,FFF:FFFFFFFFFFF
FFF,FFFFFF:::,:
@A00721:190:HGJW7DSXY:4:2355:4634:17534 2:N:0:TCTGCAAG+GCTGCACT
AGTTTTTCCACTTCTAGATGGACCTTCGAGTATCAGACTCTTGGTCCTGTAAGGTCTGGACTCAGGTG
AGAATATATTATGGGTCACCCAATACTGTAACTCGTGTGGAACAACGAATGATTGTGGATCGAACGGC
GATAGATATTCTTGT

```
+
FFFFFFFFFFFFFFFF:FFFFFFF:FF:FFFFFFFFFFFFFFFFFFFFFFFFFFFFFFFFFFFFFFF:FF
FFFFFFFFFFFFF:F:FFFFFFF:FF:FFFFFF:FFFFFFFFFFF:FFFF:F:FFFFFFFFFFFFFFF
FFFFFFFFFFFFFFF
@A00721:190:HGJW7DSXY:4:2526:1226:15922 2:N:0:TCTGCAAG+GCTGCACT
AGTTTTTCCACTTCTAGATGGACCTTCGAGTATCAGACTCTTGGTCCTGTAAGGTCTGGACTCAGGTG
AGAATATATTATGGGTCACCCAATACTGTAACTCGTGTGGAACAACGAATGATTGTGGATCGAACGGC
GATAGATATTCTTGT
+
FFFFFFFFFF:FFFFFFFFFFFFFFFFFFFFFFFFF:FF:FFFFFFFFFFFFFFFFFFFFFFFFFFFFF
FFF:FFFFFFFFFFFFFFFFFFFF,FFFFFFFFFF:FFFFFFFFFFFFFFFFFFFFFFFFFFF:FFFFFF
FFFFFFFFFF:FF:F
@A00721:190:HGJW7DSXY:4:2648:28094:19961 2:N:0:TCTGCAAG+GCTGCACT
AGTTTTTCCACTTCTAGATGGACCTTCGAGTATCAGACTCTTGGTCCTGTAAGGTCTGGACTCAGGTG
AGAATATATTATGGGTCACCCAATACTGTAACTCGTGTGGAACAACGAATGAGTGTGGATCGAACGGC
GATAGATATTCTTGT
+
FFFFFFFFFFFFFFFFFFFFFFFFFFFFFFFFFFFFFFFFFFFFFFFF,FFFFFFFFF:FFFFFFFFFF
FFFFFFFFFFFFFFFFFFFFFFFFFFFFFFFFFFFFFFFFFFFFFFFFFFFF,FFFFFFFFFFFFFFF
FFFFFF:FFFFFFFF
@A00721:190:HGJW7DSXY:4:2655:7455:24455 2:N:0:TCTGCAAG+GCTGCACT
AGTTTTTCCACTTCTAGATGGACCTTCGAGTATCAGACTCTTGGTCCTGTAAGGTCTGGACTCAGGTG
AGAATATATTATGGGTCACCCAATACTGTAACTCGTGTGGAACAACGAATGATTGTGGATCGAACGGC
GATAGATATTCTTGT
+
FFFFFFFFFFFFFFFFFFFFFFFFFFFFFFFFFFFFFFFFFFF:FFFFFFFFFFFFFF:FFFFFFFFF
FFF:FFFFFFFFFFFFFFFFFFFFFFFFFFFFFFFFFFFFFFFFFFFFFFFFF:FFFFFFFFFFFFFFF
FFFFFFFF:FFFFFF
@A00721:190:HGJW7DSXY:4:1128:29559:20744 2:N:0:TCTGCAAG+GCTGCACT
TACAAGAATATCTATCGCCGTTCGATCCACAATCATTCGTTGTTCCACAAGAGTTACAGTATTGGGTG
ACCCATAATATATTCTCACCTGAGTCCAGACCTTACAGGACCAAGAGTCTGATACTCGAAGGTCCATC
TAGAAGTGGAAAAAC
+
FFFFFFFFFFFFFFFFFFFFFFFFFFFFFFFFFF:FFFFFFFFFFFF:FFFFFF:FFFFFFFFFFFFFF
FFFFFF:FFFFFFFFFFFFFFFFFFFFFFFFFFFFFFFFFFFFFFFFFFFFFFFFFFFFFFFFFFFFFF
FFFFFFFFFF:FFFF
@A00721:190:HGJW7DSXY:4:1170:25753:19413 2:N:0:TCTGCAAG+GCTGCACT
TACAAGAATATCTATCGCCGTTCGATCCACAATCATTCGTTGTTCCACAAGAGTTACAGTATTGGGTG
ACCCATAATATATTCTCACCTGAGTCCAGACCTTACAGGACCAAGAGTCTGATACTCGAAGGTCCATC
TAGAAGTGGAAAAAC
+
FFFFFFFFFFFFFFFFFFFFFFFFFFFFFFFFFF:FFFFFFFFFFFFFFFFFFFFFFFFFFFFFFFFFFF
FFFFFFFFFFFFFFFFFFFFFFFFFFFFFFFFFFFFFFFFFFFFFFF:FFFFFFFFFFFFFFFFFFF:FF
FFFFFFFFFFFFF:F
@A00721:190:HGJW7DSXY:4:1258:29387:23891 2:N:0:TCTGCAAG+GCTGCACT
TACAAGAATATCTATCGCCGTTCGATCCACAATCATTCGTTGTTCCACAAGAGTTACAGTATTGGGTG
ACCCATAATATATTCTCACCTGAGTCCAGACCTTACAGGACCAAGAGTCTGATACTCGAAGGTCCATC
TAGAAGTGGAAAAAC
+
F,FFFFFFFFFFFFFFFFFFFFFFFFFFFFFFF:FFFFFFFFFFFFFFFFFFFFFFFFFFFFFFFFFFF
FFFFFFFFFFFFFFFFFFF:FFFFFFFFFFFFFFFFFFFFFFFFFFFFFFFFFFFFFFFFFFFFFFFFF
FFFFFFFFFFFFFFF
@A00721:190:HGJW7DSXY:4:1575:17309:28025 2:N:0:TCTGCAAG+GCTGCACT
TACAAGAATATCTATCGCCGTTCGATCCACAATCATTCGTTGTTCCACAAGAGTTACAGTATTGGGTG
```

ACCCATAATATATTCTCACCTGAGTCCAGACCTTACAGGACCAAGAGTCTGATACTCGAAGGTCCATC
TAGAAGTGGAAAAAC
+
FFFFFFFFF:FFFFFFFFFFFFFFFFFFFFFFFFFFFFFFFFFFFFFFFF,FFFFFFFFFF:FFFFFF
FFFFFFFFFFFFFFFFFFFFFFFFFFFFFFFFFFFFFFFFFFFFFFFFFFFFFFFFFFFFFFFFFFFF
FFFFFFFFFFFFFF:
@A00721:190:HGJW7DSXY:4:1604:15501:11741 2:N:0:TCTGCAAG+GCTGCACT
TACAAGAATATCTATCGCCGTTCGATCCACAATCATTCGTTGTTCCACAAGAGTTACAGTATTGGGTG
ACCCATAATATATTCTCACCTGAGTCCAGACCTTACAGGACCAAGAGTCTGATACTCGAAGGTCCATC
TAGAAGTGGAAAAAC
+
FFFFFFFFFFFFFFFFFFFFFFFFFFFFFFF:F,FFFFFFFFFFFFFFFFFFFFFFFFF:FFFFFFFF
FFFFFFFFFFFFFFFFFF:FFFFFFF:FFFFFFFFFF:FFFFFFFFFFFFFF:FFFFFFFFFFFFFFF
FFFFFFFFFFFFFF:
@A00721:190:HGJW7DSXY:4:2262:10890:8985 2:N:0:TCTGCAAG+GCTGCACT
TACAAGAATATCTATCGCCGTTCGATCCACAATCATTCGTTGTTCCACAAGAGTTACAGTATTGGGTG
ACCCATAATATATTCTCACCTGAGTCCAGACCTTACAGGACCAAGAGTCTGATACTCGAAGGTCCATC
TAGAAGTGGAAAAAC
+
FFFFFFFFFFFFFFFFFFFFFFFFFFFFFFFFF,FFFFFFFFFFFFFFFFFFF:FFFFFFFFFFFFFF
FFFFFFFFFFFFFFFFFF:FFFFFFFFFFFFFFFFFFFFFFFFFFFFFFFFFFFFFFFFFFFFFFFFF
FFFFFFFFFFFFFFF
@A00721:190:HGJW7DSXY:4:2345:9308:18740 2:N:0:TCTGCAAG+GCTGCACT
TACAAGAATATCTATCGCCGTTCGATCCACAATCATTCGTTGTTCCACAAGAGTTACAGTATTGGGTG
ACCCATAATATATTCTCACCTGAGTCCAGACCTTACAGGACCAAGAGTCTGATACTCGAAGGTCCATC
TAGAAGTGGAAAAAA
+
FFFFFFFFFFFFFFFFFFFFFFFFFFFFFFFFFFFFFFFF::FFFFFFFF,F:FFF:F:FFFFF:FF:
F:FFFFFFFFFFFF:F:F:F::F,:,FF:F::,FF:F,:F::FF,F,:::,FFF:,::FF,,:,:F::
,F,FF,:::FFFFFF
@A00721:190:HGJW7DSXY:4:2530:11849:19695 2:N:0:TCTGCAAG+GCTGCACT
AGTAATTCCACTACTAGAAGCACCTTCGAGTATCAGACTCTTGGTCCTGTAAGGTCTGGACTCAGGTG
AGAATATATTATGGGTCACCCAATACTGTAACACGACAGGAACAACGAAAGATTCTGGATCGAACGGC
GATAGATATAC
+
FF,,,:F,FF:F,:,FFF,:,:F,,F::F,:F:FF,:F,:,:,,:FF,,,FF,,,F,:FFF:FF,,,,
FFF,,F:F,FF,,,:,FFFF,FF,F,,,FF:F,:,,,,,::,:F:,,FF,FFF:,:,,FF:,FFF:,F
,F,FFF,,:,:
@A00721:190:HGJW7DSXY:4:1166:20799:9737 2:N:0:TCTGCAAG+GCTGCACT
CCAGAACCAATACAAGAATATCTATCGCCGTTCGATCCACAATCATTCGTTGTTCCACAAGAGTTACA
GTATTGGGTGACCCATAATATATTCTCACCTGAGTCCAGACCTTACAGGACCAAGAGTCTGATACTCG
AAGGTCCATCTAGAA
+
FFFFFFFFFFFFFFFFFFFFFFFFFFFFFFFFFFFFFFFF:FF:FFFFFFFFFFFFFFFFFFFFFFFF
FF:FFFFFFFFFFFFFFFFFFFFFFFFFFFFFFFFFFFFFFFFFFFFFFFFFFFFFFFFFFFFFFFFF
FFFFFFFFFFFFFFF
@A00721:190:HGJW7DSXY:4:1169:3784:6355 2:N:0:TCTGCAAG+GCTGCACT
CCAGAACCAATACAAGAATATCTATCGCCGTTCGATCCACAATCATTCGTTGTTCCACAAGAGTTACA
GTATTGGGTGACCCATAATATATTCTCACCTGAGTCCAGACCTTACAGGACCAAGAGTCTGATACTCG
AAGGTCCATCTAGAA
+
FFFFFFFFFFFFFFFFFFFFFFFFFFFFFFFFFFFFFFFFFFF:FFFFFFFFFFFFFFFFFFFFFFFF
FFFFFFFFFFFFFFFFFFFFFFFFFFFFFFFFFFFFFFFFFFFFFFFFF:FFFFFFFFFFFFFFFFFF
FFFFFFFFFFFFFFF

```
@A00721:190:HGJW7DSXY:4:1262:29432:21371 2:N:0:TCTGCAAG+GCTGCACT
CCAGAACCAATACAAGAATATCTATCGCCGTTCGATCCACAATCATTCGTTGTTCCACAAGAGTTACA
GTATTGGGTGACCCATAATATATTCTCACCTGAGTCCAGACCTTACAGGACCAAGAGTCTGATACTCG
AAGGTCCATCTAGAA
+
F,FFFFFFFFFFFFF:FFFFFFFFFFFFF:FFFFFFFFFFFFFFFFFFF:FF:FFFFFFFFFFF:FFFFF
FF:FFFFFFFFFFFFFFFFFFFF,:F:F:F::FFFFFFFFF::FFFFFF:F:FFFFFFFFFFFFFFFFFF
FFF,FFFFF::FFFF
@A00721:190:HGJW7DSXY:4:1320:6280:32158 2:N:0:TCTGCAAG+GCTGCACT
CCAGAACCAATACAAGAATATCTATCGCCGTTCGATCCACAATCATTCGTTGTTCCACAAGAGTTACA
GTATTGGGTGACCCATAATATATTCTCACCTGAGTCCAGACCTTACAGGACCAAGAGTCTGATACTCG
AAGGTCCATCTAGAA
+
FFFFFFFFFFFFFFFFFFFFFFFFFFFFFFFFFFFFFFFFFFFFFFFFFFFFFFFFFFFFFFFFFFFFFF
FFFFFFFFFFFFFFFFFFFFFFFFFFFFFFFFFFFFFFFFFFFFFFFFFFFFFFFFFFFFFFFFFFFFFF
FFFFFFFFFFFFFFF
@A00721:190:HGJW7DSXY:4:1342:28827:27023 2:N:0:TCTGCAAG+GCTGCACT
CCAGAACCAATACAAGAATATCTATCGCCGTTCGATCCACAATCATTCGTTGTTCCACAAGAGTTACA
GTATTGGGTGACCCATAATATATTCTCACCTGAGTCCAGACCTTACAGGACCAAGAGTCTGATACTCG
AAGGTCCATCTAGAA
+
FFFFF:FFFFFFFFFFFFF:FFFFFFFFFFFFF:FFFFFFFFF:FFFFFFFFFFFFFFFFFFFFFFFFFF
FFFFFFFFFFFFFFFFFFFFF:FFFFFF:F:FFFFFFFFFFFFFFFFFFFFFFFFFFFFFFFFFFFFFFF
FFFFFFFFFFFFFFF
@A00721:190:HGJW7DSXY:4:1363:21206:2895 2:N:0:TCTGCAAG+GCTGCACT
CCAGAACCAATACAAGAATATCTATCGCCGTTCGATCCACAATCATTCGTTGTTCCACAAGAGTTACA
GTATTGGGTGACCCATAATATATTCTCACCTGAGTCCAGACCTTACAGGACCAAGAGTCTGATACTCG
AAGGTCCATCTAGAA
+
FFFFFFFFFFFFFFFFFFFFFFFFFFFFFFFFFFFFFFFFFFFFFFFFFFFFFFFFFFFFFFFFFFFFFF
FFFFFFFFFFFFFFFFFFFFFFFFFFFFFFFFFFFFFFFFFFFFFFFFFFFFFFFFFFFFFFFFFFFFFF
FFFFFFFFFFFFFFF
@A00721:190:HGJW7DSXY:4:1639:26006:27398 2:N:0:TCTGCAAG+GCTGCACT
CCAGAACCAATACAAGAATATCTATCGCCGTTCGATCCACAATCATTCGTTGTTCCACAAGAGTTACA
GTATTGGGTGACCCATAATATATTCTCACCTGAGTCCAGACCTTACAGGACCAAGAGTCTGATACTCG
AAGGTCCATCTAGAA
+
FFFFFFFFFFFFFFFFFFFFFFFFFFFFFFFFFFFFFFFFFFFF:FFFFFFFFFFFFFFFFFFFFFFFFF
FFFFFFFFFFFFFF:F:FFFFFFFFFFFFFFFFFFFFFFFFFFFFFFFFFFFFFFFFFFFFFFFFFFFFF
FFFFFFFFFFFFFFF
@A00721:190:HGJW7DSXY:4:1643:6388:24893 2:N:0:TCTGCAAG+GCTGCACT
CCAGAACCAATACAAGAATATCTATCGCCGTTCGATCCACAATCATTCGTTGTTCCACAAGAGTTACA
GTATTGGGTGACCCATAATATATTCTCACCTGAGTCCAGACCTTACAGGACCAAGAGTCTGATACTCG
AAGGTCCATCTAGAA
+
FFFFFFFFFFFFFFFFFFFFFFFFFFFFFFFFFFFFFFFFFFFF:FFFFFFFFFFFFFFFFFFFFFFFFF
FFFFFFFFFFFFFFFFFFFFFFFFFFFFFFFFFFFFFFFFFFFFFFFFFFFFFFFFFFFFFFFFFFFFFF
FFFFFFFFFFFFFFF
@A00721:190:HGJW7DSXY:4:2147:4797:12931 2:N:0:TCTGCAAG+GCTGCACT
CCAGAACCAATACAAGAATATCTATCGCCGTTCGATCCACAATCATTCGTTGTTCCACAAGAGTTACA
GTATTGGGTGACCCATAATATATTCTCACCTGAGTCCAGACCTTACAGGACCAAGAGTCTGATACTCG
AAGGTCCATCTAGAA
+
FFFFFFFFFFFFFFFFFFFFFFFFFFFFFFFFFFFFFFFFFFFF,FFFFFFFFFFFFFFFF,FFFFFFFF
```

FFFFFFFFFFFFFFFFFFFFFFFFFFFF:FFFFFFFFFFFFFFFFFFFFFFFFFFFFFFFF:FFFFFF
FFFFFFFFFFFFFFF
@A00721:190:HGJW7DSXY:4:2151:21649:28526 2:N:0:TCTGCAAG+GCTGCACT
CCAGAACCAATACAAGAATATCTATCGCCGTTCGATCCACAATCATTCGTTGTTCCACAAGAGTTACA
GTATTGGGTGACCCATAATATATTCTCACCTGAGTCCAGACCTTACAGGACCAAGAGTCTGATACTCG
AAGGTCCATCTAGAA
+
FFFFFFFFFFFFFFFFFFFFFFFFFFFFFFFFFFFFFFFFFFF:FFFFFFFFF:FFFFFFFFFFFFFF
FFFFFFFFFFFFFFFFFFFFFFFFFFFF:FFFFFFFFFFFFFFFFFFFFFFFFFFFFFFFFFFFFFFF
FFFFFFFFFFFFFFF
@A00721:190:HGJW7DSXY:4:2215:22634:7623 2:N:0:TCTGCAAG+GCTGCACT
CCAGAACCAATACAAGAATATCTATCGCCGTTCGATCCACAATCATTCGTTGTTCCACAAGAGTTACA
GTATTGGGTGACCCATAATATATTCTCACCTGAGTCCAGACCTTACAGGACCAAGAGTCTGATACTCG
AAGGTCCATCTAGAA
+
FFFFFFFFFFFFFFFFFFFFFFFFFFFFFFFFFFFFFFFFFFF,FFFFFFFFFFFFFFFFFFFFFFFF
FFFFFFFFFFFFFFFFFFFFFF:FFFFF:FFFFFFFFFFFFFFFFFFFFFFFFFFFFFFFFFFFFFFF
FFFFFFFFFFFFFFF
@A00721:190:HGJW7DSXY:4:2221:11668:5196 2:N:0:TCTGCAAG+GCTGCACT
CCAGAACCAATACAAGAATATCTATCGCCGTTCGATCCACAATCATTCGTTGTTCCACAAGAGTTACA
GTATTGGGTGACCCATAATATATTCTCACCTGAGTCCAGACCTTACAGGACCAAGAGTCTGATACTCG
AAGGTCCATCTAGAA
+
FFFFFFFFFFFFFFFFFFFFFFFFFFFFFFFFFFFFFFFFFFF,FFFFFFFFFFFFFFFFFFFFFFFF
FFFFFFFFFFFFFFFFFFFFFFFFFFFF:FFFFFFFFFFFFFFFFFFFFFFFFFFFFFFFFFFFFFFF
FFFFFFFFFFFFFFF
@A00721:190:HGJW7DSXY:4:2405:6885:21966 2:N:0:TCTGCAAG+GCTGCACT
CCAGAACCAATACAAGAATATCTATCGCCGTTCGATCCACAATCAATCGTTGTTCCACAAGAGTTACA
GTATTGGGTGACCCATAATATATTCTCACCTGAGTCCAGACCTTACAGGACCAAGAGTCTGATACTCG
AAGGTCCATCTAGAA
+
F:F:FFFFFFFFFFF:FFFFFFFFFFFFFFFF:FFFFFFFFFF,F,FFFFF:FFF:FFFFFFFFFFFF
F:FFFFFFFFFFFFFFFFFFFFFFFF:F,FFFFFF:FF,F,FFFFFF:,FFFFFFFF:FFFF:FFFFF
FF:FFF:FFFFFFFF
@A00721:190:HGJW7DSXY:4:2428:23475:33505 2:N:0:TCTGCAAG+GCTGCACT
CCAGAACCAATACAAGAATATCTATCGCCGTTCGATCCACAATCATTCGTTGTTCCACAAGAGTTACA
GTATTGGGTGACCCATAATATATTCTCACCTGAGTCCAGACCTTACAGGACCAAGAGTCTGATACTCG
AAGGTCCATCTAGAA
+
FFFFFFFFFFFFFFFFFFFFFFFFFFFFFFFFFFFFFFFFFFF,FFFFFFFFFFFFFFFFFFFFFFFF
FFFFFFFFFF:FFFFFFFFFFFFFFFFF:FFFFFFFFFFFF:FFFFFF:FFFFFFFF::FFFFFFFFF
FFFFFFFFFF,FFFF
@A00721:190:HGJW7DSXY:4:2442:11035:8140 2:N:0:TCTGCAAG+GCTGCACT
CCAGAACCAATACAAGAATATCTATCGCCGTTCGATCCACAATCATTCGTTGTTCCACAAGAGTTACA
GTATTGGGTGACCCATAATATATTCTCACCTGAGTCCAGACCTTACAGGACCAAGAGTCTGATACTCG
AAGGTCCATCTAGAA
+
FFFFFFFFFFFFFFFFFFFFFFFFFFFFFFFFFFFFFFFFFFF,FFFFFFFFFFFFFFFFFFFFFFFF
FFFFFFFFFFFFFFFFFFFFFFFFFFFF:F:FFFFFFFFFFFFFFFFFFFFFFFFFFFF:FFFFFFFF
FFFFFFFFFFFFFFF
@A00721:190:HGJW7DSXY:4:2578:7979:13056 2:N:0:TCTGCAAG+GCTGCACT
CCAGAACCAATACAAGAATATCTATCGCCGTTCGATCCACAATCATTCGTTGTTCCACAAGAGTTACA
GTATTGGGTGACCCATAATATATTCTCACCTGAGTCCAGACCTTACAGGACCAAGAGTCTGATACTCG
AAGGTCCATCTAGAA

```
+
FFFFFFFFFFFFF:FFFFFFFFFFFFFFFFFFFFFFFFFFFFFF,FFFFFFFFFFFFFF:FFF:FFFF
FFFFFF:FFFFFFFFFFFFFFFFFFFFFFFFFFFFF:FFFFFFFFFFFFFFFFFFFFFFFF:FFFFFF
FFFFFFFFFFFF,FF
@A00721:190:HGJW7DSXY:4:2622:31665:36229 2:N:0:TCTGCAAG+GCTGCACT
CCAGAACCAATACAAGAATATCTATCGCCGTTCGATCCACAATCATTCGTTGTTCCACAAGAGTTACA
GTATTGGGTGACCCATAATATATTCTCACCTGAGTCCAGACCTTACAGGACCAAGAGTCTGATACTCG
AAGGTCCATCTAGAA
+
FFFFFFFFFFFFFFFFFFFFFFFFFFFFFFFFFFFFFFFFFFF:FFFFFFFFFFFFFFFFFFFFFFFF
FFFFFFFFFFFFFFFFFFFFFFFFFFFFFF:FFFFFFFFFFFFFFFFFFFFFFFFFFFFFFFFFF:FFF
FF:FFFFFFFFFFFF
@A00721:190:HGJW7DSXY:4:2658:23665:21339 2:N:0:TCTGCAAG+GCTGCACT
CCAGAACCAATACAAGAATATCTATCGCCGTTCGATCCACAATCATTCGTTGTTCCACAAGAGTTACA
GTATTGGGTGACCCATAATATATTCTCACCTGAGTCCAGACCTTACAGGACCAAGAGTCTGATACTCG
AAGGTCCATCTAGAA
+
FFFFFFFFFFFFFFFFFFFFFFFFFFFFFFFFFFFFFFFFFFF,FFFFFFFFFFFFFFFFFFFFFFFF
FFFFFFFFFFFFFFFFFFFFFF:FFFFFF:FFFFFFFFFFFFFFFFFFFFFFFFFFFFFFFFFFFFFFF
FFFFFFFFFFFFFFF
@A00721:190:HGJW7DSXY:4:2663:3287:13354 2:N:0:TCTGCAAG+GCTGCACT
CCAGAACCAATACAAGAATATCTATCGCCGTTCGATCCACAATCATTCGTTGTTCCACAAGAGTTACA
GTATTGGGTGACCCATAATATATTCTCACCTGAGTCCAGACCTTACAGGACCAAGAGTCTGATACTCG
AAGGTCCATCTAGAA
+
FFFFFFFFFFFFFFFFFFFFFFFFFFFFFFFFFFFFFFFFFFF,FFFFFFFFFFFFFFFFFFFFFFFF
FFFFFFFFFFFFFFFFFFFFFFFFFFFFF:FFFFFFFFFFFFFFFFFFFFFFFFFFFFFFFFFFFFFFF
FFFFFFFFFFFFFFF
@A00721:190:HGJW7DSXY:4:2668:3007:5196 2:N:0:TCTGCAAG+GCTGCACT
CCAGAACCAATACAAGAATATCTATCGCCGTTCGATCCACAATCATTCGTTGTTCCACAAGAGTTACA
GTATTGGGTGACCCATAATATATTCTCACCTGAGTCCAGACCTTACAGGACCAAGAGTCTGATACTCG
AAGGTCCATCTAGAA
+
FFFFFFFFFFFFFFFFFFFFFFFFFFFFFFFFFFFFFFFFFFF,FFFFF:FFFFFFFFF,FFF:FFFF
FF:FFFFFFFFFFFFFF:FFFFFFFFFF:FFFFFFFFFFFFFFFFFFFFFFFFFFFFFFFFFFFFFFF
FFFFFFFFFFFFFFF
@A00721:190:HGJW7DSXY:4:1107:15185:28385 2:N:0:TCTGCAAG+GCTGCACT
TCCAGAACCAATACAAGAATATCTATCGCCGTTCGATCCACAATCATTCGTTGTTCCACAAGAGTTAC
AGTATTGGGTGACCCATAATATATTCTCACCTGAGTCCAGACCTTACAGGACCAAGAGTCTGATACTC
GAAGGTCCATCTAGA
+
FFFFFFFFFFFFFFFFFFFFFFFFFFFFFFFFFFFFFFFFFFF:FFFF:FFFFFFFFFFFFFFFFFF
FFFFFFFFFFFFF:FFFFFFFFFFFFFFFFFFFFFFFFFF:FFFFFFFFF:FF:FFFFFF:FFFFF:FF
FFFFFFFFFFFFF:F
@A00721:190:HGJW7DSXY:4:2128:12644:16313 2:N:0:TCTGCAAG+GCTGCACT
TCCAGAACCAATACAAGAATATCTATCGCCGTTCGATCCACAATCATTCGTTGTTCCACAAGAGTTAC
AGTATTGGGTGACCCATAATATATTCTCACCTGAGTCCAGACCTTACAGGACCAAGAGTCTGATACTC
GAAGGTCCATCTAGA
+
FFFFFFFFFFFFFFFFFFFFFFFFFFFFFFFFFFFF,FFFFFFF,FF,,F,FFFFFFFFFF:FFFFFF
FFFFFFF:FFF:F:FFFFFFF:FFFFFFFFFFFFFFFFFFFFFFFFFFFFFFFFFFFFFFFF:FFFFFF
FFFFFFFFF:FF:FF
@A00721:190:HGJW7DSXY:4:2204:29071:3458 2:N:0:TCTGCAAG+GCTGCACT
TCCAGAACCAATACAAGAATATCTATCGCCGTTCGATCCACAATCATTCGTTGTTCCACAAGAGTTAC
```

AGTATTGGGTGACCCATAATATATTCTCACCTGAGTCCAGACCTTACAGGACCAAGAGTCTGATACTC
GAAGGTCCATCTAGA
+
FFFFF,FFFFFFFFFFF:FF:FFFFFFFFFF::FFFFFFFFFFF,FFFFF:FFFFFFFF:FFFFFFFF
FFFFFFFFFFFFF:FFFFFFFF:FFFFFF:FFFFFFFFFFFFF,F,FFFFFFFFFFFFFFFFFFFFFF
FFFFFFFF:FFFFFF
@A00721:190:HGJW7DSXY:4:2357:23827:6057 2:N:0:TCTGCAAG+GCTGCACT
TCCAGAACCAATACAAGAATATCTATCGCCGTTCGATCCACAATCATTCGTTGTTCCACAAGAGTTAC
AGTATTGGGTGACCCATAATATATTCTCACCTGAGTCCAGACCTTACAGGACCAAGAGTCTGATACTC
GAAGGTCCATCTAGA
+
FFFFFFFFFFFFFF:FFFFFFFFFFFFFFFFFFFFFFFFFFFFFF:FFFFFFFFFFFFFFFFFFFFFFF
FFF:FFFFFFFFF,FFFF:FFFFFFFFFF::FFFFFFFFFFFFFFFFFFFFFFFFFFFFFFF:FF:FFF
FFF:FFF:F:FFFFF
@A00721:190:HGJW7DSXY:4:1123:6705:9032 2:N:0:TCTGCAAG+GCTGCACT
ATTTCCAGAACCAATACAAGAATATCTATCGCCGTTCGATCCACAATCATTCGTTGTTCCACAAGAGT
TACAGTATTGGGTGACCCATAATATATTCTCACCTGAGTCCAGACCTTACAGGACCAAGAGTCTGATA
CTCGAAGGTCCATCT
+
FFFFFFFFFFFFFFFFFF,FFFFFFFFFFFFFFFFFFFFFFFFFFFFF,FFFFFFFFFFFFFFFFFFFF
FFFFFFFFFFFFFFFFFFFFFFFFFFFFFFFF:FFFFFFFFFFFFF:FFFFFFFFFFFFFFFFFFFFF
FFFFFFFFFFFFFFF
@A00721:190:HGJW7DSXY:4:1231:1081:31704 2:N:0:TCTGCAAG+GCTGCACT
ATTTCCAGAACCAATACAAGAATATTTATCGCCGTTCGATCCACAATCATTCGTTGTTCCACAAGAGT
TACAGTATTGGGTGACCCATAATATATTCTCACCTGAGTCCAGACCTTACAGGACCAAGAGTCTGATA
CTCGAAGGTCCATCT
+
FFFFFFFF:FF,FFFFF:FFFFFFFFF,FFF:FFFFFFFFFFFFFFF,FFFFFFFFFFFFFFFFFFFF
FFFFFFFFFFF,:FFF:FFFFFFFFFFFFF:FFFFFFFFF,FFFFFFFFFFF,FFFFFFFFFFFFFFF
FFFFFFFFFF:FF::
@A00721:190:HGJW7DSXY:4:1306:8766:13542 2:N:0:TCTGCAAG+GCTGCACT
ATTTCCAGAACCAATACAAGAATATCTATCGCCGTTCGATCCACAATCATTCGTTGTTCCACAAGAGT
TACAGTATTGGGTGACCCATAATATATTCTCACCTGAGTCCAGACCTTACAGGACCAAGAGTCTGATA
CTCGAAGGTCCATCT
+
FFFFFFFFFFFFFFFFFFFFFFFFFFFFFFFFFFFFFFFFFFFFFFFFF,FFFFFFFFFFFFFFFFFFFF
FFFFFFFFFFFFFF:FFFFFFFFFFFFFFFFF:FFFFFFFFFFFFFFFFFFFFFFFFFFFFFFFFFFFF
:FFFFFFFFFFFF:F
@A00721:190:HGJW7DSXY:4:1312:15799:34867 2:N:0:TCTGCAAG+GCTGCACT
ATTTCCAGAACCAATACAAGAATATCTATCGCCGTTCGATCCACAATCATTCGTTGTTCCACAAGAGT
TACAGTATTGGGTGACCCATAATATATTCTCACCTGAGTCCAGACCTTACAGGACCAAGAGTCTGATA
CTCGAAGGTCCATCT
+
FFFFFFFFFFFFFFFFFFFFFF:F:FFFFFFFFFFFFFFFFFFFFFF,F:FFFFFFFFF:FFFFFFF:
FFFFFFFFFFFFFFFFFFFF:F:FFFFFFFFFFFFFFFFFFFFFFFFFFFFFFFFFFFFFFFFFFFFF
FFFFFFFFFFFFFFF
@A00721:190:HGJW7DSXY:4:1466:18087:34475 2:N:0:TCTGCAAG+GCTGCACT
ATTTCCAGAACCAATACAAGAATATCTATCGCCGTTCGATCCACAATCATTCGTTGTTCCACAAGAGT
TACAGTATTGGGTGACCCATAATATATTCTCACCTGAGTCCAGACCTTACAGGACCAAGAGTCTGGTA
CTCGAAGGTCCATCT
+
FFFFFFFFFFFFFFFFFFFFFFFFFF:FFFFFFFFFFFFFFFFFFFF:FFFFFFFFFFFFFFFFFFFF
F:FFFFFFFFFFFFFFFFF,FF:FFFFFFFFFFFFFFFFFFFFFFFFFF:FFFFFFFFFFFFFFFF,FF
FFFFFFFFFFFFFFF

@A00721:190:HGJW7DSXY:4:1536:8549:20932 2:N:0:TCTGCAAG+GCTGCACT
ATTTCCAGAACCAATACAAGAATATCTATCGCCGTTCGATCCACAATCATTCGTTGTTCCACAAGAGT
TACAGTATTGGGTGACCCATAATATATTCTCACCTGAGTCCAGACCTTACAGGACCAAGAGTCTGATA
CTCGAAGGTCCATCT
+
FFFFFFFFFFFFFFFFFFFFFFFFFFFFFFFFFFFFFFFFFFFFFFF:FFFFFFFFFFF:FFFFFFFF
FFFFFFFFFFFFFFFF:FFFFFFFFFFFFFFFFFFFFFFFFFFFFFFFFFFFFFFFFFFFFFFFFFFF
FFF:FFFFFFFFFFF
@A00721:190:HGJW7DSXY:4:1650:17915:8281 2:N:0:TCTGCAAG+GCTGCACT
ATTTCCAGAACCAATACAAGAATATTTATCGCCGTTCGATCCACAATCATTCGTTGTTCCACAAGAGT
TACAGTATTGGGTGACCCATAATATATTCTCACCTGAGTCCAGACCTTACAGGACCAAGAGTCTGATA
CTCGAAGGTCCATCT
+
FFFFFFFFFFFFFFFFFFFFFFFFFFFFFFFFFFFFFFFFFFFFFFF:FFFFFFFFFFFFFFFFFFFF
FFFFFFFFFFFFFFFF:FFFFFFFFFFFFFFFF:FFFFFFFF:FFFFFFFFFFFFFFFFFFFFFFFFF
FFFFFFFFFFFFFFF
@A00721:190:HGJW7DSXY:4:1673:30544:16877 2:N:0:TCTGCAAG+GCTGCACT
ATTTCCAGAACCAATACAAGAATATTTATCGCCGTTCGATCCACAATCATTCGTTGTTCCACAAGAGT
TACAGTATTGGGTGACCCATAATATATTCTCACCTGAGTCCAGACCTTACAGGACCAAGAGTCTGATA
CTCGAAGGTCCATCT
+
FFFFFFFFFFFFFFFFFFFFFFFFFFFFFFFFFFFFFFFFFFFFFFF:FFFFFFFFFFFFFFFFFFFF
FFFFFFFF:FFFFFFF,FFFFFFFFFFFFFFF:FFFFFFFFFFFFFFFFFFFFFFFFFFFFFFFFFFF
FFFFFFFFFFFFFFF
@A00721:190:HGJW7DSXY:4:1673:31259:18490 2:N:0:TCTGCAAG+GCTGCACT
ATTTCCAGAACCAATACAAGAATATTTATCGCCGTTCGATCCACAATCATTCGTTGTTCCACAAGAGT
TACAGTATTGGGTGACCCATAATATATTCTCACCTGAGTCCAGACCTTACAGGACCAAGAGTCTGATA
CTCGAAGGTCCATCT
+
FFFFFFFFFFFFFFFFFFFFFFFFFFFFFFFFFFFFFFFFFFFFFFFF,FFF:FF,FFFFFFFFFFFF
FFFFFFFFFFFF:FFF:FFFFFFFFFF:FFFFFFFFFFFFFFFFFFFFFFFFFFFFFFFFFF,FFF:FF
FFFFFFFFFFFFF:F
@A00721:190:HGJW7DSXY:4:2133:29152:21292 2:N:0:TCTGCAAG+GCTGCACT
ATTTCCAGAACCAATACAAGAATATTTATCGCCGTTCGATCCACAATCATTCGTTGTTCCACAAGAGT
TACAGTCTTGGGTGACCCATAATATATTCTCACCTGAGTCCAGACCTTACAGGACCAAGAGTCTGATA
CTCGAAGGTCCATCT
+
FFFFFFFFFFFFFFFFFFFFFFFFF,FFFFFFFFFFFFFFFFFFFF:F,FFFFF:FFFFFFFFFFFFF,
FFFFF:,FFFFFFFFF:FFFFFF:FFFFFFFFFFFFFFFFFFFFFFFFFFFFFFFFFFFFFFFFFFFF
FFFFFF:FFFFFFFF
@A00721:190:HGJW7DSXY:4:2201:6840:27430 2:N:0:TCTGCAAG+GCTGCACT
ATTTCCAGAACCAATACAAGAATATCTATCGCCGTTCGATCCACAATCATTCGTTGTTCCACAAGAGT
TACAGTATTGGGTGACCCATAATATATTCTCACCTGAGTCCAGACCTTACAGGACCAAGAGTCTGATA
CTCGAAGGTCCATCT
+
FFFFFFFFFFFFFFFFF:FFFFFFFFFFFFFFFFFFFFFFFFFFFFFF,FFFFFFFFFFF:FFFFFF:
FFFFFFFFFFFFFFFFFFFFFFFFFFFFFFFFF:FFFFFFFFFFFFFFFFFFF:F:,FFFFFFFFF,FF
FFFFFFFFFFFFFFF
@A00721:190:HGJW7DSXY:4:2206:21757:9580 2:N:0:TCTGCAAG+GCTGCACT
ATTTCCAGAACCAATACAAGAATATTTATCGCCGTTCGATCCACAATCATTCGTTGTTCCACAAGAGT
TACAGTATTGGGTGACCCATAATATATTCTCACCTGAGTCCAGACCTTACAGGACCAAGAGTCTGATA
CTCGAAGGTCCATCT
+
FFFFFFFFFFFFFF,FFFFF:FFFFFFFFFFF:FFFFFFFFFFFFF,FFFF:FFFFFFFFFFFFFF:

```
FFF::FFFFFF:F:FF:FFFFF:FFFFFFFFF:FFFFFFFF:FFFFFFFFF:FFFFFFFFFFFF::FF
FFF:FFFFFFFFFFF
@A00721:190:HGJW7DSXY:4:2222:6135:27054 2:N:0:TCTGCAAG+GCTGCACT
ATTTCCAGAACCAATACAAGAATATCTATCGCCGTTCGATCCACAATCATTCGTTGTTCCACAAGAGT
TACAGTATTGGGTGACCCATAATATATTCTCACCTGAGTCCAGACCTTACAGGACCAAGAGTCTGATA
CTCGAAGGTCCATCT
+
FFFFFFFFFFFFFFFFFFFFFFFFFFFFFFFFFFFFFFFFFFFFFFF,FFFFFFFFFFFFFFFFFFFF
FFFFFFFFFFFFFFFFFFF:FFFFFFFFFFFFFFFFFFFFFFFFFFFFFFFFFFFFFFFFFFFFFFFF
FFFFFFFFFFFFFFF
@A00721:190:HGJW7DSXY:4:2263:24171:32675 2:N:0:TCTGCAAG+GCTGCACT
ATTTCCAGAACCAATACAAGAATATCTATCGCCGTTCGATCCACAATCATTCGTTGTTCCACAAGAGT
TACAGTATTGGGTGACCCATAATATATTCTCACCTGAGTCCAGACCTTACAGGACCAAGAGTCTGATA
CTCGAAGGTCCATCT
+
FFFFFFFFFFFFFFFFFFFFFFFFFFFFFFFFFFFFFFFFFFFFFFFFFFFFFFFFFFFFFFFFFFFF
FFFFFFFFFFFFFFFFFFFFFFFFFFFFFFF:F:FFFFFFFFFFFFFFFFFFFFFFFFFFFFFFFFFF
FFFFFFF:FFFFFFF
@A00721:190:HGJW7DSXY:4:2314:26268:6997 2:N:0:TCTGCAAG+GCTGCACT
ATTTCCAGAACCAATACAAGAATATCTATCGCCGTTCGATCCACAATCATTCGTTGTTCCACAAGAGT
TACAGTATTGGGTGACCCATAATATATTCTCACCTGAGTCCAGACCTTACAGGACCAAGAGTCTGATA
CTCGAAGGTCCATCT
+
FFFFFFFFFFFFFFFFFFFFFFFFFFFFFFFFFFFFFFFFFFFFFFFFFFFFFFFFFFFFFFFFFFFF
FFFFFFFFFFFFFFFFFFFFFFFFFFFFFFFFFFFFFFFFFFFFFFFFFFFFFFFFFFFFFFFFFFFF
FFFFFFFFFFFFFFF
@A00721:190:HGJW7DSXY:4:2545:23909:20697 2:N:0:TCTGCAAG+GCTGCACT
ATTTCCAGAACCAATACAAGAATATCTATCGCCGTTCGATCCACAATCATTCGTTGTTCCACAAGAGT
TACAGTATTGGGTGACCCATAATATATTCTCACCTGAGTCCAGACCTTACAGGACCAAGAGTCTGATA
CTCGAAGGTCCATCT
+
FFFFFFFFFFFFFFFFFFFFFFFFFFFFFFFFFFFFFFFFFFFFFFF,FFFFFFFFFFFFFFFFFFFF
FFFFFFFFFFFFFFFFFFFF:FFFFFFFFFFF:FFFFFFFFFFFFFFFFFFFFFFFFFFFFFFFFFFF
FFFFFFFFFFFFFFF
@A00721:190:HGJW7DSXY:4:2556:26268:29262 2:N:0:TCTGCAAG+GCTGCACT
ATTTCCAGAACCAATACAAGAATATCTATCGCCGTTCGATCCACAATCATTCGTTGTTCCACAAGAGT
TACAGTATTGGGTGACCCATAATATATTCTCACCTGAGTCCAGACCTTACAGGACCAAGAGTCTGATA
CTCGAAGGTCCATCT
+
FFFFFF,FFFFFFFFFFFFFFFFFFFFFFFFFFFFFFF:FFFFFFFF,FFFFFFFFFFFFFFFFFFFF
FFFFFFFFFFFFFFFFFFFFFFFFFFFFFFFF:FFFFFFFFFFFFFF,FFFFFFFFFFFFFFFFFFFF
FFFFFFFFFFFF:FF
@A00721:190:HGJW7DSXY:4:2616:12590:18975 2:N:0:TCTGCAAG+GCTGCACT
ATTTCCAGAACCAATACAAGAATATCTATCGCCGTTCGATCCACAATCATTCGTTGTTCCACAAGAGT
TACAGTATTGGGTGACCCATAATATATTCTCACCTGAGTCCAGACCTTACAGGACCAAGAGTCTGATA
CTCGAAGGTCCATCT
+
FFFFFFFFFFFFFFFFFFFFFFFFFFFFFFFFFFFFFFFFFFFFFFF,FFFFFFFFFFFFFFFFFFFF
FFFFFFFFFFFFFFFFFFFFFFFFFFFFFFFF:FFFFFFFFFFFFFFFFFFFFFFFFFFFFFFFFFFF
FFFFFFFFFFFFFFF
@A00721:190:HGJW7DSXY:4:1128:11903:2159 2:N:0:TCTGCAAG+GCTGCACT
TTATTTCCAGAACCAATACAAGAATATCTATCGCCGTTCGATCCACAATCATTCGTTGTTCCACAAGA
GTTACAGTATTGGGTGACCCATAATATATTCTCACCTGAGTCCAGACCTTACAGGACCAAGAGTCTGA
TACTCGAAGGTCCAT
```

+
FFFFFFFFFFFFFFFFFFFFFFFFFFFFFFFFFFFFFFFFFFFFFFFFFFFFFFFFFFFFFFFFFFFFFF
FFF:FFFFFFFFFFFFFFFFFFFFFFFFFFFFFF:FFFFFFFFFFFFFFFFFFFFFFFFFFFFFFFFFF:
FFFFFFFFFFFFFF:
@A00721:190:HGJW7DSXY:4:1144:23619:2033 2:N:0:TCTGCAAG+GCTGCACT
TTATTTCCAGAACCAATACAAGAATATCTATCGCCGTTCGATCCACAATCATTCGTTGTTCCACAAGA
GTTACAGTATTGGGTGACCCATAATATATTCTCACCTGAGTCCAGACCTTACAGGACCAAGAGTCTGA
TACTCGAAGGTCCAT
+
FFFFFFFFFFFFFFFFFFFFFFFFFFFFFFFFFFFF:FFFFFFFFFFFFFFFFFFFFFFFFFFFFFFFF
FFFFFFFFFFFFFFFFFFFFF,FFFFFFFFFFFFFF:FFFFFFFFFFFFFFFFFFFFFFFFFFFFFFFFF
FFFFFFFFFFFFFFF
@A00721:190:HGJW7DSXY:4:1425:26838:3599 2:N:0:TCTGCAAG+GCTGCACT
TTATTTCCAGAACCAATACAAGAATATCTATCGCCGTTCGATCCACAATCATTCGTTGTTCCACAAGA
GTTACAGTATTGGGTGACCCATAATATATTCTCACCTGAGTCCAGACCTTACAGGACCAAGAGTCTGA
TACTCGAAGGTCCAT
+
FFFFFFFFFFFFFFF:FFFFFFFFFFFFFFFFFFFFFFFFF:FFFFFFFFFFFFFF:FFFFFFFFFF:F
FFFFFF,FFFFF:FFFFF:FFFFFFFFFFFFFFFFFF:FFFFF,FFFF,FFFFFFFFFFFFFFFFFF:F
FFFFFFFFFFFFFFF
@A00721:190:HGJW7DSXY:4:1641:12798:18490 2:N:0:TCTGCAAG+GCTGCACT
TTATTTCCAGAACCAATACAAGAATATCTATCGCCGTTCGATCCACAATCATTCGTTGTTCCACAAGA
GTTACAGTATTGGGTGACCCATAATATATTCTCACCTGAGTCCAGACCTTACAGGACCAAGAGTCTGA
TACTCGAAGGTCCAT
+
FFFFFFFFFFFFFFFFFFFFFFFFFFFFFFFFFFFFFFFFFFFFFFFFFFFFFFFFFFFFFFFFFFFFFF
FFFFFFFFFFFFFFFFFFFFFFFFFFFFFFFFFFFFFFFFFFFFFFFFFFFFFFFFFFFFFFFFFFFFFF
FFFFF:FFFFFFF:F
@A00721:190:HGJW7DSXY:4:2149:29414:34303 2:N:0:TCTGCAAG+GCTGCACT
TTATTTCCAGAACCAATACAAGAATATCTATCGCCGTTCGATCCACAATCATTCGTTGTTCCACAAGA
GTTACAGTATTGGGTGACCCATAATATATTCTCACCTGAGTCCAGACCTTACAGGACCAAGAGTCTGA
TACTCGAAGGTCCAT
+
FFFFFFFFFFFFFFFFFFFFFFFFFFFFFFFFFFFFFFFFFFFFFFFFFFFFFFFFFFFFFFFFFFFFFF
FFFFFFFFFFFFFFFFFFFFFFFFFFFFFFFFFFFFFFFFFFFFFFFFFFFFFFFFFFFFFFFFFFFFFF
FFFFFFFFFFFFFFF
@A00721:190:HGJW7DSXY:4:2154:21902:9799 2:N:0:TCTGCAAG+GCTGCACT
TTATTTCCAGAACCAATACAAGAATATCTATCGCCGTTCGATCCACAATCATTCGGTGTTCCACAAGA
GTTACAGTATTGGGTGACCCATAATATATTCTCACCTGAGTCCAGACCTTACAGGACCAAGAGTCTGA
TACTCGAAGGTCCAT
+
FFFF:FFFFFFFFFFFFFF,FFFFFFFFFFFFFFFFFFFFFFFFFFFFFFFFFFFFF,:FFFFFFFF:FF
FFFFFFFFFFFFFFFFFFFFFFFFFFFFFFFFFFFFFFFFFFFFFFFFFFFFFF:FFFFFFF,FFFFFFFF
FFFF:FFFF:FFFFF
@A00721:190:HGJW7DSXY:4:2308:8477:10410 2:N:0:TCTGCAAG+GCTGCACT
TTATTTCCAGAACCAATACAAGAATATCTATCGCCGTTCGATCCACAATCATTCGTTGTTCCACAAGA
GTTACAGTATTGGGTGACCCATAATATATTCTCACCTGAGTCCAGACCTTACAGGACCAAGAGTCTGA
TACTCGAAGGTCCAT
+
FFFFFFFFFFFFFFFFFFFFFFFFFFFFFFFFFFFFFFFFFFFFFFFFFFFFFFFFFFFFFFFFFFFFFF
FFFFFFFFFFFFFFFFFFFFFFFFFFFFFFFFFFFFFFFFFFFFFFFFFFFFFFFFFFFFFFFFFFFFFF
FFFFFFFFFFFFFF:
@A00721:190:HGJW7DSXY:4:2510:26142:18865 2:N:0:TCTGCAAG+GCTGCACT
TTATTTCCAGAACCAATACAAGAATATCTATCGCCGTTCGATCCACAATCATTCGTTGTTCCACAAGA

```
GTTACAGTATTGGGTGACCCATAATATATTCTCACCTGAGTCCAGACCTTACAGGACCAAGAGTCTGA
TACTCGAAGGTCCAT
+
FFFFFFFFFFFFFFFFFFFFFFFFFF:FFFFFFFFFFFFFFFFFFFFFFFFFFFFFFFFFFFFFFFF:F
FFFFFFFFFFF::FFFFF:FFFFFFFFFFFFFFFFF,FFFFFFFFF::FFFFFFFFFFFFFFFFFFFF
FF,FFFFFF:F:FFF
@A00721:190:HGJW7DSXY:4:1231:30382:21010 2:N:0:TCTGCAAG+GCTGCACT
CAGATAGATTATTTCCAGAACCAATACAAGAATATCTATCGCCGTTCGATCCACAATCATTCGTTGTT
CCACAAGAGTTACAGTATTGGGTGACCCATAATATATTCTCACCTGAGTCCAGACCTTACAGGACCAA
GAGTCTGATACTCGA
+
FFFFFFFFFFFFFFFFFFFFFFFFFFFFFFFFFFFFFFFFFFFFFFFFFFFFFFFFFFFFFFFFFFFF
FFFFFFFFFFFFFFFFFF:FFFFFFFFFF:FFFFFFFFFFFFFFFFFFFFFFFFFFF:FFFFFFFFFF
FFFFFFFFFFFFFFF
@A00721:190:HGJW7DSXY:4:1564:5348:21527 2:N:0:TCTGCAAG+GCTGCACT
CAGATAGATTATTTCCAGAACCAATACAAGAATATCTATCGCCGTTCGATCCACAATCATTCGTTGTT
CCACAAGAGTTACAGTATTGGGTGACCCATAATATATTCTCACCTGAGTCCAGACCTTACAGGACCAA
GAGTCTGATACTCGA
+
FFFFFFFFFFFFFFFFFFFFFFFFFF:F:FFFFFFFFFFFFFFFFFFFFFFFFFFFFFFFFFFFFFFF
FFFFFFFFFFFFFFFFFFFFFFFFFFFFFFF,FFFFFFFFFFF:FFFFFFFFFFFFFFFFFFFFFFFFF
:FFFFFFFFFFFFFF
@A00721:190:HGJW7DSXY:4:1676:16396:32988 2:N:0:TCTGCAAG+GCTGCACT
CAGATAGATTATTTCCAGAACCAATACAAGAATATCTATCGCCGTTCGATCCACAATCATTCGTTGTT
CCACAAGAGTTACAGTATTGGGTGACCCATAATATATTCTCACCTGAGTCCAGACCTTACAGGACCAA
GAGTCTGATACTCGA
+
FFFFFFFFFFFFFFFFFFFFFFFFFF:FFFFFFFFFFFFFFFFFFFFFFFFFFFFFFFFFFFFF:FFFF
FFFFFFFFFF:FFFFFFFFFFFFFFFFFFFFFFFFFFFFFFFFFFFFFFFFFFFFFFFFFFFFFFFFF
FFFFFFFFFFFFFFF
@A00721:190:HGJW7DSXY:4:2212:4227:7091 2:N:0:TCTGCAAG+GCTGCACT
CAGATAGATTATTTCCAGAACCAATACAAGAATATCTATCGCCGTTCGATCCACAATCATTCGTTGTT
CCACAAGAGTTACAGTATTGGGTGACCCATAATATATTCTCACCTGAGTCCAGACCTTACAGGACCAA
GAGTCTGATACTCGA
+
FFFFFFFFFFFFFFFFFFFFFFFFFFFFFFFFFFFFFFFFFFFFFFFFFFFFFFFFFFFFFFFFFFFF
FFFFFFFFFFFFFFFFF:FFFFFFFFFFF:FF:FFFFFFFFFFFFFFFFFFFFFFFFFFFFFFFFFFF
FFFFFFFFFFFFFFF
@A00721:190:HGJW7DSXY:4:2654:22670:13009 2:N:0:TCTGCAAG+GCTGCACT
CAGATAGATTATTTCCAGAACCAATACAAGAATATCTATCGCCGTTCGATCCACAATCATTCGTTGTT
CCACAAGAGTTACAGTATTGGGTGACCCATAATATATTCTCACCTGAGTCCAGACCTTACAGGACCAA
GAGTCTGATACTCGA
+
FFFFF:FFFFFFFFFFFFFFFFFFFFFFF,FFFFFFFFFFFFFFFFFFFFFFFFFFFFFFFFFFFFFF
FFFFFFFFFFFFFFFFFFFFFFFFFFFFFFFFFFFFFFFFFF:FFFFFFFFFFFFFFFFFFFFFFFFF
FFFFFFFFFFFFFFF
@A00721:190:HGJW7DSXY:4:1676:26576:3521 2:N:0:TCTGCAAG+GCTGCACT
TGCAGATAGATTATTTCCAGAACCAATACAAGAATATCTATCGCCGTTCGATCCACAATCATTCGTTG
TTCCACAAGAGTTACAGTATTGGGTGACCCATAATATATTCTCACCTGAGTCCAGACCTTACAGGACC
AAGAGTCTGATACTC
+
FFFFFFFFFFFFFFFFFFFFFFFFFFFFFFFFFFFFFFFFFFFFFFFFFFFFFFFFFFFF:FFFFFFFF
FFFFFFFFFFFFFFFFFFFFFFFFFFFFFFFFFFFFFF:FFFFFFFFFFFFFFFFFFFFFFFFFFFFFFF
FFFFFFFFFFFFFFF
```

```
@A00721:190:HGJW7DSXY:4:2135:16758:26819 2:N:0:TCTGCAAG+GCTGCACT
TGCAGATAGATTATTTCCAGAACCAATACAAGAATATCTATCGCCGTTCGATCCACAATCATTCGTTG
TTCCACAAGAGTTACAGTATTGGGTGACCCATAATATATTCTCACCTGAGTCCAGACCTTACAGGACC
AAGAGTCTGATACTC
+
FFFFFFFFFF:FFFFFFFFFFFFFFFFFFFFFFFFFFFFFFFFFFFFFFFFFF:FFFF:FFF
FFFFFFFFFFFFFFFFFFFFFFFFFFFFFFF:FF:FFFFFFFFFFFFFFFFFFFFFFFFFFFFFFFFFFF
FFFFFFFFFFFFFFF
@A00721:190:HGJW7DSXY:4:2164:18656:15107 2:N:0:TCTGCAAG+GCTGCACT
TGCAGATAGATTATTTCCAGAACCAATACAAGAATATCTATCGCCGTTCGATCCACAATCATTCGTTG
TTCCACAAGAGTTACAGTATTGGGTGACCCATAATATATTCTCACCTGAGTCCAGACCTTACAGGACC
AAGAGTCTGATA
+
F:FFFFFFFFF:FFF,FFFF:FFFF,FFFFF:FFFF::FFF:FFFFFF:FFFF,:FFFF:,,,FFFF,
FFF:FFFFFFFFFFFF,,F:FF:FFFFFFFFFFF:FFF:,,FFFFFFFFFFF:FFFFF:FFFFF:FFF
FF,F:FFFFFF:
@A00721:190:HGJW7DSXY:4:2303:1443:28698 2:N:0:TCTGCAAG+GCTGCACT
CAGACTCTTGGTCCTGTAAGGTCTGGACTCAGGTGAGAATATATTATGGGTCACCCAATACTGTAACT
CTTGTGGAACAACGAATGATTGTGGATCGAACGGCGATAGATATTCTTGTATTGGTTCTGGAAATAAT
CTATCTGCATTTGCA
+
FFFFFFFFFFFFFFFFFFFFFFFFFFFFFFFFFFFFFFFFFFFFFFFFFFFFFFFFFFFFFFFFFFFFFF
FFFFFFFFFFFFFFFFFFFFF:FFFFFFFFFFFFFFFFFFFFFF:FFFFFFFFFFFFFFFFFFFFFFFFF
FFFFFFFFFFFFFFF
@A00721:190:HGJW7DSXY:4:2537:16432:3020 2:N:0:TCTGCAAG+GCTGCACT
CAGACTCTTGGTCCTGTAAGGTCTGGACTCAGGTGAGAATATATTATGGGTCACCCAATACTGTAACT
CTTGTGGAACAACGAATGATTGTGGATCGAACGGCGATAGATATTCTTGTATTGGTTCTGGAAATAAT
CTATCTGCATTTGCA
+
FFFFFFFFFFFFFF:FFFFFFFFFFFFFFFFFFFFFFFFFFFFFFFFFFFFFFFFFFFFFFFFFFFF:FF
FFFFFFFFFFFFFFFFFFFFFFFF:FFFFFFFFFFFFFFFFFFF:FFFFFFFFFFFFFFFFFFFFFFFFF
FFFFFFFFFFFFFFF
@A00721:190:HGJW7DSXY:4:1101:9453:17957 2:N:0:TCTGCAAG+GCTGCACT
GTAAGGTCTGGACTCAGGTGAGAATATATTATGGGTCACCCAATACTGTAACTCTTGTGGAACAACGA
ATGATTGTGGATCGAACGGCGATAGATATTCTTGTATTGGTTCTGGAAATAATCTATCTGCATTTGCA
GATATGTTGTGAAGA
+
F:FFFFFFFFFFFFFFFFFFFFFFFFFFFFFFFFFFFFFFFFFFFFFFFFFFFFFFFFFFFFFFFFFFFF
FFFF:FFFFFFFFFFFFFFFFFFFFFFFFFFFF:F:FFFFFFFFFFFFFFFFFFFFFFFFFFFFFFFFFF
FF:FFFFFFFFFF:F
@A00721:190:HGJW7DSXY:4:2207:14362:6417 2:N:0:TCTGCAAG+GCTGCACT
GTAAGGTCTGGACTCAGGTGAGAATATATTATGGGTCACCCAATACTGTAACTCTTGTGGAACAACGA
ATGATTGTGGATCGAACGGCGATAGATATTCTTGTATTGGTTCTGGAAATAATCTATCTGCATTTGCA
GATATGTTGTGAAGA
+
FFFFFFFFFFFFFFFFFFFFFFFFFFFFFFFFFFFFFFFFFFFFFFFFFFFFFFFFFFFFFFFFFFFFFF
FFFFFFFFFFFFFFFFFFFFFFFFFFFF:FFFFFFFFFFFFFFFFFFFFFFFFFFFFFFFFFFFFFFFFF
FFFFFFFFFFFFFFF
@A00721:190:HGJW7DSXY:4:1277:26106:9596 2:N:0:TCTGCAAG+GCTGCACT
CTTCAGCATCACAACATATCTGCAAATGCAGATAGATTATTTCCAGAACCAATACAAGAATATCTATC
GCCGTTCGATCCACAATCATTCGTTGTTCCACAAGAGTTACAGTATTGGGTGACCCATAATATATTCT
CACCTGAGTCCAGAC
+
FFFFFFFFFFFFFFFFFFFFFFFFFFFFFFFFFFFFFFFFFFFFFFFFFFFFF:FFFFFFFFF:FFFFFF
```

```
FFFFFFFFFFFFFFF:F:F:FFFFFFFFFFFFFFFFFFFFFFFF,:FFFFFFFFFF,F::FFFFFFFF
FFFFFFFFFFFFFFF
@A00721:190:HGJW7DSXY:4:1460:18349:14481 2:N:0:TCTGCAAG+GCTGCACT
CTTCAGCATCACAACATATCTGCAAATGCAGATAGATTATTTCCAGAACCAATACAAGAATATCTATC
GCCGTTCGATCCACAATCATTCGTTGTTCCACAAGAGTTACAGTATTGGGTGACCCATAATATATTCT
CACCTGAGTCCAGAC
+
FFFFFFFFFFFFFFFFFFFFFFFFFFFFFFFFFFF:FFFFFF:FFFFFFFFFFFFFFF:FFFFFFF
FFFFF:FFFFFFFFFFF,FFFFFF:FFFFFFFFFFFFFFFFFFFFFFFFFFFFF:FFFFFFFFFFFF
FFFFFFFFFFFFFFF
@A00721:190:HGJW7DSXY:4:2308:30192:9627 2:N:0:TCTGCAAG+GCTGCACT
CTTCAGCATCACAACATATCTGCAAATGCAGATAGATTATTTCCAGAACCAATACAAGAATATCTATC
GCCGTTCGATCCACAATCATTCGTTGTTCCACAAGAGTTACAGTATTGGGTGACCCATAATATATTCT
CACCTGAGTCCAGAC
+
FFFFFFFFFFFFFFFFFFFFFFFFFFFFFFFFFFFFFFFFFFFFFFFFFFFFF:F:FFFFFFFFFF
FFFFFFFFFFFFFFFFF,FFFFFFFFFFFF::FFFF:FFFFFFFFFFFFFFF,FFFFFFFFFFFF
F,FFFFFFFFFFFFF
@A00721:190:HGJW7DSXY:4:2544:10411:1141 2:N:0:TCTGCAAG+GCTGCACT
CTTCAGCATCACAACATATCTGCAAATGCAGATAGATTATTTCCAGAACCAATACAAGAATATCTATC
GCCGTTCGATCCACAATCATTCGTTGTTCCACAAGAGTTACAGTATTGGGTGACCCATAATATATTCT
CACCTGAGTCCAGAC
+
FFFFFFFFFFFFFFFFFFFFFFFFFFFFF:FFFFFFFFFFFFFFFFFFFFFFFFFFFFFFFFFFFFF
:FFF:FFFFFFFFFFF,FFFFFFFFFFFFFFFFFFFFFFFFFFF:FFFFFFFFFFFFFFFFFFFFFFFF
FFFFFFFFF:FFFFF
@A00721:190:HGJW7DSXY:4:2634:10646:15828 2:N:0:TCTGCAAG+GCTGCACT
CTTCAGCATCACAACATATCTGCAAATGCAGATAGATTATTTCCAGAACCAATACAAGAATATCTATC
GCCGTTCGATCCACAATCATTCGTTGTTCCACAAGAGTTACAGTATTGGGTGACCCATAATATATTCT
CACCTGAGTCCAGAC
+
FFFFFFFFFFFFFFFFFFFFFFFFFFFFFFFFFFFFFFFFFFFFFFFFFFFFFF:FFFFFFFFFFF
FFFFFFFFFFFFFFFFF,FFFFFFFFFFFFFF:FFFFFFFFFFFFFFFFFFFFFFFFFFFFFFFFFFF
FFF:FFFFFFFFFFF
@A00721:190:HGJW7DSXY:4:1127:12436:2456 2:N:0:TCTGCAAG+GCTGCACT
TCTGGACTCAGGTGAGAATATATTATGGGTCACCCAATACTGTAACTCGTGTGGAACAACGAATGATT
GTGGATCGAACGGCGATAGATATTCTTGTATTGGTTCTGGAAATAATCTATCTGCATTTGCAGATATG
TTGTGATGCTGAAGG
+
FFF:FFFFFFFFFFFFFFFFFFFFFFFFFFFFFFFFFFFFFFFFFFFFFFFFFFFF,FFFFFFFFFFF
FFFFFFFFFFFFFFFFFFFF:FFFFF:FFFFFF:FFFFFFFFFFFFFF,FFFFF:FFFFFFFFFFFFF
FFFFFFFFFFFFFFF
@A00721:190:HGJW7DSXY:4:1231:13223:8422 2:N:0:TCTGCAAG+GCTGCACT
TCTGGACTCAGGTGAGAATATATTATGGGTCACCCAATACTGTAACTCGTGTGGAACAACGAATGATT
GTGGATCGAACGGCGATAGATATTCTTGTATTGGTTCTGGAAATAATCTATCTGCATTTGCAGATATG
TTGTGATGCTGAAGG
+
FFFFFFFFFFFFFFFFF:FFFFF:FFFFFFFFFFFFFFFFFFFFFFFFFFFFFFFFFFF:FFFFFFFFFF
FFFFFFFFFFFFFFFFFFFFFFFFFFFFFFFFFFFFFFFFFF:FFFFFFFFFFFFFFFFFFFFF,F:
FFFFFFFFFFFFFFF
@A00721:190:HGJW7DSXY:4:1426:15148:18239 2:N:0:TCTGCAAG+GCTGCACT
TCTGGACTCAGGTGAGAATATATTATGGGTCACCCAATACTGTAACTCGTGTGGAACAACGAATGATT
GTGGATCGAACGGCGATAGATATTCTTGTATTGGTTCTGGAAATAATCTATCTGCATTTGCAGATATG
TTGTGATGCTGAAGG
```

```
+
FFFFFFFFFFFFFFFFFFFFFFFFFFFFFFFFFFFFFFFFFFFFFFFFFFFFFFF:FFFFFFFFFFFF
FFFFFFFFFFFFFFFFFFFFFFFFFFFFFFFFFFFFFFFFFFFFFFFFFFFFFFFFFFFFFFFFFFFF
FFFFFFFFFFF:FFF
@A00721:190:HGJW7DSXY:4:1460:14904:35383 2:N:0:TCTGCAAG+GCTGCACT
TCTGGACTCAGGTGAGAATATATTATGGGTCACCCAATACTGTAACTCGTGTGGAACAACGAATGATT
GTGGATCGAACGGCGATAGATATTCTTGTATTGGTTCTGGAAATAATCTATCTGCATTTGCAGATATG
TTGTGATGCTGAAGG
+
FFFFFFFFFFFFFFFFFFFFFFFFFFFFFFFFFFFFF:F:FFFFF:FFFFFFFFFFFFFFFFFFFF:F
FFFFFFFFFFFFFFFFFFFF,FFFFFFFFFFFFFFFFFFFFFFFFFFFFFFFFFFFFFFFFFFFFFFF
FFFFFFFFFFFFFFF
@A00721:190:HGJW7DSXY:4:1541:16767:30123 2:N:0:TCTGCAAG+GCTGCACT
TCTGGACTCAGGTGAGAATATATTATGGGTCACCCAATACTGTAACTCTTGTGGAACAACGAATGATT
GTGGATCGAACGGCGATAGATATTCTTGTATTGGTTCTGGAAATAATCTATCTGCATTTGCAGATATG
TTGTGATGCTGAAGG
+
FFFFFFFFFFFFFFFFFFFFFFFFFFFFFFFFFFFFFFFFFFFFFFFFFFFFFFF,FFFFFFFFFFFF
FFFFFFFFFFFFFFFFFFFFF:FFFFFF:FFFFFFFFFFFFFFFFFFFFFFFFFFFFFFFFFFFF:FF
FFFFFFFFFFFFFFF
@A00721:190:HGJW7DSXY:4:2348:30734:1611 2:N:0:TCTGCAAG+GCTGCACT
TCTGGACTCAGGTGAGAATATATTATGGGTCACCCAATACTGTAACTCGTGTGGAACAACGAATGATT
GTGGATCGAACGGCGATAGATATTCTTGTATTGGTTCTGGAAATAATCTATCTGCATTTGCAGATATG
TTGTGATGCTGAAGG
+
FFFFFFFFFFFFFFFFFFFFFFFFFFFFFFFFFFFFFFFFFFFFFFFFFFFFF:FFFFFFFFFFF::F
FF:FFFFFFFF,FFFFFFFFFF:FFFFFFFFFFFFFFFFFFFFFFFFFFFFFFFFFFFFFFFFFFFFF
FFFFF::FFF::F:F
@A00721:190:HGJW7DSXY:4:2430:29378:28040 2:N:0:TCTGCAAG+GCTGCACT
TCTGGACTCAGGTGAGAATATATTATGGGTCACCCAATACTGTAACTCGTGTGGAACAACGAATGATT
GTGGATCGAACGGCGATAGATATTCTTGTATTGGTTCTGGAAATAATCTATCTGCATTTGCAGATATG
TTGTGATGCTGAAGG
+
FFFFFFFFFFFFFFFFFFFFFFFFFF:FFFFFFFFFFFFFFFFFFFFFFFFFFFF:FFFFFFFFFFFF
FFFFFFFFFFFFFFFFFFFF:F:FFFFFFFFFFFFFFF,FFFFFFF:FFFFF:FFFFFFFF:FFFFFF
FFFFF:FFFFFFF,F
@A00721:190:HGJW7DSXY:4:2471:4725:14810 2:N:0:TCTGCAAG+GCTGCACT
TCTGGACTCAGGTGAGAATATATTATGGGTCACCCAATACTGTAACTCGTGTGGAACAACGAATGATT
GTGGATCGAACGGCGATAGATATTCTTGTATTGGTTCTGGAAATAATCTATCTGCATTTGCAGATATG
TTGTGATGCTGAAGG
+
FFFFFFFFFFFF:FFFFFFFFFFFFFFFFFFFFFFFFFFFFFFFFFFFFFFFFFFFFFFFFFFFFFF
FFFFFFFFFFFFFFFFFFFF:FFFFFFFFFFFFFFFFFFFFFFFFFFFFFFFFFFFFFFFFFF:FFFFF
FFFFFFFFFFFFFFF
@A00721:190:HGJW7DSXY:4:2512:23619:11866 2:N:0:TCTGCAAG+GCTGCACT
TCTGGACTCAGGTGAGAATATATTATGGGTCACCCAATACTGTAACTCGTGTGGAACAACGAATGATT
GTGGATCGAACGGCGATAGATATTCTTGTATTGGTTCTGGAAATAATCTATCTGCATTTGCAGATATG
TTGTGATGCTGAAGG
+
FFFFFFFFFFFFFFFFF,FFFFFFFFFFFFFFFFFFFFFFFFFFFFFFFFFFFFF,FF:FFFFFFFFF
FFFFFFFFFFFFFFFFFFFFFFF:,FFFFFFFFFFFFFFFFFFFFFFFFFFFFFFFFFFFFFFFFFFF
:F:FFFFFFFFFFFF
@A00721:190:HGJW7DSXY:4:2541:18502:35008 2:N:0:TCTGCAAG+GCTGCACT
TCTGGACTCAGGTGAGAATATATTATGGGTCACCCAATACTGTAACTCTTGTGGAACAACGAATGATT
```

```
GTGGATCGAACGGCGATAGATATTCTTGTATTGGTTCTGGAAATAATCTATCTGCATTTGCAGATATG
TTGTGATGCTGAAGG
+
FFFFFFFFFFFF:FFFF:FFF:FFFFFFFFFFFFFFFFFFFFFFFFFFFFFFFFFFFFFFFFFFFFFFFF
FFFFFFFFFFFFFFFFFFFF:,FFF:FFFFFFFFFFFFFFFFFFFF,FFFFFFFFFFFFF:FFFFFFFFF
F:FFFFFFFFFFFFF
@A00721:190:HGJW7DSXY:4:2631:4146:2503 2:N:0:TCTGCAAG+GCTGCACT
TCTGGACTCAGGTGAGAATATATTATGGGTCACCCAATACTGTAACTCGTGTGGAACAACGAATGATT
GTGGATCGAACGGCGATAGATATTCTTGTATTGGTTCTGGAAATAATCTATCTGCATTTGCAGATATG
TTGTGATGCTGAAGG
+
FFFFFFFFFF,F:FFFFFFFFFFFF:FFFFFFFFFF:FFFFFF::FF:FF:,FFFFFF::F::FFF:FFF
FFFFFF:FF:::FFFFFF:FFFFFFFFFFFFFFFFFFFFF,FFFFFF:FFFF::,FFFF:FFFFF:FF,
:F:FFFF,FF,:F:F
@A00721:190:HGJW7DSXY:4:1111:18213:23046 2:N:0:TCTGCAAG+GCTGCACT
CAAGAACGTTCTACCTTCAGCATCACAACATATCTGCAAATGCAGATAGATTATTTCCAGAACCAATA
CAAGAATATCTATCGCCGTTCGATCCACAATCATTCGTTGTTCCACAAGAGTTACAGTATTGGGTGAC
CCATAATATATTCTC
+
FFFF:FFFFFFFFFFFFFFFFFFFFFFFFFFFFFFFFFFFFFFFFFFFFFFFFFFFFFFFFFFFFF
F:FFFFFFFFFFFFFFFFFFFF:FFFFFFFFF:FFFF:FFFFFFFFFFFFFFFFFFFFFF:FFFFFFF:F
:FFFFFFFF,FFFFF
@A00721:190:HGJW7DSXY:4:1124:2555:9768 2:N:0:TCTGCAAG+GCTGCACT
CAAGAACGTTCTACCTTCAGCATCACAACATATCTGCAAATGCAGATAGATTATTTCCAGAACCAATA
CAAGAATATCTATCGCCGTTCGATCCACAATCATTCGTTGTTCCACAAGAGTTACAGTATTGGGTGAC
CCATAATATATTCTC
+
FFFFFFFFFFFFFFFFFFFFFFFFFFFFFFFFFFFFFFFFFFFFFFFFFFFFFFFFFFFFFFFFFFFFFF
FFFFFFFFFFFFF:FFFFFF:FFFFFFFFFFF:,FF:F:FFFFFFFFFF:FFF::FFFFF:FFFFFFFFF
FFF::FF:FFFFFFF
@A00721:190:HGJW7DSXY:4:1421:16523:25504 2:N:0:TCTGCAAG+GCTGCACT
CAAGAACGTTCTACCTTCAGCATCACAACATATCTGCAAATGCAGATAGATTATTTCCAGAACCAATA
CAAGAATATCTATCGCCGTTCGATCCACAATCATTCGTTGTTCCACAAGAGTTACAGTATTGGGTGAC
CCATAATATATTCTC
+
FFFFFFFFFFFFFFFFFFFFFFFFFFFFFFFFFFFFFFFFFFFFFFFFFFFFFFFFFFFFFFFFFFFFFF
FFFFFFFFFFFFFFFFFFFFFFFFFFFFFFFF,FFFFFFFFFFFFFFFFFFF,FFFFFFFFFFFFFFFFF
FFFFFFFFFFFFFFF
@A00721:190:HGJW7DSXY:4:2626:24062:31234 2:N:0:TCTGCAAG+GCTGCACT
CAAGAACGTTCTACCTTCAGCATCACAACATATCTGCAAATGCAGATAGATTATTTCCAGAACCAATA
CAAGAATATCTATCGCCGTTCGATCCACAATCATTCGTTGTTCCACAAGAGTTACAGTATTGGGTGAC
CCATAATATATTCTC
+
FFFFFFFFFFFFFFFFFFFFFFFFFFFFFFFFFFFFFFFFFFFFFFFFFFFFFFFFF:FFFFFFFFFFFF
FF:FFFFFFFFFFFFFF:FFFFFFFFFFFFFF,FFFFFFFFFFFF:FFFFFFFF:FF:FFF:FFFFF:FF
FFFFFFFFFFFFFFF
@A00721:190:HGJW7DSXY:4:2626:23267:30921 2:N:0:TCTGCAAG+GCTGCACT
CAAGAACGTTCTACCTTCAGCATCACAACATATCTGCAAATGCAGATAGATTATTTCCAGAACCAATA
CAAGAATATCTATCGCCGTTCGATCCACAATCATTCGTTGTTCCACAAGAGTTACAGTATTGGGTGAC
CCATAATATATTCT
+
FFFFFFFFFFFFFFFFFFFFFFFFFFFFFFFFFFFFFFFFFFFFFFFFFFFFFFFFFFFFFFFFFFFFFF
FFFFFFFFFFFFFFFFFFFFFFFFFFFFFFFF,FFFFFFFFFFFFFFFFFFFFFFFFFFFFFFFFFFFFF
FFFFFFFFFFFFFF
```

```
@A00721:190:HGJW7DSXY:4:1145:29948:14685 2:N:0:TCTGCAAG+GCTGCACT
GGAAGAACGATCCAAGAACGTTCTACCTTCAGCATCACAACATATCAGCAAATGCAGATAGATTATTT
CCAGAACCAATACAAGAATATCTATCGCCGTTCGATCCACAATCATTCGTTGTTCCACAAGAGTTACA
GTATTGGGTGACCCA
+
FF:FFFFFFFFFFFFFFFFFFFFFF:FFFFFFFFFFFFFFFFFFFF,FFFFFFFFFFFFFFFFFFFF
FFFFFFFFFFFFFFFFFFFFFFFFFF:FFFFFFFFFF:FFFFFFFFFFF,FFFFFFFFFFFFF:FF:F
FFFFFFFFFFFFFFF
@A00721:190:HGJW7DSXY:4:1334:17879:22936 2:N:0:TCTGCAAG+GCTGCACT
GGAAGAACGATCCAAGAACGTTCTACCTTCAGCATCACAACATATCTGCAAATGCAGATAGATTATTT
CCAGAACCAATACAAGAATATCTATCGCCGTTCGATCCACAATCATTCGTTGTTCCACAAGAGTTACA
GTATTGGGTGACCCA
+
FFFFFFFFFFFFFFFFFFFFFFFFFFFFFFFFFFFFFFFFFFFFFFFFFFFFFFFFFF:FFFFFFFFF
FFFFFF:FF:FFFFFFFFFFFFFFFFFFFFFFFF:FFFFFFFFFFFFFFFFFFFFFFFFFFFFF,FFFFF
FFFFFF::FFFF:FF
@A00721:190:HGJW7DSXY:4:1503:11351:34585 2:N:0:TCTGCAAG+GCTGCACT
GGAAGAACGATCCAAGAACGTTCTACCTTCAGCATCACAACATATCTGCAAATGCAGATAGATTATTT
CCAGAACCAATACAAGAATATCTATCGCCGTTCGATCCACAATCATTCGTTGTTCCACAAGAGTTACA
GTATTGGGTGACCCA
+
FFFFFFFFFFFFFFFFFFFFFFFFFFFFFFFFFFFFFFFFFFFFFFFFFFFFFFFFFFFFFFFFFFFF
FFFFFFFFFFFFFFFFFFFFFFFFFFFFFFFFFFFFFFFFFFFFFFFFFFFFFFF:FFFFFFFFFFFF
FFFFFFFFFFFFFF:
@A00721:190:HGJW7DSXY:4:2313:7030:24283 2:N:0:TCTGCAAG+GCTGCACT
GGAAGAACGATCCAAGAACGTTCTACCTTCAGCATCACAACATATCTGCAAATGCAGATAGATTATTT
CCAGAACCAATACAAGAATATCTATCGCCGTTCGATCCACAATCATTCGTTGTTCCACAAGAGTTACA
GTATTGGGTGACCCA
+
FFFFFFFFFFFFFFFFFFFFFFFFFFFFFFFFFFFFFFFFFFFFFFFFFFFFFFFFFFFFFFFFFF:F
FFFFF,FFF:FFFFFFFFFFFFFFFF:FFFFFF:F:FFFFFFFFFFFFFFFFFFFFFFFFFFFF:FFF
FFFF:FFFFFFFFFF
@A00721:190:HGJW7DSXY:4:1450:1380:35321 2:N:0:TCTGCAAG+GCTGCACT
TAGAAATAATCCGGAAGAACGATCCAAGAACGTTCTACCTTCAGCATCACAACATATCTGCAAATGCA
GATAGATTATTTCCAGAACCAATACAAGAATATCTATCGCCGTTCGATCCACAATCATTCGTTGTTCC
ACAAGAGTTACAGTA
+
FFFFFFFFFFFFFFFFFFFFFFFFFFFFFFFFFFFFFFFFFFFFFFFFFFFFFFFFFFFFFFFFFFF:
FFFFFFFFFFFFFFFFFFFFFFFFFFFFFFFFFFFFFFFFFFFF:FFFFFFFF,FFFFFFFFFFFF
FFF:FFFFF:FFFF:
@A00721:190:HGJW7DSXY:4:1473:4191:15045 2:N:0:TCTGCAAG+GCTGCACT
TAGAAATAATCCGGAAGAACGATCCAAGAACGTTCTACCTTCAGCATCACAACATATCTGCAAATGCA
GATAGATTATTTCCAGAACCAATACAAGAATATCTATCGCCGTTCGATCCACAATCATTCGTTGTTCC
ACAAGAGTTACAGTA
+
FFFFFFFFF:FFFFFFFFFFFFFFFFFFFFFFF,:FFFFFFFFF:FFFFFFFFFF:FFFFFFFFFFF:F
FFFFFFFFFFFFFFFFFFFFFFFFFFFFFFFFFFFFFFF:FFFFFFFFFFFFFFFF::F,FFFFFFFFFF
FFFFFF:FFFFFFF:
@A00721:190:HGJW7DSXY:4:1538:5927:4147 2:N:0:TCTGCAAG+GCTGCACT
TAGAAATAATCCGGAAGAACGATCCAAGAACGTTCTACCTTCAGCATCACAACATATCTGCAAATGCA
GATAGATTATTTCCAGAACCAATACAAGAATATCTATCGCCGTTCGATCCACAATCATTCGTTGTTCC
ACAAGAGTTACAGTA
+
FFFFFFFFFFFFFFFFFFFFFFFFFFFFFFFFFFFFFFFFFFFFFFFFFFFFFFFFFFFFFFFFFFFF
```

FFFFFFFFFFFFFFFFFFFFFFFFFFFFFFFFFFFFFFFFFFFFFFFFFFFFFFFFF:FFFFFFFFFFFF
FFFFFFFFFFFFFFF
@A00721:190:HGJW7DSXY:4:2145:27100:31892 2:N:0:TCTGCAAG+GCTGCACT
CTAGAAATAATCCGGAAGAACGATCCAAGAACGTTCTACCTTCAGCATCACAACATATCTGCAAATGC
AGATAGATTATTTCCAGAACCAATACAAGAATATCTATCGCCGTTCGATCCACAATCATTCGTTGTTC
CACAA
+
FFFFFFFFFFFFFFFFFFFFFFFFFFFFFFFFFFFFFFFFFFFFFFFFFFFFFFFFFFF:FFFFFFFFFF
FFFFFFFFFFFFFFFFFFFFFFFFFFFFFFF:FFFFFFFFFFFFFFFFFFFFFFFFFFF:FFF::FFFFF
FFFFF
@A00721:190:HGJW7DSXY:4:2248:19533:9142 2:N:0:TCTGCAAG+GCTGCACT
CTAGAAATAATCCGGAAGAACGATCCAAGAACGTTCTACCTTCAGCATCACAACATATCTGCAAATGC
AGATAGATTATTTCCAGAACCAATACAAGAATATCTATCGCCGTTCGATCCACAATCATTCGTTGTTC
CACAA
+
FFFFFFFFF:FFFFFFFF:FFFFFFF:F:FFFFFFFFFFFFFFFFFFFFFFFFFFFFFFFFFFFFFFFFF
FFFFFFFFFFFFFFFFFFFFFFFFFFF,FFFF:FFFFFFFF:FFFFFFFFFFFFFFFF,FFFF:FFFFFF
FFFF:
@A00721:190:HGJW7DSXY:4:1338:19759:4867 2:N:0:TCTGCAAG+GCTGCACT
GCTTTATCAGCTAGGTCAAAGACAGAGTCTCTAGAAATAATCCGGAAGAACGATCCAAGAACGTTCTA
CCTTCAGCATCACAACATATCTGCAAATGCAGATAGATTATTTCCAGAACCAATACAAGAATATCTAT
CGCCGTTCGATCCAC
+
FFFFFFFFFFFFFFFFFFFFFFFFFFFFFFFFFFFFFFFFFFFFFFFFFFFFFFFFFFFFFFFFFFFFFF
FFFFFFFFFFFFFFFFFFFFFFF:FFFFFFFFFFFFFFFFFFFFFFFFFFFFFFFFFFFFF:FFFFFFFF
FFFFF,FFFFFFFFF
@A00721:190:HGJW7DSXY:4:1419:5475:25755 2:N:0:TCTGCAAG+GCTGCACT
GCTTTATCAGCTAGGTCAAAGACAGAGTCTCTAGAAATAATCCGGAAGAACGATCCAAGAACGTTCTA
CCTTCAGCATCACAACATATCTGCAAATGCAGATAGATTATTTCCAGAACCAATACAAGAATATCTAT
CGCCGTTCGATCCAC
+
FFFFFFFFFFFFFFFFFFFFFFFFFFFFFFFFFFFFFFFFFFFFFFFFFFFFFFFFFFFFFFFFFFFFFF
FFFFFFFFFFFFFFFFFFFFFFFFFFFFFFFFFFFFFFFFFFFFFFFFFFFFFFFFFFFFFFFFFFFFFF
:FFFFFFFFFFFFFF
@A00721:190:HGJW7DSXY:4:1423:31403:14638 2:N:0:TCTGCAAG+GCTGCACT
GCTTTATCAGCTAGGTCAAAGACAGAGTCTCTAGAAATAATCCGGAAGAACGATCCAAGAACGTTCTA
CCTTCAGCATCACAACATATCTGCAAATGCAGATAGATTATTTCCAGAACCAATACAAGAATATCTAT
CGCCGTTCGATCCAC
+
FFFFFFFFFFFFFFFFFFFFFFFFFFFFFFFFFFFFFFFFFFFFFFFFFFFFFFFFFFFFFFFFFFFFFF
FFFFFFF:FFFFFFFFFFFF:FFFFFFFF:FFFFFFFFFFFFFFFFFFFFFFFFFFFFFFFFFFFFFFFF
F:FFFFFFFFFFFFF
@A00721:190:HGJW7DSXY:4:1526:6479:31093 2:N:0:TCTGCAAG+GCTGCACT
GCTTTATCAGCTAGGTCAAAGACAGAGTCTCTAGAAATAATCCGGAAGAACGATCCAAGAACGTTCTA
CCTTCAGCATCACAACATATCTGCAAATGCAGATAGATTATTTCCAGAACCAATACAAGAATATCTAT
CGCCGTTCGATCCAC
+
FFFFFFFFFFFFFFFFFFFFFFFFFFFFFFFFFFFFFFFFFFFFFFFFFFFFFFFFFFFFFFFFFFFFFF
FF:FFFFFFFFFFFFFFFFFFFFFFFFFFFFFFFFFFFFFFFFFFFFFFFFFFFFFFFFFFFFFFFFFFF
FFFFFFFFFFFFFFF
@A00721:190:HGJW7DSXY:4:1576:16595:25567 2:N:0:TCTGCAAG+GCTGCACT
GCTTTATCAGCTAGGTCAAAGACAGAGTCTCTAGAAATAATCCGGAAGAACGATCCAAGAACGTTCTA
CCTTCAGCATCACAACATATCTGCAAATGCAGATAGATTATTTCCAGAACCAATACAAGAATATCTAT
CGCCGTTCGATCCAC

```
+
FFF,FFFFFFF:FFFFFFFFFFFF,FFFFFFFFFFFFFFFFFFFFFFFFFFFFFFFFFFFFFFF:FFFFF
FFFFFFFFFFFFFFFFF:FFFFFFFFFFFFFFFFFFFFFFFFFFFFFFFFFFFFFFFFFFFFFFFFFFF
FFFFFFFFFFFFFFF
@A00721:190:HGJW7DSXY:4:2278:20790:10848 2:N:0:TCTGCAAG+GCTGCACT
GCTTTATCAGCTAGGTCAAAGACAGAGTCTCTAGAAATAATCCGGAAGAACGATCCAAGAACGTTCTA
CCTTCAGCATCACAACATATCTGCAAATGCAGATAGATTATTTCCAGAACCAATACAAGAATATCTAT
CGCCGTTCGATCCAC
+
FFFFFFFFFFFFFFFFFFFFFFFFFFFFFFFFFFFFFFFFFFFFFFFFFFFFFFFFFFFFFFFFFFFFF
FFFFFF:FFFFFFFFFFFFFFFFFFFFFFFFFFFFFFFFFFFFFFFFFFFFFF:FFFFFF:FFFFFF
FFFFF:FFFFFFFFF
@A00721:190:HGJW7DSXY:4:2516:18430:28776 2:N:0:TCTGCAAG+GCTGCACT
GCTTTATCAGCTAGGTCAAAGACAGAGTCTCTAGAAATAATCCGGAAGAACGATCCAAGAACGTTCTA
CCTTCAGCATCACAACATATCTGCAAATGCAGATAGATTATTTCCAGAACCAATACAAGAATATCTAT
CGCCGTTCGATCCAC
+
FFFFFFFFFFFFFFFFFFFFFFFFFFFFFFFFFFFFFFFFFFFFFFF:FFFFFFFFFFFFFFFFFFFFF
FFFFFFF:FFFFFFFFFFFFFFF:FFFFF:FFFFFFFFFFFFFF,FFFFFFFFFF:FFFFFFFFFFFF
:,FFF:FF,FF:,FF
@A00721:190:HGJW7DSXY:4:2622:1551:12947 2:N:0:TCTGCAAG+GCTGCACT
GCTTTATCAGCTAGGTCAAAGACAGAGTCTCTAGAAATAATCCGGAAGAACGATCCAAGAACGTTCTA
CCTTCAGCATCACAACATATCTGCAAATGCAGATAGATTATTTCCAGAACCAATACAAGAATATCTAT
CGCCGTTCGATCCAC
+
FFFFFFFFFFFFFFFFFFFFFFFFFFFFFFFFFFFFFFFFFFFFFFFFFFFFFFFFFFFFFFFFFFFFF
FFFFFFFFFFFFFFFFFFFFFFFFFFFFFFFFFFFFFFFFFFFFFFFFFFFFFFFFFFFFFFFFFFFFF
:FFFFFFFFFFFFFF
@A00721:190:HGJW7DSXY:4:1362:25057:18270 2:N:0:TCTGCAAG+GCTGCACT
AAAGCTTTATCAGCTAGGTCAAAGACAGAGTCTCTAGAAATAATCCGGAAGAACGATCCAAGAACGTT
CTACCTTCAGCATCACAACATATCTGCAAATGCAGATAGATTATTTCCAGAACCAATACAAGAATATC
TATCGCCGTTCGATC
+
FFFFFFFFFFFFFFFFFFFFFFFFFFFFFFFFFFFFFFFFFFFFFFFFFFFFFFFFFFF:FFFFFFFFF
FFFFFFFFFF:FFFFFFFFFFFFFF:FFF:FFFFFFFFFFFFFFFFFFFFFFFFFFFFFFF,FFFFFF
:FFFFFFFFFFFFFF
@A00721:190:HGJW7DSXY:4:1444:10357:9878 2:N:0:TCTGCAAG+GCTGCACT
AAAGCTTTATCAGCTAGGTCAAAGACAGAGTCTCTAGAAATAATCCGGAAGAACGATCCAAGAACGTT
CTACCTTCAGCATCACAACATATCTGCAAATGCAGATAGATTATTTCCAGAACCAATACAAGAATATC
TATCGCCGTTCGATC
+
FFFFFFFFFFFFFFFFFFFFFFFFFFFFFFFFFFFFFF:FFFFFFFFFFFFFFFFFFFFFFFFFFFFFF
FFFFFFFFF::FFFFFFFFFFFFFFFFFFFFFFFFFFFFFFFFFFF:FFFFFFFFFFFFFFFFFFFFF
FFFFFFFFFFFFFFF
@A00721:190:HGJW7DSXY:4:1610:20428:5149 2:N:0:TCTGCAAG+GCTGCACT
AAAGCTTTATCAGCTAGGTCAAAGACAGAGTCTCTAGAAATAATCCGGAAGAACGATCCAAGAACGTT
CTACCTTCAGCATCACAACATATCTGCAAATGCAGATAGATTATTTCAAGAACCAATACAAGAATATC
TATCGCCGTTCGATC
+
FFFF:F:F:FFFF,FFF:FFFF,FFFFFFFFFFFFFFFFFFFFFFFFF:FFFFFF:FF:FFF,FFFFFF
:FFF:FFFF:FFF::FFFFF,FFF:F,FF,FFFFFFFFFFF:FFFFF,FFFFFFFFFFFFFFFFFFF,
FFFF,F::FF:,FFF
@A00721:190:HGJW7DSXY:4:2211:16731:23390 2:N:0:TCTGCAAG+GCTGCACT
AAAGCTTTATCAGCTAGGTCAAAGACAGAGTCTCTAGAAATAATCCTGAAGAACGATCCAAGAACGTT
```

```
CTACCTTCAGCAACACAACATATATGCAAATGCAGAGATATTATTTCCAGAACCAATACAAGAATAAC
TATCGCCGTTCGATC
+
:FFFFF:,:FFFFF,FF:FFFFFFF:FFFF,FFFFF:FFFFFFFFF,FFF,FFFFF:F,FF,FF,FF:
FFFF,F,FFF,F,,:FF:,F,FF,F,FFFFF,FF,F,F,FF:FFFF:FFFF,,FF:F:,::,F,F,,:
FF,FFFF,,FFFFFF
@A00721:190:HGJW7DSXY:4:1104:1127:19132 2:N:0:TCTGCAAG+GCTGCACT
TTTATAATAAAGCTTTATCAGCTAGGTCAAAGACAGAGTCTCTAGAAATAATCCGGAAGAACGATCCA
AGAACGTTCTACCTTCAGCATCACAACATATCTGCAAATGCAGATAGATTATTTCCAGAACCAATACA
AGAATATCTATCGCC
+
FFFFFFFFFFFFFFFFFFFFFFFFFFFFFFFFFFFFFFFFFFFFFFFFFFFFFFFFFFFFFFFFFFFF
FFFFFFFFFFFFFFFFFFFFFFFFFFFFFFFFFFFFFFFFFFFFFFFFFFFFFFFFFFFFFFFFFFFF
F:FFFFFFFFFFFFF
@A00721:190:HGJW7DSXY:4:1258:8278:6872 2:N:0:TCTGCAAG+GCTGCACT
TTTATAATAAAGCTTTATCAGCTAGGTCAAAGACAGAGTCTCTAGAAATAATCCGGAAGAACGATCCA
AGAACGTTCTACCTTCAGCATCACAACATATCTGCAAATGCAGATAGATTATTTCCAGAACCAATACA
AGAATATCTATCGCC
+
FFFFFFFFFFFFFFFFFFFFFFFFFFFFFFFFFFFFFFFFFFFFFFFFFFFFFFFFFFFFFFFFFFFF
FF:FFFFFFFFFFFFFFFFFFFFFFFFF:FFFFFFFFFFFFFFFFFFFFFFFFFFFFFFFFFFFFFFFF
FFF:FFF:FFFFFFF
@A00721:190:HGJW7DSXY:4:1339:21278:2362 2:N:0:TCTGCAAG+GCTGCACT
TTTATAATAAAGCTTTATCAGCTAGGTCAAAGACAGAGTCTCTAGAAATAATCCGGAAGAACGATCCA
AGAACGTTCTACCTTCAGCATCACAACATATCTGCAAATGCAGATAGATTATTTCCAGAACCAATACA
AGAATATCTATCGCC
+
FFFFFF:FFFFFFFFFFFFFFFFFFFFFFFFFFFFFFFFFFFFFFFFF:FFFFFFFFFFFFFFFFFFF
:FFFFFFFFFFFFF:FFFFFFFFFFFFFFFF:FF:FFFFFFFFFFFFFFFFFFFFFFFFFFFFFFFFF
FFF:FFFF:FF,,FF
@A00721:190:HGJW7DSXY:4:1402:1877:15327 2:N:0:TCTGCAAG+GCTGCACT
TTTATAATAAAGCTTTATCAGCTAGGTCAAAGACAGAGTCTCTAGAAATAATCCGGAAGAACGATCCA
AGAACGTTCTACCTTCAGCATCACAACATATCTGCAAATGCAGATAGATTATTTCCAGAACCAATACA
AGAATATCTATCGCC
+
:FFFFFFFFFFFFFFFFFFFFFFFFFFFFF:FFFFFFFFFF:FFFFFFFFFFFFFFFFFF:FFFFFFFF
FFFFFFFFFFFFFFFFFF:F:FFFFFFFFFF:F:F:FFFF:FFFF:FF:FFFFFF,FFFFFFFFFFFF
F:FF:FFFFFFF,FF
@A00721:190:HGJW7DSXY:4:2152:10022:6073 2:N:0:TCTGCAAG+GCTGCACT
TTTATAATAAAGCTTTATCAGCTAGGTCAAAGACAGAGTCTCTAGAAATAATCCGGAAGAACGATCCA
AGAACGTTCTACCTTCAGCATCACAACATATCTGCAAATGCAGATAGATTATTTCCAGAACCAATACA
AGAATATCTATCGCC
+
FFFFFFFFFFFFFFFFFFFFFFFFFFFFFFFFFFFFFFFFFFFFFFFFFFFFFFFFFFFFFFFFFFFF
FFFFFFFFFFFFFFFFFFFFFFFFFFFFFFFFFFFFFFFFFFFFFFFFFFFFFFFFFFF:FFFFFFFFF
FFFFFFFFFFFFFFF
@A00721:190:HGJW7DSXY:4:2175:6714:3662 2:N:0:TCTGCAAG+GCTGCACT
TTTATAATAAAGCTTTATCAGCTAGGTCAAAGACAGAGTCTCTAGAAATAATCCGGAAGAACGATCCA
AGAACGTTCTACCTTCAGCATCACAACATATCTGCAAATGCAGATAGATTATTTCCAGAACCAATACA
AGAATATCTATCGCC
+
:FFFFFFFFFFFFFFFFFFFFFFFFFFFFFFF:FFFFF:FFFFFFFFFFFFFFFFFFFFFFFFFFFFFF
FFFFFFFFFFFFFFFFF:FF:FFFFF,F,F:FFFFFFFFFFF:FFFFFF:FFF:F:F:FF:FFFFFFF
F,FFFFFFFFF::F
```

@A00721:190:HGJW7DSXY:4:2222:1579:9204 2:N:0:TCTGCAAG+GCTGCACT
TTTATAATAAAGCTTTATCAGCTAGGTCAAAGACAGAGTCTCTAGAAATAATCCGGAAGAACGATCCA
AGAACGTTCTACCTTCAGCATCACAACATATCTGCAAATGCAGATAGATTATTTCCAGAACCAATACA
AGAATATCTATCGCC
+
FFFFFFFFFFFFFFFFFFFFFFFFFFFFFFFFFFFFFFFFFFFFFFFFFFFFFFFFFFFFFFFFFFFF
FFFFFFFFF:FFFFFFFFFFFFFFFFFFFFFFFFFFFFFFFFFFFFFFFFFFFFFFFFFFFFF:FFFF
FFFFFFFFF:FFFFF
@A00721:190:HGJW7DSXY:4:2222:1732:9721 2:N:0:TCTGCAAG+GCTGCACT
TTTATAATAAAGCTTTATCAGCTAGGTCAAAGACAGAGTCTCTAGAAATAATCCGGAAGAACGATCCA
AGAACGTTCTACCTTCAGCATCACAACATATCTGCAAATGCAGATAGATTATTTCCAGAACCAATACA
AGAATATCTATCGCC
+
FFFFFFFFFFFFFFFFFFFFFFFFFFFFFFFFFFFFFFFFFFFFFFFFFFFFFFFFFFFFFFFFFFFF
FFFFFFFFFFFFFFFFFFFFFFFFFFFFFFFFFFFFFFFFFFFFFFFFFFFFFFFFFFFFFFFF,FFF
FFFFFFFFFFFFFFF
@A00721:190:HGJW7DSXY:4:2222:1461:9752 2:N:0:TCTGCAAG+GCTGCACT
TTTATAATAAAGCTTTATCAGCTAGGTCAAAGACAGAGTCTCTAGAAATAATCCGGAAGAACGATCCA
AGAACGTTCTACCTTCAGCATCACAACATATCTGCAAATGCAGATAGATTATTTCCAGAACCAATACA
AGAATATCTATCGCC
+
FFFFFFF:F:FFF,FFFFFFFFFFFFFFFFFFFFFFFFFFFFFFFFFFFFFF:FFFFFFFFFFFFFFF
FFFFFFFFFFFFFFFFFFFFFFFFFFFF:FFFFFFFFFFF:FFFFFFFFFF:F:FFFFFFFFFFF:FF
FFFF:FFFF:FFFFF
@A00721:190:HGJW7DSXY:4:2376:2546:12540 2:N:0:TCTGCAAG+GCTGCACT
TTTATAATAAAGCTTTATCAGCTAGGTCAAAGACAGAGTCTCTAGAAATAATCCGGAAGAACGATCCA
AGAACGTTCTACCTTCAGCATCACAACATATCTGCAAATGCAGATAGATTATTTCCAGAACCAATACA
AGAATATCTATCGCC
+
FFFFFFFFFFFFFFFFFFFF:FFFFF:FFFFFFFFFFFFFFF:FFFFFFFFFFFFFFFFFFFFFFFFF
FFFFFFFFFFFFFFFFFFFFFFFFFFFFFFFFFFFFFFFFFFFFFFFFFF:FFFFFFFFFFFFFFFFF
FFFFFFFFFFF:FFF
@A00721:190:HGJW7DSXY:4:2453:28763:19366 2:N:0:TCTGCAAG+GCTGCACT
TTTATAATAAAGCTTTATCAGCTAGGTCAAAGACAGAGTCTCTAGAAATAATCCGGAAGAACGATCCA
AGAACGTTCTACCTTCAGCATCACAACATATCTGCAAATGCAGATAGATTATTTCCAGAACCAATACA
AGAATATCTATCGCC
+
FFFFFFFFFFFFFFFFFFFFFFFFFFFFFFFFFFFFFFFFFFFFFFFFFFFFFFFFFFFFFFFFFFFF
FFFFFFFFFFFFFFFFFFFFFFFFFFFFFFFFFFFFFFFFFFFFFFFFFFFFFFFFFFFFFFFFFFFF
F:FFFFFFFFFFFFF
@A00721:190:HGJW7DSXY:4:1518:27724:17785 2:N:0:TCTGCAAG+GCTGCACT
TATTGGTTCTGGAAATAATCTATCTGCATTTGCAGATATGTTGTGATGCTGAAGGTAGAACGTTCTTG
GATCGTTCTTCCGGATTATTTCTAGAGACTCTGTCTTTGACCTAGCTGATAAAGCTTTATTATAAACA
ACGTCTAGTTC
+
FFFFFFFFFFFFFFFFFFFFFFFFFFFFFFFFFFFFF,FFFFFFFFFFFFFFFFF::FFFFFFFFFFF
FF:FFFFFFFFFFFFFFFFFFFFFFF:FFFFFFFFFFFFFFFFFFFFFFFF:FFFFFFFFFFFFFFFF
FFFFFFFFFFF
@A00721:190:HGJW7DSXY:4:1650:32334:10238 2:N:0:TCTGCAAG+GCTGCACT
TATTGGTTCTGGAAATAATCTATCTGCATTTGCAGATATGTTGTGATGCTGAAGGTAGAACGTTCTTG
GATCGTTCTTCCGGATTATTTCTAGAGACTCTGTCTTTGACCTAGCTGATAAAGCTTTATTATAAACA
ACGTCTAGTTC
+
FFFFFFFFFFFFFFFFFFFFFFFFFFFFFFFFFFFFFFFFFFFFFFFFFFFFFFFFFFFFFFFFFFFF

F:FFFFFFFFFFFFFFFFFFFFFFFFFFFFFFFFFFFFFFFFFF:FFFFFFF::FFFFFFF:FFFFFFFF
FFFFFFFFFFF
@A00721:190:HGJW7DSXY:4:2245:17969:33646 2:N:0:TCTGCAAG+GCTGCACT
TATTGGTTCTGGAAATAATCTATCTGCATTTGCAGATATGTTGTGATGCTGAAGGTAGAACGTTCTTG
GATCGTTCTTCCGGATTATTTCTAGAGACTCTGTCTTTGACCTAGCTGATAAAGCTTTATTATAAACA
ACGTCTAGTTC
+
FFFFFFFFFFFFFFFFFFFFFFFFFFFFFFFFFFFFFFFFFFFFFFFFFFFFFFFFFFFFFFFFFFFFFF
FFFF:FFFFFFFFFFFFFFFFFFFFFFFFFFFFFFFFFFFFFFFFFFFFFFFFFFFFFFFFFFFFF
F,FFFFFFF:F
@A00721:190:HGJW7DSXY:4:2604:31213:30217 2:N:0:TCTGCAAG+GCTGCACT
TATTGGTTCTGGAAATAATCTATCTGCATTTGCAGATATGTTGTGATGCTGAAGGTAGAACGTTCTTG
GATCGTTCTTCCGGATTATTTCTAGAGACTCTGTCTTTGACCTAGCTGATAAAGCTTTATTATAAACA
ACGTCTAGTTC
+
FFFFFF:FFFFFFFFFFFFFF:FFFFFFFFFFFFFFFFFFFFFFFFFFFFFFFFFFFF,:FFFFFFFFFF
FFFFFFFFFFFFFFFFFFFFFFFFFFF:FFFFFFFFFFF:FFFFFFFFFFFFFF:FFFFFFFFFFFFF:,
F,FFFFF,FF:
@A00721:190:HGJW7DSXY:4:1252:4924:24799 2:N:0:TCTGCAAG+GCTGCACT
GATCGTATCGAACTAGACGTTGTTTATAATAAAGCTTTATCAGCTAGGTCAAAGACAGAGTCTCTAGA
AATAATCCGGAAGAACGATCCAAGAACGTTCTACCTTCAGCATCACAACATATCTGCAAATGCAGATA
GATTATTTCCA
+
FFFFFFFFFFFFFFFFFFFFFFFFFFFFFFFFFFFFFFFFFFFFFFFFFFFFFFFFFFFFFFFFFFFFFF
FFFFFFFFFFFFFFFFF:FFFFFFFFFFFFFFFFFFFFFFFFFFFFFFFFFFFFFFFFFFFFFFFFFF
FFFFFFFF:FF
@A00721:190:HGJW7DSXY:4:1444:27453:20854 2:N:0:TCTGCAAG+GCTGCACT
GATCGTATCGAACTAGACGTTGTTTATAATAAAGCTTTATCAGCTAGGTCAAAGACAGAGTCTCTAGA
AATAATCCGGAAGAACGATCCAAGAACGTTCTACCTTCAGCATCACAACATATCTGCAAATGCAGATA
GATTATTTCCA
+
FFFFF,FFFFFFFFFFFFFFFFFF:FFF,,F::FF:F,FFFFFFFF:F:FFFFFFFFFFFFFFFFFFFFF
F:FFFFFFFFFF:FFFFFFFFFFFFFFFFF,FFF:FFFF:FFFF:F::FFFF:F:FF:F:FFFFFFFF:
F:FFFFFFFFF
@A00721:190:HGJW7DSXY:4:1610:15483:25300 2:N:0:TCTGCAAG+GCTGCACT
GATCGTATCGAACTAGACGTTGTTTATAATAAAGCTTTATCAGCTAGGTCAAAGACAGAGTCTCTAGA
AATAATCCGGAAGAACGATCCAAGAACGTTCTACCTTCAGCATCACAACATATCTGCAAATGCAGATA
GATTATTTCCA
+
FFFFFFFFFFFFFFFFFFFFFFFFFFF:FFFFFFFFFFFFFFFFFFFFFFFFFFFFFFFFFFFFFFFFFF
FFFFFFFFFFFFF:FFFFFFF:FFFFFFFFFFF:FFFFFFFFFFFFFFFFF:FFFFFFFFFFFFFFFFFF
F:FFFFFFFFF
@A00721:190:HGJW7DSXY:4:1657:20103:18051 2:N:0:TCTGCAAG+GCTGCACT
GATCGTATCGAACTAGACGTTGTTTATAATAAAGCTTTATCAGCTAGGTCAAAGACAGAGTCTCTAGA
AATAATCCGGAAGAACGATCCAAGAACGTTCTACCTTCAGCATCACAACATATCTGCAAATGCAGATA
GATTATTTCCA
+
FFFFFFF:FFFFFFFFFFFFF:FFF:FFFFFFFFFFFFFFF:FFFFFFFF:FFFFFFFFFFFFFFF:FFF
FFFFFFF:,FFFFFFF:FFFFFFFFFFFFFFFFFFFFFFFFFFFFFFFFFFFFFFFFFFFFFFF:FFFFF
F:FFF:FFFFF
@A00721:190:HGJW7DSXY:4:2228:6397:18959 2:N:0:TCTGCAAG+GCTGCACT
GATCGTATCGAACTAGACGTTGTTTATAATAAAGCTTTATCAGCTAGGTCAAAGACAGAGTCTCTAGA
AATAATCCGGAAGAACGATCCAAGAACGTTCTACCTTCAGCATCACAACATATCTGCAAATGCAGATA
GATTATTTCCA

```
+
FFFFFFFFFFFFFFFFFFFF:FFFFFFFFFFFFFFFFFFFFFFFFFFFFFFFFFFFFFFFFFFFFFFF
FFFFFF:F:FFFFFFFFFFFFFFFFFFF:FFFFFFFFFFFFFFFFFFFFFFFFFFFFFFFFFFFF:FF:
FFFFFFFFFFF
@A00721:190:HGJW7DSXY:4:2257:24279:33144 2:N:0:TCTGCAAG+GCTGCACT
GATCGTATCGAACTAGACGTTGTTTATAATAAAGCTTTATCAGCTAGGTCAAAGACAGAGTCTCTAGA
AATAATCCGGAAGAACGATCCAAGAACGTTCTACCTTCAGCATCACAACATATCTGCAAATGCAGATA
GATTATTTCCA
+
FFFFFFFFFFFFFFFFFFFF:F:F:F,FF,FFFFFFF:FFFFF::FFFFFFFFFFFFFFFFFFFF:FF
FFFFFFF,F:FFFFFFFFF:FFFFFFFF::FFFFFF,FFF,F,,FFFFFFFFF,FFFFFFFFFF,FFF
,F::FFF:,FF
@A00721:190:HGJW7DSXY:4:2328:1452:31908 2:N:0:TCTGCAAG+GCTGCACT
GATCGTATCGAACTAGACGTTGTTTATAATAAAGCTTTATCAGCTAGGTCAAAGACAGAGTCTCTAGA
AATAATCCGGAAGAACGATCCAAGAACGTTCTACCTTCAGCATCACAACATATCTGCAAATGCAGATA
GATTATTTCCA
+
FFFFFFFFFFFFFFFFFFFFFFFFFFFFFFFFFFFFFFFFFFFFFFFFFFFFFFFFFFFFFFFFFFFF
FFFFFFFFFFFFFFFFFFFFFFFFFFFFFFFFFFFFFFFFFFFFFFFFFFFFFF:FFFFFFFFFFFFF
FFFFFFFFFFF
@A00721:190:HGJW7DSXY:4:2438:12418:18521 2:N:0:TCTGCAAG+GCTGCACT
GATCGTATCGAACTAGACGTTGTTTATAATGAAGCTTTATCAGCTAGGTCAAAGACAGAGTCTCTAGA
AATAATCCGGAAGAACGATCCAAGAACGTTCTACCTTCAGCATCACAACATATCTGCAAATGCAGATA
GATTATTTCCA
+
FFFFFFFFFFFFFFFFFFFFFFFFFFFFFF,::FFFFFFFFFFFFFFFFFFFFFFFFFFFFFFFFFFF
FFFFFFF:FFFFFFFF:FF:FFFFFFFFFFFFFFF,FFFFFFFFFFFFFFFFFFFFFFFFF,FFFF,F
:FFFFFFF:FF
@A00721:190:HGJW7DSXY:4:2464:29134:10833 2:N:0:TCTGCAAG+GCTGCACT
GATCGTATCGAACTAGACGTTGTTTATAATAAAGCTTTATCAGCTAGGTCAAAGACAGAGTCTCTAGA
AATAATCCGGAAGAACGATCCAAGAACGTTCTACCTTCAGCATCACAACATATCTGCAAATGCAGATA
GATTATTTCCA
+
FFFFFFFFFFFFFFFFFFFFFFFFFFFFFFFFFFFFFFFFFFFFFFFFFFFFFFFFFFFFFFFFFFFF
FFFFFFFFFFFFFFFFFFFFFFFFFFFFFFFFFFFFFFF:FFFFFFFFFFFFFFFFFFFFFFFF:FFF
FFFFFFFFFFF
@A00721:190:HGJW7DSXY:4:2520:28456:1861 2:N:0:TCTGCAAG+GCTGCACT
GATCGTATCGAACTAGACGTTGTTTATAATAAAGCTTTATCAGCTAGGTCAAAGACAGAGTCTCTAGA
AATAATCCGGAAGAACGATCCAAGAACGTTCTACCTTCAGCATCACAACATATCTGCAAATGCAGATA
GATTATTTCCA
+
FFFFFFFFFFFFFFFFFFFFFFFFFFFFFFFFFFFFFFFFFFFFFFFFFFFFFFFFFFFFFFFFFFFF
FFFFFFFFFFFFFFFFFFFFFFFFF::FFFFFFFFFFFFFFFFFFFFFFFFFFFFFFF:FFFFFFFFFF
FFFFFFFFFFF
@A00721:190:HGJW7DSXY:4:2539:26377:20619 2:N:0:TCTGCAAG+GCTGCACT
GATCGTATCGAACTAGACGTTGTTTATAATAAAGCTTTATCAGCTAGGTCAAAGACAGAGTCTCTAGA
AATAATCCGGAAGAACGATCCAAGAACGTTCTACCTTCAGCATCACAACATATCTGCAAATGCAGATA
GATTATTTCCA
+
FFF:FFFFFFFFFFFFFFFFFFFFFFFFFFFFFFFFFFFFFFFFFFFFFFFFFFFFFFFFFFFFFFFF
FFFFFF:F::FFFFFF:FFFFFFFFFFFFFFFFFF:F:F:FFFFFFFFFF:FFFFFFFFF:F:F,FFF
F:FFFFFFF,F
@A00721:190:HGJW7DSXY:4:2542:29767:13244 2:N:0:TCTGCAAG+GCTGCACT
GATCGTATCGAACTAGACGTTGTTTATAATAAAGCTTTATCAGCTAGGTCAAAGACAGAGTCTCTAGA
```

```
AATAATCCGGAAGAACGATCCAAGAACGTTCTACCTTCAGCATCACAACATATCTGCAAATGCAGATA
GATTATTTCCA
+
FFFFFFFFFFFFFFFFFFFFFFFFFFFFFFFFFFFFFFFFFFFFFFFFFFFFFFFFFFFFF:FFFFFFFF
FFFFFFFFFFFFFFFFFFFFFFFFFFFFFFFFFF:FFFFFFFFFFFF:FFFFFFF:FFFFFFFFFFFFFF
F:FFFFFFFFF
@A00721:190:HGJW7DSXY:4:1262:18204:6277 2:N:0:TCTGCAAG+GCTGCACT
GATCGTATCGAACTAGACGTTGTTTATAATAAAGCTTTATCAGCTAGGTCAAAGACAGAGTCTCTAGA
AATAATCCGGAAGAACGATCCAAGAACGTTCTACCTTCAGCATCACAACATATCTGCAAATGCAGATA
GATTATTTCC
+
FFFFFFFFFFFFFFFFFFFFFFFFFFFFFFFFFFFFFFFFFFFFFFFFFFFFFFFFFFFFFFFFFFFFFF
FFFFFFFFFFFFFFFFFFFFFFFFFFFFFFFFFFFFFFFFFFFFFFFFFFFFFFFFFFFFFFF:FFFFFF
FFFFFFFFFF
@A00721:190:HGJW7DSXY:4:1173:22010:31375 2:N:0:TCTGCAAG+GCTGCACT
CGTATCGAACTAGACGTTGTTTATAATAAAGCTTTATCAGCTAGGTCAAAGACAGAGTCTCTAGAAAT
AATCCGGAAGAACGATCCAAGAACGTTCTACCTTCAGCATCACAACATATCTGCAAATGCAGATAGAT
TATT
+
FFFFFFFFFFFFFFFFFFFFFFFFFF:FF:FFFFFFFFFFFFFFFFFFFFFFFFFFFFFFFFFFFFFFFF
FFFFFFFFFFFFFFFFFFFFFFFFFFFFFFFFFFFFFFFFFFFFFFFFFFFFFFFFFFFFFFFFFFFFFF
FFFF
@A00721:190:HGJW7DSXY:4:1173:22073:33458 2:N:0:TCTGCAAG+GCTGCACT
CGTATCGAACTAGACGTTGTTTATAATAAAGCTTTATCAGCTAGGTCAAAGACAGAGTCTCTAGAAAT
AATCCGGAAGAACGATCCAAGAACGTTCTACCTTCAGCATCACAACATATCTGCAAATGCAGATAGAT
TATT
+
FFFFFFFFF:FFFFFF::FF,:F:FFFFFFF:FFFFFFFFFFFFFFFFF:FFFFFFFF:FFFFFFFFFFF
FFFFFFFFFFFFF,F,FFFF:FFF:::FFFFFFFFFFFFFFFFFFFFFF,FFF:FFFFFFFF::FFFFFF
FFF:
@A00721:190:HGJW7DSXY:4:1321:9588:11365 2:N:0:TCTGCAAG+GCTGCACT
CGTATCGAACTAGACGTTGTTTATAATAAAGCTTTATCAGCTAGGTCAAAGACAGAGTCTCTAGAAAT
AATCCGGAAGAACGATCCAAGAACGTTCTACCTTCAGCATCACAACATATCTGCAAATGCAGATAGAT
TATT
+
FFFFFFFFFFFFFFFFFFFF:FFF:FFFFFFFFFFFFFFFFFFFFFFFFFF:FFFFFFFFFFFFFFFFFF
FFFFFFFFFFFFFFFFFFFFFFFFFFFFFFFFFFFFFFF:FFFFFFFFFFFFFFFFFFFFFFFFF:FFFF
FFFF
@A00721:190:HGJW7DSXY:4:1353:4915:28917 2:N:0:TCTGCAAG+GCTGCACT
CGTATCGAACTAGACGTTGTTTATAATAAAGCTTTATCAGCTAGGTCAAAGACAGAGTCTCTAGAAAT
AATCCGGAAGAACGATCCAAGAACGTTCTACCTTCAGCATCACAACATATCTGCAAATGCAGATAGAT
TATT
+
FFFFFFFFFFFFFFFFFFFF:FFFFFFFFFFFFFFFFFFFFFFFFFFFFFFFFFFFFFFFFFFFFFFFFF
FFFFFFFFFFFFFFFFFFFFFFFFFFFFFFFFFFFFFFFFFFFFFF:FFFFFFFFFFFFFFFFFFFFFFF
FFFF
@A00721:190:HGJW7DSXY:4:1501:24406:24878 2:N:0:TCTGCAAG+GCTGCACT
CGTATCGAACTAGACGTTGTTTATAATAAAGCTTTATCAGCTAGGTCAAAGACAGAGTCTCTAGAAAT
AATCCGGAAGAACGATCCAAGAACGTTCTACCTTCAGCATCACAACATATCTGCAAATGCAGATAGAT
TATT
+
FFFFFFFFFFFFFFFFFFFFFFFFFFFFFFFFFFFFFFFFFFFFF:FFFFFFFFFFFFFFFFFFFFFFFF
FFFFFFFF:FFFFFFFFFFFF:FFFFFFFFFFFFFFFFFFFFFFFFFFFFFFFF,FFFFFF:FFFFFFFF
FFFF
```

```
@A00721:190:HGJW7DSXY:4:2307:24071:7200 2:N:0:TCTGCAAG+GCTGCACT
CGTATCGAACTAGACGTTGTTTATAATAAAGCTTTATCAGCTAGGTCAAAGACAGAGTCTCTAGAAAT
AATCCGGAAGAACGATCCAAGAACGTTCTACCTTCAGCATCACAACATATCTGCAAATGCAGATAGAT
TATT
+
F,,FFFFFFFFFF:FF:FFFFFFFFFFFFF,FF:FFFFFFFFFFFFFFFFFFFFFFFF,FFF::FFFF,
FFFF,,FF,,FFF,FFF:FFFFFFF,::FFF:F,FF:FF:FFFFFFFFFFFFFFFFFFFFFFFFF,F:F
,F:F
@A00721:190:HGJW7DSXY:4:2443:13376:12947 2:N:0:TCTGCAAG+GCTGCACT
CGTATCGAACTAGACGTTGTTTATAATAAAGCTTTATCAGCTAGGTCAAAGACAGAGTCTCTAGAAAT
AATCCGGAAGAACGATCCAAGAACGTTCTACCTTCAGCATCACAACATATCTGCAAATGCAGATAGAT
TATT
+
FFFFFFFFFFFFFFFFFFFF:FFFFFFFFFFFFFFFFFFFFFFFFFFFFFFFFFFFFFFFFFFFFFFF
FFFFFFFFFFFFFFFFFFFFFFFFFFFFFFFFFFFFFFFFFFFFFFFFFFFFFFFFFFFFFFFFFFF,
FFFF
@A00721:190:HGJW7DSXY:4:2559:20844:6621 2:N:0:TCTGCAAG+GCTGCACT
CGTATCGAACTAGACGTTGTTTATAATAAAGCTTTATCAGCTAGGTCAAAGACAGAGTCTCTAGAAAT
AATCCGGAAGAACGATCCAAGAACGTTCTACCTTCAGCATCACAACATATCTGCAAATGCAGATAGAT
TATT
+
FFFFFFFFFFFFFFFFFFFFFFFFFFFFFFFFFFFFFFFFFFFFFFFFFFFFFFFFFFFFFFFFFFFF
FFFFFFFFFFFFFFFFFFFFFFFFFFFFFFFFFFFFFFFFFFFFFFFFFFFFFFFFFFFFFFFFFFFF
FFFF
@A00721:190:HGJW7DSXY:4:1115:13521:21496 2:N:0:TCTGCAAG+GCTGCACT
GCCGGAAAAGAAAGTGTGATCGTATCGAACTAGACGTTGTTTATAATAAAGCTTTATCAGCTAGGTCA
AAGACAGAGTCTCTAGAAATAATCCGGAAGAACGATCCAAGAACGTTCTACCTTCAGCATCACAACAT
ATCTGCAAATGCAGA
+
FFFFFFFFFFFFFFFFFFFFFFFFFFFFFFFFFFFFFFFFFFFFFFFFFFFFFFFFFFFFFFFF:FFF
FFFFFFFFFFFFFFFFFFFFFFFFFFFFFFFFFFFFFFFFF:F:FFFFFFFFFFFFFFFFFFFFFFF:
FF:FFFFFFFFFFFF
@A00721:190:HGJW7DSXY:4:1218:19723:23156 2:N:0:TCTGCAAG+GCTGCACT
GCCGGAAAAGAAAGTGTGATCGTATCGAACTAGACGTTGTTTATAATAAAGCTTTATCAGCTAGGTCA
AAGACAGAGTCTCTAGAAATAATCCGGAAGAACGATCCAAGAACGTTCTACCTTCAGCATCACAACAT
ATCTGCAAATGCAGA
+
FFFFFF:F:FFF:FFFFFFFFFFFFFFFFFFFFFFFFF:FFFFFFFFFFFFFFFFFFFFFFFFFFFFFF
FFFFFFFFFFFFFFFFFFFFFFFFFFFFFFFFFFFFFFFFFFFFFFFFFFFFFFFFFFFFFFFFFFFF
FFFFF,FFFFFFF:F
@A00721:190:HGJW7DSXY:4:1231:1579:14121 2:N:0:TCTGCAAG+GCTGCACT
GCCGGAAAAGAAAGTGTGATCGTATCGAACTAGACGTTGTTTATAATAAAGCTTTATCAGCTAGGTCA
AAGACAGAGTCTCTAGAAATAATCCGGAAGAACGATCCAAGAACGTTCTACCTTCAGCATCACAACAT
ATCTGCAAATGCAGA
+
FFFFFFFFFFFFFFFF:FFFFFF:FFFFFFFFFF:F,:F:F:FFFFFFFFFF:FFFF::FFFFFFFFF
FFFFFFFFFFFFFFFFFFFFFFFFFFFFFFFFFFFFFFFFFFFFFFFFFFFFFFFFFF::FFFFFFFFF
FFFFFFFFF::FFFF
@A00721:190:HGJW7DSXY:4:1321:4354:22874 2:N:0:TCTGCAAG+GCTGCACT
GCCGGAAAAGAAAGTGTGATCGTATCGAACTAGACGTTGTTTATAATAAAGCTTTATCAGCTAGGTCA
AAGACAGAGTCTCTAGAAATAATCCGGAAGAACGATCCAAGAACGTTCTACCTTCAGCATCACAACAT
ATCTGCAAATGCAGA
+
FFFFFFFFFFFFFFFFFFFFFFFFFF:FFFFFFFFFF:FFFFFFFFFFFFFFFFFF:FFFFFFFFFFF
```

FFFFFFFFFFFFFFF:FFFFFFFFFFFFFFFFFFFFFFFFFF:FFF,FF:FFFFFFFFFFFFFFFFFFFF
FFFFFFFFFFFFFFF
@A00721:190:HGJW7DSXY:4:1373:4887:7670 2:N:0:TCTGCAAG+GCTGCACT
GCCGGAAAAGAAAGTGTGATCGTATCGAACTAGACGTTGTTTATAATAAAGCTTTATCAGCTAGGTCA
AAGACAGAGTCTCTAGAAATAATCCGGAAGAACGATCCAAGAACGTTCTACCTTCAGCATCACAACAT
ATCTGCAAATGCAGA
+
FFFFFFFFFFFFFFFFFFFFFFFFFFFFFFFFFFFFF:FFFFFFFFFFFFFFFFFFFFFFFFFFFFFFFF
FFFFFFFFFFFFFFFFFFFFFFFFFFFFFFFFFFFFFFFFFFFFFFFFFFFFFFFFFF,FFFFFFFFFFF
FFFFFFFFFFFFFFF
@A00721:190:HGJW7DSXY:4:1420:24957:15405 2:N:0:TCTGCAAG+GCTGCACT
GCCGGAAAAGAAAGTGTGATCGTATCGAACTAGACGTTGTTTATAATAAAGCTTTATCAGCTAGGTCA
AAGACAGAGTCTCTAGAAATAATCCGGAAGAACGATCCAAGAACGTTCTACCTTCAGCATCACAACAT
ATCTGCAAATGCAGA
+
FFFF:FFFFFFFFFFFFFFFFFFFFFFFFFFFFFFFFFFFFFFFFFFFFFFFFFFFFFFFF:FFFF:FF
FFFFFFFFFFFFFFFFFFFFFFFFFFFFFF:FFFFFFFFFFFFFFFFFFFFFFFFFFFFFFFFFFFFFFF
FFFFFFFFFF:FFFF
@A00721:190:HGJW7DSXY:4:1502:17381:29684 2:N:0:TCTGCAAG+GCTGCACT
GCCGGAAAAGAAAGTGTGATCGTATCGAACTAGACGTTGTTTATAATAAAGCTTTATCAGCTAGGTCA
AAGACAGAGTCTCTAGAAATAATCCGGAAGAACGATCCAAGAACGTTCTACCTTCAGCATCACAACAT
ATCTGCAAATGCAGA
+
FFFFFFFFFFFFFFFFFFFFFF:FFFFFFFFFFFFFFF:FFFFFFFFFFFF,FFFFFFFFFFFFFF:FFF
FFFFFFFFFFFFFFFFFFFFFFFFFFFFFFFFFF:FFFFFFFFFFFFFFFFFFFFFFFFFFFFFFFFFFF
FFFFFFFFFF:FFFF
@A00721:190:HGJW7DSXY:4:1529:15383:4398 2:N:0:TCTGCAAG+GCTGCACT
GCCGGAAAAGAAAGTGTGATCGTATCGAACTAGACGTTGTTTATAATAAAGCTTTATCAGCTAGGTCA
AAGACAGAGTCTCTAGAAATAATCCGGAAGAACGATCCAAGAACGTTCTACCTTCAGCATCACAACAT
ATCTGCAAATGCAGA
+
FFFFFF,FFFFFFFFFFFFFFFFFFFFFFFFFFFFF::FFFFFFFFFFFFFFFFFFFFFFFFFFFFFFFF
FFFFFFFFFFFFFFFFFFFFFFFFFFFFFFFFFFFFFFFFFF,FFFFFFFFFFFFFFFFFFFFFFFFFFF
:FFFFFFFF:FFFFF
@A00721:190:HGJW7DSXY:4:2102:10936:34522 2:N:0:TCTGCAAG+GCTGCACT
GCCGGAAAAGAAAGTGTGATCGTATCGAACTAGACGTTGTTTATAATAAAGCTTTATCAGCTAGGTCA
AAGACAGAGTCTCTAGAAATAATCCGGAAGAACGATCCAAGAACGTTCTACCTTCAGCATCACAACAT
ATCTGCAAATGCAGA
+
FFFFFFFFFFFFFFFFFFFFFFFFFFFFFFFFFFFFFFFFFFFFFFFFFFFFFFFFFFFFFFFFFFFFFF
FFFFFFFFFFFFFFFFFFFFFFFFFFFFFFFFFFFFFFFFFFFFFFFFFFFFFFFFFFFFFFFFFFFF:F
FFFFFFFFFF:FFFF
@A00721:190:HGJW7DSXY:4:2109:30517:7592 2:N:0:TCTGCAAG+GCTGCACT
GCCGGAAAAGAAAGTGTGATCGTATCGAACTAGACGTTGTTTATAATAAAGCTTTATCAGCTAGGTCA
AAGACAGAGTCTCTAGAAATAATCCGGAAGAACGATCCAAGAACGTTCTACCTTCAGCATCACAACAT
ATCTGCAAATGCAGA
+
FFFF:FFFFFFFFFFFFFFFFFFFFFFFFFFF:FFFFF:FFFFFFFFFFFF,FFFFFFFFFFFFFFF:FF
F,F:FFFF,FFFFFF:FFFFFFFFFF:,FFF::FFFFFFFFFFFFFF:FFF,FFF:FFFFF:F:FFFFFF
FFFF:FF:FF,FFFF
@A00721:190:HGJW7DSXY:4:2109:30463:7780 2:N:0:TCTGCAAG+GCTGCACT
GCCGGAAAAGAAAGTGTGATCGTATCGAACTAGACGTTGTTTATAATAAAGCTTTATCAGCTAGGTCA
AAGACAGAGTCTCTAGAAATAATCCGGAAGAACGATCCAAGAACGTTCTACCTTCAGCATCACAACAT
ATCTGCAAATGCAGA

```
+
FFFFFFFFFFFFFFFFFFFFFFFFFFFFFFFFFFFFFFFFFFFFFFFFFFFFFFFFFFFFFFFFFFFFFF
FFF:FFFFFFFFFFFFFFFFFFFF:FFFFFFFFFF:FFFFFFFFFFFFFFFFFFFFFFFFFFFFFFFFFF
FFFFFFFFFFFFFFF
@A00721:190:HGJW7DSXY:4:2343:17372:35462 2:N:0:TCTGCAAG+GCTGCACT
GCCGGAAAAGAAAGTGTGATCGTATCGAACTAGACGTTGTTTATAATAAAGCTTTATCAGCTAGGTCA
AAGACAGAGTCTCTAGAAATAATCCGGAAGAACGATCCAAGAACGTTCTACCTTCAGCATCACAACAT
ATCTGCAAATGCAGA
+
FFFFFFFF:FFFFF:FFFFFFFFFFFFFFFFFFFFFFFFFFF:FFFFFFFFFFFFFFFFFFFFFFFFFFF
FFFFFFF:FFFFFFFFFFFFFFF:FFFFFFFFFFFFFFFFFFFFFFFFFFFFF:FFFFFF:FFFFFFFFF
FFFFF:FFFFFFFFF
@A00721:190:HGJW7DSXY:4:2349:20998:8985 2:N:0:TCTGCAAG+GCTGCACT
GCCGGAAAAGAAAGTGTGATCGTATCGAACTAGACGTTGTTTATAATAAAGCTTTATCAGCTAGGTCA
AAGACAGAGTCTCTAGAAATAATCCGGAAGAACGATCCAAGAACGTTCTACCTTCAGCATCACAACAT
ATCTGCAAATGCAGA
+
FFFFFFFFFFFFFFFFFFFFFFFFFFFFFFFFFFFFFFFFFFFFFFFFFFFFFFFFFFFFFFFFFFFFFF
FFFFFFFFFFFFFFFFFFFFFFFFFFFF,FFFFFFFFFFFFFFFFFFFFFFFFFFFFFFFFFFFFFFFFF
FFFFFFFFFFF:FFF
@A00721:190:HGJW7DSXY:4:2349:21007:9001 2:N:0:TCTGCAAG+GCTGCACT
GCCGGAAAAGAAAGTGTGATCGTATCGAACTAGACGTTGTTTATAATAAAGCTTTATCAGCTAGGTCA
AAGACAGAGTCTCTAGAAATAATCCGGAAGAACGATCCAAGAACGTTCTACCTTCAGCATCACAACAT
ATCTGCAAATGCAGA
+
FFFFF:FFFFFFFFFFFFFFFFFFFFFFFFFFFFFF:FFFFFFFFFFFFFFFFFFFFFFFFFFFFFFFFF
FFFFFFFFFFFFFFFFFFFFFFFFFFFFFF:FFFFFFFFFFFFFFFFFFFFFFFFFFFF:FFFFFF:FFF
FFF:F:FFF:F:FFF
@A00721:190:HGJW7DSXY:4:2452:28637:25347 2:N:0:TCTGCAAG+GCTGCACT
GCCGGAAAAGAAAGTGTGATCGTATCGAACTAGACGTTGTTTATAATAAAGCTTTATCAGCTAGGTCA
AAGACAGAGTCTCTAGAAATAATCCGGAAGAACGATCCAAGAACGTTCTACCTTCAGCATCACAACAT
ATCTGCAAATGCAGA
+
F:FFFFFFFFFFFFFFFFFFFFFFFFFFFFFFFFFFFFFFFFFFFFFFFFFFFFFFFFFFF,FFFF:FFF
F:F:FFFFFFFFFFFFFFFFFFFFFF::FFFFFFFFFFFFF,FFFFFFFFFFFF:FFFFFFF:FFFFFF,
FFF,F,FFF:FFFF:
@A00721:190:HGJW7DSXY:4:2533:18430:11459 2:N:0:TCTGCAAG+GCTGCACT
GCCGGAAAAGAAAGTGTGATCGTATCGAACTAGACGTTGTTTATAATAAAGCTTTATCAGCTAGGTCA
AAGACAGAGTCTCTAGAAATAATCCGGAAGAACGATCCAAGAACGTTCTACCTTCAGCATCACAACAT
ATCTGCAAATGCAGA
+
FFFFFFFFFFFFFFFFFFFFFFFFFFFFFFFFFFFFF:FFF:FFFFFFFFFFFFFFFFFFFFFFFFFFFF
F:FFFFFFFFFFFFFFFFFFFFFFFFFFFFF:FFFFFFFFFFFFFF:FFFFFFFFFFFFFFFFFFFFFFF
FFFFFFFFF:::FFF
@A00721:190:HGJW7DSXY:4:2539:2311:5431 2:N:0:TCTGCAAG+GCTGCACT
GCCGGAAAAGAAAGTGTGATCGTATCGAACTAGACGTTGTTTATAATAAAGCTTTATCAGCTAGGTCA
AAGACAGAGTCTCTAGAAATAATCCGGAAGAACGATCCAAGAACGTTCTACCTTCAGCATCACAACAT
ATCTGCAAATGCAGA
+
FFFFFFFFFFFFFFFFFFFFFFFFFFFFFFFFFFFFFFFFFFFFFFFFFFFFFFFFFFFFFFFFFFFFFF
FFFFFFFFFFFFFFFFFFFFFFFFFFFFFF:FFFFFFFF:FFFFFFFFFFFFFFFFFFF:FFFFFFFFFF
:FFFFFFFFFFFFFF
@A00721:190:HGJW7DSXY:4:2619:24659:13135 2:N:0:TCTGCAAG+GCTGCACT
GCCGGAAAAGAAAGTGTGATCGTATCGAACTAGACGTTGTTTATAATAAAGCTTTATCAGCTAGGTCA
```

```
AAGACAGAGTCTCTAGAAATAATCCGGAAGAACGATCCAAGAACGTTCTACCTTCAGCATCACAACAT
ATCTGCAAATGCAGA
+
FFFFFFF,FFFFFFFFFFFFFFFFFFFFFFFFFFFFFFF,,:FF,FFFFFFFFF:F,FFFFFFFFFFF
FFFFF,FFFFFFFFFF:FFFFFFFFFFFFFFFFFFFFFFFFFFFFFFFFFFFFFFFFFFFFFFFFFF:
FFFFFFFFFFF:FFF
@A00721:190:HGJW7DSXY:4:1207:5990:11741 2:N:0:TCTGCAAG+GCTGCACT
TTTCAAGAAGACGGCCGGAAAAGAAAGTGTGATCGTATCGAACTAGACGTTGTTTATAATAAAGCTTT
ATCAGCTAGGTCAAGACAGAGTCTCTAGAAATAATCCGGAAGAACGATCCAAGAACGTTCTACCTTCA
GCATCACAACATATC
+
FFFFFFFFFFFFFFFFFFFFFFFFFFFFFFFFFFFFFFFFFFFFFFFFFFFFFFFFFFFFFFFFFFFF
FFFFFFFFFFFFFFFFFFFFFFFFFFFFFFFFFFFFFFFFFFFFFFFFFF,FFFFFFFFFFFFFFFFF
FFFFFFFFFFFFFFF
@A00721:190:HGJW7DSXY:4:2508:4725:25989 2:N:0:TCTGCAAG+GCTGCACT
TTTCAAGAAGACGGCCGGAAAAGAAAGTGTGATCGTATCGAACTAGACGTTGTTTATAATAAAGCTTT
ATCAGCTAGGTCAAGACAGAGTCTCTAGAAATAATCCGGAAGAACGATCCAAGAACGTTCTACCTTCA
GCATCACAACATATC
+
FFFFFFFFFF:FFFFFFFFFFFFFFFFFFFFFFFFFFFFFFFFFFFFFFFF:FFFFFFFFFFFFFFFFF
FFFFFFFFFFFFFFFFFFFFFFFFFFFFFFFFFFFFFF:FFFFFFFFFFFFFFFFFFFFFFFFFFFFF
FFFFFFFFFFFFFFF
@A00721:190:HGJW7DSXY:4:2534:29342:9846 2:N:0:TCTGCAAG+GCTGCACT
TTTCAAGAAGACGGCCGGAAAAGAAAGTGTGATCGTATCGAACTAGACGTTGTTTATAATAAAGCTTT
ATCAGCTAGGTCAAGACAGAGTCTCTAGAAATAATCCGGAAGAACGATCCAAGAACGTTCTACCTTCA
GCATCACAACATATC
+
FFFFFFFFFFFFFFFFFFFFFFFFFFFFFFFFFFFFFFFFFFFFFFFFFF::FFFFFFFFFFFFFFFF
FFFFFFFFFFFFFFFFFFFFFFFFFFFFFFFFFFFFFFFFFFFFFFFFFFFFF:FFFFFFFFFFFFFF
FFFF:FFFFFFFFFF
@A00721:190:HGJW7DSXY:4:1160:6985:36542 2:N:0:TCTGCAAG+GCTGCACT
ATATTGATCACGGTCAATTTCAAGAAGACGGCCGGAAAAGAAAGTGTGATCGTATCGAACTAGACGTT
GTTTATAATAAAGCTTTATCAGCTAGGTCAAAGACAGAGTCTCTAGAAATAATCCGGAAGAACGATCC
AAGAACGTTCTACCT
+
FF,FFFFF:F:FFFFFF:FFF:FFFFFFFF,F,FFF:,,FFFFFFFFFFF,FFFF:FFF,FF:F,F,F
FF:FFFFFFFFFF:FFF:F:FF:FFFFF,FFFFFFFFF:FFFFFFFF:FFFFF:FFFFF,FFFFFF:F
:FF,FFFFFFF:F,F
@A00721:190:HGJW7DSXY:4:1335:5927:27696 2:N:0:TCTGCAAG+GCTGCACT
ATATTGATCACGGTCAATTTCAAGAAGACGGCCGGAAAAGAAAGTGTGATCGTATCGAACTAGACGTT
GTTTATAATAAAGCTTTATCAGCTAGGTCAAAGACAGAGTCTCTAGAAATAATCCGGAAGAACGATCC
AAGAACGTTCTACCT
+
FFFFFFFFFFFFFFFFFFFFFFFFFFFFFFFFFFFFFFFFFFFFFFFFFFFFFFFFFFFFFFFFFFFF
F:FFFFFFFFFFFFFFFFFFFFFFFFFFFFFFFFFFFFFFFFFFFFFFFFFFFFFFFFFFFFFFFFFF
FFFFFFFFFFFFFFF
@A00721:190:HGJW7DSXY:4:1446:5801:7404 2:N:0:TCTGCAAG+GCTGCACT
ATATTGATCACGGTCAATTTCAAGAAGACGGCCGGAAAAGAAAGTGTGATCGTATCGAACTAGACGTT
GTTTATAATAAAGCTTTATCAGCTAGGTCAAAGACAGAGTCTCTAGAAATAATCCGGAAGAACGATCC
AAGAACGTTCTACCT
+
FFFFFFFFFFFFFFFFFFFFFF:FFFF:FFFFFFFFFF:FF:FFFFFFFFFFFFFFFFFFFFFFFFF:
FF::FFFFFFFFFFFFFF:FFFFFFFFFFFFFFFFFFFFFFFFFFFFFFFFFFFFFFFFFF,FFFFFFF
FFF:FFFFFFFFFFF
```

```
@A00721:190:HGJW7DSXY:4:1456:9534:15499 2:N:0:TCTGCAAG+GCTGCACT
ATATTGATCACGGTCAATTTCAAGAAGACGGCCGGAAAAGAAAGTGTGATCGTATCGAACTAGACGTT
GTTTATAATAAAGCTTTATCAGCTAGGTCAAAGACAGAGTCTCTAGAAATAATCCGGAAGAACGATCC
AAGAACGTTCTACCT
+
FFFFFFFFFFFFFFFFFFFFFFFFFFFFFFFFFFFFFFFFFFFFFFFFFFFFFFFFFFFFFFFFFFFF
FFFFF:FFFFFFFFFF:FFFFFFFFFFFFFFFFFFFFFFFFFFFFFFFFFFFFFFFFFFFFFFFFFFF
FFFFFFFFFFFFFFF
@A00721:190:HGJW7DSXY:4:2154:6732:6950 2:N:0:TCTGCAAG+GCTGCACT
ATATTGATCACGGTCAATTTCAAGAAGACGGCCGGAAAAGAAAGTGTGATCGTATCGAACTAGACGTT
GTTTATAATAAAGCTTTATCAGCTAGGTCAAAGACAGAGTCTCTAGAAATAATCCGGAAGAACGATCC
AAGAACGTTCTACCT
+
FFF:FFFFFFFFFFFFFFFFFFFFFFFFFFFFFFFFFFFFF:FFFFFFFFFFFFFFFFFFFFFFFFF:
FFFFF:FFFFFFFFFFFFFFFFF:FFFFFFFFFFF:FFFFFFFFFFFFFFFFF:FFFFFFFFFFFFFF
FFFFFFFFFFFFFFF
@A00721:190:HGJW7DSXY:4:2221:10140:27352 2:N:0:TCTGCAAG+GCTGCACT
ATATTGATCACGGTCAATTTCAAGAAGACGGCCGGAAAAGAAAGTGTGATCGTATCGAACTAGACGTT
GTTTATAATAAAGCTTTATCAGCTAGGTCAAAGACAGAGTCTCTAGAAATAATCCGGAAGAACGATCC
AAGAACGTTCTACCT
+
FFFFFFFFFFFFFFFFFFFFFFFFFFFFFFFFFFFFFFFFFFFFFFFFFFFFFFFFFFFFFFFFFFF:
FFFFFFFFFFFFFFFFFFFFFFFFFFFFFFFFFFFFFFFFFFFFFFFFFFFFFFFFFFFFFFFFFFFF
FFFFFFFFFFFFF:F
@A00721:190:HGJW7DSXY:4:2446:16477:7420 2:N:0:TCTGCAAG+GCTGCACT
ATATTGATCACGGTCAATTTCAAGAAGACGGCCGGAAAAGAAAGTGTGATCGTATCGAACTAGACGTT
GTTTATAATAAAGCTTTATCAGCTAGGTCAAAGACAGAGTCTCTAGAAATAATCCGGAAGAACGATCC
AAGAACGTTCTACCT
+
FFFFFF:FFFFFFFFFFFFFF:FFFF::FFFFFF:FFF:FFF:FFFFFFFFFFFFFFFF:FFFF:F,F
F:FFFF:FFFFFFFFFFFFFFFFFFFFFFFFFFF:,FFFFFFFF:FFFFFFFF,FF:FFFF:F,FFF:
FFFFF,FFFFF:F:F
@A00721:190:HGJW7DSXY:4:2560:27877:29888 2:N:0:TCTGCAAG+GCTGCACT
ATATTGATCACGGTCAATTTCAAGAAGACGGCCGGAAAAGAAAGTGTGATCGTATCGAACTAGACGTT
GTTTATAATAAAGCTTTATCAGCTAGGTCAAAGACAGAGTCTCTAGAAATAATCCGGAAGAACGATCC
AAGAACGTTCTACCT
+
FFFFFFFFFFFFFFFFFFFFFFFFFFF,FFFFFFFFFF:FFFFFFFFFFFFFFFFFFFFFFFFFFFFF
FF,FFFFFFFFFFFFFFF:FFFFFFFFFFFFFFFFFFFFFFFFFFFFFFFFFFFFFFFFFFFFFFFF,
FF:FFF:FFFFFFFF
@A00721:190:HGJW7DSXY:4:2628:25807:6198 2:N:0:TCTGCAAG+GCTGCACT
ATATTGATCACGGTCAATTTCAAGAAGACGGCCGGAAAAGAAAGTGTGATCGTATCGAACTAGACGTT
GTTTATAATAAAGCTTTATCAGCTAGGTCAAAGACAGAGTCTCTAGAAATAATCCGGAAGAACGATCC
AAGAACGTTCTACCT
+
FFFFFFFFFFFFFFFFFFFFFFFFFFFFFFFFFFFFFFFFFFFFFFFFFFFFFFFFFFFFFFFFFFFF
FFFFFFFFFFFFFFFFFFFFFFFFFFFFFFFFFFFFFFFFFFFFFFFFFFFFFFFFFFFFFFFFFFFF
FFFFFFFFFFFFFFF
@A00721:190:HGJW7DSXY:4:2655:7093:32972 2:N:0:TCTGCAAG+GCTGCACT
ATATTGATCACGGTCAATTTCAAGAAGACGGCCGGAAAAGAAAGTGTGATCGTATCGAACTAGACGTT
GTTTATAATAAAGCTTTATCAGCTAGGTCAAAGACAGAGTCTCTAGAAATAATCCGGAAGAACGATCC
AAGAACGTTCTACCT
+
FFFFFFFFFFFFFFFFFFFFFFFFFFFFFFFFFFFFFFFFFFFFFFFFFFFFFFFFFFFFFFFFFF:F
```

FFF::FFFFFFFFF::FFFFFFFFFFFFFFFFFFFFFFFFFFFFFFFFFFFFFFFFFFFFFFFFFF
FFFFFFFFFFFFFFF
@A00721:190:HGJW7DSXY:4:2656:1488:12618 2:N:0:TCTGCAAG+GCTGCACT
ATATTGATCACGGTCAATTTCAAGAAGACGGCCGGAAAAGAAAGTGTGATCGTATCGAACTAGACGTT
GTTTATAATAAAGCTTTATCAGCTAGGTCAAAGACAGAGTCTCTAGAAATAATCCGGAAGAACGATCC
AAGAACGTTCTACCT
+
FFFFFFFFFFFFFFFFFFFFFFFFFFFF:FFFFFFFFFFFFFFFFFFFFFFFFFFFFFFFFFFFFFFF:
F:F:FF:FFFFFFFFFFF:FFFFF:FFFFFFFFFFFFFFFFFFFFFFFFFFFFFFFFFFFFFFFFFFF
FFFFFFFFFFFFFFF
@A00721:190:HGJW7DSXY:4:1128:27525:25019 2:N:0:TCTGCAAG+GCTGCACT
CTTATATTGATCACGGTCAATTTCAAGAAGACGGCCGGAAAAGAAAGTGTGATCGTATCGAACTAGAC
GTTGTTTATAATAAAGCTTTATCAGCTAGGTCAAAGACAGAGTCTCTAGAAATAATCCGGAAGAACGA
TCCAAGAACGTTCTA
+
FFFFF:FFFFFFFFFFFFFFFFFFFFFFFF::FFFFFFFFFFFFFFFFFFFFFFFFFF:FFFFFFFFFFF
FFFFF:,FF:FFFFFFFFFFFFFFFFFFFFFFFFFFFFFFFFFFFFFFFFFFFFFFFFFFFFFFF:FFF
FFFFFFFFFFFFFFF
@A00721:190:HGJW7DSXY:4:1136:19705:25504 2:N:0:TCTGCAAG+GCTGCACT
CTTATATTGATCACGGTCAATTTCAAGAAGACGGCCGGAAAAGAAAGTGTGATCGTATCGAACTAGAC
GTTGTTTATAATAAAGCTTTATCAGCTAGGTCAAAGACAGAGTCTCTAGAAATAATCCGGAAGAACGA
TCCAAGAACGTTCTA
+
FFFFFFFFFFFFF:FFFFFFFFFFFFFFFFFFF:FFFFFFFFFFFFFFFFFFFFFFFFFFFFFFFFF:FF
FF:F:FFFFFFFFFFF::FF:FFFFFFFFFFFFFFFFFF:FFFFFFF:FFFFFFFFF,FFFFFFFFFF
FFFFFFFF,F,FFF:
@A00721:190:HGJW7DSXY:4:1136:20256:26052 2:N:0:TCTGCAAG+GCTGCACT
CTTATATTGATCACGGTCAATTTCAAGAAGACGGCCGGAAAAGAAAGTGTGATCGTATCGAACTAGAC
GTTGTTTATAATAAAGCTTTATCAGCTAGGTCAAAGACAGAGTCTCTAGAAATAATCCGGAAGAACGA
TCCAAGAACGTTCTA
+
FFFFFFFFFFFFFFFFFFFFFFFFFFFFFFFFFFFFFFFFFFFFF:FFFFFFFFFFFFFFFFFFFFFFF
FFFFFFFFFFFFFFFFFFFFFFFFFFFFFFFFFFFFFFFFFFFFFFFFFFFFFFFFFFFFFFFFFFFFF
FFFFFFFFFF:FFFF
@A00721:190:HGJW7DSXY:4:1263:26096:11960 2:N:0:TCTGCAAG+GCTGCACT
CTTATATTGATCACGGTCAATTTCAAGAAGACGGCCGGAAAAGAAAGTGTGATCGTATCGAACTAGAC
GTTGTTTATAATAAAGCTTTATCAGCTAGGTCAAAGACAGAGTCTCTAGAAATAATCCGGAAGAACGA
TCCAAGAACGTTCTA
+
FFFFFFFFFFFFFFFFFFFFFFFFFFFFFFF:FFFFFFFFFFFFFFFFFFFFFFFFFFFFFFFFFFFFFF
FF:FFFFFFFFFFFFFFFFFFFFFFFFFFFFFFFFFFFFFFFFFFFFFFFFFFFFFFFFFFFFFF:FFF
FFFFFFFFFFFFFFF
@A00721:190:HGJW7DSXY:4:1308:2383:35274 2:N:0:TCTGCAAG+GCTGCACT
CTTATATTGATCACGGTCAATTTCAAGAAGACGGCCGGAAAAGAAAGTGTGATCGTATCGAACTAGAC
GTTGTTTATAATAAAGCTTTATCAGCTAGGTCAAAGACAGAGTCTCTAGAAATAATCCGGAAGAACGA
TCCAAGAACGTTCTA
+
FFFFFFFFFFFFFFFFFFFFFFFFFFFFFFFFFFFFFFFFF:FFFFFFFFFFFFF:FFFFFFFFFFFFFF
F:FFFFFFFFFFFFFFF:FFFFFFFFFFFFFFFFFFFFFFFFFFFFFFFFFFFFFFFFFFF,FFFFFF
FFFFF,FFFFFFFFF
@A00721:190:HGJW7DSXY:4:1310:14778:25175 2:N:0:TCTGCAAG+GCTGCACT
CTTATATTGATCACGGTCAATTTCAAGAAGACGGCCGGAAAAGAAAGTGTGATCGTATCGAACTAGAC
GTTGTTTATAATAAAGCTTTATCAGCTAGGTCAAAGACAGAGTCTCTAGAAATAATCCGGAAGAACGA
TCCAAGAACGTTCTA

```
+
FFFFFFFFFFFFFFFFFFFFFFFFFFFFFFFFFFFFFFFFFFF,FFFFFFFFFFFFFFFFFFFFFFFF
FF:FF:FFFFFFFFFFFFFFF:FFFFFFFFFFFFFFFFFFFFFFFFFFFFFFFFFFFFFFFFFFFFFF
FFFFFFFFFFFFFFF
@A00721:190:HGJW7DSXY:4:1336:3332:32659 2:N:0:TCTGCAAG+GCTGCACT
CTTATATTGATCACGGTCAATTTCAAGAAGACGGCCGGAAAAGAAAGTGTGATCGTATCGAACTAGAC
GTTGTTTATAATAAAGCTTTATCAGCTAGGTCAAAGACAGAGTCTCTAGAAATAATCCGGAAGAACGA
TCCAAGAACGTTCTA
+
FFFFFFFFFFFFFFFFFFFFFFFFFFFFFFFFFFFFFFFFFFFFFFFFFFFFFFFFFFFFF:FFFFFF
FFFFFFF:FFFFFFFFFFFFFFFFFFFFFFFFFFFFFFFFFFFFFFFFFFFFFFFFFFFFFFF:FFFF
FF,FFFFFFFFFFFF
@A00721:190:HGJW7DSXY:4:1547:7328:36605 2:N:0:TCTGCAAG+GCTGCACT
CTTATATTGATCACGGTCAATTTCAAGAAGACGGCCGGAAAAGAAAGTGTGATCGTATCGAACTAGAC
GTTGTTTATAATAAAGCTTTATCAGCTAGGTCAAAGACAGAGTCTCTAGAAATAATCCGGAAGAACGA
TCCAAGAACGTTCTA
+
FFFFFFFFFFFFFFFFFFFFFFFFFFFFFFFFFFFFFFFFFFFFFFFFFFFFFFFFFFFFF:FFFFFF
FFFF:FFFFFF,FFFFFFFFFFFFFFFFFFFFFFF::FFFFFFFFFFFFFFFFFFFFFFFFFFFFFFF
FFF:FFFFFFFFFFF
@A00721:190:HGJW7DSXY:4:1645:19027:21230 2:N:0:TCTGCAAG+GCTGCACT
CTTATATTGATCACGGTCAATTTCAAGAAGACGGCCGGAAAAGAAAGTGTGATCGTATCGAACTAGAC
GTTGTTTATAATAAAGCTTTATCAGCTAGGTCAAAGACAGAGTCTCTAGAAATAATCCGGAAGAACGA
TCCAAGAACGTTCTA
+
FFFFFFFFFFFFFFFFFFFFFFFFFFFF:FFFFFFFFFFFFFFFFFFFFFFFFFFFFFFFFFFFFFFF
FFFFFFFFFFFFFFFFFFFFFFFFFFF:FFFFFFFFFFFFFF:FFF:FFFFFFFFFFF:FFFFFFFFF
FFFFFFFFFFFFFFF
@A00721:190:HGJW7DSXY:4:2130:31611:23234 2:N:0:TCTGCAAG+GCTGCACT
CTTATATTGATCACGGTCAATTTCAAGAAGACGGCCGGAAAAGAAAGTGTGATCGTATCGAACTAGAC
GTTGTTTATAATAAAGCTTTATCAGCTAGGTCAAAGACAGAGTCTCTAGAAATAATCCGGAAGAACGA
TCCAAGAACGTTCTA
+
FFFFFFFFFFFFFFFFFFFFFFFFFFFFFFFFFFFFFFFFFFFFFFFFFFFFFFFFFFFFFFFFFFFF
FFFFFFFFFFFFFFFFFFFFFFFFFFFFFFFFFFFFFFFFFFFFFFFFFFFFFFFFF:FFFFFFFFFF
F:FFFFFFFFFFFFF
@A00721:190:HGJW7DSXY:4:2529:22643:33285 2:N:0:TCTGCAAG+GCTGCACT
CTTATATTGATCACGGTCAATTTCAAGAAGACGGCCGGAAAAGAAAGTGTGATCGTATCGAACTAGAC
GTTGTTTATAATAAAGCTTTATCAGCTAGGTCAAAGACAGAGTCTCTAGAAATAATCCGGAAGAACGA
TCCAAGAACGTTCTA
+
FFFFFFFFFFFFFFFFFFFFFFFFFFFFFFFFFFFFFFFFFFFFFFFFFFFFFFFFFFFFFFFFFFFF
FFFFFFFFFFFFFFFFFFFFFFFFFFFFFFFFFFFF:FFFFFFFFFFFFFFFFFFF::FFFFFFFFFF
FFFFFFFFFF:FFFF
@A00721:190:HGJW7DSXY:4:2539:14507:36730 2:N:0:TCTGCAAG+GCTGCACT
CTTATATTGATCACGGTCAATTTCAAGAAGACGGCCGGAAAAGAAAGTGTGATCGTATCGAACTAGAC
GTTGTTTATAATAAAGCTTTATCAGCTAGGTCAAAGACAGAGTCTCTAGAAATAATCCGGAAGAACGA
TCCAAGAACGTTCTA
+
FFFFFFFFFFFFFFFFFFFFFFFFFFFFFFFFFFFFFFFFFFFFFFFFFFFFFFFFFFFFFFFFFFFF
F:FFFFFFFFFFFFFFFFFFFFFFFFFFFFFFFFFFFFF:FFFFFFFFFFFFFFFFFFFFFFFFFFFF
FFFFFFFFFFFFFFF
@A00721:190:HGJW7DSXY:4:2604:6506:32362 2:N:0:TCTGCAAG+GCTGCACT
CTTATATTGATCACGGTCAATTTCAAGAAGACGGCCGGAAAAGAAAGTGTGATCGTATCGAACTAGAC
```

```
GTTGTTTATAATAAAGCTTTATCAGCTAGGTCAAAGACAGAGTCTCTAGAAATAATCCGGAAGAACGA
TCCAAGAACGTTCTA
+
FFFFFFFFFFFFFFFFFFFFFFFFFFFFFFFFFFFFFFFFFFFFFFFFFFFFFFFFFFFFFFFFFFFF
FFFFFFFFFFFFFFFFFFFFFFFFFFFFFFFFFFFFFFFFFFFFFFFFFFFFFFFFFFFFFFFFFF:F
FFFFFFFFFFFFFFF
@A00721:190:HGJW7DSXY:4:2611:10375:1673 2:N:0:TCTGCAAG+GCTGCACT
CTTATATTGATCACGGTCAATTTCAAGAAGACGGCCGGAAAAGAAAGTGTGATCGTATCGAACTAGAC
GTTGTTTATAATAAAGCTTTATCAGCTAGGTCAAAGACAGAGTCTCTAGAAATAATCCGGAAGAACGA
TCCAAGAACGTTCTA
+
FFFFFFFFFFFFFFFFFFFFFFFFFFFFFFFFFFFFFFFFFFFFFFFFFFFFFFFFF:FFFFFFFFFF
FFFF:,F,:FFFFFFFFFFFFFFFFFFFFFFFFFFFFFFFFFFFFFFFFFF:FFFFFFFFFFFFFFFF
FFFFFFFFFFFFFFF
@A00721:190:HGJW7DSXY:4:1262:31430:35571 2:N:0:TCTGCAAG+GCTGCACT
CTTATATTGATCACGGTCAATTTCAAGAAGACGGCCGGAAAAGAAAGTGTGATCGTATCGAACTAGAC
GTTGTTTATAATAAAGCTTTATCAGCTAGGTCAAAGACAGAGTCTCTAGAAATAATCCGGAAGAACGA
TCCAAGAACGTTCT
+
FFFFFFFFFFFFFFFFFFFFFFFFFFFF,FFFFFFFFFFFFFFFFFFFFFFFFFFFFFFFFFFFFFFF
FFFFF,FFF:FFFFFFFFFFFFFFFFFFFFFFFFFFFFFFFFFFF:FFFFFFFFFFFFFFFFFF:,FF:
FFFFFF,FFFFFFF
@A00721:190:HGJW7DSXY:4:1554:27968:19617 2:N:0:TCTGCAAG+GCTGCACT
CTTATATTGATCACGGTCAATTTCAAGAAGACGGCCGGAAAAGAAAGTGTGATCGTATCGAACTAGAC
GTTGTTTATAATAAAGCTTTATCAGCTAGGTCAAAGACAGAGTCTCTAGAAATAATCCGGAAGAACGA
TCCAAGAACGTTCT
+
FFFFFFFFFFFFFFFFFF:FFFFFFFFFFFFFFFFFFFF:FFFFFFFFFFFFFFFF:FFFFFFFFFFF
FF:FFFF:F:FF:FFFFFFFFFFFFFF:FFFFFFFFFFFFFFFFFFFFFF:F:FFFFFFFFFFFFFFF
FFFFFFF:FFFFFF
@A00721:190:HGJW7DSXY:4:1436:22941:3176 2:N:0:TCTGCAAG+GCTGCACT
AAAAGGAGGATCTTATATTGATCACGGTCAATTTCAAGAAGACGGCCGGAAAAGAAAGTGTGATCGTA
TCGAACTAGACGTTGTTTATAATAAAGCTTTATCAGCTAGGTCAAAGACAGAGTCTCTAGAAATAATC
CGGAAGAACGATCCA
+
FF,:FFFFFFFFFFFFFFFFFF:FFFFFFFFFFFFFFFFFFFFFFFFFFFFFFFFFFFFFFFFFFFFF
FFFFFFFFFFFFFFFFFF:FFFFFFFFFFFFFFFFFF:FFFFFFFFFFFFFFFFFFFFFFFFFFFFFF
F:FFFFFFFFFFFFF
@A00721:190:HGJW7DSXY:4:1625:5990:25958 2:N:0:TCTGCAAG+GCTGCACT
AAAAGGAGGATCTTATATTGATCACGGTCAATTTCAAGAAGACGGCCGGAAAAGAAAGTGTGATCGTA
TCGAACTAGACGTTGTTTATAATAAAGCTTTATCAGCTAGGTCAAAGACAGAGTCTCTAGAAATAATC
CGGAAGAACGATCCA
+
FF:FFFFFFFFFFFFFFFFFFFFFFFFFFFFFFFFFFFFFFFF,FFFFFFFFFFFFFFFFFFFFFFFFFF
FFFFFFFFFF:FFFF:FFFFFFFFFFFFFFFFFFFFFFFFFFFFFFFFFFFFFFFFFFFFFFFFFFFF
FFFFFF:FFFFFFFF
@A00721:190:HGJW7DSXY:4:2325:16640:13150 2:N:0:TCTGCAAG+GCTGCACT
AAAAGGAGGATCTTATATTGATCACGGTCAATTTCAAGAAGACGGCCGGAAAAGAAAGTGTGATCGTA
TCGAACTAGACGTTGTTTATAATAAAGCTTTATCAGCTAGGTCAAAGACAGAGTCTCTAGAAATAATC
CGGAAGAACGATCCA
+
FFFFFFFFFFFFFFFFFFFFF:FFFFFFFFFFFFFF:FFFFFFFFFFFFFFFFFFFF:FFFFFFFFFFF
FFFFFFFFFF,FFFFF,FFFFFFFFF:FF:FF,FFFFFFFFFFFFFFFFFFFFFFF:FFFFFFFFFFFF
FFFFFFFFFFFFFFF
```

@A00721:190:HGJW7DSXY:4:2325:16532:13338 2:N:0:TCTGCAAG+GCTGCACT
AAAAGGAGGATCTTATATTGATCACGGTCAATTTCAAGAAGACGGCCGGAAAAGAAAGTGTGATCGTA
TCGAACTAGACGTTGTTTATAATAAAGCTTTATCAGCTAGGTCAAAGACAGAGTCTCTAGAAATAATC
CGGAAGAACGATCCA
+
FFFFFFFFFFFFFFFFFFFFFFFFFFFFFFFFFFFFFFFFFFFFFFFFFFFF:FFFFF:FFFFFFFFFFF
FFFFFFFFF:FFFFF::FFFFFFFFFFFF,FFFFFFFFFFFFFFFFFFFFFFFFFFFFFF:FF,FFFFFF
FFFFFFFFFFFF:FF
@A00721:190:HGJW7DSXY:4:2417:24288:6856 2:N:0:TCTGCAAG+GCTGCACT
AAAAGGAGGATCTTATATTGATCACGGTCAATTTCAAGAAGACGGCCGGAAAAGAAAGTGTGATCGTA
TCGAACTAGACGTTGTTTATAATAAAGCTTTATCAGCTAGGTCAAAGACAGAGTCTCTAGAAATAATC
CGGAAGAACGATCCA
+
FFFFFFFFFFFFFFFFFFFFFFFFFFFFFFFFFFFFFFFFFFFFFFFFFFFFFFFFFFFFFFFFFFFFFF
FFFFFFFFFFFFFFFFFFFFFFFFFF:FFFFFFFFFFF:FFFFFFFFFFFFFFFFFFFFFFFFFFFFFFF
FFFFFFFFF:FFFFF
@A00721:190:HGJW7DSXY:4:2608:17951:2644 2:N:0:TCTGCAAG+GCTGCACT
AAAAGGAGGATCTTATATTGATCACGGTCAATTTCAAGAAGACGGCCGGAAAAGAAAGTGTGATCGTA
TCGAACTAGACGTTGTTTATAATAAAGCTTTATCAGCTAGGTCAAAGACAGAGTCTCTAGAAATAATC
CGGAAGAACGATCCA
+
FFFFFFFFFFFFFFFFFFFFFFFFFFFFFFFFFFFFFFFFFFFFFFFFFFFF:FFFFFFFFFFFFFFFFF
FFFFFFFFFFFFFFFFFFFFFFFFFFFFFFFFFFFFFF:FFFFFFFF:FFFFFFFFFFFFFFFFFFFFFF
FFFFFFFFFFFFFFF
@A00721:190:HGJW7DSXY:4:2677:24044:35055 2:N:0:TCTGCAAG+GCTGCACT
AAAAGGAGGATCTTATATTGATCACGGTCAATTTCAAGAAGACGGCCGGAAAAGAAAGTGTGATCGTA
TCGAACTAGACGTTGTTTATAATAAAGCTTTATCAGCTAGGTCAAAGACAGAGTCTCTAGAAATAATC
CGGAAGAACGATCCA
+
FFFFFFFFFFFFF,F:FFFFFFFFFFFFFFFFFFFFFFFFFF,FFFFFFFFFF,FFFFFFFFFFFFFFFF
FFFFFFFFFF,FFFF:F,:FFF,FFFFFFFFFF:FFF:FFFFFFFFF:FFFFFFFFFFFFFF:FFFF:
FFFFFFFFFFFFF:F
@A00721:190:HGJW7DSXY:4:1131:23258:20603 2:N:0:TCTGCAAG+GCTGCACT
CACAAAAGGAGGATCTTATATTGATCACGGTCAATTTCAAGAAGACGGCCGGAAAAGAAAGTGTGATC
GTATCGAACTAGACGTTGTTTATAATAAAGCTTTATCAGCTAGGTCAAAGACAGAGTCTCTAGAAATA
ATCCGGAAGAACGAT
+
FFFFFFFFFFFFFFFFFFFFFFFFFFFFFFFFFFFFFFFFFFFFFFFFFFFFFFFFFFFFFFFFFFFFFF
FFFFFFFFFFFFFFF:FFFFFFFFFFFFFFFFFFFFFFFFFFFFFFFFFF:FFFFFFFFFFFFFFFFFFF
FFFFFFFFFFFFFFF
@A00721:190:HGJW7DSXY:4:1248:5547:35994 2:N:0:TCTGCAAG+GCTGCACT
CACAAAAGGAGGATCTTATATTGATCACGGTCAATTTCAAGAAGACGGCCGGAAAAGAAAGTGTGATC
GTATCGAACTAGACGTTGTTTATAATAAAGCTTTATCAGCTAGGTCAAAGACAGAGTCTCTAGAAATA
ATCCGGAAGAACGAT
+
FFFFFFFFFFFFFFFFFFFFFFFFFFFFFFFFFFFFFFFFFFFFFFFFFFFFFFFFF,FFFFFFFFFFFF
FFFFFFFFFFFFFFFFFFFFFFFFFFFFFFFFF:FFFFFFFFFFFFFFFFFFFFFFFFFFFFFF:FFFFF
FFF:FF:FFFF:FFF
@A00721:190:HGJW7DSXY:4:1347:12590:3286 2:N:0:TCTGCAAG+GCTGCACT
CACAAAAGGAGGATCTTATATTGATCACGGTCAATTTCAAGAAGACGGCCGGAAAAGAAAGTGTGATC
GTATCGAACTAGACGTTGTTTATAATAAAGCTTTATCAGCTAGGTCAAAGACAGAGTCTCTAGAAATA
ATCCGGAAGAACGAT
+
FFFFFFFFFFFFFFF,FFFFFFFFFFFFFFFF:FFFFFFFFFFF,FFFFFFFFFFFFFFFFF,FFFFFFF

FFFFFFFFFFFFFFF:FFFFFF:FFFFFFFFF:FFFFFFFFFFFFFFFFFFFFFFFFFFFFFFFFFF
FFFFFFFFFFFFFFF
@A00721:190:HGJW7DSXY:4:1348:26684:22623 2:N:0:TCTGCAAG+GCTGCACT
CACAAAAGGAGGATCTTATATTGATCACGGTCAATTTCAAGAAGACGGCCGGAAAAGAAAGTGTGATC
GTATCGAACTAGACGTTGTTTATAATAAAGCTTTATCAGCTAGGTCAAAGACAGAGTCTCTAGAAATA
ATCCGGAAGAACGAT
+
FFFFFFFFFFFFFFFFFFFFFFFFFFFFFFFFFFFFFFFFFFFFF,FFFFFFFFFFFFFFFFFFFFFFFF
FFFFFFFFFFFFFFFFFFFFFFFFFFFFFF:FFFFFFFF:FFFFFFFFFF:FFFFFFFFFFFFFFFFFF
FFFF:FFFFFFFFFF
@A00721:190:HGJW7DSXY:4:1358:18050:24925 2:N:0:TCTGCAAG+GCTGCACT
CACAAAAGGAGGATCTTATATTGATCACGGTCAATTTCAAGAAGACGGCCGGAAAAGAAAGTGTGATC
GTATCGAACTAGACGTTGTTTATAATAAAGCTTTATCAGCTAGGTCAAAGACAGAGTCTCTAGAAATA
ATCCGGAAGAACGAT
+
FFFFFFFFFFFFFFFFFFFFFFFFFFFFFFFFFFFFFFFFFFFFF:FFFFFFFFFF:FFFFFFFFFFFF
FFFFFFFFFFFFFFFFFFFFFFFF:FFFFFFFFFFFFFFFFFFFFFFFFFFFFFFFFFFFFFFFFFFFFF
FFFFFFFFFFFFF:F
@A00721:190:HGJW7DSXY:4:1411:6180:3082 2:N:0:TCTGCAAG+GCTGCACT
CACAAAAGGAGGATCTTATATTGATCACGGTCAATTTCAAGAAGACGGCCGGAAAAGAAAGTGTGATC
GTATCGAACTAGACGTTGTTTATAATAAAGCTTTATCAGCTAGGTCAAAGACAGAGTCTCTAGAAATA
ATCCGGAAGAACGAT
+
FFFFFFFFFFFFFFFFFFFFFFFFFFFFFFFFFFFFFFFFFFFFFFFFFFFFFFFF:FFFFFFFFFFF
FFFFFFFFFFFFFF:FFFFFFFFFFFFFFFFFFFFFFFFFFFFFFFFFFFFFFFFFFFFFFFFFFFFFF
FFFFFFFFFFFFFFF
@A00721:190:HGJW7DSXY:4:1421:7997:10363 2:N:0:TCTGCAAG+GCTGCACT
CACAAAAGGAGGATCTTATATTGATCACGGTCAATTTCAAGAAGACGGCCGGAAAAGAAAGTGTGATC
GTATCGAACTAGACGTTGTTTATAATAAAGCTTTATCAGCTAGGTCAAAGACAGAGTCTCTAGAAATA
ATCCGGAAGAACGAT
+
FFFFFFFFFFFFFFFFFFFFFFFFFFFFFFFFFFFFFFFFFFFFFFFFFFFFFFFFFFFFFFFFFFFFF
FFFFFFFFFFFFFFFFFFFFFFFFFFFFF:FFFFFFFFFF,FFFFFFFFFFFFFFFFFFFFFFFFFFFFF
FFFFFFF:FFFFFFF
@A00721:190:HGJW7DSXY:4:1443:19271:12320 2:N:0:TCTGCAAG+GCTGCACT
CACAAAAGGAGGATCTTATATTGATCACGGTCAATTTCAAGAAGACGGCCGGAAAAGAAAGTGTGATC
GTATCGAACTAGACGTTGTTTATAATAAAGCTTTATCAGCTAGGTCAAAGACAGAGTCTCTAGAAATA
ATCCGGAAGAACGAT
+
:FFFFFFFFF,:FFFFFFFF,:FFF:FF:FFFFFFFFFFFFFF:FFFF:F:FF:::,FFF:FFFFFFF
FFFF,FFFFFFFFFF:,FF:F::FF:FFF,FF,FFFFF,F:F:FFF:FF:FFFFFFFFFFFFFFFFFF
FF,,:FFFF:FF:F:
@A00721:190:HGJW7DSXY:4:1563:9182:33332 2:N:0:TCTGCAAG+GCTGCACT
CACAAAAGGAGGATCTTATATTGATCACGGTCAATTTCAAGAAGACGGCCGGAAAAGAAAGTGTGATC
GTATCGAACTAGACGTTGTTTATAATAAAGCTTTATCAGCTAGGTCAAAGACAGAGTCTCTAGAAATA
ATCCGGAAGAACGAT
+
FFFFFFFFFFFFFFFFFFFFFFFFFFF:FFFFFFFFFFFFFFFFFFFFFFFFFFFFFFFFFFFFFFFFFF
FFFFFFFFFFFFFFFF,FFFFFFFFFFFF:FFFFFFFFFFFFFFFFFFFFFFFFFFFFFFFFFFFFFFFF
FFF,FFF:FFFFFFF
@A00721:190:HGJW7DSXY:4:2103:9462:18035 2:N:0:TCTGCAAG+GCTGCACT
CACAAAAGGAGGATCTTATATTGATCACGGTCAATTTCAAGAAGACGGCCGGAAAAGAAAGTGTGATC
GTATCGAACTAGACGTTGTTTATAATAAAGCTTTATCAGCTAGGTCAAAGACAGAGTCTCTAGAAATA
ATCCGGAAGAACGAT

```
+
:FFFF:FFFFFFFFFFFFFFFF:FFFFFFFFFFFFFFFFFFFFF:FFFFFFFF:FFFFFFFFFFFFFF
FFFFFFFFFFFFFFF:FFFFFF:FFFFFFFFFFFFFFFFFFFFFFFFFFFFFFFFFFFFFFFFFFFFF
FFFFFFF:FFFF:FF
@A00721:190:HGJW7DSXY:4:2130:28745:1767 2:N:0:TCTGCAAG+GCTGCACT
CACAAAAGGAGGATCTTATATTGATCACGGTCAATTTCAAGAAGACGGCCGGAAAAGAAAGTGTGATC
GTATCGAACTAGACGTTGTTTATAATAAAGCTTTATCAGCTAGGTCAAAGACAGAGTCTCTAGAAATA
ATCCGGAAGAACGAT
+
FFFFFF,FFFFFFFFFFFFFFFFFFFFFFFFFFFFFFFFFFFFFFFFFFFFFFFF:FFFFFFFFFF
FFFFFFFFFFFFFFF::FF:FFFFFFFFFFFF,FFFFFFFFFFFFFFFFFFFFFFFFFFFFFFFFFFF
FFFFFFFFFFFFFFF
@A00721:190:HGJW7DSXY:4:2139:13639:14904 2:N:0:TCTGCAAG+GCTGCACT
CACAAAAGGAGGATCTTATATTGATCACGGTCAATTTCAAGAAGACGGCCGGAAAAGAAAGTGTGATC
GTATCGAACTAGACGTTGTTTATAATAAAGCTTTATCAGCTAGGTCAAAGACAGAGTCTCTAGAAATA
ATCCGGAAGAACGAT
+
F:FFFFFFFFFFFFFFFFFFFFFFFFFFFFFFFFFFFFFFFFFFFFFFFFFFFFFFFFFFFFFFFFFF
FFFFFFFFFFF:FFFF:FFFFFFFFFFFFFFFFFFFFFFFFFFFFFFF:FFFFFFFFFFFFFFFFFFF
FFFF:FFFFFFFFFF
@A00721:190:HGJW7DSXY:4:2143:15293:15608 2:N:0:TCTGCAAG+GCTGCACT
CACAAAAGGAGGATCTTATATTGATCACGGTCAATTTCAAGAAGACGGCCGGAAAAGAAAGTGTGATC
GTATCGAACTAGACGTTGTTTATAATAAAGCTTTATCAGCTAGGTCAAAGACAGAGTCTCTAGAAATA
ATCCGGAAGAACGAT
+
FFFFFFFFFFF:FFFFFFFFFFFFFFFFFFFFFFFFFFFFFFFFF,,FFFFFFFFFFFFFFFFFFFFFFF
F:FFFFFF:FFFFFFF,FF:FFFFFFFFFFFFFFFFFFFFFFFFFFFFFFFFFFFFFFFFFFFF:F:F
FFFFFFFFFFFFFFF
@A00721:190:HGJW7DSXY:4:2153:18502:24236 2:N:0:TCTGCAAG+GCTGCACT
CACAAAAGGAGGATCTTATATTGATCACGGTCAATTTCAAGAAGACGGCCGGAAAAGAAAGTGTGATC
GTATCGAACTAGACGTTGTTTATAATAAAGCTTTATCAGCTAGGTCAAAGACAGAGTCTCTAGAAATA
ATCCGGAAGAACGAT
+
FFFFFFFFFFFFFFFFFFFFFFFFFFFFFFFFFFFFFFFFFFFFF,FFFFFFFFFFFFFFFFFFFFFFF
FFFFFFFFFFFFFFFFFFF:FF:FFFFFFFFF,F:FFFFFFFFFFFFF::FFFFFFFFFFFFFF:FFF
FFF:FFFFFFFF:FF
@A00721:190:HGJW7DSXY:4:2161:8305:27774 2:N:0:TCTGCAAG+GCTGCACT
CACAAAAGGAGGATCTTATATTGATCACGGTCAATTTCAAGAAGACGGCCGGAAAAGAAAGTGTGATC
GTATCGAACTAGACGTTGTTTATAATAAAGCTTTATCAGCTAGGTCAAAGACAGAGTCTCTAGAAATA
ATCCGGAAGAACGAT
+
FFFF,FFFFFFFFFFFFFFFFFFFFFFFFFFFFFFFFFFFFFFFFFFFFFFFFFFFFFFFFFFFFFFF
FFFFFFFFFFFFFFF:FFFFFFFFFFFFFFFFFFFFFFFFFFFFFFFFFF:FFFFFFFFFFFFFFFFF
FFFF:FFFFFFFFFF
@A00721:190:HGJW7DSXY:4:2228:9625:22482 2:N:0:TCTGCAAG+GCTGCACT
CACAAAAGGAGGATCTTATATTGATCACGGTCAATTTCAAGAAGACGGCCGGAAAAGAAAGTGTGATC
GTATCGAACTAGACGTTGTTTATAATAAAGCTTTATCAGCTAGGTCAAAGACAGAGTCTCTAGAAATA
ATCCGGAAGAACGAT
+
FFFFFFFFFFFFFFFFFFFFFFFFFFFFFFFFFFFFFFFFFFFFFFFFFFFFFFFFFFFFFFFFFFFF
FFFFFFFFFFFFFFFFFFFFFFFFFFFFFFFFFFFFFFFFFFFFFFFFFFFFFFFFFFFFFFFFFFFF
FFFFFFFFFFFFF:F
@A00721:190:HGJW7DSXY:4:2240:11008:16266 2:N:0:TCTGCAAG+GCTGCACT
CACAAAAGGAGGATCTTATATTGATCACGGTCAATTTCAAGAAGACGGCCGGAAAAGAAAGTGTGATC
```

GTATCGAACTAGACGTTGTTTATAATAAAGCTTTATCAGCTAGGTCAAAGACAGAGTCTCTAGAAATA
ATCCGGAAGAACGAT
+
:FFFFFFFFFFFFFFFFFFF:FFFFFFFFF,FFFFFFFFFFFFF,FFF:FFFFFFFFFFFFFFFFF,F
F:FFFFFFFFFFF:F,:FFF:F:FFFFFFFFFFFFFFFFFFFFFFFFFFF:FFFFFFFFFFFFFFFFF
FFFFFFFFFFFFFFF
@A00721:190:HGJW7DSXY:4:2356:8739:22858 2:N:0:TCTGCAAG+GCTGCACT
CACAAAAGGAGGATCTTATATTGATCACGGTCAATTTCAAGAAGACGGCCGGAAAAGAAAGTGTGATC
GTATCGAACTAGACGTTGTTTATAATAAAGCTTTATCAGCTAGGTCAAAGACAGAGTCTCTAGAAATA
ATCCGGAAGAACGAT
+
FFFFFFFFFFFFFFFFFFFFFFFFFFFFFFFFFFFFFFFFFFFF:FFFFFFFFFFFFFFFFFFFFFFF
FFFFFFFFFFFFFFF,FFFFFFFFFFFFFFFFFFFFFFFFFFFFFFFFFFFFFFFFFFFFFFFFFFFF
FFFFFFFFFFFFFFF
@A00721:190:HGJW7DSXY:4:2456:30915:32017 2:N:0:TCTGCAAG+GCTGCACT
CACAAAAGGAGGATCTTATATTGATCACGGTCAATTTCAAGAAGACGGCCGGAAAAGAAAGTGTGATC
GTATCGAACTAGACGTTGTTTATAATAAAGCTTTATCAGCTAGGTCAAAGACAGAGTCTCTAGAAATA
ATCCGGAAGAACGAT
+
FFFFF:FFFFFFFFFFFFFFFFFF:FFFFFFFFFFFFFFFFFFFFFFFFFFF:FF:FFFFFFFFFFFFFF
FFFFFFFFFFFFFFFFFFFFF,FFFFFFFFFFFFFFFFFFFFF:FFFFFFF:FFFFFFFFFFF,FFFFF
FFFFFFFFFFFFFFF
@A00721:190:HGJW7DSXY:4:2460:21757:12117 2:N:0:TCTGCAAG+GCTGCACT
CACAAAAGGAGGATCTTATATTGATCACGGTCAATTTCAAGAAGACGGCCGGAAAAGAAAGTGTGATC
GTATCGAACTAGACGTTGTTTATAATAAAGCTTTATCAGCTAGGTCAAAGACAGAGTCTCTAGAAATA
ATCCGGAAGAACGAT
+
FFFFFF:FFFFFFFFFFFFFFFFFFFFFFFFFFFFFFFFFFFFFFFFFFFFFFFFFFFFFFFFFFFFF
F:FFFFFFFFFFFFFFFFFFFFFFFFFFFFFFF,FFFFFFFFFFFFFFFFFFFFFFFFFFFFFFF:FFFF
FF:FFFFFFFFFFFF
@A00721:190:HGJW7DSXY:4:2475:12454:4648 2:N:0:TCTGCAAG+GCTGCACT
CACAAAAGGAGGATCTTATATTGATCACGGTCAATTTCAAGAAGACGGCCGGAAAAGAAAGTGTGATC
GTATCGAACTAGACGTTGTTTATAATAAAGCTTTATCAGCTAGGTCAAAGACAGAGTCTCTAGAAATA
ATCCGGAAGAACGAT
+
FFFFFFFFFFFFFFFFFFFFFFFFFFFF:FFFFFFFFFFFFFFFFFFFFFFFFFFFFFFFFFFFFFFF
FFFFFFFFFFFFFFFFFFFFFFFFFFFFFFFFFFFFFFFFFFFFFFFFFFFFFFFFFFFFFF,FFFFFF
FFFFFFFFFFFFFFF
@A00721:190:HGJW7DSXY:4:2549:3965:26396 2:N:0:TCTGCAAG+GCTGCACT
CACAAAAGGAGGATCTTATATTGATCACGGTCAATTTCAAGAAGACGGCCGGAAAAGAAAGTGTGATC
GTATCGAACTAGACGTTGTTTATAATAAAGCTTTATCAGCTAGGTCAAAGACAGAGTCTCTAGAAATA
ATCCGGAAGAACGAT
+
FFFFFFFFFFFFFFFFFFFFFFFFFFFFFFFFFFFFFFFFFFFFFFFFFFFFFFFFFFFFFFFFFFFF
FFFFF:FFFFFFFFFFFFF:FF,FFFFFFFFFFFFFFFFFFFFFFFFFF,FF:FFFFFFFFFFFFFFF
FFFFFFFFFF:FFFF
@A00721:190:HGJW7DSXY:4:2553:4209:14888 2:N:0:TCTGCAAG+GCTGCACT
CACAAAAGGAGGATCTTATATTGATCACGGTCAATTTCAAGAAGACGGCCGGAAAAGAAAGTGTGATC
GTATCGAACTAGACGTTGTTTATAATAAAGCTTTATCAGCTAGGTCAAAGACAGAGTCTCTAGAAATA
ATCCGGAAGAACGAT
+
FFFFFFFFFFFFFFFFFFFFFFFFFFFFFFFFFFFFFFFFFFFFFFFFFFFFFFFFFFFFFFFFFFFF
FFFFFFFFFFFFFFF:FFFFFFFFFFFFFFFFFFFFFFFFFFFFFFFFFFFFFFFFFFFFFFFFFFFF
FFF:FFF:FFF:FF:

```
@A00721:190:HGJW7DSXY:4:2607:27091:7921 2:N:0:TCTGCAAG+GCTGCACT
CACAAAAGGAGGATCTTATATTGATCACGGTCAATTTCAAGAAGACGGCCGGAAAAGAAAGTGTGATC
GTATCGAACTAGACGTTGTTTATAATAAAGCTTTATCAGCTAGGTCAAAGACAGAGTCTCTAGAAATA
ATCCGGAAGAACGAT
+
F:FFFFFFFFFFFFF,FFFFFFFFFFFFFF,FFFFFFFFFFFFFFFFFFFFFFFFF,FFFFFFFF:FFFF
FF,FFFFFFFFFFFF:FF::FFF:FFFFFFFF:FFFFFFFFF:FFFFFFFFFFFFFFFFFFF,FFFFFFF
FFFFFFF:FFFFFFF
@A00721:190:HGJW7DSXY:4:2622:9579:19335 2:N:0:TCTGCAAG+GCTGCACT
CACAAAAGGAGGATCTTATATTGATCACGGTCAATTTCAAGAAGACGGCCGGAAAAGAAAGTGTGATC
GTATCGAACTAGACGTTGTTTATAATAAAGCTTTATCAGCTAGGTCAAAGACAGAGTCTCTAGAAATA
ATCCGGAAGAACGAT
+
FFFFFFFFFFFFFFFFFFFFFFFFFFFFFFFFFFFFFFFFFFFFFF:FFFFFFFFFF:FFFFFFFFFFFF
FFFFF:FFFFFFFFFF::FF,FFFFFFFFFFFFFFFFFFFFFFFFFFFFFFFFFFFFFFFFFFFFFFFFF
FFFFFFFFFF,FFFF
@A00721:190:HGJW7DSXY:4:1230:11812:4914 2:N:0:TCTGCAAG+GCTGCACT
CTTCCGGATTATTTCTAGAGACTCTGTCTTTGACCTAGCTGATAAAGCTTTATTATAAACAACGTCTA
GTTCGATACGATCACACTTTCTTTTCCGGCCGTCTTCTTGAAATTGACCGTGATCAATATAAGATCCT
CCTTTTGTGATATA
+
F:FFFFFFFFFFFFFFFFFFFFFFFFFFFFFFFFFFFFFF:FFFFFFFFFFFFFFFFFFFFFFFFFFF:F
FFFFFFFFFFFFFFFFFFFFFFFFFFFFFFFFFFFFFFFFFFFFFFFFFFFFFFFFFFFFFFFF::FFFF
FFFFFFFFFFFFFFF
@A00721:190:HGJW7DSXY:4:1248:8305:3787 2:N:0:TCTGCAAG+GCTGCACT
CTTCCGGATTATTTCTAGAGACTCTGTCTTTGACCTAGCTGATAAAGCTTTATTATAAACAACGTCTA
GTTCGATACGATCACACTTTCTTTTCCGGCCGTCTTCTTGAAATTGACCGTGATCAATATAAGATCCT
CCTTTTGTGATATAA
+
FFFFFFFFFFFFFFFFFFFFFFFFFFFFFFFFFFFFFFFFFFFFFFFFFFFFFFFFFFFFFFFFFFFFFF
FFFFFFFFF:FFFFFFFF:FFFFFFFF:FFFFFFFFFFFFFFFFFFFFFFFFFFFFFFFF:FFFFFFFFF
FFF:FFFFFFFFFFF
@A00721:190:HGJW7DSXY:4:1402:18213:22983 2:N:0:TCTGCAAG+GCTGCACT
CTTCCGGATTATTTCTAGAGACTCTGTCTTTGACCTAGCTGATAAAGCTTTATTATAAACAACGTCTA
GTTCGATACGATCACACTTTCTTTTCCGGCCGTCTTCTTGAAATTGACCGTGATCAATATAAGATCCT
CCTTTTGTGATATAA
+
FFFFFFFFFFFFFFFFFFFFFFFFFFFFFFFFFFFFFFFFFFFFFFF:FFFFFFFFFFFFFFFFFFFFFF
FFFFF:FFFFFFFFFFFFFFFFFFFFFFFFFF:FFFFFFFF:FFFFF:FFFFFFFF:FFFFFFF,FFFFF
FFFFFFFFFFFFFFF
@A00721:190:HGJW7DSXY:4:1406:15456:34272 2:N:0:TCTGCAAG+GCTGCACT
CTTCCGGATTATTTCTAGAGACTCTGTCTTTGACCTAGCTGATAAAGCTTTATTATAAACAACGTCTA
GTTCGATACGATCACACTTTCTTTTCCGGCCGTCTTCTTGAAATTGACCGTGATCAATATAAGATCCT
CCTTTTGTGATATAA
+
FFFFFFFFFFFFFFFFFFFFFFFFFFFFFFFFFFFFFFFFFFFFFFFFFFFFFFFFFFFFFFFFFFFFFF
FFFFFFFFFFFFFFFFFFFFFFFFFFFFFFFFFFFFFFFFFFFFFFFFFFFFFFFFFFFFFFFFFFFFFF
FFFFFFFFFFFFFFF
@A00721:190:HGJW7DSXY:4:1512:1325:27023 2:N:0:TCTGCAAG+GCTGCACT
CTTCCGGATTATTTCTAGAGACTCTGTCTTTGACCTAGCTGATAAAGCTTTATTATAAACAACGTCTA
GTTCGATACGATCACACTTTCTTTTCCGGCCGTCTTCTTGAAATTGACCGTGATCAATATAAGATCCT
CCTTTTGTGATATAA
+
FFFFFFFFFFFFFFFFFFFFFFFFFFFFFFFFFFFFFFFFFF:FFFFFFFFFFFFFFFFFFF:FFFFFFF
```

```
:FFFFFFFFFFFFFFFFFFFFFFFFFFFFFFFFFFFFFFFF:FFFFFFFFFFFFFFFFF:FFFFFFFFFF
FFFFFFFFFFFFFFF
@A00721:190:HGJW7DSXY:4:2233:18548:4711 2:N:0:TCTGCAAG+GCTGCACT
CTTCCGGATTATTTCTAGAGACTCTGTCTTTGACCTAGCTGATAAAGCTTTATTATAAACAACGTCTA
GTTCGATACGATCACACTTTCTTTTCCGGCCGTCTTCTTGAAATTGACCGTGATCAATATAAGATCCT
CCTTTTGTGATATAA
+
FFFFFFFFFFFFFFFFFFFFFFFFFFFFFFFFFFFFFFFFFFFFFFFFFFFFFFFFFFFFFFFFFFFFFF
FFFFFFFF:FFFFFFFFFFFFFF:FF:FFFFFFFFFFFFFFFFFFF:FFFFFFFFFFFFFFFFF:FF::F
FFFFFFFF:FFFFFF
@A00721:190:HGJW7DSXY:4:2574:30490:32377 2:N:0:TCTGCAAG+GCTGCACT
CTTCCGGATTATTTCTAGAGACTCTGTCTTTGACCTAGCTGATAAAGCTTTATTATAAACAACGTCTA
GTTCGATACGATCACACTTTCTTTTCCGGCCGTCTTCTTGAAATTGACCGTGATCAATATAAGATCCT
CCTTTTGTGATATAA
+
FFFFFFFFFFFFFFF:FFFFFFFF:FF,FFFFF:FFFFFFFFF:F::FFFFFFFFFFFFF:FFFFFF:FF
FFFFF,FFFFFFFFF:FFFFFFFF:FFFF,FF,FFFFFFFFFFFFFFF,FFFFFFFFFFFFFFFFFF,FF
::F:F:FFFFFFFFF
@A00721:190:HGJW7DSXY:4:2613:1398:18693 2:N:0:TCTGCAAG+GCTGCACT
CTTCCGGATTATTTCTAGAGACTCTGTCTTTGACCTAGCTGATAAAGCTTTATTATAAACAACGTCTA
GTTCGATACGATCACACTTTCTTTTCCGGCCGTCTTCTTGAAATTGACCGTGATCAATATAAGATCCT
CCTTTTGTGATATAA
+
FFFFFFFFFFFFFFFFFFFFFFFFFFFFFFFFFFFFFFFF:FFFFFFFFFFFFFFFFFFFFFFFFFFFFF
FFFFFFFFFFFFFFFFFFFFFFFFFFF,FFFFFFFFFFFFFFFFFFFFFFFFFFFFFFFFFFFFFF:FFF
F:FFFFFFFFFFFFF
@A00721:190:HGJW7DSXY:4:1110:16477:29559 2:N:0:TCTGCAAG+GCTGCACT
CACAAAAGGAGGATCTTATATTGATCACGGTCAATTTCAAGAAGACGGCCGGAAAAGAAAGTGTGATC
GTATCGAACTAGACGTTGTTTATAATAAAGCTTTATCAGCTAGGTCAAAGACAGAGTCTCTAGAAATA
ATCCGGAGATCGGAA
+
FFFFFFFFFFFFFFFFFFFFFFFFFFFFFFFFFFFFFFFFFFFFFFFFFFFFFFFFFFFFFFFFFFFFFF
FFFFFFFFFFFFFFFFFFFFFFFFFFFFFFFFFFFFFFFFFFFFFFFFFFFFFFFFFFFFFFFFFFFFFF
FFFFFFFFFFFFFFF
@A00721:190:HGJW7DSXY:4:1322:16016:5368 2:N:0:TCTGCAAG+GCTGCACT
CACAAAAGGAGGATCTTATATTGATCACGGTCAATTTCAAGAAGACGGCCGGAAAAGAAAGTGTGATC
GTATCGAACTAGACGTTGTTTATAATAAAGCTTTATCAGCTAGGTCAAAGACAGAGTCTCTAGAAATA
ATCCGGAGATCGGAA
+
FFFFFFFFFFFFFFFFFFFFFFFFFFFFFFFFFFFFFFFFFFFFFFFFFFFFFFFFFFFFFFFFFFFFFF
FFFFFFF:FFFFFFFF,FFF,FFFFFFFFFFFFFFFFFFFFFFFFFFFFFFFFFFFFFFFFFFFFFFFFF
FFFFFFFFFFFFFF:
@A00721:190:HGJW7DSXY:4:1549:8540:10676 2:N:0:TCTGCAAG+GCTGCACT
CACAAAAGGAGGATCTTATATTGATCACGGTCAATTTCAAGAAGACGGCCGGAAAAGAAAGTGTGATC
GTATCGAACTAGACGTTGTTTATAATAAAGCTTTATCAGCTAGGTCAAAGACAGAGTCTCTAGAAATA
ATCCGGAGATCGGAA
+
FFFFFFFFFFFFFFFFFFFFFFFFFFFFFFFFFFFFFFFFFFFFFFFFFFFFFFFFF:FFFFFFFFFFFF
F:FFFFFFFFFFFFFFFFFFFFFFFFFFFFFFFFFFFFFFFFFFFFFFFFFFFFFFFFFFFFFFFFFFFF
FFFFFFFFFFFFFFF
@A00721:190:HGJW7DSXY:4:2522:6976:21433 2:N:0:TCTGCAAG+GCTGCACT
CACAAAAGGAGGATCTTATATTGATCACGGTCAATTTCAAGAAGACGGCCGGAAAAGAAAGTGTGATC
GTATCGAACTAGACGTTGTTTATAATAAAGCTTTATCAGCTAGGTCAAAGACAGAGTCTCTAGAAATA
ATCCGGAGATCGGAA
```

```
+
FFFFFFFFFFFFFFFFFFFFFFFFFFFFFFFFFFFFFFFFFFFFFFFFFFFFFF::FFFFFFFFFF:F
FFFFFFFFFFFFF:FFFFFFFF:FFFFFF:FFFFFFFFFFFFF:FFFFF,FFFFFFFFFFFFFFFFFF
FFFFFFFFFFF:FFF
@A00721:190:HGJW7DSXY:4:1120:27868:21512 2:N:0:TCTGCAAG+GCTGCACT
GTCGCCGATTATATCACAAAAGGAGGATCTTATATTGATCACGGTCAATTTCAAGAAGACGGCCGGAA
AAGAAAGTGTGATCGTATCGAACTAGACGTTGTTTATAATAAAGCTTTATCAGCTAGGTCAAAGACAG
AGTCTCTAGAAATAA
+
FFFFFFFF:FFFFFFFFF:FFFFFFFFFFFFFFFFFFFFFFFFFFFFFFFFFFFFF:FFFFFFFFFF,
FFFFFFFFFFFFFFFFFFFFFF:F,FFFF:FFF,:F:F,FFFFFFF,:F,FFFFF:FFFFFFF,FFFF
FF:FFFFFFFFF,FF
@A00721:190:HGJW7DSXY:4:1126:26585:9987 2:N:0:TCTGCAAG+GCTGCACT
GTCGCCGATTATATCACAAAAGGAGGATCTTATATTGATCACGGTCAATTTCAAGAAGACGGCCGGAA
AAGAAAGTGTGATCGTATCGAACTAGACGTTGTTTATAATAAAGCTTTATCAGCTAGGTCAAAGACAG
AGTCTCTAGAAATAA
+
FFFFFFFFFFFFFFFFFFFFFFFFFFFFFFFFFFFFFFFFFFFFFFFFFFFFFF:FFF:FFFFFFFFF
FFFFFFFFFFFFFFFFFFFFFFFFFFFFFFFFFFFFFFFFFFFFFFFFFFFF:FFFFFFFFF::FFFF
F,FFFFFFFFFFFFF
@A00721:190:HGJW7DSXY:4:1146:30924:25426 2:N:0:TCTGCAAG+GCTGCACT
GTCGCCGATTATATCACAAAAGGAGGATCTTATATTGATCACGGTCAATTTCAAGAAGACGGCCGGAA
AAGAAAGTGTGATCGTATCGAACTAGACGTTGTTTATAATAAAGCTTTATCAGCTAGGTCAAAGACAG
AGTCTCTAGAAATAA
+
FFFFFFFFFFFFFFFFFFF:FFFFFFFFFFFFFFFFF:FFFFFFFFFFFFFFFFFFFFFFFFFFFFFF
FFFFFFFFFFFFFFFFFFFFFFFFFFFFFFFFFFFFFFFFFFFFFFFFFFFFFFFFFFFFFFF,FFFF
FFFFFFFFFFFFFFF
@A00721:190:HGJW7DSXY:4:1156:25880:24925 2:N:0:TCTGCAAG+GCTGCACT
GTCGCCGATTATATCACAAAAGGAGGATCTTATATTGATCACGGTCAATTTCAAGAAGACGGCCGGAA
AAGAAAGTGTGATCGTATCGAACTAGACGTTGTTTATAATAAAGCTTTATCAGCTAGGTCAAAGACAG
AGTCTCTAGAAATAA
+
F:FFFFFFFFFFFFFFFFFFFFFFFFFFFFFFFFFFFFFFFFFFFFFFFFFFFFFFF:FFFFFFFFFF
FFFFFFFFFFFFFFFFFFFFFFFFFFFFF:FFFFFFFFFFFFF:FFFFFFFFFFFFFFFFFFFFFFFF
F:FFFFFF:FFFFFF
@A00721:190:HGJW7DSXY:4:1670:26467:21934 2:N:0:TCTGCAAG+GCTGCACT
GTCGCCGATTATATCACAAAAGGAGGATCTTATATTGATCACGGTCAATTTCAAGAAGACGGCCGGAA
AAGAAAGTGTGATCGTATCGAACTAGACGTTGTTTATAATAAAGCTTTATCAGCTAGGTCAAAGACAG
AGTCTCTAGAAATAA
+
FFFFFFFFFFFFFFFFFFFFFFFFFFFFFFFFFFFFFFFFFFFFFFFFFFFFFFFFFFFFFFFFFFFF
FFFFFFFFFFFFFFFFFFFFFFFFFFFFFF,FF:FFFFFFFFFFFFFFFFFFFFFFFFFFFFFFFFFF
FFFFFFFFFFFFFFF
@A00721:190:HGJW7DSXY:4:2272:3025:15812 2:N:0:TCTGCAAG+GCTGCACT
GTCGCCGATTATATCACAAAAGGAGGATCTTATATTGATCACGGTCAATTTCAAGAAGACGGCCGGAA
AAGAAAGTGTGATCGTATCGAACTAGACGTTGTTTATAATAAAGCTTTATCAGCTAGGTCAAAGACAG
AGTCTCTAGAAATAA
+
FFFFFFFFFFFFFFFFFFFFFFFFFFFFFFFFFFFFFFFFFFFFFFFFFFFFFFFFFF,FFFFFFFFF
FFFFFFFFFFFFFFFFFFFFFFFFFFFFFFFFF:FF:FF:FFFFFFF,FFFFFFFFFFFFFFFFFFFF
FFFFFFFFFFFFFFF
@A00721:190:HGJW7DSXY:4:2326:2899:3349 2:N:0:TCTGCAAG+GCTGCACT
GTCGCCGATTATATCACAAAAGGAGGATCTTATATTGATCACGGTCAATTTCAAGAAGACGGCCGGAA
```

```
AAGAAAGTGTGATCGTATCGAACTAGACGTTGTTTATAATAAAGCTTTATCAGCTAGGTCAAAGACAG
AGTCTCTAGAAATAA
+
FFFFFFFFFFFFFFFFFFFFFFFFFFFFFFFFFFFFFFFFFFFFFFFFFFFFFFFFFFFFFFFFFFFF
FFFF:FFFFFFF:FFFFFFFFFFFFFFFFFFF:FFFFFFFFFFFFFFFFFFFFFFFFFFFFFFFFF:F
FFFFFF:FFFFFFFF
@A00721:190:HGJW7DSXY:4:2377:12346:23218 2:N:0:TCTGCAAG+GCTGCACT
GTCGCCGATTATATCACAAAAGGAGGATCTTATATTGATCACGGTCAATTTCAAGAAGACGGCCGGAA
AAGAAAGTGTGATCGTATCGAACTAGACGTTGTTTATAATAAAGCTTTATCAGCTAGGTCAAAGACAG
AGTCTCTAGAAATAA
+
FFFFFFFFFFFFFFFFFFFFFFFFFFFFFFFFFFFFFFFFFFFFFFFFFFFFFFFFFFFFFFFFFFFF
FFFFFFFFFFFFFFFFFFFFFFFFFFFFFF:FFFFFFFFFFFFFFFFFFFFFFFFFFFFFFFFFFFFF
FFFFFFFFFFFFFFF
@A00721:190:HGJW7DSXY:4:2377:10456:23829 2:N:0:TCTGCAAG+GCTGCACT
GTCGCCGATTATATCACAAAAGGAGGATCTTATATTGATCACGGTCAATTTCAAGAAGACGGCCGGAA
AAGAAAGTGTGATCGTATCGAACTAGACGTTGTTTATAATAAAGCTTTATCAGCTAGGTCAAAGACAG
AGTCTCTAGAAATAA
+
FFFFFFFFFFFFFFFFFFFFFFFFFFFFFFFFFFFFFFFFFFFFFFFFFFFFFFFFFF:FFFFFFFFF
FFFFFFFFFFFFFFFFFFFFFFFFFFFFFFFFF:FFFFFFFFFFFFFFFFFFFFFFFFFFFFFFFFFF
FFFFFFFFFFFFFFF
@A00721:190:HGJW7DSXY:4:2405:27606:34992 2:N:0:TCTGCAAG+GCTGCACT
GTCGCCGATTATATCACAAAAGGAGGATCTTATATTGATCACGGTCAATTTCAAGAAGACGGCCGGAA
AAGAAAGTGTGATCGTATCGAACTAGACGTTGTTTATAATAAAGCTGTATCAGCTAGGTCAAAGACAG
AGTCTCTAGAAATAA
+
FFFFFFFF,FFFFFFFFFFFFFFFFFFFFFFFFFFFFFFFFFFFFFFFFF:FFFFFFFFF
FFFF:FF:FFFF:FFFFFFFF:FFFFFFF:,FFF:F:FFFFFFFFF,F:FFF:FFFFFFFFFFFFFFF
FF:FFFFFFFFFFFF
@A00721:190:HGJW7DSXY:4:2446:1344:14434 2:N:0:TCTGCAAG+GCTGCACT
GTCGCCGATTATATCACAAAAGGAGGATCTTATATTGATCACGGTCAATTTCAAGAAGACGGCCGGAA
AAGAAAGTGTGATCGTATCGAACTAGACGTTGTTTATAATAAAGCTTTATCAGCTAGGTCAAAGACAG
AGTCTCTAGAAATAA
+
:F:FFFFFFFFFFFFFFFFFFFFFFFFFFFFF:FFFFF,FFFFFFFFFFFFFFFF:F:FFFFFFFFFF
FFFFFF,FFF:FFFFF:FFFFFFF::FFF,FFF::FF:FFFFFFF::FFFFF:FFFF:FFFFF,FFFF
FFFFFFFFFF:FFFF
@A00721:190:HGJW7DSXY:4:2556:7988:14544 2:N:0:TCTGCAAG+GCTGCACT
GTCGCCGATTATATCACAAAAGGAGGATCTTATATTGATCACGGTCAATTTCAAGAAGACGGCCGGAA
AAGAAAGTGTGATCGTATCGAACTAGACGTTGTTTATAATAAAGCTTTATCAGCTAGGTCAAAGACAG
AGTCTCTAGAAATAA
+
FFFFFFFFFFF:FF:FFFF:FFFFFFFFFFFFFFF:FFFFFFFFFFFF,FFFFF:FF,FFF:FFFFFF
FF,FF:FFFFFFF,FFFF,:FFFFFFF,F:,:F,FF:FFFFFFFFFFFFF,F:FFFF:FFF:F:FFF,
F:FFFF:FFFFF:,F
@A00721:190:HGJW7DSXY:4:2662:4679:13135 2:N:0:TCTGCAAG+GCTGCACT
GTCGCCGATTATATCACAAAAGGAGGATCTTATATTGATCACGGTCAATTTCAAGAAGACGGCCGGAA
AAGAAAGTGTGATCGTATCGAACTAGACGTTGTTTATAATAAAGCTTTATCAGCTAGGTCAAAGACAG
AGTCTCTAGAAATAA
+
FFFFFFFFFFFFFFFFFFFFFFFFFFFFFFFFFFF:FFFFFFFFFFFFFFFFFFFFFF,FFFFFFFFF
FFFFFFFFFFFFFFFFFFFFFFFFFFFFFFFFFFFFFFFFFFFFFFFFFFFFFFFFFFFFFF:FFFFF
FFFFFFFFFFFFFFF
```

```
@A00721:190:HGJW7DSXY:4:1327:27236:16219 2:N:0:TCTGCAAG+GCTGCACT
GACGTCGCCGATTATATCACAAAAGGAGGATCTTATATTGATCACGGTCAATTTCAAGAAGACGGCCG
GAAAAGAAAGTGTGATCGTATCGAACTAGACGTTGTTTATAATAAAGCTTTATCAGCTAGGTCAAAGA
CAGAGTCTCTAGAAA
+
FFFFFFFFFFFFFFFFFFFFF:FFFFFFFF,FFFFFFFFFFFFFFFFFFFFFFFFFFF:FF:FFF:FF
FF,F,FFF,FFFFFF:FFFF:FFFFFFFFFFF::FFFFFFF,FFFFFF:FF,FFFFFF,F:F:FFFFF
F:FFF:FFFFFFF::
@A00721:190:HGJW7DSXY:4:1347:18683:27649 2:N:0:TCTGCAAG+GCTGCACT
GACGTCGCCGATTATATCACAAAAGGAGGATCTTATATTGATCACGGTCAATTTCAAGAAGACGGCCG
GAAAAGAAAGTGTGATCGTATCGAACTAGACGTTGTTTATAATAAAGCTTTATCAGCTAGGTCAAAGA
CAGAGTCTCTAGAAA
+
FFFFFFFFFFFFFFFFFFFFFFFFFFFFFFFFFFFF:FFFFFFFFFFFFFFFFFFFFFFFF:FFFFFF
FFFFFF::FFFFFFFFFFFFFFFFFFFF:FF::FFFFF,FFFFFFFFFFFFFFFFFFFFFFFFFFFFF
F:FFFFFFFFFFFFF
@A00721:190:HGJW7DSXY:4:1410:27462:4617 2:N:0:TCTGCAAG+GCTGCACT
GACGTCGCCGATTATATCACAAAAGGAGGATCTTATATTGATCACGGTCAATTTCAAGAAGACGGCCG
GAAAAGAAAGTGTGATCGTATCGAACTAGACGTTGTTTATAATAAAGCTTTATCAGCTAGGTCAAAGA
CAGAGTCTCTAGAAA
+
FFFFFFFFFFFFFFFFFFFFFFF,FFFFFFFFF,FF:FFFFFFFFFFFFFFFFFFFFFFFFFFFFFFF
F:FFFFFFFFFFFFFFFF::FFFFFFFFFFFF,,FFFFFFFFFFFFFFFFFFFFFFF,:FFFFFFFFF
F,FFF:FFFFFFFFF
@A00721:190:HGJW7DSXY:4:1640:27868:20760 2:N:0:TCTGCAAG+GCTGCACT
GACGTCGCCGATTATATCACAAAAGGAGGATCTTATATTGATCACGGTCAATTTCAAGAAGACGGCCG
GAAAAGAAAGTGTGATCGTATCGAACTAGACGTTGTTTATAATAAAGCTTTATCAGCTAGGTCAAAGA
CAGAGTCTCTAGAAA
+
FFFFFFFFFFFFFFFFFFFFFFFFFFFFFFFFFFFFFFFFFFFFFFFFFFFFFFFFFFFFF:FFFFFF
FFFFFFFFFFFFFFFFFF,FFFFFFFFFFFFF::F,,FFFFFFFFFFFF:FF:FFFF:FFFFFFF:FF
FFFFFFFFFFFFFFF
@A00721:190:HGJW7DSXY:4:2225:31439:18239 2:N:0:TCTGCAAG+GCTGCACT
GACGTCGCCGATTATATCACAAAAGGAGGATCTTATATTGATCACGGTCAATTTCAAGAAGACGGCCG
GAAAAGAAAGTGTGATCGTATCGAACTAGACGTTGTTTATAATAAAGCTTTATCAGCTAGGTCAAAGA
CAGAGTCTCTAGAAA
+
FFFFFFFFFFFFFFFFFFFFFFFFFFFFFFFFFFFFFFFFFFFFFFFFFFFFFFFFFFFFFFFFFFFF
FFFFFFFFFFFFFFFFFFFFFFFFFFFFFFFFFFFF,FFFFFFFFFFFFFFFFFFFFFFFFFFFFFFF
FFFFFFFFFFF:FFF
@A00721:190:HGJW7DSXY:4:2434:28013:9455 2:N:0:TCTGCAAG+GCTGCACT
GACGTCGCCGATTATATCACAAAAGGAGGATCTTATATTGATCACGGTCAATTTCAAGAAGACGGCCG
GAAAAGAAAGTGTGATCGTATCGAACTAGACGTTGTTTATAATAAAGCTTTATCAGCTAGGTCAAAGA
CAGAGTCTCTAGAAA
+
FFFFFFFFFFFFF,FFFFFFFF,FFFFFFFFFFFFFFFFFFFFFFFFFFFFFFFFFFFFFF,FFFFFF
F:FF:FFFFF:FFF:FFFFFFFFFFFFFFFFF:FFFF:FFFFFFFFF:FFFFFFFFF,FFFFFFFFFF
FFFFFFFFFFFFFFF
@A00721:190:HGJW7DSXY:4:2434:27688:11835 2:N:0:TCTGCAAG+GCTGCACT
GACGTCGCCGATTATATCACAAAAGGAGGATCTTATATTGATCACGGTCAATTTCAAGAAGACGGCCG
GAAAAGAAAGTGTGATCGTATCGAACTAGACGTTGTTTATAATAAAGCTTTATCAGCTAGGTCAAAGA
CAGAGTCTCTAGAAA
+
FFFFFFFFFFFFFFFFFF:FFFF:FFFFFFFFFFFFFFFFFFFFFFFFFFFFFFFFFFFFFFFFFFFF
```

FFF:FFFFFFFFFFFFFFFFFFFFFFFFFFFFFF:FF::F,FFFFFFFFFFFFF:FFFFF,FFFFFFFFF
FFFFFFFFFFFFFFF
@A00721:190:HGJW7DSXY:4:2530:4083:13354 2:N:0:TCTGCAAG+GCTGCACT
GACGTCGCCGATTATATCACAAAAGGAGGATCTTATATTGATCACGGTCAATTTCAAGAAGACGGCCG
GAAAAGAAAGTGTGATCGTATCGAACTAGACGTTGTTTATAATAAAGCTTTATCAGCTAGGTCAAAGA
CAGAGTCTCTAGAAA
+
F::FFFFFFFFFFFFFFFFFFFFFFFFFFFFFFFFFFFFFFFFFFFFFFFFFFFFFFFFFFFFFFFFFFF
FFFFFFFFFFFFFFFFFFFFFFFFFFFFFFFFFFFFFFFFFFFFFFFFFFFFFFFFFFFFFFFFFFFFFF
FFFFFFFFFFFFFFF
@A00721:190:HGJW7DSXY:4:2622:22697:7639 2:N:0:TCTGCAAG+GCTGCACT
GACGTCGCCGATTATATCACAAAAGGAGGATCTTATATTGATCACGGTCAATTTCAAGAAGACGGCCG
GAAAAGAAAGTGTGATCGTATCGAACTAGACGTTGTTTATAATAAAGCTTTATCAGCTAGGTCAAAGA
CAGAGTCTCTAGAAA
+
FFFFFFFFFFFFFFFFFFFFFFFF:FFFFF:FFFFFFFFFFFFFFFFFFFFFFFFFFFFFFFF:FFFFFF
FFFFFFFFFFFFFFFFFFFFFFFFFFFFFFFFFF:FFFFFFFFFFFFFFFFFFFFFFFFFFFFFFFFFFF
FFFFFFFFFFFFFFF
@A00721:190:HGJW7DSXY:4:2666:9715:11052 2:N:0:TCTGCAAG+GCTGCACT
GACGTCGCCGATTATATCACAAAAGGAGGATCTTATATTGATCACGGTCAATTTCAAGAAGACGGCCG
GAAAAGAAAGTGTGATCGTATCGAACTAGACGTTGTTTATAATAAAGCTTTATCAGCTAGGTCAAAGA
CAGAGTCTCTAGAAA
+
FFFFFFFFFFFFFFFFFFFFFFFFFFFFFFFFFFFFFFFFFFFFFFFFFFFFFFFFFFFFFFFFFFFFFF
FFFFFF:FFFFFFFFFFFFFFFFFFFFFFFF:FF:F:,F,FFFFFFFFFFFFFFFFFFFFFFFFFFFFFF
:,FFFFFFFFFFFFF
@A00721:190:HGJW7DSXY:4:1131:14163:25144 2:N:0:TCTGCAAG+GCTGCACT
AATGACGTCGCCGATTATATCACAAAAGGAGGATCTTATATTGATCACGGTCAATTTCAAGAAGACGG
CCGGAAAAGAAAGTGTGATCGTATCGAACTAGACGTTGTTTATAATAAAGCTTTATCAGCTAGGTCAA
AGACAGAGTCTCTAG
+
FFFFFFFFFFFFFFFFFFFFFFFFFF,FFFFFFFFFFFFFFFFFFFF:FFFFFFFFFFFFFFFFFF:FFF
FFFFFFFFFFFFFFFFFFFFFFFFFFFFFFFFFFFFFFFF,FFFFFFFFFFFFFFFFFFFFFFFFFFFFF
:F:FFFFFFFFFFFF
@A00721:190:HGJW7DSXY:4:1427:16504:18834 2:N:0:TCTGCAAG+GCTGCACT
AATGACGTCGCCGATTATATCACAAAAGGAGGATCTTATATTGATCACGGTCAATTTCAAGAAGACGG
CCGGAAAAGAAAGTGTGATCGTATCGAACTAGACGTTGTTTATAATAAAGCTTTATCAGCTAGGTCAA
AGACAGAGTCTCTAG
+
FFFFFFFFFFFFFFFFFFFFFFFFFFFFFFFFFFFFFFFFFFFFFFFFFFFFFFFFFFFFFFFFFFFFFF
FFFFFFF:FFFFFFFFFFFFFFFFFFFFFFFFFF,FFFFFFFFFFFFFFFFFFFFFFFFFFF:FFFFFFF
FFFFFFFFFFFF:FF
@A00721:190:HGJW7DSXY:4:1563:19081:9549 2:N:0:TCTGCAAG+GCTGCACT
AATGACGTCGCCGATTATATCACAAAAGGAGGATCTTATATTGATCACGGTCAATTTCAAGAAGACGG
CCGGAAAAGAAAGTGTGATCGTATCGAACTAGACGTTGTTTATAATAAAGCTTTATCAGCTAGGTCAA
AGACAGAGTCTCTAG
+
FFFFFFFFFFFFFFFFFFFFFFFFFFFFFFFFFFFFFFFFFFFFFFFFFFFFFFFFFFFFFFFFFFFFF:
FFFFFFFF:FFFFFFFFFFFFFFFFFFFFFFFFFFFFFFFFFFFFFFFFF:FFFFFFFFFFFFFFFFFF
FFFFF:,FFFFFFFF
@A00721:190:HGJW7DSXY:4:1651:25057:12070 2:N:0:TCTGCAAG+GCTGCACT
AATGACGTCGCCGATTATATCACAAAAGGAGGATCTTATATTGATCACGGTCAATTTCAAGAAGACGG
CCGGAAAAGAAAGTGTGATCGTATCGAACTAGACGTTGTTTATAATAAAGCTTTATCAGCTAGGTCAA
AGACAGAGTCTCTAG

```
+
FF:FFF:FFFFFFF:FF:FFFFFFFFFFFF:FFFFFF:FFFFFFFFFFFFFFFFFFFFFFFFFF:FFFF
FFFFFFFFFFFFFFFFFFFF,FFFFFFFFFFFFFF:FFFFFFFFFFFFFFFFFFFFFFFFFFFFFFFFF
FF:FFFFFFFFF:F:
@A00721:190:HGJW7DSXY:4:2335:20292:28902 2:N:0:TCTGCAAG+GCTGCACT
ACTGACGTCGCCGATTATATCACAAAAGGAGGATCTTATATTGATCACGGTCAATTTCAAGAAGACGG
CCGGAAAAGAAAGTGTGATCGTATCGAACTAGACGTTGTTTATAATAAAGCTTTATCAGCTAGGTCAA
AGACAGAGTCTCTAG
+
F,FFF:FFFFFFFFFFFFFFF:F:FF,FFFFFFFFFFFFFFFF:FFFFFFFFFFFFFFFFFFF:FFF
FFFFF,FFFFFFFFFFFF:F:FFFF:FFF,FFFFF:FFFFFFFFFFFFFFFFFFF:FFFFFFFFFFFF
FFFFFFFFFFFFFF:
@A00721:190:HGJW7DSXY:4:2340:19922:22529 2:N:0:TCTGCAAG+GCTGCACT
AATGACGTCGCCGATTATATCACAAAAGGAGGATCTTATATTGATCACGGTCAATTTCAAGAAGACGG
CCGGAAAAGAAAGTGTGATCGTATCGAACTAGACGTTGTTTATAATAAAGCTTTATCAGCTAGGTCAA
AGACAGAGTCTCTAG
+
FFFFFFFFFFFFFFFFFFFFFFFFFF:FFFFFFFFFFFFFFFFFFFFFFFFFFFFFFFFFFFFFF:FFF
FFFFFFF:FFFFFFFFFFFFFFFFFFFFFFFFFFFFFFFFFFFFFFFFFFFFFFFFFFFFFFFFFFFFF
FFFFFFFFFFFFFFF
@A00721:190:HGJW7DSXY:4:2604:7699:24283 2:N:0:TCTGCAAG+GCTGCACT
AATGACGTCGCCGATTATATCACAAAAGGAGGATCTTATATTGATCACGGTCAATTTCAAGAAGACGG
CCGGAAAAGAAAGTGTGATCGTATCGAACTAGACGTTGTTTATAATAAAGCTTTATCAGCTAGGTCAA
AGACAGAGTCTCTAG
+
FFFFFFFFFFFFFFFFFFFFFFFFF:FFFFFFFFFFFFFFFFFFFFFFFFFFFFFFFFFFFFFFF:FFF
FFFFFFFFFFFFFFFFFFFFFFFFFFFFFFFF:FFFFFFFFFFFFFFFFFFFFFFFFFFFFFFFFFFFF
FFFFFFFFFFFFFFF
@A00721:190:HGJW7DSXY:4:2627:1561:25394 2:N:0:TCTGCAAG+GCTGCACT
AATGACGTCGCCGATTATATCACAAAAGGAGGATCTTATATTGATCACGGTCAATTTCAAGAAGACGG
CCGGAAAAGAAAGTGTGATCGTATCGAACTAGACGTTGTTTATAATAAAGCTTTATCAGCTAGGTCAA
AGACAGAGTCTCTAG
+
FFFFFFFFFFFFFFFFFF:FFFFFFFFFFFFFFFFFFFFFFFFFFFFFFFFFFFFFF:FFFFFFFF:F:F
FFFFFFFFFFFFFFFFFF:FFFFFFFFFFFFFFFF,FF:F:FFFFFFFFFFFFFFFFFFF:FF:FFFF
:FFFFFFFFFFFFFF
@A00721:190:HGJW7DSXY:4:1119:2311:13166 2:N:0:TCTGCAAG+GCTGCACT
GAATGACGTCGCCGATTATATCACAAAAGGAGGATCTTATATTGATCACGGTCAATTTCAAGAAGACG
GCCGGAAAAGAAAGTGTGATCGTATCGAACTCGACGTTGTTTATAATAAAGCTTTATCAGCTAGGTCA
AAGACAGAGTCTCTA
+
FFFFFF:FFFFFFFF::FFFFFFFFFFFFFFFFFFFFFFFFFFFFFFFFFFFFFFFFFFFFFFFFFFFF
FFFFFF:FFFFFFFFFFFFFFFFF,:FFFFF,FFFFFFFFF:F,FFFFFFFFFFFFFFFFFF:FFFF:
FFFFFF:FFFFFFFF
@A00721:190:HGJW7DSXY:4:1248:22001:1548 2:N:0:TCTGCAAG+GCTGCACT
GAATGACGTCGCCGATTATATCACAAAAGGAGGATCTTATATTGATCACGGTCAATTTCAAGAAGACG
GCCGGAAAAGAAAGTGTGATCGTATCGAACTAGACGTTGTTTATAATAAAGCTTTATCAGCTAGGTCA
AAGACAGAGTCTCTA
+
FFFFFFFFFFFFFFFFFFFFFFFFFFF::FFFFFFFFFFFFFFFFFFFFFFFFFFFFFFFFFFFFFFFF
FFFFFFFFFFFFF:FFFFFFFFFFFFFFFFFFFFFFFFFFFFFFFFFFFFFFFFFFFFFFFFFFFFFFF
FF,FFFFFFFFFFFF
@A00721:190:HGJW7DSXY:4:1514:32389:3850 2:N:0:TCTGCAAG+GCTGCACT
GAATGACGTCGCCGATTATATCACAAAAGGAGGATCTTATATTGATCACGGTCAATTTCAAGAAGACG
```

GCCGGAAAAGAAAGTGTGATCGTATCGAACTAGACGTTGTTTATAATAAAGCTTTATCAGCTAGGTCA
AAGACAGAGTCTCTA
+
FFFFFFFFFFFFFFFFFFFFFFFFFFFFFFFFFFFFFFFFFFFFFFFFFFFFFFFFFFFFFFFFFFFF
FFFFFFFFFF:FFFFFFFFFFF,FFFF:FFFFFFFFFFF:FFFFFFFFFFF:FFFFFFF:FFFFFF
FFFFFFFFFFFFFFF
@A00721:190:HGJW7DSXY:4:1639:2058:31955 2:N:0:TCTGCAAG+GCTGCACT
GAATGACGTCGCCGATTATATCACAAAAGGAGGATCTTATATTGATCACGGTCAATTTCAAGAAGACG
GCCGGAAAAGAAAGTGTGATCGTATCGAACTAGACGTTGTTTATAATAAAGCTTTATCAGCTAGGTCA
AAGACAGAGTCTCTA
+
FFFFFFFFFFFFFFFFFFF:FFFFFF,FFFFFFFFFFFFFFFFFFFFFFFFFFFFFFFFFFFFFFFFF
FFFFFF:FFFFFFFFFFFFFFFFFFFFFFFFFFFFFFFFF:FFF:FF:FFFFFFFFFFFFFFFFFFFF
FFFFFFFFFFFFFFF
@A00721:190:HGJW7DSXY:4:2119:3197:16642 2:N:0:TCTGCAAG+GCTGCACT
GAATGACGTCGCCGATTATATCACAAAAGGAGGATCTTATATTGATCACGGTCAATTTCAAGAAGACG
GCCGGAAAAGAAAGTGTGATCGTATCGAACTAGACGTTGTTTATAATAAAGCTTTATCAGCTAGGTCA
AAGACAGAGTCTCTA
+
F:FFFFFFFFFFFFFFFFFFFFFFFFFFFFFFFFFFFFFFFFFFFFFFFFFFFFFFFFFFFFFFFFFF
FFFFFFFFFFFFFFFFFFFFFFFFFFFFFFFFFFFFFFFFFFFFFFFFFFFFFFFFFFFFFFFFFFFF
FFFFFFFFFFFFFFF
@A00721:190:HGJW7DSXY:4:2253:25581:11036 2:N:0:TCTGCAAG+GCTGCACT
GAATGACGTCGCCGATTATATCACAAAAGGAGGATCTTATATTGATCACGGTCAATTTCAAGAAGACG
GCCGGAAAAGAAAGTGTGATCGTATCGAACTAGACGTTGTTTATAATAAAGCTTTATCAGCTAGGTCA
AAGACAGAGTCTCTA
+
F:FFFFFFFFFFFFFFFFFFFFFFFFFFFFFFFFFFFFFFFFFFFFFFFFFFFFFFFFFFFFFFFFFF
FFFFFFFFFFFFFFFFFFFFFFFFFFFFFFFFFFFFFFFFFFFFFFFFFFFFFFFFFFFFFFFFFFFF
FFFFFFFFFFFFFFF
@A00721:190:HGJW7DSXY:4:2457:24668:25739 2:N:0:TCTGCAAG+GCTGCACT
GAATGACGTCGCCGATTATATCACAAAAGGAGGATCTTATATTGATCACGGTCAATTTCAAGAAGACG
GCCGGAAAAGAAAGTGTGATCGTATCGAACTAGACGTTGTTTATAATAAAGCTTTATCAGCTAGGTCA
AAGACAGAGTCTCTA
+
FFFFFFFFFFFFFFFFFFFFFFFFFFFFFFFFFFFFFFFFFFFFFFFFFFFFFFFFFFFFFFFFFFFF
FFFFFFFFFFFFFFFFFFF:FFFFFFFFFFFFFFF::FFFFFF:FFFFFFFFFFFFFFFFFFFFFFFF
FFFFFFFFFFFFF:F
@A00721:190:HGJW7DSXY:4:2457:28339:25989 2:N:0:TCTGCAAG+GCTGCACT
GAATGACGTCGCCGATTATATCACAAAAGGAGGATCTTATATTGATCACGGTCAATTTCAAGAAGACG
GCCGGAAAAGAAAGTGTGATCGTATCGAACTAGACGTTGTTTATAATAAAGCTTTATCAGCTAGGTCA
AAGACAGAGTCTCTA
+
F:FFFFFFFFFFFFFFFFFFFFFFFFFFFFFFFFFFFFFFFFFFFFFFFFFFFFFFFFFFFFFFFFFF
FFFFFFFFFFFFFFFFFFFFFFFFFFFFFFFFFFFFFFFFFFFFFFFFFFFFFFFFFFFFFFFFFFFF
FFFFFFFFFFFFFFF
@A00721:190:HGJW7DSXY:4:2627:1434:25300 2:N:0:TCTGCAAG+GCTGCACT
AATGACGTCGCCGATTATATCACAAAAGGAGGATCTTATATTGATCACGGTCAATTTCAAGAAGACGG
CCGGAAAAGAAAGTGTGATCGTATCGAACTAGACGTTGTTTATAATAAAGCTTTATCAGCTAGGTCAA
AGACAGAGTCTCTA
+
FFFFFFFFFFFFFFFFFFFFFFFFFFFFFFFFFFFFFFFFFF:FFFFFF:FFFFFFFFFFFF:FFF
FFFFF,FFFFFFFFFFFFFFFFFFFFFFFFFFFFF,FFF,:FFFFFFFFFFF::FFFFFFFFFFFFFF
FFFFFFFFFFFFFF

```
@A00721:190:HGJW7DSXY:4:1166:24261:32330 2:N:0:TCTGCAAG+GCTGCACT
CCCAAATATTCAAAGTGCGAGATCGGCGACTGACGTCGCCGATTATATCACAAAAGGAGGATCTTATA
TTGATCACGGTCAATTTCAAGAAGACGGCCGGAAAAGAAAGTGTGATCGTATCGAACTAGACGTTGTT
TATAATAAAGCTTTA
+
FFFFFFFFFFFFFFFFFFFFFFFFFFFF:FFFFFFFFFFFFFFF:FFFFFFFF,FFFFFFFFFFFFFF
FFFFFFFFFFFFFFFFFFFFFFF::FFFFFFFFFFFFFFFFFFFFFFFFFFFFFFFFFFFFFFFFFFF
FFFFFFFFFFFFFFF
@A00721:190:HGJW7DSXY:4:1278:31132:35399 2:N:0:TCTGCAAG+GCTGCACT
CCCAAATATTCAAAGTGCGAGATCGGCGACTGACGTCGCCGATTATATCACAAAAGGAGGATCTTATA
TTGATCACGGTCAATTTCAAGAAGACGGCCGGAAAAGAAAGTGTGATCGTATCGAACTAGACGTTGTT
TATAATAAAGCTTTA
+
FFFFFFFFFFFFFFFFFFFFFFFFFFFFFFFFFFFFFFFFFFFFFFFFFFFFF,FFFFFFFFFFFFFF
FFFFFFFFFFFFFFFFFFFFFFFFFFFFFFFFFFF:FFFFFFFFFFFFFF:FFFFFFFFF:FFF:FFF
FFFFFFFFFFFFFFF
@A00721:190:HGJW7DSXY:4:1305:10303:19147 2:N:0:TCTGCAAG+GCTGCACT
CCCAAATATTCAAAGTGCGAGATCGGCGACTGACGTCGCCGATTATATCACAAAAGGAGGATCTTATA
TTGATCACGGTCAATTTCAAGAAGACGGCCGGAAAAGAAAGTGTGATCGTATCGAACTAGACGTTGTT
TATAATAAAGCTTTA
+
FFFFFFFFFFFFFFFFFFFFFFFFFFFF:FFFFFFFFFFFFFFFFFFF:F:FFFFFFFFF:FFFFFFF
FF,FF:FFFFF:FFFFF,FFFFF:FFFF:FFF:FFF,FFF:FFFFFF,FFFF,FFF:FF,FFF,FF:F
,:FFFFFFFFFFFFF
@A00721:190:HGJW7DSXY:4:1578:13575:23688 2:N:0:TCTGCAAG+GCTGCACT
CCCAAATATTCAAAGTGCGAGATCGGCGACTGACGTCGCCGATTATATCACAAAAGGAGGATCTTATA
TTGATCACGGTCAATTTCAAGAAGACGGCCGGAAAAGAAAGTGTGATCGTATAGAACTAGAAGTTGTT
TATAATAAATCTTTA
+
FFFFFFFFFFFF,FFFFFFFF::FF,F,F:F,FF,FFFF:FFF:FFFFFFFFFFFF,FFFFF,FFFFF
,FFFF:F:FF,FFF,:FFFF,FF,,FFF,FFFFF:FF:FFFF,:,FF:,F:F,,FF,FF,F,FF,:F:
FFF:FFFFF,F,:F:
@A00721:190:HGJW7DSXY:4:2135:18376:30561 2:N:0:TCTGCAAG+GCTGCACT
CCAAATATTCAAAGTGCGAGATCGGCGACTGACGTCGCCGATTATATCACAAAAGGAGGATCTTATAT
TGATCACGGTCAATTTCAAGAAGACGGCCGGAAAAGAAAGTGTGATCGTATAGAACTAGACGTTGTTT
ATAATAAAGATTTA
+
F,FFFFFFFFFFFFF:FFFFFFF:F,,F,FFF,:FF::FFFF,FFFFFFF,FFFF:FF:FFFFFF,F,
,FF::FF,F:FF:F,FFF,FFF:::,:F,:FF,::FFFFF:FF:FF:,FFF,FFF:FFFFF,FF:FFF
FFFFFFFF,,F,FF
@A00721:190:HGJW7DSXY:4:2633:10917:28228 2:N:0:TCTGCAAG+GCTGCACT
CCCAAATATTCAAAGTGCGAGATCGGCGACTGACGTCGCCGATTATATCACAAAAGGAGGATCTTATA
TTGATCACGGTCAATTTCAAGAAGACGGCCGGAAAAGAAAGTGTGATCGTATCGAACTAGACGTTGTT
TATAATAAAGCTTTA
+
F:FFFFFFFFFFFFFFFFFFFFFFFFFFFFFFF:F:FFFFFFFF:FFF,:FFFFFFFFFFFFF,FFFF
FF,,FFFFFFFFFFFFFFFFFFFFFFFF:FFFFFFFFFFFFFFFFFFFFFF:FFFFFFFFFFF,FF:F
FF:FFFFFFF:FFFF
@A00721:190:HGJW7DSXY:4:1176:4237:26334 2:N:0:TCTGCAAG+GCTGCACT
CTTTTCACCCAAATATTCAAAGTGCGAGATCGGCGAATGACGTCGCCGATTATATCACAAAAGGAGGA
TCTTATATTGATCACGGTCAATTTCAAGAAGACGGCCGGAAAAGAAAGTGTGATCGTATCGAACTAGA
CGTTGTTAATAATAA
+
FFFFFFFFFFFFFFF,FFFFFFFFFFFFFFFFFFFFFFFFFFFFFFFFFFFFFFFFFFFFFFFFFFFF
```

```
FFFFFFFF:FFFFFFFFFFFFFFFFFFFFFFFFFFFFFFFF,FF,:F:,FFFFFFFFF:FFFFFFFFF
F:F,FFF,F::F:FF
@A00721:190:HGJW7DSXY:4:1370:32931:22075 2:N:0:TCTGCAAG+GCTGCACT
CTTTTCACCCAAATATTCAAAGTGCGAGATCGGCGAATGACGTCGCCGATTATATCACAAAAGGAGGA
TCTTATATTGATCACGGTCAATTTCAAGAAGACGGCCGGAAAAGAAAGTGTGATCGTATCGAACTAGA
CGTTGTTTATAATAA
+
FFFFFFFFFFFFFFFFFFFFFFFFFFFFFFFFFFFF:FFFFFFFFFFFFF,F:FFFFFFFFF:FFFFFF
FFFFFFF:FFFFF:FFFFFFFFFFFFF,F:FFFFFFFFFF,FFFF:FF:FFFFFF,FFFFFFFFF:FF
FFFFF,FF:FFFFFF
@A00721:190:HGJW7DSXY:4:2143:24451:29997 2:N:0:TCTGCAAG+GCTGCACT
CTTTTCACCCAAATATTCAAAGTGCGAGATCGGCGAATGACGTCGCCGATTATATCACAAAAGGAGGA
TCTTATATTGATCACGGTCAATTTCAAGAAGACGGCCGGAAAAGAAAGTGTGATCGTATCGAACTAGA
CGTTGTTTATAATAA
+
FFFFFFF:FFFFFFFFFFF:FFFFF:FFFFFFFFFFFFFFFFFFFFFFFFFFFFFFFFFFFFFFF:FFF
FFFFFFFFFFFFFFFFFFFFFF:FFFFFFFFFFFFFFFFFFFFFFFF,FFFFFFFFFFFFFFF:FFF
:FF:FFFF,FFF:FF
@A00721:190:HGJW7DSXY:4:2234:24297:26162 2:N:0:TCTGCAAG+GCTGCACT
CTTTTCACCCAAATATTCAAAGTGCGAGATCGGCGAATGACGTCGCCGATTATATCACAAAAGGAGGA
TCTTATATTGATCACGGTCAATTTCAAGAAGACGGCCGGAAAAGAAAGTGTGATCGTATCGAACTAGA
CGTTGTTTATAATAA
+
FFFFFFFFFFFFFFFFFFFFFFFFFFFFFFFF:,FFFFFFFFFFF:F:FFFFFFFFFFFFFFF:FFF:F
FFFFFFFF:FFFFFFFFFFFFFF:FFFFFF:FFF:FF:FFFFF:FFF:F,,,FFFFFFF,:FF,FF,F
F:,FF:FFFFFFFFF
@A00721:190:HGJW7DSXY:4:1517:29658:19507 2:N:0:TCTGCAAG+GCTGCACT
AATCTTTTCACCCAAATATTCAAAGTGCGAGATCGGCGAATGACGTCGCCGATTATATCACAAAAGGA
GGATCTTATATTGATCACGGTCAATTTCAAGAAGACGGCCGGAAAAGAAAGTGTGATCGTATCGAACT
AGACGTTGTTTATAA
+
FFFFFFFFFFFFFFFFFFFFFFFFFFFFFFFFFFFFFFFFFFFFFFFFFFFFFFFFFFFFFFFF::FFF
FFFFFF:FFFFFFFF:FFFFFFFFFFFFFFFFFF:FFFFFFFFFFFF,F,FFFFFFFFFF,FFFFFFF
FF:FFF:FFF:F:FF
@A00721:190:HGJW7DSXY:4:1547:13865:20494 2:N:0:TCTGCAAG+GCTGCACT
AATCTTTTCACCCAAATATTCAAAGTGCGAGATCGGCGAATGACGTCGCCGATTATATCACAAAAGGA
GGATCTTATATTGATCACGGTCAATTTCAAGAAGACGGCCGGAAAAGAAAGTGTGATCGTATCGAACT
AGACGTTGTTTATAA
+
FFFFFFFFFFFFFFFFFFFFFFFFFFFFFFFFFFFFFFFFFFFFFFFFFFFFFFFFFFFFFFFFFFFFF
FFFFFFFFFFFFFFFFFFFFFFFFFFFFFFFF:FF::FFFFFFFFFF:FFFFFFFFFFFFFFFFFFFFFF
FFFFFFFFFFFFFFF
@A00721:190:HGJW7DSXY:4:2255:23529:22952 2:N:0:TCTGCAAG+GCTGCACT
AATCTTTTCACCCAAATATTCAAAGTGCGAGATCGGCGAATGACGTCGCCGATTATATCACAAAAGGA
GGATCTTATATTGATCACGGTCAATTTCAAGAAGACGGCCGGAAAAGAAAGTGTGATCGTATCGAACT
AGACGTTGTTTATAA
+
FFFFF:FFFFFFFFFFFFFFFFFFFF:FFFFFFFFFFFFFF:FFFFFFFFFFFF:FFFFFFF:FFFFF
FFFFFFFFFFFFFFFFFFFFFFFFFFFFFFFFF:FFFFFFFFFFFFF,FFFFFFFFFFFFFFFFFFFF
FFFFFFFFFFFFFFF
@A00721:190:HGJW7DSXY:4:2434:14253:33880 2:N:0:TCTGCAAG+GCTGCACT
AATCTTTTCACCCAAATATTCAAAGTGCGAGATCGGCGAATGACGTCGCCGATTATATCACAAAAGGA
GGATCTTATATTGATCACGGTCAATTTCAAGAAGACGGCCGGAAAAGAAAGTGTGATCGTATCGAACT
AGACGTTGTTTATAA
```

+
FFFFFFFFFFFFFFFFFFFFFFFFFFFFFFFFFFFFFFFFFFFFFFFFFFFFFFFFFFFFFFFFFFFFFF
FFFFFFFFFFFFFFFFFFFFFFFFFFFFFFFFFFFFFFFFFFFFFFFFFFFFFFFFFFFFFFFFFFFFFF
FFF:FFFFF:FFFFF
@A00721:190:HGJW7DSXY:4:2478:1289:15875 2:N:0:TCTGCAAG+GCTGCACT
AATCTTTTCACCCAAATATTCAAAGTGCGAGATCGGCGAATGACGTCGCCGATTATATCACAAAAGGA
GGATCTTATATTGATCACGGTCAATTTCAAGAAGACGGCCGGAAAAGAAAGTGTGATCGTATCGAACT
AGACGTTGTTTATAA
+
FFFFFFFFFFFFFFFF:FFFFFFFFFFFFFFFFFFFFFFFFFFFFFFFFFFFFFFFFFFFFFFFFFFFFF
FFFFFFFFFFF:FFFFFFFFFFFFFFFFFFFFFF:FFFFFFFFFFFFF:FFFFFFFFFFFFFFFFFFFFF
FFFFFFFFFFFFFFF
@A00721:190:HGJW7DSXY:4:2522:24017:3881 2:N:0:TCTGCAAG+GCTGCACT
AATCTTTTCACCCAAATATTCAAAGTGCGAGATCGGCGAATGACGTCGCCGATTATATCACAAAAGGA
GGATCTTATATTGATCACGGTCAATTTCAAGAAGACGGCCGGAAAAGAAAGTGTGATCGTATCGAACT
AGAAGTTGTTTATAA
+
FFFFF:F::FF,FF,F::FFFFF:F,:F:F,FFF:F:FFF:FFFF:FFF:FFFFFFFFF,FFF:,:FF
:FFFFFFFFFF::FF::,::FF,FFFF:FFF,FF:FF:F:FFFFF,FFFFFF,FFF:,F:FF:,FF,F
FFF,,,FF,FFFFF:
@A00721:190:HGJW7DSXY:4:1129:10746:14841 2:N:0:TCTGCAAG+GCTGCACT
CGAATCTTTTCACCCAAATATTCAAAGTGCGAGATCGGCGAATGACGTCGCCGATTATATCACAAAAG
GAGGATCTTATATTGATCACGGTCAATTTCAAGAAGACGGCCGGAAAAGAAAGTGTGATCGTATCGAA
CTAGACGTTGTTTAT
+
:FFFFFFFFFFFFFFFFFFFFFFFFFFFFFFFFFFFFFFFFFFFFFFFFFFFFFFFFFFFFFFFFFFFFF
FFFFFFFFFFFFFFFFFFFFFFFFFFFFFFFFFFFF:FFFFFFFFFFFFFFFFFFFFFFFFFFFFFFFFF
FFFFFFFFFFFFFFF
@A00721:190:HGJW7DSXY:4:1322:7084:23218 2:N:0:TCTGCAAG+GCTGCACT
CGAATCTTTTCACCCAAATATTCAAAGTGCGAGATCGGCGAATGACGTCGCCGATTATATCACAAAAG
GAGGATCTTATATTGATCACGGTCAATTTCAAGAAGACGGCCGGAAAAGAAAGTGTGATCGTATCGAA
CTAGACGTTGTTTAT
+
FFFFFFFFFFFFFFFFFFFFFFFFFFFFFF:FFFFF:FFFFFFFFFF:FFFFFFFFFFFFFFFFFF,FF
:F::FF,FFFFFF:FFFFF,F:FFFFFFFFFF:FF:F:F,FF:FFFFFFFFFF:FFFFF::FFFFFFF
FFFFF:FFFFFFFFF
@A00721:190:HGJW7DSXY:4:2204:15637:28385 2:N:0:TCTGCAAG+GCTGCACT
CGAATCTTTTCACCCAAATATTCAAAGTGCGAGATCGGCGAATGACGTCGACGATTATATCACAAAAG
GAGGATCTTATATTGATCACCGTCAATTTCAAGAAGACTGCCGGAAAAGAAAGTGTGATCTTATCGAA
CTAGACGTTGATTAT
+
FF:FFF,,,,,F,FFFFF::F,:FF:,:FF,F,F::,F,FF:F:FF,FF,,FFF:FF::FFF,FFFF:
,,FFFFF,:F:FF,,F,:FF,:,,F:,,FFFFFFF,FF,F,,::F,::FFFF:F,,,FFF,:FF:FF:
FFFF,F::F,,FF:F
@A00721:190:HGJW7DSXY:4:2474:12337:31031 2:N:0:TCTGCAAG+GCTGCACT
CGAATCTTTTCACCCAAATATTCAAAGTGCGAGATCGGCGAATGACGTCGCCGATTATATCACAAAAG
GAGGATCTTATATTGATCACGGTCAATTTCAAGAAGACGGCCGGAAAAGAAAGTGTGATCGTATCGAA
CTAGACGTTGTTTAT
+
FFFFFFFFFFFFFFFFFFFFFFFFFFFFFFFFFFFFFFFFFFFFFFFFFFFFFFF:FFFFFFFFFFFFFF
FFFFFFFFFFFFF:FFFFFFFFFFFFFFFFFFFFFFF,FFFFFFFFFF:FFFFFFFFFFFFFFFFFFFFF
FFFFFFFFFFFFFFF
@A00721:190:HGJW7DSXY:4:1101:21133:3145 2:N:0:TCTGCAAG+GCTGCACT
CGACGAATCTTTTCACCCAAATATTCAAAGTGCGAGATCGGCGAATGACGTCGCCGATTATATCACAA

```
AAGGAGGATCTTATATTGATCACGGTCAATTTCAAGAAGACGGCCGGAAAAGAAAGTGTGATCGTATC
GAACTAGACGTTGTT
+
FFFFFFFFFFFFFFFFFFFFFFFFFFFFFFFFFFFFFFFFFFFFFFFFFFFFFFFFFFFFFFFFFFFFFF
:FFFFFFFFFFFFFFFFFFFFFFFFFFFFFFFFFFFFFFF:FFFFFFFF,FFFFF:FFFFFFFFFFFFFF
FFFFFFFFFFF:FFF
@A00721:190:HGJW7DSXY:4:1118:11614:2879 2:N:0:TCTGCAAG+GCTGCACT
CGACGAATCTTTTCACCCAAATATTCAAAGTGCGAGATCGGCGAATGACGTCGCCGATTATATCACAA
AAGGAGGATCTTATATTGATCACGGTCAATTTCAAGAAGACGGCCGGAAAAGAAAGTGTGATCGTATC
GAACTAGACGTTGTT
+
FFFFFFFFFFFFFFFFFFFFFFFFFFFFFFFFFFFFFFFFFFFFFFFFFFFFFFFFFFFFFFFFFFFFFF
FFFFFFFFFFFFFF:FFFFFFFFFFFFFFFFFFFFFFFFFFFFFFFFFFFFFFFFFFFFFFFFFFFFFFF
FFFFFFFFFFFFFFF
@A00721:190:HGJW7DSXY:4:1138:18909:22153 2:N:0:TCTGCAAG+GCTGCACT
CGACGAATCTTTTCACCCAAATATTCAAAGTGCGAGATCGGCGAATGACGTCGCCGATTATATCACAA
AAGGAGGATCTTATATTGATCACGGTCAATTTCAAGAAGACGGCCGGAAAAGAAAGTGTGATCGTATC
GAACTAGACGTTGTT
+
FFFFFFFFFFFFFFFFFFFFFFFF:FFFFFFFFFFFFFFFFFFFFFFFFFFFFFFFFFFFFFFFFFFFF
F:FFFFFFFFFFFFFFFFFFFFFFFFFFFFFFFFFFFFFFFFFFFFFFFFFFFFFFFFFFFFFFFFFFFF
FFFFFFFFFFFFFFF
@A00721:190:HGJW7DSXY:4:1447:13422:3537 2:N:0:TCTGCAAG+GCTGCACT
CGACGAATCTTTTCACCCAAATATTCAAAGTGCGAGATCGGCGAATGACGTCGCCGATTATATCACAA
AAGGAGGATCTTATATTGATCACGGTCAATTTCAAGAAGACGGCCGGAAAAGAAAGTGTGATCGTATC
GAACTAGACGTTGTT
+
FFFFFFFFFFFFFFFFFFFFFFFFFFFFFFFFFFFFFFFFFFFFFFFFFFFFFFFFFFFFFFFFFFFFF:
FFFFFFFFFFFFFFFFFFFFFFFFFFFFFFFFFFFFFFFFFFFFFFFFFFFFFFFFFFFFFFFFF:FFFF
FFFFFFFFFFFFFFF
@A00721:190:HGJW7DSXY:4:1603:11333:28886 2:N:0:TCTGCAAG+GCTGCACT
CGACGAATCTTTTCACCCAAATATTCAAAGTGCGAGATCGGCGAATGACGTCGCCGATTATATCACAA
AAGGAGGATCTTATATTGATCACGGTCAATTTCAAGAAGACGGCCGGAAAAGAAAGTGTGATCGTATC
GAACTAGACGTTGTT
+
FFF,FFF:FFFFFFFFFF:F:FFFF,FFFFFF:FFFFFFF::FFFFFFFFFFFFFFFFFF:F,FFFF:FF
:FFFF:FFFFFFFFFFFFFFF:FFF,F:FFFFF:FF,FF,FFF,,FF,FFF,FFF:,FFFFFF,FFFFF
,FFFFF,:FFF,:FF
@A00721:190:HGJW7DSXY:4:1619:26386:31000 2:N:0:TCTGCAAG+GCTGCACT
CGACGAATCTTTTCACCCAAATATTCAAAGTGCGAGATCGGCGAATGACGTCGCCGATTATATCACAA
AAGGAGGATCTTATATTGATCACGGTCAATTTCAAGAAGACGGCCGGAAAAGAAAGTGTGATCGTATC
GAACTAGACGTTGTT
+
FFFFFFFFFFFFFFFFFFFFFFFFFFFFFFFFFFFFFFFFFFFFFFFFFFFFFFFFFFFFFFFFFFFFFF
FFFFFFFFFFFFFFFFFFFFFFFFFFFFFFFFFFFFFFFFFFFFFFFF:FFFFFFFFFFFFFFFFFFFFF
FFFFFFFFFFFFFFF
@A00721:190:HGJW7DSXY:4:2127:28311:35837 2:N:0:TCTGCAAG+GCTGCACT
CGACGAATCTTTTCACCCAAATATTCAAAGTGCGAGATCGGCGAATGACGTCGCCGATTATATCACAA
AAGGAGGATCTTATATTGATCACGGTCAATTTCAAGAAGACGGCCGGAAAAGAAAGTGTGATCGTATC
GAACTAGACGTTGTT
+
FFFFFFFFFFFFFFFFFFFFFFFFFFFFFFFFFFFFFFFFFFFFFFFFFFFFFFFFFFFFFFFFFFFFFF
FFFFFFFFFFFFFFFFFFFFFFFFFFFFFFFFFFFFFFFFF:FFFFFFFFFFFFFFF:FFFFFFFFFFFFFF
FFFFFFFFFFFFFFF
```

@A00721:190:HGJW7DSXY:4:2221:23321:12258 2:N:0:TCTGCAAG+GCTGCACT
CGACGAATCTTTTCACCCAAATATTCAAAGTGCGAGATCGGCGAATGACGTCGCCGATTATATCACAA
AAGGAGGATCTTATATTGATCACGGTCAATTTCAAGAAGACGGCCGGAAAAGAAAGTGTGATCGTATC
GAACTAGACGTTGTT
+
FFFFFFFFFFFFFFFFFFFFFFFFFFFFFFFFFFFFFFFFFFFFFFFFFFFFFFFFFFFFFFFFFFFFFF
FFFFFFFFFFFFFFFFFFFFFFFFFFFFFFFFFFFFFFFFFFFFFFFFFFFFFFFFFFFFFFFFFFFFFF
FFFFFFFFFFFFFFF
@A00721:190:HGJW7DSXY:4:2521:31304:18067 2:N:0:TCTGCAAG+GCTGCACT
CGACGAATCTTTTCACCCAAATATTCAAAGTGCGAGATCGGCGAATGACGTCGCCGATTATATCACAA
AAGGAGGATCTTATATTGATCACGGTCAATTTCAAGAAGACGGCCGGAAAAGAAAGTGTGATCGTATC
GAACTAGACGTTGTT
+
FF:FF,FFFFFFFFFFFFFFFFFFFFFFFFFFFFFFFFFFFFFFFFFFFFFFFFFFFFFFFFFFFFFFFF
FFFFFFFFFF:FFFFFFFFFFFFFFFFFFFFFFFFFFFFFFFFFFFFFFF,FF:FFFFFFFFFFFFFF,F
FFFFF:FF:FFFF::
@A00721:190:HGJW7DSXY:4:2562:28971:7357 2:N:0:TCTGCAAG+GCTGCACT
CGACGAATCTTTTCACCCAAATATTCAAAGTGCGAGATCGGCGAATGACGTCGCCGATTATATCACAA
AAGGAGGATCTTATATTGATCACGGTCAATTTCAAGAAGACGGCCGGAAAAGAAAGTGTGATCGTATC
GAACTAGACGTTGTT
+
FFFFFFFFFFFFFFFFFFFFFFFFFFFFFFFFFFFFFFFFFFFFFFFFFFFFFFFFFFFFFFFFFFFFFF
FFFFFFFFFFFFFFFFFFFFFFFFFFFFFFFFFFFFFFFFFFFFFFFFFFFFFFFFFFFFFFF:FFFFFF
FFFFFFFFFFFFF:F
@A00721:190:HGJW7DSXY:4:2634:17535:19304 2:N:0:TCTGCAAG+GCTGCACT
CGACGAATCTTTTCACCCAAATATTCAAAGTGCGAGATCGGCGAATGACGTCGCCGATTATATCACAA
AAGGAGGATCTTATATTGATCACGGTCAATTTCAAGAAGACGGCCGGAAAAGAAAGTGTGATCGTATC
GAACTAGACGTTGTT
+
FFFFFFFFFFFFFFFFFFFFFFFFFFFFFFFFFFFFFFFFFFFFFFFFFFFFFFFFFFFFFFFFFFFFFF
FFFFFF:FFFFFFFFFFFFFFFFFFFFFFFFFFFFFFFFFFFFFFFFFFFFFFF:FFFFFFFFFF:FFFF
FFFFFFFFFFFFF,F
@A00721:190:HGJW7DSXY:4:1112:26106:14951 2:N:0:TCTGCAAG+GCTGCACT
CGACGAATCTTTTCACCCAAATATTCAAAGTGCGAGATCGGCGAATGACGTCGCCGATTATATCACAA
AAGGAGGATCTTATATTGATCACGGTCAATTTCAAGAAGACGGCCGGAAAAGAAAGTGTGATCGTATC
GAACTAGACGTTGT
+
FFFFFFFFFF:FFFFFFFFF:FFFFFFFFFFFFF:FF:FFFFFFFFFFFFFF:FFFFFF:FFFFFFFFFF
FFFFFFFFFFFFFFFF,FFFFF,FFFFFFFFFFF:FFFFFFFFF,FFFFFFFFFFFFF:FFFFFFFFFF:
FFFFFF:FFFFF:F
@A00721:190:HGJW7DSXY:4:1551:8901:1658 2:N:0:TCTGCAAG+GCTGCACT
CGACGAATCTTTTCACCCAAATATTCAAAGTGCGAGATCGGCGAATGACGTCGCCGATTATATCACAA
AAGGAGGATCTTATATTGATCACGGTCAATTTCAAGAAGACGGCCGGAAAAGAAAGTGTGATCGTATC
GAACTAGACGTTGT
+
FFFFF:FFFFFFFF:FFFFFFFFFFFFFFFFFFFFFFFFF:FFFFFFFFF:FFFFFFFFFFFFFFFFFFF
:FFFFFFFFFFFFFFFFFFFFFFFFFFFFFFFFFFFFFFFFFFFFFFFF:FFFFFFFFFFFFF,FFFFFF
FFFFFFFFFFFFFF
@A00721:190:HGJW7DSXY:4:2404:28058:11068 2:N:0:TCTGCAAG+GCTGCACT
CGACGAATCTTTTCACCCAAATATTCAAAGTGCGAGATCGGCGAATGACGTCGCCGATTATATCACAA
AAGGAGGATCTTATATTGATCACGGTCAATTTCAAGAAGACGGCCGGAAAAGAAAGTGTGATCGTATC
GAACTAGACGTTGT
+
FFFFFFFFFFFFFFFFFFFFFFFFFFFFFFFFFFFFFFFFFFFFFFFF:FFFFFFFFFF,FFFFFFFFFF

```
F,FFFFFFFF,FFFFFFFFFF:FFFFFFFFFFFFFFFFF:FFFFFFF,FFFFFFFFFFFFFFFFFFFF
FFFFFFFF:FFF:F
@A00721:190:HGJW7DSXY:4:1463:14570:32581 2:N:0:TCTGCAAG+GCTGCACT
TCCGACGAATCTTTTCACCCAAATATTCAAAGTGCGAGATCGGCGAATGACGTCGCCGATTATATCAC
AAAAGGAGGATCTTATATTGATCACGGTCAATTTCAAGAAGACGGCCGGAAAAGAAAGTGTGATCGTA
TCGAACTAGACGTTG
+
:F:FFFFFFFFF::FFFFF:FFFFFF:FFFFFFF:FFFFFFFF,FFF:FFFFF:FFFFFFFFFFFFFF
FFFFFFFFFFFFFFF:FFFFFFFFFFF,FFF:FFFFFFF:FFFFFF,,FFFFFFFFF:FF,FFFF,FF
F:FFFFFF,FF:,FF
@A00721:190:HGJW7DSXY:4:1560:15510:16141 2:N:0:TCTGCAAG+GCTGCACT
TCCGACGAATCTTTTCACCCAAATATTCAAAGTGCGAGATCGGCGAATGACGTCGCCGATTATATCAC
AAAAGGAGGATCTTATATTGATCACGGTCAATTTCAAGAAGACGGCCGGAAAAGAAAGTGTGATCGTA
TCGAACTAGACGTTG
+
FFFFFFFFFFFFFFFFFFFFFFFFFFFFFFFFFFFFFFFFFFFFFFFFFFFFFFFFFFFFFFFFFFFF
FFFFFFFFFFFFFFFFFFFFFFFFFFFFFFFFFFFFFFFF:F:FFFFFFFFFFFFFFFFFFFFFFFFF
FFFFFFFFFFFFFFF
@A00721:190:HGJW7DSXY:4:2173:22471:2487 2:N:0:TCTGCAAG+GCTGCACT
TCCGACGAATCTTTTCACCCAAATATTCAAAGTGCGAGATCGGCGAATGACGTCGCCGATTATATCAC
AAAAGGAGGATCTTATATTGATCACGGTCAATTTCAAGAAGACGGCCGGAAAAGAAAGTGTGATCGTA
TCGAACTAGACGTTG
+
FFFFFFFFFFFFFFFFFFFFFFFFFFFFFFFF:FFFFFFFFFFFFFFFFFFFFFFFFFFFF:FFFFFF:
FFFFFFFFFFFFFFFFFFFFFFFFFFFFFFFFFFFFFFFFFFFFFFFFFF,FFF,FFFFFFFFFFFF
FFFFFFFFFFFFF,:
@A00721:190:HGJW7DSXY:4:1209:12572:6699 2:N:0:TCTGCAAG+GCTGCACT
GACGAATCTTTTCACCCAAATATTCAAAGTGCGAGATCGGCGAATGACGTCGCCGATTATATCACAAA
AGGAGGATCTTATATTGATCACGGTCAATTTCAAGAAGACGGCCGGAAAAGAAAGTGTGATCGTATCG
AACTAGACAGATCGG
+
FFFFFFFFFFFFFFF:FFFFFFFFFFFFFFFFFFFFFFFFFFFFFFFFFFFFFFFFFFFFFFFFFFFF
FFFFFFFFFFFFFFF:FFFFFFFFFFFFFFFFFFFFFFFFFFFFFFFFFFFFFFFF:FFFFFFFFFFF
FFFFFF:FFFFFFFF
@A00721:190:HGJW7DSXY:4:1244:2862:29121 2:N:0:TCTGCAAG+GCTGCACT
GACGAATCTTTTCACCCAAATATTCAAAGTGCGAGATCGGCGAATGACGTCGCCGATTATATCACAAA
AGGAGGATCTTATATTGATCACGGTCAATTTCAAGAAGACGGCCGGAAAAGAAAGTGTGATCGTATCG
AACTAGACAGATCGG
+
FFFFFFFFFFFFFFFFFFFFFFFFFFFFFFFFFFFFFFFFFFFFFFFFFFFFFFFFFF:FFFFFFFFF
FFFFFFFFFFFFFFF:FFFFFFFFFFFFFFFFFFFFFFFFFFFFFFFFFFFFFFFFFFFFFFFFFFFF
FFFFFFFFFFFFFFF
@A00721:190:HGJW7DSXY:4:1318:5782:13041 2:N:0:TCTGCAAG+GCTGCACT
GACGAATCTTTTCACCCAAATATTCAAAGTGCGAGATCGGCGAATGACGTCGCCGATTATATCACAAA
AGGAGGATCTTATATTGATCACGGTCAATTTCAAGAAGACGGCCGGAAAAGAAAGTGTGATCGTATCG
AACTAGACAGATCGG
+
FFFFFFFFFFFFFFFFFF:FFFFFFFFFFFFFFFFFFFFFFFFFFFFFFFFFFFFFFFFFFFFFFFFF
FFFFFFFFFFFFFFFFFFFFFFFFFFFFFFFFFFFFFFF:FFFFFFFFFF,FFFFFFFFFFFFFFFFF
FFFFFFFFFFFFFFF
@A00721:190:HGJW7DSXY:4:1520:20383:12743 2:N:0:TCTGCAAG+GCTGCACT
GACGAATCTTTTCACCCAAATATTCAAAGTGCGAGATCGGCGAATGACGTCGCCGATTATATCACAAA
AGGAGGATCTTATATTGATCACGGTCAATTTCAAGAAGACGGCCGGAAAAGAAAGTGTGATCGTATCG
AACTAGACAGATCGG
```

+
FFFFFFFFFFFFFFFFFFFFFFFFFFFFFFFFFFFFFFFFFFFFFFFFFFFFFFFF:FFFFFFFFFFF
FFFFFFFFFFFFFFFFFFFFFFFFFFFFFFFFFFFFFFFFFFFFFFFFFFFFFFFFFFFFFFFFFFFF
FFFFF:FFFFFFFFF
@A00721:190:HGJW7DSXY:4:1520:25084:17660 2:N:0:TCTGCAAG+GCTGCACT
GACGAATCTTTTCACCCAAATATTCAAAGTGCGAGATCGGCGAATGACGTCGCCGATTATATCACAAA
AGGAGGATCTTATATTGATCACGGTCAATTTCAAGAAGACGGCCGGAAAAGAAAGTGTGATCGTATCG
AACTAGACAGATCGG
+
FFFFFFFFFFFFFF:FFFFFFFFFFFFFFFFFFFFFFFFFFFFFFFFFFFFFFFFFFFFFFFFFFFFF
:FFFFFFFFFFFFFFFFFFF:FFFFFFF:FFFFFFFFFFFFFFFFFF::FFFFFFFFFFFFFFFFFFF
FFFFFFFFFFFFFFF
@A00721:190:HGJW7DSXY:4:1670:16957:8625 2:N:0:TCTGCAAG+GCTGCACT
GACGAATCTTTTCACCCAAATATTCAAAGTGCGAGATCGGCGAATGACGTCGCCGATTATATCACAAA
AGGAGGATCTTATATTGATCACGGTCAATTTCAAGAAGACGGCCGGAAAAGAAAGTGTGATCGTATCG
AACTAGACAGATCGG
+
FFFFFFFFFFFFFFFFFFFFFFFFFFFFFFFFFFFFFFFFFFFFFFFFFFFFFFFFFF:FFFFFFFFF
FFF:FFFFFFFFFFFFFFFFFFFFFFFFF:FFFFFFFF:FFFFFFFFFFFFFFFFFFFFFFFFFFFFF
FFFFFFFFFFFFFFF
@A00721:190:HGJW7DSXY:4:2111:4155:28573 2:N:0:TCTGCAAG+GCTGCACT
GACGAATCTTTTCACCCAAATATTCAAAGTGCGAGATCGGCGAATGACGTCGCCGATTATATCACAAA
AGGAGGATCTTATATTGATCACGGTCAATTTCAAGAAGACGGCCGGAAAAGAAAGTGTGATCGTATCG
AACTAGACAGATCGG
+
FFFFFFFFFFFFFFFFFFFFFF:FFFFFFFFFFFFFFFFFFFFFFFFFFFFFFFFFFFFFFFFFFFFF
FFFFFFFFFF:FFFFFFFFFFFFFFFFFFFFFFFFFFFFFFFFFFFFFFFFF:FFFFFFFFFFFFFFF
FFFFFFFFFFFFFFF
@A00721:190:HGJW7DSXY:4:2217:28348:22435 2:N:0:TCTGCAAG+GCTGCACT
GACGAATCTTTTCACCCAAATATTCAAAGTGCGAGATCGGCGAATGACGTCGCCGATTATATCACAAA
AGGAGGATCTTATATTGATCACGGTCAATTTCAAGAATACGGCCGGAAAAGAAAGTGTGATCGTATCG
AACTAGACAGATCGG
+
FFFFFF:FFFFFFF:FFFFF:FFFFFFFF,:FFFFFFFFF::FFFFFFFFFF:FFFFFFFFFFFFFFF
FFFF:FFFFF:FF:F:FFFFFFFFFFFFFF:FFFF:F,FFFFFFFFF:F,FFFFFFFFFFFFFFFFFF
FFFFFFFFF:FFF,F
@A00721:190:HGJW7DSXY:4:2529:1561:31720 2:N:0:TCTGCAAG+GCTGCACT
GACGAATCTTTTCACCCAAATATTCAAAGTGCGAGATCGGCGAATGACGTCGCCGATTATATCACAAA
AGGAGGATCTTATATTGATCACGGTCAATTTCAAGAAGACGGCCGGAAAAGAAAGTGTGATCGTATCG
AACTAGACAGATCGG
+
FFFFFFFFFFFFFFFFFFFFFFFFFFFFFFFFFFFFFFFFFFFFFFFFFFFFFFFF:FFFFFFFFFFF
FFFFFFFFFFFFFFFFFFFFFFFFFFFFFFFFFFFFFFFFFFFFFFF,FFFFFFFFFFFFFFFFFFFF
FFFFFFFFFFFFFFF
@A00721:190:HGJW7DSXY:4:2561:22860:25927 2:N:0:TCTGCAAG+GCTGCACT
GACGAATCTTTTCACCCAAATATTCAAAGTGCGAGATCGGCGAATGACGTCGCCGATTATATCACAAA
AGGAGGATCTTATATTGATCACGGTCAATTTCAAGAAGACGGCCGGAAAAGAAAGTGTGATCGAATCG
AACTAGACAGATCGG
+
FFFF:F,FF:FF,,FF:,,FFFFFFFFFF,,F:FFFF:FFF,F:,::FF:FFFF,FFFFFF:FF,FF,
FF:FFFFF:FFFFF:,FFFFF,FFF:FFFFFFF:FFF:F,F,FF,F,FFF,FFF:,,FFF,F,,FFFF
FFFFFFFFF:,FFF:
@A00721:190:HGJW7DSXY:4:2667:14082:2675 2:N:0:TCTGCAAG+GCTGCACT
GACGAATCTTTTCACCCAAATATTCAAAGTGCGAGATCGGCGAATGACGTCGCCGATTATATCACAAA

AGGAGGATCTTATATTGATCACGGTCAATTTCAAGAAGACGGCCGGAAAAGAAAGTGTGATCGTATCG
AACTAGACAGATCGG
+
FFFFFFFFFFFFFFFFFFFFFFFFFFFFFFFFFFFFFFFFFFFFFFFFFFFFFFFFFFFFFFFFFFFF
FFFFFFFFFFFFFFFFFFFFFFFFFFFFFFFFFFFFFFFFFFFFFFFFFFFFFFFFFFFFFFFFFFFF
FFFFFFFFFFFFFFF
@A00721:190:HGJW7DSXY:4:1278:29387:36886 2:N:0:TCTGCAAG+GCTGCACT
AGATTCCGACTAATCTTTTCACCCAAATATTCAAAGTGCGAGATCGGCGAATGACGTCGCCGATTATA
TCACAAAAGGAGGATCTTATATTGATCACGGTCAATTTCAAGAAGACGGCCGGAAAAGAAAGTGTGAT
CGTATCGAACTAGAC
+
F:F:FFF::FFFFFFFFFFFFF:FFFFFFFFFFFFFFFFFFFFFFFFFFFFF:FFFFFFFF:FFFFFF
FFFFFF:FFFFFFFFFFFFFF::FFF,FFFFFFFFFFFFFFF:F::F:FFFFFFFFFFFFF:FFFFFF
FFFFFF,FFFFFFFF
@A00721:190:HGJW7DSXY:4:2236:14868:1752 2:N:0:TCTGCAAG+GCTGCACT
AGATTCCGACTAATCTTTTCACCCAAATATTCAAAGTGCGAGATCGGCGAATGACGTCGCCGATTATA
TCACAAAAGGAGGATCTTATATTGATCACGGTCAATTTCAAGAAGACGGCCGGAAAAGAAAGTGTGAT
CGTATCGAACTAGAC
+
FFFFFFFFFFFFFFFFFFFFFFFFFFF,:FFFFFF:FFFFFFFFFFFFFFFFFFF::FFFFFF:FFFF
FFFFFF:,FFFFFFFFFFFFFFFFFFFFF:FFFF:FFFFFFFFFF:FFFFFFFFF::FF:FFFFFFF:
FFFFFFFF:F::FF:
@A00721:190:HGJW7DSXY:4:2303:16947:1783 2:N:0:TCTGCAAG+GCTGCACT
AGATTCCGACTAATCTTTTCACCCAAATATTCAAAGTGCGAGATCGGCGAATGACGTCGCCGATTATA
TCACAAAAGGAGGATCTTATATTGATCACGGTCAATTTCAAGAAGACGGCCGGAAAAGAAAGTGTGAT
CGTATCGAACTAGAC
+
FFFFFFFF::F,FFFF:FFFFFFF:FFFFF,FFFFF:FF:FF,FF:F:FFFFF:FF:FFFF:FF,FFF
FFFF,F:FF,FFFFF,:,FF:FFFFFFFFF:FFFFFFFFFFFFF,FFFFFFFF:F,FFFFF::F,FF:
,FFF:FFF::F:FFF
@A00721:190:HGJW7DSXY:4:2453:16785:6605 2:N:0:TCTGCAAG+GCTGCACT
AGATTCCGACTAATCTTTTCACCCAAATATTCAAAGTGCGAGATCGGCGAATGACGTCGCCGATTATA
TCACAAAAGGAGGATCTTATATTGATCACGGTCAATTTCAAGAAGACGGCCGGAAAAGAAAGTGTGAT
CGTATCGAACTAGAC
+
FFFFFFFFFFFFFFFFFFFFFFFFFFFFFFFFFFFFFFFFFFFFFFFFFFFFFFFFFFFFFFFFFFFF
FFFFFF:FFFFFFFF:FFFFFFFFFFFFFFFFFFFFFFFFFFFFF:FFFF:F:FF,FFFFFFFFFFFF
F:FFFFFFFFFFFFF
@A00721:190:HGJW7DSXY:4:2453:17065:6684 2:N:0:TCTGCAAG+GCTGCACT
AGATTCCGACTAATCTTTTCACCCAAATATTCAAAGTGCGAGATCGGCGAATGACGTCGCCGATTATA
TCACAAAAGGAGGATCTTATATTGATCACGGTCAATTTCAAGAAGACGGCCGGAAAAGAAAGTGTGAT
CGTATCGAACTAGAC
+
FFFFFFFFFFFFFFFFFFFFFFFFFFFFFFFFFFFFFFFFFFFFFFFFFFFFFFFFFFFFFFF:FFFF
F:FFFFFFFFFFFFFFFFFFFFFFFFFFFFFFFFFFFFFFFFF,FFFFFFFFF:FFFF:FFFFFFFFF
FFFFFFFFFFFFFFF
@A00721:190:HGJW7DSXY:4:2569:26539:14356 2:N:0:TCTGCAAG+GCTGCACT
AGATTCCGACTAATCTTTTCACCCAAATATTCAAAGTGCGAGATCGGCGAATGACGTCGCCGATTATA
TCACAAAAGGAGGATCTTATATTGATCACGGTCAATTTCAAGAAGACGGCCGGAAAAGAAAGTGTGAT
CGTATCGAACTAGAC
+
FFFFFFFFFFFFFFFFFFFFFFFFF,FF:FFFFFFFFFFFFFFFFFFFFFFFFFFFFFFFFFFFFFFF
FFFFF,FFFFFFFFFFFFFFFFFF,FFFFFFFFFFFFFFFFFFFFFFFFFFFFF,:,FFFFFFFFFFF
FFFFFFFFFFFFFF:

```
@A00721:190:HGJW7DSXY:4:1206:6488:28635 2:N:0:TCTGCAAG+GCTGCACT
CAGATTCCGACGAATCTTTTCACCCAAATATTCAAAGTGCGAGATCGGCGACTGACGTCGCCGATTAT
ATCACAAAAGGAGGATCTTATATTGATCACGGTCAATTTCAAGAAGACGGCCGGAAAAGAAAGTGTGA
TCGTATCGAACTAGA
+
FFFFFFFFFFFFFFFFFFFFFFFFFFFFFFFFFFFFFFFFFFFFFFFFFFFFFFFFFFFFFFFFFFFF
FFFFFF:FFFFFFFFFFFFFFFFFFFF:FFFFFFFFFFFFFFFFFFFFFFFFFFFFFFFFFF:FFFFF
FFFFF:FFFFFFFFF
@A00721:190:HGJW7DSXY:4:1243:28989:19883 2:N:0:TCTGCAAG+GCTGCACT
CAGATTCCGACGAATCTTTTCACCCAAATATTCAAAGTGCGAGATCGGCGACTGACGTCGCCGATTAT
ATCACAAAAGGAGGATCTTATATTGATCACGGTCAATTTCAAGAAGACGGCCGGAAAAGAAAGTGTGA
TCGTATCGAACTAGA
+
FFFFFFFFFFFFFFFFFFFFFFFFFFFFFFFFFFFFFFFFFFFFFFFFFFFFFFFFFFFFFFFFFFFF
:F,FFFF,FFFFFFFFFFFFFFFFFFF:FFFFF:FFFFFFFFFF:FFFFF:FFF:FFFF,F::FFFF:
::FFFFFFFFFFFF:
@A00721:190:HGJW7DSXY:4:1317:5882:8735 2:N:0:TCTGCAAG+GCTGCACT
CAGATTCCGACGAATCTTTTCACCCAAATATTCAAAGTGCGAGATCGGCGACTGACGTCGCCGATTAT
ATCACAAAAGGAGGATCTTATATTGATCACGGTCAATTTCAAGAAGACGGCCGGAAAAGAAAGTGTGA
TCGTATCGAACTAGA
+
FFFFFFFFFFFFFFFFFFFFFFFFFFFFFFFFFFFFFFFFFFFFFFF:FFFFFFFFFFF:FFFF:FFF
FF:FFFFFFFFF,FFFFFFFFFFFFFFFFF:FFFFFFFF:FF:FFF,F:,FF:FF,FFFFFF:FF:FF
F::FFF:FFFFFF:F
@A00721:190:HGJW7DSXY:4:1371:15863:36260 2:N:0:TCTGCAAG+GCTGCACT
CAGATTCCGACGAATCTTTTCACCCAAATATTCAAAGTGCGAGATCGGCGACTGACGTCGCCGATTAT
ATCACAAAAGGAGGATCTTATATTGATCACGGTCAATTTCAAGAAGACGGCCGGAAAAGAAAGTGTGA
TCGTATCGAACTAGA
+
FFFFFFFFFFFFFFFFFFFFFFFFFFFFFFFFFFFFFFFFFFFFFFFFFFFFFFFFF:FFFFFFFFFF
FFFFFFF,FFFFFFFFFFFFFFFFFFFFFFFFFFFFFFFFFFFFF:FFFFFFFFFFFFFFFFFFFFFF
FFFFFFFFFFFFFFF
@A00721:190:HGJW7DSXY:4:1445:13485:17237 2:N:0:TCTGCAAG+GCTGCACT
CAGATTCCGACGAATCTTTTCACCCAAATATTCAAAGTGCGAGATCGGCGAATGACGTCGCCGATTAT
ATCACAAAAGGAGGATCTTATATTGATCACGGTCAATTTCAAGAAGACGGCCGGAAAAGAAAGTGTGA
TCGTATCGAACTAGA
+
FFFFFFFFFFFFFFFFFFFFFFFFFFFFF:FFFFFFFFFFFFFFFFFFFFFFFFFFFFFFFFFFF:FF
FFFFFFFFFFFFFFFFFFFFFFFFFFFFFFFFFFFFFFFFFFFFFFFFFFFFFFF:FFFFFFFFFFFF
FFFFFFFFFFFFFFF
@A00721:190:HGJW7DSXY:4:1449:13132:18505 2:N:0:TCTGCAAG+GCTGCACT
CAGATTCCGACGAATCTTTTCACCCAAATATTCAAAGTGCGAGATCGGCGAATGACGTCGCCGATTAT
ATCACAAAAGGAGGATCTTATATTGATCACGGTCAATTTCAAGAAGACGGCCGGAAAAGAAAGTGTGA
TCGTATCGAACTAGA
+
FFFFFFFFFFFFFFFFFFFFFFFFFFFF:FFFFFFFFFFFFFFF:FFFFFFFFFFFFFFFFFFFFFFF
FFFFFFFFFFFFFFFFFFFFFFFFFFFFFFFFFFFFFFFFFFFFFFFFFFFFFFFFFFFFFFFFFFFF
FFFFFFFFFFFFFFF
@A00721:190:HGJW7DSXY:4:1458:9561:27445 2:N:0:TCTGCAAG+GCTGCACT
CAGATTCCGACGAATCTTTTCACCCAAATATTCAAAGTGCGAGATCGGCGACTGACGTCGCCGATTAT
ATCACAAAAGGAGGATCTTATATTGATCACGGTCAATTTCAAGAAGACGGCCGGAAAAGAAAGTGTGA
TCGTATCGAACTAGA
+
FFFFFFFFFFFFFFFFFFFFFFFFFFFFFFFFFFF:FFFFFFFFFFFFFFFFFFFFFFFFFFFFFFFF
```

```
FFFF:FFFFFF:FFFFFFFFFFFFF:FFF::FFFFFFFFFFFF,FFFFFFF:FFFFF:F:FFFFFFFF
FFFFFFFFFFFF:FF
@A00721:190:HGJW7DSXY:4:1476:15456:25191 2:N:0:TCTGCAAG+GCTGCACT
CAGATTCCGACGAATCTTTTCACCCAAATATTCAAAGTGCGAGATCGGCGACTGACGTCGCCGATTAT
ATCACAAAAGGAGGATCTTATATTGATCACGGTCAATTTCAAGAAGACGGCCGGAAAAGAAAGTGTGA
TCGTATCGAACTAGA
+
FFFFFFFFFFFFFFFFFFFFFFF:,FFFFFF:FFFFFFFFFFF:FFFFFFFFFF:,FFFFFFFF:FFFF
FF,FFFF:FF:FFFFFF:FFFFFF,FF,:FFFFFFFF:::FF,FF:,:F::F:FF:::FFFFF:FFFF
FFF::F,FF::FF,F
@A00721:190:HGJW7DSXY:4:1510:17002:24236 2:N:0:TCTGCAAG+GCTGCACT
CAGATTCCGACGAATCTTTTCACCCAAATATTCAAAGTGCGAGATCGGCGAATGACGTCGCCGATTAT
ATCACAAAAGGAGGATCTTATATTGATCACGGTCAATTTCAAGAAGACGGCCGGAAAAGAAAGTGTGA
TCGTATCGAACTAGA
+
FFFFFFFFFFFFFFFFFFFFFFFFFFFFFFFFFFFFF,FFFFFFFFFFFFFFFFFFFFFFFFFFF:FFF
FFFFFFFF:FFFFFFFFFFFFFFFFFFFFFFFFFFFFFFFFFFFFF:FFFFFFFFFFFFFF:FFFFFF
FFFFFFFFFFFFFFF
@A00721:190:HGJW7DSXY:4:2510:1271:14935 2:N:0:TCTGCAAG+GCTGCACT
CAGATTCCGACGAATCTTTTCACCCAAATATTCAAAGTGCGAGATCGGCGACTGACGTCGCCGATTAT
ATCACAAAAGGAGGATCTTATATTGATCACGGTCAATTTCAAGAAGACGGCCGGAAAAGAAAGTGTGA
TCGTATCGAACTAGA
+
FFFFFFFFFFFFFFFFFFFFFFFFFFFFFFFFFFFFFFFFFFFFFFFFFFFFFF:FFFFFFFFFFFFF
FFFFFFFFFFFFFFFFFFFFFFFFF:FFFFFFFFFFFFFFFFFFFFFFFFFFFFFFFFFFFFFFFFFF
F:FFFFFFFFFFFFF
@A00721:190:HGJW7DSXY:4:2538:12509:32142 2:N:0:TCTGCAAG+GCTGCACT
CAGATTCCGACGAATCTTTTCACCCAAATATTCAAAGTGCGAGATCGGCGAATGACGTCGCCGATTAT
ATCACAAAAGGAGGATCTTATATTGATCACGGTCAATTTCAAGAAGACGGCCGGAAAAGAAAGTGTGA
TCGTATCGAACTAGA
+
FFFFFFFFFFFFFFFFFFFFFFFFFFFFFFFFFFFFFFFFFFFFFFFFFFFFFFFFFFFFFFFFFFFF
FFFFFFFFFFFFFFFFFFFFFFFFFFFFFFFFFFFFFFFFFFFFFFFFFFFFFFFFFFFFFFFFFFFF
FFFFFFFFFFFFFFF
@A00721:190:HGJW7DSXY:4:2518:2935:14967 2:N:0:TCTGCAAG+GCTGCACT
CATAATCAACCCAGATTCCGACGAATCTTTTCACCCAAATATTCAAAGTGCGAGATCGGCGAATGACG
TCGCCGATTATATCACAAAAGGAGGATCTTATATTGATCACGGTCAATTTCAAGAAGACGGCCGGAAA
AGAAAGTGTGATCGT
+
FFFFFFFFFFFFFFFFFFFFFFFFFFFFFFFFFFFFFFFFFFFFFFFFFFFFFF:FFFFFFFFFFFF
FFFFFFFFFFFFFFFFFFFFFFFFFFFFFFFFFFFFFFFFFFFFFFFFFFFFFFFFFFFFFFFFFFF,
FFF:FFFFFFFFFFF
@A00721:190:HGJW7DSXY:4:1123:27425:9846 2:N:0:TCTGCAAG+GCTGCACT
ACATAATCAACCCAGATTCCGACGAATCTTTTCACCCAAATATTCAAAGTGCGAGATCGGCGAATGAC
GTCGCCGATTATATCACAAAAGGAGGATCTTATATTGATCACGGTCAATTTCAAGAAGACGGCCGGAA
AAGAAAGTGTGATCG
+
FFFFFFFFFFFFFFFFFFFFFFFFFF:FFFFFFFFFFFFFFFFFFFFFFFFFFFFFFF:FFFFFFFFFF
FFFFFFFFFFFFFFFFFFFF:FFFFFFFFFFFFFFFFFFFFFFFFFFFFFFFFFFFFFFFFFFFFFFF
FFFFFFF:FFFFFF:
@A00721:190:HGJW7DSXY:4:1149:9941:26850 2:N:0:TCTGCAAG+GCTGCACT
ACATAATCAACCCAGATTCCGACGAATCTTTTCACCCAAATATTCAAAGTGCGAGATCGGCGAATGAC
GTCGCCGATTATATCACAAAAGGAGGATCTTATATTGATCACGGTCAATTTCAAGAAGACGGCCGGAA
AAGAAAGTGTGATCG
```

+
FFFFFFFFFFFFFFFFFFFFFFFFFFFFFFFFFFFFFFFFFFFFFFFFFFFFFFFFFFFFFFFFFFFF
FFFFFFFFFFFFFFFFFFF:FFFFFFFFFFFFFFFFFFFFFFFFFFFFFFFFFFFFFFFFFFFFFFF:
FFFFFFFFFFFFFFF
@A00721:190:HGJW7DSXY:4:1447:6072:19961 2:N:0:TCTGCAAG+GCTGCACT
CGATCACACTTTCTTTTCCGGCCGTCTTCTTGAAATTGACCGTGATCAATATAAGATCCTCCTTTTGT
GATATAATCGGCGACGTCATTCGCCGATCTCGCACTTTGAATATTTGGGTGAAAAGATTCGTCGGAAT
CTGGGTTGATTATGT
+
FFFFFFFFFFFFFFFFFFFFFFFFFFFFFFFFFFFFFFFFFFFF::FFFFFFFFFFFFFFFFF,,FFF
FFFFFFFFFFFFFFFFFFFFFFFFFFFFFFFFFFFFFFFF:FFFFFFFFFFFFFFFFFFFFFFFFF,F
FFFFF,FFFFFFFFF
@A00721:190:HGJW7DSXY:4:1548:8856:8061 2:N:0:TCTGCAAG+GCTGCACT
CGATCACACTTTCTTTTCCGGCCGTCTTCTTGAAATTGACCGTGATCAATATAAGATCCTCCTTTTGT
GATATAATCGGCGACGTCATTCGCCGATCTCGCACTTTGAATATTTGGGTGAAAAGATTCGTCGGAAT
CTGGGTTGATTATGT
+
FFFFFFFFFFFFFFFFFFFFFFFFFFF:FFFFFFFFFFFFFFFFFFFFFFFFFFFFFFFFFFFFFFFF
FFFFFFFFFFFFFFFFFFFFFFFFFFFFFFFFFFFFFFFFFFFFFFFFFFFFFFFFFFFFFFFFFF:F
FFFFFFFFFFFFFFF
@A00721:190:HGJW7DSXY:4:1621:10655:36417 2:N:0:TCTGCAAG+GCTGCACT
ACATAATCAACCCAGATTCCGACGAATCTTTTCACCCAAATATTCAAAGTGCGAGATCGGCGAATGAC
GTCGCCGATTATATCACAAAAGGAGGATCTTATATTGATCACGGTCAATTTCAAGAAGACGGCCGGAA
AAGAAAGTGTGATCG
+
FF:FFFFFFFFFFFFFFFFFFFFFFFFFFFFFFFFFFFFFFFFFFFFFFFFFFFFF:FFFFFFFFFFF
FFFFFFFFFF:FFFFFFFFFFFFFFFFFFFFFFFFFFFFFFF:FFFFFFFFFFFF:FFFFFFFFFF:F
FFFFFFFFFFFFFFF
@A00721:190:HGJW7DSXY:4:2319:29225:18317 2:N:0:TCTGCAAG+GCTGCACT
ACATAATCAACCCAGATTCCGACGAATCTTTTCACCCAAATATTCAAAGTGCGAGATCGGCGAATGAC
GTCGCCGATTATATCACAAAAGGAGGATCTTATATTGATCACGGTCAATTTCAAGAAGACGGCCGGAA
AAGACAGTGTGATCG
+
FFFFFFFFFFFFFFFFFFFFFFFFFFFFFFFFFFFFFFFFFFFFFFFFFFFFFFFFFFFFFFFFFFFF
FFFFFFFFFFFFFFFFFFFFFFFFFFFFFFFFFFFFFFFFFFFFFFFFFFFFFFFFFFFF::FFFFF:
FFFF,FFFFFFFFFF
@A00721:190:HGJW7DSXY:4:2332:12870:13886 2:N:0:TCTGCAAG+GCTGCACT
CGATCACACTTTCTTTTCCGGCCGTCTTCTTGAAATTGACCGTGATCAATATAAGATCCTCCTTTTGT
GATATAATCGGCGACGTCATTCGCCGATCTCGCACTTTGAATATTTGGGTGAAAAGATTCGTCGGAAT
CTGGGTTGATTATGT
+
FFFFFFFFFFFFFFFFFFFFFFFFFFFFFFFFFFFFFFFFFFFFF:FFF,FFFFFFFFFFFFFFFFFF
FFFFFFFFFFFFFFFFFFFFFFFFFFFFFFFFFFFFFFFFFFF:FFFFFFFFFF:FFFF:FFFFFFFF
FFFFFFFFFFFFFFF
@A00721:190:HGJW7DSXY:4:2361:16477:16219 2:N:0:TCTGCAAG+GCTGCACT
CGATCACACTTTCTTTTCCGGCCGTCTTCTTGAAATTGACCGTGATCAATATAAGATCCTCCTTTTGT
GATATAATCGGCGACGTCATTCGCCGATCTCGCACTTTGAATATTTGGGTGAAAAGATTCGTCGGAAT
CTGGGTTGATTATGT
+
FFFFFFFFFFFFFFFFFFFFFFFFFFFFFFFFFFFFFFFFFFFFFFFFFFFFFFFF:F:FFFFF:FFF
FFFFFFFFFFFFFFFFFF:FFFFFFFFFFFFFFFFFFFF:,FFFFFFFFFFFF:FFFFFFFFFFFFF:
FFFFFF:FFFFFFFF
@A00721:190:HGJW7DSXY:4:1107:1452:18850 2:N:0:TCTGCAAG+GCTGCACT
ACATAATCAACCCAGATTCCGACGAATCTTTTCACCCAAATATTCAAAGTGCGAGATCGGCGAATGAC

```
GTCGCCGATTATATCACAAAAGGAGGATCTTATATTGATCACGGTCAATTTCAAGAAGACGGCCGGAA
AAGAAAGTGTGATC
+
FFFFFFF:FF:FFFFFFFFFFFFFFFFFFFFF:FFFFFFFFFFFFFFF,FFFFF:FFF:FFFFFFFFF
FFF:FFFFFFFFFF:FFFFFF::F:,FFFFFFFFFF,FF:F,::FFFF:FFFFF,FF,FF:F:F:FFF
FF:FFF,::F:FF:
@A00721:190:HGJW7DSXY:4:2169:22435:19460 2:N:0:TCTGCAAG+GCTGCACT
ACATAATCAACCCAGATTCCGACGAATCTTTTCACCCAAATATTCAAAGTGCGAGATCGGCGAATGAC
GTCGCCGATTATATCACAAAAGGAGGATCTTATATTGATCACGGTCAATTTCAAGAAGACGGCCGGAA
AAGAAAGTGTGATC
+
FFFFFFFFFFFFFFFFFFFFFFFFFFFFFFFFFFFFFFFFFFFFFFFFFFFFFFFFFFFFFFFFFFFF
FFFFFFFFFFFFFFFFFFFFFFFFFFFFF:FFFFFFFFFFFFFFFFFFFFFFFFFFFFFFFFFFFFFF
FFFFFFFFFFFFFF
@A00721:190:HGJW7DSXY:4:1255:7889:21637 2:N:0:TCTGCAAG+GCTGCACT
CGACATAATCAACCCAGATTCCGACGAATCTTTTCACCCAAATATTCAAAGTGCGAGATCGGCGAATG
ACGTCGCCGATTATATCACAAAAGGAGGATCTTATATTGATCACGGTCAATTTCAAGAAGACGGCCGG
AAAAGAAAGTGTGAT
+
FFFFFFFFFFFFFFFFFFFFFFFFFFFFFFFFFFFFFFFFFFFF:FFFFFFFFFFFFFFFFFFFFFFF
FFFFFFFFFFFFFFFFFFFFFFFFFFFFFFFFFFFFFFFFFFFFFFFFFFFFFFFFFFFFFFFFFFFF
FFFFFFFFFFFFFFF
@A00721:190:HGJW7DSXY:4:1320:24108:17879 2:N:0:TCTGCAAG+GCTGCACT
CGACATAATCAACCCAGATTCCGACGAATCTTTTCACCCAAATATTCAAAGTGCGAGATCGGCGAATG
ACGTCGCCGATTATATCACAAAAGGAGGATCTTATATTGATCACGGTCAATTTCAAGAAGACGGCCGG
AAAAGAAAGTGTGAT
+
FFFFFFFFFFFFFFFFFFFFFFFFFFFFFFFFFFF:FFFF,FFFFFFFFFFFFFFFFFFFFFFFFFFF
FFFFFFFFFFFFFFFFFFFFFF:FFFFFFFFFFFFFFFFFFFFFFFFFFFFFFFFFFFFFFF:FFFFFFF
FFFFFFFFFFFFFFF
@A00721:190:HGJW7DSXY:4:1410:12997:19022 2:N:0:TCTGCAAG+GCTGCACT
CGACATAATCAACCCAGATTCCGACGAATCTTTTCACCCAAATATTCAAAGTGCGAGATCGGCGAATG
ACGTCGCCGATTATATCACAAAAGGAGGATCTTATATTGATCACGGTCAATTTCAAGAAGACGGCCGG
AAAAGAAAGTGTGAT
+
FFFFFFFFFFFFFFFFFFFFFFFFFFFFFFFFFFFFFFFFFFFFFFFFFFFFFFFFFFFFFFFFFFFF
FFFFFFFFFFFFFFFFFFF::F:FFFFFFFFFFFFFFFFFFFFFFFFFFFFFFFFFFFFFFFFFFFFF
F,F:FFFFFFFFFFF
@A00721:190:HGJW7DSXY:4:1551:12174:35978 2:N:0:TCTGCAAG+GCTGCACT
CGACATAATCAACCCAGATTCCGACGAATCTTTTCACCCAAATATTCAAAGTGCGAGATCGGCGAATG
ACGTCGCCGATTATATCACAAAAGGAGGATCTTATATTGATCACGGTCAATTTCAAGAAGACGGCCGG
AAAAGAAAGTGTGAT
+
FFFFFFF,FFFFFFFFFFFFFFFFFFFFFFFFFFFFFFFF:FF:FFFFFFFFFFFFFFFFFFFFFFFFF
FFFFFFFFFFFF:FFFFFFFFFFFFFFFFFFFFFFFFFFFFFFFFFFFFFFFFFFFFFFFF:FFFFFF
FFF:FFFFFFFFFFF
@A00721:190:HGJW7DSXY:4:1650:14552:19867 2:N:0:TCTGCAAG+GCTGCACT
CGACATAATCAACCCAGATTCCGACGAATCTTTTCACCCAAATATTCAAAGTGCGAGATCGGCGAATG
ACGTCGCCGATTATATCACAAAAGGAGGATCTTATATTGATCACGGTCAATTTCAAGAAGACGGCCGG
AAAAGAAAGTGTGAT
+
FFFFFFFFFFFFFFFFFFFFFFFFFFFFFFFFFFFFFFFFFFFFFFFFFFFFFFFFFFFFFFFFFFFF
FFFFFFFFFFFFFFFFFFFFFFF:FFFFFFFFFFFFFFFFFFFFFFFFFFFFFFFFFFFFFFFF:FFF
FFFFFFFFF,FFFFF
```

```
@A00721:190:HGJW7DSXY:4:1669:29921:19147 2:N:0:TCTGCAAG+GCTGCACT
CGACATAATCAACCCAGATTCCGACGAATCTTTTCACCCAAATATTCAAAGTGCGAGATCGGCGAATG
ACGTCGCCGATTATATCACAAAAGGAGGATCTTATATTGATCACGGTCAATTTCAAGAAGACGGCCGG
AAAAGAAAGTGTGAT
+
FFFFFFFFFFF:FFFFFFFFFFFFFFFFFFFFFFFFFFFFFFFFFFFFFFFFFFFFFFFF:FFFFFFF
FFFFFFFFF:FFFFFFFFFF,:FFFFFFFFFFFFFFFFFFFFFFFFFFFFFFFFFFFF:FFFFFFFFF
F,FFFFFFFFFFFF:
@A00721:190:HGJW7DSXY:4:2152:29270:35963 2:N:0:TCTGCAAG+GCTGCACT
CGACATAATCAACCCAGATTCCGACGAATCTTTTCACCCAAATATTCAAAGTGCGAGATCGGCGAATG
ACGTCGCCGATTCTAGCACAAAAGGAGGATCTTATATTGATCACGGTCAATTTCAAGAAGACGGCCGG
AAAAGAAAGTGTGAT
+
FFF::FFFF:FFFFFFFFFFFFF,:F:FFFF:,:FFF:F,FF,,,FF:FFFFFFFFFFFF:FF:FFF,
FF::FFFFFFFF,:,,FFFFF:FFFFFFFFFF:FF,FF:FFFFFFF,:FFFFFFFFFFFFFFF,F:F:
F:,:FFFF:FFFFFF
@A00721:190:HGJW7DSXY:4:1108:9878:4038 2:N:0:TCTGCAAG+GCTGCACT
CGACATAATCAACCCAGATTCCGACGAATCTTTTCACCCAAATATTCAAAGTGCGAGATCGGCGAATG
ACGTCGCCGATTATATCACAAAAGGAGGATCTTATATTGATCACGGTCAATTTCAAGAAGACGGCCGG
AAAAGAAAGTGTGA
+
FFFFFFFFFFFFFFFFFFFFFFFFFFFFFFFFFFFFFFFFFFFFFFFFFFFFFFFFFFFFFFFFF,FF
FFFFFFFFFF:FFFFFFFFFFFFFFFFFFFFFFFFFFFFF:FF:FFFFFFFFFFFFFFFFF:FFFFFFF
FFFFFFFFFFFFFF
@A00721:190:HGJW7DSXY:4:1428:15022:20243 2:N:0:TCTGCAAG+GCTGCACT
CTTCGACATAATCAACCCAGATTCCGACGAATCTTTTCACCCAAATATTCAAAGTGCGAGATCGGCGA
ATGACGTCGCCGATTATATCACAAAAGGAGGATCTTATATTGATCACGGTCAATTTCAAGAAGACGGC
CGGAAAAGAAAGTGT
+
FFFFF:FFFFFFFFFFFFFF:FFFFFFFFFFFFFFFFFFF,FFFFFFFF:FF,FFFFFFFFFF:FFFF
FFFFFFFFFFFFF:FFFFFFFFFF:FFFFFFFFFFFFF,FFFFFFFF:FFFFFFFFF,F,FFFFFFFF
FFFF:F:FFFFFFFF
@A00721:190:HGJW7DSXY:4:1444:23873:26897 2:N:0:TCTGCAAG+GCTGCACT
CTTCGACATAATCAACCCAGATTCCGACGAATCTTTTCACCCAAATATTCAAAGTGCGAGATCGGCGA
ATGACGTCGCCGATTATATCACAAAAGGAGGATCTTATATTGATCACGGTCAATTTCAAGAAGACGGC
CGGAAAAGAAAGTGT
+
FFFFFFFFFFFFFFFFFFFFFFFFFFFFFFFFFFFFFFFFFFFFFFFFFFFFFFFFFFFFFFFFFFFF
FFFFFFFFFFFF,FFFFFFFFFFFFFFFFFFFFFFFFFF,FFF:FFFFFFFFFFFFFFFFFFFFFFFF:F
FFFF,F,FFF:FFFF
@A00721:190:HGJW7DSXY:4:1527:14742:20885 2:N:0:TCTGCAAG+GCTGCACT
CTTCGACATAATCAACCCAGATTCCGACGAATCTTTTCACCCAAATATTCAAAGTGCGAGATCGGCGA
ATGACGTCGCCGATTATATCACAAAAGGAGGATCTTATATTGATCACGGTCAATTTCAAGAAGACGGC
CGGAAAATAAAGTGT
+
::F,:F,F:::,FFFF,,:FFFFFF:FFFFFFFFFFFFF:,FFF:F:F,F,FFFFFFFFFFFFF,F:F
F:,FFFFFF,FFF,FFFFFFFFFFFF,FF,FFFFFF:FFFF:FFFFF:,FFF:FF:F,F,:FFF::F,
F:FFFF,,,FF:::F
@A00721:190:HGJW7DSXY:4:2148:8784:32330 2:N:0:TCTGCAAG+GCTGCACT
CTTCGACATAATCAACCCAGATTCCGACGAATCTTTTCACCCAAATATTCAAAGTGCGAGATCGGCGA
ATGACGTCGCCGATTATATCACAAAAGGAGGATCTTATATTGATCACGGTCAATTTCAAGAAGACGGC
CGGAAAAGAAAGTGT
+
FFFFFFFFFFFFFFFFFFFFFFFFFFFFFFFFFFFFFFFFFFFFFFFFFFFFFFFFFFFFFFFFFFFF
```

FFFFFFFFFFFFFFFFFFFFFFFFFFFFFFFFFFFFFFFFFFFFFFFFFFFFFFFFFFFFFFFFFFFF
FFF:FFFFF:FFFFF
@A00721:190:HGJW7DSXY:4:2148:8820:32863 2:N:0:TCTGCAAG+GCTGCACT
CTTCGACATAATCAACCCAGATTCCGACGAATCTTTTCACCCAAATATTCAAAGTGCGAGATCGGCGA
ATGACGTCGCCGATTATATCACAAAAGGAGGATCTTATATTGATCACGGTCAATTTCAAGAAGACGGC
CGGAAAAGAAAGTGT
+
FFFFFFFFFFFFFFFFFFFFFFFFFFFFFFFFFFFFFFFFFFFFFFFFFFFFFFFFFFFFFFFFFFFF
FFFFFFFFFFFF:FFFFFFFFFFFFFFFFFFFFFFFFFFFFFFFFFFFFFFFFFFFFFFFFFFFFFF
FFFF:FFFFF,FFFF
@A00721:190:HGJW7DSXY:4:2654:6596:27539 2:N:0:TCTGCAAG+GCTGCACT
CTTCGACATAATCAACCCAGATTCCGACGAATCTTTTCACCCAAATATTCAAAGTGCGAGATCGGCGA
ATGACGTCGCCGATTATATCACAAAAGGAGGATCTTATATTGATCACGGTCAATTTCAAGAAGACGGC
CGGAAAAGAAAGTGT
+
FFFFFFFFFFFFFFFFFFFFFFFFFFFFFFFFFFFFFFFFFFFFF:FFFFFFFFFFFFFFFFFFFFFF
FFFFFFFFFFFFFFFFFFFFF:FFFF,FFFFFFFFFFFFFFFFFFFFFFFFFFFFFFFFFFFFFFFFF
FFFFFFFFFFFFFFF
@A00721:190:HGJW7DSXY:4:1151:23430:32111 2:N:0:TCTGCAAG+GCTGCACT
GGTTCTTCGACATAATCAACCCAGATTCCGACGAATCTTTTCACCCAAATATTCAAAGTGCGAGATCG
GCGAATGACGTCGCCGATTATATCACAAAAGGAGGATCTTATATTGATCACGGTCAATTTCAAGAAGA
CGGCCGGAAAAGAAA
+
FFFFFFFFFFFFFFFFFFFFFFFFFFFFFFFFFFFFFFF:FFFFFFFFFFFFFFFFFFFFFFFFFFFF
FFFFFFFFFFFFFFFFFFFFFFFFFF:FFFFFFFFFFFFFFFFFFFFFFFFFFFFFFFFFF:FFFFFF::
F:F:FFFFFFFFFFF
@A00721:190:HGJW7DSXY:4:1173:9634:3333 2:N:0:TCTGCAAG+GCTGCACT
GGTTCTTCGACATAATCAACCCAGATTCCGACGAATCTTTTCACCCAAATATTCAAAGTGCGAGATCG
GCGAATGACGTCGCCGATTATATCACAAAAGGAGGATCTTATATTGATCACGGTCAATTTCAAGAAGA
CGGCCGGAAAAGAAA
+
::FFFFFFFFFFFFFFFFFFFFFFFFFFFFFFFFFFFFFFFFFFFFFFFFFFFFFFFFFFFFFFFFFF
FFFFFFFFFFFFFFFFFFFFFFFFFFFFFFFFFFFFFFFFFFFFFFFFFFFFFFFFFFFFFFFFFFFF
FF:FFFFFFFFFFFF
@A00721:190:HGJW7DSXY:4:1564:26006:33912 2:N:0:TCTGCAAG+GCTGCACT
GGTTCTTCGACATAATCAACCCAGATTCCGACGAATCTTTTCACCCAAATATTCAAAGTGCGAGATCG
GCGAATGACGTCGCCGATTATATCACAAAAGGAGGATCTTATATTGATCACGGTCAATTTCAAGAAGA
CGGCCGGAAAAGAAA
+
FFFFFFFFFFFFFFFFFFFFFFFFFFFFFFFFFFFFFFFFFFFFFFFFFFFFFFFFFFFFFFFFFFFF
FFFFFFFFFFFFFFFFFFFFFFFFFFFFFFFFFFFFFFFFFFFFFFFFFFFFFFFFFFFFFFFFFFFF
FFFFFFFFFFFFFFF
@A00721:190:HGJW7DSXY:4:1643:29993:13354 2:N:0:TCTGCAAG+GCTGCACT
GGTTCTTCGACATAATCAACCCAGATTCCGACGAATCTTTTCACCCAAATATTCAAAGTGCGAGATCG
GCGAATGACGTCGCCGATTATATCACAAAAGGAGGATCTTATATTGATCACGGTCAATTTCAAGAAGA
CGGCCGGAAAAGAAA
+
FFFFFFFFFFF,FFFFFFFFFFFFFFFFFFFFFFFFFFFFFFFFFFFF:FFFFFFFFFFFFFFFF,F:
FFFFFFFFFFFFFFFFFF,FFFFFFFFFFFFFFFFFFFFFFFFFF:FFFFFFFFFFF:FFFFFFFFFF,:
FFF:FFFF,FFFFFF
@A00721:190:HGJW7DSXY:4:2130:13160:36057 2:N:0:TCTGCAAG+GCTGCACT
GGTTCTTCGACATAATCAACCCAGATTCCGACGAATCTTTTCACCCAAATATTCAAAGTGCGAGATCG
GCGAATGACGTCGCCGATTATATCACAAAAGGAGGATCTTATATTGATCACGGTCAATTTCAAGAAGA
CGGCCGGAAAAGAAA

```
+
FFFFFFFFFFFFFFFFFFFFFFFFFFFFFFFFFFFFFFFFFFFFFFFFF,FFFFFFFFFFFFFFFFFF
FFFFFFFFFFFFFFFFF:FFF:FFFFF:FFFFFFFF:FFFFFFFFFF:FFFFFFFFFFFFFFFFFFF,
FFFFFFFF,FFFFFF
@A00721:190:HGJW7DSXY:4:2554:31602:7623 2:N:0:TCTGCAAG+GCTGCACT
GGTTCTTCGACATAATCAACCCAGATTCCGACGAATCTTTTCACCCAAATATTCAAAGTGCGAGATCG
GCGAATGACGTCGCCGATTATATCACAAAAGGAGGATCTTATATTGATCACGGTCAATTTCAAGAAGA
CGGCCGGAAAAGAAA
+
FF,FFFFFFFFFFFFFFFFFFFFFFFFFFFFFFFFFFFFFFFFFFFFFFFFFFFFFFFFFFFFFFF:F
FFFFFFFFFFFFFFFF::FFFFFFF,FFFFFFFFFFFFFFFFFFFFFF:FF,FFFFFFFFF:FF,:FF
:FF,:FFFFFFFFF:
@A00721:190:HGJW7DSXY:4:2614:24632:4319 2:N:0:TCTGCAAG+GCTGCACT
GGTTCTTCGACATAATCAACCCAGATTCCGACGAATCTTTTCACCCAAATATTCAAAGTGCGAGATCG
GCGAATGACGTCGCCGATTATATCACAAAAGGAGGATCTTATATTGATCACGGTCAATTTCAAGAAGA
CGGCCGGAAAAGAAA
+
:FFFFFFF:FFFFF:FFF,FFFFFFFFFFFFFFFFFFFFFFFF:FFFFFFFFFFFFFFFFFFFFFFFF
FFFFFFFFFFFFFFFFF::FFFFFFFFFF,FFFFFFFFFFFFFFFFFFFFFFFFFF,FFFFFFFFFFF
FFFFFFFF,FFFFF:
@A00721:190:HGJW7DSXY:4:2618:25735:3223 2:N:0:TCTGCAAG+GCTGCACT
GGTTCTTCGACATAATCAACCCAGATTCCGACGAATCTTTTCACCCAAATATTCAAAGTGCGAGATCG
GCGAATGACGTCGCCGATTATATCACAAAAGGAGGATCTTATATTGATCACGGTCAATTTCAAGAAGA
CGGCCGGAAAAGAAA
+
FFFFFFFFFFFFFFFFFFFFFFFFFFFFFFFFFFFFFFFFFFFFFFFF:FF:FFFFFFFFFFF:FFF
FFFFFFFFFF,FFFFFFFFFFFF:FFFFFFF,FFFFFFFFFFFFFFFFFFF:FFFFFFFFFFF:FFFF
F:FFFFFFFFFFFFF
@A00721:190:HGJW7DSXY:4:1120:28113:10473 2:N:0:TCTGCAAG+GCTGCACT
CGGAATCCAAGGTTCTTCGACATAATCAACCCAGATTCCGACGAATCTTTTCACCCAAATATTCAAAG
TGCGAGATCGGCGAATGACGTCGCCGATTATATCACAAAAGGAGGATCTTATATTGATCACGGTCAAT
TTCAAGAAGACGGCC
+
FFFFFFFFFFFFFFFFFFFFFFFFFFFFFFFFFFFFFFFFFFFFFFFFFFFFFFFFFFFFFFFFFFFF
FFFFFFFFFFFFFFFFFFFFFFFFFFFFFFFFFFFFFFFFFFFFFFFFFFFFFFFFFFFFFFFFFFFF
FFFFF:FFFFFFF:F
@A00721:190:HGJW7DSXY:4:1330:20971:21809 2:N:0:TCTGCAAG+GCTGCACT
CGGAATCCAAGGTTCTTCGACATAATCAACCCAGATTCCGACGAATCTTTTCACCCAAATATTCAAAG
TGCGAGATCGGCGAATGACGTCGCCGATTATATCACAAAAGGAGGATCTTATATTGATCACGGTCAAT
TTCAAGAAGACGGCC
+
FFFFFFFF:FFFFFFFFFFFFFFFFFFFFFFFFFFFFFFFFFFFFFFFFFFFFFFFFFF:FFFFFFFF
FFFFFFFFFFFFFFFFFFFFFFFFFFFFFFFFFFFFFFFFFFFFFFFFFFF,FFF,FF:FFFFFFFFFF
FFF:FFFFFFFF:FF
@A00721:190:HGJW7DSXY:4:1513:13991:31391 2:N:0:TCTGCAAG+GCTGCACT
CGGAATCCAAGGTTCTTCGACATAATCAACCCAGATTCCGACGAATCTTTTCACCCAAATATTCAAAG
TGCGAGATCGGCGAATGACGTCGCCGATTATATCACAAAAGGAGGATCTTATATTGATCACGGTCAAT
TTCAAGAAGACGGCC
+
FFFF:FFFFFFFFFFFFFFFFFFFFFFFFFFFFFFFFFFFFFFFFFFFFFFFFFFFFFFF:FFF::FF
FFFFFFFFFFFFFFFFFFFFFFFFFFFFFFFFFFFFFFFFFFFFFFFFFFFFFFFFFFFFFFFFFFFF
FFFFFFFFFFF:FFF
@A00721:190:HGJW7DSXY:4:1517:30273:11177 2:N:0:TCTGCAAG+GCTGCACT
CGGAATCCAAGGTTCTTCGACATAATCAACCCAGATTCCGACGAATCTTTTCACCCAAATATTCAAAG
```

```
TGCGAGATCGGCGAATGACGTCGCCGATTATATCACAAAAGGAGGATCTTATATTGATCACGGTCAAT
TTCAAGAAGACGGCC
+
FFFFFFFFFFFFFFFFFFFFFFFFFFFFFFFFFFFFFFFFFF:FFFFFFFFFFFFFFFFFFFFFFFFFF
FFFFFFFFFFFFFFFFFFFFFFFFFFFFFFFFFFFFFFFFFFFFFFFFFFFFFFFFFFFFFFFFFFFFF
FFFFFFFF::FFFFF
@A00721:190:HGJW7DSXY:4:2134:3568:26835 2:N:0:TCTGCAAG+GCTGCACT
CGGAATCCAAGGTTCTTCGACATAATCAACCCAGATTCCGACGAATCTTTTCACCCAAATATTCAAAG
TGCGAGATCGGCGAATGACGTCGCCGATTATATCACAAAAGGAGGATCTTATATTGATCACGGTCAAT
TTCAAGAAGACGGCC
+
FFFFFFFFFFFFFFFFFFFFFFFFFFFFFFFFFFFFFFFFFFFFFFFFFFFFFF:FFFFFFFFF:FFF
FF:FFFFFFFFFFFFFFFFFFFFFFFFFFFFFFFFFFFFFFFFFFFF:FFFFFFFFFFFFFF:FFFFFF
FFFFFFFFFFFFFFF
@A00721:190:HGJW7DSXY:4:2650:17743:15875 2:N:0:TCTGCAAG+GCTGCACT
CGGAATCCAAGGTTCTTCGACATAATCAACCCAGATTCCGACGAATCTTTTCACCCAAATATTCAAAG
TGCGAGATCGGCGAATGACGTCGCCGATTATATCACAAAAGGAGGATCTTATATTGATCACGGTCAAT
TTCAAGAAGACGGCC
+
FFFFFFFFFFFFFFFFFFFFFFFF:FFFFFFFFFFFFFFFFFFFFF,FF:FFFFFFFFFF:FFFFFFFFF
FFFFFFFFFFFFFFFFFF:FFFFFFFF:FFFFFFFFFFFFFFF:FFF:FFFFFFFF:FFFFFFFFFFFFFF
FFFFF:FFFFFFFFF
@A00721:190:HGJW7DSXY:4:1154:30463:18771 2:N:0:TCTGCAAG+GCTGCACT
TCGGAATCCAAGGTTCTTCGACATAATCAACCCAGATTCCGACGAATCTTTTCACCCAAATATTCAAA
GTGCGAGATCGGCGAATGACGTCGCCGATTATATCACAAAAGGAGGATCTTATATTGATCACGGTCAA
TTTCAAGAAGACGGC
+
FFFFFFFFFFFFFFFFFFFFFFFFFFFFFFFFFFFFFFFFFFFFFFFFFFFFFFFFFFFFFFFFFF:FF
FFFFFFFFFF:FFFFFFFFFFFFFFFFFFFFFFFFFFFFF:FFFFF,FFF:FFFFFFFFFF:FFFFFFF
FFFFFFFF,FF:FFF
@A00721:190:HGJW7DSXY:4:1232:22761:3051 2:N:0:TCTGCAAG+GCTGCACT
TCGGAATCCAAGGTTCTTCGACATAATCAACCCAGATTCCGACGAATCTTTTCACCCAAATATTCAAA
GTGCGAGATCGGCGAATGACGTCGCCGATTATATCACAAAAGGAGGATCTTATATTGATCACGGTCAA
TTTCAAGAAGACGGC
+
FFFFFFFFFFFFFFFFFFFFFFFFFFFFFFFFFFFFFFFFFFFFFFFFFFFFFFFFFFFFFFFFFFFFF
FFFFFFFFFFFFFFFFFFFFFFFFFFFFFFFFFFFFFFFFFFFFFFFFFFFFFFFFFFFFFFFFFFFFF
FFFFFFFFFFFFFFF
@A00721:190:HGJW7DSXY:4:1259:31774:2848 2:N:0:TCTGCAAG+GCTGCACT
TCGGAATCCAAGGTTCTTCGACATAATCAACCCAGATTCCGACGAATCTTTTCACCCAAATATTCAAA
GTGCGAGATCGGCGAATGACGTCGCCGATTATATCACAAAAGGAGGATCTTATATTGATCACGGTCAA
TTTCAAGAAGACGGC
+
FFFFFFFF::FFFFFFFFFF:FFFFFFFFFFFFFFFFFFFFFFFFFFFFFFFFFFFFFFFFFFFFFFFF
FFFFFFFFFFFFFFFFFFFF:FFFFFFFFFFFFFFFFFFFFFFFFFFFFF::FFFFFFF:FFF:FFFFFF
FFFF:FFFF,F:FFF
@A00721:190:HGJW7DSXY:4:1303:25473:13604 2:N:0:TCTGCAAG+GCTGCACT
TCGGAATCCAAGGTTCTTCGACATAATCAACCCAGATTCCGACGAATCTTTTCACCCAAATATTCAAA
GTGCGAGATCGGCGAATGACGTCGCCGATTATATCACAAAAGGAGGATCTTATATTGATCACGGTCAA
TTTCAAGAAGACGGC
+
FFFFFFFFFF:F:F:FFFFFFFFFFFFFFFFFFFFFF:FFFFFFFFFFFF,FFFFFFFFFFF:FFFFF
:FF:FFFFFFFFFF:FFFFFF:FFFFFF,FFFFFFFFFF::F:FFFF,F,F:FFFFF:FFFFF:FFFF
FF:FFF:FF::FFFF
```

@A00721:190:HGJW7DSXY:4:1328:3947:24643 2:N:0:TCTGCAAG+GCTGCACT
TCGGAATCCAAGGTTCTTCGACATAATCAACCCAGATTCCGACGAATCTTTTCACCCAAATATTCAAA
GTGCGAGATCGGCGAATGACGTCGCCGATTATATCACAAAAGGAGGATCTTATATTGATCACGGTCAA
TTTCAAGAAGACGGC
+
FF:FFFFFFFFFFFFFFFFFFFFFFFFFFFFFFFFFFFFFFFFFFF:FFFFFFFFFFFFFFFFFFFFF
FFF:FFFFFFFFFFFFFFFFFFFFFFFFFFFFFFFFFFFFFFFFFFFFFFFFFFFFFFFFFFFFFFFF
FFFFF:FFFFFFFFF
@A00721:190:HGJW7DSXY:4:1335:6605:3975 2:N:0:TCTGCAAG+GCTGCACT
TCGGAATCCAAGGTTCTTCGACATAATCAACCCAGATTCCGACGAATCTTTTCACCCAAATATTCAAA
GTGCGAGATCGGCGAATGACGTCGCCGATTATATCACAAAAGGAGGATCTTATATTGATCACGGTCAA
TTTCAAGAAGACGGC
+
FFFFFF,FFFFFFFFFFFFFFFFFFFFFFFFFFFFFFFFFFFFFFFFFFFFFFFFFFFFFFFFFFFFF
FFF:FFFFFFFFFFFFFFFFFFFFFFFFFFFFFFFFFFFFFFFFFFFFFFF:FFFFFFF,FFFFFFFF
FFFFFF:FFFFFFFF
@A00721:190:HGJW7DSXY:4:1402:12834:24001 2:N:0:TCTGCAAG+GCTGCACT
TCGGAATCCAAGGTTCTTCGACATAATCAACCCAGATTCCGACGAATCTTTTCACCCAAATATTCAAA
GTGCGAGATCGGCGAATGACGTCGCCGATTATATCACAAAAGGAGGATCTTATATTGATCACGGTCAA
TTTCAAGAAGACGGC
+
FFFFFFFFFFFFFFFFFFFFFFFFFFFFFFFFFFFFFFFFFFFFFFFFFFFFFFFFFFFFFFFFFFFF
FFFFFFFFFFFFFFFFFFFFFFFFFFFFFFFFFFFFFFFFFFFFFFF:FFFFFFFFFFFFFFFFFFFF
FFFFFFFFFFFFFFF
@A00721:190:HGJW7DSXY:4:1402:13060:24205 2:N:0:TCTGCAAG+GCTGCACT
TCGGAATCCAAGGTTCTTCGACATAATCAACCCAGATTCCGACGAATCTTTTCACCCAAATATTCAAA
GTGCGAGATCGGCGAATGACGTCGCCGATTATATCACAAAAGGAGGATCTTATATTGATCACGGTCAA
TTTCAAGAAGACGGC
+
FFFFF:FFFFFFFFFFFFFFFFFFFFFFFFFFFFFFFFFFFFFFFFFFFFFFFFFFFFFFFFFFFFFF
FFFFFFFFFFFFFFFFFFFFFFFFFFFFF:FFFFFFFFFFFFFFFFFFFFFFFFFFFFFFFFFFFFFF
FFFFFFFFFFFFFFF
@A00721:190:HGJW7DSXY:4:2140:6696:25989 2:N:0:TCTGCAAG+GCTGCACT
TCGGAATCCAAGGTTCTTCGACATAATCAACCCAGATTCCGACGAATCTTTTCACCCAAATATTCAAA
GTGCGAGATCGGCGAATGACGTCGCCGATTATATCACAAAAGGAGGATCTTATATTGATCACGGTCAC
TTTCAAGAAGACGGC
+
FFFFFFFFF:FFFFFFFFFFFF:FF,FFFFFFFF:FFFFFFFFFFFFF::FFFFFFFFFFFFFFFFFF
FFFFFFFFFFFFFFF:FFFFFFFFFFF,FF:FFFF:FFFFFFFFFFFFFFFFF:FFFFFFFFFFFFF,
F:FF:FFF,FFFFF:
@A00721:190:HGJW7DSXY:4:2176:15393:31469 2:N:0:TCTGCAAG+GCTGCACT
TCGGAATCCAAGGTTCTTCGACATAATCAACCCAGATTCCGACGAATCTTTTCACCCAAATATTCAAA
GTGCGAGATCGGCGAATGACGTCGCCGATTATATCACAAAAGGAGGATCTTATATTGATCACGGTCAA
TTTCAAGAAGACGGC
+
FFFFFFFFFFFFFFFFFFFFFFFFFFFFFFFFFFFFFFFFFFFFFFFFFFFFFFFFFFFFFFFFFFFF
FFFFFFFFFFFFFFFFFFFFFFFFFFFFFFFFFFFFFFFFFFFFFFFFFFFFFFFFFFFFFFFFFFFF
FFFFFF:FFFFFFFF
@A00721:190:HGJW7DSXY:4:2360:22480:21950 2:N:0:TCTGCAAG+GCTGCACT
TCGGAATCCAAGGTTCTTCGACATAATCAACCCAGATTCCGACGAATCTTTTCACCCAAATATTCAAA
GTGCGAGATCGGCGAATGACGTCGCCGATTATATCACAAAAGGAGGATCTTATATTGATCACGGTCAA
TTTCAAGAAGACGGC
+
FFFFFFFFFFFFFFFFFFFFFFFFFFFFFFFFFFFFFFFFFFFFFFFFFFFFFFFFFFF:FFFFFFFF

FFFFFFFFFFFFFFFFFFFFFFFFFFFFFFFFFFFFFFFFFFFFFFFFFFFFFF:FFFFFFFFFFF
FFFFFFFFFFFF:FF
@A00721:190:HGJW7DSXY:4:2369:5764:15922 2:N:0:TCTGCAAG+GCTGCACT
TCGGAATCCAAGGTTCTTCGACATAATCAACCCAGATTCCGACGAATCTTTTCACCCAAATATTCAAA
GTGCGAGATCGGCGAATGACGTCGCCGATTATATCACAAAAGGAGGATCTTATATTGATCACGGTCAA
TTTCAAGAAGACGGC
+
FFFFFFFFFFFFFFFFFFFFFFFFFFFFFFFFFFFFFFFFFFFFFFFFFFFFFFFFFFFFFFFFFFFF
FFFFFFFFFFFFFFFFFFFFFFFFFFFFFFFFFFFFFFFFFFFFFFFFFFFFFFFFFFFFFFFFFFFF
FFFFFFFFFFFFFFF
@A00721:190:HGJW7DSXY:4:1121:4915:9627 2:N:0:TCTGCAAG+GCTGCACT
GTCGGAATCCAAGGTTCTTCGACATAATCAACCCAGATTCCGACGAATCTTTTCACCCAAATATTCAA
AGTGCGAGATCGGCGAATGACGTCGCCGATTATATCACAAAAGGAGGATCTTATATTGATCACGGTCA
ATTTCAAGAAGACGG
+
FFFFFFFFFFFFFFFFFFFFFFFFFFFFFFFF:FFFFFFFFFFFFFFFFFFFFFFFFFFFFFFFFFFF
FFFFFFFFFFFFFFFFFFFFFFFFFFFFFFFFF:FFFF::FFFFFFFFFFFFFFFFFFFFFFFFFFFF
FFFFFFFFFF:FF:F
@A00721:190:HGJW7DSXY:4:1252:16694:35352 2:N:0:TCTGCAAG+GCTGCACT
GTCGGAATCCAAGGTTCTTCGACATAATCAACCCAGATTCCGACGAATCTTTTCACCCAAATATTCAA
AGTGCGAGATCGGCGAATGACGTCGCCGATTATATCACAAAAGGAGGATCTTATATTGATCACGGTCA
ATTTCAAGAAGACGG
+
FFFFFFFFFFFFFFFFFFFFFFFFFF:FFFFFFFFFFFFFFFFFFFFFFFFFFFFFFFFFFFFFFFFF
FFFFFFFFFFFFFFFFFFFFFFFFFFFFFFFFFFFFFFFFFFFFFFFFFFFFFFFFFFFFFFFFFFFF
FFFFFFFFFFFFFFF
@A00721:190:HGJW7DSXY:4:1676:22435:18114 2:N:0:TCTGCAAG+GCTGCACT
GTCGGAATCCAAGGTTCTTCGACATAATCAACCCAGATTCCGACGAATCTTTTCACCCAAATATTCAA
AGTGCGAGATCGGCGAATGACGTCGCCGATTATATCACAAAAGGAGGATCTTATATTGATCACGGTCA
ATTTCAAGAAGACGG
+
FFFFFF,FFFFFFFF:FFFFFFFFFFFFFFFFFFFFFFFFFFFFFFFFFFFFFFFFFFFFFFFFFFFF
:FFFFFFFFFFFFFFFFFFFFFFFFFFFF,FFFFFFFFFF:,FFFFFFFFFFFFFFF::FFFFFFFFF
FFFFFFF::FFFFFF
@A00721:190:HGJW7DSXY:4:2176:28067:20541 2:N:0:TCTGCAAG+GCTGCACT
GTCGGAATCCAAGGTTCTTCGACATAATCAACCCAGATTCCGACGAATCTTTTCACCCAAATATTCAA
AGTGCGAGATCGGCGAATGACGTCGCCGATTATATCACAAAAGGAGGATCTTATATTGATCACGGTCA
ATTTCAAGAAGACGG
+
FFFFFFFFFFFFFFFFFFFFFFFFFFFFFFFFFFFFFFFFFFFFFFFFFFFFFFFFFFFFFFFFFFFF
FFFFFFFFFFFFFFFFFFFF:FFFFFFFFFFFFFFFFFFFFFFFFFFFFFFFFFFFFFFFFFFFFFFF
:FFFFFFFFFFFFFF
@A00721:190:HGJW7DSXY:4:2357:29704:15828 2:N:0:TCTGCAAG+GCTGCACT
GTCGGAATCCAAGGTTCTTCGACATAATCAACCCAGATTCCGACGAATCTTTTCACCCAAATATTCAA
AGTGCGAGATCGGCGAATGACGTCGCCGATTATATCACAAAAGGAGGATCTTATATTGATCACGGTCA
ATTTCAAGAAGACGG
+
FFFFFFFFFF:FFFFFFFFFFFFFFFFFFFFFFFFFFFFFFFFFFFFFFFF:FFFFFFFFFFFFFFFFF
FFFFFFFFFFFFFFFFFFFFFFFFFFFFFFFFFFFFFFFFFFFF:FFFFFF:FFFF:F:FFF:FFFFF
FFFF:FFFFF,FFFF
@A00721:190:HGJW7DSXY:4:2357:29722:16016 2:N:0:TCTGCAAG+GCTGCACT
GTCGGAATCCAAGGTTCTTCGACATAATCAACCCAGATTCCGACGAATCTTTTCACCCAAATATTCAA
AGTGCGAGATCGGCGAATGACGTCGCCGATTATATCACAAAAGGAGGATCTTATATTGATCACGGTCA
ATTTCAAGAAGACGG

```
+
FFFFFFFFFF:FFFFFFFFFFFFFFFFFFFFFFFFFFFFFFFFFFFFFFFFFFFFFFFFFFFFFFFFF
FFFFFFFFFFFFFFFFFFFFFFFFFFFFFFFFFFFFFFFFF,FFFFF::FFFFFFFFFFFFFFFFFFF
FFFFF:,FFFFFFFF
@A00721:190:HGJW7DSXY:4:2415:6533:26459 2:N:0:TCTGCAAG+GCTGCACT
GTCGGAATCCAAGGTTCTTCGACATAATCAACCCAGATTCCGACGAATCTTTTCACCCAAATATTCAA
AGTGCGAGATCGGCGAATGACGTCGCCGATTATATCACAAAAGGAGGATCTTATATTGATCACGGTCA
ATTTCAAGAAGACGG
+
FFFFFF:FF:,FF,FFFFFFFFFFFFF:FFFF:,FFFFFFF:FFFFFFF:,F:F:FFF:FF:FFFFFF
FF,F:,FFFFF:F:FFFF:FF:FFFFFFFFFFF:FFFFFF:F:,,F,FF::FF,FFF,F:,:F,FFFF
F:F,FF:F,FFFFFF
@A00721:190:HGJW7DSXY:4:1377:31394:36542 2:N:0:TCTGCAAG+GCTGCACT
GTCTTCTTGAAATTGACCGTGATCAATATAAGATCCTCCTTTTGTGATATAATCGGCGACGTCATTCG
CCGATCTCGCACTTTGAATATTTGGGTGAAAAGATTCGTCGGAATCTGGGTTGATTATGTCGAAGAAC
CTTGGATTCCGACAT
+
FFFFFFFFFFFFFFFFFFFFFFFFFFFFFF,FFFFFFFFFFFFFFFFFFFFFFFFFFFFFFFFFFFFF
FFFFFFFFFFFFFFFFFFFFFFFFFFFFF:FFFFFFFFFFFFF:F:FFFFFFFFFFFFFFFFFF::FF
F:FFFFFFFFF:FFF
@A00721:190:HGJW7DSXY:4:2167:15094:15139 2:N:0:TCTGCAAG+GCTGCACT
GTCTTCTTGAAATTGACCGTGATCAATATAAGATCCTCCTTTTGTGATATAATCGGCGACGTCATTCG
CCGATCTCGCACTTTGAATATTTGGGTGAAAAGATTCGTCGGAATCTGGGTTGATTATGTCGAAGAAC
CTTGGATTCCGACAT
+
FFFFFFFFFFFFFFFFFFFFF:FFFFFFFFFFFFFFFFFFFFFFFFFFFFFFFFFFF:,FFFFFFFFF
FFFFFFFFFFFFFFFFFFFFFF:FFFFFFF:FFFFFFFFFFFFFFFFFFFFFFFFFFFFFF:FFFFFF
FFF:FF:FFFFFFFF
@A00721:190:HGJW7DSXY:4:2167:13693:16595 2:N:0:TCTGCAAG+GCTGCACT
GTCTTCTTGAAATTGACCGTGATCAATATAAGATCCTCCTTTTGTGATATAATCGGCGACGTCATTCG
CCGATCTCGCACTTTGAATATTTGGGTGAAAAGATTCGTCGGAATCTGGGTTGATTATGTCGAAGAAC
CTTGGATTCCGACAT
+
F:FFFFFFFFFFFFFFFFFFFFFFFFFFFFFFFFFFFFFFFFFFFFFFFFFFFFFFFFFFFFFFFFFF
FFFFFFFFFFFFFFFFFFFFFFFF:FFFFFFFFFFFFFFFFFFFFFFFFFFFFFFFFFFFFFFFFFFF
FFFFFFFFFFFFFFF
@A00721:190:HGJW7DSXY:4:2218:12084:15937 2:N:0:TCTGCAAG+GCTGCACT
GTCTTCTTGAAATTGACCGTGATCAATATAAGATCCTCCTTTTGTGATATAATCGGCGACGTCATTCG
CCGATCTCGCACTTTGAATATTTGGGTGAAAAGATTCGTCGGAATCTGGGTTGATTATGTCGAAGAAC
CTTGGATTCCGACAT
+
FFFFFFFFFFFFFFFFFFFFFFFFFFFFFFFFFFFFFFFFFF:FFFFFFFFF:FFFFFFFFFFFFFFF
FFFFFFFFFFFFFFFFFFFFFF,FFFFFFFF,FFFFFFFFFFFFFFFFFFFFFFFFFFFFFFFFFFFF
FFFFFF:FFFFFFFF
@A00721:190:HGJW7DSXY:4:2457:16360:3583 2:N:0:TCTGCAAG+GCTGCACT
GTCTTCTTGAAATTGACCGTGATCAATATAAGATCCTCCTTTTGTGATATAATCGGCGACGTCATTCG
CCGATCTCGCACTTTGAATATTTGGGTGAAAAGATTCGTCGGAATCTGGGTTGATTATGTCGAAGAAC
CTTGGATTCCGACAT
+
FFFFFFFFFFFFFFFFFFFFFFFFFFFFFFFFFFFFFFFF:FFFFFFFFFFFFFFFFFFFFFFFFFFF
:FF:FFFFFFFFFFFFFF:FFF,FFFFF:F:FFFFFFFFFFFF,FFFFFF:FFFFFFFFFFFFFF:F:
:,,FF::FFFFFFFF
@A00721:190:HGJW7DSXY:4:2415:6280:27336 2:N:0:TCTGCAAG+GCTGCACT
GTTCTTCGACATAATCAACCCAGATTCCGACGAAAATTTTCACAAAAATATACAAAGTGCGAGATCGT
```

CGAATCACGTCGCCTATTAAATCAAAAAAGGAGGATCATATATTGTTAAAGGTCAATTTAAAGAACA
+
FFF:,,::FF:,FF,FF:F,FF,F,:,F,F:FFF,,,,:,,,,,,F,,,FF,:FF,,::::FF,FF,
F,FF:,:,,F,F:,,:F,F,FFFF,FF,,F,F:,F,,,F:FFFF,,,,,,,,FF:FF:,,FF,FF,F
@A00721:190:HGJW7DSXY:4:1129:5330:10661 2:N:0:TCTGCAAG+GCTGCACT
TAAATGTCGGAATCCAAGGTTCTTCGACATAATCAACCCAGATTCCGACGAATCTTTTCACCCAAATA
TTCAAAGTGCGAGATCGGCGAATGACGTCGCCGATTATATCACAAAAGGAGGATCTTATATTGATCAC
GGTCAATTTCAAGA
+
:FFFFFFFFFFFFFFFF:FFFFF:FFF:F:FF:FFFF:FFFFFFFFFFFFFFFFF::FFFFFFFFFFF
:FFFFFFFF::FFF,F:FFFFFFFFFF:FF:FFFFFFFF:FFFFFFFFFFFFFFFFFFFFF:FFFFFF
,FFFFFFF:FFFFF
@A00721:190:HGJW7DSXY:4:1129:5692:11130 2:N:0:TCTGCAAG+GCTGCACT
GTAAATGTCGGAATCCAAGGTTCTTCGACATAATCAACCCAGATTCCGACGAATCTTTTCACCCAAAT
ATTCAAAGTGCGAGATCGGCGAATGACGTCGCCGATTATATCACAAAAGGAGGATCTTATATTGATCA
CGGTCAATTTCAAGA
+
FFFFFFFFFFFFFFFFFFFFFFFFFFFFFFFFFFFFFFFFFFFFFFFFFFFFFFFFFFFFFFFFFFFF
FFFFFFFFFFFFFFFFFFFFFFFFFFFFFFFFFFFFFFFFFFFFFFFFFFFFFFFFFFFFFFFFFFFF
FFFFFFFFFFFFFFF
@A00721:190:HGJW7DSXY:4:1220:30689:20885 2:N:0:TCTGCAAG+GCTGCACT
GTAAATGTCGGAATCCAAGGTTCTTCGACATAATCAACCCAGATTCCGACGAATCTTTTCACCCAAAT
ATTCAAAGTGCGAGATCGGCGAATGACGTCGCCGATTATATCACAAAAGGAGGATCTTATATTGATCA
CGGTCAATTTCAAGA
+
FFFFFFFFFFFFFFFFFFFFFFFFFFFFFFFFFFFFFFFFFFFFFFFFFFFFFFFFFFFFFFFFFFFF
FFFFFFFFFFFFFFF:FFFFFFFFFFFFFFFFFFFFFFFFFFFFFFFFFFFFFFFFFFFFFFFFFFFF
FFFFF:FFFFFFFFF
@A00721:190:HGJW7DSXY:4:1355:18457:1391 2:N:0:TCTGCAAG+GCTGCACT
GTAAATGTCGGAATCCAAGGTTCTTCGACATAATCAACCCAGATTCCGACGAATCTTTTCACCCAAAT
ATTCAAAGTGCGAGATCGGCGAATGACGTCGCCGATTATATCACAAAAGGAGGATCTTATATTGATCA
CGGTCAATTTCAAGA
+
FFFF:FFFFFFFFFFFFFFFFFFFFFFFFFFFFFFFFFFFFFFFFFFFFFFFFFFFFFFFFFFFFFFF
FFFFFFFFFFFFFFFFFFFFFFFFFFFFFFFFFFFFFFFFFFFFFF::FFFFFFFFFFFFFFFFFFFF
FFFFFFFFFFFFFFF
@A00721:190:HGJW7DSXY:4:1377:31602:36338 2:N:0:TCTGCAAG+GCTGCACT
TCTTGAAATTGACCGTGATCAATATAAGATCCTCCTTTTGTGATATAATCGGCGACGTCATTCGCCGA
TCTCGCACTTTGAATATTTGGGTGAAAAGATTCGTCGGAATCTGGGTTGATTATGTCGAAGAACCTTG
GATTCCGACAT
+
FFFF,F:F,,FF:FFFFF,F:FF::FF:FF,FFF:,FF,:FFFFFF,FF:F:FFFFFFFFF:,FFF:F
,FFFFF:::FFFF:,FF,FFFFFFFFFF,:FFFFFFF:F:FF,,FFFFF,:FF,:FFFFFF:FFFFFF
FF:FF,F::FF
@A00721:190:HGJW7DSXY:4:1461:22227:18630 2:N:0:TCTGCAAG+GCTGCACT
GTAAATGTCGGAATCCAAGGTTCTTCGACATAATCAACCCAGATTCCGACGAATCTTTTCACCCAAAT
ATTCAAAGTGCGAGATCGGCGAATGACGTCGCCGATTATATCACAAAAGGAGGATCTTATATTGATCA
CGGTCAATTTCAAGA
+
FFFFFFFFFFFFFFFFFFFFFFFFFFFFFFFFFFFFFFFFFFFFFFFFFFFFFFFFFFFFFFFFFFFF
FFFFFFFFFFFFFFFFFFFFFFFFFFFFFFFFFFFFFFFFFFFFFFFFFFFFFFFFFFFFFFFFFFFF
FFFFFFFFFFFFFFF
@A00721:190:HGJW7DSXY:4:1526:29179:14325 2:N:0:TCTGCAAG+GCTGCACT
GTAAATGTCGGAATCCAAGGTTCTTCGACATAATCAACCCAGATTCCGACGAATCTTTTCACCCAAAT

ATTCAAAGTGCGAGATCGGCGAATGACGTCGCCGATTATATCACAAAAGGAGGATCTTATATTGATCA
CGGTCAATTTCAAGA
+
FFFFFFFFFFFFFFFFFFFFFFFFFFFFFFFFFFFFFFFFFFFFFFFFFFFFFFFFFFFF:FFFFFFF
FFFFFFFFFFFFFFFFFFFFFFFFFFFFFFFFFFFFFFFFFFFFFFFFFFFFFFFFFFFFFFFFFFFF
FFFFFFFFFFFFFFF
@A00721:190:HGJW7DSXY:4:1534:22110:35149 2:N:0:TCTGCAAG+GCTGCACT
GTAAATGTCGGAATCCAAGGTTCTTCGACATAATCAACCCAGATTCCGACGAATCTTTTCACCCAAAT
ATTCAAAGTGCGAGATCGGCGAATGACGTCGCCGATTATATCACAAAAGGAGGATCTTATATTTATCA
CGGTCAATTTCAAGA
+
FFFFF,FFFFFFFFFFF:F,FFFFFFF:FFFF::F,FFFFFFF:::,FFFFFFFFF,F,FF::F:FFF
FFFFF,FFFFFFFFF,FF:FFFFFFFFFF:F:FFFF,FFFFFF:FFFF:F,F:FFFF:FFFF,,FF,:
::,:FFF,F:FF:FF
@A00721:190:HGJW7DSXY:4:1669:18141:28401 2:N:0:TCTGCAAG+GCTGCACT
GTAAATGTCGGAATCCAAGGTTCTTCGACATAATCAACCCAGATTCCGACGAATCTTTTCACCCAAAT
ATTCAAAGTGCGAGATCGGCGAATGACGTCGCCGATTATATCACAAAAGGAGGATCTTATATTGATCA
CGGTCAATTTCAAGA
+
FFFFFFFFFFFF:FFFFFFFFFFFFFFFFFFFFFFFFFFFFFFFFFFFFFFFFFFFFFFFFFFFFFFF
FFFFFFFFFFFFFFFFFFFFFFFFFFFFFFFFFFFFFFFFFFFFFFFFFFFFFFFFFFFFFFFFFFFF
F,FFFFFFFFFFF,F
@A00721:190:HGJW7DSXY:4:2332:1624:25003 2:N:0:TCTGCAAG+GCTGCACT
GTAAATGTCGGAATCCAAGGTTCTTCGACATAATCAACCCAGATTCCGACGAATCTTTTCACCCAAAT
ATTCAAAGTGCGAGATCGGCGAATGACGTCGCCGATTATATCACAAAAGGAGGATCTTATATTGATCA
CGGTCAATTTCAAGA
+
FFFFFFFFFFFFFFFFFFFFFFFF:FFFFFFFFFFFFFFFFFFFFFFFFFFF,FFFFFF:FFFFFFF,
FFFFFF,FFFFFFFFFF:FFFFFFFFFFFFFFFFFFFFFFFFFFFFFFFFFFFFFFFFFF:FF:FFFFF
F::FFFFFFFF:FFF
@A00721:190:HGJW7DSXY:4:2645:3956:1517 2:N:0:TCTGCAAG+GCTGCACT
GTAAATGTCGGAATCCAAGGTTCTTCGACATAATCAACCCAGATTCCGACGAATCTTTTCACCCAAAT
ATTCAAAGTGCGAGATCGGCGAATGACGTCGCCGATTATATCACAAAAGGAGGATCTTATATTGATCA
CGGTCAATTTCAAGA
+
FFFFFFFFFFFFFFFFFFFFFFFFFFFFFFFFFFFFFFFFFFFFFFFFFFFFFFFFFFFFFFFFFFFF
F:FFFFFFFFFFFFFFFFFFFF:FFFFFFFFFFFFFFFFFFFFFFFFFFFFFFFFFFFFFFFFFFFFF
FFFFFFFFFFFFFFF
@A00721:190:HGJW7DSXY:4:1109:2293:29512 2:N:0:TCTGCAAG+GCTGCACT
TATAAATGTCGGAATCCAAGGTTCTTCGACATAATCAACCCAGATTCCGACGAATCTTTTCACCCAAA
GATTCAAAGTGCGAGATCGGCGAATGACGTCGTCGATTATATCACAAAAGGAGGATCTTATATTGATC
ACGGTCAATTTCAAG
+
:FFFF:FFFFFFFFFFF:FFFFFFFFFFFFF:FFFFFFFFFFFFFFFFFFFFFFFFFFFFFFFFF,FF
,FFFFFFFFFFFFFFFFFFFFFFFFFFFFFFFFFFFF,FFFFFFF,:F:FF,FFFFFF:FF:FF:FFF
,:FFFFFFFFFFFFF
@A00721:190:HGJW7DSXY:4:2310:7175:23688 2:N:0:TCTGCAAG+GCTGCACT
TATAAATGTCGGAATCCAAGGTTCTTCGACATAATCAACCCAGATTCCGACGAATCTTTTCACCCAAA
TATTCAAAGTGCGAGATCGGCGAATGACGTCGTCGATTATATCACAAAAGGAGGATCTTATATTGATC
ACGGTCAATTTCAAG
+
FFFFFFFFFFFFFFFFFFFFFFFFFFFFFFFFFFFFFFFFFFFFFFFFFFFFFFFFF,FFFFFFFFF:
FFFFFFFFFFFFFFFFFFFFFFFFFFFFFFFFFFFF,FFFFFFFFFF,FFFFFFFFFFFFFFFFFFFFF
FFFFFFFFFFFFFFF

@A00721:190:HGJW7DSXY:4:2311:9471:4272 2:N:0:TCTGCAAG+GCTGCACT
TATAAATGTCGGAATCCAAGGTTCTTCGACATAATCAACCCAGATTCCGACGAATCTTTTCACCCAAA
TATTCAAAGTGCGAGATCGGCGAATGACGTCGTCGATTATATCACACAAGGAGGATCTTATATTGATC
ACGGTCAATTTCAAG
+
FFFFFFFFFFFFFFFFFFFFFFFFFFFFFF:FFFFFFFFFFFFFFFFFFFFFFFFFFFFFF:FFFFFF
FFFFFFF:FFFFFFFFFFFFFFFFFFFFFFFFFFFFFF,FFFFFFF,F:FFFFFF,,:FFFFF:FFFF
F:FF::FFF,FFFF:
@A00721:190:HGJW7DSXY:4:2312:28673:31923 2:N:0:TCTGCAAG+GCTGCACT
TATAAATGTCGGAATCCAAGGTTCTTCGACATAATCAACCCAGATTCCGACGAATCTTTTCACCCAAA
TATTCAAAGTGCGAGATCGGCGAATGACGTCGTCGATTATATCACAAAAGGAGGATCTTATATTGATC
ACGGTCAATTTCAAG
+
FFFFFFFFFFFFFFFFFFFFFFFFFFFFFFFFFFFFFFFFFFFF:FFFFFFFFFFFFFFF,FFF:F:
FFFFFFFFFFFFFFFFFFFFFFFFFFF:FFFFFFFFFFFFFFFFFFFF:FFFFFF:F,FFFFFFFF:FF
FFFFF,FFFFFFFFF
@A00721:190:HGJW7DSXY:4:2678:22092:36276 2:N:0:TCTGCAAG+GCTGCACT
TATAAATGTCGGAATCCAAGGTTCTTCGACATAATCAACCCAGATTCCGACGAATCTTTTCACCCAAA
TATTCAAAGTGCGAGATCGGCGAATGACGTCGTCGATTATATCACAAAAGGAGGATCTTATATTGATC
ACGGTCAATTTCAAG
+
FFFFF,FFFFFFFFFFFFFFFFFFFFFFFFFFFFFFFFFFFFFFFFFFFFFFFFFFFFFFFFFFFFFF
FFFFFF:FFFFFFFFFFFFFFFFF:FFFFFFFFFFFFFFFFFFFFFFFFF:FFFFFF,FFFFFF,FFF
FFFFFFF:FFFFFFF
@A00721:190:HGJW7DSXY:4:1428:1787:34710 2:N:0:TCTGCAAG+GCTGCACT
GTATAAATGTCGGAATCCAAGGTTCTTCGACATAATCAACCCAGATTCCGACGAATCTTTTCACCCAA
ATATTCAAAGTGCGAGATCGGCGACTGACGTCGCCGATTATATCACAAAAGGAGGATCTTATATTGAT
CACGGTCAATTTCAA
+
FFFFFFFFFFFFFFFFFFFFFFFFFFFFFFFFFFFFFFFFFFFFFFFFFFFFFFFFFFFFFFFFFFFF
,FFFFFFFFFFFFFFFFFFFFFFFFFFFFFFFFFFFFFFF:FFFFFFFFFFFFF,FFFFFFFFFFF:F
F::FFFFFFFFFF:F
@A00721:190:HGJW7DSXY:4:1532:16966:29121 2:N:0:TCTGCAAG+GCTGCACT
GTATAAATGTCGGAATCCAAGGTTCTTCGACATAATCAACCCAGATTCCGACGAATCTTTTCACCCAA
ATATTCAAAGTGCGAGATCGGCGACTGACGTCGCCGATTATATCACAAAAGGAGGATCTTATATTGAT
CACGGTCAATTTCAA
+
::,FFFF,FFFFFFFFFFFFFF:FFFFFFFFFFFFFFFFF:FFFFFFFFFF:FFFFFFFF:FFFFFFF
F:FFFFFFFF:FFFFFFFFFFFFFFFF:FFFFFFFFF,FFF,FFFFFFFF:FFF:FF:FFFFFFFFFF
FFF,FFFFFF:F,FF
@A00721:190:HGJW7DSXY:4:1566:28501:35916 2:N:0:TCTGCAAG+GCTGCACT
GTATAAATGTCGGAATCCAAGGTTCTTCGACATAATCAACCCAGATTCCGACGAATCTTTTCACCCAA
ATATTCAAAGTGCGAGATCGGCGACTGACGTCGCCGATTATATCACAAAAGGAGGATCTTATATTGAT
CACGGTCAATTTCAA
+
FFFFFF:FFFFFFFFFFFF:FFFFFFFFFFFFFFFFFFFFFFFFFFFFFFFFFFFFFFFFFFFFFFFF
FFFFFFFFFFFFFFFFFFFFFFFFFFFFFFFFFFFFFFFFFFFFFFFFFFFFFFFFFF:FFFFFFFFF
:FFFFFFFFFFFF:F
@A00721:190:HGJW7DSXY:4:2441:16224:7889 2:N:0:TCTGCAAG+GCTGCACT
GTATAAATGTCGGAATCCAAGGTTCTTCGACATAATCAACCCAGATTCCGACGAATCTTTTCACCCAA
ATATTCAAAGTGCGAGATCGGCGACTGACGTCGCCGATTATATCACAAAAGGAGGATCTTATATTGAT
CACGGTCAATTTCAA
+
FFFFFFFFFFFFFFFFFFF:FFFFFFF:FFF:FFFFFFFFFFFFFFFFFFFFFFFFFFFFFFFFF:FFF

FFFFFFFFFFFFFFFFFFFF:F:F:FFFFFF:FFFFFFFFFFF,F:FFFF,,F:FFFFFFFFF::,FF
,F,F:F:FFF:F:FF
@A00721:190:HGJW7DSXY:4:2441:16052:8124 2:N:0:TCTGCAAG+GCTGCACT
ATAAATGTCGGAATCCAAGGTTCTTCGACATAATCAACCCAGATTCCGACGAATCTTTTCACCCAAAT
ATTCAAAGTGCGAGATCGGCGACTGACGTCGCCGATTATATCACAAAAGGAGGATCTTATATTGATCA
CGGTCAATTTCAA
+
F:FFFFFF:F:FF:FFFFFFFFFFFFFFFFFFFFFFFFFFFFFFFFFFFFFFFFFFFFFFFF:FFFF:
F:FFFFFFFFFFFFFFFFFFFFFFF:FFFFF:FFFFFFFFFFFF:F:F:FFF:FFFFFFFFF::FF,,F
F:FFFFFF::FFF
@A00721:190:HGJW7DSXY:4:2678:23918:37027 2:N:0:TCTGCAAG+GCTGCACT
TATAAATGTCGGAATCCAAGGTTCTTCGACATAATCAACCCAGATTCCGACGAATCTTTTCACCCAAA
TATTCAAAGTGCGAGATCGGCGAATGACGTCGTCGATTATATCACAAAAGGAGGATCTTATATTGATC
ACGGTCAATTTCAA
+
FF:FFFFFFFFFF:FFFFFFFFFFFFFFFFFFFFFFFFFFFFFFFFFFFFFFFFFFFFFFFFFFFFFF
FFFFFFFFFFFFFFF::FFF,FFFFFFFFFFFFF:FFFFFFFFFFFFFFFFFFFFFFFFFFFFF:FFF
FFFFFFFFFFFFFF
@A00721:190:HGJW7DSXY:4:1235:3486:21997 2:N:0:TCTGCAAG+GCTGCACT
GCAAGTATAAATGTCGGAATCCAAGGTTCTTCGACATAATCAACCCAGATTCCGACGAATCTTTTCAC
CCAAATATTCAAAGTGCGAGATCGGCGAATGACGTCGCCGATTATATCACAAAAGGAGGATCTTATAT
TGATCACGGTCAATT
+
FFFFFFFFFFFFFFFFFFFFFFFFFFFFFFFFFFFFFFFFFFFFFFFFFFFFFFFFFFFFFFFFFFFF
FFFFFFFFFFFFFFFFFFFFFFFFFFFFFFFFFFFFFFFFFFFFFFFFFFFFFFFFFFFFFFFFFFFF
FFFFFFFFFFFFFFF
@A00721:190:HGJW7DSXY:4:1244:25437:23437 2:N:0:TCTGCAAG+GCTGCACT
GCAAGTATAAATGTCGGAATCCAAGGTTCTTCGACATAATCAACCCAGATTCCGACGAATCTTTTCAC
CCAAATATTCAAAGTGCGAGATCGGCGAATGACGTCGCCGATTATATCACAAAAGGAGGATCTTATAT
TGATCACGGTCAATT
+
FFFFFFFFFFFFFFFFFFFFFFFFFFFFFFFFFFFFFFFFFFFFFFFFFFFFFFFFFFFFFF:FFFFF
FFFFFFFFFFFFFFFFFFFFFFFFFFFFFFFFFFFFFFFFFFFFFFFFFFFFFFFFFFFFFFFFFFFF
FFFFFFFFFFFFFFF
@A00721:190:HGJW7DSXY:4:1262:12011:17785 2:N:0:TCTGCAAG+GCTGCACT
GCAAGTATAAATGTCGGAATCCAAGGTTCTTCGACATAATCAACCCAGATTCCGACGAATCTTTTCAC
CCAAATATTCAAAGTGCGAGATCGGCGAATGACGTCGCCGATTATATCACAAAAGGAGGATCTTATAT
TGATCACGGTCAATT
+
FFFFFFFFFF,FFFFFFFFFFFF:FFFFFFFFFFFFFFFFFFFFFFFFFFFFFFFFFFFFFFFFFFFF
FFFFFFFFFFFFFFFFFFFFFFFF:FFFFFFFFFFFFFFFFFFFFFFFFFFFFF,FFFFFFFFFFF,FFF
FFFFFFFFFFFFFFF
@A00721:190:HGJW7DSXY:4:2229:5638:6965 2:N:0:TCTGCAAG+GCTGCACT
CAAGAATAAATGTCGGAATCCAAGGTTCTTCGACATAATCAACCCAGATTCCGACGAATCTTTTCACC
CAAATATTCAAAGTGCGAGATCGGCGAATGACGACGCCGATAATATAACAAAAGGAGGATCTTATATT
GATCACGGTCAATT
+
:FFF,FFFFF:FF,FFFFF,,FF,FF,FF:,FF,F:FFFFFFFFFF,FFFF:FF:FFFFFFF:F,F,F
:FFFFF,,FFFF:FF,FFFF,,FF,FFFFFFF,,:F,F,:F,:F,F,,:FF:FF,FF,FFFF,FFFFF
,F,:FFF:FFFFFF
@A00721:190:HGJW7DSXY:4:2611:8124:32033 2:N:0:TCTGCAAG+GCTGCACT
GCAAGTATAAATGTCGGAATCCAAGGTTCTTCGACATAATCAACCCAGATTCCGACGAATCTTTTCAC
CCAAATATTCAAAGTGCGAGATCGGCGAATGACGTCGCCGATTATATCACAAAAGGAGGATCTTATAT
TGATCACGGTCAATT

+
FFFF:F:FFF,FFFFFFF:FFFFFFFFFFFFFFFFFFFFFF:FFFFFFFFFFFFFFFFFFFFFFFF:F
:FFFF:FFFFFFFFFFFFFFFFFFFFFFFFFFFFFFFFFFF:FFFFFFFFFFFF:F:FFFFFFFFFFF
FFFFFFFFFFFFFFF
@A00721:190:HGJW7DSXY:4:2635:8847:28745 2:N:0:TCTGCAAG+GCTGCACT
GCAAGTATAAATGTCGGAATCCAAGGTTCTTCGACATAATCAACCCAGATTCCGACGAATCTTTTCAC
CCAAATATTCAAAGTGCGAGATCGGCGAATGACGTCGCCGATTATATCACAAAAGGAGGATCTTATAT
TGATCACGGTCAATT
+
FFFFFFFFFFFFFFFFFFFFFFFFFFFFFFFFFFFFFFFFFFFFFFFFFFFFFFFFFFFFF:FFFFFF
FFFFFFFFFFFFFFFFFFFFFFFFFFFFFFFFFFFFFFFFFFFFFFF:FFFFFFFF:FFFFFFFFFFF
F:FFFFFFFFFFFFF
@A00721:190:HGJW7DSXY:4:1163:20302:15421 2:N:0:TCTGCAAG+GCTGCACT
GGCAAGTATAAATGTCGGAATCCAAGGTTCTTCGACATAATCAACCCAGATTCCGACGAATCTTTTCA
CCCAAATATTCAAAGTGCGAGATCGGCGAATGACGTCGCCGATTATATCACAAAAGGAGGATCTTATA
TTGATCACGGTCAAT
+
FFFFFFFFFFF:FFFFFFFFFFFFFFFFFFFFFFFFFFFFFFFFFFFFFFFFFFFFFFFFFFFFFFFF
FFF:FFFFFFFFFFFFFFFFFFFFFFFFFFFFFFFFFFFFFFFF:FFFFFFFFFFFFFFFFFFFFFFF
FFF:FFFFFFFFFFF
@A00721:190:HGJW7DSXY:4:1167:26530:12085 2:N:0:TCTGCAAG+GCTGCACT
GCAAGTATAAATGTCGGAATCCAAGGTTCTTCGACATAATCAACCCAGATTCCGACGAATCTTTTCAC
CCAAATATTAAAAGTGCGATATCGGCGAATGACGTCGCCGATTATATCACAAAAGGAGGATCTTATAT
TGATCACGGTCAAT
+
:,FFF:FFFF:FF::,FFFF,FF::,FF,FFF,F:F:FFFFFFFFFFFFFF:F:F:FFFFFFFFF:FF
F,FFFFFFF,FFFFF:F:F,F:,:F:,FFF,FFF:F,F,:FFFFFFFFF,FFF:F:FF:FF,::FFFF
F:FF,FF,FFFFFF
@A00721:190:HGJW7DSXY:4:1335:21197:16031 2:N:0:TCTGCAAG+GCTGCACT
GGCAAGTATAAATGTCGGAATCCAAGGTTCTTCGACATAATCAACCCAGATTCCGACGAATCTTTTCA
CCCAAATATTCAAAGTGCGAGATCGGCGAATGACGTCGCCGATTATATCACAAAAGGAGGATCTTATA
TTGATCACGGTCAAT
+
FFFFFFFFFFFFFFFFFFFFFFFF:FFFFFFFFFFFFFFFFFFFFFFFFFFFFFFFFFFFFFFFFFFF
FFF,FFFFFFFFFFFFFFFFFFFFFFFFFFFFFFFFFFFFFFFFFFFFF:FFF:FF:FFFFFFFFFFF
FFFFFF::FFF::FF
@A00721:190:HGJW7DSXY:4:1432:29243:26146 2:N:0:TCTGCAAG+GCTGCACT
GGCAAGTATAAATGTCGGAATCCAAGGTTCTTCGACATAATCAACCCAGATTCCGACGAATCTTTTCA
CCCAAATATTCAAAGTGCGAGATCGGCGAATGACGTCGCCGATTATATCACAAAAGGAGGATCTTATA
TTGATCACGGTCAAT
+
FFFFFFFFFFFFFFFFFFFFFFFFFFFFFFFFFFFF:FFFFFFFFFFFFFFFFFFFFFFFFFFFFFFF
FFFFFFFFFFFFFFFFFFFFFFFFFFFFFFFFFFFFFFFFFFFF:FFFFFFFF:FFFFFFFFFFFFFF
FFF:FFFFFFFFFFF
@A00721:190:HGJW7DSXY:4:1646:14172:6840 2:N:0:TCTGCAAG+GCTGCACT
GGCAAGTATAAATGTCGGAATCCAAGGTTCTTCGACATAATCAACCCAGATTCCGACGAATCTTTTCA
CCCAAATATTCAAAGTGCGAGATCGGCGAATGACGTCGCCGATTATATCACAAAAGGAGGATCTTATA
TTGATCACGGTCAAT
+
FFFFFFFFFFFFFFFFFFFFFFFFFFFFFFFFFFFFFFFFFFFFFFFFFFFFFFFFFFFFFFFFFFFF
FFFFFFFFFFFFFFFFFFFFFFFFFFFFFFFFFFFFFFFFFFFFFFFFFFFFFFFFFFFFFFFFFFFF
FFFFFFFF:FFFFFF
@A00721:190:HGJW7DSXY:4:2154:23222:33379 2:N:0:TCTGCAAG+GCTGCACT
GCAAGTATAAATGTCGGAATCCAAGGTTCTTCGACATAATCAACCCAGATTCCGACGAATCTTTTCAC

```
CCAAATATTCAAAGTGCGAGATCGGCGAATGACGTCGCCGATTATATCACAAAAGGAGGATCTTATAT
TGATCACGGTCAAT
+
FF:FFFFFF:FFF:FFFFFFFFFFFFFFFFFFFFF,FFFFFFFFFFFFFFFF:FFFFFFFFFFFFFFF
FFFFF:FFFFFFFF:FFFFFFFFFFFFFFF:FFFFFFFFFF::FF,FFFFF:FFFFFFFFFFFFFF:F
:F:,FF:F:FFFFF
@A00721:190:HGJW7DSXY:4:2237:20636:32847 2:N:0:TCTGCAAG+GCTGCACT
GGCAAGTATAAATGTCGGAATCCAAGGTTCTTCGACATAATCAACCCAGATTCCGACGAATCTTTTCA
CCCAAATATTCAAAGTGCGAGATCGGCGAATGACGTCGCCGATTATATCACAAAAGGAGGATCTTATA
TTGATCACGGTCAAT
+
FFFFFFFFFFFFFFFFFFFFFFFFFFFFFF:FFFFFFFFFFFFFFFFFFFFFFFFFFFFFFFFFFFFF
FFFFF:FFFFFFFFFFFFFFFF:FFFFFFFFFFFF:FFFFFFFFFFFFFFF,FF:FF:FFFFFFFF:F
FFFFF::FFFFFFFF
@A00721:190:HGJW7DSXY:4:2339:31069:20760 2:N:0:TCTGCAAG+GCTGCACT
GGCAAGTATAAATGTCGGAATCCAAGGTTCTTCGACATAATCAACCCAGATTCCGACGAATCTTTTCA
CCCAAATATTCAAAGTGCGAGATCGGCGAATGACGTCGCCGATTATATCACAAAAGGAGGATCTTATA
TTGATCACGGTCAAT
+
FFFFFFFFFFF:FFFFFFFFFFFFFFFFFFFFFFFFFFFFFFFFFFFFFFFFFFFFFFFFFFFFFFFF
FFFFFFFFFFFFFFFFFFFFFFFFFFFFFFFFFFFFFFFFFFFFFFFF:FFFFFFFFFFFFFFFFFFF
FFFFFFFFFFFFFFF
@A00721:190:HGJW7DSXY:4:2372:13006:14841 2:N:0:TCTGCAAG+GCTGCACT
GGCAAGTATAAATGTCGGAATCCAAGGTTCTTCGACATAATCAACCCAGATTCCGACGAATCTTTTCA
CCCAAATATTCAAAGTGCGAGATCGGCGAATGACGTCGCCGATTATATCACAAAAGGAGGATCTTATA
TTGATCACGGTCAAT
+
FFFFFFFFFFFFFFFFFFFFFFFFFFF:::FFFFFFFFFFFFFFFFFFFFFFFFFFFFFFFF:FF:F:
FFFFFF:FFF:FFFFFFFFFFFFFFFFFFF:FFF:FFFFFFFFFFFFFFFFFF,FFFFFFFFF:FFFF
FF,FFFFF,FFFFF:
@A00721:190:HGJW7DSXY:4:2373:14326:14403 2:N:0:TCTGCAAG+GCTGCACT
GGCAAGTATAATTGTCGGAATCCAAGGTTCTTCGACATAATCAACCCAGATTCCGACGAATCTTTTCA
CCCAAATATTCAAAGTGCGAGATCGGCGAATGACGTCGCCGATTATATCACAAAAGGAGGATCTTATA
TTGATCACGGTCAAT
+
FFFFFFFFFFF,FFFFFFF,FFFFFFFFFFFFFFFFFFFFFFFFFFFFFFFFFFFFFFFFFFFFFFFF
:FFF::FFFFFFFFFFFFF:FFFF::FFFF,FFFFFFFFFFFFFFFFFF:FFFFFFFFFFFFFFFFFF
FFFFFFFFFFFFFFF
@A00721:190:HGJW7DSXY:4:2417:10339:11913 2:N:0:TCTGCAAG+GCTGCACT
GGCAAGTATAAATGTCGGAATCCAAGGTTCTTCGACATAATCAACCCAGATTCCGACGAATCTTTTCA
CCCAAATATTCAAAGTGCGAGATCGGCGAATGACGTCGCCGATTATATCACAAAAGGAGGATCTTATA
TTGATCACGGTCAAT
+
FFFFFFFFFFFFFFFFFFFFFFFFFFFFFFFFFFFFFFFFFFFFFFFFFFFFFFFFFFFFFFFFFFFF
FFFF,F:FFFFFFFFFFFFFFFFFFFFFFFFFFFFFFFFFFFFFFF,FFFFFFFFF:FF:FFFFFFFF
FFFFFFFFFFFFFFF
@A00721:190:HGJW7DSXY:4:1439:19804:8985 2:N:0:TCTGCAAG+GCTGCACT
CTCCAATTTTCCGGCAAGTATAAATGTCGGAATCCAAGGTTCTTCGACATAATCAACCCAGATTCCGA
CGAATCTTTTCACCCAAATATTCAAAGTGCGAGATCGGCGAATGACGTCGCCGATTATATCACAAAAG
GAGGATCTTATATTG
+
FFFFFFFFFF:FFFFFFFFFFFFFFFFFFFFFFFFFFFF:FFFFFFFFFFFFFFFFFFFFFFFFFFFF
FFFFFFFFFFFFFFF:FFFFFFFFFFFFFFFFFFFFFFFFFFFFFFFFFFFFFF,FFFFFFF::FFFF
FFFFFFFFFFFFFFF
```

```
@A00721:190:HGJW7DSXY:4:2311:7211:1391 2:N:0:TCTGCAAG+GCTGCACT
CTCCAATTTTCCGGCAAGTATAAATGTCGGAATCCAAGGTTCTTCGACATAATCAACCCAGATTCCGA
CGAATCTTTTCACCCAAATATTCAAAGTGCGAGATCGGCGAATGACGTCGCCGATTATATCACAAAAG
GAGGATCTTATATTG
+
FFFFFFFFFFFFFFFFFFFFFFFFFFFFFFFFFFFFFFFFFFF:FFFFFFFFFFFFFFFFFFFFFFFF
FFFFFFFFFFFFFFFFFFFFFFFFFF:F:FFFFFFFF:F:FFFFFFFFFF:FFFFFFFFFFFFF:FFF
FFFFFFFF:FFFF::
@A00721:190:HGJW7DSXY:4:2341:23231:15546 2:N:0:TCTGCAAG+GCTGCACT
CTCCAATTTTCCGGCAAGTATAAATGTCGGAATCCAAGGTTCTTCGACATAATCAACCCAGATTCCGA
CGAATCTTTTCACCCAAATATTCAAAGTGCGAGATCGGCGAATGACGTCGCCGATTATATCACAAAAG
GAGGATCTTATATTG
+
FFFFFFFFFFFFFFFFFFFFFFFFFFFFFFFFFFFFFFF:FFFFFFFFFFFFFFFFFFFFFFFFFFFF
F,FFFFFFFFFFFFFFFFFFFFFFFFFFFFFFFF:FFFFFFFFFFFFFFFFFFF:F,F,FFFFFF::F
:FFFFFFF:FFFFFF
@A00721:190:HGJW7DSXY:4:2639:18132:25222 2:N:0:TCTGCAAG+GCTGCACT
CTCCAATTTTCCGGCAAGTATAAATGTCGGAATCCAAGGTTCTTCGACATAATCAACCCAGATTCCGA
CGAATCTTTTCACCCAAATATTCAAAGTGCGAGATCGGCGAATGACGTCGCCGATTATATCACAGAAG
GAGGATCTTATATTG
+
FFFFFFFFF:FFFFFFFFFFFFFFFFFFFFFFFFFFFFFFFFFFFFFFFFFFFF,FFFFFFFFFFFFF
FFFFFFF:FFFF::F:FFFFF:FFFFF:FFF:FFFFFFFFF:FFFFFFFFFFFF,FFFFFFFFF,F,F
F,FFFFF:FFFF:FF
@A00721:190:HGJW7DSXY:4:2676:1886:16595 2:N:0:TCTGCAAG+GCTGCACT
CTCCAATTTTCCGGCAAGTATAAATGTCGGAATCCAAGGTTCTTCGACATAATCAACCCAGATTCCGA
CGAATCTTTTCACCCAAATATTCAAAGTGCGAGATCGGCGAATGACGTCGCCGATTATATCACAAAAG
GAGGATCTTATATTG
+
FFFFFFFFFFFFFFFFFFFFFFF,FFFFFFFFFFFFFFFFFFFFFFFFFFFFFFFFFFFFFFFFFFFF
FFFFFFFFFFFFFFFFFFFFFFFFFFFFFFFFFFFFFFFFFFFFFFFFFFFFF:FFFFFFFFFF,FF
FFFFFFFFFFFFFFF
@A00721:190:HGJW7DSXY:4:1202:4010:19554 2:N:0:TCTGCAAG+GCTGCACT
CCTTCACTGTCTTCTCCAATTTTCCGGCAAGTATAAATGTCGGAATCCAAGGTTCTTCGACATAATCA
ACCCAGATTCCGACGAATCTTTTCACCCAAATATTCAAAGTGCGAGATCGGCGACTGACGTCGCCGAT
TATATCACAAAAGGA
+
FFFFFFFFFFFFFFFFFFFFFFFFFFFFFFFFFFFFFFFFFFFFFFFFFFFFFFFFFFFFFFFFFFFF
FFFFFFFFFFFFFFFFFFFFFFFFFFFFFFFFFFFFFFFFFFFFFFFFFFFFFFFFFFFF:FFFFFFF
FFFFFFFFFFFF:FF
@A00721:190:HGJW7DSXY:4:1274:32298:28338 2:N:0:TCTGCAAG+GCTGCACT
CCTTCACTGTCTTCTCCAATTTTCCGGCAAGTATAAATGTCGGAATCCAAGGTTCTTCGACATAATCA
ACCCAGATTCCGACGAATCTTTTCACCCAAATATTCAAAGTGCGAGATCGGCGACTGACGTCGCCGAG
TATATCACAAAAGGA
+
FFFFFFFFFFFFFFFFFFFFFFFFFFFFFFFFFFFF:FFFFFFFFFFFFFFFFFF:FFFFFFFFFFFF
FFFFFFFFFFFFFFFFFFFFFFFFFFFFFF:F:FFFFFFFFFFF:FFFFFFFFFFFFFFF:FFF:FF,
FFFFFFFFF,,FFF:
@A00721:190:HGJW7DSXY:4:1336:8513:9189 2:N:0:TCTGCAAG+GCTGCACT
CCTTCACTGTCTTCTCCAATTTTCCGGCAAGTATAAATGTCGGAATCCAAGGTTCTTCGACATAATCA
ACCCAGATTCCGACGAATCTTTTCACCCAAATATTCAAAGTGCGAGATCGGCGACTGACGTCGCCGAT
TATATCACAAAAGGA
+
FFFFFFFFFFFFFFFFFFFFFFFFFFFFFFFFFFFFFFFFFFFFFFFFFFFFFFFFFFFFFFFFFFFF
```

FFFFFFFFFFFFFFFFFFFFFFFFFFFFFFFFFFFFFFFFFFFFFFFFFFFFFFFFFFFFFFFFFFFF
,FFFFFFFFFFFFFF
@A00721:190:HGJW7DSXY:4:2314:24080:7968 2:N:0:TCTGCAAG+GCTGCACT
CCTTCACTGTCTTCTCCAATTTTCCGGCAAGTATAAATGTCGGAATCCAAGGTTCTTCGACATAATCA
ACCCAGATTCCGACGAATCTTTTCACCCAAATATTCAAAGTGCGAGATCGGCGACTGACGTCGCCGAT
TATATCACAAAAGGA
+
FFFFFFFFFFFFFFFFFFFFFFFFFFFFFFFFFFFFFFFFFFFFFFFFFFFFFFFFFFFFFFFFFFFF
FFFFFFFFFFFFFFFFFFFFFFFFFFFFFFFFFFFFFFFFFFFFFFFFFFFFFFFFFFFFFFFFFFFF
:FFFFFFFFFFFFFF
@A00721:190:HGJW7DSXY:4:2338:19298:25989 2:N:0:TCTGCAAG+GCTGCACT
CCTTCACTGTCTTCTCCAATTTTCCGGCAAGTATAAATGTCGGAATCCAAGGTTCTTCGACATAATCA
ACCCAGATTCCGACGAATCTTTTCACCCAAATATTCAAAGTGCGAGATCGGCGACTGACGTCGCCGAT
TATATCACAAAAGGA
+
FFFFFFFFFFFFFFFFFFFFFFFFFFFFFFFFFFFFFFFFFFFFFFFFFFFFFFFFFFFFFFFFFFFF
FFFFFFFFFFFFFFFFFFFFFFFFFFFFFFFFFFFFFFFFFFFFFFFFFFFFFFFFFFFFFFFFFFFF
FFFFFFFFFFFFF:F
@A00721:190:HGJW7DSXY:4:2338:19913:26428 2:N:0:TCTGCAAG+GCTGCACT
CCTTCACTGTCTTCTCCAATTTTCCGGCAAGTATAAATGTCGGAATCCAAGGTTCTTCGACATAATCA
ACCCAGATTCCGACGAATCTTTTCACCCAAATATTCAAAGTGCGAGATCGGCGACTGACGTCGCCGAT
TATATCACAAAAGGA
+
FFFFFFFFFFFFFFFFFFFFFFFFFFFFFFFFFFFFFFFFFFFFFFFFFFFFFFFFFFFFFFFFFFFF
FFFFFFFFFFFFFFFFFFFFFFFFFFFFFFFFFFFFFFFFFFFFFFFFFFFFFFFFFFFFFFFFFFF:
FFFFFFFFFFFFFFF
@A00721:190:HGJW7DSXY:4:2602:14036:17848 2:N:0:TCTGCAAG+GCTGCACT
CCTTCACTGTCTTCTCCAATTTTCCGGCAAGTATAAATGTCGGAATCCAAGGTTCTTCGACATAATCA
ACCCAGATTCCGACGAATCTTTTCACCCAAATATTCAAAGTGCGAGATCGGCGACTGACGTCGCCGAT
TATATCACAAAAGGA
+
FFFFFFFFFFFFFFFFFFFFFFFFFFFFFFFFFFFFFFFFFFFFFFFFFF:FF,FFFF,FFFFFFFFF
FFFFFF:FFFFFFFFFFFFFFFFFFFFFFF:FFFF:FFFFFFFFFFFFFFFF,FF:FF:F,,F::FF,
FFFFF,F:FFFF:FF
@A00721:190:HGJW7DSXY:4:2330:14371:1391 2:N:0:TCTGCAAG+GCTGCACT
CCTTCACTGTCTTCTCCAATTTTCCGGCAAGTATAAATGTCGGAATCCAAGGTTCTTCGACATAATCA
ACCCAGATTCCGACGAATCTTTTCACCCAAATATTCAAAGTGCGAGATCGGCGACTGACGTCGCCGAT
TATATCACAAAAGG
+
FFFFFFFFFFFFFFFFFFFFFFFFFFFFFFFFFFFFFFFFFFFFFFFFFFFFFFFF:FFFFFFFFFFF
F,FFF:FFFFFFFFFFFFFF:FFFFFFFFF,FFFFFFFFFFFFFFFF:FFFFFFFFF:FFFFFFFF:F
FFFFFF:FFFFFFF
@A00721:190:HGJW7DSXY:4:1153:25491:20588 2:N:0:TCTGCAAG+GCTGCACT
CACCTTCACTGTCTTCTCCAATTTTCCGGCAAGTATAAATGTCGGAATCCAAGGTTCTTCGACATAAT
CAACCCAGATTCCGACGAATCTTTTCACCCAAATATTCAAAGTGCGAGATCGGCGAATGACGTCGCCG
ATTATATCACAAAAG
+
FFFFFFFFFFFFFFFFFFFFFFFFFFFFFFFFFFFFFF,FFFFFFFFFFFFFFFFFFF:FFFFFFFFF
FFFFFFFFFFFFFFFFFFFFFFFFFFFF:FFFFFFFFFFFFFFFFFFFFFFFFFFFFFFFFFFFFFFF
:FFFF:::FFFFFFF
@A00721:190:HGJW7DSXY:4:1552:24144:27211 2:N:0:TCTGCAAG+GCTGCACT
CACCTTCACTGTCTTCTCCAATTTTCCGGCAAGTATAAATGTCGGAATCCAAGGTTCTTCGACATAAT
CAACCCAGATTCCGACGAATCTTTTCACCCAAATATTCAAAGTGCGAGATCGGCGAATGACGTCGCCG
ATTATATCACAAAAG

+
FFFFF:FFF:FFFFFFFFFFFFFFFFFFFFFFF,FFFF:,FFFFFFFFFFFFFF,FFF:FFFFFFFFF
F:FF:FFFFFFFFFFFFFFFFFFF::FFFFFF:FFFFF:FFF:FFFFF:FFFFFFFFFFFFFFFFFFF
FF:FFFFFFFFFFFF
@A00721:190:HGJW7DSXY:4:2105:3920:3991 2:N:0:TCTGCAAG+GCTGCACT
CACCTTCACTGTCTTCTCCAATTTTCCGGCAAGTATAAATGTCGGAATCCAAGGTTCTTCGACATAAT
CAACCCAGATTCCGACGAATCTTTTCACCCAAATATTCAAAGTGCGAGATCGGCGAATGACGTCGCCG
ATTATATCACAAAAG
+
FFFFFFFFFFFFFFFFFFFFFFFFFFFFFFFFFFF:FFFFFFFFFFFFFFFFFFFFFFFFFFFFFFFF
FFFFFFFFFFFFFFFFFFFFFFFFFFFFFFFF:FFFFFFFFFFFFFFFFFFFFFFFFFFFFFFFFFFF
F:FF:FFFFFF:FFF
@A00721:190:HGJW7DSXY:4:2231:23312:5478 2:N:0:TCTGCAAG+GCTGCACT
CACCTTCACTGTCTTCTCCAATTTTCCGGCAAGTATAAATGTCGGAATCCAAGGTTCTTCGACATAAT
CAACCCAGATTCCGACGAATCTTTTCACCCAAATATTCAAAGTGCGAGATCGGCGAATGACGTCGCCG
ATTATATCACAAAAG
+
FFFFFFFFFFFFFFFFFFFFFFFFFFFFFFFFFFFFFFFFFFFFFFFFFFFFFFFFFFFFFFFFFFFF
FFFFFFFFFFFFFFFFFFFFFF:FFFFFFFFFFFFFFFFFFFFFFFFFFFFFFFFFFFFFFFFFFFFF
:FFFFFFFFFFFF,F
@A00721:190:HGJW7DSXY:4:2643:29351:25739 2:N:0:TCTGCAAG+GCTGCACT
CACCTTCACTGTCTTCTCCAATTTTCCGGCAAGTATAAATGTCGGAATCCAAGGTTCTTCGACATAAT
CAACCCAGATTCCGACGAATCTTTTCACCCAAATATTCAAAGTGCGAGATCGGCGAATGACGTCGCCG
ATTATATCACAAAAG
+
FFFFFFFFFFFFFFFFFFFFFFFFFFFFFFFFFFFFFFFFFFFFFFFFFFFFFFFFFFFFFFFFFFFF
FFFFFFFFFFFFFFFFFFFFFFFFFFFFFF,F:F:FFFFFFFFFFFFFFFFFFFFFFFFFFFFFFFFF
FF:FFFFFFFFFFFF
@A00721:190:HGJW7DSXY:4:1350:11749:19711 2:N:0:TCTGCAAG+GCTGCACT
AATCACCTTCACTGTCTTCTCCAATTTTCCGGCAAGTATAAATGTCGGAATCCAAGGTTCTTCGACAT
AATCAACCCAGATTCCGACGAATCTTTTCACCCAAATATTCAAAGTGCGAGATCGGCGAATGACGTCG
CCGATTATATCACAA
+
FFFF:FFFFFFFFFFFFFFFFFFFFFFFFFFFF:FFFFFFFFFFFFFFFFFFFFFFFFF:FFFFFFFF
FFFFFFFFFFFFFFFFFFFFFFFFFFFFFFFFFFFFFFFFFFF:FF:FFFFFF:FFFFFFFFFF:FFF
FFFFFFFFFFFFFFF
@A00721:190:HGJW7DSXY:4:1366:28944:14356 2:N:0:TCTGCAAG+GCTGCACT
AATCACCTTCACTGTCTTCTCCAATTTTCCGGCAAGTATAAATGTCGGAATCCAAGGTTCTTCGACAT
AATCAACCCAGATTCCGACGAATCTTTTCACCCAAATATTCAAAGTGCGAGATCGGCGAATGACGTCG
CCGATTATATCACAA
+
FFFFFFFFFFFFFFFFFFFFFFFFFFFFFFFFFFFFFFFFF:FFFFFFFFFFFFFFFFFFFFFFFFFF
FFFF:FFFFFFFFFFFFFFFFFFFF:FFFFFF:FFFFFFFFFFFFFFFFFF:FFFFFF:FFFFFFFFF
FFF,:FFFFFFFFFF
@A00721:190:HGJW7DSXY:4:1501:1344:22138 2:N:0:TCTGCAAG+GCTGCACT
AATCACCTTCACTGTCTTCTCCAATTTTCCGGCAAGTATAAATGTCGGAATCCAAGGTTCTTCGACAT
AATCAACCCAGATTCCGACGAATCTTTTCACCCAAATATTCAAAGTGCGAGATCGGCGAATGACGTCG
CCGATTATATCACAA
+
FFFF:FFFFFFFFFFFF:FFFFF:FFFFF:F,FFFFFFFFF:F,FF:FFFFFFFFF:F,FFFFFFF,F
FFFF:FFFFFFFFFFFFFFFFF:F,:FFFFFFFFFFFFFFFFFFF:FF:FFFFF:F,:,,FFF:FFFF
F,FFFFFFFFF:FFF
@A00721:190:HGJW7DSXY:4:1513:14579:2942 2:N:0:TCTGCAAG+GCTGCACT
AATCACCTTCACTGTCTTCTCCAATTTTCCGGCAAGTATAAATGTCGGAATCCAAGGTTCTTCGACAT

AATCAACCCAGATTCCGACGAATCTTTTCACCCAAATATTCAAAGTGCGAGATCGGCGAATGACGTCG
CCGATTATATCACAA
+
F,FFFFFF:FFFFFFFFFFFFF:FFFFFFFFFFFFFFF:FFFFFFFFFFFFFFFFFFFFFFFFFFFFF
FFFFFFFFFFFFFFFFFFFFFFFF:FFFFFFFF:FFFFFFFFFFFFFFFFFFFFFFFFFFF:FFFF:
FFFFFFFFFFFFFFF
@A00721:190:HGJW7DSXY:4:1556:18701:15812 2:N:0:TCTGCAAG+GCTGCACT
AATCACCTTCACTGTCTTCTCCAATTTTCCGGCAAGTATAAATGTCGGAATCCAAGGTTCTTCGACAT
AATCAACCCAGATTCCGACGAATCTTTTCACCCAAATATTCAAAGTGCGAGATCGGCGAATGACGTCG
CCGATTATATCACAA
+
FFFFFFFFFFFFFFFFFFFFFFFFFFFFFFFFFFFFFFFFFFFFFFFFFFFFFFFFFFFFFFFFFFFF
FFFFFFFFFFFFFFFFFFFFFFFFFFFFFFFFFF:FFFFFFFFFFFFFFFFFFFFFFFFFFFFFFFFF
FFF,,FFFFFFFFFF
@A00721:190:HGJW7DSXY:4:2106:27968:14074 2:N:0:TCTGCAAG+GCTGCACT
AATCACCTTCACTGTCTTCTCCAATTTTCCGGCAAGTATAAATGTCGGAATCCAAGGTTCTTCGACAT
AATCAACCCAGATTCCGACGAATCTTTTCACCCAAATATTCAAAGTGCGAGATCGGCGAATGACGTCG
CCGATTATATCACAA
+
FFFFFFFFFFFFFFFFFFFFFFFFFFFFFFFFFFFFFFFFF:FFFFFFFFFFFFFFFFFFFFFFFFFF
FFFFFFFFFFFFFFFFFFFFFFFFFFFFFFFFFFFFFFFFFFFFFFFFFFFFFFFFFFFFFFFFFFFF
FFF:FFFFFFFFFFF
@A00721:190:HGJW7DSXY:4:2254:3495:2660 2:N:0:TCTGCAAG+GCTGCACT
AATCACCTTCACTGTCTTCTCCAATTTTCCGGCAAGTATAAATGTCGGAATCCAAGGTTCTTCGACAT
AATCAACCCAGATTCCGACGAATCTTTTCACCCAAATATTCAAAGTGCGAGATCGGCGAATGACGTCG
CCGATTATATCACAA
+
FFFFFFFFFFFFFFFFFFFFFFFFFFFFFFFFFFFFFFFFFFFFFFFFF,FFFFFFFFFFFFFFFFFF
FFFFFFFFFFFFFFFFFFFFFFFFFFFFFFFFFFFFF:FFFFFFFF:FFFFFFFFFFFFFFFFFFFFFF
FFFFF:FFFFFFFF:
@A00721:190:HGJW7DSXY:4:2303:3766:18349 2:N:0:TCTGCAAG+GCTGCACT
AATCACCTTCACTGTCTTCTCCAATTTTCCGGCAAGTATAAATGTCGGAATCCAAGGTTCTTCGACAT
AATCAACCCAGATTCCGACGAATCTTTTCACCCAAATATTCAAAGTGCGAGATCGGCGAATGACGTCG
CCGATTATATCACAA
+
FFFFFFFFFFFFFFFFFFFFFFFFFFFFFFFFFFFFFFFFFFF,FFFFFFFFFFFFFFFFFFFFFFFFF
FFFFFFFFFFFFFFFFFFFFFFFFFFFF:FFF:FFFFFFFFFFFFFFFFFFFFFFFFFFFFFFFFFFF
FFFFFFFFFF:FFFF
@A00721:190:HGJW7DSXY:4:2308:24288:21449 2:N:0:TCTGCAAG+GCTGCACT
AATCACCTTCACTGTCTTCTCCAATTTTCCGGCAAGTATAAATGTCGGAATCCAAGGTTCTTCGACAT
AATCAACCCAGATTCCGACGAATCTTTTCACCCAAATATTCAAAGTGCGAGATCGGCGAATGACGTCG
CCGATTATATCACAA
+
FFFFFFFFFFFFFFFFFFFFFFFFFFFFFFFFFF:FFFFFFFFFFFFFFFFFFFFFFFFFFFFFFFFF
FFFFFFFFFFFFFFFFFFFFFFFFFFFFFFFFFF:FFFFFFFFFFFFFFFFFFFFFFFFF:FFFFFFFF
FFFFFFFF:FFFFFF
@A00721:190:HGJW7DSXY:4:2418:3314:16971 2:N:0:TCTGCAAG+GCTGCACT
AATCACCTTCACTGTCTTCTCCAATTTTCCGGCAAGTATAAATGTCGGAATCCAAGGTTCTTCGACAT
AATCAACCCAGATTCCGACGAATCTTTTCACCCAAATATTCAAAGTGCGAGATCGGCGAATGACGTCG
CCGATTATATCACAA
+
FFFFFFFFFFFFFFFFFFFFFFFFFFFFFFFFFFFFFFFFFFFFFFFFFFFFFFFFFFFFFFFFFFFF
FFFFFFFFFFFFFFFFFFFFFFFFFF:FFFFFFFFFFFFFFFFFFFFFFFFFFFFFF:FFFFFFFFFFFFF
FFFF:FFF:FFFFFF

```
@A00721:190:HGJW7DSXY:4:2549:23674:26490 2:N:0:TCTGCAAG+GCTGCACT
AATCACCTTCACTGTCTTCTCCAATTTTCCGGCAAGTATAAATGTCGGAATCCAAGGTTCTTCGACAT
AATCAACCCAGATTCCCACGAATCTTTTCACCCAAATATTCAAAGTGCGAGATCGGCGAATGACGTCG
CCGATTATATCACAA
+
FFFFFFFFFFFFFFFFFFFFFFFFFFFFFFFFFFFFFFFFFFFFFFFFFFFFFFFFFFFFFFFFFFFFFF
FFFFFF::FFFFFF,,FFF:FFFFF::FFFFFFFFFFFFF:FFFFF,FFF:FFF,FF:FFFF::FF:,FF
FF,:FFFFF,FF:FF
@A00721:190:HGJW7DSXY:4:1120:14136:29105 2:N:0:TCTGCAAG+GCTGCACT
TTCAGACGGTGATAATCACCTTCACTGTCTTCTCCAATTTTCCGGCAAGTATAAATGTCGGAATCCAA
GGTTCTTCGACATAATCAACCCAGATTCCGACGAATCTTTTCACCCAAATATTCAAAGTGCGAGATCG
GCGAATGACGTCGCC
+
FFFFFFFFFFFFFFFFFFFFFFFFFFFFFFFFFFFFFFFFFFFFFFFFF:FFFFFFFFFFFFFFFFFFFF
FFFFFFFFFFFFFFFFFFFFFFFFFFFFFFFFFFFFFFFFFFFFFFFFFFFFFFFFFF,FFFFFFFFFFF
FFFFFFFFFFFFFFF
@A00721:190:HGJW7DSXY:4:1523:19217:19022 2:N:0:TCTGCAAG+GCTGCACT
TTCAGACGGTGATAATCACCTTCACTGTCTTCTCCAATTTTCCGGCAAGTATAAATGTCGGAATCCAA
GGTTCTTCGACATAATCAACCCAGATTCCGACGAATCTTTTCACCCAAATATTCAAAGTGCGAGATCG
GCGAATGACGTCGCC
+
FFFFFFFFFFFFFFFFFFFFFFFFFFFFFFFFFFFFFFFFFFFFFFFF::FFFFFFFFFFFFFFFFFFFF
FFFFFFFFFFFFFFFFFFFFFFFFFFFFFFFFFFFFFFFFFFFFFFFFFFFFFFFFFFFFFFFFFFFFFF
FFFFFFFFFFFFFFF
@A00721:190:HGJW7DSXY:4:1632:6605:28213 2:N:0:TCTGCAAG+GCTGCACT
TTCAGACGGTGATAATCACCTTCACTGTCTTCTCCAATTTTCCGGCAAGTATAAATGTCGGAATCCAA
GGTTCTTCGACATAATCAACCCAGATTCCGACGAATCTTTTCACCCAAATATTCAAAGTGCGAGATCG
GCGAATGACGTCGCC
+
FFFFFFFFFFFFFFFFFFFFFFFFFFFFFFFFF,FFFFFFFFFFFFFFFFFFFFFFFFFFFFFF:FFF,F
FFFFFFFFFFFFFFFFFFFFFFFFFFFFFFFFFFFFFFFFFFFFFFFFFFFFFFFFFFFFFFFFFFFFFF
FFFFFFFFFFFFFFF
@A00721:190:HGJW7DSXY:4:2306:32380:30953 2:N:0:TCTGCAAG+GCTGCACT
TTCAGACGGTGATAATCACCTTCACTGTCTTCTCCAATTTTCCGGCAAGTATAAATGTCGGAATCCAA
GGTTCTTCGACATAATCAACCCAGATTCCGACGAATCTTTTCACCCAAATATTCAAAGTGCGAGATCG
GCGAATGACGTCGCC
+
FFFFFFFFFFFFFFFFFFFFFFFFFFFFFFFFFFFFFFFFFFFFFFFFFFFFFFFF:FFFFFFFFFFFFF
FFFFFFFF:FFFFFFFFFFFFFFFFFFFFFFFFFFFFFFFFFFF:FF:FFFFFFFFFFF:FFFFFF:FFF
FFFFFFFFFFFFFFF
@A00721:190:HGJW7DSXY:4:2310:29631:9878 2:N:0:TCTGCAAG+GCTGCACT
TTCAGACGGTGATAATCACCTTCACTGTCTTCTCCAATTTTCCGGCAAGTATAAATGTCGGAATCCAA
GGTTCTTCGACATAATCAACCCAGATTCCGACGAATCTTTTCACCCAAATATTCAAAGTGCGAGATCG
GCGAATGACGTCGCC
+
FFFFFFFFFFFFFFFFFFFFFFFFFFFFFFFFFFFFFFFFFFFFFFFFF:FFFF,FFFFFFFFFFFFFFF
FFFFFFFFFFFFFFFFFFFFFFFFFFFFFFFFFFFFFFFFFFFFFFFFFF:F:FFFFFFFFF,FF:FFFF
F:FFFFFFFFFFFFF
@A00721:190:HGJW7DSXY:4:2642:4562:31626 2:N:0:TCTGCAAG+GCTGCACT
TTCAGACGGTGATAATCACCTTCACTGTCTTCTCCAATTTTCCGGCAAGTATAAATGTCGGAATCCAA
GGTTCTTCGACATAATCAACCCAGATTCCGACGAATCTTTTCACCCAAATATTCAAAGTGCGAGATCG
GCGAATGACGTCGCC
+
FFFFFFFFFFFFFFFFFFFFFFFFFFFFFFFFFFFFFFFFFFFFFFFFFFFFFFFFFFFFFFFFFFFFFF
```

```
FFFFFFFFFFFFFFFFFFFFFFFFFFFFFFFFFFFFFFFFFFFF:FFFFFFFFFFFFFFFFFFFFFFFFF
FFFFFFFFFFFFFFF
@A00721:190:HGJW7DSXY:4:1119:26196:23813 2:N:0:TCTGCAAG+GCTGCACT
ATTCAGACGGTGATAATCACCTTCACTGTCTTCTCCAATTTTCCGGCAAGTATAAATGTCGGAATCCA
AGGTTCTTCGACATAATCAACCCAGATTCCGACGAATCTTTTCACCCAAATATTCAAAGTGCGAGATC
GGCGAATGACGTCGC
+
FFFFFFFFFFFFFFFFFFFFFFFFFFFFFFFFFFFFFF:FFFFFFFFFFFFFFFFFFFFFFFFFFFFFFF
FFFFFFFFFFFFFFFFFFFFFFFFFFFFFFFFFFFFFFFFFFFF:FF:FFFF:FFFFFFFFFF,FFFFFF
FFFFFFFFFF:FFFF
@A00721:190:HGJW7DSXY:4:1426:7518:20369 2:N:0:TCTGCAAG+GCTGCACT
ATTCAGACGGTGATAATCACCTTCACTGTCTTCTCCAATTTTCCGGCAAGTATAAATGTCGGAATCCA
AGGTTCTTCGACATAATCAACCCAGATTCCGACGAATCTTTTCACCCAAATATTCAAAGTGCGAGATC
GGCGAATGACGTCGC
+
FFFFFFFFFFFFFFFFFFFFFFFFFFFFFFFFFFFFFFFFFFFFFFFFF,FFFFFFFFFFFFFFFFFFFF
FFFFFFFFFFFFFFFFFFFFFFFFFFFFFFFFFFFFFFFFFFFFFFFFFFFFFFFFFFFFFFFFFFFFFF
FFFFFFFFFFFFFFF
@A00721:190:HGJW7DSXY:4:1426:23068:32111 2:N:0:TCTGCAAG+GCTGCACT
ATTCAGACGGTGATAATCACCTTCACTGTCTTCTCCAATTTTCCGGCAAGTATAAATGTCGGAATCCA
AGGTTCTTCGACATAATCAACCCAGATTCCGACGAATCTTTTCACCCAAATATTCAAAGTGCGAGATC
GGCGAATGACGTCGC
+
FFFFFFFFFFFFFFFFFFFFFFFFFFFFFFFFFFFFFFFFFFFFFFFFFFFFFFFFFFFFFFFFFFFFFF
FFFFFFFFFFFFFFFFFFFFFFFFFFFFFFFFFFFFFFFFFFFFFFFFFFF:F,FFFFFFFFFF:FFFFF
F:FFFFFF:FFFFFF
@A00721:190:HGJW7DSXY:4:1572:27688:1658 2:N:0:TCTGCAAG+GCTGCACT
ATTCAGACGGTGATAATCACCTTCACTGTCTTCTCCAATTTTCCGGCAAGTATAAATGTCGGAATCCA
AGGTTCTTCGACATAATCAACCCAGATTCCGACGAATCTTTTCACCCAAATATTCAAAGTGCGAGATC
GGCGAATGACGTCGC
+
FFFFFFFFFF:FFFFFFFFFFFFFFFFFFFFFFFFFFFFFFFF:FFFFFF:FFFFFF,FFFFFFF:FFFF
FFFFFFFFFFFFFFFFFFFFFFFFFFFFFFFFFFFFFFFFFFFFFFFFFFF,FFFFFFF,FFFFFFFFFF
FFFFFF:FFFFFFFF
@A00721:190:HGJW7DSXY:4:1624:24053:8578 2:N:0:TCTGCAAG+GCTGCACT
ATTCAGACGGTGATAATCACCTTCACTGTCTTCTCCAATTTTCCGGCAAGTATAAATGTCGGAATCCA
AGGTTCTTCGACATAATCAACCCAGATTCCGACGAATCTTTTCACCCAAATATTCAAAGTGCGAGATC
GGCGAATGACGTCGC
+
FFFFFFFFFFFFFFFFFFFFFFFFFFFFFFFFFFFFFFFFFFFFFF:FFFFFFFFFFFFFFFFFFFFFFF
F:F:FFFFFFFFFFFFFFFFFFFFFFFFFFFFFFFFFFFFFFFFFFFFFFFFFFFFFFFFFFFF:FFFFF
FFF:FFF:FFFFFFF
@A00721:190:HGJW7DSXY:4:2111:25834:9157 2:N:0:TCTGCAAG+GCTGCACT
ATTCAGACGGTGATAATCACCTTCACTGTCTTCTCCAATTTTCCGGCAAGTATAAATGTCGGAATCCA
AGGTTCTTCGACATAATCAACCCAGATTCCGACGAATCTTTTCACCCAAATATTCAAAGTGCGAGATC
GGCGAATGACGTCGC
+
FFFFFFFFFFFFFFFFFFFFFFFFFFFFFFFFFFF:FFFFFFFFFFFF:FFFFFFFFFFFFFFFFFFFFF
FFFFFFFFFF:FFFFFFFFFFFFFFFFFF,FFFFFFFFFFFFFFFFF:FFFFFFFFFFFFFFFFF:F,F:
F:FF:FFF:FFFF,F
@A00721:190:HGJW7DSXY:4:2139:12915:1971 2:N:0:TCTGCAAG+GCTGCACT
ATTCAGACGGTGATAATCACCTTCACTGTCTTCTCCAATTTTCCGGCAAGTATAAATGTCGGAATCCA
AGGTTCTTCGACATAATCAACCCAGATTCCGACGAATCTTTTCACCCAAATATTCAAAGTGCGAGATC
GGCGAATGACGTCGC
```

+
FFFFFFFFFFFFFFFFFFFFFFFF:FFFFFFFFFFFFFFFFFFFFFFF:FFFFFFFFFFFFFFFFFFF
FFFFFFFFFFFFFFFFFFFFFFFFFFFFFF:FFFFFFFFFFFFFFFFFFFFFFFFFFFFFFF:FFFFF
FFFFFFFFFFFFFFF
@A00721:190:HGJW7DSXY:4:2202:8323:26396 2:N:0:TCTGCAAG+GCTGCACT
ATTCAGACGGTGATAATCACCTTCACTGTCTTCTCCAATTTTCCGGCAAGTATAAATGTCGGAATCCA
AGGTTCTTCGACATAATCAACCCAGATTCCGACGAATCTTTTCACCCAAATATTCAAAGTGCGAGATC
GGCGAATGACGTCGC
+
FFFFFFFFFFFFFFFFFFFFFFFFFFFFFFFFFFFFFFFFFFFFFFFFFFFF,FFFFFFFFFFFFFFF
FFFFFFFFFFFFFFFFFFFFFFFFFFFFFFFFFFFFFFFFFFFFF:FFFFFFFFFFFFFFFFFFFFFF
FFFFFF,FFFFFFFF
@A00721:190:HGJW7DSXY:4:2510:10321:30765 2:N:0:TCTGCAAG+GCTGCACT
ATTCAGACGGTGATAATCACCTTCACTGTCTTCTCCAATTTTCCGGCAAGTATAAATGTCGGAATCCA
AGGTTCTTCGACATAATCAACCCAGATTCCGACGAATCTTTTCACCCAAATATTCAAAGTGCGAGATC
GGCGAATGACGTCGC
+
FFFF:FFFFFFFFFFFFFFFFFFFFFFFFFFFFFFFFFFFFFFFFFFFFFFFFFFFFFFFFFFFFFFF
FFFFFFFFFFFFFFFFFFFFFFFFFFF:FFFFFFF,FFFFFFFFF:FFFFFFFFFFFFFFFFFFFFFF
,FFFFFFFFF::FFF
@A00721:190:HGJW7DSXY:4:2510:10095:31532 2:N:0:TCTGCAAG+GCTGCACT
ATTCAGACGGTGATAATCACCTTCACTGTCTTCTCCAATTTTCCGGCAAGTATAAATGTCGGAATCCA
AGGTTCTTCGACATAATCAACCCAGATTCCGACGAATCTTTTCACCCAAATATTCAAAGTGCGAGATC
GGCGAATGACGTCGC
+
FFFFFFFFFFFFFFFFFFFFFFFFFFFFFFFFFFFFFFFFFFFFFFFFFFFFFFFFFFFFFFFFFFFF
FFFFFFFFFFFFFFFFFFFFFFFFFFFFFFFFFFFFFFFFFFFFF:FFFFFFFFFFFFFFFFFFFFFF
FFFFF:FFFFFFFFF
@A00721:190:HGJW7DSXY:4:1404:3125:12884 2:N:0:TCTGCAAG+GCTGCACT
CAACATTCAGACGGTGATAATCACCTTCACTGTCTTCTCCAATTTTCCGGCAAGTATAAATGTCGGAA
TCCAAGGTTCTTCGACATAATCAACCCAGATTCCGACGAATCTTTTCACCCAAATATTCAAAGTGCGA
GATCGGCGAATGACG
+
FFFFFF:FFFFFF:F:F,FFFFF:F:FFFFFFFFFFFFFFFFFFFF,FFFFFFFFFFFFFFFFFFFFF
FFFFF,FFFFFFF,FFF:FFFFFFF:FFFFF:FF:FFFFF::,FFFFFFF:FFFFFF:FFFF,FFF:F
,FFFFFFFFFF:FFF
@A00721:190:HGJW7DSXY:4:1440:30852:13683 2:N:0:TCTGCAAG+GCTGCACT
CAACATTCAGACGGTGATAATCACCTTCACTGTCTTCTCCAATTTTCCGGCAAGTATAAATGTCGGAA
TCCAAGGTTCTTCGACATAATCAACCCAGATTCCGACGAATCTTTTCACCCAAATATTCAAAGTGCGA
GATCGGCGAATGACG
+
FFFFFFFFFFFFFFFFFFFFFFFFFFFFF::FF:FFFFFFFFFFFFFFFFFF:FFFFFFFFFFFFF:F
FFFFFFFFFFFFFFFFFFFFFFFFFFFFFFFFFFFFFFFFFFFFF:FFFFFF,FFFFFFF:FFFFFFF
FFFFFFFFF:FFFF:
@A00721:190:HGJW7DSXY:4:1601:16477:11177 2:N:0:TCTGCAAG+GCTGCACT
CAACATTCAGACGGTGATAATCACCTTCACTGTCTTCTCCAATTTTCCGGCAAGTATAAATGTCGGAA
TCCAAGGTTCTTCGACATAATCAACCCAGATTCCGACGAATCTTTTCACCCAAATATTCAAAGTGCGA
GATCGGCGAATGACG
+
:FFFFFFFFFFFFF:FFFFFFFFFFFF:FFFFFF:FFFFF:FFFFFFFFFFF,FFFFF:FFFFFFFFF
FFFFFF:FFFFFFFFFFFFFF:FFF:FFFFFFFFFFFFF:FFFFFFFFFFFFFFFFFFFFFFFFF:FF
FFFFFFFFFFFFFFF
@A00721:190:HGJW7DSXY:4:1603:7835:15468 2:N:0:TCTGCAAG+GCTGCACT
CAACATTCAGACGGTGATAATCACCTTCACTGTCTTCTCCAATTTTCCGGCAAGTATAAATGTCGGAA

```
TCCAAGGTTCTTCGACATAATCAACCCAGATTCCGACGAATCTTTTCACCCAAATATTCAAAGTGCGA
GATCGGCGAATGACG
+
FF:FFFFFFFFFFFFFFFFFFFFFFFFFFFFFFFFFFFFFFFFFFFFFFFFFFFFF:F,FFFFFFFFF
,FFFFFF:FF:FFFFFFFFFFFFFFFFFFFFFFFFFFFFFFFFFFFFFFFFF:F:FFFFFFFFFFFFF
FFFFFFFFFFFFFFF
@A00721:190:HGJW7DSXY:4:1617:11903:19633 2:N:0:TCTGCAAG+GCTGCACT
CAACATTCAGACGGTGATAATCACCTTCACTGTCTTCTCCAATTTTCCGGCAAGTATAAATGTCGGAA
TCCAAGGTTCTTCGACATAATCAACCCAGATTCCGACGAATCTTTTCACCCAAATATTCAAAGTGCGA
GATCGGCGAATGACG
+
FFFFFFFFFFFFFFFFFFFFFFFFFFFFF:FFFFFFFFFFFFFFFFFFF:FFFFFFFFFF:FF:FFFF
FFFFFFFFFFFFFFFFFFFFFFFFFF:FFFFFFFFFFFFFFFFFFFFFF::FFFFFFF,FFFFFFF:F
FFFFF:FFFFFFFF:
@A00721:190:HGJW7DSXY:4:1634:32669:15765 2:N:0:TCTGCAAG+GCTGCACT
CAACATTCAGACGGTGATAATCACCTTCACTGTCTTCTCCAATTTTCCGGCAAGTATAAATGTCGGAA
TCCAAGGTTCTTCGACATAATCAACCCAGATTCCGACGAATCTTTTCACCCAAATATTCAAAGTGCGA
GATCGGCGAATGACG
+
FFF:FFFFFFFFFFFFFFFFFFFFFFFFFFFFFFFFFFFFFFFFFFFFFFFF:FFFFFFFFFFFFFFF
FFFFFFFFFFFFFFFFFFFFFFFFFFFFFFFFFFFFFFFFFFFFFFFFF:FFFFFFFFFFFF::FFFF
FFFFFFFFFFFFFFF
@A00721:190:HGJW7DSXY:4:2151:17390:7373 2:N:0:TCTGCAAG+GCTGCACT
CAACATTCAGACGGTGATAATCACCTTCACTGTCTTCTCCAATTTTCCGGCAAGTATAAATGTCGGAA
TCCAAGGTTCTTCGACATAATCAACCCAGATTCCGACGAATCTTTTCACCCAAATATTCAAAGTGCGA
GATCGGCGAATGACG
+
FFFFFF:F:FFFFFF:FFFFFFFFFFFFFFF:FF:FFFFFFF:,FFF:,FFFFF:FFFF:FFF::FFF
FFFFF::FFF,:FFF:,FFFFFFFFF,:::,,:FFFFFF,:FFFFF:FF,F,FF:F,F:FFFFF,:,F
:F:FFFFFFF,FFFF
@A00721:190:HGJW7DSXY:4:2357:19831:33959 2:N:0:TCTGCAAG+GCTGCACT
CAACATTCAGACGGTGATAATCACCTTCACTGTCTTCTCCAATTTTCCGGCAAGTATAAATGTCGGAA
TCCAAGGTTCTTCGACATAATCAACCCAGATTCCGACGAATCTTTTCACCCAAATATTCAAAGTGCGA
GATCGGCGAATGACG
+
FFFFFFFFFF:FFFFFFFFFFFFFFFFFFFFFFFFFFFFFFFFFFFFFFFFFFFFFFFF,FFFFFFFF
FFFFFFFFFFFFF:FFFFFFFFFFFFFFFFFFFFF:FFFFFFFFFFFFF,FFFFFFFFFFFF:FFFFF
FFFFFFFFFFFF:FF
@A00721:190:HGJW7DSXY:4:1540:3595:31798 2:N:0:TCTGCAAG+GCTGCACT
CAACATTCAGACGGTGATAATCACCTTCACTGTCTTCTCCAATTTTCCGGCAAGTATAAATGTCGGAA
TCCAAGGTTCTTCGACATAATCAACCCAGATTCCGACGAATCTTTTCACACAAATATTCAAAGTGCGA
GATCGGCGAATGA
+
:FFFFF:F:FFF::FF:FFF:FF,,FF::FFF,FFF,F:FFF:FF:F:FFFFF,FF::FFFFFFF:F:
FFFFFF,FFFFF::F::,FFFFFF,FFFFFFFFFFFFFF,,:FFF,:FF,FFFF,F,,FFFF::,,FF
FF,:,F:FF:F,F
@A00721:190:HGJW7DSXY:4:1112:13774:24095 2:N:0:TCTGCAAG+GCTGCACT
GAACAACATTCAGACGGTGATAATCACCTTCACTGTCTTCTCCAATTTTCCGGCAAGTATAAATGTCG
GAATCCAAGGTTCTTCGACATAATCAACCCAGATTCCGACGAATCTTTTCACCCAAATATTCAAAGTG
CGAGATCGGCGAATG
+
FFF:FFFFFFFFFFFFFFFFFFFFFFFFFFFFFFFFFFFFFFFFFFFFFFFFFFFFFFFFFFFFFFFF
FFFFFFFFFFFFFFFFFFFFFFFFFFFFFFFFF:FFFFFFFFFFFFFFFFFFFFF:FFFFF,FF:FF:
FFFFFFFFFFFFFFF
```

```
@A00721:190:HGJW7DSXY:4:1217:4797:17033 2:N:0:TCTGCAAG+GCTGCACT
GAACAACATTCAGACGGTGATAATCACCTTCACTGTCTTCTCCAATTTTCCGGCAAGTATAAATGTCG
GAATCCAAGGTTCTTCGACATAATCAACCCAGATTCCGACGAATCTTTTCACCCAAATATTCAAAGTG
CGAGATCGGCGAATG
+
FFFFFFFFFFFFFFFFFFFFFFFFFFFFFFFFFFFFFFFFFFFFFFFFFFFFFFFF:FFFFFFFFFFF
FF,FFFFFFFFFFFFFFFFFFFFFFFFFFFFFF:FFFFFFFFFFFFFFFFFFFFFFFFFFFFFFFFFF
F:FFFFF:FFFFFFF
@A00721:190:HGJW7DSXY:4:1231:27489:30060 2:N:0:TCTGCAAG+GCTGCACT
GAACAACATTCAGACGGTGATAATCACCTTCACTGTCTTCTCCAATTTTCCGGCAAGTATAAATGTCG
GAATCCAAGGTTCTTCGACATAATCAACCCAGATTCCGACGAATCTTTTCACCCAAATATTCAAAGTG
CGAGATCGGCGAATG
+
FFFFFFFF:FFFFFFFFFFFFFFFFFFFFFFFFFFFFFFFFFFFFFFFFFFFFFF,FFFFFFFFFFFF
FFFFFFFFFFFFFFFFFFFFFFFFFFFFFF:FFFFFFFFFFFFFFFFFFFFFFFFFFFFFFFFFFFFF
FFFFFFFFFFFF:FF
@A00721:190:HGJW7DSXY:4:1231:26051:32268 2:N:0:TCTGCAAG+GCTGCACT
GAACAACATTCAGACGGTGATAATCACCTTCACTGTCTTCTCCAATTTTCCGGCAAGTATAAATGTCG
GAATCCAAGGTTCTTCGACATAATCAACCCAGATTCCGACGAATCTTTTCACCCAAATATTCAAAGTG
CGAGATCGGCGAATG
+
FFFFFFFFFFFFFFFFFFFFFFFFFFFFFFFFF:FFFFFFFFFFFFFFFFFFFFFFFFFFFFFFFFFF
FFFFFFFFFFFFFFFFFFFFFFFFFFFFFFFFFFFFFFFFFFFFFFFFFFFFFFFFFFFFFFFFFFFF
FFFFFFFFFFFFFFF
@A00721:190:HGJW7DSXY:4:1252:10257:21574 2:N:0:TCTGCAAG+GCTGCACT
GAACAACATTCAGACGGTGATAATCACCTTCACTGTCTTCTCCAATTTTCCGGCAAGTATAAATGTCG
GAATCCAAGGTTCTTCGACATAATCAACCCAGATTCCGACGAATCTTTTCACCCAAATATTCAAAGTG
CGAGATCGGCGAATG
+
FFFFFFFF:FFFFFFFFFFFFFFFFFFFFFFFFFFFFFFFFFFFFFFFFFFFFFFFFFFFFFFFFFFF
FFFFFFFFFFFFFFFFFFFFFFFFFFFFFFFFFFFFFFFFFFFFFFFFFFFFFFFF:FFFFFF:FFFF
FFFFFFFFFFFFF:F
@A00721:190:HGJW7DSXY:4:1261:2175:14528 2:N:0:TCTGCAAG+GCTGCACT
GAACAACATTCAGACGGTGATAATCACCTTCACTGTCTTCTCCAATTTTCCGGCAAGTATAAATGTCG
GAATCCAAGGTTCTTCGACATAATCAACCCAGATTCCGACGAATCTTTTCACCCAAATATTCAAAGTG
CGAGATCGGCGAATG
+
FF:FFFFFFFFFFFFFFFFFFFFFFFFFFFFFFFFFFFFFFFFFFFFFFFFFFFFFFFFFFFFFFFFF
FF:FFFFFFFFFFFFFFFFFFFFFFFFFFFFFFFFFFFFFFF:FFFFFFFFFFFFFFFFFFFFFFFFF
FFFFFFFFFFFFFFF
@A00721:190:HGJW7DSXY:4:1278:22236:13761 2:N:0:TCTGCAAG+GCTGCACT
GAACAACATTCAGACGGTGATAATCACCTTCACTGTCTTCTCCAATTTTCCGGCAAGTATAAATGTCG
GAATCCAAGGTTCTTCGACATAATCAACCCAGATTCCGACGAATCTTTTCACCCAAATATTCAAAGTG
CGAGATCGGCGAATG
+
FFFFFFFFFFFFFFFFFFFFFFFFFFFFFFFFFFFFFFFFFFFFFFFFFFFFFFFFFFFFFFFFFFFF
FFFFFFFFFFFFFFFFFFFFFFFFFFFFFFFFFFFFFF,FFFFFFFFFFFFFFFFFFFFFFFFFFFFF
FFFFFFF,FFFFFFF
@A00721:190:HGJW7DSXY:4:1348:25663:24831 2:N:0:TCTGCAAG+GCTGCACT
GAACAACATTCAGACGGTGATAATCACCTTCACTGTCTTCTCCAATTTTCCGGCAAGTATAAATGTCG
GAATCCAAGGTTCTTCGACATAATCAACCCAGATTCCGACGAATCTTTTCACCCAAATATTCAAAGTG
CGAGATCGGCGAATG
+
FF:FFFFFFFFFFFFFFFFFFFFFFFFFFFFFFFFFFFFFFFFFFFFFFFFFFFFFFFFFFFFFFFFF
```

```
FF,FFFFFFFFFFFFFFFFFFF,FFFFFFFFFFFFFFFFFFFFFFFFFFFFFFFFFFFFFFFFF:FFF
FFFFFFFFFFFFFFF
@A00721:190:HGJW7DSXY:4:1401:29559:26913 2:N:0:TCTGCAAG+GCTGCACT
GAACAACATTCAGACGGTGATAATCACCTTCACTGTCTTCTCCAATTTTCCGGCAAGTATAAATGTCG
GAATCCAAGGTTCTTCGACATAATCAACCCAGATTCCGACGAATCTTTTCACCCAAATATTCAAAGTG
CGAGATCGGCGAATG
+
FFFFFFFFFFFFFFFFFFFFFFFFFFFFFFFFFFFFFFFFFFFFFFFFFFFFFF:FFFFFFF:FFFFF
FFFFFFFFF:FFFFFFFFFFFFFFFFFFFFFFFFFFFFFFFFFFF:FFFF:FFFFFFFFFFFFFFFFF
FFFFFFFFFFF:FF:
@A00721:190:HGJW7DSXY:4:1410:27272:35947 2:N:0:TCTGCAAG+GCTGCACT
GAACAACATTCAGACGGTGATAATCACCTTCACTGTCTTCTCCAATTTTCCGGCAAGTATAAATGTCG
GAATCCAAGGTTCTTCGACATAATCAACCCAGATTCCGACGAATCTTTTCACCCAAATATTCAAAGTG
CGAGATCGGCGAATG
+
FFFFFFFFFFFFFFFFFFFFFFFFFFFFFFFFFFFFFFFFFFFFFFFFFFFFFFFFFFFFFFFFFFFF
FFFFFFFFFFFFFFFFFFFFFFFFFFFFFFFFFFFFFFFFFFFFFFFFFFFFFFFFFFFFFFFFFFFF
FFFFFFFFFFFFFFF
@A00721:190:HGJW7DSXY:4:1430:11595:28870 2:N:0:TCTGCAAG+GCTGCACT
GAACAACATTCAGACGGTGATAATCACCTTCACTGTCTTCTCCAATTTTCCGGCAAGTATAAATGTCG
GAATCCAAGGTTCTTCGACATAATCAACCCAGATTCCGACGAATCTTTTCACCCAAATATTCAAAGTG
CGAGATCGGCGAATG
+
FFFFFFFFFFFFFFFFFFFFFFFFFFFFFFFFFFFFFFFFFFFFFFFFFFFFFFFFFFFFFF:FFFFF
FFFFFFFFFFFFFFFFFFFFFFFFFFFFFFFFFFFFFFFFFFFFFFFFFFFFFFFFFFFFFFFFFFFF
FFFFFFFFFFFFFFF
@A00721:190:HGJW7DSXY:4:1541:2908:25003 2:N:0:TCTGCAAG+GCTGCACT
GAACAACATTCAGACGGTGATAATCACCTTCACTGTCTTCTCCAATTTTCCGGCAAGTATAAATGTCG
GAATCCAAGGTTCTTCGACATAATCAACCCAGATTCCGACGAATCTTTTCACCCAAATATTCAAAGTG
CGAGATCGGCGAATG
+
FFFFFFFFFFFFFFFFFFFFFFFFFFFFFFFFFFFFFFFFFFFFFFFFFFFFFF:FFFFFFFFFFFFF
FF:FFFFFFFFFFFFFFFFFFFFFFFFFFFFFFFFFFFFFFF:FFFFFFFFFFFFFFFFFFFFFFFFF
FF:FFFFFFFFF,FF
@A00721:190:HGJW7DSXY:4:1573:28239:28072 2:N:0:TCTGCAAG+GCTGCACT
GAACAACATTCAGACGGTGATAATCACCTTCACTGTCTTCTCCAATTTTCCGGCAAGTATAAATGTCG
GAATCCAAGGTTCTTCGACATAATCAACCCAGATTCCGACGAATCTTTTCACCCAAATATTCAAAGTG
CGAGATCGGCGAATG
+
FFFFFFFFFFFFFFFFFFFFFFFFFFFFFFFFFFFFFFFFFFFFFFFFFFFFFFFFFFFFFFFFFFFF
FFFFFFFFFFFFFFFFFFFFFFFFFFFFFFFFFFFFFFFFFFFFFFFFFFFFFFFFF:FFFFFFFFFF
FFFFFFFFFFFFFFF
@A00721:190:HGJW7DSXY:4:1637:6831:5494 2:N:0:TCTGCAAG+GCTGCACT
GAACAACATTCAGACGGTGATAATCACCTTCACTGTCTTCTCCAATTTTCCGGCAAGTATAAATGTCG
GAATCCAAGGTTCTTCGACATAATCAACCCAGATTCCGACGAATCTTTTCACCCAAATATTCAAAGTG
CGAGATCGGCGAATG
+
::FFFFFFFFFFFFFFFFFFFFFFFFFFFFFFFFFFFFFFFFFFFFFFFFFFFFFFFFFFFFFFFFFF
FFFFFFFFFFFFFFFFFFFFFFFFFFFFFFFFFFFFFFFFFFFFFFFFFFFFFFFFFFFFFFFFFF:F
FFFFFFFFFFFFFFF
@A00721:190:HGJW7DSXY:4:1672:32750:17221 2:N:0:TCTGCAAG+GCTGCACT
GAACAACATTCAGACGGTGATAATCACCTTCACTGTCTTCTCCAATTTTCCGGCAAGTATAAATGTCG
GAATCCAAGGTTCTTCGACATAATCAACCCAGATTCCGACGAATCTTTTCACCCAAATATTCAAAGTG
CGAGATCGGCGAATG
```

+
FFFFFFFFFFFFFFFFFFFFFFFFFFFFFFFFFFFFFFFFFFFFFFFFFFFFFFFFFFFFFFFF:FFFFF
FFFFFFFFFFFFFFFFFFFFFFFFF:F:FFFFFFFFFFFFFFFFFFFFFFFFFFFFFFFFFFFFFFFFF,
FFFFFFFFFFFFFFF
@A00721:190:HGJW7DSXY:4:2116:31792:21887 2:N:0:TCTGCAAG+GCTGCACT
GAACAACATTCAGACGGTGATAATCACCTTCACTGTCTTCTCCAATTTTCCGGCAAGTATAAATGTCG
GAATCCAAGGTTCTTCGACATAATCAACCCAGATTCCGACGAATCTTTTCACCCAAATATTCAAAGTG
CGAGATCGGCGAATG
+
FFFFFFFFFFFFFFFFFFFFFFFFFFFFFFFFFFFFFFFFFFFFFFFFFFFFFFFFFFFFFFFFFFFFFF
FFFFFFFFFFFFFFFFFFF:FFFFFFFFFFFFFFFFFFFFFFFFFFFFFFFF:FFFFFFF:FFFFFF:FF
FFFFFFFFFFFFFFF
@A00721:190:HGJW7DSXY:4:2208:29234:33051 2:N:0:TCTGCAAG+GCTGCACT
GAACAACATTCAGACGGTGATAATCACCTTCACTGTCTTCTCCAATTTTCCGGCAAGTATAAATGTCG
GAATCCAAGGTTCTTCGACATAATCAACCCAGATTCCGACGAATCTTTTCACCCAAATATTCAAAGTG
CGAGATCGGCGAATG
+
FFFFFFFFFFFFFFFFFFF,FFFFFFFFFFFFFFFFFFFFFFFFFFFFFFFFFFFFF,F:FFFFFFF:F:
FFFFFFFFFF,FFFFFFFFFFFFFF,FFFFFFFFFFFFFFFFFFFFFFF:FFFFFFFFFFFFFFFFFFFF
FFFFFFFFFFFFFFF
@A00721:190:HGJW7DSXY:4:2241:30590:31516 2:N:0:TCTGCAAG+GCTGCACT
GAACAACATTCAGACGGTGATAATCACCTTCACTGTCTTCTCCAATTTTCCGGCAAGTATAAATGTCG
GAATCCAAGGTTCTTCGACATAATCAACCCAGATTCCGACGAATCTTTTCACCCAAATATTCAAAGTG
CGAGATCGGCGAATG
+
FFFFFFFFFFFFFFFFFFFFFFFFFFFFFFFFFFFFFFFFFFFFFFFFFFFFFFFFFFFFFFFFFFFFFF
FFFFFFFFFFFFFFFFFFFFFFFFFFFFFFFFFFFFFFFFFFFFFFFFFFFFFFFFFFFFFFFFFFF:FF
FFFFFFFFFFFFFFF
@A00721:190:HGJW7DSXY:4:2303:15040:3928 2:N:0:TCTGCAAG+GCTGCACT
GAACAACATTCAGACGGTGATAATCACCTTCACTGTCTTCTCCAATTTTCCGGCAAGTATAAATGTCG
GAATCCAAGGTTCTTCGACATAATCAACCCAGATTCCGACGAATCTTTTCACCCAAATATTCAAAGTG
CGAGATCGGCGAATG
+
FFFFFFFF:FFFFFFFFFFFFFFFFFFFFFFFFFFFFFFFFFFFFFFFFFFFFFFFFFFFF:FFFFFFF
FFFFFFFFFFFFFFFFFFFF,FFFFFFFFFFFFFFFFF:FFFFF:FFFFFFFFFFFFFFFFFFFFFFFFF
FFFFFFFFFFFFFFF
@A00721:190:HGJW7DSXY:4:2343:29324:26882 2:N:0:TCTGCAAG+GCTGCACT
GAACAACATTCAGACGGTGATAATCACCTTCACTGTCTTCTCCAATTTTCCGGCAAGTATAAATGTCG
GAATCCAAGGTTCTTCGACATAATCAACCCAGATTCCGACGAATCTTTTCACCCAAATATTCAAAGTG
CGAGATCGGCGAATG
+
FFFFFFFFFFFFFFFFFFFFFFFFFFFFFFFFFFFFFFFFFFFFFFFFFFFFFFFFF,FFFFFFFFFFFF
FFFFFFFFFFFFFFFFFFFFFFFFFFFFFFFFFFFFFFFFFFFFFFFFFFFFFFFFF:FFF:FFFFFFFF
FFF:FFFFFFFFFFF
@A00721:190:HGJW7DSXY:4:2428:30752:13197 2:N:0:TCTGCAAG+GCTGCACT
GAACAACATTCAGACGGTGATAATCACCTTCACTGTCTTCTCCAATTTTCCGGCAAGTATAAATGTCG
GAATCCAAGGTTCTTCGACATAATCAACCCAGATTCCGACGAATCTTTTCACCCAAATATTCAAAGTG
CGAGATCGGCGAATG
+
FFFFFFFFFFFFFFFFFFFF:FFFFFFFFFFFFFFFFFFFFFFFFF:FFFFFFFF:FFFFFFFFFFFFFF
FFFFFFFFFFFFFFFFF:FFFFFFFFFFFFFFFFFFFFFFFFFFFFFFFFFFF,:FFFFFFFFF:F,FFF
FFFFFFFFFFFF,FF
@A00721:190:HGJW7DSXY:4:2433:20971:13510 2:N:0:TCTGCAAG+GCTGCACT
GAACAACATTCAGACGGTGATAATCACCTTCACTGTCTTCTCCAATTTTCCGGCAAGTATAAATGTCG

GAATCCAAGGTTCTTCGACATAATCAACCCAGATTCCGACGAATCTTTTCACCCAAATATTCAAAGTG
CGAGATCGGCGAATG
+
FFFFFFFFFFFFFFFFFFFFFFFFFFFFFFFFFFFFFFFFFFFFFFFFFFFFFFFFFFFFFFFFFFFFFF
FF:FFFFFFFFFFFFFFFFFFFFFFFFFFFFFFFFFFFFFFFFFFFFFFFFFFFFFFFFFFFFFFFFFFF
FFFFFFFFFFFFFFF
@A00721:190:HGJW7DSXY:4:2447:32533:33098 2:N:0:TCTGCAAG+GCTGCACT
GAACAACATTCAGACGGTGATAATCACCTTCACTGTCTTCTCCAATTTTCCGGCAAGTATAAATGTCG
GAATCCAAGGTTCTTCGACATAATCAACCCAGATTCCGACGAATCTTTTCACCCAAATATTCAAAGTG
CGAGATCGGCGAATG
+
FFFFFFFFFFFFFFFFFFFFFFFFFFFFFFFFFFFFFFFFFFFFFFFFFFFFFFFFFFFFFFFFFFFFFF
FFFFFFFFFFFFFFFFFFFFFFFFFFFFFFFFFFFFFFF:FFFFFFFFFFFFFFFFFFFFFFFFFFFFFF
FFFFFFF:FFFF:FF
@A00721:190:HGJW7DSXY:4:2501:4616:27148 2:N:0:TCTGCAAG+GCTGCACT
GAACAACATTCAGACGGTGATAATCACCTTCACTGTCTTCTCCAATTTTCCGGCAAGTATAAATGTCG
GAATCCAAGGTTCTTCGACATAATCAACCCAGATTCCGACGAATCTTTTCACCCAAATATTCAAAGTG
CGAGATCGGCGAATG
+
FF:FFFFFFFFFFFFFFFFFFFFFFFFFFFFFFFFFFFFFFFFFFFFFFFFFFFFFFFFFFFFFFFFFFF
FFFFFFFFFFFFFFFFFFFFFFF:FFFFFFFFFFFFFFFFFFFFFFFFFFFFFFFFFFFFFFFFFFFFFF
FFFFFFF:FFFF,F:
@A00721:190:HGJW7DSXY:4:2501:4652:27179 2:N:0:TCTGCAAG+GCTGCACT
GAACAACATTCAGACGGTGATAATCACCTTCACTGTCTTCTCCAATTTTCCGGCAAGTATAAATGTCG
GAATCCAAGGTTCTTCGACATAATCAACCCAGATTCCGACGAATCTTTTCACCCAAATATTCAAAGTG
CGAGATCGGCGAATG
+
FF:FFFFFFFFFFFFFFF:FFFFFFFFFFFFFFFFFFFFFFFFFFFFFFFFFFFFFFFFFF:FFFFFFFF
FFFFFFFFFFFFFFFFFFFFFFFFFFFFFFFFFF:FF:FFFFFFFFFFFFFFFFFFFFFFFFFFFFFFFF
FFFFFFFFFFFF:FF
@A00721:190:HGJW7DSXY:4:2648:28818:28228 2:N:0:TCTGCAAG+GCTGCACT
GAACAACATTCAGACGGTGATAATCACCTTCACTGTCTTCTCCAATTTTCCGGCAAGTATAAATGTCG
GAATCCAAGGTTCTTCGACATAATCAACCCAGATTCCGACGAATCTTTTCACCCAAATATTCAAAGTG
CGAGATCGGCGAATG
+
FFFFFFFFFFFFFFFFFFFFFFFFFFFFFFFFFFFFFFFFFFFFFFFFFFFFFFFFFFFFFFFFFFFFFF
FFFFFFFFFFFFFFFFFFFFFF:FFFFFF:FFFFFFFFFFF:FFFFFFFFF:F:FFFFFFFFFFFFFFFF
FFFFF:FFFFFFFFF
@A00721:190:HGJW7DSXY:4:1110:15700:3724 2:N:0:TCTGCAAG+GCTGCACT
CGCCGATCTCGCACTTTGAATATTTGGGTGAAAAGATTCGTCGGAATCTGGGTTGATTATGTCGAAGA
ACCTTGGATTCCGACATTTATACTTGCCGGAAAATTGGAGAAGACAGTGAAGGTGATTATCACCGTCT
GAATGTTGTTCCTGA
+
FFFFFFFFFFFFFFFFFFFFFFF:FFFFFFFFFFFFFFFFFFFFFFFFFFFFFFFFFFFFFFFFFFFFFF
FFFFFFFFFFFFFFFFFFFFFFFFFFFFFFFFFFFFFFFFFFFFFFFFFFFFFFFFFF:FFFFFFFFFFF
FFFFFFFFFFFFFFF
@A00721:190:HGJW7DSXY:4:1619:12834:33270 2:N:0:TCTGCAAG+GCTGCACT
CGCCGATCTCGCACTTTGAATATTTGGGTGAAAAGATTCGTCGGAATCTGGGTTGATTATGTCGAAGA
ACCTTGGATTCCGACATTTATACTTGCCGGAAAATTGGAGAAGACAGTGAAGGTGATTATCACCGTCT
GAATGTTGTTCCTGA
+
FFFFFFFFFFFFFFFFFFFFFFF:FFFFFFFFFFFFFFFFFFFFFF:FFFFFFFFFFFFFFFFFFFFFFF
FFFFFFFFFFFFFFFFFFFFFFFFFFFFFFFFFFFFFFFFFFFFFFFFFFFFFFFFFFFFFFFFFFFFFF
FFFFFFFFFFFFFFF

```
@A00721:190:HGJW7DSXY:4:1675:10022:24486 2:N:0:TCTGCAAG+GCTGCACT
CGCCGATCTCGCACTTTGAATATTTGGGTGAAAAGATTCGTCGGAATCTGGGTTGATTATGTCGAAGA
ACCTTGGATTCCGACATTTATACTTGCCGGACAATTGGAGAAGACAGTGAAGGTGATTATCACCGTCT
GAATGTTGTTCCTGA
+
FFFFFFFFFFFFF:FF:FFFFFF:FFFFFFFFFFFF:FFFFFFFFFFFFFFFFFFFFFFFFFFFF,FF
FFF:FFFFFFFFFFFFF,FFFFFFFFFFFFF,FF:FFFFFFFFFFFFFFFFFFFFFFFFFFFFFFFFF
FFFFFFFFFFFFFFF
@A00721:190:HGJW7DSXY:4:1675:6813:24533 2:N:0:TCTGCAAG+GCTGCACT
CGCCGATCTCGCACTTTGAATATGTGGGTGAAAAGATTCGTCGGAATCTGGGTTGATTATGTCGAAGA
ACCTTGGATTCCGACATTTATACTTGCCGGAAAATTGGAGAAGACAGTGAAGGTGATTATCACCGTCT
GAATGTTGTTCCTGA
+
FFFFFFFFFFFFFFFFFFFFFFF,FFFFFFFFFFFFFFFFFFFFFFFFFFFFFFFFFFFFFFFFF:FF
FFFFFFFFFFFFFFFFFFFF:FFFFFFFFFFFFFFFFFFFFFFFFFFFFFFFFFFFFFFFFFFFFFFF
FFFFFFFFFFFFFFF
@A00721:190:HGJW7DSXY:4:2171:18421:29074 2:N:0:TCTGCAAG+GCTGCACT
CGCCGATCTCGCACTTTGAATATTTGGGTGAAAAGATTCGTCGGAATCTGGGTTGATTATGTCGAAGA
ACCTTGGATTCCGACATTTATACTTGCCGGAAAATTGGAGAAGACAGTGAAGGTGATTATCACCGTCT
GAATGTTGTTCCTGA
+
FFFFFFFF:FFFFFFFFFFFFFFFFFFFFFFFFFFFFFFFFFFFFFFFFFFFFFFFFFFFFFFFFFFF
FFF:FFFFFFFFFFFFFFFFFFFFFFFFFFF:FFFFFFFFFFFFFFFFFFFFFFFFFFFFFFFFFFFF
FFFFFFFFFFFFFFF
@A00721:190:HGJW7DSXY:4:1503:9236:16423 2:N:0:TCTGCAAG+GCTGCACT
GGAACAACATTCAGACGGTGATAATCACCTTCACTGTCTTCTCCAATTTTCCGGCAAGTATAAATGTC
GGAATCCAAGGTTCTTCGACATAATCAACCCAGATTCCGACGAATCTTTTCACCCAAATATTCAAAGT
GCGAGATCGGC
+
FFFFFFFFFFFFFFFFFFFFFFFFFFFFFFFFFFFFFFFFFFFFFFFFFFFFFFFFFFFFFFFFFFFF
FF:FFFFFFFFFFFFFFFFFFFFFFFFFFFFFFFFFFFFFFFFFFFFFFFFFFFFFFFFFFF:FFFFF
FFFFFFF:FFF
@A00721:190:HGJW7DSXY:4:1659:13485:31986 2:N:0:TCTGCAAG+GCTGCACT
GGAACAACATTCAGACGGTGATAATCACCTTCACTGTCTTCTCCAATTTTCCGGCAAGTATAAATGTC
GGAATCCAAGGTTCTTCGACATAATCAACCCAGATTCCGACGAATCTTTTCACCCAAATATTCAAAGT
GCGAGATCGGC
+
FFF,FFFFFFFFFFFFFFFFFFFFFFFFFFFFFFFFFFFFFFFFFFFFFFFFFFFFFFFFFFFFFFFF
FFFFFFFFFFFFFFFFFFFFFFFFFFFFFFFFFFFFFFFFFFFFFFFFFFFFFFFFFFF,FFFFFFFF
FFFFFFFFFFF
@A00721:190:HGJW7DSXY:4:2205:26078:32315 2:N:0:TCTGCAAG+GCTGCACT
GGAACAACATTCAGACGGTGATAATCACCTTCACTGTCTTCTCCAATTTTCCGGCAAGTATAAATGTC
GGAATCCAAGGTTCTTCGACATAATCAACCCAGATTCCGACGAATCTTTTAACCCAAATATTCAAAGT
GCGAGATAGGC
+
,FFFFFF:,FFFF:FFFFFFFFFFF,FFFFFFF:FFF:FF,FF:FFFFFF:FF:FFFFFFF,FFFFFF
,FFFF:FFF,::FFF:F:F:FF,FFFFFF,FFFFFFFFFF,FFFFFFFFF,F,F:FFFFF::FFFFFF
:,FF:FF,F,F
@A00721:190:HGJW7DSXY:4:2315:22381:28823 2:N:0:TCTGCAAG+GCTGCACT
GGAACAACATTCAGACGGTGATAATCACCTTCACTGTCTTCTCCAATTTTCCGGCAAGTATAAATGTC
GGAATCCAAGGTTCTTCGACATAATCAACCCAGATTCCGACGAATCTTTTCACCCAAATATTCAAAGT
GCGAGATCGGC
+
FFFFFFFFFFFFFFFFFFFFFFFFFFFFFFFFFFFFFFFFFFFFFFFFFFFFFFFF,FFFFFFFFFFF
```

FFFFFFFFFFFFFFFFFFFFFFFFFFFFFFFFFFFFFFFFFFFFFFFFFFFFFFFFFFFFFFFFFFFFFF
F::FFFFFFFF
@A00721:190:HGJW7DSXY:4:2504:14570:5368 2:N:0:TCTGCAAG+GCTGCACT
GGAACAACATTCAGACGGTGATAATCACCTTCACTGTCTTCTCCAATTTTCCGGCAAGTATAAATGTC
GGAATCCAAGGTTCTTCGACATAATCAACCCAGATTCCGACGAATCTTTTCACCCAAATATTCAAAGT
GCGAGATCGGC
+
FFFFFFFFFFFFFFFFFFFFFFFFFFFFFFFFFFFFFFFFFFFFFFF,FF:FF:F:FFFFFFFFFFF
FFFFFFFFFFFFFF:FF:FFF,FFFFFF:FFFFFF:FFFFFFFFFFF:F:FFFFFFFFFFFF:FFFFF
FF:FFFFFFFF
@A00721:190:HGJW7DSXY:4:2510:31078:33270 2:N:0:TCTGCAAG+GCTGCACT
GGAACAACATTCAGACGGTGATAATCACCTTCACTGTCTTCTCCAATTTTCCGGCAAGTATAAATGTC
GGAATCCAAGGTTCTTCGACATAATCAACCCAGATTCCGACGAATCTTTTCACCCAAATATTCAAAGT
GCGAGATCGGC
+
FFFFFFFFFFFFFFFFFFFFFFFFFFFFFFFFFFFFFFFFFFFFFFFFFFFFFFFFFFFFFFFFFFFFFF
FFF:FFFFFFFFFFFFFFFFFFFFFFFFFFFFFFFFFFF:FFFFFFFFFFFFF:FFFFFFFFFFF:FF
FFFFFFFFFFF
@A00721:190:HGJW7DSXY:4:2560:23737:16611 2:N:0:TCTGCAAG+GCTGCACT
GGAACAACATTCAGACGGTGATAATCACCTTCACTGTCTTCTCCAATTTTCCGGCAAGTATAAATGTC
GGAATCCAAGGTTCTTCGACATAATCAACCCAGATTCCGACGAATCTTTTCACCCAAATATTCAAAGT
GCGAGATCGGC
+
FFFFFFFFFFFFFFFFFFFFFFFFFFFFFFFFFFFFFFFFFFFFFFFFFFFFFFFFFFFFFFFFFFFFFF
FFFFFFFFFFFFFFFFFFFFFFFFFFFFFFFFFFFFF:FFFFFFFFFFFFFFFFFFFFFFFFFFFFFFF
FFFFFFFFFFF
@A00721:190:HGJW7DSXY:4:1377:32108:31516 2:N:0:TCTGCAAG+GCTGCACT
GTCAGGAACAACATTCAGACGGTGATAATCACCTTCACTGTCTTCTCCAATTTTCCGGCAAGTATAAA
TGTCGGAATCCAAGGTTCTTCGACATAATCAACCCAGATTCCGACGAATCTTTTCACCCAAATATTCA
AAGTGCGA
+
FFFFFFFFFFFFFFFFFFFFFFFF:FFFFFFFFFFFFFFFFFFFFFFFFFFFFFFFFFFFFFFFFFFF,
FFFFFFFFFFFFFFFFFFFFFFFFFFFFFFFFFFFFFFFFFFFFFFFFFFFFFFFFFFFFFFFFFFFFF
FFFF:FFF
@A00721:190:HGJW7DSXY:4:1478:11803:31548 2:N:0:TCTGCAAG+GCTGCACT
GTCAGGAACAACATTCAGACGGTGATAATCACCTTCACTGTCTTCTCCAATTTTCCGGCAAGTATAAA
TGTCGGAATCCAAGGTTCTTCGACATAATCAACCCAGATTCCGACGAATCTTTTCACCCAAATATTCA
AAGTGCGA
+
FFFFFFFFFFFFFFFFFFFFFFFFFFFFFFFFFFFFFFFFFF:FFFFFF:FFFFFFFFF:FFFFFFF:
FFFFFFFFFFFFFFFFFFFFFFF,FFFFFFFFFFFFFFFFFFFFFFFFFFFFFFFFFFFFFFFFFFFFF
FFFFFFFF
@A00721:190:HGJW7DSXY:4:1670:11794:34851 2:N:0:TCTGCAAG+GCTGCACT
GTCAGGAACAACATTCAGACGGTGATAATCACCTTCACTGTCTTCTCCAATTTTCCGGCAAGTATAAA
TGTCGGAATCCAAGGTTCTTCGACATAATCAACCCAGATTCCGACGAATCTTTTCACCCAAATATTCA
AAGTGCGA
+
FFFFFFFFFFFFFFFFFFFFFFFFFFF:FFFFFFFFFFFFFFFFFFFFFFFFFFFFFFFFFFFFF:FF,
FFFFFFFFFFFFFFFFFFFFFFFFFFFFFFFFFFFFFFFFFFFFF,FFFFFFFFFFFFFFFFFFFFFFF
FFFFFFFF
@A00721:190:HGJW7DSXY:4:2117:22146:32988 2:N:0:TCTGCAAG+GCTGCACT
GTCAGGAACAACATTCAGACGGTGATAATCACCTTCACTGTCTTCTCCAATTTTCCGGCAAGTATAAA
TGTCGGAATCCAAGGTTCTTCGACATAATCAACCCAGATTCCGACGAATCTTTTCACCCAAATATTCA
AAGTGCGA

```
+
FFFFFFFFFFFFFFFFFFFFFFFFFFFFFFFFFFFFFFFFFFFFFFFFFFFFFFFFFFFF:FFFFFFF
FFFF:FFFFFFFFFFFFFFFFFFFFFFFFFFFFFFFFFFFFFFFFFFFFFFFFFFFF:F:F:FFFFFF
FFFFFFFF
@A00721:190:HGJW7DSXY:4:2120:1136:16705 2:N:0:TCTGCAAG+GCTGCACT
GTCAGGAACAACATTCAGACGGTGATAATCACCTTCACTGTCTTCTCCAATTTTCCGGCAAGTATAAA
TGTCGGAATCCAAGGTTCTTCGACATAATCAACCCAGATTCCGACGAATCTTTTCACCCAAATATTCA
AAGTGCGA
+
FFFFFFFFFFFFFFFFFFFFFFFFFFFFFFFFFFFFFFFFFFFF:FFFFFFFFFFFFF:FFFFFFFFF
FFFFF:FFF:FFF,FFFFFFFFF:FFFFFFFFFFFFFFFF:FFFFFFFFFF,FF:FFFFF:FFFF:,F
FF,FF::F
@A00721:190:HGJW7DSXY:4:2161:13765:28745 2:N:0:TCTGCAAG+GCTGCACT
GTCAGGAACAACATTCAGACGGTGATAATCACCTTCACTGTCTTCTCCAATTTTCCGGCAAGTATAAA
TGTCGGAATCCAAGGTTCTTCGACATAATCAACCCAGATTCCGACGAATCTTTTCACCCAAATATTCA
AAGTGCGA
+
FFFFFFFFFFFFFFFFFFFFFF:FFFFFFFFFFFFFFFFFFFFFFFFFFFFFFFF:FFFFFFFFFFFF
F:FFFFFFFFFFFFFFFFFFFFFFFFFFF:FFFF:FFFFFFFFFFFFFFFFFFFFFFFFF:FFFFF:F
FFFFFFFF
@A00721:190:HGJW7DSXY:4:2238:20121:21872 2:N:0:TCTGCAAG+GCTGCACT
GTCAGGAACAACATTCAGACGGTGATAATCACCTTCACTGTCTTCTCCAATTTTCCGGCAAGTATAAA
TGTCGGAATCCAAGGTTCTTCGACATAATCAACCCAGATTCCGACGAATCTTTTCACCCAAATATTCA
AAGTGCGA
+
FFF:FFFFFFF:FFFFFFFF:,:FFFFFFFFF:F,FF:FF,,F:FF::FF:FF:FFF:FFFFFFFFFF
FFF,FFFFFFFFF::FF,,F,,FFFFFFF:FF:F,F,FF,:::FFFFFFFFFFF::,,FFFFFFFFFF
FF:F:FFF
@A00721:190:HGJW7DSXY:4:2318:1371:35023 2:N:0:TCTGCAAG+GCTGCACT
GTCAGGAACAACATTCAGACGGTGATAATCACCTTCACTGTCTTCTCCAATTTTCCGGCAAGTATAAA
TGTCGGAATCCAAGGTTCTTCGACATAATCAACCCAGATTCCGACGAATCTTTTCACCCAAATATTCA
AAGTGCGA
+
F:FFFFFFFFF:FFFFF:FFFFF:FFFFFFFFFFFFFFFFFFFFFFFFFFFFFFFFFFFFFFFFFFFF
FFFFFFFFFF:FFFFFFFFFFFFFF:FFFFF:FFFFFFF:FFF::FFFFFFFFFF,FFFFFFFFFFFF
FFFFFFFF
@A00721:190:HGJW7DSXY:4:2357:5963:1172 2:N:0:TCTGCAAG+GCTGCACT
GTCAGGAACAACATTCAGACGGTGATAATCACCTTCACTGTCTTCTCCAATTTTCCGGCAAGTATAAA
TGTCGGAATCCAAGGTTCTTCGACATAATCAACCCAGATTCCGACGAATCTTTTCACCCAAATATTCA
AAGTGCGA
+
FFFFFFFFFFFFFFFFFFFFFFFFFFFFFFFFFFFFFFFFFFFFFFFFFFFFFFFFFFFFFFFFFFFF
FFFFFFFFFFFFFFFFFFFFFFFFFFFFFFFFFFFFFFFFFFFFFFFFFFFFFFFFFFFFFFFFFFFF
FFFFFFFF
@A00721:190:HGJW7DSXY:4:2407:28402:3333 2:N:0:TCTGCAAG+GCTGCACT
GTCAGGAACAACATTCAGACGGTGATAATCACCTTCACTGTCTTCTCCAATTTTCCGGCAAGTATAAA
TGTCGGAATCCAAGGTTCTTCGACATAATCAACCCAGATTCCGACGAATCTTTTCACCCAAATATTCA
AAGTGCGA
+
FFFFFFFFFFFFFFFFFF:FFFFFFFFFFFFFFFFFFFFFFFFFFFFFFFFFFFFFFFFFFFFFFFFF
FFFFFFFFFFFFFFFFFFFFFFFFFFFFFFFFFFFFFFFFFFFFFFFFFFFFFFFF:FFFFFFFFFFF
:FFFFFFF
@A00721:190:HGJW7DSXY:4:2562:22019:8813 2:N:0:TCTGCAAG+GCTGCACT
GTCAGGAACAACATTCAGACGGTGATAATCACCTTCACTGTCTTCTCCAATTTTCCGGCAAGTATAAA
```

TGTCGGAATCCAAGGTTCTTCGACATAATCAACCCAGATTCCGACGAATCTTTTCACCCAAATATTCA
AAGTGCGA
+
FFFFFFFFFFFFFFFFFFFFFFFFFFFFFFFFFFFFFFFFFFFFFFFFFFFFFFFFFFFFFFFFFF:FFF
FFFFFFFFFFFFFFFFFFFFFFFFFFFFFFFFFFFFFFFFFFFFFFFFFFFFFFFFFFFFF:FFFFFFFF
FFFFFFFF
@A00721:190:HGJW7DSXY:4:1108:26720:26631 2:N:0:TCTGCAAG+GCTGCACT
CAGAGTATGTCAGGAACAACATTCAGACGGTGATAATCACCTTCACTGTCTTCTCCAATTTTCCGGCA
AGTATAAATGTCGGAATCCAAGGTTCTTCGACATAATCAACCCAGATTCCGACGAATCTTTTCACCCA
AATATTCAAAGTGCG
+
FFFFFFFFFFFFFFFFFFFFFFFFFFFFFFFFFFFFFFFFFFFFFFFFFFFFFFFFFFFFFFFFFFFFFF
FFFFFFFFFFFFFFFFFFFFFFFFFFFFFFFFFFFFFFFFFF:FFFFFFFFFFF:FFFFFFFFFFFFF
FFFFFFFFFFFFFFF
@A00721:190:HGJW7DSXY:4:1168:7798:35070 2:N:0:TCTGCAAG+GCTGCACT
CAGAGTATGTCAGGAACAACATTCAGACGGTGATAATCACCTTCACTGTCTTCTCCAATTTTCCGGCA
AGTATAAATGTCGGAATCCAAGGTTCTTCGACATAATCAACCCAGATTCCGACGAATCTTTTCACCCA
AATATTCAAAGTGCG
+
FFFFFFFFFFFFFFFFFFFFFFFFFFFFFFFFFFFFFFFFFFFFFFFFFFFFFFFFFFF:FFFFFFFFFF
FFFFFFF,FFFFFFFFFFFFFFFFFFFFFFFFFFFF:FFFFFFFFFFFFFFFFFFFFFFFFFFFFFFFFF
FFFFFFFFFFFFFFF
@A00721:190:HGJW7DSXY:4:1168:8034:35665 2:N:0:TCTGCAAG+GCTGCACT
CAGAGTATGTCAGGAACAACATTCAGACGGTGATAATCACCTTCACTGTCTTCTCCAATTTTCCGGCA
AGTATAAATGTCGGAATCCAAGGTTCTTCGACATAATCAACCCAGATTCCGACGAATCTTTTCACCCA
AATATTCAAAGTGCG
+
FFFFFFFFFFFFFFFFFFFFFFFFFFFFFFFFFFFFFFFFFFFFFFFFFFFFFFFFFFFFFFFFFFFFFF
FFFFFFF,F:FFFFFFFFFFFFFFFFFFFFFFFFFFFFFFFFFFFFF:FFFFFFFFFFFFFFFFFF,FF
FFFFFFFFFFFFFFF
@A00721:190:HGJW7DSXY:4:1249:4707:23422 2:N:0:TCTGCAAG+GCTGCACT
CAGAGTATGTCAGGAACAACATTCAGACGGTGATAATCACCTTCACTGTCTTCTCCAATTTTCCGGCA
AGTATAAATGTCGGAATCCAAGGTTCTTCGACATAATCAACCCAGATTCCGACGAATCTTTTCACCCA
AATATTCAAAGTGCG
+
FFFFFFFFFFF:FFFFFFFFFFFFFFFFFFFFFFFFFFFFFFFFFFFFFFFF:FFFFFFFFFFFFFFFFF
FFFF,FF:FFFFFFF:FFFF,FFFFF:FFFFFFFFFFFFFFFFFFF:FFFFFFFFFFFFFFFFFFFFFFF
FFF:FFFFFFFFFFF
@A00721:190:HGJW7DSXY:4:1249:4670:23547 2:N:0:TCTGCAAG+GCTGCACT
CAGAGTATGTCAGGAACAACATTCAGACGGTGATAATCACCTTCACTGTCTTCTCCAATTTTCCGGCA
AGTATAAATGTCGGAATCCAAGGTTCTTCGACATAATCAACCCAGATTCCGACGAATCTTTTCACCCA
AATATTCAAAGTGCG
+
FFFFFFFFFFFFFFFFFFFFFFFFFFFFFFFFFFFFFFF:FFFFFFFFFFF:FFFFFFFFFFFFFFFF:
FF:FFFFFFFFFFFFFFFFFFFFFFFFFFFFFFFFFF:FFFFFFFFFFFFFFFFFFFFFFFF,FFFFFFF:
FF:FFFFF:FFFFFF
@A00721:190:HGJW7DSXY:4:1263:8567:28667 2:N:0:TCTGCAAG+GCTGCACT
CGCACTTTGAATATTTGGGTGAAAAGATTCGTCGGAATCTGGGTTGATTATGTCGAAGAACCTTGGAT
TCCGACATTTATACTTGCCGGAAAATTGGAGAAGACAGTGAAGGTGATTATCACCGTCTGAATGTTGT
TCCTGACATACTCTG
+
FFFFFFFFFFFFFFFFFFFFFFFFFFFFFFFFFFFFFFFFFFFFFFFFFFFFFFFFFFFF,FFFFFFFFF
FFFF:FFFFF:FFFFFFFFFFFF:F:FFFFF:FFFFFFFFFFFFFFFFFFFFFFFFFFFFFFFFFFFFFF
FFFFFFFFFFF:FFF

```
@A00721:190:HGJW7DSXY:4:1306:27724:14090 2:N:0:TCTGCAAG+GCTGCACT
CAGAGTATGTCAGGAACAACATTCAGACGGTGATAATCACCTTCACTGTCTTCTCCAATTTTCCGGCA
AGTATAAATGTCGGAATCCAAGGTTCTTCGACATAATCAACCCAGATTCCGACGAATCTTTTCACCCA
AATATTCAAAGTGCG
+
FFFFFF:FFFFFFFF:FFFFFFFFFFFFFFFFFFFFFFFFFFFFFFFFFFFFFFFFFFFFFFFFFFFFFF
:FFFFFFFFFFFFFFFFFFFFFFFFFFFFFFFFFFFFFFFFFFFFFFFFFFFFFF:FFFFFFFFFFFFFF
:FFFFFFFFFFFFFF
@A00721:190:HGJW7DSXY:4:1306:28610:14215 2:N:0:TCTGCAAG+GCTGCACT
CAGAGTATGTCAGGAACAACATTCAGACGGTGATAATCACCTTCACTGTCTTCTCCAATTTTCCGGCA
AGTATAAATGTCGGAATCCAAGGTTCTTCGACATAATCAACCCAGATTCCGACGAATCTTTTCACCCA
AATATTCAAAGTGCG
+
FFFFFFFFFFFFFFFFFFFFFFFFFFFFF:FFFFFFFFFFFFFFFFFFFFFFFFFFFFFFFFFFFFFFFF
FFFFFFFFFFFFFFFFFFFFFFFFFFFFFFFFFFFFFFFFFFFFFFFFFFFFFFFFFFFFFFFFFFFFFF
FFFFFFFFF:FFFFF
@A00721:190:HGJW7DSXY:4:1309:12310:13135 2:N:0:TCTGCAAG+GCTGCACT
CGCACTTTGAATATTTGGGTGAAAAGATTCGTCGGAATCTGGGTTGATTATGTCGAAGAACCTTGGAT
TCCGACATTTATACTTGCCGGAAAATTGGAGAAGACAGTGAAGGTGATTATCACCGTCTGAATGTTGT
TCCTGACATACTCTG
+
FFFFFFFFF:FFFFFFFFFFFFFFFFFFFFFFFFFFFFFFFFFFFFFFFFFFFFFFFFFFFFFFFFFFFF
FFFFFFFFFF:FFFFFFFFF:F:FFFFFFF,FFFFFFF:FFFFF:FFFFFFFFFFFFFFFFFFFFFFFFF
FFFFFFFFFFFFFFF
@A00721:190:HGJW7DSXY:4:1338:10592:23437 2:N:0:TCTGCAAG+GCTGCACT
CGCACTTTGAATATTTGGGTGAAAAGATTCGTCGGAATCTGGGTTGATTATGTCGAAGAACCTTGGAT
TCCGACATTTATACTTGCCGGAAAATTGGAGAAGACAGTGAAGGTGATTATCACCGTCTGAATGTTGT
TCCTGACATACTCTG
+
FFFFFFFFFFFFFF:FFFFFFFFFFFFFFFFF:FFFFF:FFFFFFFFFFFFFFFFFFFFFFFFFFFFFFF
FFFFFFFFFFFFFFFFFFFFFFFFFFFFFFFFFFFFFFFFFFFFFFFFFFFFFFFFFFFFFF:FFFFFFF
FFFFFFFFFFFFFFF
@A00721:190:HGJW7DSXY:4:1457:30761:1470 2:N:0:TCTGCAAG+GCTGCACT
CGCACTTTGAATATTTGGGTGAAAAGATTCGTCGGAATCTGGGTTGATTATGTCGAAGAACCTTGGAT
TCCGACATTTATACTTGCCGGAAAATTGGAGAAGACAGTGAAGGTGATTATCACCGTCTGAATGTTGT
TCCTGACATACTCTG
+
FFFFFFFFFFFFFFFFFFFFFFFFFFFFFF:FFFFFFFFFFFFFFFFFFFFFFFFFFFFFFFFFFFFFFF
FFFFFFFFFFFFFFFFF,FFFFFFFFFFFFFFFFFFFFFFFFFFFFFFFFFFFFFFFFFFFFFFFFFFFF
FFFFFFFFFFFFFFF
@A00721:190:HGJW7DSXY:4:1505:28203:37027 2:N:0:TCTGCAAG+GCTGCACT
CGCACTTTGAATATTTGGGTGAAAAGATTCGTCGGAATCTGGGTTGATTATGTCGAAGAACCTTGGAT
TCCGACATTTATACTTGCCGGAAAATTGGAGAAGACAGTGAAGGTGATTATCACCGTCTGAATGTTGT
TCCTGACATACTCTG
+
FFFFFFFFFFFFFFFFFFFFFFFFFFFFFFFFFFFFFFFFFFFFFFFFFFFFFFFFFFFFFFFFFFFFFF
FFFFFFFFFFFFFFFFFFFFFFFFFFFFFFFFFFFFFFFFFFFFFFFFFFFFFFFFFFFFFFFFFFFFFF
FFFFFFFFFFFFFFF
@A00721:190:HGJW7DSXY:4:1539:26756:21465 2:N:0:TCTGCAAG+GCTGCACT
CAGAGTATGTCAGGAACAACATTCAGACGGTGATAATCACCTTCACTGTCTTCTCCAATTTTCCGGCA
AGTATAAATGTCGGAATCCAAGGTTCTTCGACATAATCAACCCAGATTCCGACGAATCTTTTCACCCA
AATATTCAAAGTGCG
+
FFFFFFFFFFFFFFFFFFFFFFFFFFFFFFFFFFFFFFFFFFFFFFFFFFFFFFFFFFFFFFFFFFFFFF
```

FFFFFFF:F:FFFFFFF:FFFFFFFFFFFF:FFFFFFFFFFFFFFFFF:FFFFFFFFFFFFF:FFFFF
FFFFFF:FFFF:FFF
@A00721:190:HGJW7DSXY:4:1648:28763:29356 2:N:0:TCTGCAAG+GCTGCACT
CGCACTTTGAATATTTGGGTGAAAAGATTCGTCGGAATCTGGGTTGATTATGTCGAAGAACCTTGGAT
TCCGACATTTATACTTGCCGGAAAATTGGAGAAGACAGTGAAGGTGATTATCACCGTCTGAATGTTGT
TCCTGACATACTCTG
+
FFFFFFFFFFFFFFFFFFFFFFFFFFFFFFFFFFFFFFFFFFFFFFFFFFFFFFFFFFFFFFF:FFFFF
FFFFFFFFFFFFFFFFFFFFFFFFFFFFFFFFFFFFFFFFFFFFFFFFFFFFFFFFFFFFFFFFFFFF
FFFFFFFFFFFFFFF
@A00721:190:HGJW7DSXY:4:1659:7075:28432 2:N:0:TCTGCAAG+GCTGCACT
CAGAGTATGTCAGGAACAACATTCAGACGGTGATAATCACCTTCACTGTCTTCTCCAATTTTCCGGCA
AGGATAAATGTCGGAATCCAAGGTTCTTCGACATAATCAACCCAGATTCCGACGAATCTTTTCACCCA
AATATTCAAAGTGCG
+
FFFFFFFFFFFFFFFFFFFFFFFFFFFFFFFFFFFFFFFFFFFFFFFFFFFFFFFFFFFFFFFFFFFF
FF,FFFF:FFFFFFFFFFFFFFFFFFFFFFFFFFFFFFFFFFFFFFFFFFFFFFFFFFFFFFFFFFFF
FFFFFFFFFFFFFFF
@A00721:190:HGJW7DSXY:4:2219:3884:3302 2:N:0:TCTGCAAG+GCTGCACT
CAGAGTATGTCAGGAACAACATTCAGACGGTGATAATCACCTTCACTGTCTTCTCCAATTTTCCGGCA
AGTATAAATGTCGGAATCCAAGGTTCTTCGACATAATCAACCCAGATTCCGACGAATCTTTTCACCCA
AATATTCAAAGTGCG
+
:F,FFF::F,FFFFF,:FFFFFFFFFFF,FFFFFFFFFFF:F,FFFFFFFFFFFFF:FFFFFFF,FF:
FFFFFFFFFF,F:,FFFFFFFFFFFFF:FFFFFF:F,FFFFFFFFFFFFFFFF::F:F:FFFF:F,FF
F,,FFFFFFF:F:F:
@A00721:190:HGJW7DSXY:4:2226:18231:35039 2:N:0:TCTGCAAG+GCTGCACT
CGCACTTTGAATATTTGGGTGAAAAGATTCGTCGGAATCTGGGTTGATTATGTCGAAGAACCTTGGAT
TCCGACATTTATACTTGCCGGAAAATTGGAGAAGACAGTGAAGGTGATTATCACCGTCTGAATGTTGT
TCCTGACATACTCTG
+
FFFFFFFFF:FFFFFFFFFFFFFFFFFFFFFFFFFFFF::FFFFFFFFFFFFFFFFFFFFFFFFFFFF
FFFF:FFFFFFFFFFFFF:FFFFFFFFFFFFFFFFFFFF:FFFFFFF::FFFFFFFFFFFFFFFFFFF
FFFFFFF:FFFFFFF
@A00721:190:HGJW7DSXY:4:2268:30698:4836 2:N:0:TCTGCAAG+GCTGCACT
CAGAGTATGTCAGGAACAACATTCAGACGGTGATAATCACCTTCACTGTCTTCTCCAATTTTCCGGCA
AGTATAAATGTCGGAATCCAAGGTTCTTCGACATAATCAACCCAGATTCCGACGAATCTTTTCACCCA
AATATTCAAAGTGCG
+
FFF:FFFFFFFFFFFFFFFFFFFFFFF:FFFFFFFFFFFFFFFFFFFFFFFFFFFFFFFFFFFFFFFF
FFFFFFFFFFFFFFFFFFFFFFFFFFFFFFFFFFFFFFFFFFFFFFFFFFFFFF:FFF:FFFFFFFF
FFFFFFFFFFFFFFF
@A00721:190:HGJW7DSXY:4:2322:27272:19852 2:N:0:TCTGCAAG+GCTGCACT
CAGAGTATGTCAGGAACAACATTCAGACGGTGATAATCACCTTCACTGTCTTCTCCAATTTTCCGGCA
AGTATAAATGTCGGAATCCAAGGTTCTTCGACATAATCAACCCAGATTCCGACGAATCTTTTCACCCA
AATATTCAAAGTGCG
+
FFFFFFFFFFFFFFFFFFFFFFFFFFFFFF:FFFFFFFFFFFFFFFFFFFFFFFFFFFFFFFFFFFFF
FFFFFFFFFFFFFFFFFFFFFFFF:FF:FFFFFFFFFFFFFFFFFFFFFFFFFFFFFFFFFFFFFFFF
FFFF:FFFFFFFFFF
@A00721:190:HGJW7DSXY:4:2451:14733:32737 2:N:0:TCTGCAAG+GCTGCACT
CGCACTTTGAATATTTGGGTGAAAAGATTCGTCGGAATCTGGGTTGATTATGTCGAAGAACCTTGGAT
TCCGACATTTATACTTGCCGGAAAATTGGAGAAGACAGTGAAGGTGATTATCACCGTCTGAATGTTGT
TCCTGACATACTCTG

```
+
FFFFFFFFFFF:F:FFFFFFFFFFFFFFFFFFFFFFFFFFFFFFFFFFFFFFFFFFFF:FFFFFFFFF
FFFFFFFFFFFFFFFFFFFFFFFFFFFFFFFFF:FFFFFF::FFFFFFFFFFFFFFFFFFFFFFFFFF
FFFFFFFFFFFFFF:
@A00721:190:HGJW7DSXY:4:2452:6696:8484 2:N:0:TCTGCAAG+GCTGCACT
CGCACTTTGAATATTTGGGTGAAAAGATTCGTCGGAATCTGGGTTGATTATGTCGAAGAACCTTGGAT
TCCGACATTTATACTTGCCGGAAAATTGGAGAAGACAGTGAAGGTGATTATCACCGTCTGAATGTTGT
TCCTGACATACTCTG
+
FFFFFFFFFFFFFFFFFFFFFFFFFFFFFFFFFFFFFFFFFFFFFFFFFFFFFFFFFFFFFFFFFFFF
FFFFFFFFFF:FFFFF:FFFFFFFFFFFFFFFFFFFFFFFFFFFFFFFFFFFFFFFFFFFFFFFFFFF
FFFFFFFFFFFFFFF
@A00721:190:HGJW7DSXY:4:2510:24894:15139 2:N:0:TCTGCAAG+GCTGCACT
CAGAGTATGTCAGGAACAACATTCAGACGGTGATAATCACCTTCACTGTCTTCTCCAATTTTCCGGCA
AGTATAAATGTCGGAATCCAAGGTTCTTCGACATAATCAACCCAGATTCCGACGAATCTTTTCACCCA
AATATTCAAAGTGCG
+
FFFFFFFFFFFFFFFFFFFFFFFFFFFFFFFFFFFFFF:FFFFFFFFFFFFFFFFFFFFFFFF:FFFF
FFFFFFF::FFF:FFFFFFFFFFFFFFFFFFFFFFFFFFF:FFFFFFFFFFFFFFFFFFFFFFFFFFF
FFFFFFFFFFFFFF:
@A00721:190:HGJW7DSXY:4:2540:25229:6104 2:N:0:TCTGCAAG+GCTGCACT
CGCACTTTGAATATTTGGGTGAAAAGATTCGTCGGAATCTGGGTTGATTATGTCGAAGAACCTTGGAT
TCCGACATTTATACTTGCCGGAAAATTGGAGAAGACAGTGAAGGTGATTATCACCGTCTGAATGTTGT
TCCTGACATACTCTG
+
FFFFFFFFFFFFFF:FFFFFFFFFFFFFFFFFFFFFFFFFFFFFFFFFFFFFFFFFFFFFFFFFFFFF
FFFFFFFFFFFFFFFFFFFFFFFFFFFFFFFFFFFFFFFFFFFFFFFFFFFFFFFFFFFFFFFFFFFF
FFFFFFFFFFFFFFF
@A00721:190:HGJW7DSXY:4:2572:25129:18928 2:N:0:TCTGCAAG+GCTGCACT
CGCACTTTGAATATTTGGGTGAAAAGATTCGTCGGAATCTGGGTTGATTATGTCGAAGAACCTTGGAT
TCCGACATTTATACTTGCCGGAAAATTGGAGAAGACAGTGAAGGTGATTATCACCGTCTGAATGTTGT
TCCTGACATACTCTG
+
FFF,FFF:FFFFF:FFFFFFFFFFFFFFFFFFFFFFFFFFFFFFFFFFFFFFFFFFFFFFFFFFFFFF
FFFFFFFFFF:FFFFFFFFFFFFFFFFFFFFFFFFFFFFFFFFFFFFFFFFFFF:FFFFF
FFFFF:FFFFFFFFF
@A00721:190:HGJW7DSXY:4:1113:25690:16830 2:N:0:TCTGCAAG+GCTGCACT
GTTAATTTGGCGTTCATCAGAGTATGTCAGGAACAACATTCAGACGGTGATAATCACCTTCACTGTCT
TCTCCAATTTTCCGGCAAGTATAAATGTCGGAATCCAAGGTTCTTCGACATAATCAACCCAGATTCCG
ACGAATCTTTTCACC
+
FFFFFFFFFFFFFFFFFFFFFFFFFFFFFFFFFFFFFFFFFFFFFFFFFFFFFFFFFFFFFFFFFFFF
FFFFFFFFFFFFFF:FFFFFFFFFFFFFFFFFFFFFFFFFFFFFFFFFFFFFFFFFFF:FFFFFFFFF
FFFFFFFFFFFFFFF
@A00721:190:HGJW7DSXY:4:1229:30888:26772 2:N:0:TCTGCAAG+GCTGCACT
GTTAATTTGGCGTTCATCAGAGTATGTCAGGAACAACATTCAGACGGTGATAATCACCTTCACTGTCT
TCTCCAATTTTCCGGCAAGTATAAATGTCGGAATCCAAGGTTCTTCGACATAATCAACCCAGATTCCG
ACGAATCTTTTCACC
+
FFFFFFFFFFFFFFFFFF:FFFFFFFFFFFFF,FFFFFFFFFFFFFFFFFFFFFFFFFFFFFFFFFFF
FFFFFFFFFFFFFFFFF:FFF:FF:FFFFFFFFFFFFFFFFFFFFFF:FFFFFFFFFFFFFFFFFFFF
FFFF:FFFFFFFFFF
@A00721:190:HGJW7DSXY:4:1410:25464:5854 2:N:0:TCTGCAAG+GCTGCACT
GTTAATTTGGCGTTCATCAGAGTATGTCAGGAACAACATTCAGACGGTGATAATCACCTTCACTGTCT
```

```
TCTCCAATTTTCCGGCAAGTATAAATGTCGGAATCCAAGGTTCTTCGACATAATCAACCCAGATTCCG
ACGAATCTTTTCACC
+
FFFFFFFFFFFF:FFFFFFFFFFFFFFFFFFFFFFFFFFFFFFFFFFFFFFFFFFFFFFFFFFFFFFF
FFFFFFFFFFFFFFFFFFFFFFFFFFFFFFFFFFFFFFFFFFFFFFFFFFFFFFFFFFFFFFFFFFFF
FFFFFFFFFFFFFFF
@A00721:190:HGJW7DSXY:4:1531:3739:6308 2:N:0:TCTGCAAG+GCTGCACT
GTTAATTTGGCGTTCATCAGAGTATGTCAGGAACAACATTCAGACGGTGATAATCACCTTCACTGTCT
TCTCCAATTTTCCGGCAAGTATAAATGTCGGAATCCAAGGTTCTTCGACATAATCAACCCAGATTCCG
ACGAATCTTTTCACC
+
FFFFFFFFFFFFFFF:FFFFFFFFFFFFFFFF:FFFFFFFFFFFFFFFFFF,FFFFFFFFFFFFFFFF
FFFFFFFFFFFF:FFFFFFFFFFFFFFFFFFFFFFFFFFFFFFFFFFFF:FFFFFFFFFFFFFFFFFF
FFFFFFFFFFFFFFF
@A00721:190:HGJW7DSXY:4:1553:13232:14794 2:N:0:TCTGCAAG+GCTGCACT
GTTAATTTGGCGTTCATCAGAGTATGTCAGGAACAACATTCAGACGGTGATAATCACCTTCACTGTCT
TCTCCAATTTTCCGGCAAGTATAAATGTCGGAATCCAAGGTTCTTCGACATAATCAACCCAGATTCCG
ACGAATCTTTTCACC
+
FFFFFFFFFFFFFFFFFFFFFF,FFFFFFFFFFFFFFFFFFFFFFFFFFFFFFFFFFFFFFFFFFFFF
FFFFFFFFFFFFFFFFFFFFFFFFFFFFFFFFF:FFFF:FFFFFF:FFFFFFFF:FFF:F:FFFFFFF
FFFF:FFFFFFFF:F
@A00721:190:HGJW7DSXY:4:1669:15474:15233 2:N:0:TCTGCAAG+GCTGCACT
GTTAATTTGGCGTTCATCAGAGTATGTCAGGAACAACATTCAGACGGTGATAATCACCTTCACTGTCT
TCTCCAATTTTCCGGCAAGTATAAATGTCGGAATCCAAGGTTCTTCGACATAATCAACCCAGATTCCG
ACGAATCTTTTCACC
+
FFFFFFFFFFFFFFFFFFFFFFFFFFFFFFFFFFFFFFFFFFFFFFFFFFFFFFFFFFFFFFFFFFFF
FFFFFFFFFFFFFFFFFFFFFFFFFFFFFFFFFFFFFFFFFFFFFFFFFFFFFFFFFFFFFFFFFFF:
FFF:FFFFFFFF:FF
@A00721:190:HGJW7DSXY:4:2167:22625:22357 2:N:0:TCTGCAAG+GCTGCACT
GTTAATTTGGCGTTCATCAGAGTATGTCAGGAACAACATTCAGACGGTGATAATCACCTTCACTGTCT
TCTCCAATTTTCCGGCAAGTATAAATGTCGGAATCCAAGGTTCTTCGACATAATCAACCCAGATTCCG
ACGAATCTTTTCACC
+
FFFFFFFFFFFFFFF,FFFFFFFFFFFFFFFFFFFFFF:FFFF:FFFFFFFFFFFFFFFFFFFFFFFF
FFFFFFFFFFFFFFFFFFF:FFFFFFFFFFFFFFFFFFFFFFF:FFFFFFFFFFFFFFFFFFFF:FFF
FFFFFFF::FFFFFF
@A00721:190:HGJW7DSXY:4:2242:2022:32612 2:N:0:TCTGCAAG+GCTGCACT
GTTAATTTGGCGTTCATCAGAGTATGTCAGGAACAACATTCAGACGGTGATAATCACCTTCACTGTCT
TCTCCAATTTTCCGGCAAGTATAAATGTCGGAATCCAAGGTTCTTCGACATAATCAACCCAGATTCCG
ACGAATCTTTTCACC
+
FFFFFFFFFFFFFFFFFFFFFFFFFFFFFFFFFFFFFFFFFFFFFFFFFFFFFFFFFFFFFFFFFFF:
FFFFFFFFFFFFFFFFF,FFFFF:FFFFFFFF:FFFFFFFFFFFFFFFFFF:FFFFFF,FFFF:FF:F
FFFFFF:FFFFFFF:
@A00721:190:HGJW7DSXY:4:2334:13865:29731 2:N:0:TCTGCAAG+GCTGCACT
GTTAATTTGGCGTTCATCAGAGTATGTCAGGAACAACATTCAGACGGTGATAATCACCTTCACTGTCT
TCTCCAATTTTCCGGCAAGTATAAATGTCGGAATCCAAGGTTCTTCGACATAATCAACCCAGATTCCG
ACGAATCTTTTCACC
+
FFFFFFFFFFFFFFFFFFFFFFF:FFFFFFFFFFFFFFFFFFFFFFFFF:F:FFFFFFFFFFFFFFFF
FFFFFFFFFFFFFFFFFFFFFFFFFFFFFFFFFFFFFFFFFFFFFFFFFFFFFFFFFFFFFFFFFFFF
FFFFFFFFFFFFFFF
```

@A00721:190:HGJW7DSXY:4:2369:2953:12117 2:N:0:TCTGCAAG+GCTGCACT
GTTAATTTGGCGTTCATCAGAGTATGTCAGGAACAACATTCAGACGGTGATAATCACCTTCACTGTCT
TCTCCAATTTTCCGGCAAGTATAAATGTCGGAATCCAAGGTTCTTCGACATAATCAACCCAGATTCCG
ACGAATCTTTTCACC
+
FFF:FFFF:FFFFFFFFF:FFFFFF:FFFFFFFFFFFFFFFFFFFFFFFFFFFFFFFFFFFFFFFFFF
FFFFFFFFFFFFFFFFFFFF::FFFFF:F:FFFFFF,:FFFFFFFF:FF,FFFFFFF,FFFFFFFFFF
FFFFFFFF,FFFFFF
@A00721:190:HGJW7DSXY:4:2444:14145:12148 2:N:0:TCTGCAAG+GCTGCACT
GTTAATTTGGCGTTCATCAGAGTATGTCAGGAACAACATTCAGACGGTGATAATCACCTTCACTGTCT
TCTCCAATTTTCCGGCAAGTATAAATGTCGGAATCCAAGGTTCTTCGACATAATCAACCCAGATTCCG
ACTAATCTTTTCACC
+
FF:FFFFF::FF,FFFFFFFFF:FFFFFF:FF::FFFFFFFFFFF:FF:F,FF:FF:FF:FFFFFFFF
FFFFFFFF:::FFFF:FFFFFFFFFFFFF::FFF:FFF:F,FFFFF:F:FFFFF:FFFF,F:FFFFF:
FF,FFF,F,:FFF,:
@A00721:190:HGJW7DSXY:4:2622:22164:36464 2:N:0:TCTGCAAG+GCTGCACT
GTTAATTTGGCGTTCATCAGAGTATGTCAGGAACAACATTCAGACGGTGATAATCACCTTCACTGTCT
TCTCCAATTTTCCGGCAAGTATAAATGTCGGAATCCAAGGTTCTTCGACATAATCAACCCAGATTCCG
ACGAATCTTTTCACC
+
FFFFFFFFFFFFFFFFFFFFFFFFFFFFFFFFFFFFFFFFFFFFFFFFFFFFFFFFFFFFFFFFFFFF
FFFFFFFFFFFFFFFFF,FFFFFFFFFFFFFF:FFFFFFFFFFFFFFFFFFF:FFFFF:FFFFFFFFF
FFFFFFFFFFFFFFF
@A00721:190:HGJW7DSXY:4:1403:2926:13385 2:N:0:TCTGCAAG+GCTGCACT
GTGAAAAGATTCGTCGGAATCTGGGTTGATTATGTCGAAGAACCTTGGATTCCGACATTTATACTTGC
CGGAAAATTGGAGAAGACAGTGAAGGTGATTATCACCGTCTGAATGTTGTTCCTGACATACTCTGATG
AACGCCAAATTAACT
+
FFFFFFFFF:FFFFFFFF:FFFFFFFFFFFFFFFFFFFFFFFFF,:FFFFFFFFFF:FFFFFFFF:FF
FFFFFFF,FFFFFFFFFFFFFFF:FFFFFFFFFFFFFFFFFFFFFFFFFFFFFFFF:FF:FFFFFFFFF
FFFFFFFFF,FFFF:
@A00721:190:HGJW7DSXY:4:1505:4725:29559 2:N:0:TCTGCAAG+GCTGCACT
GTGAAAAGATTCGTCGGAATCTGGGTTGATTATGTCGAAGAACCTTGGATTCCGACATTTATACTTGC
CGGAAAATTGGAGAAGACAGTGAAGGTGATTATCACCGTCTGAATGTTGTTCCTGACATACTCTGATG
AACGCCAAATTAACT
+
FFFFFFFF:FFFFFFFFFFFFFFFFFF:FFFFFFFFFFFFFFFFFFFFFFFFFFFFFFFFFFFFFF:F
FFFFFFFFFFFFFFFFFFFFFFFF:FFFFFF:FFFFFFFFFFFFFFFFFFFFFFFFFFFFFFF,FF:F
FFFFFFFFF,FFFFF
@A00721:190:HGJW7DSXY:4:1304:10294:17816 2:N:0:TCTGCAAG+GCTGCACT
AAAATTTTAGTTTTCCAGTTAATTTGGCGTTCATCAGAGTATGTCAGGAACAACATTCAGACGGTGAT
AATCACCTTCACTGTCTTCTCCAATTTTCCGGCAAGTATAAATGTCGGAATCCAAGGTTCTTCGACAT
AATCAACCCAGATTC
+
FFFFFFFFFFFFFFFFFFFFFFFFFFFFFFFFFFFFFFFFFFFFFFFFF,FFFFFFFFFFFFFFFFFF
FFFFFFFFFFFFFFFFFFFFFFFFFFFFFFFFFFFFFFFFFFFFFFFFFFFFFFF,FFFFFFFFFF:F
FFFFFFFF:FFFFFF
@A00721:190:HGJW7DSXY:4:1356:31078:31673 2:N:0:TCTGCAAG+GCTGCACT
AAAATTTTAGTTTTCCAGTTAATTTGGCGTTCATCAGAGTATGTCAGGAACAACATTCAGACGGTGAT
AATCACCTTCACTGTCTTCTCCAATTTTCCGGCAAGTATAAATGTCGGAATCCAAGGTTCTTCGACAT
AATCAACCCAGATTC
+
FFFFFFFFFFFFFFFFFFFFFFFFFFFFFFFFFFFFFFFFFFFFFFFFFFFFFFFFFFFFF:FFFFFFF

FFFFFFFFFFFFFFFFFFFFFFFF:FFFFFFFF:FFFFFFFF:FFFF:FFFFFFFFFFFFFFFFFFFFFF
FFFFFFFFFFFFFFF
@A00721:190:HGJW7DSXY:4:1374:23619:14403 2:N:0:TCTGCAAG+GCTGCACT
AAAATTTTAGTTTTCCAGTTAATTTGGCGTTCATCAGAGTATGTCAGGAACAACATTCAGACGGTGAT
AATCACCTTCACTGTCTTCTCCAATTTTCCGGCAAGTATAAATGTCGGAATCCAAGGTTCTTCGACAT
AATCAACCCAGATTC
+
:FF:FFFFFFFFFFFFFFFFFFFF:FFFFFFFFFFFFFFFFFFFFFFFFFFFFFFFFFFFFFFFFFFFFF
FFFFFFFFFFFFFFFFFFFFFFFF:FFFFFFFFFFFFFFFFF,,FFFFF:FFFFFFFFFFFFFFFFFF:F
F:FFFFFFFFFF:FF
@A00721:190:HGJW7DSXY:4:1420:32723:23688 2:N:0:TCTGCAAG+GCTGCACT
AAAATTTTAGTTTTCCAGTTAATTTGGCGTTCATCAGAGTATGTCAGGAACAACATTCAGACGGTGAT
AATCACCTTCACTGTCTTCTCCAATTTTCCGGCAAGTATAAATGTCGGAATCCAAGGTTCTTCGACAT
AATCAACCCAGATTC
+
:F:FFF:FFFFFFFFFFFFFFFFFFFFFFFFFFFFFFFFFFFFFFFFFFFF:FFFFFFFFFFFFFFFFFF
FFFFFFFFFFFFFFFFFFFFFFFFFFFFFFFFFFFFFFF,FFFFFFFFFFFFFFFF:FFFF:FFFFFF:,
FFFFFFFF::FFFFF
@A00721:190:HGJW7DSXY:4:1509:15076:26819 2:N:0:TCTGCAAG+GCTGCACT
AAAATTTTAGTTTTCCAGTTAATTTGGCGTTCATCAGAGTATGTCAGGAACAACATTCAGACGGTGAT
AATCACCTTCACTGTCTTCTCCAATTTTCCGGCAAGTATAAATGTCGGAATCCAAGGTTCTTCGACAT
AATCAACCCAGATTC
+
FFFFFFFFFFFFFFFFFFFFFFFFFFFFFFFFFFFFFFFFFFFFFFFFFFFFFFFFFFFFFFFFFFFFFF
FFFFFFFFFFFFFFFFFFFFFFFFFFFFFFFFFFFFFFFFFF:FFFFFFFFFFFFFFFFFFFFFFFFFFF
FFFFFFFFFFFFFFF
@A00721:190:HGJW7DSXY:4:2149:14018:34319 2:N:0:TCTGCAAG+GCTGCACT
AAAATTTTAGTTTTCCAGTTAATTTGGCGTTCATCAGAGTATGTCAGGAACAACATTCAGACGGTGAT
AATCACCTTCACTGTCTTCTCCAATTTTCCGGCAAGTATAAATGTCGGAATCCAAGGTTCTTCGACAT
AATCAACCCAGATTC
+
FFFFFF:,FFFF:,FFFFFFFFFFF,FFFFFFFFFFFFFFF,F,FFFFFFF,FFFFFFF:F:FFFF,FFF
FFF,F:::FFFF,,FFFFF:FFFF,,F:FFFFFFFFFF,FFFFFF,F,:,F:FFFFFF:FFFFFFFFFFF
:FF,FFF:,FFFFFF
@A00721:190:HGJW7DSXY:4:2222:11279:7842 2:N:0:TCTGCAAG+GCTGCACT
AAAATTTTAGTTTTCCAGTTAATTTGGCGTTCATCAGAGTATGTCAGGAACAACATTCAGACGGTGAT
AATCACCTTCACTGTCTTCTCCAATTTTCCGGCAAGTATAAATGTCGGAATCCAAGGTTCTTCGACAT
AATCAACCCAGATTC
+
FFFFFFFFFFFFFFFFFFFFFFFFFFFFFFFFFFFFFFFFFFFFFFFFFFFFFFFFF:FFFFFFFFFFFF
:FFFFFFFFFFFFFFFFF:FFFFFFFFFFF::FFF,:FFFFF:FFFFFFFFFF:FFFFFFFFF:FFFFFF
FFFFF:F:FFFFFFF
@A00721:190:HGJW7DSXY:4:1154:7184:16376 2:N:0:TCTGCAAG+GCTGCACT
AAAAATTTTAGTTTTCCAGTTAATTTGGCGTTCATCAGAGTATGTCAGGAACAACATTCAGACGGTGA
TAATCACCTTCACTGTCTTCTCCAATTTTCCGGCAAGTATAAATGTCGGAATCCAAGGTTCTTCGACA
TAATCAACCCAGATT
+
:FFFFFFFFFFFFFFFFFFFFFFFFFFFFFFFFFFFFFFFFFFFFFFFFFF:FFFFFFFFFFFFFFFFFF
FFFFFFFFFFFFFFFFFFFFFFFFFFFFFFFFFFFF:FFFFFFFFFFFFFFFFFFFFFFFFFFFFFFFFF
FF:FFFFFFFFFFF:
@A00721:190:HGJW7DSXY:4:1170:29288:15515 2:N:0:TCTGCAAG+GCTGCACT
AAAAATTTTAGTTTTCCAGTTAATTTGGCGTTCATCAGAGTATGTCAGGAACAACATTCAGACGGTGA
TAATCACCTTCACTGTCTTCTCCAATTTTCCGGCAAGTATAAATGTCGGAATCCAAGGTTCTTCGACA
TAATCAACCCAGATT

```
+
FFFFFFFFFFFFFFFFFFFFFFFFFFFFFFFFFFFFFFFFFFFFFFFFFFFFFFF:FFFFFFFFFFFF
FFFFFFFFFFFFFFFFFFFFFFFFFFFFFFFFFFFFFFFFFFF:FFFFFFFFFFFFFFFFFFFFFFFF
FFFFFFFFFFFFFF:
@A00721:190:HGJW7DSXY:4:1278:5665:16689 2:N:0:TCTGCAAG+GCTGCACT
AAAAATTTTAGTTTTCCAGTTAATTTGGCGTTCATCAGAGTATGTCAGGAACAACATTCAGACGGTGA
TAATCACCTTCACTGTCTTCTCCAATTTTCCGGCAAGTATAAATGTCGGAATCCAAGGTTCTTCGACA
TAATCAACCCAGATT
+
FFFFFFFFFFFFFFFFFFFFFFFFFFFFFFFFFFFFFFFFFFFFFFFF:FFFFFFFFFFFFFFFFFFF
FFFFFFFFFFFFFFFFFFFFFFFFFFFFFFFFFFFFFFFFFF,FFFFFFF:FFFFFFFFFFFFFFFFF
F:FFFFFFFFFFFFF
@A00721:190:HGJW7DSXY:4:1319:5150:23281 2:N:0:TCTGCAAG+GCTGCACT
AAAAATTTTAGTTTTCCAGTTAATTTGGCGTTCATCAGAGTATGTCAGGAACAACATTCAGACGGTGA
TAATCACCTTCACTGTCTTCTCCAATTTTCCGGCAAGTATAAATGTCGGAATCCAAGGTTCTTCGACA
TAATCAACCCAGATT
+
FFFFFFFFFFFFFFFFFFFFF:FFFFFFFFFFFFFFFFFFFFFFFFFFFFFFFFFFFFFF:FFFFFFF
FFFFFFFFFFFFFFFFFFFFFFFFFF:FFFFFFFF:FFFFFFFFFFFFFFFFFFFFFFFFFFFF,FFF
FFFFFFFF:FFFFFF
@A00721:190:HGJW7DSXY:4:1617:17400:11866 2:N:0:TCTGCAAG+GCTGCACT
AAAAATTTTAGTTTTCCAGTTAATTTGGCGTTCATCAGAGTATGTCAGGAACAACATTCAGACGGTGA
TAATCACCTTCACTGTCTTCTCCAATTTTCCGGCAAGTATAAATGTCGGAATCCAAGGTTCTTCGACA
TAATCAACCCAGATT
+
FFFFFFFFFFFF:FFFFFFFFFFFFFFFFFFFFFFFFFFFFFFFFFFFFF:FFFFFFFFFFFFFFFFF
FFFFFFFFFFFFFFFFFFFFFFFFFFFFFFFFFFFFFFFFFF:FFFFFFFFFFFFFFFFFFFFFFFFF
FFFF:FFFFFFFFFF
@A00721:190:HGJW7DSXY:4:1476:11577:21449 2:N:0:TCTGCAAG+GCTGCACT
AAAAATTTTAGTTTTCCAGTTAATTTGGCGTTCATCAGAGTATGTCAGGAACAACATTCAGACGGTGA
TAATCACCTTCACTGTCTTCTCCAATTTTCCGGCAAGTATAAATGTCGGAATCCAAGGTTCTTCGACA
TAATCAACCCAGAT
+
FFFFF:FFFFFFFFFFFFFFFFFFFFFFFFFFFFFFFFFFFFFFFFFFFFFFFFFFFFFFF:FFFFFF
FFFFFFFFFFFFFFFFFFFFFFFFFFFFFFFFFFFFFFFFFF,:FFFFFFFFF:FFFFFFFFFFFFFF
FFFFFFFFFFFFFF
@A00721:190:HGJW7DSXY:4:1132:20265:13009 2:N:0:TCTGCAAG+GCTGCACT
CTCGACCAAATTAAAAATTTTAGTTTTCCAGTTAATTTGGCGTTCATCAGAGTATGTCAGGAACAACA
TTCAGACGGTGATAATCACCTTCACTGTCTTCTCCAATTTTCCGGCAAGTATAAATGTCGGAATCCAA
GGTTCTTCGACATAA
+
FFFFFFFFFFFF:FFFF:FFFFFFFFFFFFFFFFFFFFF:FFFFFFF:FFFFFFFFFFFFFFFFFFF:
F:FFF:FFFF:FFFFFFFFFFFFFFFFF,FFFFFFF:FFFFFFFFFFFFFFFFF::FFFF:F,FFFFF
,FFFFFFFFFFFFFF
@A00721:190:HGJW7DSXY:4:1166:12689:28040 2:N:0:TCTGCAAG+GCTGCACT
CTCGACCAAATTAAAAATTTTAGTTTTCCAGTTAATTTGGCGTTCATCAGAGTATGTCAGGAACAACA
TTCAGACGGTGATAATCACCTTCACTGTCTTCTCCAATTTTCCGGCAAGTATAAATGTCGGAATCCAA
GGTTCTTCGACATAA
+
FFFFFFFFFFFFFFFFFFF:FFFFFFFFFFFFF:FFFFFFFFFFFFFFFFFFF:FFFFFFFFFFFFFF
FFFFFFFFFFFFFFFFFFFFFFFFFFFFFFFFFFFFFFFFFFFFFFF:FFFFFFFFFFFFFFFFFFFF
FFFFFFFFFFFFFFF
@A00721:190:HGJW7DSXY:4:1253:23312:35008 2:N:0:TCTGCAAG+GCTGCACT
CTCGACCAAATTAAAAATTTTAGTTTTCCAGTTAATTTGGCGTTCATCAGAGTATGTCAGGAACAACA
```

TTCAGACGGTGATAATCACCTTCACTGTCTTCTCCAATTTTCCGGCAAGTATAAATGTCGGAATCCAA
GGTTCTTCGACATAA
+
FFFFFFFFFFFFFFFFFFFFFFFFFFFFFFFFFF:FFFFFFFFFFFFFFFFFFFFFFFF:FFFFFFFF
FFFFFFFFFFFFFFFFFFFFFFFFFFFFFFFFFFF:FFFFFFFFFFFFFF:FFFFFFFFFFF,FFFFF
FFFFFFFFFFFFFFF
@A00721:190:HGJW7DSXY:4:1269:3938:33865 2:N:0:TCTGCAAG+GCTGCACT
CTCGACCAAATTAAAAATTTTAGTTTTCCAGTTAATTTGGCGTTCATCAGAGTATGTCAGGAACAACA
TTCAGACGGTGATAATCACCTTCACTGTCTTCTCCAATTTTCCGGCAAGTATAAATGTCGGAATCCAA
GGTTCTTCGACATAA
+
FFFFFFFFFFFFFFFFFFFFFFFFFFFFFFFFFFFFFFFFFFFFFFFFFFFFFFFFFFFFFFFFFFFF
FFFFFFFFFFF:FFFFFFFFFFFFFFFFFFFFFFFFFFFFFFFFFFFFFFFFFFF:FFFFFFFFFFFF
F:FFFF::FFFFFFF
@A00721:190:HGJW7DSXY:4:1317:30969:1110 2:N:0:TCTGCAAG+GCTGCACT
CTCGACCAAATTAAAAATTTTAGTTTTCCAGTTAATTTGGCGTTCATCAGAGTATGTCAGGAACAACA
TTCAGACGGTGATAATCACCTTCACTGTCTTCTCCAATTTTCCGGCAAGTATAAATGTCGGAATCCAA
GGTTCTTCGACATAA
+
FFFFFFFFFFFFFFFFFFFFFFFFFFFFFFFFFFFFFFFFF:FFFFFFFFFFFFFFFFFFFFFFFFF:
FF:FF:FFFFFFFFFFFFFFFFFF:FFFFFF:FFFFFFFFF:FFFFFFFFFFFFFFFFFFFFFFF::F
FFFFFFFF:FFFFFF
@A00721:190:HGJW7DSXY:4:1613:9679:5102 2:N:0:TCTGCAAG+GCTGCACT
CTCGACCAAATTAAAAATTTTAGTTTTCCAGTTAATTTGGCGTTCATCAGAGTATGTCAGGAACAACA
TTCAGACGGTGATAATCACCTTCACTGTCTTCTCCAATTTTCCGGCAAGTATAAATGTCGGAATCCAA
GGTTCTTCGACATAA
+
FFFFFFFFFFFFFFFFFFFFFFFFFFFFFFFFFFFFFFFFFFFFFFFFFF:FFFFFFFFFFFFFFFFF
FFFFFFFFFFFFFFFFFFFFFFFFFFFFFFFFFFFFFFFFFFFFFFFFFF:FFFFFFFF:FFFFFFFF
FFFFFFFFFFFFFFF
@A00721:190:HGJW7DSXY:4:1613:9534:5321 2:N:0:TCTGCAAG+GCTGCACT
CTCGACCAAATTAAAAATTTTAGTTTTCCAGTTAATTTGGCGTTCATCAGAGTATGTCAGGAACAACA
TTCAGACGGTGATAATCACCTTCACTGTCTTCTCCAATTTTCCGGCAAGTATAAATGTCGGAATCCAA
GGTTCTTCGACATAA
+
FFFFFFFFFFFFFFFFFFFFFFFFFFFFFFFFFFFFFFFFFFFFFFFFFFFFFFFFFFFFFFFFFFFF
FFFFFFFFFFFFFFFFFFFFFFFFFFFFFFFFFFFFFFFFFFFFFFFFFFFFFFFFF,FFFFFFFFFF
FFFFFFF:FFFFFFF
@A00721:190:HGJW7DSXY:4:2174:31973:32283 2:N:0:TCTGCAAG+GCTGCACT
CTCGACCAAATTAAAAATTTTAGTTTTCCAGTTAATTTGGCGTTCATCAGAGTATGTCAGGAACAACA
TTCAGACGGTGATAATCACCTTCACTGTCTTCTCCAATTTTCCGGCAAGTATAAATGTCGGAATCCAA
GGTTCTTCGACATAA
+
FFFFFFFFFFFFFFFFFFFFFFFFFFFFFFFFFFFFFFFFFFFFFFFFFFF:FFFFFFFFFFFFFFFF
FFFFF:FFFFFFFFFFFFFFFFFFFFFFFFFFFFFFFFFFF:FFFFFFFFFFF:,FFFF:FFFFF:FF
FFFFFFFFF,FFFFF
@A00721:190:HGJW7DSXY:4:2231:5113:31203 2:N:0:TCTGCAAG+GCTGCACT
CTCGACCAAATTAAAAATTTTAGTTTTCCAGTTAATTTGGCGTTCATCAGAGTATGTCAGGAACAACA
TTCAGACGGTGATAATCACCTTCACTGTCTTCTCCAATTTTCCGGCAAGTATAAATGTCGGAATCCAA
GGTTCTTCGACATAA
+
FFFFFFFFFFFFFFFFFFFFFF:FFFFFFFFFFFFFFF:FFFFFFFFFFFFFFFFFFFFFFFFFFFFF
FFFFFFFF:FFFFFFFFFFFFFFFFFFFFFFFFFFFFFFFF:FFFFFFFFFFFFFFFFFFFFFFFFFF
FFFFFFF:FFF:FFF

```
@A00721:190:HGJW7DSXY:4:2370:2917:28401 2:N:0:TCTGCAAG+GCTGCACT
CTCGACCAAATTAAAAATTTTAGTTTTCCAGTTAATTTGGCGTTCATCAGAGTATGTCAGGAACAACA
TTCAGACGGTGATAATCACCTTCACTGTCTTCTCCAATTTTCCGGCAAGTATAAATGTCGGAATCCAA
GGTTCTTCGACATAA
+
FFFFFFFFFFFFFFFFFFFFF:FFFFFFFFFFF:FFFFFFFFFFFFFFFFFFFFFFFFFFFFFFFFFF
FFFFFFFFFFFFFFFFFFFFFFFFFFFFFFFFFFFFFFFFFFFFFFFFFFFFFF,FFFFFFFFFFFFF
FFFFFFFFFFFFFFF
@A00721:190:HGJW7DSXY:4:2470:12192:29716 2:N:0:TCTGCAAG+GCTGCACT
CTCGACCAAATTAAAAATTTTAGTTTTCCAGTTAATTTGGCGTTCATCAGAGTATGTCAGGAACAACA
TTCAGACGGTGATAATCACCTTCACTGTCTTCTCCAATTTTCCGGCAAGTATAAATGTCGGAATCCAA
GGTTCTTCGACATAA
+
FFFFFFFFFFFFFFFFFFFFFFFFFFFFFFFFFFFFFFFFFFFFFFFFFFFFFFFFFFFFFFFFFFFF
FFFFFFFFFFFFFFFFFFFFFFFFFFFFFFFFFFFFFFFFFFFF:FFFFFFFFFFFFFFFF:FFFFFF
:FFF:FFFFFF:FFF
@A00721:190:HGJW7DSXY:4:1458:15564:11130 2:N:0:TCTGCAAG+GCTGCACT
TGTCGAAGAACCTTGGATTCCGACATTTATACTTGCCGGAAAATTGGAGAAGACAGTGAAGGTGATTA
TCACCGTCTGAATGTTGTTCCTGACATACTCTGATGAACGCCAAATTAACTGGAAAACTAAAATTTTT
AATTTGGTCGAGAGA
+
FFFFFFFFFFFFFFFFFFFFFFFFFFFFFFFFFFFFFFFFFFFFFFFFFFFFFF:FFFFFFFFFFFFF
FFFFFFFFFFFFFFFFFFFFFFFFFFFFFFFFFFFFFFFFFFFFFFFFFFFFFFFFFFFFFFFFFFFF
FFFFFFFFFFFFFFF
@A00721:190:HGJW7DSXY:4:2218:29080:11898 2:N:0:TCTGCAAG+GCTGCACT
TGTCGAAGAACCTTGGATTCCGACATTTATACTTGCCGGAAAATTGGAGAAGACAGTGAAGGTGATTA
TCACCGTCTGAATGTTGTTCCTGACATACTCTGATGAACGCCAAATTAACTGGAAAACTAAAATTTTT
AATTTGGTCGAGAGA
+
FFFFFFFFFFFFFFFFFFFFFFFFFFFFFFFFFFFFFFFFFFFFFFFFFFFFFFFFFFFF:FFFFFFF
F:FFFFFFFFFFFFFFFFFF:FFFFFFFFFFFFFF:FFF:FFFFFFFFFFFFFFFFFFFFFFFFFFFF
FFFFFFFFFFFFF:F
@A00721:190:HGJW7DSXY:4:2533:12852:6214 2:N:0:TCTGCAAG+GCTGCACT
TGTCGAAGAACCTTGGATTCCGACATTTATACTTGCCGGAAAATTGGAGAAGACAGTGAAGGTGATTA
TCACCGTCTGAATGTTGTTCCTGACATACTCTGATGAACGCCAAATTAACTGGAAAACTAAAATTTTT
AATTTGGTCGAGAGA
+
FFFFFFFFFFF:FFFFFFFFFFFFFFFFFFFFFFFFFFFFFFFFFFFFFFF,FFFFFFFFFFFFFFFF
FFFFFFFFF:FFF:FFFFFF,FFFFFFFFFF:FFFFFFFFFFFFFFFFFFF,FFFFFFFFFFFFFFFF
FFFFF:,FFFFFFF:
@A00721:190:HGJW7DSXY:4:1151:21612:35947 2:N:0:TCTGCAAG+GCTGCACT
ATTGTTCTCGACCAAATTAAAAATTTTAGTTTTCCAGTTAATTTGGCGTTCATCAGAGTATGTCAGGA
ACAACATTCAGACGGTGATAATCACCTTCACTGTCTTCTCCAATTTTCCGGCAAGTATAAATGTCGGA
ATCCAAGGTTCTTCG
+
FFFFFFFFFFFFFFFFFFFFFFFFFFFFFFFFFFFFFFFFF,FFFFFFFFFFFFFFFFFFFFFFFFFF
FFFFFFFFFFFFFFFFFFFFFFFFFFFFFFFFFFFFFFFFFFFFFFFFFFFF,FFFFFFFFFFFFFFF
FFFFFFFFFFFFFFF
@A00721:190:HGJW7DSXY:4:1204:1841:5431 2:N:0:TCTGCAAG+GCTGCACT
ATTGTTCTCGACCAAATTAAAAATTTTAGTTTTCCAGTTAATTTGGCGTTCATCAGAGTATGTCAGGA
ACAACATTCAGACGGTGATAATCACCTTCACTGTCTTCTCCAATTTTCCGGCAAGTATAAATGTCGGA
ATCCAAGGTTCTTCG
+
FFFFFFFFFFFFFFFFFFFFFFFFFFFFFFFFFFFFFFFFFFFFFFFFFFFFFF:FFFFFFFFFFFF
```

```
:FF:FFF,FFFFFFFFFFFFFFFFFFFFFFFFFFFFFFFFFFFFFFFFFFFFF,FFFFFFFFFFFFFF
,FFFF:FFFFFFFFF
@A00721:190:HGJW7DSXY:4:2241:18114:23249 2:N:0:TCTGCAAG+GCTGCACT
ATTGTTCTCGACCAAATTAAAAATTTTAGTTTTCCAGTTAATTTGGCGTTCATCAGAGTATGTCAGGA
ACAACATTCAGACGGTGATAATCACCTTCACTGTCTTCTCCAATTTTCCGGCAAGTATAAATGTCGGA
ATCCAAGGTTCTTCG
+
FFFFFFFFFFFFFFFFFFFFFFFFFFFFFFFFFFFFFFFFFFFFFFFFFFFFFFFFFFF:FFFFFFFF
FFFFFFFFFFF:FFFFFFFFFFFFFFFFFFFFFFFFFFFFFFFFFFFFFFFFFFFFFFFFFFFFFFFF
FFFFFF:FFFFFFFF
@A00721:190:HGJW7DSXY:4:2241:17815:23359 2:N:0:TCTGCAAG+GCTGCACT
ATTGTTCTCGACCAAATTAAAAATTTTAGTTTTCCAGTTAATTTGTCGTTCATCAGAGTATGTCAGGA
ACAACATTCAGACGGTGATAATCACCTTCACTGTCTTCTCCAATTTTCAGGCAAGTATAAATGTCGGA
ATCCAAGGTTCTTCG
+
FFFFFF:FFFFFFFFF:,:FFFF:FFFFFF::FFFF,FFFFFF:F,FF,FFFF:FFFF:FFFFFF:FF
FFFFFFFFFFFFFFFFFFFFFFFFFFFFFF:FFFFFFFFFFFFFFF::,FFFFFFFF:FF:FFFF::F
FFFFFF:FFFFFFFF
@A00721:190:HGJW7DSXY:4:1403:11867:7921 2:N:0:TCTGCAAG+GCTGCACT
CCAATTTCCAAAGAAATTGTTCTCGACCAAATTAAAAATTTTAGTTTTCCAGTTAATTTGGCGTTCAT
CAGAGTATGTCAGGAACAACATTCAGACGGTGATAATCACCTTCACTGTCTTCTCCAATTTTCCGGCA
AGTATAAATGTCGGA
+
FFFFFFFFFFFFF:FFFFFFFFFFFFFFFFFFFFFFFFFFFFFFFFFFFFFFFFFFFFFFFFFFFFFF
FFFFFFFFFFFFFFFFFFFFFFFFFFFFFFFFFFFFFFFFFFFFFFFFFFFFFFFFFF:FFFF,FFF:
FFFFFFFF:FFFFFF
@A00721:190:HGJW7DSXY:4:1457:4345:28463 2:N:0:TCTGCAAG+GCTGCACT
CCAATTTCCAAAGAAATTGTTCTCGACCAAATTAAAAATTTTAGTTTTCCAGTTAATTTGGCGTTCAT
CAGAGTATGTCAGGAACAACATTCAGACGGTGATAATCACCTTCACTGTCTTCTCCAATTTTCCGGCA
AGTATAAATGTCGGA
+
FFFFFFFFFF,FFFFF:FFFFFFFFFFFFF,FFFFFFFFF:FFFF:FFFFFFFFFFFFFFFFF:FFFF
FFFFF:FFFFFFFFFFFFFFFFFFFFFFFFFFFFFFFFF:FFFFFFFFFFFFFFFF:FFFFFFFFFFF
FFFFFFFFFFFF:FF
@A00721:190:HGJW7DSXY:4:1561:4354:34209 2:N:0:TCTGCAAG+GCTGCACT
CCAATTTCCAAAGAAATTGTTCTCGACCAAATTAAAAATTTTAGTTTTCCAGTTAATTTGGCGTTCAT
CAGAGTATGTCAGGAACAACATTCAGACGGTGATAATCACCTTCACTGTCTTCTCCAATTTTCCGGCA
AGTATAAATGTCGGA
+
FFFFFFFFFF:FFFFF::FFFFFFFFF::,F,:F,F:F,FFFFFFF,:F:FFFFF::FFFFF:FFFFF
F:FFFFFFFFFFFFFF,F,FF,,F:F:F,:::FFFF,FF:F,FFFFFFFF:FF,:F:FFFFFF,:F::
F,FFFFFFFFFF:,F
@A00721:190:HGJW7DSXY:4:2130:2374:17378 2:N:0:TCTGCAAG+GCTGCACT
CCAATTTCCAAAGAAATTGTTCTCGACCAAATTAAAAATTTTAGTTTTCCAGTTAATTTGGCGTTCAT
CAGAGTATGTCAGGAACAACATTCAGACGGTGATAATCACCTTCACTGTCTTCTCCAATTTTCCGGCA
AGTATAAATGTCGGA
+
FFFFFFFFFFFFFFFFFFFFFFFFFFFFFFFFFFFFFF:FFFFFFFFFFFFFFFFFFFFFFFFFFF:F
FFFFFFFFFFFFFFFFFFFFFFFFFFFFFFFFFFF:FFFFFFFFFFFFFFFFFFFF:FFFFFFFFFFF
FFFF:FFFFFFFFFF
@A00721:190:HGJW7DSXY:4:2251:11740:33599 2:N:0:TCTGCAAG+GCTGCACT
CCAATTTCCAAAGAAATTGTTCTCGACCAAATTCAAAATTTTAGTTTTCCAGTTAATTTGGCGTTCAT
CAGAGTATGTCAGGAACAACATTCAGACGGTGATAATCACCTTCACTGTCTTCTCCAATTTTCCGGCA
AGTATAAATGTCGGA
```

```
+
FFFFFFFFFFF,FFFFFFF:FFFFFFFFFF:FF,:F:FFFFF:FFF,FFF:FF::FFFFFFFFFFFFF
FFFFFF,FFFFFFFFFFFFFFF,FFFFFFF,FFFFFFFFFFFFFFFFFFFFFFFFFFFF:FFFFFFFF
FFFFFFFFFFFFFFF
@A00721:190:HGJW7DSXY:4:2370:12545:25723 2:N:0:TCTGCAAG+GCTGCACT
CCAATTTCCAAAGAAATTGTTCTCGACCAAATTAAAAATTTTAGTTTTCCAGTTAATTTGGCGTTCAT
CAGAGTATGTCAGGAACAACATTCAGACGGTGATAATCACCTTCACTGTCTTCTCCAATTTTCCGGCA
AGTATAATTGTCGGA
+
FFFFFFFFFFFFFFFFFFFFFFFFFFFFFFFFFFF:FFFFFFFFFFFFFFFFFFF:FFFFFFFFFFFF
FFFFFFFFFFF:FFFFFFFFFFFFFFFFFFFFFFFFFFFFFFFFFFFFFFFFFFFFFF:FFFFFFFF:
FFFFFFF,FFFFFFF
@A00721:190:HGJW7DSXY:4:2404:24017:1219 2:N:0:TCTGCAAG+GCTGCACT
CCAATTTCCAAAGAAATTGTTCTCGACCAAATTAAAAATTTTAGTTTTCCAGTTAATTTGGCGTTCAT
CAGAGTATGTCAGGAACAACATTCAGACGGTGATAATCACCTTCACTGTCTTCTCCAATTTTCCGGCA
AGAATAAATGTCGGA
+
FFFFFFFF:FFFFF:,FFF:FFFFFFFFFFFFFFFFFF:FFFFFFFFFFFFFFFFF:FFFF:F:FFFF
FFFFFFFFFFFFFFFF,FFFFFFFFFFFFF:FFFFF:FFFFFFFFFFFFF:FFFFFFFF:FFF:FFF,
FF,FFFFFFFFFFFF
@A00721:190:HGJW7DSXY:4:2457:5556:24079 2:N:0:TCTGCAAG+GCTGCACT
CCAATTTCCAAAGAAATTGTTCTCGACCAAATTAAAAATTTTAGTTTTCCAGTTAATTTGGCGTTCAT
CAGAGTATGTCAGGAACAACATTCAGACGGTGATAATCACCTTCACTGTCTTCTCCAATTTTCCGGCA
AGTATAAATGTCGGA
+
FFFFFFFFFFFFFFFF:FFFFFFFFFFFFF:FFFFFFFF:FFFF:F:FFFFFFFFFFF:FFFFFFFFF
FFFFFFFFF:FFFFFF:FFFF:,F:FFFF:FFF:FFFFFFFFFFFF,FFFFFFFFFFFFFFFF:FFFF
FFFF,FFF,FFFFFF
@A00721:190:HGJW7DSXY:4:2516:3631:12633 2:N:0:TCTGCAAG+GCTGCACT
CCAATTTCCAAAGAAATTGTTCTCGACCAAATTAAAAATTTTAGTTTTCCAGTTAATTTGGCGTTCAT
CAGAGTATGTCAGGAACAACATTCAGACGGTGATAATCACCTTCACTGTCTTCTCCAATTTTCCGGCA
AGTATAAATGTCGGA
+
FFFFFFFFFFFFFFFFFFFFFFFFFFFFFFFFFFF:FFFFFFF:FFFF:FFFFFFFFFFFFFFFFFFF
FFFFFFFFFFFFFFFFFFFFFFFFFFFF:FFFFFFFFFFFFFFFFFFFFFFFFFFFFFFFFFFFF:FF
FFFFFFFFFFFFFFF
@A00721:190:HGJW7DSXY:4:2543:16785:14559 2:N:0:TCTGCAAG+GCTGCACT
CCAATTTCCAAAGAAATTGTTCTCGACCAAATTAAAAATTTTAGTTTTCCAGTTAATTTGGCGTTCAT
CAGAGTATGTCAGGAACAACATTCAGACGGTGATAATCACCTTCACTGTCTTCTCCAATTTTCCGGCA
AGTATAAATGTCGGA
+
FFFFFFFFFFFFFFFFFFFFFFFFFFFFFFFFFFFFFF:FFFFFFFFFFFFFFFF:FFFFFFFFFFFF
FFFF,FFFFFFFF:FFFFFFFFFFFFFFFFFFFFF:FFFFFFFFFFFFFFFFFFFFFFFFFFFFFFFF
,FF:FFFFFFFFFFF
@A00721:190:HGJW7DSXY:4:1537:1199:19601 2:N:0:TCTGCAAG+GCTGCACT
TCAAGATGTCCAATTTCCAAAGAAATTGTTCTCGACCAAATTAAAAATTTTAGTTTTCCAGTTAATTT
GGCGTTCATCAGAGTATGTCAGGAACAACATTCAGACGGTGATAATCACCTTCACTGTCTTCTCCAAT
TTTCCGGCAAGTATA
+
FFFFFFFFFFFFFFFFFFFFFFFFFFFFFFFFFFFFFFFFFFFFFFFFFFFFFFFFFFFFFFFFFFFF
FFFFFFFFFFFFFFFFFFFFFFFFFFFFFFFFFFFFFFFFFFFFFF:FFFFFFFFFFFFFFFFFFF:F
FFFF:FFFFFFFFFF
@A00721:190:HGJW7DSXY:4:2344:4038:12273 2:N:0:TCTGCAAG+GCTGCACT
TCAAGATGTCCAATTTCCAAAGAAATTGTTCTCGACCAAATTAAAAATTTTAGTTTTCCAGTTAATTT
```

```
GGCGTTCATCAGAGTATGTCAGGAACAACATTCAGACGGTGATAATCACCTTCACTGTCTTCTCCAAT
TTTCCGGCAAGTATA
+
FFFFFFFFFFFFFFFFFFFFFFFFFFFFFFFFFFFFFFFFFFFFFFFFFFFFFFFFFFFFFFFFFFFFF
:FFFFFF:FFFFFFF:FFFFFFFFFFFFFFFFFFFFFFFFFF:FFFFFFFFFF::FFFFFFFFFFF
FFFFFFFFFFFFFFF
@A00721:190:HGJW7DSXY:4:2439:13702:15608 2:N:0:TCTGCAAG+GCTGCACT
TCAAGATGTCCAATTTCCAAAGAAATTGTTCTCGACCAAATTAAAAATTTTAGTTTTCCAGTTAATTT
GGCGTTCATCAGAGTATGTCAGGAACAACATTCAGACGGTGATAATCACCTTCACTGTCTTCTCCAAT
TTTCCGGCAAGTATA
+
FFFFFFFFFFFFFFFFFFFFFFFFFFFFFFFFFFFFFFFFFF,FFFFFFFFFFFFFFFFFFFFFFFFFF
FFFFFFFFFFFFFFFFFFFFFFFFFFF,FFF:FFFFFFFFFFFFFFFFFFFFFFFFFFFFFFFFFFFFFF
FFFFFFFFFFFFFFF
@A00721:190:HGJW7DSXY:4:2551:11894:19774 2:N:0:TCTGCAAG+GCTGCACT
TCAAGATGTCCAATTTCCAAAGAAATTGTTCTCGACCAAATTAAAAATTTTAGTTTTCCAGTTAATTT
GGCGTTCATCAGAGTATGTCAGGAACAACATTCAGACGGTGATAATCACCTTCACTGTCTTCTCCAAT
TTTCCGGCAAGTATA
+
FFFFFFFFFFFFFFFFFFFFFFFF,FFFF:FFFFFFFFFFFFFFFFFFFFFFFFFFFFFFFFFFFFFFFF
FFFFFFFFFFFFFFFFFFFFFFFFFFFFFFFFFFFF:FFFFFFFFFFFFFFFFFFFFFFFFFFFFFFFFF
FFFF:FFFFFFFFFF
@A00721:190:HGJW7DSXY:4:2644:8413:12117 2:N:0:TCTGCAAG+GCTGCACT
TCAAGATGTCCAATTTCCAAAGAAATTGTTCTCGACCAAATTAAAAATTTTAGTTTTCCAGTTAATTT
GGCGTTCATCAGAGTATGTCAGGAACAACATTCAGACGGTGATAATCACCTTCACTGTCTTCTCCAAT
TTTCCGGCAAGTATA
+
FFFFFFFFFFFFFFFFFFFFFFFFFFFFFFFFFFFFFFFFFFFFFFFFFFFFFFFFFFFFFFFFFFFFF
:FFFFFFFFFFFFFFFFFFFFFFFFFFFFFFFFFFFFFFFFFFFFFFFFFFFFFFFFFFFFFFFFFFFF
FFFFFFFFFFFFFFF
@A00721:190:HGJW7DSXY:4:1312:25192:31720 2:N:0:TCTGCAAG+GCTGCACT
CTTAACTTATTCAAGATGTCCAATTTCCAAAGAAATTGTTCTCGACCAAATTAAAAATTTTAGTTTTC
CAGTTAATTTGGCGTTCATCAGAGTATGTCAGGAACAACATTCAGACGGTGATAATCACCTTCACTGT
CTTCTCCAATTTTCC
+
FFFFFFFFFFFFFFFFFFFFFFFFFFFFFFFFFFFFFFFFFFFFFFFFFFFFFFFFFFFFFFFFFFFFF
FFFFFFFFFFFFFFFFFFFFFFFFFFFFFFFFFFFFF:FFFFFFFFFFFFFFFFFFFFFFFFFFFFFFF
FFFFFFFFFFFFFFF
@A00721:190:HGJW7DSXY:4:1377:31250:35446 2:N:0:TCTGCAAG+GCTGCACT
CTTAACTTATTCAAGATGTCCAATTTCCAAAGAAATTGTTCTCGACCAAATTAAAAATTTTAGTTTTC
CAGTTAATTTGGCGTTCATCAGAGTATGTCAGGAACAACATTCAGACGGTGATAATCACCTTCACTGT
CTTCTCCAATTTTCC
+
FFFFF,FFFFFF:FFFFFFFFFFFFFFFFFFFFFFFFFFFFFFFFFFFFFFFFFF:FFFFFFFFF:FF
FFFF:FFFFFFFFFFFFFFFFFFFFFFFFFFFFFFFFFFFFFFF,F:F,FFF:FFFF,:F:FFFFFFFF
FFFFFF,,FFFFFFF
@A00721:190:HGJW7DSXY:4:1620:9362:20462 2:N:0:TCTGCAAG+GCTGCACT
CTTAACTTATTCAAGATGTCCAATTTCCAAAGAAATTGTTCTCGACCAAATTAAAAATTTTAGTTTTC
CAGTTAATTTGGCGTTCATCAGAGTATGTCAGGAACAACATTCAGACGGTGATAATCACCTTCACTGT
CTTCTCCAATTTTCC
+
FFFFFF,FFFFFFFFFFFFFFFFFFFFFFFFFFFFFFFFFFFFFFFFFFFFFFFFFFFFFFFFFFFF:F
FFFFFFFFFFFFFFFFFFFFFFFFFFFFF:FFFFFFFFFFFFFFFFFFFFFF:FFFFFFFFFFFFFFFF
FFFFFFFFFFFFFFF
```

@A00721:190:HGJW7DSXY:4:2101:24017:5071 2:N:0:TCTGCAAG+GCTGCACT
CTTAACTTATTCAAGATGTCCAATTTCCAAAGAAATTGTTCTCGACCAAATTAAAAATTTTAGTTTTC
CAGTTAATTTGGCGTTCATCAGAGTATGTCAGGAACAACATTCAGACGGTGATAATCACCTTCACTGT
CTTCTCCAATTTTCC
+
FFFFFFFFFFFFFFFFFFFFFFFFFFFFFFFFFFFFFFFFFFFFFFFFFFFFFFF:FFFFFFFFFFFFFF
FFFFFFFFFFFFFFFFFFFFFFFFFFFFFFFFFFFFFFFFFFFFFFFFFFFFFFFFFFFFFFFFFFFFFF
FFFFFFFFFFFFF:F
@A00721:190:HGJW7DSXY:4:2157:23204:9486 2:N:0:TCTGCAAG+GCTGCACT
CTTAACTTATTCAAGATGTCCAATTTCCAAAGAAATTGTTCTCGACCAAATTAAAAATTTTAGTTTTC
CAGTTAATTTGGCGTTCATCAGAGTATGTCAGGAACAACATTCAGACGGTGATAATCACCTTCACTGT
CTTCTCCAATTTTCC
+
FFFFF:FFFFFFFFFFFFFFFFFFFFFFFFFFFFFFFFFFFFFFFFFFFFFFFFFFFFFFFFFFFFFFF:
FFFFFFF:FFFFFFFFFFFFFFFFFF:FFFFFFFFFFFFFFFFFFFFFFF::FFF:FFFFFF,FFFFFFF
FFFFFFFFFFFFFFF
@A00721:190:HGJW7DSXY:4:2311:4092:11397 2:N:0:TCTGCAAG+GCTGCACT
CTTAACTTATTCAAGATGTCCAATTTCCAAAGAAATTGTTCTCGACCAAATTAAAAATTTTAGTTTTC
CAGTTAATTTGGCGTTCATCAGAGTATGTCAGGAACAACATTCAGACGGTGATAATCACCTTCACTGT
CTTCTCCAATTTTCC
+
FFFFFFFFFFFFFFFFFFFFFFFFFFFFFFFFFFF:FFF:FFFFFFFFFFFFFFF:FFF:FFFFFFFFFF
FFFFFFFFFFFFFFFFFFFFFFFFFFFFFFFFFFFFFFF:FFFFFFFFFFFFFFFFFFFFFFFFFFFFFF
FFFFFFFFFFFFFFF
@A00721:190:HGJW7DSXY:4:1104:28257:8155 2:N:0:TCTGCAAG+GCTGCACT
TATTTCTTAACTTATTCAAGATGTCCAATTTCCAAAGAAATTGTTCTCGACCAAATTAAAAATTTTAG
TTTTCCAGTTAATTTGGCGTTCATCAGAGTATGTCAGGAACAACATTCAGACGGTGATAATCACCTTC
ACTGTCTTCTCCAAT
+
FFFFFFFFFFFFFFFFFFFFFFFFFFFFFFFFFFFFFFFFFFFFFFFFFFFFFFFFFFFFFFFFFFFFFF
FF:FFFFFFFFFFFFFFFFFFFFFFFFFFFFFFFFFFFFFFFFFFFFFFFFFFFFFFFFFFFFFFFFFFF
FFFFFF:FFFFFFFF
@A00721:190:HGJW7DSXY:4:1175:29532:13275 2:N:0:TCTGCAAG+GCTGCACT
TATTTCTTAACTTATTCAAGATGTCCAATTTCCAAAGAAATTGTTCTCGACCAAATTAAAAATTTTAG
TTTTCCAGTTAATTTGGCGTTCATCAGAGTATGTCAGGAACAACATTCAGACGGTGATAATCACCTTC
ACTGTCTTCTCCAAT
+
FFFFFFFFFFFFFFFFFFFF,FFFFFFFFFFFFFFFFFFFFFFFFFFFFFFFFFFFFFFFFFFFFFFFFF
FFFFFFFFFFFF:FFFFFFFFFFFFFF:FFFFFFFFFFF:FFFFFFFFF,FFFFFFFFFFFFFFFFFFFF
:FFFFFFFFFFFF:F
@A00721:190:HGJW7DSXY:4:1261:11921:13369 2:N:0:TCTGCAAG+GCTGCACT
TATTTCTTAACTTATTCAAGATGTCCAATTTCCAAAGAAATTGTTCTCGACCAAATTAAAAATTTTAG
TTTTCCAGTTAATTTGGCGTTCATCAGAGTATGTCAGGAACAACATTCAGACGGTGATAATCACCTTC
ACTGTCTTCTCCAAT
+
FFFFFFFFFFFFFFFFFFFFFFFFFFFFFFFFFFFFFFF:FFFF:FFFFFFFFFFFFFFFFFFFFFFFFF
FFFFFFFFFFFFFFFFFFFFFFFFFFFFFFFFFFFFFFFFFFFFFFFFFFFFFFFFFFFFFFFFFFFFFF
FFFFFFFFFFFFFFF
@A00721:190:HGJW7DSXY:4:1273:10945:35728 2:N:0:TCTGCAAG+GCTGCACT
TATTTCTTAACTTATTCAAGATGTCCAATTTCCAAAGAAATTGTTCTCGACCAAATTAAAAATTTTAG
TTTTCCAGTTAATTTGGCGTTCATCAGAGTATGTCAGGAACAACATTCAGACGGTGATAATCACCTTC
ACTGTCTTCTCCAAT
+
FFFFFFFFFFFFFFFFFFFFFFFFFFFFFFFFFF:FFFFFFFFFFFFFFFFFFFFFFFFFFFFFFF:FFF

FFFFF:FFF:FFFF,F:FFFFFFFF,FFFFFFFFF:FFFFFFFFFFFFFFFF:FF,FFFFFFFFFFFFF,
FFFFFFFFFFF:FFF
@A00721:190:HGJW7DSXY:4:1316:20166:3599 2:N:0:TCTGCAAG+GCTGCACT
TATTTCTTAACTTATTCAAGATGTCCAATTTCCAAAGAAATTGTTCTCGACCAAATTAAAAATTTTAG
TTTTCCAGTTAATTTGGCGTTCATCAGAGTATGTCAGGAACAACATTCAGACGGTGATAATCACCTTC
ACTGTCTTCTCCAAT
+
FFFFFFFFFFF:FFF:FFFFFFFFFFFFFFFFFFFFFFFFFFFFFFFFF:FFF:FFF:FFFFFFFFFFF:
FFFFFFFFFFFFFFF::FFFFFFFFFFFFFFFF:FFFF,FF,FFFFFF:FFFFFFFFFFFFFFFF:FFFF
FFF,FFFFFFFF,FF
@A00721:190:HGJW7DSXY:4:1316:20365:4288 2:N:0:TCTGCAAG+GCTGCACT
TATTTCTTAACTTATTCAAGATGTCCAATTTCCAAAGAAATTGTTCTCGACCAAATTAAAAATTTTAG
TTTTCCAGTTAATTTGGCGTTCATCAGAGTATGTCAGGAACAACATTCAGACGGTGATAATCACCTTC
ACTGTCTTCTCCAAT
+
FFFFFFFFFFFFFFFFFFFFFFFFFFFFFFFFFFFFFFFFFFFFFFFFFFFFFFFFFFFFFFFFFFFFFF
FFFFFFFFFFFFFFFFFFFFFFFFFFFFFFFFFFFFFFFFFFFFFFFFFFFFFFFFFFFFFFFFFFFFFF
FFFFFFFFFFFFFFF
@A00721:190:HGJW7DSXY:4:1324:3215:20055 2:N:0:TCTGCAAG+GCTGCACT
TATTTCTTAACTTATTCAAGATGTCCAATTTCCAAAGAAATTGTTCTCGACCAAATTAAAAATTTTAG
TTTTCCAGTTAATTTGGCGTTCATCAGAGTATGTCAGGAACAACATTCAGACGGTGATAATCACCTTC
ACTGTCTTCTCCAAT
+
FFFFFFFFFFFFFFFFFFFFFFFFFFFFFFFFFFFFFFFFFFFFFFFFFFFFFFFFFFFFFFFFFF:FFF
FF:FFFFFFFFFFFFFFFFFFFFFFFFFFFFFFFFFF::FFFFFF:FFFFFFFFFFFFFFFFFFFFFFFF
FFFFFFFFFFFFFFF
@A00721:190:HGJW7DSXY:4:1330:11270:20322 2:N:0:TCTGCAAG+GCTGCACT
TATTTCTTAACTTATTCAAGATGTCCAATTTCCAAAGAAATTGTTCTCGACCAAATTAAAAATTTTAG
TTTTCCAGTTAATTTGGCGTTCATCAGAGTATGTCAGGAACAACATTCAGACGGTGATAATCACCTTC
ACTGTCTTCTCCAAT
+
FFFFFFFFFFFFFFFFFFFFFFFFFFFFFFFFFFFFFFFFFFFFFFFFFFFFFFFFFFFFFFFFFFFFFF
FFFFFFFFFFFFFFF:FFFFF:FFFFFFFFFFFFFFFFFFFFFFFFFFFFFFFFFFFFFFFFFFFFFFFF
FFFFFFFFFFFFFFF
@A00721:190:HGJW7DSXY:4:1438:4571:14857 2:N:0:TCTGCAAG+GCTGCACT
TATTTCTTAACTTATTCAAGATGTCCAATTTCCAAAGAAATTGTTCTCGACCAAATTAAAAATTTTAG
TTTTCCAGTTAATTTGGCGTTCATCAGAGTATGTCAGGAACAACATTCAGACGGTGATAATCACCTTC
ACTGTCTTCTCCAAT
+
FFFFFFFFF,FFFF:FFFFFFFFFFFFFFFFFFFFF:F,FF:FF:FFFFFFFF:FFFFF:FFFFFFFFFF
FFFFFFFFFFFFFFFFFFFFFFFFFFFFFFFFFFFFFFFFFFFFF:FFFFF:FFFFFFFFFF:FFFFFFF
FFFFFFFFFFFFF:F
@A00721:190:HGJW7DSXY:4:1452:8865:18818 2:N:0:TCTGCAAG+GCTGCACT
TATTTCTTAACTTATTCAAGATGTCCAATTTCCAAAGAAATTGTTCTCGACCAAATTAAAAATTTTAG
TTTTCCAGTTAATTTGGCGTTCATCAGAGTATGTCAGGAACAACATTCAGACGGTGATAATCACCTTC
ACTGTCTTCTCCAAT
+
FFFFFFFFFFFFFFFFFFF:FFFFFFFFFFFFFFFFFFFFFFFFFFFFFFFFFFFFFFFFFFFFFF:FFF
FFFFFFFFFFFFFFFFFFFFFFFFFFFFFFFFFFFFFFFFFFFFFFFFF:,:FFF:FFFFFFFFFF:FFF
FFFFFFFFFFFFFFF
@A00721:190:HGJW7DSXY:4:1549:29930:13964 2:N:0:TCTGCAAG+GCTGCACT
TATTTCTTAACTTATTCAAGATGTCCAATTTCCAAAGAAATTGTTCTCGACCAAATTAAAAATTTTAG
TTTTCCAGTTAATTTGGCGTTCATCAGAGTATGTCAGGAACAACATTCAGACGGTGATAATCACCTTC
ACTGTCTTCTCCAAT

```
+
FFFFFFFFFFFFFFFFFFFFFFFFFFFFFFFFFFFFFFFFFFFFFFFFFFFFFFFFFFFFFFFFFFFFFF
FFFFFFFFFFFFFFFFFFFFFFFFFFFFFFFFFFFFFFF,FFFFFFFF:F:FFFFFFFFFFFFFFFFF
FFFFFFFFFFFFFFF
@A00721:190:HGJW7DSXY:4:1665:27181:27649 2:N:0:TCTGCAAG+GCTGCACT
TATTTCTTAACTTATTCAAGATGTCCAATTTCCAAAGAAATTGTTCTCGACCAAATTAAAAATTTTAG
TTTTCCAGTTAATTTGGCGTTCATCAGAGTATGTCAGGAACAACATTCAGACGGTGATAATCACCTTC
ACTGTCTTCTCCAAT
+
FFFFFFFFFFFFFFFFFFFFFFFFFFFFFFFF:FFFFFFFFFFFFFFFFFFFFFFFF:FFFFF:FFFF
FFFFFFF:FFFFFFFFFFFFFFFF:FFFFFFFFFFFFFFFFFFFFFFFFFF:FFFFFFFFFFFFFFFFF
FFFFFFFFFFF,FFF
@A00721:190:HGJW7DSXY:4:2538:18493:19241 2:N:0:TCTGCAAG+GCTGCACT
TATTTCTTAACTTATTCAAGATGTCCAATTTCCAAAGAAATTGTTCTCGACCAAATTAAAAATTTTAG
TTTTCCAGTTAATTTGGCGTTCATCAGAGTATGTCAGGAACAACATTCAGACGGTGATAATCACCTTC
ACTGTCTTCTCCAAT
+
FFFFFFFFFFFFFFFFFFF:FFFFFFFFFFFFFFFFFFFFFFFFFFFF:FFFFFFFFFFFFFFFFFFF
FFFFFFFFFFFFFFFF,FFFFFFFFFFFFFFFFFFFFFFFFFFFFFFFFFFFFFFFFFFFFFFFFFF
FFFFFFFFFFFFFFF
@A00721:190:HGJW7DSXY:4:2568:21450:11459 2:N:0:TCTGCAAG+GCTGCACT
TATTTCTTAACTTATTCAAGATGTCCAATTTCCAAAGAAATTGTTCTCGACCAAATTAAAAATTTTAG
TTTTCCAGTTAATTTGGCGTTCATCAGAGTATGTCAGGAACAACATTCAGACGGTGATAATCACCTTC
ACTGTCTTCTCCAAT
+
FFFFFFFFFFFFFFFFFFFFFFFFFFFF:FFFFFFFFFFFFFFFFFFFFFFFFFFF,FFFF,FFFFF
FFFFFFFFFFFFFFFFFFFFFFFFFFFFFFFFFFFF:FFFFFFFFFFFFFFFFFFFFFFFFFFFFFFFF
FFFFFFFFFFFF:FF
@A00721:190:HGJW7DSXY:4:2669:10945:23923 2:N:0:TCTGCAAG+GCTGCACT
TATTTCTTAACTTATTCAAGATGTCCAATTTCCAAAGAAATTGTTCTCGACCAAATTAAAAATTTTAG
TTTTCCAGTTAATTTGGCGTTCATCAGAGTATGTCAGGAACAACATTCAGACGGTGATAATCACCTTC
ACTGTCTTCTCCAAT
+
FFFFFFFFFFFFFFFFFFFFFFFFFFFF:FFFFFFFFFFFFFFFFFFFFFFFFFFFFFFFFFFFFFF:FF
FFFFFFFFFFFFFFFFFFFFFFFFFFFFFFFFFFFFFFFFFFFFFFFFFFFFFFFFFFFFFFFFFFFFFF
FFF,FFFFFFFFFFF
@A00721:190:HGJW7DSXY:4:1575:32443:12994 2:N:0:TCTGCAAG+GCTGCACT
GAATTATTTCTTAACTTATTCAAGATGTCCAATTTCCAAAGAAATTGTTCTCGACCAAATTAAAAATT
TTAGTTTTCCAGTTAATTTGGCGTTCATCAGAGTATGTCAGGAACAACATTCAGACGGTGATAATCAC
CTTCACTGTCTTCTC
+
F:FFFFFFFFFFF:FFFFFFFFFFFFFFFFFFFFFFFFFFFF,FFFFFFFFFFFFFFFFFFFFFFFFFFF
FFFFFFFF:FFF:FFFFFF,FF:FFFFFFFFFFFFFFFFFFFFFFFFFFFFF:F:FFFFFFFFFFF:F:
FFFFF:FFF:FFFFF
@A00721:190:HGJW7DSXY:4:2166:24460:29418 2:N:0:TCTGCAAG+GCTGCACT
GAATTATTTCTTAACTTATTCAAGATGTCCAATTTCCAAAGAAATTGTTCTCGACCAAATTAAAAATT
TTAGTTTTCCAGTTAATTTGGCGTTCATCAGAGTATGTCAGGAACAACATTCAGACGGTGATAATCAC
CTTCACTGTCTTCTC
+
FFFFFFFFFFFFFFFFFFFFFFFFFFFFFFFFFFFFFFFFFFFFFFFFFFFFFFFFFFFFFFFFFFFF:F
FFFFFFFFFFFFFFFFFFFFFFFFFFFFFFFFFFFFFFFFFFFFFFFFFFFFFFFFFFFFFFFFFFFFFF
FFFFFFFFFFFFFFF
@A00721:190:HGJW7DSXY:4:2226:32461:1908 2:N:0:TCTGCAAG+GCTGCACT
GAATTATTTCTTAACTTATTCAAGATGTCCAATTTCCAAAGAAATTGTTCTCGACCAAATTAAAAATT
```

TTAGTTTTCCAGTTAATTTGGAGTTCATCAGAGTATGTCAGGAACAAAATTCAGACGGTGATAATCAC
CTTCACTGTCTTCTC
+
FF:FFFFFFF:FFFFFFFFF,FF,FFFFFFFFFFF:,FFFF:FFFF,FF,F:FF,:FF:FFFFFFFFFF
FFFFFFF::F,F:FF:FFFFF,FFF,,F:FF,FFFFF:F:FFFFF,F,FFFF:F:FFFFFFFFF:F:F:
:,FF::::F,FF:F:
@A00721:190:HGJW7DSXY:4:2375:11460:36151 2:N:0:TCTGCAAG+GCTGCACT
GAATTATTTCTTAACTTATTCAAGATGTCCAATTTCCAAAGAAATTGTTCTCGACCAAATTAAAAATT
TTAGTTTTCCAGTTAATTTGGCGTTCATCAGAGTATGTCAGGAACAACATTCAGACGGTGATAATCAC
CTTCACTGTCTTCTC
+
FFFFFFFFFFFFFFFFFFFFFFFFFFFFFFFFFFFFFFFFFFFFFFFFFFFFFFFFFFFFFFFFFFFFF
FFFFFFFFFFFFFFFFFFFFFFFFFFFFFFFFFFFFFFFFFFFFFFFFFFFFFFFFFFFFFFFFFFFFF
FFFFFFFFFFFFFFF
@A00721:190:HGJW7DSXY:4:2406:21748:11976 2:N:0:TCTGCAAG+GCTGCACT
GAATTATTTCTTAACTTATTCAAGATGTCCAATTTCCAAAGAAATTGTTCTCGACCAAATTAAAAATT
TTAGTTTTCCAGTTAATTTGGCGTTCATCAGAGTATGTCAGGAACAACATTCAGACGGTGATAATCAC
CTTCACTGTCTTCTC
+
FFFFFFFFFFFFFFFFFFFFFFFFFFFFFFFFFFFFFFFFFFFFFFFFFFFFFFFFFFFFFFFFFFFFF
FFFFFFFFFFFFFFFFFFFFFFFFFFFFFFFFFFFFFFFFFFFFFFFFFFFFFFFFFFFFFFFFFFFFF
FFFFFFFFFFFFFFF
@A00721:190:HGJW7DSXY:4:2417:9932:27211 2:N:0:TCTGCAAG+GCTGCACT
GAATTATTTCTTAACTTATTCAAGATGTCCAATTTCCAAAGAAATTGTTCTCGACCAAATTAAAAATT
TTAGTTTTCCAGTTAATTTGGCGTTCATCAGAGTATGTCAGGAACAACATTCAGACGGTGATAATCAC
CTTCACTGTCTTCTC
+
FFFFFFFFFFFFFFFFFFFFFFFFFFFFFFFFFFFFFFFFFFFFFFFFFFFFF:FFFFFFFFFFFFFFF
FFFFFFFFF:FFFFFFFFFFFFFFFFFFFFFFFFFFFFFFFFFFFFFFFFFFF:FFFFFFFFFFFFFFF
FFFFFFFFFFFFFFF
@A00721:190:HGJW7DSXY:4:2572:29622:1094 2:N:0:TCTGCAAG+GCTGCACT
GAATTATTTCTTAACTTATTCAAGATGTCCAATTTCCAAAGAAATTGTTCTCGACCAAATTAAAAATT
TTAGTTTTCCAGTTAATTTGGCGTTCATCAGAGTATGTCAGGAACAACATTCAGACGGAGATAATCAC
CTTCACTGTCTTCTC
+
:F::FF:FFF:FF:FFFFFFFFFFFFFFFFFFFFFFFFFFFFFF,FFFFFFFF:FFFFFFFFFFFFF:F
FFFFFFFFFFFFF:F:FFF::F,FF:FFFFFFFFF:FFFFFFFFFFFFFF,FFFFFFFF,FFFFFFFFF
FFFFFF::FFFFFFF
@A00721:190:HGJW7DSXY:4:2669:26051:1861 2:N:0:TCTGCAAG+GCTGCACT
GAATTATTTCTTAACTTATTCAAGATGTCCAATTTCCAAAGAAATTGTTCTCGACCAAATTAAAAATT
TTAGTTTTCCAGTTAATTTGGCGTTCATCAGAGTATGTCAGGAACAACATTCAGACGGTGATAATCAC
CTTCACTGTCTTCTC
+
F:FFFFFFFFFFFFFFFFFFFFFFFFFFFFFFFFFFFFFFFFFFFFFFFFFFFFFFFFFFFFFFFFFFF
FFFFFFFFFFFFFFFFFFFFFFFFFFFFFFFFFFFFFFFFFFFFFFFFFFFFFFFFF:FFFFFFFFFFF
FFFFFFFFFFFFFFF
@A00721:190:HGJW7DSXY:4:1372:13241:25676 2:N:0:TCTGCAAG+GCTGCACT
GCGAAGAATTATTTCTTAACTTATTCAAGATGTCCAATTTCCAAAGAAATTGTTCTCGACCAAATTAA
AAATTTTAGTTTTCCAGTTAATTTGGCGTTCATCAGAGTATGTCAGGAACAACATTCAGACGGTGATA
ATCACCTTCACTGTC
+
FFFFFFFFFFFFFFFFFFFFFFFFFFFFFFFFFFFFFFFFFFFFFFFFFFFFFFFFFFFFFFFFFFFFF
F:FFFFFFFFFFFFFFFFFFFFFFF:FFFFF:FFFFFFF:FFFFFFFFFFFFFFFFFFFFFFFFFFFFF
FFFFFFFFFFFFFFF

```
@A00721:190:HGJW7DSXY:4:2224:27760:5760 2:N:0:TCTGCAAG+GCTGCACT
GCGAAGAATTATTTCTTAACTTATTCAAGATGTCCAATTTCCAAAGAAATTGTTCTCGACCAAATTAA
AAATTTTAGTTTTCCAGTTAATTTGGCGTTCATCAGAGTATGTCAGGAACAACATTCAGACGGTGATA
ATCACCTTCACTGTC
+
FFF,FF:FFFFFFFFFFFFFFFFFFFFFFF:FFFFFFFF:FFFFFFFFFFFFFFFFFFFF:FFFFFF:F
FFFF:FFFFFFFFFFFFFFFFFFFFFF::F:FFFFFFFFFFFFF:FFFFF:FFFFFFFFFFFFFFF:FF
FFFF:FFFFFFFFFF
@A00721:190:HGJW7DSXY:4:2403:8232:18286 2:N:0:TCTGCAAG+GCTGCACT
GCGAAGAATTATTTCTTAACTTATTCAAGATGTCCAATTTCCAAAGAAATTGTTCTCGACCAAATTAA
AAATTTTAGTTTTCCAGTTAATTTGGCGTTCATCAGAGTATGTCAGGAACAACATTCAGACGGTGATA
ATCACCTTCACTGTC
+
FFFFFFFFFFFFFFFFFF:FFFFFFF:FFFFFFFFFFFFFFFF:FFFFFFFFFFFFFFFFFFFFFFFFF
FF::FFFFFFFFFFF:FFFFFFFFFFFFFFFFFFFFFFFFFFFFFFFFFF:FFFFFFFFFFFFFFFF::
FFF:FFFFFFFFFFF
@A00721:190:HGJW7DSXY:4:2254:32298:10770 2:N:0:TCTGCAAG+GCTGCACT
GAAGGTGATTATAGCCGTCTGAATGTTGTTCCTGACATACTCTGATGAACGCCAAATTAACTGGAAAA
CTAAAATTTTTAATTTGGTCGAGAACAATGTCTTTGGAAATTGGACATCTAGAATAAGTTAATAAATA
ATACTTCGAGTTA
+
FFFF:::F::FF,:F,F:F,:FF,F:::,:FF:FFFFFFF,FFFF:,FF:,FFFFFF,FFF,,,FFFF
,:FFFFF,FF:F:F::,,FF:FF:FFF:F,FF,:F,,FF,F,,FFFF,,F,,F:FFF,FF,F,FFFFF
:F,F:FFF::F:F
@A00721:190:HGJW7DSXY:4:1212:23936:33364 2:N:0:TCTGCAAG+GCTGCACT
AACTGGAAAACTAAAATTTTTAATTTGGTCGAGAACAATTTCTTTGGAAATTGGACATCTTGAATAAG
TTAAGAAATAATTCTTCGCGTTAATGCAGAAACGCGATGAAGAAGATGAAGGAGTCCCCATTACTGTT
AAATCAGGGACATTC
+
FFFFFFFFFFFFFFFFFFFFFFFFFFFFFFFFFFF:FFFFFFFFFFFFFFFFFFFFFFFFFFFFFFFFF
FFFFFFFFFFFFFFFFFFFFFFFF:FFFFFFFFFFFFFFFFFFFFF::F:FFFFFFF:FFFFFFFFFFF
FF,FFFF::FFFFFF
@A00721:190:HGJW7DSXY:4:1351:12825:11490 2:N:0:TCTGCAAG+GCTGCACT
AACTGGAAAACTAAAATTTTTAATTTGGTCGAGAACAATTTCTTTGGAAATTGGACATCTTGAATAAG
TTAAGAAATAATTCTTCGCGTTAATGCAGAAACGCGATGAAGAAGATGAAGGAGTCCCCATTACTGTT
AAATCAGGGACATTC
+
FFFFFFFFFFFFFFF,FFFFFFFFFFFFFFFFFFFFFFFFFFFFF,:FFF,F:FFFFFFFFFFFFFF:FF
FFFFF:FFFFFFFFFFFFFFFFFFFFFFFFFFFFFFFFFFFFFFFFFFFFFFFFFFFFFFFFFFFFFFF
FFFFFFFFFFFFFFF
@A00721:190:HGJW7DSXY:4:2136:30382:23641 2:N:0:TCTGCAAG+GCTGCACT
AACTGGAAAACTAAAATTTTTAATTTGGTCGAGAACAATTTCTTTGGAAATTGGACATCTTGAATAAG
TTAAGAAATAATTCTTCGCGTTAATGCAGAAACGCGATGAAGAAGATGAAGGAGTCCCCATTACTGTT
AAATCAGGGACATTC
+
FFFFFFFF:FFFFFFFF:FFFF:FFFF:FFFFFFFFFFFFFFFF,FFFFFFFFFFFFFFFFFFF:FFF
FFF:FFFFFFFFFFFFFFFFFFFFFFFFF:FFFFFFFFFFFFFFF,FFFFFFFFFFFFFF,FF,FFF
FFFFFFFFFFFFFFF
@A00721:190:HGJW7DSXY:4:2262:31964:24815 2:N:0:TCTGCAAG+GCTGCACT
AACTGGAAAACTAAAATTTTTAATTTGGTCGAGAACAATTTCTTTGGAAATTGGACATCTTGAATAAG
TTAAGAAATAATTCTTCGCGTTAATGCAGAAACGCGATGAAGAAGATGAAGGAGTCCCCATTACTGTT
AAATCAGGGACATTC
+
FFFFFFFFF:FFFFF:FFFFFFFFFFFFFFFFFFFFFFFFFFFFFFFFFF:FFFFFFFFF:FFF:FF:,F
```

F,FFF:F::FFFFFFFFFFFFFFFFFFFFFFFFFFFFF,:FF,FFFFFFFFFFFFFFFF,F,FF:::FFF
FFFFFFFF:FFFFFF
@A00721:190:HGJW7DSXY:4:2536:8784:4022 2:N:0:TCTGCAAG+GCTGCACT
AACTGGAAAACTAAAATTTTTAATTTGGTCGAGAACAATTTCTTTGGAAATTGGACATCTTGAATAAG
TTAAGAAATAATTCTTCGCGTTAATGCAGAAACGCGATGAAGAAGATGAAGGAGTCCCCATTACTGTT
AAATCAGGGACATTC
+
FFFFFFFFFFFFFFFFFFFFFFFFFFFFFFFFFFFFFFFFFFFFFFFFFFFFFFFFFFFFFFFFFFFFFF
FFFFFFFFFFFFFFFFFFFFFFFFFFFFFFFFFFFFFFFFFFFFFFFFFFFFFFFFFFFFFFFFFFFFFF
FFFFFFFFFFFFFF:
@A00721:190:HGJW7DSXY:4:1133:12066:21574 2:N:0:TCTGCAAG+GCTGCACT
GGAAAACTAAAATTTTTAATTTGGTCGAGAACAATTTCTTTGGAAATTGGACATCTTGAATAAGTTAA
GAAATAATTCTTCGCGTTAATGCAGAAACGCGATGAAGAAGATGAAGGAGTCCCCATTACTGTTAAAT
CAGGGACATTCGCAT
+
FFF:FFFFFFFFFFFFFFFFFFFFFFFFFFFFFFFFFFFFF:FFFF:FF:FFFFFFFFFFFFFFFFFFFF
FFFFFFFFFFFFFFFFFFFFFFF:FFF:FFFFFFFFFFFFFFFF:FFFFFFFFFFF:FFFFFFFFFFFFF
FFFFFFFFFFFFFFF
@A00721:190:HGJW7DSXY:4:1277:23122:32800 2:N:0:TCTGCAAG+GCTGCACT
GGAAAACTAAAATTTTTAATTTGGTCGAGAACAATTTCTTTGGAAATTGGACATCTTGAATAAGTTAA
GAAATAATTCTTCGCGTTAATGCAGAAACGCGATGAAGAAGATGAAGGAGTCCCCATTACTGTTAAAT
CAGGGACATTCGCAT
+
FFFFFFFFFFFFFFFFFFFFFFFFFFFFFFFFFFFFFFFFFFFFFF,FFFFFFFFFFF:FFFFFFFFFFF
FFFFFFFFFFFFFFFFFFFFFFFFFFFFFFFFFFFFFF:FFFFFFFFFFFFFFFFFFFFFFFFFFFFFFF
FFFFFFFFFFFFFF:
@A00721:190:HGJW7DSXY:4:1347:23421:11397 2:N:0:TCTGCAAG+GCTGCACT
GGAAAACTAAAATTTTTAATTTGGTCGAGAACAATTTCTTTGGAAATTGGACATCTTGAATAAGTTAA
GAAATAATTCTTCGCGTTAATGCAGAAACGCGATGAAGAAGATGAAGGAGTCCCCATTACTGTTAAAT
CAGGGACATTCGCAT
+
FFFFF,FFFFFFFFFFFFFFFFFFFFFFFFFFFFFFFFFFFFFFFFFFFFFFFFFFFFFFFFFFFFFFFF
FFFFFFFFFFFFFFFFFFFFFFFFFFFFFFFFFFFFFFFFFFFFFFFFFFFFFFFFFFFFFFFFFFFFFF
FFFFFFFFFFFFFFF
@A00721:190:HGJW7DSXY:4:1374:15908:28447 2:N:0:TCTGCAAG+GCTGCACT
GGAAAACTAAAATTTTTAATTTGGTCGAGAACAATTTCTTTGGAAATTGGACATCTTGAATAAGTTAA
GAAATAATTCTTCGCGTTAATGCAGAAACGCGATGAAGAAGATGAAGGAGTCCCCATTACTGTTAAAT
CAGGGACATTCGCAT
+
FFFFFFFFFFFFFFFFFFFFFFFFFFFFFFFFFFFFFFFFF,FFFF,FFFFFFFFFFFFFFFFFFFFFFF
FFFFFFFFFFFFFFFFFFFFFFFFFFFFFFFFFFF:FFFFFFFFFFFFFFFFFFFFFFFFFFFFFFFFFF
FFFFFFFFFFFFFFF
@A00721:190:HGJW7DSXY:4:1435:19199:32643 2:N:0:TCTGCAAG+GCTGCACT
GGAAAACTAAAATTTTTAATTTGGTCGAGAACAATTTCTTTGGAAATTGGACATCTTGAATAAGTTAA
GAAATAATTCTTCGCGTTAATGCAGAAACGCGATGAAGAAGATGAAGGAGTCCCCATTACTGTTAAAT
CAGGGACATTCGCAT
+
FFFFFFFFFFFFFFFFFFFFFFFFFFFFFFFFFFFFFFFFFFFFFFFFFFFFFFFFFFFFF:FFFFFFFF
FFFFFFFFFFFFFFFFFFFFFFFFFFFFFFFFFFFFFFFFFFFFFFFFFFFFFFFFFFFFFFFFFFFF:F
FFFFFFFFFFFFFFF
@A00721:190:HGJW7DSXY:4:1539:19488:29074 2:N:0:TCTGCAAG+GCTGCACT
GGAAAACTAAAATTTTTAATTTGGTCGAGAACAATTTCTTTGGAAATTGGACATCTTGAATAAGTTAA
GAAATAATTCTTCGCGTTAATGCAGAAACGCGATGAAGAAGATGAAGGAGTCCCCATTACTGTTAAAT
CAGGGACATTCGCAT

```
+
FFFFFFFFFFFFFFFFFFFFFFFFFFFFFFFFFFFFFFFFFFFFF,FFFFFFFFFFFFFFFFFFFFFFFF
F:FFFFFFFFFFFFFFFFFFFFFFFFFFFFFFFFFFFFFFFFFFFFFFFFFFFFFFFFFFFFFFFFFFFF
FFFFFFFFFFFFFFF
@A00721:190:HGJW7DSXY:4:2666:21730:26318 2:N:0:TCTGCAAG+GCTGCACT
GGAAAACTAAAATTTTTAATTTGGTCGAGAACAATTTCTTTGGAAATTGGACATCTTGAATAAGTTAA
GAAATAATTCTTCGCGTTAATGCAGAAACGCGATGAAGAAGATGAAGGAGTCCCCATTACTGTTAAAT
CAGGGACATTCGCAT
+
FF:FFFFFFFFFFFFFFFFFFFFFFFFFFFFFFFFFFFFFFFFFFFFFFFFFFFFFFFFFFFFFFFFFF
F:FFFFFFFFFFFFFFFFFFFFFFFFFFFFFFFFFFFFFFFF:FFFFFFFFFFFFFFFFFFFFFFFFFF
FFFFFFFFFFFFFFF
@A00721:190:HGJW7DSXY:4:1103:26377:25034 2:N:0:TCTGCAAG+GCTGCACT
ATAATGCTTAATATGCGAATGTCCCTGATTTAACAGTAATGGGGACTCCTTCATCTTCTTCATCGCGT
TTCTGCATTAACGCGAAGAATTATTTCTTAACTTATTCAAGATGTCCAATTTCCAAAGAAATTGTTCT
CGACCAAATTAAAAA
+
FFFFFFFFFFFFFFFFFFFFFFFFFFFFFFFFFFFFFFFFFFFFFFFFFFFFFFFFFFFFFFFFFFFF
FFFFFFFFFFFFFFFFFFFFFFFFFFFFFFFFFFFFFFFFFFFFFFFFF:FFF:FFFFFFFFFFFFFF
FFFF:FFFFFFFFFF
@A00721:190:HGJW7DSXY:4:1228:14407:20462 2:N:0:TCTGCAAG+GCTGCACT
ATAATGCTTAATATGCGAATGTCCCTGATTTAACAGTAATGGGGACTCCTTCATCTTCTTCATCGCGT
TTCTGCATTAACGCGAAGAATTATTTCTTAACTTATTCAAGATGTCCAATTTCCAAAGAAATTGTTCT
CGACCAAATTAAAAA
+
FFFFFFFFFFFFFFFFFFFFFFFFFFFFFFFFFFF:FFF:FFFFFFFFFFFFFFFFFFFFFFFFFFFFF
FFFFFFFFFFFFFFFFFFFFFFFFFFFFFFFFFFFFFFFFFFFFFFFFFFFFFFFFFFFFFFFFFFFF
FFFFFF::FFFFFFF
@A00721:190:HGJW7DSXY:4:1255:19633:7748 2:N:0:TCTGCAAG+GCTGCACT
ATAATGCTTAATATGCGAATGTCCCTGATTTAACAGTAATGGGGACTCCTTCATCTTCTTCATCGCGT
TTCTGCATTAACGCGAAGAATTATTTCTTAACTTATTCAAGATGTCCAATTTCCAAAGAAATTGTTCT
CGACCAAATTAAAAA
+
FFFFFFFFFFF:FFFFFFFFFFFFFFFFFFFF:FFFFFFFFFFFFFFFFFFFFFFFFFFFFFFFFFFF
FFFFFFFFF:FFFFFFFFFFFFFFFFFFFFFFFFFFFFFFFFFFFFFFFFFFFFFFFFFF:FFFFFFF
FFFFFFFFFF,FFF:
@A00721:190:HGJW7DSXY:4:1510:25003:16924 2:N:0:TCTGCAAG+GCTGCACT
ATAATGCTTAATATGCGAATGTCCCTGATTTAACAGTAATGGGGACTCCTTCATCTTCTTCATCGCGT
TTCTGCATTAACGCGAAGAATTATTTCTTAACTTATTCAAGATGTCCAATTTCCAAAGAAATTGTTCT
CGACCAAATTAAAAA
+
FFFFFFFFFFFFFFFFFFFFFFFFFFFFFFFFFFFFFFFFFFFFFFFFFFFFFFFFFFFFFFFF:FFF
FFFFFFFFFFFFFFFFFFFFFFFFFFFFFFFFFFFFFFFFFFFFFFFFFFFF:FFF:FFFFFFFFFF
FFFFFFFFFFFFFFF
@A00721:190:HGJW7DSXY:4:1551:3034:25316 2:N:0:TCTGCAAG+GCTGCACT
ATAATGCTTAATATGCGAATGTCCCTGATTTAACAGTAATGGGGACTCCTTCATCTTCTTCATCGCGT
TTCTGCATTAACGCGAAGAATTATTTCTTAACTTATTCAAGATGTCCAATTTCCAAAGAAATTGTTCT
CGACCAAATTAAAAA
+
FFFFFFFFFFFFFFFFFFFFFFFFFFFFFFFFFFFFFFFFFFFFFFFFFFFFFFFFFFFFFFFFFFFF
FFFFFFFFFFFFFFFFFFF,FFFFFFFFFFFFFFFFFFFFFFFFFFFFFFFFFFFFF:FFFFFFFFFF
FFFFFFFFFFFFFFF
@A00721:190:HGJW7DSXY:4:1554:14877:33238 2:N:0:TCTGCAAG+GCTGCACT
ATAATGCTTAATATGCGAATGTCCCTGATTTAACAGTAATGGGGACTCCTTCACCTTCTTCATCGCGT
```

TTCTGCATTAACGCGAAGAATTATTTCTTAACTTATTCAAGATGTCCAATTTCCAAAGAAATTGTTCT
CGACCAAATTAAAAA
+
FFFFFFFFFFFFFFFFFFFFFFFFFFFFFFFF:FFFFFFFFFFFFFFFFFFFFFFFFFFFFFFFFFFFF
FFFFFFFFFFFFFFFFFFFFFFFFFFFFFFFFFFFFFFFFFFFFFFFFFFFFFFFFFFFF:FFFFFFFF
:FFFFFF:FFFF:FF
@A00721:190:HGJW7DSXY:4:1571:14407:8938 2:N:0:TCTGCAAG+GCTGCACT
ATAATGCTTAATATGCGAATGTCCCTGATTTAACAGTAATGGGGACTCCTTCATCTTCTTCATCGCGT
TTCTGCATTAACGCGAAGAATTATTTCTTAACTTATTCAAGATGTCCAATTTCCAAAGAAATTGTTCT
CGACCAAATTAAAAA
+
FFFFFFFFFFFFFFFFFFFFFFFFFFFFFFFF:FFFFFFFFFFFFFFFFFFFFFFFFFFFFFFFFFFFF
FFFFFFFFFFFFFFFFFFFFFFFFFFFFFFFFFFFFFFF:FFFFFFFFFFFFFFFFFFFFFFFFFFFFF
F,,FFFFFFFF,FFF
@A00721:190:HGJW7DSXY:4:1620:14326:5008 2:N:0:TCTGCAAG+GCTGCACT
ATAATGCTTAATATGCGAATGTCCCTGATTTAACAGTAATGGGGACTCCTTCATCTTCTTCATCGCGT
TTCTGCATTAACGCGAAGAATTATTTCTTAACTTATTCAAGATGTCCAATTTCCAAAGAAATTGTTCT
CGACCAAATTAAAAA
+
FFFFFFFFFFFFFFFFFFFFFFFFFFFFFFFFFFFFF:FFFFFFFFFFFFFFFFFFFFFFFFFFFFFFF
FFFFFFFFFFFFFFFFFFFFFFFFFFFFFFFFFFFFFFFFFFFFFFFFFFFFFFFFFFFFFFFFFFFFF
FFFFFFFFFFFFFFF
@A00721:190:HGJW7DSXY:4:1239:14859:17613 2:N:0:TCTGCAAG+GCTGCACT
CCCCAATATAATGCTTAATATGCGAATGTCCCTGATTTAACAGTAATGGGGACTCCTTCATCTTCTTC
ATCGCGTTTCTGCATTAACGCGAAGAATTATTTCTTAACTTATTCAAGATGTCCAATTTCCAAAGAAA
TTGTTCTCGACCAAA
+
FFFFFFFFFF,FFFF,FFFFFFFFFFF:FFFFFFFFFFFFFFFFFFFF:FFFFFFFFFFFFFFFFFFF
FFFFFFFFFFFFFFFFFFFFFFFFFFFFFFFF:FFFFFFFFFFF:FFFFFFFFFFFFFFF:FFFFF,F
FFF:FFFFFFFFFFF
@A00721:190:HGJW7DSXY:4:1460:12192:27712 2:N:0:TCTGCAAG+GCTGCACT
CCCCAATATAATGCTTAATATGCGAATGTCCCTGATTTAACAGTAATGGGGACTCCTTCATCTTCTTC
ATCGCGTTTCTGCATTAACGCGAAGAATTATTTCTTAACTTATTCAAGATGTCCAATTTCCAAAGAAA
TTGTTCTCGACCAAA
+
FFFFFFFFFFFFFFFFFFFFFFFFF:FFFFFFFFFFFFFFFFFFFFFFFFFFFFFFFFFFFFFFFFFFF
FFF:FFFFFFFFFFFFFFFFFF:FFFFFFFFFFFFFFFFFFFFFFFFFFFFFFFFFFFFFFFFFFFFFF
FFFFFFFFFFFFFFF
@A00721:190:HGJW7DSXY:4:1475:32217:5588 2:N:0:TCTGCAAG+GCTGCACT
CCCCAATATAATGCTTAATATGCGAATGTCCCTGATTTAACAGTAATGGGGACTCCTTCATCTTCTTC
ATCGCGTTTCTGCATTAACGCGAAGAATTATTTCTTAACTTATTCAAGATGTCCAATTTCCAAAGAAA
TTGTTCTCGACCAAA
+
FFFFFFFFFFFFFFFFFFFFFFFFFFFFFFFFFFFFFFFFFFFFFFFFFFFFFFFFF:FFFFFFFFFFF
FFFFF:FFFFFFFFFFFFFFFFFFFFFFFFFFFFFFFF::FFFFFFFFFFFFF:FFFFFFF:F,FFFF:
FFFFFFFF:FF,FFF
@A00721:190:HGJW7DSXY:4:1507:23981:15718 2:N:0:TCTGCAAG+GCTGCACT
CCCCAATATAATGCTTAATATGCGAATGTCCCTGATTTAACAGTAATGGGGACTCCTTCATCTTCTTC
ATCGCGTTTCTGCATTAACGCGAAGAATTATTTCTTAACTTATTCAAGATGTCCAATTTCCAAAGAAA
TTGTTCTCGACCAAA
+
FFFFFFFFFFFFFFFFFFFFFFFFFFFFFFFFFFFFFFFFFFFFFFFFFFFFFFFFFFFFFFFFFFFFF
FFF:FFFFFFFFFFFFFFFFFFFFFFFFFFFFFFFFFFFFFFFFFFFFFFFFFFFFFFFFFFFFFFFFF
FFFFFFFFFFFFFFF

```
@A00721:190:HGJW7DSXY:4:1535:19470:26694 2:N:0:TCTGCAAG+GCTGCACT
CCCCAATATAATGCTTAATATGCGAATGTCCCTGATTTAACAGTAATGGGGACTCCTTCATCTTCTTC
ATCGCGTTTCTGCATCAACGCGAAGAATTATTTCTTAACTTATTCAAGATGTCCAATTTCCAAAGAAA
TTGTTCTCGACCAAA
+
FFFFFFFFFFFFFFFFFFFFFFFFFFFFFFFFFFFFFFFFFFF:FFFFFFFFFFFFFFFFFFFFFFFFFF
FFFFFFFFFFFFFF,FFFFFFFFFF:FFFFFFFFFFFFFFFFFFFFFFFF,FFFFFF:FFFF:FFFF,F:
FFFFF:FFFFFFFFF
@A00721:190:HGJW7DSXY:4:2650:28031:10050 2:N:0:TCTGCAAG+GCTGCACT
CCCCAATATAATGCTTAATATGCGAATGTCCCTGATTTAACAGTAATGGGGACTCCTTCATCTTCTTC
ATCGCGTTTCTGCATTAACGCGAAGAATTATTTCTTAACTTATTCAAGATGTCCAATTTCCAAAGAAA
TTGTTCTCGACCAAA
+
FFFFFFFFFFFFFFFFFFFFFFFFFFFFFFFFFFFFFFFFFFFFFFFFFFFFFFFFFFFFFFFFFFFFFF
FFFFFFFFFFFFFFFFFFFFFFFFFFFFFFFFFFFFFFFFFFFFFFFFFF:FFFFFFFFFFFFFFFFFFF
FFFFFFFF,FFFFFF
@A00721:190:HGJW7DSXY:4:1136:24505:32659 2:N:0:TCTGCAAG+GCTGCACT
TTTCTTTGGAAATTGGACATCTTGAATAAGTTAAGAAATAATTCTTCGCGTTAATGCAGAAACGCGAT
GAAGAAGATGAAGGAGTCCCCATTACTGTTAAATCAGGGACATTCGCATATTAAGCATTATATTGGGG
ACTGGGGACCTATAT
+
FFFFFFFFFFFFFFFFFFFFFFFFFFFFF:FFFFFFFFFFFFFFFFFFFFFFF:FF,FFF:FFFFFF:FF
FFFFFFFFFFFFFFFFF:FFFFFFF,::FFFFFF:FF:FFFFFFFFFF:FFF:FFF,FFFF:FFF::,:F
:,FFF:FFFFFFFFF
@A00721:190:HGJW7DSXY:4:1213:15076:32612 2:N:0:TCTGCAAG+GCTGCACT
TTTCTTTGGAAATTGGACATCTTGAATAAGTTAAGAAATAATTCTTCGCGTTAATGCAGAAACGCGAT
GAAGAAGATGAAGGAGTCCCCATTACTGTTAAATCAGGGACATTCGCATATTAAGCATTATATTGGGG
ACTGGGGACCTATAT
+
FF:FF:FFFFF:FFFFFFFFFFFFFFFFFFFFFFFFFFFFFFF::FFFFFFFFFFF:FFFFFF:FFFFF:
FFFF:FFFFFFFFFFFFFFF:FFFFFFFFFFFFFFFFFF::FFFFFF:F:FFFFFF,FFFF,FFFFFFFF
F:FFFFFFFFF,FFF
@A00721:190:HGJW7DSXY:4:1264:29234:26475 2:N:0:TCTGCAAG+GCTGCACT
TTTCTTTGGAAATTGGACATCTTGAATAAGTTAAGAAATAATTCTTCGCGTTAATGCAGAAACGCGAT
GAAGAAGATGAAGGAGTCCCCATTACTGTTAAATCAGGGACATTCGCATATTAAGCATTATATTGGGG
ACTGGGGACCTATAT
+
FFFFFFFFFFFFFFFFFFFFFFFFFFFFFFFFFFFFFFFFFFFFFFFFFFFFFFFFFFFFFFFFFFFFFF
FFFFFFFFFF:FFFFFFFFFFFFFFFFFFFFFFFFFFFFFFFF,FFFFFFFFFFF:FFFFFFFFFFFFFF
FFFFFFFFFFFFFFF
@A00721:190:HGJW7DSXY:4:1342:13557:26944 2:N:0:TCTGCAAG+GCTGCACT
TTTCTTTGGAAATTGGACATCTTGAATAAGTTAAGAAATAATTCTTCGCGTTAATGCAGAAACGCGAT
GAAGAAGATGAAGGAGTCCCCATTACTGTTAAATCAGGGACATTCGCATATTAAGCATTATATTGGGG
ACTGGGGACCTATAT
+
FFFFFFFFFFFFFFFFFFFFFFFFFFFFFFFFFFFFFFFFFFFFFFFFFFFFFFFFFFFFFFFFFFFFFF
FFFFFFFFFFFFFFFFFFFFFFFFFFFFFFFFFFFFFFFFFFFFFFFFFFFFFFFFFFFFF:FFFFF:FF
FFFFFFFFFFFFFFF
@A00721:190:HGJW7DSXY:4:1545:10601:21543 2:N:0:TCTGCAAG+GCTGCACT
TTTCTTTGGAAATTGGACATCTTGAATAAGTTAAGAAATAATTCTTCGCGTTAATGCAGAAACGCGAT
GAAGAAGATGAAGGAGTCCCCATTACTGTTAAATCAGGGACATTCGCATATTAAGCATTATATTGGGG
ACTGGGGACCTATAT
+
FFFFF:FFFF:FF:FF:FFFFFFFFFFFFFFFFFFFFFFFFFFFFFFFFFFFFFFFFFFFFF:FFFFFF:
```

FFFFFFFF,FF:FFFFFFFFFFFFFFFFFFFFFFFFFFFFFFFFFFFF::FFFFFFF:FFFFFFFFFF
FFFFFFFFFFFFFFF
@A00721:190:HGJW7DSXY:4:2519:29821:5979 2:N:0:TCTGCAAG+GCTGCACT
TTTCTTTGGAAATTGGACATCTTGAATAAGTTAAGAAATAATTCTTCGCGTTAATGCAGAAACGCGAT
GAAGAAGATGAAGGAGTCCCCATTACTGTTAAATCAGGGACATTCGCATATTAAGCATTATATTGGGG
ACTGGGGACCTATAT
+
FFFFFFFF:FFFFFFFFFFFFFFFFFFFFFFFFFFFFFFFF:FFFFF:FFFFFF:FFFFFFF::FFFF
FFFFFFFFFFFFF,FFFF:FFFFFFFFFFFFFFFFF:FFFFFFFF,FFFFFFFFFFFFFFFFF:FFFF
FFFFFFFFFFFFFFF
@A00721:190:HGJW7DSXY:4:1510:26395:35117 2:N:0:TCTGCAAG+GCTGCACT
GGAAATTGGACATCTTGAATAAGTTAAGAAATAATTCTTCGCGTTAATGCAGAAACGCGATGAAGAAG
ATGAAGGAGTCCCCATTACTGTTAAATCAGGGACATTCGCATATTAAGCATTATATTGGGGACTGGGG
ACCTATATATATGTA
+
FFFFFFFFFFF:FFFFFFFFFFFFFFFFFFFFFFFFFFFFFFFFFFFFFF:FFFFFFFFFFFFFFFFF
FFFFFFFFFFFFFFFFFFFFF:FFFFFFFFFFFFFFFFFFFFFFFFFFFFFFFFFF:FFFFFFFFF:FF
:FFFFFFFFFFFFFF
@A00721:190:HGJW7DSXY:4:1556:12500:6386 2:N:0:TCTGCAAG+GCTGCACT
GGAAATTGGACATCTTGAATAAGTTAAGAAATAATTCTTCGCGTTAATGCAGAAACGCGATGAAGAAG
ATGAAGGAGTCCCCATTACTGTTAAATCAGGGACATTCGCATATTAAGCATTATATTGGGGACTGGGG
ACCTATATATATGTA
+
:FFFF:FFFFF:FFFFFFF,FFF:FFFFFFFFFFFFFFFFFFFFFFFFFFFFFFFFFFFFFFFFFF:F
FFFFFFFF:FFFFFFFFFFFFFFFFFFFF:FFFFFF:,,FFF:FFFFF,FFFFF,FFFFFF:FFFFFF
FFFFFFFFFFFFFFF
@A00721:190:HGJW7DSXY:4:2660:9715:10050 2:N:0:TCTGCAAG+GCTGCACT
GGAAATTGGACATCTTGAATAAGTTAAGAAATAATTCTTCGCGTTAATGCAGAAACGCGATGAAGAAG
ATGAAGGAGTCCCCATTACTGTTAAATCAGGGACATTCGCATATTAAGCATTATATTGGGGACTGGGG
ACCTATATATATGTA
+
:F:FFFFFFFFFFFFFFFFFFFFFFFFFFFFFFFFFFFFFFFFFFFFFFFFFFFFFFFFFFFFFFFFF
FFFFFFFFFFFFFFFFFFFFFFFFFFFFFFFFFFFFFFFFFFFFFFFFFFFFFFFFFF:FFFFFFFFF
FFFFFFFFFFFFFFF
@A00721:190:HGJW7DSXY:4:1512:10556:28573 2:N:0:TCTGCAAG+GCTGCACT
ATTCCTCCGGATTACTCTATACATATATATAGGTCCCCAGTCCCCAATATAATGCTTAATATGCGAAT
GTCCCTGATTTAACAGTAATGGGGACTCCTTCATCTTCTTCATCGCGTTTCTGCATTAACGCGAAGAA
TTATTTCTTAACTTA
+
FFFFFFFFFFFFFFFFF,FFFFFFFFFFFFFFFFFFFFFFFFFFFFFFFFFFFFFFFFFFFFFFFFFF
FFF:FFFFFFFFF:FFFFFFF:FFFFFFFFFFFFFFFFFFFFFFFFFFFFFFFFFFFFFFFFFFFFFF
FFFFFFFFFFFFFFF
@A00721:190:HGJW7DSXY:4:1607:25265:16438 2:N:0:TCTGCAAG+GCTGCACT
ATTCCTCCGGATTACTCTATACATATATATAGGTCCCCAGTCCCCAATATAATGCTTAATATGCGAAT
GTCCCTGATTTAACAGTAATGGGGACTCCTTCATCTTCTTCATCGCGTTTCTGCATTAACGCGAAGAA
TTATTTCTTAACTTA
+
FFF::FFFFFFFFFFFF:FFFFFFFF:FFFFF:FFFFFFFFF:FFFFFFFFF,F:FFFFFFF:F:FFF
FFF::FFFFFFFFFFFFFFFF:FFF::F,F:FFF:FFFFFFFFFF:F,:F::FFF:FFFFF:FFF,FF
FFF:FF,FFFFF:FF
@A00721:190:HGJW7DSXY:4:2302:14416:26021 2:N:0:TCTGCAAG+GCTGCACT
ATTCCTCCGGATTACTCTATACATATATATAGGTCCCCAGTCCCCAATATAATGCTTAATATGCGAAT
GTCCCTGATTTAACAGTAATGGGGACTCCTTCATCTTCTTCATCGCGTTTCTGCATTAACGCGAAGAA
TTATTTCTTAACTTA

+
F:FFFFFFFFFFFFFFFFFFFFFFFFFFFFFFFFFFFFFFFFFFFFFFFFFFFFFFFFFFFFFFFFFF
FFFFFFFF:FFFFFFFFFFFFFFFFFFFFFFFFFFFFFFFFFFFFF:FFFFFFFF,FF:FFFFFFFFF
FFFFFF:FFFFFFFF
@A00721:190:HGJW7DSXY:4:2549:18295:28635 2:N:0:TCTGCAAG+GCTGCACT
ATTCCTCCGGATTACTCTATACATATATATAGGTCCCCAGTCCCCAATATAATGCTTAATATGCGAAT
GTCCCTGATTTAACAGTAATGGGGACTCCTTCATCTTCTTCATCGCGTTTCTGCATTAACGCGAAGAA
TTATTTCTTAACTTA
+
FFFFFFFFFFF:FFFFFFFFFFFFFFFFFFFFFFFFFFFFFFFFFFFFFFFFFFFFFFFF
FFFFFFFFFFFFFFFFFFFFFFFFFFFFFF:F:FFFFFFFFFFFFFFF:FFFFFFFFFFFFFFFFFFF
FFFFFFFFFF:FFFF
@A00721:190:HGJW7DSXY:4:1129:23610:36714 2:N:0:TCTGCAAG+GCTGCACT
GATTCCTCCGGATTACTCTATACATATATATAGGTCCCCAGTCCCCAATATAATGCTTAATATGCGAA
TGTCCCTGATTTAACAGTAATGGGGACTCCTTCATCTTCTTCATCGCGTTTCTGCATTAACGCGAAGA
ATTATTTCTTAACTT
+
FFFFFFFFFFFFFFFFFFFFFFFFFFFFFFFFFFFFFFFFFFFFFFFFFFFFFFFFFFFFFFFFFFFF
FFFFFFFFFFFFFFFFFFFFFFFFFFFFFFFFFFFFFFFFFFFFFFFFFFFFFFFFFFFFF:FFFFFF
FFFFFFFFFFFFFFF
@A00721:190:HGJW7DSXY:4:1202:25880:25739 2:N:0:TCTGCAAG+GCTGCACT
GATTCCTCCGGATTACTCTATACATATATATAGGTCCCCAGTCCCCAATATAATGCTTAATATGCGAA
TGTCCCTGATTTAACAGTAATGGGGACTCCTTCATCTTCTTCATCGCATTTCTGCATTAACGCGAAGA
ATTATTTCTTAACTT
+
FFFFFFFFFFFFFFFFFFFFFFFFFFFFFFF:FFFFFFFFFFFFFFFFFFFFFFFFFFFFFFFFFFFF
FFFFFFFFF:FFFFFFFF,::FFFFFFFFFFFFFFFFFFFFFFFFFFFFFFFFFFFFFF:FFFFFFFF
F:FFFFFFFFFFFFF
@A00721:190:HGJW7DSXY:4:2527:21703:13777 2:N:0:TCTGCAAG+GCTGCACT
ATTCCTCCGGATTACTCTATACATATATATAGGTCCCCAGTCCCCAATATAATGCTTAATATGCGAAT
GTCCCTGATTGAACAGTAATGGGGACTCCTTCATCTTCTTCATCGCGTTTCTGCATTAACGCGAAGAA
TTATTTCTTAACTT
+
F:FF,,:,:FFF:FF,F,F,FFF::F:,FFF:F,:::FFFFFF,FFFFF,FF:F:F:FF:FF:FF::F
:FF,:FFF,,,F,:F,,,F,::,::FFF:,FFFFF::F,:FFF,FFF,,:F,:FFFFFF,,FFF,F:F
F,F:::F:,FF:::
@A00721:190:HGJW7DSXY:4:2606:11333:23437 2:N:0:TCTGCAAG+GCTGCACT
GATTCCTCCGGATTACTCTATACATATATATAGGTCCCCAGTCCCCAATATAATGCTTAATATGCGAA
TGTCCCTGATTTAACAGTAATGGGGACTCCTTCATCTTCTTCATCGCATTTCTGCATTAACGCGAAGA
ATTATTTCTTAACTT
+
FFFFFFFFFFF,FFFFFFFFF:FF:FFFFF:FF:FFFFFFFFFFFFFFFFFFFFFFFFF:FFF,F:FF
FFFF:FFFFFF:FFFFFFFFFFFFFFFFFFFFFFFFFF,FF,,FFFFF:FFFF:,FFFF::F:FF:FF
FFFFFFFFFFFFFF:
@A00721:190:HGJW7DSXY:4:1212:28212:23140 2:N:0:TCTGCAAG+GCTGCACT
AAATAATTCTTCGCGTTAATGCAGAAACGCGATGAAGAAGATGAAGGAGTCCCCATTACTGTTAAATC
AGGGACATTCGCATATTAAGCATTATATTGGGGACTGGGGACCTATATATATGTATAGAGTAATCCGG
AGGAATCTATCCCCA
+
FFFFFFFFFFFFF:FFFFFFFFFFFFFFFFF:,FF::FFFF,FFFFFFFFF:FFF:FFF,FFFFFFFF
FFFFFFFFFFFFFFFFFFFFFFFFFF:FFFFFF:FFFFFFFFF:,F,FFFFFFFF:FFFFFFFFFFFF
FFFF,FFFFFFFFFF
@A00721:190:HGJW7DSXY:4:1240:8983:33301 2:N:0:TCTGCAAG+GCTGCACT
AAATAATTCTTCGCGTTAATGCAGAAACGCGATGAAGAAGATGAAGGAGTCCCCATTACTGTTAAATC

```
AGGGACATTCGCATATTAAGCATTATATTGGGGACTGGGGACCTATATATATGTATAGAGTAATCCGG
AGGAATCTATCCCCA
+
FFFFFFFFFFFFFFFFFFFFFFFFFFFFFFFFFFFFFFFFFFFFFFFFFFFFFFFFFFFFFFFFFFFFFF
FFFFFFFFFFFFFFFFFF:FFFFFFFFFFFFFFFFFFFFFFFFF:FFFFFFFFFFFFFFFFFFFFFFFFF
FFFFFFFFFFFFFFF
@A00721:190:HGJW7DSXY:4:1304:14443:22467 2:N:0:TCTGCAAG+GCTGCACT
AAATAATTCTTCGCGTTAATGCAGAAACGCGATGAAGAAGATGAAGGAGTCCCCATTACTGTTAAATC
AGGGACATTCGCATATTAAGCATTATATTGGGGACTGGGGACCTATATATATGTATAGAGTAATCCGG
AGGAATCTATCCCCA
+
FFFFFFFFFFFFFFFFFFFFFF:F:FFFFFFFFFFFFF:::FFFFFFFFFFFFFFF:FFFFFFFFFF:FF
:FFFFFFFFFFFFFFFFF:,FFFFFFFFFFFFFFFFFFF:FFFFFFFFFFFFFFFFFFFFFFFFFFFFFF
FFFFFFFFFFF:FFF
@A00721:190:HGJW7DSXY:4:1415:14543:33160 2:N:0:TCTGCAAG+GCTGCACT
AAATAATTCTTCGCGTTAATGCAGAAACGCGATGAAGAAGATGAAGGAGTCCCCATTACTGTTAAATC
AGGGACATTCGCATATTAAGCATTATATTGGGGACTGGGGACCTATATATATGTATAGAGTAATCCGG
AGGAATCTATCCCCA
+
FFFFFFFFFFFFFFFFFFFFFFFFFFFFFFFFFFFFFFFFF:FFFF:FFFFFFFFFFFFFFFFFFFFFFF
FFFFFFFFFFFFFF,FFFFFFFFFFFFFFFFFFFFFFFFFFFFFFFFFFF,FFF:FFFFFFFFFFFFFFF
FFFF,FFF:FFF:FF
@A00721:190:HGJW7DSXY:4:1673:19199:11099 2:N:0:TCTGCAAG+GCTGCACT
AAATAATTCTTCGCGTTAATGCAGAAACGCGATGAAGAAGATGAAGGAGTCCCCATTACTGTTAAATC
AGGGACATTCGCATATTAAGCATTATATTGGGGACTGGGGACCTATATATATGTATAGAGTAATCCGG
AGGAATCTATCCCCA
+
FFFFFFFFFFFFFFFFFFFFFFFFFFFFFFFFFFFF:FFFFFFFFFFFFFFFFFFFFFFFFFFFFFFFFF
FFFFFFFFFFFFFFF:FFFFF:FFFFFFFFFFFFFFFFFFFFFFF,FFFFF:FFFFFFFFFFFF:FFFFF
FFFFFFFFFFF:FFF
@A00721:190:HGJW7DSXY:4:2443:14009:31735 2:N:0:TCTGCAAG+GCTGCACT
AAATAATTCTTCGCGTTAATGCAGAAACGCGATGAAGAAGATGAAGGAGTCCCCATTACTGTTAAATC
AGGGACATTCGCATATTAAGCATTATATTGGGGACTGGGGACCTATATATATGTATAGAGTAATCCGG
AGGAATCTATCCCCA
+
FFFFFFFFFFFFFFFFFFFFFFFF:FFFFFFFFFF,FFFFFFFFFFFFFFFFFFFFFFFFFFFFFFFFFF
FFFFFFFFFFFFFFFFFFFFFFFFFFFFFFFFFFFFFFFFFFFFFF:FFFFFFFFFF:FFFFFFFFFFFF
FFFFFFFFFFFFFFF
@A00721:190:HGJW7DSXY:4:2671:9896:20854 2:N:0:TCTGCAAG+GCTGCACT
AAATAATTCTTCGCGTTAATGCAGAAACGCGATGAAGAAGATGAAGGAGTCCCCATTACTGTTAAATC
AGGGACATTCGCATATTAAGCATTATATTGGGGACTGGGGACCTATATATATGTATAGAGTAATCCGG
AGGAATCTATCCCCA
+
FFFFFFFFFFFFFFFFFFFFFFFFFFFFFFFFFFFFFFFFFFFFFFFFFFFFFFFFFFFFFFFFFFFFFF
FFFFFFFFFFFFFFFFFFFFFFFFFFFFFFFFFFFFFFFFFFFFF:FFFFFFFFFFFFFFFFFFFFFFFF
FFFF,FFFFFFFFFF
@A00721:190:HGJW7DSXY:4:2671:9905:21183 2:N:0:TCTGCAAG+GCTGCACT
AAATAATTCTTCGCGTTAATGCAGAAACGCGATGAAGAAGATGAAGGAGTCCCCATTACTGTTAAATC
AGGGACATTCGCATATTAAGCATTATATTGGGGACTGGGGACCTATATATATGTATAGAGTAATCCGG
AGGAATCTATCCCCA
+
FFFFFFFFFFFFFFFFFFFFFFFFFFFFFFFFFFFFFFFFFFF,FFFFFFFFFFFF:FFFFFFFFFFFFF
FFFFFFFFFFFF,FF,FFFFFFFFFFFFFFFFFFFFFFFFFFFFFFFFFFFFFFFFFFFFFFFFFFFFFF
FFFF,FFFFFFFFFF
```

```
@A00721:190:HGJW7DSXY:4:1111:14986:12853 2:N:0:TCTGCAAG+GCTGCACT
CTTCGCGTTAATGCAGAAACGCGATGAAGAAGATGAAGGAGTCCCCATTACTGTTAAATCAGGACAT
TCGCATATTAAGCATTATATTGGGGACTGGGGACCTATATATATGTATAGAGTAATCCGGAGGAATCT
ATCCCCAATTGGAAT
+
FFFFFFFFFFFFFFFFFFFFFFFFFFFFFFFFFFFFFFFFFFFFFFF:F:FFFFFFFFFFFFFFFFFF
FFFF,FFFFF,FFFFFFFFFFFFFFFFFFFFFFFFFFFFFFFFFFFF:FFFFFFFFFFFFFFFFFFFF
FFFFFFFFFFFFF:F
@A00721:190:HGJW7DSXY:4:2242:18837:25285 2:N:0:TCTGCAAG+GCTGCACT
CTTCGCGTTAATGCAGAAACGCGATGAAGAAGATGAAGGAGTCCCCATTACTGTTAAATCAGGACAT
TCGCATATTAAGCATTATATTGGGGACTGGGGACCTATATATATGTATAGAGTAATCCGGAGGAATCT
ATCCCCAATTGGAAT
+
FFFFFFFFFFFFFFFFFFFFFFFFFFFFFF:FFFFFFFFFFFFFFFFFFFFFFFFFFFFFFFFF:FFF
FFFFFFFFFFFFFFFFFFFFFFFFFFFFFFF:FFFFFFFFFFFFFF,F::FFFFFFFFFFFFFF:FFF
FFFFFFFFFFFFFFF
@A00721:190:HGJW7DSXY:4:2457:1253:6355 2:N:0:TCTGCAAG+GCTGCACT
CTTCGCGTTAATGCAGAAACGCGATGAAGAAGATGAAGGAGTCCCCATTACTGTTAAATCAGGACAT
TCGCATATTAAGCATTATATTGGGGACTGGGGACCTATATATATGTATAGAGTAATCCGGAGGAATCT
ATCCCCAATTGGAAT
+
:FFFFFFFFFFFFFFFFFFFFFFFFFFFFFFFFFFFFFFFFFFFFFFFFFFFFFFFFFFFFFFFFFFF
FFFFFFFFFFFFFFFFFFFFFFFFFFFFFFFFFFFFFFFFFFFFFFFFFFFFFFFFFFFFFFFFFF:F
FFFFFFFFFFFFFFF
@A00721:190:HGJW7DSXY:4:2548:14787:14262 2:N:0:TCTGCAAG+GCTGCACT
CTTCGCGTTAATGCAGAAACGCGATGACGAAGATGAAGGAGTCCCCATTACTGTTAAATCAGGACAT
TCGCATATTAAGCATTATATTGGGGACTGGGGACCTATATATATGTATAGAGTAATCCGGAGGAATCT
ATCCCCAATTGGAAT
+
FFFFFFFFFFFFFFFFFFFFFFF:,FF,FFFF:FFFFFFFFFFFFFFFFFFFFFFFFFFFFFFFFFFF
FFFFFFF,F:FFFFFFFFF:FFFFFFFFFFFFFFFFFFFFFFFFFFFF:F:FFFFFFFFFFFFFFFFF
FFFFFFFFFFFFFFF
@A00721:190:HGJW7DSXY:4:1336:14733:8876 2:N:0:TCTGCAAG+GCTGCACT
CAATATAATGCTTAATATGCGGATGTCCCTGATTTAACAGTAATGGGGACTCCTTCATCTTCTTCATC
GCGTTTCTCCATTAACGCTAACATCATATTCAGAAATTTACTTTCATCTCTTCCCGATGAAATCAAAC
ATATCATCAAGGATA
+
FFFFFFFFFFFFFFFFFFFFFFFFFFFFFFFFFFFFFFFFFFF:FFFFFFFFFFFFFFFFFFFFFFFF
FFFFFFFFFFFFFFFFFFFFFFFFFFFFFFFFFFFFFFFFFFFFFFFFFFFFFFFFFFFFFFFFFF,F
FFFFFFF:FFFFFFF
@A00721:190:HGJW7DSXY:4:1512:13313:27054 2:N:0:TCTGCAAG+GCTGCACT
CAAAATAATGCTTAATATGCGGATGTCCCTGATTTAACAGTAATGGGGACTCCTTCATCTTCTTCATC
GCGTTTCTCCATTAACGCTAACATCATATTCAGAAATTTACTTTCATCTCTTCCCGATGAAATCAAAC
ATATCATCAAGGATA
+
FFF,FFFFFFF,,:,::FF:FFFFFFFFF:,FF,,F:FF:FFFF:,FFF,F:F:FFFFFFFF::FFFF
FFF::FF:FFF:,FFFFF:FFF,FFFFF:FFF,FF:,F:FFFFF:FFFFF,F:F:FF:,FFFFFF:FF
FFFFFF,FFF::F,F
@A00721:190:HGJW7DSXY:4:2331:28221:3333 2:N:0:TCTGCAAG+GCTGCACT
CAATATAATGCTTAATATGCGGATGTCCCTGATTTAACAGTAATGGGGACTCCTTCATCTTCTTCATC
GCGTTTCTCCATTAACGCTAACATCATATTCAGAAATTTACTTTCATCTCTTCCCGATGAAATCAAAC
ATATCATCAAGGATA
+
FFF:FFFF:FFFFFFFFFFFFFFFFFFFF:F:F:F:FFFF:FFFFFFFFFFFFFFFF:F:FFFFFFFF
```

FFF::FFFFFFF:F:FFFF:FFFFFFF:FFFFFFFFFFFFFFFFFFFFFFFFFFFFFFFFFFFFFFFF
FFFFFF:FFFFFFFF
@A00721:190:HGJW7DSXY:4:2549:2094:11443 2:N:0:TCTGCAAG+GCTGCACT
CAATATAATGCTTAATATGCGGATGTCCCTGATTTAACAGTAATGGGGACTCCTTCATCTTCTTCATC
GCGTTTCTCCATTAACGCTAACATCATATTCAGAAATTTACTTTCATCTCTTCCCGATGAAATCAAAC
ATATCATCAAGGATA
+
FFFFFFFFFFFFFFFFFFFFFFFFFFFFFFFFFFFFFFFFFFFFFFFFFFFFFFFFFFFFFFFFFFFF
FFFFFFFFFFFFFFFFFFFFFFFFFFFFFFFFFFFFFFFFFFFFFFFFFFFFFFFFFFFFFFFFFFFF
FFFFFFFFFFFFFFF
@A00721:190:HGJW7DSXY:4:1153:20509:6668 2:N:0:TCTGCAAG+GCTGCACT
CCAATATAATGCTTAATATGCGGATGTCCCTGATTTAACAGTAATGGGGACTCCTTCATCTTCTTCAT
CGCGTTTCTCCATTAACGCTAACATCATATTCAGAAATTTACTTTCATCTCTTCCCGATGAAATCAAA
CATATCATCAAGGAT
+
FFFFFFFFFFFFFFFFFFFFFFFFFFFFFFFFFFFFFFFFFFFFFFFFFFFFFFFFFFFFFFFFFFFF
FFFFFFFFFFFFFFFFFFFFF:FFFFFFFFFFFFFFFFFFFFFFFFFFFFFFFFFFFFFFFFFFFFF,
FFFFFFFFFFFFFFF
@A00721:190:HGJW7DSXY:4:1266:23032:30608 2:N:0:TCTGCAAG+GCTGCACT
CCAATATAATGCTTAATATGCGGATGTCCCTGATTTAACAGTAATGGGGACTCCTTCATCTTCTTCAT
CGCGTTTCTCCATTAACGCTAACATCATATTCAGAAATTTACTTTCATCTCTTCCCGATGAAATCAAA
CATATCATCAAGGAT
+
FFFFFFFFFFFFFFFFFFFFFFFFFFFFFFFFFFFFFFFFFFF:FFFFFFFFFFFFFFFFFFFFFFFF
FFFFFFFFFFFFFFFFFFFFFFFFFF::FF:FFFFFFFFFFFFFFFFFFFFFFFFFFFFFFFFFFFFF
FFFFFFFFFFFFFFF
@A00721:190:HGJW7DSXY:4:1409:26811:9095 2:N:0:TCTGCAAG+GCTGCACT
CCAATATAATGCTTAATATGCGGATGTCCCTGATTTAACAGTAATGGGGACTCCTTCATCTTCTTCAT
CGCGTTTCTCCATTAACGCTAACATCATATTCAGAAATTTACTTTCATCTCTTCCCGATGAAATCAAA
CATATCATCAAGGAT
+
FFFFFFFFFFFFFFFFFFFFFFFFFFFFFFFFFFFFF:FFFFF:FFFFFFFFFFFFFFFFFFFFFFFF
FFFF:FFFFFFF:FF:FFFFFFFFFF,F:FFFFFF:FFFFFFFFFFFFFFFFFFFFFFFFFFFFFFFF
FFFFFFFFFFFFFFF
@A00721:190:HGJW7DSXY:4:1418:25970:21167 2:N:0:TCTGCAAG+GCTGCACT
CCAATATAATGCTTAATATGCGGATGTCCCTGATTTAACAGTAATGGGGACTCCTTCATCTTCTTCAT
CGCGTTTCTCCATTAACGCTAACATCATATTCAGAAATTTACTTTCATCTCTTCCCGATGAAATCAAA
CATATCATCAAGGAT
+
FFFFFFFFFFFFFFFFFFFFFFFFFFFFF,FFFFFFFFF:FFFFFFFFFFFFFFFFFFFFFFFF:FFF
FFFFFFFFFFFFFFF:FFFF,FFFFFFFF,FFFFF:FFFFFFFFFFFFFFFFFFFFFFFFFFFFFFFF
FFF,FFFFFFFFFFF
@A00721:190:HGJW7DSXY:4:1445:17246:34585 2:N:0:TCTGCAAG+GCTGCACT
CCAATATAATGCTTAATATGCGGATGTCCCTGATTTAACAGTAATGGGGACTCCTTCATCTTCTTCAT
CGCGTTTCTCCATTAACGCTAACATCATATTCAGAAATTTACTTTCATCTCTTCCCGATGAAATCAAA
CATATCATCAAGGAT
+
FFFFFFF:FFFFFFFFFFFFFFFFFFFFFFFFFFFFFFFFFFF:F,FFFFFFFF:FFFFFFFFFFFFF
FFFFFFFFFFFFFFFFFFFFFFFFFFF:FFFFFFFFFFFFFFFFFFFFFFFFFFFFFFFFFF:FFFFFF
FFFFFFFFF,FFFF:
@A00721:190:HGJW7DSXY:4:2310:9525:27946 2:N:0:TCTGCAAG+GCTGCACT
CCAATATAATGCTTAATATGCGGATGTCCCTGATTTAACAGTAATGGGGACTCCTTCATCTTCTTCAT
CGCGTTTCTCCATTAACGCTAACATCATATTCAGAAATTTACTTTCATCTCTTCCCGATGAAATCAAA
CATATCATCAAGGAT

+
FFFFFFFFFFFFFFFFFFFFFFFFFFFFFFFFFFFFFFFFFFF:FFFFFFFFFFFFFFFFFFFFFFFFF
FFFFFFFFFFFFFFFFFFFFFFFFF:FFFF:FF:FFFFFFFFFFFFF:FFFFFFFFFFFFFFFFFFFFF
FFFF:FFFFFFFFFF
@A00721:190:HGJW7DSXY:4:2365:5475:33458 2:N:0:TCTGCAAG+GCTGCACT
CCAATATAATGCTTAATATGCGGATGTCCCTGATTTAACAGTAATGGGGACTCCTTCATCTTCTTCAT
CGCGTTTCTCCATTAACGCTAACATCATATTCAGAAATTTACTTTCATCTCTTCCCGATGAAATCAAA
CATATCATCAAGGAT
+
FFFFFFFFFFFFFFFFFFFFFFFFFFFFFFFFFFFFFFFFFFFFFFFFFFFFFFFFFFFFFFFFFFFFF
FFFFFFFFFFFFFFFFFFFFFFFFFFFFFFFFFFFFFFFFFFFFFFFFFFFFFFFFFFFFFFFFFFFFF
FFFFF:FF:FFFFFF
@A00721:190:HGJW7DSXY:4:2372:19868:26506 2:N:0:TCTGCAAG+GCTGCACT
CCAATATAATGCTTAATATGCGGATGTCCCTGATTTAACAGTAATGGGGACTCCTTCATCTTCTTCAT
CGCGTTTCTCCATTAACGCTAACATCATATTCAGAAATTTACTTTCATCTCTTCCCGATGAAATCAAA
CATATCATCAAGGAT
+
FFFFFFFFFFFFFFFFFFFFFFFFFFFFFFFFFFFFFFFFFFFFFFFFFFFFFFF:FFFFFFFFFFFFF
FFFFFFFFFFFFFFFFF:FFFFFFFFFFFFFFFFFFFFFFFFFFFFFFFFFFFFFFFFFFFFFFFFFFF
FFFFFF:FFFF:FFF
@A00721:190:HGJW7DSXY:4:2411:29903:8876 2:N:0:TCTGCAAG+GCTGCACT
CCAATATAATGCTTAATATGCGGATGTCCCTGATTTAACAGTAATGGGGACTCCTTCATCTTCTTCAT
CGCGTTTCTCCATTAACGCTAACATCATATTCAGAAATTTACTTTCATCTCTTCCCGATGAAATCAAA
CATATCATCAAGGAT
+
FFFFFFFF:FFFFFFFFFFFFFFFFFFFFFFFFFFFFFFFFFFFFFFFFFFFFFFFFFFFFFFFFFFFF
FFFFFFFFFFFFFFF:FFFFFFFFFFFFF:FFFFFFFFFFFFFFFF:FFFF,FFFFFF:FF:FFFFFF
FFFF:FFFFFFFFFF
@A00721:190:HGJW7DSXY:4:2674:29966:13213 2:N:0:TCTGCAAG+GCTGCACT
CCAATATAATGCTTAATATGCGGATGTCCCTGATTTAACAGTAATGGGGACTCCTTCATCTTCTTCAT
CGCGTTTCTCCATTAACGCTAACATCATATTCATAAATTTACTTTCATCTCTTCCCGATGAAATCAAA
CATATCATCAAGGAT
+
FFFFFFFFF,F:FFFF,F:F,FFFFFFFF:F:FFFFFFFF:FFFF::FFF,F:F,F:FFFFF,F::FF
FFFF:FF,FFFF,FF:,,FFFF:FF,F:FFF,F,FFF,FFF,:,F,FF,FFFF::F:FF:FFFF:FFF
FFFFF:F:FFF,FFF
@A00721:190:HGJW7DSXY:4:1163:32407:35697 2:N:0:TCTGCAAG+GCTGCACT
TGAAAGTAAATTTCTGAATATGATGTTAGCGTTAATGGAGAAACGCGATGAAGAAGATGAAGGAGTCC
CCATTACTGTTAAATCAGGGACATCCGCATATTAAGCATTATATTGGGGACTGGGGACCTATATATAT
GTATAGAGTAATCCG
+
FFF:FFFFFFFFFFFFFFFFFFFFFFFFFFFFFFFFFFFFFF,FFFFFFFFFFFFF,FFFFFFFFFFF
FFFFFFF:FFFFFFFFFFFFFFFFFFFFFFFFF:FFFFFFFFFFFFFFFFF:FFFFFFFFFFFFF:::F
FFF:FFFFFFFFFFF
@A00721:190:HGJW7DSXY:4:1325:12861:10958 2:N:0:TCTGCAAG+GCTGCACT
TGAAAGTAAATTTCTGAATATGATGTTAGCGTTAATGGAGAAACGCGATGAAGAAGATGAAGGAGTCC
CCATTACTGTTAAATCAGGGACATCCGCATATTAAGCATTATATTGGGGACTGGGGACCTATATATAT
GTATAGAGTAATCCG
+
FFFFFFFFFFF,FFFFFFFF:FFFFFFFFFF:FFFFFFFFFFFFFFFFFFF:FFFFFFFFFFFFFFFF
FFFFFFFFFFFFFFFFFFFFFFFFFFFFFFFFFFFFFFFFFFFFFFFFFFFFFFFFFFFFFFFFF:F,F
FF,FFFFFFFFFFFF
@A00721:190:HGJW7DSXY:4:1326:30165:25958 2:N:0:TCTGCAAG+GCTGCACT
TGAAAGTAAATTTCTGAATATGATGTTAGCGTTAATGGAGAAACGCGATGAAGAAGATGAAGGAGTCC

CCATTACTGTTAAATCAGGGACATCCGCATATTAAGCATTATATTGGGGACTGGGGACCTATATATAT
GTATAGAGTAATCCG
+
FFFFFFFFFFF:FFFFFFFFFFFFFFFFFFFFFFF,FFFFFFFFFFFF:FFF:FFFFFFFFFFFFFFF
FFFFFFFFFFFFFFFFFFFFFFFFFFFFFFFFFFFFFFFFFFFFFFF:FFFFFFFFFFFFFFFFFFFF
FFFFF:FFFFFFFFF
@A00721:190:HGJW7DSXY:4:1331:2239:14982 2:N:0:TCTGCAAG+GCTGCACT
TGAAAGTAAATTTCTGAATATGACGTTAGCGTTAATGGAGAAACGCGATGAAGAAGATGAAGGAGTCC
CCATTACTGTTAAATCAGGGACATCCGCATATTAAGCATTATATTGGGGACTGGGGACCTATATATAT
GTATAGAGTAATCCG
+
FFFFFFFFFFFFFFFFFFFFFFFFFFFFFFFFFFFFFFFFFFFFFFFF,:FFFFFFFFFFFFFFFFFF
FFFFFFFFFFFFFFFFFFFFFFFFFFFFFFFFFFFFFFFFFFFFFF:F:FFFFFFFFFFFFFFFFFF,
,F:FF,FFFFFFFFF
@A00721:190:HGJW7DSXY:4:1433:22914:19132 2:N:0:TCTGCAAG+GCTGCACT
TGAAAGTAAATTTCTGAATATGATGTTAGCGTTAATGGAGAAACGCGATGAAGAAGATGAAGGAGTCC
CCATTACTGTTAAATCAGGGACATCCGCATATTAAGCATTATATTGGGGACTGGGGACCTATATATAT
GTATAGAGTAATCCG
+
FFFFFFFFFFFFFFFFFFFFFFFFFFFFFFFFFFFFFFFFFFFFFFFFFFFFFFFFFFFFFFF:FFFF
FFFFFFFFFFFFFFFFFFFFFFFFFFFFFFFFFFFFFFFFFFFFFFFFFFFFFFFFFFFFFFFFFFFF
FFFFFFFFFFFFFFF
@A00721:190:HGJW7DSXY:4:1464:22010:3630 2:N:0:TCTGCAAG+GCTGCACT
TGAAAGTAAATTTCTGAATATGATGTTAGCGTTAATGGAGAAACGCGATGAAGAAGATGAAGGAGTCC
CCATTACTGTTAAATCAGGGACATCCGCATATTAAGCATTATATTGGGGACTGGGGACCTATATATAT
GTATAGAGTAATCCG
+
FFFFFFFFFFFFFFFFFFFFFFFFFFFFFFFFFFFFFFFFFFFFFFFFFFFFFFFFFF:FFFFFFFFF
FFFFFFFFFF:FFFFFFF:FFFFF:FFFFFFF:FFF:FFFFFFFFFFFFFFFFFFFFFFFFFFFFFFF
FFFFFFFFFFFFFFF
@A00721:190:HGJW7DSXY:4:1521:31250:26209 2:N:0:TCTGCAAG+GCTGCACT
TGAAAGTAAATTTCTGAATATGATGTTAGCGTTAATGGAGAAACGCGATGAAGAAGATGAAGGAGTCC
CCATTACTGTTAAATCAGGGACATCCGCATATTAAGCATTATATTGGGGACTGGGGACCTATATATAT
GTATAGAGTAATCCG
+
FFF,FFFFFFFFFFFFFFFFFFFFFFFFFFFFFFFFFFFFFFFFFFFFFFFFFFFFFFFFFFFFFFFF
FFFFFFFFFFFFFFFFFFFFFFFFFFFFFFFFFFFFFFFFFFFFFFFFFFFFFFFFFFFFFFFFFFFF
FFFFFFFFFFFFF:F
@A00721:190:HGJW7DSXY:4:1542:5520:18756 2:N:0:TCTGCAAG+GCTGCACT
TGAAAGTAAATTTCTGAATATGATGTTAGCGTTAATGGAGAAACGCGATGAAGAAGATGAAGGAGTCC
CCATTACTGTTAAATCAGGGACATCCGCATATTAAGCATTATATTGGGGACTGGGGACCTATATATAT
GTATAGAGTAATCCG
+
FFFFFFFFFFFFFFFFFFFFFFF:FFFFFFFFFFFFFFFFFFFFFFFFFFFFFFFFFFFFFFFFFFFF
FFFFFFFFFFFFFFFFFFFFFFFFFFFFFFFFF:FFFFFFFFFFFFFFFFFFFFFFFFFFFFFFFFFF
FFFFF:FFFFFFFFF
@A00721:190:HGJW7DSXY:4:2253:1072:20791 2:N:0:TCTGCAAG+GCTGCACT
TGAAAGTAAATTTCTGAATATGATGTTAGCGTTAATGGAGAAACGCGATGAAGAAGATGAAGGAGTCC
CCATTACTGTTAAATCAGGGACATCCGCATATTAAGCATTATATTGGGGACTGGGGACCTATATATAT
GTATAGAGTAATCCG
+
FFFFF,FFFFFFFF:FFF:FFFFFFFFFFFFFFFF,FFFFFFF,FFFFFFFFFFFFFF,FFFFFF:FF
,FFFFFFFFFFFFFFFFFF,F,FFF:F:FFFFFFFF:FF:FF:FFFF,FFFFFFFFFFFFFFFFFFFF
FFFFF:FFFFFF:FF

```
@A00721:190:HGJW7DSXY:4:2365:24017:26115 2:N:0:TCTGCAAG+GCTGCACT
TGAAAGTAAATTTCTGAATATGATGTTAGCGTTAATGGAGAAACGCGATGAAGAAGATGAAGGAGTCC
CCATTACTGTTAAATCAGGGACATCCGCATATTAAGCATTATATTGGGGACTGGGGACCTATATATAT
GTATAGAGTAATCCG
+
FFF:FFFFFFFFFFFFFFFFFFFFFFFFFFFFFFFFFFFFFFFFFFFFFFFFFFFFFFFFFFFFFFFFFF
FFFFFFFFFFFFFFFFFFFFFFFFFFFFFFFF:FFFFFFFFFF,FFFFFFFFFFFFFFFFFFFFFFFFFF
FFFFFFFFFFFFFFF
@A00721:190:HGJW7DSXY:4:2528:16098:19351 2:N:0:TCTGCAAG+GCTGCACT
TGAAAGTAAATTTCTGAATATGATGTTAGCGTTAATGGAGAAACGCGATGAAGAAGATGAAGGAGTCC
CCATTACTGTTAAATCAGGGACATCCGCATATTAAGCATTATATTGGGGACTGGGGACCTATATATAT
GTATAGAGTAATCCG
+
FFFFFFFFFFFFFFFFFFFFFFFFFFFFFFFFFFFFFFFFFFFFFFFFFFFFFFFFFFFFFFFFFFFFFF
FFFFFFFFFFFFFFFFFFFFFFFFFFFFFFFFFFFFFFFFFFFFFFFFFFFFFFFFFFFFFFFFFFFFFF
:FFFFFFFFFFFFFF
@A00721:190:HGJW7DSXY:4:2565:21838:17237 2:N:0:TCTGCAAG+GCTGCACT
TGAAAGTAAATTTCTGAATATGATGTTAGCGTTAATGGAGAAACGCGATGAAGAAGATGAAGGAGTCC
CCATTACTGTTAAATCAGGGACATCCGCATATTAAGCATTATATTGGGGACTGGGGACCTATATATAT
GTATAGAGTAATCCG
+
FFFFFFFFFFFFFFFFFFFFFFFFFFFFFFF:FFFFFFFFFFFFFFFFFFFFFFFFFFFFFFFFFFFFFF
FFFFFFFFFFFFFFFFFFFFFFFFFFFFFFFFFFFF,FFFFFFFFFFFF:FFFFF:FFFF:FFFFFFFFF
FFFFFFFFFFFFFFF
@A00721:190:HGJW7DSXY:4:2575:21495:25160 2:N:0:TCTGCAAG+GCTGCACT
TGAAAGTAAATTTCTGAATATGATGTTAGCGTTAATGGAGAAACGCGATGAAGAAGATGAAGGAGTCC
CCATTACTGTTAAATCAGGGACATCCGCATATTAAGCATTATATTGGGGACTGGGGACCTATATATAT
GTATAGAGTAATCCG
+
FF:FFFFFFFFFFFFFFFFFFFFFFFFFFFFFFFFFFFFFFFF:FFFFFFFFFFFFFFFFFFFFFFFFFF
FFFFFFFFFFFFFFFFFFFFFFFFFFFFFFFFFFFFFFFFFFFFFFFFFFFFFFFFFFFFFFFF:F,FFF
FFFFFFFF:FFFFFF
@A00721:190:HGJW7DSXY:4:2674:21169:16799 2:N:0:TCTGCAAG+GCTGCACT
TGAAAGTAAATTTCTGAATATGATGTTAGCGTTAATGGAGAAACGCGATGAAGAAGATGAAGGAGTCC
CCATTACTGTTAAATCAGGGACATCCGCATATTAAGCATTATATTGGGGACTGGGGACCTATATATAT
GTATAGAGTAATCCG
+
FFFFFFFFFFFFFFFFFFFFFFFFFFFFFFFFFFFFFFFFFFFFFFFFFFFFFFFFFFFFFFFFFFFFFF
FFFFFFFFFFFFF:FFFFFFFFFFFFFFFFFFFFFFFFFF:FFFF:FFFFFFFFFFFFFFFF:FFFFFFF
FFFFFFFFFFFFFFF
@A00721:190:HGJW7DSXY:4:1341:2266:1626 2:N:0:TCTGCAAG+GCTGCACT
AAATTTCTGAATATGATGTTAGCGTTAATGGAGAAACGCGATGAAGAAGATGAAGGAGTCCCCATTAC
TGTTAAATCAGGGACATCCGCATATTAAGCATTATATTGGGGACTGGGGACCTATATATATGTATAGA
GTAATCCGGAGATCG
+
FFFFFFFFFFFFFFFFFFFFFFFFFFFFFFFFFFFFFFFFFFFFFFFFFFFFFFFFFFFFFFFFFFFFFF
FFFFFFFFFFFFFFFFFFFFFFFFFFFFFFFFFFFFFFFFFFFFFFFFFFFFFFFFF:FFF,FFFFFFFF
FF:FFFFFFFFFF:F
@A00721:190:HGJW7DSXY:4:2417:14163:36542 2:N:0:TCTGCAAG+GCTGCACT
AAATTTCTGAATATGATGTTAGCGTTAATGGAGAAACGCGATGAAGAAGATGAAGGAGTCCCCATTAC
TGTTAAATCAGGGACATCCGCATATTAAGCATTATATTGGGGACTGGGGACCTATATATATGTACAGA
GTAATCCGGAGATCG
+
FFFFF:FFFFFFFFFFFFFFFFFFFFFFFFFFFFFFFFFFFF::FFFFFFFFFFFF:FFFFFF:FF:FFF
```

```
:FFFFFFFFFFF,FFFFFF:FFFF:FFFFFF:FF,FF:F:FF:FFFFFFFFFFFFFF,FFFF:FFFFF
FFFFFFFFFFFFFFF
@A00721:190:HGJW7DSXY:4:2443:9381:3834 2:N:0:TCTGCAAG+GCTGCACT
AAATTTCTGAATATGATGTTAGCGTTAATGGAGAAACGCGATGAAGAAGATGAAGGAGTCCCCATTAC
TGTTAAATCAGGGACATCCGCATATTAAGCATTATATTGGGGACTGGGGACCTATATATATGTATAGA
GTAATCCGGAGATCG
+
FFFFFFFFFFFFFFFFFFFFFFFFFFFFFFFFFFFFFFFFFFFFFFFFFFFFFFFFFFFFFFFFFFFF
FFFFFFFFFFFFFFFFFFFFFFFFFFFFFFFFFFFFFFFFFFFFFFFFFFFFFFFFFFFFFFFFFFFF
FFFFFFFFFFFFFFF
@A00721:190:HGJW7DSXY:4:2445:11505:5415 2:N:0:TCTGCAAG+GCTGCACT
AAATTTCTGAATATGATGTTAGCGTTAATGGAGAAACGCGATGAAGAAGATGAAGGAGTCCCCATTAC
TGTTAAATCAGGGACATCCGCATATTAAGCATTATATTGGGGACTGGGGACCTATATATATGTATAGA
GTAATCCGGAGATCG
+
F:FFFFFFFFFFF:FFFFFFFFFFFFFFFFFFFFFFFFFFFFFFFFFFFFFFFFFFFFFFFFFFFFFF
FFFFFFFFFFFFFFFFFFFFFFFFFFFFFFFFF,FFFFFFFFFFFFF:FFFFFFF,FFFFFFFFFF:F
FFFFF:FFFFFFFFF
@A00721:190:HGJW7DSXY:4:2466:11098:25003 2:N:0:TCTGCAAG+GCTGCACT
AAATTTCTGAATATGATGTTAGCGTTAATGGAGAAACGCGATGAAGAAGATGAAGGAGTCCCCATTAC
TGTTAAATCAGGGACATCCGCATATTAAGCATTATATTGGGGACTGGGGACCTATATATATGTATAGA
GTAATCCGGAGATCG
+
FFFFFFFFFFFFFFFFFFFFFFFFFFFFFFFFFFF:FFFFFFFFF:FFFFFFFFFFFFFFFFFFFFFF
FFFFFFFFFFFFFFFFFFFFFFFFFFFF:FFFFFFFFFFFFFFFFFFFFFFFFFFFFFFFFFFFFFFF
FFFFFFFFFFFFFFF
@A00721:190:HGJW7DSXY:4:2368:5358:34006 2:N:0:TCTGCAAG+GCTGCACT
TGGGGATAGATTCCTCCGGATTACTCTATACATATATATAGGTCCCCAGTCCCCAATATAATGCTTAA
TATGCGGATGTCCCTGATTTAACAGTAATGGGGACTCCTTCATCTTCTTCATCGCGTTTCTCCATTAA
CGCTAACATCAGAGA
+
FFFFFFFFFFFFFFFFFFFFFFFFFFFFFFFFFFFFFFFFFFFFFFFFFFFFFFFFFFFFFFFFFFF:
FFFFFFFFFFFFFFFFF:FFFFFFFFFFFFFFFFFFFFFFFFFFFFFFFFFF:FFFFFFFFFFFFFFF
FFFFFFFFFFFFFFF
@A00721:190:HGJW7DSXY:4:2370:17770:30358 2:N:0:TCTGCAAG+GCTGCACT
TGGGGATAGATTCCTCCGGATTACTCTATACATATATATAGGTCCCCAGTCCCCAATATAATGCTTAA
TATGCGGATGTCCCTGATTTAACAGTAATGGGGACTCCTTCATCTTCTTCATCGCGTTTCTCCATTAA
CGCTAACATCAGAGA
+
FFFFFFFFFFFFFFFFFFFFFFFFFFFFFFFFFFFFFFFFF:FFFFFFFFFFFFFFFFFFFFFFFFFF
FFFFFFFFFFFFFFFFF:FFFFFFFF:F:FFFFFFFFFFFFFFFFFFFFFFFFFFFFFFFFFFFFFFF
FFFFFFFFFFFFFFF
@A00721:190:HGJW7DSXY:4:2475:24804:8844 2:N:0:TCTGCAAG+GCTGCACT
TGGGGATAGATTCCTCCGGATTACTCTATACATATATATAGGTCCCCAGTCCCCAATATAATGCTTAA
TATGCGGATGTCCCTGATTTAACAGTAATGGGGACTCCTTCATCTTCTTCATCGCGTTTCTCCATTAA
CGCTAACATCAGAGA
+
FFFFFFFFFFFFFFFFFFFFFFFFFFFFFFFFFFFFFFFFFFFFFFFFFFFFFFFFFFFFFFFFFFFF
FFFFFFFFFFFFFFFFFFFFFFFFFFFFFFFFFFFFFFFFFFFFFFFFFFFFFFFF:FFFFFFFFFFF
FFFFFFFFFFFFFFF
@A00721:190:HGJW7DSXY:4:2535:9715:22608 2:N:0:TCTGCAAG+GCTGCACT
TGGGGATAGATTCCTCCGGATTACTCTATACATATATATAGGTCCCCAGTCCCCAATATAATGCTTAA
TATGCGGATGTCCCTGATTTAACAGTAATGGGGACTCCTTCATCTTCTTCATCGCGTTTCTCCATTAA
CGCTAACATCAGAGA
```

+
FFFFFFFFFFFFFFFFFFFFFFFFFFFFFFFFFFFFFFFFFFFFFFFFFFFFFFFFFFFFFFFFFFFFFF
FFFFFFFFFFFFFFFFFFFFFFFFFFFFFFFFFFFFFFFFFFFFFFFFFFFFFFF:FFFFFFFFFFFFFF
F:FFFFFFFFFFFFF
@A00721:190:HGJW7DSXY:4:2613:1461:23469 2:N:0:TCTGCAAG+GCTGCACT
TGGGGATAGATTCCTCCGGATTACTCTATACATATATATAGGTCCCCAGTCCCCAATATAATGCTTAA
TATGCGGATGTCCCTGATTTAACAGTAATGGGGACTCCTTCATCTTCTTCATCGCGTTTCTCCATTAA
CGCTAACATCAGAGA
+
FFFFFFFFFFFFFFFFFFFFFFFFFFFFFFFFFFFFFFFFFFFFFFFFFFFFFFFFFFFFFFFFFFFFFF
FFFFFFFFFFFFFFFFFFFFFFFFFFFFFFFFFFFFFFFFFFFFFFFFFFFFFFFFFFFFFFFFFFFFFF
FFFFFFFFFFFFFFF
@A00721:190:HGJW7DSXY:4:2654:5891:16172 2:N:0:TCTGCAAG+GCTGCACT
TGGGGATAGATTCCTCCGGATTACTCTATACATATATATAGGTCCCCAGTCCCCAATATAATGCTTAA
TATGCGGATGTCCCTGATTTAACAGTAATGGGGACTCCTTCATCTTCTTCATCGCGTTTCTCCATTAA
CGCTAACATCAGAGA
+
FFFFFFFFFFFFFFFFFFFFFFFFFFFFFFF,FFFFFFFFFFFFFFFFF:FFF:FFFFFFFFFFFFFFFF
FFFFFFFFFFF:F:FFFFFFFFFFFFFFFFFFFFFFFF::FFFFFFFFFFFF:F:FFF:FFFFF,FFFFF
:FFFFFFF:FFFFFF
@A00721:190:HGJW7DSXY:4:1214:22996:14606 2:N:0:TCTGCAAG+GCTGCACT
CGCGATGAAGAAGATGAAGGAGTCCCCATTACTGTTAAATCAGGGACATCCGCATATTAAGCATTATA
TTGGGGACTGGGGACCTATATATATGTATAGAGTAATCCGGAGGAATCTATCCCCAATTGGAATAGTA
ATGATTTCAAATTTC
+
FFFFFFFFFFFFF,:FFFFFFFFFFFFFFFFFFFFFFFFFFFFFFFFFFFFFFFFFFFFFFFFFFFFFFF
FFFFFFFFFFFFFFFFFFFFFFFFFFFF,FFFFFFFFFFFFFFFFFFFFFFFFFFFFFFFFFFFFFF:FF
FFFFFFFFFFFFFFF
@A00721:190:HGJW7DSXY:4:1244:17463:10347 2:N:0:TCTGCAAG+GCTGCACT
CGCGATGAAGAAGATGAAGGAGTCCCCATTACTGTTAAATCAGGGACATCCGCATATTAAGCATTATA
TTGGGGACTGGGGACCTATATATATGTATAGAGTAATCCGGAGGAATCTATCCCCAATTGGAATAGTA
ATGATTTCAAATTTC
+
FFFFFFFFFFFFFFFFFFFFFFFFFFFFFFFFFFFFFFFFFFFFFFFFFFFFFFFFFFFFFFFFFFFFFF
FFFFFFFFFFFFFFFFFFFFFFFFFFFFFFFFFFFFFFFFFFFFFFFFFFFFFFFFFFFFF:FFFFFFFF
FFFFF:FFFF:FFFF
@A00721:190:HGJW7DSXY:4:1674:29116:17472 2:N:0:TCTGCAAG+GCTGCACT
CGCGATGAAGAAGATGAAGGAGTCCCCATTACTGTTAAATCAGGGACATCCGCATATTAAGCATTATA
TTGGGGACTGGGGACCTATATATATGTATAGAGTAATCCGGAGGAATCTATCCCCAATTGGAATAGTA
ATGATTTCAAATTTC
+
FFFFFFFFFF:FFFFFFFFFFFFFFFFFFFFFFFFFFFFFFFFFFFFF:FFFFFFFFFFFFFFFFFFFFF
:FFFFFFFFFFFFFFFF:FFFFFFFFFFFFFFFFFFFFFFFFFFFF,FFFFFFFFFFFFFFFFFFFFFFF
FFFFFFFFFFFFFFF
@A00721:190:HGJW7DSXY:4:2536:3794:34898 2:N:0:TCTGCAAG+GCTGCACT
CGCGATGAAGAAGATGAAGGAGTCCCCATTACTGTTAAATCAGGGACATCCGCATATTAAGCATTATA
TTGGGGACTGGGGACCTATATATATGTATAGAGTAATCCGGAGGAATCTATCCCCAATTGGAATAGTA
ATGATTTCAAATTTC
+
FFFFFFFFFFFFFFFFFFFFFFFFFFFFFFFFFFFFFFFFFFFFFFFFFFFFFFFFFFFFFFFFFFFFF:
FFFFFFFFFFFFFFFFFFFFFFFFFFFFFFFFFFFFFFFFFFFFFFFFFF:FFFFFFFFFFFFFFFFFFF
FFFFFFFFFF:FFFF
@A00721:190:HGJW7DSXY:4:1656:14814:8672 2:N:0:TCTGCAAG+GCTGCACT
CGGGATATTTTCAAATTTGAAATTTGAAATCATTACTATTCCAATTGGGGATAGATTCCTCCGGATTA

CTCTATACATATATATAGGTCCCCAGTCCCCAATATAATGCTTAATATGCGGATGTCCCTGATTTAAC
AGTAATGGGGACTCC
+
FFFFFFFFFFFFFFFFFFFFFFFFFFFFFFFFFFFFFFFFFFFFFFFFFFFFFFF,FFFFFFFFFFFF
FFFFFFFFF:FFFFFFFFFFFFFFFFFFFFFFFFFFFFFFFFFF:FFFFFFFFFFFFFFFFFFFF:FF
:FFFF:F:FFFFFFF
@A00721:190:HGJW7DSXY:4:1656:15311:9627 2:N:0:TCTGCAAG+GCTGCACT
CGGGATATTTTCAAATTTGAAATTTGAAATCATTACTATTCCAATTGGGGATAGATTCCTCCGGATTA
CTCTATACATATATATAGGTCCCCAGTCCCCAATATAATGCTTAATATGCGGATGTCCCTGATTTAAC
AGTAATGGGGACTCC
+
FFFFFFFFFFFFFFFFFFFFFFFFFFFFFFFFFFF:F:FFFFFFFFFFFFFFFFFFFFFFFFFFFFFF
FFFFFFFFFFFF:FFFFFFFFFFFFFFFFFFFFFFFFFFFFFFFFFFFFFFFFFFFFFFFFFFF:FF
FFF:FFFFFFFFF,:
@A00721:190:HGJW7DSXY:4:2265:17445:18208 2:N:0:TCTGCAAG+GCTGCACT
CGGGATATTTTCAAATTTGAAATTTGAAATCATTACTATTCCAATTGGGGATAGATTCCTCCGGATTA
CTCTATACATATATATAGGTCCCCAGTCCCCAATATAATGCTTAATATGCGGATGTCCCTGATTTAAC
AGTAATGGGGACTCC
+
FFFFFFFFFFFFFFFFFFFFFFFFFFFFFFFFFFFFFFFFFFFFFFFFFFFFFFFFFFFFFFFFFFFF
FFFFFFFFFFFFFFFFFFFFFFFFFFFFFFFFFFFFFFFFFFFFFFFFFFFFFFFFFFFFFFFFFFFF
FFFFFFFFFFFFFF:
@A00721:190:HGJW7DSXY:4:2601:1353:36401 2:N:0:TCTGCAAG+GCTGCACT
CGGGATATTTTCAAATTTGAAATTTGAAATCATTACTATTCCAATTGGGGATAGATTCCTCCGGATTA
CTCTATACATATATATAGGTCCCCAGTCCCCAATATAATGCTTAATATGCGGATGTCCCTGATTTAAC
AGTAATGGGGACTCC
+
FFFFFFFFFFFFFFFFFFFFFF:FFFFFFFFFFFFFFF,F:FF:F:F,FFFFF:FF:FFFFF:F:FFF
FFFFFFF:FFFFFFFFFFFFFFFFF,FFFF:FFFFFF,FFFFFFFFFFF:FFFFFFF,FFFF,FFFFF
FF:FFFFF:FF,FFF
@A00721:190:HGJW7DSXY:4:1216:30273:30091 2:N:0:TCTGCAAG+GCTGCACT
TTTGAATTGGTATACTGTAGGGCGGGATATTTTCAAATTTGAAATTTGAAATCATTACTATTCCAATT
GGGGATAGATTCCTCCGGATTACTCTATACATATATATAGGTCCCCAGTCCCCAATATAATGCTTAAT
ATGCGGATGTCCCTG
+
FFFFFFFFFFFFFFF,FFF:FFFFFFFFFFFFFFFF,FFFFF:FFFFFFFFFFFFFFFFFFFFFFFFF
F:FF,FFFFFFFFFFFFFFFFFFFFF:FFFFFFFF,FFFFFFFFFFF:FFFFFF:FFFFFFFFFFFFF
FFFFFFFFFFFFFFF
@A00721:190:HGJW7DSXY:4:1221:11767:18364 2:N:0:TCTGCAAG+GCTGCACT
TTTGAATTGGTATACTGTAGGGCGGGATATTTTCAAATTTGAAATTTGAAATCATTACTATTCCAATT
GGGGATAGATTCCTCCGGATTTCTCTATACATATATATAGGTCCCCAGTCCCCAATATAATGCTTAAT
ATGCGAATGTCCCTG
+
F:FFFFFFFFFFFFFFFFFFFFFFFFFFFFFFFFFFFFFFFFFFFFFFFFFFFFFFFFFFFFFFFFFF
FFFFFFFFFFFFFFFFFFFFFFFFFFFFFFFFFFFFFFF::FFFFFFFFFFFFFFFFFFFFFFFFFFF
FFFFFFFFF:F,FFF
@A00721:190:HGJW7DSXY:4:1329:12509:31172 2:N:0:TCTGCAAG+GCTGCACT
TTTGAATTGGTATACTGTAGGGCGGGATATTTTCAAATTTGAAATTTGAAATCATTACTATTCCAATT
GGGGATAGATTCCTCCGGATTACTCTATACATATATATAGGTCCCCAGTCCCCAATATAATGCTTAAT
ATGCGGATGTCCCTG
+
FFFFFFF:FFF:FFFFFFFFF:FFFF,FFFFFFFFFFFFFFFFFFFFFFFFFFF:F:FFFFFFF:FF:
FFFFFFFFFFFFFFFFFFFFFFFFFFFFFFFFFFFFFFFFFFF:FFFFF:FFFFFFFF:FFF,FF,FF
FF,FFFFF:FFF:FF

@A00721:190:HGJW7DSXY:4:1455:17372:5306 2:N:0:TCTGCAAG+GCTGCACT
TTTGAATTGGTATACTGTAGGGCGGGATATTTTCAAATTTGAAATTTGAAATCATTACTATTCCAATT
GGGGATAGATTCCTCCGGATTACTCTATACATATATATAGGTCCCCAGTCCCCAATATAATGCTTAAT
ATGCGGATGTCCCTG
+
FFFFFFF:FFFFFFF:FFFFFFFFFFFFFFFFFFFFFFFFFFFFFFFFFFFFFFFFFFFFFFF,FFFFFF
FFFFFF,FFFFFFFFFFFFFFFFFFFFFFFFF:FFFFFF:FFFFFFFFFFFFFFFFFFFFFFFFFFFFFF
FFFFFFFFFFFFF,F
@A00721:190:HGJW7DSXY:4:1455:16342:8719 2:N:0:TCTGCAAG+GCTGCACT
TTTGAATTGGTATACTGTAGGGCGGGATATTTTCAAATTTGAAATTTGAAATCATTACTATTCCAATT
GGGGATAGATTCCTCCGGATTACTCTATACATATATATAGGTCCCCAGTCCCCAATATAATGCTTAAT
ATGCGGATGTCCCTG
+
FFFFFFFFFFFFFFFFFFFFFFFFFFFFFFFFFFFFFFFFFFFFFFFFFFFFFFFFFFFFFFFFFFFFFF
:FFFFFFFFFFFFFFFFFF:FFFFFFFFFFFFFFFFFFFFFFFFFFFFFFFFFFFFFFFFFFF:FFFFFF
FFFFFFFFFFFFFFF
@A00721:190:HGJW7DSXY:4:1542:17336:4460 2:N:0:TCTGCAAG+GCTGCACT
TTTGAATTGGTATACTGTAGGGCGGGATATTTTCAAATTTGAAATTTGAAATCATTACTATTCCAATT
GGGGATAGATTCCTCCGGATTACTCTATACATATATATAGGTCCCCAGTCCCCAATATAATGCTTAAT
ATGCGGATGTCCCTG
+
FFFFFFFFFFFFFFFFFFFFFFFFF:FFFFFFFFFFFFFFFFFFFFFFFFFFFFFFFFFFFFFFFFFFFF
FFFFFFFFFFFFFFFFF:FFFFFFFFFFFFFFFFFFFF,FFFFFFFF,FFFFFFFFFFFFFFFFFFFFFF
FFFFFFFFFFFFFF:
@A00721:190:HGJW7DSXY:4:2149:19199:32800 2:N:0:TCTGCAAG+GCTGCACT
TTTGAATTGGTATACTGTAGGGCGGGATATTTTCAAATTTGAAATTTGAAATCATTACTATTCCAATT
GGGGATAGATTCCTCCGGATTTCTCTATACATATATATAGGTCCCCAGTCCCCAATATAATGCTTAAT
ATGCGAATGTCCCTG
+
FFFFFFFFFFFFFFFFFFFFFFFFFFFFFFFFFFFFFFFFFFFFFFFFFFFFFFFFFFFFFFFFFFFFFF
FFFFFFFFFFFFFFFFFFFFFFFFFFFFFFFFFFFFFFFFFFFFFFFFFFFFFFFFFF:FFFFFFF:FFF
FFF:,FFFF::,FFF
@A00721:190:HGJW7DSXY:4:2356:3929:9549 2:N:0:TCTGCAAG+GCTGCACT
TTTGAATTGGTATACTGTAGGGCGGGATATTTTCAAATTTGAAATTTGAAATCATTACTATTCCAATT
GGGGATAGATTCCTCCGGATTTCTCTATACATATATATAGGTCCCCAGTCCCCAATATAATGCTTAAT
ATGCGAATGTCCCTG
+
FFFFFFFFFFFFFFFFFFFFFFFFFFFFFFFFFFFFFFFFFFFFFFFFFFFFFFFFFFFFFFFFFFFFFF
FFFFFFFFFFFFFFFFFFFFFFFFFFFFFFFFFFFFFFFF,FFF:FFFFFFFFFFFFFFFFFFFFFFFFF
FFFFFFFFFFFFFFF
@A00721:190:HGJW7DSXY:4:2361:11397:18411 2:N:0:TCTGCAAG+GCTGCACT
TTTGAATTGGTATACTGTAGGGCGGGATATTTTCAAATTTGAAATTTGAAATCATTACTATTCCAATT
GGGGATAGATTCCTCCGGATTACTCTATACATATATATAGGTCCCCAGTCCCCAATATAATGCTTAAT
ATGCGGATGTCCCTG
+
FFFFFFFFFFFFFFFFFFFFFFFFFFFFFFFFFFFFFFFFFFFFFFFFFFFFFFFFFFFFFFFFFFFFFF
FFFFFFFFFFFFFFFFFFFFFFFFFFFFFFFFFFFFFFFFFF:FFFFFFFFFFFFFFFFFFFFFFFFFFF
FFFFFFFFFFFFFFF
@A00721:190:HGJW7DSXY:4:2404:12165:34460 2:N:0:TCTGCAAG+GCTGCACT
TTTGAATTGGTATACTGTAGGGCGGGATATTTTCAAATTTGAAATTTGAAATCATTACTATTCCAATT
GGGGATAGATTCCTCCGGATTACTCTATACATATATATAGGTCCCCAGTCCCCAATATAATGCTTAAT
ATGCGGATGTCCCTG
+
FFFFFFFFFFFFFFFFFFFFFFFFFFFFFFFFFFFFFFFFFFFFFFFFFFFFFFFFFFFFFFFF:FFFFFFFF

FFFFFFFFFFFFFFFFFFFFFFFFFFFFFFFFFF:FFFFFF::FF:FFFF:F:FFFF:FFFFF:FFFFFF
FF,F:FFFFFFF:FF
@A00721:190:HGJW7DSXY:4:2618:5773:23390 2:N:0:TCTGCAAG+GCTGCACT
TTTGAATTGGTATACTGTAGGGCGGGATATTTTCAAATTTGAAATTTGAAATCATTACTATTCCAATT
GGGGATAGATTCCTCCGGATTTCTCTATACATATATATAGGTCCCCAGTCCCCAATATAATGCTTAAT
ATGCGAATGTCCCTG
+
FFFFFFFFFFFFFFFFFFFFFFFFFFFFFFFFFFFFFFFFFFFFFFFFFFFFFFFFFFFFFFFFFFFFFF
FFFFFFFFFFFFFFFFFFFFFFFFFFFFFFFFFFFFFFFFFFFFFFFFFFFFFFFFFFFFFFFFFFFFFF
FFF,FFFFFFFFFFF
@A00721:190:HGJW7DSXY:4:1347:18954:30123 2:N:0:TCTGCAAG+GCTGCACT
CGGAGGAATCTATCCCCAATTGGAATAGTAATGATTTTCAAATTTGAAATTTGAAATCATTACTATTC
CAATTGGGGATAGATTCCTCCGGATTACTCTATACATATATATAGGTCCCCAGTCCCCAATATAATGC
TTAATATGCGATAGA
+
FFFFFFFFFFFFFFFFFFFFFFFFFFFFFFFFFFFFFFFFFFFFFF:FFFFFFFFFFFFFFFFFFFFFFF
FFFFFFFFFFFFFFFFFFFFFFFFFFFFFFFFFFFFFFFFFFFFFFFFFFFFFFFFFFFFFFFFFFFFFF
FFFFFFFFF:FFFFF
@A00721:190:HGJW7DSXY:4:1431:18358:3693 2:N:0:TCTGCAAG+GCTGCACT
CGGAGGAATCTATCCCCAATTGGAATAGTAATGATTTTCAAATTTGAAATTTGAAATCATTACTATTC
CAATTGGGGATAGATTCCTCCGGATTACTCTATACATATATATAGGTCCCCAGTCCCCAATATAATGC
TTAATATGCGATAGA
+
FFFFFFFFFFFFFFFFFF:FFFFFFFFFFFFFFFFFFFFFFFFFFFFFFFFFFFFFFFFFFFFFFFF:FF
F:FFFFFFFFFFFFFFFFFFFFFFFFFFFFFFFFFFFFFFFFFFFFFFFFFFFFFFFFFFFFFFFFFFFF
FFFFFFFFFFFFF:F
@A00721:190:HGJW7DSXY:4:2446:1787:6746 2:N:0:TCTGCAAG+GCTGCACT
AGGAATCTATCCCCAATTGGAATAGTAATGATTTTCAAATTTGAAATTTGAAATCATTACTATTCCAA
TTTGGGATAGATTCCTCCGGATTACTCTATACATATATATAGGTCCCCAGTCCCCAATATAATGCTTA
ATATGCGATAGA
+
FFFFF:F,FFF:FFFF:F:,F,FFFFF,F,FFFFF,FFF:F::FFFFFFFF:F:FFFFFFF,F::F:F
,,,FFFFFFFF:,FFFFF,FFFFF::F:F:FF,F::F,FFF,FFFFFFFF,FFFFFFFFFFFFFFFF
F:F,F::FFFFF
@A00721:190:HGJW7DSXY:4:2573:15275:27414 2:N:0:TCTGCAAG+GCTGCACT
CGCATATTAAGCATTATATTGGGGACTGGGGACCTATATATATGTATAGAGTAATCCGGAGGAATCTA
TCCCCAATTGGAATAGTAATGATTTCAAATTTCAAATTTGAAAATATCCCGCCCTACAGTATACCAAT
TCAAATTCAAATTCA
+
FFFFFFFFFFFFFFFFFFFFFFFFFFFFFFFFFFFFFFFFFFFFFFFFFFFFFFF:FFFFFFFFF:FFFF
FFFFFFFFFF:FFFFFFFFFFFFFFFFFFFFFFFFFFFF:FFFFFFFFFFFFFFFFFFF,:FF:FFFFFF
,FFFFFFFFFFFFFF
@A00721:190:HGJW7DSXY:4:2660:18973:4633 2:N:0:TCTGCAAG+GCTGCACT
CGCATATTAAGCATTATATTGGGGACTGGGGACCTATATATATGTATAGAGTAATCCGGAGGAATCTA
TCCCCAATTGGAATAGTAATGATTTCAAATTTCAAATTTGAAAATATCCCGCCCTACAGTATACCAAT
TCAAATTCAAATTCA
+
FFF:FFFFFFFFFFFFFFFFFFFFFFFFFFFFFFFFFFFFFFFFFFFFFF:FFF::FFFFFFFFF,FFFF
FFFFFFFFFFFFFFFFFFFFFFFFFFFFFFF:FFFFFFFFFFFFFFFFFF:F,FFFFF:FFFFFFFFFFF
F:FFFFFFFFFFF,F
@A00721:190:HGJW7DSXY:4:1346:30129:22326 2:N:0:TCTGCAAG+GCTGCACT
TGGGGACTGGGGACCTATATATATGTATAGAGTAATCCGGAGGAATCTATCCCCAATTGGAATAGTAA
TGATTTCAAATTTCAAATTTGAAAATATCCCGCCCTACAGTATACCAATTCAAATTCAAATTCAAATA
GAATTCCATAGGCCC

```
+
FFFFFFFFFFF:FFFFFFFFFFFFFFFFFFFFFFFFFFFFFFFFFFFFFF:FFFFFFFFFFFFFFFFFFF
FFFFFFFFFFFFFFFFFFFFFFFFFFFFFFFFFFFFFFFFFFFFFFFFFFFFFFFFFFFFFFFFFFFFFF
FFFFFFFFFFFFFFF
@A00721:190:HGJW7DSXY:4:1351:19560:3677 2:N:0:TCTGCAAG+GCTGCACT
TGGGGACTGGGGACCTATATATATGTATAGAGTAATCCGGAGGAATCTATCCCCAATTGGAATAGTAA
TGATTTCAAATTTCAAATTTGAAAATATCCCGCCCTACAGTATACCAATTCAAATTCAAATTCAAATA
GAATTCCATAGGCCC
+
FFFFFFFFFFFFFFF:FFFFFFFFFFFFFFFFFFFFFFFFFFFFFFFFFFFFFFFFFFFFFFFFFFFFFF
FFFFFFFFFFFFFFFFFFFFFFFFFFFFFFFFFFFFFFFFFFFFFFFFFF:FFFFFFFFF:FFFFFFFFF
FF:FFF:FFFFFFFF
@A00721:190:HGJW7DSXY:4:1564:11478:14700 2:N:0:TCTGCAAG+GCTGCACT
TGGGGACTGGGGACCTATATATATGTATAGAGTAATCCGGAGGAATCTATCCCCAATTGGAATAGTAA
TGATTTCAAATTTCAAATTTGAAAATATCCCGCCCTACAGTATACCAATTCAAATTCAAATTCAAATA
GAATTCCATAGGCCC
+
FFFFFFFFFFFFFFFFFFFFFFFFFFFFFFFFFFFFFFFFFFFFFFFF:FFFFFFFFFFFFFFFFFFFFF
FFFFFFFFFFFFFFFFFFFFFFFFFFFFFFFFFFFFF:FFFF:FFFFFFFFFFFFFFFFFFFFFFFFFFF
FFFFFFFFFFFFFFF
@A00721:190:HGJW7DSXY:4:2130:29035:30953 2:N:0:TCTGCAAG+GCTGCACT
TGGGGACTGGGGACCTATATATATGTATAGAGTAATCCGGAGGAATCTATCCCCAATTGGAATAGTAA
TGATTTCAAATTTCAAATTTGAAAATATCCCGCCCTACAGTATACCAATTCAAATTCAAATTCAAATA
GAATTCCATAGGCCC
+
FFFFFFFFFFFFFFFFFFFFFFFFFFFFF:FFFFFFFFFFFFFFFFFFFFFFFFFFFFFFFFFFFFFF,F
FFFFFFFFFFFFFFFFFFFFFFF:FFF:F,FFFFFFFFFFFFFFFFFFFFFFFFFF:FFFFFFFFFFFFF
FFFFFFFFFFF:FFF
@A00721:190:HGJW7DSXY:4:2221:32117:31876 2:N:0:TCTGCAAG+GCTGCACT
TGGGGACTGGGGACCTATATATATGTATAGAGTAATCCGGAGGAATCTATCCCCAATTGGAATAGTAA
TGATTTCAAATTTCAAATTTGAAAATATCCCGCCCTACAGTATACCAATTCAAATTCAAATTCAAATA
GAATTCCATAGGCCC
+
FFFFFFFFFFFFFFFFFFFFFFFFFF:F,FFFFFFFFFFF:FFFF::FFFFFFFFFFFFFF,FFFFFFFF
FFFFFFFFFFFFFFFF:FFFFFFF:FFFFFFFFFFFFFFF:FFFFFFFFF:F:FF:FF:FFFFFFFFFFF
,FFFFF,FFFF:,FF
@A00721:190:HGJW7DSXY:4:2656:3821:23265 2:N:0:TCTGCAAG+GCTGCACT
TGGGGACTGGGGACCTATATATATGTATAGAGTAATCCGGAGGAATCTATCCCCAATTGGAATAGTAA
TGATTTCAAATTTCAAATTTGAAAATATCCCGCCCTACAGTATACCAATTCAAATTCAAATTCAAATA
GAATTCCATAGGCCC
+
FFFFFFFFFFFFFFFFFFFFFFFFFFFFFFFFFFFFFFFFFFFF:FFFFFFFFFFFFFFFFF:FFFFFF
FFFFFFFFFFFFFFFFFFFFFFFFFFFFFFFFFFFFFFFFFFFFFFFFFFFFFFFFFFFFFFFFF::FF,
FF:FFFFFF:FFFFF
@A00721:190:HGJW7DSXY:4:1259:26449:18646 2:N:0:TCTGCAAG+GCTGCACT
CTGGGGACCTATATATATGTATAGAGTAATCCGGAGGAATCTATCCCCAATTGGAATAGTAATGATTT
CAAATTTCAAATTTGAAAATATCCCGCCCTACAGTATACCAATTCAAATTCAAATTCAAATAGAATTC
CATAGGCCCCGCAGG
+
FFFFFFFF:FFFFFF:FFFFFFFFFFFFFFFFFFFFFFFFFFFFFFFFFFFFFFFFF:FFFFFFFFFFFF
FFFFFFFFFFFFFFFFFFFFFFFFFFFFFFFFFFFFFFFFFFFFFFFFFFFFFFFFFFFF:FF:FFFFFF
FFFFFFFFFFFFFFF
@A00721:190:HGJW7DSXY:4:1413:26621:28463 2:N:0:TCTGCAAG+GCTGCACT
CTGGGGACCTATATATATGTATAGAGTAATCCGGAGGAATCTATCCCCAATTGGAATAGTAATGATTT
```

CAAATTTCAAATTTGAAAATATCCCGCCCTACAGTATACCAATTCAAATTCAAATTCAAATAGAATTC
CATAGGCCCCGCAGG
+
FFFFFFFFFFFFFFFFFFFFFFFFFFFFFFFFFFFFFFFFFFFFFFFFFFFFFFFFFFFFFFFFFFFFFF
FFFFFFFFFFFFFFFFFFFFFFFFFFFFFFFFFFFFFFFFFFFFFFFFFFFFFFFFFFFFFFFFFFFF:F
FFFFFFFFFFFFFFF
@A00721:190:HGJW7DSXY:4:2366:25256:34961 2:N:0:TCTGCAAG+GCTGCACT
CTGGGGACCTATATATATGTATAGAGTAATCCGGAGGAATCTATCCCCAATTGGAATAGTAATGATTT
CAAATTTCAAATTTGAAAATATCCCGCCCTACAGTATACCAATTCAAATTCAAATTCAAATAGAATTC
CATAGGCCCCGCAGG
+
FFFFFFFFFFFFFFFFFFFFFFFFFFFFFFF:FFFFFFFFFFFFFFFFFFFFFFFFFFFFFFFFFFFFFF
FFFFFFFFFFFFFFFFFFFFFFFF:FFFFFFFFFFFFFFFFFFFF,FFFF:FFFFFFFFFFFFFFFFF:
FF:FFFFFF:FFFFF
@A00721:190:HGJW7DSXY:4:2366:24587:35994 2:N:0:TCTGCAAG+GCTGCACT
CTGGGGACCTATATATATGTATAGAGTAATCCGGAGGAATCTATCCCCAATTGGAATAGTAATGATTT
CAAATTTCAAATTTGAAAATATCCCGCCCTACAGTATACCAATTCAAATTCAAATTCAAATAGAATTC
CATAGGCCCCGCAGG
+
FFFFFFFFFFFFFFFFFFFFFFFFFFFFFFFFFFFFFFFFFFFFFFFFFFFFFFFFFFFFFFFFFFFFFF
FFFFFFFFFFFFFFFFFFFFFFFFFFFFFFFFFFFF,FFFF,FFFFFFFFFFFFFFFFFF:FFFFFFFF
:FFFFFFFFFFFFFF
@A00721:190:HGJW7DSXY:4:1123:2618:33896 2:N:0:TCTGCAAG+GCTGCACT
GGGACCTATATATATGTATAGAGTAATCCGGAGGAATCTATCCCCAATTGGAATAGTAATGATTTCAA
ATTTCAAATTTGAAAATATCCCGCCCTACAGTATACCAATTCAAATTCAAATTCAAATAGAATTCCAT
AGGCCCCGCAGGGGC
+
FFFFFFFFFFFFFFFFFFFFFFFFFFFFFFFFFFFFFFFFFFFFFFFFFFFFFFFF:FFFFFFFFFFFFF
FFFFFFFFFFFFFFFFFFFFFFFFFFFFFFFFFFFFFFFFFFFFFFFFFFFFFFFFFFFFFFFFFF,FFF
FF:FFFFFFFFFFFF
@A00721:190:HGJW7DSXY:4:1142:27263:14387 2:N:0:TCTGCAAG+GCTGCACT
GGGACCTATATATATGTATAGAGTAATCCGGAGGAATCTATCCCCAATTGGAATAGTAATGATTTCAA
ATTTCAAATTTGAAAATATCCCGCCCTACAGTATACCAATTCAAATTCAAATTCAAATAGAATTCCAT
AGGCCCCGCAGGGGC
+
:F:FFFFFFFFFFFFFFFFFFF:FFFFFFFFFFFF:FFFFFFFF:FFFFFFFFFF:FFFFFFF:FFFF
FFFFFFF:FFFFFFFFFFFFFFFFFFFFFFFF:FFFFFFF,FFFF:FFFFFFFFFFFFFFFFFFF:F:F
FF:FFFFFFFFFFFF
@A00721:190:HGJW7DSXY:4:2362:28357:34757 2:N:0:TCTGCAAG+GCTGCACT
GGGACCTATATATATGTATAGAGTAATCCGGAGGAATCTATCCCCAATTGGAATAGTAATGATTTCAA
ATTTCAAATTTGAAAATATCCCGCCCTACAGTATACCAATTCAAATTCAAATTCAAATAGAATTCCAT
AGGCCCCGCAGGGGC
+
FFFFFFFFFFFFFFFFFFFFFFFFFF:FFFFFFFFFFFFFFFFFFFFFFFFFFFFFFFFFFFFFFFFFFFF
FFFFFFFFFFFF:FFFFFFFFFFFFFFFFFFFFFFFFFFFFFFFFFFFFFFF,FFFFF:FFF:,FFFFFF
FFFFFFFFF,FFFFF
@A00721:190:HGJW7DSXY:4:2412:30689:25332 2:N:0:TCTGCAAG+GCTGCACT
GGGACCTATATATATGTATAGAGTAATCCGGAGGAATCTATCCCCAATTGGAATAGTAATGATTTCAA
ATTTCAAATTTGAAAATATCCCGCCCTACAGTATACCAATTCAAATTCAAATTCAAATAGAATTCCAT
AGGCCCCGCAGGGGC
+
FFFFFFFFFFFFFFFFFFFFFFFFFFFFFFFFFFFF:FFFFFFFFFFFFFFFFFFFFFFFFFFFFFFFFF
FFFFFFFFFFFFFFFFFFFFFFFFFFFFFFFFFFFFFFFFFFF:::FFFFFFFFFFFF:FFFFFFFFFF
FF:FFFFFF:FFFFF

@A00721:190:HGJW7DSXY:4:2441:26666:24251 2:N:0:TCTGCAAG+GCTGCACT
GGGACCTATATATATGTATAGAGTAATCCGGAGGAATCTATCCCCAATTGGAATAGTAATGATTTCAA
ATTTCAAATTTGAAAATATCCCGCCCTACAGTATACCAATTCAAATTCAAATTCAAATAGAATTCCAT
AGGCCCCGCAGGGGC
+
FFFFFFFFFFFFFFFFFFFFFFFFFFFFFFFFFFFFFFFFFFFFFFF:FF:FFFFFFFFFFFFFFFFF
F:FFFFFFFFFF:FFFFFFFFFFFFFFFFFFFFFFFFFF,FFFFFFFFFFFFF:,FFF:F:FFFF,FFF
FF:FFFFFFFF:FFF
@A00721:190:HGJW7DSXY:4:1222:2492:22811 2:N:0:TCTGCAAG+GCTGCACT
GATGCCCCTGCGGGGCCTATGGAATTCTATTTGAATTTGAATTTGAATTGGTATACTGTAGGGCGGGA
TATTTTCAAATTTGAAATTTGAAATCATTACTATTCCAATTGGGGATAGATTCCTCCGGATTACTCTA
TACATATATATAGGT
+
FFFFFFFFFFFFFFFFF:FFFFF:F:FFFF:FFFFFFFFFFFFFFFFFFFFFFFFF:FFFFFFFFFFF
F:FFFFFFFFFFFF:FFFFFFFFFFFFFFFFF,FFFFFF,FFFFFFFFFFFFFFFFFFFFFFFFFFFF
FFFFFFFFF:FFFFF
@A00721:190:HGJW7DSXY:4:2216:32280:35477 2:N:0:TCTGCAAG+GCTGCACT
GATGCCCCTGCGGGGCCTATGGAATTCTATTTGAATTTGAATTTGAATTGGTATACTGTAGGGCGGGA
TATTTTCAAATTTGAAATTTGAAATCATTACTATTCCAATTGGGGATAGATTCCTCCGGATTACTCTA
TACATATATATAGGT
+
F:FFFFFFFFFFFFFFFFFFFFFFFFFFFFFFFFFFFFFFFFFFFFFFFFFFFFFFFFFFFFFFFFFF
FFFFFFFFFFFFFFFFFFFFFFFFFFFFFF:FFFFFFFFFFF:FFFFFFFFFFFFFFFFFFFFFFFFF
FFFFFFFFFFFFF,F
@A00721:190:HGJW7DSXY:4:1436:6017:29481 2:N:0:TCTGCAAG+GCTGCACT
GTAAGATGCCCCTGCGGGGCCTATGGAATTCTATTTGAATTTGAATTTGAATTGGTATACTGTAGGGC
GGGATATTTTCAAATTTGAAATTTGAAATCATTACTATTCCAATTGGGGATAGATTCCTCCGGATTAC
TCTATACATATATAT
+
FFFFFFFFFFFFFFFFFFFFF:FFFFF:FF::FFFFFFFFFFFFFFFFFFFFFFFFFFFFFFFFFFFF
FFFFFFFFFFFF:FFFFFFFFFFFFF,FFF,FFFFFFFFFFFFFFFFFFFFFFF:FFFFFFFFFFFFF
FFF,FFFFFFFFFFF
@A00721:190:HGJW7DSXY:4:1606:21043:24408 2:N:0:TCTGCAAG+GCTGCACT
GTAAGATGCCCCTGCGGGGCCTATGGAATTCTATTTGAATTTGAATTTGAATTGGTATACTGTAGGGC
GGGATATTTTCAAATTTGAAATTTGAAATCATTACTATTCCAATTGGGGATAGATTCCTCCGGATTAC
TCTATACATATATAT
+
FFFFFFFFFFFFFFFFFFFFFF:FFFFF,FFFFFFFFFFFFFFFFFFFFFFFFFFFFFFFFFFFFFFF
FFFFFFFFFFFFFFFFFFFFFFFFFF:FFFFFFFFFFFFFFFFFFFFFFFFFFF:FFFFFFFFFFFFF
FFF,F,FFFF:FFFF
@A00721:190:HGJW7DSXY:4:1651:23927:18004 2:N:0:TCTGCAAG+GCTGCACT
GTAAGATGCCCCTGCGGGGCCTATGGAATTCTATTTGAATTTGAATTTGAATTGGTATACTGTAGGGC
GGGATATTTTCAAATTTGAAATTTGAAATCATTACTATTCCAATTGGGGATAGATTCCTCCGGATTAC
TCTATACATATATAT
+
FFFFFFFFFFFFFFFFFFFFF:FFFFFF:FFFF:FFFFF:FFFFFFFFFFFFFFFFFFFFFFFFFFFF
FFFFFFFFFFFFFFFFFF:FFFFFFFFFFFFFFFFFFFFFFFFFFF:FFFFFFF:FFFFFFFF,FFFF
FFFFFFFFFFFFFFF
@A00721:190:HGJW7DSXY:4:2525:7708:18192 2:N:0:TCTGCAAG+GCTGCACT
GTAAGATGCCCCTGCGGGGCCTATGGAATTCTATTTGAATTTGAATTTGAATTGGTATACTGTAGGGC
GGGATATTTTCAAATTTGAAATTTGAAATCATTACTATTCCAATTGGGGATAGATTCCTCCGGATTAC
TCTATACATATATAT
+
FFFFFFFFFFFFFFFFFFFFF:FFFFFFFFFF:F:FFFFFFFFFFFFFFF:FFFFFFFFFFFFFFFFF

FFFFFFFFFFFFFFFFFFFFFFFFFFFFFFFFFFFFFFFFFFFFFFFFF:FFFFFFFFFFFFFFFFF
FFFFFFFFFFFFFFF
@A00721:190:HGJW7DSXY:4:1357:30698:19617 2:N:0:TCTGCAAG+GCTGCACT
CACGGTCTGCGGTAAGATGCCCCTGCGGGGCCTATGGAATTCTATTTGAATTTGAATTTGAATTGGTA
TACTGTAGGGCGGGATATTTTCAAATTTGAAATTTGAAATCATTACTATTCCAATTGGGGATAGATTC
CTCCGGATTACTCTA
+
FFFFFFFFFFFFFFFFFFFFFFFFFFFFFFFFFFFFF:FFFFFFFFFFFFFFFFFFFFFFFFFFFFFF
FFFFFFFFFFFFFFFFFFFFFFFFF:FFFF:FFFFFFFFFFFFFFFFFFFFFFFFFFFFFFFFFFFFF
FFFFFFF,FFFFFFF
@A00721:190:HGJW7DSXY:4:1471:23294:3411 2:N:0:TCTGCAAG+GCTGCACT
CACGGTCTGCGGTAAGATGCCCCTGCGGGGCCTATGGAATTCTATTTGAATTTGAATTTGAATTGGTA
TACTGTAGGGCGGGATATTTTCAAATTTGAAATTTGAAATCATTACTATTCCAATTGGGGATAGATTC
CTCCGGATTACTCTA
+
FFF:FFFFFFFFFFFF,FFFFFFFFFFFFFFFFFFFFF:FFFFFFFFFFFFFFFFF:FFFFFFFFFFF
FFFFFF:F:FFFFFFFFFFFFFFFFFFF:FFFFFFFFFFF,FFFFFFFFFF,FFFFF,:,FFFFFFFF
FFFFF:FFFFFFFFF
@A00721:190:HGJW7DSXY:4:2177:32045:21136 2:N:0:TCTGCAAG+GCTGCACT
CACGGTCTGCGGTAAGATGCCCCTGCGGGGCCTATGGAATTCTATTTGAATTTGAATTTGAATTGGTA
TACTGTAGGGCGGGATATTTTCAAATTTGAAATTTGAAATCATTACTATTCCAATTGGGGATAGATTC
CTCCGGATTACTCTA
+
FFFFFFFFFFFFFFFFFFFFFFFFFFFFFFFFFFFFFFFFFFFFF:FFFFFFFFFFFFFF:FFFFFFF
F:FFFFFFFFFFFFFFFFFFFFFFFFFFFFFFFFFFF:,FFFFFFFFFFFF:FFFF:FFFFFFFFFF:
FFFFFFFFFFFFFFF
@A00721:190:HGJW7DSXY:4:2201:10809:17143 2:N:0:TCTGCAAG+GCTGCACT
CACGGTCTGCGGTAAGATGCCCCTGCGGGGCCTATGGAATTCTATTTGAATTTGAATTTGAATTGGTA
TACTGTAGGGCGGGATATTTTCAAATTTGAAATTTGAAATCATTACTATTCCAATTGGGGATAGATTC
CTCCGGATTACTCTA
+
FFFFFFFFFFFF,FFFFFFFFFFFFFFFFFFFFFFFFFFFFFFFFFFFFFFF:FFF:FFFFFF:FFFF:
FFFFFFFFF:FFFFFFFFFFFFFFFFFFFFFFFFFFFFFFFFFFFFFFFFFFFFFFFF:FFFFFFFF
F:FFFFFFFFFFFFF
@A00721:190:HGJW7DSXY:4:1418:10004:3568 2:N:0:TCTGCAAG+GCTGCACT
CCACGGTCTGCGGTAAGATGCCCCTGCGGGGCCTATGGAATTCTATTTGAATTTGAATTTGAATTGGT
ATACTGTAGGGCGGGATATTTTCAAATTTGAAATTTGAAATCATTACTATTCCAATTGGGGATAGATT
CCTCCGGATTACTCT
+
F:FFFFFFFFF:FFFFFFFFFFFFFFFFFFFFFFFFFFFFFFFFFFFFFFFFFFFFFFFFFFFFFF:F
FFFFFFFFFFFFFFFFFFFFFF:FFFFFFF,FFFFFFFFFFFFFFFFFFFFFFFFFFFFFFFFFFFFF
FFFFFFFF:FFFFFF
@A00721:190:HGJW7DSXY:4:1525:7880:20525 2:N:0:TCTGCAAG+GCTGCACT
CCACGGTCTGCGGTAAGATGCCCCTGCGGGGCCTATGGAATTCTATTTGAATTTGAATTTGAATTGGT
ATACTGTAGGGCGGGATATTTTCAAATTTGAAATTTGAAATCATTACTATTCCAATTGGGGATAGATT
CCTCCGGATTACTCT
+
FFFFFFFFFFFFFFFFFFFFFFFFFFFFFFFFFFFFFFFFFFFFFFFFFFFFFFFFF:FFFFFFFFFF
FFFFFFFFFFFFFFFFFFFFFFFFFFFFFFFFFFFFFFFFFFFFFFFFFFFFFFFFFFFFFFFFFFFF
FFFFFFFFFFFFFFF
@A00721:190:HGJW7DSXY:4:1526:9317:11960 2:N:0:TCTGCAAG+GCTGCACT
CCACGGTCTGCGGTAAGATGCCCCTGCGGGGCCTATGGAATTCTATTTGAATTTGAATTTGAATTGGT
ATACTGTAGGGCGGGATATTTTCAAATTTGAAATTTGAAATCATTACTATTCCAATTGGGGATAGATT
CCTCCGGATTACTCT

+
FFFFFFFFFFFFFFFFFFFFFFFFFFFFFFFFFFFFFFFFFFFFFFFFFFFFFFFFFFFFFFFFFFFF
FFFFFFFFFFFFFFFFFFFFFFFFFFFFFFFFFFFFFFFFFFFFFFFF:FFFFFFFFFFFFFFFFFFF
FFFFFFFFFFFFFFF
@A00721:190:HGJW7DSXY:4:1529:9091:8594 2:N:0:TCTGCAAG+GCTGCACT
CCACGGTCTGCGGTAAGATGCCCCTGCGGGGCCTATGGAATTCTATTTGAATTTGAATTTGAATTGGT
ATACTGTAGGGCGGGATATTTTCAAATTTGAAATTTGAAATCATTACTATTCCAATTGGGGATAGATT
CCTCCGGATTACTCT
+
FFFFFFFFFFFFFFFFFFFFFFFFFFF:FFFFFFFFFFFFFFFFFFFFFFFFFFFFFFFFFFFFFFFF
FFFFFFFFFFFFFFFFFFFFFFFFFFFFFF:FFFFFF:FFFFFFFFFFFFFFFFFFFFFFFFFF:FFF
FFFFFFFFFFFFFFF
@A00721:190:HGJW7DSXY:4:1547:18900:14309 2:N:0:TCTGCAAG+GCTGCACT
CCACGGTCTGCGGTAAGATGCCCCTGCGGGGCCTATGGAATTCTATTTGAATTTGAATTTGAATTGGT
ATACTGTAGGGCGGGATATTTTCAAATTTGAAATTTGAAATCATTACTATTCCAATTGGGGATAGATT
CCTCCGGATTACTCT
+
FFFFFFFFFFFFFFF:FFFFFFFFFFFF:FFFFFFFFFFFFFFFFFF::FFF::FFFFFFFFFFFFF:
FFFFFFFF:FFFFFFFFFF,FFFFFFFF,FFFFFFFFFFFFFFFFFFFFF:F:FFFFFF:FFFFFFFF
F:FFFFFFFFF:FFF
@A00721:190:HGJW7DSXY:4:1565:11686:23109 2:N:0:TCTGCAAG+GCTGCACT
CCACGGTCTGCGGTAAGATGCCCCTGCGGGGCCTATGGAATTCTATTTGAATTTGAATTTGAATTGGT
ATACTGTAGGGCGGGATATTTTCAAATTTGAAATTTGAAATCATTACTATTCCAATTGGGGATAGATT
CCTCCGGATTACTCT
+
FFFFFFFFFFFFFFFFFFFFFFFFFFFFFFFFFFFFFFFFFFFFFF:FFFFFFFF:FFFFFFFFFFFF
FFFFFFFFFFFFFFFFFFFFFFFFFFFFFFFFFFFFFFFFFFFFFFFFFFFFFFFFFF:FFFFFFFFF
FFFFFFFFFFFFFFF
@A00721:190:HGJW7DSXY:4:1162:21649:31970 2:N:0:TCTGCAAG+GCTGCACT
GTAATCCGGAGGAATCTATCCCCAATTGGAATAGTAATGATTTCAAATTTCAAATTTGAAAATATCCC
GCCCTACAGTATACCAATTCAAATTCAAATTCAAATAGAATTCCATAGGCCCCGCAGGGGCATCTTAC
CGCAGACCGTGGGGC
+
FFFFFFFFFFFFFFFFFFFFFFFFFFFFFFFFFFFFFFFF:FFFFFFFFFFFFFFFFFF:FFFFFFFF
FFFFF:FFFFFFFFFFFFFFFF,FFFFFFFFFFF:FFFFFFFFFFFFFF:FFF:FFFFFFFFFFFFFF
FFFFFFF:FFFFFFF
@A00721:190:HGJW7DSXY:4:1223:7346:19038 2:N:0:TCTGCAAG+GCTGCACT
GTAATCCGGAGGAATCTATCCCCAATTGGAATAGTAATGATTTCAAATTTCAAATTTGAAAATATCCC
GCCCTACAGTATACCAATTCAAATTCAAATTCAAATAGAATTCCATAGGCCCCGCAGGGGCATCTTAC
CGCAGACCGTGGGGC
+
FFFFFFFFFFFFFFFFFFFFFFFFFFFFFFFFFFFFFFFFFFFFFFFFFFFFFFFFFFFFFFFFFFFF
FFFFFFFFFFFFFFFFFFFFFFFFFFFFFFFFFFFFF::FFFFFFFFFFFFFFFFFFFFFFFFFFFFF
FFFFFFFFFFFFFFF
@A00721:190:HGJW7DSXY:4:1257:2745:13761 2:N:0:TCTGCAAG+GCTGCACT
GTAATCCGGAGGAATCTATCCCCAATTGGAATAGTAATGATTTCAAATTTCAAATTTGAAAATATCCC
GCCCTACAGTATACCAATTCAAATTCAAATTCAAATAGAATTCCATAGGCCCCGCAGGGGCATCTTAC
CGCAGACCGTGGGGC
+
FFFFFFFFFFFFFFFFFFFFFFFFF,FFFF,,FFFFFFFFFFFFFFFFFFFFFF:FFF:FFFFFFFFF
FFFFFFFFFFFFFFFFFFFFFF:FFFFF:FFFFFFFFFFFFFFFFFFFFFFFFFF:FFFFFFFFFFFF
FFFFFFFFFFFFFFF
@A00721:190:HGJW7DSXY:4:1348:27850:2597 2:N:0:TCTGCAAG+GCTGCACT
GTAATCCGGAGGAATCTATCCCCAATTGGAATAGTAATGATTTCAAATTTCAAATTTGAAAATATCCC

```
GCCCTACAGTATACCAATTCAAATTCAAATTCAAATAGAATTCCATAGGCCCCGCAGGGGCATCTTAC
CGCAGACCGTGGGGC
+
FF,FFFFFFFFFFFFFFFFFFFFFFFFFFFFFFFFFFFFFFFFFFFFFFFFFFFFFFFFFFFFFFFFF
FFFFFFFFFFFFFFF:FFFFFFFFFFFFFFFFFFFFFFF,FFFFFFFFFFFFFFFFFFFFFFFFFFFF
FFFFFFFFFFFFFFF
@A00721:190:HGJW7DSXY:4:1451:3414:23312 2:N:0:TCTGCAAG+GCTGCACT
GTAATCCGGAGGAATCTATCCCCAATTGGAATAGTAATGATTTCAAATTTCAAATTTGAAAATATCCC
GCCCTACAGTATACCAATTCAAATTCAAATTCAAATAGAATTCCATAGGCCCCGCAGGGGCATCTTAC
CGCAGACCGTGGGGC
+
FFF:FFFFFFFFFFFFFFFFFFFFFFFFFFFFFFFFFFFFFFFFFFFFFFFFFFFFFFFFFFFFFFFF
FFFFFFF:FFFFFFFFFFFFFFFFFFFFFFFFFFFFFFFFFFFFFFFFFFFFFFFFFFFF:FFFFFF
FFFFFFFFFFFFFFF
@A00721:190:HGJW7DSXY:4:2142:13304:34240 2:N:0:TCTGCAAG+GCTGCACT
GTAATCCGGAGGAATCTATCCCCAATTGGAATAGTAATGATTTCAAATTTCAAATTTGAAAATATCCC
GCCCTACAGTATACCAATTCAAATTCAAATTCAAATAGAATTCCATAGGCCCCGCAGGGGCATCTTAC
CGCAGACCGTGGGGC
+
FFFFFFFFFFFFF,FFF,FFFFFFFFFFFFFFFFFFFFFFFFFFFFFFF,FFFF,FFFF:FF:FFFFF
FFFFFFF,FF:F,FFF:FFFFFF:FFFF,:FFF,::FFF,FFFFF,:FFFFFFFF,FFFFF,FFFFF:
FFFFFFFFF,FFFFF
@A00721:190:HGJW7DSXY:4:2571:32931:26772 2:N:0:TCTGCAAG+GCTGCACT
GTAATCCGGAGGAATCTATCCCCAATTGGAATAGTAATGATTTCAAATTTCAAATTTGAAAATATCCC
GCCCTACAGTATACCAATTCAAATTCAAATTCAAATAGAATTCCATAGGCCCCGCAGGGGCATCTTAC
CGCAGACCGTGGGGC
+
FFFFFFFFFFFFFFFFFFFFFFFFFFFFF:FFFFFFFFFFFFFFF:FFFFFFFFFFF:FFFFFFFFF
FFFFFFF:FFFFFFFFFFFFFF,FFFFFFFFFFF:FFFFFFFFFFFFFF:FFFFFFFFFFFFFFFFF:
FFFFFFFFF:FFFFF
@A00721:190:HGJW7DSXY:4:1503:30056:17253 2:N:0:TCTGCAAG+GCTGCACT
GAGGAATCTATCCCCAATTGGAATAGTAATGATTTCAAATTTCAAATTTGAAAATATCCCGCCCTACA
GTATACCAATTCAAATTCAAATTCAAATAGAATTCCATAGGCCCCGCAGGGGCATCTTACCGCAGACC
GTGGGGCAAGCCAAT
+
FFFFFFFFFFFFFFFFFFFFFFFFFFFFF:FFFFFFFFFFFFFFFFFFFFFFFFFFFFFFFFFFFFFFF
FFFFFFFFFFFFFFFFFFFFFFFFFFFFFFFFFFFFFFFFFFFFFFFFFFFFFFFFFFFFFFFFFFFF
F:FFFFF,FFFFFFF
@A00721:190:HGJW7DSXY:4:2225:18376:12273 2:N:0:TCTGCAAG+GCTGCACT
GAGGAATCTATCCCCAATTGGAATAGTAATGATTTCAAATTTCAAATTTGAAAATATCCCGCCCTACA
GTATACCAATTCAAATTCAAATTCAAATAGAATTCCATAGGCCCCGCAGGGGCATCTTACCGCAGACC
GTGGGGCAAGCCAAT
+
FFFFF,FFFFFFFFFFFFFFFFFFFFFFFFFFFFFFFFFFFFFFFFFFFFFFFFFFFFFFFFFFFFFF
FFFFFFFFFFFFFFFFFFFFFFFFFFFFFFFFFFFFFFFFFFFFFFFFFFFFFFFFFFFFFFFFFFFF
FFFFFFFFFFFFFFF
@A00721:190:HGJW7DSXY:4:2264:32832:25974 2:N:0:TCTGCAAG+GCTGCACT
GAGGAATCTATCCCCAATTGGAATAGTAATGATTTCAAATTTCAAATTTGAAAATATCCCGCCCTACA
GTATACCAATTCAAATTCAAATTCAAATAGAATTCCATAGGCCCCGCAGGGGCATCTTACCGCAGACC
GTGGGGCAAGCCAAT
+
FFFFFFFFFFFFFFFFFFFFFFFFFFFFFFFFFFFFFFF:FFFFFF,FFFFFFF:FFFFFFFFFFFFF
FFFFFFFFFFFFF:FFFFFF,FFFFF,FFFFFFFFFFFF:FFFFFF:FFFFFFFFFFFFFFFFF:FFF
F:FFFF:F:FFFFFF
```

@A00721:190:HGJW7DSXY:4:2664:30346:14372 2:N:0:TCTGCAAG+GCTGCACT
GAGGAATCTATCCCCAATTGGAATAGTAATGATTTCAAATTTCAAATTTGAAAATATCCCGCCCTACA
GTATACCAATTCAAATTCAAATTCAAATAGAATTCCATAGGCCCCGCAGGGGCATCTTACCGCAGACC
GTGGGGCAAGCCAAT
+
FFFFFFFFFFFFFFFFFFFFFFFFFFFFFFFFFFFFFFFFFFFFFFFFFFFFFFFFFF:F:F::FF:F
:FFFFFFFFFFFFFFFFFFFFFFFFFFFFFFF:FFFFF,FFFFFF:FFFFFFFFFFF::FFFFFFF
FFFFFFFF,FFFFF:
@A00721:190:HGJW7DSXY:4:2647:29288:9251 2:N:0:TCTGCAAG+GCTGCACT
ATATTGGCTTGCCCCACGGTCTGCGGTAAGATGCCCCTGCGGGGCCTATGGAATTCTATTTGAATTTG
AATTTGAATTGGTATACTGTAGGGCGGGATATTTTCAAATTTGAAATTTGAAATCATTACTATTCCAA
TTGGGGATAGATTCC
+
FFFFFFFFFFFFFFFFFFFFFFFFFFFFFFFFFFFFFFFFFFFFFFFFFFFFFFFFF,FFFFFFFFF
FF,FFFFF:FFFFFFFFFFFFFFFFFFFFFFFFFFFFFFFFFFFFFFFFFFFFFFFFFFFFF:FF:F
FFFFFFFFFFFFFFF
@A00721:190:HGJW7DSXY:4:1226:10963:36104 2:N:0:TCTGCAAG+GCTGCACT
ATATTGGCTTGCCCCACGGTCTGCGGTAAGATGCCCCTGCGGGGCCTATGGAATTCTATTTGAATTTG
AATTTGAATTGGTATACTGTAGGGCGGGATATTTTCAAATTTGAAATTTGAAATCATTACTATTCCAA
TTGGGGATAGATTC
+
FFFFFFFFF:FFFFFFFFFFF:FFFFFFFFFFFFFFFFFFFFFFFF,F:FFFFFF,FFFFFF:F:FFF
FFFFFFFFFFFFF:F:FFFFFFFFFFFFFFFFFFFFFF:FFFFFFFFFFFFFFFFFFFFFFFFFFFFF
FFFFFFF:FFF:F:
@A00721:190:HGJW7DSXY:4:1234:20184:12712 2:N:0:TCTGCAAG+GCTGCACT
ATATTGGCTTGCCCCACGGTCTGCGGTAAGATGCCCCTGCGGGGCCTATGGAATTCTATTTGAATTTG
AATTTGAATTGGTATACTGTAGGGCGGGATATTTTCAAATTTGAAATTTGAAATCATTACTATTCCAA
TTGGGGATAGATTC
+
:FFFFFFFFFFFFFFFFFFFFFFFFFFFFFFFFFFFFFFFFFFFFFFFFFFFFFFFFFFFFFFFFFFF
FFFFFFFFFFFFFFFFFFFFFFFFFFFFFFFFFFFFFFFFFFFFFFFFFFFFFFFFFFFFFFFFFFFF
FFFFFF:FFFFFFF
@A00721:190:HGJW7DSXY:4:2334:23665:19774 2:N:0:TCTGCAAG+GCTGCACT
ATATTGGCTTGCCCCACGGTCTGCGGTAAGATGCCCCTGCGGGGCCTATGGAATTCTATTTGAATTTG
AATTTGAATTGGTATACTGTAGGGCGGGATATTTTCAAATTTGAAATTTGAAATCATTACTATTCCAA
TTGGGGATAGATTC
+
FFFFFFFFFFFFFFFFFFFFFFFFFFFFFFFFFFFFFFFFFFFFFFFF,FFFFFFFFFFFFFFFFFFFF
FFFFFFFFFFFFFFFFFFFFFFFFFFFFFFFFFFFFFFFFFFFFFFFFFFFFFFFF:FFFF:FF:FFF
FFFFFFF,FFFFFF
@A00721:190:HGJW7DSXY:4:1265:32108:30013 2:N:0:TCTGCAAG+GCTGCACT
ATTATATTGGCTTGCCCCACGGTCTGCGGTAAGATGCCCCTTCGGGGCCAATGGAATTCTATTTGAAT
TTGAATTTGAATTGGTATACTGTAGGGCGGGATATTTTCAAATTTGAAATTTTAAATCATTACTATTC
CAATTGGGGATAGAT
+
FFFFFF,,FFFFFF:FFF:F,FF:F,,F,FFF,FF:FF:::,FFFF,,:,FFFFFFFF:F:FF:F,FF
FF:FFFFFFF:F,F,FFF,,:F::F:F,F,FFFFFFFF,F,FFF:FFFFFFF,:,FF,FFFF:,F:F,
:FFFF,F:F:FFFFF
@A00721:190:HGJW7DSXY:4:1519:3143:27383 2:N:0:TCTGCAAG+GCTGCACT
ATTATATTGGCTTGCCCCACGGTCTGCGGTAAGATGCCCCTGCGGGGCCTATGGAATTCTATTTGAAT
TTGAATTTGAATTGGTATACTGTAGGGCGGGATATTTTCAAATTTGAAATTTGAAATCATTACTATTC
CAATTGGGGATAGAT
+
FFFFFFFFFFFFFFFFFFFFFFFFFFFFFFFFFFFFFFFFFFFFFFFFFFFFFFFFFFFFFFFFFFF:

```
FFFFFFFFFFF:FFFFFFFFFFFFFFFFFFFFFFFFFFFF,F::FFFFFF:FFFFFFFFF:FFFFFFFFF
FFFFFFFFFFFFFFF
@A00721:190:HGJW7DSXY:4:2110:4779:14058 2:N:0:TCTGCAAG+GCTGCACT
ATTATATTGGCTTGCCCCACGGTCTGCGGTAAGATGCCCCTGCGGGGCCTATGGAATTCTATTTGAAT
TTGAATTTGAATTGGTATACTGTAGGGCGGGATATTTTCAAATTTGAAATTTGAAATCATTACTATTC
CAATTGGGGATAGAT
+
FFFFFFFFFFFFFFFFFFFFFFFFFFFFFF:FFFFFFFFFFFFFFFFFFFFFFFFFFFF:FFFF,FFFF:
FFFFFFFFFFFFFFFFFFFFFFFFFFFFFFFFFF:FFFFFFFFFFFFFFFFFFFFFFFFFFFFFFFFFFF
FFF:FFFFFFFFFFF
@A00721:190:HGJW7DSXY:4:2110:2700:16157 2:N:0:TCTGCAAG+GCTGCACT
ATTATATTGGCTTGCCCCACGGTCTGCGGTAAGATGCCCCTGCGGGGCCTATGGAATTCTATTTGAAT
TTGAATTTGAATTGGTATACTGTAGGGCGGGATATTTTCAAATTTGAAATTTGAAATCATTACTATTC
CAATTGGGGATAGAT
+
FFFFFFFFFFF:FFF:FFFFFFFFFFFFFFFFFFFFFFFFFFFFFFFFFFFFFFFFF:FFF:F,:FFFFF
FFFFFFFFFFF:FFFFFFFFFFFFFFFFF:FFFFFFFFFFFFFFFFFFFFFFFFFFF:FFFFFFFFFFFF
FFFFFFFFFFFFFFF
@A00721:190:HGJW7DSXY:4:2241:10773:23531 2:N:0:TCTGCAAG+GCTGCACT
ATTATATTGGCTTGCCCCACGGTCTGCGGTAAGATGCCCCTGCGGGGCCTATGGAATTCTATTTGAAT
TTGAATTTGAATTGGTATACTGTAGGGCGGGATATTTTCAAATTTGAAATTTGAAATCATTACTATTC
CAATTGGGGATAGAT
+
F,FFFFFFFFFFFFFFFFFFFFFFF:FFFFFFFFFFFFFFFFFFFFFFFFFFF:FFFFFFFFFF:FFFF:
FFFFF:FF:FFFFFFFFFFFFFFFFFFFFFFFFFFFFFFFFFFFFFFFFFFFFFFFFFFFFFFFF,FFF
F:F::FFFFFFFFFF
@A00721:190:HGJW7DSXY:4:2406:28076:8155 2:N:0:TCTGCAAG+GCTGCACT
ATTATATTGGCTTGCCCCACGGTCTGCGGTAAGATGCCCCTGCGGGGCCTATGGAATTCTATTTGAAT
TTGAATTTGAATTGGTATACTGTAGGGCGGGATATTTTCAAATTTGAAATTTGAAATCATTACTATTC
CAATTGGGGATAGAT
+
F:FFFFFFF:FF,FF:FFFFFFFFF:FFFFFFFFFFFFFFFFFF:FFFFFFFFFFFFFFF:FF::FFFFF
FFFFFFFFFF:FFFF:FFF:FFF:F:FFFFFFFF:F:FFFFFFF::FF,FFFF:F:,,:F,:F:FFFF,F
,:,FFF,FF,:FFFF
@A00721:190:HGJW7DSXY:4:2418:31159:20791 2:N:0:TCTGCAAG+GCTGCACT
ATTATATTGGCTTGCCCCACGGTCTGCGGTAAGATGCCCCTGCGGGGCCTATGGAATTCTATTTGAAT
TTGAATTTGAATTGGTATACTGTAGGGCGGGATATTTTCAAATTTGAAATTTGAAATCATTACTATTC
CAATTGGGGATAGAT
+
FFFFFFFFFFFFFFFFFFFFFFFFFFFFFFFFFFFFFFFFFFFFFFFFFFFFFFFFFFFFFFFFFFFFFF
FFFFFFFFFFFFFFFFFFFFFFFFFFFFFFFFFFFFFFFFFFFFFF,FFFFFF:FFFF:FFFFFFFFFFF
FFFFFFF::FFF,FF
@A00721:190:HGJW7DSXY:4:2437:23448:23813 2:N:0:TCTGCAAG+GCTGCACT
ATTATATTGGCTTGCCCCACGGTCTGCGGTAAGATGCCCCTGCGGGGCCTATGGAATTCTATTTGAAT
TTGAATTTGAATTGGTATACTGTAGGGCGGGATATTTTCAAATTTGAAATTTGAAATCATTACTATTC
CAATTGGGGATAGAT
+
FFFFFFFFFFFFFFFFFFFFFFFFFFFFFFFFFFFFFFFFFFFFFFFFFF::FFFFFFFFFFFFFFFFF:
FFFFFFFFFFFFFFFFFFFFFFFFFFFFFFFFFFFFFFFFFFFFFFFFFF,FFFFFFFFFFFFFFFFFF:
FFFFFFFFFFFFFFF
@A00721:190:HGJW7DSXY:4:2441:19379:23657 2:N:0:TCTGCAAG+GCTGCACT
ATTATATTGGCTTGCCCCACGGTCTGCGGTAAGATGCCCCTGCGGGGCCTATGGAATTCTATTTGAAT
TTGAATTTGAATTGGTATACTGTAGGGCGGGATATTTTCAAATTTGAAATTTGAAATCATTACTATTC
CAATTGGGGATAGAT
```

```
+
FFFFFFFFFFFFFFFFFFFFFFFFFFFFFFFFFFFFFFFFFFFFFFFFFFFFFFFFFFFFFF:FFF:FF
FFFFFFFFFFFFFFFF,FFFFFFFFFFFFFFFFFFFFFFFFFFFFFFFFFFFF:FFFFF:FFFFFFFF
:FFFFFFFFFFFFF:
@A00721:190:HGJW7DSXY:4:1148:19994:35994 2:N:0:TCTGCAAG+GCTGCACT
AGTAATATTATATTGGCTTGCCCCACGGTCTGCGGTAAGATGCCCCTGCGGGGCCTATGGAATTCTAT
TTGAATTTGAATTTGAATTGGTATACTGTAGGGCGGGATATTTTCAAATTTGAAATTTGAAATCATTA
CTATTCCAATTGGGG
+
FFFFFFFFFFFFFFFFFFFFFFFFFFFFFFFFFFFFFFFFFFFFFFFFFFFFFFFFFFFFFFFFFF::
FFFF:FFFFFFFFFF:FFF:FFFFFFFFFF:FFFFFFFFFFFFFFFFFFFFFFFFFFFFFFFFFFFFF
FFFFFFFFFFFFFFF
@A00721:190:HGJW7DSXY:4:1168:24306:15060 2:N:0:TCTGCAAG+GCTGCACT
AGTAATATTATATTGGCTTGCCCCACGGTCTGCGGTAAGATGCCCCTGCGGGGCCTATGGAATTCTAT
TTGAATTTGAATTTGAATTGGTATACTGTAGGGCGGGATATTTTCAAATTTGAAATTTGAAATCATTA
CTATTCCAATTGGGG
+
FFFFFFFFFFFFFFFFFFFFFFFFFFFFFFFFFFFFFFFFFFFFFFFFFFFFFFFFFFFFFF:FFFFFF
FFFFF,FFFFFFFFFFFFFFFFFFFFFFFFFFFFFFFFFFFFFFFFFFFFFFFFFFFFFFFFFFFFFF
FFFFFFFFFFFFFF:
@A00721:190:HGJW7DSXY:4:1178:23294:28494 2:N:0:TCTGCAAG+GCTGCACT
AGTAATATTATATTGGCTTGCCCCACGGTCTGCGGTAAGATGCCCCTGCGGGGCCTATGGAATTCTAT
TTGAATTTGAATTTGAATTGGTATACTGTAGGGCGGGATATTTTCAAATTTGAAATTTGAAATCATTA
CTATTCCAATTGGGG
+
FFFFFFFFFFFFFFFFFFFF:FFFFFFFFFFFFFFFFFFFFFFFFFFFFFFFFFFFFFFFFFFFFFFF
FFFFFFFFFFFFFFFFFFFFFFFFFFFFFFFFFFFFFFFFFFFFFFFFFFFFFFFFFFFFFFFFFFFF
FFFFFFFFF:FF:F:
@A00721:190:HGJW7DSXY:4:1228:6632:16861 2:N:0:TCTGCAAG+GCTGCACT
AGTAATATTATATAGGCTTGCCCCACGGTCTGCGGTAAGATGCCCCTGCGGGGCCTATGGAATTCTAT
TTGAATTTGAATTTGAATTGGTATACTGTAGGGCGGGATATTTTCAAATTTGAAATTTGAAATCATTA
ATATTCCAATTGGGG
+
F:,FFFF,FFFF,,:FFF,F:F,,FF:FFF,F,:F,FFFFFF:,F,FFFFF,FF,FF:FFFFFFFF,,
FFFFF:,:FFF:FFF:F:,F:FFFFFF:FFFF,FF:FFFF:FFFFFFFF,F::FFFFF::FFFF,FFF
,F,FFFFFF:FFF:F
@A00721:190:HGJW7DSXY:4:1233:26304:13416 2:N:0:TCTGCAAG+GCTGCACT
AGTAATATTATATTGGCTTGCCCCACGGTCTGCGGTAAGATGCCCCTGCGGGGCCTATGGAATTCTAT
TTGAATTTGAATTTGAATTGGTATACTGTAGGGCGGGATATTTTCAAATTTGAAATTTGAAATCATTA
CTATTACAATTGGGG
+
FFFFFFFFFFFFFFFFF,FFFFFFFFF,FFF:F,F:FFFFFF,FFFFF,F:,FFFFF:FFF:FFFFFF
,FFFFFF:F,,:FFFFFF,F:FF,F,FF::F:FFFFF::F:FFFFF,FF,FFF:FFFFFF:FFFFF:F
:,FF,::FF:F,F:F
@A00721:190:HGJW7DSXY:4:1252:21323:29559 2:N:0:TCTGCAAG+GCTGCACT
AGTAATATTATATTGGCTTGCCCCACGGTCTGCGGTAAGATGCCCCTGCGGGGCCTATGGAATTCTAT
TTGAATTTGAATTTGAATTGGTATACTGTAGGGCGGGATATTTTCAAATTTGAAATTTGAAATCATTA
CTATTCCAATTGGGG
+
FFFFFFFFF:FFFFFFFFFFFFFFFFFFFFFFFFFFFFFFFFFFFFFFFFFFFFFFFFFFFFFFFFFF
FFFFFFFFFFFFFFFF:FFFFFFFFFFFFFFFFFFFFFFFFFFFFFFFFFFFFFFFFFFFFFFFFFFF
FFFFFFF:FFFFFFF
@A00721:190:HGJW7DSXY:4:1312:31069:14152 2:N:0:TCTGCAAG+GCTGCACT
AGTAATATTATATTGGCTTGCCCCACGGTCTGCGGTAAGATGCCCCTGCGGGGCCTATGGAATTCTAT
```

```
TTGAATTTGAATTTGAATTGGTATACTGTAGGGCGGGATATTTTCAAATTTGAAATTTGAAATCATTA
CTATTCCAATTGGGG
+
FFFFFFFFFFFFFFFFFFFFFFFFFFFFFFFFFFFFFFFFFFFFFFFFFFFFFFFFFFFFFFFF:FFF
FFFFFFFFFFFFFFFFF:FFFFFFFFFFFFFFFFFFFFFFFFFFFFFFFFFF:FFFFFFFF:FFFFFF
FFFFF:FFF,FFFFF
@A00721:190:HGJW7DSXY:4:1424:7093:20196 2:N:0:TCTGCAAG+GCTGCACT
AGTAATATTATATTGGCTTGCCCCACGGTCTGCGGTAAGATGCCCCTGCGGGGCCTATGGAATTCTAT
TTGAATTTGAATTTGAATTGGTATACTGTAGGGCGGGATATTTTCAAATTTGAAATTTGAAATCATTA
CTATTCCAATTGGGG
+
FFFFFFFFF:FFFFFFFFFFFFFFFFFFFFFFFFFFFFFFFFFFFFFFFFFFFFFFFFFFFFFFFF:F
FFFFFFFFFFFFFFFFFFFFFFFFFFFFFFFFFFFFFFFFFFFFFFFFFFFFFFFFFFFFFFFFFFFF
FFFFFFFFFFFFFFF
@A00721:190:HGJW7DSXY:4:1574:10619:4413 2:N:0:TCTGCAAG+GCTGCACT
AGTAATATTATATTGGCTTGCCCCACGGTCTGCGGTAAGATGCCCCTGCGGGGCCTATGGAATTCTAT
TTGAATTTGAATTTGAATTGGTATACTGTAGGGCGGGATATTTTCAAATTTGAAATTTGAAATCATTA
CTATTCCAATTGGGG
+
FFFFFFFFFFFFFFFFFFFFFFFFFFFFFFFFFFFFFFFFFFFFFFFFFFF:FFFFFFFFFFFFFFFF
FFFFFFFFFFFFFFFFFFFFFFFFFFFFFFFFFFFFFFFFFFFFFFFFFFFFFFFFFFFFF:FFFFFF
FFF:FFFFFFFFFFF
@A00721:190:HGJW7DSXY:4:1574:10619:4633 2:N:0:TCTGCAAG+GCTGCACT
AGTAATATTATATTGGCTTGCCCCACGGTCTGCGGTAAGATGCCCCTGCGGGGCCTATGGAATTCTAT
TTGAATTTGAATTTGAATTGGTATACTGTAGGGCGGGATATTTTCAAATTTGAAATTTGAAATCATTA
CTATTCCAATTGGGG
+
FFFFFFFFFFFFFFF:FFFFFFFFFFFFFFFFFFFFFFFFFFFFFFFFFFFFFFFFFFFFF:FFFFFF
FFFFF:FFFFFFFFFFFFFFFFFFFFFFFFFFFFFFFFFFFFFFFFFFFFFFFFFFFFFFFFFFFFF,
FFFFFFFFFFFFFFF
@A00721:190:HGJW7DSXY:4:1668:32307:27007 2:N:0:TCTGCAAG+GCTGCACT
AGTAATATTATATTGGCTTGCCCCACGGTCTGCGGTAAGATGCCCCTGCGGGGCCTATGGAATTCTAT
TTGAATTTGAATTTGAATTGGTATACTGTAGGGCGGGATATTTTCAAATTTGAAATTTGAAATCATTA
CTATTCCAATTGGGG
+
FFFFFFFFFFFFFFFFFFFFFFFFFFFFFFFFFFFFFFFFFFFFFFFFFFFFFFFFFFFFFFFFFFFF
FFFFFFFFFFFFFFFFFFFFFFFFFF:F:FFFFFFFFFFFFFFFFFFFFFFFFFFFFFF:F:FFFFFF
FFFFFFFFF:FFFFF
@A00721:190:HGJW7DSXY:4:2209:1081:11475 2:N:0:TCTGCAAG+GCTGCACT
AGTAATATTATATTGGCTTGCCCCACGGTCTGCGGTAAGATGCCCCTGCGGGGCCTATGGAATTCTAT
TTGAATTTGAATTTGAATTGGTATACTGTAGGGCGGGATATTTTCAAATTTGAAATTTGAAATCATTA
CTATTCCAATTGGGG
+
FFFFFFFFFFFFFFFFFFFFFFFFFFFFFFFFFFFFFFFFFFFFFFFFFFFFFFFFFFFFFFFFFFFF
FFFFFFFFFFFFFFFFFFFFFFFFFFFFFFFFFFFFFFFFFFFFFFFFFFFFFFFFFFFFFFFFFFFF
FFFFFF:FFFFFFFF
@A00721:190:HGJW7DSXY:4:2223:9001:1705 2:N:0:TCTGCAAG+GCTGCACT
AGTAATATTATATTGGCTTGCCCCACGGTCTGCGGTAAGATGCCCCTGCGGGGCCTATGGAATTCTAT
TTGAATTTGAATTTGAATTGGTATACTGTAGGGCGGGATATTTTCAAATTTGAAATTTGAAATCATTA
CTATTCCAATTGGGG
+
FFFF:FFFFF:FFFFFFF:FFFFFFFFFFFFFFFFFFFF:FFFFFFF,FFFFFF:FFFFFFFFF,F:F
FFFFFFFFF:FFFFF:FFFF::FFFFFFFFFFFFF:FFFFFFFFFF:FFFFFFFFFFF:FFFF:FFFF
:FFFFFFFFFFF,FF
```

```
@A00721:190:HGJW7DSXY:4:2223:10167:2597 2:N:0:TCTGCAAG+GCTGCACT
AGTAATATTATATTGGCTTGCCCCACGGTCTGCGGTAAGATGCCCCTGCGGGGCCTATGGAATTCTAT
TTGAATTTGAATTTGAATTGGTATACTGTAGGGCGGGATATTTTCAAATTTGAAATTTGAAATCATTA
CTATTCCAATTGGGG
+
FFFFFFFFFFFFFFFFFFFFFFFFFFFFFFFFFFFFFFFFFFFFFFFFFFFFFFFFFFFFFFFFFFFFFF
FFFFFFFFFFFFFFFFFFFFFFFFFFFFFFFFFFFFFFFFFFFFFFFFFFFFFFFFFFFFFFFF:FFFFF
FFFFFFFFFFFF:FF
@A00721:190:HGJW7DSXY:4:2226:6298:14559 2:N:0:TCTGCAAG+GCTGCACT
AGTAATATTATATTGGCTTGCCCCACGGTCTGCGGTAAGATGCCCCTGCGGGGCCTATGGAATTCTAT
TTGAATTTGAATTTGAATTGGTATACTGTAGGGCGGGATATTTTCAAATTTGAAATTTGAAATCATTA
CTATTCCAATTGGGG
+
FFFFFFFFFFFFFFFFFFFFFFFFFFFFFFFFFFFFFFFFFFFFFFFFFFFFFFFFFFFFFFFFFFFFFF
FFFFFFFFFFFFFFFFFFFF:FFFFFFFFFFFFFFFFFFF:FFFFFFFFFFFFFFFFFFFFFFFFFFFFF
FFFFFF:FFFFFFFF
@A00721:190:HGJW7DSXY:4:2314:20509:12900 2:N:0:TCTGCAAG+GCTGCACT
AGTAATATTATATTGGCTTGCCCCACGGTCTGCGGTAAGATGCCCCTGCGGGGCCTATGGAATTCTAT
TTGAATTTGAATTTGAATTGGTATACTGTAGGGCGGGATATTTTCAAATTTGAAATTTGAAATCATTA
CTATTCCAATTGGGG
+
FFFFFFFFFFFFFFFFFFFFFFFFFFFFFFFFFFFFFFFFFFFFFFFFFFFFFFFFFFFFFFFFFFFF:F
FFFF:FFFFFFFFFFF:FFFFFFFFFFFFFFFFFFFFFFFFFFFFFFFFFFFFFFFFFFFFFFFFFFFFF
FFFFFFFFF:FFFFF
@A00721:190:HGJW7DSXY:4:1313:29785:22639 2:N:0:TCTGCAAG+GCTGCACT
CCCAATTGGAATAGTAATGATTTCAAATTTCAAATTTGAAAATATCCCGCCCTACAGTATACCAATTC
AAATTCAAATAGAATTCCATAGGCCCCGCAGGGGCATCTTACCGCAGACCGTGGGGCAAGCCAATATC
ATATTACTGGCTTGC
+
FFFFFFFF:FF:FFFFFFFFFFFFFFFFFFFFFFFFFFF:FFFFFFFFFFFFFFFFF,FFFFFFF:FFFF
FFFFFFFFFFFFFFFFFFFFFFFFFFFFFFFFFFFFF:FFFFFFFFFFFFFFFFFFFFF,FFFFFFFFF,
FFFFFFFFFFFFFFF
@A00721:190:HGJW7DSXY:4:1424:21793:12085 2:N:0:TCTGCAAG+GCTGCACT
CCCAATTGGAATAGTAATGATTTCAAATTTCAAATTTGAAAATATCCCGCCCTACAGTATACCAATTC
AAATTCAAATAGAATTCCATAGGCCCCGCAGGGGCATCTTACCGCAGACCGTGGGGCAAGCCAATATA
ATATTACTGGCTTGC
+
FFFFFFFFFFFFFFFFFFFFFFFFFFFFFFFFFFFFFFFFFFFFFFFFFFFFFFFFFFFFFFFFFFFFFF
FFFFFFFFFFFFFFFFFFFFFFFFFFFFFFFFFFFFFFFFFFFFFFFFFFFFFFFFFFFFFFFFFFFF:F
FFFFFFFFFFFFFFF
@A00721:190:HGJW7DSXY:4:1536:32786:27806 2:N:0:TCTGCAAG+GCTGCACT
CCCAATTGGAATAGTAATGATTTCAAATTTCAAATTTGAAAATATCCCGCCCTACAGTATACCAATTC
AAATTCAAATAGAATTCCATAGGCCCCGCAGGGGCATCTTACCGCAGACCGTGGGGCAAGCCAATATA
ATATTACTGGCTTGC
+
FFFFFFFFFFFFFFFFFFFFFFFFFFFFFFFFFF:FFFFFFF:F:FFFFFFFFFFF,FFFFFFFFFFFFF
FFFFF:FFFF,FFFFFFFFF:FFFFFFFFFFFFFFFFFFFFF:FFFFFFFFFFFFFFFFFFFFFFFF:F:
FFFFFFFFFFF,FFF
@A00721:190:HGJW7DSXY:4:1537:22625:19914 2:N:0:TCTGCAAG+GCTGCACT
CCCAATTGGAATAGTAATGATTTCAAATTTCAAATTTGAAAATATCCCGCCCTACAGTATACCAATTC
AAATTCAAATAGAATTCCATAGGCCCCGCAGGGGCATCTTACCGCAGACCGTGGGGCAAGCCAATATA
ATATTACTGGCTTGC
+
FFFFFFFFFFFFFFFFFFFFFFFFFFFFFFFFFFFFFFFFFFFFFFFFFFFFFFFFFFFFFFFFFFFFFF
```

FFFFFFFFFFFFFFFFFFFFFFFFFFFFFFFFFFFFFFFFFFFFF:FFFFFFFFFFFFF:FFF:FFFFF
FFFF:FFFFFFFFFF
@A00721:190:HGJW7DSXY:4:1565:12780:26068 2:N:0:TCTGCAAG+GCTGCACT
CCCAATTGGAATAGTAATGATTTCAAATTTCAAATTTGAAAATATCCCGCCCTACAGTATACCAATTC
AAATTCAAATAGAATTCCATAGGCCCCGCAGGGGCATCTTACCGCAGACCGTGGGGCAAGCCAATATA
ATATTACTGGCTTGC
+
FFFFFFFFFFFFFFFFFFFFFFFFFFFFFFFFFF:FFFFFFFF:FFFFFFFFFFFFFFFFFFFFFFFFF
FFFFFFFFFFFFFFFFFF:FFFFFFFFFFFFFFFFFFFFFFFFFFFFFFFFFFFFFFFFFFFFFFFFFF
F,FFFF:FFFFFFFF
@A00721:190:HGJW7DSXY:4:2278:23873:2848 2:N:0:TCTGCAAG+GCTGCACT
CCCAATTGGAATAGTAATGATTTCAAATTTCAAATTTGAAAATATCCCGCCCTACAGTATACCAATTC
AAATTCAAATAGAATTCCATAGGCCCCGCAGGGGCATCTTACCGCAGACCGTGGGGCAAGCCAATATA
ATATTACTGGCTTGC
+
FFFFFFFFFFFFFFFFFFFFFFFFFFFFFFFFFFFFFFFFFFFFFFFFFFFFFFFFFF:FFFFFFFFF
FFFFFFFFFFFFFFFFFFFFFFFFFFFFFFFFFFFFFFFFFFFFFFFFFFFFFFFFFFFFFFFFFFFF
FFFFFFFFFFFFFFF
@A00721:190:HGJW7DSXY:4:2344:31873:27007 2:N:0:TCTGCAAG+GCTGCACT
CCCAATTGGAATAGTAATGATTTCAAATTTCAAATTTGAAAATATCCCGCCCTACAGTATACCAATTC
AAATTCAAATAGAATTCCATAGGCCCCGCAGGGGCATCTTACCGCAGACCGTGGGGCAAGCCAATATA
ATATTACTGGCTTGC
+
FFFFFFFFFFFFFFFFFFFFFFFFFFF:FFFFFF:FFFFFFFFFFFFFFFFFFFFFFFFFFFFFFFFFF
,FFFFFFFFF::FFFFF:FFFFF::FFFFFFFFFFFFFFFFFFFFFFFFFFFFFFFFFFF:FFFFFFF
FFFFF,FFF,FFFFF
@A00721:190:HGJW7DSXY:4:2355:20971:13479 2:N:0:TCTGCAAG+GCTGCACT
CCCAATTGGAATAGTAATGATTTCAAATTTCAAATTTGAAAATATCCCGCCCTACAGTATACCAATTC
AAATTCAAATAGAATTCCATAGGCCCCGCAGGGGCATCTTACCGCAGACCGTGGGGCAAGCCAATATA
ATATTACTGGCTTGC
+
FFFFF:FFFFFFFFFFFFFFFFFFFFFFFFFFFFFFFFFFFFFFFFFFFFFFFFFFFFFFFFFFFFFF
FFFFFFFFFFFFFFFFFFFFFFFFFFFFFFFFFFFFFFFFFFFFFFFFFFFFF:FFFFFFFFFFFFFFF
FFFFFFFFFFF::FF
@A00721:190:HGJW7DSXY:4:2665:7862:7185 2:N:0:TCTGCAAG+GCTGCACT
CCCAATTGGAATAGTAATGATTTCAAATTTCAAATTTGAAAATATCCCGCCCTACAGTATACCAATTC
AAATTCAAATAGAATTCCATAGGCCCCGCAGGGGCATCTTACCGCAGACCGTGGGGCAAGCCAATATA
ATATTACTGGCTTGC
+
FFFFFFFFFFFFFFFFFFFFFFFFFFFFFFFFFFFFFFFFFFFFFFFFFFFFFFFFFFFFFFFFFFFF
FFFFFFFFFFFFF:FFF:FFFFFFFFFFFFFFFFFFFFFFFFFFFFFFFFFFFFFFFF:FFFFFFFF:
FFFFFFFFFFFFFFF
@A00721:190:HGJW7DSXY:4:1528:31403:36432 2:N:0:TCTGCAAG+GCTGCACT
CGGGCAAGCCAGTAATATTATATTGGCTTGCCCCACGGTCTGCGGTAAGATGCCCCTGCGGGGCCTAT
GGAATTCTATTTGAATTTGAATTTGAATTGGTATACTGTAGGGCGGGATATTTTCAAATTTGAAATTT
GAAATCATTACTATT
+
FFFFFFFFFFFFFFFFFFFFFFFFFFFFFFFFFFFF:FFFFFFFFFFFFFFFFFFFFFFFFFFFFFFF
FFFFFFFFFFFFFFFFFFFFFFFFFFFF:FFFF:FFFFFFFFFFFFFFFFFFFFFFFFFFFFFFFFFF
FFFFFFFFFFFFFFF
@A00721:190:HGJW7DSXY:4:1650:16269:12414 2:N:0:TCTGCAAG+GCTGCACT
CGGGCAAGCCAGTAATATTATATTGGCTTGCCCCACGGTCTGCGGTAAGATGCCCCTGCGGGGCCGAT
GGAATTCTATTTGAATTTGAATTTGAATTGGTATACTGTAGGGCGGGATATTTTCAAATTTGAAATTT
GAAATCATTACTATT

+
FFFFFFFFFFFFFFFFFFFFFFFFFFFFFFFFFFFFFFFFFFFFFFFFFFFFFFFFFFFFFFFFFFF,FF
FFF:F,FFF,FFFF,F:FFFFFFFFFFFFFFFFF:FFF,FFFFFFF:FFFFFFFFFFFFFFFFFFFFF
FFFFFFFFFFFF:FF
@A00721:190:HGJW7DSXY:4:2465:22146:8938 2:N:0:TCTGCAAG+GCTGCACT
CGGGCAAGCCAGTAATATTATATTGGCTTGCCCCACGGTCTGCGGTAAGATGCCCCTGCGGGGCCTAT
GGAATTCTATTTGAATTTGAATTTGAATTGGTATACTGTAGGGCGGGATATTTTCAAATTTGAAATTT
GAAATCATTACTATT
+
FFFFFF:FFFFFFFFF:FFFFFFFFFFFFF:FFFFFFFFF:FFFFFFFFFFFFFFFFFFFFFFFFFFF
FFFFFFFFFFFFFFFFFFFFFFFFFFFFFFFFF:FFFFFFFFFFFFFFFFFFFFFFFFFFFFFFFFFF
FFFFFFFFF:FFFFF
@A00721:190:HGJW7DSXY:4:1140:6280:1783 2:N:0:TCTGCAAG+GCTGCACT
AGCGTACGGAGGTGTCCCCGGCGCGGGCAAGCCAGTAATATTATATTGGCTTGCCCCACGGTCTGCGG
TAAGATGCCCCTGCGGGGCCTATGGAATTCTATTTGAATTTGAATTTGAATTGGTATACTGTAGGGCG
GGATATTTTCAAATT
+
FFFFFF:FFFFFFFFFFFFFFFFFFFFFFFFFFFFFFFFFF,FFFF:FFF:FFFFFFFF:FFFFFF:FF
FFFFFFFFFFFFFFFFFFF::FFFFFFFF,:FF:FF:FFFFF:FFFF,FFFFFFFFFFFFFFFFFFFF
FFFFFFFFFFFFFFF
@A00721:190:HGJW7DSXY:4:1239:2926:9815 2:N:0:TCTGCAAG+GCTGCACT
AGCGTACGGAGGTGTCCCCGGCGCGGGCAAGCCAGTAATATTATATTGGCTTGCCCCACGGTCTGCGG
TAAGATGCCCCTGCGGGGCCTATGGAATTCTATTTGAATTTGAATTTGAATTGGTATACTGTAGGGCG
GGATATTTTCAAATT
+
FFFFFFFFFFFFFFFFFFFFFFFFFFFFFFFFFFFFFFFFFFFFFFFFFFFFFFFFFFFFFF,F,FFFF
FFFF:FFFFFFFFFFFFFFF:FFFFFFFFFFF:FFFFFFFFFFF:FFFFFFFFFFF:FFFFFFFFFFF
FFF::FFFFF:FF,F
@A00721:190:HGJW7DSXY:4:1301:1389:25441 2:N:0:TCTGCAAG+GCTGCACT
AGCGTACGGAGGTGTCCCCGGCGCGGGCAAGCCAGTAATATTATATTGGCTTGCCCCACGGTCTGCGG
TAAGATGCCCCTGCGGGGCCTATGGAATTCTATTTGTATTTGAATTTGAATTGGTATACTGTAGGGCG
GGATATTTTCAAATT
+
FFFFFFFFFFFFFFFFFFFFFFFFFFFFFFF:F:FFFF::FFF,FFF:FFFFFFFFF,FFF:,:FFFF
FFFF:FFFFFF:FFFFFF,FF,FFFFFFF,F,F:FF,F,FFF,:FFF:FFFFFFF,F,FFFFF:FF:F
F:FF:FFFF:F:F,F
@A00721:190:HGJW7DSXY:4:1301:16278:35790 2:N:0:TCTGCAAG+GCTGCACT
AGCGTACGGAGGTGTCCCCGGCGCGGGCAAGCCAGTAATATTATATTGGCTTGCCCCACGGTCTGCGG
TAAGATGCCCCTGCGGGGCCTATGGAATTCTATTTGAATTTGAATTTGAATTGGTATACTGTAGGGCG
GGATATTTTCAAATT
+
FFFFFFFFFFFFFFFFFFFFFFFFFFFFFF::FFFFFFFFFFFFF:F:FF,F,FFFFFFFFF:FFFFF
F,FFFFFFFFFFFFFFFF:::FFFFFF:FFF,FFFF,:FFFF:FFFF:FFFFFFFFFF,FFFFFFFFF
FFFFFFFFF:FFFFF
@A00721:190:HGJW7DSXY:4:1310:21206:18270 2:N:0:TCTGCAAG+GCTGCACT
AGCGTACGGAGGTGTCCCCGGCGCGGGCAAGCCAGTAATATTATATTGGCTTGCCCCACGGTCTGCGG
TAAGATGCCCCTGCGGGGCCTATGGAATTCTATTTGAATTTGAATTTGAATTGGTATACTGTAGGGCG
GGATATTTTCAAATT
+
FFFFFFFFFFFFFFFFFFFFFFFFFFFFFF,FFFFFFFFFFFFFFFFFFFFFFFFFFFFFFFFFFFFF
:FFFFF:FFFFFFFFFFFFFFFFFFFFFFFFFFFFFFFFFFFFFFFFFFF:FFFFFFFFFFFFFFF
FFFFFFFFFFFFFFF
@A00721:190:HGJW7DSXY:4:1612:22869:5963 2:N:0:TCTGCAAG+GCTGCACT
AGCGTACGGAGGTGTCCCCGGCGCGGGCAAGCCAGTAATATTATATTGGCTTGCCCCACGGTCTGCGG

```
TAAGATGCCCCTGCGGGGCCTATGGAATTCTATTTGAATTTGAATTTGAATTGGTATACTGTAGGGCG
GGATATTTTCAAATT
+
FFFFFFFFFFFFFFFFFFFFFFFFFFFFFFFFF:FFFFFF,FFFFFFFFFFFFFFFFFFFFFF:FFFF
FFFFFFF:FFFFFFFFFFFF,FFFFFFFF:FFFF,F:FFFFFFFFFFFFFFFFFFFFFFFFFFFFFFF
FFFF:FFFFFFFFFF
@A00721:190:HGJW7DSXY:4:2145:16947:17065 2:N:0:TCTGCAAG+GCTGCACT
AGCGTACGGAGGTGTCCCCGGCGCGGGCAAGCCAGTAATATTATATTGGCTTGCCCCACGGTCTGCGG
TAAGATGCCCCTGCGGGGCCTATGGAATTCTATTTGAATTTGAATTTGAATTGGTATACTGTAGGGCG
GGATATTTTCAAATT
+
FFFFFFFFFFFFFFFFFFFFFFFFFFFFF:FFFFFFFFFF:FFFFFFFFFFFFFFFFFFFFFFFFFFF
:FFFFFFFFFFFFFFFFFFF,FFFFF,FFFFFFFFFFFFFF:FFFFFFFFFFFFFFFFFFFFFFFFFF
FFFFFFFFFFFFF:F
@A00721:190:HGJW7DSXY:4:2169:19307:31955 2:N:0:TCTGCAAG+GCTGCACT
AGCGTACGGAGGTGTCCCCGGCGCGGGCAAGCCAGTAATATTATATTGGCTTGCCCCACGGTCTGCGG
TAAGATGCCCCTGCGGGGCCTATGGAATTCTATTTGAATTTGAATTTGAATTGGTATACTGTAGGGCG
GGATATTTTCAAATT
+
FFFFFFFFFFFFFFFFFFFFFFFFFFFFFFFFFFF,F:FF:FFFFFFFF:FFFFFFFFFFFFFFFFFF
:FFFFFFF:FFFFFFFFFFFFFFFFFFFFFFFFFFFF:FFFFFFFFFFFFFFFFFFFFFFFFFFFFFF
FFF:FFFFFF:FF:F
@A00721:190:HGJW7DSXY:4:2336:22968:27023 2:N:0:TCTGCAAG+GCTGCACT
AGCGTACGGAGGTGTCCCCGGCGCGGGCAAGCCAGTAATATTATATTGGCTTGCCCCACGGTCTGCGG
TAAGATGCCCCTGCGGGGCCTATGGAATTCTATTTGAATTTGAATTTGAATTGGTATACTGTAGGGCG
GGATATTTTCAAATT
+
FFFFFFFFFFFFFFFFFFFFFFFFFFFFFFFFF:FFFFF:FFFFFFFFFFFFFFFFFFFFFFFF,FFFF
FFFFFFFFFFFFFFFFFFFFFFFFFFFFFFFFF:FFFF::FFFFFFFFFFF,F,F:FFFF:F:FF,:F,
FF,FFFFFFFFF::F
@A00721:190:HGJW7DSXY:4:2544:24153:5494 2:N:0:TCTGCAAG+GCTGCACT
AGCGTACGGAGGTGTCCCCGGCGCGGGCAAGCCAGTAATATTATATTGGCTTGCCCCACGGTCTGCGG
TAAGATGCCCCTGCGGGGCCTATGGAATTCTATTTGAATTTGAATTTGAATTGGTATACTGTAGGGCG
GGATATTTTCAAATT
+
FFFFFFFFFFFFFFFFFFFFFFFFFFFFFFFFFFFFFFFFFFFFFFFFFFFFFFFFF:FFFFFFFFFF
FFFF:FFFFFFF,FFFFFFFFFFFFFFFFF,F:F:FFF:FFFFFFFFFFFFFFFF,FFF:FFFFFFFF
FFFF,FFFFFFFFFF
@A00721:190:HGJW7DSXY:4:2544:27697:5682 2:N:0:TCTGCAAG+GCTGCACT
AGCGTACGGAGGTGTCCCCGGCGCGGGCAAGCCAGTAATATTATATTGGCTTGCCCCACGGTCTGCGG
TAAGATGCCCCTGCGGGGCCTATGGAATTCTATTTGAATTTGAATTTGAATTGGTATACTGTAGGGCG
GGATATTTTCAAATT
+
FFFFFFFFFFFFFFFFFFFFFFFFFFFFF:FFFFFFFFFFFFFFFFFFFFFFFFFFFFFFFFFFFFFF
FFFFFFFFFFFFFFFFFFFFFFFFFFFFFFFFFFFFFFFFFFFFFFFFFFFFFFFFFFFFFFFFFFFF
FFFFFFFFFFFFFFF
@A00721:190:HGJW7DSXY:4:1110:21540:10332 2:N:0:TCTGCAAG+GCTGCACT
CGTAAGCGTACGGAGGTGTCCCCGGCGCGGGCAAGCCAGTAATATTATATTGGCTTGCCCCACGGTCT
GCGGTAAGATGCCCCTGCGGGGCCTATGGAATTCTATTTGAATTTGAATTTGAATTGGTATACTGTAG
GGCGGGATATTTTCA
+
FFFFFFFFFFFFFFFFFFFFFFFFFFFFFFFFFF:FFF,FFFFFFFFFFFFF:FFFF,FFFFFFFFFF:
FFFFFFFF:FFFFFFFFFFFFFFF,FFFFFFFFFFFF:FFFF,FFFFFFFFFFFFFFFFFFFF,FFFFF
F:FFFFFFFFFFFFF
```

@A00721:190:HGJW7DSXY:4:1120:28022:24815 2:N:0:TCTGCAAG+GCTGCACT
CGTAAGCGTACGGAGGTGTCCCCGGCGCGGGCAAGCCAGTAATATTATATTGGCTTGCCCCACGGTCT
GCGGTAAGATGCCCCTGCGGGGCCTATGGAATTCTATTTGAATTTGAATTTGAATTGGTATACTGTAG
GGCGGGATATTTTCA
+
FFFFFFFFFFFFFFFFFFFFFFFFFFFFFFFFFF:FFF:F:FFFFFFFFFFF:FFFFFFFFFFFFFFFFF
FFFFFFFFFFFFFFFFFFFFFFFF,F,FFFFFFFF:FFFFFFFFFFFF:FFFFFFFFFFFFFF,FFFF
FFFFFFFFFFFFFFF
@A00721:190:HGJW7DSXY:4:1301:8196:9048 2:N:0:TCTGCAAG+GCTGCACT
CGTAAGCGTACGGAGGTGTCCCCGGCGCGGGCAAGCCATTAATATTATATTGGCTTGCCCCACGGTCT
GCGGTAAGATGCCCCTGCGGGGCCTATGGAATTCTATTTGAATTTGAATTTGAATTGGTATACTGTAG
GGCGGGATATTTTCA
+
FFFF:FFFFFFF:FFF:FFFFFFFFFFFFFFFFF,FFF,,FFFF,:FFF:FFF:,FF:FF,F:FFF:F
F,FF,FFFFF:FFFF,:,,F:F::::F,FF:F:,,F,FFF:,,FFF,::F:F:FFFF:,F:F,:F,F,
FF,F:F,FFFFFF::
@A00721:190:HGJW7DSXY:4:1531:17472:19570 2:N:0:TCTGCAAG+GCTGCACT
CGTAAGCGTACGGAGGTGTCCCCGGCGCGGGCAAGCCAGTAATATTATATTGGCTTGCCCCACGGTCT
GCGGTAAGATGCCCCTGCGGGGCCTATGGAATTCTATTTGAATTTGAATTTGAATTGGTATACTGTAG
GGCGGGATATTTTCA
+
FFFFFFFFFFFFF:FFFFFFFFFFFFFFFFFFFFFFFFFFFFFFFFFFFFFFF:FFFFFFFFFFFFFF
FFFFFFFFFFF:FFFFFFFFFFFFFF,FFF:FFFFF:FFFFFFFFFFFFFFFFFFFFFFFFFFFFFFF:
FFFFFFFFFFFFFFF
@A00721:190:HGJW7DSXY:4:1641:27218:24205 2:N:0:TCTGCAAG+GCTGCACT
CGTAAGCGTACGGAGGTGTCCCCGGCGCGGGCAAGCCAGTAATATTATATTGGCTTGCCCCACGGTCT
GCGGTAAGATGCCCCTGCGGGGCCTATGGAATTCTATTTGAATTTGAATTTGAATTGGTATACTGTAG
GGCGGGATATTTTCA
+
FFFFFFFFFFFFFFFFFFFFFFFFFFFFFFFFFFFFFFFFFFFFFFF:FFFFFFFFFFFFFFFFFFFF
FFFFFFFF:FFFFFFFFFFFFFFFFFFFFFF,FF:FF,FFFF:F:FFF:F:F:FFFFFFF,FFFFFFFF
FFFFFFFFFFFFFFF
@A00721:190:HGJW7DSXY:4:2259:26449:7279 2:N:0:TCTGCAAG+GCTGCACT
CGTAAGCGTACGGAGGTGTCCCCGGCGCGGGCAAGCCAGTAATATTATATTGGCTTGCCCCACGGTCT
GCGGTAAGATGCCCCTGCGGGGCCTATGGAATTCTATTTGAATTTGAATTTGAATTGGTCTACTGTAG
GGCGGGATATTTTCA
+
FFFFFFFFFFFFF::FFFFF:F:FFF,F:FFFFFFFFFFFFF:FFFFFFF:FFF:FFFFF:FFFFFF,
:F,FFFFFFF,FFFFF:F:FFFF:FFFFFFFF:,:F:F,,:F,F,,FFFFFFFFFF::F,F:,FFFFF
,FFFFFFFFFFFFF:
@A00721:190:HGJW7DSXY:4:1541:22037:30514 2:N:0:TCTGCAAG+GCTGCACT
CGTAAGCGTACGGAGGTGTCCCCGGCGCGGGCAAGCCAGTAATATTATATTGGCTTGCCCCACGGTCT
GCGGTAAGATGCCCCTGCGGGGCCTATGGAATTCTATTTGAATTTGAATTTGAATTGGTATACTGTAG
GGCGGGATATTTTC
+
FFFFFFFFFFFFFFFFFFFFFFFFFFFFFFFFFFFFFFFFFFFFFFF,F,FFFF,FFFFFF:FFFFFF
F:FFFFFFFFFFFFFFFFFFFFFFFFFFFFF:FF:FFFFFF,FFFFFFFFFFFFF,FFFFFFF:FFF:F
FFFFFFFFFFFFFF
@A00721:190:HGJW7DSXY:4:1201:6406:36824 2:N:0:TCTGCAAG+GCTGCACT
TGACAAATCGACACGTAAGCGTACGGAGGTGTCCCCGGCGCGGGCAAGCCAGTAATATTATATTGGCT
TGCCCCACGGTCTGCGGTAAGAAGCCCCAGCGGGGCCTATGGAATTCTATTTGAATTTGAATTTGAAT
TGGTATACTGTAGGG
+
FFFFFFFFFFFFFFFFFFFFFFFFFFFFFFFFFFFFF:FFFFFFFFFFFFFFFFFFFFFFFFFFFF:F,

F,FFFFF,FFFFFFFFF:FFFF,FFFFF,FFFFFFFFFFFF:F:FFF:F:,FFFF,FF:F::FFFFF,
FFFF:FF:,FFFF,F
@A00721:190:HGJW7DSXY:4:1228:23647:17989 2:N:0:TCTGCAAG+GCTGCACT
TGACAAATCGACACGTAAGCGTACGGAGGTGTCCCCGGCGCGGGCAAGCCAGTAATAATATATTTGCT
TGCCCCACGGTCTGCGGGAAGATGCACCTGCGGGGCCTATGGAATTCTATTTGAATTTGAATTTTAAT
TGGTATACTGTAGGG
+
FFFFFFFF:FF,FFFFFFF::FF,F:FFFFF:F:F,F:FFFFF,FFF,F:F,FFF:F,FF,FFF,FFF
F:,:FF:FF,:,F,,F:,FF:FFF:,FF:FFFF::FF,:,:FFF:FFFF:FF,FFFFFFFF,FF,FF,
F,:FF,:F:FFF,:F
@A00721:190:HGJW7DSXY:4:1305:31286:30655 2:N:0:TCTGCAAG+GCTGCACT
TGACAAATCGACACGTAAGCGTACGGAGGTGTCCCCGGCGCGGGCAAGCCAGTAATATTATATTGGCT
TGCCCCACGGTCTGCGGTAAGATGCCCCTGCGGGGCCTATGGAATTCTATTTGAATTTGAATTTGAAT
TGGTATACTTTAGGG
+
FF:FFFFFFF:F,:FFFFFFFFFFF,FFFFFFFFF:,FFFF:FFF:F:FFFFF,FFFFFFF,FF,:FF
F:,FFFFFF:FFF:::F,,FF:F::FFF,FFF:FF::,F,FFFF:F,FFFFFF:F,:::F:FFF:FF,
F:FF,FF,,,FFF,F
@A00721:190:HGJW7DSXY:4:1430:28971:2816 2:N:0:TCTGCAAG+GCTGCACT
TGACAAATCGACACGTAAGCGTACGGAGGTGTCCCCGGCGCGGGCAAGCCAGTAATATTATATTGGCT
TGCCCCACGGTCTGCGGTAAGATGCCCCTGCGGGGCCTATGGAATTCTATTTGAATTTGAATTTGAAT
TGGTATACTGTAGGG
+
FFFFFFFFFF:FFFFFFFFFFFFF,FFFFFFFFFFF:FFFFFFFFFFFFFFFFFFFFFFFFFFFFFFF
F:FFFFFFFFF:,FFFFFFFFFFFFFFFFFFFFFFFFFFFFFFFFF:FFFFFFFFFFFF:FFFFFFFF
FFF:FFF:FFFFFFF
@A00721:190:HGJW7DSXY:4:1472:9751:28401 2:N:0:TCTGCAAG+GCTGCACT
TGACAAATCGACACGTAAGCGTACGGAGGTGTCCCCGGCGCGGGCAAGCCAGTAATATTATATTGGCT
TGCCCCACGGTCTGCGGTAAGATGCCCCTGCGGGGCCTATGGAATTCTATTTGAATTTGAATTTGAAT
TGGTATACTGTAGGG
+
FFFFFFFFFFFFFFFFFFFFFFFFFFFFFFFFFFFFFFFFFFFFFFFFFFFFFFFFFFFFFF,FFFFF
FFFFFFFFFFFFFFFFF:FFF:FFFFFF,:FFFFFFF:,FFFFFFFFFFFFF:FFFFFFFFFFFFFFF
FFFFFF:FFFFFFFF
@A00721:190:HGJW7DSXY:4:1475:29469:6684 2:N:0:TCTGCAAG+GCTGCACT
TGACAAATCGACACGTAAGCGTACGGAGGTGTCCCCGGCGCGGGCAAGCCAGTAATATTATATTGGCT
TGCCCCACGGTCTGCGGTAAGATGCCCCTGCGGGGCCTATGGAATTCTATTTGAATTTGAATTTGAAT
TGGTATACTGTAGGG
+
FFFFFFFFFFFF:FFFFFFFFFFFFFFFFFFFFFFFFFFFFFFFFF::F,FFFFFF:FFFFFFFFFFF
FFFFFFFFFFFFFFFFF:FFFFFFFFFFFFFFFFFFF,FFFFFFFFFFFFFFFFFFFFF:FFFFFFFF
F:FFFFFF:FFFFFF
@A00721:190:HGJW7DSXY:4:1577:21169:5431 2:N:0:TCTGCAAG+GCTGCACT
TGACAAATCGACACGTAAGCGTACGGAGGTGTCCCCGGCGCGGGCAAGCCAGTAATATTATATTGGCT
TGCCCCACGGTCTGCGGTAAGATGCCCCTGCGGGGCCTATGGAATTCTATTTGAATTTGAATTTGAAT
TGGTATACTGTAGGG
+
:FFFFFFFFFFFFFFFFF:FFFFFF:FFFFFFF:FFFFFFFFFFFFF:FFFFFFFFFFFFFFFFFFFF
F:FFFFFFFFFFFFFFF:FFFFFFF:FFFFFFFFFF:FFFFFF:FFFFF,F:FFFFFFFFFFFFFFFF
FFFFFFF:FFFFF,:
@A00721:190:HGJW7DSXY:4:1635:8205:22874 2:N:0:TCTGCAAG+GCTGCACT
TGACAAATCGACACGTAAGCGTACGGAGGTGTCCCCGGCGCGGGCAAGCCCGTAATATTATATTGGCT
TGCCCCACGGTCTGCGGTAAGATGCCCCTGCGGGGCCTATGGAATTCTATTTGAATTTGAATTTGAAT
TGGTATACTGTAGGG

+
FFFFFFFFFFFFFFFFFFFFFFFFFFFFF:FFFFFFFFFFFFFFFFFFFF,FFFF,FFF,FFFFFFFF
FFFFFFFFFFF,,F,FFF,FFFFFFFFFFFFFFFFFF::FFFFFFFFFFFFFFFF:F,FF,FFFFFFF
FFFF:FFFFFFFF:F
@A00721:190:HGJW7DSXY:4:2108:14425:34554 2:N:0:TCTGCAAG+GCTGCACT
TGACAAATCGACACGTAAGCGTACGGAGGTGTCCCCGGCGCGGGCAAGCCAGTAATATTATATTGGCT
TGCCCCACGGTCTGCGGTAAGATGCCCCTGCGGGGCCTATGGAATTCTATTTGAATTTGAATTTGAAT
TGGTATACTGTAGGG
+
FFFFFFFFFFFFFFFFFFFFFFFFFFFFFFFFFFFFFFFFFFFFFFFFFFFFFFFFFFFFFFFFFFFF
FFFFFFFFFFFFFFFFFFFFFF:FFFFFFFFFFFFFFF,FFFFFFFFFFFF,FFFFFFFFFFFFFFFF:
FFFFFFFFFFFF:FF
@A00721:190:HGJW7DSXY:4:2211:19777:13135 2:N:0:TCTGCAAG+GCTGCACT
TGACAAATCGACACGTAAGCGTACGGAGGTGTCCCCGGCGCGGGCAAGCCAGTAATATTATATTGGCT
TGCCCCACGGTCTGCGGTAAGATGCCCCTGCGGGGCCTATGGAATTCTATTTGAATTTGAATTTGAAT
TGGTATACTGTAGGG
+
FFFFFFFFFFFFFFFFFFFFF:FFFFFFFFFFFFFFFFFFFFFFFFFF:FFFFFFFF:FFFF:,FFFF:F
FFFFFFFF:FFFFFFFFFFFFFFFFFFFFFFFFFFFFFFFFFFFF:FFFFFFFFFF::FFFFFFFFFFF:
FFFFFF:F,FFFFFF
@A00721:190:HGJW7DSXY:4:2222:9281:5760 2:N:0:TCTGCAAG+GCTGCACT
TGACAAATCGACACGTAAGCGTACGGAGGTGTCCCCGGCGCGGGCAAGCCAGTAATATTATATTGGCT
TGCCCCACGGTCTGCGGTAAGATGCCCCTGCGGGGCCTATGGAATTCTATTTGAATTTGAATTTGAAT
TGGTATACTGTAGGG
+
FFFFFFFFFFFFFFFFFFFFFFFFFFFFFFFFFFFFFFFFFFFFFFFFFFFFFFFFFFFFFF:FFFFFF
FFFFFFFFFFFFFFFFFFFFFFFFFFFFFFFFFFFFFFFFFFFFFFFFFFFFFFFF:FF:FFFF:FFFFFF
FFFFFFF:FFFF:FF
@A00721:190:HGJW7DSXY:4:2226:21025:23030 2:N:0:TCTGCAAG+GCTGCACT
TGACAAATCGACACGTAAGCGTACGGAGGTGTCCCCGGCGCGGGCAAGCCAGTAATATTATATTGGCT
TGCCCCACGGTCTGCGGTAAGATGCCCCTGCGGGGCCTATGGAATTCTATTTGAATTTGAATATGAAT
TGGTATACTGTAGGG
+
FFFFFFFFF,FFFFFFFFFF:FFFF:FFFFFF:FFFFFF:FFFFFFF:FFF::FFFF:,FFFF:FF:F
F:,FFFFF,,FF,::FFFFFFFFFF:FFF:FF:FF:,FFFF:FF,,,:FF:FFFFF:,:FF:,FFFF:
:,,:,FF,FF:FF,F
@A00721:190:HGJW7DSXY:4:2236:6207:29277 2:N:0:TCTGCAAG+GCTGCACT
TGACAAATCGACACGTAAGCGTACGGAGGTGTCCCCGGCGCGGGCAAGCCAGTAATATTATATTGGCT
TGCCCCACGGTCTGCGGTAAGATGCCCCTGCGGGGCCTATGGAATTCTATTTGAATTTGAATTTGAAT
TGGTATAATGTAGGG
+
FFFFFFFFFFFFF:FFFFFF,FFFF:FFFFFF:FFFFF:FFFF:FFFFFFFFFFF,F,FFFFFF:::F
FF:FFFF:FF:FFF,:FF:FFFFF:FFF:FFFFFFFFFF:FFFFFF,FFF:FFF:FFF,F:FFFF,F,
F::FFF:,:FFFF,F
@A00721:190:HGJW7DSXY:4:2655:29053:6339 2:N:0:TCTGCAAG+GCTGCACT
TGACAAATCGACACGTAAGCGTACGGAGGTGTCCCCGGCGCGGGCAAGCCAGTAATATTATATTGGCT
TGCCCCACGGTCTGCGGTAAGATGCCCCTGCGGGGCCTATGGAATTCTATTTGAATGTGAATTTGAAT
TGGTATACTGTAGGG
+
FFFFFFFFFFFFFFFFFFFFFFF:FFFFFFFFFFFFFFFFFFFFFFFFFF:FFFFFFFFF::FFF,FFFFF
FFFFFFFFFFFFFFFFFFFFFFFFFFFFFFFFFFFFFFFFFFFFFFFFF::F,FFFFF,FFFFFFF,FFF
F,FFFFFFFFFFF:F
@A00721:190:HGJW7DSXY:4:1336:25464:1720 2:N:0:TCTGCAAG+GCTGCACT
GACAAATCGACACGTAAGCGTACGGAGGTGTCCCCGGCGCGGGCAAGCCAGTAATATTATATTGGCTT

```
GCCCCACGGTCTGCGGTAAGATGCCCCTGCGGGGCCTATGGAATTCTATTTGAATTTGAATTTGAATT
GGTATACTGTAGGAG
+
FFFFFFFFFFFFFFFFFFFFFFFFFFFFFFFFFFFFFFFFFFFFFFFFFFFFFFFFFFFFFFFFFFFF
FFFFFFFFFFFFF:FFFFFF:FFFFFFFFFFFFFFFFF:FFFFFF:FFFFFFFFF:FFFFFFFFFFFF
FFFFFF,FFFFFFFF
@A00721:190:HGJW7DSXY:4:1371:16676:26678 2:N:0:TCTGCAAG+GCTGCACT
GACAAATCGACACGTAAGCGTACGGAGGTGTCCCCGGCGCGGGCAAGCCAGTAATATTATATTGGCTT
GCCCCACGGTCTGCGGTAAGATGCCCCTGCGGGGCCTATGGAATTCTATTTGAATTTGAATTTGAATT
GGTATACTGTAGGAG
+
FFFFFFFFFFFFFFFFFFFFFFFFFFFFFFFFFFFFFFFFFFFFFFFFFFFFFFFF:FFFFFFFFFFF
FFFFFFFFFFFFFFFFFFFFFFFFFFFFFFFFFFFF:FFFFFFFFFFFFFFFFFFFFFFFFFFFFFFF
FFFFFFFFFFFFFFF
@A00721:190:HGJW7DSXY:4:1430:18927:10567 2:N:0:TCTGCAAG+GCTGCACT
GACAAATCGACACGTAAGCGTACGGAGGTGTCCCCGGCGCGGGCAAGCCAGTAATATTATATTGGCTT
GCCCCACGGTCTGCGGTAAGATGCCCCTGCGGGGCCTATGGAATTCTATTTGAATTTGAATTTGAATT
GGTATACTGTAGGAG
+
FFFFFFFFFFFFFFFFFFFFFFFFFFFFFFFFFFFFFFFFFFFFFFFFFFFFFFFF:FFFFFFFFFFF
FFFFFFFFFF:FFFFFFFFFFFFFFFFFFFFFFFFFFFFFFFFFF,F:FFFFFFFFFF,FFFFFFFFF
:FF:FF,FFFFFFFF
@A00721:190:HGJW7DSXY:4:1530:3938:2487 2:N:0:TCTGCAAG+GCTGCACT
GACAAATCGACACGTAAGCGTACGGAGGTGTCCCCGGCGCGGGCAAGCCAGTAATATTATATTGGCTT
GCCCCACGGTCTGCGGTAAGATGCCCCTGCGGGGCCTATGGAATTCTATTTGAATTTGAATTTGAATT
GGTATACTGTAGGAG
+
FFFFFFFFFFFFFFFFFFFFFFFFFFFFFFFFFFFFFFFFFFFFFFFFFFFFFFFFFFFFFFFFFFFF
FFFFFFFFFFFFFFFF:FFFFFFFFFFFFFFFFFFFF::FFFFFFFFFFFFF::FFFF:FFFFFFFFF
FFFFFFFFFFFFFFF
@A00721:190:HGJW7DSXY:4:1677:20799:11522 2:N:0:TCTGCAAG+GCTGCACT
GACAAATCGACACGTAAGCGTACGGAGGTGTCCCCGGCGCGGGCAAGCCAGTAATATTATATTGGCTT
GCCCCACGGTCTGCGGTAAGATGCCCCTGCGGGGCCTATGGAATTCTATTTGAATTTGAATTTGAATT
GGTATACTGTAGGAG
+
FFFFFFFFFFFFFFFFFFFFFFFFFFFFFFFFFFFFFFFFFFFFFFFFFFFFFFFFFFFFFFFFFFFF
FFFFFFFFFFF,FFFFF:FFFFFFFFFFFFFFFFFF,FFFFF::FFFFF::FFFFFFFFFFFFFFFFF
FFFFFFFFFFFFFFF
@A00721:190:HGJW7DSXY:4:2458:3224:24142 2:N:0:TCTGCAAG+GCTGCACT
GACAAATCGACACGTAAGCGTACGGAGGTGTCCCCGGCGCGGGCAAGCCAGTAATATTATATTGGCTT
GCCCCACGGTCTGCGGTAAGATGCCCCTGCGGGGCCTATGGAATTCTATTTGAAGTTGAATTTGAATT
GGTATACTGTAGGAG
+
FFFFFF:FFFFFFFFFFFFFFFFF:FFFFFFF:FF:FFFFFFF:FFF:F::F:F:,:F:,:,F:FFFF
,,FFF:FFF:F,FFFF:F::F:F,FFFFFFFFFFFFFF,FFF,,FFF:F,FF::,FFFFF:,FFF::F
FFF,F:FFFFFFFFF
@A00721:190:HGJW7DSXY:4:1237:4661:7310 2:N:0:TCTGCAAG+GCTGCACT
GACAAATCGACACGTAAGCGTACGGAGGTGTCCCCGGCGCGGGCAAGCCAGTAATATTATATTGGCTT
GCCCCACGGTATGCGGTAAGATGCCCCTGCGGGGCCTATGGAATTCTATTTGAATTTGAATTTGAATT
GGTATACTGTAGGA
+
FFFFFFFFFFFFFFFFFFFF:FFF:FFFFFFFFFFFFFFFFFFFFFF:FF,FF:FFFFF,FFFFFFF,
,FF:FFFFFF,FFFFFFF:FF::FFFFFFFFFFFF:F:FFFFFF::FF,FFF:F,F:FFF,FFFFF:F
FFF:,::,F,:FFF
```

@A00721:190:HGJW7DSXY:4:1325:24822:5431 2:N:0:TCTGCAAG+GCTGCACT
AATGACAAATCGACACGTAAGCGTACGGAGGTGTCCCCGGCGCGGGCAAGCCAGTAATATTATATTGG
CTTGCCCCACGGTCTGCGGTAAGATGCCCCTGCGGGGCCTATGGAATTCTATTTGAATTTGAATTTGA
ATTGGTATACTGTAG
+
FFFFFFFFFFFFFFFFFFFFFFFFFFFFFFFFFFFFFFFFFFFFFFFFFFFFFFFFFFFFFFFFFFFF
FFFFFFFFFFFFFF:FFFFFFFFFFFFFFFFFFFFFFFF,:FFFFFFFFFFFFFFFFFFFFFFFFFFF
F,FFFFFFFFFFFFF
@A00721:190:HGJW7DSXY:4:2369:7148:3223 2:N:0:TCTGCAAG+GCTGCACT
AATGACAAATCGACACGTAAGCGTACGGAGGTGTCCCCGGCGCGGGCAAGCCAGTAATATTATATTGG
CTTGCCCCACGGTCTGCGGTAAGATGCCCCTGCGGGGCCTATGGAATTCTATTTGAATTTGAATTTGA
ATTGGTATACTGTAG
+
FFFFFFFFFFFFFFFFFFFFFFFFFFFFFFFFFFFFFFFFFFFFFFFFFFFFFFFFF:FFFFFFFFFF
FFFFFFFFFFFFFFFFFFF:FFFFFFFFFFFFFFFFFFF,F,FFFFFFF:FFFFFFFFFFFFF:FFFF
FFFF:FFFFFFFFFF
@A00721:190:HGJW7DSXY:4:1504:2862:4946 2:N:0:TCTGCAAG+GCTGCACT
CTAATGACAAATCGACACGTAAGCGTACGGAGGTGTCCCCGGCGCGGGCAAGCCAGTAATATTATATT
GGCTTGCCCCACGGTCTGCGGTAAGATGCCCCTGCGGGGCCTATGGAATTCTATTTGAATTCGAATTT
GAATTGGTATACTGT
+
FFFFFFFFFFFFFFFFFFFF:FFFFFFFFFFFFFFFFFFFFFFFFFFFFFF::FF,,FFFFF::::FF
,FFFF:FFFFFFFFFFFF,FF,FFFFFFFFFF,FFFFFFFFF:FFFFFFFF,F,:FFFFFFFF:FFFF
FFFFF:FFFFFF:FF
@A00721:190:HGJW7DSXY:4:2120:31611:18693 2:N:0:TCTGCAAG+GCTGCACT
CTAATGACAAATCGACACGTAAGCGTACGGAGGTGTCCCCGGCGCGGGCAAGCCAGTAATATTATAGT
GGCTTGCCCCACGGTCTGCGGTAAGATGCCCCTGCGGGGCCTATGGAATTCTATTTGAATTCGAATTT
GAATTGGTATACTGT
+
FFFFFFFFFF:FFFFFFFFFFFFFFFFFFF:FFFFFFFFFFFFFFFFFFFFFFFFFFF:F::F:FF,F
FFFFFFFFFFFFFF:F:FFFFFF:F,F::FFFFFFF:FFF:FFFFFFF:F:,FF:FFFF:FFF:,FFF
FFFFFFFFFFFFFFF
@A00721:190:HGJW7DSXY:4:2242:18105:7482 2:N:0:TCTGCAAG+GCTGCACT
CTAATGACCAATCGACACGTAAGCGTACGGAGGTGTCCCCGGCGCGGGCAAGCCAGTAATATTATATT
GGCTTGCCCCACGGTCTGCGGTAAGATGCCCCTGCGGGGCCTATGGAATTCTATTTGACTTCGAATTT
GAATTGGTATACTGT
+
FFFFFFFF,:FFFFFFFFFFFFFF:FFFFFFFFFFFFFFFF,FFFFFFFF,FFF,F:F:F,:F,FF:F
F:FFFFFFFFFFFF:F,F,FFF:FFFFFFFFF:FFFFFFFF:FFFFF,:FF,FF,F:F,::FFF,FFF
FFF,,,FF:FFFFFF
@A00721:190:HGJW7DSXY:4:2316:30120:26694 2:N:0:TCTGCAAG+GCTGCACT
CTAATGACAAATCGACACGTAAGCGTACGGAGGTGTCCCCGGCGCGGGCAAGCCAGTAATATTATATT
GGCTTGCCCCACGGTCTGCGGTAAGATGCCCCTGCGGGGCCTATGGAATTCTATTTGAATTCGAATTT
GAATTGGTATACTGT
+
FFFFFFFFFFFFFFFFFFFFFFFFFFFFFFFFFFFFFFFFFFFFFFFFFFFFFF,FFFFF,FFF:FFF
FFFFFFFFFFFFFFF:FFFFFFFFFFFFFFFFF:FFFFFFFFFFFFFFFFFFF:FFFFFFFFFFFFFF
FFFFFFFFFFFFFFF
@A00721:190:HGJW7DSXY:4:2641:6515:33818 2:N:0:TCTGCAAG+GCTGCACT
CTAATGACAAATCGACACGTAAGCGTACGGAGGTGTCCCCGGCGCGGGCAAGCCAGTAATATTATATT
GGCTTGCCCCACGGTCTGCGGTAAGATGCCCCTGCGGGGCCTATGGAATTCTATTTGAATTCGAATTT
GAATTGGTATACTGT
+
FFFFFFFFFFFFFFFFFFFFFFFFFFFFFFFFFFFFFFFFFFFFFFFFFF,FFFF,FFFFFFFFF:FF

FFFFFFFFFFFFFFFFFFFFFFFFFFFFFFFFFFFFFFFF:FFFFFFF:F:::FFFFFFFFFFFFFF
FFFF:FF:FFFFFFF
@A00721:190:HGJW7DSXY:4:2677:17833:23797 2:N:0:TCTGCAAG+GCTGCACT
CTAATGACAAATCGACACGTAAGCGTACGGAGGTGTCCCCGGCGCGGGCAAGCCAGTAATATTATATT
GGCTTGCCCCACGGTCTGCGGTAAGATGCCCCTGCGTGGCCTATGGAATTCTATTTGAATTAGAATTT
GAATTGTTATACTGT
+
FFFFF,F,FFFFF:FFFF,:,F,F:,FF,FFFFF,F,,FFF:F,F:::,FF:FF:F:FF,FF,FFFFF
,FF:,:FFFFFFFFF,,,FFFFF,FF,:::F::,F:,F:FF:FF,FFFFFFFFFF:FFF,,,F,,,FF
,FFFFF,FFF:,FFF
@A00721:190:HGJW7DSXY:4:2677:17815:23954 2:N:0:TCTGCAAG+GCTGCACT
CTAATGACAAATCGACACGTAAGCGTACGGAGGTGTCCCCGGCGCGGGCAAGCCAGTAATATTATATT
GGCTTGCCCCACGGTCTGCGGTAAGATGCCCCTGCGGGGCCTATGGAATTCTATTTGAATTCGAATTT
GAATTGGTATACTGT
+
FFFFFFFFFFFFFFFFFFFFFFFFFFFFFFFFFFFFFFFFFFFFFFFFFFFFFFFFFFFFFFFFFFFF
FFFFFFFFFFFFFFFFFFFFFFFFF:FFFFFFFFFFFFFFFFFFFFFFFFFFFFFFFFF:FFFFFFFFF
F:FFFFFFFFFFFFF
@A00721:190:HGJW7DSXY:4:1341:7229:5274 2:N:0:TCTGCAAG+GCTGCACT
TCTAATGACAAATCGACACGTAAGCGTACGGAGGTGTCCCCGGCGCGGGCAAGCCAGTAATATTATAT
TGGCTTGCCCCACGGTCTGCGGTAAGATGCCCCTGCGGGGCCTATGGAATTCTATTTGAATTTGAATT
TGAATTGGTATACTG
+
FFFFFFFFFFFFFFFFFFFFFFFFFFFFFFFFFFFFFFFFFFFFFFFFFFFFFF,F:F:FFFFF,F:
FFFFFFFFFFF,FFFFFFFFFFFFFFFFFFFFFFFFFFFFFFF:F:FFFF:FFF:F:FFF::FFF:FFF
FFF:FFFFFFFFFFF
@A00721:190:HGJW7DSXY:4:1403:10529:8547 2:N:0:TCTGCAAG+GCTGCACT
TCTAATGACAAATCGACACGTAAGCGTACGGAGGTGTCCCCGGCGCGGGCAAGCCAGGAATATTATAT
TGGCTTGCCCCACGGTCTGCGGTAAGATGCCCCTGCGGGGCCTATGGAATTCTATTTGAATTTGAATT
TGAATTGGTATACTG
+
F:FFFFFFFFFFFFFFFFFFFFFFFFFFFFFFFFFFFFFFFFFF:FFFFF,F:FF:F,FF:F,FF:F:
F,FFFFFFFFF:FFFF,,FFFFF,:FFFF:FFFFFFFFFF:F:FF:FFF,F,FF,FFFFFFF::FFFF
FF,:FFFFFFFFFFF
@A00721:190:HGJW7DSXY:4:1545:20302:6402 2:N:0:TCTGCAAG+GCTGCACT
TCTAATGACAAATCGACACGTAAGCGTACGGAGGTGTCCCCGGCGCGGGCAAGCCAGTAATATTATAT
TGGCTTGCCCCACGGTCTGCGGTAAGATGCCCCTGCGGGGCCTATGGAATTCTATTTGAATTTGAATT
TGAATTGGTATACTG
+
FFFFFFFFFFFFFFFFFFFFFF:FFFFFFFFFFFFFFFFFFFFFFFFFFFFFFFFFFFFFFF:FFFF:
FFFFFFFFFFFFFFFF::F,FFFFFFFFFFFFFFFFFFFFFF:FFFFFF:F,FFF,FFF:FF:FF,FF
FF:F:FFFFFFFFFF
@A00721:190:HGJW7DSXY:4:2148:22905:16611 2:N:0:TCTGCAAG+GCTGCACT
TCTAATGACAAATCGACACGTAAGCGTACGGAGGTGTCCCCGGCGCGGGCAAGCCAGTAATATTATAT
TGGCTTGCCCCACGGTCTGCGGTAAGATGCCCCTGCGGGGCCTATGGAATTCTATTTGAATTTGAATT
TGAATTGGTATACTG
+
FFFFFFFFFFFFFFFFFFFFFFFFFFFFFFFFFFFFFFFFFFFFFFFFFFFFFFFFFF,FFFFF:FFF
FFFF:FFFFFFFFFFFFFFFFFFFFF:FFFFFFFFFFFFFFFFFFFFF,FFFFFFFFFFFFFFFFF:F
FFFF:FFFFFFFFFF
@A00721:190:HGJW7DSXY:4:2506:8377:16219 2:N:0:TCTGCAAG+GCTGCACT
TCTAATGACAAATCGACACGTAAGCGTACGGAGGTGTCCCCGGCGCGGGCAAGCCAGTAATATTATAT
TGGCTTGCCCCACGGTCTGCGGTAAGATGCCCCTGCGGGGCCTATGGAATTCTATTTGAATTTGAATT
TGAATTGGTATACTG

```
+
FFFFFFFFFFFFFFFFFFFFFFFFFFFFFFFFFFFFFFFFFFFFFFFFFFFFFFFFFFFFFFFFFFFFFFFFF
FFFF:F:FFFFFFFFF:FFFFFFFFFF::FFFF:FFFFFFFF,F:FFFFFFFFF:FFFFFFFFFFFFF
F,:FFF,FFFFFFFF
@A00721:190:HGJW7DSXY:4:1344:4562:35133 2:N:0:TCTGCAAG+GCTGCACT
GTCTAATGACAAATCGACACGTAAGCGTACGGAGGTGTCCCCGGCGCGGGCAAGCCAGTAATATTATA
TTGGCTTGCCCCACGGTCTGCGGTAAGATGCCCCTGCGGGGCCTATGGAATTCTATTTGAATTTGAAT
TTGAATTGGTAAGAT
+
FFFFFFFFFFFFFFFFFFFFFFFFFFFFFFFFFFFFFFFFFFFFFFFFF,FFFFFFFFFF::FFF
FFFFFFFFFFFFFFFFFFFFFFFFFFFFFFFFFFFFFFFFFF:,FFFFFFF:F,F,FFFF,F:FF:F
FFFFF,FFFFFF:FF
@A00721:190:HGJW7DSXY:4:1648:5692:33144 2:N:0:TCTGCAAG+GCTGCACT
GTCTAATGACAAATCGACACGTAAGCGTACGGAGGTGTCCCCGGCGCGGGCAAGCCAGTAATATTATA
TTGGCTTGCCCCACGGTCTGCGGTAAGATGCCCCTGCGGGGCCTATGGAATTCTATTTGAATTTGAAT
TTGAATTGGTAAGAT
+
FFFFFFFFFFFFFFFFFFFFFF:FFFFFFFFFFFFFFFFFFFFFFFFFFFFFFFFFFFFFFFFFFFFF
,FFFFFFFFFFFFFFFFFFFFFFF:FF,FFFFFFFFFFFFFFF:FFFFF,FFFFF:FFF:FF,FFF,F
FFFF::FFFFFFFFF
@A00721:190:HGJW7DSXY:4:2648:7780:27336 2:N:0:TCTGCAAG+GCTGCACT
GTCTAATGACAAATCGACACGTAAGCGTACGGAGGTGTCCCCGGCGCGGGCAAGCCAGTAATATTATA
TTGGCTTGCCCCACGGTCTGCGGTAAGATGCCCCTGCGGGGCCTATGGAATTCTATTTGAATTTGAAT
TTGAATTGGTAAGAT
+
FFFFFFFFFFFFFFFFFFFFFFFFFFFFFFFFFFFFFFFFFFFFFFFFFFFFFFFFFFFF,FFFFFFF
FFFFFFFFFFFFFFFFFFFFFFFFFFFFFFFFFFFFFFFFFFFFF,FFFFFFFFFFFFFFFF:FFFFFFF
FFFFFFFFFFFFFFF
@A00721:190:HGJW7DSXY:4:2239:9869:26976 2:N:0:TCTGCAAG+GCTGCACT
CGCGTGGTCTAATGACAAATCGACACGTAAGCGTACGGAGGTGTCCCCGGCGCGGGCAAGCCAGTAAT
ATTATATTGGCTTGCCCCACGGTCTGCGGTAAGATGCCCCTGCGGGGCCTATGGAATTCTATTTGAAT
TTGAATTTGAATTGG
+
FFFFFFFFFFFFFFFFFFFFFFFFFFFFFFFFFFFFF:FFFFFFFFFFFFFFFFFFFFFFFFFFFFFFF
FFFFFFFFFFFFFFFFFFFFFFFFF:FFFFFFFFFFFFFFFFFFFFFFFFFFFFFFF,FFFFFFFFFFF
F:FF:FFF:FFFFFF
@A00721:190:HGJW7DSXY:4:2639:6433:6214 2:N:0:TCTGCAAG+GCTGCACT
CGCGTGGTCTAATGACAAATCGACACGTAAGCGTACGGAGGTGTCCCCGGCGCGGGCAAGCCAGTAAT
ATTATATTGGCTTGCCCCACGGTCTGCGGTAAGATGCCCCTGCGGGGCCTATGGAATTCTATTTGAAT
TTGAATTTGAATTGG
+
FFFF:FFFFFFFFFFFFFFFFFFFFFFFFFFFFFFFFFFFFFFFFFFFFFFFFFFFF,FFFFFFF:F
FFFF:FFFFFFFFFFFFFFFFFFFFFFFFFFFFFFFFFFFFFFFFFFFFF,F,FFFF:FFFF:,FFF,F
FFFFFF:FFFFFFFF
@A00721:190:HGJW7DSXY:4:1128:6958:6026 2:N:0:TCTGCAAG+GCTGCACT
GTGACGCGTGGTCTAATGACAAATCGACACGTAAGCGTACGGAGGTGTCCCCGGCGCGGGCAAGCCAG
TAATATTATATTGGCTTGCCCCACGGTCTGCGGTAAGATGCCCCTGCGGGGCCTATGGAATTCTATTT
GAATTTGAATTTGAA
+
FFFFFFFFFFFFFFFFFFFFFFFFFFFFFFFFFFFFFFFFFFFFFFFFFFFFFFFFFFFFFF:FFFFFF
FFFFFFFFFFFF,FFFFFFFFFFFFFF:FFFFF:FFFFFFFFFFFFFFFFFFFF,F,FFF:FFFF:FFF
FFFFFFFFFFFFFFF
@A00721:190:HGJW7DSXY:4:1435:2220:11475 2:N:0:TCTGCAAG+GCTGCACT
GTGACGCGTGGTCTAATGACAAATCGACACGTAAGCGTACGGAGGTGTCCCCGGCGCGGGCAAGCCAG
```

```
TAATATTATATTGGCTTGCCCCACGGTCTGCGGTAAGATGCCCCTGCGGGGCCTATGGAATTCTATTT
GAATTTGAATTTGAA
+
FFFFFFFFFFFFFFFFFFFFFFFFFFFFFFFFFFFFFFFFFFFFFFFFFFFFFFFFFFFFFFFFFFF:F
FFFFFF:FFFFFFFFFFFFFFFFFFFFF,FFFFFFFFFFFFFFFFFFFFFFFFFFFFFFFFFFFFF:FF
FFFFFFFFF,FFFFF
@A00721:190:HGJW7DSXY:4:1471:23493:29684 2:N:0:TCTGCAAG+GCTGCACT
GTGACGCGTGGTCTAATGACAAATCGACACGTAAGCGTACGGAGGTGTCCCCGGCGCGGGCAAGCCAG
TAATATTATATTGGCTTGCCCCACGGTCTGCGGTAAGATGCCCCTGCGGGGCCTATGGAATTCTATTT
GAATTTGAATTTGAA
+
FFFFFFFFFFFFFFFFFFFFFFFFFFFFFFFFFFFFFFFFFFFFFFFFFFFFFFFFFFFFFFFFFFFFF
FFFFFF,F:FFFFFFFFFFFFFFFFFFF,FFFFFFFFF:FFFFFFFFFFFFFFFFFFFF,FFFFFFFF
:F:FFFFFFFFFFFF
@A00721:190:HGJW7DSXY:4:1641:5231:35790 2:N:0:TCTGCAAG+GCTGCACT
GTGACGCGTGGTCTAATGACAAATCGACACGTAAGCGTACGGAGGTGTCCCCGGCGCGGGCAAGCCAG
TAATATTATATTGGCTTGCCCCACGGTCTGCGGTAAGATGCCCCTGCGGGGCCTATGGAATTCTATTT
GAATTTGAATTTGAA
+
FFF:FFFFFFFFFFFFFFFFFFFFFFFFFFFFFFFFFFFFFFFFFFFFFFFFFFFFFFFFFFFFFFFFF
FFF:FF,FFFFFFFFFFFFFFFFFFFFFFFFFFFFFFFFFFFFFFFFFFFFFFFF:FFF:,F,F:FF:
FF:FFFFFFFFFFFF
@A00721:190:HGJW7DSXY:4:2133:1542:32409 2:N:0:TCTGCAAG+GCTGCACT
GTGACGCGTGGTCTAATGACAAATCGACACGTAAGCGTACGGAGGTGTCCCCGGCGCGGGCAAGCCAG
TAATATTATATTGGCTTGCCCCACGGTCTGCGGTAAGATGCCCCTGCGGGGCCTATGGAATTCTATTT
GAATTTGAATTTGAA
+
FFFFFFFFFFFFFFFFFFFFFFFFFFFFFFFFFFFFFFFFFFFFFFFFFFFFFFFFFFFFFFFFFFFFF
FFFFFFFFFFFFFFFFFFFFFFFFFFFFFFFFFFFFFFFFFFFFFFFFFFFFFFFFFFFFFFFFFFFFF
FFFFFFFFFFFFFFF
@A00721:190:HGJW7DSXY:4:2455:26775:12477 2:N:0:TCTGCAAG+GCTGCACT
GTGACGCGTGGTCTAATGACAAATCGACACGTAAGCGTACGGAGGTGTCCCCGGCGCGGGCAAGCCAG
TAATATTATATTGGCTTGCCCCACGGTCTGCGGTAAGATGCCCCTGCGGGGCCTATGGAATTCTATTT
GAATTTGAATTTGAA
+
FFFFFFFFFFFFFFFFFFFFF:FFFFFFFFFFFFFFFFFFFFFFFFFFFFFFFFFFFFFFFF:FFFFFF
,FFFF:FFFFFFFFF,FFFFFFFFFFFFFFFFF:FFFFFF:FFFFFFFFFFFF,,:FFF,F:FFF:FF
F:::FFF:FFF,FFF
@A00721:190:HGJW7DSXY:4:2242:28782:13698 2:N:0:TCTGCAAG+GCTGCACT
TACCAAATCAAATTCAAAAAGAAATCCATAGGCACCGCAGGGGCATATTACCGCAGACCGTGGGGCAA
GCCAATATAAAATAACAGGCTAGCCCGCGCCGGGGACACCTCCGTACGCTTACGTGTCGATTTGTCAT
TAGACCACGCGTCA
+
FFF:FF,,FFFFFFFFF:,F:FF,,FFFFFFFF:FFFF:FFF:F,,,FFFFFFFFF,F,:,FFFF:,F
FFFF,,:FFF,FF:FF,:FFF,F,F:F,FF:F:FFFFFFFFFFFF,FFFF:F:FFFFFFF::FFF,FF
,FFFFFFFFFFFF:
@A00721:190:HGJW7DSXY:4:2123:10601:31000 2:N:0:TCTGCAAG+GCTGCACT
TACCAATTCAAATTCAAATAGAATTCCATAGGCCCCGCAGGGGCATCTTACCGCAGACCGTGGGGCAA
GCCAATATAATATTACTGGCTTGCCCGCGCCGGGGACACCTCCGTACGCTTACGTGTCGATTTGTCAT
TAGACCACGCGTCAC
+
FFFFFFFFFFFFFFFFFFFFFFFFFFFFF,F:FFFFFFFFFFF:FFFFFFFFFFF:FFFFFFFFFFFF
FFF:FFFFF:FFFFFFF,FFF:FFFFFFFFFFFFFFFFFFFFFFF:F:FF,F:FFFFFFFF:FFFFFFF
FFFFFFFFFFF:FF:
```

@A00721:190:HGJW7DSXY:4:2123:11333:32518 2:N:0:TCTGCAAG+GCTGCACT
TACCAATTCAAATTCAAATAGAATTCCATAGGCCCCGCAGGGGCATCTTACCGCAGACCGTGGGGCAA
GCCAATATAATATTACTGGCTTGCCCGCGCCGGGGACACCTCCGTACGCTTACGTGTCGATTTGTCAT
TAGACCACGCGTCAC
+
FFFFFFFFFFFFFFFFFFFFFFFFFFFFFFFFFFFFFFFFFFFFFFFFFFFFFFFFFFFFFFFFFFFFFF
FFFFFFFFFF:FFFFFFFFF:FFFFFFFFFFFFFFFFFFFFFFFFFFFFFFFFF:FFFFF,FFFFFFF
FFFFFFFFF:FFFFF
@A00721:190:HGJW7DSXY:4:2124:31882:13964 2:N:0:TCTGCAAG+GCTGCACT
TACCAATTCAAATTCAAATAGAATTCCATAGGCCCCGCAGGGGCATCTTACCGCAGACCGTGGGGCAA
GCCAATATAATATTACTGGCTTGCCCGCGCCGGGGACACCTCCGTACGCTTACGTGTCGATTTGTCAT
TAGACCACGCGTCAC
+
FFFFFFFFFFFFFFFFFFFFF:FFFFFFFFFFFFFF:FFFFFFFFFFFFFFFFFFFFFFFFFFFFFFFF
FFFFFFFFFFFFFFFFFFFFFFFFFFFFFFFFFFFFFFFFFFFFFFFFFFFF:FFFFFFFFFFFFFFF
FFF:FFFFFFFFFFF
@A00721:190:HGJW7DSXY:4:2145:7690:19914 2:N:0:TCTGCAAG+GCTGCACT
TACCAATTCAAATTCAAATAGAATTCCATAGGCCCCGCAGGGGCATCTTACCGCAGACCGTGGGGCAA
GCCAATATAATATTACTGGCTTGCCCGCGCCGGGGACACCTCCGTACGCTTACGTGTCGATTTGTCAT
TAGACCACGCGTCAC
+
FFFFFFFFF:FF,FFFFFFFFF:FFFFFFFFFFFFFFFFFFFFFFFFFFFFFFFFFFFFFFFFFFFFFFF
FFFFFFFFFFFFFFFFFFFFFFFFFFFFFFFFFFFFFFFFFFFFFFFFFFFFFFFFFFFFF:FFFFFF,
FFFFFFFFFFFFFFF
@A00721:190:HGJW7DSXY:4:2344:2356:11240 2:N:0:TCTGCAAG+GCTGCACT
TACCAATTCAAATTCAAATAGAATTCCATAGGCCCCGCAGGGGCATCTTACCGCAGACCGTGGGGCAA
GCCAATATAATATTACTGGCTTGCCCGCGCCGGGGACACCTCCGTACGCTTACGTGTCGATTTGTCAT
TAGACCACGCGTCAC
+
FFFFFFFFFFFFFFF,FFFFFFFFFFFFFFFFFFFFFFFFFFFFFFFFFFFFFFFFFFFF:FFFFFFF
FFFFF:FFFFFFFFFFFFFFFFFFFFFFFFFFFFFFFFFFFFFFFFFF:FFFFFFFFFF:FFFFFFF
FF:FFFFFF:FFFFF
@A00721:190:HGJW7DSXY:4:2355:29559:26788 2:N:0:TCTGCAAG+GCTGCACT
TACCAATTCAAATTCAAATAGAATTCCATAGGCCCCGCAGGGGCATCTTACCGCAGACCGTGGGGCAA
GCCAATATAATATTACTGGCTTGCCCGCGCCGGGGACACCTCCGTACGCTTACGTGTCGATTTGTCAT
TAGACCACGCGTCAC
+
FFFFFFFFFFFFFFFFFFFFFFF:FFFFFFFFF:FFFFFFFFFFFFFFFFFFFFFFFFFFFFFFFFFFF,
FFFFFFFFFFFFFFFFFFFFFFFFFFFF:FFFFFFFFFFFFFFFFFFF::FFFFFFFFFFFFFFFFF:
FFFFFFFFFFFFFFF
@A00721:190:HGJW7DSXY:4:1344:30373:13917 2:N:0:TCTGCAAG+GCTGCACT
GATCGTGACGCGTGGTCTAATGACAAATCGACACGTAAGCGTACGGAGGTGTCCCCGGCGCGGGCAAG
CCAGTAATATTATATTGGCTTGCCCCACGGTCTGCGGTAAGATGCCCCTGCGGGGCCTATGGAATTCT
ATTTGAATTTGAATT
+
FFFFFFFFFFFFFFFFFFFFFFFFFFFFFFFFFFFFFFFFFFFFFFFFFFFFFFFFFFFFFFFFFFFFFF
FFFFFFFFFFFFFFFFFFFFFFFFFFFFFF::FFFFFFFFFFF:FFFFFFFFFFFFFFFFFFF,FF:F
:F:FFF:FFFFFFFF
@A00721:190:HGJW7DSXY:4:1362:16559:8155 2:N:0:TCTGCAAG+GCTGCACT
GATCGTGACGCGTGGTCTAATGACAAATCGACACGTAAGCGTACGGAGGTGTCCCCGGCGCGGGCAAG
CCAGTAATATTATATTGGCTTGCCCCACGGTCTGCGGTAAGATGCCCCTGCGGGGCCTATGGAATTCT
ATTTGAATTTGAATT
+
FFFF:FFFFF:F,FFFF:FF:FFFFFFFFFFFFFF:FFFFFFFFFFFFFFFFF:FFFFFFF:FFFFF:

```
FFFF,:F,FF,FFFFFFFFFFFFFFFFFFFFFFFFFFFFFFFF,FFFFFFFFFFF:FFFFFFFFFFFF
FFFF:FFFFFFFF:F
@A00721:190:HGJW7DSXY:4:1432:15374:8359 2:N:0:TCTGCAAG+GCTGCACT
GATCGTGACGCGTGGTCTAATGACAAATCGACACGTAAGCGTACGGAGGTGTCCCCGGCGCGGGCAAG
CCAGTAATATTATATTTGCTTGCCCCACGGTCTGCGGTAAGATGCCCCTGCGGGGCCTATGGAATTCT
ATTTGAATTTGAATT
+
FFF:FFFF,F,,F:FFFFFFF:FFFFF:FFFF:FFFFF::FFFFF,FF:FFFFFFF,:F:FF:F:FFF
FF::FF:FF,:FFFFF,F,:FFFFF,FF:F,:FF,F,FFFF:,,F:FFFFFF:FFFFF::,F:FFFFF
FF:FFFF,F,,FFFF
@A00721:190:HGJW7DSXY:4:1668:7374:34240 2:N:0:TCTGCAAG+GCTGCACT
GATCGTGACGCGTGGTCTAATGACAAATCGACACGTAAGCGTACGGAGGTGTCCCCGGCGCGGGCAAG
CCAGTAATATTATATTGGCTTGCCCCACGGTCTGCGGTAAGATGCCCCTGCGGGGCCTATGGAATTCT
ATGTGAATTTGAATT
+
FFFFFFFF:FFF:FFFFFFFFFFFFFFFFFFFFFFFFFFFFFFFFFFFFFFFFFFFFFFFFFFFFFFF
FFFFFFFF:FFFF:FFFFFFFFFFFFFFFFFFFFFFFFFFFFFFFFFFFFFFFFFFFF:FFFFFFFF
FF,FFFF,:FFF:FF
@A00721:190:HGJW7DSXY:4:1677:32208:4758 2:N:0:TCTGCAAG+GCTGCACT
GATCGTGACGCGTGGTCTAATGACAAATCGACACGTAAGCGTACGGAGGTGTCCCCGGCGCGGGCAAG
CCAGTAATATTATATTGGCTTGCCCCACGGTCTGCGGTAAGATGCCCCTGCGGGGCCTATGGAATTCT
ATTTGAATTTGAATT
+
FFFFFFFFFFFFFFFFFFFFFFFFFFFFFFFFFFFFFFFFFFFFFFFFFFFFFFFFFFFFFFFFFFFF
FFFFFFFFFFFFFFFFFFFFFFFFFFFFFFFFFF,FFFFFFFFFFFFFFFFFFFFFFFF,FFFFF:FFFF
:FFFFFFFFFFFF:F
@A00721:190:HGJW7DSXY:4:2174:5267:34601 2:N:0:TCTGCAAG+GCTGCACT
GATCGTGACGCGTGGTCTAATGACAAATCGACACGTAAGCGTACGGAGGTGTCCCCGGCGCGGGCAAG
CCAGTAATATTATATTGGCTTGCCCCACGGTCTGCGGTAAGATGCCCCTGCGGGGCCTATGGAATTCT
ATTTGAATTTGAATT
+
FFFFFFF,FFFFFFFFFFFFFF:FFFFFFFFFFFFFF,FFF:FFFFFFFFFFFFFFFFFFFFFFFF,F
FFFFFFFFFFFFFFFFFFFFFFFFFFFFFF:FFFFFFF:FFFFFFFFFFFFFFFFFF,F,FFF,FFFF
F::FFF:FFFFFFFF
@A00721:190:HGJW7DSXY:4:2267:5575:17534 2:N:0:TCTGCAAG+GCTGCACT
GATCGTGACGCGTGGTCTAATGACAAATCGACACGTAAGCGTACGGAGGTGTCCCCGGCGCGGGCAAG
CCAGTAATATTATATTGGCTTGCCCCACGGTCTGCGGTAAGATGCCCCTGCGGGGCCTATGGAATTCT
ATTTGAATTTGAATT
+
FFFFFFFFFFFFFFFFFFFFFFFFFFFFFFFFFFFF:FFFFFFFFFFFFFFFFFFFFFFFFFFFFFFF
FFFFFFFFFFFFFFFFFFFFFFFFFFFFFFFFFFFFFFFFFFFFFFFFFFFFFFF:FFFFF,FFFF
FFFFFFFFF,FF,:F
@A00721:190:HGJW7DSXY:4:2327:10077:6324 2:N:0:TCTGCAAG+GCTGCACT
GATCGTGACGCGTGGTCTAATGACAAATCGACACGTAAGCGTACGGAGGTGTCCCCGGCGCGGGCAAG
CCAGTAATATTATATTGGCTTGCCCCACGGTCGGCGGTAAGATGCCCCTGCGGGGCCTATGGAATTCT
ATTTGAATTTGAATT
+
FFFFFFF:FFFFFFFFFFFFFFFFFFFFFFFFFFFF:FFFFFFFFFFFFFFFFFFFFFFFFFFFFFF:F
FFFF,FF:FFFF,:FFFF:FFFFFFF:FFFFF,FFFF:,FFFF:FFFF,FFFFFFFFFF:FFFFFF:F
FFFFFF,FFFFFFFF
@A00721:190:HGJW7DSXY:4:2533:6506:11600 2:N:0:TCTGCAAG+GCTGCACT
GATCGTGACGCGTGGTCTAATGACAAATCGACACGTAAGCGTACGGAGGTGTCCCCGGCGCGGGCAAG
CCAGTAATATTATATTGGCTTGCCCCACGGTCTGCGGTAAGATGCCCCTGCGGGGCCTATGGAATTCT
ATTTGAATTTGAATT
```

```
+
FFFFFFFFFFFFFFFFFFFFFFFFFFFFFFFFFFFFFFFFFFFFFFFFFFFFFFFFFFFFFFFFFFFFF:F
FFFFFFFFFF:FFFFFFFFFF:FFFFFFFFFFFFFFFFFFF:FF:FFFFFFFFFFFF,FFFFF:FFFF
F,FFFFFFFFF::FF
@A00721:190:HGJW7DSXY:4:2670:10131:26647 2:N:0:TCTGCAAG+GCTGCACT
GATCGTGACGCGTGGTCTAATGACAAATCGACACGTAAGCGTACGGAGGTGTCCCCGGCGCGGGCAAG
CCAGTAATATTATATTGGCTTGCCCCACGGTCTGCGGTAAGATGCCCCTGCGGGGCCTATGGAATTCT
ATTTGAATTTGAATT
+
FFFFFFFFFFFFFFFFFFFFFFFFFF:FFFFFFFFFF:FFFFFFFFFFFFFFFFFFFFFFFFFFFF:FF
FFFFFF:FFFFFFF:FFFFFFFFFFFFFFFFFFFFFFFFFFFFFFFFFFFFFFFFFF,FFFFF,FFFF
F,F:FFF,FFFFFFF
@A00721:190:HGJW7DSXY:4:1611:23149:18004 2:N:0:TCTGCAAG+GCTGCACT
ATGATCGTGACGCGTGGTCTAATGACAAATCGACACGTAAGCGTACGGAGGTGTCCCCGGCGCGGGCA
AGCCAGTAATATTATATTGGCTTGCCCCACGGTCTGCGGTAAGATGCCCCTGCGGGGCCTATGGAATT
CTATTTGAATTTGAA
+
FFFFFFFFFFFFFFFFFFFFFFFFFFF:FFFFFFFFFFFFFFFFFFFFFFFFFFFFFFFFFFFFFFFFF
FFFFFFFFFFFFFFFFFFFFFFFFFFFFF:FFFFF,FFFFFFFFFFFFFFFFFFFFFFFFF,:FFFFFF,
,F,FFFF:FFFFFFF
@A00721:190:HGJW7DSXY:4:2216:14642:25629 2:N:0:TCTGCAAG+GCTGCACT
ATGATCGTGACGCGTGGTCTAATGACAAATCGACACGTAAGCGTACGGAGGTGTCCCCGGCGCGGGCA
AGCCAGTAATATTATATTGGCTTGCCCCACGGTCTGCGGTAAGATGCCCCTGCGGGGCCTATGGAATT
CTATTTGAATTTGAA
+
FFFFFFFFFFFFFFFFFFFFFFFFFFFFFFFFFFFFFFFFFFFFFFFFFFFFFFFFFFFFFFFFFFFFF
FFFFFFFFFFFFFFFFFFFFFFFFFFF:FFFFFFFFFFFFFFFFFFFFFFFFFFFFFFFFFFFF:FFFFF
FFFFFFFFFF:FFFF
@A00721:190:HGJW7DSXY:4:1128:5855:25598 2:N:0:TCTGCAAG+GCTGCACT
ATTTTTTAGATGGTCATGATCGTGACGCGTGGTCTAATGACAAATCGACACGTAAGCGTACGGAGGTG
TCCCCGGCGCGGGCAAGCCAGTAATATTATATTGGCTTGCCCCACGGTCTGCGGTAAGATGCCCCTGC
GGGGCCTATGGAATT
+
FFFFFFFFFF:FFFFFFFFFFFFFFFFFFFFFFFFFFFFFFFFFF:FFFFFFF::FF:FFFFF:FFFFFF
FF:FFFFFFFFFFFF,:FFF:FFFFFFFFFFFFF:FFF,,FFFFFFFFFFFFFFFFFFFFFFFFF::FF
FFFFFFFFFFFFFFF
@A00721:190:HGJW7DSXY:4:1128:3540:35931 2:N:0:TCTGCAAG+GCTGCACT
ATTTTTTAGATGGTCATGATCGTGACGCGTGGTCTAATGACAAATCGACACGTAAGCGTACGGAGGTG
TCCCCGGCGCGGGCAAGCCAGTAATATTATATTGGCTTGCCCCACGGTCTGCGGTAAGATGCCCCTGC
GGGGCCTATGGAATT
+
FFF:FFFF:FFFFFFFFFFFFFFFFFFFFFFFFFFFFFFFFFFFFFFFFFFFFFFFFFFFFFFFFFFFFF
FF,FFFFFFFFFFFFFFFFFFFF:FFFFFFFFFFFFFFFFFFFFFFFFFFFFFFFF:FFFFFFFFFFFF
FFFFFFFFFFFFFFF
@A00721:190:HGJW7DSXY:4:1172:20961:16344 2:N:0:TCTGCAAG+GCTGCACT
ATTTTTTAGATGGTCATGATCGTGACGCGTGGTCTAATGACAAATCGACACGTAAGCGTACGGAGGTG
TCCCCGGCGCGGGCAAGCCAGTAATATTATATTGGCTTGCCCCACGGTCTGCGGTAAGATGCCCCTGC
GGGGCCTATGGAATT
+
FFFFFFFFFFFFFFFFFFFFFFFFFFFFFFFFFFFFFFFF,FFFFFFFFFFFFFFFFFFFFFFFFFFFF:F
FFFFFFFFFFFFFFFFFFF,FFF:FF,FFFFFFFFFFFFFFFFFFFFFFF:FFFFFFFFFFFFFFFF:F
FFFFFFFFFFFFFF:
@A00721:190:HGJW7DSXY:4:1253:25129:27007 2:N:0:TCTGCAAG+GCTGCACT
ATTTTTTAGATGGTCATGATCGTGACGCGTGGTCTAATGACAAATCGACACGTAAGCGTACGGAGGTG
```

TCCCCGGCGCGGGCAAGCCAGTAATATTATATTGGCTTGCCCCACGGTCTGCGGTAAGATGCCCCTGC
GGGGCCTATGGAATT
+
FFFFFFFFFFFFFFFFFFFFFFFFFFFFFFFFFFFFFFFFFFFFFFFFFFFFFFFFFFFFFFFFFFFF
FFFFFFFFFFFFFFFFFFF:FFFFFFFFFFFFFFFFFFFFFFFFFFFFFFFFFFFFFFFFFFFFFFFF
FFFFFFFFFFFF,FF
@A00721:190:HGJW7DSXY:4:1255:23628:12633 2:N:0:TCTGCAAG+GCTGCACT
ATTTTTTAGATGGTCATGATCGTGACGCGTGGTCTAATGACAAATCGACACGTAAGCGTACGGAGGTG
TCCCCGGCGCGGGCAAGCCAGTAATATTATATTGGCTTGCCCCACGGTCTGCGGTAAGATGCCCCTGC
GGGGCCTATGGAATT
+
FFFFFF:FFF:FFFFFFFFFFFFFFFFFFFFFFFFFFFFFFFFFFFFFFFFFFFFFFFFFFFFFFFFF
FFFFFFFFFFFFFFF:FFFFFFFFFFFFFFFFFFFFFFFFFFFFFFFFFFFFFFFFFFFFF:FFFFFF
FFFFFFFFFFFF:FF
@A00721:190:HGJW7DSXY:4:1263:30581:8766 2:N:0:TCTGCAAG+GCTGCACT
ATTTTTTAGATGGTCATGATCGTGACGCGTGGTCTAATGACAAATCGACACGTAAGCGTACGGAGGTG
TCCCCGGCGCGGGCAAGCCAGTAATATTATATTGGCTTGCCCCACGGTCTGCGGTAAGATGCCCCTGC
GGGGCCTATGGAATT
+
FFF:FFFFFFFFFFFFFFFFFFFFFFFFFFFFFFFFFFFFFFFFFFFFFFFFFFF:FFFFFFFFFFFF
FFFFFFFFFFFFFFFFFFFFFFF:FFFFFFFFFFFFFFF:FFFFFFFFFFFFFFF:FFFFFFFFFFFF
FFFFFFFFFFFFFFF
@A00721:190:HGJW7DSXY:4:1317:17535:17738 2:N:0:TCTGCAAG+GCTGCACT
ATTTTTTAGATGGTCATGATCGTGACGCGTGGTCTAATGACAAATCGACACGTAAGCGTACGGAGGTG
TCCCCGGCGCGGGCAAGCCAGTAATATTATATTGGCTTGCCCCACGGTCTGCGGTAAGATGCCCCTGC
GGGGCCTATGGAATT
+
FFFFF,FFFFFFFFFFFFFFFFFFFFFFFFFFFFFFFFFFFFFFFFFFFFFFFFFFFFFFFFFFFFFF
FFFFFFFFFFFFFFFFFFFFFFFFFFFFFFFFFFFFFFFF:FFFFFFFFFFFFFFFFFFFFFFFFFFF
FFFFFF:FFFFFFFF
@A00721:190:HGJW7DSXY:4:1460:12391:12680 2:N:0:TCTGCAAG+GCTGCACT
ATTTTTTAGATGGTCATGATCGTGACGCGTGGTCTAATGACAAATCGACACGTAAGCGTACGGAGGTG
TCCCCGGCGCGGGCAAGCCAGTAATATTATATTGGCTTGCCCCACGGTCTGCGGTAAGATGCCCCTGC
GGGGCCTATGGAATT
+
FFFF:F:FFFFFFFFFFFFFFFFFFFFFFFFFFFFFFFFFFFFFFFFFF:FFFFFFFFFFFFFFFFFF
FFFFFFFFFFFFFFFFFF:,FFFFFFFFFFFFFFFFFFFFFFFFFFFFFFFFFFFFFFFFFFFFFFFF
FFFFFFFFFFFFFFF
@A00721:190:HGJW7DSXY:4:2655:7229:20838 2:N:0:TCTGCAAG+GCTGCACT
ATTTTTTAGATGGTCATGATCGTGACGCGTGGTCTAATGACAAATCGACACGTAAGCGTACGGAGGTG
TCCCCGGCGCGGGCAAGCCAGTAATATTATATTGGCTTGCCCCACGGTCTGCGGTAAGATGCCCCTGC
GGGGCCTATGGAATT
+
FFFFFFFFFFFFFFFFFFFFFFFFFFF:FFFFFFFFFFFFFFFFF:FFFFFFFFFFFFFFFFFF,F,F
FFFFFFFFFFF:FFFFFFF:FFF:FFFFFFFF,FFFFFFFFFFFFF:F:F:FF:FFFFFF:FFFFFFF
FF:FFFFFFFFFFFF
@A00721:190:HGJW7DSXY:4:2673:31810:15374 2:N:0:TCTGCAAG+GCTGCACT
ATTTTTTAGATGGTCATGATCGTGACGCGTGGTCTAATGACAAATCGACACGTAAGCGTACGGAGGTG
TCCCCGGCGCGGGCAAGCCAGTAATATTATATTGGCTTGCCCCACGGTCTGCGGTAAGATGCCCCTGC
GGGGCCTATGGAATT
+
FFFFFFFFFFFFFFFFFFFFFFFFFFFFFFFFFFFFFFFFFFFFFFFFFFFFFFFFFFFFFFFFFFFF
FFFFFFFFFFFFFFFFFF:FFFFF,FFFFFFFFFFFFFFFFFFFFFFFFFFFFFFFFFFFFFFFFFFF
FFFFFFFF:FFFFFF

```
@A00721:190:HGJW7DSXY:4:1268:16324:5431 2:N:0:TCTGCAAG+GCTGCACT
TTCATTTTTTAGATGGTTATGATCGTGACGCGTGGTCTAATGACAAATCGACACGTAAGCGTACGGAG
GTGTCCCCGGCGCGGGCAAGCCAGTAATATTATATTGGCTTGCCCCACGGTCTGCGGTAAGATGCCCC
TGCGGGGCCTATGGA
+
FFFFFFFFFFFFF:FFFFFFFFFFFFFFFFFF:FFFFFFFFFFFFFFFFFFFFFFFFF:FFFFFFFFF
FFFFFFFFFFFFFFFFFFFFFFFFF:FFFFFFFFFFFFFFFFFFFFFFFFFFFFFFFFFFFFFFFFFF
FFFFFFFFF:FFFFF
@A00721:190:HGJW7DSXY:4:1433:1036:3380 2:N:0:TCTGCAAG+GCTGCACT
TTCATTTTTTAGATGGTTATGATCGTGACGCGTGGTCTAATGACAAATCGACACGTAAGCGTACGGAG
GTGTCCCCGGCGCGGGCAAGCCAGTAATATTATATTGGCTTGCCCCACGGTCTGCGGTAAGATGCCCC
TGCGGGGCCTATGGA
+
FFFFFFFFFFFFFFFFFFFFFFFFFFFFFFFFFFFFFFFFFFFFFFFFFFFFFFFFFFFFFFFFFFFF
FFFFFFFFFFFFFFFFFFFFF:FFFFFFFFFFFFFFFFFFFFFFFFFFFFFFFFFFFFFFF,FFFFFF
FFFFFFF::FF:FFF
@A00721:190:HGJW7DSXY:4:1457:6470:2832 2:N:0:TCTGCAAG+GCTGCACT
TTCATTTTTTAGATGGTTATGATCGTGACGCGTGGTCTAATGACAAATCGACACGTAAGCGTACGGAG
GTGTCCCCGGCGCGGGCAAGCCAGTAATATTATATTGGCTTGCCCCACGGTCTGCGGTAAGATGCCCC
TGCGGGGCCTATGGA
+
FFFFFFFFFFFFF,FFFFFFFFFFFFFFFFFFFFFFFFFFFFFFFFFFFFFFFFFFFFFFFFFFFFFF
FFFFFFFFFFFFFFFFFFFFFFFFFFFFFFFF:FFFFFFFFFFFFFFFFFF:FF:FFFFFFFFFFFFF
FFFFFFFFFFFFFFF
@A00721:190:HGJW7DSXY:4:1464:1280:7498 2:N:0:TCTGCAAG+GCTGCACT
TTCATTTTTTAGATGGTTATGATCGTGACGCGTGGTCTAATGACAAATCGACACGTAAGCGTACGGAG
GTGTCCCCGGCGCGGGCAAGCCAGTAATATTATATTGGCTTGCCCCACGGTCTGCGGTAAGATGCCCC
TGCGGGGCCTATGGA
+
FFFFFFFF,FFFFFFFFFFFFFFFFFFFFFFF:FFFFFFFFFFFF:FFFFFFFFFFFFFFFFFFFFFF
FFFFFFFFFFFFFFFFFFFFFFFFFFFFF:FFFFFFFFF:FFFFFFFFFFFF::FFFFFFFFFFFFFF
FFFFFFF:FFFFFFF
@A00721:190:HGJW7DSXY:4:1535:17942:12868 2:N:0:TCTGCAAG+GCTGCACT
TTCATTTTTTAGATGGTTATGATCGTGACGCGTGGTCTAATGACAAATCGACACGTAAGCGTACGGAG
GTGTCCCCGGCGCGGGCAAGCCAGTAATATTATATTGGCTTGCCCCACGGTCTGCGGTAAGATGCCCC
TGCGGGGCCTATGGA
+
FFFFFFFF:FFFFFFFFFFFFFFFFFFFFFFFF::F:FFFFFFFFF:FF:FFFFFFFFFF:FFFF:FF
FFFFFFFFFFFFFFFFFFFFFFFFFFFFFFFFFFFFFFFF:F:FF:FFFFF:FF:FFFFF:FF:FF:F
FF:FFFFFFFFFFFF
@A00721:190:HGJW7DSXY:4:2202:15863:18975 2:N:0:TCTGCAAG+GCTGCACT
TTCATTTTTTAGATGGTTATGATCGTGACGCGTGGTCTAATGACAAATCGACACGTAAGCGTACGGAG
GTGTCCCCGGCGCGGGCAAGCCAGTAATATTATATTGGCTTGCCCCACGGTCTGCGGTAAGATGCCCC
TGCGGGGCCTATGGA
+
FFFFFF:FFFFFFFFFFFFFFFFFFFFFFFFFFFFFFFFFFFFFFFFFFFFFFFFFFFFFFFFFFFFF
FFFFFFFFFFFFFFFFFF:FFF:FFF:FFFFFFF:F,:FFF,FFFFFFFFFF:F:FFF:FFFF,FFFF
,FFFFF:FF,FFFFF
@A00721:190:HGJW7DSXY:4:2230:26250:3176 2:N:0:TCTGCAAG+GCTGCACT
TTCATTTTTTAGATGGTTATGATCGTGACGCGTGGTCTAATGACAAATCGACACGTAAGCGTACGGAG
GTGTCCCCGGCGCGGGCAAGCCAGTAATATTATATTGGCTTGCCCCACGGTCTGCGGTAAGATGCCCC
TGCGGGGCCTATGGA
+
FFFFFFFFFFFFFFFFFFFFF:FFFFFFFFFFFFFFFFFFFF:FFFFFFFFFFFFF:FFFFFFFFFFF
```

FFFFFFFFFF:FFFFFFFFFFFFFFFFFFFFFFFFFF:FFFFFFFFFFFFFFFFFFFFFFFFFFFFFF
FFFFFFF:FFFFFFF
@A00721:190:HGJW7DSXY:4:2658:18132:9502 2:N:0:TCTGCAAG+GCTGCACT
TTCATTTTTTAGATGGTTATGATCGTGACGCGTGGTCTAATGACAAATCGACACGTAAGCGTACGGAG
GTGTCCCCGGCGCGGGCAAGCCAGTAATATTATATTGGCTTGCCCCACGGTCTGCGGTAAGATGCCCC
TGCGGGGCCTATGGA
+
FFFFFFFFFFFFFFFFFFFFFFFFFFFFFFFFFFFFFFFFFFFFFFFFFFFFFFFFFFFFFFFFFFFF
FFFFFFFFFFFFFFFFFFFFFFFFFFFFFFFFFFFFFFFFFFFFFFFFFFFFFFFFFFFFFFFFFFFF
FFFFFFFFFFFFFFF
@A00721:190:HGJW7DSXY:4:1176:29514:28432 2:N:0:TCTGCAAG+GCTGCACT
ATTCATTTTTTAGATGGTTATGATCGTGACGCGTGGTCTAATGACAAATCGACACGTAAGCGTACGGA
GGTGTCCCCGGCGCGGGCAAGCCAGTAATATTATATTGGCTTGCCCCACGGTCTGCGGTAAGATGCCC
CTGCAGGGCCAGATC
+
FFFFFFFFFFFFFFFFFFFFFFFFFFFFFFFFFFFFFFFFFFFFFFF:FF:FFF::F:F:FFFFFFF
FFFFFFFFFFFFFFFFFFFFFFFFFFFFFF,FFFFFFFFFFF,FFF:FFFFFF,FFFFF:FFFF,FFF
FFFFFFFFFFFFF,F
@A00721:190:HGJW7DSXY:4:1509:27407:7247 2:N:0:TCTGCAAG+GCTGCACT
ATTCATTTTTTAGATGGTTATGATCGTGACGCGTGGTCTAATGACAAATCGACACGTAAGCGTACGGA
GGTGTCCCCGGCGCGGGCAAGCCAGTAATATTATATTGGCTTGCCCCACGGTCTGCGGTAAGATGCCC
CTGCAGGGCCAGATC
+
FFFFFFFFFFFFFFFFFFFFFFFFFFFFFFFFFFFFFFFFFFFFFFFFFFFFFFFFFFFFFFFFFFFF
FFFFFFFFFFFFFFFFFF::FFFFFFF:FFFFFFFFFFFFFFFFFFFFFFFFFF,FFFF:FFFFFFFFF
FFFFFFFFFFFFFFF
@A00721:190:HGJW7DSXY:4:1551:13467:17143 2:N:0:TCTGCAAG+GCTGCACT
ATTCATTTTTTAGATGGTTATGATCGTGACGCGTGGTCTAATGACAAATCGACACGTAAGCGTACGGA
GGTGTCCCCGGCGCGGGCAAGCCAGTAATATTATATTGGCTTGCCCCACGGTCTGCGGTAAGATGCCC
CTGCAGGGCCAGATC
+
FFFFFFFFFFFFFFFFFFFFFFFFFFFFFFFFFFFFFFFFFFFFFFFFFFFFFFFFFFFFFFFFFFFF
FFFFFFFFFFFFFFFFFFFFFFFFFFFFFFFFFFFFFFFFFFFFFFFFFFFF:FFFFFFFFFFFFFFF
FFFFFFFFFFFFFFF
@A00721:190:HGJW7DSXY:4:1636:10791:3677 2:N:0:TCTGCAAG+GCTGCACT
ATTCATTTTTTAGATGGTTATGATCGTGACGCGTGGTCTAATGACAAATCGACACGTAAGCGTACGGA
GGTGTCCCCGGCGCGGGCAAGCCAGTAATATTATATTGGCTTGCCCCACGGTCTGCGGTAAGATGCCC
CTGCAGGGCCAGATC
+
FFFFFFFFFFFFFF:FFFFFFFFFFFFFFFFFFFFFFFFFFFFFFFFFFFFFFFFFFFFFFFFFFFFF
FFFFFFFFFFFFFFFFFFFFFFFFFFFFFFFFFFFFFFFFFFFFFFFFFFFFFFFFFFFF:FFFFFFF
F:FFFFFFFFFFFFF
@A00721:190:HGJW7DSXY:4:2123:15637:25535 2:N:0:TCTGCAAG+GCTGCACT
ATTCATTTTTTAGATGGTTATGATCGTGACGCGTGGTCTAATGACAAATCGACACGTAAGCGTACGGA
GGTGTCCCCGGCGCGGGCAAGCCAGTAATATTATATTGGCTTGCCCCACGGTCTGCGGTAAGATGCCC
CTGCAGGGCCAGATC
+
FFFFFFFFFFFFFFFFFFFFFFFFFFFFFFFFFFFFFFFFFFFFFFFFFFFFFFFFFFFFFFFFFFFF
FFFFFFFFFFFFFFFFFFFF:FFFFFFFFFFFFFFFFFFFFFFFFFFFFFFFFFFFFFFFFFFFFFFF
FFFFFFFFFFFFFFF
@A00721:190:HGJW7DSXY:4:2123:14362:26209 2:N:0:TCTGCAAG+GCTGCACT
ATTCATTTTTTAGATGGTTATGATCGTGACGCGTGGTCTAATGACAAATCGACACGTAAGCGTACGGA
GGTGTCCCCGGCGCGGGCAAGCCAGTAATATTATATTGGCTTGCCCCACGGTCTGCGGTAAGATGCCC
CTGCAGGGCCAGATC

```
+
:FFFFFF:FFFFFFFFFFF:FFFFFFFFFFFFFFFFFFFFFFFFFFFFFFFFFFFFFFFF:FFFFFFFFF
FFFFFFFFFFFFFFFFFFFFFFFFFFFFFFFFFFFFFFFFFFFFFFFFFFFFFFFFFFFFFFFFFFFFFF
FFFF:FFFFFFFFFF
@A00721:190:HGJW7DSXY:4:2150:27154:17519 2:N:0:TCTGCAAG+GCTGCACT
ATTCATTTTTTAGATGGTTATGATCGTGACGCGTGGTCTAATGACAAATCGACACGTAAGCGTACGGA
GGTGTCCCCGGCGCGGGCAAGCCAGTAATATTATATTGGCTTGCCCCACGGTCTGCGGTAAGATGCCC
CTGCAGGGCCAGATC
+
FFFFFFFFFFFFFFFFFFFFFFFFFFFFFFFFFFFFFFFFFFFFFFFFFFFFFFFFFFFFFFFFFFFFFF
FFFFFFFFFFFFFFFFFFFF:FFFFFFFFFFFFF:FFFFFFFFFFFFFFFFFFFFFFFFFFFFFFFF:FF
FFFFFFFFFFFFFFF
@A00721:190:HGJW7DSXY:4:2355:14805:9283 2:N:0:TCTGCAAG+GCTGCACT
ATTCATTTTTTAGATGGTTATGATCGTGACGCGTGGTCTAATGACAAATCGACACGTAAGCGTACGGA
GGTGTCCCCGGCGCGGGCAAGCCAGTAATATTATATTGGCTTGCCCCACGGTCTGCGGTAAGATGCCC
CTGCAGGGCCAGATC
+
FFFFFFFFF:FFFF:FFFFFFFFFFFFFFFFFFFFFFFFFFFFFFFFFFFFFFFFFFFFFFFFFFFFFF
FFFFFFFFFFFFFFFFFFFFFFFFFFFFFFFFFFFFFFFFFFFFFFFFFFFFFFFFFFFFFF:FFF:FFF
FFFFF:FFFFFFF:F
@A00721:190:HGJW7DSXY:4:2374:19334:20979 2:N:0:TCTGCAAG+GCTGCACT
ATTCATTTTTTAGATGGTTATGATCGTGACGCGTGGTCTAATGACAAATCGACACGTAAGCGTACGGA
GGTGTCCCCGGCGCGGGCAAGCCAGTAATATTATATTGGCTTGCCCCACGGTCTGCGGTAAGATGCCC
CTGCAGGGCCAGATC
+
FFFFFFFFFFFFFFFFFFFFFFFFFFFFFFFFFFFFFFFFFFFFFFFFFFFFFFFFFFFFFFFFFFFFF,
FFFFFFFFFFFFFFFFFFFFFFFF:FFFFFFFFFFF:FFFFFFFFFFFFFFFFFFF:FFFFFFFFFFFF
FFFFFFFFFFFFFFF
@A00721:190:HGJW7DSXY:4:2673:13584:25989 2:N:0:TCTGCAAG+GCTGCACT
ATTCATTTTTTAGATGGTTATGATCGTGACGCGTGGTCTAATGACAAATCGACACGTAAGCGTACGGA
GGTGTCCCCGGCGCGGGCAAGCCAGTAATATTATATTGGCTTGCCCCACGGTCTGCGGTAAGATGCCC
CTGCAGGGCCAGATC
+
FFFFFFFFFFFFFFFFFFFFFFFFFFFFFFFFFFFFFFFFFFFFFFFFFFFFFFFFFFFFFFFFFFFFFF
:FFFFFFFFFFFFFFFFFFFFFFFFFFFFFFFFFFFFF:FFFFFFFFFFFFFFFFFFFFFFFFFFFFFFF
FFFFFFFFFFFFFFF
@A00721:190:HGJW7DSXY:4:1272:10520:25347 2:N:0:TCTGCAAG+GCTGCACT
ATAGAATTCATTTTTTAGATGGTTATGATCGTGACGCGTGGTCTAATGACAAATCGACACGTAAGCGT
ACGGAGGTGTCCCCGGCGCGGGCAAGCCAGTAATATTATATTGGCTTGCCCCACGGTCTGCGGTAAGA
TGCCCCTGCGGGGCC
+
:FFFFFFFFFFFFFFFFFFFFFFFFFFFFFFFFF:FFFFFFFFFFFFFFFFFFF:FFFFFFFFFFFFFF
FFFFFFFFFFFFFFFFFFFFFFFFFFFFFFFFFFFFFFFFFFFFFFFFFFF:F:F,FF:FFFFFFFFFFFF
FFFFFFFFFFFFFFF
@A00721:190:HGJW7DSXY:4:1343:26096:2253 2:N:0:TCTGCAAG+GCTGCACT
ATAGAATTCATTTTTGAGATGGTTATGATCGTGACGCGTGGTCTAATGACAAATCGACACGTAAGCGT
ACGGAGGTGTCCCCGGCGCGGGCAAGCCAGTAATATTATATTGGCTTGCCCCACGGTCTGCGGTAAGA
TGCCCCTGCGGGGCC
+
FFFFFF::FFFFF:F,FFFFF:FFF:F,,FFF,:F:FFFFFFF:FFFFFFFFFF:,F:FF,FFFF,FF
:,,F:F,F,FF:FFFFF,FFFFF:FF:F:FFFFFF::FFFFF,FFFFFFFF:,FFFF:::,F:FFF:F
FF:FFF,F:,:F:::
@A00721:190:HGJW7DSXY:4:1406:24831:26209 2:N:0:TCTGCAAG+GCTGCACT
ATAGAATTCATTTTTTAGATGGTTATGATCGTGACGCGTGGTCTAATGACAAATCGACACGTAAGCGT
```

ACGGAGGTGTCCCCGGCGCGGGCAAGCCAGTAATATTATATTGGCTTGCCCCACGGTCTGCGGTAAGA
TGCCCCTGCGGGGCC
+
FFFF:FFFFFFFFFFFFFFFFFFFFFFFFFFFFFFFFFFFFFFFFFFFFFFFFFFFFFFFFFFFFFFF:F
FF:FFFFFFFF,FFFFFF:FFFF:F,FFF:FF:FFFFFF,F:F,:FFF:FFFF:FFFF:,FFFF,FFFF
F:FFFFFFFFFF,F:
@A00721:190:HGJW7DSXY:4:1460:9109:2300 2:N:0:TCTGCAAG+GCTGCACT
ATAGAATTCATTTTTTAGATGGTCATGATCGTGACGCGTGGTCTAATGACAAATCGACACGTAAGCGT
ACGGAGGTGTCCCCGGCGCGGGCAAGCCAGTAATATTATATTGGCTTGCCCCACGGTCTGCGGTAAGA
TGCCCCTGCGGGGCC
+
FFFFF:FFFFFFFFFF:FFFFFFFFFFFFFFFFFFFFFFFFFFFFFFFFFFFFFFFFFFFFFFFFFFFFF
FFFFFFFFFFFFFFFFFFFFFFFFFFFFFFFFFFFFFFFFFFFFFFFFFFFFFFFFFFFFFFFFF:FFFF
FFFFFFFFFFFFFFF
@A00721:190:HGJW7DSXY:4:2158:27769:23124 2:N:0:TCTGCAAG+GCTGCACT
ATAGAATTCATTTTTTAGATGGTCATGATCGTGACGCGTGGTCTAATGACAAATCGACACGTAAGCGT
ACGGAGGTGTCCCCGGCGCGGGCAAGCCAGTAATATTATATTGGCTTGCCCCACGGTCTGCGGTAAGA
TGCCCCTGCGGGGCC
+
FFFFFFFFFFFFFFFFFFFFFFFFFFFFFFFFFFFFFFFFFFFFFFFFFFFFFFFFFFFFFFF:FFFFFF
FFFFFF:FFFFFFFFFFFFFFFFFFFFFFFFFFFFFFFFFFFFFFFFFFFFFFFFFFFFFFFFFFFFFFF
FFFFFFFFFFFFFFF
@A00721:190:HGJW7DSXY:4:2326:7093:34976 2:N:0:TCTGCAAG+GCTGCACT
ATAGAATTCATTTTTTAGATGGTCATGATCGTGACGCGTGGTCTAATGACAAATCGACACGTAAGCGT
ACGGAGGTGTCCCCGGCGCGGGCAAGCCAGTAATATTATATTGGCTTGCCCCACGGTCTGCGGTAAGA
TGCCCCTGCGGGGCC
+
FF,FFFFFFFFFFFFFFFFFF:FFFFFFFFFFFFFFFFFFFFFFFFFFFFFFFFFFFFFFFFFFFFFFFF
FFFFFFFFFFFFFFFFFFFFFFFFFFFFFFF:F:FFFFF,FFFFFFFF:FFFFFFFFFFFFFFFFF,FFF
FFFFFFFFFFFFFFF
@A00721:190:HGJW7DSXY:4:2349:24713:12446 2:N:0:TCTGCAAG+GCTGCACT
ATAGAATTCATTTTTTAGATGGTTATGATCGTGACGCGTGGTCTAATGACAAATCGACACGTAAGCGT
ACGGAGGTGTCCCCGGCGCGGGCAAGCCAGTAATATTATATTGGCTTGCCCCACGGTCTGCGGTAAGA
TGCCCCTGCGGGGCC
+
F:FFFFFFFFFFF:FFFF:FFFFFFFFFFFFFFFFFFFFFFFFF:FFFFFFF:FFFFFFF:FFFFFFFFF
FFFFFFF:FFFFFFFFFFFFFFFFF:FFFFFFFFFFFFFFFFF:FFFFF,FFFFFFFFFFFFFFFFFFFF
F:FFFFF:FFFFFFF
@A00721:190:HGJW7DSXY:4:2358:25238:16141 2:N:0:TCTGCAAG+GCTGCACT
ATAGAATTCATTTTTTAGATGGTCATGATCGTGACGCGTGGTCTAATGACAAATCGACACGTAAGCGT
ACGGAGGTGTCCCCGGCGCGGGCAAGCCAGTAATATTATATTGGCTTGCCCCACGGTCTGCGGTAAGA
TGCCCCTGCGGGGCC
+
FFFFFFFFFFFFFFFFF,FFFFFFFFFFFFFFFFF:FFFF:FFFFFFF:FFFFFF:FFFFFFFFFF::FF
::FFFFFFFFFFFFFFFFFFFFFFFFFFFFFF:FFFFFF:FFFFFFFFFFFFFFFFFFFFFFFFF,FFFF
FFF,FFFFFFFFFFF
@A00721:190:HGJW7DSXY:4:2555:13810:25285 2:N:0:TCTGCAAG+GCTGCACT
ATAGAATTCATTTTTTAGATGGTCATGATCGTGACGCGTGGTCTAATGACAAATCGACACGTAAGCGT
ACGGAGGTGTCCCCGGCGCGGGCAAGCCAGTAATATTATATTGGCTTGCCCCACGGTCTGCGGTAAGA
TGCCCCTGCGGGGCC
+
FFFFFFFFFFFFFFFFFF:FFFFFFFFFFFFFFFFFFFFFFFFFFFFFFFFFFF:FFFFFFFFFF:FFFF
FFFFFFFFFFFFFFFFFFFFFFFFF::FF:FFFFFFFFFFF,FFFFFFF:FFFFFFFFFFFFFFFFFFFF
FFFFFFFFFFFFFFF

@A00721:190:HGJW7DSXY:4:2636:18765:28072 2:N:0:TCTGCAAG+GCTGCACT
ATAGAATTCATTTTTTAGATGGTTATGATCGTGACGCGTGGTCTAATGACAAATCGACACGTAAGCGT
ACGGAGGTGTCACCGGCGCGGGCAAGCCAGTAATATTATATTGGCTTGCCCCACGGTCTGCGGTAAGA
TGCCCCTGCGGGGCC
+
FFFFFFFFFFFF:FFFFFFFF:FF:FFFFF,FFFF,FF:F,F:FFF,FFFFFFFF:F:F::FFFFF::
FF,FFFFF,FF,:FF,F,:FFFFFFF,:F,,:FFFF,FFF:,,FF,FFFF,FF:,FFFF:F,FFF::F
F:FF:FFF,FF,FFF
@A00721:190:HGJW7DSXY:4:2651:2257:27508 2:N:0:TCTGCAAG+GCTGCACT
ATAGAATTCATTTTTTAGATGGTCATGATCGTGACGCGTGGTCTAATGACAAATCGACACGTAAGCGT
ACGGAGGTGTCCCCGGCGCGGGCAAGCCAGTAATATTATATTGGCTTGCCCCACGGTCTGCGGTAAGA
TGCCCCTGCGGGGCC
+
F:FFFFFFFFFFFFFFFFFFFFFFFFFFFFFFFFFFFFFFFFFFFFFFFFFFFFFFFFFFFFF:FFFF
FFFFFFFFFFFFFFFF:FFFF:FFFFFFFFFFFFFFFFFFFFFFFFFFF,FF:FFFFFFFFFFFFFFF
FF:FFFFFFFFFFFF
@A00721:190:HGJW7DSXY:4:1343:26675:1971 2:N:0:TCTGCAAG+GCTGCACT
ATAGAATTCATTTTTTAGATGGTTATGATCGTGACGCGTGGTCTAATGACAAATCGACACGTAAGCGT
ACGGAGGTGTCCCCGGCGCGGGCAAGCCAGTAATATTATATTGGCTTGCCCCACGGTCTGCGGTAAGA
TGCCCCTGCGGGGC
+
FFFFFFFFFFFFFFFFFFFFFFFFFFFFFFFFFFFFFFFFFFFFFFFFFFFFFFFFFFFFFFFFFFFF
FFFFFFFFFFFFFFFFFFFFFFFFFFFFFFFFFFFFFFFFFFFFFFFFFFFFFFF:FF:FFFFFFF
FFFFFFFFFFFFFF
@A00721:190:HGJW7DSXY:4:2461:3812:17362 2:N:0:TCTGCAAG+GCTGCACT
ATAGAATTCATTTTTTAGATGGTCATGATCGTGACGCGTGGTCTAATGACAAATCGACACGTAAGCGT
ACGGAGGTGTCCCCGGCGCGGGCAAGCCAGTAATATTATATTGGCTTGCCCCACGGTCTGCGGTAAGA
TGACCCTGCGGGGC
+
FFFFFFFFFFFFFFFFFFFF,FFFFFFFFFFFFFFFFFFFF:FF,FFFFFFF::FFFF:,FFFF::
F:FFFF:FFFFF,FF:,::FFFFF::F,FFFFFFFFFF:FF,,F:FFF,FFFFFF,FF,,F,:FFFFF
FF,F,FFFFFFFFF
@A00721:190:HGJW7DSXY:4:1418:16685:14168 2:N:0:TCTGCAAG+GCTGCACT
CTATAGAATTCATTTTTTAGATGGTCATGATCGTGACGCGTGGTCTAATGACAAATCGACACGTAAGC
GTACGGAGGTGTCCCCGGCGCGGGCAAGCCAGTAATATTATATTGGCTTGCCCCACGGTCTGCGGTAA
GATGCCCCTGC
+
FFFFFFFFFFFFFFFFFFFFFFFFFFFFFFFFFFFFFFFFFFFFFFFFFFFFFFFFFFFFFFFFFFFF
FFFFFFFFFFFFFFFFFFFFFFFFFFFFFFFFFFFF,:FFFFFFFFFFFFFFFFFFFFF:FFFFFFF
FFFFFFFFFFF
@A00721:190:HGJW7DSXY:4:1613:24795:2879 2:N:0:TCTGCAAG+GCTGCACT
CTATAGAATTCATTTTTTAGATGGTCATGATCGTGACGCGTGGTCTAATGACAAATCGACACGTAAGC
GTACGGAGGTGTCCCCGGCGCGGGCAAGCCAGAAATATTATATTGGCATGCCCCACGGTCTGCGGTAA
GATGCCCCTGC
+
FFFF,FFF:FFFFF,FFFFFFFFFFFFFFFFFFF:FFFF:FFF::F:FFFFFFFFF:FFFFFFFF,FF
FFFFFFFFFFFFFFFF:FF:FF:F::F:FF,F,FF:FF::FF,FFFF,:F:FFFFF,:FF,FF:FFFF
:FF,FFFFF::
@A00721:190:HGJW7DSXY:4:2240:17481:27759 2:N:0:TCTGCAAG+GCTGCACT
CTATAGAATTCATTTTTTAGATGGTCATGATCGTGACGCGTGGTCTAATGACAAATCGACACGTAAGC
GTACGGAGGTGTCCCCGGCGCGGGCAAGCCAGTAATATTATATTGGCTTGCCCCACGGTCTGCGGTAA
GATGCCCCTGC
+
FFFFFFFFFFFFFFFFFFFFFFFFFFFFFFFFFFFFFFFFFFFFFFFFFFFFFFFFFFFFFFFFFFFF

FFFFFFFFFFFFFFFFFFFFFFFFFFFFFFFFFFFFFFFFFFFFFFFFFFFFFFFFFFFFFFFFFFFFFFF
FFFFFFFFFFF
@A00721:190:HGJW7DSXY:4:2240:14769:29074 2:N:0:TCTGCAAG+GCTGCACT
CTATAGAATTCATTTTTTAGATGGTCATGATCGTGACGCGTGGTCTAATGACAAATCGACACGTAAGC
GTACGGAGGTGTCCCCGGCGCGGGCAAGCCAGTAATATTATATTGGCTTGCCCCACGGTCTGCGGTAA
GATGCCCCTGC
+
FFFFFFFFFFFFFFFF:FFFFFFFFFFFFFFFFFFFFFFFFFFFFFFFFFFFFFFFFFFFFFFFFFFFFF
FFFFFFFFFFFFFFFFFFFFFFFFFFFFFFFFFFFFFFFFFFFFFFFFFFFFFFFFFFFFFFFFFFFFFFF
FFFFFFFFFFF
@A00721:190:HGJW7DSXY:4:2307:20925:27492 2:N:0:TCTGCAAG+GCTGCACT
CTATAGAATTCATTTTTTAGATGGTCATGATCGTGACGCGTGGTCTAATGACAAATCGACACGTAAGC
GTACGGAGGTGTCCCCGGCGCGGGCAAGCCAGTAATATTATATTGGCTTGCCCCACGGTCTGCGGTAA
GATGCCCCTGC
+
FFFFFFFFFFFFFFFFFFFFFFFFFFFFFFFFFFFF:FFFFFFFFFFFFFFFFFFFFFFFFFFFFFFFFFF
FFFFFFFFFFFFFFFFFFFFFFFFFFFFFFFFFFFFFFFFFFFFFFFFFFFFFFFFFFFFF:FFF:FFFFF
FFF:FFFF:FF
@A00721:190:HGJW7DSXY:4:2446:10321:9220 2:N:0:TCTGCAAG+GCTGCACT
CTATAGAATTCATTTTTTAGATGGTCATGATCGTGACGCGTGGTCTAATGACAAATCGACACGTAAGC
GTACGGAGGTGTCCCCGGCGCGGGCAAGCCAGTAATAGTATATTGGCTTGCCCCACGGTCTGCGGTAA
GATGCCCCTGC
+
FFFFFFFFF:FFFFFFFFFFFFFFFFFFFFFFFFFFFFFFFFFFFFFFFFFFFFFFFFFFFFFFFFFFFFF
FFFFFFFFFFFFFFFFFFFFFFFFFFF,FFFFFFFFF:,FFFFF:FFFFFFFFFFFFFFFFFFFFF,F:
FFFFFFFFFFF
@A00721:190:HGJW7DSXY:4:2519:28230:22044 2:N:0:TCTGCAAG+GCTGCACT
CTATAGAATTCATTTTTTAGATGGTCATGATCGTGACGCGTGGTCTAATGACAAATCGACACGTAAGC
GTACGGAGGTGTCCCCGGCGCGGGCAAGCCAGTAATATTATATTGGCTTGCCCCACGGTCTGCGGTAA
GATGCCCCTGC
+
FFFF:F:FFFFFFFFFFFFFFFFFFFFFFFFFFFFFFFFFFFFFFFFFFFFFFFFFFFFFFFFFFFFFFFF
FFFFFFFFFFFFFFFFFFFFFFFFFFFFFFFFFFFFFFFFFFFFFFFFFFFFFFFFFFFFFFFFFFFFF,F
FFFFF,FFFFF
@A00721:190:HGJW7DSXY:4:2653:17544:34194 2:N:0:TCTGCAAG+GCTGCACT
CTATAGAATTCATTTTTTAGATGGTCATGATCGTGACGCGTGGTCTAATGACAAATCGACACGTAAGC
GTACGGAGGTGTCCCCGGCGCGGGCAAGCCAGTAATATTATATTGGCTTGCCCCACGGTCTGCGGTAA
GATGCCCCTGC
+
FFFFFFF,FFFFFFFFFFFFFFFFFFFFFFFFFFFFFFFFFFFFFFFFFFFFFFFFFFFFFFFFFFFF:FF
FFFFFFFFFFFFFFFFFFFFFFFFFFFFFFFFFFF,F:FFFFFFFFFFFFFFFFFFFFFFFFFFFFFF:FF
FFFFFFFFF:F
@A00721:190:HGJW7DSXY:4:2653:17852:34225 2:N:0:TCTGCAAG+GCTGCACT
CTATAGAATTCATTTTTTAGATGGTCATGATCGTGACGCGTGGTCTAATGACAAATCGACACGTAAGC
GTACGGAGGTGTCCCCGGCGCGGGCAAGCCAGTAATATTATATTGGCTTGCCCCACGGTCTGCGGTAA
GATGCCCCTGC
+
FFFFFFFFFFFFFFFFFFFFFFFFFFFFFFFFFFFFFFFFFFFFFFFFFFFFFFFFFFFFFFFFFFFFFFF
FFFFFFFFFFFFFFFFFFFFFFFFFFFFFFFFFFFFFFFFFFFFF:FFFFFFFFFFFFFFFFFFFFFFFFF
FFFFFFFFFFF
@A00721:190:HGJW7DSXY:4:1119:8558:22200 2:N:0:TCTGCAAG+GCTGCACT
TGCGTCACTATAGAATTCATTTTTTAGATGGTTATGATCGTGACGCGTGGTCTAATGACAAATCGACA
CGTAAGCGTACGGAGGTGTCCCCGGCGCGGGCAAGCCAGTAATATTATATTGGCTTGCCCCACGGTCT
GCGGTAAGATGCCCC

```
+
FFFFFFFFFFFFFFFFFFFFFFFF,FFF:FFFFFFFFFFFFFFFFFFFFFFFFFFFFFFFF:FFFFFF
FFFFFFFFFFFFFFFFFFFFFFFFFFFFFFFFFFFFF,:FFFFFFF::FFFFFFFFFFFFFFFFFFFF
F:FFFFFFFFFFFFF
@A00721:190:HGJW7DSXY:4:1647:11939:23703 2:N:0:TCTGCAAG+GCTGCACT
TGCGTCACTATAGAATTCATTTTTTAGATGGTTATGATCGTGACGCGTGGTCTAATGACAAATCGACA
CGTAAGCGTACGGAGGTGTCCCCGGCGCGGGCAAGCCAGTAATATTATATTGTCTTGCCCCACGGTCT
GCGGTAAGATGCCCC
+
FFFF,FFFF,FFFFFFFFFFF:::FFFF,FF:FFFFF:FFF:FFFFF:F:FFF,FFFFFFFFFFFFFF
FFFFFFFFFF:F,FF:,FFFFFFFFFFFFF:FF:,FFFF,FFFFFFFFFFF:,FFF,F:FFFF::FFF
FFF:,F::FF:FFF:
@A00721:190:HGJW7DSXY:4:2664:18177:3317 2:N:0:TCTGCAAG+GCTGCACT
TGCGTCACTATAGAATTCATTTTTTAGATGGTTATGATCGTGACGCGTGGTCTAATGACAAATCGACA
CGTAAGCGTACGGAGGTGTCCCCGGCGCGGGCAAGCCAGTAATATTATATTGGCTTGCCCCACGGTCT
GCGGTAAGATGCCCC
+
FFFFFFFFFFFFFFFFFFFFFFF,FFFFFFFFFFFFFFFFFFFFFFF,FFFFFFFFFFFFFF:FFFFF
FFFFFFFFFFFFFFFFFFFFFFFFFFFFFFFFFFFFFFFFFFF:FFFFFFFFFFFFF:FFFFFFFFFF
FFFFFFFFFFFFFFF
@A00721:190:HGJW7DSXY:4:1214:3179:35180 2:N:0:TCTGCAAG+GCTGCACT
ATTCTACTGCGTCACTATAGAATTCATTTTTTAGATGGTTATGATCGTGACGCGTGGTCTAATGACAA
ATCGACACGTAAGCGTACGGAGGTGTCCCCGGCGCGGGCAAGCCAGTAATATTATATTGGCTTGCCCC
ACGGTCTGCGGTAAG
+
F:FFFFFFFFFFFFFFFFFFFFFFFFFFF:FF:FF,FFFFFFFFFFFFFFFFFFFFFFFFFFFFFFFF
FFFFFFFFFFFFFFFFFFFFFFFFFFF:FFFFFFFFFFF:FFFFFFFFFFFFF,FFFFFFFFFFFFFF
FFFFFF:FFFFFFFF
@A00721:190:HGJW7DSXY:4:1417:10936:12884 2:N:0:TCTGCAAG+GCTGCACT
ATTCTACTGCGTCACTATAGAATTCATTTTTTAGATGGTTATGATCGTGACGCGTGGTCTAATGACAA
ATCGACACGTAAGCGTACGGAGGTGTCCCCGGCGCGGGCAAGCCAGTAATATTATATTGGCTTGCCCC
ACGGTCTGCGGTAAG
+
FFFFFFFFFFFFFFFFFFFFFFFFFFFFFF:FFF::FFFFFFFFFFFFFFFFFFFFFFFFFFFFFFF:
FFFFFFFFFFF:FFFFFFFFFFFFFFFFFFFFFFFFFFFFFFFFFFFFF,FFFFFFFFFFFFFFFFFF
FFFFFF,FFFF:F:F
@A00721:190:HGJW7DSXY:4:1621:25852:28729 2:N:0:TCTGCAAG+GCTGCACT
ATTCTACTGCGTCACTATAGAATTCATTTTTTAGATGGTTATGATCGTGACGCGTGGTCTAATGACAA
ATCGACACGTAAGCGTACGGAGGTGTCCCCGGCGCGGGCAAGCCAGTAATATTATATTGGCTTGCCCC
ACGGTCTGCGGTAAG
+
FFFFFFFFFFFFFFFFFFFFFFFFFFFFFFFFFFFFFFFFFFFFFFFFFFFFFFFFFFFFFFFFFFFF
FFFFFFFFFFFFFFFFFFFFFFFFFFFFFFFFFFFFFFFFFFFFFFFFFFFFFFFFFFFFFFFFFFFF
FFFFFFFFFFFFFF:
@A00721:190:HGJW7DSXY:4:1656:13892:10770 2:N:0:TCTGCAAG+GCTGCACT
ATTCTACTGCGTCACTATAGAATTCATTTTTTAGATGGTTATGATCGTGACGCGTGGTCTAATGACAA
ATCGACACGTAAGCGTACGGAGGTGTCCCCGGCGCGGGCAAGCCAGTAATATTATATTGGCTTGCCCC
ACGGTCTGCGGTAAG
+
FFFFFFFFFFFFFFFFFFFFFFFFFFFFFFFFFFFFFFFFFFFFFFFFFFFFFFFFFFFFFFFFFFF:
FFFFFFFFFFFFFFFFF:FFFFFFFFFFFFFFFFFFFFFFFFFFFFFFFFFFFFFFFFFFFFFFFFFF
FFFFFFFFFFFFFFF
@A00721:190:HGJW7DSXY:4:2253:9100:6104 2:N:0:TCTGCAAG+GCTGCACT
ATTCTACTGCGTCACTATAGAATTCATTTTTTAGATGGTTATGATCGTGACGCGTGGTCTAATGACAA
```

```
ATCGACACGTAAGCGTACGGAGGTGTCCCCGGCGCGGGCAAGCCAGTAATATTATATTGGCTTGCCCC
ACGGTCTGCGGTAAG
+
FFFFFFFFFFFFFFFFFFFF:FFFFFFFFFFFFFFFF,FFFFFFF:FFFFFFF:FF:FF:FFFF:FFF
FFF,FFFFFFFF::FFFFFFFF:FFFFFFFFFFFFFFFFF,FF,FF,:FFFFFFFF,FFFFFFF,FFF
:F:F,FFFFFFFFFF
@A00721:190:HGJW7DSXY:4:1128:23402:8923 2:N:0:TCTGCAAG+GCTGCACT
CGATTTTACTGCGTCACTATAGAATTCATTTTTTAGATGGTCATGATCGTGACGCGTGGTCTAATGAC
AAATCGACACGTAAGCGTACGGAGGTGTCCCCGGCGCGGGCAAGCCAGTAATATTATATTGGCTTGCC
CCACGGTCTGCGGTA
+
FFFF,FFFFF:FFFFFFFFFFFFFFFFFFFFF:FFFFFF,FFF:FFFF:FFFFFFF:FFFFFFF,FFF
FFFFFFF,FFFF:F,FFFFF:FFF:,FFFFFFF::F:::FFFF:FFFFFFF:FFFFFFFF,FFFFFFF
F:::,F,FFFFF::F
@A00721:190:HGJW7DSXY:4:1231:6415:31391 2:N:0:TCTGCAAG+GCTGCACT
CGATTTTACTGCGTCACTATAGAATTCATTTTTTAGATGGTCATGATCGTGACGCGTGGTCTAATGAC
AAATCGACACGTAAGCGTACGGAGGTGTCCCCGGCGCGGGCAAGCCAGTAATATTATATTGGCTTGCC
CCACGGTCTGCGGTA
+
FFFFFFFFFFFFFFFFFFF,FF,FFFFFFFFFFFFFFFFFFFFFFFFFFFFFFFFFFFFFFFFFFFFF
FFFFFFFFFFFFFFFF:FFFFFFFFFFFFFFFFFFFFFFFF:FFFF,FFFFFFFFFFFFFFFFFFFFF
FF:FFFFFFFFFFFF
@A00721:190:HGJW7DSXY:4:1644:11451:13088 2:N:0:TCTGCAAG+GCTGCACT
CGATTTTACTGCGTCACTATAGAATTCATTTTTTAGATGGTCATGATCGTGACGCGTGGTCTAATGAC
AAATCGACACGTAAGCGTACGGAGGTGTCCCCGGCGCGGGCAAGCCAGTAATATTATATTGGCTTGCC
CCACGGTCTGCGGTA
+
FFF,:FFFFFFFFFFFFFFF,FF,FFFFFFFFFFFFFFF:FFFFFFFFFFFFFFFFFFFFFFFFFFFF
F,FFFFFFFFFFFFFFFFFFFFFFFFFF:FFFFFFFFFFFFFFFFFFFFFFFFFFFFFFFFFFFFFFF
FF:FFFFFFFFFFFF
@A00721:190:HGJW7DSXY:4:1338:16685:19899 2:N:0:TCTGCAAG+GCTGCACT
ATTTTACTGCGTCACTATAGAATTCATTTTTTAGATGGTCATGATCGTGACGCGTGGTCTAATGACAA
ATCGACACGTAAGCGTACGGAGGTGTCCCCGGCGCGGGCAAGCCAGTAATATTATATTGGCTTGCCCC
ACGGTCTGCGGAGAT
+
FFFFFFFFFFFF:FFFFF:FFFFFFFFFF:FFFFFFFFFFFFFFFFFFFFFFFFFFFFFFFFFFFFFF
FFFFFFFFFFFFFFFFFFFFFF:FFFFFFFFFFFFFFFFFFFFF:FFFFFFFFFFFFFFFFF:FFFFF
FFFFFF::FFFFFFF
@A00721:190:HGJW7DSXY:4:2424:28022:34679 2:N:0:TCTGCAAG+GCTGCACT
ATTTTACTGCGTCACTATAGAATTCATTTTTTAGATGGTCATGATCGTGACGCGTGGTCTAATGACAA
ATCGACACGTAAGCGTACGGAGGTGTCCCCGGCGCGGGCAAGCCAGTAATATTATATTGGCTTGCCCC
ACGGTCTGCGGAGAT
+
F:FFFFFFFFFFFFFFFFFFF:FFFFFFFFFFFFFFFFFFFFFFFFFFFFFFFFFFFFFFFFFFFFFF
FFFFFFFFFFFFFFFFFFFFFFFFFFFFFFFFFFFFFFFFFFFFFFF:F,FF:FFFFFFFFFFFFFFF
FFFFFFFFFFFFFFF
@A00721:190:HGJW7DSXY:4:2504:9290:10128 2:N:0:TCTGCAAG+GCTGCACT
ATTTTACTGCGTCACTATAGAATTCATTTTTTAGATGGTCATGATCGTGACGCGTGGTCTAATGACAA
ATCGACACGTAAGCGTACGGAGGTGTCCCCGGCGCGGGCAAGCCAGTAATATTATATTGGCTTGCCCC
ACGGTCTGCGGAGAT
+
FFFFFFFFFFF:F:FF,FFFFFFFFFFFFFFFFFFFFFFFF:FFFFFFFFFFFFFF,FFFFFFFFF:F
FFFFFFFFFFFFFFFFFFFFFFFFFFFFFFFFFFFFFFFFFF:F:FFFF,F,F,FFFF,FFFFFFFFF
:FFFFF:FFFFFFFF
```

```
@A00721:190:HGJW7DSXY:4:2522:31864:13088 2:N:0:TCTGCAAG+GCTGCACT
ATTTTACTGCGTCACTATAGAATTCATTTTTTAGATGGTCATGATCGTGACGCGTGGTCTAATGACAA
ATCGACACGTAAGCGTACGGAGGTGTCCCCGGCGCGGGCAAGCCAGTAATATTATATTGGCTTGCCCC
ACGGTCTGCGGAGAT
+
FFFFFF:FFFFFFFFFFF,FFFFFFFFFFFFFFFFFFFFFFFFFFFFFFF:FFFFFFFFFFFF:FFF
FFFF:FFFFFFFFFFFFFFFFFFFFFFFFFFFFFFFFFFF:FFFFF,FFFFFFFFFFF,FFFFFFFFF
FFFFFFFFFFFF:FF
@A00721:190:HGJW7DSXY:4:1110:23321:23406 2:N:0:TCTGCAAG+GCTGCACT
GTCGATTCTACTGCGTCACTATAGAATTCATTTTTTAGATGGTTATGATCGTGACGCGTGGTCTAATG
ACAAATCGACACGTAAGCGTACGGAGGTGTCCCCGGCGCGGGCAAGCCAGTAATATTATATTGGCTTG
CCCCACGGTCTGCGG
+
FFFFFFFFFFFFFFFFF,FFFFFFF:FFFFFFFFFFFFF,FFFFFFFFFFFFFFFFFFFFFFFFFFFF
FFFFFFFFFFFFFFFFFFFFFFFFFFFFFFF:FFFFFFFFFFF:,FFFFFF:FF:FFFFFFF:FF:FF
FFFF,FFFFFFFFFF
@A00721:190:HGJW7DSXY:4:2320:20745:14027 2:N:0:TCTGCAAG+GCTGCACT
GTCGATTCTACTGCGTCACTATAGAATTCATTTTTTAGATGGTTATGATCGTGACGCGTGGTCTAATG
ACAAATCGACACGTAAGCGTACGGAGGTGTCCCCGGCGCGGGCAAGCCAGTAATATTATATTGGCTTG
CCCCACGGTCTGCGG
+
FFFFFFFFFFFFFFFFFFFF,F,FFFFFFFFFFF:FFFFFFFFFFFFFFFFFFFFFFF:FFFFFFFFF
FFFFFFF:FFFFFFFFFFFFFFFFFFFFFFFF:FFFFFFFFFFFFFFFFFFFFFFFFFFFFFFFFFFF
FFFFFFFFFFFFFFF
@A00721:190:HGJW7DSXY:4:2331:11668:20447 2:N:0:TCTGCAAG+GCTGCACT
GTCGATTCTACTGCGTCACTATAGAATTCATTTTTTAGATGGTTATGATCGTGACGCGTGGTCTAATG
ACAAATCGACACGTAAGCGTACGGAGGTGTCCCCGGCGCGGGCAAGCCAGTAATATTATATTGGCTTG
CCCCACGGTCTGCGG
+
FFFFFFFFFFFFFFFFFFFFF:,FFFFFFFFFFFFFFFFFFFFFFFFFFFFFFFFFFF:FFFFF:FFF
FFFFFFFFFFFFFFFF:FFFFFFF:FFFFFFFFFFFFFFFFFFF:FFFFFFFFFFFFFFFFFF,FFFF
:FFFFFFFFFFFFFF
@A00721:190:HGJW7DSXY:4:2472:18331:9377 2:N:0:TCTGCAAG+GCTGCACT
GTCGATTCTACTGCGTCACTATAGAATTCATTTTTTAGATGGTTATGATCGTGACGCGTGGTCTAATG
ACAAATCGACACGTAAGCGTACGGAGGTGTCCCCGGCGCGGGCAAGCCAGTAATATTATATTGGCTTG
CCCCACGGTCTGCGG
+
FF:FFFFFFFFFFFFFFFFFFFFFFF,:FFFFFFFFFFFFFFFFFF::FFFFFF:FFFFFFFFFFFFF
FFFFFFFFFFFFFFFFFF:FF:F:FFFFFFFFF:FFFFFFFFFFF,:FFFFFFFFFFFFF:FFFF:F
FFFFFFFFFFFFFFF
@A00721:190:HGJW7DSXY:4:2472:18285:9424 2:N:0:TCTGCAAG+GCTGCACT
GTCGATTCTACTGCGTCACTATAGAATTCATTTTTTAGATGGTTATGATCGTGACGCGTGGTCTAATG
ACAAATCGACACGTAAGCGTACGGAGGTGTCCCCGGCGCGGGCAAGCCAGTAATATTATATTGGCTTG
CCCCACGGTCTGCGG
+
FFFFFFFFFFFFFFFFFFFFFFFFFFFFFFFFFFFFFFFFFFFFFFFFFFFFFFFFFFFFFFFFFFFF
FFFFFFFFFFFFFFFFFFFFFFFFFFFFFFFFFFFFFFFFFFFFF:FFFFF:FFFF:FFFFFFFFFFF
FFFFFFFFFFFFFFF
@A00721:190:HGJW7DSXY:4:1528:12816:22467 2:N:0:TCTGCAAG+GCTGCACT
CGTCGATTCTACTGCGTCACTATAGAATTCATTTTTTAGATGGTTATGATCGTGACGCGTGGTCTAAT
GACAAATCGACACGTAAGCGTACGGAGGTGTCCCCGGCGCGGGCAAGCCAGTAATATTATATTGGCTT
GCCCCACGGTCTGCG
+
FFFFFFFFFFFFFFFFFFFF:,FFFFFFFFFFFFFFFFFFFFFFFFFFFFFFFFFFFFFFFF:FFFFF
```

```
FFFFFFFFFFFFFFFFFFFFFFFFFFFFFFFFFFFFFFFFFFFFFFFFFFFFFFFFFFFFFFFFFFFFF
:FFFFFFFFFFFFFF
@A00721:190:HGJW7DSXY:4:2123:22923:15327 2:N:0:TCTGCAAG+GCTGCACT
CGTCGATTCTACTGCGTCACTATAGAATTCATTTTTTAGATGGTTATGATCGTGACGCGTGGTCTAAT
GACAAATCGACACGTAAGCGTACGGAGGTGTCCCCGGCGCGGGCAAGCCAGTAATATTATATTGGCTT
GCCCCACGGTCTGCG
+
FF:FFFFFFFFFFFFF:FFFF:FFFF,FFFFFFFFFFFFF:FFFFFFFFFFFFFFFFFFFFFFFFFFFF
FFFFFFFFFFFFFFFFFFFFFFFFFFFFFFFFFFFFFFFFFFFFFFF:FFFFFFFFFFFFFFFFF:FFF
FFF:FFFFFFFFFFF
@A00721:190:HGJW7DSXY:4:2167:3106:31579 2:N:0:TCTGCAAG+GCTGCACT
CGTCGATTCTACTGCGTCACTATAGAATTCATTTTTTAGATGGTTATGATCGTGACGCGTGGTCTAAT
GACAAATCGACACGTAAGCGTACGGAGGTGTCCCCGGCGCGGGCAAGCCAGTAATATTATATTGGCTT
GCCCCACGGTCTGCG
+
FFFFFFFFFFFFFFFFFFFFFFF:FFFFFFFFFFFFFFF:FFFFFFFFFFFFFFFFFFFFFFFFFFFFF
FFFFFFFFFFFFFFFFFFFFFFFFFFFFFFFFFFFFFFFFFFFFFFFFFFFFFFFF::FFFFFFFFFFF
FFFFFFFFFFF:FFF
@A00721:190:HGJW7DSXY:4:2167:4200:32847 2:N:0:TCTGCAAG+GCTGCACT
CGTCGATTCTACTGCGTCACTATATAATTCAAATATTATATGGTTATGATCGTGACGCGTGGTCTAAT
GACAAATCGACACGAAAGCGAACGGAGGTCTCACCGGCGCGGGCAAGCCAGAAAAATTATAAAGGCTT
GCCCCACGGACTGC
+
FFFF,FFFFFF:FFF,F::F,FF:,F:,FFF,,,,,,:,F,,,FF:,FFFF,:,FFFFFF,F:FF:F:
FFF:FFFF:FF:,:,,,,F,,FF,,F:F:,,F,FF,:F,FF,,F,:,FFF,,:,,FF,:,F,,,:FF,
,::,,:F,:,FFF:
@A00721:190:HGJW7DSXY:4:1311:4851:26396 2:N:0:TCTGCAAG+GCTGCACT
ATTGACCGTCGATTCTACTGCGTCACTATAGAATTCATTTTTTAGATGGTTATGATCGTGACGCGTGG
TCTAATGACAAATCGACACGTAAGCGTACGGAGGTGTCCCCGGCGCGGGCAAGCCAGTAATATTATAT
TGGCTTGCCCCACGG
+
FFFFFFFFFFFF,FFFF:F:FFF:FFFF,:FFFFFFFFFFFFFFF:FFFFFFFF:FFFFFFFFFF,FF
FFFFFFFFFFFFFFFFF:FFFFFFFFFF:FFFFFFFF:FFFFFFFFFFFF:FFFFFFFFFFF:FFFF:
FFFFFFFFFFFFFFF
@A00721:190:HGJW7DSXY:4:1616:6894:26710 2:N:0:TCTGCAAG+GCTGCACT
ATTGACCGTCGATTCTACTGCGTCACTATAGAATTCATTTTTTAGATGGTTATGATCGTGACGCGTGG
TCTAATGACAAATCGACACGTAAGCGTACGGAGGTGTCCCCGGCGCGGGCACGCCAGTAATATTATAT
TGGCTTGCCCCACGG
+
FFFFFFFFFFFFFFFFFFFFFFF,FFF:FFFFFFFFFFF:FF,FFFFFFFFFFFFFFFFF:FFFFFFF
,FFFFFFFFFFFFFFF::FF:FFFFFFFFFFFFFFFFFFFFFFFFFFFFFF,FF:FFF,FFFF,FFFF
F,FFFF,FFFFFFFF
@A00721:190:HGJW7DSXY:4:2206:16794:32675 2:N:0:TCTGCAAG+GCTGCACT
ATTGACCGTCGATTCTACTGCGTCACTATAGAATTCATTTTTTAGATGGTTATGATCGTGACGCGTGG
TCTAATGACAAATCGACACGTAAGCGTACGGAGGTGTCCCCGGCGCGGGCAAGCCAGTAATATTATAT
TGGCGTGCCCCACGG
+
FFFFFFFFFFFFF:FFFFFFFFFF,FF,FFFFFFF,FFFFFF:FF::FFFFFF:FFFFFF:FFFFFFF
FFFFF:FFFFFFFFFFF:F:FFFFF:,FFFFFFFFFFFFFFFFF:FFFFF,:FFFF,,:F:::FFFFF
FFFF:FFFFFFFFFF
@A00721:190:HGJW7DSXY:4:2304:12084:33254 2:N:0:TCTGCAAG+GCTGCACT
ATTGACCGTCGATTCTACTGCGTCACTATAGAATTCATTTTTTAGATGGTTATGATCGTGACGCGTGG
TCTAATGACAAATCGACACGTAAGCGTACGGAGGTGTCCCCGGCGCGGGCAAGCCAGTAATATTATAT
TGGCTTGCCCCACGG
```

```
+
FFFFFFFFFFFFFFFFFFFFFFFFFFF:FFFF:FFFFFFFFFFFFF:FFFFFFF:FFFFFF:FFFFFFF
FFFFFFFFFFFFFFFFFFFFFFFFFFFFFFFFFFFFFFFFFFFFFFFFF:FFFF:FF:FFFFFF:
FFFFFFFFFFFFF:F
@A00721:190:HGJW7DSXY:4:2509:10384:30686 2:N:0:TCTGCAAG+GCTGCACT
ATTGACCGTCGATTCTACTGCGTCACTATAGAATTCATTTTTAGATGGTTATGATCGTGACGCGTGG
TCTAATGACAAATCGACACGTAAGCGTACGGAGGTGTCCCCGGCGCGGGCAAGCCAGTAATATTATAT
TGGCTTGCCCCACGG
+
FFFFFFFFFFFF:FFFFFF:FFFFFFFF:,F:FFFFFFFFF,FFF:FFFFFFFF:FFFFFFFFFFFFF
:FFFFFFFF:FFFFFFFFFFFFFFFFFFFFFFFFFFFFFFFFFFFFFFFFFFFFFFFFFFFFFFFFFFF
FF,FFFFFFFFFFFF
@A00721:190:HGJW7DSXY:4:1103:9064:11866 2:N:0:TCTGCAAG+GCTGCACT
TGAATTTCAAAATTGACCGTCGATTCTACTGCGTCACTATAGAATTCATTTTTAGATGGTTATGATC
GTGACGCGTGGTCTAATGACAAATCGACACGTAAGCGTACGGAGGTGTCCCCGGCGCGGGCAAGCCAG
TAATATTATATTGGC
+
FFFFFFFFFFFFFFFFF:FFFFFFFFFFFFFFFFFFFFFF:FFFFFFFFFFFFFFFFFFFFFFFF,FF
FFF:FFFFFFFFFFFFFF:FFFFFFFFFFFFFF:FFFFFFFF,FFFFFFFFFFFFFFFFFF,:FFFFF
,FFF:FFFF:FFF,F
@A00721:190:HGJW7DSXY:4:1166:12554:2284 2:N:0:TCTGCAAG+GCTGCACT
TGAATTTCAAAATTGACCGTCGATTCTACTGCGTCACTATAGAATTCATTTTTAGATGGTTATGATC
GTGACGCGTGGTCTAATGACAAATCGACACGTAAGCGTACGGAGGTGTCCCCGGCGCGGGCAAGCCAG
TAATATTATATTGGC
+
FFFFFFFFFFFFF:FFFFFFFFFFFFFFFFFFFFFFFFFFFFFFFFFFFFFFF:FFFFFFFFFFFFFFF
FFFFFFFF:FFFFFFFFFFFFFFFFFFFFFFFFFFFFFFFFFFFFFFF:FFFFF:FFFFFFFFFFFF:F
F:FFFFFFFFFF,:F
@A00721:190:HGJW7DSXY:4:1331:22110:18020 2:N:0:TCTGCAAG+GCTGCACT
TGAATTTCAAAATTGACCGTCGATTCTACTGCGTCACTATAGAATTCATTTTTAGATGGTTATGATC
GTGACGCGTGGTCTAATGACAAATCGACACGTAAGCGTACGGAGGTGTCCCCGGCGCGGGCAAGCCAG
TAATATTATATTGGC
+
FF:FFFFFFFFFFFF:FFFFFFFFFFFFFFFFFFFFFFFFFFFFFFFFFFFFFFFFFFFFFFFFFFFF
FFFFFFFFFFFFFFFFFFFFFFFFFFFFFFFFFFFFFFFFFFFFFFFFFFFFFF:FFFFFFFFFFFFF
F:FFFFFFFFFFFF:
@A00721:190:HGJW7DSXY:4:1622:28718:24893 2:N:0:TCTGCAAG+GCTGCACT
TGAATTTCAAAATTGACCGTCGATTCTACTGCGTCACTATAGAATTCATTTTTAGATGGTTATGATC
GTGACGCGTGGTCTAATGACAAATCGACACGTAAGCGTACGGAGGTGTCCCCGGCGCGGGCAAGCCAG
TAATATTATATTGGC
+
FFFFFFFFFFFFFFFFFFFFFFFFFFFFFFFFFFFFFF:FFF:,FFFFFFFFFFFFFFFFFFFFFFFFF
FFFFFFFFFF:FFFFFFF:FFFFFF:FFFFFFFFFFFF,F:F:FFFFFFFFFFFFFFFFFFF:FFFFF
FFFFFFFFFFFF,:F
@A00721:190:HGJW7DSXY:4:2456:4625:10191 2:N:0:TCTGCAAG+GCTGCACT
TGAATTTCAAAATTGACCGTCGATTCTACTGCGTCACTATAGAATTCATTTTTAGATGGTTATGATC
GTGACGCGTGGTCTAATGACAAATCGACACGTAAGCGTACGGAGGTGTCCCCGGCGCGGGCAAGCCAG
TAATATTATATTGGC
+
FFFFFFFFFFFFFFFFFFFFFFFFFFFFFFFFFFFFFFFF:FFFFFFFFFFFFFFFF:FFFFFFFFFF
FFFFFFFF,FFFFFFFFF:FFFFFFFFFFFFFFFFFFFFFFFFFFFFFFFFFFFFFFFFFF,F:F,F
,FFFFFF::FFFF,F
@A00721:190:HGJW7DSXY:4:2510:11116:9001 2:N:0:TCTGCAAG+GCTGCACT
TGAATTTCAAAATTGACCGTCGATTCTACTGCGTCACTATAGAATTCATTTTTAGATGGTTATGATC
```

```
GTGACGCGTGGTCTAATGACAAATCGACACGTAAGCGTACGGAGGTGTCCCCGGCGCGGGCAAGCCAG
TAATATTATATTTGC
+
FF:FFF::FFFFFFFFFF:FFFF:F::FFFFFF:FFF:F::F,FF::F,FF,:FFFFFFFFF:FFFFF
FF,,F:FF,FFFFFF,FFFFFFFF,FFFFFFFFF:F:FFFFFFF:FFFFF:FFFFFFFFF:FF:FFF,
,,F:F,F::,FF,FF
@A00721:190:HGJW7DSXY:4:2673:9715:5447 2:N:0:TCTGCAAG+GCTGCACT
TGAATTTCAAAATTGACCGTCGATTCTACTGCGTCACTATAGAATTCATTTTTTAGATGGTTATGATC
GTGACGCGTGGTCTAATGACAAATCGACACGTAAGCGTACGGAGGTGTCCCCGGCGCGGGCAAGCCAG
TAATATTATATTGGC
+
FFF:FFFFFFFFFFFFFFFFFFFFFFFFFFFFFFFFFFFF:FFFFFFFFFFFFFFFFFFFFFFFFFFF
FFFFFFFFFFFFFFFFFFFFFFFFFFFFFFFFFFFFFFFFFFFFFFFF:FFFFFFFFFFFFFF:FFFF
FFFFFFFFFFFFFFF
@A00721:190:HGJW7DSXY:4:1516:32452:16423 2:N:0:TCTGCAAG+GCTGCACT
TTTGAATTTCAAAATTGACCGTCGATTCTACTGCGTCACTATAGAATTCATTTTTTAGATGGTTATGA
TCGTGACGCGTGGTCTAATGACAAATCGACACGTAAGCGTACGGAGGTGTCCCCGGCGCGGGCAAGCC
AGTAATATTATATTG
+
FFFFFFFFFFFFFFFFFFFFFFFFFFFFF:FFFFFFF:FFFF,F:FFFFFFFFFFFFF,FFFFFFFFF
FFFFFFFFFFFFFFFFFFFFF,F:FFFFFFFFFFFFF:FFFFFFFFFFFFFFFFF:FFFFFFFFFF,F
,FF,FFFFFF:FFFF
@A00721:190:HGJW7DSXY:4:2205:16224:6574 2:N:0:TCTGCAAG+GCTGCACT
TTTGAATTTCAAAATTGACCGTCGATTCTACTGCGTCACTATAGAATTCATTTTTTAGATGGTTATGA
TCGTGACGCGTGGTCTAATGACAAATCGACACGTAAGCGTACGGAGGTGTCCCCGGCGCGGGCAAGCC
AGTAATATTATATTG
+
FFFFFFFFFFFF:FFFFFFFFFFF:FFFFFFFFFFFFFFFFFFFFFFFFFFFFFFFFF,FFFFFFFFF
FFFFFFFFFFFFFFFF,:FFFFFFF,FFFFFFFFFF:FFFFFFFFFFFFFFFFFFFFFFFFFF,FFFF
FFFFFFF,FFFF,FF
@A00721:190:HGJW7DSXY:4:2324:28085:34757 2:N:0:TCTGCAAG+GCTGCACT
TTTGAATTTCAAAATTGACCGTCGATTCTACTGCGTCACTATAGAATTCATTTTTTAGATGGTTATGA
TCGTGACGCGTGGTCTAATGACAAATCGACACGTAAGCGTACGGAGGTGTCCCCGGCGCGGGCAAGCC
AGTAATATTATATTG
+
FFFFFFFFFFFFFFFFFFFFFFFFFFFFFFF,:F:FFFFFFFFFFFFF:FFF:FFFFFFFF,FFFFFF
FFFFFF:FFFFFFFFFFFFFFFFFFFF:FFFF:F:FFF:,FFFFFFFF,FFFFFFFFFFFFFFFFFFF
:FFFF,F:FFFFFFF
@A00721:190:HGJW7DSXY:4:2435:25780:27539 2:N:0:TCTGCAAG+GCTGCACT
TTTGAATTTCAAAATTGACCGTCGATTCTACTGCGTCACTATAGAATTCATTTTTTAGATGGTTATGA
TCGTGACGCGTGGTCTAATGACAAATCGACACGTAAGCGTACGGAGGTGTCCCCGGCGCGGGCAAGCC
AGTAATATTATATTG
+
FFFFFFF:FFFF::FFFFFFFFFFFFFFFFFF:FFFFFFFFFFFFFFFFFFFFFFFFFFFFFFFFFF:
FFFFF:FFFF:FFFFFFFFFFF:FFF,FFFFF,F,FFFFFFFF:F:FF:FFF:FFFFFFFFFF:F:F:
:FFFF:FFFFFFFFF
@A00721:190:HGJW7DSXY:4:2471:28176:12868 2:N:0:TCTGCAAG+GCTGCACT
TTTGAATTTCAAAATTGACCGTCGATTCTACTGCGTCACTATAGAATTCATTTTTTAGATGGTTATGA
TCGTGACGCGTGGTCTAATGACAAATCGACACGTAAGCGTACGGAGGTGTCCCCGGCGCGGGCAAGCC
AGTAATATTATATTG
+
FFFFFFFFFFFFFFFFFFFFFFFFFFFFFFFFFFFFFFFFFF,F:FFFFFFF:F:FFFFFFFFFFFFF
FFFFFFFFFFFFFFFFFFFFFFFFFFFFFFFFFFFFFFFFFFFFFFFFFFFFFFFFFFFFFFFFFFFF
FFFFFFFFFFFFFFF
```

@A00721:190:HGJW7DSXY:4:2521:8865:25488 2:N:0:TCTGCAAG+GCTGCACT
TTTGAATTTCAAAATTGACCGTCGATTCTACTGCGTCACTATAGAATTCATTTTTTAGATGGTTATGA
TCGTGACGCGTGGTCTAATGACAAATCGACACGTAAGCGTACGGAGGTGTCCCCGGCGCGGGCAAGCC
AGTAATATTATATTG
+
FFFFFFFF:FFFFFFFFFFFFFFFFF:FF:FFFFFFFFFFFFF:,FFF:FFFFFFFFFF:FFFFFFFF
FFFFFFFFFFFFFFFFFF:FFFFFFF:FFFFFFFFF:FFFFFFFFFFFFFFFFFFFFFFFFFFF:FFF
FFFF:FFF,FF::FF
@A00721:190:HGJW7DSXY:4:2647:30680:3239 2:N:0:TCTGCAAG+GCTGCACT
TTTGAATTTCAAAATTGACCGTCGATTCTACTGCGTCACTATAGAATTCATTTTTTAGATGGTTATGA
TCGTGACGCGTGGTCTAATGACAAATCGACACGTAAGCGTACGGAGGTGTCCCCGGCGCGGGCAAGCC
AGTAATATTATATTG
+
FFFFFFF:FFFFF:FFFFFFFFFFFFFFFFFFFFFFFFFFFF,FF:FFFFFFFF,FFFFFFFFFFFFF
FFFFFFFFFFFFFFFFFFFFFFFFFFFFFFFFFFFFFFFFFFFFFFFFFFF:FFFFFFFFFFF:FFFF
FFFFFFF,FFFF:FF
@A00721:190:HGJW7DSXY:4:1176:19732:36855 2:N:0:TCTGCAAG+GCTGCACT
ATAAATTTTGAATTTCAAAATTGACCGTCGATTCTACTGCGTCACTATAGAATTCATTTTTTAGATGG
TTATGATCGTGACGCGTGGTCTAATGACAAATCGACACGTAAGCGTACGGAGGTGTCCCCGGCGCGGG
CAAGCCAGTAATATT
+
FFFFFFF:FFFFFFFFFFFFFFFFFFFFFFF:FFFFFFFFFFFFFFFFFFFFFFFFFFFFFFFFFFFF
FFFFFFFFFFFFFFFFFFFFFFFFFF:FFF:FFFF:FFFF,FFFFFFFFFFFFFFFFFFFFFFFFFFF
FFFFFFFFFFFFFFF
@A00721:190:HGJW7DSXY:4:1427:21414:12994 2:N:0:TCTGCAAG+GCTGCACT
ATAAATTTTGAATTTCAAAATTGACCGTCGATTCTACTGCGTCACTATAGAATTCATTTTTTAGATGG
TTATGATCGTGACGCGTGGTCTAATGACAAATCGACACGTAAGCGTACGGAGGTGTCCCCGGCGCGGG
CAAGCCAGTAATATT
+
FFFFFFFFFFFFFFFFFFFFFFFFFFFFFFF:FFFFFFFFFFFFFF:FFFFFFFFFFFFFFFFFFF:F
FFFFFFFFFFFFFFFFFFFFFFFFFFFF:FFF:FFFFFFFFFFFFFFFFFFFFFFF:FFFFFFFFFFF
FFFFFF:FF:F:FFF
@A00721:190:HGJW7DSXY:4:1464:12906:5682 2:N:0:TCTGCAAG+GCTGCACT
ATAAATTTTGAATTTCAAAATTGACCGTCGATTCTACTGCGTCACTATAGAATTCATTTTTTAGATGG
TTATGATCGTGACGCGTGGTCTAATGACAAATCGACACGTAAGCGTACGGAGGTGTCCCCGGCGCGGG
CAAGCCAGTAATATT
+
FFFFFFFFFFFF:FFFFFFFFFFFFFFFFFFFFFFFFFFFFF,:,FFFFFF:FFFF:FF,FFFFF::F
FFFFF:FF:,,FFFFFF:FFFFFFFF::FFFFFF:FFF:FFFFFFFFFFFFFFFFFFFFFFFFFFFFF
FF:FFFFFFF:FF:F
@A00721:190:HGJW7DSXY:4:2254:4363:28745 2:N:0:TCTGCAAG+GCTGCACT
ATAAATTTTGAATTTCAAAATTGACCGTCGATTCTACTGCGTCACTATAGAATTCATTTTTTAGATGG
TTATGATCGTGACGCGTGGTCTAATGACAAATCGACACGTAAGCGTACGGAGGTGTCCCCGGCGCGGG
CAAGCCAGTAATATT
+
FFFFFFFFFFFF:FFFFFFFFFFFFFFFFFFFFFFFFFFFFF:FF,FFFFF:FF:FFFF:FFFFFFFF
FF:FFFFFFFF:FFFF,FFFFFFFFFFFFFFFFFFFFFFF,FFFFFFFFFFFFFFFFFFFFFFFFFFF
FF,FFFFFFFFFF,F
@A00721:190:HGJW7DSXY:4:2605:23701:12226 2:N:0:TCTGCAAG+GCTGCACT
ATAAATTTTGAATTTCAAAATTGACCGTCGATTCTACTGCGTCACTATAGAATTCATTTTTTAGATGG
TTATGATCGTGACGCGTGGTCTAATGACAAATCGACACGTAAGCGTACGGAGGTGTCCCCGGCGCGGG
CAAGCCAGTAATATT
+
FFFFFFFFFFFFFFFFFFFFFFFFFFFFFFF:FFFFFFFFFFFFFFFFFF:FFFFFFFFFFFFF:F:F

FFFFFFFFFFFFFFFFFFF,FFFFFFFFFFFF:FFFFFFFFFFFFFFFFFFFFFFFFFFFFFFFFFFFF
FFFF:F,FFFFFF:F
@A00721:190:HGJW7DSXY:4:2678:15383:9157 2:N:0:TCTGCAAG+GCTGCACT
ATAAATTTTGAATTTCAAAATTGACCGTCGATTCTACTGCGTCACTATAGAATTCATTTTTTAGATGG
TTATGATCGTGACGCGTGGTCTAATGACAAATCGACACGTAAGCGTACGGAGGTGTCCCCGGCGCGGG
CAAGCCAGTAATATT
+
FFFFFFFFFFFFFFFFFF:FFFFFFFFFFFFFFFFFFFFFFF,FFF:F:FFFFFFFFFFFFFFFFFFFF
FFFFFFFFFFFFFFFFFFFFFFFF:FFFFFFF:FFFFFFFFFFFFFFFFFFFFFFFFFFFFFFFFFFFF
FFFFFFFFFFFFFFF
@A00721:190:HGJW7DSXY:4:1147:31837:24972 2:N:0:TCTGCAAG+GCTGCACT
TATAAATTTTGAATTTCAAAATTGACCGTCGATTCTACTGCGTCACTATAGAATTCATTTTTTAGATG
GTTATGATCGTGACGCGTGGTCTAATGACAAATCGACACGTAAGCGTACGGAGGTGTCCCCGGCGCGG
GCAAGCCAGTAATAT
+
FFFFFFF::FFFF:FFFFFFFFFFFFFFFFFFFFFFFFF:,FFF:F:FFF,FFFF::FFFFFFFF,:F:
FFF,FF::FFF:F,FFF:FFFFFFFF:FFFFFFF,FFFFFF:F,,FFFFF:::FFFF,FFFFFFFFFF
FFF,F,FFFFFF:F:
@A00721:190:HGJW7DSXY:4:1147:31783:25160 2:N:0:TCTGCAAG+GCTGCACT
TATAAATTTTGAATTTCAAAATTGACCGTCGATTCTACTGCGTCACTATAGAATTCATTTTTTAGATG
GTTATGATCGTGACGCGTGGTCTAATGACAAATCGACACGTAAGCGTACGGAGGTGTCCCCGGCGCGG
GCAAGCCAGTAATAT
+
FFFFFFFF:FFFFFFFFFFFFFFFFFFFFFFFFFFFFFFFFFF::FFFF,FFFFFFFFFFFFFF:F::F
FFFFFFFFFFFFFFFFFFFFFFFFFFFFFFFFFFFFFFFFFFFFFFFFFFFFFFFFFFFFFFFFFFFF
FFF:FFFFFFFF,FF
@A00721:190:HGJW7DSXY:4:1526:22272:35462 2:N:0:TCTGCAAG+GCTGCACT
TATAAATTTTGAATTTCAAAATTGACCGTCGATTCTACTGCGTCACTATAGAATTCATTTTTTAGATG
GTTATGATCGTGACGCGTGGTCTAATGACAAATCGACACGTAAGCGTACGGAGGTGTCCCCGGCGCGG
GCAAGCCAGTAATAT
+
FFFFFFFFFFFFFFFFFFFFFFFFFFFFFFFFFFFFFFFFFFFFFFFF:FFFFFFFFFFFFFFFF:FF
FFFFFF:FFFFFFFFFFFFFFFFFF:FF:FFFFFFFFFFFFFFFFFFFFFFFFFFFFFFFFFFFFFFFF
FFFFFFF,FFFFFFF
@A00721:190:HGJW7DSXY:4:1620:30427:20337 2:N:0:TCTGCAAG+GCTGCACT
TATAAATTTTGAATTTCAAAATTGACCGTCGATTCTACTGCGTCACTATAGAATTCATTTTTTAGATG
GTTATGATCGTGACGCGTGGTCTAATGACAAATCGACACGTAAGCGTACGGAGGTGTCCCCGGCGCGG
GCAAGCCAGTAATAT
+
FFFFFFFFFFFFFFFFFFFFFFFFFFFFFFFFFFFFFFFFFFF::FFFFF:FFFFFFFFFFFFFFFFFF
FFFFFFFFFFFFFFFFFFFFFFFFFFFFFFFFFFFFFFFFFFFFFFFF:FF:FFFFFFFFFFFFFFFFF
FFFFFFFFFFFFF:F
@A00721:190:HGJW7DSXY:4:1209:13440:29277 1:N:0:TCTGCAAG+GCTGCACT
ACTGGCTTGCCCGCGCCGGGGACACCTCCGTACGCTTACGTGTCGATTTGTCATTAGACCACGCGTCA
CGATCATGACCATCTAAAAAATGAATTCTATAGTGACGCAGTAAAATCGACGGCAGATGATATGTTAT
ATCATCGTCTTTTTG
+
FFFFFFFFFFFFFFFFFFFFFFFFFFFFFFFFFFFFFFFFFFFFFFFF:FFFFFFFFFFFFFFFFFFFF
FFFFF:FFFFFFFFFFFFF::FFFF,F::FF:FFFFFFFFFFFFFFFFFFFFFFFFFFFFFFFFFFFFF
:FFFFFFFFFFFFFF
@A00721:190:HGJW7DSXY:4:1544:8648:17347 1:N:0:TCTGCAAG+GCTGCACT
ACTGGCTTGCCCGCGCCGGGGACACCTCCGTACGCTTACGTGTCGATTTGTCATTAGACCACGCGTCA
CGATCATGACCATCTAAAAAATGAATTCTATAGTGACGCAGTAAAATCGACGGCAGATGATATGTTAT
ATCATCGTCTTTTTG

+
FFFFFFFFFFFFFFFFFFFFFFFFFFFFFFFFFFFFFFFFFFFFFFFFFFFFFFFF:FFFFFFFFFFF
FFFFFFFFFFFFFFFFFFFFFFFFFFFFFFFFFFFFFFFFFFFFFFFFFFFFFFFFFFFFFF:FF:F:
FFFFFFFFFFFFFFF
@A00721:190:HGJW7DSXY:4:1554:13738:4022 1:N:0:TCTGCAAG+GCTGCACT
ACTGGCTTGCCCGCGCCGGGGACACCTCCGTACGCTTACGTGTCGATTTGTCATTAGACCACGCGTCA
CGATCATAACCATCTAAAAAATGAATTCTATAGTGACGCAGTAGAATCGACGGTCAATTTTGAAATTC
AAAATTTATATTTAT
+
FFFFFFFFFFFFFFFFFFFFFFFFFFFFFFFFFFFFFFFFFFFFFFFFFFFFFFFFFFFFFFFFFFFF
FFFFFFFFFFFFFFFFFFFFFFFFFFFFFFFFFFFFFFFFFFFFFFFF,FFFFFFFFFFFFFFFF:,,:FF
,:,FFF:FF:F:,FF
@A00721:190:HGJW7DSXY:4:1602:7129:24893 1:N:0:TCTGCAAG+GCTGCACT
ACTGGCTTGCCCGCGCCGGGGACACCTCCGTACGCTTACGTGTCGATTTGTCATTAGACCACGCGTCA
CGATCATGACCATCTAAAAAATGAATTCTATAGTGACGCAGTAAAATCGACGGCAGATGATATGTTAT
ATCATCGTCTTTTTG
+
FFFFFFFFFFFFFFFFFFFFFFFFFFFFFFFFFFF:FFFFFFFFFF:FFFFFFFFFFFFFFFFFFFFF
FFF:FFFFFFFFFFFFFF:FFFFFFF,FFF:FFFFFFFFFFFFFFFFFFFFFFFFFFFFFFFFFFF:F:
F:FFFFFFFFFFFFF
@A00721:190:HGJW7DSXY:4:2158:26187:28369 1:N:0:TCTGCAAG+GCTGCACT
ACTGGCTTGCCCGCGCCGGGGACACCTCCGTACGCTTACGTGTCGATTTGTCATTAGACCACGCGTCA
CGATCATAACCATCTAAAAAATGAATTCTATAGTGACGCAGTAGAATCGACGGTCAATTTTGAAATTC
AAAATTTATATTTAT
+
FFFFFFFFFFFFFFFFFFFFFFFFFFFFFFFFFFFFFFFFFFFFFFFFFFFFFFFFFFFFFFFFFFFF
FFFFFFFFFFFFFFFFFFFF:FFFFFFFFFFFFFFFFFFFFFFFFFFFFFFFFFFFFFFFFFFF:F:FFF
:,F:FFFFFF,FFFF
@A00721:190:HGJW7DSXY:4:2229:11749:12070 1:N:0:TCTGCAAG+GCTGCACT
ACTGGCTTGCCCGCGCCGGGGACACCTCCGTACGCTTACGTGTCGATTTGTCATTAGACCACGCGTCA
CGATCATAACCATCTAAAAAATGAATTCTATAGTGACGCAGTAGAATCGACGGTCAATTTTGAAATTC
AAAATTTATATTTAT
+
FFFFFFFFFFFFFFFFFFFFFFFFFFFFFFFFFFFFFFFFFFFFFFFFFFFFFFFFFFFFFFFFFFFF
FFFFFFFFFFFFFFFFFFFFFFFFFFFFF:FFFFFFFFFFFFFFFFFFFFFFFFFFFFF:FFFFF:FFFF
,:,:F,FFFFFF:,F
@A00721:190:HGJW7DSXY:4:2340:16504:6840 1:N:0:TCTGCAAG+GCTGCACT
ACTGGCTTGCCCGCGCCGGGGACACCTCCGTACGCTTACGTGTCGATTTGTCATTAGACCACGCGTCA
CGATCATGACCATCTAAAAAATGAATTCTATAGTGACGCAGTAAAATCGACGGCAGATGATATGTTAT
ATCATCGTCTTTTTG
+
FFFFFFFFFFFFFFFFFFFFFFFFFFFFFFFFFFFFFFFFFFFFFFFFFFFFFFFFFFFFFFFFFFFF
FFFFFFFFFFFFFFFFFFFF:FFF:FFF::FFFFFFFFFFFFFFFFFFFFFFFFFFFFFFFFFFFFFF
FFFFFFFFFFFF:FF
@A00721:190:HGJW7DSXY:4:2462:10303:26287 1:N:0:TCTGCAAG+GCTGCACT
ACTGGCTTGCCCGCGCCGGGGACACCTCCGTACGCTTACGTGTCGATTTGTCATTAGACCACGCGTCA
CGATCATGACCATCTAAAAAATGAATTCTATAGTGACGCAGTAAAATCGACGGCAGATGATATGTTAT
ATCATCGTCTTTTTG
+
FFFFFFFFFFFFFFFFFFFFFFFFFFFFFFFFFFFFFFFFFFFFFFFFFFFFF:FFFFFFFFFFFFFF
FFFFFFFFFFFFFF:F:FFFFFF:FFF:F:,,FFFFFFFFFFFFFFFFFFFFFFFFFFFFFFFFFFFF
FFFF,FFFFFFFFFF
@A00721:190:HGJW7DSXY:4:2475:21206:6057 1:N:0:TCTGCAAG+GCTGCACT
ACTGGCTTGCCCGCGCCGGGGACACCTCCGTACGCTTACGTGTCGATTTGTCATTAGACCACGCGTCA

CGATCATAACCATCTAAAAAATGAATTCTATAGTGACGCAGTAGAATCGACGGTCAATTTTGAAATTC
AAAATTTATATTTAT
+
FFFFFFFFFFFFFFFFFFFFFFFFFFFFFFFFFFFFFFFFFFFFFFFFFFFFFFFFFFFFFFFFFFFFFF
FFFFFFFFFFFFFFFFFFFFFFFFFFFFF:FFFFFFFFFFFFFFFFFFFFFFFFFFFFFFFFFFF,::FF
F:FFF,FFFF:,FFF
@A00721:190:HGJW7DSXY:4:2553:13602:11929 1:N:0:TCTGCAAG+GCTGCACT
ACTGGCTTGCCCGCGCCGGGGACACCTCCGTACGCTTACGTGTCGATTTGTCATTAGACCACGCGTCA
CGATCATGACCATCTAAAAAATGAATTCTATAGTGACGCAGTAAAATCGACGGCAGATGATATGTTAT
ATCATCGTCTTTTTG
+
FFFFFFFFFFFFFFFFFFFFFFFFFFFFFFFFFFFFFFFFFFFFFFFFFFFFFFFFFFFFFFFFFFFFFF
FFFFFFFFFFFFFFFFFFFFFFFFFF:FFFFFFFFFFFFFFFFFFFFFFFFFFFFFFFFFFFFFFFF:FFF
FFF:FFFFFFFFFFF
@A00721:190:HGJW7DSXY:4:1138:13376:9345 1:N:0:TCTGCAAG+GCTGCACT
GCTGGCTTGCCCGCGCCGGGGACACCTCCGTACGCTTACGTGTCGATTTGTCATTAGACCACGCGTCA
CGATCATAACCATCTAAAAAATGAATTCTATAGTGACGCAGTAGAATCGACGGTCAATTTTGAAATTC
AAAATTTATAGTTAT
+
FFFFFFFFFFFFFFFFFFFFFFFFFFFFFFFFFFFF,FFFFFFFFFFFFFFFFFFFFFFFFFFFFFFFFF
FFFFFFFFFFFFFFFFFFFFFFFFF,FFFFFFFF:FFFFFFFFFFFFFFFFFFFFFFFFFF:FFFFFFFF
FFF:FFFF,F,F:FF
@A00721:190:HGJW7DSXY:4:1160:23439:19883 1:N:0:TCTGCAAG+GCTGCACT
GCTGGCTTGCCCGCGCCGGGGACACCTCCGTACGCTTACGTGTCGATTTGTCATTAGACCACGCGTCA
CGATCATAACCATCTAAAAAATGAATTCTATAGTGACGCAGTAGAATCGACGGTCAATTTTGAAATTC
AAAATTTATATTTAT
+
FFFFFFFFFFFFFFFFFFFFFFFFFFFFFFFFFFFFFFFFFFFFFFFFFFFFFFFFFFFFFFFFFFFFFF
FFFFFFF:FFFFFFFFFFF:FFFFFFFFFFFFFFFFFFFFFFFFFFFFFFFFFFFFFFFFFFFFFFFFFF
FFFFFFFFFFF:FFF
@A00721:190:HGJW7DSXY:4:2162:13928:7983 1:N:0:TCTGCAAG+GCTGCACT
GCTGGCTTGCCCGCGCCGGGGACACCTCCGTACGCTTACGTGTCGATTTGTCATTAGACCACGCGTCA
CGATCATAACCATCTAAAAAATGAATTCTATAGTGACGCAGTAGAATCGACGGTCAATTTTGAAATTC
AAAATTTATATTTAT
+
FFFFFFFFFFFFFFFFFFFFFFFFFFFFFFFFFFFFFFFFFFFFFFFFFFFFFFFFFFFFFFFFFFFFFF
FFFFFFFFFFFFFF:FFFFFFFFFFFFFFFFFFFFFFFFFFFFFFFFFFFFFFFFFFFFFFFF:FFFFFF
FFF:FFFFFF:FFFF
@A00721:190:HGJW7DSXY:4:2162:13865:8750 1:N:0:TCTGCAAG+GCTGCACT
GCTGGCTTGCCCGCGCCGGGGACACCTCCGTACGCTTACGTGTCGATTTGTCATTAGACCACGCGTCA
CGATCATAACCATCTAAAAAATGAATTCTATAGTGACGCAGTAGAATCGACGGTCAATTTTGAAATTC
AAAATTTATATTTA
+
FFFFFFFFFFFFFFFFFFFFFFFFFFFFFFFFFFFFFFFFFFFFFFFFFFFFFFFFFFFFFFFFFFFFFF
FFFFFFFFFFFFFFFFFFFFFFFFFFFFFFFFFFF:FFFFFFFFFFFFFFFFFFFFFFFFFFFFFFFFFF
FFFFFFFFFF,FFF
@A00721:190:HGJW7DSXY:4:2354:6153:9737 1:N:0:TCTGCAAG+GCTGCACT
GCTGGCTTGCCCGCGCCGGGGACACCTCCGTACGCTTACGTGTCGATTTGTCATTAGACCACGCGTCA
CGATCATAACCATCTAAAAAATGAATTCTAGAGTGACGCAGTAGAATCGACGGTCAATTTTGAAATTC
AAAATTTAGATTTAT
+
FFFFFFFFFFFFFFFFFFFFFFFFFFFFFFFFFFFF:FFFFFFFFFFFFFFFFFFFFF:FF:FFFFFFF
FFFFFFFFFFFFFF,FFFF,FFFFFFF,:F,FFFFFFFFFFFFFFFFFFFFFFFFFFFFFFFF::FFFF
:F:F,FFF,:F::FF

```
@A00721:190:HGJW7DSXY:4:2447:10719:17112 1:N:0:TCTGCAAG+GCTGCACT
GCTGGCTTGCCCGCGCCGGGGACACCTCCGTACGCTTACGTGTCGATTTGTCATTAGACCACGCGTCA
CGATCATAACCATCTAAAAAATGAATTCTATAGTGACGCAGTAGAATCGACGGTCAATTTTGAAATTC
AAAATTTATATTTA
+
F:FFFFFFFFFFFFFFFFFFFFFFFFFFFFFFFFF:FFFFFFFFFF:FFFFF::FFFFFFFFFFFFFF
FFFFFFFFFFFFFFF,:FFF,FFFFFFF::FFFFF,FFF,FFFFFFFFFF:FFFFFF::FFFFFFFF:
:F,,FF:FF,FFFF
@A00721:190:HGJW7DSXY:4:2612:19560:36057 1:N:0:TCTGCAAG+GCTGCACT
GCTGGCTTGCCCGCGCCGGGGACACCTCCGTACGCTTACGTGTCGATTTGTCATTAGACCACGCGTCA
CGATCATAACCATCTAAAAAATGAATTCTATAGTGACGCAGTAGAATCGACGGTCAATTTTGAAATTC
AAAATTTATATTTAT
+
FFFFFFFFFFFFFFFFFFFFFFFFFFFFFFFFFFFFFFFFFFFFFFFFFFFFFFFFFFFFFFFFFFFF
FFFFFFFFFFFFFFFFFFFFFFFFFFFFFFFFFFF:FFFFFFF:FFFFFFFFFFFFFFFFFFFFFFFF
FFFFFFF:FFFFFF:
@A00721:190:HGJW7DSXY:4:1423:27778:13401 1:N:0:TCTGCAAG+GCTGCACT
GGCTTGCCCGCGCCGGGGACACCTCCGTACGCTTACGTGTCGATTTGTCATTAGACCACGCGTCACGA
TCATGACCATCTAAAAAATGAATTCTATAGTGACGCAGTAAAATCGACGGCAGATGATATGTTATATC
ATCGTCTTTTTGATA
+
FFFFFFFFFFFFFFFFFFFFFFFFFFFFFFFF:FFFFFFFFFFFFFFFFFFFFFFFFFFFFFFFFFFF
FFF:FFFFFFFFFFFFF,FFF,FFFFF:FFFFFFFFFFFFFFFFFFFFFFFFFFFFFFFFFFFF:FFFF
FFFFFFFFFFFFFFF
@A00721:190:HGJW7DSXY:4:2130:6894:19758 1:N:0:TCTGCAAG+GCTGCACT
GGCTTGCCCGCGCCGGGGACACCTCCGTACGCTTACGTGTCGATTTGTCATTAGACCACGCGTCACGA
TCATGACCATCTAAAAAATGAATTCTATAGTGACGCAGTAAAATCGACGGCAGATGATATGTTATATC
ATCGTCTTTTTGATA
+
FFFFFFFFFFFFFFF:FFFFFFF:FFFFFFFFFFFFFFFFF:FFFFFFFF::FFFFFFFFFFFFFFFF
,FFF,FFFFFFFFFFFF:F,FFFFFFFFF:FFF:FFFFFFFFFFFFFFFF:FFFF:FFFF:FFFFF,F
FFFFFFF:FFFFFFF
@A00721:190:HGJW7DSXY:4:2433:21612:6355 1:N:0:TCTGCAAG+GCTGCACT
GGCTTGCCCGCGCCGGGGACACCTCCGTACGCTTACGTGTCGATTTGTCATTAGACCACGCGTCACGA
TCATGACCATCTAAAAAATGAATTCTATAGTGACGCAGTAAAATCGACGGCAGATGATATGTTATATC
ATCGTCTTTTTGATA
+
FFFFFFFFFFFFFFFFFFFFFFFFFFFFFFFFFFFFFFFFFFFFFFFFFFFFFFFFFFFFFFFFFFFF
FFFFFFFFFFFFFFFFF:FFF,FFFF:FFFFFFFFFFFFFFFFFFFFFFFFFFFFFFFFFFFFFFFFF
FFFFFFFFFFFFFFF
@A00721:190:HGJW7DSXY:4:2522:6849:27978 1:N:0:TCTGCAAG+GCTGCACT
GGCTTGCCCGCGCCGGGGACACCTCCGTACGCTTACGTGTCGATTTGTCATTAGACCACGCGTCACGA
TCATGACCATCTAAAAAATGAATTCTATAGTGACGCAGTAAAATCGACGGCAGATGATATGTTATATC
ATCGTCTTTTTGATA
+
FFFFFFFFFFFFFFFFFFFFFFFFF:FFFFFFFFFFFFFFFFFFFFFFFFFFFFFFFFFFFFFFFFFF:
:FFFFFFFFFFFFFFFFFFF,F:FF:FFFFFFFFFFFFFFFFFFFFFFFFFFFFFFFFFF:FFFFFFFFF
FFFFFFF:F,F,:FF
@A00721:190:HGJW7DSXY:4:1168:3197:6903 1:N:0:TCTGCAAG+GCTGCACT
CGCCCGCGCCGGGGACACCTCCGTACGCTTACGTGTCGATTTGTCATTAGACCACGCGTCACGATCAT
AACCATCTAAAAAATGAATTCTATAGTGACGCAGTAGAATCGACGGTCAATTTTGAAATTCAAAATTT
ATAAATATTGTGGGT
+
FFFFFFFFFFFFFFFFFFFFFFFFFFFFFF:FFFFFFFFFFFFFFFFFFFFFFFFFFFFFFFFFFF:FFF
```

```
FFFFFFF:FFFFFFFF:,FFFFF,FFFFFFFFFFFFFFF:FFFFFFFFFFFF,FF,FFFFFF,FFFFF
F:,FFF:F:FFFFFF
@A00721:190:HGJW7DSXY:4:1203:23836:35352 1:N:0:TCTGCAAG+GCTGCACT
CGCCCGCGCCGGGGACACCTCCGTACGCTTACGTGTCGATTTGTCATTAGACCACGCGTCACGATCAT
AACCATCTAAAAAATGAATTCTATAGTGACGCAGTAGAATCGACGGTCAATTTTGAAATTCAAAATTT
ATAAATATTGTGGGT
+
FFFFFFFFFFFFFFFFFFFFFFFFFFFF:F:FFFF:FFFFFFFF:F:FFFFF:FFFFFFFFFFFFFFF
FFFFFFF,FFFFFFFF:,,FFFFFFFFFFFFFFFFFFFFFFFFFF:FFFF,FF::FFFFFFFFFFFFF
FF:FF:FFF:F:,FF
@A00721:190:HGJW7DSXY:4:1351:24297:1329 1:N:0:TCTGCAAG+GCTGCACT
CGCCCGCGCCGGGGACACCTCCGTACGCTTACGTGTCGATTTGTCATTAGACCACGCGTCACGATCAT
AACCATCTAAAAAATGAATTCTATAGTGACGCAGTAGAATCGACGGTCAATTTTGAAATTCAAAATTT
ATAAATATTGTGGGT
+
FFFFFFFFFFFFFFFFFFFFFFFFFFFFFFFFFFFFFFFFFFFFFFFFFFFFFFFFFFFFFFFFFFFF
FFFFFFFFFFFFFFFFFF,F:F:FFFFFFFFFFFF,FFFFFFFFFFFFFFF:FFFFFFFFFFF:FF,F
FFF:FFFFFF:FFFF
@A00721:190:HGJW7DSXY:4:1571:31024:20243 1:N:0:TCTGCAAG+GCTGCACT
CGCCCGCGCCGGGGACACCTCCGTACGCTTACGTGTCGATTTGTCATTAGACCACGCGTCACGATCAT
AACCATCTAAAAAATGAATTCTATAGTGACGCAGTAGAATCGACGGTCAATTTTGAAATTCAAAATTT
ATAAATATTGTGGGT
+
F:FFFFFFFFFF:FFFFFFFFFF:F:FFFFFFFFFFFFFFFFF:FFFFFFFFFFFFFFFFFFFFFFFF
FFFFFFFFFFFFF:FFFFFFF:FFFF:FFFFFFFFFFFFFFFF:FFFFFFF,FF:FFF:FFFFFFFFF
FFFFFFFF:FFFFFF
@A00721:190:HGJW7DSXY:4:1643:9805:8797 1:N:0:TCTGCAAG+GCTGCACT
CGCCCGCGCCGGGGACACCTCCGTACGCTTACGTGTCGATTTGTCATTAGACCACGCGTCACGATCAT
AACCATCTAAAAAATGAATTCTATAGTGACGCAGTAGAATCGACGGTCAATTTTGAAATTCAAAATTT
ATAAATATTGTGGGT
+
FFFFFFFFFFFFFFFFFFFFFFFFFFFFFFFFFFFFFFFFFFFFFFFFFFFFFFFFFFFFFFFFFFFF
FFFFFFFFFFFFFFFFFFFFFFFF,FFFFFFFFFFFFFFFFFFFFFFFFFFFFFFFFFFFFFFFFFFF
FFFFFFFFFFFFFFF
@A00721:190:HGJW7DSXY:4:1657:2338:7858 1:N:0:TCTGCAAG+GCTGCACT
CGCCCGCGCCGGGGACACCTCCGTACGCTTACGTGTCGATTTGTCATTAGACCACGCGTCACGATCAT
AACCATCTAAAAAATGAATTCTATAGTGACGCAGTAGAATCGACGGTCAATTTTGAAATTCAAAATTT
ATAAATATTGTGGGT
+
FFFFFFFFFFFFFFFFFFFFFFFFFFFFFFFFFFFFFFFFFFFFF:FFFFFFFFFFFFFFFFFFFFFF
FFFFFFFFFFFFFFFFFF:FF,F,FFFFFFFFFFFFFFFFFFFFFFFFFFFFFFF:FFFFFFFFFFFF
FFFFF::FFFFFFFF
@A00721:190:HGJW7DSXY:4:2238:21070:33098 1:N:0:TCTGCAAG+GCTGCACT
CGCCCGCGCCGGGGACACCTCCGTACGCTTACGTGTCGATTTGTCATTAGACCACGCGTCACGATCAT
AACCATCTAAAAAATGAATTCTATAGTGACGCAGTAGAATCGACGGTCAATTTTGAAATTCAAAATTT
ATAAATATTGTGGGT
+
FFFFFFFFFFFFFFFFFFFFFFFFFFFFFFFFFFFFFFFFFFFFFFFFFFFFFFFFFFFFFFFFFFFF
FFFFFFFFFFFFFFFFF:FFF,FFFFFF:FFFFFFFFF:FFFFFFFFFFFFFFFF:FFFFFF:FFFFF
F:FFFFFFFFFFFFF
@A00721:190:HGJW7DSXY:4:2238:21730:33270 1:N:0:TCTGCAAG+GCTGCACT
CGCCCGCGCCGGGGACACCTCCGTACGCTTACGTGTCGATTTGTCATTAGACCACGCGTCACGATCAT
AACCATCTAAAAAATGAATTCTATAGTGACGCAGTAGAATCGACGGTCAATTTTGAAATTCAAAATTT
ATAAATATTGTGGGT
```

```
+
FFFFFFFFFFFFFFFFFFFFFFFFF:FF:,FFFFFFFFFFFFFFF:FFFFFFFFFFFFFFFFF:FFF:
F:FFFFF,FFFFF,FFF:::F,F,FFFFFFFFFFFFFFFFFFFFFFFFFFFFFFFF,FFFFF:FFFFF
FFF:FFF::F,FFFF
@A00721:190:HGJW7DSXY:4:2258:20745:15593 1:N:0:TCTGCAAG+GCTGCACT
CGCCCGCGCCGGGGACACCTCCGTACGCTTACGTGTCGATTTGTCATTAGACCACGCGTCACGATCAT
AACCATCTAAAAAATGAATTCTATAGTGACGCAGTAGAATCGACGGTCAATTTTGAAATTCAAAATTT
ATAAATATTGTGGGT
+
FFFFFFFFFFFFFFFFFFFFFFFFFFFFFFFFFFFFFFFFFFFFFFFFFFFFFFFFFFFFFFFFFFFF
FFFFFFFFFFFFF:FFFFFFFFFFFFFFFFFFFFF:FFFFFFFFFFFFFFFFFFFFFFFFFFFFFFFF
FFF:FFFFFFFFFF:
@A00721:190:HGJW7DSXY:4:2312:19325:5525 1:N:0:TCTGCAAG+GCTGCACT
CGCCCGCGCCGGGGACACCTCCGTACGCTTACGTGTCGATTTGTCATTAGACCACGCGTCACGATCAT
AACCATCTAAAAAATGAATTCTATAGTGACGCAGTAGAATCGACGGTCAATTTTGAAATTCAAAATTT
ATAAATATTGTGGGT
+
FFFFFFFFFFFFFFFFFFFFFFFFFFFFFFFFFFFFFFFFFFFFFFFFFFFFFFFFFFFFFFFFFFFF
FFFFFFFFFFFFFFFFFFFFFFFFFFFFFFFFFFF:FFFFFFFFFFFFFFFFFFFFFFFFFFFFFFFF
FFFFFFFFFFFFFFF
@A00721:190:HGJW7DSXY:4:2413:27905:9893 1:N:0:TCTGCAAG+GCTGCACT
CGCCCGCGCCGGGGACACCTCCGTACGCTTACGTGTCGATTTGTCATTAGACCACGCGTCACGATCAT
AACCATCTAAAAAATGAATTCTATAGTGACGCAGTAGAATCGACGGTCAATTTTGAAATTCAAAATTT
ATAAATATTGTGGGT
+
FFFFFFFFFFFFFFFFFFFFFFFFFFFFFFFFFFFFFFFFFFFFFFFFFFFFFFFFFFFFFFFFFFFF
FFFFFFFFFFFFFFFFFFFFFFFFFFFFFFFFFFFFFFFFFFFFFFFFFFFFFFF:FFFFFFFFFFFF
FFFFFFFFFFF:FFF
@A00721:190:HGJW7DSXY:4:2413:27968:10974 1:N:0:TCTGCAAG+GCTGCACT
CGCCCGCGCCGGGGACACCTCCGTACGCTTACGTGTCGATTTGTCATTAGACCACGCGTCACGATCAT
AACCATCTAAAAAATGAATTCTATAGTGACGCAGTAGAATCGACGGTCAATTTTGAAATTCAAAATTT
ATAAATATTGTGGGT
+
FFFFFFFFFFFFFFFFFFFFFFFFFFFFF:FFFFFFFFFFFFFFFFFFFFFFFFFFFFFFFFFFFFFF
FFFFFFFFFFFFFFFFFFFFFFFFFFFF:FFFFFFFFFFFFFFFFFFFFFFFFFFFFFFFFFFFFFFF
FFFFFFFFFFFFFFF
@A00721:190:HGJW7DSXY:4:1109:9941:23688 1:N:0:TCTGCAAG+GCTGCACT
GCCCGCGCCGGGGACACCTCCGTACGCTTACGTGTCGATTTGTCATTAGACCACGCGTCACGATCATA
ACCATCTAAAAAAAGAATTCTATAGTGACGCAGTAGAATCGACGGTCAATTTTGAAATTCAAAATTTA
TATTTATTGTGTTGA
+
FFFFFFFFFFFFFFFFFFFFFFFFFFFFFFFFFFFFFFFFFFFFFFFFFFFFFFFFFFFFFFFFFFFF
FFFFFFFF:FFFFFFFFFFFFFFFF:FFFFFFFFFFFFFFF:FFFFFFFFFFFFFFFFFFFFF:FFF
FFF:FFFFF:FF:FF
@A00721:190:HGJW7DSXY:4:1419:15917:16720 1:N:0:TCTGCAAG+GCTGCACT
GCCCGCGCCGGGGACACCTCCGTACGCTTACGTGTCGATTTGTCATTAGACCACGCGTCACGATCATA
ACCATCTAAAAAAAGAATTCTATAGTGACGCAGTAGAATCGACGGTCAATTTTGAAATTCAAAATTTA
TATTTATTGTGTTGA
+
FFFFFFFFFFFFFFFFFFFFFFFFFFFFFFFFFFFFFFFFFFFFFFFFFFFFFFFFFFFFFFFFFFFF
FFFFFFFFFFFFFFFFFFFF:FFFFFFFFFFFFFFFFFFFFFFFFFFFFFFFFFFFFFFFFFFFFF:FFFF
F::FFFFFFFFF,F:
@A00721:190:HGJW7DSXY:4:1525:20573:2362 1:N:0:TCTGCAAG+GCTGCACT
GCCCGCGCCGGGGACACCTCCGTACGCTTACGTGTCGATTTGTCATTAGACCACGCGTCACGATCATA
```

ACCATCTAAAAAAAGAATTCTATAGTGACGCAGTAGAATCGACGGTCAATTTTGAAATTCAAAATTTA
TATTTATTGTGTTG
+
FFFFFFFFFFFFFFFFFFFFFFFFFFFFFFFFFFFFFFFFFFFFFFFFFFFFFFFFFFFFFFFFFFFF
FFFFFFFFFFFFFFFFFFFFFFFFFFFFFFFFFFFFFFFFFFFFFFFFFFFFFFFFFFFFFFF:FFFF
FF:FFFFFFFFF:F
@A00721:190:HGJW7DSXY:4:2505:15067:21167 1:N:0:TCTGCAAG+GCTGCACT
GCCCGCGCCGGGGACACCTCCGTACGCTTACGTGTCGATTTGTCATTAGACCACGCGTCACGATCATA
ACCATCTAAAAAAAGAATTCTATAGTGACGCAGTAGAATCGACGGTCAATTTTGAAATTCAAAATTTA
TATTTATTGTGTTGA
+
FFFFFFFFFFFFFFFFFFFFFFFFFFFFFFFFFFFFFFFFFFFFFFFFFFFFFFFFFFFFFFFFFFFF
FFF:FFFFFFF:FFFF,FFF:F:FFFFFFFFFFFFFFFFFFFFFFFFFFFFFF:FFFFFFF:FF:F,F
FFFFFFFFFFFFFF:
@A00721:190:HGJW7DSXY:4:1104:5593:22357 1:N:0:TCTGCAAG+GCTGCACT
CCCGCGCCGGGGACACCTCCGTACGCTTACGTGTCGATTTGTCATTAGACCACGCGTCACGATCATAA
CCATCTAAAAAATGAATTCTATAGTGACGCAGTAGAATCGACGGTCAATTTTGAAATTCAAAATTTAT
ATTTATT
+
FFFFFFFFFFFFFFFFFFFFFFFFFFFFFFFFFFFFFFFFFFFFFFFFFFFFFFFFFFFFFFFFFFFF
FFFFFFFFFFFFFFF:FFFFFFFFFFFFFFFFFFFFFFFFFFFFFFFFFFFFFFFF:FFFFFFFFFFFF
FFFFFFF
@A00721:190:HGJW7DSXY:4:1122:6153:21386 1:N:0:TCTGCAAG+GCTGCACT
CCCGCGCCGGGGACACCTCCGTACGCTTACGTGTCGATTTGTCATTAGACCACGCGTCACGATCATGA
CCATCTAAAAAATGAATTCTATAGTGACGCAGTAAAATCGACGGCAGATGATATGTTATATCATCGTC
TTTTTGA
+
FFFFFFFFFFFFFFFFFFFFFFFFFFFFFFFFFFFFFFFFFFFFFFFFFFFFFFFFFFFFFFFFFFFF
FFFFFFFFFFFFFFFFFFFFFFFFFFFFFFFFFFFFFFFFFFFFFFFFFFFFFFFFFFFFFFFFFFFF
FFFFFFF
@A00721:190:HGJW7DSXY:4:1159:3676:24142 1:N:0:TCTGCAAG+GCTGCACT
CCCGCGCCGGGGACACCTCCGTACGCTTACGTGTCGATTTGTCATTAGACCACGCGTCACGATCATGA
CCATCTAAAAAATGAATTCTATAGTGACGCAGTAAAATCGACGGCAGATGATATGTTATATCATCGTC
TTTTTGA
+
FFFFFFFFFFFFFFFFFFFFFFFFFF,FFFFFFFFFFFFFFFFFFFFFFFFFFFFFFFFFFFFFFFFF
FFFFFFFFFFFFFFF:FFFFFFFFFFFFFFFFFFFFFFFFFFFFFFFFFFFFFFF,FFF:FFFFFFFFF
FFFFFFF
@A00721:190:HGJW7DSXY:4:1163:25925:9533 1:N:0:TCTGCAAG+GCTGCACT
CCCGCGCCGGGGACACCTCCGTACGCTTACGTGTCGATTTGTCATTAGACCACGCGTCACGATCATGA
CCATCTAAAAAATGAATTCTATAGTGACGCAGTAAAATCGACGGCAGATGATATGTTATATCATCGTC
TTTTTGA
+
FFFFFFFFFFFF:FFFFFFFFFFFFF:FFFFFFFFFF:FFFFF:FFFFFFFFFFFFFFFFFFFFFFFF
FFFFFFFFFFF::FF,,FF:::,FFFFFFFFFFFFFFFFFFFFFFFFFFFFFF:FFFFFFFFFFFFFF
FFFFFFF
@A00721:190:HGJW7DSXY:4:1215:27733:12352 1:N:0:TCTGCAAG+GCTGCACT
CCCGCGCCGGGGACACCTCCGTACGCTTACGTGTCGATTTGTCATTAGACCACGCGTCACGATCATAA
CCATCTAAAAAATGAATTCTATAGTGACGCAGTAGAATCGACGGTCAATTTTGAAATTCAAAATTTAT
ATTTATTGTG
+
FFFFFFFFFFFFFFFFFFFFFFFFFFFFFFFFFFFFFFFFFFFFFFFFFFFFFFFFFFFFFFFF:FFF
FFFFF:FFFFFFFFFF:FFF:FFFFFFFFFFFFFFFFFFFFFFFFFFFFFFFFFFFFFFFFFFFFFFF
FFFFFFFFFF

```
@A00721:190:HGJW7DSXY:4:1224:13937:19774 1:N:0:TCTGCAAG+GCTGCACT
CCCGCGCCGGGGACACCTCCGTACGCTTACGTGTCGATTTGTCATTAGACCACGCGTCACGATCATGA
CCATCTAAAAAATGAATTCTATAGTGACGCAGTAAAATCGACGGCAGATGATATGTTATATCATCGTC
TTTTTGA
+
FFFFFFFFFFFFFFFFFFFFFFFFFFFFFFFFFFFFFFFFFFFFFFFFFFFFFFFFFFFFFFFFFFFF
FFFFFFFFFFFFFFFFFFFF:FFFFFFFFFFFFFF:FFFFFFFFFFFFFFFFFFFF,FFFFFF:FFFFFFF
FFFF:FF
@A00721:190:HGJW7DSXY:4:1246:20329:22858 1:N:0:TCTGCAAG+GCTGCACT
CCCGCGCCGGGGACACCTCCGTACGCTTACGTGTCGATTTGTCATTAGACCACGCGTCACGATCATGA
CCATCTAAAAAATGAATTCTATAGTGACGCAGTAAAATCGACGGCAGATGATATGTTATATCATCGTC
TTTTTGA
+
FFFFFFFFFFFFFFFFFFFFFFFFFFF:FFFFFFFFFFFFFFFFFFFFFFFFFFFFFFFFFFFFFFFFF
FFFFFFFFFFFFF:FF,FFF,F:FFFF:FFFFFFFFFFFFFFFFFFFFFFFFFFFFFFFF:FFFFFFFFFF
FFFFFFF
@A00721:190:HGJW7DSXY:4:1316:24686:18474 1:N:0:TCTGCAAG+GCTGCACT
CCCGCGCCGGGGACACCTCCGTACGCTTACGTGTCGATTTGTCATTAGACCACGCGTCACGATCATGA
CCATCTAAAAAATGAATTCTATAGTGACGCAGTAAAATCGACGGCAGATGATATGTTATATCATCGTC
TTTTTGA
+
FFFFFFFFFFFFFFFFFFFFFFFFFFFFFFFFFFFFFFFFFFFFFFFFFFFFFFFFFFFFFFFFFFFF
FFFFFFFFFFFFFFF:F:FFFFFFFFFFFFFFFFFFFFFFFFFFFFFFFFFFFFFFFFFFFFFFFFFF
FFFFFFF
@A00721:190:HGJW7DSXY:4:1325:14606:11725 1:N:0:TCTGCAAG+GCTGCACT
CCCGCGCCGGGGACACCTCCGTACGCTTACGTGTCGATTTGTCATTAGACCACGCGTCACGATCATGA
CCATCTAAAAAATGAATTCTATAGTGACGCAGTAAAATCGACGGCAGATGATATGTTATATCATCGTC
TTTTTGA
+
FFFFFFFFFFFFFFFFFFFFFFFFFFFFFFFFFFFFFFFFFFFFFFFFFFFFFFFFFFFFFFFFFFFF
FFFFFFFFFFFFFFFFFFFFFFFFFFF:FFFFFFFFFFFFFFFFFFFFFFFFFFFFFFFFFFFFFFFFF
FFFFFF:
@A00721:190:HGJW7DSXY:4:1331:21504:6731 1:N:0:TCTGCAAG+GCTGCACT
CCCGCGCCGGGGACACCTCCGTACGCTTACGTGTCGATTTGTCATTAGACCACGCGTCACGATCATAA
CCATCTAAAAAATGAATTCTATAGTGACGCAGTAGAATCGACGGTCAATTTTGAAATTCAAAATTTAT
ATTTATTGTG
+
FF:::F:FF:F::FFFFFFFFFFFFFF:FFFFFFFFFF,FFFF:F:F:FFFFF:FF:FFFFFFFFFFF
FFFFFFFFFF::FFF:FF::F,FFFFF::FFFFF,FFFFFFFF,FFFF,FF:FFFF:FFFFFF,:FFF
FF,FFFFF::
@A00721:190:HGJW7DSXY:4:1339:12255:29951 1:N:0:TCTGCAAG+GCTGCACT
CCCGCGCCGGGGACACCTCCGTACGCTTACGTGTCGATTTGTCATTAGACCACGCGTCACGATCATAA
CCATCTAAAAAATGAATTCTATAGTGACGCAGTAGAATCGACGGTCAATTTTGAAATTCAAAATTTAT
ATTTATT
+
FFFFFFFFFFFFFFFFFFFFFFFFFFFFFFFFFFFFFFFFFFFF,FFFFFFFFFFFFFFFFFFFFFFFFF
FFFFFFFFFFFFFFFF:FF:FFFFFFFFFFFFFF:FFFFFFFFFFFFFFFFFFFFFFFFFFFFFFFFFF
FFFFFFF
@A00721:190:HGJW7DSXY:4:1363:18954:8641 1:N:0:TCTGCAAG+GCTGCACT
CCCGCGCCGGGGACACCTCCGTACGCTTACGTGTCGATTTGTCATTAGACCACGCGTCACGATCATGA
CCATCTAAAAAAGGAATTCTATAGTGACGCAGTAAAATCGACGGCAGATGATATGTTATATCATCGTC
TTTTTGA
+
:FFFF,FFF:FFFFFFFFFFF,FFFFFFF:FFF,F:FFFFFFFFF:FFFFFF:FFFFFFFFFFFFFFF
```

FFFFFFF:FFF,,FF:,F:,FF,FFFFFF:FFFFFFFFF:FFFFFFFFFFF,FFF,FF:FFFFFFFFF
F,F,FFF
@A00721:190:HGJW7DSXY:4:1414:13449:4961 1:N:0:TCTGCAAG+GCTGCACT
CCCGCGCCGGGGACACCTCCGTACGCTTACGTGTCGATTTGTCATTAGACCACGCGTCACGATCATGA
CCATCTAAAAAATGAATTCTATAGTGACGCAGTAAAATCGACGGCAGATGATATGTTATATCATCGTC
TTTTTGA
+
FFFFFFFFFFFFFFFFFFFFFFFFFFFFFFFFFFFFFFFFFFFFFFFFFFFFFFFFFFFFFFFFFFFF
FFFFFFFFFFFFFFFFFFFFFFFFFFFFFFFFFFFFFFFFFFFFFFFFFFFFFFFFFFFFFFFFFFFF
FFFFFFF
@A00721:190:HGJW7DSXY:4:1427:1642:12853 1:N:0:TCTGCAAG+GCTGCACT
CCCGCGCCGGGGACACCTCCGTACGCTTACGTGTCGATTTGTCATTAGACCACGCGTCACGATCATAA
CCATCTAAAAAATGAATTCTATAGTGACGCAGTAGAATCGACGGTCAATTTTGAAATTCAAAATTTAT
ATTTATTGTG
+
FFFFFFFFFFFFFFFFFFFFFFFFFFFFFFFFFFFFFFFFFFFFFFFFFFFFFFFFFFFFFFFFFFFF
FFFFFFFFFFFFFFFFFFF:FFFFFFFFFFFFFFFFFFFFFFFFFFFFFFFFFFFFFFFFF:FFFFFF:
F:F::FFFFF
@A00721:190:HGJW7DSXY:4:1442:18403:17268 1:N:0:TCTGCAAG+GCTGCACT
CCCGCGCCGGGGACACCTCCGTACGCTTACGTGTCGATTTGTCATTAGACCACGCGTCACGATCATAA
CCATCTAAAAAATGAATTCTATAGTGACGCAGTAGAATCGACGGTCAATTTTGAAATTCAAAATTTAT
ATTTATTGTGT
+
FFFFFFFF:F:FFFF:FF:F:FFFFF,FFFFF:FFFF:FFF,FFF,,FFF:FF,FFFFFF,FFFFFF,
FFFFFFFFFFFF,:FF:FF:F:F:,F,FFFFFFFFFFFFFFFFFFFFFFFFFFFFFFFFFFFF,FF::
:FFFF:F::FF
@A00721:190:HGJW7DSXY:4:1447:22878:28588 1:N:0:TCTGCAAG+GCTGCACT
CCCGCGCCGGGGACACCTCCGTACGCTTACGTGTCGATTTGTCATTAGACCACGCGTCACGATCATGA
CCATCTAAAAAATGAATTCTATAGTGACGCAGTAAAATCGACGGCAGATGATATGTTATATCATCGTC
TTTTTGA
+
FFFFFFFFFFFFFFFFFFFFFFFFFF,FFFFFFFFFFFFFFF:FFFFFFFFFFFFFF:FFFFF:FFFFF
FFFFFFFFFFFFFF:,FF:F,FFFFFFFFFFFFFFFFFFFFFFFFFFFFFFFFF:FFFF,FFFFFFFFFF
FFFFFFF
@A00721:190:HGJW7DSXY:4:1448:14796:13996 1:N:0:TCTGCAAG+GCTGCACT
CCCGCGCCGGGGACACCTCCGTACGCTTACGTGTCGATTTGTCATTAGACCACGCGTCACGATCATAA
CCATCTAAAAAATGAATTCTATAGTGACGCAGTAGAATCGACGGTCAATTTTGAAATTCAAAATTTAT
ATTTATTGTGT
+
FFFFFFFFFFFFFFFFFFFFFFFFFFFFFFFFFFFFFFFFFFFF:FFFFFFFFFFFFFFFFFFFF:FFFFF
FFFFFFFFFFFFFFFFF:FFFF:FFFFFFFFFFFFFFFFFFFFFFFFFFFFFFFFFFFFFFFF::FFFFFF
FFFFFFFFFFF
@A00721:190:HGJW7DSXY:4:1512:4869:5541 1:N:0:TCTGCAAG+GCTGCACT
CCCGCGCCGGGGACACCTCCGTACGCTTACGTGTCGATTTGTCATTAGACCACGCGTCACGATCATAA
CCATCTAAAAAATGAATTCTATAGTGACGCAGTAGAATCGACGGTCAATTTTGAAATTCAAAATTTAT
ATTTATTGT
+
FFF:FFFFFFFFFFFFFFFFFFFFFFFFFFFFFFFFFFFFFF,F,FFFFFF:FFFFFFFFFFFFFFF:FF
FFFFFFFFFFF,FF:FFFFFFFFFFFFFF,FFFFF,FF:FFFFFFFFFFFFFF:FFFFFFFFFFFFFFF
FFFFFFFFF
@A00721:190:HGJW7DSXY:4:1512:16477:34381 1:N:0:TCTGCAAG+GCTGCACT
CCCGCGCCGGGGACACCTCCGTACGCTTACGTGTCGATTTGTCATTAGACCACGCGTCACGATCATAA
CCATCTAAAAAATGAATTCTATAGTGACGCAGTAGAATCGACGGTCAATTTTGAAATTCAAAATTTAT
ATTTATTGTG

+
FFFFFFFFFFFFFFFFFFFFFFFFFFFFFFFFFFFFFFFFFFFFFFFFFFFFFFFFFFFFFFFFFFFF
FFFFFFFFFFFFFFFFF:FF:,FFFFFFFFFFFFFFFFFFFFFFFFFFFFFFFFFFFFFFFFFFFFFF
F:FFFFFF:F
@A00721:190:HGJW7DSXY:4:1512:18584:35117 1:N:0:TCTGCAAG+GCTGCACT
CCCGCGCCGGGGACACCTCCGTACGCTTACGTGTCGATTTGTCATTAGACCACGCGTCACGATCATAA
CCATCTAAAAAATGAATTCTATAGTGACGCAGTAGAATCGACGGTCAATTTTGAAATTCAAAATTTAT
ATTTATTGTG
+
FFFFFFFFFFFFFFFFFFFFFFFFFFF:FFFFFFFFFFFFFFFFFFFFFF:FFFFFFFFFFFFFFFFF
FFFFFFFFFFFFFFFFFFFFFFFFFFFFFFFFFFFFFFFFFFFFFFFFFFFFFFFFFFFFFFF:FFFF
FFF,FFFFFF
@A00721:190:HGJW7DSXY:4:1523:32036:18584 1:N:0:TCTGCAAG+GCTGCACT
CCCGCGCCGGGGACACCTCCGTACGCTTACGTGTCGATTTGTCATTAGACCACGCGTCACGATCATGA
CCATCTAAAAAATGAATTCTATAGTGACGCAGTAAAATCGACGGCAGATGATATGTTATATCATCGTC
TTTTTGA
+
FFFFFFF::F:FFFFFFFFFFFFFFFFFFFFFFFFFFFF:FFFF::FFFFFFF,FFFF:FFFF,F:,F
FFFFFFFFFFFFFFFFFF::FFFFFFFFFFFFFFFFFFF,FFFF,FFFFFFFF:,FFFFFFFFF,FFF
FF:FFFF
@A00721:190:HGJW7DSXY:4:1530:2510:35650 1:N:0:TCTGCAAG+GCTGCACT
CCCGCGCCGGGGACACCTCCGTACGCTTACGTGTCGATTTGTCATTAGACCACGCGTCACGATCATGA
CCATCTAAAAAATGAATTCTATAGTGACGCAGTAAAATCGACGGCAGATGATATGTTATATCATCGTC
TTTTTGA
+
FFFFFFFFFFFFFFFFFFFFFFFFFFFF:FFFFFFFFFFF:FF:FFFFFFFFFFFFFFF:FFFFFF
FFFFF:FFFF::,FFFFFF:F:FFFFFFFFFFFFFFFFFFFFFFFFFFFFFF,FFFF,F:FFFFFFFF
FFF,FFF
@A00721:190:HGJW7DSXY:4:1569:31024:3615 1:N:0:TCTGCAAG+GCTGCACT
CCCGCGCCGGGGACACCTCCGTACGCTTACGTGTCGATTTGTCATTAGACCACGCGTCACGATCATGA
CCATCTAAAAAATGAATTCTATAGTGACGCAGTAAAATCGACGGCAGATGATATGTTATATCATCGTC
TTTTTGA
+
FFFFFFFFFFFFFFFFFFFFFFFFFFFFFFFFFFFFFFFFFFFFFFFFFFFFFFFFFFFFFFFFF:FF
FFFFFFFFFFFFFF:FFFFFFFFFFFFFFFFFFFFFFFFFFFFFFFFFFFFFFFFFFFFFFFFFFFFF
FFFFFFF
@A00721:190:HGJW7DSXY:4:1645:5873:25347 1:N:0:TCTGCAAG+GCTGCACT
CCCGCGCCGGGGACACCTCCGTACGCTTACGTGTCGATTTGTCATTAGACCACGCGTCACGATCATAA
CCATCTAAAAAATGAATTCTATAGTGACGCAGTAGAATCGACGGTCAATTTTGAAATTCAAAATTTAT
ATTTATT
+
FFFFFFFFFFFFFFFFFFFFFFFFFFFFFFFFFFFFFFFFFFFFFFFFFFFFFFFFFFFFFFFFFFFF
FFFFFFFFFFFFFFFFFFFFFFFFFFFFFFFFF:FFFFFFFFFFFFFFFFFFFFFFFFFFFFFFFFFF
FFFFFFF
@A00721:190:HGJW7DSXY:4:1650:3866:34616 1:N:0:TCTGCAAG+GCTGCACT
CCCGCGCCGGGGACACCTCCGTACGCTTACGTGTCGATTTGTCATTAGACCACGCGTCACGATCATAA
CCATCTAAAAAATGAATTCTATAGTGACGCAGTAGAATCGACGGTCAATTTTGAAATTCAAAATTTAT
ATTTATTGTG
+
FFFFFFFFFFFFFFFFFFFFFFFFFFFFFFFFFFFFFFFFFFFFFFFFFFFFFFFFFFFFFFFFFFF:
FFFFFFFFFFFFFFFFFFFFFFFFFFFFFFFFFFFFFFFFFFFFFFFFFFFFFFFFFFFFFF:FFFFFF
FFFFFFFFFF
@A00721:190:HGJW7DSXY:4:1663:9842:32534 1:N:0:TCTGCAAG+GCTGCACT
CCCGCGCCGGGGACACCTCCGTACGCTTACGTGTCGATTTGTCATTAGACCACGCGTCACGATCATGA

CCATCTAAAAAATGAATTCTATAGTGACGCAGTAAAATCGACGGCAGATGATATGTTATATCATCGTC
TTTTTGA
+
FFFFFFFFFFFFFFFFFFFFFFFFFFFFFFFFFFFFFFFFFFFFFFFFFFFFFFFFFFFFFFFFFFFF
FFFFFFFFFFFF:FFFFFFFFFFFFFFFFFFFFFFFFFFFFFFFFFFFFFFFFFFFFFFFFFFFFFF
FFFFFFF
@A00721:190:HGJW7DSXY:4:2138:10447:24565 1:N:0:TCTGCAAG+GCTGCACT
CCCGCGCCGGGGACACCTCCGTACGCTTACGTGTCGATTTGTCATTAGACCACGCGTCACGATCATGA
CCATCTAAAAAATGAATTCTATAGTGACGCAGTAAAATCGACGGCAGATGATATGTTATATCATCGTC
TTTTTGA
+
FFFFFF:FFFFFFFFFFFFFF:FFFFFFFFFFFFFFFFFFFFF:F:FFFFFFFFFFFFFFFFFFFFFF:F
FFFFFFFFFFFFFFF:FFFFFFFFFFFF:FFFFFFFFFFFFFFFFFFFFFFFFFF,F:FFFFFFFFF,F
F:FFFFF
@A00721:190:HGJW7DSXY:4:2159:14588:17895 1:N:0:TCTGCAAG+GCTGCACT
CCCGCGCCGGGGACACCTCCGTACGCTTACGTGTCGATTTGTCATTAGACCACGCGTCACGATCATAA
CCATCTAAAAAATGAATTCTATAGTGACGCAGTAGAATCGACGGTCAATTTTGAAATTCAAAATTTAT
ATTTATTGTGT
+
FFFFFFFFFFFFFFFFFFFFFFFFFFFFFFFFFFFFFFFFFFFFFFFFFFFFFFFFFFFFFFFFFFFF
FFFFFFFFFFFFFFFFFFFFFFFFFFF:FFFFFFFFFFFFFFFFFFFFFFF:FFFFFFFFFFFFFFFFFF
FFFFFFFFFFF
@A00721:190:HGJW7DSXY:4:2175:12057:9502 1:N:0:TCTGCAAG+GCTGCACT
CCCGCGCCGGGGACACCTCCGTACGCTTACGTGTCGATTTGTCATTAGACCACGCGTCACGATCATAA
CCATCTAAAAAATGAATTCTATAGTGACGCAGTAGAATCGACGGTCAATTTTGAAATTCAAAATTTAT
ATTTATT
+
FFFFFFFFFFFFFFFFFFFFFFFFFFFFFFFFFFFFFFFFFFFFFFFFFFFFFFFFFFFFFFFFFFFF
FFFFFFFFFFFFFFFFFFFFFFFFFFFFFFFFFFFFF:FFFFFFFFFFFFFFFFFFFFFFFFFFFFFFF
FFFFFFF
@A00721:190:HGJW7DSXY:4:2205:8612:13839 1:N:0:TCTGCAAG+GCTGCACT
CCCGCGCCGGGGACACCTCCGTACGCTTACGTGTCGATTTGTCATTAGACCACGCGTCACGATCATAA
CCATCTAAAAAATGAATTCTATAGTGACGCAGTAGAATCGACGGTCAATTTTGAAATTCAAAATTTAT
ATTTATTGTG
+
FFFFFFFFFFFFFFFFFFFFFFFFFFFFFFFFFFFFFFFFFFFFFFFFFFFFFFFFFFFFFFFFFFFF
FFFFFFFFFFFFFFFFF:FF,FFFFFFFFFFFFFFFFFFF:FFFFFFFF:FFFFFFFFFFFF:FFFFF
FFFFF:FFFF
@A00721:190:HGJW7DSXY:4:2249:4824:21840 1:N:0:TCTGCAAG+GCTGCACT
CCCGCGCCGGGGACACCTCCGTACGCTTACGTGTCGATTTGTCATTAGACCACGCGTCACGATCATGA
CCATCTAAAAAATGAATTCTATAGTGACGCAGTAAAATCGACGGCAGATGATATGTTATATCATCGTC
TTTTTGA
+
FFFFFFFFFFFFFFFFFFFFFFFFFFFFFFFFFFFFFFFFFFFFFFFFFFFFFFFFFFFFFFFFFFFF
FFFFFFFFFFFFFFFFFFFFFFFFFFFFFFFFFFFF:FFFFFFFFFFFFFFFFFFFFFFFFFFFFFF:FF
FFFFFFF
@A00721:190:HGJW7DSXY:4:2258:9932:28025 1:N:0:TCTGCAAG+GCTGCACT
CCCGCGCCGGGGACACCTCCGTACGCTTACGTGTCGATTTGTCATTAGACCACGCGTCACGATCATGA
CCATCTAAAAAATGAATTCTATAGTGACGCAGTAAAATCGACGGCAGATGATATGTTATATCATCGTC
TTTTTG
+
FFFFFFFFFFFFFFFFFFFFFFFFFFFFFFFFFFFFFFFFFFFFFFFFFFFFFFFFFFFFFFFFFFFF
FFFFFFFFFFFFFFFFFFFFFF,FFFFFFFFFFFFFFFFFFFFFFFFFFFFFFF:FFFFFFFFFFFFFFFF
FFFFFF

```
@A00721:190:HGJW7DSXY:4:2341:32398:22310 1:N:0:TCTGCAAG+GCTGCACT
CCCGCGCCGGGGACACCTCCGTACGCTTACGTGTCGATTTGTCATTAGACCACGCGTCACGATCATGA
CCATCTAAAAAATGAATTCTATAGTGACGCAGTAAAATCGACGGCAGATGATATGTTATATCATCGTC
TTTTTGA
+
FFFFFFFFFFFFFFFFFFFFFFFFFFFFFFFFFFFFFFFFFFFFFFFFFFFFFFFFFFFFFFFFFFFFFF
FFFFFFFFFFFFFFFFFFFFFFFFFFFFFFFFFFFFFFFFFFFFFFFFFFFFFFFFFFFFF:FFFFFFFF
FFFFFFF
@A00721:190:HGJW7DSXY:4:2359:27995:11083 1:N:0:TCTGCAAG+GCTGCACT
CCCGCGCCGGGGACACCTCCGTACGCTTACGTGTCGATTTGTCATTAGACCACGCGTCACGATCATGA
CCATCTAAAAAATGAATTCTATAGTGACGCAGTAAAATCGACGGCAGATGATATGTTATATCATCGTC
TTTTTGA
+
FFFFFFFFFFFFFFFFFFFFFFFFFFFFFFFFFFFFFFFFFFFFFFFFFFFFFFFFFFFFFFF,FFFFFF
FFFFFFFFFFFFFFFFFFFFFFFFFFFFFFFFFFFFFFFF:FFFFFFFFFFFFF:FFFF:FFFFFFFFFF
F,FFFFF
@A00721:190:HGJW7DSXY:4:2365:20329:10488 1:N:0:TCTGCAAG+GCTGCACT
CCCGCGCCGGGGACACCTCCGTACGCTTACGTGTCGATTTGTCATTAGACCACGCGTCACGATCATGA
CCATCTAAAAAATGAATTCTATAGTGACGCAGTAAAATCGACGGCAGATGATATGTTATATCATCGTC
TTTTTGA
+
FFFFFFFFFFFFFFFFFFFFFFFFFFFFFFFFFFFFFFF:FFFFFFFFFFFFFFFFFFFFFFFFFFF:F:
FFFFFFF:FFFFFFFFFFFFFFFFFFFFFFFFFFFFFFFFFFFFFFFFFFFFFFFFFFFFFFFFFFFFFF
FFFFFFF
@A00721:190:HGJW7DSXY:4:2445:12762:23688 1:N:0:TCTGCAAG+GCTGCACT
CCCGCGCCGGGGACACCTCCGTACGCTTACGTGTCGATTTGTCATTAGACCACGCGTCACGATCATAA
CCATCTAAAAAATGAATTCTATAGTGACGCAGTAGAATCGACGGTCAATTTTGAAATTCAAAATTTAT
ATTTATTGTG
+
FFFFFFFFFFFFFFFFFFFFFFFFFFFFFFFFFFFFFFFFFFFFFFFFFFFFFFFFFFFFFFFFFFFFFF
FFFFFFFFFFFFFFFF:FFFFFFFFFFFFFFFFFFFFFFFFFFFFFFFFFF:FFFFFFFFFFFFFFFFF:
F:::FFFFFF
@A00721:190:HGJW7DSXY:4:2447:6641:25989 1:N:0:TCTGCAAG+GCTGCACT
CCCGCGCCGGGGACACCTCCGTACGCTTACGTGTCGATTTGTCATTAGACCACGCGTCACGATCATAA
CCATCTAAAAAATGAATTCTATAGTGACGCAGTAGAATCGACGGTCAATTTTGAAATTCAAAATTTAT
ATTTATTGTG
+
FFFFFFFFFFFFFFFFFFFFFFFFFFFFFFFFFFFFFFFFFFFFFFFFFFFFFFFFFFFFFFFFFFFFFF
FFFFFFFFFFFFFFFFFFFFFFFFFFFFFFFFFFFFFFFFFFFFFFFFFFFFFFFFFFFFFFFFFFFFFF
FFFFFFFF:F
@A00721:190:HGJW7DSXY:4:2450:20003:1376 1:N:0:TCTGCAAG+GCTGCACT
CCCGCGCCGGGGACACCTCCGTACGCTTACGTGTCGATTTGTCATTAGACCACGCGTCACGATCATAA
CCATCTAAAAAATGAATTCTATAGTGACGCAGTAGAATCGACGGTCAATTTTGAAATTCAAAATTTAT
ATTTATTGTG
+
FFFFFFFFFFFFFFFFFFFFFFFFFFFFFFFFFFFFFFFFFFFFFFFFFFFFFFFFFFFFFFFFFFFFFF
FFFFFFFFFFFFFFFFFFFFFFFFFF:FFFFFFFFFFFFFFFFFFFFFFFFFFFFFFFFFFFF:F:FFFF
FFFFFFFFFF
@A00721:190:HGJW7DSXY:4:2468:5367:7623 1:N:0:TCTGCAAG+GCTGCACT
CCCGCGCCGGGGACACCTCCGTACGCTTACGTGTCGATTTGTCATTAGACCACGCGTCACGATCATAA
CCATCTAAAAAATGAATTCTATAGTGACGCAGTAGAATCGACGGTCAATTTTGAAATTCAAAATTTAT
ATTTATTGTG
+
FFFFFFFFFFFFFFFFFFFFFFFFFFFFFFFFFFFFFFFFFFFFFFFFFFFFFFFFFFFFFFFFFFFFFF
```

FFFFFFFFFFFFFFFFFFFFF:FFFFFFFFFFFFFFFFFFFFFFFFFFFFFFF:FF:FFFFFFFFFFF
FFFFFFFF:F
@A00721:190:HGJW7DSXY:4:2502:26829:2738 1:N:0:TCTGCAAG+GCTGCACT
CCCGCGCCGGGGACACCTCCGTACGCTTACGTGTCGATTTGTCATTAGACCACGCGTCACGATCATAA
CCATCTAAAAAATGAATTCTATAGTGACGCAGTAGAATCGACGGTCAATTTTGAAATTCAAAATTTAT
ATTTATTGT
+
FFFFFFFFFFFFFFFFFFFFFFFFFFFFFFFFFFFFFFFFFFFFFFFFFFFFFFFFFFFFFFFFFFFF
FFFFFFFFFFFF:FFF:FF,FFFFFFFFFFFFFFFFFFFFFFFFFFFFFFFFF:FFFFFFFFFFFFFF
FFFFFFF,F
@A00721:190:HGJW7DSXY:4:2517:6894:16908 1:N:0:TCTGCAAG+GCTGCACT
CCCGCGCCGGGGACACCTCCGTACGCTTACGTGTCGATTTGTCATTAGACCACGCGTCACGATCATGA
CCATCTAAAAAATGAATTCTATAGTGACGCAGTAAAATCGACGGCAGATGATATGTTATATCATCGTC
TTTTTGA
+
FFFFFFFFFFFFFFFFFFFFFFFFFFFFFFFFFFFFFFFFFFFFFFFFFFFFFFFFFFFFFFFFFFFF
FFFFFFFFFFFFFF:FFFFFFFFFFFFF:FFFFFFFFFFFFFFFFFFFFFFFFFFFFFFFFFFFFFFF
FFFFFFF
@A00721:190:HGJW7DSXY:4:2530:30779:5353 1:N:0:TCTGCAAG+GCTGCACT
CCCGCGCCGGGGACACCTCCGTACGCTTACGTGTCGATTTGTCATTAGACCACGCGTCACGATCATGA
CCATCTAAAAAATGAATTCTATAGTGACGCAGTAAAATCGACGGCAGATGATATGTTATATCATCGTC
TTTTTGA
+
FFFFFFFFFFFFFFFFFFFFFFFFFFFFFFFFFFFFFFFFFFFFFFFFFFFFFFFFFFFFFFFFFFFF
FFFFFFFFFFF:FFFFFFFFFFFFFF:FFFFFFFFFFFFFFFFFFFFFFFFFFFFFFFFFFFFFFFFF
FFFFFFF
@A00721:190:HGJW7DSXY:4:2533:5801:32017 1:N:0:TCTGCAAG+GCTGCACT
CCCGCGCCGGGGACACCTCCGTACGCTTACGTGTCGATTTGTCATTAGACCACGCGTCACGATCATGA
CCATCTAAAAAATGAATTCTATAGTGACGCAGTAAAATCGACGGCAGATGATATGTTATATCATCGTC
TTTTTGA
+
FFFFFFFFFFFFFFFFFFFFFFFFFFFFFFFFFFFFF,FFFFFFFFFFFFFFFFFFFFFFFFFFFFFF
FFFFFFFFFFFFFFFFFFFFFFFFFFFFFFFFFFFFFFFFFFFFFFFFFFFFFFFFFFFF:FFFFFFF
FFFFFFF
@A00721:190:HGJW7DSXY:4:2543:12491:31735 1:N:0:TCTGCAAG+GCTGCACT
CCCGCGCCGGGGACACCTCCGTACGCTTACGTGTCGATTTGTCATTAGACCACGCGTCACGATCATGA
CCATCTAAAAAATGAATTCTATAGTGACGCAGTAAAATCGACGGCAGATGATATGTTATATCATCGTC
TTTTTGA
+
FFFFFFFFFFFFFFFFFFFFFFFFFFFFFFFFFFFFFFFFFFFFFFFFFFFFFFFFFFFFFFFFF:FF
FFFFFFFFFFFFFFFFFFF:::FFFFFFFFFFFFFF:FFFF:FFFFFFFFFFFFFFFFFFFFFFFFFF
FFFFFF:
@A00721:190:HGJW7DSXY:4:2544:4227:36495 1:N:0:TCTGCAAG+GCTGCACT
CCCGCGCCGGGGACACCTCCGTACGCTTACGTGTCGATTTGTCATTAGACCACGCGTCACGATCATGA
CCATCTAAAAAATGAATTCTATAGTGACGCAGTAAAATCGACGGCAGATGATATGTTATATCATCGTC
TTTTTGA
+
FF:F:FFFFF,FFFFFF:F:F:F:FF,FFF:F:FFFF:FFF,FF::F,FF,FFFFFF::FF:FFFF:F
FFFFFFFFFF:F:,:FFF::FF:FFFFF:FFF,F::FFFFFF:FF:,FFFFF,FFF,FFFF,FFFFFF
F,FFFF:
@A00721:190:HGJW7DSXY:4:2545:15881:17096 1:N:0:TCTGCAAG+GCTGCACT
CCCGCGCCGGGGACACCTCCGTACGCTTACGTGTCGATTTGTCATTAGACCACGCGTCACGATCATAA
CCATCTAAAAAATGAATTCTATAGTGACGCAGTAGAATCGACGGTCAATTTTGAAATTCAAAATTTAT
ATTTATTGTG

```
+
FFFFFFFFFFFFFFFFFFFFFFFFFFF:FFFFFFFFFFFFFFFFF,FFFFFFFFFFFFFFFFFFFFFFFF
FFFFFFFFFFFFFFFFFFFFFFFFFFFFFFFFFFFFFFFFFFFFFFFFFFFFF:FFFFFF:FFFFF
F,:,FFFF:F
@A00721:190:HGJW7DSXY:4:2614:5330:18584 1:N:0:TCTGCAAG+GCTGCACT
CCCGCGCCGGGGACACCTCCGTACGCTTACGTGTCGATTTGTCATTAGACCACGCGTCACGATCATGA
CCATCTAAAAAATGAATTCTATAGTGACGCAGTAAAATCGACGGCAGATGATATGTTATATCATCGTC
TTTTTGA
+
FFFFFFFFFFFFFFFFFFFFFFFFFFFF:FFFFFFFFFFFFFFFFFFFFFFFFFFFFFFFFFFFFFFFF
FFFFFFFFFFFFFFFFFFFFFFFFFFFFFFFFFFFFFFFFFFFFFFFFFFFFFFFFFFFFFFFFFFFF
FFFFFFF
@A00721:190:HGJW7DSXY:4:2618:30897:21590 1:N:0:TCTGCAAG+GCTGCACT
CCCGCGCCGGGGACACCTCCGTACGCTTACGTGTCGATTTGTCATTAGACCACGCGTCACGATCATAA
CCATCTAAAAAATGAATTCTATAGTGACGCAGTAGAATCGACGGTCAATTTTGAAATTCAAAATTTAT
ATTTATTGTGT
+
FFFFFFFFFFFFFFFFFFFFFFFFFFFFFFFFFFFFFFFFFFFFF:FFFFFFFFFFFFFFFFFFFFFFF
FFFFFFFFFFFFFFFFFFFFFFFFFFFFFFFFFF:FFFFFFFFFFFFFFFFFFFFFFFFFFFFF,FF:
FFFFFFFFFFF
@A00721:190:HGJW7DSXY:4:2631:25626:1846 1:N:0:TCTGCAAG+GCTGCACT
CCCGCGCCGGGGACACCTCCGTACGCTTACGTGTCGATTTGTCATTAGACCACGCGTCACGATCATGA
CCATCTAAAAAATGAATTCTATAGTGACGCAGTAAAATCGACGGCAGATGATATGTTATATCATCGTC
TTTTTGA
+
FFFFFFFFFFFFFFFFFFFFFFFFFFFFFFFFFFFFFFFFFFFFFFFFFFFFFFFFFFFFFFFFFFFF
FFFFFFFFFFFFFFF:FFFFFFFFFFFFFFFFFFFFFFFFFFFFFFFFFFFFFFFFFF,FFFFFFFFFFF
FFFFFF:
@A00721:190:HGJW7DSXY:4:2677:8793:23265 1:N:0:TCTGCAAG+GCTGCACT
CCCGCGCCGGGGACACCTCCGTACGCTTACGTGTCGATTTGTCATTAGACCACGCGTCACGATCATAA
CCATCTAAAAAATGAATTCTATAGTGACGCAGTAGAATCGACGGTCAATTTTGAAATTCAAAATTTAT
ATTTATTGTGT
+
FFFFFFFFFFFFFFFFFFFFFFFFFFFFFFFFFFFFFFFFFFFFFFFF:FFFFFFFFFFFFFFFFFFFF
FFFFFFFFFFFFFFFFFFFFFFFFFFF:FFFFFFFFFFFFFFFFFFFFFFFF:FFFFFFFFFFFFFFFFF
FFFFF:FFFF:
@A00721:190:HGJW7DSXY:4:2677:8730:24158 1:N:0:TCTGCAAG+GCTGCACT
CCCGCGCCGGGGACACCTCCGTACGCTTACGTGTCGATTTGTCATTAGACCACGCGTCACGATCATAA
CCATCTAAAAAATGAATTCTATAGTGACGCAGTAGAATCGACGGTCAATTTTGAAATTCAAAATTTAT
ATTTATTGTGT
+
FFFFFFFFFF:FFFFFFFFFFFFFFFFFFF:FFFFFFFFFFFFFFFFFFFFFFFFFFFFFFFFFFFFFFF
FFFF:FFFFFFFFFFFFFF:FFFFF:,FFFFFFFFFFFFFFFFFFFFFFFFFFFFF,FFFFFFFFFFFF
F:FFFFFFFFF
@A00721:190:HGJW7DSXY:4:1168:13883:7153 1:N:0:TCTGCAAG+GCTGCACT
ACCTCCGTACGCTTACGTGTCGATTTGTCATTAGACCACGCGTCACGATCATAACCATCTAAAAAATG
AAATCTATAGTGACGCAGTAGAATCGACGGTCAATTTTGAAATTCAAAATTTATATTTATTGTGTTGA
TGACTGCTTTAGCAG
+
F,FFFF,FFFFFF,FFFF,FF:FFF:F,,F:FF,F::F,,:FFFF:FFFFF,FFFFF,F:FFFFFFF:
FF,,,FFFF:,:FF::FF:FFFFF:FFF,:,FFF,FF,,FFFF::FFFFFF,F:F,:FF,F,,,FFFF
,,FFFF,,FFF,,FF
@A00721:190:HGJW7DSXY:4:1304:11912:1297 1:N:0:TCTGCAAG+GCTGCACT
ACCTCCGTACGCTTACGTGTCGATTTGTCATTAGACCACGCGTCACGATCATAACCATCTAAAAAATG
```

AATTCTATAGTGACGCAGTAGAATCGACGGTCAATTTTGAAATTCAAAATTTATATTTATTGTGTTGA
TGACTGCTTTAGCAG
+
FFFFFFFFFFFF:FFFFFFFFFFFFF:FFFFFFFFFFFFFFFFFFFFFFFF:FFFFFFFFFFF::FF
FF:FFFFFFFFF:FFFFFFFFFFFFFFFFFFFFFFFFFFFFFFFFFFFFFFFFF:FFFF,,FFFFF
FFFFFFFFFFFFFFF
@A00721:190:HGJW7DSXY:4:1622:31665:13056 1:N:0:TCTGCAAG+GCTGCACT
ACCTCCGTACGCTTACGTGTCGATTTGTCATTAGACCACGCGTCACGATCATAACCATCTAAAAAATG
AATTCTATAGTGACGCAGTAGAATCGACGGTCAATTTTGAAATTCAAAATTTATATTTATTGTGTTGA
TGACTGCTTTAGCAG
+
FFFFFFFFFFFFFFFFFFFFFFFFFFFFFFFFFFFFFFFFFFFFFFFFFFFFFFFFFFFFFFFF:FFF
:FFFFFFFFFFF:FFFFFFFFFFFFFFFFFFFFFFFFFFFFFFFFFF:FF:FFFFFFFFFFFFFFFFF
FFFFFFFFFF:F:FF
@A00721:190:HGJW7DSXY:4:2168:15510:14512 1:N:0:TCTGCAAG+GCTGCACT
ACCTCCGTACGCTTACGTGTCGATTTGTCATTAGACCACGCGTCACGATCATAACCATCTAAAAAATG
AATTCTATAGTGACGCAGTAGAATCGACGGTCAATTTTGAAATTCAAAATTTATATTTATTGTGTTGA
TGACTGCTTTAGCAG
+
FFFFFFFFFFFFFFFFFF:FFFFFF::FFFFFFFFF:FFFFFFFFFFFFFFFFFFFFFFFFFF,FFFF
FFFFFFF:FFF:FFFFFFFFFFF:FFFFFFFFFFF:FFFFFFFFFFFFFFFFF:FFFFF:FFFF:FFF
FFF:FFFFFFF:F,F
@A00721:190:HGJW7DSXY:4:2611:14977:25520 1:N:0:TCTGCAAG+GCTGCACT
ACCTCCGTACGCTTACGTGTCGATTTGTCATTAGACCACGCGTCACGATCATAACCATCTAAAAAATG
AATTCTATAGTGACGCAGTAGAATCGACGGTCAATTTTGAAATTCAAAATTTATATTTATTGTGTTGA
TGACTGCTTTAGCAG
+
FFFFFFFFFFFF:FFFFFFFFFFFFFFFFFFFFFFFFFFFFFFFFFFFFFFFFFFFFFFFFFFFF:FF
FFFFFFFFFFFFFFFFFFFFFFFFFFF:FFFFFFFFFFFFFF:FFFFFFFFFF:F,FFFFFFFFFFFF:
F::FFFFFFFFFFFF
@A00721:190:HGJW7DSXY:4:2340:11767:31172 1:N:0:TCTGCAAG+GCTGCACT
CCTCTGTACGCTTACGTGTCGATTTGTCATTAGACCACGCGTCACGATCATGACCATCTAAAAAATGA
ATTCTATAGTGACGCAGTAAAATCGACGGCAGATGATATGTTATATCATCGTCTTTTTGATAACCAAT
AGACTGTTAAAAGGT
+
FFFFFFFFFFFFFFFFFFFFFFFFFFFFFFFFFFFFFFFFFFFFFFFFFFFFFFFFFFFFFFFF:FFFF
FFFF:F:FFFF:FFFFFFFFFFFFFFF,FFFFFFF:FFFFFFFFFFFFF::FFFFFFFFFFF:FFFFF
F,FFFFFFFFFFFFF
@A00721:190:HGJW7DSXY:4:1370:4544:5729 1:N:0:TCTGCAAG+GCTGCACT
CTCCGTACGCTTACGTGTCGATTTGTCATTAGACCACGCGTCACGATCATGACCATCTAAAAAATGAA
TTCTATAGTGACGCAGTAAAATCGACGGCAGATGATATGTTATATCATCGTCTTTTTGATAACCAATA
GACTGTTAAAAGGTC
+
FFFFFFFFFFFFFFFFFFFFFFFFFFFFFFFFFFFFFFFFFFFFFFFFFFFFFFFFFFFFFFFFFFFF
:FF::FFFFFFFFFFFFFF:FFFFFFFFFFFFFFF:FFFFFFFFFFFFFFFFF::FFFFF:FFFFFFF
FFFFFFFF::FFFFF
@A00721:190:HGJW7DSXY:4:1436:24496:15890 1:N:0:TCTGCAAG+GCTGCACT
CTCCGTACGCTTACGTGTCGATTTGTCATTAGACCACGCGTCACGATCATGACCATCTAAAAAATGAA
TTCTATAGTGACGCAGTAAAATCGACGGCAGATGATATGTTATATCATCGTCTTTTTGATAACCAATA
GACTGTTAAAAGGTC
+
FFFFFFFFFFFFFFFFFFFFFFFFFFFFFFFFFFFFFFFFFFFFFFFFFFFFFFFFFFFFFFFFFFFF
FFFFFFFFFFFFFF:FFFFFFFFFFFFFFFFFFFFFFFFFFFFFFFFFFFFFFFFFFFFFFFFFFFFF
FFFFFFFFFF:FFFF

```
@A00721:190:HGJW7DSXY:4:1445:1597:29810 1:N:0:TCTGCAAG+GCTGCACT
CTCCGTACGCTTACGTGTCGATTTGTCATTAGACCACGCGTCACGATCATGACCATCTAAAAAATGAA
TTCTATAGTGACGCAGTAAAATCGACGGCAGATGATATGTTATATCATCGTCTTTTTGATAACCAATA
GACTGTTAAAAGGTC
+
FFFF:FFFFFFFFFFFFFFFFFFFFFFFFFFF:FFFFFFFFF,F:FFF:F:FFFFFF:F:FFFF:FFFFF
,FFF,FFFFFFFFFFFF:FFFFF:FFFFFFF:F,:F,F:FFFFFFFFFFFFFFFF:FFFFFF::FFFFF:
:F:FFFFFF:,:FFF
@A00721:190:HGJW7DSXY:4:1478:26503:16423 1:N:0:TCTGCAAG+GCTGCACT
CTCCGTACGCTTACGTGTCGATTTGTCATTAGACCACGCGTCACGATCATGACCATCTAAAAAATGAA
TTCTATAGTGACGCAGTAAAATCGACGGCAGATGATATGTTATATCATCGTCTTTTTGATAACCAATA
GACTGTTAAAAGGTC
+
FFFFFFFFFFFFFFFFFFFFFFF,FFFFFFFFFFFFFFFFFFFFFFFFFFFFFFFFFFFFFFFFFFF:FF
FFFFF:FF:FFFFFFFFFFFFFF,FFF:FFFF:F:FFFFFF:FFFFF:FFFFFFFFFFFFF:FF:FFFFF
FFFF,F:FFFFF:FF
@A00721:190:HGJW7DSXY:4:1627:11379:36229 1:N:0:TCTGCAAG+GCTGCACT
CTCCGTACGCTTACGTGTCGATTTGTCATTAGACCACGCGTCACGATCATGACCATCTAAAAAATGAA
TTCTATAGTGACGCAGTAAAATCGACGGCAGATGATATGTTATATCATCGTCTTTTTGATAACCAATA
GACTGTTAAAAGGTC
+
FFFFFFFFFFFFFFFFFFFFFFFFFFFFFFFFFFFFFFFFFFFFFFFFFFFFFFFFFFFFFFFFFFFFF:
FF:FFFFFFFFFFFFFFFFFFFFFFFFFFFFFFFFFFF:FFFFFFFFFFFFF:FF:FFFFFFFFFFFFF:
FFFFFFFFFFFFFFF
@A00721:190:HGJW7DSXY:4:2125:6325:8907 1:N:0:TCTGCAAG+GCTGCACT
CTCCGTACGCTTACGTGTCGATTTGTCATTAGACCACGCGTCACGATCATGACCATCTAAAAAATGAA
TTCTATAGTGACGCAGTAAAATCGACGGCAGATGATATGTTATATCATCGTCTTTTTGATAACCAATA
GACTGTTAAAAGGTC
+
FFFFFFFFFFFFFFFFFFFFF:FFFFFFFFFFFFFFFFFFFFFFFFFFFFFFFFFFFFFFFFFFF:FFFF
FFF:,FFFFFFFFF:FFF:FFFFFFFFFFFFFFFFFFFFFFFFFFFFFFFFFFFF:FFFFFFFFFFFFFF
FFFFFFFFFFFFFFF
@A00721:190:HGJW7DSXY:4:2160:17815:16313 1:N:0:TCTGCAAG+GCTGCACT
CTCCGTACGCTTACGTGTCGATTTGTCATTAGACCACGCGTCACGATCATGACCATCTAAAAAATGAA
TTCTATAGTGACGCAGTAAAATCGACGGCAGATGATATGTTATATCATCGTCTTTTTGATAACCAATA
GACTGTTAAAAGGTC
+
FFFFFFFFFFFFFFFFFFFFFFFFFFFFFFFFFFFFFFFFFFFFFFFFFFFFFFFFFFFFFFFFFFFFFF
FFFFFFFFFFFFFFFFFFFFFFFFFFFFFFFFFFFFFFFFFFFFFFFFFFFFFFFFFFFFFFFFFFFFFF
FFFFFFFFFFFFFFF
@A00721:190:HGJW7DSXY:4:2547:23692:10645 1:N:0:TCTGCAAG+GCTGCACT
CTCCGTACGCTTACGTGTCGATTTGTCATTAGACCACGCGTCACGATCATGACCATCTAAAAAATGAA
TTCTATAGTGACGCAGTAAAATCGACGGCAGATGATATGTTATATCATCGTCTTTTTGATAACCAATA
GACTGTTAAAAGGTC
+
FFFFFFFFFFFFFFFFFFFFFFFFFFFF:FFFFFFFFFFFFFFFFFFFFFFFFFFFFFFFFFFFFFFFFF
:FFFF:FFFFFFFFFFF:FFFFFFFFFFFFFFFFFFFFFFFFFFFFFFFFFFFFFFFFFFFFFFFFFFFF
FFFFFFFF:F:FFFF
@A00721:190:HGJW7DSXY:4:1153:20030:5838 1:N:0:TCTGCAAG+GCTGCACT
ACGCTTACGTGTCGATTTGTCATTAGACCACGCGTCACGATCATAACCATCTAAAAAATGAATTCTAT
AGTGACGCAGTAGAATCGACGGTCAATTTTGAAATTCAAAATTTATTGTGTTGATGACTGCTTTAGCA
GTTGATTGATGGATT
+
FFFFFFFFFFFFFFFFFFFFFFFFFFFFFFFFFFFFFFFFFFFFFFFFFFFF:FFFFFFFFFFFFFFF,F
```

FFFFFFFFFFFFFFFFFFFFFFFFFFFFFFFFFFFFFFFFFFFFFFFF:FFFF,FFFFFFFFFFFFFF
FFFFFFFFFFFFFFF
@A00721:190:HGJW7DSXY:4:1309:18295:35462 1:N:0:TCTGCAAG+GCTGCACT
ACGCTTACGTGTCGATTTGTCATTAGACCACGCGTCACGATCATAACCATCTAAAAAATGAATTCTAT
AGTGACGCAGTAGAATCGACGGTCAATTTTGAAATTCAAAATTTATTGTGTTGATGACTGCTTTAGCA
GTTGATTGATGGATT
+
FFFFFFFFFFFFFFFFFFFFFFFFFFFFFFFFFFFFFFFFFFFFFFFFFFFFFFFFF,FFFFFFFFFF
FFFFFFFFFFFFFFFFFFFFFFFFFFFFFFFFFFFFFFFFFFFFFFFFFFFFFFFFFFFFFFFFFFFF
FF::FFFFFFF:FFF
@A00721:190:HGJW7DSXY:4:1331:21667:36417 1:N:0:TCTGCAAG+GCTGCACT
ACGCTTACGTGTCGATTTGTCATTAGACCACGCGTCACGATCATAACCATCTAAAAAATGAATTCTAT
AGTGACGCAGTAGAATCGACGGTCAATTTTGAAATTCAAAATTTATTGTGTTGATGACTGCTTTAGCA
GTTGATTGATGGATT
+
FFFFFFFFFFFFFFFFFFFFFFFFFFFFFFFFFFFFFFFFFFFFFFFFFFFFFFFFFFFFFFFFFFFF
FFFFFFFFFFFFFFFFFFFFFFFFFFFFFFFFFFFFFFF:FFFFFFFFFFFFFFFFFFFFFFFFFFFF
F:FFFFFFFFFFFFF
@A00721:190:HGJW7DSXY:4:1332:32678:4789 1:N:0:TCTGCAAG+GCTGCACT
ACGCTTACGTGTCGATTTGTCATTAGACCACGCGTCACGATCATAACCATCTAAAAAATGAATTCTAT
AGTGACGCAGTAGAATCGACGGTCAATTTTGAAATTCAAAATTTATTGTGTTGATGACTGCTTTAGCA
GTTGATTGATGGATT
+
FFFFFFFFFF:FFFFFFFFFFFFFFFFFFFFFFFFFFF,FFF:FFFF:F:,FFFFFF,FFFF:FFFF:
FFFFFFFFFFFFFFFFFFFF,FFFFFFFFFFF:FF,FF::FFF:,F:FFFF:FFFFF,FFFFFFF:FF
FFFF:FFFFFFFFFF
@A00721:190:HGJW7DSXY:4:2271:13015:4210 1:N:0:TCTGCAAG+GCTGCACT
ACGCTTACGTGTCGATTTGTCATTAGACCACGCGTCACGATCATAACCATCTAAAAAATGAATTCTAT
AGTGACGCAGTAGAATCGACGGTCAATTTTGAAATTCAAAATTTATTGTGTTGATGACTGCTTTAGCA
GTTGATTGATGGATT
+
FFFFFFFFFFFFFFFFFFFFFFFFFFFFFFFFFFFFFFFFFFFFFFFFFFFFFFFFFFFFFFFFFFF:
FFFFFFFFFFFFFFFFFFFFFFFFFFFFFFFFFFFFFFF:FFFFFFFFFFFFFFFFFFFFFFFFFFFF
F:FFFFFF:FFFFFF
@A00721:190:HGJW7DSXY:4:2642:29559:11475 1:N:0:TCTGCAAG+GCTGCACT
ACGCTTACGTGTCGATTTGTCATTAGACCACGCGTCACGATCATAACCATCTAAAAAATGAATTCTAT
AGTGACGCAGTAGAATCGACGGTCAATTTTGAAATTCAAAATTTATTGTGTTGATGACTGCTTTAGCA
GTTGATTGATGGATT
+
FFFFFFFFFFFFFFFFFFFFFFFFFFFFFFFFFFFFFFFFFFFFFFFFFFFFFFFFFFFFFFFFFFFF
FFFFFFFFFFFFFFFFFFFFFFFFFFFFFFFFFFFFFFFFFFFFFFFFFFFFFFFFFFFFFFFFFFFF
FFFFFFFFFF:FFFF
@A00721:190:HGJW7DSXY:4:1249:23041:12587 1:N:0:TCTGCAAG+GCTGCACT
GCTTACGTGTCGATTTGTCATTAGACCACGCGTCACGATCATAACCATCTAAAAAATGAATTCTATAG
TGACGCAGTAGAATCGACGGTCAATTTTGAAATTCAAAATTTATATTTATTGTGTTGATGACTGCTTT
AGCAGTTGATTGATG
+
:FFFFFFFFFFFFFFFFFFFFFFFFFFFFFFFFFFFFFFFFFFFFFFFFFFFFFFFFFFFFFFFFFFF
FFFFFFFFFFFFFFFFFFFFFFFFFFFFFFFFFFFFFFFFFFFFFF:FFFFFFFFFFFF:FFFFFFFF
F:FFFFF:FFFFFFF
@A00721:190:HGJW7DSXY:4:1253:8983:29042 1:N:0:TCTGCAAG+GCTGCACT
GCTTACGTGTCGATTTGTCATTAGACCACGCGTCACGATCATAACCATCTAAAAAATGAATTCTATAG
TGACGCAGTAGAATCGACGGTCAATTTTGAAATTCAAAATTTATATTTATTGTGTTGATGACTGCTTT
AGCAGTTGATTGATG

```
+
FFFFFFFFFFFFFFFFFFFFFFFFFFFFFFFFFFFFFFFFFFFFFFFFFFFFFFFFFFFFFFFFFFFFFF
FF:FFFFFFFFFFFF:FFFFFFFFFFFFFFFFFFFFFFFFFFFFF:FFFFFFFFFFFFF:FF:FFFFFF
FFFFF:FFFFFFFFF
@A00721:190:HGJW7DSXY:4:1253:8802:29074 1:N:0:TCTGCAAG+GCTGCACT
GCTTACGTGTCGATTTGTCATTAGACCACGCGTCACGATCATAACCATCTAAAAAATGAATTCTATAG
TGACGCAGTAGAATCGACGGTCAATTTTGAAATTCAAAATTTATATTTATTGTGTTGATGACTGCTTT
AGCAGTTGATTGATG
+
FFFFFFFFFFFFFFFFFFFFFFFFFFFFFFFFFFFFFFFFFFFFFFFFFFFFFFFFFFFF:FFFFFFFF
FFFF:FFFFFFFFFFFFFFFFFFFFFFFFFFFFFFFFFFFFFFF:F:FFFFF:FFFFFFFFFFFFFFF
FFFFFFFFFFFFFFF
@A00721:190:HGJW7DSXY:4:1430:27127:3192 1:N:0:TCTGCAAG+GCTGCACT
GCTTACGTGTCGATTTGTCATTAGACCACGCGTCACGATCATAACCATCTAAAAAATGAATTCTATAG
TGACGCAGTAGAATCGACGGTCAATTTTGAAATTCAAAATTTATATTTATTGTGTTGATGACTGCTTT
AGCAGTTGATTGATG
+
FFFFFFFFFFFFFFFFFFFFFFFFFFFFFFFFFFFFFFFFFFFFFFFFFFFFFFFFFFFFF::FFFFFFF
FFFFFFFFFFFFFFFFFFFFFFFFFFFFFF:FFFFF:FFF:FFFFFFFFF:F:FF:FFFFFFFFFFFF
FFFFFF:FF:FFFFF
@A00721:190:HGJW7DSXY:4:1457:4101:3427 1:N:0:TCTGCAAG+GCTGCACT
GCTTACGTGTCGATTTGTCATTAGACCACGCGTCACGATCATAACCATCTAAAAAATGAATTCTATAG
TGACGCAGTAGAATCGACGGTCAATTTTGAAATTCAAAATTTATATTTATTGTGTTGATGACTGCTTT
AGCAGTTGATTGATG
+
FFFFFFFFFFFFFFFFFFFFFFFFFFFFFFFFFFFFFFFFFFFFFFFFFFFFFFFFFFFFF:FFFFFFF
FFFFFFFFFFFFFFFFFFFFFFFFFFFFFFFFFFFFFFFFFFFF,F,F:F:FFFFFFFFFFFFFFFFFF
FFFFFFFFFFFFFFF
@A00721:190:HGJW7DSXY:4:1619:11785:6151 1:N:0:TCTGCAAG+GCTGCACT
GCTTACGTGTCGATTTGTCATTAGACCACGCGTCACGATCATAACCATCTAAAAAATGAATTCTATAG
TGACGCAGTAGAATCGACGGTCAATTTTGAAATTCAAAATTTATATTTATTGTGTTGATGACTGCTTT
AGCAGTTGATTGATG
+
FFFFFFFFFFFFFFFFFFFFFFFFFFFFFFFFFFFFFFFFFFFFFFFFFFFFFFFFFFFFF:FFFFFFF
FFFFFFFFFFFFFFFFFFFFFFFFFFFFFFFFFFFFFFFFFFFFFFF,FFFFF:FFFFFFFFFFF:FFF
FF:FFFFFFFFFFFF
@A00721:190:HGJW7DSXY:4:2108:15917:19946 1:N:0:TCTGCAAG+GCTGCACT
GCTTACGTGTCGATTTGTCATTAGACCACGCGTCACGATCATAACCATCTAAAAAATGAATTCTATAG
TGACGCAGTAGAATCGACGGTCAATTTTGAAATTCAAAATTTATATTTATTGTGTTGATGACTGCTTT
AGCAGTTGATTGATG
+
FFFFFFFFFFFFFFFFFFFFFFFFFFFFFFF:FFFFFFFFFFFFFFFFF:FFFFFFFFFFFFFFFFFF
FFFFFFFFFFFFFFFFFFFFFFFFFFFFFFFFFFF,FFFF,FFFFFFFFF,FF,FFFFFFFF:FFFFFF
FFF:FF,F:FFF:FF
@A00721:190:HGJW7DSXY:4:2132:10755:12164 1:N:0:TCTGCAAG+GCTGCACT
GCTTACGTGTCGATTTGTCATTAGACCACGCGTCACGATCATAACCATCTAAAAAATGAATTCTATAG
TGACGCAGTAGAATCGACGGTCAATTTTGAAATTCAAAATTTATATTTATTGTGTTGATGACTGCTTT
AGCAGTTGATTGATG
+
FFFFFFFFFFFFFFFFFFFFFFFFFFFFFFFFFFFFFFFFFFFFFFFFFFFFFFFFFFFFFFFFFFFFFF
FFFFFFFFFFFFFFFFFFFFFFFFFFFFFFFFFFFFFFFFFFFFFFFFFFFFF:FFFF:FFFFFFFFF
FF:FFFFFFFFFFFF
@A00721:190:HGJW7DSXY:4:2138:16523:35243 1:N:0:TCTGCAAG+GCTGCACT
GCTTACGTGTCGATTTGTCATTAGACCACGCGTCACGATCATAACCATCTAAAAAATGAATTCTATAG
```

TGACGCAGTAGAATCGACGGTCAATTTTGAAATTCAAAATTTATATTTATTGTGTTGATGACTGCTTT
AGCAGTTGATTGATG
+
FFF,FFFF::FFF:FF:FFFF:FFFF:FFFFFFFFFFFF:FFFFFFFFFFFFFFF:F:FF,FFF:F:F
FFFFFFFFFFFFFFFFFF,FFFFFFFFFFFFFFFFFFF:FFFFF,FFFFFFFFF:FFFFFF,F,FFFF
FF:FFF,::,FFFFF
@A00721:190:HGJW7DSXY:4:1315:15691:1924 1:N:0:TCTGCAAG+GCTGCACT
CTTACGTGTCGATTTGTCATTAGACCACGCGTCACGATCATAACCATCTAAAAAATGAATTCTATAGT
GACGCAGTAGAATCGACGGTCAATTTTGAAATTCAAAATTTATATTTATTGTGTTGATGACTGCTTTA
GCAGTTGATTGATGG
+
FFFFFFFFFFFFFFFFFFFFFFFFFFFFFFFFFFFFFFFFFFFFFFFFFFFFFFF:FF:FFFFFFFF
F:F:FFFFFFFFFFFFFFF:FFFFFFFFFFFFFFFFFFFFFFFFFFFFFFFFFFFFFFFFFFFFFFF
FF:FFFFFFFFFFFF
@A00721:190:HGJW7DSXY:4:1350:24749:14544 1:N:0:TCTGCAAG+GCTGCACT
CTTACGTGTCGATTTGTCATTAGACCACGCGTCACGATCATAACCATCTAAAAAATGAATTCTATAGT
GACGCAGTAGAATCGACGGTCAATTTTGAAATTCAAAATTTATATTTATTGTGTTGATGACTGCTTTA
GCAGTTGATTGATGG
+
FFFFFFFFFFFFFFFFFFFFFFFFFFFFFFFFFFFFFFFFFFFFFFFFFFFFFFFFFFFFFFFFFFFF
FFFFFFFFFFFFFFFFFFFFFFFFFFFFFFFFFFFFFFFFFFFFFFFFFFFFFFFFFFFFFFFFFFFF
FFFFFFFFFFFFFFF
@A00721:190:HGJW7DSXY:4:1370:28058:17801 1:N:0:TCTGCAAG+GCTGCACT
CTTACGTGTCGATTTGTCATTAGACCACGCGTCACGATCATAACCATCTAAAAAATGAATTCTATAGT
GACGCAGTAGAATCGACGGTCAATTTTGAAATTCAAAATTTATATTTATTGTGTTGATGACTGCTTTA
GCAGTTGATTGATGG
+
FFFFFFFFFFFFFFFFFFFFFFFFFFFFFFFFFFFFFFFF:FFFF:FFFFFFFF:FFFFFFFFFF:FF
FFFFFFFF:FFFFFFFFFFFFFFFFFFFF:FFFFFFFF:FF:FF:,FFFFFFFFFFFFFFFFFFFFFF
FFFFFFFFFFF:FFF
@A00721:190:HGJW7DSXY:4:1531:6090:35931 1:N:0:TCTGCAAG+GCTGCACT
CTTACGTGTCGATTTGTCATTAGACCACGCGTCACGATCATAACCATCTAAAAAATGAATTCTATAGT
GACGCAGTAGAATCGACGGTCAATTTTGAAATTCAAAATTTATATTTATTGTGTTGATGACTGCTTTA
GCAGTTGATTGATGG
+
FFFFFFFFFFFFFFFFFFFFFFFFFFFFFFFFFFFFFFFFFFFFFFFFFFFFFFFFFFFFFFF:FFFF
FFF:FFFFFFFFFFFFFFFFFFFFFFFFFFFFFFFFFFFFFFFF:FFFFFF:F:FFFFFFFFFFFFFF
FFFFFFFFFFFFFFF
@A00721:190:HGJW7DSXY:4:2322:10881:19366 1:N:0:TCTGCAAG+GCTGCACT
CTTACGTGTCGATTTGTCATTAGACCACGCGTCACGATCATAACCATCTAAAAAATGAATTCTATAGT
GACGCAGTAGAATCGACGGTCAATTTTGAAATTCAAAATTTATATTTATTGTGTTGATGACTGCTTTA
GCAGTTGATTGATGG
+
FFFFFFFFFFFFFFFFFFFFFFFFFFFFFFFFFFFFFFFFFFFFFFFFFFFFFFFFFFFFFFFFFFFF
F:FFFFFFFFFFFFFFFFFFFFFFFFFFFFFFFFFFFFFFFFFFFFFFFFF:FFFFFFFFFFFFFFFF
F:FFFFFF:FFFFFF
@A00721:190:HGJW7DSXY:4:2475:30544:13651 1:N:0:TCTGCAAG+GCTGCACT
CTTACGTGTCGATTTGTCATTAGACCACGCGTCACGATCATAACCATCTAAAAAATGAATTCTATAGT
GACGCAGTAGAATCGACGGTCAATTTTGAAATTCAAAATTTATATTTATTGTGTTGATGACTGCTTTA
GCAGTTGATTGATGG
+
FFFFFFFFFFFFFFFFFFFFFFFFFFFFFFFFFFFFFFFFFFFFFFFFFFFFFFFFFFFFFFFFFFFF
FFFFFFFFFFFFFFFFFF:FFFFFFFFFF:FFFFFFFFFFFFFF,FFFFFFFFFFFFFF:FFFFFFFF
FFFFFFFFFFFFFFF

```
@A00721:190:HGJW7DSXY:4:2615:25337:20729 1:N:0:TCTGCAAG+GCTGCACT
CTTACGTGTCGATTTGTCATTAGACCACGCGTCACGATCATAACCATCTAAAAAATGAATTCTATAGT
GACGCAGTAGAATCGACGGTCAATTTTGAAATTCAAAATTTATATTTATTGTGTTGATGACTGCTTTA
GCAGTTGATTGATGG
+
FFFFFFFFFFFFFFFFFFFFFFFFFFFFFFFFFFFFF:FFFFFFFFFFFFFFFFFFFFFFFFFF:FFF
FFFFFFFFF:FFFFFFFFFFFFFFFFFFF,FFFFFFFFFFF,FFF:FFFFFFFFFFFFFFFFFF,FFF
::FF:FF,FFFFFFF
@A00721:190:HGJW7DSXY:4:2203:5837:2863 1:N:0:TCTGCAAG+GCTGCACT
CCGGCGGTCCTTTTACCACTTCCTCTTCTTGGAAGACGAATCCATAAATCAACTGCTAAAGCAGACAA
CAACACAATAAATATAAATTTTGAATTTAAAAATTGACAGTCGATTCTACTGAGTCAATATATAATTC
ATAGATAGTAATAG
+
::F:F,F:FFFF:FFF,FFF,,F,,,F::FF,FF,FF,FFFFF:,,:FFFFFFF,F:FFFFFF,,,F,
FFFFF,FF,FFFFF,FFFFFF:,:F,,,FFFF:F:F,,,:F,:F,:FF,F::FF,F,,F:F,FFF,,
F,::FF,F,F:,FF
@A00721:190:HGJW7DSXY:4:1439:4987:19993 1:N:0:TCTGCAAG+GCTGCACT
TGACGCAGTAGAATCGACGGTCAATTTTGAAATTCAAAATTTATATTTATTGTGTTGATGACTGCTTT
AGCAGTTGATTGATGGATTCGTCTTCCAAGAAGAGGAAGTGGTCAGGCGGTCCGTTTAGGACCGCCGG
TTATCGTAAAACACG
+
FFFFFFFFFFFFFFFFFFFFFFFFFFFFFFFFFFFFFFFFFFF:FFFFFFFFFFFFFFFFFFFFFFFF
FFFFF,FFFFFFFFFFFFFF:FFFFFFFFFFFFFFFFFFFFFFFFFFFFFFFFFFFFFFFFFFFFFFF
FFFFFFFFFF,FFF:
@A00721:190:HGJW7DSXY:4:2138:24795:32002 1:N:0:TCTGCAAG+GCTGCACT
TGACGCAGTAGAATCGACGGTCAATTTTGAAATTCAAAATTTATATTTATTGTGTTGATGACTGCTTT
AGCAGTTGATTGATGGATTCGTCTTCCAAGAAGAGGAAGTGGTCAGGCGGTCCGTTTAGGACCGCCGG
TTATCGTAAAACAC
+
F:,,FFF:::FF,:F:FFF:FFFFF:,,F:FFF,FFF:F,F,F:F,,FF,,,,,,::FFF:F,:,:FF
FF,FFF,:FFFFF,:FFFFF:FF:FF,F::FF,FF:FF,FFF:FFFFFFF,F,F,:FF:FFFF:FF,:
,:FF::,,FFF:FF
@A00721:190:HGJW7DSXY:4:2356:5873:16986 1:N:0:TCTGCAAG+GCTGCACT
TGACGCAGTAGAATCGACGGTCAATTTTGAAATTCAAAATTTATATTTATTGTGTTGATGACTGCTTT
AGCAGTTGATTGATGGATTCGTCTTCCAAGAAGAGGAAGTGGTCAGGCGGTCCGTTTAGGACCGCCGG
TTATCGTAAAACACG
+
FFFFFFFFFFFFFFFFFFFFFFFFFFFFFFF:FFFFFFFFFFFFFFFFFFFFFFFFFFFFFFFFFFFF
FFFFF,FFFFFFFFFFFFFFFFFFFFFFFFFFFFFFFF:FFFFFFFFFFFFFFFFFFFFFFFFFFFFF
FFFFFFFFFFFFF:F
@A00721:190:HGJW7DSXY:4:2516:22489:9314 1:N:0:TCTGCAAG+GCTGCACT
TGACGCAGTAGAATCGACGGTCAATTTTGAAATTCAAAATTTATATTTATTGTGTTGATGACTGCTTT
AGCAGTTGATTGATGGATTCGTCTTCCAAGAAGAGGAAGTGGTCAGGCGGTCCGTTTAGGACCGCCGG
TTATCGTAAAACACG
+
FFFFFFFFFFFFFFFFFFFFFFFFFFFFFFFFFFFFFFFFFFFFFFFF:FFFFFFFFFFFFFFFFFFF
FFFFFFFF:FFF:FFFFFFFFFFFFFFFFFFFFFFFFFFFFFFFFFFFFF:FFFFFF,FFFFFFFFFF
,FFFFFFFFFFFFFF
@A00721:190:HGJW7DSXY:4:1114:31132:21465 1:N:0:TCTGCAAG+GCTGCACT
GGCGCAGTAGAATCGACGGTCAATTTTGAAATTCAAAATTTATATTTATTGTGTTGATGACTGCTTTA
GCAGTTGATTGATGGATTCGTCTTCCAAGAAGAGGAAGTGGTCAGGCGGTCCGTTTAGGACCGCCGGT
TATCGTAAAACACGA
+
FFFFFFFFFFFFFFFFFFFFFFFFFFFF,,,FF:,,,:FFF::FF::FFFFFFFFFFFFFFFF:FFFF
```

FFFFFFF:FFFFFFFFFFFFFFFFFFFFFFFFFFFFFFFFFF,FFFFFFFFFFF:FFFFFFFFFFFFFF,
FFFFFFFFFFFFFFF
@A00721:190:HGJW7DSXY:4:1552:27751:28855 1:N:0:TCTGCAAG+GCTGCACT
GGCGCAGTAGAATCGACGGTCAATTTTGAAATTCAAAATGTATATTTATTGTGTTGATGACTGCTTTA
GCAGTTGATTGATGGATTCGTCTTCCAAGAAGAGGAAGTGGTCAGGCGGTCCGTTTAGGACCGCCGGT
TATCGTAAAACACGA
+
FFFFFFFFFFFFFFFFFFFFFFFFFFFFFF,::FF:,,,:,,F:F,FFFFFFFFFFFF:FFFFFFFFFFF
FFFFFFFF:FFFFFFFFFFFFFFFFFFFFFF:FFFFF,FFFFFFFFFFFFFFFFFFFFFFFFFFFFFFFF
FFFFFFFFFFFFFFF
@A00721:190:HGJW7DSXY:4:2119:19054:35493 1:N:0:TCTGCAAG+GCTGCACT
GGCGCAGTAGAAACGACGGTCAATTTTGTAATTCAAAATTTATATTTATTGTGTTGAATACTGCTTTA
TCAGTTGATTGATGGATTCTTCTTCAAATAAGAGGAAGTGGACATGCGGTCCGTTTATGACCGACTGT
TATCGTAAAAAACGA
+
:F:FFFFFF::F,:F:,F:FFFF,FF,,:FF:F,::F,,:FFFFFF,FFFFF,FF,F,,F:F:,FFFF
,FF,F,,FFFFFFFFF,F:,,:,,,,F:,FF,FFFFF,F,F,,F,,::,:,F,,FF:,,,,F,,F,F,
:F:,,,FFF:,FF,F
@A00721:190:HGJW7DSXY:4:2234:19135:2190 1:N:0:TCTGCAAG+GCTGCACT
GGCGCAGTAGAATCGACGGTCAATTTTGAAATTCAAAATTTATATTTATTGTGTTGATGACTGCTTTA
GCAGTTGATTGATGGATTCGTCTTCCAAGAAGAGGAAGTGGTCAGGCGGTCCGTTTAGGACCGCCGGT
TATCGTAAAACACGA
+
FFFFFFFFFFFFFFFFFFFFFFFFFFFFF,,:FFF,,:,FFF:FF:FFFFFFFFFFFFFFFFFFFFFFFF
FFFFFFFFFFFFFFFFFFF,FFFFFFFFFFFFFFFFFFFFFFFFF:FFF::FF:F:F:FFFFFFFFFF
FFFFFFFFFFFFF:F
@A00721:190:HGJW7DSXY:4:2402:11451:15969 1:N:0:TCTGCAAG+GCTGCACT
GGCGCAGTAGAATCGACGGTCAATTTTGAAATTCAAAATTTATATTTATTGTGTTGATGACTGCTTTA
GCAGTTGATTGATGGATTCGTCTTCCAAGAAGAGGAAGTGGTCAGGCGGTCCGTTTAGGACCGCCGGT
TATCGTAAAACACGA
+
FFFFFFFFFFFF:FFFFFFFFFFFFFF:,::FF,:F:,:FFFFF,FFFFF:FFFFFFFFF:FFFFFFF
:FFFFFFFFFFFFFFFFFFFF:FFF:FFFFFFFFFFFFFF,FFFFFFFFF:,FFFF::FF:FFFFFF,
FFFFFFFFFF,F:,F
@A00721:190:HGJW7DSXY:4:1539:31765:17487 1:N:0:TCTGCAAG+GCTGCACT
CGCAGTAGAATCGACGGTCAATTTTGAAATTCAAAATTTATATTTATTGTGTTGATGACTGCTTTAGC
AGTTGATTGATGGATTCGTCTTCCAAGAAGAGGAAGTGGTCAGGCGGTCCGTTTAGGACCGCCGGTTA
TCGTAAAACACGACG
+
FFFFFFFFFFFFFFFFFFFFFFFFFF::FFFFFF:FFFFFFF:FFFFFFFFFFFFFFFFFFFFFFFF:F
FFFFFF:FF:FFFFFFFFFFFFFF:FFFFF,FFFFFFFFFFFFFFFFFFFFFFFF:FFFFFFFFFFFFF
FFFFFFFFFF,FFFF
@A00721:190:HGJW7DSXY:4:1271:10809:9878 1:N:0:TCTGCAAG+GCTGCACT
GTAGAATCGACGGTCAATTTTGAAATTCAAAATTTATATTTATTGTGTTGATGACTGCTTTAGCAGTT
GATTGATGGATTCGTCTTCCAAGAAGAGGAAGTGGTCAGGCGGTCCGTTTAGGACCGCCGGTTATCGT
AAAACACGACGAGTG
+
FFFFFFFFFFFFFFFFFFFFFFFF:FFFFFFFFFFFFFFFFFFFFFFFFFFFFFFFFFFFFFFFFFFFF
FFFFFFFFFFFFFFFFFFFFFFFFFFFFFFFFFFFFFFFFFFFFFFFFFFFFFFFFFFFFFFFFFFFFFF
FFFFFFFFFFFFFFF
@A00721:190:HGJW7DSXY:4:1559:12255:16986 1:N:0:TCTGCAAG+GCTGCACT
GTAGAATCGACGGTCAATTTTGAAATTCAAAATTTATATTTATTGTGTTGATGACTGCTTTAGCAGTT
GATTGATGGATTCGTCTTCCAAGAAGAGGAAGTGGTCAGGCGGTCCGTTTAGGACCGCCGGTTATCGT
AAAACACGACGAGTG

```
+
FFFFFFFFFFFFFFFFFFFFFFFFFFFFFFFF:FFFFFF:FF:FFFF:FFFFFFFFFFFFFFFFFFFF
FFFFFFFFFFFFFFFFFFFFFF:FFFFF:FFFFFFFFFFFFFFFF:FFFFFFFFFFFFFFFF:FFFFFF
FFFFFFFFFFFFFFF
@A00721:190:HGJW7DSXY:4:1635:28501:35571 1:N:0:TCTGCAAG+GCTGCACT
GTAGAATCGACGGTCAATTTTGAAATTCAAAATTTATATTTATTGTGTTGATGACTGCTTTAGCAGTT
GATTGATGGATTCGTCTTCCAAGAAGAGGAAGTGGTCAGGCGGTCCGTTTAGGACCGCCGGTTATCGT
AAAACACGACGAGTG
+
FFFFFFFFFFFFFFFFFFFFFFFFFFFFFFFFFFFFFFFF:FFFFF:FFFFFFFFFFFFFFFFFFFFFFF
FFFFFFFFFFFFFFFFFFFFFFFFFFFFFFFFFFFFFFFFFFFFFFFFFFFFFFFFFFFFFFFFFFFF
FFFFFFFFF:FFFFF
@A00721:190:HGJW7DSXY:4:2106:25961:33708 1:N:0:TCTGCAAG+GCTGCACT
GTAGAATCGACGGTCAATTTTGAAATTCAAAATTTATATTTATTGTGTTGATGACTGCTTTAGCAGTT
GATTGATGGATTCGTCTTCCAAGAAGAGGAAGTGGTCAGGCGGTCCGTTTAGGACCGCCGGTTATCGT
AAAACACGACGAGTG
+
FFFFFFFFFFFFFFFFFFFFFFFFFFFFFFFF:FFFFFFFFFFFFFFFFFFFFFFFFFFFFFFFFFFF
FF:FFFFFFFFFFFFFFFFFFFFFFFFFFFF:FFFFFFFFFFFFFF:FFFFFF:FFFFFFF:FFF:F:FF
:F:FFFFFFF:FFFF
@A00721:190:HGJW7DSXY:4:2212:9182:6245 1:N:0:TCTGCAAG+GCTGCACT
GTAGAATCGACGGTCAATTTTGAAATTCAAAATTTATATTTATTGTGTTGATGACTGCTTTAGCAGTT
GATTGATGGATTCGTCTTCCAAGAAGAGGAAGTGGTCAGGCGGTCCGTTTAGGACCGCCGGTTATCGT
AAAACACGACGAGTG
+
FFFFFFFFFFFFFFFFFFFFFFFFFFFFFFF:FFFFFFFFFFF:FFFFFFFFFFFFFFFFFFFFFFFFF
FF:FFFFFFFFFFFFFFFFFFFFFFFFFFFF:FFFFFFFFFFFFFFFF:::FFF::FFF:FFFFFFFFF
FFFFFFFFFF:FFFF
@A00721:190:HGJW7DSXY:4:2261:19343:28886 1:N:0:TCTGCAAG+GCTGCACT
GTAGAATCGACGGTCAATTTTGAAATTCAAAATTTATATTTATTGTGTTGATGACTGCCTTAGCAGTT
GATTGATGGATTCGTCTTCCAAGAAGAGGAAGTGGTCAGGCGGTCCGTTTAGGACCGCCGGTTATCGT
AAAACACGACGAGTG
+
FFFFFFFFFFFFFFFFFFFFFFFFFFFFFFF:F,FFFFFFFFFFFFFFFFFFFFFFFFFF:FFFFFFFFF
FFFFFFFFFFFFFFFFFFFFFFFFFFFFFFF,FFFFFFFFFFFFFF:FFFFFFFFFFFFFFFFFFFFFF
FFFFFFFFFFFFFFF
@A00721:190:HGJW7DSXY:4:2618:12635:32769 1:N:0:TCTGCAAG+GCTGCACT
GTAGAATCGACGGTCAATTTTGAAATTCAAAATTTATATTTATTGTGTTGATGACTGCTTTAGCAGTT
GATTGATGGATTCGTCTTCCAAGAAGAGGAAGTGGTCAGGCGGTCCGTTTAGGACCGCCGGTTATCGT
AAAACACGACGAGTG
+
FFFFFFFFFFFFFFFFFFFFFFFFFFFFFFF:F:FFFFFFFFFFFFFFFFFFFFFFFFFFFFFFFFFFF
FFFFFFFFFFFFFFFFFFFFFFFFFFFFFFFFFFFFFFFFFFFFFF:FFFFFFFFFFFFFFFF:FFFFF,
FFFFFF::F:FFFFF
@A00721:190:HGJW7DSXY:4:2618:12626:32784 1:N:0:TCTGCAAG+GCTGCACT
GTAGAATCGACGGTCAATTTTGAAATTCAAAATTTATATTTATTGTGTTGATGACTGCTTTAGCAGTT
GATTGATGGATTCGTCTTCCAAGAAGAGGAAGTGGTCAGGCGGTCCGTTTAGGACCGCCGGTTATCGT
AAAACACGACGAGTG
+
FFFFFFFFFFFFFFFFFFFFFFFFF,FFFFF,FFFFFF:FFFFF:FFFFFFFFFFFFFFFFFFFFFF::F
FFFFF:FFFFFFFFFFFFFFFF:FFFFF:FFFFFFFFFFF,FFFF,F,FFF,FFFFFFF:F:FFFF:F
:FFF,FFFFF,:::F
@A00721:190:HGJW7DSXY:4:1540:7835:19758 1:N:0:TCTGCAAG+GCTGCACT
TAGAATCGACGGTCAATTTTGAAATTCAAAATTAATATTAATTGTGTTGATGACTGCTTTAGCAGTTG
```

ATTGATGGATTCGTCTTCCAAGAAGAGGAAGTGGTCAGGCGGTCCGTTTAGGACCGCCGGTTAAAGTA
AAACACGACGAGTG
+
FF:FFFF:FF,F,,FFF,:,:FF:,FFFF:F,:,F:FFF,FFFF,FFFFFFFFFFF,F:,F:,,F,,F
,:F:F,:,FFF,FFFFF:FF::FFFFF:FFFFF::FF:,F,,F:,:F,,,,FFF,FF,:,,FF,,:,F
F:F,F:,FF,F,FF
@A00721:190:HGJW7DSXY:4:1155:8350:14450 1:N:0:TCTGCAAG+GCTGCACT
CCGACGGTCAATTTTGAAATTCAAAATTTATATTTATTGTGTTGATGACTGCTTTAGCAGTTGATTGA
TGGATTCGTCTTCCAAGAAGAGGAAGTGGTCAGGCGGTCCGTTTAGGACCGCCGGTTATCGTAAAACA
CGACGAGTGTTGCAA
+
FFFFFFFFFFFFFFFFFFFFFFFFFFFFFFFFFFFFFFFFFFFFFFFFFFFFFFFFFFFFFF:FFFFFFF
FFFFFFFFFFFFFFFFFFFFFFFFFFFFFFFFFFFFFFFFFFFFFFFFFFFFFFFFFFFFFFFFFFFFFF
:FFFFFFFFFFFFFF
@A00721:190:HGJW7DSXY:4:2669:22878:31626 1:N:0:TCTGCAAG+GCTGCACT
CCGACGGTCAATTTTGAAATTCAAAATTTATATTTATTGTGTTGATGACTGCTTTAGCAGTTGATTGA
TGGATTCGTCTTCCAAGAAGAGGAAGTGGTCAGGCGGTCCGTTTAGGACCGCCGGTTATCGTAAAACA
CGACGAGTGTTGCAA
+
FFFFFFFFFFFFF:FFFF:F,FFFFF:::,F:F:F,FFFFFF:FFFF::FFF,FFFFFFFFF,F:FFFFF
FFFFFFFFFFF,FF:F:FFFF::FFFF:FFFFF:F:FFF:::FFFF:F:,FFFFF:,FF:F:FFFFFF
FFFF:F,FFFFFFFF
@A00721:190:HGJW7DSXY:4:1345:8513:19022 1:N:0:TCTGCAAG+GCTGCACT
CGACGGTCAATTTTGAAATTCAAAATTTATATTTATTGTGTTGATGACTGCTTTAGCAGTTGATTGAT
GGATTCGTCTTCCAAGAAGAGGAAGTGGTCAGGCGGTCCGTTTAGGACCGCCGGTTATCGTAAAACAC
GACGAGTGTTGCAAA
+
FFFFFFFFFFFFFFFFFFFFFFF,FFFFFFFF:FFF:FFFFF:FFFFFFFFFFFFFFFFFFFFFFFFFFF
FFFFFFFFFFFFFFFFFFFFFFFFFFFFFFFFFFFFFFF:FFF,FFFFFFFFFFFFFF:F:FFFFFFFFF
FFFFFFFFFFFFFFF
@A00721:190:HGJW7DSXY:4:1351:28221:11663 1:N:0:TCTGCAAG+GCTGCACT
CGACGGTCAATTTTGACATTCAAAATTTATATTTATTGTGTTGATGACTGCTTTAGCAGTTGATTGAT
GGATTCGTCTTCCAAGAAGAGGAAGTGGTCAGGCGGTCCGTTTAGGACCGCCGGTTATCGTAAAACAC
GACGAGTGTTGCAAT
+
FFFFFFFFFFFFF:FFF,,FFF:::FFFFF:FF:FFFFFFFFFFFFFFFFFFFFFFFFFFFFFFFFFFFF
FFFFFFFFFFFFFFFFFFFFFFFFFFFFFFFFFFFFFFFFFFFFFFFFFFFFFFFF,FFFFFFFF,FFFF
FFFFFF:FFF:FFFF
@A00721:190:HGJW7DSXY:4:1448:29161:6590 1:N:0:TCTGCAAG+GCTGCACT
CGACGGTCAATTTTGAACTTCAAAATTTATATTTATTGTGTTGATGACTGCTTTAGCAGTTGATTGAT
GGATTCGTCTTCCAAGAAGAGGAAGTGGTCAGGCGGTCCGTTTAGGACCGCCGGTTATCGTAAAACAC
GACGAGTGTTGCAAT
+
FFFFFFFFFFFFFFFF:,,FFFF:FFFFFFFFFF:FFF,FFFFFFFFFFFFFFFFFFFFFF:FFFFFFFF
FFFFFFFFFFFFFF:F,FFFFFFFFFFFFFFFFFFFFF:FFFFFFFFFFF:FFFFFFFFFFF,FFF:FFF
FFF,FFFFFFF:FF:
@A00721:190:HGJW7DSXY:4:1539:18159:22013 1:N:0:TCTGCAAG+GCTGCACT
CGACGGTCAATTTTGAAATTCAAAATTTATATTTATTGTGTTGATGACTGCTTTAGCAGTTGATTGAT
GGATTCGTCTTCCAAGAAGAGGAAGTGGTCAGGCGGTCCGTTTAGGACCGCCGGTTATCGTAAAACAC
GACGAGTGTTGCAAA
+
FFFFFFFFFFFFFFFFFFFFFFF:F,FFF:FFFFFFFFFFFFFFFFFFFFFFFFFFFFFFFFFFFFFFFF
FFFFFFFFFFFFFFFFFFFFFFFFFFFFFFFFFFFFFFFFFF,FFFFFF:FFFFFF:FFFFFFFFFF:FF
FFF:FFFFFFFFFFF

```
@A00721:190:HGJW7DSXY:4:2153:11930:29888 1:N:0:TCTGCAAG+GCTGCACT
CGACGGTCAATTTTGAAATTCAAAATTTATATTTATTGTGTTGATGACTGCTTTAGCAGTTGATTGAT
GGATTCGTCTTCCAAGAAGAGGAAGTGGTCAGGCGGTCCGTTTAGGACCGCCGGTTATCGTAAAACAC
GACGAGTGTTGCAAA
+
FFFFFFFFFFFFFFFFFFFFFFFFFFFFFFFFF:FFFFFFFFFFFFFFFFFFFFFFFFFFFFFFFFFF
FFFFFFFFFFFFFFFFF:FFFFFFFFFFFFFFFFFFFFFFF:FFFFFFFFFFFFFFFFFFFFFFFFFFF
FFFFFFFFFF,FFFF
@A00721:190:HGJW7DSXY:4:2153:10728:30311 1:N:0:TCTGCAAG+GCTGCACT
CGACGGTCAATTTTGAAATTCAAAATTTATATTTATTGTGTTGATGACTGCTTTAGCAGTTGATTGAT
GGATTCGTCTTCCAAGAAGAGGAAGTGGTCAGGCGGTCCGTTTAGGACCGCCGGTTATCGTAAAACAC
GACGAGTGTTGCAAA
+
FFFFFFFFFFFFFFFFFFFFFFFFFFFFFFF:FFFFFFFFFFFFFFFFFFFFFFFFFFFFFFFFFFFF
FFFFFFFFFFFFFFFFFFFFFFFFFFFFFFFFFFFFFF:FF,FFFFFFFFFFFFFFFFFFFFFFFF,F:
FF:FFFFFFFFFFFF
@A00721:190:HGJW7DSXY:4:2217:18322:16094 1:N:0:TCTGCAAG+GCTGCACT
CGACGGTCAATTTTGAAATTCAAAATTTATATTTATTGTGTTGATGACTGCTTTAGCAGTTGATTGAT
GGATTCGTCTTCCAAGAAGAGGACGTGGTCAGGCGGTCCGTTTAGGACCGCCGGTTATCGTAAAACAC
GACGAGTGTTGC
+
FFFFFFFFFF::FFF,FFFFFFFFFFF,FFF,F,FFFF,,,:FFFFFFFFFF:FFF:FF,FFFFF:F:
:FFFFF:FFF::FFFFFF,F,F,,FFFFFFF:FFFFF:F,,,:,:F,:F,:FFF,FF,F::,FFF,FF
,F:F,,,,FF::
@A00721:190:HGJW7DSXY:4:2332:18087:11616 1:N:0:TCTGCAAG+GCTGCACT
CGACGGTCAATTTTGAAATTCAAAATTTATATTTATTGTGTTGATGACTGCTTTAGCAGTTGATTGAT
GGATTCGTCTTCCAAGAAGAGGAAGTGGTCAGGCGGTCCGTTTAGGACCGCCGGTTATCGTAAAACAC
GACGAGTGTTGCAAT
+
FFFFF:FFFFFFFFFFFFFFFFFFF:FFFFFFFFF:FF:FFFFFFFFFFFFFFFFFFFFFFFFFFFFF
FFFFFFFFFFFFFFFFFFFFFF:FFFFFFFFFFFFFF:::F,FFF:FFFFFFFFF,FFFFFFFFFF:FF
,F:,F:,FFF:FFFF
@A00721:190:HGJW7DSXY:4:2563:11207:28291 1:N:0:TCTGCAAG+GCTGCACT
CGACGGTCAATTTTGAAATTCAAAATTTATATTTATTGTGTTGATGACTGCTTTAGCAGTTGATTGAT
GGATTCGTCTTCCAAGAAGAGGAAGTGGTCAGGCGGTCCGTTTAGGACCGCCGGTTATCGTAAAACAC
GACGAGTGTTGCAAA
+
FFFFFF:FFFFFFFFFFFFFFFFFFF:FFFFF,F:FFFFFFFFFFFFFFFFFFFFF:FFFFFFFFFFF
FFFFFFFFFFFFFFFFFFF:FFF:FFFFFFFFFFFFFFFFF,FFFFFFFFFFFF:FFFFFFFFFFFFF
FFFFFFFFFFFFF,F
@A00721:190:HGJW7DSXY:4:2611:16333:6668 1:N:0:TCTGCAAG+GCTGCACT
CGACGGTCAATTTTGAAATTCAAAATTTATATTTATTGTGTTGATGACTGCTTTAGCAGTTGATTGAT
GGATTCGTCTTCCAAGAAGAGGAAGTGGTCAGGCGGTCCGTTTAGGACCGCCGGTTATCGTAAAAAAA
GACGAGTGTTGCAA
+
:FFFFFFFFFFFFFF,,FFFF:FFF:FF:FFF:FFF:FFFF:FFF,FFF:FFF:F:FFF,:FFFFFFF
FFFFFF,:FFF,:FFFFF,,,FFFFF::FFFF,:F:F:F:FFFFFFF,::::FF,FF,,:FFFFF,F,
,F,,F:F:,FF:FF
@A00721:190:HGJW7DSXY:4:2614:18105:11710 1:N:0:TCTGCAAG+GCTGCACT
CGACGGTCAATTTTGAAATTCAAAATTTATATTTATTGTGTTGATGACTGCTTTAGCAGTTGATTGAT
GGATTCGTCTTCCAAGAAGAGGAAGTGGTCAGGCGGTCCGTTTAGGACCGCCGGTTATAGTAAAACAC
GACGAGTGTTGCAAA
+
F:F:FFFFFFFFFFFFFFF:FFFFFFFFF:F,FFF:FFFF:FFFF:FFFFF:FFFFFF:FFFFFF:FF
```

```
:FFFFFFFFFF:FFF:FF:F:FFFFFFFFFFFFF:FF:,,FFFFF,F,F,FF:,:FF,,,,FFFF:F,
:FF:F,:,,F,,FFF
@A00721:190:HGJW7DSXY:4:2348:8052:22420 1:N:0:TCTGCAAG+GCTGCACT
GCACGGTCAATTTTGAAATTCAAAATTTATATTTATTGTGTTGATGACTGCTTTAGCAGTTGATTGAT
GGATTCGTCTTCCAAGAAGAGGAAGTGGTCAGGCGGTCCGTTTAGGACCGCCGGTTATCGTAAAACAC
GACGAGTGTTGCAAT
+
FF:FFFFFFFFFFFFFF,FFFFF:::FFFFFF,FFF:FFF:,FFFFFFFFFFFFFFFFFF,FFF,FFF
FFFFFFFFFFFFFF:F:FFFFF,FFFFFFFFFFFFFFFFF:FFFFFFFFFFFF,FFFFFFFFFFFFF
FFFFFFFFFFFFF,F
@A00721:190:HGJW7DSXY:4:1260:24894:29794 1:N:0:TCTGCAAG+GCTGCACT
GGTCAATTTTGAAATTCAAAATTTATATTTATTGTGTTGATGACTGCTTTAGCAGTTGATTGATGGAT
TCGTCTTCCAAGAAGAGGAAGTGGTCAGGCGGTCCGTTTAGGACCGCCGGTTATCGTAAAACACGACG
AGTGTTGCAATTCAA
+
FFFFFFFFFFFFF::FFF:FFFFFFFFFFFFFFFFFFFFFFFFFFFFFFFFFFFFFFFFFFFFFFFFF
FFFFFFFFFFFFFFFFFFFFFFFFFFFFFFFFFFFFFFFFFFFFFFFFFFFFFFFFFFFF:FFFFFFFF
FF:FFFFFFFFFFFF
@A00721:190:HGJW7DSXY:4:1260:23845:29951 1:N:0:TCTGCAAG+GCTGCACT
GGTCAATTTTGACATTCAAAATTTATATTTATTGTGTTGATGACTGCTTTAGCAGTTGATTGATGGAT
TCGTCTTCCAAGAAGAGGAAGTGGTCAGGCGGTCCGTTTAGGACCGCCGGTTATCGTAAAACACGACG
AGTGTTGCAATTCAA
+
FFFFFFFFFFF:,,FF:,:F:FFFFF:F:FF,FFFFF:FFFFFFFFFFFFFFFFFFFFFFFFFFFFFF
F:FFFFFF:FFFF:FF,FFFF:FFFFFFFFFFFFFFFFFFFFFFF:FFFFFFFFFFFFFFFF:FFFFFF
FFF,:FFFFFFFFFF
@A00721:190:HGJW7DSXY:4:1305:29704:20588 1:N:0:TCTGCAAG+GCTGCACT
GGTCAATTTTGAAATTCAAAATTTATATTTATTGTGTTGATGACTGCTTTAGCAGTTGATTGATGGAT
TCGTCTTCCAAGAAGAGGAAGTGGTCAGGCGGTCCGTTTAGGACCGCCGGTTATCGTAAAACACGACG
AGTGTTGCAATTCAA
+
FFFFFFFFFFF:FFFFFFF:FFFFFFFFFFFFFFFFFFFFFFFFFFFFFFFFFFFFF:FFFFFF:FFFFF
FFFFFFFFFFFFFFFFFFF:FFFFFFFFFFFFFFFF:FF:FFFFFFFFFF:FFFFFFFFFF,:,FFF:
FF:FFFFFFF:FFFF
@A00721:190:HGJW7DSXY:4:2120:12373:15781 1:N:0:TCTGCAAG+GCTGCACT
GGTCAATTTTGAAATTCAAAATTTATATTTATTGTGTTGATGACTGCTTTAGCAGTTGATTGATGGAT
TCGTCTTCCAAGAAGAGGAAGTGGTCAGGCGGTCCGTTTAGGACCGCCGGTTATCGTAAAACACGACG
AGTGTTGCAATTCAA
+
FFFFFFFFFFFFF,FFFF::FF:FFFFFFFFFFFFFFFFFF:FFFFFFFFFFFFFFFFFFFFFF:FFFF
FFFFFFFFFFF,FFFFF,FFFFFFFFFFFF:FFFFFFFFF,FFFFFFFFFFFFF::FFF:FFF:FFF:
FFFFFF,FFFFF,FF
@A00721:190:HGJW7DSXY:4:2120:11867:17347 1:N:0:TCTGCAAG+GCTGCACT
GGTCAATTTTGAAATTCAAAATTTATATTTATTGTGTTGATGACTGCTTTAGCAGTTGATTGATGGAT
TCGTCTTCCAAGAAGAGGAAGTGGTCAGGCGGTCCGTTTAGGACCGCCGGTTATCGTAAAACACGACG
AGTGTTGCAATTCAA
+
FFFFFFFFFFF,,,:FF,,FFFFFFFFFFFFFFFFFFFFFFFFFFFFFFFFF:FFFFFFFFFFFFFFF
F:FFFFFF:FFFFFFFFFFFFFFFFFFFFFFFFFFFFFFFF,FFFF:FFFFFFFF:FFFFFF:F:,FFF
FFFFFF::FFFFFFF
@A00721:190:HGJW7DSXY:4:2219:4716:16673 1:N:0:TCTGCAAG+GCTGCACT
GGTCAATTTTGAAATTCAAAATTTATATTTATTGTGTTGATGACTGCTTTAGCAGTTGATTGATGGAT
TCGTCTTCCAAGAAGAGGAAGTGGTCAGGCGGTCCGTTTAGGACCGCCGGTTATCGTAAAACACGACG
AGTGTTGCAATTCAA
```

```
+
FFFFFFFFFFF:F,FFFFF::FFFFFFFFFFFFFFFFFF:FFFFFFFFFFFFFFFFFFFFFFFFFFFFFF
FFFFFFFFFFFF:FFFFFFFFFFFFFFFFFFFFFFFFFFFFFFFFFFFFFFFFFFFFFFFFFFFF:FFF
FFFFFFFFFFFF,FF
@A00721:190:HGJW7DSXY:4:2529:5791:13808 1:N:0:TCTGCAAG+GCTGCACT
GGTCAATTTTGAAATTCAAAATTTATATTTATTGTGTTGATGACTGCTTTAGCAGTTGATTGATGGAT
TCGTCTTCCAAGAAGAGGAAGTGGTCAGGCGGTCCGTTTAGGACCGCCGGTTATCGTAAAACACGACG
AGTGTTGAAATTCAA
+
:FFFFFFFFFFF::FFF::FFFFFFFFFF:FFFFFFFFFFF:FF:FFFFFFFFFF:FFFFF:FFFFFF
FF,FFFFF:FF:FFFFFFFFFFFFFFFF:F:FFFFFF,FFF,FF,,FF,,FFF::FFF:FFFF,:FF,
F:FFF,,,FF,:,:F
@A00721:190:HGJW7DSXY:4:2629:13847:8625 1:N:0:TCTGCAAG+GCTGCACT
GGTCAATTTTGAAATTCAAAATTTATATTTATTGTGTTGATGACTGCTTTAGCAGTTGATTGATGGAT
TCGTCTTCCAAGAAGAGGAAGTGGTCAGGCGGTCCGTTTAGGACCGCCGGTTATCGTAAAACACGACG
AGTGTTGCAATTCAA
+
FFFFFFFFFFFFF:FFF,:FFFFFFFFF:FFFFFFFFFFFFFFFFFFFFFFFFFF:FFFFFFFFFFFF
FFFFFFFFFFFFFFFFFFFF,FFFFFFFFFFFFFFFFFFFF:FFFFFFFFFFFFFFFFFFFFFF:FFFF
FF:FFFFFFF:FFFF
@A00721:190:HGJW7DSXY:4:1223:13847:2957 1:N:0:TCTGCAAG+GCTGCACT
CTGCTGAGTCCTGTTGAATTGCAACACTCGTCGTGTTTTACGATAACCGGCGGTCCTTTTACCACTTC
CTCTTCTTGGAAGACGAATCCATCAATCAACTGCTAAAGCAGTCATCAACACAATAAATATAAATTTT
GAATTTCAAAATTGA
+
FFFFFFFFFFFFFFFFFFFFFFFFFFFFFFFFFFFFFFFFFFFFFFFFFFFFFFFFFFFFFFFFFFFF
FFFFFFFFFFFFFFFFFFFFFFFFFFFFFFFFFFFFFF:FFFFF:FF:FFFFF,FFFFFFFFFFFFFF
,:FFFFFFFFFFF:F
@A00721:190:HGJW7DSXY:4:1647:24288:27085 1:N:0:TCTGCAAG+GCTGCACT
CTGCTGAGTCCTGTTGAATTGCAACACTCGTCGTGTTTTACGATAACCGGCGGTCCTTTTACCACTTC
CTCTTCTTGGAAGACGAATCCATCAATCAACTGCTAAAGCAGTCATCAACACAATAAATATAAATTTT
GAATTTCAAAATTGA
+
FFFFFFFFFFFFFFFFFFFFFFFFFFFFFFFFFFFFFFFFFFFFFFFFFFFFFFFFFFFFFFFFFFFF
FFFFFFFFFFFFFFFFFFFFFFFFFFFFFFFFFFFFFFFFFFFFFFFFFFFFFFFFFFFFFFFFFFFF
FFFFFFFFFFFFFFF
@A00721:190:HGJW7DSXY:4:2102:22697:10363 1:N:0:TCTGCAAG+GCTGCACT
CTGCTGAGTCCTGTTGAATTGCAACACTCGTCGTGTTTTACGATAACCGGCGGTCCTTTTACCACTTC
CTCTTCTTGGAAGACGAATCCATCAATCAACTGCTAAAGCAGTCATCAACACAATAAATATAAATTTT
GAATTTCAAAATTGA
+
FFFFFFFFFFFFFFFFFFFFFFFFFFFFFFFFFFFFFFFFFFFFFFFFFFFFFFFFFFFFFFFFFFFF
FFFFFFFFFFFFFFFFFFFFFFFFFFFFFFFFFFFFFFFFFFFFFFFFFFFFFF,,FFFFFFFFFFFF
FFFFFFFFFFFFFFF
@A00721:190:HGJW7DSXY:4:2231:29089:5682 1:N:0:TCTGCAAG+GCTGCACT
CTGCTGAGTCCTGTTGAATTGCAACACTCGTCGTGTTTTACGATAACCGGCGGTCCTTTTACCACTTC
CTCTTCTTGGAAGACGAATCCATCAATCAACTGCTAAAGCAGTCATCAACACAATAAATATAAATTTT
GAATTTCAAAATTGA
+
FFFFFFFFFFFF:FFFFFFFFFFFFFFFFFFFFFFFFFFFFFFFFFFFFFFFFFFFFFFFFFFFFFFF
FFFFFFFFFFFF:FFFFFFFFFFFFFFFFFFFFFFFFFFFFFFFFFFF:FFFFFFF:FFFFFFFFFFFFF
,FFFFFFFFFFF::F
@A00721:190:HGJW7DSXY:4:2413:25925:4210 1:N:0:TCTGCAAG+GCTGCACT
CTGCTGAGTCCTGTTGAATTGCAACACTCGTCGTGTTTTACGATAACCGGCGGTCCTTTTACCACTTC
```

CTCTTCTTGGAAGACGAATCCATCAATCAACTGCTAAAGCAGTCATCAACACAATAAATATAAATTTT
GAATTTCAAAATTGA
+
FFFF,FFFFFFFFFFFFFFFFFFFFFFFFFFFFFFFFFFFFFFFFFFFFFFFFFFFFFFFFFFFFFFFF
FFFFFFFFFFFFFFFFFFFFFFFFFFFFFFFFFFFFFFF,FFFFFF:FFFFFFFFFFFFFFFFFF:FFF
,FFFFF,FFFFFF,F
@A00721:190:HGJW7DSXY:4:2465:19786:16501 1:N:0:TCTGCAAG+GCTGCACT
CTGCTGAGTCCTGTTGAATTGCAACACTCGTCGTGTTTTACGATAACCGGCGGTCCTTTTACCACTTC
CTCTTCTTGGAAGACGAATCCATCAATCAACTGCTAAAGCAGTCATCAACACAATAAATATAAATTTT
GAATTTCAAAATTGA
+
FFFFFFFFFFFFFFFFFFFFFFFFFFFFFFFFFFFFF,FF:FFFFFF:FFFFFFFF:FFFFFFFF:FF
FFFFFFF:FFFFFFFFFFFFFF:FFFFFFFFFF:FFFF:FFFFFFFFFFFFFF,FFFFFFFFF:FFFF
:FFFFFFFFFFFFFF
@A00721:190:HGJW7DSXY:4:2518:7129:2347 1:N:0:TCTGCAAG+GCTGCACT
CTGAGTCCTGTTGAATTGCAACACTCGTCGTGTTTTACGATAACCGGCGGTCCTTTTAACACTTCCTC
TTCTTGGAAGACGAATCCATCAATCAACTTCAAAAGCAGTCATCAACACAATAAATATAAATTTTGAA
TTTCAAAAATGA
+
FFFF:,FF:,F::FFFF:,F:,FF,,F,F,,F,:,FF,,F,FF,,,,F,:FFF,F,,F,,FF,,:FFF
:F:FF:,FF:F:F:FFF,FFFFF:,FF,F,F,FFF:,FFF,F,:FFFF,F:FF:FFF,FF::,FF,FF
F,F,,FFF,F,F
@A00721:190:HGJW7DSXY:4:2154:14832:16376 1:N:0:TCTGCAAG+GCTGCACT
CTGCTGAGTCCTGTTGAATTGCAACACTCGTCGTGTTTTACGATAACCGGCGGTCCTTTTACCACTTC
CTCTTCTTGGAAGACGAATCCATCAATCAACTGCTAAAGCAGTCATCAACACAATAAATATAAATTTT
GAATTTCAAAATTG
+
FFFFFFFFFFFFFFFFFFFFFFFFFFFFFFFFFFFFFFFFFFFFFFFFFFFFFFFFFFFFFFFFFFFF
FFFFFFFFFFFFFFFFFFFFFFFFFFFFFFFFFFFFFFFFFFFFFFFFFFFFFFFFFFFFFFFF:FFF
:FF:FFFFFFFFFF
@A00721:190:HGJW7DSXY:4:1374:32145:4679 1:N:0:TCTGCAAG+GCTGCACT
CCACTGAACCGGCAGCTGTGACCGCTGAGTCCTGTTGAATTGCAACACTCGTCGTGTTTTACGATAAC
CGGCGGTCCTTTTACCACTTCCTCTTCTTGGAAGACGAATCCATCAATCAACTGCTAAAGCAGTCATC
AACACAATAAATATA
+
FFFFFFFFFFFFFFFFFFFFFFFFFFFFFFFFFFFFFFFFFFFFFFFFFFFFFFFFFFFFFFFFFFFF
FFFFFFFFFFFFFFFFFFFFFFFFFFFFFFFFFFFFFFFFFFFFFFFFFFFFFFFFF:FFFFFFFFFF
F::FFFFFFFFFFFF
@A00721:190:HGJW7DSXY:4:1501:32407:31125 1:N:0:TCTGCAAG+GCTGCACT
CCACTGAACCGGCAGCTGTGACCGCTGAGTCCTGTTGAATTGCAACACTCGTCGTGTTTTACGATAAC
CGGCGGTCCTTTTACCACTTCCTCTTCTTGGAAGACGAATCCATCAATCAACTGCTAAAGCAGTCATC
AACACAATAAATATA
+
FFFFFFFFFFFFFFFFFFFFFFFFFFFFFFFFFFFFFFFFFFFFFFFFFFFFFFFFFFFFFFFFFFFF
FFFFFFFFFFFFF:FFFFFFFFFFFFFFFFFFFFFFFFF:FFFFFFFFFFFF:FFFFFF:FFF:FFFFF
FFFFFFFFFFFFFFF
@A00721:190:HGJW7DSXY:4:1130:32009:16908 1:N:0:TCTGCAAG+GCTGCACT
TGTTGATGACTGCTTTAGCAGTTGATTGATGGATTCGTCTTCCAAGAAGAGGAAGTGGTCAGGCGGTC
CGTTTAGGACCGCCGGTTATCGTAAAACACGACGAGTGTTGCAATTCAACAGGACTCAGCGGTCACAG
CTGCCGGTTCAGTGG
+
F:FFFFFFFFFFFFFFFFFFFFFFFFFFFFFFFFFFFFFFFFFFFFFFFFFFFFFFFFFFFFFFFFFF:
FF:FFFFFFFFFFFFFFFFFFFFFFFFFFFF:FFFFFFFFFFFFFFFFFFFFFFFFFFFFF:FFFFFFFFF
FF:FFFFFF:FFFFF

@A00721:190:HGJW7DSXY:4:1501:25753:13557 1:N:0:TCTGCAAG+GCTGCACT
TGTTGATGACTGCTTTAGCAGTTGATTGATGGATTCGTCTTCCAAGAAGAGGAAGTGGTCAGGCGGTC
CGTTTAGGACCGCCGGTTATCGTAAAACACGACGAGTGTTGCAATTCAACAGGACTCAGCGGTCACAG
CTGCCGGTTCAGTGG
+
FFFFFFFFFFFFFFFFFFFFFFFFFFFFFFFFFFFFFFFFFFFFFFFFFFFF:FFFFFFFFFFFFF:
FFFFF:FFF:FFFFFFFFFF:::FFFFFF:FF,FF:F:FFFFFFFF,FFFF,FFF::F:FFFFFFFF:
F:F,FFFFF,::,FF
@A00721:190:HGJW7DSXY:4:1611:15745:21903 1:N:0:TCTGCAAG+GCTGCACT
TGTTGATGACTGCTTTAGCAGTTGATTGATGGATTCGTCTTCCAAGAAGAGGAAGTGGTCAGGCGGTC
CGTTTAGGACCGCCGGTTATCGTAAAACACGACGAGTGTTGCAATTCAACAGGACTCAGCGGTCACAG
CTGCCGGTTCAGTGG
+
FFFFFFFFFFFFFFFFFFFFFFFFFFFFFFFFFFFFFFFFFFFFFFFFFFFFFFFFFFFFFFFFFFF:
FFF:FFFF,FFFFFFFFFFFFFFFFFFFFFFFFF:FFFF:FFFF:FFFFFFFFFFFFFFFFFFFF,FFF
F:FFFFFFFFFFFFF
@A00721:190:HGJW7DSXY:4:1647:1732:13135 1:N:0:TCTGCAAG+GCTGCACT
TGTTGATGACTGCTTTAGCAGTTGATTGATGGATTCGTCTTCCAAGAAGAGGAAGTGGTCAGGCGGTC
CGTTTAGGACCGCCGGTTATCGTAAAACACGACGAGTGTTGCAATTCAACAGGACTCAGCGGTCACAG
CTGCCGGTTCAGTGG
+
FFFFFFFFFFFFFFFFFFFFFFFFFFFFFFFFFFFFFFFFFFFFFFFFFFFF::FFFFFFFFFFFFFF
FFFFFFFFFFFFFFFFFFFFFFFFFFFFFFFFFFFFFFFFFFFFFFFFFFFF::FFFFFFFFFFFFFF
FFFFFFFFFF:FFFF
@A00721:190:HGJW7DSXY:4:2303:6460:6793 1:N:0:TCTGCAAG+GCTGCACT
TGTTGATGACTGCTTTAGCAGTTGATTGATGGATTCGTCTTCCAAGAAGAGGAAGTGGTCAGGCGGTC
CGTTTAGGACCGCCGGTTATCGTAAAACACGACGAGTGTTGCAATTCAACAGGACTCAGCGGTCACAG
CTGCCGGTTCAGTGG
+
FFFFFFFFFFFFFFFFFFFFFFFFFFFFFFFFFFFFFFFFFFFFFFFFFFFFFFFFFFFFFFFFFFFF
FFFFFF:FFFFFFFFF,FFFFFFFFFFFF,:FF,F:FFFF:FF:FF,FFFFFFF:FFFFFFFFFFFFF
F:FFF:F:FFFFFFF
@A00721:190:HGJW7DSXY:4:2303:7310:7858 1:N:0:TCTGCAAG+GCTGCACT
TGTTGATGACTGCTTTAGCAGTTGATTGATGGATTCGTCTTCCAAGAAGAGGAAGTGGTCAGGCGGTC
CGTTTAGGACCGCCGGTTATCGTAAAACACGACGAGTGTTGCAATTAAACAGGACTCAGCGGTCACAG
CTGCCGGTTCAGTGG
+
FFFFFFFFFFFFFFFFFFFFFFFFFFFFFFFFFFFFFFFFFFFFFFFFFFFFFFFFFFFFFFFFFFFF
FFFFF:::FFF,FF:F,FFF,:FFFFFFF,:F,FF,F:FFFFFF:F,FFFFFFFFFFF,FFF:FF:::
F:F:FFF,F,FFF,F
@A00721:190:HGJW7DSXY:4:2469:30463:23406 1:N:0:TCTGCAAG+GCTGCACT
TGTTGATGACTGCTTTAGCAGTTGATTGATGGATTCGTCTTCCAAGAAGAGGAAGTGGTCAGGCGGTC
CGTTTAGGACCGCCGGTTATCGTAAAACACGACGAGTGTTGCAATTCAACAGGACTCAGCGGTCACAG
CTGCCGGTTCAGTGG
+
FFFFFFFFFFFFFFFFFFFFFFFFFFFFFFFFFFFFFFFFFFFFFFFFFFFFFFFFFFFFFFFFFFFF
FFFFFFFFFFFFFFFFFFFFFFFFFFFFFFFFFFFFFFFFFFFFFFFFFFFFFFFFFFFFFFFFFFFF
FFFFFFFFFFFFFFF
@A00721:190:HGJW7DSXY:4:2469:30816:24173 1:N:0:TCTGCAAG+GCTGCACT
TGTTGATGACTGCTTTAGCAGTTGATTGATGGATTCGTCTTCCAAGAAGAGGAAGTGGTCAGGCGGTC
CGTTTAGGACCGCCGGTTATCGTAAAACACGACGAGTGTTGCAATTCAACAGGACTCAGCGGTCACAG
CTGCCGGTTCAGTGG
+
FFFFFFFFFFFFFFFFFFFFFFFFFFFFF:FFFFFFFFFFFFFFFFFFFFFFFFFFFFFFFFFFFFFF

FFFFFFFFFFFFFFFFFFFFFFFFFFFFFFFFFFFFFFFFFFFFFFFFFFFFFFFFFFFFFFFFFFFF
FFFFFFFFFFFFFFF
@A00721:190:HGJW7DSXY:4:2617:24144:15217 1:N:0:TCTGCAAG+GCTGCACT
TGTTGATGACTGCTTTAGCAGTTGATTGATGGATTCGTCTTCCAAGAAGAGGAAGTGGTCAGGCGGTC
CGTTTAGGACCGCCGGTTATCGTAAAACACGACGAGTGTTGCAATTCAACAGGACTCAGCGGTCACAG
CTGCCGGTTCAGTGG
+
FFFFFFFFFFFFFFFFFFFFFFFFFFFFFFFFFFFFFFFFFFFFFFFFFFFFF:FFFFFFFFFFFFFF
FFFFFFFFFFFFFFFFFFFFFFFFFFFFF:FFFFFFFFFFFFF:FFFFFFFFFFFFFFFFFFFFFFFF
FFFFFFFFFFFFFF:
@A00721:190:HGJW7DSXY:4:1132:11071:4131 1:N:0:TCTGCAAG+GCTGCACT
GTTGATGACTGCTTTAGCAGTTGATTGATGGATTCGTCTTCCAAGAAGAGGAAGTGGTCAGGCGGTCC
GTTTAGGACCGCCGGTTATCGTAAAACACGACGAGTGTTGCAATTCAACAGGACTCAGCGGTCACAGC
TGCCGGTTCAGTGGT
+
FFFFFFFFFFFFFFFFFFFFFFFF:FFFFFFFFFFFFFFFFFFFFFFFFFFFFFFFFFFFFFFFFFFF
FFFFFFFFFFFFFFFFFFFFFFFFF:FFFFFFFFFFFFFFFFFFFFFFFFFFFFFFFFFFFFFFFFFF
FFFFFFFFFFFFFFF
@A00721:190:HGJW7DSXY:4:1135:17282:30514 1:N:0:TCTGCAAG+GCTGCACT
GTTGATGACTGCTTTAGCAGTTGATTGATGGATTCGTCTTCCAAGAAGAGGAAGTGGTCAGGCGGTCC
GTTTAGGACCGCCGGTTATCGTAAAACACGACGAGTGTTGCAATTCAACAGGACTCAGCGGTCACAGC
TGCCGGTTCAGTGGT
+
FFFFFFFFFFFFFFFFFFFFFFFFFFFFFFFFFFFFFFFFFFFFFFFFFFFFFFFFFFFFFFFFFFFF
FF:FFFFF:FFFFFFFFFFFFFFFFFFFFFFFFFFFFFFFFFFFFFFFFFFFFFFFFFFF:FF,FFFF
:FFFFFFFFFFFFFF
@A00721:190:HGJW7DSXY:4:1156:15998:24878 1:N:0:TCTGCAAG+GCTGCACT
GTTGATGACTGCTTTAGCAGTTGATTGATGGATTCGTCTTCCAAGAAGAGGAAGTGGTCAGGCGGTCC
GTTTAGGACCGCCGGTTATCGTAAAACACGACGAGTGTTGCAATTCAACAGGACTCAGCGGTCACAGC
TGCCGGTTCAGTGGT
+
FFFFFFFFFFFFFFFFFFFFFFFF:FFFFFFFFFFFFFFFFFFFFFFFFFFFFFFFFFFFFFFFFFFF
FFFFFFFFFFFFFFFFFFFFFFFFF:FF:FFFFFFFFFFFFFFFF:FFFFFFFFFFFFFFFFFFFFFF
FFFFFFFFFFFFFFF
@A00721:190:HGJW7DSXY:4:1447:6189:34413 1:N:0:TCTGCAAG+GCTGCACT
GTTGATGACTGCTTTAGCAGTTGATTGATGGATTCGTCTTCCAAGAAGAGGAAGTGGTCAGGCGGTCC
GTTTAGGACCGCCGGTTATCGTAAAACACGACGAGTGTTGCAATTCAACAGGACTCAGCGGTCACAGC
TGCCGGTTCAGTGGT
+
FFFFFFFFFFFFFFFFFFFFFFFFFFFFFFFFFFFFFFFFFFFFFFFFFFFFFF:FFFFFFFFFFFFF
FFFFFFFFFFFFFFFFFFFFFFFFFFFFFFFFFFFFFFFFFFFFFFFFFFFFFFFFFFFFFFFFF,FF
FFFFFFFFFFFFF:F
@A00721:190:HGJW7DSXY:4:1519:8983:35368 1:N:0:TCTGCAAG+GCTGCACT
GTTGATGACTGCTTTAGCAGTTGATTGATGGATTCGTCTTCCAAGAAGAGGAAGTGGTCAGGCGGTCC
GTTTAGGACCGCCGGTTATCGTAAAACACGACGAGTGTTGCAATTCAACAGGACTCAGCGGTCACAGC
TGCCGGTTCAGTGGT
+
FFFFFFFFFFFFFFFFFFFFFFFF:FFFFFFFFFFFFFFFFFFFFFFFFFFFFFFFFFFFFFFFFFFF
FFFFFFFFFFFFFFFFFFFFFFFFFFFFFFFFFFFFFFFFFFFFFFFFFFFFFFFFFFFFFFFFFFFF
FF:FFF:FFFFFFFF
@A00721:190:HGJW7DSXY:4:1608:18973:5447 1:N:0:TCTGCAAG+GCTGCACT
GTTGATGACTGCTTTAGCAGTTGATTGATGGATTCGTCTTCCAAGAAGAGGAAGTGGTCAGGCGGTCC
GTTTAGGACCGCCGGTTATCGTAAAACACGACGAGTGTTGCAATTCAACAGGACTCAGCGGTCACAGC
TGCCGGTTCAGTGGT

+
FFFFFFFFFFFFFFFFFFFFFFFFFFFFFFFFFFFFFFFFFFFFFFFFFFFFFFFFFFFFFFFFFFFF:F
F:FFFFFFFFFFFFF:FFF,FFFFFFFFF:FFFF,FFFFFFFFFFFFFFF,FF,FFFFFFFFF:FFFF
FF:FFFFFFFFFFFF
@A00721:190:HGJW7DSXY:4:1634:12726:19429 1:N:0:TCTGCAAG+GCTGCACT
GTTGATGACTGCTTTAGCAGTTGATTGATGGATTCGTCTTCCAAGAAGAGGAAGTGGTCAGGCGGTCC
GTTTAGGACCGCCGGTTATCGTAAAACACGACGAGTGTTGCAATTCAACAGGACTCAGCGGTCACAGC
TGCCGGTTCAGTGGT
+
FFFFFFFFFFFFFFFFFFFF,FFFFFFFFFFFFFFFFFFFFFFFFFFFFFFFFFFFFFFFFFFFFFFF
FFFFFFFFFFFFFFFFFFFFFFFFFFF:FFFFFFFFFFFFFFFFFFFFFFFFFFFF:FFFFFFFFFFFFF
FFFFFFFFF:FFFFF
@A00721:190:HGJW7DSXY:4:1644:17427:28354 1:N:0:TCTGCAAG+GCTGCACT
GTTGATGACTGCTTTAGCAGTTGATTGATGGATTCGTCTTCCAAGAAGAGGAAGTGGTCAGGCGGTCC
GTTTAGGACCGCCGGTTATCGTAAAACACGACGAGTGTTGCAATTCAACAGGACTCAGCGGTCACAGC
TGCCGGTTCAGTGGT
+
FFFFFFFFFFFFFFFFFFFF:FFFFFFFFFFFFFFFFFFFFFFFFFFFFFFFF:FFFFFFFFFFFFFFF
FFFFFFFFFFFFFFFFFFFFFFFFF:FFF:FFFFFFFFFFFFFFFFFFFFFFFFFFF:FF:FFFFFFF:FF
FFFFFFFFFFF:FFF
@A00721:190:HGJW7DSXY:4:2152:1172:10911 1:N:0:TCTGCAAG+GCTGCACT
GTTGATGACTGCTTTAGCAGTTGATTGATGGATTCGTCTTCCAAGAAGAGGAAGTGGTCAGGCGGTCC
GTTTAGGACCGCCGGTTATCGTAAAACACGACGAGTGTTGCAATTCAACAGGACTCAGCGGTCACAGC
TGCCGGTTCAGTGGT
+
FFFFFFFFFFFFFFFFFFFFFFFFFFFFFFFFFFFFFFFFFFFFFFFFFFFFFFFFFFFFFFFFFFFF:F
FFFFFFFFFFFFFFFFFFFFFFFFFFF:FFF:FFFFFFFFFFFFFF:FFFFFFFFFFFFFFFFFFFFFFF
FFFFFFFFFFFFFFF
@A00721:190:HGJW7DSXY:4:2418:14380:33692 1:N:0:TCTGCAAG+GCTGCACT
GTTGATGACTGCTTTAGCAGTTGATTGATGGATTCGTCTTCCAAGAAGAGGAAGTGGTCAGGCGGTCC
GTTTAGGACCGCCGGTTATCGTAAAACACGACGAGTGTTGCAATTCAACAGGACTCAGCGGTCACAGC
TGCCGGTTCAGTGGT
+
FFFFFFFFFFFFFFFFFFFFFFFFFFFFFFFFFFFFFFFFFFFFFFFFFFFFFFFFFFFFFFFFFFFF:F
FF,FF:FFFFFFFFFFFFF:FFFFFFFFFFFF,FFF,FF:FFFFF:FF:F,FFF:,F:FFFFF:FFFF
FFFFFFFFFF:FFFF
@A00721:190:HGJW7DSXY:4:2548:26368:10802 1:N:0:TCTGCAAG+GCTGCACT
GTTGATGACTGCTTTAGCAGTTGATTGATGGATTCGTCTTCCAAGAAGAGGAAGTGGTCAGGCGGTCC
GTTTAGGACCGCCGGTTATCGTAAAACACGACGAGTGTTGCAATTCAACAGGACTCAGCGGTCACAGC
TGCCGGTTCAGTGGT
+
FFFFFFFFFFFFFFFFFFFFFFFFFFFFFFFFFFFFFFFFFFFFFFFFFFF:FFFFFFFFFFFFFFFFFF:
FF:FFFFFFFFFFFFFFFFFFFFFFFFFFFFFFFFFFFFFF:FFFFFFFFFFFFFFFFFFFFFFFFFFFFF
FFFFFFFFFFFFFFF
@A00721:190:HGJW7DSXY:4:2557:4092:21355 1:N:0:TCTGCAAG+GCTGCACT
GTTGATGACTGCTTTAGCAGTTGATTGATGGATTCGTCTTCCAAGAAGAGGAAGTGGTCAGGCGGTCC
GTTTAGGACCGCCGGTTATCGTAAAACACGACGAGTGTTGCAATTCAACAGGACTCAGCGGTCACAGC
TGCCGGTTCAGTGGT
+
FFFFFFFFFFFFFFFFFFFFFFFFFFFFFFFFFFFFFFFFFFFFFFFFFFFF:FFFFFFFFFFFFFFF
FFFFFFFFFFFFFFFFFF:FFFFFFFFFF:FFFFFFFFFFFFFFFFFFFFFFFFFFFFFFFFFFFFFFFF
FFFFFFFFFFFFFFF
@A00721:190:HGJW7DSXY:4:2562:25211:33912 1:N:0:TCTGCAAG+GCTGCACT
TTGATGACTGCTTTAGCAGTTGATTGATGGATTCGTCTTCCAAGAAGAGGAAGTGGTCAGGCGGTCCG

```
TTTAGGACCGCCGGTTATCGTAAAACACGACGAGTGTTGCAATTCAACAGGACTCAGCGGTCACAGCT
GCCGGTTCAGTGGT
+
F,FFFFFF,FFF:FF:FFFFFFFF,FFFFFFFFFFFFFFFFFFFFFFFFFF::F,F:FFF,FFFF,:F:
FF:FF::FFFFFF,F:F:FFFFFFFFFFFFFFFFFFFFFFFFFF::FFFFFFFFFFF,:F:FF:FFFFFF
:,FFF,FFF,,::F
@A00721:190:HGJW7DSXY:4:1136:31991:13463 1:N:0:TCTGCAAG+GCTGCACT
TGATGACTGCTTTAGCAGTTGATTGATGGATTCGTCTTCCAAGAAGAGGAAGTGGTCAGGCGGTCCGT
TTAGGACCGCCGGTTATCGTAAAACACGACGAGTGTTGCAATTCAACAGGACTCAGCGGTCACAGCTG
CCGGTTCAGTG
+
FFFFFFFFFFFFFFFFFFFFFFFFFFFFFFFFFFFFFFFFFFF:FFFFFFFFFFFFFFFFFFFFFFFFFF
FFFFFFFFFFFFFFFFFFFFFFF,FFFFFFFFFFFFFFFFFFFFFFFFFFFFFFFFFFFFFFFFFFFFFF
FFFFFF:FFFF
@A00721:190:HGJW7DSXY:4:1166:14253:23484 1:N:0:TCTGCAAG+GCTGCACT
TGATGACTGCTTTAGCAGTTGATTGATGGATTCGTCTTCCAAGAAGAGGAAGTGGTCAGGCGGTCCGT
TTAGGACCGCCGGTTATCGTAAAACACGACGAGTGTTGCAATTCAACAGGACTCAGCGGTCACAGCTG
CCGGTTCAGTG
+
FFFFFFFFFFFFFFFFFFFFFFFFFFFFFFFFFFFFFFFFFFFFFFFFFF:FFFFFFFFFFFFFFFFFFF
FFFFFFFFFFFFF:FFFFFFFFFFFFFFFFFFFF:FFF:FFFFFFFFFFFFFFFFFFFFFFFFFFFFFFF
FFFFFFF:FFF
@A00721:190:HGJW7DSXY:4:1228:30761:31876 1:N:0:TCTGCAAG+GCTGCACT
TGATGACTGCTTTAGCAGTTGATTGATGGATTCGTCTTCCAAGAAGAGGAAGTGGTCAGGCGGTCCGT
TTAGGACCGCCGGTTATCGTAAAACACGACGAGTGTTGCAATTCAACAGGACTCAGCGGTCACAGCTG
CCGGTTCAGTG
+
FFFFFFFFFFFFFFFFFFFFFFFFFFFFFF:FFFFFFFFFFFFFFFFFFFFF::FFFFFFFFFFFFFFF:
FFFFFF,F:FF,FFFFFFFFFFFF,F:,FF,F:FFFFFFFF,FFFF,F,FFFFFFFFFF:::F:,:,FFF
FF:,FF:FFF:
@A00721:190:HGJW7DSXY:4:1243:8793:26209 1:N:0:TCTGCAAG+GCTGCACT
TGATGACTGCTTTAGCAGTTGATTGATGGATTCGTCTTCCAAGAAGAGGAAGTGGTCAGGCGGTCCGT
TTAGGACCGCCGGTTATCGTAAAACACGACGAGTGTTGCAATTCAACAGGACTCAGCGGTCACAGCTG
CCGGTTCAGTG
+
FFFFFFFFFFFFFFFFFFFFFFFFFFFFFFFFFFFFFFFFFFFFFFFFFFFFFFFFFFFFFFFFFFFFF,
FFFFFFFFFFFFFFFFFFFFFFFFFFFFFFFFFFFFFFFFFFFFFFFFFFFFFFFFFFFFFFF,FFFF:F
FFFFFFFFFFF
@A00721:190:HGJW7DSXY:4:1272:1805:1986 1:N:0:TCTGCAAG+GCTGCACT
TGATGACTGCTTTAGCAGTTGATTGATGGATTCGTCTTCCAAGAAGAGGAAGAAAGGACCGCCGGTTA
TCGTAAAACACGACGAGTGTTGCAATTCAACAGGACTCAGCGGTCACAGCTGCCGGTTCAGTGGTCGA
GGTCTCTGAGA
+
FFFFFFFFFFFFFFFFFFFFFFFFFFFFFFFFFFFFFFFFFFFFFFFFFFFFFFFFFFFFFFFFFFFFFF
FFFFFFFFFFFFFFFFFFFFFFFFFFFFFFFFFFFFFFFFFFFFFFFFFFFFFFFFFFFFFFFFFFFFFF
FFFFFFFFFFF
@A00721:190:HGJW7DSXY:4:1315:10583:24017 1:N:0:TCTGCAAG+GCTGCACT
TGATGACTGCTTTAGCAGTTGATTGATGGATTCGTCTTCCAAGAAGAGGAAGTGGTCAGGCGGTCCGT
TTAGGACCGCCGGTTATCGTAAAACACGACGAGTGTTGCAATTCAACAGGACTCAGCGGTCACAGCTG
CCGGTTCAGTG
+
FFFFFFFFFFFFFFFFFF:FFFFFFFFFFFFFFFFFFFFFFFFFFFFFFFFF:FFFFFFFFFFFFFFFFF
F:FFFFFFFFFFFFFFFFFFFFFFFFFFFFFFFFFFFFFFFF:FFFFFFFFFFF:FFFFFFFFFFFFFFF
FFFFFFFFFFF
```

```
@A00721:190:HGJW7DSXY:4:1337:14027:27289 1:N:0:TCTGCAAG+GCTGCACT
TGATGACTGCTTTAGCAGTTGATTGATGGATTCGTCTTCCAAGAAGAGGAAGTGGTCAGGCGGTCCGT
TTAGGACCGCCGGTTATCGTAAAACACGACGAGTGTTGCAATTCAACAGGACTCAGCGGTCCCAGCTG
CCGGTTCAGTG
+
FFFFFFFFFFFFFFFFFF:FFFFFFFFFFFFFFFFFFFFFFFFFFFFFFFFFFFFFFFFFFFFFFFFF
FFFFFFFFFFFFF,FFFFFFFFF:FFFFFFFFFFFFFFFFFFF:FFFFFFFFFFFFFFFFF,FFFFFF
FFFFFFFFFFF
@A00721:190:HGJW7DSXY:4:1348:8350:9032 1:N:0:TCTGCAAG+GCTGCACT
TGATGACTGCTTTAGCAGTTGATTGATGGATTCGTCTTCCAAGAAGAGGAAGTGGTCAGGCGGTCCGT
TTAGGACCGCCGGTTATCGTAAAACACGACGAGTGTTGCAATTCAACAGGACTCAGCGGTCACAGCTG
CCGGTTCAGTG
+
FFFFFFFFFFFFFFFFFFFFFFFFFFFFFFFFFFFFFFFFFFFFFFFFFFFFFFFFFFFFFFFFFFFF
FFFFFFFFFFFFFFFFFFFFFFFFF:FFFFF:FFFFFFFFFFFFFFFFFFFFFFFFFFFFFFFFFFFF
FFFFFFFFFFF
@A00721:190:HGJW7DSXY:4:1411:7102:1799 1:N:0:TCTGCAAG+GCTGCACT
TGATGACTGCTTTAGCAGTTGATTGATGGATTCGTCTTCCAAGAAGAGGAAGAAAGGACCGCCGGTTA
TCGTAAAACACGACGAGTGTTGCAATTCAACAGGACTCAGCGGTCACAGCTGCCGGTTCAGTGGTCGA
GGTCTCTGAGA
+
FFFFFFFFFFFFFFFFFF:FFFFFFFFFFFFFFFFFFFFFFFFFFFFFFFFFFFFFFFFFFFFFFFFF
FFFFFFFFFFFFFFFFFFFFFFFFFFFFFFFFFFFFFFFFFFFFFFFFFFFFFFFFFFFFFFFFFFFF
FFFFFFFFFFF
@A00721:190:HGJW7DSXY:4:1412:32705:34272 1:N:0:TCTGCAAG+GCTGCACT
TGATGACTGCTTTAGCAGTTGATTGATGGATTCGTCTTCCAAGAAGAGGAAGTGGTCAGGCGGTCCGT
TTAGGACCGCCGGTTATCGTAAAACACGACGAGTGTTGCAATTCAACAGGACTCAGCGGTCACAGCTG
CCGGTTCAGTG
+
FFFFFFFFFFFFFFFFFFFFFFFFFFFFFFFFFF:FFFFFFFFFFFFFFFFFFFFFFFFFFFFFFFFF
FFFFFFFFFFFFFFFFFFFFFFFFFFF::FFFFFF:FF:FFFF:FFFFF:::FF:FFFFFFFFFFFFF
FFFFFFFFFFF
@A00721:190:HGJW7DSXY:4:1435:12355:29559 1:N:0:TCTGCAAG+GCTGCACT
TGATGACTGCTTTAGCAGTTGATTGATGGATTCGTCTTCCAAGAAGAGGAAGAAAGGACCGCCGGTTA
TCGTAAAACACGACGAGTGTTGCAATTCAACAGGACTCAGCGGTCACAGCTGCCGGTTCAGTGGTCGA
GGTCTCTGAGA
+
FFFFFFFF,FF:FFFFF:,F,FFFFFFF:F:FFFFF:FF:FFFFF,FFFFFFFFFF:FFFFF,FFFFF
F:FFFF:FFFF::,FF:F:FF,FFFFFFFF:FFFFFFFFFFFFFFFFF,F::FF:FF,F,FFFFFFFF
FFFFFFFFF,F
@A00721:190:HGJW7DSXY:4:1456:23439:9111 1:N:0:TCTGCAAG+GCTGCACT
TGATGACTGCTTTAGCAGTTGATTGATGGATTCGTCTTCCAAGAAGAGGAAGAAAGGACCGCCGGTTA
TCGTAAAACACGACGAGTGTTGCAATTCAACAGGACTCAGCGGTCACAGCTGCCGGTTCAGTGGTCGA
GGTCTCTGAGA
+
FFFFFFFFFF:FFFFFFFFFFFFFFFFFFFFFFFFFFFFFFFFFFFFFFFFFFFFFFFFFFFFFFFFF
FFFFFFFFFFFFFFFFFFFFFFFFFFFFFFFFFFFFFFFFFFFFFFFFFFFFFFFFFFFFFFFFFFFF
FFFFFFFFFFF
@A00721:190:HGJW7DSXY:4:1460:25654:17362 1:N:0:TCTGCAAG+GCTGCACT
TGATGACTGCTTTAGCAGTTGATTGATGGATTCGTCTTCCAAGAAGAGGAAGTGGTCAGGCGGTCCGT
TTAGGACCGCCGGTTATCGTAAAACACGACGAGTGTTGCAATTCAACAGGACTCAGCGGTCACAGCTG
CCGGTTCAGTG
+
FFFFFFFFFFFFFFFFFFFFFFFFFFFFFFFFFFFFFFFFFFFFFFFFFFFFFFFFFFFFFFFFFFFF
```

```
FFFFFFFFFFFFFFFFFFFFFFF:FFFFFFFFFFFFFFFFFFFFFFFFFFFFFFFFFFFFFFFFF
FFFFFFFFFFF
@A00721:190:HGJW7DSXY:4:1463:4752:12978 1:N:0:TCTGCAAG+GCTGCACT
TGATGACTGCTTTAGCAGTTGATTGATGGATTCGTCTTCCAAGAAGAGGAAGTGGTCAGGCGGTCCGT
TTAGGACCGCCGGTTATCGTAAAACACGACGAGTGTTGCAATTCAACAGGACTCAGCGGTCACAGCTG
CCGGTTCAGTG
+
FFFFFFFFFFFFFFFFFFFFFFFFFFFFFFFFFFFFFFFFFFFFFFFFFFFFFFFFFFFFFFFFFFFF
FFFFFFFFFFFFFFFFFFFFFFFFFFFFFFFFFFFFFFFFFFFFFFFFFFFFFFFFF:FFFFFFFFFF
FFFFFFFFFFF
@A00721:190:HGJW7DSXY:4:1465:32588:13777 1:N:0:TCTGCAAG+GCTGCACT
TGATGACTGCTTTAGCAGTTGATTGATGGATTCGTCTTCCAAGAAGAGGAAGTGGTCAGGCGGTCCGT
TTAGGACCGCCGGTTATCGTAAAACACGACGAGTGTTGCAATTCAACAGGACTCAGCGGTCACAGCTG
CCGGTTCAGTG
+
FFFFFFFFFFFFFFFFFFFFFFFFFFFFFFFFFFFFFFFFFFFFFFFFFFFFF:FFFFFFFFF:FFF
FFFFFFFFFFFFFFFFFFFFFFFFFFFFFFFFF:FFFFF:FFFFFF,FFFF:FFFFFFFFFFFFFFFF
FFFFFFFFFFF
@A00721:190:HGJW7DSXY:4:1465:32570:13902 1:N:0:TCTGCAAG+GCTGCACT
TGATGACTGCTTTAGCAGTTGATTGATGGATTCGTCTTCCAAGAAGAGGAAGTGGTCAGGCGGTCCGT
TTAGGACCGCCGGTTATCGTAAAACACGACGAGTGTTGCAATTCAACAGGACTCAGCGGTCACAGCTG
CCGGTTCAGTG
+
FFFFFFFFFFFFFFFFFFFFFFFFFF:FFFFFFFFFFFFFFFFFFFFFFFFFFFFFFFFFFFFFF:FFF
FFFFFFFFFFFFFFFFFFFFFFFFFFFFFFFFFFFFFFFFFFFFFFFFFFFFFFFFFFFFFFFFFFF:
FFFFFFFFFFF
@A00721:190:HGJW7DSXY:4:1469:26096:13964 1:N:0:TCTGCAAG+GCTGCACT
TGATGACTGCTTTAGCAGTTGATTGATGGATTCGTCTTCCAAGAAGAGGAAGTGGTCAGGCGGTCCGT
TTAGGACCGCCGGTTATCGTAAAACACGACGAGTGTTGCAATTCAACAGGACTCAGCGGTCACAGCTG
CCGGTTCAGTG
+
FFFFFFFFFFFFFFFFFFFFFF:FFFFFFFFFFFFFFFFFFFFFFFF:FFFFFFFFFFFFFFFFFFFF
FFFFFFFFFFFFFFFFFFFFFFFFFFFFFFFFFFFFFFFFFFFFFFFFFFFFFFFFFFFFF:FFFFFF
FFFFFFFFFFF
@A00721:190:HGJW7DSXY:4:1513:27444:5024 1:N:0:TCTGCAAG+GCTGCACT
TGATGACTGCTTTAGCAGTTGATTGATGGATTCGTCTTCCAAGAAGAGGAAGTGGTCAGGCGGTCCGT
TTAGGACCGCCGGTTATCGTAAAACACGACGAGTGTTGCAATTCAACAGGACTCAGCGGTCACAGCTG
CCGGTTCAGTG
+
FFFFFFFFFFFFFFFFFF:FFF,FFF:FFFFFFFFFFFFFFFFFFFFFFFFFFFFFFFFFFFFFF::FFF
:FFFFFFFFFF:F,FFFFFFFFFF:FFFFFFFFFFFFFFFFFFFFFFFFFFFFFFFFFFFFFFF,:FFFFF
FFFF:FFFF,F
@A00721:190:HGJW7DSXY:4:1531:18204:21465 1:N:0:TCTGCAAG+GCTGCACT
TGATGACTGCTTTAGCAGTTGATTGATGGATTCGTCTTCCAAGAAGAGGAAGTGGTCAGGCGGTCCGT
TTAGGACCGCCGGTTATCGTAAAACACGACGAGTGTTGCAATTCAACAGGACTCAGCGGTCACAGCTG
CCGGTTCAGTG
+
FFFFFFFFFFFFFFFFFFFFFFFFFFFFFFFFFFFFFFFFFFFFFFFFFFFFFFFFFFFFFFFFFFFF
FFFFFFFFFFFFF:FFFFFFFFFF:FFFFFFFFFFFF:FFFFFFFFFF:FFFFFFFFFFFFFFFFFFF
FFFFFFFF:FF
@A00721:190:HGJW7DSXY:4:1536:15989:34663 1:N:0:TCTGCAAG+GCTGCACT
TGATGACTGCTTTAGCAGTTGATTGATGGATTCGTCTTCCAAGAAGAGGAAGTGGTCAGGCGGTCCGT
TTAGGACCGCCGGTTATCGTAAAACACGACGAGTGGTGCAATTCAACAGGACTCAGCGGTCACAGCTG
CCGGTTCAGTG
```

```
+
F:F,FF:FF::FFFF:FFFFFFF:FF::FFFFF,FF:F:FFF,FF:FFFFFFFF:FFF:FFF:FF:F,
FFF,,FFFF,F:,FFF,FF:F:FFFF,,FF,FFF:,:::F:FF,FFFF,FFFFFF:FFFFFFFF:,:F
,FFF:FFFF,F
@A00721:190:HGJW7DSXY:4:1561:10059:18255 1:N:0:TCTGCAAG+GCTGCACT
TGATGACTGCTTTAGCAGTTGATTGATGGATTCGTCTTCCAAGAAGAGGAAGTGGTCAGGCGGTCCGT
TTAGGACCGCCGGTTATCGTAAAACACGACGAGTGTTGCAATTCAACAGGACTCAGCGGTCACAGCTG
CCGGTTCAGTG
+
FFFFFFFFFFFFFFFFFFFFFFFFFFFFFFFFFFFFFFFFFFFFFFFFFFFFFFFFFFFFFFFFFFFF
FFFFF:FFFFFFF:FFFFFFFFFFFFFFFFFFFFFFFFFFFFFFFFFFFFFFFFFFFFFF:FFFFFF
FFFFFFFFFFF
@A00721:190:HGJW7DSXY:4:1611:9082:35790 1:N:0:TCTGCAAG+GCTGCACT
TGATGACTGCTTTAGCAGTTGATTGATGGATTCGTCTTCCAAGAAGAGGAAGTGGTCAGGCGGTCCGT
TTAGGACCGCCGGTTATCGTAAAACACGACGAGTGTTGCAATTCAACAGGACTCAGCGGTCACAGCTG
CCGGTTCAGTG
+
FFFFFFFFFFFFFFFFFF:FFFFFFFFF:FFFFFFFFFFFFF::FFFFFFFFFFFFFFFFFFF::FFF
FF:,FFFF:FFFFFFFF:FFFFFF:F::FFFF:F,FFFFFFFF:FFFF:FFF:FF:FFFF,FFFFFFF
FFFF,F,FFF:
@A00721:190:HGJW7DSXY:4:1616:17327:9361 1:N:0:TCTGCAAG+GCTGCACT
TGATGACTGCTTTAGCAGTTGATTGATGGATTCGTCTTCCAAGAAGAGGAAGAAAGGACCGCCGGTTA
TCGTAAAACACGACGAGTGTTGCAATTCAACAGGACTCAGCGGTCACAGCTGCCGGTTCAGTGGTCGA
GGTCTCTGAGA
+
FFFFFFFFF:FFFFFFF:FFFFFFFF,FFFFFFF:FFFFFFFFFFFF:FFFFFFFF:FFF:FF:FFFF
FFFFFFFFFFFFF:FF,:F::FFFFF,FFFFFF:FFFFFFFFF,FFFF,F::FF,FF,FFFFFFF:FF
FFF:F::FFF:
@A00721:190:HGJW7DSXY:4:1618:15989:26991 1:N:0:TCTGCAAG+GCTGCACT
TGATGACTGCTTTAGCAGTTGATTGATGGATTCGTCTTCCAAGAAGAGGAAGTGGTCAGGCGGTCCGT
TTAGGACCGCCGGTTATCGTAAAACACGACGAGTGTTGCAATTCAACAGGACTCAGCGGTCACAGCTG
CCGGTTCAGTG
+
F:FFFFFFFFFFFFFFFFFFFFFFFFFFFFFFFFFFFFFFFFFFFFFFFFFFFFFFFFFFFFFFFFFF
FF:,FFFFFFFFFFFFFFFFFFF:F:FFFFF,FFFFFFFFF::FF:F:FFFFFFFFFFFF:FFFFFF
FFFFFFFF,FF
@A00721:190:HGJW7DSXY:4:1618:17933:28604 1:N:0:TCTGCAAG+GCTGCACT
TGATGACTGCTTTAGCAGTTGATTGATGGATTCGTCTTCCAAGAAGAGGAAGTGGTCAGGCGGTCCGT
TTAGGACCGCCGGTTATCGTAAAACACGACGAGTGTTGCAATTCAACAGGACTCAGCGGTCACAGCTG
CCGGTTCAGTG
+
FFFFFFFFFFFFFFFFFFFFFFFFFFFFFFFFFFFFFFFFFFFFFFFFFFFFFFFFFFFFFFFFFFFF
FFFFFFFFFFFFFFFFFFFFFFFFFFFFFFFFFFFFFFFF:FFFFFF:FFFFFFFFFFFFFFFFFFF,F
FFFFFFFF:FF
@A00721:190:HGJW7DSXY:4:1618:17951:28667 1:N:0:TCTGCAAG+GCTGCACT
TGATGACTGCTTTAGCAGTTGATTGATGGATTCGTCTTCCAAGAAGAGGAAGTGGTCAGGCGGTCCGT
TTAGGACCGCCGGTTATCGTAAAACACGACGAGTGTTGCAATTCAACAGGACTCAGCGGTCACAGCTG
CCGGTTCAGTG
+
FFFFFF:FFFFFFFFFFF,FFFFFFFFFFFFFFFFFFFFFFFFFFFFFFFFF,FFFFFFFFFFFFFFFF,
FFF:FFFF:FFFF:FFF,FFFFFF,FFFFFFFFFF,FFFFFF::FFFFFFFF,FFF::FFFFFFFFFF
FFFF:FFFFFF
@A00721:190:HGJW7DSXY:4:1618:31259:33865 1:N:0:TCTGCAAG+GCTGCACT
TGATGACTGCTTTAGCAGTTGATTGATGGATTCGTCTTCCAAGAAGAGGAAGTGGTCAGGCGGTCCGT
```

```
TTAGGACCGCCGGTTATCGTAAAACACGACGAGTGTTGCAATTCAACAGGACTCAGCGGTCACAGCTG
CCGGTTCAGTG
+
FFFFFFFFFFFFFFFFFFFFFFFFFFFFFFFFFFFFFFFFFFFFFFFFF:FF,FFFFFFFFF,,F:F
FFFFFFFFFFFFFFFFFFFFFFFFF,FFFFFFFFFFFFFFFFFFF,FFF:FFFFFFFFFFFFFFFFFF
F,FFFFFFFFF
@A00721:190:HGJW7DSXY:4:1658:27914:31610 1:N:0:TCTGCAAG+GCTGCACT
TGATGACTGCTTTAGCAGTTGATTGATGGATTCGTCTTCCAAGAAGAGGAAGTGGTCAGGCGGTCCGT
TTAGGACCGCCGGTTATCGTAAAACACGACGAGTGTTGCAATTCAACAGGACTCAGCGGTCACAGCTG
CCGGTTCAGTG
+
FFFFFFFFFFFFFFFFFFFFFFFFFFFFFFFFFFFFFFFFFFFFFFFFFFFFFFFFFFFFFFFFFFFF
FFFFFFFFFFFFFFFFFFFFFFFFFFFFFFFFFFFFFFFFFFFFFFFFFFFFFFFFFFFFF:FFFFFF
FFFFFFFFFFF
@A00721:190:HGJW7DSXY:4:1659:17336:27038 1:N:0:TCTGCAAG+GCTGCACT
TGATGACTGCTTTAGCAGTTGATTGATGGATTCGTCTTCCAAGAAGAGGAAGTGGTCAGGCGGTCCGT
TTAGGACCGCCGGTTATCGTAAAACACGACGAGTGTTGCAATTCAACAGGACTCAGCGGTCACAGCTG
CCGGTTCAGTG
+
FFFFFFFFFFFFFFFFFF:FFFFFFFFFFFFFFFFFFFFFFFFFFFFFFFFFFFFFFFFFFFFFFFFF:
FFFFFFFFFFFFFFFFFFFFFFFFFFFFFFFFFFFFFFFFFFFFFFFFFFFFFFFFFFFFFFFFFFFF
FFFFFFFFFFF
@A00721:190:HGJW7DSXY:4:2112:6705:13949 1:N:0:TCTGCAAG+GCTGCACT
TGATGACTGCTTTAGCAGTTGATTGATGGATTCGTCTTCCAAGAAGAGGAAGTGGTCAGGCGGTCCGT
TTAGGACCGCCGGTTATCGTAAAACACGACGAGTGTTGCAATTCAACAGGACTCAGCGGTCACAGCTG
CCGGTTCAGTG
+
FFFFFFFFF:FFFFFFFFFFFFFFFFFFFFFFFFFFFFFFFFFFFFF:FFFFFFFFFFFFFFFFFFFF
FFFFFFFFFFFFF,FFFFFF:FFFF,FFFFFFFFF:FFFF,FFFF,FFFFFFFF:FFFFF,FFFF:FF
,FFF,FF:F,F
@A00721:190:HGJW7DSXY:4:2140:1850:21386 1:N:0:TCTGCAAG+GCTGCACT
TGATGACTGCTTTAGCAGTTGATTGATGGATTCGTCTTCCAAGAAGAGGAAGTGGTCAGGCGGTCCGT
TTAGGACCGCCGGTTATCGTAAAACACGACGAGTGTTGCAATTCAACAGGACTCAGCGGTCACAGCTG
CCGGTTCAGTG
+
F:F:FFFFFFFF:F:FFFFFFFFFFFFFFFFFFFFFFF:,FFFFFFFFFFFFFFFFFFFFFFFFFFFF
FFF,FFF,F,FFFFFFFFFFFFF:FF,FF::F,FFFF,FFFF::FF,FF,FFF:F:,F:FFFFF:,,F
FFF:F,,FFFF
@A00721:190:HGJW7DSXY:4:2172:28899:8140 1:N:0:TCTGCAAG+GCTGCACT
TGATGACTGCTTTAGCAGTTGATTGATGGATTCGTCTTCCAAGAAGAGGAAGAAAGGACCGCCGGTTA
TCGTAAAACACGACGAGTGTTGCAATTCAACAGGACTCAGCGGTCACAGCTGCCGGTTCAGTGGTCGA
GGTCTCTGAGA
+
FFFFFFFFFFFFFFFFFFFFFFFFFFFFFFFFFFFFFFFFFFFFFFFFFFFFFFFFFFFFFFFFFFFF
FFFFFFFFFFF:FFFFFFFFFFFFFFFFFFFFFFFFFFFFFFFFFFFFFFFFFFFFFFFFFFFFFFFF
FFFFFFFFFFF
@A00721:190:HGJW7DSXY:4:2172:29152:8328 1:N:0:TCTGCAAG+GCTGCACT
TGATGACTGCTTTAGCAGTTGATTGATGGATTCGTCTTCCAAGAAGAGGAAGAAAGGACCGCCGGTTA
TCGTAAAACACGACGAGTGTTGCAATTCAACAGGACTCAGCGGTCACAGCTGCCGGTTCAGTGGTCGA
GGTCTCTGAGA
+
FFFFFFFFFFFFFFFFFFFFFFFFFFFFFFFFFFFFFFFFFFFFFFFFFFF:FFFFFFFFFFFFFFFF
FFFFFFFFFFFFFFFFFFFFFFFFFFFFFFFFFFFFFFFFFFFFFF:FFFFFFFFFFFFFFFFFFFFF
FFFFFFFFFFF
```

@A00721:190:HGJW7DSXY:4:2207:20193:31391 1:N:0:TCTGCAAG+GCTGCACT
TGATGACTGCTTTAGCAGTTGATTGATGGATTCGTCTTCCAAGAAGAGGAAGTGGTCAGGCGGTCCGT
TTAGGACCGCCGGTTATCGTAAAACACGACGAGTGTTGCAATTCAACAGGACTCAGCGGTCACAGCTG
CCGGTTCAGTG
+
FFFFFFFFFFFFFFFF:F:FFFFFFFFFFFFFFFFFFFFFFFFFFFFFFF:FFFFFFFFFFFFF:FFF
FFFFFF,FFFFFF:FFFFFFFFFFFFF,:::F:FFFFF:FF,F:FF:F,FFFFFF:FFFFF,FF,F,:
FFFFFFF,:,F
@A00721:190:HGJW7DSXY:4:2217:17219:24643 1:N:0:TCTGCAAG+GCTGCACT
TGATGACTGCTTTAGCAGTTGATTGATGGATTCGTCTTCCAAGAAGAGGAAGTGGTCAGGCGGTCCGT
TTACGACCCCCCGTTATCGTAAAAAACCACCAGAGTTTCAATTAAACACGACACATCGCTAACAACGG
CCGGTTCAG
+
FFF,FF:FFF,FFF:FFFF,FFFFFFFFFF:FFFFFFFFFF:,FF,F,FFFF,,,FF,,:F,,:FF:F
,F,,,F:F,:F,F,,,FF,:FF:F,::,:F,FF,,,,FFFF:F,:FFF,,FFFF,,,F,,,FFF,,,F
,F,:F::F:
@A00721:190:HGJW7DSXY:4:2232:26250:9032 1:N:0:TCTGCAAG+GCTGCACT
TGATGACTGCTTTAGCAGTTGATTGATGGATTCGTCTTCCAAGAAGAGGAAGTGGTCAGGCGGTCCGT
TTAGGACCGCCGGTTATCGTAAAACACGACGAGTGTTGCAATTCAACAGGACTCAGCGGTCACAGCTG
CCGGTTCAGTG
+
FFFFFFFFFFFFFFFFFFFFFFFFFFFFFFFFFFFFFFFFFFFFFFFFFF,FFFFFFFFFFFFFFFFF
FFFFFFFFFFFFFFFFFFFFFFFFFFFFFFFFFFFFFFF:FFFFFFFFFFFFFF:FFFFFFFFFFFFF
FFFFFFFFF,F
@A00721:190:HGJW7DSXY:4:2242:17635:29904 1:N:0:TCTGCAAG+GCTGCACT
TGATGACTGCTTTAGCAGTTGATTGATGGATTCGTCTTCCAAGAAGAGGAAGTGGTCAGGCGGTCCGT
TTAGGACCGCCGGTTATCGTAAAACACGACGAGTGTTGCAATTCAACAGGACTCAGCGGTCACAGCTG
CCGGTTCAGTG
+
FFFFFFFFFFFFFFFFFF:FFFFFFFFFFFFFFFFFFFFFFFFFFFFFFFFFFFFFFFFFFFFFFFFF
FFFFFFFFFFFFFFFFFFFFFFFFFF:FFFFFFFFFFFFFFFFFFF:F:FFFFFFFFFFFF,FFFFFF
FF:FFFFFFFF
@A00721:190:HGJW7DSXY:4:2245:1913:3302 1:N:0:TCTGCAAG+GCTGCACT
TGATGACTGCTTTAGCAGTTGATTGATGGATTCGTCTTCCAAGAAGAGGAAGTGGTCAGGCGGTCCGT
TTAGGACCGCCGGTTATCGTAAAACACGACGAGTGTTGCAATTCAACAGGACTCAGCGGTCACAGCTG
CCGGTTCAGTG
+
FFFFFFFFFFFFFFFFFFFFFFFFFFFFFFFFFFFFFFFFFFFFFFFFFFFFFFFFFFFFFFFFFFFF
FFFFFFFFFFFFF,FFFFFFFFFFFFFFFFFFFFFFFFFFFFFFFFFFFFFFFFFFFFFFFFFFFFFF
FFFFFFFFFFF
@A00721:190:HGJW7DSXY:4:2246:17110:2785 1:N:0:TCTGCAAG+GCTGCACT
TGATGACTGCTTTAGCAGTTGATTGATGGATTCGTCTTCCAAGAAGAGGAAGAAAGGACCGCCGGTTA
TCGTAAAACACGACGAGTGTTGCAATTCAACAGGACTCAGCGGTCACAGCTGCCGGTTCAGTGGTCGA
GGTCTCTGAGA
+
FFFFFFFFFFFFFFFFFFFFFFFFFFFFFF:FFFFFFFFFFFFFFFFFFFFFFFFFFFFFFFFFFFFF
FFFFFFFFFFFFFFFFFFFFFFFFFFFFFFFFFFFFFFFFFFFFFFFFFFFFFFFFFFFFFFFFFFFF
FFFFFFFFFFF
@A00721:190:HGJW7DSXY:4:2247:11505:31939 1:N:0:TCTGCAAG+GCTGCACT
TGATGACTGCTTTAGCAGTTGATTGATGGATTCGTCTTCCAAGAAGAGGAAGTGGTCAGGCGGTCCGT
TTAGGACCGCCGGTTATCGTAAAACACGACGAGTGTTGCAATTCAACAGGACTCAGCGGTCACAGCTG
CCGGTTCAGTG
+
FFFFFFFFFFFFFFFFFFFFFFFFFFFFFFFFFFFFFFFFFFFFFFF:FFFFFFFFFFFFFFFFFFFF

FFFF:FFFFFFFFFFFFFFFFFFFFFF:FFFFFFFFFFFFFFFFFFFFFFFFFFFFFFFFFFFFFFFFF
FFFFFFFFFFF
@A00721:190:HGJW7DSXY:4:2265:21197:29121 1:N:0:TCTGCAAG+GCTGCACT
TGATGACTGCTTTAGCAGTTGATTGATGGATTCGTCTTCCAAGAAGAGGAAGTGGTCAGGCGGTCCGT
TTAGGACCGCCGGTTATCGTAAAACACGACGAGTGTTGCAATTCAACAGGACTCAGCGGTCACAGCTG
CCGGTTCAGTG
+
FFFFFFFFFFFFFFFFFFFFFFFFFFFFFFFFFFFFFFFFFFFFFFFFFFF:FFFFFFFFFFFFFFFF
:FFFFFFFFFFFFFFFFFFFFFFF:FFFFFFFFFFFFFFFFFFFFFFFFFFFFFFFFFFFFF,FFFFFF
FFFFFFFFFFF
@A00721:190:HGJW7DSXY:4:2275:20916:20776 1:N:0:TCTGCAAG+GCTGCACT
TGATGACTGCTTTAGCAGTTGATTGATGGATTCGTCTTCCAAGAAGAGGAAGTGGTCAGGCGGTCCGT
TTAGGACCGCCGGTTATCGTAAAACACGACGAGTGTTGCAAAACAACAGGACTCAGCGGTCACAGCTG
CCGGTTCAGTG
+
,:F,FFFFFFF,FF:FF,,F,FF,FFFF:FFF,FFFF:FFFF:,,FF:F:F:FFF,FFF,:FFFF:FF
,::FFFF,FFFFFF,FF,F,FFF,FF,,:FFF,:FFF,FFF,,F:FFF:FF,,FFFF:FFF,FF:,,F
FF,,,FFF::F
@A00721:190:HGJW7DSXY:4:2308:7437:34381 1:N:0:TCTGCAAG+GCTGCACT
TGATGACTGCTTTAGCAGTTGATTGATGGATTCGTCTTCCAAGAAGAGGAAGTGGTCAGGCGGTCCGT
TTAGGACCGCCGGTTATCGTAAAACACGACGAGTGTTGCAATTCAACAGGACTCAGCGGTCACAGCTG
CCGGTTCAGTG
+
FFFFFFFFFFFFFFFFFFF:FF,FFFFFFFFFFFFFFFFFFFFFFFFFFFFFFFFFFFFFFF:FFFFF:
FF::FFFFFFFFF:FF:,:FFFFFFF:FF,:FFFFFFFFFF,F:FF:F:FFF,:FFFFFFFFFF:FFF
FFFFFFFFFFF
@A00721:190:HGJW7DSXY:4:2335:19452:18584 1:N:0:TCTGCAAG+GCTGCACT
TGATGACTGCTTTAGCAGTTGATTGATGGATTCGTCTTCCAAGAAGAGGAAGTGGTCAGGCGGTCCGT
TTAGGACCGCCGGTTATCGTAAAACACGACGAGTGTTGCAATTCAACAGGACTCAGCGGTCACAGCTG
CCGGTTCAGTG
+
FFFFFFFFFFFFFFFFFFFFFFFFFFFFFFFFFFFFFFFFFFFFFFFFFFFFFFFFFFFFFFFFFFFF:
FFFFFFFFFFFFFFFFFFFFFFFFFFFFFFF:FFFFFFFFFFFFFFFFFFFFFFFFFFFFFFFFFFFFF
FFFFFFFFFFF
@A00721:190:HGJW7DSXY:4:2335:19895:18818 1:N:0:TCTGCAAG+GCTGCACT
TGATGACTGCTTTAGCAGTTGATTGATGGATTCGTCTTCCAAGAAGAGGAAGTGGTCAGGCGGTCCGT
TTAGGACCGCCGGTTATCGTAAAACACGACGAGTGTTGCAATTCAACAGGACTCAGCGGTCACAGCTG
CCGGTTCAGTG
+
FFFFFFFFFFFFFFFFFFFFFFFFFFFFFFFFFFFFFFFFFFFFFFFFF:FFFFFFFFFFFFFFFFFFF
:FFFFFFFFFFFFFFFFFFFFFFFFFFFFFF:FFFFFFFFFFFFFFFFFFFFFFFFFFFFFFFFFFFFF
FFFFFFFFFFF
@A00721:190:HGJW7DSXY:4:2362:3314:34037 1:N:0:TCTGCAAG+GCTGCACT
TGATGACTGCTTTAGCAGTTGATTGATGGATTCGTCTTCCAAGAAGAGGAAGTGGTCAGGCGGTCCGT
TTAGGACCGCCGGTTATCGTAAAACACGACGAGTGTTGCAATTCAACAGGACTCAGAGGTCACAGCTG
CCGGTTCAGTG
+
FFFFFFFFFFFFFFF:FFFFFFFFFFFFFFFFFFFFFFFF:FF:FFFFFFFFFFFF:FFFFFFFFFFF:
F:FFFFFF:FFFFFFFFFFFFFFF:F:FF,FF:FF:,,FFFFFFFF:F:FFFFFF,,F:FFFFF,FFF
FF,FFFFF:,F
@A00721:190:HGJW7DSXY:4:2369:1163:23954 1:N:0:TCTGCAAG+GCTGCACT
TGATGACTGCTTTAGCAGTTGATTGATGGATTCGTCTTCCAAGAAGAGGAAGTGGTCAGGCGGTCCGT
TTAGGACCGCCGGTTATCGTAAAACACGACGAGTGTTGCAATTCAACAGGACTCAGCGGTCACAGCTG
CCGGTTCAGTG

+
FFFFFFFFFFF:FF:FFFFFFFFFFFFFFFFFFFFFFFFFFFFFFFFFFFF:FFFFFFFFFFFFFFFF
FFFFFFFFFFFFFFFFFFFFFFFFFF:FFFFFFF:FF:FFFFFFFFFFF,FFFFFFFFFFFFFFFF,F
FFFFFFFFFFF
@A00721:190:HGJW7DSXY:4:2413:7726:29089 1:N:0:TCTGCAAG+GCTGCACT
TGATGACTGCTTTAGCAGTTGATTGATGGATTCGTCTTCCAAGAAGAGGAAGTGGTCAGGCGGTCCGT
TTATGACCGCCGGTTATCGTAAAACACGACGAGTGTTGCAATTCAACAGGACTCAGCGGTCACAGCTG
CCGGTTCAGTG
+
FFFF:FF:FFFFFF:FFFFFFFFFFF,,FFFFFFFFFFFF:FFF:F,FFF:::FFFFF:F:,F,FF:
FFF,FFFF:FF,F:,F,FFFFFFF:FF:FF:FFFFFF,,FF:F,FFFF:FFFF,F:FF:,FFFFFFF:
F,,:FFFF:FF
@A00721:190:HGJW7DSXY:4:2427:24858:14011 1:N:0:TCTGCAAG+GCTGCACT
TGATGACTGCTTTAGCAGTTGATTGATGGATTCGTCTTCCAAGAAGAGGAAGTGGTCAGGCGGTCCGT
TTAGGACCGCCGGTTATCGTAAAACACGACGAGTGTTGCAATTCAACAGGACTCAGCGGTCACAGCTG
CCGGTTCAGTG
+
FFFFFFFFFFFFFFFFFF,FFFFFFFFFFFFFFFFFFFFFFFFFFFFFFFFFFFFFFFFFFFFFF:FF
FFF:FF:FFFFFFFFF:FF:FFFFFF:,FFFFFFFFFFFFF:FFFFFF:FF,::F:FFFFFFFF:FF:
FFFFFFFFF:F
@A00721:190:HGJW7DSXY:4:2447:2049:26459 1:N:0:TCTGCAAG+GCTGCACT
TGATGACTGCTTTAGCAGTTGATTGATGGATTCGTCTTCCAAGAAGAGGAAGAAAGGACCGCCGGTTA
TCGTAAAACACGACGAGTGTTGCAATTCAACAGGACTCAGCGGTCACAGCTGCCGGTTCAGTGGTCGA
GGTCTCTGAGA
+
FFFFFFFFFFFFFFFFFFFFFFFFFFFFFFFFFFFFFFFFFFFFFFFFFFFFFFFFFFFFFFFFFFFF
FFFFFFFFFFFFFFFFFFFFFFFFFFFFFFFFFFFFFFFFFFFFFF:FFFFFFFFFFFFFFFFFFFFF
FFFFFFFFFFF
@A00721:190:HGJW7DSXY:4:2463:4001:26804 1:N:0:TCTGCAAG+GCTGCACT
TGATGACTGCTTTAGCAGTTGATTGATGGATTCGTCTTCCAAGAAGAGGAAGAAAGGACCGCCGGTTA
TCGTAAAACACGACGAGTGTTGCAATTCAACAGGACTCAGCGGTCACAGCTGCCGGTTCAGTGGTCGA
GGTCTCTGAGA
+
FFFFFFFFFFFFFFFFFFFFFFFFFFFFFFFFFFFFFFFFFFFFFFFFFFF:FFFFFFFFFFFFFFFF
FFFFFFFFFFFFFFFFFFFFFFFFFFFFFFFFFFFFFFFFFFFFFFFFFFFFFFFFFFFFFFFFFFFF
FFFFFF,FFFF
@A00721:190:HGJW7DSXY:4:2505:16984:13714 1:N:0:TCTGCAAG+GCTGCACT
TGATGACTGCTTTAGCAGTTGATTGATGGATTCGTCTTCCAAGAAGAGGAAGTGGTCAGGCGGTCCGT
TTAGGACCGCCGGTTATCGTAAAACACGACGAGTGTTGCAATTCAACAGGACTCATCGGTCACAGCTG
CCGGTTCAGTG
+
FFFFFFFFFFFFFFFFFFFFFFFFFFFFFFFFFFFFFFFFFFFFFFFFFFFF:FFFFFFFFFFFFFF:F::
:F,FFFFF:FF,:,FF::,FFFFF:F:,:::,FF,FF,FFF,F,FF,F,FF,,:F,F,FF::FF::,:
:F,FFF,,FFF
@A00721:190:HGJW7DSXY:4:2523:9878:23390 1:N:0:TCTGCAAG+GCTGCACT
TGATGACTGCTTTAGCAGTTGATTGATGGATTCGTCTTCCAAGAAGAGGAAGTGGTCAGGCGGTCCGT
TTAGGACCGCCGGTTATCGTAAAACACGACGAGTGTTGCAATTCAACAGGACTCAGCGGTCACAGCTG
CCGGTTCAGTG
+
FFFFFFFFFFFFFFFFFFFFFFFFFFFFFFFFFFFFFFFFFFFFFFFFF:FFFFFFFFFFFFFFFFF:FFF
FF:FFFFFFFFFF::FFFFFFFFFFF::FFFFFFFFFFFFFFFFFF:FFF::FFFFFFFF:FFFFF:F
FFFF:FFF:FF
@A00721:190:HGJW7DSXY:4:2524:27489:13463 1:N:0:TCTGCAAG+GCTGCACT
TGATGACTGCTTTAGCAGTTGATTGATGGATTCGTCTTCCAAGAAGAGGAAGTGGTCAGGCGGTCCGT

```
TTAGGACCGCCGGTTATCGTAAAACACGACGAGTGTTGCAATTCAACAGGACTCAGCGGTCACAGCTG
CCGGTTCAGTG
+
FFFFFFFFFFFFFFFFFF,FFFFFFFFFFFFFFFFFFFFFFFFFFFFFFFF:FFFFFFFFFFFFFFFF
FFFFFFFFFFFFFFFFFFFFFFFFFF:FFFFFFFFFFFFF:FFFFFFF:FFFFFF:FFFFF:FFFF,F
FFFFFFFFFFF
@A00721:190:HGJW7DSXY:4:2554:17345:20885 1:N:0:TCTGCAAG+GCTGCACT
TGATGACTGCTTTAGCAGTTGATTGATGGATTCGTCTTCCAAGAAGAGGAAGTGGTCAGGCGGTCCGT
TTAGGACCGCCGGTTATCGTAAAACACGACGAGTGTTGCAATTCAACAGGACTCAGCGGTCACAGCTG
CCGGTTCAGTG
+
FFFFFFFFFFFFFFFFFFFFFFFFFFFFFFFFFFFFFFFFFFFFFFFFFFFFFFFFFFFFFFFFFFFF
FFFFFFFFFFFFFFFFFFFFFFFFFFFFFFFFFFFFFFFFFFFFFFFFFFFFFFFFFFFFFFFFFFFF
FFFFFFFFFFF
@A00721:190:HGJW7DSXY:4:2601:29686:21527 1:N:0:TCTGCAAG+GCTGCACT
TGATGACTGCTTTAGCAGTTGATTGATGGATTCGTCTTCCAAGAAGAGGAAGAAAGGACCGCCGGTTA
TCGTAAAACACGACGAGTGTTGCAATTCAACAGGACTCAGCGGTCACAGCTGCCGGTTCAGTGGTCGA
GGTCTCTGAGA
+
FFFFFFFFFFFFFFFFFF:FFFFFFFFFFFFFFFFFFFFFFFFFFFFFFFFFFFFFFFFFFFFFFFFF
FFFFFF:FFFFFFFFFFFFFFFFFFFFFFFFFFFFFFFFFFFFFFF:FFFFFFFFFFFFFFFFFFFFFF
FFFFFFFFFFF
@A00721:190:HGJW7DSXY:4:2627:24749:30890 1:N:0:TCTGCAAG+GCTGCACT
TGATGACTGCTTTAGCAGTTGATTGATGGATTCGTCTTCCAAGAAGAGGAAGAAAGGACCGCCGGTTA
TCGTAAAACACGACGAGTGTTGCAATTCAACAGGACTCAGCGGTCACAGCTGCCGGTTCAGTGGTCGA
GGTCTCTGAGA
+
FFFFFFFFFFFFFFFFFFFFFFFF:FFFFFFFFFFFFFFFFFFFFFFFFFFFFFFFFFFFFFFFFFFF
FFFFFFFFFFFFFFFFFFFFFFFFFFFFFFFFFF,FFFFFFFFFFFFFFFFFF:FFFFFFFFF:FFFFF
FFFFFFFFF:F
@A00721:190:HGJW7DSXY:4:2637:30318:36276 1:N:0:TCTGCAAG+GCTGCACT
TGATGACTGCTTTAGCAGTTGATTGATGGATTCGTCTTCCAAGAAGAGGAAGTGGTCAGGCGGTCCGT
TTAGGACCGCCGGTTATCGTAAAACACGACGAGTGTTGCAATTCAACAGGACTCAGCGGTCACAGCTG
CCGGTTCAGTG
+
FFFFFFFFFFFFFFFFFFFFFFFFFFFFFFFFFFFFFFFFFFFFFFFFFFFFFFFFFFFFFFFFFFFF
FFFFFFFFFFFFFFFFFFFFFFFFFFF:FFFFF:F:FFFFFFFFFFFFF,FFFFFFFFFFFF:FFFF,F
FFFFFFFFFFF
@A00721:190:HGJW7DSXY:4:2665:32606:32315 1:N:0:TCTGCAAG+GCTGCACT
TGATGACTGCTTTAGCAGTTGATTGATGGATTCGTCTTCCAAGAAGAGGAAGTGGTCAGGCGGTCCGT
TTAGGACCGCCGGTTATCGTAAAACACGACGAGTGTTGCAATTCAACAGGACTCAGCGGTCACAGCGG
CCGGTTCAGTG
+
FFFFFFFFFFFFFFFFFFFFFFFFFFFFFFFFFFFFFFFFFFFF:FFFFFFFFFFFFFFFFFFFFFFF:
FFFFFFFFFFFFFFFFFFFFFFFFFFFFFFFFFFFFFFFFFFFF:FFFFFFFFFFFFFFFFFFFF,F
FFFFFFFFFFF
@A00721:190:HGJW7DSXY:4:1243:19678:25144 1:N:0:TCTGCAAG+GCTGCACT
AGCAGTTGATTGATGGATTCGTCTTCCAAGAAGAGGAAGTGGTCAGGCGGTCCGTTTAGGACCGCCGG
TTATCGTAAAACACGACGAGTGTTGCAATTCAACAGGACTCAGCGGTCACAGCTGCCGGTTCAGTGGT
CGAGGTCTCTGAGAT
+
FFFFFF,:FFFFFFFFFFF,FFFFF:FFFF:FFFFFFFFFF:FFFFFF::FFFF,,FF,:F:::FFFF
FFFF,FFFFFFFF::F,:FFFF:F,FFF:FFFFFFF:FF::F,FF:FFFFF:F,FFFFFFFFFFF:FF
:,F,:FFFFFFFFFF
```

@A00721:190:HGJW7DSXY:4:2303:12924:20807 1:N:0:TCTGCAAG+GCTGCACT
AGCAGTTGATTGATGGATTCGTCTTCCAAGAAGAGGAAGTGGTCAGGCGGTCCGTTTAGGACCGCCGG
TTATCGTAAAACACGACGAGTGTTGCAATTCAACAGGACTCAGCGGTCACAGCTGCCGGTTCAGTGGT
CGAGGTCTCTGAGAT
+
FFFFFFFFFFFFFFFFFFFFFFFFFFFFFFFFFFFFFFFF:FFFFFFFFFFFFFF:FFFFFFFFFFFF:F
FFFFFFFFFFFF,FF:FFF:FF:FF:FFF:F,FF:FFFFFF:F:FFFFF:F::FFFFFFFFFF:FFFFFF
FF,FFF,F::FFFFF
@A00721:190:HGJW7DSXY:4:2476:23999:18255 1:N:0:TCTGCAAG+GCTGCACT
AGCAGTTGATTGATGGATTCGTCTTCCAAGAAGAGGAAGTGGTCAGGCGGTCCGTTTAGGACCGCCGG
TTATCGTAAAACACGACGAGTGTTGCAATTCAACAGGACTCAGCGGTCACAGCTGCCGGTTCAGTGGT
CGAGGTCTCTGAGAT
+
FFFFFF:FFFFFFFFFFFFFFFFFFFFFFFFFFFFFFFFFFFFFFFFFFFFFFFF:FFFFFFFFFFFFFF
FFFFFFFFFFFFFFFFFFFFFFFFFFFFFFFFFFFFFFFFFFFFFFFFF,FFFFFFFFFFF:FFFFFFFF
FFFFFFFFFFFFFF:
@A00721:190:HGJW7DSXY:4:1320:9525:18646 1:N:0:TCTGCAAG+GCTGCACT
GCAGTTGATTGATGGATTCGTCTTCCAAGAAGAGGAAGTGGTCAGGCGGTCCGTTTAGGACCGCCGGT
TATCGTAAAACACGACGAGTGTTGCAATTCAACAGGACTCAGCGGTCACAGCTGCCGGTTCAGTGGTC
GAGGTCTCTGAGATA
+
FFFFFFFFFFFFFF:FFFFFFFFFFFFFFF::FFFFFFFFFFFFFFFFFFFFFFF,FFFFFFFFFFFFFF
FF:FFFFFFFFFFF:FFFFFFFFFFFFFFF:FFFFFFFF:FFFFFFFFF:FFFF:F:FFFFF:FFFFFFF
FFFFFFF,FFFFFFF
@A00721:190:HGJW7DSXY:4:1535:26377:8124 1:N:0:TCTGCAAG+GCTGCACT
GCAGTTGATTGATGGATTCGTCTTCCAAGAAGAGGAAGTGGTCAGGCGGTCCGTTTAGGACCGCCGGT
TATCGTAAAACACGACGAGTGTTGCAATTCAACAGGACTCAGCGGTCACAGCTGCCGGTTCAGTGGTC
GAGGTCTCTGAGATA
+
FFFFFFFFFFFFFFFFFFFFFFFFFFFFFFFFFFFFFFFFFFFFFFFFFFFFFFFFFFFFFFFFFFFFFF
FFFFFFFFFFFFFFFFFFFFFFFFFFFFFFFFFFFFFFFFFFFFFFFFFFFFFFFFFFFFFFFFFFFFFF
FFFFFFFFFFFFFFF
@A00721:190:HGJW7DSXY:4:1615:12210:4946 1:N:0:TCTGCAAG+GCTGCACT
GCAGTTGATTGATGGATTCGTCTTCCAAGAAGAGGAAGTGGTCAGGCGGTCCGTTTAGGACCGCCGGT
TATCGTAAAACACGACGAGTGTTGCAATTCAACAGGACTCGGCGGTCACAGCTGCCGGTTCAGTGGTC
GAGGTCTCTGAGATA
+
FFFFFFFFFFFFFFFFFFFFFFFFFFFFFFFFFFFFFFF:FFFFFFFFFFFFFFFFFFFFFFFFFFFFFF
FFFFFFFFFFFFFFFFFFFFFFFFFFFFFFFFFFFFFFFFF,FFFFFFFFFFFF,FFFFFFFFF:FFFFF
FFFFFFFFF:F:FFF
@A00721:190:HGJW7DSXY:4:1615:13747:5822 1:N:0:TCTGCAAG+GCTGCACT
GCAGTTGATTGATGGATTCGTCTTCCAAGAAGAGGAAGTGGTCAGGCGGTCCGTTTAGGACCGCCGGT
TATCGTAAAACACGACGAGTGTTGCAAATCAACAGGACTCAGCGGTCACAGCTGCCGGTTCAGTGGTC
GAGGTCTCTGAGATA
+
FFFFFFFFFFF:FFFFFFFFFFFFFFFFFFFFFFFFFFFFFFFFFF:FFFFFFFFFFFFFFFFFFFFFFF
FFFFFFFFFF:FF:FFFFFFFFFF:FFF,FFFFFFFFFFFFFFFFFFF:FFFFF:FFFFFFFF:FFFFFF
FFFFFFFFFFFFFFF
@A00721:190:HGJW7DSXY:4:1630:9082:19601 1:N:0:TCTGCAAG+GCTGCACT
GCAGTTGATTGATGGATTCGTCTTCCAAGAAGAGGAAGTGGTCAGGCGGTCCGTTTAGGACCGCCGGT
TATCGTAAAACACGACGAGTGTTGCAATTCAACAGGACTCAGCGGTCACAGCTGCCGGTTCAGTGGTC
GAGGTCTCTGAGATA
+
FFFF:FFFFFFFFFFFFFFFFFFFFFFFFFFFFFFFFFFFFFFFFFFFFFFFFFFFFFFFFFFFFFFFFF

FF:FFFFFFFFF:FFFFFFFFFF,FFFFFFFFFF:FFF:FFFFFFFFFFFFF:FFFFF:FFFFFFFFF
FFFFFFFFFFFFFFF
@A00721:190:HGJW7DSXY:4:1657:7744:21167 1:N:0:TCTGCAAG+GCTGCACT
GCAGTTGATTGATGGATTCGTCTTCCAAGAAGAGGAAGTGGTCAGGCGGTCCGTTTAGGACCGCCGGT
TATCGTAAAACACGACGAGTGTTGCAATTCAACAGGACTCAGCGGTCACAGCTGCCGGTTCAGTGGTC
GAGGTCTCTGAGATA
+
FFFFFFFFFFFFFFFFFFFFFFFFFFFFF,FFFFFFFFFFFFFFFFFFFFFFFFFFFFFFFFFFFFFF
FFFFFFFFFFFFFFFFFFFFFFF:FFFFFFFFFFFFFFFFFFFFFFFF:FFFFFFFFFFFFFFFFFFF
FFFFFFFF:FFFFFF
@A00721:190:HGJW7DSXY:4:1670:19045:18599 1:N:0:TCTGCAAG+GCTGCACT
GCAGTTGATTGATGGATTCGTCTTCCAAGAAGAGGAAGTGGTCAGGCGGTCCGTTTAGGACCGCCGGT
TATCGTAAAACACGACGAGTGTTGCAATTCAACAGGACTCAGCGGTCACAGCTGCCGGTTCAGTGGTC
GAGGTCTCTGAGATA
+
FFFF:FFFFFFFFFFFFFFFFFFFFFFFFFFFFFFFFFFFFFFFFFFFFFFFFFFFFFFFFFFFFFFF
FFFFFFFFFFFFFFFFFFFFFFFFFFFFFFFFFFFFFFFFFFFFFFFFFFFFFFFFFFFFFFFFFFFF
F:FFFFFFFFFFFFF
@A00721:190:HGJW7DSXY:4:2203:25500:14434 1:N:0:TCTGCAAG+GCTGCACT
GCAGTTGATTGATGGATTCGTCTTCCAAGAAGAGGAAGTGGTCAGGCGGTCCGTTTAGGACCGCCGGT
TATCGTAAAACACGACGAGTGTTGCAATTCAACAGGACTCAGCGGTCACAGCTGCCGGTTCAGTGGTC
GAGGTCTCTGAGATA
+
FFFFFFFFFFFFFFFFFFFFFFFFFFFFFFFFFFFF:FFFFFFFFFFFFF,FFFFFF:FFFF,FF:F:
FFF,FFFFFF:F:,FF:F,FFFF:FFF,F,FFFF:FFF:FF,FFFFFFF:,F,F:F,F:FF,:FFFFF
F,,F:,:,,F:F::F
@A00721:190:HGJW7DSXY:4:2246:26838:11428 1:N:0:TCTGCAAG+GCTGCACT
GCAGTTGATTGATGGATTCGTCTTCCAAGAAGAGGAAGTGGTCAGGCGGTCCGTTTAGGACCGCCGGT
TATCGTAAAACACGACGAGTGTTGCAATTCAACAGGACTCAGCGGTCACAGCTGCCGGTTCAGTGGTC
GAGGTCTCTGAGATA
+
FFFFFFFFFFFFFFFFFFFFFFFFFFFFFFFFFFFFFFFFFFFFFFFFFFFFF:F:FF:FFFFFFFFFF
FFFFFFFFFFFF,FFFF:FFFFFF,FFFFFFFFF:FFFFFF:FFFFFFFFFF:FFFFFFFFFF:FFFF
F:FFFFFFFFFFFFF
@A00721:190:HGJW7DSXY:4:2251:22535:18818 1:N:0:TCTGCAAG+GCTGCACT
GCAGTTGATTGATGGATTCGTCTTCCAAGAAGAGGAAGTGGTCAGGCGGTCCGTTTAGGACCGCCGGT
TATCGTAAAACACGACGAGTGTTGCAATTCAACAGGACTCAGCGGTCACAGCTGCCGGTTCAGTGGTC
GAGGTCTCTGAGATA
+
FFFFFFFFFFFFFFFFFFFFFFFFFFFFFFFFFFFFFFFFFFFFFFFFFFF:FFFFFFFFFFFFFFFF
FFFFFFFFFFFFFFFF:FFFFFFFFFFFF:FFFF:FFFFFFFFFFFFFFFFFFFFFFFFFFFFFFFFF
FFFFFFFFFFFFFFF
@A00721:190:HGJW7DSXY:4:2307:9136:15781 1:N:0:TCTGCAAG+GCTGCACT
GCAGTTGATTGATGGATTCGTCTTCCAAGAAGAGGAAGTGGTCAGGCGGTCCGTTTAGGACCGCCGGT
TATCGTAAAACACGACGAGTGTTGCAATTAAACAGGACTCAGCGGTCACAGCTGCCGGTTCAGTGGTC
GAGGTCTCTGAGATA
+
FFFFFFFFFFFFFFFFFFFFFFFFFFFFFFFFFFFFFFFFFFFFFFFFFFF:FFFFF:,FFFF,FFFFF
FFF,:FFFFF:F,,F::FFFFFF,F:F:F,FF,F,FFF::F:FF:::::F,FF,FFFFFF,FFFFFF:
:,,:FFFFFFFFFFF
@A00721:190:HGJW7DSXY:4:2339:8820:10347 1:N:0:TCTGCAAG+GCTGCACT
GCAGTTGATTGATGGATTCGTCTTCCAAGAAGAGGAAGTGGTCAGGCGGTCCGTTTAGGACCGCCGGT
TATCGTAAAACACGACGAGTGTTGCAATTCAACAGGACTCAGCGGTCACAGCTGCCGGTTCAGTGGTC
GAGGTCTCTGAGATA

```
+
FFFFFFFFFFFFFFFFFFFFFFFFFFFFFFFFFFFFFFFFFFFFFFFFFFFFFFFFFFF:FFFFFFFFFF
FFF:FFFFF:FF:FFF:F:FFFF:FFFFFFFFFFFFFFFFFFFFFFFFFFF:FFFFFFFFFFFFF:FFF
FFFFF:FFFF:FFFF
@A00721:190:HGJW7DSXY:4:2346:4110:31219 1:N:0:TCTGCAAG+GCTGCACT
GCAGTTGATTGATGGATTCGTCTTCCAAGAAGAGGAAGTGGTCAGGCGGTCCGTTTAGGACCGCCGGT
TATCGTAAAACACGACGAGTGTTTCAATTCAACAGGACTCAGCGGTCACAGCTGCCGGTTCAGTGGTC
GAGGTCTCTGAGATA
+
FFFF,FFF:FFFFFF,F:FFFFFF:,,FF:F:FF:F,:F,F::,FFF,:,FF,FF:,FF,:F,::,F,,:
FFF,:FFFFFF,FF,F::F,::,F,FFF,F,FF,F,F:,FF:,F,FFFFFFFFF,,,,::FF:F,FF:FF
F,F,:F,:,FF,FFF
@A00721:190:HGJW7DSXY:4:2346:4137:31234 1:N:0:TCTGCAAG+GCTGCACT
GCAGTTGATTGATGGATTCGTCTTCCAAGAAGAGGAAGTGGTCAGGCGGTCCGTTTAGGACCGCCGGT
TATCGTAAAACACGACGAGTGTTGCAATTCAACAGGACTCAGCGGTCACAGCTGCCGGTTCAGTGGTC
GAGGTCTCTGAGATA
+
FFFF,FFFFFFFFFFFFFFFFFFFFFFFFFFFFFFFFFFFFFFFFFFFFFFFF:FFFFFFFFFFFFFFF
FFFFFFFFFF,FFFFFFFFFFFFFFFFFFFFFFFFFFFFFFF:FFFFFFFFFF::FFFFFFFFFFFFFFF
FFFFFFFFFFFFFFF
@A00721:190:HGJW7DSXY:4:2420:17571:14606 1:N:0:TCTGCAAG+GCTGCACT
GCAGTTGATTGATGGATTCGTCTTCCAAGAAGAGGAAGTGGTCAGGCGGTCCGTTTAGGACCGCCGGT
TATCGTAAAACACGACGAGTGTTGCAATTCAACAGGACTCAGCGGTCACAGCTGCCGGTTCAGTGGTC
GAGGTCTCTGATATA
+
FFFFFFFFFFFF:FFFFFFFFF:FFFFF:FFFFFFFFFFFFFFFFF:FFFFFF::,FFF:FFFF,FFFFF
FFF,FFFFFF,F,,:F,:,:,FFF:FFFFFFF:FFFF,,FF:F,FF:FFF:F,FFF,FFFFFFFFFF,
FF:F:F:F:FF,FFF
@A00721:190:HGJW7DSXY:4:2476:7591:1579 1:N:0:TCTGCAAG+GCTGCACT
GCAGTTGATTGATGGATTCGTCTTCCAAGAAGAGGAAGTGGTCAGGCGGTCCGTTTAGGACCGCCGGT
TATCGTAAAACACGACGAGTGTTGCAATTCAACAGGACTCAGCGGTCACAGCTGCCGGTTCAGTGGTC
GAGGTCTCTGAGATA
+
FFFFFFFFFFFFFFFFFFFFFFFFFFFFFFFFFFFFFFFFFFFFFFFFFFFFFF:FFFFFFFFFFFFFFF
FFFFFFFFFFFFFFFFFFFFFFFFFFFFFFFFFFF:FFFFFFFFFFFFFFFFFFFFFFFFFFFFFFFFFF
FFFFFFFFFFFFFFF
@A00721:190:HGJW7DSXY:4:2523:28863:25864 1:N:0:TCTGCAAG+GCTGCACT
GCAGTTGATTGATGGATTCGTCTTCCAAGAAGAGGAAGTGGTCAGGCGGTCCGTTTAGGACCGCCGGT
TATCGTAAAACACGACGAGTGTTGCAATTCAACAGGACTCAGCGGTCACAGCTGCCGGTTCAGTGGTC
GAGGTCTCTGAGATA
+
FFFFFFFFFFFFFFFFFFFFFFFFFFFFFFFFFFFFF:FFFFFFFFFFFFF:FFFFF:FFFFFFFFFFFF
FFFF:FFFFFFFFFFFFF:FFFFFFFFF,FFFFFF:FFFFFFFFFFFFFFFFFF,F:FFFFFFFF:FF:F
FFFFFFFF:FFFFFF
@A00721:190:HGJW7DSXY:4:2535:28628:11804 1:N:0:TCTGCAAG+GCTGCACT
GCAGTTGATTGATGGATTCGTCTTCCAAGAAGAGGAAGTGGTCAGGCGGTCCGTTTAGGACCGCCGGT
TATCGTAAAACACGACGAGTGTTGCAATTCAACAGGACTCAGCGGTCACAGCTGCCGGTTCAGTGGTC
GAGGTCTCTGAGATA
+
FFFFFFFFFFFFFFFFFFFFFFFFFFFFFFFFFFFFFFFFFFFFFFFFFFFFFF:FFFFFFFFFFFFFFF
FFFFFFFFFFFFFFFFFFFFFFFFFFFFFFFFFFFFFFFFFFFFFFFFFFFFFF,FFFFFFFFFFFFFFF
FFFFFFFFFFFFFFF
@A00721:190:HGJW7DSXY:4:2536:23411:33802 1:N:0:TCTGCAAG+GCTGCACT
GCAGTTGATTGATGGATTCGTCTTCCAAGAAGAGGAAGTGGTCAGGCGGTCCGTTTAGGACCGCCGGT
```

```
TATCGTAAAACACGACGAGTGTTGCAATTCAACAGGACTCAGCGGTCACAGCTGCCGGTTCAGTGGTC
GAGGTCTCTGAGATA
+
FFFFFFFFFFFFFFFFFFFFFFFFFFFFFFFFFFFFFFFFFFFFFFFFFFFFFFFFF:FFFFFFFFFF
FFFFFFFFFFFF:FFFFFFFFFF,FFFFFFFF:FFFFFFFF:FFFFFFF:FFFFFFFFFFFFFFFFFF
FFFFFFFFFFFFFFF
@A00721:190:HGJW7DSXY:4:2538:5882:14403 1:N:0:TCTGCAAG+GCTGCACT
GCAGTTGATTGATGGATTCGTCTTCCAAGAAGAGGAAGTGGTCAGGCGGTCCGTTTAGGACCGCCGGT
TATCGTAAAACACGACGAGTGTTGCAATTCAACAGGACTCAGCGGTCACAGCGGCCGGTTCAGTGGTC
GAGGTCTCTGAGATA
+
FFFFFFFFFFFFFFFFFFFFFFFFFFFFFFFFFFFFFFFFFFFFFFFFFFFFFF:FFFFFFFFFFFFF
FFFFFFFFFFFFF:FFFFFFFFFFFFFFFFFFFFFFFFFFFFFFFFFFFFFFFF,FFFFFFFFF:FFFFF
FFFFFFFFFFFFFFF
@A00721:190:HGJW7DSXY:4:2618:30445:3396 1:N:0:TCTGCAAG+GCTGCACT
GCAGTTGATTGATGGATTCGTCTTCCAAGAAGAGGAAGTGGTCAGGCGGTCCGTTTAGGACCGCCGGT
TATCGTAAAACACGACGAGTGTTGCAATTCAACAGGACTCAGCGGTCACAGCTGCCGGTTCAGTGGTC
GAGGTCTCTGAGATA
+
FFFFFFFFFFFFFFFFFFFFFFFFFFFFFFFFFFFFFFFFFFFFFFFFFFFFFFFFFFFFFFFFFFFF
FFFF:FFFFFFF,FFF:FFF,FFFFFFFFFFFFF:FFFFF,:FFFFFFFFFFFFFFFFFFFFF:FFFF
:,FFFFFFFF,FFFF
@A00721:190:HGJW7DSXY:4:1162:5041:17738 1:N:0:TCTGCAAG+GCTGCACT
GTTGATTGATGGATTCGTCTTCCAAGAAGAGGAAGTGGTCAGGCGGTCCGTTTAGGACCGCCGGTTAT
CGTAAAACACGACGAGTGTTGCAATTCAACAGGACTCAGCGGTCACAGCTGCCGGTTCAGTGGTCGAG
GTCTCTGAGATATAA
+
FFFFFFFFFFFFFFFFFFFFFFFFFFFFFFFFFFFFFFFFFFFFFFFFFFFFFFFFFFFFFFFF:FFF
FFFFFFFFFFFFFFFFFFFFFFFFFFFFFFFFFFFFFFFFF:FFFFFFFFF:FFFFFFFFFFFFFFFFF
FFFFFFFFFFFF:FF
@A00721:190:HGJW7DSXY:4:1162:25681:35196 1:N:0:TCTGCAAG+GCTGCACT
GTTGATTGATGGATTCGTCTTCCAAGAAGAGGAAGTGGTCAGGCGGTCCGTTTAGGACCGCCGGTTAT
CGTAAAACACGACGAGTGTTGCAATTCAACAGGACTCAGCGGTCACAGCTGCCGGTTCAGTGGTCGAG
GTCTCTGAGATATAA
+
F:FFFFFFFFFFFFFFFFFFFFFFFFFFFFFFFFFFFFFFFFFFFFFFFFFFFFFFF:FFFFFFFFFF
FFFFFFFFFFFFFFFFFFFFFFFFFFFFFFFFFFFFFFFFFFFFFFFFFF,FFFFFFFFFFFFFFFFF:
FFFFFFFFFFFF:FF
@A00721:190:HGJW7DSXY:4:2224:11930:35055 1:N:0:TCTGCAAG+GCTGCACT
GTTGATTGATGGATTCGTCTTCCAAGAAGAGGAAGTGGTCAGGCGGTCCGTTTAGGACCGCCGGTTAT
CGTAAAACACGACGAGTGTTGCAATTCAACAGGACTCAGCGGTCACAGCTGCCGGTTCAGTGGTCGAG
GTCTCTGAGATATAA
+
FFFFFFFFFFFFFFFFFFFFFFFFFFFFFFFFFFFFFFFFFFFFFFFFFFFFFFFFFFFFFFFFFFFF
FF:FFFF:F:FFFFFFFFFFFFF:FFFFFFFFFFFFFFFFFFFFFFFFFFFFFFFFFFFFFFFFFFFFF
FFFFFFFFFFFFFFF
@A00721:190:HGJW7DSXY:4:2240:5620:31266 1:N:0:TCTGCAAG+GCTGCACT
GTTGATTGATGGATTCGTCTTCCAAGAAGAGGAAGTGGTCAGGCGGTCCGTTTAGGACCGCCGGTTAT
CGTAAAACACGACGAGTGTTGCAATTCAACAGGACTCAGCGGTCACAGCTGCCGGTTCAGTGGTCGAG
GTCTCTGAGATATAA
+
FFFFFFFFFFFFFFFFFFFFFFFFFFFFFFFFFFFFFFFFFFFFFFFFFFFFFFFFFFFFFFFFFFFF
FFFFFFFFFFFFFFFFFFFFFFFFFFFFFFF:FFFFFFFFFFFFFF:FFFFFFFFFFFFFFFFFFFFFF
FFFFFFFFFFFFFFF
```

```
@A00721:190:HGJW7DSXY:4:2248:9444:7889 1:N:0:TCTGCAAG+GCTGCACT
GTTGATTGATGGATTCGTCTTCCAAGAAGAGGAAGTGGTCAGGCGGTCCGTTTAGGACCGCCGGTTAT
CGTAAAACACGACGAGTGTTGCAATTCAACAGGACTCAGCGGTCACAGCTGCCGGTTCAGTGGTCGAG
GTCTCTGAGATATAA
+
FFFFFFFFFFFFFFFFFFFFFFFFFFFFFFFFFFFFFFFFFFFFFFFFFFFFFFFFFFFFFFFFFFFF
FFFFFFFFFFFFFFFFFFFFFFFFFFFFFFFFFFFFFFFFFFFFFFFFFFFFFFFFFFFFFFFFFFFF
FFFFFFFFFFFFFFF
@A00721:190:HGJW7DSXY:4:2254:8359:18818 1:N:0:TCTGCAAG+GCTGCACT
GTTGATTGATGGATTCGTCTTCCAAGAAGAGGAAGTGGTCAGGCGGTCCGTTTAGGACCGCCGGTTAT
CGTAAAACACGACGAGTGTTGCAATTCAACAGGACTCAGCGGTCACAGCTGCCGGTTCAGTGGTCGAG
GTCTCTGAGATATAA
+
FFFFFFFFFFFFFFFFFFFFFFFFFFFFFFFFFFFFFFFFFFFFFFFFFFFFFFFFFFFFFFFFFFFF
FFFFFFFFFFFFFFFFFFFFFFFFFFFFFFFFFFFFFFFFFFFFFFFFFFFFFFFFFFFFFFFFFF:F
FFFFFFFFFFFFFFF
@A00721:190:HGJW7DSXY:4:2463:5692:3865 1:N:0:TCTGCAAG+GCTGCACT
GTTGATTGATGGATTCGTCTTCCAAGAAGAGGAAGTGGTCAGGCGGTCCGTTTAGGACCGCCGGTTAT
CGTAAAACACGACGAGTGTTGCAATTCAACAGGACTCAGCGGTCACAGCTGCCGGTTCAGTGGTCGAG
GTCTCTGAGATATAA
+
FFFFFFFFFFFFFFFFFFFFFFFFFFFFFFFFFFFFFFFFFFFFFFFFFFFF:FFFFFFFFFFFFFFF
FFFFFFFFFFFFFFFFFFFFFFFFFFFFFFFFFFFFFFFFFFFFFFFFFFFFFFFFFFFFFFFFFFFF
FFFFFFFFFFFFFFF
@A00721:190:HGJW7DSXY:4:2532:2456:2519 1:N:0:TCTGCAAG+GCTGCACT
GTTGATTGATGGATTCGTCTTCCAAGAAGAGGAAGTGGTCAGGCGGTCCGTTTAGGACCGCCGGTTAT
CGTAAAACACGACGAGTGTTGCAATTCAACAGGACTCAGCGGTCACAGCTGCCGGTTCAGTGGTCGAG
GTCTCTGAGATATAA
+
FFFFFFFFFFFFFFFFFFFFFFFFFFFFFFFFFFFFFFFFFFFFFFFFFFFFFFFFFFFFFFFFFFFF
FFFFFFF,F:FFFFFFFFFFFFFFFFFFFFF:FFFFFFFFFFFF:FFFF:FFFFFFFFFFFFFFFFFF
F:FFFFFFFFFFFFF
@A00721:190:HGJW7DSXY:4:2672:7708:24361 1:N:0:TCTGCAAG+GCTGCACT
GTTGATTGATGGATTCGTCTTCCAAGAAGAGGAAGTGGTCAGGCGGTCCGTTTAGGACCGCCGGTTAT
CGTAAAACACGACGAGTGTTGCAATTCAACAGGACTCAGCGGTCACAGCTGCCGGTTCAGTGGTCGAG
GTCTCTGAGATATAA
+
FFFFFFFFFFFFFFFFFFFFFFFFFFFFFFFFFFFFFFFFFFFFFFFFFF:FFFFFFFFFFFFFFFFF
FFFFFFFFFFFFFFFFFFFFFFFFFFFFFFFFFFFFFFFFFFFFFFFFFFFFFFFFFFFFFFFFFFFF
FFFFFFFFFFFFFFF
@A00721:190:HGJW7DSXY:4:1101:21432:30405 1:N:0:TCTGCAAG+GCTGCACT
GATGGATTCGTCTTCCAAGAAGAGGAAGTGGTCAGGCGGTCCGTATAGTAACGACCGTTATCGTAAAA
CACGACGATTGTTGAAATACAACAGGACTCATCGGTCACAGCTGCCGGTTAACTTGTAGAGTTATCTG
AGATATAATAAAAGA
+
FF,,:F:FFF,F:F,FFF:FFF::,FFFF:F,F:,FF,F,:F:F,,F,,:,:,,F,:,,FF:,FFFFF
,F:,F::F,F,:F:,FFF,::F,:,,,F,,:,F,:,,,,F,F,:F::F,,,,,,:F,,FF,,,,:,,F
F,F,,,FFF:,FF,F
@A00721:190:HGJW7DSXY:4:1105:27326:11960 1:N:0:TCTGCAAG+GCTGCACT
GATGGATTCGTCTTCCAAGAAGAGGAAGTGGTCAGGCGGTCCGTTTAGGACCGCCGGTTATCGTAAAA
CACGACGAGTGTTGCAATTCAACAGGACTCAGCGGTCACAGCTGCCGGTTCAGTGGTCGAGGTCTCTG
AGATATAATCGAAGA
+
FFFFFFFFFFFFFFFFFFFFFFFFFFFFFFFFFFFFFFFFFFFF,FF,FFFFFFFFF:FFF,FFFFFF
```

```
FFFFFFFFFFFFFFFFFFFFFF:FFFFFF:FFFFFFFFFF::,F,F:FFF:FFFFFFFF:FFF,FFFF
:F:FFFFFF:FFF,F
@A00721:190:HGJW7DSXY:4:1169:5448:31939 1:N:0:TCTGCAAG+GCTGCACT
GATGGATTCGTCTTCCAAGAAGAGGAAGTGGTCAGGCGGTCCGTTTAGGACCGCCGGTTATCGTAAAA
CACGACGAGTGTTGCAATTCAACAGGACTCAGCGGTCACAGCTGCCGGTTCAGTGGTCGAGGTCTCTG
AGATATAATCGAAGA
+
FFFFFFFFFFFFFFFFFFFFFFFFFFFFFFFFFFFFFFFFFFFFFFFFFFFFFFFFFFFFFFFFFFFF
FFFFFFFFFFFFFFFFFFFFFFFFFFFFFFFFFFFFFFFFFFFFFFFF:FFFFFFFFFFFFFFFFFFF
FFFFFFFFFFFFFFF
@A00721:190:HGJW7DSXY:4:1203:17689:8735 1:N:0:TCTGCAAG+GCTGCACT
GATGGATTCGTCTTCCAAGAAGAGGAAGTGGTCAGGCGGTCCGTTTAGGACCGCCGGTTATCGTAAAA
CACGACGAGTGTTGCAATTCAACAGGACTCAGCGGTCACAGCTGCCGGTTCAGTGGTCGAGGTCTCTG
AGATATAATCGAAGA
+
FFFFFFFFFFFFFFFFFFFFFFFFFFFFFFFFFFFFFFFFFF:FFFF:FF:FFFFFF:FFFF:FFFFF
,F:FFFFFFFFFF:FFFFFFFF,F:FFFFF:FF:FFFFFFFF,FFF:FFFFFF,FFF:FFFF,FF,:F
FFFFFFFF:,FFF,F
@A00721:190:HGJW7DSXY:4:1256:31367:15045 1:N:0:TCTGCAAG+GCTGCACT
GATGGATTCGTCTTCCAAGAAGAGGAAGTGGTCAGGCGGTCCGTTTAGGACCGCCGGTTATCGTAAAA
CACGACGAGTGTTGCAATTCAACAGGACTCAGCGGTCACAGCTGCCGGTTCAGTGGTCGAGGTCTCTG
AGATATAATCGAAGA
+
FFFFFFFFFFFFFFFFFFFFFFFFFFFFFFFFFFFFFFFFFFFFFFFFFFFFFFFFFFFFFFFFFFFF
FFFFFFFFFFFFFFFFF:FFFF:FFFFFFFFFFFFFFFFFFF,FFFFFFF:FFFFFFFF:FFFFF:FF
FFFFFFFFFFFFF:F
@A00721:190:HGJW7DSXY:4:1311:11406:23312 1:N:0:TCTGCAAG+GCTGCACT
GATGGATTCGTCTTCCAAGAAGAGGAAGTGGTCAGGCGGTCCGTTTAGGACCGCCGGTTATCGTAAAA
CACGACGAGTGTTGCAATTCAACAGGACTCAGCGGTCACAGCTGCCGGTTCAGTGGTCGAGGTCTCTG
AGATATAATCGAAGA
+
FFFFFFFFFFFFFFFFFFFFFFFFFFFFFFFFFFFFFFFF:FFFFFFFFFFFFFFFFFFFFFFFFFFF
,F::FFFFFFFFFFFFF,FFFF:FFFFFFFFFFFFFFFFF:F,FFFFFFF:FF:FFFFF:FFFFFF:F
FFFFFFFFFFFFFFF
@A00721:190:HGJW7DSXY:4:1349:28691:32111 1:N:0:TCTGCAAG+GCTGCACT
GATGGATTCGTCTTCCAAGAAGAGGAAGTGGTCAGGCGGTCCGTTTAGGACCGCCGGTTATCGTAAAA
CACGACGAGTGTTGCAATTCAACAGGACTCAGCGGTCACAGCTGCCGGTTCAGTGGTCGAGGTCTCTG
AGATATAATCGAAGA
+
FFFFFFFFFFFFFFFFFFFFFFFFFFFFFFFFFFFFFFFFFFFFFFFFFFFFFFFFFFFFFFFFFFFF
FFFFFFFFFFFFFFFFFFFFFFFFFFFFFFFFFFFFF:FFFFFFFFFFFFF,FFFFFFFFFFFFFFFF
FFFFFFFFFFFFFFF
@A00721:190:HGJW7DSXY:4:1441:8504:20259 1:N:0:TCTGCAAG+GCTGCACT
GATGGATTCGTCTTCCAAGAAGAGGAAGTGGTCAGGCGGTCCGTTTAGGACCGCCGGTTATCGTAAAA
CACGACGAGTGTTGCAATTCAACAGGACTCAGCGGTCACAGCTGCCGGTTCAGTGGTCGAGGTCTCTG
AGATATAATCGAAGA
+
FFFFFFFFFFFFFFFFFFFFFFFFFFFFFFFFFFFFFFFFFFFFFFFFFFFFFFFFFFFFFFFFFFFF
FFFFFFFFFFFFFFFF:FFFFFFFFFFFFFFFFFFFFFFFFF,FFFFFFFFFFFFFFFF:FFFF:FFF
FFFFFFFFFFFFFFF
@A00721:190:HGJW7DSXY:4:1452:20681:19116 1:N:0:TCTGCAAG+GCTGCACT
GATGGATTCGTCTTCCAAGAAGAGGAAGTGGTCAGGCGGTCCGTTTAGGACCGCCGGTTATCGTAAAA
CACGACGAGTGTTGCAATTCAACAGGACTCAGCGGTCACAGCTGCCGGTTCAGTGGTCGAGGTCTCTG
AGATATAATCGAAGA
```

```
+
FFFFFFFFFFFFFFFFFFFFFFFFFFFFFFFFFFFFFFFFFFFFFFFFFFFFFFFFFFFFFFFFFFFFFF
FFFFFFFFFFFFFFFFFFFFFFFF:FFFFFFFFFFFFFFFFFFFFFFFFFFFFFF:FFFFFFFF:FFF:F
FFFFFFFFFFFFFFF
@A00721:190:HGJW7DSXY:4:1601:22896:32064 1:N:0:TCTGCAAG+GCTGCACT
GATGGATTCGTCTTCCAAGAAGAGGAAGTGGTCAGGCGGTCCGTTTAGGACCGCCGGTTATCGTAAAA
CACGACGAGTGTTGCAATTCAACAGGACTCAGCGGTCACAGCTGCCGGTTCAGTGGTCGAGGTCTCTG
AGATATAATCGAAGA
+
FFFFFFFFFFFFFFFFFFFFFFFFFFFFFFFFFFFFFFFFF,FF,FF:,F:FF:FFFFFFFF,,FFFFFF
:F::FF:F:FFFFFFFFF,F,FFFF,FFF,:F,FFFF:,FFFF:FFFFFFFF:F:FFFF,F,FF::F:FF
,F:FFFFFF:F:F:F
@A00721:190:HGJW7DSXY:4:1672:28275:3396 1:N:0:TCTGCAAG+GCTGCACT
GATGGATTCGTCTTCCAAGAAGAGGAAGTGGTCAGGCGGTCCGTTTAGGACCGCCGGTTATCGTAAAA
CACGACGAGTGTTGCAATTCAACAGGACTCAGCGGTCACAGCTGCCGGTTCAGTGGTCGAGGTCTCTG
AGATATAATCGAAGA
+
FFFFFFFFFFFFFFFFFFFFFFFFFFFFFFFFFFFFFFFFFFFF:FFFFFFFFFFFFFFFF:FFFFFFFF
FFFFFFFFFFFFFFFFFFFFFFFFFFFFFFFFFFFFFF,FFFF:FFFFFFFFFFFFFFFFFFFFFFFF:F
FFFFFFFFFFFFFFF
@A00721:190:HGJW7DSXY:4:2214:18954:2597 1:N:0:TCTGCAAG+GCTGCACT
GATGGATTCGTCTTCCAAGAAGAGGAAGTGGTCAGGCGGTCCGTTTAGGACCGCCGGTTATCGTAAAA
CACGACGAGTGTTGCAATTCAACAGGACTCAGCGGTCACAGCTGCCGGTTCAGTGGTCGAGGTCTCTG
AGATATAATCGAAGA
+
FFFFFFFFFFFFFFFFFFFFFFFFFFFFFFFFFFFFFFFFFFFF:FFFFFFFFFFFFFF:FFFFFFFFFF
,FFFFF:FFFF:FFFFF:F:FF,FFFF:F:FFFFFFFFFFFFFFFFFFFFFFFFFFFFF:FFFFFFFFFF
FFFFFFFFFFFFF,F
@A00721:190:HGJW7DSXY:4:2216:14724:27211 1:N:0:TCTGCAAG+GCTGCACT
GATGGATTCGTCTTCCAAGAAGAGGAAGTGGTCAGGCGGTCCGTTTAGGACCGCCGGTTATCGTAAAA
CACGACGAGTGTTGCAATTCAACAGGACTCAGCGGTCACAGCTGCCGGTTCAGTGGTCGAGGTCTCTG
AGATATAATCGAAGA
+
:FFFFF:FFFFFFF,FFFFFFF:FFFFF:FFFFF,FFFFF,F:FF,FFFFF:FFF,F,FF:FFFFFFF
FFFFFF:F,FFFFF,FF,:FFFFF:FFFFFFFFFFF,,,F,FF::,,,,FFFF,,FFFFFF:F:F:,F
FF:FF,FFFF:F,:F
@A00721:190:HGJW7DSXY:4:2217:25690:7560 1:N:0:TCTGCAAG+GCTGCACT
GATGGATTCGTCTTCCAAGAAGAGGAAGTGGTCAGGCGGTCCGTTTAGGACCGCCGGTTATCGTAAAA
CACGACGAGTGTTGCAATTCAACAGGACTCAGCGGTCACAGCTGCCGGTTCAGTGGTCGAGGTCTCTG
AGATATAATCGAATA
+
FFFFFFFFFFFFFFFFFFFFFFFFFF:FFFFFFFFFFFFFFFFF:FFF,FFFFFFFFFFFFFF:FFFFFF
,F:FF:FFFFFFF:FFF,F,:F:F,F,F::F,:FF:F,FFFF::FFFFFFFF,FFFF,F:FFF:FFFF
FFFFFFFFF:FFF,F
@A00721:190:HGJW7DSXY:4:2240:1136:12352 1:N:0:TCTGCAAG+GCTGCACT
GATGGATTCGTCTTCCAAGAAGAGGAAGTGGTCAGGCGGTCCGTTTAGGACCGCCGGTTATCGTAAAA
CACGACGAGTGTTGCAATTCAACAGGACTCAGCGGTCACAGCTGCCGGTTCAGTGGTCGAGGTCTCTG
AGATATAATCGAAGA
+
FFFFFFFFFFFFFFFFFFFFFFFFFFFFFFFFFFFFFFFFFFFFFFFFFFFFFFFFFFFFFFFFFFFFFF
:F:FFFFFFFFFFFFFFFFFFFFFFFFFFFFFFFFFFFFFFFFFFFFFFFFFFFFFFFFFF,FFFFFFFF
FFFFFFFFFFFFFFF
@A00721:190:HGJW7DSXY:4:2308:22498:35571 1:N:0:TCTGCAAG+GCTGCACT
GATGGATTCGTCTTCCAAGAAGAGGAAGTGGTCAGGCGGTCCGTTTAGGACCGCCGGTTATCGTAAAA
```

CACGACGAGTGTTGCAATTCAACAGGACTCAGCGGTCACAGCTGCCGGTTCAGTGGTCGAGGTCTCTG
AGATATAATCGAAGA
+
FFFFFFFFFFF:FFFFFFFFFFFFFFFFFFFFFFFFFFFFFFF:FF:F:FFFF,FFFF,FF,,F,FFFF
,F,,FF,:,FF:F,FFF:F:FFFFFFFF,FFFF:FF:,FF:::FFFFFFF,FF,:FFFF:FFFFFFF:
FFFFFFFF:::FF,F
@A00721:190:HGJW7DSXY:4:2353:19054:22404 1:N:0:TCTGCAAG+GCTGCACT
GATGGATTCGTCTTCCAAGAAGAGGAAGTGGTCAGGCGGTCCGTTTAGGACCGCCGGTTATCGTAAAA
CACGACGAGTGTTGCAATTCAACAGGACTCAGCGGTCACAGCTGCCGGTTCAGTGGTCGAGGTCTCTG
AGATATAATCGAAGA
+
FFFFFFFFFFFFFFFFFFF:FFFFFFFFFFFFFFFFFFFFFFFFFFFFFFFFFFFFFFFFFFFFFFFFF
FFFFFFFFFFFFFFFFFFFFFFFFFFFFFFFFFFFFFFFFFFFFFFFFFFFFFFFFFFFFFFFFFFFFF
F:FFFFFFFFFFFFF
@A00721:190:HGJW7DSXY:4:2402:12644:5415 1:N:0:TCTGCAAG+GCTGCACT
GATGGATTCGTCTTCCAAGAAGAGGAAGTGGTCAGGCGGTACGTTTATGACCGCCGGTTATAGTAAAA
CACGAAGAGTGTTGCAATTAAACAGGAAACAGCCGTCACAGCTGCCGGTTCACTGTTCGAGGTCTCTG
AGAAATAATAGAAGA
+
:FF::F,:FF,FFFFFFFFFFFFF:FFFFF:FFFFFFFF,,F:,F,F,,F,,,:F:::FF:,:FFFFF
,F,,F,:::FF:F,,FF,F,FFFF,:F,:,F,:,:FF,,F,F,F,F,,FF,F,::,F,,:,FFFF,,,
::F,F,FF,F,FF,F
@A00721:190:HGJW7DSXY:4:2574:3667:29293 1:N:0:TCTGCAAG+GCTGCACT
GATGGATTCGTCTTCCAAGAAGAGGAAGTGGTCAGGCGGTCCGTTTAGGACCGCCGGTTATCGTAAAA
CACGACGAGTGTTGCAATTCAACAGGACTCAGCGGTCACAGCGGCCGGTTCAGTGGTCGAGGTCTCTG
AGATATAATCGAAGA
+
FF:FFFFFF:FFFFFFFFF:FFFFFF:FFFFFFFFFFFFFFFFFFFFFF:FFFFFFFFFFFFFFFFFF
FFFFFF:FFFFFFFFFFFFFFFFFFFFFFFFFFFFFFFFFFFF,FFFFFF:FFFFFFFFFF:FFFFF:F
FFFFFFFFFFFFF,F
@A00721:190:HGJW7DSXY:4:2637:18331:14888 1:N:0:TCTGCAAG+GCTGCACT
GATGGATTCGTCTTCCAAGAAGAGGAAGTGGTCAGGCGGTCCGTTTAGGACCGCCGGTTATCGTAAAA
CACGACGAGTGTTGCAATTCAACAGGACTCAGCGGTCACAGCTGCCGGTTCAGTGGTCGAGGTCTCTG
AGATATAATCGAAGA
+
FFFFFFFFFFFFFFFFFFFFFFFFFFFFFFFFFFFFFFFFFFF:FFFFFFFFFFFFFFFFFFFFFFFFF
FF:FFFFFFFFF:FFFFFFFFFFFFFFFFFFFFFFFF:,FFFF,FFFFFFFFFFFFFFFF:FFFFFF:F
FFFFFFFFFFFFFFF
@A00721:190:HGJW7DSXY:4:2231:29351:22075 1:N:0:TCTGCAAG+GCTGCACT
ATGGATTCGTCTTCCAAGAAGAGGAAGTGGTCAGGCGGTCCGTTTAGGACCGCCGGTTATCGTAAAAC
ACGACGAGTGTTGCAATTCAACAGGACTCAGCGGTCACAGCTGCCGGTTCAGTGGTCGAGGTCTCTGA
GATATAATCGAAGAC
+
FFFFFFFFFFFFFFFFFFFFFFFFFFFFFFFFFFFFFFFFFFF:FFFFFFFFFFFFF:FFFFFFFFFF:
FFFFFFFFFFFFFFFFFFFFFFFFF:FFFFFFFFFFFFFFFFFFFFFFFFFFFFFFFFFF:FFF:FF:FF
F:FFFFFFFFFFFFF
@A00721:190:HGJW7DSXY:4:2231:28483:22795 1:N:0:TCTGCAAG+GCTGCACT
ATGGATTCGTCTTCCAAGAAGAGGAAGTGGTCAGGCGGTCCGTTTTGGACCCCAGGTTATAGTAAAAC
ACGACCAGTGAAGCAATACAAAATGACACAACGGTCACAGCAGCCGGATCAGTGTTCTAGGACTCGGA
GATATAATCTAACAC
+
F,:,,:F:,FFFF,F:FFFF,:F:F:::FF:FF:F,,FF,::,FF,,,FFF,F,,,FFF,,:::FFFF
FF,F,FF:,,,,,:FFF,,FF,:,,,,,,F,F,,FFFF,,,,,:F,F,,F,,F,,,F,,F:,F,:,F:
,F,FFFFF,,FF,FF

```
@A00721:190:HGJW7DSXY:4:2660:21504:32409 1:N:0:TCTGCAAG+GCTGCACT
ATGGATTCGTCTTCCAAGAAGAGGAAGTGGTCAGGCGGTCCGTTTAGGACCGCCGGTTATCGTAAAAC
ACGACGAGTGTTGCAATTCAACAGGACTCAGCGGTCACAGCTGCCGGTTCAGTGGTCGAGGTCTCTGA
GATATAATCGAAGAC
+
FFFFFFFFFFFFFFFFFFFFFFFFF:FFFFFFFFFFFFFFFFFFFFFFFFFFFFFFFFFFFFFFFFFF
FFFFFFFFFFFFFFFFFFFFFFFFFFFFFFFFFFFFFFFFFFFFFFFFFFFFFFFFFFFFFFFFF,FF
FFFFFFFFFFFF:FF
@A00721:190:HGJW7DSXY:4:1353:19153:9862 1:N:0:TCTGCAAG+GCTGCACT
TGGATTCGTCTTCCAAGAAGAGGAAGTGGTCAGGCGGTCCGTTTAGGACCGCCGGTTATCGTAAAACA
CGACGAGTGTTGCAATTCAACAGGACTCAGCGGTCACAGCTGCCGGTTCAGTGGTCGAGGTCTCTGAG
ATATAATCGAAGACT
+
FFFFFFFFFFFFFFFFFFFFFFFFFFFFFFFFFFFFFFFF,FFFFFFFFFFFFF:FFFFFFFFF:FF
FF,FFFFFFFFFFFFFFFFFFFFFFFFFFFFFFFFFFFFFFFFFFFF:FFFFFFFFFFFFFF::FFF
FFFFFFFF:FFFFFF
@A00721:190:HGJW7DSXY:4:1513:15718:28494 1:N:0:TCTGCAAG+GCTGCACT
TGGATTCGTCTTCCAAGAAGAGGAAGTGGTCAGGCGGTCCGTTTAGGACCGCCGGTTATCGTAAAACA
CGACGAGTGTTGCAATTCAACAGGACTCAGCGGTCACAGCTGCCGGTTCAGTGGTCGAGGTCTCTGAG
ATATAATCGAAGACT
+
FFFFFFFFFFFFFF:FF:FFFFFFFFFFFFFFFFFFFF:FF:FFFFFFFFFFFFFFFFFFFFFFFFFF
FF:FFFFFFFFFFFF:FFFFFFFF:FFFFFFFFFF,FFFF,FFFFFFFFFFFFFFFFFFFF:FF,FFF
,,FFFFFFFFFFFFF
@A00721:190:HGJW7DSXY:4:2407:8223:32988 1:N:0:TCTGCAAG+GCTGCACT
TGGATTCGTCTTCCAAGAAGAGGAAGTGGTCAGGCGGTCCGTTTAGGACCGCCGGTTATCGTAAAACA
CGACGAGTGTTGCAATTCAACAGGACTCAGCGGTCACAGCTGCCGGTTCAGTGGTCGAGGTCTCTGAG
ATATAATCGAAGA
+
FFFFFFFFFFFFFFFFFFFFFFFFFFFFFFFFFFFFF,FFF,:FFFFFFF:FF:F:FFF:F:FFFFFF
:,F,FFFFFF:FFFFFF,FF:FFFF:FFF:FFFFF:F:,FFF:F:FF:,:F::FF:FFFFFFF::F:F
FFFFFF:FFFF:F
@A00721:190:HGJW7DSXY:4:2518:31340:24236 1:N:0:TCTGCAAG+GCTGCACT
TGGATTCGTCTTCCAAGAAGAGGAAGTGGTCAGGCGGTCCGTTTAGGACCGCCGGTTATCGTAAAACA
CGACGAGTGTTGCAATTCAACAGGACTCAGCGGTCACAGCTGCCGGTTCAGTGGTCGAGGTCTCTGAG
ATATAATCGAAGACT
+
FFFFFFFFFFFFFFFFFFFFFFFFFFFFFFFFFFFFFF,FFFFFFFF:FFFFFFFFFFF,FFFFFF:F
FF:F:FFFFFFFFFFFF:FFFF:FF:FFFFFFFFFF:FFF,FFFFFFFF,FFFFFFFFFFF:F:,F,F
FFFFFFF,FF:F,FF
@A00721:190:HGJW7DSXY:4:2576:15167:19805 1:N:0:TCTGCAAG+GCTGCACT
TGGATTCGTCTTCCAAGAAGAGGAAGTGGTCAGGCGGTCCGTTTAGGACCGCCGGTTATCGTAAAACA
CGACGAGTGTTGCAATTCAACAGGACTCAGCGGTCACAGCTGCCGGTTCAGTGGTCGAGGTCTCTGAG
ATATAATCGAAGACT
+
FFFFFFFFFFFFFFFFFFFFFFFFFFFFFFFFFFFFFFFFFF:FFFFFFFFFFFFFFFFFFFFFFFFFF
FFFFF:FFFFFFFFFFFFFFFFFFFFFFFFFFFFFFF:FFFFFFFFFFFFF:FFFFFFFFFFFFFFFFFF
FFFFFFFFFFFFFFF
@A00721:190:HGJW7DSXY:4:1418:13819:21167 1:N:0:TCTGCAAG+GCTGCACT
GGCGTGAACACACCAAGTCTTCGATTATATCTCAGAGACCTCGACCACTGAACCGGCAGCTGTGACCG
CTGAGTCCTGTTGAATTGCAACACTCGTCGTGTTTTACGATAACCGGCGGTCCTAAACGGACCGCCTG
ACCACTTCCTCTTCT
+
FFFFFFFFFFFFFFFFFFFFFFFFFFFFFFFFFFFFFFFFFFFFFFFFFFFFFFFFFFFFFFFFFFFF
```

FFFFFFFFFFFFFFFFFFFFFFFFFFFFFFFFFFFFFFFFFFFFF:FFFFFF,FFFF:FFFFFFFFFF
F:FFFFFFFFFFFFF
@A00721:190:HGJW7DSXY:4:1617:21920:26804 1:N:0:TCTGCAAG+GCTGCACT
GGCGTGAACACACCAAGTCTTCGATTATATCTCAGAGACCTCGACCACTGAACCGGCAGCTGTGACCG
CTGAGTCCTGTTGAATTGCAACACTCGTCGTGTTTTACGATAACCGGCGGTCCTAAACGGACCGCCTG
ACCACTTCCTCTTCT
+
FFFFFFFFF:FFFFFF:FF:FFFFFFFFFFFFFFFFFFFFFFFFFFFFF:FFFF:FFF:FFFFFFFFF
FFFFFFFFF:,FFFFFFFFFFFFFFFFFFF:FF,FFF:FFFFFFF:FFF,FFFFFFFF:FF:FFFF:F
FFF:FFFFFFFFFFF
@A00721:190:HGJW7DSXY:4:1233:6289:28103 1:N:0:TCTGCAAG+GCTGCACT
ACTCCGTCTTGTACTGCATAGGAGGCGTGAACACACCAAGTCTTCGATTATATCTCAGAGACCTCGAC
CACTGAACCGGCAGCTGTGACCGCTGAGTCCTGTTGAATTGCAACACTCGTCGTGTTTTACGATAACC
GGCGGTCCTAAACGG
+
FFFFFFFFFFFFFFFFFFFFFFFFFFFFFFFFFFFFFFFFFFFFFFFFFFFFFFFFFF:FFFFFFFFF
FFFFFFFFFFFFFFFFFFFFFFFFFFFFFFFFFFFFFFFFFFFFFFFFFFFFFFFFFFFFFFFFFFFF
FFFFFFFFFFFFFFF
@A00721:190:HGJW7DSXY:4:1247:31937:35540 1:N:0:TCTGCAAG+GCTGCACT
ACTCCGTCTTGTACTGCATAGGAGGCGTGAACACACCAAGTCTTCGATTATATCTCAGAGACCTCGAC
CACTGAACCGGCAGCTGTGACCGCTGAGTCCTGTTGAATTGCAACACTCGTCGTGTTTTACGATAACC
GGCGGTCCTAAACGG
+
FFFFFFFFFFFFFFFFFFFFFFFFFFFFFFFFFFFFFFFFFFFFFFFFFFFFFFFFFFFFFFFFFFFF
FFFFFFFFFFFFFFFFFFFFFFFFFFFFFFFFFFFFFFFF:FFFFFFFFFFFFFFFFFFFFFFFFFFF
FFFFFFFFFFF,FFF
@A00721:190:HGJW7DSXY:4:1258:31304:27179 1:N:0:TCTGCAAG+GCTGCACT
ACTCCGTCTTGTACTGCATAGGAGGCGTGAACACACCAAGTCTTCGATTATATCTCAGAGACCTCGAC
CACTGAACCGGCAGCTGTGACCGCTGAGTCCTGTTGAATTGCAACACTCGTCGTGTTTTACGATAACC
GGCGGTCCTAAACGG
+
FFFFFFFFFFFFFFFFFFFFFFFFFFFFFFFFFFFFFFFFFFFFFFFFFFFFFFFFFF:FFFFFFFFF
FFFFFFFFFFFFFFFFFFFFFFFFFFFFFFFFFFFFFFFFFFFFFFFFFFFFFFFFFFFFFFFFFFFF
FFFFFFFFFFFFFFF
@A00721:190:HGJW7DSXY:4:1336:31891:11287 1:N:0:TCTGCAAG+GCTGCACT
ACTCCGTCTTGTACTGCATAGGAGGCGTGAACACACCAAGTCTTCGATTATATCTCAGAGACCTCGAC
CACTGAACCGGCAGCTGTGACCGCTGAGTCCTGTTGAATTGCAACACTCGTCGTGTTTTACGATAACC
GGCGGTCCTAAACGG
+
FFFFFFFFFFFFFFFFFFFFFFFFFFFFFFFFFFFFFFFFFFFFFFFFFFFFFFFFFFFFFFFFFFFF
FFFFFFFFFFFFFFFFFFFFFFFFFFFFFFFFFFFFFFFF:FFFFFFFFFFFFFFFFFFFFFFFFFFF
FFFFFFFFFFFFFFF
@A00721:190:HGJW7DSXY:4:1536:31358:2096 1:N:0:TCTGCAAG+GCTGCACT
ACTCCGTCTTGTACTGCATAGGAGGCGTGAACACACCAAGTCTTCGATTATATCTCAGAGACCTCGAC
CACTGAACCGGCAGCTGTGACCGCTGAGTCCTGTTGAATTGCAACACTCGTCGTGTTTTACGATAACC
GGCGGTCCTAAACGG
+
FFFFFFFFFFFFFFFFFFFFFFFFFFFFFFFFFFFFFFFFFFFFFFFFFFFFFFFFFFFFFFFFFFFF
FFFFFFFFFFFFFFFFFFFFFFFFFFFFFFFFFFFFFFFFF:FFFFFFFFFFFFFFFFFF:FFFFFFF
FFFFFFFFFFFFFFF
@A00721:190:HGJW7DSXY:4:1536:31792:2190 1:N:0:TCTGCAAG+GCTGCACT
ACTCCGTCTTGTACTGCATAGGAGGCGTGAACACACCAAGTCTTCGATTATATCTCAGAGACCTCGAC
CACTGAACCGGCAGCTGTGACCGCTGAGTCCTGTTGAATTGCAACACTCGTCGTGTTTTACGATAACC
GGCGGTCCTAAACGG

+
FFFFFFFFFFFFFFFFFFFFFFFFFFFFFFF:FFFFFFFFFFFFFFFFFFFFFFFFFFFFFFFFFFFFFF
FFFFFFFFFFFFFFFFFFFFFFFFFFFFFFFFFFFFFFFFFFFFFFFFFFF:FFFFFFFFFFFFFFFFFF
FFFFFFFFFFFFFFF
@A00721:190:HGJW7DSXY:4:1655:26359:30232 1:N:0:TCTGCAAG+GCTGCACT
ACTCCGTCTTGTACTGCATAGGAGGCGTGAACACACCAAGTCTTCGATTATATCTCAGAGACCTCGAC
CACTGAACCGGCAGCTGTGACCGCTGAGTCCTGTTGAATTGCAACACTCGTCGTGTTTTACGATAACC
GGCGGTCCTAAACGG
+
FFFFFFFFFFFFFFFFFFFFFFFFFFFFFFFFFFFFFFFFFFFFFFFFFFFFFFFFFFFFFFFFFFFFFF
FFFFF:FFFFFFFFFFFFFFFFFFFFFFFFFFFFFFFFFFFFFFFFFFFFFFFFFFFFFFFFFFFFFFFF
FFFFFFFFFFFFFFF
@A00721:190:HGJW7DSXY:4:2160:16396:12915 1:N:0:TCTGCAAG+GCTGCACT
ACTCCGTCTTGTACTGCATAGGAGGCGTGAACACACCAAGTCTTCGATTATATCTCAGAGACCTCGAC
CACTGAACCGGCAGCTGTGACCGCTGAGTCCTGTTGAATTGCAACACTCGTCGTGTTTTACGATAACC
GGCGGTCCTAAACGG
+
FFFFFFFFFFFFFFFFFFFFFFFFFFFFFFFFFFFFFFFFFFFFFFFFFFFFFFFFFFFFFFFFFFFFFF
FFFFFFFFFFFFFFFFFFFFFFFFFFFFFFFFFFFFFFFFFFFFFFFFFFFFFFFFF:FFFFFFFFFFF:
FFFFFFFFFFFFFFF
@A00721:190:HGJW7DSXY:4:2373:25807:4507 1:N:0:TCTGCAAG+GCTGCACT
ACTCCGTCTTGTACTGCATAGGAGGCGTGAACACACCAAGTCTTCGATTATATCTCAGAGACCTCGAC
CACTGAACCGGCAGCTGTGACCGCTGAGTCCTGTTGAATTGCAACACTCGTCGTGTTTTACGATAACC
GGCGGTCCTAAACGG
+
FFF:FFFFFFF:FFFFFFF:FFFFFFFFFFFFFFF,FFF,FF:FFFF,,FF::FFFFFFF:F:FFFF
FFFFFFFFFFFFF:F,FFFFFFFF:FFFFFFFF:F:FFFF,FFFFFFFFFFFF,FFFFFFF:F:FFFF
F:FFFFFFFFFFFF:
@A00721:190:HGJW7DSXY:4:2545:10691:29747 1:N:0:TCTGCAAG+GCTGCACT
ACTCCGTCTTGTACTGCATAGGAGGCGTGAACACACCAAGTCTTCGATTATATCTCAGAGACCTCGAC
CACTGAACCGGCAGCTGTGACCGCTGAGTCCTGTTGAATTGCAACACTCGTCGTGTTTTACGATAACC
GGCGGTCCTAAACGG
+
FFFFFFFFFFFFFFFFFFFFFFFFFFFFFFFFFFFFFFFFFFFFFFFFFFFFFFFFFFFFFFFFFFFFFF
FFFFFFFFFFFFFFFFFFFFFFFFFFFFFFFFFFFFFFFFFFFFFFFFFFFFFFFFFFFFFFFFFFFFF:
FFFFFFFFFFFFFFF
@A00721:190:HGJW7DSXY:4:2666:25970:31657 1:N:0:TCTGCAAG+GCTGCACT
ACTCCGTCTTGTACTGCATAGGAGGCGTGAACACACCAAGTCTTCGATTATATCTCAGAGACCTCGAC
CACTGAACCGGCAGCTGTGACCGCTGAGTCCTGTTGAATTGCAACACTCGTCGTGTTTTACGATAACC
GGCGGTCCTAAACGG
+
FFFFFFFFFFFFFFFFFFFFFFFFFFFFFFFFFFFFFFFFFFFFFFFFFFFFFFFFFFFFFF:FFFFFF
FFFFFFFFFFFFFFFFFFFFFFFFFFFFFFFFFFFFFFFFFFFFFFFFFFFFFFFFFFFFFFFFFFFFFF
FFFFFFFFFFFFFFF
@A00721:190:HGJW7DSXY:4:1241:29143:1047 1:N:0:TCTGCAAG+GCTGCACT
CTGACAAACTCCGTCTTGTACTGCATAGGAGGCGTGAACACACCAAGTCTTCGATTATATCTCAGAGA
CCTCGACCACTGAACCGGCAGCTGTGACCGCTGAGTCCTGTTGAATTGCAACACTCGTCGTGTTTTAC
GATAACCGGCGGTCC
+
FFFFFFFFFFFFFFFFFFFFFFFFFFFFFFFFFFFFFFFFFFFFFFFFFFF:FFFFFFFFFFFFFFFFFF
FFFFFFFFFFFFFFFFFF:FFFFFFFFFFFFFFFFFFFFFFFFFFFF,FFFFFFFFFFFFFFFFFFFFFFFF:
FFFF:FFFFF:FFFF
@A00721:190:HGJW7DSXY:4:1241:29857:2597 1:N:0:TCTGCAAG+GCTGCACT
CTGACAAACTCCGTCTTGTACTGCATAGGAGGCGTGAACACACCAAGTCTTCGATTATATCTCAGAGA

```
CCTCGACCACTGAACCGGCAGCTGTGACCGCTGAGTCCTGTTGAATTGCAACACTCGTCGTGTTTTAC
GATAACCGGCGGTCC
+
FFFFFFFFFFFFFFFFFFFFFFFFFFFFFFFFFFFFFFFFFFFFFFFFFFFFFFFFFFFFFFFFFFFF
FFFFFFFFFFFFFFFFFFFFFFFFFFFFFFFFFFFFFFFFFFFFFFFFFFFFFFFFFFFFFFFFFFFF
FFFFFFFFFFFFFFF
@A00721:190:HGJW7DSXY:4:1264:10700:21684 1:N:0:TCTGCAAG+GCTGCACT
CTGACAAACTCCGTCTTGTACTGCATAGGAGGCGTGAACACACCAAGTCTTCGATTATATCTCAGAGA
CCTCGACCACTGAACCGGCAGCTGTGACCGCTGAGTCCTGTTGAATTGCAACACTCGTCGTGTTTTAC
GATAACCGGCGGTCC
+
FFFFFFFFFFFFFFFFFFFFFFFFFFFFFFFFFFFFFFFFFFFFFFFFFFFFFFF:F:FFFFFFFFFFF
FFFFFFFFFFFFFFFFFFFFFFFFFFFFFFFFFFFFFFFFFFFFFFFFFFFFFFFFFFFFFFFF,FFFF
FFFFFFFFFFFFFFF
@A00721:190:HGJW7DSXY:4:1546:29261:12712 1:N:0:TCTGCAAG+GCTGCACT
CTGACAAACTCCGTCTTGTACTGCATAGGAGGCGTGAACACACCAAGTCTTCGATTATATCTCAGAGA
CCTCGACCACTGAACCGGCAGCTGTGACCGCTGAGTCCTGTTGAATTGCAACACTCGTCGTGTTTTAC
GATAACCGGCGGTCC
+
FFFFFFFFFFF,FFFFFFFFFFF:FFFFFFFFFFFFFFFFFFFFFFF,FFFFFFF:FF:FFF,FFFFF:
FFFFFFFFFFFFFFFF,FFFFF::FFFFFFF::F:FFFFFFF:FF,FFFFFFFFFFFFF,,FFFFFFF
FFFFFFFFFF:F::F
@A00721:190:HGJW7DSXY:4:1634:3604:11960 1:N:0:TCTGCAAG+GCTGCACT
CTGACAAACTCCGTCTTGTACTGCATAGGAGGCGTGAACACACCAAGTCTTCGATTATATCTCAGAGA
CCTCGACCACTGAACCGGCAGCTGTGACCGCTGAGTCCTGTTGAATTGCAACACTCGTCGTGTTTTAC
GATAACCGGCGGTCC
+
FFFFFFFFFFFFFFFFFFFFFFFFFFFFFFFFFFFFFFFFFFFFFFFFFFFFFFFFFFFFFFFFFFFF
FFFFFFFFFFFFFFFFFFFFFFFFFFFFFFFFFFFFFFFFFFFFFFFFFFFFFFFFFFFFFFFFFFFF
FFFFFFFFFFFFFFF
@A00721:190:HGJW7DSXY:4:2329:5728:30890 1:N:0:TCTGCAAG+GCTGCACT
CTGACAAACTCCGTCTTGTACTGCATAGGAGGCGTGAACACACCAAGTCTTCGATTATATCTCAGAGA
CCTCGACCACTGAACCGGCAGCTGTGACCGCTGAGTCCTGTTGAATTGCAACACTCGTCGTGTTTTAC
GATAACCGGCGGTCC
+
FFFFFF:FFFFFFF:FFFFFFFFFFFFFFFFFFFFFFFFFFFFFFFFFFFFFFFF:FFFFFFFFFFFF
FFFFFFFFFFFFFFFFFFFFFFFFFFFFFFFFFFFFFFFF,FFFF:FFFFFFFFFFFFFFFFF:FFFF
FFFFFFFFFFFFFFF
@A00721:190:HGJW7DSXY:4:1129:17056:6073 1:N:0:TCTGCAAG+GCTGCACT
CCACAATATTATTAACTCTGACAAACTCCGTCTTGTACTGCATAGGAGGCGTGAACACACCAAGTCTT
CGATTATATCTCAGAGACCTCGACCACTGAACCGGCAGCTGTGACCGCTGAGTCCTGTTGAAATGCAA
CACTCGTCGTGATTT
+
FFF,:F,:::F,FFFFF:FFFF:FFF,FF:F,FF,:FF,,FFFF:,F,FF::,FFFFFFFFFFFFFFF
FFF,:FFF::FFF,FFFFF,F:FF:F,,FFFFF:FFFFFFF,,F,FF:FFF:,:FF,,FFFF,F,FFF
:FF::::FF,F,,FF
@A00721:190:HGJW7DSXY:4:1175:21278:28855 1:N:0:TCTGCAAG+GCTGCACT
CCACAATATTATTAACTCTGACAAACTCCGTCTTGTACTGCATAGGAGGCGTGAACACACCAAGTCTT
CGATTATATCTCAGAGACCTCGACCACTGAACCGGCAGCTGTGACCGCTGAGTCCTGTTGAATTGCAA
CACTCGTCGTGTTTT
+
FFFFFFFFFFFFFFFFFFFFFFFFFFFFFFFFFFFFFFFFFFFFFFFFFFFFFFFFFFFFFFFFFFFF
FFFFFFFFFFFFFFFFFFFFFFFFFFFFFFFFFFFFFFFFFFFFFFFFFFFFFFFFFFFFFFFFFFFF
FFFFFFFFF:FFFFF
```

@A00721:190:HGJW7DSXY:4:1219:23809:2988 1:N:0:TCTGCAAG+GCTGCACT
CCACAATATTATTAACTCTGACAAACTCCGTCTTGTACTGCATAGGAGGCGTGAACACACCAAGTCTT
CGATTATATCTCAGAGACCTCGACCACTGAACCGGCAGCTGTGACCGCTGAGTCCTGTTGAATTGCAA
CACTCGTCGTGTTTT
+
FFFFFFFFFFFFFFFFFFFFFFFFFFFFFFFFFFFFFFFFFFFFFFFFFFFFFFFFFFFFFFFFFFFFFF
FFFFFFFFFFFFFFFFFFFFFFFFFFFFFFFFFFFFFFFFFFFFFFFFFFFFFFFFFFFFFFFFFFFFFF
FFFFFFFFFFFFFFF
@A00721:190:HGJW7DSXY:4:1219:23475:3161 1:N:0:TCTGCAAG+GCTGCACT
CCACAATATTATTAACTCTGACAAACTCCGTCTTGTACTGCATAGGAGGCGTGAACACACCAAGTCTT
CGATTATATCTCAGAGACCTCGACCACTGAACCGGCAGCTGTGACCGCTGAGTCCTGTTGAATTGCAA
CACTCGTCGTGTTTT
+
FFFFFFFFFFFFFFFFFFFFFFFFFFFFFFFFFFFFFFFFFFFFFFFFFFFFFFFFFFFFFFFFFFFFFF
FFFFFFFFFFFFFFFFFFFFFFFFFFFFFFFFFFFFFFFFFFFFFFFFFFFFFFFFFFFFFFFFFFFFFF
FFFFFFFFFFFFFFF
@A00721:190:HGJW7DSXY:4:1337:23791:1799 1:N:0:TCTGCAAG+GCTGCACT
CCACAATATTATTAACTCTGACAAACTCCGTCTTGTACTGCATAGGAGGCGTGAACACACCAAGTCTT
CGATTATATCTCAGAGACCTCGACCACTGAACCGGCAGCTGTGACCGCTGAGTCCTGTTGAATTGCAA
CACTCGTCGTGTTTT
+
FFFFFFF:FFFFFFFF:FFFF:F::FFFFF:FFFFFFF:FF,:FFFFFFFFFFFFFFFFFFFFFFFFFF:
FF,FFFFFFFFF:FFFFF,FFFFFFFFF:FFF,FFFFF:FFF,F:FFFF:FFFFFFFFFFFFFFFFFFFF
:FFFFFFFFFFFF:F
@A00721:190:HGJW7DSXY:4:1347:11939:8829 1:N:0:TCTGCAAG+GCTGCACT
CCACAATATTATTAACTCTGACAAACTCCGTCTTGTACTGCATAGGAGGCGTGAACACACCAAGTCTT
CGATTATATCTCAGAGACCTCGACCACTGAACCGGCAGCTGTGACCGCTGAGTCCTGTTGAATTGCAA
CACTCGTCGTGTTTT
+
FFFFFFFFFFFFFFFFFFFFFFFFFFFFFFFFFFFFFFFFFFFFFFFFFFFFFFFFFFFFFFFFFFFFFF
FFFFFFFFFFFFFFFFFFFFFFFFFFFFFFFFFFFFFFFFFFFFFFFFFFFFFFFFFFF:FFFFFFFFFF
FFFFFFFFFFFFFFF
@A00721:190:HGJW7DSXY:4:1412:32181:12884 1:N:0:TCTGCAAG+GCTGCACT
CCACAATATTATTAACTCTGACAAACTCCGTCTTGTACTGCATAGGAGGCGTGAACACACCAAGTCTT
CGATTATATCTCAGAGACCTCGACCACTGAACCGGCAGCTGTGACCGCTGAGTCCTGTTGAATTTCAA
CACTCGTCGTGTTTT
+
FF::FF:FFFFFFFFFFF:FF,FFFF:F,FF,F,FFFF:F:,F:F::,F:F:::F,FFF:FF:FFFFF
,FFFFFFFFFFFFFF,F:F:,,F:,FFF,FFF::F:F:F:FFFFFF,F::FF,FFFF:FF::FF,FFF
,FF,F,FF::FF,FF
@A00721:190:HGJW7DSXY:4:2111:12084:16783 1:N:0:TCTGCAAG+GCTGCACT
CCACAATATTATTAACTCTGACAAACTCCGTCTTGTACTGCATAGGAGGCGTGAACACACCAAGTCTT
CGATTATATCTCAGAGACCTCGACCACTGAACCGGCAGCTGTGACCGCTGAGTCCTGTTGAATTGCAA
CACTCGTCGTGTTTT
+
FFFFFFFFFFFFFFFFFFFFFFFFFFFFFFFFFFFFFFFFFFFFFFFFFFFFFFFFFFFFFFFFFFFFFF
FFFFFFFFFFFFFFFFFFFFFFFFFFFFFFFFFFFFFFFFFFFFFFFFFFFFFFFFFFFFFFFFFFFFFF
FFFFFFFFFFFFFFF
@A00721:190:HGJW7DSXY:4:2222:22806:18380 1:N:0:TCTGCAAG+GCTGCACT
CCACAATATTATTAACTCTGACAAACTCCGTCTTGTACTGCATAGGAGGCGTGAACACACCAAGTCTT
CGATTATATCTCAGAGACCTCGACCACTGAACCGGCAGCTGTGACCGCTGAGTCCTGTTGAATTGCAA
CACTCGTCGTGTTTT
+
FFFFFFFFFFFFFFFFFFFFFFFFFFFFFFFFFFFFFFFFFFFFFFFFFFFFFFFFFFFFFFFFFFFFFF

```
FFFFFFFFFFFFFFFFFFFFFFFFFFFFFFFFFFFFFFFFFFFFFFFFFFFFFFFFFFFFFFFFFFFFFF
FFF::FFFFFFFFFF
@A00721:190:HGJW7DSXY:4:2370:18412:3662 1:N:0:TCTGCAAG+GCTGCACT
CCACAATATTATTAACTCTGACAAACTCCGTCTTGTACTGCATAGGAGGCGTGAACACACCAAGTCTT
CGATTATATCTCAGAGACCTCGACCACTGAACCGGCAGCTGTGACCGCTGAGTCCTGTTGAATTGCAA
CACTCGTCGTGTTTT
+
FFFFFFFFFFFFFFFFFFFFFFFFFFFFFFFFFFFFFFFFF:FFFFFFFFFFFFFFFFFFFFFFFFFFFF
FFFFFFFFFFFFFFFFFFFFFFFFFFFFFFFFFFFFFFFFFFFFFFFFFFFFFFFFFFFFFFFFFFFFFF
FFFFFFFFFFFFFFF
@A00721:190:HGJW7DSXY:4:1102:5909:18771 1:N:0:TCTGCAAG+GCTGCACT
GCCACAATATTATTAACTCTGACAAACTCCGTCTTGTACTGCATAGGAGGTGTGAACACACCAAGTCT
TCGATTATATCTCAGAGACCTCGACCACTGAACCGGCAGCTGTGACCGCTGAGTCCTGTTGAATTGCA
ACACTCGTCGTGTTT
+
FFFFFFFFFFFFFFFFFFFFFFFFFFFFFFFFFFFFFFFFFFFFFFFFFFFFFFFFFFFFFFFFFFFFFF
FFFFFFFFFFFFFFFFFFFFFFFFFFFFFFFFFFFFFFFFFFFFFFFFFFFFFFFFFFFFFFFFFFFFFF
FFFFFFFFFFFFF:F
@A00721:190:HGJW7DSXY:4:1151:12255:8061 1:N:0:TCTGCAAG+GCTGCACT
GCCACAATATTATTAACTCTGACAAACTCCGTCTTGTACTGCATAGGAGGTGTGAACACACCAAGTCT
TCGATTATATCTCAGAGACCTCGACCACTGAACCGGCAGCTGTGACCGCTGAGTCCTGTTGAATTGCA
ACACTCGTCGTGTTT
+
FFFFFFFFFFFFFFFFFFFFFFFFFFFFFFFFFFFFFFFFFFFFFFFFFFFFFFFFFFFFFFFFFFFFFF
FFFFFFFFFFFFFFFFFFFFFFFFFFFFFFFFFFFFFFFFFFFFFFFFFFFFFFFFFFFFFFFFFFFFFF
FFFFFFFFFFFFFFF
@A00721:190:HGJW7DSXY:4:1232:16306:25347 1:N:0:TCTGCAAG+GCTGCACT
GCCACAATATTATTAACTCTGACAAACTCCGTCTTGTACTGCATAGGAGGTGTGAACACACCAAGTCT
TCGATTATATCTCAGAGACCTCGACCACTGAACCGGCAGCTGTGACCGCTGAGTCCTGTTGAATTGCA
ACACTCGTCGTGTTT
+
FFFFFFFFFFFFFFFFFFFFFFFFFFFFFFFFFFFFFFFFFFFFFFFFFFFFFFFFFFFFFFFFFFFFFF
FFFFFFFFFFFFFFFFFFFFFFFFFFFFFFFFFFFFFFFFFFFFFFFFFFFFFFFFFFFFFFFFFFFFFF
FFFFFFFFFFFFFFF
@A00721:190:HGJW7DSXY:4:2158:4499:12571 1:N:0:TCTGCAAG+GCTGCACT
CCACAAAATTATTAACTCTGACAAACTCAGTCTTGTACTGCATAGGAGGCGTGAACACACCAAGTCTT
CTATTATATCTCAGAGACCTCGACCACTGAACCGGCAGCTGTGACCGCTGAGTCCTGTAGAATTGCAA
CACTCGTCGTGTTT
+
FFF,FF,F,,FFFFFFFFFFFFFFF,F,,:F:F::FFFF::FFF,:F,:FF,FFF,F:FF:FF,,:FF
,,FFFFFFFFFFF:F,FFF::,F:,F,,:FFF:,,FF:F:,FFFF:FF,FFF::FF:F,FFF,:F,FF
FF:FF,,::,,::F
@A00721:190:HGJW7DSXY:4:2223:3911:5071 1:N:0:TCTGCAAG+GCTGCACT
GCCACAATATTATTAACTCTGACAAACTCCGTCTTGTACTGCATAGGAGGTGTGAACACACCAAGTCT
TCGATTATATCTCAGAGACCTCGACCACTGAACCGGCAGCTGTGACCGCTGAGTCCTGTTGAATTGCA
ACACTCGTCGTGTTT
+
FFFFFFFFF:FFFFFFFFFFFFFFFFFFFFFFFFFFFFFF:FFFFFFFFFFFFFFFFFFFFF:FFFFFF
FFFFFFFF:FFFFFFFFFF:FFFFFFFFFFFFFFFFFFFFF:FFFFFFFF:FFFFFFFFFFFFFFF:F
:FFFF::FFF:F:FF
@A00721:190:HGJW7DSXY:4:2359:16984:29653 1:N:0:TCTGCAAG+GCTGCACT
GCCACAATATTATTAACTCTGACAAACTCCGTCTTGTACTGCATAGGAGGTGTGAACACACCAAGTCT
TCGATTATATCTCAGAGACCTCGACCACTGAACCGGCAGCTGTGACCGCTGAGTCCTGTTGAATTGCA
ACACTCGTCGTGTTT
```

```
+
FFFFFFFFFFFFFFFFFFFFFFFFFFFFFFFFFFFF:FFFFFFFFFFFFFFFFFFFFFFFFFFFFFFFFF
FFFFFFFFFFFFFFFFFFFFFFFFFFFFFFFFFFFFFFFFFFFFFFFFFFFFFFFFFFFFFFFFFFFF:F
FF:FFFFFFFFFFF:
@A00721:190:HGJW7DSXY:4:2628:4038:10833 1:N:0:TCTGCAAG+GCTGCACT
GCCACAATATTATTAACTCTGACAAACTCCGTCTTGTACTGCATAGGAGGTGTGAACACACCAAGTCT
TCGATTATATCTCAGAGACCTCGACCACTGAACCGGCAGCTGTGACCGCTGAGTCCTGTTGAATTGCA
ACACTCGTCGTGTTT
+
FFFFFF:FFFFFFFFFFFFFFFFFF:FFFFFFFFFFFFFFFFFFFFFFFFFFFFFFFFFFFFFFFFFFFF
FFFF:FFFFFFFFFFFFFFFFFFFFFFFFFFFFFFFFF:FFFFFFFFFFFFFFFFFFFFFF,FF:FFFFF
FFFFFF:FFFFFFFF
@A00721:190:HGJW7DSXY:4:1435:17445:29418 1:N:0:TCTGCAAG+GCTGCACT
CCACAATATTATTAACTCTGACAAACTCCGTCTTGTACTGCATAGGAGGTGTGAACACACCAAGTCTT
CGATTATATCTCAGAGACCTCGACCACTGAACCGGCAGCTGTGACCGCTGAGTCCTGTTGAATTGCAA
CACTCGTCGTGTT
+
F:FFFFFFF:FF,FFFFFFFF:F:FF:::,F:FF:FFFF::FFFF,F:,:,FFFF:FFFFFFFF:FFF
F,F:FFFFF:F:FFFFFFF::FFFFF:FFFFF,F:FFF:,FFFFFFFF,FFF::FF:F,FFFFFF:FF
FFFF:::FFF,FF
@A00721:190:HGJW7DSXY:4:2155:17038:31250 1:N:0:TCTGCAAG+GCTGCACT
ACACGACGAGTGTTGCAATTCAACAGGACTCAGCGGTCACAGCTGCCGGTTCAGTGGTCGAGGTCTCT
GAGATATAATCGAAGACTTGGTGTGTTCACACCTCCTATGCAGTACAAGACGGAGTTTGTCAGAGTTA
ATAATAGTGTGGACG
+
FFFFFFFFFFFFFFFFFFFFFFFFFFFFFFFFFFFFFFFFFFFF,FFFFFFFFFFFFFFFFFFFFFFFF
FFFFFFFFFFFFFFFFFFFFFFFFFFFFFFFFFFFFFFFFFFFFFFFFFFFFFFFFFFFFFFFF:FFFF
F,FFFF,FFFFFFFF
@A00721:190:HGJW7DSXY:4:2352:18792:31814 1:N:0:TCTGCAAG+GCTGCACT
ACACGACGAGTGTTGCAATTCAACAGGACTCAGCGGTCACAGCTGCCGGTTCAGTGGTCGAGGTCTCT
GAGATATAATCGAAGACTTGGTGTGTTCACACCTCCTATGCAGTACAAGACGGAGTTTGTCAGAGTTA
ATAATATTGTGGACG
+
FFFFFFFFFFFFFFFFFFFFFFFFFFFFFFFFFFFFFFFFFFFFFFFFFFFFFFFFFFFFFFFFFFFF
FFFFFFFFFFFFFFFFFFFFFFFFFFFFFFFFFFFFFFFFFFFFFFFFFFFFFFFFFFFFFFFFFFFF
FFFFFFFFFFFFFFF
@A00721:190:HGJW7DSXY:4:2433:9824:11146 1:N:0:TCTGCAAG+GCTGCACT
ACACGACGAGTGTTGCAATTCAACAGGACTCAGCGGTCACAGCTGCCGGTTCAGTGGTCGAGGTCTCT
GAGATATAATCGAAGACTTGGTGTGTTCACACCTCCTATGCAGTACAAGACGGAGTTTGTCAGAGTTA
ATAATATTGTGGACG
+
FFFFFFFFFFFFFFFFFFFFFFFFFFFFFFFFFFFFFFFFFFFFFFFFFFFFFFFFFFFFFFFFFFFF
FFFFFFF:FFFFFFFFFFFFFFFFFFFFFFFFFFFFFFFFFFFFFFFFFFFFFFFFFFFF:FFFFF:
FFFFF:FFFFFFFFF
@A00721:190:HGJW7DSXY:4:2622:9643:14278 1:N:0:TCTGCAAG+GCTGCACT
ACACGACGAGTGTTGCAATTCAACAGGACTCAGCGGTCACAGCTGCCGGTTCAGTGGTCGAGGTCTCT
GAGATATAATCGAAGACTTGGTGTGTTCACACCTCCTATGCAGTACAAGACGGAGTTTGTCAGAGTTA
ATAATATTGTGGACG
+
FFFFFFFFFFFF:FFFFFFFFFFFFFFFFFFFFFFFFFFFFFFFFFFFFFFFF:FFFFFFFFFFFFFF:
FFFF:FFFFFFFFFFFFFFFFFFFFFFFF:FFFFFFFFFFFFFFFFFFFFFFFFFFFFFFFFFFFFFFF
FFF:FFFFFFFFFFF
@A00721:190:HGJW7DSXY:4:1167:28501:19852 1:N:0:TCTGCAAG+GCTGCACT
CGACGAGTGTTGCAATTCAACAGGACTCAGCGGTCACAGCTGCCGGTTCAGTGGTCGAGGTCTCTGAG
```

ATATAATCGAAGACTTGGTGTGTTCACACCTCCTATGCAGTACAAGACGGAGTTTGTCAGAGTTAATA
ATATTGTGGACGTTG
+
FFFFFFFFFFFFFFFFFFFFFFFFFFFFFFFFFFF:FFFFFFFFFFFFFFFFFFFFFFFFFFFFFFFFF
FFFFFFFFFFFF:FFFFFFFFFFFFFFFFFFFF:FFFFFFFFFFFFFFFFFFFFFFFFFFFFFFFF::F
FFFFFFFFFFFFFFF
@A00721:190:HGJW7DSXY:4:1256:1895:6183 1:N:0:TCTGCAAG+GCTGCACT
CGACGAGTGTTGCAATTCAACAGGACTCAGCGGTCACAGCTGCCGGTTCAGTGGTCGAGGTCTCTGAG
ATATAATCGAAGACTTGGTGTGTTCACACCTCCTATGCAGTACAAGACGGAGTTTGTCAGAGTTAATA
ATATTGTGGACGTTG
+
FFFFFFFFFF:FFFFFFFFFFFFFFFFFFFFFFFFFFFFFFFFFFFFFFFFFFFFFFFFFFFFFFFFFF
FFFFFFFFFFFFFFFFFFFFFFFFFFFFFFFFFFFFFFFFFFFFFFFFFFFFFFFFFFFFFFFFF:FFF
F,FFFFFFFFFFFFF
@A00721:190:HGJW7DSXY:4:1268:4869:5635 1:N:0:TCTGCAAG+GCTGCACT
CGACGAGTGTTGCAATTCAACAGGACTCAGCGGTCACAGCTGCCGGTTCAGTGGTCGAGGTCTCTGAG
ATATAATCGAAGACTTGGTGTGTTCACACCTCCTATGCAGTACAAGACGGAGTTTGTCAGAGTTAATA
ATATTGTGGACGTTG
+
FFFFFFFFFFFFFFFFFFFFFFFFFFFFFFFFFFF,F:FFFFFFFFFFFFFFFFFFFFFFFFFFF:FFFF
FFFFFFFFFFFFFFFFFFFFFFFFFFFFFFFFFFFFFFFFFFFFFFFFFFFFFFFFFFFFFFFFFFFFF
:FF,FFFFFFFFFFF
@A00721:190:HGJW7DSXY:4:1317:10429:32142 1:N:0:TCTGCAAG+GCTGCACT
CGACGAGTGTTGCAATTCAACAGGACTCAGCGGTCACAGCTGCCGGTTCAGTGGTCGAGGTCTCTGAG
ATATAATCGAAGACTTGGTGTGTTCACACCTCCTATGCAGTACAAGACGGAGTTTGTCAGAGTTAATA
ATATTGTGGACGTTG
+
FFFFFFFFFFFFFFFFFFFFFFFFFFFFFFFFFFFFFFFFFFFFFFFFFFFFFFFFFFFFFFFF::FFF
FFFFFFFFFFFF:FFFFFFFFFF,FFFFFFFFFFFFFFFFFFFFFFFFFFFFFFFFFFFFFFFFFFF:F
F,F:FFFFFFFFFFF
@A00721:190:HGJW7DSXY:4:1672:27371:21339 1:N:0:TCTGCAAG+GCTGCACT
CGACGAGTGTTGCAATTCAACAGGACTCAGCGGTCACAGCTGCCGGTTCAGTGGTCGAGGTCTCTGAG
ATATAATCGAAGACTTGGTGTGTTCACACCTCCTATGCAGTACAAGACGGAGTTTGTCAGAGTTAATA
ATATTGTGGACGTTG
+
FFFFFFFFFFFFFFFFFFFFFFFFFFFFFFFFFFFFFFFFFFFFFFFFFFFF:FFFFFFFFFFFFFFFF
FFFFFFFFFFFFFFFFFFFFFFFFFFFFFFFFFFFFFFFFFFFFFFFFFFFFFFFFFFFFFFFFFFFFF
FFFFFFFFFFFFFFF
@A00721:190:HGJW7DSXY:4:1117:10357:30295 1:N:0:TCTGCAAG+GCTGCACT
GCGAGTGTTGCAATTCAACAGGACTCAGCGGTCACAGCTGCCGGTTCAGTGGTCGAGGTCTCTGAGAT
ATAATCGAAGACTTGGTGTGTTCACACCTCCTATGCAGTACAAGACGGAGTTTGTCAGAGTTAATAAT
ATTGTGGACGTTGGC
+
FFFFFFFFFFFFFFFFFFFFFFFFFFFFFFFFFFFFFFFFFFFFFFFFFFFFFFFFFFFFFFFFFFFFF
FFFFFFFFFFFFFFFFFFFFFFFFFFFFFFFFFFFFFFFFFFFFFFFFFFFFFFFFFFFFFFFFFF:F,
FFFFFFFFFFFFFFF
@A00721:190:HGJW7DSXY:4:1169:24885:34507 1:N:0:TCTGCAAG+GCTGCACT
GCGAGTGTTGCAATTCAACAGGACTCAGCGGTCACAGCTGCCGGTTCAGTGGTCGAGGTCTCTGAGAT
ATAATCGAAGACTTGGTGTGTTCACACCTCCTATGCAGTACAAGACGGAGTTTGTCAGAGTTAATAAT
ATTGTGGACGTTGGC
+
FFFFFFFFFFFFFFFFFFFFFFFFFFFFFFFFFFFFFFFFFFFFFFFFFFFFFFFFFFFFFFFFFFFFF
FFFFFFFFFFFFFFFFFFFFFFFFFFFFFFFFFFFFFFFFFFFFFFFFFFFFFFFFFFFFFFFFFFFFF
:FFFFFFFFFFFFFF

@A00721:190:HGJW7DSXY:4:1442:28980:18239 1:N:0:TCTGCAAG+GCTGCACT
GCGAGTGTTGCAATTCAACAGGACTCAGCGGTCACAGCTGCCGGTTCAGTGGTCGAGGTCTCTGAGAT
ATAATCGAAGACTTGGTGTGTTCACACCTCCTATGCAGTACAAGACGGAGTTTGTCAGAGTTAATAAT
ATTGTGGACGTTGGC
+
FFFFFFFFFFFFFFFFFFFFFFFFFFFFFFFFFFFFFFFFFFFFFFFFFFFFFFFFFFFFFFFFFFFF
FFFFFFFFFFFFFFFFFFFFFFFFFFFFFFFFFFFFFFFFFFFFFFFFFFFFFFFFFFFFFFFFFFFF
FFFFFFFFFFFFFFF
@A00721:190:HGJW7DSXY:4:1620:26386:6480 1:N:0:TCTGCAAG+GCTGCACT
GCGAGTGTTGCAATTCAACAGGACTCAGCGGTCACAGCTGCCGGTTCAGTGGTCGAGGTCTCTGAGAT
ATAATCGAAGACTTGGTGTGTTCACACCTCCTATGCAGTACAAGACGGAGTTTGTCAGAGTTAATAAT
ATTGTGGACGTTGGC
+
FFFFFFFFFFFFFFFFFFFFFFFFFFFFFFFFFFFFFFFFFFFFFFF:F:FFFFFFFFF:FFFFFFFF
FFFFFFFFFFFFFFFFFFFFFFFFFF:FFFFFFFFFFFFFFFFFFFFFFFFFFFFF:FFFFFFF:FFF
FFFF:FFFFFFFFF:
@A00721:190:HGJW7DSXY:4:2642:5484:14278 1:N:0:TCTGCAAG+GCTGCACT
GCGAGTGTTGCAATTCAACAGGACTCAGCGGTCACAGCTGCCGGTTCAGTGGTCGAGGTCTCTGAGAT
ATAATCGAAGACTTGGTGTGTTCACACCTCCTATGCAGTACAAGACGGAGTTTGTCAGAGTTAATAAT
ATTGTGGACGTTGGC
+
FFFFFFFFFFFFFFFFFFFFFFFFFFFFFFFFF:FFFFFFFFFFFFFFFFFFFFFFFFFFFFFFFFFF
FFFFFFFFFFFFFFFFFFFFFFFFFFFFFFFFFFFFFFFFFFFFFFFFFFFFFFFFFFFFFFFF:FFF
FFFFFF:FFFFFFFF
@A00721:190:HGJW7DSXY:4:2677:3992:36589 1:N:0:TCTGCAAG+GCTGCACT
GCGAGTGTTGCAATTCAACAGGACTCAGCGGTCACAGCTGCCGGTTCAGTGGTCGAGGTCTCTGAGAT
ATAATCGAAGACTTGGTGTGTTCACACCTCCTATGCAGTACAAGACGGAGTTTGTCAGAGTTAATAAT
ATTGTGGACGTTGGC
+
FFFFFFF:FFFFFFFFFFFFFFFFFFFFFFFFFFFFFFFFFFFFFFFFFFFFFFFFFFFFFFFFFFFF
FFFFFFFFFFFFFFFFFFFFFFFFFFFFFFFFFFFFFFFFFFFFFFFFFFFFFFFFFFFF,F:FFFFF
FFFFFFFFFFFFFFF
@A00721:190:HGJW7DSXY:4:1344:22679:12524 1:N:0:TCTGCAAG+GCTGCACT
GGCCAACGTCCACAATATTATTAACTCTGACAAACTCCGTCTTGTACTGCATAGGAGGCGTGAACACA
CCAAGTCTTCGATTATATCTCAGAGACCTCGACCACTGAACCGGCAGCTGTGACCGCTGAGTCCTGTT
GAATTGCAACACTCG
+
FFFFFFFFFFFFFFF:FFFFFFFFFFFFFFFF:FFFFFFFFFFFFFFFFFFFFFFFFFFFFFFFFFFF
FFFFFFFFFF:FFFFFFFFFFFFFFFFFFFFFFFFFFFFFFFFFFFFFFFFFFFFFFFFFFFFFFFFF
FFFFFFFFFFFFFFF
@A00721:190:HGJW7DSXY:4:1411:18602:30201 1:N:0:TCTGCAAG+GCTGCACT
GGCCAACGTCCACAATATTATTAACTCTGACAAACTCCGTCTTGTACTGCATAGGAGGCGTGAACACA
CCAAGTCTTCGATTATATCTCAGAGACCTCGACCACTGAACCGGCAGCTGTGACCGCTGAGTCCTGTT
GAATTGCAACACTCG
+
FFFFFFFFFFFFFFFFFFFFFFFFFFFFFFFFFFFFFFFFFFFFFFFFFFFFFFFFFFFFFFFFFFFF
FFFFFFFFFFFFFFFFFFFFFFFFFFFFFFFFFFFFFFFFFFFFFFF:FFFFFFFFFFFFFFFFFFFF
FFFFFFFFFFFFFFF
@A00721:190:HGJW7DSXY:4:2514:9435:22091 1:N:0:TCTGCAAG+GCTGCACT
GGCCAACGTCCACAATATTATTAACTCTGACAAACTCCGTCTTGTACTGCATAGGAGGCGTGAACACA
CCAAGTCTTCGATTATATCTCAGAGACCTCGACCACTGAACCGGCAGCTGTGACCGCTGAGTCCTGTT
GAATTGCAACACTCG
+
FFFFFFFFFFFFFFFFFFFFFFFFFFFFFFFFFFFFFFFFFFFFFFFFFFFFFFFFFFFFFFFFFFFF

```
FFFFFFFFFFFFFFFFFFFFFFFFFFFFFFFFFFFFFFFFFFFFFFFFFFFFFFFFFFFFFFFFFFFFFF
F:FFFFFFFFFFFF:
@A00721:190:HGJW7DSXY:4:1126:32235:25347 1:N:0:TCTGCAAG+GCTGCACT
TGCAATTCAACAGGACTCAGCGGTCACAGCTGCCGGTTCAGTGGTCGAGGTCTCTGAGATATAATCGA
AGACTTGGTGTGTTCACACCTCCTATGCAGTACAAGACGGAGTTTGTCAGAGTTAATAATATTGTGGA
CGTTGGCAG
+
FFFFFFFFFFFFFFFFFFFFFFFFF:FFFF:FFFFFFFFFFFFFFFFFFFFFFFF:FFFFFFFFFFFFF
FFFFFFFFFFFFFFFFFFFFFFFFFFFFFFFFFFFFFFFFFFFFFFFFFFF:FFFF:FFF,FFFFFF
FFFFFFFFF
@A00721:190:HGJW7DSXY:4:1211:32642:23860 1:N:0:TCTGCAAG+GCTGCACT
TGCAATTCAACAGGACTCAGCGGTCACAGCTGCCGGTTCAGTGGTCGAGGTCTCTGAGATATAATCGA
AGACTTGGTGTGTTCACACCTCCTATGCAGTACAAGACGGAGTTTGTCAGAGTTAATAATATTGTGGA
CGTTGGCAG
+
FFFFFFFFFFFFFFFFFFFFFFFFFFFFFFFFFFFFFFFFFFFFFFFFFFFFFFFFFFFFFFFFFFFFFF
FFFFFFFFFFFFFFFFFFFFFFFFFFFFFFFFFFFFFFFFFFFFFFFFFFFFFFFFFFFFFFFFFFFFFF
FFFFFFFFF
@A00721:190:HGJW7DSXY:4:1228:20066:7122 1:N:0:TCTGCAAG+GCTGCACT
TGCAATTCAACAGGACTCAGCGGTCACAGCTGCCGGTTCAGTGGTCGAGGTCTCTGAGATATAATCGA
AGACTTGGTGTGTTCACATCTCCTATGCAGTACAAGACGGAGTTTGTCAGAGTTAATAATATTGTGGA
CGTTGGAGATCGGAA
+
FFFFFFFFFFFFFFFFFFFFFFFFFFFFFFFFFFFFFFFFFFFFFFFFFFFFFFFFFFFFFFFFFFFFFF
FFFFFFFFFFFFFFFFFFFFFFFFFFFFFFFFFFFFFFFFFFFFFFFFFFFFFFFFFFFFFFFFFFFFFF
FFF,FFFFFFFFFFF
@A00721:190:HGJW7DSXY:4:1228:20410:7247 1:N:0:TCTGCAAG+GCTGCACT
TGCAATTCAACAGGACTCAGCGGTCACAGCTGCCGGTTCAGTGGTCGAGGTCTCTGAGATATAATCGA
AGACTTGGTGTGTTCACATCTCCTATGCAGTACAAGACGGAGTTTGTCAGAGTTAATAATATTGTGGA
CGGTGGAGATCGGAA
+
FFFFFFFFFFFFFFFFFFFFFFFFFFFFFFFFFFFFFFFFFFFFFFFFFFFFFFFFFFFFFFFFFFFFFF
FFFFFFFFFFFFFFFFFFFFFFFFFFFFFFFFFFFFFFFFFFFFFFFFFFFFFF,FFFFFFFFFFFFF
FF,FFFFFFFFFFFF
@A00721:190:HGJW7DSXY:4:1304:9525:26788 1:N:0:TCTGCAAG+GCTGCACT
TGCAATTCAACAGGACTCAGCGGTCACAGCTGCCGGTTCAGTGGTCGAGGTCTCTGAGATATAATCGA
AGACTTGGTGTGTTCACATCTCCTATGCAGTACAAGACGGAGTTTGTCAGAGTTAATAATATTGTGGA
CGTTGGAGATCGGAA
+
FFFFFFFFFFFFFFFFFFFFFFFFFFFFFFF:FFFFFFFFFFFFFFFFFFFFFFFFFFFFFFFFFFFFF:
FFFFFFFFFFFFFFFFFFFFFFFFFFFFFFFFFFFFFFFFFFFFFFFFFFFFFFFFFFFFFFFFFFFFFF
FFF,FFFFFFFFFF:
@A00721:190:HGJW7DSXY:4:1339:6488:2174 1:N:0:TCTGCAAG+GCTGCACT
TGCAATTCAACAGGACTCAGCGGTCACAGCTGCCGGTTCAGTGGTCGAGGTCTCTGAGATATAATCGA
AGACTTGGTGTGTTCACACCTCCTATGCAGTACAAGACGGAGTTTGTCAGAGTTAATAATATTGTGGA
CGTTGGCAG
+
FFFFFFFFFFFFFFFFFFFFFFFFFFFFFFF,FFFFFFFF:FFFFFFFFFFFFFF:FFFFFFFFFFFFF
FF:FFFFFFFFFFFFFFFFFFFFFFFFFFFFFFFFFFFFFFFFFFFFFFFFFFFF,FFFFFFFFFFFF
FFFFFFFFF
@A00721:190:HGJW7DSXY:4:1375:5195:9236 1:N:0:TCTGCAAG+GCTGCACT
TGCAATTCAACAGGACTCAGCGGTCACAGCTGCCGGTTCAGTGGTCGAGGTCTCTGAGATATAATCGA
AGACTTGGTGTGTTCACATCTCCTATGCAGTACAAGACGGAGTTTGTCAGAGTTAATAATATTGTGGA
CGTTGGAGATCGGAA
```

+
FFFFFFFFFFFFFFFFFFFFFFFFFFFFFFFFFFFFFFFFFFFFFFFFFFFFFFFFFFFFFFFFFFFF
FFFFFFFFFFFFFFFFFFFFFFFFFFFFFFFFFFFFFFFFFFFFFFFFFFFFFFFFFFFFFFFFFFFF
FF::FFFFFFFFFFF
@A00721:190:HGJW7DSXY:4:1441:28754:18693 1:N:0:TCTGCAAG+GCTGCACT
TGCAATTCAACAGGACTCAGCGGTCACAGCTGCCGGTTCAGTGGTCGAGGTCTCTGAGATATAATCGA
AGACTTGGTGTGTTCACATCTCCTATGCAGTACAAGACGGAGTTTGTCAGAGTTAATAATATTGTGGA
CGTTGGAGATCGGAA
+
FFFFFFFFFFFFFFFFFFFFFFFFFFFFFFFFFFFFFFFFFFFFFFFFFFFFFFFFFFFFFFFFFFFF
FFFFFFFFFFFFFFFFFFFFFFFFFFFFFFFFFFFFFFFFFFFFFFFFFFFFFFFFFFFFFFFFFFFF
FF:FFFFFFFFFFFF
@A00721:190:HGJW7DSXY:4:1464:18014:19914 1:N:0:TCTGCAAG+GCTGCACT
TGCAATTCAACAGGACTCAGCGGTCACAGCTGCCGGTTCAGTGGTCGAGGTCTCTGAGATATAATCGA
AGACTTGGTGTGTTCACACCTCCTATGCAGTACAAGACGGAGTTTGTCAGAGTTAATAATATTGTGGA
CGTTGGCAG
+
FFFFFFFFFFFFFFFFFFFFFFFFF:FFFF,FFFFFFFFFFFFFFFFFFFFFFFFFFFF:FFFFFFFF
FFFFFFFFFFFFFFFFFFFFFFFFFFFFFFFFFFFFFFFFFFFFFFFFFF:FFFFFFFFFFFFFFFFF
FFFFFFFFF
@A00721:190:HGJW7DSXY:4:1633:14154:7999 1:N:0:TCTGCAAG+GCTGCACT
TGCAATTCAACAGGACTCAGCGGTCACAGCTGCCGGTTCAGTGGTCGAGGTCTCTGAGATATAATCGA
AGACTTGGTGTGTTCACATCTCCTATGCAGTACAAGACGGAGTTTGTCAGAGTTAATAATATTGTGGA
CGTTGGAGATCGGAA
+
FFFFFFFFFFFFFFFFFFF:FFFFFFFFFFFFFFFFFFFFFFFFFFF,FFFFFFFFFFFFFFFFFFFF
FFFFFFFFFF:F:FFFFF::FFFFFFFFFFFFFFFFF:FFFFFF:FFFFFFFFFFFFFF,FFFFFFFF
FFF:FFF:FFF,FFF
@A00721:190:HGJW7DSXY:4:2202:14615:10207 1:N:0:TCTGCAAG+GCTGCACT
TGCAATTCAACAGGACTCAGCGGTCACAGCTGCCGGTTCAGTGGTCGAGGTCTCTGAGATATAATCGA
AGACTTGGTGTGTTCACACCTCCTATGCAGTACAAGACGGAGTTTGTCAGAGTTAATAATATTGTGGA
CGTTGGCAG
+
FF,FF:FFFFFFF:F:FFFFFFFFF,F,FFF,F:FFFFFF,FF:FF:FFF,FF,:FFFFFFFFFFFF:
FFFFFF:FF,FF:FFF,F:,FFF:FF:,FFFFFFFFFFF:FFFFFFFF:FFFFFFFFFFFFF:,FFFF
F,:F,FFFF
@A00721:190:HGJW7DSXY:4:2268:19063:26960 1:N:0:TCTGCAAG+GCTGCACT
TGCAATTCAACAGGACTCAGCGGTCACAGCTGCCGGTTCAGTGGTCGAGGTCTCTGAGATATAATCGA
AGACTTGGTGTGTTCACACCTCCTATGCAGTACAAGACGGAGTTTGTCAGAGTTAATAATATTGTGGA
CGTTGGCAG
+
FFFFFFFFFFFFFFFFFFFFFFFFFFFFFF,FFFFFFFFFFFFFFFFFFFFFFFFFFFFFFFFFFFFF
FFFFFFFFFFFFFFFFFFFF:FFFFFFFFFFFFFFFFFFFFFFFFFFF,FFFFFFFFFFFFFFFFFFF
FFFFFFFFF
@A00721:190:HGJW7DSXY:4:2345:30436:26303 1:N:0:TCTGCAAG+GCTGCACT
TGCAATTCAACAGGACTCAGCGGTCACAGCTGCCGGTTCAGTGGTCGAGGTCTCTGAGATATAATCGA
AGACTTGGTGTGTTCACATCTCCTATGCAGTACAAGACGGAGTTTGTCAGAGTTAATAATATTGTGGA
CGTTGGAGATCGGAA
+
FFFFFFFFFFFFFFFFFFFFFFFFFFFFFFFFFFFFFFFFFFFFFFFFFFFFFFFFFFFF:FFFFFFF
FFFFFFFFFFFFFFFFF:FFFFFFFFFFFFFFFFFFFFFFFFFFFFFFFFFFFFF:FF:FFFFFFFFF
FFF:FFFFFFFFFFF
@A00721:190:HGJW7DSXY:4:2508:1416:9987 1:N:0:TCTGCAAG+GCTGCACT
TGCAATTCAACAGGACTCAGCGGTCACAGCTGCCGGTTCAGTGGTCGAGGTCTCTGAGATATAATCGA

```
AGACTTGGTGTGTTCACATCTCCTATGCAGTACAAGACGGAGTTTGTCAGAGTTAATAATATTGTGGA
CGTTGGAGATCGGAA
+
FFFFFFFFFFFFFFFFFFFFFFFFFFFFFFFFFFFFFFFFFFFFFFFFFFFFFFFFFFFFFFFFFFFF
FFFFFFFFFFFFFFFFFFFFFFFFF:FFFF:FFFFFFF:FFFFFFFFFFFFFFFF:FFFFFFFFFFFF
FF::F:FFFFFFFFF
@A00721:190:HGJW7DSXY:4:2104:3821:14184 1:N:0:TCTGCAAG+GCTGCACT
GCAATTCAACAGGACTCAGCGGTCACAGCTGCCGGTTCAGTGGTCGAGGTCTCTGAGATATAATCGAA
GACTTGGTGTGTTCACACCTCCTATGCAGTACAAGACGGAGTTTGTCAGAGTTAATAATATTGTGGAC
GTTGGCAGTATCGTT
+
FFFFFFFFFFFFFFFFFFFFFFFF,FFFFFFFFFFFFFFFFFFFFFFFFFFFFFFFFFFFFFFFF,FF
FFFFFFFFFFFFFFFFFFFFFFFFFFFFFFFFFFFFFFFFFFFFFFFFFFFFFFFFFF:FFFFFFFFF
FFFFFFFFFFFFFFF
@A00721:190:HGJW7DSXY:4:2109:25979:23187 1:N:0:TCTGCAAG+GCTGCACT
GCAATTCAACAGGACTCAGCGGTCACAGCTGCCGGTTCAGTGGTCGAGGTCTCTGAGATATAATCGAA
GACTTGGTGTGTTCACACCTCCTATGCAGTACAAGACGGAGTTTGTCAGAGTTAATAATATTGTGGAC
GTTGGCAGTATCGTT
+
FFFFFFFFFFFFFFFFFFFFFFFF:FFFFFFFFFFFFFFFFFFFFFFFFFFFFFFFFFFFFFFFFFFF
FFFFFFFFFFFFFFFFFFFFFFFFFFFFFFFFFFFFFFFFFFFFFFFFFFFFFFFFFFFFFFFFFFFF
FFFFFFFFFFFFFFF
@A00721:190:HGJW7DSXY:4:2348:14199:19163 1:N:0:TCTGCAAG+GCTGCACT
GCAATTCAACAGGACTCAGCGGTCACAGCTGCCGGTTCAGTGGTCGAGGTCTCTGAGATATAATCGAA
GACTTGGTGTGTTCACACCTCCTATGCAGTACAAGACGGAGTTTGTCAGAGTTAATAATATTGTGGAC
GTTGGCAGTATCGTT
+
FFFFFFFFFFFFFFFFFFFFFFFFFFFFF,FFFFFFFFFFFFFFFFFFFFFFFFFFFFFFFFFFFFFF
FFFFFFFFFFFFFFFFFFFFFFFFFFFFFFFFFFFFFFFFFFFFFFFFF:FFFFFFFF,FFFFFFFFF
FFFFFFFFFFFFFFF
@A00721:190:HGJW7DSXY:4:2349:13349:22200 1:N:0:TCTGCAAG+GCTGCACT
GCAATTCAACAGGACTCAGCGGTCACAGCTGCCGGTTCAGTGGTCGAGGTCTCTGAGATATAATCGAA
GACTTGGTGTGTTCACACCTCCTATGCAGTACAAGACGGAGTTTGTCAGAGTTAATAATATTGTGGAC
GTTGGCAGTATCGTT
+
FFFFFFFFFFFFFFFFFFFFFFFF:FFFFFFFFFFFFF:FFFFFFFFFFFFFFFFFFFFFFFFFFFFF
FFFFFFFFFFFFFFFFFFFFFFFFFFFFFFFFFFFFFFFFFFFFFFFFFFFFF:FFFFFFFFFFFFFF
FFFFFFFFFFFFFFF
@A00721:190:HGJW7DSXY:4:2444:19605:23797 1:N:0:TCTGCAAG+GCTGCACT
GCAATTCAACAGGACTCAGCGGTCACAGCTGCCGGTTCAGTGGTCGAGGTCTCTGAGATATAATCGAA
GACTTGGTGTGTTCACACCTCCTATGCAGTACAAGACGGAGTTTGTCAGAGTTAATAATATTGTGGAC
GTTGGCAGTATCGTT
+
FFFFFFFFFFFFFFFFFFFFFFFFFFFFFFFFFFFFFFFFFFFFFFFFFFFF:FFFFFFFFFFFFFFF
FFFFFFFFFFF:FFFFFFFFFFFFFFFFFFFFFFFFFFFFFFFFFFFFFFFFFFFFFFFF:FFFFFFF
FFFFFFFFFFFFFFF
@A00721:190:HGJW7DSXY:4:2461:1877:7592 1:N:0:TCTGCAAG+GCTGCACT
GCAATTCAACAGGACTCAGCGGTCACAGCTGCCGGTTCAGTGGTCGAGGTCTCTGAGATATAATCGAA
GACTTGGTGTGTTCACACCTCCTATGCAGTACAAGACGGAGTTTGTCAGAGTTAATAATATTGTGGAC
GTTGGCAGTATCGTT
+
FFFFFFFFFFFFFFFFFFFFFFFFFFFFF:FFFFFFFFFFFFFFFFFFFFFFF:FFFFFFFFFFFFFF
FFFFFFFFFFFFFFFFFFFFFFFFFFFFFFFFFFFFFFFFFFFFFFFFFFFFFFFFFFFFFFFFFFFF
FFFFFFFFFFFFFFF
```

```
@A00721:190:HGJW7DSXY:4:2669:19569:18630 1:N:0:TCTGCAAG+GCTGCACT
GCAATTCAACAGGACTCAGCGGTCACAGCTGCCGGTTCAGTGGTCGAGGTCTCTGAGATATAATCGAA
GACTTGGTGTGTTCACACCTCCTATGCAGTACAAGACGGAGTTTGTCAGAGTTAATAATATTGTGGAC
GTTGGCAGTATCGTT
+
FFFFFFFFFFFFFFFFFFFFFFFFFFFFFFFFFFFFFFFFFFFFFFFFFFFFFFFFFFFFFFFFFFFF
FFFFFFFFFFFFFFFFFFFFFFFFFFFFFFFFFFFFFFFFFFFFFFFFFFF:FFFFFFFFFFFFFFFF
FFFFFFFFFFFFFFF
@A00721:190:HGJW7DSXY:4:2441:2085:8171 1:N:0:TCTGCAAG+GCTGCACT
CAATTCAACAGGACTCAGCGGTCACAGCTGCCGGTTCAGTGGTCGAGGTCTCTGAGATATAATCGAAG
ACTTGGTGTGTTCACACCTCCTATGCAGTACAAGACGGAGTTTGTCAGAGTTAATAATATTGTGGACG
TTGGCAGTATCGTT
+
FFFFFFFF:FFFFFFFF,F:FFF:F:FF::FFFFFFFFF:F:FFFFFFFFFFFFFFFFFFFFFFF:FF:
FFFF:FFFFFFF:FFFFFFFFFFFFFFFFFFFFFFFF:FFFFFFFFFFFFF:FFFFFFFF,:F:FFFF:
FFFFFFFFFFFFF:
@A00721:190:HGJW7DSXY:4:1375:31403:12132 1:N:0:TCTGCAAG+GCTGCACT
ATTCAACAGGACTCAGCGGTCACAGCTGCCGGTTCAGTGGTCGAGGTCTCTGAGATATAATCGAAGAC
TTGGTGTGTTCACATCTCCTATGCAGTACAAGACGGAGTTTGTCAGAGTTAATAATATTGTGGACGTT
GGCAGTATCGTTTGT
+
FFFFFFFFFFFFFFFFFFF:FFFFFFFFFFFFFFFFFFFFFFFFFFFFFFFFFFFFFFFFFFFFFFFF
FFFFFFFFFFFFFFFFFFFFFFFFFFFFFFFFF:FFFFFFFFFFFFFFFFFFFFFF:FFFFFFFFFFFFF
FFFFFF:FFFF,FFF
@A00721:190:HGJW7DSXY:4:1557:25464:7106 1:N:0:TCTGCAAG+GCTGCACT
ATTCAACAGGACTCAGCGGTCACAGCTGCCGGTTCAGTGGTCGAGGTCTCTGAGATATAATCGAAGAC
TTGGTGTGTTCACATCTCCTATGCAGTACAAGACGGAGTTTGTCAGAGTTAATAATATTGTGGACGTT
GGCAGTATCGTTTGT
+
FFFFFFFFFFFFFFFFFFFFFFFFFFFFFFFFFFFFFFFFFFFFFFFFFFFFFFFFFFFFFFFFFF:F
FFFFFFFFFFFFFFFFFFFFFFFFFFFFFFFFFFFFFFFFFFFFFFFFFF:FF:FFFFFFFFFFFFFFFF
FFFFFFFFFFF:FFF
@A00721:190:HGJW7DSXY:4:1308:7030:20087 1:N:0:TCTGCAAG+GCTGCACT
TGGACTCAGCGGTCACAGCTGCCGGTTCAGTGGTCGAGGTCTCTGAGATATAATCGAAGACTTGGTGT
GTTCACACCTCCTATGCAGTACAAGACGGAGTTTGTCAGAGTTAATAATATTGTGGACGTTGGCAGTA
TCGTTTGTATAAATG
+
FFFFFFFFFFFFFFFFFFFFFFFFFFFFFFFFFFFFFFFFFFFFFFFFFFFFFFFFFFFFFFFFFFFF
FFFFFFFFFFFFFFFFFFFFFFFFFFFFFFFFFFFFFFFFFFFFFFFFFFFFFFFFFFFFFFFFFFFF
FFFF:FFFFFFFFFF
@A00721:190:HGJW7DSXY:4:1308:8106:20854 1:N:0:TCTGCAAG+GCTGCACT
TGGACTCAGCGGTCACAGCTGCCGGTTCAGTGGTCGAGGTCTCTGAGATATAATCGAAGACTTGGTGT
GTTCACACCTCCTATGCAGTACAAGACGGAGTTTGTCAGAGTTAATAATATTGTGGACGTTGGCAGTA
TCGTTTGTATAAATG
+
FFFFFFFFFFFFFFFFFFFFFFFFFFFFFFFFFFFFFFFFFFFFFFFFFFFFFFFFFFFFFFFFFFFF
FFFFFFFFFFFFFFFFFFFFFFFFFFFFFFFFFFFFFFFFFFFFFFF:FFFFF:FFFFFFFFFFFFFFFF
FFFFFFFFFFFFFFF
@A00721:190:HGJW7DSXY:4:1356:24478:34773 1:N:0:TCTGCAAG+GCTGCACT
TGGACTCAGCGGTCACAGCTGCCGGTTCAGTGGTCGAGGTCTCTGAGATATAATCGAAGACTTGGTGT
GTTCACACCTCCTATGCAGTACAAGACGGAGTTTGTCAGAGTTAATAATATTGTGGACGTTGGCAGTA
TCGTTTGTATAAAT
+
FFFFFF,:,FFF:FF,FFFFF:FFFFF:FFF,:FFFFF:F:FFFFFFFFF,FFFFFFFFF,F::FFFF
```

FFFFFFFFFFF:FFFFFFFFFFFF:F:FFFF,,FFFFFFFFFFFFFFFF:FFFFF:FFFFFFFFFF,F
FFFF:FFF:FFFFF
@A00721:190:HGJW7DSXY:4:1516:6876:35227 1:N:0:TCTGCAAG+GCTGCACT
TGGACTCAGCGGTCACAGCTGCCGGTTCAGTGGTCGAGGTCTCTGAGATATAATCGAAGACTTGGTGT
GTTCACACCTCCTATGCAGTACAAGACGGAGTTTGTCAGAGTTAATAATATTGCGGACGTTGGCAGTA
TCGTTTGTATAAATG
+
FFFFFFFFFFFFFFFFFFFFFFFFFFFFFFFFFFFFFFFFFFFFFFFFFFFFFFFFFFFFFFFFFFFF
FFFFFFFFFFFFFFFFFFFFFFFFFFFFFFFFFFFFFFFFFFFFFFFFFFFFFFFFFFFFFFFFFFFF
FFFF,FFFFFFFFFF
@A00721:190:HGJW7DSXY:4:2420:10945:9173 1:N:0:TCTGCAAG+GCTGCACT
TGGACTCAGCGGTCACAGCTGCCGGTTCAGTGGTCGAGGTCTCTGAGATATAATCGAAGACTTGGTGT
GTTCACACCTCCTATGCAGTACAAGACGGAGTTTGTCAGAGTTAATAATATTGTGGACGTTGGCAGTA
TCGTTTGTATAAATG
+
FFFFFFFFFFFFFFFFFFFFFFFFFFFFFFFFFFFFFFFFFFFFFFFFFFFFFFFFFFFFFFFFFFFF
FFFFFFFFFFFFFFFFFFFFFFFFFFFFFFFFFFFFFFFFFFFFFFFFFFFFFFFFFFFFFFFFFFFF
FFFFFFFFF:FF:FF
@A00721:190:HGJW7DSXY:4:2465:1145:26303 1:N:0:TCTGCAAG+GCTGCACT
TGGACTCAGCGGTCACAGCTGCCGGTTCAGTGGTCGAGGTCTCTGAGATATAATCGAAGACTTGGTGT
GTTCACACCTCCTATGCAGTACAAGACGGAGTTTGTCAGAGTTAATAATATTGTGGACGTTGGCAGTA
TCGTTTGTATAAATG
+
FFFFFFFFFFFFFFFFFFFFFFFFFFFFFFFFFFFFFFFFFFFFFFFFFFFFFFFFFFFFFFFFFFFF
FFFFFFFFFFFFFFFFFFFFFFFFFFFFFFFFFFFFFFFFFFFFFFFFFFFFFFFFFFFFFFFFFFFF
FFFFFFFFFFFFFFF
@A00721:190:HGJW7DSXY:4:1445:9570:1939 1:N:0:TCTGCAAG+GCTGCACT
CAGCTGCCGGTTCAGTGGTCGAGGTCTCTGAGATATAATCGAAGACTTGGTGTGTTCACACCTCCTAT
GCAGTACAAGACGGAGTTTGTCAGAGTTAATAATATTGTGGACGTTGGCAGTATCGTTTGTATAAATG
ATTGTCCTACTGGA
+
FFFFFFFFFFFFFFFFFFFFFFFFFFFFFFFFFFFFFFFFFFFFFFFFFFFFFFFFFFFFFFFFFFFF
FFFFFFFFFFFFFFFFFFFFFFFFFFFFFFFFFFFFFFFFFFFFFFFFFFFFF:FFFFFFFFFFFFFF
FFFFFFFFFFFFFF
@A00721:190:HGJW7DSXY:4:2240:2672:35963 1:N:0:TCTGCAAG+GCTGCACT
CAGCTGCCGGTTCAGTGGTCGAGGTCTCTGAGATATAATCGAAGACTTGGTGTGTTCACACCTCCTAT
GCAGTACAAGACGGAGTTTGTCAGAGTTAATAATATTGTGGACGTTGGCAGTATCGTTTGTATAAATG
ATTGTCCTACTGGAA
+
FFFFFFFFFFFFFFFFFFFFFFFFFFFFFFFFFFFFFFFFFFFFFFFFFFFFFFFFFFFFFFFFFFFF
FFFFFFFFFFFFFFFFFFFFFFFFFFFFFFFFFFFFFFFFFFFFFFFFFFFFFFFFFFFFFFFFFFFF
FFFFFFFFFFFFFF:
@A00721:190:HGJW7DSXY:4:2464:10746:6605 1:N:0:TCTGCAAG+GCTGCACT
CAGCTGCCGGTTCAGTGGTCGAGGTCTCTGAGATATAATCGAAGACTTGGTGTGTTCACACCTCCTAT
GCAGTACAAGACGGAGTTTGTCAGAGTTAATAATATTGTGGACGTTGGCAGTATCGTTTGTATAAATG
ATTGTCCTACTGGAA
+
FFFFFFFFFFFFFFFFFFFFFFFFFFFFFFFFFFFFFFFFFFFFFFFFFFFFFFFFFFFFFFFFFFFF
FFFFFFFFFFFFFFFFFFFFFFFFFFFFFFFFFFFFFFFFFFFFFFFFFFFFF:FFFFFFFFFFFFFF
FFFFFFFFFFF:FFF
@A00721:190:HGJW7DSXY:4:2464:6171:37012 1:N:0:TCTGCAAG+GCTGCACT
CAGCTGCCGGTTCAGTGGTCGAGGTCTCTGAGATATAATCGAAGACTTGGTGTGTTCACACCTCCTAT
GCAGTACAAGACGGAGTTTGTCAGAGTTAATAATATTGTGGACGTTGGCAGTATCGTTTGTATAAATG
ATTGTCCTACTGGAA

```
+
FFFFFFFFFFFFFFFFFFFFFFFFFFFFFFFFFFFFFFFFFFFFFFFFFFFFFFFFFFFFFFFFFFFFFF
FFFFFFFFFFFFFFFFFFFFFFFFFFFFFFFFFFFFFFFFFFFFFFFFFFFFFFFFFFFFFFFFFFFFFF
FFFFFFFFFFFFFF:
@A00721:190:HGJW7DSXY:4:2623:19388:29340 1:N:0:TCTGCAAG+GCTGCACT
CAGCTGCCGGTTCAGTGGTCGAGGTCTCTGAGATATAATCGAAGACTTGGTGTGTTCACACCTCCTAT
GCAGTACAAGACGGAGTTTGTCAGAGTTAATAATATTGTGGACGTTGGCAGTATCGTTTGTATAAATG
ATTGTCCTACTGGAA
+
FFFFFFFFFFFFFFFFFFFFFFFFFFFFFFFFFFFFFFFFFFFFFFFFFFFFFFFFFFFFFFFFFFFFFF
FFFFFFFFFFFFFFFFFFFFFFFFFFFFF:FFFFFFFFFFFFFFFFFFFFFFFFF:FF::FFFFFFFFFFF
FFFFFFFFFFFFFFF
@A00721:190:HGJW7DSXY:4:2645:15736:20040 1:N:0:TCTGCAAG+GCTGCACT
CAGCTGCCGGTTCAGTGGTCGAGGTCTCTGGGATATAATCGAAGACTTGGTGTGTTCACACCTCCTAT
GCAGTACAAGACGGAGTTTGTCAGAGTTAATAATATTGTGGACGTTGGCAGTATCGTTTGTATAAATG
ATTGTCCTACTGGAA
+
FFFFFFFFFFFFFFFFFFFFFFFFFFFFFFFFFFFFFFFFFFFFFFFFFFFFFFFFFFFFFFFFFFFFFF
FFFFFFFFFFFFFFFFFFFFFFFFFFFFFFFFFFFFFFFFFFFFFFFFFFFFFFFFFFF,FFFFFFFFFF
FFFFFFFFFFFFFFF
@A00721:190:HGJW7DSXY:4:2650:13602:7012 1:N:0:TCTGCAAG+GCTGCACT
CAGCTGCCGGTTCAGTGGTCGAGGTCTCTGAGATATAATCGAAGACTTGGTGTGTTCACACCTCCTAT
GCAGTACAAGACGGAGTTTGTCAGAGTTAATAATATTGTGGACGTTGGCAGTATCGTTTGTATAAATG
ATTGTCCTACTGGAA
+
FFFFFFFFFFFFFFFFFFFFFFFFFFFFF:FFFFFFFF:FFFFFFFFFFFF:FFFFFFFFFFFFFFFF
FFFFFFFFFFFFFFFF:FFFFFFFFFFFFFFFFFF:FFFFFFF:F:FFFFFFFFFFFFFFFFFFFFFFF
FFFFFF:FFF:FFFF
@A00721:190:HGJW7DSXY:4:1420:12933:17315 1:N:0:TCTGCAAG+GCTGCACT
GGCCGGTTCAGTGGTCGAGGTCTCTGAGATATAATCGAAGACTTGGTGTGTTCACACCTCCTATGCAG
TACAAGACGGAGTTTGTCAGAGTTAATAATATTGTGGACGTTGGCAGTATCGTTTGTATAAATGATTG
TCCTACTGGAACCTC
+
FFFFFFFFFFFFFFFFFFFFFFFFFFFFFFFFFFFFFFFFFFFFFFFFFFFFFF:FFFFFFFFFFFFFF
FFFFFFFFFFFFFFFFFF:FFF:FFF:FFFFFFFFFFFFFFFFFFFFFFFFFFFFFFFFFFFFFFFFFFF
FFFFFFFFFFFFFFF
@A00721:190:HGJW7DSXY:4:1456:32868:28134 1:N:0:TCTGCAAG+GCTGCACT
GGCCGGTTCAGTGGTCGAGGTCTCTGAGATATAATCGAAGACTTGGTGTGTTCACACCTCCTATGCAG
TACAAGACGGAGTTTGTCAGAGTTAATAATATTGTGGACGTTGGCAGTATCGTTTGTATAAATGATTG
TCCTACTGGAACCTC
+
FFFFFFFFFFFFFFFFFFFFFFFF:FFFFFFFFFFFFFFFFFFFFFF:FFFFFFFFFFFFFFFFFFFFFFF
FFFFFFFFFFFFFFFFFFFFF,FFFFFFFFFFFFFFFFFFFFFFFFFFFFFFF:FFFFF:FFFFFFFFFFF
FFFFFFF:FFFFFFF
@A00721:190:HGJW7DSXY:4:2458:5936:5290 1:N:0:TCTGCAAG+GCTGCACT
GGCCGGTTCAGTGGTCGAGGTCTCTGAGATATAATCGAAGACTTGGTGTGTTCACACCTCCTATGCAG
TACAAGACGGAGTTTGTCAGAGTTAATAATATTGTGGACGTTGGCAGTATCGTTTGTATAAATGATTG
TCCTACTGGAACCTC
+
FFFFFFFFFFFFFFFFFFFFFFFFFFFFFFFFFFFFFFFFFFFFFFFFFFFFFFFFFFFFFFFFFFFFFF
FFFFFFFFFFFFFFFFFFFFFFFFFFFFFFFFFFFFFFFFFFFFFFFFFFFFFFFFFFFFFFFFFFFFFF
FFFFFFFFFFFFFFF
@A00721:190:HGJW7DSXY:4:2609:5285:5228 1:N:0:TCTGCAAG+GCTGCACT
GGCCGGTTCAGTGGTCGAGGTCTCTGAGATATAATCGAAGACTTGGTGTGTTCACACCTCCTATGCAG
```

TACAAGACGGAGTTTGTCAGAGTTAATAATATTGTGGACGTTGGCAGTATCGTTTGTATAAATGATTG
TCCTACTGGAACCTC
+
FFFFFFFFFFFFFFFFFFFFFFFFFFFFFFFFFFFFFFFFFFFFFFFFFFFFFFFFFFFFFFFFFFFFFF
FFFFFFFFFFFFFFFFFFFFFFFFFF:FFFFFFFFFFFFFFF:FFFFFFFFFFFFFFFFFFF:FFFFF
FFFFFFFFFFFFFFF
@A00721:190:HGJW7DSXY:4:2639:4851:1407 1:N:0:TCTGCAAG+GCTGCACT
GGCCGGTTCAGTGGTCGAGGTCTCTGAGATATAATCGAAGACTTGGTGTGTTCACACCTCCTATGCAG
TACAAGACGGAGTTTGTCAGAGTTAATAATATTGTGGACGTTGGCAGTATCGTTTGTATAAATGATTG
TCCTACTGGAACCTC
+
FFFFFFFFFFFFFFFFFFFFFFFFFFFFFFFFFFFFFFFFFFFFFFFFFFFFFFFFFFFFFFFFFFFFFF
FFFFFFFFFFFFFFFFFFFFFFFFFFFFFFFFFFFFFFFFFFFFFFFFFFFFFFFFFFFFFFFFFF:FFF
FFFFFFFFFFFFFFF
@A00721:190:HGJW7DSXY:4:1328:31358:20791 1:N:0:TCTGCAAG+GCTGCACT
GCCGGTTCAGTGGTCGAGGTCTCTGAGATATAATCGAAGACTTGGTGTGTTCACACCTCCTATGCAGT
ACAAGACGGAGTTTGTCAGAGTTAAGAATATTGTGGACGTTGGCAGTATCGTTTGTATAAATGATTGT
CCTACTGGAACCTCG
+
FFFFFFFFFFFFFFFFFFFFFFFFFFFF:FFFFFFFFFFFFFFFFFFFFFFFFFFFFFFFFFFFFFFFFF
FFFFFFFFFFFFFFFFF:FFFFFFFF,FFFFFFFFFFFF:FFFFFFFFFFFFFFFFFF:FFF:FFFFFF
F,FFFFFFFFFFFFF
@A00721:190:HGJW7DSXY:4:1547:4942:23484 1:N:0:TCTGCAAG+GCTGCACT
GCCGGTTCAGTGGTCGAGGTCTCTGAGATATAATCGAAGACTTGGTGTGTTCACACCTCCTATGCAGT
ACAAGACGGAGTTTGTCAGAGTTAATAATATTGTGGACGTTGGCAGTATCGTTTGTATAAATGATTGT
CCTACTGGAACCTCG
+
FFFFFFFFFFFFFFFFFFFFFFFFFFFFFFFFFFFFFFFFFFFFFFFFFFFFFFFFFFFFFFFFFFFFFF
FFFFFFFFFFFFFFFFFFFFFFFFFFFFFFFFFFFFFFFFFFFFFFFFFFFFFFFFFFFFFFFFFFFFFF
FFFFFFFFFFFFFFF
@A00721:190:HGJW7DSXY:4:1675:26142:20275 1:N:0:TCTGCAAG+GCTGCACT
GCCGGTTCAGTGGTCGAGGTCTCTGAGATATAATCGAAGACTTGGTGTGTTCACACCTCCTATGCAGT
ACAAGACGGAGTTTGTCAGAGTTAATAATATTGTGGACGTTGGCAGTATCGTTTGTATAAATGATTGT
CCTACTGGAACCTCG
+
FFFFFFFFFFFFFFFFFFFFFFFFFFFFFFFFFFFFFFFFFFFFFFFFFFFFFFFFFFFFFFFFFFFFFF
FFFFFFFFFFFFFFFFFFFFFFFFFFFFFFFFFFFFFFFFFFFFFFFFFFFFFFFFFFFFFFFFFFFFFF
FFFFFFFFFFFFFFF
@A00721:190:HGJW7DSXY:4:2278:7808:13197 1:N:0:TCTGCAAG+GCTGCACT
GCCGGTTCAGTGGTCGAGGTCTCTGAGATATAATCGAAGACTTGGTGTGTTCACACCTCCTATGCAGT
ACAAGACGGAGTTTGTCAGAGTTAATAATATTGTGGACGTTGGCAGTATCGTTTGTATAAATGATTGT
CCTACTGGAACCTCG
+
FFFFFFFFFFFFFFFFFFFFFFFFFFFFFFFFFFFFFFFFFFFFFFFFFFFFFFFFFFFFFFFFFFFFFF
FFFFFFFFFFFFFFFFFFFFFFFFFFFFF:FFFFFFFFFFFFFFFFFFFFFFFFFFFFFFFFFFFFFFFF
FFFFFFFFFFFFFF:
@A00721:190:HGJW7DSXY:4:2451:19931:25676 1:N:0:TCTGCAAG+GCTGCACT
GCCGGTTCAGTGGTCGAGGTCTCTGAGATATAATCGAAGACTTGGTGTGTTCACACCTCCTATGCAGT
ACAAGACGGAGTTTGTCAGAGTTAATAATATTGTGGACGTTGGCAGTATCGTTTGTATAAATGATTGT
CCTACTGGAACCTCG
+
FFFFFFFFFFFFFFFFFFFFFFFFFFFFFFFFFFFFFFFFFFFFFFFFFFFFFFFFFFFFFFFFFFFFFF
FFFFFFFFFFFFFFFFFFFFFFFFFFFFFFFFFFFFFFFFFFFFFFFFFFFFFFFFFFFFFFFFFFFFFF
FFFFFFFFFFFFFFF

```
@A00721:190:HGJW7DSXY:4:2514:4779:32534 1:N:0:TCTGCAAG+GCTGCACT
GCCGGTTCAGTGGTCGAGGTCTCTGAGATATAATCGAAGACTTGGTGTGTTCACACCTCCTATGCAGT
ACAAGACGGAGTTTGTCAGAGTTAATAATATTGTGGACGTTGGCAGTATCGTTTGTATAAATGATTGT
CCTACTGGAACCTCG
+
FFFFFFFFFFFFFFFFFFFFFFFFFFFFFFFFFFFFFFFFFFFFFFFFFFFFFFFFFFFFFFFFFFFFFF
FFFFFFFFFFFFFFFFFFFFFFFFFFFFFFFFFFFFFFFFFFFFFFFFFFFFFFFFFFFFFFFFFFFFFF
FFFFFFFFFFFFFFF
@A00721:190:HGJW7DSXY:4:1357:6424:15781 1:N:0:TCTGCAAG+GCTGCACT
CCGGTTCAGTGGTCGAGGTCTCTGAGATATAATCGAAGACTTGGTGTGTTCACACCTCCTATGCAGTA
CAAGACGGAGTTTGTCAGAGTTAATAATATTGTGGACGTTGGCAGTATCGTTTGTATAAATGATTGTC
CTACTGGAACCTCGG
+
FFFFFFFFFFFFFFFFFFFFFFFFFFFFFFFFFFFFFFFFFFFFFFFFFFFFFFFFFFFFFFFFFFFFFF
FFFFFFFFFFFFFFFFFFFFFFFFFFFFFFFFFFFFFFFFFFFFFFFFFFFFFFFFFFFFFFFFFFFFFF
FFFFFFFFFFFFFFF
@A00721:190:HGJW7DSXY:4:1404:8775:13902 1:N:0:TCTGCAAG+GCTGCACT
CCGGTTCAGTGGTCGAGGTCTCTGAGATATAATCGAAGACTTGGTGTGTTCACACCTCCTATGCAGTA
CAAGACGGAGTTTGTCAGAGTTAATAATATTGTGGACGTTGGCAGTATCGTTTGTATAAATGATTGTC
CTACTGGAACCTCGG
+
FFFFFFFFFFFFFFFFFFFFFFFFFFFFFFFFFFFFFFFFFFFFFFFFFFFFFFFFFFFFFFFFFFFFFF
FFFFFFFFFFFFFFFFFFFFFFFFFFFFFFFFFFFFFFFFFFFFFFFFFFFFFFFFFFFFFFFFFFFFFF
FFF:FF:FFFFFFFF
@A00721:190:HGJW7DSXY:4:1564:17644:19586 1:N:0:TCTGCAAG+GCTGCACT
CCGGTTCAGTGGTCGAGGTCTCTGAGATATAATCGAAGACTTGGTGTGTTCACACCTCCTATGCAGTA
CAAGACGGAGTTTGTCAGAGTTAATAATATTGTGGACGTTGGCAGTATCGTTTGTATAAATGATTGTC
CTACTGGAACCTCGG
+
FFFFFFFFFFFFFFFFFFFFFFFFFFFFFFFFFFFFFFFFFFFFFFFFFFFFFFFFFFFFFFFFFFFFFF
FFFFFFFFFFFFFFFFFFFFFFFFFFFFFFFFFFFFFFFFFF:FFFFFFFFFFFFFFFFFFFFFFFFFFF
FFFFFFFFFFFFFFF
@A00721:190:HGJW7DSXY:4:1164:1163:17973 1:N:0:TCTGCAAG+GCTGCACT
GTTCAGTGGTCGAGGTCTCTGAGATATAATCGAAGACTTGGTGTGTTCACATCTCCTATGCAGTACAA
GACGGAGTTTGTCAGAGTTAATAATATTGTGGACGTTGGCAGTATCGTTTGTATAAATGATTGTCCTA
CTGGAACCTCGGTCG
+
FFFFFFFFFFFFFFFFFFFFFFFFFFFFFFFFFFFFFFFFFFFFFFFFFFFFFFFFFF:FFFFFFFFFFF
FFFFFFFFFFFFFFFFFFFFFFFFFFF:FFFFFFFFFFFFFFFFF:FFFFFFFFFFFFFFFFFFFFFFFF
FFFFF:FFFFFFFFF
@A00721:190:HGJW7DSXY:4:1619:24985:30358 1:N:0:TCTGCAAG+GCTGCACT
GTTCAGTGGTCGAGGTCTCTGAGATATAATCGAAGACTTGGTGTGTTCACATCTCCTATGCAGTACAA
GACGGAGTTTGTCAGAGTTAATAATATTGTGGACGTTGGCAGTATCGTTTGTATAAATGATTGTCCTA
CTGGAACCTCGGTCG
+
FFFFFFFFFFFFFFFFFFFFFFFFFFFFFFFFFFFFFFFFFFFFFFFFFFFFFFFFFFFFFFFFFFFFFF
FFFFFFFFFFFFFFFFFFFFFFFFFFFFFFFFFFFFFFFFFFFFFFFF,FFFFFFFFFFFFFFFFFFFFF
FFFFF:FFFFFFFFF
@A00721:190:HGJW7DSXY:4:2134:24379:30530 1:N:0:TCTGCAAG+GCTGCACT
GTTCAGTGGTCGAGGTCTCTGAGATATAATCGAAGACTTGGTGTGTTCACATCTCCTATGCAGTACAA
GACGGAGTTTGTCAGAGTTAATAATATTGTGGACGTTGGCAGTATCGTTTGTATAAATGATTGTCCTA
CTGGAACCTCGGTCG
+
FFFFFFFFFFFFFFFFFFFFFFFFFFFFFFFFFFFFFFFFFFFFFFFFFFFFFFFFFFFFFFFFFFFFFF
```

FFFFFFFFFFFFFFFFFFFFFFFFFFFFFFFFFFFFFFFFFFFFFFFFFFFFFFFFFFFFFF,F:FFF
FFFFF,,FFFFFFFF
@A00721:190:HGJW7DSXY:4:2458:22480:2190 1:N:0:TCTGCAAG+GCTGCACT
GTTCAGTGGTCGAGGTCTCTGAGATATAATCGAAGACTTGGTGTGTTCACATCTCCTATGCAGTACAA
GACGGAGTTTGTCAGAGTTAATAATATTGTGGACGTTGGCAGTATCGTTTGTATAAATGATTGTCCTA
CTGGAACCTCGGTCG
+
FFFFFFFFFFFFFFFFFFFFFFFFFFFFFFFFFFFFFFFFFFFFFFFFFFFFFFFFFFFFFFFFFFFF
FFFFFFFFFFFFFFFFFFF:FFFFFFFFFFFFFFFFFFFFFFFFFFFFFFFFFFFFFFFFFFFFFFFF
FFFFFFFFFFFFFFF
@A00721:190:HGJW7DSXY:4:1106:12897:18223 1:N:0:TCTGCAAG+GCTGCACT
CTGTGGTCGAGGTCTCTGAGATATAATCGAAGACTTGGTGTGTTCACATCTCCTATGCAGTACAAGAC
GGAGTTTGTCAGAGTTAATAATATTGTGGACGTTGGCAGTATCGTTTGTATAAATGATTGTCCTACTG
GAACCTCGGTCGATA
+
FFFFFFFFFFFFFFFFFFFFFFFF,FFFFFFFFF:FFFFFFFFFFFFFFFFFFFFFFFFFFFFFFFFF
FFFFFFFFFFFFFFFFFFFFFFFFFFFFFFFFFFF:FFFFFFFFFFFFFFFFFFFFFFFFFFFFFFFF
FFFFFFFFFFFFFFF
@A00721:190:HGJW7DSXY:4:1564:21775:10394 1:N:0:TCTGCAAG+GCTGCACT
CTGTGGTCGAGGTCTCTGAGATATAATCGAAGACTTGGTGTGTTCACATCTCCTATGCAGTACAAGAC
GGAGTTTGTCAGAGTTAATAATATTGTGGACGTTGGCAGTATCGTTTGTATAAATGATTGTCCTACTG
GAACCTCGGTCGATA
+
FF:FFFFFFFFFFFFFFFFFFFFF:FFFFFFFFFFFFFFFFFFFFFFFFFFFFFFFFFFFFFFFFFFF
FFFFFFFFFFFFFFFFFFFF:FFFFFFFFFFFFFFFFFFFFFFFFFFFFFFFFFFFFFFFFFFFFFFF
FFFFFFFFFFFFFFF
@A00721:190:HGJW7DSXY:4:2217:30572:10128 1:N:0:TCTGCAAG+GCTGCACT
CTGTGGTCGAGGTCTCTGAGATATAATCGAAGACTTGGTGTGTTCACATCTCCTATGCAGTACAAGAC
GGAGTTTGTCAGAGTTAATAATATTGTGGACGTTGGCAGTATCGTTTGTATAAATGATTGTCCTACTG
GAACCTCGGTCGATA
+
FFFFFFFFFFFFFFFFFFFFFFFFFFFFFFFFFFFFFFFFFFFFFFFFFFFFFFFFFFFFFFFFFFFF
FFFFFFFFFFFFFFFFFFFFFFFFFFFFFFFFFFFFFFFFFFFFFFFFFFFFFFFFFFFFFFFFFFFF
FFFFFFFFFFFFFFF
@A00721:190:HGJW7DSXY:4:2301:11921:4852 1:N:0:TCTGCAAG+GCTGCACT
CTGTGGTCGAGGTCTCTGAGATATAATCGAAGACTTGGTGTGTTCACATCTCCTATGCAGTACAAGAC
GGAGTTTGTCAGAGTTAATAATATTGTGGACGTTGGCAGTATCGTTTGTATAAATGATTGTCCTACTG
GAACCTCGGTCGATA
+
FFFFFFFFFFFFFFFFFFFFFFFFFFFFFFFFFFFFFFFFFFFFFFFFFFFFFFFFFFFFFFFFFFFF
FFFFFFFFFFFFFFFFFF:FFFFFFFFFFFFFFFFFFFFFFFFFFFFFFFFFFFFFFFFFFFFFFFFF
FFFFFFFFFFFFFFF
@A00721:190:HGJW7DSXY:4:2325:27299:6308 1:N:0:TCTGCAAG+GCTGCACT
CTGTGGTCGAGGTCTCTGAGATATAATCGAAGACTTGGTGTGTTCACATCTCCTATGCAGTACAAGAC
GGAGTTTGTCAGAGTTAATAATATTGTGGACGTTGGCAGTATCGTTTGTATAAATGATTGTCCTACTG
GAACCTCGGTCGATA
+
FFFFFFFFFFFFFFFFFFFFFFFFFFFFFFFFFFFFFFFFFFFFFFFFFFFFFFFFFFFFFFFFFFFF
FFFFFFFFFFFFFFFFFFFF:FFFFFFFFFFFFFFFFFFFFFFFFFFFFFFFFFFFFFFFFFFFFFFF
FFFFFFFFFFFFFF:
@A00721:190:HGJW7DSXY:4:2603:28890:22686 1:N:0:TCTGCAAG+GCTGCACT
CTGTGGTCGAGGTCTCTGAGATATAATCGAAGACTTGGTGTGTTCACATCTCCTATGCAGTACAAGAC
GGAGTTTGTCAGAGTTAATAATATTGTGGACGTTGGCAGTATCGTTTGTATAAATGATTGTCCTACTG
GAACCTCGGTCGATA

```
+
FFFFFFFFFFFFFFFFFFFFFFFFFFFFFFFFFFFFFFFFFFFFFFFFFFFFFFFFFFFFFFFFFFFFFF
FFFFFFFFFFFFFFFFFFFFFFFFFFFFFFFFFFFFFFFFFFFFFFFFFFFFFFFFFFFFFFFFFFFFFF
FFFFFFF:FFFFFFF
@A00721:190:HGJW7DSXY:4:2258:22697:31939 1:N:0:TCTGCAAG+GCTGCACT
CTCGTATCGACCGAGGTTCCAGTAGGACAATCATTTATACAAACGATACTGCCAACGTCCACAATATT
ATTAACTCTGACAAACTCCGTCTTGTACTGCATAGGAGGCGTGAACACACCAAGTCTTCGATTATATC
TCAGAGACCTCGACA
+
FFFFFFFFFFFFFFFFFFFFFFFFFFFFFFFFFFFFFFFFFFFFFFFFFFFFFFFFFFFFFFFFFFFFFF
FFFFFFFFFFFFFFFFFFFFFFFFFFFFFFFFFFFFFFFFFFFFFFFFFFFFFFFFFFFFFFFFFFFFFF
FF:FFFFFFFFFFFF
@A00721:190:HGJW7DSXY:4:2614:8133:35493 1:N:0:TCTGCAAG+GCTGCACT
CTCGTATCGACCGAGGTTCCAGTAGGACAATCATTTATACAAACGATACTGCCAACGTCCACAATATT
ATTAACTCTGACAAACTCCGTCTTGTACTGCATAGGAGGCGTGAACACACCAAGTCTTCGATTATATC
TCAGAGACCTCGACA
+
FFFFFFFFFF:FFFFFFFF:FFFFFFFFFFFFFFFFFFFFF:FFF:FFFFFFFFFFF::FFFFFFFF:FF
FF:FFFF:FFF:FFFFFFFFF:FFF:FFF:FFFFFFFFFFFFFFFFFFF:FF:FFFFFFFFFF,FFFF:F
FF:FFFFFFFFFFFF
@A00721:190:HGJW7DSXY:4:1156:15121:17534 1:N:0:TCTGCAAG+GCTGCACT
GTCGAGGTCTCTGAGATATAATCGAAGACTTGGTGTGTTCACACCTCCTATGCAGTACAAGACGGAGT
TTGTCAGAGTTAATAATATTGTGGACGTTGGCAGTATCGTTTGTATAAATGATTGTCCTACTGGAACC
TCGGTCGATACGAGA
+
FFFFFFFFFFFFFFFFFFFFFF:FFFFFFFFFF:FFFFFFFFFFF:FFF:FFFF:FF:FFFF::FFF:FF
FFFFFFFF::FFFFFF:FFF,FFFFF,:FF:FFFFFFFFFFFFFF:FFFFFFFFFFFFFFFFFFF:F,FF
FFFFF:FFFFFFFFF
@A00721:190:HGJW7DSXY:4:2431:9534:14246 1:N:0:TCTGCAAG+GCTGCACT
GTCGAGGTCTCTGAGATATAATCGAAGACTTGGTGTGTTCACACCTCCTATGCAGTACAAGACGGAGT
TTGTCAGAGTTAATAATATTGTGGACGTTGGCAGTATCGTTTGTATAAATGATTGTCCTACTGGAACC
TCGGTCGATACGAGA
+
FFFFFFFFFFFFFFFFFFFFFFFFFFFFFFFFFFFFFFFFFFFFFFFFFFFFFFFFFFFFFFFFFFFFFF
FFFFFFFFFFFFFFFFFFFFFFFFFFFFFFFFFFFFFFFFFFFFFFFFFFFFFFFF:FF:FFFFFFF:FF
FFFFFFFFFFFFFFF
@A00721:190:HGJW7DSXY:4:2551:24596:33348 1:N:0:TCTGCAAG+GCTGCACT
GTCGAGGTCTCTGAGATATAATCGAAGACTTGGTGTGTTCACACCTCCTATGCAGTACAAGACGGAGT
TTGTCAGAGTTAATAATATTGTGGACGTTGGCAGTATCGTTTGTATAAATGATTGTCCTACTGGAACC
TCGGTCGATACGAGA
+
FFFFFFFFFFFFFFFFFFFFFFFFFFFFFFFFFFFFFFFFFFFFFFFFFFFFFFFFFFFFFFFFFFFFFF
FFFFFFFFFFFFFFFFFFFFFFFFFFFFFFFFFFFFFFFF:FFFFFFFFFF:FFF:FFFFFFFFFFFFFF
FFFFFFFFFFFFFFF
@A00721:190:HGJW7DSXY:4:1160:19443:33974 1:N:0:TCTGCAAG+GCTGCACT
GTCTCTGAGATATAATCGAAGACTTGGTGTGTTCACACCTCCTATGCAGTACAAGACGGAGTTTGTCA
GAGTTAATAATATTGTGGACGTTGGCAGTATCGTTTGTATAAATGATTGTCCTACTGGAACCTCGGTC
GATACGAGACATACC
+
FFFFFFFFFFFFFFFFFFFFFFFFFFFFFFFFFFFFFFFFFFFFFFFFFFFFFFFFFFFFFFFFFFFFFF
FFFFFFFFFFFFFFFFFFFFFFFFFFFFFFFFFFFFFFFFFFFFFFFFFFFFFFFFFFFFFFFFFFFFFF
FFFFFFFFFFFFFFF
@A00721:190:HGJW7DSXY:4:1417:10574:21746 1:N:0:TCTGCAAG+GCTGCACT
GTCTCTGAGATATAATCGAAGACTTGGTGTGTTCACACCTCCTATGCAGTACAAGACGGAGTTTGTCA
```

GAGTTAATAATATTGTGGACGTTGGCAGTATCGTTTGTATAAATGATTGTCCTACTGGAACCTCGGTC
GATACGAGACATACC
+
FFFFFFFFFFFFFFFFFFFFFFFFFFFFFFFFFFFFFFFFFFFFFFFFFFFFFFFFFFFFFFFFFFFF
FFFFFFFFFFFFFFFFFFFFFFFFFFFFFFFFFFFFFFFFFFFFFFFFFFFFFFFFFFFFFFFF:FFF
FFFF,FFFFFFFF:F
@A00721:190:HGJW7DSXY:4:1467:15329:25034 1:N:0:TCTGCAAG+GCTGCACT
GTCTCTGAGATATAATCGAAGACTTGGTGTGTTCACACCTCCTATGCAGTACAAGACGGAGTTTGTCA
GAGTTAATAATATTGTGGACGTTGGCAGTATCGTTTGTATAAATGATTGTCCTACTGGAACCTCGGTC
GATACGAGACATACC
+
FFFFFFFFFFFFFFFFFFFFFFFFFFFFFFFFFFFFFFFFFFFFFFFFFFFFFFFFFFFFFFFFFFFF
FFFFFFFFFFFFFFFFFFFFFFFFFFFFFFFFFFFFFFFFFFFFFFFFFFFFFFFFFFFFFFFFFFFF
FFFFFFFFFFFFFFF
@A00721:190:HGJW7DSXY:4:1621:22381:31078 1:N:0:TCTGCAAG+GCTGCACT
GTCTCTGAGATATAATCGAAGACTTGGTGTGTTCACACCTCCTATGCAGTACAAGACGGAGTTTGTCA
GAGTTAATAATATTGTGGACGTTGGCAGTATCGTTTGTATAAATGATTGTCCTACTGGAACCTCGGTC
GATACGAGACATACC
+
FFFFFFFFFFFFFFFFFFFFFFFFFFFFFFFFFFFFFFFFFFFFFFFFFFFFFFFFFFFFFFFFFFFF
FFFFFFFF:FFFFFFFFFFFFFFFFFFFF:FFF:FFFFFFFFFFFFFFFFFFFFFFFFF,FFFFFFFF
FFFFFFF:FFFFFFF
@A00721:190:HGJW7DSXY:4:1657:17607:33144 1:N:0:TCTGCAAG+GCTGCACT
GTCTCTGAGATATAATCGAAGACTTGGTGTGTTCACACCTCCTATGCAGTACAAGACGGAGTTTGTCA
GAGTTAATAATATTGTGGACGTTGGCAGTATCGTTTGTATAAATGATTGTCCTACTGGAACCTCGGTC
GATACGAGACATACC
+
FFFFFFFFFFFFFFFFFFFFFFFFFFFFFFFFFFFFFFFFFFFFFFFFFFFFFFFFFFFFFFFFFFFF
FFFFFFF:FFFFFFFFFFFFFFFFFFFFFFFFFFFFFFFFFFFFFFFFFFFFFFFFFFFFFFFFFFFF
FFFFFFFFFFFFFFF
@A00721:190:HGJW7DSXY:4:2227:15718:5040 1:N:0:TCTGCAAG+GCTGCACT
GTCTCTGAGATATAATCGAAGACTTGGTGTGTTCACACCTCCTATGCAGTACAAGACGGAGTTTGTCA
GAGTTAATAATATTGTGGACGTTGGCAGTATCGTTTGTATAAATGATTGTCCTACTGGAACCTCGGTC
GATACGAGACATACC
+
FFFFFFFFFFFFFFFFFFFFF:FFFFFFFFFFFFFFFFFFFFFFFFFFFFFFFFFFFFFFFFFFFFFF
FFFFFFFFFFFFFFFFFFFFFFFFFFFFFFFFF:FFFFFFFFFFFFFFFFFFFFFFFFFFFFFFFFFF
FFFFFFFFFFFFFFF
@A00721:190:HGJW7DSXY:4:2503:8088:31939 1:N:0:TCTGCAAG+GCTGCACT
GTCTCTGAGATATAATCGAAGACTTGGTGTGTTCACACCTCCTATGCAGTACAAGACGGAGTTTGTCA
GAGTTAATAATATTGTGGACGTTGGCAGTATCGTTTGTATAAATGATTGTCCTACTGGAACCTCGGTC
GATACGAGACATAC
+
FFFFFF:FFFFFFFFFFFFFFFFFFFFFFFFFFFFFFFFFFFFFFFF:FFFFFF:FFFFFFFFFFFFF
FFFFFFFFFFFFFFFFFFFFFFF,FFF:FFF:F:FFFFFFFFFFFFFF:F:FFFFFFFFFF:FF:FF:
FFFFF,F:FFF:FF
@A00721:190:HGJW7DSXY:4:2572:15483:5822 1:N:0:TCTGCAAG+GCTGCACT
GTCTCTGAGATATAATCGAAGACTTGGTGTGTTCACACCTCCTATGCAGTACAATACGGAGTTTGTCA
GAGTTAATAATATTGTGGACGTTGGCAGTATCGTTTGTATAAATGATTGTCCTACTGGAACCTCGGTC
GATACGAGACATAC
+
FFFFF,FFFFFF,FFFFFFFFFFFFFFF:,FFFF:FF,F:FFFF:FFF,FFF:F,FFFFFFF,FF,,F
FFFFFFFFFFFF:::FFFFFFFFFF:FFFFFF:FFFFFFFFFFF:FFFFF:FFFFFFFFF:FFF:F,F
FF,FF,FFFFF:FF

@A00721:190:HGJW7DSXY:4:1304:3423:18255 1:N:0:TCTGCAAG+GCTGCACT
CTCTGAGATATAATCGAAGACTTGGTGTGTTCACACCTCCTATGCAGTAAAAGACGGAGTTTGTCAGA
GTTAATAAAATTGTGGACGTTGGCAGTATCGTTTGTATAAAAGATTGTCCTACTGGAACCTCGGTCGA
TACCAGACATACCCA
+
FFFF,F:F:F::F:F,FF:FFFF,FFFFF,FFF:FF,FFFFFFF,FFF,F,FFFFFF,FFFF,,F::FFF
FFFF::F,,FFF,FFFFFF,FF:,FFFFFF,:F:FFF,F:F:F,,F,F:F,:FFFFF:F,,FFF,,,FFF
FF,,,FFFFFF,,F:
@A00721:190:HGJW7DSXY:4:1304:2980:18333 1:N:0:TCTGCAAG+GCTGCACT
CTCTGAGATATAATCGAAGACTTGGTGTGTTCACACCTCCTATGCAGTACAAGACGGAGTTTGTCAGA
GTTAATAATATTGTGGACGTTGGCAGTATCGTTTGTATAAATGATTGTCCTACTGGAACCTCGGTCGA
TACGAGACATACCCA
+
FFFFFFFFFFFFFFFFFFFFFFFFFFFFFFFFFFFFFFFFFFFFFFFFFFFFFFFFFFFFFFFFFFFFFF
FFFFFFFFFFFFFFFFFFFFFFFFFFFFFFFF:FFFFFFFFFFFFFFFFFFFFFFFFFFFFFFFFFFFFF
F:FFFFFFFFFFFFF
@A00721:190:HGJW7DSXY:4:1346:10963:35102 1:N:0:TCTGCAAG+GCTGCACT
CTCTGAGATATAATCGAAGACTTGGTGTGTTCACACCTCCTATGCAGTACAAGACGGAGTTTGTCAGA
GTTAATAATATTGTGGACGTTGGCAGTATCGTTTGTATAAATGATTGTCCTACTGGAACCTCGGTCGA
TACGAGACATACCCA
+
FFFFFFFFFFFFFFFFFFFFFFFFFFFFFFFFFFFFFFFFFFFFFFFFFFFFFFFFFFFFFFFFFFFFFF
FFFFFFFFFFFFFFFFFFFFFFFFFFFFFFF::FFFFFF:FFFFFFFFFFFFFFFFFFFFFFFFFFFFFF
FFFFFFFFFFFFFFF
@A00721:190:HGJW7DSXY:4:1467:1814:7764 1:N:0:TCTGCAAG+GCTGCACT
CTCTGAGATATAATCGAAGACTTGGTGTGTTCACACCTCCTATGCAGTACAAGACGGAGTTTGTCAGA
GTTAATAATATTGTGGACGTTGGCAGTATCGTTTGTATAAATGATTGTCCTACTGGAACCTCGGTCGA
TACGAGACATACCCA
+
FFFFFFFFF:FFFFFFFFFFFFFFFFFFFFFFFFFFFFFFFFFFFFFFFFFFFFFFFFFFFFFFFFFFFF
FFFFFFFFFFFFFFFFFFFFFFFFFFFFF:FFF:FFFFFFFFFFFFFFFFFFFFFFFFFFFFFFFFFFFF
FFFFFFFFFFFFFFF
@A00721:190:HGJW7DSXY:4:1658:25491:33646 1:N:0:TCTGCAAG+GCTGCACT
CTCTGAGATATAATCGAAGACTTGGTGTGTTCACACCTCCTATGCAGTACAAGACGGAGTTTGTCAGA
GTTAATAATATTGTGGACGTTGGCAGTATCGTTTGTATAAATGATTGTCCTACTGGAACCTCGGTCGA
TACGAGACATACCCA
+
FFFFFFFFFFFFFFFFFFFFFFF,FFFFFFFFFFFFFFFFFFFFFFFFFFFFFFFF,FFFF,FFFFFFFF
FFFFFFFFFF:FFFFFFFFF,FFFFFF:FF:FF:::FFFFFFFFFFFFF:FFFFFFFFFFFFFFFFFFFF
FFFFFFFFFFFF,FF
@A00721:190:HGJW7DSXY:4:2402:12807:25363 1:N:0:TCTGCAAG+GCTGCACT
CTCTGAGATATAATCGAAGACTTGGTGTGTTCACACCTCCTATGCAGTACAAGACGGAGTTTGTCAGA
GTTAATAATATTGTGGACGTTGGCAGTATCGTTTGTATAAATGATTGTCCTACTGGAACCTCGGTCGA
TACGAGACATACCCA
+
FFFFFFFFFFFFFFFFFFFFFFFFFFFFFFFFFFFFFFFFFFFFFFFFFFFFFFFFFF:FFFFFFFFFFF
FFFFFF:FFFF:FFFFFFFFF:FFFFFFF:FF:FFFFFFFFFFFFFFFFFFFFFFF:FFFFFFFFFFF:F
FFF:F:FFFFFF:FF
@A00721:190:HGJW7DSXY:4:2577:15221:4993 1:N:0:TCTGCAAG+GCTGCACT
CTCTGAGATATAATCGAAGACTTGGTGTGTTCACACCTCCTATGCAGTACAAGACGGAGTTTGTCAGA
GTTAATAATATTGTGGACGTTGGCAGTATCGTTTGTATAAATGATTGTCCTACTGGAACCTCGGTCGA
TACGAGACATACCCA
+
FFFFFFFFFFFFFFFFFFFFFFFFFFFFFFFFFFFFFFFFFFFFFFFFFFFFFFFFFFFFFFFFFFFFFF

```
FFFFFFFFFFFFFFFFFFFFFFFFFFFFFFFFFFFFFFFFFFFFFFFFFFFFFFFFFFFFFFFFFFFFFF
FFF:FFFFFFFFFFF
@A00721:190:HGJW7DSXY:4:1244:12825:8234 1:N:0:TCTGCAAG+GCTGCACT
CTGAGATATAATCGAAGACTTGGTGTGTTCACACCTCCTATGCAGTACAAGACGGAGTTTGTCAGAGT
TAATAATATTGTGGACGTTGGCAGTATCGTTTGTATAAATGATTGTCCTACTGGAACCTCGGTCGATA
CGAGACATACCCAGA
+
FFFFFFFFFFFFFFFFFFFFFFFFFFFFFFFFFFFFFFFFFFFFFFFFFFFFFFFFFFFFFFFFFFFFFF
FFFFFFFFFFFFFFFFFFFFFFFFFFFF:FFFFFFFFFFFFFFFFFFFFFFFFFFFFFFFFFFFFFFFFF
FFFFFFFFFFFFFFF
@A00721:190:HGJW7DSXY:4:1641:27959:20384 1:N:0:TCTGCAAG+GCTGCACT
CTGAGATATAATCGAAGACTTGGTGTGTTCACACCTCCTATGCAGTACAAGACGGAGTTTGTCAGAGT
TAATAATATTGTGGACGTTGGCAGTATCGTTTGTATAAATGATTGTCCTACTGGAACCTCGGTCGATA
CGAGACATACCCAGA
+
FFFFFFFFFFFFFFFFFFFFFFFFFFFFFFFFFFFFFFFFFFFFFFFFFFFFFFFFFFFFFFFFFFFFF,
FFFFFFFFFFFFFFFFFFFFFFFFFFFFFFFFFFF,FFFFFFFFFF:FF,FFFFFFFFFFFFFFFFFF
FFFFFFFFFFFFFFF
@A00721:190:HGJW7DSXY:4:1674:12843:14747 1:N:0:TCTGCAAG+GCTGCACT
CTGAGATATAATCGAAGACTTGGTGTGTTCACACCTCCTATGCAGTACAAGACGGAGTTTGTCAGAGT
TAATAATATTGTGGACGTTGGCAGTATCGTTTGTATAAATGATTGTCCTACTGGAACCTCGGTCGATA
CGAGACATACCCAGA
+
FFFFFFFFFFFFFFFFFFFFFFFFFFFFFFFFFFFFFFFFFFFFFFFFFFFFFFFFFFFFFFFFFFFFFF
FFFFFFFFFFFFFFFFFFFFFFFFFFF:FFFFFFFFFFFFFFFFFFFFFFFFFFFFFFFFFFFFFFFFFF
FFFFFFFFFFFFFFF
@A00721:190:HGJW7DSXY:4:2102:22996:10848 1:N:0:TCTGCAAG+GCTGCACT
CTGAGATATAATCGAAGACTTGGTGTGTTCACACCTCCTATGCAGTACAAGACGGAGTTTGTCAGAGT
TAATAATATTGTGGACGTTGGCAGTATCGTTTGTATAAATGATTGTCCTACTGGAACCTCGGTCGATA
CGAGACATACCCAGA
+
FFFFFFFFFFFFFFFFFFFFFFFFFFFFFFFFFFFFFFFFFFFFFFFFFFFFFFFFFFFFFFFFFFFFFF
FFFFFFFFFFFFFFFFFFFFFFFFFFFFFFF,FFFFFFFFFFFFFFFFFFFFFFFFFFFFFFFFFFFFFF
FFFFFFFFFFFFFFF
@A00721:190:HGJW7DSXY:4:2139:27227:14606 1:N:0:TCTGCAAG+GCTGCACT
CTGAGATATAATCGAAGACTTGGTGTGTTCACACCTCCTATGCAGTACAAGACGGAGTTTGTCAGAGT
TAATAATATTGTGGACGTTGGCAGTATCGTTTGTATAAATGATTGTCCTACTGGAACCTCGGTCGATA
CGAGACATACCCAGA
+
FFFFFFFFFFFFFFFFFFFFFFFFFFFFFFFFFFFFFFFFFFFFFFFFFFFFFFFFFFFFFFFFFF:FFF
FFFFFFFFFFFFFFFFFFFFFFFFFFFFFFFFFFFFFFFFFFFFFFFF:FFFFFFFFFFFFFFFFFFFFF
FFFFFFFFFFFFFFF
@A00721:190:HGJW7DSXY:4:1349:1136:6402 1:N:0:TCTGCAAG+GCTGCACT
AGATATAATCGAAGACTTGGTGTGTTCACACCTCCTATGCAGTACAAGACGGAGTTTGTCAGAGTTAA
TAATATTGTGGACGTTGGCAGTATCGTTTGTATAAATGATTGTCCTACTGGAACCTCGGTCGATACGA
GACATACCCAGA
+
F:F,F:FFFF,FFFFFFFFFFF:FFFFFFFFFFFFFFFFFFFFFFFFFF:F:FFFFFFFFFFFF:FFFF
FFFFFF:FFFFFFFFF,FFFFFFFFFFF:FFFFFFF,FF:FFF:FF,FFFFFFFFFF:FFFFFFF,FF
FF:FFFFF:FFF
@A00721:190:HGJW7DSXY:4:1578:17219:19351 1:N:0:TCTGCAAG+GCTGCACT
AGATATAATCGAAGACTTGGTGTGTTCACACCTCCTATGCAGTACAAGACGGAGTTTGTCAGAGTTAA
TAATATTGTGGACGTTGGCAGTATCGTTTGTATAAATGATTGTCCTACTGGAACCTCGGTCGATACGA
GAGATCTGAAGAG
```

+
F,F,F:FF:F:F:FF:FF:FF:FF,FFF:FF,FFFFFFF,FFFFFFFFF,FFF,FFF:FF,FFFFFFFFF
FFFFFFF:F:FFFFFFFFF,:FFF:FFFFFFFFFFFFF,FFFF:F,FF::F,FFFFFFF,FFFFFFFFFF
:::FFF,FFFF:F
@A00721:190:HGJW7DSXY:4:1126:21296:12101 1:N:0:TCTGCAAG+GCTGCACT
CTCACTTTCTGGGTATGTCTCGTATCGACCGAGGTTCCAGTAGGACAATCATTTATACAAACGATACT
GCCAACGTCCACAATATTATTAACTCTGACAAACTCCGTCTTGTACTGCATAGGAGGTGTGAACACAC
CAAGTCTTCGATTAT
+
FFFFFFFFFFFFFFFFFFFFFFFFFFFFFFFFFFFFFFFFFFFFFFFFFFFFFFFFFFFFFFFFFFFFFF
FFFFFFFFFFFFFFFFFFFFFFFFFFFFFFFFFFFFFFFFFFFFFFFFFFFFFFFFFFFFFFFFFFFFFF
FFFFFFFFFFFFFFF
@A00721:190:HGJW7DSXY:4:1509:9824:33004 1:N:0:TCTGCAAG+GCTGCACT
CTCACTTTCTGGGTATGTCTCGTATCGACCGAGGTTCCAGTAGGACAATCATTTATACAAACGATACT
GCCAACGTCCACAATATTATTAACTCTGACAAACTCCGTCTTGTACTGCATAGGAGGTGTGAACACAC
CAAGTCTTCGATTAT
+
FFFFFFFFFFFFFFFFFFFFFFFFFFFFFFFFFFFFFFFF:FFFFFFFFFFFFFFFFFFFFFFFFFFFFF
FFFFFFFFFFFFFFFFFFFFFFFFFFFFFFF:FFFFFFFFFFFFFFFFFFF:FFFFFFFFFFFFFFFFFF
FFFFFFFFFFFFFFF
@A00721:190:HGJW7DSXY:4:2455:3947:28244 1:N:0:TCTGCAAG+GCTGCACT
CTCACTTTCTGGGTATGTCTCGTATCGACCGAGGTTCCAGTAGGACAATCATTTATACAAACGATACT
GCCAACGTCCACAATATTATTAACTCTGACAAACTCCGTCTTGTACTGCATAGGAGGTGTGAACACAC
CAAGTCTTCGATTAT
+
FFFFFFFFFFFFFFFFFFFFFFFFFFFFFFFFFFFFFFFFFFFFFFFFFFFFFFFFFFFFFFFFFFFFFF
FFFFFFFFFFFF:FFFFFF,FFFFFFFFFFFFFFFFFFFFFFFFFFFFFFFFFFFFFFFFFFFFFFFFFF
FFFFFFFFFFF:FFF
@A00721:190:HGJW7DSXY:4:2527:16685:21245 1:N:0:TCTGCAAG+GCTGCACT
CTCACTTTCTGGGTATGTCTCGTATCGACCGAGGTTCCAGTAGGACAATCATTTATACAAACGATACT
GCCAACGTCCACAATATTATTAACTCTGACAAACTCCGTCTTGTACTGCATAGGAGGTGTGAACACAC
CAAGTCTTCGATTAT
+
FFFFFFFFFFFFFFFFFFFFFFFFFFFFFFFFFFFFFFFFFFFFFFFFFFFFFFFFFFFFFFFFFFFFFF
FFFFFFFFFFFFFFFFFFFFFFFFFFFFFFFFFFFFFFFFFFFFFFFFFFFFFFFFFFFFFFFFFFFFFF
FFFFFFFFFFFFFFF
@A00721:190:HGJW7DSXY:4:2550:31548:1924 1:N:0:TCTGCAAG+GCTGCACT
CTCACTTTCTGGGTATGTCTCGTATCGACCGAGGTTCCAGTAGGACAATCATTTATACAAACGATACT
GCCAACGTCCACAATATTATTAACTCTGACAAACTCCGTCTTGTACTGCATAGGAGGTGTGAACACAC
CAAGTCTTCGATTAT
+
FFFFFFFFFFFFFFFFFFFFFFFFFFFFFFFFFFFFFFFFFFFFFFFFFFFFFFFFFFFFFFFFFFFFFF
FFFFFFFFFFFFFFFFFFFFFFFFFFFFFFFFFFFFFFFFFFFFFFFFFFFFFFFFFFFFFFFFFFFFFF
FFFFFFFFFFFFFFF
@A00721:190:HGJW7DSXY:4:2601:4526:31501 1:N:0:TCTGCAAG+GCTGCACT
CTCACTTTCTGGGTATGTCTCGTATCGACCGAGGTTCCAGTAGGACAATCATTTATACAAACGATACT
GCCAACGTCCACAATATTATTAACTCTGACAAACTCCGTCTTGTACTGCATAGGAGGTGTGAACACAC
CAAGTCTTCGATTAT
+
FFFFFFFFFFFFFFFFFFFFFFFFFFFFFFFFFFFFFFFFFFFFFFFFFFFFFFFFFFFFFFFFFFFFFF
FFFF:FFFFFFFFFFFFFF:FFFFFFFFFFFF:FFFFFFFFFFFFFFFFFF:FFFFFFFFFFFFFFFFFF
FFFFFFFFFFF:FFF
@A00721:190:HGJW7DSXY:4:1102:8847:6261 1:N:0:TCTGCAAG+GCTGCACT
CGAAGACTTGGTGTGTTCACATCTCCTATGCAGTACAAGACGGAGTTTGTCAGAGTTAATAATATTGT

```
GGACGTTGGCAGTATCGTTTGTATAAATGATTGTCCTACTGGAACCTCGGTCGATACGAGACATACCC
AGAAAGTGAGATGGA
+
FFFFFFF:FFFFFFFFFFFFFFFFFFFFFFFFFFFFFFFFFFFFFFFFFFFFFFFFFFFFFFFFFFFF
FFFFFFFFFFFFFFFFF:FFFFFF:FFFFFFFFFFFFFFFFF:FFFFFFFFFFFFFFFFFFFFFFFFF
FFF:FF:FFFFFFFF
@A00721:190:HGJW7DSXY:4:1206:28248:5478 1:N:0:TCTGCAAG+GCTGCACT
CGAAGACTTGGTGTGTTCACATCTCCTATGCAGTACAAGACGGAGTTTGTCAGAGTTAATAATATTGT
GGACGTTGGCAGTATCGTTTGTATAAATGATTGTCCTACTGGAACCTCGGTCGATACGAGACATACCC
AGAAAGTGAGATGGA
+
FFFFFFFFFFFFFFFFFFFFFFFFFFFFFFFFFFFFFFFFFFFFFFFFFFFFFFFFFFFF:FFFFFFF
FFFFFFFFFFFFFFFFFFFFFFFFFFFFFFFFFFFFFFFFFFFFFFFFFFFFFFFFFFFF:FFFFFFF
FFFFFFFFFFFFF:F
@A00721:190:HGJW7DSXY:4:1267:18493:6402 1:N:0:TCTGCAAG+GCTGCACT
CGAAGACTTGGTGTGTTCACATCTCCTATGCAGTACAAGACGGAGTTTGTCAGAGTTAATAATATTGT
GGACGTTGGCAGTATCGTTTGTATAAATGATTGTCCTACTGGAACCTCGGTCGATACGAGACATACCC
AGAAAGTGAGATGGA
+
FFFFFFFFFFFFFFFFFFFFFFFFFFFFFFFFFFFFFFFFFFFFFFFFFFFFFFFFFFFFFFFFFFFF
FFFFFFFFFFFFFFFFFF:FFFFFFFFFFFFFFFFFFFFFFFFFFFFFFFFFFFFFFFFFFFFFFFFF
FFFFFF:FFFFFFFF
@A00721:190:HGJW7DSXY:4:1644:29948:7326 1:N:0:TCTGCAAG+GCTGCACT
TGTGTTCACATCTCCTATGCAGTACAAGACGGAGTTTGTCAGAGTTAATAATATTGTGGACGTTGGCA
GTATCGTTTGTATAAATGATTGTCCTACTGGAACCTCGGTCGATACGAGACATACCCAGAAAGTGAGA
TGGAATCGAATTGTG
+
FFFFFFFFFFFF,FFFFFFFF,:FFFF:FF,FFFFFFFFFFFFFF:FFFFF:F,,F,F:FF::FF:FF
FF,FFFF:FFFF,F:FFFFFFF:FFF::FFFF,FFFF:F,:FFFFFFFFFFFFFFF:,FFFFFFFFFF
F,FFFFFFF::::FF
@A00721:190:HGJW7DSXY:4:2118:5629:13996 1:N:0:TCTGCAAG+GCTGCACT
TGTGTTCACATCTCCTATGCAGTACAAGACGGAGTTTGTCAGAGTTAATAATATTGTGGACGTTGGCA
GTATCGTTTGTATAAAAGATTGTCCTACTGGAACCTCGGTCGATACGAGACATACCCAGAAAGTGAGA
TGGAATCGAATTGTG
+
:F,FF,FFF:FF,FFFFFF::FFF,FFFFFF:FFFFFFFFFFFF,FFFFFF,FF,,:F:FFFFF,FFF
F:FFF,FF,:,FF,F,,:F,FFF,FFFFF:FF:FFFF,::FFFFF,,:,FFFFF::,F,FFFF,,FFF
:,,FFF:,FF,,F,:
@A00721:190:HGJW7DSXY:4:2121:11496:9815 1:N:0:TCTGCAAG+GCTGCACT
TGTGTTCACATCTCCTATGCAGTACAAGACGGAGTTTGTCAGAGTTAATAATATTGTGGACGTTGGCA
GTATCGTTTGTATAAATGATTGTCCTACTGGAACCTCGGTCGATACGAGACATACCCAGAAAGTGAGA
TGGAATCGAATTGTG
+
FFFFFFFFFFFFFFFFFFFFFFFFFFFFFFFFFFFFFFFFFFFFFFFFFFFFFFFFFFFFFFFFFFFF
FFFFFFFFFFFFFFFFFFFFFFFFFFF:FFFFFFFFFFFFFFFFFFFFFFFFFFFFFFFFF:FFFFFFF
FFFFFFFFFF:FFFF
@A00721:190:HGJW7DSXY:4:2635:16604:25081 1:N:0:TCTGCAAG+GCTGCACT
TGTGTTCACATCTCCTATGCAGTACAAGACGGAGTTTGTCAGAGTTAATAATATTGTGGACGTTGGCA
GTATCGTTTGTATAAATGATTGTCCTACTGGAACCTCGGTCGATACGAGACATACCCAGAAAGTGAGA
TGGAATCGAATTGTG
+
FFFFFFFFFFFFFFFFFFFFFFFFFFFFFFFFFFFFFFFFFFFFFFFFFFFFF:FFFFFFFFFFFFFFF
FFFFFFFFFFFFFFFFFFFFFFFFFFFFFFFFFFFFFFFFFFFFFFFFFFFFFFFFFFFFFFFFFFFF
FFFFFFFFFFFFFFF
```

```
@A00721:190:HGJW7DSXY:4:1549:12536:22733 1:N:0:TCTGCAAG+GCTGCACT
GTTCACACCTCCTATGCAGTACAAGACGGAGTTTGTCAGAGTTAATAATATTGTGGACGTTGGCAGTA
TCGTTTGTATAAATGATTGTCCTACTGGAACCTCGGTCGATACGAGACATACCCAGAAAGTGAGATGG
AATCGAATTGT
+
FFFFFFFFFFFFFFFFFFFFFFFFFFFFFFFFFFFFFFFFFFFFFFFFFFFFFFFFFFFFFFFFFFFFFF
FFFFFFFFFFFFFFFFFFFFFFFFFFFFFFFFFFFFFFFFFFFFFFFFFFFFFFFFFFFFFFFFFFFFFF
FFFFFFFFFFF
@A00721:190:HGJW7DSXY:4:2403:1353:22623 1:N:0:TCTGCAAG+GCTGCACT
GTTCACACCTCCTATGCAGTACAAGACGGAGTTTGTCAGAGTTAATAATATTGTGGACGTTGGCAGTA
TCGTTTGTATAAATGATTGTCCTACTGGAACCTCGGTCGATACGAGACATACCCAGAAAGTGAGATGG
AATCGAATTGT
+
FFFFFFFFFFFFFFFFFFFFFFFFFFFFFFFFFFFFFFFFFFFFFFFFFFFFFFFFFFFFFFFFFFFFFF
FFFFFFFFFFFFFFFFFFFFFFFFFFFFFFFFFFFFFFFFFFFFFFFFFFFFFFFFFFFFFFFFFFFFFF
FFFFFFFFFFF
@A00721:190:HGJW7DSXY:4:2420:26106:29951 1:N:0:TCTGCAAG+GCTGCACT
GTTCACACCTCCTATGCAGTACAAGACGGAGTTTGTCAGAGTTAATAATATTGTGGACGTTGGCAGTA
TCGTTTGTATAAATGATTGTCCTACTGGAACCTCGGTCGATACGAGACATACCCAGAAAGTGAGATGG
AATCGAATTGT
+
FFFFFFFFFFFFFFFFFFFFFFFFFFFFFFFFFFFFFFFFFFFFFFFFFFFFFFFFFFFFFFFFFFFFFF
FFF:FFFFF:FFFFFFFFFFFFFFFFFFFFFFFFFFFFFFFFFFFFFFFFFFFFFFFFFFFFFFFFFFFF
F:FFFFFFFFF
@A00721:190:HGJW7DSXY:4:1357:5448:31219 1:N:0:TCTGCAAG+GCTGCACT
CCTCCTATGCAGTACAAGACGGAGTTTGTCAGAGTTAATAATATTGTGGACGTTGGCAGTATCGTTTG
TATAAATGATTGTCCTACTGGAACCTCGGTCGATACGAGACATACCCAGAAAGTGAGATGGAATCGAA
TTGTGCTTAACGGTT
+
FFFFFFFFFFFFFFFFFFFFFFFFFFFFFFF:FFFFFFFFFFFFFFFFFFFFFFFFFFFFFFFFFFFFFF
FFFFFFFFFFFFFFFFFFFFFFF,FFFFFFFFFFFFF,FFFFFFFFFFFFFFFFFFFFFFFFFFFFFFFF
FFFFFFFFFFFFFFF
@A00721:190:HGJW7DSXY:4:2109:28980:14575 1:N:0:TCTGCAAG+GCTGCACT
CCTCCTATGCAGTACAAGACGGAGTTTGTCAGAGTTAATAATATTGTGGACGTTGGCAGTATCGTTTG
TATAAATGATTGTCCTACTGGAACCTCGGTCGATACGAGACATACCCAGAAAGTGAGATGGAATCGAA
TTGTGCTTAACGGTT
+
FFFFFFFFFFFFFFFFFFFFFFFFFFFFFFFFFFFFFFFFFFFFFFFFFFFFFFFFFFFFFFFFFFFFFF
FFFFFFFFFFFFFFFFFFFFFFFFFFFFFFFFFFFFFFFFFFFFFFFFFFFFFFFFFFFFFFFFFFFFFF
FFFFFFFFFFFFF:F
@A00721:190:HGJW7DSXY:4:2248:11207:34303 1:N:0:TCTGCAAG+GCTGCACT
CCTCCTATGCAGTACAAGACGGAGTTTGTCAGAGTTAATAATATTGTGGACGTTGGCAGTATCGTTTG
TATAAATGATTGTCCTACTGGAACCTCGGTCGATACGAGACATACCCAGAAAGTGAGATGGAATCGAA
TTGTGCTTAACGGTT
+
FFFFFFFFFFFFFFFFFFFFFFFFFFFFFFFFFFF,FFFFFFFFFFFFFFFFFFFFFFFFFFFFFFFFFF
FFFF:FFFFFFFFFFFFFFFFF:FFFFFFFFFFFFFFFFFFFFFFFFFFFFFFFFFFFFFFFFF,FFFFF
FFFFFFFFFFF,FFF
@A00721:190:HGJW7DSXY:4:2650:11776:11209 1:N:0:TCTGCAAG+GCTGCACT
CCTCCTATGCAGTACAAGACGGAGTTTGTCAGAGTTAATAATATTGTGGACGTTGGCAGTATCGTTTG
TATAAATGATTGTCCTACTGGAACCTCGGTCGATACGAGACATACCCAGAAAGTGAGATGGAATCGAA
TTGTGCTTAACGGTT
+
FFFFFFFFFFFFFFFFFFFFFFFFFF:FFF:FFFFFFFFFFFFFFFFFFFFFFFFFFFFFFFFFFF,FFF
```

FFFFF:FFFFFFFFFFFFFFFF:FFFFFFFFFFFFFFFFFFFFFFFFFFFFFFFFFFFFFFFF,FFFFF
FFFFFFFFFFFFFF:
@A00721:190:HGJW7DSXY:4:1303:7491:30843 1:N:0:TCTGCAAG+GCTGCACT
ATGCAGTACAAGACGGAGTTTGTCAGAGTTAATAATATTGTGGACGTTGGCAGTATCGTTTGTATAAA
TGATTGTCCTACTGGAACCTCGGTCGATACGAGACATACCCAGAAAGTGAGATGGAATCGAATTGTGC
TTAACGGTTCAGTTA
+
FFFFFFFFFFFFFFFFFFFFFFFFFFFFFFFFFFFFFFFFFFFFFFFFFFFFFFFFFF,FFFFFFFFF
FFFFFFFFFFFFFFFFFFFFFFFFFFFFFFFFFFF:FFFFF,FFFFFFFFFFFFFF:FFFFFFF:FFF
FFFFFFFFFFFF:FF
@A00721:190:HGJW7DSXY:4:1346:23628:3521 1:N:0:TCTGCAAG+GCTGCACT
ATGCAGTACAAGACGGAGTTTGTCAGAGTTAATAATATTGTGGACGTTGGCAGTATCGTTTGTATAAA
TGATTGTCCTACTGGAACCTCGGTCGATACGAGACATACCCAGAAAGTGAGATGGAATCGAATTGTGC
TTAACGGTTCAGTTA
+
FFFFFFFFFFFFFFFFFFFFFFFFFFFFFF:FFFFFFFFFFFFFFFFFFFFFFFFFFFFFFFFFFFFF
FFFFFFFFFFFFFFFFFFFFFFFFFFFFFFFFFFFFFFFFFFFFFFFFFFF:FFFFFFFFFFFFFFFF
FFFFFFFFFFFFFFF
@A00721:190:HGJW7DSXY:4:1554:19425:13526 1:N:0:TCTGCAAG+GCTGCACT
ATGCAGTACAAGACGGAGTTTGTCAGAGTTAATAATATTGTGGACGTTGGCAGTATCGTTTGTAGAAA
TGATTGTCCTACTGGAACCTCGGTCGATACGAGACATACCCAGAAAGTGAGATGGAATCGAATTGTGC
TTAACGGTTCAGTTA
+
FFFFFFFFFFFFFFFFFFFFFFFFFFFFFFFF:FFFFFFFFFFFFFFFFFFFFFFFFFFFFFFFF,FFF
FFFFFFFFFFFFFFFFFFFFFFFFFFFFFFFFFFFFFFFFFFFFFFFFFFFFFFFFF:FFFFFFFFFFF
FFFFFFFFFFFFFFF
@A00721:190:HGJW7DSXY:4:2337:14253:29121 1:N:0:TCTGCAAG+GCTGCACT
ATGCAGTACAAGACGGAGTTTGTCAGAGTTAATAATATTGTGGACGTTGGCAGTATCGTTTGTATAAA
TGATTGTCCTACTGGAACCTCGGTCGATACGAGACATACCCAGAAAGTGAGATGGAATCGAATTGTGC
TTAACGGTTCAGTTA
+
FFFFFFFFFFFFFFFFFFFFFFFFFFFFFFFFFFFFFFFFFFFFFFFFFFFFFFFFFFFFFFFFFFFF
FFFFFFFFFFFFFFFFFFFFFFFFFFFFFFFFFFFFFFFFFFFFFFFFFFFFFFFFFFFFFFFFFFFF
FFFFF:F:FFFFFFF
@A00721:190:HGJW7DSXY:4:2459:5204:17174 1:N:0:TCTGCAAG+GCTGCACT
ATGCAGTACAAGACGGAGTTTGTCAGAGTTAATAATATTGTGGACGTTGGCAGTATCGTTTGTATAAA
TGATTGTCCTACTGGAACCTCGGTCGATACGAGACATACCCAGAAAGTGAGATGGAATCGAATTGTGC
TTAACGGTTCAGTTA
+
FFFFFFFFFFFFFFFFFFFFFFFFFFFFFFFFFFFFFF:FFF,F:FFFFFFFFF:FFF:FFFFFFFFF
FFFFFFFFFFFFFFFFFFFFFFFFFFFFFFFFFFFFFFFFFFFFF:FFFF,FFFFFFF:FFFFFF
FFFFFF:FF:FFFFF
@A00721:190:HGJW7DSXY:4:1268:19840:21668 1:N:0:TCTGCAAG+GCTGCACT
ACAAGACGGAGTTTGTCAGAGTTAATAATATTGTGGACGTTGGCAGTATCGTTTGTATAAATGATTGT
CCTACTGGAACCTCGGTCGATACGAGACATACCCAGAAAGTGAGATGGAATCGAATTGTGCTTAACGG
TTCAGTTAAGTTGAC
+
FFFFFF,FFFFF,FFFFFFF:FF:FFFFFFFFFFF:FFFFFF:FFFFFFFFFFFFFF,FFFFFFFFFF
FFFFFFF:FF:FFF:FFFFFFFFFFFFFFFFFFFFF:FFFFFFFFFFF:F:FFFFFFFFFF:FFFFFF,
:FFFF:FFFFFFFFF
@A00721:190:HGJW7DSXY:4:1336:19678:31782 1:N:0:TCTGCAAG+GCTGCACT
ACAAGACGGAGTTTGTCAGAGTTAATAATATTGTGGACGTTGGCAGTATCGTTTGTATAAATGATTGT
CCTACTGGAACCTCGGTCGATACGAGACATACCCAGAAAGTGAGATGGAATCGAATTGTGCTTAACGG
TTCAGTTAAGTTGAC

```
+
FFFFFFFFFFFFFFFFFFF:FFFFFFFFFFFFFFFFFFFFFFFFFFFFFFFFFFFFFFFFFFFFFFFFF
FFFFFFFFF,FFFFFFFFFFFFFFFFFFFFFFFFFFFFFFFFFFFFFFFFFFFFFFFFFFFFFFFFFFF
FFFFFFFFFFFF:FF
@A00721:190:HGJW7DSXY:4:1577:10285:24158 1:N:0:TCTGCAAG+GCTGCACT
ACAAGACGGAGTTTGTCAGAGTTAATAATATTGTGGACGTTGGCAGTATCGTTTGTATAAATGATTGT
CCTACTGGAACCTCGGTCGATACGAGACATACCCAGAAAGTGAGATGGAATCGAATTGTGCTTAACGG
TTCAGTTAAGTTGAC
+
FFFFFFFFFFFFFFFFFFFFFFFFFFFFFFFFFFFFFFFFFFFFFFFFFFFFFFFFFFFFFFFFFFFFF
FFFFFFFFF,FFFFFFFFFFFFFFF:FFFFFFFFFFFFFFFFFFFFFFFFFFFFFFFFFFFFFFFFFFF
FFF:FFFFFFFFFFF
@A00721:190:HGJW7DSXY:4:1627:26051:6496 1:N:0:TCTGCAAG+GCTGCACT
ACAAGACGGAGTTTGTCAGAGTTAATAATATTGTGGACGTTGGCAGTATCGTTTGTATAAATGATTGT
CCTACTGGAACCTCGGTCGATACGAGACATACCCAGAAAGTGAGATGGAATCGAATTGTGCTTAACGG
TTCAGTTAAGTTGAC
+
FFFF:FFFFFFFFFFFFFF,:FFFFFFFFFFFFFFFFFFFFFFFFF:FF:FFF:FFF:F:FF::F:,FF
FF,FFFFF:,FFF:FFFFFFFFF:FFFF:FFFFFFFFFFFFFFFFFF:FF,FFFFF:FFFFFFFFFFFF
:FFFFF:FFFFF,FF
@A00721:190:HGJW7DSXY:4:1627:25319:7545 1:N:0:TCTGCAAG+GCTGCACT
ACAAGACGGAGTTTGTCAGAGTTAATAATATTGTGGACGTTGGCAGTATCGTTTGTATAAATGATTGT
CCTACTGGAACCTCGGTCGATACGAGACATACCCAGAAAGTGAGATGGAATCGAATTGTGCTTAACGG
TTCAGTTAAGTTGAC
+
FFFFFFFFFFFFFFFFFFFFFFFFFFFFFFFFFFFFFFFFFFFFFFFFFFFFFFFFFFFFFFFFFFFFF
FFFFFFFFFFFFFFFFFFFFFFF:FFFFFFFFFFFFFFFFFFFFFFFFFFF:FFFFF,FFFFFFFFFFFF
:FFFFFFFFFFFFFF
@A00721:190:HGJW7DSXY:4:2207:5457:11412 1:N:0:TCTGCAAG+GCTGCACT
ACAAGACGGAGTTTGTCAGAGTTAATAATATTGTGGACGTTGGCAGTATCGTTTGTATAAATGATTGT
CCTACTGGAACCTCGGTCGATACGAGACATACCCAGAAAGTGAGATGGAATCGAATTGTGCTTAACGG
TTCAGTTAAGTTGAC
+
F:FFFFF:FFFF:F:FFFFF:FFFFFFFFFFFFFFFFFF::FF:F,:F:FFFFFFFFFFFFFFFFFFF
::FFFFFFFFFFFF,FFFFFFFF:F:FFFFFFFFF,FFFF:FFFFFFFFFFFFFFFF,F,FF,FFFFF
FF:F,FFFF::F,F:
@A00721:190:HGJW7DSXY:4:2216:7102:1548 1:N:0:TCTGCAAG+GCTGCACT
ACAAGACGGAGTTTGTCAGAGTTAATAATATTGTGGACGTTGGCAGTATCGTTTGTATAAATGATTGT
CCTACTGGAACCTCGGTCGATACGAGACATACCCAGAAAGTGAGATGGAATCGAATTGTGCTTAACGG
TTCAGTTAAGTTGAC
+
FFFFFFFFFFFFFFFFFFFFFF:FFFFFFFFFFFFFFFFF:FFFFFFFFFF:FFFFFFFFFFFFFF:F
FFFFFFFFFFF:FFF,FFFFFFF:F:FFFFFFFF::FF:FFFFF:FFFFFFFFFFFFFF:FFFFFFFF
F,:FFFFFFFFFFFF
@A00721:190:HGJW7DSXY:4:2274:31394:14434 1:N:0:TCTGCAAG+GCTGCACT
ACAAGACGGAGTTTGTCAGAGTTAATAATATTGTGGACGTTGGCAGTATCGTTTGTATAAATGATTGT
CCTACTGGAACCTCGGTCGATACGAGACATACCCAGAAAGTGAGATGGAATCGAATTGTGCTTAACGG
TTCAGTTAAGTTGAC
+
FFFFFFFFFFFFFFFFFFFF:FFFFFFFFFFFFFFFFFF:FFFFFFFFFFFFFFFFFFFFFFFFFFFFFF
:FFFFFFFFFFFFFFFFFFFFFFFFFFFFFFFFFFFFFFFFFFFFFFFFFFFF,FFFFFFFFFFFFF
::,FFFFFFFFFFF:
@A00721:190:HGJW7DSXY:4:2325:9091:12289 1:N:0:TCTGCAAG+GCTGCACT
ACAAGACGGAGTTTGTCAGAGTTAATAATATTGTGGACGTTGGCAGTATCGTTTGTATAAATGATTGT
```

```
CCTACTGGAACCTCGGTCGATACGAGACATACCCAGAAAGTGAGATGGAATCGAATTGTGCTTAACGG
TTCAGTTAAGTTGAC
+
FFFFFFFFFFFFFFFFFFFFFFFFFFFFFFFFFFFFFFFFFFFFFFFFFFFFFFFFFFFFFFFFFFFF
FFFFFFFFFFFFFFFFFFFFFFFFF,FFFFFFFFFFFFFFFF:FFFFFFFFFFF:F:FFFFFFFFFFF
FFFFFFFFF:FFFFF
@A00721:190:HGJW7DSXY:4:2466:17635:23985 1:N:0:TCTGCAAG+GCTGCACT
ACAAGACGGAGTTTGTCAGAGTTAATAATATTGTGGACGTTGGCAGTATCGTTTGTATAAATGATTGT
CCTACTGGAACCTCGGTCGATACGAGACATACCCAGAAAGTGAGATGGAATCGAATTGTGCTTAACGG
TTCAGTTAAGTTGAC
+
FFFFFFFFFFFFFFFFFFFFFFFFFFFFFFFFFFFFFFFFFFFFFFFFFFFFFFFFFFFFFFFFFFFF
FFFFFFFFFFFFFFFFFFFFFFFFFFFFFFFFFFFFFFFFFFFFFFFFFFFFFFFFFFFFFFFFFFFF
FFFFFF:FFFFFFFF
@A00721:190:HGJW7DSXY:4:2576:7482:32080 1:N:0:TCTGCAAG+GCTGCACT
ACAAGACGGAGTTTGTCAGAGTTAATAATATTGTGGACGTTGGCAGTATCGTTTGTATAAATGATTGT
CCTACTGGAACCTCGGTCGATACGAGACATACCCAGAAAGTGAGATGGAATCGAATTGTGCTTAACGG
TTCAGTTAAGTTGAC
+
FFFFFFFFFFFFFFFFFFFFFFFFFFFFFFFFFFFFFFFFFFFFFFFFFFFFFFFFFFFFFFFFFFFF
FFFFFFFFFFFFFFFFFFFFFFFFFFFFFFFFFFFFFFFFFFFFFFFFF:FFFFFFFFFFFFFFFFFF
FFFFF:FFFFFFFFF
@A00721:190:HGJW7DSXY:4:2634:1859:26631 1:N:0:TCTGCAAG+GCTGCACT
ACAAGACGGAGTTTGTCAGAGTTAATAATATTGTGGACGTTGGCAGTATCGTTTGTATAAATGATTGT
CCTACTGGAACCTCGGTCGATACGAGACATACCCAGAAAGTGAGATGGAATCGAATTGTGCTTAACGG
TTCAGTTAAGTTGAC
+
FFFFFFFFFFFFFFFFFFFFFFFFFFFFFFFFFFFFFFFFFFFFFFFFFFF:FFFFFFFFFFFFFFFF
FFFFFFFFF:FFFFFFFFFFFFFFF:FFFFFFFFFFFFFFFFFFFFFFFFFFFFFF:FFFFFFFFFFF
FFFFFFFFFFFF:FF
@A00721:190:HGJW7DSXY:4:1551:20726:2534 1:N:0:TCTGCAAG+GCTGCACT
ACTTAACTGAACCGTTAAGCACAATTCGATTCCATCTCACTTTCTGGGTATGTCTCGTATCGACCGAG
GTTCCAGTAGGACAATCATTTATACAAACGATACTGCCAACGTCCACAATATTATTAACTCTGACAAA
CTCCGTCTTG
+
FFFFFFFFFFFFFFFFFFFFFFF:FFFFFFFFFFFFFFFFFFFFFFFFFFFFFFFFF:FFFFFFFFFF
FFFFFFFFFFFFF:FFFFFFFFFFFFFFFFFFFFFFFFFFFFFFFFFF,,F:FFFFFF:FFFFFFFFF
FFFFFFFFFF
@A00721:190:HGJW7DSXY:4:1619:5810:6574 1:N:0:TCTGCAAG+GCTGCACT
ACTTAACTGAACCGTTAAGCACAATTCGATTCCATCTCACTTTCTGGGTATGTCTCGTATCGACCGAG
GTTCCAGTAGGACAATCATTTATACAAACGATACTGCCAACGTCCACAATATTATTAACTCTGACAAA
CTCCGTCTTG
+
FFFFFFFFFFFFFFFFFFFFFFFFFFFFFFFFFFFFFFFFFFFFFFFFFFFFFFFFFFFFFFFFFFFF
FFFFFFFFFFFFFFFFFFFFFFFFFFFFFFFFFFFFFFFFFFFFFFFFFFFFFFFFFFFFFFFFFF:F
FFFFFFFFFF
@A00721:190:HGJW7DSXY:4:1643:21269:16157 1:N:0:TCTGCAAG+GCTGCACT
ACTTAACTGAACCGTTAAGCACAATTCGATTCCATCTCACTTTCTGGGTATGTCTCGTATCGACCGAG
GTTCCAGTAGGACAATCATTTATACAAACGATACTGCCAACGTCCACAATATTATTAACTCTGACAAA
CTCCGTCTTG
+
FFFFFFFFFFFFFFFFFFFFFFFFFFFFFFFFFFFFFFFFFFFFFFFFFFFFFFFFFFFFFFFFFFFF
FFFFFFFFFFFFFFFFFFFFFFFFFFFFFFFFFFFFFFFFFFFFFFFFFFFFFFFFFFFFFFFFFFFF
FFFFFFFFFF
```

```
@A00721:190:HGJW7DSXY:4:1125:20699:22185 1:N:0:TCTGCAAG+GCTGCACT
GACGGAGTTTGTCAGAGTTAATAATATTGTGGACGTTGGCAGTATCGTTTGTATAAATGATTGTCCTA
CTGGAACCTCGGTCGATACGAGACATACCCAGAAAGTGAGATGGAATCGAATTGTGCTTAACGGTTCA
GTTAAGTTGACAGAT
+
FFFFFFFFFFFFFFFFFFFFFFFFFFFFFFFFFFFFFFFFFFFFFFFFFFFFFFFFFFFFFFFFFFFFFF
:FFFF:FFFFFFFFFFFFFFFFFFFFFFFFFFFFFFFFFFFFFFFFFFFFFFFFFFFFFFFFFFFFFFFF
FFFFF:FFFFFFFFF
@A00721:190:HGJW7DSXY:4:1126:15474:25160 1:N:0:TCTGCAAG+GCTGCACT
GACGGAGTTTGTCAGAGTTAATAATATTGTGGACGTTGGCAGTATCGTTTGTATAAATGATTGTCCTA
CTGGAACCTCGGTCGATACGAGACATACCCAGAAAGTGAGATGGAATCGAATTGTGCTTAACGGTTCA
GTTAAGTTGACAGAT
+
FFFFFFFFFFFFFFFFFFFFFFFFFFFFFFFFFFFFFFFFFFFFFFFFFFFFFFFFFFFFFFFFFFF,FF
FF:FF:FFFFFFFFFFFFFF:FFFFFFFFF:FFFFFFFFFFFFFFF,FFFFFF:,:FFFFFFFFFFFFFF
:FFFFFFFFFFFFFF
@A00721:190:HGJW7DSXY:4:1136:1398:19883 1:N:0:TCTGCAAG+GCTGCACT
GCTAGTCAACTTAACTGAACCGTTAAGCACAATTCGATTCCATCTCACTTTCTGGGTATGTCTCGTAT
CGACCGAGGTTCCAGTAGGACAATCATTTATACAAACGATACTGCCAACGTCCACAATATTATTAACT
CTGACAAACTCCGTC
+
FFFFFFFFFFFFFFFFFFFFFFFFFFFFFFFFFFFFFFFFFFFFFFFFFFFFFFFFFFFFFFFFFFFFFF
FFFFFFFFFFFFFFFFFFFFFFFFFFFFFFFFFFFF:FFFFFFFFFFFFF::FFFFFFFFFFFFFFFFFF
FFFFFFFFFFFFFFF
@A00721:190:HGJW7DSXY:4:1148:18521:18630 1:N:0:TCTGCAAG+GCTGCACT
GCTAGTCAACTTAACTGAACCGTTAAGCACAATTCGATTCCATCTCACTTTCTGGGTATGTCTCGTAT
CGACCGAGGTTCCAGTAGGACAATCATTTATACAAACGATACTGCCAACGTCCACAATATTATTAACT
CTGACAAACTCCGTC
+
F,FF:FFFFFFFFF,F:FFFFFF:FFFFF:FFFFFFFFFFFFFFFF,:FFFFFFFFFFFFFFFF,FFFFF
FF,FF:FF:F::FFF,,FFFFF,FFFFFFFF:FFFF:F,FFF:F:,FFFF::F:FFFFFFFFFFFFFF:F
:FFFFFFFFFF,FFF
@A00721:190:HGJW7DSXY:4:1355:6352:28056 1:N:0:TCTGCAAG+GCTGCACT
GACGGAGTTTGTCAGAGTTAATAATATTGTGGACGTTGGCAGTATCGTTTGTATAAATGATTGTCCTA
CTGGAACCTCGGTCGATACGAGACATACCCAGAAAGTGAGATGGAATCGAATTGTGCTTAACGGTTCA
GTTAAGTTGACAGAT
+
FFFFFFFFFFFFFFFFFFF:FFFFFFFFF:FF:FFFFFFFFFFFFF:FFF,FFFFFFFFFFFFFF:FFFF
FFFFFFFFFF::FFFFFFFFFFFFFF:FF:FFF:FFFFFFFFFFFFFFFFFF:F:FFFFFFFF::FFFFF
,FFFFFFF:FFFFFF
@A00721:190:HGJW7DSXY:4:1359:20184:27743 1:N:0:TCTGCAAG+GCTGCACT
GCTAGTCAACTTAACTGAACCGTTAAGCACAATTCGATTCCATCTCACTTTCTGGGTATGTCTCGTAT
CGACCGAGGTTCCAGTAGGACAATCATTTATACAAACGATACTGCCAACGTCCACAATATTATTAACT
CTGACAAACTCCGTC
+
FFFFFFFFFFFFFFFFFFFFFFFFFFFFFFFFFFFFFFFFFFFFFFFFFFFFFFFFFFFFFFFFFFF:FF
FFFFFFFFFFFFFFFFFFFFFFFFFFF:FFFFFFFFFFFFFFFFFF:FFFFFFFFFFFF,FFFFFFFFFF
FFFFFFFFFFFFFFF
@A00721:190:HGJW7DSXY:4:1359:19569:28902 1:N:0:TCTGCAAG+GCTGCACT
GCTAGTCAACTTAACTGAACCGTTAAGCACAATTCGATTCCATCTCACTTTCTGGGTATGTCTCGTAT
CGACCGAGGTTCCAGTAGGACAATCATTTATACAAACGATACTGCCAACGTCCACAATATTATTAACT
CTGACAAACTCCGTC
+
FFFFFFFFFFFFFFFFFFFFFFFFFFFFFFFFFFFFFFFFFFFF:FFFFFFFFFFFFFFFFFFFFFFFFF
```

```
FFFFFFFFFFFFFFFFFFFFFFFFFFFFFFFFFFFF:FFFFFFFFFFFFFF:FFFFFF:FFFFFFFFFF
FFFFFFFFFFFFFFF
@A00721:190:HGJW7DSXY:4:1510:12843:36104 1:N:0:TCTGCAAG+GCTGCACT
GACGGAGTTTGTCAGAGTTAATAATATTGTGGACGTTGGCAGTATCGTTTGTATAAATGATTGTCCTA
CTGGAACCTCGGTCGATACGAGACATACCCAGAAAGTGAGATGGAATCGAATTGTGCTTAACGGTTCA
GTTAAGTTGACAGAT
+
FFFFFFFFFFFFFFFFFFFFFFFFFFFFFFFFFFFFFFFFFFFFFFFFFFFFFFFFFFFFFFFFFFFF
FFFFFFFFFFFFFFFFFFFFFFFFFFFFFFFFFFFFFFFFFFFFFFFFFFFFFFFFFFFFFF,FFFFFF
FFFFFFFFFFFFFFF
@A00721:190:HGJW7DSXY:4:1574:15203:9502 1:N:0:TCTGCAAG+GCTGCACT
GCTAGTCAACTTAACTGAACCGTTAAGCACAATTCGATTCCATCTCACTTTCTGGGTATGTCTCGTAT
CGACCGAGGTTCCAGTAGGACAATCATTTATACAAACGATACTGCCAACGTCCACAATATTATTAACT
CTGACAAACTCCGTC
+
FFFFFFFFFFFFFFFFFFFFFFFFFFFFFFFFFFFFFFFFFF:FFFFFFFFFFFFFFFFFFFFFFFFF
FFFFFFFFFFFFFFFFFFFFFFFFFFFFFFFFFFFF:FFFFFFFFFFFFFFFFFFFFFFFFFFFFFFF
FFFFFFFFFFFFF:F
@A00721:190:HGJW7DSXY:4:2142:18159:22420 1:N:0:TCTGCAAG+GCTGCACT
GACGGAGTTTGTCAGAGTTAATAATATTGTGGACGTTGGCAGTATCGTTTGTATAAATGATTGTCCTA
CTGGAACCTCGGTCGATACGAGACATACCCAGAAAGTGAGATGGAATCGAATTGTGCTTAACGGTTCA
GTTAAGTTGACAGAT
+
FFFFFFFFFFFFFFFFFFFFFFFFFFFFFFFFFFFFFFFFFFFFFFFFFFFFFFFFFFFFFFFFFFFF
FFFFFFFFFFFFFFFFFFFFFFFFFFFFFFFFFFF:FFFFFFFFFFFFFFFFFFFFFFFFFFFFFFFF
FFFFFFFFFFFFFFF
@A00721:190:HGJW7DSXY:4:2253:29713:5415 1:N:0:TCTGCAAG+GCTGCACT
GACGGAGTTTGTCAGAGTTAATAATATTGTGGACGTTGGCAGTATCGTTTGTATAAATGATTGTCCTA
CTGGAACCTCGGTCGATACGAGACATACCCAGAAAGTGAGATGGAATCGAATTGTGCTTAACGGTTCA
GTTAAGTTGACAGAT
+
FFFFFFFFFFFFFFFFFFFFFFFFFFFFFFFFFFFFFFFFFFFFFFFFFFFFFFFFFFFFFFFFFFFF
FFFFFFFFFFFFFFFFFFFFFFFFFFFFFFFFFFFFFFFFFFFFFFFFFFFFFFFFFFFFFFFFFFFF
FFFFFFFFFFFFFFF
@A00721:190:HGJW7DSXY:4:2477:14877:9032 1:N:0:TCTGCAAG+GCTGCACT
GACGGAGTTTGTCAGAGTTAATAATATTGTGGACGTTGGCAGTATCGTTTGTATAAATGATTGTCCTA
CTGGAACCTCGGTCGATACGAGACATACCCAGAAAGTGAGATGGAATCGAATTGTGCTTAACGGTTCA
GTTAAGTTGACAGAT
+
FFFFFFFFFFFFFFFFFF,FFFFFFFFFFFFFFFFFFFFFFFFFFFFFFFFFFFFFFFFFFFFFFFFF
FFFFFFFFFFFFFFFFFFFFFFFFF:FFFFFFFFFFFFFFFFFFFFF:FFFFFFFFFFFFFFFFFFF:FFF
FFFFFFF:FFFFFFF
@A00721:190:HGJW7DSXY:4:2665:7473:31031 1:N:0:TCTGCAAG+GCTGCACT
GACGGAGTTTGTCAGAGTTAATAATATTGTGGACGTTGGCAGTATCGTTTGTATAAATGATTGTCCTA
CTGGAACCTCGGTCGATACGAGACATACCCAGAAAGTGAGATGGAATCGAATTGTGCTTAACGGTTCA
GTTAAGTTGACAGAT
+
FFFFFFFFFFFFFFFFFFFFFFFFFFFFFFFFFFFFFFFFFFFFFFFFFFFFFFFFFFFF:FFFF:FFF
FFFFFFFFFFFFFFFFFFFFFFFFFFFFFFFFFFFFFFFFFF:FFFFFFFFFFFFFFFFFFFF,FFFFF
FFFFFFFFFFFFFFF
@A00721:190:HGJW7DSXY:4:2372:18304:36542 1:N:0:TCTGCAAG+GCTGCACT
CACGGAGTTTGTCAGAGTTAATAATATTGTGGACGTTGGCAGTATCGTTTGTATAAATGATTGTCCTA
CTGGAACCTCGGTCGATACGAGACATACCCAGAAAGTGAGATGGAATCGAATTGTGCTTAACGGTTCA
GTTAAGTTGACTAGC
```

+
FFFFFFFFFFFFFFFFFFFFF:FFFFFFFFFFF:FFFFFFFFFFFFFFFFF:FFFFFFFFFFFFFFF:
FFFFFFFFFFFFFFFFFFFFF:FFFFFFFFFFFFFFFFFFFFF:FFFFF:FFFFFFFFFFFFFFFFFFF
:FFFFFFF:FFFF,F
@A00721:190:HGJW7DSXY:4:2372:18222:36558 1:N:0:TCTGCAAG+GCTGCACT
CACGGAGTTTGTCAGAGTTAATAATATTGTGGACGTTGGCAGTATCGTTTGTATAAATGATTGTCCTA
CTGGAACCTCGGTCGATACGAGACATACCCAGAAAGTGAGATGGAATCGAATTGTGCTTAACGGTTCA
GTTAAGTTGACTAGC
+
FFFFFFFFFFFFFFFFFFFFFFFFFFFFFFFFFFFFFFFFFFFFFFFFFFFFFFFFFFFFFFFFFFFF
FFFFFFFFFFFFFFFFFFFFFFFFFFFFFFFFFFFFFFFFFFFFFFFFFFFFFFFFFFFFFFFFFFFF
FFFFFFFFFFFFFFF
@A00721:190:HGJW7DSXY:4:1163:32850:2049 1:N:0:TCTGCAAG+GCTGCACT
GGAGTTTGTCAGAGTTAATAATATTGTGGACGTTGGCAGTATCGTTTGTATAAATGATTGTCCTACTG
GAACCTCGGTCGATACGAGACATACCCAGAAAGTGAGATGGAATCGAATTGTGCTTAACGGTTCAGTT
AAGTTGACTAGCGAA
+
FFFFFFFFFFFFFFFFFFFFFFFFFFFFFFFFFFFFFFFFFFFFFFFFFFFFFFFFFFFFFFFFFFFF
FFFFFFFFFFFFFFFFFFFFFFFFFFFFFFFFFFFFFFFFFFFFFFFFFFFFFFFFFFFFFFFF:FFF
FFFFFFFFFFFFF:F
@A00721:190:HGJW7DSXY:4:1337:11650:28964 1:N:0:TCTGCAAG+GCTGCACT
GGAGTTTGTCAGAGTTAATAATATTGTGGACGTTGGCAGTATCGTTTGTATAAATGATTGTCCTACTG
GAACCTCGGTCGATACGAGACATACCCAGAAAGTGAGATGGAATCGAATTGTGCTTAACGGTTCAGTT
AAGTTGACTAGCGAA
+
FFFFFFFFFFFFFFFFFFFFFFFFFFFFFFFFFFFFFFFFFFFFFFFFFFFFFFFFFFFFFFFFFFFF
FFFFFFFFFFFFFFFFFF:FFFFFFFFFFFFFFFFFFFFFFFFFFFFFFFFFFFFFFFFFFFFFFFFF
FFFFFFFFFFFF:FF
@A00721:190:HGJW7DSXY:4:2152:32823:34225 1:N:0:TCTGCAAG+GCTGCACT
GGAGTTTGTCAGAGTTAATAATATTGTGGACGTTGGCAGTATCGTTTGTATAAATGATTGTCCTACTG
GAACCTCGGTCGATACGAGACATACCCAGAAAGTGAGATGGAATCGAATTGTGCTTAACGGTTCAGTT
AAGTTGACTAGCGAA
+
FFFFFFFFFFFFFFFFFFFFFFFFFFFFFFFFFFFFFFFFFFFFF:FFFFFFFFFFFFFFFFFFFFFF
FFFFFFFFFFFFFFFFF:FFFFFFFFFFF:FFFFFFFFFF:F:FFFFFFFFFFFFFFFFFFFFFFFFF
FFFFFF,FFFFFFFF
@A00721:190:HGJW7DSXY:4:1337:4616:2754 1:N:0:TCTGCAAG+GCTGCACT
CAGAGTTAATAATATTGTGGACGTTGGCAGTATCGTTTGTATAAATGATTGTCCTACTGGAACCTCGG
TCGATACGAGACATACCCAGAAAGTGAGATGGAATCGAATTGTGCTTAACGGTTCAGTTAAGTTGACT
AGCGAAGCCAATGTT
+
FFFFFFFFFFFFFFFFFFFFFFFFFFFFFFFFFFFFFFFFFFFFFFFFFFFFFFFFFFFFFFFFFFFF
FFFFFFFFFFFFFFFFFFFFFFFFFFFFFFFFFF:FFFF:FFFFFFFFFFFFFFFFFFFFFFFFFFFF
FFFFFF:FFFFFFFF
@A00721:190:HGJW7DSXY:4:1350:24659:17644 1:N:0:TCTGCAAG+GCTGCACT
CAGAGTTAATAATATTGTGGACGTTGGCAGTATCGTTTGTATAAATGATTGTCCTACTGGAACCTCGG
TCGATACGAGACATACCCAGAAAGTGAGATGGAATCGAATTGTGCTTAACGGTTCAGTTAAGTTGACT
AGCGAAGCCAATGTT
+
FFFFFFFFFFFFFFFFFFFFFFFFFFFFFFFFFFFFFFFFFFFFFFFFFFFFFFFFFFFFFFFFFFFF
FFFFFFFFFFFFFFFFFFFFFFFFFFFFFFFFFFFFFFFFFF:FFFFFFFFFFFFFFF:FFFFFFFFF
F:FFFFFFFFFFFFF
@A00721:190:HGJW7DSXY:4:1548:21920:27899 1:N:0:TCTGCAAG+GCTGCACT
CAGAGTTAATAATATTGTGGACGTTGGCAGTATCGTTTGTATAAATGATTGTCCTACTGGAACCTCGG

TCGATACGAGACATACCCAGAAAGTGAGATGGAATCGAATTGTGCTTAACGGTTCAGTTAAGTTGACT
AGCGAAGCCAATGTT
+
FF:FFFFFFFFFFFFFFFFFFFFFFFFFFFFFFFFFFFFFFFFFFFFFFFFFFFFFFFFFFFFFFFFFFF
FFFFFFFFFFFFFFFFFFFFFFFFFFFFFFFFFFFFFFFFFFFFFFFFFFFFFFFFFFFFFFFFFFFFF,
F:FFFFFFFFFF:FF
@A00721:190:HGJW7DSXY:4:1578:15546:17425 1:N:0:TCTGCAAG+GCTGCACT
CAGAGTTAATAATATTGTGGACGTTGGCAGTATCGTTTGTATAAATGATTGTCCTACTGGAACCTCGG
TCGATACGAGACATACCCAGAAAGTGAGATGGAATCGAATTGTGCTTAACGGTTCAGTTAAGTTGACT
AGCGAAGCCAATGTT
+
FFFFFFFFFFFFFFFFFFFFFF,FFFFFFFFFFFFFFFFFFFFFFFFFFFFFFFFFFFFFFFFFFFFFFF
FFFFFFFFFFFFFFFFFFFFFFFFFFFFFFFFFFFFFFFFFFFFFFFFFFFFFFFFFFFFFFFFFFF:FF
FFFFFFFFFFFFFFF
@A00721:190:HGJW7DSXY:4:1612:7048:14450 1:N:0:TCTGCAAG+GCTGCACT
CAGAGTTAATAATATTGTGGACGTTGGCAGTATCGTTTGTATAAATGATTGTCCTACTGGAACCTCGG
TCGATACGAGACATACCCAGAAAGTGAGATGGAATCGAATTGTGCTTAACGGTTCAGTTAAGTTGACT
AGCGAAGCCAATGTT
+
FFFFFFFFFFFFFFFFFFFFFFFFFFFFFFFFFFF:FFFFFFFFFFFFFFFFFFFFFFFFFFFFFFFFFF
FFFFFFFFFFFFFFFFFFFFFFFFFFFFFFFFFFFFFFFFFFFFFFFFFFFFFFFFFFFFFFFFFFFFFF
F:FFFF:FFFFFFFF
@A00721:190:HGJW7DSXY:4:2109:32714:25770 1:N:0:TCTGCAAG+GCTGCACT
CAGAGTTAATAATATTGTGGACGTTGGAAGTATCGTTTGTATAAATGATATTCCTACTTGAACCTCGG
TCGATACGAGACATACCCAGAAAGTGAGATGGAATCGAATTTTGCTTAACGTTTCAGTTAAGTTTACT
AGCGAAGCCAA
+
FFFFF:FFFFFF,FFF,,F,,F:FF::,F:FF:,FFF:,FFFF:FF,:F,,,FF:F:,,,F:F,FF,,
F,FFFF,,F,F,FFFF,FFF:FFF:,::FFF:F:FF,FF,,:F::,,FFFF,,F:F,:FFF,,,,,,F
F:,F,F:FFFF
@A00721:190:HGJW7DSXY:4:2169:4761:3693 1:N:0:TCTGCAAG+GCTGCACT
CAGAGTTAATAATATTGTGGACGTTGGCAGTATCGTTTGTATAAATGATTGTCCTACTGGAACCTCGG
TCGATACGAGACATACCCAGAAAGTGAGATGGAATCGAATTGTGCTTAACGGTTCAGTTAAGTTGACT
AGCGAAGCCAATGTT
+
FFFFFFFFFFFFFFFFFFFFFFFFFFFFFFFFFFFFFFFFFFFFFFFFFFFFFFFFFFFFFFFFFFFFFF
FFFFFFFFFFFFFFFF:FFFFFFFFFFFFFFFFFFFFFFFFFFFFFFFFFFFFFFFFFFFFFFFFFFFF
F:FFFFFFFFFFFFF
@A00721:190:HGJW7DSXY:4:2237:5394:16877 1:N:0:TCTGCAAG+GCTGCACT
CAGAGTTAATAATATTGTGGACGTTGGCAGTATCGTTTGTATAAATGATTGTCCTACTGGAACCTCGG
TCGATACGAGACATACCCAGAAAGTGAGATGGAATCGAATTGTGCTTAACGGTTCAGTTAAGTTGACT
AGCGAAGCCAATGTT
+
FFFFFFFFFFFFFFFFFFFFFFFFFFFFFFFFFFFFFFFFFFFFFFFFFFFFFFFFFFFFFFFFFFFFFF
FFFFFFFFFFFFFFFFFFFFFFFFFFFFFFFFFF,FFFFFFFFFFFFFFFFFFFFFFFF,FFFFFFFFFF
FFFFFFFFFFFFFFF
@A00721:190:HGJW7DSXY:4:2271:4227:34053 1:N:0:TCTGCAAG+GCTGCACT
CAGAGTTAATAATATTGTGGACGTTGGCAGTATCGTTTGTATAAATGATTGTCCTACTGGAACCTCGG
TCGATACGAGACATACCCAGAAAGTGAGATGGAATCGAATTGTGCTTAACGGTTCAGTTAAGTTGACT
AGCGAAGCCAATGTT
+
FFFFFFFFFFFFFFFFFFFFFFFFFFFFFFFFFFFFFFFFFFFFFFFFFFFFFFFFFFFFFFFFFFFFFF
FFFFFFFFFFFFFFFFFFFFFFFFFFFFFFFFFFFFFFFFFFFFFFFFFFFFFFFFFFFFFFFFFFFFFF
FFFFFFFFFFFFFFF

```
@A00721:190:HGJW7DSXY:4:2377:14091:20917 1:N:0:TCTGCAAG+GCTGCACT
CAGAGTTAATAATATTGTGGACGTTGGCAGTATCGTTTGTATAAATGATTGTCCTACTGGAACCTCGG
TCGATACGAGACATACCCAGAAAGTGAGATGGAATCGAATTGTGCTTAACGGTTCAGTTAAGTTGACT
AGCGAAGCCAATGTT
+
FFFFFFFFFFFFFFFFFFFFFFFFFFFFFFFFFFFFFFFFFFFFFFFFFFFFFFFFFFFFFFFFFFFF
FFFFFFFFFFFFFFFFFFFFFFFFFFFFFFFFFFFFFFFFFFFFFFFFFFFFFFFFFFFFFFFFFFFF
FFFF:F:FFFFFFFF
@A00721:190:HGJW7DSXY:4:2438:27380:6511 1:N:0:TCTGCAAG+GCTGCACT
CAGAGTTAATAATATTGTGGACGTTGGCAGTATCGTTTGTATAAATGATTGTCCTACTGGAACCTCGG
TCGATACGAGACATACCCAGAAAGTGAGATGGAATCGAATTGTGCTTAACGGTTCAGTTAAGTTGACT
AGCGAAGCCAATGTT
+
FFFFFFFFFFFFFFFFFFFFFFFFFFFFFFFFFFFFFFFFFFFFFFFFFFFFF:FFFFFFFFFFFFFF
FFFFFFFFFFFFFFFFFFFFFFFFFFFFFFFFFF:FFFFFFFFFFFFFFF:FFFFFFFFFFFFFFFFFF
FF:FFFFFFF:FFFF
@A00721:190:HGJW7DSXY:4:2508:4643:6151 1:N:0:TCTGCAAG+GCTGCACT
CAGAGTTAATAATATTGTGGACGTTGGCAGTATCGTTTGTATAAATGATTGTCCTACTGGAACCTCGG
TCGATACGAGACATACCCAGAAAGTGAGATGGAATCGAATTGTGCTTAACGGTTCAGTTAAGTTGACT
AGCGAAGCCAATGTT
+
FFFFFFFFFFFFFFFFFFFFFFFFFFFFFFFFFFFFFFFFFFFFFFFFFFFFFFFFFFFFF:FFFFFF
FFFFFFFFF,FFFFFFFFFFFFFFFFFFFFFFFFFFFFFFFFFFFFFFFFFFFFFFFFFFFFFFFFFF
F:FFFFFFF:F:FFF
@A00721:190:HGJW7DSXY:4:2543:21007:23437 1:N:0:TCTGCAAG+GCTGCACT
CAGAGTTAATAATATTGTGGACGTTGGCAGTATCGTTTGTATAAATGATTGTCCTACTGGAACCTCGG
TCGATACGAGACATACCCAGAAAGTGAGATGGAATCGAATTGTGCTTAACGGTTCAGTTAAGTTGACT
AGCGAAGCCAATGTT
+
FFFFFFFFFFFFFFFFFFFFFFFFFFFFFFFFFFFFFFFFFFFFFFFFFFFFFFFFFFFFFFFFFFFF
FFFFFFFFFFFF:FFFFFFFFFFFFFFFFFFFFFFFFFFFFFFFFFFFFFFFFFFFFFFFFFFFFFFF
FFFFFFFFFFFFFFF
@A00721:190:HGJW7DSXY:4:2614:12979:1141 1:N:0:TCTGCAAG+GCTGCACT
CAGAGTTAATAATATTGTGGACGTTGGCAGTATCGTTTGTATAAATGATTGTCCTACTGGAACCTCGG
TCGATACGAGACATACCCAGAAAGTGAGATGGAATCGAATTGTGCTTAACGGTTCAGTTAAGTTGACT
AGCGAAGCCAATGTT
+
FFFFFFFFFFFFFFFFFFFFFFFFF:FFF:FFFFF:FFFFFFFFFF:FF:FFFFFFFF:FFF:FFFFF
FF,FFFF,F,F,:FF:FF:FFFFFFFFFFFFFFFFFFFF,FFFFFFFFFFFFFF,FFFFFFFF:,FFF
F,FFFF:FF:FF:FF
@A00721:190:HGJW7DSXY:4:1456:16929:20102 1:N:0:TCTGCAAG+GCTGCACT
AGAGTTAATAATATTGTGGACGTTGGCAGTATCGTTTGTATAAATGATTGTCCTACTGGAACCTCGGT
CGATACGAGACATACCCAGAAAGTGAGATGGAATCGAATTGTGCTTAAAGGTTAAGTTAAGTTGACTA
GCGAAGCCAATGTT
+
FFFF,FFFFFFFFF,FF,FF::FFF::F:FFF:FFFF:FFFFFFFF:F:F:F,FFFFFF:FF:F:F::
FFFFF,FFFF,FFFFFFFFFFFFF,FFF,:,FF:F:F,FFFFF,FFFF,F::F,F,FFFF:FFFFFFF
,FFFFF,FFFF,FF
@A00721:190:HGJW7DSXY:4:1578:18530:7341 1:N:0:TCTGCAAG+GCTGCACT
AGAGTTAATAATATTGTGGACGTTGGCAGTATCGTTTGTATAAATGATTGTCCTACTGGAACCTCGGT
CGATACGAGACATACCCAGAAAGTGAGATGGAATCGAATTGTGCTTAACGGTTCAGTTAAGTTGACTA
GCGAAGCCAATGTTC
+
FFFFFFFFFFFFFFFFFFFFFFFFFFFFFF:FFFFFFFFFFFF:FFFFFFFFFFFFFFFFFFFFFF:F
```

FFFFFFFFFFFFFFFFFFFFFFFFFFFFFFFF,FFFFFFFFFFFFFFFFFFFFFFFFFFFFFFFFFFF
FFFFFFFFFFFFFFF
@A00721:190:HGJW7DSXY:4:1610:19768:23140 1:N:0:TCTGCAAG+GCTGCACT
AGAGTTAATAATATTGTGGACGTTGGCAGTATCGTTTGTATAAATGATTGTCCTACTGGAACCTCGGT
CGATACGAGACATACCCAGAAAGTGAGATGGAATCGAATTGTGCTTAACGGTTCAGTTAAGTTGACTA
GCGAAGCCAATGTTC
+
FFFFFFFFFFF:FFFFFFFFFFFFFFFFFFFFFFF:FFFFFFFFFFFFF:FFFFFFFFFFFFFFFFFF
FFFFFFFFFFFFFFFFFFFFFFFFFFFFFFFF:FFFFFFF:FFFFFFFFFFFFFFFFFFFFFFFFFFF
FFFFFFFFFF:FFFF
@A00721:190:HGJW7DSXY:4:2660:11605:2300 1:N:0:TCTGCAAG+GCTGCACT
AGAGTTAATAATATTGTGGACGTTGGCAGTATCGTTTGTATAAATGATTGTCCTACTGGAACCTCGGT
CGATACGAGACATACCCAGAAAGTGAGATGGAATCGAATTGTGCTTAACGGTTCAGTTAAGTTGACTA
GCGAAGCCAATGTTC
+
FFFFFFFFFFFFFFFFFFFFFFFFFFFFFFFFFFFFFFFFFFFFFFFFFFFFFFFFFFFFFFFFFFFF
FFFFFFFFFFFFFFFFFFFFFFFFFFFFFFFFFFFFFFFFFFFFFFFFFFFFFFFFFFFF:FFFFFFF
FFFFFFFFFFFFFFF
@A00721:190:HGJW7DSXY:4:1107:21459:4867 1:N:0:TCTGCAAG+GCTGCACT
GAGTTAATAATATTGTGGACGTTGGCAGTATCGTTTGTATAAATGATTGTCCTACTGGAACCTCGGTC
GATACGAGACATACCCAGAAAGTGAGATGGAATCGAATTGTGCTTAACGGTTCAGTTAAGTTGACTAG
CGAAGCCAATG
+
FFFFFFF:FFFF,FFFFFFFFFFFFFFFFFFFFFFF:FFFFFFFFFFFFFFFFFFFFFFFFFFFFFFF
FFFFFFFFFFFFFFFFFFFFF:FFFFFF:FF:F:FFFFFFFFFFFFF:FFFFFFF,FFFFFFFFFFFF
,FFFF:FFFF:
@A00721:190:HGJW7DSXY:4:1328:32199:18364 1:N:0:TCTGCAAG+GCTGCACT
GAGTTAATAATATTGTGGACGTTGGCAGTATCGTTTGTATAAATGATTGTCCTACTGGAACCTCGGTC
GATACGAGACATACCCAGAAAGTGAGATGGAATCGAATTGTGCTTAACGGTTCAGTTAAGTTGACTAG
CGAAGCCAATG
+
FFFFFFFFFFFFFFFFFFFFFFFFFFFFFFFFFFFFFFFFFFFFFFFFFFFFFFFFFFFFFFFFFFFF
FFFFFFFFFFFF:FFFFFFFFFFFFF:FFFFFFFFFFFFFFFFFFFFFFFFFFFFFFFFFFFFFFFFF
FFFFFFFFFFF
@A00721:190:HGJW7DSXY:4:1418:32145:21809 1:N:0:TCTGCAAG+GCTGCACT
GAGTTAATAATATTGTGGACGTTGGCAGTATCGTTTGTATAAATGATTGTCCTACTGGAACCTCGGTC
GATACGAGACATACCCAGAAAGTGAGATGGAATCGAATTGTGCTTAACGGTTCAGTTAAGTTGACTAG
CGAAGCCAAT
+
FFFFFFFFFFFFFFFFFFFFFFFFFFFFFFFFFFFF,FFFFFFFFFFFFF:FFFFFFFFF,FFFFFFF
FFFFFFFFFFFFFFFFFFFFFFFFFFFF:FFFFFFFFF::FFFFFFFFFFFF,FFFFFFFFFFFFFF:
,FFF:FFFFF
@A00721:190:HGJW7DSXY:4:1505:18014:1877 1:N:0:TCTGCAAG+GCTGCACT
GAGTTAATAATATTGTGGACGTTGGCAGTATCGTTTGTATAAATGATTGTCCTACTGGAACCTCGGTC
GATACGAGACATACCCAGAAAGTGAGATGGAATCGAATTGTGCTTAACGGTTCAGTTAAGTTGACTAG
CGAAGCCAATG
+
FFFFFFFFFFFFFFFFFFFFFFFFFFFFFFFFFFFFFFFFFFFFFFFFF:FFF:FFFFFFFFFFFFFF
FFFFFFFFFFFFFFFFF:FFFFFFF:FFFFF::FFFFFFFFF:FFFFF:FFFFFFFFFFFFF,FFFF:
FFFF:FFFFF:
@A00721:190:HGJW7DSXY:4:1556:31801:17738 1:N:0:TCTGCAAG+GCTGCACT
GAGTTAATAATATTGTGGACGTTGGCAGTATCGTTTGTATAAATGATTGTCCTACTGGAACCTCGGTC
GATACGAGACATACCCAGAAAGTGAGATGGAATCGAATTGTGCTTAACGGTTCAGTTAAGTTGACTAG
CGAAGCCAATG

+
FFFFFFFFFFFFFFFFFFFFFFFFFFFFFFFFFFFFFFFFFFFFFFFFFFFFFFFFFFFFFFFFFFFFFF
FFFFFFFFFFFFFFFFFFFFFFFFFFFFFFFFFFFFFFFF:FFFFFFFFFFFFFFFFFFFFFFFFFFFFF
FFFFFFFFFF:
@A00721:190:HGJW7DSXY:4:1571:2184:9345 1:N:0:TCTGCAAG+GCTGCACT
GAGTTAATAATATTGTGGACGTTGGCAGTATCGTTTGTATAAATGATTGTCCTACTGGAACCTCGGTC
GATACGAGACATACCCAGAAAGTGAGATGGAATCGAATTGTGCTTAACGGTTCAGTTAAGTTGACTAG
CGAAGCCAATG
+
FFFFFFFFFFFFFFFFFFFFFFFF:FFFFFFFFFFFFFFFFFFFFFFFFFFFFFFFFFFFFFFFFFFFF
FFFF:FFFFFFFFFFFFFFFFFFFFFFFFF:FFFFFFF:FFFFFFFFFFF:FFFFFFFFFF,FFF:FF,
FFFF:F:FFFF
@A00721:190:HGJW7DSXY:4:1601:14407:7122 1:N:0:TCTGCAAG+GCTGCACT
GAGTTAATAATATTGTGGACGTTGGCAGTATCGTTTGTATAAATGATTGTCCTACTGGAACCTCGGTC
GATACGAGACATACCCAGAAAGTGAGATGGAATCGAATTGTGCTTAACGGTTCAGTTAAGTTGACTAG
CGAAGCCAATG
+
FFFFFFF:FFFFFFFFFFFFFFFFFFFFFFFFFFFFFFFFFFFFFFFFFFFFFFF:FFFFFFFFFFFF
FFFFFFFFFFFFFFFFFFF:FFFFFFFFFFF:FFFFF:FFFFFFFFFFFFFFFFF:FFFFFFFFFFFF
FFFFFFFF,FF
@A00721:190:HGJW7DSXY:4:1614:27317:18208 1:N:0:TCTGCAAG+GCTGCACT
GAGTTAATAATATTGTGGACGTTGGCAGTATCGTTTGTATAAATGATTGTCCTACTGGAACCTCGGTC
GATACGAGACATACCCAGAAAGTGAGATGGAATCGAATTGTGCTTAACGGTTCAGTTAAGTTGACTAG
CGAAGCCAATG
+
FFFFFFFFFFFFFFFFFFFFFFFFFFFFFFFFFFFFFFFFFFFFFFFFFFFFFFFFFFFFFFFFFFFF
FFFFFFFFFFFFFFFFFFFFFFFFFFFFFFFFFFFFFFFFFFFFFFF:FFFFFFFFFFFFFFF:FFFF:
FFFF:FFFFFF
@A00721:190:HGJW7DSXY:4:2152:5231:36949 1:N:0:TCTGCAAG+GCTGCACT
GAGTTAATAATATTGTGGACGTTGGCAGTATCGTTTGTATAAATGATTGTCCTACTGGAACCTCGGTC
GATACGAGACATACCCAGAAAGTGAGATGGAATCGAATTGTGCTTAACGGTTCAGTTAAGTTGACTAG
CGAAGCCAATG
+
FFFFFFFFFFFFFFFFFFFFFFFFFFFFFFFFFFFFFFFFFFFF:FFFFFFFFFFFFFFFFFFFFFFFF
FFFFFFFFFFFFFFFFFFFFFFFFFFFFFFFFFFFFFFFFFFFFFFFFFFFFF:FFFFFFFFFFFFFF
FFFFFFFFFFF
@A00721:190:HGJW7DSXY:4:2160:11153:7059 1:N:0:TCTGCAAG+GCTGCACT
GAGTTAATAATATTGTGGACGTTGGCAGTATCGTTTGTATAAATGATTGTCCTACTGGAACCTCGGTC
GATACGAGACATACCCAGAAAGTGAGATGGAATCGAATTGTGCTTAACTGTTCAGTTAAGTTGACTAG
CGAAGCCAATG
+
FFFFFFFFFFFFFFF:FFFF:FFFFFFFFFFFF,,::::FFFF,:FF,FFF,FFF:FFFFFF:FFFFF
FFFFFFFF::FFF::FFFFFFFFFF,:FFFF:FFFFFFFF:FFFF:FF,,FFFFFFFFFFF,,FFFFF
:FFFFF,FFF:
@A00721:190:HGJW7DSXY:4:2368:30725:12524 1:N:0:TCTGCAAG+GCTGCACT
GAGTTAATAATATTGTGGACGTTGGCAGTATCGTTTTTATAAATGATTGTCCTACTGGAAACTCGGTC
GATAAGAGACATACCCAGAAAGTGAGATGGAATCGAATTGTGCTTAACGGTTCAGTTAAGTTGACTAT
CGAAGACAATG
+
F,F:FFF:F::FFFFF:FFFF,F,::F,,FF,:::,,FFFFFF:FF,F:F,,FF,F,,F,,:F,F,FF
:FFF,FF,F:F:FF,,F,FFF:F:FFFFF,F,FFF,FF,,,:,F,FFF,F:F,F,F:F:FFFFF,:F,
:FFF:,FFF:F
@A00721:190:HGJW7DSXY:4:2368:31458:13072 1:N:0:TCTGCAAG+GCTGCACT
GAGTTAATAATATTGTGGACGTTGGCAGTATCGTTTGTATAAATGATTGTCCTACTGGAACCTCGGTC

GATACGAGACATACCCAGAAAGTGAGATGGAATCGAATTGTGCTTAACGGTTCAGTTAAGTTGACTAG
CGAAGCCAATG
+
FFFFFFFFFFFFFFFFFFFFFFFFFFFFFFFFFFFFFFFFFFFFFFFFFFFFFFF:FFFFFFFFFFFFF
FFFFFFFFFFFFF:FFFFFFFFFFFFFFFF:FFFF:FFFFFF:FFFFFFFFFFFFFFFFFF:FFFFFF
:FFF,FFFFFF
@A00721:190:HGJW7DSXY:4:2417:3956:32612 1:N:0:TCTGCAAG+GCTGCACT
GAGTTAATAATATTGTGGACGTTGGCAGTATCGTTTGTATAAATGATTGTCCTACTGGAACCTCGGTC
GATACGAGACATACCCAGAAAGTGAGATGGAATCGAATTGTGCTTAACGGTTCAGTTAAGTTGACTAG
CGAAGCCAATG
+
FFFFFFFFFFFFFFFFFFFFFFFFFFFFFFFFFFFFFFFFFFFFFFFFFFFFFFFFFFFFFFFFFFFF:
FFFFFFFFFFFFFFFFFFFFFFFFFFFFFFFF:FFFFFFFFFFFFFFF:FFFFFFFFFFFFFFFFFFFF
FFFFFFFFFFF
@A00721:190:HGJW7DSXY:4:2432:23149:26897 1:N:0:TCTGCAAG+GCTGCACT
GAGTTAATAATATTGTGGACGTTGGCAGTATCGTTTGTATAAATGATTGTCCTACTGGAACCTCGGTC
GATACGAGACATACCCAGAAAGTGAGATGGAATCGAATTGTGCTTAACGGTTCAGTTAAGTTGACTAG
CGAAGCCAATG
+
FFFFFFFFFFFFFFFFFFFFFFFFFFFFFFFFFFFFFFFFFFFFFFFFFFFFFFFFFFFFFFFFFFFFF
FFFFFFFFFFFFFFFFFFFFFFFFFFFFFFFFFFFFFFFFFFFFFFFFFFFFFFFFFFFFFFFFFFFFF
FFFFFFFFFFF
@A00721:190:HGJW7DSXY:4:2447:28366:8375 1:N:0:TCTGCAAG+GCTGCACT
GAGTTAATAATATTGTGGACGTTGGCAGTATCGTTTGTATAAATGATTGTCCTACTGGAACCTCGGTC
GATACGAGACATACCCAGAAAGTGAGATGGAATCGAATTGTGCTTAACGGTTCAGTTAAGTTGACTAG
CGAAGCCAATG
+
FFFFFFFFFFFFFFFFFFFFFFFFFFFFFFFFFFFFFFFFFFFFFFFFFFFFFFFFFFFFFFFFFFFFF
FFFFFFFFFFFFFFFFFFFFFFFFFFFFFFFF,FFFFFFFFFFFFFFFFFFFFFFFFFFFFFF:FFFFF
FFFFFFFFFFF
@A00721:190:HGJW7DSXY:4:2577:12409:26428 1:N:0:TCTGCAAG+GCTGCACT
GAGTTAATAATATTGTGGACGTTGGCAGTATCGTTTGTATAAATGATTGTCCTACTGGAACCTCGGTC
GATACGAGACATACCCAGAAAGTGAGATGGAATCGAATTGTGCTTAACGGTTCAGTTAAGTTGACTAG
CGAAGCCAATG
+
FFFFFFFFFFFFFFFFFFFFFFFFFFFFFFFFFFFFFFFFFFFFFFFFFFFFFFFFFFFFFFFFFFFFF
FFFFFFFFFFFFFFFFFFFFFFFFFFFFFFFFFFFFFFFFFFFFFFFFFFFFF:FFFFFFFFFFFFFF
FFFFFFFFFFF
@A00721:190:HGJW7DSXY:4:2614:28465:10582 1:N:0:TCTGCAAG+GCTGCACT
GAGTTAATAATATTGTGGACGTTGGCAGTATCGTTTGTATAAATGATTGTCCTACTGGAACATCGGTC
GATAAGATACATACCCAGAAAGTGAGATGGAATCGAATTGTGCTTAACGGTTCAGTTAAGTTGACTAG
CGAAGCCAATG
+
F,,FFFFFFFFFFFFFF:FFFFF,,FF:FFFF,FFFF:,,F::FFFFFFF,:FF,FFFFF:,,F:FFF
FFFF,,F:FFFFF,,FFFF,F,FFFFFF,:F,F,,:FFFF:F,FFFF:FFFF,F:FF:FF:FFF:FFF
,FFFFFFFF,F
@A00721:190:HGJW7DSXY:4:2670:14326:35822 1:N:0:TCTGCAAG+GCTGCACT
GAGTTAATAATATTGTGGACGTTGGCAGTATCGTTTGTATAAATGATTGTCCTACTGGAACCTCGGTC
GATACGAGACATACCCAGAAAGTGAGATGGAATCGAATTGTGCTTAACGGTTCAGTTAAGTTGACTAG
CGAAGCCAATG
+
FFFFFFFFFFFFFFFFFFFFFFFFFFFFFFFFFFFFFFFFFFFFFFFFFFFFFFFFFFFFF:FFFFFFFF
FFFFFFFFFFFFFFFFFFFFFFFFFFFFFFFFFFFFFFFFFFFFFFFFFFFFFFFFFFFFFFFFFFFFF
FFFFFFFFFFF

@A00721:190:HGJW7DSXY:4:1355:12798:10661 1:N:0:TCTGCAAG+GCTGCACT
GGGTTAATAATATTGTGGACGTTGGCAGTATCGTTTGTATAAATGATTGTCCTACTGGAACCTCGGTC
GATACGAGACATACCCAGAAAGTGAGATGGAATCGAATTGTGCTTAACGGTTCAGTTAAGTTGACTAG
CGAAGCCAATG
+
FFF:FFFFFFFFFFFFFFFF:FFFFFF:FF::FFFFFF:FFFFFFFFFFFFFFFFFFFFFF:FFFF:F
FFFFFFFFFFFFFFFFFFFFFF:FFFFFFFFFFFFF:FFFFFFFFFFF,FFFFFFFFFF:FFFFFFFF
F:FFF:FFFFF
@A00721:190:HGJW7DSXY:4:1514:32606:25676 1:N:0:TCTGCAAG+GCTGCACT
GGGTTAATAATATTGTGGACGTTGGCAGTATCGTTTGTATAAATGATTGTCCTACTGGAACCTCGGTC
GATACGAGACATACCCAGAAAGTGAGATGGAATCGAATTGTGCTTAACGGTTCAGTTAAGTTGACTAG
CGAAGCCAATG
+
FFFFFFFFFFFFFFFFFFFFFFFFFFFFFFFFFFFFFFFFFFFFFFFFFFFFFFFFFFFFFFFFFFFF
FFFFFFFFFFFFFFFFFFFFFFFFFFFFFFF:FFFFFFFFFFFFFFF:FFFF:FFFFFF:FFFF:FF:
FF:FFFFFFFF
@A00721:190:HGJW7DSXY:4:1573:1723:30686 1:N:0:TCTGCAAG+GCTGCACT
GGGTTAATAATATTGTGGACGTTGGCAGTATCGTTTGTATAAATGATTGTCCTACTGGAACCTCGGTC
GATACGAGACATACCCAGAAAGTGAGATGGAATCGAATTGTGCTTAACGGTTCAGTTAAGTTGACTAG
CGAAGCCAATG
+
FFFFFFFFFFFFFFFFFFFFFFFFFFFFF:FFFFFFFFFFFFFFFFFFFFFFFFFFFFF,FFFFFFFF
FFFFFFFFFFFFFFFFFFFFFFFFFFFFFFF,FFFFFFFFFFFFFFFFFFFFFFFFFFFFFFFFFF:F
FFF:FFFFFFF
@A00721:190:HGJW7DSXY:4:2157:4833:12399 1:N:0:TCTGCAAG+GCTGCACT
GGGTTAATAATATTGTGGACGTTGGCAGTATCGTTTGTATAAATGATTGTCCTACTGGAACCTCGGTC
GATACGAGACATACCCAGAAAGTGAGATGGAATCGAATTGTGCTTAACGGTTCAGTTAAGTTGACTAG
CGAAGCCAATG
+
FFFFFFFFFFFFFFFFFFFFFFFFFFFFFFFFFFFFFFFFFFFFFFFFFFFFFFFFFF:FFFFFFFFF
FFFFFFFFFF:FFFFFFFFFFFFFFFFFFFFFFFFFFFFFFFFFFFFFFFFFFFFFFFFFFFFFFFFF
FFFFFFFFFFF
@A00721:190:HGJW7DSXY:4:2409:22896:27962 1:N:0:TCTGCAAG+GCTGCACT
GGGTTAATAATATTGTGGACGTTGGCAGTATCGTTTGTATAAATGATTGTCCTACTGGAACCTCGGTC
GATACGAGACATACCCAGAAAGTGAGATGGAATCGAATTGTGCTTAACGGTTCAGTTAAGTTGACTAG
CGAAGCCA
+
FFFFFFFFFFFFFFFFFF::FFFFFFFFFFFFFF:FFFFFFFFFFFFFF:FFFFFF:,F,FFFF,FFF
:F:F:FFFFFFFFF:FFFFFFF:FFFF,FFF,FFFFF,F:F:FFFFF:F,FF,F,FFFF,F,FFFFFF
,FFF:FFF
@A00721:190:HGJW7DSXY:4:2430:15185:17801 1:N:0:TCTGCAAG+GCTGCACT
GGGTTAATAATATTGTGGACGTTGGCAGTATCGTTTGTATAAATGATTGTCCTACTGGAACCTCGGTC
GATACGAGACATACCCAGAAAGTGAGATGGAATCGAATTGTGCTTAACGGTTCAGTTAAGTTGACTAG
CGAAGCCAATG
+
FFFFFFFFFFFFFFFFFFFFFFFFFFFFFFFFFFFFFFFFFFFFFFFFFFFFFFFFFFF:FFFFFFFF
FFFFFFFFFFFFFFFFFFFFFFFFFFFFF:FFFFFFFFFFFFFFFFFFFFFFFFFFFFFFFFFFFFFF
:FFFFFFFFFF
@A00721:190:HGJW7DSXY:4:2430:14850:18693 1:N:0:TCTGCAAG+GCTGCACT
GGGTTAATAATATTGTGGACGTTGGCAGTATCGTTTGTATAAATGATTGTCCTACTGGAACCTCGGTC
GATACGAGACATACCCAGAAAGTGAGATGGAATCGAATTGTGCTTAACGGTTCAGTTAAGTTGACTAG
CGAAGCCAATG
+
,FFFFFFFF,FFFF::FFFFFFFFF:FFFFF:::FFFFFFFFFFFFFFFFF:FFFF,FF::FFFFFFF

FFF::FF:F,:FFFFFFFFFFFFFFFFFFFF::F:FF,:FF,FFFFFF:FF:,F,FFFFFF:FFFFFF
:FFFFFFFFF:
@A00721:190:HGJW7DSXY:4:1311:23710:28244 1:N:0:TCTGCAAG+GCTGCACT
AGTGGACGTTGGCAGTATCGTTTGTATAAATGATTGTCCTACTGGAACCTCGGTCGATACGAGACATA
CCCAGAAAGTGAGATGGAATCGAATTGTGCTTAACGGTTCAGTTAAGTTGACTAGCGAAGCCAATGTT
CATAATATGAAGCAG
+
FFFFFFFFFFFFFFFFFFFFFFFFF:FF,FFFFFFFFFFFFFFFFFFFFFFFFFFFFFFFFFFFFFFF
FFF,FFFFFFFFFFFFFFFFFFFFFFFFFFFFFFFFFFFFFFFFFFFFFFFFFF:FFFF:FFFFFFFF
FFFFF:FFFFFFFFF
@A00721:190:HGJW7DSXY:4:1643:29948:13369 1:N:0:TCTGCAAG+GCTGCACT
AGTGGACGTTGGCAGTATCGTTTGTATAAATGATTGTCCTACTGGAACCTCGGTCGATACGAGACATA
CCCAGAAAGTGAGATGGAATCGAATTGTGCTTAACGGTTCAGTTAAGTTGACTAGCGAAGCCAATGTT
CATAATATGAAGCAG
+
FFFFFFFFFFFFFFFFFFFFFFFFFFFFFFFFFFFFFFFFFFFFFF,FFFFFFFFFFFFFFFFFFFFF
FFFFFFFFFFFFFFFFFF,FFFF,FFFFFFFFFFFFFFFFF:F:FFFFFFFFFFFFFFF:FFFFFFFF
FF:FFFFFFFFF:FF
@A00721:190:HGJW7DSXY:4:2212:17833:27868 1:N:0:TCTGCAAG+GCTGCACT
AGTGGACGTTGGCAGTATCGTTTGTATAAATGATTGTCCTACTGGAACCTCGGTCGATACGAGACATA
CCCATAAAGTGAGATGGAATCGAATTGTGCTTAACGGTTCAGTTAAGTTGACTAGCGAAGCCAATGTT
CATAATATGAAGCAG
+
FFFFFFFFFF:FFFF:FF:FFFF:FFFFFFF,,FFFFF::FFFF:FFFFFFFFFF:FFFFFF,FFFFF
:,:F,FFF:F:F,FF::FF,FFFFFF,F,FFFFFF,F:FF:,,FFFFFF,F,FF,F,FF,,FFFFFF:
:FFFFFFF,FFF:F:
@A00721:190:HGJW7DSXY:4:2215:27633:11021 1:N:0:TCTGCAAG+GCTGCACT
AGTGGACGTTGGCAGTATCGTTTGTATAAATGATTGTCCTACTGGAACCTCGGTCGATACGAGACATA
CCCAGAAAGTGAGATGGAATCGAATTGTGCTTAACGGTTCAGTTAAGTTGACTAGCGAAGCCAATGTT
CATAATATGAAGCA
+
FFFFFFFFFFF:FFFFFFF:,FF:FFFFFF:FFFFFFFFFF:FFFF:FFFFFFF:FFFFFFF,FFFFF
::FFFFFF:F:FFFF:FFF,,FFFFFFFFFF:FFF,FFF:FFFFFFFFFFF,FFFFFFF:::,FF:FF
:FFFFFFFFFFF,F
@A00721:190:HGJW7DSXY:4:2413:28311:7968 1:N:0:TCTGCAAG+GCTGCACT
AGTGGACGTTGGCAGTATCGTTTGTATAAATGATTGTCCTACTGGAACCTCGGTCGATACGAGACATA
CCCAGAAAGTGAGATGGAATCGAATTGTGCTTAACGGTTCAGTTAAGTTGACTAGCGAAGCCAATGTT
CATAATATGAAGCAG
+
FFFFFFFFFFFFFFFFFFFFFFFFFFFFFFFFFFFFFFFFFFFFFFFFFFFFFFFFFFFFFFFFFFFF
FFFFFFFFFFFFFFFFFFFFFFFFFFFFFFFFFFFFFFFFFFFFFFFFFFFFFFFFFFF:FFFFFFFF
FFFFFFFFF:FFFFF
@A00721:190:HGJW7DSXY:4:2426:9444:30311 1:N:0:TCTGCAAG+GCTGCACT
AGTGGACGTTGGCAGTATCGTTTGTATAAATGATTGTCCTACTGGAACCTCGGTCGATACGAGACATA
CCCAGAAAGTGAGATGGAATCGAATTGTGCTTAACGGTTCAGTTAAGTTGACTAGCGAAGCCAATGTT
CATAATATGAAGCAG
+
FFFFFFFFFFFFFFFFFFFFF:FFFFFFFFFFFFFFFFFFFFFFFF:FFFFFFFFFFFFFFFFFFFFF
FFFFFFFFFFFFFFFFFFFFFFFFFFFFFFFFFFFFFF:FFFFFFFFFFFFFFFFFFFFFFFFFFFFF
FFFFFFFFFFF:FFF
@A00721:190:HGJW7DSXY:4:2426:9362:30859 1:N:0:TCTGCAAG+GCTGCACT
AGTGGACGTTGGCAGTATCGTTTGTATAAATGATTGTCCTACTGGAACCTCGGTCGATACGAGACATA
CCCAGAAAGTGAGATGGAATCGAATTGTGCTTAACGGTTCAGTTAAGTTGACTAGCGAAGCCAATGTT
CATAATATGAAGCAG

```
+
FFFFFFFFFFFFFFFFFFFFFFFFFFF:FFFFFFFFFFFFFFFFFF:FFFFFFFFFFFFFFFFFFFFF
FFFFFFFFFFFFFFFFFFFFFFFFFFFFFFFFFFFFFFFFFFFFFFFFFFFFFFFFFFFFFFFFFFFF
FFFFFFFFFFFF:FF
@A00721:190:HGJW7DSXY:4:2605:23285:10567 1:N:0:TCTGCAAG+GCTGCACT
AGTGGACGTTGGCAGTATCGTTTGTATAAATGATTGTCCTACTGGAACCTCGGTCGATACGAGACATA
CCCAGAAAGTGAGATGGAATCGAATTGTGCTTAACGGTTCAGTTAAGTTGACTAGCGAAGCCAATGTT
CATAATATGAAGCAG
+
FFFFFFFFFFFFFFFFFFFFFFFFFFFFFFFFFFFFFFFFFFFFFFFFFFFFFFFFFFFFFFFFFFFF
FFFFFFFFFFFFFFFFFF:FFF:FFFFFFFFFFF:FFFFFFFFFFF:FFF:FFFFFFFFF:FF:FFFF
FFFFFFFFFFF:FFF
@A00721:190:HGJW7DSXY:4:1273:6361:29356 1:N:0:TCTGCAAG+GCTGCACT
GTGGACGTTGGCAGTATCGTTTGTATAAATGATTGTCCTACTGGAACCTCGGTCGATACGAGACATAC
CCAGAAAGTGAGATGGAATCGAATTGTGCTTAACGGTTCAGTTAAGTTGACTAGCGAAGCCAATGTTC
ATAATATGAAGCAGT
+
FFFFFFFFFFFFFFFFFFFFFFFFFFFFFFFFFFFFFFFFFFFFFFFFFFFFFFFFFFFFFFFFFFFF
FFFFFFFFFFFFFFFFFFFFFFFFFFFFFFFFFFFFFFFFFFFFFFFFFFFFFFFFFF,FFFFFFFFF
FFFFFFFFFF::FFF
@A00721:190:HGJW7DSXY:4:1404:20401:34914 1:N:0:TCTGCAAG+GCTGCACT
GTGGACGTTGGCAGTATCGTTTGTATAAATGATTGTCCTACTGGAACCTCGGTCGATACGAGACATAC
CCAGAAAGTGAGATGGAATCGAATTGTGCTTAACGGTTCAGTTAAGTTGACTAGCGAAGCCAATGTTC
ATAATATGAAGCAGT
+
FFFFFFFFFFFFFFFFFFFFFFFFFFFFFFFFFFFFFFFFFFFF::FFF:FFFFFFF:FFFFFFFFFF
FFF:FFFFFFFF:FFFFFF:FFFFF:FFFFFFF,FFFFFFFFFFF,FFFFFFFF:FFFF:FFFFFFFF
FFFF:FFFFF,:FFF
@A00721:190:HGJW7DSXY:4:2122:13937:36808 1:N:0:TCTGCAAG+GCTGCACT
GTGGACGTTGGCAGTATCGTTTGTATAAATGATTGTCCTACTGGAACCTCGGTCGATACGAGACATAC
CCAGAAAGTGAGATGGAATCGAATTGTGCTTAACGGTTCAGTTAAGTTGACTAGCGAAGCCAATGTTC
ATAATATGAAGCAGT
+
FFFFFFFFFFF:FFFFFFFFFFFFFFFFFFFFFFFFFFFFFFF:FFFFF:FFFFFFFFFFFFF,FFFFF
FF:FFFF,FFFF:FF:FFFFFFFFF,FFFFFFFFFFFFFF:FFF:FF,FFFFFFFFFFF:F,FFFF
FFFFFFF:FFF:F,:
@A00721:190:HGJW7DSXY:4:1137:21775:33348 1:N:0:TCTGCAAG+GCTGCACT
CACTGCTTCATATTATGAACATTGGCTTCGCTAGTCAACTTAACTGAACCGTTAAGCACAATTCGATT
CCATCTCACTTTCTGGGTATGTCTCGTATCGACCGAGGTTCCAGTAGGACAATCATTTATACAAACGA
TACTGCCAACGTCCA
+
FFFFFFFFFFFFFFFFFFFFFFFFFFFFFFFFFFFFFFFFFFFFFFFFFFFFFF,FFFFFFFFFFFFF
FFFFFFFFFFFFFFFFFFFFFFFFFFFFFFFFFFFFFFFFFFFFFFFFFFFFFFF:FFFFFFFFFFFF
FFFFFFFFFFFFFF:
@A00721:190:HGJW7DSXY:4:1161:25744:3803 1:N:0:TCTGCAAG+GCTGCACT
TGGACGTTGGCAGTATCGTTTGTATAAATGATTGTCCTACTGGAACCTCGGTCGATACGAGACATACC
CAGAAAGTGAGATGGAATCGAATTGTGCTTAACGGTTCAGTTAAGTTGACTAGCGAAGCCAATGTTCA
TAATATGAAGCAGTG
+
FFFFFFFFFFFFFFFFFFFFFF:FFFFFFFFFFFF,F:FF:FFFFFFFFFFFFF:F:FFFFFFFFFF:F
F,FFFFFFFFFFF:FFFFFFFFFFFFF:FFFFF,:FFFFFFFFFF:FFFFFFFFFF:F:FFFF:F,FF
FFFFFF,FFFFFFFF
@A00721:190:HGJW7DSXY:4:1166:17833:19445 1:N:0:TCTGCAAG+GCTGCACT
TGGACGTTGGCAGTATCGTTTGTATAAATGATTGTCCTACTGGAACCTCGGTCGATACGAGACATACC
```

CAGAAAGTGAGATGGAATCGAATTGTGCTTAACGGTTCAGTTAAGTTGACTAGCGAAGCCAATGTTCA
TAATATGAAGCAGTG
+
FFFFFFFFFFFFFFFFFFF:FFFFFFFFFFFFFFFFFFFFFFFFFFFFFFFFFFFFFFFFFFFFFFFF
FFFFFFFFFFFFFFFFFFFFFFFFFFFFFFFFFFFFFFFFFFFF:FFFFFFF:FFFFFFFFFFFFFFF
FFFFFFFFFFFFFFF
@A00721:190:HGJW7DSXY:4:1241:23583:18223 1:N:0:TCTGCAAG+GCTGCACT
TGGACGTTGGCAGTATCGTTTGTATAAATGATTGTCCTACTGGAACCTCGGTCGATACGAGACATACC
CAGAAAGTGAGATGGAATCGAATTGTGCTTAACGGTTCAGTTAAGTTGACTAGCGAAGCCAATGTTCA
TAATATGAAGCAGTG
+
FFFFFFFFFFFFFFFFFFFFFFFFFFFFFFFFFFFFFFFFFFFFFFFFFFFFFFFFFFFFFFFFFF:F
FFFFFFFFFFFF:FFFFFFFFFFFFFFFFFFFFFFFFFFFFFFFFFFFFFFFFFFF:FFFFFFF:FF
FFFFFFFFFF:FFFF
@A00721:190:HGJW7DSXY:4:1413:24451:17503 1:N:0:TCTGCAAG+GCTGCACT
CACTGCTTCATATTATGAACATTGGCTTCGCTAGTCAACTTAACTGAACCGTTAAGCACAATTCGATT
CCATCTCACTTTCTGGGTATGTCTCGTATCGACCGAGGTTCCAGTAGGACAATCATTTATACAAACGA
TACTGCCAACGTCCA
+
FFFFFFFFFFFFFFFFFFFFFFFFFFFFFFFFFFFFFFFFFFFFFFFFFFFFFFFFFFFFFFFFFFFF
FFFFFFFFFFFFFFFFFFFFFFFFFFFFFFFFFFFFFFFFFFFFFFFFFFFFFFFFFFFFFFFFFFFF
FFFFFFFF:FFFFFF
@A00721:190:HGJW7DSXY:4:1514:4589:7467 1:N:0:TCTGCAAG+GCTGCACT
TGGACGTTGGCAGTATCGTTTGTATAAATGATTGTCCTACTGGAACCTCGGTCGATACGAGACATACC
CAGAAAGTGAGATGGAATCGAATTGTGCTTAACGGTTCAGTTAAGTTGACTAGCGAAGCCAATGTTCA
TAATATGAAGCAGTG
+
FFFFFFFFFFFFFFFFFFF:FFFFFFFFFFFFFFFFFFFFFFFFFFFFFFFFFFFFFFFFFFFFFFFF
FFFFFFFFFFFFFFFFFFFFFFFFFFFFFFFFFFFFFFFFFFFFFFFFFFFFFFFF:FFFF:FFFFFF
FFFFFFFFFFFFFFF
@A00721:190:HGJW7DSXY:4:1529:16179:20682 1:N:0:TCTGCAAG+GCTGCACT
CACTGCTTCATATTATGAACATTGGCTTCGCTAGTCAACTTAAATGAACCGTTAAGCACAATTCGATT
CCATCTCACTTTCTGGGTATGTCTCGTATCGACCGAGGTTCCAGTAGGACAATCATTTATACAAACGA
TACTGCCAACGTCCA
+
FFFF,,FF:FFFF,F:,FF,FFF:F,FF:FFFFFF,FF,,FFF,F,F:,FF:FF:,FFFF:,,F,FFF
F:FFFFFFF::FFFF:FFFF,FFF::FFF,,:,F,F,:FFFFFFF:F:F,F::FF,FFF,F:FFFF,F
:F::,::F:F,:::F
@A00721:190:HGJW7DSXY:4:1578:3314:7357 1:N:0:TCTGCAAG+GCTGCACT
TGGACGTTGGCAGTATCGTTTGTATAAATGATTGTCCTACTGGAACCTCGGTCGATACGAGACATACC
CAGAAAGTGAGATGGAATCGAATTGTGCTTAACGGTTCAGTTAAGTTGACTAGCGAAGCCAATGTTCA
TAATATGAAGCAGTG
+
FFFFFF:FFFFFFFFFFF,FFFFFFFFFFFFFFFFFFFFFFFFFFFFFFFFFFFFFFFFF,FFFFFFF
FFFFFFFFFFFFFFFFFFFFFFFFFFFFFFFFFFFFFFFFFFFFFFFFFFFFF:FFFFFFFFFFFFFF
FFFFFFFFFF::FFF
@A00721:190:HGJW7DSXY:4:1653:31675:5807 1:N:0:TCTGCAAG+GCTGCACT
TGGACGTTGGCAGTATCGTTTGTATAAATGATTGTCCTACTGGAACCTCGGTCGATACGAGACATACC
CAGAAAGTGAGATGGAATCGAATTGTGCTTAACGGTTCAGTTAAGTTGACTAGCGAAGCCAATGTTCA
TAATATGAAGCAGTG
+
FFFFFFFFFFFFFFFFFFFFFFFFFFFFFFFFFFFFFFFFFFFFFFFFFFFFFFFFFFFFFFFFFFFF
FFFFFFFFFFFFFFFFFFFFFFFFFFFFFFF:FFFFFFFFFFFFFFFFFFFFFFFF:FFFFFFFFFFF
:FFFFFFFFFFFFFF

@A00721:190:HGJW7DSXY:4:1673:29903:23563 1:N:0:TCTGCAAG+GCTGCACT
TGGACGTTGGCAGTATCGTTTGTATAAATGATTGTCCTACTGGAACCTCGGTCGATACGAGACATACC
CAGAAAGTGAGATGGAATCGAATTGTGCTTAACGGTTCAGTTAAGTTGACTAGCGAAGCCAATGTTCA
TAATATGAAGCAGTG
+
FFFFFFFFFFFFFFFFFFFFFFFFFFFFFFFFFFFFFFFFFFFF,FFFFFFFFFFFFFFFFFFFFFFF
FFFFFFFFFFFFFFFF:FFFFFFFFFFFFFFFFFFFFFFFFFFFFFFFFFFFFFFF:FFFFFFFFFF
FFFFFFFFFFFFFFF
@A00721:190:HGJW7DSXY:4:2204:17824:20369 1:N:0:TCTGCAAG+GCTGCACT
TGGACGTTGGCAGTATCGTTTGTATAAATGATTGTCCTACTGGAACCTCGGTCGATACGAGACATACC
CAGAAAGTGAGATGGAATCGAATTGTGCTTAACGGTTCAGTTAAGTTGACTAGCGAAGCCAATGTTCA
TAATATGAAGCAGTG
+
FFFFFFFFFFFFFFFFFFFFFFFFFFFFFFFFFFFFFFFFFFFFFFFFFFFFFFFFFFFFFFFFFFFF
F,FF:FFFFFFFFFFFFFFFFFFF:FFFFFFFFFFFFFFFFFFFFFFFFFFFF:FFFFFFFFFFFFFF
FFFFFF,FFFFFFFF
@A00721:190:HGJW7DSXY:4:2243:32334:31845 1:N:0:TCTGCAAG+GCTGCACT
TGGACGTTGGCAGTATCGTTTGTATAAATGATTGTCCTACTGGAACCTCGGTCGATACGAGACATACC
CAGAAAGTGAGATGGAATCGAATTGTGCTTAACGGTTCAGTTAAGTTGAATAGCGAAGCCAATGTTCA
TAATATGAAGCA
+
FF:FFFFFFFFFFFFFFFF:FF:FFFFFF,FFFF:FFFFFFFFFFFFFFFFFF:FFFFFFFFFF,FF:
FFFFFF:FFF,:FFFFFFFF::FFFFF,FFFF,FFFF:FFF:FF,:FF:,FF,,FFFF,FFF::FFFF
FFFFFFFFFFFF
@A00721:190:HGJW7DSXY:4:2340:32859:18724 1:N:0:TCTGCAAG+GCTGCACT
CACTGCTTCATATTATGAACATTGGCTTCGCTAGTCAACTTAACTGAACCGTTAAGCACAATTCGATT
CCATCTCACTTTCTGGGTATGTCTCGTATCGACCGAGGTTCCAGTAGGACAATCATTTATACAAACGA
TACTGCCAACGTCCA
+
FFF,F,,,FFFFFFFF:FFFFFFF::FF:F,FF:FFFFFF:FFFF:FF:,FF:FFFFFFFF:FF:FF:
FFFFFFFF,FFF,:FFFFFFFF,FFFFFFF,:F:FFFFFFFFF,FFF:FF:FFFFFFFFFF:FFFF:F
FFF,F:,F:F,FF,F
@A00721:190:HGJW7DSXY:4:2368:22553:4914 1:N:0:TCTGCAAG+GCTGCACT
TGGACGTTGGCAGTATCGTTTGTATAAATGATTGTCCTACTGGAACCTCGGTCGATACGAGACATACC
CAGAAAGTGAGATGGAATCGAATTGTGCTTAACGGTTCAGTTAAGTTGACTAGCGAAGCCAATGTTCA
TAATATGAAGCAGTG
+
F:FFFFFFFFFFFFFFFFFFFFFFFFFFFFFFFFFFFFFFFFFFFFFFFFFFFFFFFFFFFFFFFFFF
FFFFFFFFFFFFFFFFFFFFFFFF:FFFFFFFFF,FFFFFFFFFFFFFFFFFFFFFF,FFF:FFFFFF
FFFFF:FFF::FFFF
@A00721:190:HGJW7DSXY:4:2378:15320:3098 1:N:0:TCTGCAAG+GCTGCACT
TGGACGTTGGCAGTATCGTTTGTATAAATGATTGTCCTACTGGAACCTCGGTCGATACGAGACATACC
CAGAAAGTGAGATGGAATCGAATTGTGCTTAACGGTTCAGTTAAGTTGACTAGCGAAGCCAATGTTCA
TAATATGAAGCAGTG
+
FFFFFFFFFFFFFFFFFFFFFFFFFFFFFFFFFFFFFFFFFFFFFFFFFFFFFFFFFFFFFFFFFFFF
FFFFFFFFFFFFFFFFFFFFFFFFFFFFFFFFFFFFFFFFFFFFFFFFFFFFFFFFFFFFFFFFFFFF
FFFFFFFFFFFFF:F
@A00721:190:HGJW7DSXY:4:2407:31955:35196 1:N:0:TCTGCAAG+GCTGCACT
TGGACGTTGGCAGTATCGTTTGTATAAATGATTGTCCTACTGGAACCTCGGTCGATACGAGACATACC
CAGAAAGTGAGATGGAATCGAATTGTGCTTAAAGGTTCAGTTAAGTTGACTATCGAAGCCAATGTTCA
TAATATGAAGCAGTG
+
FFFFFFFF,FFFFFFFFFFFFFFFFFFFFFFFF,F,FFFFFFFFFFFFFF:FFFFFF:FFFFFFFF:F

```
:FFFFF:FFFFFFFFFFF,FFFF::FFFFFFF,FFFFF::FFFF:FFFFFFF,FFFF,F,FFFFFF,F
FFFFF:::F,FF::F
@A00721:190:HGJW7DSXY:4:2509:14371:19053 1:N:0:TCTGCAAG+GCTGCACT
TGGACGTTGGCAGTATCGTTTGTATAAATGATTGTCCTACTGGAACCTCGGTCGATACGAGACATACC
CAGAAAGTGAGATGGAATCGAATTGTGCTTAACGGTTCAGTTAAGTTGACTAGCGAAGCCAATGTTCA
TAATATGAAGCAGTG
+
FFFFFFFFFFFFFFFFFFFFFFFFFFFFFFFFFFFFFFFFFFFFFFFFFFFFFFFFFFFF:FFF:FFF
FFFFFFFFFFFFFFFFFFFFFFFFFFFFFFFFFFFFFFFFFFFFFFFFFFFFFFFFF:FFFFFFFFFF
FFFFFFFFFF,FF:F
@A00721:190:HGJW7DSXY:4:2569:17996:35227 1:N:0:TCTGCAAG+GCTGCACT
TGGACGTTGGCAGTATCGTTTGTATAAATGATTGTCCTACTGGAACCTCGGTCGATACGAGACATACC
CAGAAAGTGAGATGGAATCGAATTGTGCTTAACGGTTCAGTTAAGTTGACTAGCGAAGCCAATGTTCA
TAATATGAAGCAGTG
+
FFFFFF:FFFFFFFFFFFFFFFFFFFFFFFFFFFFFFFFFFFFFFFFFFFFFFFFFFFFFFFFFFFFF
FFFFFFFFFFFFFFFF:FFFFFFFFFFFFFFFFFFFFFFFFFFFFFFFFFFFFFFFFFFFFFFFFFFF
FFFFFFFFFF:FFFF
@A00721:190:HGJW7DSXY:4:1148:10366:3317 1:N:0:TCTGCAAG+GCTGCACT
CGGACGTTGGCAGTATCGTTTGTATAAATGATTGTCCTACTGGAACCTCGGTCGATACGAGACATACC
CAGAAAGTGAGATGGAATCGAATTGTGCTTAACGGTTCAGTTAAGTTGACTAGCGAAGCCAATGTTCA
TAATATGAAGCAGTG
+
FFFFFFFFFFFFFFFFFF:FFFFF:FFFFFFFFFFFFFFFFFFFFFFFFFFFFFFFFFFFFFFFFFFF
FFFFFFFFFF:F:FFFFFFFFFFFFFFFFFFFFFFFFFFFFFFFFFF:FFFFFFFFFFFFFFFFFFFF
FFFFFFFFFF:FFFF
@A00721:190:HGJW7DSXY:4:1444:25536:23923 1:N:0:TCTGCAAG+GCTGCACT
CGGACGTTGGCAGTATCGTTTGTATAAATGATTGTCCTACTGGAACCTCGGTCGATACGAGACATACC
CAGAAAGTGAGATGGAATCGAATTGTGCTTAACGGTTCAGTTAAGTTGACTAGCGAAGCCAATGTTCA
TAATATGAAGCAGTG
+
FFFFFFFFFFFFFFFFFFFFFFFFFFFFFFFF:FFFFFFFFFFFFFFFF:FFFFFFF:FFFFFFFFFF
FFFFFFF::FFFFFFFFFFFFFFF,FFFFFFFF,FFFFFF:FFFFFF:FFFF:FFFFFF::F:FFFFF
FF:FFFFFFF:FFFF
@A00721:190:HGJW7DSXY:4:1512:17906:22889 1:N:0:TCTGCAAG+GCTGCACT
CGGACGTTGGCAGTATCGTTTGTATAAATGATTGTCCTACTGGAACCTCGGTCGATACGAGACATACC
CAGAAAGTGAGATGGAATCGAATTGTGCTTAACGGTTCAGTTAAGTTGACTAGCGAAGCCAATGTTCA
TAATATGAAGCAGTG
+
FFFFFFFFFFFFFFFFFFFFFFFFFFFFFFFFFFFFFFFFFFFFFFFFFFFFFFFFFFFFFFFFFF:F
FFFFFFFFFFFFFFFFFFFFFFFFFFFFFFFFFFFFF:FFFFFFFFFFFFFFFFFFFFFFFFFFFFFF
FFFFFF:FF,FFFFF
@A00721:190:HGJW7DSXY:4:2131:14705:33505 1:N:0:TCTGCAAG+GCTGCACT
CGGACGTTGGCAGTATCGTTTGTATAAATGATTGTCCTACTGGAACCTCGGTCGATACGAGACATACC
CAGAAAGTGAGATGGAATCGAATTGTGCTTAACGGTTCAGTTAAGTTGACTAGCGAAGCCAATGTTCA
TAATATGAAGCAGTG
+
FFFFFFFFFFF:FFFFFFFFF:FFFFFFFFFFFFFFFFFF:FFFF,FFFFFFFFFFF:FFFFFFFFFF
F:FFFFFFFFFFFFFFFFFFFFFFFFFFFFFF:FFFF:FFFFFFFFF,FFFF:FFFF:FFFFFFFFFF
FFFFF:FF:F,FFF:
@A00721:190:HGJW7DSXY:4:2225:12102:27461 1:N:0:TCTGCAAG+GCTGCACT
CGGACGTTGGCAGTATCGTTTGTATAAATGATTGTCCTACTGGAACCTCGGTCGATACGAGACATACC
CAGAAAGTGAGATGGAATCGAATTGTGCTTAACGGTTCAGTTAAGTTGACTAGCGAAGCCAATGTTCA
TAATATTAAGCAGTG
```

```
+
FFFFFFFFFFFFFFFFFFFFFFFFFFFFFFFFFFFFFFFFFFFFFFFFFFFFFFFFFFFFFFFFFFF,
FFFFFFFFFFFFFFFFFFFFFF:FFFFFFFFF,F:FFFFFFFFF,FF:FFFF:FFFF::FFFFFFF:F
FFFFFF,FF,FF:FF
@A00721:190:HGJW7DSXY:4:2244:13901:9095 1:N:0:TCTGCAAG+GCTGCACT
CGGACGTTGGCAGTATCGTTTGTATAAATGATTGTCCTACTGGAACCTCGGTCGATACGAGACATACC
CAGAAAGTGAGATGGAATCGAATTGTGCTTAACGGTTCAGTTAAGTTGACTAGCGAAGCCAATGTTCA
TAATATGAAGCAGTG
+
F:FFFFFF:FFF:FFFF,F:FFFFFFFFF:FFF:FFFFFFFFFFFFF:F:FFF:F,F::FFFFFFFFF
,FFFFF,F:F,FF:FF,F:,,FFF,F,F:FFFFFF,F,:FFFFFF:F,F:FF,F,F:,FFFF,FF:,F
FFFFFF:FFF,:,:F
@A00721:190:HGJW7DSXY:4:2265:6108:36119 1:N:0:TCTGCAAG+GCTGCACT
CGGACGTTGGCAGTATCGTTTGTATAAATGATTGTCCTACTGGAACCTCGGTCGATACGAGACATACC
CAGAAAGTGAGATGGAATCGAATTGTGCTTAACGGTTCAGTTAAGTTGACTAGCGAAGCCAATGTTCA
TAATATGAAGCAGTG
+
FFFFFFFFFFFFFFFFFFFFFFFFFFFFFFFFFFFFFFFFFFFFFFFFFFFFFFFFFFFFFFFFFFFF
FFFFFFFFFFFFFFFFFFFFFFFFF:FFFFFFFFFFFFFFFFFFFFFFFFFFFFFFFFFFFFFFFFF:
FFFFFFFFFF,FFFF
@A00721:190:HGJW7DSXY:4:2265:6551:36323 1:N:0:TCTGCAAG+GCTGCACT
CGGACGTTGGCAGTATCGTTTGTATAAATGATTGTCCTACTGGAACCTCGGTCGATACGAGACATACC
CAGAAAGTGAGATGGAATCGAATTGTGCTTAACGGTTCAGTTAAGTTGACTAGCGAAGCCAATGTTCA
TAATATGAAGCAGTG
+
FFFFFF:FFFFFFFFFFF:FFFFFFFFFFFFFFFFFFFFFFFFF:FFFFFFFFFFFFFFFFFFFFFFF
FFFFFFFFFFFFFFFFFFFFFFFFFFFFFFFFFFFFFFFFFFFFFFFFFFFFFFFFFFFFFFF:FFFF
FFFFFFFFFF:FFFF
@A00721:190:HGJW7DSXY:4:2431:9498:35289 1:N:0:TCTGCAAG+GCTGCACT
CGGACGTTGGCAGTATCGTTTGTATAAATGATTGTCCTACTGGAACCTCGGTCGATACGAGACATACC
CAGAAAGTGAGATGGAATCGAATTGTGCTTAACGGTTCAGTTAAGTTGACTAGCGAAGCCAATGTTCA
TAATATGAAGCAGTG
+
FFFFFFFFFFFFFFFFFFFFFFFFFFFFFFFFFFFFFFFFFFFFFFFFFFFFFFFFFFFFFFFFFFFF
FFFFFFFFFFFFFFFFFFFFFFFFFFFFFFFFFFFFFFFFFFFFFFFFFFFFF:FFFFFFFFF:FFFF
FFFFFFFFFF:FFFF
@A00721:190:HGJW7DSXY:4:2467:1434:20603 1:N:0:TCTGCAAG+GCTGCACT
CGGACGTTGGCAGTATCGTTTGTATAAATGAATGACCTACTGGAACCTCGTACGATACGAGACATACC
CAGAAAGTGAGATGGAATCGAATTGTGCTTAACGGTTCAGTTAAGTTGACTAGCGAAGCCAATGTTCA
TAATATGAAGCAG
+
F,FFFFF:,:FFFF:,FF:FFFFFFFF:FFF,,F,,FFFF,,FFFF,,,,,,FFFFFF:FFFFF,F,F
,::FFFFFFFFFF,FFFF,FFFF:,FFF,FFFF,:FF::F,FFF,FF,FF::FFFF:,FFFF,,:F:F
FFF,FFFFF::F:
@A00721:190:HGJW7DSXY:4:2649:10402:35634 1:N:0:TCTGCAAG+GCTGCACT
CGGACGTTGGCAGTATCGTTTGTATAAATGATTGTCCTACTGGAACCTCGGTCGATACGAGACATACC
CAGAAAGTGAGATGGAATCGAATTGTGCTTAACGGTTCAGTTAAGTTGACTAGCGAAGCCAATGTTCA
TAATATGAAGCAGTG
+
FFFFFFFFFFFFFFFFFF,FFFFFFFFFFFFFFFFFFFFFFFFFFFFFFFFFFFFFFFFFFFFFFFFF
FFFFFFFFFFFFFFFFFFFFFFFFFFFFFFFFFFFFFFFFFFFFFFFFFFFFFFFFFFFFFFFFFFFF
FFFFFFFFFFFFFFF
@A00721:190:HGJW7DSXY:4:1159:14271:29716 1:N:0:TCTGCAAG+GCTGCACT
TGGCAGTATCGTTTGTATAAATGATTGTCCTACTGGAACCTCGGTCGATACGAGACATACCCAGAAAG
```

TGAGATGGAATCGAATTGTGCTTAACGGTTCAGTTAAGTTGACTAGCGAAGCCAATGTTCATAATATG
AAGCAGTGGCTGTGG
+
FFFFFFFFFFFFFFFFFFFFFFFFFFFFFFFFFFFFFFFFFFFFFFFFFFFFFFFFFFFFFFFFFFFFFF
FFFFFFFFFFFFFFFFFFFFFFFFFFFFFFFFFFFFFFFFFFFFFFFF:FFFFFFFFFFFFFFFFFFFFF
FF::FFFF:FFFFFF
@A00721:190:HGJW7DSXY:4:1234:3577:22936 1:N:0:TCTGCAAG+GCTGCACT
TGGCAGTATCGTTTGTATAAATGATTGTCCTACTGGAACCTCGGTCGATACGAGACATACCCAGAAAG
TGAGATGGAATCGAATTGTGCTTAACGGTTCAGTTAAGTTGACTAGCGAAGCCAATGTTCATAATATG
AAGCAGTGGCTGTGG
+
FFFFFFFFFFFFFFFFFFFFFFFFFFFFFFFFFFFFFFFFFFFFFFFFFFFFFFFFFFFFFFFFFFFFFF
FFFFFFFFFFFFFFFFFFFFFFFFFFFFFFFFFFFFFFFFFFFFFFFFFFFFFFFFFFFFFFFFFFFFFF
FFFFFFFFFFFFFFF
@A00721:190:HGJW7DSXY:4:1635:4083:16329 1:N:0:TCTGCAAG+GCTGCACT
TGGCAGTATCGTTTGTATAAATGATTGTCCTACTGGAACCTCGGTCGATACGAGACATACCCAGAAAG
TGAGATGGAATCGAATTGTGCTTAACGGTTCAGTTAAGTTGACTAGCGAAGCCAATGTTCATAATATG
AAGCAGTGGCTGTGG
+
FFFFFFFFFFFFFFFFFFFFFFFFFFFFFFFFFFFFFFFFFFFFFFFFFFFFFFFFFFFFFFFFFFFFFF
FFFFFFFFFFFFFFFFFFFFFFFFFFFFFFFFFFFFFFFFFFFFFFF,FFFFFFFFFFFFFFFFFFFFFF
FFFFFFFFFF:FFFF
@A00721:190:HGJW7DSXY:4:2137:18765:1110 1:N:0:TCTGCAAG+GCTGCACT
TGGCAGTATCGTTTGTATAAATGATTGTCCTACTGGAACCTCGGTCGATACGAGACATACCCAGAAAG
TGAGATGGAATCGAATTGTGCTTAACGGTTCAGTTAAGTTGACTAGCGAAGCCAATGTTCATAATATG
AAGCAGTGGCTGTGG
+
FFFFFFFFFFFFFFFFFFFFFFFFFFFFFFFFFFFFFFFFFFFFFFFFFFFFFFFFFFFFFFFFFFFFFF
FFFFFFFFFFFFFFFFFFFFFFFFFFFFFFFFFFFFFFFFFFFFFFFFFFFFFFFFFFFFFFFFFFFFFF
FF:,FFFFFFFFFFF
@A00721:190:HGJW7DSXY:4:2259:13268:3834 1:N:0:TCTGCAAG+GCTGCACT
TGGCAGTATCGTTTGTATAAATGATTGTCCTACTGGAACCTCGGTCGATACGAGACATACCCAGAAAG
TGAGATGGAATCGAATTGTGCTTAACGGTTCAGTTAAGTTGACTAGCGAAGCCAATGTTCATAATATG
AAGCAGTGGCTGTGG
+
FFFFFFFFFFFFFFFFFFFFFFFFFFFFFFFFFFFFFFFFFFFFFFFFFFFFFFF:FFFFFFFFFFFFFF
FFFFFFFFFFFFFFFFFFFFFFFFFFFFFFFFFFFFFFFFFFFFFFFFFFFFFFFFFFFFFFFFFFFFFF
FFF:F:FF:FFFFFF
@A00721:190:HGJW7DSXY:4:2352:2248:17347 1:N:0:TCTGCAAG+GCTGCACT
TGGCAGTATCGTTTGTATAAATGATTGTCCTACTGGAACCTCGGTCGATACGAGACATACCCAGAAAG
TGAGATGGAATCGAATTGTGCTTAACGGTTCAGTTAAGTTGACTAGCGAAGCCAATGTTCATAATATG
AAGCAGTGGCT
+
F::FFFFFF:F,,:FFFFFFFFF:FFF:FF:FFFFF,FF:,F::FF,FFFFFFF,FF:,FFFFFFFFF,
,,FFFFFFFFF:FFF::FFFFFFFFFFFFFFFFFFFFFFF,F:FFF:,FFF:,FFF:FF::,FF:FFF:F
FFF:F,:F:F:
@A00721:190:HGJW7DSXY:4:2440:21802:29794 1:N:0:TCTGCAAG+GCTGCACT
TGGCAGTATCGTTTGTATAAATGATTGTCCTACTGGAACCTCGGTCGATACGAGACATACCCAGAAAG
TGAGATGGAATCGAATTGTGCTTAACGGTTCAGTTAAGTTGACTAGCGAAGCCAATGTTCATAATATG
AAGCAGTGGCTGTGG
+
FFFFFFFFFFFF,FFFFFFFFFFFFFFFFFFFFFFFFFFFFFFFFFFFFFFFFFFFFFFFFFFFFFFFFF
FFFFFFFF:FFFFFFFFFFFFFFFFFFFFFFFFFFFFF:FFFFFFFFFFFFFFFFFFFFFFFFFFFFFF:
FFF:FFFFFFFFFFF

```
@A00721:190:HGJW7DSXY:4:2450:31467:20290 1:N:0:TCTGCAAG+GCTGCACT
TGGCAGTATCGTTTGTATAAATGATTGTCCTACTGGAACCTCGGTCGATACGAGACATACCCAGAAAG
TGAGATGGAATCGAATTGTGCTTAACGGTTCAGTTAAGTTGACTAGCGAAGCCAATGTTCATAATATG
AAGCAGTGGCTGTGG
+
FFFFFFFFFFFFFFFFFFFFFFFFFFFFFFFFFFFFFFFFFFFFFFFFFFFFFFFFFFFFFFFFFFFF
FFFFFFFFFFFFFFFFF:FFFFFFFFFFFFFFFFFFFFFFFFFFFFFFFFFFF,FFFF:FFFFFFFFFF
FF:FFFFFF:FFFFF
@A00721:190:HGJW7DSXY:4:1217:3631:30170 1:N:0:TCTGCAAG+GCTGCACT
CCGGCAGTATCGTTTGTATAAATGATTGTCCTACTGGAACCTCGGTCGATACGAGACATACCCAGAAA
GTGAGATGGAATCGAATTGTGCTTAACGGTTCAGTTAAGTTGACTAGCGAAGCCAATGTTCATAATAT
GAAGCAGTGGCTGTG
+
FFFFFFFFFFFFFFFFFFFFFFFFFFFFFFFFFFFFFFFFFFFFFFFFFFFF:FFFFFFFFFFFFFFF
FFFFFFFFFFFFFFFFFF:FFFFFFFFFF:FFFFFFFFFFFFFFFFFFFFFFF:FFFFFF:FFFFFF,FF
FFFFFFFFFFFFFFF
@A00721:190:HGJW7DSXY:4:2278:15429:25238 1:N:0:TCTGCAAG+GCTGCACT
CCGGCAGTATCGTTTGTATAAATGATTGTCCTACTGGAACCTCGGTCGATACGAGACATACCCAGAAA
GTGAGATGGAATCGAATTGTGCTTAACGGTTCAGTTAAGTTGACTAGCGAAGCCAATGTTCATAATAT
GAAGCAGTGGCTGTG
+
FFFFFFFFFFFFFFFFFFFFFFFFFFFFFFFFFFFFFFFFFFFFFFFFFFFFFFFFFFFFFFFFFFFF
FFFFFFFFFFFFFFFFFFFFFFFFFFFFFFFFFFFFFFFFFFFFFFFFFFFFFFFFFFFFFF:FFFFF
FFFFFFFFFFFFFFF
@A00721:190:HGJW7DSXY:4:1101:25979:11381 1:N:0:TCTGCAAG+GCTGCACT
AGGCAGTATCGTTTGTATAAATGATTGTCCTACTGGAACCTCGGTCGATACGAGACATACCCAGAAAG
TGAGATGGAATCGAATTGTGCTTAACGGTTCAGTTAAGTTGACTAGCGAAGCCAATGTTCATAATATG
AAGCAGTGGCTGTGG
+
FFFFFFFFFFFFFFFFFFFFFFFFFFFFFFFFFFFFF:FFFFFFFFFFFFFFFFFFFFFFFFFFFFFF
FFFFFFFFFFFFFFFFFFFFFFFFFFFFFFFFFFFFFFFFFFFFFFFFFFFFFFFFFFFFFFFFFFFF
FFF:FFFF:FFFFFF
@A00721:190:HGJW7DSXY:4:2212:27679:10254 1:N:0:TCTGCAAG+GCTGCACT
AGGCAGTATCGTTTGTATAAATGATTGTCCTACTGGAACCTCGGTCGATACGAGACATACCCAGAAAG
TGAGATGGAATCGAATTGTGCTTAACGGTTCAGTTAAGTTGACTAGCGAAGCCAATGTTCATAATATG
AAGCAGTGGCTGTGG
+
FFFFFFFFFFFFFFFFFFFFFFFFFFFFFFFFFFFF:FFFFFFFFFFFFFFF:FFFFFFFFFFFFFF
FFFFFFFFFFFFFFFFFFFFFFFFFFFFFFFFFFFF:FFFFFFF:FFFFFFFFFFFFFFFFFFFFFF:
FFF:F:FFFFFFFFF
@A00721:190:HGJW7DSXY:4:1118:31114:28698 1:N:0:TCTGCAAG+GCTGCACT
GGCAGTATCGTTTGTATAAATGATTGTCCTACTGGAACCTCGGTCGATACGAGACATACCCAGAAAGT
GAGATGGAATCGAATTGTGCTTAACGGTTCAGTTAAGTTGACTAGCGAAGCCAATGTTCATAATATGA
AGCAGTGGCTGTGGA
+
FFFFFFFFFFFFFFFFFFFFFFFFFFFFFFFFFFFFFFFFFFFFFFFFFFFFFFFFFFF:FFFFFFFF
FFFFFFFFFF:FFFFFFFFFFFFFFFFFFFFFFFFFFFFF:FFFFFFFFFFFFFFFFFFFFFFFFFFF,F
FF:FF:FFFFFF,FF
@A00721:190:HGJW7DSXY:4:1161:12066:9298 1:N:0:TCTGCAAG+GCTGCACT
GGCAGTATCGTTTGTATAAATGATTGTCCTACTGGAACCTCGGTCGATACGAGACATACCCAGAAAGT
GAGATGGAATCGAATTGTGCTTAACGGTTCAGTTAAGTTGACTAGCGAAGCCAATGTTCATAATATGA
AGCAGTGGCTGTGGA
+
FFF,FFFFFFFFFFFFFFFFFFFFFFFFFFFFFFFFFFFFFFFFFFFFFFFF:FFFFFFFFFFFFFFF
```

FFFFFFFFFFFFFFFFFFFFFFFFFFFFFFFFFFFFFFFFFFFFFFFFFFFFFFFFFFFFFFFFFFFF
FFFFFFFFFFFFFFF
@A00721:190:HGJW7DSXY:4:1209:17861:14606 1:N:0:TCTGCAAG+GCTGCACT
GGCAGTATCGTTTGTATAAATGATTGTCCTACTGGAACCTCGGTCGATACGAGACATACCCAGAAAGT
GAGATGGAATCGAATTGTGCTTAACGGTTCAGTTAAGTTGACTAGCGAAGCCAATGTTCATAATATGA
AGCAGTGGCTGTGGA
+
FFFFF:FFF,:,:,FFFFFF::FFFFF:F:F,FFFF:,FFF:F,F:F:FFFF,F:F,F,F:FFFFF:F
FF,FFF:FFFFFFFFF,FFF:,F:F,FFFFF:FFFFF,FFF,FF,FFF,F:F,FFFF,FF,FF,F:,F
FF:FF,:FFFF,:,:
@A00721:190:HGJW7DSXY:4:1261:19054:26005 1:N:0:TCTGCAAG+GCTGCACT
GGCAGTATCGTTTGTATAAATGATTGTCCTACTGGAACCTCGGTCGATACGAGACATACCCAGAAAGT
GAGATGGAATCGAATTGTGCTTAACGGTTCAGTTAAGTTGACTAGCGAAGCCAATGTTCATAATATGA
AGCAGTGGCTGTGGA
+
FFFFFFFFFFFFFFFFFFFFFFFFFFFFFFFFFFFFFFFFFFFFFFFFFFFFFFFFFFFFFFFFFFF:
FF:FFFFF::FFFFFFFFFFFFFFFFFFFFFFFFFFFFFFFFFFFFFFFFFFFFFFFFFFFFFFFFFF
FFFFFFFFFFFFFFF
@A00721:190:HGJW7DSXY:4:1313:28872:5901 1:N:0:TCTGCAAG+GCTGCACT
GGCAGTATCGTTTGTATAAATGATTGTCCTACTGGAACCTCGGTCGATACGAGACATACCCAGAAAGT
GAGATGGAATCGAATTGTGCTTAACGGTTCAGTTAAGTTGACTAGCGAAGCCAATGTTCATAATATGA
AGCAGTGGCTGTGGA
+
FFFFFFFFFFFFFFFFFFFFFFFFFFFFFFFFFFFFFFFFFFFFFFFFFFFFFFFFFFFFFFFFFFFF
FFFFFFFFFFFFFFFFFFFFFFFFFFFFFFFFFFFFFFFFFFFF:FFFFFFFFFFFFFFFFFFFFFFF
FF:FFFFFFFFFFFF
@A00721:190:HGJW7DSXY:4:1365:18864:34507 1:N:0:TCTGCAAG+GCTGCACT
GGCAGTATCGTTTGTATAAATGATTGTCCTACTGGAACCTCGGTCGATACGAGACATACCCAGAAAGT
GAGATGGAATCGAATTGTGCTTAACGGTTCAGTTAAGTTGACTAGCGAAGCCAATGTTCATAATATGA
AGCAGTGGCTGTGGA
+
FFFFFFFFFFF:FFFFFFFFFFFFFFFFFFFFFFFFFFFFFFFFFFFFFFFFFFFFFFFFFFFFFFFF
FFF:FFFFFFFFFFFFFFFFFFFFF:FFFFFFFFFFFFFFFFFFFFFFFFFF:FFFFFFFFFFFFFFF
FFFFFFFFFFF:FFF
@A00721:190:HGJW7DSXY:4:1444:30029:25410 1:N:0:TCTGCAAG+GCTGCACT
GGCAGTATCGTTTGTATAAATGATTGTCCTACTGGAACCTCGGTCGATACGAGACATACCCAGAAAGT
GAGATGGAATCGAATTGTGCTTAACGGTTCAGTTAAGTTGACTAGCGAAGCCAATGTTCATAATATGA
AGCAGTGGCTGTGGA
+
FFFFFFFFFFFFFFFFFFF:FFFFF:FFFFFFFFFFFFFFFFFFFFFFFFFFFFFFFFFFFFFFFFFF
FFFFFFFFFFFFFFF:FFFFFFFFFFFFFFFFFFFFFFF:FFFF:FFFFFFFFFF::FFFF,FFFFFF
FF:FFF,FFFFFFFF
@A00721:190:HGJW7DSXY:4:1638:28546:20181 1:N:0:TCTGCAAG+GCTGCACT
GGCAGTATCGTTTGTATAAATGATTGTCCTACTGGAACCTCGGTCGATACGAGACATACCCAGAAAGT
GAGATGGAATCGAATTGTGCTTAACGGTTCAGTTAAGTTGACTAGCGAAGCCAATGTTCATAATATGA
AGCAGTGGCTGTGGA
+
FFFFFFFFFFFFFFFFFFFFFFFFFFFFFFFFFFFF:FFFFFFFFFFFFFFFFFFFFFFFFFFFFFFF
FFFFFFFFFFFFFF:FFFFFFFFFF:FFFFFFFFFFFFFFFFFF:FFFFFFFFFFFFFFFFFFFFFF:
FFFFFFFFFFFFFFF
@A00721:190:HGJW7DSXY:4:1646:9498:23672 1:N:0:TCTGCAAG+GCTGCACT
GGCAGTATCGTTTGTATAAATGATTGTCCTACTGGAACCTCGGTCGATACGAGACATACCCAGAAAGT
GAGATGGAATCGAATTGTGCTTAACGGTTCAGTTAAGTTGACTAGCGAAGCCAATGTTCATAATATGA
AGCAGTGGCTGTGGA

```
+
FFFFFFFFFFFFFFFFFFF:FFFFFFFFFFFFFFFFFFFFFFFFFFFFFFFFFFFFFFFFFFFFFFFF
FFFFFFFFFFFFFFFFFFFFFFFFFF:FFFFFFFFFFFFFFFFFFFFFFFFFFFFFFF:FFFFFFFFF
F,:FFFFFFFFFFFF
@A00721:190:HGJW7DSXY:4:2139:4544:6762 1:N:0:TCTGCAAG+GCTGCACT
GGCAGTATCGTTTGTATAAATGATTGTCCTACTGGAACCTCGGTCGATACGAGACATACCCAGAAAGT
GAGATGGAATCGAATTGTGCTTAACGGTTCAGTTAAGTTGACTAGCGAAGCCAATGTTCATAATATGA
AGCAGTGGCTGTGGA
+
FFFFFFFFFF::FFFFFFFFFFFFFFFFFFFFFFFFFFFFFFFFFFFFFFFFFFFFFFFFFFFF:FFF
FFFFFFFFFFFFFFFFFFFFFFFFFF:FFFFFF:FFFFFFFFFFFFFFFFFFFFFFFFFFFFFFFF:F
F,:FFFFF:FFFFFF
@A00721:190:HGJW7DSXY:4:2204:30165:35258 1:N:0:TCTGCAAG+GCTGCACT
GGCAGTATCGTTTGTATAAATGATTGTCCTACTGGAACCTCGGTCGATACGAGACATACCCAGAAAGT
GAGATGGAATCGAATTGTGCTTAACGGTTCAGTTAAGTTGACTAGCGAAGCCAATGTTCATAATATGA
AGCAGTGGCTGTGGA
+
FFFFFFFFFFFFF:FFFFFFFFFFFFF:FFFFFFFFFFFFFFFFF:FFF:,F:FFF,F:F:FFFFF::
FF:FFFFFFFFFFF,F:FF,,FFF::FFFFF,FFFF:FFFFFFFF:FFF:FF,F,FFFFFFFF,FF,F
F:,F:F:FFFFFFFF
@A00721:190:HGJW7DSXY:4:2206:1434:31751 1:N:0:TCTGCAAG+GCTGCACT
GGCAGTATCGTTTGTATAAATGATTGTCCTACTGGAACCTCGGTCGATACGAGACATACCCAGAAAGT
GAGATGGAATCGAATTGTGCTTAACGGTTCAGTTAAGTTGACTAGCGAAGCCAATGTTCATAATATGA
AGCAGTGGCTGTGGA
+
FFFFFFFFFFFFFFFFFFFFFFFFFFFFFFFFFFFFFFFFFFFFFFF:FFFFFFFFFFFFFFFFFFFF
FFFFFFFFFFFFFFFFFFFFFFFF,FFFFFFFFFFFFF:FFFFFFFFFF:FFFFFFFFFFFFFFFFF:
FF:FFFFFFFFFFFF
@A00721:190:HGJW7DSXY:4:2260:15257:31454 1:N:0:TCTGCAAG+GCTGCACT
GGCAGTATCGTTTGTATAAATGATTGTCCTACTGGAACCTCGGTCGATACGAGACATACCCAGAAAGT
GAGATGGAATCGAATTGTGCTTAACGGTTCAGTTAAGTTGACTAGCGAAGCCAATGTTCATAATATGA
AGCAGTGGCTGTGGA
+
FFFFFFFFFFFFFFFFFF:FFFFFFFFFFFFFFFFFFFFFFFFFFFFFFFFFFFFFFFFFFFFFFFFF
FFFFFFFFFFFFFFFFFFFFFFFFFFFFFFFFFFFFFFFFFFFFFFFFFFFFFFFFFFFFFFF:FFFF
FF:FFFFFFFFFFFF
@A00721:190:HGJW7DSXY:4:2349:12572:11052 1:N:0:TCTGCAAG+GCTGCACT
GGCAGTATCGTTTGTATAAATGATTGTCCTACTGGAACCTCGGTCGATACGAGACATACCCAGAAAGT
GAGATGGAATCGAATTGTGCTTAACGGTTCAGTTAAGTTGACTAGCGAAGCCAATGTTCATAATATGA
AGCAGTGGCTGTGGA
+
FFFFFFFFFF:FFFFFFF:FFFFFFFFFFFFFFFFFFFFFFFFFFFFFFFFFFFFFFFFFFFFFFFFF
FFFFFFFFFFFFFFFFFFFFFFFFFFFFFFFFFFFFFFF:FFFFFFFFFFFF,FFFFFFFFFFFFFFF
FF:FFFFFFFFFFFF
@A00721:190:HGJW7DSXY:4:2621:16215:3646 1:N:0:TCTGCAAG+GCTGCACT
GGCAGTATCGTTTGTATAAATGATTGTCCTACTGGAACCTCGGTCGATACGAGACATACCCAGAAAGT
GAGATGGAATCGAATTGTGCTTAACGGTTCAGTTAAGTTGACTAGCGAAGCCAATGTTCATAATATGA
AGCAGTGGCTGTGGA
+
FFFFFFFFFFFFFF,FFFFFFFFFFFFFFFFFFFFFFFFFFFFFFFFFFFFF:FFFFFFFFFFFFFFF
FF:FFFFFFFFFFFFFFFFFFFFFFF:FF:FFFFFFFFFFFFFFFFFFFFFFFFFFFFFFFFFFFFFF
F,FFFFFFFFFFFFF
@A00721:190:HGJW7DSXY:4:2645:14561:12211 1:N:0:TCTGCAAG+GCTGCACT
GGCAGTATCGTTTGTATAAATGATTGTCCTACTGGAACCTCGGTCGATACGAGACATACCCAGAAAGT
```

```
GAGATGGAATCGAATTGTGCTTAACGGTTCAGTTAAGTTGACTAGCGAAGCCAATGTTCATAATATGA
AGCAGTGGCTGTGGA
+
FFFFFFFFFFFFFFFFFFFFFFFFFFFFFFFFFFFFFFFFFFFFFFFFFFFFFFFFFFFFFFFFFFFF
FFFFFFFFFFFFFFFFFFFFFFFFFFFFFFFFFFFFFFFFFFFFFFFFFFFFFFFFFFFFFFFFFFFF
FF:FFFFFFFFFFFF
@A00721:190:HGJW7DSXY:4:2645:14235:12461 1:N:0:TCTGCAAG+GCTGCACT
GGCAGTATCGTTTGTATAAATGATTGTCCTACTGGAACCTCGGTCGATACGAGACATACCCAGAAAGT
GAGATGGAATCGAATTGTGCTTAACGGTTCAGTTAAGTTGACTAGCGAAGCCAATGTTCATAATATGA
AGCAGTGGCTGTGGA
+
FFFFFFF:FFFFFFFFFFFFFFFFFFFFFFFFFFFFF,FFFF:FFFFFFFFFFFFFFFFFFFFFFFFFF
FFFFFFFFFFFFFFFFFFFFFFFFFFFFFFFFF:FFFFFFFFFFFFFFF:FFFFFFFFFFFFFFFFFF
F,:FFFFFFFFFFFF
@A00721:190:HGJW7DSXY:4:1530:18837:32487 1:N:0:TCTGCAAG+GCTGCACT
ATCGTTTGTATAAATGATTGTCCTACTGGAACCTCGGTCGATACGAGACATACCCAGAAAGTGAGATG
GAATCGAATTGTGCTTAACGGTTCAGTTAAGTTGACTAGCGAAGCCAATGTTCATAATATGAAGCAGT
GGCTGTGGATGTGGC
+
FFFFFFFFFFFFFFFFFFFFFFFFFFFFFFFFF:F:FFFFFFFFFFFFFFFFFFF:FFFFFFFFFFFF,
FFF,FFFFF::FF:FFFFF:F,FF,FFF:FFFFFFFFFF:FFFFFFFFFF:FFF:FFFFFFFFFFFFF
FFFFF,FFFFFFFFF
@A00721:190:HGJW7DSXY:4:2242:22815:21402 1:N:0:TCTGCAAG+GCTGCACT
ATCGTTTGTATAAATGATTGTCCTACTGGAACCTCGGTCGATACGAGACATACCCAGAAAGTGAGATG
GAATCGAATTGTGCTTAACGGTTCAGTTAAGTTGACTAGCGAAGCCAATGTTCATAATATGAAGCAGT
GGCTGTGGATGTGGC
+
FFFFFFF:FFFFFFFFFFFFFFFFFFFFFFFFFFFFFFFFFFFFFFFFFFFFFFFFFFFFFFFFFFFF
FFFFFFFFFFFFFFFFFFFFFFFFFFFFFFFFFFFF:FFFFFFFFFFFFFFFFFFFFFFFFFFFFF:FFF
FFFFFFFFFFFFFFF
@A00721:190:HGJW7DSXY:4:2457:12138:23422 1:N:0:TCTGCAAG+GCTGCACT
ATCGTTAGTATAAATGATTGTCCTACTGGAACCTCGGTCGATACGAGACATACCCAGAAAGTGAGATG
GAATCGAATTGTGCTTAACGGTTCAGTTAAGTTGACTAGCGAAGCCAATGTTCATAATATGAAGCAGT
GGCTGTGGATGTGGC
+
FFFFFF,:,FFFFF:FFFFF,F:FF::F:F:FFFFFFFF,::FF:F:FFFFF,FFF:FFFF,FF:FFF
FFFF,FFFFF:FFFFFFFF,FFF:FF:FFF:::FFFFFFFFF:FF::FF,FFFF:FF,,,:F::FFFF
F,:FFFFFFF:F:,F
@A00721:190:HGJW7DSXY:4:1165:21233:6981 1:N:0:TCTGCAAG+GCTGCACT
CGTTTGTATAAATGATTGTCCTACTGGAACCTCGGTCGATACGAGACATACCCAGAAAGTGAGATGGA
ATCGAATTGTGCTTAACGGTTCAGTTAAGTTGACTAGCGAAGCCAATGTTCATAATATGAAGCAGTGG
CTGTGGATGTGGCTC
+
FFFFFFFFFFFFFFFFFFFFFFFFFFFFFFFFFFFFFFFFFFFFFFFFFFFFFFFFFFFFFFFFFFFF
FFFFFFFFFFFFFFFFFFFFFFFFFFFFFFFFFFFFFFFFFFFFFFFFFFF:F,FF:FFFFFFFFF
FFFFFFFFFFFFFFF
@A00721:190:HGJW7DSXY:4:1375:24930:18646 1:N:0:TCTGCAAG+GCTGCACT
CGTTTGTATAAATGATTGTCCTACTGGAACCTCGGTCGATACGAGACATACCCAGAAAGTGAGATGGA
ATCGAATTGTGCTTAACGGTTCAGTTAAGTTGACTAGCGAAGCCAATGTTCATAATATGAAGCAGTGG
CTGTGGATGTGGCT
+
F:FFFFFFFFFFFFFFF::FFFFF::FFFFFFFFFFFFFFFFFFFFFFFFFFFF:F:FF:,FFF:FFFFF
FFFFFFFF,,FF::FFF:FFFFF:FFFFFFFFFFFFFFFFFFFFFFF:FFFFFFFFFFFFFFF,,FFFFF
:FF,,:FFFFFFFF
```

```
@A00721:190:HGJW7DSXY:4:1545:4408:17268 1:N:0:TCTGCAAG+GCTGCACT
CGTTTGTATAAATGATTGTCCTACTGGAACCTCGGTCGATACGAGACATACCCAGAAAGTGAGATGGA
ATCGAATTGTGCTTAACGGTTCAGTTAAGTTGACTAGCGAAGCCAATGTTCATAATATGAAGCAGTGG
CTGTGGATGTGGCTC
+
FFFFFFFFFFFFFFFFFFFFFFFFFFFFFFFFFFFFFFFFFFFFFFFFFFFFFFFFFFFFFFFFFFFFFF
FFFFFFFFFFFFFFFFFFFFFFFFFFFFFFFFFFFFFFFFFFFFFFFFFFFFFFFFFFFFFFFFFFFFFF
FFFFFFFFFFFFFFF
@A00721:190:HGJW7DSXY:4:1638:17381:4382 1:N:0:TCTGCAAG+GCTGCACT
CGTTTGTATAAATGATTGTCCTACTGGAACCTCGGTCGATACGAGACATACCCAGAAAGTGAGATGGA
ATCGAATTGTGCTTAACGGTTCAGTTAAGTTGACTAGCGAAGCCAATGTTCATAATATGAAGCAGTGG
CTGTGGATGTGGCTC
+
FFFFFFFFFFFFFFFFFFFFFFFFFFFFFFFFFFFFFFFFFFFFFF:FFFFFFFFFFFFFFFFFFFFFFF
FFFFFF:FFFFFFFFFFFFFFFFFFFFFFFFFFFFFFFFFFFFFFFFFFFFFFFFFFFFFFFFFFFFFFF
FFFFFFFFFFF:FFF
@A00721:190:HGJW7DSXY:4:2169:14362:6324 1:N:0:TCTGCAAG+GCTGCACT
CGTTTGTATAAATGATTGTCCTACTGGAACCTCGGTCGATACGAGACATACCCAGAAAGTGAGATGGA
ATCGAATTGTGCTTAACGGTTCAGTTAAGTTGACTAGCGAAGCCAATGTTCATAATATGAAGCAGTGG
CTGTGGATGTGGCTC
+
FFFFFFFFFFFFFFFFFFFFFFFFFFFF,FFFFFFFFFFFFFF:FFFFFFFFFF:FFFFFFFFFFFFFFF
FFFFFF:FFFFFFFFFFFFFFFFFFFFFFFF:FFFFFFFFFFFFFFFFFFFFFFFFFFFFFFF:FF:FF:
F:FFFFF:FFFFFFF
@A00721:190:HGJW7DSXY:4:2358:22083:34851 1:N:0:TCTGCAAG+GCTGCACT
CGTTTGTATAAATGATTGTCCTACTGGAACCTCGGTCGATACGAGACATACCCAGAAAGTGAGATGGA
ATCGAATTGTGCTTAACGGTTCAGTTAAGTTGACTAGCGAAGCCAATGTTCATAATATGAAGCAGTGG
CTGTGGATGTGGCTC
+
FFFFFFFFFFFFFFFFFFFFFFFFFFFFFFFFFFFFFFFFFFFFFFFFFFFFFFFFFFFFFFFFFFFFFF
:FFFFFFFFFFFFFFFFFFFFFFF:FFFFFFFFFFFFFFFFFFFFFFFFFFFFFFFFFFFFFFFFFFFFF
FFFFFFFFFFFFFFF
@A00721:190:HGJW7DSXY:4:2543:5448:7451 1:N:0:TCTGCAAG+GCTGCACT
CGTTTGTATAAATGATTGTCCTACTGGAACCTCGGTCGATACGAGACATACCCAGAAAGTGAGATGGA
ATCGAATTGTGCTTAACGGTTCAGTTAAGTTGACTAGCGAAGCCAATGTTCATAATATGAAGCAGTGG
CTGTGGATGTGGCTC
+
FFFFFFFFFFFFFFFFFFFFFFFFFFFFFFFFFFFFFFFFFFFFFFFFFFFFFFFFFFFFFFFFFFFFFF
FFFFFFFFFFFFFFFFFFFFFFFFFFFFFFFFFFFFFFFFFFFFFFFFFFFFFFFFFFFFFFF::FFFFF
FFFFFFFFFFFFFFF
@A00721:190:HGJW7DSXY:4:2628:18747:17080 1:N:0:TCTGCAAG+GCTGCACT
CGTTTGTATAAATGATTGTCCTACTGGAACCTCGGTCGATACGAGACATACCCAGAAAGTGAGATGGA
ATCGAATTGTGCTTAACGGTTCAGTTAAGTTGACTAGCGAAGCCAATGTTCATAATATGAAGCAGTGG
CTGTGGATGTGGCTC
+
FF,:FFFFFFFFFFF:FFFFFFFFFFFFFFFFFFFFFFFFFFFFFF,FFFFFFFFFFFFFFFFFFFFFFF
FF,FFFFFFFFFFFFF:FFFFFFFFFFFFFFFFFFFFF:FFFFFFFFFFFFFFFFFF:FF:FF:FFFFFF
FFFFFFFFFFFFF:F
@A00721:190:HGJW7DSXY:4:1145:2157:8891 1:N:0:TCTGCAAG+GCTGCACT
GTTTGTATAAATGATTGTCCTACTGGAACCTCGGTCGATACGAGACATACCCAGAAAGTGAGATGGAA
TCGAATTGTGCTTAACGGTTCAGTTAAGTTGACTAGCGAAGCCAATGTTCATAATATGAAGCAGTGGC
TGTGGATGTGGCTCG
+
::FFF:,FFFFFFFFFFFFFFFFF:FF::FFFFFFFFFFFFFFFFFFFFFFFFF::FF:FFFFFFF:FF,
```

F:FFFFF:FFFFFFFFFFF:FFF,FFFFFFFF:FFFFFFFFF,FFFFFFFF:FF,FFFFF::FFFFFF
FFFFFFF:FFFFFF:
@A00721:190:HGJW7DSXY:4:1222:18566:6684 1:N:0:TCTGCAAG+GCTGCACT
GTTTGTATAAATGATTGTCCTACTGGAACCTCGGTCGATACGAGACATACCCAGAAAGTGAGATGGAA
TCGAATTGTGCTTAACGGTTCAGTTAAGTTGACTAGCGAAGCCAATGTTCATAATATGAAGCAGTGGC
TGTGGATGTGGCTCG
+
FFFFFFFFFFFFFFFFFFFFFFFFFFFFFFFFFFFFFFFFFFFFFFFFFFFFFFFFFFFFFFFFFFF:
FFFFFFFFF:FFFFFFFFFFFFFFFFFFFFFFFFFFFFF,FFFFFFFFFFF,FFFFF:FFFFFFFFFF
FFFFFFFFFFFFFF:
@A00721:190:HGJW7DSXY:4:1313:12066:26271 1:N:0:TCTGCAAG+GCTGCACT
GTTTGTATAAATGATTGTCCTACTGGAACCTCGGTCGATACGAGACATACCCAGAAAGTGAGATGGAA
TCGAATTGTGCTTAACGGTTCAGTTAAGTTGACTAGCGAAGCCAATGTTCATAATATGAAGCAGTGGC
TGTGGATGTGGCTCG
+
FFFFFFFFFFFFFFFFFFFFFFFFFFF:FFFFFFFFFFFFFFFFFFFFFFFFFFFFFFFFFFFFFFFF
FFFFFFFFFFFFFFFFFFFFFFFFFFFFFFFFFFFFFFFFFFFF:FFFFFFFFFFFFFFFFFFFFFFF
FFFFFFFFFFFFFFF
@A00721:190:HGJW7DSXY:4:1330:30002:24236 1:N:0:TCTGCAAG+GCTGCACT
GTTTGTATAAATGATTGTCCTACTGGAACCTCGGTCGATACGAGACATACCCAGAAAGTGAGATGGAA
TCGAATTGTGCTTAACGGTTCAGTTAAGTTGACTAGCGAAGCCAATGTTCATAATATGAAGCAGTGGC
TGTGGATGTGGCTCG
+
FFFFFFFFFFFFFFFFFFFFFFFFFFF,FFFFFFFFFFFFFFFFFFFFFFFFFFFFFF:FFFFFFFFF
FFFFFFFFFFFFFFFFFFFFFFFFFFFFFFFFFFFFFFFFFFFFFFFFFFFFFF:FFFFFFFFFFFFF
FFFFFFFFFFFFFFF
@A00721:190:HGJW7DSXY:4:1426:16993:28761 1:N:0:TCTGCAAG+GCTGCACT
GTTTGTATAAATGATTGTCCTACTGGAACCTCGGTCGATACGAGACATACCCAGAAAGTGAGATGGAA
TCGAATTGTGCTTAACGGTTCAGTTAAGTTGACTAGCGAAGCCAATGTTCATAATATGAATCAGTGGC
TGTGGATGTGGCTCG
+
FFF,FFFFFFFF:FFF::F,FF:F,:FFFFF:::FFFFFF:FF:FFFFFF:F,,FFF:F:FFFFFFFF
,:FFF,FFF:,F,F:FFFF,FFFF:FFF:,F:FFFFF,FFF,,FF,:F:FFF,F:F,:FF,:FFFF,,
FFFF:FFFF,:,F:F
@A00721:190:HGJW7DSXY:4:1515:23882:12070 1:N:0:TCTGCAAG+GCTGCACT
GTTTGTATAAATGATTGTCCTACTGGAACCTCGGTCGATACGAGACATACCCAGAAAGTGAGATGGAA
TCGAATTGTGCTTAACGGTTCAGTTAAGTTGACTAGCGAAGCCAATGTTCAGAATATGAAGCAGTGGC
TGTGGATGTGGCTCG
+
FF,F,FFFFFF,FFF:FFFFFFFFF:FFF::FFFFFFFFFFFFFFFF,FF:FFFFFFFF:F,FFFFFF
F,,FFFF,F:,:FFFF,,F:,FFFFFF:FF:FFFF,,FFFFFFFFFF,FFF,FF,FFFFF,,FF,:FF
F,,:FFFFFFFFFFF
@A00721:190:HGJW7DSXY:4:1543:8133:35399 1:N:0:TCTGCAAG+GCTGCACT
GTTTGTATAAATGATTGTCCTACTGGAACCTCGGTCGATACGAGACATACCCAGAAAGTGAGATGGAA
TCGAATTGTGCTTAACGGTTCAGTTAAGTTGACTAGCGAAGCCAATGTTCATAATATGAAGCAGTGGC
TGTGGATGTGGCTCG
+
FFFFFFFFFFFFFFFFFFFFFFFFFFFF:FFFFFFFFFFFFFFFF:FFFFFFFFFFFFF,FFFFFFFFF
FFFFFFFFFFFFFFFFFFFFFFFFFFFFFFFFFFFFFFFFFFFFFFFFFFF,FFFFFFFFFFFFFFFF
FFFFFFFFFFFFFFF
@A00721:190:HGJW7DSXY:4:1664:16776:26256 1:N:0:TCTGCAAG+GCTGCACT
GTTTGTATAAATGATTGTCCTACTGGAACCTCGGTCGATACGAGACATACCCAGAAAGTGAGATGGAA
TCGAATTGTGCTTAACGGTTCAGTTAAGTTGACTAGCGAAGCCAATGTTCATAATATGAAGCAGTGGC
TGTGGATGTGGCTCG

+
FFFFFFFFFFFFFFFFFFFFFFFFFFFFFFFFFFFFFFFFFFFFFFFFFFFFFFFFFFFFFFFFFFFFFF
FFFFFFFF:FFFFFFFFFFFFFFFFFFFFFFFFFFFFF:FFFFFFFFFFFFFFFFFFFFFFFFFFFFFFF
FFFFFFFFFFFFFFF
@A00721:190:HGJW7DSXY:4:1676:14724:13745 1:N:0:TCTGCAAG+GCTGCACT
GTTTGTATAAATGATTGTCCTACTGGAACCTCGGTCGATACGAGACATACCCAGAAAGTGAGATGGAA
TCGAATTGTGCTTAACGGTTCAGTTAAGTTGACTAGCGAAGCCAATGTTCATAATATGAAGCAGTGGC
TGTGGATGTGGCTCG
+
FFFFF:FFF:FFFFFFFFFFFFFFFFFFFFFFFFFFFFFFFFFFFFFFFFFFFFFFFFFFFFFFFFFFFF
FFFFFFFFFFFFFFFFFFFF:FFFFFFFFFFFFFFFFFFFFFFFF:FFFFFFF:FFFFFFFFFFFFFFFF
FFFFFFFFFFFFFF:
@A00721:190:HGJW7DSXY:4:2117:16812:19930 1:N:0:TCTGCAAG+GCTGCACT
GTTTGTATAAATGATTGTCCTACTGGAACCTCGGTCGATACGAGACATACCCAGAAAGTGAGATGGAA
TCGAATTGTGCTTAACGGTTCAGTTAAGTTGACTAGCGAAGCCAATGTTCATAATATGAAGCAGTGGC
TGTGGATGTGGCTCG
+
FF:,FFFFFFFFFF:FFFFFFFFFFFF,FFFFFFFFFFFFFFFFFFFFFFFFFFFFFFFFFFFFFFFFF:
FFFFF:FFFFFFFFFFFFFFFFFFFFFFFFFFFFFFFFFFFFFFFFFFF:FF:FFFFFFFFFF:FFFFFF
FFFFFFFFFFFFFFF
@A00721:190:HGJW7DSXY:4:2169:5258:33614 1:N:0:TCTGCAAG+GCTGCACT
GTTTGTATAAATGATTGTCCTACTGGAACCTCGGTCGATACGAGACATACCCAGAAAGTGAGATGGAA
TCGAATTGTGCTTAACGGTTCAGTTAAGTTGACTAGCGAAGCCAATGTTCATAATATGAAGCAGTGGC
TGTGGATGTGGCTCG
+
FF:FFFFFFFFFFFFFFFFFFFFFFFFFFFFFFFFFFFFFFFFFFFFFFFFFFFFFFFF:FFFFFFFFF
FFFFFFFFFFFFFFFFFFFFFFFFFFFFFFFFFFFFFFFFFFFFFFFFFFFFFFFFFFFFFFFFFFFFFF
FFFFFFFFFFFFFFF
@A00721:190:HGJW7DSXY:4:2264:31250:35383 1:N:0:TCTGCAAG+GCTGCACT
GTTTGTATAAATGATTGTCCTACTGGAACCTCGGTCGATACGAGACATACCCAGAAAGTGAGATGGAA
TCGAATTGTGCTTAACGGTTCAGTTAAGTTGACTAGCGAAGCCAATGTTCATAATATGAAGCAGTGGC
TGTGGATGTGGCTCG
+
FFFFFFFFFFFFFFFFFFFFFFFFFFFFFFFFFFFFFFFFFFFFFFFFFFFFFFFFFFFF:FFFFFFFF:
FFFFFFFFFFFFFFFFFFFFFFFFFFFFFFFFFFFFFFFFFFFFFFFFFFFFFFFFFFFFFFFFFFFFFF
FFFFFFFFF:FFFFF
@A00721:190:HGJW7DSXY:4:2264:31376:35916 1:N:0:TCTGCAAG+GCTGCACT
GTTTGTATAAATGATTGTCCTACTGGAACCTCGGTCGATACGAGACATACCCAGAAAGTGAGATGGAA
TCGAATTGTGCTTAACGGTTCAGTTAAGTTGACTAGCGAAGCCAATGTTCATAATATGAAGCAGTGGC
TGTGGATGTGGCTCG
+
FFFFFFFFF:F:FFFFFFFFFFFFFFFFFF:FFFFFFFFFFFFFFFFFFFFFFFFFFFFFFFFFFFFFFF
FFFFFFFFFFFFFFF:FFFFFFFFFFFFFFFFFFFFFFFFF:FFFFFFFFFFFFFFFFFFFF::FFFFFF
F:FFFFFFFF,FFFF
@A00721:190:HGJW7DSXY:4:2267:8422:12320 1:N:0:TCTGCAAG+GCTGCACT
GTTAGTATAAATGATTGTCCTACTGGAACCTCGGTCGATACGAGACATACCCAGAAAGTGAGATGGAA
TCGAATTGTGCTTAACGGTTCAGTTAAGTTGACTAGCGAAGCCAATGTTCATAATATGAAGCAGTGGC
TGTGGATGTGGCTCG
+
FFF,FFFFFFFFFFFFFFFFFFF:FFFFFFFFFFFFFFFFFFFFFFFFFFFFFFFFFFF,FFFFFFFFFF
FFFFFFFFFFFFFFFFFFFFFFFFFFFFFFFFFFFFFFFFFFFFFFFFFFFFFFFFFFFFFFF:FFFFF:
FFFFFFFFFFFFFFF
@A00721:190:HGJW7DSXY:4:2317:12400:11099 1:N:0:TCTGCAAG+GCTGCACT
GTTTGTATAAATGATTGTCCTACTGGAACCTCGGTCGATACGAGACATACCCAGAAAGTGAGATGGAA

```
TCGAATTGTGCTTAACGGTTCAGTTAAGTTGACTAGCGAAGCCAATGTTCATAATATGAAGCAGTGGC
TGTGGATGTGGCTCG
+
FFFFFFF:FFF::FFFF:FFFF:FFFFFFFFFFF:FFFFFFFFFFFFFF:FFFFFFFFFFF:,FF:
F:FFFFFFFF:FFFFFF:FF:FFFFFFFFFFFFFFFFFFFFFFFF:FFFFFFF,,FFF,FFFF,FF
FFFF:FF:F:FFFFF
@A00721:190:HGJW7DSXY:4:2408:8250:6825 1:N:0:TCTGCAAG+GCTGCACT
GTTTGTATAAATGATTGTCCTACTGGAACCTCGGTCGATACGAGACATACCCAGAAAGTGAGATGGAA
TCGAATTGTGCTTAACGGTTCAGTTAAGTTGACTAGCGAAGCCAATGTTCATAATATGAAGCAGTGGC
TGTGGATGTGGCTCG
+
F:FFFFFFFFFFFFFFFFFFFFFFFFFFFFFFFFFFFFFFFFFFFFFFFFFFFFFF:FFFFFFFFF
FFFFFFFFFFFFFFFFFFFF:FFFFFFFFFFFFFFFFFFFFFFFFFFFF:FFFFFFFFF:FFFFFF
FFFFFFFFFFFFFFF
@A00721:190:HGJW7DSXY:4:2652:26576:7435 1:N:0:TCTGCAAG+GCTGCACT
GTTTGTATAAATGATTGTCCTACTGGAACCTCGGTCGATACGAGACATACCCAGAAAGTGAGATGGAA
TCGAATTGTGCTTAACGGTTCAGTTAAGTTGACTAGCGAAGCCAATGTTCATAATATGAAGCAGTGGC
TGTGGATGTGGCTCG
+
FFFFFFFFFFFFFFFFFFFFFFFFFFFFFFFFFFFFFFFFFFFFFFFFFFFFFFFFFFFFFFFFFF
FFFFFFFFFFFFFFFFFFFFFF,FFFFFFFFFFFFFFFFFFFFFFFFFFFFFFFFFFFF:FFFFFF
FFFFFFFFFFFFFFF
@A00721:190:HGJW7DSXY:4:2656:23773:24158 1:N:0:TCTGCAAG+GCTGCACT
GTTTGTATAAATGATTGTCCTACTGGAACCTCGGTCGATACGAGACATACCCAGAAAGTGAGATGGAA
TCGAATTGTGCTTAACGGTTCAGTTAAGTTGACTAGCGAAGCCAATGTTCATAATATGAAGCAGTGGC
TGTGGATGTGGCTCG
+
FFFFFFFFFFFFFFFFFFFFFFFFFFFFFFFFFFFFFFFFFFFFFFFFFFFFFFFFFFFFFFFFFF
FFFFFFFFFFFFFFFFFFFFFFFFFFFFFFFFFFFFFFFFFFFFFFFFFFFFFFFFFFF:FFFFFF
FFFFFFFFFFFFFFF
@A00721:190:HGJW7DSXY:4:2656:23909:24236 1:N:0:TCTGCAAG+GCTGCACT
GTTTGTATAAATGATTGTCCTACTGGAACCTCGGTCGATACGAGACATACCCAGAAAGTGAGATGGAA
TCGAATTGTGCTTAACGGTTCAGTTAAGTTGACTAGCGAAGCCAATGTTCATAATATGAAGCAGTGGC
TGTGGATGTGGCTCG
+
FFFFFFFFFFFFFFFFFFFFFFFFFFFFFFFFFFFFFFFFFFFFFFFFFFFFFFFFFFFFFFFFFF
FFFFFFFFFFFFFFFFFFFFFFFFFFFFFFFFFFFFFFFFFF:FFFFFFFFFFFFFFFFFFFFFFF
FFFFFFFFFFFFFFF
@A00721:190:HGJW7DSXY:4:1274:16315:24706 1:N:0:TCTGCAAG+GCTGCACT
TTTGAATAAATGATTGTCCTACTGGAACCTCGGTCGATACGAGACATACCCAGAAAGTGAGATGGAAT
CGAATTGTTCTTAACGGTTCAGTTAAGTTGACTAGCGAAGCCAATGTTCATAATATTAAGCAGTGGCT
GTGGATGTGGCTCGT
+
::FF,FFFFFFFF,F,FFFFFF::FFF:FFF,:FFFFFFFFFFFFFFFFF:FFF:FFF,FFF:F::
,,F:::F,FFFFF,FF,FFF,FF:FFF:,F::F:FFFF:FFFFF:,FFFFFFF:,FFFFF:FFF,F
,FF:FF:FFF:FF,:
@A00721:190:HGJW7DSXY:4:1330:30454:11083 1:N:0:TCTGCAAG+GCTGCACT
TTTGTATAAATGATTGTCCTACTGGAACCTCGGTCGATACGAGACATACCCAGAAAGTGAGATGGAAT
CGAATTGTGCTTAACGGTTCAGTTAAGTTGACTAGCGAAGCCAATGTTCATAATATGAAGCAGTGGCT
GTGGATGTGGCTCGT
+
FFFFFFFFFFFFFFFFFFFFFFFFF:FFFFFFFFFFFFFFFFFFFFFFFFFFFFFFFFFFFFFFFF
FFF:FFFFFFFFFFFFFFF:FFFFFFFFFFFFFFFFFFFFFFFFFFFFFFFFFFFFFFFFFFFFFF
FFFFFFFFFFFFFFF
```

```
@A00721:190:HGJW7DSXY:4:1337:13711:8171 1:N:0:TCTGCAAG+GCTGCACT
TTTGTATAAATGATTGTCCTACTGGAACCTCGGTCGATACGAGACATACCCAGAAAGTGAGATGGAAT
CGAATTGTGCTTAACGGTTCAGTTAAGTTGACTAGCGAAGCCAATGTTCATAATATGAAGCAGTGGCT
GTGGATGTGGCTCGT
+
FFFFFFFFFFFFFFFFFFFFFFFFFF,FFFFFFFFFFFFFFFFFFFFFFFFFFFFFFFFFFFFFFFFF:F
FFFFFFFFFFFFFFFFFFFFFFFFFFFFFFFFFFFFFFFFFFFFFFFFFFFFFFFFFFFFFFFFF:FFFF
FFFFFFFFFFFFFFF
@A00721:190:HGJW7DSXY:4:1376:17056:1626 1:N:0:TCTGCAAG+GCTGCACT
TTTGTATAAATGATTGTCCTACTGGAACCTCGGTCGATACGAGACATACCCAGAAAGTGAGATGGAAT
CGAATTGTGCTTAACGGTTCAGTTAAGTTGACTAGCGAAGCCAATGTTCATAATATGAAGCAGTGGCT
GTGGATGTGGCTCGT
+
FFFFFFFFFFFFFFFFFFFFFFFFFFFFFFFFFFFFFFFFFFFFFFFFFFFFFFFFFFF:FFFFFFFFFF
FFFFFFFFFFFFFFFFFFFFFFFFFFFFFFFFFFFFFFFFFFF,FFFFFFFFFF,FFFFFFFFFFFFFFF
FFFFFFFFFFF:FFF
@A00721:190:HGJW7DSXY:4:1376:16288:2080 1:N:0:TCTGCAAG+GCTGCACT
TTTGTATAAATGATTGTCCTACTGGAACCTCGGTCGATACGAGACATACCCAGAAAGTGAGATGGAAT
CGAATTGTGCTTAACGGTTCAGTTAAGTTGACTAGCGAAGCCAATGTTCATAATATGAAGCAGTGGCT
GTGGATGTGGCTCGT
+
FFFFFFFFFFFFFFFFFFFFFFFFFFFFFFFFFFFFFFFFFFFFFFFFFFFFFFFFFFF:FFFFFFFFFF
FFFF,FFFFFFFFFFFFFFFFFFFFFFFFFFF:FFFFFFFFFFFFFFFFFFFFF:FFFFFFFFFFFFF:FF
FFFFFFFFFFFFFFF
@A00721:190:HGJW7DSXY:4:1502:11026:4554 1:N:0:TCTGCAAG+GCTGCACT
TTTGTATAAATGATTGTCCTACTGGAACCTCGGTCGATACGAGACATACCCAGAAAGTGAGATGGAAT
CGAATTGTGCTTAACGGTTCAGTTAAGTTGACTAGCGAAGCCAATGTTCATAATATGAAGCAGTGGCT
GTGGATGTGGCTCGT
+
FFFFFFFFFFFFFFFFFFFFFFFFFF,FFFFFFFFFFFFFFFFFFFFFFFFFFFFFFFFFFFFFFFF:,F
FFFFFFFFFFFFFFFFFFFFFFFFFFFFFFFFFFFFFFFFFFFFFFFFFFFFFFFFFFFFF:FFFFFFFF
FFFFFFFFFFFFFFF
@A00721:190:HGJW7DSXY:4:1663:19352:11397 1:N:0:TCTGCAAG+GCTGCACT
TTTGTATAAATGATTGTCCTACTGGAACCTCGGTCGATACGAGACATACCCAGAAAGTGAGATGGAAT
CGAATTGTGCTTAACGGTTCAGTTAAGTTGACTAGCGAAGCCAATGTTCATAATATGAAGCAGTGGCT
GTGGATGTGGCTCGT
+
FFFFFFFFFFFFFFFFFFFFFFFFFFFFFFFFFFFFFFFFFFFFFFFFFFFFFFFFF,FFFFFFFFFFFF
F:FFFFFFFFFFFFFFFFFFFFFFFFFFFFFFFFFFFFFFFFFFFFFFFFFFFFFFFFFFFFFFFFFFFF
FFFFFFFFFFFFFFF
@A00721:190:HGJW7DSXY:4:2328:3043:7294 1:N:0:TCTGCAAG+GCTGCACT
TTTGTATAAATGATTGTCCTACTGGAACCTCGGTCGATACGAGACATACCCAGAAAGTGAGATGGAAT
CGAATTGTGCTTAACGGTTCAGTTAAGTTGACTAGCGAAGCCAATGTTCATAATATGAAGCAGTGGCT
GTGGATGTGGCTCG
+
FF:FFFFFFFFFFFFFFFFFFFFFFFFF:FFFFFFFFFFFFFFFFFFFFFFF:FF:FF:FFFFFFFFFFF
F:FFFFFFFFFFFFFFF:FFFF:FFFFFFFFFFFFFFFFFF:FFFFF:FFFF:FFFFF:FFF:FFFFFFF
F:FFFFFFFFFFFF
@A00721:190:HGJW7DSXY:4:2507:21558:13589 1:N:0:TCTGCAAG+GCTGCACT
TTTGTATAAATGATTGTCCTACTGGAACCTCGGTCGATACGAGACATACCCAGAAAGTGAGATGGAAT
CGAATTGTGCTTAACGGTTCAGTTAAGTTGACTAGCGAAGCCAATGTTCATAATATGAAGCAGTGGCT
GTGGATGTGGCTCGT
+
FFFFFFFFFFFFFFFFFFFFFFFFFF:FFFFFFFFFFFFFFFFFFFFFFFFFFFFF:FFFFFFFFFFFFF
```

```
F::FFFFFFFFFFFFFFFFFFFFFFFFFFFFFFFFFFFFFFFF:FFFFFFFFFFFFFFFFFF:FFFFFFF
FFFFF,FFFFFFF,:
@A00721:190:HGJW7DSXY:4:2507:31186:15953 1:N:0:TCTGCAAG+GCTGCACT
TTTGTATAAATGATTGTCCTACTGGAACCTCGGTCGATACGAGACATACCCAGAAAGTGAGATGGAAT
CGAATTGTGCTTAACGGTTCAGTTAAGTTGACTAGCGAAGCCAATGTTCATAATATGAAGCAGTGGCT
GTGGATGTGGCTCGT
+
FFFFFFFFFFFFFFFFFFFFFFFFFFF,FFFFFFFFFFFFFFFFFFFFFFFFFFFFFFFFFFFFFFFFFF
FFFFFFFFFFFFFFFFFFFFFFFFFFFFFFFFFFFFFFFFFFFFFFFFFFFFF:FFFFFFFFFFFFFFFF
FFFFFFFFFFFFFFF
@A00721:190:HGJW7DSXY:4:2615:2166:23156 1:N:0:TCTGCAAG+GCTGCACT
TTTGTATAAATGATTGTCCTACTGGAACCTCGGTCGATACGAGACATACCCAGAAAGTGAGATGGAAT
CGAATTGTGCTTAACGGTTCAGTTAAGTTGACTAGCGAAGCCAATGTTCATAATATGAAGCAGTGGCT
GTGGATGTGGCTCG
+
FFFFFFFFFFFFFFFFFFFFF:FFFFF:FFFFFFFFFFFFFFFFFFFFFFFFFFFFFFFFFFFFFFFF:F
FFFFFFFFFFFFFFFFFFFFFFFF:FFFFFFFFFFFFFFFFFFFFFFFFFFFFFF,FFFFFF:FFFFFFF
FFFFFFFFFFFFFF
@A00721:190:HGJW7DSXY:4:2626:9091:31704 1:N:0:TCTGCAAG+GCTGCACT
TTTGTATAAATGATTGTCCTACTGGAACCTCGGTCGATACGAGACATACCCAGAAAGTGAGATGGAAT
CGAATTGTGCATAACGGTTCAGTTAAGTTGACTAGCGAAGCCAATGTTCATAATATGAAGCAGTGGCT
GTGGATGTGGCTCGT
+
FFFFFFFFFFFFFFFFFFFFFFFFFFFFFFFFFFFFFFFFFFFFFFFFFFFFFFFFFFFFFFFFFFFFFF
FFFFFFFFFF,FFFFFFFFFFFFFFFFFFFFFFFFFFFFFFFFFFFFFFFFFFFFFFFFFFF:FFFFFFF
FFFFFFFFFFFFFFF
@A00721:190:HGJW7DSXY:4:2635:30626:8719 1:N:0:TCTGCAAG+GCTGCACT
TTTGTATAAATGATTGTCCTACTGGAACCTCGGTCGATACGAGACATACCCAGAAAGTGAGATGGAAT
CGAATTGTGCTTAACGGTTCAGTTAAGTTGACTAGCGAAGCCAATGTTCATAATATGAAGCAGTGGCT
GTGGATGTGGCTCGT
+
FFFFFFFFFFFFFFFFFFFFFFFFFFF:FFFFFFFFFFFFFFFFFFFFFFFFFFFFFFFFFFFFFFFFFF
FFFFFFFFFFFFFFFFFFFFF:FFFFFFFFFFFFFFFFFFFFFFFFFFFFFFFFFFFF:FF,FFFFFFFF
FFFFFFFFFFFFFFF
@A00721:190:HGJW7DSXY:4:2635:32705:9095 1:N:0:TCTGCAAG+GCTGCACT
TTTGTATAAATGATTGTCCTACTGGAACCTCGGTCGATACGAGACATACCCAGAAAGTGAGATGGAAT
CGAATTGTGCTTAACGGTTCAGTTAAGTTGACTAGCGAAGCCAATGTTCATAATATGAAGCAGTGGCT
GTGGATGTGGCTCG
+
FFFFFFFFFFFFFFFFFFFFFFFFFFFFFFFFFFFFFFFFFFFFFFFFFFFFFFFFFFFFFFFFFFFFFF
FFFFFFFFFFFFF:FFFFFF:FFFFFFFFFFF,FFFFFFFFFFFFF:F:F::F:F:F:FFFFFFFFFF::
FFFFF:FFFF:::F
@A00721:190:HGJW7DSXY:4:1336:5575:34287 1:N:0:TCTGCAAG+GCTGCACT
GTATAAATGATTGTCCTACTGGAACCTCGGTCGATACGAGACATACCCAGAAAGTGAGATGGAATCGA
ATTGTGCTTAACGGTTCAGTTAAGTTGACTAGCGAAGCCAATGTTCATAATATGAAGCAGTGGCTGTG
GATGTGGCTCGTGTA
+
FFFFFFFFFFFFFFFFFFFFFF::FFFFFFFFFFFFFFFFFFFFFFFFFFFF:FFFFFFFFFFFFFFFFF
FFFFFFFFFFF:FFFFF:F,FFFFFFFFFFFFFFFFFFFFFFFFFFFFFFFFFFFFFFFFFFFFFFFFFF
FFFFFFFFFFFFFFF
@A00721:190:HGJW7DSXY:4:1625:5213:31344 1:N:0:TCTGCAAG+GCTGCACT
GTATAAATGATTGTCCTACTGGAACCTCGGTCGATACGAGACATACCCAGAAAGTGAGATGGAATCGA
ATTGTGCTTAACGGTTCAGTTAAGTTGACTAGCGAAGCCAATGTTCATAATATGAAGCAGTGGCTGTG
GATGTGGCTCGTGTA
```

```
+
FFFFFFFFFFFFFFFFFFFFFF:FFFFFFFFFFFFFFFFFFFFFFFFFFFFFFFFFFFFFFFFFFF:FFFF
FFFFFFFFFFFFFFFFFFFFFFFFFFFFFFFFFFFFFFFFFFFFFF,FFFFFFFFFFFFFFFFFFFFFF
FFFFFFFF:FFFFFF
@A00721:190:HGJW7DSXY:4:2102:12888:7467 1:N:0:TCTGCAAG+GCTGCACT
GTATAAATGATTGTCCTACTGGAACCTCGGTCGATACGAGACATACCCAGAAAGTGAGATGGAATCGA
ATTGTGCTTAACGGTTCAGTTAAGTTGACTAGCGAAGCCAATGTTCATAATATGAAGCAGTGGCTGTG
GATGTGGCTCGTGTA
+
FFFFFFFFFFFFFFFFFFFFFFFFFFFFFFFFFFFFFFFFFFFFFFFFFFFFFFFFFFFFFFFFFFFF
FFFFFFFFFFFFFFFFFFFFFFFFFFFFFFFFFFF:FFFFFFFFFFFFFFFFFFFFF:FFFFFFFFFF
FFFFFFFFFFFFFFF
@A00721:190:HGJW7DSXY:4:2108:10565:9674 1:N:0:TCTGCAAG+GCTGCACT
GTATAAATGATTGTCCTACTGGAACCTCGGTCGATACGAGACATACCCAGAAAGTGAGATGGAATCGA
ATTGTGCTTAACGGTTCAGTTAAGTTGACTAGCGAAGCCAATGTTCATAATATGAAGCAGTGGCTGTG
GATGTGGCTCGTGTA
+
FFFFFFFFFFFFFFFFFFFFFFF,FFFFFFFFFFFFFFFFFFFFFFFF,FFFFFFFFFFFFFFFFFFF
:FFFFFFFFFFFFFFFF:FFFFFFFFFFFFFFFFFFFFFFFFFFFFFF,FFFFFFFFFFFFFFFFFFFF
FFFFFFFFFFFFFFF
@A00721:190:HGJW7DSXY:4:2531:19768:35102 1:N:0:TCTGCAAG+GCTGCACT
GTATAAATGATTGTCCTACTGGAACCTCGGTCGATACGAGACATACCCAGAAAGTGAGATGGAATCGA
ATTGTGCTTAACGGTTCAGTTAAGTTGACTAGCGAAGCCAATGTTCATAATATGAAGCAGTGGCTGTG
GATGTGGCTCGTGTA
+
FFFFFFFFFFFFFFFFFFFFFF::FFFFFFFFFFFFFFFFFFFFFF:FFFFFFFFFFFFFFFFFFFFF
FFFFFFFFFFFFFFFFFFFFFFFFFFFF:FFFFFFFFFFFFFFF:FFF:FFFFFFFFF:FFFFFF:F:F
:FFFFFFF:F:FFFF
@A00721:190:HGJW7DSXY:4:2625:6090:26506 1:N:0:TCTGCAAG+GCTGCACT
GTATAAATGATTGTCCTACTGGAACCTCGGTCGATACGAGACATACCCAGAAAGTGAGATGGAATCGA
ATTGTGCTTAACGGTTCAGTTAAGTTGACTAGCGAAGCCAATGTTCATAATATGAAGCAGTGGCTGTG
GATGTGGCTCGTGTA
+
FFFFFFFFFFFFFFFFFFFFFFFFFFFFFFFFFFFFFFFFFFFFFFFFFFFFFFFFFFFFFFFFFFFF
FFFFFFFFFFFFFFFFFFFFFFFFFFFFFFFFFFFFFFFFFFFFFFFFFFFFFFFFFFFFFFFFFFFF
FFFFFFFFFFF:FFF
@A00721:190:HGJW7DSXY:4:1271:29080:7733 1:N:0:TCTGCAAG+GCTGCACT
GTCCTACTGGAACCTCGGTCGATACGAGACATACCCAGAAAGTGAGATGGAATCGAATTGTGCTTAAC
GGTTCAGTTAAGTTGACTAGCGAAGCCAATGTTCATAATATGAAGCAGTGGCTGTGGATGTGGCTCGT
GTATGATAGACGTCC
+
FFFFFFFFFF:,FFFFFFFFFFFFFFFF:FFFFFFFFFFF:FFFFFFFFFFFFFFFF:FFFFFFFFFF
FFFFFFFFFFFFFFFFFFFFFFFFFFFFFFFFFF:FFFFFFFFFFFFFFFFFF:FFFFFFFFFFFFF:F
FFFFFFFF,FF:FFF
@A00721:190:HGJW7DSXY:4:1324:4878:27508 1:N:0:TCTGCAAG+GCTGCACT
GTCCTACTGGAACCTCGGTCGATACGAGACATACCCAGAAAGTGAGATGGAATCGAATTGTGCTTAAC
GGTTCAGTTAAGTTGACTAGCGAAGCCAATGTTCATAATATGAAGCAGTGGCTGTGGATGTGGCTCGT
GTATGATAAGATCGG
+
FFFFFFFFFFFFFFFFFFFFFFFFFFFFFFFFFFFFFFFFFFFFFFFFFFFFFFFFFFFFFFFFFFFF
FFFFFFFFFFFFFFFFFFFFFFFFFFFFFFFFFFFFFFFFFFFFFFFFFFFFFFFFFFFFFFFFFFFF
FFFF:FFFFFFFFFF
@A00721:190:HGJW7DSXY:4:1342:13485:29481 1:N:0:TCTGCAAG+GCTGCACT
GTCCTACTGGAACCTCGGTCGATACGAGACATACCCAGAAAGTGAGATGGAATCGAATTGTGCTTAAC
```

GGTTCAGTTAAGTTGACTAGCGAAGCCAATGTTCATAATATGAAGCAGTGGCTGTGGATGTGGCTCGT
GTATGATAAGATCGG
+
FFFFFFFFFFFFFFFFFFFFFFFFFFFFFFFFFFFFFFFFFFFFFFFFFFFFF,FFFFF:FFFFFFFFFF
FFFFFFFFFFFFFFFFFFFFFFFFFFFFFFFFFFFFFFFFFFFFFFFFFFFFFFFFFFFFFFFFFFFFF:
F,FFFFFFFFFFFFF
@A00721:190:HGJW7DSXY:4:1367:28565:27696 1:N:0:TCTGCAAG+GCTGCACT
GTCCTACTGGAACCTCGGTCGATACGAGACATACCCAGAAAGTGAGATGGAATCGAATTGTGCTTAAC
GGTTCAGTTAAGTTGACTAGCGAAGCCAATGTTCATAATATGAAGCAGTGGCTGTGGATGTGGCTCGT
GTATGATAAGATCGG
+
FFFFFFFFFFFFFFFFFFFFFFFFFFFFFFFFFFFFFFFFFFFFFFFFFFFFFFFFFFFFFFFFFFFFFF
FFFFFFFFFFFFFFFFFFFFFFFFFFFFFFFFFFFFFFFFFFFFFFFFFFFFFFFFFFFFFFFFFFFFF:
FFFFFFFFFFFFFFF
@A00721:190:HGJW7DSXY:4:1372:25355:14967 1:N:0:TCTGCAAG+GCTGCACT
GTCCTACTGGAACCTCGGTCGATACGAGACATACCCAGAAAGTGAGATGGAATCGAATTGTGCTTAAC
GGTTCAGTTAAGTTGACTAGCGAAGCCAATGTTCATAATATGAAGCAGTGGCTGTGGATGTGGCTCGT
GTATGATAGACGTCC
+
FFFFFFFFFFFFFFFFFFFFFFFFFFFFFFFFFFFFFFF:FFFFFFFFFFFFFFFFFFFFFFFFFFFFFF
FFFFFFFFFFFFFFFFFFFFFFFFFFFFFFFFFFFFFFFFFFFFFFFFFFFFFFFFFFFFFFFFFFFFFF
FFFFFFFFFFFFFFF
@A00721:190:HGJW7DSXY:4:1374:27624:8093 1:N:0:TCTGCAAG+GCTGCACT
GTCCTACTGGAACCTCGGTCGATACGAGACATACCCAGAAAGTGAGATGGAATCGAATTGTGCTTAAC
GGTTCAGTTAAGTTGACTAGCGAAGCCAATGTTCATAATATGAAGCAGTGGCTGTGGATGTGGCTCGT
GTATGATAAGATCGG
+
FFFFFFFFFFFFFFFFFFFFFFFFFFFFFFFFFFFFFFFFFFFFFFF:FFFFFFFFFFFFFFFFFFFFFF
FFFFFFFFFFFFFFFFFFFFFFF,FFFFFFFFFFFFF:FFFFFFFFFFFFFFFFFFFFFFFFFFFF:FF,
F:FFFFFFFFFFFFF
@A00721:190:HGJW7DSXY:4:1455:10691:34914 1:N:0:TCTGCAAG+GCTGCACT
GTCCTACTGGAACCTCGGTCGATACGAGACATACCCAGAAAGTGAGATGGAATCGAATTGTGCTTAAC
GGTTCAGTTAAGTTGACTAGCGAAGCCAATGTTCATAATATGAAGCAGTGGCTGTGGATGTGGCTCGT
GTATGATAAGATCGG
+
FFFFFFFFFFFFFFFFFFFFFFFFFFFFFFFFFFFFFFFFFFF:FFFFFFFFFFFFFFFFFFFFFFFFFF
FFFFFFFFFFFFFFFFFFFFFFFFFFFFFFFFFFFFFFFFFFFFFFFFFFFFFFFFFFFFFFFFFFFFFF
FFFFFFFFFFFFFFF
@A00721:190:HGJW7DSXY:4:2208:8648:2440 1:N:0:TCTGCAAG+GCTGCACT
GTCCTACTGGAACCTCGGTCGATACGAGACATACCCAGAAAGTGAGATGGAATCGAATTGTGCTTAAC
GGTTCAGTTAAGTTGACTAGCGAAGCCAATGTTCATAATATGAAGCAGTGGCTGTGGATGTGGCTCGT
GTATGATAAGATCGG
+
FFFFFFFFFFFFFFFFFFFFFFFFFFFF:FFFFFFFFFFFFFFFFFFFFFFFF:FFFFFFFFFFFFFFFF
FFFFFFFFFFFFFFFFFFFFFFFFFFFFFFFFFFFFFFFFFFFFFF:FFFFFFFFFFFFFFFFFFFFFFF
FFFFFFFFFFFFFFF
@A00721:190:HGJW7DSXY:4:2402:16559:5181 1:N:0:TCTGCAAG+GCTGCACT
GTCCTACTGGAACCTCGGTCGATACGAGACATACCCAGAAAGTGAGATGGAATCGAATTGTGCTTAAC
GGTTCAGTTAAGTTGACTAGCGAAGCCAATGTTCATAATATGAAGCAGTGGCTGTGGATGTGGCTCGT
GTATGATAGACGTCC
+
FFFFFFFFFFF:FFFFFFFFFFFFFFFFFFFFFFFFFFFFFFF:FFFFFFFFFFFFFFFFFFFFFFFFFF
FFFFFFFFFFFFFFFFFFFFFFFFFFFFFFFFFFF:FFFFFFFFF:FFFFFFFFFFFFFFFFFFFFFFFF
FFFFFFFFFFFFFFF

```
@A00721:190:HGJW7DSXY:4:2557:2184:7811 1:N:0:TCTGCAAG+GCTGCACT
GTCCTACTGGAACCTCGGTCGATACGAGACATACCCAGAAAGTGAGATGGAATCGAATTGTGCTTAAC
GGTTCAGTTAAGTTGACTAGCGAAGCCAATGTTCATAATATGAAGCAGTGGCTGTGGATGTGGCTCGT
GTATGATAAGATCGG
+
FFFFFFFFFFF:FFFFFFFFFFFFFFFFFFFFFFFFFFFFFFFFFFFFFFFFFFFFFFFFFFFFFFFF
FFFFFFFFFFFFFFFFFFFFFFFFFFFFF:FFFFFFFFFFFFFFFFFFFFFFFFFFFFFFFFFFFFFF:
FFFFFFFFFFFFFFF
@A00721:190:HGJW7DSXY:4:2170:2618:18176 1:N:0:TCTGCAAG+GCTGCACT
TCCTACTGGAACCTCGGTCGATACGAGACATACCCAGAAAGTGAGATGGAATCGAATTGTGCTTAACG
GTTCAGTTAAGTTGACTAGCGAAGCCAATGTTCATAATATGAAGCAGTGGCTGTGGATGTGGCTCGTG
TATGATAGACGTCCC
+
FFFFFFFFFFFFFFFFFFFFFFFFFFFFFFFFFFFFFFFFF,FFFFFFFFFFFFFFFFFFFFFFFFFF
FFFFFFFFFFFFFFFFFFFFFFFFFFFFF:FFFFF,FFFFFFFFFFFFFFFF:FFFFFFFFFFF:FFFF
FFFFFFFFFFFFFFF
@A00721:190:HGJW7DSXY:4:2411:17888:10833 1:N:0:TCTGCAAG+GCTGCACT
TCCTACTGGAACCTCGGTCGATACGAGACATACCCAGAAAGTGAGATGGAATCGAATTGTGCTTAACG
GTTCAGTTAAGTTGACTAGCGAAGCCAATGTTCATAATATGAAGCAGTGGCTGTGGATGTGGCTCGTG
TATGATAGACGTCCC
+
FFFFFFFFFFFFFFFFFFFFFFFFFFFFFFFFFFFFFFFFFFFFFFFFFFFFF:F:FFFFFFFFF,F
FFFFFFFFFFFFFFFFFFFF:FFFFFFFFFFF:F,FFFFFFFFF,FFF:FFFFF:FF:F:FFFFFFFF
FFFFFFF:FFFFFFF
@A00721:190:HGJW7DSXY:4:2525:30653:31876 1:N:0:TCTGCAAG+GCTGCACT
TCCTACTGGAACCTCGGTCGATACGAGACATACCCAGAAAGTGAGATGGAATCGAATTGTGCTTAACG
GTTCAGTTAAGTTGACTAGCGAAGCCAATGTTCATAATATGAAGCAGTGGCTGTGGATGTGGCTCGTG
TATGATAGACGTCCC
+
FFFFFFFFFFFFFFFFFFFFFFFFFFFFFFFFFFFFFFFFFFFFFFFFFFFFFFFFFFFFFFFFFFFF
FFFFFFFFFFFFFFFFFFFFFFFFFFFFFFFFFFFFFFFFFFFFFFFFFFFFFFFFFFFFFFFFFFFF
FFFF,FFFFFFFFFF
@A00721:190:HGJW7DSXY:4:2247:10420:35759 1:N:0:TCTGCAAG+GCTGCACT
CCTACTGGAACCTCGGTCGATACGAGACATACCCAGAAAGTGAGATGGAATCGAATTGTGCTTAACGG
TTCAGTTAAGTTGACTAGCGAAGCCAATGTTCATAATATGAAGCAGTGGCTGTGGATGTGGCTCGTGT
ATGATAAGATCGG
+
FFFFF:FFF::FFFF::FFFFFF:FFFFFFFFFFF:FFFFFFFFFFFFF:FF::FFFFF:,FFFFFF
:,,:,FFFFFF,FFFFFFFFFFFFFFFFFFFFF:F:FFFFFFF:F,,FFF:F:FFF:F,:,FFFF,FF
FF,FFFF,F:F,F
@A00721:190:HGJW7DSXY:4:1131:12897:27430 1:N:0:TCTGCAAG+GCTGCACT
CTGGAACCTCGGTCGATACTAGACATACCCAGAAATTGAGATTGAAAAGAATTGTGCTTCACGGTTCA
GTTAAGTGGACTAGCGAAGCCAATGTTCATAATATGAAGCAGTGGCTGTGGATGTGGCTCGTGTATGA
TAGACGTCCCGGTAA
+
FFFF::FFFFFFFFFFFFF,FFFFFFFFFFFFFFFF:FFFFF,F:,,,FF,FF,FFFFF:FFFFFFFF
F:FFFFFFFFFFFFFFFFFFFFFFFFFFFFFFFFFFFFFFFFFFFFFFFFFF:FFFFFFFFFFFFFFFF
FFF:FFFFFFFFFFF
@A00721:190:HGJW7DSXY:4:1248:14561:27398 1:N:0:TCTGCAAG+GCTGCACT
CTGGAACCTCGGTCGATACGAGACATACCCAGAAAGTGAGATGGAATCGAATTGTGCTTAACGGTTCA
GTTAAGTTGACTAGCGAAGCCAATGTTCATAATATGAAGCAGTGGCTGTGGATGTGGCTCGTGTATGA
TAGACGTCCCGGTAA
+
FFFF::FFFFFFFFFFFFFFFFFFFFFFFFFFFFFF,FFFFFFFF,FFFFFFFFFFFFFFFFFFFFFF
```

```
FF:FFFFFFFFFFFFFFFFFFFFFFFFF:FFFFFFFFFFFFFFFFFFFFFFFFFFFFFFFFFFFFFFFF:F
FFFFFFFFFFFFFFF
@A00721:190:HGJW7DSXY:4:1564:3522:1454 1:N:0:TCTGCAAG+GCTGCACT
CTGGAACCTCGGTCGATACGAGACATACCCAGAAAGTGAGATGGAATCGAATTGTGCTTAACGGTTCA
GTTAAGTTGACTAGCGAAGCCAATGTTCATAATATGAAGCAGTGGCTGTGGATGTGGCTCGTGTATGA
TAGACGTCCCGGTAA
+
FFFFFFFFFFFFFFFFFFFFFFFFFFFFFFFFFFFF,FFFFFFFFFFFFFFFFFFFFFFFFFFFFF:FF
FFFFFFFFFFFFFFFFFFFFFFFFFFFFFFFFFFFFFFFFFFFFFFFFFFFFFFFFFFFFFFFFFFFF
FFFFFFFFFFFFFFF
@A00721:190:HGJW7DSXY:4:2231:21187:30232 1:N:0:TCTGCAAG+GCTGCACT
CTGGAACCTCGGTCGATACGAGACATACCCAGAAAGTGAGATGGAATCGAATTGTGCTTAACGGTTCA
GTTAAGTTGACTAGCGAAGCCAATGTTCATAATATGAAGCAGTGGCTGTGGATGTGGCTCGTGTATGA
TAGACGTCCCGGTAA
+
FFFFF:FFFFFFFFFFFFFFFFFFFFFFFFFFFFFF:FFFFFFFFFFFFFFFFFFFFFFFFFFFFFFF
FFFFFFFFFFFFFFFFFFFFFFFFFFFFFFFFFFFFFFFFFFFFFFFFFFFFFFFFFFFFFFFFFFFF
FFFFFFFFFFFFFFF
@A00721:190:HGJW7DSXY:4:2621:31295:22122 1:N:0:TCTGCAAG+GCTGCACT
CTGGAACCTCGGTCGATACGAGACATACCCAGAAAGTGAGATGGAATCGAATTGTGCTTAACGGTTCA
GTTAAGTTGACTAGCGAAGCCAATGTTCATAATATGAAGCAGTGGCTGTGGATGTGGCTCGTGTATGA
TAGACGTCCCGGTAA
+
FFFFFFFFFFFFFFFFFFFFFFFFFFFFFFFFFFFF:FFFFFFFFFFFFFFFFFFFFFFFFFFFFFFF
FFFFFFFFFFFFFFFFFFFFFFFFFFFFFFFFFFFFFFFFFFFFFFFFFFFFFFFFFFFFFFFFFFF:
FFFFFFFFFFFFFFF
@A00721:190:HGJW7DSXY:4:2666:30011:8061 1:N:0:TCTGCAAG+GCTGCACT
CTGGAACCTCGGTCGATACGAGACATACCCAGAAAGTGAGATGGAATCGAATTGTGCTTAACGGTTCA
GTTAAGTTGACTAGCGAAGCCAATGTTCATAATATGAAGCAGTGGCTGTGGATGTGGCTCGTGTATGA
TAGACGTCCCGGTAA
+
FFFFF:FFFFFFFFFFFFFFFFFFFFFFFFF,FFFFF:FFFFFFFFFFFFFFFFFFFFFFFFFFFFFF,
FFFFFFFFFFFFFFFFFFFFFFFFFFFFFFFFF,FFFFFFFFFFFFFFFFFFFFFFFFFFFFFFFFFF
FFFFFFFFFFFFFFF
@A00721:190:HGJW7DSXY:4:2219:24099:10535 1:N:0:TCTGCAAG+GCTGCACT
CTCGGTCGATACGAGACATACCCAGAAAGTGAGATGGAATCGAATTGTGCTTAACGGTTCAGTTAAGT
TGACTAGCGAAGCCAATGTTCATAATATGAAGCAGTGGCTGTGGATGTGGCTCGTGTATGATAGACGT
CCCGGTAATTCTAAT
+
FFFFFFFFFFFFFFFFFFFFFFFFFFFFFFFFFFFFFFFFFFFFFFFFFFFFFFFFFFFFFFFFFFFF
FFFFFFFFFFFFFFFFFFFFFFFFFFFFFFFFFFFFFFFFFFFFFFFFFFFFFFFFFFFFFFF:FFFF
FFFFFFFFFFFFF:F
@A00721:190:HGJW7DSXY:4:2239:21215:34350 1:N:0:TCTGCAAG+GCTGCACT
CTCGGTCGATACGAGACATACCCAGAAAGTGAGATGGAATCGAATTGTGCTTAACGGTTCAGTTAAGT
TGACTAGCGAAGCCAATGTTCATAATATGAAGCAGTGGCTGTGGATGTGGCTCGTGTATGATAGACGT
CCCGGTAATTCTAAT
+
FFFFFFFFFFFFFFFFFFFFFFFFFFFFFFFFFFFFFFFFFFFFFFFFFFFFFFFFFFFF:FFFFFFF
FFFFFFFFFFFFFFFFFFFFFF:FFFFFFFFFFFFFFFFFF:FFFFFFFFFFF,FFFF,FFFFFF:FFFF
FFFFFF:FFFFFFFF
@A00721:190:HGJW7DSXY:4:1306:13612:22561 1:N:0:TCTGCAAG+GCTGCACT
TCGGTCGATACGAGACATACCCAGAAAGTGAGATGGAATCGAATTGTGCTTAACGGTTCAGTTAAGTT
GACTAGCGAAGCCAATGTTCATAATATGAAGCAGTGGCTGTGGATGTGGCTCGTGTATGATAGACGTC
CCGGTAATTCTAATC
```

+
FFFFFFFFFFFFFFFFFFFFFFFFFFFFFF:FFFFFFFFFFFFFFFFFFFFFFFFFFFFFFFFFFFFFFF
FFFFFFFFFFFFFFFFFFFFFFFFFFFFFFFFFFFFFFFFFFFFFFFFFFFFFFFFF:FFFFFFFFFFFF
FFFFFFFFFFFFFFF
@A00721:190:HGJW7DSXY:4:2119:11559:25708 1:N:0:TCTGCAAG+GCTGCACT
TCGGTCGATACGAGACATACCCAGAAAGTGAGATGGAATCGAATTGTGCTTAACGGTTCAGTTAAGTT
GACTAGCGAAGCCAATGTTCATAATATGAAGCAGTGGCTGTGGATGTGGCTCGTGTATGATAGACGTC
CCGGTAATTCTAATC
+
FFFFFFFFFFFFFFFFF:FFFFFFFFFFFFFFFFFFFFFFFFF:FF:FFFFFFFF:FFFFFFFFFFFFFF
FFFFFFFFFFFFFFFF,F:FFF,FFFFFFFFFFFFFFFFFFFFFFFFFF:F:FFFFF:FFFFFFFFFFFF
FFFFFF,::FFFFFF
@A00721:190:HGJW7DSXY:4:2333:17363:25207 1:N:0:TCTGCAAG+GCTGCACT
TCGGTCGATACGAGACATACCCAGAAAGTGAGATGGAATCGAATTGTGCTTAACGGTTCAGTTAAGTT
GACTAGCGAAGCCAATGTTCATAATATGAAGCAGTGGCTGTGGATGTGGCTCGTGTATGATAGACGTC
CCGGTAATTCTAATC
+
FFFFFFFFFFFFFFFFFFFFFFFFFFFFFFFFFFFFFFF:FFFFFFFFFFFFFFFFFFFFFFFFFFFFFF
FFFFFFFFFFFFFFFFFFFFF:FFFFFFFFFFFFFFFFFFFFFFFFFFFFFFFFFFF,FFFFFFFFFFFF
FFFFFFFFFFFFFFF
@A00721:190:HGJW7DSXY:4:2464:5764:26256 1:N:0:TCTGCAAG+GCTGCACT
TCGGACGATACGAGACATACCCAGAAAGTGAGATGGAATCGAATTGTGCTTAACGGTTCAGTTAAGTT
GACTAGCGAAGCCAATGTTCATAATATGAAGCAGTGGCTGTGGATGTGGCTCGTGTATGATATACGTC
ACGGTAATTCTAATC
+
FF,F,FFFFFF::FFF,:FF,FF:FF,F:FFFF,::FFF:FF:FFF,F:FFF:F:F:FFFFFFFFF:FF
,F,F,:FFFFFFFFFF:FFFF,F:,FFF:FF:FFF,:FF,FFFFFF:,F,F:::,FF:FFF,,FF,:F
,,,,,FFFF:FFF,F
@A00721:190:HGJW7DSXY:4:1316:28646:10645 1:N:0:TCTGCAAG+GCTGCACT
GTCGATACGAGACATACCCAGAAAGTGAGATGGAATCGAATTGTGCTTAACGGTTCAGTTAAGTTGAC
TAGCGAAGCCAATGTTCATAATATGAAGCAGTGGCTGTGGATGTGGCTCGTGTATGATAGACGTCCCG
GTAATTCTAATCCCA
+
:FFF:FFFFF:F:F:FF:FFFFF:FFFFFFF,:F:FFFFFFFFF,,F:FF:,F,FFFF,:FF:,F,FF
:F,F:FFF:FFFFFFFFFF,FFF:,FF,,F,,F,FF:,FFFF:FFFFFFFFFF:FFF:F:FFFFFF,F
:FFFFFFFF,FF:FF
@A00721:190:HGJW7DSXY:4:1454:26729:33098 1:N:0:TCTGCAAG+GCTGCACT
GTCGATACGAGACATACCCAGAAAGTGAGATGGAATCGAATTGTGCTTAACGGTTCAGTTAAGTTGAC
TAGCGAAGCCAATGTTCATAATATGAAGCAGTGGCTGTGGATGTGGCTCGTGTATGATAGACGTCCCG
GTAATTCTAATCCCA
+
FFFFFFFFFFFFFFFFFFFFFFFFFFFFFFFFFFF:FFFFFFFFFFFFFFFFFFFFFFFFFFFFFFFFFF
FFFFFFFFFFFFFFFFFFFFFFFFFFFFFFFFFFFFFFFFFFFFFFFFFFFFFFFFFFFFFFFFFFFFFF
FFFFFFFFFFFFFFF
@A00721:190:HGJW7DSXY:4:1512:1678:22060 1:N:0:TCTGCAAG+GCTGCACT
GTCGATACGAGACATACCCAGAAAGTGAGATGGAATCGAATTGTGCTTAACGGTTCAGTTAAGTTGAC
TAGCGAAGCCAATGTTCATAATATGAAGCAGTGGCTGTGGATGTGGCTCGTGTATGATAGACGTCCCG
GTAATTCTAATCCCA
+
FFFFFFFFFFFFFFFFFFF:FFFFFFFFFFFFFFF,FFFFFFFFFFFFFFFFFFFFFFFFFFFFFFFFFF
FFFFFFFFFFFFFFFFFFFFFFFFFFFFFFFFFFFFFFFFFFFFFFFFFFFFFFFFFFFFFFFFFFFFFF
FF:FFFFFFFFFFFF
@A00721:190:HGJW7DSXY:4:1571:16794:29262 1:N:0:TCTGCAAG+GCTGCACT
GTCGATACGAGACATACCCAGAAAGTGAGATGGAATCGAATTGTGCTTAACGGTTCAGTTAAGTTGAC

TAGCGAAGCCAATGTTCATAATATGAAGCAGTGGCTGTGGATGTGGCTCGTGTATGATAGACGTCCCG
GTAATTCTAATCCCA
+
FFFFFFFFFFFFFFFFFFFFFFFFFFFFFFFFFF,FFFFFFFFFFFFFFFFFF,FFFFFFFFFFFFFF
FFFFFFFFFFFFFFFFFF:FFFFFFFFFFFFFFFFFFFFFFFFFFF:FFFFFFFFFFFFFFFFFFFFF
FFFFFFFFFFFFFFF
@A00721:190:HGJW7DSXY:4:2624:12355:24799 1:N:0:TCTGCAAG+GCTGCACT
GTCGATACGAGACATACCCAGAAAGTGAGATGGAATCGAATTGTGCTTAACGGTTCAGTTAAGTTGAC
TAGCGAAGCCAATGTTCATAATATGAAGCAGTGGCTGTGGATGTGGCTCGTGTATGATAGACGTCCCG
GTAATTCTAATCCCA
+
FFFFFFFFFFFFFFFFFFFFFFFFFFFFFFFFFFFFFFFFFFFFFFFFFFFFFFFFFFFFFFFFFFFF
FFFFFFFFFFFFFFFFFF:FFFFFFFFF:FFFFFFFFFFFFFFFFFFFFFFFFFFFFFFFFFFFFFFF
F:FFFFFFFFF:FFF
@A00721:190:HGJW7DSXY:4:2638:16812:15358 1:N:0:TCTGCAAG+GCTGCACT
GTCGATACGAGACATACCCAGAAAGTGAGATGGAATCGAATTGTGCTTAACGGTTCAGTTAAGTTGAC
TAGCGAAGCCAATGTTCATAATATGAAGCAGTGGCTGTGGATGTGGCTCGTGTATGATAGACGTCCCG
GTAATTCTAATCCCA
+
FFF:FFFFFF:FFFFFFFFFFFFFFFFFFF::FFFF:FFF:FFFFFFFFFFF:FF:FFFFFFFFFFF,
F,FFFFF,FFFF:FFFF:FFF:FFFFFF:FF:F,FFFFF:FFFFFF:FFFFFFFFFFFF:FF:FFFFF
F:FFFFFFFFF,F:F
@A00721:190:HGJW7DSXY:4:2660:22327:25379 1:N:0:TCTGCAAG+GCTGCACT
GTCGATACGAGACATACCCAGAAAGTGAGATGGAATCGAATTGTGCTTAACGGTTCAGTTAAGTTGAC
TAGCGAAGCCAATGTTCATAATATGAAGCAGTGGCTGTGGATGTGGCTCGTGTATGATAGACGTCCCG
GTAATTCTAATCCCA
+
FFFFF,FFFFFF:FFFF:FF:FFFFFFFFFF:FFF,:FFFF::F:F:FFFFF::FFFFF:FFF:,:FF
FF:FFFF,F:FFF,,FFFFFFFFFFFF::F:,:,FF:::FFFFF:::F,:FFFFF,FFFFFFFF::F:
,:FF::::FF,:::F
@A00721:190:HGJW7DSXY:4:1140:9688:20619 1:N:0:TCTGCAAG+GCTGCACT
CTGGGATTAGAATTACCGGGACGTCTATCATACACGAGCCACATCCACAGCCACTGCTTCATATTATG
AACATTGGCTTCGCTAGTCAACTTAACTGAACCGTTAAGCACAATTCGATTCCATCTCACTTTCTGGG
TATGTCTCGTATCGA
+
FFFFFFFFFFFFFFFFFFFFFFFFFFFFFFFFFFFFFFFFFFFFFFFFFFFFFFFFFFFFFFFFFFFF
FFFFFFFFFFFFFFFFFFFFFFFFFFFFFFFFFFFFFFFFFFFFFFFFFFFFFFFFFFFFFFFFFFFF
FFFFFFFFFFFFFFF
@A00721:190:HGJW7DSXY:4:1357:27299:21402 1:N:0:TCTGCAAG+GCTGCACT
CTGGGATTAGAATTACCGGGACGTCTATCATACACGAGCCACATCCACAGCCACTGCTTCATATTATG
AACATTGGCTTCGCTAGTCAACTTAACTGAACCGTTAAGCACAATTCGATTCCATCTCACTTTCTGGG
TATGTCTCGTATCGA
+
FFFFFFFFFFFFFFFFFFFFFFFFFFFFFFFFFFFFFFFFFFFFFFFFFFFFFFFFFFFFFFFFFFFF
FFFFFFFFFFFFFFFFFFFFFFFFFFFFFFFFFFFFFFFFFFFFFFFFFFFFFFFFFFFFFFFFFFFF
FFFFFFFFFFFFFFF
@A00721:190:HGJW7DSXY:4:1460:18050:4789 1:N:0:TCTGCAAG+GCTGCACT
CTGGGATTAGAATTACCGGGACGTCTAACATACACGAGCCACATCCACAGCCACTGCTTCATATTATG
AACATTGGCTTCGCTAGTCAACTTAACTGAACCGTTAAGCACAATTCGATTCCATCTCACTTTCTGGG
TATGTCTCGTATCGA
+
FFFFFFFFFFFFFF:FFFFFFFFFFFF,FFFFFFFFFFFFFFFFFFFFFFFFFFFFFFFFFFF:FFFF
FFFFFFFFFFFFFFFFFFFFFFFFFFFFFFFFFFFFFFFFFF::FFFFFFFFFFFFFFFFFFFFFFFF
FFFFFFFFFFFFFFF

```
@A00721:190:HGJW7DSXY:4:2215:18412:33536 1:N:0:TCTGCAAG+GCTGCACT
CTGGGATTAGAATTACCGGGACGTCTATCATACACGAGCCACATCCACAGCCACTGCTTCATATTATG
AACATTGGCTTCGCTAGTCAACTTAACTGAACCGTTAAGCACAATTCGATTCCATCTCACTTTCTGGG
TATGTCTCGTATCGA
+
FFFFFFFFFFFFFFFFFFFFFFFFFFFFFFFFFFFFFFFFFFFFFFFFFFFFFFFFFFFFFFFFFFFFFF
FFFFFFFFFFFFFFFFFFFFFFFFFFFFFFFFFFFFFFFFFFFFFFFFFFFFFFFFFFFFFFFFFFFFFF
FFFFFFFFFFFFFFF
@A00721:190:HGJW7DSXY:4:2249:15447:11459 1:N:0:TCTGCAAG+GCTGCACT
CTGGGATTAGAATTACCGGGACGTCTATCATACACGAGCCACATCCACAGCCACTGCTTCATATTATG
AACATTGGCTTCGCTAGTCAACTTAACTGAACCGTTAAGCACAATTCGATTCCATCTCACTTTCTGGG
TATGTCTCGTATCGA
+
FFFFFFFFFFFFFFFFFFFFFFFFFFFFFFFFFFFFFFFFFFFFFFFFFFFFFFFFFFFFFFFFFFFFFF
FFFFFFFFFFFFFFFFFFFFFFFFFFFFFFFFFFFFFFFFFFFFFFFFFFFFFFFFFFFFFFFFFFFFFF
FFFFFFFFFFFFFFF
@A00721:190:HGJW7DSXY:4:1104:4960:3944 1:N:0:TCTGCAAG+GCTGCACT
CTGGGATTAGAATTACCGGGACGTCTATCATACACGAGCCACATCCACAGCCACTGCTTCATATTATG
AACATTGGCTTCGCTAGTCAACTTAACTGAACCGTTAAGCACAATTCGATTCCATCTCACTTTCTGGG
TATGTCTCGTAGATC
+
FFFFFFFFFFFFFFFFFFFFFFFFFFFFFFFFFFFFFFFFFFFFFFFFFFFFFFFFFFFFFFFFFFFFFF
FFFF:FFFFFFFFFFFFFFFFFFFFFFFFFFF:FFFFFFFFFFFF:FFFF:FFFF:FFFFFFFFFFFFFF
F:FFFFFFFFFFFFF
@A00721:190:HGJW7DSXY:4:1104:4381:4006 1:N:0:TCTGCAAG+GCTGCACT
CTGGGATTAGAATTACCGGGACGTCTATCATACACGAGCCACATCCACAGCCACTGCTTCATATTATG
AACATTGGCTTCGCTAGTCAACTTAACTGAACCTTTAAGCACAATTCGATTCCATCTCACTTTCTGGG
TATGTCTCGTAGATC
+
FF:FF:,,FFFFFFF,FF:FFF,,,FFFFF,FFF:,FFFFFFFF:FF,FFF:FF,,FFFFF:F,FF,,
FFF:FF:,F:FF,F,FFFFFF,,FFF,F,FFFF,FFFF,F:FF,,FF:FFFF:FFFFFF,FFF,F,:,
F,:,F,FF,FF,FFF
@A00721:190:HGJW7DSXY:4:1104:5005:4022 1:N:0:TCTGCAAG+GCTGCACT
CTGGGATTAGAATTACCGGGACGTCTATCATACACGAGCCACATCCACAGCCACTGCTTCATATTATG
AACATTGGCTTCGCTAGTCAACTTAACTGAACCGTTAAGCACAATTCGATTCCATCTCACTTTCTGGG
TATGTCTCGTAGATC
+
FFFFFFFFFFFFFFF:FFFFFFFFFFFFFFFFFFFFFFFFFFFFFFFFFFFFFFFFFFFFFFFFFFFFFF
FFF:FFFFF:FFFFFFFFFFFFFFFFFFFFFFFFFFFFFFFFFFFFFF:FFFFFFFFFFFFFFFFFFFFF
FFFFFFFFFFFFFFF
@A00721:190:HGJW7DSXY:4:1253:2944:30013 1:N:0:TCTGCAAG+GCTGCACT
CTGGGATTAGAATTACCGGGACGTCTATCATACACGAGCCACATCCACAGCCACTGCTTCATATTATG
AACATTGGCTTCGCTAGTCAACTTAACTGAACCGTTAAGCACAATTCGATTCCATCTCACTTTCTGGG
TATGTCTCGTAGATC
+
FFFFFFFFFFFFFFFFFFFFFFFFFFFFFFFFFFFFFFFFFFFFFFFFFFFFFFFFFFFFFFFFFFFFFF
FFFFFFFFFFFFFFF,FFF,FFFFFFFFFFFFFFFFFFFFFFFFFFFFFFFFFFFFFFFFFFFFFFFFFF
FFFFFFFFFFFFFFF
@A00721:190:HGJW7DSXY:4:1421:9914:32158 1:N:0:TCTGCAAG+GCTGCACT
CTGGGATTAGAATTACCGGGACGTCTATCATACACGAGCCACATCCACAGCCACTGCTTCATATTATG
AACATTGGCTTCGCTAGTCAACTTAACTGAACCGTTAAGCACAATTCGATTCCATCTCACTTTCTGGG
TATGTCTCGTAGATC
+
FFFFFFFFFFFFFFFFFFFFFFFFFFFFFFFFFFFFFFFFFFFFFFFFFFFFFFFFFFFFFFFFFFFFFF
```

FFFFFFFFFFFFFFFFFFFFFFFFFFFFFFFFFFFFFFFFFFFFFFFFFFFFFFFFFFFFFFFFFFFF
FFFFFFFFFFFFFFF
@A00721:190:HGJW7DSXY:4:1421:10520:32831 1:N:0:TCTGCAAG+GCTGCACT
CTGGGATTAGAATTACCGGGACGTCTATCATACACGAGCCACATCCACAGCCACTGCTTCATATTATG
AACATTGGCTTCGCTAGTCAACTTAACTGAACCGTTAAGCACAATTCGATTCCATCTCACTTTATGGG
TATGTCTAGTAGATC
+
FFFF,FFFF,FF:FF,FFFFFF:FFFF:FF,FFF:FFFF:F,FF,,FFFF:FFFF,FF::FF:F,FFF
FFFFF:,FFF:FFFFFF:FFFFFFFFF,FF:,F,,,FF,:F,FF,F:,FFFFFF:::FFF,:F,F,FF
F,FF:::,:FF:F:F
@A00721:190:HGJW7DSXY:4:1569:20754:19398 1:N:0:TCTGCAAG+GCTGCACT
CTGGGATTAGAATTACCGGGACGTCTATCATACACGAGCCACATCCACAGCCACTGCTTCATATTATG
AACATTGGCTTCGCTAGTCAACTTAACTGAACCGTTAAGCACAATTCGATTCCATCTCACTTTCTGGG
TATGTCTCGTAGATC
+
FFFFFFFFFFFFFFFFFFFFFFFFFFFFFFFFFFFFFFFFFFFFFFFFFFFFFFFFFFFFFFFFFFFF
FFFFFFFFFFFFFFFFFFFFFFFFFFFFFFFFFFFFFFFFFFF:FFFFFFFFFFFFFFFFFFFFFFFF
FFFFFFFFFFFFFFF
@A00721:190:HGJW7DSXY:4:1668:31647:4413 1:N:0:TCTGCAAG+GCTGCACT
CTGGGATTAGAATTACCGGGACGTCTATCATACACGAGCCACATCCACAGCCACTGCTTCATATTATG
AACATTGGCTTCGCTAGTCAACTTAACTGAACCGTTAAGCACAATTCGATTCCATCTCACTTTCTGGG
TATGTCTCGTAGATC
+
FFFFFFFFFFFFFFFFFFFFFFFFFFFFFFFFFFFFF:FFFFFFFFFFFFFFFFFFFFFFFFFFFFFF
FFFFFFFFFFFFFFFFFFFFFFFFFFFFFFFFFFFFFFFFFFFFFFFFFFFFFFFFFFFFFFFFFFFF
F:FFFFFFFFFFFFF
@A00721:190:HGJW7DSXY:4:2516:6931:5822 1:N:0:TCTGCAAG+GCTGCACT
CTGGGATTAGAATTACCGGGACGTCTATCATACACGAGCCACATCCACAGCCACTGCTTCATATTATG
AACATTGGCTTCGCTAGTCAACTTAACTGAACCGTTAAGCACAATTCGATTCCATCTCACTTTCTGGG
TATGTCTCGTAGATC
+
FFFFFFFFFFFFFFFFFFFFFFFFFFFFFFFFFFFFFFFFFFFFFFFFFFFFFFFFFFFFFFFFFFFF
FFFFFFFFFFFFFFFFFFF:FFFFFFFFFFFFFFFFFFFFFFFFFFFFFFFFFFFFFFFFFFFFFFFF
FFFFFFFF:FF,FFF
@A00721:190:HGJW7DSXY:4:1140:31186:26162 1:N:0:TCTGCAAG+GCTGCACT
CACCCAGAAAGTGAGATGGAATCGAATTGTGCTTAACGGTTCAGTTAAGTTGACTAGCGAAGCCAATG
TTCATAATATGAAGCAGTGGCTGTGGATGTGGCTCGTGTATGATAGACGTCCCGGTAATTCTAATCCC
AGAGTGAATGAGATA
+
FFFFFFFFFFFFFFFFFFFFFFFFFFFFFFFFFFFFF:FFFFFFFFFFFFFFFFFFFFFFFFFFFFFF
FFFFFFFFFFFFF:FFFFFFFFFFFFFFFFFFFFFFFFFFFFFFFFFFFFFFFFFFFFFFFFFFFFFF
FFFFFFFFFFFFFFF
@A00721:190:HGJW7DSXY:4:1143:28501:9361 1:N:0:TCTGCAAG+GCTGCACT
CACCCAGAAAGTGAGATGGAATCGAATTGTGCTTAACGGTTCAGTTAAGTTGACTAGCGAAGCCAATG
TTCATAATATGAAGCAGTGGCTGTGGATGTGGCTCGTGTATGATAGACGTCCCGGTAATTCTAATCCC
AGAGTGAATGAGATA
+
FFFFFFFFFFFFFFFFFFFF:FFFFFFFFFFFFFFFFFFFFFFFFFFFFFFFFFFFFFFFFFFFFFFF
FFFF,FFFFFFFFFFFFFFFFFFFFFFFFFFFFFFFFFFFFFFFFFFFFFFFFFFFFFFFFFFFFFFF
FFFFFFFFFFFFFFF
@A00721:190:HGJW7DSXY:4:1177:19569:18630 1:N:0:TCTGCAAG+GCTGCACT
CACCCAGAAAGTGAGATGGAATCGAATTGTGCTTAACGGTTCAGTTAAGTTGACTAGCGAAGCCAATG
TTCATAATATGAAGCAGTGGCTGTGGATGTGGCTCGTGTATGATAGACGTCCCGGTAATTCTAATCCC
AGAGTGAATGAGATA

```
+
FFFFFFFFFFFFFFFFFFFFFFFFFFFFFFFFFFFFFFFFFFFFFFFFFFFFFFFFFFFFFFFFFFFF
FFFFFFFFFFFFFFFFFFFFF:FFFFFFFFFFFFFFFFFFFFFFFFFFFFFFFFFFFFFFFFFFFFFF
FFFFFFF:FFFFFFF
@A00721:190:HGJW7DSXY:4:1221:30337:31109 1:N:0:TCTGCAAG+GCTGCACT
CACCCAGAAAGTGAGATGGAATCGAATTGTGCTTAACGGTTCAGTTAAGTTGACTAGCGAAGCCAATG
TTCATAATATGAAGCAGTGGCTGTGGATGTGGCTCGTGTATGATAGACGTCCCGGTAATTCTAATCCC
AGAGTGAATGAGATA
+
FFFFFFFFFFFFFFFFFFFFFFFFFFFFFFFFFFFFFFFFFFFFFFFFFFFFFFFFFFFFFFFFFFFF
FFFFFFFFFFFFFFFFFFFFFFFFFFFFFFFFFFFFFFFFFFFFFFFFFFFFFFFFF,FFFFFFFFFF
FFFFFFFFFFFFFFF
@A00721:190:HGJW7DSXY:4:1239:16532:3035 1:N:0:TCTGCAAG+GCTGCACT
CACCCAGAAAGTGAGATGGAATCGAATTGTGCTTAACGGTTAAGTTAAGATGACTAGCGAAGCCAATG
TTCATAATATGAAGCAGTGGCTGTGGATGTGGCTCGTGTATGAAATACTACCCGCTCATTCAAATCCC
AGAGTGAATGAGATA
+
FFFFFF:FFF:,,:,FF,F:,,F,F::,FF,F:,F:FFFF,,F,,FF:F,,:,F,FFF:FFF,FFF:,
F:,F,FFFFFFFF,FFF,F,FFF,,FFF,F,,F,F,,FFFFFF,F,F,,,FFF,,F,::FF,F,FFFF
F,FFFFF,F,F,,FF
@A00721:190:HGJW7DSXY:4:1273:17192:7529 1:N:0:TCTGCAAG+GCTGCACT
CACCCAGAAAGTGAGATGGAATCGAATTGTGCTTAACGGTTCAGTTAAGTTGACTAGCGAAGCCAATG
TTCATAATATGAAGCAGTGGCTGTGGATGTGGCTCGTGTATGATAGACGTCCCGGTAATTCTAATCCC
AGAGTGAATGAGATA
+
FFFFFFFFFFFFFFFFFFFFFFFFFFFFFFFFFFFF:FFFFFFFFFFFFFFFFFFFFFFFFFFFFFFF
FFFFFFFFFFFFFFFFFFFFFFFFFFFFFFFFFFFFFFFFFFFFFFFFFFFFFFFFFFFFFFFFFFFF
FFFFFFFFFFFFFFF
@A00721:190:HGJW7DSXY:4:1314:31295:7435 1:N:0:TCTGCAAG+GCTGCACT
CACCCAGAAAGTGAGATGGAATCGAATTGTGCTTAACGGTTCAGTTAAGTTGACTAGCGAAGCCAATG
TTCATAATATGAAGCAGTGGCTGTGGATGTGGCTCGTGTATGATAGACGTCCCGGTAATTCTAATCCC
AGAGTGAATGAGATA
+
FFFFFFFFFFFFFFFFFFFFFFFFFFFFFFFFFFFFFFFFFFFFFFFFFFFFFFFFFFFFFFFFFFFF
FFFFFFFFFFFFFFFFFFFFFFFFFFFFFFFFFF:FFFFFFF:FFFFFFFF:FFFF:FFFFFFFFF:F
FFFFFFFFFFFFFFF
@A00721:190:HGJW7DSXY:4:1315:22525:20870 1:N:0:TCTGCAAG+GCTGCACT
CACCCAGAAAGTGAGATGGAATCGAATTGTGCTTAACGGTTCAGTTAAGTTGACTAGCGAAGCCAATG
TTCATAATATGAAGCAGTGGCTGTGGATGTGGCTCGTGTATGATAGACGTCCCGGTAATTCTAATCCC
AGAGTGAATGAGATA
+
FFFFFFFFFFFFFFFFFFFFFFFFFFFFFFFFFFFFFFFFFFFFFFFFFFFFFFFFFFFFFFFFFFFF
FFFFFFFFFFFFFFFFFFFFFFFFFFFFFFFFFFFFFFFFFFFF:FFFFFFFFFFFFFFFFFFFFFFF
FFFFFFFFFFFFFFF
@A00721:190:HGJW7DSXY:4:1408:13865:27727 1:N:0:TCTGCAAG+GCTGCACT
CACCCAGAAAGTGAGATGGAATCGAATTGTGCTTAACGGTTCAGTTAAGTTGACTAGCGAAGCCAATG
TTCATAATATGAAGCAGTGGCTGTGGATGTGGCTCGTGTATGATAGACGTCCCGGTAATTCTAATCCC
AGAGTGAATGAGATA
+
FFFFFFFFFFFFFFFFFFFF:FFFFFFFFFFFFFFFFFFFFFFFFFFFFFFFFFFFFFFFFFFFFFFF
:FFF:FFFFFFFFF:FFFFFFFFFFFFFFFFFFFFFFFFFFFFFFFFFFFFFFFFFF,FFFFF:F:FFF
FFFFFFFFFFFFFFF
@A00721:190:HGJW7DSXY:4:2148:8757:34976 1:N:0:TCTGCAAG+GCTGCACT
CACCCAGAAAGTGAGATGGAATCGAATTGTGCTTAACGGTTCAGTTAAGTTGACTAGCGAAGCCAATG
```

```
TTCATAATATGAAGCAGTGGCTGTGGATGTGGCTCGTGTATGATAGACGTCCCGGTAATTCTAATCCC
AGAGTGAATGAGATA
+
FFFFFFFFFFFFFFFFFFFFFFFFFFFFFFFFFFFFFFFFFFFFFFFFFFFFFFFFFFFFFFFFFFFFFF
FFFFFFFFFFFFFFFFFFFFFFFFFFFFFFFFFFFFFFFFFFFFFFFFFFFFFFFFFFFFFFFFFFFFFF
FFFFFFFFFFFFFFF
@A00721:190:HGJW7DSXY:4:2148:11704:35728 1:N:0:TCTGCAAG+GCTGCACT
CACCCAGAAAGTGAGATGGAATCGAATTGTGCTTAACGGTTCAGTTAAGTTGACTAGCGAAGCCAATG
TTCATAATATGAAGCAGTGGCTGTGGATGTGGCTCGTGTATGATAGACGTCCCGGTAATTCTAATCCC
AGAGTGAATGAGATA
+
FFFFFFFFFFFFFFFFFFFFFFFFFFFFFFFFFFFFFFFFFFFFFFFFFFFFFFFFFFFFFFFFFFFFFF
FFFFFFF:FFFFFFFFFFFFFFFFFFFFFFFFFFFFFFFFFFFFFFFFFFFFFFFFFFFFFFFFFFFFFF
FFFFFFFFFFFFFFF
@A00721:190:HGJW7DSXY:4:2177:16215:21496 1:N:0:TCTGCAAG+GCTGCACT
CACCCAGAAAGTGAGATGGAATCGAATTGTGCTTAACGGTTCAGTTAAGTTGACTAGCGAAGCCAATG
TTCATAATATGAAGCAGTGGCTGTGGATGTGGCTCGTGTATGATAGACGTCCCGGTAATTCTAATCCC
AGAGTGAATGAGATA
+
FFFFFFFFFFFFFFFF:FFFFFFFFFFFFFFFFFFFFFFFFFFFFFFFFFFFFFFFFFFFFFFFFFFFFF
FFFFFFFFFFFFFFFFFFFFFFFFFFFFFFFFFFFFFFFFFFFFFFFFFFFFFFFFFF:FFFFFF:FFFF
FFFFFFFFFFFFFFF
@A00721:190:HGJW7DSXY:4:2238:16541:31704 1:N:0:TCTGCAAG+GCTGCACT
CACCCAGAAAGTGAGATGGAATCGAATTGTGCTTAACGGTTCAGTTAAGTTGACTAGCGAAGCCAATG
TTCATAATATGAAGCAGTGGCTGTGGATGTGGCTCGTGTATGATAGACGTCCCGGTAATTCTAATCCC
AGAGTGAATGAGATA
+
FFFFFFFFFFFFFFFFFFFF,FFFFFFFFFFFFFFFFFFFFFFFFFF:FFFFFFFFFFFFFFFFFFFFFF
FFFFFFFF,FFFFF:FFFFFFFFFFFFFFFFFFF:FFFFFFFFFFFFFFFFFFFFFFF,FFFFF,FFF:F
FFFFFFFFFFFFFFF
@A00721:190:HGJW7DSXY:4:2307:6623:9424 1:N:0:TCTGCAAG+GCTGCACT
CACCCAGAAAGTGAGATGGAATCGAATTGTGCTTAACGGTTCAGTTAAGTTGACTAGCGAAGCCAATG
TTCATAATATGAAGCAGTGGCTGTGGATGTGGCTCGTGTATGATAGACGTCCCGGTAATTCTAATCCC
AGAGTGAATGAGATA
+
FFFFFFFFFFFFFFFFFFFFFFFFFFFFFFFFFFFFFFFFFFFFFFFFFFFFFFFFFFFFFFFFFFFFFF
FFFF:FFFFFFFFF:FFFFFFFFFFFFFFFFFFF:FFFFFFFFFFFFFFFFFFFFFFF:FFFFFFFFFFF
FFFFFFFFFFFFFFF
@A00721:190:HGJW7DSXY:4:2349:20139:1830 1:N:0:TCTGCAAG+GCTGCACT
CACCCAGAAAGTGAGATGGAATCGAATTGTGCTTAACGGTTCAGTTAAGTTGACTAGCGAAGCCAATG
TTCATAATATGAAGCAGTGGCTGTGGATGTGGCTCGTGTATGATAGACGTCCCGGTAATTCTAATCCC
AGAGTGAATGAGATA
+
FFFFFFFFFFFFFFFFFFFF:FFFFFFFFFFFFFFFFFFFFFFFFFFFFFFFFFFFFFFFFFFFFFFFFF
FFFFFFFFFFFFFFFFFFFFFFFFFFFFFFFFFFFFFFFFFFFFFFFFFFFFFFFFFFFFFFFFFFFFFF
FFFFFFFFFFFFFFF
@A00721:190:HGJW7DSXY:4:2357:24641:5149 1:N:0:TCTGCAAG+GCTGCACT
CACCCAGAAAGTGAGATGGAATCGAATTGTGCTTAACGGTTCAGTTAAGTTGACTAGCGAAGCCAATG
TTCATAATATGAAGCAGTGGCTGTGGATGTGGCTCGTGTATGATAGACGTCCCGGTAATTCTAATCCC
AGAGTGAATGAGATA
+
FFFFFFFFFFFFFFFFFFFFFFFFFFFFFFFFFFFFFFFFFFFFFFFFFFFFFFFFFFFFFFFFFFFFFF
F:FFFFFFFFFFFFFFFFFFFFFFFFFFFFFFFFFFFFFFFFFFFFFFFFFFFFFFFFFFFFFF:FFFFF
FFFFFFFFFFFFFFF
```

```
@A00721:190:HGJW7DSXY:4:2601:17318:4617 1:N:0:TCTGCAAG+GCTGCACT
CACCCAGAAAGTGAGATGGAATCGAATTGTGCTTAACGGTTCAGTTAAGTTGACTAGCGAAGCCAATG
TTCATAATATGAAGCAGTGGCTGTGGATGTGGCTCGTGTATGATAGACGTCCCGGTAATTCTAATCCC
AGAGTGAATGAGATA
+
FFFFFFFFFFFFFFFFFFFFFFFFFFFFFFFFFFFFFFFFFFFFFFFFFFFFFFFFFFFFFFFFFFFF
FFFFFFFFFFFFFF:FFFFF:FFFFFFFFFF:FFFFFFFFFFFFFFFFFFFFFFFFFFFFFFFFFFFF
FFFF:FFFFFFFFFF
@A00721:190:HGJW7DSXY:4:2601:17128:4633 1:N:0:TCTGCAAG+GCTGCACT
CACCCAGAAAGTGAGATGGAATCGAATTGTGCTTAACGGTTCAGTTAAGTTGACTAGCGAAGCCAATG
TTCATAATATGAAGCAGTGGCTGTGGATGTGGCTCGTGTATGATAGACGTCCCGGTAATTCTAATCCC
AGAGTGAATGAGATA
+
FFFFFFFFFFFFFFFFFFFFFFFFFFFFFFFFFFFFFFFFFFFFFFFFFFFFFFFFFFFFFFFFFFFF
FFFFFFFFFFFFFFFFFFFFFFFFFFFFFFFFFFFFFFFFFFF:FFFFFFFFFFFFFFFFFFFFFFFF
FFFFFFF:FFFFFFF
@A00721:190:HGJW7DSXY:4:2664:18014:21010 1:N:0:TCTGCAAG+GCTGCACT
CACCCAGAAAGTGAGATGGAATCGAATTGTGCTTAACGGTTCAGTTAAGTTGACTAGCGAAGCCAATG
TTCATAATATGAAGCAGTGGCTGTGGATGTGGCTCGTGTATGATAGACGTCCCGGTAATTCTAATCCC
AGAGTGAATGAGATA
+
FFFFFFFFFFFFF:FFFFFF:FFFFFFFFFFFFFFFF,FFFFFFFFFFFFFFFFFFFFFFFFFFFFFF
FFFF:FFFFFFFFF:FFFFFFFFFFFFFFFFF:FFFFFFFFFFFFFFFFFFFFFFFFFFFFFFFFFFF
FFF:FFFFFFFFF,F
@A00721:190:HGJW7DSXY:4:2677:3549:36104 1:N:0:TCTGCAAG+GCTGCACT
CACCCAGAAAGTGAGATGGAATCGAATTGTGCTTAACGGTTCAGTTAAGTTGACTAGCGAAGCCAATG
TTCATAATATGAAGCAGTGGCTGTGGATGTGGCTCGTGTATGATAGACGTCCCGGTAATTCTAATCCC
AGAGTGAATGAGATA
+
FFFFFFFFFFFFFFFFFFFF:FFFFFFFFFFFFFFFFFFFFFFFFFFFFFFFFFFFFFFFFFFFFFFF
FFFFFFFFFFF:FFFFFFFFFFFFFFFFFFFFFFFFFFFFFF:FFFFFFFFFFFFFFFFFFFFFFFFF
FFFFFFFFFFFFFFF
@A00721:190:HGJW7DSXY:4:1263:21802:34115 1:N:0:TCTGCAAG+GCTGCACT
ACCCAGAAAGTGAGATGGAATCGAATTGTGCTTAACGGTTCAGTTAAGTTGACTAGCGAAGCCAATGT
TCATAATATGAAGCAGTGGCTGTGGATGTGGCTCGTGTATGATAGACGTCCCGGTAATTCTAATCCCA
GAGTGAATGAGATAT
+
FFFFFFFFFFFFFFFFFFFFFFFFFFFFFFFFFFFFFFFFFFFFFFFFFFFFFFFFFFFFFFFFFFFF
FFF,FF:FFFFFFFFFFFFFFFFF:FFFFFFFFFFFFFFFFFFFFFFFFFFFFFFFFFFFFFFFFFFF
FFFFFFFFFFFFFFF
@A00721:190:HGJW7DSXY:4:1329:14669:33348 1:N:0:TCTGCAAG+GCTGCACT
ACCCAGAAAGTGAGATGGAATCGAATTGTGCTTAACGGTTCAGTTAAGTTGACTAGCGAAGCCAATGT
TCATAATATGAAGCAGTGGCTGTGGATGTGGCTCGTGTATGATAGACGTCCCGGTAATTCTAATCCCA
GAGTGAATGAGATAT
+
FFFFFFFFFF:FF:FFFFF:FFFFFFFFFFFFFFF:F:FFFFFFFFFFFFFFFFFFFFFFFFFFFFFF
F,,F:FF:FFFFFF:FFFF:FFFF::FFFF,FFFFFFFFF,FFFF:F:FFFFFFFFFF:FFFFFFFFF
FF:FFFFFFFFF:FF
@A00721:190:HGJW7DSXY:4:1377:11984:33614 1:N:0:TCTGCAAG+GCTGCACT
ACCCAGAAAGTGAGATGGAATCGAATTGTGCTTAACGGTTCAGTTAAGTTGACTAGCGAAGCCAATGT
TCATAATATGAAGCAGTGGCTGTGGATGTGGCTCGTGTATGATAGACGTCCCGGTAATTCTAATCCCA
GAGTGAATGAGATAT
+
FFFFFFFFFFFFFFFFFFFFFFFFFFFFFFFFFFFFFFFFFFFFFFFFFFFFFFFFFFFFFFFFFFFF
```

FFF:FFFFFFFFFFFFFFFFFFFFFFFFFFFFFFFFFFFFF:FFFFFFFFFFFFFFFFFFFFFFFFFFF
FFFFFFFFFFFFFFF
@A00721:190:HGJW7DSXY:4:1522:3830:24627 1:N:0:TCTGCAAG+GCTGCACT
ACCCAGAAAGTGAGATGGAATCGAATTGTGCTTAACGGTTCAGTTAAGTTGACTAGCGAAGCCAATGT
TCATAATATGAAGCAGTGGCTGTGGATGTGGCTCGTGTATGATAGACGTCCCGGTAATTCTAATCCCA
GAGTGAATGAGATAT
+
FFFFFFFFFFFFFFFFFFFFFFFFFFFFFFFFFFF:FFF::FFFFFFFFFFFFFFFFFFFFFFFFFFFF
FFFFFFFFFFFFFFFFFFFFFFFFFFFFFFFFFFF:FFFFFFFF:FFFFFFFFFFFFFFFFFFFFFFFF
FFFFFFFFFFFFFFF
@A00721:190:HGJW7DSXY:4:1530:3884:18270 1:N:0:TCTGCAAG+GCTGCACT
ACCCAGAAAGTGAGATGGAATCGAATTGTGCTTAACGGTTCAGTTAAGTTGACTAGCGAAGCCAATGT
TCATAATATGAAGCAGTGGCTGTGGATGTGGCTCGTGTATGATAGACGTCCCGGTAATTCTAATCCCA
GAGTGAATGAGATAT
+
FFFFFFFFFFFFFFFF:FFFFFFFFFFFFFFFFFFFF:FFFFFFFFFFFFFFFF:FFFFFFFFFFFFF
FFFFFFFFFFFFFFFFFFFFFFFFFFFFFFF:FFFFFFFFFFFFFFFFFFFFFFFFF,FFFFFFFFFFF
FFFFFFFFFFFFFFF
@A00721:190:HGJW7DSXY:4:1530:5746:20087 1:N:0:TCTGCAAG+GCTGCACT
ACCCAGAAAGTGAGATGGAATCGAATTGTGCTTAACGGTTCAGTTAAGTTGACTAGCGAAGCCAATGT
TCATAATATGAAGCAGTGGCTGTGGATGTGGCTCGTGTATGATAGACGTCCCGGTAATTCTAATCCCA
GAGTGAATGAGATAT
+
FFFFFFFFFFFFFFFFFFFFF:FFFFFFFFFFFFFFFFFF:FFFFFFFFFFFFFFFF:FFFFFFFFFFFFF
F:FFFFFFFFFFFFFFFFFFF:FFFFFFFFFFFFFFFFFF:FFFFFFFF,FFFFFFFFFFFFFFF:FFF
FFF:FF:F:FFFFFF
@A00721:190:HGJW7DSXY:4:1540:14904:7921 1:N:0:TCTGCAAG+GCTGCACT
ACCCAGAAAGTGGGATGGAATCGAATTGTGCTTAACGGTTCAGTTAAGTTGACTAGCGAAGCCAATGT
TCATAATATGAAGCAGTGGCTGTGGATGTGGCTCGTGTATGATAGACGTCCCGGTAATTCTAATCCCA
GAGTGAATGAGATAT
+
FFFFFFFFFFFF:FFFFFF,FFFFFFFFFFFFFFFFFFFFFFFFFFFFF:FFFFFFFFFFFFFFFFFFF
FFF:FFFFFFFFFFFFFFFFFFFFFFFFFFFFFFFFFFFFFFFFFFFFF:FF:F:F:FFFFFFFFFFFFF
FFFFFFFFF,FFFFF
@A00721:190:HGJW7DSXY:4:2372:5095:24377 1:N:0:TCTGCAAG+GCTGCACT
ACCCAGAAAGTGAGATGGAATCGAATTGTGCTTAACGGTTCAGTTAAGTTGACTAGCGAAGCCAATGT
TCATAATATGAAGCAGTGGCTGTGGATGTGGCTCGTGTATGATAGACGTCCCGGTAATTCTAATCCCA
GAGTGAATGAGATAT
+
FFFFFFFFFFFFFFFFFFFFFFFFFFFFFFFFFFFFFFFFFFFFFFFFFFFFFFF:FFFFFFFFFFFFF
FFFFFFFFFFFFFF:FFFFFFFFFFFFFFFFFFFFFFFFFFFFFFFFFFFFFFFFF:FFFFFFFFFFF
FFFFFFFFFFFFFFF
@A00721:190:HGJW7DSXY:4:2420:28926:18646 1:N:0:TCTGCAAG+GCTGCACT
ACCCAGAAAGTGAGATGGAATCGAATTGTGCTTAACGGTTCAGTTAAGTTGACTAGCGAAGCCAATGT
TCATAATATGAAGCAGTGGCTGTGGATGTGGCTCGTGTATGATAGACGTCCCGGTAATTCTAATCCCA
GAGTGAATGAGATAT
+
FFFFFFFFFFFFFFFFFFFFFFFFFF:FFFFFFFFFFFFFFFFFFFFFFFFFFFFFFFFFFFFFFFFFF
FFFFFFFFFFFFFFFFFFFFFFFFFFFFFFFFFFFFFFFFFFFFFFFFFFFFFFFFF:FFFFFFFFFFF
FFFFFF:FFFFFFFF
@A00721:190:HGJW7DSXY:4:2503:5764:24283 1:N:0:TCTGCAAG+GCTGCACT
ACCCAGAAAGTGAGATGGAATCGAATTGTGCTTAACGGTTCAGTTAAGTTGACTAGCGAAGCCAATGT
TCATAATATGAAGCAGTGGCTGTGGATGTGGCTCGTGTATGATAGACGTCCCGGTAATTCTAATCCCA
GAGTGAATGAGATAT

```
+
FFFFFFFFFFFFFFFFFFF:FFFFFFFFFFFFFFFFFFFFFFFFFFFFFFFFFFFFFFFFFFFFFFFF:FF
FFFFFFFFFFFFF:FFFFFFFFFFFFFFFFFFFFFFFFFFFFFFFFFFFFFFFFFFFFFFFFFFFFF,FFF
FFFFFF:FFFFFFFF
@A00721:190:HGJW7DSXY:4:1447:22354:9204 1:N:0:TCTGCAAG+GCTGCACT
CCCCAGAAAGTGAGATGGAATCGAATTGTGCTTAACGGTTCAGTTAAGTTGACTAGCGAAGCCAATGT
TCATAATATGAAGCAGTGGCTGTGGATGTGGCTCGTGTATGATAGACGTCCCGGTAATTCTAATCCCA
GAGTGAATGAGATAT
+
FFFFFFFFFFFFFFFFFFFFFFFFFFFFFFFFFFFFFFFFFFFFFFFFFFFFFFFFFFFFFFFFFFFFFF
FFFFFFFFFFFFFFFFFFFF:FFFFFFFFFFFFFFFFFFFFFFFFFFFFFFFFFFFFFFFFFFFFFFFFF
FFFFFF,FFFFFFFF
@A00721:190:HGJW7DSXY:4:2278:32108:21840 1:N:0:TCTGCAAG+GCTGCACT
CCCCAGAAAGTGAGATGGAATCGAATTGTGCTTAACGGTTCAGTTAAGTTGACTAGCGAAGCCAATGT
TCATAATATGAAGCAGTGGCTGTGGATGTGGCTCGTGTATGATAGACGTCCCGGTAATTCTAATCCCA
GAGTGAATGAGATAT
+
:FFFF,FFFFFFFFFFFFFFFFFFFFFFFFF,FFFFFFFFFFFFFFFF,FFFFFFFFFFFF:FFFFFFF
FFFFFFFFFFFF::FFFFFFF:FFFFFFFF::FFFFFFFFFFFF:FFFFFFF:FFFFFFFFFF:FFFF
FFF:FFFFFFFFFFF
@A00721:190:HGJW7DSXY:4:2374:24361:26428 1:N:0:TCTGCAAG+GCTGCACT
CCCCAGAAAGTGAGATGGAATCGAATTGTGCTTAACGGTTCAGTTAAGTTGACTAGCGAAGCCAATGT
TCATAATATGAAGCAGTGGCTGTGGATGTGGCTCGTGTATGATAGACGTCCCGGTAATTCTAATCCCA
GAGTGAATGAGATAT
+
FFFFFFFFFFFFFFFFFFF:FFFFFFFFFFFFFFFFFFFFFFFFFFFFFFFFFFFFFFFFFFFFFFFFFF
FFFF:FFFFFFFF:FFFFFFFFFFFFFFFFFFFFFFFFFFFFFFF:FFFFFFFFFFFFFFFFFFFFFFFF
FFFFFFFFFFFFFFF
@A00721:190:HGJW7DSXY:4:1153:28836:3239 1:N:0:TCTGCAAG+GCTGCACT
CCAGAAAGTGAGATGGAATCGAATTGTGCTTAACGGTTCAGTTAAGTTGACTAGCGAAGCCAATGTTC
ATAATATGAAGCAGTGGCTGTGGATGTGGCTCGTGTATGATAGACGTCCCGGTAATTCTAATCCCAGA
GTGAATGAGATATTC
+
FFFFFFFFFFFFFFFFFFFFFFFFFFFFFFFFFFFFFFFFFFFFFFFFFFFFFFFFFFFFFFFFFFFFFF
FFFFFFFFFFFFFFFFFFFFFFFFFFFFFFFFFFFFFFFFFFFFFFFFFFFFFFFFFFFFFFFFFFFFFF
FFFFFFFFFFFFFFF
@A00721:190:HGJW7DSXY:4:1266:2193:33536 1:N:0:TCTGCAAG+GCTGCACT
CCAGAAAGTGAGATGGAATCGAATTGTGCTTAACGGTTCAGTTAAGTTGACTAGCGAAGCCAATGTTC
ATAATATGAAGCAGTGGCTGTGGATGTGGCTCGTGTATGATAGACGTCCCGGTAATTCTAATCCCAGA
GTGAATGAGATATTC
+
FFFFFFFFFFFFF:FFF:FFFFFFFFFFFFFFFFFFFFFFFFFFFFFFFFFFFFFFFF::FFFFFFFFF
FFFFFFFFFFFFFFFFFFFFF:FFFFFFFFFFF,FFFFFFFF:FFFFFFFFFFFFFFFFFFFFFFFFFF
FFFFFFFFFFFF:FF
@A00721:190:HGJW7DSXY:4:1362:22073:28260 1:N:0:TCTGCAAG+GCTGCACT
CCAGAAAGTGAGATGGAATCGAATTGTGCTTAACGGTTCAGTTAAGTTGACTAGCGAAGCCAATGTTC
ATAATATGAAGCAGTGGCTGTGGATGTGGCTCGTGTATGATAGACGTCCCGGTAATTCTAATCCCAGA
GTGAATGAGATATTC
+
FFFFFFFFFFFFFFFFFFFFFFFFFFFFFFFFFFFFFFFFFFFFFFFFFFFFFFFFFFFFFFFFFFFFF:
FFFFFFFFFFFFFF:FFFFFFFFFFFFFFFFFFFFFFFFFFFFFFFFFFFFFFFFFFFFFFFFFFFFFF
FFFFFFFFFFFFFFF
@A00721:190:HGJW7DSXY:4:1411:32280:29183 1:N:0:TCTGCAAG+GCTGCACT
CCAGAAAGTGAGATGGAATCGAATTGTGCTTAACGGTTCAGTTAAGTTGACTAGCGAAGCCAATGTTC
```

```
ATAATATGAAGCAGTGGCTGTGGATGTGGCTCGTGTATGATAGACGTCCCGGTAATTCTAATCCCAGA
GTGAATGAGATATTC
+
FFFFFFFFFFFFFFFFFFFFFFFFFFFFFFFFFFFFFFFFF:FFFFFFFFFFFFFFFFFFFFFFFFFF
FFFFFFFFFFFFFFFFFFFFFFFFFFFFFFFFFFFFFFFFFFFFFFFFFFFFFFFFFFFFFFFFFFFF
F:FFFFFFFFFFFFF
@A00721:190:HGJW7DSXY:4:1421:18349:36151 1:N:0:TCTGCAAG+GCTGCACT
CCAGAAAGTGAGATGGAATCGAATTGTGCTTAACGGTTCAGTTAAGTTGACTAGCGAAGCCAATGTTC
ATAATATGAAGCAGTGGCTGTGGATGTGGCTCGTGTATGATAGACGTCCCGGTAATTCTAATCCCAGA
GTGAATGAGATATTC
+
FFFFFFFFFFFFFFFFFFFFFFFFFFFFFFFFFFFFFFFFFFFFFFFFFFFFFFFFFFFFFFFFFFFF
FFFFFFF:FFFFFFFFFFFFFFFFFFFFFFFFFFFFFFFFFFFFFFFFFFFFFFFFFFFFFFFFFFFF
FFFFFFFFFFFFFFF
@A00721:190:HGJW7DSXY:4:1429:30427:26725 1:N:0:TCTGCAAG+GCTGCACT
CCAGAAAGTGAGATGGAATCGAATTGTGCTTAACGGTTCAGTTAAGTTGACTAGCGAAGCCAATGTTC
ATAATATGAAGCAGTGGCTGTGGATGTGGCTCGTGTATGATAGACGTCCCGGTAATTCTAATCCCAGA
GTGAATGAGATATTC
+
FFFFFFFFFFFFFFFFFFFFFFFFFFFFFFFFF:FFFFFFFFFFFFFFFFFFFFFFFFFFFFFFFFFF
FFFFFFFFFF:FFFFFFFFFFFFFFFFFFFFFFFFFFFFFFFFFFFFFFFFFFFFFFFFFFFFFFFFF
FFFFFFFFFFFFFFF
@A00721:190:HGJW7DSXY:4:1441:20048:8437 1:N:0:TCTGCAAG+GCTGCACT
CCAGAAAGTGAGATGGAATCGAATTGTGCTTAACGGTTCAGTTAAGTTGACTAGCGAAGCCAATGTTC
ATAATATGAAGCAGTGGCTGTGGATGTGGCTCGTGTATGATAGACGTCCCGGTAATTCTAATCCCAGA
GTGAATGAGATATTC
+
FFFFFFFFFFFFFFFFFFFFFFFFFFFFFFFFFFFFFFFFFFFFFFFFFFFFFFFFFFFFFFFFFFFF
FFFFFFFFFFF:FFFFFF:FFFFFFFFFFFFFFFFFFFFFFFFFFFFFFFFFFFFFFFFFFFFFFFFF
FFFFFFFFFFFFFFF
@A00721:190:HGJW7DSXY:4:1474:11867:36229 1:N:0:TCTGCAAG+GCTGCACT
CCAGAAAGTGAGATGGAATCGAATTGTGCTTAACGGTTCAGTTAAGTTGACTAGCGAAGCCAATGTTC
ATAATATGAAGCAGTGGCTGTGGATGTGGCTCGTGTATGATAGACGTCCCGGTAATTCTAATCCCAGA
GTGAATGAGATATTC
+
FFFFFFFFFFFFFFFFFFFFFF:FFFFFFFFFFFFFFFFFFFFFFFFFFFFFFFFFFFFFF:FFFFFF
FFFFFFFFFFFFFFFFFFFFFFFFFFFFFFFFFFFFFFFFFFFFFFFFFFFFFFFFFFFFFFFFFFFF
FFFFFFFFFFFFFFF
@A00721:190:HGJW7DSXY:4:1543:19886:23750 1:N:0:TCTGCAAG+GCTGCACT
CCAGAAAGTGAGATGGAATCGAATTGTGCTTAACGGTTCAGTTAAGTTGACTAGCGAAGCCAATGTTC
ATAATATGAAGCAGTGGCTGTGGATGTGGCTCGTGTATGATAGACGTCCCGGTAATTCTAATCCCAGA
GTGAATGAGATATTC
+
FFFFFFFFFFFFFFFFF,FFFFFFFFFFFFFFFFFFFFFFFFFFFFFFFFFFFFFFFFFFFFFFFFFF
FFFFFFFFFFF:FFFFFFFFFFFFFFFFFFFFFFFFFFFFFFFFFFFFFFFFFFFFFFFFFFFFFFFF
FFFFFFFFFFFFFFF
@A00721:190:HGJW7DSXY:4:1653:21983:30639 1:N:0:TCTGCAAG+GCTGCACT
CCAGAAAGTGAGATGGAATCGAATTGTGCTTAACGGTTCAGTTAAGTTGACTAGCGAAGCCAATGTTC
ATAATATGAAGCAGTGGCTGTGGATGTGGCTCGTGTATGATAGACGTCCCGGTAATTCTAATCCCAGA
GTGAATGAGATATTC
+
FFFFFFFFFFFFFFFFFFFFFFFFFFFFFFFFFFFFFFFFFFFFFFFFFFFFFFFFFFFFFFFFFFFF
FFFFFFFFFFFFFFFFFFFF:FFFFFFFFFFFFFFFFFFFFFFFFFFFFFFFFFFFFFFFFFFFFFFF
FFFF,FFFFFFFFFF
```

```
@A00721:190:HGJW7DSXY:4:2253:2085:21887 1:N:0:TCTGCAAG+GCTGCACT
CCAGAAAGTGAGATGGAATCGAATTGTGCTTAACGGTTCAGTTAAGTTGACTAGCGAAGCCAATGTTC
ATAATATGAAGCAGTGGCTGTGGATGTGGCTCGTGTATGATAGACGTCCCGGTAATTCTAATCCCAGA
GTGAATGAGATATTC
+
FFF,FFFF,FFFFFFFF,FFFFF:FFFFFFFFFFFFFFFFFFFFFFFFFFF:FFFFFFFFFFF::FFF
FFFFFFFFFF:FFFFFFF:FFFFFFFFFFFFFFFFFFFFFF::FFFFFF:FFFFFFFFF:F,FFFFFF
FFFFFF:FFFFFFFF
@A00721:190:HGJW7DSXY:4:2265:18349:30170 1:N:0:TCTGCAAG+GCTGCACT
CCAGAAAGTGAGATGGAATCGAATTGTGCTTAACGGTTCAGTTAAGTTGACTAGCGAAGCCAATGTTC
ATAATATGAAGCAGTGGCTGTGGATGTGGCTCGTGTATGATAGACGTCCCGGTAATTCTAATCCCAGA
GTGAATGAGATATTC
+
FFFFFFFFFFFFF,FFFFFFFFFFFFFFFFFFFFFFFFFFFFFFFFFFFFFFFFFFFFFFFFFFFFFF
FFFFFFFFFFFFFFFFFF:FFFFFFFFF:FFFFFFFFFFFF:FFFFFFFFFFFFFFFFFFFFFFFFFF
FFFFFFFFFFFFFFF
@A00721:190:HGJW7DSXY:4:2336:9706:8312 1:N:0:TCTGCAAG+GCTGCACT
CCAGAAAGTGAGATGGAATCGAATTGTGCTTAACGGTTCAGTTAAGTTGACTAGCGAAGCCAATGTTC
ATAATATGAAGCAGTGGCTGTGGATGTGGCTCGTGTATGATAGACGTCCCGGTAATTCTAATCCCAGA
GTGAATGAGATATTC
+
FFFFFFFFFFFFFFFFFFFFFFFFFFFFFFFFFFFFFFFFFFFFFFFFFFFFFFFFFFFFFFFFFFFF
FFFFFFFFFFF:FFFFFF:FFFFFFFFFFFFFFFFFFFFFFFFFFFFFFFFFF:FFFFFFFFF:FFFF
FFFFFFF:FFFFFFF
@A00721:190:HGJW7DSXY:4:2338:12816:2926 1:N:0:TCTGCAAG+GCTGCACT
CCAGAAAGTGAGATGGAATCGAATTGTGCTTAACGGCTCAGTTAAGTTGACTAGCGAAGCCAATGTTC
ATAATATGAAGCAGTGGCTGTGGATGTGGCTCGTGTATGATAGACGTCCCGGTAATTCTAATCCCAGA
GTGAATGAGATATTC
+
FFFFFFFFFFFFFFFFFFFFFFFFFFFFFFFFFFFFFFFFF:FFFFFFFFFFFFFFFFFFF:FFFFFF
FFFFFFFFFFFFFFFFFFFFFFFFFFFFFFFFFFFF,FFFFFFFFFFFFFFFFFFFFFFFFFFFFFFF
FFFFFFFFFFFFFFF
@A00721:190:HGJW7DSXY:4:2338:12807:2942 1:N:0:TCTGCAAG+GCTGCACT
CCAGAAAGTGAGATGGAATCGAATTGTGCTTAACGGTTCAGTTAAGTTGACTAGCGAAGCCAATGTTC
ATAATATGAAGCAGTGGCTGTGGATGTGGCTCGTGTATGATAGACGTCCCGGTAATTCTAATCCCAGA
GTGAATGAGATATTC
+
FFFFFFFFFFFFFFFFFFFFFFFFFFFFFFFFFFFFFFFFFFFFFFFFFFFFFFFFFFFFFFFFFFFF
FFFFFFFFFFFFFFFFFFFFFFFFFFFFFFFFFFFFFFFFFFFFFFFFFFFFFFFFFFFFFFFFFFFF
FFFFFFFFFFFFFFF
@A00721:190:HGJW7DSXY:4:2507:11966:28823 1:N:0:TCTGCAAG+GCTGCACT
CCAGAAAGTGAGATGGAATCGAATTGTGCTTAACGGTTCAGTTAAGTTGACTAGCGAAGCCAATGTTC
ATAATATGAAGCAGTGGCTGTGGATGTGGCTCGTGTATGATAGACGTCCCGGTAATTCTAATCCCAGA
GTGAATGAGATATTC
+
FFFFFFFFFFFFFFFFFFFFFFF:FFFFFFFFFFFF:FFFFFFFFFFFFFFFFFFFFFFFFFFFFFFF
FFFFFFFFFFF:FFFFFFFFFFFFFFFFFFFFFFFFFFFFFFF:FFFFFFFFFFFFFFFFFFFFFFFF
FFFF:FFFFFFFFFF
@A00721:190:HGJW7DSXY:4:2658:18258:26318 1:N:0:TCTGCAAG+GCTGCACT
CCAGAAAGTGAGATGGAATCGAATTGTGCTTAACGGTTCAGTTAAGTTGACTAGCGAAGCCAATGTTC
ATAATATGAAGCAGTGGCTGTGGATGTGGCTCGTGTATGATAGACGTCCCGGTAATTCTAATCCCAGA
GTGAATGAGATATTC
+
FFFFFFFFFFFFFFFFF:FFFFFFFFFFFFFFFFFFFFFFFFFFFFFFFFFFFFFFFFFFFFFFFFFF
```

FFFFFFFFFFF:FFFFFFFFFFFFFFFFFFFFFFFFFFFFFFFFFFFFFFFFFFFFFFFFFFFF
FFFFFFFFFFFFFFF
@A00721:190:HGJW7DSXY:4:1126:7744:7075 1:N:0:TCTGCAAG+GCTGCACT
CAGAAAGTGAGATTGAATCGAATTGTGCTTAACGGTTCAGTTAAGTTGACTAGCGAAGCCAATGTTCA
TAATATGAATCAGAGGCTGTGGATGTGGCTCGTTTATGATAGACGTCCCGGTAATTCTAATCCCAGAG
TGAATGAGATATT
+
:F:FFFFF:FFFF,FFF:F,FFFFFF,:FFFF:::FF,F,,FFF,:F,:F,FFFFFF:F:FFF,,FFF
FFFF:FFFF,FF,,,,,:,:,,FF,F,,F:,,F,FF,:F:F,F:FFF,F,,:F:FF::FFFF,,FFF:
,FFFFFFF:F:F:
@A00721:190:HGJW7DSXY:4:1163:29903:11318 1:N:0:TCTGCAAG+GCTGCACT
CAGAAAGTGAGATGGAATCGAATTGTGCTTAACGGTTCAGTTAAGTTGACTAGCGAAGCCAATGTTCA
TAATATGAAGCAGTGGCTGTGGATGTGGCTCGTGTATGATAGACGTCCCGGTAATTCTAATCCCAGAG
TGAATGAGATATTCA
+
FF:FFFFFFFFFFFFF:FFFFFFFFFFFFFFFFFFFFFFFFFFFFFFFFFFFFFFFFFFFFFFFFFFF
FFFFFFFFFFFFFFFFFFFFF,FFFFFFFFFFFFFFFFFFF:F:FFF:FFFFFFFFFFFFFFFFFFF
FFFFFFFFFFFFFFF
@A00721:190:HGJW7DSXY:4:1202:14660:18615 1:N:0:TCTGCAAG+GCTGCACT
CAGAAAGTGAGATGGAATCGAATTGTGCTTAACGGTTCAGTTAAGTTGACTAGCGAAGCCAATGTTCA
TAATATGAAGCAGTGGCTGTGGATGTGGCTCGTGTATGATAGACGTCCCGGTAATTCTAATCCCAGAG
TGAATGAGATATTCA
+
FFFFFFFFFFFFFFFFFFFFFFFFFFFFFFFFFFFFFFFFFFFFFFFFFFFFFFFFFFFFFFFFFFF:
FFFFFFFFFFFFFFFFFFFFFFFFFFFFFFFFFFFFFFFFFFFFFFFFFFF:FFFFF:FFFFFFFF
FFFFFFFFFFFFFFF
@A00721:190:HGJW7DSXY:4:1353:18005:7780 1:N:0:TCTGCAAG+GCTGCACT
CAGAAAGTGAGATGGAATCGAATTGTGCTTAACGGTTCAGTTAAGTTGACTAGCGAAGCCAATGTTCA
TAATATGAAGCAGTGGCTGTGGATGTGGCTCGTGTATGATAGACGTCCCGGTAATTCTAATCCCAGAG
TGAATGAGATATTCA
+
FFFFFFFFFFFFFFFFFFFFFFFFFFFFFFFFFFFFFFFFFFFFFFFFFFFFFFFFFFFFFFFFFFFF
FFFFFFFFFFFFFFFFFFFFFFFFFFFFFFFFFFFFFFFFFFFFFFFFFFFFFFFFFFFFFFFFFFFF
FFFFFFFFFFFFFFF
@A00721:190:HGJW7DSXY:4:1634:8757:23422 1:N:0:TCTGCAAG+GCTGCACT
CAGAAAGTGAGATGGAATCGAATTGTGCTTAACGGTTCAGTTAAGTTGACTAGCGAAGCCAATGTTCA
TAATATGAAGCAGTGGCTGTGGATGTGGCTCGTGTATGATAGACGTCCCGGTAATTCTAATCCCAGAG
TGAATGAGATATTCA
+
FFFFFFF:FFFF:F:FFF:FFFFFFFFFFFFFFFFF:FF:FFFFF,FFF:FF:FFFFF,FFF:,FFFF
:FFFFF:FFFFFFFFFFFF:F:F,FFFFF::F:FFFFFFFF:FF,FF,FF:FFFF,FFFF,FFFFFF,
:FFFFFFFFFFF::F
@A00721:190:HGJW7DSXY:4:2134:4372:26443 1:N:0:TCTGCAAG+GCTGCACT
CAGAAAGTGAGATGGAATCGAATTGTGCTTAACGGTTCAGTTAAGTTGACTAGCGAAGCCAATGTTCA
TAATATGAAGCAGTGGCTGTGGATGTGGCTCGTGTAAGATAGACGTCCCGGTAATTCTAATCCCAGAG
TGAATGAGATATTCA
+
FFFFFFFFFFFFFFFFFFFFFFFFFFFFFFFFFFFFFFFFFFFFFFFFFFFFFFFFFFFFFFFFFFFF
FFFFFFFFFF:FFFFFFFFFFFFFFFFFFFFFFFFFF,FFFFFFFFFFFFFFFFFFFFFFFFF:FFFFFF
FFFFFFFFFFFFFFF
@A00721:190:HGJW7DSXY:4:2134:1452:27461 1:N:0:TCTGCAAG+GCTGCACT
CAGAAAGTGAGATGGAATCGAATTGTGCTTAACGGTTCAGTTAAGTTGACTAGCGAAGCCAATGTTCA
TAATATGAAGCAGTGGCTGTGGATGTGGCTCGTGTATGATAGACGTCCCGGTAATTCTAATCCCAGAG
TGAATGAGATATTCA

+
FFFFFFFFFFFFFFFFFFFFFFFFFFFFFFFFFFFFFFFFFFFFFFFFFFFFFFFFFFFFFFFFFFFFFF
FFFFFFFFFF:FFFFFFFFFFFFFFFFFFFFFFFFFFFFFFFFFFF:FFFFFFFFFFFFFFFFFFFFFFF
FFFFFFFFFFFFFFF
@A00721:190:HGJW7DSXY:4:2278:1705:20572 1:N:0:TCTGCAAG+GCTGCACT
CAGAAAGTGAGATGGAATCGAATTGTGCTTAACGGTTCAGTTAAGTTGACTAGCGAAGCCAATGTTCA
TAATATGAAGCAGTGGCTGTGGATGTGGCTCGTGTATGATAGACGTCCCGGTAATTCTAATCCCAGAG
TGAATGAGAAGATC
+
FF:FFFF,FFFFF:FFFF:FFF,FF,F,FFFFFFFFFFFFF::FFF,FFFFF,FFFFFFFFFFF:FFFFF
FFFFFFFFFF:FF,F:FFFFF:FFFF:F,FF:FFFFFFFFFF,FFFF::FF:F:F:FFFFFFF,FFFFF
F,FF,FFFFF:FFF
@A00721:190:HGJW7DSXY:4:2348:27805:17926 1:N:0:TCTGCAAG+GCTGCACT
CAGAAAGTGAGATGGAATCGAATTGTGCTTAACGGTTCAGTTAAGTTGACTAGCGAAGCCAATGTTCA
TAATATGAAGCAGTGGCTGTGGATGTGGCTCGTGTATGATAGACGTCCCGGTAATTCTAATCCCAGAG
TGAATGAGATATTCA
+
:FFFFFFFFFFFFFFFFFFFFFFFFFFFFFFFFFFFFF:FF:FFFFF:FF:FFFF:F:FFFFFF:,FF:F
:FFFFFFFFFFFFFFFFFF,FFFFFFFFFFF,FFFFFFFFFFFFFFFFFFFFFF:FFFFF:FFFFFFFF:
FFFFFFF:FFFFFFF
@A00721:190:HGJW7DSXY:4:2416:10294:25582 1:N:0:TCTGCAAG+GCTGCACT
CAGAAAGTGAGATGGAATCGAATTGTGCTTAACGGTTCAGTTAAGTTGACTAGCGAAGCCAATGTTCA
TAATATGAAGCAGTGGCTGTGGATGTGGCTCGTGTATGATAGACGTCCCGGTAATTCTAATCCCAGAG
TGAATGAGATATTCA
+
FFFFFFFFFFFFFFFFFFFFFF:FFFFFFFFFFFFFFFFFFFFFFFFFFFFFFFFFFFFFFFFFFFFFFF
FFFFFFFFFFFFFFFFFFFFFFFFFFFFFFFFFFFFFFFFFFFFFFFFFFFFFFFFFFFFFFFFFFFFFF
FFFFFFFFFFFFFFF
@A00721:190:HGJW7DSXY:4:2446:10872:32597 1:N:0:TCTGCAAG+GCTGCACT
CAGAAAGTGAGATGGAATCGAATTGTGCTTAACGGTTCAGTTAAGTTGACTAGCGAAGCCAATGTTCA
TAATATGAAGCAGTGGCTGTGGATGTGGCTCGTGTATGATAGACGTCCCGGTAATTCTAATCCCAGAG
TGAATGAGATATTCA
+
FFFFFFFFFFFFFFFFF:FFFFFFFFFFFFFFFFFFFFFFFFFFFFFFFFFFFFFFFFFFFFFFFFFFFF
FFFFFFFFFFFFFFFFFFFFFFFFFFFFFFFFF:FFFFFFFFFFFFFFFFFFFFFFFFFFFFFFFFFFFF
FFFFFFFFFFFFFFF
@A00721:190:HGJW7DSXY:4:2539:27145:19758 1:N:0:TCTGCAAG+GCTGCACT
CAGAAAGTGAGATGGAATCGAATTGTGCTTAACGGTTCAGTTAAGTTGACTAGCGAAGCCAATGTTCA
TAATATGAAGCAGTGGCTGTGGATGTGGCTCGTGTATGATAGACGTCCCGGTAATTCTAATCCCAGAG
TGAATGAGAAGATCG
+
FFFFFFFFFFFFFFFFFFFFFFFFFFFFFFFFFFFFFFFFFFFFFFFFFFFFFFFFFFFFFFFFFFFFFF
FFFF:FFFFF:FFFFFFFFFFFFFFFFFFFFFFFFFFFFFFFFFFFFFFFFFFFFFFFFFFFFFFFFFFF
FFFFFFFFFFFFFFF
@A00721:190:HGJW7DSXY:4:2633:6705:24283 1:N:0:TCTGCAAG+GCTGCACT
CAGAAAGTGAGATGGAATCGAATTGTGCTTAACGGTTCAGTTAAGTTGACTAGCGAAGCCAATGTTCA
TAATATGAAGCAGTGGCTGTGGATGTGGCTCGTGTATGATAGACGTCCCGGTAATTCTAATCCCAGAG
TGAATGAGAAGATCG
+
FFFFFFFFFFFFFFFFFFFFFFFFFFFFFFFFFFFFFFFFF:FFFFFFFFFFFF:FFFFFFFFFFFFFFF
FFFFFFFFFFFFFFFFFFFFFFFFFFFFFFFFFFFFFFFFFFFFFFFFFFFFFFFF:FFFFFFFFFFFFF
FFFFFFFFFFFFF,F
@A00721:190:HGJW7DSXY:4:2643:32334:27899 1:N:0:TCTGCAAG+GCTGCACT
CAGAAAGTGAGATGGAATCGAATTGTGCTTAACGGTTCAGTTAAGTTGACTAGCGAAGCCAATGTTCA

TAATATGAAGCAGTGGCTGTGGATGTGGCTCGTGTATGATAGACGTCCCGGTAATTCTAATCCCAGAG
TGAATGAGATATTCA
+
FFFFFFFFFFFFF:FFFFFFFFFFFFFFFFFFFFFFFFFFFFFFFFFFFFFFFF:FFFFFFFFFFF:FFF
FFFFFFFFFFFFFFFFFFFFFFFFFFFFFFFFFFFFFFFFFFFF:F:FFFFFFFFFFFFFFFFFFFFFFFF
FF,FFFFFFFFFFFF
@A00721:190:HGJW7DSXY:4:2673:29197:9502 1:N:0:TCTGCAAG+GCTGCACT
CAGAAAGTGAGATGGAATCGAATTGTGCTTAACGGTTCAGTTAAGTTGACTAGCGAAGCCAATGTTCA
TAATATGAAGCAGTGGCTGTGGATGTGGCTCGTGTATGATAGACGTCCCGGTAATTCTAATCCCAGAG
TGAATGAGATATTCA
+
FFFFFFFFFFFFFFFFFFFFFFFFFFFFFFFFFFFFFFFFFFFFFFFFFFFFFFFFFFFFFFFFFFFFFF
FFFFFFFFFFFFFFFFFFFFFFFFFFFFFFFFFFF:FFFFFFFFFFFFFFFFFFFFFFFFFFFFFFFFFF
FFFFFFFFFFFFFFF
@A00721:190:HGJW7DSXY:4:1612:21151:16078 1:N:0:TCTGCAAG+GCTGCACT
CGTGAGATGGAATCGAATTGTGCTTAACGGTTCAGTTAAGTTGACTAGCGAAGCCAATGTTCATAATA
TGAAGCAGTGGCTGTGGATGTGGCTCGTGTATGATAGACGTCCCGGTAATTCTAATCCCAGAGTGAAT
GAGATATTCATCGGG
+
FFFFFFFFFFF:FFFFFFFFFFFFFF:FFFFFFFFFFFFFFFFFFFFFFFFF,FFFFFFFFFFFFFFFF
FFFFF:FFFFFFFFFFFFFFFFFFFFFFFFFFFFFFFFFFFFFFFFFFFFFFFFFF:FFFFFFFFFF
FFFFFFFFFFFFFFF
@A00721:190:HGJW7DSXY:4:2119:16251:8531 1:N:0:TCTGCAAG+GCTGCACT
CGTGAGATGGAATCGAATTGTGCTTAACGGTTCAGTTAAGTTGACTAGCGAAGCCAATGTTCATAATA
TGAAGCAGTGGCTGTGGATGTGGCTCGTGTATGATAGACGTCCCGGTAATTCTAATCCCAGAGTGAAT
GAGATATTCATCGGG
+
FFFFFFFFFFFFFFFFFFFFFFFFFFFFFFFFFFF:FFFFFFFFFFFFFFFFFFFFFFFFFFFFFFFFF
FFFFF:FFFFFFFFFFFFFFFFFFFFFFFFFFFFFFFFFFFFFFFFFFFFFFFFFF:FFFFFFFFFF
FFFFFFFFFFFFFFF
@A00721:190:HGJW7DSXY:4:2654:30228:30326 1:N:0:TCTGCAAG+GCTGCACT
CGTGAGATGGAATCGAATTGTGCTTAACGTTTCAGTTAAGTTGACTAGAGAAGCCAATGTTCATAATA
TGAAGCAGTGGCTGTGGATGTGGCTCGTGTATGATAGACGTCCCGGTAATTCTAATCCCAGAGTGAAT
TAGATATTCATC
+
FFF,F:F:,:FFFFFFFFFF,::FFFFFF,FF:FFFFFF,,,,F,,F,,FFFFF:FFFF:,,F,FFFF
F:FFF:FF,F,:F:F,F:::FFF:FF:FFFFFFF,FFF,FFFFF::,FFF,,FFF:,F,F:FF,,FFF
,FFFFFF:FF,F
@A00721:190:HGJW7DSXY:4:1122:3830:26913 1:N:0:TCTGCAAG+GCTGCACT
CCCCGATGAATATCTCATTCACTCTGGGATTAGAATTACCGGGACGTCTATCATACACGAGCCACATC
CACAGCCACTGCTTCATATTATGAACATTGGCTTCGCTAGTCAACTTAACTGAACCGTTAAGCACAAT
TCGATTCCATCTCAC
+
FFFFFFFFF:F:FF:FFFFFFF:FFFFFFFFFFFFFFFF:::FFFFFFFFFFFFFFFFFFFF,FFFFF
FFFFFFF:F::FFFFF:FFF:FFFFFF,:F,FFFFFFFF:FFFFFFFFFFFFFFFFFFFFFFFFFFFF:
FF:FFFF:F:FFFFF
@A00721:190:HGJW7DSXY:4:1161:3215:19460 1:N:0:TCTGCAAG+GCTGCACT
CCCCGATGAATATCTCATTCACTCTGGGATTAGAATTACCGGGACGTCTATCATACACGAGCCACATC
CACAGCCACTGCTTCATATTATGAACATTGGCTTCGCTAGTCAACTTAACTGAACCGTTAAGCACAAT
TCGATTCCATCTCAC
+
FFFFFFFFFFFFFFFFFFFFFFFFFFFFFFFFFFFFFFFFFFFFFFFFFFFFFFFFFFFFFFFFFFFFFF
FFFFFFFFFFFFFFFFFFFFFFFFFFFFFFFFFFFFFFFFFFFFFFFFFFFFF,FFFFFFFFFFFFFF
FFFFFFFFFFFFFFF

```
@A00721:190:HGJW7DSXY:4:1204:3414:8970 1:N:0:TCTGCAAG+GCTGCACT
CCCCGATGAATATCTCATTCACTCTGGGATTAGAATTACCGGGACGTCTATCATACACGAGCCACATC
CACAGCCACTGCTTCATATTATGAACATTGGCTTCGCTAGTCAACTTAACTGAACCGTTAAGCACAAT
TCGATTCCATCTCAC
+
FFFFFFFFFFFFFFF:FFFFFF:FFFFFFFFFFFFFFFFFFFFFFFFFFFFFFFF:FFFFFFFFFFFFFF
FFFFFFFFFFFF:FFFFFFFFFFFF:FFFFFFFFFFFFFFFFFFFFF:FFFFFFFFFFFFFFFFFFFFF:
:FFFFF:FFF::FFF
@A00721:190:HGJW7DSXY:4:1223:27905:35853 1:N:0:TCTGCAAG+GCTGCACT
CCCCGATGAATATCTCATTCACTCTGGGATTAGAATTACCGGGACGTCTATCATACACGAGCCACATC
CACAGCCACTGCTTCATATTATGAACATTGGCTTCGCTAGTCAACTTAACTGAACCGTTAAGCACAAT
TCGATTCCATCTCAC
+
FFFFFFFFFFFFFFFFFFFFFFFFFFFFFFFFFFFFFFFFFFFFFFFFFFFFFFFFFFFFFFFFFFFFFF
FFFFFFFFFFFFFFFFFF:FFFFFFFFFFFFF:FFFFFFFFFFFFFFFFFFFFF::FFFFFFFFFFFFF:
FFFFFFFFFFF:FFF
@A00721:190:HGJW7DSXY:4:1662:13449:28197 1:N:0:TCTGCAAG+GCTGCACT
CCCCGATGAATATCTCATTCACTCTGGGATTAGAATTACCGGGACGTCTATCATACACGAGCCACATC
CACAGCCACTGCTTCATATTATGAACATTGGCTTCGCTAGTCAACTTAACTGAACCGTTAAGCACAAT
TCGATTCCATCTCAC
+
FFFFFFFFFFFFFFFFFFFFFFFFFFFFFFFFFFFFFFFFFFFFFFFFFFFFFFFFFFFFFFFFFFFFFF
FFFFFFFFFFFFFFFFFFFFFFFFFFFFFFFFFFFFFFFFFFFFFFFFFFFFFFFFFFFFFFFFFFFFFF
FFFFFFFFFFFFFFF
@A00721:190:HGJW7DSXY:4:1665:29152:14747 1:N:0:TCTGCAAG+GCTGCACT
CCCCGATGAATATCTCATTCACTCTGGGATTAGAATTACCGGGACGTCTATCATACACGAGCCACATC
CACAGCCACTGCTTCATATTATGAACATTGGCTTCGCTAGTCAACTTAACTGAACCGTTAAGCACAAT
TCGATTCCATCTCAC
+
FFFFFFFFFFFFFFFFFFFFFFFFFFFFFFFFFFFFFFFFFFFFFFFFFFFFFFFFFFFFFFFFFFFFFF
FFFFFFFFFFFFFFFFFFFFFFFFFFFFFFFFFFFFFFFFFFFFFFFFFFFFFFFFFFFFFFFFFFFFFF
FFFFFFFFFFFFFFF
@A00721:190:HGJW7DSXY:4:2178:17635:23328 1:N:0:TCTGCAAG+GCTGCACT
GTGAGATGGAATCGAATTGTGCTTAACGGTTCAGTTAAGTTGACTAGCGAAGCCAATGTTCATAATAT
GAAGCAGTGGCTGTGGATGTGGCTCGTGTATGATAGACGTCCCGGTAATTCTAATCCCAGAGTGAATG
AGATATTCATCGGGG
+
FFFFFFFFFFFFFFFFFFFFFFFFFFFFFFFFFFFFFFFFFFFFFFFFFFFFFFFFFFFFFFFFFFFFFF
FFFFFFFFFFFFFFFFFFFFFFFFFFFFFFFFFFFFFFFFFFFFFFFFFFFFFFFFFFFFFFFFFFFF::
FFFFFFFFFFFFFFF
@A00721:190:HGJW7DSXY:4:2434:1316:4147 1:N:0:TCTGCAAG+GCTGCACT
GTGAGATGGAATCGAATTGTGCTTAACGGTTCAGTTAAGTTGACTAGCGAAGCCAATGTTCATAATAT
GAAGCAGTGGCTGTGGATGTGGCTCGTGTATGATAGACGTCCCGGTAATTCTAATCCCAGAGTGAATG
AGATATTCATCGGGG
+
F:FFFFFFFFFFFFFFFFFFFFFF:FFFFF:FFFFFFFF:F:FFFFFFFFFFFF,FFFFFFFFFFFFFF:
:FFFFFFFFFFFFFFFFFFFFFFFFFFFFFFFF:FFFFF:FFF,FFFF,:FFFFFFFFFFFFFF,FFFFF
FFFFFFF:FFFFF:F
@A00721:190:HGJW7DSXY:4:2509:11306:9173 1:N:0:TCTGCAAG+GCTGCACT
GTGAGATGGAATCGAATTGTGCTTAACGGTTCAGTTAAGTTGACTAGCGAAGCCAATGTTCATAATAT
GAAGCAGTGGCTGTGGATGTGGCTCGTGTATGATAGACGTCCCGGTAATTCTAATCCCAGAGTGAATG
AGATATTCATCGGGG
+
FFFFFFFFFFFFFFFFFFFFFFFFFFFFFF:FFFFFFFFFFFFFFFFFFFFFFFFFFFFFFFFFFFFFFF
```

FFFF:FFFFFFFFFFFFFFFFFFFFFFFFFFFFFFFFFFFFFFF,FFFFFFFFFFFFFFFFFFFFF
FFFFFFFFFFF:FFF
@A00721:190:HGJW7DSXY:4:2528:15420:18333 1:N:0:TCTGCAAG+GCTGCACT
GTGAGATGGAATCGAATTGTGCTTAACGGTTCAGTTAAGTTGACTAGCGAAGCCAATGTTCATAATAT
GAAGCAGTGGCTGTGGATGTGGCTCGTGTATGATAGACGTCCCGGTAATTCTAATCCCAGAGTGAATG
AGATATTCATCGGGG
+
FFFFFFFFFFFFFFFFFFFFFFFFFFFFFFFFFFFFFFFFFFFFFFFFFFFFFFFFFFFFFFFFFFFF
FFFF:FFFFFFFFFFFFFFFFFFFFFFFFFFFFFFFFFFFFFFFFFFFFFFFFFFFFFFFFFFFFFFF
FFFFFFFFFFFF:FF
@A00721:190:HGJW7DSXY:4:2610:15971:16376 1:N:0:TCTGCAAG+GCTGCACT
GTGAGATGGAATCGAATTGTGCTTAACGGTTCAGTTAAGTTGACTAGCGAAGCCAATGTTCATAATAT
GAAGCAGTGGCTGTGGATGTGGCTCGTGTATGATAGACGTCCCGGTAATTCTAATCCCAGAGTGAATG
AGATATTCATCGGGG
+
FFFFFFFFFFFFFFFFFFFFFFFFFFFFFFFFFFFFFFFFFFFFFFFFFFFFF:FFFFFFFFFFFFFF
FFFF:FFFFFFFFFFFFFFFF:FFFFFFF:FFFFFFFFFFFFFFFFFFFFFFFFFFFFFFFFFFFFFFF
FFFFFFFFFFFFFFF
@A00721:190:HGJW7DSXY:4:2627:14045:29606 1:N:0:TCTGCAAG+GCTGCACT
CCCCGATGAATATCTCATTCACTCTGGGATTAGAATTACCGGGACGTCTATCATACACGAGCCACATC
CACAGCCACTGCTTCATATTATGAACATTGGCTTCGCTAGTCAACTTAACTGAACCGTTAAGCACAAT
TCGATTCCATCTCAC
+
FFFFFFFFFFFFFFFFFFFFFFFFFFFFFFFFFFFFFFFFFFFFFFFFFFFFFFFFFFFFFFFFFFFF
FFFFFFFFFFFFF:FFFFFFFFFFFFFFFFFFFFFF:FFFFFFFFFFFFFFFFFFFFFFFFFFFFFFFF
FFFFFFFFFFFFFFF
@A00721:190:HGJW7DSXY:4:2675:28980:35493 1:N:0:TCTGCAAG+GCTGCACT
CCCCGATGAATATCTCATTCACTCTGGGATTAGAATTACCGGTACTTCTATCATACACGAGCCACATC
CACAGCCACTGCTTCATATTATGAACATTGGCTTCGCTAGTCAACTTAACAGAACCGTTAAGAACAAA
TCGATTCCATCTCAC
+
FFFF:FFFF:FF,:,FF:,FF::,,F::FFFFF::F:FFFF,,F:,F,,FFFF:FFFF,FF:FFFF,:
,FFFF,F:,F:F,FFFFFFFFF:FFFF,,,F,FF:FFFFFFFFF:F,FFF,:FF:F::FFFF,FFF:,
FFFFFF,FFFFFFFF
@A00721:190:HGJW7DSXY:4:2678:25265:34037 1:N:0:TCTGCAAG+GCTGCACT
CCCCGATGAATATCTCATTCACTCTGGGATTAGAATTACCGGGACGTCTATCATACACGAGCCACATC
CACAGCCACTGCTTCATATTATGAACATTGGCTTCGCTAGTCAACTTAACTGAACCGTTAAGCACAAT
TCGATTCCATCTCAC
+
FFFFFFFFF:FFFFFFFFFFFFFFFFFFFFFFFFFFFFFFFFFFFFFFFFFFFFFFFFFFFFFFFFFF
FFFFFFFFFFFFFFFFFFFFFFFFFFFFFFFFFFFFFFFFFFFFFFFFFFFFFFFFFFFFFFFFFFFF
FFFFFFFFFFFFFFF
@A00721:190:HGJW7DSXY:4:1270:29432:33708 1:N:0:TCTGCAAG+GCTGCACT
GAGGTGGAATCGAATTGTGCTTAACGGTTCAGTTAAGTTGACTAGCGAAGCCAATGTTCATAATATGA
AGCAGTGGCTGTGGATGTGGCTCGTGTATGATAGACGTCCCGGTAATTCTAATCCCAGAGTGAATGAG
ATATTCATCGGGGAA
+
FFFFFFFFFFFFFFFFFFFFFFFFFFFF:FFFFFFFFFFFFFFFFFFFFFFFFFFFFFFFFFFFFFFF
FFFFFFFFFFFFFFFFFFFFFFFFFFFFFFFFFFFFFFFFFFFFFFFFFFFFFFFFFFFFFFFFFFFF
FFFFFFFFFFFFFF:
@A00721:190:HGJW7DSXY:4:1450:20808:8437 1:N:0:TCTGCAAG+GCTGCACT
GAGGTGGAATCGAATTGTGCTTAACGGTTCAGTTAAGTTGACTAGCGAAGCCAATGTTCATAATATGA
AGCAGTGGCTGTGGATGTGGCTCGTGTATGATAGACGTCCCGGTAATTCTAATCCCAGAGTGAATGAG
ATATTCATCGGGGAA

```
+
FFFFFFFFFFFFFFFFFFFFFFFFFFFFFFFFFFFFFFFFFFFFFFFFFFFFFFFFFFFFFFFFFFFF
FFFFFFFFFFFFFFFFFFFFFFFFFFFFFFFFFFFFFFFFFFFFFFFFFFFFFFFFFFFFFFFFFFFF
FFFFFFFFFFFFFFF
@A00721:190:HGJW7DSXY:4:1566:12635:7874 1:N:0:TCTGCAAG+GCTGCACT
GAGGTGGAATCGAATTGTGCTTAACGGTTCAGTTAAGTTGACTAGCGAAGCCAATGTTCATAATATGA
AGCAGTGGCTGTGGATGTGGCTCGTGTATGATAGACGTCCCGGTAATTCTAATCCCAGAGTGAATGAG
ATATTCATCGGGGAA
+
FFFFFFFFFFFFFFFFFFFFFFFFFFF:FFFFFFFFFFFFFFFFFFFFFFFFFFFFFFFFF,FFFFFFF
FFFFFFFFFFFFFFFFFFFFFFFF:FFFFFFFFFF:FFFFFFFFFFF:FFFFFFFFFFFFFFFF:FFFF
FFF:FFFFFFFFFF:
@A00721:190:HGJW7DSXY:4:1675:31304:34914 1:N:0:TCTGCAAG+GCTGCACT
GAGGTGGAATCGAATTGTGCTTAACGGTTCAGTTAAGTTGACTAGCGAAGCCAATGTTCATAATATGA
AGCAGTGGCTGTGGATGTGGCTCGTGTATGATAGACGTCCCGGTAATTCTAATCCCAGAGTGAATGAG
ATATTCATCGGGGAA
+
F:FFFFFFFFFFFFFFFFFFFFFFF,FFFFFFFFFFFFFFFFFF,FFFFFFFFFFFFFFFFFFFFFFFF
F,FFFFFFFFFFFFFFFFFFFFFFFFFFFFFFFFFFFFFFFFFFFFFFFFF:FFFFFFFFFFFFFFFF
FFFFFFFFFFFFFFF
@A00721:190:HGJW7DSXY:4:2138:30553:24815 1:N:0:TCTGCAAG+GCTGCACT
GAGGTGGAATCGAATTGTGCTTAACGGTTCAGTTAAGTTGACTAGCGAAGCCAATGTTCATAATATGA
AGCAGTGGCTGTGGATGTGGCTCGTGTATGATAGACGTCCCGGTAATTCTAATCCCAGAGTGAATGAG
ATATTCATCGGGGAA
+
FFFF:FFFFFFFFFFFFFFFFFFFFFFFFFFFFFFFFFFFFFFFFFFFFFFFFFFFFFFFFFFFFF:FF
FF:FFFFFFFFFFFFFFFFFFFFFFFF:FFFFFFFFFFFFFFFFFFFFFFFFFFFFFFFFFFFFF:FFFF
FFFFFFFFF,FFFF:
@A00721:190:HGJW7DSXY:4:2314:20003:20572 1:N:0:TCTGCAAG+GCTGCACT
GAGGTGGAATCGAATTGTGCTTAACGGTTCAGTTAAGTTGACTAGCGAAGCCAATGTTCATAATATGA
AGCAGTGGCTGTGGATGTGGCTCGTGTATGATAGACGTCCCGGTAATTCTAATCCCAGAGTGAATGAG
ATATTCATCGGGGAA
+
FFFFFFFFFFFFFF:FFFFFFFFFFFFFFFFFFFFFFFFFFFFFFFFFFFFFFFFFFFFFFFFFFFFF
FFFFFFFFFFFFFFFFFFFFFFFFFFFFFFFFFFFFFFFFFFFFFFFFFFFFFFFFFFFFFFFFFFFF
FFFFFFFFFFFFFFF
@A00721:190:HGJW7DSXY:4:2666:12626:35258 1:N:0:TCTGCAAG+GCTGCACT
GAGGTGGAATCGAATTGTGCTTAACGGTTCAGTTAAGTTGACTAGCGAAGCCAATGTTCATAATATGA
AGCAGTGGCTGTGGATGTGGCTCGTGTATGATAGACGTCCCGGTAATTCTAATCCCAGAGTGAATGAG
ATATTCATCGGGGAA
+
FFFFFFFFF:FFFFFFFFFFFFFFFFFFF:FFFFFFFFFFFFFFFFFFFFFFFFFFFFFFFFFFFFFFF
FFFFFFFFFFFFFFFFFFFFFFFFFFFFFFFFFFFFFFFFFFFFFFFFFFFFFFFFFFFFFFFFFFFF
FFFFFFF:FFFFFFF
@A00721:190:HGJW7DSXY:4:1174:8422:16517 1:N:0:TCTGCAAG+GCTGCACT
ATGGAATCGAATTGTGCTTAACGGTTCAGTTAAGTTGACTAGCGAAGCCAATGTTCATAATATGAAGC
AGTGGCTGTGGATGTGGCTCGTGTATGATAGACGTCCCGGTAATTCTAATCCCAGAGTGAATGAGATA
TTCATCGGGGAAAGT
+
FFFFFFFFFFFFFFFFFFFFFFFFF:FFFFFFFFFFFFFFFFFFFFFFFFFFFFFFFFFFFFFFFFFF
FFFFFFFFFFFFFFFFFFFFFFFFFFFFFFFFFFFFFFFFFFFFFFFFFFFFFFFFFFFFFFFFFFFF
FFFFFFFFFFFFFFF
@A00721:190:HGJW7DSXY:4:1220:18927:34585 1:N:0:TCTGCAAG+GCTGCACT
ATGGAATCGAATTGTGCTTAACGGTTCAGTTAAGTTGACTAGCGAAGCCAATGTTCATAATATGAAGC
```

```
AGTGGCTGTGGATGTGGATCGTGTATGATAGACGTCCAGGTAATTCTAATACCAGAGTGAATGAGATA
TTCATCGGGGAAAGT
+
FF,:FFF,FFF:,::FFFFFFFF:::FFFFFFF::F,FFFFFF:FF,F:FFF,F:FFFFFFFFFFF,,
FF:,,FFF:,FFFF:,F,F:,FFFFF,:FFFFFFF:,,::FFF,FFFFFF,,FF,FF:FFFFFFFFFF
FFFFF:FFFFFFFFF
@A00721:190:HGJW7DSXY:4:1222:9390:22263 1:N:0:TCTGCAAG+GCTGCACT
ATGGAATCGAATTGTGCTTAACGGTTCAGTTAAGTTGACTAGCGAAGCCAATGTTCATAATATGAAGC
AGTGGCTGTGGATGTGGCTCGTGTATGATAGACGTCCCGGTAATTCTAATCCCAGAGTGAATGAGATA
TTCATCGGGGAAAGT
+
F:F,FFFFFFFFF:FFFFFFFFF:FFFFFFFFFFFFFFFFF:FFFFFFFFFFF:FFFFFFFFF,FFFF
FFFFFFFFFFF:FFFF:FFFFFFFFFFFFFFFFFFF:FFF:FFFFFFFFFFFFFFFFF,FFFFFFF:F
FFFF::FFF:FFFFF
@A00721:190:HGJW7DSXY:4:1225:19786:20572 1:N:0:TCTGCAAG+GCTGCACT
ATGGAATCGAATTGTGCTTAACGGTTCAGTTAAGTTGACTAGCGAAGCCAATGTTCATAATATGAAGC
AGTGGCTGTGGATGTGGCTCGTGTATGATAGACGTCCCGGTAATTCTAATCCCAGAGTGAATGAGATA
TTCATCGGGGAAAG
+
FFFFFFFFFFFFFFF,F:FFF:FFFFFFFFFFFFFFFFFFFFFFFFFFFFFFFFFFFFFFFFFFFFF:
FFFFFFFFFFFFFFFFFFFF:FFFFFFFFFFFF:FFFFFFFFFFFFFFFFFFFFFFFFFF:FFFFFFF
FFFFFFFFFFFFFF
@A00721:190:HGJW7DSXY:4:1234:23981:32471 1:N:0:TCTGCAAG+GCTGCACT
ATGGAATCGAATTGTGCTTAACGGTTCAGTTAAGTTGACTAGCGAAGCCAATGTTCATAATATGAAGC
AGTGGCTGTGGATGTGGCTCGTGTATGATAGACGTCCCGGTAATTCTAATCCCAGAGTGAATGAGATA
TTCATCGGGGAAAGT
+
FFFFF:FFFFFFFFFFFFFFFFFFFFFFFFFFFFFFFFFFFFFFFFFFFFFFFFFFFFFFFFFFFFF:
FFFFFFFF:FFFFFFFFFFFFFFFFFFFFFFFFFFFFFFFFFFFFFFFFFFFFFFFFFF:FFFFFFF
FFFFFFFFFFFFFFF
@A00721:190:HGJW7DSXY:4:1268:11162:20666 1:N:0:TCTGCAAG+GCTGCACT
ATGGAATCGAATTGTGCTTAACGGTTCAGTTAAGTTGACTAGCGAAGCCAATGTTCATAATATGAAGC
AGTGGCTGTGGATGTGGCTCGTGTATGATAGACGTCCCGGTAATTCTAATCCCAGAGTGAATGAGATA
TTCATCGGGGAAAGT
+
FFFFF:FFFFFFFFFFFFFFFFFFFFFFFFFFFFFFFFFFFFFFFFFFFFFFFFFFF,FFFFFFFFFF
FFFFFFFFFFFFFFFFFFFFFFFFFFFFFFFFFFFFFFFFFFFFFFFFFFFFFFF:FFFFFFFFFFFF
FFFFFFFFFFFFFFF
@A00721:190:HGJW7DSXY:4:1345:2157:5071 1:N:0:TCTGCAAG+GCTGCACT
ATGGAATCGAATTGTGCTTAACGGTTCAGTTAAGTTGACTAGCGAAGCCAATGTTCATAATATGAAGC
AGTGGCTGTGGATGTGGCTCGTGTATGATAGACGTCCCGGTAATTCTAATCCCAGAGTGAATGAGATA
TTCATCGGGGAAAGT
+
FFFFF:FFFFFFFFFFFFFFFFFF:FFFFFFFFFFFFFFFFFFFFFFFFFFFFFFFF,FFFFFFFFF:
FFFFFFFFFFFFFFFFFFFFFFFFFFFFFFFFFFFFFFFFFF:FFFF:FFFFFFFFFFFFFFFFFFFF
FFFFFFFFFF::FFF
@A00721:190:HGJW7DSXY:4:1363:16984:21480 1:N:0:TCTGCAAG+GCTGCACT
ATGGAATCGAATTGTGCTTAACGGTTCAGTTAAGTTGACTAGCGAAGCCAATGAACATAATATGAAGC
AGTGGCTGTGGATGTGGCTCGTGTATTATAGACGTCACGGTAATTCTAATCCCAGAGTGAATGAGATA
TTCATCTGGGAAAGT
+
FF::F,,,FFFFF,FF:FFFF,F,::FF,FFFF:FF:F:FF,,,FF,FFFFF,,,FFFFFF,:,FF:F
FFF,F::,,F,FFF:FFF,,F,F:FF,F,FFF,F,F,F,,FFFF,,FFF,F:FFFF,,:FF,FFFFFF
:,,F,F,:::FFF:F
```

@A00721:190:HGJW7DSXY:4:1501:29722:19711 1:N:0:TCTGCAAG+GCTGCACT
ATGGAATCGAATTGTGCTTAACGGTTCAGTTAAGTTGACTAGCGAAGCCAATGTTCATAATATGAAGC
AGTGGCTGTGGATGTGGCTCGTGTATGATAGACGTCCCGGTAATTCTAATCCCAGAGTGAATGAGATA
TTCATCGGGGAAAGT
+
FFFFFFFFFFFFFFFFFFFFFFFFFFFFFFFFFFFFFFFFFFFFFFFFFFFFFFFFFFFFFFFFFFFF
FFFFFFFFFFFFFFFFFFFFFFFFFFFFFFFFFFFFFFFFFFFFFFFFFFF:FFFFFFFFFFFFFFFF
FFFFFF:FFFFFFFF
@A00721:190:HGJW7DSXY:4:1564:26341:4554 1:N:0:TCTGCAAG+GCTGCACT
ATGGAATCGAATTGTGCTTAACGGTTCAGTTAAGTTGACTAGCGAAGCCAATGTTCATAATATGAAGC
AGTGGCTGTGGATGTGGCTCGTGTATGATAGACGTCCCGGTAATTCTAATCCCAGAGTGAATGAGATA
TTCATCGGGGAAAGT
+
FFFFFFFFFFFFFFFFFFFFFFFFFFFFFFFFFFFFFFFFFFFFFFFFFFFFFFFFFFFFFFFFFFFF
FFFFFFFFFFFFFFFFFFFFFFFFFFFFFFFFFF:FFFFFFFFFFFFFFFFFFFFFFFFFFFFFFFFF
FF:FFFFFFFFFFFF
@A00721:190:HGJW7DSXY:4:1621:12057:13730 1:N:0:TCTGCAAG+GCTGCACT
ATGGAATCGAATTGTGCTTAACGGTTCAGTTAAGTTGACTAGCGAAGCCAATGTTCATAATATGAAGC
AGTGGCTGTGGATGTGGCTCGTGTATGATAGACGTCCCGGTAATTCTAATCCCAGAGTGAATGAGATA
TTCATCGGGGAAAGT
+
FFFFFFFFFFFFFFFFFFFFFFFFFFFFFFFFFFFFFFFFFFFFFFFFFFFFFFFFFFFFFFFFFFF:
FFFFFFFFFFFFFFFFFFFFFFFFFFFFFFFFFFFFFFFFFFFFFFFFFFFFFFFFFFFFFFFFFFFF
FFFFFFFFFFFFFFF
@A00721:190:HGJW7DSXY:4:1626:5981:6590 1:N:0:TCTGCAAG+GCTGCACT
ATGGAATCGAATTGTGCTTAACGGTTCAGTTAAGTTGACTAGCGAAGCCAATGTTCATAATATGAAGC
AGTGGCTGTGGATGTGGCTCGTGTATGATAGACGTCCCGGTAATTCTAATCCCAGAGTGAATGAGATA
TTCATCGGGGAAAGT
+
F:FFF:FFFFFF:FFFFFFFFFFF:FFFFFFFFF:FFFFFFFFFF:FFFFF:FFFF::FF,FF:FFFF
FFFFFFFFFFFFFFFFFFFFF:FFFFFFFF:FFFFFFFFFFF:FFFFFFFFFFFFFFFFFFFFFFFFF
FFFFFFFFFFF:FF:
@A00721:190:HGJW7DSXY:4:2128:24876:15358 1:N:0:TCTGCAAG+GCTGCACT
ATGGAATCGAATTGTGCTTAACGGTTCAGTTAAGTTGACTAGCGAAGCCAATGTTCATAATATGAAGC
AGTGGCTGTGGATGTGGCTCGTGTATGATAGACGTCCCGGTAATTCTAATCCCAGAGTGAATGAGATA
TTCATCGGGGAAAGT
+
FFFFFFFFFFFFFFFFFFFFFFFFFFFFFFFFFFFFFFFFFFFFFFFFFFFFFFFFFFFFFFFFFFFF
FFFFF:FFFFFFFFFFFFFFFFFFFFFFFFFFFFFFFFFFFFFFFFFFFFFFFFFFFFFFFFFFFFFF
FFFFFFFFFFFFF:F
@A00721:190:HGJW7DSXY:4:2213:18349:22811 1:N:0:TCTGCAAG+GCTGCACT
ATGGAATCGAATTGTGCTTAACGGTTCAGTTAAGTTGACTAGCGAAGCCAATGTTCATAATATGAAGC
AGTGGCTGTGGATGTGGCTCGTGTATGATAGACGTCCCGGTAATTCTAATCCCAGAGTGAATGAGATA
TTCATCGGGGAAAGT
+
FFFFF:FFFFFFFFFFFFFFFFFFFFFFFFFFFFFFFFFFFFFFFFFFFFFFFFFFFFFFFFFFFFF:
FFFFFFFFFFFFFFFF:FFFFFFFFFFFFFFFFFFFFFFFFFFFFFFFFFFFFFFFFFFFFFFFFFFF
FFFFFFFFFFFFFFF
@A00721:190:HGJW7DSXY:4:2213:7708:26334 1:N:0:TCTGCAAG+GCTGCACT
ATGGAATCGAATTGTGCTTAACGGTTCAGTTAAGTTGACTAGCGAAGCCAATGTTCATAATATGAAGC
AGTGGCTGTGGATGTGGCTCGTGTATGATAGACGTCCCGGTAATTCTAATCCCAGAGTGAATGAGATA
TTCATCGGGGAAAGT
+
F:FFFFFFFFFFFFFFFFFFFFFFFFFFFFFFFFFFFFFFFFFFFFFFFFFFFFFFF:FFFFFFFFF:

```
FF:FFF:FFFFFFFFFFFFFFFFFFFFFFFFFFFFFFFFFFFFFFFFF:FFFFFFFFFFFFFFFFFFFFF
FFFFFFFFFFFFFFF
@A00721:190:HGJW7DSXY:4:2227:12608:4852 1:N:0:TCTGCAAG+GCTGCACT
ATGGAATCGAATTGTGCTTAACGGTTCAGTTAAGTTGACTAGCGAAGCCAATGTTCATAATATGAAGC
AGTGGCTGTGGATGTGGCTCGTGTATGATAGACGTCCCGGTAATTCTAATCCCAGAGTGAATGAGATA
TTCATCGGGGAAAGT
+
FFFFFFFFFFFFFFFFFFFFFFFFFFFFFFFFFFFFFFFFFFFFFFFFFFFFFFFFFF:FFFFFFFFFF
FFFFFFFFFFF:FFFFFFFFFFFFFFFFFFFFFFFFFFFFFFFFFFFFFFFF:FFFFFFF,FFFFFFFF
FFFFFFFFFFFFFFF
@A00721:190:HGJW7DSXY:4:2271:4182:29465 1:N:0:TCTGCAAG+GCTGCACT
ATGGAATCGAATTGTGCTTAACGGTTCAGTTAAGTTGACTAGCGAAGCCAATGTTCATAATATGAAGC
AGTGGCTGTGGATGTGGCTCGTGTATGATAGACGTCCCGGTAATTCTAATCCCAGAGTGAATGAGATA
TTCATCGGGGAAAGT
+
FFFFF:FFFFFFFFFFFFFFFFFFFFFFFFFFFFFFFFFFFFFFFFFFFFFFFFFFFFFFFFFFFFFFFF
FFFFFFFFFFFFFFFFFFFFFFFFFFFFFFFFFFFFFFFFFFFFFFFFFFFFFFFFFFFFFFFFFFFFFF
FFFFFFFFFFF:FF:
@A00721:190:HGJW7DSXY:4:2301:5873:25911 1:N:0:TCTGCAAG+GCTGCACT
ATGGAATCGAATTGTGCTTAACGGTTCAGTTAAGTTGACTAGCGAAGCCAATGTTCATAATATGAAGC
AGTGGCTGTGGATGTGGCTCGTGTATGATAGACGTCCCGGTAATTCTAATCCCAGAGTGAATGAGATA
TTCATCGGGGAAAGT
+
FFFFFFFFFFFFFFFFFFFFFFFFFFF:FFFFFFFFFFFFFFFFFFFFFFFFFF:FFFFFFFFFFFFFFF
FFFFFF:FFFFFFFFFFFFFFFFFFFFFFFFFFFFFFFFFFFFFFFFFFFFFFFFFFFFFFFFFFFFFFF
FFFFFFFFFFFFFFF
@A00721:190:HGJW7DSXY:4:2348:9010:35446 1:N:0:TCTGCAAG+GCTGCACT
ATGGAATCGAATTGTGCTTAACGGTTCAGTTAAGTTGACTAGCGAAGCCAATGTTCATAATATGAAGC
AGTGGCTGTGGATGTGGCTCGTGTATGATAGACGTCCCGGTAATTCTAATCCCAGAGTGAATGAGATA
TTCATCGGGGAAAGT
+
FFFFFFFFFFFFFFFFFFFFFFFFFFFFFFFFFFFFFFFFFFFFFFFFFFFFFFFFFFF:FFFFFFFFFF
FFFFFFFFFFFFFFFFFFFFFFFFFFFFFFF:FFFFFFFFFFFFFFFFFFFFFFFFFFFFFFFFFFFFFF
FFFFFFFFFFF:FFF
@A00721:190:HGJW7DSXY:4:2527:4553:5149 1:N:0:TCTGCAAG+GCTGCACT
ATGGAATCGAATTGTGCTTAACGGTTCAGTTAAGTTGACTAGCGAAGCCAATGTTCATAATATGAAGC
AGTGGCTGTGGATGTGGCTCGTGTATGATAGACGTCCCGGTAATTCTAATCCCAGAGTGAATGAGATA
TTCATCGGGGGAAAG
+
FFFFFFFFFFFFFFFFFFFFFFFFFF:FFFFFFFFFFFFFFFFFFFFFFFFFFFFFFFF,FFFFFFFFF:
FFFFFFFFFFFFFFFFFFFFFF:FFFFFFFFFFFFFFFFFFFFFFFFFFFFFFFFFFFFFFFFFFFFFFF
FFFFFF:FFFFFFFF
@A00721:190:HGJW7DSXY:4:2533:1154:28040 1:N:0:TCTGCAAG+GCTGCACT
ATGGAATCGAATTGTGCTTAACGGTTCAGTTAAGTTGACTAGCGAAGCCAATGTTCATAATATGAAGC
AGTGGCTGTGGATGTGGCTCGTGTATGATAGACGTCCCGGTAATTCTAATCCCAGAGTGAATGAGATA
TTCATCGGGGAAAGT
+
FF:FFFF:FFFFFF,FF:FFFFF,:F:FFFFFFFFFFFFFFF,F:FF,F,F::,F,FF:FF,:FFFFFF
FFF:FFFFFF:FFFF:FFFFFFFFFFF,FFFFFFFF,FFFFFFFFFFFFFF::FFF:F,F:FFFFFFF:F
FFFF:FF:,FFFFFF
@A00721:190:HGJW7DSXY:4:2543:12753:33254 1:N:0:TCTGCAAG+GCTGCACT
ATGGAATCGAATTGTGCTTAACGGTTCAGTTAAGTTGACTAGCGAAGCCAATGTTCATAATATGAAGC
AGTGGCTGTGGATGTGGCTCGTGTATGATAGACGTCCCGGTAATTCTAATCCCAGAGTGAATGAGATA
TTCATCGGGGAAAGT
```

+
FFFFF:FFFFFFFFFFFFFFFFFFFFFFFFFFFFFFFFFFFFFFFFFFFFFFFFFFFFFFFFFFFFFFF
FFFFFFFFFFFFFFFFFFFFFFFFFFFFF:FFFFFFFFFFFFFFFFFFFF:FFFFFFFFFFFF:FFFFF
FFFFFFFFFFFFFFF
@A00721:190:HGJW7DSXY:4:2655:25482:12399 1:N:0:TCTGCAAG+GCTGCACT
ATGGAATCGAATTGTGCTTAACGGTTCAGTTAAGTTGACTAGCGAAGCCAATGTTCATAATATGAAGC
AGTGGCTGTGGATGTGGCTCGTGTATGATAGACGTCCCGGTAATTCTAATCCCAGAGTGAATGAGATA
TTCATCGGGGAAAGT
+
FFFFF:FFFFFFFFFFFFFFFFFFFFFFFFFFFFFFFFFFFFFFFFFFFFFFFFFFF:FFFFFFF:F
FFFFFFFFFFFFFFFFFFFFFFFFFFFFFFFFFFFFFFFFFFFFFFFFFFFFFFFFFFF:FFFFFFF
FFFFFFFFFFF:FFF
@A00721:190:HGJW7DSXY:4:1150:22589:32315 1:N:0:TCTGCAAG+GCTGCACT
CAATCGAATTGTGCTTAACGGTTCAGTTAAGTTGACTAGCGAAGCCAATGTTCATAATATGAAGCAGT
GGCTGTGGATGTGGCTCGTGTATGATAGACGTCCCGGTAATTCTAATCCCAGAGTGAATGAGATATTC
ATCGGGGAAAGTCGG
+
FFFFFFFFFFFFFFFFFFF:FFFFFF:FFFFFFFFFFFFFFFFFFFFFFFFFFFFFFFFFFFFFFFFFF
F:FFFFFFFFFFFFFFF:FFFFFFFFFFFFFFFFFFFFFFFFFFFFFF:FFFFFFFFFFFFFFFFFFFF
FFFFFFFF:FFFFFF
@A00721:190:HGJW7DSXY:4:1609:24587:30921 1:N:0:TCTGCAAG+GCTGCACT
CAATCGAATTGTGCTTAACGGTTCAGTTAAGTTGACTAGCGAAGCCAATGTTCATAATATGAAGCAGT
GGCTGTGGATGTGGCTCGTGTATGATAGACGTCCCGGTAATTCTAATCCCAGAGTGAATGAGATATTC
ATCGGGGAAAGTCGG
+
FFFFFFFFFFFFFFFFFFFFFFFFFFFFFFFFFFFFFFFFFFFFFFFFFFFFFFF,F:FFFFFFFFFFF
FFFFFFFFFFFFFFFFFFFFFFFFFFFFFFFFFF:FFFFFFFFFFFFFFFFFFFFFFFFFFFFFFFFFF
FFFFFFFFFFFFFFF
@A00721:190:HGJW7DSXY:4:2102:28772:18129 1:N:0:TCTGCAAG+GCTGCACT
CAATCGAATTGTGCTTAACGGTTCAGTTAAGTTGACTAGCGAAGCCAATGTTCATAATATGAAGCAGT
GGCTGTGGATGTGGCTCGTGTATGATAGACGTCCCGGTAATTCTAATCCCAGAGTGAATGAGATATTC
ATCGGGGAAAGTCGG
+
FFFFFFFFFFFFFFFFFFFFFFFFFFFFFFFFFFFFF:FFFFF:FFFFFFFFF:FFFFFFFFFFFFFF
FFFFFFFFFFFFFFFFFFFFFFFFFFFFFFFFFFFFFFFFF:FFFFF:FFFFFFFFFFFFFFFFFFFFF
:FF,FFFFF,FFFFF
@A00721:190:HGJW7DSXY:4:2115:25093:25817 1:N:0:TCTGCAAG+GCTGCACT
CAATCGAATTGTGCTTAACGGTTCAGTTAAGTTGACTAGCGAAGCCAATGTTCATAATATGAAGCAGT
GGCTGTGGATGTGGCTCGTGTATGATAGACGTCCCGGTAATTCTAATCCCAGAGTGAATGAGATATTC
ATCGGGGAAAGTCGG
+
FFFFFFFFFFFFFFFFFFFFFFFFFFFFFFFFFFFFFFFFFFFFFFFFFFFFFF:FFFFFFFFFFFFFF
FFF:FFFFFFFFFFFFFFFFFFFFFFFFFFFFFFFFFFFFFFFFFFFFFFFFFFFFF,FFFFFFFFFF
FFFFFFFFFFFFFFF
@A00721:190:HGJW7DSXY:4:2133:23240:18035 1:N:0:TCTGCAAG+GCTGCACT
CAATCGAATTGTGCTTAACGGTTCAGTTAAGTTGACTAGCGAAGCCAATGTTCATAATATGAAGCAGT
GGCTGTGGATGTGGCTCGTGTATGATAGACGTCCCGGTAATTCTAATCCCAGAGTGAATGAGATATTC
ATCGGGGAAAGTCGG
+
FFFFFFFFFFFFFFFFFFFFFFFFFFFFFFFFFFFFFFFFFFFFFFFFFFFFFFF:F,FFFFFFF:FFF
FFFFFFFFFFFFFFFFFFFFFFFFFFFFFFFFFFFFFFFFFFFFFFFFFFFFFFFFF,FFFFFFFFFF
FFFFFFFFFFFFFFF
@A00721:190:HGJW7DSXY:4:2512:30572:36839 1:N:0:TCTGCAAG+GCTGCACT
CAATCGAATTGTGCTTAACGGTTCAGTTAAGTTGACTAGCGAAGCCAATGTTCATAATATGAAGCAGT

GGCTGTGGATGTGGCTCGTGTATGATAGACGTCCCGGTAATTCTAATCCCAGAGTGAATGAGATATTC
ATCGGGGAAAGTCGG
+
FFFFFFFFFFFFFFFFFFFFFFFFFFFFFFFFFFFFFFFFFFFFFFFFFFFFFFFFFFFFFFFFFFFF
FFFFFFFFFFFFFFFFFFFFFFFFFFFFFFFFFFFFFFFFFFFFFFFFFFFFFFFFFFFFFFFFFFFF
FFFFFFFFFFFFFFF
@A00721:190:HGJW7DSXY:4:1250:30563:5415 1:N:0:TCTGCAAG+GCTGCACT
GGTGCTTAACGGTTCAGTTAAGTTGACTAGCGAAGCCAATGTTCATAATATGAAGCAGTGGCTGTGGA
TGTGGCTCGTGTATGATAGACGTCCCGGTAATTCTAATCCCAGAGTGAATGAGATATTCATCGGGGAA
AGTCGGGATCCAGAT
+
FFFFFFFFFFFFFFFFFFFFFFFFFFFFFFFFFFFFFFFFFFFFFFFFFFFFFFFFFFFFFFFFFFFF
FFFFFFFFFFFFFFFFFFFFFFFFFFFFFFFFFFFFFFFFFFFFFFFFFFFFFFFFFFFFFFF:FFFF
FFFFFFFFFFFFFFF
@A00721:190:HGJW7DSXY:4:1668:19253:9721 1:N:0:TCTGCAAG+GCTGCACT
GGTGCTTAACGGTTCAGTTAAGTTGACTAGCGAAGCCAATGTTCATAATATGAAGCAGTGGCTGTGGA
TGTGGCTCGTGTATGATAGACGTCCCGGTAATTCTAATCCCAGAGTGAATGAGATATTCATCGGGGAA
AGTCGGGATCCAGAT
+
FFFFFFFFFFFFFFFFFFFFFFFFFFFFFFFFFFFFFFFFFFFFFFFFFFFFFFFFFFFFFFFFFFFF
FFFF:FFFFFFFFFFFFFFFFFFFFFFFFFFFFFFFFFFFFFFFFFFFFFFFFFFFFFFFFFFFF::
FFFFFFFFFFFFFFF
@A00721:190:HGJW7DSXY:4:1668:18900:9799 1:N:0:TCTGCAAG+GCTGCACT
GGTGCTTAACGGTTCAGTTAAGTTGACTAGCGAAGCCAATGTTCATAATATGAAGCAGTGGCTGTGGA
TGTGGCTCGTGTATGATAGACGTCCCGGTAATTCTAATCCCAGAGTGAATGAGATATTCATCGGGGAA
AGTCGGGATCCAGAT
+
F:FFFFFFFFFFFFFFF:FFFF:FFFFFFFFFFFFFFFFF:FFFFFFFFFF:FFFFFFFFFF:::FFF
FFFFFFFF,FFFFFFFFFFFFFFFFF:FFFFFFFFFFFFFFFFFFFFF,FFFFFFFF:FFFFFF:FFF
FF:FFFFFFF:F,FF
@A00721:190:HGJW7DSXY:4:2220:22914:10708 1:N:0:TCTGCAAG+GCTGCACT
GGTGCTTAACGGTTCAGTTAAGTTGACTAGCGAAGCCAATGTTCATAATATGAAGCAGTGGCTGTGGA
TGTGGCTCGTGTATGATAGACGTCCCGGTAATTCTAATCCCAGAGTGAATGAGATATTCATCGTGGAA
AGTCGGGATCCAGAT
+
FF:FF:FFFFFFFFF:FF,FF::F:FFFF,FFFFFFFFFFF:F,FF:F,FFFFFFFF:FF:FFF,,:F
FFF,:FFFFF:F:::F,F:FFFFF::FFF:FF:,FFFFF:::,:F::F,:FFFFFFFFFFFFF,FF::
,,F:FFFFFFFFFFF
@A00721:190:HGJW7DSXY:4:2249:31286:3599 1:N:0:TCTGCAAG+GCTGCACT
GGTGCTTAACGGTTCAGTTAAGTTGACTAGCGAAGCCAATGTTCATAATATGAAGCAGTGGCTGTGGA
TGTGGCTCGTGTATGATAGACGTCCCGGTAATTCTAATCCCAGAGTGAATGAGATATTCATCGGGGAA
AGTCGGGATCCAGAT
+
FFFFFFFFFFFFFFFFFFFFFFFFFFFFFFFFFFFFFFFFFFFFFFFFFFFFFFFFFFFFFFFFFFFF
FFFFFFFFFFFFFFFFFFFFFFFFFFFFFFFFFFFFFFFFFFFFFFFFFFFFFFFFFFFFFFFFFFFF
FFFFFFFFFF:FFFF
@A00721:190:HGJW7DSXY:4:2375:12120:6480 1:N:0:TCTGCAAG+GCTGCACT
GGTGCTTAACGGTTCAGTTAAGTTGACTAGCGAAGCCAATGTTCATAATATGAAGCAGTGGCTGTGGA
TGTGGCTCGTGTATGATAGACGTCCCGGTAATTCTAATCCCAGAGTGAATGAGATATTCATCGGGGAA
AGTCGGGATCCAGAT
+
FFFFFFFFFFFFFFFFFFFFFFFFFFFFFFFFFFFFFFFFFFFFFFFFFFFFFFFF:FFFFFFFFFFF
FFFFFFFFFFFFFFFFFFFFFFFFFFFFFFFFFFFFFFFFFFFFFFFFFFFFFFFFFFFFFFFFFFF:
FFFFFFFFFFFFFFF

```
@A00721:190:HGJW7DSXY:4:2453:28944:14199 1:N:0:TCTGCAAG+GCTGCACT
GGTGCTTAACGGTTCAGTTAAGTTGACTAGCGAAGCCAATGTTCATAATATGAAGCAGTGGCTGTGGA
TGTGGCTCGTGTATGATAGACGTCCCGGTAATTCTAATCCCAGAGTGAATGAGATATTCATCGGGGAA
AGTCGGGATCCAGAT
+
FFFFFFFFFFFFFFFFFFFFFFFFFFFFFFFFFFFFFFFFFFFFFFFFFFFFFFFFFFFFFFFFFFFFFF
FFFFFFFFFFFFFFFFFFFFFFFFFFFFFFFFFFFFFFFFFFFFFFFFFFFFFFFFFFFFFFFF:FFFFF
FFFFFFFFFFFFFFF
@A00721:190:HGJW7DSXY:4:2570:25093:21151 1:N:0:TCTGCAAG+GCTGCACT
GGTGCTTAACGGTTCAGTTAAGTTGACTAGCGAAGCCAATGTTCATAATATGAAGCAGTGGCTGTGGA
TGTGGCTCGTGTATGATAGACGTCCCGGTAATTCTAATCCCAGAGTGAATGAGATATTCATCGGGGAA
AGTCGGGATCCAGAT
+
:FFFFF:FFFFFFFFFFFFFFFF:FFFFFFFFFFFF:FFFFFFFFF:FFFFFFFFF::F:F::FFFF:FF
FFFFFFFFFFFFF:FFFF:FFFFFF::FFFFFFFFFFFFFFFFFF::FFFFFFFFFFFFFFFFF:,FFFF
FFFFFFFFFFFFFFF
@A00721:190:HGJW7DSXY:4:1377:20943:29027 1:N:0:TCTGCAAG+GCTGCACT
TGCTTAACGGTTCAGTTAAGTTGACTAGCGAAGCCAATGTTCATAATATGAAGCAGTGGCTGTGGATG
TGGCTCGTGTATGATAGACGTCCCGGTAATTCTAATCCCAGAGTGAATGAGATATTCATCGGGGAAAG
TCGGGATCCAGATAC
+
FFFFFFFFFFFFFFFFFFFFFFFFFFFFFFFFFFFFFFFFFFFFFFFFFFFFFFFFFFFFFFFFFFFFFF
FFFFFFFFFFFFFFFFFFFFFFFFFFFFFFFFFFFFFFFFFFFFFFFFFFFFFFFFFFFFFF:FFFFFFF
FFFFFFFFFFFFFFF
@A00721:190:HGJW7DSXY:4:1637:13792:16736 1:N:0:TCTGCAAG+GCTGCACT
TGCTTAACGGTTCAGTTAAGTTGACTAGCGAAGCCAATGTTCATAATATGAAGCAGTGGCTGTGGATG
TGGCTCGTGTATGATAGACGTCCCGGTAATTCTAATCCCAGAGTGAATGAGATATTCATCGGGGAAAG
TCGGGATCCAGATAC
+
FFFFFFFFFFFFFFFFFFFFFFFFFFFFFFFFFFFFFFFFFFFFFFFFFFFFFFFFFFFFFFFFFFFFFF
FFFFFFFFFFFFFFFFF:FFFFFFFFFFFFFFFFFFFFFFFFFFFFFFFFFFFFFFFFFFFFFFFFFFFF
FFFFFFFFFFFFFFF
@A00721:190:HGJW7DSXY:4:2158:2338:21104 1:N:0:TCTGCAAG+GCTGCACT
TGCTTAACGGTTCAGTTAAGTTGACTAGCGAAGCCAATGTTCATAATATGAAGCAGTGGCTGTGGATG
TGGCTCGTGTATGATAGACGTCCCGGTAATTCTAATCCCAGAGTGAATGAGATATTCATCGGGGAAAG
TCGGGATCCAGATAC
+
FFFFFFFFFFFFFFFFFFFFFFFFFFFFFFFFFFFFFFFFFFFFFFF:FFFFFFF:FFFFFFFFFFFFFF
FFFFFFFFFFFFFFFFFFFFFFFFFFFF:FFFFFFFFFFFFFFFFFFFFFFFFFFFFFF:FFFF:FFFFF
FFFFFFFFFFFFFFF
@A00721:190:HGJW7DSXY:4:2470:13621:29340 1:N:0:TCTGCAAG+GCTGCACT
TGCTTAACGGTTCAGTTAAGTTGACTAGCGAAGCCAATGTTCATAATATGAAGCAGTGGCTGTGGATG
TGGCTCGTGTATGATAGACGTCCCGGTAATTCTAATCCCAGAGTGAATGAGATATTCATCGGGGAAAG
TCGGGATCCAGATAC
+
FFFFFFFFFFFFFFFFFFFFFFFFFFFFFFFFFFFFFFFFFFFFFFFFFFFFFFFFFFFFFFFFFFFFFF
FFFFFFF:FFFFFFFFFFFFFFFFFFFFFFFFFFFFFFFFFFFFFFFFFFFFFFFFFFFFFFFFFFFFFF
FFFFFFFFFFFFFFF
@A00721:190:HGJW7DSXY:4:1251:19162:13291 1:N:0:TCTGCAAG+GCTGCACT
CGGTTCAGTTAAGTTGACTAGCGAAGCCAATGTTCATAATATGAAGCAGTGGCTGTGGATGTGGCTCG
TGTATGATAGACGTCCCGGTAATTCTAATCCCAGAGTGAATGAGATATTCATCGGGGAAAGTCGGGAT
CCAGATACTTGGATG
+
FFFFFFFFFFFFFFFFFFFFFFFFFFFFFFFFFFFFFFFFFFFFFFFF:FFFFFF:FFFFFFFFFFFFFF
```

FFFFFFFFFFFFFFFFFFFFFFFFFFFFFFFFFFFFFFFFFFFFFFFFFFFFFFFFFFFFFFFFFFFFFF
FFFFFFFFF:FFFFF
@A00721:190:HGJW7DSXY:4:1472:4797:20760 1:N:0:TCTGCAAG+GCTGCACT
CGGTTCAGTTAAGTTGACTAGCGAAGCCAATGTTCATAATATGAAGCAGTGGCTGTGGATGTGGCTCG
TGTATGATAGACGTCCCGGTAATTCTAATCCCAGAGTGAATGAGATATTCATCGGGGAAAGTCGGGAT
CCAGATACTTGGATG
+
FFF:FFFFFFFFFFFFFFFFFFFFFFFFFFFFFFFFFFFFFFFFFFFFFFFFFFFFFFFFFFFFFFFFFF
FFFFFFFFFFFFFFFFFFFFFFFFFFFFFFFFFFFFFFFFFFFFFFFFFFFFFFFFFFFFFFFFFFFFFF
FFFFFFFFFFFFFFF
@A00721:190:HGJW7DSXY:4:1472:5864:21449 1:N:0:TCTGCAAG+GCTGCACT
CGGTTCAGTTAAGTTGACTAGCGAAGCCAATGTTCATAATATGAAGCAGTGGCTGTGGATGTGGCTCG
TGTATGATAGACGTCCCGGTAATTCTAATCCCAGAGTGAATGAGATATTCATCGGGGAAAGTCGGGAT
CCAGATACTTGGATG
+
FFFFFFFFFFFFFFFFFFFFFFFFFFFFFFFFFFFFFFFFFFFFFFFFF:FFFFFFFFFFFFFFFFFFFF
FFFFFFFFFFFFFFFFFFFFFFFFFFFFFFFFFFFFFFFFFFFFFFFFFFFFFF:FFFFFFFFFFFFFFF
FFFFFFFFFFFFFF:
@A00721:190:HGJW7DSXY:4:1618:17409:21183 1:N:0:TCTGCAAG+GCTGCACT
CGGTTCAGTTAAGTTGACTAGCGAAGCCAATGTTCATAATATGAAGCAGTGGCTGTGGATGTGGCTCG
TGTATGATAGACGTCCCGGTAATTCTAATCCCAGAGTGAATGAGATATTCATCGGGGAAAGTCGGGAT
CCAGATACTTGGATG
+
FFFFFFFFFFFFFFFFFFFFFFFFFFFFFFFFFFFFF:FFFFFFFFFFFFFFFFFFFFFFFFFFFFFFFF
FFFFFFFFFFFFFFFFFFFFFFFFFFFFFFFFFFFFFFFFF:FFFFFFFFFFFFFF:FFF:FFFFFFF:F
FFFFFFFFFFFFFFF
@A00721:190:HGJW7DSXY:4:2341:2745:19648 1:N:0:TCTGCAAG+GCTGCACT
CGGTTCAGTTAAGTTGACTAGCGAAGCCAATGTTCATAATATGAAGCAGTGGCTGTGGATGTGGCTCG
TGTATGATAGACGTCCCGGTAATTCTAATCCCAGAGTGAATGAGATATTCATCGGGGAAAGTCGGGAT
CCAGATACTTGGATG
+
FFFFFFFFFFFFFFFFFFFFFFFFF:FFFFFFFFFFFFFF:FFFFFF:FFFFFFFFFFFFFFFFFFFFFF
FFFFFFFFFFFFFFFFFFFFF:FFFFFFFFFFF:FFFFFFFFFFFFFFFFFFFFFFFFFFFFFFFFFFFF
FFFFF:FFFFFFFFF
@A00721:190:HGJW7DSXY:4:1152:4679:15984 1:N:0:TCTGCAAG+GCTGCACT
GGTTCAGTTAAGTTGACTAGCGAAGCCAATGTTCATAATATGAAGCAGTGGCTGTGGATGTGGCTCGT
GTATGATAGACGTCCCGGTAATTCTAATCCCAGAGTGAATGAGATATTCATCGGGGAAAGTCGGGATC
CAGATACTTGGATGA
+
FFFFFFFFF:FFFFFFFFFFFFFFFFFFFFFFFFFFFFFFFFFFFFF:FFFFFF:FFFFFFFFFFFFFFF
FFFFFFFFFFFFFFFFFFFFFFFFFFFFFFFFFFFFFFF:FFFFFFFFFFFFFFFFFFF:FFFFFFFFFF
FFFFFFFFFFFFFFF
@A00721:190:HGJW7DSXY:4:2303:31593:23422 1:N:0:TCTGCAAG+GCTGCACT
GGTTCAGTTAAGTTGACTAGCGAAGCCAATGTTCATAATATGAAGCAGTGGCTGTGGATGTGGCTCGT
GTATGATAGACGTCCCGGTAATTCTAATCCCAGAGTGAATGAGATATTCATCGGGGAAAGTCGGGATC
CAGATACTTGGATGA
+
FFFFFFFFFFFFFFFFFFFFFFFFFFFFFFFFFFFFFFFFFFFFFFFFFFFFFFFFFFFFFFFFFFFFFF
FFFFFFFFFFFFFFFFFFFFF:FFFFFFFFFFFFFFFFF,FFFFFFFFFFFFFFFFFFFFFFFFFFFFFF
FFFFFF:FFFFFFF:
@A00721:190:HGJW7DSXY:4:2314:6686:26224 1:N:0:TCTGCAAG+GCTGCACT
GGTTCAGTTAAGTTGACTAGCGAAGCCAATGTTCATAATATGAAGCAGTGGCTGTGGATGTGGCTCGT
GTATGATAGACGTCCCGGTAATTCTAATCCCAGAGTGAATGAGATATTCATCGGGGAAAGTCGGGATC
CAGATACTTGGATGA

```
+
FFFFFFFFFFFFFFFFFFFFFFFFFFFFFFFFFFFFFFFFFFFFFFFFFFFFFFFFFFFFFFFFFFFF
FFFFFFFFFFFFFFFFFFFFFFFFFFFFFFFFFFFFFF:FFFFFFFFFFFFFFFFFFFFFFFFFFFFF
FFFFFFFFFFFFFFF
@A00721:190:HGJW7DSXY:4:2372:29080:36104 1:N:0:TCTGCAAG+GCTGCACT
GGTTCAGTTAAGTTGACTAGCGAAGCCAATGTTCATAATATGAAGCAGTGGCTGTGGATGTGGCTCGT
GTATGATAGACGTCCCGGTAATTCTAATCCCAGAGTGAATGAGATATTCATCGGGGAAAGTCGGGATC
CAGATACTTGGATGA
+
FFFFFFFFFFFFFFFFFFFFFFFFFFFFFFFFFFFFFFFFFFF:F:FFFFFFFFFFFFFFFFFFFFFF
FFFFFFFFFFFFFFFFFFF:FFFFFFFFFFFFFFFFFFFFFFFFFFFFFFFFFFFFFFFFFFFFF:FF
FFFFFFF:FFFFFFF
@A00721:190:HGJW7DSXY:4:1472:29161:27821 1:N:0:TCTGCAAG+GCTGCACT
GTTCAGTTAAGTTGACTAGCGAAGCCAATGTTCATAATATGAAGCAGTGGCTGTGGATGTGGCTCGTG
TATGATAGACGTCCCGGTAATTCTAATCCCAGAGTGAATGAGATATTCATCGGGGAAAGTCGGGATCC
AGATACTTGGATGAT
+
FFFFFFFFFFFFFFFFFFFFFF:FFF:FFFFFFFFFFFFFFFFFFFFFFFF,FFFFFFFFFFFFFFFF
FFFFFFFFFFFFFFFFFFF:FFFFFFFFFFFFFFFFFFFFFFFFFFFFFFFFFFFFFFFFFFFFFFFF
FFF:FFFFFFFFFFF
@A00721:190:HGJW7DSXY:4:1516:28610:20635 1:N:0:TCTGCAAG+GCTGCACT
GTTCAGTTAAGTTGACTAGCGAAGCCAATGTTCATAATATGAAGCAGTGGCTGTGGATGTGGCTCGTG
TATGATAGACGTCCCGGTAATTCTAATCCCAGAGTGAATGAGATATTCATCGGGGAAAGTCGGGATCC
AGATACTTGGATGAT
+
FFFFFFFFFFFFFFFFFFFFFFFFFFFFFFFFFFFFFFFFFFFFFFFFFFFFFFFFF:FFFFFFFFFF
FFFFFFFFFFFFFFFFFFFFFFFFFFFFFFFFFFFFFFFFFFFFFFFFFFFFF:FFFFFFFFFFFFFF
FFFFFFFFFFFFFFF
@A00721:190:HGJW7DSXY:4:1103:32660:8578 1:N:0:TCTGCAAG+GCTGCACT
GATCATCCAAGTATCTGGATCCCGACTTTCCCCGATGAATATCTCATTCACTCTGGGATTAGAATTAC
CGGGACGTCTATCATACACGAGCCACATCCACAGCCACTGCTTCATATTATGAACATTGGCTTCGCTA
GTCAACTTAACTGAA
+
FFFFFFFFFFFFFFFFFFFFFFFFFFFFFFFFFFFFFFFFFFFFFFF:FFFFFFFFFF:F,FFFFF:F
FFFFFFFFFFFFFFFFFFFFFFFFFFF:FFFFFFFFFFFFFFFFFFFFFFFFFFFFFFFFFFFFFFFF
FFFFFFFFF:FFFFF
@A00721:190:HGJW7DSXY:4:1204:22435:29011 1:N:0:TCTGCAAG+GCTGCACT
TTCAGTTAAGTTGACTAGCGAAGCCAATGTTCATAATATGAAGCAGTGGCTGTGGATGTGGCTCGTGT
ATGATAGACGTCCCGGTAATTCTAATCCCAGAGTGAATGAGATATTCATCGGGGAGAGTCGGGATCCA
GATACTTGGATGATC
+
FFFFFFFFFFFFFFFFFFFFFFFFF:FFFFFFF:F:FFFFFFFFFFFFFFFFFFFFFFFFFFFFFFFF
FFFFFFFFFFFFFF:FFFFFFFFFFFFFFFFFFFFFFFFFFFFFFFFFFF:FFFF,FFFFFFF:FFFF
FFFFFFFFFFFFFFF
@A00721:190:HGJW7DSXY:4:1273:13584:29590 1:N:0:TCTGCAAG+GCTGCACT
TTCAGTTAAGTTGACTAGCGAAGCCAATGTTCATAATATGAAGCAGTGGCTGTGGATGTGGCTCGTGT
ATGATAGACGTCCCGGTAATTCTAATCCCAGAGTGAATGAGATATTCATCGGGGAAAGTCGGGATCCA
GATACTTGGATGATC
+
FFFFFFFFFFFFFFFFFFFFFFFFFFFFFFFFFFFFFFFFFFFFFFFFFFFFFFFFFFFFFFFFFFFF
FFFFFFFFFFFFFFFFF:FFFFFFFFFFFFFFFFFFFFFFFFFFFFFFFFFFFFF:F:FFFFFFFFFF
FFFFFFFFFFFFFFF
@A00721:190:HGJW7DSXY:4:1355:14778:30499 1:N:0:TCTGCAAG+GCTGCACT
GATCATCCAAGTATCTGGATCCCGACTTTCCCCGATGAATATCTCATTCACTCTGGGATTAGAATTAC
```

CGGGACGTCTATCATACACGAGCCACATCCACAGCCACTGCTTCATATTATGAACATTGGCTTCGCTA
GTCAACTTAACTGAA
+
FFFFFFFFFFFFFFFFFFFFFFFFFFFFFFFFFFFFFFFFFFFFFFFFFFFFFFFFFFFFFFFFFFFFFF
FFFFFFFFFFFFFFFFFFFFFFFFFFFFFFFFFFFFFFFFFFFFFFFFFFFFFFFFFFFFFFFFFFFFFF
FFFFFFFFFFFFFFF
@A00721:190:HGJW7DSXY:4:1565:23095:11052 1:N:0:TCTGCAAG+GCTGCACT
GATCATCCAAGTATCTGGATCCCGACTTTCCCCGATGAATATCTCATTCACTCTGGGATTAGAATTAC
CGGGACGTCTATCATACACGAGCCACATCCACAGCCACTGCTTCATATTATGAACATTGGCTTCGCTA
GTCAACTTAACTGAA
+
FFFFFFFFFFFFFFFFFFFFFFFFFFFFFFFFFFFFFFFFFFFFFFFFFFFFFFFFFFFFFFFFFFFFFF
FFFFFFFFFFFFFFFFFFFFFFFFFFFFFFFFFFFFFFFFFFFFFFFFFFFFFFFFFFFFFFFFFFFFFF
FFFFFFFFFFFFFFF
@A00721:190:HGJW7DSXY:4:2101:31656:29293 1:N:0:TCTGCAAG+GCTGCACT
GATCATCCAAGTATCTGGATCCCGACTTTCCCCGATGAATATCTCATTCACTCTGGGATTAGAATTAC
CGGGACGTCTATCATACACGAGCCACATCCACAGCCACTGCTTCATATTATGAACATTGGCTTCGCTA
GTCAACTTAACTGAA
+
FFFFFFFFFFFFFFFFFFFFFFFFFFFFFFFFFFFFFFFFFFFFFFFFFFFFFFFFFFFFFFFFFFFFFF
FFFFFFFFFFFFFFFFFFFFFF,FFFFFFFFFFFFFFFFFFFFFFFFFFFFF:FFF:FFFFFFFF:FF
FFFFF:FFFFFFF:F
@A00721:190:HGJW7DSXY:4:2167:6831:17581 1:N:0:TCTGCAAG+GCTGCACT
GATCATCCAAGTATCTGGATCCCGACTTTCCCCGATGAATATCTCATTCACTCTGGGATTAGAATTAC
CGGGACGTCTATCATACACGAGCCACATCCACAGCCACTGCTTCATATTATGAACATTGGCTTCGCTA
GTCAACTTAACTGAA
+
FFFFFFFFFFFFFFFFFFFFFFFFFFFFFFFFFFFFFFFFFFFFFFFFFFFFFFFFFFFFFFFFFFFFFF
FFFFFFFFFFFFFFFFFFFFFFFFFFFFFFFFFFFFFFFFFFFFFFFFFFFFFFFFFFFFFFFFFFFFFF
FFFFFFFFFFFFFFF
@A00721:190:HGJW7DSXY:4:2308:19126:24439 1:N:0:TCTGCAAG+GCTGCACT
TTCAGTTAAGTTGACTAGCGAAGCCAATGTTCATAATATGAAGCAGTGGCTGTGGATGTGGCTCGTGT
ATGATAGACGTCCCGGTAATTCTAATCCCAGAGTGAATGAGATATTCATCGGGGAAAGTCGGGATCCA
GATACTTGGATGATC
+
FFFFFFFFFFFFFFFFFFFFFFFFFFFFFFFFFFFFFFFFFFFFF:FFFFFFFFFFFFFFFFFFFFFFFF
FFFFF:FFFFFFFFFFFFFFFFFFFFFFFFFFFFF:FF,FFFFFFFFFFFFFFFFFFFFFFFFFFFFFFFF
FFFFFFFFFFFFFFF
@A00721:190:HGJW7DSXY:4:1658:31322:2879 1:N:0:TCTGCAAG+GCTGCACT
GATCATCCAAGTATCTGGATCCCGACTTTCCCCGATGAATATCTCATTCACTCTGGGATTAGAATTAC
CGGGACGTCTATCATACACGAGCCACATCCACAGCCACTGCTTCATATTATGAACATTGGCTTCGCTA
GTCAACTTAACTGA
+
FFFFFFFFFFFFFFFFFFFFFFFFFFF:F:FFFFFFFFFFFFFFFFFFFFFFFFFF:F,FFFFFF:FFF
FFFFFFFFFFFFFFFFFFFFFFFFFFF:FFFFFFFFFFFFFFFFFFFFFFFFFFFF:FFF:FFFF:FFF
FFFFFFFFFF:FFF
@A00721:190:HGJW7DSXY:4:1601:27814:36323 1:N:0:TCTGCAAG+GCTGCACT
CAGTTAAGTTGACTAGCGAAGCCAATGTTCATAATATGAAGCAGTGGCTGTGGATGTGGCTCGTGTAT
GATAGACGTCCCGGTAATTCTAATCCCAGAGTGAATGAGATATTCATCGGGGAAAGTCGGGATCCAGA
TACTTGGATGAT
+
FFFFFFFFFFFFFFFFFFFFFFF:FFFFFF,F:FFF:FFFFFFFFFFFFFFFFFFF::FFFFFFFFFF
FFFFFFFFFFFFFFF,FFFFF:FFFF:FFFFFFFFFFFFFFFFFFFFFF:FFFFFFFFFFFFFFFFFFF
FFFFFFFFFFFF

```
@A00721:190:HGJW7DSXY:4:1176:1072:8234 1:N:0:TCTGCAAG+GCTGCACT
TTAAGTTGACTAGCGAANCCAATGTTCATAATATGAAGCAGTGGCTGTGGATGTGGCTCGTGTATGAT
AGACGTCCCGGTAATTCTAATCCCAGAGTGAATGAGATATTCA
+
FFFFFFFF:FFFFFFFF#FF:FFFFFFFFFFFFFF:FFFFFFFFF:FFFFFFFFFFFFFFFFFFFFFF
FFFFFFFFFFFFFFFFFFFFFFFFFFFFFFFFFFFFFFFFFFF
@A00721:190:HGJW7DSXY:4:1142:23818:9518 1:N:0:TCTGCAAG+GCTGCACT
GTTGACTAGCGAAGCCAATGTTCATAATATGAAGCAGTGGCTGTGGATGTGGCTCGTGTATGATAGAC
GTCCCGGTAATTCTAATCCCAGAGTGAATGAGATATTCATCGGGGAAAGTCGGGATCCAGATACTTGG
ATGATCAACATGGAT
+
FFFFFF:FFFFFFFFFFFFFFFFFFFFFFFFFFF:FFFFFFFF:FFFFFFFFFFFFFFFFFFFFFFFF
FFFFFFFFFFFFFFFFFFFFFFFFFFFFFFFFFFFFFFFFFFFFFFFFFFFFFFFFFFFFFFFFFFFF
FFFFF:FFFFFFFFF
@A00721:190:HGJW7DSXY:4:1349:18475:10504 1:N:0:TCTGCAAG+GCTGCACT
GTTGACTAGCGAAGCCAATGTTCATAATATGAAGCAGTGGCTGTGGATGTGGCTCGTGTATGATAGAC
GTCCCGGTAATTCTAATCCCAGAGTGAATGAGATATTCATCGGGGAAAGTCGGGATCCAGATACTTGG
ATGATCAACATGGAT
+
FFFFFFFFFFFFFFFFFFFFFFFFFFF:FFFFFFFFFFFFFFFFFFFFFFFFFFFFFFFFFFFFFFFF
FFFFFFFFFFFFFFFFFFFFFFFFFFFFFFFFFFFFFFFFFFFFFFFFFFFFFFFFFFFFFFFFF:FF
FFFFFFFFFFFFFFF
@A00721:190:HGJW7DSXY:4:1566:7319:21778 1:N:0:TCTGCAAG+GCTGCACT
GTTGACTAGCGAAGCCAATGTTCATAATATGAAGCAGTGGCTGTGGATGTGGCTCGTGTATGATAGAC
GTCCCGGTAATTCTAATCCCAGAGTGAATGAGATATTCATCGGGGAAAGTCGGGATCCAGATACTTGG
ATGATCAACATGGAT
+
FFFFFFFFFFFFFFFFFFFFFFFFFFFFFFFFFFFFFFFFFFFFFFFFFFFFFFFFFFFFFFFFFFFF
FFFFFFFFFFFFFFFFFFFFFFFFFFFFFFFFFFFFFFFFFFFFFFFFFFFFFFFFFFFFFFFFFFFF
FFFFFFFFFFFFFFF
@A00721:190:HGJW7DSXY:4:1604:6460:19633 1:N:0:TCTGCAAG+GCTGCACT
GTTGACTAGCGAAGCCAATGTTCATAATATGAAGCAGTGGCTGTGGATGTGGCTCGTGTATGATAGAC
GTCCCGGTAATTCTAATCCCAGAGTGAATGAGATATTCATCGGGGAAAGTCGGGATCCAGATACTTGG
ATGATCAACATGGAT
+
F,FFFFFFFFFFF:FFFFFFFFFFFFF:FF:FFF:FFFF,F,F:FFF::FFFF:,,FFF:FFFFFFF,
FF:FFFFFFF:F,FFFF:FFF,FFFFFFF:FFFFFFFFFFFF,F,FFF,FFFFFFFFFFF::F,,FF:
FF,FFF::F:F,FFF
@A00721:190:HGJW7DSXY:4:2137:27471:24298 1:N:0:TCTGCAAG+GCTGCACT
GTTGACTAGCGAAGCCAATGTTCATAATATGAAGCAGTGGCTGTGGATGTGGCTCGTGTATGATAGAC
GTCCCGGTAATTCTAATCCCAGAGTGAATGAGATATTCATCGGGGAAAGTCGGGATCCAGATACTTGG
ATGATCAACATGGAT
+
F:FFFFFFFFFFFFFFFFFFFFFF:FFFFFFFFF:FFFFFF:FFFFFFFFFFFFFFFFFFFFFFFFFF
FFFFFFFF:FFFFFFFF:FFFFFFFFF,FFFFFFFFFFFFFFFFFF,FFFFFFFFFFFFFF,FF,FFF
FFFFFFFFFFFFFFF
@A00721:190:HGJW7DSXY:4:2142:15564:10347 1:N:0:TCTGCAAG+GCTGCACT
GTTGACTAGCGAAGCCAATGTTCATAATATGAAGCAGTGGCTGTGGATGTGGCTCGTGTATGATAGAC
GTCCCGGTAATTCTAATCCCAGAGTGAATGAGATATTCATCGGGGAAAGTCGGGATCCAGATACTTGG
ATGATCAACATGGAT
+
FFFFFFFFFFFFFFFFFFFFFFF:F,FFFFFFFFFFFFFFF:FFFFFFFFFFFFFFFFFFFFFFFFFF
FFFFFFFFF:FFFF:FFFFFFFFFFFF:FFFFFFFFFFFFFFFFFFFFFFFFFFFFFFFFFFFFFFFF
FFFFFFFFFF:FFFF
```

@A00721:190:HGJW7DSXY:4:2301:18177:13463 1:N:0:TCTGCAAG+GCTGCACT
GTTGACTAGCGAAGCCAATGTTCATAATATGAAGCAGTGGCTGTGGATGTGGCTCGTGTATGATAGAC
GTCCCGGTAATTCTAATCCCAGAGTGAATGAGATATTCATCGGGGAAAGTCGGGATCCAGATACTTGG
ATGATCAACATGGAT
+
FFFFFFFFFFFFF:FFFFFFFFFFFFFFFFFFFFF:FFFFFFFFFFFFFFFFFFFFFFFFFFFFFFFFFF
FFFFFFFFFFFFFFFFFFF:FFFFFFFFFFFFFFFFFF:FFFFFFFFFF:FF:FFFFFFFFFFFFFFFFF
FFFFFFFFFFFFFFF
@A00721:190:HGJW7DSXY:4:2352:1226:9408 1:N:0:TCTGCAAG+GCTGCACT
GTTGACTAGCGAAGCCAATGTTCATAATATGAAGCAGTGGCTGTGGATGTGGCTCGTGTATGATAGAC
GTCCCGGTAATTCTAATCCCAGAGTGAATGAGATATTCATCGGGGAAAGTCGGGATCCAGATACTTGG
ATGATCAACATGGAT
+
FFFFFFFFFFFFFFFFFFFFFFFFFFFFFFFFFFFFFFFFFFFFFFFFFFFFFFFFFFFFFFFFFFFFFF
FFFFFFFFFFFFFFFFFFFFFFFFFFFFFFFFFFFFFFFFFFFFFFFFFFFFFFFFFFFFFFFFFFFFFF
FFFFFFFFFFFFFFF
@A00721:190:HGJW7DSXY:4:2628:14642:36307 1:N:0:TCTGCAAG+GCTGCACT
GTTGACTAGCGAAGCCAATGTTCATAATATGAAGCAGTGGCTGTGGATGTGGCTCGTGTATGATAGAC
GTCCCGGTAATTCTAATCCCAGAGTGAATGAGATATTCATCGGGGAAAGTCGGGATCCAGATACTTGG
ATGATCAACATGGAT
+
FFFFFFFFFFFFFFFFFFFFFFFFFFFFFFFFFFFFFFFFFFFFFFFFFFFFFFFFFFFFFFFFFFFFFF
FFFFFFFFFFFFFFFFFFFFFFFFFFFF:FFFFFFFFFFFFFFFFFFFFFFFFFFFFFFFFFFFFFFFFF
FFFFFFFFFFFFFFF
@A00721:190:HGJW7DSXY:4:1121:9100:22232 1:N:0:TCTGCAAG+GCTGCACT
AGCGAAGCCAATGTTCATAATATGAAGCAGTGGCTGTGGATGTGGCTCGTGTATGATAGACGTCCCGG
TAATTCTAATCCCAGAGTGAATGAGATATTCATCGGGGAAAGTCGGGATCCAGATACTTGGATGATCA
ACATGGATAATGTTG
+
FFFFFFFFFFFFFFFFFFFF:FFFFF:FFF:FFFFF,FFFFFFFFFFFFFFFFFFF:FFFFFFFFF::FF
F,FFFFFFFFFFFFFFF:FFFFFFFFFFFFFFFFF:FF:FF:FF:FF:FFFFFFFFFFF:FFFFFFF:FF
FF:FFFFFF:F:FFF
@A00721:190:HGJW7DSXY:4:1144:5963:1673 1:N:0:TCTGCAAG+GCTGCACT
AGCGAAGCCAATGTTCATAATATGAAGCAGTGGCTGTGGATGTGGCTCGTGTATGATAGACGTCCCGG
TAATTCTAATCCCAGAGTGAATGAGATATTCATCGGGGAAAGTCGGGATCCAGATACTTGGATGATCA
ACATGGATAATGTTG
+
FFFFFFFFFFFFFFFFFFFFFFFFFFFFFFFFFFFFFFFFFFFFFFFFFFFFFFFFFFFFFFFFFFFFFF
FFFFFFFFFFFFFFFFFFFFFFFFFFFFFFFFFFFFFFFFFFFFFFFFFFFFFFFFFFFFFFFFFFFFFF
FFFFFFFFFFFFFFF
@A00721:190:HGJW7DSXY:4:1361:31801:3865 1:N:0:TCTGCAAG+GCTGCACT
AGCGAAGCCAATGTTCATAATATGAAGCAGTGGCTGTGGATGTGGCTCGTGTATGATAGACGTCCCGG
TAATTCTAATCCCAGAGTGAATGAGATATTCATCGGGGAAAGTCGGGATCCAGATACTTGGATGATCA
ACATGGATAATGTTG
+
FFFFFFFFFFFFFFFFFFFFFFFFFFFFF:FFFFFFFFFFFFFFFFFFFFFFFFFFFFFFFFFFFFFFFF
F:FFFFFFFFFFFFFFFFFFFFFFFFFFFFFFFFF:FFFFFF::FFFFFFFFFFFFFF:FFFFFFFFFFF
F::,FFFFFF:FF:F
@A00721:190:HGJW7DSXY:4:1361:4381:5791 1:N:0:TCTGCAAG+GCTGCACT
AGCGAAGCCAATGTTCATAATATGAAGCAGTGGCTGTGGATGTGGCTCGTGTATGATAGACGTCCCGG
TAATTCTAATCCCAGAGTGAATGAGATATTCATCGGGGAAAGTCGGGATCCAGATACTTGGATGATCA
ACATGGATAATGTTG
+
FFFFFFFFFFFFFFFFFFFFFFFFFFFFFFFFFF:FFFFFFFFFFFFFFFFFFFFFFFFFFFFFFFFFFF

FFFFFFFFFFFFFFFFFF:FFFFFFFFFFFFFFFF:FFFFFFFFFFFFFF:F:FFF:FF:FFFFFF:F
FFFFF,FFFFFFFFF
@A00721:190:HGJW7DSXY:4:1466:28592:12587 1:N:0:TCTGCAAG+GCTGCACT
AGCGAAGCCAATGTTCATAATATGAAGCAGTGGCTGTGGATGTGGCTCGTGTATGATAGACGTCCCGG
TAATTCTAATCCCAGAGTGAATGAGATATTCATCGGGGAAAGTCGGGATCCAGATACTTGGATGATCA
ACATGGATAATGTTG
+
FFFFFFFFFFFFFFFFFFFFFFFFFFFFFFFFFFFFFFFFFFFFFFFFFFFFFFFFFFFFFFFFFFFF
FFFFFFFFFFFFFFFFFFFFFFFFFFFFFFFFFFFFFFF:FFFFFFFFFFFFFFFFFFFFFFFFFFFF
FFFFFFFFFFFFFFF
@A00721:190:HGJW7DSXY:4:1652:17155:1548 1:N:0:TCTGCAAG+GCTGCACT
AGCGAAGCCAATGTTCATAATATGAAGCAGTGGCTGTGGATGTGGCTCGTGTATGATAGACGTCCCGG
TAATTCTAATCCCAGAGTGAATGAGATATTCATCGGGGAAAGTCGGGATCCAGATACTTGGATGATCA
ACATGGATAATGTTG
+
FFFFFFFFFFFFFFFFFFFFFFFFFFFFFFFFFFFFFFFFFFFFFFFFFFFFFFFFFFFFFFFFFFFF
FFFFFFFFFFFFFFFFFFFF:FFFFFFFFFFFFFFFFFFFFFFFFFF:FFFFFFFFFFFFFFFFFFFF
FFFFFFFFFFFFFFF
@A00721:190:HGJW7DSXY:4:1652:17381:2002 1:N:0:TCTGCAAG+GCTGCACT
AGCGAAGCCAATGTTCATAATATGAAGCAGTGGCTGTGGATGTGGCTCGTGTATGATAGACGTCCCGG
TAATTCTAATCCCAGAGTGAATGAGATATTCATCGGGGAAAGTCGGGATCCAGATACTTGGATGATCA
ACATGGATAATGTTG
+
FFFFFFFFFFFFFFFFFFFFFFFFFFFFFFF:FFFFFFFFFFFFFFFFFFFFFFFFFFFFFFFFFFFF
FFFFFFFFFFFFFFFFFFFFFFFFFFFFFFFFFFFFFFFFFFFFFFFFFFFFFFFFFFFFFFFFFFFF
FFFFFFFFFFFFFFF
@A00721:190:HGJW7DSXY:4:2116:19199:21527 1:N:0:TCTGCAAG+GCTGCACT
AGCGAAGCCAATGTTCATAATATGAAGCAGTGGCTGTGGATGTGGCTCGTGTATGATAGACGTCCCGG
TAATTCTAATCCCAGAGTGAATGAGATATTCATCGGGGAAAGTCGGGATCCAGATACTTGGATGATCA
ACATGGATAATGTTG
+
FFFFFFFFFFFFFFFFFFFFFFFFFFFFFFFFFFFFFFFFFFFFFFFFFFFFFFFFFFFFFFFFFFFF
F:FFFFFFFFFFFFFFFFFFFFFFFFFFFFFFFFFFFFFFFFFFFFF:FFFFFFFFFFFFFFFFFFFF
FF::FFFFFFFF:FF
@A00721:190:HGJW7DSXY:4:2116:19298:22451 1:N:0:TCTGCAAG+GCTGCACT
AGCGAAGCCAATGTTCATAATATGAAGCAGTGGCTGTGGATGTGGCTCGTGTATGATAGACGTCCCGG
TAATTCTAATCCCAGAGTGAATGAGATATTCATCGGGGAAAGTCGGGATCCAGATACTTGGATGATCA
ACATGGATAATGTTG
+
FFFFFFFFFFFFFFFFFFFFFFFFFFFFFFFFFFFFFFFFFFFFFFFFFFFFFFFFFFFFFFFFFFFF
FFFFFFFFFFFFFFFFFFFFFFFFFFFFFFFFFFFFFFFFFFFFFFFFFFFFFFFFFFFFFFFFFFFF
FFF:FFF:FFFF:FF
@A00721:190:HGJW7DSXY:4:2172:8205:26475 1:N:0:TCTGCAAG+GCTGCACT
AGCGAAGCCAATGTTCATAATATGAAGCAGTGGCTGTGGATGTGGCTCGTGTATGATAGACGTCCCGG
TAATTCTAATCCCAGAGTGAATGAGATATTCATCGGGGAAAGTCGGGATCCAGATACTTGGATGATCA
ACATGGATAATGTTG
+
FFFFFFFFFFFFFFFFFFFFFFFFFFFFFFFFFFFFFFFFFFFFFF:FFFFFFFFFFFFFFFFFFFFF
FFFFFFFFFFFFFFFFFFFFFFFFFFFFFFFFFFFFFFFFFFFFFFFFFFFFFFFFFFFFF:FFFFFF
FFFFFFF:FFFF:F:
@A00721:190:HGJW7DSXY:4:2233:25201:17863 1:N:0:TCTGCAAG+GCTGCACT
AGCGAAGCCAATGTTCATAATATGAAGCAGTGGCTGTGGATGTGGCTCGTGTATGATAGACGTCCCGG
TAATTCTAATCCCAGAGTGAATGAGATATTCATCGGGGAAAGTCGGGATCCAGATACTTGGATGATCA
ACATGGATAATGTTG

+
FFFFFFFFFFFFFFFFFFFFFFFFFFF:FFFFFFFFFFFFFFFFFFFFFFFFFFFFFFFFFFFFFFFFFF
FFFFFFFFFFFFFFFFFFFFFFFFFFFFFFFFFFFFFFFFFFFFFFFFFFFFFFFFFFFFFFFFFFFFFF
FFFFFFFFFFFFFFF
@A00721:190:HGJW7DSXY:4:2234:5376:19727 1:N:0:TCTGCAAG+GCTGCACT
AGCGAAGCCAATGTTCATAATATGAAGCAGTGGCTGTGGATGTGGCTCGTGTATGATAGACGTCCCGG
TAATTCTAATCCCAGAGTGAATGAGATATTCATCGGGGAAAGTCGGGATCCAGATACTTGGATGATCA
ACATGGATAATGTTG
+
FFFFFFFFFFF:FFFFFF:FFFFFFFF:FFFFFFFFFFFFFFFFFFFFFFFFFFFFFFFFFFFFFFFFFF
FFFFFFFFFFFFFFFFFFFFFFFFFFFFFFFFFFFFFFFFFFFFFFFFFFFFFFFFFFFFFFFFFFF:FF
FFFFFFF:F::FFFF
@A00721:190:HGJW7DSXY:4:2238:10303:29199 1:N:0:TCTGCAAG+GCTGCACT
AGCGAAGCCAATGTTCATAATATGAAGCAGTGGCTGTGGATGTGGCTCGTGTATGATAGACGTCCCGG
TAATTCTAATCCCAGAGTGAATGAGATATTCATCGGGGAAAGTCGGGATCCAGATACTTGGATGATCA
ACATGGATAATGTTG
+
FFFFFFFFFFFFFFFFF:FF:FFFFFFFFFFFFFFFFFFFFFFFFFFFFFFFFFFFFFFFFFFFFFFFFF
FFFFFFFFFFFFFFFFFFFFFFFFFFFFFFFFFFFFFFFFFFFFFFFFFFFFFFFFFFFFFFFFFFFFFF
FFF:FFF:FFFFFFF
@A00721:190:HGJW7DSXY:4:2271:14082:19836 1:N:0:TCTGCAAG+GCTGCACT
AGCGAAGCCAATGTTCATAATATGAAGCAGAGGCTGTGGATGTGGCTCGTGTATGATAGACGTCCCGG
TAATTCTAATCCCAGAGTGAATGAGATATTCATCGGGGAAAGTCGGGATCCAGATACTTGGATGATCA
ACATGGATAATGTT
+
F:FFFF:FFFF,FFFF:FFF:F:FFF,FF,,F,F::FF,F::,,F,FF,::FFFFFFF,FF:FFFFF,
FFFF,,,FF,FFFFFF,,FFF::FFFFFFFFFF,:,FFFF:,FF,:,FFFFFFF,::FFF,FF:FFFF
,F:,,:FFFF,:,F
@A00721:190:HGJW7DSXY:4:2340:22733:34851 1:N:0:TCTGCAAG+GCTGCACT
AGCGAAGCCAATGTTCATAATATGAAGCAGTGGCTGTGGATGTGGCTCGTGTATGATAGACGTCCCGG
TAATTCTAATCCCAGAGTGAATGAGATATTCATCGGGGAAAGTCGGGATCCAGATACTTGGATGATCA
ACATGGATAATGTTG
+
FFFFFFFFFFFFFFFFFF:FFFFFFFFFFFFFFFFFFFFFFFFFFFFFFFFFFFFFFFFFFFFFFFFFFF
FFFFFFFFFFFFFFFFFFFFFFFFFFFFFFFFFFFFFFFFFFFFFFFFFFFFFFFFFFFFFFFFFFFFFF
FFFFFFF:FFFFFFF
@A00721:190:HGJW7DSXY:4:2574:10764:24799 1:N:0:TCTGCAAG+GCTGCACT
AGCGAAGCCAATGTTCATAATATGAAGCAGTGGCTGTGGATGTGGCTCGTGTATGATAGACGTCCCGG
TAATTCTAATCCCAGAGTGAATGAGATATTCATCGGGGAAAGTCGGGATCCAGATACTTGGATGATCA
ACATGGATAATGTTG
+
FFFFFFFFF:FFFFFFF:FFFFFFFFFFFFFFFFFFFFFFFFFFFFFFFFFFFFFFFFFFFFFFFFFFFF
FFFFFFFFFFFFFFFFFFFF:FFFFFFFFFFFFFFFFFFFFFFFFFFFFFFFFFFFFFFFFFFFFFFFFF
FFFFFFF:FFFF:FF
@A00721:190:HGJW7DSXY:4:1244:15130:31735 1:N:0:TCTGCAAG+GCTGCACT
AAACCTATCAACATTATCCATGTTGATCATCCAAGTATCTGGATCCCGACTTTCCCCGATGAATATCT
CATTCACTCTGGGATTAGAATTACCGGGACGTCTATCATACACGAGCCACATCCACAGCCACTGCTTC
ATATTATGAACATTG
+
FFFFFFFFFFFFFFFFFFFFFFFFFFFFFFFFFFFFFFFFFFFFFFFFFFFFFFFFFFFFFFFFFFFFFF
FFFFFFFFFFFFFFFFFFFFFFFFFFFFFFFFFFFFFFFFFFFFFFFFFFFFFFFFFFFFFFFFFFFFFF
FFFFFFFFFFFFFFF
@A00721:190:HGJW7DSXY:4:1312:29568:13088 1:N:0:TCTGCAAG+GCTGCACT
AAACCTATCAACATTATCCATGTTGATCATCCAAGTATCTGGATCCCGACTTTCCCCGATGAATATCT

CATTCACTCTGGGATTAGAATTACCGGGACGTCTATCATACACGAGCCACATCCACAGCCACTGCTTC
ATATTATGAACATTG
+
FFFFFFFFFFFFFFFFFFFFFFFFFFFFFFFFFFFFFFFFFFFFFFFFFFFFFFFFFFFFFFFFFFFFFF
FFFFFFFFFFFFFF:FFFFFFFFFFFFFFFFFFFFFFFFFFFFFFFFFFFFFFFFFFFFFFF:FFFFFFF
FFFFFFFFFFFFFFF
@A00721:190:HGJW7DSXY:4:1444:13503:16454 1:N:0:TCTGCAAG+GCTGCACT
AAACCTATCAACATTATCCATGTTGATCATCCAAGTATCTGGATCCCGACTTTCCCCGATGAATATCT
CATTCACTCTGGGATTAGAATTACCGGGACGTCTATCATACACGAGCCACATCCACAGCCACTGCTTC
ATATTATGAACATTG
+
FFFFFFFFFFFFF:FFFFFFFFFFFFFFFFFFFFFFFFFFFFFFFFFFFFFFFFFFFFFFFFFFF:FFFF
FFFFFFFFFFFFFFFFFFFFFFFFFFFFFFFFFFFFFFFFFFFFFFF:FFFFFFFFFFFFFFFFFFFFFF
F:FFFFFFFFFFFFF
@A00721:190:HGJW7DSXY:4:2104:18466:29684 1:N:0:TCTGCAAG+GCTGCACT
AAACCTATCAACATTATCCATGTTGATCATCCAAGTATCTGGATCCCGACTATCCCCGATGAATATCT
CATTCACTCTGGGATTAGAATTACCGGGACGTCTATCATACACGAGCCACATCCACAGCCACTGCTTC
ATATAATGAACATTG
+
FFFFFFFF,FFFFFFFFF::,:FF:FFFFFF,FF:FF,,F:,FFFFFFF,F,:FFF:FFFFFF,FFFF
FFF,FFF,,:,,,FFFFFFFFF:FF:F:FF:FFFF::FFFFFFFFFFF:FFFFFF:F:FFFFFF,F:F
FF:::F,:FFFF,FF
@A00721:190:HGJW7DSXY:4:2228:5321:17660 1:N:0:TCTGCAAG+GCTGCACT
AAACCTATCAACATTATCCATGTTGATCATCCAAGTATCTGGATCCCGACTTTCCCCGATGAATATCT
CATTCACTCTGGGATTAGAATTACCGGGACGTCTATCATACACGAGCCACATCCACAGCCACTGCTTC
ATATTATGAACATTG
+
FFFFFFFFFFFFF:F:FFFFFFFFFFFFFFFFFFFFFFFFFFFFFFFFFFFFFFFFFFFFFFFFFFFFFF
FF:FFFFFFFFFFFFFFFFFFFFFFFFFFFFFFFFFFFFFFFFFFFFFFFFFFFFFFFFFFFFFFFFF:F
F:FFFFFFFFFFF:F
@A00721:190:HGJW7DSXY:4:2265:15293:30358 1:N:0:TCTGCAAG+GCTGCACT
AAACCTATCAACATTATCCATGTTGATCATCCAAGTATCTGGATCCCGACTTTCCCCGATGAATATCT
CATTCACTCTGGGATTAGAATTACCGGGACGTCTATCATACACGAGCCACATCCACAGCCACTGCTTC
ATATTATGAACATTG
+
FFFFFFFFFFFFFFFFFFFFFFFFFFFFFFFFFFFFFFFFFFFFFFFFFFFFFFFFFFFFFFFFFFFFFF
FFFFFFFFFFFFFFFFFFFFFFFFFFFFFFFFFFFFFFFFFFFFFFFFFFFFFFFFFFFFFFFFFFFFFF
FFFFFFFFFFFFFFF
@A00721:190:HGJW7DSXY:4:2571:14687:18756 1:N:0:TCTGCAAG+GCTGCACT
AAACCTATCAACATTATCCATGTTGATCATCCAAGTATCTGGATCCCGACTTTCCCCGATGAATATCT
CATTCACTCTGGGATTAGAATTACCGGGACGTCTATCATACACGAGCCACATCCACAGCCACTGCTTC
ATATTATGAACATTG
+
FFFFFFFFFFFFFFFFFFFFFFFFFFFFFFFFFFFFFFFFFFFFFFFFFFFFFFFFFFFFFFFFFFFFFF
FFFFFFFFFFFFFF:FFFFFFFFFFFFFFFFFFFFFFFFFFFFFFFFFFFFFFFFFFFFFFFFFFFFFFF
FFFFFFFFFFFFFFF
@A00721:190:HGJW7DSXY:4:1368:8892:28635 1:N:0:TCTGCAAG+GCTGCACT
GAAGCAGTGGCTGTGGATGTGGCTCGTGTATGATAGACGTCCCGGTAATTCTAATCCCAGAGTGAATG
AGATATTCATCGGGGAAAGTCGGGATCCAGATACTTGGATGATCAACATGGATAATGTTGATAGGTTT
AGTGTCGAGATCGGA
+
FFF:FF:,F:F:F:F,F:F,:F,FF:FFFFFFFFFFFFFFFFFF,FFFFFFFFFFFFFF:F,F,FFFF
FFFFF::FFFF:,FFFFF:,,FFFFFFF:,,,,,,::FF::FFFF:FFF::FFFF:,FFF,F:,F:::
FF,:FFFFFFFF:,F

```
@A00721:190:HGJW7DSXY:4:2402:18719:26835 1:N:0:TCTGCAAG+GCTGCACT
GAAGCAGTGGCTGTGGATGTGGCTCGTGTATGATAGACGTCCCGGTAATTCTAATCCCAGAGTGAATG
AGATATTCATCGGGGAAAGTCGGGATCCAGATACTTGGATGATCAACATGGATAATGTTGATAGGTTT
AGTGTCGAGATCGGA
+
FFFFFFFFFFFFFFFFFFFFFFFFFFFFFFFFFFFFFFFFFFFFFFFFFFFFFFFFFFFFFFFFFFFF
FFFFFFFFFFFFFFFFFFFFFFFFFFFFFFFFFF:FFFFFFFFFFFFFFFFFFF:FFFFFFFFFFFFF
FFFFFFFFFFFFFFF
@A00721:190:HGJW7DSXY:4:2454:30129:5415 1:N:0:TCTGCAAG+GCTGCACT
GAAGCAGTGGCTGTGGATGTGGCTCGTGTATGATAGACGTCCCGGTAATTCTAATCCCAGAGTGAATG
AGATATTCATCGGGGAAAGTCGGGATCCAGATACTTGGATGATCAACATGGATAATGTTGATAGGTTT
AGTGTCGAGATCGGA
+
FFFFFFFFFFF:FFFFFFFFFFFFFFFFFFFFFFFFFFFFFFFFFFFFFFFFFFFFFFFFFFFFFFFF
FFFFFFFFFFFFFFFF:FFFFFFFFFFFFFFFFF:FFFFFFFFFFFFFFFFFF,FFFFFFFFFFFFFF
FFFFFFFFFFFFFFF
@A00721:190:HGJW7DSXY:4:2628:5005:9251 1:N:0:TCTGCAAG+GCTGCACT
GAAGCAGTGGCTGTGGATGTGGCTCGTGTATGATAGACGTCCCGGTAATTCTAATCCCAGAGTGAATG
AGATATTCATCGGGGAAAGTCGGGATCCAGATACTTGGATGATCAACATGGATAATGTTGATAGGTTT
AGTGTCGAGATCGGA
+
FFFFFFFFFFFFFFFFFFFFFFFFFFFFFFFFFFFFFFFFFFFFFFFFFFFFFFFFFFFFFFFFF:FF
FFFFFFFFFFFFFFFFFFFFFFFFFFFFFFFFFFF,FFFFFFFFFFFF:FFFFF:FFFFFFFFFFFFF
F:FFFFFFFFFFFFF
@A00721:190:HGJW7DSXY:4:1224:16360:5901 1:N:0:TCTGCAAG+GCTGCACT
TGGCTGTGGATGTGGCTCGTGTATGATAGACGTCCCGGTAATTCTAATCCCAGAGTGAATGAGATATT
CATCGGGGAAAGTCGGGATCCAGATACTTGGATGATCAACATGGATAATGTTGATAGGTTTAGTGTCG
TTTGTGAAAAA
+
FFFFFFFFFFFFFFFFFFFFFFFFFFFFFFFFFFFFFFFFFFFFFFFFFFFFFFFFFFFFFFFFFFFF
FFFFFFFFFFFFFFFFFFFFFFFFFFFFFFFFFFFFFFFFFFFFFFFFFFFFFFFFFFFFFFFFFFFF
F,FFFFFFFFF
@A00721:190:HGJW7DSXY:4:1301:6876:6104 1:N:0:TCTGCAAG+GCTGCACT
TGGCTGTGGATGTGGCTCGTGTATGATAGACGTCCCGGTAATTCTAATCCCAGAGTGAATGAGATATT
CATCGGGGAAAGTCGGGATCCAGATACTTGGATGATCAACATGGATAATGTTGATAGGTTTAGTGTCG
TGTGTGAAAAACGA
+
:FFF:FFFFFFFFFFFFFFFFFFFFFFFFFFFFFFFFFFFFFFFFFF:FFFFFFFFFFFFFFFFFFFF
FFFFFFFFF:FFFFFFFFFFFFFF,FFFFFFF:FFFFFFFFFFF,:FFFFFFFF,FFFFFFFFFFFFF
F,F,F:FFF,FF:F
@A00721:190:HGJW7DSXY:4:1301:6180:6277 1:N:0:TCTGCAAG+GCTGCACT
TGGCTGTGGATGTGGCTCGTGTATGATAGACGTCCCGGTAATTCTAATCCCAGAGTGAATGAGATATT
CATCGGGGAAAGTCGGGATCCAGATACTTGGATGATCAACATGGATAATGTTGATAGGTTTAGTGTCG
TTTGTGAAAAACGAT
+
FFFFFFFFFFFFFFFFFFFFFFFFFFFFFFFFFFFFFFFFFFFFFFFFFFFFFFFFFFFFFFFFFFFF
FFFFFFFFFFFFFFFFFFFFFFFFFFF,FFFFFFFFFFFFFFFFFFFFFFFFFFFF:FFFFFFFFFFF
F,FFF:FFFFF,,FF
@A00721:190:HGJW7DSXY:4:1334:9679:21668 1:N:0:TCTGCAAG+GCTGCACT
TGGCTGTGGATGTGGCTCGTGTATGATAGACGTCCCGGTAATTCTAATCCCAGAGTGAATGAGATATT
CATCGGGGAAAGTCGGGATCCAGATACTTGGATGATCAACATGGATAATGTTGATAGGTTTAGTGTCG
TTTGTGAAAAACGAT
+
FFFFFFFFFFFFFFFFFFFFFFFFFFFFFFFFFFFFFFFFFFFFFFFFFFFFFFFFFFFFFFFFFFFF
```

FFFFFFFFFFFFFFFFFFFFFFFFFFFFFFFFFFFFFFFFFFFFFFFFFFFFFFFFFFFFFFFFFFFF
F:FFF,:FFFFF:F:
@A00721:190:HGJW7DSXY:4:1467:23990:21026 1:N:0:TCTGCAAG+GCTGCACT
TGGCTGTGGATGTGGCTCGTGTATGATAGACGTCCCGGTAATTCTAATCCCAGAGTGAATGAGATATT
CATCGGGGAAAGTCGGGATCCAGATACTTGGATGATCAACATGGATAATGTTGATAGGTTTAGTGTCG
TTTGTGAAAAACGA
+
FFFFFFFFFFFFFFFFFFFFFFFFFFFFFFFFFFFFFFFFFFFFFFFFFFFFFFFFFFFFFFFFFFFF
FFFFFFFFFFFFFFFFFFFFFFFFFFFFFFFFFFFFFFFFFFFFFFFFFFFFFFF:FFFFFFFFFFFF
FFF:FFFFFFF:FF
@A00721:190:HGJW7DSXY:4:1537:6081:21668 1:N:0:TCTGCAAG+GCTGCACT
TGGCTGTGGATGTGGCTCGTGTATGATAGACGTCCCGGTAATTCTAATCCCAGAGTGAATGAGATATT
CATCGGGGAAAGTCGGGATCCAGATACTTGGATGATCAACATGGATAATGTTGATAGGTTTAGTGTCG
TTTGTGAAAAACGA
+
FFFFFFFFFFFFFFFFFFFFFFF:FFFFFFFFFFFFFFFFFFFFFFFFFFFFFFFFFFFFFFFFFFFF
FFFFFFFFF:FFFFFFFFFFF:FFFFFFFFFFFFFFF:FFF:FFFFFFFFFFFF:FFFFFFFFFFFFF
FFFFFFFFFFF,FF
@A00721:190:HGJW7DSXY:4:2136:31882:11490 1:N:0:TCTGCAAG+GCTGCACT
TGGCTGTGGATGTGGCTCGTGTATGATAGACGTCCCGGTAATTCTAATCCCAGAGTGAATGAGATATT
CATCGGGGAAAGTCGGGATCCAGATACTTGGATGATCAACATGGATAATTTTGATAGGTTTAGTGTCG
TTTGTGAAAA
+
FFFFFFFFFFFFFFFFFFFFFFFFF,F:FFFF:FFFFFFFFFFFFFFF:FFFFFFF:FFFFFFFFFFFF
:FFFFFF:FFFFF:FFFFF:,FFFFFFF:FFFFFFFFFF:FFFFFFFFF,FF:FFFF,FFFFFF,FF,
FFF,F,,FFF
@A00721:190:HGJW7DSXY:4:2608:29053:25848 1:N:0:TCTGCAAG+GCTGCACT
TGGCTGTGGATGTGGCTCGTGTATGATAGACGTCCCGGTAATTCTAATCCCAGAGTGAATGAGATATT
CATCGGGGAAAGTCGGGATCCAGATACTTGGATGATCAACATGGATAATGTTGATAGGTTTAGTGTCG
TTTGTGAAAAAAACAT
+
FFFFFFFFFFFFFFFFFFFFFFFFFFFFFFFFFFFFFFFFFFFFFFFFFFFFFFFFFFFFFFFFFFFF
FFFFFFFFFFFFFFFFFFFFFFFFFFFFFFFFFFFFFFFFFFFFFFFFFFFFFFFFFFFFFFFFFFFF
F:F,F::FFFF,,F:
@A00721:190:HGJW7DSXY:4:1366:3775:8249 1:N:0:TCTGCAAG+GCTGCACT
CTGTGGATGTGGCTCGTGTATGATAGACGTCCCGGTAATTCTAATCCCAGAGTGAATGAGATATTCAT
CGGGGAAAGTCGGGATCCAGATACTTGGATGATCAACATGGATAATGTTGATAGGTTTAGTGTCGTTT
GTGAAAAACGATACG
+
FFFFFFFFFFFFFFFFFFFFFFFFFFFFFFFFFFFFFFFFFFFFFFFFFFFFFFFFF:FFFFFFFFFF
FFFFFFFFFFFFFFFFFFFFFFFFFFFFFFFFFFFFFFFFFFFFFF:FFFFFFFFFFFFFFFFFFF:FFF
,FFFFFFF:FFFFFF
@A00721:190:HGJW7DSXY:4:1537:30129:9204 1:N:0:TCTGCAAG+GCTGCACT
CTGTGGATGTGGCTCGTGTATGATAGACGTCCCGGTAATTCTAATCCCAGAGTGAATGAGATATTCAT
CGGGGAAAGTCGGGATCCAGATACTTGGATGATCAACATGGATAATGTTGATAGGTTTAGTGTCGTTT
GTGAAAAACGATACG
+
FFFFFFF:FFFFFFFFFFFFFFFFFFFFFFFFFFFFFFFFFFFFFFFF:FFFFFFFFFFFFFFFFFFF
FFFFFFFFFFFFFFFFFFFFFFFFFFFFFFFFFFFFFFFFFFFF:FFFFFFFFFFFFFFFFFF:FFFFFF
,FFFFFFFFFFFFFF
@A00721:190:HGJW7DSXY:4:1567:3432:1110 1:N:0:TCTGCAAG+GCTGCACT
CTGTGGATGTGGCTCGTGTATGATAGACGTCCCGGTAATTCTAATCCCAGAGTGAATGAGATATTCAT
CGGGGAAAGTCGGGATCCAGATACTTGGATGATCAACATGGATAATGTTGATAGGTTTAGTGTCGTTT
GTGAAAAACGATACG

```
+
FFFFFFFFFFFFFFFFFFFFFFFFFFFFFFFFFFFFFFFFFFFFF:FFFFFFFFFF:FFFFFFFFFFF
F::FFFFFFFFFFFFFF:FFF:FFFFFFFFFFFFFFFFFFF,FFFFFFFFFFFFFFF:F:FFFFFFFF
,FFFFFFF,:FFFF:
@A00721:190:HGJW7DSXY:4:1601:30942:19695 1:N:0:TCTGCAAG+GCTGCACT
CTGTGGATGTGGCTCGTGTATGATAGACGTCCCGGTAATTCTAATCCCAGAGTGAATGAGATATTCAT
CGGGGAAAGTCGGGATCCAGATACTTGGATGATCAACATGGATAATGTTGATAGGTTTAGTGTCGTTT
GTGAAAAAAGATACG
+
FFFFFFFFFFFFFFFFFFFFFFFFFFFFFFFFFFFFFFFFFFFFFFFFFFFFFFFFFFFFFFFFFFFF
FFFFFFFFFFFFFFFFFFFFFFFFFFFFFFFFFFFFFFFFFFFFFF:FFFFFFFFFFFFFFFFFFFF,F
FFF:FFFF::F,:FF
@A00721:190:HGJW7DSXY:4:2154:20546:18255 1:N:0:TCTGCAAG+GCTGCACT
CTGTGGATGTGGCTCGTGTATGATAGACGTCCCGGTAATTCTAATCCCAGAGTGAATGAGATATTCAT
CGGGGAAAGTCGGGATCCAGATACTTGGATGATCAACATGGATAATGTTGATAGGTTTAGTGTCGTTT
GTGAAAAACGATACG
+
F:FFFFFFFF:FFFFFFFFFFFFFFFFFF:FFFFFFFFFFFFFF,FFFFFFFFFF:FFFFFFFFFFF
FFFFFFFFFFFFFFFFFFFFFFFFFF:FFFFFFFFFFFFFFFFFFFFFFFFFFFFFFFFFFFF:FFFFFFF
FFFFFFFF::F,F,:
@A00721:190:HGJW7DSXY:4:2531:13566:22326 1:N:0:TCTGCAAG+GCTGCACT
CTGTGGATGTGGCTCGTGTATGATAGACGTCCCGGTAATTCTAATCCCAGAGTGAATGAGATATTCAT
CGGGGAAAGTCGGGATCCAGATACTTGGATGATCAACATGGATAATGTTGATAGGTTTAGTGTCGTTT
GTGAAAAACGATAC
+
FFFFFFFFFFFFFFFFFFFFFFFFFFFFFFFFFFFFFFFFFFFFFFFFFFFFFFFFFFFFFFFFFFFF
FFFFFFFFFFFFFFFFFFFFFFFFFFFFFFFFFF:FFFFFFFF,FF:FF:FF:FFFFFFFFFFFFFFFF
FFFFFFFF::F,FF
@A00721:190:HGJW7DSXY:4:2544:6479:34131 1:N:0:TCTGCAAG+GCTGCACT
CTGTGGATGTGGCTCGTGTATGATAGACGTCCCGGTAATTCTAATCCCAGAGTGAATGAGATATTCAT
CGGGGAAAGTCGGGATCCAGATACTTGGATGATCAACATGGATAATGTTGATAGGTTTAGTGTCGTTT
GTGAAAAAGATAGGA
+
FFFFFFFFFFFFFFFFFFFFFFFFFFFFFFFFFFFFFFFFFFFFFFFFFFFFFFFFFFFFFFFFFFFF
FFFFFFFFFFFFFFFFFFFFFFFFFFFFFFFFFFFFFFFFFFFFFFFFFFFFFFFFFFFFFFFF:FFF
:F:FFFFF,FF,FFF
@A00721:190:HGJW7DSXY:4:2556:31675:4805 1:N:0:TCTGCAAG+GCTGCACT
CTGTGGATGTGGCTCGTGTATGATAGACGTCCCGGTAATTCTAATCCCAGAGTGAATGAGATATTCAT
CGGGGAAAGTCGGGATCCAGATACTTGGATGATCAACATGGATAATGTTGATAGGTTTAGTGTCGTTT
GTGAAAAAGATCGGA
+
FFFFFFFFFFFFFFFFFFFFFFFFFFFFFFFFFFFFFFFFFFFFFFFFFFFFFFFFFFFFFFFFFFFF
FFFFFFFFFFFFFFFFFFFFFFFF:FFFFFFFFFFFFFFFFFFFFFFFFFFFFFFFFFFFFFFFFFFF
FFFFFFFF,FF,FFF
@A00721:190:HGJW7DSXY:4:2656:7536:20932 1:N:0:TCTGCAAG+GCTGCACT
CTGTGGATGTGGCTCGTGTATGATAGACGTCCCGGTAATTCTAATCCCAGAGTGAATGAGATATTCAT
CGGGGAAAGTCGGGATCCAGATACTTGGATGATCAACATGGATAATGTTGATAGGTTTAGTGTCGTTT
GTGAAAAAGATCGGA
+
FFFFFF:FFFFFFFFFFFFFFFFFFFFFFFFFFFFF:FFFFFFFF:FFFFFFFFFFFFFFFFFFFFFFF
FFFFF::FFFFFFF:FFFFFFFFFFFFFFFFFFFFFFFFFFFFFFFFFFFFFFF:FFFFFFFFFFFF,F
FF,FFFFF,FF:,FF
@A00721:190:HGJW7DSXY:4:2676:9326:17581 1:N:0:TCTGCAAG+GCTGCACT
CTGTGGATGTGGCTCGTGTATGATAGACGTCCCGGTAATTCTAATCCCAGAGTGAATGAGATATTCAT
```

CGGGGAAAGTCGGGATCCAGATACTTGGATGATCAACATGGATAATGTTGATAGGTTTAGTGTCGTTT
GTGAAAAACGATACG
+
FFFFFFFFFFFFFFFFFFFFFFFF:FFFFFFFFFFFFFFFFFFFFFFFFFFFF:FFFFFFFFFFFFF:
FFFFFFFFFFFFFFFFFFFFFFFFFFFFFFFFFFFFFFFFFFFFFFFFFFFFFFFFFFFFFFFFFFFF:F
FFFFF:FF,::FF,F
@A00721:190:HGJW7DSXY:4:1102:30544:36072 1:N:0:TCTGCAAG+GCTGCACT
GTGGATGTGGCTCGTGTATGATAGACGTCCCGGTAATTCTAATCCCAGAGTGAATGAGATATTCATCG
GGGAAAGTCGGGATCCAGATACTTGGATGATCAACATGGATAATGTTGATAGGTTTAGTGTCGTTTTT
GAAAAAAGATACGTT
+
FFFFFFFFFFFFFFFFFFFFFFFFFFFFFFFFFFFFFFFFFFFFFFFFFFFFFFFFFFFFFFFFFFFFFF
FFFFFFFFFFFFFFFFFF:FFFFFFFFFFFFFFFFFFFFFFFFFFFFFFFFFFFFFFFFFFFFFFF:F,F
,FFFFF,::,FF,FF
@A00721:190:HGJW7DSXY:4:1163:31060:2832 1:N:0:TCTGCAAG+GCTGCACT
GTGGATGTGGCTCGTGTATGATAGACGTCCCGGTAATTCTAATCCCAGAGTGAATGAGATATTCATCG
GGGAAAGTCGGGATCCAGATACTTGGATGATCAACATGGATAATGTTGATAGGTTTAGTGTCGTTTGT
GAAAAACGATACGTT
+
FFFFFFFFFFFFFFFFFFFFFFFFFFFFFFFFFFFFFFFFFFFFFFFFFFFFFFFFFFFFFFFFFFFFFF
FFFF:FFFFFFFFFFFFFFFFFFFFFFFFFFFFFFFFFFFFFF:FFFFFFFFFFFFFFFFFFFFFFFFF
,FFFFF:FFFFFFFF
@A00721:190:HGJW7DSXY:4:1237:32949:14622 1:N:0:TCTGCAAG+GCTGCACT
GTGGATGTGGCTCGTGTATGATAGACGTCCCGGTAATTCTAATCCCAGAGTGAATGAGATATTCATCG
GGGAAAGTCGGGATCCAGATACTTGGATGATCAACATGGATAATGTTGATAGGTTTAGTGTCGTTTGT
GAAAAACGATACGTT
+
FFFFFFFFFFFFFFFF:FFFFFFFFFFFFFFFFFFFFFFFFFFFFFFFF:FFFFFFFFFFFFFFFFFFF
FFFFFFFFFFFFFFFFFFFFFF:FF:FFFFFFFFFFFFFF:F,FFFFFFF:FFFFFFFFFFFFFF:F,F
:,F:FF,:FFFFF:F
@A00721:190:HGJW7DSXY:4:1358:8693:6558 1:N:0:TCTGCAAG+GCTGCACT
GTGGATGTGGCTCGTGTATGATAGACGTCCCGGTAATTCTAATCCCAGAGTGAATGAGATATTCATCG
GGGAAAGTCGGGATCCAGATACTTGGATGATCAACATGGATAATGTTGATAGGTTTAGTGTCGTTTGT
GAAAAACGATAAGTT
+
FFFFFFFFFFFFFFFF:FFFFF::FFFFFF:FFFFFF::FFFFFFFFFFFF:FFFFFFF:FF:FFFFF
:::FFF:,FFFF:FFFFFFFFFFF:FF:FFFFFFFFF:FF:FFFFFFFF:FF,FFFFFFFFF:F:FFF
FFFFFFF,:FF,,FF
@A00721:190:HGJW7DSXY:4:2160:20952:21371 1:N:0:TCTGCAAG+GCTGCACT
GTGGATGTGGCTCGTGTATGATAGACGTCCCGGTAATTCTAATCCCAGAGTGAATGAGATATTCATCG
GGGAAAGTCGGGATCCAGATACTTGGATGATCAACATGGATAATGTTGATAGGTTTAGTGTCGTTTGT
GAAAAACGATACGTT
+
FFFFFFFFFFFFFFFFFFFFFFFFFFFFFFFFFFFFFFFFFFF:FFFFFFFFFFFFFFFFFFFFFFFFFF
FFFFFFFFFFFFFFFFFFFFFFFFFFFFFFFFFFFFFFFFFFFFFF:FFFFFFFFFFFFFFFFFFFF,F
FFFFFF,F::F::FF
@A00721:190:HGJW7DSXY:4:2449:20419:4100 1:N:0:TCTGCAAG+GCTGCACT
GTGGATGTGGCTCGTGTATGATAGACGTCCCGGTAATTCTAATCCCAGAGTGAATGAGATATTCATCG
GGGAAAGTCGGGATCCAGATACTTGGATGATCAACATGGATAATGTTGATAGGTTTAGTGTCGTTTGT
GAAAAACGATACGTT
+
FFFFFFFFFFFFFFFFFFFFFFFFFFFFFFFFFFFFFFFFFFFFFFFFFFFFFFFFFFFFFFFFFFFFFF
FFFFFFFFFFFFFFFFFFFFFFFFFFFFFFFFFFFFFFFFFFFF:FF:FFFFFFFFFFFFFFFFFFFFFFF
FFFFFF,:FFFFFFF

```
@A00721:190:HGJW7DSXY:4:2556:8070:2065 1:N:0:TCTGCAAG+GCTGCACT
GTGGATGTGGCTCGTGTATGATAGACGTCCCGGTAATTCTAATCCCAGAGTGAATGAGATATTCATCG
GGGAAAGTCGGGATCCAGATACTTGGATGATCAACATGGATAATGTTGATAGGTTTAGTGTCGTTTGT
GAAAAACGATACGTT
+
FFFFFFFFFFFFFFFFFFFFFFFFFFFFFFFFFFFFFFFFFFFFFFFFFFFF:FFFFFFFFFFFFFFFFF
FFFF:FFFFFFFFFFFFFFFFFFFFFFFFFFFFFFFFFFFFFFFFFFFFFFFFFFFFFFFFFFFFFFF,F
FFFFFFFFFFFFFFF
@A00721:190:HGJW7DSXY:4:2607:32877:10113 1:N:0:TCTGCAAG+GCTGCACT
GTGGATGTGGCTCGTGTATGATAGACGTCCCGGTAATTCTAATCCCAGAGTGAATGAGATATTCATCG
GGGAAAGTCGGGATCCAGATACTTGGATGATCAACATGGATAATGTTGATAGGTTTAGTGTCGTTTGT
GAAAAAAGATAAGTT
+
FFFFFFFFFFFFFFFFFFFFFFFFFFFFFFFFFFFFFFFFFFFFFFFFF:FFFFFFFFFFFFFFFFFFFF
:FFFFFFFFFFFFFFFFFFFFFFFFF:FFFFFFFFFFFF:FFFFFFFFFFFFFF:FF::FFFFFFF,F,F
,,FFFF,F:::,,FF
@A00721:190:HGJW7DSXY:4:1167:17589:12571 1:N:0:TCTGCAAG+GCTGCACT
GGCTCGTGTATGATAGACGTCCCGGTAATTCTAATCCCAGAGTGAATGAGATATTCATCGGGGAAAGT
CGGGATCCAGATACTTGGATGATCAACATGGATAATGTTGATAGGTTTAGTGTCGTTTGTGAAAAACG
ATACGTTCTAGAAGG
+
FFFFFFFFFFFFFFFFFFFFFFFFFFFFFFFFFFFFFFFFFFFFFFFFFFFFFFFFFFFFFFFFFF:FFF
FFFFFFFFFFFFFFFFFFFFFFFFFFFFFFFFFFFFFF:FFFFFFFFFFFFFFFFFFFFFFFFFFFFF:F
:FFFF,FFFF:FFFF
@A00721:190:HGJW7DSXY:4:1245:5430:36104 1:N:0:TCTGCAAG+GCTGCACT
GGCTCGTGTATGATAGACGTCCCGGTAATTCTAATCCCAGAGTGAATGAGATATTCATCGGGGAAAGT
CGGGATCCAGATACTTGGATGATCAACATGGATAATGTTGATAGGTTTAGTGTCGTTTGTGAAAAACG
ATACGTTCTAGAAGG
+
FFFFFFFFFFFFFFFFFFFFFFFFFFFFFFFFFFFFFFFFFFFFFFFFFFFFFFFFFFFFFFFFFFFFFF
FFFFFFFFFFFFFFFFFFFFFFFFFFFFFFFFFFFFFFFFFFFFFFFF:FFFFFFFFFF:,F:FFFFF:F
FFF:F:FFFF:FF::
@A00721:190:HGJW7DSXY:4:1326:3640:18443 1:N:0:TCTGCAAG+GCTGCACT
GGCTCGTGTATGATAGACGTCCCGGTAATTCTAATCCCAGAGTGAATGAGATATTCATCGGGGAAAGT
CGGGATCCAGATACTTGGATGATCAACATGGATAATGTTGATAGGTTTAGTGTCGTTTGTGAAAAACG
ATACGTTCTAGAAG
+
F:FFFFFFFFFFFFFFFFFFFFFFFFFFFFFFFFFFFFFFFFFFFFFFFFFFFFFFFFFFFFFFFFFFFF
FFFFFFFFFFFF:F:FFFFFFFFFFFFFFFFFFF:FF:FFFFF,FFFFFFFFFFFFFF,F,:,:FFFF,,
::FFFFF,:F,FF:
@A00721:190:HGJW7DSXY:4:1448:29197:17331 1:N:0:TCTGCAAG+GCTGCACT
GGCTCGTGTATGATAGACGTCCCGGTAATTCTAATCCCAGAGTGAATGAGATATTCATCGGGGAAAGT
CGGGATCCAGATACTTGGATGATCAACATGGATAATGTTGATAGGTTTAGTGTCGTTTGTGAAAAACG
ATACGTTCTAGAAGG
+
FFFFFFFFFFFFFFFFFFFFFFFFFFFF:FFFFFFFFFFFFFFFFFFFFFFFFFFFFF:FFFFFFFFFFF
FFFFFFFFFFFFFFFFFFFFFFFFFFFFFFFFFF:FFFFFFFFFFFFFFFFFFFFFFFFF:FFFFFFFF:
FFF,:FFFFF,FF,:
@A00721:190:HGJW7DSXY:4:1524:9706:16047 1:N:0:TCTGCAAG+GCTGCACT
GGCTCGTGTATGATAGACGTCCCGGTAATTCTAATCCCAGAGTGAATGAGATATTCATCGGGGAAAGT
CGGGATCCAGATACTTGGATGATCAACATGGATAATGTTGATAGGTTTAGTGTCGTTTGTGAAAAACG
ATACGTTCTAGAAGG
+
FFFFFFFFFFFFFFFFFFFFFFFFFFFFFFFFFFFFFFFFFFFFFFFFFFFFFFFFFFFFFFFFFFFFFF
```

```
FFFFFFFFFFFFFFFFFFFFFFFFFFFFFFFFFFFFFFFFFFFFFFFFFFFFFFFFFF,FFFF,FFFF::
:FF,FFF:FF,FF:F
@A00721:190:HGJW7DSXY:4:1630:24668:33692 1:N:0:TCTGCAAG+GCTGCACT
GGCTCGTGTATGATAGACGTCCCGGTAATTCTAATCCCAGAGTGAATGAGATATTCATCGGGGAAAGT
CGGGATCCAGATACTTGGATGATCAACATGGATAATGTTGATAGGTTTAGTGTCGTTTGTGAAAAACG
ATACGTTCTAGAAGG
+
FFFFFFFFFFFFFFFFFFFFFFFFFFFFFFFFFFFFFFFFFFFFFFFFFFFFFFFFFFFFFFFFFFFFFF
FFFFFFFFFFFFFFFFFFFFFFFFFFFF:FFFFFFFFFFFFFFFFFFFFFFFFFFFFFFFFFF:FF,:,F
FFF:F,F,FF:FF,:
@A00721:190:HGJW7DSXY:4:2222:16758:7592 1:N:0:TCTGCAAG+GCTGCACT
GGCTCGTGTATGATAGACGTCCCGGTAATTCTAATCCCAGAGTGAATGAGATATTCATCGGGGAAAGT
CGGGATCCAGATACTTGGATGATCAACATGGATAATGTTGATAGGTTTAGTGTCGTTTGTGAAAAACG
ATAAGTTCTAGAAGG
+
FFFFFFFFFFFFFFFFFFFFFFFFFF:FFFFFFFFFFFFFFFFFFF:FFFFFFFFFFFFFFFFFFFFFFF
FFFFFFFFFFF:FFFFFFFFFFFFFFFFFFFFFFFFFFFFFFFFFFFFFFFFFFFFFF,FFF,FFFFF,:
F:F,,FF,:F,:F,F
@A00721:190:HGJW7DSXY:4:2271:25364:26537 1:N:0:TCTGCAAG+GCTGCACT
GGCTCGTGTATGATAGACGTCCCGGTAATTCTAATCCCAGAGTGAATGAGATATTCATCGGGGAAAGT
CGGGATCCAGATACTTGGATGATCAACATGGATAATGTTGATAGGTTTAGTGTCGTTTGTGAAAAACG
ATACGTTCTAGAAGG
+
FFFFFFFFFFFFFFFFFFFFFFFFFFFFFFFFFFFFFFFFFFFFFFFFFFFFFFFFFFFFFFFFFF:FFF
FFFFFFFFFFFFFFFFFFFFFFFFFFFFFFFFFFFFFFFFFFFFFFFFFFFFFFFFFFFF:FFFFFFFFF
FFFFF:F,:FFFF,F
@A00721:190:HGJW7DSXY:4:2315:13630:8249 1:N:0:TCTGCAAG+GCTGCACT
GGCTCGTGTATGATAGACGTCCCGGTAATTCTAATCCCAGAGTGAATGAGATATTCATCGGGGAAAGT
CGGGATCCAGATACTTGGATGATCAACATGGATAATGTTGATAGGTTTAGTGTCGTTTGTGAAAAAAG
ATAAGTTCTAGAAGG
+
FFFFFFFFFFFFFFFFFFFFFFFFFFFFFFFFFFFFFFFFFFFFFFFFFFFFFFFFFFFFFFFFFFFFFF
FFFFFFFFFFFFFFFFFFFFFFFFFFFFFFFFFFFFFFFFFFFFFFFFFFFFFFFFFF:FFF:FFFFF,:
FFF,,:F,FF,,F,F
@A00721:190:HGJW7DSXY:4:2433:22019:6840 1:N:0:TCTGCAAG+GCTGCACT
GGCTCGTGTATGATAGACGTCCCGGTAATTCTAATCCCAGAGTGAATGAGATATTCATCGGGGAAAGT
CGGGATCCAGATACTTGGATGATCAACATGGATAATGTTGATAGGTTTAGTGTCGTTTGTGAAAAACG
ATACGTTCTAGAAGG
+
FFFFFFFFFFFFFFFFFFFFFFFFFFFFFFFFFFFFFFFFFFFFFFFFFFFFFFFFFFFFFF:FFFFF:F
FFFFFFFFFFFFFF:FFFFFFFFFFFFFFFFFFFF:FFFFFFFFFFFFFFFFFFFFFF:FFF:FFFFF,,
:FFF,FF,FF,FF,:
@A00721:190:HGJW7DSXY:4:2449:10050:10128 1:N:0:TCTGCAAG+GCTGCACT
GGCTCGTGTATGATAGACGTCCCGGTAATTCTAATCCCAGAGTGAATGAGATATTCATCGGGGAAAGT
CGGGATCCAGATACTTGGATGATCAACATGGATAATGTTGATAGGTTTAGTGTCGTTTGTGAAAAACG
ATACGTTCTAGAAGG
+
FFFFFFFFFFFFFFFFFFFFFFFFFFFFFFFFFFFFFFFFFFFFFFFFFFFFFFFFFFFFFFFFFFFFFF
FFFFFFFFFFFFFFF:FFFFFFFFFFFFFFFFFFFFFFFFFFFFFFFFFFFFFFFFFF,FFFFFFFFF,F
FFF:FFF:FFFFF,F
@A00721:190:HGJW7DSXY:4:2462:14235:6981 1:N:0:TCTGCAAG+GCTGCACT
GGCTCGTGTATGATAGACGTCCCGGTAATTCTAATCCCAGAGTGAATGAGATATTCATCGGGGAAAGT
CGGGATCCAGATACTTGGATGATCAACATGGATAATGTTGATAGGTTTAGTGTCGTTTGTGAAAAACG
ATACGTTCTAGAAGG
```

+
FFFFFFFFFFFFFFFFFFFFFFFFFFFFFFFFFFFFFFFFFFFFFFFFFFFFFFFFFFFFFFFFFFFFFF
FFFFFFFFFFFFFFFFFFFFFFFFFFFFFFFFFFFFFFFFFFFFFFFFFFFFFFFFFFF:FFFFFFFF::
FFF,FFFFFF,FF:F
@A00721:190:HGJW7DSXY:4:2513:30201:15186 1:N:0:TCTGCAAG+GCTGCACT
GGCTCGTGTATGATAGACGTCCCGGTAATTCTAATCCCAGAGTGAATGAGATATTCATCGGGGAAAGT
CGGGATCCAGATACTTGGATGATCAACATGGATAATGTTGATAGGTTTAGTGTCGTTTGTGAAAAACG
ATAAGTTCTATAA
+
FFFFFFFFFFFFFFFFFFFFFFFFFFFFFFFFFFFFFFFFFFFFFFFFFFFFFFFFFFFFFFFFFFFFFF
FFFFFFFFFFFFFFF:FFFFFFFFFFFFFFFFFFFFFFFFFFFFFF:FFFFFFFFFF,F,FF,FFFF,,
FFF,FF:,FF,F:
@A00721:190:HGJW7DSXY:4:2549:11903:30029 1:N:0:TCTGCAAG+GCTGCACT
GGCTCGTGTATGATAGACGTCCCGGTAATTCTAATCCCAGAGTGAATGAGATATTCATCGGGGAAAGT
CGGGATCCAGATACTTGGATGATCAACATGGATAATGTTGATAGGTTTAGTGTCGTTTGTGAAAAACG
ATACGTTCTAGAAGG
+
FFFFFFFFFFFFFFFFFFFFFFFFFFFFFFFFFFFFFFFFFFFFFFFFFFFFFFFFFFFFFFFFFFFFFF
FFFFFFFFFFFFFFFFFFFFFFFFFFFFFFFFFFFFFFFFFFFFFFFFFFFFFFFF,FFFFFFFFFFF
FF:::FFFFFFFFFF
@A00721:190:HGJW7DSXY:4:2636:4960:20979 1:N:0:TCTGCAAG+GCTGCACT
GGCTCGTGTATGATAGACGTCCCGGTAATTCTAATCCCAGAGTGAATGAGATATTCATCGGGGAAAGT
CGGGATCCAGATACTTGGATGATCAACATGGATAATGTTGATAGGTTTAGTGTCGTTTGTGAAAAACG
ATACGTTCTAGAAGG
+
FFFFFFFFFFFFFFFFFFFFFFFFFFFFFFFFFFFFFFFFFFFFFFFFFFFFFFFFFFFFFFFFFFFFFF
FFFFFFFFFFFFFFFFFFFFFFFFFFFFFFFFFF:FFFFFFFFF:FFFFFFFFFFFFF:F,F::FFFFF:
FFFF:FF:FF,FF:F
@A00721:190:HGJW7DSXY:4:1266:29939:23500 1:N:0:TCTGCAAG+GCTGCACT
AGCTCGTGTATGATAGACGTCCCGGTAATTCTAATCCCAGAGTGAATGAGATATTCATCGGGGAAAGT
CGGGATCCAGATACTTGGATGATCAACATGGATAATGTTGATAGGTTTAGTGTCGTTTGTGAAAAACG
ATACGTTCTAGAAGG
+
FFFFFFFFFFFFFFFFFFFFFFFFFFFFFFFFFFFFFFFFFFFFFFFFFFFFFFFFFFFFFFFFFFFFFF
FFFFFFFFFFFFFFFFFFFFFFFFFFFFFFFFFFFFFFFFFFFFFF:FFFFFFFFFF:FFFFFFFFF,F
FFF:FFFFFF:FF:F
@A00721:190:HGJW7DSXY:4:1371:30454:33567 1:N:0:TCTGCAAG+GCTGCACT
AGCTCGTGTATGATAGACGTCCCGGTAATTCTAATCCCAGAGTGAATGAGATATTCATCGGGGAAAGT
CGGGATCCAGATACTTGGATGATCAACATGGATAATGTTGATAGGTTTAGTGTCGTTTGTGAAAAACG
ATACGTTCTAGAAGG
+
FFFFFFFFFFFFFFFFFFFFFFFFFFFFFFFFFFFFFFFFFFFFFFFFFFFFFFFFFFFFFFFFF:FF:
FFFFFFFFFFFFFF::FFF:FFFFFFFFFFFFFFF:FFFFFFFFFFFFFFFFFFF:FFFFF:FFFF,F
:F:FFFF:FFFFFFF
@A00721:190:HGJW7DSXY:4:1662:11017:13213 1:N:0:TCTGCAAG+GCTGCACT
AGCTCGTGTATGATAGACGTCCCGGTAATTCTAATCCCAGAGTGAATGAGATATTCATCGGGGAAAGT
CGGGATCCAGATACTTGGATGATCAACATGGATAATGTTGATAGGTTTAGTGTCGTTTGTGAAAAACG
ATACGTTCTAGAAGG
+
FFFFFFFFFFFFFFFFFFFFFFFFFFFFFFFFFFFFFFFFFFFFFFFFFFFFFFFFFFFFFFFFFFFFFF
FFFFFFFFFFF:FFFFFFFFFFFFFFFFFFFFFFFFFFF:FFFFFFFFFFFFFFFFFFFF,FFFFFFF:F
FFFFFFFFFFFFFFF
@A00721:190:HGJW7DSXY:4:2243:20103:2769 1:N:0:TCTGCAAG+GCTGCACT
AGCTCGTGTATGATAGACGTCCCGGTAATTCTAATCCCAGAGTGAATGAGATATTCATCGGGGAAAGT

CGGGATCCAGATACTTGGATGATCAACATGGATAATGTTGATAGGTTTAGTGTCGTGTGTGAAAAACG
ATACGTTCTAGAAGG
+
FFFFFFFFFFFFFFFFFFFFFFFFFFFFFFFFFFFFFFFFFFFFFFFFFFFFFFFFFFFFFFFFFFFF
FFFFFFFFFFF:FFFFFFFFFFFFFFFFFFFFFFFFFF:FFFFFFFFFFFFFFFFF,FF,F:FFFF,F
FFF:,FF:FF:FFF:
@A00721:190:HGJW7DSXY:4:2374:8874:26193 1:N:0:TCTGCAAG+GCTGCACT
AGCTCGTGTATGATAGACGTCCCGGTAATTCTAATCCCAGAGTGAATGAGATATTCATCGGGGAAAGT
CGGGATCCAGATACTTGGATGATCAACATGGATAATGTTGATAGGTTTAGTGTCGTTTGTGAAAAACG
ATACGTTCTAGAAG
+
FF:,:,FFFF,:FFF:F,FFFFF::FFFFFFFFFFFFFF:FF::F,:FFFFFFFFFFFF::FFF:::,
FFF,FF,,FFF,FFFF:FF:FFFFF:,:,FFF,:F:,:F,FF:FFF,FFF,,FFF,,,::F:FFFF:,
F,:F:,,FF:,FF:
@A00721:190:HGJW7DSXY:4:2433:13865:33458 1:N:0:TCTGCAAG+GCTGCACT
AGCTCGTGTATGATAGACGTCCCGGTAATTCTAATCCCAGAGTGAATGAGATATTCATCGGGGAAAGT
CGGGATCCAGATACTTGGATGATCAACATGGATAATGTTGATAGGTTTAGTGTCGTTTGTGAAAAACG
ATACGTTCTAGAAGG
+
FFFFFFFFFFFFFFFFFFFFFFFFFFFFFFFFFFFFFFFFFFFFFFFFFFFFFFFFFFFFFFFFFFFF
FFFFFFFFFFFFFFFFFFFFFFFFFFFFFFFFF:FFFFFFFFFFFFFFFFFFFFFFFF:::,FFFF,:
::FF:,F:FF,FFFF
@A00721:190:HGJW7DSXY:4:1120:15899:32252 1:N:0:TCTGCAAG+GCTGCACT
CTCGTGTATGATAGACGTCCCGGTAATTCTAATCCCAGAGTGAATGAGATATTCATCGGGGAAAGTCG
GGATCCAGATACTTGGATGATCAACATGGATAATGTTGATAGGTTTAGTGTCGTTTGTGTAAAACGAT
ACGTTCTAGAAGGTC
+
FFFFFFFFFFFFFFFFFFFFFFFFFFFFFFFFFFFFFFFFFFFFFFFFFFFFFFFFFFFFFFFFFFFF
FFFFFFFFFF:FFFFFFFFFFFFFFFFFFF:FF,FFFFFFFFFFFFFFFFFFFF,FFFF,FFFF,::F
FF:,F:FF::F:F::
@A00721:190:HGJW7DSXY:4:1145:23710:2347 1:N:0:TCTGCAAG+GCTGCACT
CTCGTGTATGATAGACGTCCCGGTAATTCTAATCCCAGAGTGAATGAGATATTCATCGGGGAAAGTCG
GGATCCAGATACTTGGATGATCAACATGGATAATGTTGATAGGTTTAGTGTCGTTTGTGAAAAACGAT
ACGTTCTAGAAGGTC
+
FFFFFFFFFFFF:FFFFFFFFFFFFFFFFFFFFFFFFFFFFFFFFFFFFFFFFFFFFFFFFFFFFFF
FFFFFFFFFFFFFFFFFFFFFFFFFFFFFFFF:FFF:FFFFFFFFFFFFFFFFF:F:FFFFFFFF:FF
F::FFFFF,FFFFF:
@A00721:190:HGJW7DSXY:4:1157:30535:35493 1:N:0:TCTGCAAG+GCTGCACT
CTCGTGTATGATAGACGTCCCGGTAATTCTAATCCCAGAGTGAATGAGATATTCATCGGGGAAAGTCG
GGATCCAGATACTTGGATGATCAACATGGATAATGTTGATAGGTTTAGTGTCGTTTGTGAAAAACGAT
ACGTTCTAGAAGGTC
+
FFFFFFFFFFFFFFFFFFFFFFFFFFFFFFFFFFFFFFFFFFFFFFFFFFFFFFFFFFFFFF:FFFFF
FFFFFFFFFFFFFFFFFFFFFFFFFFFFFFFFFFFFFFFFFFFFFFFFFFFFFF,FFFF:FFFFFFF:
FFFFF:FFFFF,FF:
@A00721:190:HGJW7DSXY:4:1250:30065:23688 1:N:0:TCTGCAAG+GCTGCACT
CTCGTGTATGATAGACGTCCCGGTAATTCTAATCCCAGAGTGAATGAGATATTCATCGGGGAAAGTCG
GGATCCAGATACTTGGATGATCAACATGGATAATGTTGATAGGTTTAGAGTCGTTTTTGAAAAACGAT
A
+
FFF:FFFFF:FFFFFFFF,F,::,F,FF:FFFFF,FFFFFF,FFFFFFFFFF:FFF:F,FFFFF:FFF
F,F:,FFF,F:,FFFF:,:FFF:FFFFFFF::FFFF:FF::FFF,FFF,FFF:F,F,FFFF:FF:,FF
F

```
@A00721:190:HGJW7DSXY:4:1259:25907:1517 1:N:0:TCTGCAAG+GCTGCACT
CTCGTGTATGATAGACGTCCCGGTAATTCTAATCCCAGAGTGAATGAGATATTCATCGGGGAAAGTCG
GGATCCAGATACTTGGATGATCAACATGGATAATGTTGATAGGTTTAGTGTCGTTTGTGAAAAACGAT
ACGTTCTAGAAGGTC
+
FFFFFFFFFFFF:FFFFFFFFFFFFFFFFFFFFFFFFFFFFFFFFFFFFFFFFFFFFFFFFF:FFFFF
FFFFFFFFFFFFFFFFFFFFFFFFFFFFFFFFFFFFFFFFFFFFFFFFFFFFFF:FFFFFFFFFF:FF
FFFFF::F:FFFFFF
@A00721:190:HGJW7DSXY:4:1369:8395:4977 1:N:0:TCTGCAAG+GCTGCACT
CTCGTGTATGATAGACGTCCCGGTAATTCTAATCCCAGAGTGAATGAGATATTCATCGGGGAAAGTCG
GGATCCAGATACTTGGATGATCAACATGGATAATGTTGATAGGTTTAGTGTCGTTTGTGAAAAACGAT
ACGTTCTAGAAGGTC
+
FFFFFFFFFFFF:FFFFFFFFFFF:FFFFFFFFFFFFFFFFFFFFFFFFFFFFFFFFFFFFFFFFFFF
FFFFFF:FFFFFFFFFFFF:FFFFFFFFFFFFFFFFFFFF:FFFFFFFFFFFFF:FFF:FFFFFFFFF
FFFFF:FF:FFFFFF
@A00721:190:HGJW7DSXY:4:1375:19560:9784 1:N:0:TCTGCAAG+GCTGCACT
CTCGTGTATGATAGACGTCCCGGTAATTCTAATCCCAGAGTGAATGAGATATTCATCGGGGAAAGTCG
GGATCCAGATACTTGGATGATCAACATGGATAATGTTGATAGGTTTAGTGTCGTTTGTGAAAAACGAT
ACGTTCTAGAAGGTC
+
FFFFFFFFFFFFFFFFFFFFFFFFFFFFFFFFFFFFFFFFFFFFFFFFFFFFFFFFFFFFFFFFFFFF
FFFFFFFFFFFFFFFFFFFFFFFFFF:FFFFFFFFFFFFFFFFFFFFFFFFFFFFFFF:FFFFFFFF:
FF:,F::F:FF:FFF
@A00721:190:HGJW7DSXY:4:1446:16098:29152 1:N:0:TCTGCAAG+GCTGCACT
CTCGTGTATGATAGACGTCCCGGTAATTCTAATCCCAGAGTGAATGAGATATTCATCGGGGAAAGTCG
GGATCCAGATACTTGGATGATCAACATGGATAATGTTGATAGGTTTAGTGTCGTTTGTGAAAAACGAT
ACGTTCTAGAAGGTC
+
FFFFFFFFFFFFFFFFFFFFFFFFFFFFFFFFFFFFFFFFFFF:FFFFFFFFFFFFFFFFFFFFFFFF
FFFFFFFFFFFFFFFFFFFFFFFFFFFFFFFFFFFFFFFFFFFFFFFFFFFFFF:FFFF:FFFFFFFF
FFF,FFFFFFF,FFF
@A00721:190:HGJW7DSXY:4:1560:6967:29528 1:N:0:TCTGCAAG+GCTGCACT
CTCGTGTATGATAGACGTCCCGGTAATTCTAATCCCAGAGTGAATGAGATATTCATCGGGGAAAGTCG
GGATCCAGATACTTGGATGATCAACATGGATAATGTTGATAGGTTTAGTGTCGTTTGTGAAAAACGAT
ACGTTCTAGAAGGTC
+
FFFFFFFFFFFFFFFFFFFFFFFFFFFFFFFFFFFFFFFFFFFFFFFFFFFFFFFFFFFFFFFFFFFF
FFFFFFFFFFFFFFFFFFFFFFFFFFFFFFFFFFFF:FFFFFFFFFFFFFFFFF,F:F:FFFFF:FFF
FF:,F:FFFFFFFFF
@A00721:190:HGJW7DSXY:4:2326:19325:32142 1:N:0:TCTGCAAG+GCTGCACT
CTCGTGTATGATAGACGTCCCGGTAATTCTAATCCCAGAGTGAATGAGATATTCATCGGGGAAAGTCG
GGATCCAGATACTTGGATGATCAACATGGATAATGTTGATAGGTTTAGTGTCGTTTGTGAAAAACGAT
ACGTTCTAGAAGGTC
+
FFFFFFFFFFFFFFFFFFFFFFFFFFFFFFFFFFFFFFFFFFFFFFFFFFFFFFFFFFFFFFFFFFFF
FFFFFFFFFFFFFFFFFFFFFFFFFFFFFFFFFFFFFFFFFFFFFFFFFFFF:F:FFFF:FFFF:::F
FF,FF,FF:FF,:F:
@A00721:190:HGJW7DSXY:4:2339:2871:6590 1:N:0:TCTGCAAG+GCTGCACT
CTCGTGTATGATAGACGTCCCGGTAATTCTAATCCCAGAGTGAATGAGATATTCATCGGGGAAAGTCG
GGATCCAGATACTTGGATGATCAACATGGATAATGTTGATAGGTTTAGTGTCGTTTGTGAAAAACGAT
ACGTTCTAGAAGGTC
+
FFFFFFFFFFFFFFF:FFFFFFFFFFFFFFFFF:FFFFFFFFFFFFFFFFFFFFFFFFFFFFFFFFFF
```

```
FF:FFFFFFFFFFFFFFFFFFFFFFFFFFFFFF:FFFFFFFFFFFFFFFFFFFFF,F,F,FFFFF:::F
F:,FF,FF,FF,:F:
@A00721:190:HGJW7DSXY:4:2341:28800:2988 1:N:0:TCTGCAAG+GCTGCACT
CTCGTGTATGATAGACGTCCCGGTAATTCTAATCCCAGAGTGAATGAGATATTCATCGGGGAAAGTCG
GGATCCAGATACTTGGATGATCAACATGGATAATGTTGATAGGTTTAGTGTCGTTTGTGAAAAACGAT
ACGTTCTAGAAGGT
+
FFFFFFFFFFFFFFFFFFFFFFFFFFFFFFFFFFFFFFFFFFFFFFFFFFFFFFFFFFFFFFFFFFFF
FFFFFFFFFFFFFFFFFFFFFFFFFFFFFFFF:FFFFFFFFFFFFFFFFFFFF,FFF,:FFFF::FF
F,::F,,F::F,FF
@A00721:190:HGJW7DSXY:4:2351:30409:30671 1:N:0:TCTGCAAG+GCTGCACT
CTCGTGTATGATAGACGTCCCGGTAATTCTAATCCCAGAGTGAATGAGATATTCATCGGGGAAAGTCG
GGATCCAGATACTTGGATGATCAACATGGATAATGTTGATAGGTTTAGTGTCGTTTGTGAAAAACGAT
ACGTTCTAGAAGGTC
+
FFFFFFFFFFFFFFFFFFFFFFFFFFFFFFFFFFFFFFFFFFFFFFFFFFFFFFFFFFFFFFF:FFFFF
FFFFFFFFFFFFFFFFFFFFFFFFFF:FFFFFFFFF:FFFFFFFFFFFFFFFF,FFFF:FFFFFF::
FF,FF,:FF:F:FFF
@A00721:190:HGJW7DSXY:4:2366:29930:32409 1:N:0:TCTGCAAG+GCTGCACT
CTCGTGTATGATAGACGTCCCGGTAATTCTAATCCCAGAGTGAATGAGATATTCATCGGGGAAAGTCG
GGATCCAGATACTTGGATGATCAACATGGATAATGTTGATAGGTTTAGTGTCGTTTGTGAAAAACGAT
ACGTTCTAGAAGGT
+
FFFFFFFFFFFFFFFFFFFFFFFFFFFFFFFFFFFFFFFFFFFFFFFFFFFFFFFFFFFFFFFFFFFF
FFFFFFFFFFFFFFFFFFFFFFFFFFFFFF:FFFFFFFF:FFFFFFFFFFFFFF,F:FFFFFFFF,:F
::F,FFF:F:FFFF
@A00721:190:HGJW7DSXY:4:2414:31521:16877 1:N:0:TCTGCAAG+GCTGCACT
CTCGTGTATGATAGACGTCCCGGTAATTCTAATCCCAGAGTGAATGAGATATTCATCGGGGAAAGTCG
GGATCCAGATACTTGGATGATCAACATGGATAATGTTGATAGGTTTAGTGTCGTTTGTTAAAAAAGAT
ACGTTATAGAAGGT
+
FFFFFFFFFFFFFFFFFFFFFFFFFF:FFFFF:FFFFFFFFFFFFFFFFFFFFFFFFFFFFFFFFFFF
FFFFFFFFFFF:FFFFFFFFFFFFFFFFFFFF,FFFFFF:FFFFFFFFFFFFFF,FFF,,FFFF,,:F
::,:F,:F,:F,::
@A00721:190:HGJW7DSXY:4:2416:14886:8609 1:N:0:TCTGCAAG+GCTGCACT
CTCGTGTATGATAGACGTCCCGGTAATTCTAATCCCAGAGTGAATGAGATATTCATCGGGGAAAGTCG
GGATCCAGATACTTGGATGATCAACATGGATAATGTTGATAGGTTTAGTGTCGTTTGTGAAAAACGAT
ACGTTATAGAAGGT
+
FFFFFFFFFFFFFFFFFFFFFFFFFFFFFFFFFFFFFFFFFFFF:FFFFFFFFFFFFFFFFFFFFFFFF
FFFFFFFFFFFFFFFFFFFFFFFFFFF:FFF:FFFFFFFFFFFFFFFFFFFFFFFF::FFF,FFFF,FF:
F,:FF,:F,FF,FF
@A00721:190:HGJW7DSXY:4:2436:6271:25974 1:N:0:TCTGCAAG+GCTGCACT
CTCGTGTATGATAGACGTCCCGGTAATTCTAATCCCAGAGTGAATGAGATATTCATCGGGGAAAGTCG
GGATCCAGATACTTGGATGATCAACATGGATAATGTTGATAGGTTTAGTGTCGTTTGTGAAAAACGAT
ACGTTCTAGAAGGTC
+
:FFFFFFFFFFFFF:FFFFFFFFFFFFFFFFFF:FFFFFFFFF,FFFFFFFFFFFFFFFFFFF:FFF:
FFFFFF:FFFF,FFFFFFFF:FFFFFFFFF,FFFFFFFF:FFFFFFFFFFFFFF::,FF,FFF,F:,F
FF,:FF:F,,F:FF:
@A00721:190:HGJW7DSXY:4:2449:31620:17456 1:N:0:TCTGCAAG+GCTGCACT
CTCGTGTATGATAGACGTCCCGGTAATTCTAATCCCAGAGTGAATGAGATATTCATCGGGGAAAGTCG
GGATCCAGATACTTGGATGATCAACATGGATAATGTTGATAGGTTTAGTGTCGTTTGTGAAAAACGAT
ACGTTCTAGAAGGT
```

+
FFFFFFFFFFFFFFFFFFFF:FFF:FFFFFFFFFFFFFFFFFFFFFFFFFFFFFFFFFFFFFFFFFFF
FFFFFFFFFFFF:FFFFFFFFFFFFF:FFFFF,FFFFFFFFFFFFFFFFFFFFF,FF:FFFFFF,F:F
FFFFF,FF,,FFFF
@A00721:190:HGJW7DSXY:4:2522:27190:32988 1:N:0:TCTGCAAG+GCTGCACT
CTCGTGTATGATAGACGTCCCGGTAATTCTAATCCCAGAGTGAATGAGATATTCATCGGGGAAAGTCG
GGATCCAGATACTTGGATGATCAACATGGATAATGTTGATAGGTTTAGTGTCGTTTGTGAAAAACGAT
ACGTTCTAGAAGGTC
+
FFFFFFFFFFFFFFFFFFFFFFFFFFFFFFFFFFFFFFFFFFFFFFFFFFFFFFFFFFFFF:FFFFFF
FF,FFFFFFFFFFFFFFFFFFFFFFFFFFFFFFFFFFFFFFFFFFFFFFFFFFF:FFFFFFFFF:FFF
FF:FF,FF,FF,F::
@A00721:190:HGJW7DSXY:4:2534:32868:2206 1:N:0:TCTGCAAG+GCTGCACT
CTCGTGTATGATAGACGTCCCGGTAATTCTAATCCCAGAGTGAATGAGATATTCATCGGGGAAAGTCG
GGATCCAGATACTTGGATGATCAACATGGATAATGTTGATAGGTTTAGTGTCGTTTGTGAAAAACGAT
ACGTTCTAGAAGGTC
+
FFFFFFFFFFFFFFFFFFFFFFFFFFFFFFFFFFFFFFFFFFFFFFFFFFFFFFFFFFFFFFFFFFFF
FFFFFFFFFFF:FFFFFFFFFFFFFFFFFFFFFFFFFFFFFFFFFFFF:FFFFFFFFFFFFFF:,FFF
F,FFF,:,,,F,FF:
@A00721:190:HGJW7DSXY:4:2644:22679:2221 1:N:0:TCTGCAAG+GCTGCACT
CTCGTGTATGATAGACGTCCCGGTAATTCTAATCCCAGAGTGAATGAGATATTCATCGGGGAAAGTCG
GGATCCAGATACTTGGATGATCAACATGGATAATGTTGATAGGTTTAGTGTCGTTTGTGAAAAACGAT
ACGTTCTAGAAGGTC
+
FFFFFFFFFFFFFFFFFFFFFFFFFFFFFFFFFFFFFFFFFFFFFFFFFFFFFFFFFFFFFFFFFFFF
FFFFFFFFFFFFFFFFFFFFFFFFFFFFFFFFF,F,FF:FFFFFFFFFFFFFFF:FFFF:F,FF,FF:
FFF:F:FF,FF:FF:
@A00721:190:HGJW7DSXY:4:2647:4101:28855 1:N:0:TCTGCAAG+GCTGCACT
CTCGTGTATGATAGACGTCCCGGTAATTCTAATCCCAGAGTGAATGAGATATTCATCGGGGAAAGTCG
GGATCCAGATACTTGGATGATCAACATGGATAATGTTGATAGGTTTAGTGTCGTTTGTGAAAAACGAT
ACGTTCTAGAAGGTC
+
FFFFFFFFFFFFFFFFFFFFFFFFFFFFFFFFFFFFFFFFFFFFFFFFFFFFFFFFFFFFFFFFFFFF
FFFFFFFFFFFFFFFFFFFFFFFFFFFFFFFFFFFFFFFFFFFFFFFFFFFFFF,:,::::FFF,F,F
F::FF::F::F:FFF
@A00721:190:HGJW7DSXY:4:2662:5204:21997 1:N:0:TCTGCAAG+GCTGCACT
CTCGTGTATGATAGACGTCCCGGTAATTCTAATCCCAGAGTGAATGAGATATTCATCGGGGAAAGTCG
GGATCCAGATACTTGGATGATCAACATGGATAATGTTGATAGGTTTAGTGTCGTTTGTGAAAAACGAT
ACGTTCTAGAAGGTC
+
FFFFFFFFFFFFFFFFFFFFFFFFFFFFFFFFFFFFFFFFFFFFFFFFFFFFFFFFFFFFFFFFFFFF
FFFFFFFFFFFFFFFFFFFFFFFFFFFFFFFFFFFFFFFFFFFFFFFFFFFFFF,F:FFFFFFF:FFF
F:FFFFFF,FF:FFF
@A00721:190:HGJW7DSXY:4:1576:25084:30029 1:N:0:TCTGCAAG+GCTGCACT
CCGGTAATTCTAATCCCAGAGTGAATGAGATATTCATCGGGGAAAGTCGGGATCCAGATACTTGGATG
ATCAACATGGATAATGTTGATAGGTTTAGTGTCGTTTGTGAAAAACGATACGTTCTAGAAGGTCGTGG
TTACGTTGATTCATC
+
FFFFFFFFFFFFFFFFFFFFFFFFFFFFFFFFFFFFFFFFFFFFFFFFFFFFFFFFF:FFF:FFFFFF
FFFFFFFFFF:,,F:FFFFFFFFF:FFFFFFFFFF,FFFF:FFF:FFFFFFF,F:FFFFFFFFFFFFF
:FFFF,FFFFFF:FF
@A00721:190:HGJW7DSXY:4:2117:14769:10285 1:N:0:TCTGCAAG+GCTGCACT
CCGGTAATTCTAATCCCAGAGTGAATGAGATATTCATCGGGGAAAGTCGGGATCCAGATACTTGGATG

ATCAACATGGAGAATGTTGATAGGTTTAGTGTCGTTTGTGAAAAAAGATAAGTTATATAAGGTAGTGG
TTAAGTTTATTAA
+
F:F,:FFF,FF:FFFFFFFFFF,FFFFF:F:FFFFF,F:FFFF,FFFFFFF,FF:FFFFF:F:F,F,F
FF,FFFF,:FF,FFFFFF,FF:,,FFFFF:FFFFFFF,F,:FFFF,F,:F,,::,FF,,F,F,,:,,F
,F:,,F,,,,F::
@A00721:190:HGJW7DSXY:4:2165:7419:29496 1:N:0:TCTGCAAG+GCTGCACT
CCGGTAATTCTAATCCCAGAGTGAATGAGATATTCATCGGGGAAAGTCGGGATCCAGATACTTGGATG
ATCAACATGGATAATGTTGATAGGTTTAGTGTCGTTTGTGAAAAACGATACGTTCTAGAAGGTCGTGG
TTACGTTGATTCATC
+
FFFFFFFFFFFFFFFFFFFFFFFFFFFFFFFFFFFFFFFFFFFFFFFFFFFFFFFFFFFFFFFFFFFF
FFFFFFFFFFFFFFFFFFFFFFFFFFFFFFFFFFFFFFFFFFFF,FFF::F:F,F:F:FFFFFFFFF
,FF:F,::,FF::,F
@A00721:190:HGJW7DSXY:4:2471:9824:28651 1:N:0:TCTGCAAG+GCTGCACT
CCGGTAATTCTAATCCCAGAGTGAATGAGATATTCATCGGGGAAAGTCGGGATCCAGATACTTGGATG
ATCAACATGGATAATGTTGATAGGTTTAGTGTCGTTTGTGAAAAACGATACGTTCTAGAAGGTCGTGG
TTACGGTGATTCATC
+
FFFFFFFFFFFFF:FFFFFFFFFFFFFFFFFFFFFFFFFFFFFFFFFFFFFFFFFFFFFFFFFFFFFF
FFFFFFFFFFFFFFFFFFFFFFFFF:FFFFFFFFFFF:FFFF:FFFFFFFFF:FF:F,FFFFFFFFFF
FFF:F,F:FFFFFF:
@A00721:190:HGJW7DSXY:4:2535:23981:15781 1:N:0:TCTGCAAG+GCTGCACT
CCGGTAATTCTAATCCCAGAGTGAATGAGATATTCATCGGGGAAAGTCGGGATCCAGATACTTGGATG
ATCAACATGGATAATGTTGATAGGTTTAGTGTCGTTTGTGAAAAACGATACGTTCTAGAAGGTCGTGG
TTACGTTGATTCAT
+
FFFFFFFFFFFFFFFFFFFFFFFFFFFFFFFFFFFFFFFFFFFFFFFFFFFFFFFFFFFF::FFFFFF
FFFFFFFFFFF,F:FFFFFFFFFFFFFFFFFFFFF:FFF:,FFFFFFFFF,FFF:FFFFF,FF:F:,F
FFF:FFF::FFFFF
@A00721:190:HGJW7DSXY:4:2630:20980:24987 1:N:0:TCTGCAAG+GCTGCACT
CCGGTAATTCTAATCCCAGAGTGAATGAGATATTCATCGGGGAAAGTCGGGATCCAGATACTTGGATG
ATCAACATGGATAATGTTGATAGGTTTAGTGTCGTGTGTGAAAAAAGATACGTTCTAGAAGGTCGTGG
TTACGTTGATTCATC
+
FFFFFFFFFFFFFFFFFFFFFFFFFFFFFFFF:FFFFFFFFFFFFFFFFFFFFFFFFFFFFFFFFFFF
FFFFFFFFFFF,FFFFFFFF,FFFF:FFFFFFFFF,FFFF,FFFF,,,F:,::F,FF:,F,FF:F,:F
FF:,FFFF:FF,:,:
@A00721:190:HGJW7DSXY:4:1457:28619:13542 1:N:0:TCTGCAAG+GCTGCACT
GGTAATTCTAATCCCAGAGTGAATGAGATATTCATCGGGGAAAGTCGGGATCCAGATACTTGGATGAT
CAACATGGATAATGTTGATAGGTTTAGTGTCGTTTGTGAAAAACGTTACGTTCTAGAAGGTCGTGGTT
ACGGTGATTCATC
+
F:FFFFF:FFFFFFFFFFFFFFFFFFFFFFF:F:F,FFFFFFFFFFFFFFFFFFFFFF:FFFFFFF:FF
:FFFF::FFF:F:FFFFF,FFFFFFFFFFFFFF,FFFFFFF:F,:,:F:,,FFFF,FF:FF,:FFFFF
F,:,F:FFFFFFF
@A00721:190:HGJW7DSXY:4:1168:12183:18427 1:N:0:TCTGCAAG+GCTGCACT
CGGGGAAAGTCGGGATCCAGATACTTGGATGATCAACATGGATAATGTTGATAGGTTTAGTGTCGTTT
GTGAAAAACGATACGTTCTAGAAGGTCGTGGTTACGTTGATTCATCCATGGATGCGGACGACGACGTT
AAGAACGCTTACTT
+
FFFFFFFFFFFFFFFFFFFFFFFFFFFFFFFFFFFFFFFFFFFFF:FFFFFFFFFFF:FFFFFFFFFF
FFFFFFFF:FFFFF:FFFFF,FFFFFFF:FFFFF:F,FF::FFFF,F:FFFFF:FFFFFFFFF:FFFF
FF,FFFF::FF:,F

```
@A00721:190:HGJW7DSXY:4:1403:5819:18584 1:N:0:TCTGCAAG+GCTGCACT
CGGGGAAAGTCGGGATCCAGATACTTGGATGATCAACATGGATAATGTTGATAGGTTTAGTGTCGTGT
GTGAAAAACGATACGTTCTAGAAGGTCGTGGTTACGTTGATTCATCCATGGATGCGGACGACGACGTT
AAGAAAGCTTA
+
FFFFFF:FFFFFFFFFFFFFFFFFFFFFFFFFFFFFFFFFFFF,FFFFFFFFFFFFFFFFFFFFF,F
:,F:FFFF,,F:F:F,F,FF,F::F::F,FF,FF:,,F,,FF,:,,F:,,F:F,:FF,::,FFFF:,F
FF:,F,FF,:F
@A00721:190:HGJW7DSXY:4:1653:32524:11506 1:N:0:TCTGCAAG+GCTGCACT
CGGGGAAAGTCGGGATCCAGATACTTGGATGATCAACATGGATAATGTTGATAGGTTTAGTGTCGTTT
GTGAAAAACGATACGTTCTAGAAGGTCGGGGTTACGTTGATTCATCCATGGATGCGGACGACGACGTT
AAGAACGCTT
+
FFFFFFFFFFFFFFFFFFF:FFFFFFFFFFFFFFFF,FFFFFFFFFFFFFFFFFFFFFFFFFFFFF
:F::FFFFFFFFF:FFFFFF::F,FFFF,FF,FFFF,FFFFFFF:,F,F:F,F,FFFFFFF:FFF::F
FF:FFFF:,F
@A00721:190:HGJW7DSXY:4:1661:16278:30436 1:N:0:TCTGCAAG+GCTGCACT
CGGGGAAAGTCGGGATCCAGATACTTGGATGATCAACATGGATAATGTTGATAGGTTTAGTGTCGTTT
GTGAAAAACGATACGTTCTAGAAGGTCGTGGTTACGTTGATTCATCCATGGATGCGGACGACGACGTT
AAGAACGCTTACTTC
+
FFFFFF:FFFFFFFFFFFFFFFFFFFFFFFFFFFFFFF,FFFFFFFFF,FFFFFF:FFFFFFFFFFF
:FFFFFFFFF:FF,FF:FFFFFF:FFFF:FF,FFFFFFFFFFFFF:FFF:FFF:FFF:FFFFFFF::F
FF:FF:FF:F:F,F:
@A00721:190:HGJW7DSXY:4:2257:19443:32064 1:N:0:TCTGCAAG+GCTGCACT
CGGGGAAAGTCGGGATCCAGATACTTGGATGATCAACATGGATAATGTTGATAGGTTTAGTGTCGTTT
GTGAAAAACGATACGGTCTAGAAGGTCGTGGTTACGTTGATTCATCCATGGATGCGGACGACGACGTT
AAGAACGCTTACTT
+
FFFFFFFFFFFFFFFFFFFFFFFFFFFFFFFFFFFFFFFFFFFFFFFFFFFFFFFFFFFFFFFFFF,F
,,:FFFFFFF::F:F,FFFF:FFFFF:F:,F:FFFF,F:FFFFF:FF,,FF:F,FFFFF:FF:FF,FF
FF,:FF:FFF:F:F
@A00721:190:HGJW7DSXY:4:2312:19271:21527 1:N:0:TCTGCAAG+GCTGCACT
CGGGGAAAGTCGGGATCCAGATACTTGGATGATCAACATGGATAATGTTGATAGGTTTAGTGTCGTGT
GTGAAAAACGATACGTTCTAGAAGGTCGTGGGTACGTTGATTCATCCATGGATGCGGACGACGACGTT
AAGAACGCTTACTT
+
FFFFFFFFFFFFFFFFFFFFFFFFFFFFFFFF:FFFFFFFFFFFFFFFFFFFFFFFFFFFFFFFFFF,F
,FF,FFFFF::,F,,:F,,F,:F,:FFF::F,FF:F:F,,:F,:,,F:,FFF,,:::FF:FF::,FF:
F:,FF:,F,:F:,F
@A00721:190:HGJW7DSXY:4:2505:8757:19789 1:N:0:TCTGCAAG+GCTGCACT
CGGGGAAAGTCGGGATCCAGATACTTGGATGATCAACATGGATAATGTTGATAGGTTTAGTGTCGTGT
GTGTAAAAAGATAAGTTCTAGAATGTCGTGGGTAAGTTGTTTAATCCATGGATGCGG
+
FFFFFFFFFFFFFFFFFFFFFFFFFFFFFFFFFFFFFFFFFFFFFFFFFFFFFFFFFFFFFFFFFF,F
,,,,FFFF,,F,,,,,F,:,,FF,::,F,:F,F,,:,F,,FF,,F,::,,:,F:,FF
@A00721:190:HGJW7DSXY:4:2634:19994:33364 1:N:0:TCTGCAAG+GCTGCACT
CGGGGAAAGTCGGGATCCAGATACTTGGATGATCAACATGGATAATGTTGATAGGTTTAGTGTCGTTT
GTGAAAAAAGAAACGTTCTAGAACGTCATGGTTAAGTTGAATAATACATGGATGCGGACGA
+
FF,::FF:F:FFFFFF,FFFFFFFF:FFF,F:FFFFFF::F:,FFFFFFFF:FFFFFFF,F:::FF:F
,FF,FFF:F,,,F,FFFF,F:FF,,,,,F:,,FF,,,F,F,:,FF,,F,,FFF,,,,FFFF
@A00721:190:HGJW7DSXY:4:1119:25174:21919 1:N:0:TCTGCAAG+GCTGCACT
GGGGAAAGTCGGGATCCAGATACTTGGATGATCAACATGGATAATGTTGATAGGTTTAGTGTCGTTTG
```

```
TGAAAAACGATACGTTCTAGAAGGTCGTGGTTACGTTGATTCATCCATGGATGCGGACGACGACGTTA
ATAACGCGTACTTC
+
FFFFFFFFFFFFFFFFFFFFFFFF:FFF,FFFFFFFF:FFFFF:FFFFFFFFFFFFFFFFFFFF,,F,
F,:FFFF,,:FF:F:F,F,:FF,FF:F:FFFFF,F::F,,F:,:FF:::F,FF,FFF:,FFFF:,,FF
F,,F,::,FF,:F:
@A00721:190:HGJW7DSXY:4:1331:22046:6355 1:N:0:TCTGCAAG+GCTGCACT
GGGGAAAGTCGGGATCCAGATACTTGGATGATCAACATGGATAATGTTGATAGGTTTAGTGTCGTTTG
TGAAAAACGATACGTTCTAGAAGGTCGTGGTTACGTTGATTCATCCATGGATGCGGACGACGACGTTA
AGAACGCTTACTT
+
FFFFFFFFFFFFFFFFFFFFFFFFFFFFFFFFFFFFFFFFFFFF::FFFFFFFFFFFFFFFFFFFFFF
F:FFFFF,F,FF::,F:FFF:F,FF::,FF,FFF:,:F,FFFFF,F:,:FFF,:FFFF:,:FFF:FFF
F:FFF:F,:F:,F
@A00721:190:HGJW7DSXY:4:1369:20012:14137 1:N:0:TCTGCAAG+GCTGCACT
GGGGAAAGTCGGGATCCAGATACTTGGATGATCAACATGGATAATGTTGATAGGTTTAGTGTCGTTTG
TGAAAAACGATACGTTCTAGAAGGTCGTGGTTACGTTGATTCATCCATGGATGCGGACGACGACGTTA
AGAACGCTTACTTCT
+
FFFFFFFFFFFFFFFFFFFFFFFFFFFFFFFFFFFFF:FFFFFFFFFFFFFFFFFFFFFFFFFFF:FF
,:,FFFFF:FF:FFFFFFFF:FFFFFF::F:FF,F,FFFFFFFF,FFFFFFFF:FFF:FFFFFFF:FF
F,:F:F::F::,FFF
@A00721:190:HGJW7DSXY:4:1408:13548:19132 1:N:0:TCTGCAAG+GCTGCACT
GGGGAAAGTCGGGATCCAGATACTTGGATGATCAACATGGATAATGTTGATAGGTTTAGTGTCGTTTG
TGAAAAACGATACGTTCTAGAAGGTCGTGGTTACGTTGATTCATCCAAGGATGCGGACGACGACGTTA
AGAACGCTTA
+
FFFFFFFFFFFFFFFFFFFFFFFFFFFFFFFFFFFFF,FFFFFFFF:FFFFFFFFFFFFFFFFFF,F,
FF,FFFF,:FFF,::F,FF:FF,F::F,FF:F,::,F:,FFFF,,FF,:FFF,,FF:,:FF,F:,,FF
F,,F:,F:FF
@A00721:190:HGJW7DSXY:4:1413:4354:11663 1:N:0:TCTGCAAG+GCTGCACT
GGGGAAAGTCGGGATCCTGATACTTGGATGATCAACATGGATAATGTTGATAGGTTTAGTGTCGTTTG
TGAAAAACGATACGTTCTATAAGGTCGTGGTTACGTTGATTCATCCATGGATGCGGACGACGACGTTA
AGAACGCTTA
+
FFFFFFFFFFFFFFFFFFFFFF:FFFFFFFFFFFFFFFFFFFFFFFFFFF:FFFFFFFFFFFFFF,F:
FFFFFFF,FFFF,FFFF::,FF:FF:FFFF,FF,FFF,:FF,FF:F::FFFF,FFFFF,FF::F:,FF
F,:F,:F,FF
@A00721:190:HGJW7DSXY:4:1451:5231:21449 1:N:0:TCTGCAAG+GCTGCACT
GGGGAAAGTCGGGATCCAGATACTTGGATGATCAACATGGATAATGTTGATAGGTTTAGTGTCGTTTG
TGAAAAACGATACGTTCTAGAAGGTCGTGGTTACGTTGATTCATCCATGGATGCGGACGACGACGTTA
AGAACGCTTACGTCT
+
FFFFFFFFFFFFFFFFF:FFFFF:FFFFFFFFFFFF::FFF:FF,FF,FFFFFFFFFFFFFFFFFFFF
F:FFFFFFF:FFFF:FFFFF:F:F,FF,FFF:FFF,FF,FFFFF:FFFFF:F,F:FFFFF:FFFF:FF
FFFF,F::,FF,F:F
@A00721:190:HGJW7DSXY:4:2112:25771:6261 1:N:0:TCTGCAAG+GCTGCACT
GGGGAAAGTCGGGATCCAGATACTTGGATGATCAACATGGATAATGTTGATAGGTTTAGTGTCGTTTG
TGTAAAACGATAAGTTCTAGAAGGTCGTGGTTACGGTGATTCATCCAAGGATGCGGACGACGACGTTA
ATAACGCTT
+
FFFFFFFFFFFFFFFFFFFFFFF:FFFFFFFFFFFFFFFFFFFFFFFFFFFFFFFFFFFFFFFFF,F,
FF,FFF,,,F,:,:,F:F::FF,FF::,FF,F,,,,F,::F,F,F,:,,F,F,,:F:FF:F,FF,:F,
F,:F,::,F
```

```
@A00721:190:HGJW7DSXY:4:2302:24297:16329 1:N:0:TCTGCAAG+GCTGCACT
GGGGAAAGTCGGGATCCAGATACTTGGATGATCAACATGGATAATGTTGATAGGTTTAGTGTCGTTTG
TGAAAAACGATACGTTCTAGAAGGTCGTGGGTACGTTGATTAATCCATGGATGCGGACGACGACGTTA
ATAA
+
FFFFFFFFFFFFFFFFFFFFFFFFFFFFFFFFFFFFFFFFFFFFFFFFFFFFFFFFFFFFFFFFF,F,
F,,FFFF:,FFF,,FF,FF,FF,FF,F,FF,F:,:,F:,FF,F::F:,:F,F:FFFF::FF:FF:,F,
F,FF
@A00721:190:HGJW7DSXY:4:2324:15609:2127 1:N:0:TCTGCAAG+GCTGCACT
GGGGAAAGTCGGGATCCAGATACTTGGATGATCAACATGGATAATGTTGATAGGTTTAGTGTCGTTTG
TGAAAAACGATACGTTCTAGAAGGTCGTGGTTACGTTGTTTCATCCATGGCTGCGGACGACGACGTTA
AGAACGCTTACT
+
FFFFFFFFFFFFFFFFFFFFFFFFFFFFFFFFFFFFFFFFFFFF:FFFFFFFFFFFFFFFFFFFF,FF
F,:FFFF,F:FF,:FF:,F,,F::,,:,FF,F,:F:F:,FFFF,,F::FF,:F::FF:FF:,F:,FFF
F,FFF:F:FF:F
@A00721:190:HGJW7DSXY:4:2452:20500:18302 1:N:0:TCTGCAAG+GCTGCACT
GGGGAAAGTCGGGATCCAGATACTTGGATGATCAACATGGATAATGTTGATAGGTTTAGTGTCGTTTG
TGAAAAACGATACGTTCTAGAAGGTCGTGGTTACGTTGATTCATCCATGGATGCGGACGACGACGTTA
AGAACGCTTACT
+
FFFFFFFFFFFFFFFFFFFFFFFFFFFFFFFFFFFFFFFFFFFFFFFFFFFFFFFFFFFFFFFFF,FF
F,FFFFFF,FFF:FFF:FF::F:FF:FFFF:F::FFF,,FF,,F:F:,FFFF:FFFFFFFFFFFFFFF
F,FFF:F,,F:F
@A00721:190:HGJW7DSXY:4:2468:14769:5243 1:N:0:TCTGCAAG+GCTGCACT
GGGGAAAGTCGGGATCCAGATACTTGGATGATCAACATGGATAATGTTGATAGGTTTAGTGTCGTTTG
TGAAAAACGATACGTTCTAGAAGGTCGTGGTTACGTTGATTCATCCATGGATGCGGACGACGACGTTA
AGAACGCTTACTTCT
+
FFFFFFFFFFFFFFFFFFFFFFFFFFFFFFFFFFFFFFFFFFFF,FFFFF,FFFFFFFFFFFFFFF::
F,,FFFF,FFFFFFFF::FF:FFFFFFFFF:F::FFF:,FFFFF,FF:FF:F,FFFF,FFFF:FF,FF
FF,FFFF:FFF:F:F
@A00721:190:HGJW7DSXY:4:2572:30942:8422 1:N:0:TCTGCAAG+GCTGCACT
GGGGAAAGTCGGGATCCTGATACTTGGATGATCAACATGGATAATGTTGATAGGTTTAGTGTCGTTTG
TGAAAAACGATACGTTCTAGAAGGTCGTGGTTACGTTGATTCATCCATGGATGCGGACGACGACGTTA
AGAACGCTTACTTCT
+
FFFFFFFFFFFFFFFFFFFFFFFFFFFFFFFFFFFFFFFFFFFFFFFFFFFFFFFFFFFFFFFFFFFF
FFFFFFF:FFFFFFFFFFF,FFFF::F:,FFFFFF:FFFFFFFFFFFF,FFFFFFFFFF:FFFFFFFF
FFFF,FF,FF:FFFF
@A00721:190:HGJW7DSXY:4:2606:11957:15718 1:N:0:TCTGCAAG+GCTGCACT
GGGGAAAGTCGGGATCCAGATACTTGGATGATCAACATGGATAATGTTGATAGGTTTAGTGTCGTTTG
TGTAAAACGATAAGTTATAGAAGGTCGTGGTTACGGTGTTTAATCCAGGGATGCGGACGACGACGTTA
ATAACGC
+
FFFFFFFFFFFFFFFFF:FFFFFFFFFFFFF:FFFFFFFFFFFFFFF:FFFFFFFFFFFFFFFFF,F,
FF,,FFFFF,F,,:,F,:FF:F,FFFF,FF,,FF,,FF,FF,F,,F,,,F:,F:FF:,FFF,,:,,F,
F,,F,:F
@A00721:190:HGJW7DSXY:4:2632:7654:8829 1:N:0:TCTGCAAG+GCTGCACT
GGGGAAAGTCGGGATCCAGATACTTGGATGATCAACATGGATAATGTTGATAGGTTTAGTGTCGTTTG
TGAAAAACGATACGTTCTAGAAGGTCGTGGTTACGTTGATTCATCCATGGATTCGGACGACGACGTTA
AGAACGCTTACTT
+
FFFFFFFFFFFFFFFFFFFFFFFF:FFFFFFFFFFFFFFFFFFFFFFFFFF:FFFFFFFFFFFFF,FF
```

FF:FFFF,,:FF,,:F::F,F:,FF,:,,F:FF,:,F:F,F::,,F:,:FF,,F:FFF:FF,F:F,FF
F,,F,F:,F:F,F
@A00721:190:HGJW7DSXY:4:2666:24686:5635 1:N:0:TCTGCAAG+GCTGCACT
GGGGAAAGTCGGGATCCAGATACTTGGATGATCAACATGGATAATGTTGATAGGTTTAGTGTCGTGTG
TGAAAAACGATACGTTCTAGAAGGTCGGGGTTACGTTGATTCATCCATGGATGCGGACGACGACGTTA
AGAACGCTTACTT
+
:FFFF:FFFFFFFFFFFFFFFFFFFFFFFFFFFFFFFF:FFFFFFFFFFFFFFFFFFFFFFFFFFFF,F:
FF:FFFF::FFFFFFF:FFFFFFFFFF,:FFFFFFFFFFFF,:FFFFF,FFFFFFFFFFFFF,FFFFF
F:FF,,F,:F:FF
@A00721:190:HGJW7DSXY:4:1201:25563:27070 1:N:0:TCTGCAAG+GCTGCACT
GGGAAAGTCGGGATCCAGATACTTGGATGATCAACATGGATAATGTTGATAGGTTTAGTGTCGTTTGT
GAAAAAAGATACGTTCTAGAAGGTCGTGGTTACGGTGTTTCATCCAAGGATGCGGACGACGACGTTAA
GAACGCGTAATTCTG
+
FFFFFFFFFFFFFFFFFFFFFF:FFFFFFFFFFFFFFFFF,FFFFFFFFFFFFFFFFFFFFFFFF,F,F
,FFFFF,,FFF,,,F,:F,,F,:F,F,F:,FF,,,:F,FF:F,FFF,:F,::,FF:,FF,:FF,,FFF
,:F,::,::,,F,:F
@A00721:190:HGJW7DSXY:4:1534:11650:28808 1:N:0:TCTGCAAG+GCTGCACT
GGGAAAGTCGGGATCCAGATACTTGGATGATCAACATGGATAATGTTGATAGGTTTAGTGTCGTTTGT
GAAAAACGATACGTTCTAGAAGGTCGTGGTTACGTTGATTCATCCATGGATGCGGACGACGACGTTAA
GAACGCTTACTTCTG
+
FFFFFFFFFFFFFFFFFFFFFF:FFFFFFFFFFFFF:FFFF::FFFF,FFFFFFFFFFFFFFFFF,FF,
FFFFFFF:F:FFFFF:FF,:F:F:,F::F,FF:FFF:,FF:,F:FFF,FFFF:FFFF:FFFF:FFFFF
,FFF:F,F:F:,,F:
@A00721:190:HGJW7DSXY:4:1633:8739:26835 1:N:0:TCTGCAAG+GCTGCACT
GGGAAAGTCGGGATCCAGATACTTGGATGATCAACATGGATAATGTTGATAGGTTTAGTGTCGTTTGT
GAAAAACGATACGTTCTAGAAGGTCGTGGTTACGTTGATTCATCCATGGATGCGGACGACGACGTTAA
GAACGCTTACTTCTG
+
FFFFFFFFFFFFFFFFFFFFFFFFFFFFFFFFFFFFFFFFFFF:FF:FFFFFFFFFFFFFFFFF,FFF
F:FFFFF:FFF,F,F::F,FF:FF:FFFFFF,,FFF:FFF:FF:::,,:FF,FFFFFFFF,:F:FFFF
:FFF:F:F:,:,,::
@A00721:190:HGJW7DSXY:4:2166:32913:14465 1:N:0:TCTGCAAG+GCTGCACT
GGGAAAGTCGGGATCCAGATACTTGGATGATCAACATGGATAATGTTGATAGGTTTAGTGTCGTTTGT
GAAAAACGATACGTTCTAGAAGGTCGTGGTTACGTTGATTCATCCATGGATGCGGACGACGACGTTAA
GAAAGCTTACTT
+
FFFFFFFFFFFFFFFFFFFFFFFFFFFFFFFFFFF:FFFF:FFFFFFFFFFF::FFFFFFFFFFFFFF
FFFFFFFFFFF:F:FFFFF,F,FF:FFFF,F:FF,FFF,F,F,FFF:F::,F::FFFFF::,FF,F::
,,F,F:,F:F,F
@A00721:190:HGJW7DSXY:4:2312:15492:6997 1:N:0:TCTGCAAG+GCTGCACT
GGGAAAGTCGGGATCCAGATACTTGGATGATCAACATGGATAATGTTGATAGGTTTAGTGTCGTTTGT
GAAAAACGATAAGTTCTAGAATGTCGTGGGTACGTTGACTCATCCATGGATTCGGACGACGACGTTAA
TAACGCTTAATTC
+
FFFFFFFFFFFFFFFF:FFFFFF:FFFFFFFFFFFFFFFFFF:FFFFFFFFFFFFFFFFFFFFF,FFF
F,FFFF,,::F,::F,FF:,F,FF,:F,F,:,,::F:,,F,F:,FF,,F,,,F:FF,::,,::::FFF
,FF,::,F:,,F:
@A00721:190:HGJW7DSXY:4:2322:18548:21245 1:N:0:TCTGCAAG+GCTGCACT
GGGAAAGTCGGGATCCAGATACTTGGATGATCAACATGGATAATGTTGATAGGTTTAGTGTCGTTTGT
GAAAAACGATACGTTCTAGAATGTCGTGGTTTCGGTGATTCATCCAAGGATGCGGACGACGACGTTAA
GAACGCGTACTT

+
FFFF:FFFFFFFFFFFFFF:FFFFFFFFFFFFFFFFFFFFFFFF::FFFFFFFFFFFFFF,FFFFF,F:F
:,FFFF,,:,:F,F,,,F,,F,F:,:FFF:F,::,:F,:F,:F,F,,,F,:F,FF::F,FFFF:,FFF
:,F:F,:::F:F
@A00721:190:HGJW7DSXY:4:2441:28854:26162 1:N:0:TCTGCAAG+GCTGCACT
GGGAAAGTCGGGATCCAGATACTTGGATGATCAACATGGATAATGTTGATAGGTTTAGTGTCGTTTGT
GAAAAACGATACGTTCTAGAAGGTCGTGGTTACGGTGATTCATCCAAGGATGCGGACGACGACGTTAA
GAACGCTTACTTCTG
+
FFFF:FFFFFFFFFFFFFFFF:FFFFFFFFFFFFFFFFFFFFFFFFFFFFFFFFFFFFFFFFFFF,F,F
:,FFFF:::FF,:FF::F:FF:FF:F::FFFFF:,FFFFF,F::F:,,FFF,F:FF::F:FFFFFFFF
,:F,FF:F:,,F:FF
@A00721:190:HGJW7DSXY:4:1104:4200:29058 1:N:0:TCTGCAAG+GCTGCACT
GTCGGGATCCAGATACTTGGATGATCAACATGGATAATGTTGATAGGTTTAGTGTCGTTTGTTAAAAA
CGATACGTTCTAGAAGGTCGTGGTTACGTTGATTCATCCAAGGATGCGGACGACGACGTTAAGAAAGC
TTAAATCTGTTATAT
+
FFFFFFFFFFFFFFFFFFFFFFFFFFFFFFFFFFFFFFFFFFFFFFFFFFFFFFFFFFFFF,,,:,,FFFF
,,F,:,::F,FF,FF,FF,::FFFF::,,F:FFF,::,F,::FF,,,:FF,:F:::,,,FFF,:F,,F
,FF,,F,,,,F::FF
@A00721:190:HGJW7DSXY:4:1104:4255:29371 1:N:0:TCTGCAAG+GCTGCACT
GTCGGGATCCAGATACTTGGATGATCAACATGGATAATGTTGATAGGTTTAGTGTCGTTTTTGAAAAA
AGATACGTTCTATAAGGTCGTGGTTAAGTTGATTCATCCAAGGATTCGGACGACGACGTTAAGAAAGC
TTAATTCTGTTATAT
+
FFFFFFFFFFFFFFFFFFFFFFFFFFFFFF:FFFFFFFFFFFFFFFFFFFFFFFFFFFF,F,F,:FFFF
,,F::,,FF,:F,:F,:,,:,FFF,:,::,F,FF,F,:::,,F:F,:FFF::F:::,,:FFF,:F,,F
,F:,,:,FF:F:FFF
@A00721:190:HGJW7DSXY:4:1134:23854:20384 1:N:0:TCTGCAAG+GCTGCACT
GTCGGGATCCAGATACTTGGATGATCAACATGGATAATGTTGATAGGTTTAGTGTCGTTTGTGAAAAA
CGATACGTTCTAGAAGGTCGTGGTTACGTTGATTCATCCATTGATGCGGACGACGACGTTAAGAACGC
TTAATTCTGTTATAT
+
FFFFFFFFFFFFFFFFFFFFFFFFFFFFFFFFFFFFFFFFFFFFFFFFFFFFFFFFFFFF,FFFF,FFFF
,FF,F,FFF,FF,FF,FFFFFFFFFFFF:FF,FF:F,,FF,,FFF,FFFF::F:FF,FFFFF,FF,:F
,FF,,FFFFFFF::F
@A00721:190:HGJW7DSXY:4:1220:1714:9439 1:N:0:TCTGCAAG+GCTGCACT
GTCGGGATCCAGATACTTGGATGATCAACATGGATAATGTTGATAGGTTTAGTGTCGTTTGTGAAAAA
CGATACGTTCTAGAAGGTCGTGGTTACGTTGATTCATCCATGGATGCGGACGACGACGTTAAGAACGC
TTACTTCTGTTATAT
+
FFFFFFFFFF:FFFFFFFFFFFFFFFFFFFFFFFFFF:FFFFFFFFFFFFFFFFFFFFFF:FFF,FFFFF
,:FFF,,FF::F,FF::F:F:FF::F:F:,FF,F,FF:FF,FF:F,FFFFFFFF,FFF,FFF,FF::F
:FFF,::FFFFFFFF
@A00721:190:HGJW7DSXY:4:1412:4227:16767 1:N:0:TCTGCAAG+GCTGCACT
GTCGGGATCCAGATACTTGGATGATCAACATGGATAATGTTGATAGGTTTAGTGTCGTTTGTGAAAAA
CGATACGTTCTAGAAGGTCGTGGTTACGTTGATTCATCCAAGGATGCGGACGACGACGTTAAGAACGC
TTACATCTGTTATAT
+
FFFFFFFFFFFFFFFFFFFFFFFFFFFFFFFFFFFFFFFFFFFFFF:FFFFFFFFFFFFFFF,,FFFF
F,:FFF,,F,FF,FF,FFF:::F::F,F,F::FFFF,,:,,FFF,,:FFFF:F,F:F:FF:F,FF::F
,FFF,F:,FFF:FFF
@A00721:190:HGJW7DSXY:4:1412:28293:17394 1:N:0:TCTGCAAG+GCTGCACT
GTCGGGATCCAGATACTTGGATGATCAACATGGATAATGTTGATAGGTTTAGTGTCGTTTGTGAAAAA

```
CGATACGTTCTAGAAGGTCGTGGTTACGTTGATTCATCCAAGGATGCGGACGACGACGTTAAGAACGC
TTACTTCTGTTATAT
+
F:FFFFFFFFFFFFFFF:FFFFFFFFFFFFFFFFFFFFFFFFFFFFFFFFFFFFFFFF:F:FFFFFFF
,FFFF:,:F,FFFFF,F,,FF:FFFF::FFF:FF:F,,F:,,F:F:FFFFF:FF,,::FFFF:FFF,:
:F:,,F,,,FFFFFF
@A00721:190:HGJW7DSXY:4:1417:31647:32440 1:N:0:TCTGCAAG+GCTGCACT
GTCGGGATCCAGATACTTGGATGATCAACATGGATAATGTTGATAGGTTTAGTGTCGTTTGTGAAAAA
CGATACGTTCTAGAAGGTCGTGGTTACGTTGATTAATCCATTGATGCGGACGACGACGTTAAGAACGC
TTACGTCTGTTATAT
+
FFFFFFFFFFFFFFFFFFFFFFFFFFFFFFFFFFF:FFFFFFFFFFFFFFFFFFFFF:F:FFFFFFF
,:F:FF:FF,FF,FF,FF,:::FFF:,::FFFFF,:FF:F,,F:F,:FFFF:FFFF,:FF:F,FF:,:
,F:,,:,,F:FFFF:
@A00721:190:HGJW7DSXY:4:1421:29188:6981 1:N:0:TCTGCAAG+GCTGCACT
GTCGGGATCCAGATACTTGGATGATCAACATGGATAATGTTGATAGGTTTAGTGTCGTTTGTGAAAAA
CGATACGTTCTAGAAGGTCGTGGTTACGTTGATTCATCCATGGATGCGGACGACGACGTTAAGAACGC
TTACTTCTGTTATAT
+
FFFFFFFFFFFFFFFFFFFFFFFFFFFFFFFFFFFFFFFFFFFFFFFFFFFFFFFFFFFFFF:F:,FFFF
F:::FFFFF,:FFFF,FFFFF,F,F:::,FF:FFFFF::F,FFFF,:FFFFFF:F,F,FFFF:FF,:F
:FF,,F:F,FFFFFF
@A00721:190:HGJW7DSXY:4:1477:10755:16579 1:N:0:TCTGCAAG+GCTGCACT
GTCGGGATCCAGATACTTGGATGATCAACATGGATAATGTTGATAGGTTTAGTGTCGTTTGTGAAAAA
CGATACGTTCTAGAAGGTCGTGGTTACGTTGATTCATCCATGGATGCGGACGACGACGTTAAGAACGC
TTACTTCTGTTATAT
+
FFFFFFFFFF:FFFFFFFFFFFFFFFFFFF:FFFFFFFFFFFF:FFFF:FFFFFFFFF:FFFFFFFFF
:FF:FF:FF,FF:FFFFF,:FFF,FFF:,FFF:FFFF:F:F:F:FFF:FFFFFFFFFF:FFF::F:F:
::FF:F:,,,FFFFF
@A00721:190:HGJW7DSXY:4:1542:9652:7686 1:N:0:TCTGCAAG+GCTGCACT
GTCGGGATCCAGATACTTGGATGATCAACATGGATAATGTTGATAGGTTTAGTGTCGTTTGTGAAAAA
CGATACGTTCTAGAAGGTCGTGGTTACGTTGATTCATCCATGGATGCGGACGACGACGTTAAGAACGC
TTACTTCTGTTATAT
+
FFFFFFFFFFFFFFFFFFFFFFFFFFFFFFFFFFFFFFFFFFF:FFFFFFFFFFFFFFFFFFF:FFFF
,FFFFF::F,FF,FF,FFFFFFF,FF,:,F,FFF:FF:FF,FF:::FFFFF,FF:FF:FFFF:FF,,F
,F:,:F,:::FFFFF
@A00721:190:HGJW7DSXY:4:1611:27254:11334 1:N:0:TCTGCAAG+GCTGCACT
GTCGGGATCCAGATACTTGGATGATCAACATGGATAATGTTGATAGGTTTAGTGTCGTTTGTGAAAAA
CGATACGTTATAGAAGGTCGTGGTTACGTTGATTCATCCAAGGATGCGGACGACGACGTTAAGAACGC
TTACTTCTGTTATAT
+
FFFFFFFFFFFFF:FFFFFFFFFFFFFFFF:FFFFFFFFFFFFFFFFFFFFFFFFFFFFF::,F,FFFFF
:,:FF,,:F,F:,FF,F:FF:,F,F,,F:F,,FFFFFFFF,FF:F:FFFFFFFFFFF::FFF:FF,:,
:F,,,F,,:,F:F,F
@A00721:190:HGJW7DSXY:4:1629:26847:4460 1:N:0:TCTGCAAG+GCTGCACT
GTCGGGATCCAGATACTTGGATGATCAACATGGATAATGTTGATAGGTTTAGTGTCGTTTGTGAAAAA
CGATACGTTCTAGAAGGTCGTGGTTACGTTGATTCATCCATGGATGCGGACGACGACGTTAAGAACGC
TTACTTCTGTTATAT
+
FFFFFFFFFFFFFFFFFFFFFFFFFFFFFFFFFFFFFFFFFFFFFFFFFFFFFFFFFF:FFFFFFFFF
FFFFFF:,F:::,FFFF,,F,FFFF:FFFFFFFFFFFFFF,FFF:F,FFFFFFFFFF,FFFF:FFF:F
:FF,FF:FF,F:FFF
```

@A00721:190:HGJW7DSXY:4:1642:30852:17503 1:N:0:TCTGCAAG+GCTGCACT
GTCGGGATCCAGATACTTGGATGATCAACATGGATAATGTTGATAGGTTTAGTGTCGTTTGTGAAAAA
CGATACGTTCTAGAAGGTCGTGGTTACGTTGATTCATCCATGGATTCGGACGACGACGTTAATAACGC
TTACTTCTGTTATAT
+
FFFFFFFFF:FFFFFF:FFFFFFFFFFFFFFFFFFFFFFFFFFFFFFFFFFFFFFF:FFFF,F,FFFFF
,,F:F,:,F:,F:FF,:F:FF:F:FF,,F::FFF,:,:FF,,FF:,FFFF,FF,,,::FFFF,FF,:F
F:F:FF,F::FFFFF
@A00721:190:HGJW7DSXY:4:2230:22905:29324 1:N:0:TCTGCAAG+GCTGCACT
GTCGGGATCCAGATACTTGGATGATCAACATGGATAATGTTGATAGGTTTAGTGTCGTTTGTGAAAAA
CGATAACTTATAGAAGTTAGCGGTTACGGTGAATAAAACATTGATTCGGACGAAGAAATTAAAAACCC
ATA
+
F::F:FFFFFFFF:FF:,FFFF:F:,FFF:F,F:FFFFFF,,F:F,F:FFFF:::FF:FF,F,FFFFF
:,F,F,,FF,FF,FF,,,,F,FF,,F,,,:FF:F:F,,F:F,:,F,,F::,,::,:,,:FFF,FF,:F
,F:
@A00721:190:HGJW7DSXY:4:2301:11207:5682 1:N:0:TCTGCAAG+GCTGCACT
GTCGGGATCCAGATACTTGGATGATCAACATGGATAATGTTGATAGGTTTAGTGTCGTTTGTGTAAAA
AGATAAGTTCTATAATGTCGTGGTTACGTTGTTTAATCCATGGATGCGGACGACGACGTTAATAACCC
TTAAATATGTTATAT
+
FFFFFF:FFFFFFFFFFFFFFFFFFFFFFFFFFFFFFFFFFFFFFFFFFFFFFFFFF,F,,,,FFFF
,,:,F,,:F,FF,FF,FF,::FFF:F,,,F,,FF:F,F:F,,:,F::,F:::FF,:,,FF:F,,FF,:
,FF,,F,:,FF,FF:
@A00721:190:HGJW7DSXY:4:2301:14633:11898 1:N:0:TCTGCAAG+GCTGCACT
GTCGGGATCCAGATACTTGGATGATCAACATGGATAATGTTGATAGGTTTAGTGTCGTTTGTGAAAAA
ACATACCTTCTAGAAGGTCGGGGTTACGTTTATTCATACAAGGATTCGGACGACGACGGTAAGAAAGC
GTACTTGTGTTATAT
+
FFFFFFFFFFFFFFFFFFFFFFFFFFFFFF::FFFFFFFFFFFFFFFFFFFFFFFFFFF:F,,:,FFFF
,,:,:,,::,FF::F,F,,,,FF:F,,,,:,,:F::,,F,,,F:,,,FF,F,,:,,:,,F,F,,F:,:
,,:F,,,:FF:,F:F
@A00721:190:HGJW7DSXY:4:2473:4643:29168 1:N:0:TCTGCAAG+GCTGCACT
GTCGGGATCCAGATACTTGGATGATCAACATGGATAATGTTGATAGGTTTAGTGTCGTTTGTGAAAAA
CGATACGTTCTAGAAGGTCGTGGTTACGTTGATTCATCCATGGATGCGGACGACGACGTTAAGAACGC
TTACTTCTGGTATAT
+
FFFFFFFFFFFFFFFF:FFFFFFFFFFFFFFFFFFFFFFFFFFFFFFFFFFFFFFFFF::FFFFFFFFF
,:FFFF,FF,,FFFF:FF:::FF:FFFFFFF,FF:F:FFF:,FFF,FFFFFFFFFFFFFFFF,FF:FF
:FFF,FFF:,FFFFF
@A00721:190:HGJW7DSXY:4:2504:24216:34507 1:N:0:TCTGCAAG+GCTGCACT
GTCGGGATCCAGATACTTGGATGATCAACATGGATAATGTTGATAGGTTTAGTGTCGTTTGTGAAAAA
AGATAAGGTCTATAAGGTCGTGGTTAAGTTGATTCATCCAAGGATGCGGACGACGACGGTAAGAACGC
GTAATTCGGTTATA
+
FFFFFFFFFFFFFFFFFFFFFFFFFFFFFFFFFFFFFFFFFFFFFFFFFFFFFFFFFFFFF,F:,FFFF
,:,:F,,,F,,F,,F,F:,:,FFFFF,,,F,:,:,F:,:,F,FFF:,,FF,,:,::,,,F,F,:FF,F
,F::,F,,F,F,:F
@A00721:190:HGJW7DSXY:4:2520:4743:1752 1:N:0:TCTGCAAG+GCTGCACT
GTCGGGATCCAGATACTTGGATGATCAACATGGATAATGTTGATAGGTTTAGTGTCGTTTGTGAAAAA
AGATACGTTGAAGAATTTCGTGGTTAACGTGATTCATCCATGGAGTCCGACGAAGACGTTTAGAACGC
GTAAATATGTTATAT
+
F:FFFFFFF,F:FFFF,::FFFFFFFFFF:,:FFFF:FFFFFF:FF,F,F:,,F,FFF,F,F,,FFFF

```
:,FFF,,F:,,F,,F,,F,,,,,FFF,,,,,F::,::,F:,FF:,,,,FFF,:,:F:,FF,:,,F:,F
,:F,,:,,::::,:F
@A00721:190:HGJW7DSXY:4:2554:24551:16861 1:N:0:TCTGCAAG+GCTGCACT
GTCGGGATCCAGATACTTGGATGATCAACATGGATAATGTTGATAGGTTTAGTGTCGTTTGTGAAAAA
CGATACGTTCTAGAAGGTCGTGGTTAAGTTGATTCATCCATGGATGCGGACGACGACGTTAAGAACGC
TTACTTCTGTTATAT
+
FFFFFFFFFFFFFFFF:FFFFFFFFFFFFFFFFFFFFFFFFFFFFFFFFFFFFFFF:FFFF:FFFF
::FFFF:FFFFF,FF,FF:FFFFFFF,F:FF:FFFF::FF,,FFF,FFFF:FFFFFFFFF:FF,FF:F
FF:,,F,:FFF:FFF
@A00721:190:HGJW7DSXY:4:2613:4887:16094 1:N:0:TCTGCAAG+GCTGCACT
GTCGGGATCCAGATACTTGGATGATCAACATGGATAATGTTGATAGTTTTAGTGTCGTTTGTGAAAAA
CGATACGTTCTAGAAGGTCGTGGTTACGTTGATTCATCCATGGATGCGGACGACGACGTTAAGAACGC
TTACTTCTGTTATAT
+
FFFFFFFFFFFFFFFFFFFFFFFFFFFFFFFFFFFFFFFFFFFFFFFFFFFFFFFF:FFF,FFFFF
,,FF:::FF,FF,FF,,F,:F::FFF,F:FF,FF,FF,::,FFFF:F:FFFFFF:FF:FFFF::F,,:
:FF,,F,F,FFFF:F
@A00721:190:HGJW7DSXY:4:2641:18249:16125 1:N:0:TCTGCAAG+GCTGCACT
GTCGGGATCCAGATACTTGGATGATCAACATGGATAATGTTGATAGGTTTAGTGTCGTTTGTGAAAAA
CGATACGTTCTATAAGGTCGTGGTTACGTTGATTCATCCATGGATGCGGACGACGACGTTAAGAACGC
TTACTTCTGTTATAT
+
FFFFFFFFFFFFFFFFFFFFFFFFFFFFFF:FFFFFFFFFFFFFFFFFFFFFFFFFFFF,F:F:FFFFF
,,FFF:::F,:F,FF,F,,F:FFFFF,:FF:,FF,FF,,F,,FF::F:FF::FF,FF::FFF,,F,::
,,,::F,:,,,FFFF
@A00721:190:HGJW7DSXY:4:2658:27995:36323 1:N:0:TCTGCAAG+GCTGCACT
GTCGGGATCCAGATACTTGGATGATCAACATGGATAATGTTGATAGGTTTAGTGTCGTTTGTGAAAAA
CGATACGTTCTAGAAGGTCGTGGTTAAGTTGATTCATCCATGGATGCGGACGACGACGTTAAGAACGC
TTACTTCTGTTATAT
+
FFFFFFFFFFFFFFFFFFFFFFFFFFFFFFFFFFFFFFFFFFFFFFFFFFFFFFFFFFFFFFF,FFFFF
,FFFF,:FF:,F,FF:FF:F::FFFF,:FFF:FF:F::::,FFFF:FFFFFFF,:F:F:FFF,FF,:F
:FF,,F,F,FFFFFF
@A00721:190:HGJW7DSXY:4:2675:19018:32080 1:N:0:TCTGCAAG+GCTGCACT
GTCGGGATCCAGATACTTGGATGATCAACATGGATAATGTTGATAGGTTTAGTGTCGTTTGTGAAAAA
CGATACGTTCTAGAAGGTCGTGGTTAAGTTGATTCATCCATGGATTCGGACGACGACGTTAAGAACGC
TTACTTCTGTTATAT
+
FFFFFFFFFFFFFFFFFFFFFFFFFFFFFFFFFFFFFFFFF:FFFFFFFFFFFFFFFFFF:FFF:FFFFF
FFFFFFF:F,FF:FF,FFFFFFFFFF,F:FFF:F:FF,F:F:FF:,F:FF,FF:FFFFFFFF,FF::F
,FFF,:,,FFFFFFF
@A00721:190:HGJW7DSXY:4:1114:29586:34068 1:N:0:TCTGCAAG+GCTGCACT
TCGGGATCCAGATACTTGGATGATCAACATGGATAATGTTGATAGGTTTAGTGTCGTTTTTGAAAAAA
GATACGTTCTATAAGGTAGTGGGTACGTTGATTAATCCATGGATGCGGACGACGACGTTAAGAACGCG
TACTTATGTTATAT
+
FFFFFFFFFFFFFFFFFFFFFFFFFFFFFFFFFFF:FFFFFFFFFFFFFFFFFFFFFFF,F,F,FFFFF,
,:FF:,FF,:F,FF:,F,,,,F,F:F,FF:::F,F,:FF:,FFF,,,F::,F::FF,FFFF,FF,,F,
FFF,F,::FFFFFF
@A00721:190:HGJW7DSXY:4:1114:29577:34366 1:N:0:TCTGCAAG+GCTGCACT
TCGGGATCCAGATACTTGGATGATCAACATGGATAATGTTGATAGGTTTAGTGTCGTTTGTGAAAAAC
GATACGTTCTAGAAGGTCGTGGTTACGGTGATTCATCCATGGATGCGGACGACGACGTTAATAACGCG
TACTTCTGTTATATG
```

```
+
FFFFFFFFFFFFFFF:FFFFFFFFFFFFFFFFFFFFFFFFFFFFFFFFFFFFFFFFFFF,F,FFFFFF:F
F,FFF,:F,FFFFF:FF,FF:F,F,,F,FFFFF,F:FFFF:,,FFFFF:FFFF::F:,F,F,FFF:F,
F::,:,,:,FFFFF:
@A00721:190:HGJW7DSXY:4:1517:1696:16767 1:N:0:TCTGCAAG+GCTGCACT
TCGGGATCCAGATACTTGGATGATCAACATGGATAATGTTGATAGGTTTAGTGTCGTTTGTGAAAAAC
GATACGTTCTAGAAGGTCGTGGTTACGTTGTTTCATCCAAGGATGCGGACGACGACGTTAAGAACGCT
TAATTCTGTTATATG
+
FFFFFFFFFFFFFFFFFFFFFFFFFFFFFFFFFFFFFF::FFFFFFF,FFF:FFFF,,FFFFFFFFF:
:FFF,,,F,:F:FF,:F::F:FFFF:FF:F,FF,F::FF,,:::,F,FFF,FF,FFF:FFF,FF:,F,
,F,,,F,FFFFFFFF
@A00721:190:HGJW7DSXY:4:1603:7139:33990 1:N:0:TCTGCAAG+GCTGCACT
TCGGGATCCAGATACTTGGATGATCAACATGGATAATGTTGATAGGTTTAGTGTCGTTTGTGAAAAAC
GATACGTTCTAGAAGGTCGTGGTTACGTTGATTCATCCAAGGATTCGGACGACGACGTTAAGAACGCT
TA
+
FFFFFFFFFFFFFFFFFFFFFFFFFFFFFFFFFFFF,FFFFFFFFFFFFFFFFFFF,F,F::FFFF,
,F,::,:F:FF,FF,:F:F,FFF:FF::FF:FF,F:,F,,:FFF,F:FFF,FF:FF,FFFF,FF,F:,
F:
@A00721:190:HGJW7DSXY:4:1652:29695:2722 1:N:0:TCTGCAAG+GCTGCACT
TCGGGATCCAGATACTTGGATGATCAACATGGATAATGTTGATAGGTTTAGTGTCGTTTGTGAAAAAC
GATACGTTCTAGAAGGTCGTGGTTACGTTGATTCATCCATGGATGCGGACGACGACGTTAAGAACGCT
TACTTCTGTTATATG
+
FFFFFFFFFFFFFFFF,FFFFFFFFFFFFFFFFFFFFFFFFFFFFFFFFFFFFFFFF:FFF:FFFFFF
FF:F,,FF::FF:FFFF:F:F:FFFFFFF,FFFFF::FF,,FFFFFFFF::FFFFFFFF:F,FFFFFF
FFF:F:FFFFFFFF:
@A00721:190:HGJW7DSXY:4:2350:22498:29309 1:N:0:TCTGCAAG+GCTGCACT
TCGGGATCCAGATACTTGGATGATCAACATGGATAATGTTGATAGGTTTAGTGTCGTTTGTGAAAAAC
GATACGTTCTAGAAGGTCGTGGTTACGTTGATTCATCCATGGATGCGGACGACGACGTTAAGAAAGCT
TACTTCTGTTATAT
+
FFFFFFFFFFFFFFFF:FFFFFFFFFFFFFFFFFFFFFFFFFFFFFFFFFFFFFFFFFF,F:FFFFFFF:
:FFF,:FF::F,FF,FFF:FFF:FFF::FF,FF,:,:FF,,:FF,::FFF,F,,FF::FFF,FF,:F:
FFF,F::,:FF::F
@A00721:190:HGJW7DSXY:4:2541:18295:32049 1:N:0:TCTGCAAG+GCTGCACT
TCGGGATCCAGATACTTGGATGATCAACATGGATAATGTTGATAGGTTTAGTGTCGTTTGTGAAAAAC
GATACGTTCTAGAAGGTCGTGGTTACGTTGATTCATCCATGGATGCGGACGACGACGTTAAGAACGCG
TACTTCTGTTA
+
FFFFFFFFFFFFFFFFFFFFFFFFFFFFFFFFFFFFFFFFFFFFFFFFFFFFFFFF:F:FFFFFFF,
:FFF,FFFF,F,FF,FFFF,FFFF:FF:F,:FF:,,FFF,:FF:,F:FFF,F:F:F::F:F,,F:F:,
FF,:F:,FFFF
@A00721:190:HGJW7DSXY:4:2611:1913:22842 1:N:0:TCTGCAAG+GCTGCACT
TCGGGATCCAGATACTTGGATGATCAACATGGATAATGTTGATAGGTTTAGTGTCGTGTTTGTAAAAA
GATACGTTCTAGAAGGTCGTGGTTACGTTGATTAATCCAAGGATTCGGACGACGACGTTAAGAACGCG
TACTTCAGTGATAT
+
FFFF,F,FFFFFFF,:FFFFFFFF:FFFFFF:FFFFFFFF:FFFFFF:FFF:,:FFF,:,FF,FFFF,
,F,F:,,F,FF,FF,,:::,::FFFF:,F:,::,F,F,F,:F:F,::FFF:F,FF:,,,:F,FF,,:,
,:FFF,,FF,F,F:
@A00721:190:HGJW7DSXY:4:2674:15429:34444 1:N:0:TCTGCAAG+GCTGCACT
TCGGGATCCAGATACTTGGATGATCAACATGGATAATGTTGATAGGTTTAGTGTCGTTTGTGAAAAAC
```

```
GATACGTTCTAGAAGGTCGTGGTTACGTTGATTCATCCATGGATGCGGACGACGACGTTAAGAACGCT
TACTTCTGTTATATG
+
FFFFFFFFFFFFFFFFFFFFFFFFFFFFFFFFFFFFFFFFFFFFFFFFFFFFFFFFFFFFFF:FFFFF
F,FF,:FF,FF,FFFFFFF::F,FFFF,F:,FFFF,FFF:FFFF:FFFFF:FF:FFF:FFF,FF,FF:
FFF:FF:F::FFFFF
@A00721:190:HGJW7DSXY:4:1220:1452:9455 1:N:0:TCTGCAAG+GCTGCACT
CGGGATCCAGATACTTGGATGATCAACATGGATAATGTTGATAGGTTTAGTGTCGTGTGTTAAAAACG
ATAAGTTCAATAAGGTCGTGGTGACGTTGATTCATACATGGAAGCGGAAGACGACGTTAAGAACGCTT
ACTTCTGTTATAT
+
F,FF:,F,F:FFFFFF::FFFFFFFFFFFFFFF,FFF::FF,F,FFFFFFFFFFFF,F:F,FFF:F,:
,:F,,,:,,F,FF,,,,F,,FF,,,,,:F:FF,F:,F,:,F:,:FF:F,F::,,,F,F,F,,FF:,:F
,,,,:,FF:,FFF
@A00721:190:HGJW7DSXY:4:2561:5394:27774 1:N:0:TCTGCAAG+GCTGCACT
CGGGATCCAGATACTTGGATGATCAACATGGATAATGTTGATAGGTTTAGTGTCGTTTGTGAAAAACG
ATACGTTCTAGAAGGTCGTGGTTACGTTGATTCATCCATGGATGCGGACGACGACGTTAATAACGCTT
ACTTCTGTTATAT
+
FFFFFFFFFFFFFFFFFFFFFFFFFFFFFFFFFFFFF:FFFFFFFFFFFFFFFFFFFFFF:FFFFF::
FFF,FFF,:F:FF:FFFF,FFFFFFF:FFFFF,FF::F::FFF:FFFF,FFFF::F:FFF,FF,:FFF
FFFF,:FFFFFFF
@A00721:190:HGJW7DSXY:4:1375:25807:1438 1:N:0:TCTGCAAG+GCTGCACT
CAGATACTTGGATGATCAACATGGATAATGTTGATAGGTTTAGTGTCGTTTGTGAAAAACGATACGTT
CTAGAAGGTCGTGGTTACGTTGATTCATCCATGGATGCGGACGACGACGTTAAGAACGCTTACTTCTG
TTATATGAGGTCTA
+
FFFFFFFFFFFFFFFFFFFFFFFFFF:FFF:FFFFFFFFFFFFFFFFF,FFFF,FFFFFFFFFFFF:F
FFF:FFFFFFF,FFFFFFFFFFFFFFFF:FF,,FFFFFFFFFFFF::FFFFFF:FFFFF:FFFFF::F
,FFFFF:F,FFFFF
@A00721:190:HGJW7DSXY:4:1504:17824:21652 1:N:0:TCTGCAAG+GCTGCACT
CAGATACTTGGATGATCAACATGGATAATGTTGATAGGTTTAGTGTCGTTTGTGAAAAAAGATACGTT
CTAGAAGGTCGTGGTTACGTTGATTCATCCATGGATGCGGACGACGACGTTAAGAACGCGTACTTCTG
TTATATGAGGTCTA
+
FFFFFFFFFFFFFFFFFFFFFFFFFFFFFFFFFFFFFFFFFFFFFFFFF:F,F::FFFF,:F,FFF:F
F:F,FF,FF,,,FF,FF:F,FF,FF,F,FFF,:F,F:FFFFFFF,,:F::FFF:FF,FF,FF,,:,,F
F,FF,F,F,FF,FF
@A00721:190:HGJW7DSXY:4:2128:11767:13228 1:N:0:TCTGCAAG+GCTGCACT
CAGATACTTGGATGATCAACATGGATAATGTTGATAGGTTTAGTGTCGTTTGTGAAAAAAGATACGTT
CTAGAAGGTCGTGGTTACGTTTATTCATCCATGGATTCGGACGACGACGTTAAGAAAGCTTACTTCTG
TTATATTAGGTCTA
+
FFFFFFFFFFFFFFFFFFFFFFFFFFFFFFFFFFFFFFFFFFFFFFFFF,F:F,,FFFF,,F::,,:F
,:F,FF,,F::,F:,FF,,,F,FFF:F,,FF,,FFF,F,F:,:F,,F,:::FF,FF,,F,:F,,F,,:
FFFFFF,FF:,FFF
@A00721:190:HGJW7DSXY:4:2132:32723:8406 1:N:0:TCTGCAAG+GCTGCACT
CAGATACTTGGATGATCAACATGGATAATGTTGATAGGTTTAGTGTCGTTTGTGAAAAACGATACGTT
CTAGAAGGTCGGGGTTACGTTGATTCATCCATGGATTCGGACGACGACGTTAAGAAAGCTTACTTCTG
TTATATGATGTCTA
+
FFFFFFFFFFFFFFFFFFFFFFFFFFFFFFF:FFFFFFFFFFFFFFFFF:F:::,FFFF,:,:FF,FF
::F:FF,:F::,,FFF,:FF:,::F,,,,FF,:F:F,FFFF,FF,::FFFFFF:FF,,F,F,,,F::F
FFFFFF,:,FF:F:
```

```
@A00721:190:HGJW7DSXY:4:2151:2446:4883 1:N:0:TCTGCAAG+GCTGCACT
CAGATACTTGGATGATCAACATGGATAATGTTGATAGGTTTAGTGTCGTTTGTGAAAAACGATACGTT
CTAGAAGGTCGTGGTTACGTTGATTCATCCATGGATGCGGACGACGACGTTAAGAACCCTTACTTCTG
TTATATTAGGTCTAG
+
FFFF:FFFFFFFFFFF:FFFF,FFFFFFFFFFFFFFFFFFFFFFFFFFF:F::,FFFFF,,:,F,,,:
,,F,FF,FFF:,,F,FF,:,FFFFF::,,,,,,FFF,FFFF:,F,,F:,:FFF,FF:,F,:F,,:,,F
,:F:F,,F,:FF,F:
@A00721:190:HGJW7DSXY:4:2269:8919:31469 1:N:0:TCTGCAAG+GCTGCACT
CAGATACTTGGATGATCAACATGGATAATGTTGATAGGTTTAGTGTCGTTTGTGAAAAACGATACGTT
CTAGAAGGTCGTGGTTACGTTGATTCATCCATGGATGCGGACGACGACGTTAAGAACGCTTACTTCTG
TTATATGAGGT
+
FFFFFFFFFFFFFFFFFFFFFFFFFFFFFFFFFFFFFFFFFFFFFFFFF:F:F:FFFF,,FFFFF:FF
:FF,:FFFFFF:FF:FFF:,F:,FFFFFFFFF:FFF,F:FFFF:FF:FF,FFF:,F,F:,FF,,F,,F
FFFFFFF:FFF
@A00721:190:HGJW7DSXY:4:2627:4887:13902 1:N:0:TCTGCAAG+GCTGCACT
CAGATACTTGGATGATCAACATGGATAATGTTGATAGGTTTAGTGTCGTTTGTGAAAAACGATACGTT
CTAGAAGGTCGTGGTTACGTTGATTAATCCAATGATGCGGACGACGACGTTAAGAACGCTTACTTCTG
TTATATGAGGTCTA
+
FFFFFFFFFFFFFFFFFFFFFFFFFFFFFFFFFFFFFFFFFFFFFFFFF,FFFFFFFFF,:::F,,:F
:FF,FF,F::F,FFFF:,FFFF,:F,FF,FF,,F:FF:,FF,FF:FF,:,FFF,FF,:F,F:,,F,,:
FFFF:F,F:FF,FF
@A00721:190:HGJW7DSXY:4:1157:24948:14199 1:N:0:TCTGCAAG+GCTGCACT
CGGTCTAGACCTCATATAACAGAAGTAAGCGTTCTTAACGTCGTCGTCCGCATCCATGGATGAATCAA
CGTAACCACGACCTTCTAGAACGTATCGTTTTTCACAAACGACACTAAACCTATCAACATTATCCATG
TTGATCATCCAAGTA
+
FFFFFFFFFFFFFFFFFFFFF:FFFFFFFFFFFF::FFF:FFF:FFFFFFFF::FF:FFFFFFFFFFF
FFFFFFFFFFFF:FFF:F:FFF,FFFFFF,:FFFFFF::FFFFFFFFFF:F:FF:FFFFFFFFFFFFF
F::FFFFF,FFFFFF
@A00721:190:HGJW7DSXY:4:1421:6253:5619 1:N:0:TCTGCAAG+GCTGCACT
CGGTCTAGACCTCATATAACAGAAGTAAGCGTTCTTAACGTCGTCGTCCGCATCCATGGATGAATCAA
CGTAACCACGACCTTCTAGAACGTATCGTTTTTCACAAACGACACTAAACCTATCAACATTATCCATG
TTGATCATCCAA
+
FFFF,FF,FFFF:FFFFFFFFFFFFFFF::FFFFFFFFFF,F:FF:FF:,FFFFFFFFFFFFFF,FFF
FFFFFFF:FFFFFFFFFFFF:FF,::FF:FF:F,FFFF,FFFFFFFFFF:FF,F:FFFF,FFFFFFF,
,F:FF::F:FFF
@A00721:190:HGJW7DSXY:4:1475:32045:31876 1:N:0:TCTGCAAG+GCTGCACT
GATGATCAACATGGATAATGTTGATAGGTTTAGTGTCGTTTGTGAAAAACGATACGTTCTAGAAGGTC
GTGGTTACGTTGATTCATCCATGGATGCGGACGACGACGTTAAGAACGCTTACTTCTGTTATATGAGG
TCTAGACCGAT
+
F:FFFFFFFFFFFFFFFFFFFFFFFFFFFFFFFFFFFFF:F:FF,FFFFFFFFFFFFFFFFF,F:FFF
FFFF:FFFF,:FFFF:FF:FF:FFFFFFFFFFFFF:FFFFFFFFFF,FF::FF:FFFFFFFFFF,F,F
FFFF,F:F,FF
@A00721:190:HGJW7DSXY:4:1571:22345:3709 1:N:0:TCTGCAAG+GCTGCACT
GATGATCAACATGGATAATGTTGATAGGTTTAGTGTCGTTTGTGAAAAACGATACGTTCTAGAAGGTC
GTGGTTACGTTGATTCATCCATGGATGCGGACGACGACGTTAATAACGCTTACATCTGTTATATGAGG
TCTAGACCGAT
+
FFFFFFFFFFFFFFFFFFFFFFFFFFFFFFFFFFFFFFFFFFF:FFFFFFFFFF,:FF:FFFFFFF:F
```

FFFFFFFFFFFFF,F:,:,F:,FFFFFF:FFFFFF:FF:FFFF,FFF,F,FFF,F,:F:FFFFFFFFF
FFFFFF,FFFF
@A00721:190:HGJW7DSXY:4:2443:28275:34867 1:N:0:TCTGCAAG+GCTGCACT
GATGATCAACATGGATAATGTTGATAGGTTTAGTGTCGTTTGTGAAAAACGATACGTTCTAGAAGGTC
GTGGTTACGTTGATTCATCCATGGATGCGGACGACGACGTTAAGAACGCTTACTTCTGTTATATGAGG
TCTAGAACGAT
+
FFFFFFFFFFFFFFFFFFFFFFFFFFFFFFFFFFFFFFFFFFF,:FFFF:,,:F,:FF,:FFFF,FF:
F:FFF:FFF,FFF:F,:,,FF,FF,F:FFFF,::FFF::FFFF,:F,:F,FF:,F,F:,FFFF:,:,F
F,:,,:,:,FF
@A00721:190:HGJW7DSXY:4:2448:29785:1814 1:N:0:TCTGCAAG+GCTGCACT
GATGATCAACATGGATAATGTTGATAGGTTTAGTGTCGTTTGTGAAAAACGATAAGTTCTAGAAGGTC
GTGGTTACGTTGATTCATCCATGGATGCGGACGACGACGTTAAGAACGCTTACGTCTGTTATATGAGG
TCTAGACCGAT
+
FFFFFFFFFFFFFFFFFFFFFFFFFFFFFFFFFFFFFFFFFFFFFFFFF:,:,F,:,FFFF:FF:FF:
F,,F,FF,F:FFFFF:F,:F:FFF:FFFFFFF::FFF,F,F:F::F:,FFF:F,:,F:FFFFFF,FF:
FF:F,F,F:F:
@A00721:190:HGJW7DSXY:4:2578:6099:8046 1:N:0:TCTGCAAG+GCTGCACT
GATGATCAACATGGATAATGTTGATAGGTTTAGTGTCGTTTGTGAAAAACGATACGTTCTAGAAGGTC
GTGGTTACGGTGATTCATCCATGGATGCGGACGACGACGTTAAGAACGCTTACTTCTGTTATATGAGG
TCTAGACCGAT
+
F:FFFFFFFFFFFFF,FFFFFFFFFFFFFFFFFFFFFFFFFFFFFFFFF,FF:F:,FFFFF,:F:FFF
FF:F:FFFF,F:FFFFF:,FF,,FFFFFFFFF:FF:FF:FFFF,FFF:F,F:FFFFFFFFFFFFFFFF
F:FF,FFFFFF
@A00721:190:HGJW7DSXY:4:1269:24234:31845 1:N:0:TCTGCAAG+GCTGCACT
ATAGGTTTAGTGTCGTTTGTGAAAAACGATACGTTCTAGAAGGTCGTGGTTACGTTGATTCATCCATG
GATGCGGACGACGACGTTAAGAACGCTTACTTCTGTTATATGAGGTCTAGACCGATTTCTGTGAGGAA
GAAACTTAGTGGACT
+
FFFFFFFFFFFFFFFFFFFFFFFFFFFFFFFF,FFF:F,FF:FFFFFFFFFF:FF:,FFF,FF:FFFF
FFFFFFFF::FFFF::FFFF,FFFFFFFF,:F:FFFF:F:F:FFFF,FF,F,FFFFFF,FFFFFFFFF
:FFF::FF:F,:FFF
@A00721:190:HGJW7DSXY:4:1312:30273:32252 1:N:0:TCTGCAAG+GCTGCACT
ATAGGTTTAGTGTCGTTTGTGAAAAAAGATACGTTATAGAAGGTCGTGGTTACGTTGATTCATCCATG
GATGCGGACGACGACGTTAAGAACGCTTACTTCTGTTATATTAGGTCTATACCGATTTATTTGAGGAA
GAAAAATATTGGAC
+
FFFFFFFFFFFFFFFF,F:F,,FFFF,:FFF,,FF,:F,FF:FF::FFF:F,:F,F:,FF:F,:F:,,
F,F::FFF,,FF,FF,FFFF,FF,:F,F,,:F:,FFFFFFF,F:FF,FF,F,FFF:FF,:,:,,,FFF
,FFF,,:F,F,F:F
@A00721:190:HGJW7DSXY:4:1509:29668:7874 1:N:0:TCTGCAAG+GCTGCACT
ATAGGTTTAGTGTCGTTTGTGAAAAACGATACGTTCTAGAAGGTCGTGGTTACGTTGATTAATCCATG
GATGCGGACGACGAAGTTAAGAACGCTTACTTCTGTTATATTAGGTCTAGACCGATTTATGTGAGGAA
GAAACTTAGTTGAC
+
FFFFFFFFFFFFFFFF,F,,,FFFFF,,F,FF,,F,FF,FF,FF,:FFF,FF,,,F::FF,F,::,,:
F:F:::FFF,FF,F,,:FFF,FF,,:,F,,,F:,FFF:FFF,,,FF,FF:,FF:F::F,:,FFFFFFF
:FFF,,FF,F,FF:
@A00721:190:HGJW7DSXY:4:1522:23873:4319 1:N:0:TCTGCAAG+GCTGCACT
ATAGGTTTAGTGTCGTTTTTGAAAAAAGATACGTTATAGAAGGTCGTGGTTACGTTGATTCAACCCAG
GATTCGGACGACGACGTTAATAACGCTTACTTCTGTTATATGAGGTCTAGACCGATTTATGTGAGGAA
TAAAAATAGTTGAC

```
+
FFFFFFFFFFFFFFF:,F,F:FFFFF,,F:F,:FF,:F,FFFFF,F,:F,FF::,FFF:F:,,:F,:,
F,F,F,FF,,:F,FF:,FFF,FF:,:,FF,,F:,FFFFFF,,F,,F,,F,F:,,FFFF,F,FFF:FFF
,FFF,::F,,,:F:
@A00721:190:HGJW7DSXY:4:1654:25211:35759 1:N:0:TCTGCAAG+GCTGCACT
ATAGGTTTAGTGTCGTTTGTGAAAAACGATACGTTCTAGAAGGTCGTGGTTACGTTGATTCATCCATG
GATGCGGACGACGACGTTAAGAACGCTTACTTCTGTTATATGAGGTCTAGACCGATTTCTGTGAGGAA
GAAACTTAGTGGACT
+
FFFFFFFFFFFFFFFFFF:FFFFFFFFFFFFF:FF:,F:FF:FFFF:FFFFFFF,FF,FFFFF:FF,F
F:F,FFFF:,FFF:FFFFFF,FF::F,FF::F:FFFF::FF,F,FF,FF,FFFFF,FF:FFFFF,FFF
,FFF:,FF,FFFFFF
@A00721:190:HGJW7DSXY:4:2266:5728:1204 1:N:0:TCTGCAAG+GCTGCACT
ATAGGTTTAGTGTCGTTTGTGAAAAACGATACGTTCTAGAAGGTCGTGGTTAAGTTGATTCATCCATG
GATGCGGACGACGACGTTAAGAACGCTTACTTCTGTTATATTAGGTCTAGACCGATTTCTGTGAGGAA
GAAACTTATTGGACT
+
F:FFFFFFFFFFFFFFFF:::FFFFF:FF:FF:FFF:F,FF,FF,::FFFFF,FFF,FFFFF:::F:F
FFFF:FFFF,FF,FF:FFFF,FF:::,FF,,F,,FFFF:FF,FFFF:FFFF,F:F:FFFFFFFF:FFF
FFFF:FFF,FFF,:F
@A00721:190:HGJW7DSXY:4:2456:5963:35274 1:N:0:TCTGCAAG+GCTGCACT
ATAGGTTTAGTGTCGTTTGTGAAAAACGATACGTTCTAGAAGGTCGTGGTTACGTTGATTCATCCATG
GATGCGGACGACGACGTTAAGAACGCTTACTTCTGTTATATGAGGTCTAGACCGATTTCTGTGAGGAA
GAAACTTAGTGGACT
+
FFFFFFFFFFFFFFF:,F,FFFFFFFF:FFF,,FFFFFF:FFFFF:,FF:FF,FFFFFFFFF,,FF:,
F:FFFFFFF:F::FF,FFFF,FFF,F:F,F:FFFFFFF,F:F:,FF:FF,FFF,:FFFF:FFFF:FFF
FFFFF,FFFF,FF::
@A00721:190:HGJW7DSXY:4:2546:21920:21042 1:N:0:TCTGCAAG+GCTGCACT
ATAGGTTTAGTGTCGTTTGTGAAAAACGATACGTTCTAGAAGGTCGTGGTTACGTTGATTCATCCATG
GATGCGGACGACGACGTTAAGAACGCTTACTTCTGTTATATGAGGTCTAGACCGATTTCTGTGAGGAA
GAAACTTAGTTGA
+
FFFFFFFFFFFFFFFF:F,FFFFFFFF::FFF,FF,FF,:FFFF,FF:F,FFFFFFF:FF:FF:::F,
FF,,FFFF:F,F:FF:FFFF,FF:FF:FFF:,F,F:FFF:F,FFFF,,FF:,FF:FFF,::FF:,FFF
,FFF,FFF,F,:F
@A00721:190:HGJW7DSXY:4:2639:14742:4100 1:N:0:TCTGCAAG+GCTGCACT
ATAGGTTTAGTGTCGTTTGTGAAAAACGATACGTTCTAGAATGTCGTGGTTACGTTGATTAATCCATG
GATTCGGACGACGACGTTAAGAAAGCTTACTTCTGTTATATGAGGTCTAGACCGATTTCTGTGAGGAA
GAAACTTAGTGGAC
+
FFFFFFFFFFFFFFFF,FFFFFFFFF,:F:FF,FF:FF,FF,FF:F:FF:FF,F:FF:FF,:,,FF:F
FF:,:,FFF,F:,FF,,F:F,FF,,:,F:,,F,,:FFFFF::F::F:FF::FF,FF,F,,,:FF:FFF
,FFF,,FF,F,FF:
@A00721:190:HGJW7DSXY:4:1265:11623:36495 1:N:0:TCTGCAAG+GCTGCACT
CTAGGTTTAGTGTCGTTTGTTAAAAACGATACGTTCTAGAAGGTCGTGGTTACGTTGATTCATCCATG
GATGCGGACGACGACGTTAAGAACGCTTACATCTGTTATATGAGGTCTAGACCGATTTCTGTTAGGAA
GAAACTTAGTGGAC
+
FFFFFFFFFFFF:FFF,FFF,FFFFF,,FFF,:FFFFFFF:,,FF,F:,FFFF,,,,:F,,::FF::,
F::,F,F:F:FF,FF:,FFF,FFF,F:FF,,,:,,FFFFF:,FFFF,:F,F::FF:F:F:,F,F:FFF
:FFFF:,F:F,:FF
@A00721:190:HGJW7DSXY:4:1448:25961:10473 1:N:0:TCTGCAAG+GCTGCACT
CTAGGTTTAGTGTCGTTTGTGAAAAACGATACGTTCTAGAAGGTCGTGGTTACGTTGATTCATCCATG
```

GATGCGGACGACGACGGTAAGTACGCGTACGTCGGTTATATGAGGTCTAGACCGATTTCTGTGAGGAA
GAAACTTAGTGGACT
+
FFFFFFFFFFFFFFFF:F,FFFFFFFF::,FFFFFFFFFFF,FFF::FFFFFF:,FF,:F:FF:F:,F
:,::FFFFFFFF:,FF,FFF:,FF:,,FF,,F:,FFFFFF:FF:F:FFF:F:FF:F:F,FF:FFFF,F
FFFFF:FF,:FF:FF
@A00721:190:HGJW7DSXY:4:2104:22263:13620 1:N:0:TCTGCAAG+GCTGCACT
CTAGGTTTAGTGTCGTTTGTGAAAAAAGATACGTTCTATAAGGTCGTGGTTACGTTTATTCAACCAAG
GATGCGGACGACGACGTTAAGAACGCTTACATATGTTATATTAGGTATAGACCGATTTATGTGAGGAA
AAAACATATTGGA
+
FFFFFFFFFFFFFFFF:F,F:,FFFF,,F,F,,:F,:F,FF,:F,:,:FFFF,,FF,::F,F,,:F,,
F,:,,:FF,,F,,F,,:F,F,FF,,:,FF:,F,,,,FFFF,,,,,:,:F,F,:,F,:F:,,F,,,F:F
,FFF,,:F,:,F:
@A00721:190:HGJW7DSXY:4:2157:18041:25598 1:N:0:TCTGCAAG+GCTGCACT
CTAGGTTTAGTGTCGTTTGTGAAAAACGATACGTTCTAGAAGGTCGTGGTTACGTTGATTCATCCATG
GATGCGGACGACGACGTTAAGAACGCTTACTTCTGTTATATGAGGTCTAGACCGATTTATGTGAGGAA
GAAACTTAGTGGACT
+
FFFFFFFFFFFFFFFF,F,::F:FFFF,FFF::FF,,F,,F,FF,F,FF:F:,FFF:F:F,F::F:,,
FFF,F:FF:FF,FFFF,FFFF:F,,::FFFFFF,FFFFFFFFF,FF,F,:::F,FF:F,F:FFF:FFF
:FFF:,FF,F,FFF:
@A00721:190:HGJW7DSXY:4:2575:30183:36385 1:N:0:TCTGCAAG+GCTGCACT
CTAGGTTTAGTGTCGTTTGTGAAAAACGATACGTTCTATAAGGTCGTGGTTACGTTGATTCATCCATG
GATTCGGACGAAGACGTTAATAACGCTTACTTCTGTTAAATGAGGTCTAGACCGATTTATGTGAGGAA
GAAAAATAGTGGA
+
F:FFFFFFFFFFFFFF:F,F,FFFFFFFF:FF::F,,F,:F,:FF:FFFFFF,,FF,:,FFF:,FF,,
:,,,FFFF:,F,,F:,:FFF,:F,,F,,:,,,:FFF:F,FF,F,:FF,F:F,F,F:,,,FF,,FF:FF
,FFF,,FF,FFFF
@A00721:190:HGJW7DSXY:4:2656:27724:5760 1:N:0:TCTGCAAG+GCTGCACT
CTAGGTTTAGTGTCGTTTGTGAAAAACGATACGTTCTAGAAGGTCGTGGTTACGTTGATTCATCCATG
GATGCGGACGACCACGTTAAGAACGCTTACTTCTGTTATATGAGGTCTAGACCGATTTCTGTGAGGAA
GAAAATTAGTGGACT
+
FFFFFFFFFFFFFFFF,FFF:FFFFF,,FFFF,:F,FF:FF,FFFFFFFFFFF:,FFFFF,F:,FF,,
FF:,F:FF::F:,F:::FFF,FFFFF:F,F,F:,::FFFFF:FFFF:FF,::F:FFFF,F:F:FFFFF
,FFF,,FF,F,,F::
@A00721:190:HGJW7DSXY:4:1144:14859:6308 1:N:0:TCTGCAAG+GCTGCACT
GGGTTTAGTGTCGTTTGTGAAAAACGAAACTTTCTAGAAGTTCGTGGTTACGTTTAATAAACCAATGA
TTCGGAAGACGACCTTAAGAACGCTTAATTCTTGTATAATAGTTAAAGACCGAATTATTTGAGGAACA
AACAAACAGGAC
+
::FFFFFFFFFF,F,::F,FFFFF,,:,F,,FF:FF,:F,,,F,,F,,,FF,,,,:,,:,,FFF:,FF
,,F,FF,FF:,,F,:FFF,FF:FF,FF,,FF,,,,FFF,,,,,:,,,,FFF:F,FF:F,F:F,,FF,F
FFF:,F,,,FF:
@A00721:190:HGJW7DSXY:4:1144:15790:6355 1:N:0:TCTGCAAG+GCTGCACT
GGGTTTAGTGTCGTTTGTGAAAAACGATACGTTCTAGAAGGTCGGGGTTACGTTGATTCATCCAAGGA
TGCGGACGACGACGTTAAGAACGCTTACTTCTGTTATATGAGGTCTAGACCGATTTCTGTGAGGAAGA
AACTTAGTGGAC
+
FFFFFF:FFFFFFF,F:F:FFFFF,,,:F:,,F,,F,:F,FFFF,:F,FFF:,F,,,F:,F:F,,::,
:,F:FF,FFF:F,,:FFF,FF,,,,FF,:F,F,,FFF:F,F,::,:F,::FFFFFF::,:::,FFF,F
FF,,FF,F,FFF

```
@A00721:190:HGJW7DSXY:4:1323:12617:11569 1:N:0:TCTGCAAG+GCTGCACT
GGGTTTAGTGTCGTTTGTGAAAAACGAAACGTTATAGAAGGTCGTGGTTACGTTGATTCATCCATGGA
TGCGGACGACGACGTTAAGAAAGCTTACTTCTGTTATATTATGTCTAGACCGATTTATGTGAGGAAGA
AAAATATTGGA
+
FFFFFFFFFFFFFF,F,FFFFFFFF:F,F:::F,FF,FF,:F:F,,FF:F,:FF:F:FF,,,:::,FF
,,,FFF,:FF,F,,FFFF,FF,,::FF:,F:,,FFFFFF,F,:F,:FFFFF,F,FF,F,F,F:F,F:F
FF,,FF,FFFF
@A00721:190:HGJW7DSXY:4:1403:32307:8281 1:N:0:TCTGCAAG+GCTGCACT
GGGTTTAGTGTCGTTTGTGAAAAAAGATAAGTTATATAATGTAGTGGTTAAGTTTATTAATACAAGGA
TTCGGAAGACGACCTTAAGAACCCTTACTTCTGTTATATGATGTGTAGACCGATTTATTTGTGGAAGA
AAAATAGT
+
FFFFFFFFFFFFFF,F:F,,FFFF,,FFF,,,F,,F,FF,:F,:,,FFFF,,,F,,:F:F,,F:,,,F
F:,FF:,,F,,FF,FF:F,:F,,:,F:,,F,,,F:,FFF,:,FF,FF,F,F,F::FF:,FF,,F:F,F
FF,,F:,:
@A00721:190:HGJW7DSXY:4:1566:9227:8265 1:N:0:TCTGCAAG+GCTGCACT
GGGTTTAGTGTCGTTTGTGAAAAACGATACGTTCTAGAAGGTCGTGGTTACGTTGATTCATCCATGGA
TGCGGACGACGACGTTAAGAACGCTTACTTCTGTTATATGAGGTCTAGACCGATTTCTGTGAGGAAGA
AACTTAGTGGACTCA
+
FFFFFFFFFFFFFFFFFFFFFFFFFFFFF,FFF,FFFFFFFFFFFFF:FFFFFFFFFFFF::FF:FFF
F,FFFFFFFFFF,FFFFF,FF,FFFFFF,F,::FFFFFF:FFFF:FF,F,FFF:FFFF,FFF,FFF,F
FFFF:FFF,FF:FFF
@A00721:190:HGJW7DSXY:4:2571:26313:11334 1:N:0:TCTGCAAG+GCTGCACT
GGGTTTAGTGTCGTTTGTGAAAAACGATACGTTCTAGAAGGTCGTGGTTACGTTGATTCATCCATGGA
TGCGGACGACGACGTTAAGAACGCTTACTTCTGTTATATTAGGTCTAGACCGATTTCTGTGAGGAAGA
AACTTAGTGGACTCA
+
FFFFFFFFFFFFFFFFFFFFFFFF,:FFFF:FF:FF:FF,FF:FF:FFFF:FFF,::F::F,F::,FF
FFFFFFF:FFFFFFFFFF,FFF::FF,:F::FFFFFFFF,::FF,FF,F,FFFFFFF,:FFFFFFF,F
FFF,FF,F,FFFF:F
@A00721:190:HGJW7DSXY:4:1103:4056:16094 1:N:0:TCTGCAAG+GCTGCACT
GTCGTTTGTGAAAAACGATACGTTCTAGAAGGTCGTGGTTACGTTGATTCATCCATGGATGCGGACGA
CGACGTTAAGAACGCTTACTTCTGTTATATGAGGTCTAGACCGATTTCTGTGAGGAAGAAACTTAGTG
GACTCACAGTTTTTA
+
FFFFFFFFFFFFFFFFFFFFFFFFFFFFFFFFFFF:FFFFFFFFFFFFFFFF:FFFFFFFFFFFFFFF
FFFFFFFFFFFFFFFFFFFFFFFFF:FFFFFFFFFFFFFFFFFFFFFFFFFFFFFFFFFFFFFFFFFF
FFFFFFFFFFFFFFF
@A00721:190:HGJW7DSXY:4:1140:4652:2660 1:N:0:TCTGCAAG+GCTGCACT
GTCGTTTGTGAAAAACGATACGTTCTAGAAGGTCGTGGTTACGTTGATTCATCCATGGATGCGGACGA
CGACGTTAAGAACGCTTACTTCTGTTATATGAGGTCTAGACCGATTTCTGTGAGGAAGAAACTTAGTG
GACTCACAGTTTTTA
+
FFFF:FFFFFFFFFFFFFFFFFFFFFFFFFFFFFFFFFFFFFFFFFFFFFFF:FFFFFFFFFFFFFFF
FFFFFFFFF:FFFFFFF:F:FFFFF:FFFFFFFFFFFFFFFFFFFFFFFFFFFFFFFFFFFFFFFFFF
FFFFFFFFFFF:FFF
@A00721:190:HGJW7DSXY:4:1315:9399:22936 1:N:0:TCTGCAAG+GCTGCACT
GTCGTTTGTGAAAAACGATACGTTCTAGAAGGTCGTGGTTACGTTGATTCATCCATGGATGCGGACGA
CGACGTTAAGAACGCTTACTTCTGTTATATGAGGTCTAGACCGATTTCTGTGAGGAAGAAACTTAGTG
GACTCACAGTTTTTA
+
FFFFFFFFFFFFFFFFFFFFFFFFFFFFFFFFFFFFFFFFFFFFFFFFFFFFFFFFFFFFFFFFFFFF
```

FFFFFFFFFFFFFFFFFFFFFFF:F:FFFFFFFFFFFFFFFFFFFFFFFFFFFFFFFFFFFFFFFFFFFF
FFFFFFFFFFFFFFF
@A00721:190:HGJW7DSXY:4:1510:17743:36292 1:N:0:TCTGCAAG+GCTGCACT
GTCGTTTGTGAAAAACGATACGTTCTAGAAGGTCGTGGTTACGTTGATTCATCCATGGATGCGGACGA
CGACGTTAAGAACGCTTACTTCTGTTATATGAGGTCTAGACCGATTTCTGTGAGGAAGAAACTTAGTG
GACTCACAGTTTTTA
+
FFFFFFFFFFFFFFFFFFFFFFFFFFFFFFFFFFFF:FFFFFFFF:FFFFFFFF:FFFFFFFFFFFFFFF
FFFFFFFFFFFFFFFFFFFFFF::FFF:FFFFFFFFFFFFFFFFF:FFFFFFFFFFFFFFFFFFFFFFFF
FFFFFFFFFFF:FFF
@A00721:190:HGJW7DSXY:4:1577:28763:31767 1:N:0:TCTGCAAG+GCTGCACT
GTCGTTTGTGAAAAACGATACGTTCTAGAAGGTCGTGGTTACGTTGATTCATCCATGGATGCGGACGA
CGACGTTAAGAACGCTTACTTCTGTTATATGAGGTCTAGACCGATTTCTGTGAGGAAGAAACTTAGTG
GACTCACAGTTTTTA
+
FFFFFFFFFFFFFFFFFFFFFFFFFFFFFFFFFFFFFFFFFFFFFFFFFFFFFFFFF:FFFFFFFFFFFF
FFFFF:FFFFFFFFFFFFF:FFFFFF,FFFFFFFFFFFFF:FFFFFFFFFFFFFFFFFFF:FFFFFFFFF
FFFFFFFFFFFFFFF
@A00721:190:HGJW7DSXY:4:1609:7500:32142 1:N:0:TCTGCAAG+GCTGCACT
GTCGTTTGTGAAAAACGATACGTTCTAGAAGGTCGTGGTTACGTTGATTCATCCATGGATGCGGACGA
CGACGTTAAGAACGCTTACTTCTGTTATATGAGGTCTAGACCGATTTCTGTGAGGAAGAAACTTAGTG
GACTCACAGTTTTTA
+
FFFFFFFF:FFFFFFFF:FF,FFFFFFFFFFFFFFFFFFFFFFFFFFFFFFFF:FFFFFFFFF:FFFFFF
FFFFFFFFF:FF:F,FFFFF::FFFFFFFF:FFFFFFF:FFFFFFFFFFF::FF:FFFFFFFFFFFFFFF
FFFFFFFFFFFFFFF
@A00721:190:HGJW7DSXY:4:2121:7374:13260 1:N:0:TCTGCAAG+GCTGCACT
GTCGTTTGTGAAAAACGATACGTTCTAGAAGGTCGTGGTTACGTTGATTCATCCATGGATGCGGACGA
CGACGTTAAGAACGCTTACTTCTGTTATATGAGGTCTAGACCGATTTCTGTGAGGAAGAAACTTAGTG
GACTCACAGTTTTTA
+
FF,FFFF::FFFFF:FFFFF:FFF,,FFFF::FF:FFFFFFF:F:,:FFFFF:FFF,FF:FFF:F,:F
F:FFFFFFF:FFF:FFFFF:FFF,FFFFFF:F,F:::F:FFFFFFFFF:F::FFFFF:FFF,:,F,FF
:FFF:FFF:,F:FFF
@A00721:190:HGJW7DSXY:4:2248:1127:23046 1:N:0:TCTGCAAG+GCTGCACT
GTCGTTTGTGAAAAACGATACGTTCTAGAAGGTCGTGGTTACGTTGATTCATCCATGGATGCGGACGA
CGACGTTAAGAACGCTTACTTCTGTTATATGAGGTCTAGACCGATTTCTGTGAGGAAGAAACTTAGTG
GACTCACAGTTTTTA
+
FFFF:F:FFFFFFFFFFFFFFF:FFFFFFFFFFFFFFFFFFFFFFFFFFFFFFF:FFFFFFFFFFFFFFF
FFFFFFFFFFFFFFFFFFFFFFFFFFFFFFFFFFFFFFFFFFFFFFFFFFFFFFFFFFFFFFFFFFFFFF
FFFFFFFFFFFFFFF
@A00721:190:HGJW7DSXY:4:2248:1307:23077 1:N:0:TCTGCAAG+GCTGCACT
GTCGTTTGTGAAAAACGATACGTTCTAGAAGGTCGTGGTTACGTTGATTCATCCATGGATGCGGACGA
CGACGTTAAGAACGCTTACTTCTGTTATATGAGGTCTAGACCGATTTCTGTGAGGAAGAAACTTAGTG
GACTCACAGTTTTTA
+
FFFFFFFFFFFFFFFFFFFFFFFFFFFFFFFFFFFFFFF:FFFFFFFFFFFFFFFFFFFFFFFFFFFFFF
FFFFFFFFFFFFFFFFFF:FFF:FFFFF:FFFFFFFFFFFFFFFFFFFFFFFFFFFFFFFFFFFFFFFFF
FFFFFFFFFFFFFFF
@A00721:190:HGJW7DSXY:4:2333:17942:29872 1:N:0:TCTGCAAG+GCTGCACT
GTCGTTTGTGAAAAACGATACGTTCTAGAAGGTCGTGGTTACGTTGATTCATCCATGGATGCGGACGA
CGACGTTAAGAACGCTTACTTCTGTTATATGAGGTCTAGACCGATTTCTGTGAGGAAGAAACTTAGTG
GACTCACAGTTTTTA

```
+
FFFFFFFFFFFFFFFFFFFFFFFFFFFFFFFFFFFFFFFFFFFFFFFFFFFF:FFFFFFFFFFFFFFF
FFFFFF:FFFFFFFFFFFFFFFFFFFFFFFFFFF:FFFFFF:FFFFFFFFF,FFFFF:FFFFFFFFFFF
FFFFFFFF:FFF:FF
@A00721:190:HGJW7DSXY:4:2365:12093:17206 1:N:0:TCTGCAAG+GCTGCACT
GTCGTTTGTGAAAAACGATACGTTCTAGAAGGTCGTGGTTACGTTGATTCATCCATGGATGCGGACGA
CGACGTTAAGAACGCTTACTTCTGTTATATGAGGTCTAGACCGATTTCTGTGAGGAAGAAACTTAGTG
GACTCACAGTTTTTA
+
FFFFFFFFFFFFFFFFFFFFFFFFFFFFFFFFFFFFFFFFFFFFFFFFFFFFFFFFFFFFFFFFFFFF
FFFFFFFFFFFFFFFFFF:FFF:F:FF:FFFFFFFFFFFFFFFFFFFFFFFFFFFFFF:FFFFFFFFF
FFFFFFFFFFFFFFF
@A00721:190:HGJW7DSXY:4:2365:32859:35665 1:N:0:TCTGCAAG+GCTGCACT
GTCGTTTGTGAAAAACGATACGTTCTAGAAGGTCGTGGTTACGTTGATTCATCCATGGATGCGGACGA
CGACGTTAAGAACGCTTACTTCTGTTATATGAGGTCTAGACCGATTTCTGTGAGGAAGAAACTTAGTG
GACTCACAGTTTTTA
+
FFFFFFFFFFFFFFFFFFFFFFFFFFFFFFFFFF:FFFFFFFFFFFFFFFFFF:FFFFFFFFFFFFFFF
FFFFFFFFFF:FFF:FFFFF:F:FFFFFFFFFFFFFFFFFFFFFFFFFFFFFFFFFFF:FFFFFFFF:
FFFFFFFFFFFFFFF
@A00721:190:HGJW7DSXY:4:2408:11008:15953 1:N:0:TCTGCAAG+GCTGCACT
GTCGTTTGTGAAAAACGATACGTTCTAGAAGGTCGTGGTTACGTTGATTCATCCATGGATGCGGACGA
CGACGTTAAGAACGCTTACTTCTGTTATATGAGGTCTAGACCGATTTCTGTGAGGAAGAAACTTAGTG
GACTCACAGTTTTTA
+
FFFFFFFF:FFFFFFFFFFFFFFFFFFFFFFFFFFFFFFFFFFFFFFFFFF:F:FFFFFFFFFFFF
FFFFFFFFFFFFFFFFFFFFFFFFFFFFFFFFFFFFFFFFFFFFFFFFFF:FFFFFFF:FFFFFFFFF
FFFFFFFFFFFFFFF
@A00721:190:HGJW7DSXY:4:2454:32940:21840 1:N:0:TCTGCAAG+GCTGCACT
GTCGTTTGTGAAAAACGATACGTTCTAGAAGGTCGTGGTTACGTTGATTCATCCATGGATGCGGACGA
CGACGTTAAGAACGCTTACTTCTGTTATATGAGGTCTAGACCGATTTCTGTGAGGAAGAAACTTAGTG
GACTCACAGTTTTTA
+
FFFFFFFFFFFFFFFFF:FFFFFFFFFFFFFFFFFFFFFFFFFFF:FFFFFFFF:FFFFFFFFFFFFFF
FFFFFFFFFFFFFFFFF:FFF:FFFFFFFFFFFFFFFFFFFFFFFFFFFFFFFFFFFFFFFFFFFFFF
FF:FFFFFFFFFFFF
@A00721:190:HGJW7DSXY:4:2574:1479:26976 1:N:0:TCTGCAAG+GCTGCACT
GTCGTTTGTGAAAAACGATACGTTCTAGAAGGTCGTGGTTACGTTGATTCATCCATGGATGCGGACGA
CGACGTTAAGAACGCTTACTTCTGTTATATGAGGTCTAGACCGATTTCTGTGAGGAAGAAACTTAGTG
GACTCACAGTTTTTA
+
FFFFFFFFFFFFFF:FFFFFFFFFFFFFFF,FFFFFFFFFFFFFFFFFFFFFF:FFFFFFFFFFFFFF
FFFFFFFFFFFFFFFFFF:FFF:FFFFF:FFFFFFFFFFFFFFFFFFFFFFFFFFFF:F:FFFFFFFFF
FFFFFFFFFFFFFFF
@A00721:190:HGJW7DSXY:4:1104:10601:22639 1:N:0:TCTGCAAG+GCTGCACT
CGTTTGTGAAAAACGATACGTTCTAGAAGGTCGTGGTTACGTTGATTCATCCATGGATGCGGACGACG
ACGTTAAGAACGCTTACGTCTGTTATATGAGGTCTAGACCGATTTCTGTGAGGAAGAAACTTAGTGGA
CTCACAGTTTT
+
FFF:FFFF:FFFFF:FFFFFFFF:FFFF,FFFFFFFFFFFFFF::FFFFF,FF::FFFF:FFFFFFF:
FF:FFFFFFF:FF::FF,F,,F,FFFFF:FFFF:FF:FFF:F:FF,FFFFFFFFFFFFFFFFF:FF:F
FF:FFF:FFFF
@A00721:190:HGJW7DSXY:4:1127:14543:20791 1:N:0:TCTGCAAG+GCTGCACT
CGTTTGTGAAAAACGATACGTTCTAGAAGGTCGTGGTTACGTTGATTCATCCATGGATGCGGACGACG
```

```
ACGTTAAGAACGCTTACTTCTGTTATATGAGGTCTAGACCGATTTCTGTGAGGAAGAAACTTAGTGGA
CTCACAGTTTT
+
FFFFFFF:FFFFFFFFFFFFFFFFFFFFFFFF:FFFFFFFFFFFFFFFFFFFFFFFFFFF:FFFFFFFFF
FFFFFFF,FFFFF:FFF:FFFFFFFFFFF,F,FFFFF,F:FFFFFFFFFFFFFFFFFFFFFF::F,FFFF
FFFFFFFFFFF
@A00721:190:HGJW7DSXY:4:1169:21829:25770 1:N:0:TCTGCAAG+GCTGCACT
CGTTTGTGAAAAACGATACGTTCTAGAAGGTCGTGGTTACGTTGATTCATCCATGGATGCGGACGACG
ACGTTAAGAACGCTTACTTCTGTTATATGAGGTCTAGACCGATTTCTGTGAGGAAGAAACTTAGTGGA
CTCACAGTTTT
+
FFFFFFFFFFFFFFFFFFFFFFFFFFFFFFFFFFFFFFFFFF:FFFFFFFFFFFFFFFFFFFFFFFFFFF
FFFFFFFFFFFFFFFF:::FFF:FFFFFFFFFFFFFFFFFFFFFFFFFFFFFFFFFFFFFFFFFFFFFFF
FFFFFFFFFFF
@A00721:190:HGJW7DSXY:4:1237:5321:24267 1:N:0:TCTGCAAG+GCTGCACT
CGTTTGTGAAAAACGATACGTTCTAGAAGGTCGTGGTTACGTTGATTCATCCATGGATGCGGACGACG
ACGTTAAGAACGCTTACTTCTGTTATATGAGGTCTAGACCGATTTCTGTGAGGAAGAAACTTAGTGGA
CTCACAGTTTT
+
FFFFFFFFFFFF:FFFFFFF:FFFFFFFFFFFFFFFF:FFFF:FFFFFFFFF:F::FFFF,FFFFFFFFF
FFFFFFF:FF:FF:FFF:FF,FFFFFF:FF:FFF:FFFFFFFFFFFFFFFFFFFFFFFFFFFFFFFFFFF
FFFFFFFFFFF
@A00721:190:HGJW7DSXY:4:1243:21486:16814 1:N:0:TCTGCAAG+GCTGCACT
CGTTTGTGAAAAACGATACGTTCTAGAAGGTCGTGGTTACGTTGATTCATCCATGGATGCGGACGACG
ACGTTAAGAACGCTTACTTCTGTTATATGAGGTCTAGACCGATTTCTGTGAGGAAGAAACTTAGTGGA
CTCACAGTTTT
+
FFF:FFFFFFFFFF:FFFFFFFFFFFFFFFFFFFFFF:FFFF,FFFFFFFFF,FFFFFFFFFFFFFFFFF
FFFFFFF:FFFFFFFFFFFF,FF:FFFFFFFFFFFFF:FFFFFFFFFFF:FFFFFFFFFFFFFFF:FFFF
FFFFFFFFFFF
@A00721:190:HGJW7DSXY:4:1323:1741:29778 1:N:0:TCTGCAAG+GCTGCACT
CGTTTGTGAAAAACGATACGTTCTAGAAGGTCGTGGTTACGTTGATTCATCCAAGGATGCGGACGACG
ACGTTAAGAACGCTTACTTCTGTTATATGAGGTCTAGACCGATTTCTGTGAGGAAGAAACTTAGTGGA
CTCACAGTTTT
+
FFFFFFF:FFFFFFFFFFFFFFF:F,FFFF:FFFFFF:FFFFF:::FF:FF,:FF,:FF::F,FFFFF:,
F,F:FFF,FF:FFFFF,,::,FFFF:FF:FF,,FFFFFFF:::FFFF:,FFFFFFFFF,,,FFF:FFF
F,FFF:,FFFF
@A00721:190:HGJW7DSXY:4:1610:23502:25723 1:N:0:TCTGCAAG+GCTGCACT
CGTTTGTGAAAAACGATACGTTCTAGAAGGTCGTGGTTACGTTGATTCATCCATGGATGCGGACGACG
ACGTTAAGAACGCTTACTTCTGTTATATGAGGTCTAGACCGATTTCTGTGAGGAAGAAACTTAGTGGA
CTCACAGTTTT
+
FF,:FFFFFFFF:F:FFFF:,F,FF:FFFFFF::FFFFF,:,F,FF:FFF:F,FFF:,FFFFFF,:F:
F,::FFF:FF:,F,FF::F,,F,,F:FFFFFFF:,F,F,F,F,FFF:F:,FF:FFFFFFFF:F:FFFF
FFFFFF,F:F:
@A00721:190:HGJW7DSXY:4:2302:2781:25254 1:N:0:TCTGCAAG+GCTGCACT
CGTTTGTGAAAAACGATACGTTCTAGAAGGTCGTGGTTACGTTGATTCATCCATGGATGCGGACGACG
ACGTTAAGAACGCTTACTTCTGTTATATGAGGTCTAGACCGATTTCTGTGAGGAAGAAACTTATTGGA
CTCACAGTTTT
+
FFF,F,F:FFFFF,:F:F::FF::F,FF,:,:F,FF,FFF:,F:FFF,F:,::,,FFF,FFFFF:F,F
FF:FFFF,FF,FF:FF::F:,F:FFFFFFF:FFF:F,FFF:FFFF,FF:FF,F,FFFFF,,FF,::FF
F,FF:F,FF:F
```

@A00721:190:HGJW7DSXY:4:1239:4209:8625 1:N:0:TCTGCAAG+GCTGCACT
CTCTACACTGCCACCAGTGGTGTCCTTAAAAACTGTGAGTCCACTAAGTTTCTTCCTCACAGAAATCG
GTCTAGACCTCATATAACAGAAGTAAGCGTTCTTAACGTCGTCGTCCGCATCCATGGATGAATCAACG
TAACCACGACCTTCT
+
FFFFFFFFFFFFFFFFFFFFFFFFFFFFFFFFFFFFFFFFFFFFFFFFFFFFFFFFFFFFFFFFFFFF
FFFFFF:FF:FFFFFFFFFFFFFFFFFFFFFFFFFFFFFFFFFFFFFFFFFFFFFFFF:FFFFFFFFF
FFFFFFFFFFFFFFF
@A00721:190:HGJW7DSXY:4:1254:21784:36902 1:N:0:TCTGCAAG+GCTGCACT
CTCTACACTGCCACCAGTGGTGTCCTTAAAAACTGTGAGTCCACTAAGTTTCTTCCTCACAGAAATCG
GTCTAGACCTCATATAACAGAAGTAAGCGTTCTTAACGTCGTCGTCCGCATCCATGGATGAATCAACG
TAACCACGACCTTCT
+
FFFFFFFFFFFFFFFFFFFFFFFFFFFFFFFFFFFFFFFFFFFFFFFFFFFFFFFFFFFFFFFFFFFF
F:FFFFFFFFFFFFFFFFFFFFFFFFFFFFFFFFFFFFFFFFFFFFFFFFFFFFFFFFF::FFFFFFF
FFFFFFFFFFFFFFF
@A00721:190:HGJW7DSXY:4:1340:18511:14606 1:N:0:TCTGCAAG+GCTGCACT
CTCTACACTGCCACCAGTGGTGTCCTTAAAAACTGTGAGTCCACTAAGTTTCTTCCTCACAGAAATCG
GTCTAGACCTCATATAACAGAAGTAAGCGTTCTTAACGTCGTCGTCCGCATCCATGGATGAATCAACG
TAACCACGACCTTCT
+
FFFFFFFFFFFFFFFFFFFFFFFFFFFFFFFFFFFFFFFFFFFFFFFFFFFFFFFFFFFFFFFFFFFF
FFFFFFFFFFFFFFFFFFFFFFFFFFFFFFFFFFFFFFFFFFFFFFFFFF::FFF:FFFFFFFFFFFF
FFFFFFFFFFFF,FF
@A00721:190:HGJW7DSXY:4:1417:31132:3803 1:N:0:TCTGCAAG+GCTGCACT
CTCTACACTGCCACCAGTGGTGTCCTTAAAAACTGTGAGTCCACTAAGTTTCTTCCTCACAGAAATCG
GTCTAGACCTCATATAACAGAAGTAAGCGTTCTTAACGTCGTCGTCCGCATCCATGGATGAATCAACG
TAACCACGACCTTCT
+
FFFFFFFFFFFFFFFFFFFFFFFFFFFFFFFFFFFFFFFFFFFFFFFFFFFFFFFFFFFFFF:FFFFF
FF:FF,FFF:FFFFFFFFFFFFFFFFFFFFFFFFFFFFFFFFFFFFFFFFFFFFFFFFFFFFF,FF:F
FFFFFFFFFFFFFFF
@A00721:190:HGJW7DSXY:4:1440:4074:6104 1:N:0:TCTGCAAG+GCTGCACT
TCTACACTGCCACCAGTGGTGTCCTTAAAAACTGTGAGTCCACTAAGTTTCTTCCTCACAGAAATCGG
TCTAGACCACATATAACAGAAGTAAGCGTTCTTAACGTCGTCGTCCGCATCCATGTATGAATCAACGT
AACCACGACCTACT
+
F:F:FFFF:F:F:FFFFF:F:::,:FFFFFF,FFFFFFFFFFF,FFFFF,FF,FF,,FFFFF:FFF:F
FF,,,:F:,,FFFFFFFFFFFF,FF::F,,FFFFFFFF::FFFFFFFFFF,F,FF,F,,FFFFF::FF
FFFFFFFFFFF:FF
@A00721:190:HGJW7DSXY:4:1524:15374:16188 1:N:0:TCTGCAAG+GCTGCACT
CTCTACACTGCCACCAGTGGTGTCCTTAAAAACTGTGAGTCCACTAAGTTTCTTCCTCACAGAAATCG
GTCTAGACCTCATATAACAGAAGTAAGCGTTCTTAACGTCGTCGTCCGCATCCATGGATGAATCAACG
TAACCACGACCTTCT
+
FFFFFFFFFFFFFFFFFFFFFFFFFFFFFFFFFFFFFFFFFFFFFFFFFFFFFFFFFFFFF:FFFFFF
FFFFFFFFFFFFFF:FFFFFFFFFFFFFFFFFFFF:FFFFFFFFFFFFFFFFFFFFFFFFFFFFFFFF
FFFFFFFFFFFF:FF
@A00721:190:HGJW7DSXY:4:1632:29143:7059 1:N:0:TCTGCAAG+GCTGCACT
CTCTACACTGCCACCAGTGGTGTCCTTAAAAACTGTGAGTCCACTAAGTTTCTTCCTCACAGAAATCG
GTCTAGACCTCATATAACAGAAGTAAGCGTTCTTAACGTCGTCGTCCGCATCCATGGATGAATCAACG
TAACCACGACCTTCT
+
FFFFFFFFFFFFFFFFFFFFFFFFFFFFFFF:FFFFFFFFFFFFFFFFFFFFFFFFFFFFFFFFFFFF

FFFFFFFFFFFFFFFFFFFFFFFFF:FFFFFFFFFFFFFFFFFFFFFFFFFFFFFFFFFFFFFFFFFF
FFFFFFFFFFFFFFF
@A00721:190:HGJW7DSXY:4:2231:20763:32346 1:N:0:TCTGCAAG+GCTGCACT
CTCTACACTGCCACCAGTGGTGTCCTTAAAAACTGTGAGTCCACTAAGTTTCTTCCTCACAGAAATCG
GTCTAGACCTCATATAACAGAAGTAAGCGTTCTTAACGTCGTCGTCCGCATCCATGGATGAATCAACG
TAACCACGACCTTCT
+
FFFFFFFFFFFFFFFFFFFFFFFFFFFFFFFFFFFFFFFFFFFF:FFFFFFFFFFFFFFFFFFFFFFF
FFFFFFFFF:FFFFFFFFFFFFFFFFFFFFFFFFFFFFFFFFFFFFFFF:FFFFFFFFFFFFFFFFFF
:FFFFFFFFFFF:FF
@A00721:190:HGJW7DSXY:4:2270:27832:31344 1:N:0:TCTGCAAG+GCTGCACT
CTCTACACTGCCACCAGTGGTGTCCTTAAAAACTGTGAGTCCACTAAGTTTCTTCCTCACAGAAATCG
GTCTAGACCTCATATAACAGAAGTAAGCGTTCTTAACGTCGTCGTCCGCATCCATGGATGAATCAACG
TAACCACGACCTTCT
+
FFFFFFFFFFFFFFFFFFFFFF:FFFFFFFFFFFFFFFFFFFFFFFFFFFF:FFFFFFFFFFFFFFFF
FFFFFFFFFFFFFFFFFFF:FFFFFFFFFFFFFFFFFFFFFFFFFFFFFFFFFFFFFFFFFFFFFFFF
FFFFFFFFFFFFFF:
@A00721:190:HGJW7DSXY:4:2321:1163:1282 1:N:0:TCTGCAAG+GCTGCACT
CTCTACACTGCCACCAGTGGTGTCCTTAAAAACTGTGAGTCCACTAAGTTTCTTCCTCACAGAGATCG
GTCTAGACCTCATATAACAGAAGTAAGCGTTCTTAACGTCGTCGTCCGCATCCATGGATGAATCAACG
TAACAACGACCTTCT
+
FFFFFFFFFFFFFFFFFFFFFF:FFFFF:FFFFFFFFFFFFFF,FFFFFFF:FFFFF,F,F:FFFFF:
FF:FF,F,FF,FFFFFF,F,FFFFFFFFFFFFFFFF::F:FFFFFFFFFFF,FFF:FFF::FFF:FFF
FFF:,F,FFFFFFFF
@A00721:190:HGJW7DSXY:4:2324:23547:30342 1:N:0:TCTGCAAG+GCTGCACT
CTCTACACTGCCACCAGTGGTGTCCTTAAAAACTGTGAGTCCACTAAGTTTCTTCCTCACAGAAATCG
GTCTAGACCTCATATAACAGAAGTAAGCGTTCTTAACGTCGTCGTCCGCATCCATGGATGAATCAACG
TAACCACGACCTTCT
+
FFFFFFFFFFFFFFFFFFFFFFFFFFFFFFFFFFFFFFFFFFFFFFFFFFFFFFFFFFFFFFF:FFFF
FFFF:FFFFFFFFFFFFFFFFFFFFFFFFFFFFFFFFFFFFFFFFFFFFFFFFFFFFFFFFFFFFFFF
FFFFFF:FFFFFFFF
@A00721:190:HGJW7DSXY:4:2350:25229:35039 1:N:0:TCTGCAAG+GCTGCACT
CTCTACACTGCCACCAGTGGTGTCCTTAAAAACTGTGAGTCCACTAAGTTTCTTCCTCACAGAAATCG
GTCTAGACCTCATATAACAGAAGTAAGCGTTCTTAACGTCGTCGTCCGCATCCATGGATGAATCAACG
TAACCACGACCTTCT
+
FFFFFFFFFFFFFFFFFFFFFFFFFFFFFFFFFFFFFFFFFFFFF:FFFFFFFFFFFFFFFFFFFFFF
FFFFFFFFFFFFFFFFFFFFFFFFFFFFFFFFFFFFFFFFFFFFFFFFFFFFFFFFFFFFFFFFFFFF
FFFF:FFFFFFFFFF
@A00721:190:HGJW7DSXY:4:2502:6903:26256 1:N:0:TCTGCAAG+GCTGCACT
CTCTACACTGCCACCAGTGGTGTCCTTAAAAACTGTGAGTCCACTAAGTTTCTTCCTCACAGAAATCG
GTCTAGACCTCATATAACAGAAGTAAGCGTTCTTAACGTCGTCGTCCGCATCCATGGATGAATCAACG
TAACCACGACCTTCT
+
FFFFFFFFFFFFFFFFFFFFFFFFFFFFFFFFFF:FFFFFFFFF,FFFFFFFFFFFFFFFFFFFFFFF
FFFFFFFFF:FFFFFFF:FFFFFFFF:FFFFFFFFFFFFFFFFFFFFFFFFFFFFFFFF,:F:FFFFF
FFF:FFF:FFF:FFF
@A00721:190:HGJW7DSXY:4:2550:32768:9392 1:N:0:TCTGCAAG+GCTGCACT
CTCTACACTGCCACCAGTGGTGTCCTTAAAAACTGTGAGTCCACTAAGTTTCTTCCTCACAGAAATCG
GTCTAGACCTCATATAACAGAAGTAAGCGTTCTTAACGTCGTCGTCCGCATCCATGGATGAATCAACG
TAACCACGACCTTCT

+
FFFFFFFFFFFFFFFFFFFFFFFFFFFFFFFFFFFFFFFFFFFFFFFFFFFFFFFFFFFFFFFFFFFFFFFF
FFFFFFFFFFFFFFFFFFFFFFFFFFFFFFFFFFFFFFFFFFFFFFFFFFFFFFFFFFFFFFFFFFFFFF:F
FFFFFFFFFFFFFFF
@A00721:190:HGJW7DSXY:4:2557:10140:4398 1:N:0:TCTGCAAG+GCTGCACT
CTCTACACTGCCACCAGTGGTGTCCTTAAAAACTGTGAGTCCACTAAGTTTCTTCCTCACAGAAATCG
GTCTAGACCTCATATAACAGAAGTAAGCGTTCTTAACGTCGTCGTCCGCATCCATGGATGAATCAACG
TAACCACGACCTTCT
+
FFFFFFFFFFFFFFFFFFFFFFFFFFFFFFFFFFFFFFFF,FFFFFFFFFFFFFFFFFFFFFFFFFFFFFFF
FFFFF::FFFFFFFFFFFFFFFFFFFFFFFFFFFFFFFFFFFFFFFFFFFFFFFFFFFFFFFFFFFFFFFFF
FFFF:FFFFFFFFFF
@A00721:190:HGJW7DSXY:4:1262:4065:5462 1:N:0:TCTGCAAG+GCTGCACT
CCTCTACACTGCCACCAGTGGTGTCCTTAAAAACTGTGAGTCCACTAAGTTTCTTCCTCACAGAAATC
GGTCTAGACCTCATATAACAGAAGTAAGCGTTCTTAACGTCGTCGTCCGCATCCATGGATGAATCAAC
GTAACCACGACCTTC
+
FFFFFFFFFFFFFFFFFFFFFFFFFFFFFFFFFFFFFFFFFFFFFFFFFFFFFFFFFFFFFFFFFFFFFFFF
FFFFFFFFFFFFFFFFFFFFFFFFFFFFFFFFFFFFFFFFFFFFFFFFFFFFFFFFFFFFFFFFFFFFFFFF
FFFFFFFFFFFFFFF
@A00721:190:HGJW7DSXY:4:1605:5584:12477 1:N:0:TCTGCAAG+GCTGCACT
CCTCTACACTGCCACCAGTGGTGTCCTTAAAAACTGTGAGTCCACTAAGTTTCTTCCTCACAGAAATC
GGTCTAGACCTCATATAACAGAAGTAAGCGTTCTTAACGTCGTCGTCCGCATCCATGGATGAATCAAC
GTAACCACGACCTTC
+
FFFFFFFFFFFFFFFFFFFFFFFF:FFFFFFFFFFFFFFFFFFFFFFFFFFFFFFFFFFFFF:FFFFFFFF
FF:F,FFFFFFF:FFFFFFFF,FFFFFFFFFFFFFFFFF:FFFFFFFFFFFFFFFFFFFFFFF:FFFFF
FFFFFFFFFFFF,FF
@A00721:190:HGJW7DSXY:4:2228:23213:12226 1:N:0:TCTGCAAG+GCTGCACT
CCTCTACACTGCCACCAGTGGTGTCCTTAAAAACTGTGAGTCCACTAAGTTTCTTCCTCACAGAAATC
GGTCTAGACCTCATATAACAGAAGTAAGCGTTCTTAACGTCGTCGTCCGCATCCATGGATGAATCAAC
GTAACCACGACCTTC
+
FF:FFFFFFFFFFFFFFFFFFFFFFFFFFFFFFFFFFFFFFFFFFFFFFFFFFFFFFFFFFFFFFFFFFFFF
FFFFFFFFFFFFFFFFFFFFFFFFFFFFFFFFFFFFFFFFFFFFFFFFFFFFFFF,FFFFFF:FFFFF
FFFFFFFF:FFFFFF
@A00721:190:HGJW7DSXY:4:2250:5466:6386 1:N:0:TCTGCAAG+GCTGCACT
CCTCTACACTGCCACCAGTGGTGTCCTTAAAAACTGTGAGTCCACTAAGTTTCTTCCTCACAGAAATC
GGTCTAGACCTCATATAACAGAAGTAAGCGTTCTTAACGTCGTCGTCCGCATCCATGGATGAATCAAC
GTAACCACGACCTTC
+
FFFFFFFFFFFFFFFFFFFFFFFFFFFFFFFFFFFFFFFFFFFFFFFFFFFFFFFFFFFFFFFFFFFFFFFF
FFFFFFFFFFFFFFFFFFFFFFFFFFFFF:FFFFFFFFFFFFFFFFFFFFFFFFFFFFFFFFFFFFFFFFF
FFFFFFFFFFFFFFF
@A00721:190:HGJW7DSXY:4:2425:1904:2347 1:N:0:TCTGCAAG+GCTGCACT
CCTCTACACTGCCACCAGTGGTGTCCTTAAAAACTGTGAGTCCACTAAGTTTCTTCCTCACAGAAATC
GGTCTAGACCTCATATAACAGAAGTAAGCGTTCTTAACGTCGTCGTCCGCATCCATGGATGAATCAAC
GTAACCACGACCTTC
+
FFFFFFFFFFFFFFFFFFFFFFFFFFFFFFFFFF:FFFFFFFFFFF:FFFFFFFFFFFFFFFFFFFFFFFF
FFFFFFFFF:FFFFFFFFFFFFFFFFFFFFFFFFF:F:FFFFFFFFFFFFFFFFFFFFFFFFFFFFFFFFF
:FFF:FFF:FFFFFF
@A00721:190:HGJW7DSXY:4:2569:22055:29199 1:N:0:TCTGCAAG+GCTGCACT
CCTCTACACTGCCACCAGTGGTGTCCTTAAAAACTGTGAGTCCACTAAGTTTCTTCCTCACAGAAATC

GGTCTAGACCTCATATAACAGAAGTAAGCGTTCTTAACGTCGTCGTCCGCATCCATGGATGAATCAAC
GTAACCACGACCTTC
+
FFFFFFFFFFFFFFFFFFFFFFFFFFFFFFFFFFFFFFFFFFFFFFFFFFFFFFFFFFFFFFFFFFFF
FFFFFFFFFFFFFFFFFFFFFFFFFFFFFFFFFFFFFFFFFFFFFFFFFFFFFFFFFFFFFFFFFFFF
FFFFFFFFFFFFFFF
@A00721:190:HGJW7DSXY:4:1143:24297:27633 1:N:0:TCTGCAAG+GCTGCACT
CGTGGTTACGTTGATTCATCCATGGATGCGGACGACGACGTTAAGAACGCTTACTTCTGTTATATGAG
GTCTAGACCGATTTCTGTGAGGAAGAAACTTAGTGGACTCACAGTTTTTAAGGACACCACTGGTGGCA
GTGTAGAGGACATGA
+
FF:FFFFFFF:FFFFFFFF:FFFFFFFFFFFFFF:FFFFFFFFFFFFFFFFFFFFFFFFFFFF:FFFF
FFFFFFFFFFFFFFFFFFFFFFFFF:FFFFFFFFFFFFFFFFFFFFFFFFFFFFFFFFFFFFFFF:FF
FFFFFFFFFFFFFFF
@A00721:190:HGJW7DSXY:4:1315:8404:27258 1:N:0:TCTGCAAG+GCTGCACT
CGTGGTTACGTTGATTCATCCATGGATGCGGACGACGACGTTAAGAACGCTTACTTCTGTTATATGAG
GTCTAGACCGATTTCTGTGAGGAAGAAACTTAGTGGACTCACAGTTTTTAAGGACACCACTGGTGGCA
GTGTAGAGGACATGA
+
FFFFFFFFFFFFFFFFFFF:FFFFFFFFFFFFFFFFFFFFFFFFFFFFFFFFFFFFFFFFFFFFFFFF
FFFFFFFFFFFFFFFFFFFFFFFFFFFFFFFFFFFFFFFFFFFFFFFFFFFFFFFFFFFFFFFFFFFF
FFFFFFFFFFFFFFF
@A00721:190:HGJW7DSXY:4:1359:12635:13416 1:N:0:TCTGCAAG+GCTGCACT
CGTGGTTACGTTGATTCATCCATGGATGCGGACGACGACGTTAAGAACGCTTACTTCTGTTATATGAG
GTCTAGACCGATTTCTGTGAGGAAGAAACTTAGTGGACTCACAGTTTTTAAGGACACCACTGGTGGCA
GTGTAGAGGACATGA
+
FFFFFFFFFFFFFFFFF:F:FFFFFFFFFFFFFFFFFFFFFFFFFFFFFFFFFFFFFFFF:,FFFFFF
FFFFFFFFFFFFFFFFFFFF:FFFFFFFFFFF:FFFFFFFFFFFFFFFFFFFFFFFFFFFF:FFFFFF
FFFFFFFFFFF:FFF
@A00721:190:HGJW7DSXY:4:1612:29333:7200 1:N:0:TCTGCAAG+GCTGCACT
CGTGGTTACGTTGATTCATCCATGGATGCGGACGACGACGTTAAGAACGCTTACTTCTGTTATATGAG
GTCTAGACCGATTTCTGTGAGGAAGAAACTTAGTGGACTCACAGTTTTTAAGGACACCACTGGTGGCA
GTGTAGAGGACATGA
+
FFFFFFFFFFFFFFFFFFF:FFFFFFFFFFFFFFFFFFFFFFFFFFFFFF,F,FFFF:FFFFFFFFFF
FFFFFFFFFFFFFFFFFFFFFFFFFFFFFFFFFFFFFFFFFFFFFFFFFFFFFFFFFFFFFFFFFFFF
FFFFFFFFFFFFFFF
@A00721:190:HGJW7DSXY:4:2134:11514:3302 1:N:0:TCTGCAAG+GCTGCACT
CGTGGTTACGTTGATTCATCCATGGATGCGGACGACGACGTTAAGAACGCTTACTTCTGTTATATGAG
GTCTAGACCGATTTCTGTGAGGAAGAAACTTAGTGGACACACAGTTTTTAAGGACACCACTGGTGGCA
GTGTAGAGGACATGA
+
FFFFFFFFFFFFFFFFFFF:FFFFFFFFFFFFFFFFFFFFFFFFFFFFFFFFFFFFFFFFFFFFFFFF
FFFFFFFFFFFFFFFFF:FFFFFFFFFFFFFFFFFFFF,FFFFFFFFFFFFFFFF:FFFFFFFFFFFF
FFFFFFFFFFFFFFF
@A00721:190:HGJW7DSXY:4:2143:27787:31548 1:N:0:TCTGCAAG+GCTGCACT
CGTGGTTACGTTGATTCATCCATGGATGCGGACGACGACGTTAAGAACGCTTACTTCTGTTATATGAG
GTCTAGACCGATTTCTGTGAGGAAGAAACTTAGTGGACTCACAGTTTTTAAGGACACCACTGGTGGCA
GTGTAGAGGACATGA
+
FFFFFFFFFFFFFFFFFFF:FFFFFFFFFFFFFFFFFFFFFFFFFFFFFFFFFFFFFFFFFFFFFFFF
FFFFFFFFFFFFFFFFFFFFFFFFFFFFFFFFFFFFFFFFFFFFFFFFFFFFFFFFFFFFFFFFFFFF
FFFFFFFFFFFFFFF

```
@A00721:190:HGJW7DSXY:4:2202:18918:5697 1:N:0:TCTGCAAG+GCTGCACT
CGTGGTTACGTTGATTCATCCATGGATGCGGACGACGACGTTAAGAACGCTTACTTCTGTTATATGAG
GTCTAGACCGATTTCTGTGAGGAAGAAACTTAGTGGACTCACAGTTTTTAAGGACACCACTGGTGGCA
GTGTAGAGGACATGA
+
FFFFFFFFFFFFFFFFFFFFFFFFFFFFFFFFFFFFFFFFFFFFFFFFFFFFFFFFFFFFFFFFFFFF
FFFFFFFFFFFFFFFFFFFFFFFFFFFFFFFFFFFFFFFFFFFFFF:FFFFFFFFFFFFFFFFFF:FF
FFF:FFFFFFFFFFF
@A00721:190:HGJW7DSXY:4:2249:6515:18944 1:N:0:TCTGCAAG+GCTGCACT
CGTGGTTACGTTGATTCATCCATGGATGCGGACGACGACGTTAAGAACGCTTACTTCTGTTATATGAG
GTCTAGACCGATTTCTGTGAGGAAGAAACTTAGTGGACTCACAGTTTTTAAGGACACCACTGGTGGCA
GTGTAGAGGACATGA
+
FFFFFFFFFFFFFFFFFFF:FFFFFFFFFFFFFFFFFFFFFFFFFFFFFFFFFFFFFFFFFFFFFFFF
FFFFF:FFFFFFFFFFFFFFFFFFFFFFFFFFFFFFFFFFFFFFFFFFFFFFFFFFFFFFFFFFFFFF
FFFFFFFFFFFFFFF
@A00721:190:HGJW7DSXY:4:2265:21802:2080 1:N:0:TCTGCAAG+GCTGCACT
CGTGGTTACGTTGATTCATCCATGGATGCGGACGACGACGTTAAGAACGCTTACTTCTGTTATATGAG
GTCTAGACCGATTTCTGTGAGGAAGAAACTTAGTGGACTCACAGTTTTTAAGGACACCACTGGTGGCA
GTGTAGAGGACATGA
+
FFFFFFFFFFFFFFFFFFFFFFFFFFFFFFFFFFFFFFFF:FFFFFFFFFFFFFFFF:FFFFFFFFFF
FFFFFFFFFFFFFFFFF:FFFFFFFFFFFFFFFFFFFFFFFFFFFF:FFFFF:FFFFFFFFFFFFFFF
FFFFFFFFFFFFFFF
@A00721:190:HGJW7DSXY:4:2405:29740:24471 1:N:0:TCTGCAAG+GCTGCACT
CGTGGTTACGTTGATTCATCCATGGATGCGGACGACGACGTTAAGAACGCTTACTTCTGTTATATGAG
GTCTAGACCGATTTCTGTGAGGAAGAAACTTAGTGGACTCACAGTTTTTAAGGACACCACTGGTGGCA
GTGTAGAGGACATGA
+
FFFFFFFFFFF:FFFFFFF:FFFFFFFFFFFFFFFFFFFFFFFFFFFFFFFFFFFFFFFFFFFFFFFF
FFFFFFFFFFFFFFFFFFFFFFFFFFFF:FFFFFFFFFFFFFFFFFFFFFFFFFF:FFFFFFFFFFFF
FFFFFFFFFFFFFFF
@A00721:190:HGJW7DSXY:4:2456:6153:2597 1:N:0:TCTGCAAG+GCTGCACT
CGTGGTTACGTTGATTCATCCATGGATGCGGACGACGACGTTAAGAACGCTTACTTCTGTTATATGAG
GTCTAGACCGATTTCTGTGAGGAAGAAACTTAGTGGACTCACAGTTTTTAAGGACACCACTGGTGGCA
GTGTAGAGGACATGA
+
FFFFFFFFFFFFFFFFFFF:FFFFFFFFFFFFFFFFFFFFFFFFFFFFFFFFFFFFFFFFFFFFFFFF
FFFFFFFFFFFFFFFFFFFFFFFFFFFFFFFFFFFFFFFFFFFFFFFFFFFFFFFFFFFFFFFFF:FF
FFFFFFFFFFFFFFF
@A00721:190:HGJW7DSXY:4:2641:22634:5744 1:N:0:TCTGCAAG+GCTGCACT
CGTGGTTACGTTGATTCATCCATGGATGCGGACGACGACGTTAAGAACGCTTACTTCTGTTATATGAG
GTCTAGACCGATTTCTGTGAGGAAGAAACTTAGTGGACTCACAGTTTTTAAGGACACCACTGGTGGCA
GTGTAGAGGACATGA
+
F::FFFFFFFFFFFFFFFF:FFFFFFFFFFFFFFFFFFFFFF:FFFFFFFFFF:FFFFFFFFFFFFFF
FFFFFFFFFFFFFFFFFFFFFFFFFFFFFFFFFFFFFFFFFFF:FFFFFFFFFFFFFFFFFFFFFFFF
FFFFFFFFFFFFFFF
@A00721:190:HGJW7DSXY:4:2678:14823:4335 1:N:0:TCTGCAAG+GCTGCACT
CGTGGTTACGTTGATTCATCCATGGATGCGGACGACGACGTTAAGAACGCTTACTTCTGTTATATGAG
GTCTAGACCGATTTCTGTGAGGAAGAAACTTAGTGGACTCACAGTTTTTAAGGACACCACTGGTGGCA
GTGTAGAGGACAAGA
+
FFFFFFFFFFFFFFF,FFF,FFFFFFFFFFFFFFFFFFFFF,FF:FFF,FFFFFFFFFFF,FFFFFFF
```

```
FFFFFFFFFFFF:FFFFF:FFFFFFFFFFF,FF:FFFFFFFFFFFFFFFFF:FFFFFFFF:FFFFF:F
FFF:FFF:FF:F,:F
@A00721:190:HGJW7DSXY:4:1511:11324:20509 1:N:0:TCTGCAAG+GCTGCACT
GCTCCTTTCATCATGTCCTCTACACTGCCACCAGTGGTGTCCTTAAAAACTGTGAGTCCACTAAGTTT
CTTCCTCACAGAAATCGGTCTAGACCTCATATAACAGAAGTAAGCGTTCTTAACGTCGTCGTCCGCAT
CCATGGATGAATCAA
+
FFFFFFFFFFFFFFFFFFFFFFFFFFFFFFFFFFFFF:FFFFFFFFFFFFFFFFFFFFFFFFFFFFFFFF
FFFFFFFFFFFFFFFFFFFFFFFFFFFFFFFFFFFFFFFFFFFF:FFFFFFFFFFFFFFFFFFFFFF:F
FFFFFFFFFFFFFFF
@A00721:190:HGJW7DSXY:4:1533:28393:13495 1:N:0:TCTGCAAG+GCTGCACT
GCTCCTTTCATCATGTCCTCTACACTGCCACCAGTGGTGTCCTTAAAAACTGTGAGTCCACTAAGTTT
CTTCCTCACAGAAATCGGTCTAGACCTCATATAACAGAAGTAAGCGTTCTTAACGTCGTCGTCCGCAT
CCATGGATGAATCAA
+
FFFFFFFFFFFFFFFFFFFFFFFFFFFFFFFFFFFFFFFFFFFFFFFFFFFFFFFFFFFFFFFFFFFFFF
FFFFFFFFFFFFFFFFFFFFFFFFFF:FFFFFFFFF:FFFFFFFFFFFFFF:FFFFFFFFFFFFFFF
FFFFFFFFFFFFFFF
@A00721:190:HGJW7DSXY:4:1637:21495:26475 1:N:0:TCTGCAAG+GCTGCACT
GCTCCTTTCATCATGTCCTCTACACTGCCACCAGTGGTGTCCTTAAAAACTGTGAGTCCACTAAGTTT
CTTCCTCACAGAAATCGGTCTAGACCTCATATAACAGAAGTAAGCGTTCTTAACGTCGTCGTCCGCAT
CCATGGATGAATCAA
+
FFFFFFFFFFFFFFFFFFFFFFFFFFFFFFFFFFFFFFFFFFFFFFFFFFFFFFFFFFFFFFFFFFFFFF
FFFFFFFFFF:FFFFF:FFFFFFFFFFFFFFFFFFF:F:FFFFFFFFFFFFFFF,FFFFFFFFFFFF
FFFF:FFFFFFFFFF
@A00721:190:HGJW7DSXY:4:2227:27507:34976 1:N:0:TCTGCAAG+GCTGCACT
GCTCCTTTCATCATGTCCTCTACACTGCCACCAGTGGTGTCCTTAAAAACTGTGAGTCCACTAAGTTT
CTTCCTCACAGAAATCGGTCTAGACCTCATATAACAGAAGTAAGCGTTCTTAACGTCGTCGTCCGCAT
CCATGGATGAATCAA
+
FFFFFFFFFFFFFFFFFFFFFFFFFFFFFFFFFFFFFFF:FFFFFFFFFFFFFFFFFFF:FFFFFFFF
FFFFFFFFFFFFFFFFFFFFFFFFFFFFFFFFFFFFFFF:FFFFFFFFFFFFF:FFFFFFFFFFFFF
FFF,FF:FFF:FFFF
@A00721:190:HGJW7DSXY:4:2474:29053:26944 1:N:0:TCTGCAAG+GCTGCACT
GCTCCTTTCATCATGTCCTCTACACTGCCACCAGTGGTGTCCTTAAAAACTGTGAGTCCACTAAGTTT
CTTCCTCACAGAAATCGGTCTAGACCTCATATAACAGAAGTAAGCGTTCTTAACGTCGTCGTCCGCAT
CCATGGATGAATCAA
+
FFFFFFFFFFFFFFFFFFFFFFFFFFFFFFFFFFFFFFFFFFFFFFFFFFFFFFFFFFFFFFFFFFFFFF
FFFFFFFFFFFF:FFFFFFFFFFFFFFFFFFFFFFFFFFFFFFFFFFFFFFFFFFFFFFFFFFFFFFFF
FFFFFFFFFFFFFFF
@A00721:190:HGJW7DSXY:4:1328:7554:7028 1:N:0:TCTGCAAG+GCTGCACT
TCCATGGATGCGGACGACGACGTTAAGAACGCTTACTTCTGTTATATGAGGTCTAGACCGATTTCTGT
GAGGAAGAAACTTAGTGGACTCACAGTTTTTAAGGACACCACTGGTGGCAGTGTAGAGGACATGATGA
AAGGAGCTTATTATT
+
F:FFFFFFFFFFFFFFFFFFFFFFFFFFFFFFFFFFF::FFFFFFFFFFFFFFFFFFFFFFFFFFFFFF
FFFFFFFFFFFFFFFFFFFFFFFFFFFFFFFFFFFFF,FFFFFFFFFFFFFFFFFFFFFFFFFFFFFFF
FFFFFFFFFFFFFFF
@A00721:190:HGJW7DSXY:4:1653:4029:25473 1:N:0:TCTGCAAG+GCTGCACT
TCCATGGATGCGGACGACGACGTTAAGAACGCTTACTTCTGTTATATGAGGTCTAGACCGATTTCTGT
GAGGAAGAAACTTAGTGGACTCACAGTTTTTAAGGACACCACTGGTGGCAGTGTAGAGGACATGATGA
AAGGAGCTTATTATT
```

+
FFFFFFFFFFFFFFFFFFFFFFFFFFFFFFFFFFFFFF:FFF::FFFFFFFFFFFFFFFFFF:FFFFFF
FFFFFFFFFFFFFFFFFFFFFFFFFFFFFFFFFFFFFFFFFFFFFFFFFFFFFFFFFFFFFFFFFFFF
FFFFFFFFFFFFFFF
@A00721:190:HGJW7DSXY:4:2216:26838:20666 1:N:0:TCTGCAAG+GCTGCACT
TCCATGGATGCGGACGACGACGTTAAGAACGCTTACTTCTGTTATATGAGGTCTAGACCGATTTCTGT
GAGGAAGAAACTTAGTGGACTCACAGTTTTTAAGGACACCACTGGTGGCAGTGTAGAGGACATGATGA
AAGGAGCTTATTATT
+
FFFFFFFFFFFFFFFFFFFFFFFFFFFFF:FFFFFF,::FFFFF:FFFFFFFFFFFFFFFFFFFFFFFF
FFFFFFFFFFFFFFFFFFFFFFFFFFFFF:FFFFFFFFFFFFFFFFFFFFFFFFFFFFFFFFFFFFFFFF
FFFFFFFF:FFFFFF
@A00721:190:HGJW7DSXY:4:1235:2293:14763 1:N:0:TCTGCAAG+GCTGCACT
GACGACGACGTTAAGAACGCTTACTTCTGTTATATGAGGTCTAGACCGATTTCTGTGAGGAAGAAACT
TAGTGGACTCACAGTTTTTAAGGACACCACTGGTGGCAGTGTAGAGGACATGATGAAAGGAGCTTATT
ATTTAGTTTTTGCGA
+
FFFFFFFFFF,FFFFFFFFFFFFFFFF,FF,F:,FFFF,FFFFFFF:FFFFFF,FFFFFFFFFFFF:F
,FF,F:FFFF:FF:,:,F,F::FFFFFFFFFFF:,FFFFFFFFFFFFFFFFFFFFFFF,F,FFFF,:FF
F,FFF:F,FFFFF:F
@A00721:190:HGJW7DSXY:4:1414:28917:10614 1:N:0:TCTGCAAG+GCTGCACT
GACGACGACGTTAAGAACGCTTACTTCTGTTATATGAGGTCTAGACCGATTTCTGTGAGGAAGAAACT
TAGTGGACTCACAGTTTTTAAGGACACCACTGGTGGCAGTGTAGAGGACATGATGAAAGGAGCTTATT
ATTTAGTTTTTGCGA
+
F:,FF:FFF:,F:FF,F:FFFFF:,F:FFFF:FFF:FFFFF,FFFF,:FFFFFF:F::F,FFF:FFF:
FFFF,FF:::F,F,FFFFFFF:FFFF,FF:FF:FFFFF:FF:,FF:FF:FF,FF:FFFFFFF,FFFFF
,FF:FFF:FF,::FF
@A00721:190:HGJW7DSXY:4:1565:25880:21543 1:N:0:TCTGCAAG+GCTGCACT
GACGACGACGTTAAGAACGCTTACTTCTGTTATATGAGGTCTAGACCGATTTCTGTGAGGAAGAAACT
TAGTGGACTCACAGTTTTTAAGGACACCACTGGTGGCAGTGTAGAGGACATGATGAAAGGAGCTTATT
ATTTAGTTTTTGCGA
+
F:FFFFFFFFFFFFFFFFFFFFFFFFFFFF,FF:FFFFFFFFFFFFFFFF:FFFFFFFFFFFFFFFFFF
FFFFFFFFFFFFFFFFFFFFFFFFFFFFFFFFFFFFFFFFFFFFFFFFFFFFFFFFFFFFFFFF:FFFF
F,:FFFFFFFFFFFF
@A00721:190:HGJW7DSXY:4:1565:24948:21840 1:N:0:TCTGCAAG+GCTGCACT
GACGACGACGTTAAGAACGCTTACTTCTGTTATATGAGGTCTAGACCGATTTCTGTGAGGAAGAAACT
TAGTGGACTCACAGTTTTTAAGGACACCACTGGTGGCAGTGTAGAGGACATGATGAAAGGAGCTTATT
ATTTAGTTTTTGCGA
+
FFFFFFFFFFFFFFFFFFFF,FFFFFFF:FFFFF,,FFF,,FFF:FFFFFFFFFF:FFFFFFFFFFFFF
FFFFFFFFFFFFFF,FFFFFFFFFFFFFFFFFF,FFFFFFFFFFFF,:FF:F:FF:FFFF:FF:FFFFF
FFFFF::FFF:F:FF
@A00721:190:HGJW7DSXY:4:2434:12952:3756 1:N:0:TCTGCAAG+GCTGCACT
GACGACGACGATAAGAACGCTTACTTCTGGTATATGAGGTCTAGACCGATTTCTGTGAGGAAGAAACT
TAGTGGACTCACAGTTTTTAAGGACACCACAGGTGGCAGTGTAGAGGACATGATGAAAGGAGATTATT
ATATAGTTTTTGCGA
+
FFF:F,FF:F,:FF:FFFFF,,FFF,F:F,:F:FFFFF,,FFF,FF,,F,FF,FFFFFFFFFFFFFFF
FF:F,:F,FFFFFF,F:FFFFFFF:FFFFF,F,,,FF:F:F,F,F,FFFF,FFF,FFF:,FF,,,FFF
FF:FFFFFFFFFFFF
@A00721:190:HGJW7DSXY:4:2514:10411:7842 1:N:0:TCTGCAAG+GCTGCACT
GACGACGACGTTAAGAACGCTTACTTCTGTTATATGAGGTCTAGACCGATTTCTGTGAGGAAGAAACT

TAGTGGACTCACAGTTTTTAAGGACACCACTGGTGGCAGTGTAGAGGACATGATGAAAGGAGCTTATT
ATTTAGTTTTTGCGA
+
FFFFFFFFFFFFFFFFFFFF:F:FF:F,F:FFF:FFFFFFFFFFFFFFFFFFFFFFFFFFFFFFFFFFFF
FFFFFFFFFFFFFFFFFFFFFFFFFFFFFFFFFFFFFFFFFFFFFFFFFFFFFFFFFFFFFFFFF,FFFF
FFFFFFFFFFFFF:F
@A00721:190:HGJW7DSXY:4:2542:9941:26537 1:N:0:TCTGCAAG+GCTGCACT
GACGACGACGTTAAGAACGCTTACTTCTGTTATATGAGGTCTAGACCGATTTCTGTGAGGAAGAAACT
TAGTGGACTCACAGTTTTTAAGGACACCACTGGTGGCAGTGTAGAGGACATGATGAAAGGAGCTTATT
ATTTAGTTTTTGCGA
+
FFFFFFFFFFFFFFFFFFFFFFFFFFFFFFFFFFFFFFFFFFFFFFFFFFFFFFFFFFFFFFFFFFFFFF
FFFFFFFFFFFFFFFF,FFFFFFFFFFFFFFFFFFFFFFFFFFFFFFFFFFFFFFFFFFFFFFFFFFFF
F:FFFFFFFFFFFFF
@A00721:190:HGJW7DSXY:4:2611:6533:4601 1:N:0:TCTGCAAG+GCTGCACT
GACGACGACGTTAAGAACGCTTACTTCTGTTATATGAGGTCTAGACCGATTTCTGTGAGGAAGAAACT
TAGTGGACTCACAGTTTTTAAGGACACCACTGGTGGCAGTGTAGAGGACATGATGAAAGGAGCTTATT
ATTTAGTTTTTGCGA
+
FFFFFFFFFFFFFFFFFFFF:FFFFFFFFFFFFFFFFFFFFFF:FFFFFFFFFFFFFFFFFFFFFFFFFF
FFFFFFFFFFFFFFFFFFFFFFFFFFFFFFFFFFFFFFFFFFFFFFFFFFFFFFFFFFFFFFFFF:FFFF
F,FFF:FFFFFFF:F
@A00721:190:HGJW7DSXY:4:2666:25961:5682 1:N:0:TCTGCAAG+GCTGCACT
GACGACGACGTTAAGAACGCTTACTTCTGTTATATGAGGTCTAGACCGATTTCTGTGAGGAAGAAACT
TAGTGGACTCACAGTTTTTAAGGACACCACTGGTGGCAGTGTAGAGGACATGATGAAAGGAGCTTATT
ATTTAGTTTTTGCGA
+
FFFFFFFFFFFFFFFFFFFFFFFFFFF:FFFFFFFFFFFFFFFFFFFFFFFFFFFFFFFFFFFFFFFFFF
FFFFFFFFFFFFFFFFFFFFFFFFFFFFFFFFFFFFFFFFFFFFFFFFFFFFFFFFF,FFFFFFFFFFF
FFFFFFFFFFFFFFF
@A00721:190:HGJW7DSXY:4:1214:4435:11334 1:N:0:TCTGCAAG+GCTGCACT
CGACGACGTTAAGAACGCTTACTTCTGTTATATGAGGTCTAGACCGATTTCTGTGAGGAAGAAACTTA
GTGGACTCACAGTTTTTAAGGACACCACTGGTGGCAGTGTAGAGGACATGATGAAAGGAGCTTATTAT
TTAGTTTTTGCGACA
+
FF:FFFFFFFFFFFFFFFF:FFFFFFF:FFFFFFFFFFFFFFFFFFFFFFFFFFFFFFFFFFFFFFFFFF
FFFFFFFFFFFFFFFFFF:FFFFFFFFFFFFFFFFFFFFFFFFFFFFFFFFF:FFFFFFFFFFFFFFFFF
FFFFFFFFFFFFFFF
@A00721:190:HGJW7DSXY:4:1275:21748:22780 1:N:0:TCTGCAAG+GCTGCACT
CGACGACGTTAAGAACGCTTACTTCTGTTATATGAGGTCTAGACCGATTTCTGTGAGGAAGAAACTTA
GTGGACTCACAGTTTTTAAGGACACCACTGGTGGCAGTGTAGAGGACATGATGAAAGGAGCTTATTAT
TTAGTTTTTGCGACA
+
FFFFFFFFFFFFFFFFFFF:FFFFF:FF,FF:FFFFFFFFFF:FFFFFFFFFFFFFFFFFFFFFFFF:F
F::FFFFFFFFFFFFFFFFFFFFFFFF:FFFFFFFFFFFF:FFFFFFFFFFFFFFFFFFFFFFFFFFFFF
FFFFFFFFF:FFFFF
@A00721:190:HGJW7DSXY:4:1446:21206:7028 1:N:0:TCTGCAAG+GCTGCACT
CGACGACGTTAAGAACGCTTACTTCTGTTATATGAGGTCTAGACCGATTTCTGTGAGGAAGAAACTTA
GTGGACTCACAGTTTTTAAGGACACCACTGGTGGCAGTGTAGAGGACATGATGAAAGGAGCTTATTAT
TTAGTTTTTGCGACA
+
FFFFFFFFFFFFFFFFFFFFFFFFFFFFFFFFFFFFFFFFFFFFFFFFFFFFFFF:FFFFFFFFFFFFFF
FFFFFFFFFFFFFFFFFFFFFFFFFFFFFFFFFFFFFFFFFFFFFFFFFFFFFFFFFFFFFFFF:FFFF,
FFF:FFFFFFFFFFF

@A00721:190:HGJW7DSXY:4:1462:3378:17331 1:N:0:TCTGCAAG+GCTGCACT
CGACGACGTTAAGAACGCTTACTTCTGTTATATGAGGTCTAGACCGATTTCTGTGAGGAAGAAACTTA
GTGGACTCACAGTTTTTAAGGACACCACTGGTGGCAGTGTAGAGGACATGATGAAAGGAGCTTATTAT
TTAGTTTTTGCGACA
+
F::FFFFFFFFFFFFFFFFFF::FFFFFFFFFFFFF:FFFFFFFFFFFFFFFFF:FFFFFFFFFFFFFFF
FFFFFFFFFFFFFFFFFFFF,FFFFFFFFFFFFFFFFFFFFF:FFFFFFF:FFFFFFFF:FFFFFFFFF:
FFF,FFFFF::,FFF
@A00721:190:HGJW7DSXY:4:1504:22978:20713 1:N:0:TCTGCAAG+GCTGCACT
CGACGACGTTAAGAACGCTTACTTCTGTTATATGAGGTCTAGACCGATTTCTGTGAGGAAGAAACTTA
GTGGACTCACAGTTTTTAAGGACACCACTGGTGGCAGTGTAGAGGACATGATGAAAGGAGCTTATTAT
TTAGTTTTTGCGACA
+
FFFFFFFFFFFFFFFFFFFFFF:FF:F,FF:FFFFFFFFFFFFFFFFFFFFFFFFFFFFFFFFFFFFFFF
FFFFFFFFFFFFFFF:FFFFFFFFFFFFFFFFFFFFFFFFFFFFFFFFFFFFFFFFF,FF:FFF,:FFFF
FFFFFFF:FFFFFFF
@A00721:190:HGJW7DSXY:4:1536:11216:36135 1:N:0:TCTGCAAG+GCTGCACT
CGACGACGTTAAGAACGCTTACTTCTGTTATATGAGGTCTAGACCGATTTCTGTGAGGAAGAAACTTA
GTGGACTCACAGTTTTTAAGGACACCACTGGTGGCAGTGTAGAGGACATGATGAAAGGAGCTTATTAT
TTAGTTTTTGCGACA
+
FFFFFFFFFFFFFFFFFFFF:F:FFFFFFFFFFFFFFFFFFFFFFFFFFFFFFF:FFFFFFFFFFFFFFF
FFFFFFFFFFFFFFFFFFFFFFFFFFFFFFFFFFFFFFFFFFFFFFFFFFFFFFFFFFFFFF:F:FFFFF
FFFFFFF:FFFFFFF
@A00721:190:HGJW7DSXY:4:1554:32280:29747 1:N:0:TCTGCAAG+GCTGCACT
CGACGACGTTAAGAACGCTTACTTCTGTTATATGAGGTCTAGACCGATTTCTGTGAGGAAGAAACTTA
GTGGACTCACAGTTTTTAAGGACACCACTGGTGGCAGTGTAGAGGACATGATGAAAGGAGCTTATTAT
TTAGTTTTTGCGACA
+
FFFFFFFFFFFFFFFFFFFFF:FFFFFFFFFFFFFFFFFFFFFFFFFFFFFFFFFFFFFFFFFFFFFFFF
FFFFFFFFFFFFFFFFFFFFFFFFFFFFFFFFFFFFFFFFFFFFFFFFFFFFFFFFFFFFFFF:FFFFFF
FFFFFFFFFFFFFFF
@A00721:190:HGJW7DSXY:4:2133:24153:5525 1:N:0:TCTGCAAG+GCTGCACT
CGACGACGTTAAGAACGCTTACTTCTGTTATATGAGGTCTAGACCGATTTCTGTGAGGAAGAAACTTA
GTGGACTCACAGTTTTTAAGGACACCACTGGTGGCAGTGTAGAGGACATGATGAAAGGAGCTTATTAT
TTAGTTTTTGCGACA
+
FFFFFFFFFFFFFFFFFFFFFFFFFFFFFFFFFFFFFFFFFFFFFFFFFFFFFFFFFFFFFFFFFFFFFF
FFFFFFFFFFFFFFFFFFFFFFFFFFFFFFFFFFFFFFFFFFFFFFFFFFFFFFFFFFFFFFFFFFFFFF
FFFFFFFFFFF:FFF
@A00721:190:HGJW7DSXY:4:2178:5095:13260 1:N:0:TCTGCAAG+GCTGCACT
CGACGACGTTAAGAACGCTTACTTCTGTTATATGAGGTCTAGACCGATTTCTGTGAGGAAGAAACTTA
GTGGACTCACAGTTTTTAAGGACACCACTGGTGGCAGTGTAGAGGACATGATGAAAGGAGCTTATTAT
TTAGTTTTTGCGACA
+
FFFFFFFFFFFFFFFFFFFFFFFFFFFFFF,FFFFFFFFFFFFFFFFFFFFFFFFFFFFFFFFFFFFFFF
FFFFFFFFFFFFFFFFFFFFFFFFFFFFFFFFFFFFFFFFFFFFFFFFFFFFFFFFFFFFFFFFFFFFFF
FFFFFFFFFFFFFFF
@A00721:190:HGJW7DSXY:4:2178:4490:14591 1:N:0:TCTGCAAG+GCTGCACT
CGACGACGTTAAGAACGCTTACTTCTGTTATATGAGGTCTAGACCGATTTCTGTGAGGAAGAAACTTA
GTGGACTCACAGTTTTTAAGGACACCACTGGTGGCAGTGTAGAGGACATGATGAAAGGAGCTTATTAT
TTAGTTTTTGCGACA
+
FFFFFFFFFFFFFFFFFFFFFFFFFFFFF:FFFFFFFFFFFFFFFFFFFFFFFFFFFFFFFFFFFFFFFF

```
FFFFFFFFFFFFFFFFFFFFFFFFFFFFFFFFFFFFFFFFFFFFFFFFFFFFFFFFFFFFFFFFFFFF
FFFFF:FFFFFFFFF
@A00721:190:HGJW7DSXY:4:2223:18059:28166 1:N:0:TCTGCAAG+GCTGCACT
CGACGACGTTAAGAACGCTTACTTCTGTTATATGAGGTCTAGACCGATTTCTGTGAGGAAGAAACTTA
GTGGACTCACAGTTTTTAAGGACACCACTGGTGGCAGTGTAGAGGACATGATGAAAGGAGCTTATTAT
TTAGTTTTTGCGACA
+
F:FFFFFFFFFFFFFFFFFFFFFFF,F:F,FFFFFFFFFFFFFFFFFFFFFFFFFFFFFFFFFFFF:F
FFFFFFFFFFFFFFFFFFFFFFFFFFFFFFFFFFFFFFFFFFFFFFFFFFF:FFFFFFFFFFFF:,F,
FFFFFFFFFFFFFFF
@A00721:190:HGJW7DSXY:4:2223:16794:29199 1:N:0:TCTGCAAG+GCTGCACT
CGACGACGTTAAGAACGCTTACTTCTGTTATATGAGGTCTAGACCGATTTCTGTGAGGAAGAAACTTA
GTGGACTCACAGTTTTTAAGGACACCACTGGTGGCAGTGTAGAGGACATGATGAAAGGAGCTTATTAT
TTAGTTTTTGCGACA
+
FFFFFFFFFFFFFFFFFFFFF:FFFFF:FFFFFFFFFFFFFFFFFFFFFFFFFFFFFFFFFFFFFFFF
FFFFFFFFFFFFFFFFFFFFFFFFFFFFFFFFFFFFFFFFFFFFFFFFFFFFFFFFFFFFF,FFFFFF
,FFFF,FFF:FFFFF
@A00721:190:HGJW7DSXY:4:2346:1479:24158 1:N:0:TCTGCAAG+GCTGCACT
CGACGACGTTAAGAACGCTTACTTCTGTTATATGAGGTCTAGACCGATTTCTGTGAGGAAGAAACTTA
GTGGACTCACAGTTTTTAAGGACACCACTGGTGGCAGTGTAGAGGACATGATGAAAGGAGCTTATTAT
TTAGTTTTTGCGACA
+
FFFFFFFFFFFFFFFFFFFFFFFFFFFFFFFFFFFFFFFFFFFFFFFFFFFFFFFFFFFFFFFFFFFF
FFFFFFFFFFFFFFFFFFFFFFFFFFFFFFFFFFFFFFFFFFFFFFFFFFFFFFFFFFFFFFFFFFFF
FFFFFFFFFFFFFFF
@A00721:190:HGJW7DSXY:4:2354:23882:36996 1:N:0:TCTGCAAG+GCTGCACT
CGACGACGTTAAGAACGCTTACTTCTGTTATATGAGGTCTAGACCGATTTCTGTGAGGAAGAAACTTA
GTGGACTCACAGTTTTTAAGGACACCACTGGTGGCAGTGTAGAGGACATGATGAAAGGAGCTTATTAT
TTAGTTTTTGCGACA
+
FFFFFFFFFFFFFFFFF:FFFFFFFFFFFFFFFFFFFFFFFFFFFFFFFFFFFFFFFFFFFFFFFFFF
FFFFFFFFFFFFFFFFFFFFFFFFFFFFFFFFF:FFFFFFFFFFFFFFFFFFFFFFFFFFFFFFFFFF
FFFFFFFFFF:FFFF
@A00721:190:HGJW7DSXY:4:2472:12970:26897 1:N:0:TCTGCAAG+GCTGCACT
CGACGACGTTAAGAACGCTTACTTCTGTTATATGAGGTCTAGACCGATTTCTGTGAGGAAGAAACTTA
GTGGACTCACAGTTTTTAAGGACACCACTGGTGGCAGTGTAGAGGACATGATGAAAGGAGCTTATTAT
TTAGTTTTTGCGACA
+
FFFFFFFFFFFFFFFFFFFFFFFFF:FFFFFFFFFFFFFFFFFFFFFFFFFFFFFFFFFFFFFFFFFF
FFFFFFFFFFFFFFFFFFFFFFFFFFFFFFFFFFFFFFFFFFFFFFFFFFFFFFFFFFFFFFFFFFFF
FFFFFFFFFFFFFFF
@A00721:190:HGJW7DSXY:4:1533:26205:15468 1:N:0:TCTGCAAG+GCTGCACT
CGCAAAAACTAAATAATAAGCTCCTTTCATCATGTCCTCTACACTGCCACCAGTGGTGTCCTTAAAAA
CTGTGAGTCCACTAAGTTTCTTCCTCACAGAAATCGGTCTAGACCTCATATAACAGAAGTAAGCGTTC
TTAACGAGATCGGAA
+
FFFFFFFFFFFFFFFFFFFFFFFFFFFFFFFFFFFFFFFFFFFFFFFFFFFFFFFFFFFFFFFFFFFF
FFFFFFFFFFFFFFFFFFFFFFFFFFFFFFFFFFFFFFFFFFFF:FFFFFFFFFFFFFFFFFFFFFFF
FFFFFFFFFFFFFFF
@A00721:190:HGJW7DSXY:4:1553:11803:5932 1:N:0:TCTGCAAG+GCTGCACT
CGCAAAAACTAAATAATAAGCTCCTTTCATCATGTCCTCTACACTGCCACCAGTGGTGTCCTTAAAAA
CTGTGAGTCCACTAAGTTTCTTCCTCACAGAAATCGGTCTAGACCTCATATAACAGAAGTAAGCGTTC
TTAACGAGATCGGAA
```

```
+
FFFFFFFFFFFFFFFFFFFFFFFFFFFFFFFFFFFFFFFFFFFFFFFFFFFFFFFFFFFFFFFFFFFFFFF
FFFFFFFFFFFFFFFFFFFFFFFFFFFFFFFFFFFFFFFFFFFFFFFFFFFFFFFFFFFFFFFFFFFFFFF
FFFFFFFFFFFFFFFF
@A00721:190:HGJW7DSXY:4:2555:25699:14246 1:N:0:TCTGCAAG+GCTGCACT
CGCAAAAACTAAATAATAAGCTCCTTTCATCATGTCCTCTACACTGCCACCAGTGGTGTCCTTAAAAA
CTGTGAGTCCACTAAGTTTCTTCCTCACAGAAATCGGTCTAGACCTCATATAACAGAAGTAAGCGTTC
TTAACGAGATCGGAA
+
FFFFFFFFFFFFFFFFFFFFFFFFFFFFFFFFFFFFFFFFFFFFFFFFFFFFFFFFFFFFFFFFFFFFFFF
FFFFFFFFFFFFFFFFFFFFFFFFFFFFFFFFFFFFFFFFFFFFFFFFFFFFFFFFFFFFFFFFFFFFFFF
FFFFFFFFFFFFFFFF
@A00721:190:HGJW7DSXY:4:2424:21242:9721 1:N:0:TCTGCAAG+GCTGCACT
CGCAAAAACTAAATAATAAGCTCCTTTCATCATGTCCTCTACACTGCCACCAGTGGTGTCCTTAAAAA
CTGTGAGTCCACTAAGTTTCTTCCTCACATAAATCGGTCTAGACCTAATATAACAGAAGTAAGCGTTC
TTAACGAGATC
+
F:FF,::F:,FFFFF:,FF::F,,,:,,F,,FFF,FF,FFFFFFFFF,F,:F:FFF,FF:,,FFFFFF
F:FFFF::FFFF,::,,F,,FFF,F:FF,,FF::FFFF,:FF,:FF,:FF:,,FFF,FFFFFFFF:FF
F,FF:FFFF,:
@A00721:190:HGJW7DSXY:4:1461:10827:9189 1:N:0:TCTGCAAG+GCTGCACT
GTTAAGAACGCTTACTTCTGTTATATGAGGTCTAGACCGATTTCTGTGAGGAAGAAACTTAGTGGACT
CACAGTTTTTAAGGACACCACTGGTGGCAGTGTAGAGGACATGATGAAAGGAGCTTATTATTTAGTTT
TTGCGACATTTCATC
+
FFFFFFFFFFFFFFFFFFFFFFFFFFFFFFFFFFFFFFFFFFFFFFFFFFFFFFFFFFFFFFFFFFFFFFF
FFF:FFFFFFFFF:FFFFFFFFFFFFFFFFFFFFFFFFFFFFFFFFFFFFFFFFFFFFFFFFFFFFFFF
FFFFFFFFFFFFFFFF
@A00721:190:HGJW7DSXY:4:1475:18756:36041 1:N:0:TCTGCAAG+GCTGCACT
GTTAAGAACGCTTACTTCTGTTATATGAGGTCTAGACCGATTTCTGTGAGGAAGAAACTTAGTGGACT
CACAGTTTTTAAGGACACCACTGGTGGCAGTGTAGAGGACATGATGAAAGGAGCTTATTATTTAGTTT
TTGCGACATTTCATC
+
FFFFFFFFFFFFFFFFFFFFFFFFFFFFFFFFFFFFFFFFFFFFFFFFFFFFF:FFFFFFFFFFFFFFFF
FFFFFFFFFFFFFFFFFFFFFFFFFFFFFFFFFFFFFFFFFFFFFF:FFFFFFFFFFFFFFFFFFFFFFFF
FFFFFFFFFFFFFFFF
@A00721:190:HGJW7DSXY:4:1558:16288:18865 1:N:0:TCTGCAAG+GCTGCACT
GTTAAGAACGCTTACTTCTGTTATATGAGGTCTAGACCGATTTCTGTGAGGAAGAAACTTAGTGGACT
CACAGTTTTTAAGGACACCACTGGTGGCAGTGTAGAGGACATGATGAAAGGAGCTTATTATTTAGTTT
TTGCGACATTTCATC
+
FFFFFFFFFFF:FFFFFFFFFFF:FFFFF:FFFFFFFF::F:FFF:F:FFFFFFFFFFFF:FF,FFFF
FFFFFFF:FFFFFFFFFFFFFFF,FFFFFFF:FFFFF,F:FFFFFFFFFF,FFFFFFFFFFFFFF:FF
FF,FFFFFF,FFFF:
@A00721:190:HGJW7DSXY:4:2268:28411:14372 1:N:0:TCTGCAAG+GCTGCACT
GTTAAGAACGCTTACTTCTGTTATATGAGGTCTAGACCGATTTCTGTGAGGAAGAAACTTAGTGGACT
CACAGTTTTTAAGGACACCACTGGTGGCAGTGTAGAGGACATGATGAAAGGAGCTTATTATTTAGTTT
TTGCGACATTTCATC
+
FFFFFFFFFFFFFFFFFFFFFFFFFFFFFFFFFFFFFFFFFFFFFFFFFFFFFFFFFFFFFFFFFFFFFFF
FFFFFFFFFFFFFFFFFFFFFFFFFFFFFFFFFFFFFFFFFFFFFFFFFFFFF:FFFFFFFFFFF:FFF
FFF:FFFFFFFFFFF
@A00721:190:HGJW7DSXY:4:2420:19651:33771 1:N:0:TCTGCAAG+GCTGCACT
GTTAAGAACGCTTACTTCTGTTATATGAGGTCTAGACCGATTTCTGTGAGGAAGAAACTTAGTGGACT
```

CACAGTTTTTAAGGACACCACTGGTGGCAGTGTAGAGGACATGATGAAAGGAGCTTATTATTTAGTTT
TTGCGACATTTCATC
+
FFFFFFFFFFFFFFFFFFFFFFFFFFFFFFFFFFFFFFFFFFFFFFFFFFFFFFFFFFFFFFFFFFFF
FFFFFFFFFFFFFFF:FFFFFFFFFFFFFFFFFFFFFFFFF:FFFFFFFFFFFFFFFFFFFFFFFFFF
FFF:FFFFFFFFFFF
@A00721:190:HGJW7DSXY:4:2565:6280:15311 1:N:0:TCTGCAAG+GCTGCACT
GTTAAGAACGCTTACTTCTGTTATATGAGGTCTAGACCGATTTCTGTGAGGAAGAAACTTAGTGGACT
CACAGTTTTTAAGGACACCACTGGTGGCAGTGTAGAGGACATGATGAAAGGAGCTTATTATTTAGTTT
TTGCGACATTTCATC
+
FF:FF,FFFFF::FFFFF,::FFFFF,FFF,FFFFFFFFFFFFF:F:F:FFFFF::FFFF,FF:F:FFF
FFFF:FFFFFFF:FFFFF:FFFFF,:FFFFFFFFF:F:FF:F,FFFFFF:,FF,FFFFFF:FFFFFFF
FFFFFF:F:FF:FF:
@A00721:190:HGJW7DSXY:4:1501:9046:2910 1:N:0:TCTGCAAG+GCTGCACT
CTTACTTCTGTTATATGAGGTCTAGACCGATTTCTGTGAGGAAGAAACTTAGTGGACTCACAGTTTTT
AAGGACACCACTGGTGGCAGTGTAGAGGACATGATGAAAGGAGCTTATTATTTAGTTTTTGCGACATT
TCATCAATATGCGAT
+
FFFFFFFFFFFFFF:FFFFFFFFFFFFFFFFFFFFFFFFFFFFF:FFFFFFFFFFFFFFFFFF:FFFF
FFFF:FFFFFFFFFFFFFFFFFFFFFFFFF::FFFFFFFFFFFFF:FFFFFFFFFFFFF:F:FFFFFF
FFFFFFFFFF:FFFF
@A00721:190:HGJW7DSXY:4:1564:8106:7388 1:N:0:TCTGCAAG+GCTGCACT
CTTACTTCTGTTATATGAGGTCTAGACCGATTTCTGTGAGGAAGAAACTTAGTGGACTCACAGTTTTT
AAGGACACCACTGGTGGCAGTGTAGAGGACATGATGAAAGGAGCTTATTATTTAGTTTTTGCGACATT
TCATCAATATGCGAT
+
FFFFF:FFFFFFFFFFFFFFFFFFFFFFFFFFFFFFFFFF:FFFFFFFFFFFFFFFFFFFFFFFFFFF
FFFFFFFFFFFFFFFFFFFFFFFFFFFFFFFFFFFFFFFFFFFFFFFFFFFFFFFFFFFFFFF:FFFFF
FFFFFFFFFFF,FFF
@A00721:190:HGJW7DSXY:4:2635:6072:29230 1:N:0:TCTGCAAG+GCTGCACT
CTTACTTCTGTTATATGAGGTCTAGACCGATTTCTGTGAGGAAGAAACTTAGTGGACTCACAGTTTTT
AAGGACACCACTGGTGGCAGTGTAGAGGACATGATGAAAGGAGCTTATTATTTAGTTTTTGCGACATT
TCATCAATATGCGAT
+
FFFFFFFFFFFFFFFFFFFFFFFFFFFFFFFFFF:FFFFFFFFFFFFFFFFFFFFFFFFFFFFFFFFF
FFFFFFFFFFFFFFFFFFFFFFFFFFFFFFF:FFFFFFFFFFFFFFFFFFFFF:FFFFFFFFFFFFFF
FFFFFFFFFFF:FFF
@A00721:190:HGJW7DSXY:4:1104:7934:30545 1:N:0:TCTGCAAG+GCTGCACT
CTTCTGTTATATGAGGTCTAGACCGATTTCTGTGAGGAAGAAACTTAGTGGACTCACAGTTTTTAAGG
ACACCACTGGTGGCAGTGTAGAGGACATGATGAAAGGAGCTTATTATTTAGTTTTTGCGACATTTCAT
CAATATGCGATGGAA
+
FFFFFFFFFFFFFFFFFFFFFFFFFFFFFFFFFFFFFFFFFFFFFFFFFFFFFFFFFFFFFFFFFFFF
FFFFFFFFFFFFFFFFFFFFFFFFFFFFFFFFFFFFFFFFFFFFFFFFFFFFFFFFFFFFFFFF:FFF
FFFFFFFFFFFFFFF
@A00721:190:HGJW7DSXY:4:1375:23204:33442 1:N:0:TCTGCAAG+GCTGCACT
CTTCTGTTATATGAGGTCTAGACCGATTTCTGTGAGGAAGAAACTTAGTGGACTCACAGTTTTTAAGG
ACACCACTGGTGGCAGTGTAGAGGACATGATGAAAGGAGCTTATTATTTAGTTTTTGCGACATTTCAT
CAATATGCGATGGAA
+
FFFFFFFFFFFFFFFFFFFFFFFFFFFFFFFFFFFFFFFFFFFFFFFFFFFFFFFFFFFFFFFFFFFF
FFFFFFFFFFFFFFFFFFFFFFFFFFFFFFFFFFFFFFFFFFFFFFFFFFFFFFFF:FFFFFFFFFFF
FFFFFF:FFFFFFFF

@A00721:190:HGJW7DSXY:4:2566:29577:33113 1:N:0:TCTGCAAG+GCTGCACT
CTTCTGTTATATGAGGTCTAGACCGATTTCTGTGAGGAAGAAACTTAGTGGACTCACAGTTTTTAAGG
ACACCACTGGTGGCAGTGTAGAGGACATGATGAAAGGAGCTTATTATTTAGTTTTTGCGACATTTCAT
CAATATGCGATGGAA
+
FFFFFFFFFFFFFFFFFFFFFFFFFFFFFFFFFFFFFFFFFFFFFFFFFFFFFFFFFFFFFFFFFFFF
FFFFFFFFFFFFFFFFFFFFFFFFFFFFFFFFFFFFFFFF:FFFFFFFFFFFFFFFFFFFFFFFFFFF
FFFFFF::FFFFFFF
@A00721:190:HGJW7DSXY:4:2660:20329:24956 1:N:0:TCTGCAAG+GCTGCACT
CTTCTGTTATATGAGGTCTAGACCGATTTCTGTGAGGAAGAAACTTAGTGGACTCACAGTTTTTAAGG
ACACCACTGGTGGCAGTGTAGAGGACATGATGAAAGGAGCTTATTATTTAGTTTTTGCGACATTTCAT
CAATATGCGATGGAA
+
FFF:FFFFFFFFFFFFFFFFFFFFFFFFFFFFFFFFFFFFFFFFFFF:FFFFFFFFFFFFFFFFFFFF
FFFFFFFFFFFFFFFFFFFFFFFFF:FFFFFFFFFFFFFFFFFFFFFFFFFFFFFFF:FFFFFFFFFF
FFFFFFFFFFF:FFF
@A00721:190:HGJW7DSXY:4:2604:4408:8281 1:N:0:TCTGCAAG+GCTGCACT
TTCTGTTATATGAGGTCTAGACCGATTTCTGTGAGGAAGAAACTTAGTGGACTCACAGTTTTTAAGGA
CACCACTGGTGGCAGTGTAGAGGACATGATGAAAGGAGCTTATTATTTAGTTTTTGCGACATTTCATC
AATATGCGATGGAA
+
F,F,FFFFF,:,FFFFFFF:FFFFFF:FF:FFFF,FFFFFFFFFFFFFFFFFFFFFFF,,,FFFFFFF
F:FFFFF:FFFFFFFFFF:FFF,F::F:F:FF:F:FFFFFFFFFFFFFF:FFFFF::FFFFF,F:FF:
FFF::F,FFFF,FF
@A00721:190:HGJW7DSXY:4:1223:26069:34178 1:N:0:TCTGCAAG+GCTGCACT
GTTATATGAGGTCTAGACCGATTTCTGTGAGGAAGAAACTTAGTGGACTCACAGTTTTTAAGGACACC
ACTGGTGGCAGTGTAGAGGACATGATGAAAGGAGCTTATTATTTAGTTTTTGCGACATTTCATCAATA
TGCGATGGAAGTTGT
+
FFFFFFFFFFFFFFFFFFFFFFFFFFFFFFFFFFFFFFFFFFFFFFFFFFFFFFFFFFFFFFFFFFFF
FFFFFFFFFFFFFFFFFFFFFFFFFFFFFFFFFFF:FFFFF:FFFFFFFFFFFFFF:FFFFFFFFFFF
FF,FFFFFFFFFFFF
@A00721:190:HGJW7DSXY:4:1304:22426:7451 1:N:0:TCTGCAAG+GCTGCACT
GTTATATGAGGTCTAGACCGATTTCTGTGAGGAAGAAACTTAGTGGACTCACAGTTTTTAAGGACACC
ACTGGTGGCAGTGTAGAGGACATGATGAAAGGAGCTTATTATTTAGTTTTTGCGACATTTCATCAATA
TGCGATGGAAGTTGT
+
FFFFFFFFFFFFFFFFFFFFFFFFFFFFFFFFFFFFFFFFFFFFFFFFFFFFFFFFFFFFFFFFFFFF
FFFFFFFFFFFFFFFFFFFFFFFFFFFFFFFFFFFF:FFFFFFFFFFFFFFFFFFFFFFFFFFFFFFF
FFFFFFFFF:FFFFF
@A00721:190:HGJW7DSXY:4:1467:30861:20181 1:N:0:TCTGCAAG+GCTGCACT
GTTATATGAGGTCTAGACCGATTTCTGTGAGGAAGAAACTTAGTGGACTCACAGTTTTTAAGGACACC
ACTGGTGGCAGTGTAGAGGACATGATGAAAGGAGCTTATTATTTAGTTTTTGCGACATTTCATCAATA
TGCGATGGAAGTTGT
+
FFFFFFFFFFFFFFFFFFFFFFFFFFFFFFFFFFF:FFFFFFFFFFFFFFFFFFFFFFFFFFFF:FFF
FFFFFFFFFFFFFFFFFFFFFFFFFFFFFFFFFFFFFFFFFFFFFFFFFFFFFFFFFFFFFFFFFFFF
FFFFFFFFF:FFFFF
@A00721:190:HGJW7DSXY:4:1519:22354:18443 1:N:0:TCTGCAAG+GCTGCACT
GTTATATGAGGTCTAGACCGATTTCTGTGAGGAAGAAACTTAGTGGACTCACAGTTTTTAAGGACACC
ACTGGTGGCAGTGTAGAGGACATGATGAAAGGAGCTTATTATTTAGTTTTTGCGACATTTCATCAATA
TGCGATGGAAGTTGT
+
FFFFFFFFFFFFFFFFFFFFFFFFFFFFFFFFFFFFFFFFFFFFFFFFFFFFFFFFFFFFFFFFFFFF

FFFFFFFFFFFFFFFFFFFFFFFFFFFFFFFFFFFFFFFFFFFFFFFFFFFFFFFFFFFFFFFFFFFF
FFFFFFFFFFFFFFF
@A00721:190:HGJW7DSXY:4:1526:15709:9659 1:N:0:TCTGCAAG+GCTGCACT
GTTATATGAGGTCTAGACCGATTTCTGTGAGGAAGAAACTTAGTGGACTCACAGTTTTTAAGGACACC
ACTGGTGGCAGTGTAGAGGACATGATGAAAGGAGCTTATTATTTAGTTTTTGCGACATTTCATCAATA
TGCGATGGAAGTTGT
+
FFFFFFFFFFFFFFFFFFFFFFFFFFFFFFFFFFFFFFFFFFFFFFFFFFFFFFFFFFFFFFFFFFFF
FFFFFFFFFFFFFFFFFFFFFFFFFFFFFFFFFFFFFFFFFFFFFFFFFFFFF:FFFFFFFFFFFFFF
FFFFFFFFF:FFFFF
@A00721:190:HGJW7DSXY:4:2347:15845:17284 1:N:0:TCTGCAAG+GCTGCACT
GTTATATGAGGTCTAGACCGATTTCTGTGAGGAAGAAACTTAGTGGACTCACAGTTTTTAAGGACACC
ACTGGTGGCAGTGTAGAGGACATGATGAAAGGAGCTTATTATTTAGTTTTTGCGACATTTCATCAATA
TGCGATGGAAGTTGT
+
FF:FFFFFFFFFFFFFFFFFFFFFFFFFFFFFFFFFFFFFFFFFFFFFFFFFFFFFFFFFFFFFFFFF
FFFFFFFFFFFFFFFFFFFFFFFFFFFFFFFFFFFFFFFFFFFFFFFFFFFFFFFFFFFFFFFFFFFF
FF:FFFFFFFFFFFF
@A00721:190:HGJW7DSXY:4:2517:13196:17550 1:N:0:TCTGCAAG+GCTGCACT
GTTATATGAGGTCTAGACCGATTTCTGTGAGGAAGAAACTTAGTGGACTCACAGTTTTTAAGGACACC
ACTGGTGGCAGTGTAGAGGACATGATGAAAGGAGCTTATTATTTAGTTTTTGCGACATTTCATCAATA
TGCGATGGAAGTTGT
+
FFFFFFFFFFFFFFFFFFFFFFFFFFFFFFFFFFFFFFFFFFFFFFFFFFFFFFFFFFFFFFFFFFFF
FFFFFFFFFFFFFFFFFFFFFFFFFFFFFFFFFFFFFFFFF:FFFFFFFFFFFFFFFFFFF:FFFFFF
FFFFFFFFFFFFFFF
@A00721:190:HGJW7DSXY:4:2663:23999:32346 1:N:0:TCTGCAAG+GCTGCACT
GTTATATGAGGTCTAGACCGATTTCTGTGAGGAAGAAACTTAGTGGACTCACAGTTTTTAAGGACACC
ACTGGTGGCAGTGTAGAGGACATGATGAAAGGAGCTTATTATTTAGTTTTTGCGACATTTCATCAATA
TGCGATGGAAGTTGT
+
FFFFFFFFFFFFFFFFFFFFFFFFFFFFFFFFFFFFFFFFFFFFFFFFFFFFFFFFFFFFFFFFFFFF
FFFFFFFFFFFFFFFFFFFFFFFFFFFFFFFFFFFFFFFFFFFFFFFFFFFFFFFFFFFFFFFFFFFF
FF:FFFFFFFFFFFF
@A00721:190:HGJW7DSXY:4:1150:2239:13730 1:N:0:TCTGCAAG+GCTGCACT
ATGAGGTCTAGACCGATTTCTGTGAGGAAGAAACTTAGTGGACTCACAGTTTTTAAGGACACCACTGG
TGGCAGTGTAGAGGACATGATGAAAGGAGCTTATTATTTAGTTTTTGCGACATTTCATCAATATGCGA
TGGAAGTTGCATTAA
+
FFFFFFFFFFFFFFFFFFFFFFFFFFFFFFFFFFFFFFFFFFFFFFFFFFFFFFFFFFFFFFFFFFFF
FFFFFFFFFFFFFFFFFFFFFFFFFFFFFFFFFFFFFFFFFFFFFFFFFFF:FFF:FFFFFFFFF:FF
FFFF:FFFFFFFFFF
@A00721:190:HGJW7DSXY:4:1266:4264:23657 1:N:0:TCTGCAAG+GCTGCACT
ATGAGGTCTAGACCGATTTCTGTGAGGAAGAAACTTAGTGGACTCACAGTTTTTAAGGACACCACTGG
TGGCAGTGTAGAGGACATGATGAAAGGAGCTTATTATTTAGTTTTTGCGACATTTCATCAATATGCGA
TGGAAGTTGCATTAA
+
FFFFFFFFFFFFFFFFFFFFFFFFFFFFFFFFFFFFFFFFFFFFFFFFFFFFFFFFFFFFFFFFFFFF
FFFFFFFFFFFFFFFFFFFFFFF:FFFFFF:FFFFFFFFFFFFFFFFFFFFFFFFFFFFFFFFFFFFF
FFFFFFFFFFFFFFF
@A00721:190:HGJW7DSXY:4:1472:13919:20400 1:N:0:TCTGCAAG+GCTGCACT
ATGAGGTCTAGACCGATTTCTGTGAGGAAGAAACTTAGTGGACTCACAGTTTTTAAGGACACCACTGG
TGGCAGTGTAGAGGACATGATGAAAGGAGCTTATTATTTAGTTTTTGCGACATTTCATCAATATGCGA
TGGAAGTTGCATTAA

+
F,FFFFFFFF,FFFFFFFFFFF,FFFFFFF:,FF,FFFFFFFFFFFFFFF:FFFFFFFFFFFFFFF:,
F:FFFF::FFFF:FFFFF:F:F:FFFFFFF:::F,F,FFF:,FF:FFFFFFFFFFFFFFFFFFFFFFFF
FFFFFF:FF,FFFFF
@A00721:190:HGJW7DSXY:4:1501:12780:15358 1:N:0:TCTGCAAG+GCTGCACT
ATGAGGTCTAGACCGATTTCTGTGAGGAAGAAACTTAGTGGACTCACAGTTTTTAAGGACACCACTGG
TGGCAGTGTAGAGGACATGATGAAAGGAGCTTATTATTTAGTTTTTGCGACATTTCATCAATATGCGA
TGGAAGTTGCATTAA
+
FFFFFFF:FF:FF:FFFFFFFFFFFFFFFFFFFFFFFFFFFFFFFFFFFFF:FFFFFFFFFFFFFFFF
FFFFFFFFFFFFFFFFFFFFFFFFFFFFFFFFFFFFFFFFFFFFFFFFFFFFFFFFFFFFFFFFFFF:F
FFFFFFFFFFFFFFF
@A00721:190:HGJW7DSXY:4:2352:32832:11631 1:N:0:TCTGCAAG+GCTGCACT
ATGAGGTCTAGACCGATTTCTGTGAGGAAGAAACTTAGTGGACTCACAGTTTTTAAGGACACCACTGG
TGGCAGTGTAGAGGACATGATGAAAGGAGCTTATTATTTAGTTTTTGCGACATTTCATCAATATGCGA
TGGAAGTTGCATTAA
+
FFFFFFFFFFFFFFFFFFFFFFFFFFFFFFFFFFFFFFFFFF:FFFFFFFFFFFFFF:FFFFFFFFFF
FFFFFFFFFFFFFFFFFFFFFFFFFFF:FF:FFFFFFFFFFFFFFFFFFFFFFFFFF::FFFF:FFFF:F
FF:FF:FFFFFFFF:
@A00721:190:HGJW7DSXY:4:2442:29695:26991 1:N:0:TCTGCAAG+GCTGCACT
ATGAGGTCTAGACCGATTTCTGTGAGGAAGAAACTTAGTGGACTCACAGTTTTTAAGGACACCACTGG
TGGCAGTGTAGAGGACATGATGAAAGGAGCTTATTATTTAGTTTTTGCGACATTTCATCAATATGCGA
TGGAAGTTGCATTAA
+
FFFFFFFFFFFFFFFFFFFFFFFFFFFFFFFFFFFFFFFFFFFFFFFFFFFFFFFFFFFFFFFFFFFFF
FFFFFFFFFFFFFFFFFFFFFFFFFFFFFFFFFFFFFFFFFFFFFFFFFFFFFFFFFFFFFFFFFFF:F
FFFFFFFFFFFFFF:
@A00721:190:HGJW7DSXY:4:2443:23601:4695 1:N:0:TCTGCAAG+GCTGCACT
ATGAGGTCTAGACCGATTTCTGTGAGGAAGAAACTTAGTGGACTCACAGTTTTTAAGGACACCACTGG
TGGCAGTGTAGAGGACATGATGAAAGGAGCTTATTATTTAGTTTTTGCGACATTTCATCAATATGCGA
TGGAAGTTGCATTAA
+
FFFFFFFFFFFFFFFFFFFFFFFFFFFFFFFFFFFFFFFFFFFFFFFFFFFFFFFFFFFFFFFFFFFFF
FFFFFFFFFFFFFFFFFFFFFFFFFFFFFFFFFFFF:FFFFFFFFFFFFFFFFFFFFFFFFF:FFFFFF
FFFFFFF:FFFFFFF
@A00721:190:HGJW7DSXY:4:1115:26341:8844 1:N:0:TCTGCAAG+GCTGCACT
GTCTGGACCGATTTCTGTGAGGAAGAAACTTAGTGGACTCACAGTTTTTAAGGACACCACTGGTGGCA
GTGTAGAGGACATGATGAAAGGAGCTTATTATTTAGTTTTTGCGACATTTCATCAATATGCGATGGAA
GTTGCATTAAATGTA
+
FFFFFFFFFFFFFFFFFFFFFFFFFFFFFFFFFFFFFFFFFFFFFFFFFFFFFFFFFFFFFFFFFFFFF
FFFFFFFFFFFFFFFFFFFFFFFFFFFFFFFFFFFFFFFFFFFFFFFFFFFFFFFFFFFFFFFFFFFFF
FFFFFF:FFFFFFFF
@A00721:190:HGJW7DSXY:4:1157:27887:31438 1:N:0:TCTGCAAG+GCTGCACT
GTCTAGACCGATTTCTGTGAGGAAGAAACTTAGTGGACTCACAGTTTTTAAGGACACCACTGGTGGCA
GTGTAGAGGACATGATGAAAGGAGCTTATTATTTAGTTTTTGCGACATTTCATCAATATGCGATGGAA
GTTGCATTAAATGTA
+
FFFFFFFFFFFFFFFFFFFFFFFFFFFFFFFFFFFFFFFFFFFFFFFFFFFFFFFFFFFFFFFFFFFFF
FFFFFFFFFFFFFFFFFF:FFFFFFFFFFFFFFFFFFFFFFFFFFFFFFFFFFFFFFFFFFFFFFFFF:
FFFFFFFFFFFFFF:
@A00721:190:HGJW7DSXY:4:1219:2781:13698 1:N:0:TCTGCAAG+GCTGCACT
GTCTAGACCGATTTCTGTGAGGAAGAAACTTAGTGGACTCACAGTTTTTAAGGACACCACTGGTGGCA

GTGTAGAGGACATGATGAAAGGAGCTTATTATTTAGTTTTTGCGACATTTCATCAATATGCGATGGAA
GTTGCATTAAATGTA
+
FFFFFFFFFFFFFFFFF:FFFFFFFFFFFFFFFFFFFFFFFFFFFFFFFFFFFFFFFFFFFFFFFFFF
FFFFFFFFFFFFFFFFFFFFFFFFFFFFFFFFFFFFFFFFFFFFFFFFFFFFFFFFFFFFFFFFFFFF
FFFFFF:FFFFFFFF
@A00721:190:HGJW7DSXY:4:1361:7346:15092 1:N:0:TCTGCAAG+GCTGCACT
GTCTAGACCGATTTCTGTGAGGAAGAAACTTAGTGGACTCACAGTTTTTAAGGACACCACTGGTGGCA
GTGTAGAGGACATGATGAAAGGAGCTTATTATTTAGTTTTTGCGACATTTCATCAATATGCGATGGAA
GTTGCATTAAATGTA
+
FFFFFFFFFFFFFFFFFFFFFFFFFFFFFFFFFFFFFFFFFFFFFFFFFF:FFFFFFFFFFFFFFFFF
FFFFFFFFFFFFFFFFFFFFFFFFFFF:FFFFFFFFFFFFFFFFFFFFFFFFFFFFFFFFFFFFF:FF
FFFFFFFF:FFFFFF
@A00721:190:HGJW7DSXY:4:1368:9173:13839 1:N:0:TCTGCAAG+GCTGCACT
GTCTAGACCGATTTCTGTGAGGAAGAAACTTAGTGGACTCACAGTTTTGAAGGACACCACTGGTGGCA
GAGTAGAGGACATGATGAAAGGAGCTTATTATTTAGTTTTAGCGACATTTCATCAAAATGCGATGGAC
GTTGCATTAAATGTA
+
F,FFF,FFF,FFFFF:F:,FF::F:FFFFFFF:FF,F,FFFFF,FFF,,:,F:FFFFFFF,,,,:,FF
,,F:,FF,F:,FF:F,,,FF,FF:FF,FFFF,,F:,FFF,,F,FFFFF:,FFFFFF,F,:,F,F:FF,
,F:FFF,,:F:FFFF
@A00721:190:HGJW7DSXY:4:1427:27796:26303 1:N:0:TCTGCAAG+GCTGCACT
GTCTAGACCGATTTCTGTGAGGAAGAAACTTAGTGGACTCACAGTTTTTAAGGACACCACTGGTGGCA
GTGTAGAGGACATGATGAAAGGAGCTTATTATTTAGTTTTTGCGACATTTCATCAATATGCGATGGAA
GTTGCATTAAATGTA
+
FFFFFFFFFFFFFFFFF:FFFFFFFFFFFFFFFFFFFFFFFFFFFFFFFFFFFFFFFFFFFFFFFFFF
FFFFFFFFFFFFFFFFFFFFFFFFFF:F:FFFFFFFFFFFFFFFFFFFFFFFFFFFFFFFFFFFFFF:
FFFFFFFFFFFFFF:
@A00721:190:HGJW7DSXY:4:1454:15465:13182 1:N:0:TCTGCAAG+GCTGCACT
GTCTGGACCGATTTCTGTGAGGAAGAAACTTAGTGGACTCACAGTTTTTAAGGACACCACTGGTGGCA
GTGTAGAGGACATGATGAAAGGAGCTTATTATTTAGTTTTTGCGACATTTCATCAATATGCGATGGAA
GTTGCATTAAATGTA
+
FFFFFFFFFFFFFFFFFFFFFFFFFFFFFFFFFFFFFFFF:FFFFFFFFFFFFFFFFFFFFFFFFFFF
FFFFFFFFFFFFFFFFFFFFFFFFFFFFFFFFFFFFFFFFFF:FFFFFFFFFFFFFFFFFFFFFFFFF
FFFFFFFFFFFFFFF
@A00721:190:HGJW7DSXY:4:2247:10276:36855 1:N:0:TCTGCAAG+GCTGCACT
GTCTAGACCGATTTCTGTGAGGAAGAAACTTAGTGGACTCACAGTTTTTAAGGACACCACTGGTGGCA
GTGTAGAGGACATGATGAAAGGAGCTTATTATTTAGTTTTTGCGACATTTCATCAATATGCGATGGAA
GTTGCATTAAATGTA
+
FFFFFFFFFFFFFFFFFFFFFFFFFFFFFFFFFFFFFFFFFFFFFFFFFFFFFFFFFFFFFFFFFFFF
FFFFFFFFFFFFFFFFFFFFFFFFFFFFFFFFFFFFFFFFFFFFFFFFFFFFFFFFFFFFF:FFFFFF
FFFFFF:F:FFFFFF
@A00721:190:HGJW7DSXY:4:2262:6361:14826 1:N:0:TCTGCAAG+GCTGCACT
GTCTGGACCGATTTCTGTGAGGAAGAAACTTAGTGGACTCACAGTTTTTAAGGACACCACTGGTGGCA
GTGTAGAGGACATGATGAAAGGAGCTTATTATTTAGTTTTTGCGACATTTCATCAATATGCGATGGAA
GTTGCATTAAATGTA
+
FFFFFFFFFFFFFFFFF:FFFFFFFFFFFFFFFFFFFFFFFFFFFFFFFFFFFFFFFFFFFFFFFFFF
FFFFFFFFFFFFFFFFFFFFFFFFFFFFFFFFFFFFFFFFFFFFFFFFFFFFFFFFFFFFFFFFF:FF
FFFFFFFFFFFFFFF

```
@A00721:190:HGJW7DSXY:4:2338:7310:12743 1:N:0:TCTGCAAG+GCTGCACT
GTCTAGACCGATTTCTGTGAGGAAGAAACTTAGTGGACTCACAGTTTTTAAGGACACCACTGGTGGCA
GTGTAGAGGACATGATGAAAGGAGCTTATTATTTAGTTTTTGCGACATTTCATCAATATGCGATGGAA
GTTGCATTAAATGTA
+
FFFFFFFFFFFFFFFFFFFFFFFFFFFFFFFFFFFFFFFFFFFFFFFFFFFFFFFFFFFFFFFFFFFF
FFFF:FFFFFFFFFFFFFFFFFFFFFFFFFFFFFFFFFFF,FFFFFFFFFFFFFFFFF:FFFFFFF
FFFFFFFFFF:FFFF
@A00721:190:HGJW7DSXY:4:2345:11704:37012 1:N:0:TCTGCAAG+GCTGCACT
GTCTAGACCGATTTCTGTGAGGAAGAAACTTAGTGNACTCACAGTTTTTAAGGACACCACTGGTGGCA
GTGTAGAGGACATGATGAAAGGAGCTTATTATTTAGTTTTTGCGACATTTCATCAATATGCGATGGAA
GTTGCATTAAATGTA
+
FFFFFFFFFFFFFFFFFFFFFFFFFFFFFFFFFFF#FFFFFFFFFFFFFFFFFFFFFFFFFFFFFFFF
FFFFFFFFFFFFFFFFFF:FFFFFFFFFFFFFFFFFFFFFFFFFFFFFFFFFFFFFFFFFFFFFFFFF:
FFFFFFFFFFFFFFF
@A00721:190:HGJW7DSXY:4:2464:22851:33270 1:N:0:TCTGCAAG+GCTGCACT
GTCTAGACCGATTTCTGTGAGGAAGAAACTTAGTGGACTCACAGTTTTTAAGGACACCACTGGTGGCA
GTGTAGAGGACATGATGAAAGGAGCTTATTATTTAGTTTTTGCGACATTTCATCAATATGCGATGGAA
GTTGCATTAAATGTA
+
FFFFFFFFFFFFFFFFFFFFFFFFFFFFFFFFFFFFFFFFFFFFFFFFFFFFFFFFFFFFFFFFFFFF
FFFFFFFFFFFFFFFFFFFFFFFFFFFFFFFFFFFFFFFFFFFFFFFFFFFFFFFFFFFFFFFFFFFF
FFFFFFFFFFFFFFF
@A00721:190:HGJW7DSXY:4:2554:4842:28823 1:N:0:TCTGCAAG+GCTGCACT
GTCTGGACCGATTTCTGTGAGGAAGAAACTTAGTGGACTCACAGTTTTTAAGGACACCACTGGTGGCA
GTGTAGAGGACATGATGAAAGGAGCTTATTATTTAGTTTTTGCGACATTTCATCAATATGCGATGGAA
GTTGCATTAAATGTA
+
F,FF::FFFFFFFFFFFFFFFFFF,FFFFF,FF::FFFFFFFFFFF:FFFFF,FFFFFFFFFFFFFFF
F,F,FFFFFFF:::FFFFFFFFFF:FFF:FFFF,F:F:FFFF:FF:FFF,FFFFFFFFFFFFFF:FFF
FFF:FF,FFFFF:FF
@A00721:190:HGJW7DSXY:4:1250:21043:1830 1:N:0:TCTGCAAG+GCTGCACT
CGTACATTTAATGCAACTTCCATCGCATATTGATGAAATGTCGCAAAAACTAAATAATAAGCTCCTTT
CATCATGTCCTCTACACTGCCACCAGTGGTGTCCTTAAAAACTGTGAGTCCACTAAGTTTCTTCCTCA
CAGAAATCGGTCTAG
+
FFFFFFFFFFFFFFFFFFFFFFFFFFFFFFFFFFFFFFFFFFFFFFFFFFFFFFFFFFFFFFFFF:FF
FFFFFFFFFFFFFFFFFFFFFFFFFFFFFFFFFFFFFFFFFFFFFFFFFFFFFFFFFFFFFFFFFFFF
FFFFFFFFFFFFFFF
@A00721:190:HGJW7DSXY:4:1251:9209:35477 1:N:0:TCTGCAAG+GCTGCACT
CGTACATTTAATGCAACTTCCATCGCATATTGATGAAATGTCGCAAAAACTAAATAATAAGCTCCTTT
CATCATGTCCTCTACACTGCCACCAGTGGTGTCCTTAAAAACTGTGAGTCCACTAAGTTTCTTCCTCA
CAGAAATCGGTCTAG
+
FFFFFFFFFFFFFFFFFFFFFFFFFFFFFFFFFFFFFFFFFFFFFFFFFFFFFFFFFFFFFFFFFF:F
FFFFFFFFFFFFFFFFFFFFFFFFFFFFFFFFFFFFFFFFFFFFFFFFFFFFFFFFFFFFFFFFFFFF
FFFFFFFFFFFFFFF
@A00721:190:HGJW7DSXY:4:1262:3784:30436 1:N:0:TCTGCAAG+GCTGCACT
CGTACATTTAATGCAACTTCCATCGCATATTGATGAAATGTCGCAAAAACTAAATAATAAGCTCCTTT
CATCATGTCCTCTACACTGCCACCAGTGGTGTCCTTAAAAACTGTGAGTCCACTAAGTTTCTTCCTCA
CAGAAATCGGTCTAG
+
FFFFFFFFFFFFFFFFFFFFFFFFFFFFFFFFFFFFFFFFFFFFFFFF,FFFFFFFFFFFFFFFFFFF
```

FFFFFFFFFFFFFFFFFFFFFFFFFFFFFFFFFFFFFFFFFFFFFFFFFFFFFFFFFFFFFFFFFFFF
FF:FFFFFFFFFFFF
@A00721:190:HGJW7DSXY:4:1347:16640:19288 1:N:0:TCTGCAAG+GCTGCACT
CGTACATTTAATGCAACTTCCATCGCATATTGATGAAATGTCGCAAAAACTAAATAATAAGCTCCTTT
CATCATGTCCTCTACACTGCCACCAGTGGTGTCCTTAAAAACTGTGAGTCCACTAAGTTTCTTCCTCA
CAGAAATCGGTCTAG
+
FFFFFFFFFFFFFFFFFFFFFFFFFFFFFFFFFFFFFFFFFFFFFFFFFFFFFFFFFFFFFFFFFFFF
FFFFFFFFFFFFFFFFFFFFFFFFFF:FFFFFFFFFFFFFFFFFFFFFFFFFFFFFFFFFFFFFFFFF
FFFFFFFFFFFFFFF
@A00721:190:HGJW7DSXY:4:2307:19958:8343 1:N:0:TCTGCAAG+GCTGCACT
CGTACATTTAATGCAACTTCCATCGCATATTGATGAAATGTCGCAAAAACTAAATAATAAGCTCCTTT
CATCATGTCCTCTACACTGCCACCAGTGGTGTCCTTAAAAACTGTGAGTCCACTAAGTTTCTTCCTCA
CAGAAATCGGTCTAG
+
FF:FFFFFFFFFFFFFF:FFFFFFFFF:FF:FFF:FFFFFFFFFFFFFFFFFFFFFFFFFFFFFFFFF
FFFFFFFFFFFFFFFFFFFFFFFFFFFFF:FFFF:FFFFFFF:FFFFFFFFFFFFFFFFFFFF,FFFF
FF,FFFFFFFFFFF:
@A00721:190:HGJW7DSXY:4:2326:19045:34601 1:N:0:TCTGCAAG+GCTGCACT
CGTACATTTAATGCAACTTCCATCGCATATTGATGAAATGTCGCAAAAACTAAATAATAAGCTCCTTT
CATCATGTCCTCTACACTGCCACCAGTGGTGTCCTTAAAAACTGTGAGTCCACTAAGTTTCTTCCTCA
CAGAAATCGGTCTAG
+
FFFFFFFFFFFFFFFFFFFFFFFFFFFFFFFFFFFFFFFFFFFFFFFFFFFFFFFFFFFFFFFFFFFF
FFFFFFFFFFFFFFFFFFFFFFFFFFFFFFFFFFFFFFFFFFFFFFFFFFFFFFFFFFFFFFFFFFFF
FF:FFFFFFFFFFFF
@A00721:190:HGJW7DSXY:4:2361:22968:14967 1:N:0:TCTGCAAG+GCTGCACT
CGTACATTTAATGCAACTTCCATCGCATATTGATGAAATGTCGCAAAAACTAAATAATAAGCTCCTTT
CATCATGTCCTCTACACTGCCACCAGTGGTGTCCTTAAAAACTGTGAGTCCACTAAGTTTCTTCCTCA
CAGAAATCGGTCTAG
+
FFFFFFFFFFFFFFFFFFFFFFFFFFFFFFFFFFFFFFFFFFFFFFFFFFFFFFFFFFFFFFFFFFFF
FFFFFFFFFFFFFFFFFFFFFFFFFFFFFFFFFFFFFFFFFFFFFFFFFF:FFFFFFFFF:FFFFFFF
FFFFFFFFFFFFFFF
@A00721:190:HGJW7DSXY:4:1102:12545:14418 1:N:0:TCTGCAAG+GCTGCACT
GACCGATTTCTGTGAGGAAGAAACTTAGTGGACTCACAGTTTTTAAGGACACCACTGGTGGCAGTGTA
GAGGACATGATGAAAGGAGCTTATTATTTAGTTTTTGCGACATTTCATCAATATGCGATGGAAGTTGC
ATTAAATGTACGAAT
+
FFFFFFFFFFFFFFFFFFFFFFFFFFFFFFFFFFFFFFFFFFFFFFFFFFFFFFFFFFFFFFFFFFFF
FFFFFFFFFFFFFFFFFFFFFFFFFFFFFFFFFFFFFFFFFFFFFFFFFFFFFFFFFFFFFFFFFFFF
FFFFFFFFFFFFFFF
@A00721:190:HGJW7DSXY:4:1232:3721:19774 1:N:0:TCTGCAAG+GCTGCACT
GACCGATTTCTGTGAGGAAGAAACTTAGTGGACTCACAGTTTTTAAGGACACCACTGGTGGCAGTGTA
GAGGACATGATGAAAGGAGCTTATTATTTAGTTTTTGCGACATTTCATCAATATGCGATGGAAGTTGC
ATTAAATGTACGAAT
+
FFFFFFFFFFFFFFFFFFFFFFFFFFFFFFFFFFFFFFFFFFFFFFFFFFFFFFFFFFFFFFFFFFFF
FFFFFFFFFFFFFFFFFFFFFFFFF:FFFFFFFFFFFFFFFFFFFFFFFFFFFFFFFFFFFFFFFFFF
FF:FFFFFFFFFFF:
@A00721:190:HGJW7DSXY:4:1275:10013:33238 1:N:0:TCTGCAAG+GCTGCACT
GACCGATTTCTGTGAGGAAGAAACTTAGTGGACTCACAGTTTTTAAGGACACCACTGGTGGCAGTGTA
GAGGACATGATGAAAGGAGCTTATTATTTAGTTTTTGCGACATTTCATCAATATGCGATGGAAGTTGC
ATTAAATGTACGAAT

```
+
FFFFFFFFFFFFFFFFFFFFFFFFFFFFFFFFFFFFFFFFFFFFFFFFFFFFFFFFFFFFFFFFFFFFFF
FFFFFFFFFFFFFFFFFFFFFFFFFFFFFFFFFFFFFFFFFFFFFFFFFFFFFFFFFFFFFFFFFFFFFF
FFFFFFFFFFFFFFF
@A00721:190:HGJW7DSXY:4:1307:20220:15280 1:N:0:TCTGCAAG+GCTGCACT
GACCGATTTCTGTGAGGAAGAAACTTAGTGGACTCACAGTTTTTAAGGACACCACTGGTGGCAGTGTA
GAGGACATGATGAAAGGAGCTTATTATTTAGTTTTTGCGACATTTCATCAATATGCGATGGAAGTTGC
ATTAAATGTACGAAT
+
FFF,FFFFFFF:FF:FFFFFFFFFF:FFFFFFFFFFFFFF:FFFF:FFFFFFFFFFFFF:FFFFFF:F:F
FFFFFFFFFFF:FFF:,FF,F,FFFF:F,FF,,FFFF,FFFFFFFFFF:FF:FFFFF:FFFFFFFFFF:F
FFFFFFFF:FF,FFF
@A00721:190:HGJW7DSXY:4:1307:19596:15389 1:N:0:TCTGCAAG+GCTGCACT
GACCGATTTCTGTGAGGAAGAAACTTAGTGGACTCACAGTTTTTAAGGACACCACTGGTGGCAGTGTA
GAGGACATGATGAAAGGAGCTTATTATTTAGTTTTTGCGACATTTCATCAATATGCGATGGAAGTTGC
ATTAAATGTACGAAT
+
FFFFFFFFFFFFFFFFFFFFFFFFFFFFFFFFFFFFFFFFF:FFFFFFFFFFFFFFFFFFFFFFFFFFFF
FFFFFFFFFFFFFFFFFFFFFFFFFFFFFFFFFFFFFFFFFFFFFFFFFFFFFFFFFFFFFFFFFF:FFF
FFFFFFFFFFFFFFF
@A00721:190:HGJW7DSXY:4:1320:9353:15499 1:N:0:TCTGCAAG+GCTGCACT
GACCGATTTCTGTGAGGAAGAAACTTAGTGGACTCACAGTTTTTAAGGACACCACTGGTGGCAGTGTA
GAGGACATGATGAAAGGAGCTTATTATTTAGTTTTTGCGACATTTCATCAATATGCGATGGAAGTTGC
ATTAAATGTACGAAT
+
FFFFFFFFFFFFFFFFFFFFFFFFFFFFFFFFFFFFFFFFFFFFFFFFFFFFFFFFFFFFFFFFFFFFFF
FFFFFFFFFFFFFFFFFFFFFFFFFFFFFFFFFFFFF:FFFFFFFFFFFFFFFFFFFFFFFFFFFFFFFF
FFFFFF:FFFFFFFF
@A00721:190:HGJW7DSXY:4:1324:13015:34021 1:N:0:TCTGCAAG+GCTGCACT
GACCGATTTCTGTGAGGAAGAAACTTAGTGGACTCACAGTTTTTAAGGACACCACTGGTGGCAGTGTA
GAGGACATGATGAAAGGAGCTTATTATTTAGTTTTTGCGACATTTCATCAATATGCGATGGAAGTTGC
ATTAAATGTACGAA
+
FFFFFFFFFFFFFFFFFF:FFFFFFFFFFFFFFFFFFFFFFFFFFFFFFFFFFFFFFFFFFFFFFFFFFF
FFFFFFFFFF:FFFFFFFFFFF,FFF,FFFFFFFFFFFFFFFFFFFFFFFFFFFFFFFFFFFFFFFFFFF
FFFFFFFFFFFFFF
@A00721:190:HGJW7DSXY:4:1360:15989:10081 1:N:0:TCTGCAAG+GCTGCACT
GACCGATTTCTGTGAGGAAGAAACTTAGTGGACTCACAGTTTTTAAGGACACCACTGGTGGCAGTGTA
GAGGACATGATGAAAGGAGCTTATTATTTAGTTTTTGCGACATTTCATCAATATGCGATGGAAGTTGC
ATTAAATGTACGAAT
+
FFFFFFFFFFFF,FFFFFFFFFFFFFFFFFFFFFFFFFFFFFFFFFFFFFFFFFFFFFFFFFFFFFFFFF
FFFFFFFFFFFFFFFFFFFFFF:FFFFFFFFFFFFFFFFFFFFFFFFFFFFFFFFFFFFFFFFF:FFFFF
FFFFFFFFFFFFFFF
@A00721:190:HGJW7DSXY:4:1406:13358:35336 1:N:0:TCTGCAAG+GCTGCACT
GACCGATTTCTGTGAGGAAGAAACTTAGTGGACTCACAGTTTTTAAGGACACCACTGGTGGCAGTGTA
GAGGACATGATGAAAGGAGCTTATTATTTAGTTTTTGCGACATTTCATCAATATGCGATGGAAGTTGC
ATTAAATGTACGAAT
+
FFFFFFFFFFFFFFFFFFFFFFFFFFFFFFFFFFFFFFFFFFFFFFFFFFFFFFFFFFFFFFFFFFFFFF
FFFFFFFFFFFFFFFFFFFFFFFFFFFFFFFFFFFFFFFFFFFFFFFFFFFFFFFFFFFFFFFFFFFFFF
FFFFFFF:FFFFFFF
@A00721:190:HGJW7DSXY:4:1411:29568:29465 1:N:0:TCTGCAAG+GCTGCACT
GACCGATTTCTGTGAGGAAGAAACTTAGTGGACTCACAGTTTTTAAGGACACCACTGGTGGCAGTGTA
```

```
GAGGACATGATGAAAGGAGCTTATTATTTAGTTTTTGCGACATTTCATCAATATGCGATGGAAGTTGC
ATTAAATGTACGAAT
+
FFFFFF:FFFFFFFF,FFF:FFFF:FF,:,FFFF:FFF:,FFFFFF,FFFFFFF::F,F,F,F:,F:F
,FF,F,FF:FF,FFF:FF,FFFF:FFFFFF:FFF:FF::FFF,FFFFF::FFF:,FFFFF,FF,:F:F
FFFFFFF:FFFFFFF
@A00721:190:HGJW7DSXY:4:1448:1118:20744 1:N:0:TCTGCAAG+GCTGCACT
GACCGATTTCTGTGAGGAAGAAACTTAGTGGACTCACAGTTTTTAAGGACACCACTGGTGGCAGTGTA
GAGGACATGATGAAAGGAGCTTATTATTTAGTTTTTGCGACATTTCATCAATATGCGATGGAAGTTGC
ATTAAATGTACGAAT
+
FFFFFFFFFFFFFFFFFFFFFFFFFFFFFFFFFFFFFFFFFFF:FFFFFFFFFFFFFFFFFFFFFFFFFF
FFFFFFFFFFFFFFFFFFFFFFFFFFFF:FFFFFFFFFFFFFFFFFFFFFFFFFFFFFFFFFFFFFFFFF
F,FFFFFFFFFFFFF
@A00721:190:HGJW7DSXY:4:1508:12066:23891 1:N:0:TCTGCAAG+GCTGCACT
GACCGATTTCTGTGAGGAAGAAACTTAGTGGACTCACAGTTTTTAAGGACACCACTGGTGGCAGTGTA
GAGGACATGATGAAAGGAGCTTATTATTTAGTTTTTGCGACATTTCATCAATATGCGATGGAAGTTGC
ATTAAATGTACGAAT
+
FFFFFFFFFFFFFFFFFFFFFFFFFFFFFFFFFFFFFFFFFFFFFFFFFFFFFFFFFFFFFFFFFFFFFF
FFFFFFFFFFFFFFFFFFFFFFFFFFFFFFFFFFFFFFFFFFFFFFFFFFFFFFFFFFFFFFFFFFFFFF
FFFFFFFFFFFFFFF
@A00721:190:HGJW7DSXY:4:2105:25663:23547 1:N:0:TCTGCAAG+GCTGCACT
GACCGATTTCTGTGAGGAAGAAACTTAGTGGACTCACAGTTTTTAAGGACACCACTGGTGGCAGTGTA
GAGGACATGATGAAAGGAGCTTATTATTTAGTTTTTGCGACATTTCATCAATATGCGATGGAAGTTGC
ATTAAATGTACGAAT
+
FFFFFFFFFFFFFFFFFFFFFFFFFFFFFFFFFFFFFFFFFFFFF:FFFFFFFFFFFFFFFFFFFFFFFF
FFFFFFFFFFFFFFFFFFFFFFFFFFFFFFFFFFFFFFFFFFFFFFFFFFFFFFFFFFFFFFFFFFFFFF
FFFFFFFFFF::FFF
@A00721:190:HGJW7DSXY:4:2126:3676:32409 1:N:0:TCTGCAAG+GCTGCACT
GACCGATTTCTGTGAGGAAGAAACTTAGTGGACTCACAGTTTTTAAGGACACCACTGGTGGCAGTGTA
GAGGACATGATGAAAGGAGCTTATTATTTAGTTTTTGCGACATTTCATCAATATGCGATGGAAGTTGC
ATTAAATGTACGAAT
+
FFFFFFFFFFFFFFFFFFFFFFFFFFFFFFFFFFFFFFFFFFFFFFFFFFFFFFFFFFFFFFFFFFFFFF
FFFFFFFFFFFFFFFFFFFFFF:FFFFFFFFFFFFFFFFFFFFFFFFFFFFFFF:FFFFFFFFFFFFFFF
FFFFFFFFFFFFF,F
@A00721:190:HGJW7DSXY:4:2212:16143:34334 1:N:0:TCTGCAAG+GCTGCACT
GACCGATTTCTGTGAGGAAGAAACTTAGTGGACTCACAGTTTTTAAGGACACCACTGGTGGCAGTGTA
GAGGACATGATGAAAGGAGCTTATTATTTAGTTTTTGCGACATTTCATCAATATGCGATGGAAGTTGC
ATTAAATGTACGAAT
+
FFFFFFFFFFFFFFFFFFFFFFFFFFFFFFFFFFFFFFFFFFFFFFFFFFFF:FFFFFFFFFFFFFFFFF
FFFFFFFFFFFFFFFFFFFFFFFFFFFFFFFFFFFFFFFFFFFFFFFFFFFFFFFFFFFFFFFFFFFFFF
FFFFFFFFFFFFFFF
@A00721:190:HGJW7DSXY:4:2334:26648:20369 1:N:0:TCTGCAAG+GCTGCACT
GACCGATTTCTGTGAGGAAGAAACTTAGTGGACTCACAGTTTTTAAGGACACCACTGGTGGCAGTGTA
GAGGACATGATGAAAGGAGCTTATTATTTAGTTTTTGCGACATTTCATCAATATGCGATGGAAGTTGC
ATTAAATGTACGAAT
+
FFFFFFFFFFFFFFFFFFFFFFFFFFFFF:FFFFFFFFFFFFFFFFFFFFFFFFFFFFFFFFFFFFFFFF
FFFFFFFFFFFFFFFFFFFFFFFFFFF::FFFFFFFFFFFFFFFFFFFFFFFFFFFFFFF:FFFFFFFFF
FF:FFFFFFFFFFF:
```

```
@A00721:190:HGJW7DSXY:4:2354:20555:12477 1:N:0:TCTGCAAG+GCTGCACT
GACCGATTTCTGTGAGGAAGAAACTTAGTGGACTCACAGTTTTTAAGGACACCACTGGTGGCAGTGTA
GAGGACATGATGAAAGGAGCTTATTATTTAGTTTTTGCGACATTTCATCAATATGCGATGGAAGTTGC
ATTAAATGTACGAAT
+
FFFFFFFFFFFFFFFFFFFFFFFFFFFFFFFFFFFFFFFFFFFFFFFFFFFFFFFFFFFFFFFFFFFF
FFFFFFFFFFFFFFFFFFFF::FFFFFFFFFFFFFFF:FFFFFFFFFFFF:FFFF:FFFFFFFFFFFF
FFFFFFFFFFFFFFF
@A00721:190:HGJW7DSXY:4:2355:28004:4398 1:N:0:TCTGCAAG+GCTGCACT
GACCGATTTCTGTGAGGAAGAAACTTAGTGGACTCACAGTTTTTAAGGACACCACTGGTGGCAGTGTA
GAGGACATGATGAAAGGAGCTTATTATTTAGTTTTTGCGACATTTCATCAATATGCGATGGAAGTTGC
ATTAAATGTACGAAT
+
FFFFFFFFFFFFF:FFFFFFFFFFFFFFFFFFFFFFFFFFFFFFFFFFFFFFFFFFFFFFFFFFFFFF
FFFFFFFFFFFFFFFFFFFFFFFFFF:FFFFFFFFF:FFFFFFFFFFFFFFFF:FFFFFFFF:F:FFF
FFFFFFFFFF:F:FF
@A00721:190:HGJW7DSXY:4:2362:28854:30295 1:N:0:TCTGCAAG+GCTGCACT
GACCGATTTCTGTGAGGAAGAGACTTAGTGGACTCACAGTTTTTAAGGACACCACTGGTGGCAGTGTA
GAGGACATGATGAAAGGAGCTTATTATTTAGTTTTTGCGACATTTCATCAATATGCGATGGAAGTTGC
ATTAAATGTACGAAT
+
FFFFFFFFFFFFFFFFFFFFFFFFFFFFFFFFFFFFFFFFFFFFFFFFFFFFFFFFFFFFFFFFFFFF
FFFFFFFFFFFFFFFFFFFF:FF:FFFFFFFFFFFFFFFFFFFFFFFFFFFFF:FFFFFFFFFFFFFF
FFFFFFFFFFF:FFF
@A00721:190:HGJW7DSXY:4:2468:23574:32550 1:N:0:TCTGCAAG+GCTGCACT
GACCGATTTCTGTGAGGAAGAAACTTAGTGGACTCACAGTTTTTAAGGACACCACTGGTGGCAGTGTA
GAGGACATGATGAAAGGAGCTTATTATTTAGTTTTTGCGACATTTCATCAATATGCGATGGAAGTTGC
ATTAAATGTACGAAT
+
FFFFFFFFFFFFFFFFFFFFFFFFFFFFFFFFFFFFFFFFFFFFF:FFFFFFFFFFFFFFFFFFFFFF
FFFFFFFFFFFFFFFFFFFFFFFFFFFFFFFFFFFFFFFFFFFFFFFFFFFFFFFFFFFFFFFFFFFF
FFFFFFFFFFFFFFF
@A00721:190:HGJW7DSXY:4:2478:18041:35712 1:N:0:TCTGCAAG+GCTGCACT
GACCGATTTCTGTGAGGAAGAAACTTAGTGGACTCACAGTTTTTAAGGACACCACTGGTGGCAGTGTA
GAGGACATGATGAAAGGAGCTTATTATTTAGTTTTTGCGACATTTCATCAATATGCGATGGAAGTTGC
ATTAAATGTACGAAT
+
FFFFFFFFFFFFFFFFF:FFFFFFFFFFFFFFFFFFFFFFFFFFFFFFFFFFFFFFFFFFFFFFFFFF
FFFFFFFFFF:FFFFFFFFF::FFFFFFFFFFFFFFFFFFFFFFFFFFFFFFFFFFFFFFFFFFFFFF
FFFF:FFFFFFFFFF
@A00721:190:HGJW7DSXY:4:2478:16731:36041 1:N:0:TCTGCAAG+GCTGCACT
GACCGATTTCTGTGAGGAAGAAACTTAGTGGACTCACAGTTTTTAAGGACACCACTGGTGGCAGTGTA
GAGGACATGATGAAAGGAGCTTATTATTTAGTTTTTGCGACATTTCATCAATATGCGATGGAAGTTGC
ATTAAATGTACGAAT
+
FFFFFFFFFFFF:FFFFFFFFFFFFFFF:FFFFFFFFFFFFFFFFFFFFFFFFFFFFFFFFFFFFFFF
FFFFFFFFFFFFFFFFFFFFFFFFFFFFFFFFFFFFFFFFFFFFFFFFFFFFFFFFFFFFFFFFFFFF
FFFFFFFFFFFFFFF
@A00721:190:HGJW7DSXY:4:2523:11116:16673 1:N:0:TCTGCAAG+GCTGCACT
GACCGATTTCTGTGAGGAAGAAACTTAGTGGACTCACAGTTTTTAAGGACACCACTGGTGGCAGTGTA
GAGGACATGATGAAAGGAGCTTATTATTTAGTTTTTGCGACATTTCATCAATATGCGATGGAAGTTGC
ATTAAATGTACGAAT
+
FFFFFF,F::FFFFFF,FFFFFFFFFF,,FFF:FFF:,FFFFFFF,:,FFF:FFFFF,FF,FFFFFFF
```

```
:,:FFFFFFF,:FFFF,:F,FFFFFF:FFFFFFFF:F,FF,F::FFFFFFFF,,F,F:F:FF:FF,:F
F::FFFFFFFFFF,F
@A00721:190:HGJW7DSXY:4:2561:14235:10207 1:N:0:TCTGCAAG+GCTGCACT
GACCGATTTCTGTGAGGAAGAAACTTAGTGGACTCACAGTTTTTAAGGACACCACTGGTGGCAGTGTA
GAGGACATGATGAAAGGAGCTTATTATTTAGTTTTTGCGACATTTCATCAATATGCGATGGAAGTTGC
ATTAAATGTACGAAT
+
FFFFFFFFFFFFFFFFFFFFFFFFFFFFFFFFFFFFFFFFFFFFF,FFFFFFFFFFFFFFFFFFFFFF
FFFFFFFFFFFFFFFFFFFFFFFFFFFFFFFFFFFFF:FFFFFFFFFFFFFFFFFFFFFFFFFFFFFF
FFFFFFFFFFFFFFF
@A00721:190:HGJW7DSXY:4:2601:5882:29089 1:N:0:TCTGCAAG+GCTGCACT
GACCGATTTCTGTGAGGAAGAAACTTAGTGGACTCACAGTTTTTAAGGACACCACTGGTGGCAGTGTA
GAGGACATGATGAAAGGAGCTTATTATTTAGTTTTTGCGACATTTCATCAATATGCGATGGAAGTTGC
ATTAAATGTACGAAT
+
FFFFFFFFFFFFFFFFFFFFFFFFFFFFFFFFFFFFFFFFFFFFFFFFFFFFFFFFFFFFFFFFFFFF
FFFFFFFFFFFFFFFF:FFFFFFFFFFFFFFFFFFFFFFFFFFFFFFFFFFFFFFFFFFF:FFFFFFF
FFFFFFFFFFF,FFF
@A00721:190:HGJW7DSXY:4:2603:13232:15076 1:N:0:TCTGCAAG+GCTGCACT
GACCGATTTCTGTGAGGAAGAAACTTAGTGGACTCACAGTTTTTAAGGACACCACTGGTGGCAGTGTA
GAGGACATGATGAAAGGAGCTTATTATTTAGTTTTTGCGACATTTCATCAATATGCGATGGAAGTTGC
ATTAAATGTACGAAT
+
FFFFFFFFFFFFFFFFFFFFFFFFFFFFFFFFFFFFFFFFFFFFFFFFFFFFFFFFFFFFFFFFFF,F
FFFFFFFFFFFFFFFFFFFFFFFFFFFFFFFFFFFFFFFFFFFFFFFFFFFFFFFFFFFFFFFFFFFF
FFFFFFFFFFFFFFF
@A00721:190:HGJW7DSXY:4:2634:22616:27477 1:N:0:TCTGCAAG+GCTGCACT
GACCGATTTCTGTGAGGAAGAAACTTAGTGGACTCACAGTTTTTAAGGACACCACTGGTGGCAGTGTA
GAGGACATGATGAAAGGAGCTTATTATTTAGTTTTTGCGACATTTCATCAATATGCGATGGAAGTTGC
ATTAAATGTACGAAT
+
FFFFFFFFFFFFFFFFFFFFFFFFFFFFFFFFFFFFFFFFFFFFFFFFFFFFFFFFFFFFFFFFFFFF
FFFFFFFFFFFFF:FFFFFFFFFFF:FFFFFFFFFFFF:FFFFFFFFFFFFFFFF:FFFFFFFFFFFF
FF:FFFFFFFFFFFF
@A00721:190:HGJW7DSXY:4:2544:23601:21167 1:N:0:TCTGCAAG+GCTGCACT
ACCGATTTCTGTGAGGAAGAAACTTAGTGGACTCACAGTTTTTAAGGACACCACTGGTGGCAGTGTAG
AGGACATGATGAAAGGAGCTGATTATTTAGTTTTTGAGACATTTCATCAATATGCTATTGAAGTTTCA
TTAAATGTACGAAT
+
FF,:FF,,FFFF:F,,FF:FF,FFFF:,,,:F,:F:F,,:FFFFF,:F,:,:FFF,F,:F:FF:FFFF
FF:FFFF,FF,FFF,FFF,F,FFF::,FF,F:FFF::F:FF,:FFFFFFFFF::,,F:,F:,F,F,FF
F,F,FF:FF,,FFF
@A00721:190:HGJW7DSXY:4:1139:9561:35837 1:N:0:TCTGCAAG+GCTGCACT
CCACATTCGTACATTTAATGCAACTTCCATCGCATATTGATGAAATGTCGCAAAAACTAAATAATAAG
CTCCTTTCATCATGTCCTCTACACTGCCACCAGTGGTGTCCTTAAAAACTGTGAGTCCACTAAGTTTC
TTCCTCACAGAAATC
+
FFFFFFFFFFFFFFFFFFFFFFFFFFFFFFFFFFFFFFFFFFFFFFFFFFFFFFFFFFFFFFFFFFFF
FFFFFFFFFFFFFFFFFFFFFFFFFFFFFFFF:FFFFFFFFFFFFFFFFFFFFFFFFFFFFFFFFFFF
FFFFFFFFFFFFFFF
@A00721:190:HGJW7DSXY:4:1276:11478:23563 1:N:0:TCTGCAAG+GCTGCACT
CCACATTCGTACATTTAATGCAACTTCCATCGCATATTGATGAAATGTCGCAAAAACTAAATAATAAG
CTCCTTTCATCATGTCCTCTACACTGCCACCAGTGGTGTCCTTAAAAACTGTGAGTCCACTAAGTTTC
TTCCTCACAGAAATC
```

```
+
FFFFFFFFFFFFFFFFFFFFFFFFFFFFF:FFFFFFFFFFFFFFFFFFFFFFFFFFFF:FFFFFFFFF
FFFFFFFFFFFFFFFFFFFFFFFFFFFFFFFFFFFFFF,FFFFFFFFFFFFFFFFFFFFFFFFFFFFF
FFFFFFFFFFFFFFF
@A00721:190:HGJW7DSXY:4:1318:3893:6637 1:N:0:TCTGCAAG+GCTGCACT
CCACATTCGTACATTTAATGCAACTTCCATCGCATATTGATGAAATGTCGCAAAAACTAAATAATAAG
CTCCTTTCATCATGTCCTCTACACTGCCACCAGTGGTGTCCTTAAAAACTGTGAGTCCACTAAGTTTC
TTCCTCACAGAAATC
+
FFFFFFFFFFFFFFFFFFFFFFFFFFFFFFFFFFFFFFFFFFFF:FFFFFFFFFFFFFFFFFFFFFFF
FFFFFFFFFFFFFFFFFFFFFFFFFFFFFFFFFFFFFFFFFFFFFFFFFFFFFFFFFFFFFFFFFFFF
FFFFFFFFFFF:FFF
@A00721:190:HGJW7DSXY:4:1462:18195:26553 1:N:0:TCTGCAAG+GCTGCACT
CCACATTCGTACATTTAATGCAACTTCCATCGCATATTGATGAAATGTCGCAAAAACTAAATAATAAG
CTCCTTTCATCATGTCCTCTACACTGCCACCAGTGGTGTCCTTAAAAACTGTGAGTCCACTAAGTTTC
TTCCTCACAGAAATC
+
FFFFFFFFFFFFFFFFFFFFFFFFFFFFFFFFFFFFFFFFFFFFFFFFFFFFFFFFFFFFFFFFFFFF
FFFFF,FFFFFFFFFFFFFFFFFFFFFF:FFFFFFFFFFFFFFFFFFFFFFFFFFFFFFFFFFFFFFF
FFFFFFFFFFFFFFF
@A00721:190:HGJW7DSXY:4:1524:19560:19241 1:N:0:TCTGCAAG+GCTGCACT
CCACATTCGTACATTTAATGCAACTTCCATCGCATATTGATGAAATGTCGCAAAAACTAAATAATAAG
CTCCTTTCATCATGTCCTCTACACTGCCACCAGTGGTGTCCTTAAAAACTGTGAGTCCACTAAGTTTC
TTCCTCACAGAAATC
+
FFFFFFFFFFFFFFFFFFFFFFFFFFFFFFFFFFFFFFFFFFFFFFFFFFFFFFFFFFFFFFFFFFFF
FFFFFFFFFFFFFFFFFFFFFFFFFFFFFFFFFFFFFFFFFFFFFFFFFFFF:FFFFFFFFFFFFFFF
FFFFFFFFFFFFFFF
@A00721:190:HGJW7DSXY:4:1549:14100:9972 1:N:0:TCTGCAAG+GCTGCACT
CCACATTCGTACATTTAATGCAACTTCCATCGCATATTGATGAAATGTCGCAAAAACTAAATAATAAG
CTCCTTTCATCATGTCCTCTACACTGCCACCAGTGGTGTCCTTAAAAACTGTGAGTCCACTAAGTTTC
TTCCTCACAGAAATC
+
FFFFFF,FFFFFF,,FFFFFF:,FFFF:FFF:FF:FFFFFFFFFFFFFFFFFFFF,FFF:FFFFFF:F
FFF,F,FFFFFFFFFFFFFFFFFF:FFFFFFF:FFFFFFFFFFF:FFFFFF:FF:FF:FFFFFFFF:F
FF:F:FFFF:FFFFF
@A00721:190:HGJW7DSXY:4:1624:7654:33599 1:N:0:TCTGCAAG+GCTGCACT
CCACATTCGTACATTTAATGCAACTTCCATCGCATATTGATGAAATGTCGCAAAAACTAAATAATAAG
CTCCTTTCATCATGTCCTCTACACTGCCACCAGTGGTGTCCTTAAAAACTGTGAGTCCACTAAGTTTC
TTCCTCACAGAAATC
+
FFFFFFFFFFFFFFFFFFFFFFFFFFFFFFFFFFFFFFFFFFFFFFFFFFFFFFF,FFFFFFFFFFFF
FFFFFFFFFFFFFFFFFFFFFFFFFFFFFFFFFFFFFFFFFFFFFFFF:FFFFFFFFFFF:FFFFFFF
FFFFFFFFFFFFFFF
@A00721:190:HGJW7DSXY:4:1656:13114:16376 1:N:0:TCTGCAAG+GCTGCACT
CCACATTCGTACATTTAATGCAACTTCCATCGCATATTGATGAAATGTCGCAAAAACTAAATAATAAG
CTCCTTTCATCATGTCCTCTACACTGCCACCAGTGGTGTCCTTAAAAACTGTGAGTCCACTAAGTTTC
TTCCTCACAGAAATC
+
FFFFFFFFFFFFFFFFFFFFFFFFFFFFFFFFFFFFFFFFFFFFFFFFFFFFFFFFFFFFFFFFFFFF
FFFFFFFFFFFFFFFFFFFFFFFFFFFFFFFFFFFFFFFFFFFFFFFFFFFFFFFF:FFFFFFFFFFF
FFFFFFFFFFFFFFF
@A00721:190:HGJW7DSXY:4:1408:15212:35446 1:N:0:TCTGCAAG+GCTGCACT
CTGTGAGGAAGAAACTTAGTGGACTCACAGTTTTTAAGGACACCACTGGTGGCAGTGTAGAGGACATG
```

ATGAAAGGAGATTATTATTTAGTTTTTGCGACATTTCATCAATATGCGATGGAAGTTGCATTAAATGT
ACGAA
+
FFFFFF:FFFFFFFF,,FFFF,FFF,FFF:,F,FFFF,F:FFFF:F:FFF,:FFFF:FF:F,FF,F,F
F,FFFFF:FF,F,F,FF::FFFFFFF,,F:F:FFFFFF:FFFFFFFF,FFF:FF,FFF:FF:FFFF:F
FF:FF
@A00721:190:HGJW7DSXY:4:1454:24460:14105 1:N:0:TCTGCAAG+GCTGCACT
CTGTGAGGAAGAAACTTAGTGGACTCACAGTTTTTAAGGACACCACTGGTGGCAGTGTAGAGGACATG
ATGAAAGGAGCTTATTATTTAGTTTTTGCGACATTTCATCAATATGCGATGGAAGTTGCATTAAATGT
ACGAATGT
+
FFFFFFFFFFFFFFFFFFFFFFFFFFFFFFFFFF:FFFFFFFFFFFFFFFFFFFFFFFFFFFFFFFFFF
F:FFFFFFFFFFFFFFFFFFFFFFFFFFFFFFFFFFFFFFFFFFFFFFFFFFFF:FFFFFFFFFFFFFF
FFFFFFFF
@A00721:190:HGJW7DSXY:4:2308:19407:11365 1:N:0:TCTGCAAG+GCTGCACT
CTGTGAGGAAGAAACTTAGTGGACTCACAGTTTTTAAGGACACCACTGGTGGCAGTGTAGAGGACATG
ATGAAAGGAGCTTATTATTTAGTTTTTGCGACATTTCATCAATATGCGATGGAAGTTGCATTAAATGT
ACGAATGT
+
FFFFFFFFFFFFFFFFFFFFFFFFFFFFFFFFFFFFFFFFFFFFFFFFFFFFFFFFFFFFFFFFFFFFF
FFFFFFFFFFF::F,FF:FFFFFFFFFFFFFFFFFFFFFFFFFFFFFFFFFFFFFFFFFF:FFFFFFF
FFFFFFFF
@A00721:190:HGJW7DSXY:4:2528:28592:36667 1:N:0:TCTGCAAG+GCTGCACT
CTGTGAGGAAGAAACTTAGTGGACTCACAGTTTTTAAGGACACCACTGGTGGCAGTGTAGAGGACATG
ATGAAAGGAGCTTATTATTTAGTTTTTGCGACATTTCATCAATATGCGATGGAAGTTGCATTAAATGT
ACGAATGT
+
FFFFFFFFFFFFFFFFFFFFFFFFFFFFFFFFFFFFFFFFFFFFFFFFFFFFFFFFFFFFFFFFFFFFF
FFFF:FFFFFF:FFFFFFFFFFFFFFFFFFFFFFF:FFFFFFFFFFFFFFFFFF:FFFFFFFFF:FFFF
FFFFFFFF
@A00721:190:HGJW7DSXY:4:2532:29993:22780 1:N:0:TCTGCAAG+GCTGCACT
CTGTGAGGAAGAAACTTAGTGGACTCACAGTTTTTAAGGACACCACTGGTGGCAGTGTAGAGGACATG
ATGAAAGGAGCTTATTATTTAGTTTTTGCGACATTTCATCAATATGCGATGGAAGTTGCATTAAATGT
ACGAATGT
+
FFFFFFFFFFFFFFFFFFFFFFFFFFFFFFFFFFFFFFFFFFFFFFFFFFFFFFFFFFFFFFFFFFFFF
FFFFFFFFFFFFFF:FFFFFFFFFFFFFFFFFFFFFFFFFFFFFFFFFFFFFFFFFFFFFFFFFFFFFF
FFFFFFFF
@A00721:190:HGJW7DSXY:4:2635:10601:18662 1:N:0:TCTGCAAG+GCTGCACT
CTGTGAGGAAGAAACTTAGTGGACTCACAGTTTTTAAGGACACCACTGGTGGCAGTGTAGAGGACATG
ATGAAAGGAGCTTATTATTTAGTTTTTGCGACATTTCATCAATATGCGATGGAAGTTGCATTAAATGT
ACGAATGT
+
FFFFFFFFFFFFFFFFFFFFFFFFFFFFFFFFFFFFFFFFFFFFFFFFFFFFFFFFFFFFFFFFFFFFF
FFFFFFFFFFFFFFFFFFFFFFFFFFFFFFFFFFFFFFFFFFFFFFFFFFFFFFFFFFFFFFFFFFFFF
FF,FFFFF
@A00721:190:HGJW7DSXY:4:1339:31051:2284 1:N:0:TCTGCAAG+GCTGCACT
TCTGAACCACATTCGTACATTTAATGCAACTTCCATCGCATATTGATGAAATGTCGCAAAAACTAAAT
AATAAGCTCCTTTCATCATGTCCTCTACACTGCCACCAGTGGTGTCCTTAAAAACTGTGAGTCCACTA
AGTTTCTTCCTCACA
+
FFFFFFFFFFFFFFFFFFFFFFFFFFFFFFFFFFFFFFFFFFFFFFFFFFFFFFFFFFFFFFFFFFFFF
FFFFFFFFFFFFFFFFFFFFFFFFFFFFFFFFFFFFFFFFFFFFFFFFFFFFFFFFFFFFFFFFFFFFF
FFFFFFFFFFFFFFF

@A00721:190:HGJW7DSXY:4:1511:2401:25848 1:N:0:TCTGCAAG+GCTGCACT
TCTGAACCACATTCGTACATTTAATGCAACTTCCATCGCATATTGATGAAATGTCGCAAAAACTAAAT
AATAAGCTCCTTTCATCATGTCCTCTACACTGCCACCAGTGGTGTCCTTAAAAACTGTGAGTCCACTA
AGTTTCTTCCTCACA
+
FFFFFFFFFFFFFFFFFFFFFFFFFFFFFFFFFFFFFFFFFFFFFFFFFFFFFFFFFFFFFFFFFFFFFF
FFFFFFFFFFFFFFFFFFFFFFFFFFFFFFFFFFFFFFFFFFFFFFFFFFFFFFFFFFFFFFFFFFFFFF
FFFFFFFFFFFFFFF
@A00721:190:HGJW7DSXY:4:2309:24460:29763 1:N:0:TCTGCAAG+GCTGCACT
TCTGAACCACATTCGTACATTTAATGCAACTTCCATCGCATATTGATGAAATGTCGCAAAAACTAAAT
AATAAGCTCCTTTCATCATGTCCTCTACACTGCCACCAGTGGTGTCCTTAAAAACTGTGAGTCCACTA
AGTTTCTTCCTCACA
+
FFFFFFFFFFFFFFFFFFFFFFFFFFFFFFFFFFFFFFFFFFFFFFFFFFFFFFFFFFFFFFF:FFFFFF
FFFFFFFFFFFFFFFFFFFFFFFFFFFFFFFFFFFFFFFFFFF:FFFFFFFFFF:FFFFFFFFFFFFFFF
FFFF,FFF:FF:FFF
@A00721:190:HGJW7DSXY:4:1201:16297:23484 1:N:0:TCTGCAAG+GCTGCACT
CTCACAGTTTTTAAGGACACCACTGGTGGCAGTGTAGAGGACATGATGAAAGGAGCTTATTATTAGTT
TTTGCGACATTTCATCAATATGCGATGGAAGTTGCATTAAATGTACGAATGTGGTTCAGAAGTGTTTA
ATAAAATGATATATA
+
FFFFFFFFFFFFFFFFFFFFFFFFFFFFFFFFFFFFFFFFFFFFFFFFFFFFFFFFFFFFFFFFFFFFFF
FFFFFFFFFFFFFFFFFFFFFFFFFFFFFFFFFFFFFFFFFFFFFFFFFFFFFFFFFFFFFFFFFFFFFF
FFFFFFF:FFFFFFF
@A00721:190:HGJW7DSXY:4:1202:22516:15624 1:N:0:TCTGCAAG+GCTGCACT
CTCACAGTTTTTAAGGACACCACTGGTGGCAGTGTAGAGGACATGATGAAAGGAGCTTATTATTAGTT
TTTGCGACATTTCATCAATATGCGATGGAAGTTGCATTAAATGTACGAATGTGGTTCAGAAGTGTTTA
ATAAAATGATATATA
+
FFFFFFFFFFFFFFFFFFFFFFFFFFFFF:FFFFFFFFFFFFFFFFFFFFFFFFFFFFFFFFFFFFFFFF
FFFFFFFFFFFFFFFFFFFFFFFFFFFFFFFFFFFFFFFFFFFFFFFF:FFFFFFFFFFFFF:FFFFFFF
FFF:FFFFF:FFFFF
@A00721:190:HGJW7DSXY:4:1503:25554:15405 1:N:0:TCTGCAAG+GCTGCACT
CTCACAGTTTTTAAGGACACCACTGGTGGCAGTGTAGAGGACATGATGAAAGGAGCTTATTATTAGTT
TTTGCGACATTTCATCAATATGCGATGGAAGTTGCATTAAATGTACGAATGTGGTTCAGAAGTGTTTA
ATAAAATGATATATA
+
FFFFFFFFFFFFFFFFFFFFFFFFFFFFFFFFFFFFFFFFFFFFFFFFFFFFFFFFFFFFFFFFFFFFFF
FFFFFFFFFFFFFFFFFFFFFFFFFFFFFFF:FFFFFFFFFFFFFFFFFF:FFFFFFFFFFFFFFFFFFF
FFFFFFFFF:FFFFF
@A00721:190:HGJW7DSXY:4:1626:1750:28792 1:N:0:TCTGCAAG+GCTGCACT
CTCACAGTTTTTAAGGACACCACTGGTGGCAGTGTAGAGGACATGATGAAAGGAGCTTATTATTAGTT
TTTGCGACATTTCATCAATATGCGATGGAAGTTGCATTAAATGTACGAATGTGGTTCAGAAGTGTTTA
ATAAAATGATATATA
+
FFFFFFFFFFFFFFFFFFF:FF:F,FFF:FFFFFFFFFFFFFFFFFFFFFFFFFFFFFFFFFFFFFF:FF
FF:FFFFFFFFFFFFFFFFFFF:FFFFFFFFFFFFFFFFFFFFFFFFF:FFFFFFFF::FFFFFFFFFFF
FFFFFFF:FFFFFFF
@A00721:190:HGJW7DSXY:4:1650:20383:8547 1:N:0:TCTGCAAG+GCTGCACT
CTCACAGTTTTTAAGGACACCACTGGTGGCAGTGTAGAGGACATGATGAAAGGAGCTTATTATTAGTT
TTTGCGACATTTCATCAATATGCGATGGAAGTTGCATTAAATGTACGAATGTGGTTCAGAAGTGTTTA
ATAAAATGATATATA
+
FFFFFFFFFFFFFFFFFFFFFFFFFFFFFFFFFFFFFFFFFFF:FFFFFFFFFFFFFFFFFFFFFFFFFF

FFFFFFFFFFFFFFFFFFFFFFFFFFFFFFFFFFFFFFFFFFFFFFFFFFFFFFFFFFFFFFFFFFFFFF
FFFFFFFFFFFFFFF
@A00721:190:HGJW7DSXY:4:1674:25265:1376 1:N:0:TCTGCAAG+GCTGCACT
CTCACAGTTTTTAAGGACACCACTGGTGGCAGTGTAGAGGACATGATGAAAGGAGCTTATTATTAGTT
TTTGCGACATTTCATCAATATGCGATGGAAGTTGCATTAAATGTACGAATGTGGTTCAGAAGTGTTTA
ATAAAATGATATATA
+
FFFFFFFFFFFFFFFFFFFFFFFFFFFFFFFFFFFFFFFFFFFF:FFFFFFFFFFFF:FFFFFFFFFF
FFFFFFFFFFFFFFFFFFFFFFFFFFFFFFFFFFFFFF:FFFFFFFF:FFFFFFFFFFFF:FFFFFFFF
FFFFFFFFFFFFFFF
@A00721:190:HGJW7DSXY:4:2220:1741:36605 1:N:0:TCTGCAAG+GCTGCACT
CTCACAGTTTTTAAGGACACCACTGGTGGCAGTGTAGAGGACATGATGAAAGGAGCTTATTATTAGTT
TTTGCGACATTTCATCAATATGCGATGGAAGTTGCATTAAATGTACGAATGTGGTTCAGAAGTGTTTA
ATAAAATGATATATA
+
FFFFFFFFFFFFFFFFFFFFFFFFFFFFFFFFFFFFFFFFFFFFFFFFFFFFFFFFFFFFFFFFFFFFFF
FFFFFFFFFFFFFFFFFFFFFFFFFFFFFFFFFFFFFFFFFFFFFFFFFFFFFFFFFFFFFFFFFFFFFF
FFFFFFFFFFFFFFF
@A00721:190:HGJW7DSXY:4:2231:29776:35556 1:N:0:TCTGCAAG+GCTGCACT
CTCACAGTTTTTAAGGACACCACTGGTGGCAGTGTAGAGGACATGATGAAAGGAGCTTATTATTAGTT
TTTGCGACATTTCATCAATATGCGATGGAAGTTGCATTAAATGTACTAATGTGGTTCAGAAGTGTTTA
ATAAAATGATATATA
+
F:FFFFFFFFFFFFFFFFFFFFFFFF:FFFFF:FF::FFF,FFFF:FFFFFFFFFFFFFFFFFF:FFFF
FF:FFFF,FF:FFFFFFFFFFFF:FF:,FFF:,FFFF,FFFFFFF,,FFF:FFFFFF,FFFFFFF:FF
:FFFFF:F,:FFFFF
@A00721:190:HGJW7DSXY:4:1162:24903:32784 1:N:0:TCTGCAAG+GCTGCACT
GGACACCACTGGTGGCAGTGTAGAGGACATGATGAAAGGAGCTTATTATTTAGTTTTTGCGACATTTC
ATCAATATGCGATGGAAGTTGTATTAAATGTACGAATGTGGTTCAGAAGTGTTTAATAAAATGATATA
TATTCATTTTCCATT
+
FFFFFFFFFFFFFFFFFFFFFFFFFFFFFFFFFFFFFFFFFFFFFFFFFFFFFFFFF:FFFFFFFFFF
FFFFFFFFFFFFFFFFFFFFFFFFFFFFFFFFFFFFFFFFFFFF:FFFFFFFF:FFFFF:F,FFFF
FFFF:FFFFFFFFFF
@A00721:190:HGJW7DSXY:4:1234:29640:34162 1:N:0:TCTGCAAG+GCTGCACT
GGACACCACTGGTGGCAGTGTAGAGGACATGATGAAAGGAGCTTATTATTTAGTTTTTGCGACATTTC
ATCAATATGCGATGGAAGTTGTATTAAATGTACGAATGTGGTTCAGAAGTGTTTAATAAAATGATATA
TATTCATTTTCCATT
+
FFFFFFFFFFFFFFFFFFFFFFFFFFFFFFFFFFFFFFFFFFFFFFFFFFFFFFFFFFFFFFFFFFFFFF
FFFFFFFFFFFFFFFFFFFFFFFFFFFFFFFFFFFFFF:F,FFFFFFFFFFFFFFFFFFFFFFFFFFFF
FFFFFFFFFFFFFFF
@A00721:190:HGJW7DSXY:4:1252:24768:14951 1:N:0:TCTGCAAG+GCTGCACT
GGACACCACTGGTGGCAGTGTAGAGGACATGATGAAAGGAGCTTATTATTTAGTTTTTGCGACATTTC
ATCAATATGCGATGGAAGTTGTATTAAATGTACGAATGTGGTTCAGAAGTGTTTAATAAAATGATATA
TATTCATTTTCCATT
+
FFFFFFFFFFFFFFFFFFFFFFFFFFFFFFFFFFFFFFFFFFFFFFFFFFFFFFFFFFFFFFFFFFFFFF
FFFFFFFFFFFFFFFFFFFFFFFF,FFF:FFFFFFFFFFFFFFFFFF,FFFFFFFFFFFFFFFFFFFFF:F:
FFFFFFFFFFFFFFF
@A00721:190:HGJW7DSXY:4:1252:24686:14967 1:N:0:TCTGCAAG+GCTGCACT
GGACACCACTGGTGGCAGTGTAGAGGACATGATGAAAGGAGCTTATTATTTAGTTTTTGCGACATTTC
ATCAATATGCGATGGAAGTTGTATTAAATGTACTAATGTGGTTCAGAAGTGTTTAATAAAATGATATA
TATTCATTTTCCA

+
F,FFF,:FF,::FF,FF::FFFFF,,FFFFF:F,,FFFFF::FFF,FF,:,F:FFFFF,F,F:F,FFF
F:,FFF,:FF:FFF,,FFFFFFF:FFF:F,FFF,:FFFFF,FFFF:FFFFF:F::FFFFFFF,FFFFF
FF,:,:FF:FFFF
@A00721:190:HGJW7DSXY:4:1254:24017:32659 1:N:0:TCTGCAAG+GCTGCACT
GGACACCACTGGTGGCAGTGTAGAGGACATGATGAAAGGAGCTTATTATTTAGTTTTTGCGACATTTC
ATCAATATGCGATGGAAGTTGTAGTAAATGTACGAATGTGGTTCAGAAGTGTTTAATAAAATGATATA
TATTCATTTTCCATT
+
FFFFFFFF:FFFFFFFFFFFFF,FFFFFF,FFFFFF:FFF:FFFFFFFFFFFFF,F:FFFFFFFFFFF
:FFFFFFF:,FFFFFFFFFF:FF,F:FFFFFFFFFFFF,F::F,F:FFF:FF:FFFFFFFFFFFFFFF
FFFFFFFFFF::FFF
@A00721:190:HGJW7DSXY:4:1303:30626:30201 1:N:0:TCTGCAAG+GCTGCACT
GGACACCACTGGTGGCAGTGTAGAGGACATGATGAAAGGAGCTTATTATTTAGTTTTTGCGACATTTC
ATCAATATGCGATGGAAGTTGTATTAAATGTACGAATGTGGTTCAGAAGTGTTTAATAAAATGATATA
TATTCATTTTCCATT
+
FFFFFFFFFFFFFFFFFFFFF:FFFFFFFFFFFFFFFFFFFFFFFFFFFFFFFFFFFFFFFFFFFFFF
FFFFFFFFFFFF:FFFFF:FFF,FFF,FFFFFFFFFFFFFFFFFFFFFFFFFFFFFFFFFFFFFFFFF
FFFFFFFFFFFFFFF
@A00721:190:HGJW7DSXY:4:1529:32163:4586 1:N:0:TCTGCAAG+GCTGCACT
GGACACCACTGGTGGCAGTGTAGAGGACATGATGAAAGGAGCTTATTATTTAGTTTTTGCGACATTTC
ATCAATATGCGATGGAAGTTGTATTAAATGTACGAATGTGGTTCAGAAGTGTTTAATAAAATGATATA
TATTCATTTTCCATT
+
FFFFFFFFF,FFFFFFFFFFFFFFFFFFFFFFFFFFFFFFFFFFF:FFFFFFFFFFFFFFFFFFFFFF
FFFFFFFFFFF::FFF:FFFFFF:F:F:FFFFFFFFFFFFFFFFFFFFFFFFFFF:FFF:FFFFFFF,
FFFFFFFFFFFFF,F
@A00721:190:HGJW7DSXY:4:1550:18105:29465 1:N:0:TCTGCAAG+GCTGCACT
GGACACCACTGGTGGCAGTGTAGAGGACATGATGAAAGGAGCTTATTATTTAGTTTTTGCGACATTTC
ATCAATATGCGATGGAAGTTGTATTAAATGTACGAATGTGGTTCAGAAGTGTTTAATAAAATGATATA
TATTCATTTTCCATT
+
FFFFFFFFFFFFFFFFFFFFFFFFFFFFFFFFFFFF:FFFFFFFF:FFFFFFFFFFFFFFFFFFF:FF
FFFFFF:FFFFFFFFFFFFFFFFF:FFFFFFFFFF,FFFFFFFFFFFFFFFFFFFFFFFFFFF:FFF
:FFFFFFFFFFFFFF
@A00721:190:HGJW7DSXY:4:1551:29333:14779 1:N:0:TCTGCAAG+GCTGCACT
GGACACCACTGGTGGCAGTGTAGAGGACATGATGAAAGGAGCTTATTATTTAGTTTTTGCGACATTTC
ATCAATATGCGATGGAAGTTGTATTAAATGTACGAATGTGGTTCAGAAGTGTTTAATAAAATGATATA
TATTCATTTTCCATT
+
FFFFFFFFF,:F:FFFFFFFFFFFFFFF:FFF:FFFFFFFFFFFFFFFFFFFF,FFFFFF:FFFFFFF
FFFFFFFF:FFFFFFFFF:FFF:FFFF:FFFFFFFFFFFF:FFFFF:FF:F,FFFF:FFFFFF:FF:F
FFF,FF:FFF:FFFF
@A00721:190:HGJW7DSXY:4:1578:23104:1736 1:N:0:TCTGCAAG+GCTGCACT
GGACACCACTGGTGGCAGTGTAGAGGACATGATGAAAGGAGCTTATTATTTAGTTTTTGCGACATTTC
ATCAATATGCGATGGAAGTTGTATTAAATGTACGAATGTGGTTCAGAAGTGTTTAATAAAATGATATA
TATTCATTTTCCATT
+
FF:FFFFFFFFFFFFFFFFF:FFFFFFFFFFFFF,FFFFFFFFFFFFFFFFFFFFFFFFFFFFFFFFF
FFFFFFFFFFFFFFFFFF:FF,:FFF:FFFFFFFFFFFFFFFFFFFFFFFFFFFFF:FFFFFFFF:FFF
FFFFFFFFFFFFFFF
@A00721:190:HGJW7DSXY:4:2647:12997:10066 1:N:0:TCTGCAAG+GCTGCACT
GGACACCACTGGTGGCAGTGTAGAGGACATGATGAAAGGAGCTTATTATTTAGTTTTTGCGACATTTC

ATCAATATGCGATGGAAGTTGTATTAAATGTACGAATGTGGTTCAGAAGTGTTTAATAAAATGATATA
TATTCATTTTCCATT
+
FFFFFFFFFFFFFFFFFFFFFFFFFFFFFFFF:FFFFFFFFFFFFFFFFFFFFFFF:FFFFFFFFFFF
FFFFFFFFFFFFFFFFFFFFFFFFFFFFFFFFFFFFFFFFFFFF:FFFFFFFFFFFFFFFFFFFFFFF
FFFFFFFFFFFFFFF
@A00721:190:HGJW7DSXY:4:1246:21811:10614 1:N:0:TCTGCAAG+GCTGCACT
CAGCTTATTATTTAGTTTTTGCGACATTTCATCAATATGCGATGGAAGTTGCATTAAATGTACGAATG
TGGTTCAGAAGTGTTTAATAAAATGATATATATTCATTTTCCATTCGATAATCTTTGATTACATGATC
TAGATTTTCCATTGC
+
FFFFFFFFFFFFFFFFFFFFFFFFFFFFFFFFFFFFFFFFFFFFFF:FFFFFFFFFFFFFFFFFFFFF
FFFFFFFFFFFFF:FFFFFFFFFFFFFFFFFFFFFFFFFFFFFFFFFFFFFF:FFFFFFFFFFFFFFF
FFFFFFFFFFFFFF:
@A00721:190:HGJW7DSXY:4:1505:24198:24017 1:N:0:TCTGCAAG+GCTGCACT
CAGCTTATTATTTAGTTTTTGCGACATTTCATCAATATGCGATGGAAGTTGCATTAAATGTACGAATG
TGGTTCAGAAGTGTTTAATAAAATGATATATATTCATTTTCCATTCGATAATCTTTGATTACATGATC
TAGATTTTCCATTGC
+
FFFFFFFFFFFFFFFFFFFFFFFFFFFFFFFFFFFFFFFFFFFFFFFFFFFFFFFFFFFFFFFFFFFF
FFFFFFFFFFFFFFFFFFFFFFFFFFFFF:FFFFFFFFFFFFFFFFFFFFFFFFFFFFFFFFFFFFFF
FFFFFFFFFFFFFF:
@A00721:190:HGJW7DSXY:4:2248:23936:33583 1:N:0:TCTGCAAG+GCTGCACT
CAGCTTATTATTTAGTTTTTGCGACATTTCATCAATATGCGATGGAAGTTGCATTAAATGTACGAATG
TGGTTCAGAAGTGTTTAATAAAATGATATATATTCATTTTCCATTCGATAATCTTTGATTACATGATC
TAGATTTTCCATTGC
+
FFFFFFFFFFFFFFFFFFFFFFFFFFFFFFFFFFFFFFFFFFFFFFFFFFFFFFFFFFFFFFFFFFFF
FFFFFFFFFFFFFFFFFFFFFFFFFFFFFFFFFFFFFFFFFFFFFFFFFFFFF:FFFFFFFFFFFFFF
FFFFFFFFFFFFFFF
@A00721:190:HGJW7DSXY:4:2264:21893:30984 1:N:0:TCTGCAAG+GCTGCACT
CAGCTTATTATTTAGTTTTTGCGACATTTCATCAATATGCGATGGAAGTTGCATTAAATGTACGAATG
TGGTTCAGAAGTGTTTAATAAAATGATATATATTCATTTTCCATTCGATAATCTTTGATTACATGATC
TAGATTTTCCATTGC
+
FFFFFFFFFFFFFFFFFFFFFFFFFFFFFFFFFFFFFFFFFFF:FFFFFFF:FFFFFFFFFFFFFFFF
FFFFFFFFFFFFFFFFFFFFFFFFFFFFFFFFFFFFFFFFFFFFFFFFFFFFFFFFFFFFFFFFFFFF
FFFFFFFFFFFFFFF
@A00721:190:HGJW7DSXY:4:2270:9625:22263 1:N:0:TCTGCAAG+GCTGCACT
CAGCTTATTATTTAGTTTTTGCGACATTTCATCAATATGCGATGGAAGTTGCATTAAATGTACGAATG
TGGTTCAGAAGTGTTTAATAAAATGATATATATTCATTTTCCATTCGATAATCTTTGATTACATGATC
TAGATTTTCCATTGC
+
FFFFFFFFFFFFFFFFFFFFFFFFFFFFFFFFFFFFFFFFFFFFFFFFFFFFFFFFFFFFFFFFFFFF
FFFFFFFFFFFFFFFFFFFFFFFFFFFFFFFFFFFFFFFFFFFFFF:FFFFFFFFFFFFFFFFFFFFF
FFFFFFFFFFFFFF:
@A00721:190:HGJW7DSXY:4:1271:25681:32503 1:N:0:TCTGCAAG+GCTGCACT
AGGGGTAATTTCAATTTATAATTTTTGTTATGCGTGGCATAAAGCAATGGAAAATCTAGATCATGTAA
TCAAAGATTATCGAATGGAAAATGAATATATATCATTTTATTAAACACTTCTGAACCACATTCGTACA
TTTAATGCAACTTCC
+
FFFFFFF:FFFFFFFFFFFFFF:FFFFFFFFFFFFFFFFFFFFF:FFFFFFFFFFFFFF,FFFFFFFF
FFFFF:FFFFF:FFFFFFFFFFFFFFFFFFFFFFFFFFFFFFFFFFFFFFFFFFFFFFFFFFF:FF,F
FFFFF:FFFFFFFF:

```
@A00721:190:HGJW7DSXY:4:1349:32280:10207 1:N:0:TCTGCAAG+GCTGCACT
AGGGGTAATTTCAATTTATAATTTTTGTTATGCGTGGCATAAAGCAATGGAAAATCTAGATCATGTAA
TCAAAGATTATCGAATGGAAAATGAATATATATCATTTTATTAAACACTTCTGAACCACATTCGTACA
TTTAATGCAACTTCC
+
FFFFFFFFFFFFFFFFFFFFFFFFFFFFFFFFFFFFFFFFFFFFFFFFFFFFF:FFFFF:FFFFFFFF
FFFF:FFFFFFFFFFFFFFFFFFFFFFFFFFFFFFFFFFFFFFF:FFFFFFFFFFFFFFFFFFFFFFF
FFFFFFFFFFFFFFF
@A00721:190:HGJW7DSXY:4:1364:26395:20588 1:N:0:TCTGCAAG+GCTGCACT
AGGGGTAATTTCAATTTATAATTTTTGTTATGCGTGGCATAAAGCAATGGAAAATCTAGATCATGTAA
TCAAAGATTATCGAATGGAAAATGAATATATATCATTTTATTAAACACTTCTGAACCACATTCGTACA
TTTAATGCAACTTCC
+
FFFFFFFFF:FFFFFFFFFFFFFFFFFFFFFFFFFFFFFFFFFFFFFFFFF,FFFFFFFFFFFFF:FF
FFFF:FFFFFFFFFFFFFFFFFFFFFFFFFFFFFFFFFFFFFFF:FFFFFFFFFFFFFFFFFFFFFFF
FFFFFFFFFFFFFF:
@A00721:190:HGJW7DSXY:4:1367:32452:31015 1:N:0:TCTGCAAG+GCTGCACT
AGGGGTAATTTCAATTTATAATTTTTGTTATGCGTGGCATAAAGCAATGGAAAATCTAGATCATGTAA
TCAAAGATTATCGAATGGAAAATGAATATATATCATTTTATTAAACACTTCTGAACCACATTCGTACA
TTTAATGCAACTTCC
+
FFFFFFFFFFFFFFFFFFFFFFFFFFFFFFFFFFFFFFFFFFFFFFFFFFFFFFFFF:F,FFFFF:FF
FFFFFF,FFFFFFFFFFFFFF:FFFFFFFFFFFFFFFFFFFFF:,FFFFFFFFFFFFFFF:FF:FF:F
F:FFFFFFFFFFF:F
@A00721:190:HGJW7DSXY:4:2137:1976:31814 1:N:0:TCTGCAAG+GCTGCACT
AGGGGTAATTTCAATTTATAATTTTTGTTATGCGTGGCATAAAGCAATGGAAAATCTAGATCATGTAA
TCAAAGATTATCGAATGGAAAATGAATATATATCATTTTATTAAACACTTCTGAACCACATTCGTACA
TTTAATGCAACTTCC
+
FFFFFFFFFFFFFFFFFFFFFFFFFFFFFFFFFFFFFFFFFFFFFFFFFFFFFFFFFFFFFFFFFFFF
FFFFFFFFFFFFFFFFFFFFFFFFFFFFFFFFFFFFFFFFFFFF:FFFFF:FFFFF:FFFFFFFFFFF
FFFFFF,FFFFFFFF
@A00721:190:HGJW7DSXY:4:2250:5367:17926 1:N:0:TCTGCAAG+GCTGCACT
AGGGGTAATTTCAATTTATAATTTTTGTTATGCGTGGCATAAAGCAATGGAAAATCTAGATCATGTAA
TCAAAGATTATCGAATGGAAAATGAATATATATCATTTTATTAAACACTTCTGAACCACATTCGTACA
TTTAATGCAACTTCC
+
FFFFFFFFFFFFFF:FFFFFFFFFFFFFFFFFFFFFFFFFFFFFFFFFFFFFFFFFFFF:FFFFFFFF
FFFFFFFFFFFFFFFFF:FFFFFFFFFF:FFFFFFFFFFFFFFF:FF:FFFFFFFFFFFFFFFFFFFF
:FFFFF:FFF:FFFF
@A00721:190:HGJW7DSXY:4:2328:4182:5979 1:N:0:TCTGCAAG+GCTGCACT
AGGGGTAATTTCAATTTATAATTTTTGTTATGCGTGGCATAAAGCAATGGAAAATCTAGATCATGTAA
TCAAAGATTATCGAATGGAAAATGAATATATATCATTTTATTAAACACTTCTGAACCACATTCGTACA
TTTAATGCAACTTC
+
FFFF,FFFFFFFFF,FFF,:FFFFFFFF:FF:FFFFFFFFFFFFFFFFFFFFFFFFF,F:FFFFFFFF
FFF,F:FFFFFFFFFFF:FFFFFFFFFFFFFFFF:FF,FFFFF::FFFF,FF,,F,FFFFFFFFFFFF
FFFF,::FFFFFFF
@A00721:190:HGJW7DSXY:4:2328:3721:6339 1:N:0:TCTGCAAG+GCTGCACT
AGGGGTAATTTCAATTTATAATTTTTGTTATGCGTGGCATAAAGCAATGGAAAATCTATATCATGAAA
TCAAAGATTATCGAATGGAAAATGAATATATATCATTTTATTAAAAACTTCTGAAACACATTCTTACA
ATTAATGCAACTTC
+
FF,:,F:F:F:FFFFFFFFFFFF:FF,FFFF:,FF,FF,FFFF,:FFF,FFFFF:FFF,,:FF,F,FF
```

```
FFFF::,F,FFFFFFF,:FFF,F:,FFFFFF:F:F:FF:FF,F,FFF:F,FF:F,,FF,FFFF,FFFF
,FFFF,,,FFFFFF
@A00721:190:HGJW7DSXY:4:2615:23104:3709 1:N:0:TCTGCAAG+GCTGCACT
AGGGGTAATTTCAATTTATAATTTTTGTTATGCGTGGCATAAAGCAATGGAAAATCTAGATCATGTAA
TCAAAGATTATCGAATGGAAAATGAATATATATCATTTTATTAAACACTTCTGAACCACATTCGTACA
TTTAATGCAACTTC
+
FF:FFFFF:,FF,FFF:FFFFFFF:FFF,F:FF,FFFFFFFFF,FFF:FFFFFFFFFF,,FFFF::FF
FFF,FF:F,FF,FFFFF,FF:FF:FFFFFFFFFFFF,FFFFFFFFFF:FFFF:FF:,FFFF:FFFF,F
F,,FFFF,FF:FFF
@A00721:190:HGJW7DSXY:4:1441:19144:22404 1:N:0:TCTGCAAG+GCTGCACT
GTACGAATGTGGTTCAGAAGTGTTTAATAAAATGATATATATTCATTTTCCATTCGATAATCTTTGAT
TACATGATCTAGATTTTCCATTGCTTTATGCCACGCATAACAAAAATTATAAATTGAAATTACCCCTA
AATTACAAAGATTTT
+
FFFFFFFFFFFFFFFFFFFFFFFFFFFFFFFFFFFFFFFFFFFFFFFFF:FFFFFFFFFFFFFFFFFF
FFFFFFFFFFFFFFFFFFFFFFFFFFFFFFFFFFFFFFFFFFFFFFFFFFFFFFFFFFFFFFFFFFFF
FFFFFFFFFFFFFFF
@A00721:190:HGJW7DSXY:4:1623:32271:15139 1:N:0:TCTGCAAG+GCTGCACT
GTACGAATGTGGTTCAGAAGTGTTTAATAAAATGATATATATTCATTTTCCATTCGATAATCTTTGAT
TACATGATCTAGATTTTCCATTGCTTTATGCCACGCATAACAAAAATTATAAATTGAAATTACCCCTA
AATTACAAAGATTTT
+
FFFFFFFFFFFFFFFFFFFFFFFFFFFFFFFFFFFFFFFFFFFFFFFFFFFFF:FFFFFFFFFFFFFF
F:FFFFFFFFFFFFFFFFFFFFFFFFFFFFFFFFF:FFFFFFFFFFFFFFFFFFFFFFFFFFFF:FFFF
FFFFFFFFFFFFFFF
@A00721:190:HGJW7DSXY:4:1642:15582:24189 1:N:0:TCTGCAAG+GCTGCACT
GTACGAATGTGGTTCAGAAGTGTTTAATAAAATGATATATATTCATTTTCCATTCGATAATCTTTGAT
TACATGATCTAGATTTTCCATTGCTTTATGCCACGCATAACAAAAATTATAAATTGAAATTACCCCTA
AATTACAAAGATTTT
+
FFFFFFFFFFFFFFFFFF:FFFFFFFFFFFFFFFFFFFFFFFFFFFFFFFFFFFFFFFFFFFFFFFFF
FFFFFFFFFFFFFFFFFFFFFFFFFFFFFFFFFFFFFF:FFFFFF:FFFFFFFFFFFFFFFFFFFFFFF
FFFFFFFFFFFFFFF
@A00721:190:HGJW7DSXY:4:1645:5611:23703 1:N:0:TCTGCAAG+GCTGCACT
GTACGAATGTGGTTCAGAAGTGTTTAATAAAATGATATATATTCATTTTCCATTCGATAATCTTTGAT
TACATGATCTAGATTTTCCATTGCTTTATGCCACGCATAACAAAAATTATAAATTGAAATTACCCCTA
AATTACAAAGATTTT
+
FFFFFFFFFFFFFFFFFFFFFFFFFFFFFFFFFFFFFFFFFFFFFFFFFFFFFFFFFFFFFFFFFFFF
F:FFFFFFFFFFFFFFFFFFFFFFFFFFFFFFFFFFFFFFFFFFFFFFFFFFFF:FFFFFFFFFF,
FFFFFFFFFFFF:FF
@A00721:190:HGJW7DSXY:4:2343:3974:9032 1:N:0:TCTGCAAG+GCTGCACT
GTACGAATGTGGTTCAGAAGTGTTTAATAAAATGATATATATTCATTTTCCATTCGATAATCTTTGAT
TACATGATCTAGATTTTCCATTGCTTTATGCCACGCATAACAAAAATTATAAATTGAAATTACCCCTA
AATTACAAAGATTTT
+
FFFFFFFFFFFFFFFFFFFFFFFFFFFFFFFFFFFFFFFFFFFFFFFFFFFFFFFFFFFFFFFFFFFF
FFFFFFFFFFFFFFFFFFFFFFFFFFFFFFFFFFFFFFFFFFFFFFFFFFFFFFFFFFFFFFFFFFFF
FFFFFFFFF:FFFFF
@A00721:190:HGJW7DSXY:4:2358:21748:2049 1:N:0:TCTGCAAG+GCTGCACT
GTACGAATGTGGTTCAGAAGTGTTTAATAAAATGATATATATTCATTTTCCATTCGATAATCTTTGAT
TACATGATCTAGATTTTCCATTGCTTTATGCCACGCATAACAAAAATTATAAATTGAAATTACCCCTA
AATTACAAAGATTTT
```

+
FFFFFFFFFFFFFFFFFFFFFFFFFFFFFFFFFFFFFFFFFFFFFFFFFFFFFFFFFFFFFFFFFFFFFF
FFFFFFFFFFFFFFFFFFFFFFFFFFFFFFFFFFFFFFFFFFFFFFFFFFFFFFFFFFFFFFFFFFFFFF
FFFFFFFFF:FFFFF
@A00721:190:HGJW7DSXY:4:2554:18891:28385 1:N:0:TCTGCAAG+GCTGCACT
GTACGAATGTGGTTCAGAAGTGTTTAATAAAATGATATATATTCATTTTCCATTCGATAATCTTTGAT
TACATGATCTAGATTTTCCATTGCTTTATGCCACGCATAACAAAAATTATAAATTGAAATTACCCCTA
AATTACAAAGATTTT
+
FFFFFFFFFFFFFFFFFFFFFFFFFFFFFFFFFFFFFFFFFFFFFFFFFFFFFFFFFFFFFFFFFFFFFF
FFFFFFFFFFFFFFFFFFFFFFFFFFFFFFFFFFFFFFFFFFFFFFFFFFFFFFFFFFFFFFFFFFFFF:
FFFFFFFFFFFFFFF
@A00721:190:HGJW7DSXY:4:1264:26539:7780 1:N:0:TCTGCAAG+GCTGCACT
TGTGGTTCAGAAGTGTTTAATAAAATGATATATATTCATTTTCCATTCGATAATCTTTGATTACATGA
TCTAGATTTTCCATTGCTTTATGCCACGCATAACAAAAATTATAAATTGAAATTACCCCTAAATTACA
AAGATTTTTATGGAT
+
FFFFFFFFFFFFFFFFFFFFFFFFFFFFFFFFFFFFFFFFFFFFFFFFFFFFFFFFFFFFFFFFFFFFFF
FFFFFFFFFFFFFFFFFFF:FFFFFFFFFFFFFFFFFFF:FFFFFFFFFFFFFFFFFFFFFFFFFFFFFF
F:FFFFFFFFFFFFF
@A00721:190:HGJW7DSXY:4:1264:26250:7968 1:N:0:TCTGCAAG+GCTGCACT
TGTGGTTCAGAAGTGTTTAATAAAATGATATATATTCATTTTCCATTCGATAATCTTTGATTACATGA
TCTAGATTTTCCATTGCTTTATGCCACGCATAACAAAAATTATAAATTGAAATTACCCCTAAATTACA
AAGATTTTTATGGAT
+
FFFFFFFFFF:FFFFFFFFF:FFFFFFFFFFFFFFFFFFFFFFFFFFFFFFFFF:FFFFFFFFFFFF:FF
FFFFFFFFFFFFFFFFFFFFFFF:FFFFFFFFFFFFFFFFFFFFFFFFFFFFFFFFFFFFF,FFFFFFFF
:FFFF:F:FF:FF,F
@A00721:190:HGJW7DSXY:4:1329:32633:6903 1:N:0:TCTGCAAG+GCTGCACT
TGTGGTTCAGAAGTGTTTAATAAAATGATATATATTCATTTTCCATTCGATAATCTTTGATTACATGA
TCTAGATTTTCCATTGCTTTATGCCACGCATAACAAAAATTATAAATTGAAATTACCCCTAAATTACA
AAGATTTTTATGGAT
+
FFFFFFFFFFFFFFFFFFFFFFFFFFFFFFFFFFFFFFFFFFFFFF:FFFFFFFFFFF,FFFF:FFF:FF
FFFFFFFFFFFFFFFFFFFFFFFFFFFF:FFFFFFFFFF:FFFFFFFFFFFFFFFFFF:FFFFF,FFFFF
FF:FFFFFFFFFFFF
@A00721:190:HGJW7DSXY:4:1466:16740:24220 1:N:0:TCTGCAAG+GCTGCACT
TGTGGTTCAGAAGTGTTTAATAAAATGATATATATTCATTTTCCATTCGATAATCTTTGATTACATGA
TCTAGATTTTCCATTGCTTTATGCCACGCATAACAAAAATTATAAATTGAAATTACCCCTAAATTACA
AAGATTTTTATGGAT
+
FFFFFFFFFFFFFFFFFFFFFFFFFFFFFFFFFFFFFFFFFFFFFFFFFFFFFFFFFFFFFFFFFFFFFF
FFFFFFFFFFFFFFFFFFFFFFFFFFFFFFFFFFFFFFFFFFFFFFFFFFFFFFFFFFFFFF:FFFFF:F
FFFFFFFFFFFFFFF
@A00721:190:HGJW7DSXY:4:2508:13286:33959 1:N:0:TCTGCAAG+GCTGCACT
TGTGGTTCAGAAGTGTTTAATAAAATGATATATATTCATTTTCCATTCGATAATCTTTGATTACATGA
TCTAGATTTTCCATTGCTTTATGCCACGCATAACAAAAATTATAAATTGAAATTACCCCTAAATTACA
AAGATTTTTATGGAT
+
FFFFFFFFFFFFFFFFFFFFFFFFFFFFF:FFFFFFFFFFFFFFFFFFFFFFFFFFFFFFFFFFFFFFFF
FFFFFFF:FFFFFFFFFFFFFFF:FFFFFFF:FFFFFFFFFFFFFFFF:FFFFFFFF,FFFFFFFFFFFF
FF:FFF::FFFF,FF
@A00721:190:HGJW7DSXY:4:1265:21866:7670 1:N:0:TCTGCAAG+GCTGCACT
GTGGTTCAGAAGTGTTTAATAAAATGATATATATTCATTTTCCATTCGATAATCTTTGATTACATGAT

```
CTAGATTTTCCATTGCTTTATGCCACGCATAACAAAAATTATAAATTGAAATTACCCCTAAATTACAA
AGATTTTTATGGATA
+
FFFFFFFFF:FFFFFFFFFFFFFFFFFFFFFFFFFFFFFFFFFFFFFFFFFFFFFFFFFFFFFFFFFFFF
FFFFFFFFFFFFFFFFFFFFFFFFFFFFFFFFFFFFFFFFFFFFFFFFFFFFFFFFFFFFFFF:FFFFFF
FFFFFFFFFFFFFFF
@A00721:190:HGJW7DSXY:4:1320:16740:26349 1:N:0:TCTGCAAG+GCTGCACT
GTGGTTCAGAAGTGTTTAATAAAATGATATATATTCATTTTCCATTCGATAATCTTTGATTACATGAT
CTAGATTTTCCATTGCTTTATGCCACGCATAACAAAAATTATAAATTGAAATTACCCCTAAATTACAA
AGATTTTTATGGATA
+
FFFFFFFFFFFFFFFFFFFFFFFFFFFFFFFFFFFFFFFFFFFFFFFFFFFFFFFFFFFFFFFFFFFFFF
FFFFFFFFFFFFFFFFFFFFFFFFFFF:FFFFFFFFFFFFFFFFFFFFFFFFFFFFF:FFFFFFFFFFFF
FF:F:FFFFFF,FFF
@A00721:190:HGJW7DSXY:4:1667:8124:13839 1:N:0:TCTGCAAG+GCTGCACT
GTGGTTCAGAAGTGTTTAATAAAATGATATATATTCATTTTCCATTCGATAATCTTTGATTACATGAT
CTAGATTTTCCATTGCTTTATGCCACGCATAACAAAAATTATAAATTGAAATTACCCCTAAATTACAA
AGATTTTTATGGATA
+
FFFFFFFFFFFFFFFFFFFFFFFFFFFFFFFFFFFFFFFFFFFFFFFFFF::FFFFFFFFF:FFFFFFFF
FFFFFFFFFFFFFFFFFFFFFFFFFFFFFFFFFFFF:FFFFFFFFFF:FFFFFFFFFFFFFFFFFFFFFF
F:FFFFFFFFFFFFF
@A00721:190:HGJW7DSXY:4:2167:31937:19758 1:N:0:TCTGCAAG+GCTGCACT
GTGGTTCAGAAGTGTTTAATAAAATGATATATATTCATTTTCCATTCGATAATCTTTGATTACATGAT
CTAGATTTTCCATTGCTTTATGCCACGCATAACAAAAATTATAAATTGAAATTACCCCTAAATTACAA
AGATTTTTATGGATA
+
FFFFFFFFFFFFFFFFFFFFFFFFFFFFFFFFFFFFFFFFFFFFFFFFFFFFFFFF,FFFFFFFFFFFFF
FFFFFFFFFFFFFFFFFFFFFFFFFFFFFFFFFFFFFFFFFFFFFFFFFFFFFFFFF,FFFFFFFFFFFF
FF:FFFFF,FFFF,F
@A00721:190:HGJW7DSXY:4:2427:19678:22388 1:N:0:TCTGCAAG+GCTGCACT
GTGGTTCAGAAGTGTTTAATAAAATGATATATATTCATTTTCCATTCGATAATCTTTGATTACATGAT
CTAGATTTTCCATTGCTTTATGCCACGCATAACAAAAATTATAAATTGAAATTACCCCTAAATTACAA
AGATTTTTATGGATA
+
FFFFFFFFFFFFFFFFFFFFFFFFFFFF:FFFFFFFFFFFFFFFFFFFFFFFFFFFFFFFF,FFFFFFFF
FFFFFFFFFFFFFFFFFFFFFFFFFFFFFF:FFFFFFFFFFFFFFFFFFFFFFFFFFFFFF:FFFFFFFF
FFFFFFFFFF:FFFF
@A00721:190:HGJW7DSXY:4:2559:15085:3662 1:N:0:TCTGCAAG+GCTGCACT
GTGGTTCAGAAGTGTTTAATAAAATGATATATATTCATTTTCCATTCGATAATCTTTGATTACATGAT
CTAGATTTTCCATTGCTTTATGCCACGCATAACAAAAATTATAAATTGAAATTACCCCTAAATTACAA
AGATTTTTATGGATA
+
FFFFFFFFF:FFFFFFFFF:FFFFFFFFFFFFFFFFFFFFFFFFFFFFFFFFFFFFFFFFFFFFFFFFFF
FFFFFFFFFFFFFFFFFFFFFFFFFFFFFFFFFFFFFFFFFFFFFFFFFFFFFFFFFFFFFFFFFFFFFF
FFFFFFFFFFFFFFF
@A00721:190:HGJW7DSXY:4:2620:7500:29919 1:N:0:TCTGCAAG+GCTGCACT
GTGGTTCAGAAGTGTTTAATAAAATGATATATATTCATTTTCCATTCGATAATCTTTGATTACATGAT
CTAGATTTTCCATTGCTTTATGCCACGCATAACAAAAATTATAAATTGAAATTACCCCTAAATTACAA
AGATTTTTATGGATA
+
FFFFFFFFFFFFFFFFFFFFFFFFFFFFFFFFFFFFFFFFFFFFFFFFFFFFFFFFFFFFFFFFFFFFFF
FFFFFFFFFFFFFFFFFFFFFFFFFFFFFFFFFFFFFFFFFFFFFFFFFFFFFFF:F:FFFFFFFFFFFF
FFFFFFFFFF,:FFF
```

```
@A00721:190:HGJW7DSXY:4:2259:9760:25786 1:N:0:TCTGCAAG+GCTGCACT
TGGTTCAGAAGTGTTTAATAAAATGATATATATACATTATCCATTCGATAATCTTTGATTACATGATC
TAGATTTTCCAATGCTTTATGCCACGCATAACAAAAATTATAAATTGAAATTACCCCTAAATTACAAA
GATTATTATGGATA
+
F:,FF:FFF:,,F:,FFF,FFFF,FFFFFF:F:,FFF,,F,F,FFF:FFFF,F,,FFF,FFFFF,FFF
,F:F,F::FFF,,,:F,FFF,F:FF,FFFFFFF:FFFFFFFFFF,F:FFF,,FFFFFF:FF,,FFFFF
FFFF,,FF,F,F,F
@A00721:190:HGJW7DSXY:4:1213:20546:10551 1:N:0:TCTGCAAG+GCTGCACT
GTTCAGAAGTGTTTAATAAAATGATATATATTCATTTTCCATTCGATAATCTTTGATTACATGATCTA
GATTTTCCATTGCTTTATGCCACGCATAACAAAAATTATAAATTGAAATTACCCCTAAATTACAAAGA
TTTTTATGGATATAA
+
FFFFFFFFFFFFFFFFFFFFFFFF:FFFFFFFFFFFFFFFFFFFFFFFFFFFFFFFFFFFFFFFFFFF
FFFFFFFFFFFFFFFFFFFFFFFFFFFFFFFFFF:FFFFFFFFFFFFFFFFFFFFFFFFFFFFFFFFF
FFFFFFFFFFFFFFF
@A00721:190:HGJW7DSXY:4:1217:29550:5854 1:N:0:TCTGCAAG+GCTGCACT
GTTCAGAAGTGTTTAATAAAATGATATATATTCATTTTCCATTCGATAATCTTTGATTACATGATCTA
GATTTTCCATTGCTTTATGCCACGCATAACAAAAATTATAAATTGAAATTACCCCTAAATTACAAAGA
TTTTTATGGATATAA
+
FFFFFFFFFFFFFFFFFFFFFFFFFFFFFFFFFFFFFFFFFFFFFFFFFFF:FFFFFFFFFFFFFFFF
FFFFFFFFFFFFFFFFFFFFFFFFFFFFFFFFFFFFFFFFFFFFFFFFFFFFFFFFFFFFFFFFFFFF
FFFFFFFFFFF:FFF
@A00721:190:HGJW7DSXY:4:1236:26060:1094 1:N:0:TCTGCAAG+GCTGCACT
GTTCAGAAGTGTTTAATAAAATGATATATATTCATTTTCCATTCGATAATCTTTGATTACATGATCTA
GATTTTCCATTGCTTTATGCCACGCATAACAAAAATTATAAATTGAAATTACCCCTAAATTACAAAGA
TTTTTATGGATATAA
+
FFFFFFFFFFFFFFFFFFFFFFFFFFFFF:FFFFFFFFF:::FFFFFFFFFF:FFFFFFFFFFFFFFF
FFFF:FFFFFFFFFFFFFF:FFFFFF:FFFFFFFF,FFFFFFFFFFFFFFF:FFFFFFF,F:,FFF:F
FFFFF,FFFFFFFFF
@A00721:190:HGJW7DSXY:4:1308:25012:21073 1:N:0:TCTGCAAG+GCTGCACT
GTTCAGAAGTGTTTAATAAAATGATATATATTCATTTTCCATTCGATAATCTTTGATTACATGATCTA
GATTTTCCATTGCTTTATGCCACGCATAACAAAAATTATAAATTGAAATTACCCCTAAATTACAAAGA
TATTTATGGATATAA
+
F:FFF:FFF:FF,FF:FFF::FF,FFF,F:FF:FF:,FFFFF:F,FFFF:F::FFFFFF,FF,F,,F:
FF:F,FFFF:F:F,FFFF:FFF:FFFFFF::FF:FF,FFFFFFF:FFFF,F,F,F,FFFFFF,FF,,F
F,F:,F,,FF:FFFF
@A00721:190:HGJW7DSXY:4:1434:10230:33332 1:N:0:TCTGCAAG+GCTGCACT
GTTCAGAAGTGTTTAATAAAATGATATATATTCATTTTCCATTCGATAATCTTTGATTACATGATCTA
GATTTTCCATTGCTTTATGCCACGCATAACAAAAATTATAAATTGAAATTACCCCTAAATTACAAAGA
TTTTTATGGATATAA
+
FFFFFFFFFFFFFFFFFFFFFFFFFFFFFFFFFFFFFFFFFFFFFFFFFFFFFFFFFFFFFFFFFFFF
FFFFFFFFFFFFFFFFFFFFFFFFFFFFFFFFFFFFFFFFFFFFFFFFFFFFFFFFFFFFFFFFFF:F
FFFFFFF:FFFFFFF
@A00721:190:HGJW7DSXY:4:1437:3938:20932 1:N:0:TCTGCAAG+GCTGCACT
GTTCAGAAGTGTTTAATAAAATGATATATATTCATTTTCCATTCGATAATCTTTGATTACATGATCTA
GATTTTCCATTGCTTTATGCCACGCATAACAAAAATTATAAATTGAAATTACCCCTAAATTACAAAGA
TTTTTATGGATATAA
+
FFFFFFFFFFFFFFFFFFFFFFFFFFFFFFFFFFFFFFFFFFFFFFFFFFFFFFFFFFFFFFFFFFFF
```

FFFFFFFFFFFFFFFFFFFFFFFFFFFFFFFFFFFFFFFFFFFFFFFFFFFFFFFFFFFFFFFFFFFF,F
FFFFFFFFFF:FFFF
@A00721:190:HGJW7DSXY:4:1504:4345:31187 1:N:0:TCTGCAAG+GCTGCACT
GTTCAGAAGTGTTTAATAAAATGATATATATTCATTTTCCATTCGATAATCTTTGATTACATGATCTA
GATTTTCCATTGCTTTATGCCACGCATAACAAAAATTATAAATTGAAATTACCCCTAAATTACAAAGA
TTTTTATGGATATAA
+
:F:FFFFFFFFFFFFFFFFF:FFFFFF:FFFFFFFFFFFFFFFFFFFFFFFFFFFFFFFFFFFFFFFFF
FFFFFFFFFFFFFFFF:FFFFFFFFFFFFFFF,FFFFFFF:FFF:FFFFFF,FFFFFFFFFFFFF:FF
FFFFFFFFFFFFFFF
@A00721:190:HGJW7DSXY:4:1617:4047:11882 1:N:0:TCTGCAAG+GCTGCACT
GTTCAGAAGTGTTTAATAAAATGATATATATTCATTTTCCATTCGATAATCTTTGATTACATGATCTA
GATTTTCCATTGCTTTATGCCACGCATAACAAAAATTATAAATTGAAATTACCCCTAAATTACAAAGA
TTTTTATGGATATAA
+
FFFFFFFFFFFFFFFFFFFFFFFFFFFF:FFFFFFFFFFFFFFFFFFFFFFFF:FFFFFFFFFFFFFFF
FFFFFFFFFFFFFFFFFFFFFFFFFFFFFFFFFF:FFFFFFFFFFFFFFFFFFFFFFFFFFFFFFFFFF
FFFFFFF::FFFF:F
@A00721:190:HGJW7DSXY:4:2132:11514:10629 1:N:0:TCTGCAAG+GCTGCACT
GTTCAGAAGTGTTTAATAAAATGATATATATTCATTTTCCATTCGATAATCTTTGATTACATGATCTA
GATTTTCCATTGCTTTATGCCACGCATAACAAAAATTATAAATTGAAATTACCCCTAAATTACAAAGA
TTTTTATGGATATAA
+
FFFFFFFFFFFFFFFFFFFFFFFFFFFFFFFFFFFFFFFFFFFFFFFFFFFFFFFFFFFFFFFFFFFFF
FFFFFFFFFFFFFFFFFFFFFFFFFFFFFFFFFFFFFFFFFFFFFFFFFFFFFF:FFFFFFFFFFFFFF
FFFFFFFF,FFFFFF
@A00721:190:HGJW7DSXY:4:2440:28763:33990 1:N:0:TCTGCAAG+GCTGCACT
GTTCAGAAGTGTTTAATAAAATGATATATATTCATTTTCCATTCGATAATCTTTGATTACATGATCTA
GATTTTCCATTGCTTTATGCCACGCATAACAAAAATTATAAATTGAAATTACCCCTAAATTACAAAGA
TTTTTATGGATATAA
+
FFFFFFFFFFFFFFFFFFFFF:FFFFFFFFFFFFFFFFFFFFFFFFFFFFFFFFFFFFFFFFFFFFFFF
FFFFFFFFFFFFFFFFFFFFFFFFFFFFFFFFFFFFFFFFFFFFFFFFFFFF:FFFFFFFFFFFFFFFF
FFFFFFFF:FFFFFF
@A00721:190:HGJW7DSXY:4:2642:23213:18552 1:N:0:TCTGCAAG+GCTGCACT
GTTCAGAAGTGTTTAATAAAATGATATATATTCATTTTCCATTCGATAATCTTTGATTACATGATCTA
GATTTTCCATTGCTTTATGCCACGCATAACAAAAATTATAAATTGAAATTACCCCTAAATTACAAAGA
TTTTTATGGATATAA
+
FFFFFFFFFFFFFFFFFFFFF:FFFFFFFFFFFFFFFFFFFFFFFFFFFFFFFFFFFFFFFFFFFFFFF
FFFFFFFFFFFFFFFFFFFFFFFFFFFFF,FFFFFFFFFFFFFFFFFFFFFFF:FFFFFFFFFFFFFFFF
FFFFFF:::FFFFFF
@A00721:190:HGJW7DSXY:4:1106:20057:33943 1:N:0:TCTGCAAG+GCTGCACT
TTCAGAAGTGTTTAATAAAATGATATATATTCATTTTCCATTCGATAATCTTTGATTACATGATCTAG
ATTTTCCATTGCTTTATGCCACGCATAACAAAAATTATAAATTGAAATTACCCCTAAATTACAAAGAT
TTTTATGGATATAAA
+
FFFFFFFFFF:FFFFFFFFFFFFFFFFFFFFFFFFFFFFFFFFFFFFFFFFFFFFFFFFFFFFFFFFFF
FFFFFFFFFFFFFFFFFF:FFFFFFF:FFFFFFFFFFFFFFFF:FFFFFFFFFFF:FFFFFFFFFFFF
FFFFFFF,FFFFFFF
@A00721:190:HGJW7DSXY:4:1302:11433:34037 1:N:0:TCTGCAAG+GCTGCACT
TTCAGAAGTGTTTAATAAAATGATATATATTCATTTTCCATTCGATAATCTTTGATTACATGATCTAG
ATTTTCCATTGCTTTATGCCACGCATAACAAAAATTATAAATTGAAATTACCCCTAAATTACAAAGAT
TTTTATGGATATAAA

```
+
FFFFFFFFFFFFFFFFFFFFFFFFFFFFFFFF:FFFFFFFFFFFFFFFFFFF:FFFFFFFFFFF:FFFF
FFFFFFFFF:F:FFFFF:FFFF:FF,FFFFFFFFFFFFFFFFF:FFFFFFF:FF:FFFFFF,FFF,FF
FF:FFFF,FFFFFFF
@A00721:190:HGJW7DSXY:4:1425:29559:20494 1:N:0:TCTGCAAG+GCTGCACT
TTCAGAAGTGTTTAATAAAATGATATATATTCATTTTCCATTCGATAATCTTTGATTACATGATCTAG
ATTTTCCATTGCTTTATGCCACGCATAACAAAAATTATAAATTGAAATTACCCCTAAATTACAAAGAT
TTTTATGGATATAAA
+
FFFFFFFFFFFFFFFFFFFFFFFF:FFFFFFFFFFFFFFFFFFFFFFFFFFFFFFFFFFFFFFFFFFF
FFFFFFFFFFFFFFFFFFFFFFFFFFFFFFFFFFFFFF:FFFFFFFFFFFFFFFFFFFFFFFFFF:FFF
FFFFFFFFFFFF:FF
@A00721:190:HGJW7DSXY:4:1450:19352:22075 1:N:0:TCTGCAAG+GCTGCACT
TTCAGAAGTGTTTAATAAAATGATATATATTCATTTTCCATTCGATAATCTTTGATTACATGATCTAG
ATTTTCCATTGCTTTATGCCACGCATAACAAAAATTATAAATTGAAATTACCCCTAAATTACAAAGAT
TTTTATGGATATAAA
+
FFFFFFFFFF:,FFFFFFFFFFFFFFFFF:FFFFF:FFFFFFFFFFFFF:FFFFFFFFFFFFFFFFFF
F:FFFFFFF,FFFF:FF,FFF:FFFFFF:FFF:FF,FFFFFFFFFFFFFF:,FFF:FFFF,FFF:FF,
:FFFFFFFF:F,FFF
@A00721:190:HGJW7DSXY:4:1521:5204:8750 1:N:0:TCTGCAAG+GCTGCACT
TTCAGAAGTGTTTAATAAAATGATATATATTCATTTTCCATTCGATAATCTTTGATTACATGATCTAG
ATTTTCCATTGCTTTATGCCACGCATAACAAAAATTATAAATTGAAATTACCCCTAAATTACAAAGAT
TTTTATGGATATAAA
+
FFFFFFFFFFFFFFFFFFFFFFFFFFFFFFFFFFFFFFFFFFFFFFFFFFFFFFFFFFFFFFFFFFFF
FFFFFFFFFFFFFFFFFFFFFFFFFFFFFFFFFFFFFFFFFFFFFFFFFFFFFFF:FFFFFFFFFFFF
FFFFFFFFFFFFFFF
@A00721:190:HGJW7DSXY:4:2178:24433:18599 1:N:0:TCTGCAAG+GCTGCACT
TTCAGAAGTGTTTAATAAAATGATATATATTCATTTTCCATTCGATAATCTTTGATTACATGATCTAG
ATTTTCCATTGCTTTATGCCACGCATAACAAAAATTATAAATTGAAATTACCCCTAAATTACAAAGAT
TTTTATGGATATAAA
+
FFFFFFFFFFFFFFFFFFFFF:FF:FFFFFFFFFFFFFFFFFFFFFFFFFFFFFFFFFFFFFFFFFFF
FFFFFFF,FFFFFFFFFFFFFFFFFFFFFFFFFFFFFFFFFFFFFFFFF:FFFFFFFFFFFFF:::F
FFFFFFF:FFFFFFF
@A00721:190:HGJW7DSXY:4:2531:19578:2300 1:N:0:TCTGCAAG+GCTGCACT
TTCAGAAGTGTTTAATAAAATGATATATATTCATTTTCCATTCGATAATCTTTGATTACATGATCTAG
ATTTTCCATTGCTTTATGCCACGCATAACAAAAATTATAAATTGAAATTACCCCTAAATTACAAAGAT
TTTTATGGATATAAA
+
FFFFFFFFFFFFFFFFFFFFFFFFFFFFFFFFFFFFFFFFFFFFFFFFFFFFFFFFFFF:FFFFFFFF
FFFFFFFFFFFFFFFFFFFFFFFFFFFFFFFFFFFFFFFFFFFFFFFFFF::FFFFFFFFFFFFF:FF
FFFFFFFFFFFFFFF
@A00721:190:HGJW7DSXY:4:2533:29568:33849 1:N:0:TCTGCAAG+GCTGCACT
TTCAGAAGTGTTTAATAAAATGATATATATTCATTTTCCATTCGATAATCTTTGATTACATGATCTAG
ATTTTCCATTGCTTTATGCCACGCATAACAAAAATTATAAATTGAAATTACCCCTAAATTACAAAGAT
TTTTATGGATATAAA
+
FFFFFFFFFFFFFFFFFFFFFFFFFFFFFFFFFFFFFFFFFFFFFFFFFFFFFFFFFFFFFFFFFFFF
FFFFFFFFFFFFFFFFFFFFFFFFFFFFFFFFFFFFFFFFFFFFF:FFFFFFFFFFFFFFFFFFFFFFF
F:FFFFFFFFFFFFF
@A00721:190:HGJW7DSXY:4:2538:12075:23750 1:N:0:TCTGCAAG+GCTGCACT
TTCAGAAGTGTTTAATAAAATGATATATATTCATTTTCCATTCGATAATCTTTGATTACATGATCTAG
```

```
ATTTTCCATTGCTTTATGCCACGCATAACAAAAATTATAAATTGAAATTACCCCTAAATTACAAAGAT
TTTTATGGATATAAA
+
FFFFFFFFFFFFFFFFFFFFFFFFFFFFFFFFFFFFFFFFFFFFFFFFFFFFFFFFFFFFFFFFFFFF
FFFFFFFFFFFFFFFFFF:FFFFFFFFFFFFFFFF:FFFFFFFFFFFFFFF,FFFFFFFFFFFFF:FF
FFFFFF:,FFFFFFF
@A00721:190:HGJW7DSXY:4:1173:16803:32409 1:N:0:TCTGCAAG+GCTGCACT
CCCATTCGATAATCTTTGATTACATGATCTAGATTTTCCATTGCTTTATGCCACGCATAACAAAAATT
ATAAATTGAAATTACCCCTAAATTACAAAGATTTTTATGGATATAAAAATAAAGCCGTCGGGATAAAG
TCCCAGAAATCATCG
+
FFFFFFFFFFFFFFFFFFFFFFFF,:FFFFFFFFFFFFFFFFFFFFFFFFFFFFFFFFFFFFFFFFFF
FFFFFFFFFFFFFFFFFFFFFFFFFFFFF:FFFFFFFFFFFFFFFFFFFFFFFFF:FF:FFFFFFFFF:F
FFFFFFFFFFFFFFF
@A00721:190:HGJW7DSXY:4:1563:20546:32440 1:N:0:TCTGCAAG+GCTGCACT
CCCATTCGATAATCTTTGATTACATGATCTAGATTTTCCATTGCTTTATGCCACGCATAACAAAAATT
ATAAATTGAAATTACCCCTAAATTACAAAGATTTTTATGGATATAAAAATAAAGCCGTCGGGATAAAG
TCCCAGAAATCATCG
+
FFFFFFFFFFFFFFFFFFFFFFFFFFFFFFFFFFFFFFFFFFFFFFFFFFFFFFFFFFFFFFFFFFFF
FFFFFFFFFFFFFFFFFFFFFFFFFFFFFFFFFFFFFFFFFFFFF:FFFFFFFFFFFFFFFFFFFFFFF
FFFFFFFFFFFFFFF
@A00721:190:HGJW7DSXY:4:2131:23773:27915 1:N:0:TCTGCAAG+GCTGCACT
CCCATTCGATAATCTTTGATTACAGGATCTAGATTTTCCATTGCTTTATGCCACGCATAACAAAAATT
ATAAATTGAAATTACCCCTAAATTACAAAGATTTTTATGGATATAAAAATAAAGCCGTCGGGATAAAG
TCCCAGAAATCATCG
+
FFFFFFFFFFFFFFFFFFFFFFFFF,F:FFFFFFFFFFFFFFFFFFFFFFFFFFFFFFFFFFFFFFFFF
FFFFFFFF:FFFFFFFFFFFFFFFFFFFFFF:FFFFF:FFFFFFFFF:FFFFFF:FFF:FFFFFFFFFF
FFFFFFF:FFFFFFF
@A00721:190:HGJW7DSXY:4:2529:22896:9267 1:N:0:TCTGCAAG+GCTGCACT
CCCATTCGATAATCTTTGATTACATGATCTAGATTTTCCATTGCTTTATGCCACGCATAACAAAAATT
ATAAATTGAAATTACCCCTAAATTACAAAGATTTTTATGGATATAAAAATAAAGCCGTCGGGATAAAG
TCCCAGAAATCATCG
+
FFFFFFFFFFFFFFFFFFFFFFFF::FFFFFFFFFFFF:FFFFFFFFFFFFFFFFFFFFFFFFFFFFFFF
FFFFFFFFFFFFFFFFFFFFFFFF:FFFFFFF:FFFF,FFFFFFFFFFFFFFFF:FFFFF,FFFFFFF:
FFF:FFFF::FFFF:
@A00721:190:HGJW7DSXY:4:2529:22968:9267 1:N:0:TCTGCAAG+GCTGCACT
CCCATTCGATAATCTTTGATTACATGATCTAGATTTTCCATTGCTTTATGCCACGCATAACAAAAATT
ATAAATTGAAATTACCCCTAAATTACAAAGATTTTTATGGATATAAAAATAAAGCCGTCGGGATAAAG
TCCCAGAAATCATCG
+
FFFFFFFFFFFFFFFFFFFFFFFFFFFFFFFFFFFFFFFFFFFFFFFFFFFFFFFFFFFFFFFFFFFF
FFFFFFFFFFFFFFFFFFFFFFFFFFFFF:FFFFFFFFFFFFFFFFFFFFFFFFFFFFFFFFFFFFFFF
F:FFFFFF:FFFFFF
@A00721:190:HGJW7DSXY:4:2633:7319:6965 1:N:0:TCTGCAAG+GCTGCACT
CCCATTCGATAATCTTTGATTACATGATCTAGATTTTCCATTGCTTTATGCCACGCATAACAAAAATT
ATAAATTGAAATTACCCCTAAATTACAAAGATTTTTATGGATATAAAAATAAAGCCGTCGGGATAAAG
TCCCAGAAATCATCG
+
FFFFFFFFFFFFFFFFFFFFFFFFFFFFFFFFFFFFFFFFFFFFFFFFFFFFFFFFFFFFFFFFFFFF
FFFFFFFFFFFFFFF,FFFFFFFFFFFFF:FFFFFFFFFFFFFFFFF:FFFFFFF:FFFFFFFFFFFFF
FFFFFFFFFFFFFFF
```

```
@A00721:190:HGJW7DSXY:4:1345:30789:9659 1:N:0:TCTGCAAG+GCTGCACT
CCATTCGATAATCTTTGATTACATGATCTAGATTTTCCATTGCTTTATGCCACGCATAACAAAAATTA
TAAATTGAAATTACCCCTAAATTACAAAGATTTTTATGGATATAAAAATAAAGCCGTCGGGATAAAGT
CCCAGAAATCATCGT
+
FF,FFFFFFFFFFFFFFFFFFFFFFFFFFFFFFFFFFFFFFFFFFFFFFFFFFFFFFFFFFFFFFFFFFF
FFFFFFFFFFFFF:FFFFFFFFFFFFFFFFFFFFFFFFFFFFFFFFFFFFFFFFFF:FFFFFFFFFFF:F
FFFFFFF:FFFFFFF
@A00721:190:HGJW7DSXY:4:1529:23737:22748 1:N:0:TCTGCAAG+GCTGCACT
CCATTCGATAATCTTTGATTACATGATCTAGATTTTCCATTGCTTTATGCCACGCATAACAAAAATTA
TAAATTGAAATTACCCCTAAATTACAAAGATTTTTATGGATATAAAAATAAAGCCGTCGGGATAAAGT
CCCAGAAATCATCGT
+
FFFFFFFFFFFFFFFFFFFFFFFFFFFFFFFFFFFFFFFFFFFFFFFFFFFFFFFFFFFFFFFFFFFFFF
FFFFFFFFFFFFFFFFFFFFFFFFFFFFFFFF:FFFFFFFFFFFFFFFFFFFFF,FFFF:FFFFFFFFFF
FFFFFFFFFFFFF::
@A00721:190:HGJW7DSXY:4:1553:5113:30326 1:N:0:TCTGCAAG+GCTGCACT
CCATTCGATAATCTTTGATTACATGATCTAGATTTTCCATTGCTTTATGCCACGCATAACAAAAATTA
TAAATTGAAATTACCCCTAAATTACAAAGATTTTTATGGATATAAAAATAAAGCCGTCGGGATAAAGT
CCCAGAAATCATCGT
+
FFFFFFFFFFFFFFFFFFF:FFFFFFFFFFFFFFFFFFFFFFFFFFFFFFFFFFFFFFFFFFFFFFFFFF
FFFFFFFFFFFFFF:FFFFFFFFFFFFFFFFFFFFFFFFFFFFF:FFFFFFFFFFFFFFFFFFFFFFFFF
FFFFFFFFFFFFF:F
@A00721:190:HGJW7DSXY:4:1578:10945:30530 1:N:0:TCTGCAAG+GCTGCACT
CCATTCGATAATCTTTGATTACATGATCTAGATTTTCCATTGCTTTATGCCACGCATAACAAAAATTA
TAAATTGAAATTACCCCTAAATTACAAAGATTTTTATGGATATAAAAATAAAGCCGTCGGGATAAAGT
CCCAGAAATCATCGT
+
FFFFFFFFFFFFFFFFFFFFFFFFFFFFFFFFFFFFFFFFFFFFFFFFFFFFFFFFFFFFFFFFFFFFFF
FFFFFFFFFFFFFFFFFFFFFFFFFFFFFFFFFFFFFFFFFFFFFFFFFFFFFFFFFFFFFFFFFFFFFF
FFFFFFFFFFFFFFF
@A00721:190:HGJW7DSXY:4:2262:20166:19100 1:N:0:TCTGCAAG+GCTGCACT
CCATTCGATAATCTTTGATTACATGATCTAGATTTTCCATTGCTTTATGCCACGCATAACAAAAATTA
TAAATTGAAATTACCCCTAAATTACAAAGATTTTTATGGATATAAAAATAAAGCCGTCGGGATAAAGT
CCCAGAAATCATCGT
+
FFFFFFFFFFFFFFFFFFFFFFFFFFFFFFFFFFFFFFFFFFFFFFFFFFFFFFFFFFFFFFFFFFFFFF
FFFFFFFFFFFFFFFFFFFFFFFFFFFFFFFFFFFFFFFFFFFFFFFFFFFFFFFFFFFFFFFFFFFFF:
FFFFFFFFFFFFF:F
@A00721:190:HGJW7DSXY:4:2553:3992:36432 1:N:0:TCTGCAAG+GCTGCACT
CCATTCGATAATCTTTGATTACATGATCTAGATTTTCCATTGCTTTATGCCACGCATAACAAAAATTA
TAAATTGAAATTACCCCTAAATTACAAAGATTTTTATGGATATAAAAATAAAGCCGTCGGGATAAAGT
CCCAGAAATCATCGT
+
FFFFFFFFFFFFFFFFFFFFFFFFFFFFFFFFFFFFFFFFFFFFFFFFFFFFFFFFFFFFFFFFFFFFFF
FFFFFFFFFFFFFFFFFFFFFFFFFFFFFFFFFFFFFFFFFFF:FFFFFFFFFFFFFFFFFFFFFFFFFF
FFFFFFFFFFFFFFF
@A00721:190:HGJW7DSXY:4:2567:2483:16501 1:N:0:TCTGCAAG+GCTGCACT
CCATTCGATAATCTTTGATTACATGATCTAGATTTTCCATTGCTTTATGCCACGCATAACAAAAATTA
TAAATTGAAATTACCCCTAAATTACAAAGATTTTTATGGATATAAAAATAAAGCCGTCGGGATAAAGT
CCCAGAAATCATCGT
+
FFFFFFFFFFFFFFFFFFFFFFFFFFFFFFFFFFFFFFFFFFFFFFFFFFFFFFFFFFFFFFFFFFFFFF
```

```
FFFFFFFFFFFFFFFFFFFFFFFFFFFF:FFFFFFFFFFFFFFFFFFFFFFFFFFFFFFFFFFFFFFFFF
FFFFFFFFFFFFFFF
@A00721:190:HGJW7DSXY:4:2607:25843:31469 1:N:0:TCTGCAAG+GCTGCACT
CCATTCGATAATCTTTGATTACATGATCTAGATTTTCCATTGCTTTATGCCACGCATAACAAAAATTA
TAAATTGAAATTACCCCTAAATTACAAAGATTTTTATGGATATAAAAATAAAGCCGTCGGGATAAAGT
CCCAGAAATCATCGT
+
FFFFFFFFFFFFFFFFFFFFFFFFFFFFFFFFFFFFFFFFFFFFFFFFFFFFFFFFFFFFFFFFFFFFFF
FFFFFFFFFFFFFFFFFFFFFFFFFFFFFFFFFFFFFFFFFFFFFF::FFFFFFFFFFFFFFFFFFFFFF
FFFFFFFFF:FFFF:
@A00721:190:HGJW7DSXY:4:1416:12635:29293 1:N:0:TCTGCAAG+GCTGCACT
ATTCGATAATCTTTGATTACATGATCTAGATTTTCCATTGCTTTATGCCACGCATAACAAAAATTATA
AATTGAAATTACCCCTAAATTACAAAGATTTTTATGGATATAAAAATAAAGCCGTCGGGATAAAGTCC
CAGAAATCATCGTCT
+
FFFFFFFFFFFFFFFFFFFFFFFFFFFFFFFFFFFFFFFFFFFFFFFFFFFFFFFFFFFFFFFF:FFFFF
FFFFFFFFFFFFFFFFFFFFFFFFFFFFFFFFFFFFFFFFFFFFFFFFFFFF:F:FFFFFFFFFFFFFFF
FFFFFFFFFFFFFFF
@A00721:190:HGJW7DSXY:4:1465:1316:13385 1:N:0:TCTGCAAG+GCTGCACT
ATTCGATAATCTTTGATTACATGATCTAGATTTTCCATTGCTTTATGCCACGCATAACAAAAATTATA
AATTGAAATTACCCCTAAATTACAAAGATTTTTATGGATATAAAAATAAAGCCGTCGGGATAAAGTCC
CAGAAATCATCGTCT
+
FFFFFFFFFFFFFFFFFFFF:FFFFFFFFFF:FFFFFFFFFFFFFFFFFFFFFFFFFFF:FFFFFFFFF
FFFFFFFFFFFFFFFFFFFFFFFFFF:FFFFFFFFFFFFFFFFFFFFFFFFFFFFFFFFFFFFFFFFFFF
FF:FFFF:FFFFFFF
@A00721:190:HGJW7DSXY:4:2450:4417:23766 1:N:0:TCTGCAAG+GCTGCACT
ATTCGATAATCTTTGATTACATGATCTAGATTTTCCATTGCTTTATGCCACGCATAACAAAAATTATA
AATTGAAATTACCCCTAAATTACAAAGATTTTTATGGATATAAAAATAAAGCCGTCGGGATAAAGTCC
CAGAAATCATCGTCT
+
FFFFFFFFFFFFFFFFFFFFFFFFFFFFFFFFFFFFFFFFFFFFFFFFFFFFFFFFFFFFFFFFFFFFFF
FFFFFFFFFFFFFFFFFFFFFFFFFFFFFFFFFFFFFFFFFFFFFFFFFFFFF:FFFFFFFFFFFFFFFF
FFFF:FFFFFF,FFF
@A00721:190:HGJW7DSXY:4:2576:14262:7122 1:N:0:TCTGCAAG+GCTGCACT
ATTCGATAATCTTTGATTACATGATCTAGATTTTCCATTGCTTTATGCCACGCATAACAAAAATTATA
AATTGAAATTACCCCTAAATTACAAAGATTTTTATGGATATAAAAATAAAGCCGTCGGGATAAAGTCC
CAGAAATCATCGTCT
+
FFFFF:FFFFFFFFFFFFFFFFFFFFFFFFFFFFFFFFFFFFFFFFFFFFFFFFFFFFFFFFFFFFFFFF
FFFFFFFFFFFFFFFFFFFFFFFFFFFFFFFFFFFFFFFFFFFFFFFFFFFFFFFFFFFFFFFFFFFFFF
FFFFFFFFFFFFFFF
@A00721:190:HGJW7DSXY:4:2662:23936:7435 1:N:0:TCTGCAAG+GCTGCACT
ATTCGATAATCTTTGATTACATGATCTAGATTTTCCATTGCTTTATGCCACGCATAACAAAAATTATA
AATTGAAATTACCCCTAAATTACAAAGATTTTTATGGATATAAAAATAAAGCCGTCGGGATAAAGTCC
CAGAAATCATCGTCT
+
FFFFFFFFFFFFFFFF:FFFFFFFFFFFFFFFFFFFFFFFFFFFFFFFFFFFFFFFF:FFFFFF,FFFFF
FFFFFFFFFFFFFFFFFFFFFFFFFFFFFFFFFFFFFFFFFFFFFFFFFFFFFFFFFFFFFFFFFFFFFF
FFFFFFFFFFFFFFF
@A00721:190:HGJW7DSXY:4:2678:27127:7889 1:N:0:TCTGCAAG+GCTGCACT
ATTCGATAATCTTTGATTACATGATCTAGATTTTCCATTGCTTTATGCCACGCATAACAAAAATTATA
AATTGAAATTACCCCTAAATTACAAAGATTTTTATGGATATAAAAATAAAGCCGTCGGGATAAAGTCC
CAGAAATCATCGTCT
```

```
+
FFFFFFFFFFFFFFFFFFFFFFFFFFFFFFFFFFFFFFFFFFFFFFFFFFFFFFFFFFFFFFFFFFFF
FFFFFFFFFFFFFFFFFFFFFFFFFFFFFFFFFFFFFFFFFFFFFFFFFF:FFFFFFFF:FFFFFFFF
FFFFFFFFFFFFFFF
@A00721:190:HGJW7DSXY:4:1246:23421:1846 1:N:0:TCTGCAAG+GCTGCACT
CTAGATTTTCCATTGCTTTATGCCACGCATAACAAAAATTATAAATTGAAATTACCCCTAAATTACAA
AGATTTTTATGGATATAAAAATAAAGCCGTCGAGATAAAGTCCCAGAAATCATCGTCTTCCATGACGG
GTTCTGCAGATCGGA
+
FFFFFFFFFFFFFFFFFFFFFFFFFFFFFFFFFFFFFFFFFFFFFFFFFFFFFFFFFFF:FFFFFFFF
FFFFFFFFFFFFFFFFFFFFFFFFFFFFF:FFFFF:FFFFFFFFFFFFFFFFFFFFFF:FFFFFFFFF
FFFFFF:FFFFFFFF
@A00721:190:HGJW7DSXY:4:1246:7084:3176 1:N:0:TCTGCAAG+GCTGCACT
CTAGATTTTCCATTGCTTTATGCCACGCATAACAAAAATTATAAATTGAAATTACCCCTAAATTACAA
AGATTTTTATGGATATAAAAATAAAGCCGTCGAGATAAAGTCCCAGAAATCATCGTCTTCCATGACGG
GTTCTGCAGATCGGA
+
FFFFFFFFFFFFFFFFFFFFFF:FFFFFFF:FFFFFFFFFFFFFFFFFFFFFFFFFFFFFFFFFFFFF
FFFFFFFFFFFFFFFFFFF,FFFFFFFFFFFFFFFFFF:FFFFFFFFFFFF:FFFFFFFFFFFF:FFF
FFFFFFFFFFFF,FF
@A00721:190:HGJW7DSXY:4:1459:16676:22983 1:N:0:TCTGCAAG+GCTGCACT
CTAGATTTTCCATTGCTTTATGCCACGCATAACAAAAATTATAAATTGAAATTACCCCTAAATTACAA
AGATTTTTATGGATATAAAAATAAAGCCGTCGAGATAAAGTCCCAGAAATCATCGTCTTCCATGACGG
GTTCTGCAGATCGGA
+
FFFFFFFFFFFFFFFFFFFFFFFFFFFFFFFFFFFFFFFFFFFFFFFFFFFFFFFFFFFFFFFFFFFF
FFFFFFFFFFFFFFFFFFFFFFFF:FFFFFFFFFFFFFFFFFFFFFFFFFFFFFFFFFFFFFFFFFFF
FFFFFFFFFFFFFFF
@A00721:190:HGJW7DSXY:4:1459:17445:23531 1:N:0:TCTGCAAG+GCTGCACT
CTAGATTTTCCATTGCTTTATGCCACGCATAACAAAAATTATAAATTGAAATTACCCCTAAATTACAA
AGATTTTTATGGATATAAAAATAAAGCCGTCGAGATAAAGTCCCAGAAATCATCGTCTTCCATGACGG
GTTCTGCAGATCGGA
+
FFFFFFF:FF::FFFFFFFF:F:FFF,FFF:FFFF:FF:FFFFFFF:F::F:,F::,F:F:FF,FF:F
F:FFF::F:FF,F,FF:F,FFF:FF,:,:F,FF,FF:F:,,:::FF,:F:,:F:,FFF,F:F,F:F,F
F,,,:F,F:FF::FF
@A00721:190:HGJW7DSXY:4:1513:8214:31219 1:N:0:TCTGCAAG+GCTGCACT
CTAGATTTTCCATTGCTTTATGCCACGCATAACAAAAATTATAAATTGAAATTACCCCTAAATTACAA
AGATTTTTATGGATATAAAAATAAAGCCGTCGAGATAAAGTCCCAGAAATCATCGTCTTCCATGACGG
GTTCTGCAGATCGGA
+
FFFFFFFFFFFFFFFFFFFFFFFFFFFFFFFFFFFFFFFFFFFFFFFFFFFFFFFFFFFFFFFFFFFF
FFFFFFFFFFFFFFFFFFFFFFFFFFFFFFFFFFFF:FFFFFFFFFFFFFFFFFFFFFFFFFFFFFFF
FFFFFF:FFFFFFFF
@A00721:190:HGJW7DSXY:4:2167:9634:30827 1:N:0:TCTGCAAG+GCTGCACT
CTAGATTTTCCATTGCTTTATGCCACGCATAACAAAAATTATAAATTGAAATTACCCCTAAATTACAA
AGATTTTTATGGATATAAAAATAAAGCCGTCGAGATAAAGTCCCAGAAATCATCGTCTTCCATGACGG
GTTCTGCAGATCGGA
+
FFFFFFFFFFFFFFFFFFFFFFFFFFFFFFFFFFFFFFFFFFFFFFFFFFFFFFFFFFFFFFFFFFFF
FFFFFFFFF:FFFF:FFFFFFFFFFFFFFFFFFFFFFFFFFFFFFFFF:FFFFFF:FFFFFFFFFFFF
FFFFFFFFFFFFFFF
@A00721:190:HGJW7DSXY:4:2216:16884:8375 1:N:0:TCTGCAAG+GCTGCACT
CTAGATTTTCCATTGCTTTATGCCACGCATAACAAAAATTATAAATTGAAATTACCCCTAAATTACAA
```

AGATTTTTATGGATATAAAAATAAAGCCGTCGAGATAAAGTCCCAGAAATCATCGTCTTCCATGACGG
GTTCTGCAGATCGGA
+
FFFFFFFFFFFFFFFFFFFFFFFFFFFFFFFFFFFFFFFFFFFFFFFFFFFFFFFFFFFFFFFFFFFF
FFFFFFFFFFFFFFFFFFFFFFFFF:FFFFFFFFFFFFFFFFFFFFFFFFFFF:FFFFFFFFFFFF::
FFFFFFFFFFFFFFF
@A00721:190:HGJW7DSXY:4:2304:11008:33332 1:N:0:TCTGCAAG+GCTGCACT
CTAGATTTTCCATTGCTTTATGCCACGCATAACAAAAATTATAAATTGAAATTACCCCTAAATTACAA
AGATTTTTATGGATATAAAAATAAAGCCGTCGAGATAAAGTCCCAGAAATCATCGTCTTCCATGACGG
GTTCTGCAGATCGGA
+
FFFFFFFFFFFFFFFFFFFFFFFFFFFFFFFFFFFFFFFFFFFFFFFFFFFFFFFFFFFFFFFFFFFF
FFFFFFFFFFFFFF:FFFFFFFFFFFFFFFFFFFFF:FFFFFFFFFFFFFFFFFFFFFFFFFFFFFFF
FFFFFFFFFFFFFFF
@A00721:190:HGJW7DSXY:4:2558:26820:12743 1:N:0:TCTGCAAG+GCTGCACT
CTAGATTTTCCATTGCTTTATGCCACGCATAACAAAAATTATAAATTGAAATTACCCCTAAATTACAA
AGATTTTTATGGATATAAAAATAAAGCCGTCGAGATAAAGTCCCAGAAATCATCGTCTTCCATGACGG
GTTCTGCAGATCGGA
+
FFFFFFFFFFFFFFFFFFFFFFFFFFFFFFFFFFFFFFFFFFFFFFFFFFFFFFFFFFFFFFFFFFFF
FFFFFFFFFFFFFFFFFFFFFFFFFFFFFFFFFFFFFFFFFFFFFFFFFFFFF:FFFFFFFFFFFFFF
FFFFFFFFFFFFFFF
@A00721:190:HGJW7DSXY:4:1118:31548:6934 1:N:0:TCTGCAAG+GCTGCACT
AGATTTTCCATTGCTTTATGCCACGCATAACAAAAATTATAAATTGAAATTACCCCTAAATTACAAAG
ATTTTTATGGATATAAAAATAAAGCCGTCGGGATAAAGTCCCAGAAATCATCGTCTTCCATGACTGGT
TCTGCAGTA
+
FFFFFF:FFF:::FFFFFFFF:FFFFFFFFFFFFFFFFF:FFFFFFFFFFFFFFFFFFFFFFF,FFF,
FFFFFFFFFFFFFFFFFFF:FFF,FFF:F,FFFFFFF:FFF,FFFFFFFF,F,::FFFFFFF:F,F:F
FFF:FF:FF
@A00721:190:HGJW7DSXY:4:1344:3143:9721 1:N:0:TCTGCAAG+GCTGCACT
AGATTTTCCATTGCTTTATGCCACGCATAACAAAAATTATAAATTGAAATTACCCCTAAATTACAAAG
ATTTTTATGGATATAAAAATAAAGCCGTCGGGATAAAGTCCCAGAAATCATCGTCTTCCATGACGGGT
TCTGCAGTA
+
FFFFFFFFFFFFFFFFFFFFFFFFFFFFFFFFFFFFFFFFFFFFFFFFFFFFFFFFFFFFFFFFFFFF
FFFFFFFFFFFFFFFFFFFFFFFFFFFFFFFFFFFFFFFFFFFFFFFFFFFF:FFFFFFFFFFFFFFF
FFFFFFFFF
@A00721:190:HGJW7DSXY:4:1567:22761:12696 1:N:0:TCTGCAAG+GCTGCACT
AGATTTTCCATTGCTTTATGCCACGCATAACAAAAATTATAAATTGAAATTACCCCTAAATTACAAAG
ATTTTTATGGATATAAAAATAAAGCCGTCGGGATAAAGTCCCAGAAATCATCGTCTTCCATGACGGGT
TCTGCAGTA
+
FFFFFFFFFFFFFFFFFFFFFFFFFFFFFFFFFFFFFFFFFFFFFFFFFFFFFFFFFFFFFFFFFFFF
FFFFFFFFFFFFFFFFFFFFFFFFFFFFFFFFFFFFFFFFFFFFFFFFFFFFFFFFFFFFFFFFFFFF
:FFFFFFFF
@A00721:190:HGJW7DSXY:4:2342:27308:7106 1:N:0:TCTGCAAG+GCTGCACT
AGATTTTCCATTGCTTTATGCCACGCATAACAAAAATTATAAATTGAAATTACCCCTAAATTACAAAG
ATTTTTATGGATATAAAAATAAAGCCGTCGGGATAAAGTCCCAGAAATCATCGTCTTCCATGACGGGT
TCTGCAGTA
+
FFFFFFFFFF:FFFFFFFFFFFFFFFFFFFFFFFFFFFFFFFFFFFFFFFFFFFFFFFFFFFFFFF:F
FFFFFFFFFFFFFFFFFFFFFFFF:FFFFFFFFFFFFFFFFFFFFFFFFFFFFFFFF:FFFFFFFFFF
FFFFFFFFF

```
@A00721:190:HGJW7DSXY:4:2357:31611:22357 1:N:0:TCTGCAAG+GCTGCACT
AGATTTTCCATTGCTTTATGCCACGCATAACAAAAATTATAAATTGAAATTACCCCTAAATTACAAAG
ATTTTTATGGATATAAAAATAAAGCCGTCGGGATAAAGTCCCAGAAATCATCGTCTTCCATGACGGGT
TCTGCAGTA
+
FFFFFFFFFFFFFFFFFFFFFFFFFFFFFFFFFFFFFFFFFFFFFFFFFFFFFFFFFFFFFFFFFFF:
FFFFFFFFFFFFFFFFF:FFFFFFFFFFFFFFFFFFFFFFFFFFFFFFFFFFFFFFFFFFFFFF:FFF
F,FFFFFFF
@A00721:190:HGJW7DSXY:4:2648:21594:16658 1:N:0:TCTGCAAG+GCTGCACT
AGATTTTCCATTGCTTTATGCCACGCATAACAAAAATTATAAATTGAAATTACCCCTAAATTACAAAG
ATTTTTATGGATATAAAAATAAAGCCGTCGGGATAAAGTCCCAGAAATCATCGTCTTCCATGACGGGT
TCTGCAGTA
+
FFFFFFFFFFFFFFFFFFFFFFFFFFFFFFFFFFFFFFFFFFFFFFFFFFFFFFFFFFFFFFFFFFFF
FFFFFFFFFFFFFFFFFFFFFFFFFFFFFFFFFFFFFFFFFFFFFFFFFFFFFFFFF:FFFFF:FFFF
FFFFFFFFF
@A00721:190:HGJW7DSXY:4:1603:18358:5635 1:N:0:TCTGCAAG+GCTGCACT
TTTCCATTGCTTTATGCCACGCATAACAAAAATTATAAATTGAAATTACCCCTAAATTACAAAGATTT
TTATGGATATAAAAATAAAGCCGTCGGGATAAAGTCCCAGAAATCATCGTCTTCCATGACGGGTTCTG
CAGTAATTCTGGGAC
+
FFFFFFFFFFFFFFFFFFFFFFFFFFFFFFFF:FFFFFFFFFFFFFFFFFFFFFFFFFFFFFFFFFFF
FFFFFFF:FFFFFFFFFFFFFFFFFFFFFFFFFFFFFFFFFFFFFFFFFFFFFFFFFFFFFFFFFFFF
FFFFFFFFFFFFFF:
@A00721:190:HGJW7DSXY:4:2318:23484:29418 1:N:0:TCTGCAAG+GCTGCACT
TTTCCATTGCTTTATGCCACGCATAACAAAAATTATAAATTGAAATTACCCCTAAATTACAAAGATTT
TTATGGATATAAAAATAAAGCCGTCGGGATAAAGTCCCAGAAATCATCGTCTTCCATGACGGGTTCTG
CAGTAATTCTGGGAC
+
FFFFFFFFFFFFFFFFFFFFFFFFFFFFFFFFFFFFFFFFFFFFFFFFFFFFFFFFFFFFFFFFFFFF
FFFFFFFFFFFFFFFFFFFFFFFFFFFFFFFFFFFFFFFFFFFFFFFFFFFFFFFFFFFFFFFFFFFF
FFFFFFFFFFF:FFF
@A00721:190:HGJW7DSXY:4:2349:5113:3020 1:N:0:TCTGCAAG+GCTGCACT
TTTCCATTGCTTTATGCCACGCATAACAAAAATTATAAATTGAAATTACCCCTAAATTACAAAGATTT
TTATGGATATAAAAATAAAGCCGTCGGGATAAAGTCCCAGAAATCATCGTCTTCCATGACGGGTTCTG
CAGTAATTCTGGG
+
FFFFFFFFFFFFFFFFFFFFFFFFFFFFFFFFFFFFF:FFFFFFFFFFFFFFFFFFFFFFFFFFFFFF
FFFFFFF,FFFFFFFFFFFF:FF:FFFFFFFFFFFFFFFFFFFFF:,FFFFFFFF:FF,FFFFFFFFF
F,F:F::FF:FFF
@A00721:190:HGJW7DSXY:4:2404:21314:35524 1:N:0:TCTGCAAG+GCTGCACT
TTTCCATTGCTTTATGCCACGCATAACAAAAATTATAAATTGAAATAACCCCTAAATTACAAAGATTT
TTATGGATATAAAAATAAAGCCGTCTGGATAAAGTCCCAGAAATCATCGTCTTCCATGACGGGTTCTG
CAGTAATACTGGGAC
+
FFFF:FFF::FF:FFFFFFF:F:FFFFFFFFFFFF::FF:FFFFFF,FFFFFFFFF:FFFFF:FFFFF
:FFFFFF:FFFFF,:FFF:FF,,,F,,:FFFFFFFF:F,:FFFFFFF:FFF,:F,F:FF,,FFFFFFF
FF::FFF,,F,,FFF
@A00721:190:HGJW7DSXY:4:2469:29957:36245 1:N:0:TCTGCAAG+GCTGCACT
TTTCCATTGCTTTATGCCACGCATAACAAAAATTATAAATTGAAATTACCCCTAAATTACAAAGATTT
TTATGGATATAAAAATAAAGCCGTCGGGATAAAGTCCCAGAAATCATCGTCTTCCATGACGGGTTCTG
CAGTAATTCTGGGAC
+
FFFFFFFFFFFFFFFFFFFFFFFFFFFFFFFF:FFFFFFFFFFFFFFFFFFFFFFFFFFFFFFFFFFF
```

FFFFFFFFFFFFFFFFFFFFFFFFF:FFFFFFFFFFFFFFFFFFFFFFFFFFFFFFFFFFFFFFFFFF
FFFFFFFFFFF:FF:
@A00721:190:HGJW7DSXY:4:2678:27597:13589 1:N:0:TCTGCAAG+GCTGCACT
TTTCCATTGCTTTATGCCACGCATAACAAAAATTATAAATTGAAATTACCCCTAAATTACAAAGATTT
TTATGGATATAAAAATAAAGCCGTCGGGATAAAGTCCCAGAAATCATCGTCTTCCATGACGGGTTCTG
CAGTAATTCTGGGAC
+
FFFFFFFFFFFFFFFFFFFFFFFFFFFFFFFFFFFFFFF:FFFFFFFFFFFFFFFFFFFFFFFFFFFF
FFFFFFFFFFFFFFFFFFFFFFFFFFFFFFFFFFFFFFFFFFFFFFFFFFFFFFFFFFFFFFFFFFFF
FFFFFFFFFFFFFFF
@A00721:190:HGJW7DSXY:4:1230:1362:22263 1:N:0:TCTGCAAG+GCTGCACT
TTCCATTGCTTTATGCCACGCATAACAAAAATTATAAATTGAAATTACCCCTAAATTACAAAGATTTT
TATGGATATAAAAATAAAGCCGTCGGGATAAAGTCCCAGAAATCATCGTCTTCCATGACGGGTTCTGC
AGTAATTCTGGGAC
+
FFFFFFFFFFFFFF:FFFFFFFFFFFFFFFF:FFFFFFFFFFFFFFFFFFFFFFFFFFFFFFFFFFFF
FFFFFFFFFFFFF,FFFFF::FFFF:FFFFFF:FF:FFFFFFFFFFF:FFFFFFFFFFFFFFFFFFFF
FFFFFFFFFFFFFF
@A00721:190:HGJW7DSXY:4:2101:10664:36338 1:N:0:TCTGCAAG+GCTGCACT
TTCCATTGCTTTATGCCACGCATAACAAAAATTATAAATTGAAATTACCCCTAAATTACAAAGATTTT
TATGGATATAAAAATAAAGCCGTCGGGATAAAGTCCCAGAAATCATCGTCTTCCATGACGGGTTCTGC
AGTAATTCTGGGACT
+
FFFFFFFFFFFFFFFFFFFFFFFFFFFFFFFFFFFFFFFFFFFFFFFFFFFFFFFFFFFFFFFFFFFF
FF:FFFFFFFFF:FFFFFFFFFFFFFFFFFFFFFF:FFFFFFFFFFFFFFFFFFFFFFFFFFFFFFFF
FFFFFFFFFFFFF,F
@A00721:190:HGJW7DSXY:4:2139:31901:32910 1:N:0:TCTGCAAG+GCTGCACT
TTCCATTGCTTTATGCCACGAATAACAAAAATTATAAATTGAAATTACCCCTAAATTACAAAGATTTT
TATGGATATAAAAATAAAGCCGTCGGGATAAAGTCCCAGAAATCATCGTCTTCCATGACGGGTTCTGC
AGTAATTCTGGGAC
+
FFFFFFFFFFFFFFFFFFFFFFFFFFFFFFFFFFFFFFFFFFFFFFFFFFFFFFFFFFFFFFFFFFFF
FFFFFFFFFFFFFFFFFFF:FFF:FFFFFFFFFFFF:FFFFFFFFFFFFFFFFFFFFFFFF:FFFFFF
FFFFFFFFFFFFFF
@A00721:190:HGJW7DSXY:4:2151:7654:4726 1:N:0:TCTGCAAG+GCTGCACT
TTCCATTGCTTTATGCCACGCATAACAAAAATTATAAATTGAAATTACCCCTAAATTACAAAGATTTT
TATGGATATAAAAATAAAGCCGTCGGGATAAAGTCCCAGAAATCATCGTCTTCCATGACGGGTTCTGC
AGTAATTCTGGGACT
+
FFFFFFFFFFFFFFFFFFFFFFFFFFFFFFFFFFFFFFFFFFFFFFFFFFFFFFFFFFFFFF,FFFFF
FFFFFFFFFFFFFFFFFFFFFFFFFFFF:FFFFFF:FFFFFFFFFFFFFFFFFFFFFF:FFFFFFFFF
FFFFFFFFFF:FFFF
@A00721:190:HGJW7DSXY:4:2260:14253:8296 1:N:0:TCTGCAAG+GCTGCACT
TTCCATTGCTTTATGCCACGAATAACAAAAATTATAAATTGAAATTACCCCTAAATTACAAAGATTTT
TATGGATATAAAAATAAAGCCGTCGGGATAAAGTCCCAGAAATCATCGTCTTCCATGACGGGTTCTGC
AGTAATTCTGGGACT
+
FFFFFFFFFFFFFFFFFFFFFFFFFFFFFF:FFFFFFFFFFFFFFFFFFFFFFFFFFFFFFFFFFFFF
FFFFFFFFFFFFFFFFFFFFFFFFFFFFFFFFFFFFFFFFFFFFFFFFFFFFF:FFFFFFFFFFFFF
FFFFFFFFFFFFF:F
@A00721:190:HGJW7DSXY:4:2310:7654:31407 1:N:0:TCTGCAAG+GCTGCACT
TTCCATTGCTTTATGCCACGCATAACAAAAATTATAAATTGAAATTACCCCTAAATTACAAAGATTTT
TATGGATATAAAAATAAAGCCGTCGGGATAAAGTCCCAGAAATCATCGTCTTCCATGACGGGTTCTGC
AGTAATTCTGGGACT

+
FFFFFFFFFFFFFFFFFFFFFFFFFFFFFFFFFFFFFFFFFFFFFFFFFFFFFFFFFFFFFFFFFFFF
FFFFFFFFFFFFFFFFFFFFFFFFFFFFFFFFFFFFFFFFFFFFFFFFFFFFFFFFFFFFFFFFFFFF
FFFFFFFFFFFFFFF
@A00721:190:HGJW7DSXY:4:2328:10474:19820 1:N:0:TCTGCAAG+GCTGCACT
TTCCATTGCTTTATGCCACGAATAACAAAAATTATAAATTGAAATTACCCCTAAATTACAAAGATTTT
TATGGATATAAAAATAAAGCCGTCGGGATAAAGTCCCAGAAATCATCGTCTTCCATGACGGGTTCTGC
AGTAATTCTGGGACT
+
FFFFFFFFFFFFFFFFFFFFFFFFFFFF:FFFFFFFFFFFFFFFFFFFFFFFFFFFFFFFFFFFFFFF
FFFFFFFFFFFFFFFFFFFFFFFFFFFFFFFFFFFFFFFFFFFFFFFFFFFFFFFFF:FFFFFFFFFF
FFFFFFFFFFFFF:F
@A00721:190:HGJW7DSXY:4:2635:18738:30592 1:N:0:TCTGCAAG+GCTGCACT
TTCCATTGCTTTATGCCACGAATAACAAAAATTATAAATTGAAATTACCCCTAAATTACAAAGATTTT
TATGGATATAAAAATAAAGCCGTCGGGATAAAGTCCCAGAAATCATCGTCTTCCATGACGGGTTCTGC
AGTAATTCTGGGACT
+
FFFFFFFFFFFFFFFFFFFFFFFFFFFFFFFFFFFFFFFFFFFFFFFFFFFFFFFFFFFFF:FFFFFF
FFFFFFFFFFFFFFFFFFFFFFFFFFFFFFFFFFFFFFFFFFFFFFFFFFFFFFFFFFFFFFFFFFFF
FFFF,FFFFFFFFFF
@A00721:190:HGJW7DSXY:4:1108:14850:35070 1:N:0:TCTGCAAG+GCTGCACT
ATCCATTGCTTTATGCCACGCATAACAAAAATTATAAATTGAAATTACCCCTAAATTACAAAGATTTT
TATGGATATAAAAATAAAGCCGTCGGGATAAAGTCCCAGAAATCATCGTCTTCCATGACGGGTTCTGC
AGTAATTCTGGGACT
+
FFFFFFFFFFFFFFFFFFFFFFFFFFFFFFFFFFFFFFFFFFFFFFFFFFFFFFFFFFFFFFFFFFFF
FFFFFFFFFFFFFFFFFFFFFFFFFFFFFFFFFFFFFFFFFFFFFFFFFFFFFFFFFFFFFFFFFFFF
FFFFFFFFFFFFFFF
@A00721:190:HGJW7DSXY:4:1202:8269:27461 1:N:0:TCTGCAAG+GCTGCACT
ATCCATTGCTTTATGCCACGCATAACAAAAATTATAAATTGAAATTACCCCTAAATTACAAAGATTTT
TATGGATATAAAAATAAAGCCGTCGGGATAAAGTCCCAGAAATCATCGTCTTCCATGACGGGTTCTGC
AGTAATTCTGGGACT
+
F:FFFFFFFFFFFFFFFFFFFFFFFFFFFFFFFFFFFFFFFFFFFFFFFFFFFFFFFFFFFFFFFFFF
FFFFFFFFFFFFFFFFFFFFFFFFFFFFFFFFFFFFFFFFFFFFFFFFFFFFFFFFFFFFFFFFFFFF
FFFFFFFFFFFFFFF
@A00721:190:HGJW7DSXY:4:1335:23583:2503 1:N:0:TCTGCAAG+GCTGCACT
ATCCATTGCTTTATGCCACGCATAACAAAAATTATAAATTGAAATTACCCCTAAATTACAAAGATTTT
TATGGATATAAAAATAAAGCCGTCGGGATAAAGTCCCAGAAATCATCGTCTTCCATGACGGGTTCTGC
AGTAATTCTGGGACT
+
FFFFFFFFFFFFFFFFFFFFFFFFFFFFFFFFFFFFFFFFFFFFFFFFFFFFFFFFFFFFFFFFFFFF
FFFFFFFFFFFFFFFFFFFFFFFFFFFFFFFFFFFFFFFFFFFFFFFFFFFFFFFFFFFFFFFFFFFF
FFFFFFFFFFFFFFF
@A00721:190:HGJW7DSXY:4:1552:8712:15984 1:N:0:TCTGCAAG+GCTGCACT
ATCCATTGCTTTATGCCACGCATAACAAAAATTATAAATTGAAATTACCCCTAAATTACAAAGATTTT
TATGGATATAAAAATAAAGCCGTCGGGATAAAGTCCCAGAAATCATCGTCTTCCATGACGGGTTCTGC
AGTAATTCTGGGACT
+
FFFFFFFFFFFFFFFFFFFFFFFFFFFFFFFFFFFFFFFFFFFFFFFFFFFFFFFFFFFFFFFFFFFF
FFFFFFFFFFFFFFFFFFFFFFF:F:FFFFFFFFFFFFFFFFFFFFFFFFFFFFFFFFFFFFFFFFFF
FFFFFFFFFFFFFFF
@A00721:190:HGJW7DSXY:4:1659:31530:24815 1:N:0:TCTGCAAG+GCTGCACT
CTCCATTGCTTTATGCCACGCATAACAAAAATTATAAATTGAAATTACCCCTAAATTACAAAGATTTT

```
TATGGATATAAAAATAAAGCCGTCGAGATAAAGTCCCAGAAATCATCGTCTTCCATGACGGGTTCTGC
AGTAATTCTGGGACT
+
FFFFFFFFFFFFFFFFFFFFFFFFFFFFFFFFFFFFFFFFFFFFFFFFFFFFFFFFFFFFFFFFFFFFFF
FF:FFFFFFFFFFFFFF:F,FFFFFFFFFFFFFFFFFFFFFF:FFFFFFFFFFFFFFFFFFFFFFFFFFF
FFFFFFFFFFFFFFF
@A00721:190:HGJW7DSXY:4:1661:26160:6778 1:N:0:TCTGCAAG+GCTGCACT
ATCCATTGCTTTATGCCACGCATAACAAAAATTATAAATTGAAATTACCCCTAAATTACAAAGATTTT
TATGGATATAAAAATAAAGCCGTCGGGATAAAGTCCCAGAAATCATCGTCTTCCATGACGGGTTCTGC
AGTAATTCTGGGACT
+
FFFFFFFFFFFFFFFFFFFFFFFFFFFFFFFFFFFFFFFFFFFFFFFFFFFFFFFFFFFFFFFFFFFFFF
FFFFFFFFFFFFFFFFFFFFFFFFFFFFFFFFFFFFFFFFFFFFFFFFFFFFFFFFFFFFFFFFFFFFFF
FFFFFFFFFFFFFFF
@A00721:190:HGJW7DSXY:4:2122:19831:4304 1:N:0:TCTGCAAG+GCTGCACT
ATCCATTGCTTTATGCCACGCATAACAAAAATTATAAATTGAAATTACCCCTAAATTACAAAGATTTT
TATGGATATAAAAATAAAGCCGTCGGGATAAAGTCCCAGAAATCATCGTCTTCCATGACGGGTTCTGC
AGTAATTCTGGGACT
+
FFFFFFFFFFFFFFFFFFFFFFFFFFFFFFFFFFFFFFFFFFFFFFFFFFFFFFFFFFFFFFFFFFFFFF
FFFFFFFFFFFFFFFFFFFFFFFFFFFFFFFFFFFFFFFFFFFFFFFFFFFFFFFFFFFFFFFFFFFFFF
FFFFFFFFFFFFF:F
@A00721:190:HGJW7DSXY:4:2207:25319:4601 1:N:0:TCTGCAAG+GCTGCACT
CTCCATTGCTTTATGCCACGCATAACAAAAATTATAAATTGAAATTACCCCTAAATTACAAAGATTTT
TATGGATATAAAAATAAAGCCGTCGAGATAAAGTCCCAGAAATCATCGTCTTCCATGACGGGTTCTGC
AGTAATTCTGGGACT
+
FFFFFFFFFFFFFFFFFFFFFFFFFFFFFFFFFFFFFFFFFFFFFFFFFFFFFFFFFFFFFFFFFFFFFF
FFFFFFFFFFFFFFFFFFFFFFFFFFFFFFFFFFFFFFFFF:FFFFFFFFFFFFFFFFFFFFFFFFFFFF
FFFFFFFFFFFFFFF
@A00721:190:HGJW7DSXY:4:2251:1814:36699 1:N:0:TCTGCAAG+GCTGCACT
CTCCATTGCTTTATGCCACGCATAACAAAAATTATAAATTGAAATTACCCCTAAATTACAAAGATTTT
TATGGATATAAAAATAAAGCCGTCGAGATAAAGTCCCAGAAATCATCGTCTTCCATGACGGGTTCTGC
AGTAATTCTGGGACT
+
FFFFFFFFFFFFFFFFFFFFFFFFFFFFFFFFFFFFFFFFFFFFFFFFFFFFFFFFFFFFFFF:FF:FFF
FFFFFFFFFFFFFFFFFFFFFFFFFFFFFFFFFFFFFFFFFFFFFFFFFFFFFFFFFFFFFFFFFFFFFF
FFFFFFFFFFFFF,F
@A00721:190:HGJW7DSXY:4:2359:18837:19492 1:N:0:TCTGCAAG+GCTGCACT
ATCCATTGCTTTATGCCACGCATAACAAAAATTATAAATTGAAATTACCCCTAAATTACAAAGATTTT
TATGGATATAAAAATAAAGCCGTCGGGATAAAGTCCCAGAAATCATCGTCTTCCATGACGGGTTCTGC
AGTAATTCTGGGACT
+
FFFFFFFFFFFFFFFFFFFFFFFFFFFFFFFF:FFFFFFFFFFFFFFFFFFFFFFFFFFFFFFFFFFFFF
FFFFFFFFF:FFFFFFFFFFFFFFFFFFFFFFFFFFFFFFFFFFFFFFFFFFFFFFFFFFFFFFFFFFFF
FFFFFFFFFFF:FFF
@A00721:190:HGJW7DSXY:4:2406:30581:5854 1:N:0:TCTGCAAG+GCTGCACT
CTCCATTGCTTTATGCCACGCATAACAAAAATTATAAATTGAAATTACCCCTAAATTACAAAGATTTT
TATGGATATAAAAATAAAGCCGTCGAGATAAAGTCCCAGAAATCATCGTCTTCCATGACGGGTTCTGC
AGTAATTCTGGGACT
+
FFFFFFFFFFFFFFFFFFFFFFFFFFFFFFFFFFFFFFFFFF:FFFFFFFFFFFFFFFFFFFFFFFFFFF
FFFFFFF:FFF:FFFFFF:FFFFFFFFFFFFFFFFF:FFFFFFFFFFFFFFFFFF:FFF:FFF:FFFFFF
FFFFFFFFFFFFF:F
```

@A00721:190:HGJW7DSXY:4:2453:30897:9940 1:N:0:TCTGCAAG+GCTGCACT
ATCCATTGCTTTATGCCACGCATAACAAAAATTATAAATTGAAATTACCCCTAAATTACAAAGATTTT
TATGGATATAAAAATAAAGCCGTCGGGATAAAGTCCCAGAAATCATCGTCTTCCATGACGGGTTCTGC
AGTAATTCTGGGACT
+
F:FFFFFFFFFFFFFFFFFFFFFFFFFFFFFFFFFFFFFFFFFFFFFFFFFFFFFFFFFFF:FFFFFF
FFFFFFFFFFFFFFFFFFFFFFFFFFFFFFFFFFFFFFFFFFFFFFFFFFFFFFFFFFFFFFFFFFFF
FFFFFFFFFFFFFFF
@A00721:190:HGJW7DSXY:4:2563:10095:34413 1:N:0:TCTGCAAG+GCTGCACT
ATCCATTGCTTTATGCCACGCATAACAAAAATTATAAATTGAAATTACCCCTAAATTACAAAGATTTT
TATGGATATAAAAATAAAGCCGTCGGGATAAAGTCCCAGAAATCATCGTCTTCCATGACGGGTTCTGC
AGTAATTCTGGGA
+
FFFFFFFFFFFFFFFFFFFFFFFFFFFFFFFFFFFFFFFFFFFFFFFFFFFFFFFFFFFFF:FFFFFF
FF:FFFFFFFFFFFFFFFFFF:FFFFFFFFFFFFFFFFFFFFFFFFFFFFFFFFFFFFFFFFFFFFFF
FFFFFFFFFFFFF
@A00721:190:HGJW7DSXY:4:2621:26341:16924 1:N:0:TCTGCAAG+GCTGCACT
ATCCATTGCTTTATGCCACGCATAACAAAAATTATAAATTGAAATTACCCCTAAATTACAAAGATTTT
TATGGATATAAAAATAAAGCCGTCGGGATAAAGTCCCAGAAATCATCGTCTTCCATGACGGGTTCTGC
AGTAATTCTGGGAC
+
FFFFFFFFFFFFFFFFFFFFFFFFFFFFFFFFFFFFFFFFFFFFFFFFFFFFFFFFFFFFF:FFFFFF
FFFFFFFFFFFFFFFFFFFFFFFFFFFFFFFFFFFFFFFFFFFFFFFFFFFFFFFFFFFFFFFFFFFF
FFFFFFFFFFFFF:
@A00721:190:HGJW7DSXY:4:2623:7337:19241 1:N:0:TCTGCAAG+GCTGCACT
CTCCATTGCTTTATGCCACGCATAACAAAAATTATAAATTGAAATTACCCCTAAATTACAAAGATTTT
TATGGATATAAAAATAAAGCCGTCGAGATAAAGTCCCAGAAATCATCGTCTTCCATGACGGGTTCTGC
AGTAATTCTGGGACT
+
FFFFFFFFFFFFFFFFFFFFFFFFFFFFFFFFFFFFFFFFFFFFFFFFFFFFFFFFFFFFFFFFFFFF
FFFFFFFFFFFFFFFFFFFFFFFFFFFFFFFFFFFFFFF:FFFFFFFFFFFFFFFFFFFFFFFFFFFF
FFFFFFFFFFFFFFF
@A00721:190:HGJW7DSXY:4:2643:6795:30201 1:N:0:TCTGCAAG+GCTGCACT
CTCCATTGCTTTATGCCACGCATAACAAAAATTATAAATTGAAATTACCCCTAAATTACAAAGATTTT
TATGGATATAAAAATAAAGCCGTCGAGATAAAGTCCCAGAAATCATCGTCTTCCATGACGGGTTCTGC
AGTAATTCTGGGACT
+
FFFFFFFFFFFFFFFFFFFFFFFFFFFFFFFFFFFFFFFFFFFFFFFFFFFFFFFFFFFFFFFFFFFF
FFFFFFFFFFFFFFFFFFFFFFFFFFFFFFFFFFFFFFFFFFFFFFFFFFFFFFFFFFFFFFFFFFFF
FFFFFFFFFFFFF:F
@A00721:190:HGJW7DSXY:4:1215:17255:19758 1:N:0:TCTGCAAG+GCTGCACT
CATTGCTTTATGCCACGCATAACAAAAATTATAAATTGAAATTACCCCTAAATTACAAAGATTTTTAT
GGATATAAAAATAAAGCCGTCGAGATAAAGTCCCAGAAATCATCGTCTTCCATGACGGGTTCTGCAGT
AATTCTGGGACTGGT
+
FFFFFFFFFFFFFFFFFFFFFFFFFFFFFFFFFFFFFFFFF:FFFFFFFFFFFFFFFFFFFFFFFFF:
FFFFFFFFF,FFFFFFFFFFFFFFFFFFFFFFFFFFFFFFFFFFFFFFFFFFFFFFFFFFFFF:FF
FFFFFFFFFFFFFFF
@A00721:190:HGJW7DSXY:4:1215:17454:20228 1:N:0:TCTGCAAG+GCTGCACT
CATTGCTTTATGCCACGCATAACAAAAATTATAAATTGAAATTACCCCTAAATTACAAAGATTTTTAT
GGATATAAAAATAAAGCCGTCGAGATAAAGTCCCAGAAATCATCGTCTTCCATGACGGGTTCTGCAGT
AATTCTGGGACTGGT
+
FFFFFFFFFFFFFFFFFFFFFFFFFFFFFFFFFFFFFFFFFFFFFFFFFFFFFFFFFFFFFFFFFFFF

FFFFFFFFFFFFFFFFFFFFFFFFFFFFFFFFFFFFFFFFFFFFFFFFFFFFFFFFFFFFFFFFFFFFFF
FFFFFFFFFFFFFFF
@A00721:190:HGJW7DSXY:4:1223:29785:8797 1:N:0:TCTGCAAG+GCTGCACT
CATTGCTTTATGCCACGCATAACAAAAATTATAAATTGAAATTACCCCTAAATTACAAAGATTTTTAT
GGATATAAAAATAAAGCCGTCGAGATAAAGTCCCAGAAATCATCGTCTTCCATGACGGGTTCTGCAGT
AATTCTGGGACTGGT
+
FFFFFFFFFFFFFFFFFFFFFFFFFFFFFFFFFFFFFFFFFFFFFFFFFFFFFFFFFFFF:FFFFFFFFF
FFFFFFFFFFFFFFFFFFFFFFFFFFFFFFFFFFFFFFFFFFFFFFFFFFF:FFFFFFFFFFFFFFFFFF
F:FFFFFFFFFFFFF
@A00721:190:HGJW7DSXY:4:1367:11153:1924 1:N:0:TCTGCAAG+GCTGCACT
CAATGCTTTATGCCACGCATAACAAAAAATATAAATTGAAAATACCCCTAAATTACAAAGATTAATAT
GGATATAAAAATAAAGCCGTCGAGATAAATTCCCAGAAATCATCGTATTCCATGACGGGTTCTGCAGT
AATTCTGGGACTGGT
+
:F,:F:FFFFF::FFF,FF,FFFFFFF:,FF,FFFFF,F,F,FFFF:,F:FFFFFFFFF,:FF,:FF,
,F:FF:FFFFF,FFF:,:::,FFFF,FFF,FF,,F,F:FF:FFFF,,FF,,F:F:F,:F,F,,:FFFF
F:F,FF,:FFFF,:F
@A00721:190:HGJW7DSXY:4:1367:10682:2237 1:N:0:TCTGCAAG+GCTGCACT
CATTGCTTTATGCCACGCATAACAAAAATTATAAATTGAAATTACCCCTAAATTACAAAGATTTTTAT
GGATATAAAAATAAAGCCGTCGAGATAAAGTCCCAGAAATCATCGTCTTCCATGACGGGTTCTGCAGT
AATTCTGGGACTGGT
+
FFFFFFFFFFFFFFFFFFFFFFFFFFFF:FFFFFFFFFFFFFFFFFFFFFFFFFFFFFFFF:FFFFFFFF
FFFFFFFFFFFFFFFFFFFFFFFFFFFFFFFFFFFFFFFFFFFFFFFFFFFFFFFFFFFFFFFFFFFFFF
F,FFFFFFFFFFFFF
@A00721:190:HGJW7DSXY:4:1375:24831:32377 1:N:0:TCTGCAAG+GCTGCACT
CATTGCTTTATGCCACGCATAACAAAAATTATAAATTGAAATTACCCCTAAATTACAAAGATTTTTAT
GGATATAAAAATAAAGCCGTCGAGATAAAGTCCCAGAAATCATCGTCTTCCATGACGGGTTCTGCAGT
AATTCTGGGACTGGT
+
FFFFFFFFFFFFFFFFFFFFFFFFFFFFFFFFFFFFFFFFFF,FFF:FFFFFFFFFFFFFFFFFFFF:FF
:FFFFFF:FFFFFFFFFFFFFFFFFFFFFFFFFF:FFFFFFFFFFFFFFF:FF::FFFFFFFFFFFFFFF
FFFFFFFFFFFFFFF
@A00721:190:HGJW7DSXY:4:1465:19316:22169 1:N:0:TCTGCAAG+GCTGCACT
CATTGCTTTATGCCACGCATAACAAAAATTATAAATTGAAATTACCCCTAAATTACAAAGATTTTTAT
GGATATAAAAATAAAGCCGTCGAGATAAAGTCCCAGAAATCATCGTCTTCCATGACGGGTTCTGCAGT
AATTCTGGGACTGGT
+
FFFFFFFFFFFFFFFFFFFFFFFF:FFFFFFFFFFFFFFFFFFFF,FFFFFFFFFFFFFFFFF:FFFFFF
FFFFFFFFFFFFFFFFFFFFFFFFFFFFFFFFFFFFFFFFFFFFFFFFFFFFFFFFFFFFFFFFFFFFFF
FFFFFFFFFFFFFFF
@A00721:190:HGJW7DSXY:4:1508:16288:34992 1:N:0:TCTGCAAG+GCTGCACT
CATTGCTTTATGCCACGCATAACAAAAATTATAAATTGAAATTACCCCTAAATTACAAAGATTTTTAT
GGATATAAAAATAAAGCCGTCGGGATAAAGTCCCAGAAATCATCGTCTTCCATGACGGGTTCTGCAGT
AATTCTGGGACTGGT
+
FFFFFFFFFFFFFFFFFFFFFFFFFFFFFFFFFFFFFFFFFFFFFFFFFFFFFFFFFFFFFFFFFFFFFF
FFFFFFFFFFFFFFFFFFFFFFFFFFFFFFFFFFFFFFFFFFFFFFFFFFFFFFFFFFFFFFFFFFFFFF
FFFFFFFFFFFFFFF
@A00721:190:HGJW7DSXY:4:1630:28122:21793 1:N:0:TCTGCAAG+GCTGCACT
CATTGCTTTATGCCACGCATAACAAAAATTATAAATTGAAATTACCCCTAAATTACAAAGATTTTTAT
GGATATAAAAATAAAGCCGTCGGGATAAAGTCCCAGAAATCATCGTCTTCCATGACGGGTTCTGCAGT
AATTCTGGGACTGGT

```
+
FFFFFFFFFFFFFFFFFFFFFFFFFFFFFFFFFFFFFFFFFFFFFFFFFFFFFFFFFFFFFFFFFFFFFF
FFFFFFFFFFFFFFFFFFFFFFFFFFFFFFFFFFFFFFFFFFFFFFFFFFFFFFFFFFFFFFFFFFFFFF
FFFFFFFFFFF:FFF
@A00721:190:HGJW7DSXY:4:1634:28474:31548 1:N:0:TCTGCAAG+GCTGCACT
CATTGCTTTATGCCACGCATAACAAAAATTATAAATTGAAATTACCCCTAAATTACAAAGATTTTTAT
GGATATAAAAATAAAGCCGTCGGGATAAAGTCCCAGAAATCATCGTCTTCCATGACGGGTTCTGCAGT
AATTCTGGGACTGGT
+
FFFFFFFFFFFFFFFFFFFFFFFFFFFFFFFFFFF:FFFFFFFFFFFFFFFFFFFFFFFFFFFFFFFFFF
FFFFF:FFF,:FFFFFFFFFFFFFFFFFFFFFFFFFFFFFFFFFFFFFFFFFFFFFFFFFFFFFFFF:FF
FFFFFFFFF:FFFFF
@A00721:190:HGJW7DSXY:4:1662:21124:13620 1:N:0:TCTGCAAG+GCTGCACT
CATTGCTTTATGCCACGCATAACAAAAATTATAAATTGAAATTACCCCTAAATTACAAAGATTTTTAT
GGATATAAAAATAAAGCCGTCGAGATAAAGTCCCAGAAATCATCGTCTTCCATGACGGGTTCTGCAGT
AATTCTGGGACTGGT
+
FFFFFFF:FFFFFFFFFFFF:FFFFFFFFFFFFFFFFFFFFFFFFFFFFFFFFFFFFFFFFFFFFFFFFFF
FFFFFFFFFFFFFFFFFFFFFFFFFFFFFFFFFFFFFFFFFFFFFFFFFFFFFFFFFFFFFFFFFFFFFF
F:FFFFFFFFFFFFF
@A00721:190:HGJW7DSXY:4:1671:5999:35274 1:N:0:TCTGCAAG+GCTGCACT
CATTGCTTTATGCCACGCATAACAAAAATTATAAATTGAAATTACCCCTAAATTACAAAGATTTTTAT
GGATATAAAAATAAAGCCGTCGAGATAAAGTCCCAGAAATCATCGTCTTCCATGACGGGTTCTGCAGT
AATTCTGTGACTGGT
+
FFFFFFFF,F:FFFFFFF:FFFFFFF:FF:FFFFF:FF:,FF::,:,FFFFFFFF,F:::FFFF,FFF
FFFFFFFFFFFFF::::F,,FFFFFFFFFF:FF,FFFFF:FFF:FFFFF:FFF:FF,,F,FFF:F,F,
FFFF:FF,FFFF,F:
@A00721:190:HGJW7DSXY:4:2327:24605:9377 1:N:0:TCTGCAAG+GCTGCACT
CATTGCTTTATGCCACGCATAACAAAAATTATAAATTGAAATTACCCCTAAATTACAAAGATTTTTAT
GGATATAAAAATAAAGCCGTCGAGATAAAGTCCCAGAAATCATCGTCTTCCATGACGGGTTCTGCAGT
AATTCTGGGACTGGT
+
FFFFFFFFFFFFFFFFFFFFFFFFFFFFFFFFFFFFFFFFFFFFFFFFFFFFFFFFFFFFF:FFFFFFFF
FFFFFFFFFFFFFFFFFFFFFFFFFFFFFFFFFFFFFFFFFFFFFFFFFFFFFFFFFFFFFFFFFFFFFF
FFFFFFFFFF:FFF:
@A00721:190:HGJW7DSXY:4:2408:8458:6997 1:N:0:TCTGCAAG+GCTGCACT
CATTGCTTTATGCCACGCATAACAAAAATTATAAATTGAAATTACCCCTAAATTACAAAGATTTTTAT
GGATATAAAAATAAAGCCGTCGAGATAAAGTCCCAGAAATCATCGTCTTCCATGACGGGTTCTGCAGT
AATTCTGGGACTGGT
+
FFFFFFFFFFFFFFFFFFFFFFFFFFFFFFFFFFFFFFFFFF:FFFFFFFFFFFFFFFFFFFFFFFFFF:F
FFFFFFFFFFFFFFFFFFFFFFFFFFFFFFFFFFFFFFFFFFFFFFFFFFFFFFF:FFFFFFFFFFFF
FFFFFFFFFFFFFFF
@A00721:190:HGJW7DSXY:4:2505:14823:21746 1:N:0:TCTGCAAG+GCTGCACT
CATTGCTTTATGCCACGCATAACAAAAATTATAAATTGAAATTACCCCTAAATTACAAAGATTTTTAT
GGATATAAAAATAAAGCCGTCGAGATAAAGTCCCAGAAATCATCGTCTTCCATGACGGGTTCTGCAGT
AATTCTGGGACTGG
+
:FFFFF,FFFFFFFFFFFF,FFFFFFFFF:FFFFF:,FF:F:FFFFFFFFFFFFFFFFFFFFFFFFFFF
:FFFF,F,FFFFFFF:FF:F::F:FFFFFFFFFFFFFF::,FFFFFFFFFFFF,FFFF:FFFFFFF::
::FFFFFFFFFF:F
@A00721:190:HGJW7DSXY:4:2603:4227:10441 1:N:0:TCTGCAAG+GCTGCACT
CATTGCTTTATGCCACGCATAACAAAAATTATAAATTGAAATTACCCCTAAATTACAAAGATTTTTAT
```

```
GGATATAAAAATAAAGCCGTCGAGATAAAGTCCCAGAAATCATCGTCTTCCATGACGGGTTCTGCAGT
AATTCTGGGACTGGT
+
FFFFFFFFFFFFFFFFFFFFFFFFFFF:FFFFFFFFFFFFF,F:FFFFFFFFFFFFFFFFFFFFFFFFF
FFFFFFFFFFFFFFFFFFFFFFFFFFFFFFF:FFFFFFFFFFFFFFFFFFFF:FFFFFFFFFFFFF
FFFFFFFFFF:FFFF
@A00721:190:HGJW7DSXY:4:2611:6262:9580 1:N:0:TCTGCAAG+GCTGCACT
CATTGCTTTATGCCACGCATAACAAAAATTATAAATTGAAATTACCCCTAAATTACAAAGATTTTTAT
GGATATAAAAATAAAGCCGTCGAGATAAAGTCCCAGAAATCATCGTCTTCCATGACGGGTTCTGCAGT
AATTCTGGGACTGGT
+
FFFFFFFFFF:FFFFFFFFFFFFFFFFFFFFFFFFFFFFFFFFFF,FFFFFFFFFFFFFFFFF,FFFFF
:FFFFFFFFFFFFFF:FFFFF:FFFFFFFF,FFFFFFFFFFFFF:,:FF,FFF:FF:FFFFFFFFFFF
FFF,FFFFFFFFFFF
@A00721:190:HGJW7DSXY:4:2678:24921:25426 1:N:0:TCTGCAAG+GCTGCACT
AATTGCTTTATGCCACGCATAACAAAAATTATAAATTGAAATTACCCCTAAATTACAAAGATTTTTAT
GGATATAAAAATAAAGCCGTCGGGATAAAGTCCCAGAAATCATCGTCTTCCATGACGGGTTCTGCAGT
AATTCTGGGACTGGT
+
FFFFFFFFFFFFFFFFFFFFFFFFFFFFFFFFFFFFFFFFFFFFFFFFFFFFFFFFFFFFFFFFFFFFF
FFFFFFF:FFFFFFFFFFFFFFFFFFFFFFFFFFFFFFFFFFFFFFFFFFFFFFFFFFFFFFFFFFFFF
FFFFFFFFFFFFFF:
@A00721:190:HGJW7DSXY:4:1176:8612:33004 1:N:0:TCTGCAAG+GCTGCACT
ATTGCTTTATGCCACGCATAACAAAAATTATAAATTGAAATTACCCCTAAATTACAAAGATTTTTATG
GATATAAAAATAAAGCCGTCGGGATAAAGTCCCAGAAATCATCGTCTTCCATGACGGGTTCTGCAGTA
ATTCTGGGACTGGTT
+
FFFFFFFFFFFFFFFFFFFFFFFFFFFFFFFFFFFFFFFFFFFFFFFFFFFFFFFFFFFFFFFFFFFFF
FFFF:FFFFFFFFFFFFFFFFFFFFFFFFFFFFFFFFFFFFFFFFFFFFFFFFFFFFFFFFFFFFFFFF
FFFFFFFFF:FFFFF
@A00721:190:HGJW7DSXY:4:1177:23204:10426 1:N:0:TCTGCAAG+GCTGCACT
ATTGCTTTATGCCACGCATAACAAAAATTATAAATTGAAATTACCCCTAAATTACAAAGATTTTTATG
GATATAAAAATAAAGCCGTCGGGATAAAGTCCCAGAAATCATCGTCTTCCATGACGGGTTCTGCAGTA
ATTCTGGGACTGGTT
+
F:FFFFFFFFFFFFFFFFFFFFFFFFFFFFFFFFFFFFFFFFFFFFFFFFFFFFFFFFFFFFFFFFFFF
FF:FFF:FFFFFFFFFFFFFFFFFFFFFFFFFFFFFFFFFFFFFFFFFFFFFFFFFFFFFFFFFFFFFF
FFFFFFFFFFFFFFF
@A00721:190:HGJW7DSXY:4:1225:18810:7294 1:N:0:TCTGCAAG+GCTGCACT
ATTGCTTTATGCCACGCATAACAAAAATTATAAATTGAAATTACCCCTAAATTACAAAGATTTTTATG
GATATAAAAATAAAGCCGTCGGGATAAAGTCCCAGAAATCATCGTCTTCCATGACGGGTTCTGCAGTA
ATTCTGGGACTGGTT
+
FFFFFFFFF:FFFFFFFFFFFFFFFFFFFFFFFFF:FFFFFFFFFF:FFFFFFFFFFFFFFFFFFFFFF
FFFFFFFFF,FFFFF:FFFFFFFFFFFFFFFF:FFFFFF:FFFFFFFFFFFFFFFFFFFFFFFFFFFFF
FFFFFFFFF:FFFFF
@A00721:190:HGJW7DSXY:4:1269:10068:35368 1:N:0:TCTGCAAG+GCTGCACT
ATTGCTTTATGCCACGCATAACAAAAATTATAAATTGAAATTACCCCTAAATTACAAAGATTTTTATG
GATATAAAAATAAAGCCGTCGGGATAAAGTCCCAGAAATCATCGTCTTCCATGACGGGTTCTGCAGTA
ATTCTGGGACTGGTT
+
FFFFFFFFFFFFFFFFFFFFFFFFFFFFFFFFFFFFFFFFFFFFFFFFFFFFFFFFFFFFFFF::FFFFF
FF:FFFFFFFFFFFFFFFFFFFFFFFFFFFFFFFFFFFFFFFFFF,FFFFFFF:FFFFFFFFFFFFFFFF
FFFFFFFFFF:FFFF
```

```
@A00721:190:HGJW7DSXY:4:1340:20799:33755 1:N:0:TCTGCAAG+GCTGCACT
ATTGCTTTATGCCACGCATAACAAAAATTATAAATTGAAATTACCCCTAAATTACAAAGATTTTTATG
GATATAAAAATAAAGCCGTCGGGATAAAGTCCCAGAAATCATCGTCTTCCATGACGGGTTCTGCAGTA
ATTCTGGGACTGGTT
+
FFFFFFFFFFFFFFFFF:FFFFFFFFFFFFFFFFFFFFFFFFFF:FFFFFFFFFFF:F:FFFFFFFFF
FF:FFFFFFFFFFFF:FFFFFFFFFFFFFFFFFFFFFFFFFFFFFFFFFFFFFFFFFFFFFFFF:FFF
FFFFFFFFFFFFFF:
@A00721:190:HGJW7DSXY:4:1340:21061:33927 1:N:0:TCTGCAAG+GCTGCACT
ATTGCTTTATGCCACGCATAACAAAAATTATAAATTGAAATTACCCCTAAATTACAAAGATTTTTATG
GATATAAAAATAAAGCCGTCGGGATAAAGTCCCAGAAATCATCGTCTTCCATGACGGGTTCTGCAGTA
ATTCTGGGACTGGTT
+
FFFFFFFFFFFFFFFFFFFFFFFFFFFFFFFFFFFFFFFFFFFFFFFFFFFFFFFFFFFFFFFFFFFF
FFFFFFFFFFFFFFFFFFFFFFFFFFFFFFFFFFFFFFFFFFFFFFFFFFFFFFFFFFFFFFFFFFFF
:FFFFFFFFF:FFFF
@A00721:190:HGJW7DSXY:4:1347:11071:15624 1:N:0:TCTGCAAG+GCTGCACT
ATTGCTTTATGCCACGCATAACAAAAATTATAAATTGAAATTACCCCTAAATTACAAAGATTTTTATG
GATATAAAAATAAAGCCGTCGGGATAAAGTCCCAGAAATCATCGTCTTCCATGACGGGTTCTGCAGTA
ATTCTGGGACTGGTT
+
FFFFFFFFFFFFFFFFFFFFFFFFFFFFFFFFFFFFFFFFFFFFFFFFFFFFFFFFFFFFFFFFFFFF
FFFFFFFFFFFFFFFFFFFFFFFFFFFFFFFFFFFFFFFFFFFFFFFFFFFF:FFFFFFFFFFFFFFF
FFFFFFFFFFFFFFF
@A00721:190:HGJW7DSXY:4:1557:5023:16454 1:N:0:TCTGCAAG+GCTGCACT
ATTGCTTTATGCCACGCATAACAAAAATTATAAATTGAAATTACCCCTAAATTACAAAGATTTTTATG
GATATAAAAATAAAGCCGTCGGGATAAAGTCCCAGAAATCATCGTCTTCCATGACGGGTTCTGCAGTA
ATTCTGGGACTGGTT
+
FFFFFFFFFFFFFFFFFFFFFFFFFFFFFFFFFFFFFFFFFFFFFFFFFFFFFFFFFFFFFFFFFFFF
FFFFFFFFFFFFFFFFFFFFFFFFFFFFFFFFFFFFFFFFFFFFFFFFFFFFFFFFFFFFFFFFFFFF
FFFFFFFFFFFFFFF
@A00721:190:HGJW7DSXY:4:1618:3296:12774 1:N:0:TCTGCAAG+GCTGCACT
ATTGCTTTATGCCACGCATAACAAAAATTATAAATTGAAATTACCCCTAAATTACAAAGATTTTTATG
GATATAAAAATAAAGCCGTCGGGATAAAGTCCCAGAAATCATCGTCTTCCATGACGGGTTCTGCAGTA
ATTCTGGGACTGGTT
+
FFFFFFFFFFFFFFFFFFFFFFFFFFFFFFFFFFFFFFFFFFFFFFFFFFFFFFFFF:FFFFFFFFFF
FFFFFFFFFFFFFFFFFFFFFFFFFFFFFFF:FFFFFFFFFFFFFFFFFFFFFFFFFFFFFFFFFF:F
FFFFFFFFFFFFF:F
@A00721:190:HGJW7DSXY:4:1664:2284:35383 1:N:0:TCTGCAAG+GCTGCACT
ATTGCTTTATGCCACGCATAACAAAAATTATAAATTGAAATTACCCCTAAATTACAAAGATTTTTATG
GATATAAAAATAAAGCCGTCGGGATAAAGTCCCAGAAATCATCGTCTTCCATGACGGGTTCTGCAGTA
ATTCTGGGACTGGTT
+
FFFFFFFFFFFFFFFFFFFFFFFFFFFFFFFFFFFFFFFFFFFFFFFFFFFFFFFFFFFFFFFFFFFF
FFFFFFFFFFFFFFFFFFFF:FFFFFFFFFFFFFFFFFFFFFFFFFFFFFFFFFFFFFFFFFFFFFFF
FFFFFFFFFFFFFFF
@A00721:190:HGJW7DSXY:4:1670:8332:12915 1:N:0:TCTGCAAG+GCTGCACT
ATTGCTTTATGCCACGCATAACAAAAATTATAAATTGAAATTACCCCTAAATTACAAAGATTTTTATG
GATATAAAAATAAAGCCGTCGGGATAAAGTCCCAGAAATCATCGTCTTCCATGACGGGTTCTGCAGTA
ATTCTGGGACTGGTT
+
FFFFFFFFFFFFFFFFFFFFFFFFFFFFFFFFFFFFFFFFFFFFFFFFFF:FFFFFFFFFFFFFFFFF
```

FFF,FFFFFFFFFFFFFFFFFFFFFFFFFFFFFFFFFFFFFFFFFFFFFFFFFFFFFFFFFFFFFFFF
FFFFFFFFF:FFFFF
@A00721:190:HGJW7DSXY:4:2157:4056:11021 1:N:0:TCTGCAAG+GCTGCACT
ATTGCTTTATGCCACGCATAACAAAAATTATAAATTGAAATTACCCCTAAATTACAAAGATTTTTATG
GATATAAAAATAAAGCCGTCGGGATAAAGTCCCAGAAATCATCGTCTTCCATGACGGGTTCTGCAGTA
ATTCTGGGACTGGTT
+
FFFFFFFFFFFFFFFFFFFFFFFFFFFFFF,FFFFFFFFFFFFFFFFFFFFFFFFFFFFFFFFFFF,F
FF:F:FFFFFFFFFFFFFFFFFFF:FFFFFFFFFFFFFFFFFFFFFFFFFFFFFFFFFFFFFFFFFFF
FFFFFFF:FFFFFFF
@A00721:190:HGJW7DSXY:4:2165:5665:32503 1:N:0:TCTGCAAG+GCTGCACT
ATTGCTTTATGCCACGCATAACAAAAATTATAAATTGAAATTACCCCTAAATTACAAAGATTTTTATG
GATATAAAAATAAAGCCGTCGGGATAAAGTCCCAGAAATCATCGTCTTCCATGACGGGTTCTGCAGTA
ATTCTGGGACTGGTT
+
FFFFFFFFFFFFFFFFFFFFFFFFFFFFFFFFFFFFFFFFFFFFFFFFFFFFFFFFFFFFFFFFFFFF
FFFFFFFFFFFFFFFFFFFFFFF:FFFFFFFFFFFFFFFFFFFFFFFFFFFFFFFFFF:FFFFFFFFF
FFFFFFFFFF:FFFF
@A00721:190:HGJW7DSXY:4:2177:24053:12837 1:N:0:TCTGCAAG+GCTGCACT
ATTGCTTTATGCCACGCATAACAAAAATTATAAATTGAAATTACCCCTAAATTACAAAGATTTTTATG
GATATAAAAATAAAGCCGTCGGGATAAAGTCCCAGAAATCATCGTCTTCCATGACGGGTTCTGCAGTA
ATTCTGGGACTGGTT
+
FFF:FFFFF:FFFFFFFFFFFFFFFFF::FFF:FF:,:FFF,FFFFF::FF:FFFFFF:::FF:FFFF
:FFFFFFFFFFFFF:F:FFFFF:FFFFFF:F,FFFFF,::FFFFF,FFF,F:FF:FFFFFFFFFFF,F
FFFFFFFFFFFFFFF
@A00721:190:HGJW7DSXY:4:2209:22209:14497 1:N:0:TCTGCAAG+GCTGCACT
ATTGCTTTATGCCACGCATAACAAAAATTATAAATTGAAATTACCCCTAAATTACAAAGATTTTTATG
GATATAAAAATAAAGCCGTCGGGATAAAGTCCCAGAAATCATCGTCTTCCATGACGGGTTCTGCAGTA
ATTCTGGGACTGGTT
+
FFFF,FFFFFFFFFFFFFFFFFFFF::FFFFFFFFFFFFFFFFFFFFFFFFFFFFFFFF:FFFFFFFF
FFF:FFF:FFFFFF::F,FFFFFFFFFFFFF:FFFFFFFFFFFFFFFFFFFFFFFF:FFFFFF:FFFF
FFFFFFFFF:FFFFF
@A00721:190:HGJW7DSXY:4:2217:3305:18145 1:N:0:TCTGCAAG+GCTGCACT
ATTGCTTTATGCCACGCATAACAAAAATTATAAATTGAAATTACCCCTAAATTACAAAGATTTTTATG
GATATAAAAATAAAGCCGTCGGGATAAAGTCCCAGAAATCATCGTCTTCCATGACGGGTTCTGCAGTA
ATTCTGGGACTGGTT
+
F:FFFFFFFF:FFFFFFFFFFFFFFFFFFFFFFFFFFFFFFFFFFFFFFFFFFFFFFFFFFFF:FFFF
FFFFFFFFFFFFFFFFFFFFFFFFFFFFF:FFFFFFFFFFFFFFFFFFF:FFFFF:FFFFFF:FFFFF
FFFFFFFFFFFFFFF
@A00721:190:HGJW7DSXY:4:2222:6605:6637 1:N:0:TCTGCAAG+GCTGCACT
ATTGCTTTATGCCACGCATAACAAAAATTATAAATTGAAATTACCCCTAAATTACAAAGATTTTTATG
GATATAAAAATAAAGCCGTCGGGATAAAGTCCCAGAAATCATCGTCTTCCATGACGGGTTCTGCAGTA
ATTCTGGGACTGGTT
+
FFF:FF,:F,:FFFFFFFFFFFFFFFF::FFFFFFFFFFFFFFFFFFFFFFFFF,FFFFFFF:FFF:F
FFFFFFFFFFFFFF:FFF:,::FF,FFFFFFF:F,FFFFFF:F,:FFFF,FFFFFF,,F::F,FFFFF
FFFFFFF:FFF,F,F
@A00721:190:HGJW7DSXY:4:2226:2555:20071 1:N:0:TCTGCAAG+GCTGCACT
ATTGCTTTATGCCACGCATAACAAAAATTATAAATTGAAATTACCCCTAAATTACAAAGATTTTTATG
GATATAAAAATAAAGCCGTCGGGATAAAGTCCCAGAAATCATCGTCTTCCATGACGGGTTCTGCAGTA
ATTCTGGGACTGGTT

```
+
FFFFFFFFFFFFFFFFFFFFFFFFFFFFFFFFFFFFFFFFFFFFFFFFFFFFFFFFFFFFFFFFFFFF
FFFFFFFFFFFFFFFFFFFFFFFFFFFFFFFFFFFFFFFFFFFFFFFFFFFFFFFFFFFFFFFFFF:F
FFFFFFFFFFFFF:F
@A00721:190:HGJW7DSXY:4:2252:27127:17315 1:N:0:TCTGCAAG+GCTGCACT
ATTGCTTTATGCCACGCATAACAAAAATTATAAATTGAAATTACCCCTAAATTACAAAGATTTTTATG
GATATAAAAATAAAGCCGTCGGGATAAAGTCCCAGAAATCATCGTCTTCCATGACGGGTTCTGCAGTA
ATTCTGGGACTGGT
+
FFFFFFFFFFFFFFFFFFFFFFFFFFFFFFFFFFFFFFFFFFFFFFFFFFFFFFFFFFFFFFFFFF:F
FFFFFFFF:FFFFFFFFFFFFFFFFFFFFFFFFFFFFFFFFFFFFFFFFFFFFFFFFFFFFFFFFFF:
FFFFFFFFF:FFFF
@A00721:190:HGJW7DSXY:4:2310:31213:17378 1:N:0:TCTGCAAG+GCTGCACT
ATTGCTTTATGCCACGCATAACAAAAATTATAAATTGAAATTACCCCTAAATTACAAAGATTTTTATG
GATATAAAAATAAAGCCGTCGGGATAAAGTCCCAGAAATCATCGTCTTCCATGACGGGTTCTGCAGTA
ATTCTGGGACTGGTT
+
FFFFFFFFFFFFFFF:FF:FFFFFFF:FFFFFFFFFFFFFFFFFFFF:FFFFFFFFFFFFFFFFFFFF
FFFFFFF:F:FFFFF:FFFFFFFFFFFFFFF:FFFFFFF:FFF::FFFFFFFFFFFFFFF:FFFFFFF
FFFFFFFFFFFFF:F
@A00721:190:HGJW7DSXY:4:2326:9833:31986 1:N:0:TCTGCAAG+GCTGCACT
ATTGCTTTATGCCACGCATAACAAAAATTATAAATTGAAATTACCCCTAAATTACAAAGATTTTTATG
GATATAAAAATAAAGCCGTCGGGATAAAGTCCCAGAAATCATCGTCTTCCATGACGGGTTCTGCAGTA
ATTCTGGGACTGGTT
+
FFFFFFFFFFFFFFFFFFFFFFFFFFFFFFFFFFFFFFFFFFFFFFFFFFFFFFFFFFFFFFFFFFFF
FFFFFFFFFFFFFFFFFFFFFFFFFFFFFFFFFFFFFFFFFFFFFFFFFFFFFFFFFFFFFFFFFFFF
FFFFFFFFFFFFFFF
@A00721:190:HGJW7DSXY:4:2635:15835:28855 1:N:0:TCTGCAAG+GCTGCACT
ATTGCTTTATGCCACGCATAACAAAAATTATAAATTGAAATTACCCCTAAATTACAAAGATTTTTATG
GATATAAAAATAAAGCCGTCGGGATAAAGTCCCAGAAATCATCGTCTTCCATGACGGGTTCTGCAGTA
ATTCTGGGACTGGTT
+
FFFFFFFFFFFFFFFFFFFFFFFFFF:FFFFFFFFFFFFFFFFFFFFFFFFFFFFFFFFFFFFFFFFF
FFFFFFFFFFFFFFFFFFF:FFFFFFFFFFF,FFFFFFFFFFFFFFFFF:FFFF:FFFFFFFFFFFFF
FFFFFF,FFF,FFFF
@A00721:190:HGJW7DSXY:4:2678:31503:30655 1:N:0:TCTGCAAG+GCTGCACT
ATTGCTTTATGCCACGCATAACAAAATTTATAAATTGAAATTACCCCTAAATTACAAAGATTTTTATG
GATATAAAAATAAAGCCGTCGGGATAAAGTCCCAGAAATCATCGTCTTCCATGACGGGTTCTGCAGTA
ATTCTGGGACTGGTT
+
FFFFFFFFFFFFFFFFFFFFFFFFFF,:FFFFFFFFFFFFFFFFFFFFFFFFFFFFFF::FFFFFFFF
FFFFFFFFFFFFF:FFFFFFFFFFFFFFFFF:FFFFFFFFFFFFFFFFFF,FFFFFFFFFFFFFFFFF
FFFFFFFF:,,FFFF
@A00721:190:HGJW7DSXY:4:1511:4264:21057 1:N:0:TCTGCAAG+GCTGCACT
GCCACGCATAACAAAAATTATAAATTGAAATTACCCCTAAATTACAAAGATTTTTATGGATATAAAAA
TAAAGCCGTCGGGATAAAGTCCCAGAAATCATCGTCTTCCATGACGGGTTCTGCAGTAATTCTGGGAC
TGGTTGTAATGTC
+
FFFFFFFFFFFFFFFFFFFFFFFFFFFFFFFFFFFFFFFFFFFFFFFFFFFFFFF:FFFFFFFFFFFF
FFFFFFFFFFFFFFFFFFFFFFFFFFFFFFFFFFFFFFFFFFFFFFFFFFFFFFFFFFFFFFFFFF:F
:FFFFFFFFFFFF
@A00721:190:HGJW7DSXY:4:1176:25066:7420 1:N:0:TCTGCAAG+GCTGCACT
TCCACGCATAACAAAAATTATAAATTGAAATTACCCCTAAATTACAAAGATTTTTATGGATATAAAAA
```

```
TAAAGCCGTCGGGATAAAGTCCCAGAAATCATCGTCTTCCATGACGGGTTCTGCAGTAATTCTGGGAC
TGGTTGTAATGTCGT
+
FFFFFFFFFFFFFFFFFFFFFFFFFFFFFFFFFFFFFFFFFFFFFFF:FFFFFFFFFFFFFFFFFFFF
FFFFFFFFFFFFFFFFFFFFFFFFFFFFFFFFFFFFF:FFFFFFFFFFFFFFFF:FFFFF:FFF:FFF
FFFFFFFFFFFFFFF
@A00721:190:HGJW7DSXY:4:1245:15908:1078 1:N:0:TCTGCAAG+GCTGCACT
TCCACGCATAACAAAAATTATAAATTGAAATTACCCCTAAATTACAAAGATTTTTATGGATATAAAAA
TAAAGCCGTCGGGATAAAGTCCCAGAAATCATCGTCTTCCATGACGGGTTCTGCAGTAATTCTGGGAC
TGGTTGTAATGTCGT
+
FFFFFFFFFFFFFFFFFFFFFFFFFFFFFFFFFFFFFFFFFFFFFFFFFFFFFFFFFFFFF:FFFFFF
FFFFFFFFFFFFFFFFFFFFFFFFFFFFFFFFFFFFFFFFFFFFFFFFFFFFFFFFFF:FFFFFFF:F
FFFFFFFFFFFFFFF
@A00721:190:HGJW7DSXY:4:2225:3685:22748 1:N:0:TCTGCAAG+GCTGCACT
TCCACGCATAACAAAAATTATAAATTGAAATTACCCCTAAATTACAAAGATTTTTATGGATATAAAAA
TAAAGCCGTCGGGATAAAGTCCCAGAAATCATCGTCTTCCATGACGGGTTCTGCAGTAATTCTGGGAC
TGGTTGTAATGTCGT
+
FFFFFFFFFFFFFFFFFFFFFFFFFFFFFFFFFFFFFFFFFFFFFFFF:FFFFFFFFFFFFFFFFFFF
FFFFF:FFFFFFFFFFFFFFFF:F:FFFFFFFFFFFF,FFFFFFFFFFF:FFFFFFFFFFFFFF:FF:
FFFFFFF:FF:FF,F
@A00721:190:HGJW7DSXY:4:2243:7943:28526 1:N:0:TCTGCAAG+GCTGCACT
TCCACGCATAACAAAAATTATAAATTGAAATTACCCCTAAATTACAAAGATTTTTATGGATATAAAAA
TAAAGCCGTCGGGATAAAGTCCCAGAAATCATCGTCTTCCATGACGGGTTCTGCAGTAATTCTGGGAC
TGGTTGTAATGTCGT
+
FFFFFFFF:FFFFFFFFFFFFFFFFFFFFFFFFFFFFFFFFFFFFFFFFFFFFFFFFFFFFFFFFFFF
FFFFF:FFFFFFFFFFFFFFFFFFFFFFFFFFFFFFFFFFFFFFFFFFFFFFFFFFFFFFFFFFFFFF
FFFFFFFFFFFFFFF
@A00721:190:HGJW7DSXY:4:2342:9570:19977 1:N:0:TCTGCAAG+GCTGCACT
TCCACGCATAACAAAAATTATAAATTGAAATTACCCCTAAATTACAAAGATTTTTATGGATATAAAAA
TAAAGCCGTCGGGATAAAGTCCCAGAAATCATCGTCTTCCATGACGGGTTCTGCAGTAATTCTGGGAC
TGGTTGTAATGTCGT
+
FFFFFFFFFFFFFFFFFFFFFFFFFFFFFFFFFFFFFFFFFFFFFFF:FFFFFFFFFFFFFFFFFFFF
FFFFFFFFFFFFFFFFFFFFFFFFFFFFFFFFFFFFFFF:FFFFFFFFFFFFFFFFFFFFFFFFFFFF
FFFFFFFFFFFFFFF
@A00721:190:HGJW7DSXY:4:2358:19524:33708 1:N:0:TCTGCAAG+GCTGCACT
TCCACGCATAACAAAAATTATAAATTGAAATTACCCCTAAATTACAAAGATTTTTATGGATATAAAAA
TAAAGCCGTCGGGATAAAGTCCCAGAAATCATCGTCTTCCATGACGGGTTCTGCAGTAATTCTGGGAC
TGGTTGTAATGTCGT
+
FFFFFFFFFFFFFFFFFFFFFFFFFFFFFFFFFFFFFFFFFFFFFFFFFFFFFF:FFFFFFFFFFFF
FFFF:FFFFFFFFFFFFFFFFFFFFFFF:FFFFFFFFFFFFFFFFFFFFFFFFFFFFFFFFFFFFFFF
FFFFFF:FFFFFFFF
@A00721:190:HGJW7DSXY:4:2554:14362:7858 1:N:0:TCTGCAAG+GCTGCACT
TCCACGCATAACAAAAATTATAAATTGAAATTACCCCTAAATTACAAAGATTTTTATGGATATAAAAA
TAAAGCCGTCGGGATAAAGTCCCAGAAATCATCGTCTTCCATGACGGGTTCTGCAGTAATTCTGGGAC
TGGTTGTAATGTCGT
+
FFFFFFFFFFFFFFFFFFFFFFFFFFFFFFFFFFFFFFFFFFFFFFFF:FFFFFFFFFFFFFFFFF,F
FFFFFFFFFFFFFFFFFFFFFFFFFFFFFFFFFFFFFF:FFFFF:FFFFFFFFFFFFFFFFFFFFFFF
FFFFFFFFFFFFFFF
```

```
@A00721:190:HGJW7DSXY:4:2665:8232:9486 1:N:0:TCTGCAAG+GCTGCACT
TCCACGCATAACAAAAATTATAAATTGAAATTACCCCTAAATTACAAAGATTTTTATGGATATAAAAA
TAAAGCCGTCGGGATAAAGTCCCAGAAATCATCGTCTTCCATGACGGGTTCTGCAGTAATTCTGGGAC
TGGTTGTAATGTCGT
+
FFFFFFFFFFFFFFFFFFFFFFFFFFFFFFFFFFFFFFFFFFFFFFFFFFFFFFFFFFFFFFFFFFFF
FFFFFFFFFFFFFFFFFFFFFFFFFFFFFFFFFFFFFFFFFFFFFFFFFFFFFFFFFFFFFFFFFFFF
:FFFFFFFFFFFFFF
@A00721:190:HGJW7DSXY:4:1257:21784:6715 1:N:0:TCTGCAAG+GCTGCACT
CACGCATAACAAAAATTATAAATTGAAATTACCCCTAAATTACAAAGATTTTTATGGATATAAAAATA
AAGCCGTCGGGATAAAGTCCCAGAAATCATCGTCTTCCATGACGGGTTCTGCAGTAATTCTGGGACTG
GTTGTAATGTCGTCC
+
FFFFFFFFFFFFFFFFFFFFFF:FFFFFFFFFFFFFFFFFFFFFFFFFFFFFFFFFFFFFFFFFF:FFF
FFFFFFFFFFFFFFFFFFFFFFFFFFFFFFFFFFF:FFFFF:FFFF:FFFFFFFFF:FF:FFFFF:,F
FFFFF:::FFFF:FF
@A00721:190:HGJW7DSXY:4:1602:10511:19507 1:N:0:TCTGCAAG+GCTGCACT
CACGCATAACAAAAATTATAAATTGAAATTACCCCTAAATTACAAAGATTTTTATGGATATAAAAATA
AAGCCGTCGGGATAAAGTCCCAGAAATCATCGTCTTCCATGACGGGTTCTGCAGTAATTCTGGGACTG
GTTGTAATGTCGTCC
+
FFFFFFFF:FFFFFFFFFFFFFFF:FFFF:FFF:FFFFFF:FFFFFFFFF:FFFFF,F:FFFFFFF:F
FFF:FFFFFFFFFFFFFFF,FFFFFFFFFF,FFFFFFFFF,FF:FF,FFFFFFFFFFFFFFF:FFF:F
:FFFF:FF:,FF,FF
@A00721:190:HGJW7DSXY:4:1602:10438:19852 1:N:0:TCTGCAAG+GCTGCACT
CACGCATAACAAAAATTATAAATTGAAATTACCCCTAAATTACAAAGATTTTTATGGATATAAAAATA
AAGCCGTCGGGATAAAGTCCCAGAAATCATCGTCTTCCAAGACGGGTTCTGCAGTAATTCTGGGACTG
GTTGTAATGTCGTC
+
FFF:FF:FFFFFFFF:FFFFFFFFFFFF,FFFFFFFFFFFFF,FF:,FFFFFFFFFFFFFFFFFFF:F
:FFFF,,FF::FFFFFFFFF,:FFFFFFFFFF:FFFFFF,:FFFF:FFFFFFFF:FFF:FF:FFF:F:
:FFFFFF:FFF:FF
@A00721:190:HGJW7DSXY:4:1602:12834:21308 1:N:0:TCTGCAAG+GCTGCACT
CACGCATAACAAAAATTATAAATTGAAATTACCCCTAAATTACAAAGATTTTTATGGATATAAAAATA
AAGCCGTCGGGATAAAGTCCCAGAAATCATCGTCTTCCATGACGGGTTCTGCAGTAATTCTGGGACTG
GTTGTAATGTCGTCC
+
FFFFFFFFFFFFFFF:FFFFFFFFFFFFFFFFFFFFFFFFFFFFFFFFFFFFFFFFFFFFFFF:FFFF
FFFFFFFFFFFFFFFFFFFFFFFFFFFFFFFFFFFFFFFFFFF:FFFFFFFFFFFFFFFFFFFFFFFF
FFFFFFFFFFFFFFF
@A00721:190:HGJW7DSXY:4:1620:17824:21966 1:N:0:TCTGCAAG+GCTGCACT
CACGCATAACAAAAATTATAAATTGAAATTACCCCTAAATTACAAAGATTTTTATGGATATAAAAATA
AAGCCGTCGGGATAAAGTCCCAGAAATCATCGTCTTCCATGACGGGTTCTGCAGTAATTCTGGGACTG
GTTGTAATGTCGTCC
+
FFFFFFFFFFFFFF:FFFFFFFFFFFFFFFFFFFFFFFFFFFFFFFFFFFFFFFFFFFFFFFFFFFFF
FFFFFFFFFFFF:FFFFFFFFFFFFFFFFFFFFFFFF:FFFFFFFFFFFFFFFFFFFFFFFFFFFFFF
FFFFFFFFFFFFFFF
@A00721:190:HGJW7DSXY:4:1649:15718:16658 1:N:0:TCTGCAAG+GCTGCACT
CACGCATAACAAAAATTATAAATTGAAATTACCCCTAAATTACAAAGATTTTTATGGATATAAAAATA
AAGCCGTCGGGATAAAGTCCCAGAAATCATCGTCTTCCATGACGGGTTCTGCAGTAATTCTGGGACTG
GTTGTAATGTCGTCC
+
FFFFFFFFFFFFFFFFFFFFFFFFFFFFFFFFFFFFFFFFFFFFFFFFFFFFFFFFFFFFFFFFFFFF
```

FFFFFFFFFFFFFFFFFFFFFFFFFFFFFFFFFFFFFFFFFFFFFFFFFFFFFFFFFFFFFFFFFFFF
FFFFFFFFFFFFFFF
@A00721:190:HGJW7DSXY:4:2167:26521:21715 1:N:0:TCTGCAAG+GCTGCACT
CACGCATAACAAAAATTATAAATTGAAATTACCCCTAAATTACAAAGATTTTTATGGATATAAAAATA
AAGCCGTCGGGATAAAGTCCCAGAAATCATCGTCTTCCATGACGGGTTCTGCAGTAATTCTGGGACTG
GTTGTAATGTCGTCC
+
FFFFFFFFFFFFFFFFFFFFFFFFFFFFFFFFFFFFFFFFFFFFFFFFFFFFFFFFFFFFFFFFFFFF
FFFFFFFFFFFFFFFFFFFFFFFFFFFFFFFFFFF:FFFFFFFFFFFFFFFFFFFFFFFFFFFFF:FF
FFFFFFFFFFFFFFF
@A00721:190:HGJW7DSXY:4:2317:29884:17738 1:N:0:TCTGCAAG+GCTGCACT
CACGCATAACAAAAATTATAAATTGAAATTACCCCTAAATTACAAAGATTTTTATGGATATAAAAATA
AAGCCGTCGGGATAAAGTCCCAGAAATCATCGTCTTCCATGACGGGTTCTGCAGTAATTCTGGGACTG
GTTGTAATGTCGTCC
+
FFFFFFFFFFFFFFFFFFFFFFFFFFFFFFFFFFFFFFFFFFFFFFFFFFFFFFFFFFFFFFFFFFFF
FFFFFFFFFFFFFFFFFFFFFFFFFFFFFFFFFFFFFFFFFFFFFFFFFFFFFFFFFFFFFFFFFFFF
FFFFFFFFFFFFFFF
@A00721:190:HGJW7DSXY:4:2362:5348:26068 1:N:0:TCTGCAAG+GCTGCACT
CACGCATAACAAAAATTATAAATTGAAATTACCCCTAAATTACAAAGATTTTTATGGATATAAAAATA
AAGCCGTCGGGATAAAGTCCCAGAAATCATCGTCTTCCATGACGGGTTCTGCAGTAATTCTGGGACTG
GTTGTAATGTCGTCC
+
FFFFFFFFFFFFFFFFFFFFFFFFFFFFFFFFFFFFFFFFFFFFFFFFFFFFFF:FFFFFFFFFFFFF
FFFFFF:FFFFFFFFFFFFFFFFFFFFFFFFFFFFFFFFFFFFFFFFFFFFFFFFFFFFFFFFFFFFF
F:FFFFFFFFFFFFF
@A00721:190:HGJW7DSXY:4:2540:9408:23516 1:N:0:TCTGCAAG+GCTGCACT
CACGCATAACAAAAATTATAAATTGAAATTACCCCTAAATTACAAAGATTTTTATGGATATAAAAATA
AAGCCGTCGGGATAAAGTCCCAGAAATCATCGTCTTCCATGACGGGTTCTGCAGTAATTCTGGGACTG
GTTGTAATGTCGTCC
+
FFFFFFFFFFFFFFF:FFFFFFF:FFFFFFFFFFF:FFFF:FFFFFFFFF,FFFFFFFFFFFFFFFFF
FFFFFF:FFFFFFFFFFFFFFFFFFFFFFFFFFFFFFFFFFFFFFFFFFFFFFFFFFFFFFFFFFFFF
FFFFFFFFFFFFFFF
@A00721:190:HGJW7DSXY:4:2601:9227:1783 1:N:0:TCTGCAAG+GCTGCACT
CACGCATAACAAAAATTATAAATTGAAATTACCCCTAAATTACAAAGATTTTTATGGATATAAAAATA
AAGCCGTCGGGATAAAGTCCCAGAAATCATCGTCTTCCATGACGGGTTCTGCAGTAATTCTGGGACTG
GTTGTAATGTCGTCC
+
FFFFFFFFFFFFFFFFFFFFFFFFFFFFFFFFFFFFFFFFFFFFFFFFFFFFFFFFFFFFFFFFFFFF
F:FFFFFFFFFFFFFFFFFF:FFFFFFFFFFFFFFFFFFFFFFFFFFFFFFFFFFFFFFFFFFFFFFF
FFFFFFFFFFFFFFF
@A00721:190:HGJW7DSXY:4:2624:15609:4069 1:N:0:TCTGCAAG+GCTGCACT
CACGCATAACAAAAATTATAAATTGAAATTACCCCTAAATTACAAAGATTTTTATGGATATAAAAATA
AAGCCGTCGGGATAAAGTCCCAGAAATCATCGTCTTCCATGACGGGTTCTGCAGTAATTCTGGGACTG
GTTGTAATGTCGTCC
+
FFFFFFFFFFFFFFFFFFFFFFFFFFFFFFFFFFFFFFFFFFFFFFFFF:FFF,FFFFFFFFFFFFFF
FFFFFFFFFFFFFFFFFFFFFFFFFFFFF:F:FFFFFFFFFFFFFFFFFFF,FFFFFFFFFFFFFFFF
FFFFFF:FFFFFFF:
@A00721:190:HGJW7DSXY:4:1574:7111:20854 1:N:0:TCTGCAAG+GCTGCACT
TACCCCTAAATTACAAAGATTTTTATGGATATAAAAATAAAGCCGTCGGGATAAAGTCCCAGAAATCA
TCGTCTTCCATGACGGGTTCTGCAGTAATTCTGGGACTGGTTGTAATGTCGTCCAGATGCGGATAGTC
CACCATGCTCGCTTT

+
FFFFFFFFFFFFFFFFFFFFFF:FFFFFFFFFFFFFFFFFFFFFFFFFFFFFFFFFFFFFFFFFFFFF
FFFFFFFFFFFFFFFFFFFFFFFFFFFFFFFFFFFFFFFFFFF:FFFFFFFFFFFFFFFFFFFFFFFF
FFFF::FFFFFFFFF
@A00721:190:HGJW7DSXY:4:2261:5457:4460 1:N:0:TCTGCAAG+GCTGCACT
TACCCCTAAATTACAAAGATTTTTATGGATATAAAAATAAAGCCGTCGGGATAAAGTCCCAGAAATCA
TCGTCTTCCATGACGGGTTCTGCAGTAATTCTGGGACTGGTTGTAATGTCGTCCAGATGCGGATAGTC
CACCATGCTCGCTTT
+
FFFFFFFFFFFFFFFF:FFFFFFFF:FFFFFFFFFFFFFFFFFFFFFFFFFFFFFFFFFFFF:FFFFF
FFFFFFFFFFF:::FFFFFF:FFFFFFFFFFFFF::,FFFFFFFFFFFFFFFFFFFFFFFFFFFFFFF
FFFFF:FFFFFFFFF
@A00721:190:HGJW7DSXY:4:2465:18141:36104 1:N:0:TCTGCAAG+GCTGCACT
TACCCCTAAATTACAAAGATTTTTATGGATATAAAAATAAAGCCGTCGGGATAAAGTCCCAGAAATCA
TCGTCTTCCATGACGGGTTCTGCAGTAATTCTGGGACTGGTTGTAATGTCGTCCAGATGCGGATAGTC
CACCATGCTCGCTTT
+
FFFFFFFFFFFFFFFFFFFFFFFFFFFFFF:FFFFF,FFFFFFFFFFFFFFFFF:FFFFFFFFFFFFF
FFF:FFFFFFFFFFFFFFFFFFFFFFFFFFFFFFFF:FFFFFFFFFFFFFFFFFFFFFFFFFFFFFFF
FFFFFFFFFFFFFFF
@A00721:190:HGJW7DSXY:4:2549:17716:36276 1:N:0:TCTGCAAG+GCTGCACT
TACCCCTAAATTACAAAGATTTTTATGGATATAAAAATAAAGCCGTCGGGATAAAGTCCCAGAAATCA
TCGTCTTCCATGACGGGTTCTGCAGTAATTCTGGGACTGGTTGTAATGTCGTCCAGATGCGGATAGTC
CACCATGCTCGCTTT
+
FFFFFFFFFFFFFFFFFFFFFFFFFFFFFFFFFFFFFFFFFFFFFFFFFFFFFFFFFFFFFFFFFFFF
FFFFFFFFFFFFFFFFFFFFFFFFFFFFFFFFFFFFFFFFF:FFFF,FFFFFFFFFFFFFFFFFFFFF
F,FF:FFFFF:FFFF
@A00721:190:HGJW7DSXY:4:1431:5141:7607 1:N:0:TCTGCAAG+GCTGCACT
ACCCCTAAATTACAAAGATTTTTATGGATATAAAAATAAAGCCGTCGGGATAAAGTCCCAGAAATCAT
CGTCTTCCATGACGGGTTCTGCAGTAATTCTGGGACTGGTTGTAATGTCGTCCAGATGCGGATAGTCC
ACCATGCTCGCTTTA
+
FFFFFFFFFFFFFFFFFFFFFFFFFFF:F:FFFFFFFFFFFFFFFFFFF:FFFFFFFFFFFFF:FFFF
FF:FFFFFFFF,FFFFFFFFFFFFFF:FFFFFFF:F:FF:FFFFFFFFFFFFFFFFFFFFF:FFFFFF
FFFFFFFFFFFFFFF
@A00721:190:HGJW7DSXY:4:1568:27055:21355 1:N:0:TCTGCAAG+GCTGCACT
ACCCCTAAATTACAAAGATTTTTATGGATATAAAAATAAAGCCGTCGGGATAAAGTCCCAGAAATCAT
CGTCTTCCATGACGGGTTCTGCAGTAATTCTGGGACTGGTTGTAATGTCGTCCAGATGCGGATAGTCC
ACCATGCTCGCTTTA
+
FFFFFFFFFFFFFFFFFFFFFFFFFFFFFFFFFFFFFFFFFFFFFFFFFFFFFFFFF:FFFFFFFFFF
FFFFFFFFFFFFFFFFFFFFFFFFFFFFFFFFFFFFFFFFFFFFFFFFFFFFF:FFFFFFFFFFFFFF
FFF,FFFFFFFFFFF
@A00721:190:HGJW7DSXY:4:2320:28492:21684 1:N:0:TCTGCAAG+GCTGCACT
ACCCCTAAATTACAAAGATTTTTATGGATATAAAAATAAAGCCGTCGGGATAAAGTCCCAGAAATCAT
CGTCTTCCATGACGGGTTCTGCAGTAATTCTGGGACTGGTTGTAATGTCGTCCAGATGCGGATAGTCC
ACCATGCTCGCTTTA
+
FFFFFFFFFFFFFFFFFFFFFFF:FFFFFFFFFFFFFFFFFFFFFFFFFFFFFFFFFFFFFFFFFFFF
FFFFFFFFFFFFFFFFFFFFFFFFF:FFFFFFFFF::FFFFFFFFFFFFFFFFFFFFFFFFFFFFFFF
FFF,FFFFFFFFFFF
@A00721:190:HGJW7DSXY:4:2477:10728:31093 1:N:0:TCTGCAAG+GCTGCACT
ACCCCTAAATTACAAAGATTTTTATGGATATAAAAATAAAGCCGTCGGGATAAAGTCCCAGAAATCAT

```
CGTCTTCCATGACGGGTTCTGCAGTAATTCTGGGACTGGTTGTAATGTCGTCCAGATGCGGATAGTCC
ACCATGCTCGCTTTA
+
FFFFFFFFFFFFFFFFFFFFFFFFFFFFFFFFFFFFFFFFFFFFFFFFFFFFFFFFFFFFFFFFFFFF
FFFFFFFFFFFFFFFFFFFFFFFFFFFFFFFFFFFFFFFFFFFFFFFFFFFFFFF:FFF:FFFFFFF
FFF:FFFFFFFFFFF
@A00721:190:HGJW7DSXY:4:1273:9435:23876 1:N:0:TCTGCAAG+GCTGCACT
CCCTAAATTACAAAGATTTTTATGGATATAAAAATAAAGCCGTCGGGATAAAGTCCCAGAAATCATCG
TCTTCCATGACGGGTTCTGCAGTAATTCTGGGACTGGTTGTAATGTCGTCCAGATGCGGATAGTCCAC
CATGCTCGCTTTATC
+
FFFFFFFFFFFFF:,FFFFFFFFFFF:FFFFFFFFFFFFFFFFFFFFFFFFF:FFFFFF:FFFFFFFFF
FFFFFFFFF:FFFFFFFFFFFFFFFFFFFFFFFF:FFF,FFF,FFFFFFFFFFFFFFFFFFFFFFFFFF
FF,FFFFFFFFFFFF
@A00721:190:HGJW7DSXY:4:1362:11180:22608 1:N:0:TCTGCAAG+GCTGCACT
CCCTAAATTACAAAGATTTTTATGGATATAAAAATAAAGCCGTCGGGATAAAGTCCCAGAAATCATCG
TCTTCCATGACGGGTTCTGCAGTAATTCTGGGAGTGGTTGTAATGTCGTCCAGATGCGGATAGTCCAC
CATGCTCGCTTTATC
+
FFFFFFFFFFFFFFFFFFFFFFFFFFFFFFFFFFFFF:FFFFFFFFFFFFF:FFFFFFFFFFFFFFFF
FFFFFFFFFFFFFFFFFFFFFFFFFFFFFFFFFFFFF:FFFFFFFFFFFFFFFFFFFFFFFF:FFFFFF
FF,FFFFFFFFFFFF
@A00721:190:HGJW7DSXY:4:1533:6379:4523 1:N:0:TCTGCAAG+GCTGCACT
CCCTAAATTACAAAGATTTTTATGGATATAAAAATAAAGCCGTCGGGATAAAGTCCCAGAAATCATCG
TCTTCCATGACGGGTTCTGCAGTAATTCTGGGAGTGGTTGTAATGTCGTCCAGATGCGGATAGTCCAC
CATGCTCGCTTTATC
+
FFFFFFFFFFFFF:FFFFFFFFFFFFFFFFFFFFFFFFFFFFFFFFFFFFFFFFFFFFFFF:FFFFFF
FFFFFFFFFFFFFFFFFFFFFFFFF:FFFFFFFFFFFFFFFFFFFFFFFFFFFFFFFFFFFFFFFFFFF
F:FFFFFFFFFFFFF
@A00721:190:HGJW7DSXY:4:2303:26802:19132 1:N:0:TCTGCAAG+GCTGCACT
CCCTAAATTACAAAGATTTTTATGGATATAAAAATAAAGCCGTCGGGATAAAGTCCCAGAAATCATCG
TCTTCCATGACGGGTTCTGCAGTAATTCTGGGAGTGGTTGTAATGTCGTCCAGATGCGGATAGTCCAC
CATGCTCGCTTTATC
+
FFFFFFFFFFFFFFFFFFFFFFFFFFFFFFFFFFFFFFFFFFFFFFFFFFFFFFFFFFFFFFFFFFFFF
FFFFFFFFFFFFFFFFFFFFFFFFFFFFFFFFFFFFFFFFFF,FFFFFFFFFFF:F:FFFFFFFF,F
F::FF:FFFFFFFFF
@A00721:190:HGJW7DSXY:4:2445:32606:23202 1:N:0:TCTGCAAG+GCTGCACT
CCCTAAATTACAAAGATTTTTATGGATATAAAAATAAAGCCGTCGGGATAAAGTCCCAGAAATCATCG
TCTTCCATGACGGGTTCTGCAGTAATTCTGGGAGTGGTTGTAATGTCGTCCAGATGCGGATAGTCCAC
CATGCTCGCTTTATC
+
FFFFFFFFFFFFFFFFFFFFFFFFF:FFFF:FFFFFFFFFFFFFFFFF:FFFFFFFFFFFFFFF:FFF
FF:FFFFFFFFFFFFFFFFFFFFFFFFFFFFFFFFF:FFF:FFFFFFFFFFFFFFFFFFFFFFFFFFFFF
FFFFFFFFFFFFF:F
@A00721:190:HGJW7DSXY:4:2570:16188:24643 1:N:0:TCTGCAAG+GCTGCACT
CCCTAAATTACAAAGATTTTTATGGATATAAAAATAAAGCCGTCGGGATAAAGTCCCAGAAATCATCG
TCTTCCATGACGGGTTCTGCAGTAATTCTGGGACTGGTTGTAATGTCGTCCAGATGCGGATAGTCCAC
CATGCTCGCTTTATC
+
FFFFFFFFFFFFFFFFFFFFFFFFFFFFFFFFFFFFFFFFFFFFFFFFFFFFFFFFFFFFFFFFFFFFF
FFFFFFFFFFFFFFFFFFFFFFFFFFFFFFFFFF:FFFFFFFFFFFFFFFFFFFFFFFFFFF::FFFF:F
F,FFFFFFFFFFFF
```

@A00721:190:HGJW7DSXY:4:1139:11523:1188 1:N:0:TCTGCAAG+GCTGCACT
CCTAAATTACAAAGATTTTTATGGATATAAAAATAAAGCCGTCTGGATAAAGTCCCAGAAATCATCGT
CTTCCATGACGGGTTCTGCAGTAATTCTGGGACTGGTTGTAATGTCGTCCAGATGCGGATAGTCCACC
ATGCTCGCTTAATCC
+
FFFF:::FFFFFFFFFF,FFF:::FF:FFF,FFFFF::F:,,F,F:F,FFFFFFFFF:FFFFFFF,,F
:FFFFF,:F,,,FFFFF,FFFFFFF:,:FF,FF:::,FFF:FF,FF,F,FFFFF,:,,FFFF,F,FFF
F:F,FF:,FF,,:F:
@A00721:190:HGJW7DSXY:4:1207:3332:13777 1:N:0:TCTGCAAG+GCTGCACT
CCTAAATTACAAAGATTTTTATGGATATAAAAATAAAGCCGTCGGGATAAAGTCCCAGAAATCATCGT
CTTCCATGACGGGTTCTGCAGTAATTCTGGGACTGGTTGTAATGTCGTCCAGATGCGGATAGTCCACC
ATGCTCGCTTTATCC
+
FFFFFFFFFFFFFFFFFFFFFF:FFFFFFFFFFFFFFFFFFFFFFFFFFFFFFFFFFFFFFFFFFFFFFF
FFFFFFFFFFFFFFFFFFFFFFFFFFFFFFFFFFFFFFFFFFFFFFFFFFFFFFFFFFFFFFFFFFFFFF
,FFFFFFFFFFF:FF
@A00721:190:HGJW7DSXY:4:1642:22191:32972 1:N:0:TCTGCAAG+GCTGCACT
CCTAAATTACAAAGATTTTTATGGATATAAAAATAAAGCCGTCGGGATAAAGTCCCAGAAATCATCGT
CTTCCATGACGGGTTCTGCAGTAATTCTGGGACTGGTTGTAATGTCGTCCAGATGCGGATAGTCCACC
ATGCTCGCTTTATCC
+
FFFFFFFFFFFFFFFFFFFFFFFFFFFFFFFFFFFFFFFFFFFFFFFFFFFFFFFFFFFFF:FFFFFFFF
FFFFFFFF:FFFFFFFFFFFFFFFFFFFFFFFFF,FFF:FFF,FFFFFFFFFFFFFFFFFFFFFFFFF:F
,FFFFFFFFFF:FFF
@A00721:190:HGJW7DSXY:4:2159:8992:21136 1:N:0:TCTGCAAG+GCTGCACT
CCTAAATTACAAAGATTTTTATGGATATAAAAATAAAGCCGTCGGGATAAAGTCCCAGAAATCATCGT
CTTCCATGACGGGTTCTGCAGTAATTCTGGGACTGGTTGTAATGTCGTCCAGATGCGGATAGTCCACC
ATGCTCGCTTTATCC
+
FFFFFFFFFFFFFFFFFFFFFFFFFF,F:FFFFFFFFFFFFFFFFFFFFFFFFFFFFFFFFFFFFFFFFF
FFFFFFFFFFFFFFFFFFFFFFFFFFFFFFFFFF:FFF:FFFFFFFFFFFFFFFFFFFFFFFFFFFF,FF
FFFFFFFFFFFFFFF
@A00721:190:HGJW7DSXY:4:2207:16369:3662 1:N:0:TCTGCAAG+GCTGCACT
CCTAAATTACAAAGATTTTTATGGATATAAAAATAAAGCCGTCGGGATAAAGTCCCAGAAATCATCGT
CTTCCATGACGGGTTCTGCAGTAATTCTGGGACTGGTTGTAATGTCGTCCAGATGCGGATAGTCCACC
ATGCTCGCTTTATCC
+
FFFFFFFFFFFFFFFFFFFFFFFFFFFFFFFFFFFFF:FFFFFFFFFFFFFFFFFFFFFFFFFFFFFFFF
FFFFFFFFFFFFFFFFFFFFFFFFFFFFFFFFF:FFF,FFFFFFFFFFFFFFFFFFFFFFFFFFFFFFFF
F,FFFFFFFFFFFFF
@A00721:190:HGJW7DSXY:4:2444:1425:23218 1:N:0:TCTGCAAG+GCTGCACT
CCTAAATTACAAAGATTTTTATGGATATAAAAATAAAGCCGTCGGGATAAAGTCCCAGAAATCATCGT
CTTCCATGACGGGTTCTGCAGTAATTCTGGGACTGGTTGTAATGTCGTCCAGATGCGGATAGTCCACC
ATGCTCGCTTTATCC
+
FFFFFFFFFFFFFFFFFFFFFFFFFFFFFFF:FFFFFFFFFFFFFFFFFFFFFFFFFFFFFFFFFFFFFF
FFFFFFFFFFFFFFFFFFFFFFFFFFFFFFFFFFFFFF:FFFFFFFFFFFFFFFFFFFFFFFFFFFFFFF
:FFFFFFFFFFFFFF
@A00721:190:HGJW7DSXY:4:2528:26386:3881 1:N:0:TCTGCAAG+GCTGCACT
CCTAAATTACAAAGATTTTTATGGATATAAAAATAAAGCCGTCGGGATAAAGTCCCAGAAATCATCGT
CTTCCATGACGGGTTCTGCAGTAATTCTGGGACTGGTTGTAATGTCGTCCAGATGCGGATAGTCCACC
ATGCTCGCTTTATCC
+
FFFFFFFFFFF:FFFFFFFFFFFFFFFFFFF:FFFFFFFFFFFFFFFFFFFFFFFFFF:FFFFFFFFFFF

FFFFFFFFFFFFFFFFFFFFFFFFFFFFF:FF:FFFFFFFF:FFFFFFF,FFFFFFFFFFFFFF,FF
,FF,::FFF:FFFF:
@A00721:190:HGJW7DSXY:4:2531:17020:31876 1:N:0:TCTGCAAG+GCTGCACT
CCTAAATTACAAAGATTTTTATGGATATAAAAATAAAGCCGTCGGGATAAAGTCCCAGAAATCATCGT
CTTCCATGACGGGTTCTGCAGTAATTCTGGGACTGGTTGTAATGTCGTCCAGATGCGGATAGTCCACC
ATGCTCGCTTTATCC
+
FFFFFFFFFFFFFFFFFFFFFFFFFFF:FFFFFFFFFFFFFFFFFFFFFFFFFFFFFFFF:FFFFFFFF
FFFFFFFFFFFFFFFFFFFFFFFFFFFFFFFFFFFFFFFFFFFFFFFFFFFFFFFFFFFFF,FFFFFFF
,FFFFFFFFFFFFFF
@A00721:190:HGJW7DSXY:4:2617:5439:21684 1:N:0:TCTGCAAG+GCTGCACT
CCTAAATTACAAAGATTTTTATGGATATAAAAATAAAGCCGTCGGGATAAAGTCCCAGAAATCATCGT
CTTCCATGACGGGTTCTGCAGTAATTCTGGGACTGGTTGTAATGTCGTCCAGATGCGGATAGTCCACC
ATGCTCGCTTTATCC
+
FFFFFFFFFFFFFFFFFFFFFFFFFFFFFFFFFFFFFFFFFFFFFFFFFFFFFFFFFFFFFFFFFFFFF
FFFFFFFFFFFFFFFFFFFFFFFFFFFFFFFFF:FFFFFFFFFFFFFFFFFFFFFFFFFFFFFFFFFFF
:FFFFFFFFFFFFFF
@A00721:190:HGJW7DSXY:4:2678:9028:28275 1:N:0:TCTGCAAG+GCTGCACT
CCTAAATTACAAAGATTTTTATGGATATAAAAATAAAGCCGTCGGGATAAAGTCCCAGAAATCATCGT
CTTCCATGACGGGTTCTGCAGTAATTCTGGGACTGGTTGTAATGTCGTCCAGATGCGGATAGTCCACC
ATGCTCGCTTTATCC
+
FFFFFFFFFFFFFFFFFFFFFFFFFFFFFFFFFFFFFFFFFFFFFFFFFFFFFFFFFFFFFFFFFFFFF
FFFFFFFFFFFFFFFFFFFFFFFFFFFFFFFFFFFFFFFFFFFFFFFFFFFFFFFFFFFFFFFFFFFFF
:FFFFFFFFFFFFFF
@A00721:190:HGJW7DSXY:4:2678:9317:31187 1:N:0:TCTGCAAG+GCTGCACT
CCTAAATTACAAAGATTTTTATGGATATAAAAATAAAGCCGTCGGGATAAAGTCCCAGAAATCATCGT
CTTCCATGACGGGTTCTGCAGTAATTCTGGGACTGGTTGTAATGTCGTCCAGATGCGGATAGTCCACC
ATGCTCGCTTTATCC
+
FFFFFFFFFFFFFFFFFFFFFFFFFFFFFFFFFFFFFFFFFFFFFFFFFFFFFFFFFFFFFFFFFFFFF
FFFFFFFFFFFFFFFFFFFFFFFFFFFFFFFFFFFFF::FFFFFFFFFFFFFFFFFFFFFFFFFFFFFF
F,FFFFFFFFFFFFF
@A00721:190:HGJW7DSXY:4:1102:21902:22013 1:N:0:TCTGCAAG+GCTGCACT
CTAAATTACAAAGATTTTTATGGATATAAAAATAAAGCCGTCGGGATAAAGTCCCAGAAATCATCGTC
TTCCATGACGGGTTCTGCAGTAATTCTGGGACTGGTTGTAATGTCGTCCAGATGCGGATAGTCCACCA
TGCTCGCTTTATCCG
+
FFFFFFFFFFFFFFFFFFFFFFFFFFFF:FFFFFFFFFFFFFFFFFFFFFFFFFFFFFFFFFFFFFFFF
FFFFFFFFFFFFFFFFFFFFFFFFFFFFFFF,:FFFFFFFFFFFF:FFFFFFFFFFFFFFFFFF,FFF
FFFFFFFF,FFFFFF
@A00721:190:HGJW7DSXY:4:1107:2953:22921 1:N:0:TCTGCAAG+GCTGCACT
CTAAATTACAAAGATTTTTATGGATATAAAAATAAAGCCGTCGGGATAAAGTCCCAGAAATCATCGTC
TTCCATGACGGGTTCTGCAGTAATTCTGGGACTGGTTGTAATGTCGTCCAGATGCGGATAGTCCACCA
TGCTCGCTTTATCCG
+
FFFFFFFFFFFFFFFFFFFFFFFFFFFFFFFFFFFFFFFFFFFFFFFFFFFFFFFFFFFFFFFFFFFFF
FFFFFFFFFFFFFFFFFFFFFFFFFFFFFFFFFFFFFFFFF:FFFFFFFFFFFFFFFFF,FFFFFFFF
FFFFFFFFFFFFFFF
@A00721:190:HGJW7DSXY:4:1109:32253:26005 1:N:0:TCTGCAAG+GCTGCACT
CTAAATTACAAAGATTTTTATGGATATAAAAATAAAGCCGTCGGGATAAAGTCCCAGAAATCATCGTC
TTCCATGACGGGTTCTGCAGTAATTCTGGGACTGGTTGTAATGTCGTCCAGATGCGGATAGTCCACCA
TGCTCGCTTTATCCG

+
FFFFFFFFFFFFFFFFFFFFF:FFFFFFFFFFFFFFFFFFFFFFFFFFFFFFFFFFFFFFFFFFFFFFFF
FFFFFFF:FFFFFFFFFF:FFFFFFFFFFFFFFFFFFFFFFFFFFFFFFFFFFFFFFFFF:FFF,FFFF
FFFFFFFFFFFFF:F
@A00721:190:HGJW7DSXY:4:1171:24361:36824 1:N:0:TCTGCAAG+GCTGCACT
CTAAATTACAAAGATTTTTATGGATATAAAAATAAAGCAGTCGGGATAAAGTCCCAGAAATCATCGTC
TTCCATGACGGGTTCTGCAGTAATTCTGGGACTGGTTGTAATGTCGTCCAGATGCGGATAGTCCACCA
TGCTCGCTTTATCCG
+
:FFFFFFFFFF:,FFF:FFF:F,FFFF:FFFFF:FFFF,,FF::FF:FFF:FFFFFF::F:FFFF,FF
FF,FF:F:FF,,:,:F,FFFFFF:FFFFF,F,F,:FF:FFFFFFF:F:FFFFF,FFFFF:,F,:,,FF
:F:FFF,FFFFF,:F
@A00721:190:HGJW7DSXY:4:1219:25861:36072 1:N:0:TCTGCAAG+GCTGCACT
CTAAATTACAAAGATTTTTATGGATATAAAAATAAAGCCGTCGGGATAAAGTCCCAGAAATCATCGTC
TTCCATGACGGGTTCTGCAGTAATTCTGGGACTGGTTGTAATGTCGTCCAGATGCGGATAGTCTGTCC
AAATCTTTAAAGATG
+
FFFFFFFFFFFFFFFFFFFFFFFFFFFFFFFFFFFFFFF:FFFFFFFFFFFFFFFFFFFFFFFFFFFF
FFFFFFFFFFFFFFFFFFFFFFFFFFFFFFFFF,FFFFFFFFFFFFFFFFFFFF:FFFFFFFFFFFFFFF
FFFFFFFFFFFFFFF
@A00721:190:HGJW7DSXY:4:1267:7111:33098 1:N:0:TCTGCAAG+GCTGCACT
CTAAATTACAAAGATTTTTATGGATATAAAAATAAAGCCGTCGGGATAAAGTCCCAGAAATCATCGTC
TTCCATGACGGGTTCTGCAGTAATTCTGGGACTGGTTGTAATGTCGTCCAGATGCGGATAGTCCACCA
TGCTCGCTTTATCCG
+
FFFFFFFFFFFFFFFFFFFFFFFFF:FFFFFFFFFFFFFFFFFFFFFFFFFFFFFFFFFFFFFFFFFFF
FFFFFFFFFFFFFFFFFFFFFFFFFFFFFFFFFF,FF:FFFFFFFFFFFFFFFFFFFFFFFFFFFFFFFF
FFFFFFFFFFFFFFF
@A00721:190:HGJW7DSXY:4:1308:24795:23453 1:N:0:TCTGCAAG+GCTGCACT
CTAAATTACAAAGATTTTTATGGATATAAAAATAAAGCCGTCGGGATAAAGTCCCAGAAATCATCGTC
TTCCATGACGGGTTCTGCAGTAATTCTGGGACTGGTTGTAATGTCGTCCAGATGCGGATAGTCTGTCC
AAATCTTTAAAGATG
+
FFFFFFFFFFFFFFFFFFFFF:FFFFFFFFFFFFFFFFFFFFFFFFFFFFFFFFFFFFFFFFFFFFFFFF
FFFFFFFFFFFFFFFFFFFFFFFFFFFFFFFFFFFFFFFFFFFFFFFFFFFFFFFFFFFF:FFFFFFFF
FFFFFFFFFF:FFFF
@A00721:190:HGJW7DSXY:4:1350:3712:18912 1:N:0:TCTGCAAG+GCTGCACT
CTAAATTACAAAGATTTTTATGGATATAAAAATAAAGCCGTCGGGATAAAGTCCCAGAAATCATCGTC
TTCCATGACGGGTTCTGCAGTAATTCTGGGACTGGTTGTAATGTCGTCCAGATGCGGATAGTCCACCA
TGCTCGCTTTATCCG
+
FFFFFFFFFFFFFFFFFFFF:FFFFFFFFFFFFFFFFFFFFFFFFFFFFFFFFFFF:FFFFFFFFFFFFF
FFFFFFFFFFFFFFFFF:FFF:FFFFFFF:FFFFF:FFFFFFFFFFFFFFFFFFFFFFFFFFF:FFFF
FFFFFFFFFFFFFFF
@A00721:190:HGJW7DSXY:4:1426:17210:22874 1:N:0:TCTGCAAG+GCTGCACT
CTAAATTACAAAGATTTTTATGGATATAAAAATAAAGCCGTCGGGATAAAGTCCCAGAAATCATCGTC
TTCCATGACGGGTTCTGCAGTAATTCTGGGACTGGTTGTAATGTCGTCCAGATGCGGATAGTCCACCA
TGCTCGCTTTATCCG
+
FFFFFFFFFFFFFFFFFFFFFFFFFFFFFFFFFFFFF:FFFFFFFFFFFFFFFFFFFFFFFFFFFFFFFF
FFFFFFFFFFFFFFFFFFFFFFFFFFFFFFFFF:FFFFFF:FFFFFFFFFFFFFFFFFFFFFFFFFFFF:
FFFFFFFFFFFFFFF
@A00721:190:HGJW7DSXY:4:1438:9923:9846 1:N:0:TCTGCAAG+GCTGCACT
CTAAATTACAAAGATTTTTATGGATATAAAAATAAAGCCGTCGGGATAAAGTCCCAGAAATCATCGTC

TTCCATGACGGGTTCTGCAGTAATTCTGGGACTGGTTGTAATGTCGTCCAGATGCGGATAGTCCACCA
TGCTCGCTTTATCCG
+
FFFFFFFFFFFFFFFFFFFFFFFFFFFFFFFFFFFFFFFFFFFFFFFFFFFFFFFFFFFFFFFFFFFF
FFFFFFFFFFFFFFFFFFFFFFFFFFFFFFFFFF:FFFFFFFFFFFFFF:FFFFFFFFFFFFFF:FFF
FFFFFFFF,FFFFFF
@A00721:190:HGJW7DSXY:4:1462:25437:7467 1:N:0:TCTGCAAG+GCTGCACT
CTAAATTACAAAGATTTTTATGGATATAAAAATAAAGCCGTCGGGATAAAGTCCCAGAAATCATCGTC
TTCCATGACGGGTTCTGCAGTAATTCTGGGACTGGTTGTAATGTCGTCCAGATGCGGATAGTCTGTCC
AAATCTTTAAAGATG
+
FFFFFFFFFFFFFFFFFFFFFFFFFFFF:FFFFFF,FFFFFFFFFFFFFFFFFFFFFFF:FFFFFFFF
FFFFFFFFFFFFFFFFFFFFFFFFFFFFFFFF:FFFFFFFFFFFFFFFFFFFFFFFFFFFFFFFFFFF
FFFFFFFFFFFFFFF
@A00721:190:HGJW7DSXY:4:1510:29875:27868 1:N:0:TCTGCAAG+GCTGCACT
CTAAATTACAAAGATTTTTATGGATATAAAAATAAAGCCGTCGGGATAAAGTCCCAGAAATCATCGTC
TTCCATGACGGGTTCTGCAGTAATTCTGGGACTGGTTGTAATGTCGTCCAGATGCGGATAGTCCACCC
TGCTCGCTTTATCCG
+
FFFFFFFFFFFFFFFFFFFFFFFFFFFFFFFFFFFFFFFFFFF:FFFFFFFFFFFFFFFFFFFFFFFF
FFFFFFFFFFFFFFFFFFFFFF,FFFFFFFFFFFFFFFFFF:FFFFFFFFFFFFFFFFFFFFFFFFF,
FFFFFFFF:FFFFFF
@A00721:190:HGJW7DSXY:4:1532:7012:11162 1:N:0:TCTGCAAG+GCTGCACT
CTAAATTACAAAGATTTTTATGGATATAAAAATAAAGCCGTCGGGATAAAGTCCCAGAAATCATCGTC
TTCCATGACGGGTTCTGCAGTAATTCTGGGACTGGTTGTAATGTCGTCCAGATGCGGATAGTCCACCA
TGCTCGCTTTATCCG
+
FFFFFFFFFFF:FFFFFFFFFFFFFFFFFFFFFFFFFFFFFFFFFFFFFFFFFFFFFFF:FFFFFFFF
FFFFFFFFFFFFFFFFFFFFFFFFFFFFFFFFFFFF:FFFFFFFF:FFFFFFFFFFFFFFFFFFFFFF
FFFFFFFFFFFFFFF
@A00721:190:HGJW7DSXY:4:1536:28971:25676 1:N:0:TCTGCAAG+GCTGCACT
CTAAATTACAAAGATTTTTATGGATATAAAAATAAAGCCGTCGGGATAAAGTCCCAGAAATCATCGTC
TTCCATGACGGGTTCTGCAGTAATTCTGGGACTGGTTGTAATGTCGTCCAGATGCGGATAGTCTGTCC
AAATCTTTAAAGATG
+
FFFFFFFFFFFF:FFFFFFFFFFFF:FFF:FFFFFFFF:FFFFFFF:FFFFFFF:FFFFFFFFFFFFF
FFFFFFFFFFFFFF:FF:,FFFFFFFFF:FF:FFFFFFFFFFFFFFFFFFFFFFFFFFFF:FFFFFF,
FFFFFFFFFFFFFF:
@A00721:190:HGJW7DSXY:4:1550:28637:16799 1:N:0:TCTGCAAG+GCTGCACT
CTAAATTACAAAGATTTTTATGGATATAAAAATAAAGCCGTCGGGATAAAGTCCCAGAAATCATCGTC
TTCCATGACGGGTTCTGCAGTAATTCTGGGACTGGTTGTAATGTCGTCCAGATGCGGATAGTCCACCA
TGCTCGCTTTATCCG
+
FFFFFFFFFFFFFFFFFFFF:FFFFFF:FFFFFFFFFFFF,FFFFFFFFFFFFFFFFF:FFFFFFFFF
FFFFFFFFFFFFFFFFFFFFFFFFFFFFFF,FFFFF:FFF:FFFFFFFFFFFFFFFFFFFFFFF,FF,
:FFFFFFFFFFFFFF
@A00721:190:HGJW7DSXY:4:1562:10827:4523 1:N:0:TCTGCAAG+GCTGCACT
CTAAATTACAAAGATTTTTATGGATATAAAAATAAAGCCGTCGGGATAAAGTCCCAGAAATCATCGTC
TTCCATGACGGGTTCTGCAGTAATTCTGGGACTGGTTGTAATGTCGTCCAGATGCGGATAGTCCACCA
TGCTCGCTTTATCCG
+
FFFFFFFFFFFFFFFFFFFFFFFFFFFFFFFFFFFFFFFFFFFFFFFFFFFFFFFFFFFFFFFFFFFF
FFFFFFFFFFFFFFFFFFFFF,FFFFFFFFFF,FFFFFFFFFFFFFFFFFFFFFFFFFFFFF:FFFF,
FFFFFFFFFFFFFFF

@A00721:190:HGJW7DSXY:4:1570:23619:7513 1:N:0:TCTGCAAG+GCTGCACT
CTAAATTACAAAGATTTTTATGGATATAAAAATAAAGCCGTCGGGATAAAGTCCCAGAAATCATCGTC
TTCCATGACGGGTTCTGCAGTAATTCTGGGACTGGTTGTAATGTCGTCCAGATGCGGATAGTCTGTCC
AAATCTTTAAAGATG
+
FFFFFFFFFFFFFFFFFFFFFFFFFFFFFFFFFFFFFFFFFFFFFFFFFFFFFFFFFFFFFFFFFFFFFF
FFFFFFFFFFFFFFFFFFFFFFFFFFFFF:FFFFFFFFFFFFFFFFFFFFFFFFFFFFFFFFFFFFFFFF
F:FFFFFFFFFFFFF
@A00721:190:HGJW7DSXY:4:1571:29631:25817 1:N:0:TCTGCAAG+GCTGCACT
CTAAATTACAAAGATTTTTATGGATATAAAAATAAAGCCGTCGGGATAAAGTCCCAGAAATCATCGTC
TTCCATGACGGGTTCTGCAGTAATTCTGGGACTGGTTGTAATGTCGTCCAGATGCGGATAGTCCACCA
TGCTCGCTTTATCCG
+
FFFFFFFFFFFFFFFFFFFFFFFFFFFFFFFF:FFFFFFFFFFFFFFFFFFFFFFFFFFFFFFFFFFFFF
FFFFFFFFFFFFFFFFFFFFFFFF:FFFFFFFF:FFFFFFFFFFFFFFFFFFFFFFFFFFFFFFFF,FF:
FFFFFFFFFFFFFFF
@A00721:190:HGJW7DSXY:4:1659:27823:3396 1:N:0:TCTGCAAG+GCTGCACT
CTAAATTACAAAGATTTTTATGGATATAAAAATAAAGCCGTCGGGATAAAGTCCCAGAAATCATCGTC
TTCCATGACGGGTTCTGCAGTAATTCTGGGACTGGTTGTAATGTCGTCCAGATGCGGATAGTCTGTCC
AAATCTTTAAAGATG
+
FFFFFFFFFFFFFFFFFFFFFFFFFF:FFFFFFFFFFFFFFFFFFFFFFFFFFFFFFFFFFFFFFFFFFF
FFFFFFFFFFFFFFFFFFFFFFFFFFFFFFFFFFFFFFFF:FFFFFFFFFFFFF:FFFFFFFFFFFFFFF
FFFFFF:F,,FFFFF
@A00721:190:HGJW7DSXY:4:1671:28284:12273 1:N:0:TCTGCAAG+GCTGCACT
CTAAATTACAAAGATTTTTATGGATATAAAAATAAAGCCGTCGGGATAAAGTCCCAGAAATCATCGTC
TTCCATGACGGGTTCTGCAGTAATTCTGGGACTGGTTGTAATGTCGTCCAGATGCGGATAGTCCACCA
TGCTCGCTTTATCCG
+
:FFFFFFFFFFFFFFFFFFFFFFFFFFFFFFFFFFFFFFFFFFFFFFF:FFFFFFFFFFFFFFF,FFFFF
FFFFFFF,FFFFFFFFFFFFFFFFF:FFFFFF:,FFFFFFF,FFFFFFFFFFFFFFFF:FFFFFFF:FFF
::FFFFFFFFFFFFF
@A00721:190:HGJW7DSXY:4:2104:15600:5932 1:N:0:TCTGCAAG+GCTGCACT
CTAAATTACAAAGATTTTTATGGATGTAAAAATAAAGCCGTCGGGATAAAGTCCCAGAAATCATCGTC
TTCCATGACGGGTTCTGCAGTAATTCTGGGACTGGTTGTAATGTCGTCCAGATGCGGATAGTCTGTCC
AAATCTTTAAAGATG
+
FFFFFFFFFFFFFFFF:FFF:FFFF,:FFFFFFFFFFFFFF:FFFFFFFFFFFFFFFFFFFFFFFFFFFF
FFFFFFFFFFFFFFFFFFFFFFFFFFFFFFFFF:FFF:FFFF,FFFFFFFFFFFFFFFF:F:FFFFFFFF
FFFFFFFFFFFFFFF
@A00721:190:HGJW7DSXY:4:2108:3070:18333 1:N:0:TCTGCAAG+GCTGCACT
CTAAATTACAAAGATTTTTATGGATATAAAAATAAAGCCGTCGGGATAAAGTCCCAGAAATCATCGTC
TTCCATGACGGGTTCTGCAGTAATTCTGGGACTGGTTGTAATGTCGTCCAGATGCGGATAGTCTGTCC
AAATCTTTAAAGATG
+
FFFFFFFFFFFFFFFFFFFFFFFFFFF:F:FFFFFFF:FFFFFFFFFFFFFFFFFFFFFFFFFFFFFFFF
FFFFFFFFFFFFFFFFFFFFFFFFFFFFFFFFFFFF,FFFFFF:FFFFFFFFF:FFFFFF:FFFF:FFFF
FFFFFFFFFFFFFFF
@A00721:190:HGJW7DSXY:4:2114:16658:22545 1:N:0:TCTGCAAG+GCTGCACT
CTAAATTACAAAGATTTTTATGGATATAAAAATAAAGCCGTCGGGATAAAGTCCCAGAAATCATCGTC
TTCCATGACGGGTTCTGCAGTAATTCTGGGACTGGTTGTAATGTCGTCCAGATGCGGATAGTCTGTCC
AAATCTTTAAAGATG
+
FFFFFFFFFFFFFFFFFFFFFFFFFFFFF,FFFFFFFFFFFFFFFFFFFFFFFFFFFFFFFFFFFFFFFF

```
FFFFFFFFFFFFFFFFFFFFFFFFFFFFFFFFFFFF:FFFFFFFFFFFFFFFFFFFFFFFFFFFFFFF
FFFFFFFFFFFFFFF
@A00721:190:HGJW7DSXY:4:2147:29640:8015 1:N:0:TCTGCAAG+GCTGCACT
CTAAATTACAAAGATTTTTATGGATATAAAAATAAAGCCGTCGGGATAAAGTCCCAGAAATCATCGTC
TTCCATGACGGGTTCTGCAGTAATTCTGGGACTGGTTGTAATGTCGTCCAGATGCGGATAGTCCACCA
TGCTCGCTTTATCCG
+
FFFFFFFFFFFFFFFFFFFFFFFFFFFFFFFFFFF:FFFFFFFFFFFFFFFFFFFFFFFFFFFFFFFF
FFFFFFFFFFFFFFFFFFFFFFFFFFFFFFF,FFFFFFFF:FFFFFFFFFFFFFFFFFFFFF:F:FFF
FFFFFFFFFFFFFFF
@A00721:190:HGJW7DSXY:4:2150:3468:4085 1:N:0:TCTGCAAG+GCTGCACT
CTAAATTACAAAGATTTTTATGGATATAAAAATAAAGCCGTCGGGATAAAGTCCCAGAAATCATCGTC
TTCCATGACGGGTTCTGCAGTAATTCTGGGACTGGTTGTAATGTCGTCCAGATGCGGATAGTCCACCA
TGCTCGCTTTATCCG
+
FFFFFFFFFFFFFFFFFFFFF:FFFFFFFFFFFFFFFFFFFF:FFFFFFFFFFFFFFFFFFFFFFFFF
FFFFFFFFFFFFFFFFFFFFFFFFFFFFFFFFFFFFFFFFFFFFF:FFFFFFFFFFFFFFFFFFFFF,
FFFFFFFFFFFFF,F
@A00721:190:HGJW7DSXY:4:2163:2971:15217 1:N:0:TCTGCAAG+GCTGCACT
CTAAATTACAAAGATTTTTATGGATATAAAAATAAAGCCGTCGGGATAAAGTCCCAGAAATCATCGTC
TTCCATGACGGGTTCTGCAGTAATTCTGGGACTGGTTGTAATGTCGTCCAGATGCGGATAGTCCACCA
TGCTCGCTTTATCCG
+
FFFFFFFFFFFFFFFFFFF,FFFF:FFFFFFFFFFFFFFFFFFFFFFFFFFFFFFFFFFFFFFFFFFF
FFFFFFFFFFFFFFFFFFFFFFFFFFFFFFFFFFF:FFFFFFFFFFFFFFFFFFFFFFFFFFFF:FF,
FFF:FFFFFFF:FFF
@A00721:190:HGJW7DSXY:4:2231:26178:29794 1:N:0:TCTGCAAG+GCTGCACT
CTAAATTACAAAGATTTTTATGGATATAAAAATAAAGCCGTCGGGATAAAGTCCCAGAAATCATCGTC
TTCCATGACGGGTTCTGCAGTAATTCTGGGACTGGTTGTAATGTCGTCCAGATGCGGATAGTCCACCA
TGCTCGCTTTATCCG
+
FFFFFFFFFFF:FFFFFFFFFFFFFFFFFFFFFFFFFFFFFFFFFFFFFFFFFFFFFFFFFFFFFFFF
FFFFFFFFFFFFFFFFFFFFFFFFFFFFFFF:FFFF:FFFFFFFFFFFFFFFFFFFFFFFFFFFFFF,
,FFFFFFFFFFFFFF
@A00721:190:HGJW7DSXY:4:2259:25066:3944 1:N:0:TCTGCAAG+GCTGCACT
CTAAATTACAAAGATTTTTATGGATATAAAAATAAAGCCGTCGGGATAAAGTCCCAGAAATCATCGTC
TTCCATGACGGGTTCTGCAGTAATTCTGGGACTGGTTGTAATGTCGTCCAGATGCGGATAGTCCACCC
TGCTCGCTTTATCCG
+
FFFFFFFFFFFFFFFFFFFFFFFFFFFFFFFFFFFFFFFFFFFFFFFFFFFFFFFFFF:FFFFFFFFF
FFFFFFFFFFFFFFFFFFFFFF:FFFFFFFFFFFFFFFFFFFFFFFFFFFFFFFFFFFF:FFFFFFF,
FFFFFFFFFFFFFFF
@A00721:190:HGJW7DSXY:4:2343:7527:22451 1:N:0:TCTGCAAG+GCTGCACT
CTAAATTACAAAGATTTTTATGGATATAAAAATAAAGCCGTCGGGATAAAGTCCCAGAAATCATCGTC
TTCCATGACGGGTTCTGCAGTAATTCTGGGACTGGTTGTAATGTCGTCCAGATGCGGATAGTCTGTCC
AAATCTTTAAAGATG
+
FFFFFFFFFFFFFFFFFFFFFFFFFFFFFFFFFFFFFFFFFFFFFFFFFFFFFFFFFFFFFFFFFFFF
FFFFFFFFFFFFFFFFFFFFFFFFFFFFFFFFFFFFFFFFFFFFFFFFFFFFFFFFFFFF:FFFFFFF
FFFFFFFFFFFFFFF
@A00721:190:HGJW7DSXY:4:2416:12834:32800 1:N:0:TCTGCAAG+GCTGCACT
CTAAATTACAAAGATTTTTATGGATATAAAAATAAAGCCGTCGGGATAAAGTCCCAGAAATCATCGTC
TTCCATGACGGGTTCTGCAGTAATTCTGGGACTGGTTGTAATGTCGTCCAGATGCGGATAGTCCACCA
TGCTCGCTTTATCCG
```

```
+
FFFFFFFFFFFFFFFFFFFFFFFFFFFFFFFFFFFFFFFFF:FFFFFFFFFFFFFFFFFFFFFFFFFFFF
FFFFFFFFFFFFFFFFFFFFFFFFFFFFFFFFFFFFFFFFFFFFFFFFFFFFFFFFFFFFFFFFFFFFFF
FFFFFFFFFFFFFFF
@A00721:190:HGJW7DSXY:4:2471:7148:18192 1:N:0:TCTGCAAG+GCTGCACT
CTAAATTACAAAGATTTTTATGGATATAAAAATAAAGCCGTCGGGATAAAGTCCCAGAAATCATCGTC
TTCCATGACGGGTTCTGCAGTAATTCTGGGACTGGTTGTAATGTCGTCCAGATGCGGATAGTCCACCA
TGCTCGCTTTATCC
+
F:FFF:FFFFF::FFFFFFFFFFF:FFFFFFFFFFF:FFF,FFFFFFFFFFFFFFFFFFFFFFFFF::F
FF:FFFFF:,FF,FFFFFFFFFFFFFFFFFF:::FFFF:FFFFFFFFFFFFF:FFFFFFFFFFFFFFFF
FFFFF,FF:FFFFF
@A00721:190:HGJW7DSXY:4:2473:30038:7420 1:N:0:TCTGCAAG+GCTGCACT
CTAAATTACAAAGATTTTTATGGATATAAAAATAAAGCCGTCGGGATAAAGTCCCAGAAATCATCGTC
TTCCATGACGGGTTCTGCAGTAATTCTGGGACTGGTTGTAATGTCGTCCAGATGCGGATAGTCCACCA
TGCTCGCTTTATCCG
+
FFFFFFFFFFFFFFFFFFFFFFFFFFF:FFF:FFFFFFFFFFFFFFFFFFFFFFFFFFFFFFFFFFFFFF
FFFFFFF:FFFFFFFFFFFFFFFFFFFFFFFFFFFF:FFFFFFFFFFFFFFFFFFFFFFFFFFFFFFFFF
FFFFFFFFFFFFFFF
@A00721:190:HGJW7DSXY:4:2503:14778:1689 1:N:0:TCTGCAAG+GCTGCACT
CTAAATTACAAAGATTTTTATGGATATAAAAATAAAGCCGTCGGGATAAAGTCCCAGAAATCATCGTC
TTCCATGACGGGTTCTGCAGTAATTCTGGGACTGGTTGTAATGTCGTCCAGATGCGGATAGTCCACCA
TGCTCGCTTTATCCG
+
FFFFFFFFFFFFFFFFFFFFFFFFFFFFFFF:FFFFFFFFFFFFFFFFFFFFFFFFFFFFFFFFFFFFFF
FFFFFFFFFFFFFFFFFFFFF:FFFFFFFFFFFFFFFFFFFFFFFFFFFFFFFFFFFFFFFFFF::FF:
FFFFFFFFFFFFFFF
@A00721:190:HGJW7DSXY:4:2503:17734:14231 1:N:0:TCTGCAAG+GCTGCACT
CTAAATTACAAAGATTTTTATGGATATAAAAATAAAGCCGTCGGGATAAAGTCCCAGAAATCATCGTC
TTCCATGACGGGTTCTGCAGTAATTCTGGGACTGGTTGTAATGTCGTCCAGATGCGGATAGTCCACCA
TGCTCGCTTTATCCG
+
FFFFFFFFFFFFFFFFFFFFFFFFF:FFFFFFFFFFFFFFFFFFFFFFFFFFFFF:FFFFFFFFFFFFFF
FFFFFFFFFFF:FFFFFFFFFFFFFFFFFFF:FFFFFFFFFFFFFFFFFFFFFFFFFFFFFFFFFFFFFF
,FFFFFFF:FFFFFF
@A00721:190:HGJW7DSXY:4:2503:12174:30217 1:N:0:TCTGCAAG+GCTGCACT
CTAAATTACAAAGATTTTTATGGATATAAAAATAAAGCCGTCGGGATAAAGTCCCAGAAATCATCGTC
TTCCATGACGGGTTCTGCAGTAATTCTGGGACTGGTTGTAATGTCGTCCAGATGCGGATAGTCTGTCC
AAATCTTTAAAGATG
+
FFFFFFFFFFFFFFFFFFFFFFFFFFFFFF:FFFFFFFFFF:FFFFFFFFFFFFFFFFFFFFFFFFFFFF
FFFFFFFFFFFFFFFFFFFFFFFFFFFFFFFFFFFFF:FFFFFFFFFFFFFFFFFFFFFFFFFFFFFFFF
FFFFFFFFFFFFFFF
@A00721:190:HGJW7DSXY:4:2570:19859:12399 1:N:0:TCTGCAAG+GCTGCACT
CTAAATTACAAAGATTTTTATGGATATAAAAATAAAGCCGTCGGGATAAAGTCCCAGAAATCATCGTC
TTCCATGACGGGTTCTGCAGTAATTCTGGGACTGGTTGTAATGTCGTCCAGATGCGGATAGTCCACCA
TGCTCGCTTTATCCG
+
FFFFFFFFFFFFFFFFFFFFFFFFFFFFFFFFFFFFFFFFFFFFFFFFFFFFFFFFFFFFFFFFFFFFFF
FFFFFFFFFFFFFFFFFFFFFFFFFFFFFFFFFFFFFFFFFFFFFFFFFFFFFFFFFFFFFFFFFFFFFF
FFFFFFFF,FFFFFF
@A00721:190:HGJW7DSXY:4:2570:20717:12602 1:N:0:TCTGCAAG+GCTGCACT
CTAAATTACAAAGATTTTTATGGATATAAAAATAAAGCCGTCGGGATAAAGTCCCAGAAATCATCGTC
```

TTCCATGACGGGTTCTGCAGTAATTCTGGGACTGGTTGTAATGTCGTCCAGATGCGGATAGTCCACCA
TGCTCGCTTTATCCG
+
FFFFFFFFFFFFFFFFFFF,:FFFFFFF:FF:FFFFFFFFFFFFFFFFFFFFFFFFFFFFFF:FFFFFF
FFFFFFFFFFFFFFFFFFFFFFFFFFFFFFFFFFF:FFFFFFFFFFFF:FFFFFFFFFFFFFFFFFFF
FFFFFFFF,FFFFFF
@A00721:190:HGJW7DSXY:4:2575:20518:21840 1:N:0:TCTGCAAG+GCTGCACT
CTAAATTACAAAGATTTTTATGGATATAAAAATAAAGCCGTCGGGATAAAGTCCCAGAAATCATCGTC
TTCCATGACGGGTTCTGCAGTAATTCTGGGACTGGTTGTAATGTCGTCCAGATGCGGATAGTCCACCA
TGCTCGCTTTATCCG
+
FFFF:FFFFFFFFFFFFFFFFFFFF,FFF:FFFFFFFFFFFFFFFFFFFFFFFFF:FFFFFFFFFFF:FF
FFFFFFFFFFFFFFFFFFFFFFFFFFFFFFFFFFFFFFFFFFFFFFFFFFFFFFFFFFFFFFFFFFFFF:
FFFFFF:F:FFFFFF
@A00721:190:HGJW7DSXY:4:2614:20717:25254 1:N:0:TCTGCAAG+GCTGCACT
CTAAATTACAAAGATTTTTATGGATATAAAAATAAAGCCGTCGGGATAAAGTCCCAGAAATCATCGTC
TTCCATGACGGGTTCTGCAGTAATTCTGGGACTGGTTGTAATGTCGTCCAGATGCGGATAGTCTGTCC
AAATCTTTAAAGATG
+
FFFFFFFFFFFFFFFFFFFFFFFFFFF:FFFFFFFFFFFFFFFFFFFFFFFFFFFFFFFFFFFFFFFFF
FFFFFFFFFFFFFFFFFFFFFFFFFFFFFFFFFFFFFFFFFFFFFFF,FFFFFFF:FFFFFFFFFFFFF
FFFFFFFFFFFFFFF
@A00721:190:HGJW7DSXY:4:2616:29143:2832 1:N:0:TCTGCAAG+GCTGCACT
CTAAATTACAAAGATTTTTATGGATATAAAAATAAAGCCGTCGGGATAAAGTCCCAGAAATCATCGTC
TTCCATGACGGGTTCTGCAGTAATTCTGGGACTGGTTGTAATGTCGTCCAGATGCGGATAGTCTGTCC
AAATCTTTAAAGATG
+
FFFFFFFFFFFFFFFFFFFFFFFFFFFFFFFFFFFFFFFFFFFFFFFFFFFFFFFFFFFFFFFFFFFFF
FFFFFFFFFFFFFFFFFFFFFFFFFFFFFFF:FFFFFFFFFFFFFFFFFFFFFFFFFFFFFFFFFFFFF
FFFFFFFFFFFFFFF
@A00721:190:HGJW7DSXY:4:2623:20564:31250 1:N:0:TCTGCAAG+GCTGCACT
CTAAATTACAAAGATTTTTATGGATATAAAAATAAAGCCGTCGGGATAAAGTCCCAGAAATCATCGTC
TTCCATGACGGGTTCTGCAGTAATTCTGGGACTGGTTGTAATGTCGTCCAGATGCGGATAGTCTGTCC
AAATCTTTAAAGATG
+
FFFFFFFFFFFFFFFFFFFFFFFFFFFFFF:FFFFFFFFFFFFFFFFFFFFFFFFFFFFF:FFFFFFFF
FFFFFFFFFFFF:FFFF:FFFFFFFFFFFF::FFFFFFFFFFFFFFFFFFFFFFFFFFFFFFFF,FFFF
FFFFFFFFFFFFFFF
@A00721:190:HGJW7DSXY:4:2632:22824:17347 1:N:0:TCTGCAAG+GCTGCACT
CTAAATTACAAAGATTTTTATGGATATAAAAATAAAGCCGTCGGGATAAAGTCCCAGAAATCATCGTC
TTCCATGACGGGTTCTGCAGTAATTCTGGGACTGGTTGTAATGTCGTCCAGATGCGGATAGTCTGTCC
AAATCTTTAAAGATG
+
FFFFFFFFFFFFFFFFFFFFFFFFFFFFFFFFFFFFFFFFFFFFFFFFFFFFFFFFFFFFFFFFFFFFF
FFFFFFFFFFFFFFFFFFFFFFFFFFFFFFFFFFFFFFFFFFFFFFFFFFFFFFFFFF:FFFFFFFFF
FFFFFFFFFFFFFF:
@A00721:190:HGJW7DSXY:4:2651:31675:13792 1:N:0:TCTGCAAG+GCTGCACT
CTAAATTACAAAGATTTTTATGGATATAAAAATAAAGCCGTCGGGATAAAGTCCCAGAAATCATCGTC
TTCCATGACGGGTTCTGCAGTAATTCTGGGACTGGTTGTAATGTCGTCCAGATGCGGATAGTCCACCA
TGCTCGCTTTATCCG
+
FFFFFFFFFFFFFFFFFFF:FFFFFFF:F:FFFFFFFFFFFFFFFFFFFFFFFFFFFF:FFFFFFFFFF
FFFFFFFFFFFFFFFFFFFFFFFFFFFFFFF:FFFFFFFFFFFFFFFFFFFFFFFFFFF:FFFFF:FF:
FFFFFFFFFFFFFFF

@A00721:190:HGJW7DSXY:4:2663:18747:3458 1:N:0:TCTGCAAG+GCTGCACT
CTAAATTACAAAGATTTTTATGGATATAAAAATAAAGCCGTCGGGATAAAGTCCCAGAAATCATCGTC
TTCCATGACGGGTTCTGCAGTAATTCTGGGACTGGTTGTAAGGTCGTCCAGATGCGGATAGTCCACCA
TGCTCGCTTTATCCG
+
FFFFFFFFFFFFFFFFFFFF:FFFFFF,F:FFFFFFFFFFFFFFF,FFFFFFF:FFFFF,FFFFFFFF
FFFFFFFFFFFFFF:FFFFF:FFFFFFFFFF,FFFFFFFF:,FFFFFFFFFFFFFFFF::FFF,FFF,
FFFFFFFFFF:FF:F
@A00721:190:HGJW7DSXY:4:1111:3097:21512 1:N:0:TCTGCAAG+GCTGCACT
GCGGATAAAGCGAGCATGGTGGACTATCCGCATCTGGACGACATTACAACCAGTCCCAGAATTACTGC
AGAACCCGTCATGGAAGACGATGATTTCTGGGACTTTATCCCGACGGCTTTATTTTTATATCCATAAA
AATCTTTGTAATTTA
+
FFFFFFFFFFFFFFFFFFFFFFFFFFFFFFFFFFFFFFFFFFFFFFFFFFFFFFFFFFFFFFFFFFFF
FFFFFFFFFFFFFFFFFFFFFFFFFFFFFFFFFFFFFFFFFFFFFFFFFFFFFFFFFFFFFFFFFFFF
FFF:FFFFFFF:FF:
@A00721:190:HGJW7DSXY:4:1130:29550:12994 1:N:0:TCTGCAAG+GCTGCACT
TAAATTACAAAGATTTTTATGGATATAAAAATAAAGCCGTCGGGATAAAGTCCCAGAAATCATCGTCT
TCCATGACGGGTTCTGCAGTAATTCTGGGACTGGTTGTAATGTCGTCCAGATGCGGATAGTCCACCAT
GCTCGCTTTATCCGC
+
FFFFFFFFFFFFFFFFFFF,FFFFFFFFFF:FFFFFFFFFFFFFFFFFFFFFFFFFFFFFFFFFFFFF
FFFFFFFFFFFFFFFFFFFFFFFFFFFFFFFFFF:FFF:FFFFFFFFFFFFFFFFFFFFFFF:FFFFF
FFFFFFFFFFFFFFF
@A00721:190:HGJW7DSXY:4:1223:9381:5650 1:N:0:TCTGCAAG+GCTGCACT
GCGGATAAAGCGAGCATGGTGGACTATCCGCATCTGGACGACATTACAACCAGTCCCAGAATTACTGC
AGAACCCGTCATGGAAGACGATGATTTCTGGGACTTTATCCCGACGGCTTTATTTTTATATCCATAAA
AATCTTTGTAATTTA
+
FFFFFFFFFFFFFFF:FFFFFFFFFFFFFFF,FFFFFFFFFFFFFFFFFFFFFFFFFFFFFFFFFFFF
FFFFFFFFFFFFFFFFFFFFFFFFFFFFFFFFFFFFFFFFFFFFFFFFFFFFFFFFFFFFFFFFFFFF
FFFFFFFFF:FFFF:
@A00721:190:HGJW7DSXY:4:1444:15537:33880 1:N:0:TCTGCAAG+GCTGCACT
GCGGATAAAGCGAGCATGGTGGACTATCCGCATCTGGACGACATTACAACCAGTCCCAGAATTACTGC
AGAACCCGTCATGGAAGACGATGATTTCTGGGACTTTATCCCGACGGCTTTATTTTTATATCCATAAA
AATCTTTGTAATTTA
+
FFFFFFFFFFFFFFFFFFFFFFFFFFFFFFFFFFFFFFFFFFFFFFFFFFFFFFFFFFFFFFFFF:FF
FFFFFFFFFFFFFFFFFFFFFFFFFFFFFFFFFFFFFFFFFFFFFFFFFFFFFFFFFFFFFFFFFFFF
FFF,F:FFFFFFFFF
@A00721:190:HGJW7DSXY:4:1447:32009:23766 1:N:0:TCTGCAAG+GCTGCACT
TAAATTACAAAGATTTTTATGGATATAAAAATAAAGCCGTCGGGATAAAGTCCCAGAAATCATCGTCT
TCCATGACGGGTTCTGCAGTAATTCTGGGACTGGTTGTAATGTCGTCCAGATGCGGATAGTCTGTCCA
AATCTTTAAAGATG
+
FFFFFFFFFF,FFFF:FFFFF:FFFFF:::FFFF,,FFFFF:F:FFFF:,FFFFFFFFF,FFFFFFF:
FFFFF:FFFFFF:F::FFFFFFF:FFF,FFFFF::,FFFFFFFFFFF:,F,FFFFFFFFF:FFF:FFF
FFFFF:FFFFFFF:
@A00721:190:HGJW7DSXY:4:1465:10438:31125 1:N:0:TCTGCAAG+GCTGCACT
TAAATTACAAAGATTTTTATGGATATAAAAATAAAGCCGTCGGGATAAAGTCCCAGAAATCATCGTCT
TCCATGACGGGTTCTGCAGTAATTCTGGGACTGGTTGTAATGTCGTCCAGATGCGGATAGTCCACCAT
GCTCGCTTTATCCGC
+
FFFFFFFFFFFFFFFFFFFFFFFFFFFFFFFFFFFFFFFFFFFFFFFFFFFFFF:FFFFFFFFFFFFF

FFFFFFFFFFFFFFFFFFFFFFFFFFFFFFFFFF:FFFFFFFFFFFFFFFFFFFFFFFFFFFFF:FF,F
FFFFFFFFFFFFFFF
@A00721:190:HGJW7DSXY:4:1530:27091:26741 1:N:0:TCTGCAAG+GCTGCACT
TAAATTACAAAGATTTTTATGGATATAAAAATAAAGCCGTCGGGATAAAGTCCCAGAAATCATCGTCT
TCCATGACGGGTTCTGCAGTAATTCTGGGACTGGTTGTAATGTCGTCCAGATGCGGATAGTCCACCAT
GCTCGCTTTATCCGC
+
FFFFFFFFFFFFFFFF,FF,FFFF::FFFFFFFFFFFFFFFFFFFFFFFFFFFFFFFFFFFF:FFFFFF
FFFFFFFFFFFFFFFFF,FFFFFFFFFFFFFFFFFFFFFFFFFFFFFFFFFFFFFFFFFFFFFFFFFF,
FFFFFFFFF:FFFFF
@A00721:190:HGJW7DSXY:4:1544:20961:31093 1:N:0:TCTGCAAG+GCTGCACT
TAAATTACAAAGATTTTTATGGATATAAAAATAAAGCCGTCGGGATAAAGTCCCAGAAATCATCGTCT
TCCATGACGGGTTCTGCAGTAATTCTGGGACTGGTTGTAATGTCGTCCAGATGCGGATAGTCCACCAT
GCTCGCTTTATCCGC
+
FFFFFFFFFFFFFFFFFF:FFFFFFFFFFF,FFFFFFFFFFFFFFFFFFFFFFFFFFFFFFFFFFFFFF
FFFFFFFFFFFFFFFFFFFFFFFFFFFFFFFFFFFFFFFFFFFFFFFFFFFFFFFFFFFFFFFFFFFFF
FFFFFFFFFFFFFFF
@A00721:190:HGJW7DSXY:4:1578:28845:29590 1:N:0:TCTGCAAG+GCTGCACT
TAAATTACAAAGATTTTTATGGATATAAAAATAAAGCCGTCGGGATAAAGTCCCAGAAATCATCGTCT
TCCATGACGGGTTCTGCAGTAATTCTGGGACTGGTTGTAATGTCGTCCAGATGCGGATAGTCCACCAT
GCTCGCTTTATCCGC
+
FFFFFFFFFFFFFFFFFF:FFFFFFFFFFF:FFF:FFFFFFFFFFFFFFFFFFFFFFFFFFFFFF:FFF
FFFFFFFFFFFFFFFFFFFFFFFFFFFFFFF:F,FFFFFFFFFFFFFFFFFFFFFFFFFFFFFFFFFFFF
[truncated: 435,473 more chars]
