# Supplementary material for: Characterization of Spanish Olive Virome by High Throughput Sequencing Opens New Insights and Uncertainties
Source: Viruses. 2021 Nov 6;13(11):2233. doi: 10.3390/v13112233 (PMC8622421; doi:10.3390/v13112233)
Supplement: Supplementary file 1 [file DNA-B reads.pdf › DNA-B reads.pdf]

@A00721:190:HGJW7DSXY:4:1123:28791:14653 2:N:0:TCTGCAAG+GCTGCACT
AATAAATATAAATTTTGAATTTCAAAATTGACCGTCGATTCTACTGCGTCACTATAGAATTCATTTTT
TAGATGGTTATGATCGTGACGCGTGGTCTAATGACAAATCGACACGTAAGCGTACGGAGGTGTCCCCG
GCGCGGGCAAGCCAG
+
FFFFFFFFFFFFFFFFFFFFFFFFFFFFFFFFFFFFFFFFFFF:FFFFFF:FFFF:FFFFFFFFFFFF
FFFFFFFFFFFF:FFFFF,:FFFFFFFFFFFF:FFFFFFFFFFFFFFFFFFFFFFFFFFFFFFFFFFF
FFFFFFFFFF:FFFF
@A00721:190:HGJW7DSXY:4:1308:4553:21715 2:N:0:TCTGCAAG+GCTGCACT
AATAAATATAAATTTTGAATTTCAAAATTGACCGTCGATTCTACTGCGTCACTATAGAATTCATTTTT
TAGATGGTTATGATCGTGACGCGTGGTCTAATGACAAATCGACACGTAAGCGTACGGAGGTGTCCCCG
GCGCGGGCAAGCCAG
+
FFF:FFFFFFFFFFFFFFFFF:FFFFFFFFFFFFFFFFFFFFFFFFFFF:FF,:FFFF,FFFFFFFFF
FFF:FFFFFFFF:FFFFFFFFFF:FF:FFFFFFFFFFFFFFFFFFFF:FFFFF:FFF,FF,FFFFFFF
FFFFFFFF:FFFF:F
@A00721:190:HGJW7DSXY:4:1367:31656:30514 2:N:0:TCTGCAAG+GCTGCACT
AATAAATATAAATTTTGAATTTCAAAATTGACCGTCGATTCTACTGCGTCACTATAGAATTCATTTTT
TAGATGGTTATGATCGTGACGCGTGGTCTAATGACAAATCGACACGTAAGCGTACGGAGGTGTCCCCG
GCGCGGGCAAGCCAG
+
FFFFF:FFFFFFFFFFFFFFFFFFFFFFFFFFFFFFFF,FFFFFFFFF:F:FF,::F::F::FFFFF,
:FF:FFFFFFFFFF:FFF:FFFFFFFFFFF:FF::FFFF,FFF:FFFFFFF,F:FFFFFF,:FFFFFF
FFFFFFFF,,::FFF
@A00721:190:HGJW7DSXY:4:1464:24804:36276 2:N:0:TCTGCAAG+GCTGCACT
AATAAATATAAATTTTGAATTTCAAAATTGACCGTCGATTCTACTGCGTCACTATAGAATTCATTTTT
TAGATGGTTATGATCGTGACGCGTGGTCTAATGACAAATCGACACGTAAGCGTACGGAGGTGTCCCCG
GCGCGGGCAAGCCAG
+
FFFFFFFFFFFFFFFFFFFFFFFFFFFFFFFFFFFFFFFFFFFFFFFFFFFFFFFFFFFFFFFFFFFF
F:FF:FFFFFFFFFFFFFFFFFF:FFFFFFFFFFFFFFFFFFFFFFFFFFFFFFFFFFFFFFFFFFFF
FFFFFFFFFFF:F,F
@A00721:190:HGJW7DSXY:4:1470:32072:7811 2:N:0:TCTGCAAG+GCTGCACT
AATAAATATAAATTTTGAATTTCAAAATTGACCGTCGATTCTACTGCGTCACTATAGAATTCATTTTT
TAGATGGTTATGATCGTGACGCGTGGTCTAATGACAAATCGACACGTAAGCGTACGGAGGTGTCCCCG
GCGCGGGCAAGCCAG
+
FFFFFFFFFFFFFFFFF,FF:FFFF,FFFFFFFFFFFFFFFFFFFFFFFFF:::::::FFF,FFFFF:
FFFF:F:FFFFFFFFFFFFFF,FFFFFFF:FFFFFFFFFFF:FFFFFFF:FFFFFFF:FFFFFFF:FF
FFFFFFFFFFFFF,F
@A00721:190:HGJW7DSXY:4:1566:24053:9893 2:N:0:TCTGCAAG+GCTGCACT
AATAAATATAAATTTTGAATTTCAAAATTGACCGTCGATTCTACTGCGTCACTATAGAATTCATTTTT
TAGATGGTTATGATCGTGACGCGTGGTCTAATGACAAATCGACACGTAAGCGTACGGAGGTGTCCCCG
GCGCGGGCAAGCCAG
+
FFFF:FFFFFFFFFFFFFFFFFFFFFFFFFFFFFFFFF:FFFFFFFFF::FFF:F,,:FFFFFFFFFF
FFFFFFFFFFFFFFFFFF:FFFFFFFFFFFFFFFFFFFFFFFFFFFFFFFFFFFFFFFFFFFFFF:FF
FFFFFFFFFFFFFF:
@A00721:190:HGJW7DSXY:4:2611:17237:2315 2:N:0:TCTGCAAG+GCTGCACT
AATAAATATAAATTTTGAATTTCAAAATTGACCGTCGATTCTACTGCGTCACTATAGAATTCATTTTT
TAGATGGTTATGATCGTGACGCGTGGTCTAATGACAAATCGACACGTAAGCGTACGGAGGTGTCCCCG
GCGCGGGCAAGCCAG
+
FFFFFFFFFFFFFFFFFFFFFFFFFFFFFFFFFFFFFFFFFFFFFFFFFFF,FF:,FF:FFFFFFFFF

FFFFFFFFFFFFFFFFFFFFFFFFFFFFFFFFFFFFFFFFFF:FFFFFFFFFFFFFFF::FFFFFFFF
FFFFFFFF:FFFF,F
@A00721:190:HGJW7DSXY:4:2612:5168:33207 2:N:0:TCTGCAAG+GCTGCACT
AATAAATATAAATTTTGAATTTCAAAATTGACCGTCGATTCTACTGCGTCACTATAGAATTCATTTTT
TAGATGGTTATGATCGTGACGCGTGGTCTAATGACAAATCGACACGTAAGCGTACGGAGGTGTCCCCG
GCGCGGGCAAGCCAG
+
F:FFFFFFFFFFFFFFFFFFFFFFFFFFFFFFFFFFFFFFFFFFFFFFFFFFFFF,:FFFFFFFFFF:
FFFF:FFFFFFFFFFFFFFFFFF,FFFFFF:FF,FFFFF:FFFFFFFFFFFFFFFFFFFF:FFFFFFF
FFFFFFFF:,FFF:F
@A00721:190:HGJW7DSXY:4:1402:4146:21793 2:N:0:TCTGCAAG+GCTGCACT
AATAAATATAAATTTTGAATTTCAAAATTGACCGTCGATTCTACTGCGTCACTATAGAATTCATTTTT
TAGATGGTTATGATCGTGACGCGTGGTCTAATGACAAATCGACACGTAAGCGTACGGAGGTGTCCCCG
GCGCGGGCAAGCCCG
+
FFFFFF:FFFFFF:FFFFFFFFFFFFFFFFFFFFFFFF::FFFFFFFFFF:F,F:FFF:FFFFFFFFF
F:FF:FFFFF:::FFFFFFFFFFFFFFFFFFFF:FFFFFFFFFFF::F::FFF:FFFFFFFFFFFFFF
FFFFFFFF::FFF,F
@A00721:190:HGJW7DSXY:4:1215:27733:12352 2:N:0:TCTGCAAG+GCTGCACT
ACACAATAAATATAAATTTTGAATTTCAAAATTGACCGTCGATTCTACTGCGTCACTATAGAATTCAT
TTTTTAGATGGTTATGATCGTGACGCGTGGTCTAATGACAAATCGACACGTAAGCGTACGGAGGTGTC
CCCGGCGCGGGCAAG
+
FFFFFFFFFFFFFFFFFFFFFFFFFFFFFFFFFFFFFFFFFF,FFFFFFFFF,FFF:FFFFFF:FFFF
FFFFFFFFFFFFFFFFFFFFFFFFFFF,FFFFFFFFFFFFFFF:FF:FFFFF,FFFFFFFFFFFFFFF
FFFFFFFFFFFFFFF
@A00721:190:HGJW7DSXY:4:1331:21504:6731 2:N:0:TCTGCAAG+GCTGCACT
ACACAATAAATATAAATTTTGAATTTCAAAATTGACCGTCGATTCTACTGCGTCACTATAGAATTCAT
TTTTTAGATGGTTATGATCGTGACGCGTGGTCTAATGACAAATCGACACGTAAGAGTACGGAGGTGTC
CCCGGCGCGGGCAAG
+
F:F:FFFFFF,F:FFFFFFFFFFFFFFFFFFFFFFFF:FFFF:FFF::F:FF:,F:FF,,:F,FFFFF
FFF,FFFF,:FF:FFFF:::F:FFFFFFFFFFFFFFFF,FFF,FFFFFFFFFF:,::FF:FF,F,:FF
:FF,FFF:F::FFF:
@A00721:190:HGJW7DSXY:4:1512:4869:5541 2:N:0:TCTGCAAG+GCTGCACT
ACACAATAAATATAAATTTTGAATTTCAAAATTGACCGTCGATTCTACTGCGTCACTATAGAATTCAT
TTTTTAGATGGTTATGATCGTGACGCGTGGTCTAATGACAAATCGACACGTAAGCGTACGGAGGTGTC
CCCGGCGCGGGCAAG
+
FFFFFFFFFFFFFFFFFFFFFFFFFFFFF:FFFFFFFFFFFF:F,FFFF:FFFFFF:F::F:FFF:FF
FFFFFFF,FFFFFFF::F:FFFFFFFFFFFFFFFFFFFFFFFF,FFFFFFFFFFFFFFFFF,FFFFF,
FFFFFFFFFFFFFFF
@A00721:190:HGJW7DSXY:4:1650:3866:34616 2:N:0:TCTGCAAG+GCTGCACT
ACACAATAAATATAAATTTTGAATTTCAAAATTGACCGTCGATTCTACTGCGTCACTATAGAATTCAT
TTTTTAGATGGTTATGATCGTGACGCGTGGTCTAATGACAAATCGACACGTAAGCGTACGGAGGTGTC
CCCGGCGCGGGCAAG
+
FFFFFFFFFFFFFFFFFFFFFFFFFFFFFFFFFFFFFFFFFFFFFFFFFFFFFFFFFFFFFFFFFFFF
FFFFFFFFFFFFFFFFFFFFFFFFFFF:FFFFFFFFFFFFFFFFFFFFFFFFFFFFFFFFFFFFFFFF
FFFFFFFFFFFFFFF
@A00721:190:HGJW7DSXY:4:2205:8612:13839 2:N:0:TCTGCAAG+GCTGCACT
ACACAATAAATATAAATTTTGAATTTCAAAATTGACCGTCGATTCTACTGCGTCACTATAGAATTCAT
TTTTTAGATGGTTATGATCGTGACGCGTGGTCTAATGACAAATCGACACGTAAGCGTACGGAGGTGTC
CCCGGCGCGGGCAAG

```
+
FFFFFFFFFFFFFFFFFFFFFFFFFFFFFFFFFFFFFFFFFFFFFFFFFFFFFFFFFF,FFFFFFFFF
FFFFFFF:FFFFFFFF,F:FFFFFFFFFFFFFFFFFFFFFFFFFFFFFFFF:FFFFFFFFF:FFFFFF
FFFFFFFFFFFFF,F
@A00721:190:HGJW7DSXY:4:2447:6641:25989 2:N:0:TCTGCAAG+GCTGCACT
ACACAATAAATATAAATTTTGAATTTCAAAATTGACCGTCGATTCTACTGCGTCACTATAGAATTCAT
TTTTTAGATGGTTATGATCGTGACGCGTGGTCTAATGACAAATCGACACGTAAGCGTACGGAGGTGTC
CCCGGCGCGGGCAAG
+
FFFFFFFFFFFFFFFFFFFFFFFFFFFFFFFFFFFFFFFFFFFFFFFFFFFFFFFFFFFFFFFFFFFF
FFFFFFFFFFFFFFFFFFFFFFFFFFFFFFFFFFFFFFFFFFFFFFFFFFFFFFFFFFFFFFFFFFFF
FFFFFFFFFFFFF,F
@A00721:190:HGJW7DSXY:4:2450:20003:1376 2:N:0:TCTGCAAG+GCTGCACT
ACACAATAAATATAAATTTTGAATTTCAAAATTGACCGTCGATTCTACTGCGTCACTATAGAATTCAT
TTTTTAGATGGTTATGATCGTGACGCGTGGTCTAATGACAAATCGACACGTAAGCGTACGGAGGTGTC
CCCGGCGCGGGCAAG
+
FFFFF,FFFFFFFFFFFFFFFFFFFFFFFFFFFFFFFFFFFFFFFFFFFFFFFFFFF:F,F:FFFFFF
FF:FFFF:FFFFFFFFFFFFFF:FFFFFFFFFFFFF:FFFF:FF:FFFFFFFFFFFFF:FFFFFFFFF
FFFFFFFFFFFFF:F
@A00721:190:HGJW7DSXY:4:2502:26829:2738 2:N:0:TCTGCAAG+GCTGCACT
ACACAATAAATATAAATTTTGAATTTCAAAATTGACCGTCGATTCTACTGCGTCACTATAGAATTCAT
TTTTTAGATGGTTATGATCGTGACGCGTGGTCTAATGACAAATCGACACGTAAGCGTACGGAGGTGTC
CCCGGCGCGGGCAAG
+
FFFFFFFFFFFFFFFFFFFFFFFFFFFFFFFFFF:FFFFFFF:FFFF:FFFF:F,:FFFFFFFFFFFF
FFFFFFFFFFFFFFFFFFFFFFFFFFF:FFFFFFFFF:FFFFFFFF:FFFFFF,FFFFFF,FFFF:F:
FFFFFFFF::FF:FF
@A00721:190:HGJW7DSXY:4:2319:23854:34569 2:N:0:TCTGCAAG+GCTGCACT
CAACACAATAAATATAAATTTTGAATTTCAAAATTGACCGTCGATTCTACTGCGTCACTATAGAATTC
ATTTTTTAGATGGTTATGATCGTGACGCGTGGTCTAATGACAAATCGACACGTAAGCGTACGGAGGTG
TCCCCGGCGCGGGCA
+
FFFFFFFFFFFFFFFFFFFFFFFFFFFFFFFFFFFFFFFFFFFF:FFFFFFFFFFFFFFF:FFFFFFF
FFFFFFFFFFFFFFFFFFFFFFFFFFFFFFFFFFFFFFF:FFFFFFFFFFFFFFFFFFFFFFFFFFFF
FFFFFFFFFFFFFFF
@A00721:190:HGJW7DSXY:4:1159:3676:24142 2:N:0:TCTGCAAG+GCTGCACT
TTTTAACAGTCTATTGGTTATCAAAAAGACGATGATATAACATATCATCTGCCGTCGATTTTACTGCG
TCACTATAGAATTCATTTTTTAGATGGTCATGATCGTGACGCGTGGTCTAATGACAAATCGACACGTA
AGCGTACGGAGGTGT
+
FFFFFFFFFFFFF,FFFFFFFFFFFFFFFFFFFFFFFFFFFFFFFFFF:FF:FFFFFFFFFFFF:FFF
FFFFFFFFFFFFFFFFFFFFFF:FFFFFFFFFFFFFFFFFFFFFFFFF:FFFFFFFF:F:FFFFFFFF
FFFFFFFFFFFFF:F
@A00721:190:HGJW7DSXY:4:1163:25925:9533 2:N:0:TCTGCAAG+GCTGCACT
TTTTAACAGTCTATTGGTTATCAAAAAGACGATGATATAACATATCATCTGCCGTCGATTTTACTGCG
TCACTATAGAATTCATTTTTTAGATGGTCATGATCGTGACGCGTGGTCTAATGACAAATCGACACGTA
AGCGTACGGAGGTGT
+
FFFFFFFFFFFFFFFFFFFFFFFFFFFFFFFFFFFFFFFFFFFFFFFFFFFFFFFFFFFFFFFFFFFF
FFFFF:FFFFFFFFFFFFFFFFFFFFFFFFFFFFFFFFFFFFFFFFFFFFFFFFFFFFF:FFFFFFFF
FFFFFFFFFFFFFFF
@A00721:190:HGJW7DSXY:4:2138:10447:24565 2:N:0:TCTGCAAG+GCTGCACT
TTTTAACAGTCTATTGGTTATCAAAAAGACGATGATATAACATATCATCTGCCGTCGATTTTACTGCG
```

```
TCACTATAGAATTCATTTTTTAGATGGTCATGATCGTGACGCGTGGTCTAATGACAAATCGACACGTA
AGCGTACGGAGGTGT
+
FFFFFFFFFFFFFFFFFFF:FFFFFFFFFFFFFFFFFFFFFFFFFFF:FFFF:FFFFFF:FFFFFFF:
FFFFFFFFFFFF,FF:FFFFFFFFF:FFFFF,FF:FFFFFFF:FFFFFFFFF:F,FFFF:F:FFFFFF
FF,FFF::FFFFFFF
@A00721:190:HGJW7DSXY:4:2249:4824:21840 2:N:0:TCTGCAAG+GCTGCACT
TTTTAACAGTCTATTGGTTATCAAAAAGACGATGATATAACATATCATCTGCCGTCGATTTTACTGCG
TCACTATAGAATTCATTTTTTAGATGGTCATGATCGTGACGCGTGGTCTAATGACAAATCGACACGTA
AGCGTACGGAGGTGT
+
FFFFFFFFFFFFFFFFFFFFFFFFFFFFFFFFFFFFFFFFFFFFFFFFFFFF:FFFFFFFFFFFFFFF
FFFFFFFFFFFFFFFFFFFFFFFFFFFFFFFFFFFFFFFFFFFFFFFFFFFFFFFFFFF:FFFFFFFF
FF:FFF:FFFFFFFF
@A00721:190:HGJW7DSXY:4:2258:9932:28025 2:N:0:TCTGCAAG+GCTGCACT
TTTTAACAGTCTATTGGTTATCAAAAAGACGATGATATAACATATCATCTGCCGTCGATTTTACTGCG
TCACTATAGAATTCATTTTTTAGATGGTCATGATCGTGACGCGTGGTCTAATGACAAATCGACACGTA
AGCGTACGGAGGTGT
+
FFFFFFFFFFFFFFFFFFFFFFFFFFFFFFFFFFFFFFFFFFFFFFFFFFFFFFFFFFFFFFFFFFFF
:FFFF:FFFFFFFFFFFFFFFFFFFFFFFFFFFFFFFFFFFFFFFFFFFFFFFFFFFFFFFFFFFFFF
FFFFFFFFFFFFFFF
@A00721:190:HGJW7DSXY:4:2543:12491:31735 2:N:0:TCTGCAAG+GCTGCACT
TTTTAACAGTCTATTGGTTATCAAAAAGACGATGATATAACATATCATCTGCCGTCGATTTTACTGCG
TCACTATAGAATTCATTTTTTAGATGGTCATGATCGTGACGCGTGGTCTAATGACAAATCGACACGTA
AGCGTACGGAGGTGT
+
F:FFFFFFFFFFFFFFFFFFFFFFFFFFFF:FFFFFFFFFFFFFFFFFFFFF:FFFFFFFFFFFFFFF
FFFFFF:FFF:FFFFFFFFFFFFFFF:FFFFFFFFFFFFFFFFFF:FFFFFFFFFFFFFFFFFF:FFF
F,FFFFFFFF:FFFF
@A00721:190:HGJW7DSXY:4:1104:5593:22357 2:N:0:TCTGCAAG+GCTGCACT
TGCTAAAGCAGTCATCAACACAATAAATATAAATTTTGAATTTCAAAATTGACCGTCGATTCTACTGC
GTCACTATAGAATTCATTTTTTAGATGGTTATGATCGTGACGCGTGGTCTAATGACAAATCGACACGT
AAGCGTACGGAGGTG
+
FFFFFFFFFFFFFFFF:FFFFFFFFFFFFFFFFFFFFFFFFFFFFFFFFFFFFFFFFFFFFFFFFFFF
FFFFFF,F:F:FFFFFFFFFFFFF,FFF:FFF:FFFFF:,FFFFFFF:FFFFFFF,FFFFFFFFFFFF
FFFFFFFFFFFFFFF
@A00721:190:HGJW7DSXY:4:1209:13440:29277 2:N:0:TCTGCAAG+GCTGCACT
CTTTTAACAGTCTATTGGTTATCAAAAAGACGATGATATAACATATCATCTGCCGTCGATTTTACTGC
GTCACTATAGAATTCATTTTTTAGATGGTCATGATCGTGACGCGTGGTCTAATGACAAATCGACACGT
AAGCGTACGGAGGTG
+
FF:FFFFFFFFFFFFFFFFFFFFFFFFFFFFF:FFFFFFFFFFFFFFFFFFFFFFFFFFFFFFFFFFF
FFFFFFFFF::FFFFFFFFFFFFFFFFFFFFF::FFF:FFFFFFFFFFFFFFFFF:FFFFFFFFFFFF
FF,FFFFFFFFF:FF
@A00721:190:HGJW7DSXY:4:1262:31051:32565 2:N:0:TCTGCAAG+GCTGCACT
CTTTTAACAGTCTATTGGTTATCAAAAAGACGATGATATAACATATCATCTGCCGTCGATTTTACTGC
GTCACTATAGAATTCATTTTTTAGATGGTCATGATCGTGACGCGTGGTCTAATGACAAATCGACACGT
AAGCGTACGGAGGTG
+
FFFFFFFFFFFFFFFFFFFFFFFFFFFFFFFFFFFFFFFFFFFFFFFFFFFFFFFFFFFFFFFFFFFF
FFFFFFFFFFFFFFFFFFFFFFFFFFFFF:FFFFFFFFFFFFFFFFFFFFFFFFFFFFFFFFFFFFFF
FFFFFFFFFFFFFFF
```

```
@A00721:190:HGJW7DSXY:4:1262:31575:33661 2:N:0:TCTGCAAG+GCTGCACT
CTTTTAACAGTCTATTGGTTATCAAAAAGACGATGATATAACATATCATCTGCCGTCGATTTTACTGC
GTCACTATAGAATTCATTTTTTAGATGGTCATGATCGTGACGCGTGGTCTAATGACAAATCGACACGT
AAGCGTACGGAGGTG
+
FFFFFFFFFFFF:FFFFFFFFFFFFFFFFFFFFFFFFFFFFFFFFFFFFFFFFF:FFFFFFFFFFFFFFF
FFFFFFFFFFFFFFFFFFFFFFFFFFFFFFFFFFFFFFFFFFFFFFFFFFFFFFFFFFFFFFFF:F:F:F
FFFFF:FFFFFFFFF
@A00721:190:HGJW7DSXY:4:1339:12255:29951 2:N:0:TCTGCAAG+GCTGCACT
TGCTAAAGCAGTCATCAACACAATAAATATAAATTTTGAATTTCAAAATTGACCGTCGATTCTACTGC
GTCACTATAGAATTCATTTTTTAGATGGTTATGATCGTGACGCGTGGTCTAATGACAAATCGACACGT
AAGCGTACGGAGGTG
+
FFF:FFFFFFFFFFFFFFFFFFFFFFFFFFFFFFFFFFFFFFFFFFFFFFFFFFFFFFFFFFFFFFFFFF
FFFFFFFF:FFFFFFFFFFF::FFFF:FFFF:FFFFFFFFFFFFFF,FFFFFFFF,FFFFFFFFFFFFFF
F:FFFFFFFFFFF::
@A00721:190:HGJW7DSXY:4:1522:2410:36793 2:N:0:TCTGCAAG+GCTGCACT
CTTTTAACAGTCTATTGGTTATCAAAAAGACGATGATATAACATATCATCTGCCGTCGATTTTACTGC
GTCACTATAGAATTCATTTTTTAGATGGTCATGATCGTGACGCGTGGTCTAATGACAAATCGACACGT
AAGCGTACGGAGGTG
+
FFFFFFFFFFFFFFFFFFFFFFFFFFFFFFFFFFFFFFFFFFFFFFFFFFFFFFFFFFFFFFFFFFFFFF
FFFFFFFFFFFFFFFFFFFFFFFFFFFFFFFFFFFFFFFFFFFFFFFFFFFFFFFFFFF:FF:FFFFFFF
F:FFFFFFFFFFFFF
@A00721:190:HGJW7DSXY:4:1544:8648:17347 2:N:0:TCTGCAAG+GCTGCACT
CTTTTAACAGTCTATTGGTTATCAAAAAGACGATGATATAACATATCATCTGCCGTCGATTTTACTGC
GTCACTATAGAATTCATTTTTTAGATGGTCATGATCGTGACGCGTGGTCTAATGACAAATCGACACGT
AAGCGTACGGAGGTG
+
FFFFFFFFFFFFFFFFFFFFFFFFFFFF:F:FFFFFFFFFFFFFFFFFFFFFFFFFFFFFFFFFFFFFF:
FFFFFFF:FFFFFFFFF:FFFFFFFFF:FFFFFFFFFFFFFFFFFFFFFFFFFFFFFF:FFFFFFFFFFF
F,,FFFFFFFFFFFF
@A00721:190:HGJW7DSXY:4:1602:7129:24893 2:N:0:TCTGCAAG+GCTGCACT
CTTTTAACAGTCTATTGGTTATCAAAAAGACGATGATATAACATATCATCTGCCGTCGATTTTACTGC
GTCACTATAGAATTCATTTTTTAGATGGTCATGATCGTGACGCGTGGTCTAATGACAAATCGACACGT
AAGCGTACGGAGGTG
+
FFFFFFFFFFFFFFFFFFFFFFFFFFFFFFFFFFFFFFFFFFFFFFFFFFFFFFFFFFFFFFFFFFFFFF
FFFFFFFFFFFFFFF,FFFFFFFFFFFFFFF:F:FFFFF:FFFFFFFFFFFFFFFFFFFFFFFFFFFFFF
FFFFFFFFFFFF:FF
@A00721:190:HGJW7DSXY:4:1645:5873:25347 2:N:0:TCTGCAAG+GCTGCACT
TGCTAAAGCAGTCATCAACACAATAAATATAAATTTTGAATTTCAAAATTGACCGTCGATTCTACTGC
GTCACTATAGAATTCATTTTTTAGATGGTTATGATCGTGACGCGTGGTCTAATGACAAATCGACACGT
AAGCGTACGGAGGTG
+
FFFFFFFFFFFFFFFFFFFFFFFFFFFFFFFFFFFFFFFFFFFFFFFFFFFFFFFFFFFFFFFFFFFFFF
FFFFFFFF:FFFFFFF:FFFFFFFF:FFFFFFFFFFF:F:FFFFFFFFFFFFF:FFFFFFFFFFFFFFFF
FFFFFFFFFF:FFFF
@A00721:190:HGJW7DSXY:4:1667:12301:20948 2:N:0:TCTGCAAG+GCTGCACT
CTTTTAACAGTCTATTGGTTATCAAAAAGACGATGATATAACATATCATCTGCCGTCGATTTTACTGC
GTCACTATAGAATTCATTTTTTAGATGGTCATGATCGTGACGCGTGGACTAATGACAAATCGACACGT
AAGCGTACGGAGGTG
+
:F:FFFFFFFFFFFF:FFFFFFFFFFFFFFFFFF:FFFFFFFFFFFF::F:FFFF:F,,:FF::FFFFFF
```

```
,FFFFFFF:F::FFFF,:FFFFF,F:::FFF::FFFF,FFFFFF,F:,FFFFF,FFFFFFFFF,FFFF
FF:FF:FFFFF:,F:
@A00721:190:HGJW7DSXY:4:2123:21151:29168 2:N:0:TCTGCAAG+GCTGCACT
CTTTTAACAGTCTATTGGTTATCAAAAAGACGATGATATAACATATCATCTGCCGTCGATTTTACTGC
GTCACTATAGAATTCATTTTTTAGATGGTCATGATCGTGACGCGTGGTCTAATGACAAATCGACACGT
AAGCGTACGGAGGTG
+
:FFFFFFFFFFFFFFFFFFFFFFFFFFFFFFFFFFFFFFFFFFFFFFFFFFFF:FFFFFFFFFFFFFF
FFFFFFFFFF:FFFFFFFFF:FFFFFF,FFFFFFFFFFFFFFFFFFFFFFFFFFFFFFFFF,FF:FF:F
:F:FFFFF,FF::F:
@A00721:190:HGJW7DSXY:4:2175:12057:9502 2:N:0:TCTGCAAG+GCTGCACT
TGCTAAAGCAGTCATCAACACAATAAATATAAATTTTGAATTTCAAAATTGACCGTCGATTCTACTGC
GTCACTATAGAATTCATTTTTTAGATGGTTATGATCGTGACGCGTGGTCTAATGACAAATCGACACGT
AAGCGTACGGAGGTG
+
FFFFFFFFFFFFFFFFFFFFFFFFFFFFF:FFFFFFFFFFFFFFFFFFFFFFFFFFFFFFFFFFFFFF
FFFFFFFFFFFFFFFFFFFFFFFFFFFFFFFFF,FFFFFF:FFFFFFFFFFFFFFFFFFFFF:FFFFFF
FFFFFFFF:FFFFFF
@A00721:190:HGJW7DSXY:4:2340:16504:6840 2:N:0:TCTGCAAG+GCTGCACT
CTTTTAACAGTCTATTGGTTATCAAAAAGACGATGATATAACATATCATCTGCCGTCGATTTTACTGC
GTCACTATAGAATTCATTTTTTAGATGGTCATGATCGTGACGCGTGGTCTAATGACAAATCGACACGT
AAGCGTACGGAGGTG
+
FFFFFFFFFFFFFFFFFFFFFFF:FFFFFF:FFFFFFFF:FFFFFFFFFFFFFFFFFFFFFFFFFFFF
FFFFFFFFFFFFFFFFFFFFFFFFFFFFFFFFFFFFFFFFFFFF:FFFFFFFFFF:FFFFFFFFFFFF
FF,F:FFFFFFFFFF
@A00721:190:HGJW7DSXY:4:2462:10303:26287 2:N:0:TCTGCAAG+GCTGCACT
CTTTTAACAGTCTATTGGTTATCAAAAAGACGATGATATAACATATCATCTGCCGTCGATTTTACTGC
GTCACTATAGAATTCATTTTTTAGATGGTCATGATCGTGACGCGTGGTCTAATGACAAATCGACACGT
AAGCGTACGGAGGTG
+
FFFFFFFFFFFFFFFFFFFFFFFFFFFFFFFFFFFFF:FFFFFFFFFFFFFFFFFFFFFFFFFFFFFF
FFFFFFFFF:FFFFFFFFFFFFFFFFFFFFFFFFFFF:FFFFFFFFFFFFFFF:FFFFFFF:FFFFFFF
FFFFFFFFFFFFFFF
@A00721:190:HGJW7DSXY:4:2553:13602:11929 2:N:0:TCTGCAAG+GCTGCACT
CTTTTAACAGTCTATTGGTTATCAAAAAGACGATGATATAACATATCATCTGCCGTCGATTTTACTGC
GTCACTATAGAATTCATTTTTTAGATGGTCATGATCGTGACGCGTGGTCTAATGACAAATCGACACGT
AAGCGTACGGAGGTG
+
FFFFFFFFFFFFFFF,FFFFFFFFFFFFFFFFFFFFFFFFFFFFFFFFFFFFFFFFFFFF:FFFFFFFF
FFFFFFFFFFFFFFFFFFFFFFFFFFFFFFFFFFFFFF:FFFFFFFFFF:FFFFFFFFF:FFFFFFF
FFFFFFFFFFFFFF:
@A00721:190:HGJW7DSXY:4:2608:5484:6887 2:N:0:TCTGCAAG+GCTGCACT
CTTTTAACAGTCTATTGGTTATCAAAAAGACGATGATATAACATATCATCTGCCGTCGATTTTACTGC
GTCACTATAGAATTCATTTTTTAGATGGTCATGATCGTGACGCGTGGTCTAATGACAAATCGACACGT
AAGCGTACGGAGGTG
+
FFFFFFFFFF:FFF:FFFFFFFFFFFFFF:FFFFFF:FFFFFFFFFFFFF:FFFFFFFFFFFFFFFF:
FF:FFF,FFFFFFFFFFFFFFFFFFF:FFF,FF:FFFF,:FFFFFFFFFFFFFFFFFFFFF:,FFFFFFF
FFFF:FFF,FF,FFF
@A00721:190:HGJW7DSXY:4:1427:1642:12853 2:N:0:TCTGCAAG+GCTGCACT
AATCAACTGCTAAAGCAGTCATCAACACAATAAATATAAATTTTGAATTTCAAAATTGACCGTCGATT
CTACTGCGTCACTATAGAATTCATTTTTTAGATGGTTATGATCGTGACGCGTGGTCTAATGACAAATC
GACACGTAAGCGTAC
```

+
FFFFFFFFFFFFFFFFFFFFFFFFFFFFFFFFFFFFFFFFFFFFFFFFFFFFFFFFFFFFFFFFFFFF
FFFFFFFFFFFFFFF:FFFFFFFFFFFFFFFFFFFFFFFFFFFFFFFFFFFFFFFFFFFFFFFFFFFF
FFF:FFFF,FFFFFF
@A00721:190:HGJW7DSXY:4:1512:16477:34381 2:N:0:TCTGCAAG+GCTGCACT
AATCAACTGCTAAAGCAGTCATCAACACAATAAATATAAATTTTGAATTTCAAAATTGACCGTCGATT
CTACTGCGTCACTATAGAATTCATTTTTTAGATGGTTATGATCGTGACGCGTGGTCTAATGACAAATC
GACACGTAAGCGTAC
+
FFFFFFFFFFFFFFFFFFFFFFFFFFFFFFFFFFFFFFFFFFFFFFFFFFFFFFFFFFFFFFFFFFFF
FFFFFFFFFFFFFFF:F:FFFFFFFFFF:FFF:FFFFFFF:FFFFFF:FFFFFFFFFFFF,FFFFFFF
FFFFFFFFFFFFFFF
@A00721:190:HGJW7DSXY:4:1512:18584:35117 2:N:0:TCTGCAAG+GCTGCACT
AATCAACTGCTAAAGCAGTCATCAACACAATAAATATAAATTTTGAATTTCAAAATTGACCGTCGATT
CTACTGCGTCACTATAGAATTCATTTTTTAGATGGTTATGATCGTGACGCGTGGTCTAATGACAAATC
GACACGTAAGCGTAC
+
FFFFFFFFFFFFFFFFFFFFFFFFFFFFFFFFFFFFFFFFFFFFFFFFFFFFFFFFFFFFFFFFFFFF
FFFFFFFFFFFFF:::FFFFFFFFFFFFFFFFFFFFFFFFFFFFFFFFFFFF:FFFFFFFFFFFFFFF
FFFFF:FFFFFFFFF
@A00721:190:HGJW7DSXY:4:2445:12762:23688 2:N:0:TCTGCAAG+GCTGCACT
AATCAACTGCTAAAGCAGTCATCAACACAATAAATATAAATTTTGAATTTCAAAATTGACCGTCGATT
CTACTGCGTCACTATAGAATTCATTTTTTAGATGGTTATGATCGTGACGCGTGGTCTAATGACAAATC
GACACGTAAGCGTAC
+
FFFFFFFFFFFFFFFFFFFFFFFFFFFFFFFFFFFFFFFFFFFFFFFFFFFFFFFFFFFFFFFFFFFF
FFFFFFFFFFFFFFFFFFFFFFFFF:FFFFFFFFFFFFFFFFFFFFFFFFFFFFFFFFFFFFFFFFFF
FFFFFFFFFFFFFFF
@A00721:190:HGJW7DSXY:4:2468:5367:7623 2:N:0:TCTGCAAG+GCTGCACT
AATCAACTGCTAAAGCAGTCATCAACACAATAAATATAAATTTTGAATTTCAAAATTGACCGTCGATT
CTACTGCGTCACTATAGAATTCATTTTTTAGATGGTTATGATCGTGACGCGTGGTCTAATGACAAATC
GACACGTAAGCGTAC
+
FFFFFFFFFFFFFFFFFFFFFFFFFFFFFFFFFFFFFFFFFFFFFFFFFFFFFFFFFFFFFFFFFFFF
FFFFFFFFFFFFFFFFFFFFFFFFFFFFF:FFFFFFFFFFFFFFFFFFFFFFFFFFFFFFFFFFFFFF
:FFFFFFFFFFFFFF
@A00721:190:HGJW7DSXY:4:2545:15881:17096 2:N:0:TCTGCAAG+GCTGCACT
AATCAACTGCTAAAGCAGTCATCAACACAATAAATATAAATTTTGAATTTCAAAATTGACCGTCGATT
CTACTGCGTCACTATAGAATTCATTTTTTAGATGGTTATGATCGTGACGCGTGGTCTAATGACAAATC
GACACGTAAGCGTAC
+
FFFFFFFFFFFFFFFFFFFFFFFFFFFFFFFFFFFFFFFFFFFFFFFFFFFFFFFFFFFFFFFFFF,F
FFFFFFFFFFFFF:FFF:FFFFFFFFFFFFF,FFFFFFFFFFFFFFFFFFFFFFFFFF:FFFFF:FFF
FFFFFFFFFFFFFFF
@A00721:190:HGJW7DSXY:4:1325:14606:11725 2:N:0:TCTGCAAG+GCTGCACT
GCCATTTATATAGACCTTTTAACAGTCTATTGGTTATCAAAAAGACGATGATATAACATATCATCTGC
CGTCGATTTTACTGCGTCACTATAGAATTCATTTTTTAGATGGTCATGATCGTGACGCGTGGTCTAAT
GACAAATCGACACGT
+
FFFFFFFFFFFFFFFFFF:FFFFFFFFFFFFFFFFFFFFFFF:FFFFFFFFFFFFFFFFFFFFFFFFF
FFFFFFFFFFFFF,FFFF:FFFFFF:FFFFFFFFFFFFFFF:FFFFFF:FFFFFFFFFFFFFFFFFFF
::FFFFF:FFFFF:F
@A00721:190:HGJW7DSXY:4:1414:13449:4961 2:N:0:TCTGCAAG+GCTGCACT
GCCATTTATATAGACCTTTTAACAGTCTATTGGTTATCAAAAAGACGATGATATAACATATCATCTGC

CGTCGATTTTACTGCGTCACTATAGAATTCATTTTTTAGATGGTCATGATCGTGACGCGTGGTCTAAT
GACAAATCGACACGT
+
FFFFFFFFFFFFFFFFFFFFFFFFFFFFFFFFFFFFFFFFFFFFFFFFFFFFFFFFFFFFFFFFFFFF
FFFFFFFFFFFFFFFFFFFFFFFFFFFFFFFFFFFFFFFFFFFFFFF,FFFFFFF:FFFFFFFFFFFF
FFFFFF:FFFFFF:F
@A00721:190:HGJW7DSXY:4:1569:31024:3615 2:N:0:TCTGCAAG+GCTGCACT
GCCATTTATATAGACCTTTTAACAGTCTATTGGTTATCAAAAAGACGATGATATAACATATCATCTGC
CGTCGATTTTACTGCGTCACTATCGAATTCATTTTTTAGATGGTCATGATCGTGACGCGTGGTCTAAT
GACAAATCGACACGT
+
F:FFFFFFFFFFFFFFFFFFFFFFFFFFFFFFFFFFFFFFFFFF,FFFFFFFFFFF,FFFFFFFFFFF
FFFFFFFFFFFFFFFFF:FFFFF,:FFFFFFFFFFFFF:FFFFFFFF:FFFFFFFFFFFFFFFF:FFF
F,:FFFFF,F:FFFF
@A00721:190:HGJW7DSXY:4:1663:9842:32534 2:N:0:TCTGCAAG+GCTGCACT
GCCATTTATATAGACCTTTTAACAGTCTATTGGTTATCAAAAAGACGATGATATAACATATCATCTGC
CGTCGATTTTACTGCGTCACTATAGAATTCATTTTTTAGATGGTCATGATCGTGACGCGTGGTCTAAT
GACAAATCGACACGT
+
FFFFFFFFFFFFFFFFFFFFFFFFFFFFFFFFFF,FFFFFFFFFFFFFFFFFFFFFFFFFFFFFFFFF
:FFFFFFFFFFFFFFFFFFFFFFF,FFFFFFFFFFFFF:FFF,FFFFF:FFFFFF:FFFFFFFFFFFF
:FFFFFFFFFFFFFF
@A00721:190:HGJW7DSXY:4:2341:32398:22310 2:N:0:TCTGCAAG+GCTGCACT
GCCATTTATATAGACCTTTTAACAGTCTATTGGTTATCAAAAAGACGATGATATAACATATCATCTGC
CGTCGATTTTACTGCGTCACTATAGAATTCATTTTTTAGATGGTCATGATCGTGACGCGTGGTCTAAT
GACAAATCGACACGT
+
FFFFFFFFFFFFFFFFFFFFFFFFFFFFFFF:FFFFFFFFFF:FFFFFFFFFFFFFFFFFFFFFFFFF
FFFFFF::FFFFFFFFFFFFFF:FFF:FFFFFFFFFFFFFFFFF:FFF,FFFFFFFFFF:FFF:FF,F
,:FFFFF,FFFFFFF
@A00721:190:HGJW7DSXY:4:2365:20329:10488 2:N:0:TCTGCAAG+GCTGCACT
GCCATTTATATAGACCTTTTAACAGTCTATTGGTTATCAAAAAGACGATGATATAACATATCATCTGC
CGTCGATTTTACTGCGTCACTATAGAATTCATTTTTTAGATGGTCATGATCGTGACGCGTGGTCTAAT
GACAAATCGACACGT
+
FFFFFFFFFFFFFFFFFFFFFFFFFFFFFFFFFFFFFFFFFFF:FFFFFFFFFFFFFFFFFFFFFFFF
:FFFFFFFFF:FFF:FFFFFFFFFFFFFFFFFFFFFFFFFFFFFFFFFFFFFFFFFFFFF:FFFFFFF
:FFFFFF,:FFFFFF
@A00721:190:HGJW7DSXY:4:2533:5801:32017 2:N:0:TCTGCAAG+GCTGCACT
GCCATTTATATAGACCTTTTAACAGTCTATTGGTTATCAAAAAGACGATGATATAACATATCATCTGC
CGTCGATTTTACTGCGTCACTATAGAATTCATTTTTTAGATGGTCATGATCGTGACGCGTGGTCTAAT
GACAAATCGACACGT
+
F:FFFFFFFFFFFFFFFFFFFFFFFFFFFF,FFFFFFFFFFFFFFFFFFFFFFFFFFFFFFFFFFFFF
FFFFFFFFFFFFFFFFFFFF,:FFF,FFFFFFFFFFFFFFFFFFFFFFFFFFFF::FFFFFFFFFFFF
FF:FFFFFFFFF:F:
@A00721:190:HGJW7DSXY:4:2544:4227:36495 2:N:0:TCTGCAAG+GCTGCACT
GCCATTTATATAGACCTTTTAACAGTCTATTGGTTATCAAAAAGACGATGATATAACATATCATCTGC
CGTCGATTTTACTGCGTCACTATAGAATTCATTTTTTAGATGGTCATGATCTTGACGCGTGGTCTAAT
GACAAATCTACACGT
+
FFFF:FFFFFFFFFFFF:FFFF,FFFFFFFFFFFFFFFFFFFFFFFFF:FFF:FFFFFFFFFFFFF:F
F:FF:F,FFFFF,::F:FFFF,,:,F,,,:FFFFF:FFFFF,:F:FF:FFF,::FF:FF::FFFFFFF
,FFFFFFF,FFFF,F

```
@A00721:190:HGJW7DSXY:4:2614:5330:18584 2:N:0:TCTGCAAG+GCTGCACT
GCCATTTATATAGACCTTTTAACAGTCTATTGGTTATCAAAAAGACGATGATATAACATATCATCTGC
CGTCGATTTTACTGCGTCACTATAGAATTCATTTTTTAGATGGTCATGATCGTGACGCGTGGTCTAAT
GACAAATCGACACGT
+
FFFFFFFFFFFFFFFFFFFFFFFFFFFFFFFFFFFFFFFFFFFFFFFFFFFFFFFFFFFFFFFFFFFF
FFFFFFFFFFFFFFFFFFFFFFFFFF:FFFFFFFFFFFFFFF:FFFFFFFFFFFFFFFFFFFFFFFFF
FFFFFFFFFFFFFFF
@A00721:190:HGJW7DSXY:4:1423:27778:13401 2:N:0:TCTGCAAG+GCTGCACT
TAGCCATTTATATAGACCTTTTAACAGTCTATTGGTTATCAAAAAGACGATGATATAACATATCATCT
GCCGTCGATTTTACTGCGTCACTATAGAATTCATTTTTTAGATGGTCATGATCGTGACGCGTGGTCTA
ATGACAAATCGACAC
+
FFFFF,FF:FFFFFF:FFF,FFFFFFFFFFFFFFF,F,FFF:FFFFFFFFF:FFF:FFFFFFFFFFFF
FFFFFFFFFFFFFFF:FFFFFFFFF,,:FFFFFFFFFFFFF:FFFFFFFFFFFFF:FFFFFFFFFFFF
FFFFFF:FF:FFFFF
@A00721:190:HGJW7DSXY:4:2130:6894:19758 2:N:0:TCTGCAAG+GCTGCACT
TAGCCATTTATATAGACCTTTTAACAGTCTATTGGTTATCAAAAAGACGATGATATAACATATCATCT
GCCGTCGATTTTACTGCGTCACTATAGAATTCATTTTTTAGATGGTCATGATCGTGACGCGTGGTCTA
ATGACAAATCGACAC
+
FFFFF::FFFFFFFFFFFFFFFFFFFFFFFFFF:F:FFFFFFFFFFFFFFFFFFFFFFFF:FFFFFFF
::,FFFF:,FFFF,F:FFFFFF:F:F:FFFF,F:FFF:FFFFF,FF:FF,FF:,::FFFFFFFFF,FF
FF:F,FFFF::FFFF
@A00721:190:HGJW7DSXY:4:2433:21612:6355 2:N:0:TCTGCAAG+GCTGCACT
TAGCCATTTATATAGACCTTTTAACAGTCTATTGGTTATCAAAAAGACGATGATATAACATATCATCT
GCCGTCGATTTTACTGCGTCACTATAGAATTCATTTTTTAGATGGTCATGATCGTGACGCGTGGTCTA
ATGACAAATCGACAC
+
FFFFFFFFFFFFFFFFFFFFFFFFFFFFFFFFFFFFFFFFFFFF:F:FFFFFFFFFFFFFFFFFFFFF
FFFFFFFFFFFFFFFFFFFFFFF:FF:FFFFFFFFFFFFFFFFFFFFFFF:F:FFFFF:FFFFFFFFF
FFF:FFFFFF:FFFF
@A00721:190:HGJW7DSXY:4:2522:6849:27978 2:N:0:TCTGCAAG+GCTGCACT
TAGCCATTTATATAGACCTTTTAACAGTCTATTGGTTATCAAAAAGACGATGATATAACATATCATCT
GCCGTCGATTTTACTGCGTCACTATAGAATTCATTTTTTAGATGGTCATGATCGTGACGCGTGGTCTA
ATGACAAATCGACAC
+
FFFFFFF:FFFFFFFFFFFFFFFFFFFFFFFFFFFFFFFFFFFF:FFFFFFFFFFFFFFFFFFFFFFF
FFFFFFFFFFFFFFFFFFFFFFFF:::FFFFFFFFFFFFFFF,FFF,FFFFF,FF:FFFFFFFFF,FF
FFFF,FFF,,,FFFF
@A00721:190:HGJW7DSXY:4:1224:13937:19774 2:N:0:TCTGCAAG+GCTGCACT
CAAAAGATAAATGGCATAGCCATTTATATAGACCTTTTAACAGTCTATTGGTTATCAAAAAGACGATG
ATATAACATATCATCTGCCGTCGATTTTACTGCGTCACTATAGAATTCATTTTTTAGATGGTCATGAT
CGTGACGCGTGGTCT
+
FFFFFFFFFFFFFFFFF,FFFFFFFFFFFFFFFFFFFFFFFFFFFF:FFFFFFFFFFFFFFFFFFFFF
FFFFFFF:FFFF,FFFFFFFFFFFFFFFFFFFFFFFFF,FFFFFFFFFFFFFFFF,FFFFFFFFFFFF
FFFFFFFFF:FFFFF
@A00721:190:HGJW7DSXY:4:1447:22878:28588 2:N:0:TCTGCAAG+GCTGCACT
CAAAAGATAAATGGCATAGCCATTTATATAGACCTTTTAACAGTCTATTGGTTATCAAAAAGACGATG
ATATAACATATCATCTGCCGTCGATTTTACTGCGTCACTATAGAATTCATTTTTTAGATGGTCATGAT
CGTGACGCGTGGTCT
+
:FF:FFFFFFFFFFFFFFFF:FFFF:FFFFFFFFFFFFFFFFFFFF,FFFFFFFFFFFFFFF,FFFFF
```

FFFFFFFFFFF:FFFFFF:FF:FFFFFFF:FFFFFFFFFF,F,FFFFFFFFFF:FFFFFFFFF:FFFF
FFFFFFFFF:FFFFF
@A00721:190:HGJW7DSXY:4:2530:30779:5353 2:N:0:TCTGCAAG+GCTGCACT
CAAAAGATAAATGGCATAGCCATTTATATAGACCTTTTAACAGTCTATTGGTTATCAAAAAGACGATG
ATATAACATATCATCTGCCGTCGATTTTACTGCGTCACTATAGAATTCATTTTTAGATGGTCATGAT
CGTGACGCGTGGTCT
+
FFF:FFFFFF:FFFFFFFFFFFFFFFFFFFFFFFFFFFFFFFFFFFFFFFFFFFFFFFFFF:FFFFFFF
FFFFFFFFFFFFFFFFFFFFFFFF:FFF:FFFFFFFFF,F:FFF:FF:FFFFFFFFFFFFFFFFFF:F
,FFFF:F:FFFFFFF
@A00721:190:HGJW7DSXY:4:2631:25626:1846 2:N:0:TCTGCAAG+GCTGCACT
CAAAAGATAAATGGCATAGCCATTTATATAGACCTTTTAACAGTCTATTGGTTATCAAAAAGACGATG
ATATAACATATCATCTGCCGTCGATTTTACTGCGTCACTATAGAATTCATTTTTAGATGGTCATGAT
CGTGACGCGTGGTCT
+
FFF:FFFFFFFFFFFFFFFFFFFFFFFFFFFFFF:FFFFFFFFFFFFFFFFFFFFFFFFFFFFFFFFF
FFFFFFFFFFFFFFFFFFFFFFFFFFFFFFFFFFFFF:FFFFF:FFFFFFFFFF:,FFFFFFFFFFF
FFFF:FFFFFFFFFF
@A00721:190:HGJW7DSXY:4:1525:28519:27430 2:N:0:TCTGCAAG+GCTGCACT
ATTCAAAAGATAAATGGCATAGCCATTTATATAGACCTTTTAACAGTCTATTGGTTATCAAAAAGACG
ATGATATAACATATCATCTGCCGTCGATTTTACTGCGTCACTATAGAATTCATTTTTAGATGGTCAT
GATCGTGACGCGTGG
+
FFFFFFFFFFFFFFFFFFFFFFFFFF,FFFFFFFFFFFFF:FFFFFF:F:FFFF:FFFFFF:F:FFFF
FF:::FFFFFFFFFFFFFFFFFFFFFFFF:FF:FFFFFF::FFF:,FF:FF:FFFFFF:FFFFFFFFFF
F:FFFF:,FFFFFFF
@A00721:190:HGJW7DSXY:4:2511:30644:30984 2:N:0:TCTGCAAG+GCTGCACT
ATTCAAAAGATAAATGGCATAGCCATTTATATAGACCTTTTAACAGTCTATTGGTTATCAAAAAGACG
ATGATATAACATATCATCTGCCGTCGATTTTACTGCGTCACTATAGAATTCATTTTTAGATGGTCAT
GATCGTGACGCGTGG
+
FFFFFF:FFFFFF:FFFFFFFFFFFFFFFFFFFF:::F,FFFFFFFFFFFFFFFFFFF:FFFFFFFFF
FFF:FFFFFFFFFF:FFF:F::FFFFFFFFFF:FFFFFFF:FF,,F,FFF,,FFFFFF:FFF:FF:FF
::F:FFF:FFFF:FF
@A00721:190:HGJW7DSXY:4:2569:12997:13448 2:N:0:TCTGCAAG+GCTGCACT
ATTCAAAAGATAAATGGCATAGCCATTTATATAGACCTTTTAACAGTCTATTGGTTATCAAAAAGACG
ATGATATAACATATCATCTGCCGTCGATTTTACTGCGTCACTATAGAATTCATTTTTAGATGGTCAT
GATCGTGACGCGTGG
+
FFFFFFFFFFFFFFFFFFFFFFFFFFFFFFFFFFFFF,FFFFFFFFF:FFFFFFFFFFFFFFFFFFF
FFFFFFFFFFFFF:FFFFFFFFFFFFFFFFFFFFFFFFFFFFFFF:FFFFFFFFFFFFFFFFFFFFFFF
FFFFFFFFFFFFFFF
@A00721:190:HGJW7DSXY:4:1114:8775:7294 2:N:0:TCTGCAAG+GCTGCACT
TTACGAAATAACATTCAAAAGATAAATGGCATAGCCATTTATATAGACCTTTTAACAGTCTATTGGTT
ATCAAAAAGACGATGATATAACATATCATCTGCCGTCGATTTTACTGCGTCACTATAGAATTCATTTT
TTAGATGGTCATGAT
+
FFFFFFFFFF,FF:FFFFFFFFFFFFFFFFFFFFFFFFFFFFFFFFFFF::FFFFFFFFFFFFFFFFF
FFFFFFF::FFFFFFFFFFFFFFFFFFFFFFFFFFFFFFF:FFFFFFFFFFF,FF::,FFF:FFFF:FF
FFFFF,FFF:F:FFF
@A00721:190:HGJW7DSXY:4:1614:4833:35790 2:N:0:TCTGCAAG+GCTGCACT
TTACGAAATAACATTCAAAAGATAAATGGCATAGCCATTTATATAGACCTTTTAACAGTCTATTGGTT
ATCAAAAAGACGATGATATAACATATCATCTGCCGTCGATTTTACTGCGTCACTATAGAATTCATTTT
TTAGATGGTCATGAT

+
FFFFFFFFFFFFFF:FFF:FFFFFFFFFFFFFFFFF:FF:FFFFFFF:FFFFFFFFFF,FFFFFFFFF
F:FFFFFFFFFFFFFFFFFFFFFFFFFFFFF:F:FFFF:F:FFFFFFFFFF:FF,F:::FFF:FF:FF
FFFFF,,FFFFFFFF
@A00721:190:HGJW7DSXY:4:2209:17635:1438 2:N:0:TCTGCAAG+GCTGCACT
TTACGAAATAACATTCAAAAGATAAATGGCATAGCCATTTATATAGACCTTTTAACAGTCTATTGGTT
ATCAAAAAGACGATGATATAACATATCATCTGCCGTCGATTTTACTGCGTCACTAGAGAATTCATTTT
TTAGATGGTCATGAT
+
FFFFFFFFFFFFFFFFFFFFFFFFFFFFFFFFFFFFFF:FFFFFFFFFFFF:FFFFFFFFFFF:FFFF
:FFFFFF::FFFFFFFF,FFFFF:FFFFFFFFFFFFF:F:FFFFFFFFF,FFF,F,:F,:,F:FFFFF
F:,FFFFFFFFFFFF
@A00721:190:HGJW7DSXY:4:2209:16911:1783 2:N:0:TCTGCAAG+GCTGCACT
TTACGAAATAACATTCAAAAGATAAATGGCATAGCCATTTATATAGACCTTTTAACAGTCTATTGGTT
ATCAAAAAGACGATGATATAACATATCATCTGCCGTCGATTTTACTGCGTCACTATAGAATTCATTTT
TTAGATGGTCATGAT
+
FFFFFFFFFFFFFFFFFFFFFFFFFFFFFFFFFFFF:FFFFFFFFFFFFF,FFFFFFFF:FF:FFF:F
FFFFFFFFFFFFFFFF:F,,FFFFFFFFFFFFFFFFFFFF:FFFFFFFFFFFFFFFFFFFFF:FFF:F
F,F:FF:FFFFFFFF
@A00721:190:HGJW7DSXY:4:2647:25852:7028 2:N:0:TCTGCAAG+GCTGCACT
TTACGAAATAACATTCAAAAGATAAATGGCATAGCCATTTATATAGACCTTTTAACAGTCTATTGGTT
ATCAAAAAGACGATGATATAACATATCATCTGCCGTCGATTTTACTGCGTCACTATAGAATTCATTTT
TTAGATGGTCATGAT
+
FFFFFFFFFFFFFFFFFF:F:FFFFFFFFFFFFFFF:FFFFFF:FFFFFFFFFFFFFFFFFFFFFFFF
FFFFFFFFFFFFFFFFFFFFFFFFFFFFFFFF:FFFFFFFFFFFFFF:::FFF,FFFF:FFFFFFFFF
FFFFFFFFFFFFF,F
@A00721:190:HGJW7DSXY:4:2340:11767:31172 2:N:0:TCTGCAAG+GCTGCACT
AATTACGAAATAACATTCAAAAGATAAATGGCATAGCCATTTATATAGACCTTTTAACAGTCTATTGG
TTATCAAAAAGACGATGATATAACATATCATCTGCCGTCGATTTTACTGCGTCACTATAGAATTCATT
TTTTAGATGGTCATG
+
FFFFFFFFF:FFFFFF:FFFF:FFFFFFFFFFFF:FFFFFFF,FFFFFFF,F:FFFFFF,F:FFFF:F
FFFFFFFFF:FFFFFFF:FFFFFFFFFFFFFFFF::FFFFFFFFFF,FFF:FFFFFFFF:FFF:FFFF
FFFFF:FF,FF,FFF
@A00721:190:HGJW7DSXY:4:1370:4544:5729 2:N:0:TCTGCAAG+GCTGCACT
CATAGATAAATTACGAAATAACATTCAAAAGATAAATGGTATAGCCATTTATATAGACCTTTTAACAG
TCTATTGGTTATCAAAAAGACGATGATATAACATATCATCTGCCGTCGATTTTACTGCGTCACTCTAG
AATTCATTTTTTAGA
+
FFFFFFFFFFFFFFFFFFF:FFFFFFFFFFFF:FFFFFFFFFFFFF::FFFFFFFFFFFFFFFFFFFF
FFFFF,F:FFF:FFFFFFFFFFFFFFFFFFFFFFFFFFFFFFFFFFFFFFFFFFFFFFFFFFFF,FFF
FFFFFFFFFFFFFFF
@A00721:190:HGJW7DSXY:4:1436:24496:15890 2:N:0:TCTGCAAG+GCTGCACT
CATAGATAAATTACGAAATAACATTCAAAAGATAAATGGTATAGCCATTTATATAGACCTTTTAACAG
TCTATTGGTTATCAAAAAGACGATGATATAACATATCATCTGCCGTCGATTTTACTGCGTCACTATAG
AATTCATTTTTTAGA
+
FFFFFFFFFFFFFFFFFFFFFFFFFFFFFFFFFFFFFFFFFFFFFFFFFFFFFFFFFFFFFFFFFFFF
FFFFFFFF,FFFFFFFFFFFFFFFFFFFFFFFFFFFFFFFF:F:FFFFFFFFFF:F:FFFFFFFFFF:
FF,FFFF:FFFFF:F
@A00721:190:HGJW7DSXY:4:1445:1597:29810 2:N:0:TCTGCAAG+GCTGCACT
CATAGATAAATTACGAAATAACATTCAAAAGATAAATGGTATAGCCATTTATATAGACCTTTTAACAG

```
TCTATTGGTTATCAAAAAGACGATGATATAACATATCATCTGCCGTCGATTTTACTGCGTCACTATAG
AATTAATTTTTTAGA
+
FFFFFFFFFFFFFFFFFFFFFFFF,FFFFFFFFFFFFFFFFFFF:FFFF:FFFFFF::FFFF:FFFFFFF
FFFF:FFF:F:FFFFFFFFFFF:FFFF,FFFFF:FFFF:FFFFFFFFFFFF:FFFFFFFFFFF,F,F,FF
FFFF,F:F:FFFF:F
@A00721:190:HGJW7DSXY:4:1478:26503:16423 2:N:0:TCTGCAAG+GCTGCACT
CATAGATAAATTACGAAATAACATTCAAAAGATAAATGGTATAGCCATTTATATAGACCTTTTAACAG
TCTATTGGTTATCAAAAAGACGATGATATAACATATCATCTGCCGTCGATTTTACTGCGTCACTATAG
AATTCATTTTTTAGA
+
FFFFFFFFFFFFFFFFFFFFFFFFFFFFFFFFFFFFFFFFFF,FFFFFF:FFFFFFFFFFFFFFFF:FFF
FFFFF,FFFFFFFFFFFFF,FFFFFFFFFFFFFFFFFFFFFFFFFFFFFFFF:FFFFFFFFFFF:FF:FF
::FFFFFFFFFFFF:
@A00721:190:HGJW7DSXY:4:1627:11379:36229 2:N:0:TCTGCAAG+GCTGCACT
CATAGATAAATTACGAAATAACATTCAAAAGATAAATGGTATAGCCATTTATATAGACCTTTTAACAG
TCTATTGGTTATCAAAAAGACGATGATATAACATATCATCTGCCGTCGATTTTACTGCGTCACTATAG
AATTCATTTTTTAGA
+
FFFFFF:FFFFFFFFFF,FFFFF,FFFFF:FFFFFFFFFFFFFF:FFFFFFFFFFFFFFFFFFFFFFFFF
FFFFF:FFFFFFFFFFFFFFFFFFFFFFFFFFFFFFFFFFFFFFFFFFFFFFFFFFFFF,:FFF:F
FFFFFFFFFF:FFF:
@A00721:190:HGJW7DSXY:4:2125:6325:8907 2:N:0:TCTGCAAG+GCTGCACT
CATAGATAAATTACGAAATAACATTCAAAAGATAAATGGTATAGCCATTTATATAGACCTTTTAACAG
TCTATTGGTTATCAAAAAGACGATGATATAACATATCATCTGCCGTCGATTTTACTGCGTCACTATAG
AATTCATTTTTTAGA
+
FFFFFFFFFFFFFFFFFFFFFFFFFFFFFFFFFFFFFFFFFFFFFFFFF:FFFFFFFFFFFFFFFF:FFF
FFFF:FFF:F:FFFFFFFFFFFFFFFFFFFFFFFFFFFFFFFFFFFFFFFFF:FFFFFFFFF:F:F:F,F
FFFFFFFFFF:FFF:
@A00721:190:HGJW7DSXY:4:2160:17815:16313 2:N:0:TCTGCAAG+GCTGCACT
CATAGATAAATTACGAAATAACATTCAAAAGATAAATGGTATAGCCATTTATATAGACCTTTTAACAG
TCTATTGGTTATCAAAAAGACGATGATATAACATATCATCTGCCGTCGATTTTACTGCGTCACTATAG
AATTCATTTTTTAGA
+
FFFFFFFFFFFFFFFFFFFFFFFFFFFFFFFF:FFFFFFFFFFFFFFFFFFFFFFFFFFFFFFFFFFFFF
FFFFFFFFFFF:FFFF:FF,FFFFFFFFFFFFFFFFFFFFFFFFFFFFFFFFFFFFFFFFFFF,FFFFFF
FFFFFFFFF:FFFFF
@A00721:190:HGJW7DSXY:4:2547:23692:10645 2:N:0:TCTGCAAG+GCTGCACT
CATAGATAAATTACGAAATAACATTCAAAAGATAAATGGTATAGCCTTTTATATAGACCTTTTAACAG
TCTATTGGTTATCAAAAAGACGATGATATAACATATCATCTGCCGTCGATTTTACTGCGTCACTATAG
AATTCATTTTTTAGA
+
FFFFFFFFFFFFFFFFFFFFFFF:FFFFFFFF:FF:FFFFFFFFFFFFFFFF:FFFFFFFFFFF,FFFFF
FFFFFFFFFF:FFFFFFFFFFFFFFFF:FFFFFFFFFFFFFFFFFFFFFFFFFFFFFFFFFFFFFFFFFF
FFFF:FFFFFFFF:F
@A00721:190:HGJW7DSXY:4:1122:6153:21386 2:N:0:TCTGCAAG+GCTGCACT
TAATTATTAACGCGTAATTCATAGATAAATTACGAAATAACATTCAAAAGATAAATGGCATAGCCATT
TATATAGACCTTTTAACAGTCTATTGGTTATCAAAAAGACGATGATATAACATATCATCTGCCGTCGA
TTTTACTGCGTCACT
+
FFFFFFFFFFFFFFFFFFFFFFFFFFFFFFFFFFFFFFFFFFFFFFFFF:FFFFFFFFFFFFFFFFFFF:
FFFFFFFFFFFFFFFFFFFFFFFFF:FFFF:FFFFFFFFFFFFFFFFF,FFFFFFFFFFFFFFFFFFFFF
:FFF:FFFFFF:FFF
```

@A00721:190:HGJW7DSXY:4:1316:24686:18474 2:N:0:TCTGCAAG+GCTGCACT
TAATTATTAACGCGTAATTCATAGATAAATTACGAAATAACATTCAAAAGATAAATGGCATAGCCATT
TATATAGACCTTTTAACAGTCTATTGGTTATCAAAAAGACGATGATATAACATATCATCTGCCGTCGA
TTTTACTGCGTCACT
+
FFFFFFFFFFFFFFFFFFFFFFFFFFFFFFFFFFFFFFFFFFFFFFFFFFFFFFFFFFFFFFFFFFFFFF
FFFFFFFFFFFFFFFFFFFFFFFF:FFFFF,FFFFFFFFFFFFFFFFFFFFFFFFFFFFFFFFFFFFFFF
:FFFFF:FFFFFFFF
@A00721:190:HGJW7DSXY:4:1363:18954:8641 2:N:0:TCTGCAAG+GCTGCACT
TAATTATTAACGCGTAATTCATAGATAAATTACGAAATAACATTCAAAAGATAAATGGCATAGCCATT
TATATAGACCTTTTAACAGTCTATTGGTTATCAAAAAGACGATGATATAACATATCATCTGCCGTCGA
TTTTACTTCGTCACT
+
FFFFFFFFFFF:FFFFFFFFFFFFFFF:FFFFFFFFFFFFFFFF:F:F:FF,F:FFFFFFFFFFFFFF:FF
FFF:FFFFFFF:FFF,FFFF:FFFF::FFFFFF:FFFFF:FFFFFFFFFFFFFFFFFFFF,FF:FFFFFF
FFFFFF:,F:FFFFF
@A00721:190:HGJW7DSXY:4:1523:32036:18584 2:N:0:TCTGCAAG+GCTGCACT
TAATTATTAACGCGTAATTCATAGATAAATTACGAAATAACATTCAAAAGATAAATGGCATAGCCATT
TATATAGACCTTTTAACAGTCTATTGGTTATCAAAAAGACGATGATATAACATATCATCTGCCGTCGA
TTTTACTGCGTCACT
+
FFFFFF:FFF::FFFFFFF,F::,FFFFFF:F::FFFFF,,F:FFFF:F,F:FFFFFFFFFF,:FFFF,
FFFFFF,FF:FFFFFFFFFFFFFFF,,F:FFFFFFFFFF,F:,F,FFF,FFF,:FFFF:,FFF,F,F,,F
:,FF::FF:,FF:,F
@A00721:190:HGJW7DSXY:4:1530:2510:35650 2:N:0:TCTGCAAG+GCTGCACT
TAATTATTAACGCGTAATTCATAGATAAATTACGAAATAACATTCAAAAGATAAATGGCATAGCCATT
TATATAGACCTTTTAACAGTCTATTGGTTATCAAAAAGACGATGATATAACATATCATCTGCCGTCGA
TTTTACTGCGTCACT
+
FFFFFFFFFFFFFFFFFFFFFFFFFFFFFFFFFFFFFFFFFFFFFFFFFFFFFFFFFFFFFFFFFFFFFF
FFFFFFFFFFFFFFFFFFFFFFFFFFFFFFFFFFFFFFFFFFFFFFFFFFFFFFFFFFFFFFFFFFFFFF
:FFFFFFFFFFFFFF
@A00721:190:HGJW7DSXY:4:2359:27995:11083 2:N:0:TCTGCAAG+GCTGCACT
TAATTATTAACGCGTAATTCATAGATAAATTACGAAATAACATTCAAAAGATAAATGGCATAGCCATT
TATATAGACCTTTTAACAGTCTATTGGTTATCAAAAAGACGATGATATAACATATCATCTGCCGTCGA
TTTTACTGCGTCACT
+
F,FFFFFF::FFFFFF,FF:FFFFFFFFFFFFFF:FFFFFF:F:FFFF,FFFFFF,FFFFFFFFFF,F:,
FFFFFFFFFFFFFFF,FFFFFF,FFFFF:FFFFFFFFFFFFFFFFFFFF:FFFFFFF,FFFFFFFFFFFF
FF,FFFFFFFFFFFF
@A00721:190:HGJW7DSXY:4:2517:6894:16908 2:N:0:TCTGCAAG+GCTGCACT
TAATTATTAACGCGTAATTCATAGATAAATTACGAAATAACATTCAAAAGATAAATGGCATAGCCATT
TATATAGACCTTTTAACAGTCTATTGGTTATCAAAAAGACGATGATATAACATATCATCTGCCGTCGA
TTTTACTGCGTCACT
+
FFFFFFFFFFFFFFFFFFFFFFFFFFFFFFFFFFFFFFFFFFFFFFFFFFFFFFFFFFFFFFFFFFFFFF
FFFFFFFFFFFFFFFFFFFFFFFFF:FFFFFFFFFFFFFFFFFFFFFFFFFFFFFFFFFFFFFFFFFF:F
FFFFFFFFFFFFFFF
@A00721:190:HGJW7DSXY:4:1246:20329:22858 2:N:0:TCTGCAAG+GCTGCACT
TAATTATTAACGCGTAATTCATAGATAAATTACGAAATAACATTCAAAAGATAAATGGCATAGCCATT
TATATAGACCTTTTAACAGTCTATTGGTTATCAAAAAGACGATGATATAACATATCATCTGCCGTCGA
TTTTACTGCGTCAC
+
FFFFFFFFFFFFFFF:FFFFFFFFFFFFFFFF:FFFFFFFFFF:FFFFFFFFFFFFFFFFFFFFFFFF,F

FFFFFFFFFFF:,FFFFFFFFF:FFFFF:FFFFFFFFFFFFFFFF:FFFFFFFFFFFFFFFFFFFFFF
::FFFFFFFF:FFF
@A00721:190:HGJW7DSXY:4:1220:26232:35524 2:N:0:TCTGCAAG+GCTGCACT
CTATAATTATTAACGCGTAATTCATAGATAAATTACGAAATAACATTCAAAAGATAAATGGCATAGCC
ATTTATATAGACCTTTTAACAGTCTATTGGTTATCAAAAAGACGATGATATAACATATCATCTGCCGT
CGATTTTACAGATCG
+
FFFFFFFFFFFFFFFFFFFFFFFFFFFFFFFFFFFFFFFFFFFFFFFFFFFFFFFFFFFFFFFFFFFF
FFFFFFFFFFFFFFFFFFFFFFFFFF:FFFFFFFFFFFFFFFFFFFFFFFFFF:FFFFFFFFFFFFFFFF
FFFFFFFFFFFFFF:F
@A00721:190:HGJW7DSXY:4:1261:1416:18630 2:N:0:TCTGCAAG+GCTGCACT
CTATAATTATTAACGCGTAATTCATAGATAAATTACGAAATAACATTCAAAAGATAAATGGCATAGCC
ATTTATATAGACCTTTTAACAGTCTATTGGTTATCAAAAAGACGATGATATAACATATCATCTGCCGT
CGATTTTACAGATCG
+
F:FFFFFFFFFFFFFFFFFFFFFFFFFF:FFFFFFFFFFFFFFFFF:FFF:FFFFFFFFFFFFFFFFF
F:FFFFFFFFFFFFFFFF,FFF:,FF,FFF::FFFFFFFFFFFFFFFFFFFFFFFFFFFFFFFFFFFF
FFFFFFFFFFFFFFF
@A00721:190:HGJW7DSXY:4:1635:26865:31203 2:N:0:TCTGCAAG+GCTGCACT
CTATAATTATTAACGCGTAATTCATAGATAAATTACGAAATAACATTCAAAAGATAAATGGCATAGCC
ATTTATATAGACCTTTTAACAGTCTATTGGTTATCAAAAAGACGATGATATAACATATCATCTGCCGT
CGATTTTACAGATCG
+
FFFFFFFFFFFFFFFFFFFFFFFFFFFFFFFFFFFFFFFFFFFFFFFFFFF:FFFFFFFFFFFFFFF:F
FFFFFFFFFFFF:FFFFF:FFFFFFFFFFFFFFFFFFF,FFFFFFFFFFFFFFFFFFFFFFFFFFFF:F
FFFFFFFFFFFFF,F
@A00721:190:HGJW7DSXY:4:2171:11803:3051 2:N:0:TCTGCAAG+GCTGCACT
CTATAATTATTAACGCGTAATTCATAGATAAATTACGAAATAACATTCAAAAGATAAATGGCATAGCC
ATTTATATAGACCTTTTAACAGTCTATTGGTTATCAAAAAGACGATGATATAACATATCATCTGCCGT
CGATTTTACAGATCG
+
FFFFFFFFFFFFFFFFFFFFFFFFFFFFFFFFFFFFFFFFFFFFFFFFFFFFFFFFFFFFFFFFFFFF
F:FFFFFFFFFFFFFFFFFFFFFFFFF,FFFFFFFFFFFFFFFFFFFFFF:FFFFFFFFFFFFFFFFFFF
FFFFFFFFFFFF:FF
@A00721:190:HGJW7DSXY:4:2473:10610:6621 2:N:0:TCTGCAAG+GCTGCACT
CTATAATTATTAACGCGTAATTCATAGATAAATTACGAAATAACATTCAAAAGATAAATGGCATAGCC
ATTTATATAGACCTTTTAACAGTCTATTGGTTATCAAAAAGACGATGATATAACATATCATCTGCCGT
CGATTTTACAGATCG
+
FFFFFF:FFFFFFFFFFFFFFFFFFFFFFFFFFFFFFFFFFFFFFFFFFFFFFFFFFFFFFFFFFFFF
FFFFFFFFFFFFFFFFFFFFFFFFFFF:FFFFFFFFFFFFFFFFFFFFFFFFFF:FFFFFFFFFFFFFFF
FFFFFFFFFFFFFFF
@A00721:190:HGJW7DSXY:4:2624:17309:32440 2:N:0:TCTGCAAG+GCTGCACT
CTATAATTATTAACGCGTAATTCATAGATAAATTACGAAATAACATTCAAAAGATAAATGGCATAGCC
ATTTATATAGACCTTTTAACAGTCTATTGGTTATCAAAAAGACGATGATATAACATATCATCTGCCGT
CGATTTTACAGATCG
+
FFFFFFFFFF:FFFFFFFFFFFFFFFFFFFFFFFFFFFFFFFFFFFFFFFFF:FFFFFFFFFFFFFFF:
FFFFFFFFFFFFFFFFFFFFFFFFFFF:FFFFF:FFF:FFFF,FFFFFF,FFFFFFF:FFFFFFFFFFF
FFF:FFFFFFFFFFF
@A00721:190:HGJW7DSXY:4:2267:32642:31375 2:N:0:TCTGCAAG+GCTGCACT
TGAAAATGTATCTCGATTTGTTCAAAACAAATCTTACAAATAATCAAGACGAAAACTATAATTATTAA
CGCGTAATTCATAGATAAATTACGAAATAACATTCAAAAGATAAATGGCATAGCCATTTATATAGACC
TTTTAACAGTCTATT

```
+
FFFFFFFFFFFFFFFFFFFFFFFFFFFFFFFFFFFFFFFFFFFFFFFF:FFFFF:FFFFFFFFFFFF:
:FFFFFFFFFFFFFFFFFFFFFFFFFFFFFFFFFFFF:FFFFFF:FFF,FFFF:FFFFFFFFFF,F:F
FFFF::FFFF:FFFF
@A00721:190:HGJW7DSXY:4:1606:26087:35524 2:N:0:TCTGCAAG+GCTGCACT
GGTCTATATAAATGGCTATGCCATTTATCTTTTGAATGTTATTTCGTAATTTATCTATGAATTACGCG
TTAATAATTATAGTTTTCGTCTTGATTATTTGTAAGATTTGTTTTGAACAAATCGAGATACATTTTCA
ATGTCCGTCGAAGGA
+
FFFFFFFFFFFFFFFFFFFFFFFFFFFFFFFF:FFFFFFFFFFFFFFFFFFFFFFFFFFFFFFFFFFF
FFFFFFFFFFFFFFFFFFFFFFFFFFFFFFFFFFFFFFFFFFFFFFFFFFFFFF:FFFFF:FFFFFFF
FFFFF,FFFFF:FFF
@A00721:190:HGJW7DSXY:4:1610:2917:28588 2:N:0:TCTGCAAG+GCTGCACT
GGTCTATATAAATGGCTATGCCATTTATCTTTTGAATGTTATTTCGTAATTTATCTATGAATTACGCG
TTAATAATTATAGTTTTCGTCTTGATTATTTGTAAGATTTGTTTTGAACAAATCGAGATACATTTTCA
ATGTCCGTCGAAGGA
+
FFFFFFFFFFF,FFFFFFFFFF,FFFFFFFFFFFFFFFFF,FFFFFFFFFFFFFFFFFFFFFFFFFFF
FFFFFFFFFFFFFFFFFFF,:,::F,FFFFFFFFFFFFFFF:FF:FFFFFFFFFFFFFFFFFFFFFFF
FFFFFFFFFFFFFFF
@A00721:190:HGJW7DSXY:4:1657:31593:31000 2:N:0:TCTGCAAG+GCTGCACT
GGTCTATATAAATGGCTATGCCATTTATCTTTTGAATGTTATTTCGTAATTTATCTATGAATTACGCG
TTAATAATTATAGTTTTCGTCTTGATTATTTGTAAGATTTGTTTTGAACAAATCGAGATACATTTTCA
ATGTCCGTCGAAGGA
+
FFFFFFFFFFFFFFFFFFFFFF:FFFFFF:FFFFFFFFFFFFFFFFFFFFFFFFFFFFFFFFFFFFFF
FFF:FFFFFFF:FFFFFFFF:FFF::FFFFFFFFFF,FFFFF:,FF:FF:FFFFFFFFFFFFFFFFF:
FFFFF:FFF,F:FFF
@A00721:190:HGJW7DSXY:4:1657:31756:31156 2:N:0:TCTGCAAG+GCTGCACT
GGTCTATATAAATGGCTATGCCATTTATCTTTTGAATGTTATTTCGTAATTTATCTATGAATTACGCG
TTAATAATTATAGTTTTCGTCTTGATTATTTGTAAGATTTGTTTTGAACAAATCGAGATACATTTTCA
ATGTCCGTCGAAGGA
+
:FFFFFFF:FF:FFFFFFFFFFFFFFFFFFFFFFFFF:F:FFFFFFFFFFFFFFFFFFFFF:FFFFFFF
,FF,FFFFFFFFFFFFF,FFF:FFFFFFFFFFF:FF,FFFFFFFFFFFFFFFFFFFFFFFFFFFFFFF
FFFFFFFFFF:FFFF
@A00721:190:HGJW7DSXY:4:2146:21504:32189 2:N:0:TCTGCAAG+GCTGCACT
GGTCTATATAAATGGCTATGCCATTTATCTTTTGAATGTTATTTCGTAATTTATCTATGAATTACGCG
TTAATAATTATAGTTTTCGTCTTGATTATTTGTAAGATTTGTTTTGAACAAATCGAGATACATTTTCA
ATGTCCGTCGAAGGA
+
FFFFFFFFFFFFFFFFF:FFFFF:,FFF:FFFFF,FFFFFF,FFFFFFF:,FFFFFFFFFFFFFFFFF
:F:FFFFFFFFF:FFFF,FFF,FFF,FFFFFFF:FFFFFF:F::FF,FFFF:FFFFFF:FFFF:FFFF
FFFFFFFFFFFFFFF
@A00721:190:HGJW7DSXY:4:1336:23809:10441 2:N:0:TCTGCAAG+GCTGCACT
ATTTATCTTTTGAATGTTATTTCGTAATTTATCTATGAATTACGCGTTAATAATTATAGTTTTCGTCT
TGATTATTTGTAAGATTTGTTTTGAACAAATCGAGATACATTTTCAATGTCCGTCGAAGGAGATCTGT
TTGATCGAGATTTTA
+
F:FFFFFFFFFFFFFFFFFFFFFFFFFF:FFFFFFFFFFFFFFFFFFFFFFFFFFFFFFFFFFFFFFF
FFFFFFFF:FFFF::FFFFFFFFFFFFFFFFFFFFFFFFFFFFFFFFFFF::,FFFFFFFFFFFFFFF
FFFFF:F:FFFFFFF
@A00721:190:HGJW7DSXY:4:1471:4444:22467 2:N:0:TCTGCAAG+GCTGCACT
ATTTATCTTTTGAATGTTATTTCGTAATTTATCTATGAATTACGCGTTAATAATTATAGTTTTCGTCT
```

```
TGATTATTTGTAAGATTTGTTTTGAACAAATCGAGATACATTTTCAATGTCCGTCGAAGGAGATCTGT
TTGATCGAGATTTTA
+
FFFFFFFFFFFFFFFFFFFFFFFFFFFFFFFFFFFFFFFFFFFFFFFFFFFFFFFFFFFFFFFFF:FFFF
FFF:F:FFFFFFFF:FFFFFFFFFFFFFFFFFFFFFFFFFFFFFFFFF:FF:FFFFFFFFFFF:FFFFFF
FFFFFF:FFFFFFFF
@A00721:190:HGJW7DSXY:4:1531:6298:6825 2:N:0:TCTGCAAG+GCTGCACT
ATTTATCTTTTGAATGTTATTTCGTAATTTATCTATGAATTACGCGTTAATAATTATAGTTTTCGTCT
TGATTATTTGTAAGATTTGTTTTGAACAAATCGAGATACATTTTCAATGTCCGTCGAAGGAGATCTGT
TTGATCGAGATTTTA
+
:F,:FFFF:FFFFFF::FFFFFFFFFFFFFFFF:FFFFF:FFFFFFFFFFFFFFFFFFFFFFFFF,FFFF
:FFFFFFFFFFFFF:FFF,FFFFFFFFFFFFFFF,FFFFFFFFFFFFFFF:F,:FF:F,FFFFFFFFFFF
FFFFFF:FFFFFFFF
@A00721:190:HGJW7DSXY:4:2158:18584:30733 2:N:0:TCTGCAAG+GCTGCACT
ATTTATCTTTTGAATGTTATTTCGTAATTTATCTATGAATTACGCGTTAATAATTATAGTTTTCGTCT
TGATTATTTGTAAGATTTGTTTTGAACAAATCGAGATACATTTTCAATGTCCGTCGAAGGAGATCTGT
TTGATCGAGATTTTA
+
FFFFF:FFFFFFFFFFFFFFFFFFFFFFFFFFFFFFFFFFFFFFFFFFFFFFFFFFFFFFFFFFFFFFFF
FFFFFFFFFFFFFFFFFFFFFFFFFFFFFFFFFFFFFFFFFFFFFFFFFFFFFFFFFFFFFFFFFFFFFF
FFFFFFFF,FFFFFF
@A00721:190:HGJW7DSXY:4:2162:14624:35086 2:N:0:TCTGCAAG+GCTGCACT
ATTTATCTTTTGAATGTTATTTCGTAATTTATCTATGAATTACGCGTTAATAATTATAGTTTTCGTCT
TGATTATTTGTAAGATTTGTTTTGAACAAATCGAGATACATTTTCAATGTCCGTCGAAGGAGATCTGT
TTGATCGAGATTTTA
+
FFFFFFFFFFFFFFFFFFFFFFFFFFFFFFFFFFFFFFFFFFFFFFFFFFFFFFFFFFFFFFFFFFFFFF
FFFFFFFFFFFFFFFFFF:FFFFFFFF:FFFFFFFFFFFFFFFFFFFFFFFFFFFFFFFFFFFFFFFFFF
FFFFFFFFFFFFFFF
@A00721:190:HGJW7DSXY:4:2335:25726:17895 2:N:0:TCTGCAAG+GCTGCACT
ATTTATCTTTTGAATGTTATTTCGTAATTTATCTATGAATTACGCGTTAATAATTATAGTTTTCGTCT
TGATTATTTGTAAGATTTGTTTTGAACAAATCGAGATACATTTTCAATGTCCGTCGAAGGAGATCTGT
TTGATCGAGATTTTA
+
FFFFFFFFFFFFFFFFFFFFFFFFFFFFFFFFFFFFFFFFFFFFFFFFFFFFFFFFFFFFFFFFFFFFFF
FFF:FFFFFFFFFF:FFFFFFFFFFFFFFFFFFFFFFFFFFFFFFFFFFFFFF:FFF:FFFFF:FFFFFF
FFFFFFFFFFFFFFF
@A00721:190:HGJW7DSXY:4:2670:8793:29027 2:N:0:TCTGCAAG+GCTGCACT
ATTTATCTTTTGAATGTTATTTCGTAATTTATCTATGAATTACGCGTTAATAATTATAGTTTTCGTCT
TGATTATTTGTAAGATTTGTTTTGAACAAATCGAGATACATTTTCAATGTCCGTCGAAGGAGATCTGT
TTGATCGAGATTTTA
+
FFFFFFFFFFFFFFFFFFFFFFFFFFFFFFFFFFFFFFFFFFFFFFFFFFFFFFFFFFFFFFFFFFFFFF
FFFFFFFFFFFFFFFFFFFFFFFFFFFFFFFFF:FFFFFFFFFFFFFFFFFFFFFFFFFFFFFFFFFFFF
FFFFFFFFFFFFFFF
@A00721:190:HGJW7DSXY:4:1146:27317:25567 2:N:0:TCTGCAAG+GCTGCACT
CGTTTTCGTCTTAAAATCTCGATCAATCAGATCTCCTTCGACGGACATTGAAAATGTATCTCGATTTG
TTCAAAACAAATCTTACAAATAATCAAGACGAAAACTATAATTATTAACGCGTAATTCATAGATAAAT
TACGAAATAACATTC
+
FFFFFFFFFFFFFFFFFFFFFFFFFF,FFFFFFFFFFFFFFFFFFFFFFFFFFFFFFFFFFFFFFFFFFF
FFFFF,:FFFFFFFFFFFFFFFFFFFFFFFFFFF:FFFFFFFFFFFFFFFFFFFFFFFFFFFFFFFF:FF
FFFFFFFFFFFFFFF
```

```
@A00721:190:HGJW7DSXY:4:1245:21106:12493 2:N:0:TCTGCAAG+GCTGCACT
CGTTTTCGTCTTAAAATCTCGATCAAACAGATCTCCTTCGACGGACATTGAAAATGTATCTCGATTTG
TTCAAAACAAATCTTACAAATAATCAAGACGAAAACTATAATTATTAACGCGTAATTCATAGATAAAT
TACGAAATAACATTC
+
FFFFFFFFFFFFFFFFFFFFFFFFFFFFFFFFFFFFFFFFFFFFFFFFFFFFFFFFFFFFFFFFFFFFFF
FFFFFFFFFFFFFFFFFFFFFFFFFFFFFFFFFFFFFFFFFFFFFFFFFFFFFFFFFFFFFFF:FFFFFF
FFFFFFFFFFFFFFF
@A00721:190:HGJW7DSXY:4:1265:26829:21151 2:N:0:TCTGCAAG+GCTGCACT
CGTTTTCGTCTTAAAATCTCGATCAAACAGATCTCCTTCGACGGACATTGAAAATGTATCTCGATTTG
TTCAAAACAAATCTTACAAATAATCAAGACGAAAACTATAATTATTAACGCGTAATTCATAGATAAAT
TACGAAATAACATTC
+
FFFFFFFFFFFFFF:FFFFFFFFFFF:FFFFFFFFFFFFFFF:FFFFFFFFFFFFFFFFFFFFFFFFFFF
FFFFFFFFFFFFFFFFFFFFFF:FFFFFFFFFFFFFFFFFFFF:FFFFFFFFFFFFFFFFFFFFFFFFFF
FFFFFFFFFFFFFFF
@A00721:190:HGJW7DSXY:4:1424:8097:25473 2:N:0:TCTGCAAG+GCTGCACT
CGTTTTCGTCTTAAAATCTCGATCAAACAGATCTCCTTCGACGGACATTGAAAATGTATCTCGATTTG
TTCAAAACAAATCTTACAAATAATCAAGACGAAAACTATAATTATTAACGCGTAATTCATAGATAAAT
TACGAAATAACATTC
+
FFFFFFFFFFFFFFFFFFFFFFFFFFFFFFFFFFFFFFFFFFFFFFFFFFFFFFFFFFFFFFFFFFFFFF
FFFFFFFFFFFFFFFFFFFFFFFFFFFFFFFFFFFFFFFFFFFFFFFFFFFFFFFFFFFFFFFFFFFFFF
FFFFFFFFFFFFFFF
@A00721:190:HGJW7DSXY:4:1443:12915:18944 2:N:0:TCTGCAAG+GCTGCACT
CGTTTTCGTCTTAAAATCTCGATCAAACAGATCTCCTTCGACGGACATTGAAAATGTATCTCGATTTG
TTCAAAACAAATCTTACAAATAATCAAGACGAAAACTATAATTATTAACGCGTAATTCATAGATAAAT
TACGAAATAACATTC
+
FFFFFFFFFFFFFFFFFFFFFFFFFFFFFFFFFFFFFFFFFFFFFFFFFFFFFFFFFFFFFFFFFFFFFF
FFFFF:FFFFFFFFFFFFFFFFFFFFFFFFFFFFFFFFFFFFFFFFFFFFFF:FFFFFFFFFFFFF:FFF
FFFFFFFFFFFFFFF
@A00721:190:HGJW7DSXY:4:1618:30879:28072 2:N:0:TCTGCAAG+GCTGCACT
CGTTTTCGTCTTAAAATCTCGATCAAACAGATCTCCTTCGACGGACATTGAAAATGTATCTCGATTTG
TTCAAAACAAATCTTACAAATAATCAAGACGAAAACTATAATTATTAACGCGTAATTCATAGATAAAT
TACGAAATAACATTC
+
FFFFFFFFFFFFFFFFFFFFFFFFFFFFFFFFFFFFFFFFFFFFFFFFFFFFFFFFFFFFFFFFFFFFFF
FFFFFFFFFFFFFFFFFFF:FFFFFFFFFFFFFF:FFFFFFFFFFFFFFFFFFFFFFFFFFFFFFFFFFF
FFFFFFFFFFFFFFF
@A00721:190:HGJW7DSXY:4:1622:16179:32957 2:N:0:TCTGCAAG+GCTGCACT
CGTTTTCGTCTTAAAATCTCGATCAAACAGATCTCCTTCGACGGACATTGAAAATGTATCTCGATTTG
TTCAAAACAAATCTTACAAATAATCAAGACGAAAACTATAATTATTAACGCGTAATTCATAGATAAAT
TACGAAATAACATTC
+
F:FFFFFF:FFFFFFFFFFFFFF:FF,FF,FFFFFFFFFFFFFF:FFFFFFFFFFFFFFFFFFFFFF:FF
FFFFFF:FFFFF:FFFFFFFFFF:FFF:FFFFFFFFFFFFFFFFFFFFFFFFFF:FFFFFFF,FFFFFFF
FFFFFFFF:FFF,F:
@A00721:190:HGJW7DSXY:4:2363:23728:20447 2:N:0:TCTGCAAG+GCTGCACT
CGTTTTCGTCTTAAAATCTCGATCAAACAGATCTCCTTCGACGGACATTGAAAATGTATCTCGATTTG
TTCAAAACAAATCTTACAAATAATCAAGACGAAAACTATAATTATTAACGCGTAATTCATAGATAAAT
TACGAAATAACATTC
+
FFFFFFFFFFFFFFFFFFFFFFFFFFFFFFFFFFFFFFFFFFFFFFFFFFFF:FFFFFFFFFFFFFFFFF
```

```
FFFFFFFFFFFFFFFFFFFFFFFFFFFFFFFFFFFFFFFFFFFFFFFFFFF:FFFFFFFFFFF:FF:FFF
FFF:FFFFFFFFFFF
@A00721:190:HGJW7DSXY:4:2376:20473:17378 2:N:0:TCTGCAAG+GCTGCACT
CGTTTTCGTCTTAAAATCTCGATCAAACAGATCTCCTTCGACGGACATTGAAAATGTATCTCGATTTG
TTCAAAACAAATCTTACAAATAATCAAGACGAAAACTATAATTATTAACGCGTAATTCATAGATAAAT
TACGAAATAACATTC
+
FFFFFFFFFFFFFFFFFFFFFF:FFFF,FFFFFFFFFFFFFFFFFFFFFFFFFFFFFFFFFFFFFFFFFF
FFF:FF:FF:FFFF,FFFFFFFFFFFFF:FFF,F,FFFFFFFFFFFFFFFFFFFFFF:FFF:FFFFFFFF
FFFFFFFFFFFF,FF
@A00721:190:HGJW7DSXY:4:2442:11388:10535 2:N:0:TCTGCAAG+GCTGCACT
CGTTTTCGTCTTAAAATCTCGATCAAACAGATCTCCTTCGACGGACATTGAAAATGTATCTCGATTTG
TTCAAAACAAATCTTACAAATAATCAAGACGAAAACTATAATTATTAACGCGTAATTCATAGATAAAT
TACGAAATAACATTC
+
FFFFFFFFFFFFFFFFFFFFFFFFFFFFFFFFFFFFFFFFFFFFFFFFFFFFFFFFFFFFFFFFFFFFFF
FFFFFFFFFF,FFFFFFFFFF::FFFFFFFFFFFF:FFFFFFFFFFFFF:FFFFFFFFFFFFFF:FFF:F
FFFFFFFF::FFFFF
@A00721:190:HGJW7DSXY:4:1223:9317:25081 2:N:0:TCTGCAAG+GCTGCACT
TTTCCATTAGCAGCAGCCTGGATCGTTTTCGTCTTAAAATCTCGATCAAACAGATCTCCTTCGACGGA
CATTGAAAATGTATCTCGATTTGTTCAAAACAAATCTTACAAATAATCAAGACGAAAACTATAATTAT
TAACGCGTAATTCAT
+
FFFFFFFFFFFFFFFFFFFFFFFFFFFFFFFFFFFFFFFFFFFFFFFFFFFFFFFFFFFFFFFFFFFFFF
FFFFFFFFFFFFFFFFFFFFFFFFFFFFFF:FFFFFFFFFFFFFFFFFFFFFFFFFFFFFFFFFFFFFFF
FFFFFFFFFFFFFFF
@A00721:190:HGJW7DSXY:4:1277:14886:7138 2:N:0:TCTGCAAG+GCTGCACT
TTTCCATTAGCAGCAGCCTGGATCGTTTTCGTCTTAAAATCTCGATCAAACAGATCTCCTTCGACGGA
CATTGAAAATGTATCTCGATTTGTTCAAAACAAATCTTACAAATAATCAAGACGAAAACTATAATTAT
TAACGCGTAATTCAT
+
FFFFFFFFFFFFFFFFFFFFFFFFFFFFFFFFFFFFFFFFFFFFFFFFFFFFFFFFFFFFFFFFFFFFFF
FFFFFFFFFFFFFFFFFFFFFFFFFFFFF,FFFFFFFFFFFFFFFFFFFFF:FFFFFFFFFFFFFFFFFF
FFFF,FFFFFFFFFF
@A00721:190:HGJW7DSXY:4:1334:2908:18396 2:N:0:TCTGCAAG+GCTGCACT
TTTCCATTAGCAGCAGCCTGGATCGTTTTCGTCTTAAAATCTCGATCAAACAGATCTCCTTCGACGGA
CATTGAAAATGTATCTCGATTTGTTCAAAACAAATCTTACAAATAATCAAGACGAAAACTATAATTAT
TAACGCGTAATTCAT
+
FFFFFFFFFFFFFFFFFFFFFFFFFFFFFFFFFFFFFFFFFFFFFFFFFFFFFFFFFFFFFFFFFFFFFF
FFFFFFFFFFFFFFFFFFFFFFFFFFFFFF:FF,FFFFFFFFFFFFFFFFFFFFFFFFFFFFFFFFFFFF
FFFFFFFFF:FFFFF
@A00721:190:HGJW7DSXY:4:1370:7916:2080 2:N:0:TCTGCAAG+GCTGCACT
TTTCCATTAGCAGCAGCCTGGATCGTTTTCGTCTTAAAATCTCGATCAAACAGATCTCCTTCGACGGA
CATTGAAAATGTATCTCGATTTGTTCAAAACAAATCTTACAAATAATCAAGACGAAAACTATAATTAT
TAACGCGTAATTCAT
+
FFFFFFFFFFFFFFFFFFFFFFFFFFFFFFFFFFFFFF:FFFFFFFFFFFFFFFFFFFFFFFFFFFFFFF
FFFFFFFFFFFFFFFFFFFFFFFFFFFFFFFFFFFFFFFFFFFFFFFFFFFFFFFFFFFFFFFFFFFFFF
FFFFFFFFFFFFFFF
@A00721:190:HGJW7DSXY:4:1510:12599:13009 2:N:0:TCTGCAAG+GCTGCACT
TTTCCATTAGCAGCAGCCTGGATCGTTTTCGTCTTAAAATCTCGATCAAACAGATCTCCTTCGACGGA
CATTGAAAATGTATCTCGATTTGTTCAAAACAAATCTTACAAATAATCAAGACGAAAACTATAATTAT
TAACGCGTAATTCAT
```

```
+
FFFFFFFFFFFFFFFFFFFFFFFFFFFFFFFFFFF:FFFFFFFFFFFFFFFFFFFFFFFFFFFFFFFFFF
FFFFFFFFFFFFFFFFFFFFFFFFFFFFFF,FFFFFFFFFFFFFFFFFFFFFFFFFFFFFFFFFFFFFFF
FF:FF:FFFFFFFFF
@A00721:190:HGJW7DSXY:4:1638:1163:10363 2:N:0:TCTGCAAG+GCTGCACT
TTTCCATTAGCAGCAGCCTGGATCGTTTTCGTCTTAAAATCTCGATCAAACAGATCTCCTTCGACGGA
CATTGAAAATGTATCTCGATTTGTTCAAAACAAATCTTACAAATAATCAAGACGAAAACTATAATTAT
TAACGCGTAATTCAT
+
FFFFFFFFFFFFFFFF:FFFFFFFFFFFFFF:FFFFFFFFFFFFFFFFFF,FFFFFFFFFFFFFFFFFFF
FFFFFFFFFFFFFFFFF:FFFFFFFFF::FFFFFFFFFFFFFFFFFFFF,FFFFF:FFFFFFFFFFFFFF
FF,F:FFFFFFFFFF
@A00721:190:HGJW7DSXY:4:2510:14452:18192 2:N:0:TCTGCAAG+GCTGCACT
TTTCCATTAGCAGCAGCCTGGATCGTTTTCGTCTTAAAATCTCGATCAAACAGATCTCCTTCGACGGA
CATTGAAAATGTATCTCGATTTGTTCAAAACAAATCTTACAAATAATCAAGACGAAAACTATAATTAT
TAACGCGTAATTCAT
+
FFFFFFFFFFFFFFFFFFFFFFFFFFFFFFFFFFFFFFFFFFFFFFFFFFFFFFFFFFFFFFFFFFFFFF
FFFFFFFFFFFF:FFFFFFFFFFFFFFFFFFFFFFFFFFFFFFFFFFFFFFFFFFFFFFFFFFFFFFFF
FFFFFFFFFFFFFFF
@A00721:190:HGJW7DSXY:4:2536:9815:10254 2:N:0:TCTGCAAG+GCTGCACT
TTTCCATTAGCAGCAGCCTGGATCGTTTTCGTCTTAAAATCTCGATCAAACAGATCTCCTTCGACGGA
CATTGAAAATGTATCTCGATTTGTTCAAAACAAATCTTACAAATAATCAAGACGAAAACTATAATTAT
TAACGCGTAATTCAT
+
FFFFFFFFFFFFFF:FFFFFFFFFFFFFFFFFFFFFFFFFFFFFFFFFFFFFFFFFFFFFFFFFF:FFFF
FFFFFFFFFFFFFFFFFFFFFFFFFFFFFFFFFFFFFFFFFFFFFFFFFFFFFFFFFFFFFFFFFFFFFF
FFFFFFFFFFFFFFF
@A00721:190:HGJW7DSXY:4:1175:5475:3302 2:N:0:TCTGCAAG+GCTGCACT
GTTTTCGTCTTGATTATTTGTAAGATTTGTTTTGAACAAATCGAGATACATTTTCAATGTCCGTCGAA
GGAGATCTGTTTGATCGAGATTTTAAGACGAAAACGATCCAGGCTGCTGCTAATGGAAATTGGGGCAA
TGTAAAGCTCCAAAT
+
FFFFFFFFFFFFFFFFFFFFFFFFF:FFFFFFFFFFFFFFFFFFFFFFFFFFFFFFFFFFFFFFFFFFFF
FFFFFFFFFFFFFFFFFFFFFFFFFF:FFFFFFFFFFFFFFFFFFFFFFFFFFFFFFFFFFFFF,FFFFF
FFFFFF:FFFFFFFF
@A00721:190:HGJW7DSXY:4:1178:24089:9173 2:N:0:TCTGCAAG+GCTGCACT
GTTTTCGTCTTGATTATTTGTAAGATTTGTTTTGAACAAATCGAGATACATTTTCAATGTCCGTCGAA
GGAGATCTGTTTGATCGAGATTTTAAGACGAAAACGATCCAGGCTGCTGCTAATGGAAATTGGGGCAA
TGTAAAGCTCCAAAT
+
FFFFFFFFFFFFFFFFFFFFFFFFF:FFFFFFFFFFFFFFFFFFFFFFFFFFFFFF:FFFFFFFFFF,FF
FFFFFFFFFF,FFFFFFFFFFFFFFFFF:FFFFFFF:FFFFFF:FFFFFFFFFFFFF:FFFFFFFF:FFF
FFFFFF,FFFF:FFF
@A00721:190:HGJW7DSXY:4:1178:23185:10019 2:N:0:TCTGCAAG+GCTGCACT
GTTTTCGTCTTGATTATTTGTAAGATTTGTTTTGAACAAATCGAGATACATTTTCAATGTCCGTCGAA
GGAGATCTGTTTGATCGAGATTTTAAGACGAAAACGATCCAGGCTGCTGCTAATGGAAATTGGGGCAA
TGTAAAGCTCCAAAT
+
FF:FFFFFFFFFFFFFFFFFFFFFFFFFFFFFFFFFFFFFFFFFFFFFFFFFFFFFFFFFFFFFFFFFFF
FFFFFFFFFFFFFFFFFFFFFFFFFFFFFFFFFFFFFFFFFFFFFFFFFFFFFFFFFFFFFFF:FFFFFF
FFFFFFFFFF:FFFF
@A00721:190:HGJW7DSXY:4:1256:2220:12727 2:N:0:TCTGCAAG+GCTGCACT
GTTTTCGTCTTGATTATTTGTAAGATTTGTTTTGAACAAATCGAGATACATTTTCAATGTCCGTCGAA
```

GGAGATCTGTTTGATCGAGATTTTAAGACGAAAACGATCCAGGCTGCTGCTAATGGAAATTGGGGCAA
TGTAAAGCTCCAAAT
+
FFFF:FFFFFFFFFFFFFFFFFFFF:FFFFFFFFFFFFFFF:FFFFFF:FFFFF:FFFFFFF:F:FFFF
FFFFFFFF,FFFFFF:FFFFFFFFFFF:FFF:FF:FFFFFFFFFFFFFFFFFFFFFFFFF:F:F:,FFF
FF:FFF,FFF,FFFF
@A00721:190:HGJW7DSXY:4:1364:28718:28902 2:N:0:TCTGCAAG+GCTGCACT
GTTTTCGTCTTGATTATTTGTAAGATTTGTTTTGAACAAATCGAGATACATTTTCAATGTCCGTCGAA
GGAGATCTGTTTGATCGAGATTTTAAGACGAAAACGATCCAGGCTGCTGCTAATGGAAATTGGGGCAA
AGTAAAGCTCCAAAT
+
FFFFFFFFFFFFFFFF:FFFFFFF:FFFFFFFFFFFFFFFFFFFFFFFFFFFFFFFFFFFFFFFFFFFF
FFFFFFFFFFFFFFFFFFF:FFFFFFFFFFFFFFFFFFF,FFFF:FFFFFFFFFFFF,FFFFF:FF:F
,FFFFFFFFFFFFFF
@A00721:190:HGJW7DSXY:4:1475:29930:28494 2:N:0:TCTGCAAG+GCTGCACT
GTTTTCGTCTTGATTATTTGTAAGATTTGTTTTGAACAAATCGAGATACATTTTCAATGTCCGTCGAA
GGAGATCTGTTTGATCGAGATTTTAAGACGAAAACGATCCAGGCTGCTGCTAATGGAAATTGGGGCAA
TGTAAAGCTCCAAAT
+
FFFFFFFFF:FFFFFFFFFFFFFF,FFFFFFFFFFFFFFFFFFFFFFFFFFFFFFF:FFFFFFFFFFF
FFFFFFFFFFFFFFFFFFFFFFFFF:FF:F:FFFFFFFF:FFFFFFFFFFFF,FFFFFFFF:FFFFFFF
FFFFFFFFFFFFFFF
@A00721:190:HGJW7DSXY:4:1516:1506:34006 2:N:0:TCTGCAAG+GCTGCACT
GTTTTCGTCTTGATTATTTGTAAGATTTGTTTTGAACAAATCGAGATACATTTTCAATGTCCGTCGAA
GGAGATCTGTTTGATCGAGATTTTAAGACGAAAACGATCCAGGCTGCTGCTAATGGAAATTGGGGCAA
TGTAAAGCTCCAAAT
+
FFFFFFFFFFFFFFFFFFFFFF:FFFF:FFFFFFFFFF:F:FFFFFFFFFF:FF:FF:FFFFFFFFFF
FFFFFF:FF:FFFFFFFF:FFFFFF:FF:FFFFFFFFFFFFFFFFFFFFFFFFFFFFFFFFFFFFFFFF
FFFFFFFFFFFFFFF
@A00721:190:HGJW7DSXY:4:1560:27624:30232 2:N:0:TCTGCAAG+GCTGCACT
GTTTTCGTCTTGATTATTTGTAAGATTTGTTTTGAACAAATCGAGATACATTTTCAATGTCCGTCGAA
GGAGATCTGTTTGATCGAGATTTTAAGACGAAAACGATCCAGGCTGCTGCTAATGGAAATTGGGGCAA
TGTAAAGCTCCAAAT
+
FFFFFFFFFFFFFFFFFF:FFFFF:FFFFFFFFFFFFF:FFFFFFFF:FFFFFFFFFFFFFFFFFFFF
FFFFFFFFFFFFFFFFFFF:FFFFFFFFFFFFFF:FFFFFFFFFFFFFFFF:FFFFF:FFFFFFF:FF
FFFF,FF:FFFFFFF
@A00721:190:HGJW7DSXY:4:1622:8630:29872 2:N:0:TCTGCAAG+GCTGCACT
GTTTTCGTCTTGATTATTTGTAAGATTTGTTTTGAACAAATCGAGATACATTTTCAATGTCCGTCGAA
GGAGATCTGTTTGATCGAGATTTTAAGACGAAAACGATCCAGGCTGCTGCTAATGGAAATTGGGGCAA
TGTAAAGCTCCAAAT
+
FFFFFFFFF:FFFFFFFFFFFFFFFFFFFFFFFFFFFFFFFFFFFFF:FFFFFF:FFFFFFFFFFFFF
FFFFFFFFFFFFFFFFFFFFFFFFFFFFFFFFFFFFFFFFFFFFFFFFFFFFFFFFFFFFFFFFFFFF
FFFFFFFFFFFFFFF
@A00721:190:HGJW7DSXY:4:1640:9905:31297 2:N:0:TCTGCAAG+GCTGCACT
GTTTTCGTCTTGATTATTTGTAAGATTTGTTTTGAACAAATCGAGATACATTTTCAATGTCCGTCGAA
GGAGATCTGTTTGATCGAGATTTTAAGACGAAAACGATCCAGGCTGCTGCTAATGGAAATTGGGGCAA
TGTAAAGCTCCAAAT
+
FFFFFFFFF:FFFFFFFFFFFFFF:FFFFFFFFFFFFFFFF:FFFFFFFFFFFF:FFFFFF:FFFF:F
F,FFFFFFFFFFFFFFFFFFFFFFFFF:F::FFFF:FFFFFFFFFFFFFFFFFFFFFFFFFFFFFFFF
FFFFFFFFF:FFFF:

```
@A00721:190:HGJW7DSXY:4:2238:32814:5744 2:N:0:TCTGCAAG+GCTGCACT
GTTTTCGTCTTGATTATTTGTAAGATTTGTTTTGAACAAATCGAGATACATTTTCAATGTCCGTCGAA
GGAGATCTGTTTGATCGAGATTTTAAGACGAAAACGATCCAGGCTGCTGCTAATGGAAATTGGGGCAA
TGTAAAGCTCCAAAT
+
FFFFFFFFFFF:FFFFF:FFFFFFFFFFFFFFFFFF:FFFFFFFFFFFFFF:FFFFFFF:FFFFF,FF
FFFFFFFFFFFF:FFFFFF:FFFFF:FF:FFFFFFFFFF:FF:FFFFFF:FFFF,FFFFFFF,FF:FF
FFFFF:F:F:FFFFF
@A00721:190:HGJW7DSXY:4:2318:25807:4539 2:N:0:TCTGCAAG+GCTGCACT
GTTTACGTCTTGAATAATTGTAATATTTGTTTTGAACAAATCGAGATACATATTCAATGTCCGTCGAA
GGAGAACTGTTTGATAGAGAATTTAAGACGAAAAAGATCCAGTCTGCTGCTAATGGAAATTGGGCCAA
TGTAAAGATCCAAA
+
:F,F,F:,F,F,F,F,,F,F:FF,,F:F,FFF:,FFFF::,,FFFFFF:::,:::F::F:FF:FFFFF
,,:FF,FFFFF,,F,,F:FF,FFFF:,FFF:FFF:FF,F:FF::FFFF,,,:FFF,FFF,,,,,,:,F
,,FFF,,,,,FFFF
@A00721:190:HGJW7DSXY:4:2503:28926:7905 2:N:0:TCTGCAAG+GCTGCACT
GTTTTCGTCTTGATTATTTGTAAGATTTGTTTTGAACAAATCGAGATACATTTTCAATGTCCGTCGAA
GGAGATCTGTTTGATCGAGATTTTAAGACGAAAACGATCCAGGCTGCTGCTAATGGAAATTGGGGCAA
TGTAAAGCTCCAAAT
+
FFFFFFFFFFFFFFFFFFFFFFFF:FF:FFFFFFFFFFFFFFFFFFFFFFFFFFFFFFF:FFFFFFFF
FFFFFF:FFFFFFFFFFFFFFFFFFFFFFF:FFF:FFFFFFFFFFFFFFFFF:FFF:FFFFFF:FFFFF
FFFFFFFFFFFFFFF
@A00721:190:HGJW7DSXY:4:2525:25319:12868 2:N:0:TCTGCAAG+GCTGCACT
GTTTTCGTCTTGATTATTTGTAAGATTTGTTTTGAACAAATCGAGATACATTTTCAATGTCCGTCGAA
GGAGATCTGTTTGATCGAGATTTTAAGACGAAAACGATCCAGGCTGCTGCTAATGGAAATTGGGGCAA
TGTAAAGCTCCAAAT
+
FFF:FFFF:FFFFFFFFFF:FFFFFFFFFFFFFFFFFFFFFFFFF,FFFF::FFF:FFFF:F:FFFFFF
FFFFFFFFFFFF:FFFFFFFFFFFFFF:FFF:FFFFFF:FFFFF:FFFFFF:FFF:F:FF:FFFF,FF
F:FF:FFFFF:F:FF
@A00721:190:HGJW7DSXY:4:2604:29613:3991 2:N:0:TCTGCAAG+GCTGCACT
GTTTTCGTCTTGATTATTTGTAAGATTTGTTTTGAACAAATCGAGATACATTTTCAATGTCCGTCGAA
GGAGATCTGTTTGATCGAGATTTTAAGACGAAAACGATCCAGGCTGCTGCTAATGGAAATTGGGGCAA
TGTAAAGCTCCAAAT
+
FFF,FFFFF:FFF,FFFF,FFFFFFFFF:FFFFFFFFFFFFF,FFFFFFFFF:FFFFFFF:FF,FFFF
F:F:F,FF:FF::FF,FFFFFFFFFF:FF,FFFF,FFF,FF,FFFFF:FFF:FFF:FFFFFFFFFFFF
:FFFFFF:FFFFFFF
@A00721:190:HGJW7DSXY:4:1201:21594:22576 2:N:0:TCTGCAAG+GCTGCACT
CTGGGCATTTGGAGCTTTACATTGCCCCAATTTCCATTAGCAGCAGCCTGGATCGTTTTCGTCTTAAA
ATCTCGATCAAACAGATCTCCTTCGACGGATATTGAAAATGTATCTCGATTTGTTCAAAACAAATCTT
ACAAATAATCAAGAC
+
FFFFFFFFFFFFFFFFFFFFFFFFFFFFFFFFFFFFFFFFFFFFFFFFFFFFFFFFFFFFFFFFFFFF
FFFFFFFFFFFFFFFFFFFFFFFFFFFFFFFFFFFFFFFFFFFFFFFFFFFFFFFFFFFFFFFFFFFF
FFFFFFFFFFFF:FF
@A00721:190:HGJW7DSXY:4:1204:15989:32377 2:N:0:TCTGCAAG+GCTGCACT
CTGGGCATTTGGAGCTTTACATTGCCCCAATTTCCATTAGCAGCAGCCTGGATCGTTTTCGTCTTAAA
ATCTCGATCAAACAGATCTCCTTCGACGGATATTGAAAATGTATCTCGATTTGTTCAAAACAAATCTT
ACAAATAATCAAGAC
+
FFFFFFFFFFFFFFFFFFFFFFFFFFFFFFFFFFFFFFFFFFFFFFFFFFFFFFFFFFFFFFFFFFFF
```

FFFFFFFFFFFFFFFFFFFFFFFFFFFFFFFFFFFFFFFFFFFFFFFFFFFFFFFFFFFFFFFFFFFF
FFFFFFFFFFFFFFF
@A00721:190:HGJW7DSXY:4:1513:9453:28980 2:N:0:TCTGCAAG+GCTGCACT
CTGGGCATTTGGAGCTTTACATTGCCCCAATTTCCATTAGCAGCAGCCTGGATCGTTTTCGTCTTAAA
ATCTCGATCAAACAGATCTCCTTCGACGGATATTGAAAATGTATCTCGATTTGTTCAAAACAAATCTT
ACAAATAATCAAGAC
+
FFFFFF::FFFFFFFFFFFFFFFFFFFFFFFFFFFFFFFFFFFFFFFFFFFFFFFFFFFFFFFFFFFF
FFFFFFFFFFFFFFFFFFFFFFFFFFFFFFFFFFFFFFFFFFFFFFFFFFFFFFFFFFF:FFFFFFFF
FFF:FFF:FFFFFF:
@A00721:190:HGJW7DSXY:4:1567:19379:25254 2:N:0:TCTGCAAG+GCTGCACT
CTGGGCATTTGGAGCTTTACATTGCCCCAATTTCCATTAGCAGCAGCCTGGATCGTTTTCGTCTTAAA
ATCTCGATCAAACAGATCTCCTTCGACGGATATTGAAAATGTATCTCGATTTGTTCAAAACAAATCTT
ACAAATAATCAAGAC
+
FFFFFFFFFFFFFFF:FFFFFFFFFFF:FFFFFFFFFFFFFFFFFFFFFFFFFFFFFFFFFFFFFFFF
FFFFFFFFFFFFFFFFFFFFFFFFFFFFFFFFF:FFFFFFFFFFFFFFFFFFFFFFFFFFFFFFFFFF
FFFFFFFFFFFF,FF
@A00721:190:HGJW7DSXY:4:1614:1958:17660 2:N:0:TCTGCAAG+GCTGCACT
CTGGGCATTTGGAGCTTTACATTGCCCCAATTTCCATTAGCAGCAGCCTGGATCGTTTTCGTCTTAAA
ATCTCGATCAAACAGATCTCCTTCGACGGATATTGAAAATGTATCTCGATTTGTTCAAAACAAATCTT
ACAAATAATCAAGAC
+
FFFFFFFFFFFFFFFFFFFFFFFFFFFFFFFFFFFFFFFFFFFFFFFFFFFFFFFFFFFFFFFFFFFF
FFFFFFFFFFFFFFFFFFFFFFFFFFFFFFFFFFFF:FFFFFFFFFFFFFFFFFFFFFFFFFFFFFF
FFFFFFFFFFFFFFF
@A00721:190:HGJW7DSXY:4:2361:24044:25160 2:N:0:TCTGCAAG+GCTGCACT
CTGGGCATTTGGAGCTTTACATTGCCCCAATTTCCATTAGCAGCAGCCTGGATCGTTTTCGTCTTAAA
ATCTCGATCAAACAGATCTCCTTCGACGGATATTGAAAATGTATCTCGATTTGTTCAAAACAAATCTT
ACAAATAATCAAGAC
+
FFFFFFFFFFFFFFFFFFFFFFFFFFFFFFFFFFFFFFFFFFFFFFFFFFFFFFFFFFFFFFFFFFFF
FFFFFFFFFFFFFFFFFFFFFFFFFFFFFFFFFFFFFFFFFFFFFFFFFFFFFFFFFFFF:FFFFFFF
FFFFFFFFFFFFF:F
@A00721:190:HGJW7DSXY:4:2650:2935:3161 2:N:0:TCTGCAAG+GCTGCACT
CTGGGCATTTGGAGCTTTACATTGCCCCAATTTCCATTAGCAGCAGCCTGGATCGTTTTCGTCTTAAA
ATCTCGATCAAACAGATCTCCTTCGACGGATATTGAAAATGTATCTCGATTTGTTCAAAACAAATCTT
ACAAATAATCAAGAC
+
FFFFFFFFFFFFFFFFFFFFFFFFFFFFFFFFFFFFFFFFFFFFFFFFFFFFFFFFFFFFFFFFFFFF
FFFFFFFFFFFFFFFFFFFFFFFFFFFFFFFFFFFFFFFFFFFFFFFFFFFFFFFFFFF:FFFFFFFF
FFFFFFFFFFFF:FF
@A00721:190:HGJW7DSXY:4:2119:24740:21574 2:N:0:TCTGCAAG+GCTGCACT
CTGGGCATTTGGAGCTTTACATTGCCCCAATTTCCATTAGCAGCAGCCTGGATCGTTTTCGTCTTAAA
ATCTCGATCAAACAGATCTCCTTCGACGGATATTGAAAATGTATCTCGATTTGTTCAAAACAAATCTT
ACAAATAATCAAGA
+
:FFFFFFFF:FFFFFFFFFFFFFFFFFFFFFFF:FFFFFFFFFFFF:FFFFF:FFFFFFFFFFFFFFF
FFFFF:FFFFFFFFFFFFFFFF:FFFFFFFFFF:FFFFFF::FFFF:FFFFF:FF,:FFF:FF,FFFF
F,FFFFFFFFFFFF
@A00721:190:HGJW7DSXY:4:1244:7509:6918 2:N:0:TCTGCAAG+GCTGCACT
TACTGGGCATTTGGAGCTTTACATTGCCCCAATTTCCATTAGCAGCAGCCTGGATCGTTTTCGTCTTA
AAATCTCGATCAAACAGATCTCCTTCGACGGACATTGAAAATGTATCTCGATTTGTTCAAAACAAATC
TTACAAATAATCAAG

+
FFFFFFFFFFFFFFFFF:FFFFFFFFFFFFFFFFFFFFFFFFFFFFFFFFFFFFFFFFFFFFFFFFFF
FFFFFFFFFFFFFFFFFFFFFFFFFFFFFFFFFFFFFFFFFFFFFFFFFFFFFFFFFFFFFFFFFFFF
FFFFFFFFFFFFFFF
@A00721:190:HGJW7DSXY:4:1616:4372:16203 2:N:0:TCTGCAAG+GCTGCACT
TACTGGGCATTTGGAGCTTTACATTGCCCCAATTTCCATTAGCAGCAGCCTGGATCGTTTTCGTCTTA
AAATCTCGATCAAACAGATCTCCTTCGACGGACATTGAAAATGTATCTCGATTTGTTCAAAACAAATC
TTACAAATAATCAAG
+
FFFFFFFFFFFFFFFFFFFFFFFFF:FFFFFFFFFFFFFFFFFFFFFF:FFFFFFFFFF:FFFFFFF:
FF:FFFFFFFFFFFFFFFFFFFFFFFFFFFFFF,FFFFFFFFFFFFFFFFFFFFFFFFFF,FFFFFFF
FFFFFFFFFFFFFFF
@A00721:190:HGJW7DSXY:4:2102:28664:28902 2:N:0:TCTGCAAG+GCTGCACT
TACTGGGCATTTGGAGCTTTACATTGCCCCAATTTCCATTAGCAGCAGCCTGGATCGTTTTCGTCTTA
AAATCTCGATCAAACAGATCTCCTTCGACGGACATTGAAAATGTATCTCGATTTGTTCAAAACAAATC
TTACAAATAATCAAG
+
FFFFFFFFFFFFFFFFFFFFFFFFFFFFFFFFFFFFFFFFFFFFFF:FF:FFFFFFFFFFFFFFFFFF
FFFFFFFFFFFFFFFFFFFFFFFFFFFFFFFFFF:FFF,,:FFFFFFFFFFFFFFFFF,::FFFF:FF
FFFFFFFF:FFF::F
@A00721:190:HGJW7DSXY:4:2673:10520:34021 2:N:0:TCTGCAAG+GCTGCACT
TACTGGGCATTTGGAGCTTTACATTGCCCCAATTTCCATTAGCAGCAGCCTGGATCGTTTTCGTCTTA
AAATCTCGATCAAACAGATCTCCTTCGACGGACATTGAAAATGTATCTCGATTTGTTCAAAACAAATC
TTACAAATAATCAAG
+
FFFFFFFFFFFFFFFFFFFFFFFFFFFFFFFFFFFFFFFFFFFFFFFFFFFFFFFFFFFFFFFFFFFF
FFFFFFFFFFFFFFFFFFFFFFFFFFFFFFFFFFFFFFFFFFFFFFFFFFFFFFFFFFFFFFFFFFFF
FFFFFFFFFFFFFF:
@A00721:190:HGJW7DSXY:4:1339:4119:9283 2:N:0:TCTGCAAG+GCTGCACT
TACTGGGCATTTGGAGCTTTACATTGCCCCAATTTCCATTAGCAGCAGCCTGGATCGTTTTCGTCTTA
AAATCTCGATCAAACAGATCTCCTTCGACGGACATTGAAAATGTATCTCGATTTGTTCAAAACAAATC
TTACAAATAATCAA
+
FFFFFFFF:FFFFFFFFFFFFFFFFFFFFFFFFFFFFFFFFFF,FFFFFFFFFFFFFFFFFFFFFFFF
FF:FFFFFFFFFFFFFFFFFFFFFFF:FFFFFFF:,FFFFFFFFFFFFFFFFFFFFFF:FFFFFFFFF
FFFFFFFFFFFFFF
@A00721:190:HGJW7DSXY:4:2257:24026:10974 2:N:0:TCTGCAAG+GCTGCACT
TACTGGGCATTTGGAGCTCTACATTGCCCCAATTTCCATTAGCAGCAGCCTGGATCGTTTTCGTCTTA
AAATCTCGATCAAACAGATCTCCTTCGACGGACATTGAAAATGTATCTCGATTTGTTCAAAACAAATC
TTACAAATAATCAA
+
FFFF:FFFFFFFFFFFFFFFFFFFFFFFFFFFFFFFFF:F:FFFFFFFF:FFFF,FFFFFFF:FF,FF
:FFFFFFFFFFFFFFFFFF:F,FFFFFFFFFFFFF,FFFFFFFFFF,F,FFFFF:FFF,FFFFFFFFF
FF::FFFFFFF,FF
@A00721:190:HGJW7DSXY:4:1504:15772:15937 2:N:0:TCTGCAAG+GCTGCACT
CCATACTGGGCATTTGGAGCTTTACATTGCCCCAATTTCCATTAGCAGCAGCCTGGATCGTTTTCGTC
TTAAAATCTCGATCAAACAGATCTCCTTCGACGGACATTGAAAATGTATCTCGATTTGTTCAAAACAA
ATCTTACAAATAATC
+
FFFFFFFFFFFFFFFFFFFFFFFFFFFFFFFFFFFFFFFFFFFFFF:FFFFFFFFFFFFFFFFFFFFF
FFFFFFFFFFFFFFFFFFFFFFFFFFFFFFFFFFFFFFFFFFFFFFFFFFFFFFFFFFFFFFF::FFF
FFFFFFFFF:FFFFF
@A00721:190:HGJW7DSXY:4:2201:12527:23218 2:N:0:TCTGCAAG+GCTGCACT
CCATACTGGGCATTTGGAGCTTTACATTGCCCCAATTTCCATTAGCAGCCGCCTGGATCGTTTTCGTC

```
TTAAAATCTCGATCAAACAGATCTCCTTCGACGGACATTGAAAATGTCTCTCGATTTGTTCAAAACAA
ATCTTACAAATAATC
+
FFFFFFFFFFFF,FFFFFFFFFFFFF,FFFFFFFFFFFFFFFFF:FFFF,F:F,:FFFFFFF:FFFFF
FF:FFFFFFFFF:FFFFFFFFFFF:FFFFFFF:FFFF:FF::FFFFF,FFFFFFFFFFFFFF,F,FFF
FFFFFFFFFFFFFFF
@A00721:190:HGJW7DSXY:4:2255:8811:17190 2:N:0:TCTGCAAG+GCTGCACT
CCATACTGGGCATTTGGAGCTTTACATTGCCCCAATTTCCATTAGCAGCAGCCTGGATCGTTTTCGTC
TTAAAATCTCGATCAAACAGATCTCCTTCGACGGACATTGAAAATGTATCTCGATTTGTTCAAAACAA
ATCTTACAAATAATC
+
FFFFFFFFFFFFFFFFFFFFFFFFFFFFFFFFFFFFFFFFFFFFFFFFFFFFFFFFFFFFFFFFFF,F
FFFFF:FFFFFFFFFFFFFFFFFFFFF:FFFFFFFFFFFFFFFFFFFFFFFFFFFFFFFFFFFF,:FF
FFFFFFFFFFFFFFF
@A00721:190:HGJW7DSXY:4:2318:20772:18490 2:N:0:TCTGCAAG+GCTGCACT
CCATACTGGGCATTTGGAGCTTTACATTGCCCCAATTTCCATTAGCAGCAGCCTGGATCGTTTTCGTC
TTAAAATCTCGATCAAACAGATCTCCTTCGACGGACATTGAAAATGTATCTCGATTTGTTCAAAACAA
ATCTTACAAATAATC
+
FFFFFFFFFFFFFFFFFFFFFFFFFFFFFFFFFF:FFFFFFFFFFFFFFFFFFFFFFFFFFFFFFFFF
F:FFFFFFFFFFFFFFFFFFFFFFFFFFFFFFFFFFFFFFFFFFFFFFFFFFFFFFFFFFFFFF,FFF
FFFF:FFFFFFFFFF
@A00721:190:HGJW7DSXY:4:2504:17472:20040 2:N:0:TCTGCAAG+GCTGCACT
CCATACTGGGCATTTGGAGCTTTACATTGCCCCAATTTCCATTAGCAGCAGCCTGGATCGTTTTCGTC
TTAAAATCTCGATCAAACAGATCTCCTTCGACGGACATTGAAAATGTATCTCGATTTGTTCAAAACAA
ATCTTACAAATAATC
+
:FFFFFFFFFFFFFFFFFFF:FFFFFFFFFFFFFFFFFFFFFFFFFFFFFFFFFFFFFFFFFFFFFFF
FFFFFFFFFFFFFFFFFFFFFFFFFFFFFFFFFFFFFFFFFFFFFFFFFFFFFFFFFFFFFFFFFFFF
FFFFFFFFFFFFFFF
@A00721:190:HGJW7DSXY:4:2609:7862:26224 2:N:0:TCTGCAAG+GCTGCACT
CCATACTGGGCATTTGGAGCTTTACATTGCCCCAATTTCCATTAGCAGCAGCCTGGATCGTTTTCGTC
TTAAAATCTCGATCAAACAGATCTCCTTCGACGGACATTGAAAATGTATCTCGATTTGTTCAAAACAA
ATCTTACAAATAATC
+
FFFFFFFFFFFFFFFFFFFFFFFFFFFFFFFFFFFFFFFFFFFFFFFFFFFFFFFFFFFFFFFFFFFF
FFFFFFFFFFFFFFFFFFFFFFFFFFFFFFFFFFFFFFFFFFFFFFFFFFFFFFFFFFFFFFFFFFFF
FFFFFFFFFFFFFFF
@A00721:190:HGJW7DSXY:4:2244:9136:8390 2:N:0:TCTGCAAG+GCTGCACT
CCATACTGGGCATTTGGAGCTTTACATTGCCCCAATTTCCATTAGCAGCAGCCTGGATCATTTTCGTC
TTAAAATCTCGATCAAACAGATCTCCTTCGACGGACATTGAAAATGTATCTCGATTTGTTCAAAACAA
ATCTTACAAATAAT
+
FFFFFFFFFFFFFFFFFFFFFFFFFFFFFFFFFFFFFFFFFFFFFFFFFFFFFFFFFFF,FFFFFFFF
FFFFFFFFFFFFFFFFFFFFFF,FFFFFFFFFFFFFFFF:FFFFF:FFFFFF:FFFF:FFFFFFFFFF
F::FFF,FFFFFF:
@A00721:190:HGJW7DSXY:4:1527:4942:29841 2:N:0:TCTGCAAG+GCTGCACT
CTTCAACTTATTGTACATCACATCGGCCATACTGGGCATTTGGAGCTTTACATTGCCCCAATTTCCAT
TAGCAGCAGCCTGGATCGTTTTCGTCTTAAAATCTCGATCAAACAGATCTCCTTCGACGGACATTGAA
AATGTATCTCGATTT
+
FFFFFFFFFFFFFFFFFFFFFFFFFFFFFFFFFFFFFFFFFFFFFFFFFFFFFFFFFFFFFFFFFFFF
FFFFFFFFFFFFFFFFFFFFFFFFFFFFFFFFFFFFFFFFFFFFFFFFFFFFFFFFFFFFFFFFFFFF
FFFFFFFFFFFFFFF
```

@A00721:190:HGJW7DSXY:4:1609:8269:6230 2:N:0:TCTGCAAG+GCTGCACT
CTTCAACTTATTGTACATCACATCGGCCATACTGGGCATTTGGAGCTTTACATTGCCCCAATTTCCAT
TAGCAGCAGCCTGGATCGTTTTCGTCTTAAAATCTCGATCAAACAGATCTCCTTCGACGGACATTGAA
AATGTATCTCGATTT
+
FFFFFFFFFFFFFFFFFFFFFFFFFFFFFF:FFFFFFFFFFFFFFFFFFFFFFFFFFFFFFFFFFFFF
FFFFFFFFFFFFFFFFFFFFFFFFFFFFFFFFFFFFFFFFFF:FFFFFFFFFFFFF:FFFFFFFFFFF
FFFFFFFFFF:FFFF
@A00721:190:HGJW7DSXY:4:1122:20582:2910 2:N:0:TCTGCAAG+GCTGCACT
AATCGAGATACATTTTCAATGTCCGTCGAAGGAGATCTGTTTGATCGAGATTTTAAGACGAAAACGAT
CCAGGCTGCTGCTAATGGAAATTGGGGCAATGTAAAGCTCCAAATGCCCAGTATGGCCGATGTGATGT
ACAATAAGTTGAAGA
+
FFFFFFFFFFFFFFFFFFFFFFFFFFFFFFFFFFFFFFFFFFFFFFFFFFFFFFFFFFFFFFFFFFFF
FFFFFFFFFFFFF:F:FFFFFFFFFFFFFFFFFFFFFFFFFFFFFF:FFFF:FFFFFFFFFFFFFFFF
FFFFF,FFFFFFFFF
@A00721:190:HGJW7DSXY:4:1160:16387:13964 2:N:0:TCTGCAAG+GCTGCACT
AATCGAGATACATTTTCAATGTCCGTCGAAGGAGATCTGTTTGATCGAGATTTTAAGACGAAAACGAT
CCAGGCTGCTGCTAATGGAAATTGGGGCAATGTAAAGCTCCAAATGCCCAGTATGGCCGATGTGATGT
ACAATAAGTTGAAGA
+
FFFFFFFFFFFFFFFFFFFFFFFFFFFFFFFFFFFFFFFFFFFFFFFFFFFFFFFFFFFFFFFFFFFF
FFFFFFFFFFFFFFFFFFFFFFFFFFFFFFFFFFF:FFFFFFFFFFFFFFFFFFFFFFFFFFFFFFFF
FFFFFFFFFFFFFFF
@A00721:190:HGJW7DSXY:4:1336:17264:23938 2:N:0:TCTGCAAG+GCTGCACT
AATCGAGATACATTTTCAATGTCCGTCGAAGGAGATCTGTTTGATCGAGATTTTAAGACGAAAACGAT
CCAGGCTGCTGCTAATGGAAATTGGGGCAATGTAAAGCTCCAAATGCCCAGTATGGCCGATGTGATGT
ACAATAAGTTGAAGA
+
FFFFFFFFFFFFFFFFFFFFFFFFFFFFFFFFFFFFFFFFFFFFFFFFFFFFFFFFFFFFFFFFFFFF
FFFFFFFFFFFFFFFFFFFFFFFFFFFFFFFFFFFFFFFFFFFFFFFFFFF:FFFFFFFFFFFFFFFF
FFF:FFFFFFFFFFF
@A00721:190:HGJW7DSXY:4:1552:8703:16783 2:N:0:TCTGCAAG+GCTGCACT
AATCGAGATACATTTTCAATGTCCGTCGAAGGAGATCTGTTTGATCGAGATTTTAAGACGAAAACGAT
CCAGGCTGCTGCTAATGGAAATTGGGGCAATGTAAAGCTCCAAATGCCCAGTATGGCCGATGTGATGT
ACAATAAGTTGAAGA
+
FFFFFFFFFFFFFFFFFFFFFFFFFFFFFFFFFFFFFFFFFFFFFFFFFFFFFFFFFFFFFFF:FFFF
FFFFFFFFFFFFFFFFFF:FFFFFFFFFFFFFFFFFFFFFFFFFFFFFFFFFFFFFFFFF,FFF::F:
FFFF:FFFFFFFFFF
@A00721:190:HGJW7DSXY:4:1608:19741:8093 2:N:0:TCTGCAAG+GCTGCACT
AATCGAGATACATTTTCAATGTCCGTCGAAGGAGATCTGTTTGATCGAGATTTTAAGACGAAAACGAT
CCAGGCTGCTGCTAATGGAAATTGGGGCAATGTAAAGCTCCAAATGCCCAGTATGGCCGATGTGATGT
ACAATAAGTTGAAGA
+
FFFFFFFFF:FFFFFFFFFFFFFFFFFFFFFFFFFFFFFFFFF:FFFFFFFFFFFFFFFFFFFFFFFF
FFFFFFFFFFFFFFFFFFFFFFFFFFFFFFFFF:FFFFFFFFFFFFFFFFFF:FFFFFFFFFFFFFFF
FFFFFFFFFFFFFFF
@A00721:190:HGJW7DSXY:4:1677:27498:11819 2:N:0:TCTGCAAG+GCTGCACT
AATCGAGATACATTTTCAATGTCCGTCGAAGGAGATCTGTTTGATCGAGATTTTAAGACGAAAACGAT
CCAGGCTGCTGCTAATGGAAATTGGGGCAATGTAAAGCTCCAAATGCCCAGTATGGCCGATGTGATGT
ACAATAAGTTGAAGA
+
FFFFFFFFFFFFFFFFFFFFFFFFFFFFFFFFFFFFFFFFFFFF:FFFFFFFFFFFFFFFFFFFFFFF

```
FFFFFFFFFFFFF:FFFFF:FFFFFFFFFFF:FFFFFFFFFFFFFFFFFFFFFFFFFFFF:FFFFFFF
FFFFFFFFFFFFF::
@A00721:190:HGJW7DSXY:4:2258:32244:9361 2:N:0:TCTGCAAG+GCTGCACT
AATCGAGATACATTTTCAATGTCCGTCGAAGGAGATCTGTTTGATCGAGATTTTAAGACGAAAACGAT
CCAGGCTGCTGCTAATGGAAATTGGGGCAATGTAAAGCTCCAAATGCCCAGTATGGCCGATGTGATGT
ACAATAAGTTGAAGA
+
FFFFFFFFFFFFFFFFFFFFFFFF:FFFFFFFFFFFFFFFFFFF:FFFFFFFFFFFFFFFFFFFFFFFF
FFFFFFFFFFFFFFFFFFFFFFFF:FFFFFFFFFFFFFFFFFFFFFF:FF:FFFFFFFFFFFFFFFFFF
F:FFFFFFFFFFFFF
@A00721:190:HGJW7DSXY:4:2258:30463:19241 2:N:0:TCTGCAAG+GCTGCACT
AATCGAGATACATTTTCAATGTCCGTCGAAGGAGATCTGTTTGATCGAGATTTTAAGACGAAAACGAT
CCAGGCTGCTGCTAATGGAAATTGGGGCAATGTAAAGCTCCAAATGCCCAGTCTGGCCGATGTGATGT
ACAATAAGTTGAAGA
+
FFFFFFFFFFFFFFFFFFFFFFFFFFFFFFFFFFFFFFFFFFFFFFFFFFFFFFF:FFFFF:FFFF
FFFFFFFFFFFFFFFFFFFFFFFFFFFFFFFFFFFFFFFFFF:FFFFFFFF,FFFFFFFFFFFF:FF
FFF:FF:FFFFFFFF
@A00721:190:HGJW7DSXY:4:2408:4508:29716 2:N:0:TCTGCAAG+GCTGCACT
AATCGAGATACATTTTCAATGTCCGTCGAAGGAGATCTGTTTGATCGAGATATTAAGACGAAAACGAT
CCAGGCTGCTGCTAATGGAAATTGGTGCAATGTAAAGCTCCAAATGCCCAGTATGGCCGATGTGATGT
ACAATAAGTTGAAGA
+
F:,FFF:FFF:F,F,FFFFF,F,F:FF,F,FFFFFF,:FFFFFFFF:F:F,,,FFF:FFFFFF:F,FF
FFFF:,,,FF:,FFFF,FFFFFFF,,FFFFFF,FFF,FF,FF:F:FF:F:FF,F:,FF,FF:,FFF,F
FFFFFFF:,::FF:F
@A00721:190:HGJW7DSXY:4:2409:8856:35117 2:N:0:TCTGCAAG+GCTGCACT
AATCGAGATACATTTTCAATGTCCGTCGAAGGAGATCTGTTTGATCGAGATTTTAAGACGAAAACGAT
CCAGGCTGCTGCTAATGGAAATTGGGGCAATGTAAAGCTCCAAATGCCCAGTATGGCCGATGTGATGT
ACAATAAGTTGAAGA
+
FFFFFFFFFFFFFFFFFFFFFFFFFFFFFFFFFFFFFFFFFFFFFFFFFFFFFFFFFFFFFFFFFFFF
FFFFFFFFFFFFFFFFFFFFFFFFFFFFFFFFFFFFFFFFFFFFFF:FFFFFFFFFFFFFFFFFFFFF
FFFFFFFFFF:FFF:
@A00721:190:HGJW7DSXY:4:2533:27037:6511 2:N:0:TCTGCAAG+GCTGCACT
AATCGAGATACATTTTCAATGTCCGTCGAAGGAGATCTGTTTGATCGAGATTTTAAGACGAAAACGAT
CCAGGCTGCTGCTAATGGAAATTGGGGCAATGTAAAGCTCCAAATGCCCAGTATGGCCGATGTGATGT
ACAATAAGTTGAAGA
+
FFFFFFFFFFFFFFFFFFFFFFFFFFFFFFFFFFFFFFFFFFFFFFFFFFF:,FFFFFFFFFFFFFFF
FFFFFFFFFFFFFFFFFFFFFFFFFFFFFF:FFFF:FFFFFFFFFFF:FFFFFFFFFFFFF,FFFFFFF
FFFFFFFF:FF:FFF
@A00721:190:HGJW7DSXY:4:1311:5864:24956 2:N:0:TCTGCAAG+GCTGCACT
CAATCATGATCGCGTGTTTCTTCAACTTATTGTACATCACATCGGCCATACTGGGCATTTGGAGCTTT
ACATTGCCCCAATTTCCATTAGCAGCAGCCTGGATCGTTTTCGTCTTAAAATCTCGATCAAACAGATC
TCCTTCGACAGATCG
+
FFFFFFFFFFFFFFFFFFFFFFFFF:FFFFFFFFFFFFFFFFFFFFFFFFFFFFFFFFFFFFFFFFFF
FFFFFFFFFFFFFFFFFFFFFFF:FFFFFFFFFFFFFFFFFFF::FFFFF:FF:FFFFFFFF,FFFFF,
F:F:FFFFFFFFFFF
@A00721:190:HGJW7DSXY:4:1662:17671:7263 2:N:0:TCTGCAAG+GCTGCACT
CAATCATGATCGCGTGTTTCTTCAACTTATTGTACATCACATCGGCCATACTGGGCATTTGGAGCTTT
ACATTGCCCCAATTTCCATTAGCAGCAGCCTGGATCGTTTTCGTCTTAAAATCTCGATCAAACAGATC
TCCTTCGACAGATCG
```

+
FFFFFFFFFFFFFFFFFFFFFFFFFFFFFFFFFFFFFFFFFFFFFFFFFFFFFFFFFFFFFFFFFFFFFF
FFFFFFFFFFFFFFFFFFFFFFFFFFFFFFFFFFFFFFFFFFFFFFFF,FFFFFFFFFFFFFF:FFFFFF
FFFFFFFFFFFFFFF
@A00721:190:HGJW7DSXY:4:2246:23891:30123 2:N:0:TCTGCAAG+GCTGCACT
CAATCATGATCGCGTGTTTCTTCAACTTATTGTACATCACATCGGCCATACTGGGCATTTGGAGCTTT
ACATTGCCCCAATTTCCATTAGCAGCAGCCTGGATCGTTTTCGTCTTAAAATCTCGATCAAACAGATC
TCCTTCGACAGATCG
+
FFFFFFFFFFFFFFFFFFFFFFFFFFFFFFFFFFFFFFFFFFFFFFFFFFFFFFFFFFFFFFFFFFFFFF
FFFFFFFFFFFFFFFFFFFFFFFFFFFFFFFFFFFFFFFFFFFFFFFFFFFFFFFFFFFFFFFFFFFFFF
FFFFFF:FFFFFFFF
@A00721:190:HGJW7DSXY:4:2276:3513:1814 2:N:0:TCTGCAAG+GCTGCACT
CAATCATGATCGCGTGTTTCTTCAACTTATTGTACATCACATCGGCCATACTGGGCATTTGGAGCTTT
ACATTGCCCCAATTTCCATTAGCAGCAGCCTGGATCGTTTTCGTCTTAAAATCTCGATCAAACAGATC
TCCTTCGACAGATCG
+
FFFFFFFFFFFFFFFFFFFFFFFFFFFFFFFFFFFFFFFFFFFFFFFFFFFFFFFFFFFFFFFFFFFFFF
FFFFFFFFFFFFFFFFFFFFFFFFFFFFFFFFFFFFFFFFFFFFFFFFFFFFFFFFFFFFFFFFFFFFFF
FFFFFFFFFFFFFFF
@A00721:190:HGJW7DSXY:4:2375:15365:9690 2:N:0:TCTGCAAG+GCTGCACT
CAATCATGATCGCGTGTTTCTTCAACTTATTGTACATCACATCGGCCATACTGGGCATTTGGAGCTTT
ACATTGCCCCAATTTCCATTAGCAGCAGCCTGGATCGTTTTCGTCTTAAAATCTCGATCAAACAGATC
TCCTTCGACAGATCG
+
FFFFFFFFFFFFFFFFFFFFFFFFFFFFFFFFFFFFFFFFFFFFFFFFFFFFFFFFFFFFFFFFFFFFFF
FFFFFFFFFFFFFFFFFFFFFFFFFFFFFFFFFFFFFFFFFFFFFFFFFFFFFFFFFFFFFFFFFFFFFF
FFFFFFFFFFFFFFF
@A00721:190:HGJW7DSXY:4:2525:31919:31688 2:N:0:TCTGCAAG+GCTGCACT
CAATCATGATCGCGTGTTTCTTCAACTTATTGTACATCACATCGGCCATACTGGGCATTTGGAGCTTT
ACATTGCCCCAATTTCCATTAGCAGCAGCCTGGATCGTTTTCGTCTTAAAATCTCGATCAAACAGATC
TCCTTCGACAGATCG
+
FFFFFFFFFFFFFFFFFFFFFFFFFFFFFFFFFFFFFFFFFFFFFFFFFFFFFFFFFFFFFFFFFFFFFF
FFFFFFFFFFFFFFFFFFFFFFFFFFFFFFFFFFFFFFFFFFFFFFFFFFFFFF:FF:FF:FFF:FFFFF
FFFFFFFFFFFFFFF
@A00721:190:HGJW7DSXY:4:1114:21269:5447 2:N:0:TCTGCAAG+GCTGCACT
CACAGCGATCAATCATGATCGCGTGTTTCTTCAACTTATTGTACATCACATCGGCCATACTGGGCATT
TGGAGCTTTACATTGCCCCAATTTCCATTAGCAGCAGCCTGGATCGTTTTCGTCTTAAAATCTCGATC
AAACAGATCTCCTTC
+
F:FFFFFFFFFFFFF:FFFFFFF:FFFFFFFFFFFF,FFFFFFFFFFFFFFFFFFFFFFFFFFFFFFFFF
FFFFFFFFFFFFFFFFFFF::FFFFFFFFFFFFFFFFFFFFFF:FFFFFFFFFFFFFFFF:FFFFFFFFF
:FFFFFFFFFFFFFF
@A00721:190:HGJW7DSXY:4:1402:9634:12633 2:N:0:TCTGCAAG+GCTGCACT
CACAGCGATCAATCATGATCGCGTGTTTCTTCAACTTATTGTACATCACATCGGCCATACTGGGCATT
TGGAGCTTTACATTGCCCCAATTTCCATTAGCAGCAGCCTGGATCGTTTTCGTCTTAAAATCTCGATC
AAACAGATCTCCTTC
+
FFFFFFFFFFFFFFFFFFFFFFFFFFFFFFFFFFFFFFFFFFFFFFFFFFF:FFFFFFFFFFFFFFFFFF
FFFFFFFFFFFFFF,FFFFFFFFFFFFFFFFFFFFFFFFFF:FFFFFFF:FFFFFFFFFFF:FFFFF:FF
FFFFFFFFFFFFFFF
@A00721:190:HGJW7DSXY:4:2101:20419:16438 2:N:0:TCTGCAAG+GCTGCACT
CACAGCGATCAATCATGATCGCGTGTTTCTTCAACTTATTGTACATCACATCGGCCATACTGGGCATT

```
TGGAGCTTTACATTGCCCCAATTTCCATTAGCAGCAGCCTGGATCGTTTTCGTCTTAAAATCTCGATC
AAACAGATCTCCTTC
+
FFFFFFFFFFFFFFFFFFFFFFFFFFFFFFFFFFFFFFFFFFFFFFFFFFFFFFFFFFFFFFFFFFFF
FFFFFFFFFFFFFFFFFFFFFFFFFFFFFFFFFFFFFFFFFFFFFFFFFFFFFFFFFFFFFFFFFFFF
FF:FFFFFFFFFFFF
@A00721:190:HGJW7DSXY:4:1251:21260:35775 2:N:0:TCTGCAAG+GCTGCACT
AGGAGATCTGTTTGATCGAGATTTTAAGACGAAAACGATCCAGGCTGCTGCTAATGGAAATTGGGGCA
ATGTAAAGCTCCAAATGCCCAGTATGGCCGATGTGATGTACAATAAGTTGAAGAAACACGCGATCATG
ATTGATCGCTGTGTT
+
FFFFFFFFFFFFFFFFFFFFFFFFFFFF:FFFFFFFFFFFFFFFFFFFFFFFFFFFFFFFFFFFFFFF
FFFFFFFFFFFFFFFFFFFFFF:FFFFFFFFFFFFFFFFFFFFFFFFFFFFFFFFFFFFFFFFFFFFF
FFFFFFFFFFFFFFF
@A00721:190:HGJW7DSXY:4:1524:1380:7388 2:N:0:TCTGCAAG+GCTGCACT
AGGAGATCTGTTTGATCGAGATTTTAAGACGAAAACGATCCAGGCTGCTGCTAATGGAAATTGGGGCA
ATGTAAAGCTCCAAATGCCCAGTATGGCCGATGTGATGTACAATAAGTTGAAGAAACACGCGATCATG
ATTGATCGCTGTGTT
+
FFFFFFFFFFFFFFFFFFFFFFFFFFFFFFFFFFFFFFFFFFFFFFFFFFFFFFFFFFFFFFFFFFFF
:FFFFFFFFFFFFFFFF:FFFFFFFFFFFFFF:FFFFFFFFFF,FFFFFFFFFFFFFFFFF:FFFFFF
FFFFFFFFFFFFFFF
@A00721:190:HGJW7DSXY:4:2170:29677:3944 2:N:0:TCTGCAAG+GCTGCACT
AGGAGATCTGTTTGATCGAGATTTTAAGACGAAAACGATCCAGGCTGCTGCTAATGGAAATTGGGGCA
ATGTAAAGCTCCAAATGCCCAGTATGGCCGATGTGATGTACAATAAGTTGAAGAAACACGCGATCATG
ATTGATCGCTGTGTT
+
FFFFFFFFFFFFFFFFFFFFFFFFFFFFFFFFFFFFFFFFFFFFFFFFFFFFFFFFFFFFFFFFFFFF
FFFFFFFFFFFFFFFFFFFFFFFFFFFFFFFFFFFFFFFFFFF:FFFFFFFFFFFFFFFFFFFFFFFF
FFFFFFFFFFFFFFF
@A00721:190:HGJW7DSXY:4:2265:20781:12493 2:N:0:TCTGCAAG+GCTGCACT
AGGAGATCTGTTTGATCGAGATTTTAAGACGAAAACGATCCAGGCTGCTGCTAATGGAAATTGGGGCA
ATGTAAAGCTCCAAATGCCCAGTATGGCCGATGTGATGTACAATAAGTTGAAGAAACACGCGATCATG
ATTGATCGCTGTGTT
+
FFFFFFFFFFFFFFFFFFFFFFFFFFFFFFFFFFFFFFFFFFFFFFFFFFFFFFFFFFFFFFFFFFFF
FFFFFFFFFFFFFFFFFFFFFFFFFF:FFFFFFFFFFFFFFFFFFFFFFFFFFFFFFFFFFFFFFFFF
FFFFFFFFFFFFFFF
@A00721:190:HGJW7DSXY:4:2343:2311:15671 2:N:0:TCTGCAAG+GCTGCACT
AGGAGATCTGTTTGATCGAGATTTTAAGACGAAAACGATCCAGGCTGCTGCTAATGGAAATTGGGGCA
ATGTAAAGCTCCAAATGCCCAGTATGGCCGATGTGATGTACAATAAGTTGAAGAAACACGCGATCATG
ATTGATCGCTGTGTT
+
FFFFFFFFFFFFFFFFFFFFFFFFFFFFFFFFFFFFFFFFFFFFFFFFFFFFFFFFFF::FFFFFFFF
FFFFF,FFFFFFFFFFFFFFFFFFFFFFFFFFFFFFFFFFFFFFFFFF:FFFFFFFFFFFFFFFFFFF
FFFFFFFFFFFFFFF
@A00721:190:HGJW7DSXY:4:2418:9588:18286 2:N:0:TCTGCAAG+GCTGCACT
CAATCATGATCGCGTGTTTCTTCAACTTATTGTACATCACATCGGCCATACTGGGCATTTGGAGCTTT
ACATTGCCCCAATTTCCATTAGCAGCAGCCTGGATCGTTTTCGTCTTAAAATATCGATAAAACAGAT
+
FFFFFFF:FF:FFFFFFFFFFFFFFFFFF:FFFFFFFFFFFFFFFFFFFFF::FF:FFFFF:F::FFF
FFFFF,::F:FFFFF,:FF:F,:F:,F:FF::,FF:,:FFF:,:FFFFFFF,,,,,F:,FFF,F,F:
@A00721:190:HGJW7DSXY:4:1117:21802:4899 2:N:0:TCTGCAAG+GCTGCACT
CTCGATAACACAGCGATCAATCATGATCGCGTGTTTCTTCAACTTATTGTACATCACATCGGCCATAC
```

TGGGCATTTGGAGCTTTACATTGCCCCAATTTCCATTAGCAGCAGCCTGGATCGTTTTCGTCTTAAAA
TCTCGATCAAACAGA
+
FFFFFFFFFFFFFFFFFFFFFFFFFFFFFFFFFFFFFFFFFFFFFFF:FFFFFFFFFFFFFFFFFFFFFF
FFFFFFFFFF:FFFFFFFFFFFFFFFFFFFFFFFFFFFFFFFFFFFFFFFFFFFFFFFFFFFFFFF,FFF
FFFFFFFFFFFFFFF
@A00721:190:HGJW7DSXY:4:1367:28366:26537 2:N:0:TCTGCAAG+GCTGCACT
CTCGATAACACAGCGATCAATCATGATCGCGTGTTTCTTCAACTTATTGTACATCACATCGGCCATAC
TGGGCATTTGGAGCTTTACATTGCCCCAATTTCCATTAGCAGCAGCCTGGATCGTTTTCGTCTTAAAA
TCTCGATCAAACAGA
+
FFFFFFFFFFFFFFFFFFFFFFFFFFFFFFFFFFFFFFFFFFF:FFFFFFFFFFFFFFFFFFFFFFFFFF
FFFFFFFFFFFFFFFFFFFFFFFFFFFFFFFFFFFFFFFFFFFFFFFFFFFFFFFFFFFFFFFFFFFFFF
FFFFFFFFFF:FFFF
@A00721:190:HGJW7DSXY:4:1549:19831:31798 2:N:0:TCTGCAAG+GCTGCACT
CTCGATAACACAGCGATCAATCATGATCGCGTGTTTCTTCAACTTATTGTACATCACATCGGCCATAC
TGGGCATTTGGAGCTTTACATTGCCCCAATTTCCATTAGCAGCAGCCTGGATCGTTTTCGTCTTAAAA
TCTCGATCAAACAGA
+
FFFFFF:FFFFFFFFFFFFFFFFFFFFFFFFFFFFFFFFFFFFFFFFFFFFFFFFFFFFFFFFFFFFFFF
FFFFFFFFFFFFFFFFFFFFFFFFFFFFFFFFFFFFFFFFFFFFFFFFFFFFFF:F:FFFFFFFFFFFFF
FFFFFFFFFF:FFFF
@A00721:190:HGJW7DSXY:4:1644:6515:24768 2:N:0:TCTGCAAG+GCTGCACT
CTCGATAACACAGCGATCAATCATGATCGCGTGTTTCTTCAACTTATTGTACATCACATCGGCCATAC
TGGGCATTTGGAGCTTTACATTGCCCCAATTTCCATTAGCAGCAGCCTGGATCGTTTTCGTCTTAAAA
TCTCGATCAAACAGA
+
FFFFFFFFFFFFFFFFFFFFFFFFFFFFFFFFFFFFFFFFFFFFFFFFFFFFFFFFFFFFFFFFFFFFFF
FFFFFFF:FFFF:FFFFFFFFFFFFFFFFFFFFFFFFFFFFFFFFFFFFFFFFFFFFFFFFFFF:FFFF
FFFFFFFFFFFFFF:
@A00721:190:HGJW7DSXY:4:2201:1714:33364 2:N:0:TCTGCAAG+GCTGCACT
CTCGATAACACAGCGATCAATCATGATCGCGTGTTTCTTCAACTTATTGTACATCACATCGGCCATAC
TGGGCATTTGGAGCTTTACATTGCCCCAATTTCCATTAGCAGCAGCCTGGATCGTTTTCGTCTTAAAA
TCTCGATCAAACAGA
+
FFF:FFFFFFFF:FFFFFFFFFFFFFFFFFFFFFFFFFFFFFFFF,FFFFFFFFFFFFFFF,FFFFFFFF
FF:FFFF:FF:FFF,:FFFFFFFF,:FFFFFFFFFFF,FF:FF:FF:FFFFFFFFFFFFFFFFFFFFFFF
FFFF:FFFFFFFFFF
@A00721:190:HGJW7DSXY:4:2208:32443:27492 2:N:0:TCTGCAAG+GCTGCACT
CTCGATAACACAGCGATCAATCATGATCGCGTGTTTCTTCAACTTATTGTACATCACATCGGCCATAC
TGGGCATTTGGAGCTTTACATTGCCCCAATTTCCATTAGCAGCAGCCTGGATCGTTTTCGTCTTAAAT
TCTCGATCAAACAGA
+
FFFFFFFFFFFFFFFFFF:FFFFFFFFFFFFFFFFFFFFFFF:FFFFFFFFFFFFFFFFFFFFFFFFFFF
F,FFFFFFFFFFFFFFFFFFFFFF:FFFFFFFF:FFFFFFFFFFFFFFFFFFFFFF:FFFFFFFFFFF:,
FFFFF:FFFF:FFFF
@A00721:190:HGJW7DSXY:4:2344:12084:3443 2:N:0:TCTGCAAG+GCTGCACT
CTCGATAACACAGCGATCAATCATGATCGCGTGTTTCTTCAACTTATTGTACATCACATCGGCCATAC
TGGGCATTTGGAGCTTTACATTGCCCCAATTTCCATTAGCAGCAGCCTGGATCGTTTTCGTCTTAAAA
TCTCGATCAAACAGA
+
FFFFFFFFFFFFFFFFFFFFFFFFFFFFFFFFFFFFFFFFFFFF,FFFFFFFFFFFFFFFFFFFFFFFFF
FFFFFFFFFFFFFFFFFFFFFFFFFFFFFFFFFFF:FFFFFFFFFFFFFFFFFFFFFFFFFFFFFFFFF:
FFFF:FFFFFFFFFF

```
@A00721:190:HGJW7DSXY:4:2529:32009:6825 2:N:0:TCTGCAAG+GCTGCACT
CTCGATAACACAGCGATCAATCATGATCGCGTGTTTCTTCAACTTATTGTACATCACATCGGCCATAC
TGGGCATTTGGAGCTTTACATTGCCCCAATTTCCATTAGCAGCAGCCTGGATCGTTTTCGTCTTAAAA
TCTCGATCAAACAGA
+
FFFFFFFFFFFFFFFFFFFFFFFFFFFFFFFFFFFFFFFFFFFFFFFFFFFFFFFFFFFFFFFFFFFFFF
FFFFFFFFFFFFFFFFFFFFFFFFFFFFFFFFFFFFFFFFFFFF:FFFFFFFFFFFFFFFFFFFF,FFFF
FFFFFFFFFFFF:FF
@A00721:190:HGJW7DSXY:4:2649:8757:4319 2:N:0:TCTGCAAG+GCTGCACT
CTCGATAACACAGCGATCAATCATGATCGCGTGTTTCTTCAACTTATTGTACATCACATCGGCCATAC
TGGGCATTTGGAGCTTTACATTGCCCCAATTTCCATTAGCAGCAGCCTGGATCGTTTTCGTCTTAAAA
TCTCGATCAAACAGA
+
FFFFFFFFFFFFFFFFFFFFFFFFFFFFFFFFFFFFFFFFFFFFFFFFFFFFFFFFFFFFFFFFFFFFFF
FFFFFFFFFFFFFFFFFFFFFFFFFFFFFFFFFFFFFFF:FFFFFFFFFFFFFFFFFFFFFFFFFFFFFF
FFFF:FFFFF:FFFF
@A00721:190:HGJW7DSXY:4:1155:31882:22263 2:N:0:TCTGCAAG+GCTGCACT
CTGGTGTCGATACTCGATAACACAGCGATCAATCATGATCGCGTGTTTCTTCAACTTATTGTACATCA
CATCGGCCATACTGGGCATTTGGAGCTTTACATTGCCCCAATTTCCATTAGCAGCAGCCTGGATCGTT
TTCGTCTTAAAATCT
+
FFFFFFFFFFFFFFFFFFFFFFFFFFFFFFFFFFFFFFFFFFFFFFFFFFFFFFFFFFFFFFFFFFFFFF
FFFFFFFFFFFFFFFFFFFFFFFFFFFFFFFFFFFFFFFFFFFFFFFFFFFFFFFFFFFFFFF:FFFFFF
FFFFFFF::FFFFFF
@A00721:190:HGJW7DSXY:4:1157:12762:10066 2:N:0:TCTGCAAG+GCTGCACT
CTGGTGTCGATACTCGATAACACAGCGATCAATCATGATCGCGTGTTTCTTCAACTTATTGTACATCA
CATCGGCCATACTGGGCATTTGGAGCTTTACATTGCCCCAATTTCCATTAGCAGCAGCCTGGATCGTT
TTCGTCTTAAAATCT
+
FFFFFFFFFFFFFFFFF:FFFFFFFFFFFFFFFFFFFFFFFFFFFFFFFFFFFFF,FFFFFFFFFFFFFF
FFFFFFFFFFFFFFFFFFFFFFFFFFFFFFFFFFFFFFFFFFFFFFFFFFFFFFFFFFFFFFFFFFFFFF
FFFFFFFFFFFFFFF
@A00721:190:HGJW7DSXY:4:1302:21233:28494 2:N:0:TCTGCAAG+GCTGCACT
CTGGTGTCGATACTCGATAACACAGCGATCAATCATGATCGCGTGTTTCTTCAACTTATTGTACATCA
CATCGGCCATACTGGGCATTTGGAGCTTTACATTGCCCCAATTTCCATTAGCAGCAGCCTGGATCGTT
TTCGTCTTAAAATCT
+
FFFFFFF:FFFFFFFFFFFFFFFFFFFFFFFFFFFFFFFFFFFFFFFFFFFFFFFFFFFFFFFFFFFFFF
FFFFFFFFFFFFFFFFFFFFFFF:FFFFFFFFFFFFFFFFFFFFFFFFFFF:FFFFFFFFFFFFF:FFFF
:FFFFFFFFFFFFFF
@A00721:190:HGJW7DSXY:4:1412:24252:33191 2:N:0:TCTGCAAG+GCTGCACT
CTGGTGTCGATACTCGATAACACAGCGATCAATCATGATCGCGTGTTTCTTCAACTTATTGTACATCA
CATCGGCCATACTGGGCATTTGGAGCTTTACATTGCCCCAATTTCCATTAGCAGCAGCCTGGATCGTT
TTCGTCTTAAAATCT
+
FFFFFFFFFFFFFFFFFFFFFFFFFFFFFFFFFFFFFFFFFFFFFFFFFFFFFFFFFFFFFFFFFFFFFF
FFFFFFFFFFFFFFFFF:FFFFFFFFFFFFFFFFFFFFFFFFFFFFFFFFFFFFFFF:FFFFFFFFFFFF
FFFFFFFFFFF,FFF
@A00721:190:HGJW7DSXY:4:1412:24885:34256 2:N:0:TCTGCAAG+GCTGCACT
CTGGTGTCGATACTCGATAACACAGCGATCAATCATGATCGCGTGTTTCTTCAACTTATTGTACATCA
CATCGGCCATACTGGGCATTTGGAGCTTTACATTGCCCCAATTTCCATTAGCAGCAGCCTGGATCGTT
TTCGTCTTAAAATCT
+
FFFFFFFFFFFFF:FFFFFFFFFFFFFFFFFFFFFFFFFFFFFFFFFFFFFFFFFFFFFFFFFFFFFFFF
```

FFFFFFFFFFFFFFFFFFFFFFFFFFFFFFFFFFFFFFFFFFFFFFFFFFFFFFFFFFFFFFFFFFFF
FFFFFFFFFFFFFFF
@A00721:190:HGJW7DSXY:4:2332:15854:17989 2:N:0:TCTGCAAG+GCTGCACT
CTGGTGTCGATACTCGATAACACAGCGATCAATCATGATCGCGTGTTTCTTCAACTTATTGTACATCA
CATCGGCCATACTGGGCATTTGGAGCTTTACATTGCCCCAATTTCCATTAGCAGCAGCCTGGATCGTT
TTCGTCTTAAAATCT
+
FFFFFFFFFFFFFFFFFFFFFFFFFFFFFFFFFFFFFFFFFFFFFFFFFFFFFFFFFFFFFFFFFFFF
FFFFFFFFFFFFFFFFFF:FFFFFFFFFFFFFFFFFFFFFFFFFFFFFFFFFFFFFFFFFFFFFFFFF
FFFFFFFFFFFFFFF
@A00721:190:HGJW7DSXY:4:2539:1371:16110 2:N:0:TCTGCAAG+GCTGCACT
CTGGTGTCGATACTCGATAACACAGCGATCAATCATGATCGCGTGTTTCTTCAACTTATTGTACATCA
CATCGGCCATACTGGGCATTTGGAGCTTTACATTGCCCCAATTTCCATTAGCAGCAGCCTGGATCGTT
TTCGTCTTAAAATCT
+
FFF:FFFFFFFFFFFFFFFFFFFFFFFFFFFFFFFFFFFFFFFFFFFFFFFFFFFFFFFFFFFFFFFF
FFFFFFFFFFFFFFFFFFFFFFFFFF:FFFFFFFFFFFFFFFFFFFFFFFFFFFFFFFFFFFFFFFFF
FFFFFFFFFFFFFFF
@A00721:190:HGJW7DSXY:4:1146:10719:34240 2:N:0:TCTGCAAG+GCTGCACT
TATTAATCGGACACTGGTGTCGATACTCGATAACACAGCGATCAATCATGATCGCGTGTTTCTTCAAC
TTATTGTACATCACATCGGCCATACTGGGCATTTGGAGCTTTACATTGCCCCAATTTCCATTAGCAGC
AGCCTGGATCGTTTT
+
FFFFFFFFFFFFFFFFFFFFFFF:FFFFFFFFFFFFFFFFFFFFFFFFFFFFFFFFFFFFFFFFFFFF
FFFFFFFFFFFFFFFFFFFFFFFFFFFFFFFFFFFFFFFFFFFFFFFFFFFFFFFFFFFFFFFFFFFF
FFFFFFFFFFF:FFF
@A00721:190:HGJW7DSXY:4:1217:26078:9768 2:N:0:TCTGCAAG+GCTGCACT
TATTAATCGGACACTGGTGTCGATACTCGATAACACAGCGATCAATCATGATCGCGTGTTTCTTCAAC
TTATTGTACATCACATCGGCCATACTGGGCATTTGGAGCTTTACATTGCCCCAATTTCCATTAGCAGC
AGCCTGGATCGTTTT
+
FFFFFFFFFFFFFFFFFFFFFFFFFFFFFFFFFFFFFFFFFFFFFFFFFFFFFFFFFFFFFFFFFFFF
FFFFFFFFFFFFFFFFFFFFF:FFFFFFFFFFFFFFFFFFFFFFFFFFFFFFFFFFFFFFFFFFFFFF
FFFFFFFFFFF:FFF
@A00721:190:HGJW7DSXY:4:1217:28311:13823 2:N:0:TCTGCAAG+GCTGCACT
TATTAATCGGACACTGGTGTCGATACTCGATAACACAGCGATCAATCATGATCGCGTGTTTCTTCAAC
TTATTGTACATCACATCGGCCATACTGGGCATTTGGAGCTTTACATTGCCCCAATTTCCATTAGCAGC
AGCCTGGATCGTTTT
+
FFFFFFFFFFFFFFFFFFFFFFFFFFFFFFFFFFFFFFFFFFFFFFFFFFFFFFFFFFFFFFFFFFFF
FFFFFFFFFFFFFFFFFFFFFFFFFFFFFFFFFFFFFFFF:FFFFFFFFFFFFFFFFFFFFFFFF:FF
FFFFFFFFFF:,,FF
@A00721:190:HGJW7DSXY:4:2269:25355:24862 2:N:0:TCTGCAAG+GCTGCACT
TATTAATCGGACACTGGTGTCGATACTCGATAACACAGCGATCAATCATGATCGCGTGTTTCTTCAAC
TTATTGTACATCACATCGGCCATACTGGGCATTTGGAGCTTTACATTGCCCCAATTTCCATTAGCAGC
AGCCTGGATCGTTTT
+
FFFFFFFFFFFFFFFFFFFFFFFFFFFFFFFFFFFFFFFFFFFFFFFFFFFFFFFFFFFFFFFFFFFF
FFFFFFFFFFFFFFFFFFFFFFFFFFFFFFFFFFF:FFF:FFFFFFFFFFFFFFFFFFFFFFFFFFFF
FFFFFFFFFFFFFFF
@A00721:190:HGJW7DSXY:4:2573:1696:11663 2:N:0:TCTGCAAG+GCTGCACT
TATTAATCGGACACTGGTGTCGATACTCGATAACACAGCGATCAATCATGATCGCGTGTTTCTTCAAC
TTATTGTACATCACATCGGCCATACTGGGCATTTGGAGCTTTACATTGCCCCAATTTCCATTAGCAGC
AGCCTGGATCGTTTT

```
+
FF:FFFFFFFFFFFFFFFFFFFFFFFFFFFFFFFF:FFFFFFFFFFFFFFFFFFFFF:FFFFFFFFF,F
FFFFFFFFFFFFFFFFFFFFFFFFFFFFFFFFFFFFFFFF:FFFF::FFFFFFFFFFFFFF:FFFFFFF
,FFFFFFFFF:,FFF
@A00721:190:HGJW7DSXY:4:1417:18982:23030 2:N:0:TCTGCAAG+GCTGCACT
TATTAATCGGACACTGGTGTCGATACTCGATAACACAGCGATCAATCATGATCGCGTGTTTCTTCAAC
TTATTGTACATCACATCGGCCATACTGGGCATTTGGAGCTTTACATTGCCCCAATTTCCATTAGCAGC
AGCCTGGATCGTTT
+
FFFFFFFFFFFFFFFFFFFFFFFFFFFFFFFFFFFFFFFFFFFFFFFFFF:FFFFF:FFFFFFFFFFF
FFFFFFFFFFFFFFFFFFFFFFFFFFFFFFFFFFFFFFFFFFFFFFFFFFFFFFFFFFFFFFFFFFFF:
FFFFFFFFFFF:FF
@A00721:190:HGJW7DSXY:4:1319:30779:11303 2:N:0:TCTGCAAG+GCTGCACT
CGGATCCCTCACTATTAATCGGACACTGGTGTCGATACTCGATAACACAGCGATCAATCATGATCGCG
TGTTTCTTCAACTTATTGTACATCACATCGGCCATACTGGGCATTTGGAGCTTTACATTGCCCCAATT
TCCATTAGCAGCAGC
+
FFFF:FFFFFFFFFFFFFFFFFFFFFFFFFFFFFFFFFFFFFFFFFFFFFFFFFFFFFFFFFFFFFFFF
FFFFFFFFFFFFFFFFFFFFFFFFFFFFFFFFFFFFFFFFFFFFF:FFFFFFFFFFFFFFFFFFFFFFFF
FFFFFFFFFFFFFFF
@A00721:190:HGJW7DSXY:4:1446:18900:31814 2:N:0:TCTGCAAG+GCTGCACT
CGGATCCCTCACTATTAATCGGACACTGGTGTCGATACTCGATAACACAGCGATCAATCATGATCGCG
TGTTTCTTCAACTTATTGTACATCACATCGGCCATACTGGGCATTTGGAGCTTTACATTGCCCCAATT
TCCATTAGCAGCAGC
+
FFFFFFFFFFFFFFFFFFFFFFFFFFFFFFFFFFF:FFFFFFFFFFFFFFFFFFF:F,FFFFFFFFFFF
FFFFFFFFFFFFFFFFFFFFFFFFFFFFFFFFFFF:FFFFFFFFFFFFF:FFFFFFFFFFFFFFFFFFF,
FFFFFFFFFFFFFFF
@A00721:190:HGJW7DSXY:4:2412:14570:9565 2:N:0:TCTGCAAG+GCTGCACT
CGGATCCCTCACTATTAATCGGACACTGGTGTCGATACTCGATAACACAGCGATCAATCATGATCGCG
TGTTTCTTCAACTTATTGTACATCACATCGGCCATACTGGGCATTTGGAGCTTTACATTGCCCCAATT
TCCATTAGCAGCAGC
+
FFFFFFFFFFFFFFFFFFFFFFFFFFFFFFFFFFFFFFFFFFFFFFFFFFFFFFFFFFFFFFFFFF:FF
FFFF:F:FFFFFFFFFFFFFFFFFFFFFFFFFFF:F:FFFFFFFFF:FFFFFFFFFFFFFFFFFFFFFF
FFFFFFFFFFFFFFF
@A00721:190:HGJW7DSXY:4:2422:20184:5822 2:N:0:TCTGCAAG+GCTGCACT
CGGATCCCTCACTATTAATCGGACACTGGTGTCGATACTCGATAACACAGCGATCAATCATGATCGCG
TGTTTCTTCAACTTATTGTACATCACATCGGCCATACTGGGCATTTGGAGCTTTACATTGCCCCAATT
TCCATTAGCAGCAGC
+
FFFFFFFFFFFFFFFFFFFFFFFFF:FFFFFFFF:FFFFFF:FFFFFFFFFFFFFFF:FFFFFFFFFFF
FFFFFFFFFFFFFFFFFFFFFFFFFFFFFFFFFFFF:FFFF:FFF:FFFFFFFFFFFFFFFFFFFFFFFF
FFFFFFFFFFFFFFF
@A00721:190:HGJW7DSXY:4:2658:20464:2801 2:N:0:TCTGCAAG+GCTGCACT
CGGATCCCTCACTATTAATCGGACACTGGTGTCGATACTCGATAACACAGCGATCAATCATGATCGCG
TGTTTCTTCAACTTATTGTACATCACATCGGCCATACTGGGCATTTGGAGCTTTACATTGCCCCAATT
TCCATTAGCAGCAGC
+
FFFFFFFFFFFFFFFFFFFFFFF:F,FFFFFFFFF,FFFFFFFFFFFFFFFFFFFFFFFFFFFFFFFFFF
FFFFFFFFFFFFFFFF:FFFFFFFFFFFFFFFFFFFFFFFFFFFFFFFFFFFFFFFFFFFFFFFFFFFF
FFFFFFFFFFFFFFF
@A00721:190:HGJW7DSXY:4:1111:11885:31594 2:N:0:TCTGCAAG+GCTGCACT
GTGCACCTGGATCCTAACGGATCCCTCACTATTAATCGGACACTGGTGTCGATACTCGATAACACAGC
```

```
GATCAATCATGATCGCGTGTTTCTTCAACTTATTGTACATCACATCGGCCATACTGGGCATTTGGAGC
TTTACATTGCCCCAA
+
FFFFFFFFFFFFFFFFFFFFFFFFFFFFFFFFFFFFFFFFFFFFFFFFFFFFFFFFFFFFFFFFFFFFFF
FFFFFFFFFFFFFFFFFFFFFFFFFFFFFFFFFFFFFFFFFFFFFFFFFFFFFFFFFFFFFFFFFFFFFF
FFFFFFFFFFFFFFF
@A00721:190:HGJW7DSXY:4:1375:30843:28009 2:N:0:TCTGCAAG+GCTGCACT
GTGCACCTGGATCCTAACGGATCCCTCACTATTAATCGGACACTGGTGTCGATACTCGATAACACAGC
GATCAATCATGATCGCGTGTTTCTTCAACTTATTGTACATCACATCGGCCATACTGGGCATTTGGAGC
TTTACATTGCCCCAA
+
FFFFFFFFFFFFFF:FFFFFFFFFFFFFFFFFFFFFFFFFFFFFFFFFFFFFFFFFFFFFFFFFFFFFFFF
FFFFFFFFFFFFFFFFFFFFFFFFFFFF,FFFFFFFFFFFFFFFFFFFFFFFFFFFFFFFFFFFFFFFFF
FFFFFFFFFFFFFFF
@A00721:190:HGJW7DSXY:4:1654:14516:2300 2:N:0:TCTGCAAG+GCTGCACT
GTGCACCTGGATCCTAACGGATCCCTCACTATTAATCGGACACTGGTGTCGATACTCGATAACACAGC
GATCAATCATGATCGCGTGTTTCTTCAACTTATTGTACATCACATCGGCCATACTGGGCATTTGGAGC
TTTACATTGCCCCAA
+
FFFFFFFFFFFFFFFFFFFFFFFF,FFF::FFFFFFFFFFFFFFFFFFFFFFFFFFFFFFFFFFFFFFFF
FFFFFFFFFFFFFFFFFFFFFFFFFFFFFFFFFFFFFFFFF:FFFF:FFFFFFFFFFFFFFFFFFFFFF,
F::FFFFFFFFFFFF
@A00721:190:HGJW7DSXY:4:2325:31114:32957 2:N:0:TCTGCAAG+GCTGCACT
GTGCACCTGGATCCTAACGGATCCCTCACTATTAATCGGACACTGGTGTCGATACTCGATAACACAGC
GATCAATCATGATCGCGTGTTTCTTCAACTTATTGTACATCACATCGGCCATACTGGGCATTTGGAGC
TTTACATTGCCCCAA
+
FFFFFFFFFFFFFFFFFFFFFFFFFFFFFFFFFFFFFFFFFFFFFFFFFFFFFFFFFFFFFFFFFFFFFF
FFFFFFFFFFFFFFFFFFFFFFFFFFFFFFFFFFFFFFFFFFFFFFFFFFFFFFFFFFFFFFFFFFFFFF
FFFFFFFFFFFFFFF
@A00721:190:HGJW7DSXY:4:1402:20482:12696 2:N:0:TCTGCAAG+GCTGCACT
GCTCCAAATGCCCAGTATGGCCGATGTGATGTACAATAAGTTGAAGAAACACGCGATCATGATTGATC
GCTGTGTTATCGAGTATCGACACCAGTGTCCGATTAATAGTGAGGGATCCGTTAGGATCCAGGTGCAC
GATAGTCGAATGATG
+
FFFFFFFFFFFFFFFFFFFFFFFFFFFFFFFFFFFFFFFFFFFFFFFFFFFFFFFFFFFFFFFFFFFFFF
FFFFFFFFFFFFFFFFFFFFFFFFFFFFFFFFFFFFFFFFFFFFFFFFFFFFFF::FFFFFFFFFFFFFF
FFFFFFFFFFFFFFF
@A00721:190:HGJW7DSXY:4:1648:31403:1485 2:N:0:TCTGCAAG+GCTGCACT
GCTCCAAATGCCCAGTATGGCCGATGTGATGTACAATAAGTTGAAGAAACACGCGATCATGATTGATC
GCTGTGTTATCGAGTATCGACACCAGTGTCCGATTAATAGTGAGGGATCCGTTAGGATCCAGGTGCAC
GATAGTCGAATGATG
+
FFFFFFFFFFFFFFFFFFFFFFFFFFFFFFFFFFFF:FFFFFFFFFFFFFFFFFFFFFFFFFFFFFFFFF
FFFFFFFFFFFFFFFFFFFFFFFFFFFFFFFFFFFFFFF,FFFFFFFFFFFFF:FFFF:FFFFFFFFFFF
FFFFFFFFFFFFFFF
@A00721:190:HGJW7DSXY:4:2133:8006:28291 2:N:0:TCTGCAAG+GCTGCACT
GCTCCAAATGCCCAGTATGGCCGATGTGATGTACAATAAGTTGAAGAAACACGCGATCATGATTGATC
GCTGTGTTATCGAGTATCGACACCAGTGTCCGATTAATAGTGAGGGATCCGTTAGGATCCAGGTGCAC
GATAGTCGAATGATG
+
FFFFFFFFFFFFF:FF,FFFFFFFFFFFFFFFFFFFFFFFFFFFFFFFFFFFFFFFFFFFFFFFFFFFFF
FFFFFF:FFFFFFFFFFFFFFFFFFFFFFFFFFF:FFFFFFFFFFFFFFFFFF:FFFFFFFFFFFFFFFF
F:FFFFFFFFFFFFF
```

```
@A00721:190:HGJW7DSXY:4:2362:11894:14450 2:N:0:TCTGCAAG+GCTGCACT
GCTCCAAATGCCCAGTATGGCCGATGTGATGTACAATAAGTTGAAGAAACACGCGATCATGATTGATC
GCTGTGTTATCGAGTATCGACACCAGTGTCCGATTAATAGTGAGGGATCCGTTAGGATCCAGGTGCAC
GATAGTCGAATGATG
+
FFFFFFFFFFFFFFFFFFFFFFFFFFFFFFFFFFFFFFFFFFFFFFFFFFFFFFFFFFFFFFFFFFFF
FFFFFFFFFFFFF,FFFFFFFFFFFFFFFFFFFFFFFFFFFFFFFFFFFFFFFFFFFFFFFFFFFFFF
FFFFFFFFFFFFFFF
@A00721:190:HGJW7DSXY:4:1523:24316:13291 2:N:0:TCTGCAAG+GCTGCACT
GCCCAGTATGGCCGATGTGATGTACAATAAGTTGAAGAAACACGCGATCATGATTGATCGCTGTGTTA
TCGAGTATCGACACCAGTGTCCGATTAATAGTGAGGGATCCGTTAGGATCCAGGTGCACGATAGTCGA
ATGATGGGAACAGCG
+
FFFFFFF,FFFFFFFFFFFFFFFFFFFFFFFFFFFFFFFFFFFFFFFFFFFFFFFFFFFFFFFFFFFF
FFFFFFFFFFFFFFFFFFFFFFFFFFFFFF:FFF:FFFFFFFFFFFFFFFFFFFFFFFFFFFFFFF:F
FFFFFFFFFFFFFFF
@A00721:190:HGJW7DSXY:4:2304:2600:23469 2:N:0:TCTGCAAG+GCTGCACT
GCCCAGTATGGCCGATGTGATGTACAATAAGTTGAAGAAACACGCGATCATGATTGATCGCTGTGTTA
TCGAGTATCGACACCAGTGTCCGATTAATAGTGAGGGATCCGTTAGGATCCAGGTGCACGATAGTCGA
ATGATGGGAACAGCG
+
FFFFFFF:FFFFFFFFFFFFFFFFFFFFFFFFFFFFFFFFFFFFFFFFFFFFFFFFFFFFFFFFFFFF
FFFFFFFFFFFFFFFFFFFFFFFF:FFFFFFFFFFFFFFFFFFFFFFFFFFFFFFFFFFFFFFFFFFF
FFFFFFFFF,FFFF:
@A00721:190:HGJW7DSXY:4:2523:20961:17848 2:N:0:TCTGCAAG+GCTGCACT
GCCCAGTATGGCCGATGTGATGTACAATAAGTTGAAGAAACACGCGATCATGATTGATCGCTGTGTTA
TCGAGTATCGACACCAGTGTCCGATTAATAGTGAGGGATCCGGTAGGATCCAGGTGCACGATAGTCGA
ATGATGGGAACAGCG
+
FFFFFFF,FFFFFFFFFFFFFFFFFFFFFFFFFFFFFFFFFFFFFFFFFFFFFFFFFFFFFFFFFFFF
F:FFFFFFFFFFFF:FFFFFFFFFFFFFFFFFFFFFFFFFFF,FFFFFFFFFFFF:FFFFFFFFFFF:
:FFFFFFFFFFFFFF
@A00721:190:HGJW7DSXY:4:1317:32027:23797 2:N:0:TCTGCAAG+GCTGCACT
TCGACTATCGTGCACCTGGATCCTAACGGATCCCTCACTATTAATCGGACACTGGTGTCGATACTCGA
TAACACAGCGATCAATCATGATCGCGTGTTTCTTCAACTTATTGTACATCACATCGGCCATACTAGAT
+
FFFFFFFFFFFFFFFFFFFFFFFFFFFFFFFFFFFFFFFFFFFFFFFFFFFFFFFFFFFFFFFFFFFF
FFFFFFFFFFFFFFFFFFFFFFFFFFFFFFFFFFFF:FFFFFFFFFFFFFFFFFFFFFFFFFFFFFFF
@A00721:190:HGJW7DSXY:4:1425:6153:31532 2:N:0:TCTGCAAG+GCTGCACT
TCGACTATCGTGCACCTGGATCCTAACGGATCCCTCACTATTAATCGGACACTGGTGTCGATACTCGA
TAACACAGCGATCAATCATGATCGCGTGTTTCTTCAACTTATTGTACATCACATCGGCCATACTAGAT
+
FFFFFFFFFFFFFFFFFFFFFFFFFFFFFFFFFFFFFFFFFFFFFFFFFFFFFFFFFFFFFFFFFFFF
FFFFFFFFFFFFFFFFFFFFFFFFFFFFFFFFFFFFFFFFFFFFFFFFFFFFFFFFFFF:FFFFFFF:
@A00721:190:HGJW7DSXY:4:1536:28619:15608 2:N:0:TCTGCAAG+GCTGCACT
TCGACTATCGTGCACCTGGATCCTAACGGATCCCTCACTATTAATCGGACACTGGTGTCGATACTCGA
TAACACAGCGATCAATCATGATCGCGTGTTTCTTCAACTTATTGTACATCACATCGGCCATACTAGAT
+
FFFFFFFFFFFFFFFFFFFFFFFFFFFFFFFFFFFFFFFFFFFFFFFFFFFFFFFFFFFFFFFFFFFF
FFFFFFFFFFFFFFFFFFFFFFFFFFFFFFFFFFFF:FFFFFFFFFFFFFFFFFFFFFFFFFFFFFFF
@A00721:190:HGJW7DSXY:4:1669:19352:20102 2:N:0:TCTGCAAG+GCTGCACT
TCGACTATCGTGCACCTGGATCCTAACGGATCCCTCACTATTAATCGGACACTGGTGTCGATACTCGA
TAACACAGCGATCAATCATGATCGCGTGTTTCTTCAACTTATTGTACATCACATCGGCCATACTAGAT
+
```

```
FFFFFFFFFFFFFFFFFFFFFFFFFFFFFFFFFFFFFFFFFFFFFFFFFFF:FFFFFFFFFFFFFFFF
FFFF:F::FFFFFFFFFFFFFFFFFFFF:FFFFFFFFFFFFFFFFFFFFFFFFFFFFFFFFFFFFFFF
@A00721:190:HGJW7DSXY:4:2611:13331:11365 2:N:0:TCTGCAAG+GCTGCACT
TCGACTATCGTGCACCTGGATCCTAACGGATCCCTCACTATTAATCGGACACTGGTGTCGATACTCGA
TAACACAGCGATCAATCATGATCGCGTGTTTCTTCAACTTATTGTACATCACATCGGCCATACTAGAT
+
FFFFFFFFFFFFFFFFFFFFFFFFFFFFFFFFFFFFFFFFFFFFFFFFFFFFFFFFFFFFFFFFFFFF
FFFFFFFFFFFFFFFFFFFFFFFFFFFFFFFFFFFFFFFFFFFFFFFFFFFFFFF:FFFFFFFFFFFF
@A00721:190:HGJW7DSXY:4:1141:30798:15843 2:N:0:TCTGCAAG+GCTGCACT
GCCGATGTGATGTACAATAAGTTGAAGAAACACGCGATCATGATTGATCGCTGTGTTATCGAGTATCG
ACACCAGTGTCCGATTAATAGTGAGGGATCCGTTAGGATCCAGGTGCACGATAGTCGAATGATGGGAA
CAGCGTCTAAACAAG
+
FFFFFFFFFFFFFFFFFFFFFFFFFFFF:FFF:FFFFFFFFFFFFFFFFFFFFFFFFFFFFFFFFFFF
FFFFFFFFFFFFFFFFFFFFFFFFFFFFFFF::FFFFFFFFFFFFFFFFFFFFFFF:FFFFFFFFF:FF
FFF:FFFFFFF:FFF
@A00721:190:HGJW7DSXY:4:1162:26359:12727 2:N:0:TCTGCAAG+GCTGCACT
GCCGATGTGATGTACAATAAGTTGAAGAAACACGCGATCATGATTGATCGCTGTGTTATCGAGTATCG
ACACCAGTGTCCGATTAATAGTGAGGGATCCGTTAGGATCCAGGTGCACGATAGTCGAATGATGGGAA
CAGCGTCTAAACAAG
+
FFFFFFFFFFFFFFFFFFFFFFFFFFFFFFFFFFFFFFFFFFFFFFFFFFFFFFFFFFFFFFFFFFFF
FFFFFFFFFFFFFFFFFFFFFFFFFFFFFFFFFFFFFFFFFFFFFFFFFFF:FFFFF:FFFFFFFFFF
FFFFFFFFF:FFFFF
@A00721:190:HGJW7DSXY:4:1413:22309:1454 2:N:0:TCTGCAAG+GCTGCACT
GCCGATGTGATGTACAATAAGTTGAAGAAACACGCGATCATGATTGATCGCTGTGTTATCGAGTATCG
ACACCAGTGTCCGATTAATAGTGAGGGATCCGTTAGGATCCAGGTGCACGATAGTCGAATGATGGGAA
CAGCGTCTAAACAAG
+
FFFFFFFF:FFFFF:FFFFFFFFFFFFFFFFFFF:FFFFFFFFFFFFFFFFFFFFFFFFFFFFFFFFF
FFFFFFFFFFFFFFFFFFFFFFFFF:FFFFFFFF:FFFFFFFFFFFF:FFFFFFFFFF:FFFFF:F::
FFFFFFFFFFFFFFF
@A00721:190:HGJW7DSXY:4:1459:1759:19664 2:N:0:TCTGCAAG+GCTGCACT
GCCGATGTGATGTACAATAAGTTGAAGAAACACGCGATCATGATTGATCGCTGTGTTATCGAGTATCG
ACACCAGTGTCCGATTAATAGTGAGGGATCCGTTAGGATCCAGGTGCACGATAGTCGAATGATGGGAA
CAGCGTCTAAACAAG
+
FFFFFFFFFFFFFFFFF:FFFFFFFFFFFFFFFFFFFFFFFFFFFFFFFFFFFFFFFFFFFFFF:FFF
FFFFFFFFFFFFFFFFFFFFFFFFFFFFFFFFFF:FFFFFFFFFFFFFFFFFFFFFFF:FFFFFFFF::
FFFFFFFFFFFFFFF
@A00721:190:HGJW7DSXY:4:1666:11867:12837 2:N:0:TCTGCAAG+GCTGCACT
GCCGATGTGATGTACAATAAGTTGAAGAAACACGCGATCATGATTGATCGCTGTGTTATCGAGTATCG
ACACCAGTGTCCGATTAATAGTGAGGGATCCGTTAGGATCCAGGTGCACGATAGTCGAATGATGGGAA
CAGCGTCTAAACAAG
+
FFFFF,FFFFFFFFFFFFF:FFFFFFF,F:F:FFFFFFFFFFFFFFFFFFFFFFFFFFFFFFFF:FFF
FFFFFFFFFFFFFFFFFFFFFFFFFFFFFFFFFFFFFFFFFFFFFFFFFFFFFFFFFFFFFFFFFF:F:
FF:,FFFF:F:FFF:
@A00721:190:HGJW7DSXY:4:2325:24505:35540 2:N:0:TCTGCAAG+GCTGCACT
GCCGATGTGATGTACAATAAGTTGAAGAAACACGCGATCATGATTGATCGCTGTGTTATCGAGTATCG
ACACCAGTGTCCGATTAATAGTGAGGGATCCGTTAGGATCCAGGTGCACGATAGTCGAATGATGGGAA
CAGCGTCTAAACAAG
+
FFFFFFFFFFFFFFFFFFFFFFFFFFFFFFFFFFFFFFFFFFFFFFFFFFFFFFFFFFFFFFFF:FFF
```

```
FFFFFFFFFFFFFFFFFFFFFFFFFFFFFFFFF:FFFFFFFFFFFFFFFFFF:FFFFFFFFFFFFFF:
FFFFFFFFFFFFFFF
@A00721:190:HGJW7DSXY:4:2362:5150:26725 2:N:0:TCTGCAAG+GCTGCACT
GCCGATGTGATGTACAATAAGTTGAAGAAACACGCGATCATGATTGATCGCTGTGTTATCGAGTATCG
ACACCAGTGTCCGATTAATAGTGAGGGATCCGTTAGGATCCAGGTGCACGATAGTCGAATGATGGGAA
CAGCGTCTAAACAAG
+
FFFFFFF:FF:FFFFFFFFFFFFF:FFFFFFFFFFFFFFFFFFFFFFFFFFFFFFFFFFFFFFFFFFF
FFFFFFFFFFFFFFFFFFFFFFFFFFFFFFFFFFFFFFFFFFFFFFFFFFFF:FFFFFFFFFFF:FFF
FFFFFFFFFFFFFFF
@A00721:190:HGJW7DSXY:4:2563:21531:21715 2:N:0:TCTGCAAG+GCTGCACT
GCCGATGTGATGTACAATAAGTTGAAGAAACACGCGATCATGATTGATCGCTGTGTTATCGAGTATCG
ACACCAGTGTCCGATTAATAGTGAGGGATCCGTTAGGATCCAGGTGCACGATAGACGAATGATGGGAA
CAGCGTCTAAACAAG
+
F:FFFFFFFF:FFFF,F:FFFFFFFFFF:FF:F:FFFFFFF:FFF,FFFFFFFFF:FFFFFFFFFFFF
FFFFFF:F,,FFFF:F,F,FF:FF:FFFFFFF,FFFFFFF:FFFF,FFFFFF,FF,FF:FFFFFFFF,
FF:FFFFFFFF::FF
@A00721:190:HGJW7DSXY:4:2641:31331:10504 2:N:0:TCTGCAAG+GCTGCACT
GCCGATGTGATGTACAATAAGTTGAAGAAACACGCGATCATGATTGATCGCTGTGTTATCGAGTATCG
ACACCAGTGTCCGATTAATAGTGAGGGATCCGTTAGGATCCAGGTGCACGATAGTCGAATGATGGGAA
CAGCGTCTAAACAAG
+
FFFFFFFFFFFFFFFFFFFFFFFFFFF:FFFFFFFFFFFFFFFFFF:FFFFFFFFFFFFFFFFFFFFF
FFFFFFFFFFFFFFFFFFFFFFFFFFFFFFFFFFFFFFFFFFFFFFFFFFFFFFFFFFFFF:FFFFFF
FFFFFFFFF:FFFFF
@A00721:190:HGJW7DSXY:4:1326:9507:10379 2:N:0:TCTGCAAG+GCTGCACT
TTTAGACGCTGTTCCCATCATTCGACTATCGTGCACCTGGATCCTAACGGATCCCTCACTATTAATCG
GACACTGGTGTCGATACTCGATAACACAGCGATCAATCATGATCGCGTGTTTCTTCAACTTATTGTAC
ATCACATCGG
+
FFFFF:FFFFFFFFFFFFFFFFFFFFFFFF:FFFFFFFFFFFFFFFFFFFFFFFFFFFFFFFFFFFFF
:FFFFFFFFFFFFFFFFFFFFFFFFFFF:FFFFFFFFFFFFFFFFFFFFFFF:FFFFFFFFFFFFFFF
FFFFFF:F:F
@A00721:190:HGJW7DSXY:4:1442:31024:36808 2:N:0:TCTGCAAG+GCTGCACT
TTTAGACGCTGTTCCCATCATTCGACTATCGTGCACCTGGATCCTAACGGATCCCTCACTATTAATCG
GACACTGGTGTCGATACTCGATAACACAGCGATCAATCATGATCGCGTGTTTCTTCAACTTATTGTAC
ATCACATCGG
+
FFFFFFFFFFFFFFFFFFFFFFFFFFFFFFFFFFFFFFFFFFFFFFFFFFFFFFFFFFFFFFFFFFFF
FFFFFFFFFFFFFFFFFFFFFFFFFFFFFFFFFFFFFFFFFFFFFFFFFFFFFFFFFFFFFFFFFFFF
FFFFFFFFFF
@A00721:190:HGJW7DSXY:4:2119:30834:30968 2:N:0:TCTGCAAG+GCTGCACT
TTTAGACGCTGTTCCCATCATTCGACTATCGTGCACCTGGATCCTAACGGATCCCTCACTATTAATCG
GACACTGGTGTCGATACTCGATAACACAGCGATCAATCATGATCGCGTGTTTCTTCAACTTATTGTAC
ATCACATCGG
+
FFFFF:FFFFFFFFFFFFFFFFFFFFFFFFFFFFFFFFFFFFFFFFFFFFFFFFFFFFFFFFFFFFFF
FFFFFFFFFFFFFFFFFFFFFFFFFFFFFFFFFFFFFFFFFFFFFFFFFFFFFFFFFFFFFFFFFFFF
FFFFFFFFFF
@A00721:190:HGJW7DSXY:4:2549:18530:31798 2:N:0:TCTGCAAG+GCTGCACT
TTTAGACGCTGTTCCCATCATTCGACTATCGTGCACCTGGATCCTAACGGATCCCTCACTATTAATCG
GACACTGGTGTCGATACTCGATAACACAGCGATCAATCATGATCGCGTGTTTCTTCAACTTATTGTAC
ATCACATCGG
```

```
+
F:,FFFF::FFFFFFFFFFFFFF,FFFF:,F:FFF,F,FFFFFFFFFFF:FFFFF::FFFFFFFFFFF
:FFFFFFFF:FF,F:FFFFFFFFFFFFFFFFFFFFFFFFFFF:FFFFFF,FFF:F::FFFFF,FF,FFFF
FFFFFFFFF:
@A00721:190:HGJW7DSXY:4:2644:25129:17550 2:N:0:TCTGCAAG+GCTGCACT
TTTAGACGCTGTTCCCATCATTCGACTATCGTGCACCTGGATCCTAACGGATCCCTCACTATTAATCG
GACACTGGTGTCGATACTCGATAACACAGCGATCAATCATGATCGCGTGTTTCTTCAACTTATTGTAC
ATCACATCGG
+
FFFFFFFFFFFFFFFFFFFFFFFFFFFFFFFFFFFFFFFFFFFFFFFFFFFFFFFFFFFFFFFF:FFFF
FFFFFFFFFFFFFFFFFFFFFFFFFFFFFFFFFFFFFFFFFFFFFFFFFFFFFFFFFFFFFFFFFFFFF
FFFFFFFFF:
@A00721:190:HGJW7DSXY:4:1439:14434:8954 2:N:0:TCTGCAAG+GCTGCACT
TTTAGACGCTGTTCCCATCATTCGACTATCGTGCACCTGGATCCTAACGGATCCCTCACTATTAATCG
GACACTGGTGTCGATACTCGATAACACAGCGATCAATCATGATCGCGTGTTTCTTCAACTTATTGTAC
ATCAC
+
FFFFFFFFFFFFFFFFFFFFFFFF:FFFFFFFFFFFFFFFFFFFFFFFFFFFFFFFFFFFFFFFFFFFF
FFFFFFFFFFFFFFFFFFFFFFFFFFFFFFFFFFFFFFFFFFFFF:FFFFFFFFFFFFFFFFF,FFFFF:
FF:FF
@A00721:190:HGJW7DSXY:4:1508:14823:3740 2:N:0:TCTGCAAG+GCTGCACT
TTTAGACGCTGTTCCCATCATTCGACTATCGTGCACCTGGATCCTAACGGATCCCTCACTATTAATCG
GACACTGGTGTCGATACTCGATAACACAGCGATCAATCATGATCGCGTGTTTCTTCAACTTATTGTAC
ATCAC
+
FFFFFFFFFFFFFFFFFFFFFFFFFFFFFFFFFFFFFFFFFFFFFFFFFFFFFFFFFFFFFFFFFFFFF
FFFFFFFFFFFFFFFFFF:FFFFFFFFFF:FFFFFFFFFFFFFFFFFFFFFFFFFFFFFFFFFFFFFFF
FF:FF
@A00721:190:HGJW7DSXY:4:2248:20356:31297 2:N:0:TCTGCAAG+GCTGCACT
TTTAGACGCTGTTCCCATCATTCGACTATCGTGCACCTGGATCCTAACGGATCCCTCACTATTAATCG
GACACTGGTGTCGATACTCGATAACACAGCGATCAATCATGATCGCGTGTTTCTTCAACTTATTGTAC
ATCAC
+
FFFFFFFFFFFFFFFFFFFFF:FFFFFFFFFFFFFFFFFFFFFFFFFFFFFFFFFFFFFFFFFFFFFFF
FFFFFFFFFFFFFFFFFFFFFF:FFFFF:FFFFFFFFFFFF:FFFFF,FFFFFFFFFFF:FFFFFFFFF
FF,FF
@A00721:190:HGJW7DSXY:4:2428:16649:2487 2:N:0:TCTGCAAG+GCTGCACT
TTTAGACGCTGTTCCCATCATTCGACTATCGTGCACCTGGATCCTAACGGATCCCTCACTATTAATCG
GACACTGGTGTCGATACTCGATAACACAGCGATCAATCATGATCGCGTGTTTCTTCAACTTATTGTAC
ATCAC
+
FFFFFFFFFFFFFFFFFFFFFFFFFFFFFFFFFFFFFFFFFFFFFFFFFFFFFFFFFFFFFFFFFFFFF
FFFFFFFFFFFFFFFFFFFFFFFFFFFFFFFFFFFFFFFFFFFFFFFFFFFFFFFFFFFFFFFFFFFFF
FF:FF
@A00721:190:HGJW7DSXY:4:2168:29957:3051 2:N:0:TCTGCAAG+GCTGCACT
TTTAGACGCTGTTCCCATCATTCGACTATCGTGCACCTGGATCCTAACGGATCCCTCACTATTAATCG
GACACTGGTGTCGATACTCGATAACACAGCGATCAATCATGATCGCGTGTTTCTTCAACTTATTGTAC
ATCA
+
FFFFFFFFFFFFFFFFFFFFFFFFFFFF,FFFFFFFFFFFFFFFFFFFFFFFFFFFFFFFFFFFFFFFF
FFFFFFFFFFFFFFFFFFFFFFFFFFFFFFFFFFFFFFFFFFFFFFFFFFFFFFFFFFFFFFF:FFFFFF
FFFF
@A00721:190:HGJW7DSXY:4:1351:3965:28087 2:N:0:TCTGCAAG+GCTGCACT
AACTGGCATTACGAATTCAGCTTGTTTAGACGCTGTTCCCATCATTCGACTATCGTGCACCTGGATCC
```

```
TAACGGATCCCTCACTATTAATCGGACACTGGTGTCGATACTCGATAACACAGCGATCAATCATGATC
GCGTGTTTCTTCAAC
+
FFFFFFFF:FFFFFF:FFFFF,:FFFFFFFFFFFFFFFFFFFFFFFFFFFFFFFFFFFFFFFFFFFFFFF
FFFFFFFFFFFFFFFFFFFFFFFFFFFFFFFFFF:FFFFFFFFFFFFFFFFFFFFFFFFFFFFFFFFFFF
FFFFFFFFF:FFFFF
@A00721:190:HGJW7DSXY:4:2122:27272:7952 2:N:0:TCTGCAAG+GCTGCACT
CGTAACTGGCATTACGAATTCAGCTTGTTTAGACGCTGTTCCCATCATTCGACTATCGTGCACCTGGA
TCCTAACGGATCCCTCACTATTAATCGGACACTGGTGTCGATACTCGATAACACAGCGATCAATCATG
ATCGCGTGTTTCTTC
+
FFFFFFFFFFFFFFFFFFFFFFFFF:FFFFFFFFFFFFFFFFFFFFFFFFFFFFFFFFFF:FFFFFFFFF
FFF::FF:FFFFFFFFFFFFFFFFFFFFFFFFFFFFFFF:FF:FFFFFFFFFF:FFFF:FF:FF,FFFFF
FFFFFFFFFFFFFFF
@A00721:190:HGJW7DSXY:4:2122:30273:8265 2:N:0:TCTGCAAG+GCTGCACT
CGTAACTGGCATTACGAATTCAGCTTGTTTAGACGCTGTTCCCATCATTCGACTATCGTGCACCTGGA
TCCTAACGGATCCCTCACTATTAATCGGACACTGGTGTCGATACTCGATAACACAGCGATCAATCATG
ATCGCGTGTTTCTTC
+
FFFFFFFFFFFFFFFFFFFFFFFFFFFFFFFFFFFFFFFFFFFFFFFFFFFFFFFFFFFFFFFFFFFFFF
FFFFFFFFFFFFFFFFFFFFFFFFFFFFFFFFFFFFFFFFFFFFFFFFFFFFFFFFFFFF:FFFFFFFFF
FFFFFFFFFFFFFFF
@A00721:190:HGJW7DSXY:4:2161:1877:36683 2:N:0:TCTGCAAG+GCTGCACT
CGTAACTGGCATTACGAATTCAGCTTGTTTAGACGCTGTTCCCATCATTCGACTATCGTGCACCTGGA
TCCTAACGGATCCCTCACTATTAATCGGACACTGGTGTCGATACTCGATAACACAGCGATCAATCATG
ATCGCGTGTTTCTTC
+
FFFFFFFFFFFFFFFFFFFFFFFFF:FFFFFFFFFFFFFFFFFFFFFFFFFFFFFFFFFFFFFFFFFFFF
FFFF:FFFFFFFFFFFFFFFFFFFFFFFFFFFFFFFFFFFFFFFFFFFFFFFFFFFFFFFFFFFFFFFFF
FFFFFFFFFFFFFFF
@A00721:190:HGJW7DSXY:4:1559:8115:13291 2:N:0:TCTGCAAG+GCTGCACT
GTTACACGTAACTGGCATTACGAATTCAGCTTGTTTAGACGCTGTTCCCATCATTCGACTATCGTGCA
CCTGGATCCTAACGGATCCCTCACTATTAATCGGACACTGGTGTCGATACTCGATAACACAGCGATCA
ATCATGATCGCGTGT
+
FFFFFFFFFFFFFFFFFFFFFFFFFFFFFFFFFFFFFFFFFFFFFFFFFFFFFFFFFFFFFFFFFFFFFF
FFFFFFFFFFFFFFFFFFFFFFFFFFFFFFFFFFFFFFFFFFFFFFFFFFFFFFFFFFFFFFFFFFFFFF
FFFFFFFFF:FFFFF
@A00721:190:HGJW7DSXY:4:1671:29686:31955 2:N:0:TCTGCAAG+GCTGCACT
GTTACACGTAACTGGCATTACGAATTCAGCTTGTTTAGACGCTGTTCCCATCATTCGACTATCGTGCA
CCTGGATCCTAACGGATCCCTCACTATTAATCGGACACTGGTGTCGATACTCGATAACACAGCGATCA
ATCATGATCGCGTGT
+
FFFFFFFFFFFFFFFFFFFFFFFFFFFFFFFFFFFFFFFFFFFFFFFFFFFFFFFFFFFFFFFFFFFFFF
FFFFFFFFFFFFFFFFFFFFFFFFFFFFFFFFFFFFFFFFFFFFFFFFFFFFFFFFFFFFFFFFFFFFFF
FFFFFFFFFFFFFFF
@A00721:190:HGJW7DSXY:4:2168:15799:20776 2:N:0:TCTGCAAG+GCTGCACT
GTTACACGTAACTGGCATTACGAATTCAGCTTGTTTAGACGCTGTTCCCATCATTCGACTATCGTGCA
CCTGGATCCTAACGGATCCCTCACTATTAATCGGACACTGGTGTCGATACTCGATAACACAGCGATCA
ATCATGATCGCGTGT
+
FFFFFFFFFFFFFFFFFFFFFFFFFFFFFFFFFFFFFFFFFFFFFFFFFFFFFFFFFFFFFFFFFFFFFF
FFFFFFFFFFFFFFFFFFFFFFFFFFFFFFFFFFFFFFFFFFFFFFFFF:FFFFFFFFFFFFFFFFFFFF
FFFFFFF:FFFFFFF
```

```
@A00721:190:HGJW7DSXY:4:2202:25229:15718 2:N:0:TCTGCAAG+GCTGCACT
GTTACACGTAACTGGCATTACGAATTCAGCTTGTTTAGACGCTGTTCCCATCATTCGACTATCGTGCA
CCTGGATCCTAACGGATCCCTCACTATTAATCGGACACTGGTGTCGATACTCGATAACACAGCGATCA
ATCATGATCGCGTGT
+
FFFFFFFFFFFFFFFFFFFFFFFFFFFFFFFFFFFFFFFFFFFFFFFFFFFFFFFFFFFFFFFFFFFFFF
FFFFFFFFFFFFFFFFFFFFFFFFFFFFFFFFFFFFFFFFFFFFFFFFFFFFFFFFFFFFFFFFFFFFFF
FFFFFFFFF:FFFFF
@A00721:190:HGJW7DSXY:4:2336:7916:2206 2:N:0:TCTGCAAG+GCTGCACT
GTTACACGTAACTGGCATTACGAATTCAGCTTGTTTAGACGCTGTTCCCATCATTCGACTATCGTGCA
CCTGGATCCTAACGGATCCCTCACTATTAATCGGACACTGGTGTCGATACTCGATAACACAGCGATCA
ATCATGATCGCGTGT
+
FFFFFFFFFFFFFFFFFFFF:FFF:FFFFF,FFFFF:FFFFFFFFFFFFFFFFF:FFF:FF:FFFFFF
FFF:FFFFFFF:FFFFFF,FFF,FFFFFFFF:FFF,FFF:FFF:FFFFFFFFFFF:FFFFFFFFFFFF
FFFFFFFFFFFFFFF
@A00721:190:HGJW7DSXY:4:2428:20039:20979 2:N:0:TCTGCAAG+GCTGCACT
GTTACACGTAACTGGCATTACGAATTCAGCTTGTTTAGACGCTGTTCCCATCATTCGACTATCGTGCA
CCTGGATCCTAACGGATCCCTCACTATTAATCGGACACTGGTGTCGATACTCGATAACACAGCGATCA
ATCATGATCGCGTGT
+
FFFFFFF::FFFFFFF:FFFFFFFFFFFFFFFFFFFFFFFFFFFFFFFFFFFFFFFFFFF:FFFFFFFF
FFFF,FFFFFFFF,FF:FFFFFFFFF:,FFFF,FFFFFFFFFFFFFF:FFF:FFF:FFFFF,FFFF:F
FF,FFFFFFF:FFFF
@A00721:190:HGJW7DSXY:4:2428:19524:22529 2:N:0:TCTGCAAG+GCTGCACT
GTTACACGTAACTGGCATTACGAATTCAGCTTGTTTAGACGCTGTTCCCATCATTCGACTATCGTGCA
CCTGGATCCTAACGGATCCCTCACTATTAATCGGACACTGGTGTCGATACTCGATAACACAGCGATCA
ATCATGATCGCGTGT
+
FFFFFFFFFFFFFFFFFFFFFFFFFFFFFF:FFFFFFFFFFFFFFFFFFFFFFFFFFFFFFFFFFFFFFF
FFFFFFFFFFFFFFFFFFFFFFFFFFFFFFFFFFFFFFFFFFFFFFFFFFFFFFFFFFFFFFFFFFFFFF
FFFFFFFFFFFFFFF
@A00721:190:HGJW7DSXY:4:2543:29640:24361 2:N:0:TCTGCAAG+GCTGCACT
GTTACACGTAACTGGCATTACGAATTCAGCTTGTTTAGACGCTGTTCCCATCATTCGACTATCGTGCA
CCTGGATCCTAACGGATCCCTCACTATTAATCGGACACTGGTGTCGATACTCGATAACACAGCGATCA
ATCATGATCGCGTGT
+
FFFFFFFFFFFFFFFFFFFFFFFFFFFFFFFFFFFFFFFFFFFFFFFFFFFFFFFFFFFFFFFFFF:FFF
FFFFFFFFFFFFFFFFFFFFFF:FFFFFFFFFFFFFFF:FFFFFFFFFFFFFFFFFFFFFFFFFFFFFFF
FFFFFFFFFFFF:FF
@A00721:190:HGJW7DSXY:4:2563:14687:3505 2:N:0:TCTGCAAG+GCTGCACT
GTTACACGTAACTGGCATTACGAATTCAGCTTGTTTAGACGCTGTTCCCATCATTCGACTATCGTGCA
CCTGGATCCTAACGGATCCCTCACTATTAATCGGACACTGGTGTCGATACTCGATAACACAGCGATCA
ATCATGATCGCGTGT
+
FFFFFFFFFFFFFFFFFFFFFFFFFFFFFFFFFFFFFFF:FFFFFFFFFFFFFFFFFFFFFFFFFFFFFF
FFFFFFFFFFFFFFFFFFFFFFFFFFFFFFFFFFFFFFFFFFFFFFFFFFFFFFFFFFFFFFFFFFFFFF
FFFFFFFFFFFFFFF
@A00721:190:HGJW7DSXY:4:2566:17852:23923 2:N:0:TCTGCAAG+GCTGCACT
GTTACACGTAACTGGCATTACGAATTCAGCTTGTTTAGACGCTGTTCCCATCATTCGACTATCGTGCA
CCTGGATCCTAACGGATCCCTCACTATTAATCGGACACTGGTGTCGATACTCGATAACACAGCGATCA
ATCATGATCGCGTGT
+
FFFFFFFFFFFFFFFFFFFFFFFFFFFFFFFFFFFFFFFFFFFFFFFFFFFFFFFFFFFFFFFFFFFFFF
```

FFFFFFFFFFFFFFFFFFFFFFFFFFFFFFFFFFFFFFFFFFFFFFFFFFFFFFFFFFFFFFFFFFFF
FFFFFFFFFFFFFFF
@A00721:190:HGJW7DSXY:4:2602:24451:1031 2:N:0:TCTGCAAG+GCTGCACT
GTTACACGTAACTGGCATTACGAATTCAGCTTGTTTAGACGCTGTTCCCATCATTCGACTATCGTGCA
CCTGGATCCTAACGGATCCCTCACTATTAATCGGACACTGGTGTCGATACTCGATAACACAGCGATCA
ATCATGATCGCGTGT
+
FFFFFFFFFFFFFF::F:F,FFFFFFFFFFFFFFF:FFF:FF:FFF:F:FFFFF,:FFFFFFFFFFFF
FFFFFFFFFFFFFF:FFFFFFFFFF::FFFFFFFFFFF,FF:FFFFFFFFFFFFFFFFFFF:FFFFFF
FFFFF:F:FFF:,FF
@A00721:190:HGJW7DSXY:4:2602:23421:2409 2:N:0:TCTGCAAG+GCTGCACT
GTTACACGTAACTGGCATTACGAATTCAGCTTGTTTAGACGCTGTTCCCATCATTCGACTATCGTGCA
CCTGGATCCTAACGGATCCCTCACTATTAATCGGACACTGGTGTCGATACTCGATAACACAGCGATCA
ATCATGATCGCGTGT
+
FFFFFFFFFFFFFFFFFFFFFFFFFFFFFF:FFFFFFFFFFFFFFFFFFFFF:FFFFFFFFFFFFFFF
FFFFFFFFFFFFFFFFFFFFFFFFFFFFFFFFFFFFFFFFFFFFFFFFFFFFFFFFFFFFFFFFFFFF
FFFFFFFFFFFFFFF
@A00721:190:HGJW7DSXY:4:1262:19651:31234 2:N:0:TCTGCAAG+GCTGCACT
ATCGCAGTTACACGTAACTGGCATTACGAATTCAGCTTGTTTAGACGCTGTTCCCATCATTCGACTAT
CGTGCACCTGGATCCTAACGGATCCCTCACTATTAATCGGACACTGGTGTCGATACTCGATAACACAG
CGATCAATCATGATC
+
FFFFFFFFFFFFFFFFFFFFFFFFFFFF:FFFF:FFFFFFFFFFFFFFFFFFFFFFFFFFFFFFFFFF
FFFFFFFFFFFFFFFFFFFFFFFFFFFFFFFFFFFFFFFFFFFFFFFFFFFFFFFFFFFFFFFFFFFF
FFFFFFFFFFFFFFF
@A00721:190:HGJW7DSXY:4:2108:25509:8594 2:N:0:TCTGCAAG+GCTGCACT
ATCGCAGTTACACGTAACTGGCATTACGAATTCAGCTTGTTTAGACGCTGTTCCCATCATTCGACTAT
CGTGCACCTGGATCCTAACGGATCCCTCACTATTAATCGGACACTGGTGTCGATACTCGATAACACAG
CGATCAATCATGATC
+
FFF:FFFFFFFFFFFFFFFFFFFFFFFFFFFFFFFF:FFFFFFFFFFFFFFFFFFFFFFFFFFFFFFF
FFFFFFFFFFFFFFFFFFFFFFFFFFFFFFFFFFFFFF:FFFFFFFFFFFFFFFFFFFFFFFFFFFFF
,FFFFFFFFFFFFFF
@A00721:190:HGJW7DSXY:4:2108:25120:9048 2:N:0:TCTGCAAG+GCTGCACT
ATCGCAGTTACACGTAACTGGCATTACGAATTCAGCTTGTTTAGACGCTGTTCCCATCATTCGACTAT
CGTGCACCTGGATCCTAACGGATCCCTCACTATTAATCGGACACTGGTGTCGATACTCGATAACACAG
CGATCAATCATGATC
+
FFFFFFFFFFFFFFFFFFFFFFFFFFFFFFFFFFF:,FFF,FFFFFFFFFFFFFFFFFFFFFFFFFFF
FFFFFFFFFFFFFFF,FFFFFFFFFFFFF:FFFFFFFFFFFFFFFFFFFFFFFFFFFFFFFF:F,FF,
FFFFFFFF::FFFFF
@A00721:190:HGJW7DSXY:4:2156:21712:5212 2:N:0:TCTGCAAG+GCTGCACT
ATCGCAGTTACACGTAACTGGCATTACGAATTCAGCTTGTTTAGACGCTGTTCCCATCATTCGACTAT
CGTGCACCTGGATCCTAACGGATCCCTCACTATTAATCGGACACTGGTGTCGATACTCGATAACACAG
CGATCAATCATGATC
+
FFFFFFFFFFFFFFFFFFFFFFFFFFFFFFFFFFF::FFFFFFFFFFFFFFFFFFFFFFFFFFFFFFF
FFFFFFFFFFFFFFFFFFFFFFFFFFFFFFFFFFFFFFFFFFFFFFFFFFFFFFFFFFFFFFFFFFFF
FFFFFFFFFFFFFFF
@A00721:190:HGJW7DSXY:4:2371:15420:11788 2:N:0:TCTGCAAG+GCTGCACT
ATCGCAGTTACACGTAACTGGCATTACGAATTCAGCTTGTTTAGACGCTGTTCCCATCATTCGACTAT
CGTGCACCTGGATCCTAACGGATCCCTCACTATTAATCGGACACTGGTGTCGATACTCGATAACACAG
CGATCAATCATGATC

```
+
FFFFFFFFFFFFFFFFFFFFFF:FF:FFFFFFFFFFFFF:FFFFFFFFFFFFFFFFFFFFFFFFFFFF
FFFFFFFFFFFFFFFFFFFFFFFFFFFFFFFFFFFFFFFFFFFFFFFFFFFFFFFFFFFFFFFFFFFF
FFFFFFFFFFFFFFF
@A00721:190:HGJW7DSXY:4:1239:21856:16955 2:N:0:TCTGCAAG+GCTGCACT
CAGATCGCAGTTACACGTAACTGGCATTACGAATTCAGCTTGTTTAGACGCTGTTCCCATCATTCGAC
TATCGTGCACCTGGATCCTAACGGATCCCTCACTATTAATCGGACACTGGTGTCGATACTCGATAACA
CAGCGATCAATCATG
+
FF:FFFFFFFFFFFFFFFFFFFFFFFF:FFFFFFFFFFFF:FFFFFFFFFFFFFFFFFFFFFFFFFFF
FFFFFFFFFFFFFFFFFFFFFFFFFFFFFFFFFFFFFFFFFFFFFFFFFFFFFFFFFFFFFFFFFFFF
FFFFFFFFFFFFFFF
@A00721:190:HGJW7DSXY:4:1473:30309:14340 2:N:0:TCTGCAAG+GCTGCACT
CAGATCGCAGTTACACGTAACTGGCATTACGAATTCAGCTTGTTTAGACGCTGTTCCCATCATTCGAC
TATCGTGCACCTGGATCCTAACGGATCCCTCACTATTAATCGGACACTGGTGTCGATACTCGATAACA
CAGCGATCAATCATG
+
FFFFFFFFFFFFFFFFFFFFFFFFFFFFFFF:FFFFFFFFFFFFFFFFFFFFFFFFFFFFFFFFFFFF
FFFFFFFFFFFFFFFFFFFFFFFFFFFFFFFFFFFFFFFFFFFFFFFFFFFFFFFFFFFFFFFFFFFF
FFFFFFFFFFFFFFF
@A00721:190:HGJW7DSXY:4:2436:7003:29622 2:N:0:TCTGCAAG+GCTGCACT
CAGATCGCAGTTACACGTAACTGGCATTACGAATTCAGCTTGTTTAGACGCTGTTCCCATCATTCGAC
TATCGTGCACCTGGATCCTAACGGATCCCTCACTATTAATCGGACACTGGTGTCGATACTCGATAACA
CAGCGATCAATCATG
+
FFFFFFFFFFFFFFFFFFFFFFFFFFFF,FFFFFFFFFF,:FF:FFFFFFFFFFFFFFFFFFFFFFFF
FFFFFFFFFFFFFFFFFFFF:FFFFFFFFFFFFFFFFFFFFFFFF:F:FFFFFFFFF,FFFFFFFFFF
FFFFFFFFF:FFFFF
@A00721:190:HGJW7DSXY:4:1524:21513:20682 2:N:0:TCTGCAAG+GCTGCACT
CGTCAGATCGCAGTTACACGTAACTGGCATTACGAATTCAGCTTGTTTAGACGCTGCTCCCATCATTC
GACTATCGTGCACCTGGATCCTAACGGATCCCTCACTATTAATCGGACACTGGTGTCGATACTCGATA
ACACAGCGATCAATC
+
:FFFFFFFFFFFFFFFFFFFFFFFFFFFFFFFFFFFFFFF:FFFFFFFF:FFF:FFFFFFFFFFFFFF
FFFFFFF:FFFFFFFF:FFFFFFFF:FFFF:,FFFFFFFFFFFF,FFFFF,FFFFFFFFFFFFFFFFF
FFFFFF,FF:FFFFF
@A00721:190:HGJW7DSXY:4:2153:18692:13980 2:N:0:TCTGCAAG+GCTGCACT
CGTCAGATCGCAGTTACACGTAACTGGCATTACGAATTCAGCTTGTTTAGACGCTGTTCCCATCATTC
GACTATCGTGCACCTGGATCCTAACGGATCCCTCACTATTAATCGGACACTGGTGTCGATACTCGATA
ACACAGCGATCAATC
+
FFFFFFFFFFFFFFFFFFFFFFFFF:FFFFFFFFFFFFFFFF,FFFFFFFFFFFFFFFFFFFFFFFFF
FFFFFFFFFFFFFFFFFFFFFFFFFFFFFF:FFFFFFFFFFFFFF:FFF:FFFFFFFFFFFFFFFFFF
FFFFFFFF:FFFFFF
@A00721:190:HGJW7DSXY:4:2252:21106:2409 2:N:0:TCTGCAAG+GCTGCACT
CGTCAGATCGCAGTTACACGTAACTGGCATTACGAATTCAGCTTGTTTAGACGCTGTTCCCATCATTC
GACTATCGTGCACCTGGATCCTAACGGATCCCTCACTATTAATCGGACACTGGTGTCGATACTCGATA
ACACAGCGATCAATC
+
FFFFFFFFFFFFFFF:FFFFFFFFFFFFFFF:FFFF:FFFFFFFFFFFFFFFFFFFFFF:FFF:FFFF
FFFFFFF:FFF:F:FFFFFF:FF::FFFFFFFFFFFFFFFFF,F:FFFFFF:FFFFFFFF::FFFF::
FFFFFFFF:FFFFF:
@A00721:190:HGJW7DSXY:4:2306:2510:23124 2:N:0:TCTGCAAG+GCTGCACT
CGTCAGATCGCAGTTACACGTAACTGGCATTACGAATTCAGCTTGTTTAGACGCTGTTCCCATCATTC
```

GACTATCGTGCACCTGGATCCTAACGGATCCCTCACTATTAATCGGACACTGGTGTCGATACTCGATA
ACACAGCGATCAATC
+
FF:FFFFFFFFFFFFFFFFFFFFFFFFFFFFFFFFFFFFFFF:FFFFFFFFFFFFF:FFFFF:FFFFF
FFFF:FFFFFFFFFF:FFFFFFF:FFFFFFFFFFFFF:FFFFFFFFFFFFFFFFFFFFFFFFFFFFFF
FFFFFFFFFFFFFFF
@A00721:190:HGJW7DSXY:4:2306:2528:23124 2:N:0:TCTGCAAG+GCTGCACT
CGTCAGATCGCAGTTACACGTAACTGGCATTACGAATTCAGCTTGTTTAGACGCTGTTCCCATCATTC
GACTATCGTGCACCTGGATCCTAACGGATCCCTCACTATTAATCGGACACTGGTGTCGATACTCGATA
ACACAGCGATCAATC
+
FFFF:FFFFFFFFFFFFFFFFFFFFFFFFFFFFF:FFFFFFF,FFFFFFFFFFFFF:FFFFFFFFFFF
FFFFFFFFFFFFFFFFFFFFFF:FFFFFFFFFFFFFFFFFFFFFFFFFFF,FFFFFFFFFFFFFFFFF
FFFFFFFFFFFFFFF
@A00721:190:HGJW7DSXY:4:2322:15872:31767 2:N:0:TCTGCAAG+GCTGCACT
CGTCAGATCGCAGTTACACGTAACTGGCATTACGAATTCAGCTTGTTTAGACGCTGTTCCCATCATTC
GACTATCGTGCACCTGGATCCTAACGGATCCCTCACTATTAATCGGACACTGGTGTCGATACTCGATA
ACACAGCGATCAATC
+
FFFFFFFFF:FFFFFFFFFFFFFFFFFFFFFFFF::FFFFF:F:F:FFFFFFFFFFFF:FFFFFFFFF
FFFFFFFFFFFFFFFFFFFFFF,FFFFFFF:FFFFFFFFFFFFFFFF:,FFFFFFFFFFFFFFFFFFF
FFFFFFFFFFFFFFF
@A00721:190:HGJW7DSXY:4:2645:10257:18724 2:N:0:TCTGCAAG+GCTGCACT
CGTCAGATCGCAGTTACACGTAACTGGCATTACGAATTCAGCTTGTTTAGACGCTGTTCCCATCATTC
GACTATCGTGCACCTGGATCCTAACGGATCCCTCACTATTAATCGGACACTGGTGTCGATACTCGATA
ACACAGCGATCAATC
+
FFFFFFFFFFFFFFFFFFFFFFFFFFFFFFFFFFFFFFFFFFFFFFFFFFFFFFFFFFFFFFFFFFFF
FFFFFFFFFFFFFFFFFFFFFFFFFFFFFFFFFFFFFFFFFFF,F:FFFFFFFFFFFFF,FFFFFFFF
FFFFFFFFFFFFFFF
@A00721:190:HGJW7DSXY:4:1149:29044:19727 2:N:0:TCTGCAAG+GCTGCACT
CGTAAAACGTCAGATCGCAGTTACACGTAACTGGCATTACGAATTCAGCTTGTTTAGACGCTGTTCCC
ATCATTCGACTATCGTGCACCTGGATCCTAACGGATCCCTCACTATTAATCGGACACTGGTGTCGATA
CTCGATAACACAGCG
+
FFFF:FFFFFFFFFFFFFFFFFFFFFFFFFF:FFFFFFFFFFFF:FFFFFFFFFFFFFFFFFFFFFFF
FFFFFFFFFFFFFF:FFFFFFFFFFFFFFFFFFFFFFFFFFFFFFFFFFFFFFFFFFFFFFFFFFF,F
FFFFFFFFFFFFFF:
@A00721:190:HGJW7DSXY:4:1249:1841:9878 2:N:0:TCTGCAAG+GCTGCACT
CGTAAAACGTCAGATCGCAGTTACACGTAACTGGCATTACGAATTCAGCTTGTTTAGACGCTGTTCCC
ATCATTCGACTATCGTGCACCTGGATCCTAACGGATCCCTCACTATTAATCGGACACTGGTGTCGATA
CTCGATAACACAGCG
+
FFFFFFFFFFFFFFFFFFFFFFFFFFF:FFFFFFFF:FFFFFFFFFFFFFFFFFFFFFFFFFFFFFFF
FFFFFFFFFFFFFFFFFFFFFFFFFFFFFFFFFFF,FFFFFFFFFFFFFFF:FFFFFFFFFFFFFFFF
FFFFFFFFFFFFFFF
@A00721:190:HGJW7DSXY:4:1530:3812:31548 2:N:0:TCTGCAAG+GCTGCACT
CGTAAAACGTCAGATCGCAGTTACACGTAACTGGCATTACGAATTCAGCTTGTTTAGACGCTGTTCCC
ATCATTCGACTATCGTGCACCTGGATCCTAACGGATCCCTCACTATTAATCGGACACTGGTGTCGATA
CTCGATAACACAGCG
+
FFFFFFFFFFFFFFFFFFFFFFFFFFFF:FFFFFFFFFFFFF:FFFFF:FFFFFFFFFFFFFFFFFFF
FFFFFFFFFFFFFFFFFFFFFFFFFFFFFFFFFFFFFFFFFFFFFFFFFFF:FFFFFFFFFFFFFFFF
FFFFFFFFFFFFFFF

```
@A00721:190:HGJW7DSXY:4:1530:3775:31610 2:N:0:TCTGCAAG+GCTGCACT
CGTAAAACGTCAGATCGCAGTTACACGTAACTGGCATTACGAATTCAGCTTGTTTAGACGCTGTTCCC
ATCATTCGACTATCGTGCACCTGGATCCTAACGGATCCCTCACTATTAATCGGACACTGGTGTCGATA
CTCGATAACACAGCG
+
FFFFFFFFFFFFFFFFFFFFFFFFFFFFFFFFFFFFFFFFFFFFFFFFFFFFFFFFFFFFFFFFFFFF
FFFFFFFFFFFFFFFFFFFFFFFFFFFFFFFFFFFFFFFFFFFFFFFFFFFFFFFFF:FFFFFFFFFF
FFFFFFF:FFFFFFF
@A00721:190:HGJW7DSXY:4:1656:10981:27962 2:N:0:TCTGCAAG+GCTGCACT
CGTAAAACGTCAGATCGCAGTTACACGTAACTGGCATTACGAATTCAGCTTGTTTAGACGCTGTTCCC
ATCATTCGACTATCGTGCACCTGGATCCTAACGGATCCCTCACTATTAATCGGACACTGGTGTCGATA
CTCGATAACACAGCG
+
FFFFFF:FFFFFFFFFFFFFFFFFFFFFFFFFFFFFFFFFFFFFFFFFFFFFFFFFFFFFFFFFFFFF
FFFFFFFFFFFFFFFFFFFFFFFFFFFFFFFFFFFFFFFFFFFFFFFFFFFFFFFFFFFFFFFF:FFF
FFFFFFFFFFFFFFF
@A00721:190:HGJW7DSXY:4:2144:10185:18161 2:N:0:TCTGCAAG+GCTGCACT
CGTAAAACGTCAGATCGCAGTTACACGTAACTGGCATTACGAATTCAGCTTGTTTAGACGCTGTTCCC
ATCATTCGACTATCGTGCACCTGGATCCTAACGGATCCCTCACTATTAATCGGACACTGGTGTCGATA
CTCGATAACACAGCG
+
FFFFFFFFFFFFFFFFFF:FFFFFFFFFFF:FFFFFFFFFFFFFFFFFFFFF,FFFFFFFFFF:FFFF
F:FFFFFFFFFFFFFFFFFFFFFFFFFFFF:FFFFFFFFFFFFFFFFFFFFFFFFFFFFFFFFFFFFF
FFFFFFFFFFFFFFF
@A00721:190:HGJW7DSXY:4:2323:16495:26897 2:N:0:TCTGCAAG+GCTGCACT
CGTAAAACGTCAGATCGCAGTTACACGTAACTGGCATTACGAATTCAGCTTGTTTAGACGCTGTTCCC
ATCATTCGACTATCGTGCACCTGGATCCTAACGGATCCCTCACTATTAATCGGACACTGGTGTCGATA
CTCGATAACACAGCG
+
FFFFFFFFFFFFFFFFFFFFFFFFFFFF:FFFFFFFFFFFFFFFFFFFFFFFFFFFFFFFFFFFFFFF
FFFFFFFFFF:FFFFFFFFFFFFFFFFFFF:FFFFFFFFFFFFFFFFFFFFFFFFFFF:FFFFFFFF:
FFFFFFFFFFFFFFF
@A00721:190:HGJW7DSXY:4:1574:8404:17926 2:N:0:TCTGCAAG+GCTGCACT
GTAAAACGTCAGATCGCAGTTACACGTAACTGGCATTACGAATTCAGCTTGTTTAGACGCTGTTCCCA
TCATTCGACTATCGTGCACCTGGATCCTAACGGATCCCTCACTATTAATCGGACACTGGTGTCGATAC
TCGATAACACAGC
+
FFFFFFFFFFFFFF:FFFFFFFFFFFFFFFFFFFFFFFFFFFFFFFFFFFFFFFFFFFFFFFFF:,FF
FFFFFFFFFFFFF:FFFFFFFFFFFFFFFFFFFFFFFFF:FFFFFFFFFFF:FFFFFFFF:FF:FFFF
FF:FFFFFFFFFF
@A00721:190:HGJW7DSXY:4:1129:1796:30906 2:N:0:TCTGCAAG+GCTGCACT
CTCCAACAACTTCCGTAAAACGTCAGATCGCAGTTACACGTAACTGGCATTACGAATTCAGCTTGTTT
AGACGCTGTTCCCATCATTCGACTATCGTGCACCTGGATCCTAACGGATCCCTCACTATTAATCGGAC
ACTGGTGTCGATACT
+
FFFFFFFFFFFFFFFFFFFFFFFFFFFFFFFFFFFFFFFFFFFFFFFFFFFFFFFFFFFFFF,:FFFF
FFFFFFFFFFFFFFFFFFFFFFFFFFFFFFFFFFFFFFFFFFFFFFFFFFFFFFFFFFFF:FFFFFFF
FFFFFFFFFFFFFFF
@A00721:190:HGJW7DSXY:4:1246:32832:21308 2:N:0:TCTGCAAG+GCTGCACT
CTCCAACAACTTCCGTAAAACGTCAGATCGCAGTTACACGTAACTGGCATTACGAATTCAGCTTGTTT
AGACGCTGTTCCCATCATTCGACTATCGTGCACCTGGATCCTAACGGATCCCTCACTATTAATCGGAC
ACTGGTGTCGATACT
+
FFFFFFFFFFF:FFFFFFF,FFFFFFFFFFFFFFFFF,FFFFFFFFFF:FFFFFF,FFF:F:FFFFFF
```

```
FFFFFFFFFFFFF:FFFFFFFFFFFFFFFFF:FFFFFFFFFFFFFFFFFFFFFFFFFFFFFFFFFFFFFF
FFFFFFFFFFFFFFF
@A00721:190:HGJW7DSXY:4:1278:32669:27289 2:N:0:TCTGCAAG+GCTGCACT
CTCCAACAACTTCCGTAAAACGTCAGATCGCAGTTACACGTAACTGGCATTACGAATTCAGCTTGTTT
AGACGCTGTTCCCATCATTCGACTATCGTGCACCTGGATCCTAACGGATCCCTCACTATTAATCGGAC
ACTGGTGTCGATACT
+
FFFFFFFFFFFFFFFFFFF:FFFFFFFFFFFFFFFFFFFFFFFFFFFFFFFFFFFFF:FFFFFF:FFFFF
FF:FFFFFFFFFFFFFFFFFFFFFFFFFFFFFFFFFFFFFFFFFFFFFF:FFFFFFFFFFFFFFFFFFFF
FFFFFFFFFFFFFFF
@A00721:190:HGJW7DSXY:4:1341:28185:25473 2:N:0:TCTGCAAG+GCTGCACT
CTCCAACAACTTCCGTAAAACGTCAGATCGCAGTTACACGTAACTGGCATTACGAATTCAGCTTGTTT
AGACGCTGTTCCCATCATTCGACTATCGTGCACCTGGATCCTAACGGATCCCTCACTATTAATCGGAC
ACTGGTGTCGATACT
+
FFFFFFFFFFFFFFFFFFFFFFFFFFFFFFFFFFFFFFFFFFFFFFFFFFF:FFFFFFFFFFFFFFFFFF
FFFFFFFFFFFFFFFFFFFFFFFFFFFFFFFFFFFFFFFFFFF:FFFFFFFFFFFFFF:FFFFFFFFFFF
FF:FFFFFFFFFFFF
@A00721:190:HGJW7DSXY:4:1412:8151:36307 2:N:0:TCTGCAAG+GCTGCACT
CTCCAACAACTTCCGTAAAACGTCAGATCGCAGTTACACGTAACTGGCATTACGAATTCAGCTTGTTT
AGACGCTGTTCCCATCATTCGACTATCGTGCACCTGGATCCTAACGGATCCCTCACTATTAATCGGAC
ACTGGTGTCGATACT
+
FFFFFFFFFFFFFFFFFFFFFFFFFFFFFFFFFFFFFFFFFFFFFFFFFFFFFFFFFFFFFFFFFFFFFF
F:FFFFFFFFFFFFFFFFFFFFFFFFFFFFFFFFFFFF:FFFFFFFFFFFFFFFFFFFFFFFFFFF:FFF
FFFFFF:FFFFFFFF
@A00721:190:HGJW7DSXY:4:1434:28094:29481 2:N:0:TCTGCAAG+GCTGCACT
CTCCAACAACTTCCGTAAAACGTCAGATCGCAGTTACACGTAACTGGCATTACGAATTCAGCTTGTTT
AGACGCTGTTCCCATCATTCGACTATCGTGCACCTGGATCCTAACGGATCCCTCACTATTAATCGGAC
ACTGGTGTCGATACT
+
FFFFFFFFFFFFFFFF:FFFFFFFFFFFFFFFFFFFFFFFFFFFFFFFFFFFFFFFFFFFFFFFF:FF:F
FFFFFFFFFFFFFFFFFFFFFFFFFFFFFFFFFFFFFFFFFFFFFFFFFFFFFFFFFFFFFFFFFFFFFF
FFFFFFFFFF:FFFF
@A00721:190:HGJW7DSXY:4:1464:15058:28917 2:N:0:TCTGCAAG+GCTGCACT
CTCCAACAACTTCCGTAAAACGTCAGATCGCAGTTACACGTAACTGGCATTACGAATTCAGCTTGTTT
AGACGCTGTTCCCATCATTCGACTATCGTGCACCTGGATCCTAACGGATCCCTCACTATTAATCGGAC
ACTGGTGTCGATACT
+
FFFFF:FFFFFFFFFFFFF,FFFFFFFFFFFFFFFFFFFFF:FFFFFFFFFFFFFF:FFFFFFFFFF::F
FFFFFFFFFFFFFFFFFFFFFFFFFFFFFFFFFFFFFFFFFFFFFFFFFFFFFFFF:FFFFFFFFFFFFF
FFFFFFFFFFFFFFF
@A00721:190:HGJW7DSXY:4:1527:11469:27774 2:N:0:TCTGCAAG+GCTGCACT
CTCCAACAACTTCCGTAAAACGTCAGATCGCAGTTACACGTAACTGGCATTACGAATTCAGCTTGTTT
AGACGCTGTTCCCATCATTCGACTATCGTGCACCTGGATCCTAACGGATCCCTCACTATTAATCGGAC
ACTGGTGTCGATACT
+
FFFFFFFFFFFFFFFFFFFFFFFFFFFFFFF::FFFFFFFFFFFFFFFFFFFFFFFFFFFFFFFFFFFFF
FFFFFFFFFFFFFFFFFFFFFFFFFFFFFFFFFFFFFF:FFFFF,FFFFFFF:FFFFFFFFFFFFFFFFF
:FFFFFFFFFFFFFF
@A00721:190:HGJW7DSXY:4:1606:26087:15107 2:N:0:TCTGCAAG+GCTGCACT
CTCCAACAACTTCCGTAAAACGTCAGATCGCAGTTACACGTAACTGGCATTACGAATTCAGCTTGTTT
AGACGCTGTTCCCATCATTCGACTATCGTGCACCTGGATCCTAACGGATCCCTCACTATTAATCGGAC
ACTGGTGTCGATACT
```

```
+
FFFFFFFFFFFFFFFFFFF:FFFFFFFFFFFFFFFFFFFFFFFFFFFFFFFFFFFFFFFFFFFFFFFF
FFFFFFFFFFFFFFFFFFFFFFFFFFFFFFFFFFFFFFFFFFFFFFFFFFFFFFFFFFFFFFFF:FFF
FFFFFFFFFFFFFFF
@A00721:190:HGJW7DSXY:4:2116:25536:36855 2:N:0:TCTGCAAG+GCTGCACT
CTCCAACAACTTCCGTAAAACGTCAGATCGCAGTTACACGTAACTGGCATTACGAATTCAGCTTGTTT
AGACGCTGTTCCCATCATTCGACTATCGTGCACCTGGATCCTAACGGATCCCTCACTATTAATCGGAC
ACTGGTGTCGATACT
+
FFFFF:FFFFFFFFFFFFF:FFFFFFFFFFFFFFFFFFFFFFFFFFFFFFF,FFFFF:,FFFF:FFFF
FFF:F,FFFFFF:FFFFFF:FFFFFFF:FF:FFFF::FFF,:FFFFFFFF:FFFFFFFF:FFFF::FF
FFFF::FFF:FFFFF
@A00721:190:HGJW7DSXY:4:2177:31864:12837 2:N:0:TCTGCAAG+GCTGCACT
CTCCAACAACTTCCGTAAAACGTCAGATCGCAGTTACACGTAACTGGCATTACGAATTCAGCTTGTTT
AGACGCTGTTCCCATCATTCGACTATCGTGCACCTGGATCCTAACGGATCCCTCACTATTAATCGGAC
ACTGGTGTCGATACT
+
FFFFFFFFFFFFFFFFFFFFFFFFFFFFFFFFFFFFFFFFFFFFFFFFFFFFFFFFFFFFFFFFFFFF
FFFFFFFFFFFFFFFFFFFFFFFFFFFFFFFFFFFFFFFFFFFFFFFFFFFFFFFFFFFFF:FFFFFF
FFFFFFFFFFFFFFF
@A00721:190:HGJW7DSXY:4:2277:24695:27414 2:N:0:TCTGCAAG+GCTGCACT
CTCCAACAACTTCCGTAAAACGCCAGATCGCAGTTACACGTAACTGGCATTACGAATTCAGCTTGTTT
AGACGCTGTTCCCATCATTCGACTATCGTGCACCTGGATCCTAACGGATCCCTCACTATTAATCGGAC
ACTGGTGTCGATACT
+
FFFFFFFFFFFFFFFFF:FFFF,FFFFFFFFFFFFFFFFFFFFFFFFFFFFFFFFFFFFFFF,FFFFF
FFFFFFFFFFFFFFFFFFFFFFFFFFFFFFFFFFFFFFFFFFFFFFFFFFFFFFFFF:FFFFFFFFFF
FFFFFFFFF:FFFFF
@A00721:190:HGJW7DSXY:4:2322:3540:23594 2:N:0:TCTGCAAG+GCTGCACT
CTCCAACAACTTCCGTAAAACGTCAGATCGCAGTTACACGTAACTGGCATTACGAATTCAGCTTGTTT
AGACGCTGTTCCCATCATTCGACTATCGTGCACCTGGATCCTAACGGATCCCTCACTATTAATCGGAC
ACTGGTGTCGATACT
+
FF:,FFFFFFFFF:FFFFFFFFFFFFFFFFFF:FFFFFFFFFFF:FFFFFFFFFFF:FFFF::FFFF,
F:FF:FFFFFF:FFFFFF,FFFF:FFFFF,FF,FF:FFFFFFFF,F:FF,FFFFFFFF::FFFFFFFF
FF,::FFFF:FFFFF
@A00721:190:HGJW7DSXY:4:2375:26368:14716 2:N:0:TCTGCAAG+GCTGCACT
CTCCAACAACTTCCGTAAAACGTCAGATCGCAGTTACACGTAACTGGCATTACGAATTCAGCTTGTTT
AGACGCTGTTCCCATCATTCGACTATCGTGCACCTGGATCCTAACGGATCCCTCACTATTAATCGGAC
ACTGGTGTCGATACT
+
FFFFFFFFFFFFFFFFFFFFFFFFFFFFFFFFFFFFFFFFFFFFFFFFFFFFFFFFFFFFFF:FFFFF
FFFFFFFFFFFFFFFFFFFFFFFFFFFFFFFFFFFFFFFFFFFFFFFFFFFFFFFFFFFFFFFFFFFF
FFFFFFFFFFFFFFF
@A00721:190:HGJW7DSXY:4:2411:23439:2347 2:N:0:TCTGCAAG+GCTGCACT
CTCCAACAACTTCCGTAAAACGTCAGATCGCAGTTACACGTAACTGGCATTACGAATTCAGCTTGTTT
AGACGCTGTTCCCATCATTCGACTATCGTGCACCTGGATCCTAACGGATCCCTCACTATTAATCGGAC
ACTGGTGTCGATACT
+
FFFFFFFFFFFFFFFFFFFFFFFFFFFFFFFFFFFFFFFFFFFFFFFFFFFFFFFFFFFFFFFFFFFF
FFFFFFFFFFFFFFFFFFFFFFFFFFFFFFFFFFFF:FFFFFFFFFFFFFFFFFFFFFFFFFFFFFFF
FFFFFFFFFFFFFFF
@A00721:190:HGJW7DSXY:4:2555:3378:19022 2:N:0:TCTGCAAG+GCTGCACT
CTCCAACAACTTCCGTAAAACGTCAGATCGCAGTTACACGTAACTGGCATTACGAATTCAGCTTGTTT
```

AGACGCTGTTCCCATCATTCGACTATCGTGCACCTGGATCCTAACGGATCCCTCACTATTAATCGGAC
ACTGGTGTCGATACT
+
FFFFFFFFFFFFFFFFFFF:FFFFFFFFFFFFFFFFFFFFFFFFFFFF:FFFFFFFFFFFFFF:F,FF
FF:FFFFFFFFFFFFFFFFFFFFFFFFFFFFFFFFFFFFFFF:FFFFFFFFFFFFFFFFFFFFFFFFF
FFFFFFFFFFFFFFF
@A00721:190:HGJW7DSXY:4:2422:25563:8813 2:N:0:TCTGCAAG+GCTGCACT
CAACAACTTCCGTAAAACGTCAGATCGCAGTTACACGTAACTGGCATTACGAATTCAGCTTGTTTAGA
CGCTGTTCCCATCATTCGACTCTCGTGCACCTGGATCCTAACGGATCCCTCACTATTAATCGGACCGA
TCGGAGGAGCGTCG
+
FFFFFFFFFFFFFFFFFFFFFFFFFFFFFF,FFF,FFFFFFFFFFFFFFFFF,F,F,F:F:FFFFFFF
FF,,FFFFFF,FFFFFFF:FF,FFFFFFFFFFFFFFFF,:::FF:FFFFFF::::FFF,F,FF,,,F,
:FFF,,F,FFFF:F
@A00721:190:HGJW7DSXY:4:1122:2998:11130 2:N:0:TCTGCAAG+GCTGCACT
TCAGACGATGCAGAACTCCAACAACTTCCGTAAAACGTCAGATCGCAGTTACACGTAACTGGCATTAC
GAATTCAGCTTGTTTAGACGCTGTTCCCATCATTCGACTATCGTGCACCTGGATCCTAACGGATCCCT
CACTATTAATCGGAC
+
FFFFFFFFF:FFFFFFFFFFFFFFFFFFFFFFFFFFFFFFFFFFFFFFFFFFFFFF:FFFFFFFFFFF
FF,FFFFFF,:FF,FFFFFFFFFFFFFFFFFFFFFFFFFFFFFFFFFFFFFFFFFFFFFFFFFFFFFF
FFFFFFFFFFFFFFF
@A00721:190:HGJW7DSXY:4:1152:21640:20776 2:N:0:TCTGCAAG+GCTGCACT
TCAGACGATGCAGAACTCCAACAACTTCCGTAAAACGTCAGATCGCAGTTACACGTAACTGGCATTAC
GAATTCAGCTTGTTTAGACGCTGTTCCCATCATTCGACTATCGTGCACCTGGATCCTAACGGATCCCT
CACTATTAATCGGAC
+
FFFFFFFFFFFFFFFFFFFFFFFFFFFFFFFFFFFFFFFFFFFFFFFFFFFFFFFFFFFFFFFFFFFF
FFFFFFFFFFFFFFFFFFFFFFFFFFFFFFFFFFFFFFFFFFFFFFFFFFFFFFF:FFFFFFFFFFFF
FFFFFFFFFFFFFFF
@A00721:190:HGJW7DSXY:4:1351:29704:11005 2:N:0:TCTGCAAG+GCTGCACT
TCAGACGATGCAGAACTCCAACAACTTCCGTAAAACGTCAGATCGCAGTTACACGTAACTGGCATTAC
GAATTCAGCTTGTTTAGACGCTGTTCCCATCATTCGACTATCGTGCACCTGGATCCTAACGGATCCCT
CACTATTAATCGGAC
+
FFFFFFFFFFFFFFFFFFFFFFFFFFFFFFFFFF:FFFFFFFFFFFFFFFFF:FFFFFFFFFFFFFFF
FFFFFFFFFFFFFFFFFFFFFFFFFFFFFFFFFFFFFFFFFF:FF:FFFFFFFFFFFFFFFFFFFFFF
FFFFFFFFFFFFFFF
@A00721:190:HGJW7DSXY:4:1474:31819:13730 2:N:0:TCTGCAAG+GCTGCACT
TCAGACGATGCAGAACTCCAACAACTTCCGTAAAACGTCAGATCGCAGTTACACGTAACTGGCATTAC
GAATTCAGCTTGTTTAGACGCTGTTCCCATCATTCGACTATCGTGCACCTGGATCCTAACGGATCCCT
CACTATTAATCGGAC
+
FFFFFFFFFFFFFFFFFFFFFFFFFFFFFFFFFFFFFFFFFFFFFFFFFFFF:FFFFFFFFFFFFFFF
FFFFFFFFFF:FFFFFFFFFFFFFFFFFFFFFFFFFFFFFFFFFFFFFFFFFFFFFFFFFFFFFFFFF
FFFFFFFFFFFFFFF
@A00721:190:HGJW7DSXY:4:1538:20021:19038 2:N:0:TCTGCAAG+GCTGCACT
TCAGACGATGCAGAACTCCAACAACTTCCGTAAAACGTCAGATCGCAGTTACACGTAACTGGCATTAC
GAATTCAGCTTGTTTAGACGCTGTTCCCATCATTCGACTATCGTGCACCTGGATCCTAACGGATCCCT
CACTATTAATCGGAC
+
FFFFFFFFFFFFFFFFFFFFFFFFFFFFFFFFFFFFFFFFFFFFFFFFFFFFFFFFFFFFFFFFFFFF
FFFFFFFFFFFFFFFFFFFFFFFFFFFFFFFFFFFFFF:FFFFFFFFFFFFFFFFFFFFFFFFFFFFF
FFFFFFFFFFFFFFF

```
@A00721:190:HGJW7DSXY:4:1559:11369:5588 2:N:0:TCTGCAAG+GCTGCACT
TCAGACGATGCAGAACTCCAACAACTTCCGTAAAACGTCAGATCGCAGTTACACGTAACTGGCATTAC
GAATTCAGCTTGTTTAGACGCTGTTCCCATCATTCGACTATCGTGCACCTGGATCCTAACGGATCCCT
CACTATTAATCGGAC
+
FFFFFFFFFFFFFFFFFFFFFFFFFFFFFFFFFFF,FFFFFFFFFFFFFFFFFFFFFFFFFFFFFFFFFF
FFFFFFFFFFFFFFFFFFFFFFFFFFFFFFFFFFFFFFFFFFFFFFFFFFFFFFFFFFFFFFFFFFFFFF
FFFFFF:FFFFFFFF
@A00721:190:HGJW7DSXY:4:2178:12689:17957 2:N:0:TCTGCAAG+GCTGCACT
TCAGACGATGCAGAACTCCAACAACTTCCGTAAAACGTCAGATCGCAGTTACACGTAACTGGCATTAC
GAATTCAGCTTGTTTAGACGCTGTTCCCATCATTCGACTATCGTGCACCTGGATCCTAACGGATCCCT
CACTATTAATCGGAC
+
FFFFFFFFFFFFFFFFFFFFFFFFFFFFFFFFFFFFFFFFFFFFFFFFFFFFFFFFFFFFFFFFFFFFFF
FFFFFFFFFF::FFFFFFFFFFFFFFFFFFFFFFFFFFFFFFFFFFFFFFFFFFFFFFFFFFFFFFFFFF
FFFFF:FFFFFFFFF
@A00721:190:HGJW7DSXY:4:2178:12183:18239 2:N:0:TCTGCAAG+GCTGCACT
TCAGACGATGCAGAACTCCAACAACTTCCGTAAAACGTCAGATCGCAGTTACACGTAACTGGCATTAC
GAATTCAGCTGGTTTAGACGCTGTTCCCATCATTCGACTATCGTGCACCTGGATCCTAACGGATCCCT
CACTATTAATCGGAC
+
FFFFFFFFFFFFFFFFFFFFFFFFFFFFFFFFFFFFFFFFFFFFFFFFFFFFFF:FFFFFFFFFFFFFFF
FFF::FFF:F,FFFFFFFFFFFFFFFFFFFFFFFFFFFFFFFFFFFFFFFFFFFFFFFFFFFFFFFFFFF
FFFFFFF:FFFFFFF
@A00721:190:HGJW7DSXY:4:2178:10791:22874 2:N:0:TCTGCAAG+GCTGCACT
TCAGACGATGCAGAACTCCAACAACTTCCGTAAAACGTCAGATCGCAGTTACACGTAACTGGCATTAC
GAATTCAGCTTGTTTAGACGCTGTTCCCATCATTCGACTATCGTGCACCTGGATCCTAACGGATCCCT
CACTATTAATCGGAC
+
FFFFFFFFFFFFFFFFFFFFFFFFFFFFFFFFFFF:FFFFFFFFFFFFFFFFFFFFFFFFFFFFFFFFFF
FFFFFFFFFFFFFFFFFFFFFFFFFFFFFFFFFFFFFFFFFFFFFFFFFFFFFFFFFFFFFFFFFFFFFF
FFFFF:FFFFFFFFF
@A00721:190:HGJW7DSXY:4:2178:10484:23312 2:N:0:TCTGCAAG+GCTGCACT
TCAGACGATGCAGAACTCCAACAACTTCCGTAAAACGTCAGATCGCAGTTACACGTAACTGGCATTAC
GAATTCAGCTTGTTTAGACGCTGTTCCCATCATTCGACTATCGTGCACCTGGATCCTAACGGATCCCT
CACTATTAATCGGAC
+
FFFFFFFFFFFFFFFFFFFFFFFFFFFFFFFFFFF,FFFFFFFFFFFFFFFFFFFFFFFFFFFFFFFFFF
FFFFFFFFFFFFFFFFFFFFFFFFFFFFFFFFFFFFFFFFFFF:FFFFFFFFFFFFFFFFFFFFFFF:FF
FFFFFFFFFFF::FF
@A00721:190:HGJW7DSXY:4:2450:24071:20791 2:N:0:TCTGCAAG+GCTGCACT
TCAGACGATGCAGAACTCCAACAACTTCCGTAAAACGTCAGATCGCAGTTACACGTAACTGGCATTAC
GAATTCAGCTTGTTTAGACGCTGTTCCCATCATTCGACTATCGTGCACCTGGATCCTAACGGATCCCT
CACTATTAATCGGAC
+
FF:F:FFFFFFFFFFFFFFFFFFFFF:FFFFFFFF:FFFFFFFFFFFFFFFFFFFFFFFFFFFFFFFFFF
FFFFFFFFF,:FFFFFFFFFFFFFFFFFFF:FFFFFFFFFFFFFFFFFFFFFFFFFFFFFFFFFFFFFFF
FFFFFFF,FFFFFFF
@A00721:190:HGJW7DSXY:4:2631:30535:12790 2:N:0:TCTGCAAG+GCTGCACT
TCAGACGATGCAGAACTCCAACAACTTCCGTAAAACGTCAGATCGCAGTTACACGTAACTGGCATTAC
GAATTCAGCTTGTTTAGACGCTGTTCCCATCATTCGACTATCGTGCACCTGGATCCTAACGGATCCCT
CACTATTAATCGGAC
+
FFFFFFFFFFFFFFFFFFFFFFFFFFFFFFFFFFF,FFFFFFFFFFFFFFFFFFFFFFFFFFFFFFFFFF
```

```
FFFFFFFFF,FFFFFFF:FFFFFFFF:FFFFFFFFFFFFFFFFFFFFFFFFFFFFFFFFFFF:FFFFF
FFFFFFFFFFFFFFF
@A00721:190:HGJW7DSXY:4:1306:24731:5368 2:N:0:TCTGCAAG+GCTGCACT
CATCTTCCAAGGCATCTCATCAGACGATGCAGAACTCCAACAACTTCCGTAAAACGTCAGATCGCAGT
TACACGTAACTGGCATTACGAATTCAGCTTGTTTAGACGCTGTTCCCATCATTCGACTATCGTGCACC
TGGATCCTAACGGAT
+
FFFFFFFFFFFFFFFFFFFFFFFFFFFFFFFFFFFFFFFFFFFFFFFFFFFFFFFFFFFFFFFFFFFF
FFFFFFFFFFFFFFFFFFFFFFFFFFFFF,FFFFFFFFFFFFFFFFFFFFFFFFFFFFFFFFFFF:FF
FFFFFFFFFFFFFF:
@A00721:190:HGJW7DSXY:4:1344:24939:10739 2:N:0:TCTGCAAG+GCTGCACT
ATCTTCCAAGGCATCTCATCAGACGATGCAGAACTCCAACAACTTCCGTAAAACGTCAGATCGCAGTT
ACACGTAACTGGCATTACGAATTCAGCTTGTTTAGACGCTGTTCCCATCATTCGACTATCGTGCACCT
GGATCCTAACTGAT
+
FFFFFF,FFF,FFFFFFFFFF,FF:FFFFF:FF,FF:FFFFFF,,FF:,FFFFF:FFFF,FF,FFFFF
F:,FFFFFFFF:FF,F::FFFF::FFFF:FF,FFFF:FFFFFFF:FF,FFFF:FFFFFFFFFFFFF,F
FFFFFFFFFF,FFF
@A00721:190:HGJW7DSXY:4:1417:5737:32283 2:N:0:TCTGCAAG+GCTGCACT
CATCTTCCAAGGCATCTCATCAGACGATGCAGAACTCCAACAACTTCCGTAAAACGTCAGATCGCAGT
TACACGTAACTGGCATTACGAATTCAGCTTGTTTAGACGCTGTTCCCATCATTCGACTATCGTGCACC
TGGATCCTAACGGAT
+
FFFFFFFFFFFFFFFFFFFFFFFFFFFFFFFFFFFFFFFFFFFFFFFFFFFF:FFFFFFFFFFFFFFF
FFFFFFF:FFFFFF:FFFFFFFFFFFFFFFFFFFFFFFFFFFFFFFFFFFFFFFFFFFFFFFFFFFFF
FFFFFFFFFFFFFFF
@A00721:190:HGJW7DSXY:4:1624:9968:25332 2:N:0:TCTGCAAG+GCTGCACT
CATCTTCCAAGGCATCTCATCAGACGATGCAGAACTCCAACAACTTCCGTAAAACGTCAGATCGCAGT
TACACGTAACTGGCATTACGAATTCAGCTTGTTTAGACGCTGTTCCCATCATTCGACTATCGTGCACC
TGGATCCTAACGGAT
+
FFFFFFFFFFFFFFFFFFFFFFFFFFFFFFFFFFFFFFFFFFFFFFFFFFFFFFFFFFFFFFFFFFFF
FFFFFF:F:FF:FFFFFFFFF:FFFFFF,FFFFFFFFFFFFFFFFFFFFFFFFF:FFFFFFFFFFFFF
FFFFFFFFFFF:FFF
@A00721:190:HGJW7DSXY:4:2608:22941:11882 2:N:0:TCTGCAAG+GCTGCACT
CATCTTCCAAGGCATCTCATCAGACGATGCAGAACTCCAACAACTTCCGTAAAACGTCAGATCGCAGT
TACACGTAACTGGCATTACGAATTCAGCTTGTTTAGACGCTGTTCCCATCATTCGACTATCGTGCACC
TGGATCCTAACGGAT
+
F:FFFFFFFFF:FFFFFFFFFFFFFFFFFFFFFFFFFFFFFFFFFFFFFFFFFFFFFF:FFFFFFFFF
FFFFFFFFFFFFFFFFFFFFFFFFFFFFF:FFFFFFFFFFFFFFFFFFFFF,FFFFFF:FFFFFFFFF
F:FFFFFFFFFFFFF
@A00721:190:HGJW7DSXY:4:1353:4634:22858 2:N:0:TCTGCAAG+GCTGCACT
TTAGGATCCAGGTGCACGATAGTCGAATGATGGGAACAGCGTCTAAACAAGCTGAATTCGTAATGCCA
GTTACGTGTAACTGCGATCTGACGTTTTACGGAAGTTGTTGGAGTTCTGCATCGTCTGATGAGATGCC
TTGGAAGATGTTGTA
+
:FFF:FFFFFFFFFFFFFFFFFFFFF:FFFFFFFFFFFFFFFFFFFFFFFFFFFFFFFFFFFFFFFF:
FFFFFFFFFFFFFF:FFFFFFFFFF,FFFFF:FFFF,FF,FFFFFFFFF::FFFFFFFF:FFFFFFFF
FFF::FF,,FF:FFF
@A00721:190:HGJW7DSXY:4:1632:24089:29747 2:N:0:TCTGCAAG+GCTGCACT
TTAGGATCCAGGTGCACGATAGTCGAATGATGGGAACAGCGTCTAAACAAGCTGAATTCGTAATGCCA
GTTACGTGTAACTGCGATCTGACGTTTTACGGAAGTTGTTGGAGTTCTGCATCGTCTGATGAGATGCC
TTGGAAGATGTTGTA
```

```
+
FFFFFFFFFFFFFFFFFFFFFFFFFFFFFFFFFFF:FFFFFFFFFFFFFFFFFFFFFFFFFFFFF:FF
FFFFFFFFFFFFF:FFFFFFFFFFFFFFFF:FFFFFFF,FF,FFFFFFFFFFFFFFFFFFFFFFFFFF
:FFFFFFFFFFFFFF
@A00721:190:HGJW7DSXY:4:2430:23222:14810 2:N:0:TCTGCAAG+GCTGCACT
TTAGGATCCAGGTGCACGATAGTCGAATGATGGGAACAGCGTCTAAACAAGCTGAATTCGTAATGCCA
GTTACGTGTAACTGCGATCTGACGTTTTACGGAAGTTGTTGGAGTTCTGCATCGTCTGATGAGATGCC
TTGGAAGATGTTGTA
+
FFFFFFFFFFFFFFFFFFFFFFFFFFFFFFFFFFF,FFFFFFFFFFFFFFFFFFFFFFFFFFFFFFFF
FFFFFFFFFFFFFFFFFFFFFFFFFFFFFFFFFFFFFFFFFFFFFFFFF:FFFFFFFFFFFFFFFFFF
FFFFFFFFFFFFFFF
@A00721:190:HGJW7DSXY:4:2543:11469:28870 2:N:0:TCTGCAAG+GCTGCACT
TTAGGATCCAGGTGCACGATAGTCGAATGATGGGAACAGCGTCTAAACAAGCTGAATTCGTAATGCCA
GTTACGTGTAACTGCGATCTGACGTTTTACGGAAGTTGTTGGAGTTCTGCATCGTCTGATGAGATGCC
TTGGAAGATGTTGTA
+
FFFFFFFFFFFFFFF:FF:FFFF:F:FFFFF:FF:F:FFF:FFFF:FFFFFFF:FF:FFFFFFFFFFF
FFFFFFFFFFFFF:FFFFF:FFFFF,FFFFFFFFF::F,,FFFFF,FFFFFFFFFFFFFFF,FFFFFF
FFFFFFFFFF:FFFF
@A00721:190:HGJW7DSXY:4:1116:9100:34225 2:N:0:TCTGCAAG+GCTGCACT
ATCCAGGTGCACGATAGTCGAATGATGGGAACAGCGTCTAAACAAGCTGAATTCGTAATGCCAGTTAC
GTGTAACTGCGATCTGACGTTTTACGGAAGTTGTTGGAGTTCTGCATCGTCTGATGAGATGCCTTGGA
AGATGTTGTATAGGT
+
FFFFFFFFFFFFFFFFFFFFFFFFFFFFFFFFFFFFFFFFFFFFFFFFFFFFFFFFFFFFFFFFFFFF
FFFFFFFFFFFFFFFFFFFFFFFFFFFFFFFFFFFFFFFFFFFFFFFFFFFFFFFFFFFFFFFFFFFF
FFFFFFFFFFFFFFF
@A00721:190:HGJW7DSXY:4:1163:8097:4742 2:N:0:TCTGCAAG+GCTGCACT
ATCCAGGTGCACGATAGTCGAATGATGGGAACAGCGTCTAAACAAGCTGAATTCGTAATGCCAGTTAC
GTGTAACTGCGATCTGACGTTTTACGGAAGTTGTTGGAGTTCTGCATCGTCTGATGAGATGCCTTGGA
AGATGTTGTATAGGT
+
FFFFFFFFFFFFFFFFFFFFFFFFFFFFFF:FFFFFFFFFFFFFFFFFFFFFFFFFFFFFFF:FFFFF
FFFFFFFFFFFFFFFFFFFFFFFFFFFFFFF:FFFFFFFFFFFFFFFFFFFFFFFFFFFFFFFFFFFF
FFFFFFFFFFFFFFF
@A00721:190:HGJW7DSXY:4:1501:12192:34976 2:N:0:TCTGCAAG+GCTGCACT
ATCCAGGTGCACGATAGTCGAATGATGGGAACAGCGTCTAAACAAGCTGAATTCGTAATGCCAGTTAC
GTGTAACTGCGATCTGACGTTTTACGGAAGTTGTTGGAGTTCTGCATCGTCTGATGAGATGCCTTGGA
AGATGTTGTATAGGT
+
FFFFFFFFFF:F:F:F,FFFFFFFFFFF,FFFFFFFFFFFFFFFFFFFFFFFFFF:FFFF:FFFF:FF
F,FFFFFF:FFFFFFFF::FFFFF,FFFFFF:FFFFFF,FFF:FFFFF,FF::FFFF:FF::,F:,FF
FFFF::F,FF:FF,F
@A00721:190:HGJW7DSXY:4:2129:14335:33489 2:N:0:TCTGCAAG+GCTGCACT
ATCCAGGTGCACGATAGTCGAATGATGGGAACAGCGTCTAAACAAGCTGAATTCGTAATGCCAGTTAC
GTGTAACTGCGATCTGACGTTTTACGGAAGTTGTTGGAGTTCTGCATCGTCTGATGAGATGCCTTGGA
AGATGTTGTATAGGT
+
FFFFFFFFFFFFFF:FFFFFFFFFFFFFFFFFFFFFFFFFFFFFFFFFFFFFFFFF:FFFFFFFFFFF
FFFFFFFFFFFFFFFFFFFFFFFFFFFFFF,FF:FFFFFFFFFFFFFFFFFFFFFFFFFFFFFFF:FF
FFFFFFFFFFFFFFF
@A00721:190:HGJW7DSXY:4:2323:10131:33379 2:N:0:TCTGCAAG+GCTGCACT
ATCCAGGTGCACGATAGTCGAATGATGGGAACAGCGTCTAAACAAGCTGAATTCGTAATGCCAGTTAC
```

GTGTAACTGCGATCTGACGTTTTACGGAAGTTGTTGGAGTTCTGCATCGTCTGATGAGATGCCTTGGA
AGATGTTGTATAGGT
+
FFFFFFFFFFFFFFFFFFFFFFFFFFFFFFFFFFFFFFFFFFFFFFFFFFFFF:FFFFFFFFFFFFFFFF
FFFF:FFFFFFFFFFFFFFFFFFFFFFFFFFFF:FFFFFFFFFFFFFFFFFFFFF:FFFFFFFFFFFFF
F:FFFFFFFFFFFFF
@A00721:190:HGJW7DSXY:4:2537:6361:8093 2:N:0:TCTGCAAG+GCTGCACT
ATCCAGGTGCACGATAGTCGAATGATGGGAACAGCGTCTAAACAAGCTGAATTCGTAATGCCAGTTAC
GTGTAACTGCGATCTGACGTTTTACGGAAGTTGTTGGAGTTCTGCATCGTCTGATGAGATGCCTTGGA
AGATGTTGTATAGGT
+
FFFF:FFFFFFFFFFFFFFFFFFFFFFFFFFFFFFFFFFF:FFFFFFFFFF:FFFFFFFFFFFFFFFFFF
F,FFFFFFFFFFFFFFFFFFFFFFFFFFFFF:FFF:FFFFFFFFFFFFFFFFFFFFFFFFFFFFFFFFFF
FFFFFFFFFFFFFFF
@A00721:190:HGJW7DSXY:4:1105:6578:8625 2:N:0:TCTGCAAG+GCTGCACT
CAGCGTCTAAACAAGCTGAATTCGTAATGCCAGTTACGTGTAACTGCGATCTGACGTTTTACGGAAGT
TGTTGGAGTTCTGCATCGTCTGATGAGATGCCTTGGAAGATGTTGTATAGGTTGGAAAATTCCAACCT
CAAAACAGGTACTCT
+
FFFFFFFFFFFFFFFFFFFFFFFFFFFFFFFFFFFFFFFFFFFFFFFFFFFFFFFFFFFFFFFFFFFFFF
FF:FFFFFFFFFFFFFFFFFFFFFFFFFFFFFFFFFFFFFFFFFFFFFFFFFFFFFFFFFFFFFFFFFFF
FFFFFFFFFFFFFFF
@A00721:190:HGJW7DSXY:4:1251:7093:19727 2:N:0:TCTGCAAG+GCTGCACT
CAGCGTCTAAACAAGCTGAATTCGTAATGCCAGTTACGTGTAACTGCGATCTGACGTTTTACGGAAGT
TGTTGGAGTTCTGCATCGTCTGATGAGATGCCTTGGAAGATGTTGTATAGGTTGGAAAATTCCAACCT
CAAAACAGGTACTCT
+
F:FFFFFFFFFFFFFFFFFFFFFFFFFFFFFFFFFFFFFFFFFFFFFFFFFFFFFFFFFFFFFFFFFFFF
FFF:FFFFFFFFFFFFFFFFFFFFFFFF:FFFFFFF:FFFFFFFFFFFFFFFFFFFFFFFFFFFFFFFFF
F:FFFFFFFFFFFFF
@A00721:190:HGJW7DSXY:4:1269:23782:27117 2:N:0:TCTGCAAG+GCTGCACT
CAGCGTCTAAACAAGCTGAATTCGTAATGCCAGTTACGTGTAACTGCGATCTGACGTTTTACGGAAGT
TGTTGGAGTTCTGCATCGTCTGATGAGATGCCTTGGAAGATGTTGTATAGGTTGGAAAATTCCAACCT
CAAAACAGGTACTCT
+
FFFFFFFFFFFFFF:FFFFFF:FFFFFFFFFFFFFFFFFFFFFFFFFFFFF:FFFFFFFFFFFFFFF,F:
FFF:FFFFFFFFFFFFFFFFFFFFFFFFFFFFFFFFFFFFFFF,FFFFFFFFFFFFFFFFFFFFFF,FFF
FFFFFFFF:F:FFFF
@A00721:190:HGJW7DSXY:4:1342:23818:34256 2:N:0:TCTGCAAG+GCTGCACT
CAGCGTCTAAACAAGCTGAATTCGTAATGCCAGTTACGTGTAACTGCGATCTGACGTTTTACGGAAGT
TGTTGGAGTTCTGCATCGTCTGATGAGATGCCTTGGAAGATGTTGTATAGGTTGGAAAATTCCAACCT
CAAAACAGGTACTCT
+
FFFFFFFFFFFFFFFFFFFFFFFFFFFFFFFFFFFFFF:FFFF:FFFFFFFFFF::FFFFFFF:FFFFFF
FFFFFFFFFFFFFFFFFFFFFFFFFF:FFFFFFFFFFFFFFFFFFFFFFF:FFFFFFFFFFFFFFFFFFF
FFFFFFF:FFFFFFF
@A00721:190:HGJW7DSXY:4:2410:24876:21057 2:N:0:TCTGCAAG+GCTGCACT
CAGCGTCTAAACAAGCTGAATTCGTAATGCCAGTTACGTGTAACTGCGATCTGACGTTTTACGGAAGT
TGTTGGAGTTCTGCATCGTCTGATGAGATGCCTTGGAAGATGTTGTATAGGTTGGAAAATTCCAACCT
CAAAACAGGTACTCT
+
FFFFFFFF:FFF,FFFFFFFFFFFFFFFFFFFFFFFFFFFFFFFFFFFFFFFFFFFFFFFFFFF:FF:FF
FFFFFFFFFFFFFFFFFFFFFFFFFFFFFFFFFFFF:FFFF:FFFFFFFFFFFFFFFFFFFFFFFFFFFF
FFFFFFF:FFFFFF:

```
@A00721:190:HGJW7DSXY:4:2511:18701:25614 2:N:0:TCTGCAAG+GCTGCACT
CAGCGTCTAAACAAGCTGAATTCGTAATGCCAGTTACGTGTAACTGCGATCTGACGTTTTACGGAAGT
TGTTGGAGTTCTGCATCGTCTGATGAGATGCCTTGGAAGATGTTGTATAGGTTGGAAAATTCCAACCT
CAAAACAGGTACTC
+
FFFFFFF::FFF:FFFFFFFFFFFFFFFFF:FFFFFFFFFFFFFFFFFFFF:FFFFFFFFFFFFFFFFF,
,F,FFFFFFFFFFFFFFFFFFFFFFFFFFFFFFFFFFFFFFFFFFFFFFFFFFF:FF::FFFFFFFFFFF
FFF,FFFFFFFFFF
@A00721:190:HGJW7DSXY:4:2615:26078:32816 2:N:0:TCTGCAAG+GCTGCACT
CAGCGTCTAAACAAGCTGAATTCGTAATGCCAGTTACGTGTAACTGCGATCTGACGTTTTACGGAAGT
TGTTGGAGTTCTGCATCGTCTGATGAGATGCCTTGGAAGATGTTGTATAGGTTGGAAAATTCCAACCT
CAAAACAGGTACTCT
+
FFFFFFFFFFFFFFFFFFFFFFFFFFFFFFFFFFFFFFFFFFFFFFFFFFFFFFFFFFFFFFFFFFFFFF
FFFFFFFFFFFFFFFFFFFFFFFFFFFFFFFFFFFFFFFFFFFFFFFFFFFFFFFFFFFFFFFFFFFFFF
FFFFFFFFFFFFFFF
@A00721:190:HGJW7DSXY:4:2676:22092:5932 2:N:0:TCTGCAAG+GCTGCACT
CAGCGTCTAAACAAGCTGAATTCGTAATGCCAGTTACGTGTAACTGCGATCTGACGTTTTACGGAAGT
TGTTGGAGTTCTGCATCGTCTGATGAGATGCCTTGGAAGATGTTGTATAGGTTGGAAAATTCCAACCT
CAAAACAGGTACTCT
+
FFFFFFFFFFFFFFFFFFFFFFFFFFFFFFFFFFFFFFFFFFFFFFFFFFFFFFFFFFFFFFFFFFFFFF
FF,FFFFFFFFFFFFFFFFFFFFFFFFFFFFFFFFFFFFFFFFFFFFFFF:FFFFFFFFFFFFFFFFFFF
FFF:FFFFFFFFFF:
@A00721:190:HGJW7DSXY:4:1118:3667:12696 2:N:0:TCTGCAAG+GCTGCACT
AAACAGAGTACCTGTTTTGAGGTTGGAATTTTCCAACCTATACAACATCTTCCAAGGCATCTCATCAG
ACGATGCAGAACTCCAACAACTTCCGTAAAACGTCAGATCGCAGTTACACGTAACTGGCATTACGAAT
TCAGCTTGTTTAGAC
+
FFFFFFFFFFFFFFFFFFFFFFFFFFFFFFFFFFFFFFFFFFFFFFFFFFFFFFFFFFFFFFFFFFFFFF
FFFFFFFFFFFFFFFFFFFFFFFFFFFFFFFFFFFFFFFFFFFFFFFFFFFFFFFFFFFFFFFFFFFFFF
FFFFFFFFFFFFFFF
@A00721:190:HGJW7DSXY:4:2105:19217:28479 2:N:0:TCTGCAAG+GCTGCACT
AAACAGAGTACCTGTTTTGAGGTTGGAATTTTCCAACCTATACAACATCTTCCAAGGCATCTCATCAG
ACGATGCAGAACTCCAACAACTTCCGTAAAACGTCAGATCGCAGTTACACGTAACTGGCATTACGAAT
TCAGCTTGTTTAGAC
+
FFFFFFFFFFFFFFFFFFFFFFFFFFFFFFFFFFFFFFFFFFFFFFFFFFFFFFFFFFFFFFFFFFFFFF
FFFFFFFFFFFFFFFFFFFFFF:FFFFFFF:FFFFFFFFFFFFFFFFFFFFFFFFFFFFFFFFFFF:FFF
FFFF:FFFFFFFFFF
@A00721:190:HGJW7DSXY:4:2350:13910:18724 2:N:0:TCTGCAAG+GCTGCACT
AAACAGAGTACCTGTTTTGAGGTTGGAATTTTCCAACCTATACAACATCTTCCAAGGCATCTCATCAG
ACGATGCAGAACTCCAACAACTTCCGTAAAACGTCAGATCGCAGTTACACGTAACTGGCATTACGAAT
TCAGCTTGTTTAGAC
+
FFFFFFFFFFFFFFF:FFFFFFFFFFFFFFFFFFFFFFFFFFFFFFFFFFFFFFFFFFFFFFFFFFFFFF
FFFFFFFFFFFFFFFFFFFFFFFFFFFFFFFFFFFFFFFFFFFFFFFFFFFFFFFFFFFFFFFFFFFFFF
FFFFFFFFFFFFFFF
@A00721:190:HGJW7DSXY:4:2576:32850:21903 2:N:0:TCTGCAAG+GCTGCACT
AAACAGAGTACCTGTTTTGAGGTTGGAATTTTCCAACCTATACAACATCTTCCAAGGCATCTCATCAG
ACGATGCAGAACTCCAACAACTTCCGTAAAACGTCAGATCGCAGTTACACGTAACTGGCATTACGAAT
TCAGCTTGTTTAGAC
+
FFFFFFFFFFFFFFF:FFFFFFFFFFFFFFFFFFFFFFFFFFFFFFFFFFFFFFFFFFFFFFFFFFFFFF
```

```
FFFFFFFFF:FFFFFFFFFFFFFFFFFFFFFFFFFFFFFFFFFF:FFFFFFFFFFFFFFFF:FFFFFF
FFFFFFFFFFFFFFF
@A00721:190:HGJW7DSXY:4:2564:30463:35524 2:N:0:TCTGCAAG+GCTGCACT
CACATAGCTTCAGACGTCCTTTGATTCTACAAAACAGAGTACCTGTTTTGAGGTTGGAATTTTCCAAC
CTATACAACATCTTCCAAGGCATCTCATCAGACGATGCAGAACTCCAACAACTTCCGTAAAACGTCAG
ATCGCAGTTACACGT
+
FFFFFFFFFFFFF:FFFFF:FFFFFFFFFFFFFFFFFFFFFFFFFFFFFFFFFFFFFFFFFFFFFFFF
FFFFFFFFFFFFFFFFFFFFFFFFFFFFFFFFFFFFFFFF:FFFFFFFFFFFFFFFFFFFFFFFFF:F
FFFFFFFFFFFFFFF
@A00721:190:HGJW7DSXY:4:1402:16152:14215 2:N:0:TCTGCAAG+GCTGCACT
AAGCACATAGCTTCAGACGTCCTTTGATTCTACAAAACAGAGTACCTGTTTTGAGGTTGGAATTTTCC
AACCTATACAACATCTTCCAAGGCATCTCATCAGACGATGCAGAACTCCAACAACTTCCGTAAAACGT
CAGATCGCAGTTACA
+
FFFFFFFFFFFFFFFFFFF:FF:FFFF:FFFFFFFFFFFFFFFFFFFFF,F:FFFFFFFFFF,FF:FFF
FFFFF,FFFFFFFFFFFFFF:FFFFFFFFFFFFFFFFFFFFFFFFFFFFFFFFFFFFFFFF,FFFFF:F
F:FFFFFFFFFFFFF
@A00721:190:HGJW7DSXY:4:1406:7057:10989 2:N:0:TCTGCAAG+GCTGCACT
AAGCACATAGCTTCAGACGTCCTTTGATTCTACAAAACAGAGTACCTGTTTTGAGGTTGGAATTTTCC
AACCTATACAACATCTTCCAAGGCATCTCATCAGACGATGCAGAACTCCAACAACTTCCGTAAAACGT
CAGATCGCAGTTACA
+
FFFFFFFFFFFFFFFFFFFFFFFFFFFFFFFFFFFFFFFFFFFFFFFFFFFFFFFFFFFFFFFFFFFF
F,FFFFFFFFFFFFFFF:FFFFFFFFFFF:FFFFFFFFFFFFFFFFFFFFFFFFFFFFFFFFFF:FFFF
FFFFFF:F,FFFFFF
@A00721:190:HGJW7DSXY:4:1451:30816:7576 2:N:0:TCTGCAAG+GCTGCACT
AAGCACATAGCTTCAGACGTCCTTTGATTCTACAAAACAGAGTACCTGTTTTGAGGTTGGAATTTTCC
AACCTATACAACATCTTCCAAGGCATCTCATCAGACGATGCAGAACTCCAACAACTTCCGTAAAACGT
CAGATCGCAGTTACA
+
FFFFFFFFFFFFFFFFFFFFFFFFFFFFFFFFFFFFFFFFFFFFFFFFFFFFFFFFFFFFFFFFFFFF
FFFFFFFFFFFFFFFFFFFFFFFFFFFFFFFFFFFFFFFFFFFFFFFFFFFFFFFFFFFFFFFFFFFF
FFFFFFFFFFFFFFF
@A00721:190:HGJW7DSXY:4:1534:24560:35383 2:N:0:TCTGCAAG+GCTGCACT
AAGCACATAGCTTCAGACGTCCTTTGATTCTACAAAACAGAGTACCTGTTTTGAGGTTGGAATTTTCC
AACCTATACAACATCTTCCAAGGCATCTCATCAGACGATGCAGAACTCCAACAACTTCCGTAAAACGT
CAGATCGCAGTTACA
+
FFFFFFFFFFFFFFFFFFFFFFFFFFFFFFFFFFFFFFFFFFFFFFFFFFFFFFFFFFFFFFFFFFFF
FFFFFFFFFFFFFFFFFFFFFFFFFFFFFFF:FFFFFFFFFFFFFFFFFFFFFFFFFFFFFFFFF:FFF
FFFFFF:FFFFFFFF
@A00721:190:HGJW7DSXY:4:2372:15591:8296 2:N:0:TCTGCAAG+GCTGCACT
AAGCACATAGCTTCAGACGTCCTTTGATTCTACAAAACAGAGTACCTGTTTTGAGGTTGGAATTTTCC
AACCTATACAACATCTTCCAAGGCATCTCATCAGACGATGCAGAACTCCAACAACTTCCGTAAAACGT
CAGATCGCAGTTACA
+
FFFFFFFFFFFFFFFFFFFFFFFFFFFFFFFFFFFFFFFFFFFFFFFFFFFFFFFFFFFFFFFFFFFF
FFFFFFFFFFFFFFFFFFFFFFFFFFFFFFFFFFFFFFFFFFFFFFFFFFFFFFFFFFFFFFFFFFFF
FFFFFFFFFFFFFFF
@A00721:190:HGJW7DSXY:4:1125:15944:21402 2:N:0:TCTGCAAG+GCTGCACT
CCTCAACGCGCTTCGAAGCACATAGCTTCAGACGTCCTTTGATTCTACAAAACAGAGTACCTGTTTTG
AGGTTGGAATTTTCCAACCTATACAACATCTTCCAAGGCATCTCATCAGACGATGCAGAACTCCAACA
AC
```

```
+
FFFFFFFFFFFFFFFFFFFFFFFFFFFFFFFFFFF:FFFFFFFFFFFFFFFFFF:FFFFFFFFFFFFFFF
FFFFFFFFFFFFFFFFFFFFFFFFFFFFFFFFFFFFFFFFFFFFFFFFFFFFFFFFFFFFFFFFFFFFFF
FF
@A00721:190:HGJW7DSXY:4:1453:31060:17487 2:N:0:TCTGCAAG+GCTGCACT
CCTCAACGCGCTTCGAAGCACATAGCTTCAGACGTCCTTTGATTCTACAAAACAGAGTACCTGTTTTG
AGGTTGGAATTTTCCAACCTATACAACATCTTCCAAGGCATCTCATCAGACGATGCAGAACTCCAACA
AC
+
FFFFFFFFFFFFFFFFFFFFFFFFFFFFFFFFFFFFFFFFFFFFFFFFFFFFFFFFFFFFFFFFFFFFFF
FFFFFFFFFFFFFFFFFFFFFFFFFFFFFFFFFFFFFFFFFFFF:F:FFFFFFFFFFFFFFFFFFFFFFF
FF
@A00721:190:HGJW7DSXY:4:1611:2844:17754 2:N:0:TCTGCAAG+GCTGCACT
CCTCAACGCGCTTCGAAGCACATAGCTTCAGACGTCCTTTGATTCTACAAAACAGAGTACCTGTTTTG
AGGTTGGAATTTTCCAACCTATACAACATCTTCCAAGGCATCTCATCAGACGATGCAGAACTCCAACA
AC
+
FFFFFFFFFFFFFFFFFFFFFFFFFFFFF,FFFFFFFFFFFFFFFFFFFFFFFFFFFFFFFFFFFF:FFF
FFFFFFFFFFFFFFFFFFFF:FFFFFFFFFFFFFFFFFFFFFFFFFFFFFFF:FFFF,FFFFFFFFFFF:
F:
@A00721:190:HGJW7DSXY:4:2115:14968:5462 2:N:0:TCTGCAAG+GCTGCACT
CCTCAACGCGCTTCGAAGCACATAGCTTCAGACGTCCTTTGATTCTACAAAACAGAGTACCTGTTTTG
AGGTTGGAATTTTCCAACCTATACAACATCTTCCAAGGCATCTCATCAGACGATGCAGAACTCCAACA
AC
+
FFFFFFFFFFFFFF:FFFFFFFFFFFFFFFFFFFFFFFF:FFFFFFFFFFFFFFFFFFFFFFFFF,FFFF
F:FFFFFFFFFFFFFFFFFFFFFFFFFFFFFFFFFFFFFFFF:F,FFFFFFFFFFFF:FFFFFFFF:FFF
FF
@A00721:190:HGJW7DSXY:4:2135:5547:3302 2:N:0:TCTGCAAG+GCTGCACT
CCTCAACGCGCTTCGAAGCACATAGCTTCAGACGTCCTTTGATTCTACAAAACAGAGTACCTGTTTTG
AGGTTGGAATTTTCCAACCTATACAACATCTTCCAAGGCATCTCATCAGACGATGCAGAACTCCAACA
AC
+
FFFFFFFFFFFFFFFFFFFFFFFFFFFFFFFFFFFFFFFFFFFFFFFFFFFFFFFFFFFFFFFFF:FFFF
FFFFFFFFFFFFFFFFFFFFFFFFFFFFFFFFFFFFFFFFFFFFFFFFFFFFFFFFFFFFF:FFFFFFFF
F:
@A00721:190:HGJW7DSXY:4:2518:9317:11115 2:N:0:TCTGCAAG+GCTGCACT
CCTCAACGCGCTTCGAAGCACATAGCTTCAGACGTCCTTTGATTCTACAAAACAGAGTACCTGTTTTG
AGGTTGGAATTTTCCAACCTATACAACATCTTCCAAGGCATCTCATCAGACGATGCAGAACTCCAACA
AC
+
FFFFFFFFFFFFFFFFFFFFFFFFFFFFFFFFFFFFFFFFFFFFFFFFFFFF:FFFF:FFFFFFFFF:FF
FFFFFFFFFFFFFFFFFFFFFFFFFFFFFFFFFFFFFFFFFFFFFFFFFFFFFFFFFFFFFFFFFFFFFF
FF
@A00721:190:HGJW7DSXY:4:1317:20202:26240 2:N:0:TCTGCAAG+GCTGCACT
TCCTGGGACTCCTGAACTCAATCTCCTCAACGCGCTTCGAAGCACATAGCTTCAGACGTCCTTTGATT
CTACAAAACAGAGTACCTGTTTTGAGGTTGGAATTTTCCAACCTATACAACATCTTCCAAGGCATCTC
ATCAGACGATGCAGA
+
FFFFFFFFFFFFFFFFFFFFFFFFFFFFFFFFFFFFFFFFFFFFFFFFFFFFFFFFFFFFFFFFFFFFFF
FFFFFFFFFFFFFFFFFFFF:FFFFFFFFFFFFFFFFFFFFFFFFFFFFFFFFFFFFFFFFFFFFFFFFF
FFFFFFFFFFFFFFF
@A00721:190:HGJW7DSXY:4:1375:32488:6621 2:N:0:TCTGCAAG+GCTGCACT
TCCTGGGACTCCTGAACTCAATCTCCTCAACGCGCTTCGAAGCACATAGCTTCAGACGTCCTTTGATT
```

```
CTACAAAACAGAGTACCTGTTTTGAGGTTGGAATTTTCCAACCTATACAACATCTTCCAAGGCATCTC
ATCAGACGATGCAGA
+
FFFFFFFFFFFFFFF:FFFFFFFFFFFFFFFFFFFFFFFF:FFFFFFFFFFFFFFFFFFFF,FFFFFFF
FFFFFFFFFFFFFFFFFFF:F,FF:F:FF:FFFFFFFFFFFFFFFFFFFFFFFFFFFFFFFFFFFFFFF
FFFFFFFFFFFF:FF
@A00721:190:HGJW7DSXY:4:1376:17671:7232 2:N:0:TCTGCAAG+GCTGCACT
TCCTGGGACTCCTGAACTCAATCTCCTCAACGCGCTTCGAAGCACATAGCTTCAGACGTCCTTTGATT
CTACAAAACAGAGTACCTGTTTTGAGGTTGGAATTTTCCAACCTATACAACATCTTCCAAGGCATCTC
ATCAGACGATGCAGA
+
FFFFFFFFFFFFFFFFFFFFFFFFFFFFFFFFFFFFFFFFFFFFFFFFFFFFFFFFFFFFFFFFFFFF
FFFFFFFFFF:FFFFFFFFFFFFFFFFFFFFFFFFFFFFFFFFFFFFFFFFFFFFFFFFFFFFFFFFFF
FFFFFFFFFFFFFFF
@A00721:190:HGJW7DSXY:4:1619:27145:24236 2:N:0:TCTGCAAG+GCTGCACT
TCCTGGGACTCCTGAACTCAATCTCCTCAACGCGCTTCGAAGCACATAGCTTCAGACGTCCTTTGATT
CTACAAAACAGAGTACCTGTTTTGAGGTTGGAATTTTCCAACCTATACAACATCTTCCAAGGCATCTC
ATCAGACGATGCAGA
+
FFFFFFFFFFFFFFFFFFFFFFFFFFFFFFFFFFFF:FFFFFFFFFFFFFFFFFFFFFFFFFFFFF,F
FFFF:F::FFFFFFFFFFFFFFFFFFFF,FFFFFFFFFFFFFFFFFFFFFFFFFFFFFFFFFFFFFFFF
FFFFFFFFFFFFFFF
@A00721:190:HGJW7DSXY:4:2223:30535:14669 2:N:0:TCTGCAAG+GCTGCACT
TCCTGGGACTCCTGAACTCAATCTCCTCAACGCGCTTCGAAGCACATAGCTTCAGACGTCCATTGATT
CTACAAAACAGAGTACCTGTTTTGAGGTTGGAATTTTCAAACCTATACAACATCTTCAAAGGCATCTC
ATCAGACGATGAAGA
+
F:FF:FFF:F,,F,FFFFFF:FFFFFFFFFF:F,:FFF,FFFFFFFFFFFFF,FFF:FFF:,:,:FFF
FFFFFF:FFFFF:FFFFFF:,FFFF:,FF:,::FFFF:,F,,FF::F:F:,FF,FF,,FFF,,F,:,:
:FFF,FF,FFF,,FF
@A00721:190:HGJW7DSXY:4:1109:6533:12211 2:N:0:TCTGCAAG+GCTGCACT
CTCTTCGACAGGATGTTGATCCTGGGACTCCTGAACTCAATCTCCTCAACGCGCTTCGAAGCACATAG
CTTCAGACGTCCTTTGATTCTACAAAACAGAGTACCTGTTTTGAGGTTGGAATTTTCCAACCTATACA
ACATCTTCCAAGGCA
+
FFFFFF:FFFFFFFFFFFFFFFFFFFFFFFFFFFFFFFFFFFFFFFFFFFFFFFFFFFFFFFFFFFFF
F:FFFFFFFFFFFFFFFFFFFFFFFFFFFFFFFFFFFFFFFFFFFFFFFFFFFFFFFFFFFFFFFFFF
FFFFFFFFFFFFFFF
@A00721:190:HGJW7DSXY:4:1126:31991:13683 2:N:0:TCTGCAAG+GCTGCACT
CTCTTCGACAGGATGTTGATCCTGGGACTCCTGAACTCAATCTCCTCAACGCGCTTCGAAGCACATAG
CTTCAGACGTCCTTTGATTCTACAAAACAGAGTACCTGTTTTGAGGTTGGAATTTTCCAACCTATACA
ACATCTTCCAAGGCA
+
FFFFFFFFFF:FFFFFFFFFFFFFFFFFFFFFFFFFFFFFFFFFFFFFFFFFFFFFFFFFFFFFFFFF
FFFFF:FFFF:FFFFFFF:FFFFF:FFFFFFFFFFFFF,FFFFFFFFFFFFFFFFFFFFFFFFFFFFFF
FFFFFFFFFFFFFFF
@A00721:190:HGJW7DSXY:4:1228:21540:13275 2:N:0:TCTGCAAG+GCTGCACT
CTCTTCGACAGGATGTTGATCCTGGGACTCCTGAACTCAATCTCCTCAACGCGCTTCGAAGCACATAG
CTTCAGACGTCCTTTGATTCTACAAAACAGAGTACCTGTTTTGAGGTTGGAATTTTCCAACCTATACA
ACATCTTCCAAGGCA
+
FFFFFFFFFFFFFFFFFFFFFFFFFFFFFFFFFFFFFFFFFFFFFFFFFFFFFFFFFFFFFFFFFFFF
FFFFFFFFFFFFFFFFFFFFFF:FFFFFFFFFFFFFFFFFFFFFFFFFFFFFFFFFFFFFFFFFFFFF
FFFFFFFFFFFFFFF
```

```
@A00721:190:HGJW7DSXY:4:1315:8730:7498 2:N:0:TCTGCAAG+GCTGCACT
CTCTTCGACAGGATGTTGATCCTGGGACTCCTGAACTCAATCTCCTCAACGCGCTTCGAAGCACATAG
CTTCAGACGTCCTTTGATTCTACAAAACAGAGTACCTGTTTTGAGGTTGGACTTTTCCAACCTATACA
ACATCTTCCAAGGCA
+
FFFFFFFFFFFFFFFFFFFFFFFFFFFFFFFFFFFFFFFFFFFFFFFFFFFFFFFFFFFFFFFF,FFFFF
FFFFFFFFFFFFFFFFFFFFFFFFFFFFFFFFFFFFFFF:FFFFFFFFFFFFF,FFFFFFFFFFFFFFFF
FFF:FFFF:F:F::F
@A00721:190:HGJW7DSXY:4:2310:3513:4695 2:N:0:TCTGCAAG+GCTGCACT
CTCTTCGACAGGATGTTGATCCTGGGACTCCTGAACTCAATCTCCTCAACGCGCTTCGAAGCACATAG
CTTCAGACGTCCTTTGATTCTACAAAACAGAGTACCTGTTTTGAGGTTGGAATTTTCCAACCTATACA
ACATCTTCCAAGGCA
+
FFF,FFFFFFFFF:FFFFFFFFFFFFFFFFFFFFFFFFFFFFFFFFFFFFFFFFFFFFFFFF:FFFFFFF
FFFFFFFFFFFF:FFFFFFFFFF:FFFFFFFFFFFFFFFF,FFFFFF:FFFFFFFFFFFFFFFFFFFFFF
FFFFFFFFFFF,FFF
@A00721:190:HGJW7DSXY:4:2355:30219:20134 2:N:0:TCTGCAAG+GCTGCACT
CTCTTCGACAGGATGTTGATCCTGGGACTCCTGAACTCAATCTCCTCAACGCGCTTCGAAGCACATAG
CTTCAGACGTCCTTTGATTCTACAAAACAGAGTACCTGTTTTGAGGTTGGAATTTTCCAACCTATACA
ACATCTTCCAAGGCA
+
FFFFFFFFFFFFFFFF:FFFFFFFFFFFFFFFFFFFFFFFFFFFFFFFFFFFFFFFFFFFFFFFFFF:FF
FFFFFFFF:FFFF,FFFFFFFFFFFFFFFFFFFFFFFFFFFFFFFFFFFFFFFF::FFFFF,FFFFFFFF
FFFF:FFFFFFFFFF
@A00721:190:HGJW7DSXY:4:2456:32759:26913 2:N:0:TCTGCAAG+GCTGCACT
CTCTTCGACAGGATGTTGATCCTGGGACTCCTGAACTCAATCTCCTCAACGCGCTTCGAAGCACATAG
CTTCAGACGTCCTTTGATTCTACAAAACAGAGTACCTGTTTTGAGGTTGGAATTTTCCAACCTATACA
ACATCTTCCAAGGCA
+
FFFFFFFFFFFFFFFFFFFFFFFFFFFFFFFFFFFFFFFFFFFFFFFFFFFFFFFFFFFFFFF:FFFFFF
FFFFFFFFFFFFFFFFFFFFFFFFFFFFFFFFFFF,FFFFFFFFFF:FFF:FFFFF:F:FFFFFFFFFFF
FFFF:FF:,FF,FFF
@A00721:190:HGJW7DSXY:4:1153:28447:29371 2:N:0:TCTGCAAG+GCTGCACT
GCCTCTTCGACAGGATGTTGATCCTGGGACTCCTGAACTCAATCTCCTCAACGCGCTTCGAAGCACAT
AGCTTCAGACGTCCTTTGATTCTACAAAACAGAGTACCTGTTTTGAGGTTGGAATTTTCCAACCTATA
CAACATCTTCCAAGG
+
FFFFFFFFFFFFFFFFFFFFFFFFFFFFFFFFFFFFFFFFFFFFFFFFFFFFFFFFFFFFFFFFFFFFFF
FFFFFFFF:FFFFFFFFFFFFFFFFFFFFFFFFFFFFFFFFFFFFFFFFFFFFFFFFFFFFFFFFFFFFF
FFFFFFFFFFFFFFF
@A00721:190:HGJW7DSXY:4:1166:1633:15248 2:N:0:TCTGCAAG+GCTGCACT
GCCTCTTCGACAGGATGTTGATCCTGGGACTCCTGAACTCAATCTCCTCAACGCGCTTCGAAGCACAT
AGCTTCAGACGTCCTTTGATTCTACAAAACAGAGTACCTGTTTTGAGGTTGGAATTTTCCAACCTATA
CAACATCTTCCAAGG
+
FFFFFFFFFFFFFFFFFFFFFFFFFFFFFFFFFFFFFFFFFFFFFFFFFFFFFFFFFFFFFFFFFF:FFF
FFFFFFFFFFFFFF:FFFFFFFFFFFFFFFFFFFFFFFFFFFFFFFFFFFFFFFFFFFFFFFFFFFFFFF
FFFFFFFFFFFFFFF
@A00721:190:HGJW7DSXY:4:1263:6759:15421 2:N:0:TCTGCAAG+GCTGCACT
GCCTCTTCGACAGGATGTTGATCCTGGGACTCCTGAACTCAATCTCCTCAACGCGCTTCGAAGCACAT
AGCTTCAGACGTCCTTTGATTCTACAAAACAGAGTACCTGTTTTGAGGTTGGAATTTTCCAACCTATA
CAACATCTTCCAAGG
+
FFFFFFFFFFFFFFF,FFFFFFFFFFFFFFFFFFFFFFFFFFFFFFFFFFFFFFFFFFFFFFFFFFFFFF
```

FFFFFFFFFFFFFFF:FFFFFFFFFF:FFFFFFFFFFFFF,FFFFFFFFFFFF,FFFFFFFFFFF:FF
FFFFFFFFFFFFFFF
@A00721:190:HGJW7DSXY:4:2467:7509:6355 2:N:0:TCTGCAAG+GCTGCACT
GCCTCTTCGACAGGATGTTGATCCTGGGACTCCTGAACTCAATCTCCTCAACGCGCTTCGAAGCACAT
AGCTTCAGACGTCCTTTGATTCTACAAAACAGAGTACCTGTTTTGAGGTTGGACTTTTCCAACCTATA
CAACATCTTCCAAGG
+
FFFFFFFFFFFFFFFFFFFFFFFFFFFFFFFFFFFFFFFFF:FFFFFFFFFFFFFFFFFFFFFFFFFF
FFFFFFFFFFFFFFF::FFFFFFFFFFFFFFFFFFFFFFFFFFFFFFFFFFFF,FFFFFFFFFFF:FF
FFFFFFFFFFFFFFF
@A00721:190:HGJW7DSXY:4:2633:2736:17409 2:N:0:TCTGCAAG+GCTGCACT
GCCTCTTCGACAGGATGTTGATCCTGGGACTCCTGAACTCAATCTCCTCAACGCGCTTCGAAGCACAT
AGCTTCAGACGTCCTTTGATTCTACAAAACAGAGTACCTGTTTTGAGGTTGGAATTTTCCAACCTATA
CAACATCTTCCAAGG
+
FFFFFF:FFFFFFFF:FFFFFFFFFFFFFFFFFFFFFFFFFFFFFFFFF:FFFFFFFFFFFFFFFFFF
FFFFFFFFFFFFFF:F,FFFFFFFFF:FFFFFFFFFFFFFFF:FFFFFFFFFFF,F:FFFFFFFFFF:
FFFFF:FFFFF:,FF
@A00721:190:HGJW7DSXY:4:2678:12382:33332 2:N:0:TCTGCAAG+GCTGCACT
GCCTCTTCGACAGGATGTTGATCCTGGGACTCCTGAACTCAATCTCCTCAACGCGCTTCGAAGCACAT
AGCTTCAGACGTCCTTTGATTCTACAAAACAGAGTACCTGTTTTGAGGTTGGAATTTTCCAACCTATA
CAACATCTTCCAAGG
+
FFFFFFFFFFFFFFFFFFFFFFFFFFFFFFFFFFFFFFFFFFFFFFFFFFFFFFFFFFFFFFFFFFFF
FFFFFFFFFFFFFFFFFFFFFFFFFFFFFFFFFFFFFFFFF:FFFFFFFFFFFFFFFFFFFF:FFFFFF
FFFFFFFFFFFFFFF
@A00721:190:HGJW7DSXY:4:2368:25771:33661 2:N:0:TCTGCAAG+GCTGCACT
GCCTCTTCGACAGGATGTTGATCCTGGGACTCCTGAACTCAATCTCCTCAACGCGCTTCGAAGCACAT
AGCTTCAGACGTCCTTTGATTCTACAAAACAGAGTACCTGTTTTGAGGTTGGAATTTTCCAACCTATA
CAACATCTTCCAAG
+
FFFFFFFFFFFFFFFFFFFFFFFFFFFFFFFFFFFFFFFFFFFFFFFFFFFFFFFFFFFFFFFFFFFF
FFFFFFFFFFFFFFFFFFFFFFFFFFFFFFFFFFFFFFFFFFFFFFFFFFFFFFFFFFFFFFFFFFFF
FFFFFFFFFF:FFF
@A00721:190:HGJW7DSXY:4:1223:30626:30264 2:N:0:TCTGCAAG+GCTGCACT
TGTATAGGTTGGAAAATTCCAACCTCAAAACAGGTACTCTGTTTTGTAGAATCAAAGGACGTCTGAAG
CTATGTGCTTCGAAGCGCGTTGAGGAGATTGAGTTCAGGAGTCCCAGGATCAACATCCTGACGAAGAG
GCATGAAGCCAAGGA
+
F,FF,F:FFFFFFFFFFFFFFFFFFFFFFF:FFF,FF::F:,FF,FF:FFF:F,FF:F:FF,F,F:FFF
F,FF,,FFFF,FFFF:FFF::F:F:FFFF,FFF,F:FF,F:FF:FFFFFFFFFFFFFFFF,FFF:FF,
FFFF:F,F,FFFFFF
@A00721:190:HGJW7DSXY:4:1268:28131:6370 2:N:0:TCTGCAAG+GCTGCACT
TGTATAGGTTGGAAAATTCCAACCTCAAAACAGGTACTCTGTTTTGTAGAATCAAAGGACGTCTGAAG
CTATGTGCTTCGAAGCGCGTTGAGGAGATTGAGTTCAGGAGTCCCAGGATCAACATCCTGTCGAAGAG
GCATGAAGCCAAGGA
+
FFFFFFFFFFFFFFFFFFFFFFFFFFFFFFFFFFFFFFFFFF:FFFFFFFFFFFFFFFFFFFFFFFFFF
F:FFFFFFFFFFFFFFFFFFFFFFFFFFFFFFFFFFFFFFFFFFFFFFFFFFFFFFFFFFFFFFFFFFF
FFFFFFFFFFFFFFF
@A00721:190:HGJW7DSXY:4:1350:9091:1705 2:N:0:TCTGCAAG+GCTGCACT
TGTATAGGTTGGAAAATTCCAACCTCAAAACAGGTACTCTGTTTTGTAGAATCAAAGGACGTCTGAAG
CTATGTGCTTCGAAGCGCGTTGAGGAGATTGAGTTCAGGAGTCCCAGGATCAACATCCTGTCGAAGAG
GCATGAAGCCAAGGA

```
+
FFFFFFFFFFFFFFFFFFFFFFFFFFFFFFFFFFFFFFFFFFFF:FFFFFFFFFFFFFFFFFFFFFFFFFF
FFFFFFFFFFFFFFFFFFFFFFFFFFFFFFFFFFFFFFFFFFFFFFFFFFFFFFFFFFFFFFFF:FFFFF
FFFFFFFFFFFFFFF
@A00721:190:HGJW7DSXY:4:1628:16676:3443 2:N:0:TCTGCAAG+GCTGCACT
TGTATAGGTTGGAAAATTCCAACCTCAAAACAGGTACTCTGTTTTGTAGAATCAAAGGACGTCTGAAG
CTATGTGCTTCGAAGCGCGTTGAGGAGATTGAGTTCAGGAGTCCCAGGATCAACATCCTGTCGAAGAG
GCATGAAGCCAAGGA
+
FFFFFFFFFFFFFFFFFFFFFFFFFFFFFFFFFFFFFFFFFFFFF:FFFFFFFFFFFFFFFFFF:FF
FFFFFFFFFFFFF,FFFFFFFFFFFFFFFFFFFFFFFFFFFFFFFFF:FFFFFFFFFFFFFFFFF:F
FFFFFFFFFFFFFFF
@A00721:190:HGJW7DSXY:4:2277:27046:21934 2:N:0:TCTGCAAG+GCTGCACT
TGTATAGGTTGGAAAATTCCAACCTCAAAACAGGTACTCTGTTTTGTAGAATCAAAGGACGTCTGAAG
CTATGTGCTTCGAAGCGCGTTGAGGAGATTGAGTTCAGGAGTCCCAGGATCAACATCCTGTCGAAGAG
GCATGAAGCCAAGGA
+
FFFFFFFFFFFFF:FFFFFFFFFFFFFFFFFFFFF,FFF,FFFFFFFFFFFFFFFFFFFFFFFFFFFF
FF:FFFFFFFFF::FFFFF,FFFFFFFF:,FFF,FFFFFFFFFFFFFFFFFFFFFFFF:FFFFF:FFF
FFFFFFFFFFF:FFF
@A00721:190:HGJW7DSXY:4:2451:30391:6778 2:N:0:TCTGCAAG+GCTGCACT
TGTATAGGTTGGAAAATTCCAACCTCAAAACAGGTACTCTGTTTTGTAGAATCAAAGGACGTCTGAAG
CTATGTGCTTCGAAGCGCGTTGAGGAGATTGAGTTCAGGAGTCCCAGGATCAACATCCTGTCGAAGAG
GCATGAAGCCAAGGA
+
FFFFFFFFFFFF:FFFFFFFFFFFFFFFFFFFFFFFFFF:FFFFFFFFFFFFFFF:FFFFFFFFF,:F
FFFFFFFFFFFF,:FFFFFFFFFFFFFFFFF,FFFFFFFFFFFFFFFFFFFF:FFFFFFFFFFFFFFF
FF:FFFFFFF:FFFF
@A00721:190:HGJW7DSXY:4:2520:10484:1235 2:N:0:TCTGCAAG+GCTGCACT
TGTATAGGTTGGAAAATTCCAACCTCAAAACAGGTACTCTGTTTTGTAGAATCAAAGGACGTCTGAAG
CTATGTGCTTCGAGGCGCGTTGAGGAGATTGAGTTCAGGAGTCCCAGGATCAACATCCTGTCGAAGAG
GCATGAAGCCAAGGA
+
FFFFFFFFFFFFFFFFFFFFFFFFFFFFF:FFFFFFFF::FFF:FFFFFFFFFFF,FFFFFFFFFFFF
FFFFFFFFFFFFF,FFFFFFFFFFF:FFFFF,F:FFFFFFFFF:FFFFFFFFFFFFFFFFFFFFFFFF
FFF:FFFFFFFFFFF
@A00721:190:HGJW7DSXY:4:2520:10529:1344 2:N:0:TCTGCAAG+GCTGCACT
TGTATAGGTTGGAAAATTCCAACCTCAAAACAGGTACTCTGTTTTGTAGAATCAAAGGACGTCTGAAG
CTATGTGCTTCGAAGCGCGTTGAGGAGATTGAGTTCAGGAGTCCCAGGATCAACATCCTGTCGAAGAG
GCATGAAGCCAAGGA
+
FFFFFFFFFFFFFFFFFFFFFFFFFFFF:FFFFFFFFF:FFFFFFF:FFFFFFFFFFFFFFFFFFF:F
F:,FFFFFFFFFFFFFFFF,FFFFFFFF:FFFFFFFFFFFFFFFFFFFFFFFFFFFFFFFFFFFFFFF
FFFFF:FFFFFFFFF
@A00721:190:HGJW7DSXY:4:1261:30400:33974 2:N:0:TCTGCAAG+GCTGCACT
TATCTATATCCTTGGCTTCATGCCTCTTCGACAGGATGTTGATCCTGGGACTCCTGAACTCAATCTCC
TCAACGCGCTTCGAAGCACATAGCTTCAGACGTCCTTTGATTCTACAAAACAGAGTACCTGTTTTGAG
GTTGGAATTTTCCAA
+
FFFFFFFFFFFFFFFFFFFFFFFFFFFFFFFFFFFFFFFFFFFFFFFFFFFFFFFFFFFFFFFFFFFF
FFFFFFFFFFFFFFFFF:FFFFFFFFFFFFFFFFF,FFF:FFFFFFF:FFFFFFFFFFFFFFFFFFFF
FFFFFF,FFFFFFFF
@A00721:190:HGJW7DSXY:4:1452:12653:23876 2:N:0:TCTGCAAG+GCTGCACT
TATCTATATCCTTGGCTTCATGCCTCTTCGACAGGATGTTGATCCTGGGACTCCTGAACTCAATCTCC
```

TCAACGCGCTTCGAAGCACATAGCTTCAGACGTCCTTTGATTCTACAAAACAGAGTACCTGTTTTGAG
GTTGGAATTTTCCAA
+
FFFFFFFFFFFFFFFFFFFFFFFFFFFFFFFFFFFFFFFFFFFFFFFFFFFFFFFF:FFFFFFFF:FF
FFFFFFFFFFFFFFFFFFFFFF:FFFF:FFFFF:F::FFFFFFFFFFFFFFF:FFFFFFFF,FFFFFF
FFFFFFFFFFFFFFF
@A00721:190:HGJW7DSXY:4:2273:1796:4914 2:N:0:TCTGCAAG+GCTGCACT
TATCTATATCCTTGGCTTCATGCCTCTTCGACAGGATGTTGATCCTGGGACTCCTGAACTCAATCTCC
TCAACGCGCTTCGAAGCACATAGCTTCAGACGTCCTTTGATTCTACAAAACAGAGTACCTGTTTTGAG
GTTGGAATTTTCCAA
+
FFFFFFFFFFFFFFFFFFFFFFFFFFFFFFFFFFFFFFFFFFFFFFFFFFFFFFFFFFFFFFFFFFFF
FFFFFFFFFFFFFFFFFFFFFFFFFFFFFFFFFFFFFFFFFFFFFFF::FFFFFFFFFFFFFFFFFFF
FFFFFF:FFFFFFFF
@A00721:190:HGJW7DSXY:4:2273:1524:5008 2:N:0:TCTGCAAG+GCTGCACT
TATCTATATCCTTGGCTTCATGCCTCTTCGACAGGATGTTGATCCTGGGACTCCTGAACTCAATCTCC
TCAACGCGCTTCGAAGCACATAGCTTCAGACGTCCTTTGATTCTACAAAACAGAGTACCTGTTTTGAG
GTTGGAATTTTCCAA
+
FFFFFFFFFFFFFFFFFFFFFFFFFFFFFFFFFFFFFFFFFFFFFFFFFFFFFFFFFF:FFFFFFFFF
FFFFFFFFFFFFFFFFFFFFFFFFFFFFFFFFFF:FFFFFFFFFFFFFFFFFFFFFFFFFF,FFFF:F
FFFFFFFFFFFFF:F
@A00721:190:HGJW7DSXY:4:2273:1362:5071 2:N:0:TCTGCAAG+GCTGCACT
TATCTATATCCTTGGCTTCATGCCTCTTCGACAGGATGTTGATCCTGGGACTCCTGAACTCAATCTCC
TCAACGCGCTTCGAAGCACATAGCTTCAGACGTCCTTTGATTCTACAAAACAGAGTACCTGTTTTGAG
GTTGGAATTTTCCAA
+
FFFFFFFFFFFFFFFFFFFFFFFFFFFFFFFF:FFFFFFFFFFFFFFFFFFFFFFFFFFFFFFFFFFF
FFFFFFFFFFFFFFFFFFFFFFFFFFFFF:FFFFF:FFFFFFFFFFFFFFFFFFFFFFFFFFFFFFFF
FFFFFFFF:FFFFFF
@A00721:190:HGJW7DSXY:4:2665:29161:32174 2:N:0:TCTGCAAG+GCTGCACT
TATCTATATCCTTGGCTTCATGCCTCTTCGACAGGATGTTGATCCTGGGACTCCTGAACTCAATCTCC
TCAACGCGCTTCGAAGCACATAGCTTCAGACGTCCTTTGATTCTACAAAACAGAGTACCTGTTTTGAG
GTTGGAATTTTCCAA
+
FFFFFFFFFFFFFFFFFFFFFFFFFFFFFFFFFFFFFFFFFFFFFFFFFFFFFFFFFFFFFFFFFFFF
FFFFFFFFFFFFFFFFFFFFFFFFFFFFFFFFFFFFFFFFFFFFFFFFFFFFFFFFFFFFFFFFFFFF
FF:F:FFFFFF:FFF
@A00721:190:HGJW7DSXY:4:1236:9652:22185 2:N:0:TCTGCAAG+GCTGCACT
GCCACATATCTATATCCTTGGCTTCATGCCTCTTCGACAGGATGTTGATCCTGGGACTCCTGAACTCA
ATCTCCTCAACGCGCTTCGAAGCACATAGCTTCAGACGTCCTTTGATTCTACAAAACAGAGTACCTGT
TTTGAGGTTGGAATT
+
FFFFFFFFFFFFFFFFFFFFFFFFFFFFFFFFFFFFFFFFFFFFFFFFFFFFFFFFFFFFFFFFFFFF
FFFFFFFFFFFFFFFFFFFFFFFFFF:FFFFFFFFFFFFFF::FFFFFFFFFFFFFFFFFFFFFFFFF
:FFFFFF:FFFFFFF
@A00721:190:HGJW7DSXY:4:2139:1624:26882 2:N:0:TCTGCAAG+GCTGCACT
GCCACATATCTATATCCTTGGCTTCATGCCTCTTCGACAGGATGTTGATCCTGGGACTCCTGAACTCA
ATCTCCTCAACGCGCTTCGAAGCACATAGCTTCAGACGTCCTTTGATTCTACAAAACAGAGTACCTGT
TTTGAGGTTGGAATT
+
:FFFFFFFFFFFFFFFFFFFFFFFFFFFFFFFFFFFFFFFFFFFFFFFFFFFFFFFFFFFFFFFFFFF
FFFFFFFFFFFFFFFFFFFFFFFFFFFFFFFFFFFFFFFFF:FFFFFFFFFFFFF:FFFFFFFFFFFF
FFFFFFFFFFFFFFF

@A00721:190:HGJW7DSXY:4:2361:22218:32111 2:N:0:TCTGCAAG+GCTGCACT
GCCACATATCTATATCCTTGGCTTCATGCCTCTTCGACAGGATGTTGATCCTGGGACTCCTGAACTCA
ATCTCCTCAACGCGCTTCGAAGCACATAGCTTCAGACGTCCTTTGATTCTACAAAACAGAGTACCTGT
TTTGAGGTTGGAATT
+
FFFFFFFFFFFFFFFFFFFFFFFFFFFFFFFFFFFFFFFFFFFFFFFFFFFFF:FFFFFFFFFFFFFFFF
FFFFFFFFFFFFFFFFFFFFFFFFFFFFFFFFFFFFFFFFFFFFFFFFFFFFFFFFFFF:FFFFFFFFFF
,FFFFFFFFFFFFFF
@A00721:190:HGJW7DSXY:4:2610:12337:1282 2:N:0:TCTGCAAG+GCTGCACT
GCCACATATCTATATCCTTGGCTTCATGCCTCTTCGACAGGATGTTGATCCTGGGACTCCTGAACTCA
ATCTCCTCAACGCGCTTCGAAGCACATAGCTTCAGACGTCCTTTGATTCTACAAAACAGAGTACCTGT
TTTGAGGTTGGAATT
+
FFFFFFFFFFFFFFFFFFFFFFFFFF:FFFFFFFFFFFFFFFFFFFFFFFFFFFFFFFFFFFFFFFFFFF
FFFFFFFFFFFFFFFFFFFFFFFFFFFFFFFFFFFFFFFFFF:FFFFF:FFFFFFFF:FFFFFFFFFFFFF
:FFFFFFFF:FFFFF
@A00721:190:HGJW7DSXY:4:2652:4906:12430 2:N:0:TCTGCAAG+GCTGCACT
GCCACATATCTATATCCTTGGCTTCATGCCTCTTCGACAGGATGTTGATCCTGGGACTCCTGAACTCA
ATCTCCTCAACGCGCTTCGAAGCACATAGCTTCAGACGTCCTTTGATTCTACAAAACAGAGTACCTGT
TTTGAGGTTGGAATT
+
FFFFFFFFFFFFFFFFFFFFFFFFFFFFFFFFFFFFFFFFFFFFFFFFFFFFFFFFFFFFFFFFFFFFFF
,FFFFFFFFFFFFFFFFFFFFFFFFFFFFFFFFFFFFFFFFFFF,,FFFFFFFFF:FFFFFFFFFFFFFF
FFFFFFFFFFFFFFF
@A00721:190:HGJW7DSXY:4:1138:18014:32221 2:N:0:TCTGCAAG+GCTGCACT
CGTGGGTTGACTAGTGGCATATTCAACATGCCACATATCTATATCCTTGGCTTCATGCCTCTTCGACA
GGATGTTGATCCTGGGACTCCTGAACTCAATCTCCTCAACGCGCTTCGAAGCACATAGCTTCAGACGT
CCTTTGATTCTACAA
+
F:FFFFFFFFFFFFFFFFF:FFFFFFFF:FFFFFFFFFFFFFFFFFFFFF:FFFFFFFFFFFFF,FFFFF
FFFFFFFFFFFFFFFFFFFFFFFFFFFFF:FFFFFFFFFFFFFFFFFFFFFF:FFFFFFFFFFFFFFF:F
FFFFFFFFFFFFFFF
@A00721:190:HGJW7DSXY:4:2203:3079:9580 2:N:0:TCTGCAAG+GCTGCACT
CGTGGGTTGACTAGTGGCATATTCAACATGCCACATATCTATATCCTTGGCTTCATGCCTCTTCGACA
GGATGTTGATCCTGGGACTCCTGAACTCAATCTCCTCAACGCGCTTCGAAGCACATAGCTTCAGACGT
CCTTTGATTCTACAA
+
FFFFF:FFFFFFFFFFFFFFFF:FFFFFFFFFFFFFFFFFF,FFFFFFFFFFFFFFFF:FFFFFFFFFFF
FFFFFFFFFFFFFFFFFFFFFFFFFFFFFFFFFF:FFFFFFFFFFFFFFFFFFFFFFFFFFFFFFFFFF:
F:F,FFFFFFFFFFF
@A00721:190:HGJW7DSXY:4:2378:12364:12477 2:N:0:TCTGCAAG+GCTGCACT
CGTGGGTTGACTAGTGGCATATTCAACATGCCACATATCTATATCCTTGGCTTCATGCCTCTTCGACA
GGATGTTGATCCTGGGACTCCTGAACTCAATCTCCTCAACGCGCTTCGAAGCACATAGCTTCAGACGT
CCTTTGATTCTACAA
+
FFFFFFFFFFFFFFFF:FFFFFFFFFFFFFFFFFFFFFFFFFFFFFFFFFFFFFFFFFFFFFF,FFFFFF
FFFFFFFFFFFFFFFFFFFFFFFFFFFFFFFFFFFFFFFFFFFFFFFFFFFFFFFFFFFFFFFFFFFFF:
FFFFFFFFFFFFFFF
@A00721:190:HGJW7DSXY:4:1328:3233:23813 2:N:0:TCTGCAAG+GCTGCACT
GTTCAACGTGGGTTGACTAGTGGCATATTCAACATGCCACATATCTATATCCTTGGCTTCATGCCTCT
TCGACAGGATGTTGATCCTGGGACTCCTGAACTCAATCTCCTCAACGCGCTTCGAAGCACATAGCTTC
AGACGTCCTTTGATT
+
FFFFFFFF:FFFFFFFFFFFFFFFFFFFFFFFFFFFFFFFFFFFFFFFFFFFFFFFFFFFFFFFFFFFF

FFFFFFFFFFFFFF:FFFFFFF:FFFF:FFFFFFFFFFFFFFFFFFFFFFFFFFFFFFFFFFFFFFFF
FFFFFFFF:FFFFFF
@A00721:190:HGJW7DSXY:4:2427:27516:28948 2:N:0:TCTGCAAG+GCTGCACT
GTTCAACGTGGGTTGACTAGTGGCATATTCAACATGCCACATATCTATATCCTTGGCTTCATGCCTCT
TCGACAGGATGTTGATCCTGGGACTCCTGAACTCAATCTCCTCAACGCGCTTCGAAGCACATAGCTTC
AGACGTCCTTTGATT
+
FFFFFFFF:FFFFFFFFFFFFFFF:FFFFFFFFFFFFFFFFFFFFFFFFFFFFFFFFFFFFFFFFFFFF
FFFFFFFFFFFFFFFFFFFFFFFFFFFFFFFFFF:FFFFFFFFFFFFFFFFF:FFFF:FFFFFF:FFF
FFFFFFFF,FFFFFF
@A00721:190:HGJW7DSXY:4:2126:11207:34773 2:N:0:TCTGCAAG+GCTGCACT
GTTCAACGTGGGTTGACTAGTGGCATATTCAACATGCCACATATCTATATCCTTGGCTTCATGCCTCT
TCGACAGGATGTTGATCCTGGGACTCCTGAACTCAATCTCCTCAACGCGCTTCGAAGCACATAGCTTC
AGACGTCCTTTGAT
+
FFFFF:FFFFFFFFFFFFFFFFFFFFF:FFFFFFFFFFFFFF,FFFFFFFFFFFFFFFFFFFFFFFFFF
FFFFFFFF:FFFFFFFFFFFFFFFFFFFFFFFFFF::FFFFFFFFFFFFFFFFFFFFFFFFFFFFFF:F
FFFFFFFF::FFFF
@A00721:190:HGJW7DSXY:4:1629:12310:16673 2:N:0:TCTGCAAG+GCTGCACT
CACGGTTCAACGTGGGTTGACTAGTGGCATATTCAACATGCCACATATCTATATCCTTGGCTTCATGC
CTCTTCGACAGGATGTTGATCCTGGGACTCCTGAACTCAATCTCCTCAACGCGCTTCGAAGCACATAG
CTTCAGACGTCCTTT
+
FFFFFFFFFFFFFFFFFFFFFFFFFFFFFFFFFFFFFFFFFFF:FFFFFFFFFFFFFFFFFFFFFFFFF
FFFFFFFFFFFFFFFFFFFFFFFFFFFFFFFFFFFFFFFFFFFFFFFFFFFFFFFFFFFFFFFFFFFFF
FFFFFFFFFFFFFFF
@A00721:190:HGJW7DSXY:4:2221:12771:9142 2:N:0:TCTGCAAG+GCTGCACT
CACGGTTCAACGTGGGTTGACTAGTGGCATATTCAACATGCCACATATCTATATCCTTGGCTTCATGC
CTCTTCGACAGGATGTTGATCCTGGGACTCCTGAACTCAATCTCCTCAACGCGCTTCGAAGCACATAG
CTTCAGACGTCCTTT
+
FFFFFFFFFFFF,FFFFFFFFFFFFFFFFFFFFFFFFFFFFFFFFFFFFFFFFFFFFFFFFFFFFF:FF
FFFFFFFFFFFFFFFFFFFFFFFFFFFFFFFFFFFFFFFFFFFFFFFFFFFFFFFFFFFFFF:FFFFFF
FFFFFFFFFFFFFFF
@A00721:190:HGJW7DSXY:4:1408:15826:23610 2:N:0:TCTGCAAG+GCTGCACT
CACGAAAGCACACGGTTCAACGTGGGTTGACTAGTGGCATATTCAACATGCCACATATCTATATCCTT
GGCTTCATGCCTCTTCGACAGGATGTTGATCCTGGGACTCCTGAACTCAATCTCCTCAACGCGCTTCG
AAGCACATAGCTTCA
+
FFFFFFFFFFFFFFFFFFFFFFF:FFFFFFFFFFFFFFFFFFFFFFFFFFFFFFFFFFFFFFFFFFFFF
FFFFFFFFFFFFFFFFFFFFFFFFFFFFFFFFFFFFFFFFFFFFFFFFFFFFF:FFFFFFFFFFFFFFF
FFFFFFFFFFF::FF
@A00721:190:HGJW7DSXY:4:1451:31159:16564 2:N:0:TCTGCAAG+GCTGCACT
CACGAAAGCACACGGTTCAACGTGGGTTGACTAGTGGCATATTCAACATGCCACATATCTATATCCTT
GGCTTCATGCCTCTTCGACAGGATGTTGATCCTGGGACTCCTGAACTCAATCTCCTCAACGCGCTTCG
AAGCACATAGCTTCA
+
FFFFFFFFFFFFFFFFFFFFFFFFFFFFFFFFFFFFFFFFFFFFFFFFFFFFFFFFFFFFFFFFFFFFF
FFFFFFFFFFFFFFFFFFFFFFF::FFFFFFFFFFFFFFFFFFFFFFFFFFFFFFFFFFFFFFFFFFFF
FFFF:FFFFFFFFFF
@A00721:190:HGJW7DSXY:4:1645:32398:15765 2:N:0:TCTGCAAG+GCTGCACT
CACGAAAGCACACGGTTCAACGTGGGTTGACTAGTGGCATATTCAACATGCCACATATCTATATCCTT
GGCTTCATGCCTCTTCGACAGGATGTTGATCCTGGGACTCCTGAACTCAATCTCCTCAACGCGCTTCG
AAGCACATAGCTTCA

+
FFFFFFFFFFFFFFFFFFFFFFFFFFFFF:FFFFFFFFFFFFFFFFFFFFFFFFFF:FFFFFFFFFFF
FFFFFFFFFFFFFFFF:F:F,FFFFFFFF:FFFFFFFFFFFFF:FFFFFFFFFF:FFFFFFFFFFFFF
FFFFFFFFFFFFFFF
@A00721:190:HGJW7DSXY:4:2271:21233:21512 2:N:0:TCTGCAAG+GCTGCACT
CACGAAAGCACACGGTTCAACGTGGGTTGACTAGTGGCATATTCAACATGCCACATATCTATATCCTT
GGCTTCATGCCTCTTCGACAGGATGTTGATCCTGGGACTCCTGAACTCAATCTCCTCAACGCGCTTCG
AAGCACATAGCTTCA
+
FFFFFFFFFFFFFFFFFFFFFFFFFFFFFFFFFFFFFFFFFFFFFFFFFFFFFFFFFFFFFFFFFFFF
FFFFFFFFFFFFFFFFFFFFFFFFFFFFFFFFFFFFFFFFFFFFFFFFFFFFFFFFFFFFFFFFFFFF
FFFFFFFFFFFFFFF
@A00721:190:HGJW7DSXY:4:2271:23023:22138 2:N:0:TCTGCAAG+GCTGCACT
CACGAAAGCACACGGTTCAACGTGGGTTGACTAGTGGCATATTCAACATGCCACATATCTATATCCTT
GGCTTCATGCCTCTTCGACAGGATGTTGATCCTGGGACTCCTGAACTCAATCTCCTCAACGCGCTTCG
AAGCACATAGCTTCA
+
FFFFFFFFFFFFFFFFFFFFFF:FFFFFFFFFFFFFFFFFFFFFFFFFFFFFFFFFFFFFFFFFFFFF
FFFFFFFFFFFFFFFFFFFFFFFFFFFFFFFFFFFFFFFFFFFFFFFFFFFFFFFFFFFFFFFFFFFF
FFFFFFFFFFFFFFF
@A00721:190:HGJW7DSXY:4:2608:6072:2268 2:N:0:TCTGCAAG+GCTGCACT
CACGAAAGCACACGGTTCAACGTGGGTTGACTAGTGGCATATTCAACATGCCACATATCTATATCCTT
GGCTTCATGCCTCTTCGACAGGATGTTGATCCTGGGACTCCTGAACTCAATCTCCTCAACGCGCTTCG
AAGCACATAGCTTCA
+
FFFFFFFFFFFFFFFFFFFFFFFFFFFFFFFFFFFFFFFFFFFFFFFFFFFFFFFFFFFFFFFFFFFF
FFFFFFFFFFFFFFFFFFFFFFFFFFFFFFFFFFFFFFFFFFFFFFFFFFFFFFFFFFFFFFFFFFFF
FFFFFFFFFFFFFFF
@A00721:190:HGJW7DSXY:4:2625:19117:10833 2:N:0:TCTGCAAG+GCTGCACT
CACGAAAGCACACGGTTCAACGTGGGTTGACTAGTGGCATATTCAACATGCCACATATCTATATCCTT
GGCTTCATGCCTCTTCGACAGGATGTTGATCCTGGGACTCCTGAACTCAATCTCCTCAACGCGCTTCG
AAGCACATAGCTTCA
+
FFFFFFFFFFFFFFFFFFFFFFFFFFFFFFFFFFFFFFFFFFFFFFFFFFFFFFF:FFFFFFFFFFFF
FFFFFFFF,FFFFFFF:FFFFFF,F:FFF:FFFFFFFFFFFFFFFFFFF,FFFFFFF:FFFF:FFFFF
FFFFFFFFFFFFFFF
@A00721:190:HGJW7DSXY:4:1238:1443:20181 2:N:0:TCTGCAAG+GCTGCACT
ACGCACGAAAGCACACGGTTCAACGTGGGTTGACTAGTGGCATATTCAACATGCCACATATCTATATC
CTTGGCTTCATGCCTCTTCGACAGGATGTTGATCCTGGGACTCCTGAACTCAATCTCCTCAACGCGCT
TCGAAGCACATAGCT
+
FFFFFFFFFFFFFFFFFFFFFFFFFFFFFFFFFFFFFFFFFFFFFFFFFFFFFFFFFFFFFFFFFFFF
FFFFFFFFFFFFFFFFFFFFFFFFFFFFFFFFFFFFFFFFFFFFFFFFFFFFFFFFFFFFFFFFFFFF
FFFFFFFFFFFFFFF
@A00721:190:HGJW7DSXY:4:1472:9055:4429 2:N:0:TCTGCAAG+GCTGCACT
ACGCACGAAAGCACACGGTTCAACGTGGGTTGACTAGTGGCATATTCAACATGCCACATATCTATATC
CTTGGCTTCATGCCTCTTCGACAGGATGTTGATCCTGGGACTCCTGAACTCAATCTCCTCAACGCGCT
TCGAAGCACATAGCT
+
FFFFFFFFF:FFFFFFFF:FFFFFFFFFFFFFFFFFFFFFFFFFFFFFFFFFFFF,FFFF:FFFFFFF
FFFFFFFFFFFFFFFFFFFFFFFFFFFFFFFFFFFFFFFFFFFFFF:FFFFFFFFFFFFFFFFFF,FF
FFFFF:FFFFFFFFF
@A00721:190:HGJW7DSXY:4:1578:25654:33708 2:N:0:TCTGCAAG+GCTGCACT
ACGCACGAAAGCACACGGTTCAACGTGGGTTGACTAGTGGCATATTCAACATGCCACATATCTATATC

CTTGGCTTCATGCCTCTTCGACAGGATGTTGATCCTGGGACTCCTGAACTCAATCTCCTCAACGCGCT
TCGAAGCACATAGCT
+
FFFFFFFFFFFFFFFFFFFFFFFFFF:FFFFFFFFFFFFFFFFFFFFFFFFFFFFFFFFFFFFFFFFFFF
FFFFFFFFFFFFFFFFFFFFFFFFFFFFFFFFFFFFFFFFFFFFFFFFFFFFFFFFFFFFFFFFFFFFFF
FFFFFFF:FFFFFFF
@A00721:190:HGJW7DSXY:4:1678:5195:33943 2:N:0:TCTGCAAG+GCTGCACT
ACGCACGAAAGCACACGGTTCAACGTGGGTTGACTAGTGGCATATTCAACATGCCACATATCTATATC
CTTGGCTTCATGCCTCTTCGACAGGATGTTGATCCTGGGACTCCTGAACTCAATCTCCTCAACGCGCT
TCGAAGCACATAGCT
+
FFFFFFFFFFFFFFFFFFFFFFFFFFFFFFFFFFFFFFFFFFFFFFFFFFFFFFFFFFFFFFFFFFFFFF
FFFFFFFFFFFFFFFFFFFFFFFFFFFFFFFFFFFFFFFFFFFFFFF:FFFFFFFFFFFFFFFFFFFFFF
FFFFFFFFFFFFFFF
@A00721:190:HGJW7DSXY:4:2107:29008:23954 2:N:0:TCTGCAAG+GCTGCACT
ACGCACGAAAGCACACGGTTCAACGTGGGTTGACTAGTGGCATATTCAACATGCCACATATCTATATC
CTTGGCTTCATGCCTCTTCGACAGGATGTTGATCCTGGGACTCCTGAACTCAATCTCCTCAACGCGCT
TCGAAGCACATAGCT
+
FFFFFFFFFFFFFFFFFFFFFFFFFFFFFFFFFFFFFFFFFFFFFFFFF:FFFFFFFFFFFFFFFFFFFF
FFFFFFFFFFFFFFFFFFFFFFFFFFFFFFFFFFFFFFFFFFFFFFFFFFFFFFFFFFFFFFF:FFFFFF
FFFFFFF:FFFFFFF
@A00721:190:HGJW7DSXY:4:2206:2230:22482 2:N:0:TCTGCAAG+GCTGCACT
ACGCACGAAAGCACACGGTTCAACGTGGGTTGACTAGTGGCATATTCAACATGCCACATATCTATATC
CTTGGCTTCATGCCTCTTCGACAGGATGTTGATCCTGGGACTCCTGAACTCAATCTCCTCAACGCGCT
TCGAAGCACATAGCT
+
FFFFFFFFFFFFFFFFFFFFFFFFFFFFFFFFFFFFFFFFFFFFFFFFFFFFFFFFFFFFFF:FFFFFFF
FFFFFFFFFFFFFFFFF:FFFFFFFFFFFFFFFFFFFFFFFFFFFFFFFFFFFFFFFFFFFFFFFFFFFF
FFFFFFF:FFFFFFF
@A00721:190:HGJW7DSXY:4:2260:12066:8797 2:N:0:TCTGCAAG+GCTGCACT
ACGCACGAAAGCACACGGTTCAACGTGGGTTGACTAGTGGCATATTCAACATGCCACATATCTATATC
CTTGGCTTCATGCCTCTTCGACAGGATGTTGATCCTGGGACTCCTGAACTCAATCTCCTCAACGCGCT
TCGAAGCACATAGCT
+
FFFFFFFFFFFFFFFFFFFFFFFFFFFFFFFFFFFFFFFFFFFFFFFFFFFFFFFFFFFFFFFFFFFFFF
FFFFFFFFFFFFFFFFFFFFFFFFFFFFFFFFFFFFFFFFFFFFFFFFFFFFFFFFFFFFFFFFFFFFFF
FFFFFFFFFFFFFFF
@A00721:190:HGJW7DSXY:4:2431:31638:11350 2:N:0:TCTGCAAG+GCTGCACT
ACGCACGAAAGCACACGGTTCAACGTGGGTTGACTAGTGGCATATTCAACATGCCACATATCTATATC
CTTGGCTTCATGCCTCTTCGACAGGATGTTGATCCTGGGACTCCTGAACTCAATCTCCTCAACGCGCT
TCGAAGCACATAGCT
+
FFFFFFFFFFFFFFFFFFFFFFF:FFFFFFFFFFFFFFFFFFFFFFFFFFFFFFF:FFFFFFFF:FFFFF
FFFFFFFFFFFFFFFFFFFFFFFFFFFFFFFFFFFFFFFFFFFFFFFFFFFFFFFFFFFFFFFFFFFFFF
FFFFF:FFFFFFFFF
@A00721:190:HGJW7DSXY:4:1474:5475:11005 2:N:0:TCTGCAAG+GCTGCACT
ATGGAATCAGAACTAAGTCTTCTCCGATCTACGCACGAAAGCACACGGTTCAACGTGGGTTGACTAGT
GGCATATTCAACATGCCACATATCTATATCCTTGGCTTCATGCCTCTTCGACAGGATGTTGATCCTGG
GACTCCTGAACTCAA
+
FFFFF,FFFFFFFFFFFFFFFFFFFFFFFFFFFFFFFFFFFFFFFFFFFFFFFFFFF:FFFFFFFFFFFF
FFFFFFFFFFFFFFFFFFFFFFFFFFFFFFFF:FFFFFFFFFFFFFFFFFFFFFFFFFFFFFFFFFFFFF
:FFFFFFFFFFFFFF

```
@A00721:190:HGJW7DSXY:4:2201:28546:21308 2:N:0:TCTGCAAG+GCTGCACT
ATGGAATCAGAACTAAGTCTTCTCCGATCTACGCACGAAAGCACACGGTTCAACGTGGGTTGACTAGT
GGCATATTCAACATGCCACATATCTATATCCTTGGCTTCATGCCTCTTCGACAGGATGTTGATCCTGG
GACTCCTGAACTCAA
+
FFF:FFF:FFFFFFFF::FFFFFFFFFFFFFFF:FFFFFF:FFFF:FF:FFFFFFFFFFFFFFFF::,
F:FFFFFFFFF:FF::FFFF:FFFFFFF:F,F:,:FFF,FF:F:FFFF:,FFF:,FFFF:FFF:FFFF
FFFFFFFFFFFFFFF
@A00721:190:HGJW7DSXY:4:2264:25292:19304 2:N:0:TCTGCAAG+GCTGCACT
ATGGAATCAGAACTAAGTCTTCTCCGATCTACGCACGAAAGCACACGGTTCAACGTGGGTTGACTAGT
GGCATATTCAACATGCCACATATCTATATCCTTGGCTTCATGCCTCTTCGACAGGATGTTGATCCTGG
GACTCCTGAACTCAA
+
FFFFFFFFFFFFFFFFFFF:FFFFFFFFFFFFFFFFFFFFFFFFFFFFFFFFFFFFFFFFFFFFFFFF
FFFFFFFFFFFFFFFFFFFFFFFFFFFFFFFFFFFFFFFFFFFFFFFFFFFFFFFFFFFFFFFFFFFF
FFFFFFFFFFFFFFF
@A00721:190:HGJW7DSXY:4:2505:29324:26193 2:N:0:TCTGCAAG+GCTGCACT
ATGGAATCAGAACTAAGTCTTCTCCGATCTACGCACGAAAGCACACGGTTCAACGTGGGTTGACTAGT
GGCATATTCAACATGCCACATCTCTATATCCTTGGCTTCATGCCTCTTCGACAGGATGTTGATCCTGG
GACTCCTGAACTCAA
+
FFFFFF,FFFFFFF,FFFFFFFFFFFFFFFFFFFFFFFFFFFFFFFFFFFFFFFF,FFFFFFFFFFFF
FFFFFFFFFFFFFFFFFFFFFF,FFF:FFFFFFFFFFFFFFFFFFFF:FFFFFFFFFFFFFFFFFFFF
FFFFFFFFFFFFFFF
@A00721:190:HGJW7DSXY:4:2561:27263:28479 2:N:0:TCTGCAAG+GCTGCACT
ATGGAATCAGAACTAAGTCTTCTCCGATCTACGCACGAAAGCACACGGTTCAACGTGGGTTGACTAGT
GGCATATTCAACATGCCACATATCTATATCCTTGGCTTCATGCCTCTTCGACAGGATGTTGATCCTGG
GACTCCTGAACTCAA
+
FFFFF,FFFFFFFFFFFFFFFF:FFFFFFFFFFFFFFFFFFFFFFFFFFFFFFFFFFFFFFFFFFFFF
FFFFFF,FFFFFFFFFFFFFFFFF,FF:FFFFFFFFFFFFFFFFFFFFFFFFFFFFF:FFFFFFFFFF
FFFFFFFFFFF:FFF
@A00721:190:HGJW7DSXY:4:2623:32235:21684 2:N:0:TCTGCAAG+GCTGCACT
ATGGAATCAGAACTAAGTCTTCTCCGATCTACGCACGAAAGCACACGGTTCAACGTGGGTTGACTAGT
GGCATATTCAACATGCCACATATCTATATCCTTGGCTTCATGCCTCTTCGACAGGATGTTGATCCTGG
GACTCCTGAACTCAA
+
FFFF,FFFFF:FFFFFFFFFFFFFFFFFFF:FFFFFFFFFFFFFFFFFFFFFFFFFFFFFFFFFFFFF
FFFFFFFFFFFF,FFFFFFFFFFFFFFFFFFFFFFFFFFFFFFFFFFFFFFFFFFFFFFFFFF:FFFF
FFFFF,,FFFFFFFF
@A00721:190:HGJW7DSXY:4:2666:8983:35806 2:N:0:TCTGCAAG+GCTGCACT
ATGGAATCAGAACTAAGTCTTCTCCGATCTACGCACGAAAGCACACGGTTCAACGTGGGTTGACTAGT
GGCATATTCAACATGCCACATATCTATATCCTTGGCTTCATGCCTCTTCGACAGGATGTTGATCCTGG
GACTCCTGAACTCAA
+
FFFF:FFFFFFFFFFFFFFFFFFFFFFFFFFFFFFFFFFFFFFFFFFFFFFFFFFFFFFFFFFFFFFF
FFFFFFFFFF:FFFFFFFFFFFFFFFFFFFFFFFFFFFFFFFFFFFFFFFFFFFFF:FFFFFFFFFFF
FFFFFFFFFFFFFFF
@A00721:190:HGJW7DSXY:4:1311:7292:27743 2:N:0:TCTGCAAG+GCTGCACT
CCGGGCAAGACCGTAATGGAATCAGAACTAAGTCTTCTCCGATCTACGCACGAAAGCACACGGTTCAA
CGTGGGTTGACTAGTGGCATATTCAACATGCCACATATCTATATCCTTGGCTTCATGCCTCTTCGACA
GGATGTTGATCCTGG
+
FFFFFFFFFFFFFFFF:FFFFFFFFFFFFFFFFFFFFFFFFFFFFFFFFFFFFFFFFFFFFFFFFFFF
```

FFFFFFFFFFFFFF:FFFFFFFFFFFFFFFFFFFF,FFFFFFFFFFFFFFFFFFFFFFFFFFFFFFFF
FFFFFFFFFFFFFFF
@A00721:190:HGJW7DSXY:4:1431:6017:3082 2:N:0:TCTGCAAG+GCTGCACT
CCGGGCAAGACCGTAATGGAATCAGAACTAAGTCTTCTCCGATCTACGCACGAAAGCACACGGTTCAA
CGTGGGTTGACTAGTGGCATATTCAACATGCCACATATCTATATCCTTGGCTTCATGCCTCTTCGACA
GGATGTTGATCCTGG
+
FFFFFFFFFFFFFFFFFFFFFFFFFFFFFFFFFFFFFFFFFFFFFFFFFFFFFFFFFFFFFFFFFFFF
FFFFFFFFFFFFFFFFFFFFFFFFFFFFFFFFFFFFFFFFFFFFFFFFFFFFFFFFFFFFFFFFFFFF
FFFFFFFFFFFFFFF
@A00721:190:HGJW7DSXY:4:2136:1226:11663 2:N:0:TCTGCAAG+GCTGCACT
CCGGGCAAGACCGTAATGGAATCAGAACTAAGTCTTCTCCGATCTACGCACGAAAGCACACGGTTCAA
CGTGGGTTGACTAGTGGCATATTCAACATGCCACATATCTATATCCTTGGCTTCATGCCTCTTCGACA
GGATGTTGATCCTGG
+
FFFFFFFFFFFFFFFF:FFF:FFFFFFFFFFFFFFFFFFFFFFFFFFFFFFFFFFFFFFFFFFFFFFF
FFFFFFFFFFFFFFFFFFFFFFFFFFFFFFFFFFFFFFFFFFFFFFFFFFFFFFFFFFFFFFFFFFFF
FFFFFFFFFFFFFFF
@A00721:190:HGJW7DSXY:4:2318:30825:15796 2:N:0:TCTGCAAG+GCTGCACT
CCGGGCAAGACCGTAATGGAATCAGAACTAAGTCTTCTCCGATCTACGCACGAAAGCACACGGTTCAA
CGTGGGTTGACTAGTGGCATATTCAACATGCCACATATCTATATCCTTGGCTTCATGCCTCTTCGACA
GGATGTTGATCCTGG
+
FFFFFFFFF:FFFFFF,FFFFFFFFFFFFFFFFFFFFFFFFFFFFFFFFFFF:FFFFFFFFFFFFFF:
FFFFFFFFFFFFFFFFFFFFFFFFFFFFFFFFFFFFFFFFFFFFFFFFFF:FFFF:FFFFFFFFF:FFF
FFFFFFFF:FF:FFF
@A00721:190:HGJW7DSXY:4:2410:8775:17942 2:N:0:TCTGCAAG+GCTGCACT
CCGGGCAAGACCGTAATGGAATCAGAACTAAGTCTTCTCCGATCTACGCACGAAAGCACACGGTTCAA
CGTGGGTTGACTAGTGGCATATTCAACATGCCACATATCTATATCCTTGGCTTCATGCCTCTTCGACA
GGATGTTGATCCTGG
+
FFFFFFFFFFFFFFFFFFFF:FFFFFFFFFFFFFFF:FFFFFFFFFFFFFFFFFFFFFFFFFFFFFF:F
FFFFFFFF:FFFFFFFFFFFFFFFFFFFFFFFFFFFFF:FF:FFFFF,:FFFFFFFFFFFFFFFFFFFF
FFFFFFFFFFFFFFF
@A00721:190:HGJW7DSXY:4:2414:3739:33082 2:N:0:TCTGCAAG+GCTGCACT
CCGGGCAAGACCGTAATGGAATCAGAACTAAGTCTTCTCCGATCTACGCACGAAAGCACACGGTTCAA
CGTGGGTTGACTAGTGGCATATTCAACATGCCACATATCTATATCCTTGGCTTCATGCCTCTTCGACA
GGATGTTGATCCTGG
+
FFFFFFFFFFFFFFFFFFFFFFFFFFFFFFFFFFFFFFFFFFFFFFFFFFFFFFFFFFFFFFFFFFFF
FFFFFFFFFFFFFFFFFFFFFFFFFFFFFFFFFFFFFFFFFFFFFFFFFFFFFFFFFFFFFFFFFFFF
FFFFFFFFFFFFFFF
@A00721:190:HGJW7DSXY:4:2427:18078:1141 2:N:0:TCTGCAAG+GCTGCACT
CCGGGCAAGACCGTAATGGAATCAGAACTAAGTCTTCTCCGATCTACGCACGAAAGCACACGGTTCAA
CGTGGGTTGACTAGTGGCATATTCAACATGCCACATATCTATATCCTTGGCTTCATGCCTCTTCGACA
GGATGTTGATCCTGG
+
FFFFFFFFFFFFFFFF:FFFFFFFFFFFFFFFFFFFFFFFFFFFFFFFFFFFFFFFFFFFFFFFFFFF
FFFFFFFFFFFFFFFFFFFFFFFFFFFFFFFFFFFFFFFFFFFFFFFFFFFFFFFFFFFFFFFFFFFF
FFFFFFFFFFFFFFF
@A00721:190:HGJW7DSXY:4:2616:32353:34413 2:N:0:TCTGCAAG+GCTGCACT
CCGGGCAAGACCGTAATGGAATCAGAACTAAGTCTTCTCCGATCTACGCACGAAAGCACACGGTTCAA
CGTGGGTTGACTAGTGGCATATTCAACATGCCACATATCTATATCCTTGGCTTCATGCCTCTTCGACA
GGATGTTGATCCTGG

```
+
FFFFFFFFFFFFFFFFFFFFFFFFFFFFFFFFFFFFFFFFFFFFFFFFFFFFFFFFFFFFFFFFFFFFFF
FFFFFFFFFFFFFFFFFFFFFFFFFFFFFFFFFFFF:FFFFFFFFFFFFFFFFFFFFFFFFFFFFFFFFF
FFFFFFFFFFFFFFF
@A00721:190:HGJW7DSXY:4:1213:6867:36902 2:N:0:TCTGCAAG+GCTGCACT
CATTCCGGGCAAGACCGTAATGGAATCAGAACTAAGTCTTCTCCGATCTACGCACGAAAGCACACGGT
TCAACGTGGGTTGACTAGTGGCATATTCAACATGCCACATATCTATATCCTTGGCTTCATGCCTCTTC
GACAGGATGTTGATC
+
FFFFFFFFFFFFFFFFFFFFFFFFF:FFFFF:FFFFFFFFFFFFFFFFFFFFFFFFFFFFFFFFFFFFFF
FFFFFF,FFFFFFFFF:FFFFF:FF:FFFFFFFFFFFFFFFFFFF:FFFFFFFFFFFFFFFFFFFFFFFF
FFFFFFF:FFFFFFF
@A00721:190:HGJW7DSXY:4:1308:22932:24893 2:N:0:TCTGCAAG+GCTGCACT
ATTCCGGGCAAGACCGTAATGGAATCAGAACTAAGTCTTCTCCGATCTACGCACGAAAGCACACGGTT
CAACGTGGGTTGACTAGTGGCATATTCAACAGGCCACATATCTATATCCTTGGCTACATGCCTCTTCG
ACAGGATGTTGATC
+
F:FFFFFFFFFFFF:FF:F,F:FF,:FFFF:::FF:FF:FFFFFFFFFF:FFFF,FFF,FFFFFFFFF
FFFFFFFF,:FFFF,:FF,:,FFFF:FFF,F,:FFFFF,FFFFFFFF:FFF:FFF,,F::FF:F,F,:
FF:,:FFF,F,FF:
@A00721:190:HGJW7DSXY:4:1669:11487:24079 2:N:0:TCTGCAAG+GCTGCACT
CATTCCGGGCAAGACCGTAATGGAATCAGAACTAAGTCTTCTCCGATCTACGCACGAAAGCACACGGT
TCAACGTGGGTTGACTAGTGGCATATTCAACATGCCACATATCTATATCCTTGGCTTCATGCCTCTTC
GACAGGATGTTGATC
+
FFFFFFFFFFFFFFFFFFFFFFFFFFFFFFFFFFFFFFFFFFFFFFFFFFFFFFFFFFFFFFFFFFFFFF
FFFFFFFFFFFFFFFFFFFFFFFFFFFFFFFFFFFFFFFFFFFFFFFFFFFFFFFFFFFFFFFFFFFFFF
FFFFFFFFFFFFFFF
@A00721:190:HGJW7DSXY:4:2228:25780:34240 2:N:0:TCTGCAAG+GCTGCACT
CATTCCGGGCAAGACCGTAATGGAATCAGAACTAAGTCTTCTCCGATCTACGCACGAAAGCACACGGT
TCAACGTGGGTTGACTAGTGGCATATTCAACATGCCACATATCTATATCCTTGGCTTCATGCCTCTTC
GACAGGATGTTGATC
+
FFFFFFFFFFFFFFFFFFF::FFFFFFFFFFFFFFFFFFFFFFFFFFFFFFFFFFFFFFFFFFFFFFFFF
FFFFFFFFFFFFFFFFFFFFFFFFFFFFFFFFFFFFFFFFFFFFFFFFFFFFFFFFFFFFFFFFFFFF,F
FFFFFFFFFFFFFFF
@A00721:190:HGJW7DSXY:4:2237:22110:19867 2:N:0:TCTGCAAG+GCTGCACT
CATTCCGGGCAAGACCGTAATGGAATCAGAACTAAGTCTTCTCCGATCTACGCACGAAAGCACACGGT
TCAACGTGGGTTGACTAGTGGCATATTCAACATGCCACATATCTATATCCTTGGCTTCATGCCTCTTC
GACAGGATGTTGATC
+
FFF:FFFF:FFF:FF:FFFF:F:FFF:FFFFFFFFF,FFF:FFFFFFFFFFFFFFFFFFFFFFFFFFFFF
FFFFF:FFFFFFFFFFFFFFFFF:F:FFFFFFFFF:FFF:F:FF:F:FF:FFFFFFFFFFFF:FFFFF
,F:FFFFF::FFFFF
@A00721:190:HGJW7DSXY:4:2261:4634:36354 2:N:0:TCTGCAAG+GCTGCACT
CATTCCGGGCAAGACCGTAATGGAATCAGAACTAAGTCTTCTCCGATCTACGCACGAAAGCACACGGT
TCAACGTGGGTTGACTAGTGGCATATTCAACATGCCACATATCTATATCCTTGGCTTCATGCCTCTTC
GACAGGATGTTGATC
+
FFFFFFFFFFFFFFFFFFFFFFFFF:FFFFFFFFFFFFFFFFFFFFFFFFFFFFFFFFFFFFFFFFFFFF
FFFFFFFFFFFFFFFFFFFFFFFFFF:FFFFFFFFFFFFFFFFFFFFFFFFFFFFFFFFFFFFFFFFFFF
FFF,FFFFFFFFFFF
@A00721:190:HGJW7DSXY:4:2337:27037:25739 2:N:0:TCTGCAAG+GCTGCACT
CATTCCGGGCAAGACCGTAATGGAATCAGAACTAAGTCTTCTCCGATCTACGCACGAAAGCACACGGT
```

```
TCAACGTGGGTTGACTAGTGGCATATTCAACATGCCACATATCTATATCCTTGGCTTCATGCCTCTTC
GACAGGATGTTGATC
+
FFFFFFFFFFF:FFFFFFFF,,FFFFFFFFFFFFFFFFFF:FFFF,:FFFFF:FFFFFFFFFFFFFFFF,
FFFFFFFFFFFFFFFFF::FFFF:F:FFFFFFFFF,:FFFFFFFFFFFFFF,FFFF:FFFFFFFFFF:FF
FFFFFFFFFFFFFFF
@A00721:190:HGJW7DSXY:4:2525:28492:12759 2:N:0:TCTGCAAG+GCTGCACT
CATTCCGGGCAAGACCGTAATGGAATCAGAACTAAGTCTTCTCCGATCTACGCACGAAAGCACACGGT
TCAACGTGGGTTGACTAGTGGCATATTCAACATGCCACATATCTATATCCTTGGCTTCATGCCTCTTC
GACAGGATGTTGATC
+
FFFFFFFFFFFFFFFFFFFFFFFFFFFFFFFFFFFFFFFFFFFFFFFFFFFFFFFFFFFFFFFFFFFFFF
FFFFFFFFFFFFFFFFFFFFFFFFFFFFFFFFFFFFFFFFFFFFFFFFFFFFFFFFFFFFFFFFFFFFFF
FFFFFFFFFFFFFFF
@A00721:190:HGJW7DSXY:4:1228:2428:1470 2:N:0:TCTGCAAG+GCTGCACT
ACACATTCCGGGCAAGACCGTAATGGAATCAGAACTAAGTCTTCTCCGATCTACGCACGAAAGCACAC
GGTTCAACGTGGGTTGACTAGTGGCATATTCAACATGCCACATATCTATATCCTTGGCTTCATGCCTC
TTCGACAGGATGTTG
+
FFFFFFFFFFFFFFFFFFFFFFFFFFFFFFFFFFFFFFFFFFFFFFFFFFFFFFFFFFFFFFFFFFFFFF
FFFFFFFFFFFFFFFFFFF:FFFFFFFFFFFFFFFFFFFFFFFFFFFFFFFFFFFFFFFFFFFFFFFFFF
FFFFFFFFFFFFFFF
@A00721:190:HGJW7DSXY:4:1267:23755:11381 2:N:0:TCTGCAAG+GCTGCACT
ACACATTCCGGGCAAGACCGTAATGGAATCAGAACTAAGTCTTCTCCGATCTACGCACGAAAGCACAC
GGTTCAACGTGGGTTGACTAGTGGCATATTCAACATGCCACATATCTATATCCTTGGCTTCATGCCTC
TTCGACAGGATGTTG
+
FFFFFFFFFFFFFFFFFFFFFFF:FFFFFFFFFFFFFFFFFFFFFFFFFFFFFFFFFFFFFFFFFFFFFF
FFFFFFFFFFFFFFFFFFFFFFFFFFFFFFFFFFFFFFFFFFFFFFFFFFFFFFFFFFFFFFFFFFFFFF
FFFFFFFFFFFFFFF
@A00721:190:HGJW7DSXY:4:1460:26946:6010 2:N:0:TCTGCAAG+GCTGCACT
ACACATTCCGGGCAAGACCGTAATGGAATCAGAACTAAGTCTTCTCCGATCTACGCACGAAAGCACAC
GGTTCAACGTGGGTTGACTAGTGGCATATTCAACATGCCACATATCTATATCCTTGGCTTCATGCCTC
TTCGACAGGATGTTG
+
FFFFFFFFFFFFFFFFFFFFFFF:FFFFFFFFFFFFFFFFFFFFFFFFFFFFFFFFFFFFFFFFFFFFFF
FFFFFFFFFFFFFFFFFF:F,FFFFFFFFFFF:FFFFFF:F:FFFFFFFFFFFFFFFFFFFFFFFFFFFF
FFFFFFFFFFFFFFF
@A00721:190:HGJW7DSXY:4:2210:29957:24126 2:N:0:TCTGCAAG+GCTGCACT
ACACATTCCGGGCAAGACCGTAATGGAATCAGAACTAAGTCTTCTCCGATCTACGCACGAAAGCACAC
GGTTCAACGTGGGTTGACTAGTGGCATATTCAACATGCCACATATCTATATCCTTGGCTTCATGCCTC
TTCGACAGGATGTTG
+
FFFFFFFFFFFFFFFFFFFFFFF:FFFFFFFFFFFFFFFFFFFFFFFFFFFFFFFFFFFFFFFFFFFFFF
FFFFFFFFFFFFFFFFFFFFFFFFFFFFFFFFFFFFFFFFFFFFFFFFFFFFFFFFFF:FFFFFFFFFFF
FFFFFFFFFF:FFFF
@A00721:190:HGJW7DSXY:4:2457:21007:36057 2:N:0:TCTGCAAG+GCTGCACT
ACACATTCCGGGCAAGACCGTAATGGAATCAGAACTAAGTCTTCTCCGATCTACGCACGAAAGCACAC
GGTTCAACGTGGGTTGACTAGTGGCATATTCAACATGCCACATATCTATATCCTTGGCTTCATGCCTC
TTCGACAGGATGTTG
+
FFFFFFFFFFFFFFFFFFFFF:FFFFFFFFFFFFFFFFFFFFFFFFFFFFFFFFFFFFFFFFFFFFFFFF
FFFFFF:FFFFFFFFFFFFFFFFFFFFFFFFFFFFFFFFFFFFFFFFFFFFFFFFFFFFFFFFFFFFFFF
FFFFFFFFFFFFFFF
```

@A00721:190:HGJW7DSXY:4:2558:21404:21057 2:N:0:TCTGCAAG+GCTGCACT
ACACATTCCGGGCAAGACCGTAATGGAATCAGAACTAAGTCTTCTCCGATCTACGCACGAAAGCACAC
GGTTCAACGTGGGTTGACTAGTGGCATATTCAACATGCCACATATCTATATCCTTGGCTTCATGCCTC
TTCGACAGGATGTTG
+
FFFFF:FFFFFFFFFFFFFFFFF:FFFFFFFFFFFFFFFFFFFFFFFFFFFFFFFFFFFFFFFFFFFFFF
FFFFFFFFFFFFFFFFFFFFFFFFFFFFFFFFFFFFFFFFFFFFFF:FFFFFFFFFFFFFFFFFFFFFFF
FFFFFFFFFFFFFFF
@A00721:190:HGJW7DSXY:4:1109:2962:30170 2:N:0:TCTGCAAG+GCTGCACT
CTGGATATCTCACTGTAACACATTCCGGGCAAGACCGTAATGGAATCAGAACTAAGTCTTCTCCGATC
TACGCACGAAAGCACACGGTTCAACGTGGGTTGACTAGTGGCATATTCAACATGCCACATATCTATAT
CCTTGGCATCATGCC
+
F:,F:,FFF,FFF,:FFFFFFF:FF,F:,FFFFFFF,,FFF:,F:,FFFFFFFFF,FFFFF:F,,F,F
FFFFFFF,FFFF,FFFF,:FFFFFFFFF,F:,:FFFF:,FFFF,F:F::FFFF:,FFFF:F,F,::FF
:F,,F,:,FFFFFFF
@A00721:190:HGJW7DSXY:4:1174:9851:4742 2:N:0:TCTGCAAG+GCTGCACT
CTGGATATCTCACTGTAACACATTCCGGGCAAGACCGTAATGGAATCAGAACTAAGTCTTCTCCGATC
TACGCACGAAAGCACACGGTTCAACGTGGGTTGACTAGTGGCATATTCAACATGCCACATATCTATAT
CCTTGGCTTCATGCC
+
FFFFFFFFFFFFFFFFFFFFFFFFFFFFFFFFFFFFFFFFFFFFFFFFFFFFFFFFFFFFFFFFFFFFFF
FFFFFFFFFFFFFFFFFFFFFFFFFFFFFFFFFFFFFFFFFFFFF:FFFFFFFFFFFFFFFFFFFFFFFF
FFFFFFFFFFFFFFF
@A00721:190:HGJW7DSXY:4:1438:17644:12759 2:N:0:TCTGCAAG+GCTGCACT
CTGGATATCTCACTGTAACACATTCCGGGCAAGACCGTAATGGAATCAGAACTAAGTCTTCTCCGATC
TACGCACGAAAGCACACGGTTCAACGTGGGTTGACTAGTGGCATATTCAACATGCCACATATCTATAT
CCTTGGCTTCATGCC
+
FFFFFFFFFFFFFFFFFFFFFFFFFFFFFFFFFFFFFFFF:FFFFFFFFFFFFFFFFFFFFFFFFFFFFF
FFFFFFFFFFFFFFFFFFFFFFFFFFFFFFFFFFFFFFFFFFFFFFFFFFFFFFFFFFFFFFFFF:FFFF
FFFFFFFFFFFFFFF
@A00721:190:HGJW7DSXY:4:2104:5900:29121 2:N:0:TCTGCAAG+GCTGCACT
CAGGATATCTCACTGTAACACATTCCGGGCAAGACCGTAATGGAATCAGAACTAAGTCTTCTCCGATC
TACGCACGAAAGCACACGGTTCAACGTGGGTTGACTAGTGGCATATTCAACATGCCACATATCTATAT
CCTTGGCTTCATGCC
+
F,FFFFFFFFFFFFFFFFFFFFFFFFFFFFFFFFFFFFFFFFFFFFFFFFFFFFFFFFFFFFFFFFFFFF
FFFFFFFFFFFFFFFFFFFFFFFFFFFFFFFFFFFFFFFFFFFFFFFFFFFFFFFFFFFFFFFFFFFFFF
FFFFFFFFFFFFFFF
@A00721:190:HGJW7DSXY:4:2109:3558:34929 2:N:0:TCTGCAAG+GCTGCACT
CTGGATATCTCACTGTAACACATTCCGGGCAAGACCGTAATGGAATCAGAACTAAGTCTTCTCCGATC
TACGCACGAAAGCACACGGTTCAACGTGGGTTGACTAGTGGCATATTCAACATGCCACATATCTATAT
CCTTGGCTTCATGCC
+
FFFFFFFFFFFFFFFFFFFFFFFFFFFFFFFFFFFFFFFF,FFFFFFFFFFFFFFFFFFFFFFFFFFFFF
FFFFFFFFFFFFFFFFFFFFFFFFFFFFFFFFFFFFFFFFFFFFFFFFFFFFFFFFFFFFFFFFFFFFFF
FFFFFFFFFFFFFFF
@A00721:190:HGJW7DSXY:4:2531:31494:28197 2:N:0:TCTGCAAG+GCTGCACT
CTGGATATCTCACTGTAACACATTCCGGGCAAGACCGTAATGGAATCAGAACTAAGTCTTCTCCGATC
TACGCACGAAAGCACACGGTTCAACGTGGGTTGACTAGTGGCATATTCAACATGCCACATATCTATAT
CCTTGGCTTCATGCC
+
FFFFFFFFFFFFFFFFFFFFFFFFFFFFFFFFFFFFFFFFFFFFFFFFFFFFFFFFFFFFFFFFFFFFFF

```
FFFFFFFFFFFFFFFFFFFFFFFFFFFFFFFFFFFFFFFFFFFFFFF:FFFFFFFFFFFFFFFFFFF
FFFFFFFFFFFFFFF
@A00721:190:HGJW7DSXY:4:1478:9950:11741 2:N:0:TCTGCAAG+GCTGCACT
CTGGATATCTCACTGTAACACATTCCGGGCAAGACCGTAATGGAATCAGAACTAAGTCTTCTCCGATC
TACGCACGAAAGCACACGGTTCAACGTGGGTTGACTAGTGGCATATTCAACATGCCACATATCTATAT
CCTTGGCTTCATGC
+
FFFFFFFFFFFFFFFFFFFFFFFFFFFFFFFFFFFFFFFFFFFF:FFFFFFFFFFFFFFFFFFFFFFFF
FFFFFFFFFFFFFFFFFFFFFFFFFFFFFFFFFFFFFFFFFFFFFFFFFFFFFFFFFFFFFFFFFFFFF
FFFFFFFFFFFFFF
@A00721:190:HGJW7DSXY:4:1157:12400:30765 2:N:0:TCTGCAAG+GCTGCACT
ATCACATTCAGCTATCGATCTTCTGGATATCTCACTGTAACACATTCCGGGCAAGACCGTAATGGAAT
CAGAACTAAGTCTTCTCCGATCTACGCACGAAAGCACACGGTTCAACGTGGGTTGACTAGTGGCATAT
TCAACATGCTACATA
+
FFFFFFFFFFFFFFFFFFFFFFFFFFFFFFFFFFFFFFFFFFFFFFFFFFFFFFFFFFFF::FFF:F
FFFFFFFFFFFFFFFFFFFFFFFFFFFFFFFFFFFFFFFFFFFF:FFFFFFFFFFFFFFFFFFFFFFFF
FFFFFFFFFFFFFFF
@A00721:190:HGJW7DSXY:4:1329:2971:23359 2:N:0:TCTGCAAG+GCTGCACT
ATCACATTCAGCTATCGATCTTCTGGATATCTCACTGTAACACATTCCGGGCAAGACCGTAATGGAAT
CAGAACTAAGTCTTCTCCGATCTACGCACGAAAGCACACGGTTCAACGTGGGTTGACTAGTGGCATAT
TCAACATGCTACATA
+
FFFFFFFFFFFFFFFFFFFF:FFFFFFFFFFFFFFFFFFFFFFFFFFFFFFFFFFFFFFF:F:FF:F:
FFFFFFFFFFFFFFFFFFFFFFFFFFFFFFFFFFFF,FFFFFFFFFFFF,FFFFFFFF::FFFFFFFFF
:FFFFFFFFFFFFFF
@A00721:190:HGJW7DSXY:4:1359:23258:21574 2:N:0:TCTGCAAG+GCTGCACT
ATCACATTCAGCTATCGATCTTCTGGATATCTCACTGTAACACATTCCGGGCAAGACCGTAATGGAAT
CAGAACTAAGTCTTCTCCGATCTACGCACGAAAGCACACGGTTCAACGTGGGTTGACTAGTGGCATAT
TCAACATGCTACATA
+
FFFFFFFFFFFFFFFFFFFFFFFFFFFFFFFFFFFFFFFFFFFFFFFFFFFFFFFFFFFFF:F:FFFFF
FFFFFFFFFFFFFFFFFFFFFFFFFFFFFFFFFFFFFFFFFFFFF:FFFFFFFFFFFFFFFFFFFFFFFF
FFFFFFFFFFFFFFF
@A00721:190:HGJW7DSXY:4:1469:24325:26115 2:N:0:TCTGCAAG+GCTGCACT
ATCACATTCAGCTATCGATCTTCTGGATATCTCACTGTAACACATTCCGGGCAAGACCGTAATGGAAT
CAGAACTAAGTCTTCTCCGATCTACGCACGAAAGCACACGGTTCAACGTGGGTTGACTAGTGGCATAT
TCAACATGCTACATA
+
FFFFFFFFFFFFFFFFFFFFFFFFFFFFF:FFFFFFFFFFFFFFFFFFFFFFFF:FFFFFFFFFFFFFFF
FFFFFFFFFFFFFFFFFFFFFFFFFFFFFFFFFFFFFFFFFFFFFFFFFFFFFFFFFFFFFFFFFFFFF
FFFFFFFFFFFFFFF
@A00721:190:HGJW7DSXY:4:2131:27380:9862 2:N:0:TCTGCAAG+GCTGCACT
ATCACATTCAGCTATCGATCTTCTGGATATCTCACTGTAACACATTCCGGGCAAGACCGTAATGGAAT
CAGAACTAAGTCTTCTCCGATCTACGCACGAAAGCACACGGTTCAACGTGGGTTGACTAGTGGCATAT
TCAACATGCTACATA
+
:FFFFFFFFFFFFFFFFFFFFFFFFFFFF:FFFFFFFFFFFFFFFFFFFFFFFFFFFFFFFFF::FFF:F
FFFFFFFFFFFFFFFFFFFFFFFFFFFFFFFFFFFFFFFF:FFFFFFF:FFFFFFFFFFFFFFFFFFFF
FFFFFFFFFF:FF:F
@A00721:190:HGJW7DSXY:4:2176:4924:32628 2:N:0:TCTGCAAG+GCTGCACT
ATCACATTCAGCTATCGATCTTCTGGATATCTCACTGTAACACATTCCGGGCAAGACCGTAATGGAAT
CAGAACTAAGTCTTCTCCGATCTACGCACGAAAGCACACGGTTCAACGTGGGTTGACTAGTGGCATAT
TCAACATGCTACATA
```

+
FFFFFFFFFFFFFFFFFFFFFFFFFFFFFFFFFFFFFFFFFFFFFFFFFFFFFFFFFFFFFFF:FF:FF
FFFFFFFFFFFFFFFFFFFFFFFFFFFF:FFFFFFFFFFFFFFFFFFFFFFFFFFFFFFFFFFFFFFFF
FFFFFFFFFFFFFFF
@A00721:190:HGJW7DSXY:4:2423:13621:9173 2:N:0:TCTGCAAG+GCTGCACT
ATCACATTCAGCTATCGATCTTCTGGATATCTCACTGTAACACATTCCGGGCAAGACCGTAATGGAAT
CAGAACTAAGTCGTCTCCGATCTACGCACGAAAGCACACGGTTCAACGGGGGTTGACTAGTGGCATAT
TCAACATGCTACATA
+
FFFFFFFFFFFFFF,FFF,FFF:FFFF,FFF:FFFFFFFFFF:F:FFF:FFF,F,FFFF:F:F,:FF:
FF,,FFFFFFFF,,FFF,FFFFFFF,FFFFF:,,FFFFFFFFFFFFFF,,FF:FFFF::FFFFF:FFF
F,FFFFFF,::FFFF
@A00721:190:HGJW7DSXY:4:2550:23375:12571 2:N:0:TCTGCAAG+GCTGCACT
ATCACATTCAGCTATCGATCTTCTGGATATCTCACTGTAACACATTCCGGGCAAGACCGTAATGGAAT
CAGAACTAAGTCTTCTCCGATCTACGCACGAAAGCACACGGTTCAACGTGGGTTGACTAGTGGCATAT
TCAACATGCTACATA
+
FFFFFFFFFFFFFFFFFFFFFFFFFFFFFFFFFFFFFFFFFFFFFFFFFFFFFFFFFFFFFFFF:FFF:F
FFFFFFFFFFFFFFFFFFFFFFFFFFFFFFFFF:FFFFFFFFFFFFFFFFFFFFFFFFF:FFFFFFFFFF
FFFFFFFFFFFFF:F
@A00721:190:HGJW7DSXY:4:2669:2537:34788 2:N:0:TCTGCAAG+GCTGCACT
ATCACATTCAGCTATCGATCTTCTGGATATCTCACTGTAACACATTCCGGGCAAGACCGTAATGGAAT
CAGAACTAAGTCTTCTCCGATCTACGCACGAAAGCACACGGTTCAACGTGGGTTGACTAGTGGCATAT
TCAACATGCTACATA
+
:FFFFFFFFFFFFFFFFFFFFFFFFFFFFFFFFFFFFFFFFFFFFFFFFFFFFFFFFFFFFFFFFFFFF
FFFFFFFFFFFFFFFFFFFFFFFFFFFFFFFFFFFFFFFFF:FFFFFFF:FFFFFFFFF,F:FFFFFFF
FFFFFF:FFFFFFFF
@A00721:190:HGJW7DSXY:4:1120:25310:26600 2:N:0:TCTGCAAG+GCTGCACT
CTCAAAACTATGTCTTCTATCACATTCAGCTATCGATCTTCTGGATATCTCACTGTAACACATTCCGG
GCAAGACCGTAATGGAATCAGAACTAAGTCTTCTCCGATCTACGCACGAAAGCACACGGTTCAACGTG
GGTTGACTAGTGGCA
+
FFF:FFFFFFFFFFFFFFFFFFFFFFFFFFFFFFFFFFFFFFFFFFFFFFFFFFFFFFFFFFFFFFFFF
FFFFFFFFFFFFFFFFFFFFFFFFFFFFFFFFFFFFFFFFFFFFFFFFFFFFFFFFFFFFFFFFFFFF:F
FFFFFFFFFFFFFFF
@A00721:190:HGJW7DSXY:4:1203:7572:31046 2:N:0:TCTGCAAG+GCTGCACT
CTCAAAACTATGTCTTCTATCACATTCAGCTATCGATCTTCTGGATATCTCACTGTAACACATTCCGG
GCAAGACCGTAATGGAATCAGAACTAAGTCTTCTCCGATCTACGCACGAAAGCACACGGTTCAACGTG
GGTTGACTAGTGGCA
+
FFFFFF:FFF:FFFFFFFFFFFFFFFFFFFFFFFFFF:FFFFFF,FFFF:F:FFFF:FFFFFFFFF,,F
FFFFFFFFFFFFF,FFFFFFFFFF:FFF:FFFF:FFFFFFFFF,FFFFFFFFFFFFF,FFFFFFFFF:
FFFF:FFFFFFF:FF
@A00721:190:HGJW7DSXY:4:1478:31873:29700 2:N:0:TCTGCAAG+GCTGCACT
CTCAAAACTATGTCTTCTATCACATTCAGCTATCGATCTTCTGGATATCTCACTGTAACACATTCCGG
GCAAGACCGTAATGGAATCAGAACTAAGTCTTCTCCGATCTACGCACGAAAGCACACGGTTCAACGTG
GGTTGACTAGTGGCA
+
FFFFFFFFFFFFFFFFFFFFFFFFFFFFFFFFF:FFFFFFFFFFFFFFFFFFFFFFFFFFFFFFFFFFF
FFFFFFFFFFFF:FF:FFFFFFFFFF:FFFFFFFFFFFFFFFFFFFFFFFFFFFFFFFFFFFFFFFFFF
FFFFFFFFFFFFFFF
@A00721:190:HGJW7DSXY:4:2270:1470:6668 2:N:0:TCTGCAAG+GCTGCACT
CTCAAAACTATGTCTTCTATCACATTCAGCTATCGATCTTCTGGATATCTCACTGTAACACATTCCGG

```
GCAAGACCGTAATGGAATCAGAACTAAGTCTTCTCCGATCTACGCACGAAAGCACACGGTTCAACGTG
GGTTGACTAGTGGCA
+
FFFFFFFFFFFFFFFFFFFFFFFFFFFFFFFFFFFFFFFFFFFFFFFFFFFFFFFFFFFFFFFFFFFF
FFFFFFFFFFFF:FFFFFFFFFFFFFFFFFFFFFFFFFFFFFF:FFFFFFFFFFFFFFFFFFFFFFFF
FFFFFFFFFFFFFFF
@A00721:190:HGJW7DSXY:4:2346:19859:31062 2:N:0:TCTGCAAG+GCTGCACT
CTCAAAACTATGTCTTCTATCACATTCAGCTATCGATCTTCTGGATATCTCACTGTAACACATTCCGG
GCAAGACCGTAATGGAATCAGAACTAAGTCTTCTCCGATCTACGCACGAAAGCACACGGTTCAACGTG
GGTTGACTAGTGGCA
+
FFFFFFFFF:FFFFFFFFFFFFFFFFFFFFFFFFFFFFFFFFFFFFFFFFFFFFFFFFFFFFFFFFFF
FFFFFFFFFFFF:FFFFFFFFFFFFFFFFFFFFFFFFFFFFFFFFFFFFFFFFFFFF:FFFFFFFF
FFFFFFFFFFFF:F:
@A00721:190:HGJW7DSXY:4:2414:29460:28338 2:N:0:TCTGCAAG+GCTGCACT
CTCAAAACTATGTCTTCTATCACATTCAGCTATCGATCTTCTGGATATCTCACTGTAACACATTCCGG
GCAAGACCGTAATGGAATCAGAACTAAGTCTTCTCCGATCTACGCACGAAAGCACACGGTTCAACGTG
GGTTGACTAGTGGCA
+
FFFFFFFFFFFFFFFFFFFFFFFFFFF:FFFFFFFFFFFFFFFFFFFFFFFFFFFFFFFFFFFFFFFF
FFFFFFFFFFFF,FFF:FFFF:FFFFFFF:FFFFFFFFFFFFFFFFFFFFFFFFFFFFFFFFFFFF:F
FFFFFFFFFFFFFFF
@A00721:190:HGJW7DSXY:4:1274:19180:2832 2:N:0:TCTGCAAG+GCTGCACT
GCAATGACGTTGCTCGATATCTCAAAACTATGTCTTCTATCACATTCAGCTATCGATCTTCTGGATAT
CTCACTGTAACACATTCCGGGCAAGACCGTAATGGAATCAGAACTAAGTCTTCTCCGATCTACGCACG
AAAGCACACGGTTCA
+
FFFFFF:FFFFFFFFFF:FFFFFFFFFFFFFFFFFFFFFFFFFFFFFFFFFFFFFFFFFFFFFFFF:F
FFFFFFFFFFFFFFFFFFFFFFFFFFFFFFFFFFFFFFFFFFFFFFFFFFFFFFFFFFFFFFFFFFFF
FFFFFFFFFFFFFFF
@A00721:190:HGJW7DSXY:4:1601:27543:28839 2:N:0:TCTGCAAG+GCTGCACT
GCAATGACGTTGCTCGATATCTCAAAACTATGTCTTCTATCACATTCAGCTATCGATCTTCTGGATAT
CTCACTGTAACACATTCCGGGCAAGACCGTAATGGAATCAGAACTAAGTCTTCTCCGATCTACGCACG
AAAGCACACGGTTCA
+
FFFF:FFFFFFFFFFFFFFFFFFFFFFFFFFFFFFFFFFFFFFFFFFFFFFFFFF:FFFFFFF:F,F
FFFFFFFFF:FFFFFFFFFFFFFFFFFFFFFFFFFFF,FFFFFFFFFFFFFFFFFF:FFFFFFFFFFFF
FFFFFFFFFFFFFFF
@A00721:190:HGJW7DSXY:4:1538:30110:20322 2:N:0:TCTGCAAG+GCTGCACT
TCAGCAATGACGTTGCTCGATATCTCAAAACTATGTCTTCTATCACATTCAGCTATCGATCTTCTGGA
TATCTCACTGTAACACATTCCGGGCAAGACCGTAATGGAATCAGAACTAAGTCTTCTCCGATCTACGC
ACGAAAGCACACGGT
+
FFFFFFFFFFFFFFFFFFFFFFFFFFFFFFFFFFFFFFFFFFFFFFFFFFFFFFFFFFFFFFFFFFFF
FFFFFFFFFFFFFFFFF:FFFFFFFFFFFFFFFFFFFFFFFFFFFFFFFFFFFFFFFFFFFFFFFFFF
FFFFFFFFFFFFFFF
@A00721:190:HGJW7DSXY:4:1653:12662:3004 2:N:0:TCTGCAAG+GCTGCACT
TCAGCAATGACGTTGCTCGATATCTCAAAACTATGTCTTCTATCACATTCAGCTATCGATCTTCTGGA
TATCTCACTGTAACACATTCCGGGCAAGACCGTAATGGAATCAGAACTAAGTCTTCTCCGATCTACGC
ACGAAAGCACACGGT
+
F:F:FFFFF::FF,FFFF:FFFFF::FFFFF,FFFFFFF:FFFFFFFFFFF,FFFFF:FFFFFFFFFF
FFFFFFFFFF:F,,FFFFFF:FFFFFFFFFF:FFFFF:F,FFF:FFFFFFFFFFFFFFF,F:FFFFFF
FF:FFFFFFFF,F,F
```

@A00721:190:HGJW7DSXY:4:2350:11532:8030 2:N:0:TCTGCAAG+GCTGCACT
TCAGCAATGACGTTGCTCGATATCTCAAAACTATGTCTTCTATCACATTCAGCTATCGATCTTCTGGA
TATCTCACTGTAACACATTCCGGGCAAGACCGTAATGGAATCAGAACTAAGTCTTCTCCGATCTACGC
ACGAAAGCACACGGT
+
FFFFFFFFFFFFFFFFFFFFFF:FFFFFFFFFFFFFFFFFFFFFFFFFFFFFFFFFFFFFFFFFFFFFFF
FFFFFFFFFFFFFFFFFF:FFFFFFFFFFFFFFFFFFFFF,FFFFFFFFFFFFFFFFFFFFFFFFFFFFF
FFFFFFFFFFFFFFF
@A00721:190:HGJW7DSXY:4:1116:18475:31328 2:N:0:TCTGCAAG+GCTGCACT
CTTCAACTTTGGCATCAGCAATGACGTTGCTCGATATCTCAAAACTATGTCTTCTATCACATTCAGCT
ATCGATCTTCTGGATATCTCACTGTAACACATTCCGGGCAAGACCGTAATGGAATCAGAACTAAGTCT
TCTCCGATCTACGCA
+
FFFFFFFFFFFFFFFFFFFFFFFFFFFFFFFFFFFFFFFFFFFFFF:FFFFFFFFFFFFFFFFFFFFFFF
FFFF:FFFFFFFFFFFFFFFFFFFFFFFFFFFFFFF:FFF:FFFFFFFFFFFFF,FFFFFFFFF,FFFFF
FFF:FFFF:FF:,FF
@A00721:190:HGJW7DSXY:4:1136:13602:5071 2:N:0:TCTGCAAG+GCTGCACT
CTTCAACTTTGGCATCAGCAATGACGTTGCTCGATATCTCAAAACTATGTCTTCTATCACATTCAGCT
ATCGATCTTCTGGATATCTCACTGTAACACATTCCGGGCAAGACCGTAATGGAATCAGAACTAAGTCT
TCTCCGATCTACGCA
+
FFFFFFFFFFFFFFFFFFFFFFFFFFFFFFFFFFFF:FFFFFFFFFFFFFFFFFFFFFFFFFFFFFFFFF
FFFFFFFFFFFFFFFFFFFFFFFFFFFFFFFFFFFFFFFFF:FFFFFFFFFFFFFFFFFFFFFFFFFFFF
FFFFFFFFFFFFFFF
@A00721:190:HGJW7DSXY:4:1167:31168:30044 2:N:0:TCTGCAAG+GCTGCACT
CTTCAACTTTGGCATCAGCAATGACGTTGCTCGATATCTCAAAACTATGTCTTCTATCACATTCAGCT
ATCGATCTTCTGGATATCTCACTGTAACACATTCCGGGCAAGACCGTAATGGAATCAGAACTAAGTCT
TCTCCGATCTACGCA
+
FFFFFFFFFFFFFFFFFFFFFFFFFFFFFFFFFFFFFFFFFFFFFFFFFFFFFFFFFFFFFFFFFFFFFF
FFFFFF:FFFFFFFFFFFFFFFFFFFFFFFFFFFFFFF:FFFF:FFFFFFFF,FF,FFFFFF,FFFFFFF
FFFFFFFFFFF:FFF
@A00721:190:HGJW7DSXY:4:2161:18041:7592 2:N:0:TCTGCAAG+GCTGCACT
CTTCAACTTTGGCATCAGCAATGACGTTGCTCGATATCTCAAAACTATGTCTTCTATCACATTCAGCT
ATCGATCTTCTGGATATCTCACTGTAACACATTCCGGGCAAGACCGTAATGGAATCAGAACTAAGTCT
TCTCCGATCTACGCA
+
FFFFFFFFFFFFFFFFFFFFFFFFFFFFFFFFFFFFFFFFFFFFFFFFFFFFFFFFFFFFFFFFFFFFFF
FFFFFFFFFFFFFFFFFFFFFFFFFFFFFFFF:FFFFFFFFFFFFFFFF:FFFFF:FFFFFFFFFFFFFF
FFFFFFF:FFFFFFF
@A00721:190:HGJW7DSXY:4:2241:23511:9612 2:N:0:TCTGCAAG+GCTGCACT
CTTCAACTTTGGCATCAGCAATGACGTTGCTCGATATCTCAAAACTATGTCTTCTATCACATTCAGCT
ATCGATCTTCTGGATATCTCACTGTAACACATTCCGGGCAAGACCGTAATGGAATCAGAACTAAGTCT
TCTCCGATCTACGCA
+
FFFFFFFFFFFFFFFFFFFFFFFFFFFFFFFFFFFFFFFFFFFFF:FFFFFFFFFFFFFFFFFFFFFFFF
FFFFFFFFFFFFFFFFFFFFFFFFFFFFFFFFFFFFFFFFFFFFFFFFFFFFFFFFFFFFFFFFFFFFFF
FFFFFFFFFFFFFFF
@A00721:190:HGJW7DSXY:4:2242:14389:18991 2:N:0:TCTGCAAG+GCTGCACT
CTTCAACTTTGGCATCAGCAATGACGTTGCTCGATATCTCAAAACTATGTCTTCTATCACATTCAGCT
ATCGATCTTCTGGATATCTCACTGTAACACATTCCGGGCAAGACCGTAATGGAATCAGAACTAAGTCT
TCTCCGATCTACGCA
+
FFFFFFFFFFFFFFFFFFFFFFFFFFFFFFFFFFFFFFFFFFFFFFFFFF:FFFFFFFFFFFFFFFFFF,

```
FFFFFFFFFFFFFFFFFFFFFFFFFFFFFFFFFFFFFFFFFFFFFFFF:FFFFFFFFFFFFFFFFFFF
FFFFFFFFFFFFFFF
@A00721:190:HGJW7DSXY:4:2367:32533:31876 2:N:0:TCTGCAAG+GCTGCACT
CTTCAACTTTGGCATCAGCAATGACGTTGCTCGATATCTCAAAACTATGTCTTCTATCACATTCAGCT
ATCGATCTTCTGGATATCTCACTGTAACACATTCCGGGCAAGACCGTAATGGAATCAGAACTAAGACT
TCTCCGATCTACGCA
+
FFFFFFFF:FFFF,FFFF:FF:FFFFF:FFFFFFFF,FFF:FFFFFFF:FFFFFFFF,FFFFFFFFFF
FFFFF,:,FFFFFFFFF:,F:FFFFFF,FFF:FF:FFFFFFFF:,FFFF:FFFFF:FFFFFF:F:,:F
F:FFF,F,FFFFFFF
@A00721:190:HGJW7DSXY:4:2558:18331:34084 2:N:0:TCTGCAAG+GCTGCACT
CTTCAACTTTGGCATCAGCAATGACGTTGCTCGATATCTCAAAACTATGTCTTCTATCACATTCAGCT
ATCGATCTTCTGGATATCTCACTGTAACACATTCCGGGCAAGACCGTAATGGAATCAGAACTAAGTCT
TCTCCGATCTACGCA
+
FFFFFFFFFFFFFFFFFFFFFFFFFFFFFFFFFFFFFFFFFFFFFFFFFFFFFFFFFFFFFFFFFFFF
FFFFFFFFFFFFFFFFFFFFFFFFFFFFFFFFFFFFFFFFFFFFFFFFFFFFFFFFFFFFFFFFFFFF
F:FFFFFFFFFF:FF
@A00721:190:HGJW7DSXY:4:2560:4707:13620 2:N:0:TCTGCAAG+GCTGCACT
CTTCAACTTTGGCATCAGCAATGACGTTGCTCGATATCTCAAAACTATGTCTTCTATCACATTCAGCT
ATCGATCTTCTGGATATCTCACTGTAACACATTCCGGGCAAGACCGTAATGGAATCAGAACTAAGTCT
TCTCCGATCTACGCA
+
FFFFFFFFFFFFFFFFFFFFFFFFFFFFFFFFFFFFFFFFFFFFFFFFFFFFFFFFFFFFFFFFFFFF
FFFFFFF:FFFFFFFFFFFFFFFFFFFF:FFFFFFFFFFFF:FFFFFFFFFFFFFFFFFFFFFFFFFFF
FF:FF:FFFFFFFFF
@A00721:190:HGJW7DSXY:4:2576:10719:25880 2:N:0:TCTGCAAG+GCTGCACT
CTTCAACTTTGGCATCAGCAATGACGTTGCTCGATATCTCAAAACTATGTCTTCTATCACATTCAGCT
ATCGATCTTCTGGATATCTCACTGTAACACATTCCGGGCAAGACCGTAATGGAATCAGAACTAAGTCT
TCTCCGATCTACGCA
+
FFFFFFFFFFFFFFFFFFFFFFFFFFFFFFFFFFFFFFFFFFFFFFFFFFFFFFFFFFFFFFFFFFFF
FFFFFFFFFFFFFFFFFFFFFFFFFFFFFFFFFFFFFFFFFFFFFFFFFFFF:,FFFFFFFFFFFFF:
FFFFFFFFFFFFFFF
@A00721:190:HGJW7DSXY:4:2670:18014:14591 2:N:0:TCTGCAAG+GCTGCACT
CTTCAACTTTGGCATCAGCAATGACGTTGCTCGATATCTCAAAACTATGTCTTCTATCACATTCAGCT
ATCGATCTTCTGGATATCTCACTGTAACACATTCCGGGCAAGACCGTAATGGAATCAGAACTAAGTCT
TCTCCGATCTACGCA
+
FFFFFFFFFFFFFFFFFFFFFFFFFFFFFFFFFFFFFFFFFFFFFFFFFFFFFFFFFFFFFFFFFFFF
FFFFFFFFFFFFFFFFFFFFFFFFFFFFFFFFFFFFFFFFFFFFFFFF,,FFFFFFFFFFFFFFFFFF:
FFFFFFFFFFFFFFF
@A00721:190:HGJW7DSXY:4:1228:7392:24189 2:N:0:TCTGCAAG+GCTGCACT
CGGGCCTAATATCTTCAACTTTGGCATCAGCAATGACGTTGCTCGATATCTCAAAACTATGTCTTCTA
TCACATTCAGCTATCGATCTTCTGGATATCTCACTGTAACACATTCCGGGCAAGACCGTAATGGAATC
AGAACTAAGTCTTCT
+
FFFFFFFFFFFFFFFFFFFFFFFFFFFFFFFFFFFF:FFFFFFFFFFFFFFFFFFFFFFFFFFFFFFFF
FF:FFFFFFFFFFFFFFFFFFFFFFFFFFFFFFFFFFFFFFFFF,FFFFFFFFFFFF:FFFFFFFFFFF
FFFFFFFFFFFFFFF
@A00721:190:HGJW7DSXY:4:1350:2745:7153 2:N:0:TCTGCAAG+GCTGCACT
CGGGCCTAATATCTTCAACTTTGGCATCAGCAATGACGTTGCTCGATATCTCAAAACTATGTCTTCTA
TCACATTCAGCTATCGATCTTCTGGATATCTCACTGTAACACATTCCGGGCAAGACCGTAATGGAATC
AGAACTAAGTCTTCT
```

+
FFFFFFFFFFFFFFFFFFFFFFFFFFFFFFFFFFFFFFFFFFFFFFFFFFFFFFFFFFFFFFFFFFFFFF
FFFFFFFFFFFFFFFFFFFFFFFFFFFFFFFFFFFFFFFFFFFFF:FFFFFFFFFFFFFFFFFFFFFFFF
FF:FFFFFFFFFFF:
@A00721:190:HGJW7DSXY:4:1350:2284:7169 2:N:0:TCTGCAAG+GCTGCACT
CGGGCCTAATATCTTCAACTTTGGCATCAGCAATGACGTTGCTCGATATCTCAAAACTATGTCTTCTA
TCACATTCAGCTATCGATCTTCTGGATATCTCACTGTAACACATTCCGGGCAAGACCGTAATGGAATC
AGAACTAAGTCTTCT
+
:FFFFFFFFFFFF:FFFFFFFFFFFFFFFFFFFFFFFFFFFFFFFFFFFFFFFFFFFFFFFFFFFFFFF
FFFFFFFFFFFFFFFFFFFFFFFFFFFFFFFFFFFFFFFFFFFFFFFFFFFFFFFFFFFFFFFFFFFFFF
FFFFFFFFFFFFFFF
@A00721:190:HGJW7DSXY:4:1362:2727:36057 2:N:0:TCTGCAAG+GCTGCACT
CGGGCCTAATATCTTCAACTTTGGCATCAGCAATGACGTTGCTCGATATCTCAAAACTATGTCTTCTA
TCACATTCAGCTATCGATCTTCTGGATATCTCACTGTAACACATTCCGGGCAAGACCGTAATGGAATC
AGAACTAAGTCTTCT
+
FFFFFFFFFFFFFFFFFFFFFFFFFFFFFFFFFFFFFFFFFFFFFFFFFFFFFFFFFFFFFFFFFFFFFF
FFFFFFFFFF:FFFFFFFFFFFFFFFFFFFFFFFFFFFFFFFFF,FFFFFFFFFFFFFFFFFFFFF:FFF
FFFFFFFFFFFFFFF
@A00721:190:HGJW7DSXY:4:1366:11867:3255 2:N:0:TCTGCAAG+GCTGCACT
CGGGCCTAATATCTTCAACTTTGGCATCAGCAATGACGTTGCTCGATATCTCAAAACTATGTCTTCTA
TCACATTCAGCTATCGATCTTCTGGATATCTCACTGTAACACATTCCGGGCAAGACCGTAATGGAATC
AGAACTAAGTCTTCT
+
FFFFFFFFFFFFFFFFFFFFFFFFFFFFFFFFFFFFFFFFFFFFFFFFFFFFFFFFFFFFFFFFFFFFFF
FFFFFFFFFFFFFFFFFFFFFFFFFFFFFFFFFFFFFFFFFFFFFFFFFFFFFFFFFFFFFFFFFFFFFF
FFFFFFFFFFFFFFF
@A00721:190:HGJW7DSXY:4:1368:29649:12164 2:N:0:TCTGCAAG+GCTGCACT
CGGGCCTAATATCTTCAACTTTGGCATCAGCAATGACGTTGCTCGATATCTCAAAACTATGTCTTCTA
TCACATTCAGCTATCGATCTTCTGGATATCTCACTGTAACACATTCCGGGCAAGACCGTAATGGAATC
AGAACTAAGTCTTCT
+
FFFFFFFFFFFFFFFFFFFFFFFFFFFFFFFFFFFFFFFFFFFFFFFFFFFFFFFFFFFFFFFFFFFFFF
FFFFFFFFFFFFFFFFFFFFFFFFFFFFFFFFFFFFFFFFFFFFFFFFFFFF:FFFFFFFFFFFFFFFFF
FFFFFFFFFFFFFFF
@A00721:190:HGJW7DSXY:4:2220:16812:31641 2:N:0:TCTGCAAG+GCTGCACT
CGGGCCTAATATCTTCAACTTTGGCATCAGCAATGACGTTGCTCGATATCTCAAAACTATGTCTTCTA
TCACATTCAGCTATCGATCTTCTGGATATCTCACTGTAACACATTCCGGGCAAGACCGTAATGGAATC
AGAACTAAGTCTTCT
+
FFFFFF:FFFFFFFFFFFFFFF:FFFFFFFF:FFFFFFFFFFFFFFFFFFFFFFFFFFFFFFFFFFFFFF
FFFFFFFFFFFFFFFFFFFFFFFFFFFFFFFFFFFFFFFFFFFFF:FFFFFFF,FFFFFF::FFFF,:FF
FFFFFFFFFFFFFFF
@A00721:190:HGJW7DSXY:4:2220:16333:32002 2:N:0:TCTGCAAG+GCTGCACT
CGGGCCTAATATCTTCAACTTTGGCATCAGCAATGACGTTGCTCGATATCTCAAAACTATGTCTTCTA
TCACATTCAGCTATCGATCTTCTGGATATCTCACTGTAACACATTCCGGGCAAGACCGTAATGGAATC
AGAACTAAGTCTTCT
+
FFFFFFFFFFFFFFFFFFFFFFFFFFFFFFFFFFFFFFFFFFFFFFFFFFFFFFFFFFFFFFFFFFFFFF
FFFFFFFFFFFFFFFFFFFFFFFFFFFFFFFFFFFFFFFFFFFFFFFFFFFFFFFFFFFFFFFFFFF,FF
FFFFFFFFFFFFFFF
@A00721:190:HGJW7DSXY:4:2302:1145:2440 2:N:0:TCTGCAAG+GCTGCACT
CGGGCCTAATATCTTCAACTTTGGCATCAGCAATGACGTTGCTCGATATCTCAAAACTATGTCTTCTA

TCACATTCAGCTATCGATCTTCTGGATATCTCACTGTAACACATTCCGGGCAAGACCGTAATGGAATC
AGAACTAAGTCTTCT
+
FFFFFFFFFFFFFFFFFFFFFFFFFFFFFFFFFFFFFFFFFFFFFFFFFFFFFFFFFFFFFFFFFFFFFF
FFFFFFFFFFFFFFFFFFFFFFFFFFFFFFFFFFFFFFFFFFFFFFF:FFFFFFFFFFFFFFFFFFFFFF
FFFFFFFFFFFFFF:
@A00721:190:HGJW7DSXY:4:2345:32678:36699 2:N:0:TCTGCAAG+GCTGCACT
CGGGCCTAATATCTTCAACTTTGGCATCAGCAATGACGTTGCTCGATATCTCAAAACTATGTCTTCTA
TCACATTCAGCTATCGATCTTCTGGATATCTCACTGTAACACATTCCGGGCAAGACCGTAATGGAATC
AGAACTAAGTCTTCT
+
FFFFFFFFFFFFFFFFFF:FFFFFFFFFFFFFFFFFFFFFFFFFFFFFFFFFFFFFFFFF:FFFFFFFFF
FFFFFFFFFFFFFFFFFFFFFF:FFFFFFFFFFFFFFFFFFFFFFFFFFFFFFF,FFFFFFFFFFFF,FF
FFFFFFFFFFFFFFF
@A00721:190:HGJW7DSXY:4:2348:26982:24424 2:N:0:TCTGCAAG+GCTGCACT
CGGGCCTAATATCTTCAACTTTGGCATCAGCAATGACGTTGCTCGATATCTCAAAACTATGTCTTCTA
TCACATTCAGCTATCGATCTTCTGGATATCTCACTGTAACACATTCCGGGCAAGACCGTAATGGAATC
AGAACTAAGTCTTCT
+
FFFFFFFFFFFFFFFFFFFFFFFFFFFFFFFFFFFFFFFFFFFFFFF:FFFFFFFFFFFFFFFFFFFFFF
FFFFFFFFFFFFFFFFFFFFFFFFFFFFFFFFFFFFFFFFFFFFFFFFFFFFFFFFFFFFFFFFFFF,FF
FFFFFFFFFFFFFFF
@A00721:190:HGJW7DSXY:4:2350:28980:21151 2:N:0:TCTGCAAG+GCTGCACT
CGGGCCTAATATCTTCAACTTTGGCATCAGCAATGACGTTGCTCGATATCTCAAAACTATGTCTTCTA
TCACATTCAGCTATCGATCTTCTGGATATCTCACTGTAACACATTCCGGGCAAGACCGTAATGGAATC
AGAACTAAGTCTTCT
+
:F:FFFFFFFFFFFFFFFFFFFFFFFFFFFFFFF:F:FFFFFFFFFFFFFFFFFFFFFFFFF:FFFFFFF
FFFFFFFFF:FFFFFFFFFFFF:FFFFFFFFFFFFFFFFF,FFF:::F,F:FFF,FF,FFFFFFFFFFFF
F:FFFFFFFFFFFFF
@A00721:190:HGJW7DSXY:4:2445:2266:29904 2:N:0:TCTGCAAG+GCTGCACT
CGGGCCTAATATCTTCAACTTTGGCATCAGCAATGACGTTGCTCGATATCTCAAAACTATGTCTTCTA
TCACATTCAGCTATCGATCTTCTGGATATCTCACTGTAACACATTCCGGGCAAGACCGTAATGGAATC
AGAACTAAGTCTTCT
+
FF:F:FFFFFFFFFFFFFFF:F:FFFFFFFFFF:FFFFFF:FFFF:FFFFFFFFFFFFFFFFFFFFFFFF
FFFFFF::FFFFFFFFFFF:FFF:FFFFF:FFFFFF:FFFFFFFF:,F,F:F:F:FFF:F::F:F:FFFF
FFFFFFFFFFFFFFF
@A00721:190:HGJW7DSXY:4:2476:23990:29606 2:N:0:TCTGCAAG+GCTGCACT
CGGGCCTAATATCTTCAACTTTGGCATCAGCAATGACGTTGCTCGATATCTCAAAACTATGTCTTCTA
TCACATTCAGCTATCGATCTTCTGGATATCTCACTGTAACACATTCCGGGCAAGACCGTAATGGAATC
AGAACTAAGTCTTCT
+
FFFFFFFFFFFFFFFFFFFFFFFFFFFFFFFFFFFFFFFFFFFFFFFFFFFFFFFFFFFFFFFFFFFFFF
FFFFFFFFFFFFFFFFFFFFFFFFFFFFFFFFFFFFFFFFFFFF::FFFFFFFFFFFFFFFF:FFFFFFF
FFFF,FFFFFFFFFF
@A00721:190:HGJW7DSXY:4:2510:15980:26725 2:N:0:TCTGCAAG+GCTGCACT
CGGGCCTAATATCTTCAACTTTGGCATCAGCAATGACGTTGCTCGATATCTCAAAACTATGTCTTCTA
TCACATTCAGCTATCGATCTTCTGGATATCTCACTGTAACACATTCCGGGCAAGACCGTAATGGAATC
AGAACTAAGTCTTCT
+
FFFFFFFFFFFFFFFFFFFFFFFFFFFFFFFFFFFFFFFFFFFFFFFFFFFFFFFFFFFFFFFFFFFFFF
FFFFFFFFFFFFFFFFFFFFFFFFFFFFFFFFFFFFFFFFFFFFFFFFFFFFFFFFFFFFFFFFFFFFFF
FFFFFFFFFFFFFFF

```
@A00721:190:HGJW7DSXY:4:2670:28601:30358 2:N:0:TCTGCAAG+GCTGCACT
CGGGCCTAATATCTTCAACTTTGGCATCAGCAATGACGTTGCTCGATATCTCAAAACTATGTCTTCTA
TCACATTCAGCTATCGATCTTCTGGATATCTCACTGTAACACATTCCGGGCAAGACCGTAATGGAATC
AGAACTAAGTCTTCT
+
FFFFFFFFFFFFFFFFFFFFFFFFFFFFFFFFFFFFFFFFFFFFFFFFFFFFFFFFFFFFFFFFFFFFFF
FFFFFFFFFFFFFFFFFFFFFFFFFFFFFFFFFFFFFFFFFFFFFFFFFFFFFFF:FFFFF:FFFFFFFF
FFFFFFFFFFFFFFF
@A00721:190:HGJW7DSXY:4:1222:24903:6668 2:N:0:TCTGCAAG+GCTGCACT
TCCATTACGGTCTTGCCCGGAATGTGTTACAGTGAGATATCCAGAAGATCGATAGCTGAATGTGATAG
AAGACATAGTTTTGAGATATCGAGCAACGTCATTGCTGATGCCAAAGTTGAAGATATTAGGCCCGAAG
ATAGTGTTAGTCAGA
+
FFFF:FFFFF:FFFFFFFF:FFFFFFFFFFFFFFFFFFFFFFFFFFFFFFFFFFFF,FFFFFFFFFFFFF
FFFF:F:FFFFFFFFFFFFFFF:FFFFFFFFFF:FFFFFFFFFFFFFF:FF:FFF:,F:F:FFFF:FF,F
FFFFFFFFFFFFFFF
@A00721:190:HGJW7DSXY:4:2163:24162:5635 2:N:0:TCTGCAAG+GCTGCACT
TCCATTACGGTCTTGCCCGGAATGTGTTACAGTGAGATATCCAGAAGATCGATAGCTGAATGTGATAG
AAGACATAGTTTTGAGATATCGAGCAACGTCATTGCTGATGCCAAAGTTGAAGATATTAGGCCCGAAG
ATAGTGTTAGTCAGA
+
FFFFFFFFFFFFFFFFFFFFFFFFFFFFFFFFFFFFFFF:FFFFFFFFFFFFFFFFFFFFF:FFFFFF,F
F:FFFFFFFFFFFFFFFFFFFFFFFFFFFFFFFFFFFFFFFFFFF,FF,FFF:F,FFFFFFFFFFFFFFF
F,FFFFFFFFFFFFF
@A00721:190:HGJW7DSXY:4:2252:24822:17519 2:N:0:TCTGCAAG+GCTGCACT
TCCATTACGGTCTTGCCCGGAATGTGTTACAGTGAGATATCCAGAAGATCGATAGCTGAATGTGATAG
AAGACATAGTTTTGAGATATCGAGCAACGTCATTGCTGATGCCAAAGTTGAAGATATTAGGCCCGAAG
ATAGTGTTAGTCAGA
+
FFFFFF:FFFFFFFFFFFFFFFFFFFFFFFFFFFFFFFFFFFFFFFFFFFFFFFFFFFFFFFF,FFF:F
F,FFFFFFFFFFFFFFFFFFFFF:FFFFFFFFFFFFFFFFFFFFFFFFFFFFFF:FF,FFFFFF:FFFFF
F,FFFFFFFFFFFFF
@A00721:190:HGJW7DSXY:4:2437:8269:5228 2:N:0:TCTGCAAG+GCTGCACT
TCCATTACGGTCTTGCCCGGAATGTGTTACAGTGAGATATCCAGAAGATCGATAGCTGAATGTGATAG
AAGACATAGTTTTGAGATATCGAGCAACGTCATTGCTGATGCCAAAGTTGAAGATATTAGGCCCGAAG
ATAGTGTTAGTCAGA
+
FFFFFFFFFFFFFFFFFFFFFFFFF:FFFFFFFFFFFFFFFFF:::FFFFFFFFFFF:FFFFFFFFFF:F
:FFFFFFFFFFF:FFFFFFFFFFFFFFFFFFFFFFFFFFFFFFFFF:FFF:F:FFFFFFF:F,FFFF:FF
FFFFFFFFF:FFFFF
@A00721:190:HGJW7DSXY:4:2611:8856:35243 2:N:0:TCTGCAAG+GCTGCACT
TCCATTACGGTCTTGCCCGGAATGTGTTACAGTGAGATATCCAGAAGATCGATAGCTGAATGTGATAG
AAGACATAGTTTTGAGATATCGAGCAACGTCATTGCTGATGCCAAAGTTGAAGATATTAGGCCCGAAG
ATAGTGTTAGTCAGA
+
FFFFFFFFFFFFFFFFFFFFFFFFFFFFFFFFFFFFFFFFFFFFFFFFFFFFFFFFFFFFFFFFFFFFFF
FFFFFF:FFFFFFFFFFFFFF:FFFFFFFFFFFFFFFFFFFFFFFFFFFFFFFFFF,F:FFFFFFFFFFF
:FFFFFFFFFFFFFF
@A00721:190:HGJW7DSXY:4:2654:5430:31187 2:N:0:TCTGCAAG+GCTGCACT
TCCATTACGGTCTTGCCCGGAATGTGTTACAGTGAGATATCCAGAAGATCGATAGCTGAATGTGATAG
AAGACATAGTTTTGAGATATCGAGCAACGTCATTGCTGATGCCAAAGTTGAAGATATTAGGCCCGAAG
ATAGTGTTAGTCAGA
+
FFFFFFFFFFFFFFFFFFFFFFFFFFFFFFFFF:FFFFFFFFFFFFFFFFFFFFF:FFFFFFFF:FF,FF
```

FFFFF:FFFFF:FFFFFFFFFFFFFFFFFFFFFFFFFFFFFFF,FFFFFFFFFFFF,F:FFFFFFFF
F::FFFF:FFFFFFF
@A00721:190:HGJW7DSXY:4:1160:12391:30843 2:N:0:TCTGCAAG+GCTGCACT
GTTACAGTGAGATATCCAGAAGATCGATAGCTGAATGTGATAGAAGACATAGTTTTGAGATATCGAGC
AACGTCATTGCTGATGCCAAAGTTGAAGATATTAGGCCCGAAGATAGTGTTAGTCAGACGTCGAGTGT
TAGACGAGACCAGTG
+
FFFFFFFFFFFFFFFFFFFFFFFFFFFFFFFFFFFFFFFFFFFFFFFFFFFFFFFFFFFFFFFFFFF
FFFFFFFFFFFFFFFFFFFFFFFFFFFFFFFFFFFFFFFFFFFFFFFFFFFFFFFFFFFFFFFFFFF
FFFFFFFFFFFFFFF
@A00721:190:HGJW7DSXY:4:2437:15691:26412 2:N:0:TCTGCAAG+GCTGCACT
GTTACAGTGAGATATCCAGAAGATCGATAGCTGAATGTGATAGAAGACATAGTTTTGAGATATCGAGC
AACGTCATTGCTGATGCCAAAGTTGAAGATATTAGGCCCGAAGATAGTGTTAGTCAGACGTCGAGTGT
TAGACGAGACCAGTG
+
FFFFFFFFFFFFFFFFFFFFFFFFFFFFFFFFFF:FFFFFFFFFFFFFFFFFFFFFFFFFFFFFFFF
F:FFFFFFFFFFFFFFFFFFFFFFFFFFFFFFFFFFFFFFFFFFF:FFFFFFFFFFFFFFFFFFFFF
FFFFFFFFFFFFFFF
@A00721:190:HGJW7DSXY:4:2437:16170:26678 2:N:0:TCTGCAAG+GCTGCACT
GTTACAGTGAGATATCCAGAAGATCGATAGCTGAATGTGATAGAAGACATAGTTTTGAGATATCGAGC
AACGTCATTGCTGATGCCAAAGTTGAAGATATTAGGCCCGAAGATAGTGTTAGTCAGACGTCGAGTGT
TAGACGAGACCAGTG
+
FFFFFFFFFFFFFFFFFFFF,FFFFFFFFFFFFFF:FFFFFFFFFFFFFFFFF:FFFFF:FFFFFFF
FFFFFFFFFFFFFFFFFFFFFFFFFFFFF,FFFFFFF:FFFFFFFFFFFF:FFFFFFFFFF:FFFFF
FFFFFFFFFFFFFFF
@A00721:190:HGJW7DSXY:4:2173:1931:6965 2:N:0:TCTGCAAG+GCTGCACT
ACAGTGAGATATCCAGAAGATCGATAGCTGAATGTGATAGAAGACATAGTTTTGAGATATCGAGCAAC
GTCATTGCTGATGCCAAAGTTGAAGATATTAGGCCCGAAGATAGTGTTAGTCAGACGTCGAGTGTTAG
ACGAGACCAGTG
+
::FFFFFFFFFF:FFFFF,F:F:FFFFFFFFFFFFFF,FFFFFF:FFF:F,,FFFFFFFFFF:FF:F
F,FF:F:FFFFFF,FFF:F,,FFF:FF,,,F,:FF,F,FFFFF,F:,,FFFFFFF::,F:FFFFFFFF
FFFFFFFFF:::
@A00721:190:HGJW7DSXY:4:1117:10041:28933 2:N:0:TCTGCAAG+GCTGCACT
ATTCATAATGCTGGTCGATCCAGCCAGACACTGGTCTCGTCTAACACTCGACGTCTGACTAACACTAT
CTTCGGGCCTAATATCTTCAACTTTGGCATCAGCAATGACGTTGCTCGATATCTCAAAACTATGTCTT
CTATCACATTCAGCT
+
FFFFFFFFFFFFFFFFFFFFFFFFFFFFFFFFFFFFFFFFFFFFFFFFFFFFFFFFFFFFFFFFFFF
FFFFFFFFFFFFFFFFFFFFFFFFFFFFFFFFFFFFFFFFFFFFFFFFFFFFFFFFFFFFFFFFFFF
FFFFFFFFFFFFFFF
@A00721:190:HGJW7DSXY:4:1275:3351:22169 2:N:0:TCTGCAAG+GCTGCACT
ATTCATAATGCTGGTCGATCCAGCCAGACACTGGTCTCGTCTAACACTCGACGTCTGACTAACACTAT
CTTCGGGCCTAATATCTTCAACTTTGGCATCAGCAATGACGTTGCTCGATATCTCAAAACTATGTCTT
CTATCACATTCAGCT
+
FFFFFFFFFFFFFFFFFFFFFFFFFFFFFFFFFFFFFFFFFFFFFFFFFFFFFFFFFFFFFFFFF:F
FFFFFFFFFFFFFFFFFFFFFFFFFFFFFFFFFFFFFFFFFFFFFFFFFFFFFFFFFFFFFFFFFFF
FFFFFFFFFFFFFFF
@A00721:190:HGJW7DSXY:4:1407:23836:24267 2:N:0:TCTGCAAG+GCTGCACT
ATTCATAATGCTGGTCGATCCAGCCAGACACTGGTCTCGTCTAACACTCGACGTCTGACTAACACTAT
CTTCGGGCCTAATATCTTCAACTTTGGCATCAGCAATGACGTTGCTCGATATCTCAAAACTATGTCTT
CTATCACATTCAGCT

```
+
FFFFFFFFFFFFFFFFFFFFFFFFFFFFFFFFFFFFFFFFFFFFFFFFFFFFFFFFFFFFFFFFFFFFFF
FFFFFFFFFFFFFFFFFFFFFFFFFFFFFFFFFFFFFFFFFFFFFFFFFFFFFFFFFFFFFFFFFFFFFF
FFFFFFFFFFFFFFF
@A00721:190:HGJW7DSXY:4:1545:20871:35916 2:N:0:TCTGCAAG+GCTGCACT
ATTCATAATGCTGGTCGATCCAGCCAGACACTGGTCTCGTCTAACACTCGACGTCTGACTAACACTAT
CTTCGGGCCTAATATCTTCAACTTTGGCATCAGCAATGACGTTGCTCGATATCTCAAAACTATGTCTT
CTATCACATTCAGCT
+
FFFFFFFFFFFFFFFFFFFFFFFFFFFFFFFFFFFFFFFFFFFFFFFFFFFFFFFFFFFFFFFFFFFFFF
FFFFFFFFFFFFFFFFFFFFFFFFFFFFFFFFFFFFFFFFFFFFFFFFFFFFFFFFFFFFFFFFFFFFFF
FFFFFFFFFFFFFFF
@A00721:190:HGJW7DSXY:4:2141:27190:2644 2:N:0:TCTGCAAG+GCTGCACT
ATTCATAATGCTGGTCGATCCAGCCAGACACTGGTCTCGTCTAACACTCGACGTCTGACTAACACTAT
CTTCGGGCCTAATATCTTCAACTTTGGCATCAGCAATGACGTTGCTCGATATCTCAAAACTATGTCTT
CTATCACATTCAGCT
+
FFFFFFFFFFFFFFFFFFFFFFFFFFFFFFFFFFFFFFFFFFFFFFFFFFFFFFFFFFFFFFFFFFFFFF
FFFFFFFFFF,FFFFFFFFFFFFFFFFFFFFFFFFFFFFFFFFFFFFFFFFFFFFFFFFFFFFFFFFFFF
FF:FFFFFFFFFFFF
@A00721:190:HGJW7DSXY:4:2334:28782:13228 2:N:0:TCTGCAAG+GCTGCACT
ATTCATAATGCTGGTCGATCCAGCCAGACACTGGTCTCGTCTAACACTCGACGTCTGACTAACACTAT
CTTCGGGCCTAATATCTTCAACTTTGGCATCAGCAATGACGTTGCTCGATATCTCAAAACTATGTCTT
CTATCACATTCAGCT
+
FFFFFFFFFFFFFFFFFFFFFFFFFFFFFFFFFFFFFFFFFFFFFFFFFFFFFFFFFFFFFFFFFFFFFF
FFFFFFFFFFFFFFFFFFFF:FFFFFFFF:FFFFFFFFFFFFFFFFFFFFFFFFFFFFFFFFFFFFFFFF
FFFF:FFFFFFFFFF
@A00721:190:HGJW7DSXY:4:2407:25880:24987 2:N:0:TCTGCAAG+GCTGCACT
ATTCATAATGCTGGTCGATCCAGCCAGACACTGGTCTCGTCTAACACTCGACGTCTGACTAACACTAT
CTTCGGGCCTAATATCTTCAACTTTGGCATCAGCAATGACGTTGCTCGATATCTCAAAACTATGTCTT
CTATCACATTCAGCT
+
FFFFFFFFFFFFFFFFFFFFFFFFFFFFFFFFFFFFFFFFFFFFFFFFFFFFFFFFFFFFFFFFFFFFFF
FFFFFFFFFFFFFFFFFFFFFFFFFFFFFFFFFFFFFFFFFFFFFFFFFFFFFFFFFFFFFFFFFFFFFF
FFFFFFFFFFF:FFF
@A00721:190:HGJW7DSXY:4:1156:12527:9815 2:N:0:TCTGCAAG+GCTGCACT
CATTCATAATGCTGGTCGATCCAGCCAGACACTGGTCTCGTCTAACACTCGACGTCTGACTAACACTA
TCTTCGGGCCTAATATCTTCAACTTTGGCATCAGCAATGACGTTGCTCGATATCTCAAAACTATGTCT
TCTATCACATTCAGC
+
FFFFFFFFFFFFFFFFFFFFFFFFFFFFFFFFFFFFFFFFFFFFFFFFFFFFFFFFFFFFFFFFFFFFFF
FFFFFFFFFFFFFFFFFFFFFFFFFFFFFFFFFFFFFFFFFFFFFFFFFFFFFFFFFFFFFFFFFFFFFF
FFFFFFFFFFFFFFF
@A00721:190:HGJW7DSXY:4:1336:31882:6918 2:N:0:TCTGCAAG+GCTGCACT
CATTCATAATGCTGGTCGATCCAGCCAGACACTGGTCTCGTCTAACACTCGACGTCTGACTAACACTA
TCTTCGGGCCTAATATCTTCAACTTTGGCATCAGCAATGACGTTGCTCGATATCTCAAAACTATGTCT
TCTATCACATTCAGC
+
FFFFFFFFFFFFFFFFFFFFFFFFFFFFFFFFFFFFFFFFFFFFFFFFFFFFFFFFFFFF:FFFFFFFFF
FFFFFFFFFFFFFFFFFFFFFFFFFFFFFFFFFFFFFFFFFFFFFFFFFFFFFFFFFFFFFFFFFFFFFF
FFFFFFFFFFFFF:F
@A00721:190:HGJW7DSXY:4:1344:31295:19460 2:N:0:TCTGCAAG+GCTGCACT
CATTCATAATGCTGGTCGATCCAGCCAGACACTGGTCTCGTCTAACACTCGACGTCTGACTAACACTA
```

TCTTCGGGCCTAATATCTTCAACTTTGGCATCAGCAATGACGTTGCTCGATATCTCAAAACTATGTCT
TCTATCACATTCAGC
+
FFFFFFFFFFFFFFFFFFFFFFFFFFFFFFFFFFFFFFFFFFFFFFFFFFFFFFFFFFFFFFFFFFFFFF
FFFFFFFFFFFFFFFFF:FFFFFFFFFFFFFFFFFFFFFFFFFFFFFFFFFFFFFFFFFFFFFFFFFFFF
FFFFFFFFFFFFFFF
@A00721:190:HGJW7DSXY:4:1414:32154:24925 2:N:0:TCTGCAAG+GCTGCACT
CATTCATAATGCTGGTCGATCCAGCCAGACACTGGTCTCGTCTAACACTCGACGTCTGACTAACACTA
TCTTCGGGCCTAATATCTTCAACTTTGGCATCAGCAATGACGTTGCTCGATATCTCAAAACTATGTCT
TCTATCACATTCAGC
+
FF:FFFFFFFFFFFFFFFFFFFFFFFFF:FFFFFFFFFFFFFFFFFFFFFFFFFFFFFFFFFFFFFFFFF
FFFFFFFFFFFFFFFFFFFFFFFF:FFFFFFFFFFFFFFFFFFFFFFFFFFFFFFFFFFFFFFFFFFFFF
FFFFFFFFFFF:FF:
@A00721:190:HGJW7DSXY:4:1442:28565:30326 2:N:0:TCTGCAAG+GCTGCACT
CATTCATAATGCTGGTCGATCCAGCCAGACACTGGTCTCGTCTAACACTCGACGTCTGACTAACACTA
TCTTCGGGCCTAATATCTTCAACTTTGGCATCAGCAATGACGTTGCTCGATATCTCAAAACTATGTCT
TCTATCACATTCAGC
+
FFFFFFFFFFFFFFFFFFFFFFFFFFFFFFFFFFFFFFFFFFFFFFFFFFFFFFFFFFFFFFFFFFFFFF
FFFFFFFFFFFFFFFFFFFFFFFFFFFFFFFFFFFFFFFFFFFFFFFFFF:FFFFFFFFFFFFFFFFFFF
FFFFFFFFFFFFFFF
@A00721:190:HGJW7DSXY:4:1464:19153:13338 2:N:0:TCTGCAAG+GCTGCACT
CATTCATAATGCTGGTCGATCCAGCCAGACACTGGTCTCGTCTAACACTCGACGTCTGACTAACACTA
TCTTCGGGCCTAATATCTTCAACTTTGGCATCAGCAATGACGTTGCTCGATATCTCAAAACTATGTCT
TCTATCACATTCAGC
+
FFFFFFFFFFFFFFFFFFFFFFFFFFFFFFFFFFFFFFFFFFFFFFFFFFFFFFFFFFFFFFFFFFFFFF
FFFFFFFFFFFFFFFFFFFFFFFFFFFFFFFFFFFFFFFFFFF:FFFFFFFFFFFFFFFFFFFFFFFFFF
FF:FFFFFFFFFFFF
@A00721:190:HGJW7DSXY:4:1651:30454:3693 2:N:0:TCTGCAAG+GCTGCACT
CATTCATAATGCTGGTCGATCCAGCCAGACACTGGTCTCGTCTAACACTCGACGTCTGACTAACACTA
TCTTCGGGCCTAATATCTTCAACTTTGGCATCAGCAATGACGTTGCTCGATATCTCAAAACTATGTCT
TCTATCACATTCAGC
+
FFFFFFFFFFFFFFFFFFFFFFFFFFFFFFFFFFFFFFFFFFFFFFFFFFFFFFFFFFFFFFFFFFFFFF
FFFFFFFFFFF:FFFFFFFFFFFFFFFFFFFFFFFFFFFFFFFFFFFFFFFFFFFFFFFFFFFFFFFFFF
FFFFFFFFFFFFFF:
@A00721:190:HGJW7DSXY:4:2229:2799:17738 2:N:0:TCTGCAAG+GCTGCACT
CATTCATAATGCTGGTCGATCCAGCCAGACACTGGTCTCGTCTAACACTCGACGTCTGACTAACACTA
TCTTCGGGCCTAATATCTTCAACTTTGGCATCAGCAATGACGTTGCTCGATATCTCAAAACTATGTCT
TCTATCACATTCAGC
+
FFFFFFFFFFFFFFFFFFFFFFFFFFFFFFFFFFFFFFFFFFFFFFF,FFFFFFFFFFFFFFFFFFFFFF
FFFFFFFFFFFFFFFFFFFFFFFF:FF,FFFFFFFFFFFFFFFFFFFFFFFFFFFFFFFFFFFFFFFFFF
FFF,FFFFFFFFFF:
@A00721:190:HGJW7DSXY:4:2241:26684:12226 2:N:0:TCTGCAAG+GCTGCACT
CATTCATAATGCTGGTCGATCCAGCCAGACACTGGTCTCGTCTAACACTCGACGTCTGACTAACACTA
TCTTCGGGCCTAATATCTTCAACTTTGGCATCAGCAATGACGTTGCTCGATATCTCAAAACTATGTCT
TCTATCACATTCAGC
+
FFFFFFFFFFFFFFFFFFFFFFFFFFFFFFFFFFFFFFFFFFFFFFFFFFFFFFFFFFFFFFFFFFFFFF
FFFFFFFFFFFFFFFFFFFFFFFFFFFFFFFFFFFFFFFFFFFFFFFFFFFFFFFFFFFFFFFFFFFFFF
FFFFF:FFFFFFFFF

```
@A00721:190:HGJW7DSXY:4:2241:31204:28948 2:N:0:TCTGCAAG+GCTGCACT
CATTCATAATGCTGGTCGATCCAGCCAGACACTGGTCTCGTCTAACACTCGACGTCTGACTAACACTA
TCTTCGGGCCTAATATCTTCAACTTTGGCATCAGCAATGACGTTGCTCGATATCTCAAAACTATGTCT
TCTATCACATTCAGC
+
FFFFFFFFFFFFFFFFFFFFFFFFFFFF:FFFFFFFFFFFFFF:FFFFFFFFFF:FFFFFFFFF,FFF
FFFFFFFFF:FF:FFFFFFFFFFFF,::FF:FFFFFFF:FFFF:FFFFFFFFF:FFFFFF:F:FFFFF
FFFFF,,FFFF,FF:
@A00721:190:HGJW7DSXY:4:2643:12536:36354 2:N:0:TCTGCAAG+GCTGCACT
CATTCATAATGCTGGTCGATCCAGCCAGACACTGGTCTCGTCTAACACTCGACGTCTGACTAACACTA
TCTTCGGGCCTAATATCTTCAACTTTGGCATCAGCAATGACGTTGCTCGATATCTCAAAACTATGTCT
TCTATCACATTCAGC
+
FFFFFFFFFFFFFFFFFFFFFFFFFFFFFFFFFFFFFFFFFFFFFFFFFFFFFFFFFFFFFFFFFFFF
FFFFFFFFFFFFFFFFFFFFFFFFFFFFFFFFFFFFFFFFFFFFFFFFFFFFFFFFFFFFFFFFFFFF
FFFFFFFFFFFFFF:
@A00721:190:HGJW7DSXY:4:1117:31946:14575 2:N:0:TCTGCAAG+GCTGCACT
GCATTCATAATGCTGGTCGATCCAGCCAGACACTGGTCTCGTCTAACACTCGACGTCTGACTAACACT
ATCTTCGGGCCTAATATCTTCAACTTTGGCATCAGCAATGACGTTGCTCGATATCTCAAAACTATGTC
TTCTATCACATTCAG
+
FFFFFFFFFFFF:FFFFFFFFFFFFFFFFFFFFFFFFFFFFFFFFFFFFFFFFFFFFFF,FFFFFFFF
FFFFFFFFFFFFFFFFF:FFFFFFFFFFFFFFFFFFFFFFFFFFFFFFFFF:FFFFFFFFFFFF,FFFF
FFFFFFFFFFFFFFF
@A00721:190:HGJW7DSXY:4:1237:29243:20322 2:N:0:TCTGCAAG+GCTGCACT
GCATTCATAATGCTGGTCGATCCAGCCAGACACTGGTCTCGTCTAACACTCGACGTCTGACTAACACT
ATCTTCGGGCCTAATATCTTCAACTTTGGCATCAGCAATGACGTTGCTCGATATCTCAAAACTATGTC
TTCTATCACATTCAG
+
::FFFFFFFFFFFFFFFFFFFFFFFFFFFFFFFFFFFFFFFFFFFFFFFFFFFFFFFFFFFFFFFFFF
FFFFFFFFFFFFFFFFFFFFFFFFFFFFFFFFFFFFFFFFFFFFFFFFFFFFFFFFFFFFFFFFFFFF
FFFFFFFFFFFFFFF
@A00721:190:HGJW7DSXY:4:1240:25780:14231 2:N:0:TCTGCAAG+GCTGCACT
GCATTCATAATGCTGGTCGATCCAGCCAGACACTGGTCTCGTCTAACACTCGACGTCTGACTAACACT
ATCTTCGGGCCTAATATCTTCAACTTTGGCATCAGCAATGACGTTGCTCGATATCTCAAAACTATGTC
TTCTATCACATTCAG
+
FFFFFFFFFFFFFFFFFFFFFFFFFFFFFFFFFFFFFFFFFFFFFFFFFFFFFFFFFFFFFFFFFFFF
:FFFFFFFFFFFFFFF:F:FFFFFFFFFFFFFFFFFFFFFFFFFFFFFFFFFFFFFFFFFFFFFFFFF
FFFFFFFFFFFFFFF
@A00721:190:HGJW7DSXY:4:1313:7129:32597 2:N:0:TCTGCAAG+GCTGCACT
GCATTCATAATGCTGGTCGATCCAGCCAGACACTGGTCTCGTCTAACACTCGACGTCTGACTAACACT
ATCTTCGGGCCTAATATCTTCAACTTTGGCATCAGCAATGACGTTGCTCGATATCTCAAAACTATGTC
TTCTATCACATTCAG
+
FFFFFFFFFFFFFFFFFFFFFFFFFFFFFFFFFFFFFFFFFFFFFFFFFFFFFFFFFFFFFFFFFFFF
FFFFFFFF:FFFFFFFFFFFFFFFFFFFFFFFFFFFFFFFFFFFFFFFFFF:FFFFFFFFFFFF:FF
FFFFFFFFFF:FFFF
@A00721:190:HGJW7DSXY:4:1424:19018:33395 2:N:0:TCTGCAAG+GCTGCACT
GCATTCATAATGCTGGTCGATCCAGCCAGACACTGGTCTCGTCTAACACTCGACGTCTTACTAACACT
ATCTTCGGGCCTAATATCTTCAACTTTGGCATCAGCAATGACGTTGCTCGATATCTCAAAACTATGTC
TTCTATCACATTCAG
+
FFFF,FF,FF:FFF,:FFFFF,FFF::FFF,FFF:FFFFF,:FFFFFFFF::FFFFF,,FFFFF:F:F
```

```
FF,FFF,F,:FFF:FFFFFFF:FFF,,:,FFFF,:FFF:,FFFFFFF:FFFF:FFFFFFFFFFFFFF:
:FFFFFF:,FFFF,F
@A00721:190:HGJW7DSXY:4:1475:5267:30185 2:N:0:TCTGCAAG+GCTGCACT
GCATTCATAATGCTGGTCGATCCAGCCAGACACTGGTCTCGTCTAACACTCGACGTCTGACTAACACT
ATCTTCGGGCCTAATATCTTCAACTTTGGCATCAGCAATGACGTTGCTCGATATCTCAAAACTATGTC
TTCTATCACATTCAG
+
FFF,FFFFFFFFFFFFFFFFFFFFFFFFFFFFFFFFFFFFFFFFFFFFFFFFFFFFFFFFFFFFFFFF
FFFFFFFFFFFFFFFFFFFFFFFFFFFFFFFFFFFFFFFFFFFFFFFFFFFFFFFFFFFFFFFFFFFF
FFFFFFFFFFFFFFF
@A00721:190:HGJW7DSXY:4:1531:2148:31892 2:N:0:TCTGCAAG+GCTGCACT
ATTCATAATGCTGGTCGATCCAGCCAGACACTGGTCTCGTCTAACACTCGACGTCTGACTAACACTAT
CTTCGGGCCTAATATCTTCAACTTTGGCATCAGCAATGACGTTGCTCGATATCTCAAAACTATGTCTT
CTATCACATTCAG
+
FF,:FFFFFFF,FFFF:FFFFFFF:FFFFFFFF:FFFFFFF:FF:F:F:FF:FFFFFFFFFFFFFFFF
FFFF:F:FFFFFFF:FF,FFFFFFFFFFFFFFF:FFF:FFFFF:FFFFFFFFFFFFFFFF:FFFFFFF
:FFF:FFFF:FFF
@A00721:190:HGJW7DSXY:4:1657:4092:18161 2:N:0:TCTGCAAG+GCTGCACT
GCATTCATAATGCTGGTCGATCCAGCCAGACACTGGTCTCGTCTAACACTCGACGTCTGACTAACACT
ATCTTCGGGCCTAATATCTTCAACTTTGGCATCAGCAATGACGTTGCTCGATATCTCAAAACTATGTC
TTCTATCACATTCAG
+
FF:FFFFFFFFFFFFFFFFFFFFFFFFFFFFFFFFFFFFFFFFFFFFFFFFFFFFFFFFFFFFFFFFF
FFFFFFFFFFFFFFFFFFFFFFFFFFFF:FFFFFFFFFFFFFFFFFFFFFFFFFFFFFFFFFFFFFFF
FFFFFFFFFFFFFFF
@A00721:190:HGJW7DSXY:4:1657:4806:19586 2:N:0:TCTGCAAG+GCTGCACT
GCATTCATAATGCTGGTCGATCCAGCCAGACACTGGTCTCGTCTAACACTCGACGTCTGACTAACACT
ATCTTCGGGCCTAATATCTTCAACTTTGGCATCAGCAATGACGTTGCTCGATATCTCAAAACTATGTC
TTCTATCACATTCAG
+
FFFFFFFFFFFFFFFFFFFFFFFFFFFFFFFFFFFFFFFFFFFFFFFFFFFFFFFFFFFFFFFFFFFF
FFFFFFFFFFFFFFFFFFFFFFFFFFFFFFFFFFFFFFFFFFFFFFFFFFFF:FFFFFFF:FFFFFFF
FFFFFFFFFFFFFFF
@A00721:190:HGJW7DSXY:4:2276:32904:15765 2:N:0:TCTGCAAG+GCTGCACT
GCATTCATAATGCTGGTCGATCCAGCCAGACACTGGTCTCGTCTAACACTCGACGTCTGACTAACACT
ATCTTCGGGCCTAATATCTTCAACTTTGGCATCAGCAATGACGTTGCTCGATATCTCAAAACTATGTC
TTCTATCACATTCAG
+
FFFFFFFFFFFFFFFFFFFFFFFFFFFFFFFFF,FFFFFFFFFFFFFFFFFFFFFFFFFFFFFFFFFF
FFFFFFFFFFFFFFFFFFFFFFFFFFFFFFFFFFFFFFFFFFFFFFFFFFFFFFFFFFFF:FFFF,FF
FFFFFFFFFFFFF:F
@A00721:190:HGJW7DSXY:4:2363:2528:2895 2:N:0:TCTGCAAG+GCTGCACT
GCATTCATAATGCTGGTCGATCCAGCCAGACACTGGTCTCGTCTAACACTCGACGTCTGACTAACACT
ATCTTCGGGCCTAATATCTTCAACTTTGGCATCAGCAATGACGTTGCTCGATATCTCAAAACTATGTC
TTCTATCACATTCAG
+
FFFFFFFFFFFFFFFFFFFFFFFFFFFFFFFFFFFFFFFFFFFFFFFFFFFFFFFFFFFFFFFFFFFF
:FFFFFFFFFFFFFFFFFFFFFFFFFFFFFFFFFFFFFFFFFFFFFFFFFFFFFFFFFFFFFFFFFFF
FFFFFFFFFFFFFFF
@A00721:190:HGJW7DSXY:4:2642:5068:7795 2:N:0:TCTGCAAG+GCTGCACT
GCATTCATAATGCTGGTCGATCCAGCCAGACACTGGTCTCGTCTAACACTCGACGTCTGACTAACACT
ATCTTCGGGCCTAATATCTTCAACTTTGGCATCAGCAATGACGTTGCTCGATATCTCAAAACTATGTC
TTCTATCACATTCAG
```

```
+
FFFFFFFFFFFFFFFFFFFFFFFFFFFFFFFFFFFFFFFFFFFFFFFFFFFFFFFFFFFFFFFFFFF:FFFF
FFFFFFFFFFFFFFFFFFFFFFFFFF:FFFFFFFFFFFFFFFFFFFFFFFFFFFFFFFFFFFFFFFFFFFFF
,FFFFFFF:FFFFFF
@A00721:190:HGJW7DSXY:4:1251:8612:3850 2:N:0:TCTGCAAG+GCTGCACT
GATCAGCATTCATAATGCTGGTCGATCCAGCCAGACACTGGTCTCGTCTAACACTCGACGTCTGACTA
ACACTATCTTCGGGCCTAATATCTTCAACTTTGGCATCAGCAATGACGTTGCTCGATATCTCAAAACT
ATGTCTTCTATCACA
+
FFFFFFFFFFFFFFFFFFFFFFFFFFFFFFFFFFFFFFFFFFFFFFFFFFFFFFFFFFFFFFFFFFFFFFFF
FFFFFFFFFFFFFFFFFFFFFFFFFFFFFFFF,FFFFFFFFFFFFFFFF:FFFFFFFFFFFFFFFFFFFFFF
FFFFFFFFFFF:FFF
@A00721:190:HGJW7DSXY:4:1537:10755:35806 2:N:0:TCTGCAAG+GCTGCACT
GATCAGCATTCATAATGCTGGTCGATCCAGCCAGACACTGGTCTCGTCTAACACTCGACGTCTGACTA
ACACTATCTTCGGGCCTAATATCTTCAACTTTGGCATCAGCAATGACGTTGCTCGATATCTCAAAACT
ATGTCTTCTATCACA
+
FFFFFFFFFFFFFFFFFFFFFFFFFFFFFFFFFFFF:FFFFFFFFFFFFFFFFFFFFFFFFFFFFFFFFFFF
FFFFFFFFFFFFFFFFFFFF:FFFFFFFFFFFFFFFFFFFFFFFFFFFFFFFFFFFFFFFF:FFFFFFFFFF
FFFFFFFFFFFFFFF
@A00721:190:HGJW7DSXY:4:1545:20347:27712 2:N:0:TCTGCAAG+GCTGCACT
GATCAGCATTCATAATGCTGGTCGATCCAGCCAGACACTGGTCTCGTCTAACACTCGACGTCTGACTA
ACACTATCTTCGGGCCTAATATCTTCAACTTTGGCATCAGCAATGACGTTGCTCGATATCTCAAAACT
ATGTCTTCTATCACA
+
FFFFFFFFFFFFFFFFFFFFFFFFFFFFFFFFFFFFFFFFFFFFFFFFFFFFFFFFFFFFFFFFFFFFFFFF
FFFFFFFFFFFFFFFFFFFFFFF:FFFFFFFFFFFFFFF:FFFFFF,FFF::,FFFFFFFFFFFFFFFFFF
FFFFF:FFFFFFFFF
@A00721:190:HGJW7DSXY:4:2321:31295:26882 2:N:0:TCTGCAAG+GCTGCACT
ATCAGCATTCATAATGCTGGTCGATCCAGCCAGACACTGGTCTCGTCTAACACTCGACGTCTGACTAA
CACTATCTTCGGGCCTAATATCTTCAACTTTGGCATCAGCAATGACGTTGCTCGATATCTCAAAACTA
TGTCTTCTATCACA
+
F,FF,FFFFFF:FFFFF,F:FF:FFFFFF::FFF,F,FF,F:F,:FFFFF,FFFFFFFFF,::FF:FF
FFF:F::FFFF,:,:FFFFFF,FF:FFFF:,,,FFFF,FFF,F:::F,FF,FF,FFFF:F::FFFFFF
F:FFFF,FFF,F,F
@A00721:190:HGJW7DSXY:4:2430:4137:2049 2:N:0:TCTGCAAG+GCTGCACT
GATCAGCATTCATAATGCTGGTCGATCCAGCCAGACACTGGTCTCGTCTAACACTCGACGTCTGACTA
ACACTATCTTCGGGCCTAATATCTTCAACTTTGGCATCAGCAATGACGTTGCTCGATATCTCAAAACT
ATGTCTTCTATCACA
+
FFFFFFFFFFFFFFFFFFFFFFFFFFFFFFFFFFFFFFFFFFFFFFFFFFFFFFFFFFFFFFFFFFFFFFFF
FFFFFFFFFFFFFFFFFFFFFFFFFFFFFFFFFF:FFFFFFFFFFFFFFFFFFFFFFFFFFFFFFFFFFFFF
FF:FFFFFFF:FFFF
@A00721:190:HGJW7DSXY:4:2535:27272:34507 2:N:0:TCTGCAAG+GCTGCACT
GATCAGCATTCATAATGCTGGTCGATCCAGCCAGACACTGGTCTCGTCTAACACTCGACGTCTGACTA
ACACTATCTTCGGGCCTAATATCTTCAACTTTGGCATCAGCAATGACGTTGCTCGATATCTCAAAACT
ATGTCTTCTATCACA
+
FFFFFFFFFFFFF,FFFFFFFFFFFFFFFFFFFFFFFFFFFFFFFFFFFFFFFFFFFFFFFFFFFFFFFFFF
FFFFF:FF:FFFFFFFF:FFFFFFFFFFFFFFFFFFFFFFFFF,FFFFFFFFFFFFFFF:FFFFFFFFF
:FFFFFFFFFFFFFF
@A00721:190:HGJW7DSXY:4:2546:6262:12493 2:N:0:TCTGCAAG+GCTGCACT
GATCAGCATTCATAATGCTGGTCGATCCAGCCAGACACTGGTCTCGTCTAACACTCGACGTCTGACTA
```

ACACTATCTTCGGGCCTAATATCTTCAACTTTGGCATCAGCAATGACGTTGCTCGATATCTCAAAACT
ATGTCTTCTATCACA
+
FFFF:FFFFFFFFFFFFFFFFF:FFFFFFFFFFFFFFFFFFFFFFFFFFFFFFFFFFFFFFFFFFFFFF
:FFFFFF:FFFFFFFFF,FFFFFFFFFFFFFFFFFFFFFFFFFFFFFFFFFFFFF:FFFFFFFF:F:
:FFFFFFFFFFFFFF
@A00721:190:HGJW7DSXY:4:2546:7591:12947 2:N:0:TCTGCAAG+GCTGCACT
GATCAGCATTCATAATGCTGGTCGATCCAGCCAGACACTGGTCTCGTCTAACACTCGACGTCTGACTA
ACACTATCTTCGGGCCTAATATCTTCAACTTTGGCATCAGCAATGACGTTGCTCGATATCTCAAAACT
ATGTCTTCTATCACA
+
FFFFFFFFFFFFFFFFFFFFFFFFFFFFFFFFFFFF:FFFFFFFFFFFFFFFFFFFFFFFFFFFFFFF
FFFFFFFFFFFFFFFFFFFFFFFFFFFFFFFFFFFF:FFFFFFFFFFFFFFFFFFFFFFFFF::FFFF
FFFFFFFFFFFFFFF
@A00721:190:HGJW7DSXY:4:1152:10375:34397 2:N:0:TCTGCAAG+GCTGCACT
CTATCCATAGCAATCTGCAATATATCGACCAGATCAGCATTCATAATGCTGGTCGATCCAGCCAGACA
CTGGTCTCGTCTAACACTCGACGTCTGACTAACACTATCTTCGGGCCTAATATCTTCAACTTTGGCAT
CAGCAATGACGTTGC
+
FFFFFFFFFFFFFFFFFFFFFFFFFFFFFFFFFFFFFFFFFFFFFFFFFFFFFFFFFFFFFFFFFFF:
FFFFFFFFFFFFFFFFFFFFFFFFFFFFFFFFFFFFFFFF:FFFFFFFFFFFFFFFFFFFFFFFFFFF
FFFFFFFFFFFFFF:
@A00721:190:HGJW7DSXY:4:1259:1542:30029 2:N:0:TCTGCAAG+GCTGCACT
CTATCCATAGCAATCTGCAATATATCGACCAGATCAGCATTCATAATGCTGGTCGATCCAGCCAGACA
CTGGTCTCGTCTAACACTCGACGTCTGACTAACACTATCTTCGGGCCTAATATCTTCAACTTTGGCAT
CAGCAATGACGTTGC
+
FFFFFFFFFFFFFFFFFFFFFFFFFFFFFFFFFFFFFFFFFFFFFFFFFFFFFFFFFFFFFFFFFFFF
FFFFFFFFFFFFFFFFFFFFFFFFFFFFFFFFFFFFFFFFFFFFFFFFFFFFFFFFFFFFFFFFFFFF
FFFFFFFFFFFFFFF
@A00721:190:HGJW7DSXY:4:2117:23529:36010 2:N:0:TCTGCAAG+GCTGCACT
CTATCCATAGCAATCTGCAATATATCGACCAGATCAGCATTCATAATGCTGGTCGATCCAGCCAGACA
CTGGTCTCGTCTAACACTCGACGTCTGACTAACACTATCTTCGGGCCTAATATCTTCAACTTTGGCAT
CAGCAATGACGTTGC
+
FFFFFFFFFFFFFFFFFFF:FFFFFFFFFFFFFFFFFFFFFFFFFFFFFFFFFFFFFFFFFFFFFFFF
FFFFFFFFFFFFFFFFFFFFFFFFFFFFFFF:FFFF,FFFFFFFFFFFFFFFFFFFFFFFFFFFFFFF
FFFFF,FFFFFFFFF
@A00721:190:HGJW7DSXY:4:2145:20735:2112 2:N:0:TCTGCAAG+GCTGCACT
CTATCCATAGCAATCTGCAATATATCGACCAGATCAGCATTCATAATGCTGGTCGATCCAGCCAGACA
CTGGTCTCGTCTAACACTCGACGTCTGACTAACACTATCTTCGGGCCTAATATCTTCAACTTTGGCAT
CAGCAATGACGTTGC
+
FFFFFFFFFFFFFFFFFFFFFFFFFFFFFFFFFFFFFFFFFFFFFFFFFFFFFFFFFFFFFFFFFFFF
FFFFFFFFFFFFFFFFFFFFFFFFFFFFFFFFFFFFFFFFFFFFFFFFFFFFFFFFFFFFFFFFFFFF
FFFFFFFFFFFFFFF
@A00721:190:HGJW7DSXY:4:2545:25997:27038 2:N:0:TCTGCAAG+GCTGCACT
CAAATATTGATTTTTTTCCCCTATCCATAGCAATCTGCAATATATCGACCAGATCAGCATTCATAATG
CTGGTCGATCCAGCCAGACACTGGTCTCGTCTAACACTCGACGTCTGACTAACACTATCTTCGGGCCT
AATATCTTCAACTTT
+
FFFFFFFFFFFFFFFFFFFFFFFFFFFFFFFFFFFFFFFFFFFFFFFFFFFFFFFFFFFFFFFFFFFF
FFFFFFFFFFFFFFFFFFFFFFFFFFFFFFFFFFFFFFFFFFFFFFFFFFFFFFFFF,FFFFFFFFFF
FFFFFFFFFFFFFFF

```
@A00721:190:HGJW7DSXY:4:2676:26115:7513 2:N:0:TCTGCAAG+GCTGCACT
CAAATATTGATTTTTTTCCCCTATCCATAGCAATCTGCAATATATCGACCAGATCAGCATTCATAATG
CTGGTCGATCCAGCCAGACACTGGTCTCGTCTAACACTCGACGTCTGACTAACACTATCTTCGGGCCT
AATATCTTCAACTTT
+
FFFFFFFFFFFFFFFFFFFFFFFFFFFFFFFFFFFFFFFFFFFFFFFFFFFFFFFFFFFFFFFFFFFF
FFFFFFFFFFFFFFFFFFFFFFFFFFFFFFFFFFFFFFFFFFFFFFFFFFFFFFFFFFFFFFFFFFFF
FFFFFFFFFFFFFFF
@A00721:190:HGJW7DSXY:4:1329:2112:8218 2:N:0:TCTGCAAG+GCTGCACT
AATATTGATTTTTTTCCCCTATCCATAGCAATCTGCAATATATCGACCAGATCAGCATTCATAATGCT
GGTCGATCCAGCCAGACACTGGTCTCGTCTAACACTCGACGTCTGACTAACACTATCTTCGGGCCTAA
TATCTTCAACT
+
FFFF:FFFFFFFFFFFFFFFFFFF:FFFFFFFFFFFF:FFFFFFFFFFFFFFFFFFFFFFFFFFFFFF
FFFF:FFFFFFFFFFFFFFFFFFFFFFFFFFFFFFFFFFFFFFFFFFFFFFFFF,FFFFFFFFFFFFF
FFFFFFFFFFF
@A00721:190:HGJW7DSXY:4:1631:5855:22999 2:N:0:TCTGCAAG+GCTGCACT
AATATTGATTTTTTTCCCCTATCCATAGCAATCTGCAATATATCGACCAGATCAGCATTCATAATGCT
GGTCGATCCAGCCAGACACTGGTCTCGTCTAACACTCGACGTCTGACTAACACTATCTTCGGGCCTAA
TATCTTCAACT
+
FFFFFFFFFFF:FFFFFFFFFFFFFFFFFFFF:FFFFFFFFFFFFFFFFFFFFFFFFFFFFFFFFFFF
FFFFFFF:FF:FFFFFFFFFFFFFFFFFFFFFFFFFFFFFFFFFFFFFFFFFFFFFFFFFFFFFFFFF
FFFFFFFFFFF
@A00721:190:HGJW7DSXY:4:2156:10050:13542 2:N:0:TCTGCAAG+GCTGCACT
AATATTGATTTTTTTCCCCTATCCATAGCAATCTGCAATATATCGACCAGATCAGCATTCATAATGCT
GGTCGATCCAGCCAGACACTGGTCTCGTCTAACACTCGACGTCTGACTAACACTATCTTCGGGCCTAA
TATCTTCAACT
+
FFF:FFFF:FFFFFFFFFFF,FFFFFFFFFF:FFFFFFFFFFFFFFFFFFFFFFFFFFFFFFFFFF:F
FFFFFFFFFFFFFFFFFFFFFFFFFF:FFFFFFFFFFFFFF,FFFFFFFFFFFFFFFFFFFFFFFFFF
,FFFFFFFFFF
@A00721:190:HGJW7DSXY:4:2350:20591:4648 2:N:0:TCTGCAAG+GCTGCACT
AATATTGATTTTTTTCCCCTATCCATAGCAATCTGCAATATATCGACCAGATCAGCATTCATAATGCT
GGTCGATCCAGCCAGACACTGGTCTCTTCTAACACTCGACGTCTGACTAACACTATCTTCGGGCCTAA
TATCTTCAACT
+
FF:FF,FFFFFFFFFFFFFFFFF:FFFFFFFFFFFF:F:FFFFFFFFFFFF,,:,FFFFFFFFFF:,F
FFFFF::FFFFF,F:FFFFF,FF:FF,FFFFFFFFFF,FFFFFF,F:FFF:FFFFFFFF,FF,F:FFF
FFF,FFFFFFF
@A00721:190:HGJW7DSXY:4:2355:2935:33442 2:N:0:TCTGCAAG+GCTGCACT
AATATTGATTTTTTTCCCCTATCCATAGCAATCTGCAATATATCGACCAGATCAGCATTCATAATGCT
GGTCGATCCAGCCAGACACTGGTCTCGTCTAACACTCGACGTCTGACTAACACTATCTTCGGGCCTAA
TATCTTCAACT
+
FFFFFFFFFFFFFFFFFFFFFFFFFFFFFFFFFFFFFFFFFFFFFFFFFFFFFFFFFFFFFFFFFFFF
FFFFFFFFFFFFFFFFFFFFFFFFFFFFFFFFFFFFFFFFFFFFFFFF:FFFFFFFFFFFFFFFFFFF
FFFFFFFFFFF
@A00721:190:HGJW7DSXY:4:2419:30346:16689 2:N:0:TCTGCAAG+GCTGCACT
AATATTGATTTTTTTCCCCTATCCATAGCAATCTGCAATATATCGACCAGATCAGCATTCATAATGCT
GGTCGATCCAGCCAGACACTGGTCTCGTCTAACACTCGACGTCTGACTAACACTATCTTCGGGCCTAA
TATCTTCAACT
+
F:FFFFFFFFFFFFFFF:FFFFFFF:FFFFFFFFFFFFFFFFFFFFFFFFFFFFFFFFFF:FFFFFFF
```

FFFFFFFFFFFFFFFFFFFFFFFFFFFFFFFFFFFFFFFFFFFFFFFFFFFFFFFFFFFF:FFFFF::
FFFFFFFFFFF
@A00721:190:HGJW7DSXY:4:2464:1994:15092 2:N:0:TCTGCAAG+GCTGCACT
AATATTGATTTTTTTCCCCTATCCATAGCAATCTGCAATATATCGACCAGATCAGCATTCATAATGCT
GGTCGATCCAGCCAGACACTGGTCTCGTCTAACACTCGACGTCTGACTAACACTATCTTCGGGCCTAA
TATCTTCAACT
+
FF:FFFFF:FFFFFFFFFFFFFFFFF:FFFFFFFFFFFFFFFFF:FFFFFFFFFFFF:FFFFFFFFFF
FFFFFFFFFFFFFFFFFFFFFFFFFFFFFFFFFFFFFFFFFFFFFFFFFFFFFFFFFFFFFFFFFFFF
FFFFFFFFFFF
@A00721:190:HGJW7DSXY:4:2513:11803:9001 2:N:0:TCTGCAAG+GCTGCACT
AATATTGATTTTTTTCCCCTATCCATAGCAATCTGCAATATATCGACCAGATCAGCATTCATAATGCT
GGTCGATCCAGCCAGACACTGGTCTCGTCTAACACTCGACGTCTGACTAACACTATCTTCGGGCCTAA
TATCTTCAACT
+
FFFFFFFFFFFFFFFFFFFFFFFFFFFFFFFFFFFFFFFFFFFFFFFFFFFFFFFFFFFFFFFFFFFF
FFFFFFFFFFFFFFFFFFFFFFFFFF:FFFFFFFFFFFFFFFFFFFFFFFFFFFFFFFFFFFFFFFFF
FFFFFFFFFFF
@A00721:190:HGJW7DSXY:4:1663:5701:34413 2:N:0:TCTGCAAG+GCTGCACT
AATAATGATTTTTATCCCCTATCCATAGCAATCTGCAATATATCGACCAGATAAGCATTCATAATGCT
GGTAGAACAAGCCAGACACTGGTCTCGTCTAACAATCGACGTCAGACTAACACTAACTTCGGGCCTAA
TATCTTCA
+
FF,F,:,FFF,F,,,F,,F,FFFFF,F,FFFFF,,,FF,F,F:FFFF,F,F,,F,F:F,,F:F:,,:F
,,F,FF,F,F,,:F,FF,F,,F:F:,F,F:FFFF,F:FFF,,,,,FF,FF,FF,F,F:,:,,,F,,FF
:F:F,:FF
@A00721:190:HGJW7DSXY:4:1262:9426:31563 2:N:0:TCTGCAAG+GCTGCACT
ATGACTGTCAAATATTGATTTTTTTCCCCTATCCATAGCAATCTGCAATATATCGACCAGATCAGCAT
TCATAATGCTGGTCGATCCAGCCAGACACTGGTCTCGTCTAACACTCGACGTCTGACTAACACTATCT
TCGGGCCTAATATCT
+
FFFFFFFFFFFFFFFFFFF:FFFFFFFFFFFFFFFFF:FFFFFFFFFFFFFFFFFFF:FFFFFFFFFF
FFFFFFFF:FFFFFFFFFFFFFF:FFFFF:FFFFFFFFF:FFFFFFFFFFFFFFFFFF:FF,FFFFFF
,FFFFFFF,F,FFFF
@A00721:190:HGJW7DSXY:4:1262:10800:32377 2:N:0:TCTGCAAG+GCTGCACT
TGACTGTCAAATATTGATTTTTTTCCCCTATCCATAGCAATCTGCAATATATCGACCAGATCAGCATT
CATAATGCTGGTCGATCCAGCCAGACACTGGTCTCGTCTAACACTCGACGTCTGACTAACACTATCTT
CGGGCCTAATATCT
+
FFFFFFFFFFFFFFFFFFFFFFFFFFFFFFFFFFFFFFFFFFFFFFFFFFFFFFFFFFFFFFFFFFFF
FFFFFFFFFFFFFF:FFFFFFFFFFFFFFFFFFFFFFFFFFFFFFFFFFFFFFFFFFFFFFFFF:FFF
FFFFFFFFFFFFFF
@A00721:190:HGJW7DSXY:4:1315:12192:12148 2:N:0:TCTGCAAG+GCTGCACT
ATGACTGTCAAATATTGATTTTTTTCCCCTATCCATAGCAATCTGCAATATATCGACCAGATCAGCAT
TCATAATGCTGGTCGATCCAGCCAGACACTGGTCTCGTCTAACACTCGACGTCTGACTAACACTATCT
TCGGGCCTAATATCT
+
FFFFFFFFFFFFF:FFFFFFFFFFFFFFFF,FFFFFFFFFFFFFFFFFFFFFFFFFFFFFFFFFFFFF
FFFFFFFFFFFFFFFFFFFFFFFFFFFFFFFFFFFFFFFFFFFFFFFFFFFFFFFFFFFFFFFFFFFF
FFFFFFFFFFFFFFF
@A00721:190:HGJW7DSXY:4:1406:17671:17503 2:N:0:TCTGCAAG+GCTGCACT
ATGACTGTCAAATATTGATTTTTTTCCCCTATCCATAGCAATCTGCAATATATCGACCAGATCAGCAT
TCATAATGCTGGTCGATCCAGCCAGACACTGGTCTCGTCTAACACTCGACGTCTGACTAACACTATCT
TCGGGCCTAATATCT

```
+
FFFFFFFFFFFFFFFFFFFFFFFFFFFFFFFFFFFFFFFFFFFFFFFFFFFFFFFFFFFFFFFFFFFFFF
FFFFFFFFFFFFFFFFFFFFFFFFFFFFFFFFFFFFFFFFFFFFFFFFFFFFFFFFFFFFFFFFFFFFFF
FFFFFFFFFFFFFFF
@A00721:190:HGJW7DSXY:4:1471:3269:1767 2:N:0:TCTGCAAG+GCTGCACT
ATGACTGTCAAATATTGATTTTTTTCCCCTATCCATAGCAATCTGCAATATATCGACCAGATCAGCAT
TCATAATGCTGGTCGATCCAGCCAGACACTGGTCTCGTCTAACACTCGACGTCTGACTAACACTATCT
TCGGGCCTAATATCT
+
FFFFF:FFF,F:FFFFFFFFFFFFFFFFFF:FFFFFFFFFFFFFFFFFFFFFFFFFFFFFFFF:F:,FFF
,FFFFFF,FFFFFFF:FFFFFFFFFFFFF,FFFFFFFFFFFFFFFFFFFFFFFFF,FFFFFFFFFFFFFF
:FFFFFF:FFFFFFF
@A00721:190:HGJW7DSXY:4:1668:4291:2472 2:N:0:TCTGCAAG+GCTGCACT
ATGACTGTCAAATATTGATTTTTTTCCCCTATCCATAGCAATCTGCAATATATCGACCAGATCAGCAT
TCATAATGCTGGTCGATCCAGCCAGACACTGGTCTCGTCTAACACTCGACGTCTGACTAACACTATCT
TCGGGCCTAATATCT
+
FFFFFFFFFFFF,F:FFFFFFFFFFFFFFFFFFFFFFFFFFFFFFFFFFFFFFFFFFFFFFFFFFFFFF:
FFFFFFFFFFFFFFFFFFFFFFFFFFFFFFFFFFFFFFFFFFFFFFFFFFFFFFFFFFFFFFFFFFFFFF
FFFFFFFF:FFFFFF
@A00721:190:HGJW7DSXY:4:2243:16721:36996 2:N:0:TCTGCAAG+GCTGCACT
ATGACTGTCAAATATTGATTTTTTTCCCCTATCCATAGCAATCTGCAATATATCGACCAGATCAGCAT
TCATAATGCTGGTCGATCCAGCCAGACACTGGTCTCGTCTAACACTCGACGTCTGACTAACACTATCT
TCGGGCCTAATATCT
+
FFFFFFFFFFFF:FFFFFFFFFFFFFFFFFFFFFFFFFFFFFFFFFFFFFFFFFFFFFFFFFFFFFFFF
FFFF:FFFFFFFFFFFFFFFFFFFFFFFFFFFFFFFFFFFFFFFFFFFFFFFFFFFFFFFFFFFFFFFF
FFFFFFFFFFFFF,F
@A00721:190:HGJW7DSXY:4:2446:16495:18067 2:N:0:TCTGCAAG+GCTGCACT
ATGACTGTCAAATATTGATTTTTTTCCCCTATCCATAGCAATCTGCAATATATCGACCAGATCAGCAT
TCATAATGCTGGTCGATCCAGCCAGACACTGGTCTCGTCTAACACTCGACGTCTGACTAACACTATCT
TCGGGCCTAATATCT
+
FFFFFFFFFFFFFFFFFFFFFFFFFFFFFFFFFFFFFFFFFFFFFFFFFFFFFFFF:FFFFFFFFFFFFF
FFFFFFFFFFFFFFFFFFFFFFFFFFFFFFFFFFFFFFFFFFFFFFFFFFFFFFFFFFFFFFFFFFFFFF
FFFFFFFFFFFFFFF
@A00721:190:HGJW7DSXY:4:2461:23484:23062 2:N:0:TCTGCAAG+GCTGCACT
ATGACTGTCAAATATTGATTTTTTTCCCCTATCCATAGCAATCTGCAATATATCGACCAGATCAGCAT
TCATAATGCTGGTCGATCCAGCCAGACACTGGTCTCGTCTAACACTCGACGTCTGACTAACACTATCT
TCGGGCCTAATATCT
+
FFFFFFFFFFFFFFFFFFFFFFFFFFFFFFFFFFFFFFFFFFFFFFFFFFFFFFFFFFFFFFFFFFFFFF
FFFFFFFFFFFFFFFFFFFFFFFFFFFFFFFFFFFFFFFFFFFFFFFFFFFFFFFFFFFFFFFFFFFFFF
FFFFFFFFFFFFFFF
@A00721:190:HGJW7DSXY:4:2528:6876:7513 2:N:0:TCTGCAAG+GCTGCACT
ATGACTGTCAAATATTGATTTTTTTCCCCTATCCATAGCAATCTGCAATATATCGACCAGATCAGCAT
TCATAATGCTGGTCGATCCAGCCAGACACTGGTCTCGTCTAACACTCGACGTCTGACTAACACTATCT
TCGGGCCTAATATCT
+
FFFFFFFFFFFFFFFFFFFFFFFFFFFFFFFFFFFFFFFFFFFFFFFFFFFFFFFFFFFFFFFFFFFFFF
FFFFFFFFFFFFFFFFFFFFFFFFFFFFFFFFFFFFFFFFFFFFFFFFFFFFFFFFFFFFFFFFFFFFFF
FFFFFFFFFFFFFFF
@A00721:190:HGJW7DSXY:4:2528:6596:7780 2:N:0:TCTGCAAG+GCTGCACT
ATGACTGTCAAATATTGATTTTTTTCCCCTATCCATAGCAATCTGCAATATATCGACCAGATCAGCAT
```

TCATAATGCTGGTCGATCCAGCCAGACACTGGTCTCGTCTAACACTCGACGTCTGACTAACACTATCT
TCGGGCCTAATATCT
+
FFFFFFFFFFFFFFFFFFFFFFFFFFFFFFFFFFFFFFFFFFFFFFFFFFFFFFFFFFFFFFFFFFFFFF
FFFFFFFFFFFFFFFFFFFFFFFFFFFFFFFFFFFFFFFFFFFFFFFFFFFFFFFFFFFFFFFFFFFFFF
FFFFFFFFFFFFFFF
@A00721:190:HGJW7DSXY:4:1147:28049:2503 2:N:0:TCTGCAAG+GCTGCACT
CCGAAGATAGTGTTAGTCAGACGTCGAGTGTTAGACGAGACCAGTGTCTGGCTGGATCGACCAGCATT
ATGAATGCTGATCTGGTCGATATATTGCAGATTGCTATGGATAGGGGAAAAAAATCAATATTTGACAG
TCATATTACTAAAGA
+
FFFFFFFF:FFFFFFFFFFFFFFFFFFFFFFFFFFFFFFFFFFFFFFFFFFFFFFFFFFFFFFFFFFF:F
FFFFFFFFFFFFFFFFFFFFFFFFFFFFFFFFFFFFFFFF:FF:FFFFF,FFFFFFFFFFFFFFFFFFFF
FFFF:FFFFFFFFFF
@A00721:190:HGJW7DSXY:4:1344:14850:8265 2:N:0:TCTGCAAG+GCTGCACT
CCGAAGATAGTGTTAGTCAGACGTCGAGTGTTAGACGAGACCAGTGTCTGGCTGGATCGACCAGCATT
ATGAATGCTGATCTGGTCGATATATTGCAGATTGCTATGGATAGGGGAAAAAAATCAATATTTGACAG
TCATATTACTAAAGA
+
FFFFFFFFFFFFFFFFFFFFFFFFFFFFFFFFFFFFFFFFFFFFFFFFFFFFFFFFFFFFFFFFFFFFFF
FFFFFFFFFF::FFFFFFFFFFFFFFFFFFFFFFFFFFFFFFFFFFFFFFFFFFFFFFFFFFFFFFFFFF
FFFFFFFFFFFFFFF
@A00721:190:HGJW7DSXY:4:1410:19777:30859 2:N:0:TCTGCAAG+GCTGCACT
CCGAAGATAGTGTTAGTCAGACGTCGAGTGTTAGACGAGACCAGTGTCTGGCTGGATCGACCAGCATT
ATGAATGCTGATCTGGTCGATATATTGCAGATTGCTATGGATAGGGGAAAAAAATCAATATTTGACAG
TCATATTACTAAAGA
+
FFFFFFFFFFFFFFFFFFFFFFFFFFFFFFFFFFFFFFFFFFFFFFFFFFFFFFFFFFFFFFFFFFFF,F
FFFFFFFFFFFFFFFFFFFFFFFFFFFFFFFFFFFF:FFFFFFF::FF:FFFFFFFFFFFFFFFFFFFFF
FF:FFFFFFFFFFFF
@A00721:190:HGJW7DSXY:4:1502:6289:22811 2:N:0:TCTGCAAG+GCTGCACT
CCGAAGATAGTGTTAGTCAGACGTCGAGTGTTAGACGAGACCAGTGTCTGGCTGGATCGACCAGCATT
ATGAATGCTGATCTGGTCGATATATTGCAGATTGCTATGGATAGGGGAAAAAAATCAATATTTGACAG
TCATATTACTAAAGA
+
FFFFF:FFFF,:FFFFFFFFFF::F:FF:FF:FFFFFF:FFFF:FFFFFFFFFFFFF:FFF:F:FFFF
::FFF:FFFFFFF:FFFFFFFFFFFFFFFFFFFF:F:::FF,FF:FFF::FFFFFFFFFF:,FF:FFFF
FFFFFFFFF,,FFFF
@A00721:190:HGJW7DSXY:4:2102:25518:15374 2:N:0:TCTGCAAG+GCTGCACT
CCGAAGATAGTGTTAGTCAGACGTCGAGTGTTAGACGAGACCAGTGTCTGGCTGGATCGACCAGCATT
ATGAATGCTGATCTGGTCGATATATTGCAGATTGCTATGGATAGGGGAAAAAAATCAATATTTGACAG
TCATATTACTAAAGA
+
FFFFFFFFFFFFFFFFFFFFFFFFFFFFFFFFFFFFFFFFFFFFFFFFFFFFFFFFFFFFFFFFFFFFFF
FFFFFFFFFFFFFFFFFFFFFFFFFFFFFFFFFFFFFFFFFFFFFFFFFFFFFFFFFFFFFFFFFFFFFF
FFFFFFFFFFFFF:F
@A00721:190:HGJW7DSXY:4:2251:32072:28886 2:N:0:TCTGCAAG+GCTGCACT
CCGAAGATAGTGTTAGTCAGACGTCGAGTGTTAGACGAGACCAGTGTCTGGCTGGATCGACCAGCATT
ATGAATGCTGATCTGGTCGATATATTGCAGATTGCTATGGATAGGGGAAAAAAATCAATATTTGACAG
TCATATTACTAAAGA
+
FFFFFFFFFFFFFFFFFFFFFFFFFFFFFFFFFFFFFFFFFFFFFFFFFFFFFFFFFFFFFFFFFFFFFF
FFFFFFFFFFFFFFFFFFFFFFFFFFFFFFFFFFFFFFFFF:F:,F,FFFFFFFFFFFFFFF:FFF
FFFFFFFFFFF::FF

```
@A00721:190:HGJW7DSXY:4:2251:32443:28933 2:N:0:TCTGCAAG+GCTGCACT
CCGAAGATAGTGTTAGTCAGACGTCGAGTGTTAGACGAGACCAGTGTCTGGCTGGATCGACCAGCATT
ATGAATGCTGATCTGGTCGATATATTGCAGATTGCTATGGATAGGGGAAAAAAATCAATATTTGACAG
TCATATTACTAAAGA
+
FFFFFFFFFFFFFFFFFFFFFFFFFFFFFFFFFFFFFFFFF:FFFFFFFFFFFFFFFFFFFFFFFFFFFF
FFFFFFFFFFFFFFFFFFFFFFFFFFFFFFFFFFFFFFFFFFFFFFFFFFFFF:FFFFFFFFFFFFFFFF
FFFFFFFFFFFFFFF
@A00721:190:HGJW7DSXY:4:2262:27977:20603 2:N:0:TCTGCAAG+GCTGCACT
CCGAAGATAGTGTTAGTCAGACGTCGAGTGTTAGACGAGACCAGTGTCTGGCTGGATCGACCAGCATT
ATGAATGCTGATCTGGTCGATATATTGCAGATTGCTATGGATAGGGGAAAAAAATCAATATTTGACAG
TCATATTACTAAAGA
+
FFFFFFFFFFFFFFFFFFFFFFFFFFFFFFFFFFFFFFFFFFFFFFFFFFFFFFFFFFFFFFFFFFFFFF
FFFFF:FFFFFFFFFFFFFFFFFFFF,FFFFFFFFFFFF:FFFF:FFFFF:FFFFFFFFFFFFFF,FFFF
F,F:FFFF:FFFF:F
@A00721:190:HGJW7DSXY:4:2372:24641:10567 2:N:0:TCTGCAAG+GCTGCACT
CCGAAGATAGTGTTAGTCAGACGTCGAGTGTTAGACGAGACCAGTGTCTGGCTGGATCGACCAGCATT
ATGAATGCTGATCTGGTCGATATATTGCAGATTGCTATGGATAGGGGAAAAAAATCAATATTTGACAG
TCATATTACTAAAGA
+
FFFFFFFFFFFFFFFFFFFFFFFFFFFFFFFFFFFFFFFFFFFFFFFFFFFFFFFFFFFFFFFFFFFFFF
FFFFFFFFFFFFFFFFFFFFFFFFFFFFFFFFFFFFFFFFFFFFFFFFFF,FFFFFFFFFFFFF:FFFFF
FFFFFFFF:FFFFFF
@A00721:190:HGJW7DSXY:4:1156:4716:25222 2:N:0:TCTGCAAG+GCTGCACT
TGTTAGTCAGACGTCGAGTGTTAGACGAGACCAGTGTCTGGCTGGATCGACCAGCATTATGAATGCTG
ATCTGGTCGATATATTGCAGATTGCTATGGATAGGGGAAAAAAATCAATATTTGACAGTCATATTACT
AAAGAAAGAAATAAG
+
FFFFFFFFFFFFFFFFFFFFFFFFFFFFFFFFFF:FFFFFFFFFFFFFFFFFFFFFFFFFFFFFFFFFFF
FFFFFFFFFFFFFFFFFF:FFFFFFFFFFFFFFFFFFFFFFFFFFFFFFFFFFFFFFFFFFFFFFFFFFF
FFFFFFFF:FFFFFF
@A00721:190:HGJW7DSXY:4:1357:31611:30248 2:N:0:TCTGCAAG+GCTGCACT
TGTTAGTCAGACGTCGAGTGTTAGACGAGACCAGTGTCTGGCTGGATCGACCAGCATTATGAATGCTG
ATCTGGTCGATATATTGCAGATTGCTATGGATAGGGGAAAAAAATCAATATTTGACAGTCATATTACT
AAAGAAAGAAATAAG
+
F,FFFFFFFF:FFF:,FFFF,FF:FFFFFF:FFFFF:FF:FFFF:FFFFFFFFFFFFFF:FFFFFF::
FFFFFFFFFFFFFFFFFFF:FFFFFFFFFFF:FFFFFFFFF:FFFF:FFFFFFFFFFFFFF:FFF::FFF
FFFFFFFFFFFFFFF
@A00721:190:HGJW7DSXY:4:2546:29496:10895 2:N:0:TCTGCAAG+GCTGCACT
TGTTAGTCAGACGTCGAGTGTTAGACGAGACCAGTGTCTGGCTGGATCGACCAGCATTATGAATGCTG
ATCTGGTCGATATATTGCA
+
:FFFFFFFFFFFFF:FFFFFFFFFFFFFFFFFFFFFFFFFFFFFFFFFFFFFFFFFFFFFFFFFFFFFFFFF
FFFFFFFFFFFFFFFFFFF
@A00721:190:HGJW7DSXY:4:1378:7265:3709 2:N:0:TCTGCAAG+GCTGCACT
GTTAGTCAGACGTCGAGTGTTAGACGAGACCAGTGTCTGGCTGGATCGACCAGCATTATGAATGCTGA
TCTGGTCGATATATTGCAGATTGCTATGGATAGGGGAAAAAAATCAATATTTGACAGTCATATTACTA
AAGAAAGAAATAAGA
+
FFFFFFFFFFFFFFFFFFFFFFFFFFFFFFFFFFFFFFFFFFFFFFFFFFFFFFFFFFFFFFFFFFFFFF
FFFFFFFFFFFFFFFFFFFFFFFFFFFFFFFFFFFFFFFFFFFFFFFFFFFF:FFFFFFFFFFFFFFFF,
FFFF,::FFFFFFFF
```

```
@A00721:190:HGJW7DSXY:4:1450:30074:4288 2:N:0:TCTGCAAG+GCTGCACT
GTTAGTCAGACGTCGAGTGTTAGACGAGACCAGTGTCTGGCTGGATCGACCAGCATTATGAATGCTGA
TCTGGTCGATATATTGCAGATTGCTATGGATAGGGGAAAAAAATCAATATTTGACAGTCATATTACTA
AAGAAAGAAATAAGA
+
FFFFFFFFFFFFFFFFFFFFFFFFFFFFFFFFFFFFFFFFFFFFFFFFFFFFFFFFFFFFFFFFFFFF
FFFFFFFFFFFFFFFFFFFFFF:FFFFFFFFFFFFFFFFFFFFFFFFFFFFFFFFFFFFFFFFFFFFF
FF:FF:FFFFFFFFF
@A00721:190:HGJW7DSXY:4:2238:20889:19445 2:N:0:TCTGCAAG+GCTGCACT
GTTAGTCAGACGTCGAGTGTTAGACGAGACCAGTGTCTGGCTGGATCGACCAGCATTATGAATGCTGA
TCTGGTCGATATATTGCAGATTGCTATGGATAGGGGAAAAAAATCAATATTTGACAGTCATATTACTA
AAGAAAGAAATAAGA
+
FFFFFFFFFFFFFFFFFFFFFFFFFFFFFFFFFFFFFFFFFFFFFFFFFFFFFFFFFFFFFFFFFFFF
FFFFFFFFFFFFFFFFFFFFFFFFFFFFFFFFFFFFFFFFFFFFFFFFFFFFFFFFFFFFFFFFFFFF
FFFFFFFFFFFFFFF
@A00721:190:HGJW7DSXY:4:2245:12689:12696 2:N:0:TCTGCAAG+GCTGCACT
GTTAGTCAGACGTCGAGTGTTAGACGAGACCAGTGTCTGGCTGGATCGACCAGCATTATGAATGCTGA
TCTGGTCGATATATTGCAGATTGCTATGGATAGGGGAAAAAAATCAATATTTGACAGTCATATTACTA
AAGAAAGAAATAAGA
+
FFFFFFFFFFFFFFFFFFFFFFFFFFFFFFFFFFFFFFFFFFFFFFFFFFFFFFFFFFFFFFFFFFFF
FFFFFFFFFFFFFFFFFFFFFFFFFFFFFFFFFFFFFFFFFFFFFFFFFFFFFFFFFFFFFFFFFFFF
,FFFFFFF:FFFFFF
@A00721:190:HGJW7DSXY:4:2308:15700:31657 2:N:0:TCTGCAAG+GCTGCACT
GTTAGTCAGACGTCGAGTGTTAGACGAGACCAGTGTCTGGCTGGATCGACCAGCATTATGAATGCTGA
TCTGGTCGATATATTGCAGATTGCTATGGATAGGGGAAAAAAATCAATATTTGACAGTCATATTACTA
AAGAAAGAAATAAGA
+
FFFFFFFFFFFFFFFFFFFFFFFFFFFFFFFFFFFFFFFFFFFFFFFFFFFFFFFFFFFFFFFFFFFF
FFFFFFFFFFFFFFFFFFFFFFFFFFFFFFFFFFFFFFFFFFFFFFFFFFFFFFFFFFFFFFFFFFFF
FFFFFFFFFFFFFFF
@A00721:190:HGJW7DSXY:4:2378:27877:13322 2:N:0:TCTGCAAG+GCTGCACT
GTTAGTCAGACGTCGAGTGTTAGACGAGACCAGTGTCTGGCTGGATCGACCAGCATTATGAATGCTGA
TCTGGTCGATATATTGCAGATTGCTATGGATAGGGGAAAAAAATCAATATTTGACAGTCATATTACTA
AAGAAAGAAATAAGA
+
FFFFFFFFFFFFFFFFFFFFFFFFFFFFFFFFFFFFFFFFFFFFFFFFFFFFFFFFFFFFFFFFFFFF
FFFFFFFFFFFFFFFFFFFFFFFFFFFFFF,FFFFFFFFFFFFFFFFFFFFFFFFFFFFFFFFFFFFF
FFFFFFFFFFFFFFF
@A00721:190:HGJW7DSXY:4:1555:32696:35916 2:N:0:TCTGCAAG+GCTGCACT
TTTTAGTCAGACTTCTAGTGTTAGACTAGACCAGTGTCTGGCTGGATCGACCAGCATTATGAATTCTG
ATCTGGTCGATATATTGCAGATTGCTATGGATAGGTGAAAAAAATAAATATTTGACAGTCATATTACT
AAAGAAAGAAATAA
+
F,::FFFFF,F,,F:,F,::F,F,F,,F:F,FF:F:F:F,,FF,,FF,,F:FFFFFFFFF,FF,,,F,
FFFF,,F,,FFFFFF,:FFFFFF,FFFF,FFFFF,,FFFFFFFFF,FF,F:FF:FFFFFFFFFFFF,F
FFF,FFF,FFFFFF
@A00721:190:HGJW7DSXY:4:1251:8531:16517 2:N:0:TCTGCAAG+GCTGCACT
ATTTTATTTCATTTCACGTCTTATTTCTTTCTTTAGTAATATGACTGTCAAATATTGATTTTTTTCCC
CTATCCATAGCAATCTGCAATATATCGACCAGATCAGCATTCATAATGCTGGTCGATCCAGCCAGACA
CTGGTCTCGTCTAAC
+
FFFFFFFFFFFFF:FFFFFFF:FFFF:FFFF,FFFFFFFFFFFFFFFFFFFFF,FFFFFFFFFFFFFF
```

FFFFFFFFFFFFFFFFFFF:FFFFFFFFFFFFFFFFFFFFFFFFFFFFFFFFFFFFFFFFFFFFFFFF
FFFFFFFFFFFFFFF
@A00721:190:HGJW7DSXY:4:1270:25409:24643 2:N:0:TCTGCAAG+GCTGCACT
ATTTTATTTCATTTCACGTCTTATTTCTTTCTTTAGTAATATGACTGTCAAATATTGATTTTTTTCCC
CTATCCATAGCAATCTGCAATATATCGACCAGATCAGCATTCATAATGCTGGTCGATCCAGCCAGACA
CTGGTCTCGTCTAAC
+
FFFFFFFFFFFFFFFFFFFFFFFFFFFFFFFFFFFFFFFFFFFFFFFFFFFFFFFFFFFFFFFFFFFF
FFFFFFFFFFFFFFFFFFFFFFFFFFFFFFFFFFF:FFFFFFFFFFFFFFFFFFFFFFFFFFFFFFFF
FFFFFFFFFFFFFFF
@A00721:190:HGJW7DSXY:4:2316:3667:28197 2:N:0:TCTGCAAG+GCTGCACT
ATTTTATTTCATTTCACGTCTTATTTCTTTCTTTAGTAATATGACTGTCAAATATTGATTTTTTTCCC
CTATCCATAGCAATCTGCAATATATCGACCAGATCAGCATTCATAATGCTGGTCGATCCAGCCAGACA
CTGGTCTCGTCTAAC
+
FFFFFFFFFFFFFFFFFFFFFFFFFFFFFFF:F:FFF:FFFFFFFFFFFFFFFFFFFFFFFFFFFFFF
FFFFFFFFFFFFFFFFFFFFFFFFF:FFFFFFFFFFFFFFFFFFFFFFFFFFFFFFFFFFFFFFFFFF
FFFFFFFFFFFFFFF
@A00721:190:HGJW7DSXY:4:2423:1913:35055 2:N:0:TCTGCAAG+GCTGCACT
ATTTTATTTCATTTCACGTCTTATTTCTTTCTTTAGTAATATGACTGTCAAATATTGATTTTTTTCCC
CTATCCATAGCAATCTGCAATATATCGACCAGATCAGCATTCATAATGCTGGTCGATCCAGCCAGACA
CTGGTCTCGTCTAAC
+
FFFFFFFFFFFFFFFFFFFFFFF:FFFFFFFFFFFFFFFFFFFFFFFFFFFFFFFFFFFFFFFFFFF:
FFFFFFFFFFFFFFFFFFFF:FFFFFFFFFFFFFFFFFFFFFFFFFFF,FFFFFFFFFFFFFFFFFFF
FFFFFFFFFFFFFFF
@A00721:190:HGJW7DSXY:4:2613:8124:12774 2:N:0:TCTGCAAG+GCTGCACT
ATTTCATTTCATTTCACGTCTTATTTCTTTCTTTAGTAATATGACTGTCAAATATTGATTTTTTTCCC
CTATCCATAGCAATCTGCAATATATCGACCAGATCAGCATTCATAATGCTGGTCGATCCAGCCAGACA
CTGGTCTCGTCTAAC
+
FFFF:FFFFFFFFFFFFFFFFF:FFF::FFFFFFFFFFFFFFFFFFFFFFFFFFFFFFFFFFFFFFF:
FFFFFFFFFFFFFFFFFFFFFFFFFFFFFFFFFFFFFFFFFFFFFFFFFFFFFFFFFFFFFFFFFFFF
F:F:FFFFFFFFFFF
@A00721:190:HGJW7DSXY:4:1135:20817:17879 2:N:0:TCTGCAAG+GCTGCACT
ATTAGATGATTAATAAAATTACAAATTTTATTTCATTTCACGTCTTATTTCTTTCTTTAGTAATATGA
CTGTCAAATATTGATTTTTTTCCCCTATCCATAGCAATCTGCAATATATCGACCAGATCAGCATTCAT
AATGCTGGTCGATCC
+
FFFFFFFFFFFFF:FFFFFFFFFFFFFFF:FFFFFFFFFFFFFFFFFFFFFFFFFFFFFFFFFFFFFF
FFFFFFFFFFFFFFFFFFFFFFFFFFFFFFFFFFFFFFFFFFFFFFFFFFFFFFFFFFFFFFFFFFFF
FFFFFFFFFFFFFFF
@A00721:190:HGJW7DSXY:4:1301:15628:24831 2:N:0:TCTGCAAG+GCTGCACT
ATTAGATGATTAATAAAATTACAAATTTTATTTCATTTCACGTCTTATTTCTTTCTTTAGTAATATGA
CTGTCAAATATTGATTTTTTTCCCCTATCCATAGCAATCTGCAATATATCGACCAGATCAGCATTCAT
AATGCTGGTCGATCC
+
FFFFFFFFFFFFFFFFFFFFF:FF:FFFFFFFFFFFFFFFFFF:FFFFFF:FFFFFFFFFFFFFFFFF
FFFF:FFFFFFFFFFFFFFFFF,::FFFFFFFFFFFFFFFFFFFFFFFFFFF:FFFFFFFF:FFFFFF
FFFF,FFFFFFFF::
@A00721:190:HGJW7DSXY:4:1301:15682:24893 2:N:0:TCTGCAAG+GCTGCACT
ATTAGATGATTAATAAAATTACAAATTTTATTTCATTTCACGTCTTATTTCTTTCTTTAGTAATATGA
CTGTCAAATATTGATTTTTTTCCCCTATCCATAGCAATCTGCAATATATCGACCAGATCAGCATTCAT
AATGCTGGTCGATCC

```
+
F:FFFFFFFFFFFFFFFF:FFFFFFFFFFFFFFFFFFFFFFFFFFFFFFFFFFFF:FFFFFFFFFFFF
FFFFFFFFFFFFFFFFFFFFFFFFFFFFFFFFF:FFFFFFFFF:FFFFFFFFFFFFFFFFFFFFFFFF
:FFFFFFFFFFFFFF
@A00721:190:HGJW7DSXY:4:1428:9833:15139 2:N:0:TCTGCAAG+GCTGCACT
ATTAGATGATTAATAAAATTACAAATTTTATTTCATTTCACGTCTTATTTCTTTCTTTAGTAATATGA
CTGTCAAATATTGATTTTTTTCCCCTATCCATAGCAATCTGCAATATATCGACCAGATCAGCATTCAT
AATGCTGGTCGATCC
+
F:FFFFFFFFFFFFFFFFFFFFFFFFFFFFFFFFFFFFFFFFFFFFFFFFFFFFFFFFFFFFFFFFFF
FFFFFFFFFFFFFFFFFFFFFFFFFFFFFFFFFFFFFFFFFFFFFFFFFF:FFFFFFFFFFFFFFFFF
F:FFFFFFFFFFFFF
@A00721:190:HGJW7DSXY:4:1576:22173:4382 2:N:0:TCTGCAAG+GCTGCACT
ATTAGATGATTAATAAAATTACAAATTTTATTTCATTTCACGTCTTATTTCTTTCTTTAGTAATATGA
CTGTCAAATATTGATTTTTTTCCCCTATCCATAGCAATCTGCAATATATCGACCAGATCAGCATTCAT
AATGCTGGTCGATCC
+
FFFFFF:FFFFFF:FFFFFFFFFFFFFFFFFFFFFFFFFFFFFFFFFFFFFFFFFFFFFFFFFFFFFF
FFFFFFFFFFFFFFFFFFFFFFFFFFFFFFFFFFFFFFFFFFFFFFFFFF:FFFFFFFFFFFFFFFFF
FFFFFFFFFFF,FFF
@A00721:190:HGJW7DSXY:4:2319:13277:26334 2:N:0:TCTGCAAG+GCTGCACT
ATTAGATGATTAATAAAATTACAAATTTTATTTCATTTCACGTCTTCTTTCTTTCTTTAGTAATATGA
CTGTCAAATATTGATTTTTTTCCCCTATCCATAGCAATCTGCAATATATCGACCAGATCAGCATTCAT
AATGCTGGTCGATCC
+
::FFFFFFFFFFFFFFFFFFFFFFFFFFFFFFFFFFFFFFFFFF::,FFFFFFFF::FFFFFFF:FFF
FFFFFFFFFFFFFFFFFFFFFFFFFF:FFFFFFFFFFF:FFFF,FFFFFFFFFFFFFFFFFFFFFFFF
FF:FFFFFFFFFFFF
@A00721:190:HGJW7DSXY:4:2458:26386:5134 2:N:0:TCTGCAAG+GCTGCACT
ATTAGATGATTAATAAAATTACAAATTTTATTTCATTTCACGTCTTATTTCTTTCTTTAGTAATATGA
CTGTCAAATATTGATTTTTTTCCCCTATCCATAGCAATCTGCAATATATCGACCAGATCAGCATTCAT
AATGCTGGTCGATCC
+
FFFFFFFFFFFFF:FFFFFFFFFFFFFFFFFFFFFFFFFFFFFFFFF:FFFFFF:FFFFFF:FFFFFF
FFFFFF::FFFFFFFFFFFFFFFFFFFFFFFFFFFFFFFFFFFFFFFFFFFFFFFFFFFFFFFFFF,F
FFFFFFFFFFFFFFF
@A00721:190:HGJW7DSXY:4:2471:14290:9580 2:N:0:TCTGCAAG+GCTGCACT
ATTAGATGATTAATAAAATTACAAATTTTATTTCATTTCACGTCTTATTTCTTTCTTTAGTAATATGA
CTGTCAAATATTGATTTTTTTCCCCTATCCATAGCAATCTGCAATATATCGACCAGATCAGCATTCAT
AATGCTGGTCGATCC
+
FFFFFFFFFFFFFFFFFFFFFFFFFFFFFFFFFFFFFFFFFFFFFFFFFFFFFFFFFFFFFFFFFFFF
FFFFFFFFFFFFFFFFFFFFFF:FFFFFFFFFFFFFFFFFFFFFFFFFFFFFFFFFFFFF:FFFFFFF
FFF:FFFFFFFFF:F
@A00721:190:HGJW7DSXY:4:2542:12988:22764 2:N:0:TCTGCAAG+GCTGCACT
ATTAGATGATTAATAAAATTACAAATTTTATTTCATTTCACGTCTTATTTCTTTCTTTAGTAATATGA
CTGTCAAATATTGATTTTTTTCCCCTATCCATAGCAATCTGCAATATATCGACCAGATCAGCATTCAT
AATGCTGGTCGATCC
+
FFFFFFFFFFFFFFFFFFFFFFFFFFFFFFFFFFFFFFFFFFFFFFFFFFFFFFFFFFFFFFFFFFFF
FFFFFFFFFFFFFFFFFFFFFFFFFFFFF:FFFFFFFFFFFFFFFFFFFFFFF:FFFFFFFFFFFFFF
FFFFFFFFFF:FF:F
@A00721:190:HGJW7DSXY:4:2666:19298:20885 2:N:0:TCTGCAAG+GCTGCACT
ATTAGATGATTAATAAAATTACAAATTTTATTTCATTTCACGTCTTATTTCTTTCTTTAGTAATATGA
```

CTGTCAAATATTGATTTTTTTCCCCTATCCATAGCAATCTGCAATATATCGACCAGATCAGCATTCAT
AATGCTGGTCGATCC
+
FFFFFF,FFFFFFFFFF:FFFFFF:FFFFFFFFFFFFFFFFFFF::,F:FFFFFF:FFFFFFFFFFF
FFFFFFF:FFFFF:FFFFFFFFFFFFFFFFFFFFF:FFFFFFFFFFFFFFFFFFFFFFFFF:FFFFFF
:FFFFFF:FFFFFFF
@A00721:190:HGJW7DSXY:4:1154:14778:25676 2:N:0:TCTGCAAG+GCTGCACT
ATTAGATGATTAATAAAATTACAAATTTTATTTCATTTCACGTCTTATTTCTTTCTTTAGTAATATGA
CTGTCAAATATTGATTTTTTTCCCCTATCCATAGCAATCTGCAATATATCGACCAGATCAGCATTCAT
AATGCTGGTCGATC
+
FFFFFFFFFFFFFFFFFFFFFFFFFFFFFFFFFFFFFFFFFFFFFFFFFF:,FFFFFFFF,FFF
FFFF:FF:FFFFFFFFFFFFFFFFF,FFFFFFFFFFFFFFFFFFFFFFFFFFFFFFFFFFFFFFFFF
FFFFFFFFFFFFFF
@A00721:190:HGJW7DSXY:4:2168:10312:4852 2:N:0:TCTGCAAG+GCTGCACT
ATTAGATGATTAATAAAATTACAAATTTTATTTCATTTCACGTCTTATTTCTTTCTTTAGTAATATGA
CTGTCAAATATTGATTTTTTTCCCCTATCCATAGCAATCTGCAATATATCGACCAGATCAGCATTCAT
AATGCTGGTCGATC
+
FFFFFFFFFFFFFFFFFFFFFFFF:FFFF:FFFFF:FFFF:FF,FF:FFFFFFF::F:FF:FFFFFFF
FFFF,F,:FFFF,:FFFFFFFFFFFFFF:FFF:F,FFFFFFFFFFFF:FFFFF:FFFF,FFFF:FF:F
FFFFFFFFFF:FFF
@A00721:190:HGJW7DSXY:4:1119:19208:12336 2:N:0:TCTGCAAG+GCTGCACT
TGTATTAGATGATTAATAAAATTACAAATTTTATTTCATTTCACGTCTTATTTCTTTCTTTAGTAATA
TGACTGTCAAATATTGATTTTTTTCCCCTATCCATAGCAATCTGCAATATATCGACCAGATCAGCATT
CATAATGCTGGTCGA
+
FFFFFFFFFFFFFFFFFFFFFFFFFFFFFFFFFFFFFFFFFFFFFFFFFFFFFFFFFFFFFFFFFFFF
FF:FFFFFFFFFFFFFFFFFFFFFFFFFFFFFFFFFFFFFFFFFFFFFFFFFFFFFFFFFFFFFFFFF
FFFFFFFFFFFFFFF
@A00721:190:HGJW7DSXY:4:2175:19407:14716 2:N:0:TCTGCAAG+GCTGCACT
TGTATTAGATGATTAATAAAATTACAAATTTTATTTCATTTCACGTCTTATTTCTTTCTTTAGTAATA
TGACTGTCAAATATTGATTTTTTTCCCCTATCCATAGCAATCTGCAATATATCGACCAGATCAGCATT
CATAATGCTGGTCGA
+
FFFFFFFFFFFFFFFFFFFFFFFFFF:FFFFFFFFFFFFFFFFFFFFFFFFFFFFFFFFFF:FFFFFFFF
FFFFFFFFFFFFFFFFFFFFFFFFFFFFFFFFFFFFFF:FFFFFFFFFFFFFFFFFFFFFFFFFFFFFF
FF:FFFFFFFFFFFF
@A00721:190:HGJW7DSXY:4:2250:30364:6104 2:N:0:TCTGCAAG+GCTGCACT
TGTATTAGATGATTAATAAAATTACAAATTTTATTTCATTTCACGTCTTATTTCTTTCTTTAGTAATA
TGACTGTCAAATATTGATTTTTTTCCCCTATCCATAGCAATCTGCAATATATCGACCAGATCAGCATT
CATAATGCTGGTCGA
+
FFFFFFFFFFFFFFFFFFFFFFFFF::FFFFFFFFFF:FFFFFFFF,:F,FFF:FFFFFFFFFFFFF:
FF:FF:FFF::F,FFFFFFFFFFFFF:FFFFF:FFFF:F:FFFFFFFFFFFFFFFFFFF:F:FFFF:F
FFFFFFF,FFFFFFF
@A00721:190:HGJW7DSXY:4:2253:24840:24815 2:N:0:TCTGCAAG+GCTGCACT
TGTATTAGATGATTAATAAAATTACAAATTTTATTTCATTTCACGTCTTATTTCTTTCTTTAGTAATA
TGACTGTCAAATATTGATTTTTTTCCCCTATCCATAGCAATCTGCAATATATCGACCAGATCAGCATT
AATAATGCTGGTCGA
+
FFFFFFFF:FFFFFFFFFFFFFFFFFF:F:FFFFF,FFFFFFFFFFFFFFFFFF:F:FFFFFFFFFFF
FFF:FFFFFFF:FFF,FFFF:FFF::F:,FFF:F,F:FFFF,F,FFFFFFFFF,FF,FFFFF:F:F::
,FFFFF::FFFF:F:

```
@A00721:190:HGJW7DSXY:4:2502:7383:6136 2:N:0:TCTGCAAG+GCTGCACT
TGTATTAGATGATTAATAAAATTACAAATTTTATTTCATTTCACGTCTTATTTCTTTCTTTAGTAATA
TGACTGTCAAATATTGATTTTTTTCCCCTATCCATAGCAATCTGCAATATATCGACCAGATCAGCATT
CATAATGCTGGTCGA
+
FFFFFFFFFFFFFFFFFFFFFFFFFFFFFFFFFFFFFFFFFFFFFFFFF,FFF:FFFFFFFFFFFFFF
FFFFFFFFFFFFFFFFFFFFFFFFFFFFFFFFFFFF:FFFFFFF:FFFFFFF:FFF:FFFFFFFFFFF
FF:FFFFFFFFFFFF
@A00721:190:HGJW7DSXY:4:1465:30544:5447 2:N:0:TCTGCAAG+GCTGCACT
TTCGATGTATTAGATGATTAATAAAATTACAAATTTTATTTCATTTCACGTCTTATTTCTTTCTTTAG
TAATATGACTGTCAAATATTGATTTTTTTCCCCTATCCATAGCAATCTGCAATATATCGACCAGATCA
GCATTCATAATGCTG
+
FFFFFFFFF:FFFFFFFFFFF:FFFFFFFFFFFFFFFFFFFFFFFFFFFFFFFFFFFFFFFFFFFFFF
FFFFFFFFFFFFFFFFFFFFFFFFFFFFFFFFFF:FFFFFFF,FFFFFFFFFFFFFFFFFFFFFFFFF
FF:FFFFFFFFFFFF
@A00721:190:HGJW7DSXY:4:2226:15745:33144 2:N:0:TCTGCAAG+GCTGCACT
TTCGATGTATTAGATGATTAATAAAATTACAAATTTTATTTCATTTCACGTCTTATTTCTTTCTTTAG
TAATATGACTGTCAAATATTGATTTTTTTCCCCTATCCATAGCAATCTGCAATATATCGACCAGATCA
GCATTCATAATGCTG
+
FFFFFFFFFFFFFFFFFFFFFFFFFFFFFFFFFFFFFFFFFFFFFFFFFFFFFFFFFFFFFFFFFFFF
FFFFFFFFFFFFFFFFFFFFFFFFFFFFFF:FFFFFFFFFFFFFFFFFFFFFFFFFFF:FFFFFFFFFF
FFFFFFFFFFFFFFF
@A00721:190:HGJW7DSXY:4:2261:23077:10864 2:N:0:TCTGCAAG+GCTGCACT
TTCGATGTATTAGATGATTAATAAAATTACAAATTTTATTTCATTTCACGTCTTATTTCTTTCTTTAG
TAATATGACTGTCAAATATTGATTTTTTTCCCCTATCCATAGCAATCTGCAATATATCGACCAGATCA
GCATTCATAATGCTG
+
FF:FFFFFF:FFFFF:FFFFFFFFFFFFFFFFFFFFFFFFF:FFFFFFFFF:FFFFF:FFF:FFFFFFF
FFFFFF:FFFFF:F:FFFF:,FFFFFF:FFFFFFFFF,FFFFFFFF:FFFFFFFFFFFFFFFFF,FFF
FFFFFFF,FFFF:FF
@A00721:190:HGJW7DSXY:4:1260:14281:21559 2:N:0:TCTGCAAG+GCTGCACT
GTTCGATGTATTAGATGATTAATAAAATTACAAATTTTATTTCATTTCACGTCTTATTTCTTTCTTTA
GTAATATGACTGTCAAATATTGATTTTTTTCCCCTATCCATAGCAATCTGCAATATATCGACCAGATC
AGCATTCATAATGCT
+
FFFFFFFFF:FFFFFFFFFFF:FFFFFFFFFFFFFFFFFFFFFFFFFFFFFFFFFFFFFFFFFFFFFF
FFFFFFFFFFFFFFFFFFFFFF:FFFFFFFF,FFFFFFFFFFFFFFFFF,FFFFFFFFFFFF:FFFFF
FFFFFFFFFFFFFFF
@A00721:190:HGJW7DSXY:4:1613:29143:13354 2:N:0:TCTGCAAG+GCTGCACT
GTTCGATGTATTAGATGATTAATAAAATTACAAATTTTATTTCATTTCACGTCTTATTTCTTTCTTTA
GTAATATGACTGTCAAATATTGATTTTTTTCCCCTATCCATAGCAATCTGCAATATATCGACCAGATC
AGCATTCATAATGCT
+
F:FFFF:FFFFFFFFFFFFFFF:F:FFFFFFFFFFFFFFFFFFFFFFFFFFFFF:F,F:FFF:FFFFFFF
F:FFFFFFFF:FFFFFF:FFFFFFFFFFFFFFFF,FFFFF,FFFFFFFFFFFFF:FFFFFFFFFFFFF
FFFFF:FFFFFFFFF
@A00721:190:HGJW7DSXY:4:2233:20907:32033 2:N:0:TCTGCAAG+GCTGCACT
GTTCGATGTATTAGATGATTAATAAAATTACAAATTTTATTTCATTTCACGTCTTATTTCTTTCTTTA
GTAATATGACTGTCAAATATTGATTTTTTTCCCCTATCCATAGCAATCTGCAATATATCGACCAGATC
AGCATTCATAATGCT
+
FFFFFFFFFFFFFFFFFFFFFFFFFFFFFFFF:FFFFFFFFFFFFFFFFFFFFFFFFFFFFFFFFFFF
```

```
FFFFFFFFFFFFFFFFFFFFFFFFFFFFFFFFFFFF:FFFFFFFFFFFFFFFFFFFFF,FF:FFFFF
FFFFFFFFFFFFFFF
@A00721:190:HGJW7DSXY:4:1233:30409:21496 2:N:0:TCTGCAAG+GCTGCACT
CGTTCGATGTATTAGATGATTAATAAAATTACAAATTTTATTTCATTTCACGTCTTATTTCTTTCTTT
AGTAATATGACTGTCAAATATTGATTTTTTTCCCCTATCCATAGCAATCTGCAATATATCGACCAGAT
CAGCATTCATAATGC
+
FFFFFFFFFFFFFFFFFFFFFFFFFFFFFFFFFFFFFFFFFFFFFFFFFFFF:FFF,FFFFFFFFF
FFFFFFFFFFFFFFFFFFFFFFFFFFFFFFFFFFFFFFFFFFFFFFFFFFFFFFFFFFFF:FFFFFFF
FFFFFFFF:FFFFFF
@A00721:190:HGJW7DSXY:4:2118:12852:15295 2:N:0:TCTGCAAG+GCTGCACT
CGTTCGATGTATTAGATGATTAATAAAATTACAAATTTTATTTCATTTCACGTCTTATTTCTTTCTTT
AGTAATATGACTGTCAAATATTGATTTTTTTCCCCTATCCATAGCAATCTGCAATATATCGACCAGAT
CAGCATTCATAATGC
+
FFFFFFFFFFFFFFFFFFFFFFFFFFFFFFFFFF:FFFFFFFFFFFFFF:FFFFFFFFFFFFFFFFFFF
FFFFFFFFFFFFFFFFFFFFFFFF:FFFFFFFFFFFFFFFFFFFFFFFFFFFFFFFFFFFF,FFFFFFF
FFFFFFFFFFFFFF:
@A00721:190:HGJW7DSXY:4:1448:3513:36793 2:N:0:TCTGCAAG+GCTGCACT
CGTTCGATGTATTAGATGATTAATAAAATTACAAATTTTATTTCATTTCACGTCTTATTTCTTTCTTT
AGTAATATGACTGTCAAATATTGATTTTTTTCCCCTATCCATAGCAATCTGCAATATATCGACCAGAT
CAGCATTCATAATG
+
FFFFFFFFFFFFFFFFFFFFFFFFFFFFFFFFFFFFFFFFFFFFFFFFFFFFFFFFFFFFFFFFFFFF
FFFFFFFFFFFFFFFFFFFFFFFFFFFFFFFF:FFFFFFFFFFFFFFFFFFFFFFFFFFFF::FFFFFFF
FF:FFFFFFFFFFF
@A00721:190:HGJW7DSXY:4:2209:18701:31720 2:N:0:TCTGCAAG+GCTGCACT
GTTCGATGTATTAGATGATTAATAAAATTACAAATTTTATTTCATTTCACGTCTTATTTCTTTCTTTA
GTAATATGACTGTCAAATATTGATTTTTTTCACCTATCCATAGCAATATGCAATATATCGACCAGATC
AGCATTCATAAGTCT
+
F,F,FFFFFFFFFFFFFFFFFFF:FFFFFFFF:FFFFFFFFFFFFFFFFFFF:FFFFFFFFFFFFFFF
:FFFFFFF:FFFFFF:FFFFF:FFFFFFFFF,FF,:FFFFFF,FFF,,F,FFFFF:F:,:FF,FFFF,
FFF:F::FFF,,,FF
@A00721:190:HGJW7DSXY:4:2261:23140:11819 2:N:0:TCTGCAAG+GCTGCACT
TCGATGTATTAGATGATTAATAAAATTACAAATTTTATTTCATTTCACGTCTTATTTCTTTCTTTAGT
AATATGACTGTCAAATATTGATTTTTTTCCCCTATCCATAGCAATCTGCAATATATCGACCAGATCAG
CATTCATAA
+
:F:FFFFFF,F:FF:FFFFF:FFFF::FFFFFFFFFFF,F:FFFFFFF,FF,FFF,F:FFFFF:FF:F
FFFFFFFFF:F,FFF:FFF,FFFFFFFFFF:F,FFF,FFFF,FFFFF,:FFF:,F:,,FFFF:FF:F:
,F,,FF:FF
@A00721:190:HGJW7DSXY:4:2646:22209:9737 2:N:0:TCTGCAAG+GCTGCACT
ATTCCAATACACCAGCCAATAGCGTTCGATGTATTAGATGATTAATAAAATTACAAATTTTATTTCAT
TTCACGTCTTATTTCTTTCTTTAGTAATATGACTGTCAAATATTGATTTTTTTCCCCTATCCATAGCA
ATCTGCAATATATCG
+
FFFFFFFFFFFFFFFFFFFFFFFFFFFFFFFFFFFFFFFFFFFFFFFFFFFFFFFFFFFFFFFFFFFF
FFFFFFF,FFFF:FFFFFFFFFFFFFFFFFF:FFFFFFFFFF::FFFFFFFFFFFFFFFFFF:FFFFFFF
:FFFFFFFFFFFFF:
@A00721:190:HGJW7DSXY:4:1365:3396:3396 2:N:0:TCTGCAAG+GCTGCACT
CATTAATTCCAATACACCAGCCAATAGCGTTCGATGTATTAGATGATTAATAAAATTACAAATTTTAT
TTCATTTCACGTCTTATTTCTTTCTTTAGTAATATGACTGTCAAATATTGATTTTTTTCCCCTATCCA
TAGCAATCTGCAATA
```

+
F:FFFFFFFFFFFFFFFFFFFF:FFF:FFFFFFFFFFF:FFFFFFFF:FFFFFFFFFFFFF:FFFFFF
FFFFFFFF:FFFF:,FFFFFFFFFFFFFFFFFFFFFFFFFFF::FFFFFFFFFFFFFFFFF:FFFFFF
FFFFFFFFFFFF:FF
@A00721:190:HGJW7DSXY:4:1436:10493:5822 2:N:0:TCTGCAAG+GCTGCACT
CATTAATTCCAATACACCAGCCAATAGCGTTCGATGTATTAGATGATTAATAAAATTACAAATTTTAT
TTCATTTCACGTCTTATTTCTTTCTTTAGTAATATGACTGTCAAATATTGATTTTTTTCCCCTATCCA
TAGCAATCTGCAATA
+
FFFFFFFFFFFFFFFFFFFFFFF,FFFFFFFFFF:FFF,:FFF:FFFFFFFFFFFFFFFFFFFFFFFF
FFFFFFFFFFFFFFFFFFFFFFFFFFFFFFFFFFFFFFFFFFFFF,FFFFFFFFFFFFFFFFFFFFFF
FFFFFFFFFFFF:FF
@A00721:190:HGJW7DSXY:4:2531:19217:19930 2:N:0:TCTGCAAG+GCTGCACT
CATTAATTCCAATACACCAGCCAATAGCGTTCGATGTATTAGATGATTAATAAAATTACAAATTTTAT
TTCATTTCACGTCTTATTTCTTTCTTTAGTAATATGACTGTCAAATATTGATTTTTTTCCCCTATCCA
TAGCAATCTGCAATA
+
FFFFFFFFFFFFFFFFFFFFFFFFFFFFFFFFFFFFFFFFFFFFFFFFFFFFFFFFFFFFFFFFFFFF
FFFFFFFFFFFFFF:FFFFFFFFFFFFFFFFFFFFFFFFFFFFFFFFFFFFF:FFFFFFFFFFFFFFF
FFFFFFFFFFF:FFF
@A00721:190:HGJW7DSXY:4:2541:15203:6590 2:N:0:TCTGCAAG+GCTGCACT
CATTAATTCCAATACACCAGCCAATAGCGTTCGATGTATTAGATGATTAATAAAATTACAAATTTTAT
TTCATTTCACGTCTTATTTCTTTCTTTAGTAATATGACTGTCAAATATTGATTTTTTTCCCCTATCCA
TAGCAATCTGCAATA
+
FF:FFFFFFFFFFFFFFFFFFFFFFFFFFFFFFFFFFFFFFFFFFFFFFFFFFFFFFFFFFFFFFFFF
FFFFFFFFF:FFFFFFFFF:FFFF:FFFFFFFFFFFFFFFFFFFFFFFFFFFFFFF:FFF:FFFFFFF
FFF:FFFFFFFFFFF
@A00721:190:HGJW7DSXY:4:1572:26277:30655 2:N:0:TCTGCAAG+GCTGCACT
GATACATTAATTCCAATACACCAGCCAATAGCGTTCGATGTATTAGATGATTAATAAAATTACAAATT
TTATTTCATTTCACGTCTTATTTCTTTCTTTAGTAATATGACTGTCAAATATTGATTTTTTTCCCCTA
TCCATAGCAATCTGC
+
FFFFFFFFFFFFFFFFFFFFFFFFFFFFFFFFFFFFFFFFFFFFFFFFFFFFFFFFFFFFFFFFFFFF
FFFFFFFFFFFFFFFFFFFFF:FFFFFFFFFFFFFFFFFFFFFFFFFFFFFFFFFFFFFFFFFFFFF:FF
FF:FFFF:FFFFFFF
@A00721:190:HGJW7DSXY:4:2361:31530:26694 2:N:0:TCTGCAAG+GCTGCACT
GATACATTAATTCCAATACACCAGCCAATAGCGTTCGATGTATTAGATGATTAATAAAATTACAAATT
TTATTTCATTTCACGTCTTATTTCTTTCTTTAGTAATATGACTGTCAAATATTGATTTTTTTCCCCTA
TCCATAGCAATCTGC
+
FFFFFFFFFFFFFFFFFFFFFFFFFFFFFFFFF:FFFFFFFFFFFFFFFFFFF:FFFFFFFFFFFFFF
FFFFFFFFFFFFFFFFF,FFFFFFFFFFFFFFFFFFFFF:FFFFFFFFFFFFF:FFFFF:FFF:::FF
FFFFFF:FFFFFF:F
@A00721:190:HGJW7DSXY:4:2572:29568:34820 2:N:0:TCTGCAAG+GCTGCACT
GATACATTAATTCCAATACACCAGCCAATAGCGTTCGATGTATTAGATGATTAATAAAATTACAAATT
TTATTTCATTTCACGTCTTATTTCTTTCTTTAGTAATATGACTGTCAAATATTGATTTTTTTCCCCTA
TCCATAGCAATCTGC
+
FFFFFFFFFFFFFFFFFFFFFFFFFFFFFFFFFFFFF:FFF:FFFFFFFFFFFF:FFFFFFFFFFFFF
FFFFFFFFFFFFFFFFFFFF,FFFF:FFF,FFFFFFFFFFFFFFFF:FF,FFFFFFFFFFFFFFFF:F
FFFFFFFFFFFFFFF
@A00721:190:HGJW7DSXY:4:1269:8486:25833 2:N:0:TCTGCAAG+GCTGCACT
ATATTTGACAGTCATATTACTAAAGAAAGAAATAAGACGTGAAATGAAATAAAATTTGTAATTTTATT

```
AATCATCTAATACATCGAACGCTATTGGCTGGTGTATTGGAATTAATGTATCTGACATCTGGTCATAG
TGACCTATTAAATAC
+
FFFFFFFFFFFFFFFFFFFFFFFFFFFFFFFFFFFFFFFFFFFFFFFFFFFFFFFFFFFFFFFFFFFFFF
FFFFFFFFFFFFFFFFFFFFFFFFFFFFFF:FFFFFFFFFFFF,FFFFFFFFFFFFFFFFFFFFFFFFFF
FFFFFFFFFFFFFFF
@A00721:190:HGJW7DSXY:4:1611:6298:24987 2:N:0:TCTGCAAG+GCTGCACT
ATATTTGACAGTCATATTACTAAAGAAAGAAATAAGACGTGAAATGAAATAAAATTTGTAATTTTATT
AATCATCTAATACATCGAACGCTATTGGCTGGTGTATTGGAATTAATGTATCTGACATCTGGTCATAG
TGACCTATTAAATAC
+
FFFFFFFFFFFFFFFFFFFFFFFFFFFFFFFF,FFFFFF:FFFFFFFFFFFFFF:FFFFFFFFFFFFFFF
FFFFFFFFFFFFFFFFFFFFFFFFFFFFF,FFFFFFFFFFFFFFFFFFFFF:FFFFFF:FFFFFFFFFF,
FFF:FFFFFFFFFFF
@A00721:190:HGJW7DSXY:4:2621:3432:10942 2:N:0:TCTGCAAG+GCTGCACT
ATATTTGACAGTCATATTACTAAAGAAAGAAATAAGACGTGAAATGAAATAAAATTTGTAATTTTATT
AATCATCTAATACATCGAACGCTATTGGCTGGTGTATTGGAATTAATGTATCTGACATCTGGTCATAG
TGACCTATTAAATAC
+
FF:FFFFFF,FFFFFFFFFFFFFFFFFFFFFFFF:FFFFFFFFFFFFFFFFFFFFF,:FFFFFFFFFFF,
FFFFFFFFFFF::F,:FFFFFFFFFFF:F:FFFFFFF:FFFFFFF:FFFFFFF:F,F:FFFFFFFFF:FF
FFFF:FFFF:FFFFF
@A00721:190:HGJW7DSXY:4:1603:2193:3881 2:N:0:TCTGCAAG+GCTGCACT
TTACTAAAGAAAGAAATAAGACGTGAAATGAAATAAAATTTGTAATTTTATTAATCATCTAATACATC
GAACGCTATTGGCTGGTGTATTGGAATTAATGTATCTGACATCTGGTCATAGTGACCTATTAAATACG
GAAAGTGATTCAGAT
+
FFFFFFFFFFFFFFFFFFFFFFFFFFF:FFFFFFF:FFFFFFFFFFFFFFFFFFFFFFFFFFFFFFFFFF
FFFFFFFFFFFFFFFFFFFFFFFFFFFFFFFFFFFFFFFFFFFFFFFFFFFFFFFFFFFFFFFFFFFFF,
FFFFFFFFFFFFFFF
@A00721:190:HGJW7DSXY:4:2155:10917:13604 2:N:0:TCTGCAAG+GCTGCACT
TTACTAAAGAAAGAAATAAGACGTGAAATGAAATAAAATTTGTAATTTTATTAATCATCTAATACATC
GAACGCTATTGGCTGGTGTATTGGAATTAATGTATCTGACATCTGGTCATAGTGACCTATTAAATACG
GAAAGTGATTCAGAT
+
FFFFFFFFFFFFFFFFFFFFFFFFFFFFFFFFFF,FFFFFFFFFFFFFFFFFFFFFFFFFF:FFFFFFFF
FFFFFFF::FFFFFFFFFFFFFFFFFFFF:FFFFFFFFFFFFFFFFFFFFFFFFFFFFFFFFFFF:FFFF
FFFFFFFFFFFFFFF
@A00721:190:HGJW7DSXY:4:1269:22706:22247 2:N:0:TCTGCAAG+GCTGCACT
AGACGAAATCGTTCTAATGTACGACTACTTTAGATATCCTCCACCAAATCCTGATCTGAATCACTTTC
CGTATTTAATAGGTCACTATGACCAGATGTCAGATACATTAATTCCAATACACCAGCCAATAGCGTTC
GATGTATTAGATGAT
+
FFFFFFFFFFFFFFFFFFFFFFFFFFFFFFFFFFFFFFFFFFFFFFFFFFFFFFFFFFFFFFFFFFFFFF
:FFFFFFFFFFFFFFFFFFFFFFFFFFFFFFFFFFFFFFFFFFFFFFFFFFFFFFFFFFFFFFFFFFFFF
FFFFFFFFFFFFFFF
@A00721:190:HGJW7DSXY:4:1530:18123:29246 2:N:0:TCTGCAAG+GCTGCACT
AGACGAAATCGTTCTAATGTACGACTACTTTAGATATCCTCCACCAAATCCTGATCTGAATCACTTTC
CGTATTTAATAGGTCACTATGACCAGATGTCAGATACATTAATTCCAATACACCAGCCAATAGCGTTC
GATGTATTAGATGAT
+
FFFFFFFFFFFFFFFFFFFFFFFFFFFFFFFFFFFFFFFFFFFFFFFFFFFFFFFFFFFFFFFFFF:FFF
FFFFFFFFFFFFFFFFFFFFFFFFFFFFFFFFFFFF:FFFFFFFFFFFFFFFFFFFFFFFFFFFFFFFFF
FFFFFFFFFFFFFFF
```

```
@A00721:190:HGJW7DSXY:4:1618:3586:16532 2:N:0:TCTGCAAG+GCTGCACT
AGACGAAATCGTTCTAATGTACGACTACTTTAGATATCCTCCACCAAATCCTGATCTGAATCACTTTC
CGTATTTAATAGGTCACTATGACCAGATGTCAGATACATTAATTCCAATACACCAGCCAATAGCGTTC
GATGTATTAGATGAT
+
FFFFFFFFFFFFFFFFFFFFFFFF:FFFFFFFF:FFFFF:FFFFFFFFFFFFFFFFFF:FFFFFFFFF
FFFFFFFFFFF::FFFFFF:FFF:FFFFFFFFFFFFFF,FFFFFF:FFF:FFFFFFFFFFFFFFFFFF
F:,FFFFFFFFFFFF
@A00721:190:HGJW7DSXY:4:2621:10791:21997 2:N:0:TCTGCAAG+GCTGCACT
AGACGAAATCGTTCTAATGTACGACTACTTTAGATATCCTCCACCAAATCCTGATCTGAATCACTTTC
CGTATTTAATAGGTCACTATGACCAGATGTCAGATACATTAATTCCAATACACCAGCCAATAGCGTTC
GATGTATTAGATGAT
+
FF:FFFFFFFFFFF:FFFFFFFFFFFFFFFFFFFFFFFFFFFFFFFFFFFF:FFFFFFFFFFFFFFFF
FFFFFFFFFF,FFFFFFFFFFFFFFFFFFFFFFFFFFFFFFFFFFFFFFFFFFFFFFFFFFFFF:FFF
FFFFFFFFFF:FFFF
@A00721:190:HGJW7DSXY:4:1135:24768:11725 2:N:0:TCTGCAAG+GCTGCACT
CTATTACGAAGAACTGGAATGCAGATGCATGGATTTCGTTGGAATAGACGAAATCGTTCTAATGTACG
ACTACTTTAGATATCCTCCACCAAATCCTGATCTGAATCACTTTCCGTATTTAATAGGTCACTATGAC
CAGATGTCAGATACA
+
FFFFFFFFFFFFFFFFFFFFFFFFF:FFFFFFFFFFFFFFFFF:FFFFFFFFFFFFFFFFFFFFFFFF
FFFFFFFFFFFFFFFFFFFFFFFFFFFFFFFFFFFFFFFFFFFFFFFFFFFFFFFFFFFFFFFFFFFF
FFFFFFFFFFFFFFF
@A00721:190:HGJW7DSXY:4:1315:31557:23860 2:N:0:TCTGCAAG+GCTGCACT
CTATTACGAAGAACTGGAATGCAGATGCATGGATTTCGTTGGAATAGACGAAATCGTTCTAATGTACG
ACTACTTTAGATATCCTCCACCAAATCCTGATCTGAATCACTTTCCGTATTTAATAGGTCACTATGAC
CAGATGTCAGATACA
+
FFFFFFFFFFFFFFFFFFFFFFFFFFFFFFFFFFFFFFFFFFFFFFFFFFFFFFFFFFFFFFFFFFFF
FFFFFFFFFFFFFFFFFFF,FFFFFFFFFFFFFFFFFFFFFFFF:FFFFFFFFFFFFFFFFFFFFFFF:
FFFFFFFFFFFFFFF
@A00721:190:HGJW7DSXY:4:1466:12554:36479 2:N:0:TCTGCAAG+GCTGCACT
CTATTACGAAGAACTGGAATGCAGATGCATGGATTTCGTTGGAATAGACGAAATCGTTCTAATGTACG
ACTACTTTAGATATCCTCCACCAAATCCTGATCTGAATCACTTTCCGTATTTAATAGGTCACTATGAC
CAGATGTCAGATACA
+
FFFFFFFFFF:FFF:FFFF:FFF,FFFFFFFFFFF::FFFF:FFFFFFFFFFFFF:FFFFFFFFFFFF
FFFF:FFFFFFF:FFFFF::FFFFFFF,FFFF,FFFFFFF,FFFFFFFFF:FFFFFFFFFFFFFFFF
FFFFFFFFFFFFFFF
@A00721:190:HGJW7DSXY:4:2111:12093:28698 2:N:0:TCTGCAAG+GCTGCACT
CTATTACGAAGAACTGGAATGCAGATGCATGGATTTCGTTGGAATAGACGAAATCGTTCTAATGTACG
ACTACTTTAGATATCCTCCACCAAATCCTGATCTGAATCACTTTCCGTATTTAATAGGTCACTATGAC
CAGATGTCAGATACA
+
FFFFFFFFFFFFFFFFFFFFFFFFF::FFFFFFFFFFFFFFFFFFFFFFFFFFFFFFFFFFFFFFFFF
FFFFFFFFFFFFFFFFFFFFFFFFFFFFFFFFFFFFFFFFFFFFFFFFFFFFFFFFFFFFFFFFFFFF
FFFFFFFFFFFFFFF
@A00721:190:HGJW7DSXY:4:2145:5647:6699 2:N:0:TCTGCAAG+GCTGCACT
CTATTACGAAGAACTGGAATGCAGATGCATGGATTTCGTTGGAATAGACGAAATCGTTCTAATGTACG
ACTACTTTAGATATCCTCCACCAAATCCTGATCTGAATCACTTTCCGTATTTAATAGGTCACTATGAC
CAGATGTCAGATACA
+
FFFFFFFFFFFFFFFFFFFFFFFFFFFFFFFFFFFFFFFFFFFFFFFFFFFFFFFFFFFFFFFFFFFF
```

FFFFFFFFFFFFFFFFFFFFFFFFFFFFFFFFFFFFFFFF:,FFFFFFFFFF:FFFFFFFFFFFFFF
FFFFFFFFFFFFFFF
@A00721:190:HGJW7DSXY:4:2239:31656:34272 2:N:0:TCTGCAAG+GCTGCACT
CTATTACGAAGAACTGGAATGCAGATGCATGGATTTCGTTGGAATAGACGAAATCGTTCTAATGTACG
ACTACTTTAGATATCCTCCACCAAATCCTGATCTGAATCACTTTCCGTATTTAATAGGTCACTATGAC
CAGATGTCAGATACA
+
FFFFFFFFFFFFFFFFFFFFFFFFFFFFFFFFFFFFFFFFFFFFFFFFFFFFFFFFFFFFFFFFFFFF
FFFFFFFFFFFFFFFFFFFFFFFFFFFFFFFFFFFFFFFFF:FFFFFFFFFFFFFFF:FFFFFFFFFF
FFFFFFFFFFFFF:F
@A00721:190:HGJW7DSXY:4:2274:24044:5650 2:N:0:TCTGCAAG+GCTGCACT
CTATTACGAAGAACTGGAATGCAGATGCATGGATTTCGTTGGAATAGACGAAATCGTTCTAATGTACG
ACTACTTTAGATATCCTCCACCAAATCCTGATCTGAATCACTTTCCGTATTTAATAGGTCACTATGAC
CAGATGTCAGATACA
+
FFFFFFFFFFFFFFFFFFFFFFFFFFFFFFFFFFFFFFFFFFFFFFFFFFFFFFFFFFFFFFFFFFFF
FFFFFFFFFFFFFFFFFFFFFFFFFFFFFFFFFFFFFFFFF:FFFFFFFFFFFFFFFFFFFFFFFFFF
FFFFFFFFFFFFFFF
@A00721:190:HGJW7DSXY:4:2420:1642:12477 2:N:0:TCTGCAAG+GCTGCACT
CTATTACGAAGAACTGGAATGCAGATGCATGGATTTCGTTGGAATAGACGAAATCGTTCTAATGTACG
ACTACTTTAGATATCCTCCACCAAATCCTGATCTGAATCACTTTCCGTATTTAATAGGTCACTATGAC
CAGATGTCAGATACA
+
FFFFFFFFFFFFFFFFFFFFFFFFFFFFFFFFFFFFFFFFFFFFFFFFFFFFFFFFFFFFFFFFFFFF
FFFFFFFFFFFFFFFFFFFFFFF:FFFFFFF:FFFFFFFFF,FFFFFFFFF:FFFFFFFFFFFFFFFF
FFFFFFFFFFFFFFF
@A00721:190:HGJW7DSXY:4:2661:21612:16908 2:N:0:TCTGCAAG+GCTGCACT
CTATTACGAAGAACTGGAATGCAGATGCATGGATTTCGTTGGAATAGACGAAATCGTTCTAATGTACG
ACTACTTTAGATATCCTCCACCAAATCCTGATCTGAATCACTTTCCGTATTTAATAGGTCACTATGAC
CAGATGTCAGATACA
+
FFFFFFFFFFFFFFFFFFFFFF:F:,FFFFFFFFFFFFFFFFFFFFFFFFFFFFFFFFFFFFFFFFFF
FFFFFFFFFFFFFFFFFFFFFFFFFFFFFFFFFFFF:FFFFFFFFFFFFFFFF:FFF:FFFFFFFFFF
FFFFFFFFFFFFFFF
@A00721:190:HGJW7DSXY:4:1315:23231:2143 2:N:0:TCTGCAAG+GCTGCACT
AATTAATGATATCGAAAACGAGTATAACTATTACGAAGAACTGGAATGCAGATGCATGGATTTCGTTG
GAATAGACGAAATCGTTCTAATGTACGACTACTTTAGATATCCTCCACCAAATCCTGATCTGAATCAC
TTTCCGTATTTAATA
+
FFFFFFFFFFFFFFFFFFFFFFFFFFFFFFFFFFFFFFFFFFFFFFFFFFFFFFFFFFFFFFFFFFFF
FFFFFFFFFF:FFFFFFFFFFFFFFFFFFFFFFFFFFFFFFFFFFFFFFFFFFFFFFFFFFFFFFFFF
FFFFFFFFFFFFFFF
@A00721:190:HGJW7DSXY:4:1441:29315:25050 2:N:0:TCTGCAAG+GCTGCACT
AATTAATGATATCGAAAACGAGTATAACTATTACGAAGAACTGGAATGCAGATGCATGGATTTCGTTG
GAATAGACGAAATCGTTCTAATGTACGACTACTTTAGATATCCTCCACCAAATCCTGATCTGAATCAC
TTTCCGTATTTAATA
+
FFFFFFFFFFFFFFFFFFFFFFF,FFFFFFFFFFFFFFFFFFFFFFFFFFFFFFFFFFFFFFFFFFFF
FFFFFFFFFFFF:FFFFFFFFFFFFFFFFF:FFFFFFFFFFFFFFFFFFFFFFFFFFFFFFFFFFFFF
FFFFFFFFFFFFFFF
@A00721:190:HGJW7DSXY:4:2139:30192:31015 2:N:0:TCTGCAAG+GCTGCACT
AATTAATGATATCGAAAACGAGTATAACTATTACGAAGAACTGGAATGCAGATGCATGGATTTCGTTG
GAATAGACGAAATCGTTCTAATGTACGACTACTTTAGATATCCTCCACCAAATCCTGATCTGAATCAC
TTTCCGTATTTAATA

```
+
FFFFFFFFFFFFFFFFFFFFFFFFFFFFFFFFFFFFFFFFFFFFFFFFFFFFFFFFFFFFFFFFFFFFFF
FFFFFFFFFFFFFFFFFFFFFFFFFFFFFFFFFFFFFFFFFFFFF:FFFFFFFFFFFFFFFFFFFFFFFF
F:FFFFFFFFFFFFF
@A00721:190:HGJW7DSXY:4:2212:17806:1141 2:N:0:TCTGCAAG+GCTGCACT
AATTAATGATATCGAAAACGAGTATAACTATTACGAAGAACTGGAATGCAGATGCATGGATTTCGTTG
GAATAGACGAAATCGTTCTAATGTACGACTACTTTAGATATCCTCCACCAAATCCTGATCTGAATCAC
TTTCCGTATTTAATA
+
FFFFFFFFFFFFFFFFFFFFFFFFFFF,FFFFFFFFFFFFFFFFFFFFFFFFFFFFFFFFFFFFFFFFFF
FFFFFFFFFFFFFFFFFFFFFFFFFFFFFFF:FFFFFFFFFFFFFFFFFFFFFFFFFFFFFFFFFFFFFF
F:FFFFFFFFFFFFF
@A00721:190:HGJW7DSXY:4:2318:17779:15405 2:N:0:TCTGCAAG+GCTGCACT
AATTAATGATATCGAAAACGAGTATAACTATTACGAAGAACTGGAATGCAGATGCATGGATTTCGTTG
GAATAGACGAAATCGTTCTAATGTACGACTACTTTAGATATCCTCCACCAAATCCGGATCTGAATCAC
TGTCCGTATTTAATA
+
FFFFF::FFFFFFFFF:FFFFFF:FFFFFFFFFFFFFFFFFFFFFFFF,F:FFFF:FFFFFFFFFFFFFF
FFFFFF:FFFF:F:FFFFFFFFFFFFFFFFFFFFF:FFFFFFFFFFFFFFFFF:FFF,FF,FFFFFFFFF
F,FFFFF:F:F:FFF
@A00721:190:HGJW7DSXY:4:2578:7618:1157 2:N:0:TCTGCAAG+GCTGCACT
AATTAATGATATCGAAAACGAGTATAACTATTACGAAGAACTGGAATGCAGATGCATGGATTTCGTTG
GAATAGACGAAATCGTTCTAATGTACGACTACTTTAGATATCCTCCACCAAATCCTGATCTGAATCAC
TTTCCGTATTTAATA
+
FFFFFFFFFFFFFFFF:FFFFFFFFFFFFFFF:FFFFFFFFF:FFFFFFFFFFFFFFFFFFFFFFFFFFF
:FFFFFFF:FFFFFFFFFFFFFFFFFFFFFFFFFFFFFFFFFFFFFFFFFFFFFFFFFFFFFFFFFFFFF
FFF:FFFFFFFFFFF
@A00721:190:HGJW7DSXY:4:2613:6551:1282 2:N:0:TCTGCAAG+GCTGCACT
AATTAATGATATCGAAAACGAGTATAACTATTACGAAGAACTGGAATGCAGATGCATGGATTTCGTTG
GAATAGACGAAATCGTTCTAATGTACGACTACTTTAGATATCCTCCACCAAATCCTGATCTGAATCAC
TTTCCGTATTTAATA
+
F:FFFFFFFFFFFFFFFFFFFFF,FFFFFFFFFFFFFFFFFFFFFFFFFFFFFFFFFFFFFFFFFFF:FF
FFFFFFFFFFFFFFFFFFFFFFFFFFFFFFFFFF:FFFFFFFFFFFFFFFFFFFFFFFFFFFFFFFFFFF
FFFFFFFFFFFFFFF
@A00721:190:HGJW7DSXY:4:1407:8793:35884 2:N:0:TCTGCAAG+GCTGCACT
ATCGTTTCGATGAAATATATAATTAATGATATCGAAAACGAGTATAACTATTACGAAGAACTGGAATG
CAGATGCATGGATTTCGTTGGAATAGACGAAATCGTTCTAATGTACGACTACTTTAGATATCCTCCAC
CAAATCCTGATCTGA
+
FFFFFFFFFFFFFFFFFFFFFFFFFFFFFFFFFFFFFFFFFFFFFFFFFFFFFFFFFFFF:FFFFFFFFF
FFFFFFFFFFFFFFFFFFFFFFFFFFFFFFFF:FFFFFFFFFFFFFF:FFFFFFFFFFFFFFFFFFFFFF
FFFFFFFFFFFFFFF
@A00721:190:HGJW7DSXY:4:1424:26576:24126 2:N:0:TCTGCAAG+GCTGCACT
ATCGTTTCGATGAAATATATAATTAATGATATCGAAAACGAGTATAACTATTACGAAGAACTGGAATG
CAGATGCATGGATTTCGTTGGAATAGACGAAATCGTTCTAATGTACGACTACTTTAGATATCCTCCAC
CAAATCCTGATCTGA
+
FFFFFFFFFFFFFFFFFFFFFFFFFFFFFFFFFFFFFFFFFFFFFFFFFFFFFFFFFFFFFFFFFFFFFF
FFFFFFFFFFFFFFFFFFFFF:FFFFFFFFFFFFFFFFFFFFFFFFFFFFFFFFFFFFFFFFFFFFFFFF
FFFFFFFFFFFFFFF
@A00721:190:HGJW7DSXY:4:1558:29134:8046 2:N:0:TCTGCAAG+GCTGCACT
ATCGTTTCGATGAAATATATAATTAATGATATCGAAAACGAGTATAACTATTACGAAGAACTGGAATG
```

```
CAGATGCATGGATTTCGTTGGAATAGACGAAATCGTTCTAATGTACGACTACTTTAGATATCCTCCAC
CAAATCCTGATCTGA
+
FFFFFFFFFFFFFFFFFFFFFFFFFFFFFFFFFFFFFFFFFFFFFFFFFFFFFFFFFFFFFFFFFFFFFF
FFFFFFFFFFFFFFFFFFFFFFFFFFFFFFFFFFFFFFFFFFFFFFFFFFFFFFFFFFFFFFFFFFFFFF
FFFFFFFFFFFFFFF
@A00721:190:HGJW7DSXY:4:2169:12038:7091 2:N:0:TCTGCAAG+GCTGCACT
ATCGTTTCGATGAAATATATAATTAATGATATCGAAAACGAGTATAACTATTACGAAGAACTGGAATG
CAGATGCATGGATTTCGTTGGAATAGACGAAATCGTTCTAATGTACGACTACTTTAGATATCCTCCAC
CAAATCCTGATCTGA
+
FFFFFFFFFFFF:FFFFFFFFFFFFFFFFFFFFFFFFFFFFFFFFFFFFFFFFFFFFFFFFFFFFFFFFF
FFFFFFFFFFFFFFFFFF:FFFFFFFFFFFFFFF:FFFFFFFFFFFFFFFFFFFFFFFFFFFFFFFFFFF
FFFFFFFFFFFFFFF
@A00721:190:HGJW7DSXY:4:2424:26693:17284 2:N:0:TCTGCAAG+GCTGCACT
ATCGTTTCGATGAAATATATAATTAATGATATCGAAAACGAGTATAACTATTACGAAGAACTGGAATG
CAGATGCATGGATTTCGTTGGAATAGACGAAATCGTTCTAATGTACGACTACTTTAGATATCCTCCAC
CAAATCCTGATCTGA
+
FFFFFFFFFFFFFFFFFFFFFFFFFFFFFF:FFF:FFFFFFFFFFFFFFFFFFFFF:FFFFFFFFFFFFF
FFFFFFFFFFFFFFFFFFFFF::FFFFFFFFFFFFFFFFFFFFFFFFFFFFFFFFFF:FFFFFFFFFFFF
FFFFFFFFFFFFFFF
@A00721:190:HGJW7DSXY:4:2571:3649:5494 2:N:0:TCTGCAAG+GCTGCACT
ATCGTTTCGATGAAATATATAATTAATGATATCGAAAACGAGTATAACTATTACGAAGAACTGGAATG
CAGATGCATGGATTTCGTTGGAATAGACGAAATCGTTCTAATGTACGACTACTTTAGATATCCTCCAC
CAAATCCTGATCTGA
+
FFFFFFFFFFFFFFFFFFFFFFFFFFFFFFFFFFFFFFFFFFFFFFFFFFFFFFFFFFFFFFFFFFFFFF
FFFFFFFFFFFFFFFFFFFFFFFFFFFFFFFFFFFFFFFFFFFFFFFFFFFFFFFFFFFFFFFFFFFFFF
FFFFFFFFFFFFFFF
@A00721:190:HGJW7DSXY:4:2603:2889:23469 2:N:0:TCTGCAAG+GCTGCACT
ATCGTTTCGATGAAATATATAATTAATGATATCGAAAACGAGTATAACTATTACGAAGAACTGGAATG
CAGATGCATGGATTTCGTTGGAATAGACGAAATCGTTCTAATGTACGACTACTTTAGATATCCTCCAC
CAAATCCTGATCTGA
+
FFFFFFFFFF:FFFFFFFFFFFFFFFFFFFFFFFFFFFFFFFFFFFFFFFFFFFFFFFFFFFFFFFFFFF
FFFFFFFFFFFFFFFFFFFFFFFFFFFFFFFFFFFFFFFFFFFFFFFFFFFF:FFFFFFFFFFFFFF:FF
FFFFFFFFFFF:FFF
@A00721:190:HGJW7DSXY:4:1277:29821:25895 2:N:0:TCTGCAAG+GCTGCACT
AATTAACCAGATCAGCATTCCCAACAGTGTGATCGTTTCGATGAAATATATAATTAATGATATCGAAA
ACGAGTATAACTATTACGAAGAACTGGAATGCAGATGCATGGATTTCGTTGGAATAGACGAAATCGTT
CTAATGTACGACTAC
+
FFFFFFFFFFFFFFFFFFFFFFFFFFFFFFFFFFFF:FFFFFFFFFFFFFFFFFFFFFFFFFFFFFFFFF
FFFFFFFFFFFFFFFFFFFFF:FFFFFFFFFFFFFFFFFFFFFFFFFFFFFFFFFFFFFFFFFFFFFFFF
FFFFFFFFFFFFFFF
@A00721:190:HGJW7DSXY:4:1277:29857:25895 2:N:0:TCTGCAAG+GCTGCACT
AATTAACCAGATCAGCATTCCCAACAGTGTGATCGTTTCGATGAAATATATAATTAATGATATCGAAA
ACGAGTATAACTATTACGAAGAACTGGAATGCAGATGCATGGATTTCGTTGGAATAGACGAAATCGTT
CTAATGTACGACTAC
+
FFFFFFFFFFFFFFFFFFFFFFFFFFFFFFFFFFFFFFFFFFFFFFFFFFFFFFFFFFFFFFFFFF:FFF
FFFFFFFFF:FFFFFFFFFFFFFFFFFFFFFFFFFFFFFFFFFFFFFFFFFFFFF,FFFFFF
FFFFFFFFF:FFFFF
```

```
@A00721:190:HGJW7DSXY:4:1339:14859:15828 2:N:0:TCTGCAAG+GCTGCACT
AATTAACCAGATCAGCATTCCCAACAGTGTGATCGTTTCGATGAAATATATAATTAATGATATCGAAA
ACGAGTATAACTATTACGAAGAACTGGAATGCAGATGCATGGATTTCGTTGGAATAGACGAAATCGTT
CTAATGTACGACTAC
+
FFFFFFFFFFFFFFFFFFFFFFFFFFFFFFFFFFF:FFFFF:F:FFFFFFFFFFFFFFFFFFFFFFFF
,FF:FFFFFFFFFFFF:FFFF,FFFFFFFFF,F:FFFF,FFF,FFFFFFFFFFFFFFFFFF:FF:FFF
FFF,FFFFFFFFFFF
@A00721:190:HGJW7DSXY:4:1460:2953:1125 2:N:0:TCTGCAAG+GCTGCACT
AATTAACCAGATCAGCATTCCCAACAGTGTGATCGTTTCGATGAAATATATAATTAATGATATCGAAA
ACGAGTATAACTATTACGAAGAACTGGAATGCAGATGCATGGATTTCGTTGGAATAGACGAAATCGTT
CTAATGTACGACTAC
+
FFFFFFFFFFFFFFFFFFFFFFFFFFFFFFFFFFFFFFFFF:FFFFFFFFFFFFFFFFFFFFFFFFFF
FFFFFFFFFFFFFFFFFFFFFFFFFFFFFFFFFFFFFFFFFFFFFFFFFFFFFFFF,FFFFFFFFFFF
FFFFFFFFF:FFFFF
@A00721:190:HGJW7DSXY:4:2176:5358:2691 2:N:0:TCTGCAAG+GCTGCACT
AATTAACCAGATCAGCATTCCCAACAGTGTGATCGTTTCGATGAAATATATAATTAATGATATCGAAA
ACGAGTATAACTATTACGAAGAACTGGAATGCAGATGCATGGATTTCGTTGGAATAGACGAAATCGTT
CTAATGTACGACTAC
+
FFF:FFFFFFF:FFFFF:FFFFFFFFFF::FFFFFFFFFFFFFF::FFFFFFFFFFFFFFFFFFFFFF
,FFFFFFFFFFF,FFFF:FFF:FFFFFFFFF,,FF:FFFF,FFFFFFF,FFFFFFFFFFF:F,FFFFF
FFFF:FFF,FF,FFF
@A00721:190:HGJW7DSXY:4:2249:23149:32690 2:N:0:TCTGCAAG+GCTGCACT
AATTAACCAGATCAGCATTCCCAACAGTGTGATCGTTTCGATGAAATATATAATTAATGATATCGAAA
ACGAGTATAACTATTACGAAGAACTGGAATGCAGATGCATGGATTTCGTTGGAATAGACGAAATCGTT
CTAATGTACGACTAC
+
FFFFFFFFFFFFFFFFFFFFFFFFFFFFFFFFFFFFFFFFFFFF:FFFFFFFFFFFFFFFFF:FFFFF
FFFFFFFFFFFF,:FFFFFFFFFFFFFFFFFFFFFFFFFFFFFFFFFFFFFFFFFFFFFFFFFFFFFF
FFFF:FFFFFFFFF:
@A00721:190:HGJW7DSXY:4:2442:15338:16250 2:N:0:TCTGCAAG+GCTGCACT
AATTAACCAGATCAGCATTCCCAACAGTGTGATCGTTTCGATGAAATATATAATTAATGATATCGAAA
ACGAGTATAACTATTACGAAGAACTGGAATGCAGATGCATGGATTTCGTTGGAATAGACGAAATCGTT
CTAATGTACGACTAC
+
FFFFFFFFFFFFFFFFFFFFFFFFF,F:F,FFFFFFFFFFF,FFFFFFFFFFFFFFFFFFFFFFFFFF
FF:FFFFFFF::FFFFFFFFFFFFFFFFFFFFFFFFFFFFFFF:F:FFF:FFFFFFF:FF,FF:FFFF
FF::FFFFFFFFFF:
@A00721:190:HGJW7DSXY:4:2570:1931:18771 2:N:0:TCTGCAAG+GCTGCACT
AATTAACCAGATCAGCATTCCCAACAGTGTGATCGTTTCGATGAAATATATAATTAATGATATCGAAA
ACGAGTATAACTATTACGAAGAACTGGAATGCAGATGCATGGATTTCGTTGGAATAGACGAAATCGTT
CTAATGTACGACTAC
+
FFFFFFFFFFFFFFFFFFFFFFFFFFFFFFFFFFFFFFFFFFFFFFFFFFFFFFFFFFFFFFFFFFFF
FFFFFFFFFFFFFFFFFFFFFFFFFFFFFFFFFFFFFFFFFFFFFFFFFFFFFFFFFFFFFFFFFFFF
FFFFFFFFFFFFFFF
@A00721:190:HGJW7DSXY:4:1146:7744:17284 2:N:0:TCTGCAAG+GCTGCACT
GACGAAGTCGTCTATACAATTAACCAGATCAGCATTCCCAACAGTGTGATCGTTTCGATGAAATATAT
AATTAATGATATCGAAAACGAGTATAACTATTACGAAGAACTGGAATGCAGATGCATGGATTTCGTTG
GAATAGACGAAATCG
+
FFFFFFFFFFFFFFFFFFFFFFFFFFFFFFFFFFFFFFFFFFFFFF:FFFFFFFFFFFFF:FFFFFFF
```

FFFFFFFFFFFFFFFFFFFFFFFFFFFFFFFFFFFFFFFFFFFFFFFFFFFFFFFFFFFFFFFFFF:FFF
FFFFFFFF:F:FFF:
@A00721:190:HGJW7DSXY:4:1150:28320:16219 2:N:0:TCTGCAAG+GCTGCACT
GACGAAGTCGTCTATACAATTAACCAGATCAGCATTCCCAACAGTGTGATCGTTTCGATGAAATATAT
AATTAATGATATCGAAAACGAGTATAACTATTACGAAGAACTGGAATGCAGATGCATGGATTTCGTTG
GAATAGACGAAATCG
+
FFFFFFFFFFFF:FFFFFFFFFFFFFFFFFFFFFFFFFFFFFFFFFFFFFFFFFFFFFFFFF,FFFFFFF
FFFFFFFFFFFFFFFFFFFFFFFFFFFFFFFFFFF:F:FFFFFFFFFFFFFFFFFFFFFFFFFFFFFFFF
FF:FF:FFFFFF:FF
@A00721:190:HGJW7DSXY:4:1165:26494:27461 2:N:0:TCTGCAAG+GCTGCACT
GACGAAGTCGTCTATACAATTAACCAGATCAGCATTCCCAACAGTGTGATCGTTTCGATGAAATATAT
AATTAATGATATCGAAAACGAGTATAACTATTACGAAGAACTGGAATGCAGATGCATGGATTTCGTTG
GAATAGACGAAATCG
+
FFFFFFFFFFFFFFFFFFFFFFFFFFFFFFFFFFFFFFFFFFFFFFFFFFFFFFFFFFFF:FFFFFFFFF
FFFFFFFFFFFFFFFFFFFFFFFFFFFFFFFFFFFFFFFFFFFFFFF:FFFFFFFFFFFFFFFFFFFFFF
FFFFFFFFFFFFFFF
@A00721:190:HGJW7DSXY:4:1304:15709:15045 2:N:0:TCTGCAAG+GCTGCACT
GACGAAGTCGTCTATACAATTAACCAGATCAGCATTCCCAACAGTGTGATCGTTTCGATGAAATATAT
AATTAATGATATCGAAAACGAGTATAACTATTACGAAGAACTGGAATGCAGATGCATGGATTTCGTTG
GAATAGACGAAATCG
+
FFFFFFFFFFFFFFFFFFFFFFFFFFFFFFFFFFFFFFFFFFFFFFFFFFFFFFFFFFFFFFFFFFFFFF
FFFFFFFFFFFFFFFFFFFFFFFFFFFFF:FFFFFFFFFF,FFFFFFFFFFFFFFFFFFFFFFFFFFFFF
FF:FFFFFFFFFFFF
@A00721:190:HGJW7DSXY:4:1643:32443:10363 2:N:0:TCTGCAAG+GCTGCACT
GACGAAGTCGTCTATACAATTAACCAGATCAGCATTCCCAACAGTGTGATCGTTTCGATGAAATATAT
AATTAATGATATCGAAAACGAGTATAACTATTACGAAGAACTGGAATGCAGATGCATGGATTTCGTTG
GAATAGACGAAATCG
+
FFFFFFFFFFFFFFFFFFFFFFFFFFFFFFFFFFFFFFFFFFFFFFFFFFF:FFFFFFFFFFFFFFFFFF
FFFFFFFFFFFFFFFFFFFFFFFFFFFFFFFFFFFFFFFFFFFFFFFFFFFFFFFFF,FFFFFFFF:FFF
FFFFFFFFFFFFFF:
@A00721:190:HGJW7DSXY:4:1472:28565:33238 2:N:0:TCTGCAAG+GCTGCACT
ATACTTTCAGACATGCACATCCGTGTAAAGGAAGTAATAGACGAAGTCGTCTATACAATTAACCAGAT
CAGCATTCCCAACAGTGTGATCGTTTCGATGAAATATATAATTAATGATATCGAAAACGAGTATAACT
ATTACGAAGAACTGG
+
FFFFFFFFFFFFFFFFFFFFFFFFFF:FFFFFFFFFFFFFFFFFFFFFFFFFFFFFFFFFFFFFFFFFFF
FFFFFFFFFFFFFFFFFFFFFFFFFFFFFF:FFFFFFFFFFFFFFFFFF:FFFFFFFFFFFFFFFFFFFF
FFFFFFFF:,FFFFF
@A00721:190:HGJW7DSXY:4:1474:15826:31657 2:N:0:TCTGCAAG+GCTGCACT
ATACTTTCAGACATGCACATCCGTGTAAAGGAAGTAATAGACGAAGTCGTCTATACAATTAACCAGAT
CAGCATTCCCAACAGTGTGATCGTTTCGATGAAATATATAATTAATGATATCGAAAACGAGTATAACT
ATTACGAAGAACTGG
+
FFFFFFFFFFFFFFFFFFFFFFFFFFFFFFFFFFFFFFFFFFFFFFFFFFFFFFFFFFFFFFFFFFFFFF
FFFFFFFFFFFFFFFFFFFFFFFFFFFFFFFFFFFFFFFFFFFFFFFFFFFFFFFFFFFFFFFFFFFFFF
FFFFFFFFFFFFFFF
@A00721:190:HGJW7DSXY:4:1521:30716:24064 2:N:0:TCTGCAAG+GCTGCACT
ATACTTTCAGACATGCACATCCGTGTAAAGGAAGTAATAGACGAAGTCGTCTATACAATTAACCAGAT
CAGCATTCCCAACAGTGTGATCGTTTCGATGAAATATATAATTAATGATATCGAAAACGAGTATAACT
ATTACGAAGAACTGG

```
+
FFFFFFFFFFFFFFFFFFFFFFFFFFFF:FFFFFFFFFFFFFFFFFFFFFFFFFFFFFFFFFFFFFFFFF
FFFFFFFFFFFFFFFFFFFFFFFFFFFFFF::FFFFFFFFFFFFFFFFFFFFFFFFFFF:FFFFFFFFFF
FFFFFF,FFFFFFFF
@A00721:190:HGJW7DSXY:4:2132:26765:27305 2:N:0:TCTGCAAG+GCTGCACT
ATACTTTCAGACATGCACATCCGTGTAAAGGAAGTAATAGACGAAGTCGTCTATACAATTAACCAGAT
CAGCATTCCCAACAGTGTGATCGTTTCGATGAAATATATAATTAATGATATCGAAAACGAGTATAACT
ATTACGAAGAACTGG
+
FFFFFFFFFFFFFFFFFFFFFFFFFFFFFFFFFFFFFFFFFFFFFFFFFFFFFFFFFFFFFFFFFFFFFF
FFFFFFFFFFFFFFFFFFFFFFFFFFFFFFFFFFFFFFFFFFF:FFFFFFFFFFFFFFFFFFFFF:FFFF
FFFFFFF:FFFFFFF
@A00721:190:HGJW7DSXY:4:2135:9833:15577 2:N:0:TCTGCAAG+GCTGCACT
ATACTTTCAGACATGCACATCCGTGTAAAGGAAGTAATAGACGAAGTCGTCTATACAATTAACCAGAT
CAGCATTCCCAACAGTGTGATCGTTTCGATGAAATATATAATTAATGATATCGAAAACGAGTATAACT
ATTACGAAGAACTGG
+
FFFFFFFFFFFFFFFFFFFFFFFFFFFFFFFFFFFFFFFFFFFFFFFFFFFF:FFFFFFFFFFFFFFFFF
FFFFFFFFFFFFFFFFF:FFFFFF:FFF::FF:F:FFFFFFFFFF:FFFFF:FFFFFFFFFFFFFFFFFF
FFFFFFFFFFFFFFF
@A00721:190:HGJW7DSXY:4:2135:12093:17425 2:N:0:TCTGCAAG+GCTGCACT
ATACTTTCAGACATGCACATCCGTGTAAAGGAAGTAATAGACGAAGTCGTCTATACAATTAACCAGAT
CAGCATTCCCAACAGTGTGATCGTTTCGATGAAATATATAATTAATGATATCGAAAACGAGTATAACT
ATTACGAAGAACTGG
+
FFFFFFFFFFFFFFFFFFFFFFFFFFFFFFFFFFFFFFFFFFFFFFFFFFFFFFFFFFFFFFFFFFFFFF
FFFFFFFFFFFFFFFFFFFFFFFFFFFFFFFFFFFFFFFFFFFFFFFFFFFFFFFFFFFFFFFFFFFFFF
FFFFFFFFFFFFFFF
@A00721:190:HGJW7DSXY:4:2249:11098:35744 2:N:0:TCTGCAAG+GCTGCACT
ATACTTTCAGACATGCACATCCGTGTAAAGGAAGTAATAGACGAAGTCGTCTATACAATTAACCAGAT
CAGCATTCCCAACAGTGTGATCGTTTCGATGAAATATATAATTAATGATATCGAAAACGAGTATAACT
ATTACGAAGAACTGG
+
FFFFFFFFFFFFFFFFFFFFFFFFFFFFFFFFFFFFFFFFFFFFFFFFFFFFFFFFFFFFFFFFFFFFFF
FFFFFFFFFFFFFFFFFFFFFFFFFF:FFFFFFF:FFFFFFFFFFFFFFFFFFFFFFFFFFFFFF,FFFF
FFFFFFFFFFFFFFF
@A00721:190:HGJW7DSXY:4:2628:14344:9048 2:N:0:TCTGCAAG+GCTGCACT
ATACTTTCAGACATGCACATCCGTGTAAAGGAAGTAATAGACGAAGTCGTCTATACAATTAACCAGAT
CAGCATTCCCAACAGTGTGATCGTTTCGATGAAATATATAATTAATGATATCGAAAACGAGTATAACT
ATTACGAAGAACTGG
+
FFFFFFFFFFFFFFFFFFFFFFFFFFFFFFFFFFFFFFFFFFFFFFFFFFFFFFFFFFF:FFFFFFFFFF
FFFFFFFFFFFFFFFFFFFFFFFFFFFFFFFFFFFFFFFFFFFFFFFFF:FFFF:FFFFFFFFFFFFFFF
FFFFFFFFFFFFFFF
@A00721:190:HGJW7DSXY:4:1569:26982:34131 2:N:0:TCTGCAAG+GCTGCACT
CTTCGTAATAGTTATACTCGGTTTCGATATCATTAATTATATATTTCATCGAAACGATCACACTGTTG
GGAATGCTGATCTGGTTAATTGTATAGACGACTTCGTCTATTACTTCCTTTACACGGATGTGCATGTC
TGAAAGTATGTCGTC
+
FFFFFFFFFFFFFFFFFFFFFFFFFFFFFFFFFFFFFFFFFFFFFFFFFFFFFFFFFFFFFFFFFFFFFF
FFFFFFFFFFFFFFFFFFFFFFFFFFFF:FFFFFFFFFFFFFFFFFFFFFFFFFFFFFFFFFFFFFFFFF
FFF:FFFFFFFFFFF
@A00721:190:HGJW7DSXY:4:2252:18502:16908 2:N:0:TCTGCAAG+GCTGCACT
CTTCGTAATAGTTATACTCGGTTTCGATATCATTAATTATATATTTCATCGAAACGATCACACTGTTG
```

GGAATGCTGATCTGGTTAATTGTATAGACGACTTCGTCTATTACTTCCTTTACACGGATGTGCATGTC
TGAAAGTATGTCGTC
+
F,FFFFF,FFFFF:FFFFFFFFFFFFFFF:FFF,FFF::FFFFFFFFFFFFFFFFFFFF:FFFFF,FF
FF,FFFFFFFFFFFFF,,FFFFF::F:FFFFFF:FFFF::FFFFFFFFF:FF:FFFFFFFFFF,:FFF
FFF:FFF:FFFFF:F
@A00721:190:HGJW7DSXY:4:2568:3613:31203 2:N:0:TCTGCAAG+GCTGCACT
CTTCGTAATAGTTATACTCGGTTTCGATATCATTAATTATATATTTCATCGAAACGATCACACTGTTG
GGAATGCTGATCTGGTTAATTGTATAGACGACTTCGTCTATTACTTCCTTTACACGGATGTGCATGTC
TGAAAGTATGTCGTC
+
F,FFFFFFF:FFFFFFFFFFF:FFFFF:FFFFFFFF:FF:FFFFFF:FFFFFFFFFFFFFFF:F:FFF
FFFFFFFFFF::FFFFFFFFFFFFFFF:FFFFFFF:FFF:FFFFFFFFFFFFFFFFFFFFFFFFFFFF
FFFFF:FFF:FFFFF
@A00721:190:HGJW7DSXY:4:2568:3794:31516 2:N:0:TCTGCAAG+GCTGCACT
CTTCGTAATAGTTATACTCGGTTTCGATATCATTAATTATATATTTCATCGAAACGATCACACTGTTG
GGAATGCTGATCTGGTTAATTGTATAGACGACTTCGTCTATTACTTCCTTTACACGGATGTGCATGTC
TGAAAGTATGTCGTC
+
FFFFFFFFFFFFFFFFFFFFFFFFFFFFFFFFFFFF:FFF:FFFFFFFFFFFFFFFFFFFFFFFFFFF
FFFFFFFFFFFFFFFFFFFFFFFFFFF:FFFFFFFFFFFFFFFFFFFFFFFFFFFFFFFFFFFFFFFF
FFFFFFFFFFFFFFF
@A00721:190:HGJW7DSXY:4:1127:16016:24659 2:N:0:TCTGCAAG+GCTGCACT
CATTAATTATATATTTCATCGAAACGATCACACTGTTGGGAATGCTGATCTGGTTAATTGTATAGACG
ACTTCGTCTATTACTTCCTTTACACGGATGTGCATGTCTGAAAGTATGTCGTCTCTGAAGAATGCGAA
GCAATTGTAATCCGG
+
FFFFFFFFFFFFFFFFFFFFFFFFFFFFFFFFFFFFFFFFFFFFFFFFFFFFFFFFFFFFFFFFF:F:
FFFFFFFFFFFFFFFFFFFFFFFFFFFFFFFFFFFFFFFFFFFFFFFFFFFFFFFFFFFFFFFFFFFF
FFF,FFFFFFFFFF:
@A00721:190:HGJW7DSXY:4:1171:12337:5822 2:N:0:TCTGCAAG+GCTGCACT
CATTAATTATATATTTCATCGAAACGATCACACTGTTGGGAATGCTGATCTGGTTAATTGTATAGACG
ACTTCGTCTATTACTTCCTTTACACGGATGTGCATGTCTGAAAGTATGTCGTCTCTGAAGAATGCGAA
GCAATTGTAATCCGG
+
FFFFFFFFFFFFFFFFFFFFFFFFFFFFFFFFFFFFFFFFF,FFFFFFFFFFFFFFF:FFFFFFF:FF
FF:FFFFFFFFFFFFFFFFFFFFFFFFF:F:FFFFFFFFFF:FFFFFFFFFFFFFFFFFFFFFFFFFF
FFFFFFFFFFFFFFF
@A00721:190:HGJW7DSXY:4:1330:26630:6621 2:N:0:TCTGCAAG+GCTGCACT
CATTAATTATATATTTCATCGAAACGATCACACTGTTGGGAATGCTGATCTGGTTAATTGTATAGACG
ACTTCGTCTATTACTTCCTTTACACGGATGTGCATGTCTGAAAGTATGTCGTCTCTGAAGAATGCGAA
GCAATTGTAATCCGG
+
F:FFFFFFFFFF:FFFFFFFFFFFFFFFFFFFFFFFFFFFFFFFF:FFFFFFFFFFFFFFFFFFF:FF
FFFFFFFFFFFFFFFFFFFFFFFFFFFFFFFFFFFFFFFFFFFFFFFFFFFFFFFFFFFFFFFFFFFF
FFFFFFF:FFFFFFF
@A00721:190:HGJW7DSXY:4:1454:24740:27398 2:N:0:TCTGCAAG+GCTGCACT
CATTAATTATATATTTCATCGAAACGATCACACTGTTGGGAATGCTGATCTGGTTAATTGTATAGACG
ACTTCGTCTATTACTTCCTTTACACGGATGTGCATGTCTGAAAGTATGTCGTCTCTGAAGAATGCGAA
GCAATTGTAATCCGG
+
FFFFFFFFFFFFFFFFFFFFFFFFFFFFFFFFF:FFFFFFFFFFFFFFFFFFFFFFFFFFFFFF::FF
FFFFFFFFFFFFFFFFFFFFFFFFFFFFFFFFFFFFFFFFFFFFFFFFFFFFFFFFFFFFFFFF,FFF
FFF:FFFFF,FFFFF

```
@A00721:190:HGJW7DSXY:4:1454:24180:27743 2:N:0:TCTGCAAG+GCTGCACT
CATTAATTATATATTTCATCGAAACGATCACACTGTTGGGAATGCTGATCTGGTTAATTGTATAGACG
ACTTCGTCTATTACTTCCTTTACACGGATGTGCATGTCTGAAAGTATGTCGTCTCTGAAGAATGCGAA
GCAATTGTAATCCGG
+
FFFFFFFFFFFFFFFFFFFFFFFFFFFFFFFFFFFFFFFFFFFFFFFFFFFFFFFFFFFFFFFFF,FF
FFFFFFFFFFFFFFFFFFFFFFFFFFFFFFFFFFFFFFFFFFFFFFFFFFFFFFFFFFFFFFFFFFFF
FFF,FFFFF,FFFFF
@A00721:190:HGJW7DSXY:4:2269:5412:16219 2:N:0:TCTGCAAG+GCTGCACT
CATTAATTATATATTTCATCGAAACGATCACACTGTTGGGAATGCTGATCTGGTTAATTGTATAGACG
ACTTCGTCTATTACTTCCTTTACACGGATGTGCATGTCTGAAAGTATGTCGTCTCTGAAGAATGCGAA
GCAATTGTAATCCGG
+
FFFFFFFFFFFFFFFFFFFFFFFFFFFFFFFFFFFFFFFFFFFFFFFFFF:FFFFFFFFFFFFFFFFF
FFFFFFFFFFFFFFFFFFFFFFFFFFFF:FFFFFFFFFFFFFFFFFFFFFFFFFFFFFFFFFFFFFFF
FFF:FFFFFFFF:FF
@A00721:190:HGJW7DSXY:4:2536:1434:33473 2:N:0:TCTGCAAG+GCTGCACT
CATTAATTATATATTTCATCGAAACGATCACACTGTTGGGAATGCTGATCTGGTTAATTGTATAGACG
ACTTCGTCTATTACTTCCTTTACACGGATGTGCATGTCTGAAAGTATGTCGTCTCTGAAGAATGCGAA
GCAATTGTAATCCGG
+
FFFFFFFFFFFFFFFFFFFFFFFFFFFFFFFFFFF,FFFFFFFFFFFFFFFFFFFFFFFFFFFFF,FF
FFFFFFFFFFFFFFFFFFFFFFFFFFFFFFFFFFFFFFFFFFFFFFFFFFFFFFFFF:FFFFFF:FFF
FFF:FFFFFFFFFFF
@A00721:190:HGJW7DSXY:4:1410:30210:30859 2:N:0:TCTGCAAG+GCTGCACT
AGCAGATATACTGGTCCAGATACGTAGAAAACGAAGCACGATTCAGAGCTGCTCTGAAAATGACGAAG
ATCATATATCCATTCTGGTGTCCGGATTACAATTGCTTCGCATTCTTCAGAGACGACATACTTTCAGA
CATGCACATCCGTGT
+
FFFFFFFFFFFFFFFFFFFFFFFFFFFFFFFFFFFFFFFFFFFFFFFFFFFFFFFFFFFFFF:FFFFF
FFFFFFFFFFFFFFFFFFFFFFFFFFFFFFFFFFFFFFFFFFFFFFFFFFFFFFF:FFFFFF:FF,FF
FFFFFFFFFFFFFFF
@A00721:190:HGJW7DSXY:4:1462:15835:2174 2:N:0:TCTGCAAG+GCTGCACT
AGCAGATATACTGGTCCAGATACGTAGAAAACGAAGCACGATTCAGAGCTGCTCTGAAAATGACGAAG
ATCATATATCCATTCTGGTGTCCGGATTACAATTGCTTCGCATTCTTCAGAGACGACATACTTTCAGA
CATGCACATCCGTGT
+
FFFFFFFFF,FFFF:FFFF:FFFFFFFFFF:FFFFFFFFFFFFFFFFFFFFFFFFFFFFFFFFFFFFF
FFF:F:FFFFFFFFFFFFFFFFFFFFFFFFFFFFFF:FFFFFFFFFFF:FFFFFFFFFFFFFFFF:FF
FFFFFF:FFF,FFF:
@A00721:190:HGJW7DSXY:4:2446:22254:25786 2:N:0:TCTGCAAG+GCTGCACT
AGCAGATATACTGGTCCAGATACGTAGAAAACGAAGCACGATTCAGAGCTGCTCTGAAAATGACGAAG
ATCATATATCCATTCTGGTGTCCGGATTACAATTGCTTCGCATTCTTCAGAGACGACATACTTTCAGA
CATGCACATCCGTGT
+
FFFFFFFFFFFFFFFFFFFFFFFFFFFFFF:FFFFFFFFFFFFFFFFFFFFFFFFFFFFFFFFFFFFF
FFFFFFFFFFFFFFFFFFFFFFFFFFFFFFFFFFFFFFFFFFFFFFFFFFF:FFF:FFFFFFFFFFFF
F,FFFFFFFFFFFFF
@A00721:190:HGJW7DSXY:4:2503:31349:21339 2:N:0:TCTGCAAG+GCTGCACT
AGCAGATATACTGGTCCAGATACGTAGAAAACGAAGCACGATTCAGAGCTGCTCTGAAAATGACGAAG
ATCATATATCCATTCTGGTGTCCGGATTACAATTGCTTCGCATTCTTCAGAGACGACATACTTTCAGA
CATGCACATCCGTGT
+
FFFFFFFFFF:FFFFFFFF:FFFFFFFFFFFFFFFFFFFFFFFFFF:FFFFFFF:FFFFFFFFFFFFF
```

FFFFFFFFFFFFFFFFFFFFFFFFFFFFFFFFFFFFFFFFFFFFFFFF:F:FFFFFFFFFFFFFFFFF
FFFFFFFFFFFFFFF
@A00721:190:HGJW7DSXY:4:2671:28610:9455 2:N:0:TCTGCAAG+GCTGCACT
AGCAGATATACTGGTCCAGATACGTAGAAAACGAAGCACGATTCAGAGCTGCTCTGAAAATGACGAAG
ATCATATATCCATTCTGGTGTCCGGATTACAATTGCTTCGCATTCTTCAGAGACGACATACTTTCAGA
CATGCACATCCGTGT
+
FFFF:FFFFFFFFFFFFFFFFFFFFFFFFFFFFFFFFFFFFFFFFFFFFFFFFF:FFFFFFF:FFFFF
FFFFFFFFFFFFFFFFFFFFFFF:FFFFFFFFFF:FFFFF:FFFFFFFFFFFFF:FFFFFFFFFFFF:
FFFFFFFFFFFFFFF
@A00721:190:HGJW7DSXY:4:1405:13015:24878 2:N:0:TCTGCAAG+GCTGCACT
AGCAGATATACTGGTCCAGATACGTAGAAAACGAAGCACGATTCAGAGCTGCTCTGAAAATGACGAAG
ATCATATATCCATTCTGGTGTCCGGATTACAATTGCTTCGCATTCTTCAGAGACGACATACTTTCAGA
CATGCACATCC
+
FFFFFFF,FFFFFFFFFFFFFFFFFFFFFFFFFFF:FFFFFFFFFFFFFFFFFFFFFFFFFFFFFFFF
FFFFFFFFFFFFFFFFFFFFFFFFFFFFFFFFFF:FFFF,FFFFFFFFFFFFFF:FFFF:FFFFFFFF
FF:FFFFFFFF
@A00721:190:HGJW7DSXY:4:1409:11822:12821 2:N:0:TCTGCAAG+GCTGCACT
AGCAGATATACTGGTCCAGATACGTAGAAAACGAAGCACGATTCAGAGCTGCTCTGAAAATGACGAAG
ATCATATATCCATTCTGGTGTCCGGATTACAATTGCTTCGCATTCTTCAGAGACGACATACTTTCAGA
CATGCACATCC
+
FFFFFFF:FFFFFFFFFFFFFFFFFFFFFFFFFFFFFFFFFFFFFFFFFFFFFFFFFFFFFFFFFFFF
FFFFFFFFFFFFFFFFFFFFFFFFFFFFFFFFFFFFFFFFFFFFFFFFFFFFFFFFFFFFFFFFFFFF
FFFFFFFFFFF
@A00721:190:HGJW7DSXY:4:1561:29369:22858 2:N:0:TCTGCAAG+GCTGCACT
AGCAGATATACTGGTCCAGATACGTAGAAAACGAAGCACGATTCAGAGCTGCTCTGAAAATGACGAAG
ATCATATATCCATTCTGGTGTCCGGATTACAATTGCTTCGCATTCTTCAGAGACGACATACTTTCAGA
CATGCACATCC
+
FFFFFFFFFFFFFFFFFFFFFFFFFFFFFFFFFFFFFFFFFFFFFFFFFFFFFFFFFFFF:FFFFFFF
FFFFFFFFFFFFFFFFFFFFFFFFFFFFFFFFFF:FFFF::FFFFFFFFFFFFFFFF:FFFFFFFFFF
FFFFFFFFFFF
@A00721:190:HGJW7DSXY:4:2202:10013:18959 2:N:0:TCTGCAAG+GCTGCACT
AGCAGATATACTGGTCCAGATACGTAGAAAACGAAGCACGATTCAGAGCTGCTCTGAAAATGACGAAG
ATCATATATCCATTCTGGTGTCCGGATTACAATTGCTTCGCATTCTTCAGAGACGACATACTTTCAGA
CATGCACATCC
+
FFFFFFFFFFFFFFFFFFFFFFFFFFFFFFFFFFFFFFFFFFFFFFFFFFFFFFFFFFFFFFFFFFFF
FFFFFFFFFFFFFFFFF,FFFFFFFFFFFFF:FFFF:F:F:FFFFFFFFFFFFFFFFF,FFFFFFFFF
FFFFF:FFFFF
@A00721:190:HGJW7DSXY:4:2407:26711:3004 2:N:0:TCTGCAAG+GCTGCACT
AGCAGATATACTGGTCCAGATACGTAGAAAACGAAGCACGATTCAGAGCTGCTCTGAAAATGACGAAG
ATCATATATCCATTCTGGTGTCCGGATTACAATTGCTTCGCATTCTTCAGAGACGACATACTTTCAGA
CATGCACATCC
+
FFFFFFF:FFFFFFFFFFFFFFFFFFFFFFFFFFFFFFFFFFFFFFFFFFFFFFFFFFFFFF:FFF:F
FFFFFF:FFFFFFFFFFFFFFFFFFFFFFFFFFFFFFFF:FFFFFFFFFFFFFFFFFFFFFFFFF,FF
F,FFFFFFFFF
@A00721:190:HGJW7DSXY:4:2427:28782:4272 2:N:0:TCTGCAAG+GCTGCACT
AGCAGATATACTGGTCCAGATACGTAGAAAACGAAGCACGATTCAGAGCTGCTCTGAAAATGACGAAG
ATCATATATCCATTCTGGTGTCCGGATTACAATTGCTTCGCATTCTTCAGAGACGACATACTTTCAGA
CATGCACATCC

```
+
FFFFFFFFFFFFFFFFFFFFFFFF,FFFFFFFFFFFFFFFFFFFFFFFFFFFFFF:FFFFFFFFFFFFFF
FFFFFFFFFF:FFFF::FFFFF:FFFFFFFFFF:FFFF::FFFFFFFFFFFFFF:FFFFFFF:FFFF:
FFFFFFFFF,F
@A00721:190:HGJW7DSXY:4:2432:18502:7733 2:N:0:TCTGCAAG+GCTGCACT
AGCAGATATACTGGTCCAGATACGTAGAAAACGAAGCACGATTCAGAGCTGCTCTGAAAATGACGAAG
ATCATATATCCATTCTGGTGTCCGGATTACAATTGCTTCGCATTCTTCAGAGACGACATACTTTCAGA
CATGCACATCC
+
FFFFFFFFFFFFFFFFFFFFFFFFFFFFFFFFFFFFFFFFFFFFFFFFFFFFFFFFFFFFFFFFFFFF
FFFFF:FFFFFFFFFFFFFFFFFFFFFFFFFFFF:FFFFFFFFFFFF:FFFFFFFFFFFFFFFFFFFF
FFFFFFFFFFF
@A00721:190:HGJW7DSXY:4:2518:21133:16642 2:N:0:TCTGCAAG+GCTGCACT
AGCAGATATACTGGTCCAGATACGTAGAAAACGAAGCACGATTCAGAGCTGCTCTGAAAATGACGAAG
ATCATATATCCATTCTGGTGTCCGGATTACAATTGCTTCGCATTCTTCAGAGACGACATACTTTCAGA
CATGCACATCC
+
FFFFFFFFFFFFFFFFFFFFFFFFFFFFFFFFFFFFFFFFFFFFFFFFFFFFFFF,FFFFFFFFFFF
FFFFFFFFFFFFFFFFFFFFFFFFFFFFF:FFFFFFFFFFFFFFFFFFFFFFFFFFFFFFFFFFFF,FF
FFFFFFFFFFF
@A00721:190:HGJW7DSXY:4:2609:9923:19711 2:N:0:TCTGCAAG+GCTGCACT
AGCAGATATACTGGTCCAGATACGTAGAAAACGAAGCACGATTCAGAGCTGCTCTGAAAATGACGAAG
ATCATATATCCATTCTGGTGTCCGGATTACAATTGCTTCGCATTCTTCAGAGACGACATACTTTCAGA
CATGCACATCC
+
FFFFFFF,F:FFFFFFFFFFFFFFFFFFFFFFFFFFFFFFFFFFFFFFFFF:FFFFFFFFFFFFFFFF
FFFFFFFFFFFFFFFFFFFFFFFFFFFFFFFFFFFFFFFFFFFFFFFFFFFFFFFFFFFFFFFFFFFF
FFFFFFFFFFF
@A00721:190:HGJW7DSXY:4:2364:13069:33614 2:N:0:TCTGCAAG+GCTGCACT
AGCAGATATACTGGTCCAGATACGTAGAAAACGAAGCACGATTCAGAGCTGCTCTGAAAATGACGAAG
ATCATATATCCATTCTGGTGTCCGGATTACAATTGCTTCGCATTCTTCAGAGACGACATACTTTCAGA
CATGCACATC
+
F,FFFFFF::F::FFF,FFFFF::FF,F:FF::FFFF::FFFFFFFFFFFFFF:FFFFFFFF:FFF,,
FFFF,,FFF,,F,,FF,:,F:F,FFF,:FFFF,F:F:FFFFF:FFFFFFFFFF:FFFFFFFFF,:FFF
:F,FF:FF:F
@A00721:190:HGJW7DSXY:4:1327:8305:36323 2:N:0:TCTGCAAG+GCTGCACT
TTGAAGCATCAAGTCAAGCAGATATACTGGTCCAGATACGTAGAAAACGAAGCACGATTCAGAGCTGC
TCTGAAAATGACGAAGATCATATATCCATTCTGGTGTCCGGATTACAATTGCTTCGCATTCTTCAGAG
ACGACATACTTTCAG
+
FFFFFFFFFFFFFFFFFFFFFFFFFFFFFFFFFFFFFFFFFFFFFFFFFFFFFFFFFFFFFFFFFFFF
FFFFFFFFFFFFFFFFFFFFFFFFFFFFFFFFFFFFFFFFFFFFFFFFFFFFFFFF:FFFFFFFFFFF:
FFFFFFFFFFFFFFF
@A00721:190:HGJW7DSXY:4:2244:17743:25113 2:N:0:TCTGCAAG+GCTGCACT
TTGAAGCATCAAGTCAAGCAGATATACTGGTCCAGATACGTAGAAAACGAAGCACGATTCAGAGCTGC
TCTGAAAATGACGAAGATCATATATCCATTCTGGTGTCCGGATTACAATTGCTTCGCATTCTTCAGAG
ACGACATACTTTCAG
+
FFFFFFFFFFFFFFFFFFFFFFFFFFFFFFFFFFFF:FFFFFFFFFFFFFFFFFFFFFFFFFFFFFFF
FFFFFFFFFFFFFFFFFFFFFFFFFFFFFFFFFFFFFFFFFFFFFFFFFFFFFFFFFFFFFFFFFFFF
FFFFFFFFFFFFFFF
@A00721:190:HGJW7DSXY:4:2334:25590:33661 2:N:0:TCTGCAAG+GCTGCACT
TTGAAGCATCAAGTCAAGCAGATATACTGGTCCAGATACGTAGAAAACGAAGCACGATTCAGAGCTGC
```

```
TCTGAAAATGACGAAGATCATATATCCATTCTGGTGTCCGGATTACAATTGCTTCGCATTCTTCAGAG
ACGACATACTTTCAG
+
FFFFFFFFFFFFFFFFFFFFFFFFFF:FFFFFFFFFFFFFFFFFFFFFFFFFFFFFFFFFFFFFFFFF
FFFFFFFFFFFFFFFFFFFFFFFFFFFFFFFFFFFFFFFFFFFFFFFFFFFFFFF,FFFFFFFFFFFF
FFFFFFFFFF:FFFF
@A00721:190:HGJW7DSXY:4:2443:8585:23469 2:N:0:TCTGCAAG+GCTGCACT
TTGAAGCATCAAGTCAAGCAGATATACTGGTCCAGATACGTAGAAAACGAAGCACGATTCAGAGCTGC
TCTGAAAATGACGAAGATCATATATCCATTCTGGTGTCCGGATTACAATTGCTTCGCATTCTTCAGAG
ACGACATACTTTCAG
+
FFFFFFFFFFFFFFFFFFFFFFFFFFFFFFFFFFFFFFFFFFFFFFFFFFFFFFFFFFFFFFFFFFFF
FFFFFFFFFFFFFFFFFFFFFFFFFFFFFFFFFFFFFFFFFFFFFFFFFFFFFFFFFFFFFFFFFFFF
FF:FFFFFFFFFFFF
@A00721:190:HGJW7DSXY:4:2443:8612:24205 2:N:0:TCTGCAAG+GCTGCACT
TTGAAGCATCAAGTCAAGCAGATATACTGGTCCAGATACGAAGAAAACGAAGCACGATTCAGAGCTGC
TCTGAAAATGACGAAGATCATATATCCATTCTGGTGTCCGGATTACAATTGCTTCGCATTCTTCAGAG
ACGACATACTTTCAG
+
FFFFFFFFFFFFFFFFFFFFFFFFFF:FFFFFFFFFFF:FFFFFFFFFFF,FFFFFFFFFFFFFFFFF
FFFFFFFFFFFFFFFFF:FFFFFFFFFFFFFFFFFFF:FFFFFFFFFFFFFFFFFFFFFFFFFFFFFF
FFFFFFFFFFFFF:F
@A00721:190:HGJW7DSXY:4:2551:23213:15264 2:N:0:TCTGCAAG+GCTGCACT
TTGAAGCATCAAGTCAAGCAGATATACTGGTCCAGATACGTAGAAAACGAAGCACGATTCAGAGCTGC
TCTGAAAATGACGAAGATCATATATCCATTCTGGTGTCCGGATTACAATTGCTTCGCATTCTTCAGAG
ACGACATACTTTCAG
+
FFFFFFFFFFFFFFFFFFFFFFFFFFFFFFFFFFFFFFFFFFFFFFFFFFFFFFFFFFFFFFFFFFFF
FFFFFFFFFFFFFFFFFFFFFFFFFFFFFFFFFFFFFFFFFFFFFF:FFFFFF:FFFFFFFFFFFFFF
FFFFF:FFFFFFFFF
@A00721:190:HGJW7DSXY:4:1438:3730:31093 2:N:0:TCTGCAAG+GCTGCACT
AAGCATCAAGTCAAGCAGATATACTGGTCCAGATACGTAGAAAACGAAGCACGATTCAGAGCTGCTCT
GAAAATGACGAAGATCATATATCCATTCTGGTGTCCGGATTACAATTGCTTCGCATTCTTCAGAGACG
ACATACTTTCA
+
FFFFFFFF:FFF:FFFFFFFF:,FFFFFFFFFFFFFFFFFFFFFFFFFFFFFFFFFFFFFFFFFFFFF
FFFFFFFFFFFFFFFFFFFFFFFFFFFFFFFFFFFFFFFFFFFFFFFFFFFF:FFFFFFFFFFFFFFF
FFFFFFFFFFF
@A00721:190:HGJW7DSXY:4:1517:19253:19210 2:N:0:TCTGCAAG+GCTGCACT
AAGCATCAAGTCAAGCAGATATACTGGTCCAGATACGTAGAAAACGAAGCACGATTCAGAGCTGCTCT
GAAAATGACGAAGATCATATATCCATTCTGGTGTCCGGATTACAATTGCTTCGCATTCTTCAGAGACG
ACATACTTTCA
+
FFFFFFFF:FFFFFFFFFFFFFFFFFFFFFFFFFFFFFFFFFFFFFFFFFFFFFFFFFFFFFFFFFFF
FFFFFFFFFFFFFFFFFFFFFFF:FFFFFFFFFFFFFFFFFFFFFF:FFFFFF:FFFFFFFFF:FFFF
FFFFFFFFFFF
@A00721:190:HGJW7DSXY:4:1524:3396:24439 2:N:0:TCTGCAAG+GCTGCACT
ACGCATCAAGTCAAGCAGATATACTGGTCCAGATACGTAGAAAACGAAGCACGATTCAGAGCTGCTCT
GAAAATGACGAAGATCATATATCCATTCTGGTGTCCGGATTACAATTGCTTCGCATTCTTCAGAGACG
ACATACTTTCA
+
F,FFFFFFFFFFFFFFFFFF,FFFFF:FFFFFFFF:FFF,FFFFFFFF,FFFFFFFFFFFFFFFFFFF
FFFFFF::FFFFFFFFFFFFFFFFFF:FFF,:FF,FFFFFFFFFFF,F,FFFFFFFFFF,FFFF:FFF
FF:FFFFFFFF
```

```
@A00721:190:HGJW7DSXY:4:1536:24451:21512 2:N:0:TCTGCAAG+GCTGCACT
AAGCATCAAGTCAAGCAGATATACTGGTCCAGATACGTAGAAAACGAAGCACGATTCAGAGCTGCTCT
GAAAATGACGAAGATCATATATCCATTCTGGTGTCCGGATTACAATTGCTTCGCATTCTTCAGAGACG
ACATACTTTCA
+
,FFFFFFFFFFFFFFFFFFFFFFFFFFFFFFFFFFFFFFFFFFFFFFFFFFFFFFFFFFFFFFFFFFFFF
FFFFFFFFFF:FFFFFFFFFFFFFFFFFFFFFFFFFFFFFFFFFFFFFFFFFFFFFFFFFFF
FFFFFFFFFFF
@A00721:190:HGJW7DSXY:4:2406:9254:16297 2:N:0:TCTGCAAG+GCTGCACT
AAGCATCAAGTCAAGCAGATATACTGGTCCAGATACGTAGAAAACGAAGCACGATTCAGAGCTGCTCT
GAAAATGACGAAGATCATATATCCATTCTGGTGTCCGGATTACAATTGCTTCGCATTCTTCAGAGACG
ACATACTTTCA
+
FFFFFFFFFFFFFFFFFFFFFFFFFFFFFFFFFFFFFFFFFFFFFFFFFFFFFFFFFFFFFFFFFFFFFF
FFFFFFFFFFFFFFFFFFFFFFFFFFFFFFFFFFFFFFFFFFFFFFFF:FFFFFFFFFFFFFFFFFFFFF
FFFFFFFFFFF
@A00721:190:HGJW7DSXY:4:2632:2880:13088 2:N:0:TCTGCAAG+GCTGCACT
AAGCATCAAGTCAAGCAGATATACTGGTCCAGATACGTAGAAAACGAAGCACGATTCAGAGCTGCTCT
GAAAATGACGAAGATCATATATCCATTCTGGTGTCCGGATTACAATTGCTTCGCATTCTTCAGAGACG
ACATACTTTCA
+
FFFFFFFFFFFFFFFFFFFFFFFFFFFFFFFFFFFFFFFFFFFFFFFFFFFFFFFFFFFFFFFFFFFFFF
FFF:FFFFFFFFFF:FFFFFFFFFFF:FFFFFFFFFFFFFFFFFFFFFFFFFFFFFFFFFFFFFFFFFFF
FFFF:FFFFFF
@A00721:190:HGJW7DSXY:4:1150:28718:16908 2:N:0:TCTGCAAG+GCTGCACT
CTACAACATTCTCCCAGATGACTTGAAGCATCAAGTCAAGCAGATATACTGGTCCAGATACGTAGAAA
ACGAAGCACGATTCAGAGCTGCTCTGAAAATGACGAAGATCATATATCCATTCTGGTGTCCGGATTAC
AATTGCTTCGCATTC
+
FFFFFFFFFFFFFFFFFFFFFFFFFFFFFFFFFFFFFFFFFFFFFFFFFFFFFFFFFFFFF:FFFFFFFF
FFFFFFFFFFF:FFFFFFFFFFFFFFFFFFFFFFFFFFFF:FFF:FFFFFFFFFFFFFFFFFFFFFFFFF
FFFFFFFFFFFFFFF
@A00721:190:HGJW7DSXY:4:1472:12057:14826 2:N:0:TCTGCAAG+GCTGCACT
CTACAACATTCTCCCAGATGACTTGAAGCATCAAGTCAAGCAGATATACTGGTCCAGATACGTAGAAA
ACGAAGCACGATTCAGAGCTGCTCTGAAAATGACGAAGATCATATATCCATTCTGGTGTCCGGATTAC
AATTGCTTCGCATTC
+
FFFFFFF:FFFFFFFFFFFFFFFFFFFFFFFFFFF,FFFFFFFFFFFFFFFFFFFFFFFFFFFFFFFFFF
FFFFFFFFFFFFFFFFFFFFFFFFFFFFFFF,FFFFFF:FFFFF:FF,FFFFFFFFFFFFFFFFFFFFFF
FFFFFFFFFFFFFFF
@A00721:190:HGJW7DSXY:4:1510:31268:4476 2:N:0:TCTGCAAG+GCTGCACT
CTACAACATTCTCCCAGATGACTTGAAGCATCAAGTCAAGCAGATATACTGGTCCAGATACGTAGAAA
ACGAAGCACGATTCAGAGCTGCTCTGAAAATGACGAAGATCATATATCCATTCTGGTGTCCGGATTAC
AATTGCTTCGCATTC
+
FFFFFFFFFFFFFFFFFFFFFFFFFFFFFFFFFFF:FFFFFFFFFFFFFFFFFFFFFFFFFFFFFFFFFF
FFFFFFFFFFFFFFFFFFFFFFFFFFFFFFF:FFFFFFFFFFF:FFFFFFFFFFFFFFFFFFFFFFFFFF
FFFFFFFFFFFFFFF
@A00721:190:HGJW7DSXY:4:1603:26458:17002 2:N:0:TCTGCAAG+GCTGCACT
CTACAACATTCTCCCAGATGACTTGAAGCATCAAGTCAAGCAGATATACTGGTCCAGATACGTAGAAA
ACGAAGCACGATTCAGAGCTGCTCTGAAAATGACGAAGATCATATATCCATTCTGGTGTCCGGATTAC
AATTGCTTCGCATTC
+
FFFFFFFFFFFFFFFFFFFFFFFFFFFFFFFFFFFFFFFFFFFFFFFFFFFFFFFFFFFFFFFFFFFFFF
```

,FFFFFFFFFFFFFFFFFFFFFFFFFFFFFFFFFFFFFFFFFFFFFFFFFFFFFFFFFFFFFFFFFFF
FFFFFFFFFFFFFFF
@A00721:190:HGJW7DSXY:4:1662:8314:13792 2:N:0:TCTGCAAG+GCTGCACT
CTACAACATTCTCCCAGATGACTTGAAGCATCAAGTCAAGCAGATATACTGGTCCAGATACGTAGAAA
ACGAAGCACGATTCAGAGCTGCTCTGAAAATGACGAAGATCATATATCCATTCTGGTGTCCGGATTAC
AATTGCTTCGCATTC
+
FFFFFFFFFFFFFFFFFFFFFFFFFFFFFFFFFFFFFFFFFFFFF:F:FFFFFFFFFF,FFFFFFFFF
FFFFFFF:FFF,FFFFFFFFFFFFFFFFFF,FFFFFFFFFFF,FFFFFFFFFFFFFFFFFFFFFFFFF
FFF:FFFFF:FFFFF
@A00721:190:HGJW7DSXY:4:1664:4517:27727 2:N:0:TCTGCAAG+GCTGCACT
CTACAACATTCTCCCAGATGACTTGAAGCATCAAGTCAAGCAGATATACTGGTCCAGATACGTAGAAA
ACGAAGCACGATTCAGAGCTGCTCTGAAAATGACGAAGATCATATATCCAGTCTGGTGTCCGGATTAC
AATTGCTTCGCATTC
+
FFFF,F:FFFFFFF:FFFF:F:FF:,FFFF,:F,FFFFF,FFFFFF:FF,:FFFFFF:FFF:,F,FFF
FFFF,FFFFFF,FFF,FFFFFFFFFFFFFFFF:,F,FFFF,:,,FFFFFFF,:,FFF:,F,F:FFFFFF
FF:,F:FFFFFF,:F
@A00721:190:HGJW7DSXY:4:2576:4951:26256 2:N:0:TCTGCAAG+GCTGCACT
CTACAACATTCTCCCAGATGACTTGAAGCATCAAGTCAAGCAGATATACTGGTCCAGATACGTAGAAA
ACGAAGCACGATTCAGAGCTGCTCTGAAAATGACGAAGATCATATATCCATTCTGGTGTCCGGATTAC
AATTGCTTCGCATTC
+
FFFFFFFFFFFFFFFFFFFFFFFFFFFFFFFFFFFFFFFFFFFFFFFFFFFFFFFFFFFFFFFFFFFF
:FFFFFFFFFFFFFFFFFFFFFFFFFFFFFFFFFFFFFFFFFFFFFFFFFFFFFFFFFFFFFFFFFFF
FFFFFFFFFFFFFFF
@A00721:190:HGJW7DSXY:4:2617:24306:17910 2:N:0:TCTGCAAG+GCTGCACT
CTACAACATTCTCCCAGATGACTTGAAGCATCAAGTCAAGCAGATATACTGGTCCAGATACGTAGAAA
ACGAAGCACGATTCAGAGCTGCTCTGAAAATGACGAAGATCATATATCCATTCTGGTGTCCGGATTAC
AATTGCTTCGCATTC
+
FFFFFFFFFFFFFFFFFFFFFFFFFFFFF:FFFFFFFFFFFFFFFFFFFFFFFFFFFFFFFF:FFFFF
FFFFFFFFFFFFFFFFFFFFFFFFFFFFFFFFFFFFFFFFFFFFFFFFFFFFFFFFFFFFFFFFFFFF
FFFFFFFFF:FFFFF
@A00721:190:HGJW7DSXY:4:1147:32434:3771 2:N:0:TCTGCAAG+GCTGCACT
GATATCTACAACATTCTCCCAGATGACTTGAAGCATCAAGTCAAGCAGATATACTGGTCCAGATACGT
AGAAAACGAAGCACGATTCAGAGCTGCTCTGAAAATGACGAAGATCATATATCCATTCTGGTGTCCGG
ATTACAATTGATTCG
+
FFFFFFFFFFFFFFFFFFFFFFFFFFFFFFFFFFFFFFFFFFFFFFFFFFFFF:FFFFFFFFFFFFFF
FFFFFFFFFFFFFF:FFFFFF,FFFFFFFFFFF:FFFFFFFFF:FFFFFFFFF:FFF:FFFFFF:FFF
FFF:FFFFFF,FF,F
@A00721:190:HGJW7DSXY:4:1168:1199:25488 2:N:0:TCTGCAAG+GCTGCACT
GATATCTACAACATTCTCCCAGATGACTTGAAGCATCAAGTCAAGCAGATATACTGGTCCAGATACGT
AGAAAACGAAGCACGATTCAGAGCTGCTCTGAAAATGACGAAGATCATATATCCATTCTGGTGTCCGG
ATTACAATTGCTTCG
+
FFFFFFFFFFFFFFFFFFFFFFFFFFFFFFFFFFFFFFFFFFFFFFFFFFFFFFFFFFFFFFFFFFFF
FFFFFFFFFFFFFFFFFFFFFFFFFFFFFFFFFFFFFFFFFFFFFFFFFFFFFFFFFFFFFFFFFFFF
FFFFFFFFFFFFFFF
@A00721:190:HGJW7DSXY:4:1209:16929:16532 2:N:0:TCTGCAAG+GCTGCACT
GATATCTACAACATTCTCCCAGATGACTTGAAGCATCAAGTCAAGCAGATATACTGGTCCAGATACGT
AGAAAACGAAGCACGATTCAGAGCTGCTCTGAAAATGACGAAGATCATATATCCATTCTGGTGTCCGG
ATTACAATTGCTTCG

+
FFFFFFFFFFFFFFFFFFFFFFFFFFFFFF:FFFFFFFFFFFFFFFFFFFFFF,FFFFFFFFFFFFFF
FFFFFFFFF:FFFFFFFFFFFFFFFFFFFFFFFFFFFFFFFFFFFFFF:FFFFFFF:FFFFFFFFFFF
FFFFFFFFFFFFFFF
@A00721:190:HGJW7DSXY:4:1269:2419:26068 2:N:0:TCTGCAAG+GCTGCACT
GATATCTACAACATTCTCCCAGATGACTTGAAGCATCAAGTCAAGCAGATATACTGGTCCAGATACGT
AGAAAACGAAGCACGATTCAGAGCTGCTCTGAAAATGACGAAGATCATATATCCATTCTGGTGTCCGG
ATTACAATTGCTTCG
+
FFFFFFFFFFFFFFFFFFFFFFFFFFFFFFFFFFFFFF::FFFFFFFFFFFFFFFFFFFFFFFFFFFF
FFFFFFFFFFFFFFFFFFFFFFFF:FFFFFFFFFFFFFF:FFFFFFFFFFFFFFF:FFFFFFFFFFFF
FFFFFFFFFFFFFFF
@A00721:190:HGJW7DSXY:4:1269:2510:26287 2:N:0:TCTGCAAG+GCTGCACT
GATATCTACAACATTCTCCCAGATGACTTGAAGCATCAAGTCAAGCAGATATACTGGTCCAGATACGT
AGAAAACGAAGCACGATTCAGAGCTGCTCTGAAAATGACGAAGATCATATATCCATTCTGGTGTCCGG
ATTACAATTGCTTCG
+
FFFFFF:FFFFFFFFFFFFFFFFFFFFFFFFFFFFFFFFFFFFFFFFFFFFF:FFFFFFFFFFFFFFF
F:FFF:FFFFFFFFFFFFFFFFFFFFFFFFFFFFFFFFFFFFFFFFFF,FFFFFF,FFFFFF,FFFFF
:FFFFFFFFF:FFFF
@A00721:190:HGJW7DSXY:4:1339:32642:29027 2:N:0:TCTGCAAG+GCTGCACT
GATATCTACAACATTCTCCCAGATGACTTGAAGCATCAAGTCAAGCAGATATACTGGTCCAGATACGT
AGAAAACGAAGCACGATTCAGAGCTGCTCTGAAAATGACGAAGATCATATATCCATTCTGGTGTCCGG
ATTACAATTGCTTCG
+
FFFFFFFFFFFFFFFFFFFFFFFFFFFFFFFFFFFFFFFFFFFFFFFFFFFFFFFFFFFFFFFFFFFF
FFFFFFFFFFFFFFFFFFFFFFFFFFFFFFFFFFFFFFFFFFFFFFFFFFFFFFFFFFFFFFFF:FFF
FFF:FFFFFFFFFFF
@A00721:190:HGJW7DSXY:4:1362:9471:7122 2:N:0:TCTGCAAG+GCTGCACT
GATATCTACAACATTCTCCCAGATGACTTGAAGCATCAAGTCAAGCAGATATACTGGTCCAGATACGT
AGAAAACGAAGCACGATTCAGAGCTGCTCTGAAAATGACGAAGATCATATATCCATTCTGGTGTCCGG
ATTACAATTGCTTCG
+
FFFFFFFFFFFFFFFFFFFFFFFFFFFFFFFFFFFFFFFFFFFFFFFFFF,FF:FFFFFFFFFFFFFF
FFFFFFFFFFFFFFFFFFFFFFFFFFFFFFFFFFFFFFFFFFFFFFFFFFFFFFFFFFFFFFFFFFFF
FFFFFFFFFFFFFFF
@A00721:190:HGJW7DSXY:4:1502:19271:32737 2:N:0:TCTGCAAG+GCTGCACT
GATATCTACAACATTCTCCCAGATGACTTGAAGCATCAAGTCAAGCAGATATACTGGTCCAGATACGT
AGAAAACGAAGCACGATTCAGAGCTGCTCTGAAAATGACGAAGATCATATATCCATTCTGGTGTCCGG
ATTACAATTGCTTCG
+
::FFFFFFFFFFFFFFFFFFFFFFFFFFFFFFFFFFFFFFFFFFFFFFFFFFFFFFFFFFFFFFFFFF
FFFFFFFFFFFFFFFFFFFFFFFFFFFFFFFFFFFFFFFFFFFFFFFFFFFFFFFFFFFFFFFFFFFF
FFFFFFFFFFFFFFF
@A00721:190:HGJW7DSXY:4:1502:18475:32831 2:N:0:TCTGCAAG+GCTGCACT
GATATCTACAACATTCTCCCAGATGACTTGAAGCATCAAGTCAAGCAGATATACTGGTCCAGATACGT
AGAAAACGAAGCACGATTCAGAGCTGCTCTGAAAATGACGAAGATCATATATCCATTCTGGTGTCCGG
ATTACAATTGCTTCG
+
FFFF:FFFFFFFFFFFFFFFFFFFFFFFFFF:FFFFFFFFFFFFFF:FFF:FFF,FFFFFFFFFFFFF
FFFFFFFFFFFFFFFFFFF:FFFFFFFFFFFFFF:FFFFF:F:F:FF:FFFFFFFFFFFFFFFFFFFF
FFFFFFFFFFFFFFF
@A00721:190:HGJW7DSXY:4:1515:31774:20447 2:N:0:TCTGCAAG+GCTGCACT
GATATCTACAACATTCTCCCAGATGACTTGAAGCATCAAGTCAAGCAGATATACTGGTCCAGATACGT

```
AGAAAACGAAGCACGATTCAGAGCTGCTCTGAAAATGACGAAGATCATATATCCATTCTGGTGTCCGG
ATTACAATTGCTTCG
+
F:FFFFFFFFFFFFFFFFFFFFFFFFFFFFFFFFFFFFFFFFFFFFFFFFFFFFFFFFFFFFFFFFFF
FFFFFFFFFFFFFFFFFFFFFFFFFFFFFFFFFFFFFFFFFFFFFFFFFFFFFFFFFFFFFFFFFFFF
FFFFFFFFFFFFFFF
@A00721:190:HGJW7DSXY:4:1558:22507:21527 2:N:0:TCTGCAAG+GCTGCACT
GATATCTACAACATTCTCCCAGATGACTTGAAGCATCAAGTCAAGCAGATATACTGGTCCAGATACGT
AGAAAACGAAGCACGATTCAGAGCTGCTCTGAAAATGACGAAGATCATATATCCATTCTGGTGTCCGG
ATTACAATTGCTTCG
+
FFFFFFFFFFFFFFFFFFFFFFFFFFFFFFFFFFFFFFFFFFFFFF:FFFF:FFFFFFFFFFFFFFFF
FFFFFFFFFFFFFFFFFFFFFFFFFFFFFFFFFFFFFFFFFFFFFFFFFFFFFFFFFFFFFFFFFFFF
FFFFFFFFFFFFFFF
@A00721:190:HGJW7DSXY:4:1614:5249:23610 2:N:0:TCTGCAAG+GCTGCACT
GATATCTACAACATTCTCCCAGATGACTTGAAGCATCAAGTCAAGCAGATATACTGGTCCAGATACGT
AGAAAACGAAGCACGATTCAGAGCTGCTCTGAAAATGACGAAGATCATATATCCATTCTGGTGTCCGG
ATTACAATTGCTTCG
+
FFFFFFFFFFFFFFFFFFFFFFFFFFFFFFFFFFFFFFFFFFFFFF:FFF:FFFFFFFFFFFFFFFFF
FFFFFFFFFFFFFFFFFFFFFFFFFFFFFFFFFFFFFFFFFFFF:FFFFFFFFFFFFFFFFFFFFFFF
F,FFFFF:FFF::FF
@A00721:190:HGJW7DSXY:4:1624:7256:15280 2:N:0:TCTGCAAG+GCTGCACT
GATATCTACAACATTCTCCCAGATGACTTGAAGCATCAAGTCAAGCAGATATACTGGTCCAGATACGT
AGAAAACGAAGCACGATTCAGAGCTGCTCTGAAAATGACGAAGATCATATATCCATTCTGGTGTCCGG
ATTACAATTGCTTCG
+
FFFFFFFFFFFFFFFFFFFFFFFFFFFFFFFFFFFFFFFFFFFFFFFFF:FF:FFFFFFFFF,FFFFF
FFFFFFFFFFFFFFFFFFFFFFFFFFFFFFFFFFFFFFFFFF:FFFFFFFFFF::FFF:FFFFFFFFF
FFFFFFFFFFFFFFF
@A00721:190:HGJW7DSXY:4:1624:8079:15452 2:N:0:TCTGCAAG+GCTGCACT
GATATCTACAACATTCTCCCAGATGACTTGAAGCATCAAGTCAAGCAGATATACTGGTCCAGATACGT
AGAAAACGAAGCACGATTCAGAGCTGCTCTGAAAATGACGAAGATCATATATCCATTCTGGTGTCCGG
ATTACAATTGCTTCG
+
FFFFFFFFFFFFFFFFFFFFFFFFFFFFFFFFFFFFFFFFFFFFFF:FFFFFFFFFFFFFFFFFFFFF
FFFFFFFFFFFFFFFFFFFFFFFFFFFFFFFFFFFFFFFFFFFFFFFFFFFFF:FFFFFFFFFFFFFF
FFFFFFFFFFFFFFF
@A00721:190:HGJW7DSXY:4:1665:32000:16485 2:N:0:TCTGCAAG+GCTGCACT
GATATCTACAACATTCTCCCAGATGACTTGAAGCATCAAGTCAAGCAGATATACTGGTCCAGATACGT
AGAAAACGAAGCACGATTCAGAGCTGCTCTGAAAATGACGAAGATCATATATCCATTCTGGTGTCCGG
ATTACAATTGCTTCG
+
FFFFFFFFFFFFFFFFFFFFFF:FFFFFFFFFFFFFFFFFFFFFFFFFFFFFFF,FFFFFFFFFFFFF
FFFFFFFFFFFFFFFFFFFFFFFFFFFFFFFFFFFFFFFFFFFFFFFFFFFFFF,FFFFFFFFFFFFF
FFFFFFFFFFFFFFF
@A00721:190:HGJW7DSXY:4:2153:23411:8750 2:N:0:TCTGCAAG+GCTGCACT
GATATCTACAACATTCTCCCAGATGACTTGAAGCATCAAGTCAAGCAGATATACTGGTCCAGATACGT
AGAAAACGAAGCACGATTCAGAGCTGCTCTGAAAATGACGAAGATCATATATCCATTCTGGTGTCCGG
ATTACAATTGCTTCG
+
FFFFFFFFFFFFFFFFFFFFFFFFFFFFFFFFFFFFFFFFFFFFFFFFFFFFFFFFFFFFFFFFFFFF
FFFFFFFFFFFFFFFFFFFFFFFFFFFFFFFFFFFFFFFFFFFFFFFFFFFFF:FFFFFFFFFFFFFF
FFFFFFFFFFFFFFF
```

```
@A00721:190:HGJW7DSXY:4:2225:5556:22200 2:N:0:TCTGCAAG+GCTGCACT
GATATCTACAACATTCTCCCAGATGACTTGAAGCATCAAGTCAAGCAGATATACTGGTCCAGATACGT
AGAAAACGAAGCACGATTCAGAGCTGCTCTGAAAATGACGAAGATCATATATCCATTCTGGTGTCCGG
ATTACAATTGCTTCG
+
FFFFFFFFFFFFFFFFFFFFFFFFFFFFFFFFFFFFFFFFFFFFFFFFFF:FFFFFFFFFFFFFFFFF
FFFFFFFFFFFFFFFFFFFFFFFFFFFFFFFFFFFFFFFFFFFFFFF:FFFFFF:FFFFFFFFFFFFF
FFFFFFFFFFFFFFF
@A00721:190:HGJW7DSXY:4:2253:6108:17362 2:N:0:TCTGCAAG+GCTGCACT
GATATCTACAACATTCTCCCAGATGACTTGAAGCATCAAGTCAAGCAGATATACTGGTCCAGATACGT
AGAAAACGAAGCACGATTCAGAGCTGCTCTGAAAATGACGAAGATCATATATCCATTCTGGTGTCCGG
ATTACAATTGCTTCG
+
FFFFFFFFFFFFFFFFF:FFFFFFFFFFFFFFFFFFF:FFFFFFFFFFFFF:FFFFFFFFFFFFFFFF
FFFFFFFFFFFFFFFFFFFFFFFFFFFFFFFF:FFFFFFFFFFFFFFFFFFFFFF,FFFFFFFFFFFF
F:FFFFFFFFFFFFF
@A00721:190:HGJW7DSXY:4:2307:19967:4633 2:N:0:TCTGCAAG+GCTGCACT
GATATCTACAACATTCTCCCAGATGACTTGAAGCATCAAGTCAAGCAGATATACTGGTCCAGATACGT
AGAAAACGAAGCACGATTCAGAGCTGCTCTGAAAATGACGAAGATCATATATCCATTCTGGTGTCCGG
ATTACAATTGCTTCG
+
FFFFFFFFFFFFFFFFFFFFFFFFFFFFFFFFFFFFFFFFFFFFFFFFFF:FFFFFFFFFFFFFFFFF
FFFFFFFFFFFFFFFFFFFFFFFFFFFFFFFFFFFFFFFFF:FF,FFFFFFFFFFFFFFFFFFFFFFF
FFFFFFF:FFFFFFF
@A00721:190:HGJW7DSXY:4:2352:5602:31923 2:N:0:TCTGCAAG+GCTGCACT
GATATCTACAACATTCTCCCAGATGACTTGAAGCATCAAGTCAAGCAGATATACTGGTCCAGATACGT
AGAAAACGAAGCACGATTCAGAGCTGCTCTGAAAATGACGAAGATCATATATCCATTCTGGTGTCCGG
ATTACAATTGCTTCG
+
:FFFFFFFFFFFFFFFFFFFFFFFFFFFFFFFFFFFFFFF,F,FFF:FFF:FFF,FFFFFFFFFFFFF
FFFFFFFFFFFFFFFFFFFFFFFF:FFFFFFFFFFFFFFFFF::,FFFFFFFFFFFFFFFFFFFFFFF
FF:FFFFFFFFFFFF
@A00721:190:HGJW7DSXY:4:2427:18213:30060 2:N:0:TCTGCAAG+GCTGCACT
GATATCTACAACATTCTCCCAGATGACTTGAAGCATCAAGTCAAGCAGATATACTGGTCCAGATACGT
AGAAAACGAAGCACGATTCAGAGCTGCTCTGAAAATGACGAAGATCATATATCCATTCTGGTGTCCGG
ATTACAATTGCTTCG
+
FFFFFFFFFFFFFFFFFFFFFFFFFFFFFFFFFFFFFFFFFF:FFFFFFFFF:FFFFFFFFFFF:FFF
FFFFFFFFFFFFFFFFFFFFFFFFFFFFFFFFFFFFFFFFFFFFFFFFFFFFFFFFFFFFFFFFFFFF
FFFFFFFFFFFFFFF
@A00721:190:HGJW7DSXY:4:2522:29803:32189 2:N:0:TCTGCAAG+GCTGCACT
GATATCTACAACATTCTCCCAGATGACTTGAAGCATCAAGTCAAGCAGATATACTGGTCCAGATACGT
AGAAAACGAAGCACGATTCAGAGCTGCTCTGAAAATGACGAAGATCATATATCCATTCTGGTGTCCGG
ATTACAATTGCTTCG
+
FFFFFFFFFFFFFFFFFFFFFFFFFFFFFFFFFFFFFFFFFFFFFFFFFF:FFFFFFFFFFFFFFFFF
FFFFFFFFFFFFFFFFFFFFFFFFFFFFFFFFFFFFFFFFFFFF:FFFFFFFFFFFFFFFFFFFFFFF
FFFFFFFFFFFFFFF
@A00721:190:HGJW7DSXY:4:2552:9607:19601 2:N:0:TCTGCAAG+GCTGCACT
GATATCTACAACATTCTCCCAGATGACTTGAAGCATCAAGTCAAGCAGATATACTGGTCCAGATACGT
AGAAAACGAAGCACGATTCAGAGCTGCTCTGAAAATGACGAAGATCATATATCCATTCTGGTGTCCGG
ATTACAATTGCTTCG
+
FFFFFFFFFFFFFFFFFFFFFFFFFFFFFFFFFFFFFFFFFFFFFFFFFFFFFF,FFFFFFFFFFFFF
```

```
FFFFFFFFFFFFFFFFFFFFFFFFFFFFFFFFFFFFFFFFFFFFFFF:FFFFFFFFFFFFFFFFFFFF
FFFFFFFFFFFFFFF
@A00721:190:HGJW7DSXY:4:2603:1145:22639 2:N:0:TCTGCAAG+GCTGCACT
GATATCTACAACATTCTCCCAGATGACTTGAAGCATCAAGTCAAGCAGATATACTGGTCCAGATACGT
AGAAAACGAAGCACGATTCAGAGCTGCTCTGAAAATGACGAAGATCATATATCCATTCTGGTGTCCGG
ATTACAATTGCTTCG
+
FFFFFFFFFFFFFFFFFFFFFFFFFFFFFFFFFFFFFFFFFFFFFFFFFFFFFFFFFFFFFF:FFFFFF
FFFFFFFFFFFFFFFFFFFFFFFFFFFFFFFFFFFFFFFFFFF:FFFFFFFFFFFFFFFFFFFFFFFFF
FFFFFFFFFFFFFFF
@A00721:190:HGJW7DSXY:4:1259:24903:19695 2:N:0:TCTGCAAG+GCTGCACT
TGTCAAATGGCGTCGAACTATGGTACTATCAAATTTGACGATATCTACAACATTCTCCCAGATGACTT
GAAGCATCAAGTCAAGCAGATATACTGGTCCAGATACGTAGAAAACGAAGCACGATTCAGAGCTGCTC
TGAAAATGACGAAGA
+
FFF:FFFFFFFFFFFFFFFFFFFFFFFFFFFFFFFFFFFFFFFFFFFFFFFFFFFFFFFFFFFFFFFF
FFFFFFFFFFFFFFFFFFFFFFFFFFFFFFFFFFFFFFFFFFFFFFFFFFFFFFFFFFFFFFFFFFFF
FFFFFFFFFFFFFFF
@A00721:190:HGJW7DSXY:4:2209:17544:36573 2:N:0:TCTGCAAG+GCTGCACT
TGTCAAATGGCGTCGAACTATGGTACTATCAAATTTGACGATATCTACAACATTCTCCCAGATGACTT
GAAGCATCAAGTCAAGCAGATATACTGGTCCAGATACGTAGAAAACGAAGCACGATTCAGAGCTGCTC
TGAAAATGACGAAGA
+
FFFFFFFFFFFFFFFFFFFFFFFF:FFFFFFFFFFFFFFFFFFFFFFFFFFFFFFFFFFFFFFFFFFF
FFFFFFFFFFFFFFFFFFFFFFFFFF:FFFFFFFFFFFFFFFFFFFFFFFFFFFFFFFFFFF:FFFFFFF
FFFFFFFFFFFFFFF
@A00721:190:HGJW7DSXY:4:1101:21965:30671 2:N:0:TCTGCAAG+GCTGCACT
CTTCGTCATTTTCAGAGCAGCTCTGAATCGTGCTTCGTTTTCTACGTATCTGGACCAGTATATCTGCT
TGACTTGATGCTTCAAGTCATCTGGGAGAATGTTGTAGATATCGTCAAATTTGATAGTACCATAGTTC
GACGCCATTTGACAC
+
FFFFFFFFFFFFFFFFFFFFFFFFFFFFFFFFFFFFFFFFFFFFFFFFFFFFFFFFFFFFFFFFFFFF
FF:FFFFFFFFFFFFFFFFFFFFFFFFFFF:FFFFFFFFFFFFF:FFFFFFFFFFFFFFFF:FFFFFFF
,FFFFFFFFFFFFFF
@A00721:190:HGJW7DSXY:4:1170:2519:33661 2:N:0:TCTGCAAG+GCTGCACT
CTTCGTCATTTTCAGAGCAGCTCTGAATCGTGCTTCGTTTTCTACGTATCTGGACCAGTATATCTGCT
TGACTTGATGCTTCAAGTCATCTGGGAGAATGTTGTAGATATCGTCAAATTTGATAGTACCATAGTTC
GACGCCATTTGACAC
+
FFFFFFFFFFFFFF:FFFFFFFFFF,FFFFFFFFFFFFFFFFFFFFFFFFFFFFFFFFFF:FFFFFFFF
F:FFFFFFFFFFFFFFFFFFFFFFF::FFFFFFFFFFFFFFFFFFFF:FFFFFFFFFFFFFF:FFFFFFF
:FFFFFFFFFFFFFF
@A00721:190:HGJW7DSXY:4:1351:27787:7279 2:N:0:TCTGCAAG+GCTGCACT
CTTCGTCATTTTCAGAGCAGCTCTGAATCGTGCTTCGTTTTCTACGTATCTGGACCAGTATATCTGCT
TGACTTGATGCTTCAAGTCATCTGGGAGAATGTTGTAGATATCGTCAAATTTGATAGTACCATAGTTC
GACGCCATTTGACAC
+
FFFFFFFFFFFFFFFFFFFFFFFFFFFF:FFFFFFFFFFFFFFFFFF:FFFFFFFFFFF:FFFFFFFF
:FFFFFFFFFFFFFFFFFFFFFFFFFFFFFFFFFFFFFFFFFFFFFFFFFFFFFFFFFFFFFFFFFFF
FFFFFFFFFFFFFFF
@A00721:190:HGJW7DSXY:4:2109:8766:11162 2:N:0:TCTGCAAG+GCTGCACT
CTTCGTCATTTTCAGAGCAGCTCTGAATCGTGCTTCGTTTTCTACGTATCTGGACCAGTATATCTGCT
TGACTTGATGCTTCAAGTCATCTGGGAGAATGTTGTAGATATCGTCAAATTTGATAGTACCATAGTTC
GACGCCATTTGACAC
```

+
FFFFFFFFFFFFFFFFFFFFFFFFFFFFFFFFFFFFFFFFFFFFFFFFFFFFFFFFFFFFFFFFFFFF
F:FFFFFFFFFFFFFFFFFFFFFFFFFFFFFFFFFFFFF,FFFFFFFF:FFFFF:FFFFFFFFFFFFF
:FF:FFF,FFF:FF:
@A00721:190:HGJW7DSXY:4:2138:18810:22858 2:N:0:TCTGCAAG+GCTGCACT
CTTCGTCATTTTCAGAGCAGCTCTGAATCGTGCTTCGTTTTCTACGTATCTGGACCAGTATATCTGCT
TGACTTGATGCTTCAAGTCATCTGGGAGAATGTTGTAGATATCGTCAAATTTGATAGTACCATAGTTC
GACGCCATTTGACAC
+
:FFFFFFFFFFFFF:FFFFFFFF:FFFFF,FFFFFFFFFFFFFFFFFFFF:F:FFFFFFFFFFFF,F:
,FFFFFFFFF,FF:FFFFFFFF:FFFFFFFFFFFFFFFFFF:FFFFFFFF:FFFFFFFFF:FF:FFFF
,FFFFFFFFFFFFFF
@A00721:190:HGJW7DSXY:4:2526:10520:22717 2:N:0:TCTGCAAG+GCTGCACT
CTTCGTCATTTTCAGAGCAGCTCTGAATCGTGCTTCGTTTTCTACGTATCTGGACCAGTATATCTGCT
TGACTTGATGCTTCAAGTCATCTGGGAGAATGTTGTAGATATCGTCAAATTTGATAGTACCATAGTTC
GACGCCATTTGACAC
+
FFFFFFFFFFFFFFFFFFFFFFFFFFFFFFFFFFFFFFFFFFFFFFFFFFFFFFFFFFF,FFFFFFFF
FFFFFFFFFFFFFFFFFFFFFFFFFFFFFFFF,FFFFFFFFFFFFF,FFFFFFFF:FFF:FFFFF:FF
FFFFFFFFFFFFFFF
@A00721:190:HGJW7DSXY:4:1310:15989:11397 2:N:0:TCTGCAAG+GCTGCACT
CAAATGGCGTCGAACTATGGTACTATCAAATTTGACGATATCTACAACATTCTCCCAGATGACTTGAA
GCATCAAGTCAAGCAGATATACTGGTCCAGATACGTAGAAAACGAAGCACGATTCAGAGCTGCTCTGA
AAATGACG
+
FFFFFFFFFFFFFFFFFFFFF:FF:FFFFFFFFFFFFFFFFFFFFFFFFFFFFFFFFFFFFFFFFFFF
FFFFFFFFFFFFFFFFFFFFFFFFFFFFFFFFFFFFFFFFFFFFFFFFFFFFFFFFFFFFFFFFFFFF
FFFFFFF:
@A00721:190:HGJW7DSXY:4:1313:19407:2002 2:N:0:TCTGCAAG+GCTGCACT
CAAATGGCGTCGAACTATGGTACTATCAAATTTGACGATATCTACAACATTCTCCCAGATGACTTGAA
GCATCAAGTCAAGCAGATATACTGGTCCAGATACGTAGAAAACGAAGCACGATTCAGAGCTGCTCTGA
AAATGACG
+
FFFFFFFFFFFFFFFFFFFFF:FFFFFFFFFFFFFFFFFFFFFFFFFFFFFFFFFFFFFFFFFFFFFF
FFFFFFFFFFFFFFFFFFFFFFFFFFFFFFFFFFFFFFFFFFFFFFFFFF:FFFFFFFFFFFFFFFFF
FFFFFFFF
@A00721:190:HGJW7DSXY:4:1436:10456:5916 2:N:0:TCTGCAAG+GCTGCACT
CAAATGGCGTCGAACTATGGTACTATCAAATTTGACGATATCTACAACATTCTCCCAGATGACTTGAA
GCATCAAGTCAAGCAGATATACTGGTCCAGATACGTAGAAAACGAAGCACGATTCAGAGCTGCTCTGA
AAATGACG
+
FFFFFFFFFFFFFFFFFFFFFFFF:FFFFFFFFFFFFFFFFFFFFFFFFFFFFFFFFFFFFFFFFFFF
FFFFFFFFFFFFFFFFFFFFFFFFFFFFFFFFFFFFFFFFFFFFFFFFFFFFFFFFFFFFFFFFFFFF
FFFFFFFF
@A00721:190:HGJW7DSXY:4:1503:3387:14841 2:N:0:TCTGCAAG+GCTGCACT
CAAATGGCGTCGAACTATGGTACTATCAAATTTGACGATATCTACAACATTCTCCCAGATGACTTGAA
GCATCAAGTCAAGCAGATATACTGGTCCAGATACGTAGAAAACGAAGCACGATTCAGAGCTGCTCTGA
AAATGACG
+
FFFFFFFFFFFFFFFFFFFFFFFF,FFFFFFFFFFFFFF:FFFFFFFFFFFFFFFFFFFFFFFFFFFF
FFFFFFFFFFFFFFFFFFFFFFFFFFFFFFFFFFFFFFFFF:FFFFFFFFFF:FFFFFFFFFF:FFFF
FF:FFFFF
@A00721:190:HGJW7DSXY:4:2377:32072:12915 2:N:0:TCTGCAAG+GCTGCACT
CAAATGGCGTCGAACTATGGTACTATCAAATTTGACGATATCTACAACATTCTCCCAGATGACTTGAA

GCATCAAGTCAAGCAGATATACTGGTCCAGATACGTAGAAAACGAAGCACGATTCAGAGCTGCTCTGA
AAATGACG
+
FFFFFFFFFFFFFFFFFFFFFFFFFFFF:FFFF:FFFFFFFFFFFFFFFFFFFFFFFFFFFFFFFFFF
FFFFFF,FFFFFFFFFFFFFFFFFFFF,FFFFFFFFFFFFFFFFFFFFFFFFFFFFFFFFFFFFFFFF
FFFFF,:F
@A00721:190:HGJW7DSXY:4:1103:3757:7905 2:N:0:TCTGCAAG+GCTGCACT
AAATGGCGTCGAACTATGGTACTATCAAATTTGACGATATCTACAACATTCTCCCAGATGACTTGAAG
CATCAAGTCAAGCAGATATACTGGTCCAGATACGTAGAAAACGAAGCACGATTCAGAGCTGCTCTGAA
AATGACAGATCGGAA
+
FFFFF:FFFFFFFFFFFFFFFFFFFFFFFFFFFFFFFFFFFFFFFFFFFFFFFFFFFFFFFFFFFFFF
FFFFFFFFFFFFFFFFFFFFFFFFFFFFFFFFFFFFFFFFFFFFFFFFFFFFFFF:FFFFFFFFFFF:
FFFFFFFFFFFFFFF
@A00721:190:HGJW7DSXY:4:1366:15356:27211 2:N:0:TCTGCAAG+GCTGCACT
AAATGGCGTCGAACTATGGTACTATCAAATTTGACGATATCTACAACATTCTCCCAGATGACTTGAAG
CATCAAGTCAAGCAGATATACTGGTCCAGATACGTAGAAAACGAAGCACGATTCAGAGCTGCTCTGAA
AATGACAGATCGGAA
+
FFFFFFFFFFFFFFFFFFFFFFF:FFFFFFFFFFFFFFFFFFFFFFFFFFFFFFFFFFFFFFFFFFFF
FFFFFFFFFFFFFFFFFFFFFFFFFFFFFFFFFFFFFFFFFFFFFFFFFFFFF:FFFFFFFFFFFFFF
FFFF:FFFFFFFFF:
@A00721:190:HGJW7DSXY:4:1654:27661:33489 2:N:0:TCTGCAAG+GCTGCACT
AAATGGCGTCGAACTATGGTACTATCAAATTTGACGATATCTACAACATTCTCCCAGATGACTTGAAG
CATCAAGTCAAGCAGATATACTGGTCCAGATACGTAGAAAACGAAGCACGATTCAGAGCTGCTCTGAA
AATGACAGATCGGAA
+
FFFFFFFFFFFF:FFFFFFF:F,,FFFFFFFFFFFFFFFFFFFFFFFFFFFFFFFFFFFFFFFFFFFF
FFFFFFFFFFFFFFFFFFFFFFFFFFFFFFFFFFFFFFFF,FFFFFFFFFFF:FFFFFFFFFFFFFFF
FFFFFF:FFFFFFF:
@A00721:190:HGJW7DSXY:4:2276:11478:33865 2:N:0:TCTGCAAG+GCTGCACT
AAATGGCGTCGAACTATGGTACTATCAAATTTGACGATATCTACAACATTCTCCCAGATGACTTGAAG
CATCAAGTCAAGCAGATATACTGGTCCAGATACGTAGAAAACGAAGCACGATTCAGAGCTGCTCTGAA
AATGACAGATCGGAA
+
FFFFFFFFFFFFFFFFFFFFFFFFFFFFFFFFFFFFFFFFFFFFFFFFFFFFFFFFFFFFFFFFFFFF
FFFFF:FFFFFFFFFFFFFFFFFFFFFFFFFFFFFFFFFFFFFFFFFFFFFFFFFFFFFFFFFFFFFF
FFFFFFFFFFFFFFF
@A00721:190:HGJW7DSXY:4:2323:1235:17065 2:N:0:TCTGCAAG+GCTGCACT
AAATGGCGTCGAACTATGGTACTATCAAATTTGACGATATCTACAACATTCTCCCAGATGACTTGAAG
CATCAAGTCAAGCAGATATACTGGTCCAGATACGTAGAAAACGAAGCACGATTCAGAGCTGCTCTGAA
AATGACAGATCGGAA
+
F:FFFFFFFFFFFFFFFFFFFF:FFFFFFFFFFFFFFFFFFFFFFFFFFFFFFFFFFFFFFFFFFFFF
FFFFFFFFFFFF:FFFFFFFFFFFFFFFFFFFFFFFFFFFFFFFFFFFFFFFFFFFFFFFFFFFFFF
FFFFFFFFFFFFFFF
@A00721:190:HGJW7DSXY:4:2468:13015:27289 2:N:0:TCTGCAAG+GCTGCACT
AAATGGCGTCGAACTATGGTACTATCAAATTTGACGATATCTACAACATTCTCCCAGATGACTTGAAG
CATCAAGTCAAGCAGATATACTGGTCCAGATACGTAGAAAACGAAGCACGATTCAGAGCTGCTCTGAA
AATGACAGATCGGAA
+
FFFFFFFFFFFFFFFFFFFFFFFFFFF:FFFFFFFFFFFFFFFFFFFFFFFFFFFFFFFFFFFFFFFF
FFFFFFFFFFFFFFFFFFFFFFFFFFFFFFFFFFFFFFFFFFFFFFFFFFFFFFFFFFFFFFFFFFF:
FFFFFFFFFFFFFFF

```
@A00721:190:HGJW7DSXY:4:2225:19732:19100 2:N:0:TCTGCAAG+GCTGCACT
AAATGGCGTCGAACTATGGTACTATAAAATTTGACGATATCTACAACATTCTCACAGATGACTTGAAG
CATCAAGTCAAGCAGATATACTGGTCCAGATACGTAGAAAACGAAGCACGATTCAGAGCTGCTCTGAA
AATGACAGATCGGA
+
FFFFF:FFFF,FFF:FF,,FF,FF::FFFFF,,F:FFFF:,,FFFFFFF,,:,,FF,FFFF:FFFFF,
:FFF,:,F:FFF,F:FFFFFF:,,F,:F,F:F:,FF::FFF:,FFF:F,:FFFFFFFFFF,FFF:FF:
FFFFFFFFF:F:FF
@A00721:190:HGJW7DSXY:4:2311:11288:17785 2:N:0:TCTGCAAG+GCTGCACT
CAAATGGCGTCGAACTATGGTACTATCAAATTTGACGATATCTACAACATTCTCCCAGATGACTTGAA
GCATCAAGTCAAGCAGATATACTGGTCCAGATACGTAGAAAACGAAGCACGATTCAGAGCTGCTCTGA
AAATGAC
+
FFFFF:FFFFFFFFFFFFFFFFFFFFFFFFF:FF:FFFFFFFFFFFFFFF:FFFFFFFFFFFFFFFFFF
FFFF:FFF,FFFFFFFF:FFFFFFFFFFFFFFFFFFFFFFFFFFFFFFFFFFFFFFFFFFFFFFFFFF
FFF::FF
@A00721:190:HGJW7DSXY:4:1310:27570:28009 2:N:0:TCTGCAAG+GCTGCACT
TTTCAGAGCAGCTCTGAATCGTGCTTCGTTTTCTACGTATCTGGACCAGTATATCTGCTTGACTTGAT
GCTTCAAGTCATCTGGGAGAATGTTGTAGATATCGTCAAATTTGATAGTACCATAGTTCGACGCCATT
TGACACACTGTTGA
+
FFFFFFFFFFFFFFFFF:FFFFFF:FFFFFFFFFFFFF,FFFFFFFFFFFFFFFFFFFFFFFFFFFFF
FFFFFFFFFFFFFFFFF:FF,FF,FF,FFFFF,FFFFFFFFFFFF:FFFFFF:FFFFFFFFFFFFF:F
FFFFFFFF:F:FFF
@A00721:190:HGJW7DSXY:4:1611:8621:13385 2:N:0:TCTGCAAG+GCTGCACT
TTTCAGAGCAGCTCTGAATCGTGCTTCGTTTTCTACGTATCTGGACCAGTATATCTGCTTGACTTGAT
GCTTCAAGTCATCTGGGAGAATGTTGTAGATATCGTCAATTTTGATAGTACCATAGTTCGACGCCATT
TGACACACTGTTGAT
+
FFFFFFFFFFFFFFFFFFFFFFFF:FFF:FFFFFFFFFFFFFFFFFFFFFFFFFFFFFFFFFFFFFFF
:FFFFFFFFFFFFFFFFFF,,FFFFFFF:FFFFFFFFFF,FFFFF:FFFFFFFFFFFFFFFFFFFFFF
FFFFFFFFFFF:FFF
@A00721:190:HGJW7DSXY:4:2109:25672:7341 2:N:0:TCTGCAAG+GCTGCACT
TTTCAGAGCAGCTCTGAATCGTGCTTCGTTTTCTACGTATCTGGACCAGTATATCTGCTTGACTTGAT
GCTTCAAGTCATCTGGGAGAATGTTGTAGATATCGTCAAATTTGATAGTACCATAGTTCGACGCCATT
TGACACACTGTTGAT
+
FFFFFFFFFFFFFFFFFFFFFFFFFFFFFFFFFFFFFFFFFFFFFFFFFFFFFFFFFFFFFFFFFFFF
FFFFFFFFFFFF:FFFFFFFFFF:FFFFFFFFFFFFFFFFFFFFFFFFFFF:FFF:,:FFFFFFFFFF
FFFFFFFFFFFFFFF
@A00721:190:HGJW7DSXY:4:2423:5556:18223 2:N:0:TCTGCAAG+GCTGCACT
TTTCAGAGCAGCTCTGAATCGTGCTTCGTTTTCTACGTATCTGGACCAGTATATCTGCTTGACTTGAT
GCTTCAAGTCATCTGGGAGAATGTTGTAGATATCGTCAAATTTGATAGTACCATAGTTCGACGCCATT
TGACACACTGTTGAT
+
FFFFFFFFFFFFFFFFFFFFFFFFFFFFFFFFFFFFFFFFFFFFFFFFFFFFFFFFFFFFFFFFFFFF
FFFFFFFFFFFF,FFFFFFFFFFFFFFFFFFFFF:FFF:FFFFFF:F:FFFFFFFFFFFFFFFFFFF,
FFFFFFFFFF:FFFF
@A00721:190:HGJW7DSXY:4:1110:24261:6120 2:N:0:TCTGCAAG+GCTGCACT
TTTACATTATATTACAAATTCATCATATCAACAGTGTGTCAAATGGCGTCGAACTATGGTACTATCAA
ATTTGACGATATCTACAACATTCTCCCAGATGACTTGAAGCATCAAGTCAAGCAGATATACTGGTCCA
GATACGTAGAAAACG
+
FFFFFFFF:FFFFFFFFFFF:FFFFFFFFFFFFFFFFFFFFFFFFFFFFFFFFFFFFFFFFFF,FFFFF
```

FFFFFF:FFF:FFFFFFFFFFFFFFFFFFFFFFFFFFFFFFFFFFFFFFF::FF,FFFFFFFFFFFFFFF
FFFFF:FFFFFFFFF
@A00721:190:HGJW7DSXY:4:1202:4978:22514 2:N:0:TCTGCAAG+GCTGCACT
TTTACATTATATTACAAATTCATCATATCAACAGTGTGTCAAATGGCGTCGAACTATGGTACTATCAA
ATTTGACGATATCTACAACATTCTCCCAGATGACTTGAAGCATCAAGTCAAGCAGATATACTGGTCCA
GATACGTAGAAAACG
+
:FFFFFFFFFFFFFFFFFFFFFF:FFFFFFFFFFF:FFFF:FFFFFFF:F:FFFFFFFFF,F:FFFFFFF
FF,FFFFFFFFFFFFFFFFFFFFFFFFFFFFFFFFFFFFF:FFF:FFFFFFFFF,FFFFFFFFF,FFF:F
FFFFFFFFFFFFF::
@A00721:190:HGJW7DSXY:4:1274:16242:4131 2:N:0:TCTGCAAG+GCTGCACT
TTTACATTATATTACAAATTCATCATATCAACAGTGTGTCAAATGGCGTCGAACTATGGTACTATCAA
ATTTGACGATATCTACAACATTCTCCCAGATGACTTGAAGCATCAAGTCAAGCAGATATACTGGTCCA
GATACGTAGAAAACG
+
FFFFFFFFFFFFFFFFFFFFFFFFFFFFFFFFFFFFFFFFFFFFFFFFFFFFFFFFFFFFFF,FFFFFFF
FFFFFFFFFFFFFFFFFFFFF:F:FFFFFFFFFFFFFFFFFF:FFFF:FFFFFFFF:FFFFFFFF:FFFF
FFFFFF:F:F:FFFF
@A00721:190:HGJW7DSXY:4:1417:17146:30373 2:N:0:TCTGCAAG+GCTGCACT
TTTACATTATATTACAAATTCATCATATCAACAGTGTGTCAAATGGCGTCGAACTATGGTACTATCAA
ATTTGACGATATCTACAACATTCTCCCAGATGACTTGAAGCATCAAGTCAAGCAGATATACTGGTCCA
GATACGTAGAAAACG
+
FFFFFF:FFFFFFFFFFFFFFFFFFFFFFFFFFFFFFFFFFFFFFFFFFFFFFFFFFFFFFFFFFFFFFF
FFFFFFFFFFFFFFFFFFFFFFFFFF:FFFFFFFFFFFFFFFFFFFFFFFFFFFFFFFFFFFFFFFFF:F
FFFFFFFFFFFF:F:
@A00721:190:HGJW7DSXY:4:1649:23529:5353 2:N:0:TCTGCAAG+GCTGCACT
TTTACATTATATTACAAATTCATCATATCAACAGTGTGTCAAATGGCGTCGAACTATGGTACTATCAA
ATTTGACGATATCTACAACATTCTCCCAGATGACTTGAAGCATCAAGTCAAGCAGATATACTGGTCCA
GATACGTAGAAAACG
+
FFFFF:FFFFFFFFFF:F:FFFFFFFFFFFFFFFFFFFFFFFFFFFFFFFFFFFFFFFFFFF:FF:FFFF
FFFFFFFFFFFFFFFFFFFFFFFFFFFFFFFFFFFFFFFFF:FFFFFFFFFFF:FFFFF,FFFFFFFFFF
FFFFF:FFF:FFFFF
@A00721:190:HGJW7DSXY:4:2304:3061:34820 2:N:0:TCTGCAAG+GCTGCACT
TTTACATTATATTACAAATTCATCATATCAACAGTGTGTCAAATGGCGTCGAACTATGGTACTATCAA
ATTTGACGATATCTACAACATTCTCCCAGATGACTTGAAGCATCAAGTCAAGCAGATATACTGGTCCA
GATACGTAGAAAACG
+
FFFFFFFFFFFFFFFFF:FFFFFFFFFFFFFFFF:FFFFFFFFFF:FFFFFFFFFFFFFFFFFFFFFFFF
FFFFFFFFFFFFFFFFF:FFFFFFFFFFFFFFFFFFFFFFFFFFFFFFFFFFFF::FFFFFFFFFFFFFF
FFFFFF:FFFFFFFF
@A00721:190:HGJW7DSXY:4:2332:17418:13777 2:N:0:TCTGCAAG+GCTGCACT
TTTACATTATATTACAAATTCATCATATCAACAGTGTGTCAAATGGCGTCGAACTATGGTACTATCAA
ATTTGACGATATCTACAACATTCTCCCAGATGACTTGAAGCATCAAGTCAAGCAGATATACTGGTCCA
GATACGTAGAAAACG
+
FFFFFFFFFFFFFFFFFFFFFFFFFFFFFFFFFFFFFFFFFFFFFFFFFFFFFFFFFFFFFF:FFFFFFF
FFFFFFFFFFFFFFFFFFFFFFFFF:FFFFFFFFFFFFFFFFFFFFFFFFFFFFFFFFFFFFFFFFFFFF
FFFFFFFF:FFFFF:
@A00721:190:HGJW7DSXY:4:2648:4472:33254 2:N:0:TCTGCAAG+GCTGCACT
TTTACATTATATTACAAATTCATCATATCAACAGTGTGTCAAATGGCGTCGAACTATGGTACTATCAA
ATTTGACGATATCTACAACATTCTCCCAGATGACTTGAAGCATCAAGTCAAGCAGATATACTGGTCCA
GATACGTAGAAAACG

+
FFFFFFFFFFFFFFF,FFFFFFFFFFFFFFFFFFFFFFFF:FFFFFFFFFF:F,FFFF:FFFFF:FFFF
FFF:FFFFFFFFFFFFFFFFFFFFFFFFFFFF:FFFFFFF::FFFFFFFFFFFFFFFFFFFFFFF:FFFF
FFFF:FFFFFFFF::
@A00721:190:HGJW7DSXY:4:1471:31458:32424 2:N:0:TCTGCAAG+GCTGCACT
TTTACATTATATTACAAATTCAGCATATCAACAGTGTGTCAAATGGCGTCGAACTATGGTACTATCAA
ATTTGACGATATCTACAACATTCTCCCAGATGACTTGAAGCATCAAGTCAAGCAGATATACTGGTCCA
GATACGTAGAAAAC
+
FFFFFFFFFFFFFFFFFFFFFF,FFFFFFFFFFFFFFFFFFFFFFFFFFFFFFFFFF:FFFFFFFFFF
FFFFFFFFFFFFFFFFFFFFFFFFFFFFFFFFFFFFFFFFFFFFFFFFFFFFFFFFFFFFFF,F:FF:FF
FFFFFFFFFFFF,F
@A00721:190:HGJW7DSXY:4:1172:12111:35368 2:N:0:TCTGCAAG+GCTGCACT
ATTTTACATTATATTACAAATTCATCATATCAACAGTGTGTCAAATGGCGTCGAACTATGGTACTATC
AAATTTGACGATATCTACAACATTCTCCCAGATGACTTGAAGCATCAAGTCAAGCAGATATACTGGTC
CAGATACGTAGAAAA
+
FFFFFFFFFFFFFFFFF:FFFFFFFFFFFFFFFFFFFFFFFFFFFFFFFFFFFF:FFFFFFFFFFFFFF
FFFFFFF:F:FFFFFFFF:FFFFFF:FFFFFFF:FFFFFFFFFFF:FFF:FFFFFFFFFFFFF:FFFF
FFFFFFFFFFFFFFF
@A00721:190:HGJW7DSXY:4:1514:17255:12900 2:N:0:TCTGCAAG+GCTGCACT
TTTTCTACGTATCTGGACCAGTATATCTGCTTGACTTGATGCTTCAAGTCATCTGGGAGAATGGTGTA
GATATCGTCAAATTTGATAGTACCATAGTTCGACGCCATTTGACACACTGTTGATATGATGAATTTGT
AATATAATGTAAAAT
+
FFFFFFFFFFF,FFFFFFFFFFF:FFFFFF::FF:FFFFFFFFFF:F,F:F:FFFF,FFFF:F,FFFF
FFFF:FFFFFFFFFF,FF:F:FF:F:F,FFFFFFFFFF:FFFF:FF,::FFFFFF:FFFF:F:::F:F
FFF:FFF:FFF:F:F
@A00721:190:HGJW7DSXY:4:1648:12608:28025 2:N:0:TCTGCAAG+GCTGCACT
ATTTTACATTATATTACAAATTCATCATATCAACAGTGTGTCAAATGGCGTCGAACTATGGTACTATC
AAATTTGACGATATCTACAACATTCTCCCAGATGACTTGAAGCATCAAGTCAAGCAGATATACTGGTC
CAGATACGTAGAAAA
+
FFFF,FFFFFFFF,FFF,F,FFFFFFFFFFFFF:FFFFFFFFFF,FFFF:FFFFFFFFFFF:FFFFFF
::FFFFFFFFF:FFFFFFFFFFFFFFFFFFFFFFFFFFFFFFFFFFFFFFFFFFFFFFFFFFFFF,:F
FFF:,FFFFFF:FFF
@A00721:190:HGJW7DSXY:4:2122:2727:36777 2:N:0:TCTGCAAG+GCTGCACT
TTTTCTACGTATCTGGACCAGTATATCTGCTTGACTTGATGCTTCAAGTCATCTGGGAGAATGTTGTA
GATATCGTCAAATTTGATAGTACCATAGTTCGACGCCATTTGACACACTGTTGATATGATGAATTTGT
AATATAATGTAAAAT
+
FFFFFFFFFFFFFFFFFFFFFFFFFFFFFFFF:FFFFFFFFFFFFFFFFFFFFFFFF:FFFF:FFFFFFF
FFF:F:FFFFFFFFFFFFFFFFFFFFFFFFFF:FFFFFF,FFFFF:FFFF:FFFFFFFFF::F:FFFFF
FFFFFFFFFFFFFFF
@A00721:190:HGJW7DSXY:4:2146:25672:16611 2:N:0:TCTGCAAG+GCTGCACT
ATTTTACATTATATTACAAATTCATCATATCAACAGTGTGTCAAATGGCGTCGAACTATGGTACTATC
AAATTTGACGATATCTACAACATTCTCCCAGATGACTTGAAGCATCAAGTCAAGCAGATATACTGGTC
CAGATACGTAGAAAA
+
FFFFFFFFFFF:FFFFFFFFFFFFFFFFFFFFFFFFF:FFFFFFF:FFFFFFFFFFFFFFFFFFFFFFFF
F,FFFFFFFFFFFFFFFFFFFFFFFFFFFFFFFFFFFFFFFFFFFFFFFFFFFFFFFFFFFFFFF::FFF
F:FFFFFFFF:FFFF
@A00721:190:HGJW7DSXY:4:2178:11975:11240 2:N:0:TCTGCAAG+GCTGCACT
ATTTTACATTATATTACAAATTCATCATATCAACAGTGTGTCAAATGGCGTCGAACTATGGTACTATC

```
AAATTTGACGATATCTACAACATTCTCCCAGATGACTTGAAGCATCAAGTCAAGCAGATATACTGGTC
CAGATACGTAGAAAA
+
FFFFFFFFFFFFFFFFFFFFFFFFFFFFFFFFFFFFFFFFFFFFFFFFFFFFFFFFFFFFFF:FFFFF
FFFFFFFFF:FFFFFFFFFFFFF:FFFFFFFFFFFFFFFFFFFFFFFFFFFFFFFFFFFFFFFFFFFF
FFFFFFFFFFFFFFF
@A00721:190:HGJW7DSXY:4:2178:12038:11256 2:N:0:TCTGCAAG+GCTGCACT
ATTTTACATTATATTACAAATTCATCATATCAACAGTGTGTCAAATGGCGTCGAACTATGGTACTATC
AAATTTGACGATATCTACAACATTCTCCCAGATGACTTGAAGCATCAAGTCAAGCAGATATACTGGTC
CAGATACGTAGAAAA
+
FFFFFFFFFFFFFFFFFFFFFFFFFFFFFFFFFFFFFFFFFFFFFFFFFFFFFFFFFFFFFFFFFFFF
FFFFFFFFFFFFFFFFFFFFFFFFFFFFFFFFFFFFFFFFFF:FFFFFFFFFFFFFFFFFFFFF:FFF
FFFFFFF:FF:FFFF
@A00721:190:HGJW7DSXY:4:2211:13078:5040 2:N:0:TCTGCAAG+GCTGCACT
ATTTTACATTATATTACAAATTCATCATATCAACAGTGTGTCAAATGGCGTCGAACTATGGTACTATC
AAATTTGACGATATCTACAACATTCTCCCAGATGACTTGAAGCATCAAGTCAAGCAGATATACTGGTC
CAGATACGTAGAAAA
+
FFF:FFFFFFFFFFFFFFFFFFFFFFFFFFFFFFFFFFFFFFFFFFFFFFFFFFFFFFFFFFFFF:FF
FFFFFFFFFFFFFFFFFFFFFFFFFFFFFFFFFFFFFFFFFFFFFFFFFFFFFFFFFFFFFFFFFFFF
FFFFFFFFFFF:FFF
@A00721:190:HGJW7DSXY:4:2421:3025:3975 2:N:0:TCTGCAAG+GCTGCACT
TTTACATTATATTAAAAATTCATCATATCAACAGTGTGTCAAATGGCGTCGAACTATGGTACTATCAA
ATTTGACGATATCTACAACAATCTCCCAGATGACTAGAAGCATCAAGTCAAGCAGATATACTGGTCCA
GATACGTAGAAAA
+
F,FFFFF::FFFFF,:FF:FFFFFF:FFFFFFFF,FFFFFFFFFFFFFFF:FFFFFFF,FFFF,FFFF
:F:F:F,FFFFF,F:,FFFF,,:FFFFFFF,,FFF,:FFF:FFFFF::FFF,FF:FFFFFF:,:FFF:
FFFF::FFFFFFF
@A00721:190:HGJW7DSXY:4:2426:3685:13761 2:N:0:TCTGCAAG+GCTGCACT
ATTTTACATTATATTACAAATTCATCATATCAACAGTGTGTCAAATGGCGTCGAACTATGGTACTATC
AAATTTGACGATATCTACAACATTCTCCCAGATGACTTGAAGCATCAAGTCAAGCAGATATACTGGTC
CAGATACGAAGAAAA
+
FFFFFFFFFFFFFFFFFF:F,FFFFFFFFFFFFF:FFFFFFFFFFFFFFFFFFFFFFFFFFFFFFF:F
FFFFFFFF:FFFFFFF:FFFFFFFFFF:FFF:FFFFFFFFF:FFF:FFFFFFFFFFFFFFFFFFF,,F
,FF,FF,:,:F,:::
@A00721:190:HGJW7DSXY:4:2528:12364:23657 2:N:0:TCTGCAAG+GCTGCACT
ATTTTACATTATATTACAAATTCATCATATCAACAGTGTGTCAAATGGCGTCGAACTATGGTACTATC
AAATTTGACGATATCTACAACATTCTCCCAGATGACTTGAAGCATCAAGTCAAGCAGATATACTGGTC
CAGATACGTAGAAAA
+
FF,F:F:FFFFFFFFFFFFFFFF,FFF,F::FFFF:FFFFFFFFF:,FFFFFFFFF:FF:FF:FFFFF
FFF,FF,FFF:FF,FFFFFFFFFFFFFF:F:F:,FF:F,:FF:FFFFF:FFFF,:FF:F:FF:F,F,F
,FFF,FF:FF,FFFF
@A00721:190:HGJW7DSXY:4:2538:23258:21543 2:N:0:TCTGCAAG+GCTGCACT
ACATTATATTACAAATTCATCATATCAACAGTGTGTCAAATGTCGTCGAACTATGGTACTATCAAATT
TGAAGATATCTACAACATTCTCCCAGATGACTTGAAGCATCAAGACAAGAAGATATACTGGTCCAGAT
ACGAAGAAAA
+
F:FFFFFF:,FFFFFFF,FFFFFF:,FF,FF,,F,F,FFFF:,,F,F:FFF,FFF,,:,FFFFFFFF:
,FF,FFFFFF,F,FF,F:,,F,,,FFF,FF,,,FFF:,FFFFF,,,FFF,F,FFFFF,F:,FF,FFFF
F,,,F,FFFF
```

@A00721:190:HGJW7DSXY:4:2571:28935:14403 2:N:0:TCTGCAAG+GCTGCACT
ATTTTACATTATATTACAAATTCATCATATCAACAGTGTGTCAAATGGCGTCGAACTATGGTACTATC
AAATTTGACGATATCTACAACATTCTCCCAGATGACTTGAAGCATCAAGTCAAGCAGATATACTGGTC
CAGATACGTAGAAAA
+
FFFFFFFFFFFFFFFFFFFFFFFFFFFFFFFFFFFFFFFFFFFFFFFFFFFFFFFFFFFFFFFFFFFF
FFFFFFFFFFFFFFFFFFFFFFFFFFFFFFFFFFFFFFFFFFFFFFFFFFFFFFFFFFFFFFFFFFFF
FFFFFF,FFFFFFFF
@A00721:190:HGJW7DSXY:4:2601:7500:17206 2:N:0:TCTGCAAG+GCTGCACT
ATTTTACATTATATTACAAATTCATCATATCAACAGTGTGTCAAATGGCGTCGAACTATGGTACTATC
AAATTTGACGATATCTACAACATTCTCCCAGATGACTTGAAGCATCAAGTCAAGCAGATATACTGGTC
CAGATACGTAGAAAA
+
FF:FFFFFFFFFFFFFFF:FFFFFFFFFFFFFFFFFFFFFFFFFFFFFFFFFFFFFFFFFFF:FF:FF
FFFFFFFFF:FFFF:FFFFFFFFFFFF:FFFFFFFFFFFFFFFFFFF:FFFF:FFFFFF:FFFFFFFF
FFFF::FFFFFFFFF
@A00721:190:HGJW7DSXY:4:2625:8024:27007 2:N:0:TCTGCAAG+GCTGCACT
TTTTCTACGTATCTGGACCAGTATATCTGCTTGACTTGATGCTTCAAGTCATCTGGGAGAATGTTGTA
GATATCGTCAAATTTGATAGTACCATAGTTCGACGCCATTTGACACACTGTTGATATGATGAATTTGT
AATATAATGTAAAAT
+
FFFFFFFFFFFF:FFFFFFFFF:FFFFFFFFFFFFFFFFFFFFFFFFFFF:FFFFFFFFFFF:FFFFF
:FFFFFFFFFFFFFF:FFFFF:FFFFFFFFF,FFFFFF,FF:FF:FFFFFFFFF:F:FFFF:FFFFFF
FFFFFFF:FFFFF:F
@A00721:190:HGJW7DSXY:4:2676:7274:28338 2:N:0:TCTGCAAG+GCTGCACT
ATTTTACATTATATTACAAATTCATCATATCAACAGTGTGTCAAATGGCGTCGAACTATGGTACTATC
AAATTTGACGATATCTACAACATTCTCCCAGATGACTTGAAGCATCAAGTCAAGCAGATATACTGGTC
CAGATACGTAGAAAA
+
FFFFFFFFFFFFFFFFFFFFFFFFFFFFFFFFFFFFFFFFFFFFFFFFFFFFFFFFFFFFFFFFFFFF
FFFFFFFFFFFFFFFFFFFFFFFFFFFFFFFFFFFFFFFFFFFFFFFFFFFFFFFFFFFFFFFFFFFF
FFFFFFFFFFFFFFF
@A00721:190:HGJW7DSXY:4:1275:18213:25426 2:N:0:TCTGCAAG+GCTGCACT
AACAAAACTCACAACACATTTTACATTATATTACAAATTCATCATATCAACAGTGTGTCAAATGGCGT
CGAACTATGGTCCTATCAAATTTGACGATATCTACAACATTCTCCCAGATGACTTGAAGCATCAAGTC
AAGCAGATATACTGG
+
FFFFFFFFFFFFFFFFFF:FFFFFFFFFFFFFFFFF::FFFFFFFFFFFFFFFFFFFFFFFFFFFFFF
FFFFFFFFFFF,FF:FFFFFFFFFFFFFFFFFFFFFFFFFFFFFFFFFFFFFFFFF:FFFFFFF:FFF
FFFFFFFFFFFFFF:
@A00721:190:HGJW7DSXY:4:1275:18141:25457 2:N:0:TCTGCAAG+GCTGCACT
AACAAAACTCACAACACATTTTACATTATATTACAAATTCATCATATCAACAGTGTGTCAAATGGCGT
CGAACTATGGTACTATCAAATTTGACGATATCTACAACATTCTCCCAGATGACTTGAAGCATCAAGTC
AAGCAGATATACTGG
+
FFFFFFFFFF:FFFFFFFFFFFFFFFFFFFFFFFFFFFFFFFFFFFFFFFFFFFFFFFFFFFFFF:FF
FFFF:FFFFFFFF:FFFFFFFFFF,FFFFFFFFFFFFFFF:FFFFFFFFFFFFF:FFFFFFFFF:F:F
FF::FFFFFFFFFFF
@A00721:190:HGJW7DSXY:4:1329:1660:12477 2:N:0:TCTGCAAG+GCTGCACT
AACAAAACTCACAACACATTTTACATTATATTACAAATTCATCATATCAACAGTGTGTCAAATGGCGT
CGAACTATGGTACTATCAAATTTGACGATATCTACAACATTCTCCCAGATGACTTGAAGCATCAAGTC
AAGCAGATATACTGG
+
F:FFFFFFFFFFFFFFFFFFFFFFFFFFFFFFFFFFFFFFFFFFFFFFFFFFFFFFFFFFFFFFFFFF

```
FFFFFFFF:FFFFF,FFFFFFFFFFF:FFFFFFFFFFFFFFFFFFFFFFFFFFFFF,FFFFFFFFFFFFF
F:FFFFFF,FFFFF:
@A00721:190:HGJW7DSXY:4:1625:29668:11412 2:N:0:TCTGCAAG+GCTGCACT
AACAAAACTCACAACACATTTTACATTATATTACAAATTCATCATATCAACAGTGTGTCAAATGGCGT
CGAACTATGGTACTATCAAATTTGACGATATCTACAACATTCTCCCAGATGACTTGAAGCATCAAGTC
AAGCAGATATACTGG
+
FFFFFFFFFFFFFFFFFFFFFFFFFF:FFF,FFFF:FFFFFFFF,FFFFFFFFFFFFFFFFF::FFFFFF
FFFFFFFFFFFFFFFFFFFFFFFFFFFFFFFFFFFFFFFFFFFFFFFFFFFFFFFFFFFFFFFFFFFFFF
FFFFFFFFFFFFFFF
@A00721:190:HGJW7DSXY:4:2129:14895:5368 2:N:0:TCTGCAAG+GCTGCACT
AACAAAACTCACAACACATTTTACATTATATTACAAATTCATCATATCAACAGTGTGTCAAATGGCGT
CGAACTATGGTACTATCAAATTTGACGATATCTACAACATTCTCCCAGATGACTTGAAGCATCAAGTC
AAGCAGATATACTGG
+
FFFFFFFFFFFFFFFFFFF:FFFFFFFFFFFFFFFFFF,FFFFFFFFFFFFFFFFFFFFFFFFFFFFFFF
FFFFFF,FFFFFFFFFFFFFFFFFFFFFFFFFFFFFFFFFFFFFFFFFFFFFFFFFFFFFFFFFF:FFFF
FFFFFFFFFFFFFFF
@A00721:190:HGJW7DSXY:4:2343:6045:13338 2:N:0:TCTGCAAG+GCTGCACT
AACAAAACTCACAACACATTTTACATTATATTACAAATTCATCATATCAACAGTGTGTCAAATGGCGT
CGAACTATGGTACTATCAAATTTGACGATATCTACAACATTCTCCCAGATGACTTGAAGCATCAAGTC
AAGCAGATATACTGG
+
FFFFFFFFFFFFFFFFFFFFFFFFFFFF:FFFFFF:,:FFFFFFFFFFFFFFFFFFFFFFFFFFFFFFFF
FFFFFFFFFFFFFFFFFFFFF:FFFFFFFFFFFFFFFFFFFFFFFFFFFFFFFF:FFFFFFFFFFFFFFF
FFFFFFFFFFFFFFF
@A00721:190:HGJW7DSXY:4:2406:1678:22153 2:N:0:TCTGCAAG+GCTGCACT
AACAAAACTCACAACACATTTTACATTATATTACAAATTCATCATATCAACAGTGTGTCAAATGGCGT
CGAACTATGGTACTATCAAATTTGACGATATCTACAACATTCTCCCAGATGACTTGAAGCATCAAGTC
AAGCAGATATACTGG
+
FFFFFF:FFFFFFFFFFFFFFFFFFF:F:FFFFFFFFF,FFF:FFFFFFF:FFFFFFF:FFFFFF:FFFF
FFFFFFFFFFFF:FFFFFFF:FFFF:FFFFFF,FFFFFFF:FFFFFF:FFFFFFFFFFFFFFFFFF::FF
FFFFF,FFFFFF::F
@A00721:190:HGJW7DSXY:4:2435:16007:35070 2:N:0:TCTGCAAG+GCTGCACT
AACAAAACTCACAACACATTTTACATTATATTACAAATTCATCATATCAACAGTGTGTCAAATGGCGT
CGAACTATGGTACTATCAAATTTGACGATATCTACAACATTCTCCCAGATGACTTGAAGCATCAAGTC
AAGCAGATATACTGG
+
FFFFFFFFFFFFFFFFFFFFFFFFFFFFFFFFFFFFFFFFFFFFFFFFFFFFFFFFFFFFFFFFFF:FFF
FFFFFFFFFFFFFFFF:FFFFFFFFFFFFFFFFFFFFFFFFFFFFFFFFFFFFFFFFFFFFFFFFFFFFF
FFFF:FFFFFFFFFF
@A00721:190:HGJW7DSXY:4:2461:4264:31485 2:N:0:TCTGCAAG+GCTGCACT
AACAAAACTCACAACACATTTTACATTATATTACAAATTCATCATATCAACAGTGTGTCAAATGGCGT
CGAACTATGGTACTATCAAATTTGACGATATCTACAACATTCTCCCAGATGACTTGAAGCATCAAGTC
AAGCAGATATACTGG
+
FFFFFFFFFFFFFFFFFFFFFFFFFFFFFFFFFFFFFFFFFFFFFFFFFFFFFFFFFFFFFFFFFFFFFF
FFFFFFFFFFF,F::FF:,FFFFFFFFFFFFFFFFFFFFFFFFFFFFFFFFFFFFFFFFFFFFFFFFFFF
FFFFFFF:F:FFFFF
@A00721:190:HGJW7DSXY:4:2435:16794:36808 2:N:0:TCTGCAAG+GCTGCACT
AACAAAACTCACAACACATTTTACATTATATTACAAATTCATCATATCAACAGTGTGTCAAATGGCGT
CGAACTATGGTACTATCAAATTTGACGATATCTACAACATTCTCCCAGATGACTTGAAGCATCAAGTC
AAGCAGATATACTG
```

```
+
FFFFFFFFFFFFFFFFFFFFFFFFFFFFFFFFFFFFFFFFFFFFFFFFFFFFFFFFFFFFFFFFFFFF
FFFFFFFFFFFF:FFFFFFFFFFFFFFFFFFFFFFFFFFFFFFFFFFFFFFFFFFFFFFFFFFFFFFF
FFFFFFFFFFFFFF
@A00721:190:HGJW7DSXY:4:1339:18032:27743 2:N:0:TCTGCAAG+GCTGCACT
GAACATAACAAAACTCACAACACATTTTACATTATATTACAAATTCATCATATCAACAGTGTGTCAAA
TGGCGTCGAACTATGGTACTATCAAATTTGACGATATCTACAACATTCTCCCAGATGACTTGAAGCAT
CAAGTCAAGCAGATA
+
FFFFFFFFFFFFFFFFFFFFFFFFFFFFFFFFFFFFFFFFFFF:FFFFFFFFFFFFF:FFFFFFFFFF
FFFF:FFFFFFFFFFFFFFF:FFFFFFFFFFFFFFFFFFFFFFFFFFFFFFFFFFF:FFFFFFFFFFF
FFFFFFFFFFFFFFF
@A00721:190:HGJW7DSXY:4:1342:7835:35196 2:N:0:TCTGCAAG+GCTGCACT
GAACATAACAAAACTCACAACACATTTTACATTATATTACAAATTCATCATATCAACAGTGTGTCAAA
TGGCGTCGAACTATGGTACTATCAAATTTGACGATATCTACAACATTCTCCCAGATGACTTGAAGCAT
CAAGTCAAGCAGATA
+
FFFFFFFFFFFFFFFFFFFFFFFFFFFFFFFFFFFFFFFFFFFFFFFFFFFFFFFFFFFFFFFFFFFF
FFFFFFFFFFFFFFFFFFFFFFFFFFFFFFFFFFFFFFFFFFFFFFFFFFFFFFFFFFFFFFFFFFFF
FFFFFFFF:FFFFFF
@A00721:190:HGJW7DSXY:4:1449:12518:21198 2:N:0:TCTGCAAG+GCTGCACT
GAACATAACAAAACTCACAACACATTTTACATTATATTACAAATTCATCATATCAACAGTGTGTCAAA
TGGCGTCGAACTATGGTACTATCAAATTTGACGATATCTACAACATTCTCCCAGATGACTTGAAGCAT
CAAGTCAAGCAGATA
+
FF:FFFFFFFFF:FFFFFFFFFFF,FFFFFFFFFFFFFFFFFFFFFFFF:FFFFFFFFF:FFFFFFFF
FF:FFFFFFFFFFF:FF:FFFFFFFFFFFFFF:FFFFFFFFFFFFFFFFFFFFFFFFFFFFFFFFFFF
FFFFFFF:FFFFFFF
@A00721:190:HGJW7DSXY:4:1526:16667:29763 2:N:0:TCTGCAAG+GCTGCACT
GAACATAACAAAACTCACAACACATTTTACATTATATTACAAATTCATCATATCAACAGTGTGTCAAA
TGGCGTCGAACTATGGTACTATCAAATTTGACGATATCTACAACATTCTCCCAGATGACTTGAAGCAT
CAAGTCAAGCAGATA
+
FFFFFFFFFFFFFFFFFFFFFFFFFFF,FFF:FFFFFFFF:F:F:FFFFFFFFFFFFFFFFFFFFFFF
FFFFFFFFFFFFFFFF:F:FFF,F:FFFFFFFFF,FFFFFFFFFFFFFFFFFFFFFFFFFFFFFFFF
FFFFFFFFFFFFF:F
@A00721:190:HGJW7DSXY:4:2242:26549:15687 2:N:0:TCTGCAAG+GCTGCACT
GAACATAACAAAACTCACAACACATTTTACATTATATTACAAATTCATCATATCAACAGTGTGTCAAA
TGGCGTCGAACTATGGTACTATCAAATTTGACGATATCTACAACATTCTCCCAGATGACTTGAAGCAT
CAAGTCAAGCAGATA
+
FFFFFFFFFFFFFFFFFFFFFFFFFFFFFFFFFFFFFFFFFFFFFFFFFFFFFFFFFFFFFFFFFFFF
FF:FFFFFFFFFFFFFFFFFFFFFFFFFFFFFFFFFFFFFFFFFFFFFFFFFFFFFFFFFFFFFFF:F
FFFFFFFFFFF:FFF
@A00721:190:HGJW7DSXY:4:2318:15456:12665 2:N:0:TCTGCAAG+GCTGCACT
GAACATAACAAAACTCACAACACATTTTACATTATATTACAAATTCATCATATCAACAGTGTGTCAAA
TGGCGTCGAACTATGGTACTATCAAATTTGACGATATCTACAACATTCTCCCAGATGACTTGAAGCAT
CAAGTCAAGCAGATA
+
FFFFFFFFFFFFFFFFFFFFFFFFFFFFFFFFFFFFFFFFFFFFFFFFFFFFFFFFFFFFFFFFFFFF
FFFFFFFFFFFFFFFFF:FFFFFFFFFFFFFF:FFFFFFFFFFFFFFFFFFFFFFFFFFFFFFFFFFF
FFFFFFFFFFFFFFF
@A00721:190:HGJW7DSXY:4:2318:15474:13416 2:N:0:TCTGCAAG+GCTGCACT
GAACATAACAAAACTCACAACACATTTTACATTATATTACAAATTCATCATATCAACAGTGTGTCAAA
```

TGGCGTCGAACTATGGTACTATCAAATTTGACGATATCTACAACATTCTCCCAGATGACTTGAAGCAT
CAAGTCAAGCAGATA
+
FFFFFFFFFFFFFFFFFFFFFFFFFFFFFFFFFFFFFFFFFFFFFFFFFFFFFFFFFFFFFFFFFFFFF
FFFFFFFFFFFFFFFFFFFFFFFFFFFFFFFFFFFFFFFFFFFFFFFFFFFFFFFFFFFFFFFFFFFFF
FFFFFFFF,FFFF::
@A00721:190:HGJW7DSXY:4:2360:24569:26412 2:N:0:TCTGCAAG+GCTGCACT
GAACATAACAAAACTCACAACACATTTTACATTATATTACAAATTCATCATATCAACAGTGTGTCAAA
TGGCGTCGAACTATGGTCCTATCAAATTTGACGATATCTACAACATTCTCCCAGATGACTTGAAGCAT
CAAGTCAAGCAGATA
+
FFFFFF:FFFFFFFFFFFFFFFFF,FFFFFFFFFFFFFFFFFFFFFFFFFFFFFFFFFFFFFFFFFFFF
FFFFFFFFFFFF:FFFF,FFFFFFFFFFFFFFFFFFFFFFFFFFFFFFFFFFFFFFFFFFFFFFFFFF
FFFFFFFF,FFFFFF
@A00721:190:HGJW7DSXY:4:2432:23149:36573 2:N:0:TCTGCAAG+GCTGCACT
GAACATAACAAAACTCACAACACATTTTACATTATATTACAAATTCATCATATCAACAGTGTGTCAAA
TGGCGTCGAACTATGGTACTATCAAATTTGACGATATCTACAACATTCTCCCAGATGACTTGAAGCAT
CAAGTCAAGCAGATA
+
FFFFFFFFFFFFFFFFFFFFFFFFFFFFFF:FFFFF:FFFFF:FFFFFFFFFFFFFFFFFFFFFFFFFF
FFFFFFFFFFFFFFFFFFFFF:FFFFFFFFFFFFFFFFFFFFFFFFFFFFFFFFFFFFFFFFFFFFFFF
FFFFFFFFFFFFFFF
@A00721:190:HGJW7DSXY:4:2464:5981:35493 2:N:0:TCTGCAAG+GCTGCACT
GAACATAACAAAACTCACAACACATTTTACATTATATTACAAATTCATCATATCAACAGTGTGTCAAA
TGGCGTCGAACTATGGTACTATCAAATTTGACGATATCTACAACATTCTCCCAGATGACTTGAAGCAT
CAAGTCAAGCAGATA
+
FFFFFFFFFFFFFFFFFFFFFFFFFFFFFFFFFFFFFFFFFFF:FFFFFFFFFFFFFFFFFFFFFFFFF
FFFFFFFFFFFFFFFFFFFFF:FFFFFFFFFFFFFFFFFFFFFFFFFFFFFFFFFFFFFFFFFFFFFFF
FFFFFFFFFFFFFFF
@A00721:190:HGJW7DSXY:4:2533:13973:25755 2:N:0:TCTGCAAG+GCTGCACT
GAACATAACAAAACTCACAACACATTTTACATTATATTACAAATTCATCATATCAACAGTGTGTCAAA
TGGCGTCGAACTATGGTACTATCAAATTTGACGATATCTACAACATTCTCCCAGATGACTTGAAGCAT
CAAGTCAAGCAGATA
+
FFFFFFFFFFFF:FFFFFFFFFFFFFFFFFF:FFFFFFFF:F,FFFFFFFFFFFFFF:F:FFFFFFFF
FFFFFFFFFFFFFFFFFFFF:,:FFFF:FFFFFFFFFFFFFFFFFFFFFFFFFFFFFFFFFFFFFFFFF
FFFFFFFFFFFFFFF
@A00721:190:HGJW7DSXY:4:2629:23303:1485 2:N:0:TCTGCAAG+GCTGCACT
GAACATAACAAAACTCACAACACATTTTACATTATATTACAAATTCATCATATCAACAGTGTGTCAAA
TGGCGTCGAACTATGGTACTATCAAATTTGACGATATCTACAACATTCTCCCAGATGACTTGAAGCAT
CAAGTCAAGCAGATA
+
FFFFFFFFFFFFFFFFFFFFFFFFFFFFFFFFFFFFFFFFFFFFFFFFFFFFFFFFFFFFFFFFFFFFF
FFFFFFFFFFFFFFFFFF:FFFFFFFFFFFFFFFFFFFFFFFFFFFFFFFFFFFFFFFFFFFFFFFFFF
FFFFFFFFFFFFFFF
@A00721:190:HGJW7DSXY:4:1417:5367:27915 2:N:0:TCTGCAAG+GCTGCACT
CATTGTATAAATGTAGTGTTGTTCGAACATAACAAAACTCACAACACATTTTACATTATATTACAAAT
TCATCATATCAACAGTGTGTCAAATGGCGTCGAACTATGGTACTATCAAATTTGACGATATCTACAAC
ATTCTCCCAGATGAC
+
FFFFFFFFFFF,FFFFFFFFF:FFFFFFFFFFFFF:FFFFFFFFFFFFFFFF:,FFFFFFFFFFFFFF
FFFFFFFFFFFFFFFF:FFFFFFFFFFFFFFFFFFFFFFFFF,FF,FFFFFFFFFFFFFFFFFFFFFFFF
FFFFFFFFFFFFFFF

@A00721:190:HGJW7DSXY:4:1467:18982:12884 2:N:0:TCTGCAAG+GCTGCACT
CATTGTATAAATGTAGTGTTGTTCGAACATAACAAAACTCACAACACAGTTTACATTATATTACAAAT
TCATCATATCAACAGTGTGTCAAATGGCGTCGAACTATGGTACTATCAAAGTTGACGATATCTACAAC
ATTCTCCCAGATGAC
+
FFFFFFFFFFFFFFFFFFFFFFFFFFFFFF:FFFFFFFFFFFFFFFFF,FFFFFFFFFFFFF:::F,,
FFFFFFFF:FFFFFFFFFFFFFF,FFFFFFFFFFFFFFFFF,FF:FF,:F,FFF:FFFFFFFFFFFFF
FFFFFFFFFFFFFFF
@A00721:190:HGJW7DSXY:4:1576:26503:33301 2:N:0:TCTGCAAG+GCTGCACT
CATTGTATAAATGTAGTGTTGTTCGAACATAACAAAACTCACAACACATTTTACATTATATTACAAAT
TCATCATATCAACAGTGTGTCAAATGGCGTCGAACTATGGTACTATCAAATTTGACGATATCTACAAC
ATTCTCCCAGATGAC
+
FFFFFFFFFF,FFFFFFFFFFFFFFF:FFFFFFFFFFFFFFFF:F:FF,FFFFF:FFFF:FFFFFFFF
FFFFF:FFFFFFFFFFFFFFFFF:FFFFFFF:FFFFFFFFFFFFFFFF:FFFFFFFFFF,FFFFFFFF
FFFFFF,FFFFFFFF
@A00721:190:HGJW7DSXY:4:1603:5484:23140 2:N:0:TCTGCAAG+GCTGCACT
CATTGTATAAATGTAGTGTTGTTCGAACATAACAAAACTCACAACACATTTTACATTATATTACAAAT
TCATCATATCAACAGTGTGTCAAATGGCGTCGAACTATGGTACTATCAAATTTGACGATATCTACAAC
ATTCTCCCAGATGAC
+
FFFFFFFFFF:FFFFFFFFFFFFFFFFFFFFFFFFFFFFFFFFFFFFFFFFFFFFFFFFFFFFF:FFF
FFFFFFFFFFFFFFFFF:FFFFFFFF:FFFFFFFFF:FFFFFFF:FFFF:FFFFFFFFFFFFFFFFFF
FFFFFFFFFFFFF:F
@A00721:190:HGJW7DSXY:4:2202:30092:3787 2:N:0:TCTGCAAG+GCTGCACT
CATTGTATAAATGTAGTGTTGTTCGAACATAACAAAACTCACAACACATTTTACATTATATTACAAAT
TCATCATATCAACAGTGTGTCAAATGGCGTCGAACTATGGTACTCTCAAATTTGACGATATCTACAAC
ATTCTCCCAGATGAC
+
FFFFFFFFFFFFFFFFFFFFFFFFF,FFFFFFFFFF:FFFFFFFFFFFFFFF::FFFF,F:FFF::,F
FF:FFF:FFFFFFFFFFFFFFF:FF:FFFFFFFFFFFFFFF,:F,FFF:F:FFF::FFF,FFF::FFF
FFFFFFFFFFFFFFF
@A00721:190:HGJW7DSXY:4:2212:3134:26303 2:N:0:TCTGCAAG+GCTGCACT
CATTGTATAAATGTAGTGTTGTTCGAACATAACAAAACTCACAACACATTTTACATTATATTACAAAT
TCATCATATCAACAGTGTGTCAAATGGCGTCGAACTATGGTACTATCAAATTTGACGATATCTACAAC
ATTCTCCCAGATGAC
+
FFFFFFFFFFFFFFFFFFFFFF:FFFF:FFFFFFFFFFFFFFFFFFFF,FFFF:F:FFFFFFFFFFFF
FFFF:FFFFFFFFFFFFFFFFFFFFFFFFFFFFFFFFFFFFFFFF:FF:FF:FFFFFFFFFFFFFFFF
FF:FFFFFFFFFFF:
@A00721:190:HGJW7DSXY:4:2367:10746:5603 2:N:0:TCTGCAAG+GCTGCACT
CATTGTATAAATGTAGTGTTGTTCGAACATAACAAAACTCACAACACATTTTACATTATATTACAAAT
TCATCATATCAACAGTGTGTCAAATGGCGTCGAACTATGGTACTATCAAATTTGACGATATCTACAAC
ATTCTCCCAGATGAC
+
FFFFFFFFFFFFFFFFFFFFFFFFFFFFFFFFFFFFFFFFFFFFFFFF:FFFFFFFFFFFFFFFFFFF
FFFFFFFFFFFFFFFFFFFFFFFFFFFFFFFFFFFFFFF:FFFF:FFFFFFFFFFFFFFFFFFF:FF:
FFFFFFFFFFFFFFF
@A00721:190:HGJW7DSXY:4:2367:11912:5744 2:N:0:TCTGCAAG+GCTGCACT
CATTGTATAAATGTAGTGTTGTTCGAACATAACAAAACTCACAACACATTTTACATTATATTACAAAT
TCATCATATCAACAGTGTGTCAAATGGCGTCGAACTATGGTACTATCAAATTTGACGATATCTACAAC
ATTCTCCCAGATGAC
+
FFFFFFFFFFFFFFFFFFFFFFFFFFFFFFFFFFFFFFFFFFFFFFFFFFFFF:FFFFF,FFFFFF,FF

```
FFF,FFFF,FFFFF:FFFFFFFF:FFFFFFFFFFFFFFFF:FFFFFFFFF:FFFF,F,FFFFFFFFF
F::FFFF:FFFF,:F
@A00721:190:HGJW7DSXY:4:1336:22498:26960 2:N:0:TCTGCAAG+GCTGCACT
AAGCAACAAGATTCTCTTATATACATATCGAATCTTAAACATTGTATAAATGTAGTGTTGTTCGAACA
TAACAAAACTCACAACACATTTTACATTATATTACAAATTCATCATATCAACAGTGTGTCAAATGGCG
TCGAACTATGGTACT
+
FFFFFFFFFFFFFFFFFFFF:FFFFFFFFFFFF:FFFFFFFFFFFFFFFFFFFFFFFFF:FFFFFFFF
F:F:FFFFFFFFFFFFF:FFFFFFFFFFFFF,FFFFFFFFFFFFFFFFFFFFFFFFFFFFFFFFFFFF
FFFFFFFFFFFFFFF
@A00721:190:HGJW7DSXY:4:2369:26033:15013 2:N:0:TCTGCAAG+GCTGCACT
AAGCAACAAGATTCTCTTATATACATATCGAATCTTAAACATTGTATAAATGTAGTGTTGTTCGAACA
TAACAAAACTCACAACACATTTTACATTATATTACAAATTCATCATATCAACAGTGTGTCAAATGGCG
TCGAACTATGGTACT
+
FFFFFFFFFFFFFFFFFFFFFFFFFFFFFFFFFFFFFFFFFFFFFFFFF:FFFFFFFFFFFFFFFFFF
FFFFFFFFFFFFFFFFFFFFFFFFFFFFFFFFFFFFF:FFFFFFFFFFFFF:FFFFFFFFF:FFFFFF
FFFFFFFFF:FFFFF
@A00721:190:HGJW7DSXY:4:2438:28619:26725 2:N:0:TCTGCAAG+GCTGCACT
AAGCAACAAGATTCTCTTATATACATATCGAATCTTAAACATTGTATAAATGTAGTGTTGTTCGAACA
TAACAAAACTCACAACACATTTTACATTATATTACAAATTCATCATATCAACAGTGTGTCAAATGGCG
TCGAACTATGGTACT
+
FFFFFFFFFFFFFFFFFFFFFFFFFFFFFFFFFFFFFFFFFFFFFFFFFFFFFFFFFFFFFFFFFFFF
FFFFFFFFFFFFFFFFFFFFFFFFFFFFFFFFFFFFFFFFFFFFFFFFFFFFFFFFFFFFFFFFFFFF
FFFFFFF:FFFF:FF
@A00721:190:HGJW7DSXY:4:2603:20708:19820 2:N:0:TCTGCAAG+GCTGCACT
AAGCAACAAGATTCTCTTATATACATATCGAATCTTAAACATTGTATAAATGTAGTGTTGTTCGAACA
TAACAAAACTCACAACACAGTTTACATTATATTACAAATTCATCATATCAACAGTGTGTCAAATGTCG
TCGATCTATGGTACT
+
FF:FFFFFF:F:F,F,:FFFF:FFFFFFFFFF,:FFFFFFFF:FFFFFFF,FFFF:FFF:FFFFF:FF
FFFFFFFFFFF,FF,F::F,FFFFFFF:FF,FFFFFF,:FFFF:FFFFFFFFFFFFF:FFF:FFF,FF
FFFF,F:FFF:FF:F
@A00721:190:HGJW7DSXY:4:1152:19208:22952 2:N:0:TCTGCAAG+GCTGCACT
AAAAGGTTAAAAGCAACAAGATTCTCTTATATACATATCGAATCTTAAACATTGTATAAATGTAGTGT
TGTTCGAACATAACAAAACTCACAACACATTTTACATTATATTACAAATTCATCATATCAACAGTGTG
TCAAATGGCGTCGAA
+
FFFFFFFFFFFFFFFFFFFFFFFFFFFFFFFFFFFFFFFFFFFFFFFFFFFFFFFFFFFFFFFFFFFF
FFFFFFFFFFFFFFFFFFFFFFFFFFFFFF:FFFFFFFFFFFFFFFFFFFFFFFFFFFFFFFFFFFFF
FFFFFFFFFFFFFFF
@A00721:190:HGJW7DSXY:4:1323:30472:21104 2:N:0:TCTGCAAG+GCTGCACT
AAAAGGTTAAAAGCAACAAGATTCTCTTATATACATATCGAATCTTAAACATTGTATAAATGTAGTGT
TGTTCGAACATAACAAAACTCACAACACATTTTACATTATATTACAAATTCATCATATCAACAGTGTG
TCAAATGGCGTCGAA
+
FFFFFFFFFFFFFFFFFFFFFFFFFFFFFFFFFFFFFFFFFFFFFFFFF,FFFFFFFFFFFFFFFFFF
FFFFF:FFFFFFFFFFFFFFFFFFFFFFFFFF:FF:FFFFFFFFFFFFFFFFFFFFFFFFFFF,FFFFF
FFFF:FFFFFFFFF:
@A00721:190:HGJW7DSXY:4:1511:1832:25676 2:N:0:TCTGCAAG+GCTGCACT
AAAAGGTTAAAAGCAACAAGATTCTCTTATATACATATCGAATCTTAAACATTGTATAAATGTAGTGT
TGTTCGAACATAACAAAACTCACAACACATTTTACATTATATTACAAATTCATCATATCAACAGTGTG
TCAAATGGCGTCGAA
```

```
+
F:FFF:FFFFFFFFFFFFFFFFFFFFFFFFFFFFFFFFFFFFFFFFFFFFFFFFFFFFF:FFFFFFFF
FFFFFFFFFFFFFFFFFFFFFFFFF:FFFFFFFFFFFFFFFFFFFFFFFFFFFFFFFFFFFFFFFFFF
FFFFFFFFFFFFFFF
@A00721:190:HGJW7DSXY:4:1672:27335:11725 2:N:0:TCTGCAAG+GCTGCACT
AAAAGGTTAAAAGCAACAAGATTCTCTTATATACATATCGAATCTTAAACATTGTATAAATGTAGTGT
TGTTCGAACATAACAAAACTCACAACACATTTTACATTATATTACAAATTCATCATATCAACAGTGTG
TCAAATGGCGTCGAA
+
FFFFFFFFFFFFFFFFFFFFFFFFFFFFFFFFFFFFFFFFFFFFFFFFFFFFFFFFFFFFFFFFFFFF
FFFFFFFFFFFFFFFFFFFFFFFFFFFFFFFFFFFFFFFFFFFFFFFFFFFFFFFFFFFFFFFFFFFF
FFFFFFFFFFFFFFF
@A00721:190:HGJW7DSXY:4:2430:15239:22529 2:N:0:TCTGCAAG+GCTGCACT
AAAAGGTTAAAAGCAACAAGATTCTCTTATATACATATCGAATCTTAAACATTGTATAAATGTAGTGT
TGTTCGAACATAACAAAACTCACAACACATTTTACATTATATTACAAATTCATCATATCAACAGTGTG
TCAAATGGCGTCGAA
+
FFFFFFFFFFFFFFFFFFFFFFFFFFFFFFFFFFFFFFFFFFFFFFFFFFFFFFFFFFFFFFFFFFFF
FFFFFFFFF:FFFFFFFFFFFFFFFFFFF:FFFFFFFFFFFFFFFFFFFFFFFFFFFFFFFFFFFFFF
FFFFFFFFFFFFFFF
@A00721:190:HGJW7DSXY:4:1259:17318:29293 2:N:0:TCTGCAAG+GCTGCACT
ATGATGAATTTGTAATATAATGTAAAATGTGTTGTGAGTTTTGTTATGTTCGAACAACACTACATTTA
TACAATGTTTAAGATTCGATATGTATATAAGAGAATCTTGTTGCTTTTAACCTTTTTTTTGAATTTTG
ACATAAAGATGAAAT
+
F:FFFFFFFF:FFFFFFF:FFFFFFFFFF:FFFFFF:FF:FFFFFF:FFFFFFFFFFFF:FFFF:FFF
,FFF,FFFFFFFFFFF::FFFFF,FFF:,FFFFFFFFFFFFF:FFF,FFFFFF::,FFFFF,FFFFFF
:FFFFF:FFFFFF:F
@A00721:190:HGJW7DSXY:4:1459:20726:2597 2:N:0:TCTGCAAG+GCTGCACT
ATGATGAATTTGTAATATAATGTAAAATGTGTTGTGAGTTTTGTTATGTTCGAACAACACTACATTTA
TACAATGTTTAAGATTCGATATGTATATAAGAGAATCTTGTTGCTTTTAACCTTTTTTTTGAATTTTG
ACATAAAGATGAAAT
+
FFFFFFFFFFFFFFFFFFFFFFFFFFFFFFFFFFFFFFFFFFFFFFFFFFFFFFFFFFFFFFFFFFFF
FFFFFFFFFFFFFFFFFFFFFFF:FFFFFFFF:FFFF:FFFFFFFFFFFFFFFFFFFFFFFFFFFFFF
FFFFFF:,FFFFFFF
@A00721:190:HGJW7DSXY:4:2169:21965:36025 2:N:0:TCTGCAAG+GCTGCACT
ATGATGAATTTGTAATATAATGTAAAATGTGTTGTGAGTTTTGTTATGTTCGAACAACACTACATTTA
TACAATGTTTAAGATTCGATATGTATATAAGAGAATCTTGTTGCTTTTAACCTTTTTTTTGAATTTTG
ACATAAAGATGAAAT
+
FFFFFFFFFFFFFFFFFFFFFFFFFFFFFFFFFFFFFF:F:FFFFFFFFFFFFFFFFFFFFFFFFFFF
FFFFFFFFFFFFFFFFFFFFFFFFFFFFFFFFFFFFFFFFFFF:FFFFFFF:FFFFFFFFFFFFFFFF
FFFFFFFFFFFFFFF
@A00721:190:HGJW7DSXY:4:2277:20636:16188 2:N:0:TCTGCAAG+GCTGCACT
ATGATGAATTTGTAATATAATGTAAAATGTGTTGTGAGTTTTGTTATGTTCGAACAACACTACATTTA
TACAATGTTTAAGATTCGATATGTATATAAGAGAATCTTGTTGCTTTTAACCTTTTTTTTGAATTTTG
ACATAAAGATGAAAT
+
FFFFFFFFFFFFFFFFFFFFFFFFFFFFF:FFFFFF:FFFFFFFFFFFFFFFFFFFFFFFFFFFFFFF
FF:FFFFFFFFFFFFFFFFFFFFFFFFFFFFFFFFFFFFFFFF:FFFFFFFFFFFFFFFFF,FFFFFF
F:FFFFF:FF:FF,F
@A00721:190:HGJW7DSXY:4:2242:18313:7404 2:N:0:TCTGCAAG+GCTGCACT
CTTTGACAGTCGATTTCATCTTTATGTCAAAATTCAAAAAAAGGTTAAAAGCAAAAAGATTCTATTAT
```

```
ATACATATCGAATCTTAAACATTGTATAAATGTAGTGTTTTTCGAACATAACAAAAATCACAACACAA
TTTACATTATATTAC
+
FF,FFF:FFF:FFFFFFF,,FFFF:F,,:FFFFFFFFFFFFFF:,F:FFF:FFF,FFFFFFFF,,FFF
:FF,FFFF,:,FFFFFFFFFF:F,FFFFFFF::F,F,FF,,FFFFF,FFFFFFF:F,FFFFFFFFFF,
::FF,FFFF,:FFF:
@A00721:190:HGJW7DSXY:4:2671:20862:3333 2:N:0:TCTGCAAG+GCTGCACT
CTTTGACAGTCGATTTCATCTTTATGTCAAAATTCAAAAAAAGGTTAAAAGCAACAAGATTCTCTTAT
ATACATATCGAATCTTAAACATTGTATAAATGTAGTGTTGTTCGAACATAACAAAACTCACAACACAT
TTTACATTATATTAC
+
FFFFFFFFFFFFFFFFFFFFFFFFFFFFFFFFFFFFFFFFFFFFFFFFFFFFFFFFFFFFFFFFFFFF
FFFFFFFFFF,FFFFFFFF:FFFFFFFFFFFFFFFFFFFFFFFFFFFFFFFFFFFFFFFFFFFFFF,F
:FF:FFFFFFFFFFF
@A00721:190:HGJW7DSXY:4:1336:31385:2832 2:N:0:TCTGCAAG+GCTGCACT
CTTTGACAGTCGATTTCATCTTTATGTCAAAATTCAAAAAAAGGTTAAAAGCAACAAGATTCTCTTAT
ATACATATCGAATCTTAAACATTGTATAAATGTAGTGTTGTTCGAACATAACAAAACTCACAACACAT
TTTACATTATATTA
+
FFFFFFFFFFFFF:FF:FF:FFFFF:F,,FFFFF:FFFFFFF,F:FF:FFFFFFFFF:FFFFF,FFFF
FFFFF:F:,:FFF,FFFF::FFF:FFFFF:F:FF:FFFFFFF:FFF:FFFF,,FFFF,FF:FFFFFF,
FFF::FFFFFFFFF
@A00721:190:HGJW7DSXY:4:2509:17210:14826 2:N:0:TCTGCAAG+GCTGCACT
CTTTGACAGTCGATTTCATCTTTATGTCAAAATTCAAAAAAAGGTTAAAAGCAACAAGATTCTCTTAT
ATACATATCGAATCTTAAACATTGTATAAATGTAGTGTTGTTCGAACATAACAAAACTCACAACACAT
TTTACATTATATTA
+
FFFFFFFFFFFFFFFFFFFFFFFFFFFF:FFFFFFFFFFFFFFFFFFFFFFFFFF:FFFFFF:F:,FFF
FFFFF,FF,FFFF,FFFFF:FFF,FFFFFFF:FF:F,FF:FF,FFFFF,FF,FFFF::FFFFF,FFF,
FFFFFF:FFFF,FF
@A00721:190:HGJW7DSXY:4:1318:16016:34679 2:N:0:TCTGCAAG+GCTGCACT
GCCGTACAAACTTTGACAGTCGATTTCATCTTTATGTCAAAATTCAAAAAAAGGTTAAAAGCAACAAG
ATTCTCTTATATACATATCGAATCTTAAACATTGTATAAATGTAGTGTTGTTCGAACATAACAAAACT
CACAACACAGTTTAC
+
FFFFFFFFFFFFFFFFFFFFFFF,FFFFFFF:F,:F:FFF:FFFFFFFFFF:FFFF,FFF:FFFFFFF
FFFFF::F:FFF:FFFFF,F:FF,F:,F,FFFF,FFFFFF,,FFFFF:F:FFFFFFFFFFFFFFFFF:
FFF,FFFFF,FFF::
@A00721:190:HGJW7DSXY:4:1352:1841:14450 2:N:0:TCTGCAAG+GCTGCACT
GCCGTACAAACTTTGACAGTCGATTTCATCTTTATGTCAAAATTCAAAAAAAGGTTAAAAGCAACAAG
ATTCTCTTATATACATATCGAATCTTAAACATTGTATAAATGTAGTGTTGTTCGAACATAACAAAACT
CACAACACATTTTAC
+
FFFFFFFFFFFFFFFFFFFFFFFFFFFFFFFFFFFFF:F:FFFFFFFFFFFFFFFFFFFFFFFFFFFFF
FFFFFFFFFFFFFFFFFFFFFFFFFFFF:FFFFFFFFFFFFFFF:FFFFFFFFFFFFFFFFFF,FFFF
FF:FFFF:F,FFFFF
@A00721:190:HGJW7DSXY:4:2117:2555:10551 2:N:0:TCTGCAAG+GCTGCACT
GCCGTACAAACTTTGACAGTCGATTTCATCTTTATGTCAAAATTCAAAAAAAGGTTAAAAGCAACAAG
ATTCTCTTATATACATATCGAATCTTAAACATTGTATAAATGTAGTGGTGTTCGAACATACCAAAACT
CACAACACAGTTTAC
+
FFFFFFFFFFFFFFFFFFFFFFFFFFFFFFFFF:F:FFF,FF:FF::FF:,FFFF,FFF:F:FFFFFFF
:F,:::,F::FFFFFFFFFF::::FF:FFF,FFF,FFF::FFFFF:,FF,F:F:FF:,,,,,,FFFF
F:FFFFFFF,:F::F
```

```
@A00721:190:HGJW7DSXY:4:2267:29369:15154 2:N:0:TCTGCAAG+GCTGCACT
GCCGTACAAACTTTGACAGTCGATTTCATCTTTATGTCAAAATTCAAAAAAAGGTTAAAAGCAACAAG
ATTCTCTTATATACATATCGAATCTTAAACATTGTATAAATGTAGTGTTGTTCGAACATAACAAAACT
CACAACACATTTTAC
+
FFFFFFFFFFFFFFFFFFFFFFFFFFFFF:FFFFFF,FFFFFFFFFFFFFFFFFFFF:FFFFFFFFF:
FFFFFFFFFFFFFFFFFF:FF:F:FFFFFFFFFFFFFFF:F,FF:FFFF,FFF::F,FFFFFFFFF:F
FFFFF:F:FF,FF:F
@A00721:190:HGJW7DSXY:4:2662:6885:1705 2:N:0:TCTGCAAG+GCTGCACT
GCCGTACAAACTTTGACAGTCGATTTCATCTTTATGTCAAAATTCAAAAAAAGGTTAAAAGCAACAAG
ATTCTCTTATATACATATCGAATCTTAAACATTGTATAAATGTAGTGTTGTTCGAACATAACAAAACT
CACAACACATTTTAC
+
FFFFFFFFFFFFFFFFFFFFFFFFFFFFFFF,FFFFFF:FFFFFFFFFFFFFFFF:FFFFFFFFFFFF
FFFFFFFFFFFFFFFFFF:FFFFFFFFFFFFFFFFFFFFFFFFFFFFFFF:FFFFFFFFFFFFFFFF:F
FFFFF,FFF:FFFF:
@A00721:190:HGJW7DSXY:4:1508:20338:16705 2:N:0:TCTGCAAG+GCTGCACT
GCCGTACAAACTTTGACAGTCGATTTCATCTTTATGTCAAAATTCAAAAAAAGGTTAAAAGCAACAAG
ATTCTCTTATATACATATCGAATCTTAAAAATTGTATAAATGTAGTGTTGTTCGAACATAACAAAAAA
CACAACACATTTTA
+
FFFFFFFFFFFFFFFFFFFFFFFFFF:FFFFFFFF::FFFFFFFFFFFFFF:FFF:FFFFFFFFFFF,
FFF:F:,FF,F:F:,FFFFFFFF:FF:FF,F,F:FFFFFFFFFFFF:FF:FF:::F:::FF,:FFF,,
,:FFFFFFF,F,FF
@A00721:190:HGJW7DSXY:4:2352:3052:19085 2:N:0:TCTGCAAG+GCTGCACT
GCCGTACAAACTTTGACAGTCGATTTCATCTTTATGTCAAAATTCAAAAAAAGGTTAAAAGCAACAAG
ATTCTCTTATATAAATATCGAATCTTAAACATTGTATAAATGTAGTGTTGTTCGAACATAACAAAACT
CACAACACAGTTTA
+
FFFFFFFFFFFF:FFFFFFFFFF,FF:FF:FFF,FFF:,FFFFFF,FF,FFFFFF,:FFF:FFFFFFF
F:F:FF:FFFFFF,FFFFFFFFFFFFFFFFFFFFFFFFFFFFFFF,F,FF,,F:F:F:FF:F,FFFFFF
:::FF,F,F,,,FF
@A00721:190:HGJW7DSXY:4:2417:4318:23124 2:N:0:TCTGCAAG+GCTGCACT
GCCGTACAAACTTTGACAGTCGATTTCATCTTTATGTCAAAATTCAAAAAAAGGTTAAAAGCAACAAG
ATTCTCTTATATACATATCGAATCTTAAACATTGTATAAATGTAGTGTTGTTCGAACATAACAAAACT
CACAACACATTTTA
+
FFFFFFFFFFFFFFFFFFFFFFFFF,FFFF:FF,:FF,F:FFFFFFFFFFF:F:F,F,FFFFFFFFFF:
F:FFF::FFFFFFFF:F:,FFFFFFFFF:FF:F:FFFFFFFFFFFF:FFF:FFF,,F:F,FFFFFF,F
FFFFF:FFF,F,FF
@A00721:190:HGJW7DSXY:4:1325:15338:8265 2:N:0:TCTGCAAG+GCTGCACT
GTTTTGTTATGTTCGAACAACACTACATTTATACAATGTTTAAGATTCGATATGTATATAAGAGAATC
TTGTTGCTTTTAACCTTTTTTTGAATTTTGACATAAAGATGAAATCGACTGTCAAAGTTTGTACGGCT
CATATTTTCAAATTT
+
FFFFFFFFFFFFFFFFFFFFFFFFFFFFFFFFFFFFFFFFFFFFFFFFFFFFFFFFFFFFFFFFFFFF
FFFFFFFFFFFFF:FFFFFFFFFFFFFFFFFFFFFFFFF:FFFFFFFFFFFFFFFFFFFFFFFFFF::FF
FFFFFFFFFFFFFFF
@A00721:190:HGJW7DSXY:4:2422:11776:30749 2:N:0:TCTGCAAG+GCTGCACT
GTTTTGTTATGTTCGAACAACACTACATTTATACAATGTTTAAGATTCGATATGTATATAAGAGAATC
TTGTTGCTTTTAACCTTTTTTTGAATTTTGACATAAAGATGAAATCGACTGTCAAAGTTTGTACGGCT
CATATTTTCAAATTT
+
FF,FF,FFF:FFFFF:FFFFFF:FFFFFFFFFF::FF::FFF,:FFFF,FFF,F:F::FFFF:FFFF,
```

```
:F:FF:FFFFFF:::F:FFF::FFFFFFFFFF:FFFF,FFFFFFF::FFFF,FFFFFF,F,FFF::F:
FF,F,FFFF:FFFFF
@A00721:190:HGJW7DSXY:4:1660:13593:14920 2:N:0:TCTGCAAG+GCTGCACT
CATTTATACAATGTTTAAGATTCGATATGTATATAAGAGAATCTTGTTGCTTTTAACCTTTTTTTGAA
TTTTGACATAAAGATGAAATCGACTGTCAAAGTTTGTACGGCTCATATTTTCAAATTTGAAATTTGAA
TTTCAAATCTATTTC
+
FFFFFFFFFFF:FFFFFFFF:FFFFFFFFFFFFFFFFFFF,FFFFFFFFFFFFFFFFFFFFFFFFFFFF
FFFFFFFFFFFFFFFFFFFFFFFFFFFFFFF:FFFFFFFF:FFFFFFFFFFFFF:FFFFFFFFFFFFF
FFFFFFFFFFFFFFF
@A00721:190:HGJW7DSXY:4:1660:13015:15421 2:N:0:TCTGCAAG+GCTGCACT
CATTTATACAATGTTTAAGATTCGATATGTATATAAGAGAATCTTGTTGCTTTTAACCTTTTTTTGAA
TTTTGACATAAAGATGAAATCGACTGTCAAAGTTTGTACGGCTCATATTTTCAAATTTGAAATTTGAA
TTTCAAATCTATTTC
+
FFFFFFFFFFF:FFFFFFFFFFFFFFFFFFFFFFFFFFFFFFFF:FFFFFFFFFFFFFFFFFFFFFFFF
FFFFFFFFFFFFFFFFFFFFFFFFFFFFFF:FFFFFFFFFFFFFFFFFFFFFFFFFFFFF:FFFFFFFF
FFFFFFFFFFFFFFF
@A00721:190:HGJW7DSXY:4:2240:12816:8437 2:N:0:TCTGCAAG+GCTGCACT
CATTTATACAATGTTTAAGATTCGATATGTATATAAGAGAATCTTGTTGCTTTTAACCTTTTTTTGAA
TTTTGACATAAAGATGAAATCGACTGTCAAAGTTTGTACGGCTCATATTTTCAAATTTGAAATTTGAA
TTTCAAATCTATTT
+
FFFFFFFFFFFFFFFFFFFFFFFFFFFFFFFFFFFFFFFFFFFFFFFFFFFFFFFFFFFFFFFFFFFF
FFFFF:FFFFFFFF,FFFFFFFFFFFFFFFFFFFFFFFFFFFFFFFFFFFFFFFFFFFFFFFFFF,FF
FFFF:FFF:FFFFF
@A00721:190:HGJW7DSXY:4:2438:11234:36229 2:N:0:TCTGCAAG+GCTGCACT
CATTTATACAATGTTTAAGATTCGATATGTCTATAAGAGAATCTTGTTGCTTTTAACCTTTTTTTGAA
TTTTGACATAAAGATGAAATCGACTGTCAAAGTTTGTACGGCTCATATTTTCAAATTTGAAATTTGAA
TTTCAAATCTATTTC
+
FFFFFFFFFF:FFF:FFFFF,FFFFF::F:,,FFF:FFFF,FFF:FFFF:F:,:FFFFFFFFF,,FFF
FFFFFFFFF:FFFF:FFFFFFFFFFFFFFF:F,FFFFFFFFFFFFFFFF:FFF:F::FFFFFFFFFFF
FFFFFF,FFFFFFFF
@A00721:190:HGJW7DSXY:4:1563:9860:14215 2:N:0:TCTGCAAG+GCTGCACT
ATTCGATATGTATATAAGAGAATCTTGTTGCTTTTAACCTTTTTTTGAATTTTGACATAAAGATGAAA
TCGACTGTCAAAGTTTGTACGGCTCATATTTTCAAATTTGAAATTTGAATTTCAAATCTATTTCAACT
GTACAAACATTTACT
+
FFFFFFFFFFFFFFFFFFFFFFFFFFFFFF:FFFFFFFFFFFFFFFFFFFFFFFFFFFFFFF,FFFFFF
FFFFFFFFFFFFFFFFFFFFFFFFFFFFFFFFFFFFFFFF:FFFFFFFFFFFFFFFF,FFFFFFFFFFFF
FFFFFFFFF:FFFFF
@A00721:190:HGJW7DSXY:4:2401:15221:27070 2:N:0:TCTGCAAG+GCTGCACT
ATTCGATATGTATATAAGAGAATCTTGTTGCTTTTAACCTTTTTTTGAATTTTGACATAAAGATGAAA
TCGACTGTCAAAGTTTGTACGGCTCATATTTTCAAATTTGAAATTTGAATTTCAAATCTATTTCAACT
GTACAAACATTTACT
+
FFFFFFFFFFFFFFFFFFFFFFFFFFFFFFFFFFFFFFFFFFFFFFFFFFFFFFFFFFFFFFFFFF:F
FFFFFFFFFFFFFFFFFFFFFFFFFFFFFFFFFFFFFFFFFFFFFFFFFFFFFFFFFFFFFFFF:FFFF
FFFFFF:FFF:FF,F
@A00721:190:HGJW7DSXY:4:1149:12029:30718 2:N:0:TCTGCAAG+GCTGCACT
CGACTGTCAAAGTTTGTACGGCTCATATTTTCAAATTTGAAATTTGAATTTCAAATCTATTTCAACTG
TACAAACATTTACTGTATACATTACTTATGATCTAAGTCAGCCACTTACATATACTTGTCATATATTT
CGAATCTACTGATTC
```

+
FFFFFFFFFF:FFFFFFFFFFFFFFFFFFFFFFFFFFFFFFFFFFFFFFFFFFFFFFFF:FFFFFFFF
FFFFFFFFFFFFFFFFFFFFFFFFFF:FFFFFFFFF:FFFFFFFFFFFFFFFFFFFFFFFFFFF:,FF
,FFFFFFFFFFFFFF
@A00721:190:HGJW7DSXY:4:1570:12536:33724 2:N:0:TCTGCAAG+GCTGCACT
CGACTGTCAAAGTTTGTACGGCTCATATTTTCAAATTTGAAATTTGAATTTCAAATCTATTTCAACTG
TACAAACATTTACTGTATACATTACTTATGATCTAAGTCAGCCACTTACATATACTTGTCATATATTT
CGAATCTACTGATTC
+
FFFFFFFFFFF:FFFFFFFFFFFFFFFFFFF:FFFFFFFFFFFF:FFFFFFFFF:F:FFFFFFFFFFF
FFFFF:FFFFFFF:FFFFFFFFFFFF,FFF:FFFFFFFF:FF:FFFFFFF,FFFFFFFFFFFFFFFFF
FFFFFFFFFFFFFFF
@A00721:190:HGJW7DSXY:4:1647:15338:33943 2:N:0:TCTGCAAG+GCTGCACT
CGACTGTCAAAGTTTGTACGGCTCATATTTTCAAATTTGAAATTTGAATTTCAAATCTATTTCAACTG
TACAAACATTTACTGTATACATTACTTATGATCTAAGTCAGCCACTTACATATACTTGTCATATATTT
CGAATCTACTGATTC
+
FFFFFF:FFFFFFFFFFFFFF:FF,FFF:FFFFFFFFFFFFF,,FFFFFFFFFFFFFFFF,FFFFFF:
FFF,FFFF::FFFF:FFFFFFF:F:FFF,FF:FF:FFFFF,FFFFFFFFFFFFFFFFFFFF,FF:FFF
F,:FFFFFFFF:FFF
@A00721:190:HGJW7DSXY:4:2162:28583:10473 2:N:0:TCTGCAAG+GCTGCACT
CGACTGTCAAAGTTTGTACGGCTCATATTTTCAAATTTGAAATTTGAATTTCAAATCTATTTCAACTG
TACAAACATTTACTGTATACATTACTTATGATCTAAGTCAGCCACTTACATATACTTGTCATATATTT
CGAATCTACTGATTC
+
FFFFFFFFFFFFFFFFFFFFF:FFFFFFFFFFFFFFFFFFFFFFFFFFFFFFFF,F:FFFFFFF:FFF
FFFFFF,FFFFFFFFFFFF:FFFF:FFFFFFFFFFF,FFFFF,FFFFFFFFFFF:FFFF:FF,:FFFF
F:FFFFFF,FFFFF:
@A00721:190:HGJW7DSXY:4:1112:16459:3317 2:N:0:TCTGCAAG+GCTGCACT
TTCAAAATTAAATGACAGTCCTCGAATCAGTAGATTCGAAATATATGACAAGTATATGTAAGTGGCTG
ACTTAGATCATAAGTAATGTATACAGTAAATGTTTGTACAGTTGAAATAGATTTGAAATTCAAATTTC
AAATTTGAAAATATG
+
FFFFFFFFFFFFFFFFFFFFFFFFFFFFFFFFFF:FFFFFFFFFFFFFFFFFFFFFFFF:FFFFFFFF
FFFFFFFFFFFFFFFFFFFFFFFFFFFFFFFFFFFFFFFFFFFFFFFFFFFFFFFFFFFFF::FFFFF
FFFFFFFFFFFF,FF
@A00721:190:HGJW7DSXY:4:1112:16405:3787 2:N:0:TCTGCAAG+GCTGCACT
TTCAAAATTAAATGACAGTCCTCGAATCAGTAGATTCGAAATATATGACAAGTATATGTAAGTGGCTG
ACTTAGATCATAAGTAATGTATACAGTAAATGTTTGTACAGTTGAAATAGATTTGAAATTCAAATTTC
AAATTTGAAAATATG
+
FFFFFFFFFFFFFFFFFFFFFFFFFFFF:FFFF:FFFFFFFFFFFFFFFFFFFFFFFFFFFFFFFFFF
FFFFFFF:FFFFFFF:FFFFFFFFFFFFFFFFFFFFFFFFFFFFF:FFFFFFFFFFFFFFFFFFFFFF
FFFFFFF:FFFFFFF
@A00721:190:HGJW7DSXY:4:1228:6659:31720 2:N:0:TCTGCAAG+GCTGCACT
TTCAAAATTAAATGACAGTCCTCGAATCAGTAGATTCGAAATATATGACAAGTATATGTAAGTGGCTG
ACTTAGATCATAAGTAATGTATACAGTAAATGTTTGTACAGTTGAAATAGATTTGAAATTCAAATTTC
AAATTTGAAAATATG
+
FFFFFFFFFFFFFFFFFFFFFFFFFFFFFFFFFFFFFFFFFFFFFFFFFFFFFFFFFFFFFFFFFFFF
FFF:FFFFFFFFFFFFFFFFFFFFFFFFFFFFFFFFFFFFFFFFFFFFFFFFFFFFFFFFFFF:FFFF
FFFFFFFFFFFFFFF
@A00721:190:HGJW7DSXY:4:1316:15917:25457 2:N:0:TCTGCAAG+GCTGCACT
TTCAAAATTAAATGACAGTCCTCGAATCAGTAGATTCGAAATATATGACAAGTATATGTAAGTGGCTG

ACTTAGATCATAAGTAATGTATACAGTAAATGTTTGTACAGTTGAAATAGATTTGAAATTCAAATTTC
AAATTTGAAAATATG
+
:FFFFFFFFFFFFFFFFFFFFFFFFFFFFFFFFFFFFFFFFFFFFFFFFFFFFFFFFFFFFFFFFFFF
FFFFFFFFFFFFFFFF:FFFFFFFFFFFFFFFFFFFFFFFFFFFFFFFFFFFFFFFFFFFFFFFFFF:
FFFFFFFFFFFFFFF
@A00721:190:HGJW7DSXY:4:1341:7075:34945 2:N:0:TCTGCAAG+GCTGCACT
TTCAAAATTAAATGACAGTCCTCGAATCAGTAGATTCGAAATATATGACAAGTATATGTAAGTGGCTG
ACTTAGATCATAAGTAATGTATACAGTAAATGTTTGTACAGTTGAAATAGATTTGAAATTCAAATTTC
AAATTTGAAAATATG
+
FFFFFFFFFFFFFFFFFFFFFFFFFFFFFFFFFFFFFFFFFFFFFFFFFFFFFFFFFFFFFFFFFFFF
FFFFFFFFFFFFFFFFFFFFFFFFFFFFFFFFFFFFFFFFFFFFFFFFFFFFFFFFFFFFFFFFFFFF
FFFFFFFFFFFFFFF
@A00721:190:HGJW7DSXY:4:1539:32145:31391 2:N:0:TCTGCAAG+GCTGCACT
TTCAAAATTAAATGACAGTCCTCGAATCAGTAGATTCGAAATATATGACAAGTATATGTAAGTGGCTG
ACTTAGATCATAAGTAATGTATACAGTAAATGTTTGTACAGTTGAAATAGATTTGAAATTCAAATTTC
AAATTTGAAAATATG
+
FFFFFFFFFFF,FFFFFFFFFFFFFFFFFFF:FFFFFFFFFFFFFFFFFFFFFFFFFFFFFFFFFFFF
FF:FFFFFFFFFFFFFFFFFFFFFFFFFFFFFFFFFFFFFFFFFFFFFFFFFFFFFFFFFFFFFFFFF
FFFFFFFF:FFF:F:
@A00721:190:HGJW7DSXY:4:1620:13783:15906 2:N:0:TCTGCAAG+GCTGCACT
TTCAAAATTAAATGACAGTCCTCGAATCAGTAGATTCGAAATATATGACAAGTATATGTAAGTGGCTG
ACTTAGATCATAAGTAATGTATACAGTAAATGTTTGTACAGTTGAAATAGATTTGAAATTCAAATTTC
AAATTTGAAAATATG
+
FFFFFFFFFFF:FFFFFFFFFFFFFFFFFFFFFFFFFFFFFF:FFFFFFFFFFFFFFFFFFFFFFFFF
FFFFFFFFFFFFFFFFFFFFFFFFFFFFFFFFFFFFFFFFFFFFFFFFFFFFFFFFFFFF:FFFFFFF
FFFFFFFFFFFFFFF
@A00721:190:HGJW7DSXY:4:1661:27136:5494 2:N:0:TCTGCAAG+GCTGCACT
TTCAAAATTAAATGACAGTCCTCGAATCAGTAGATTCGAAATATATGACAAGTATATGTAAGTGGCTG
ACTTAGATCATAAGTAATGTATACAGTAAATGTTTGTACAGTTGAAATAGATTTGAAATTCAAATTTC
AAATTTGAAAATATG
+
FFFFFFFFFFFFFFFFFFFFFFFFFFFFFFFFFF:FFFFFFFFFFFFFFFFFFFFFFFFFFFFFFFFF
FF:FFFFFF:FFFFFFFFFF:FFFFFFFFFFFFFFFFFFFFF:FFFFFFFFFFFFFFFFFFF:FFFFF
FFFF,FFFFFFFFFF
@A00721:190:HGJW7DSXY:4:2113:17933:9220 2:N:0:TCTGCAAG+GCTGCACT
TTCAAAATTAAATGACAGTCCTCGAATCAGTAGATTCGAAATATATGACAAGTATATGTAAGTGGCTG
ACTTAGATCATAAGTAATGTATACAGTAAATGTTTGTACAGTTGAAATAGATTTGAAATTCAAATTTC
AAATTTGAAAATATG
+
FFFFFFFFFFFFFFFFFFFFFFFFFFFFFFFFFF:FFFFFFFFFFFFFFFFFFFFFFFFFFFFFFFFF
FFFFFFFFFF:FFFFF:FFF:FFFFFFFFFFFFFFF::FFFFFFFFFFFFF:FFFFFFFF:F:F:FFF
FFFFFFFF:FFFFF:
@A00721:190:HGJW7DSXY:4:2122:1994:10269 2:N:0:TCTGCAAG+GCTGCACT
TTCAAAATTAAATGACAGTCCTCGAATCAGTAGATTCGAAATATATGACAAGTATATGTAAGTGGCTG
ACTTAGATCATAAGTAATGTATACAGTAAATGTTTGTACAGTTGAAATAGATTTGAAATTCAAATTTC
AAATTTGAAAATATG
+
FFFFFFFFFFFFFFFFFFFFFFFFFFFFFF,FFF:FFFFFFFFFFFFFFFFFFFFFFFFFFFFFFFFF
F:FFFFFFFFFFFFFFFFFFFFFFFFFFFFFFFFFFFFFFFFFFFFFFFFFFFFFFFFFFFFFFFFFF
FFFFFFFFFFFFFFF

@A00721:190:HGJW7DSXY:4:2463:20998:31093 2:N:0:TCTGCAAG+GCTGCACT
TTCAAAATTAAATGACAGTCCTCGAATCAGTAGATTCGAAATATATGACAAGTATATGTAAGTGGCTG
ACTTAGATCATAAGTAATGTATACAGTAAATGTTTGTACAGTTGAAATAGATTTGAAATTCAAATTTC
AAATTTGAAAATATG
+
FFFFFFFFFFFFFFFFFFFFFFFFFFFFFFFFFFFFFFFFFFFFFFFFFFFFFFFFFFFFFFFFFFFFFF
FFFFFFFFFFFFFFFFFFFFFFFFFFFFFFFFFFFFFFFFFFFFFFFFFFFFFFFFFFFFFFFFFFFFFF
FFFFFFFFFFFFFFF
@A00721:190:HGJW7DSXY:4:2463:21278:32205 2:N:0:TCTGCAAG+GCTGCACT
TTCAAAATTAAATGACAGTCCTCGAATCAGTAGATTCGAAATATATGACAAGTATATGTAAGTGGCTG
ACTTAGATCATAAGTAATGTATACAGTAAATGTTTGTACAGTTGAAATAGATTTGAAATTCAAATTTC
AAATTTGAAAATATG
+
FFFFFFFFFFF:FFFFFFFFFFFFFFFF:FFFFFFFFFFFFFFFFFFFFFFFFFFFFFFFFFFFFFFFFF
FFFFFFFFFFFFFFFFFFFFFFFFFFFFFFFFFFFFFFFFFFFFFFFFFFFFFFF,FFFFFF:FFFFFF
FFFFFFF:FFFFFF:
@A00721:190:HGJW7DSXY:4:1122:3613:15796 2:N:0:TCTGCAAG+GCTGCACT
TGATATCAGATCTTTCATTATTCAAAATTAAATGACAGTCCTCGAATCAGTAGATTCGAAATATATGA
CAAGTATATGTAAGTGGCTGACTTAGATCATAAGTAATGTATACAGTAAATGTTTGTACAGTTGAAAT
AGATTTGAAATTCAA
+
FFFFFFFFFFFFFFFFFFFFFFFFFFFFFFFFFFFFFFFFFFFFFFFFFFFFFFFFFFFFFFFFFFFFFF
FFFFFFFFFFFFFFFFFFFFFFFFFFFFFFFFFFFFFFFFFFFFFFFFFFFFFFFFFFFFFFFFFFFFFF
FFFFFFFFFFFFFFF
@A00721:190:HGJW7DSXY:4:1122:4643:18771 2:N:0:TCTGCAAG+GCTGCACT
TGATATCAGATCTTTCATTATTCAAAATTAAATGACAGTCCTCGAATCAGTAGATTCGAAATATATGA
CAAGTATATGTAAGTGGCTGACTTAGATCATAAGTAATGTATACAGTAAATGTTTGTACAGTTGAAAT
AGATTTGAAATTCAA
+
FFF:FFFFFFFF,FFFFF:FFFFFFFFFFFFFFFFFFFFFFFFFFFFFFFFFFFFFFFFFFFFFFFFFF:
FFFFFFFFFFFFFFFF:FFFFFF,FFFFF:FFFFFF:FFFFFFFFFFFFFFFFFFFFFFFFFF:FFFFFF
F:FFFFFFFFFFFFF
@A00721:190:HGJW7DSXY:4:1153:22173:3223 2:N:0:TCTGCAAG+GCTGCACT
TGATATCAGATCTTTCATTATTCAAAATTAAATGACAGTCCTCGAATCAGTAGATTCGAAATATATGA
CAAGTATATGTAAGTGGCTGACTTAGATCATAAGTAATGTATACAGTAAATGTTTGTACAGTTGAAAT
AGATTTGAAATTCAA
+
FFFFFFFFFFFFFFFFFFFFFFFFFFFFFFFFFFFFFFFFFFFFFFFFFFFFFFFF:FFFFFFFFFFFFF
FFFFFFFFFFFFFFFFFFFFFFFFFFFFFFFFFF:FF,FFFFFFFFFFFFFFFFFFFFFF,FFFFFFFFF
F:FFFF,FFFFF,FF
@A00721:190:HGJW7DSXY:4:1547:16161:26162 2:N:0:TCTGCAAG+GCTGCACT
TGATATCAGATCTTTCATTATTCAAAATTAAATGACAGTCCTCGAATCAGTAGATTCGAAATATATGA
CAAGTATATGTAAGTGGCTGACTTAGATCATAAGTAATGTATACAGTAAATGTTTGTACAGTTGAAAT
AGATTTGAAATTCAA
+
FFFFFFFFFFFFF:FFFFFFFFFFFFFFFFFFFFFFFFFFFFFFFFFFFFFFFFFFFFFFFFFFFFFFFF
FFFFFFFFFFFFFFFFFFFFFFFF:FFFFF:FFFFFFFFFFFFFFFFFFFFFFFFFFFFFFFF::FFFFF
FFFFFFFFFFFFFFF
@A00721:190:HGJW7DSXY:4:1630:18394:8484 2:N:0:TCTGCAAG+GCTGCACT
TGATATCAGATCTTTCATTATTCAAAATTAAATGACAGTCCTCGAATCAGTAGATTCGAAATATATGA
CAAGTATATGTAAGTGGCTGACTTAGATCATAAGTAATGTATACAGTAAATGTTTGTACAGTTGAAAT
AGATTTGAAATTCAA
+
FFFFFFFFFFFFFFFFFFFFFFFFFFFFFFFFFFFFFFFFFFFFFFFFFFFFFFFF:FFFFFFFFFFFFF

FFFFFFFFFFFF:FFFFFFFFFF:FFFFF:FFFFFFFFFFFFFFFFFFFFFFFFFFFFFFFFF:FFFFFF
FFFFFFFFFFFFFFF
@A00721:190:HGJW7DSXY:4:1668:20772:21026 2:N:0:TCTGCAAG+GCTGCACT
TGATATCAGATCTTTCATTATTCAAAATTAAATGACAGTCCTCGAATCAGTAGATTCGAAATATATGA
CAAGTATATGTAAGTGGCTGACTTAGATCATAAGTAATGTATACAGTAAATGTTTGTACAGTTGAAAT
AGATTTGAAATTCAA
+
FFFFFFFFFFFFFFFFFFFFFFFFFFFFFFFFFFFFFFFFFFFFFFFFFFFFFFFF:FFFFFFFFFFFFF
FFFFFFFFFFFFFFFFFFFFFFFFFFFFFFFFFFFFFFFFF,FFFFFFFFFFFFFFFFFFFFFFFFFFFF
FFFFFFFFFFFFFFF
@A00721:190:HGJW7DSXY:4:2174:2365:7592 2:N:0:TCTGCAAG+GCTGCACT
TGATATCAGATCTTTCATTATTCAAAATTAAATGACAGTCCTCGAATCAGTAGATTCGAAATATATGA
CAAGTATATGTAAGTGGCTGACTTAGATCATAAGTAATGTATACAGTAAATGTTTGTACAGTTGAAAT
AGATTTGAAATTCAA
+
FFFFFFFFFFFFFFFFFFFFFFFFFFFFFFFFFFFFFFFFFFFFFFFFFFF,FFFFFFFFFFFFFFF:FF
FFFFFFFFFFFFFFFFFFFFFFFFFFFFFFFF:FFFF,FFFFFFFFFFFFFFFFFFFFFFFFFFFFF:FF
FFF:FFFFFFFFFFF
@A00721:190:HGJW7DSXY:4:2525:18882:26177 2:N:0:TCTGCAAG+GCTGCACT
TGATATCAGATCTTTCATTATTCAAAATTAAATGACAGTCCTCGAATCAGTAGATTCGAAATATATGA
CAAGTATATGTAAGTGGCTGACTTAGATCATAAGTAAGGTATACAGTAAATGTTTGTACAGTTGAAAT
AGATTTGAAATTCAA
+
FFFFFFFFFFFFFFFFFFFFFFFFFF:FFFFFFFFFFFFFFFFFFFFFFFFFFFFFFFFFFFFFFFFFFF
F,FFFFF,FFFFFFFFFFFFFFF::FFFFFFFF:FFF,:FFFFFFFFFFFFFFFFFFFFFF,:FFFF,FF
F:FFFFFF:FFFF:F
@A00721:190:HGJW7DSXY:4:2547:13087:32330 2:N:0:TCTGCAAG+GCTGCACT
TGATATCAGATCTTTCATTATTCAAAATTAAATGACAGTCCTCGAATCAGTAGATTCGAAATATATGA
CAAGTATATGTAAGTGGCTGACTTAGATCATAAGTAATGTATACAGTAAATGTTTGTACAGTTGAAAT
AGATTTGAAATTCAA
+
FFFFFFFFFFFFFFFFFFFF,FFFFFFFFFFFFFFFFFFFF:FFFFFFFF,FFFFFFFFFFFFFFFFFFF
:FFFFFFFFFFFFFFFFFFFFFFF:FFFFFFF:FF:FFFF:FFFFFFFFF:FFFFF:FFFFF:FFFFFFF
FFFFFFFFFFFFFFF
@A00721:190:HGJW7DSXY:4:2654:18493:3646 2:N:0:TCTGCAAG+GCTGCACT
TGATATCAGATCTTTCATTATTCAAAATTAAATGACAGTCCTCGAATCAGTAGATTCGAAATATATGA
CAAGTATATGTAAGTGGCTGACTTAGATCATAAGTAATGTATACAGTAAATGTTTGTACAGTTGAAAT
AGATTTGAAATTCAA
+
FFFFFFFFFFFFFFFFFFFFFFFFFFFFFFFFFFFFFFFFFFFFFFFFFFFFFFFFFFFFFFFFFFFFFF
FFFFF:FFFFFFFFFFFFFFFFFFFFFFFFFFFFFFFFFFFFFFFFFFFFFFFFFFFFFFFFFFFFFFFF
FFFFFFFFFFFFF:F
@A00721:190:HGJW7DSXY:4:1117:1533:23249 2:N:0:TCTGCAAG+GCTGCACT
GTTCTAACACGTCGTTTGAAAGATGATATCAGATCTTTCATTATTCAAAATTAAATGACAGTCCTCGA
ATCAGTAGATTCGAAATATATGACAAGTATATGTAAGTGGCTGACTTAGATCATAAGTAATGTATACA
GTAAATGTTTGTACA
+
FFFFFFFFFF:FFFFFFFFFFF:FFFFFFFFFFFFFFFFFF,F:FFFFFF,FFFF,,FFFFFFFFFFF
FF:FF,FFFFFF:FFFFFFFF:FFFFFFFFFFF,FFFFFF:FFFFF,FFFFFFFFF,FF:FF:FF:FF
FFFFF:FFFFFF,FF
@A00721:190:HGJW7DSXY:4:1241:11568:20650 2:N:0:TCTGCAAG+GCTGCACT
GTTCTAACACGTCGTTTGAAAGATGTTATCAGATCTTTCATTATTCAAAATTAAATGACAGTCCTCGA
ATCAGTAGATTCGAAATATATGACAAGTATATGTAAGTGGCTGACTTAGATCATAAGTAATGTATACA
GTAAATGTTTGTACA

+
FFFFFFFFFFFFFFFFFFFFFFFFFFFFFFFFFFFFFFFFFFFFFFFFFFFFFFFFFFFFFFFFFFFF
FF:FFFFFFFFFFFFFFFFFFFFFFFFFFFFFFFFFFFFFFFFFFFFF:FFFFFFFFFFFFFFFFFFF
FFFFFFFFFF:FFFF
@A00721:190:HGJW7DSXY:4:1241:11794:20729 2:N:0:TCTGCAAG+GCTGCACT
GTTCTAACACGTCGTTTGAAAGATGTTATCAGATCTTTCATTATTCAAAATTAAATGACAGTCCTCGA
ATCAGTAGATTCGAAATATATGACAAGTATATGTAAGTGGCTGACTTAGATCATAAGTAATGTATACA
GTAAATGTTTGTACA
+
FFFFFFFFFFF:FFFFFFFFFFFFFFFFFFFFFFFFFFFFFFFFFFFFFFFFFFFFFFFFF:FFFFFF
FFFFF::FFFFFFFFFFFFFFFFFFFFFFFFFFFFFFFFFFFFFFFFFFFFFFFFFFFFFFFFFFFFF
FFFFFFFFFFF:FFF
@A00721:190:HGJW7DSXY:4:1427:24985:35618 2:N:0:TCTGCAAG+GCTGCACT
GTTCTAACACGTCGTTTGAAAGATGTTATCAGATCTTTCATTATTCAAAATTAAATGACAGTCCTCGA
ATCAGTAGATTCGAAATATATGACAAGTATATGTAAGTGGCTGACTTAGATCATAAGTAATGTATACA
GTAAATGTTTGTACA
+
FFFFFFFFFFF:FFFFFFFFFFFFFFFFFFFFFFFFFFFFFFFFFFFFFFFFFFFFFFFFFFFFFFFF
FFFFFFFFFFFF:FFFFFFFFFFFFFFFFFFFFFFFFFF:FFFFFFFFFFFFFFFF,FFFFFFFFFFF
FFFFFFFFFFFFFFF
@A00721:190:HGJW7DSXY:4:1549:1678:5337 2:N:0:TCTGCAAG+GCTGCACT
GTTCTAACACGTCGTTTGAAAGATGATATCAGATCTTTCATTATTCAAAATTAAATGACAGTCCTCGA
ATCAGTAGATTCGAAATATATGACAAGTATATGTAAGTGGCTGACTTAGATCATAAGTAATGTATACA
GTAAATGTTTGTACA
+
FFFFFFFFFFFFFFFFFFFFFFF:FFFFFFFFFFFFFFFFFFFFFFFFFFFFFFFFFFFFFFFFFFFF
FFFFFFFFFFFFFFFFFFFFFFFFFFFFFFFFFFFFFFFFFFFFF,FFFFFFFFFFFFFFFFFFFFFF
FFFFFFFFFFFFFFF
@A00721:190:HGJW7DSXY:4:1624:23556:19022 2:N:0:TCTGCAAG+GCTGCACT
GTTCTAACACGTCGTTTGAAAGATGTTATCAGATCTTTCATTATTCAAAATTAAATGACAGTCCTCGA
ATCAGTAGATTCGAAATATATGACAAGTATATGTAAGTGGCTGACTTAGATCATAAGTAATGTATACA
GTAAATGTTTGTACA
+
FFFFFFFFFFFFFFFFFFFFFF:FFFFFFFFFFFFFFFFFFFFFFFFFFFFFFFFFFFFFFFFFFFFF
FF:FF:FFFFF:FFFFFFFFFFFFFFFFFFFFFFFFFF,FFFFFFFFFFFFFFFFFFFFFFFFFFFFF
FFFFFF,FFFFFF:F
@A00721:190:HGJW7DSXY:4:1626:30346:7451 2:N:0:TCTGCAAG+GCTGCACT
GTTCTAACACGTCGTTTGAAAGATGTTATCAGATCTTTCATTATTCAAAATTAAATGACAGTCCTCGA
ATCAGTAGATTCGAAATATATGACAAGTATATGTAAGTGGCTGACTTAGATCATAAGTAATGTATACA
GTAAATGTTTGTACA
+
FFFFFFFFFFFFFFFFFFFFFFFFFFFFFFFF:FFFFFFFFFFFFFFFFFFFFF:FFFFFFFFFFFFF
FFFFFFFFFFFFFFFFFFFFF:FFFFFFFFFFFFFFFFFFFFFFFFFFFFFFFFFFFFFFFFFFFFFF
FFFFFFFFFFFFFF:
@A00721:190:HGJW7DSXY:4:2224:20039:5228 2:N:0:TCTGCAAG+GCTGCACT
GTTCTAACACGTCGTTTGAAAGATGATATCAGATCTTTCATTATTCAAAATTAAATGACAGTCCTCGA
ATCAGTAGATTCGAAATATATGACAAGTATATGTAAGTGGCTGACTTAGATCATAAGTAATGTATACA
GTAAATGTTTGTACA
+
FFFFFFFFFFFFFFFFFFFFFF:FFFFFFFFFFFFFFFFFFF,FFFFFFFFFFFFFFFFFFFFFFFFF
FFFFFFFFFFF:FFFFFFFFFFFFFFFFFFFFFFFFFFFFFFFFFFFFFFFFF:FFFFFFFF,FFFFF
FFFFFFFFFFFFF:F
@A00721:190:HGJW7DSXY:4:2536:1859:14512 2:N:0:TCTGCAAG+GCTGCACT
GTTCTAACACGTCGTTTGAAAGATGTTATCAGATCTTTCATTATTCAAAATTAAATGACAGTCCTCGA

ATCAGTAGATTCGAAATATATGACAAGTATATGTAAGTGGCTGACTTAGATCATAAGTAATGTATACA
GTAAATGTTTGTACA
+
:FFFFFFFFFFFFFFFFFFFFFFFFFFFFFFFFFFFFFFFFFFFFFFFFFFFFF:FFFFFFFFFFFF
FFFFFFFFFFFFFFFFFFFFFFFFFFFFFFFFFFFFFFFFFFFFFFFFFFFFFFFFFFFFFFFFFFFF
FF:F,FFFFFFFFFF
@A00721:190:HGJW7DSXY:4:1134:23936:3270 2:N:0:TCTGCAAG+GCTGCACT
CTGGTTTCTACACCGAAATGGATATACGTCACAGATAACCAGTTCTAACACGTCGTTTGAAAGATGAT
ATCAGATCTTTCATTATTCAAAATTAAATGACAGTCCTCGAATCAGTAGATTCGAAATATATGACAAG
TATATGTAAGTGAGA
+
FFFFFFFFFFFFFFFFFFFFFFFFFFFFFFFFFFFFFFFFFFFFFFFFFFFFFFFFFFFFFFFFFFFF
FFFFFFFFFFFFFFFFFFFFFFFFFFFFFFFFFFFFFFFFFFFFFFFFFFFFFFFFFFFFFFFFFFFF
FFFFFFFFFFFFFFF
@A00721:190:HGJW7DSXY:4:1436:9570:3693 2:N:0:TCTGCAAG+GCTGCACT
CTGGTTTCTACACCGAAATGGATATACGTCACAGATAACCAGTTCTAACACGTCGTTTGAAAGATGAT
ATCAGATCTTTCATTATTCAAAATTAAATGACAGTCCTCGAATCAGTAGATTCGAAATATATGACAAG
TATATGTAAGTGAGA
+
FFFFFFFFFFFFFFFFFF:FFFFFFFFFFFFFFFFFFFFFFFFFFFFFFFFFFFFFFFFFFFFFFFFF
FFFFFFFFFFFFFFFFFFFFFFFFFFFFFFFFFFFFFFFFFF:FFFFFFFFFFFFFFFFFFFFFFFFF
FFFFFFFFF:FFFFF
@A00721:190:HGJW7DSXY:4:1436:9634:3771 2:N:0:TCTGCAAG+GCTGCACT
CTGGTTTCTACACCGAAATGGATATACGTCACAGATAACCAGTTCTAACACGTCGTTTGAAAGATGAT
ATCAGATCTTTCATTATTCAAAATTAAATGACAGTCCTCGAATCAGTAGATTCGAAATATATGACAAG
TATATGTAAGTGAGA
+
FFFFFFFFFFFFFFFFFFFFFFFFFFFFFFFFFFFFFFFFFFFFFFFFFFFFFFFFFFFFFFFFFFFF
FFFFFFFFFFFFFFFFFFFFFFFFFFFFFFFFFFFFFFFFFFFFFFFFFFFFFFFFFFFFFFFFFFFF
FFFFFFFFFFFFFFF
@A00721:190:HGJW7DSXY:4:1456:26756:3458 2:N:0:TCTGCAAG+GCTGCACT
CTGGTTTCTACACCGAAATGGATATACGTCACAGATAACCAGTTCTAACACGTCGTTTGAAAGATGAT
ATCAGATCTTTCATTATTCAAAATTAAATGACAGTCCTCGAATCAGTAGATTCGAAATATATGACAAG
TATATGTAAGTGAGA
+
FFFFFFFFFFFFFFFFFFFFFFFFFFFFFFFFFFFFFFFFFFFFFFFFFFFFFFFFFFFFFFFFFFFF
FFFFFFFFFFFFFFFFFFFFFFFFFFFF:FFFFFFFFFFFFFFFFFFFFFFFFFFFFFFFFFFFFFF:F
FFFFFFFFFFFFFFF
@A00721:190:HGJW7DSXY:4:1540:6849:4366 2:N:0:TCTGCAAG+GCTGCACT
CTGGTTTCTACACCGAAATGGATATACGTCACAGATAACCAGTTCTAACACGTCGTTTGAAAGATGAT
ATCAGATCTTTCATTATTCAAAATTAAATGACAGTCCTCGAATCAGTAGATTCGAAATATATGACAAG
TATATGTAAGTGAGA
+
FFFFFFFFFFFFFFFFFFFFFFFFFFFFFFFFFFFFFFFFFFFFFFFFFFFFFFFFFFFFFFFFFFFF
FFFFFFFFFFFFFFFF,FFFFFFFFFFFFFFFFFFFFF:FFFFFFFFFF:FFFFFFFFFFFFFFFFFF
FFFFFFFFFFFFFFF
@A00721:190:HGJW7DSXY:4:2512:25346:29105 2:N:0:TCTGCAAG+GCTGCACT
CTGGTTTCTACACCGAAATGGATATACGTCACAGATAACCAGTTCTAACACGTCGTTTGAAAGATGAT
ATCAGATCTTTCATTATTCAAAATTAAATGACAGTCCTCGAATCAGTAGATTCGAAATATATGACAAG
TATATGTAAGTGAGA
+
FFFFFFFFFFFFFFFFFFFFFFFFFFFFFFFFFFFFFFFFFFFFFFFFFFFFFFFFFFFFFFFFFFFF
FFFFFFFFFFFFFFFFFFFFFFFFFFFFFFFFFFFFFFFFFFFFFFFFFF:FFFFFFFFFFFFFFFFF
FFFFFFFFFFFFFFF

```
@A00721:190:HGJW7DSXY:4:1157:19488:8061 2:N:0:TCTGCAAG+GCTGCACT
CTTACATATACTTGTCATATATTTCGAATCTACTGATTCGAGGACTGTCATTTAATTTTGAATAATGA
AAGATCTGATATCATCTTTCAAACGACGTGTTAGAACTGGTTATCTGTGACGTATATCCATTTCGGTG
TAGAAACCAGAATCC
+
FFFFFFFFFFFFFFFFFFFFFFFFFFFFFFFFFFFFFF,FFFFFFFFFFFFFFFFFFFFFFFFFFFFFFF
FFF:FFFFFFFFFFFFFFFFFFFFFFFFFFFFFFFFFFFFFFFFFFFFFFFFFFFFFFFFFFFFFFF,FF
FFFFFFFFFFFFFFF
@A00721:190:HGJW7DSXY:4:1263:31295:6433 2:N:0:TCTGCAAG+GCTGCACT
CTTACATATACTTGTCATATATTTCGAATCTACTGATTCGAGGACTGTCATTTAATTTTGAATAATGA
AAGATCTGATATCATCTTTCAAACGACGTGTTAGAACTGGTTATCTGTGACGTATATCCATTTCGGTG
TAGAAACCAGAATCC
+
FFFFFFFFFFFFFFFFFFFFFFFFFFFFFFFFFFFFFFFFFFFFFFFFFFFFFFFFFFFFFFFFFFFFFF
FFF:FFFFFFFFFFFFFFFFFF:FFFFFFFFFFFFFFFFFFFFFFFFFFFFFFFFFFFFFFFFFFFFFFF
FFFFFFFFFFFFFF:
@A00721:190:HGJW7DSXY:4:1632:12301:13808 2:N:0:TCTGCAAG+GCTGCACT
CTTACATATACTTGTCATATATTTCGAATCTACTGATTCGAGGACTGTCATTTAATTTTGAATAATGA
AAGATCTGATATCATCTTTCAAACGACGTGTTAGAACTGGTTATCTGTGACGTATATCCATTTCGGTG
TAGAAACCAGAATCC
+
FFFFFFFFFFFFFFFFFFFFFFFFFFFFFFFFFFFFFFFFFFFFFFFFFFFFFFFFFFFFFFFFFFFFFF
FFFFFFFFFFFFFFFFFFFFFFFFFFFFFF,FFFFFFFFFFFFFFFF:FFFFFFFFFFFFFFFFFFFFFF
FFFFFFFFFFF:FFF
@A00721:190:HGJW7DSXY:4:2173:10402:28244 2:N:0:TCTGCAAG+GCTGCACT
CTTACATATACTTGTCATATATTTCGAATCTACTGATTCGAGGACTGTCATTTAATTTTGAATAATGA
AAGATCTGATATCATCTTTCAAACGACGTGTTAGAACTGGTTATCTGTGACGTATATCCATTTCGGTG
TAGAAACCAGAATCC
+
FFFFFFFFFFFFFFFFF,FFFFFFFFFFFFFFFFFFFFFFFFFFFFFFFFFFFFFFFFFFFFFFFFFFFF
FFFFFFFFFFFFFFFFFFFFFF,FFFFFFFFFFFFFFFFFFFFFFFFFFFFFFFFFFFFFFFFFFFFFFF
FF:FFFFFFFFFFFF
@A00721:190:HGJW7DSXY:4:1447:6244:14372 2:N:0:TCTGCAAG+GCTGCACT
CGAATCTACTGATTCGAGGACTGTCATTTAATTTTGAATAATGAAAGATCTGATATCATCTTTCAAAC
GACGTGTTAGAACTGGTTATCTGTGACGTATATCCATTTCGGTGTAGAAACCAGAATCCTTCAGATCG
GCAATGAATTCCGAT
+
FFFFFFFFFFFFFFFFFFFFFFFFFFFFFFFFFFFFFFFFFFFFFFFFFFFFFFFFFFFFFFFFFFFFFF
FFFFFFFFFFFFFFFFFFFFFFFFFFFFFFFFFFFFFFFFFF::FFFF:FFFFFFFFFFFFFFFFF:FFF
FFF:FF:FFFFFFFF
@A00721:190:HGJW7DSXY:4:2522:7645:5588 2:N:0:TCTGCAAG+GCTGCACT
CGAATCTACTGATTCGAGGACTGTCATTTAATTTTGAATAATGAAAGATCTGATATCATCTTTCAAAC
GACGTGTTAGAACTGGTTATCTGTGACGTATATCCATTTCGGTGTAGAAACCAGAATCCTTCAGATCG
GCAATGAATTCCGAT
+
FFFF,FFFFFFFFFFFFFFFF:FFF:FFFFFF,,FFFFFFFFFFFFFFFFFFFFFFFFFFFFFFFFFFFF
FFFFFFFFFFFFFFFFFFFFFFFF:FFFFFFFFFFFFFFFFFFFF:FF,FFF:FFFFFFFFFFFFFFFFF
FFF,FFF:FFF:FFF
@A00721:190:HGJW7DSXY:4:1452:3052:24095 2:N:0:TCTGCAAG+GCTGCACT
ATCGATAGATCCTCATCGGAATTCATTGCCGATCTGAAGGATTCTGGTTTCTACACCGAAATGGATAT
ACGTCACAGATAACCAGTTCTAACACGTCGTTTGAAAGATGATATCAGATCTTTCATTATTCAAAATT
AAATGACAGTCCTCG
+
FFFFFFFFFFFFFFFFFFFFFFFFFFFFFFFFFFFFFFFFFFFFFFFFFFFFFFFFFFFFFFFFFFFFFF
```

```
FFFFFFFFFFFFFFFFFFFFFFFFFFFFFFF:FFFFFFFFFFFFFFFFFFFFFFFFFFFFFFFFFFFFFF
FFFFFFFFFFFFFFF
@A00721:190:HGJW7DSXY:4:1555:14986:27070 2:N:0:TCTGCAAG+GCTGCACT
ATCGATAGATCCTCATCGGAATTCATTGCCGATCTGAAGGATTCTGGTTTCTACACCGAAATGGATAT
ACGTCACAGATAACCAGTTCTAACACGTCGTTTGAAAGATGATATCAGATCTTTCATTATTCAAAATT
AAATGACAGTCCTCG
+
FF:FFFFFFFF:F:FFFFFFF:FFFFFFFFF:F,,FFF,FFFFFFFFFFFF:FFFFFF:FFFFFFFFFFF
:FFFFFFFFFFFFF:,FFFFFFFFFFF:FFFFFFFFFF:FFFFFFFFF:FFF:FFFFFFFFF:FFFFFFF
FFF,FF,F,:FFFFF
@A00721:190:HGJW7DSXY:4:2153:22354:22670 2:N:0:TCTGCAAG+GCTGCACT
ATCGATAGATCCTCATCGGAATTCATTGCCGATCTGAAGGATTCTGGTTTCTACACCGAAATGGATAT
ACGTCACAGATAACCAGTTCTAACACGTCGTTTGAAAGATGATATCAGATCTTTCATTATTCAAAATT
AAATGACAGTCCTCG
+
FFFFFFFFFFFFFFFFFFFFFFFFFFFFFFFFFFFFFFFFFFFFFFFFFFFFFFFFFF:FFF:FFFFFFF
FFFFFFFFFFFFFF:FFFFFFFFFFFFFFFFFFFFFFFFFFFFFFFFFFFFFFFF:FFFFFFFFFFFFFF
FFFFFF:FFFFFF:F
@A00721:190:HGJW7DSXY:4:2513:5258:18114 2:N:0:TCTGCAAG+GCTGCACT
ATCGATAGATCCTCATCGGAATTCATTGCCGATCTGAAGGATTCTGGTTTCTACACCGAAATGGATAT
ACGTCACAGATAACCAGTTCTAACACGTCGTTTGAAAGATGATATCAGATCTTTCATTATTCAAAATT
AAATGACAGTCCTCG
+
FFFFFFFFFFFFFFFFFFFFFFFFFFFFFFFFFFFFFFFFFFFFFFFFFFFFFFFFFFFFFFFFFFFFFF
FFFFFFFFFFFFFFFFFFFFFFFFFFFFFFFFFFFFFFFFFFFFFFFFFFFFFFFFFF:F:FFFFFFFFF
FFFFFFFFFFFFFFF
@A00721:190:HGJW7DSXY:4:2629:13865:4648 2:N:0:TCTGCAAG+GCTGCACT
ATCGATAGATCCTCATCGGAATTCATTGCCGATCTGAAGGATTCTGGTTTCTACACCGAAATGGATAT
ACGTCACAGATAACCAGTTCTAACACGTCGTTTGAAAGATGATATCAGATCTTTCATTATTCAAAATT
AAATGACAGTCCTCG
+
FFFFFFFFFFFFFFFFFFFFFFFFFFFFFFFFFFFFFFFFFFFFFFFFFFFFFFFFFFFFFFFFFFFFFF
FFFFFFFFFFFFFFFFFFFFFFFFFFFFFFFFFFFFFFFFFFFFFFFFFFFFFFFFFFFFFFFFFFFFFF
FFFFF:FFFFFFFFF
@A00721:190:HGJW7DSXY:4:1339:22625:19570 2:N:0:TCTGCAAG+GCTGCACT
CAGAAGTGAAGATCGATAGATCCTCATCGGAATTCATTGCCGATCTGAAGGATTCTGGTTTCTACACC
GAAATGGATATACGTCACAGATAACCAGTTCTAACACGTCGTTTGAAAGATGATATCAGATCTTTCAT
TATTCAAAATTAAAT
+
FF:FFFFFFFFFFFFFFFFFFFFFFFFFFFFFFFFFFFFFFFFFFFFFFFFFFFFFFFFFFFFFFFFFFF
FFFFFFFFFFFFFFFFFFFFFFFFFFFFFFFFFFFFFFFFFFFFFFFFFFFFF:FFFFFFFFFFFFFFFF
FFFFFFFFFFFFFFF
@A00721:190:HGJW7DSXY:4:1462:12961:10347 2:N:0:TCTGCAAG+GCTGCACT
CAGAAGTGAAGATCGATAGATCCTCATCGGAATTCATTGCCGATCTGAAGGATTCTGGTTTCTACACC
GAAATGGATATACGTCACAGATAACCAGTTCTAACACGTCGTTTGAAAGATGATATCAGATCTTTCAT
TATTCAAAATTAAAT
+
FFFFFFFFFFFF,FFFFF:FFFFFFFFFFFFFFFFFFFFFFFFFFFFFFFFFFFFFFFFFFFFFFFFFFF
FFFFFFF:FFFFFFFFFFFFFFFFFFFFFFFFFFFFFFFFFFFFFFFF::FFFFF:FFFFFFFFFFFFF,
FFFFFFFFFFFFFFF
@A00721:190:HGJW7DSXY:4:1669:21594:4413 2:N:0:TCTGCAAG+GCTGCACT
CAGAAGTGAAGATCGATAGATCCTCATCGGAATTCATTGCCGATCTGAAGGATTCTGGTTTCTACACC
GAAATGGATATACGTCACAGATAACCAGTTCTAACACGTCGTTTGAAAGATGATATCAGATCTTTCAT
TATTCAAAATTAAAT
```

+
FFFFFFFFF,FFFFFFFFFFFFFFFFFFFFFFFFFFFFFFFFFFFFFFFFFFFFFFFFFFFFFFFFFF
FFFFFFFFFFFFFFFFFFFFFFF:FFFFFFFFFFFFFFFFFFFFFFFFFFFFFF:FFFFF:FFFFFFF
FFFFFFFFFFFFFFF
@A00721:190:HGJW7DSXY:4:2123:2275:27164 2:N:0:TCTGCAAG+GCTGCACT
CAGAAGTGAAGATCGATAGATCCTCATCGGAATTCATTGCCGATCTGAAGGATTCTGGTTTCTACACC
GAAATGGATATACGTCACAGATAACCAGTTCTAACACGTCGTTTGAAAGATGATATCAGATCTTTCAT
TATTCAAAATTAAAT
+
FFFFFFFFFFFFFFFFFFFFFFFFFFFFFFFFFFFFFFFFFFFFFFFFFFFFFFFFFFFFFFFFFFFF
FFFFFFFFFFFFFFFFFFFFFFFFFFFFFFFFFFFFFFFFFFFFFFFFFFFFFFFFFFFFF,FFFFFF
FFFFFFFFFFFFFFF
@A00721:190:HGJW7DSXY:4:2250:4499:29293 2:N:0:TCTGCAAG+GCTGCACT
CAGAAGTGAAGATCGATAGATCCTCATCGGAATTCATTGCCGATCTGAAGGATTCTGGTTTCTACACC
GAAATGGATATACGTCACAGATAACCAGTTCTAACACGTCGTTTGAAAGATGATATCAGATCTTTCAT
TATTCAAAATTAAAT
+
FFFFFFFFFFFFFFFFFFFFFFFFFFFFFFFFFFFFFFFFFFFFFFFFFFFFFFFFFF:FFFFFFFFF
FFFFFFFFFFFFFFFFFFFFFFFFFFFFFFFFFFFFFFFFFFFFFFFFFFFFF:FFFFFFFFFFFF:F
FFFFFFFFFFFFFFF
@A00721:190:HGJW7DSXY:4:2353:32018:29011 2:N:0:TCTGCAAG+GCTGCACT
CAGAAGTGAAGATCGATAGATCCTCATCGGAATTCATTGCCGATCTGAAGGATTCTGGTTTCTACACC
GAAATGGATATACGTCACAGATAACCAGTTCTAACACGTCGTTTGAAAGATGATATCAGATCTTTCAT
TATTCAAAATTAAAT
+
FFFFFFFFFFFFFFFFFFFFFFFFFFFFFFFFFFFFFFFFFFFFFFFFFFFFFFFFFFFFFFFFF:FF
FFFFFFFFFFFFFFFFFFFFFFFFFFFFFFFFF:FFFFFFFFFFFFFFFFFFFFFFFFFFFFFFFF:F
FFFFF:FFFFF:FFF
@A00721:190:HGJW7DSXY:4:2657:15628:1157 2:N:0:TCTGCAAG+GCTGCACT
CAGAAGTGAAGATCGATAGATCCTCATCGGAATTCATTGCCGATCTGAAGGATTCTGGTTTCTACACC
GAAATGGATATACGTCACAGATAACCAGTTCTAACACGTCGTTTGAAAGATGATATCAGATCTTTCAT
TATTCAAAATTAAAT
+
FFFFFFFFFFFFFFFFFFFFFFFFFF:FFFFFFFFFFFFFFFFFFFFFFFFFFFFFFFFFFFFFFFFF
FFFFFFFFFFFFFFFFFFFFFFFFFFFFFFFFFFFFFFFFFFFFFFFFFFFFFFFFFFFFFFFFFFF:
FFF,FFFFFFFFF:F
@A00721:190:HGJW7DSXY:4:1214:21856:8907 2:N:0:TCTGCAAG+GCTGCACT
CGTCGACAATAGACAGAAGTGAAGATCGATAGATCCTCATCGGAATTCATTGCCGATCTGAAGGATTC
TGGTTTCTACACCGAAATGGATATACGTCACAGATAACCAGTTCTAACACGTCGTTTGAAAGATGATA
TCAGATCTTTCATTA
+
FFFFFFFFFFFFFFFFFFFFFFFFFFFFFFFFFFFFFFFFFFFFFFFFFFFFFFFFFFFFFFFFFFFF
FFF:FFFFFFFFFFFFFFFFFFFFFFFFFFFFFFFFFFFFFFFFFFFFFFFFFFFFFFFFFFFFFFF,
FFFFFFFFFFFF:FF
@A00721:190:HGJW7DSXY:4:1331:25500:17597 2:N:0:TCTGCAAG+GCTGCACT
CGTCGACAATAGACAGAAGTGAAGATCGATAGATCCTCATCGGAATTCATTGCCGATCTGAAGGATTC
TGGTTTCTACACCGAAATGGATATACGTCACAGATAACCAGTTCTAACACGTCGTTTGAAAGATGATA
TCAGATCTTTCATTA
+
FFFFFFFFFFFFFFF:FFFFFFFFFFFFFFFFFFFFFFFFFFFFFFFFFFFFFFFFFFF:FFFFFFFF
FFFFFFFFFFFFFFFFFFFFFFFFFFFFFFFFFFFFFFFFFFFFFFFFFFF:FFFFFFFFFFFFFFFF
FFFFFFFFFFF:::F
@A00721:190:HGJW7DSXY:4:1506:18602:21183 2:N:0:TCTGCAAG+GCTGCACT
CGTCGACAATAGACAGAAGTGAAGATCGATAGATCCTCATCGGAATTCATTGCCGATCTGAAGGATTC

TGGTTTCTACACCGAAATGGATATACGTCACAGATAACCAGTTCTAACACGTCGTTTGAAAGATGATA
TCAGATCTTTCATTA
+
FFFFFFFFFFFFFFFFFFFFFFFFFFFFFFFFFFFFFFFFFFFFFFFFFFFFFFFFFFFFF:FFFFFF
FFFFFFFFFFFFFFFFFFFFFFFFFFFFFFFFFFFF:FFFFFFFFFFFFFFFFFFFFFFFFFFFFFFF
FFFFFFFFFFFFFFF
@A00721:190:HGJW7DSXY:4:1558:21829:14779 2:N:0:TCTGCAAG+GCTGCACT
CGTCGACAATAGACAGAAGTGAAGATCGATAGATCCTCATCGGAATTCATTGCCGATCTGAAGGATTC
TGGTTTCTACACCGAAATGGATATACGTCACAGATAACCAGTTCTAACACGTCGTTTGAAAGATGATA
TCAGATCTTTCATTA
+
FFFFFFFFFFFFFFFFFFFFFFFFFFFFFFFFFFFFFFFFFFFFFFFFFFFFFFFFFFFFFFFFFFFF
FFFFFFFFFFFFFFFFFFFFFFFFFFFFFFFFFFF:FFFFFFFFFFFFFFFFFFFFFF:FFFFFFFFF
FFFFFFFFFFFFFFF
@A00721:190:HGJW7DSXY:4:1617:5376:32503 2:N:0:TCTGCAAG+GCTGCACT
CGTCGACAATAGACAGAAGTGAAGATCGATAGATCCTCATCGGAATTCATTGCCGATCTGAAGGATTC
TGGTTTCTACACCGAAATGGATATACGTCACAGATAACCAGTTCTAACACGTCGTTTGAAAGATGATA
TCAGATCTTTCATTA
+
FFFFFFFFFFFFFFFFFFFFFFFFFFFFFFFFFFFFFFFFFFF:FFFFFFFFFFF,FFFFFFFFF:FF
FFFFFFFFFFFFFFFFFFFFFFFFFFFFFFFFFFFFFFFFFFFFFFFFFFFFFFFFFFFFFFFFFFFF
FFFFFFFFFFFFFFF
@A00721:190:HGJW7DSXY:4:2258:9561:9846 2:N:0:TCTGCAAG+GCTGCACT
CGTCGACAATAGACCGAAGTGAAGATCGATAGATCCTCATCGGAATTCATTGCCGATCTGAAGGATTC
TGGTTTCTACACCGAAATGGATATACGTCACAGATAACCAGTTCTAACACGTCGTTTGAAAGATGATA
TCAGATCTTTCATTA
+
FFFFF:FFFFFF:F,:FFFFFFFFFFFFFFFFFFFFFFFFFFFFFFFFFFFFFFFFFFFF:FFFF:FF
FFFFFFFFFFFFFFFFFFF:FFFFFFFFFFFFFFFFFFFFFFFFFFFFFFFFFFFFFFFFFFFFFFF:
FFFFFFFFFFFFFFF
@A00721:190:HGJW7DSXY:4:2261:24153:11913 2:N:0:TCTGCAAG+GCTGCACT
CGTCGACAATAGACAGAAGTGAAGATCGATAGATCCTCATCGGAATTCATTGCCGATCTGAAGGATTC
TGGTTTCTACACCGAAATGGATATACGTCACAGATAACCAGTTCTAACACGTCGTTTGAAAGATGATA
TCAGATCTTTCATTA
+
FFFFFFFFFFFFFFFFF:FFFFFFFFFFFFFFFFFFFFFFFFFFFFFFFFFFFFFFFFFFFFFFFFFF
FFFFFFFFFFFFFFFFFFFFFFFFFFFFFFFFFFFFFFFFFFFFFFFFFFFFF:FFFFFFF:FFFFF
FFFFFFFFFFFFFFF
@A00721:190:HGJW7DSXY:4:2333:4020:20509 2:N:0:TCTGCAAG+GCTGCACT
CGTCGACAATAGACAGAAGTGAAGATCGATAGATCCTCATCGGAATTCATTGCCGATCTGAAGGATTC
TGGTTTCTACACCGAAATGGATATACGTCACAGATAACCAGTTCTAACACGTCGTTTGAAAGATGATA
TCAGATCTTTCATTA
+
FFFFFFFFFFFFFFFFFFFFFFFFFFFFFFFFFFFFFFFFFFFFFFFFFFFFFFFFFFFFFFFFFFFF
FFFFFFFFFFFFFFFFFFFFFFFFFFFFFFFFFFFFFFFFFFFFFFFFFFFFFFFFFFFFFFFFFFFF
FFFFFFFFFFFFFFF
@A00721:190:HGJW7DSXY:4:2514:6388:13150 2:N:0:TCTGCAAG+GCTGCACT
CGTCGACAATAGACAGAAGTGAAGATCGATAGATCCTCATCGGAATTCATTGCCGATCTGAAGGATTC
TGGTTTCTACACCGAAATGGATATACGTCACAGATAACCAGTTCTAACACGTCGTTTGAAAGATGATA
TCAGATCTTTCATTA
+
FFFFFFFF:FFFFFFFFFFFFFFFFFFFFFFFFFFFFFFFFFFFFFFFFFFFFFFFFFFFFFFFFFFF
FFFFFFFFFFFFFFFFFFFFFFFFFFFFFFFFFFFFFFFFFFFFFFFFFF:FFFFFFFFFFFFFFFFF
FFFFFFFFFFFFFFF

@A00721:190:HGJW7DSXY:4:2533:15790:32033 2:N:0:TCTGCAAG+GCTGCACT
CGTCGACAATAGACAGAAGTGAAGATCGATAGATCCTCATCGGAATTCATTGCCGATCTGAAGGATTC
TGGTTTCTACACCGAAATGGATATACGTCACAGATAACCAGTTCTAACACGTCGTTTGAAAGATGATA
TCAGATCTTTCATTA
+
FFFFFFFFFFFFFFFFFFFFFFFFFFFFFFFFFFFFFFFFFFFFFFFFFFFFFFFFF:FFFFFFFFFF
FFFFFFFFFFFFFFFFFFFFFFFFFF:FFFFFFFFFFFFFFFFFFFFFFFFFFFFFFFFFFFFFFFFF
FFFFFFFFFFFFFFF
@A00721:190:HGJW7DSXY:4:2629:14959:1251 2:N:0:TCTGCAAG+GCTGCACT
CGTCGACAATAGACAGAAGTGAAGATCGATAGATCCTCATCGGAATTCATTGCCGATCTGAAGGATTC
TGGTTTCTACACCGAAATGGATATACGTCACAGATAACCAGTTCTAACACGTCGTTTGAAAGATGATA
TCAGATCTTTCATTA
+
FFFFFFFFFFFFFFFF,FFFFFFFFFFFFFFFFFFFFFFFFFFFFFFFFFFFFFFFFFFFFFFFFFFF
FFFF:FFFFFFFFFFFFFFFFFFFFFFFFFFFFFFFFFFFF:FFFFF:FFFFFFFFFFFFFFFFF:FF
FFFFFFFFFFFFFFF
@A00721:190:HGJW7DSXY:4:2546:19804:25614 2:N:0:TCTGCAAG+GCTGCACT
CGTCGACAATAGACAGAAGTGAAGATCGATAGATCCTCATCGGAATTCATTGACTATCTGAAGGATTC
TGGTTTCTACACAGAAATGGATATACGTCACAGATAACCAGTTCTAAAACGTCGTTTGAAAGATGATA
TCAGATCTATCA
+
F:FF::FFFFFFF,FFFFFF,F:::FF:FFFFF:FFFF:FFFFFFFF:FF,,,F,FF,F,FF:F:FFF
FF:F:F:FFFF,,FFF:,,FF,FFFFFFFFF:FF,FFFF:F:FF:F,FFF,F,,FF:FFF:FF,F,F
FFFFFF:,,FF:
@A00721:190:HGJW7DSXY:4:1248:18783:10598 2:N:0:TCTGCAAG+GCTGCACT
TAACATTCCCAGCAGCGCATATCGTCGACAATAGACAGAAGTGAAGATCGATAGATCCTCATCGGAAT
TCATTGCCGATCTGAAGGATTCTGGTTTCTACACCGAAATGGATATACGTCACAGATAACCAGTTCTA
ACACGTCGTTTGAAA
+
FFFFFFFFFFFFFFFFFFFFFFFFFFFFFFFFFFFFFFFFFFFFFFFFFFFFFFFFFFFFFFFFFFFF
FFF:FFFFFFFFFFFFFFFFFFFFFFFFFFFFFFFFFFFFFFFF:FFFFFFFFFFFFFFFFFFFFFFF
FFFFFFFFFFFFFFF
@A00721:190:HGJW7DSXY:4:2636:6433:27195 2:N:0:TCTGCAAG+GCTGCACT
TAACATTCCCAGCAGCGCATATCGTCGACAATAGACAGAAGTGAAGATCGATAGATCCTCATCGGAAT
TCATTGCCGATCTGAAGGATTCTGGTTTCTACACCGAAATGGATATACGTCACAGATAACCAGTTCTA
ACACGTCGTTTGAAA
+
FFFFFFFFFFFFF:FFFFFFFFFFFFFFFFFFFFFFFFFFFFFFFFFFFFFFFFFFFFFFFFFFFFFF
FFFFFFFFFFFF:F:FFFFFFFFFF,FFFFFFFFFFFF:FFFF,:FFFF:FFF:FFF:FFFFFFFFFF
FFFFFFFFFFFFFFF
@A00721:190:HGJW7DSXY:4:1432:28791:2910 2:N:0:TCTGCAAG+GCTGCACT
CGAACATGAATCGTCGAATACATAACATTCCCGGCAGCGCATATCGTCGACAATAGACAGAAGTGAAG
ATCGATAGATCCTCATCGGAATTCATTGCCGATCTGAAGGATTCTGGTTTCTACACCGAAATGGATAT
ACGTCACAGATAACC
+
FFFFFFFFFFFFFFFFFFFFFFFFFFFFFFFFFFF,FFFFFFFFFFFFFFFFFFFFFFFFF,FFFFFF
FFFFFFFFFFFFFFFFFFFFFFFFFFFFFFFFFFFFFFFFFFFFFFFFFFFFFFFFFFFFFFFFFFFF
FFFFFFFFFFFFFFF
@A00721:190:HGJW7DSXY:4:1625:23466:24940 2:N:0:TCTGCAAG+GCTGCACT
CGAACATGAATCGTCGAATACATAACATTCCCAGCAGCGCATATCGTCGACAATAGACAGAAGTGAAG
ATCGATAGATCCTCATCGGAATTCATTGCCGATCTGAAGGATTCTGGTTTCTACACCGAAATGGATAT
ACGTCACAGATAACC
+
FFFFFFFFFFFFFFFFFFFFFFFFFFFFFFFFFFFFFFFFFFFFFFFFFFFFFFFFFFFFFFFFFFFF

FFFFFFFFFFFFFFFFFFFFFFFFFFFFFFFFFFFFFFFFFFFFFFFFFFFFFFFFFFFFFFFFFFFF
FFFFFFFFFFFFFFF
@A00721:190:HGJW7DSXY:4:1646:27001:15875 2:N:0:TCTGCAAG+GCTGCACT
CGAACATGAATCGTCGAATACATAACATTCCCAGCAGCGCATATCGTCGACAATAGACAGAAGTGAAG
ATCGATAGATCCTCATCGGAATTCATTGCCGATCTGAAGGATTCTGGTTTCTACACCGAAATGGATAT
ACGTCACAGATAACC
+
FFFFFFFFFFFFFFFFFFFFFFFFFFFFFFFFFFFF,FFFFFFFFFFFFFFFFFFFFFFFFFFFFFFF
FFFFFFFFFFFFFFFFFFFFFFFFFFFFFFFFFFFFFFFFFFFFFFFFFFFFFFFFFFFFFFFFFFFF
FFFFFFFFFFFFFFF
@A00721:190:HGJW7DSXY:4:1114:32579:9220 2:N:0:TCTGCAAG+GCTGCACT
AAGCGAACATGAATCGTCGAATACATAACATTCCCAGCAGCGCATATCGTCGACAATAGACAGAAGTG
AAGATCGATAGATCCTCATCGGAATTCATTGCCGATCTGAAGGATTCTGGTTTCTACACCGAAATGGA
TATACGTCACAGATA
+
FFFFFFFFFFFFFFFFFFFFFFFFFFFFFFFFFFFFF:FFFFFFFFFFFFFFFFFFFFFFFFFFFFFF
FFFFFFFFFFFFFFFFFFFFFF:FFFFFFFFFFFFFFFFFFFFF:FFFF,FFFFFFF,FFFFFFFFF:
F:F:FFFFFFFFFFF
@A00721:190:HGJW7DSXY:4:1129:5755:3850 2:N:0:TCTGCAAG+GCTGCACT
AAGCGAACATGAATCGTCGAATACATAACATTCCCAGCAGCGCATATCGTCGACAATAGACAGAAGTG
AAGATCGATAGATCCTCATCGGAATTCATTGCCGATCTGAAGGATTCTGGTTTCTACACCGAAATGGA
TATACGTCACAGATA
+
FFFFFFFFFFFFFFFFFFFFFFFFFF::FFFFFFFFFFFFFFFFFFFFFFFFFFF:FFFFFFFFFFF:
FFFFFFFFFF:FFFFFFFFF:FFFFFFFFFFFFFF:FFFFFFFFFFFFFF,FFFFFFFFFFFFFFFFF
FFFFFFFFFFFFFFF
@A00721:190:HGJW7DSXY:4:1349:24433:7545 2:N:0:TCTGCAAG+GCTGCACT
AAGCGAACATGAATCGTCGAATACATAACATTCCCAGCAGCGCATATCGTCGACAATAGACAGAAGTG
AAGATCGATAGATCCTCATCGGAATTCATTGCCGATCTGAAGGATTCTGGTTTCTACACCGAAATGGA
TATACGTCACAGATA
+
FFFFFFFFFFFFFFFFFFFFFFFFFFFFFFFFFFFFFFFFFFFFFFFFFFFFFFFFFFFFFFFFFFFF
FFFFFFFFFFFFFFFFFFFFFFFFFFFFFFFFFFFFFFFFFFFFFFFFFFF:FFFFFFFFFFFFFFFF
FFFFFFFFFFFFFFF
@A00721:190:HGJW7DSXY:4:1607:21206:24220 2:N:0:TCTGCAAG+GCTGCACT
AAGCGAACATGAATCGTCGAATACATAACATTCCCAGCAGCGCATATCGTCGACAATAGACAGAAGTG
AAGATCGATAGATCCTCATCGGAATTCATTGCCGATCTGAAGGATTCTGGTTTCTACACCGAAATGGA
TATACGTCACAGATA
+
FFFFFFFFFFFFFFFFFFFFFFFFFFFFFFFFFFFFFFFFFFFFFFFFFFFFF:FFFFFFFFFFFFFF
FFFFFFFFFFFFFFFFFFFFFFFFFFFFFFFFFFFFFFFFFFFFFFFFFFFFFFFFFFFFFFFFFFFF
FFFFFFFFFFFFFFF
@A00721:190:HGJW7DSXY:4:1670:1253:15248 2:N:0:TCTGCAAG+GCTGCACT
AAGCGAACATGAATCGTCGAATACATAACATTCCCAGCAGCGCATATCGTCGACAATAGACAGAAGTG
AAGATCGATAGATCCTCATCGGAATTCATTGCCGATCTGAAGGATTCTGGTTTCTACACCGAAATGGA
TATACGTCACAGATA
+
FFFFFFFFFFFFFFFFFFFFFFFFFFFFFFFFFFFFFFFFFFFFFFFFFFFFFFFFFFFFFFFFFFFF
FFFFFFFFFFFFFFFFFFFFFFFFFFFFFFFFFFFFFFFFFFFFFFFFFFFFFFFFFFFFFF:FFFFF
FFFFFFFFFFFFFFF
@A00721:190:HGJW7DSXY:4:2529:7536:20400 2:N:0:TCTGCAAG+GCTGCACT
AAGCGAACATGAATCGTCGAATACATAACATTCCCAGCAGCGCATATCGTCGACAATAGACAGAAGTG
AAGATCGATAGATCCTCATCGGAATTCATTGCCGATCTGAAGGATTCTGGTTTCTACACCGAAATGGA
TATACGTCACAGATA

+
FFFFFFFFFFFFFFFFFFFFFFFFFFFFFFFFFFFFFFFFFFFFFFFFFFFFFFFFFFFFFFFFFFFFFF
FFFFFFFFF:FFFFFFFFFFFFFFFFFFFFFFFFFFFFFFFFFFFFFFFFFFFFFFFFFFFFFFFFFFFF
FFFFFFFFFFFFFFF
@A00721:190:HGJW7DSXY:4:1119:11550:8938 2:N:0:TCTGCAAG+GCTGCACT
ACGAAAGCGAACATGAATCGTCGAATACATAACATTCCCAGCAGCGCATATCGTCGACAATAGACAGA
AGTGAAGATCGATAGATCCTCATCGGAATTCATTGCCGATCTGAAGGATTCTGGTTTCTACACCGAAA
TGGATATACGTCACA
+
FFFFFFFFFFFFFFFFFFFFFFFFFFFFFFFFFFFFFFFFFFFFFFFFFFFFFFFFFFFFF:FFFFFFFF
FFFFFFFFFFFFFFFFFFFFFFFFFFFFFFFFFFFFFFFFFFFFFFFFFFFFFFFFFFFFFFFFFFFFFF
FFFFFFFFFFFFFFF
@A00721:190:HGJW7DSXY:4:1237:4499:34992 2:N:0:TCTGCAAG+GCTGCACT
ACGAAAGCGAACATGAATCGTCGAATACATAACATTCCCAGCAGCGCATATCGTCGACAATAGACAGA
AGTGAAGATCGATAGATCCTCATCGGAATTCATTGCCGATCTGAAGGATTCTGGTTTCTACACCGAAA
TGGATATACGTCACA
+
FFFFFFFFFFFFFFFFFFFFFFFFFFFF:FFFFFFFFFFFFF:FFFFFFFFFFFFFFFFFFFFFFFFFF:
FFFFFFFFFFFF:FFFFFFFFFFFFFFFFFFFFFFFFFFFFFFFFFFFFFFFFFFFFFFFFFFFFFFFFF
FF:FFFFFFFFFFFF
@A00721:190:HGJW7DSXY:4:1306:10402:36949 2:N:0:TCTGCAAG+GCTGCACT
ACGAAAGCGAACATGAATCGTCGAATACATAACATTCCCAGCAGCGCATATCGTCGACAATAGACAGA
AGTGAAGATCGATAGATCCTCATCGGAATTCATTGCCGATCTGAAGGATTCTGGTTTCTACACCGAAA
TGGATATACGTCACA
+
FFFFFFFFFFFFFFFFFFFFFFFF:FFFFFFFFFFFFFFFFFFFFFFFFFFFFFFFFFFFFFFFFFFFFF
FFFFFFFFFFFFFFFFFFFFFFFFFFFF:FFFFFFFFFFFFFFFFFFFFFFFFFF:FFFFFFFFFFFFFF
FFFFFFFFFFFFFF:
@A00721:190:HGJW7DSXY:4:1311:4860:31360 2:N:0:TCTGCAAG+GCTGCACT
ACGAAAGCGAACATGAATCGTCGAATACATAACATTCCCAGCAGCGCATATCGTCGACAATAGACAGA
AGTGAAGATCGATAGATCCTCATCGGAATTCATTGCCGATCTGAAGGATTCTGGTTTCTACACCGAAA
TGGATATACGTCACA
+
FFFFFFFFFFFFFFFFFFFFFFFF:FFFFFFFFFFFFFFFFF,FFFFFFFFFFFFFFFFFFFFFFFFFFF
FFFFFFFFFFFFFFFFFFFFFFFFFFFFFFFFFFF:FFFFFFFFFFFFFFFFFFFFFFFFFFFFFFFF:F
FFFFFFFFFFFFFFF
@A00721:190:HGJW7DSXY:4:1442:24903:20102 2:N:0:TCTGCAAG+GCTGCACT
ACGAAAGCGAACATGAATCGTCGAATACATAACATTCCCAGCAGCGCATATCGTCGACAATAGACAGA
AGTGAAGATCGATAGATCCTCATCGGAATTCATTGCCGATCTGAAGGATTCTGGTTTCTACACCGAAA
TGGATATACGTCACA
+
FFFFFFFFFFFFFFFFFFFFFFFFFFFFFFF:FFFFFFFFFFFFFFFFFFFFFFFFFFFFFF:FFFFFFFF
FFFFF:FFFFFFFFFFFFFFFFFF:FFFFFFFFFFFFFFFFFFFFFFFFFFFFFFFFFFFFFFFFFFFFF
F:FFFFFFFFFFFFF
@A00721:190:HGJW7DSXY:4:1654:20374:36933 2:N:0:TCTGCAAG+GCTGCACT
ACGAAAGCGAACATGAATCGTCGAATACATAACATTCCCAGCAGCGCATATCGTCGACAATAGACAGA
AGTGAAGATCGATAGATCCTCATCGGAATTCATTGCCGATCTGAAGGATTCTGGTTTCTACACCGAAA
TGGATATACGTCACA
+
FFFFFFFFFFFFFFFFFFFFFFFFFFFFFFFFFFFFFFFFFFFFFFFFFFFFFFFFFFFFFFFFFFF:FF
:FFFFFFFFFFFF,FFFFFFFFFFFFFFFFFFFFFFFFFFFFFFFFFFFFFFFFFFFFFFFFFFFFFFFF
FFFFFFFFFFFFFFF
@A00721:190:HGJW7DSXY:4:2229:5746:34053 2:N:0:TCTGCAAG+GCTGCACT
ACGAAAGCGAACATGAATCGTCGAATACATAACATTCCCAGCAGCGCATATCGTCGACAATAGACAGA

```
AGTGAAGATCGATAGATCCTCATCGGAATTCATTGCCGATCTGAAGGATTCTGGTTTCTACACCGAAA
TGGATATACGTCACA
+
FFFFFFFFFFFFFFFFFFFFFFFFFFFFFFFFFFF:FFFFFFF:FFFFFFFFFFFFFFFFFFFFFFFFFF
FFFFFFFFFFFFFFFFFFFFFFFFFFFF,FFFFFFFFFFFFFFFFFFFFFFFFFFFFFFFFFFFFFFF:F
:FFFFFFFFFFFFF:
@A00721:190:HGJW7DSXY:4:2254:3586:35415 2:N:0:TCTGCAAG+GCTGCACT
ACGAAAGCGAACATGAATCGTCGAATACATAACATTCCCAGCAGCGCATATCGTCGACAATAGACAGA
AGTGAAGATCGATAGATCCTCATCGGAATTCATTGCCGATCTGAAGGATTCTGGTTTCTACACCGAAA
TGGATATACGTCACA
+
FFFFFFFFFFFFFFFFFFFFFFFFFFFFFFFFFFFFFFFFFFFFFFFFFFFFFFFFFFFFFFFFFFFFFF
FFFFFFFFFFFFFF:FFFFFFFFFFFFFFFFFFFFFFFFFFFFFFFFFFFFFFFFFFFFFFFFFFFFFFF
FFFFFFFFFFFFFFF
@A00721:190:HGJW7DSXY:4:2264:2157:33567 2:N:0:TCTGCAAG+GCTGCACT
ACGAAAGCGAACATGAATCGTCGAATACATAACATTCCCAGCAGCGCATATCGTCGACAATAGACAGA
AGTGAAGATCGATAGATCCTCATCGGAATTCATTGCCGATCTGAAGGATTCTGGTTTCTACACCGAAA
TGGATATACGTCACA
+
FFFFFFFFFFFFFFFFFFFF:FFFFF:FF:FFFFFFFFFFFFF,FFFFFFFFFFFFFFFFFF:FFFFFFF
FFFFFFFFFFFFFFFFFFFFFFFFFFFF,FFFFFFFFFFFFFFFFFFFFFFFFFFFFFFFFFFFFFFFFF
FFFFF:FFF:FFFFF
@A00721:190:HGJW7DSXY:4:2325:10999:13025 2:N:0:TCTGCAAG+GCTGCACT
ACGAAAGCGAACATGAATCGTCGAATACATAACATTCCCAGCAGCGCATATCGTCGACAATAGACAGA
AGTGAAGATCGATAGATCCTCATCGGAATTCATTGCCGATCTGAAGGATTCTGGTTTCTACACCGAAA
TGGATATACGTCACA
+
FFFFFFFFFFFFFFFFFFFFFFFFFFFFFFFFFFFFFFFFFFFFFFFFFFFFFFFFFFFFFFFFFFFFFF
FFFFFFFFFFFFFFFFFFFFFFFFFFF:FFFFFFFFFFFFFFFFFFFFFFFFFFFFFFFFFFFFFFFFFF
FFFFFFFFFFFFFFF
@A00721:190:HGJW7DSXY:4:2615:4264:10974 2:N:0:TCTGCAAG+GCTGCACT
ACGAAAGCGAACATGAATCGTCGAATACATAACATTCCCAGCAGCGCATATCGTCGACAATAGACAGA
AGTGAAGATCGATAGATCCTCATCGGAATTCATTGCCGATCTGAAGGATTCTGGTTTCTACACCGAAA
TGGATATACGTCACA
+
FFFFF:FFFFFFFFFFFFFFFFFFFFFFFFFFFFFFFFFFFFFFFFFFFF:FFFFFFFFFFFFFFFFFFF
FFFFFFFFFFFFFFFFFFFFFFFFFFFFFFFFFFFFFFFF:FFFFFFFFFFFFFFFFFFFFFFFFFFFF:
FFFFFFFFFFFF:FF
@A00721:190:HGJW7DSXY:4:1219:16468:28980 2:N:0:TCTGCAAG+GCTGCACT
CGAATTCAGAACTTTATTGTTCTACAATAACGAAAGCGAACATGAATCGTCGAATACATAACATTCCC
AGCAGCGCATATCGTCGACAATAGACAGAAGTGAAGATCGATAGATCCTCATCGGAATTCATTGCCGA
TCTGAAGGATTCTGG
+
FFFFFFFFFFFFFFFFFFFFFFFFFFFFFFFFFFFFFFFFFFFFFFFFFFFFFFFFFFFF,FFFFFFFFF
FFF:FFFFFFFFFFFFFFFFFFFFFFFFFFFFFFFFFFFFFF:FFFFFFFFFFFFFFF,FFFFFFFFFFF
FFFFFFFFFFFFFFF
@A00721:190:HGJW7DSXY:4:1303:28076:29951 2:N:0:TCTGCAAG+GCTGCACT
CGAATTCAGAACTTTATTGTTCTACAATAACGAAAGCGAACATGAATCGTCGAATACATAACATTCCC
AGCAGCGCATATCGTCGACAATAGACAGAAGTGAAGATCGATAGATCCTCATCGGAATTCATTGCCGA
TCTGAAGGATTCTGG
+
FFFFFFFFFFFFFFFFFFFFFFFFFFFFFFFFFFFFFFFFFFFFFFFFFFFFFFFFFFF:FFFFFFFFFF
FFF,FFFFFFFFFFFFFFFFFFFFFFFFFFFFFF:FF:FFFFFFFFFFFFFFFFFFFFFFFF:FFFFFFF
FFFFFFFFF:FFFFF
```

@A00721:190:HGJW7DSXY:4:1678:10954:35305 2:N:0:TCTGCAAG+GCTGCACT
CGAATTCAGAACTTTATTGTTCTACAATAACGAAAGCGAACATGAATCGTCGAATACATAACATTCCC
AGCAGCGCATATCGTCGACAATAGACAGAAGTGAAGATCGATAGATCCTCATCGGAATTCATTGCCGA
TCTGAAGGATTCTGG
+
FFFFFFFFFFFFFFFFFFFFFFFFFFFFFFFFFFFFFFFFFFFFFFFFFFFFFFFFFFFFFFFFFFFFFF
FFF,FFFFFFFFFFFFFFFFFFFFFFFFFFFFFFFFFFFFFFFFFFFFFFFFFFFFFFFFFFFFFFFFFF
FFFFFFFFFFFFFFF
@A00721:190:HGJW7DSXY:4:2116:25075:19554 2:N:0:TCTGCAAG+GCTGCACT
CGAATTCAGAACTTTATTGTTCTACAATAACGAAAGCGAACATGAATCGTCGAATACATAACATTCCC
AGCAGCGCATATCGTCGACAATAGACAGAAGTGAAGATCGATAGATCCTCATCGGAATTCATTGCCGA
TCTGAAGGATTCTGG
+
FFFFFFFFFFFFFFFFFFFFFFFFFFFFFFFFFFFFFFFFFFFFFFFFFFFFFFFFF:F:FFFFFFFFFF
:FF:FFFFFFFFFFFFFFF,FFF:FFFFFFFFFFFFFFF:FFFFFFFFFFFFFFFFFFFFFFFFFFFFFF
FFFFFFFFFFFFFFF
@A00721:190:HGJW7DSXY:4:2203:28031:25394 2:N:0:TCTGCAAG+GCTGCACT
CGAATTCAGAACTTTATTGTTCTACAATAACGAAAGCGAACATGAATCGTCGAATACATAACATTCCC
AGCAGCGCATATCGTCGACAATAGACAGAAGTGAAGATCGATAGATCCTCATCGGAATTCATTGCCGA
TCTGAAGGATTCTGG
+
FFFFFFFFFFFFFFFFFFFFFFFFFFFFFFFFFFFFFFFFFFFFFFFFFFFFFFFF:FFFFFFFFFFFFF
FFFFFFFFFFFFFFFFFFFFFFFFFFFFFFFFFFFFFFFFFFFFFFFFFFFFFFFFFF,FFFFFFFFFFF
FFFFFFFFFFFFFFF
@A00721:190:HGJW7DSXY:4:2269:6885:31454 2:N:0:TCTGCAAG+GCTGCACT
CGAATTCAGAACTTTATTGTTCTACAATAACGAAAGCGAACATGAATCGTCGAATACATAACATTCCC
AGCAGCGCATATCGTCGACAATAGACAGAAGTGAAGATCGATAGATCCTCATCGGAATTCATTGCCGA
TCTGAAGGATTCTGG
+
FFFFFFFFFFFFFFFFFFFFFFFFFFFFFFFFFFFFFFFFFFFFFFFFFFFFFFFFFFFFFFFFFFFFFF
FFFFFFFFFFFFFFFFFFFFFFFFFFFFFFFFFFFFFFFFFFFFFFFFFFFFFFFFFFFFFFFFFFFFFF
FFFFFFFFFFFFFF:
@A00721:190:HGJW7DSXY:4:1216:18973:11115 2:N:0:TCTGCAAG+GCTGCACT
TAACATTCTGGTGTGACGAATTCAGAACTTTATTGTTCTACAATAACGAAAGCGAACATGAATCGTCG
AATACATAACATTCCCAGCAGCGCATATCGTCGACAATAGACAGAAGTGAAGATCGATAGATCCTCAT
CGGAATTCATTGCCG
+
FFFFFFFFFFFFFFFFFFFFFFFFFFFFFFFFFFFFFFFFFFFFFFFFFFFFFFFFFFFFFFFFFFFFFF
FFFFFFFFFFFFFFFFFFFFFFFFFFFFFFFFFFFFFFFFFFFFFFFFFFFFFFFFFFFFFFFFFFFFFF
FFFFFFFFFFFFFFF
@A00721:190:HGJW7DSXY:4:1219:25473:32863 2:N:0:TCTGCAAG+GCTGCACT
TAACATTCTGGTGTGACGAATTCAGAACTTTATTGTTCTACAATAACGAAAGCGAACATGAATCGTCG
AATACATAACATTCCCAGCAGCGCATATCGTCGACAATAGACAGAAGTGAAGATCGATAGATCCTCAT
CGGAATTCATTGCCG
+
FFFFFFFFFFFFFFFFFFFFFFFFFFFFFFFFFFFF,FFFFFFFFFFFFFFFFFFFFFFFFFFFFFFFFF
FFFFFFFFFFFFFFFFFFFFFFFFFFFFFFFFFFFFFFFFFFFFFFFFFFFFFFFFFFFFFFFFFFFFFF
FFFFFFFFFFFFFFF
@A00721:190:HGJW7DSXY:4:1445:19416:1861 2:N:0:TCTGCAAG+GCTGCACT
TAACATTCTGGTGTGACGAATTCAGAACTTTATTGTTCTACAATAACGAAAGCGAACATGAATCGTCG
AATACATAACATTCCCAGCAGCGCATATCGTCGACAATAGACAGAAGTGAAGATCGATAGATCCTCAT
CGGAATTCATTGCCG
+
FFFFFFFFFFF,FFFFFFFF,FFFFFFFFFFFFFFFFFFFFF:FFFFF,FFFFFFFFFFF:FFFFFF:F

```
FFFFF:FFFFFFFFFFFFFFFFFFFFFFFF:FFFFFFFFFFFFF:FF,FFFFFFFFFFFFFFF,FFFF,
FF:FFFFFF:F,:FF
@A00721:190:HGJW7DSXY:4:2326:3477:12148 2:N:0:TCTGCAAG+GCTGCACT
TAACATTCTGGTGTGACGAATTCAGAACTTTATTGTTCTACAATAACGAAAGCGAACATGAATCGTCG
AATACATAACATTCCCAGCAGCGCATATCGTCGACAATAGACAGAAGTGAAGATCGATAGATCCTCAT
CGGAATTCATTGCC
+
FFFFFFFFFFFFFFFFFFFFFFFFFFFFFFFFFFFFFFFFFFFFFFFFFFFFFFFFFFFFFFFFFFFF
FFFFFFFFF:FFFFFFFFFFFFFFFFFFFFFFFFFFFFFFFFFFFFFF:FFF:FFF:FFFFFFF:F:FF
:FFFFF::FF::FF
@A00721:190:HGJW7DSXY:4:1614:31729:16799 2:N:0:TCTGCAAG+GCTGCACT
GTTCTAATAACATTCTGGTGTGACGAATTCAGAACTTTATTGTTCTACAATAACGAAAGCGAACATGA
ATCGTCGAATACATAACATTCCCAGCAGCGCATATCGTCGACAATAGACAGAAGTGAAGATCGATAGA
TCCTCATCGGAATTC
+
FFFFFFFFFFFF:FFFFFFFFFFFFFFFFFFFFFFFFFFFFFFFFFFFFFFFFFFFFFFFF:FFFFFF
FFFFFFFFFFFFFFFFFFFFFFFFFFFFFFFFFFFFFFFFFFFFFFFFFFF:FFFFFFFFFFFFFFFF
FFFFFFFF:FFFFFF
@A00721:190:HGJW7DSXY:4:2506:18710:10723 2:N:0:TCTGCAAG+GCTGCACT
GTTCTAATAACATTCTGGTGTGACGAATTCAGAACTTTATTGTTCTACAATAACGAAAGCGAACATGA
ATCGTCGAATACATAACATTCCCAGCAGCGCATATCGTCGACAATAGACAGAAGTGAAGATCGATAGA
TCCTCATCGGAATTC
+
FFFFFFFFFFFFFFFFFFFFFFFFFFFFFFFFFFFFFFFFFFFFFFFFFFFFFFFFFFFFFFFFFFFF
FFFFFFFFFFFFFFFFFFFFFFFFFFFFFFFFFFFFFFFFFFFFFFFFFFFFFFFFFFFFFFFFFFFF
FFFFFFFFFFFFFFF
@A00721:190:HGJW7DSXY:4:2513:26359:28354 2:N:0:TCTGCAAG+GCTGCACT
GTTCTAATAACATTCTGGTGTGACGAATTCAGAACTTTATTGTTCTACAATAACGAAAGCGAACATGA
ATCGTCGAATACATAACATTCCCAGCAGCGCATATCGTCGACAATAGACAGAAGTGAAGATCGATAGA
TCCTCATCGGAATTC
+
FFFFFFFFFFFFFFFFFFFFFFFFFFFFFFFFFFFFFFFFFFFFFFFFFFFFFFFFFFFFFFFFFFFF
FFFFFFFFFFFFFFFFFFFFFFFFFFFFFFFFFFFFFFFFFFFFFF:FFFFFFFFFFFFFFFFFFFFF
FFFFFFFFFFFFFFF
@A00721:190:HGJW7DSXY:4:1155:16947:24173 2:N:0:TCTGCAAG+GCTGCACT
CAGAATGACGTCAGAGGTTCTAATAACATTCTGGTGTGACGAATTCAGAACTTTATTGTTCTACAATA
ACGAAAGCGAACATGAATCGTCGAATACATAACATTCCCAGCAGCGCATATCGTCGACAATAGACAGA
AGTGAAGATCGATAG
+
FFFFFFFFFFFFFFFFFFFFFFFFFFFFFFFFFFFFFFFFFFFFFFFFFFFFFFFFFF:FFFFFFFF:
FFFFFFFFFFFFFFFFFFFFFFFFFFFFFFFFFFFFFFFFFFFFF:FF:F:FFFFFFFFFFFFFFFFF
FFFFFFFFFF:FFF:
@A00721:190:HGJW7DSXY:4:1157:26476:18944 2:N:0:TCTGCAAG+GCTGCACT
CAGAATGACGTCAGAGGTTCTAATAACATTCTGGTGTGACGAATTCAGAACTTTATTGTTCTACAATA
ACGAAAGCGAACATGAATCGTCGAATACATAACATTCCCAGCAGCGCATATCGTCGACAATAGACAGA
AGTGAAGATCGATAG
+
FFFFFFFFFFFFFFFFFFFFFFFFFFFFFFFFFFFFFFFFFFFFFFFFFFFFFFFFFFFFFFFFFFFF
FFFFFFFFFFFFFFFFFFFFFFFF:FFFFFFFFFFFFFFFFFFFFFFFFFFFFFFFFFFFFFFFFFFF
FFFFFFFFFFFFFF:
@A00721:190:HGJW7DSXY:4:1157:26883:19648 2:N:0:TCTGCAAG+GCTGCACT
CAGAATGACGTCAGAGGTTCTAATAACATTCTGGTGTGACGAATTCAGAACTTTATTGTTCTACAATA
ACGAAAGCGAACATGAATCGTCGAATACATAACATTCCCAGCAGCGCATATCGTCGACAATAGACAGA
AGTGAAGATCGATAG
```

```
+
FFFFFFFFFFFFFFFFFFFFFFFFFFFFFFFFF:FFFFFFFFFFFFFFFFFFFFFFFFFFFFFFFFFF
FFFFFFF:FFFFFFFFFFF:FFFFFFFFFFFFFFFFFFFFFFFFF::FFFFFFFF:FF:FFF,FFFFF
FFFFFF:FFFFFFFF
@A00721:190:HGJW7DSXY:4:1165:26711:8891 2:N:0:TCTGCAAG+GCTGCACT
CAGACTGACGTCAGAGGTTCTAATAACATTCTGGTGTGACGAATTCAGAACTTTATTGTTCTACAATA
ACGAAAGCGAACATGAATCGTCGAATACATAACATTCCCAGCAGCGCATATCGTCGACAATAGACAGA
AGTGAAGATCGATAG
+
FFFF,FFFFFFFFFFFF:FFFFFFFFFFFFF:FFFFFFFFFFFFFFFFFFFFFFFFFFFFFFFFFFFF
FFFFFFFFFFFFFFFFFFFFFFFF:FFFFFFFFFFFFFFFFF,FFFFFFFFFFFFFFFFFFFFFFFFF
FFFFFFFFFFFFFFF
@A00721:190:HGJW7DSXY:4:1205:13141:30608 2:N:0:TCTGCAAG+GCTGCACT
CAGAATGACGTCAGAGGTTCTAATAACATTCTGGTGTGACGAATTCAGAACTTTATTGTTCTACAATA
ACGAAAGCGAACATGAATCGTCGAATACATAACATTCCCAGCAGCGCATATCGTCGACAATAGACAGA
AGTGAAGATCGATAG
+
FFFFFFFFFFFFFFFFFFFFFFFFFFFFFFFFFFFFFFFFFF:FFFFFFFFFFFFFFFFFFFFFFFFF
FFFFFFFFFFFFFFFFFFFFFFFFFFFFFFFFFFFFFFFFFFFFFFF:FF:FFFFF:FF:FFFFF:FF
FFFFFFFFFFFFFFF
@A00721:190:HGJW7DSXY:4:1325:31458:9752 2:N:0:TCTGCAAG+GCTGCACT
CAGAATGACGTCAGAGGTTCTAATAACATTCTGGTGTGACGAATTCAGAACTTTATTGTTCTACAATA
ACGAAAGCGAACATGAATCGTCGAATACATAACATTCCCAGCAGCGCATATCGTCGACAATAGACAGA
AGTGAAGATCGATAG
+
FF:FFFFFFFFFFFFFFFFFFFFFFFFFFFFFFFFFFFFFFFFFFFFFFFFFFFFF:FFFFFFFFFF
FFFFFFFFFFFFFFFFFFFFFFFFFFFFFFFFFFFFFFFFFF:FFFFF:FFFFFFFFFFFFFFFFFFF
FFFFFFF:FFFFFFF
@A00721:190:HGJW7DSXY:4:1360:29152:3255 2:N:0:TCTGCAAG+GCTGCACT
CAGAATGACGTCAGAGGTTCTAATAACATTCTGGTGTGACGAATTCAGAACTTTATTGTTCTACAATA
ACGAAAGCGAACATGAATCGTCGAATACATAACATTCCCAGCAGCGCATATCGTCGACAATAGACAGA
AGTGAAGATCGATAG
+
FFFFFFFFFFFFFF:FFFFFFFFFFFFFFFFFFFFFFFFFFFFFFFFFFFFFFFFFFFFFFFFFFFFF
FFFFFFFFFFFFFFFFFFFFFFFFFFFFFFFFFFFFFFFFFF:FFFFFFFFFF:FFFFFFFFFFFFFF
,FFFFFFFFF,FFFF
@A00721:190:HGJW7DSXY:4:1557:13304:5556 2:N:0:TCTGCAAG+GCTGCACT
CAGAATGACGTCAGAGGTTCTAATAACATTCTGGTGTGACGAATTCAGAACTTTATTGTTCTACAATA
ACGAAAGCGAACATGAATCGTCGAATACATAACATTCCCAGCAGCGCATATCGTCGACAATAGACAGA
AGTGAAGATCGATAG
+
FFFF:FFFFFFFFFFFFFFFFFFFFFFFFFFFFFFFFFFFFFFFFFFFFFFFFFFFFF:FFFFFFFFF
FFFFFF:FFFFFFFFFFFFFFFFFFFFFFFFFFFFFFFFFFFFFFFFFFFFFFFFFFFFFFFFFFFFF
F:FFFFFFFFFFFFF
@A00721:190:HGJW7DSXY:4:2232:17861:15139 2:N:0:TCTGCAAG+GCTGCACT
CAGAATGACGTCAGAGGTTCTAATAACATTCTGGTGTGACGAATTCAGAACTTTATTGTTCTACAATA
ACGAAAGCGAACATGAATCGTCGAATACATAACATTCCCAGCAGCGCATATCGTCGACAATAGACAGA
AGTGAAGATCGATAG
+
FFFFFFFFFFFFFFFFFFFFFFFFFFFFFFFFFFFFFFFFFFFFFFFFFFFFFFFFFFFFFFFFFFFF
FFFFFFFFFFFFFFFFFFFFFFFFFFFFFFFFFFFFFFFFFFFFFFFFFFFFFFFFFFFFFFFFFFFF
F:FFF,FFFFFFFFF
@A00721:190:HGJW7DSXY:4:2306:13431:19179 2:N:0:TCTGCAAG+GCTGCACT
CAGAATGACGTCAGAGGTTCTAATAACATTCTGGTGTGACGAATTCAGAACTTTATTGTTCTACAATA
```

ACGAAAGCTAAAATGAATCGTCGAATACATAACATTCCCAGCAGCGCATATCGTCGACAATAGACAGA
AGTGAAGATCTATAG
+
FFFFFFFF,FFFF,:FF:FFFFFFFFFFFFFFF,FFF:FFFFFFF:F:FF:FFFFFFFFF,FFFFFFF
FF,FFFFF,FF,FFFFFF,FFF:FFFFFFFFF,FFFF,:FF:FF,FFFFFF,,FF,F,FFFFFFFFF:
FFF:FFFFF,,FFF:
@A00721:190:HGJW7DSXY:4:2369:11369:4805 2:N:0:TCTGCAAG+GCTGCACT
CAGAATGACGTCAGAGGTTCTAATAACATTCTGGTGTGACGAATTCAGAACTTTATTGTTCTACAATA
ACGAAAGCGAACATGAATCGTCGAATACATAACATTCCCAGCAGCGCATATCGTCGACAATAGACAGA
AGTGAAGATCGATAG
+
FFFF:FFFFFFFFFFFFFFFFFFFFFFFFFFFFFFFFFFFFFFFFFFFFFFFFFFFFFFFFFFFFFFF
FFFFFFFFFFFFFFFFFFFFFFFFFFFFFFFFFFFFFFFFFFFFFFFFFFFFFFFFFFFFFFFFFFFF
FFFFFFFFFFFFFF:
@A00721:190:HGJW7DSXY:4:2469:12328:6339 2:N:0:TCTGCAAG+GCTGCACT
CAGAATGACGTCAGAGGTTCTAATAACATTCTGGTGTGACGAATTCAGAACTTTATTGTTCTACAATA
ACGAAAGCGAACATGAATCGTCGAATACATAACATTCCCAGCAGCGCATATCGTCGACAATAGACAGA
AGTGAAGATCGATAG
+
FFFF:FFFFFFFFFFFFFFFFFFFFFFFFFFFFFFFFFFFFFFFFFFFFFFFFFFFFFFFFFFFFFFF
FFFFFFFFFFFFFFFFFFFFFFFFFFFFFFFFFFFFFFFFFF:FFFFFFFFFFFFFFFFFFFFFF,FF
FFFFF:FFFFFFFFF
@A00721:190:HGJW7DSXY:4:2666:20157:22874 2:N:0:TCTGCAAG+GCTGCACT
CAGAATGACGTCAGAGGTTCTAATAACATTCTGGTGTGACGAATTCAGAACTTTATTGTTCTACAATA
ACGAAAGCGAACATGAATCGTCGAATACATAACATTCCCAGCAGCGCATATCGTCGACAATAGACAGA
AGTGAAGATCGATAG
+
FFFFFFFFFFFFFFFFFFFFFFFFFFFFFFFFFFFFFFFFFFFFFFFFFFFFFFFFFFFFFFFFFFFF
FFFFFFFFFFFFFFFFFFFFFFFFFFFFFFFFFFFFFFFFFFFFFFFFFFFFFFFFFFFFFFFFFFFF
FFFFFFFFFFFFFFF
@A00721:190:HGJW7DSXY:4:1123:27100:26005 2:N:0:TCTGCAAG+GCTGCACT
CAGAATGACGTCAGAGGTTCTAATAACATTCTGGTGTGACGAATTCAGAACTTTATTGTTCTACAATA
ACGAAAGCGAACATGAATCGTAGAATACATAACATTCCCAGCAGCGCATATCGTCGACAATAGACAGA
AGTGAAGATCTATA
+
:F:F:F,FF,:,F:FFFFFF:FFFFF:FF,:,FFF,F:FFFFFFF,FFF,FF::FFFFFF:FFFFFFF
F,:F:FFFFFF,FF:FFFFF,,FFFFFFFFFFFFFF,:FFFFFFFFFFFFF,FFF,FFFFFF,FFF,F
FFFFFF::,F:FFF
@A00721:190:HGJW7DSXY:4:2344:16215:28009 2:N:0:TCTGCAAG+GCTGCACT
CAGAATGACGTCAGAGGTTCTAATAACATTCTGGTGTGACGAATTCAGAACTTTATTGTTCTACAATA
ACGAAAGCGAACATGAATCGTCGAATACATAACATTCCCAGCAGCGCATATCGTCGACAATAGACAGA
AGTGAAGATCGATA
+
FFFF,FFFFFFFFFFFFF:FFFFFFFFFFFFFFFFFFFFFFFFFFFFFFFFFFFFFFFFFF:FFFFFF
FFFFFFFFFFFFFFFFFFFFFFFFFFFFFFFFFFFF:FFFFFFFFFFFFFFFFFFFFFFFFFFFFFFF
FFFFFFFFFFFFFF
@A00721:190:HGJW7DSXY:4:1119:20817:2660 2:N:0:TCTGCAAG+GCTGCACT
ATGATATGAGACAATTGATGCGGACCACCAGAATGACGTCAGAGGTTCTAATAACATTCTGGTGTGAC
GAATTCAGAACTTTATTGTTCTACAATAACGAAAGCGAACATGAATCGTCGAATACATAACATTCCCA
GCAGCAAGATCGGAA
+
FFFFFFFFFFFFFFFFFFFFFFFFFFFFFFFFFFFFFFFFFFFFFFFFFFFFFFFFFFFFFFFFFFFF
FFFFFFFFFFFFFFFFFFFFFFFFFFFFF,FFFFFFFFFFFFFFFFF:FFFFFFFFFFFFFFFFFFFF
FFFFFFFFFFFFF:F

@A00721:190:HGJW7DSXY:4:1173:22318:20259 2:N:0:TCTGCAAG+GCTGCACT
ATGATATGAGACAATTGATGCGGACCACCAGAATGACGTCAGAGGTTCTAATAACATTCTGGTGTGAC
GAATTCAGAACTTTATTGTTCTACAATAACGAAAGCGAACATGAATCGTCGAATACATAACATTCCCA
GCAGCAAGATCGGAA
+
FFFFFFFFFFFFFFFFFFFFFFFFFFFFFFFFFFFFFFFFFFFFFFFFFFFFFFFFFFFFFFFFFFFF
FFFFFFFFFFFFFFFFFFFFFFFFFFFFFFFFFFFFFFFFFFFFFFFFFFFFFFFF::FFFFFFFFFF
FFFFFFFFFFFFFFF
@A00721:190:HGJW7DSXY:4:1415:4689:35477 2:N:0:TCTGCAAG+GCTGCACT
ATGATATGAGACAATTGATGCGGACCACCAGAATGACGTCAGAGGTTCTAATAACATTCTGGTGTGAC
GAATTCAGAACTTTATTGTTCTACAATAACGAAAGCGAACATGAATCGTCGAATACATAACATTCCCA
GCAGCAAGATCGGAA
+
FFFFF,F:FFFFFF:FFFFFF:FFFFFF:FFFFFFFFFFFFFFFFFFFFFFFFFFFF:FF,FFFFFFF
,FF,FFFFFFFF:FFFFFFF:FF,FF:FFFFFFFFFFFFFF::FF:F:,FFFFFFFFFFF,:F:FFFF
FFF,FFFFF,FFFFF
@A00721:190:HGJW7DSXY:4:1445:21395:27618 2:N:0:TCTGCAAG+GCTGCACT
ATGATATGAGACAATTGATGCGGACCACCAGAATGACGTCAGAGGTTCTAATAACATTCTGGTGTGAC
GAATTCAGAACTTTATTGTTCTACAATAACGAAAGCGAACATGAATCGTCGAATACATAACATTCCCA
GCAGCAAGATCGGAA
+
FFFFFFFFFFFFFFFFFFFFFFFFFFFFFFFFFFFFFFFFFFFFFFFFFFFFFFFFFFF:FFFFFFFF
FFFFFFFFFFFFFFFFFFFFFFFFFFFFFF:FFFFFFFFFFFFFFFFFFFFFFFFFFFFFFFFFFFFF
FFFFFF:FFFFFFFF
@A00721:190:HGJW7DSXY:4:1503:19605:5885 2:N:0:TCTGCAAG+GCTGCACT
ATGATATGAGACAATTGATGCGGACCACCAGAATGACGTCAGAGGTTCTAATAACATTCTGGTGTGAC
GAATTCAGAACTTTATTGTTCTACAATAACGAAAGCGAACATGAATCGTCGAATACATAACATTCCCA
GCAGCAAGATCGGAA
+
FFFFFFFFFFFFFFFFFFFFFFFFFFFFFFFFFFFFFFFFFFFFFFFFFFFFFFFFFFFFFFFFFFFF
FFFFFFFFFFFFFFFFFFFFFFFFFFFFFFFFFFFFFFFFFFFFFFFFFFFFFFFFFFFFFFFFFFFF
FFFFFFFFFFF:FF:
@A00721:190:HGJW7DSXY:4:1649:6831:35117 2:N:0:TCTGCAAG+GCTGCACT
ATGATATGAGACAATTGATGCGGACCACCAGAATGACGTCAGAGGTTCTAATAACATTCTGGTGTGAC
GAATTCAGAACTTTATTGTTCTACAATAACGAAAGCGAACATGAATCGTCGAATACATAACATTCCCA
GCAGCAAGATCGGAA
+
FFFFFFFFFFFFFFFFFFFFFFFFFFFFFFFFFFFFFFFFFFFFFFFFFFFFFFFFFFFFFFFFFFFF
FFFFFFFFFFFFFFFFFFFFFFFFFFFFFFFFFFFFFFFFFFFFFFFFFFFFFFFFFFFFFFFFFFFF
FFFFFFFFFFFFFFF
@A00721:190:HGJW7DSXY:4:2137:8558:30373 2:N:0:TCTGCAAG+GCTGCACT
ATGATATGAGACAATTGATGCGGACCACCAGAATGACGTCAGAGGTTCTAATAACATTCTGGTGTGAC
GAATTCAGAACTTTATTGTTCTACAATAACGAAAGCGAACATGAATCGTCGAATACATAACATTCCCA
GCAGCAAGATCGGAA
+
FFFFFFFFFFFFFFFFFFFFFFFFFFFFFFFF:FFFFFFFFFFFFFFFFFFFFFFFFFFFFFFFFFFF
FF:FFFFFFFFFFFFFFFF:F:FFFFFFFFFFFFFFFFFFFFFFFFFFFFFFFFFFFFFFFFFFFFFF
FFFFF:,FFFFFFFF
@A00721:190:HGJW7DSXY:4:2223:22544:20118 2:N:0:TCTGCAAG+GCTGCACT
ATGATATGAGACAATTGATGCGGACCACCAGAATGACGTCAGAGGTTCTAATAACATTCTGGTGTGAC
GAATTCAGAACTTTATTGTTCTACAATAACGAAAGCGAACATGAATCGTCGAATACATAACATTCCCA
GCAGCAAGATCGGAA
+
FFFFFFFFFFFFFFFFFFFFFFFFFFFFFFFFFFFFFFFFFFFFFFFFFFFFFFFFFFFFFFFFFFFF

```
FFFFFFFFFFFFFFFFFFFFFFFFFFFFFFFFFFFFFFFFFFFFFFFFFFFFFFFFFFFFFFFFFFFF
FFFFFFFFFFFFFFF
@A00721:190:HGJW7DSXY:4:2269:3341:34491 2:N:0:TCTGCAAG+GCTGCACT
ATGATATGAGACAATTGATGCGGACCACCAGAATGACGTCAGAGGTTCTAATAACATTCTGGTGTGAC
GAATTCAGAACTTTATTGTTCTACAATAACGAAAGCGAACATGAATCGTCGAATACATAACATTCCCA
GCAGCAAGATCGGAA
+
FFFFFFFFFFFFFFFFFFFFFFFFFFFFFFFFFFFFFFFFFFFFFFFFFFFFFFFFFFFFFFFFFFFF
FFF:FFFFFFFFFFFFFFFFFFFFFFFFFFFFFFFFFFFFFFFFFFFFFFFFFFFFFFFFFFFFFFFF
FFFFF:FFFFFFFF:
@A00721:190:HGJW7DSXY:4:2378:10059:3975 2:N:0:TCTGCAAG+GCTGCACT
ATGATATGAGACAATTGATGCGGACCACCAGAATGACGTCAGAGGTTCTAATAACATTCTGGTGTGAC
GAATTCAGAACTTTATTGTTCTACAATAACGAAAGCGAACATGAATCGTCGAATACATAACATTCCCA
GCAGCAAGATCGGAA
+
FFFFFFFFFFFFFFFFFFFFFFFFFFFFFFFFFFFFFFFFFFFFFFFFFFFFFFFFFFFFFFFFFFFF
FFFFFFFFFFFFFFFFFFFFFFFFFFFFFFFFFFFFF:FFFFFFFFFFFFFFFFFFFFFFFFFFFFFF
FFFFFFFFFFFFFFF
@A00721:190:HGJW7DSXY:4:2378:9968:7482 2:N:0:TCTGCAAG+GCTGCACT
ATGATATGAGACAATTGATGCGGACCACCAGAATGACGTCAGAGGTTCTAATAACATTCTGGTGTGAC
GAATTCAGAACTTTATTGTTCTACAATAACGAAAGCGAACATGAATCGTCGAATACATAACATTCCCA
GCAGCAAGATCGGAA
+
FFFFFFFFFFFFF::FFFFFFFFFFFFFFFFFFFFFFFFFFF:FFFFFFFFFFFFF,FFFFFFFFFFF
:FFFFFFFFFFFFFFFFFFFFFFFFFFF:FFFFFFFFFFFFFFFFFFFFFFFFFFFFFFFFFFFFFFF
FFFFFFFFFFFFFFF
@A00721:190:HGJW7DSXY:4:2563:19416:34585 2:N:0:TCTGCAAG+GCTGCACT
ATGATATGAGACAATTGATGCGGACCACCAGAATGACGTCAGAGGTTCTAATAACATTCTGGTGTGAC
GAATTCAGAACTTTATTGTTCTACAATAACGAAAGCGAACATGAATCGTCGAATACATAACATTCCCA
GCAGCAAGATCGGAA
+
FFFFFFFFFFFFF:FFFFFFFFFFFFFFFFFFFFFFFFFFFFFFFFFFFFFFFFFFFFFFFFFFFFFF
FFFFFFFFFFFFFFFFFFFFFFFFFFFFFFFFFFFFFFFFFFFFFFFFFF:FFFFFFFFFFFFFFFFF
FFFFFFFFFFFFF:F
@A00721:190:HGJW7DSXY:4:2618:10140:14011 2:N:0:TCTGCAAG+GCTGCACT
ATGATATGAGACAATTGATGCGGACCACCAGAATGACGTCAGAGGTTCTAATAACATTCTGGTGTGAC
GAATTCAGAACTTTATTGTTCTACAATAACGAAAGCGAACATGAATCGTCGAATACATAACATTCCCA
GCAGCAAGATCGGAA
+
FFFFFFFFFFFFF:FFFFFFFFFFFFFFFFFFFFFFFFFFFFFFFFFFFFFFFFFFFFFFFFFFFFFF
FFFFFFFFFFFFFFFFFFFFFFFFFFFFFFFFFFFFFFFFFFFFFFFFFFFFFFFFFFFFFFFFF:FF
FFFFFF:FFFFFFFF
@A00721:190:HGJW7DSXY:4:2133:32832:11443 2:N:0:TCTGCAAG+GCTGCACT
ATGATATGAGACAATTGATGCGGACCACCAGAATGACGTCAGAGGTTCTAATAACATTCTGGTGTGAC
GAATTCAGAACTTTATTGTTCTACAATAACGAAAGCGAACATGAATCGTCGAATACATAACATTCCCA
GCAGCAAGATCGGA
+
FFFFFFFFFFFFFFFFFFFFFFFFFFFFFFFFFFFFFFFFFFFFFFFFFFFFFFFFFFFFFFFFFFFF
FFFFFFFFFFFFFFFFFFFFFFFFFFFFFFFFFFFF:FFFFFFFFFF:FFF,FFFFFFFFFFFFFF:F
FFFFFFFF:FFF:F
@A00721:190:HGJW7DSXY:4:1345:10438:16470 2:N:0:TCTGCAAG+GCTGCACT
TCATTAACTAAATGATATGAGACAATTGATGCGGACCACCAGAATGACGTCAGAGGTTCTAATAACAT
TCTGGTGTGACGAATTCAGAACTTTATTGTTCTACAATAACGAAAGCGAACATGAATCGTCGAATACA
TAACATTCCCAGCAG
```

```
+
FFFFFFFFFFFFFFFFFFFFFFFFFFFFFFFFFFFFFFFFFFFFFFFFFFFFFFFFFFFFFFFFFFFFFFFFFFFFFFFFF
FFFFFFFFFFFFFFFFFFFFFFFFFFFFFFFFFFFFFFFFFFFFFFFFFFFFFFFFFFFFFFFFFFFFFFFFFFFFFFFFF
FFFFFFFFFF,FF:F
@A00721:190:HGJW7DSXY:4:1569:10854:33442 2:N:0:TCTGCAAG+GCTGCACT
TCATTAACTAAATGATATGAGACAATTGATGCGGACCACCAGAATGACGTCAGAGGTTCTAATAACAT
TCTGGTGTGACGAATTCAGAACTTTATTGTTCTACAATAACGAAAGCGAACATGAATCGTCGAATACA
TAACATTCCCAGCAG
+
FFFFFFFFFFFFFFFFFFFFFFFFFFFFFFFF:FFFFFFFFFFFFFFFFFFFFFFFFFFFFFFFFFFF
FFFFFFFFFFFFFFFFFFFFFFFFFFFFFFFFFFFFFFFFFFFFFFFFFFFFFFFFFFFFFFFFFFFF
:FFFFFFFFFFFFFF
@A00721:190:HGJW7DSXY:4:2158:13991:36808 2:N:0:TCTGCAAG+GCTGCACT
TCATTAACTAAATGATATGAGACAATTGATGCGGACCACCAGAATGACGTCAGAGGTTCTAATAACAT
TCTGGTGTGACGAATTCAGAACTTTATTGTTCTACAATAACGAAAGCGAACATGAATCGTCGAATACA
TAACATTCCCAGCAG
+
FFFFFFFFFFFFFFFFFFFFFFFFFFFFFFFFFFFFFFFFFFFFFFFFFFFFF:FFFFFFFFFFFFFF
FFFFFFFFFFFFFFFFFFFFFFFFFFFFFFFFFFF:FFFFFFFFFF:FFFFFFFFFFFFFFFFFFFFF
FFFFFFFFFFFFFFF
@A00721:190:HGJW7DSXY:4:2369:26304:18114 2:N:0:TCTGCAAG+GCTGCACT
TCATTAACTAAATGATATGAGACAATTGATGCGGACCACCAGAATGACGTCAGAGGTTCTAATAACAT
TCTGGTGTGACGAATTCAGAACTTTATTGTTCTACAATAACGAAAGCGAACATGAATCGTCGAATACA
TAACATTCCCAGCAG
+
FFFFFFFFFFFFFFFFFFFFFFFFFFFFFFFFFFFFFFFFFFFF,FFFF:FFFFFFFFFFFFFFFFFFF
FFFFFFFFFFFFFFFFFFFFFFFFFFFFFFFFFFFFFFFFFFF:FFFFFFFFFFFFFFFFFFFFFFFF
F:FFFFFFFFFFFFF
@A00721:190:HGJW7DSXY:4:2578:15673:28635 2:N:0:TCTGCAAG+GCTGCACT
TCATTAACTAAATGATATGAGACAATTGATGCGGACCACCAGAATGACGTCAGAGGTTCTAATAACAT
TCTGGTGTGACGAATTCAGAACTTTATTGTTCTACAATAACGAAAGCGAACATGAATCGTCGAATACA
TAACATTCCCAGCAG
+
FFF,FFFFF:FFFFFFFFFFFFFF:FFFFFFFFFFFFFFFFFFFFFFFFFFFFFFFFFFFFFFFFFFF
FFFFFFFFFFFFF:FFFFFFFFFFFFFFFFFFFFFFFFFFFF:FFFFFFFFFFFFFFFF:FFFF:FFFF
FFFFFFFFFFFFFFF
@A00721:190:HGJW7DSXY:4:2661:24758:27993 2:N:0:TCTGCAAG+GCTGCACT
TCATTAACTAAATGATATGAGACAATTGATGCGGACCACCAGAATGACGTCAGAGGTTCTAATAACAT
TCTGGTGTGACGAATTCAGAACTTTATTGTTCTACAATAACGAAAGCGAACATGAATCGTCGAATACA
TAACATTCCCAGCAG
+
FFFFFFFFFFF::FFFFFFF,FFFFFFFFFFFFFFFFFFFFFFFFFFFFFFFFFFFFFFFFF,FFFFFF
FFFFFFFFFFFFFFFFFFFFFFFFFFFFFFFFFFFFFFFF:F,FFFF:FFFFFFFFFFFFFF,F::F:,
FFFFFFFF,FF:FFF
@A00721:190:HGJW7DSXY:4:1220:3504:22780 2:N:0:TCTGCAAG+GCTGCACT
TCCTCATTAACTAAATGATATGAGACAATTGATGCGGACCACCAGAATGACGTCAGAGGTTCTAATAA
CATTCTGGTGTGACGAATTCAGAACTTTATTGTTCTACAATAACGAAAGCGAACATGAATCGTCGAAT
ACATAACATTCCCAG
+
FFFFFFFFFFFFFFFFFF:FFFFFFFFFFFFFFFFFFFFFFFFFFFFFFFFFFFFFFFFFFFFFFFFFF
FFFFFFFFFFFFFFFFFFFFFFFFFFFFFF::FFFFFFFFFFFFFFFFFFFF:FFFFFFFFFFFFFFF:
FFFFFFFFFFFFFFF
@A00721:190:HGJW7DSXY:4:1238:4056:32878 2:N:0:TCTGCAAG+GCTGCACT
TCCTCATTAACTAAATGATATGAGACAATTGATGCGGACCACCAGAATGACGTCAGAGGTTCTAATAA
```

CATTCTGGTGTGACGAATTCAGAACTTTATTGTTCTACAATAACGAAAGCGAACATGAATCGTCGAAT
ACATAACATTCCCAG
+
FFFFFFFFFFFFFFFFFFFFFFFFFFFFFFFFFFFFFFFFFFFFFF,FFFFFFFFFFFFFFFFFFFFF
FFFFFFFFFFFFFFFFFF,FFFFFFFFFFFFFFFFFFFFFFFFFFFFFFFFFFFFFFFFFFFFFFFFF
FFFFFFFFFFFFFFF
@A00721:190:HGJW7DSXY:4:1365:6316:2221 2:N:0:TCTGCAAG+GCTGCACT
TCCTCATTAACTAAATGATATGAGACAATTGATGCGGACCACCAGAATGACGTCAGAGGTTCTAATAA
CATTCTGGTGTGACGAATTCAGAACTTTATTGTTCTACAATAACGAAAGCGAACATGAATCGTCGAAT
ACATAACATTCCCAG
+
FFFFFFFFFFFFFFFFFFFFFFFFFFFFFFFFFFFFFFFFFFFF:FFFFFFFFFFFFFFFFFFFFFFF
FFFFFFFFFFFFFFFFFFFFFFFFFFFFFFFFFFFFFFFFFFFFFFFFFFFFFFFFFFFFFFFFFFFF
FFFFFFFFFFFFFFF
@A00721:190:HGJW7DSXY:4:1453:18683:12023 2:N:0:TCTGCAAG+GCTGCACT
TCCTCATTAACTAAATGATATGAGACAATTGATGCGGACCACCAGAATGACGTCAGAGGTTCTAATAA
CATTCTGGTGTGACGAATTCAGAACTTTATTGTTCTACAATAACGAAAGCGAACATGAATCGTCGAAT
ACATAACATTCCCAG
+
FFFFFFFFFFFFFFFFFFFFFFFFFFFFFFFFFFFFFFFFFFFFFFFFFFFFFFFFFFFFFFFFFFFF
FFFFFFFFFFFFFFFFFFFFFFFFFFFFFFFF,FFFFFFFFFFFFFFFFFFFFFFFFFFFFFFFFFFF
FFFFFFFFFFFFF,F
@A00721:190:HGJW7DSXY:4:1656:29206:27868 2:N:0:TCTGCAAG+GCTGCACT
TCCTCATTAACTAAATGATATGAGACAATTGATGCGGACCACCAGAATGACGTCAGAGGTTCTAATAA
CATTCTGGTGTGACGAATTCAGAACTTTATTGTTCTACAATAACGAAAGCGAACATGAATCGTCGAAT
ACATAACATTCCCAG
+
FFFFFFFFFFFFFFFFFFFFFFFFFFFFFFFFFFFFFFFFFFFFFFFFFFFFFFFFFFFFFFFFFFFF
FFFFFFFFFFFFFF:FFFFFF:FFFFFFFF:FFFFFFFFFFFFFFFFFFFFFFFFFFFFFFFFFFF:F
FFFFFFFFFFFFFFF
@A00721:190:HGJW7DSXY:4:2549:5348:22968 2:N:0:TCTGCAAG+GCTGCACT
TCCTCATTAACTAAATGATATGAGACAATTGATGCGGACCACCAGAATGACGTCAGAGGTTCTAATAA
CATTCTGGTGTGACGAATTCAGAACTTTATTGTTCTACAATAACGAAAGCGAACATGAATCGTCGAAT
ACATAACATTCCCAG
+
FFF:FFFFFFFFFFFFFFFFFFFFFFFFFFFFFFFFFFFFFFFFFFFFFFFFFFFFFFF:FFFFFFFF
FFFFFFF:F:FFFF:FF,FFFFFFFFFFF:FFFFFFFFFFFFFF:FFFFFFFFFFFFFFFFFFFFFF:
FFFFFFFF::FFFFF
@A00721:190:HGJW7DSXY:4:1147:19723:33833 2:N:0:TCTGCAAG+GCTGCACT
ATTATCCTCATTAACTAAATGATATGAGACAATTGATGCGGACCACCAGAATGACGTCAGAGGTTCTA
ATAACATTCTGGTGTGACGAATTCAGAACTTTATTGTTCTACAATAACGAAAGCGAACATGAATCGTC
GAATACATAACATTC
+
FFFFFFFFFFFFFFFFFFFFFFF:FFFFFFFFFFFFFFFFFFFFFFFFFFFFFFFFFFFFFFFFFFFF
FFFFFFFFFFFFFFFFFFFFFFFFFFFFFFFFFFFFFFFFFFFFFFFFFFFFFFFFFFFFFFFFFFFF
FFFFFFFFFFFFFFF
@A00721:190:HGJW7DSXY:4:1449:10257:32064 2:N:0:TCTGCAAG+GCTGCACT
ATTATCCTCATTAACTAAATGATATGAGACAATTGATGCGGACCACCAGAATGACGTCAGAGGTTCTA
ATAACATTCTGGTGTGACGAATTCAGAACTTTATTGTTCTACAATAACGAAAGAGAACATGAATCGTC
GAATACATAACATTC
+
FFF,FFFFFFFFF,FFFFFFFF,FF:FFFF:F:FFFFF,FFFFFFFFFFF:FFFFFFFFFFFFFF::F
FFFFFF:FFFFFF:FF:FFFF:FFFFFFFFF:FFF:FFFFF,FF:FFFFFFFF,FFFFFF:FFFFFFF
:F:FFFFFF:FFF:F

```
@A00721:190:HGJW7DSXY:4:1502:4472:33442 2:N:0:TCTGCAAG+GCTGCACT
ATTATCCTCATTAACTAAATGATATGAGACAATTGATGCGGACCACCAGAATGACGTCAGAGGTTCTA
ATAACATTCTGGTGTGACGAATTCAGAACTTTATTGTTCTACAATAACGAAAGCGAACATGAATCGTC
GAATACATAACATTC
+
FFFFFFFFF:FF:FFFFFFFFFFFFFFFFFFFFFFFFFFFFFFFFFFFFF,FFFFFFFFFFFFFFFFFFF
FFFFFFFFFFFFFFFFFFFFFFFFFFFFFFFFFFFFF,FFFFFFFFFFFFFFFFFFFFFFFFFFFFFFFF
FFFFFFFFFFFFFFF
@A00721:190:HGJW7DSXY:4:1526:4273:16626 2:N:0:TCTGCAAG+GCTGCACT
ATTATCCTCATTAACTAAATGATATGAGACAATTGATGCGGACCACCAGAATGACGTCAGAGGTTCTA
ATAACATTCTGGTGTGACGAATTCAGAACTTTATTGTTCTACAATAACGAAAGCGAACATGAATCGTC
GAATACATAACATTC
+
FFFFFFFFFFFFFFFFFFFFFFFFFFFFFF:FFFFFFFFFFFFFFFFFFFFFFFFFFFFFFFFFF,FF:F
FFFFFFFFFFFFFFFFFFFF:FFFFFFFFFFFFF:FFFF:FFFFFFFFFFFFF:FFF:FFFFFFFFFFFF
FFFFFFFFFFFFFFF
@A00721:190:HGJW7DSXY:4:1604:31033:23735 2:N:0:TCTGCAAG+GCTGCACT
ATTATCCTCATTAACTAAATGATATGAGACAATTGATGCGGACCACCAGAATGACGTCAGAGGTTCTA
ATAACATTCTGGTGTGACGAATTCAGAACTTTATTGTTCTACAATAACGAAAGCGAACATGAATCGTC
GAATACATAACATTC
+
FFFFFFFFFFFFFFFFFFFFFFFFFFFFFFFFFFFFFFFFFFFFFFFFFFFFFFFFFFFFFFFFFFFFFF
:FFFFFFFFFFFFFFFFFFFFFFFFFFFFFFFFFFFFFFFFFFFFFFFFFFFFF:FFFFFFFFFFFFFF
FFFFFFFFFFFFFFF
@A00721:190:HGJW7DSXY:4:2106:16441:30686 2:N:0:TCTGCAAG+GCTGCACT
ATTATCCTCATTAACTAAATGATATGAGACAATTGATGCGGACCACCAGAATGACGTCAGAGGTTCTA
ATAACATTCTGGTGTGACGAATTCAGAACTTTATTGTTCTACAATAACGAAAGCGAACATGAATCGTC
GAATACATAACATTC
+
FFF:FFFFFFFFFFFFFFFFFF,FFFFFFFF:FFFFFFFFFFFFFFFFFF,FFFFFFFFFFFFFFFFFFF
FFFFFFFFF:FFFFFFFFFFFFFFFFF:FF:F:FFFFFFFFFFFFFFFFFFFFFFFFFFFFFFFFFFFFF
:FFFFFFF,FFFFFF
@A00721:190:HGJW7DSXY:4:2453:22137:29872 2:N:0:TCTGCAAG+GCTGCACT
ATTATCCTCATTAACTAAATGATATGAGACAATTGATGCGGACCACCAGAATGACGTCAGAGGTTCTA
ATAACATTCTGGTGTGACGAATTCAGAACTTTATTGTTCTACAATAACGAAAGCGAACATGAATCGTC
GAATACATAACATTC
+
FFFFFFFFFFFFFFFFFFFFFFFFFFFFFFFFFFFFFFFFFFFFFFFFFFFFFFFFFFFFFFFFFFFFFF
FFFFFFFFFFFFFFFFFFFFFFFFFFFFFFFFFFFFFFFFFFFFFFFFFFFFFFFFFFFFFFFFFFFFFF
FFFFFFFFFFFFFFF
@A00721:190:HGJW7DSXY:4:1261:28031:26146 2:N:0:TCTGCAAG+GCTGCACT
ATATTATCCTCATTAACTAAATGATATGAGACAATTGATGCGGACCACCAGAATGACGTCAGAGGTTC
TAATAACATTCTGGTGTGACGAATTCAGAACTTTATTGTTCTACAATAACGAAAGCGAACATGAATCG
TCGAATACATAACAT
+
FFFFFFFFFFFFFFFFFFFFFFFF,:FFFFFFFFFFFFFFFFFFFFFFFFFFF:FFFFFFFFFFFFF:FF
FFFFFFFFFFF,FFFFFFFFF:FFFFFFFFFFFFFFFFFFFFFF,:F:FFFFFFF,FFFF:FF:FFFFFF
FFFFFFFFFFFFFFF
@A00721:190:HGJW7DSXY:4:1367:29776:35775 2:N:0:TCTGCAAG+GCTGCACT
ATATTATCCTCATTAACTAAATGATATGAGACAATTGATGCGGACCACCAGAATGACGTCAGAGGTTC
TAATAACATTCTGGTGTGACGAATTCAGAACTTTATTGTTCTACAATAACGAAAGCGAACATGAATCG
TCGAATACATAACAT
+
FFF:FFFFFFFFFFFFFFFFFFFFFFFFFFFFFF:FFFFFFFFFFFFFFFFFFF,FFFFFFFFFFFFF:F
```

FFFFFFFFFFFFFFFFFFF:FFF:FFFFFFFFFFFFFFF:FFFF:FF,FFFFF:FFFFFF:FFFFFF,
FFFFFFFFFF:FFFF
@A00721:190:HGJW7DSXY:4:1529:30147:9706 2:N:0:TCTGCAAG+GCTGCACT
ATATTATCCTCATTAACTAAATGATATGAGACAATTGATGCGGACCACCAGAATGACGTCAGAGGTTC
TAATAACATTCTGGTGTGACGAATTCAGAACTTTATTGTTCTACAATAACGAAAGCGAACATGAATCG
TCGAATACATAACAT
+
FFFFFFFFFFFFFFFFFFFFFFFF,FFFFFFFFFFFFFFFFFFFFFFFFFFFFFFFFFFFFFFFFFFF
FFFFFFFFFFFFFFFFFFFFFFFFFFFFFFFFFFFFFFFFFFFFFFFF:FFFFFFFFFFFFFFFFFFF
FFFFFFFFFFFFF:F
@A00721:190:HGJW7DSXY:4:1535:31548:6057 2:N:0:TCTGCAAG+GCTGCACT
ATATTATCCTCATTAACTAAATGATATGAGACAATTGATGCGGACCACCAGAATGACGTCAGAGGTTC
TAATAACATTCTGGTGTGACGAATTCAGAACTTTATTGTTCTACAATAACGAAAGCGAACATGAATCG
TCGAATACATAACAT
+
FFFFFFFFFFFFFFFFFFFFFFFFFFFFFFFFFFFFFFFFFFFFFFFFFFFFFFFFFFFFFFFFFFFF
FFFFFFFFFFFFFFFFFFFFFFFFFFFFFFFFFFFFFFFFFFFFFFFFFFFFFFF:FFFFFFFFFFFF
FFFFFFFFFFFFFFF
@A00721:190:HGJW7DSXY:4:2208:24795:7357 2:N:0:TCTGCAAG+GCTGCACT
ATATTATCCTCATTAACTAAATGATATGAGACAATTGATGCGGACCACCAGAATGACGTCAGAGGTTC
TAATAACATTCTGGTGTGACGAATTCAGAACTTTATTGTTCTACAATAACGAAAGCGAACATGAATCG
TCGAATACATAACAT
+
FFFFFFFFFFFFFFFFFFFFFFFFFFFFFFFFFFFFFFFFFF:FFFFFFFFFFFFF:FFFFFFFFFFF
FFFFFFFFFFFFFFFFF:FFFFFFFFFFFFFFFFFFFFFFFFFFFFFFFFFFFFFFF,FFFFFFFFFFF
FFFFFFFFFFFFFFF
@A00721:190:HGJW7DSXY:4:2278:4842:29512 2:N:0:TCTGCAAG+GCTGCACT
ATATTATCCTCATTAACTAAATGATATGAGACAATTGATGCGGACCACCAGAATGACGTCAGAGGTTC
TAATAACATTCTGGTGTGACGAATTCAGAACTTTATTGTTCTACAATAACGAAAGCGAACATGAATCG
TCGAATACATAACAT
+
FFFFFFFFFFFFFFFFFFFFFFFFFFFFFFFFFFFFFFFFFFFFFFFFFFFFFFFFFFFF:FFFFFFF
F:FFFFFFFFFFFFFFFFFFFFFFFFFFFFFFFFFFFFFFFFFFF:FFFFFFFFF:FFFFFFFFFFFF
FFFF,FFFFF:FFFF
@A00721:190:HGJW7DSXY:4:1129:20817:7827 2:N:0:TCTGCAAG+GCTGCACT
CAAACATATCATCAAGGATAAATATTATCCTCATTAACTAAATGATATGAGACAATTGATGCGGACCA
CCAGAATGACGTCAGAGGTTCTAATAACATTCTGGTGTGACGAATTCAGAACTTTATTGTTCTACAAT
AACGAAAGCGAACAT
+
FFFFFFFFFFFFFFFFFFFFFFFFF:FFFFFFFFFFFFFFFFFFFFFFFFFFFFFFFFFFFFFFFFFF
FFFFFFFFFFFFFFFFFFF:FFFFFFFFFFFFFFFFFFFFFFFFF:FFFFFFFFFFFFFFFFFFFFFF
FFFFFFFFFFFFFFF
@A00721:190:HGJW7DSXY:4:2174:21703:11459 2:N:0:TCTGCAAG+GCTGCACT
CAAACATATCATCAAGGATAAATATTATCCTCATTAACTAAATGATATGAGACAATTGATGCGGACCA
CCAGAATGACGTCAGAGGTTCTAATAACATTCTGGTGTGACGAATTCAGAACTTTATAGTTCTACAAT
ACCGAAAGCGAACAT
+
FFFFFFFFFFF,F,FFFFFFFFFF,FFFFFFFFFFFFFFFFFF:F:FFFFF:FFF,FFFFFFF:FF:F
:FFFF:,FFF:FFFFFFFF::FF:FFFF:FFFFFF:FFFFF,F:FFFFFFFF:FFF,,F:FFFFFFFF
F,F,:FFF:F:FFFF
@A00721:190:HGJW7DSXY:4:2404:30897:10598 2:N:0:TCTGCAAG+GCTGCACT
CAAACATATCATCAAGGATAAATATTATCCTCATTAACTAAATGATATGAGACAATTGATGCGGACCA
CCAGAATGACGTCAGAGGTTCTAATAACATTCTGGTGTGACGAATTCAGAACTTTATTGTTCTACAAT
AACGAAAGCGAACAT

+
FFF:FFFFFFFFFFFFFF:FFFFFFFFFFFFFFFFFFFFFFFFFFFFFFFFFFFFFFFFFFFFFFFFF
FFFFFFFFFFFFFFFFFFFFFFF:FFFFFFFFFFFFFFFFFFFFFFFFFFFFFFFFFFFFFFFFFFFF
F:FFF:FFF:FFFFF
@A00721:190:HGJW7DSXY:4:2413:3568:17347 2:N:0:TCTGCAAG+GCTGCACT
CAAACATATCATCAAGGATAAATATTATCCTCATTAACTAAATGATATGAGACAATTGATGCGGACCA
CCAGAATGACGTCAGAGGTTCTAATAACATTCTGGTGTGACGAATTCAGAACTTTATTGTTCTACAAT
AACGAAAGCGAACAT
+
FFFFFFFFFFFFFFFFFFFFFFFFFFFFFFFFFFFFFFFFFFFFFFFFFFFFFFFFFFFFFFFFFFFF
FFFFF:FFFFFFFFFFFFFFFFFFFFFFFFFFFFFFFFFFFFFFFFFFFFFFFFFFFFFFFFFFFFFF
FFFFFFFFFFFFFFF
@A00721:190:HGJW7DSXY:4:2469:31385:5588 2:N:0:TCTGCAAG+GCTGCACT
CAAACATATCATCAAGGATAAATATTATCCTCATTAACTAAATGATATGAGACAATTGATGCGGACCA
CCAGAATGACGTCAGAGGTTCTAATAACATTCTGGTGTGACGAATTCAGAACTTTATTGTTCTACAAT
AACGAAAGCGAACAT
+
FFFFFFFFFFFFFF:FFFFFFFFFFFFFFFFFFFFFFFFFFFFFFFFFFFFFFFFFFFFFFFFFFFFF
:FFFFFFF:FFFFFFFFFFFFFFFFFFFFFFFFFFFFFFFFFFFFFFFFFF:FFFFFFFFFFFFFFFF
FFFFF:F:FFFFFFF
@A00721:190:HGJW7DSXY:4:2477:3007:11898 2:N:0:TCTGCAAG+GCTGCACT
CAAACATATCATCAAGGATAAATATTATCCTCATTAACTAAATGATATGAGACAATTGATGCGGACCA
CCAGAATGACGTCAGAGGTTCTAATAACATTCTGGTGTGACGAATTCAGAACTTTATTGTTCTACAAT
AACGAAAGCGAACAT
+
F::FFFFFFFFFFFFFFFFFFFFFFFFFFFFFF,FFFFFFFFFFFF:FFFFFFF:FFFFFFFFFFFFF
FFFFFFFFFFFFFFFFFF:FFFFFFFFFFFFF:FFFFFFFFFFF:FFFFFFFFFFFFFF,FFFFFFFF
FFF:FF:FFFFFFFF
@A00721:190:HGJW7DSXY:4:2535:19452:26162 2:N:0:TCTGCAAG+GCTGCACT
CAAACATATCATCAAGGATAAATATTATCCTCATTAACTAAATGATATGAGACAATTGATGCGGACCA
CCAGAATGACGTCAGAGGTTCTAATAACATTCTGGTGTGACGAATTCAGAACTTTATTGTTCTACAAT
AACGAAAGCGAACAT
+
:FFFFFFFFFFFFFFFFFFFFFFFFFFFFFFFFFFFFFFFFFFFFFFFFFFFFFFF:FFFFFFFFFFF
FFFFFFF:FFFFFFFFFFFFFFFFFFFFFFFFFFFFFFFFFFFFFFFFFFFFFFFFFFFFFFFFFFFF
FFF:FFF:FFFFFF:
@A00721:190:HGJW7DSXY:4:2560:17797:32628 2:N:0:TCTGCAAG+GCTGCACT
CAAACATATCATCAAGGATAAATATTATCCTCATTAACTAAATGATATGAGACAATTGATGCGGACCA
CCAGAATGACGTCAGAGGTTCTAATAACATTCTGGTGTGACGAATTCAGAACTTTATTGTTCTACAAT
AACGAAAGCGAACAT
+
FFFFFFFFFFFFFFFFFFFFFFFFFFFFFFFFFFFFFFFFFFFFFFFFFFF:FFFFFFFFFFFFFFFF
FFFFFFFFFFFFFFFFFFFFFFFFFFFFFFFFFFFFFF:FFFFFFFFFFFFFFFFF:FFFFFFFFFFF
:FFFF:FFFFFFFFF
@A00721:190:HGJW7DSXY:4:2641:7889:29747 2:N:0:TCTGCAAG+GCTGCACT
CAAACATATCATCAAGGATAAATATTATCCTCATTAACTAAATGATATGAGACAATTGATGCGGACCA
CCAGAATGACGTCAGAGGTTCTAATAACATTCTGGTGTGACGAATTCAGAACTTTATTGTTCTACAAT
AACGAAAGCGAACAT
+
FFFFFFFFFFFFFFFFFFFFFFFFFFFFFFFFFFFFFFF:FFFFFFFFFFFFFFFFFFFFFFFFFFFF
FFFFF:FFFFFFFFFFFF:FF:FFFFFFFFFFFFFFFFFFFFFF:FFFFFFFFFFFFFFFFFFFFFFF
FFFFF:FFFFFFFFF
@A00721:190:HGJW7DSXY:4:1159:28926:7905 2:N:0:TCTGCAAG+GCTGCACT
AAACATATCATCAAGGATAAATATTATCCTCATTAACTAAATGATATGAGACAATTGATGCGGACCAC

```
CAGAATGACGTCAGAGGTTCTAATAACATTCTGGTGTGACGAATTCAGAACTTTATTGTTCTACAATA
ACGAAAGCGAACAGA
+
FFFFFFFFFFFFFFFFFFFFFFFFFFFFFFFFFFF:FFFFFFFFFFFFFFFFFFFFFFFFFFFFFFFF
FFFFFFFFFFFFFFFFFFFFFFFFFFFFFFFFFFFFFFFFFFFFFFFFFFFFFFFFFFFFFFFFFFFF
FFFFFFFFFFFFFF:
@A00721:190:HGJW7DSXY:4:1401:25355:8891 2:N:0:TCTGCAAG+GCTGCACT
AAACATATCATCAAGGATAAATATTATCCTCATTAACTAAATGATATGAGACAATTGATGCGGACCAC
CAGAATGACGTCAGAGGTTCTAATAACATTCTGGTGTGACGAATTCAGAACTTTATTGTTCTACAATA
ACGAAAGCGAACAGA
+
FFFFFFFFFFFFFFFFFFFFFFFFFF::FFFFFFFFF:FFFFFFFFFFF:F:FFFF:FF:FFFFF:FF
FFFFFFFFFFFFFFF:FFFFFFFFFFFFFFFFFFFF:FFFFFFFFFFFFFF:FFFF::FFFFFFFFFF
F:,FFFF::F:,FFF
@A00721:190:HGJW7DSXY:4:1435:26458:32002 2:N:0:TCTGCAAG+GCTGCACT
AAACATATCATCAAGGATAAATATTATCCTCATTAACTAAATGATATGAGACAATTGATGCGGACCAC
CAGAATGACGTCAGAGGTTCTAATAACATTCTGGTGTGACGAATTCAGAACTTTATTGTTCTACAATA
ACGAAAGCGAACAGA
+
FFFFFFFFFFFFFFFFFFFFFFFFFFFFFFFFFFFFFFFFFFFFFFFFFFFFFFFFFFFFFFFFFFFF
FFFF:FFFFFFFFFFFFFFFFFFFFFFFFFFFFFFFFFFFFFFFFFFFFFFFFFFFFFFFFFFFFFFF
FF:FFFFFFF:FFFF
@A00721:190:HGJW7DSXY:4:2260:4453:23829 2:N:0:TCTGCAAG+GCTGCACT
AAACATATCATCAAGGATAAATATTATCCTCATTAACTAAATGATATGAGACAATTGATGCGGACCAC
CAGAATGACGTCAGAGGTTCTAATAACATTCTGGTGTGACGAATTCAGAACTTTATTGTTCTACAATA
ACGAAAGCGAACAGA
+
FFFFFFFFFFFFFFFFFFFFFFFFFFFFFFFFFFFFFFFFFFFFFFFFFFFFFFFFFFFFFFFFFFFF
FFFFFFFFFFFFFFFFFFFFFFFFFFFFFFFFFFFFFFFFFFFFFFFFFFFFFFFFFFFFFFFFFFFF
FFFFF:FFFFFFFFF
@A00721:190:HGJW7DSXY:4:1106:17653:10802 2:N:0:TCTGCAAG+GCTGCACT
ATCAAACATATCATCAAGGATAAATATTATCCTCATTAACTAAATGATATGAGACAATTGATGCGGAC
CACCAGAATGACGTCAGAGGTTCTAATAACATTCTGGTGTGACGAATTCAGAACTTTATTGTTCTACA
ATAACGAAAGCGAAC
+
FFFFFFFFFFFFFFFFFFFF:FFFFFFFFFFFFFFFFFFFF:FFFFFF:FFFFFF:FFFFFFFFFFFF
FFFFFFF:FFFFFFFFFFFFFFFFFFFFFFFFFFFFFFFFFFFFFFFFFF:FFFFFFFFFFFFFFFFF
FFFFFFFFFFFFFFF
@A00721:190:HGJW7DSXY:4:1132:21450:16094 2:N:0:TCTGCAAG+GCTGCACT
ATCAAACATATCATCAAGGATAAATATTATCCTCATTAACTAAATGATATGAGACAATTGATGCGGAC
CACCAGAATGACGTCAGAGGTTCTAATAACATTCTGGTGTGACGAATTCAGAACTTTATTGTTCTACA
ATAACGAAAGCGAAC
+
FFFFF,FFFFFFFFFFFFFFFFFFFFFFFFFFFFFFFFFFFFFFFFFFFFF,FFFFFFFFFFFFFFFF
FFFFFF:FFFFFFFFFF,FFF:FFFFFFFFFFFFFFFFFFFFFFFFFFFFFFFFFFFFFFFFFFFFFF
::FFFFF:FFFF,FF
@A00721:190:HGJW7DSXY:4:1146:19651:22153 2:N:0:TCTGCAAG+GCTGCACT
ATCAAACATATCATCAAGGATAAATATTATCCTCATTAACTAAATGATATGAGACAATTGATGCGGAC
CACCAGAATGACGTCAGAGGTTCTAATAACATTCTGGTGTGACGAATTCAGAACTTTATTGTTCTACA
ATAACGAAAGCGAAC
+
FFFFFFFFFFFFFFFFFFFF:FFFFFFFFFFFFFF:FFFFFFFFFF:FFFFFFFFFFFFFFFFFFFFF
FFFFFFFFFFFFFFFFFFFFFFFFF:FFFFFFFFFFFFFFFFFFFFFFFFFFFFFFFFFFFFFFFFFF
FFFFFFFFFFFFFFF
```

```
@A00721:190:HGJW7DSXY:4:1152:26449:18552 2:N:0:TCTGCAAG+GCTGCACT
ATCAAACATATCATCAAGGATAAATATTATCCTCATTAACTAAATGATATGAGACAATTGATGCGGAC
CACCAGAATGACGTCAGAGGTTCTAATAACATTCTGGTGTGACGAATTCAGAACTTTATTGTTCTACA
ATAACGAAAGCGAAC
+
FFFFFFFFFFFFFFFFFFFFFFFFFFFFFFFFFFFFFFFFFFFFFFFF:FFFFFFFFFFFFFFFFFFFFF
FFFFFFF,FFFFFFFFFFFFFFFFFFFFFFFFFFFFFFFFFFFFFFFFFFFFFFFFFFFFFFFFFFFFFF
FFFFFFFFFFFFF:F
@A00721:190:HGJW7DSXY:4:1173:11523:1251 2:N:0:TCTGCAAG+GCTGCACT
ATCAAACATATCATCAAGGATAAATATTATCCTCATTAACTAAATGATATGAGACAATTGATGCGGAC
CACCAGAATGACGTCAGAGGTTCTAATAACATTCTGGTGTGACGAATTCAGAACTTTATTGTTCTACA
ATAACGAAAGCGAAC
+
FFFFFFFFFFFFFFFFFFFFFFFFFFFFFFFFFFFFFFFFFFFFFFFFFFFFFFFFFFFFFFFFFFFFFF
FFFFFFFFFFFFFFFFFFFFFFFFFFFFFFFFFFFFFFFFFFFFFFFFFFFFFFFFFFFFFFFFFFFFFF
FFFFF:FFFFFFFFF
@A00721:190:HGJW7DSXY:4:1268:15176:1344 2:N:0:TCTGCAAG+GCTGCACT
ATCAAACATATCATCAAGGATAAATATTATCCTCATTAACTAAATGATATGAGACAATTGATGCGGAC
CACCAGAATGACGTCAGAGGTTCTAATAACATTCTGGTGTGACGAATTCAGAACTTTATTGTTCTACA
ATAACGAAAGCGAAC
+
FFFFFFFFFFFFFFFFFFFFFFFFFFFFFFFFFFFFFFFFFFFFFFFFFFFFFFFFFFFFFFFFFFFFFF
FFFFFFFFFFFFFFFFFFFFFFFFFFFFFFFFFFFFFFFFFFFFFFFFFFFFFFFFFFFFFFFFFFFFFF
FFFFFFFFFFFFFFF
@A00721:190:HGJW7DSXY:4:1310:32000:20556 2:N:0:TCTGCAAG+GCTGCACT
ATCAAACATATCATCAAGGATAAATATTATCCTCATTAACTAAATGATATGAGACAATTGATGCGGAC
CACCAGAATGACGTCAGAGGTTCTAATAACATTCTGGTGTGACGAATTCAGAACTTTATTGTTCTACA
ATAACGAAAGCGAAC
+
FFFFFFFFFFFFFFFFFFFFFFFFFFFFFFFF,FFFFFFFFFFFFFFFFFFFFFFFFFFFFFFFFFFFFF
FFFFFFFFFFFFFFFFFFFFF:FFFFFFFFFFFF,FFFFFFFFFFFFFFFFFFFFFFFFFFFFFFFFFFF
FFFFFFFFFFFFFFF
@A00721:190:HGJW7DSXY:4:1317:5755:26960 2:N:0:TCTGCAAG+GCTGCACT
ATCAAACATATCATCAAGGATAAATATTATCCTCATTAACTAAATGATATGAGACAATTGATGCGGAC
CACCAGAATGACGTCAGAGGTTCTAATAACATTCTGGTGTGACGAATTCAGAACTTTATTGTTCTACA
ATAACGAAAGCGAAC
+
FFFFFFFFFFFFFFFFFFFFFFFFFFFFFFFFFFFFFFFFFFFFFFFFFFFFFFFFFFFFFFFFFF:FFF
FFFFFFFFFFFFFFFFFFFFFFFFFFFFFFFFFFFFFFFFFFFFFFFFFFFFF:FFFFFFFFFFFFFFFF
FFFFF:FFFF:FFFF
@A00721:190:HGJW7DSXY:4:1320:8901:9925 2:N:0:TCTGCAAG+GCTGCACT
ATCAAACATATCATCAAGGATAAATATTATCCTCATTAACTAAATGATATGAGACAATTGATGCGGAC
CACCAGAATGACGTCAGAGGTTCTAATAACATTCTGGTGTGACGAATTCAGAACTTTATTGTTCTACA
ATAACGAAAGCGAAC
+
FFFFF:FFFFFFFFFFFFFFFFFFFFFFFFFFFFFFFFFFFFFFFFF:FFFFFFFFFFFFFFFFFFFFFF
FFFFFFFFFFFFFFFFFFFFFFFF::FFFFFFF:FFFFFFFFFFFFFFFFFFF:FFFFFFFFFF:FFFFF
FFF:FFFFFFFFFFF
@A00721:190:HGJW7DSXY:4:1328:15709:6903 2:N:0:TCTGCAAG+GCTGCACT
ATCAAACATATCATCAAGGATAAATATTATCCTCATTAACTAAATGATATGAGACAATTGATGCGGAC
CACCAGAATGACGTCAGAGGTTCTAATAACATTCTGGTGTGACGAATTCAGAACTTTATTGTTCTACA
ATAACGAAAGCGAAC
+
FFFFFFFFFFFFFFFFFFFF:FFFFFFFFFFFFFFFFFFFFFFFFFFFFFFFFFFFFFFFFFFFFFFFFF
```

FFFFFFFFFFFFFFFFF:FFFFFFFFFFFFFFFFFFFFFFFFFFFF:FFFFFFFFFFFFFFFFFFFFFF
FFFFFFFFFFFFFFF
@A00721:190:HGJW7DSXY:4:1471:26793:35336 2:N:0:TCTGCAAG+GCTGCACT
ATCAAACATATCATCAAGGATAAATATTATCCTCATTAACTAAATGATATGAGACAATTGATGCGGAC
CACCAGAATGACGTCAGAGGTTCTAATAACATTCTGGTGTGACGAATTCAGAACTTTATTGTTCTACA
ATAACGAAAGCGAAC
+
FFFFFFFFFFFFFFFFFFFFFFFFFFFFFFFFFFFFFFFFFFFFFFFFFFFFFFFFFFFFFFFFFFFF
FFFFFFFFFFFFFFFFFFFFFFFFFFFFFFFFFFFFFFFFFFFFFFFFFFFFFFFFFFFFFFFFFFFF
FFF:FFFFFFFFFFF
@A00721:190:HGJW7DSXY:4:1611:18611:9987 2:N:0:TCTGCAAG+GCTGCACT
ATCAAACATATCATCAAGGATAAATATTATCCTCATTAACTAAATGATATGAGACAATTGATGCGGAC
CACCAGAATGACGTCAGAGGTTCTAATAACATTCTGGTGTGACGAATTCAGAACTTTATTGTTCTACA
ATAACGAAAGCGAAC
+
FFFFFFFFFFFFFFFFFFFFFFFFFFFFFFFFFFFFFFFFFFFFFFFFFFFFFFFFFFFFFFFFFFFF
FFFFFFFFFFFFFFFFFFFFFFFFFFFFFFFFFFFFFFFFFFFFFFFFFFFFFFFFFFFFFFFFFFFF
FFFFFFFFFFFFFF:
@A00721:190:HGJW7DSXY:4:1611:20012:10504 2:N:0:TCTGCAAG+GCTGCACT
ATCAAACATATCATCAAGGATAAATATTATCCTCATTAACTAAATGATATGAGACAATTGATGCGGAC
CACCAGAATGACGTCAGAGGTTCTAATAACATTCTGGTGTGACGAATTCAGAACTTTATTGTTCTACA
ATAACGAAAGCGAAC
+
FFFFF:FFFFFFFFFFFFFFFFFF:FFFFFFFFFFFFFFFFFFFFFFFFFFFFFFFFFFFFFFFFFFF
FFFFFFFFFFFFFFFFFFFFFFFFFFFFFFFFFFFFFFFFFFFFFFFFFFFFFFFFFFFFFFFFFFFF
FFFFFFFFFFFFFFF
@A00721:190:HGJW7DSXY:4:1660:3305:18959 2:N:0:TCTGCAAG+GCTGCACT
ATCAAACATATCATCAAGGATAAATATTATCCTCATTAACTAAATGATATGAGACAATTGATGCGGAC
CACCAGAATGACGTCAGAGGTTCTAATAACATTCTGGTGTGACGAATTCAGAACTTTATTGTTCTACA
ATAACGAAAGCGAAC
+
FFFFFFFFFFFFFFFFFFFFFFFFFFFFFFFFFFFF:FFFFFFFFFFFFFFFFFFFFFFFFFFFFFFF
FFFFFFFFFFFFFFFFF:FFFFFFFFFFFFFFFFFFFFFFFFFFFFFFFFFFFFFFFFFFFFFFFFFF
FFFFFFFFFFFFFFF
@A00721:190:HGJW7DSXY:4:2143:6732:33223 2:N:0:TCTGCAAG+GCTGCACT
ATCAAACATATCATCAAGGATAAATATTATCCTCATTAACTAAATGATATGAGACAATTGATGCGGAC
CACCAGAATGACGTCAGAGGTTCTAATAACATTCTGGTGTGACGAATTCAGAACTTTATTGTTCTACA
ATAACGAAAGCGAAC
+
FFFFFFFFFFFFFFFFFFFFFFFFFFFFFFFFFFFFFFFFFFFFFFFFFFFFFFFFFFFFFFFFFFFF
FFFFFFF,FFFFFFFFFFFFFFFFFFFFFFFFFFFFFFFFFFFFFF:FFFFFFFFFFFFFFFFFFFFF
FFFFFFF,FFFFFFF
@A00721:190:HGJW7DSXY:4:2153:8196:27868 2:N:0:TCTGCAAG+GCTGCACT
ATCAAACATATCATCAAGGATAAATATTATCCTCATTAACTAAATGATATGAGACAATTGATGCGGAC
CACCAGAATGACGTCAGAGGTTCTAATAACATTCTGGTGTGACGAATTCAGAACTTTATTGTTCTACA
ATAACGAAAGCGAAC
+
FFFFFFFFFFFFFFFFFFFF,FFFFFFFFFFFFFFFFFFFFFFFFFFFFFFFFFFFFFF:FFFFFFFFF
:FFFFFFFFFFFFFFFFFFFFFFFFFFFFFFFFFFFFFFFFFFF,FFFFFFFFFF:FFFFFFFFFFF
FFFFF,FFFF,FFFF
@A00721:190:HGJW7DSXY:4:2233:11234:29590 2:N:0:TCTGCAAG+GCTGCACT
ATCAAACATATCATCAAGGATAAATATTATCCTCATTAACTAAATGATATGAGACAATTGATGCGGAC
CACCAGAATGACGTCAGAGGTTCTAATAACATTCTGGTGTGACGAATTCAGAACTTTATTGTTCTACA
ATAACGAAAGCGAAC

```
+
FFFFFFFFFFFFFFFFFFFFFFFFFFFFFFFFFFFFFFFFFFFFFFFFFFFFFFFFFFFFFFFFFFFF
FFFFFFFFFFFFFFFFFFFFFFFFFFFFFFFFFFFFFFFFFFFFFFFFFFFFFFFFFFFFFFFFFFFF
FFFFFFFFFF:FFFF
@A00721:190:HGJW7DSXY:4:2257:7563:21386 2:N:0:TCTGCAAG+GCTGCACT
ATCAAACATATCATCAAGGATAAATATTATCCTCATTAACTAAATGATATGAGACAATTGATGCGGAC
CACCAGAATGACGTCAGAGGTTCTAATAACATTCTGGTGTGACGAATTCAGAACTTTATTGTTCTACA
ATAACGAAAGCGAAC
+
:FFFFFFFFFFFFFFFFFFFFFFFFFFFFFFFFFFFFFFFFFFFFFFFFFFFFFFFFFFFFFFF:FFF
FFFFFFFFFFFFFFFF:FFFFFFFFFFFFFFFFFF:FFFF:FF:FFFF:F:FF:F,FFFFFFFFFFFF
FFFF,:FFFF:FFFF
@A00721:190:HGJW7DSXY:4:2404:8404:16078 2:N:0:TCTGCAAG+GCTGCACT
ATCAAACATATCATCAAGGATAAATATTATCCTCATTAACTAAATGATATGAGACAATTGATGCGGAC
CACCAGAATGACGTCAGAGGTTCTAATAACATTCTGGTGTGACGAATTCAGAACTTTATTGTTCTACA
ATAACGAAAGCGAAC
+
FFFFFFFFFFFFFFFFFFFFFFFFFFFFFFFFFFFFFFFFFFFFFFFFFFFFFFFFFFFFFFFFFFFF
FFFFFFFFFFFFFFFFFFFFFFFFFFFFFFFFFFFFFFFFFFFFFFFFFFFFFFFFFFFFFFFFFFFF
FFFFFFFFF:FFFFF
@A00721:190:HGJW7DSXY:4:2523:27661:35775 2:N:0:TCTGCAAG+GCTGCACT
ATCAAACATATCATCAAGGATAAATATTATCCTCATTAACTAAATGATATGAGACAATTGATGCGGAC
CACCAGAATGACGTCAGAGGTTCTAATAACATTCTGGTGTGACGAATTCAGAACTTTATTGTTCTACA
ATAACGAAAGCGAAC
+
FFFFFFFFFFFFFFFFFFFFFFFFFFFFFFFFFFFFFFFFFFFFFFFF:FFFFFFFFFFFFFFFFFFF
FFFFFFF,FFFFFFFFFFFFFFFFFFFFFFFFFFFFFFFFFFFFF,:FFFFFFFFFFFF:FFFF:FF,
FFFFFFFFFFFFFFF
@A00721:190:HGJW7DSXY:4:2528:14154:21026 2:N:0:TCTGCAAG+GCTGCACT
ATCAAACATATCATCAAGGATAAATATTATCCTCATTAACTAAATGATATGAGACAATTGATGCGGAC
CACCAGAATGACGTCAGAGGTTCTAATAACATTCTGGTGTGACGAATTCAGAACTTTATTGTTCTACA
ATAACGAAAGCGAAC
+
FFFFFFFFFFFFFFFFFFFFFFFFFFFFFFFFFFFFFFFFFFFFFFFFFFFFFFFFFFFFFFFFFFFF
FFFFFFFFFFFFFFFFFFFFFFFFFFFFFFFFFFFFFFFFFFFFFFFFFFFFFFFFFFFFFFFFFFFF
FFFFFFFFFFFFFFF
@A00721:190:HGJW7DSXY:4:2548:17481:1329 2:N:0:TCTGCAAG+GCTGCACT
ATCAAACATATCATCAAGGATAAATATTATCCTCATTAACTAAATGATATGAGACAATTGATGCGGAC
CACCAGAATGACGTCAGAGGTTCTAATAACATTCTGGTGTGACGAATTCAGAACTTTATTGTTCTACA
ATAACGAAAGCGAAC
+
FFFFFFFFFFFFFFFFFFFFFFFFFFFFFFFFFFFFFFFFFFFFFFFFFFFFFFFFFFFFFFFFFFFF
FFFFFFF:FFFFFFFFFFFFFFFFFFFFFFFFFFFFFFFFFFFFFFFFFFFFFFFFFFFFFFFFFFFF
FFFFFFFFFFFFFFF
@A00721:190:HGJW7DSXY:4:2652:16839:32910 2:N:0:TCTGCAAG+GCTGCACT
ATCAAACATATCATCAAGGATAAATATTATCCTCATTAACTAAATGATATGAGACAATTGATGCGGAC
CACCAGAATGACGTCAGAGGTTCTAATAACATTCTGGTGTGACGAATTCAGAACTTTATTGTTCTACA
ATAACGAAAGCGAAC
+
:FFFFFFFFFFFFFFFFFFFFFFFFFFFFFFFFFF:FFFFFFFFFFFFFFFFFFFFFFFFFFFFFFFF
FFFFFFFFFFFFFFFFFFFFFFFFFFFFFFFFFFFFFFFFFFFFFFFFFFFFFFFFFFFFFFFFFFFF
FFFFFFFFFFFFFFF
@A00721:190:HGJW7DSXY:4:2661:1163:12774 2:N:0:TCTGCAAG+GCTGCACT
ATCAAACATATCATCAAGGATAAATATTATCCTCATTAACTAAATGATATGAGACAATTGATGCGGAC
```

CACCAGAATGACGTCAGAGGTTCTAATAACATTCTGGTGTGACGAATTCAGAACTTTATTGTTCTACA
ATAACGAAAGCGAAC
+
FFFFFFFFFFFFFFFFFFFFFFFFFFFFFFFFFFFFFFFFFFFFFFFFFFFFFFFFFFFFFFFFFFFF
FFFFFFFFFFFFFFFFFFFFFFFFFFFFFFFFFFFFFFFFFFFFFFFFFFFFFFFFFFFFFFFFFFFF
FFFFFFFFFF:FFFF
@A00721:190:HGJW7DSXY:4:2676:31385:26318 2:N:0:TCTGCAAG+GCTGCACT
ATCAAACATATCATCAAGGATAAATATTATCCTCATTAACTAAATGATATGAGACAATTGATGCGGAC
CACCAGAATGACGTCAGAGGTTCTAATAACATTCTGGTGTGACGAATTCAGAACTTTATTGTTCTACA
ATAACGAAAGCGAAC
+
FFFFFFFFFFFFFFFFFFFFFFFFFFFFFFFFFFFFFFFFFFFFFFFFFFFFFFFFFFFFFFFFFF:F
FFFFFFFFFFFFFFFFFFFFFFFFFFFFFF:FFFFFFFFFFFFFFFFFFFFFFFFFFFFFF:FFF:FF
:FF:FFFF,FFFFFF
@A00721:190:HGJW7DSXY:4:1564:9534:15123 2:N:0:TCTGCAAG+GCTGCACT
CGATGAAATCAAACATATCATCAAGGATAAATATTATCCTCATTAACTAAATGATATGAGACAATTGA
TGCGGACCACCAGAATGACGTCAGAGGTTCTAATAACATTCTGGTGTGACGAATTCAGAACTTTATTG
TTCTACAATAACGAA
+
FFFFFFFFFFFFFFFFFFFFFFFFFFFFFFFFFFFFFFFFFFFFFFFFFFFFFFFFFFFFFFFFFFFF
FFFFFFFFFFFFFF:FFFFFFFFFFFFFFFFFFFFFFFFFFFFFFFFFFFFFFFFFFFFFFFFFFFFF
FFFFFFFFFFFFFFF
@A00721:190:HGJW7DSXY:4:2227:15564:20181 2:N:0:TCTGCAAG+GCTGCACT
ATCAAACATATCATCAAGGATAAATATTATCCTCATTAACTAAATGATATGAGACAATTGATGCGGAC
CACCAGAATGACGTCAGAGGTTCTAATAACATTCTGGTGTGACGAATTCAGAACTTTATTGTTCTACA
ATAACGAGATCGGAA
+
FFFFFFFFFFFFFFFFFFFFFFFFFFFFFFFFFFFFFFFFFFFFFFFFFFFFFFFFFFFFFFFFFFFF
FFFFFFFFFFFFFFFFFFFFFFFFFFFFFFFFFFFFFFFFFFFFFFFFFFFFFFFFFFFFFFFFFFFF
FFFFFFFFFFFFFFF
@A00721:190:HGJW7DSXY:4:2530:12789:7169 2:N:0:TCTGCAAG+GCTGCACT
ATCAAACATATCATCAAGGATAAATATTATCCTCATTAACTAAATGATATGAGACAATTGATGCGGAC
CACCAGAATGACGTCAGAGGTTCTAATAACATTCTGGTGTGACGAATTCAGAACTTTATTGTTCTACA
ATAACGAGATCGGAA
+
FFFFF:FFFFFFFFFFFFFFFFFFFFFFFFFFFFFFFFFFFFFFFFFFFFFFFFFFFFFFFFFFFFFF
FFFFFFFFFFFFFFFFFFFFFFFFFFFFFFFFFFFFFFFFFFFFFFFFFFFFFFFFFFFFFFFFFFFF
FFFFFFFFFFF:FFF
@A00721:190:HGJW7DSXY:4:1127:4978:36511 2:N:0:TCTGCAAG+GCTGCACT
CTCTTCCCGATGAAATCAAACATATCATCAAGGATAAATATTATCCTCATTAACTAAATGATATGAGA
CAATTGATGCGGACCACCAGAATGACGTCAGAGGTTCTAATAACATTCTGGTGTGACGAATTCAGAAC
TTTATTGTTCTACAA
+
FFFFFFFFFFFFFFFFFFF:FFFFFFFFFFFFFFFFFFFFFFFFFFFFFFFFFFFFFFFFF:FFFFFF
FF:FFFFFFFFFFFFFFFFFFFFFFFFFFFFFFF::FFFFFFFFFFFF:FFFFFFFFFFF:FFFFFFF
FFFF:FFFFFFFFFF
@A00721:190:HGJW7DSXY:4:1206:15890:12226 2:N:0:TCTGCAAG+GCTGCACT
CTCTTCCCGATGAAATCAAACATATCATCAAGGATAAATATTATCCTCATTAACTAAATGATATGAGA
CAATTGATGCGGACCACCAGAATGACGTCAGAGGTTCTAATAACATTCTGGTGTGACGAATTCAGAAC
TTTATTGTTCTACAA
+
FFFFFFFFFFFFFFFFFFFFFFFFFFFFFFFFFFFFFFFFFFFFFFFFFFFFFFFFFFFFFFFFFFFF
FFFFFFFFFFFFFFFFFFFFF:FFFFFFFFFFFFFFFFFFFFFFFFFFFFFFFFFFFFFFFFFFFFFF
FFFFFFFFFFFFFFF

```
@A00721:190:HGJW7DSXY:4:1206:15420:15702 2:N:0:TCTGCAAG+GCTGCACT
CTCTTCCCGATGAAATCAAACATATCATCAAGGATAAATATTATCCTCATTAACTAAATGATATGAGA
CAATTGATGCGGACCACCAGAATGACGTCAGAGGTTCTAATAACATTCTGGTGTGACGAATTCAGAAC
TTTATTGTTCTACAA
+
FFFFFFFFFFFFFFFFFFFFFFFFFFFFFFFFFFFFFFFFFFFFFFFFFFFFFFFFF,FFFF:FFFFF
FFF:FFFFFFFFFFFFFFFFF,FFFFFFFFFFFFFFFFFF:FFFFFFFFFFFFFFFFFF,FFFFFFFF
FFFFFFFFFFFFFFF
@A00721:190:HGJW7DSXY:4:1313:5095:12195 2:N:0:TCTGCAAG+GCTGCACT
CTCTTCCCGATGAAATCAAACATATCATCAAGGATAAATATTATCCTCATTAACTAAATGATATGAGA
CAATTGATGCGGACCACCAGAATGACGTCAGAGGTTCTAATAACATTCTGGTGTGACGAATTCAGAAC
TTTATTGTTCTACAA
+
FFFFFFFFFFFFFFFFFFFFFFFFFFFFFFFFFFFFFFFFFFFFFFFFFFFFFFFFFFFFFFFFFFFF
FFFFFFFFFFFFFFFFFFFFFFFFFFFFFFFFFFFFFFFFFFFFFFFFFFFFFFFFFFFFFFFFFFFF
FFFFFFFFFFFFFFF
@A00721:190:HGJW7DSXY:4:1538:29324:8500 2:N:0:TCTGCAAG+GCTGCACT
CTCTTCCCGATGAAATCAAACATATCATCAAGGATAAATATTATCCTCATTAACTAAATGATATGAGA
CAATTGATGCGGACCACCAGAATGACGTCAGAGGTTCTAATAACATTCTGGTGTGACGAATTCAGAAC
TTTATTGTTCTACAA
+
FFFFFFFFFFFFFFFFFFFFFFFFFFFFFFFFFFFFFFFFFFFFFFFFFFFFFFFFFFFFFFFFFFFF
FFFFFFFFFFFFFFFFFFFFFFFFFFFFFFFFFFFFFFFFFFFFFFFFFFFFFFFFFFFFFFFFFF,F
FFFFFFFFFFFFFFF
@A00721:190:HGJW7DSXY:4:2167:18078:17394 2:N:0:TCTGCAAG+GCTGCACT
CTCTTCCCGATGAAATCAAACATATCATCAAGGATAAATATTATCCTCATTAACTAAATGATATGAGA
CAATTGATGCGGACCACCAGAATGACGTCAGAGGTTCTAATAACATTCTGGTGTGACGAATTCAGAAC
TTTATTGTTCTACAA
+
FFFFFFFFFFFFFF:FFFF:FFFFFFFFF:FFFFFFFFFFFFFFFFFFFFFFFFFFFFFFFFFFFFFF
FFFFFFFFFFFFFFFFFFF:FF:FFFFFFFFFFFFFFFFFFFFFF:FFFFFFFFFFFFFFFFFFFFFF
FFFFFFFFFFFFFFF
@A00721:190:HGJW7DSXY:4:2167:20772:19272 2:N:0:TCTGCAAG+GCTGCACT
CTCTTCCCGATGAAATCAAACATATCATCAAGGATAAATATTATCCTCATTAACTAAATGATATGAGA
CAATTGATGCGGACCACCAGAATGACGTCAGAGGTTCTAATAACATTCTGGTGTGACGAATTCAGAAC
TTTATTGTTCTACAA
+
FFFFFFFFFFFFFFFFFFFFFFFFFFFFFFFFFFFFFFFFFFFFFFFFFFFFFFFFFFFFFFFFFFFF
FF:FFFFFFFFFFFFFFFFFF:FFFFFFFFFFFFFFFFFFFFFFFFFFFFFFFFFFFFFFFFF,FFFF
FFFFFFFFFFFFFFF
@A00721:190:HGJW7DSXY:4:2356:13819:21856 2:N:0:TCTGCAAG+GCTGCACT
CTCTTCCCGATGAAATCAAACATATCATCAAGGATAAATATTATCCTCATTAACTAAATGATATGAGA
CAATTGATGCGGACCACCAGAATGACGTCAGAGGTTCTAATAACATTCTGGTGTGACGAATTCAGAAC
TTTATTGTTCTACAA
+
FFFFFFFFFFFFFFFFFFF:FFFFFFFFFFFFFFFFFFFFFFFFFFFFFFFFFFFFFFFFFFFFFFFF
FFFFFFFFFFFFFFFFFFFFF:FFFFFFFFFFFFFFFFFFFFFFFFFFFFFFFFFFFFFFFFF:FFFF
FFFFFFFFFFFFFFF
@A00721:190:HGJW7DSXY:4:2418:27335:33395 2:N:0:TCTGCAAG+GCTGCACT
CTCTTCCCGATGAAATCAAACATATCATCAAGGATAAATATTATCCTCATTAACTAAATGATATGAGA
CAATTGATGCGGACCACCAGAATGACGTCAGAGGTTCTAATAACATTCTGGTGTGACGAATTCAGAAC
TTTATTGTTCTACAA
+
FFFFFFFFFFFFFFFFFFFFFFFFFFFFFFFFFFFFFFFFFFFFFFFFFFFFFFFFFFFFFFFFFFFF
```

FFFFFFFFFFFFFFFFFFFFFFFFFFFFFFFFFFFF:FFFFFFFFFF:FFFFFFFFFFFFFFFFFFFFFF
FFF:FFFFFFFFFFF
@A00721:190:HGJW7DSXY:4:1111:26521:30138 2:N:0:TCTGCAAG+GCTGCACT
CTTTCATCTCTTCCCGATGAAATCAAACATATCATCAAGGATAAATATTATCCTCATTAACTAAATGA
TATGAGACAATTGATGCGGACCACCAGAATGACGTCAGAGGTTCTAATAACATTCTGGTGTGACGAAT
TCAGAACTTTATTGT
+
FFFFFFFFFFFFFFFFFFFFFFFFFFFFFFFFFFFFFFFFFFFFFFF:FFFFFFFFFFFFFFFFFFFF:F
FFFFFFFFFFFFFFFFFFFFF:FFFFFFF:FFFFFFFFFFFFFFFFFFFFFFFFFFFFFFFFF:FFFFF:
FFFFFFFFFFFFFFF
@A00721:190:HGJW7DSXY:4:1346:31720:24017 2:N:0:TCTGCAAG+GCTGCACT
CTTTCATCTCTTCCCGATGAAATCAAACATATCATCAAGGATAAATATTATCCTCATTAACTAAATGA
TATGAGACAATTGATGCGGACCACCAGAATGACGTCAGAGGTTCTAATAACATTCTGGTGTGACGAAT
TCAGAACTTTATTGT
+
FFFF,FFFFFFFF:FFFFFFFFFFFFFFFFFFFFFFFF:FF,,FFFFFFFFFFFFFFFFF,FFFFFFFFF
FFFFFFFFF:FFF:FF:FFF:FF,FF::FFFFFFFFFFFFFF:FFFFF,FFF:FFFF,:,:FFFFFFFFF
FFF,FF:FF,FFFFF
@A00721:190:HGJW7DSXY:4:1452:32163:29982 2:N:0:TCTGCAAG+GCTGCACT
CTTTCATCTCTTCCCGATGAAATCAAACATATCATCAAGGATAAATATTATCCTCATTAACTAAATGA
TATGAGACAATTGATGCGGACCACCAGAATGACGTCAGAGGTTCTAATAACATTCTGGTGTGACGAAT
TCAGAACTTTATTGT
+
FFFFF:FFFFFFFFFFFFFFFFFFFFFFFFFFFFFFFFF:FFFF:,FFFFFFFFFFFFFFFFFFFFFFFF
FFFFFFFFFFFFF:FFFFFFFFFFFFFF:FFF:F:FFFFFFFFFFFFFFFFFFFFFFFFFFFFF:FFFFF
FFFFFFFFFFFF,FF
@A00721:190:HGJW7DSXY:4:1666:18304:4539 2:N:0:TCTGCAAG+GCTGCACT
CTTTCATCTCTTCCCGATGAAATCAAACATATCATCAAGGATAAATATTATCCTCATTAACTAAATGA
TATGAGACAATTGATGCGGACCACCAGAATGACGTCAGAGGTTCTAATAACATTCTGGTGTGACGAAT
TCAGAACTTTATTGT
+
FFFFFFFFFFFFFFFFFFFFFFFFFFFFFFFFFFFFFF:FFFFFFFFFFFFFFFFFFFFFFFFFFFFFFF
FFFFFFFFFFFFFFFFFF:FFFFFFFFFFFFFFFFFFFFFFFFFFFFFFFFFFFFFFFFFFFFFFFFFFF
F:FFFFFFFF:FFFF
@A00721:190:HGJW7DSXY:4:2663:16776:12101 2:N:0:TCTGCAAG+GCTGCACT
CTTTCATCTCTTCCCGATGAAATCAAACATATCATCAAGGATAAATATTATCCTCATTAACTAAATGA
TATGAGACAATTGATGCGGACCACCAGAATGACGTCAGAGGTTCTAATAACATTCTGGTGTGACGAAT
TCAGAACTTTATTGT
+
FFFFFFFFFFFFFFFFFFFFFFFFFFFFFFFFFFFF:FFFFFFFFFFFFFFFFFFFFFFFF:FFFFFFFF
FFFFFFFFFFFFFFFFFFFFFFFFFFFFFFFFFFFFFFFFFFFFFFFFFFFFFFFFFFFFFFFFFFFFFF
FFFFFFFFFFFFFFF
@A00721:190:HGJW7DSXY:4:1412:8413:1720 2:N:0:TCTGCAAG+GCTGCACT
ATTTACTTTCATCTCTTCCCGATGAAATCAAACATATCATCAAGGATAAATATTATCCTCATTAACTA
AATGATATGAGACAATTGATGCGGACCACCAGAATGACGTCAGAGGTTCTAATAACATTCTGGTGTGA
CGAATTCAGAACTTT
+
FFFFFFFFFFFFFFFFFFFFFFFFFFFFFFFFFFFFFFFFFFFFFFFFFFFFFFFFFFFFFFFFFFFFFF
FFFFFFFFFFFFFFFFFFFFFFFFFFFFFFFFFFFFFFFFFFFFFFFFFFFFFFFFFFFFFFFFFFFFFF
FFFFF:FFFFFFFFF
@A00721:190:HGJW7DSXY:4:2452:17598:7576 2:N:0:TCTGCAAG+GCTGCACT
ATTTACTTTCATCTCTTCCCGATGAAATCAAACATATCATCAAGGATAAATATTATCCTCATTAACTA
AATGATATGAGACAATTGATGCGGACCACCAGAATGACGTCAGAGGTTCTAATAACATTCTGGTGTGA
CGAATTCAGAACTTT

```
+
FFFFFFFFFFFFFFFFFFFFFFFFFFFFFFF,FFFFFFFFFFFFFFFFFFFFFFFFFFFFFFFFFFFFF
FFFFFFFFFFFFFFFFFFFFFFFF:FFFFFFFFFFFFFFFFFFFFFFFFFFFFFFFFF:FFFFFFFFFF
F:F,FFFFFFFFFFF
@A00721:190:HGJW7DSXY:4:1247:15899:34131 2:N:0:TCTGCAAG+GCTGCACT
AATTTACTTTCATCTCTTCCCGATGAAATCAAACATATCATCAAGGATAAATATTATCCTCATTAACT
AAATGATATGAGACAATTGATGCGGACCACCAGAATGACGTCAGAGGTTCTAATAACATTCTGGTGTG
ACGAATTCAGAACTT
+
FFFFFFFFFFFFFFFFFFFFFFFFFFFFFFFFFFFFFFFFFFFFFFFFFFFFFFFFFFFFFFFFFFFFF
FFFFFFFFFFFFFFFFFFFFFFFFFFFFFFFFFFFFFFFFFFFFFFFFFFFFFFFFFFFFFFFFFFFFF
FFFFFFFFFFFFFFF
@A00721:190:HGJW7DSXY:4:1423:7048:22811 2:N:0:TCTGCAAG+GCTGCACT
AATTTACTTTCATCTCTTCCCGATGAAATCAAACATATCATCAAGGATAAATATTATCCTCATTAACT
AAATGATATGAGACAATTGATGCGGACCACCAGAATGACGTCAGAGGTTCTAATAACATTCTGGTGTG
ACGAATTCAGAACTT
+
FFFFFFFFFFFFFFFFFFFFFFFFFFFFFFF:FFFFFFFFFFFFFFFFFF:FFFFFFFFFFFFFFFFFF
FFFFFF:FFFFFFFFFFFFFFFFFFFFFFFFFFF,FFFFFFFFFFFFFFFFFF:FFFFFFFFFFFFFFF
FFFFFFFFFF:FFFF
@A00721:190:HGJW7DSXY:4:1510:28339:33755 2:N:0:TCTGCAAG+GCTGCACT
AATTTACTTTCATCTCTTCCCGATGAAATCAAACATATCATCAAGGATAAATATTATCCTCATTAACT
AAATGATATGAGACAATTGATGCGGACCACCAGAATGACGTCAGAGGTTCTAATAACATTCTGGTGTG
ACGAATTCAGAACTT
+
F,FFFFFFFFFFFFFFFFFFFFFFFFFFFFFFFFFFFFFFFFFFFFFFFFFFFFFFFFFFFFFFFFFFF
FFFFFFFFFFFFFFFFFFFFFFFFFFFFFFFFFFFFF:FFFFFFFFFFFFFFFF:FFFFFFFFFFFF:F
:F:FFF:F:FFFFFF
@A00721:190:HGJW7DSXY:4:1662:19569:29215 2:N:0:TCTGCAAG+GCTGCACT
AATTTACTTTCATCTCTTCCCGATGAAATCAAACATATCATCAAGGATAAATATTATCCTCATTAACT
AAATGATATGAGACAATTGATGCGGACCACCAGAATGACGTCAGAGGTTCTAATAACATTCTGGTGTG
ACGAATTCAGAACTT
+
FFFFFFFFFFFFFFFFF,FFFFFFFFFFFFFFFFFFFFFFFFFFFFFF:FFFFFFFFFFFFFFF:FFFF
FFFFFFFFFFFFFFF:FFFFFFFFFFFFFFFFFFFFFF:FFFFFFFFFFFFFFFFFFFF:FFFFFFFFF
FFFFF,FFFFFFFFF
@A00721:190:HGJW7DSXY:4:2344:6506:8343 2:N:0:TCTGCAAG+GCTGCACT
AATTTACTTTCATCTCTTCCCGATGAAATCAAACATATCATCAAGGATAAATATTATCCTCATTAACT
AAATGATATGAGACAATTGATGCGGACCACCAGAATGACGTCAGAGGTTCTAATAACATTCTGGTGTG
ACGAATTCAGAACTT
+
FFFFFFFFFFFFFFFFFFFFFFFFFFFFFFFFF:FFFFFFFFFFFFFFFFFFFFFFFFFFFFF:FFFFF
F:FFFFFFFFFFFFFFFFFFFFFFFFFFFFFFFFFFFFFFFFFFFFFFF,FF,FFFFFFF,:,FFFFFF
FFFFFFFFFFFFFFF
@A00721:190:HGJW7DSXY:4:2476:16179:18740 2:N:0:TCTGCAAG+GCTGCACT
AATTTACTTTCATCTCTTCCCGATGAAATCAAACATATCATCAAGGATAAATATTATCCTCATTAACT
AAATGATATGAGACAATTGATGCGGACCACCAGAATGACGTCAGAGGTTCTAATAACATTCTGGTGTG
ACGAATTCAGAACT
+
FFFF:FFFFFFFF,FFFFFFFFFFF:FF::FFFFFFFFFFFFFFFF,FFFFF:FFFFFF:FFFFFF:FF
FFFFFF:FFFF:FFFFFFFFF:FFFFFFFF:FFFFFFFFF:FFFFFFFFFFFFFFFFFF,:FFFFFFFF
FFFFF:FFFF:FFF
@A00721:190:HGJW7DSXY:4:1116:22869:30859 2:N:0:TCTGCAAG+GCTGCACT
CAGAAATTTACTTTCATCTCTTCCCGATGAAATCAAACATATCATCAAGGATAAATATTATCCTCATT
```

```
AACTAAATGATATGAGACAATTGATGCGGACCACCAGAATGACGTCAGAGGTTCTAATAACATTCTGG
TGTGACGAATTCAGA
+
FFFFFFFFFFF:::FFFFFF::FFFFF:FFFFFFFFFFFFFF:FFFFFFFF:FF:,FFFF:F:FFFF,
FFFFFFFF:FFFFFFFFF:F:FFFFFFFFFFFFFFFF:F,FFFFFFFFFFFFFFFFFFFFFFFFFFF
,FFF:FFFFFFFF,F
@A00721:190:HGJW7DSXY:4:1317:19226:31908 2:N:0:TCTGCAAG+GCTGCACT
CAGAAATTTACTTTCATCTCTTCCCGATGAAATCAAACATATCATCAAGGATAAATATTATCCTCATT
AACTAAATGATATGAGACAATTGATGCGGACCACCAGAATGACGTCAGAGGTTCTAATAACATTCTGG
TGTGACGAATTCAGA
+
FFFFFFFFFFFFFFFFFFFFFFFFFFFFFFFFFFFFFFFFFFFFFFFFFFFFFFFFFFFFFFFFFFFF
FFFFFFFFFFFFFFFFFFFFFFFFFFFFFFFFFFFFFFF,FFFFFFFFFFFFFFFFFFFFFFFFFFFF
FFFFFFFFFFFFFFF
@A00721:190:HGJW7DSXY:4:1412:23023:33943 2:N:0:TCTGCAAG+GCTGCACT
CAGAAATTTACTTTCATCTCTTCCCGATGAAATCAAACATATCATCAAGGATAAATATTATCCTCATT
AACTAAATGATATGAGACAATTGATGCGGACCACCAGAATGACGTCAGAGGTTCTAATAACATTCTGG
TGTGACGAATTCAGA
+
F:FFFFFFFFFFFFFFFFFFFFFFFFFFFFFFFFFFFFFFFFFFFFFFFFFFFFFFFFFFFFFFFFFF
FFFFFFFFFF:FFFFF:FFFFFFFFFFFFFFFFFFF:FFFFFFFFFFFFFFFFFFFFFFFFFFFFF:F
FFF:FF,FFFFFFFF
@A00721:190:HGJW7DSXY:4:1505:14235:10300 2:N:0:TCTGCAAG+GCTGCACT
CAGAAATTTACTTTCATCTCTTCCCGATGAAATCAAACATATCATCAAGGATAAATATTATCCTCATT
AACTAAATGATATGAGACAATTGATGCGGACCACCAGAATGACGTCAGAGGTTCTAATAACATTCTGG
TGTGACGAATTCAGA
+
FFFFFFFFFFFFFFFFFFFFFFFFFFFFFFFFFFFF:FFFFFFFFFFFFFFFFFFFFFFFFFFFFFFF
FFFFFFFFFFFFFFF:FFFFFFFFFFFFFFFFFFFFFFFF,FFFFFFFFFFFFFFFFFFFFFFFFFFF
FFFFFFFFFFFFFFF
@A00721:190:HGJW7DSXY:4:1521:6677:10833 2:N:0:TCTGCAAG+GCTGCACT
CAGAAATTTACTTTCATCTCTTCCCGATGAAATCAAACATATCATCAAGGATAAATATTATCCTCATT
AACTAAATGATATGAGACAATTGATGCGGACCACCAGAATGACGTCAGAGGTTCTAATAACATTCTGG
TGTGACGAATTCAGA
+
FFFFFFFFFFFFFFFFFFFFFFFFFFFFFFFFFFFFFFFFFFFFFFFFFFFFFFFFFFFFFFFFFFFF
FFFFFFFFFFFFFFFFFFF,FF:FFFFFFFFFFFFFFFFFFFFFFFFFFFFFFFFFFFFFFFFFFFFF
FFFF:FFFFFFFFFF
@A00721:190:HGJW7DSXY:4:1554:25491:5274 2:N:0:TCTGCAAG+GCTGCACT
CAGAAATTTACTTTCATCTCTTCCCGATGAAATCAAACATATCATCAAGGATAAATATTATCCTCATT
AACTAAATGATATGAGACAATTGATGCGGACCACCAGAATGACGTCAGAGGTTCTAATAACATTCTGG
TGTGACGAATTCAGA
+
FFFFFFFFFFFFFFFFFFFFFFFFFFFFFFFFFFFF::FFFFFFFFFFFFFFFFF:FFFFFFFFFFFFF
FFFFFFFFFFFFFFFFFFFFFFFFFFFFFFFFFFFFFFFFFFFFFFFFFFF:FFFF:FFF:FFFFFFF
FFFFFFFFFFFFFFF
@A00721:190:HGJW7DSXY:4:1603:32705:11788 2:N:0:TCTGCAAG+GCTGCACT
CAGAAATTTACTTTCATCTCTTCCCGATGAAATCAAACATATCATCAAGGATAAATATTATCCTCATT
AACTAAATGATATGAGACAATTGATGCGGACCACCAGAATGACGTCAGAGGTTCTAATAACATTCTGG
TGTGACGAATTCAGA
+
FFFFFFFFFFFFF::FFFFFFFFFFFFFFFFFFFFFFFFFFFFFFFFFF:FF,FFFFF:F:FFFFFFFF
FFFF:FFFFFF:FFFFFFFFFFF,FFFFFFFFFFFFF:FFFFFFFFF::F:FFFFFFFFFFFF::FFF
FFFFFFFFFFFF:FF
```

```
@A00721:190:HGJW7DSXY:4:2263:5791:18944 2:N:0:TCTGCAAG+GCTGCACT
CAGAAATTTACTTTCATCTCTTCCCGATGAAATCAAACATATCATCAAGGATAAATATTATCCTCATT
AACTAAATGATATGAGACAATTGATGCGGACCACCAGAATGACGTCAGAGGTTCTAATAACATTCTGG
TGTGACGAATTCAGA
+
FFFFFFFFFFFFFFFFFFFFFFFFFFFFFFFFFFFF:FFFFFFFFFFFFFFFFFFFFFFFFFFFFFFF
FFFFFFFFFFFFFFFFFFF,FFFFFFFFFFFFFFFFFFFFFFFFFFFFFFFFFFFFFFFFFFF:FFFFF
FFFFFFFFFFFFFFF
@A00721:190:HGJW7DSXY:4:2268:30472:1282 2:N:0:TCTGCAAG+GCTGCACT
CAGAAATTTACTTTCATCTCTTCCCGATGAAATCAAACATATCATCAAGGATAAATATTATCCTCATT
AACTAAATGATATGAGACAATTGATGCGGACCACCAGAATGACGTCAGAGGTTCTAATAACATTCTGG
TGTGACGAATTCAGA
+
FFF:FFFFFFFFFFFFFFFF:FFFFFFFFFFFFFFF:FFFFFFFFFFF:F::FFFFFFFFFFFF:FF:
F:FFFFFFFFFFFFFFFFFFFFFF:FFFFFFF:FFFFFF:FF:FFFF,F:FFFFFF:FFFFF:FF,FFF
FFFF,FFFFFFFFFF
@A00721:190:HGJW7DSXY:4:2313:3866:30765 2:N:0:TCTGCAAG+GCTGCACT
CAGAAATTTACTTTCATCTCTTCCCGATGAAATCAAACATATCATCAAGGATAAATATTATCCTCATT
AACTAAATGATATGAGACAATTGATGCGGACCACCAGAATGACGTCAGAGGTTCTAATAACATTCTGG
TGTGACGAATTCAGA
+
FFFFFFFFFFFFFFFFFFFFFFFFFFFFFFFFFFFF::FFFFFFFFFFFFFFFFFFFF,FFFFFFFFF:
F:FFFFFFFF:FFFFFFFF:FFFFFFFFFF:FFFFFFFFFFFFFFFFFFFFFFFFFFFFFFFF:FFFF:
FFFFFFFF:FFFFFF
@A00721:190:HGJW7DSXY:4:2373:19551:20885 2:N:0:TCTGCAAG+GCTGCACT
CAGAAATTTACTTTCATCTCTTCCCGATGAAATCAAACATATCATCAAGGATAAATATTATCCTCATT
AACTAAATGATATGAGACAATTGATGCGGACCACCAGAATGACGTCAGAGGTTCTAATAACATTCTGG
TGTGACGAATTCAGA
+
FFFF:FFFFFFF:FFFFFFFFFFFFFFFFFFF:F:FFFFFFFF:FFFFFFFFFFFFFFFF:FFFF:F
FFFFFFFFFFFFFFFFFF:FFFF:FFFFFFFFFFFFFF,FFFFFFFFFFFFFFFFFFFFFFFFF:FFF
FFFFFF:FFFFFFFF
@A00721:190:HGJW7DSXY:4:2377:17336:18082 2:N:0:TCTGCAAG+GCTGCACT
CAGAAATTTACTTTCATCTCTTCCCGATGAAATCAAACATATCATCAAGGATAAATATTATCCTCATT
AACTAAATGATATGAGACAATTGATGCGGACCACCAGAATGACGTCAGAGGTTCTAATAACATTCTGG
TGTGACGAATTCAGA
+
FFFFFFFFFFFF:FFFFFFFFFFFFFFFFFFFFFF:FFFF:FFFFFFFFFFF:FFFFFF:FFFFFFFF:
,FFFFFFFFF::FFFFFFFFFFFFFFFFFFFFFFFFFFF:FFFFFFFFFFFF,FFFF,FFFFFFFF:FF
FFFFFFF:FFFFFFF
@A00721:190:HGJW7DSXY:4:2505:8558:8985 2:N:0:TCTGCAAG+GCTGCACT
CAGAAATTTACTTTCATCTCTTCCCGATGAAATCAAACATATCATCAAGGATAAATATTATCCTCATT
AACTAAATGATATGAGACACTTGATGCGGACCACCAGAATGACGTCAGAGGTTCTAATAACATTCTGG
TGTGACGAATTCAGA
+
FF,FFFFFF,F:FFFF:FFFFFF:FFF,:FFFFFFF:FFFFFFFFFFFF,F,FFFF:FFFFFF::FFF
FFF,FF,FFFFFF:FFFFF,:FFF:FFFFFF:FFFFFFFF:FFFFFFFFFFFFF:FFFFFFFF,F::F
:FF::FFFFFFF::F
@A00721:190:HGJW7DSXY:4:2541:32190:22357 2:N:0:TCTGCAAG+GCTGCACT
CAGAAATTTACTTTCATCTCTTCCCGATGAAATCAAACATATCATCAAGGATAAATATTATCCTCATT
AACTAAATGATATGAGACAATTGATGCGGACCACCAGAATGACGTCAGAGGTTCTAATAACATTCTGG
TGTGACGAATTCAGA
+
FFF:FFFFF:FFFFFFFFFFFFF:F:FFF:FFFFFF:FFFFFFFFF:FFFFFFFFFFFFFFFFFFFFF
```

```
F:FFFFFFFFFFF:FFFFFFFF:FFFFF,FFF:F,FFFFF,FF,FFFFF:FFFFFF:FFFFFFF,FFF
FFFFFFFFFFF:FFF
@A00721:190:HGJW7DSXY:4:2602:11315:6590 2:N:0:TCTGCAAG+GCTGCACT
CAGAAATTTACTTTCATCTCTTCCCGATGAAATCAAACATATCATCAAGGATAAATATTATCCTCATT
AACTAAATGATATGAGACAATTGATGCGGACCACCAGAATGACGTCAGAGGTTCTAATAACATTCTGG
TGTGACGAATTCAGA
+
FFFFFFFFFFFFFFFFFFFFFFFFFFFFFFFFFFF,FFFFF:FFFFFFFFFFFFFFFFFFFFFF:FF
FFFFFFFFFFF:FFFFFFF,FFFFFFFFFFFFFFFFFFF:FFFFFFFFFFFFFFFFFFFFFFFFFFF
FFFFFF:FFFFFFF:
@A00721:190:HGJW7DSXY:4:1254:12002:24377 2:N:0:TCTGCAAG+GCTGCACT
CATATTCAGAAATTTACTTTCATCTCTTCCCGATGAAATCAAACATATCATCAAGGATAAATATTATC
CTCATTAACTAAATGATATGAGACAATTGATGCGGACCACCAGAATGACGTCAGAGGTTCTAATAACA
TTCTGGTGTGACGAA
+
FFFFFFFFFFFFFFFFFFFFFFFFFFFFFFFFFFFFFFFFFFFFFFFFFFFFFFF,FFFFFFFFFFF
FFFFFFFFFFFFFFFFF:FFFFFFFFFFFFFFFF:FFFFFFFFFFFFFFFFF:FFFFFFFFFFFFF
FFFFFFFFFFFFFFF
@A00721:190:HGJW7DSXY:4:1261:19307:15170 2:N:0:TCTGCAAG+GCTGCACT
CATATTCAGAAATTTACTTTCATCTCTTCCCGATGAAATCAAACATATCATCAAGGATAAATATTATC
CTCATTAACTAAATGATATGAGACAATTGATGCGGACCACCAGAATGACGTCAGAGGTTCTAATAACA
TTCTGGTGTGACGAA
+
FFFFFFFFFF:FFFFFFFFFFFFFFFFFFFFFFFFFFFFFFFFFFFFFF::FFFFFFFFFFFFFFFFF
FFFFFFFFFFFFFFFFFFFFFFFFFFFFFFFFFFFFFFFFFFFFFFFFF:FFF,FFFFFFFFFFFFF
FFFFFFFFFFFFFFF
@A00721:190:HGJW7DSXY:4:1434:8612:5572 2:N:0:TCTGCAAG+GCTGCACT
CATATTCAGAAATTTACTTTCATCTCTTCCCGATGAAATCAAACATATCATCAAGGATAAATATTATC
CTCATTAACTAAATGATATGAGACAATTGATGCGGACCACCAGAATGACGTCAGAGGTTCTAATAACA
TTCTGGTGTGACGAA
+
FFFFFFFFFFFFFFFFFFFFFFFFFFFFFFFFFFFFFFFFFFFFFFFFFFFFFFFFFFFFFFFFFFFF
FFFFFFFFFFFFFFFFFFFFFFFFFF:FFFFFFF:FFFFFFFFFFFFFF:FFFF::FFFFFF,FFFFF
FFFFFFFFFFFFFFF
@A00721:190:HGJW7DSXY:4:2319:3468:18552 2:N:0:TCTGCAAG+GCTGCACT
CATATTCAGAAATTTACTTTCATCTCTTCCCGATGAAATCAAACATATCATCAAGGATAAATATTATC
CTCATTAACTAAATGATATGAGACAATTGATGCGGACCACCAGAATGACGTCAGAGGTTCTAATAACA
TTCTGGTGTGACGAA
+
FFFFFFFFFF:FFFFFFFFFFFFFFFFFFFFFFFFFFFFFF:FFFFFFFFFFFFFFFFF:FFFFFFFFF
FFFFFFFFFFFFFFFF:FFFFFFFFFFFFFFFFFFFFFFFFFFFF,:FFFFFFFFFFFFFFF:FFFFFF
FFFFFFFFFFFFFFF
@A00721:190:HGJW7DSXY:4:2418:14326:12179 2:N:0:TCTGCAAG+GCTGCACT
CATATTCAGAAATTTACTTTCATCTCTTCCCGATGAAATCAAACATATCATCAAGGATAAATATTATC
CTCATTAACTAAATGATATGAGACAATTGATGCGGACCACCAGAATGACGTCAGAGGTTCTAATAACA
TTCTGGTGTGACGAA
+
FFFF:FFFFFFFFFFFFFFFFFFFFFFFFFF:FFFFFFFFFFFFFFFFFFFFFFFFFFFFFFFFFFFFF
,FFFF,FFFFFFFFFFF,FFFFFFFFF:FFFFFFFFFFFFFFFFFFFFFFFFFFFFFFFFFFF:FFFFF
FFFFFFFFFFFFFFF
@A00721:190:HGJW7DSXY:4:2450:17626:33520 2:N:0:TCTGCAAG+GCTGCACT
CATATTCAGAAATTTACTTTCATCTCTTCCCGATGAAATCAAACATATCATCAAGGATAAATATTATC
CTCATTAACTAAATGATATGAGACAATTGATGCGGACCACCAGAATGACGTCAGAGGTTCTAATAACA
TTCTGGTGTGACGAA
```

+
FFFFFF,FFFFFFFFF:FF:FFFFFF:FFF:FFFFFFFFFFFFFFFFFFFFFFFFFFFFFFFFFFFFFF
FFFFFFFFFFFFFFFFFFF:FFFFFFFF:FFFFFFFF,FF:FFFFFFFFFFFFFFFFFFFFFFFFFFFF
:F,F:FFFFFFF:FF
@A00721:190:HGJW7DSXY:4:1125:27651:35164 2:N:0:TCTGCAAG+GCTGCACT
CGCTAACATCATATTCAGAAATTTACTTTCATCTCTTCCCGATGAAATCAAACATATCATCAAGGATA
AATATTATCCTCATTAACTAAATGATATGAGACAATTGATGCGGACCACCAGAATGACGTCAGAGGTT
CTAATAACATTCTGG
+
FFFFFFFFFFFFFFF:FFF,:FFFFFFFFFFFFFFFFFFFFFFFFFFFFFFFFFF:F:FFFFFFFFFF
FF:FFFFFFFFFF:FFFFFFFFFFFFFFFF,FF,FFFFFFF:FFFFFFFFF,F,FFFFFFFFFFFFFF
FF:FFFFFFFFFF::
@A00721:190:HGJW7DSXY:4:2474:13919:24283 2:N:0:TCTGCAAG+GCTGCACT
CGCTAACATCATATTCAGAAATTTACTTTCATCTCTTCCCGATGAAATCAAACATATCATCAAGGATA
AATATTATCCTCATTAACTAAATGATATGAGACAATTGATGCGGACCACCAGAATGACGTCAGAGGTT
CTAATAACATTCTGG
+
FFFFFFFFFFFFFFFFFFFFFFFFFFFFFFFFFFFFFFFFFFFFFFFFFFF:FFFFFFFFFFFFFFFF
FFFFFFFFFFFFFFFFFFFFFFFFFFFFFFFF,FFFFFFFFFFFFFFFFFFFF:FFFFFFFFFFFFFF
FFFFFF:FFF:FFFF
@A00721:190:HGJW7DSXY:4:2545:25880:10269 2:N:0:TCTGCAAG+GCTGCACT
CGCTAACATCATATTCAGAAATTTACTTTCATCTCTTCCCGATGAAATCAAACATATCATCAAGGATA
AATATTATCCTCATTAACTAAATGATATGAGACAATTGATGCGGACCACCAGAATGACGTCAGAGGTT
CTAATAACATTCTGG
+
FFFFFFF:FFFFFFFFFFFFFFFFFFFFFFFFFFFFFFFFFFFFFFFFFFF:FFFFFFFFFFFFFFFF
FFFFFFFFFFFFFFFFFFFF:FFFFFFFF:FFFFFFFFFFFFFFFFFFFFFFFFFFFFFFFFFFFFFF
FFF:FFFFFFFFFF:
@A00721:190:HGJW7DSXY:4:1135:28989:13808 2:N:0:TCTGCAAG+GCTGCACT
GTCCGCATCAATTGTCTCATATCATTTAGTTAATGAGGATAATATTTATCCTTGATGAAATCAAACAT
ATCATCAAGGATAAATATTATCCTCATTAACTAAATGATATGAGACAATTGATGCGGACCACCAGAAT
GACGTCAGAGGTTCT
+
FFFFFFFFFFFFFFFFFFFFFFFFFFFFFFFFFFFFFFFFFF:FFFFF:FFFFFFFFFF:F,,F:F
FFFFFFFFFFFFF:FFFFFFFF,FFF,FFFFFFFFFFFFFFFFF:FF,FFFFFFFFFFFFFF:FFF,F
FFFFFFFF,FFFFFF
@A00721:190:HGJW7DSXY:4:1271:2682:27054 2:N:0:TCTGCAAG+GCTGCACT
GTCCGCATCAATTGTCTCATATCATTTAGTTAATGAGGATAATATTTATCCTTGATGAAATCAAACAT
ATCATCAAGGATAAATATTATCCTCATTAACTAAATGATATGAGACAATTGATGCGGACCACCAGAAT
GACGTCAGAGGTTCT
+
FFFFFFFFFF:FFFFFFFFFFFFFFFFFFFFFFFFFFFFFFFFFFFFFFFFFFFFFFFFFFFF,,FFF
FFFFFFFFFFFFFFFFFFFFFFFF,FFFF:FFFFFFFFFFFFFFFFFFFFFFFFFFFFFFFFFFFFFF
FF:FFFFFFFFFFFF
@A00721:190:HGJW7DSXY:4:1639:31087:14027 2:N:0:TCTGCAAG+GCTGCACT
GTCCGCATCAATTGTCTCATATCATTTAGTTAATGAGGATAATATTTATCCTTGATGAAATCAAACAT
ATCATCAAGGATAAATATTATCCTCATTAACTAAATGATATGAGACAATTGATGCGGACCACCAGAAT
GACGTCAGAGGTTCT
+
FFFFFFFFFFFFFFFFFF:FFFFFFFFFFFFFFFFFFFFFFFFFFFFFFFFFFFFFFFFFFFFF,FFF
FFFFFFFFFFFFFFFFFFF,FFFFFF:F:FFFFFFFFFFFFFFFFFFFFFFFFFFFFFFFFFFFF:FF
FFFFFFFFFFFFFFF
@A00721:190:HGJW7DSXY:4:2150:25943:25034 2:N:0:TCTGCAAG+GCTGCACT
GTCCGCATCAATTGTCTCATATCATTTAGTTAATGAGGATAATATTTATCCTTGATGAAATCAAACAT

ATCATCAAGGATAAATATTATCCTCATTAACTAAATGATATGAGACAATTGATGCGGACCACCAGAAT
GACGTCAGAGGTTCT
+
FFFFFFFFFFFFFFFFFFFFFFFFFFFFFFFFFFFFFFFFFFFFFFFFFFFFFF::FFFFFF::,FF,
FFFFFFFFFFFF,,FFFFFFFF:F:FFFFFFFFFF:FFFFF:FFFFFF,FFFF:FFFFFFFFFFFFFF
FFFFFFFFFFF:FFF
@A00721:190:HGJW7DSXY:4:2466:11550:8187 2:N:0:TCTGCAAG+GCTGCACT
GTCCGCATCAATTGTCTCATATCATTTAGTTAATGAGGATAATATTTATCCTTGATGAAATCAAACAT
ATCATCAAGGATAAATATTATCCTCATTAACTAAATGATATGAGACAATTGATGCGGACCACCAGAAT
GACGTCAGAGGTTCT
+
FFFFFFFFFF:FFFFFFFFFFFFFFFFFFFFFFFFFFFFFFFFFFFFFFFFFFF:FFFF:FFFF:FF:
FFFFFF::FFFFF,FFFFFFFF:FFFFFF:FFFF:FF:FFFFF:,FFFFFFFFFFFFF:FFFFFFF:F
FFFFFF:FFFF::FF
@A00721:190:HGJW7DSXY:4:1362:11523:35509 2:N:0:TCTGCAAG+GCTGCACT
CTCCTTCATCTTCTTCATCGCGTTTCTCCATTAACGCTAACATCATATTCAGAAATTTACTTTCATCT
CTTCCCGATGAAATCAAACATATCATCAAGGATAAATATTATCCTCATTAACTAAATGATATGAGACA
ATTGATGCGGACCAC
+
FFFFFFFFFFFFFFFFFFFFFFFFFFFFFFFFFFFFFFFFFFFFFFFF,FFFF:FFFFFFFFFFFFFF
FFFFFFFFFFFFFFFFFFFFFFF:FFFFFFFFFFFFFFFFFFFFFFFFFFFFFFFFFFFFFFFFFFFF
FFFFFFFFFFFFFFF
@A00721:190:HGJW7DSXY:4:1440:3604:6981 2:N:0:TCTGCAAG+GCTGCACT
CTCCTTCATCTTCTTCATCGCGTTTCTCCATTAACGCTAACATCATATTCAGAAATTTACTTTCATCT
CTTCCCGATGAAATCAAACATATCATCAAGGATAAATATTATCCTCATTAACTAAATGATATGAGACA
ATTGATGCGGACCAC
+
FFFFFFFFFFFFFFFFFFFFFFFFFFFFFFFFF:FFFFFFFFFFFFFFFFFFFFFFFFFFFFFFFFFF
FF:FFFFFFFFFFFFFF:FFFFFFFFFFFFFFFFFFFFFFFFFFFFFFFFFFFFFFFFFFFFFFFFFF
FFFFFF:FFFFFFFF
@A00721:190:HGJW7DSXY:4:1510:10791:26506 2:N:0:TCTGCAAG+GCTGCACT
CTCCTTCATCTTCTTCATCGCGTTTCTCCATTAACGCTAACATCATATTCAGAAATTTACTTTCATCT
CTTCCCGATGAAATCAAACATATCATCAAGGATAAATATTATCCTCATTAACTAAATGATATGAGACA
ATTGATGCGGACCAC
+
FFFFFFFFFFFFFFFFFFFFFFFFFFFFFFFFFFFFFFFFFFFFFFFFFFFFFFFFFFFFFFFFFFFF
FFFFFFFFFFFFFFFFFF:FFFFFFFFFFFFFFFFFFFFFFFFFFFFFFFFFFFFFFFFFFFFFFFFF
FFFFFFFFFFFF:FF
@A00721:190:HGJW7DSXY:4:1511:21034:24424 2:N:0:TCTGCAAG+GCTGCACT
CTCCTTCATCTTCTTCATCGCGTTTCTCCATTAACGCTAACATCATATTCAGAAATTTACTTTCATCT
CTTCCCGATGAAATCAAACATATCATCAAGGATAAATATTATCCTCATTAACTAAATGATATGAGACA
ATTGATGCGGACCAC
+
FFFFFFFFFFFFFFFFFFFFF:FFFFFFFFFFF:FFFFFFFFFFFFFFFFFFFFFFFFFFFFFFFFFF
FFFFFFFFFFFFFFFFF:FFFFFFFFFFFFFFFFFFFFFFFFFFFFFFFF,FFFFFFFFFFFFFFFFF
FFFFFFFFFFFFFFF
@A00721:190:HGJW7DSXY:4:1630:1931:22435 2:N:0:TCTGCAAG+GCTGCACT
CTCCTTCATCTTCTTCATCGCGTTTCTCCATTAACGCTAACATCATATTCAGAAATTTACTTTCATCT
CTTCCCGATGAAATCAAACATATCATCAAGGATAAATATTATCCTCATTAACTAAATGATATGAGACA
ATTGATGCGGACCAC
+
FFFFFFFFFFFFFFFFFFFFFFFFFFFFFFFFFFFFFFFFFFFF:FFFFFFFFFFFFFFFFFFFFFFF
FFFFFFFFFFFF:FFFFFFFFFFFFFFFFFFFFFFFFFFFFFF:FFFFFFFFFFFFF:FFFFFFFFFF
FFFFFFFFFFFFFFF

```
@A00721:190:HGJW7DSXY:4:1661:16532:15342 2:N:0:TCTGCAAG+GCTGCACT
CTCCTTCATCTTCTTCATCGCGTTTCTCCATTAACGCTAACATCATATTCAGAAATTTACTTTCATCT
CTTCCCGATGAAATCAAACATATCATCAAGGATAAATATTATCCTCATTAACTAAATGATATGAGACA
ATTGATGCGGACCAC
+
FFFFFFFFFFFFFFFFFFFFFFFFFFFFFFFFFFFFFFFFFFFFFFFFFFFFFFFFF:FFFFFFFFFF
FFF:FFFFFFFFFFFFFFFFFFFFFFFFFFFFFFFFFFFFFFFFFFFFFFFFFFFFFFFFFFFFFFFF
FFFFFF,F,FFFFFF
@A00721:190:HGJW7DSXY:4:2106:1768:19523 2:N:0:TCTGCAAG+GCTGCACT
CTCCTTCATCTTCTTCATCGCGTTTCTCCATTAACGCTAACATCATATTCAGAAATTTACTTTCATCT
CTTCCCGATGAAATCAAACATATCATCAAGGATAAATATTATCCTCATTAACTAAATGATATGAGACA
ATTGATGCGGACCAC
+
FFFFFFFFFFFFFFFFFFFFFFFFFFFFFFFFFFFFFFFFFFFFFFFFFFFFFFFFFFFFFFFFFFFF
FFFFF:FFFFFFFFFFFFFFFFFFFFFFFFFFFFFFFFFFFFFFFFFFFFFFFFFFFFFFFFFFFFFF
F::FFFF::FFFFFF
@A00721:190:HGJW7DSXY:4:2242:28167:14450 2:N:0:TCTGCAAG+GCTGCACT
CTCCTTCATCTTCTTCATCGCGTTTCTCCATTAACGCTAACATCATATTCAGAAATTTACTTTCATCT
CTTCCCGATGAAATCAAACATATCATCAAGGATAAATATTATCCTCATTAACTAAATGAAATGAGACA
ATTGATGCGGACCAC
+
FFFFFFFFFFFF,FFFF,FFFFFFFFFFFF,FFFFFF:FFFFFF::FFFFFFFFFFF:FFF:FFFFFF
F:FFFFFF,FFFFFFFF,FF,FFFFFFF:FFFFFFFFF:FF,F::FFF,F,F:F:FFF:,FFFFFFFF
F:,FFF:FFF::FFF
@A00721:190:HGJW7DSXY:4:2342:28330:16266 2:N:0:TCTGCAAG+GCTGCACT
CTCCTTCATCTTCTTCATCGCGTTTCTCCATTAACGCTAACATCATATTCAGAAATTTACTTTCATCT
CTTCCCGATGAAATCAAACATATCATCAAGGATAAATATTATCCTCATTAACTAAATGATATGAGACA
ATTGATGCGGACCAC
+
FFFFFFFFFFFFFFFFFFFFFFFFFFFFFFFFFFFFFFFFFFFFFFFFFFFFFFFFFFFFFFFFFFFF
FFFFFFFFFFFFFFFFFFFFFFFFFFFFFFFFFFFFFFFFFF:FF:FFFFFFFFFFFFFFFFFFFFFF
FFFF,FF:,FFFFFF
@A00721:190:HGJW7DSXY:4:2420:31629:15718 2:N:0:TCTGCAAG+GCTGCACT
CTCCTTCATCTTCCTCATCGCGTTTCTCCATTAACGCTAACATCATATTCAGAAATTTACTTTCATCT
CTTCCCGATGAAATCAAACATATCATCAAGGATAAATATTATCCTCATTAACTAAATGATATGAGACA
ATTGATGCGGACCAC
+
FFFFFFFFFFFFFFFFFFFFFFFFFFFFFFFFFFFFFFFFFFFFFFFFFFFFFFFFFFFFFFFFFFFF
FFFFFFFFFFFFFFFFFFFFFFFFFFFFFFFFFFFFFFFFFF,:FFFFFFFFFFFFF,FFFFFFFFFF
FFF,FF,:,FFF:FF
@A00721:190:HGJW7DSXY:4:2420:30626:17206 2:N:0:TCTGCAAG+GCTGCACT
CTCCTTCATCTTCTTCATCGCGTTTCTCCATTAACGCTAACATCATATTCAGAAATTTACTTTCATCT
CTTCCCGATGAAATCAAACATATCATCAAGGATAAATATTATCCTCATTAACTAAATGATATGAGACA
CTTGATGCGGACCAC
+
FFFFFFFFFFFFFFFFFFFFFFFFFFFFFFFFFFFFFFFFFFFFFFFFF:FFFFFFFFFFFFFFFFFF
FFFFFFFFFFFFFFFFF:FFFFFFFFFFF:FFFFF:FFFFFFFFFFFFFF:FFF:FFFFFFFFFFFFF
,FFF:FFFFFFF,FF
@A00721:190:HGJW7DSXY:4:2551:4553:8406 2:N:0:TCTGCAAG+GCTGCACT
CTCCTTCATCTTCTTCATCGCGTTTCTCCATTAACGCTAACATCATATTCAGAAATTTACTTTCATCT
CTTCCCGATGAAATCAAACATATCATCAAGGATAAATATTATCCTCATTAACTAAATGATATGAGACA
ATTGATGCGGACCAC
+
FFFFFFFFFFFFFFFFFFFFFF,FFFFFFFFFFFFFFFFFFFFFFFFFFFFFFFFFFFFFFFFFFFFF
```

```
FFFFFFFFFFFFFFFFF:FFFFFFFFFFFFFFFFFFFFFFFFF:FFFFFFFFFFFFF:FFFFFFFFFF
FFFFFFFF:FFFFFF
@A00721:190:HGJW7DSXY:4:2609:25400:25723 2:N:0:TCTGCAAG+GCTGCACT
CTCCTTCATCTTCTTCATCGCGTTTCTCCATTAACGCTAACATCATATTCAGAAATTTACTTTCATCT
CTTCCCGATGAAATCAAACATATCATCAAGGATAAATATTATCCTCATTAACTAAATGATATGAGACA
ATTGATGCGGACCAC
+
FFFFFFFFFFFFFFFFFFFFFFFFFFFFFFFFFFFFFFFFFFFFFFFFFFFFFFFFFFFFFFFFFFFF
FFFFFFFFFFFFFFFFF,FFFFFFFFFFFFFFFFFFFFFFFFF:FFFFFFFFFF:FFFFFF:FFFFF
FFFFFFFFFFF:FFF
@A00721:190:HGJW7DSXY:4:2642:4571:28761 2:N:0:TCTGCAAG+GCTGCACT
CTCCTTCATCTTCTTCATCGCGTTTCTCCATTAACGCTAACATCATATTCAGAAATTTACTTTCATCT
CTTCCCGATGAAATCAAACATATCATCAAGGATAAATATTATCCTCATTAACTAAATGATATGAGACA
ATTGATGCGGACCAC
+
FFFFFFFFFFFFFFFFFFFFFFFFFFFFFFFFFFFFFFFFFFFFFFFFFFFFFFFFFFFFFFFFFFFF
FFFFFFFFFFFFFFFFFFFFFFFFFFFFFFFFFFFFFFFFFFF:FFFFFFFFFFFFFFFFFFFFFFFF
FFFFFFFFFFFFFFF
@A00721:190:HGJW7DSXY:4:2675:26232:30452 2:N:0:TCTGCAAG+GCTGCACT
CTCCTTCATCTTCTTCATCGCGTTTCTCCATTAACGCTAACATCATATTCAGAAATTTACTTTCATCT
CTTCCCGATGAAATCAAACATATCATCAAGGATAAATATTATCCTCATTAACTAAATGATATGAGACA
ATTGATGCGGACCAC
+
FFFFFFFFFFFFFFFFFFFFFFFFFFFFFFFFFFFFFFFFFFFFFFFFFFFFFFFFFFFFFFFFFFFF
FFFFFFFFFFFFFFFFF:FFFFFFFFFFFFFFFFFFFFFFFFFFFFFFFFF:FFFFFFFFFFFFFFFF
FFFFFFFF:FFFFFF
@A00721:190:HGJW7DSXY:4:1342:26241:2848 2:N:0:TCTGCAAG+GCTGCACT
CCTGATTTAACAGTAATGGGGACTCCTTCATCTTCTTCATCGCGTTTCTCCATTAACGCTAACATCAT
ATTCAGAAATTTACTTTCATCTCTTCCCGATGAAATCAAACATATCATCAAGGATAAATATTATCCTC
ATTAACTAAATGATA
+
FFFFFFFFFFFFFFFFFFFFFFFFFFFFFFFFFFFFFFFFFFFFFFFFFFFFFFFFFFFFFFFFFFFF
FFFFFFFFFFFFFFFFFFFFFFFFFFFFFFFFFFFFFFFFFFFFFFFFFFF:FFFFFFFFFFFFFFFF
FFFFFFFFFFFFFFF
@A00721:190:HGJW7DSXY:4:1366:26449:36245 2:N:0:TCTGCAAG+GCTGCACT
CCTGATTTAACAGTAATGGGGACTCCTTCATCTTCTTCATCGCGTTTCTCCATTAACGCTAACATCAT
ATTCAGAAATTTACTTTCATCTCTTCCCGATGAAATCAAACATATCGTCAAGGATAAATATTATCCTC
ATTAACTAAATGATA
+
FFFFFFFFFFFFFFFFFFFFFFFFFFFFFFFFFFFFFFFFFFFFFFFFFFFFFFFFFFFFFFFFFFFF
FFFFFFFFFFFFFFFFFFFFFFFFFFFFFFFFFF:FFFFFFFFFFFFFFFFFFFFFFFFFFFFFFFFF
FFFFFFFFFFFFFFF
@A00721:190:HGJW7DSXY:4:1561:1732:17206 2:N:0:TCTGCAAG+GCTGCACT
CCTGATTTAACAGTAATGGGGACTCCTTCATCTTCTTCATCGCGTTTCTCCATTAACGCTAACATCAT
ATTCAGAAATTTACTTTCATCTCTTCCCGATGAAATCAAACATATCATCAAGGATAAATATTATCCTC
ATTAACTAAATGATA
+
FFFFFFFFFFFFFFFFFFFFFFFFFFFFFFFFFFFFFFFFFFFFFFFFFFFFFFFFFFFFFF:FF:FFF
FFFFFFFFFFFFFFFFFFFFFFFFFFFFFFFFFFFFFFFFFFF:FFFFFFFFFFFFF:FFFFFFFFFFF
FFFFFFFFFFFFFFF
@A00721:190:HGJW7DSXY:4:1565:16080:31814 2:N:0:TCTGCAAG+GCTGCACT
CCTGATTTAACAGTAATGGGGACTCCTTCATCTTCTTCATCGCGTTTCTCCATTAACGCTAACATCAT
ATTCAGAAATTTACTTTCATCTCTTCCCGATGAAATCAAACATATCATCAAGGATAAATATTATCCTC
ATTAACTAAATGATA
```

+
FFFFFFFFFFFFFFF:FFFFFFFFFFFFFFFFFFFFFFFFFFFFFFFFFFFFFFFFFFFFFFFFFFFF
FFFFFFFFFFFFFFFFFFFFFFFFFFF:FFFFFFFFFFF:FFFFFFFFFFFFFF:FFF:FFFFFFFFF
FFFFFFFFFFFFF:F
@A00721:190:HGJW7DSXY:4:1645:12870:29324 2:N:0:TCTGCAAG+GCTGCACT
CCTGATTTAACAGTAATGGGGACTCCTTCATCTTCTTCATCGCGTTTCTCCATTAACGCTAACATCAT
ATTCAGAAATTTACTTTCATCTCTTCCCGATGAAATCAAACATATCATCAAGGATAAATATTATCCTC
ATTAACTAAATGATA
+
FFFFFFFFFFFFFFFFFFFFFFFFFFFFFFFFFFFFFFFFFFFFFFFFFFFFFFFFFFFFFFFFFFFF
FFFFFFF,FFFFFFFFFFFFFFFFFFFFFFFFFFFFFFFFFFFFFFFFFFFFFFFFFFFFFFFFFFFF
FFFF:FFFFFFFFFF
@A00721:190:HGJW7DSXY:4:1648:15094:20932 2:N:0:TCTGCAAG+GCTGCACT
CCTGATTTAACAGTAATGGGGACTCCTTCATCTTCTTCATCGCGTTTCTCCATTAACGCTAACATCAT
ATTCAGAAATTTACTTTCATCTCTTCCCGATGAAATCAAACATATCATCAAGGATAAATATTATCCTC
ATTAACTAAATGATA
+
FFFFFFFFFFFFFFFFFFFFFFFFFFFFFFFFFFFFFFFFFFFFFFFFFFFFFFFFFFFFFFF,FFFF
FFFFFFFFFFFFFFFFFFFFFFFFFFFFFFFFFFFFFFFFFFFFFFFFFFFFFFFFFFFFFFFFFFFF
FFFFFFFFFFFFFFF
@A00721:190:HGJW7DSXY:4:1670:14335:34648 2:N:0:TCTGCAAG+GCTGCACT
CCTGATTTAACAGTAATGGGGACTCCTTCATCTTCTTCATCGCGTTTCTCCATTAACGCTAACATCAT
ATTCAGAAATTTACTTTCATCTCTTCCCGATGAAATCAAACATATCATCAAGGATAAATATTATCCTC
ATTAACTAAATGATA
+
FFFFFFFFFFFFFFFFFFFFFFFFFFFFFFFFFFFFFFFFFFFFFFFFFFFFFFF,FFFFFFFFFFF:
FFFFFFFFFF:FFFFFFFFFFFFFFF:FFFFFFFFFFFFFFFFFF:FFFFFF,FFFFFFFFFFFF:FF
FFFFFFF:FFFFFFF
@A00721:190:HGJW7DSXY:4:2167:32226:35289 2:N:0:TCTGCAAG+GCTGCACT
CCTGATTTAACAGTAATGGGGACTCCTTCATCTTCTTCATCGCGTTTCTCCATTAACGCTAACATCAT
ATTCAGAAATTTACTTTCATCTCTTCCCGATGAAATCAAACATATCATCAAGGATAAATATTATCCTC
ATTAACTAAATGATA
+
FFFFFFFFFFFFFFFFFFFFFFFFFFFFFFFFFFFFFFFFFFFFFFFFFFFFFFF:FFFFFFFFFFFF
FFFFFFFFFFFFFFFFFFFFFFFFFFFFFFFFFFFFFFF:FFFFFFFFFF:FFFFFF:FFFFFFF:FF
FFFFFFFFF::,FFF
@A00721:190:HGJW7DSXY:4:2173:32786:22858 2:N:0:TCTGCAAG+GCTGCACT
CCTGATTTAACAGTAATGGGGACTCCTTCATCTTCTTCATCGCGTTTCTCCATTAACGCTAACATCAT
ATTCAGAAATTTACTTTCATCTCTTCCCGATGAAATCAAACATATCATCAAGGATAAATATTATCCTC
ATTAACTAAATGATA
+
FFFF:FFFFFFFFFFFFFFFFFFFFFFFFFFFFFFFFF:FFFFFFFFFFFFFFFF,FFFFF:::FFFF
FFFFFFFFFFFFFFFFFFFFFFFFFFFFFFFFFFFFFFFFFFFFFFFFFFFFFFFF,:FFFFFFFFF:
FFFF,FFFFFFF,FF
@A00721:190:HGJW7DSXY:4:2249:24026:5400 2:N:0:TCTGCAAG+GCTGCACT
CCTGATTTAACAGTAATGGGGACTCCTTCATCTTCTTCATCGCGTTTCTCCATTAACGCTAACATCAT
ATTCAGAAATTTACTTTCATCTCTTCCCGATGAAATCAAACATATCATCAAGGATAAATATTATCCTC
ATTAACTAAATGATA
+
FFFFFFFFFFFFFFFFFFFFFFFFFFFFFFFFFFFFFFFFF:FF:FFFFFFF:FFFFFFFFFFFFFFF
FFFFFFFFFFFFFFFFFFFFFFFFFFFFFFFFFFFFFFFFFFFFF:FFFFFFFFFFFFFFFFFF:,FF
FFFFFFFF:FFFFFF
@A00721:190:HGJW7DSXY:4:2460:1633:3067 2:N:0:TCTGCAAG+GCTGCACT
CCTGATTTAACAGTAATGGGGACTCCTTCATCTTCTTCATCGCGTTTCTCCATTAACGCTAACATCAT

```
ATTCAGAAATTTACTTTCATCTCTTCCCGATGAAATCAAACATATCATCAAGGATAAATATTATCCTC
ATTAACTAAATGATA
+
FFFFFFFFFFFFFFFFFFFFFFFFFFFFFFFFFFFFFFFFFFFFFFFFFFFFFFFFFFFFFFFFFFFF
FFFFFFFFFFFFFFFFFFFFFFFFFFFFFFFFFFFFFFFFFFFFFFFFFFFFFFFFFFFFFFFFFFFF
FFFFFFFFFFFFFFF
@A00721:190:HGJW7DSXY:4:2508:18059:7373 2:N:0:TCTGCAAG+GCTGCACT
CCTGATTTAACAGTAATGGGGACTCCTTCATCTTCTTCATCGCGTTTCTCCATTAACGCTAACATCAT
ATTCAGAAATTTACTTTCATCTCTTCCCGATGAAATCAAACATATCATCAAGGATAAATATTATCCTC
ATTAACTAAATGATA
+
F:FFFFFFFFFFFFFFFFFFFFFFFFFFFFFFFFFFFFFFFFFFFFFFFFFFFFFFFFFFFFFFFFFF:
FFFFFFFFFFFFFFFFFFFFFFFFFFFFFFFFFFFFFFFFFFFFFFFFFFFFFFFFFFFFFFFFFFFF
FFFFFFFFFFF:FFF
@A00721:190:HGJW7DSXY:4:2539:24632:15029 2:N:0:TCTGCAAG+GCTGCACT
CCTGATTTAACAGTAATGGGGACTCCTTCATCTTCTTCATCGCGTTTCTCCATTAACGCTAACATCAT
ATTCAGAAATTTACTTTCATCTCTTCCCGATGAAATCAAACATATCATCAAGGATAAATATTATCCTC
ATTAACTAAATGATA
+
FFFFFFFFFFFFFFFFFFFFFFFFFFFFFFFFFFFFFFFFFFFFFFFFFFFFFFFFFFFFF:FFFFFFF
F:FFFFFFFFFFFFFFFFFFFFFFFFFFFFFFFFFFFFFFFFFFFFF:FFFFFF:FFFFFFFFFFF:FFF
FFFFFFFFFFFFFFF
@A00721:190:HGJW7DSXY:4:1517:25382:25598 2:N:0:TCTGCAAG+GCTGCACT
CCCTGATTTAACAGTAATGGGGACTCCTTCATCTTCTTCATCGCGTTTCTCCATTAACGCTAACATCA
TATTCAGAAATTTACTTTCATCTCTTCCCGATGAAATCAAACATATCATCAAGGATAAATATTATCCT
CATTAACTAAATGAT
+
F:FFFFFFFFFFFFFFFFFFFFFFFFFFFFFFFFFFFFFFFFFFFFFFFFFFFFFF:FFFFFFFFFFF
FFFFFFFFFFFFFFFFFFFFFFFFFFFFFFFFFFFFFFFFF:FFFFFFFFFFFFFF:FFFFFFFFF,FF
FFFFFFFFFFFFFFF
@A00721:190:HGJW7DSXY:4:1616:16559:14512 2:N:0:TCTGCAAG+GCTGCACT
CCCTGATTTAACAGTAATGGGGACTCCTTCATCTTCTTCATCGCGTTTCTCCATTAACGCTAACATCA
TATTCAGAAATTTACTTTCATCTCTTCCCGATGAAATCAAACATATCATCAAGGATAAATATTATCCT
CATTAACTAAATGAT
+
FFFFFFFFFFFFFFFFFFFFFFFFFFFFFFFFFFFFFFFFFFFFFFFFFFFFFFFFFFFFFFFFFFFF
FFFFFFFFFFFFFFFFFFFFFFFFFFFFFFFFFFFFFFFFF:FFFFFFFFFFFFFFFFFFFFFFFFFFF
FFFFFFFFFFFFFFF
@A00721:190:HGJW7DSXY:4:2147:28384:7780 2:N:0:TCTGCAAG+GCTGCACT
CCCTGATTTAACAGTAATGGGGACTCCTTCATCTTCTTCATCGCGTTTCTCCATTAACGCTAACATCA
TATTCAGAAATTTACTTTCATCTCTTCCCGATGAAATCAAACATATCATCAAGGATAAATATTATCCT
CATTAACTAAATGAT
+
FFFFFFFFFFFFFFFF:FFFFFFFFFFFF:F:F:FFFFFFFFFFFFF:FFF,FFFFFFFFFFFFFFFF
F:FFFFFFFFFFFFFFFFFFFFFFFFFFFFFFFFFFFFFFFFFFFFF,FFFFFFFFFFFFFF:FFFFFF
F:FFF,FFFFFFFF:
@A00721:190:HGJW7DSXY:4:2360:4255:23703 2:N:0:TCTGCAAG+GCTGCACT
CCCTGATTTAACAGTAATGGGGACTCCTTCATCTTCTTCATCGCGTTTCTCCATTAACGCTAACATCA
TATTCAGAAATTTACTTTCATCTCTTCCCGATGAAATCAAACATATCATCAAGGATAAATATTATCCT
CATTAACTAAATGAT
+
FFFFFFFFFFFFFFFFFFFFFFFFFFFFFFFFFFF:FFFFFFFFFFFFFFFFFFFFFFFFFFFFFFFFF
FFFFFFFF::FFFFFFFFFFFFFFFFFFFFFFFFFFFFFFFFFFFFFFFFFFFFFFFFFFFFFFFFFFF
FFFF::FFFFFF:FF
```

```
@A00721:190:HGJW7DSXY:4:1336:14733:8876 2:N:0:TCTGCAAG+GCTGCACT
CAATATAATGCTTAATATGCGGATGTCCCTGATTTAACAGTAATGGGGACTCCTTCATCTTCTTCATC
GCGTTTCTCCATTAACGCTAACATCATATTCAGAAATTTACTTTCATCTCTTCCCGATGAAATCAAAC
ATATCATCAAGGATA
+
FFFFFFFFFFFFFFFFFFFFFFFFFFFFFFFFFFFFFFFFFFF:FFFFFFFFFFFFFFFFFFFFFFFF
FFFFFFFFFFFFFFFFFFFFFFFFFFFFFFFFFFFFFFFFFFFFFFFFFFFFFFFFFFFFFFFFFF,F
FFFFFFF:FFFFFFF
@A00721:190:HGJW7DSXY:4:1512:13313:27054 2:N:0:TCTGCAAG+GCTGCACT
CAAAATAATGCTTAATATGCGGATGTCCCTGATTTAACAGTAATGGGGACTCCTTCATCTTCTTCATC
GCGTTTCTCCATTAACGCTAACATCATATTCAGAAATTTACTTTCATCTCTTCCCGATGAAATCAAAC
ATATCATCAAGGATA
+
FFF,FFFFFFF,,:,::FF:FFFFFFFFF:,FF,,F:FF:FFFF:,FFF,F:F:FFFFFFFF::FFFF
FFF::FF:FFF:,FFFFF:FFF,FFFFF:FFF,FF:,F:FFFFF:FFFFF,F:F:FF:,FFFFFF:FF
FFFFFF,FFF::F,F
@A00721:190:HGJW7DSXY:4:2331:28221:3333 2:N:0:TCTGCAAG+GCTGCACT
CAATATAATGCTTAATATGCGGATGTCCCTGATTTAACAGTAATGGGGACTCCTTCATCTTCTTCATC
GCGTTTCTCCATTAACGCTAACATCATATTCAGAAATTTACTTTCATCTCTTCCCGATGAAATCAAAC
ATATCATCAAGGATA
+
FFF:FFFF:FFFFFFFFFFFFFFFFFFFF:F:F:F:FFFF:FFFFFFFFFFFFFFFF:F:FFFFFFFF
FFF::FFFFFFF:F:FFFF:FFFFFFF:FFFFFFFFFFFFFFFFFFFFFFFFFFFFFFFFFFFFFFFF
FFFFFF:FFFFFFFF
@A00721:190:HGJW7DSXY:4:2549:2094:11443 2:N:0:TCTGCAAG+GCTGCACT
CAATATAATGCTTAATATGCGGATGTCCCTGATTTAACAGTAATGGGGACTCCTTCATCTTCTTCATC
GCGTTTCTCCATTAACGCTAACATCATATTCAGAAATTTACTTTCATCTCTTCCCGATGAAATCAAAC
ATATCATCAAGGATA
+
FFFFFFFFFFFFFFFFFFFFFFFFFFFFFFFFFFFFFFFFFFFFFFFFFFFFFFFFFFFFFFFFFFFF
FFFFFFFFFFFFFFFFFFFFFFFFFFFFFFFFFFFFFFFFFFFFFFFFFFFFFFFFFFFFFFFFFFFF
FFFFFFFFFFFFFFF
@A00721:190:HGJW7DSXY:4:1153:20509:6668 2:N:0:TCTGCAAG+GCTGCACT
CCAATATAATGCTTAATATGCGGATGTCCCTGATTTAACAGTAATGGGGACTCCTTCATCTTCTTCAT
CGCGTTTCTCCATTAACGCTAACATCATATTCAGAAATTTACTTTCATCTCTTCCCGATGAAATCAAA
CATATCATCAAGGAT
+
FFFFFFFFFFFFFFFFFFFFFFFFFFFFFFFFFFFFFFFFFFFFFFFFFFFFFFFFFFFFFFFFFFFF
FFFFFFFFFFFFFFFFFFFFF:FFFFFFFFFFFFFFFFFFFFFFFFFFFFFFFFFFFFFFFFFFFFF,
FFFFFFFFFFFFFFF
@A00721:190:HGJW7DSXY:4:1266:23032:30608 2:N:0:TCTGCAAG+GCTGCACT
CCAATATAATGCTTAATATGCGGATGTCCCTGATTTAACAGTAATGGGGACTCCTTCATCTTCTTCAT
CGCGTTTCTCCATTAACGCTAACATCATATTCAGAAATTTACTTTCATCTCTTCCCGATGAAATCAAA
CATATCATCAAGGAT
+
FFFFFFFFFFFFFFFFFFFFFFFFFFFFFFFFFFFFFFFFFFF:FFFFFFFFFFFFFFFFFFFFFFFF
FFFFFFFFFFFFFFFFFFFFFFFFFF::FF:FFFFFFFFFFFFFFFFFFFFFFFFFFFFFFFFFFFFF
FFFFFFFFFFFFFFF
@A00721:190:HGJW7DSXY:4:1409:26811:9095 2:N:0:TCTGCAAG+GCTGCACT
CCAATATAATGCTTAATATGCGGATGTCCCTGATTTAACAGTAATGGGGACTCCTTCATCTTCTTCAT
CGCGTTTCTCCATTAACGCTAACATCATATTCAGAAATTTACTTTCATCTCTTCCCGATGAAATCAAA
CATATCATCAAGGAT
+
FFFFFFFFFFFFFFFFFFFFFFFFFFFFFFFFFFFFF:FFFFF:FFFFFFFFFFFFFFFFFFFFFFFF
```

FFFF:FFFFFFF:FF:FFFFFFFFFF,F:FFFFFF:FFFFFFFFFFFFFFFFFFFFFFFFFFFFFFFFFF
FFFFFFFFFFFFFFF
@A00721:190:HGJW7DSXY:4:1418:25970:21167 2:N:0:TCTGCAAG+GCTGCACT
CCAATATAATGCTTAATATGCGGATGTCCCTGATTTAACAGTAATGGGGACTCCTTCATCTTCTTCAT
CGCGTTTCTCCATTAACGCTAACATCATATTCAGAAATTTACTTTCATCTCTTCCCGATGAAATCAAA
CATATCATCAAGGAT
+
FFFFFFFFFFFFFFFFFFFFFFFFFFFFF,FFFFFFFFF:FFFFFFFFFFFFFFFFFFFFFFFFFF:FFF
FFFFFFFFFFFFFFF:FFFF,FFFFFFFF,FFFFF:FFFFFFFFFFFFFFFFFFFFFFFFFFFFFFFFFF
FFF,FFFFFFFFFFF
@A00721:190:HGJW7DSXY:4:1445:17246:34585 2:N:0:TCTGCAAG+GCTGCACT
CCAATATAATGCTTAATATGCGGATGTCCCTGATTTAACAGTAATGGGGACTCCTTCATCTTCTTCAT
CGCGTTTCTCCATTAACGCTAACATCATATTCAGAAATTTACTTTCATCTCTTCCCGATGAAATCAAA
CATATCATCAAGGAT
+
FFFFFFF:FFFFFFFFFFFFFFFFFFFFFFFFFFFFFFFFFFF:F,FFFFFFFF:FFFFFFFFFFFFFFF
FFFFFFFFFFFFFFFFFFFFFFFFFFF:FFFFFFFFFFFFFFFFFFFFFFFFFFFFFFFFFFF:FFFFFF
FFFFFFFFF,FFFF:
@A00721:190:HGJW7DSXY:4:2310:9525:27946 2:N:0:TCTGCAAG+GCTGCACT
CCAATATAATGCTTAATATGCGGATGTCCCTGATTTAACAGTAATGGGGACTCCTTCATCTTCTTCAT
CGCGTTTCTCCATTAACGCTAACATCATATTCAGAAATTTACTTTCATCTCTTCCCGATGAAATCAAA
CATATCATCAAGGAT
+
FFFFFFFFFFFFFFFFFFFFFFFFFFFFFFFFFFFFFFFFFFF:FFFFFFFFFFFFFFFFFFFFFFFFFF
FFFFFFFFFFFFFFFFFFFFFFFFF:FFFF:FF:FFFFFFFFFFFFF:FFFFFFFFFFFFFFFFFFFFFF
FFFF:FFFFFFFFFF
@A00721:190:HGJW7DSXY:4:2365:5475:33458 2:N:0:TCTGCAAG+GCTGCACT
CCAATATAATGCTTAATATGCGGATGTCCCTGATTTAACAGTAATGGGGACTCCTTCATCTTCTTCAT
CGCGTTTCTCCATTAACGCTAACATCATATTCAGAAATTTACTTTCATCTCTTCCCGATGAAATCAAA
CATATCATCAAGGAT
+
FFFFFFFFFFFFFFFFFFFFFFFFFFFFFFFFFFFFFFFFFFFFFFFFFFFFFFFFFFFFFFFFFFFFFF
FFFFFFFFFFFFFFFFFFFFFFFFFFFFFFFFFFFFFFFFFFFFFFFFFFFFFFFFFFFFFFFFFFFFFF
FFFFF:FF:FFFFFF
@A00721:190:HGJW7DSXY:4:2372:19868:26506 2:N:0:TCTGCAAG+GCTGCACT
CCAATATAATGCTTAATATGCGGATGTCCCTGATTTAACAGTAATGGGGACTCCTTCATCTTCTTCAT
CGCGTTTCTCCATTAACGCTAACATCATATTCAGAAATTTACTTTCATCTCTTCCCGATGAAATCAAA
CATATCATCAAGGAT
+
FFFFFFFFFFFFFFFFFFFFFFFFFFFFFFFFFFFFFFFFFFFFFFFFFFFFFFF:FFFFFFFFFFFFF
FFFFFFFFFFFFFFFFF:FFFFFFFFFFFFFFFFFFFFFFFFFFFFFFFFFFFFFFFFFFFFFFFFFFFF
FFFFFF:FFFF:FFF
@A00721:190:HGJW7DSXY:4:2411:29903:8876 2:N:0:TCTGCAAG+GCTGCACT
CCAATATAATGCTTAATATGCGGATGTCCCTGATTTAACAGTAATGGGGACTCCTTCATCTTCTTCAT
CGCGTTTCTCCATTAACGCTAACATCATATTCAGAAATTTACTTTCATCTCTTCCCGATGAAATCAAA
CATATCATCAAGGAT
+
FFFFFFFF:FFFFFFFFFFFFFFFFFFFFFFFFFFFFFFFFFFFFFFFFFFFFFFFFFFFFFFFFFFFFF
FFFFFFFFFFFFFFF:FFFFFFFFFFFFFF:FFFFFFFFFFFFFFFF:FFFF,FFFFFF:FF:FFFFFFF
FFFF:FFFFFFFFFF
@A00721:190:HGJW7DSXY:4:2674:29966:13213 2:N:0:TCTGCAAG+GCTGCACT
CCAATATAATGCTTAATATGCGGATGTCCCTGATTTAACAGTAATGGGGACTCCTTCATCTTCTTCAT
CGCGTTTCTCCATTAACGCTAACATCATATTCATAAATTTACTTTCATCTCTTCCCGATGAAATCAAA
CATATCATCAAGGAT

+
FFFFFFFFF,F:FFFF,F:F,FFFFFFFF:F:FFFFFFFF:FFFF::FFF,F:F,F:FFFFF,F::FF
FFFF:FF,FFFF,FF:,,FFFF:FF,F:FFF,F,FFF,FFF,:,F,FF,FFFF::F:FF:FFFF:FFF
FFFFF:F:FFF,FFF
@A00721:190:HGJW7DSXY:4:1163:32407:35697 2:N:0:TCTGCAAG+GCTGCACT
TGAAAGTAAATTTCTGAATATGATGTTAGCGTTAATGGAGAAACGCGATGAAGAAGATGAAGGAGTCC
CCATTACTGTTAAATCAGGGACATCCGCATATTAAGCATTATATTGGGGACTGGGGACCTATATATAT
GTATAGAGTAATCCG
+
FFF:FFFFFFFFFFFFFFFFFFFFFFFFFFFFFFFFFFFFFF,FFFFFFFFFFFFF,FFFFFFFFFFF
FFFFFFF:FFFFFFFFFFFFFFFFFFFFFFFF:FFFFFFFFFFFFFFFFF:FFFFFFFFFFFFFF::F
FFF:FFFFFFFFFFF
@A00721:190:HGJW7DSXY:4:1325:12861:10958 2:N:0:TCTGCAAG+GCTGCACT
TGAAAGTAAATTTCTGAATATGATGTTAGCGTTAATGGAGAAACGCGATGAAGAAGATGAAGGAGTCC
CCATTACTGTTAAATCAGGGACATCCGCATATTAAGCATTATATTGGGGACTGGGGACCTATATATAT
GTATAGAGTAATCCG
+
FFFFFFFFFFF,FFFFFFFF:FFFFFFFFFF:FFFFFFFFFFFFFFFFFFF:FFFFFFFFFFFFFFFF
FFFFFFFFFFFFFFFFFFFFFFFFFFFFFFFFFFFFFFFFFFFFFFFFFFFFFFFFFFFFFFFF:F,F
FF,FFFFFFFFFFFF
@A00721:190:HGJW7DSXY:4:1326:30165:25958 2:N:0:TCTGCAAG+GCTGCACT
TGAAAGTAAATTTCTGAATATGATGTTAGCGTTAATGGAGAAACGCGATGAAGAAGATGAAGGAGTCC
CCATTACTGTTAAATCAGGGACATCCGCATATTAAGCATTATATTGGGGACTGGGGACCTATATATAT
GTATAGAGTAATCCG
+
FFFFFFFFFFF:FFFFFFFFFFFFFFFFFFFFFFF,FFFFFFFFFFFF:FFF:FFFFFFFFFFFFFFF
FFFFFFFFFFFFFFFFFFFFFFFFFFFFFFFFFFFFFFFFFFFFFFF:FFFFFFFFFFFFFFFFFFFF
FFFFF:FFFFFFFFF
@A00721:190:HGJW7DSXY:4:1331:2239:14982 2:N:0:TCTGCAAG+GCTGCACT
TGAAAGTAAATTTCTGAATATGACGTTAGCGTTAATGGAGAAACGCGATGAAGAAGATGAAGGAGTCC
CCATTACTGTTAAATCAGGGACATCCGCATATTAAGCATTATATTGGGGACTGGGGACCTATATATAT
GTATAGAGTAATCCG
+
FFFFFFFFFFFFFFFFFFFFFFFFFFFFFFFFFFFFFFFFFFFFFFFF,:FFFFFFFFFFFFFFFFFF
FFFFFFFFFFFFFFFFFFFFFFFFFFFFFFFFFFFFFFFFFFFFFF:F:FFFFFFFFFFFFFFFFFF,
,F:FF,FFFFFFFFF
@A00721:190:HGJW7DSXY:4:1433:22914:19132 2:N:0:TCTGCAAG+GCTGCACT
TGAAAGTAAATTTCTGAATATGATGTTAGCGTTAATGGAGAAACGCGATGAAGAAGATGAAGGAGTCC
CCATTACTGTTAAATCAGGGACATCCGCATATTAAGCATTATATTGGGGACTGGGGACCTATATATAT
GTATAGAGTAATCCG
+
FFFFFFFFFFFFFFFFFFFFFFFFFFFFFFFFFFFFFFFFFFFFFFFFFFFFFFFFFFFFFFF:FFFF
FFFFFFFFFFFFFFFFFFFFFFFFFFFFFFFFFFFFFFFFFFFFFFFFFFFFFFFFFFFFFFFFFFFF
FFFFFFFFFFFFFFF
@A00721:190:HGJW7DSXY:4:1464:22010:3630 2:N:0:TCTGCAAG+GCTGCACT
TGAAAGTAAATTTCTGAATATGATGTTAGCGTTAATGGAGAAACGCGATGAAGAAGATGAAGGAGTCC
CCATTACTGTTAAATCAGGGACATCCGCATATTAAGCATTATATTGGGGACTGGGGACCTATATATAT
GTATAGAGTAATCCG
+
FFFFFFFFFFFFFFFFFFFFFFFFFFFFFFFFFFFFFFFFFFFFFFFFFFFFFFFFFF:FFFFFFFFF
FFFFFFFFFF:FFFFFFF:FFFFF:FFFFFFF:FFF:FFFFFFFFFFFFFFFFFFFFFFFFFFFFFFF
FFFFFFFFFFFFFFF
@A00721:190:HGJW7DSXY:4:1521:31250:26209 2:N:0:TCTGCAAG+GCTGCACT
TGAAAGTAAATTTCTGAATATGATGTTAGCGTTAATGGAGAAACGCGATGAAGAAGATGAAGGAGTCC

CCATTACTGTTAAATCAGGGACATCCGCATATTAAGCATTATATTGGGGACTGGGGACCTATATATAT
GTATAGAGTAATCCG
+
FFF,FFFFFFFFFFFFFFFFFFFFFFFFFFFFFFFFFFFFFFFFFFFFFFFFFFFFFFFFFFFFFFFF
FFFFFFFFFFFFFFFFFFFFFFFFFFFFFFFFFFFFFFFFFFFFFFFFFFFFFFFFFFFFFFFFFFFF
FFFFFFFFFFFFF:F
@A00721:190:HGJW7DSXY:4:1542:5520:18756 2:N:0:TCTGCAAG+GCTGCACT
TGAAAGTAAATTTCTGAATATGATGTTAGCGTTAATGGAGAAACGCGATGAAGAAGATGAAGGAGTCC
CCATTACTGTTAAATCAGGGACATCCGCATATTAAGCATTATATTGGGGACTGGGGACCTATATATAT
GTATAGAGTAATCCG
+
FFFFFFFFFFFFFFFFFFFFFFF:FFFFFFFFFFFFFFFFFFFFFFFFFFFFFFFFFFFFFFFFFFFF
FFFFFFFFFFFFFFFFFFFFFFFFFFFFFFFFF:FFFFFFFFFFFFFFFFFFFFFFFFFFFFFFFFFF
FFFFF:FFFFFFFFF
@A00721:190:HGJW7DSXY:4:2253:1072:20791 2:N:0:TCTGCAAG+GCTGCACT
TGAAAGTAAATTTCTGAATATGATGTTAGCGTTAATGGAGAAACGCGATGAAGAAGATGAAGGAGTCC
CCATTACTGTTAAATCAGGGACATCCGCATATTAAGCATTATATTGGGGACTGGGGACCTATATATAT
GTATAGAGTAATCCG
+
FFFFF,FFFFFFFF:FFF:FFFFFFFFFFFFFFFF,FFFFFFF,FFFFFFFFFFFFFF,FFFFFF:FF
,FFFFFFFFFFFFFFFFFF,F,FFF:F:FFFFFFFF:FF:FF:FFFF,FFFFFFFFFFFFFFFFFFFF
FFFFF:FFFFFF:FF
@A00721:190:HGJW7DSXY:4:2365:24017:26115 2:N:0:TCTGCAAG+GCTGCACT
TGAAAGTAAATTTCTGAATATGATGTTAGCGTTAATGGAGAAACGCGATGAAGAAGATGAAGGAGTCC
CCATTACTGTTAAATCAGGGACATCCGCATATTAAGCATTATATTGGGGACTGGGGACCTATATATAT
GTATAGAGTAATCCG
+
FFF:FFFFFFFFFFFFFFFFFFFFFFFFFFFFFFFFFFFFFFFFFFFFFFFFFFFFFFFFFFFFFFFF
FFFFFFFFFFFFFFFFFFFFFFFFFFFFFFF:FFFFFFFFFF,FFFFFFFFFFFFFFFFFFFFFFFFF
FFFFFFFFFFFFFFF
@A00721:190:HGJW7DSXY:4:2528:16098:19351 2:N:0:TCTGCAAG+GCTGCACT
TGAAAGTAAATTTCTGAATATGATGTTAGCGTTAATGGAGAAACGCGATGAAGAAGATGAAGGAGTCC
CCATTACTGTTAAATCAGGGACATCCGCATATTAAGCATTATATTGGGGACTGGGGACCTATATATAT
GTATAGAGTAATCCG
+
FFFFFFFFFFFFFFFFFFFFFFFFFFFFFFFFFFFFFFFFFFFFFFFFFFFFFFFFFFFFFFFFFFFF
FFFFFFFFFFFFFFFFFFFFFFFFFFFFFFFFFFFFFFFFFFFFFFFFFFFFFFFFFFFFFFFFFFFF
:FFFFFFFFFFFFFF
@A00721:190:HGJW7DSXY:4:2565:21838:17237 2:N:0:TCTGCAAG+GCTGCACT
TGAAAGTAAATTTCTGAATATGATGTTAGCGTTAATGGAGAAACGCGATGAAGAAGATGAAGGAGTCC
CCATTACTGTTAAATCAGGGACATCCGCATATTAAGCATTATATTGGGGACTGGGGACCTATATATAT
GTATAGAGTAATCCG
+
FFFFFFFFFFFFFFFFFFFFFFFFFFFFFF:FFFFFFFFFFFFFFFFFFFFFFFFFFFFFFFFFFFFF
FFFFFFFFFFFFFFFFFFFFFFFFFFFFFFFFFF,FFFFFFFFFFFF:FFFFF:FFFF:FFFFFFFFF
FFFFFFFFFFFFFFF
@A00721:190:HGJW7DSXY:4:2575:21495:25160 2:N:0:TCTGCAAG+GCTGCACT
TGAAAGTAAATTTCTGAATATGATGTTAGCGTTAATGGAGAAACGCGATGAAGAAGATGAAGGAGTCC
CCATTACTGTTAAATCAGGGACATCCGCATATTAAGCATTATATTGGGGACTGGGGACCTATATATAT
GTATAGAGTAATCCG
+
FF:FFFFFFFFFFFFFFFFFFFFFFFFFFFFFFFFFFFFFFF:FFFFFFFFFFFFFFFFFFFFFFFFF
FFFFFFFFFFFFFFFFFFFFFFFFFFFFFFFFFFFFFFFFFFFFFFFFFFFFFFFFFFFFFFFF:F,FFF
FFFFFFFF:FFFFFF

```
@A00721:190:HGJW7DSXY:4:2674:21169:16799 2:N:0:TCTGCAAG+GCTGCACT
TGAAAGTAAATTTCTGAATATGATGTTAGCGTTAATGGAGAAACGCGATGAAGAAGATGAAGGAGTCC
CCATTACTGTTAAATCAGGGACATCCGCATATTAAGCATTATATTGGGGACTGGGGACCTATATATAT
GTATAGAGTAATCCG
+
FFFFFFFFFFFFFFFFFFFFFFFFFFFFFFFFFFFFFFFFFFFFFFFFFFFFFFFFFFFFFFFFFFFFFF
FFFFFFFFFFFFFF:FFFFFFFFFFFFFFFFFFFFFFFF:FFFF:FFFFFFFFFFFFFFFFF:FFFFFFF
FFFFFFFFFFFFFFF
@A00721:190:HGJW7DSXY:4:1341:2266:1626 2:N:0:TCTGCAAG+GCTGCACT
AAATTTCTGAATATGATGTTAGCGTTAATGGAGAAACGCGATGAAGAAGATGAAGGAGTCCCCATTAC
TGTTAAATCAGGGACATCCGCATATTAAGCATTATATTGGGGACTGGGGACCTATATATATGTATAGA
GTAATCCGGAGATCG
+
FFFFFFFFFFFFFFFFFFFFFFFFFFFFFFFFFFFFFFFFFFFFFFFFFFFFFFFFFFFFFFFFFFFFFF
FFFFFFFFFFFFFFFFFFFFFFFFFFFFFFFFFFFFFFFFFFFFFFFFFFFFFFFFF:FFF,FFFFFFFF
FF:FFFFFFFFFF:F
@A00721:190:HGJW7DSXY:4:2417:14163:36542 2:N:0:TCTGCAAG+GCTGCACT
AAATTTCTGAATATGATGTTAGCGTTAATGGAGAAACGCGATGAAGAAGATGAAGGAGTCCCCATTAC
TGTTAAATCAGGGACATCCGCATATTAAGCATTATATTGGGGACTGGGGACCTATATATATGTACAGA
GTAATCCGGAGATCG
+
FFFFF:FFFFFFFFFFFFFFFFFFFFFFFFFFFFFFFFFFF::FFFFFFFFFFFFF:FFFFFF:FF:FFF
:FFFFFFFFFFF,FFFFFF:FFFF:FFFFFF:FF,FF:F:FF:FFFFFFFFFFFFFFFF,FFFF:FFFFF
FFFFFFFFFFFFFFF
@A00721:190:HGJW7DSXY:4:2443:9381:3834 2:N:0:TCTGCAAG+GCTGCACT
AAATTTCTGAATATGATGTTAGCGTTAATGGAGAAACGCGATGAAGAAGATGAAGGAGTCCCCATTAC
TGTTAAATCAGGGACATCCGCATATTAAGCATTATATTGGGGACTGGGGACCTATATATATGTATAGA
GTAATCCGGAGATCG
+
FFFFFFFFFFFFFFFFFFFFFFFFFFFFFFFFFFFFFFFFFFFFFFFFFFFFFFFFFFFFFFFFFFFFFF
FFFFFFFFFFFFFFFFFFFFFFFFFFFFFFFFFFFFFFFFFFFFFFFFFFFFFFFFFFFFFFFFFFFFFF
FFFFFFFFFFFFFFF
@A00721:190:HGJW7DSXY:4:2445:11505:5415 2:N:0:TCTGCAAG+GCTGCACT
AAATTTCTGAATATGATGTTAGCGTTAATGGAGAAACGCGATGAAGAAGATGAAGGAGTCCCCATTAC
TGTTAAATCAGGGACATCCGCATATTAAGCATTATATTGGGGACTGGGGACCTATATATATGTATAGA
GTAATCCGGAGATCG
+
F:FFFFFFFFFFF:FFFFFFFFFFFFFFFFFFFFFFFFFFFFFFFFFFFFFFFFFFFFFFFFFFFFFFFF
FFFFFFFFFFFFFFFFFFFFFFFFFFFFFFFFFF,FFFFFFFFFFFFF:FFFFFFF,FFFFFFFFFFF:F
FFFFF:FFFFFFFFF
@A00721:190:HGJW7DSXY:4:2466:11098:25003 2:N:0:TCTGCAAG+GCTGCACT
AAATTTCTGAATATGATGTTAGCGTTAATGGAGAAACGCGATGAAGAAGATGAAGGAGTCCCCATTAC
TGTTAAATCAGGGACATCCGCATATTAAGCATTATATTGGGGACTGGGGACCTATATATATGTATAGA
GTAATCCGGAGATCG
+
FFFFFFFFFFFFFFFFFFFFFFFFFFFFFFFFFFFF:FFFFFFFFF:FFFFFFFFFFFFFFFFFFFFFFF
FFFFFFFFFFFFFFFFFFFFFFFFFFFFF:FFFFFFFFFFFFFFFFFFFFFFFFFFFFFFFFFFFFFFFF
FFFFFFFFFFFFFFF
@A00721:190:HGJW7DSXY:4:2368:5358:34006 2:N:0:TCTGCAAG+GCTGCACT
TGGGGATAGATTCCTCCGGATTACTCTATACATATATATAGGTCCCCAGTCCCCAATATAATGCTTAA
TATGCGGATGTCCCTGATTTAACAGTAATGGGGACTCCTTCATCTTCTTCATCGCGTTTCTCCATTAA
CGCTAACATCAGAGA
+
FFFFFFFFFFFFFFFFFFFFFFFFFFFFFFFFFFFFFFFFFFFFFFFFFFFFFFFFFFFFFFFFFFFFF:
```

```
FFFFFFFFFFFFFFFFF:FFFFFFFFFFFFFFFFFFFFFFFFFFFFFFFFFFFFF:FFFFFFFFFFFFFF
FFFFFFFFFFFFFFF
@A00721:190:HGJW7DSXY:4:2370:17770:30358 2:N:0:TCTGCAAG+GCTGCACT
TGGGGATAGATTCCTCCGGATTACTCTATACATATATATAGGTCCCCAGTCCCCAATATAATGCTTAA
TATGCGGATGTCCCTGATTTAACAGTAATGGGGACTCCTTCATCTTCTTCATCGCGTTTCTCCATTAA
CGCTAACATCAGAGA
+
FFFFFFFFFFFFFFFFFFFFFFFFFFFFFFFFFFFFFFFFFFF:FFFFFFFFFFFFFFFFFFFFFFFFFF
FFFFFFFFFFFFFFFFF:FFFFFFFF:F:FFFFFFFFFFFFFFFFFFFFFFFFFFFFFFFFFFFFFFFFF
FFFFFFFFFFFFFFF
@A00721:190:HGJW7DSXY:4:2475:24804:8844 2:N:0:TCTGCAAG+GCTGCACT
TGGGGATAGATTCCTCCGGATTACTCTATACATATATATAGGTCCCCAGTCCCCAATATAATGCTTAA
TATGCGGATGTCCCTGATTTAACAGTAATGGGGACTCCTTCATCTTCTTCATCGCGTTTCTCCATTAA
CGCTAACATCAGAGA
+
FFFFFFFFFFFFFFFFFFFFFFFFFFFFFFFFFFFFFFFFFFFFFFFFFFFFFFFFFFFFFFFFFFFFFF
FFFFFFFFFFFFFFFFFFFFFFFFFFFFFFFFFFFFFFFFFFFFFFFFFFFFFFFFFF:FFFFFFFFFFF
FFFFFFFFFFFFFFF
@A00721:190:HGJW7DSXY:4:2535:9715:22608 2:N:0:TCTGCAAG+GCTGCACT
TGGGGATAGATTCCTCCGGATTACTCTATACATATATATAGGTCCCCAGTCCCCAATATAATGCTTAA
TATGCGGATGTCCCTGATTTAACAGTAATGGGGACTCCTTCATCTTCTTCATCGCGTTTCTCCATTAA
CGCTAACATCAGAGA
+
FFFFFFFFFFFFFFFFFFFFFFFFFFFFFFFFFFFFFFFFFFFFFFFFFFFFFFFFFFFFFFFFFFFFFF
FFFFFFFFFFFFFFFFFFFFFFFFFFFFFFFFFFFFFFFFFFFFFFFFFFFFFFF:FFFFFFFFFFFFFF
F:FFFFFFFFFFFFF
@A00721:190:HGJW7DSXY:4:2613:1461:23469 2:N:0:TCTGCAAG+GCTGCACT
TGGGGATAGATTCCTCCGGATTACTCTATACATATATATAGGTCCCCAGTCCCCAATATAATGCTTAA
TATGCGGATGTCCCTGATTTAACAGTAATGGGGACTCCTTCATCTTCTTCATCGCGTTTCTCCATTAA
CGCTAACATCAGAGA
+
FFFFFFFFFFFFFFFFFFFFFFFFFFFFFFFFFFFFFFFFFFFFFFFFFFFFFFFFFFFFFFFFFFFFFF
FFFFFFFFFFFFFFFFFFFFFFFFFFFFFFFFFFFFFFFFFFFFFFFFFFFFFFFFFFFFFFFFFFFFFF
FFFFFFFFFFFFFFF
@A00721:190:HGJW7DSXY:4:2654:5891:16172 2:N:0:TCTGCAAG+GCTGCACT
TGGGGATAGATTCCTCCGGATTACTCTATACATATATATAGGTCCCCAGTCCCCAATATAATGCTTAA
TATGCGGATGTCCCTGATTTAACAGTAATGGGGACTCCTTCATCTTCTTCATCGCGTTTCTCCATTAA
CGCTAACATCAGAGA
+
FFFFFFFFFFFFFFFFFFFFFFFFFFFFFF,FFFFFFFFFFFFFFFFFFF:FFF:FFFFFFFFFFFFFFF
FFFFFFFFFFF:F:FFFFFFFFFFFFFFFFFFFFFFF::FFFFFFFFFFFF:F:FFF:FFFFFF,FFFFF
:FFFFFFF:FFFFFF
@A00721:190:HGJW7DSXY:4:1239:14859:17613 2:N:0:TCTGCAAG+GCTGCACT
CCCCAATATAATGCTTAATATGCGAATGTCCCTGATTTAACAGTAATGGGGACTCCTTCATCTTCTTC
ATCGCGTTTCTGCATTAACGCGAAGAATTATTTCTTAACTTATTCAAGATGTCCAATTTCCAAAGAAA
TTGTTCTCGACCAAA
+
FFFFFFFFFF,FFFF,FFFFFFFFFFF:FFFFFFFFFFFFFFFFFFFFFF:FFFFFFFFFFFFFFFFFFF
FFFFFFFFFFFFFFFFFFFFFFFFFFFFFFFF:FFFFFFFFFFFF:FFFFFFFFFFFFFFFF:FFFFF,F
FFF:FFFFFFFFFFF
@A00721:190:HGJW7DSXY:4:1460:12192:27712 2:N:0:TCTGCAAG+GCTGCACT
CCCCAATATAATGCTTAATATGCGAATGTCCCTGATTTAACAGTAATGGGGACTCCTTCATCTTCTTC
ATCGCGTTTCTGCATTAACGCGAAGAATTATTTCTTAACTTATTCAAGATGTCCAATTTCCAAAGAAA
TTGTTCTCGACCAAA
```

```
+
FFFFFFFFFFFFFFFFFFFFFFFFF:FFFFFFFFFFFFFFFFFFFFFFFFFFFFFFFFFFFFFFFFFFFF
FFF:FFFFFFFFFFFFFFFFFF:FFFFFFFFFFFFFFFFFFFFFFFFFFFFFFFFFFFFFFFFFFFFFFF
FFFFFFFFFFFFFFF
@A00721:190:HGJW7DSXY:4:1475:32217:5588 2:N:0:TCTGCAAG+GCTGCACT
CCCCAATATAATGCTTAATATGCGAATGTCCCTGATTTAACAGTAATGGGGACTCCTTCATCTTCTTC
ATCGCGTTTCTGCATTAACGCGAAGAATTATTTCTTAACTTATTCAAGATGTCCAATTTCCAAAGAAA
TTGTTCTCGACCAAA
+
FFFFFFFFFFFFFFFFFFFFFFFFFFFFFFFFFFFFFFFFFFFFFFFFFFFFFFF:FFFFFFFFFFFF
FFFFF:FFFFFFFFFFFFFFFFFFFFFFFFFFFFFFFFF::FFFFFFFFFFFFF:FFFFFFF:F,FFFF:
FFFFFFFF:FF,FFF
@A00721:190:HGJW7DSXY:4:1507:23981:15718 2:N:0:TCTGCAAG+GCTGCACT
CCCCAATATAATGCTTAATATGCGAATGTCCCTGATTTAACAGTAATGGGGACTCCTTCATCTTCTTC
ATCGCGTTTCTGCATTAACGCGAAGAATTATTTCTTAACTTATTCAAGATGTCCAATTTCCAAAGAAA
TTGTTCTCGACCAAA
+
FFFFFFFFFFFFFFFFFFFFFFFFFFFFFFFFFFFFFFFFFFFFFFFFFFFFFFFFFFFFFFFFFFFF
FFF:FFFFFFFFFFFFFFFFFFFFFFFFFFFFFFFFFFFFFFFFFFFFFFFFFFFFFFFFFFFFFFFF
FFFFFFFFFFFFFFF
@A00721:190:HGJW7DSXY:4:1535:19470:26694 2:N:0:TCTGCAAG+GCTGCACT
CCCCAATATAATGCTTAATATGCGAATGTCCCTGATTTAACAGTAATGGGGACTCCTTCATCTTCTTC
ATCGCGTTTCTGCATCAACGCGAAGAATTATTTCTTAACTTATTCAAGATGTCCAATTTCCAAAGAAA
TTGTTCTCGACCAAA
+
FFFFFFFFFFFFFFFFFFFFFFFFFFFFFFFFFFFFFFFFFF:FFFFFFFFFFFFFFFFFFFFFFFFFF
FFFFFFFFFFFFFF,FFFFFFFFFF:FFFFFFFFFFFFFFFFFFFFFFF,FFFFFF:FFFF:FFFF,F:
FFFFF:FFFFFFFFF
@A00721:190:HGJW7DSXY:4:2650:28031:10050 2:N:0:TCTGCAAG+GCTGCACT
CCCCAATATAATGCTTAATATGCGAATGTCCCTGATTTAACAGTAATGGGGACTCCTTCATCTTCTTC
ATCGCGTTTCTGCATTAACGCGAAGAATTATTTCTTAACTTATTCAAGATGTCCAATTTCCAAAGAAA
TTGTTCTCGACCAAA
+
FFFFFFFFFFFFFFFFFFFFFFFFFFFFFFFFFFFFFFFFFFFFFFFFFFFFFFFFFFFFFFFFFFFF
FFFFFFFFFFFFFFFFFFFFFFFFFFFFFFFFFFFFFFFFFFFFFFFFF:FFFFFFFFFFFFFFFFFF
FFFFFFFF,FFFFFF
@A00721:190:HGJW7DSXY:4:1136:24505:32659 2:N:0:TCTGCAAG+GCTGCACT
TTTCTTTGGAAATTGGACATCTTGAATAAGTTAAGAAATAATTCTTCGCGTTAATGCAGAAACGCGAT
GAAGAAGATGAAGGAGTCCCCATTACTGTTAAATCAGGGACATTCGCATATTAAGCATTATATTGGGG
ACTGGGGACCTATAT
+
FFFFFFFFFFFFFFFFFFFFFFFFFFFFF:FFFFFFFFFFFFFFFFFFFFFF:FF,FFF:FFFFFF:FF
FFFFFFFFFFFFFFFFF:FFFFFFF,::FFFFFF:FF:FFFFFFFF:FFF:FFF,FFFF:FFF::,:F
:,FFF:FFFFFFFFF
@A00721:190:HGJW7DSXY:4:1213:15076:32612 2:N:0:TCTGCAAG+GCTGCACT
TTTCTTTGGAAATTGGACATCTTGAATAAGTTAAGAAATAATTCTTCGCGTTAATGCAGAAACGCGAT
GAAGAAGATGAAGGAGTCCCCATTACTGTTAAATCAGGGACATTCGCATATTAAGCATTATATTGGGG
ACTGGGGACCTATAT
+
FF:FF:FFFFF:FFFFFFFFFFFFFFFFFFFFFFFFFFFFFFF::FFFFFFFFFFF:FFFFFF:FFFFF:
FFFF:FFFFFFFFFFFFFFF:FFFFFFFFFFFFFFF::FFFFFF:F:FFFFFF,FFFF,FFFFFFFF
F:FFFFFFFFF,FFF
@A00721:190:HGJW7DSXY:4:1264:29234:26475 2:N:0:TCTGCAAG+GCTGCACT
TTTCTTTGGAAATTGGACATCTTGAATAAGTTAAGAAATAATTCTTCGCGTTAATGCAGAAACGCGAT
```

GAAGAAGATGAAGGAGTCCCCATTACTGTTAAATCAGGGACATTCGCATATTAAGCATTATATTGGGG
ACTGGGGACCTATAT
+
FFFFFFFFFFFFFFFFFFFFFFFFFFFFFFFFFFFFFFFFFFFFFFFFFFFFFFFFFFFFFFFFFFFF
FFFFFFFFFF:FFFFFFFFFFFFFFFFFFFFFFFFFFFFFFF,FFFFFFFFFFF:FFFFFFFFFFFFF
FFFFFFFFFFFFFFF
@A00721:190:HGJW7DSXY:4:1342:13557:26944 2:N:0:TCTGCAAG+GCTGCACT
TTTCTTTGGAAATTGGACATCTTGAATAAGTTAAGAAATAATTCTTCGCGTTAATGCAGAAACGCGAT
GAAGAAGATGAAGGAGTCCCCATTACTGTTAAATCAGGGACATTCGCATATTAAGCATTATATTGGGG
ACTGGGGACCTATAT
+
FFFFFFFFFFFFFFFFFFFFFFFFFFFFFFFFFFFFFFFFFFFFFFFFFFFFFFFFFFFFFFFFFFFF
FFFFFFFFFFFFFFFFFFFFFFFFFFFFFFFFFFFFFFFFFFFFFFFFFFFFFFFFFFF:FFFFF:FF
FFFFFFFFFFFFFFF
@A00721:190:HGJW7DSXY:4:1545:10601:21543 2:N:0:TCTGCAAG+GCTGCACT
TTTCTTTGGAAATTGGACATCTTGAATAAGTTAAGAAATAATTCTTCGCGTTAATGCAGAAACGCGAT
GAAGAAGATGAAGGAGTCCCCATTACTGTTAAATCAGGGACATTCGCATATTAAGCATTATATTGGGG
ACTGGGGACCTATAT
+
FFFFF:FFFF:FF:FF:FFFFFFFFFFFFFFFFFFFFFFFFFFFFFFFFFFFFFFFFFFF:FFFFFF:
FFFFFFFF,FF:FFFFFFFFFFFFFFFFFFFFFFFFFFFFFFFFFFFF::FFFFFFF:FFFFFFFFFF
FFFFFFFFFFFFFFF
@A00721:190:HGJW7DSXY:4:2519:29821:5979 2:N:0:TCTGCAAG+GCTGCACT
TTTCTTTGGAAATTGGACATCTTGAATAAGTTAAGAAATAATTCTTCGCGTTAATGCAGAAACGCGAT
GAAGAAGATGAAGGAGTCCCCATTACTGTTAAATCAGGGACATTCGCATATTAAGCATTATATTGGGG
ACTGGGGACCTATAT
+
FFFFFFFF:FFFFFFFFFFFFFFFFFFFFFFFFFFFFFFFF:FFFFF:FFFFFF:FFFFFFF::FFFF
FFFFFFFFFFFFF,FFFF:FFFFFFFFFFFFFFFFF:FFFFFFFF,FFFFFFFFFFFFFFFFF:FFFF
FFFFFFFFFFFFFFF
@A00721:190:HGJW7DSXY:4:1510:26395:35117 2:N:0:TCTGCAAG+GCTGCACT
GGAAATTGGACATCTTGAATAAGTTAAGAAATAATTCTTCGCGTTAATGCAGAAACGCGATGAAGAAG
ATGAAGGAGTCCCCATTACTGTTAAATCAGGGACATTCGCATATTAAGCATTATATTGGGGACTGGGG
ACCTATATATATGTA
+
FFFFFFFFFFF:FFFFFFFFFFFFFFFFFFFFFFFFFFFFFFFFFFFFFFF:FFFFFFFFFFFFFFFF
FFFFFFFFFFFFFFFFFFFFF:FFFFFFFFFFFFFFFFFFFFFFFFFFFFFFFFF:FFFFFFFFF:FF
:FFFFFFFFFFFFFF
@A00721:190:HGJW7DSXY:4:1556:12500:6386 2:N:0:TCTGCAAG+GCTGCACT
GGAAATTGGACATCTTGAATAAGTTAAGAAATAATTCTTCGCGTTAATGCAGAAACGCGATGAAGAAG
ATGAAGGAGTCCCCATTACTGTTAAATCAGGGACATTCGCATATTAAGCATTATATTGGGGACTGGGG
ACCTATATATATGTA
+
:FFFF:FFFFF:FFFFFFF,FFF:FFFFFFFFFFFFFFFFFFFFFFFFFFFFFFFFFFFFFFFFFF:F
FFFFFFFF:FFFFFFFFFFFFFFFFFFFF:FFFFFF:,,FFF:FFFFF,FFFFF,FFFFFF:FFFFFF
FFFFFFFFFFFFFFF
@A00721:190:HGJW7DSXY:4:2660:9715:10050 2:N:0:TCTGCAAG+GCTGCACT
GGAAATTGGACATCTTGAATAAGTTAAGAAATAATTCTTCGCGTTAATGCAGAAACGCGATGAAGAAG
ATGAAGGAGTCCCCATTACTGTTAAATCAGGGACATTCGCATATTAAGCATTATATTGGGGACTGGGG
ACCTATATATATGTA
+
:F:FFFFFFFFFFFFFFFFFFFFFFFFFFFFFFFFFFFFFFFFFFFFFFFFFFFFFFFFFFFFFFFFF
FFFFFFFFFFFFFFFFFFFFFFFFFFFFFFFFFFFFFFFFFFFFFFFFFFFFFFFFF:FFFFFFFFFF
FFFFFFFFFFFFFFF

```
@A00721:190:HGJW7DSXY:4:1512:10556:28573 2:N:0:TCTGCAAG+GCTGCACT
ATTCCTCCGGATTACTCTATACATATATATAGGTCCCCAGTCCCCAATATAATGCTTAATATGCGAAT
GTCCCTGATTTAACAGTAATGGGGACTCCTTCATCTTCTTCATCGCGTTTCTGCATTAACGCGAAGAA
TTATTTCTTAACTTA
+
FFFFFFFFFFFFFFFFF,FFFFFFFFFFFFFFFFFFFFFFFFFFFFFFFFFFFFFFFFFFFFFFFFFF
FFF:FFFFFFFFF:FFFFFFF:FFFFFFFFFFFFFFFFFFFFFFFFFFFFFFFFFFFFFFFFFFFFFF
FFFFFFFFFFFFFFF
@A00721:190:HGJW7DSXY:4:1607:25265:16438 2:N:0:TCTGCAAG+GCTGCACT
ATTCCTCCGGATTACTCTATACATATATATAGGTCCCCAGTCCCCAATATAATGCTTAATATGCGAAT
GTCCCTGATTTAACAGTAATGGGGACTCCTTCATCTTCTTCATCGCGTTTCTGCATTAACGCGAAGAA
TTATTTCTTAACTTA
+
FFF::FFFFFFFFFFFF:FFFFFFFF:FFFFF:FFFFFFFFF:FFFFFFFFF,F:FFFFFFF:F:FFF
FFF::FFFFFFFFFFFFFFFF:FFF::F,F:FFF:FFFFFFFFFF:F,:F::FFF:FFFFF:FFF,FF
FFF:FF,FFFFF:FF
@A00721:190:HGJW7DSXY:4:2302:14416:26021 2:N:0:TCTGCAAG+GCTGCACT
ATTCCTCCGGATTACTCTATACATATATATAGGTCCCCAGTCCCCAATATAATGCTTAATATGCGAAT
GTCCCTGATTTAACAGTAATGGGGACTCCTTCATCTTCTTCATCGCGTTTCTGCATTAACGCGAAGAA
TTATTTCTTAACTTA
+
F:FFFFFFFFFFFFFFFFFFFFFFFFFFFFFFFFFFFFFFFFFFFFFFFFFFFFFFFFFFFFFFFFFF
FFFFFFFF:FFFFFFFFFFFFFFFFFFFFFFFFFFFFFFFFFFFFF:FFFFFFFF,FF:FFFFFFFFF
FFFFFF:FFFFFFFF
@A00721:190:HGJW7DSXY:4:2549:18295:28635 2:N:0:TCTGCAAG+GCTGCACT
ATTCCTCCGGATTACTCTATACATATATATAGGTCCCCAGTCCCCAATATAATGCTTAATATGCGAAT
GTCCCTGATTTAACAGTAATGGGGACTCCTTCATCTTCTTCATCGCGTTTCTGCATTAACGCGAAGAA
TTATTTCTTAACTTA
+
FFFFFFFFFFF:FFFFFFFFFFFFFFFFFFFFFFFFFFFFFFFFFFFFFFFFFFFFFFFFFFFFFFFF
FFFFFFFFFFFFFFFFFFFFFFFFFFFFFF:F:FFFFFFFFFFFFFFF:FFFFFFFFFFFFFFFFFFF
FFFFFFFFFF:FFFF
@A00721:190:HGJW7DSXY:4:1129:23610:36714 2:N:0:TCTGCAAG+GCTGCACT
GATTCCTCCGGATTACTCTATACATATATATAGGTCCCCAGTCCCCAATATAATGCTTAATATGCGAA
TGTCCCTGATTTAACAGTAATGGGGACTCCTTCATCTTCTTCATCGCGTTTCTGCATTAACGCGAAGA
ATTATTTCTTAACTT
+
FFFFFFFFFFFFFFFFFFFFFFFFFFFFFFFFFFFFFFFFFFFFFFFFFFFFFFFFFFFFFFFFFFFF
FFFFFFFFFFFFFFFFFFFFFFFFFFFFFFFFFFFFFFFFFFFFFFFFFFFFFFFFFFFFF:FFFFFF
FFFFFFFFFFFFFFF
@A00721:190:HGJW7DSXY:4:1202:25880:25739 2:N:0:TCTGCAAG+GCTGCACT
GATTCCTCCGGATTACTCTATACATATATATAGGTCCCCAGTCCCCAATATAATGCTTAATATGCGAA
TGTCCCTGATTTAACAGTAATGGGGACTCCTTCATCTTCTTCATCGCATTTCTGCATTAACGCGAAGA
ATTATTTCTTAACTT
+
FFFFFFFFFFFFFFFFFFFFFFFFFFFFFFF:FFFFFFFFFFFFFFFFFFFFFFFFFFFFFFFFFFFF
FFFFFFFFF:FFFFFFFF,::FFFFFFFFFFFFFFFFFFFFFFFFFFFFFFFFFFFFFF:FFFFFFFF
F:FFFFFFFFFFFFF
@A00721:190:HGJW7DSXY:4:2527:21703:13777 2:N:0:TCTGCAAG+GCTGCACT
ATTCCTCCGGATTACTCTATACATATATATAGGTCCCCAGTCCCCAATATAATGCTTAATATGCGAAT
GTCCCTGATTGAACAGTAATGGGGACTCCTTCATCTTCTTCATCGCGTTTCTGCATTAACGCGAAGAA
TTATTTCTTAACTT
+
F:FF,,:,:FFF:FF,F,F,FFF::F:,FFF:F,:::FFFFFF,FFFFF,FF:F:F:FF:FF:FF::F
```

:FF,:FFF,,,F,:F,,,F,::,::FFF:,FFFFF::F,:FFF,FFF,,:F,:FFFFFF,,FFF,F:F
F,F:::F:,FF:::
@A00721:190:HGJW7DSXY:4:2606:11333:23437 2:N:0:TCTGCAAG+GCTGCACT
GATTCCTCCGGATTACTCTATACATATATATAGGTCCCCAGTCCCCAATATAATGCTTAATATGCGAA
TGTCCCTGATTTAACAGTAATGGGGACTCCTTCATCTTCTTCATCGCATTTCTGCATTAACGCGAAGA
ATTATTTCTTAACTT
+
FFFFFFFFFFF,FFFFFFFFF:FF:FFFFF:FF:FFFFFFFFFFFFFFFFFFFFFFFFF:FFF,F:FF
FFFF:FFFFFF:FFFFFFFFFFFFFFFFFFFFFFFFFF,FF,,FFFFF:FFFF:,FFFF::F:FF:FF
FFFFFFFFFFFFFF:
@A00721:190:HGJW7DSXY:4:1212:28212:23140 2:N:0:TCTGCAAG+GCTGCACT
AAATAATTCTTCGCGTTAATGCAGAAACGCGATGAAGAAGATGAAGGAGTCCCCATTACTGTTAAATC
AGGGACATTCGCATATTAAGCATTATATTGGGGACTGGGGACCTATATATATGTATAGAGTAATCCGG
AGGAATCTATCCCCA
+
FFFFFFFFFFFFF:FFFFFFFFFFFFFFFFF:,FF::FFFF,FFFFFFFFF:FFF:FFF,FFFFFFFF
FFFFFFFFFFFFFFFFFFFFFFFFFF:FFFFFF:FFFFFFFFF:,F,FFFFFFFF:FFFFFFFFFFFF
FFFF,FFFFFFFFFF
@A00721:190:HGJW7DSXY:4:1240:8983:33301 2:N:0:TCTGCAAG+GCTGCACT
AAATAATTCTTCGCGTTAATGCAGAAACGCGATGAAGAAGATGAAGGAGTCCCCATTACTGTTAAATC
AGGGACATTCGCATATTAAGCATTATATTGGGGACTGGGGACCTATATATATGTATAGAGTAATCCGG
AGGAATCTATCCCCA
+
FFFFFFFFFFFFFFFFFFFFFFFFFFFFFFFFFFFFFFFFFFFFFFFFFFFFFFFFFFFFFFFFFFFF
FFFFFFFFFFFFFFFFFF:FFFFFFFFFFFFFFFFFFFFFFF:FFFFFFFFFFFFFFFFFFFFFFFFF
FFFFFFFFFFFFFFF
@A00721:190:HGJW7DSXY:4:1304:14443:22467 2:N:0:TCTGCAAG+GCTGCACT
AAATAATTCTTCGCGTTAATGCAGAAACGCGATGAAGAAGATGAAGGAGTCCCCATTACTGTTAAATC
AGGGACATTCGCATATTAAGCATTATATTGGGGACTGGGGACCTATATATATGTATAGAGTAATCCGG
AGGAATCTATCCCCA
+
FFFFFFFFFFFFFFFFFFFFFF:F:FFFFFFFFFFFF:::FFFFFFFFFFFFFF:FFFFFFFFFF:FF
:FFFFFFFFFFFFFFFFF:,FFFFFFFFFFFFFFFFF:FFFFFFFFFFFFFFFFFFFFFFFFFFFFFF
FFFFFFFFFFF:FFF
@A00721:190:HGJW7DSXY:4:1415:14543:33160 2:N:0:TCTGCAAG+GCTGCACT
AAATAATTCTTCGCGTTAATGCAGAAACGCGATGAAGAAGATGAAGGAGTCCCCATTACTGTTAAATC
AGGGACATTCGCATATTAAGCATTATATTGGGGACTGGGGACCTATATATATGTATAGAGTAATCCGG
AGGAATCTATCCCCA
+
FFFFFFFFFFFFFFFFFFFFFFFFFFFFFFFFFFFFFFF:FFFF:FFFFFFFFFFFFFFFFFFFFFFF
FFFFFFFFFFFFFF,FFFFFFFFFFFFFFFFFFFFFFFFFFFFFFFFF,FFF:FFFFFFFFFFFFFFF
FFFF,FFF:FFF:FF
@A00721:190:HGJW7DSXY:4:1673:19199:11099 2:N:0:TCTGCAAG+GCTGCACT
AAATAATTCTTCGCGTTAATGCAGAAACGCGATGAAGAAGATGAAGGAGTCCCCATTACTGTTAAATC
AGGGACATTCGCATATTAAGCATTATATTGGGGACTGGGGACCTATATATATGTATAGAGTAATCCGG
AGGAATCTATCCCCA
+
FFFFFFFFFFFFFFFFFFFFFFFFFFFFFFFFFFF:FFFFFFFFFFFFFFFFFFFFFFFFFFFFFFFF
FFFFFFFFFFFFFFF:FFFFF:FFFFFFFFFFFFFFFFFFFFF,FFFFF:FFFFFFFFFFFF:FFFFF
FFFFFFFFFFF:FFF
@A00721:190:HGJW7DSXY:4:2443:14009:31735 2:N:0:TCTGCAAG+GCTGCACT
AAATAATTCTTCGCGTTAATGCAGAAACGCGATGAAGAAGATGAAGGAGTCCCCATTACTGTTAAATC
AGGGACATTCGCATATTAAGCATTATATTGGGGACTGGGGACCTATATATATGTATAGAGTAATCCGG
AGGAATCTATCCCCA

+
FFFFFFFFFFFFFFFFFFFFFFFFF:FFFFFFFFF,FFFFFFFFFFFFFFFFFFFFFFFFFFFFFFFFFF
FFFFFFFFFFFFFFFFFFFFFFFFFFFFFFFFFFFFFFFFFFFFFF:FFFFFFFFFF:FFFFFFFFFFFF
FFFFFFFFFFFFFFF
@A00721:190:HGJW7DSXY:4:2671:9896:20854 2:N:0:TCTGCAAG+GCTGCACT
AAATAATTCTTCGCGTTAATGCAGAAACGCGATGAAGAAGATGAAGGAGTCCCCATTACTGTTAAATC
AGGGACATTCGCATATTAAGCATTATATTGGGGACTGGGGACCTATATATATGTATAGAGTAATCCGG
AGGAATCTATCCCCA
+
FFFFFFFFFFFFFFFFFFFFFFFFFFFFFFFFFFFFFFFFFFFFFFFFFFFFFFFFFFFFFFFFFFFFFF
FFFFFFFFFFFFFFFFFFFFFFFFFFFFFFFFFFFFFFFFFFFFF:FFFFFFFFFFFFFFFFFFFFFFFF
FFFF,FFFFFFFFFF
@A00721:190:HGJW7DSXY:4:2671:9905:21183 2:N:0:TCTGCAAG+GCTGCACT
AAATAATTCTTCGCGTTAATGCAGAAACGCGATGAAGAAGATGAAGGAGTCCCCATTACTGTTAAATC
AGGGACATTCGCATATTAAGCATTATATTGGGGACTGGGGACCTATATATATGTATAGAGTAATCCGG
AGGAATCTATCCCCA
+
FFFFFFFFFFFFFFFFFFFFFFFFFFFFFFFFFFFFFFFFFFFF,FFFFFFFFFFFF:FFFFFFFFFFFF
FFFFFFFFFFFF,FF,FFFFFFFFFFFFFFFFFFFFFFFFFFFFFFFFFFFFFFFFFFFFFFFFFFFFFF
FFFF,FFFFFFFFFF
@A00721:190:HGJW7DSXY:4:1111:14986:12853 2:N:0:TCTGCAAG+GCTGCACT
CTTCGCGTTAATGCAGAAACGCGATGAAGAAGATGAAGGAGTCCCCATTACTGTTAAATCAGGGACAT
TCGCATATTAAGCATTATATTGGGGACTGGGGACCTATATATATGTATAGAGTAATCCGGAGGAATCT
ATCCCCAATTGGAAT
+
FFFFFFFFFFFFFFFFFFFFFFFFFFFFFFFFFFFFFFFFFFFFFFFFF:F:FFFFFFFFFFFFFFFFFF
FFFF,FFFFF,FFFFFFFFFFFFFFFFFFFFFFFFFFFFFFFFFFFFFF:FFFFFFFFFFFFFFFFFFFF
FFFFFFFFFFFFF:F
@A00721:190:HGJW7DSXY:4:2242:18837:25285 2:N:0:TCTGCAAG+GCTGCACT
CTTCGCGTTAATGCAGAAACGCGATGAAGAAGATGAAGGAGTCCCCATTACTGTTAAATCAGGGACAT
TCGCATATTAAGCATTATATTGGGGACTGGGGACCTATATATATGTATAGAGTAATCCGGAGGAATCT
ATCCCCAATTGGAAT
+
FFFFFFFFFFFFFFFFFFFFFFFFFFFFFFF:FFFFFFFFFFFFFFFFFFFFFFFFFFFFFFFFFF:FFF
FFFFFFFFFFFFFFFFFFFFFFFFFFFFFFF:FFFFFFFFFFFFFF,F::FFFFFFFFFFFFFF:FFF
FFFFFFFFFFFFFFF
@A00721:190:HGJW7DSXY:4:2457:1253:6355 2:N:0:TCTGCAAG+GCTGCACT
CTTCGCGTTAATGCAGAAACGCGATGAAGAAGATGAAGGAGTCCCCATTACTGTTAAATCAGGGACAT
TCGCATATTAAGCATTATATTGGGGACTGGGGACCTATATATATGTATAGAGTAATCCGGAGGAATCT
ATCCCCAATTGGAAT
+
:FFFFFFFFFFFFFFFFFFFFFFFFFFFFFFFFFFFFFFFFFFFFFFFFFFFFFFFFFFFFFFFFFFFFF
FFFFFFFFFFFFFFFFFFFFFFFFFFFFFFFFFFFFFFFFFFFFFFFFFFFFFFFFFFFFFFFFFFFF:F
FFFFFFFFFFFFFFF
@A00721:190:HGJW7DSXY:4:2548:14787:14262 2:N:0:TCTGCAAG+GCTGCACT
CTTCGCGTTAATGCAGAAACGCGATGACGAAGATGAAGGAGTCCCCATTACTGTTAAATCAGGGACAT
TCGCATATTAAGCATTATATTGGGGACTGGGGACCTATATATATGTATAGAGTAATCCGGAGGAATCT
ATCCCCAATTGGAAT
+
FFFFFFFFFFFFFFFFFFFFFFFF:,FF,FFFF:FFFFFFFFFFFFFFFFFFFFFFFFFFFFFFFFFFFF
FFFFFFF,F:FFFFFFFFF:FFFFFFFFFFFFFFFFFFFFFFFFFFFFF:F:FFFFFFFFFFFFFFFFFF
FFFFFFFFFFFFFFF
@A00721:190:HGJW7DSXY:4:1214:22996:14606 2:N:0:TCTGCAAG+GCTGCACT
CGCGATGAAGAAGATGAAGGAGTCCCCATTACTGTTAAATCAGGGACATCCGCATATTAAGCATTATA

TTGGGGACTGGGGACCTATATATATGTATAGAGTAATCCGGAGGAATCTATCCCCAATTGGAATAGTA
ATGATTTCAAATTTC
+
FFFFFFFFFFFFF,:FFFFFFFFFFFFFFFFFFFFFFFFFFFFFFFFFFFFFFFFFFFFFFFFFFFFFFF
FFFFFFFFFFFFFFFFFFFFFFFFFFFF,FFFFFFFFFFFFFFFFFFFFFFFFFFFFFFFFFFFFFF:FF
FFFFFFFFFFFFFFF
@A00721:190:HGJW7DSXY:4:1244:17463:10347 2:N:0:TCTGCAAG+GCTGCACT
CGCGATGAAGAAGATGAAGGAGTCCCCATTACTGTTAAATCAGGGACATCCGCATATTAAGCATTATA
TTGGGGACTGGGGACCTATATATATGTATAGAGTAATCCGGAGGAATCTATCCCCAATTGGAATAGTA
ATGATTTCAAATTTC
+
FFFFFFFFFFFFFFFFFFFFFFFFFFFFFFFFFFFFFFFFFFFFFFFFFFFFFFFFFFFFFFFFFFFFFF
FFFFFFFFFFFFFFFFFFFFFFFFFFFFFFFFFFFFFFFFFFFFFFFFFFFFFFFFFFFFF:FFFFFFFF
FFFFF:FFFF:FFFF
@A00721:190:HGJW7DSXY:4:1674:29116:17472 2:N:0:TCTGCAAG+GCTGCACT
CGCGATGAAGAAGATGAAGGAGTCCCCATTACTGTTAAATCAGGGACATCCGCATATTAAGCATTATA
TTGGGGACTGGGGACCTATATATATGTATAGAGTAATCCGGAGGAATCTATCCCCAATTGGAATAGTA
ATGATTTCAAATTTC
+
FFFFFFFFFF:FFFFFFFFFFFFFFFFFFFFFFFFFFFFFFFFFFFFF:FFFFFFFFFFFFFFFFFFFFF
:FFFFFFFFFFFFFFFFF:FFFFFFFFFFFFFFFFFFFFFFFFFFF,FFFFFFFFFFFFFFFFFFFFFFF
FFFFFFFFFFFFFFF
@A00721:190:HGJW7DSXY:4:2536:3794:34898 2:N:0:TCTGCAAG+GCTGCACT
CGCGATGAAGAAGATGAAGGAGTCCCCATTACTGTTAAATCAGGGACATCCGCATATTAAGCATTATA
TTGGGGACTGGGGACCTATATATATGTATAGAGTAATCCGGAGGAATCTATCCCCAATTGGAATAGTA
ATGATTTCAAATTTC
+
FFFFFFFFFFFFFFFFFFFFFFFFFFFFFFFFFFFFFFFFFFFFFFFFFFFFFFFFFFFFFFFFFFFFF:
FFFFFFFFFFFFFFFFFFFFFFFFFFFFFFFFFFFFFFFFFFFFFFFFFFF:FFFFFFFFFFFFFFFFFF
FFFFFFFFFF:FFFF
@A00721:190:HGJW7DSXY:4:1656:14814:8672 2:N:0:TCTGCAAG+GCTGCACT
CGGGATATTTTCAAATTTGAAATTTGAAATCATTACTATTCCAATTGGGGATAGATTCCTCCGGATTA
CTCTATACATATATATAGGTCCCCAGTCCCCAATATAATGCTTAATATGCGGATGTCCCTGATTTAAC
AGTAATGGGGACTCC
+
FFFFFFFFFFFFFFFFFFFFFFFFFFFFFFFFFFFFFFFFFFFFFFFFFFFFFFFFF,FFFFFFFFFFFF
FFFFFFFFF:FFFFFFFFFFFFFFFFFFFFFFFFFFFFFFFFFFFF:FFFFFFFFFFFFFFFFFFFF:FF
:FFFF:F:FFFFFFF
@A00721:190:HGJW7DSXY:4:1656:15311:9627 2:N:0:TCTGCAAG+GCTGCACT
CGGGATATTTTCAAATTTGAAATTTGAAATCATTACTATTCCAATTGGGGATAGATTCCTCCGGATTA
CTCTATACATATATATAGGTCCCCAGTCCCCAATATAATGCTTAATATGCGGATGTCCCTGATTTAAC
AGTAATGGGGACTCC
+
FFFFFFFFFFFFFFFFFFFFFFFFFFFFFFFFFFFF:F:FFFFFFFFFFFFFFFFFFFFFFFFFFFFFFF
FFFFFFFFFFFF:FFFFFFFFFFFFFFFFFFFFFFFFFFFFFFFFFFFFFFFFFFFFFFFFFFFFFF:FF
FFF:FFFFFFFFF,:
@A00721:190:HGJW7DSXY:4:2265:17445:18208 2:N:0:TCTGCAAG+GCTGCACT
CGGGATATTTTCAAATTTGAAATTTGAAATCATTACTATTCCAATTGGGGATAGATTCCTCCGGATTA
CTCTATACATATATATAGGTCCCCAGTCCCCAATATAATGCTTAATATGCGGATGTCCCTGATTTAAC
AGTAATGGGGACTCC
+
FFFFFFFFFFFFFFFFFFFFFFFFFFFFFFFFFFFFFFFFFFFFFFFFFFFFFFFFFFFFFFFFFFFFFF
FFFFFFFFFFFFFFFFFFFFFFFFFFFFFFFFFFFFFFFFFFFFFFFFFFFFFFFFFFFFFFFFFFFFFF
FFFFFFFFFFFFFF:

@A00721:190:HGJW7DSXY:4:2601:1353:36401 2:N:0:TCTGCAAG+GCTGCACT
CGGGATATTTTCAAATTTGAAATTTGAAATCATTACTATTCCAATTGGGGATAGATTCCTCCGGATTA
CTCTATACATATATATAGGTCCCCAGTCCCCAATATAATGCTTAATATGCGGATGTCCCTGATTTAAC
AGTAATGGGGACTCC
+
FFFFFFFFFFFFFFFFFFFFFFF:FFFFFFFFFFFFFFFF,F:FF:F:F,FFFFF:FF:FFFFF:F:FFF
FFFFFFF:FFFFFFFFFFFFFFFFFF,FFFF:FFFFFF,FFFFFFFFFFFF:FFFFFFF,FFFF,FFFFF
FF:FFFFF:FF,FFF
@A00721:190:HGJW7DSXY:4:1216:30273:30091 2:N:0:TCTGCAAG+GCTGCACT
TTTGAATTGGTATACTGTAGGGCGGGATATTTTCAAATTTGAAATTTGAAATCATTACTATTCCAATT
GGGGATAGATTCCTCCGGATTACTCTATACATATATATAGGTCCCCAGTCCCCAATATAATGCTTAAT
ATGCGGATGTCCCTG
+
FFFFFFFFFFFFFFF,FFF:FFFFFFFFFFFFFFFFF,FFFFF:FFFFFFFFFFFFFFFFFFFFFFFFFF
F:FF,FFFFFFFFFFFFFFFFFFFFFF:FFFFFFFF,FFFFFFFFFFF:FFFFFF:FFFFFFFFFFFFFF
FFFFFFFFFFFFFFF
@A00721:190:HGJW7DSXY:4:1221:11767:18364 2:N:0:TCTGCAAG+GCTGCACT
TTTGAATTGGTATACTGTAGGGCGGGATATTTTCAAATTTGAAATTTGAAATCATTACTATTCCAATT
GGGGATAGATTCCTCCGGATTTCTCTATACATATATATAGGTCCCCAGTCCCCAATATAATGCTTAAT
ATGCGAATGTCCCTG
+
F:FFFFFFFFFFFFFFFFFFFFFFFFFFFFFFFFFFFFFFFFFFFFFFFFFFFFFFFFFFFFFFFFFFFF
FFFFFFFFFFFFFFFFFFFFFFFFFFFFFFFFFFFFFFFF::FFFFFFFFFFFFFFFFFFFFFFFFFFFF
FFFFFFFFF:F,FFF
@A00721:190:HGJW7DSXY:4:1329:12509:31172 2:N:0:TCTGCAAG+GCTGCACT
TTTGAATTGGTATACTGTAGGGCGGGATATTTTCAAATTTGAAATTTGAAATCATTACTATTCCAATT
GGGGATAGATTCCTCCGGATTACTCTATACATATATATAGGTCCCCAGTCCCCAATATAATGCTTAAT
ATGCGGATGTCCCTG
+
FFFFFFF:FFF:FFFFFFFFFF:FFFF,FFFFFFFFFFFFFFFFFFFFFFFFFFFF:F:FFFFFFF:FF:
FFFFFFFFFFFFFFFFFFFFFFFFFFFFFFFFFFFFFFFFFFFFF:FFFFF:FFFFFFFF:FFF,FF,FF
FF,FFFFF:FFF:FF
@A00721:190:HGJW7DSXY:4:1455:17372:5306 2:N:0:TCTGCAAG+GCTGCACT
TTTGAATTGGTATACTGTAGGGCGGGATATTTTCAAATTTGAAATTTGAAATCATTACTATTCCAATT
GGGGATAGATTCCTCCGGATTACTCTATACATATATATAGGTCCCCAGTCCCCAATATAATGCTTAAT
ATGCGGATGTCCCTG
+
FFFFFFF:FFFFFFF:FFFFFFFFFFFFFFFFFFFFFFFFFFFFFFFFFFFFFFFFFFFFFFF,FFFFFF
FFFFFF,FFFFFFFFFFFFFFFFFFFFFFFFF:FFFFFF:FFFFFFFFFFFFFFFFFFFFFFFFFFFFFF
FFFFFFFFFFFFF,F
@A00721:190:HGJW7DSXY:4:1455:16342:8719 2:N:0:TCTGCAAG+GCTGCACT
TTTGAATTGGTATACTGTAGGGCGGGATATTTTCAAATTTGAAATTTGAAATCATTACTATTCCAATT
GGGGATAGATTCCTCCGGATTACTCTATACATATATATAGGTCCCCAGTCCCCAATATAATGCTTAAT
ATGCGGATGTCCCTG
+
FFFFFFFFFFFFFFFFFFFFFFFFFFFFFFFFFFFFFFFFFFFFFFFFFFFFFFFFFFFFFFFFFFFFFF
:FFFFFFFFFFFFFFFFFF:FFFFFFFFFFFFFFFFFFFFFFFFFFFFFFFFFFFFFFFFFFF:FFFFFF
FFFFFFFFFFFFFFF
@A00721:190:HGJW7DSXY:4:1542:17336:4460 2:N:0:TCTGCAAG+GCTGCACT
TTTGAATTGGTATACTGTAGGGCGGGATATTTTCAAATTTGAAATTTGAAATCATTACTATTCCAATT
GGGGATAGATTCCTCCGGATTACTCTATACATATATATAGGTCCCCAGTCCCCAATATAATGCTTAAT
ATGCGGATGTCCCTG
+
FFFFFFFFFFFFFFFFFFFFFFFFF:FFFFFFFFFFFFFFFFFFFFFFFFFFFFFFFFFFFFFFFFFFFF

```
FFFFFFFFFFFFFFFFF:FFFFFFFFFFFFFFFFFF,FFFFFFFF,FFFFFFFFFFFFFFFFFFFFFF
FFFFFFFFFFFFFF:
@A00721:190:HGJW7DSXY:4:2149:19199:32800 2:N:0:TCTGCAAG+GCTGCACT
TTTGAATTGGTATACTGTAGGGCGGGATATTTTCAAATTTGAAATTTGAAATCATTACTATTCCAATT
GGGGATAGATTCCTCCGGATTTCTCTATACATATATATAGGTCCCCAGTCCCCAATATAATGCTTAAT
ATGCGAATGTCCCTG
+
FFFFFFFFFFFFFFFFFFFFFFFFFFFFFFFFFFFFFFFFFFFFFFFFFFFFFFFFFFFFFFFFFFFF
FFFFFFFFFFFFFFFFFFFFFFFFFFFFFFFFFFFFFFFFFFFFFFFFFFFFFF:FFFFFFF:FFF
FFF:,FFFF::,FFF
@A00721:190:HGJW7DSXY:4:2356:3929:9549 2:N:0:TCTGCAAG+GCTGCACT
TTTGAATTGGTATACTGTAGGGCGGGATATTTTCAAATTTGAAATTTGAAATCATTACTATTCCAATT
GGGGATAGATTCCTCCGGATTTCTCTATACATATATATAGGTCCCCAGTCCCCAATATAATGCTTAAT
ATGCGAATGTCCCTG
+
FFFFFFFFFFFFFFFFFFFFFFFFFFFFFFFFFFFFFFFFFFFFFFFFFFFFFFFFFFFFFFFFFFFF
FFFFFFFFFFFFFFFFFFFFFFFFFFFFFFFFFFFFFFF,FFF:FFFFFFFFFFFFFFFFFFFFFFFF
FFFFFFFFFFFFFFF
@A00721:190:HGJW7DSXY:4:2361:11397:18411 2:N:0:TCTGCAAG+GCTGCACT
TTTGAATTGGTATACTGTAGGGCGGGATATTTTCAAATTTGAAATTTGAAATCATTACTATTCCAATT
GGGGATAGATTCCTCCGGATTACTCTATACATATATATAGGTCCCCAGTCCCCAATATAATGCTTAAT
ATGCGGATGTCCCTG
+
FFFFFFFFFFFFFFFFFFFFFFFFFFFFFFFFFFFFFFFFFFFFFFFFFFFFFFFFFFFFFFFFFFFF
FFFFFFFFFFFFFFFFFFFFFFFFFFFFFFFFFFFFFFFF:FFFFFFFFFFFFFFFFFFFFFFFFFFF
FFFFFFFFFFFFFFF
@A00721:190:HGJW7DSXY:4:2404:12165:34460 2:N:0:TCTGCAAG+GCTGCACT
TTTGAATTGGTATACTGTAGGGCGGGATATTTTCAAATTTGAAATTTGAAATCATTACTATTCCAATT
GGGGATAGATTCCTCCGGATTACTCTATACATATATATAGGTCCCCAGTCCCCAATATAATGCTTAAT
ATGCGGATGTCCCTG
+
FFFFFFFFFFFFFFFFFFFFFFFFFFFFFFFFFFFFFFFFFFFFFFFFFFFFFFFFFFFF:FFFFFFFF
FFFFFFFFFFFFFFFFFFFFFFFFFFFFFFFFF:FFFFFF::FF:FFFF:F:FFFF:FFFFF:FFFFFF
FF,F:FFFFFFF:FF
@A00721:190:HGJW7DSXY:4:2618:5773:23390 2:N:0:TCTGCAAG+GCTGCACT
TTTGAATTGGTATACTGTAGGGCGGGATATTTTCAAATTTGAAATTTGAAATCATTACTATTCCAATT
GGGGATAGATTCCTCCGGATTTCTCTATACATATATATAGGTCCCCAGTCCCCAATATAATGCTTAAT
ATGCGAATGTCCCTG
+
FFFFFFFFFFFFFFFFFFFFFFFFFFFFFFFFFFFFFFFFFFFFFFFFFFFFFFFFFFFFFFFFFFFF
FFFFFFFFFFFFFFFFFFFFFFFFFFFFFFFFFFFFFFFFFFFFFFFFFFFFFFFFFFFFFFFFFFFF
FFF,FFFFFFFFFFF
@A00721:190:HGJW7DSXY:4:1347:18954:30123 2:N:0:TCTGCAAG+GCTGCACT
CGGAGGAATCTATCCCCAATTGGAATAGTAATGATTTTCAAATTTGAAATTTGAAATCATTACTATTC
CAATTGGGGATAGATTCCTCCGGATTACTCTATACATATATATAGGTCCCCAGTCCCCAATATAATGC
TTAATATGCGATAGA
+
FFFFFFFFFFFFFFFFFFFFFFFFFFFFFFFFFFFFFFFFFFFF:FFFFFFFFFFFFFFFFFFFFFFF
FFFFFFFFFFFFFFFFFFFFFFFFFFFFFFFFFFFFFFFFFFFFFFFFFFFFFFFFFFFFFFFFFFFF
FFFFFFFFF:FFFFF
@A00721:190:HGJW7DSXY:4:1431:18358:3693 2:N:0:TCTGCAAG+GCTGCACT
CGGAGGAATCTATCCCCAATTGGAATAGTAATGATTTTCAAATTTGAAATTTGAAATCATTACTATTC
CAATTGGGGATAGATTCCTCCGGATTACTCTATACATATATATAGGTCCCCAGTCCCCAATATAATGC
TTAATATGCGATAGA
```

```
+
FFFFFFFFFFFFFFFFFF:FFFFFFFFFFFFFFFFFFFFFFFFFFFFFFFFFFFFFFFFFFFFFFF:FF
F:FFFFFFFFFFFFFFFFFFFFFFFFFFFFFFFFFFFFFFFFFFFFFFFFFFFFFFFFFFFFFFFFFFF
FFFFFFFFFFFFF:F
@A00721:190:HGJW7DSXY:4:2446:1787:6746 2:N:0:TCTGCAAG+GCTGCACT
AGGAATCTATCCCCAATTGGAATAGTAATGATTTTCAAATTTGAAATTTGAAATCATTACTATTCCAA
TTTGGGATAGATTCCTCCGGATTACTCTATACATATATATAGGTCCCCAGTCCCCAATATAATGCTTA
ATATGCGATAGA
+
FFFFF:F,FFF:FFFF:F:,F,FFFFF,F,FFFFF,FFF:F::FFFFFFFF:F:FFFFFFF,F::F:F
,,,FFFFFFFF:,FFFFF,FFFFF::F:F:FF,F::F,FFF,FFFFFFFF,FFFFFFFFFFFFFFFFF
F:F,F::FFFFF
@A00721:190:HGJW7DSXY:4:2573:15275:27414 2:N:0:TCTGCAAG+GCTGCACT
CGCATATTAAGCATTATATTGGGGACTGGGGACCTATATATATGTATAGAGTAATCCGGAGGAATCTA
TCCCCAATTGGAATAGTAATGATTTCAAATTTCAAATTTGAAAATATCCCGCCCTACAGTATACCAAT
TCAAATTCAAATTCA
+
FFFFFFFFFFFFFFFFFFFFFFFFFFFFFFFFFFFFFFFFFFFFFFFFFFFFF:FFFFFFFFF:FFFF
FFFFFFFFFF:FFFFFFFFFFFFFFFFFFFFFFFFFFFFF:FFFFFFFFFFFFFFFFFF,:FF:FFFFFF
,FFFFFFFFFFFFFF
@A00721:190:HGJW7DSXY:4:2660:18973:4633 2:N:0:TCTGCAAG+GCTGCACT
CGCATATTAAGCATTATATTGGGGACTGGGGACCTATATATATGTATAGAGTAATCCGGAGGAATCTA
TCCCCAATTGGAATAGTAATGATTTCAAATTTCAAATTTGAAAATATCCCGCCCTACAGTATACCAAT
TCAAATTCAAATTCA
+
FFF:FFFFFFFFFFFFFFFFFFFFFFFFFFFFFFFFFFFFFFFFFFFFF:FFF::FFFFFFFFF,FFFF
FFFFFFFFFFFFFFFFFFFFFFFFFFFFFFF:FFFFFFFFFFFFFFFFF:F,FFFFF:FFFFFFFFFFF
F:FFFFFFFFFFF,F
@A00721:190:HGJW7DSXY:4:1346:30129:22326 2:N:0:TCTGCAAG+GCTGCACT
TGGGGACTGGGGACCTATATATATGTATAGAGTAATCCGGAGGAATCTATCCCCAATTGGAATAGTAA
TGATTTCAAATTTCAAATTTGAAAATATCCCGCCCTACAGTATACCAATTCAAATTCAAATTCAAATA
GAATTCCATAGGCCC
+
FFFFFFFFFFF:FFFFFFFFFFFFFFFFFFFFFFFFFFFFFFFFFFFFF:FFFFFFFFFFFFFFFFFFF
FFFFFFFFFFFFFFFFFFFFFFFFFFFFFFFFFFFFFFFFFFFFFFFFFFFFFFFFFFFFFFFFFFFFF
FFFFFFFFFFFFFFF
@A00721:190:HGJW7DSXY:4:1351:19560:3677 2:N:0:TCTGCAAG+GCTGCACT
TGGGGACTGGGGACCTATATATATGTATAGAGTAATCCGGAGGAATCTATCCCCAATTGGAATAGTAA
TGATTTCAAATTTCAAATTTGAAAATATCCCGCCCTACAGTATACCAATTCAAATTCAAATTCAAATA
GAATTCCATAGGCCC
+
FFFFFFFFFFFFFFF:FFFFFFFFFFFFFFFFFFFFFFFFFFFFFFFFFFFFFFFFFFFFFFFFFFFFF
FFFFFFFFFFFFFFFFFFFFFFFFFFFFFFFFFFFFFFFFFFFFFFFFFFF:FFFFFFFFF:FFFFFFFF
FF:FFF:FFFFFFFF
@A00721:190:HGJW7DSXY:4:1564:11478:14700 2:N:0:TCTGCAAG+GCTGCACT
TGGGGACTGGGGACCTATATATATGTATAGAGTAATCCGGAGGAATCTATCCCCAATTGGAATAGTAA
TGATTTCAAATTTCAAATTTGAAAATATCCCGCCCTACAGTATACCAATTCAAATTCAAATTCAAATA
GAATTCCATAGGCCC
+
FFFFFFFFFFFFFFFFFFFFFFFFFFFFFFFFFFFFFFFFFFFFFFFF:FFFFFFFFFFFFFFFFFFFF
FFFFFFFFFFFFFFFFFFFFFFFFFFFFFFFFFFFFFF:FFFF:FFFFFFFFFFFFFFFFFFFFFFFFFF
FFFFFFFFFFFFFFF
@A00721:190:HGJW7DSXY:4:2130:29035:30953 2:N:0:TCTGCAAG+GCTGCACT
TGGGGACTGGGGACCTATATATATGTATAGAGTAATCCGGAGGAATCTATCCCCAATTGGAATAGTAA
```

TGATTTCAAATTTCAAATTTGAAAATATCCCGCCCTACAGTATACCAATTCAAATTCAAATTCAAATA
GAATTCCATAGGCCC
+
FFFFFFFFFFFFFFFFFFFFFFFFFFFFF:FFFFFFFFFFFFFFFFFFFFFFFFFFFFFFFFFFFF,F
FFFFFFFFFFFFFFFFFFFFFFF:FFF:F,FFFFFFFFFFFFFFFFFFFFFFFF:FFFFFFFFFFFFF
FFFFFFFFFFF:FFF
@A00721:190:HGJW7DSXY:4:2221:32117:31876 2:N:0:TCTGCAAG+GCTGCACT
TGGGGACTGGGGACCTATATATATGTATAGAGTAATCCGGAGGAATCTATCCCCAATTGGAATAGTAA
TGATTTCAAATTTCAAATTTGAAAATATCCCGCCCTACAGTATACCAATTCAAATTCAAATTCAAATA
GAATTCCATAGGCCC
+
FFFFFFFFFFFFFFFFFFFFFFFFFF:F,FFFFFFFFF:FFFF::FFFFFFFFFFFFFF,FFFFFFFF
FFFFFFFFFFFFFFFF:FFFFFFF:FFFFFFFFFFFFF:FFFFFFFFF:F:FF:FF:FFFFFFFFFFF
,FFFFF,FFFF:,FF
@A00721:190:HGJW7DSXY:4:2656:3821:23265 2:N:0:TCTGCAAG+GCTGCACT
TGGGGACTGGGGACCTATATATATGTATAGAGTAATCCGGAGGAATCTATCCCCAATTGGAATAGTAA
TGATTTCAAATTTCAAATTTGAAAATATCCCGCCCTACAGTATACCAATTCAAATTCAAATTCAAATA
GAATTCCATAGGCCC
+
FFFFFFFFFFFFFFFFFFFFFFFFFFFFFFFFFFFFFFFFFFFF:FFFFFFFFFFFFFFFF:FFFFFF
FFFFFFFFFFFFFFFFFFFFFFFFFFFFFFFFFFFFFFFFFFFFFFFFFFFFFFFFFFFFFFF::FF,
FF:FFFFFF:FFFFF
@A00721:190:HGJW7DSXY:4:1259:26449:18646 2:N:0:TCTGCAAG+GCTGCACT
CTGGGGACCTATATATATGTATAGAGTAATCCGGAGGAATCTATCCCCAATTGGAATAGTAATGATTT
CAAATTTCAAATTTGAAAATATCCCGCCCTACAGTATACCAATTCAAATTCAAATTCAAATAGAATTC
CATAGGCCCCGCAGG
+
FFFFFFFF:FFFFFF:FFFFFFFFFFFFFFFFFFFFFFFFFFFFFFFFFFFFFFF:FFFFFFFFFFFF
FFFFFFFFFFFFFFFFFFFFFFFFFFFFFFFFFFFFFFFFFFFFFFFFFFFFFFFFFF:FF:FFFFFF
FFFFFFFFFFFFFFF
@A00721:190:HGJW7DSXY:4:1413:26621:28463 2:N:0:TCTGCAAG+GCTGCACT
CTGGGGACCTATATATATGTATAGAGTAATCCGGAGGAATCTATCCCCAATTGGAATAGTAATGATTT
CAAATTTCAAATTTGAAAATATCCCGCCCTACAGTATACCAATTCAAATTCAAATTCAAATAGAATTC
CATAGGCCCCGCAGG
+
FFFFFFFFFFFFFFFFFFFFFFFFFFFFFFFFFFFFFFFFFFFFFFFFFFFFFFFFFFFFFFFFFFFF
FFFFFFFFFFFFFFFFFFFFFFFFFFFFFFFFFFFFFFFFFFFFFFFFFFFFFFFFFFFFFFFFFF:F
FFFFFFFFFFFFFFF
@A00721:190:HGJW7DSXY:4:2366:25256:34961 2:N:0:TCTGCAAG+GCTGCACT
CTGGGGACCTATATATATGTATAGAGTAATCCGGAGGAATCTATCCCCAATTGGAATAGTAATGATTT
CAAATTTCAAATTTGAAAATATCCCGCCCTACAGTATACCAATTCAAATTCAAATTCAAATAGAATTC
CATAGGCCCCGCAGG
+
FFFFFFFFFFFFFFFFFFFFFFFFFFFFFFF:FFFFFFFFFFFFFFFFFFFFFFFFFFFFFFFFFFFF
FFFFFFFFFFFFFFFFFFFFFFF:FFFFFFFFFFFFFFFFFFFF,FFFF:FFFFFFFFFFFFFFFFF:
FF:FFFFFF:FFFFF
@A00721:190:HGJW7DSXY:4:2366:24587:35994 2:N:0:TCTGCAAG+GCTGCACT
CTGGGGACCTATATATATGTATAGAGTAATCCGGAGGAATCTATCCCCAATTGGAATAGTAATGATTT
CAAATTTCAAATTTGAAAATATCCCGCCCTACAGTATACCAATTCAAATTCAAATTCAAATAGAATTC
CATAGGCCCCGCAGG
+
FFFFFFFFFFFFFFFFFFFFFFFFFFFFFFFFFFFFFFFFFFFFFFFFFFFFFFFFFFFFFFFFFFFF
FFFFFFFFFFFFFFFFFFFFFFFFFFFFFFFFFFF,FFFF,FFFFFFFFFFFFFFFFFF:FFFFFFFF
:FFFFFFFFFFFFFF

@A00721:190:HGJW7DSXY:4:1123:2618:33896 2:N:0:TCTGCAAG+GCTGCACT
GGGACCTATATATATGTATAGAGTAATCCGGAGGAATCTATCCCCAATTGGAATAGTAATGATTTCAA
ATTTCAAATTTGAAAATATCCCGCCCTACAGTATACCAATTCAAATTCAAATTCAAATAGAATTCCAT
AGGCCCCGCAGGGGC
+
FFFFFFFFFFFFFFFFFFFFFFFFFFFFFFFFFFFFFFFFFFFFFFFFFFFFF:FFFFFFFFFFFFFF
FFFFFFFFFFFFFFFFFFFFFFFFFFFFFFFFFFFFFFFFFFFFFFFFFFFFFFFFFFFFFFFF,FFF
FF:FFFFFFFFFFFF
@A00721:190:HGJW7DSXY:4:1142:27263:14387 2:N:0:TCTGCAAG+GCTGCACT
GGGACCTATATATATGTATAGAGTAATCCGGAGGAATCTATCCCCAATTGGAATAGTAATGATTTCAA
ATTTCAAATTTGAAAATATCCCGCCCTACAGTATACCAATTCAAATTCAAATTCAAATAGAATTCCAT
AGGCCCCGCAGGGGC
+
:F:FFFFFFFFFFFFFFFFFFF:FFFFFFFFFFFF:FFFFFFFF:FFFFFFFFFF:FFFFFFF:FFFF
FFFFFFF:FFFFFFFFFFFFFFFFFFFFFFF:FFFFFFF,FFFF:FFFFFFFFFFFFFFFFFFF:F:F
FF:FFFFFFFFFFFF
@A00721:190:HGJW7DSXY:4:2362:28357:34757 2:N:0:TCTGCAAG+GCTGCACT
GGGACCTATATATATGTATAGAGTAATCCGGAGGAATCTATCCCCAATTGGAATAGTAATGATTTCAA
ATTTCAAATTTGAAAATATCCCGCCCTACAGTATACCAATTCAAATTCAAATTCAAATAGAATTCCAT
AGGCCCCGCAGGGGC
+
FFFFFFFFFFFFFFFFFFFFFFFFF:FFFFFFFFFFFFFFFFFFFFFFFFFFFFFFFFFFFFFFFFFF
FFFFFFFFFFFF:FFFFFFFFFFFFFFFFFFFFFFFFFFFFFFFFFFFFF,FFFFF:FFF:,FFFFFF
FFFFFFFFF,FFFFF
@A00721:190:HGJW7DSXY:4:2412:30689:25332 2:N:0:TCTGCAAG+GCTGCACT
GGGACCTATATATATGTATAGAGTAATCCGGAGGAATCTATCCCCAATTGGAATAGTAATGATTTCAA
ATTTCAAATTTGAAAATATCCCGCCCTACAGTATACCAATTCAAATTCAAATTCAAATAGAATTCCAT
AGGCCCCGCAGGGGC
+
FFFFFFFFFFFFFFFFFFFFFFFFFFFFFFFFFFFF:FFFFFFFFFFFFFFFFFFFFFFFFFFFFFFF
FFFFFFFFFFFFFFFFFFFFFFFFFFFFFFFFFFFFFFFFFFF::FFFFFFFFFFFF:FFFFFFFFFF
FF:FFFFFF:FFFFF
@A00721:190:HGJW7DSXY:4:2441:26666:24251 2:N:0:TCTGCAAG+GCTGCACT
GGGACCTATATATATGTATAGAGTAATCCGGAGGAATCTATCCCCAATTGGAATAGTAATGATTTCAA
ATTTCAAATTTGAAAATATCCCGCCCTACAGTATACCAATTCAAATTCAAATTCAAATAGAATTCCAT
AGGCCCCGCAGGGGC
+
FFFFFFFFFFFFFFFFFFFFFFFFFFFFFFFFFFFFFFFFFFFFFFF:FF:FFFFFFFFFFFFFFFFF
F:FFFFFFFFFF:FFFFFFFFFFFFFFFFFFFFFFFFFF,FFFFFFFFFFFF:,FFF:F:FFFF,FFF
FF:FFFFFFFF:FFF
@A00721:190:HGJW7DSXY:4:1222:2492:22811 2:N:0:TCTGCAAG+GCTGCACT
GATGCCCCTGCGGGGCCTATGGAATTCTATTTGAATTTGAATTTGAATTGGTATACTGTAGGGCGGGA
TATTTTCAAATTTGAAATTTGAAATCATTACTATTCCAATTGGGGATAGATTCCTCCGGATTACTCTA
TACATATATATAGGT
+
FFFFFFFFFFFFFFFFF:FFFFF:F:FFFF:FFFFFFFFFFFFFFFFFFFFFFFFF:FFFFFFFFFFF
F:FFFFFFFFFFFF:FFFFFFFFFFFFFFFFF,FFFFFF,FFFFFFFFFFFFFFFFFFFFFFFFFFFF
FFFFFFFFF:FFFFF
@A00721:190:HGJW7DSXY:4:2216:32280:35477 2:N:0:TCTGCAAG+GCTGCACT
GATGCCCCTGCGGGGCCTATGGAATTCTATTTGAATTTGAATTTGAATTGGTATACTGTAGGGCGGGA
TATTTTCAAATTTGAAATTTGAAATCATTACTATTCCAATTGGGGATAGATTCCTCCGGATTACTCTA
TACATATATATAGGT
+
F:FFFFFFFFFFFFFFFFFFFFFFFFFFFFFFFFFFFFFFFFFFFFFFFFFFFFFFFFFFFFFFFFFF

FFFFFFFFFFFFFFFFFFFFFFFFFFFFFFF:FFFFFFFFFFF:FFFFFFFFFFFFFFFFFFFFFFFFFF
FFFFFFFFFFFFF,F
@A00721:190:HGJW7DSXY:4:1436:6017:29481 2:N:0:TCTGCAAG+GCTGCACT
GTAAGATGCCCCTGCGGGGCCTATGGAATTCTATTTGAATTTGAATTTGAATTGGTATACTGTAGGGC
GGGATATTTTCAAATTTGAAATTTGAAATCATTACTATTCCAATTGGGGATAGATTCCTCCGGATTAC
TCTATACATATATAT
+
FFFFFFFFFFFFFFFFFFFFFF:FFFFF:FF::FFFFFFFFFFFFFFFFFFFFFFFFFFFFFFFFFFFF
FFFFFFFFFFFF:FFFFFFFFFFFFF,FFF,FFFFFFFFFFFFFFFFFFFFFFFF:FFFFFFFFFFFFF
FFF,FFFFFFFFFFF
@A00721:190:HGJW7DSXY:4:1606:21043:24408 2:N:0:TCTGCAAG+GCTGCACT
GTAAGATGCCCCTGCGGGGCCTATGGAATTCTATTTGAATTTGAATTTGAATTGGTATACTGTAGGGC
GGGATATTTTCAAATTTGAAATTTGAAATCATTACTATTCCAATTGGGGATAGATTCCTCCGGATTAC
TCTATACATATATAT
+
FFFFFFFFFFFFFFFFFFFFFF:FFFFF,FFFFFFFFFFFFFFFFFFFFFFFFFFFFFFFFFFFFFFFF
FFFFFFFFFFFFFFFFFFFFFFFFFF:FFFFFFFFFFFFFFFFFFFFFFFFFFFF:FFFFFFFFFFFFF
FFF,F,FFFF:FFFF
@A00721:190:HGJW7DSXY:4:1651:23927:18004 2:N:0:TCTGCAAG+GCTGCACT
GTAAGATGCCCCTGCGGGGCCTATGGAATTCTATTTGAATTTGAATTTGAATTGGTATACTGTAGGGC
GGGATATTTTCAAATTTGAAATTTGAAATCATTACTATTCCAATTGGGGATAGATTCCTCCGGATTAC
TCTATACATATATAT
+
FFFFFFFFFFFFFFFFFFFFFF:FFFFFF:FFFF:FFFFF:FFFFFFFFFFFFFFFFFFFFFFFFFFFF
FFFFFFFFFFFFFFFFFFF:FFFFFFFFFFFFFFFFFFFFFFFFFFFFF:FFFFFFF:FFFFFFFF,FFFF
FFFFFFFFFFFFFFF
@A00721:190:HGJW7DSXY:4:2525:7708:18192 2:N:0:TCTGCAAG+GCTGCACT
GTAAGATGCCCCTGCGGGGCCTATGGAATTCTATTTGAATTTGAATTTGAATTGGTATACTGTAGGGC
GGGATATTTTCAAATTTGAAATTTGAAATCATTACTATTCCAATTGGGGATAGATTCCTCCGGATTAC
TCTATACATATATAT
+
FFFFFFFFFFFFFFFFFFFFFF:FFFFFFFFFF:F:FFFFFFFFFFFFFFF:FFFFFFFFFFFFFFFFF
FFFFFFFFFFFFFFFFFFFFFFFFFFFFFFFFFFFFFFFFFFFFFFFFFFF:FFFFFFFFFFFFFFFFF
FFFFFFFFFFFFFFF
@A00721:190:HGJW7DSXY:4:1357:30698:19617 2:N:0:TCTGCAAG+GCTGCACT
CACGGTCTGCGGTAAGATGCCCCTGCGGGGCCTATGGAATTCTATTTGAATTTGAATTTGAATTGGTA
TACTGTAGGGCGGGATATTTTCAAATTTGAAATTTGAAATCATTACTATTCCAATTGGGGATAGATTC
CTCCGGATTACTCTA
+
FFFFFFFFFFFFFFFFFFFFFFFFFFFFFFFFFFFFFFFF:FFFFFFFFFFFFFFFFFFFFFFFFFFFFF
FFFFFFFFFFFFFFFFFFFFFFFFFF:FFFF:FFFFFFFFFFFFFFFFFFFFFFFFFFFFFFFFFFFFFF
FFFFFFF,FFFFFFF
@A00721:190:HGJW7DSXY:4:1471:23294:3411 2:N:0:TCTGCAAG+GCTGCACT
CACGGTCTGCGGTAAGATGCCCCTGCGGGGCCTATGGAATTCTATTTGAATTTGAATTTGAATTGGTA
TACTGTAGGGCGGGATATTTTCAAATTTGAAATTTGAAATCATTACTATTCCAATTGGGGATAGATTC
CTCCGGATTACTCTA
+
FFF:FFFFFFFFFFFF,FFFFFFFFFFFFFFFFFFFFFF:FFFFFFFFFFFFFFFFF:FFFFFFFFFFF
FFFFFF:F:FFFFFFFFFFFFFFFFFFFF:FFFFFFFFFFFF,FFFFFFFFFF,FFFFF,:,FFFFFFFF
FFFFF:FFFFFFFFF
@A00721:190:HGJW7DSXY:4:2177:32045:21136 2:N:0:TCTGCAAG+GCTGCACT
CACGGTCTGCGGTAAGATGCCCCTGCGGGGCCTATGGAATTCTATTTGAATTTGAATTTGAATTGGTA
TACTGTAGGGCGGGATATTTTCAAATTTGAAATTTGAAATCATTACTATTCCAATTGGGGATAGATTC
CTCCGGATTACTCTA

+
FFFFFFFFFFFFFFFFFFFFFFFFFFFFFFFFFFFFFFFFFFFFFFF:FFFFFFFFFFFFFF:FFFFFFF
F:FFFFFFFFFFFFFFFFFFFFFFFFFFFFFFFFFFFF:,FFFFFFFFFFFFF:FFFF:FFFFFFFFFFF:
FFFFFFFFFFFFFFF
@A00721:190:HGJW7DSXY:4:2201:10809:17143 2:N:0:TCTGCAAG+GCTGCACT
CACGGTCTGCGGTAAGATGCCCCTGCGGGGCCTATGGAATTCTATTTGAATTTGAATTTGAATTGGTA
TACTGTAGGGCGGGATATTTTCAAATTTGAAATTTGAAATCATTACTATTCCAATTGGGGATAGATTC
CTCCGGATTACTCTA
+
FFFFFFFFFFFF,FFFFFFFFFFFFFFFFFFFFFFFFFFFFFFFFFFFFFFFF:FFF:FFFFFF:FFFF:
FFFFFFFFF:FFFFFFFFFFFFFFFFFFFFFFFFFFFFFFFFFFFFFFFFFFFFFFFF:FFFFFFFF
F:FFFFFFFFFFFFF
@A00721:190:HGJW7DSXY:4:1418:10004:3568 2:N:0:TCTGCAAG+GCTGCACT
CCACGGTCTGCGGTAAGATGCCCCTGCGGGGCCTATGGAATTCTATTTGAATTTGAATTTGAATTGGT
ATACTGTAGGGCGGGATATTTTCAAATTTGAAATTTGAAATCATTACTATTCCAATTGGGGATAGATT
CCTCCGGATTACTCT
+
F:FFFFFFFFF:FFFFFFFFFFFFFFFFFFFFFFFFFFFFFFFFFFFFFFFFFFFFFFFFFFFFFFF:F
FFFFFFFFFFFFFFFFFFFFFF:FFFFFFF,FFFFFFFFFFFFFFFFFFFFFFFFFFFFFFFFFFFFFF
FFFFFFFF:FFFFFF
@A00721:190:HGJW7DSXY:4:1525:7880:20525 2:N:0:TCTGCAAG+GCTGCACT
CCACGGTCTGCGGTAAGATGCCCCTGCGGGGCCTATGGAATTCTATTTGAATTTGAATTTGAATTGGT
ATACTGTAGGGCGGGATATTTTCAAATTTGAAATTTGAAATCATTACTATTCCAATTGGGGATAGATT
CCTCCGGATTACTCT
+
FFFFFFFFFFFFFFFFFFFFFFFFFFFFFFFFFFFFFFFFFFFFFFFFFFFFFFFFFF:FFFFFFFFFF
FFFFFFFFFFFFFFFFFFFFFFFFFFFFFFFFFFFFFFFFFFFFFFFFFFFFFFFFFFFFFFFFFFFFF
FFFFFFFFFFFFFFF
@A00721:190:HGJW7DSXY:4:1526:9317:11960 2:N:0:TCTGCAAG+GCTGCACT
CCACGGTCTGCGGTAAGATGCCCCTGCGGGGCCTATGGAATTCTATTTGAATTTGAATTTGAATTGGT
ATACTGTAGGGCGGGATATTTTCAAATTTGAAATTTGAAATCATTACTATTCCAATTGGGGATAGATT
CCTCCGGATTACTCT
+
FFFFFFFFFFFFFFFFFFFFFFFFFFFFFFFFFFFFFFFFFFFFFFFFFFFFFFFFFFFFFFFFFFFFF
FFFFFFFFFFFFFFFFFFFFFFFFFFFFFFFFFFFFFFFFFFFFFFFFF:FFFFFFFFFFFFFFFFFFF
FFFFFFFFFFFFFFF
@A00721:190:HGJW7DSXY:4:1529:9091:8594 2:N:0:TCTGCAAG+GCTGCACT
CCACGGTCTGCGGTAAGATGCCCCTGCGGGGCCTATGGAATTCTATTTGAATTTGAATTTGAATTGGT
ATACTGTAGGGCGGGATATTTTCAAATTTGAAATTTGAAATCATTACTATTCCAATTGGGGATAGATT
CCTCCGGATTACTCT
+
FFFFFFFFFFFFFFFFFFFFFFFFFFF:FFFFFFFFFFFFFFFFFFFFFFFFFFFFFFFFFFFFFFFFF
FFFFFFFFFFFFFFFFFFFFFFFFFFFFFF:FFFFFF:FFFFFFFFFFFFFFFFFFFFFFFFFFF:FFF
FFFFFFFFFFFFFFF
@A00721:190:HGJW7DSXY:4:1547:18900:14309 2:N:0:TCTGCAAG+GCTGCACT
CCACGGTCTGCGGTAAGATGCCCCTGCGGGGCCTATGGAATTCTATTTGAATTTGAATTTGAATTGGT
ATACTGTAGGGCGGGATATTTTCAAATTTGAAATTTGAAATCATTACTATTCCAATTGGGGATAGATT
CCTCCGGATTACTCT
+
FFFFFFFFFFFFFFF:FFFFFFFFFFFF:FFFFFFFFFFFFFFFFF::FFF::FFFFFFFFFFFFFF:
FFFFFFFF:FFFFFFFFFF,FFFFFFFF,FFFFFFFFFFFFFFFFFFFFFF:F:FFFFFF:FFFFFFFF
F:FFFFFFFFF:FFF
@A00721:190:HGJW7DSXY:4:1565:11686:23109 2:N:0:TCTGCAAG+GCTGCACT
CCACGGTCTGCGGTAAGATGCCCCTGCGGGGCCTATGGAATTCTATTTGAATTTGAATTTGAATTGGT

```
ATACTGTAGGGCGGGATATTTTCAAATTTGAAATTTGAAATCATTACTATTCCAATTGGGGATAGATT
CCTCCGGATTACTCT
+
FFFFFFFFFFFFFFFFFFFFFFFFFFFFFFFFFFFFFFFFFFFFFF:FFFFFFFF:FFFFFFFFFFFF
FFFFFFFFFFFFFFFFFFFFFFFFFFFFFFFFFFFFFFFFFFFFFFFFFFFFFFFFFF:FFFFFFFFF
FFFFFFFFFFFFFFF
@A00721:190:HGJW7DSXY:4:1162:21649:31970 2:N:0:TCTGCAAG+GCTGCACT
GTAATCCGGAGGAATCTATCCCCAATTGGAATAGTAATGATTTCAAATTTCAAATTTGAAAATATCCC
GCCCTACAGTATACCAATTCAAATTCAAATTCAAATAGAATTCCATAGGCCCCGCAGGGGCATCTTAC
CGCAGACCGTGGGGC
+
FFFFFFFFFFFFFFFFFFFFFFFFFFFFFFFFFFFFFFFF:FFFFFFFFFFFFFFFFFF:FFFFFFFF
FFFFF:FFFFFFFFFFFFFFFF,FFFFFFFFFFF:FFFFFFFFFFFFFF:FFF:FFFFFFFFFFFFFF
FFFFFFF:FFFFFFF
@A00721:190:HGJW7DSXY:4:1223:7346:19038 2:N:0:TCTGCAAG+GCTGCACT
GTAATCCGGAGGAATCTATCCCCAATTGGAATAGTAATGATTTCAAATTTCAAATTTGAAAATATCCC
GCCCTACAGTATACCAATTCAAATTCAAATTCAAATAGAATTCCATAGGCCCCGCAGGGGCATCTTAC
CGCAGACCGTGGGGC
+
FFFFFFFFFFFFFFFFFFFFFFFFFFFFFFFFFFFFFFFFFFFFFFFFFFFFFFFFFFFFFFFFFFFF
FFFFFFFFFFFFFFFFFFFFFFFFFFFFFFFFFFFFFF::FFFFFFFFFFFFFFFFFFFFFFFFFFFF
FFFFFFFFFFFFFFF
@A00721:190:HGJW7DSXY:4:1257:2745:13761 2:N:0:TCTGCAAG+GCTGCACT
GTAATCCGGAGGAATCTATCCCCAATTGGAATAGTAATGATTTCAAATTTCAAATTTGAAAATATCCC
GCCCTACAGTATACCAATTCAAATTCAAATTCAAATAGAATTCCATAGGCCCCGCAGGGGCATCTTAC
CGCAGACCGTGGGGC
+
FFFFFFFFFFFFFFFFFFFFFFFFF,FFFF,,FFFFFFFFFFFFFFFFFFFFFF:FFF:FFFFFFFFF
FFFFFFFFFFFFFFFFFFFFFF:FFFFF:FFFFFFFFFFFFFFFFFFFFFFFFFF:FFFFFFFFFFFF
FFFFFFFFFFFFFFF
@A00721:190:HGJW7DSXY:4:1348:27850:2597 2:N:0:TCTGCAAG+GCTGCACT
GTAATCCGGAGGAATCTATCCCCAATTGGAATAGTAATGATTTCAAATTTCAAATTTGAAAATATCCC
GCCCTACAGTATACCAATTCAAATTCAAATTCAAATAGAATTCCATAGGCCCCGCAGGGGCATCTTAC
CGCAGACCGTGGGGC
+
FF,FFFFFFFFFFFFFFFFFFFFFFFFFFFFFFFFFFFFFFFFFFFFFFFFFFFFFFFFFFFFFFFFF
FFFFFFFFFFFFFFF:FFFFFFFFFFFFFFFFFFFFFFF,FFFFFFFFFFFFFFFFFFFFFFFFFFFF
FFFFFFFFFFFFFFF
@A00721:190:HGJW7DSXY:4:1451:3414:23312 2:N:0:TCTGCAAG+GCTGCACT
GTAATCCGGAGGAATCTATCCCCAATTGGAATAGTAATGATTTCAAATTTCAAATTTGAAAATATCCC
GCCCTACAGTATACCAATTCAAATTCAAATTCAAATAGAATTCCATAGGCCCCGCAGGGGCATCTTAC
CGCAGACCGTGGGGC
+
FFF:FFFFFFFFFFFFFFFFFFFFFFFFFFFFFFFFFFFFFFFFFFFFFFFFFFFFFFFFFFFFFFFF
FFFFFFF:FFFFFFFFFFFFFFFFFFFFFFFFFFFFFFFFFFFFFFFFFFFFFFFFFFFFF:FFFFFF
FFFFFFFFFFFFFFF
@A00721:190:HGJW7DSXY:4:2142:13304:34240 2:N:0:TCTGCAAG+GCTGCACT
GTAATCCGGAGGAATCTATCCCCAATTGGAATAGTAATGATTTCAAATTTCAAATTTGAAAATATCCC
GCCCTACAGTATACCAATTCAAATTCAAATTCAAATAGAATTCCATAGGCCCCGCAGGGGCATCTTAC
CGCAGACCGTGGGGC
+
FFFFFFFFFFFFF,FFF,FFFFFFFFFFFFFFFFFFFFFFFFFFFFFFF,FFFF,FFFF:FF:FFFFF
FFFFFFF,FF:F,FFF:FFFFFF:FFFF,:FFF,::FFF,FFFFF,:FFFFFFFF,FFFFF,FFFFF:
FFFFFFFFF,FFFFF
```

@A00721:190:HGJW7DSXY:4:2571:32931:26772 2:N:0:TCTGCAAG+GCTGCACT
GTAATCCGGAGGAATCTATCCCCAATTGGAATAGTAATGATTTCAAATTTCAAATTTGAAAATATCCC
GCCCTACAGTATACCAATTCAAATTCAAATTCAAATAGAATTCCATAGGCCCCGCAGGGGCATCTTAC
CGCAGACCGTGGGGC
+
FFFFFFFFFFFFFFFFFFFFFFFFFFFFF:FFFFFFFFFFFFFFFF:FFFFFFFFFFF:FFFFFFFFF
FFFFFFF:FFFFFFFFFFFFFF,FFFFFFFFFFF:FFFFFFFFFFFFFF:FFFFFFFFFFFFFFFFF:
FFFFFFFFF:FFFFF
@A00721:190:HGJW7DSXY:4:1503:30056:17253 2:N:0:TCTGCAAG+GCTGCACT
GAGGAATCTATCCCCAATTGGAATAGTAATGATTTCAAATTTCAAATTTGAAAATATCCCGCCCTACA
GTATACCAATTCAAATTCAAATTCAAATAGAATTCCATAGGCCCCGCAGGGGCATCTTACCGCAGACC
GTGGGGCAAGCCAAT
+
FFFFFFFFFFFFFFFFFFFFFFFFFFFF:FFFFFFFFFFFFFFFFFFFFFFFFFFFFFFFFFFFFFFF
FFFFFFFFFFFFFFFFFFFFFFFFFFFFFFFFFFFFFFFFFFFFFFFFFFFFFFFFFFFFFFFFFFFF
F:FFFFF,FFFFFFF
@A00721:190:HGJW7DSXY:4:2225:18376:12273 2:N:0:TCTGCAAG+GCTGCACT
GAGGAATCTATCCCCAATTGGAATAGTAATGATTTCAAATTTCAAATTTGAAAATATCCCGCCCTACA
GTATACCAATTCAAATTCAAATTCAAATAGAATTCCATAGGCCCCGCAGGGGCATCTTACCGCAGACC
GTGGGGCAAGCCAAT
+
FFFFF,FFFFFFFFFFFFFFFFFFFFFFFFFFFFFFFFFFFFFFFFFFFFFFFFFFFFFFFFFFFFFF
FFFFFFFFFFFFFFFFFFFFFFFFFFFFFFFFFFFFFFFFFFFFFFFFFFFFFFFFFFFFFFFFFFFF
FFFFFFFFFFFFFFF
@A00721:190:HGJW7DSXY:4:2264:32832:25974 2:N:0:TCTGCAAG+GCTGCACT
GAGGAATCTATCCCCAATTGGAATAGTAATGATTTCAAATTTCAAATTTGAAAATATCCCGCCCTACA
GTATACCAATTCAAATTCAAATTCAAATAGAATTCCATAGGCCCCGCAGGGGCATCTTACCGCAGACC
GTGGGGCAAGCCAAT
+
FFFFFFFFFFFFFFFFFFFFFFFFFFFFFFFFFFFFFF:FFFFFF,FFFFFFF:FFFFFFFFFFFFFF
FFFFFFFFFFFFF:FFFFFF,FFFFF,FFFFFFFFFFFF:FFFFFF:FFFFFFFFFFFFFFFFF:FFF
F:FFFF:F:FFFFFF
@A00721:190:HGJW7DSXY:4:2664:30346:14372 2:N:0:TCTGCAAG+GCTGCACT
GAGGAATCTATCCCCAATTGGAATAGTAATGATTTCAAATTTCAAATTTGAAAATATCCCGCCCTACA
GTATACCAATTCAAATTCAAATTCAAATAGAATTCCATAGGCCCCGCAGGGGCATCTTACCGCAGACC
GTGGGGCAAGCCAAT
+
FFFFFFFFFFFFFFFFFFFFFFFFFFFFFFFFFFFFFFFFFFFFFFFFFFFFFFFFFF:F:F::FF:F
:FFFFFFFFFFFFFFFFFFFFFFFFFFFFFFFFF:FFFFF,FFFFFF:FFFFFFFFFF::FFFFFFFF
FFFFFFFF,FFFFF:
@A00721:190:HGJW7DSXY:4:2647:29288:9251 2:N:0:TCTGCAAG+GCTGCACT
ATATTGGCTTGCCCCACGGTCTGCGGTAAGATGCCCCTGCGGGGCCTATGGAATTCTATTTGAATTTG
AATTTGAATTGGTATACTGTAGGGCGGGATATTTTCAAATTTGAAATTTGAAATCATTACTATTCCAA
TTGGGGATAGATTCC
+
FFFFFFFFFFFFFFFFFFFFFFFFFFFFFFFFFFFFFFFFFFFFFFFFFFFFFFFFFF,FFFFFFFFF
FF,FFFFF:FFFFFFFFFFFFFFFFFFFFFFFFFFFFFFFFFFFFFFFFFFFFFFFFFFFFFF:FF:F
FFFFFFFFFFFFFFF
@A00721:190:HGJW7DSXY:4:1226:10963:36104 2:N:0:TCTGCAAG+GCTGCACT
ATATTGGCTTGCCCCACGGTCTGCGGTAAGATGCCCCTGCGGGGCCTATGGAATTCTATTTGAATTTG
AATTTGAATTGGTATACTGTAGGGCGGGATATTTTCAAATTTGAAATTTGAAATCATTACTATTCCAA
TTGGGGATAGATTC
+
FFFFFFFFF:FFFFFFFFFFF:FFFFFFFFFFFFFFFFFFFFFFFF,F:FFFFFF,FFFFFF:F:FFF

```
FFFFFFFFFFFFF:F:FFFFFFFFFFFFFFFFFFFFFF:FFFFFFFFFFFFFFFFFFFFFFFFFFFFF
FFFFFFF:FFF:F:
@A00721:190:HGJW7DSXY:4:1234:20184:12712 2:N:0:TCTGCAAG+GCTGCACT
ATATTGGCTTGCCCCACGGTCTGCGGTAAGATGCCCCTGCGGGGCCTATGGAATTCTATTTGAATTTG
AATTTGAATTGGTATACTGTAGGGCGGGATATTTTCAAATTTGAAATTTGAAATCATTACTATTCCAA
TTGGGGATAGATTC
+
:FFFFFFFFFFFFFFFFFFFFFFFFFFFFFFFFFFFFFFFFFFFFFFFFFFFFFFFFFFFFFFFFFFF
FFFFFFFFFFFFFFFFFFFFFFFFFFFFFFFFFFFFFFFFFFFFFFFFFFFFFFFFFFFFFFFFFFFF
FFFFFF:FFFFFFF
@A00721:190:HGJW7DSXY:4:2334:23665:19774 2:N:0:TCTGCAAG+GCTGCACT
ATATTGGCTTGCCCCACGGTCTGCGGTAAGATGCCCCTGCGGGGCCTATGGAATTCTATTTGAATTTG
AATTTGAATTGGTATACTGTAGGGCGGGATATTTTCAAATTTGAAATTTGAAATCATTACTATTCCAA
TTGGGGATAGATTC
+
FFFFFFFFFFFFFFFFFFFFFFFFFFFFFFFFFFFFFFFFFFFFFFF,FFFFFFFFFFFFFFFFFFFF
FFFFFFFFFFFFFFFFFFFFFFFFFFFFFFFFFFFFFFFFFFFFFFFFFFFFFFFF:FFFF:FF:FFF
FFFFFFF,FFFFFF
@A00721:190:HGJW7DSXY:4:1265:32108:30013 2:N:0:TCTGCAAG+GCTGCACT
ATTATATTGGCTTGCCCCACGGTCTGCGGTAAGATGCCCCTTCGGGGCCAATGGAATTCTATTTGAAT
TTGAATTTGAATTGGTATACTGTAGGGCGGGATATTTTCAAATTTGAAATTTTAAATCATTACTATTC
CAATTGGGGATAGAT
+
FFFFFF,,FFFFFF:FFF:F,FF:F,,F,FFF,FF:FF:::,FFFF,,:,FFFFFFFF:F:FF:F,FF
FF:FFFFFFF:F,F,FFF,,:F::F:F,F,FFFFFFFF,F,FFF:FFFFFFF,:,FF,FFFF:,F:F,
:FFFF,F:F:FFFFF
@A00721:190:HGJW7DSXY:4:1519:3143:27383 2:N:0:TCTGCAAG+GCTGCACT
ATTATATTGGCTTGCCCCACGGTCTGCGGTAAGATGCCCCTGCGGGGCCTATGGAATTCTATTTGAAT
TTGAATTTGAATTGGTATACTGTAGGGCGGGATATTTTCAAATTTGAAATTTGAAATCATTACTATTC
CAATTGGGGATAGAT
+
FFFFFFFFFFFFFFFFFFFFFFFFFFFFFFFFFFFFFFFFFFFFFFFFFFFFFFFFFFFFFFFFFFF:
FFFFFFFFFFF:FFFFFFFFFFFFFFFFFFFFFFFFFFF,F::FFFFFF:FFFFFFFF:FFFFFFFFF
FFFFFFFFFFFFFFF
@A00721:190:HGJW7DSXY:4:2110:4779:14058 2:N:0:TCTGCAAG+GCTGCACT
ATTATATTGGCTTGCCCCACGGTCTGCGGTAAGATGCCCCTGCGGGGCCTATGGAATTCTATTTGAAT
TTGAATTTGAATTGGTATACTGTAGGGCGGGATATTTTCAAATTTGAAATTTGAAATCATTACTATTC
CAATTGGGGATAGAT
+
FFFFFFFFFFFFFFFFFFFFFFFFFFFFF:FFFFFFFFFFFFFFFFFFFFFFFFFFF:FFFF,FFFF:
FFFFFFFFFFFFFFFFFFFFFFFFFFFFFFFFF:FFFFFFFFFFFFFFFFFFFFFFFFFFFFFFFFFF
FFF:FFFFFFFFFFF
@A00721:190:HGJW7DSXY:4:2110:2700:16157 2:N:0:TCTGCAAG+GCTGCACT
ATTATATTGGCTTGCCCCACGGTCTGCGGTAAGATGCCCCTGCGGGGCCTATGGAATTCTATTTGAAT
TTGAATTTGAATTGGTATACTGTAGGGCGGGATATTTTCAAATTTGAAATTTGAAATCATTACTATTC
CAATTGGGGATAGAT
+
FFFFFFFFFFF:FFF:FFFFFFFFFFFFFFFFFFFFFFFFFFFFFFFFFFFFFFF:FFF:F,:FFFFF
FFFFFFFFFFF:FFFFFFFFFFFFFFFF:FFFFFFFFFFFFFFFFFFFFFFFFFF:FFFFFFFFFFFF
FFFFFFFFFFFFFFF
@A00721:190:HGJW7DSXY:4:2241:10773:23531 2:N:0:TCTGCAAG+GCTGCACT
ATTATATTGGCTTGCCCCACGGTCTGCGGTAAGATGCCCCTGCGGGGCCTATGGAATTCTATTTGAAT
TTGAATTTGAATTGGTATACTGTAGGGCGGGATATTTTCAAATTTGAAATTTGAAATCATTACTATTC
CAATTGGGGATAGAT
```

```
+
F,FFFFFFFFFFFFFFFFFFFFFFF:FFFFFFFFFFFFFFFFFFFFFFFFFFF:FFFFFFFFFF:FFFF:
FFFFF:FF:FFFFFFFFFFFFFFFFFFFFFFFFFFFFFFFFFFFFFFFFFFFFFFFFFFFFFF,FFF
F:F::FFFFFFFFFF
@A00721:190:HGJW7DSXY:4:2406:28076:8155 2:N:0:TCTGCAAG+GCTGCACT
ATTATATTGGCTTGCCCCACGGTCTGCGGTAAGATGCCCCTGCGGGGCCTATGGAATTCTATTTGAAT
TTGAATTTGAATTGGTATACTGTAGGGCGGGATATTTTCAAATTTGAAATTTGAAATCATTACTATTC
CAATTGGGGATAGAT
+
F:FFFFFFF:FF,FF:FFFFFFFF:FFFFFFFFFFFFFFFFFF:FFFFFFFFFFFFFF:FF::FFFFF
FFFFFFFFFF:FFFF:FFF:FFF:F:FFFFFFF:F:FFFFFF::FF,FFFF:F:,,:F,:F:FFFF,F
,:,FFF,FF,:FFFF
@A00721:190:HGJW7DSXY:4:2418:31159:20791 2:N:0:TCTGCAAG+GCTGCACT
ATTATATTGGCTTGCCCCACGGTCTGCGGTAAGATGCCCCTGCGGGGCCTATGGAATTCTATTTGAAT
TTGAATTTGAATTGGTATACTGTAGGGCGGGATATTTTCAAATTTGAAATTTGAAATCATTACTATTC
CAATTGGGGATAGAT
+
FFFFFFFFFFFFFFFFFFFFFFFFFFFFFFFFFFFFFFFFFFFFFFFFFFFFFFFFFFFFFFFFFFFF
FFFFFFFFFFFFFFFFFFFFFFFFFFFFFFFFFFFFFFFFFFFFFFFF,FFFFFF:FFFF:FFFFFFFFFF
FFFFFFF::FFF,FF
@A00721:190:HGJW7DSXY:4:2437:23448:23813 2:N:0:TCTGCAAG+GCTGCACT
ATTATATTGGCTTGCCCCACGGTCTGCGGTAAGATGCCCCTGCGGGGCCTATGGAATTCTATTTGAAT
TTGAATTTGAATTGGTATACTGTAGGGCGGGATATTTTCAAATTTGAAATTTGAAATCATTACTATTC
CAATTGGGGATAGAT
+
FFFFFFFFFFFFFFFFFFFFFFFFFFFFFFFFFFFFFFFFFFFFFFFFFFF::FFFFFFFFFFFFFFFF:
FFFFFFFFFFFFFFFFFFFFFFFFFFFFFFFFFFFFFFFFFFFFFFFFFFF,FFFFFFFFFFFFFFFFF:
FFFFFFFFFFFFFFF
@A00721:190:HGJW7DSXY:4:2441:19379:23657 2:N:0:TCTGCAAG+GCTGCACT
ATTATATTGGCTTGCCCCACGGTCTGCGGTAAGATGCCCCTGCGGGGCCTATGGAATTCTATTTGAAT
TTGAATTTGAATTGGTATACTGTAGGGCGGGATATTTTCAAATTTGAAATTTGAAATCATTACTATTC
CAATTGGGGATAGAT
+
FFFFFFFFFFFFFFFFFFFFFFFFFFFFFFFFFFFFFFFFFFFFFFFFFFFFFFFFFFFFFFF:FFF:FF
FFFFFFFFFFFFFFFF,FFFFFFFFFFFFFFFFFFFFFFFFFFFFFFFFFFFFF:FFFFF:FFFFFFFF
:FFFFFFFFFFFFF:
@A00721:190:HGJW7DSXY:4:1148:19994:35994 2:N:0:TCTGCAAG+GCTGCACT
AGTAATATTATATTGGCTTGCCCCACGGTCTGCGGTAAGATGCCCCTGCGGGGCCTATGGAATTCTAT
TTGAATTTGAATTTGAATTGGTATACTGTAGGGCGGGATATTTTCAAATTTGAAATTTGAAATCATTA
CTATTCCAATTGGGG
+
FFFFFFFFFFFFFFFFFFFFFFFFFFFFFFFFFFFFFFFFFFFFFFFFFFFFFFFFFFFFFFFFFFFF::
FFFF:FFFFFFFFFF:FFF:FFFFFFFFFF:FFFFFFFFFFFFFFFFFFFFFFFFFFFFFFFFFFFFFF
FFFFFFFFFFFFFFF
@A00721:190:HGJW7DSXY:4:1168:24306:15060 2:N:0:TCTGCAAG+GCTGCACT
AGTAATATTATATTGGCTTGCCCCACGGTCTGCGGTAAGATGCCCCTGCGGGGCCTATGGAATTCTAT
TTGAATTTGAATTTGAATTGGTATACTGTAGGGCGGGATATTTTCAAATTTGAAATTTGAAATCATTA
CTATTCCAATTGGGG
+
FFFFFFFFFFFFFFFFFFFFFFFFFFFFFFFFFFFFFFFFFFFFFFFFFFFFFFFFFFFFFF:FFFFFF
FFFFF,FFFFFFFFFFFFFFFFFFFFFFFFFFFFFFFFFFFFFFFFFFFFFFFFFFFFFFFFFFFFFFF
FFFFFFFFFFFFFF:
@A00721:190:HGJW7DSXY:4:1178:23294:28494 2:N:0:TCTGCAAG+GCTGCACT
AGTAATATTATATTGGCTTGCCCCACGGTCTGCGGTAAGATGCCCCTGCGGGGCCTATGGAATTCTAT
```

TTGAATTTGAATTTGAATTGGTATACTGTAGGGCGGGATATTTTCAAATTTGAAATTTGAAATCATTA
CTATTCCAATTGGGG
+
FFFFFFFFFFFFFFFFFFFF:FFFFFFFFFFFFFFFFFFFFFFFFFFFFFFFFFFFFFFFFFFFFFFF
FFFFFFFFFFFFFFFFFFFFFFFFFFFFFFFFFFFFFFFFFFFFFFFFFFFFFFFFFFFFFFFFFFFF
FFFFFFFFF:FF:F:
@A00721:190:HGJW7DSXY:4:1228:6632:16861 2:N:0:TCTGCAAG+GCTGCACT
AGTAATATTATATAGGCTTGCCCCACGGTCTGCGGTAAGATGCCCCTGCGGGGCCTATGGAATTCTAT
TTGAATTTGAATTTGAATTGGTATACTGTAGGGCGGGATATTTTCAAATTTGAAATTTGAAATCATTA
ATATTCCAATTGGGG
+
F:,FFFF,FFFF,,:FFF,F:F,,FF:FFF,F,:F,FFFFFF:,F,FFFFF,FF,FF:FFFFFFFF,,
FFFFF:,:FFF:FFF:F:,F:FFFFFF:FFFF,FF:FFFF:FFFFFFFF,F::FFFFF::FFFF,FFF
,F,FFFFFF:FFF:F
@A00721:190:HGJW7DSXY:4:1233:26304:13416 2:N:0:TCTGCAAG+GCTGCACT
AGTAATATTATATTGGCTTGCCCCACGGTCTGCGGTAAGATGCCCCTGCGGGGCCTATGGAATTCTAT
TTGAATTTGAATTTGAATTGGTATACTGTAGGGCGGGATATTTTCAAATTTGAAATTTGAAATCATTA
CTATTACAATTGGGG
+
FFFFFFFFFFFFFFFFF,FFFFFFFFF,FFF:F,F:FFFFFF,FFFFF,F:,FFFFF:FFF:FFFFFF
,FFFFFF:F,,:FFFFFF,F:FF,F,FF::F:FFFFF::F:FFFFF,FF,FFF:FFFFFF:FFFFF:F
:,FF,::FF:F,F:F
@A00721:190:HGJW7DSXY:4:1252:21323:29559 2:N:0:TCTGCAAG+GCTGCACT
AGTAATATTATATTGGCTTGCCCCACGGTCTGCGGTAAGATGCCCCTGCGGGGCCTATGGAATTCTAT
TTGAATTTGAATTTGAATTGGTATACTGTAGGGCGGGATATTTTCAAATTTGAAATTTGAAATCATTA
CTATTCCAATTGGGG
+
FFFFFFFFF:FFFFFFFFFFFFFFFFFFFFFFFFFFFFFFFFFFFFFFFFFFFFFFFFFFFFFFFFFF
FFFFFFFFFFFFFFFF:FFFFFFFFFFFFFFFFFFFFFFFFFFFFFFFFFFFFFFFFFFFFFFFFFFF
FFFFFFF:FFFFFFF
@A00721:190:HGJW7DSXY:4:1312:31069:14152 2:N:0:TCTGCAAG+GCTGCACT
AGTAATATTATATTGGCTTGCCCCACGGTCTGCGGTAAGATGCCCCTGCGGGGCCTATGGAATTCTAT
TTGAATTTGAATTTGAATTGGTATACTGTAGGGCGGGATATTTTCAAATTTGAAATTTGAAATCATTA
CTATTCCAATTGGGG
+
FFFFFFFFFFFFFFFFFFFFFFFFFFFFFFFFFFFFFFFFFFFFFFFFFFFFFFFFFFFFFFFF:FFF
FFFFFFFFFFFFFFFFF:FFFFFFFFFFFFFFFFFFFFFFFFFFFFFFFFFF:FFFFFFFF:FFFFFF
FFFFF:FFF,FFFFF
@A00721:190:HGJW7DSXY:4:1424:7093:20196 2:N:0:TCTGCAAG+GCTGCACT
AGTAATATTATATTGGCTTGCCCCACGGTCTGCGGTAAGATGCCCCTGCGGGGCCTATGGAATTCTAT
TTGAATTTGAATTTGAATTGGTATACTGTAGGGCGGGATATTTTCAAATTTGAAATTTGAAATCATTA
CTATTCCAATTGGGG
+
FFFFFFFFF:FFFFFFFFFFFFFFFFFFFFFFFFFFFFFFFFFFFFFFFFFFFFFFFFFFFFFFFF:F
FFFFFFFFFFFFFFFFFFFFFFFFFFFFFFFFFFFFFFFFFFFFFFFFFFFFFFFFFFFFFFFFFFFF
FFFFFFFFFFFFFFF
@A00721:190:HGJW7DSXY:4:1574:10619:4413 2:N:0:TCTGCAAG+GCTGCACT
AGTAATATTATATTGGCTTGCCCCACGGTCTGCGGTAAGATGCCCCTGCGGGGCCTATGGAATTCTAT
TTGAATTTGAATTTGAATTGGTATACTGTAGGGCGGGATATTTTCAAATTTGAAATTTGAAATCATTA
CTATTCCAATTGGGG
+
FFFFFFFFFFFFFFFFFFFFFFFFFFFFFFFFFFFFFFFFFFFFFFFFFFF:FFFFFFFFFFFFFFFF
FFFFFFFFFFFFFFFFFFFFFFFFFFFFFFFFFFFFFFFFFFFFFFFFFFFFFFFFFFFFF:FFFFFF
FFF:FFFFFFFFFFF

```
@A00721:190:HGJW7DSXY:4:1574:10619:4633 2:N:0:TCTGCAAG+GCTGCACT
AGTAATATTATATTGGCTTGCCCCACGGTCTGCGGTAAGATGCCCCTGCGGGGCCTATGGAATTCTAT
TTGAATTTGAATTTGAATTGGTATACTGTAGGGCGGGATATTTTCAAATTTGAAATTTGAAATCATTA
CTATTCCAATTGGGG
+
FFFFFFFFFFFFFFF:FFFFFFFFFFFFFFFFFFFFFFFFFFFFFFFFFFFFFFFFFFFFF:FFFFFF
FFFFF:FFFFFFFFFFFFFFFFFFFFFFFFFFFFFFFFFFFFFFFFFFFFFFFFFFFFFFFFFFFFF,
FFFFFFFFFFFFFFF
@A00721:190:HGJW7DSXY:4:1668:32307:27007 2:N:0:TCTGCAAG+GCTGCACT
AGTAATATTATATTGGCTTGCCCCACGGTCTGCGGTAAGATGCCCCTGCGGGGCCTATGGAATTCTAT
TTGAATTTGAATTTGAATTGGTATACTGTAGGGCGGGATATTTTCAAATTTGAAATTTGAAATCATTA
CTATTCCAATTGGGG
+
FFFFFFFFFFFFFFFFFFFFFFFFFFFFFFFFFFFFFFFFFFFFFFFFFFFFFFFFFFFFFFFFFFFF
FFFFFFFFFFFFFFFFFFFFFFFFFF:F:FFFFFFFFFFFFFFFFFFFFFFFFFFFFFF:F:FFFFFF
FFFFFFFFF:FFFFF
@A00721:190:HGJW7DSXY:4:2209:1081:11475 2:N:0:TCTGCAAG+GCTGCACT
AGTAATATTATATTGGCTTGCCCCACGGTCTGCGGTAAGATGCCCCTGCGGGGCCTATGGAATTCTAT
TTGAATTTGAATTTGAATTGGTATACTGTAGGGCGGGATATTTTCAAATTTGAAATTTGAAATCATTA
CTATTCCAATTGGGG
+
FFFFFFFFFFFFFFFFFFFFFFFFFFFFFFFFFFFFFFFFFFFFFFFFFFFFFFFFFFFFFFFFFFFF
FFFFFFFFFFFFFFFFFFFFFFFFFFFFFFFFFFFFFFFFFFFFFFFFFFFFFFFFFFFFFFFFFFFF
FFFFFF:FFFFFFFF
@A00721:190:HGJW7DSXY:4:2223:9001:1705 2:N:0:TCTGCAAG+GCTGCACT
AGTAATATTATATTGGCTTGCCCCACGGTCTGCGGTAAGATGCCCCTGCGGGGCCTATGGAATTCTAT
TTGAATTTGAATTTGAATTGGTATACTGTAGGGCGGGATATTTTCAAATTTGAAATTTGAAATCATTA
CTATTCCAATTGGGG
+
FFFF:FFFFF:FFFFFFF:FFFFFFFFFFFFFFFFFFFF:FFFFFFF,FFFFFF:FFFFFFFFF,F:F
FFFFFFFFF:FFFFF:FFFF::FFFFFFFFFFFFF:FFFFFFFFFF:FFFFFFFFFFF:FFFF:FFFF
:FFFFFFFFFFF,FF
@A00721:190:HGJW7DSXY:4:2223:10167:2597 2:N:0:TCTGCAAG+GCTGCACT
AGTAATATTATATTGGCTTGCCCCACGGTCTGCGGTAAGATGCCCCTGCGGGGCCTATGGAATTCTAT
TTGAATTTGAATTTGAATTGGTATACTGTAGGGCGGGATATTTTCAAATTTGAAATTTGAAATCATTA
CTATTCCAATTGGGG
+
FFFFFFFFFFFFFFFFFFFFFFFFFFFFFFFFFFFFFFFFFFFFFFFFFFFFFFFFFFFFFFFFFFFF
FFFFFFFFFFFFFFFFFFFFFFFFFFFFFFFFFFFFFFFFFFFFFFFFFFFFFFFFFFFFFF:FFFFF
FFFFFFFFFFFF:FF
@A00721:190:HGJW7DSXY:4:2226:6298:14559 2:N:0:TCTGCAAG+GCTGCACT
AGTAATATTATATTGGCTTGCCCCACGGTCTGCGGTAAGATGCCCCTGCGGGGCCTATGGAATTCTAT
TTGAATTTGAATTTGAATTGGTATACTGTAGGGCGGGATATTTTCAAATTTGAAATTTGAAATCATTA
CTATTCCAATTGGGG
+
FFFFFFFFFFFFFFFFFFFFFFFFFFFFFFFFFFFFFFFFFFFFFFFFFFFFFFFFFFFFFFFFFFFF
FFFFFFFFFFFFFFFFFFF:FFFFFFFFFFFFFFFFFFF:FFFFFFFFFFFFFFFFFFFFFFFFFFFF
FFFFFF:FFFFFFFF
@A00721:190:HGJW7DSXY:4:2314:20509:12900 2:N:0:TCTGCAAG+GCTGCACT
AGTAATATTATATTGGCTTGCCCCACGGTCTGCGGTAAGATGCCCCTGCGGGGCCTATGGAATTCTAT
TTGAATTTGAATTTGAATTGGTATACTGTAGGGCGGGATATTTTCAAATTTGAAATTTGAAATCATTA
CTATTCCAATTGGGG
+
FFFFFFFFFFFFFFFFFFFFFFFFFFFFFFFFFFFFFFFFFFFFFFFFFFFFFFFFFFFFFFFFFF:F
```

FFFF:FFFFFFFFFFF:FFFFFFFFFFFFFFFFFFFFFFFFFFFFFFFFFFFFFFFFFFFFFFFFF
FFFFFFFFF:FFFFF
@A00721:190:HGJW7DSXY:4:1313:29785:22639 2:N:0:TCTGCAAG+GCTGCACT
CCCAATTGGAATAGTAATGATTTCAAATTTCAAATTTGAAAATATCCCGCCCTACAGTATACCAATTC
AAATTCAAATAGAATTCCATAGGCCCCGCAGGGGCATCTTACCGCAGACCGTGGGGCAAGCCAATATC
ATATTACTGGCTTGC
+
FFFFFFFF:FF:FFFFFFFFFFFFFFFFFFFFFFFFFF:FFFFFFFFFFFFFFFF,FFFFFFF:FFFF
FFFFFFFFFFFFFFFFFFFFFFFFFFFFFFFFFFFFF:FFFFFFFFFFFFFFFFFFFF,FFFFFFFFF,
FFFFFFFFFFFFFFF
@A00721:190:HGJW7DSXY:4:1424:21793:12085 2:N:0:TCTGCAAG+GCTGCACT
CCCAATTGGAATAGTAATGATTTCAAATTTCAAATTTGAAAATATCCCGCCCTACAGTATACCAATTC
AAATTCAAATAGAATTCCATAGGCCCCGCAGGGGCATCTTACCGCAGACCGTGGGGCAAGCCAATATA
ATATTACTGGCTTGC
+
FFFFFFFFFFFFFFFFFFFFFFFFFFFFFFFFFFFFFFFFFFFFFFFFFFFFFFFFFFFFFFFFFFFF
FFFFFFFFFFFFFFFFFFFFFFFFFFFFFFFFFFFFFFFFFFFFFFFFFFFFFFFFFFFFFFFFFF:F
FFFFFFFFFFFFFFF
@A00721:190:HGJW7DSXY:4:1536:32786:27806 2:N:0:TCTGCAAG+GCTGCACT
CCCAATTGGAATAGTAATGATTTCAAATTTCAAATTTGAAAATATCCCGCCCTACAGTATACCAATTC
AAATTCAAATAGAATTCCATAGGCCCCGCAGGGGCATCTTACCGCAGACCGTGGGGCAAGCCAATATA
ATATTACTGGCTTGC
+
FFFFFFFFFFFFFFFFFFFFFFFFFFFFFFFFFF:FFFFFFF:F:FFFFFFFFFFF,FFFFFFFFFFF
FFFFF:FFFF,FFFFFFFFF:FFFFFFFFFFFFFFFFFFFF:FFFFFFFFFFFFFFFFFFFFFFFF:F:
FFFFFFFFFFF,FFF
@A00721:190:HGJW7DSXY:4:1537:22625:19914 2:N:0:TCTGCAAG+GCTGCACT
CCCAATTGGAATAGTAATGATTTCAAATTTCAAATTTGAAAATATCCCGCCCTACAGTATACCAATTC
AAATTCAAATAGAATTCCATAGGCCCCGCAGGGGCATCTTACCGCAGACCGTGGGGCAAGCCAATATA
ATATTACTGGCTTGC
+
FFFFFFFFFFFFFFFFFFFFFFFFFFFFFFFFFFFFFFFFFFFFFFFFFFFFFFFFFFFFFFFFFFFF
FFFFFFFFFFFFFFFFFFFFFFFFFFFFFFFFFFFFFFFFFFFFFF:FFFFFFFFFFFFF:FFF:FFFFF
FFFF:FFFFFFFFFF
@A00721:190:HGJW7DSXY:4:1565:12780:26068 2:N:0:TCTGCAAG+GCTGCACT
CCCAATTGGAATAGTAATGATTTCAAATTTCAAATTTGAAAATATCCCGCCCTACAGTATACCAATTC
AAATTCAAATAGAATTCCATAGGCCCCGCAGGGGCATCTTACCGCAGACCGTGGGGCAAGCCAATATA
ATATTACTGGCTTGC
+
FFFFFFFFFFFFFFFFFFFFFFFFFFFFFFFFFF:FFFFFFFF:FFFFFFFFFFFFFFFFFFFFFFFFF
FFFFFFFFFFFFFFFFF:FFFFFFFFFFFFFFFFFFFFFFFFFFFFFFFFFFFFFFFFFFFFFFFFFF
F,FFFF:FFFFFFFF
@A00721:190:HGJW7DSXY:4:2278:23873:2848 2:N:0:TCTGCAAG+GCTGCACT
CCCAATTGGAATAGTAATGATTTCAAATTTCAAATTTGAAAATATCCCGCCCTACAGTATACCAATTC
AAATTCAAATAGAATTCCATAGGCCCCGCAGGGGCATCTTACCGCAGACCGTGGGGCAAGCCAATATA
ATATTACTGGCTTGC
+
FFFFFFFFFFFFFFFFFFFFFFFFFFFFFFFFFFFFFFFFFFFFFFFFFFFFFFFFFF:FFFFFFFFF
FFFFFFFFFFFFFFFFFFFFFFFFFFFFFFFFFFFFFFFFFFFFFFFFFFFFFFFFFFFFFFFFFFFF
FFFFFFFFFFFFFFF
@A00721:190:HGJW7DSXY:4:2344:31873:27007 2:N:0:TCTGCAAG+GCTGCACT
CCCAATTGGAATAGTAATGATTTCAAATTTCAAATTTGAAAATATCCCGCCCTACAGTATACCAATTC
AAATTCAAATAGAATTCCATAGGCCCCGCAGGGGCATCTTACCGCAGACCGTGGGGCAAGCCAATATA
ATATTACTGGCTTGC

+
FFFFFFFFFFFFFFFFFFFFFFFFFFF:FFFFFF:FFFFFFFFFFFFFFFFFFFFFFFFFFFFFFFFFFF
,FFFFFFFFF::FFFFF:FFFFF::FFFFFFFFFFFFFFFFFFFFFFFFFFFFFFFFFFF:FFFFFFF
FFFFF,FFF,FFFFF
@A00721:190:HGJW7DSXY:4:2355:20971:13479 2:N:0:TCTGCAAG+GCTGCACT
CCCAATTGGAATAGTAATGATTTCAAATTTCAAATTTGAAAATATCCCGCCCTACAGTATACCAATTC
AAATTCAAATAGAATTCCATAGGCCCCGCAGGGGCATCTTACCGCAGACCGTGGGGCAAGCCAATATA
ATATTACTGGCTTGC
+
FFFFF:FFFFFFFFFFFFFFFFFFFFFFFFFFFFFFFFFFFFFFFFFFFFFFFFFFFFFFFFFFFFFF
FFFFFFFFFFFFFFFFFFFFFFFFFFFFFFFFFFFFFFFFFFFFFFFFFFF:FFFFFFFFFFFFFFFF
FFFFFFFFFFF::FF
@A00721:190:HGJW7DSXY:4:2665:7862:7185 2:N:0:TCTGCAAG+GCTGCACT
CCCAATTGGAATAGTAATGATTTCAAATTTCAAATTTGAAAATATCCCGCCCTACAGTATACCAATTC
AAATTCAAATAGAATTCCATAGGCCCCGCAGGGGCATCTTACCGCAGACCGTGGGGCAAGCCAATATA
ATATTACTGGCTTGC
+
FFFFFFFFFFFFFFFFFFFFFFFFFFFFFFFFFFFFFFFFFFFFFFFFFFFFFFFFFFFFFFFFFFFF
FFFFFFFFFFFFF:FFF:FFFFFFFFFFFFFFFFFFFFFFFFFFFFFFFFFFFFFFF:FFFFFFFF:
FFFFFFFFFFFFFFF
@A00721:190:HGJW7DSXY:4:1528:31403:36432 2:N:0:TCTGCAAG+GCTGCACT
CGGGCAAGCCAGTAATATTATATTGGCTTGCCCCACGGTCTGCGGTAAGATGCCCCTGCGGGGCCTAT
GGAATTCTATTTGAATTTGAATTTGAATTGGTATACTGTAGGGCGGGATATTTTCAAATTTGAAATTT
GAAATCATTACTATT
+
FFFFFFFFFFFFFFFFFFFFFFFFFFFFFFFFFFF:FFFFFFFFFFFFFFFFFFFFFFFFFFFFFFFF
FFFFFFFFFFFFFFFFFFFFFFFFFFF:FFFF:FFFFFFFFFFFFFFFFFFFFFFFFFFFFFFFFFFF
FFFFFFFFFFFFFFF
@A00721:190:HGJW7DSXY:4:1650:16269:12414 2:N:0:TCTGCAAG+GCTGCACT
CGGGCAAGCCAGTAATATTATATTGGCTTGCCCCACGGTCTGCGGTAAGATGCCCCTGCGGGGCCGAT
GGAATTCTATTTGAATTTGAATTTGAATTGGTATACTGTAGGGCGGGATATTTTCAAATTTGAAATTT
GAAATCATTACTATT
+
FFFFFFFFFFFFFFFFFFFFFFFFFFFFFFFFFFFFFFFFFFFFFFFFFFFFFFFFFFFFFFFFF,FF
FFF:F,FFF,FFFF,F:FFFFFFFFFFFFFFFFF:FFF,FFFFFFF:FFFFFFFFFFFFFFFFFFFFF
FFFFFFFFFFFF:FF
@A00721:190:HGJW7DSXY:4:2465:22146:8938 2:N:0:TCTGCAAG+GCTGCACT
CGGGCAAGCCAGTAATATTATATTGGCTTGCCCCACGGTCTGCGGTAAGATGCCCCTGCGGGGCCTAT
GGAATTCTATTTGAATTTGAATTTGAATTGGTATACTGTAGGGCGGGATATTTTCAAATTTGAAATTT
GAAATCATTACTATT
+
FFFFFF:FFFFFFFFF:FFFFFFFFFFFFF:FFFFFFFFF:FFFFFFFFFFFFFFFFFFFFFFFFFFF
FFFFFFFFFFFFFFFFFFFFFFFFFFFFFFFFF:FFFFFFFFFFFFFFFFFFFFFFFFFFFFFFFFFF
FFFFFFFFF:FFFFF
@A00721:190:HGJW7DSXY:4:1140:6280:1783 2:N:0:TCTGCAAG+GCTGCACT
AGCGTACGGAGGTGTCCCCGGCGCGGGCAAGCCAGTAATATTATATTGGCTTGCCCCACGGTCTGCGG
TAAGATGCCCCTGCGGGGCCTATGGAATTCTATTTGAATTTGAATTTGAATTGGTATACTGTAGGGCG
GGATATTTTCAAATT
+
FFFFFF:FFFFFFFFFFFFFFFFFFFFFFFFFFFFFFFFFF,FFFF:FFF:FFFFFFFF:FFFFFF:FF
FFFFFFFFFFFFFFFFFFF::FFFFFFFF,:FF:FF:FFFFF:FFFF,FFFFFFFFFFFFFFFFFFFF
FFFFFFFFFFFFFFF
@A00721:190:HGJW7DSXY:4:1239:2926:9815 2:N:0:TCTGCAAG+GCTGCACT
AGCGTACGGAGGTGTCCCCGGCGCGGGCAAGCCAGTAATATTATATTGGCTTGCCCCACGGTCTGCGG

```
TAAGATGCCCCTGCGGGGCCTATGGAATTCTATTTGAATTTGAATTTGAATTGGTATACTGTAGGGCG
GGATATTTTCAAATT
+
FFFFFFFFFFFFFFFFFFFFFFFFFFFFFFFFFFFFFFFFFFFFFFFFFFFFFFFFFFFFF,F,FFFF
FFFF:FFFFFFFFFFFFFFF:FFFFFFFFFFF:FFFFFFFFFFF:FFFFFFFFFFF:FFFFFFFFFFF
FFF::FFFFF:FF,F
@A00721:190:HGJW7DSXY:4:1301:1389:25441 2:N:0:TCTGCAAG+GCTGCACT
AGCGTACGGAGGTGTCCCCGGCGCGGGCAAGCCAGTAATATTATATTGGCTTGCCCCACGGTCTGCGG
TAAGATGCCCCTGCGGGGCCTATGGAATTCTATTTGTATTTGAATTTGAATTGGTATACTGTAGGGCG
GGATATTTTCAAATT
+
FFFFFFFFFFFFFFFFFFFFFFFFFFFFFFF:F:FFFF::FFF,FFF:FFFFFFFFF,FFF:,:FFFF
FFFF:FFFFFF:FFFFFF,FF,FFFFFFF,F,F:FF,F,FFF,:FFF:FFFFFFF,F,FFFFF:FF:F
F:FF:FFFF:F:F,F
@A00721:190:HGJW7DSXY:4:1301:16278:35790 2:N:0:TCTGCAAG+GCTGCACT
AGCGTACGGAGGTGTCCCCGGCGCGGGCAAGCCAGTAATATTATATTGGCTTGCCCCACGGTCTGCGG
TAAGATGCCCCTGCGGGGCCTATGGAATTCTATTTGAATTTGAATTTGAATTGGTATACTGTAGGGCG
GGATATTTTCAAATT
+
FFFFFFFFFFFFFFFFFFFFFFFFFFFFFF::FFFFFFFFFFFFF:F:FF,F,FFFFFFFFF:FFFFF
F,FFFFFFFFFFFFFFFF:::FFFFFF:FFF,FFFF,:FFFF:FFFF:FFFFFFFFFF,FFFFFFFFF
FFFFFFFFF:FFFFF
@A00721:190:HGJW7DSXY:4:1310:21206:18270 2:N:0:TCTGCAAG+GCTGCACT
AGCGTACGGAGGTGTCCCCGGCGCGGGCAAGCCAGTAATATTATATTGGCTTGCCCCACGGTCTGCGG
TAAGATGCCCCTGCGGGGCCTATGGAATTCTATTTGAATTTGAATTTGAATTGGTATACTGTAGGGCG
GGATATTTTCAAATT
+
FFFFFFFFFFFFFFFFFFFFFFFFFFFFF,FFFFFFFFFFFFFFFFFFFFFFFFFFFFFFFFFFFFFF
:FFFFF:FFFFFFFFFFFFFFFFFFFFFFFFFFFFFFFFFFFFFFFFFFFFF:FFFFFFFFFFFFFFF
FFFFFFFFFFFFFFF
@A00721:190:HGJW7DSXY:4:1612:22869:5963 2:N:0:TCTGCAAG+GCTGCACT
AGCGTACGGAGGTGTCCCCGGCGCGGGCAAGCCAGTAATATTATATTGGCTTGCCCCACGGTCTGCGG
TAAGATGCCCCTGCGGGGCCTATGGAATTCTATTTGAATTTGAATTTGAATTGGTATACTGTAGGGCG
GGATATTTTCAAATT
+
FFFFFFFFFFFFFFFFFFFFFFFFFFFFFFFFF:FFFFFF,FFFFFFFFFFFFFFFFFFFFFF:FFFF
FFFFFFF:FFFFFFFFFFFF,FFFFFFFF:FFFF,F:FFFFFFFFFFFFFFFFFFFFFFFFFFFFFFF
FFFF:FFFFFFFFFF
@A00721:190:HGJW7DSXY:4:2145:16947:17065 2:N:0:TCTGCAAG+GCTGCACT
AGCGTACGGAGGTGTCCCCGGCGCGGGCAAGCCAGTAATATTATATTGGCTTGCCCCACGGTCTGCGG
TAAGATGCCCCTGCGGGGCCTATGGAATTCTATTTGAATTTGAATTTGAATTGGTATACTGTAGGGCG
GGATATTTTCAAATT
+
FFFFFFFFFFFFFFFFFFFFFFFFFFFFF:FFFFFFFFFF:FFFFFFFFFFFFFFFFFFFFFFFFFFF
:FFFFFFFFFFFFFFFFFFF,FFFFF,FFFFFFFFFFFFFFF:FFFFFFFFFFFFFFFFFFFFFFFFF
FFFFFFFFFFFFF:F
@A00721:190:HGJW7DSXY:4:2169:19307:31955 2:N:0:TCTGCAAG+GCTGCACT
AGCGTACGGAGGTGTCCCCGGCGCGGGCAAGCCAGTAATATTATATTGGCTTGCCCCACGGTCTGCGG
TAAGATGCCCCTGCGGGGCCTATGGAATTCTATTTGAATTTGAATTTGAATTGGTATACTGTAGGGCG
GGATATTTTCAAATT
+
FFFFFFFFFFFFFFFFFFFFFFFFFFFFFFFFFF,F:FF:FFFFFFFF:FFFFFFFFFFFFFFFFFFF
:FFFFFFF:FFFFFFFFFFFFFFFFFFFFFFFFFFF:FFFFFFFFFFFFFFFFFFFFFFFFFFFFFFF
FFF:FFFFFF:FF:F
```

```
@A00721:190:HGJW7DSXY:4:2336:22968:27023 2:N:0:TCTGCAAG+GCTGCACT
AGCGTACGGAGGTGTCCCCGGCGCGGGCAAGCCAGTAATATTATATTGGCTTGCCCCACGGTCTGCGG
TAAGATGCCCCTGCGGGGCCTATGGAATTCTATTTGAATTTGAATTTGAATTGGTATACTGTAGGGCG
GGATATTTTCAAATT
+
FFFFFFFFFFFFFFFFFFFFFFFFFFFFFFFFF:FFFFF:FFFFFFFFFFFFFFFFFFFFFFF,FFFF
FFFFFFFFFFFFFFFFFFFFFFFFFFFFFFF:FFFF::FFFFFFFFFFFF,F,F:FFFF:F:FF,:F,
FF,FFFFFFFFF::F
@A00721:190:HGJW7DSXY:4:2544:24153:5494 2:N:0:TCTGCAAG+GCTGCACT
AGCGTACGGAGGTGTCCCCGGCGCGGGCAAGCCAGTAATATTATATTGGCTTGCCCCACGGTCTGCGG
TAAGATGCCCCTGCGGGGCCTATGGAATTCTATTTGAATTTGAATTTGAATTGGTATACTGTAGGGCG
GGATATTTTCAAATT
+
FFFFFFFFFFFFFFFFFFFFFFFFFFFFFFFFFFFFFFFFFFFFFFFFFFFFFFFFFF:FFFFFFFFF
FFFF:FFFFFFF,FFFFFFFFFFFFFFFFF,F:F:FFF:FFFFFFFFFFFFFFFF,FFF:FFFFFFFF
FFFF,FFFFFFFFFF
@A00721:190:HGJW7DSXY:4:2544:27697:5682 2:N:0:TCTGCAAG+GCTGCACT
AGCGTACGGAGGTGTCCCCGGCGCGGGCAAGCCAGTAATATTATATTGGCTTGCCCCACGGTCTGCGG
TAAGATGCCCCTGCGGGGCCTATGGAATTCTATTTGAATTTGAATTTGAATTGGTATACTGTAGGGCG
GGATATTTTCAAATT
+
FFFFFFFFFFFFFFFFFFFFFFFFFFFF:FFFFFFFFFFFFFFFFFFFFFFFFFFFFFFFFFFFFFFF
FFFFFFFFFFFFFFFFFFFFFFFFFFFFFFFFFFFFFFFFFFFFFFFFFFFFFFFFFFFFFFFFFFFF
FFFFFFFFFFFFFFF
@A00721:190:HGJW7DSXY:4:1110:21540:10332 2:N:0:TCTGCAAG+GCTGCACT
CGTAAGCGTACGGAGGTGTCCCCGGCGCGGGCAAGCCAGTAATATTATATTGGCTTGCCCCACGGTCT
GCGGTAAGATGCCCCTGCGGGGCCTATGGAATTCTATTTGAATTTGAATTTGAATTGGTATACTGTAG
GGCGGGATATTTTCA
+
FFFFFFFFFFFFFFFFFFFFFFFFFFFFFFFFF:FFF,FFFFFFFFFFFFF:FFFF,FFFFFFFFFF:
FFFFFFFF:FFFFFFFFFFFFFFF,FFFFFFFFFFFF:FFFF,FFFFFFFFFFFFFFFFFFF,FFFFF
F:FFFFFFFFFFFFF
@A00721:190:HGJW7DSXY:4:1120:28022:24815 2:N:0:TCTGCAAG+GCTGCACT
CGTAAGCGTACGGAGGTGTCCCCGGCGCGGGCAAGCCAGTAATATTATATTGGCTTGCCCCACGGTCT
GCGGTAAGATGCCCCTGCGGGGCCTATGGAATTCTATTTGAATTTGAATTTGAATTGGTATACTGTAG
GGCGGGATATTTTCA
+
FFFFFFFFFFFFFFFFFFFFFFFFFFFFFFFFF:FFF:F:FFFFFFFFFFF:FFFFFFFFFFFFFFFF
FFFFFFFFFFFFFFFFFFFFFFFF,F,FFFFFFFF:FFFFFFFFFFFF:FFFFFFFFFFFFFF,FFFF
FFFFFFFFFFFFFFF
@A00721:190:HGJW7DSXY:4:1301:8196:9048 2:N:0:TCTGCAAG+GCTGCACT
CGTAAGCGTACGGAGGTGTCCCCGGCGCGGGCAAGCCATTAATATTATATTGGCTTGCCCCACGGTCT
GCGGTAAGATGCCCCTGCGGGGCCTATGGAATTCTATTTGAATTTGAATTTGAATTGGTATACTGTAG
GGCGGGATATTTTCA
+
FFFF:FFFFFFFF:FFF:FFFFFFFFFFFFFFFF,FFF,,FFFF,:FFF:FFF:,FF:FF,F:FFF:F
F,FF,FFFFF:FFFF,:,,F:F::::F,FF:F:,,F,FFF:,,FFF,::F:F:FFFF:,F:F,:F,F,
FF,F:F,FFFFFF::
@A00721:190:HGJW7DSXY:4:1531:17472:19570 2:N:0:TCTGCAAG+GCTGCACT
CGTAAGCGTACGGAGGTGTCCCCGGCGCGGGCAAGCCAGTAATATTATATTGGCTTGCCCCACGGTCT
GCGGTAAGATGCCCCTGCGGGGCCTATGGAATTCTATTTGAATTTGAATTTGAATTGGTATACTGTAG
GGCGGGATATTTTCA
+
FFFFFFFFFFFFF:FFFFFFFFFFFFFFFFFFFFFFFFFFFFFFFFFFFFFF:FFFFFFFFFFFFFFF
```

FFFFFFFFFFF:FFFFFFFFFFFFFF,FFF:FFFFF:FFFFFFFFFFFFFFFFFFFFFFFFFFFFFF:
FFFFFFFFFFFFFFF
@A00721:190:HGJW7DSXY:4:1641:27218:24205 2:N:0:TCTGCAAG+GCTGCACT
CGTAAGCGTACGGAGGTGTCCCCGGCGCGGGCAAGCCAGTAATATTATATTGGCTTGCCCCACGGTCT
GCGGTAAGATGCCCCTGCGGGGCCTATGGAATTCTATTTGAATTTGAATTTGAATTGGTATACTGTAG
GGCGGGATATTTTCA
+
FFFFFFFFFFFFFFFFFFFFFFFFFFFFFFFFFFFFFFFFFFFFFF:FFFFFFFFFFFFFFFFFFFFF
FFFFFFFF:FFFFFFFFFFFFFFFFFFFFF,FF:FF,FFFF:F:FFF:F:F:FFFFFFF,FFFFFFFF
FFFFFFFFFFFFFFF
@A00721:190:HGJW7DSXY:4:2259:26449:7279 2:N:0:TCTGCAAG+GCTGCACT
CGTAAGCGTACGGAGGTGTCCCCGGCGCGGGCAAGCCAGTAATATTATATTGGCTTGCCCCACGGTCT
GCGGTAAGATGCCCCTGCGGGGCCTATGGAATTCTATTTGAATTTGAATTTGAATTGGTCTACTGTAG
GGCGGGATATTTTCA
+
FFFFFFFFFFFFF::FFFFF:F:FFF,F:FFFFFFFFFFFFF:FFFFFFF:FFF:FFFFF:FFFFFF,
:F,FFFFFFF,FFFFF:F:FFFF:FFFFFFFF:,:F:F,,:F,F,,FFFFFFFFFF::F,F:,FFFFF
,FFFFFFFFFFFFF:
@A00721:190:HGJW7DSXY:4:1541:22037:30514 2:N:0:TCTGCAAG+GCTGCACT
CGTAAGCGTACGGAGGTGTCCCCGGCGCGGGCAAGCCAGTAATATTATATTGGCTTGCCCCACGGTCT
GCGGTAAGATGCCCCTGCGGGGCCTATGGAATTCTATTTGAATTTGAATTTGAATTGGTATACTGTAG
GGCGGGATATTTTC
+
FFFFFFFFFFFFFFFFFFFFFFFFFFFFFFFFFFFFFFFFFFFFFFF,F,FFFF,FFFFFF:FFFFFF
F:FFFFFFFFFFFFFFFFFFFFFFFFFFFFF:FF:FFFFFF,FFFFFFFFFFFFF,FFFFFFF:FFF:F
FFFFFFFFFFFFFF
@A00721:190:HGJW7DSXY:4:1201:6406:36824 2:N:0:TCTGCAAG+GCTGCACT
TGACAAATCGACACGTAAGCGTACGGAGGTGTCCCCGGCGCGGGCAAGCCAGTAATATTATATTGGCT
TGCCCCACGGTCTGCGGTAAGAAGCCCCAGCGGGGCCTATGGAATTCTATTTGAATTTGAATTTGAAT
TGGTATACTGTAGGG
+
FFFFFFFFFFFFFFFFFFFFFFFFFFFFFFFFFFFFF:FFFFFFFFFFFFFFFFFFFFFFFFFFFF:F,
F,FFFFF,FFFFFFFFF:FFFF,FFFFF,FFFFFFFFFFFF:F:FFF:F:,FFFF,FF:F::FFFFF,
FFFF:FF:,FFFF,F
@A00721:190:HGJW7DSXY:4:1228:23647:17989 2:N:0:TCTGCAAG+GCTGCACT
TGACAAATCGACACGTAAGCGTACGGAGGTGTCCCCGGCGCGGGCAAGCCAGTAATAATATATTTGCT
TGCCCCACGGTCTGCGGGAAGATGCACCTGCGGGGCCTATGGAATTCTATTTGAATTTGAATTTTAAT
TGGTATACTGTAGGG
+
FFFFFFFF:FF,FFFFFFF::FF,F:FFFFF:F:F,F:FFFFF,FFF,F:F,FFF:F,FF,FFF,FFF
F:,:FF:FF,:,F,,F:,FF:FFF:,FF:FFFF::FF,:,:FFF:FFFF:FF,FFFFFFFF,FF,FF,
F,:FF,:F:FFF,:F
@A00721:190:HGJW7DSXY:4:1305:31286:30655 2:N:0:TCTGCAAG+GCTGCACT
TGACAAATCGACACGTAAGCGTACGGAGGTGTCCCCGGCGCGGGCAAGCCAGTAATATTATATTGGCT
TGCCCCACGGTCTGCGGTAAGATGCCCCTGCGGGGCCTATGGAATTCTATTTGAATTTGAATTTGAAT
TGGTATACTTTAGGG
+
FF:FFFFFFF:F,:FFFFFFFFFFF,FFFFFFFFF:,FFFF:FFF:F:FFFFF,FFFFFFF,FF,:FF
F:,FFFFFF:FFF:::F,,FF:F::FFF,FFF:FF::,F,FFFF:F,FFFFFF:F,:::F:FFF:FF,
F:FF,FF,,,FFF,F
@A00721:190:HGJW7DSXY:4:1430:28971:2816 2:N:0:TCTGCAAG+GCTGCACT
TGACAAATCGACACGTAAGCGTACGGAGGTGTCCCCGGCGCGGGCAAGCCAGTAATATTATATTGGCT
TGCCCCACGGTCTGCGGTAAGATGCCCCTGCGGGGCCTATGGAATTCTATTTGAATTTGAATTTGAAT
TGGTATACTGTAGGG

```
+
FFFFFFFFFF:FFFFFFFFFFFFF,FFFFFFFFFFF:FFFFFFFFFFFFFFFFFFFFFFFFFFFFFFF
F:FFFFFFFFF:,FFFFFFFFFFFFFFFFFFFFFFFFFFFFFFFFF:FFFFFFFFFFFF:FFFFFFFF
FFF:FFF:FFFFFFF
@A00721:190:HGJW7DSXY:4:1472:9751:28401 2:N:0:TCTGCAAG+GCTGCACT
TGACAAATCGACACGTAAGCGTACGGAGGTGTCCCCGGCGCGGGCAAGCCAGTAATATTATATTGGCT
TGCCCCACGGTCTGCGGTAAGATGCCCCTGCGGGGCCTATGGAATTCTATTTGAATTTGAATTTGAAT
TGGTATACTGTAGGG
+
FFFFFFFFFFFFFFFFFFFFFFFFFFFFFFFFFFFFFFFFFFFFFFFFFFFFFFFFFFFFFFF,FFFFF
FFFFFFFFFFFFFFFFF:FFF:FFFFFF,:FFFFFFF:,FFFFFFFFFFFFFF:FFFFFFFFFFFFFF
FFFFFF:FFFFFFFF
@A00721:190:HGJW7DSXY:4:1475:29469:6684 2:N:0:TCTGCAAG+GCTGCACT
TGACAAATCGACACGTAAGCGTACGGAGGTGTCCCCGGCGCGGGCAAGCCAGTAATATTATATTGGCT
TGCCCCACGGTCTGCGGTAAGATGCCCCTGCGGGGCCTATGGAATTCTATTTGAATTTGAATTTGAAT
TGGTATACTGTAGGG
+
FFFFFFFFFFFF:FFFFFFFFFFFFFFFFFFFFFFFFFFFFFFFFFFF::F,FFFFFF:FFFFFFFFFF
FFFFFFFFFFFFFFFFF:FFFFFFFFFFFFFFFFFFF,FFFFFFFFFFFFFFFFFFFFF:FFFFFFFF
F:FFFFFF:FFFFFF
@A00721:190:HGJW7DSXY:4:1577:21169:5431 2:N:0:TCTGCAAG+GCTGCACT
TGACAAATCGACACGTAAGCGTACGGAGGTGTCCCCGGCGCGGGCAAGCCAGTAATATTATATTGGCT
TGCCCCACGGTCTGCGGTAAGATGCCCCTGCGGGGCCTATGGAATTCTATTTGAATTTGAATTTGAAT
TGGTATACTGTAGGG
+
:FFFFFFFFFFFFFFFFF:FFFFFF:FFFFFFF:FFFFFFFFFFFF:FFFFFFFFFFFFFFFFFFFF
F:FFFFFFFFFFFFFFF:FFFFFFF:FFFFFFFFFF:FFFFFF:FFFFF,F:FFFFFFFFFFFFFFFF
FFFFFFF:FFFFF,:
@A00721:190:HGJW7DSXY:4:1635:8205:22874 2:N:0:TCTGCAAG+GCTGCACT
TGACAAATCGACACGTAAGCGTACGGAGGTGTCCCCGGCGCGGGCAAGCCCGTAATATTATATTGGCT
TGCCCCACGGTCTGCGGTAAGATGCCCCTGCGGGGCCTATGGAATTCTATTTGAATTTGAATTTGAAT
TGGTATACTGTAGGG
+
FFFFFFFFFFFFFFFFFFFFFFFFFFFFF:FFFFFFFFFFFFFFFFFFFF,FFFF,FFF,FFFFFFFF
FFFFFFFFFFF,,F,FFF,FFFFFFFFFFFFFFFFFF::FFFFFFFFFFFFFFFF:F,FF,FFFFFFF
FFFF:FFFFFFFF:F
@A00721:190:HGJW7DSXY:4:2108:14425:34554 2:N:0:TCTGCAAG+GCTGCACT
TGACAAATCGACACGTAAGCGTACGGAGGTGTCCCCGGCGCGGGCAAGCCAGTAATATTATATTGGCT
TGCCCCACGGTCTGCGGTAAGATGCCCCTGCGGGGCCTATGGAATTCTATTTGAATTTGAATTTGAAT
TGGTATACTGTAGGG
+
FFFFFFFFFFFFFFFFFFFFFFFFFFFFFFFFFFFFFFFFFFFFFFFFFFFFFFFFFFFFFFFFFFFF
FFFFFFFFFFFFFFFFFFFFFF:FFFFFFFFFFFFFFF,FFFFFFFFFFFF,FFFFFFFFFFFFFFFF:
FFFFFFFFFFFF:FF
@A00721:190:HGJW7DSXY:4:2211:19777:13135 2:N:0:TCTGCAAG+GCTGCACT
TGACAAATCGACACGTAAGCGTACGGAGGTGTCCCCGGCGCGGGCAAGCCAGTAATATTATATTGGCT
TGCCCCACGGTCTGCGGTAAGATGCCCCTGCGGGGCCTATGGAATTCTATTTGAATTTGAATTTGAAT
TGGTATACTGTAGGG
+
FFFFFFFFFFFFFFFFFFFF:FFFFFFFFFFFFFFFFFFFFFFFFFF:FFFFFFFF:FFFF:,FFFF:F
FFFFFFFF:FFFFFFFFFFFFFFFFFFFFFFFFFFFFFFFFFFFF:FFFFFFFFFF::FFFFFFFFFFF:
FFFFFF:F,FFFFFF
@A00721:190:HGJW7DSXY:4:2222:9281:5760 2:N:0:TCTGCAAG+GCTGCACT
TGACAAATCGACACGTAAGCGTACGGAGGTGTCCCCGGCGCGGGCAAGCCAGTAATATTATATTGGCT
```

TGCCCCACGGTCTGCGGTAAGATGCCCCTGCGGGGCCTATGGAATTCTATTTGAATTTGAATTTGAAT
TGGTATACTGTAGGG
+
FFFFFFFFFFFFFFFFFFFFFFFFFFFFFFFFFFFFFFFFFFFFFFFFFFFFFFFFFFFFFFF:FFFFFF
FFFFFFFFFFFFFFFFFFFFFFFFFFFFFFFFFFFFFFFFFFFFFFFFFFFFFFF:FF:FFFF:FFFFFF
FFFFFFF:FFFF:FF
@A00721:190:HGJW7DSXY:4:2226:21025:23030 2:N:0:TCTGCAAG+GCTGCACT
TGACAAATCGACACGTAAGCGTACGGAGGTGTCCCCGGCGCGGGCAAGCCAGTAATATTATATTGGCT
TGCCCCACGGTCTGCGGTAAGATGCCCCTGCGGGGCCTATGGAATTCTATTTGAATTTGAATATGAAT
TGGTATACTGTAGGG
+
FFFFFFFFF,FFFFFFFFFF:FFFF:FFFFFF:FFFFFF:FFFFFFF:FFF::FFFF:,FFFF:FF:F
F:,FFFFF,,FF,::FFFFFFFFFF:FFF:FF:FF:,FFFF:FF,,,:FF:FFFFF:,:FF:,FFFF:
:,,:,FF,FF:FF,F
@A00721:190:HGJW7DSXY:4:2236:6207:29277 2:N:0:TCTGCAAG+GCTGCACT
TGACAAATCGACACGTAAGCGTACGGAGGTGTCCCCGGCGCGGGCAAGCCAGTAATATTATATTGGCT
TGCCCCACGGTCTGCGGTAAGATGCCCCTGCGGGGCCTATGGAATTCTATTTGAATTTGAATTTGAAT
TGGTATAATGTAGGG
+
FFFFFFFFFFFFF:FFFFFF,FFFF:FFFFFF:FFFFF:FFFF:FFFFFFFFFFF,F,FFFFFF:::F
FF:FFFF:FF:FFF,:FF:FFFFF:FFF:FFFFFFFFFF:FFFFFF,FFF:FFF:FFF,F:FFFF,F,
F::FFF:,:FFFF,F
@A00721:190:HGJW7DSXY:4:2655:29053:6339 2:N:0:TCTGCAAG+GCTGCACT
TGACAAATCGACACGTAAGCGTACGGAGGTGTCCCCGGCGCGGGCAAGCCAGTAATATTATATTGGCT
TGCCCCACGGTCTGCGGTAAGATGCCCCTGCGGGGCCTATGGAATTCTATTTGAATGTGAATTTGAAT
TGGTATACTGTAGGG
+
FFFFFFFFFFFFFFFFFFFFFF:FFFFFFFFFFFFFFFFFFFFFFFF:FFFFFFFFF::FFF,FFFFF
FFFFFFFFFFFFFFFFFFFFFFFFFFFFFFFFFFFFFFFFFFFFFFF::F,FFFFF,FFFFFFF,FFF
F,FFFFFFFFFFF:F
@A00721:190:HGJW7DSXY:4:1336:25464:1720 2:N:0:TCTGCAAG+GCTGCACT
GACAAATCGACACGTAAGCGTACGGAGGTGTCCCCGGCGCGGGCAAGCCAGTAATATTATATTGGCTT
GCCCCACGGTCTGCGGTAAGATGCCCCTGCGGGGCCTATGGAATTCTATTTGAATTTGAATTTGAATT
GGTATACTGTAGGAG
+
FFFFFFFFFFFFFFFFFFFFFFFFFFFFFFFFFFFFFFFFFFFFFFFFFFFFFFFFFFFFFFFFFFFF
FFFFFFFFFFFFF:FFFFFF:FFFFFFFFFFFFFFFF:FFFFFF:FFFFFFFFF:FFFFFFFFFFFF
FFFFFF,FFFFFFFF
@A00721:190:HGJW7DSXY:4:1371:16676:26678 2:N:0:TCTGCAAG+GCTGCACT
GACAAATCGACACGTAAGCGTACGGAGGTGTCCCCGGCGCGGGCAAGCCAGTAATATTATATTGGCTT
GCCCCACGGTCTGCGGTAAGATGCCCCTGCGGGGCCTATGGAATTCTATTTGAATTTGAATTTGAATT
GGTATACTGTAGGAG
+
FFFFFFFFFFFFFFFFFFFFFFFFFFFFFFFFFFFFFFFFFFFFFFFFFFFFFFF:FFFFFFFFFFF
FFFFFFFFFFFFFFFFFFFFFFFFFFFFFFFFFFF:FFFFFFFFFFFFFFFFFFFFFFFFFFFFFFFF
FFFFFFFFFFFFFFF
@A00721:190:HGJW7DSXY:4:1430:18927:10567 2:N:0:TCTGCAAG+GCTGCACT
GACAAATCGACACGTAAGCGTACGGAGGTGTCCCCGGCGCGGGCAAGCCAGTAATATTATATTGGCTT
GCCCCACGGTCTGCGGTAAGATGCCCCTGCGGGGCCTATGGAATTCTATTTGAATTTGAATTTGAATT
GGTATACTGTAGGAG
+
FFFFFFFFFFFFFFFFFFFFFFFFFFFFFFFFFFFFFFFFFFFFFFFFFFFFFFFF:FFFFFFFFFFF
FFFFFFFFFF:FFFFFFFFFFFFFFFFFFFFFFFFFFFFFFFFFFFF,F:FFFFFFFFFF,FFFFFFFFF
:FF:FF,FFFFFFFF

```
@A00721:190:HGJW7DSXY:4:1530:3938:2487 2:N:0:TCTGCAAG+GCTGCACT
GACAAATCGACACGTAAGCGTACGGAGGTGTCCCCGGCGCGGGCAAGCCAGTAATATTATATTGGCTT
GCCCCACGGTCTGCGGTAAGATGCCCCTGCGGGGCCTATGGAATTCTATTTGAATTTGAATTTGAATT
GGTATACTGTAGGAG
+
FFFFFFFFFFFFFFFFFFFFFFFFFFFFFFFFFFFFFFFFFFFFFFFFFFFFFFFFFFFFFFFFFFFF
FFFFFFFFFFFFFFFF:FFFFFFFFFFFFFFFFFFF::FFFFFFFFFFFFF::FFFF:FFFFFFFFFF
FFFFFFFFFFFFFFF
@A00721:190:HGJW7DSXY:4:1677:20799:11522 2:N:0:TCTGCAAG+GCTGCACT
GACAAATCGACACGTAAGCGTACGGAGGTGTCCCCGGCGCGGGCAAGCCAGTAATATTATATTGGCTT
GCCCCACGGTCTGCGGTAAGATGCCCCTGCGGGGCCTATGGAATTCTATTTGAATTTGAATTTGAATT
GGTATACTGTAGGAG
+
FFFFFFFFFFFFFFFFFFFFFFFFFFFFFFFFFFFFFFFFFFFFFFFFFFFFFFFFFFFFFFFFFFFF
FFFFFFFFFFF,FFFFF:FFFFFFFFFFFFFFFFFF,FFFFF::FFFFF::FFFFFFFFFFFFFFFFF
FFFFFFFFFFFFFFF
@A00721:190:HGJW7DSXY:4:2458:3224:24142 2:N:0:TCTGCAAG+GCTGCACT
GACAAATCGACACGTAAGCGTACGGAGGTGTCCCCGGCGCGGGCAAGCCAGTAATATTATATTGGCTT
GCCCCACGGTCTGCGGTAAGATGCCCCTGCGGGGCCTATGGAATTCTATTTGAAGTTGAATTTGAATT
GGTATACTGTAGGAG
+
FFFFFF:FFFFFFFFFFFFFFFFF:FFFFFFF:FF:FFFFFFF:FFF:F::F:F:,:F:,:,F:FFFF
,,FFF:FFF:F,FFFF:F::F:F,FFFFFFFFFFFFFF,FFF,,FFF:F,FF::,FFFFF:,FFF::F
FFF,F:FFFFFFFFF
@A00721:190:HGJW7DSXY:4:1237:4661:7310 2:N:0:TCTGCAAG+GCTGCACT
GACAAATCGACACGTAAGCGTACGGAGGTGTCCCCGGCGCGGGCAAGCCAGTAATATTATATTGGCTT
GCCCCACGGTATGCGGTAAGATGCCCCTGCGGGGCCTATGGAATTCTATTTGAATTTGAATTTGAATT
GGTATACTGTAGGA
+
FFFFFFFFFFFFFFFFFFFF:FFF:FFFFFFFFFFFFFFFFFFFFFF:FF,FF:FFFFF,FFFFFFF,
,FF:FFFFFF,FFFFFFF:FF::FFFFFFFFFFFF:F:FFFFFF::FF,FFF:F,F:FFF,FFFFF:F
FFF:,::,F,:FFF
@A00721:190:HGJW7DSXY:4:1325:24822:5431 2:N:0:TCTGCAAG+GCTGCACT
AATGACAAATCGACACGTAAGCGTACGGAGGTGTCCCCGGCGCGGGCAAGCCAGTAATATTATATTGG
CTTGCCCCACGGTCTGCGGTAAGATGCCCCTGCGGGGCCTATGGAATTCTATTTGAATTTGAATTTGA
ATTGGTATACTGTAG
+
FFFFFFFFFFFFFFFFFFFFFFFFFFFFFFFFFFFFFFFFFFFFFFFFFFFFFFFFFFFFFFFFFFFF
FFFFFFFFFFFFFF:FFFFFFFFFFFFFFFFFFFFFFFF,:FFFFFFFFFFFFFFFFFFFFFFFFFFF
F,FFFFFFFFFFFFF
@A00721:190:HGJW7DSXY:4:2369:7148:3223 2:N:0:TCTGCAAG+GCTGCACT
AATGACAAATCGACACGTAAGCGTACGGAGGTGTCCCCGGCGCGGGCAAGCCAGTAATATTATATTGG
CTTGCCCCACGGTCTGCGGTAAGATGCCCCTGCGGGGCCTATGGAATTCTATTTGAATTTGAATTTGA
ATTGGTATACTGTAG
+
FFFFFFFFFFFFFFFFFFFFFFFFFFFFFFFFFFFFFFFFFFFFFFFFFFFFFFFFF:FFFFFFFFFF
FFFFFFFFFFFFFFFFFFF:FFFFFFFFFFFFFFFFFFF,F,FFFFFFF:FFFFFFFFFFFFF:FFFF
FFFF:FFFFFFFFFF
@A00721:190:HGJW7DSXY:4:1504:2862:4946 2:N:0:TCTGCAAG+GCTGCACT
CTAATGACAAATCGACACGTAAGCGTACGGAGGTGTCCCCGGCGCGGGCAAGCCAGTAATATTATATT
GGCTTGCCCCACGGTCTGCGGTAAGATGCCCCTGCGGGGCCTATGGAATTCTATTTGAATTCGAATTT
GAATTGGTATACTGT
+
FFFFFFFFFFFFFFFFFFFF:FFFFFFFFFFFFFFFFFFFFFFFFFFFFFF::FF,,FFFFF::::FF
```

```
,FFFF:FFFFFFFFFFFF,FF,FFFFFFFFFF,FFFFFFFFF:FFFFFFFF,F,:FFFFFFFF:FFFF
FFFFF:FFFFFF:FF
@A00721:190:HGJW7DSXY:4:2120:31611:18693 2:N:0:TCTGCAAG+GCTGCACT
CTAATGACAAATCGACACGTAAGCGTACGGAGGTGTCCCCGGCGCGGGCAAGCCAGTAATATTATAGT
GGCTTGCCCCACGGTCTGCGGTAAGATGCCCCTGCGGGGCCTATGGAATTCTATTTGAATTCGAATTT
GAATTGGTATACTGT
+
FFFFFFFFFF:FFFFFFFFFFFFFFFFFFF:FFFFFFFFFFFFFFFFFFFFFFFFFFF:F::F:FF,F
FFFFFFFFFFFFFF:F:FFFFFF:F,F::FFFFFFF:FFF:FFFFFFF:F:,FF:FFFF:FFF:,FFF
FFFFFFFFFFFFFFF
@A00721:190:HGJW7DSXY:4:2242:18105:7482 2:N:0:TCTGCAAG+GCTGCACT
CTAATGACCAATCGACACGTAAGCGTACGGAGGTGTCCCCGGCGCGGGCAAGCCAGTAATATTATATT
GGCTTGCCCCACGGTCTGCGGTAAGATGCCCCTGCGGGGCCTATGGAATTCTATTTGACTTCGAATTT
GAATTGGTATACTGT
+
FFFFFFFF,:FFFFFFFFFFFFFF:FFFFFFFFFFFFFFFF,FFFFFFFF,FFF,F:F:F,:F,FF:F
F:FFFFFFFFFFFF:F,F,FFF:FFFFFFFFF:FFFFFFFF:FFFFF,:FF,FF,F:F,::FFF,FFF
FFF,,,FF:FFFFFF
@A00721:190:HGJW7DSXY:4:2316:30120:26694 2:N:0:TCTGCAAG+GCTGCACT
CTAATGACAAATCGACACGTAAGCGTACGGAGGTGTCCCCGGCGCGGGCAAGCCAGTAATATTATATT
GGCTTGCCCCACGGTCTGCGGTAAGATGCCCCTGCGGGGCCTATGGAATTCTATTTGAATTCGAATTT
GAATTGGTATACTGT
+
FFFFFFFFFFFFFFFFFFFFFFFFFFFFFFFFFFFFFFFFFFFFFFFFFFFF,FFFFF,FFF:FFF
FFFFFFFFFFFFFFF:FFFFFFFFFFFFFFFFF:FFFFFFFFFFFFFFFFFFF:FFFFFFFFFFFFFF
FFFFFFFFFFFFFFF
@A00721:190:HGJW7DSXY:4:2641:6515:33818 2:N:0:TCTGCAAG+GCTGCACT
CTAATGACAAATCGACACGTAAGCGTACGGAGGTGTCCCCGGCGCGGGCAAGCCAGTAATATTATATT
GGCTTGCCCCACGGTCTGCGGTAAGATGCCCCTGCGGGGCCTATGGAATTCTATTTGAATTCGAATTT
GAATTGGTATACTGT
+
FFFFFFFFFFFFFFFFFFFFFFFFFFFFFFFFFFFFFFFFFFFFFFFFFF,FFFF,FFFFFFFFFF:FF
FFFFFFFFFFFFFFFFFFFFFFFFFFFFFFFFFFFFFFFFFF:FFFFFFF:F:::FFFFFFFFFFFFFF
FFFF:FF:FFFFFFF
@A00721:190:HGJW7DSXY:4:2677:17833:23797 2:N:0:TCTGCAAG+GCTGCACT
CTAATGACAAATCGACACGTAAGCGTACGGAGGTGTCCCCGGCGCGGGCAAGCCAGTAATATTATATT
GGCTTGCCCCACGGTCTGCGGTAAGATGCCCCTGCGTGGCCTATGGAATTCTATTTGAATTAGAATTT
GAATTGTTATACTGT
+
FFFFF,F,FFFFF:FFFF,:,F,F:,FF,FFFFF,F,,FFF:F,F:::,FF:FF:F:FF,FF,FFFFF
,FF:,:FFFFFFFFF,,,FFFFF,FF,:::F::,F:,F:FF:FF,FFFFFFFFFF:FFF,,,F,,,FF
,FFFFF,FFF:,FFF
@A00721:190:HGJW7DSXY:4:2677:17815:23954 2:N:0:TCTGCAAG+GCTGCACT
CTAATGACAAATCGACACGTAAGCGTACGGAGGTGTCCCCGGCGCGGGCAAGCCAGTAATATTATATT
GGCTTGCCCCACGGTCTGCGGTAAGATGCCCCTGCGGGGCCTATGGAATTCTATTTGAATTCGAATTT
GAATTGGTATACTGT
+
FFFFFFFFFFFFFFFFFFFFFFFFFFFFFFFFFFFFFFFFFFFFFFFFFFFFFFFFFFFFFFFFFFFF
FFFFFFFFFFFFFFFFFFFFFFFFF:FFFFFFFFFFFFFFFFFFFFFFFFFFFFFFFFF:FFFFFFFFF
F:FFFFFFFFFFFFF
@A00721:190:HGJW7DSXY:4:1341:7229:5274 2:N:0:TCTGCAAG+GCTGCACT
TCTAATGACAAATCGACACGTAAGCGTACGGAGGTGTCCCCGGCGCGGGCAAGCCAGTAATATTATAT
TGGCTTGCCCCACGGTCTGCGGTAAGATGCCCCTGCGGGGCCTATGGAATTCTATTTGAATTTGAATT
TGAATTGGTATACTG
```

```
+
FFFFFFFFFFFFFFFFFFFFFFFFFFFFFFFFFFFFFFFFFFFFFFFFFFFFFF,F:F:FFFFF,F:
FFFFFFFFFFF,FFFFFFFFFFFFFFFFFFFFFFFFFFFFFF:F:FFFF:FFF:F:FFF::FFF:FFF
FFF:FFFFFFFFFFF
@A00721:190:HGJW7DSXY:4:1403:10529:8547 2:N:0:TCTGCAAG+GCTGCACT
TCTAATGACAAATCGACACGTAAGCGTACGGAGGTGTCCCCGGCGCGGGCAAGCCAGGAATATTATAT
TGGCTTGCCCCACGGTCTGCGGTAAGATGCCCCTGCGGGGCCTATGGAATTCTATTTGAATTTGAATT
TGAATTGGTATACTG
+
F:FFFFFFFFFFFFFFFFFFFFFFFFFFFFFFFFFFFFFFFFFF:FFFFF,F:FF:F,FF:F,FF:F:
F,FFFFFFFFF:FFFF,,FFFFF,:FFFF:FFFFFFFFFF:F:FF:FFF,F,FF,FFFFFFF::FFFF
FF,:FFFFFFFFFFF
@A00721:190:HGJW7DSXY:4:1545:20302:6402 2:N:0:TCTGCAAG+GCTGCACT
TCTAATGACAAATCGACACGTAAGCGTACGGAGGTGTCCCCGGCGCGGGCAAGCCAGTAATATTATAT
TGGCTTGCCCCACGGTCTGCGGTAAGATGCCCCTGCGGGGCCTATGGAATTCTATTTGAATTTGAATT
TGAATTGGTATACTG
+
FFFFFFFFFFFFFFFFFFFFFFF:FFFFFFFFFFFFFFFFFFFFFFFFFFFFFFFFFFFFFFF:FFFF:
FFFFFFFFFFFFFFFF::F,FFFFFFFFFFFFFFFFFFFFFFF:FFFFFF:F,FFF,FFF:FF:FF,FF
FF:F:FFFFFFFFFF
@A00721:190:HGJW7DSXY:4:2148:22905:16611 2:N:0:TCTGCAAG+GCTGCACT
TCTAATGACAAATCGACACGTAAGCGTACGGAGGTGTCCCCGGCGCGGGCAAGCCAGTAATATTATAT
TGGCTTGCCCCACGGTCTGCGGTAAGATGCCCCTGCGGGGCCTATGGAATTCTATTTGAATTTGAATT
TGAATTGGTATACTG
+
FFFFFFFFFFFFFFFFFFFFFFFFFFFFFFFFFFFFFFFFFFFFFFFFFFFFFFFFFF,FFFFF:FFF
FFFF:FFFFFFFFFFFFFFFFFFFFFF:FFFFFFFFFFFFFFFFFFFFFF,FFFFFFFFFFFFFFFFF:F
FFFF:FFFFFFFFFF
@A00721:190:HGJW7DSXY:4:2506:8377:16219 2:N:0:TCTGCAAG+GCTGCACT
TCTAATGACAAATCGACACGTAAGCGTACGGAGGTGTCCCCGGCGCGGGCAAGCCAGTAATATTATAT
TGGCTTGCCCCACGGTCTGCGGTAAGATGCCCCTGCGGGGCCTATGGAATTCTATTTGAATTTGAATT
TGAATTGGTATACTG
+
FFFFFFFFFFFFFFFFFFFFFFFFFFFFFFFFFFFFFFFFFFFFFFFFFFFFFFFFFFFFFFFFFFFF
FFFF:F:FFFFFFFFF:FFFFFFFFFF::FFFF:FFFFFFFF,F:FFFFFFFFF:FFFFFFFFFFFF
F,:FFF,FFFFFFFF
@A00721:190:HGJW7DSXY:4:1344:4562:35133 2:N:0:TCTGCAAG+GCTGCACT
GTCTAATGACAAATCGACACGTAAGCGTACGGAGGTGTCCCCGGCGCGGGCAAGCCAGTAATATTATA
TTGGCTTGCCCCACGGTCTGCGGTAAGATGCCCCTGCGGGGCCTATGGAATTCTATTTGAATTTGAAT
TTGAATTGGTAAGAT
+
FFFFFFFFFFFFFFFFFFFFFFFFFFFFFFFFFFFFFFFFFFFFFFFFF,FFFFFFFFFFF::FFF
FFFFFFFFFFFFFFFFFFFFFFFFFFFFFFFFFFFFFFFFFFFFF:,FFFFFFF:F,F,FFFF,F:FF:F
FFFFF,FFFFFF:FF
@A00721:190:HGJW7DSXY:4:1648:5692:33144 2:N:0:TCTGCAAG+GCTGCACT
GTCTAATGACAAATCGACACGTAAGCGTACGGAGGTGTCCCCGGCGCGGGCAAGCCAGTAATATTATA
TTGGCTTGCCCCACGGTCTGCGGTAAGATGCCCCTGCGGGGCCTATGGAATTCTATTTGAATTTGAAT
TTGAATTGGTAAGAT
+
FFFFFFFFFFFFFFFFFFFFFFF:FFFFFFFFFFFFFFFFFFFFFFFFFFFFFFFFFFFFFFFFFFFF
,FFFFFFFFFFFFFFFFFFFFFFFF:FF,FFFFFFFFFFFFFFF:FFFFF,FFFFF:FFF:FF,FFF,F
FFFF::FFFFFFFFF
@A00721:190:HGJW7DSXY:4:2648:7780:27336 2:N:0:TCTGCAAG+GCTGCACT
GTCTAATGACAAATCGACACGTAAGCGTACGGAGGTGTCCCCGGCGCGGGCAAGCCAGTAATATTATA
```

```
TTGGCTTGCCCCACGGTCTGCGGTAAGATGCCCCTGCGGGGCCTATGGAATTCTATTTGAATTTGAAT
TTGAATTGGTAAGAT
+
FFFFFFFFFFFFFFFFFFFFFFFFFFFFFFFFFFFFFFFFFFFFFFFFFFFFFFFFFFFF,FFFFFFF
FFFFFFFFFFFFFFFFFFFFFFFFFFFFFFFFFFFFFFFFFFFF,FFFFFFFFFFFFFFF:FFFFFFF
FFFFFFFFFFFFFFF
@A00721:190:HGJW7DSXY:4:2239:9869:26976 2:N:0:TCTGCAAG+GCTGCACT
CGCGTGGTCTAATGACAAATCGACACGTAAGCGTACGGAGGTGTCCCCGGCGCGGGCAAGCCAGTAAT
ATTATATTGGCTTGCCCCACGGTCTGCGGTAAGATGCCCCTGCGGGGCCTATGGAATTCTATTTGAAT
TTGAATTTGAATTGG
+
FFFFFFFFFFFFFFFFFFFFFFFFFFFFFFFFFFFF:FFFFFFFFFFFFFFFFFFFFFFFFFFFFFFF
FFFFFFFFFFFFFFFFFFFFFFFFF:FFFFFFFFFFFFFFFFFFFFFFFFFFFFFF,FFFFFFFFFFF
F:FF:FFF:FFFFFF
@A00721:190:HGJW7DSXY:4:2639:6433:6214 2:N:0:TCTGCAAG+GCTGCACT
CGCGTGGTCTAATGACAAATCGACACGTAAGCGTACGGAGGTGTCCCCGGCGCGGGCAAGCCAGTAAT
ATTATATTGGCTTGCCCCACGGTCTGCGGTAAGATGCCCCTGCGGGGCCTATGGAATTCTATTTGAAT
TTGAATTTGAATTGG
+
FFFF:FFFFFFFFFFFFFFFFFFFFFFFFFFFFFFFFFFFFFFFFFFFFFFFFFFFFF,FFFFFFF:F
FFFF:FFFFFFFFFFFFFFFFFFFFFFFFFFFFFFFFFFFFFFFFFFFFFFF,F,FFFF:FFFF:,FFF,F
FFFFFF:FFFFFFFF
@A00721:190:HGJW7DSXY:4:1128:6958:6026 2:N:0:TCTGCAAG+GCTGCACT
GTGACGCGTGGTCTAATGACAAATCGACACGTAAGCGTACGGAGGTGTCCCCGGCGCGGGCAAGCCAG
TAATATTATATTGGCTTGCCCCACGGTCTGCGGTAAGATGCCCCTGCGGGGCCTATGGAATTCTATTT
GAATTTGAATTTGAA
+
FFFFFFFFFFFFFFFFFFFFFFFFFFFFFFFFFFFFFFFFFFFFFFFFFFFFFFFFFFFFFF:FFFFFF
FFFFFFFFFFFF,FFFFFFFFFFFFFF:FFFFF:FFFFFFFFFFFFFFFFFFF,F,FFF:FFFF:FFF
FFFFFFFFFFFFFFF
@A00721:190:HGJW7DSXY:4:1435:2220:11475 2:N:0:TCTGCAAG+GCTGCACT
GTGACGCGTGGTCTAATGACAAATCGACACGTAAGCGTACGGAGGTGTCCCCGGCGCGGGCAAGCCAG
TAATATTATATTGGCTTGCCCCACGGTCTGCGGTAAGATGCCCCTGCGGGGCCTATGGAATTCTATTT
GAATTTGAATTTGAA
+
FFFFFFFFFFFFFFFFFFFFFFFFFFFFFFFFFFFFFFFFFFFFFFFFFFFFFFFFFFFFFFFFFF:F
FFFFFF:FFFFFFFFFFFFFFFFFFFFFF,FFFFFFFFFFFFFFFFFFFFFFFFFFFFFFFFFFFF:FF
FFFFFFFFF,FFFFF
@A00721:190:HGJW7DSXY:4:1471:23493:29684 2:N:0:TCTGCAAG+GCTGCACT
GTGACGCGTGGTCTAATGACAAATCGACACGTAAGCGTACGGAGGTGTCCCCGGCGCGGGCAAGCCAG
TAATATTATATTGGCTTGCCCCACGGTCTGCGGTAAGATGCCCCTGCGGGGCCTATGGAATTCTATTT
GAATTTGAATTTGAA
+
FFFFFFFFFFFFFFFFFFFFFFFFFFFFFFFFFFFFFFFFFFFFFFFFFFFFFFFFFFFFFFFFFFFF
FFFFFF,F:FFFFFFFFFFFFFFFFFFF,FFFFFFFFF:FFFFFFFFFFFFFFFFFFFF,FFFFFFFF
:F:FFFFFFFFFFFF
@A00721:190:HGJW7DSXY:4:1641:5231:35790 2:N:0:TCTGCAAG+GCTGCACT
GTGACGCGTGGTCTAATGACAAATCGACACGTAAGCGTACGGAGGTGTCCCCGGCGCGGGCAAGCCAG
TAATATTATATTGGCTTGCCCCACGGTCTGCGGTAAGATGCCCCTGCGGGGCCTATGGAATTCTATTT
GAATTTGAATTTGAA
+
FFF:FFFFFFFFFFFFFFFFFFFFFFFFFFFFFFFFFFFFFFFFFFFFFFFFFFFFFFFFFFFFFFFF
FFF:FF,FFFFFFFFFFFFFFFFFFFFFFFFFFFFFFFFFFFFFFFFFFFFFF:FFF:,F,F:FF:
FF:FFFFFFFFFFFF
```

```
@A00721:190:HGJW7DSXY:4:2133:1542:32409 2:N:0:TCTGCAAG+GCTGCACT
GTGACGCGTGGTCTAATGACAAATCGACACGTAAGCGTACGGAGGTGTCCCCGGCGCGGGCAAGCCAG
TAATATTATATTGGCTTGCCCCACGGTCTGCGGTAAGATGCCCCTGCGGGGCCTATGGAATTCTATTT
GAATTTGAATTTGAA
+
FFFFFFFFFFFFFFFFFFFFFFFFFFFFFFFFFFFFFFFFFFFFFFFFFFFFFFFFFFFFFFFFFFFFFF
FFFFFFFFFFFFFFFFFFFFFFFFFFFFFFFFFFFFFFFFFFFFFFFFFFFFFFFFFFFFFFFFFFFFFF
FFFFFFFFFFFFFFF
@A00721:190:HGJW7DSXY:4:2455:26775:12477 2:N:0:TCTGCAAG+GCTGCACT
GTGACGCGTGGTCTAATGACAAATCGACACGTAAGCGTACGGAGGTGTCCCCGGCGCGGGCAAGCCAG
TAATATTATATTGGCTTGCCCCACGGTCTGCGGTAAGATGCCCCTGCGGGGCCTATGGAATTCTATTT
GAATTTGAATTTGAA
+
FFFFFFFFFFFFFFFFFFFFF:FFFFFFFFFFFFFFFFFFFFFFFFFFFFFFFFFFFFFFFFF:FFFFFF
,FFFF:FFFFFFFFF,FFFFFFFFFFFFFFFFFF:FFFFFF:FFFFFFFFFFFFF,,:FFF,F:FFF:FF
F:::FFF:FFF,FFF
@A00721:190:HGJW7DSXY:4:2242:28782:13698 2:N:0:TCTGCAAG+GCTGCACT
TACCAAATCAAATTCAAAAAGAAATCCATAGGCACCGCAGGGGCATATTACCGCAGACCGTGGGGCAA
GCCAATATAAAATAACAGGCTAGCCCGCGCCGGGGACACCTCCGTACGCTTACGTGTCGATTTGTCAT
TAGACCACGCGTCA
+
FFF:FF,,FFFFFFFFFF:,F:FF,,FFFFFFFF:FFFF:FFF:F,,,FFFFFFFFF,F,:,FFFF:,F
FFFF,,:FFF,FF:FF,:FFF,F,F:F,FF:F:FFFFFFFFFFFF,FFFF:F:FFFFFFF::FFF,FF
,FFFFFFFFFFFF:
@A00721:190:HGJW7DSXY:4:2123:10601:31000 2:N:0:TCTGCAAG+GCTGCACT
TACCAATTCAAATTCAAATAGAATTCCATAGGCCCCGCAGGGGCATCTTACCGCAGACCGTGGGGCAA
GCCAATATAATATTACTGGCTTGCCCGCGCCGGGGACACCTCCGTACGCTTACGTGTCGATTTGTCAT
TAGACCACGCGTCAC
+
FFFFFFFFFFFFFFFFFFFFFFFFFFFFFF,F:FFFFFFFFFFFF:FFFFFFFFFFF:FFFFFFFFFFFF
FFF:FFFFF:FFFFFFF,FFF:FFFFFFFFFFFFFFFFFFFFFFFF:F:FF,F:FFFFFFFF:FFFFFFF
FFFFFFFFFFF:FF:
@A00721:190:HGJW7DSXY:4:2123:11333:32518 2:N:0:TCTGCAAG+GCTGCACT
TACCAATTCAAATTCAAATAGAATTCCATAGGCCCCGCAGGGGCATCTTACCGCAGACCGTGGGGCAA
GCCAATATAATATTACTGGCTTGCCCGCGCCGGGGACACCTCCGTACGCTTACGTGTCGATTTGTCAT
TAGACCACGCGTCAC
+
FFFFFFFFFFFFFFFFFFFFFFFFFFFFFFFFFFFFFFFFFFFFFFFFFFFFFFFFFFFFFFFFFFFFFF
FFFFFFFFFF:FFFFFFFFF:FFFFFFFFFFFFFFFFFFFFFFFFFFFFFFFFFFF:FFFFF,FFFFFFF
FFFFFFFFF:FFFFF
@A00721:190:HGJW7DSXY:4:2124:31882:13964 2:N:0:TCTGCAAG+GCTGCACT
TACCAATTCAAATTCAAATAGAATTCCATAGGCCCCGCAGGGGCATCTTACCGCAGACCGTGGGGCAA
GCCAATATAATATTACTGGCTTGCCCGCGCCGGGGACACCTCCGTACGCTTACGTGTCGATTTGTCAT
TAGACCACGCGTCAC
+
FFFFFFFFFFFFFFFFFFFFF:FFFFFFFFFFFFFFF:FFFFFFFFFFFFFFFFFFFFFFFFFFFFFFFF
FFFFFFFFFFFFFFFFFFFFFFFFFFFFFFFFFFFFFFFFFFFFFFFFFFFFFFFFF:FFFFFFFFFFFF
FFF:FFFFFFFFFFF
@A00721:190:HGJW7DSXY:4:2145:7690:19914 2:N:0:TCTGCAAG+GCTGCACT
TACCAATTCAAATTCAAATAGAATTCCATAGGCCCCGCAGGGGCATCTTACCGCAGACCGTGGGGCAA
GCCAATATAATATTACTGGCTTGCCCGCGCCGGGGACACCTCCGTACGCTTACGTGTCGATTTGTCAT
TAGACCACGCGTCAC
+
FFFFFFFFF:FF,FFFFFFFFF:FFFFFFFFFFFFFFFFFFFFFFFFFFFFFFFFFFFFFFFFFFFFFFF
```

```
FFFFFFFFFFFFFFFFFFFFFFFFFFFFFFFFFFFFFFFFFFFFFFFFFFFFFFFFFFFFFF:FFFFFF,
FFFFFFFFFFFFFFF
@A00721:190:HGJW7DSXY:4:2344:2356:11240 2:N:0:TCTGCAAG+GCTGCACT
TACCAATTCAAATTCAAATAGAATTCCATAGGCCCCGCAGGGGCATCTTACCGCAGACCGTGGGGCAA
GCCAATATAATATTACTGGCTTGCCCGCGCCGGGGACACCTCCGTACGCTTACGTGTCGATTTGTCAT
TAGACCACGCGTCAC
+
FFFFFFFFFFFFFFF,FFFFFFFFFFFFFFFFFFFFFFFFFFFFFFFFFFFFFFFFFFFFFF:FFFFFFF
FFFFF:FFFFFFFFFFFFFFFFFFFFFFFFFFFFFFFFFFFFFFFFFFFFF:FFFFFFFFFF:FFFFFFF
FF:FFFFFF:FFFFF
@A00721:190:HGJW7DSXY:4:2355:29559:26788 2:N:0:TCTGCAAG+GCTGCACT
TACCAATTCAAATTCAAATAGAATTCCATAGGCCCCGCAGGGGCATCTTACCGCAGACCGTGGGGCAA
GCCAATATAATATTACTGGCTTGCCCGCGCCGGGGACACCTCCGTACGCTTACGTGTCGATTTGTCAT
TAGACCACGCGTCAC
+
FFFFFFFFFFFFFFFFFFFFFFFF:FFFFFFFFF:FFFFFFFFFFFFFFFFFFFFFFFFFFFFFFFFFF,
FFFFFFFFFFFFFFFFFFFFFFFFFFFFF:FFFFFFFFFFFFFFFFFFFF::FFFFFFFFFFFFFFFFF:
FFFFFFFFFFFFFFF
@A00721:190:HGJW7DSXY:4:1344:30373:13917 2:N:0:TCTGCAAG+GCTGCACT
GATCGTGACGCGTGGTCTAATGACAAATCGACACGTAAGCGTACGGAGGTGTCCCCGGCGCGGGCAAG
CCAGTAATATTATATTGGCTTGCCCCACGGTCTGCGGTAAGATGCCCCTGCGGGGCCTATGGAATTCT
ATTTGAATTTGAATT
+
FFFFFFFFFFFFFFFFFFFFFFFFFFFFFFFFFFFFFFFFFFFFFFFFFFFFFFFFFFFFFFFFFFFFFF
FFFFFFFFFFFFFFFFFFFFFFFFFFFFFFF::FFFFFFFFFFF:FFFFFFFFFFFFFFFFFFFF,FF:F
:F:FFF:FFFFFFFF
@A00721:190:HGJW7DSXY:4:1362:16559:8155 2:N:0:TCTGCAAG+GCTGCACT
GATCGTGACGCGTGGTCTAATGACAAATCGACACGTAAGCGTACGGAGGTGTCCCCGGCGCGGGCAAG
CCAGTAATATTATATTGGCTTGCCCCACGGTCTGCGGTAAGATGCCCCTGCGGGGCCTATGGAATTCT
ATTTGAATTTGAATT
+
FFFF:FFFFF:F,FFFF:FF:FFFFFFFFFFFFFFF:FFFFFFFFFFFFFFFFF:FFFFFFF:FFFFFF:
FFFF,:F,FF,FFFFFFFFFFFFFFFFFFFFFFFFFFFFFFFFF,FFFFFFFFFFF:FFFFFFFFFFFFF
FFFF:FFFFFFFF:F
@A00721:190:HGJW7DSXY:4:1432:15374:8359 2:N:0:TCTGCAAG+GCTGCACT
GATCGTGACGCGTGGTCTAATGACAAATCGACACGTAAGCGTACGGAGGTGTCCCCGGCGCGGGCAAG
CCAGTAATATTATATTTGCTTGCCCCACGGTCTGCGGTAAGATGCCCCTGCGGGGCCTATGGAATTCT
ATTTGAATTTGAATT
+
FFF:FFFF,F,,F:FFFFFFFF:FFFFF:FFFF:FFFFF::FFFFF,FF:FFFFFFFF,:F:FF:F:FFF
FF::FF:FF,:FFFFF,F,:FFFFF,FF:F,:FF,F,FFFF:,,F:FFFFFFF:FFFFF::,F:FFFFF
FF:FFFF,F,,FFFF
@A00721:190:HGJW7DSXY:4:1668:7374:34240 2:N:0:TCTGCAAG+GCTGCACT
GATCGTGACGCGTGGTCTAATGACAAATCGACACGTAAGCGTACGGAGGTGTCCCCGGCGCGGGCAAG
CCAGTAATATTATATTGGCTTGCCCCACGGTCTGCGGTAAGATGCCCCTGCGGGGCCTATGGAATTCT
ATGTGAATTTGAATT
+
FFFFFFFF:FFF:FFFFFFFFFFFFFFFFFFFFFFFFFFFFFFFFFFFFFFFFFFFFFFFFFFFFFFFF
FFFFFFFF:FFFF:FFFFFFFFFFFFFFFFFFFFFFFFFFFFFFFFFFFFFFFFFFFFFFF:FFFFFFFF
FF,FFFF,:FFF:FF
@A00721:190:HGJW7DSXY:4:1677:32208:4758 2:N:0:TCTGCAAG+GCTGCACT
GATCGTGACGCGTGGTCTAATGACAAATCGACACGTAAGCGTACGGAGGTGTCCCCGGCGCGGGCAAG
CCAGTAATATTATATTGGCTTGCCCCACGGTCTGCGGTAAGATGCCCCTGCGGGGCCTATGGAATTCT
ATTTGAATTTGAATT
```

```
+
FFFFFFFFFFFFFFFFFFFFFFFFFFFFFFFFFFFFFFFFFFFFFFFFFFFFFFFFFFFFFFFFFFFF
FFFFFFFFFFFFFFFFFFFFFFFFFFFFFFFF,FFFFFFFFFFFFFFFFFFFFFFFF,FFFFF:FFFF
:FFFFFFFFFFFF:F
@A00721:190:HGJW7DSXY:4:2174:5267:34601 2:N:0:TCTGCAAG+GCTGCACT
GATCGTGACGCGTGGTCTAATGACAAATCGACACGTAAGCGTACGGAGGTGTCCCCGGCGCGGGCAAG
CCAGTAATATTATATTGGCTTGCCCCACGGTCTGCGGTAAGATGCCCCTGCGGGGCCTATGGAATTCT
ATTTGAATTTGAATT
+
FFFFFFF,FFFFFFFFFFFFFF:FFFFFFFFFFFFFF,FFF:FFFFFFFFFFFFFFFFFFFFFFFF,F
FFFFFFFFFFFFFFFFFFFFFFFFFFFFFFF:FFFFFFF:FFFFFFFFFFFFFFFFFF,F,FFF,FFFF
F::FFF:FFFFFFFF
@A00721:190:HGJW7DSXY:4:2267:5575:17534 2:N:0:TCTGCAAG+GCTGCACT
GATCGTGACGCGTGGTCTAATGACAAATCGACACGTAAGCGTACGGAGGTGTCCCCGGCGCGGGCAAG
CCAGTAATATTATATTGGCTTGCCCCACGGTCTGCGGTAAGATGCCCCTGCGGGGCCTATGGAATTCT
ATTTGAATTTGAATT
+
FFFFFFFFFFFFFFFFFFFFFFFFFFFFFFFFFFFFF:FFFFFFFFFFFFFFFFFFFFFFFFFFFFFF
FFFFFFFFFFFFFFFFFFFFFFFFFFFFFFFFFFFFFFFFFFFFFFFFFFFFFFFFF:FFFFF,FFFF
FFFFFFFFF,FF,:F
@A00721:190:HGJW7DSXY:4:2327:10077:6324 2:N:0:TCTGCAAG+GCTGCACT
GATCGTGACGCGTGGTCTAATGACAAATCGACACGTAAGCGTACGGAGGTGTCCCCGGCGCGGGCAAG
CCAGTAATATTATATTGGCTTGCCCCACGGTCGGCGGTAAGATGCCCCTGCGGGGCCTATGGAATTCT
ATTTGAATTTGAATT
+
FFFFFFF:FFFFFFFFFFFFFFFFFFFFFFFFFFF:FFFFFFFFFFFFFFFFFFFFFFFFFFFFFF:F
FFFF,FF:FFFF,:FFFF:FFFFFFF:FFFFF,FFFF:,FFFF:FFFF,FFFFFFFFFF:FFFFFF:F
FFFFFF,FFFFFFFF
@A00721:190:HGJW7DSXY:4:2533:6506:11600 2:N:0:TCTGCAAG+GCTGCACT
GATCGTGACGCGTGGTCTAATGACAAATCGACACGTAAGCGTACGGAGGTGTCCCCGGCGCGGGCAAG
CCAGTAATATTATATTGGCTTGCCCCACGGTCTGCGGTAAGATGCCCCTGCGGGGCCTATGGAATTCT
ATTTGAATTTGAATT
+
FFFFFFFFFFFFFFFFFFFFFFFFFFFFFFFFFFFFFFFFFFFFFFFFFFFFFFFFFFFFFFFFFF:F
FFFFFFFFFF:FFFFFFFFFF:FFFFFFFFFFFFFFFFFFF:FF:FFFFFFFFFFFF,FFFFF:FFFF
F,FFFFFFFFF::FF
@A00721:190:HGJW7DSXY:4:2670:10131:26647 2:N:0:TCTGCAAG+GCTGCACT
GATCGTGACGCGTGGTCTAATGACAAATCGACACGTAAGCGTACGGAGGTGTCCCCGGCGCGGGCAAG
CCAGTAATATTATATTGGCTTGCCCCACGGTCTGCGGTAAGATGCCCCTGCGGGGCCTATGGAATTCT
ATTTGAATTTGAATT
+
FFFFFFFFFFFFFFFFFFFFFFFFFF:FFFFFFFFFF:FFFFFFFFFFFFFFFFFFFFFFFFFFFF:FF
FFFFFF:FFFFFFF:FFFFFFFFFFFFFFFFFFFFFFFFFFFFFFFFFFFFFFFFFF,FFFFF,FFFF
F,F:FFF,FFFFFFF
@A00721:190:HGJW7DSXY:4:1611:23149:18004 2:N:0:TCTGCAAG+GCTGCACT
ATGATCGTGACGCGTGGTCTAATGACAAATCGACACGTAAGCGTACGGAGGTGTCCCCGGCGCGGGCA
AGCCAGTAATATTATATTGGCTTGCCCCACGGTCTGCGGTAAGATGCCCCTGCGGGGCCTATGGAATT
CTATTTGAATTTGAA
+
FFFFFFFFFFFFFFFFFFFFFFFFFFFF:FFFFFFFFFFFFFFFFFFFFFFFFFFFFFFFFFFFFFFF
FFFFFFFFFFFFFFFFFFFFFFFFFFFFF:FFFFF,FFFFFFFFFFFFFFFFFFFFFFFF,:FFFFFF,
,F,FFFF:FFFFFFF
@A00721:190:HGJW7DSXY:4:2216:14642:25629 2:N:0:TCTGCAAG+GCTGCACT
ATGATCGTGACGCGTGGTCTAATGACAAATCGACACGTAAGCGTACGGAGGTGTCCCCGGCGCGGGCA
```

```
AGCCAGTAATATTATATTGGCTTGCCCCACGGTCTGCGGTAAGATGCCCCTGCGGGGCCTATGGAATT
CTATTTGAATTTGAA
+
FFFFFFFFFFFFFFFFFFFFFFFFFFFFFFFFFFFFFFFFFFFFFFFFFFFFFFFFFFFFFFFFFFFF
FFFFFFFFFFFFFFFFFFFFFFFFFF:FFFFFFFFFFFFFFFFFFFFFFFFFFFFFFFFFFF:FFFFF
FFFFFFFFFF:FFFF
@A00721:190:HGJW7DSXY:4:1128:5855:25598 2:N:0:TCTGCAAG+GCTGCACT
ATTTTTTAGATGGTCATGATCGTGACGCGTGGTCTAATGACAAATCGACACGTAAGCGTACGGAGGTG
TCCCCGGCGCGGGCAAGCCAGTAATATTATATTGGCTTGCCCCACGGTCTGCGGTAAGATGCCCCTGC
GGGGCCTATGGAATT
+
FFFFFFFFFF:FFFFFFFFFFFFFFFFFFFFFFFFFFFFFFFF:FFFFFFF::FF:FFFFF:FFFFFF
FF:FFFFFFFFFFFF,:FFF:FFFFFFFFFFFFF:FFF,,FFFFFFFFFFFFFFFFFFFFFFFF::FF
FFFFFFFFFFFFFFF
@A00721:190:HGJW7DSXY:4:1128:3540:35931 2:N:0:TCTGCAAG+GCTGCACT
ATTTTTTAGATGGTCATGATCGTGACGCGTGGTCTAATGACAAATCGACACGTAAGCGTACGGAGGTG
TCCCCGGCGCGGGCAAGCCAGTAATATTATATTGGCTTGCCCCACGGTCTGCGGTAAGATGCCCCTGC
GGGGCCTATGGAATT
+
FFF:FFFF:FFFFFFFFFFFFFFFFFFFFFFFFFFFFFFFFFFFFFFFFFFFFFFFFFFFFFFFFFFF
FF,FFFFFFFFFFFFFFFFFFFF:FFFFFFFFFFFFFFFFFFFFFFFFFFFFF:FFFFFFFFFFFFFF
FFFFFFFFFFFFFFF
@A00721:190:HGJW7DSXY:4:1172:20961:16344 2:N:0:TCTGCAAG+GCTGCACT
ATTTTTTAGATGGTCATGATCGTGACGCGTGGTCTAATGACAAATCGACACGTAAGCGTACGGAGGTG
TCCCCGGCGCGGGCAAGCCAGTAATATTATATTGGCTTGCCCCACGGTCTGCGGTAAGATGCCCCTGC
GGGGCCTATGGAATT
+
FFFFFFFFFFFFFFFFFFFFFFFFFFFFFFFFFFFFFFF,FFFFFFFFFFFFFFFFFFFFFFFFFF:F
FFFFFFFFFFFFFFFFFFF,FFF:FF,FFFFFFFFFFFFFFFFFFFFFF:FFFFFFFFFFFFFFFF:F
FFFFFFFFFFFFFF:
@A00721:190:HGJW7DSXY:4:1253:25129:27007 2:N:0:TCTGCAAG+GCTGCACT
ATTTTTTAGATGGTCATGATCGTGACGCGTGGTCTAATGACAAATCGACACGTAAGCGTACGGAGGTG
TCCCCGGCGCGGGCAAGCCAGTAATATTATATTGGCTTGCCCCACGGTCTGCGGTAAGATGCCCCTGC
GGGGCCTATGGAATT
+
FFFFFFFFFFFFFFFFFFFFFFFFFFFFFFFFFFFFFFFFFFFFFFFFFFFFFFFFFFFFFFFFFFFF
FFFFFFFFFFFFFFFFFFF:FFFFFFFFFFFFFFFFFFFFFFFFFFFFFFFFFFFFFFFFFFFFFFFF
FFFFFFFFFFFF,FF
@A00721:190:HGJW7DSXY:4:1255:23628:12633 2:N:0:TCTGCAAG+GCTGCACT
ATTTTTTAGATGGTCATGATCGTGACGCGTGGTCTAATGACAAATCGACACGTAAGCGTACGGAGGTG
TCCCCGGCGCGGGCAAGCCAGTAATATTATATTGGCTTGCCCCACGGTCTGCGGTAAGATGCCCCTGC
GGGGCCTATGGAATT
+
FFFFFF:FFF:FFFFFFFFFFFFFFFFFFFFFFFFFFFFFFFFFFFFFFFFFFFFFFFFFFFFFFFFF
FFFFFFFFFFFFFFF:FFFFFFFFFFFFFFFFFFFFFFFFFFFFFFFFFFFFFFFFFFFFF:FFFFFF
FFFFFFFFFFFF:FF
@A00721:190:HGJW7DSXY:4:1263:30581:8766 2:N:0:TCTGCAAG+GCTGCACT
ATTTTTTAGATGGTCATGATCGTGACGCGTGGTCTAATGACAAATCGACACGTAAGCGTACGGAGGTG
TCCCCGGCGCGGGCAAGCCAGTAATATTATATTGGCTTGCCCCACGGTCTGCGGTAAGATGCCCCTGC
GGGGCCTATGGAATT
+
FFF:FFFFFFFFFFFFFFFFFFFFFFFFFFFFFFFFFFFFFFFFFFFFFFFFFFFF:FFFFFFFFFFF
FFFFFFFFFFFFFFFFFFFFFFFF:FFFFFFFFFFFFFF:FFFFFFFFFFFFFFFF:FFFFFFFFFFF
FFFFFFFFFFFFFFF
```

```
@A00721:190:HGJW7DSXY:4:1317:17535:17738 2:N:0:TCTGCAAG+GCTGCACT
ATTTTTTAGATGGTCATGATCGTGACGCGTGGTCTAATGACAAATCGACACGTAAGCGTACGGAGGTG
TCCCCGGCGCGGGCAAGCCAGTAATATTATATTGGCTTGCCCCACGGTCTGCGGTAAGATGCCCCTGC
GGGGCCTATGGAATT
+
FFFFF,FFFFFFFFFFFFFFFFFFFFFFFFFFFFFFFFFFFFFFFFFFFFFFFFFFFFFFFFFFFF
FFFFFFFFFFFFFFFFFFFFFFFFFFFFFFFFFFFFFFFFFF:FFFFFFFFFFFFFFFFFFFFFFFFFFF
FFFFFF:FFFFFFFF
@A00721:190:HGJW7DSXY:4:1460:12391:12680 2:N:0:TCTGCAAG+GCTGCACT
ATTTTTTAGATGGTCATGATCGTGACGCGTGGTCTAATGACAAATCGACACGTAAGCGTACGGAGGTG
TCCCCGGCGCGGGCAAGCCAGTAATATTATATTGGCTTGCCCCACGGTCTGCGGTAAGATGCCCCTGC
GGGGCCTATGGAATT
+
FFFF:F:FFFFFFFFFFFFFFFFFFFFFFFFFFFFFFFFFFFFFFFFFFFF:FFFFFFFFFFFFFFFFFF
FFFFFFFFFFFFFFFFFF:,FFFFFFFFFFFFFFFFFFFFFFFFFFFFFFFFFFFFFFFFFFFFFFFFFF
FFFFFFFFFFFFFFF
@A00721:190:HGJW7DSXY:4:2655:7229:20838 2:N:0:TCTGCAAG+GCTGCACT
ATTTTTTAGATGGTCATGATCGTGACGCGTGGTCTAATGACAAATCGACACGTAAGCGTACGGAGGTG
TCCCCGGCGCGGGCAAGCCAGTAATATTATATTGGCTTGCCCCACGGTCTGCGGTAAGATGCCCCTGC
GGGGCCTATGGAATT
+
FFFFFFFFFFFFFFFFFFFFFFFFFFF:FFFFFFFFFFFFFFFFFF:FFFFFFFFFFFFFFFFFFF,F,F
FFFFFFFFFFF:FFFFFFFFF:FFF:FFFFFFFF,FFFFFFFFFFFFF:F:F:FF:FFFFFF:FFFFFFF
FF:FFFFFFFFFFFF
@A00721:190:HGJW7DSXY:4:2673:31810:15374 2:N:0:TCTGCAAG+GCTGCACT
ATTTTTTAGATGGTCATGATCGTGACGCGTGGTCTAATGACAAATCGACACGTAAGCGTACGGAGGTG
TCCCCGGCGCGGGCAAGCCAGTAATATTATATTGGCTTGCCCCACGGTCTGCGGTAAGATGCCCCTGC
GGGGCCTATGGAATT
+
FFFFFFFFFFFFFFFFFFFFFFFFFFFFFFFFFFFFFFFFFFFFFFFFFFFFFFFFFFFFFFFFFFFFFF
FFFFFFFFFFFFFFFFFFF:FFFFF,FFFFFFFFFFFFFFFFFFFFFFFFFFFFFFFFFFFFFFFFFFFF
FFFFFFFF:FFFFFF
@A00721:190:HGJW7DSXY:4:1268:16324:5431 2:N:0:TCTGCAAG+GCTGCACT
TTCATTTTTTAGATGGTTATGATCGTGACGCGTGGTCTAATGACAAATCGACACGTAAGCGTACGGAG
GTGTCCCCGGCGCGGGCAAGCCAGTAATATTATATTGGCTTGCCCCACGGTCTGCGGTAAGATGCCCC
TGCGGGGCCTATGGA
+
FFFFFFFFFFFFF:FFFFFFFFFFFFFFFFFFF:FFFFFFFFFFFFFFFFFFFFFFFFFF:FFFFFFFFF
FFFFFFFFFFFFFFFFFFFFFFFFFF:FFFFFFFFFFFFFFFFFFFFFFFFFFFFFFFFFFFFFFFFFFF
FFFFFFFFF:FFFFF
@A00721:190:HGJW7DSXY:4:1433:1036:3380 2:N:0:TCTGCAAG+GCTGCACT
TTCATTTTTTAGATGGTTATGATCGTGACGCGTGGTCTAATGACAAATCGACACGTAAGCGTACGGAG
GTGTCCCCGGCGCGGGCAAGCCAGTAATATTATATTGGCTTGCCCCACGGTCTGCGGTAAGATGCCCC
TGCGGGGCCTATGGA
+
FFFFFFFFFFFFFFFFFFFFFFFFFFFFFFFFFFFFFFFFFFFFFFFFFFFFFFFFFFFFFFFFFFFFFF
FFFFFFFFFFFFFFFFFFFFFF:FFFFFFFFFFFFFFFFFFFFFFFFFFFFFFFFFFFFFFFF,FFFFFF
FFFFFFF::FF:FFF
@A00721:190:HGJW7DSXY:4:1457:6470:2832 2:N:0:TCTGCAAG+GCTGCACT
TTCATTTTTTAGATGGTTATGATCGTGACGCGTGGTCTAATGACAAATCGACACGTAAGCGTACGGAG
GTGTCCCCGGCGCGGGCAAGCCAGTAATATTATATTGGCTTGCCCCACGGTCTGCGGTAAGATGCCCC
TGCGGGGCCTATGGA
+
FFFFFFFFFFFFF,FFFFFFFFFFFFFFFFFFFFFFFFFFFFFFFFFFFFFFFFFFFFFFFFFFFFFFFF
```

```
FFFFFFFFFFFFFFFFFFFFFFFFFFFFFFFF:FFFFFFFFFFFFFFFFFF:FF:FFFFFFFFFFFFF
FFFFFFFFFFFFFFF
@A00721:190:HGJW7DSXY:4:1464:1280:7498 2:N:0:TCTGCAAG+GCTGCACT
TTCATTTTTTAGATGGTTATGATCGTGACGCGTGGTCTAATGACAAATCGACACGTAAGCGTACGGAG
GTGTCCCCGGCGCGGGCAAGCCAGTAATATTATATTGGCTTGCCCCACGGTCTGCGGTAAGATGCCCC
TGCGGGGCCTATGGA
+
FFFFFFFF,FFFFFFFFFFFFFFFFFFFFFFF:FFFFFFFFFFFF:FFFFFFFFFFFFFFFFFFFFFF
FFFFFFFFFFFFFFFFFFFFFFFFFFFFF:FFFFFFFFF:FFFFFFFFFFF::FFFFFFFFFFFFFFF
FFFFFFF:FFFFFFF
@A00721:190:HGJW7DSXY:4:1535:17942:12868 2:N:0:TCTGCAAG+GCTGCACT
TTCATTTTTTAGATGGTTATGATCGTGACGCGTGGTCTAATGACAAATCGACACGTAAGCGTACGGAG
GTGTCCCCGGCGCGGGCAAGCCAGTAATATTATATTGGCTTGCCCCACGGTCTGCGGTAAGATGCCCC
TGCGGGGCCTATGGA
+
FFFFFFFF:FFFFFFFFFFFFFFFFFFFFFFFF::F:FFFFFFFFF:FF:FFFFFFFFFF:FFFF:FF
FFFFFFFFFFFFFFFFFFFFFFFFFFFFFFFFFFFFFFFF:F:FF:FFFFF:FF:FFFFF:FF:FF:F
FF:FFFFFFFFFFFF
@A00721:190:HGJW7DSXY:4:2202:15863:18975 2:N:0:TCTGCAAG+GCTGCACT
TTCATTTTTTAGATGGTTATGATCGTGACGCGTGGTCTAATGACAAATCGACACGTAAGCGTACGGAG
GTGTCCCCGGCGCGGGCAAGCCAGTAATATTATATTGGCTTGCCCCACGGTCTGCGGTAAGATGCCCC
TGCGGGGCCTATGGA
+
FFFFFF:FFFFFFFFFFFFFFFFFFFFFFFFFFFFFFFFFFFFFFFFFFFFFFFFFFFFFFFFFFFFF
FFFFFFFFFFFFFFFFFF:FFF:FFF:FFFFFFF:F,:FFF,FFFFFFFFFF:F:FFF:FFFF,FFFF
,FFFFF:FF,FFFFF
@A00721:190:HGJW7DSXY:4:2230:26250:3176 2:N:0:TCTGCAAG+GCTGCACT
TTCATTTTTTAGATGGTTATGATCGTGACGCGTGGTCTAATGACAAATCGACACGTAAGCGTACGGAG
GTGTCCCCGGCGCGGGCAAGCCAGTAATATTATATTGGCTTGCCCCACGGTCTGCGGTAAGATGCCCC
TGCGGGGCCTATGGA
+
FFFFFFFFFFFFFFFFFFFFF:FFFFFFFFFFFFFFFFFFFF:FFFFFFFFFFFFF:FFFFFFFFFFF
FFFFFFFFFF:FFFFFFFFFFFFFFFFFFFFFFFFFF:FFFFFFFFFFFFFFFFFFFFFFFFFFFFFF
FFFFFFF:FFFFFFF
@A00721:190:HGJW7DSXY:4:2658:18132:9502 2:N:0:TCTGCAAG+GCTGCACT
TTCATTTTTTAGATGGTTATGATCGTGACGCGTGGTCTAATGACAAATCGACACGTAAGCGTACGGAG
GTGTCCCCGGCGCGGGCAAGCCAGTAATATTATATTGGCTTGCCCCACGGTCTGCGGTAAGATGCCCC
TGCGGGGCCTATGGA
+
FFFFFFFFFFFFFFFFFFFFFFFFFFFFFFFFFFFFFFFFFFFFFFFFFFFFFFFFFFFFFFFFFFFF
FFFFFFFFFFFFFFFFFFFFFFFFFFFFFFFFFFFFFFFFFFFFFFFFFFFFFFFFFFFFFFFFFFFF
FFFFFFFFFFFFFFF
@A00721:190:HGJW7DSXY:4:1176:29514:28432 2:N:0:TCTGCAAG+GCTGCACT
ATTCATTTTTTAGATGGTTATGATCGTGACGCGTGGTCTAATGACAAATCGACACGTAAGCGTACGGA
GGTGTCCCCGGCGCGGGCAAGCCAGTAATATTATATTGGCTTGCCCCACGGTCTGCGGTAAGATGCCC
CTGCAGGGCCAGATC
+
FFFFFFFFFFFFFFFFFFFFFFFFFFFFFFFFFFFFFFFFFFFFFFFF:FF:FFF::F:F:FFFFFFF
FFFFFFFFFFFFFFFFFFFFFFFFFFFFFF,FFFFFFFFFFF,FFF:FFFFFF,FFFFF:FFFF,FFF
FFFFFFFFFFFFF,F
@A00721:190:HGJW7DSXY:4:1509:27407:7247 2:N:0:TCTGCAAG+GCTGCACT
ATTCATTTTTTAGATGGTTATGATCGTGACGCGTGGTCTAATGACAAATCGACACGTAAGCGTACGGA
GGTGTCCCCGGCGCGGGCAAGCCAGTAATATTATATTGGCTTGCCCCACGGTCTGCGGTAAGATGCCC
CTGCAGGGCCAGATC
```

```
+
FFFFFFFFFFFFFFFFFFFFFFFFFFFFFFFFFFFFFFFFFFFFFFFFFFFFFFFFFFFFFFFFFFFFFFFF
FFFFFFFFFFFFFFFFFF::FFFFFFF:FFFFFFFFFFFFFFFFFFFFFFFFFF,FFFF:FFFFFFFFF
FFFFFFFFFFFFFFF
@A00721:190:HGJW7DSXY:4:1551:13467:17143 2:N:0:TCTGCAAG+GCTGCACT
ATTCATTTTTTAGATGGTTATGATCGTGACGCGTGGTCTAATGACAAATCGACACGTAAGCGTACGGA
GGTGTCCCCGGCGCGGGCAAGCCAGTAATATTATATTGGCTTGCCCCACGGTCTGCGGTAAGATGCCC
CTGCAGGGCCAGATC
+
FFFFFFFFFFFFFFFFFFFFFFFFFFFFFFFFFFFFFFFFFFFFFFFFFFFFFFFFFFFFFFFFFFFFFFFF
FFFFFFFFFFFFFFFFFFFFFFFFFFFFFFFFFFFFFFFFFFFFFFFFFFFFFF:FFFFFFFFFFFFFF
FFFFFFFFFFFFFFF
@A00721:190:HGJW7DSXY:4:1636:10791:3677 2:N:0:TCTGCAAG+GCTGCACT
ATTCATTTTTTAGATGGTTATGATCGTGACGCGTGGTCTAATGACAAATCGACACGTAAGCGTACGGA
GGTGTCCCCGGCGCGGGCAAGCCAGTAATATTATATTGGCTTGCCCCACGGTCTGCGGTAAGATGCCC
CTGCAGGGCCAGATC
+
FFFFFFFFFFFFFF:FFFFFFFFFFFFFFFFFFFFFFFFFFFFFFFFFFFFFFFFFFFFFFFFFFFFFF
FFFFFFFFFFFFFFFFFFFFFFFFFFFFFFFFFFFFFFFFFFFFFFFFFFFFFFFFFFFFF:FFFFFFF
F:FFFFFFFFFFFFF
@A00721:190:HGJW7DSXY:4:2123:15637:25535 2:N:0:TCTGCAAG+GCTGCACT
ATTCATTTTTTAGATGGTTATGATCGTGACGCGTGGTCTAATGACAAATCGACACGTAAGCGTACGGA
GGTGTCCCCGGCGCGGGCAAGCCAGTAATATTATATTGGCTTGCCCCACGGTCTGCGGTAAGATGCCC
CTGCAGGGCCAGATC
+
FFFFFFFFFFFFFFFFFFFFFFFFFFFFFFFFFFFFFFFFFFFFFFFFFFFFFFFFFFFFFFFFFFFFFFFF
FFFFFFFFFFFFFFFFFFFF:FFFFFFFFFFFFFFFFFFFFFFFFFFFFFFFFFFFFFFFFFFFFFFFF
FFFFFFFFFFFFFFF
@A00721:190:HGJW7DSXY:4:2123:14362:26209 2:N:0:TCTGCAAG+GCTGCACT
ATTCATTTTTTAGATGGTTATGATCGTGACGCGTGGTCTAATGACAAATCGACACGTAAGCGTACGGA
GGTGTCCCCGGCGCGGGCAAGCCAGTAATATTATATTGGCTTGCCCCACGGTCTGCGGTAAGATGCCC
CTGCAGGGCCAGATC
+
:FFFFFF:FFFFFFFFFFF:FFFFFFFFFFFFFFFFFFFFFFFFFFFFFFFFFFFFFFFF:FFFFFFFFF
FFFFFFFFFFFFFFFFFFFFFFFFFFFFFFFFFFFFFFFFFFFFFFFFFFFFFFFFFFFFFFFFFFFFFFFF
FFFF:FFFFFFFFFF
@A00721:190:HGJW7DSXY:4:2150:27154:17519 2:N:0:TCTGCAAG+GCTGCACT
ATTCATTTTTTAGATGGTTATGATCGTGACGCGTGGTCTAATGACAAATCGACACGTAAGCGTACGGA
GGTGTCCCCGGCGCGGGCAAGCCAGTAATATTATATTGGCTTGCCCCACGGTCTGCGGTAAGATGCCC
CTGCAGGGCCAGATC
+
FFFFFFFFFFFFFFFFFFFFFFFFFFFFFFFFFFFFFFFFFFFFFFFFFFFFFFFFFFFFFFFFFFFFFFFF
FFFFFFFFFFFFFFFFFFFF:FFFFFFFFFFFFF:FFFFFFFFFFFFFFFFFFFFFFFFFFFFFFF:FF
FFFFFFFFFFFFFFF
@A00721:190:HGJW7DSXY:4:2355:14805:9283 2:N:0:TCTGCAAG+GCTGCACT
ATTCATTTTTTAGATGGTTATGATCGTGACGCGTGGTCTAATGACAAATCGACACGTAAGCGTACGGA
GGTGTCCCCGGCGCGGGCAAGCCAGTAATATTATATTGGCTTGCCCCACGGTCTGCGGTAAGATGCCC
CTGCAGGGCCAGATC
+
FFFFFFFFF:FFFF:FFFFFFFFFFFFFFFFFFFFFFFFFFFFFFFFFFFFFFFFFFFFFFFFFFFFFF
FFFFFFFFFFFFFFFFFFFFFFFFFFFFFFFFFFFFFFFFFFFFFFFFFFFFFFFFFFFFFF:FFF:FFF
FFFFF:FFFFFFF:F
@A00721:190:HGJW7DSXY:4:2374:19334:20979 2:N:0:TCTGCAAG+GCTGCACT
ATTCATTTTTTAGATGGTTATGATCGTGACGCGTGGTCTAATGACAAATCGACACGTAAGCGTACGGA
```

GGTGTCCCCGGCGCGGGCAAGCCAGTAATATTATATTGGCTTGCCCCACGGTCTGCGGTAAGATGCCC
CTGCAGGGCCAGATC
+
FFFFFFFFFFFFFFFFFFFFFFFFFFFFFFFFFFFFFFFFFFFFFFFFFFFFFFFFFFFFFFFFFFFFF,
FFFFFFFFFFFFFFFFFFFFFFFF:FFFFFFFFFFF:FFFFFFFFFFFFFFFFFFFF:FFFFFFFFFFFF
FFFFFFFFFFFFFFF
@A00721:190:HGJW7DSXY:4:2673:13584:25989 2:N:0:TCTGCAAG+GCTGCACT
ATTCATTTTTTAGATGGTTATGATCGTGACGCGTGGTCTAATGACAAATCGACACGTAAGCGTACGGA
GGTGTCCCCGGCGCGGGCAAGCCAGTAATATTATATTGGCTTGCCCCACGGTCTGCGGTAAGATGCCC
CTGCAGGGCCAGATC
+
FFFFFFFFFFFFFFFFFFFFFFFFFFFFFFFFFFFFFFFFFFFFFFFFFFFFFFFFFFFFFFFFFFFFFF
:FFFFFFFFFFFFFFFFFFFFFFFFFFFFFFFFFFFFFF:FFFFFFFFFFFFFFFFFFFFFFFFFFFFFF
FFFFFFFFFFFFFFF
@A00721:190:HGJW7DSXY:4:1272:10520:25347 2:N:0:TCTGCAAG+GCTGCACT
ATAGAATTCATTTTTTAGATGGTTATGATCGTGACGCGTGGTCTAATGACAAATCGACACGTAAGCGT
ACGGAGGTGTCCCCGGCGCGGGCAAGCCAGTAATATTATATTGGCTTGCCCCACGGTCTGCGGTAAGA
TGCCCCTGCGGGGCC
+
:FFFFFFFFFFFFFFFFFFFFFFFFFFFFFFFFF:FFFFFFFFFFFFFFFFFFFF:FFFFFFFFFFFFFF
FFFFFFFFFFFFFFFFFFFFFFFFFFFFFFFFFFFFFFFFFFFFFFFFF:F:F,FF:FFFFFFFFFFFFF
FFFFFFFFFFFFFFF
@A00721:190:HGJW7DSXY:4:1343:26096:2253 2:N:0:TCTGCAAG+GCTGCACT
ATAGAATTCATTTTTGAGATGGTTATGATCGTGACGCGTGGTCTAATGACAAATCGACACGTAAGCGT
ACGGAGGTGTCCCCGGCGCGGGCAAGCCAGTAATATTATATTGGCTTGCCCCACGGTCTGCGGTAAGA
TGCCCCTGCGGGGCC
+
FFFFFF::FFFFF:F,FFFFF:FFF:F,,FFF,:F:FFFFFFFFF:FFFFFFFFFF:,F:FF,FFFF,FF
:,,F:F,F,FF:FFFFF,FFFFF:FF:F:FFFFFF::FFFFFF,FFFFFFFFF:,FFFF:::,F:FFF:F
FF:FFF,F:,:F:::
@A00721:190:HGJW7DSXY:4:1406:24831:26209 2:N:0:TCTGCAAG+GCTGCACT
ATAGAATTCATTTTTTAGATGGTTATGATCGTGACGCGTGGTCTAATGACAAATCGACACGTAAGCGT
ACGGAGGTGTCCCCGGCGCGGGCAAGCCAGTAATATTATATTGGCTTGCCCCACGGTCTGCGGTAAGA
TGCCCCTGCGGGGCC
+
FFFF:FFFFFFFFFFFFFFFFFFFFFFFFFFFFFFFFFFFFFFFFFFFFFFFFFFFFFFFFFFFFFF:F
FF:FFFFFFFFF,FFFFFFF:FFFF:F,FFF:FF:FFFFF,F:F,:FFF:FFFF:FFFF:,FFFF,FFFF
F:FFFFFFFFFF,F:
@A00721:190:HGJW7DSXY:4:1460:9109:2300 2:N:0:TCTGCAAG+GCTGCACT
ATAGAATTCATTTTTTAGATGGTCATGATCGTGACGCGTGGTCTAATGACAAATCGACACGTAAGCGT
ACGGAGGTGTCCCCGGCGCGGGCAAGCCAGTAATATTATATTGGCTTGCCCCACGGTCTGCGGTAAGA
TGCCCCTGCGGGGCC
+
FFFFF:FFFFFFFFFFF:FFFFFFFFFFFFFFFFFFFFFFFFFFFFFFFFFFFFFFFFFFFFFFFFFFFF
FFFFFFFFFFFFFFFFFFFFFFFFFFFFFFFFFFFFFFFFFFFFFFFFFFFFFFFFFFFFFFFFF:FFFF
FFFFFFFFFFFFFFF
@A00721:190:HGJW7DSXY:4:2158:27769:23124 2:N:0:TCTGCAAG+GCTGCACT
ATAGAATTCATTTTTTAGATGGTCATGATCGTGACGCGTGGTCTAATGACAAATCGACACGTAAGCGT
ACGGAGGTGTCCCCGGCGCGGGCAAGCCAGTAATATTATATTGGCTTGCCCCACGGTCTGCGGTAAGA
TGCCCCTGCGGGGCC
+
FFFFFFFFFFFFFFFFFFFFFFFFFFFFFFFFFFFFFFFFFFFFFFFFFFFFFFFFFFFFFFF:FFFFFF
FFFFFF:FFFFFFFFFFFFFFFFFFFFFFFFFFFFFFFFFFFFFFFFFFFFFFFFFFFFFFFFFFFFFFF
FFFFFFFFFFFFFFF

```
@A00721:190:HGJW7DSXY:4:2326:7093:34976 2:N:0:TCTGCAAG+GCTGCACT
ATAGAATTCATTTTTTAGATGGTCATGATCGTGACGCGTGGTCTAATGACAAATCGACACGTAAGCGT
ACGGAGGTGTCCCCGGCGCGGGCAAGCCAGTAATATTATATTGGCTTGCCCCACGGTCTGCGGTAAGA
TGCCCCTGCGGGGCC
+
FF,FFFFFFFFFFFFFFFFF:FFFFFFFFFFFFFFFFFFFFFFFFFFFFFFFFFFFFFFFFFFFFFFF
FFFFFFFFFFFFFFFFFFFFFFFFFFFFFF:F:FFFFF,FFFFFFFF:FFFFFFFFFFFFFFFF,FFF
FFFFFFFFFFFFFFF
@A00721:190:HGJW7DSXY:4:2349:24713:12446 2:N:0:TCTGCAAG+GCTGCACT
ATAGAATTCATTTTTTAGATGGTTATGATCGTGACGCGTGGTCTAATGACAAATCGACACGTAAGCGT
ACGGAGGTGTCCCCGGCGCGGGCAAGCCAGTAATATTATATTGGCTTGCCCCACGGTCTGCGGTAAGA
TGCCCCTGCGGGGCC
+
F:FFFFFFFFFFF:FFFF:FFFFFFFFFFFFFFFFFFFFFFFF:FFFFFFF:FFFFFF:FFFFFFFFF
FFFFFFF:FFFFFFFFFFFFFFFF:FFFFFFFFFFFFFFFF:FFFFF,FFFFFFFFFFFFFFFFFFFF
F:FFFFF:FFFFFFF
@A00721:190:HGJW7DSXY:4:2358:25238:16141 2:N:0:TCTGCAAG+GCTGCACT
ATAGAATTCATTTTTTAGATGGTCATGATCGTGACGCGTGGTCTAATGACAAATCGACACGTAAGCGT
ACGGAGGTGTCCCCGGCGCGGGCAAGCCAGTAATATTATATTGGCTTGCCCCACGGTCTGCGGTAAGA
TGCCCCTGCGGGGCC
+
FFFFFFFFFFFFFFFFF,FFFFFFFFFFFFFFFF:FFFF:FFFFFFF:FFFFFF:FFFFFFFFF::FF
::FFFFFFFFFFFFFFFFFFFFFFFFFFFFF:FFFFFF:FFFFFFFFFFFFFFFFFFFFFFFF,FFFF
FFF,FFFFFFFFFFF
@A00721:190:HGJW7DSXY:4:2555:13810:25285 2:N:0:TCTGCAAG+GCTGCACT
ATAGAATTCATTTTTTAGATGGTCATGATCGTGACGCGTGGTCTAATGACAAATCGACACGTAAGCGT
ACGGAGGTGTCCCCGGCGCGGGCAAGCCAGTAATATTATATTGGCTTGCCCCACGGTCTGCGGTAAGA
TGCCCCTGCGGGGCC
+
FFFFFFFFFFFFFFFFFF:FFFFFFFFFFFFFFFFFFFFFFFFFFFFFFFFF:FFFFFFFFFF:FFFF
FFFFFFFFFFFFFFFFFFFFFFFF::FF:FFFFFFFFFFF,FFFFFFF:FFFFFFFFFFFFFFFFFFF
FFFFFFFFFFFFFFF
@A00721:190:HGJW7DSXY:4:2636:18765:28072 2:N:0:TCTGCAAG+GCTGCACT
ATAGAATTCATTTTTTAGATGGTTATGATCGTGACGCGTGGTCTAATGACAAATCGACACGTAAGCGT
ACGGAGGTGTCACCGGCGCGGGCAAGCCAGTAATATTATATTGGCTTGCCCCACGGTCTGCGGTAAGA
TGCCCCTGCGGGGCC
+
FFFFFFFFFFFF:FFFFFFFF:FF:FFFFF,FFFF,FF:F,F:FFF,FFFFFFFF:F:F::FFFFF::
FF,FFFFF,FF,:FF,F,:FFFFFFF,:F,,:FFFF,FFF:,,FF,FFFF,FF:,FFFF:F,FFF::F
F:FF:FFF,FF,FFF
@A00721:190:HGJW7DSXY:4:2651:2257:27508 2:N:0:TCTGCAAG+GCTGCACT
ATAGAATTCATTTTTTAGATGGTCATGATCGTGACGCGTGGTCTAATGACAAATCGACACGTAAGCGT
ACGGAGGTGTCCCCGGCGCGGGCAAGCCAGTAATATTATATTGGCTTGCCCCACGGTCTGCGGTAAGA
TGCCCCTGCGGGGCC
+
F:FFFFFFFFFFFFFFFFFFFFFFFFFFFFFFFFFFFFFFFFFFFFFFFFFFFFFFFFFFFFF:FFFF
FFFFFFFFFFFFFFFF:FFFF:FFFFFFFFFFFFFFFFFFFFFFFFFF,FF:FFFFFFFFFFFFFFFF
FF:FFFFFFFFFFFF
@A00721:190:HGJW7DSXY:4:1343:26675:1971 2:N:0:TCTGCAAG+GCTGCACT
ATAGAATTCATTTTTTAGATGGTTATGATCGTGACGCGTGGTCTAATGACAAATCGACACGTAAGCGT
ACGGAGGTGTCCCCGGCGCGGGCAAGCCAGTAATATTATATTGGCTTGCCCCACGGTCTGCGGTAAGA
TGCCCCTGCGGGGC
+
FFFFFFFFFFFFFFFFFFFFFFFFFFFFFFFFFFFFFFFFFFFFFFFFFFFFFFFFFFFFFFFFFFFF
```

FFFFFFFFFFFFFFFFFFFFFFFFFFFFFFFFFFFFFFFFFFFFFFFFFFFFFFFFF:FF:FFFFFFF
FFFFFFFFFFFFFF
@A00721:190:HGJW7DSXY:4:2461:3812:17362 2:N:0:TCTGCAAG+GCTGCACT
ATAGAATTCATTTTTTAGATGGTCATGATCGTGACGCGTGGTCTAATGACAAATCGACACGTAAGCGT
ACGGAGGTGTCCCCGGCGCGGGCAAGCCAGTAATATTATATTGGCTTGCCCCACGGTCTGCGGTAAGA
TGACCCTGCGGGGC
+
FFFFFFFFFFFFFFFFFFFFF,FFFFFFFFFFFFFFFFFFFFF:FF,FFFFFFF::FFFF:,FFFF::
F:FFFF:FFFFF,FF:,::FFFFF::F,FFFFFFFFFF:FF,,F:FFF,FFFFFF,FF,,F,:FFFFF
FF,F,FFFFFFFFF
@A00721:190:HGJW7DSXY:4:1418:16685:14168 2:N:0:TCTGCAAG+GCTGCACT
CTATAGAATTCATTTTTTAGATGGTCATGATCGTGACGCGTGGTCTAATGACAAATCGACACGTAAGC
GTACGGAGGTGTCCCCGGCGCGGGCAAGCCAGTAATATTATATTGGCTTGCCCCACGGTCTGCGGTAA
GATGCCCCTGC
+
FFFFFFFFFFFFFFFFFFFFFFFFFFFFFFFFFFFFFFFFFFFFFFFFFFFFFFFFFFFFFFFFFFFF
FFFFFFFFFFFFFFFFFFFFFFFFFFFFFFFFFFFF,:FFFFFFFFFFFFFFFFFFFFFF:FFFFFFF
FFFFFFFFFFF
@A00721:190:HGJW7DSXY:4:1613:24795:2879 2:N:0:TCTGCAAG+GCTGCACT
CTATAGAATTCATTTTTTAGATGGTCATGATCGTGACGCGTGGTCTAATGACAAATCGACACGTAAGC
GTACGGAGGTGTCCCCGGCGCGGGCAAGCCAGAAATATTATATTGGCATGCCCCACGGTCTGCGGTAA
GATGCCCCTGC
+
FFFF,FFF:FFFFF,FFFFFFFFFFFFFFFFFFF:FFFF:FFF::F:FFFFFFFFF:FFFFFFFF,FF
FFFFFFFFFFFFFFFF:FF:FF:F::F:FF,F,FF:FF::FF,FFFF,:F:FFFFF,:FF,FF:FFFF
:FF,FFFFF::
@A00721:190:HGJW7DSXY:4:2240:17481:27759 2:N:0:TCTGCAAG+GCTGCACT
CTATAGAATTCATTTTTTAGATGGTCATGATCGTGACGCGTGGTCTAATGACAAATCGACACGTAAGC
GTACGGAGGTGTCCCCGGCGCGGGCAAGCCAGTAATATTATATTGGCTTGCCCCACGGTCTGCGGTAA
GATGCCCCTGC
+
FFFFFFFFFFFFFFFFFFFFFFFFFFFFFFFFFFFFFFFFFFFFFFFFFFFFFFFFFFFFFFFFFFFF
FFFFFFFFFFFFFFFFFFFFFFFFFFFFFFFFFFFFFFFFFFFFFFFFFFFFFFFFFFFFFFFFFFFF
FFFFFFFFFFF
@A00721:190:HGJW7DSXY:4:2240:14769:29074 2:N:0:TCTGCAAG+GCTGCACT
CTATAGAATTCATTTTTTAGATGGTCATGATCGTGACGCGTGGTCTAATGACAAATCGACACGTAAGC
GTACGGAGGTGTCCCCGGCGCGGGCAAGCCAGTAATATTATATTGGCTTGCCCCACGGTCTGCGGTAA
GATGCCCCTGC
+
FFFFFFFFFFFFFFFFF:FFFFFFFFFFFFFFFFFFFFFFFFFFFFFFFFFFFFFFFFFFFFFFFFFF
FFFFFFFFFFFFFFFFFFFFFFFFFFFFFFFFFFFFFFFFFFFFFFFFFFFFFFFFFFFFFFFFFFFF
FFFFFFFFFFF
@A00721:190:HGJW7DSXY:4:2307:20925:27492 2:N:0:TCTGCAAG+GCTGCACT
CTATAGAATTCATTTTTTAGATGGTCATGATCGTGACGCGTGGTCTAATGACAAATCGACACGTAAGC
GTACGGAGGTGTCCCCGGCGCGGGCAAGCCAGTAATATTATATTGGCTTGCCCCACGGTCTGCGGTAA
GATGCCCCTGC
+
FFFFFFFFFFFFFFFFFFFFFFFFFFFFFFFFFFF:FFFFFFFFFFFFFFFFFFFFFFFFFFFFFFFF
FFFFFFFFFFFFFFFFFFFFFFFFFFFFFFFFFFFFFFFFFFFFFFFFFFFFFFFFFF:FFF:FFFFF
FFF:FFFF:FF
@A00721:190:HGJW7DSXY:4:2446:10321:9220 2:N:0:TCTGCAAG+GCTGCACT
CTATAGAATTCATTTTTTAGATGGTCATGATCGTGACGCGTGGTCTAATGACAAATCGACACGTAAGC
GTACGGAGGTGTCCCCGGCGCGGGCAAGCCAGTAATAGTATATTGGCTTGCCCCACGGTCTGCGGTAA
GATGCCCCTGC

```
+
FFFFFFFFF:FFFFFFFFFFFFFFFFFFFFFFFFFFFFFFFFFFFFFFFFFFFFFFFFFFFFFFFFFFFF
FFFFFFFFFFFFFFFFFFFFFFFFFFF,FFFFFFFFF:,FFFFF:FFFFFFFFFFFFFFFFFFFFFF,F:
FFFFFFFFFFF
@A00721:190:HGJW7DSXY:4:2519:28230:22044 2:N:0:TCTGCAAG+GCTGCACT
CTATAGAATTCATTTTTTAGATGGTCATGATCGTGACGCGTGGTCTAATGACAAATCGACACGTAAGC
GTACGGAGGTGTCCCCGGCGCGGGCAAGCCAGTAATATTATATTGGCTTGCCCCACGGTCTGCGGTAA
GATGCCCCTGC
+
FFFF:F:FFFFFFFFFFFFFFFFFFFFFFFFFFFFFFFFFFFFFFFFFFFFFFFFFFFFFFFFFFFFFFF
FFFFFFFFFFFFFFFFFFFFFFFFFFFFFFFFFFFFFFFFFFFFFFFFFFFFFFFFFFFFFFFFFFFF,F
FFFFF,FFFFF
@A00721:190:HGJW7DSXY:4:2653:17544:34194 2:N:0:TCTGCAAG+GCTGCACT
CTATAGAATTCATTTTTTAGATGGTCATGATCGTGACGCGTGGTCTAATGACAAATCGACACGTAAGC
GTACGGAGGTGTCCCCGGCGCGGGCAAGCCAGTAATATTATATTGGCTTGCCCCACGGTCTGCGGTAA
GATGCCCCTGC
+
FFFFFFF,FFFFFFFFFFFFFFFFFFFFFFFFFFFFFFFFFFFFFFFFFFFFFFFFFFFFFFFFFFF:FF
FFFFFFFFFFFFFFFFFFFFFFFFFFFFFFFFFF,F:FFFFFFFFFFFFFFFFFFFFFFFFFFFFFF:FF
FFFFFFFFF:F
@A00721:190:HGJW7DSXY:4:2653:17852:34225 2:N:0:TCTGCAAG+GCTGCACT
CTATAGAATTCATTTTTTAGATGGTCATGATCGTGACGCGTGGTCTAATGACAAATCGACACGTAAGC
GTACGGAGGTGTCCCCGGCGCGGGCAAGCCAGTAATATTATATTGGCTTGCCCCACGGTCTGCGGTAA
GATGCCCCTGC
+
FFFFFFFFFFFFFFFFFFFFFFFFFFFFFFFFFFFFFFFFFFFFFFFFFFFFFFFFFFFFFFFFFFFFFF
FFFFFFFFFFFFFFFFFFFFFFFFFFFFFFFFFFFFFFFFFFFFF:FFFFFFFFFFFFFFFFFFFFFFFF
FFFFFFFFFFF
@A00721:190:HGJW7DSXY:4:1119:8558:22200 2:N:0:TCTGCAAG+GCTGCACT
TGCGTCACTATAGAATTCATTTTTTAGATGGTTATGATCGTGACGCGTGGTCTAATGACAAATCGACA
CGTAAGCGTACGGAGGTGTCCCCGGCGCGGGCAAGCCAGTAATATTATATTGGCTTGCCCCACGGTCT
GCGGTAAGATGCCCC
+
FFFFFFFFFFFFFFFFFFFFFFFFF,FFF:FFFFFFFFFFFFFFFFFFFFFFFFFFFFFFFFFF:FFFFFF
FFFFFFFFFFFFFFFFFFFFFFFFFFFFFFFFFFFFFFFF,:FFFFFFF::FFFFFFFFFFFFFFFFFFFF
F:FFFFFFFFFFFFF
@A00721:190:HGJW7DSXY:4:1647:11939:23703 2:N:0:TCTGCAAG+GCTGCACT
TGCGTCACTATAGAATTCATTTTTTAGATGGTTATGATCGTGACGCGTGGTCTAATGACAAATCGACA
CGTAAGCGTACGGAGGTGTCCCCGGCGCGGGCAAGCCAGTAATATTATATTGTCTTGCCCCACGGTCT
GCGGTAAGATGCCCC
+
FFFF,FFFF,FFFFFFFFFFF:::FFFF,FF:FFFFF:FFF:FFFFF:F:FFF,FFFFFFFFFFFFFF
FFFFFFFFFF:F,FF:,FFFFFFFFFFFFF:FF:,FFFF,FFFFFFFFFFF:,FFF,F:FFFF::FFF
FFF:,F::FF:FFF:
@A00721:190:HGJW7DSXY:4:2664:18177:3317 2:N:0:TCTGCAAG+GCTGCACT
TGCGTCACTATAGAATTCATTTTTTAGATGGTTATGATCGTGACGCGTGGTCTAATGACAAATCGACA
CGTAAGCGTACGGAGGTGTCCCCGGCGCGGGCAAGCCAGTAATATTATATTGGCTTGCCCCACGGTCT
GCGGTAAGATGCCCC
+
FFFFFFFFFFFFFFFFFFFFFFFF,FFFFFFFFFFFFFFFFFFFFFFFF,FFFFFFFFFFFFFF:FFFFF
FFFFFFFFFFFFFFFFFFFFFFFFFFFFFFFFFFFFFFFFFFF:FFFFFFFFFFFFF:FFFFFFFFFF
FFFFFFFFFFFFFFF
@A00721:190:HGJW7DSXY:4:1214:3179:35180 2:N:0:TCTGCAAG+GCTGCACT
ATTCTACTGCGTCACTATAGAATTCATTTTTTAGATGGTTATGATCGTGACGCGTGGTCTAATGACAA
```

```
ATCGACACGTAAGCGTACGGAGGTGTCCCCGGCGCGGGCAAGCCAGTAATATTATATTGGCTTGCCCC
ACGGTCTGCGGTAAG
+
F:FFFFFFFFFFFFFFFFFFFFFFFFFFF:FF:FF,FFFFFFFFFFFFFFFFFFFFFFFFFFFFFFFF
FFFFFFFFFFFFFFFFFFFFFFFFFFF:FFFFFFFFFFF:FFFFFFFFFFFFF,FFFFFFFFFFFFFF
FFFFFF:FFFFFFFF
@A00721:190:HGJW7DSXY:4:1417:10936:12884 2:N:0:TCTGCAAG+GCTGCACT
ATTCTACTGCGTCACTATAGAATTCATTTTTTAGATGGTTATGATCGTGACGCGTGGTCTAATGACAA
ATCGACACGTAAGCGTACGGAGGTGTCCCCGGCGCGGGCAAGCCAGTAATATTATATTGGCTTGCCCC
ACGGTCTGCGGTAAG
+
FFFFFFFFFFFFFFFFFFFFFFFFFFFFFF:FFF::FFFFFFFFFFFFFFFFFFFFFFFFFFFFFFF:
FFFFFFFFFFF:FFFFFFFFFFFFFFFFFFFFFFFFFFFFFFFFFFFFF,FFFFFFFFFFFFFFFFFF
FFFFFF,FFFF:F:F
@A00721:190:HGJW7DSXY:4:1621:25852:28729 2:N:0:TCTGCAAG+GCTGCACT
ATTCTACTGCGTCACTATAGAATTCATTTTTTAGATGGTTATGATCGTGACGCGTGGTCTAATGACAA
ATCGACACGTAAGCGTACGGAGGTGTCCCCGGCGCGGGCAAGCCAGTAATATTATATTGGCTTGCCCC
ACGGTCTGCGGTAAG
+
FFFFFFFFFFFFFFFFFFFFFFFFFFFFFFFFFFFFFFFFFFFFFFFFFFFFFFFFFFFFFFFFFFFF
FFFFFFFFFFFFFFFFFFFFFFFFFFFFFFFFFFFFFFFFFFFFFFFFFFFFFFFFFFFFFFFFFFFF
FFFFFFFFFFFFFF:
@A00721:190:HGJW7DSXY:4:1656:13892:10770 2:N:0:TCTGCAAG+GCTGCACT
ATTCTACTGCGTCACTATAGAATTCATTTTTTAGATGGTTATGATCGTGACGCGTGGTCTAATGACAA
ATCGACACGTAAGCGTACGGAGGTGTCCCCGGCGCGGGCAAGCCAGTAATATTATATTGGCTTGCCCC
ACGGTCTGCGGTAAG
+
FFFFFFFFFFFFFFFFFFFFFFFFFFFFFFFFFFFFFFFFFFFFFFFFFFFFFFFFFFFFFFFFFFF:
FFFFFFFFFFFFFFFFF:FFFFFFFFFFFFFFFFFFFFFFFFFFFFFFFFFFFFFFFFFFFFFFFFFF
FFFFFFFFFFFFFFF
@A00721:190:HGJW7DSXY:4:2253:9100:6104 2:N:0:TCTGCAAG+GCTGCACT
ATTCTACTGCGTCACTATAGAATTCATTTTTTAGATGGTTATGATCGTGACGCGTGGTCTAATGACAA
ATCGACACGTAAGCGTACGGAGGTGTCCCCGGCGCGGGCAAGCCAGTAATATTATATTGGCTTGCCCC
ACGGTCTGCGGTAAG
+
FFFFFFFFFFFFFFFFFFFF:FFFFFFFFFFFFFFFF,FFFFFFF:FFFFFFF:FF:FF:FFFF:FFF
FFF,FFFFFFFF::FFFFFFFF:FFFFFFFFFFFFFFFFF,FF,FF,:FFFFFFFF,FFFFFFF,FFF
:F:F,FFFFFFFFFF
@A00721:190:HGJW7DSXY:4:1128:23402:8923 2:N:0:TCTGCAAG+GCTGCACT
CGATTTTACTGCGTCACTATAGAATTCATTTTTTAGATGGTCATGATCGTGACGCGTGGTCTAATGAC
AAATCGACACGTAAGCGTACGGAGGTGTCCCCGGCGCGGGCAAGCCAGTAATATTATATTGGCTTGCC
CCACGGTCTGCGGTA
+
FFFF,FFFFF:FFFFFFFFFFFFFFFFFFFFF:FFFFFF,FFF:FFFF:FFFFFFF:FFFFFFF,FFF
FFFFFFF,FFFF:F,FFFFF:FFF:,FFFFFFF::F:::FFFF:FFFFFFF:FFFFFFFF,FFFFFFF
F:::,F,FFFFF::F
@A00721:190:HGJW7DSXY:4:1231:6415:31391 2:N:0:TCTGCAAG+GCTGCACT
CGATTTTACTGCGTCACTATAGAATTCATTTTTTAGATGGTCATGATCGTGACGCGTGGTCTAATGAC
AAATCGACACGTAAGCGTACGGAGGTGTCCCCGGCGCGGGCAAGCCAGTAATATTATATTGGCTTGCC
CCACGGTCTGCGGTA
+
FFFFFFFFFFFFFFFFFFF,FF,FFFFFFFFFFFFFFFFFFFFFFFFFFFFFFFFFFFFFFFFFFFFF
FFFFFFFFFFFFFFFF:FFFFFFFFFFFFFFFFFFFFFFFF:FFFF,FFFFFFFFFFFFFFFFFFFFF
FF:FFFFFFFFFFFF
```

```
@A00721:190:HGJW7DSXY:4:1644:11451:13088 2:N:0:TCTGCAAG+GCTGCACT
CGATTTTACTGCGTCACTATAGAATTCATTTTTTAGATGGTCATGATCGTGACGCGTGGTCTAATGAC
AAATCGACACGTAAGCGTACGGAGGTGTCCCCGGCGCGGGCAAGCCAGTAATATTATATTGGCTTGCC
CCACGGTCTGCGGTA
+
FFF,:FFFFFFFFFFFFFFF,FF,FFFFFFFFFFFFFFF:FFFFFFFFFFFFFFFFFFFFFFFFFFFFF
F,FFFFFFFFFFFFFFFFFFFFFFFFFFF:FFFFFFFFFFFFFFFFFFFFFFFFFFFFFFFFFFFFFFFF
FF:FFFFFFFFFFFF
@A00721:190:HGJW7DSXY:4:1338:16685:19899 2:N:0:TCTGCAAG+GCTGCACT
ATTTTACTGCGTCACTATAGAATTCATTTTTTAGATGGTCATGATCGTGACGCGTGGTCTAATGACAA
ATCGACACGTAAGCGTACGGAGGTGTCCCCGGCGCGGGCAAGCCAGTAATATTATATTGGCTTGCCCC
ACGGTCTGCGGAGAT
+
FFFFFFFFFFFF:FFFFF:FFFFFFFFFF:FFFFFFFFFFFFFFFFFFFFFFFFFFFFFFFFFFFFFFF
FFFFFFFFFFFFFFFFFFFFFF:FFFFFFFFFFFFFFFFFFFFF:FFFFFFFFFFFFFFFFF:FFFFF
FFFFFF::FFFFFFF
@A00721:190:HGJW7DSXY:4:2424:28022:34679 2:N:0:TCTGCAAG+GCTGCACT
ATTTTACTGCGTCACTATAGAATTCATTTTTTAGATGGTCATGATCGTGACGCGTGGTCTAATGACAA
ATCGACACGTAAGCGTACGGAGGTGTCCCCGGCGCGGGCAAGCCAGTAATATTATATTGGCTTGCCCC
ACGGTCTGCGGAGAT
+
F:FFFFFFFFFFFFFFFFFFF:FFFFFFFFFFFFFFFFFFFFFFFFFFFFFFFFFFFFFFFFFFFFFF
FFFFFFFFFFFFFFFFFFFFFFFFFFFFFFFFFFFFFFFFFFFFF:F,FF:FFFFFFFFFFFFFFF
FFFFFFFFFFFFFFF
@A00721:190:HGJW7DSXY:4:2504:9290:10128 2:N:0:TCTGCAAG+GCTGCACT
ATTTTACTGCGTCACTATAGAATTCATTTTTTAGATGGTCATGATCGTGACGCGTGGTCTAATGACAA
ATCGACACGTAAGCGTACGGAGGTGTCCCCGGCGCGGGCAAGCCAGTAATATTATATTGGCTTGCCCC
ACGGTCTGCGGAGAT
+
FFFFFFFFFFF:F:FF,FFFFFFFFFFFFFFFFFFFFFFFF:FFFFFFFFFFFFFF,FFFFFFFFF:F
FFFFFFFFFFFFFFFFFFFFFFFFFFFFFFFFFFFFFFFFFF:F:FFFF,F,F,FFFF,FFFFFFFFF
:FFFFF:FFFFFFFF
@A00721:190:HGJW7DSXY:4:2522:31864:13088 2:N:0:TCTGCAAG+GCTGCACT
ATTTTACTGCGTCACTATAGAATTCATTTTTTAGATGGTCATGATCGTGACGCGTGGTCTAATGACAA
ATCGACACGTAAGCGTACGGAGGTGTCCCCGGCGCGGGCAAGCCAGTAATATTATATTGGCTTGCCCC
ACGGTCTGCGGAGAT
+
FFFFFF:FFFFFFFFFFF,FFFFFFFFFFFFFFFFFFFFFFFFFFFFFFFF:FFFFFFFFFFFFF:FFF
FFFF:FFFFFFFFFFFFFFFFFFFFFFFFFFFFFFFFFFFF:FFFFF,FFFFFFFFFFF,FFFFFFFFF
FFFFFFFFFFFF:FF
@A00721:190:HGJW7DSXY:4:1110:23321:23406 2:N:0:TCTGCAAG+GCTGCACT
GTCGATTCTACTGCGTCACTATAGAATTCATTTTTTAGATGGTTATGATCGTGACGCGTGGTCTAATG
ACAAATCGACACGTAAGCGTACGGAGGTGTCCCCGGCGCGGGCAAGCCAGTAATATTATATTGGCTTG
CCCCACGGTCTGCGG
+
FFFFFFFFFFFFFFFFF,FFFFFFF:FFFFFFFFFFFFF,FFFFFFFFFFFFFFFFFFFFFFFFFFFFF
FFFFFFFFFFFFFFFFFFFFFFFFFFFFFFFF:FFFFFFFFFFF:,FFFFFF:FF:FFFFFFF:FF:FF
FFFF,FFFFFFFFFF
@A00721:190:HGJW7DSXY:4:2320:20745:14027 2:N:0:TCTGCAAG+GCTGCACT
GTCGATTCTACTGCGTCACTATAGAATTCATTTTTTAGATGGTTATGATCGTGACGCGTGGTCTAATG
ACAAATCGACACGTAAGCGTACGGAGGTGTCCCCGGCGCGGGCAAGCCAGTAATATTATATTGGCTTG
CCCCACGGTCTGCGG
+
FFFFFFFFFFFFFFFFFFFF,F,FFFFFFFFFFF:FFFFFFFFFFFFFFFFFFFFFFFF:FFFFFFFFF
```

```
FFFFFFF:FFFFFFFFFFFFFFFFFFFFFFF:FFFFFFFFFFFFFFFFFFFFFFFFFFFFFFFFFFFF
FFFFFFFFFFFFFFF
@A00721:190:HGJW7DSXY:4:2331:11668:20447 2:N:0:TCTGCAAG+GCTGCACT
GTCGATTCTACTGCGTCACTATAGAATTCATTTTTTAGATGGTTATGATCGTGACGCGTGGTCTAATG
ACAAATCGACACGTAAGCGTACGGAGGTGTCCCCGGCGCGGGCAAGCCAGTAATATTATATTGGCTTG
CCCCACGGTCTGCGG
+
FFFFFFFFFFFFFFFFFFFF:,FFFFFFFFFFFFFFFFFFFFFFFFFFFFFFFFFFFF:FFFFF:FFF
FFFFFFFFFFFFFFFF:FFFFFFF:FFFFFFFFFFFFFFFFFFF:FFFFFFFFFFFFFFFFFF,FFFF
:FFFFFFFFFFFFFF
@A00721:190:HGJW7DSXY:4:2472:18331:9377 2:N:0:TCTGCAAG+GCTGCACT
GTCGATTCTACTGCGTCACTATAGAATTCATTTTTTAGATGGTTATGATCGTGACGCGTGGTCTAATG
ACAAATCGACACGTAAGCGTACGGAGGTGTCCCCGGCGCGGGCAAGCCAGTAATATTATATTGGCTTG
CCCCACGGTCTGCGG
+
FF:FFFFFFFFFFFFFFFFFFFFFFF,:FFFFFFFFFFFFFFFFFF::FFFFFF:FFFFFFFFFFFFF
FFFFFFFFFFFFFFFFFF:FF:F:FFFFFFFFF:FFFFFFFFFFF,:FFFFFFFFFFFFFF:FFFF:F
FFFFFFFFFFFFFFF
@A00721:190:HGJW7DSXY:4:2472:18285:9424 2:N:0:TCTGCAAG+GCTGCACT
GTCGATTCTACTGCGTCACTATAGAATTCATTTTTTAGATGGTTATGATCGTGACGCGTGGTCTAATG
ACAAATCGACACGTAAGCGTACGGAGGTGTCCCCGGCGCGGGCAAGCCAGTAATATTATATTGGCTTG
CCCCACGGTCTGCGG
+
FFFFFFFFFFFFFFFFFFFFFFFFFFFFFFFFFFFFFFFFFFFFFFFFFFFFFFFFFFFFFFFFFFFF
FFFFFFFFFFFFFFFFFFFFFFFFFFFFFFFFFFFFFFFFFFFF:FFFFF:FFFF:FFFFFFFFFFFF
FFFFFFFFFFFFFFF
@A00721:190:HGJW7DSXY:4:1528:12816:22467 2:N:0:TCTGCAAG+GCTGCACT
CGTCGATTCTACTGCGTCACTATAGAATTCATTTTTTAGATGGTTATGATCGTGACGCGTGGTCTAAT
GACAAATCGACACGTAAGCGTACGGAGGTGTCCCCGGCGCGGGCAAGCCAGTAATATTATATTGGCTT
GCCCCACGGTCTGCG
+
FFFFFFFFFFFFFFFFFFFF:,FFFFFFFFFFFFFFFFFFFFFFFFFFFFFFFFFFFFFFFF:FFFFF
FFFFFFFFFFFFFFFFFFFFFFFFFFFFFFFFFFFFFFFFFFFFFFFFFFFFFFFFFFFFFFFFFFFF
:FFFFFFFFFFFFFF
@A00721:190:HGJW7DSXY:4:2123:22923:15327 2:N:0:TCTGCAAG+GCTGCACT
CGTCGATTCTACTGCGTCACTATAGAATTCATTTTTTAGATGGTTATGATCGTGACGCGTGGTCTAAT
GACAAATCGACACGTAAGCGTACGGAGGTGTCCCCGGCGCGGGCAAGCCAGTAATATTATATTGGCTT
GCCCCACGGTCTGCG
+
FF:FFFFFFFFFFFFF:FFFF:FFFF,FFFFFFFFFFFFF:FFFFFFFFFFFFFFFFFFFFFFFFFFF
FFFFFFFFFFFFFFFFFFFFFFFFFFFFFFFFFFFFFFFFFFFFF:FFFFFFFFFFFFFFFFFF:FFF
FFF:FFFFFFFFFFF
@A00721:190:HGJW7DSXY:4:2167:3106:31579 2:N:0:TCTGCAAG+GCTGCACT
CGTCGATTCTACTGCGTCACTATAGAATTCATTTTTTAGATGGTTATGATCGTGACGCGTGGTCTAAT
GACAAATCGACACGTAAGCGTACGGAGGTGTCCCCGGCGCGGGCAAGCCAGTAATATTATATTGGCTT
GCCCCACGGTCTGCG
+
FFFFFFFFFFFFFFFFFFFFFFF:FFFFFFFFFFFFFFF:FFFFFFFFFFFFFFFFFFFFFFFFFFFF
FFFFFFFFFFFFFFFFFFFFFFFFFFFFFFFFFFFFFFFFFFFFFFFFFFFFFFFF::FFFFFFFFFF
FFFFFFFFFFF:FFF
@A00721:190:HGJW7DSXY:4:2167:4200:32847 2:N:0:TCTGCAAG+GCTGCACT
CGTCGATTCTACTGCGTCACTATATAATTCAAATATTATATGGTTATGATCGTGACGCGTGGTCTAAT
GACAAATCGACACGAAAGCGAACGGAGGTCTCACCGGCGCGGGCAAGCCAGAAAAATTATAAAGGCTT
GCCCCACGGACTGC
```

```
+
FFFF,FFFFFF:FFF,F::F,FF:,F:,FFF,,,,,,:,F,,,FF:,FFFF,:,FFFFFF,F:FF:F:
FFF:FFFF:FF:,:,,,,F,,FF,,F:F:,,F,FF,:F,FF,,F,:,FFF,,:,,FF,:,F,,,:FF,
,::,,:F,:,FFF:
@A00721:190:HGJW7DSXY:4:1311:4851:26396 2:N:0:TCTGCAAG+GCTGCACT
ATTGACCGTCGATTCTACTGCGTCACTATAGAATTCATTTTTTAGATGGTTATGATCGTGACGCGTGG
TCTAATGACAAATCGACACGTAAGCGTACGGAGGTGTCCCCGGCGCGGGCAAGCCAGTAATATTATAT
TGGCTTGCCCCACGG
+
FFFFFFFFFFFF,FFFF:F:FFF:FFFF,:FFFFFFFFFFFFFFF:FFFFFFFF:FFFFFFFFFF,FF
FFFFFFFFFFFFFFFFF:FFFFFFFFFF:FFFFFFFF:FFFFFFFFFFFF:FFFFFFFFFFF:FFFF:
FFFFFFFFFFFFFFF
@A00721:190:HGJW7DSXY:4:1616:6894:26710 2:N:0:TCTGCAAG+GCTGCACT
ATTGACCGTCGATTCTACTGCGTCACTATAGAATTCATTTTTTAGATGGTTATGATCGTGACGCGTGG
TCTAATGACAAATCGACACGTAAGCGTACGGAGGTGTCCCCGGCGCGGGCACGCCAGTAATATTATAT
TGGCTTGCCCCACGG
+
FFFFFFFFFFFFFFFFFFFFFFFF,FFF:FFFFFFFFFFF:FF,FFFFFFFFFFFFFFFFF:FFFFFFF
,FFFFFFFFFFFFFFF::FF:FFFFFFFFFFFFFFFFFFFFFFFFFFFFFFF,FF:FFF,FFFF,FFFF
F,FFFF,FFFFFFFF
@A00721:190:HGJW7DSXY:4:2206:16794:32675 2:N:0:TCTGCAAG+GCTGCACT
ATTGACCGTCGATTCTACTGCGTCACTATAGAATTCATTTTTTAGATGGTTATGATCGTGACGCGTGG
TCTAATGACAAATCGACACGTAAGCGTACGGAGGTGTCCCCGGCGCGGGCAAGCCAGTAATATTATAT
TGGCGTGCCCCACGG
+
FFFFFFFFFFFFF:FFFFFFFFFF,FF,FFFFFFF,FFFFFF:FF::FFFFFF:FFFFFF:FFFFFFF
FFFFF:FFFFFFFFFFF:F:FFFFF:,FFFFFFFFFFFFFFFFF:FFFFF,:FFFF,,:F:::FFFFF
FFFF:FFFFFFFFFF
@A00721:190:HGJW7DSXY:4:2304:12084:33254 2:N:0:TCTGCAAG+GCTGCACT
ATTGACCGTCGATTCTACTGCGTCACTATAGAATTCATTTTTTAGATGGTTATGATCGTGACGCGTGG
TCTAATGACAAATCGACACGTAAGCGTACGGAGGTGTCCCCGGCGCGGGCAAGCCAGTAATATTATAT
TGGCTTGCCCCACGG
+
FFFFFFFFFFFFFFFFFFFFFFFFFF:FFFF:FFFFFFFFFFFFF:FFFFFFF:FFFFFF:FFFFFFF
FFFFFFFFFFFFFFFFFFFFFFFFFFFFFFFFFFFFFFFFFFFFFFFFFFF:FFFF:FF:FFFFFF:
FFFFFFFFFFFFF:F
@A00721:190:HGJW7DSXY:4:2509:10384:30686 2:N:0:TCTGCAAG+GCTGCACT
ATTGACCGTCGATTCTACTGCGTCACTATAGAATTCATTTTTTAGATGGTTATGATCGTGACGCGTGG
TCTAATGACAAATCGACACGTAAGCGTACGGAGGTGTCCCCGGCGCGGGCAAGCCAGTAATATTATAT
TGGCTTGCCCCACGG
+
FFFFFFFFFFFF:FFFFFF:FFFFFFFF:,F:FFFFFFFFF,FFF:FFFFFFFF:FFFFFFFFFFFFF
:FFFFFFFF:FFFFFFFFFFFFFFFFFFFFFFFFFFFFFFFFFFFFFFFFFFFFFFFFFFFFFFFFFFF
FF,FFFFFFFFFFFF
@A00721:190:HGJW7DSXY:4:1103:9064:11866 2:N:0:TCTGCAAG+GCTGCACT
TGAATTTCAAAATTGACCGTCGATTCTACTGCGTCACTATAGAATTCATTTTTTAGATGGTTATGATC
GTGACGCGTGGTCTAATGACAAATCGACACGTAAGCGTACGGAGGTGTCCCCGGCGCGGGCAAGCCAG
TAATATTATATTGGC
+
FFFFFFFFFFFFFFFFF:FFFFFFFFFFFFFFFFFFFFFFF:FFFFFFFFFFFFFFFFFFFFFFFFF,FF
FFF:FFFFFFFFFFFFFF:FFFFFFFFFFFFFF:FFFFFFFF,FFFFFFFFFFFFFFFFFF,:FFFFF
,FFF:FFFF:FFF,F
@A00721:190:HGJW7DSXY:4:1166:12554:2284 2:N:0:TCTGCAAG+GCTGCACT
TGAATTTCAAAATTGACCGTCGATTCTACTGCGTCACTATAGAATTCATTTTTTAGATGGTTATGATC
```

GTGACGCGTGGTCTAATGACAAATCGACACGTAAGCGTACGGAGGTGTCCCCGGCGCGGGCAAGCCAG
TAATATTATATTGGC
+
FFFFFFFFFFFFF:FFFFFFFFFFFFFFFFFFFFFFFFFFFFFFFFFFFFF:FFFFFFFFFFFFFFFF
FFFFFFFF:FFFFFFFFFFFFFFFFFFFFFFFFFFFFFFFFFFFFFF:FFFFF:FFFFFFFFFFFF:F
F:FFFFFFFFFF,:F
@A00721:190:HGJW7DSXY:4:1331:22110:18020 2:N:0:TCTGCAAG+GCTGCACT
TGAATTTCAAAATTGACCGTCGATTCTACTGCGTCACTATAGAATTCATTTTTTAGATGGTTATGATC
GTGACGCGTGGTCTAATGACAAATCGACACGTAAGCGTACGGAGGTGTCCCCGGCGCGGGCAAGCCAG
TAATATTATATTGGC
+
FF:FFFFFFFFFFFF:FFFFFFFFFFFFFFFFFFFFFFFFFFFFFFFFFFFFFFFFFFFFFFFFFFFF
FFFFFFFFFFFFFFFFFFFFFFFFFFFFFFFFFFFFFFFFFFFFFFFFFFFFF:FFFFFFFFFFFFFF
F:FFFFFFFFFFFF:
@A00721:190:HGJW7DSXY:4:1622:28718:24893 2:N:0:TCTGCAAG+GCTGCACT
TGAATTTCAAAATTGACCGTCGATTCTACTGCGTCACTATAGAATTCATTTTTTAGATGGTTATGATC
GTGACGCGTGGTCTAATGACAAATCGACACGTAAGCGTACGGAGGTGTCCCCGGCGCGGGCAAGCCAG
TAATATTATATTGGC
+
FFFFFFFFFFFFFFFFFFFFFFFFFFFFFFFFFFFFFF:FFF:,FFFFFFFFFFFFFFFFFFFFFFFF
FFFFFFFFFF:FFFFFFF:FFFFFF:FFFFFFFFFFFF,F:F:FFFFFFFFFFFFFFFFFFF:FFFFF
FFFFFFFFFFFF,:F
@A00721:190:HGJW7DSXY:4:2456:4625:10191 2:N:0:TCTGCAAG+GCTGCACT
TGAATTTCAAAATTGACCGTCGATTCTACTGCGTCACTATAGAATTCATTTTTTAGATGGTTATGATC
GTGACGCGTGGTCTAATGACAAATCGACACGTAAGCGTACGGAGGTGTCCCCGGCGCGGGCAAGCCAG
TAATATTATATTGGC
+
FFFFFFFFFFFFFFFFFFFFFFFFFFFFFFFFFFFFFFFF:FFFFFFFFFFFFFFFF:FFFFFFFFFF
FFFFFFFF,FFFFFFFFF:FFFFFFFFFFFFFFFFFFFFFFFFFFFFFFFFFFFFFFFFFFF,F:F,F
,FFFFFF::FFFF,F
@A00721:190:HGJW7DSXY:4:2510:11116:9001 2:N:0:TCTGCAAG+GCTGCACT
TGAATTTCAAAATTGACCGTCGATTCTACTGCGTCACTATAGAATTCATTTTTTAGATGGTTATGATC
GTGACGCGTGGTCTAATGACAAATCGACACGTAAGCGTACGGAGGTGTCCCCGGCGCGGGCAAGCCAG
TAATATTATATTTGC
+
FF:FFF::FFFFFFFFFF:FFFF:F::FFFFFF:FFF:F::F,FF::F,FF,:FFFFFFFFF:FFFFF
FF,,F:FF,FFFFFF,FFFFFFFF,FFFFFFFFF:F:FFFFFFF:FFFFF:FFFFFFFFF:FF:FFF,
,,F:F,F::,FF,FF
@A00721:190:HGJW7DSXY:4:2673:9715:5447 2:N:0:TCTGCAAG+GCTGCACT
TGAATTTCAAAATTGACCGTCGATTCTACTGCGTCACTATAGAATTCATTTTTTAGATGGTTATGATC
GTGACGCGTGGTCTAATGACAAATCGACACGTAAGCGTACGGAGGTGTCCCCGGCGCGGGCAAGCCAG
TAATATTATATTGGC
+
FFF:FFFFFFFFFFFFFFFFFFFFFFFFFFFFFFFFFFFF:FFFFFFFFFFFFFFFFFFFFFFFFFFF
FFFFFFFFFFFFFFFFFFFFFFFFFFFFFFFFFFFFFFFFFFFFFFFF:FFFFFFFFFFFFFF:FFFF
FFFFFFFFFFFFFFF
@A00721:190:HGJW7DSXY:4:1516:32452:16423 2:N:0:TCTGCAAG+GCTGCACT
TTTGAATTTCAAAATTGACCGTCGATTCTACTGCGTCACTATAGAATTCATTTTTTAGATGGTTATGA
TCGTGACGCGTGGTCTAATGACAAATCGACACGTAAGCGTACGGAGGTGTCCCCGGCGCGGGCAAGCC
AGTAATATTATATTG
+
FFFFFFFFFFFFFFFFFFFFFFFFFFFFF:FFFFFFF:FFFF,F:FFFFFFFFFFFFF,FFFFFFFFF
FFFFFFFFFFFFFFFFFFFFF,F:FFFFFFFFFFFFF:FFFFFFFFFFFFFFFFF:FFFFFFFFFF,F
,FF,FFFFFF:FFFF

```
@A00721:190:HGJW7DSXY:4:2205:16224:6574 2:N:0:TCTGCAAG+GCTGCACT
TTTGAATTTCAAAATTGACCGTCGATTCTACTGCGTCACTATAGAATTCATTTTTAGATGGTTATGA
TCGTGACGCGTGGTCTAATGACAAATCGACACGTAAGCGTACGGAGGTGTCCCCGGCGCGGGCAAGCC
AGTAATATTATATTG
+
FFFFFFFFFFFF:FFFFFFFFFFF:FFFFFFFFFFFFFFFFFFFFFFFFFFFFFFFFF,FFFFFFFFF
FFFFFFFFFFFFFFFF,:FFFFFFF,FFFFFFFFFF:FFFFFFFFFFFFFFFFFFFFFFFFFF,FFFF
FFFFFFF,FFFF,FF
@A00721:190:HGJW7DSXY:4:2324:28085:34757 2:N:0:TCTGCAAG+GCTGCACT
TTTGAATTTCAAAATTGACCGTCGATTCTACTGCGTCACTATAGAATTCATTTTTAGATGGTTATGA
TCGTGACGCGTGGTCTAATGACAAATCGACACGTAAGCGTACGGAGGTGTCCCCGGCGCGGGCAAGCC
AGTAATATTATATTG
+
FFFFFFFFFFFFFFFFFFFFFFFFFFFFFFF,:F:FFFFFFFFFFFFF:FFF:FFFFFFFF,FFFFFF
FFFFFF:FFFFFFFFFFFFFFFFFFFF:FFFF:F:FFF:,FFFFFFFF,FFFFFFFFFFFFFFFFFFF
:FFFF,F:FFFFFFF
@A00721:190:HGJW7DSXY:4:2435:25780:27539 2:N:0:TCTGCAAG+GCTGCACT
TTTGAATTTCAAAATTGACCGTCGATTCTACTGCGTCACTATAGAATTCATTTTTAGATGGTTATGA
TCGTGACGCGTGGTCTAATGACAAATCGACACGTAAGCGTACGGAGGTGTCCCCGGCGCGGGCAAGCC
AGTAATATTATATTG
+
FFFFFFF:FFFF::FFFFFFFFFFFFFFFFFF:FFFFFFFFFFFFFFFFFFFFFFFFFFFFFFFFFF:
FFFFF:FFFF:FFFFFFFFFFF:FFF,FFFFF,F,FFFFFFFF:F:FF:FFF:FFFFFFFFFF:F:F:
:FFFF:FFFFFFFFF
@A00721:190:HGJW7DSXY:4:2471:28176:12868 2:N:0:TCTGCAAG+GCTGCACT
TTTGAATTTCAAAATTGACCGTCGATTCTACTGCGTCACTATAGAATTCATTTTTAGATGGTTATGA
TCGTGACGCGTGGTCTAATGACAAATCGACACGTAAGCGTACGGAGGTGTCCCCGGCGCGGGCAAGCC
AGTAATATTATATTG
+
FFFFFFFFFFFFFFFFFFFFFFFFFFFFFFFFFFFFFFFFF,F:FFFFFFF:F:FFFFFFFFFFFF
FFFFFFFFFFFFFFFFFFFFFFFFFFFFFFFFFFFFFFFFFFFFFFFFFFFFFFFFFFFFFFFFFFFF
FFFFFFFFFFFFFFF
@A00721:190:HGJW7DSXY:4:2521:8865:25488 2:N:0:TCTGCAAG+GCTGCACT
TTTGAATTTCAAAATTGACCGTCGATTCTACTGCGTCACTATAGAATTCATTTTTAGATGGTTATGA
TCGTGACGCGTGGTCTAATGACAAATCGACACGTAAGCGTACGGAGGTGTCCCCGGCGCGGGCAAGCC
AGTAATATTATATTG
+
FFFFFFFF:FFFFFFFFFFFFFFFFFF:FF:FFFFFFFFFFFF:,FFF:FFFFFFFFFF:FFFFFFFF
FFFFFFFFFFFFFFFFFF:FFFFFFF:FFFFFFFFF:FFFFFFFFFFFFFFFFFFFFFFFFFFF:FFF
FFFF:FFF,FF::FF
@A00721:190:HGJW7DSXY:4:2647:30680:3239 2:N:0:TCTGCAAG+GCTGCACT
TTTGAATTTCAAAATTGACCGTCGATTCTACTGCGTCACTATAGAATTCATTTTTAGATGGTTATGA
TCGTGACGCGTGGTCTAATGACAAATCGACACGTAAGCGTACGGAGGTGTCCCCGGCGCGGGCAAGCC
AGTAATATTATATTG
+
FFFFFFF:FFFFF:FFFFFFFFFFFFFFFFFFFFFFFFFFFFF,FF:FFFFFFFF,FFFFFFFFFFFF
FFFFFFFFFFFFFFFFFFFFFFFFFFFFFFFFFFFFFFFFFFFFFFFFFF:FFFFFFFFFFF:FFFF
FFFFFFF,FFFF:FF
@A00721:190:HGJW7DSXY:4:1176:19732:36855 2:N:0:TCTGCAAG+GCTGCACT
ATAAATTTTGAATTTCAAAATTGACCGTCGATTCTACTGCGTCACTATAGAATTCATTTTTAGATGG
TTATGATCGTGACGCGTGGTCTAATGACAAATCGACACGTAAGCGTACGGAGGTGTCCCCGGCGCGGG
CAAGCCAGTAATATT
+
FFFFFFF:FFFFFFFFFFFFFFFFFFFFFFF:FFFFFFFFFFFFFFFFFFFFFFFFFFFFFFFFFFFF
```

```
FFFFFFFFFFFFFFFFFFFFFFFFFF:FFF:FFFF:FFFF,FFFFFFFFFFFFFFFFFFFFFFFFFFF
FFFFFFFFFFFFFFF
@A00721:190:HGJW7DSXY:4:1427:21414:12994 2:N:0:TCTGCAAG+GCTGCACT
ATAAATTTTGAATTTCAAAATTGACCGTCGATTCTACTGCGTCACTATAGAATTCATTTTTTAGATGG
TTATGATCGTGACGCGTGGTCTAATGACAAATCGACACGTAAGCGTACGGAGGTGTCCCCGGCGCGGG
CAAGCCAGTAATATT
+
FFFFFFFFFFFFFFFFFFFFFFFFFFFFFFF:FFFFFFFFFFFFFF:FFFFFFFFFFFFFFFFFFF:F
FFFFFFFFFFFFFFFFFFFFFFFFFFFF:FFF:FFFFFFFFFFFFFFFFFFFFFFFF:FFFFFFFFFF
FFFFFF:FF:F:FFF
@A00721:190:HGJW7DSXY:4:1464:12906:5682 2:N:0:TCTGCAAG+GCTGCACT
ATAAATTTTGAATTTCAAAATTGACCGTCGATTCTACTGCGTCACTATAGAATTCATTTTTTAGATGG
TTATGATCGTGACGCGTGGTCTAATGACAAATCGACACGTAAGCGTACGGAGGTGTCCCCGGCGCGGG
CAAGCCAGTAATATT
+
FFFFFFFFFFFF:FFFFFFFFFFFFFFFFFFFFFFFFFFFFF,:,FFFFFF:FFFF:FF,FFFFF::F
FFFFF:FF:,,FFFFFF:FFFFFFFF::FFFFFF:FFF:FFFFFFFFFFFFFFFFFFFFFFFFFFFFF
FF:FFFFFFF:FF:F
@A00721:190:HGJW7DSXY:4:2254:4363:28745 2:N:0:TCTGCAAG+GCTGCACT
ATAAATTTTGAATTTCAAAATTGACCGTCGATTCTACTGCGTCACTATAGAATTCATTTTTTAGATGG
TTATGATCGTGACGCGTGGTCTAATGACAAATCGACACGTAAGCGTACGGAGGTGTCCCCGGCGCGGG
CAAGCCAGTAATATT
+
FFFFFFFFFFFF:FFFFFFFFFFFFFFFFFFFFFFFFFFFFF:FF,FFFFF:FF:FFFF:FFFFFFFF
FF:FFFFFFFF:FFFF,FFFFFFFFFFFFFFFFFFFFFFF,FFFFFFFFFFFFFFFFFFFFFFFFFFF
FF,FFFFFFFFFF,F
@A00721:190:HGJW7DSXY:4:2605:23701:12226 2:N:0:TCTGCAAG+GCTGCACT
ATAAATTTTGAATTTCAAAATTGACCGTCGATTCTACTGCGTCACTATAGAATTCATTTTTTAGATGG
TTATGATCGTGACGCGTGGTCTAATGACAAATCGACACGTAAGCGTACGGAGGTGTCCCCGGCGCGGG
CAAGCCAGTAATATT
+
FFFFFFFFFFFFFFFFFFFFFFFFFFFFFFF:FFFFFFFFFFFFFFFFFF:FFFFFFFFFFFFF:F:F
FFFFFFFFFFFFFFFFFFF,FFFFFFFFFFFF:FFFFFFFFFFFFFFFFFFFFFFFFFFFFFFFFFFF
FFFF:F,FFFFFF:F
@A00721:190:HGJW7DSXY:4:2678:15383:9157 2:N:0:TCTGCAAG+GCTGCACT
ATAAATTTTGAATTTCAAAATTGACCGTCGATTCTACTGCGTCACTATAGAATTCATTTTTTAGATGG
TTATGATCGTGACGCGTGGTCTAATGACAAATCGACACGTAAGCGTACGGAGGTGTCCCCGGCGCGGG
CAAGCCAGTAATATT
+
FFFFFFFFFFFFFFFFFF:FFFFFFFFFFFFFFFFFFFFFF,FFF:F:FFFFFFFFFFFFFFFFFFFF
FFFFFFFFFFFFFFFFFFFFFFF:FFFFFFF:FFFFFFFFFFFFFFFFFFFFFFFFFFFFFFFFFFFF
FFFFFFFFFFFFFFF
@A00721:190:HGJW7DSXY:4:1147:31837:24972 2:N:0:TCTGCAAG+GCTGCACT
TATAAATTTTGAATTTCAAAATTGACCGTCGATTCTACTGCGTCACTATAGAATTCATTTTTTAGATG
GTTATGATCGTGACGCGTGGTCTAATGACAAATCGACACGTAAGCGTACGGAGGTGTCCCCGGCGCGG
GCAAGCCAGTAATAT
+
FFFFFFF::FFFF:FFFFFFFFFFFFFFFFFFFFFFFF:,FFF:F:FFF,FFFF::FFFFFFFF,:F:
FFF,FF::FFF:F,FFF:FFFFFFFF:FFFFFFF,FFFFFF:F,,FFFFF:::FFFF,FFFFFFFFFF
FFF,F,FFFFFF:F:
@A00721:190:HGJW7DSXY:4:1147:31783:25160 2:N:0:TCTGCAAG+GCTGCACT
TATAAATTTTGAATTTCAAAATTGACCGTCGATTCTACTGCGTCACTATAGAATTCATTTTTTAGATG
GTTATGATCGTGACGCGTGGTCTAATGACAAATCGACACGTAAGCGTACGGAGGTGTCCCCGGCGCGG
GCAAGCCAGTAATAT
```

```
+
FFFFFFFF:FFFFFFFFFFFFFFFFFFFFFFFFFFFFFFFFFF::FFFF,FFFFFFFFFFFFFF:F::F
FFFFFFFFFFFFFFFFFFFFFFFFFFFFFFFFFFFFFFFFFFFFFFFFFFFFFFFFFFFFFFFFFFFFF
FFF:FFFFFFFF,FF
@A00721:190:HGJW7DSXY:4:1526:22272:35462 2:N:0:TCTGCAAG+GCTGCACT
TATAAATTTTGAATTTCAAAATTGACCGTCGATTCTACTGCGTCACTATAGAATTCATTTTTTAGATG
GTTATGATCGTGACGCGTGGTCTAATGACAAATCGACACGTAAGCGTACGGAGGTGTCCCCGGCGCGG
GCAAGCCAGTAATAT
+
FFFFFFFFFFFFFFFFFFFFFFFFFFFFFFFFFFFFFFFFFFFFFFF:FFFFFFFFFFFFFFFF:FF
FFFFFF:FFFFFFFFFFFFFFFFFF:FF:FFFFFFFFFFFFFFFFFFFFFFFFFFFFFFFFFFFFFFF
FFFFFFF,FFFFFFF
@A00721:190:HGJW7DSXY:4:1620:30427:20337 2:N:0:TCTGCAAG+GCTGCACT
TATAAATTTTGAATTTCAAAATTGACCGTCGATTCTACTGCGTCACTATAGAATTCATTTTTTAGATG
GTTATGATCGTGACGCGTGGTCTAATGACAAATCGACACGTAAGCGTACGGAGGTGTCCCCGGCGCGG
GCAAGCCAGTAATAT
+
FFFFFFFFFFFFFFFFFFFFFFFFFFFFFFFFFFFFFFFFFFF::FFFFF:FFFFFFFFFFFFFFFFFF
FFFFFFFFFFFFFFFFFFFFFFFFFFFFFFFFFFFFFFFFFFFFFFFFF:FF:FFFFFFFFFFFFFFFF
FFFFFFFFFFFFF:F
@A00721:190:HGJW7DSXY:4:1209:13440:29277 1:N:0:TCTGCAAG+GCTGCACT
ACTGGCTTGCCCGCGCCGGGGACACCTCCGTACGCTTACGTGTCGATTTGTCATTAGACCACGCGTCA
CGATCATGACCATCTAAAAAATGAATTCTATAGTGACGCAGTAAAATCGACGGCAGATGATATGTTAT
ATCATCGTCTTTTTG
+
FFFFFFFFFFFFFFFFFFFFFFFFFFFFFFFFFFFFFFFFFFFFFFF:FFFFFFFFFFFFFFFFFFFFF
FFFFF:FFFFFFFFFFFFF::FFFF,F::FF:FFFFFFFFFFFFFFFFFFFFFFFFFFFFFFFFFFFFF
:FFFFFFFFFFFFFF
@A00721:190:HGJW7DSXY:4:1544:8648:17347 1:N:0:TCTGCAAG+GCTGCACT
ACTGGCTTGCCCGCGCCGGGGACACCTCCGTACGCTTACGTGTCGATTTGTCATTAGACCACGCGTCA
CGATCATGACCATCTAAAAAATGAATTCTATAGTGACGCAGTAAAATCGACGGCAGATGATATGTTAT
ATCATCGTCTTTTTG
+
FFFFFFFFFFFFFFFFFFFFFFFFFFFFFFFFFFFFFFFFFFFFFFFFFFFFFFFFF:FFFFFFFFFFF
FFFFFFFFFFFFFFFFFFFFFFFFFFFFFFFFFFFFFFFFFFFFFFFFFFFFFFFFFFFFFFF:FF:F:
FFFFFFFFFFFFFFF
@A00721:190:HGJW7DSXY:4:1554:13738:4022 1:N:0:TCTGCAAG+GCTGCACT
ACTGGCTTGCCCGCGCCGGGGACACCTCCGTACGCTTACGTGTCGATTTGTCATTAGACCACGCGTCA
CGATCATAACCATCTAAAAAATGAATTCTATAGTGACGCAGTAGAATCGACGGTCAATTTTGAAATTC
AAAATTTATATTTAT
+
FFFFFFFFFFFFFFFFFFFFFFFFFFFFFFFFFFFFFFFFFFFFFFFFFFFFFFFFFFFFFFFFFFFFF
FFFFFFFFFFFFFFFFFFFFFFFFFFFFFFFFFFFFFFFFFFFFFFFF,FFFFFFFFFFFFFFF:,,:FF
,:,FFF:FF:F:,FF
@A00721:190:HGJW7DSXY:4:1602:7129:24893 1:N:0:TCTGCAAG+GCTGCACT
ACTGGCTTGCCCGCGCCGGGGACACCTCCGTACGCTTACGTGTCGATTTGTCATTAGACCACGCGTCA
CGATCATGACCATCTAAAAAATGAATTCTATAGTGACGCAGTAAAATCGACGGCAGATGATATGTTAT
ATCATCGTCTTTTTG
+
FFFFFFFFFFFFFFFFFFFFFFFFFFFFFFFFFFFF:FFFFFFFFFF:FFFFFFFFFFFFFFFFFFFFFF
FFF:FFFFFFFFFFFFFF:FFFFFFF,FFF:FFFFFFFFFFFFFFFFFFFFFFFFFFFFFFFFFFF:F:
F:FFFFFFFFFFFFF
@A00721:190:HGJW7DSXY:4:2158:26187:28369 1:N:0:TCTGCAAG+GCTGCACT
ACTGGCTTGCCCGCGCCGGGGACACCTCCGTACGCTTACGTGTCGATTTGTCATTAGACCACGCGTCA
```

CGATCATAACCATCTAAAAAATGAATTCTATAGTGACGCAGTAGAATCGACGGTCAATTTTGAAATTC
AAAATTTATATTTAT
+
FFFFFFFFFFFFFFFFFFFFFFFFFFFFFFFFFFFFFFFFFFFFFFFFFFFFFFFFFFFFFFFFFFFF
FFFFFFFFFFFFFFFFFFF:FFFFFFFFFFFFFFFFFFFFFFFFFFFFFFFFFFFFFFFFFF:F:FFF
:,F:FFFFFF,FFFF
@A00721:190:HGJW7DSXY:4:2229:11749:12070 1:N:0:TCTGCAAG+GCTGCACT
ACTGGCTTGCCCGCGCCGGGGACACCTCCGTACGCTTACGTGTCGATTTGTCATTAGACCACGCGTCA
CGATCATAACCATCTAAAAAATGAATTCTATAGTGACGCAGTAGAATCGACGGTCAATTTTGAAATTC
AAAATTTATATTTAT
+
FFFFFFFFFFFFFFFFFFFFFFFFFFFFFFFFFFFFFFFFFFFFFFFFFFFFFFFFFFFFFFFFFFFF
FFFFFFFFFFFFFFFFFFFFFFFFFFFF:FFFFFFFFFFFFFFFFFFFFFFFFFFFF:FFFFF:FFFF
,:,:F,FFFFFF:,F
@A00721:190:HGJW7DSXY:4:2340:16504:6840 1:N:0:TCTGCAAG+GCTGCACT
ACTGGCTTGCCCGCGCCGGGGACACCTCCGTACGCTTACGTGTCGATTTGTCATTAGACCACGCGTCA
CGATCATGACCATCTAAAAAATGAATTCTATAGTGACGCAGTAAAATCGACGGCAGATGATATGTTAT
ATCATCGTCTTTTTG
+
FFFFFFFFFFFFFFFFFFFFFFFFFFFFFFFFFFFFFFFFFFFFFFFFFFFFFFFFFFFFFFFFFFFF
FFFFFFFFFFFFFFFFFFFF:FFF:FFF::FFFFFFFFFFFFFFFFFFFFFFFFFFFFFFFFFFFFFF
FFFFFFFFFFFF:FF
@A00721:190:HGJW7DSXY:4:2462:10303:26287 1:N:0:TCTGCAAG+GCTGCACT
ACTGGCTTGCCCGCGCCGGGGACACCTCCGTACGCTTACGTGTCGATTTGTCATTAGACCACGCGTCA
CGATCATGACCATCTAAAAAATGAATTCTATAGTGACGCAGTAAAATCGACGGCAGATGATATGTTAT
ATCATCGTCTTTTTG
+
FFFFFFFFFFFFFFFFFFFFFFFFFFFFFFFFFFFFFFFFFFFFFFFFFFFFF:FFFFFFFFFFFFFF
FFFFFFFFFFFFFF:F:FFFFFF:FFF:F:,,FFFFFFFFFFFFFFFFFFFFFFFFFFFFFFFFFFFF
FFFF,FFFFFFFFFF
@A00721:190:HGJW7DSXY:4:2475:21206:6057 1:N:0:TCTGCAAG+GCTGCACT
ACTGGCTTGCCCGCGCCGGGGACACCTCCGTACGCTTACGTGTCGATTTGTCATTAGACCACGCGTCA
CGATCATAACCATCTAAAAAATGAATTCTATAGTGACGCAGTAGAATCGACGGTCAATTTTGAAATTC
AAAATTTATATTTAT
+
FFFFFFFFFFFFFFFFFFFFFFFFFFFFFFFFFFFFFFFFFFFFFFFFFFFFFFFFFFFFFFFFFFFF
FFFFFFFFFFFFFFFFFFFFFFFFFFFF:FFFFFFFFFFFFFFFFFFFFFFFFFFFFFFFFFF,::FF
F:FFF,FFFF:,FFF
@A00721:190:HGJW7DSXY:4:2553:13602:11929 1:N:0:TCTGCAAG+GCTGCACT
ACTGGCTTGCCCGCGCCGGGGACACCTCCGTACGCTTACGTGTCGATTTGTCATTAGACCACGCGTCA
CGATCATGACCATCTAAAAAATGAATTCTATAGTGACGCAGTAAAATCGACGGCAGATGATATGTTAT
ATCATCGTCTTTTTG
+
FFFFFFFFFFFFFFFFFFFFFFFFFFFFFFFFFFFFFFFFFFFFFFFFFFFFFFFFFFFFFFFFFFFF
FFFFFFFFFFFFFFFFFFFFFFFF:FFFFFFFFFFFFFFFFFFFFFFFFFFFFFFFFFFFFFF:FFF
FFF:FFFFFFFFFFF
@A00721:190:HGJW7DSXY:4:1138:13376:9345 1:N:0:TCTGCAAG+GCTGCACT
GCTGGCTTGCCCGCGCCGGGGACACCTCCGTACGCTTACGTGTCGATTTGTCATTAGACCACGCGTCA
CGATCATAACCATCTAAAAAATGAATTCTATAGTGACGCAGTAGAATCGACGGTCAATTTTGAAATTC
AAAATTTATAGTTAT
+
FFFFFFFFFFFFFFFFFFFFFFFFFFFFFFFFFFF,FFFFFFFFFFFFFFFFFFFFFFFFFFFFFFFF
FFFFFFFFFFFFFFFFFFFFFFFF,FFFFFFFF:FFFFFFFFFFFFFFFFFFFFFFFFF:FFFFFFFF
FFF:FFFF,F,F:FF

```
@A00721:190:HGJW7DSXY:4:1160:23439:19883 1:N:0:TCTGCAAG+GCTGCACT
GCTGGCTTGCCCGCGCCGGGGACACCTCCGTACGCTTACGTGTCGATTTGTCATTAGACCACGCGTCA
CGATCATAACCATCTAAAAAATGAATTCTATAGTGACGCAGTAGAATCGACGGTCAATTTTGAAATTC
AAAATTTATATTTAT
+
FFFFFFFFFFFFFFFFFFFFFFFFFFFFFFFFFFFFFFFFFFFFFFFFFFFFFFFFFFFFFFFFFFFF
FFFFFFF:FFFFFFFFFFF:FFFFFFFFFFFFFFFFFFFFFFFFFFFFFFFFFFFFFFFFFFFFFFFF
FFFFFFFFFFF:FFF
@A00721:190:HGJW7DSXY:4:2162:13928:7983 1:N:0:TCTGCAAG+GCTGCACT
GCTGGCTTGCCCGCGCCGGGGACACCTCCGTACGCTTACGTGTCGATTTGTCATTAGACCACGCGTCA
CGATCATAACCATCTAAAAAATGAATTCTATAGTGACGCAGTAGAATCGACGGTCAATTTTGAAATTC
AAAATTTATATTTAT
+
FFFFFFFFFFFFFFFFFFFFFFFFFFFFFFFFFFFFFFFFFFFFFFFFFFFFFFFFFFFFFFFFFFFF
FFFFFFFFFFFFFF:FFFFFFFFFFFFFFFFFFFFFFFFFFFFFFFFFFFFFFFFFFFFFF:FFFFFF
FFF:FFFFFF:FFFF
@A00721:190:HGJW7DSXY:4:2162:13865:8750 1:N:0:TCTGCAAG+GCTGCACT
GCTGGCTTGCCCGCGCCGGGGACACCTCCGTACGCTTACGTGTCGATTTGTCATTAGACCACGCGTCA
CGATCATAACCATCTAAAAAATGAATTCTATAGTGACGCAGTAGAATCGACGGTCAATTTTGAAATTC
AAAATTTATATTTA
+
FFFFFFFFFFFFFFFFFFFFFFFFFFFFFFFFFFFFFFFFFFFFFFFFFFFFFFFFFFFFFFFFFFFF
FFFFFFFFFFFFFFFFFFFFFFFFFFFFFFFFFFFF:FFFFFFFFFFFFFFFFFFFFFFFFFFFFFFF
FFFFFFFFFF,FFF
@A00721:190:HGJW7DSXY:4:2354:6153:9737 1:N:0:TCTGCAAG+GCTGCACT
GCTGGCTTGCCCGCGCCGGGGACACCTCCGTACGCTTACGTGTCGATTTGTCATTAGACCACGCGTCA
CGATCATAACCATCTAAAAAATGAATTCTAGAGTGACGCAGTAGAATCGACGGTCAATTTTGAAATTC
AAAATTTAGATTTAT
+
FFFFFFFFFFFFFFFFFFFFFFFFFFFFFFFFFFFF:FFFFFFFFFFFFFFFFFFFF:FF:FFFFFFF
FFFFFFFFFFFFFF,FFFF,FFFFFFF,:F,FFFFFFFFFFFFFFFFFFFFFFFFFFFFFFF::FFFF
:F:F,FFF,:F::FF
@A00721:190:HGJW7DSXY:4:2447:10719:17112 1:N:0:TCTGCAAG+GCTGCACT
GCTGGCTTGCCCGCGCCGGGGACACCTCCGTACGCTTACGTGTCGATTTGTCATTAGACCACGCGTCA
CGATCATAACCATCTAAAAAATGAATTCTATAGTGACGCAGTAGAATCGACGGTCAATTTTGAAATTC
AAAATTTATATTTA
+
F:FFFFFFFFFFFFFFFFFFFFFFFFFFFFFFFFFF:FFFFFFFFFF:FFFFF::FFFFFFFFFFFFF
FFFFFFFFFFFFFFF,:FFF,FFFFFFF::FFFFF,FFF,FFFFFFFFFF:FFFFFF::FFFFFFFF:
:F,,FF:FF,FFFF
@A00721:190:HGJW7DSXY:4:2612:19560:36057 1:N:0:TCTGCAAG+GCTGCACT
GCTGGCTTGCCCGCGCCGGGGACACCTCCGTACGCTTACGTGTCGATTTGTCATTAGACCACGCGTCA
CGATCATAACCATCTAAAAAATGAATTCTATAGTGACGCAGTAGAATCGACGGTCAATTTTGAAATTC
AAAATTTATATTTAT
+
FFFFFFFFFFFFFFFFFFFFFFFFFFFFFFFFFFFFFFFFFFFFFFFFFFFFFFFFFFFFFFFFFFFF
FFFFFFFFFFFFFFFFFFFFFFFFFFFFFFFFFFFF:FFFFFFF:FFFFFFFFFFFFFFFFFFFFFFF
FFFFFFF:FFFFFF:
@A00721:190:HGJW7DSXY:4:1423:27778:13401 1:N:0:TCTGCAAG+GCTGCACT
GGCTTGCCCGCGCCGGGGACACCTCCGTACGCTTACGTGTCGATTTGTCATTAGACCACGCGTCACGA
TCATGACCATCTAAAAAATGAATTCTATAGTGACGCAGTAAAATCGACGGCAGATGATATGTTATATC
ATCGTCTTTTTGATA
+
FFFFFFFFFFFFFFFFFFFFFFFFFFFFFFFFF:FFFFFFFFFFFFFFFFFFFFFFFFFFFFFFFFFF
```

FFF:FFFFFFFFFFFFF,FFF,FFFFF:FFFFFFFFFFFFFFFFFFFFFFFFFFFFFFFFFFF:FFFF
FFFFFFFFFFFFFFF
@A00721:190:HGJW7DSXY:4:2130:6894:19758 1:N:0:TCTGCAAG+GCTGCACT
GGCTTGCCCGCGCCGGGGACACCTCCGTACGCTTACGTGTCGATTTGTCATTAGACCACGCGTCACGA
TCATGACCATCTAAAAAATGAATTCTATAGTGACGCAGTAAAATCGACGGCAGATGATATGTTATATC
ATCGTCTTTTTGATA
+
FFFFFFFFFFFFFFF:FFFFFFF:FFFFFFFFFFFFFFFFF:FFFFFFFF::FFFFFFFFFFFFFFFF
,FFF,FFFFFFFFFFFF:F,FFFFFFFFF:FFF:FFFFFFFFFFFFFFF:FFFF:FFFF:FFFFF,F
FFFFFFF:FFFFFFF
@A00721:190:HGJW7DSXY:4:2433:21612:6355 1:N:0:TCTGCAAG+GCTGCACT
GGCTTGCCCGCGCCGGGGACACCTCCGTACGCTTACGTGTCGATTTGTCATTAGACCACGCGTCACGA
TCATGACCATCTAAAAAATGAATTCTATAGTGACGCAGTAAAATCGACGGCAGATGATATGTTATATC
ATCGTCTTTTTGATA
+
FFFFFFFFFFFFFFFFFFFFFFFFFFFFFFFFFFFFFFFFFFFFFFFFFFFFFFFFFFFFFFFFFFFF
FFFFFFFFFFFFFFFFF:FFF,FFFF:FFFFFFFFFFFFFFFFFFFFFFFFFFFFFFFFFFFFFFFFF
FFFFFFFFFFFFFFF
@A00721:190:HGJW7DSXY:4:2522:6849:27978 1:N:0:TCTGCAAG+GCTGCACT
GGCTTGCCCGCGCCGGGGACACCTCCGTACGCTTACGTGTCGATTTGTCATTAGACCACGCGTCACGA
TCATGACCATCTAAAAAATGAATTCTATAGTGACGCAGTAAAATCGACGGCAGATGATATGTTATATC
ATCGTCTTTTTGATA
+
FFFFFFFFFFFFFFFFFFFFFFFF:FFFFFFFFFFFFFFFFFFFFFFFFFFFFFFFFFFFFFFFFFFF:
:FFFFFFFFFFFFFFFFFFF,F:FF:FFFFFFFFFFFFFFFFFFFFFFFFFFFFFFFFF:FFFFFFFFF
FFFFFFF:F,F,:FF
@A00721:190:HGJW7DSXY:4:1168:3197:6903 1:N:0:TCTGCAAG+GCTGCACT
CGCCCGCGCCGGGGACACCTCCGTACGCTTACGTGTCGATTTGTCATTAGACCACGCGTCACGATCAT
AACCATCTAAAAAATGAATTCTATAGTGACGCAGTAGAATCGACGGTCAATTTTGAAATTCAAAATTT
ATAAATATTGTGGGT
+
FFFFFFFFFFFFFFFFFFFFFFFFFFFFFF:FFFFFFFFFFFFFFFFFFFFFFFFFFFFFFFFFF:FFF
FFFFFFF:FFFFFFFF:,FFFFF,FFFFFFFFFFFFFFF:FFFFFFFFFFFF,FF,FFFFFF,FFFFF
F:,FFF:F:FFFFFF
@A00721:190:HGJW7DSXY:4:1203:23836:35352 1:N:0:TCTGCAAG+GCTGCACT
CGCCCGCGCCGGGGACACCTCCGTACGCTTACGTGTCGATTTGTCATTAGACCACGCGTCACGATCAT
AACCATCTAAAAAATGAATTCTATAGTGACGCAGTAGAATCGACGGTCAATTTTGAAATTCAAAATTT
ATAAATATTGTGGGT
+
FFFFFFFFFFFFFFFFFFFFFFFFFFFFF:F:FFFF:FFFFFFFF:F:FFFFF:FFFFFFFFFFFFFFF
FFFFFFF,FFFFFFFF:,,FFFFFFFFFFFFFFFFFFFFFFFFFFF:FFFF,FF::FFFFFFFFFFFFF
FF:FF:FFF:F:,FF
@A00721:190:HGJW7DSXY:4:1351:24297:1329 1:N:0:TCTGCAAG+GCTGCACT
CGCCCGCGCCGGGGACACCTCCGTACGCTTACGTGTCGATTTGTCATTAGACCACGCGTCACGATCAT
AACCATCTAAAAAATGAATTCTATAGTGACGCAGTAGAATCGACGGTCAATTTTGAAATTCAAAATTT
ATAAATATTGTGGGT
+
FFFFFFFFFFFFFFFFFFFFFFFFFFFFFFFFFFFFFFFFFFFFFFFFFFFFFFFFFFFFFFFFFFFF
FFFFFFFFFFFFFFFFFF,F:F:FFFFFFFFFFFF,FFFFFFFFFFFFFFF:FFFFFFFFFFF:FF,F
FFF:FFFFFF:FFFF
@A00721:190:HGJW7DSXY:4:1571:31024:20243 1:N:0:TCTGCAAG+GCTGCACT
CGCCCGCGCCGGGGACACCTCCGTACGCTTACGTGTCGATTTGTCATTAGACCACGCGTCACGATCAT
AACCATCTAAAAAATGAATTCTATAGTGACGCAGTAGAATCGACGGTCAATTTTGAAATTCAAAATTT
ATAAATATTGTGGGT

```
+
F:FFFFFFFFFF:FFFFFFFFFF:F:FFFFFFFFFFFFFFFFF:FFFFFFFFFFFFFFFFFFFFFFFFF
FFFFFFFFFFFFF:FFFFFFF:FFFF:FFFFFFFFFFFFFFFF:FFFFFFF,FF:FFF:FFFFFFFFF
FFFFFFFF:FFFFFF
@A00721:190:HGJW7DSXY:4:1643:9805:8797 1:N:0:TCTGCAAG+GCTGCACT
CGCCCGCGCCGGGGACACCTCCGTACGCTTACGTGTCGATTTGTCATTAGACCACGCGTCACGATCAT
AACCATCTAAAAAATGAATTCTATAGTGACGCAGTAGAATCGACGGTCAATTTTGAAATTCAAAATTT
ATAAATATTGTGGGT
+
FFFFFFFFFFFFFFFFFFFFFFFFFFFFFFFFFFFFFFFFFFFFFFFFFFFFFFFFFFFFFFFFFFFF
FFFFFFFFFFFFFFFFFFFFFFFFF,FFFFFFFFFFFFFFFFFFFFFFFFFFFFFFFFFFFFFFFFFF
FFFFFFFFFFFFFFF
@A00721:190:HGJW7DSXY:4:1657:2338:7858 1:N:0:TCTGCAAG+GCTGCACT
CGCCCGCGCCGGGGACACCTCCGTACGCTTACGTGTCGATTTGTCATTAGACCACGCGTCACGATCAT
AACCATCTAAAAAATGAATTCTATAGTGACGCAGTAGAATCGACGGTCAATTTTGAAATTCAAAATTT
ATAAATATTGTGGGT
+
FFFFFFFFFFFFFFFFFFFFFFFFFFFFFFFFFFFFFFFFFFFFF:FFFFFFFFFFFFFFFFFFFFFF
FFFFFFFFFFFFFFFFFF:FF,F,FFFFFFFFFFFFFFFFFFFFFFFFFFFFFFF:FFFFFFFFFFFF
FFFFF::FFFFFFFF
@A00721:190:HGJW7DSXY:4:2238:21070:33098 1:N:0:TCTGCAAG+GCTGCACT
CGCCCGCGCCGGGGACACCTCCGTACGCTTACGTGTCGATTTGTCATTAGACCACGCGTCACGATCAT
AACCATCTAAAAAATGAATTCTATAGTGACGCAGTAGAATCGACGGTCAATTTTGAAATTCAAAATTT
ATAAATATTGTGGGT
+
FFFFFFFFFFFFFFFFFFFFFFFFFFFFFFFFFFFFFFFFFFFFFFFFFFFFFFFFFFFFFFFFFFFF
FFFFFFFFFFFFFFFFF:FFF,FFFFFF:FFFFFFFFF:FFFFFFFFFFFFFFFF:FFFFFF:FFFFF
F:FFFFFFFFFFFFF
@A00721:190:HGJW7DSXY:4:2238:21730:33270 1:N:0:TCTGCAAG+GCTGCACT
CGCCCGCGCCGGGGACACCTCCGTACGCTTACGTGTCGATTTGTCATTAGACCACGCGTCACGATCAT
AACCATCTAAAAAATGAATTCTATAGTGACGCAGTAGAATCGACGGTCAATTTTGAAATTCAAAATTT
ATAAATATTGTGGGT
+
FFFFFFFFFFFFFFFFFFFFFFFFF:FF:,FFFFFFFFFFFFFF:FFFFFFFFFFFFFFFFF:FFF:
F:FFFFF,FFFFF,FFF:::F,F,FFFFFFFFFFFFFFFFFFFFFFFFFFFFFFFF,FFFFF:FFFFF
FFF:FFF::F,FFFF
@A00721:190:HGJW7DSXY:4:2258:20745:15593 1:N:0:TCTGCAAG+GCTGCACT
CGCCCGCGCCGGGGACACCTCCGTACGCTTACGTGTCGATTTGTCATTAGACCACGCGTCACGATCAT
AACCATCTAAAAAATGAATTCTATAGTGACGCAGTAGAATCGACGGTCAATTTTGAAATTCAAAATTT
ATAAATATTGTGGGT
+
FFFFFFFFFFFFFFFFFFFFFFFFFFFFFFFFFFFFFFFFFFFFFFFFFFFFFFFFFFFFFFFFFFFF
FFFFFFFFFFFFF:FFFFFFFFFFFFFFFFFFFFF:FFFFFFFFFFFFFFFFFFFFFFFFFFFFFFFF
FFF:FFFFFFFFFF:
@A00721:190:HGJW7DSXY:4:2312:19325:5525 1:N:0:TCTGCAAG+GCTGCACT
CGCCCGCGCCGGGGACACCTCCGTACGCTTACGTGTCGATTTGTCATTAGACCACGCGTCACGATCAT
AACCATCTAAAAAATGAATTCTATAGTGACGCAGTAGAATCGACGGTCAATTTTGAAATTCAAAATTT
ATAAATATTGTGGGT
+
FFFFFFFFFFFFFFFFFFFFFFFFFFFFFFFFFFFFFFFFFFFFFFFFFFFFFFFFFFFFFFFFFFFF
FFFFFFFFFFFFFFFFFFFFFFFFFFFFFFFFFFFF:FFFFFFFFFFFFFFFFFFFFFFFFFFFFFFF
FFFFFFFFFFFFFFF
@A00721:190:HGJW7DSXY:4:2413:27905:9893 1:N:0:TCTGCAAG+GCTGCACT
CGCCCGCGCCGGGGACACCTCCGTACGCTTACGTGTCGATTTGTCATTAGACCACGCGTCACGATCAT
```

AACCATCTAAAAAATGAATTCTATAGTGACGCAGTAGAATCGACGGTCAATTTTGAAATTCAAAATTT
ATAAATATTGTGGGT
+
FFFFFFFFFFFFFFFFFFFFFFFFFFFFFFFFFFFFFFFFFFFFFFFFFFFFFFFFFFFFFFFFFFFFFF
FFFFFFFFFFFFFFFFFFFFFFFFFFFFFFFFFFFFFFFFFFFFFFFFFFFFFFFFF:FFFFFFFFFFFF
FFFFFFFFFFF:FFF
@A00721:190:HGJW7DSXY:4:2413:27968:10974 1:N:0:TCTGCAAG+GCTGCACT
CGCCCGCGCCGGGGACACCTCCGTACGCTTACGTGTCGATTTGTCATTAGACCACGCGTCACGATCAT
AACCATCTAAAAAATGAATTCTATAGTGACGCAGTAGAATCGACGGTCAATTTTGAAATTCAAAATTT
ATAAATATTGTGGGT
+
FFFFFFFFFFFFFFFFFFFFFFFFFFFFFF:FFFFFFFFFFFFFFFFFFFFFFFFFFFFFFFFFFFFFFF
FFFFFFFFFFFFFFFFFFFFFFFFFFFFF:FFFFFFFFFFFFFFFFFFFFFFFFFFFFFFFFFFFFFFFF
FFFFFFFFFFFFFFF
@A00721:190:HGJW7DSXY:4:1109:9941:23688 1:N:0:TCTGCAAG+GCTGCACT
GCCCGCGCCGGGGACACCTCCGTACGCTTACGTGTCGATTTGTCATTAGACCACGCGTCACGATCATA
ACCATCTAAAAAAAGAATTCTATAGTGACGCAGTAGAATCGACGGTCAATTTTGAAATTCAAAATTTA
TATTTATTGTGTTGA
+
FFFFFFFFFFFFFFFFFFFFFFFFFFFFFFFFFFFFFFFFFFFFFFFFFFFFFFFFFFFFFFFFFFFFFF
FFFFFFFF:FFFFFFFFFFFFFFFFF:FFFFFFFFFFFFFFF:FFFFFFFFFFFFFFFFFFFFFF:FFF
FFF:FFFFF:FF:FF
@A00721:190:HGJW7DSXY:4:1419:15917:16720 1:N:0:TCTGCAAG+GCTGCACT
GCCCGCGCCGGGGACACCTCCGTACGCTTACGTGTCGATTTGTCATTAGACCACGCGTCACGATCATA
ACCATCTAAAAAAAGAATTCTATAGTGACGCAGTAGAATCGACGGTCAATTTTGAAATTCAAAATTTA
TATTTATTGTGTTGA
+
FFFFFFFFFFFFFFFFFFFFFFFFFFFFFFFFFFFFFFFFFFFFFFFFFFFFFFFFFFFFFFFFFFFFFF
FFFFFFFFFFFFFFFFFFFF:FFFFFFFFFFFFFFFFFFFFFFFFFFFFFFFFFFFFFFFFFFFF:FFFF
F::FFFFFFFFF,F:
@A00721:190:HGJW7DSXY:4:1525:20573:2362 1:N:0:TCTGCAAG+GCTGCACT
GCCCGCGCCGGGGACACCTCCGTACGCTTACGTGTCGATTTGTCATTAGACCACGCGTCACGATCATA
ACCATCTAAAAAAAGAATTCTATAGTGACGCAGTAGAATCGACGGTCAATTTTGAAATTCAAAATTTA
TATTTATTGTGTTG
+
FFFFFFFFFFFFFFFFFFFFFFFFFFFFFFFFFFFFFFFFFFFFFFFFFFFFFFFFFFFFFFFFFFFFFF
FFFFFFFFFFFFFFFFFFFFFFFFFFFFFFFFFFFFFFFFFFFFFFFFFFFFFFFFFFFFFFFFF:FFFF
FF:FFFFFFFFF:F
@A00721:190:HGJW7DSXY:4:2505:15067:21167 1:N:0:TCTGCAAG+GCTGCACT
GCCCGCGCCGGGGACACCTCCGTACGCTTACGTGTCGATTTGTCATTAGACCACGCGTCACGATCATA
ACCATCTAAAAAAAGAATTCTATAGTGACGCAGTAGAATCGACGGTCAATTTTGAAATTCAAAATTTA
TATTTATTGTGTTGA
+
FFFFFFFFFFFFFFFFFFFFFFFFFFFFFFFFFFFFFFFFFFFFFFFFFFFFFFFFFFFFFFFFFFFFFF
FFF:FFFFFFF:FFFF,FFF:F:FFFFFFFFFFFFFFFFFFFFFFFFFFFFFFF:FFFFFFF:FF:F,F
FFFFFFFFFFFFFF:
@A00721:190:HGJW7DSXY:4:1104:5593:22357 1:N:0:TCTGCAAG+GCTGCACT
CCCGCGCCGGGGACACCTCCGTACGCTTACGTGTCGATTTGTCATTAGACCACGCGTCACGATCATAA
CCATCTAAAAAATGAATTCTATAGTGACGCAGTAGAATCGACGGTCAATTTTGAAATTCAAAATTTAT
ATTTATT
+
FFFFFFFFFFFFFFFFFFFFFFFFFFFFFFFFFFFFFFFFFFFFFFFFFFFFFFFFFFFFFFFFFFFFFF
FFFFFFFFFFFFFFF:FFFFFFFFFFFFFFFFFFFFFFFFFFFFFFFFFFFFFFF:FFFFFFFFFFFFFF
FFFFFFF

```
@A00721:190:HGJW7DSXY:4:1122:6153:21386 1:N:0:TCTGCAAG+GCTGCACT
CCCGCGCCGGGGACACCTCCGTACGCTTACGTGTCGATTTGTCATTAGACCACGCGTCACGATCATGA
CCATCTAAAAAATGAATTCTATAGTGACGCAGTAAAATCGACGGCAGATGATATGTTATATCATCGTC
TTTTTGA
+
FFFFFFFFFFFFFFFFFFFFFFFFFFFFFFFFFFFFFFFFFFFFFFFFFFFFFFFFFFFFFFFFFFFF
FFFFFFFFFFFFFFFFFFFFFFFFFFFFFFFFFFFFFFFFFFFFFFFFFFFFFFFFFFFFFFFFFFFF
FFFFFFF
@A00721:190:HGJW7DSXY:4:1159:3676:24142 1:N:0:TCTGCAAG+GCTGCACT
CCCGCGCCGGGGACACCTCCGTACGCTTACGTGTCGATTTGTCATTAGACCACGCGTCACGATCATGA
CCATCTAAAAAATGAATTCTATAGTGACGCAGTAAAATCGACGGCAGATGATATGTTATATCATCGTC
TTTTTGA
+
FFFFFFFFFFFFFFFFFFFFFFFFFF,FFFFFFFFFFFFFFFFFFFFFFFFFFFFFFFFFFFFFFFFF
FFFFFFFFFFFFFFF:FFFFFFFFFFFFFFFFFFFFFFFFFFFFFFFFFFFFF,FFF:FFFFFFFFFF
FFFFFFF
@A00721:190:HGJW7DSXY:4:1163:25925:9533 1:N:0:TCTGCAAG+GCTGCACT
CCCGCGCCGGGGACACCTCCGTACGCTTACGTGTCGATTTGTCATTAGACCACGCGTCACGATCATGA
CCATCTAAAAAATGAATTCTATAGTGACGCAGTAAAATCGACGGCAGATGATATGTTATATCATCGTC
TTTTTGA
+
FFFFFFFFFFFF:FFFFFFFFFFFFF:FFFFFFFFFF:FFFFF:FFFFFFFFFFFFFFFFFFFFFFFF
FFFFFFFFFFF::FF,,FF:::,FFFFFFFFFFFFFFFFFFFFFFFFFFFFFF:FFFFFFFFFFFFFF
FFFFFFF
@A00721:190:HGJW7DSXY:4:1215:27733:12352 1:N:0:TCTGCAAG+GCTGCACT
CCCGCGCCGGGGACACCTCCGTACGCTTACGTGTCGATTTGTCATTAGACCACGCGTCACGATCATAA
CCATCTAAAAAATGAATTCTATAGTGACGCAGTAGAATCGACGGTCAATTTTGAAATTCAAAATTTAT
ATTTATTGTG
+
FFFFFFFFFFFFFFFFFFFFFFFFFFFFFFFFFFFFFFFFFFFFFFFFFFFFFFFFFFFFFFFF:FFF
FFFFF:FFFFFFFFFF:FFF:FFFFFFFFFFFFFFFFFFFFFFFFFFFFFFFFFFFFFFFFFFFFFF
FFFFFFFFFF
@A00721:190:HGJW7DSXY:4:1224:13937:19774 1:N:0:TCTGCAAG+GCTGCACT
CCCGCGCCGGGGACACCTCCGTACGCTTACGTGTCGATTTGTCATTAGACCACGCGTCACGATCATGA
CCATCTAAAAAATGAATTCTATAGTGACGCAGTAAAATCGACGGCAGATGATATGTTATATCATCGTC
TTTTTGA
+
FFFFFFFFFFFFFFFFFFFFFFFFFFFFFFFFFFFFFFFFFFFFFFFFFFFFFFFFFFFFFFFFFFFF
FFFFFFFFFFFFFFFFFFF:FFFFFFFFFFFFF:FFFFFFFFFFFFFFFFFFF,FFFFFF:FFFFFFF
FFFF:FF
@A00721:190:HGJW7DSXY:4:1246:20329:22858 1:N:0:TCTGCAAG+GCTGCACT
CCCGCGCCGGGGACACCTCCGTACGCTTACGTGTCGATTTGTCATTAGACCACGCGTCACGATCATGA
CCATCTAAAAAATGAATTCTATAGTGACGCAGTAAAATCGACGGCAGATGATATGTTATATCATCGTC
TTTTTGA
+
FFFFFFFFFFFFFFFFFFFFFFFFFF:FFFFFFFFFFFFFFFFFFFFFFFFFFFFFFFFFFFFFFFFF
FFFFFFFFFFFF:FF,FFF,F:FFFF:FFFFFFFFFFFFFFFFFFFFFFFFFFFFFF:FFFFFFFFFF
FFFFFFF
@A00721:190:HGJW7DSXY:4:1316:24686:18474 1:N:0:TCTGCAAG+GCTGCACT
CCCGCGCCGGGGACACCTCCGTACGCTTACGTGTCGATTTGTCATTAGACCACGCGTCACGATCATGA
CCATCTAAAAAATGAATTCTATAGTGACGCAGTAAAATCGACGGCAGATGATATGTTATATCATCGTC
TTTTTGA
+
FFFFFFFFFFFFFFFFFFFFFFFFFFFFFFFFFFFFFFFFFFFFFFFFFFFFFFFFFFFFFFFFFFFF
```

FFFFFFFFFFFFFFF:F:FFFFFFFFFFFFFFFFFFFFFFFFFFFFFFFFFFFFFFFFFFFFFFFFFF
FFFFFFF
@A00721:190:HGJW7DSXY:4:1325:14606:11725 1:N:0:TCTGCAAG+GCTGCACT
CCCGCGCCGGGGACACCTCCGTACGCTTACGTGTCGATTTGTCATTAGACCACGCGTCACGATCATGA
CCATCTAAAAAATGAATTCTATAGTGACGCAGTAAAATCGACGGCAGATGATATGTTATATCATCGTC
TTTTTGA
+
FFFFFFFFFFFFFFFFFFFFFFFFFFFFFFFFFFFFFFFFFFFFFFFFFFFFFFFFFFFFFFFFFFFF
FFFFFFFFFFFFFFFFFFFFFFFFFFF:FFFFFFFFFFFFFFFFFFFFFFFFFFFFFFFFFFFFFFFF
FFFFFF:
@A00721:190:HGJW7DSXY:4:1331:21504:6731 1:N:0:TCTGCAAG+GCTGCACT
CCCGCGCCGGGGACACCTCCGTACGCTTACGTGTCGATTTGTCATTAGACCACGCGTCACGATCATAA
CCATCTAAAAAATGAATTCTATAGTGACGCAGTAGAATCGACGGTCAATTTTGAAATTCAAAATTTAT
ATTTATTGTG
+
FF:::F:FF:F::FFFFFFFFFFFFFF:FFFFFFFFFF,FFFF:F:F:FFFFF:FF:FFFFFFFFFFF
FFFFFFFFFF::FFF:FF::F,FFFFF::FFFFF,FFFFFFFF,FFFF,FF:FFFF:FFFFFF,:FFF
FF,FFFFF::
@A00721:190:HGJW7DSXY:4:1339:12255:29951 1:N:0:TCTGCAAG+GCTGCACT
CCCGCGCCGGGGACACCTCCGTACGCTTACGTGTCGATTTGTCATTAGACCACGCGTCACGATCATAA
CCATCTAAAAAATGAATTCTATAGTGACGCAGTAGAATCGACGGTCAATTTTGAAATTCAAAATTTAT
ATTTATT
+
FFFFFFFFFFFFFFFFFFFFFFFFFFFFFFFFFFFFFFFFFFF,FFFFFFFFFFFFFFFFFFFFFFFF
FFFFFFFFFFFFFFFF:FF:FFFFFFFFFFFFFF:FFFFFFFFFFFFFFFFFFFFFFFFFFFFFFFFF
FFFFFFF
@A00721:190:HGJW7DSXY:4:1363:18954:8641 1:N:0:TCTGCAAG+GCTGCACT
CCCGCGCCGGGGACACCTCCGTACGCTTACGTGTCGATTTGTCATTAGACCACGCGTCACGATCATGA
CCATCTAAAAAAGGAATTCTATAGTGACGCAGTAAAATCGACGGCAGATGATATGTTATATCATCGTC
TTTTTGA
+
:FFFF,FFF:FFFFFFFFFFFF,FFFFFFF:FFF,F:FFFFFFFFF:FFFFFF:FFFFFFFFFFFFFFF
FFFFFFF:FFF,,FF:,F:,FF,FFFFFF:FFFFFFFFF:FFFFFFFFFFF,FFF,FF:FFFFFFFFF
F,F,FFF
@A00721:190:HGJW7DSXY:4:1414:13449:4961 1:N:0:TCTGCAAG+GCTGCACT
CCCGCGCCGGGGACACCTCCGTACGCTTACGTGTCGATTTGTCATTAGACCACGCGTCACGATCATGA
CCATCTAAAAAATGAATTCTATAGTGACGCAGTAAAATCGACGGCAGATGATATGTTATATCATCGTC
TTTTTGA
+
FFFFFFFFFFFFFFFFFFFFFFFFFFFFFFFFFFFFFFFFFFFFFFFFFFFFFFFFFFFFFFFFFFFF
FFFFFFFFFFFFFFFFFFFFFFFFFFFFFFFFFFFFFFFFFFFFFFFFFFFFFFFFFFFFFFFFFFFF
FFFFFFF
@A00721:190:HGJW7DSXY:4:1427:1642:12853 1:N:0:TCTGCAAG+GCTGCACT
CCCGCGCCGGGGACACCTCCGTACGCTTACGTGTCGATTTGTCATTAGACCACGCGTCACGATCATAA
CCATCTAAAAAATGAATTCTATAGTGACGCAGTAGAATCGACGGTCAATTTTGAAATTCAAAATTTAT
ATTTATTGTG
+
FFFFFFFFFFFFFFFFFFFFFFFFFFFFFFFFFFFFFFFFFFFFFFFFFFFFFFFFFFFFFFFFFFFF
FFFFFFFFFFFFFFFFFFF:FFFFFFFFFFFFFFFFFFFFFFFFFFFFFFFFFFFFFFFF:FFFFFF:
F:F::FFFFF
@A00721:190:HGJW7DSXY:4:1442:18403:17268 1:N:0:TCTGCAAG+GCTGCACT
CCCGCGCCGGGGACACCTCCGTACGCTTACGTGTCGATTTGTCATTAGACCACGCGTCACGATCATAA
CCATCTAAAAAATGAATTCTATAGTGACGCAGTAGAATCGACGGTCAATTTTGAAATTCAAAATTTAT
ATTTATTGTGT

```
+
FFFFFFFF:F:FFFF:FF:F:FFFFF,FFFFF:FFFF:FFF,FFF,,FFF:FF,FFFFFF,FFFFFF,
FFFFFFFFFFFF,:FF:FF:F:F:,F,FFFFFFFFFFFFFFFFFFFFFFFFFFFFFFFFFFFF,FF::
:FFFF:F::FF
@A00721:190:HGJW7DSXY:4:1447:22878:28588 1:N:0:TCTGCAAG+GCTGCACT
CCCGCGCCGGGGACACCTCCGTACGCTTACGTGTCGATTTGTCATTAGACCACGCGTCACGATCATGA
CCATCTAAAAAATGAATTCTATAGTGACGCAGTAAAATCGACGGCAGATGATATGTTATATCATCGTC
TTTTTGA
+
FFFFFFFFFFFFFFFFFFFFFFFFF,FFFFFFFFFFFFFF:FFFFFFFFFFFFFF:FFFFF:FFFFF
FFFFFFFFFFFFFF:,FF:F,FFFFFFFFFFFFFFFFFFFFFFFFFFFFFFFF:FFFF,FFFFFFFFFF
FFFFFFF
@A00721:190:HGJW7DSXY:4:1448:14796:13996 1:N:0:TCTGCAAG+GCTGCACT
CCCGCGCCGGGGACACCTCCGTACGCTTACGTGTCGATTTGTCATTAGACCACGCGTCACGATCATAA
CCATCTAAAAAATGAATTCTATAGTGACGCAGTAGAATCGACGGTCAATTTTGAAATTCAAAATTTAT
ATTTATTGTGT
+
FFFFFFFFFFFFFFFFFFFFFFFFFFFFFFFFFFFFFFFF:FFFFFFFFFFFFFFFFFFFF:FFFFF
FFFFFFFFFFFFFFFF:FFFF:FFFFFFFFFFFFFFFFFFFFFFFFFFFFFFFFFFFFFF::FFFFFF
FFFFFFFFFFF
@A00721:190:HGJW7DSXY:4:1512:4869:5541 1:N:0:TCTGCAAG+GCTGCACT
CCCGCGCCGGGGACACCTCCGTACGCTTACGTGTCGATTTGTCATTAGACCACGCGTCACGATCATAA
CCATCTAAAAAATGAATTCTATAGTGACGCAGTAGAATCGACGGTCAATTTTGAAATTCAAAATTTAT
ATTTATTGT
+
FFF:FFFFFFFFFFFFFFFFFFFFFFFFFFFFFFFFFFFF,F,FFFFFF:FFFFFFFFFFFFFF:FF
FFFFFFFFFFF,FF:FFFFFFFFFFFFF,FFFFF,FF:FFFFFFFFFFFFFF:FFFFFFFFFFFFFFF
FFFFFFFFF
@A00721:190:HGJW7DSXY:4:1512:16477:34381 1:N:0:TCTGCAAG+GCTGCACT
CCCGCGCCGGGGACACCTCCGTACGCTTACGTGTCGATTTGTCATTAGACCACGCGTCACGATCATAA
CCATCTAAAAAATGAATTCTATAGTGACGCAGTAGAATCGACGGTCAATTTTGAAATTCAAAATTTAT
ATTTATTGTG
+
FFFFFFFFFFFFFFFFFFFFFFFFFFFFFFFFFFFFFFFFFFFFFFFFFFFFFFFFFFFFFFFFFFFF
FFFFFFFFFFFFFFFFF:FF:,FFFFFFFFFFFFFFFFFFFFFFFFFFFFFFFFFFFFFFFFFFFFFF
F:FFFFFF:F
@A00721:190:HGJW7DSXY:4:1512:18584:35117 1:N:0:TCTGCAAG+GCTGCACT
CCCGCGCCGGGGACACCTCCGTACGCTTACGTGTCGATTTGTCATTAGACCACGCGTCACGATCATAA
CCATCTAAAAAATGAATTCTATAGTGACGCAGTAGAATCGACGGTCAATTTTGAAATTCAAAATTTAT
ATTTATTGTG
+
FFFFFFFFFFFFFFFFFFFFFFFFFFFF:FFFFFFFFFFFFFFFFFFFFFFF:FFFFFFFFFFFFFFFF
FFFFFFFFFFFFFFFFFFFFFFFFFFFFFFFFFFFFFFFFFFFFFFFFFFFFFFFFFFFFFFF:FFFF
FFF,FFFFFF
@A00721:190:HGJW7DSXY:4:1523:32036:18584 1:N:0:TCTGCAAG+GCTGCACT
CCCGCGCCGGGGACACCTCCGTACGCTTACGTGTCGATTTGTCATTAGACCACGCGTCACGATCATGA
CCATCTAAAAAATGAATTCTATAGTGACGCAGTAAAATCGACGGCAGATGATATGTTATATCATCGTC
TTTTTGA
+
FFFFFFF::F:FFFFFFFFFFFFFFFFFFFFFFFFFFFFF:FFFF::FFFFFFF,FFFF:FFFF,F:,F
FFFFFFFFFFFFFFFFFF::FFFFFFFFFFFFFFFFFFF,FFFF,FFFFFFFF:,FFFFFFFFF,FFF
FF:FFFF
@A00721:190:HGJW7DSXY:4:1530:2510:35650 1:N:0:TCTGCAAG+GCTGCACT
CCCGCGCCGGGGACACCTCCGTACGCTTACGTGTCGATTTGTCATTAGACCACGCGTCACGATCATGA
```

```
CCATCTAAAAAATGAATTCTATAGTGACGCAGTAAAATCGACGGCAGATGATATGTTATATCATCGTC
TTTTTGA
+
FFFFFFFFFFFFFFFFFFFFFFFFFFFF:FFFFFFFFFFFF:FF:FFFFFFFFFFFFFFFF:FFFFFF
FFFFF:FFFF::,FFFFFF:F:FFFFFFFFFFFFFFFFFFFFFFFFFFFFFF,FFFF,F:FFFFFFFF
FFF,FFF
@A00721:190:HGJW7DSXY:4:1569:31024:3615 1:N:0:TCTGCAAG+GCTGCACT
CCCGCGCCGGGGACACCTCCGTACGCTTACGTGTCGATTTGTCATTAGACCACGCGTCACGATCATGA
CCATCTAAAAAATGAATTCTATAGTGACGCAGTAAAATCGACGGCAGATGATATGTTATATCATCGTC
TTTTTGA
+
FFFFFFFFFFFFFFFFFFFFFFFFFFFFFFFFFFFFFFFFFFFFFFFFFFFFFFFFFFFFFFFFF:FF
FFFFFFFFFFFFFF:FFFFFFFFFFFFFFFFFFFFFFFFFFFFFFFFFFFFFFFFFFFFFFFFFFFFF
FFFFFFF
@A00721:190:HGJW7DSXY:4:1645:5873:25347 1:N:0:TCTGCAAG+GCTGCACT
CCCGCGCCGGGGACACCTCCGTACGCTTACGTGTCGATTTGTCATTAGACCACGCGTCACGATCATAA
CCATCTAAAAAATGAATTCTATAGTGACGCAGTAGAATCGACGGTCAATTTTGAAATTCAAAATTTAT
ATTTATT
+
FFFFFFFFFFFFFFFFFFFFFFFFFFFFFFFFFFFFFFFFFFFFFFFFFFFFFFFFFFFFFFFFFFFF
FFFFFFFFFFFFFFFFFFFFFFFFFFFFFFFF:FFFFFFFFFFFFFFFFFFFFFFFFFFFFFFFFFFF
FFFFFFF
@A00721:190:HGJW7DSXY:4:1650:3866:34616 1:N:0:TCTGCAAG+GCTGCACT
CCCGCGCCGGGGACACCTCCGTACGCTTACGTGTCGATTTGTCATTAGACCACGCGTCACGATCATAA
CCATCTAAAAAATGAATTCTATAGTGACGCAGTAGAATCGACGGTCAATTTTGAAATTCAAAATTTAT
ATTTATTGTG
+
FFFFFFFFFFFFFFFFFFFFFFFFFFFFFFFFFFFFFFFFFFFFFFFFFFFFFFFFFFFFFFFFFFF:
FFFFFFFFFFFFFFFFFFFFFFFFFFFFFFFFFFFFFFFFFFFFFFFFFFFFFFFFFFFFF:FFFFFF
FFFFFFFFFF
@A00721:190:HGJW7DSXY:4:1663:9842:32534 1:N:0:TCTGCAAG+GCTGCACT
CCCGCGCCGGGGACACCTCCGTACGCTTACGTGTCGATTTGTCATTAGACCACGCGTCACGATCATGA
CCATCTAAAAAATGAATTCTATAGTGACGCAGTAAAATCGACGGCAGATGATATGTTATATCATCGTC
TTTTTGA
+
FFFFFFFFFFFFFFFFFFFFFFFFFFFFFFFFFFFFFFFFFFFFFFFFFFFFFFFFFFFFFFFFFFFF
FFFFFFFFFFFF:FFFFFFFFFFFFFFFFFFFFFFFFFFFFFFFFFFFFFFFFFFFFFFFFFFFFFFF
FFFFFFF
@A00721:190:HGJW7DSXY:4:2138:10447:24565 1:N:0:TCTGCAAG+GCTGCACT
CCCGCGCCGGGGACACCTCCGTACGCTTACGTGTCGATTTGTCATTAGACCACGCGTCACGATCATGA
CCATCTAAAAAATGAATTCTATAGTGACGCAGTAAAATCGACGGCAGATGATATGTTATATCATCGTC
TTTTTGA
+
FFFFFF:FFFFFFFFFFFFFF:FFFFFFFFFFFFFFFFFFFF:F:FFFFFFFFFFFFFFFFFFFFF:F
FFFFFFFFFFFFFFF:FFFFFFFFFFFF:FFFFFFFFFFFFFFFFFFFFFFFFFF,F:FFFFFFFFF,F
F:FFFFF
@A00721:190:HGJW7DSXY:4:2159:14588:17895 1:N:0:TCTGCAAG+GCTGCACT
CCCGCGCCGGGGACACCTCCGTACGCTTACGTGTCGATTTGTCATTAGACCACGCGTCACGATCATAA
CCATCTAAAAAATGAATTCTATAGTGACGCAGTAGAATCGACGGTCAATTTTGAAATTCAAAATTTAT
ATTTATTGTGT
+
FFFFFFFFFFFFFFFFFFFFFFFFFFFFFFFFFFFFFFFFFFFFFFFFFFFFFFFFFFFFFFFFFFFF
FFFFFFFFFFFFFFFFFFFFFFFFFF:FFFFFFFFFFFFFFFFFFFFFFF:FFFFFFFFFFFFFFFFFF
FFFFFFFFFFF
```

```
@A00721:190:HGJW7DSXY:4:2175:12057:9502 1:N:0:TCTGCAAG+GCTGCACT
CCCGCGCCGGGGACACCTCCGTACGCTTACGTGTCGATTTGTCATTAGACCACGCGTCACGATCATAA
CCATCTAAAAAATGAATTCTATAGTGACGCAGTAGAATCGACGGTCAATTTTGAAATTCAAAATTTAT
ATTTATT
+
FFFFFFFFFFFFFFFFFFFFFFFFFFFFFFFFFFFFFFFFFFFFFFFFFFFFFFFFFFFFFFFFFFFFFF
FFFFFFFFFFFFFFFFFFFFFFFFFFFFFFFFFFFFFF:FFFFFFFFFFFFFFFFFFFFFFFFFFFFFFF
FFFFFFF
@A00721:190:HGJW7DSXY:4:2205:8612:13839 1:N:0:TCTGCAAG+GCTGCACT
CCCGCGCCGGGGACACCTCCGTACGCTTACGTGTCGATTTGTCATTAGACCACGCGTCACGATCATAA
CCATCTAAAAAATGAATTCTATAGTGACGCAGTAGAATCGACGGTCAATTTTGAAATTCAAAATTTAT
ATTTATTGTG
+
FFFFFFFFFFFFFFFFFFFFFFFFFFFFFFFFFFFFFFFFFFFFFFFFFFFFFFFFFFFFFFFFFFFFFF
FFFFFFFFFFFFFFFFF:FF,FFFFFFFFFFFFFFFFFFFFF:FFFFFFFF:FFFFFFFFFFFF:FFFFF
FFFFF:FFFF
@A00721:190:HGJW7DSXY:4:2249:4824:21840 1:N:0:TCTGCAAG+GCTGCACT
CCCGCGCCGGGGACACCTCCGTACGCTTACGTGTCGATTTGTCATTAGACCACGCGTCACGATCATGA
CCATCTAAAAAATGAATTCTATAGTGACGCAGTAAAATCGACGGCAGATGATATGTTATATCATCGTC
TTTTTGA
+
FFFFFFFFFFFFFFFFFFFFFFFFFFFFFFFFFFFFFFFFFFFFFFFFFFFFFFFFFFFFFFFFFFFFFF
FFFFFFFFFFFFFFFFFFFFFFFFFFFFFFFFFFFF:FFFFFFFFFFFFFFFFFFFFFFFFFFFFFF:FF
FFFFFFF
@A00721:190:HGJW7DSXY:4:2258:9932:28025 1:N:0:TCTGCAAG+GCTGCACT
CCCGCGCCGGGGACACCTCCGTACGCTTACGTGTCGATTTGTCATTAGACCACGCGTCACGATCATGA
CCATCTAAAAAATGAATTCTATAGTGACGCAGTAAAATCGACGGCAGATGATATGTTATATCATCGTC
TTTTTG
+
FFFFFFFFFFFFFFFFFFFFFFFFFFFFFFFFFFFFFFFFFFFFFFFFFFFFFFFFFFFFFFFFFFFFFF
FFFFFFFFFFFFFFFFFFFFFF,FFFFFFFFFFFFFFFFFFFFFFFFFFFFFF:FFFFFFFFFFFFFFFF
FFFFFF
@A00721:190:HGJW7DSXY:4:2341:32398:22310 1:N:0:TCTGCAAG+GCTGCACT
CCCGCGCCGGGGACACCTCCGTACGCTTACGTGTCGATTTGTCATTAGACCACGCGTCACGATCATGA
CCATCTAAAAAATGAATTCTATAGTGACGCAGTAAAATCGACGGCAGATGATATGTTATATCATCGTC
TTTTTGA
+
FFFFFFFFFFFFFFFFFFFFFFFFFFFFFFFFFFFFFFFFFFFFFFFFFFFFFFFFFFFFFFFFFFFFFF
FFFFFFFFFFFFFFFFFFFFFFFFFFFFFFFFFFFFFFFFFFFFFFFFFFFFFFFFFFFFF:FFFFFFFF
FFFFFFF
@A00721:190:HGJW7DSXY:4:2359:27995:11083 1:N:0:TCTGCAAG+GCTGCACT
CCCGCGCCGGGGACACCTCCGTACGCTTACGTGTCGATTTGTCATTAGACCACGCGTCACGATCATGA
CCATCTAAAAAATGAATTCTATAGTGACGCAGTAAAATCGACGGCAGATGATATGTTATATCATCGTC
TTTTTGA
+
FFFFFFFFFFFFFFFFFFFFFFFFFFFFFFFFFFFFFFFFFFFFFFFFFFFFFFFFFFFFFFF,FFFFFF
FFFFFFFFFFFFFFFFFFFFFFFFFFFFFFFFFFFFFFF:FFFFFFFFFFFFFF:FFFF:FFFFFFFFFF
F,FFFFF
@A00721:190:HGJW7DSXY:4:2365:20329:10488 1:N:0:TCTGCAAG+GCTGCACT
CCCGCGCCGGGGACACCTCCGTACGCTTACGTGTCGATTTGTCATTAGACCACGCGTCACGATCATGA
CCATCTAAAAAATGAATTCTATAGTGACGCAGTAAAATCGACGGCAGATGATATGTTATATCATCGTC
TTTTTGA
+
FFFFFFFFFFFFFFFFFFFFFFFFFFFFFFFFFFFFFF:FFFFFFFFFFFFFFFFFFFFFFFFFFFF:F:
```

FFFFFFF:FFFFFFFFFFFFFFFFFFFFFFFFFFFFFFFFFFFFFFFFFFFFFFFFFFFFFFFFFFF
FFFFFFF
@A00721:190:HGJW7DSXY:4:2445:12762:23688 1:N:0:TCTGCAAG+GCTGCACT
CCCGCGCCGGGGACACCTCCGTACGCTTACGTGTCGATTTGTCATTAGACCACGCGTCACGATCATAA
CCATCTAAAAAATGAATTCTATAGTGACGCAGTAGAATCGACGGTCAATTTTGAAATTCAAAATTTAT
ATTTATTGTG
+
FFFFFFFFFFFFFFFFFFFFFFFFFFFFFFFFFFFFFFFFFFFFFFFFFFFFFFFFFFFFFFFFFFFF
FFFFFFFFFFFFFFFF:FFFFFFFFFFFFFFFFFFFFFFFFFFFFFFFFF:FFFFFFFFFFFFFFFF:
F:::FFFFFF
@A00721:190:HGJW7DSXY:4:2447:6641:25989 1:N:0:TCTGCAAG+GCTGCACT
CCCGCGCCGGGGACACCTCCGTACGCTTACGTGTCGATTTGTCATTAGACCACGCGTCACGATCATAA
CCATCTAAAAAATGAATTCTATAGTGACGCAGTAGAATCGACGGTCAATTTTGAAATTCAAAATTTAT
ATTTATTGTG
+
FFFFFFFFFFFFFFFFFFFFFFFFFFFFFFFFFFFFFFFFFFFFFFFFFFFFFFFFFFFFFFFFFFFF
FFFFFFFFFFFFFFFFFFFFFFFFFFFFFFFFFFFFFFFFFFFFFFFFFFFFFFFFFFFFFFFFFFFF
FFFFFFFF:F
@A00721:190:HGJW7DSXY:4:2450:20003:1376 1:N:0:TCTGCAAG+GCTGCACT
CCCGCGCCGGGGACACCTCCGTACGCTTACGTGTCGATTTGTCATTAGACCACGCGTCACGATCATAA
CCATCTAAAAAATGAATTCTATAGTGACGCAGTAGAATCGACGGTCAATTTTGAAATTCAAAATTTAT
ATTTATTGTG
+
FFFFFFFFFFFFFFFFFFFFFFFFFFFFFFFFFFFFFFFFFFFFFFFFFFFFFFFFFFFFFFFFFFFF
FFFFFFFFFFFFFFFFFFFFFFFFFFF:FFFFFFFFFFFFFFFFFFFFFFFFFFFFFFFFFFF:F:FFFF
FFFFFFFFFF
@A00721:190:HGJW7DSXY:4:2468:5367:7623 1:N:0:TCTGCAAG+GCTGCACT
CCCGCGCCGGGGACACCTCCGTACGCTTACGTGTCGATTTGTCATTAGACCACGCGTCACGATCATAA
CCATCTAAAAAATGAATTCTATAGTGACGCAGTAGAATCGACGGTCAATTTTGAAATTCAAAATTTAT
ATTTATTGTG
+
FFFFFFFFFFFFFFFFFFFFFFFFFFFFFFFFFFFFFFFFFFFFFFFFFFFFFFFFFFFFFFFFFFFF
FFFFFFFFFFFFFFFFFFFFFF:FFFFFFFFFFFFFFFFFFFFFFFFFFFFFFFFF:FF:FFFFFFFFFF
FFFFFFFF:F
@A00721:190:HGJW7DSXY:4:2502:26829:2738 1:N:0:TCTGCAAG+GCTGCACT
CCCGCGCCGGGGACACCTCCGTACGCTTACGTGTCGATTTGTCATTAGACCACGCGTCACGATCATAA
CCATCTAAAAAATGAATTCTATAGTGACGCAGTAGAATCGACGGTCAATTTTGAAATTCAAAATTTAT
ATTTATTGT
+
FFFFFFFFFFFFFFFFFFFFFFFFFFFFFFFFFFFFFFFFFFFFFFFFFFFFFFFFFFFFFFFFFFFF
FFFFFFFFFFFF:FFF:FF,FFFFFFFFFFFFFFFFFFFFFFFFFFFFFFFFFFF:FFFFFFFFFFFFF
FFFFFFF,F
@A00721:190:HGJW7DSXY:4:2517:6894:16908 1:N:0:TCTGCAAG+GCTGCACT
CCCGCGCCGGGGACACCTCCGTACGCTTACGTGTCGATTTGTCATTAGACCACGCGTCACGATCATGA
CCATCTAAAAAATGAATTCTATAGTGACGCAGTAAAATCGACGGCAGATGATATGTTATATCATCGTC
TTTTTGA
+
FFFFFFFFFFFFFFFFFFFFFFFFFFFFFFFFFFFFFFFFFFFFFFFFFFFFFFFFFFFFFFFFFFFF
FFFFFFFFFFFFFF:FFFFFFFFFFFFFF:FFFFFFFFFFFFFFFFFFFFFFFFFFFFFFFFFFFFFFF
FFFFFFF
@A00721:190:HGJW7DSXY:4:2530:30779:5353 1:N:0:TCTGCAAG+GCTGCACT
CCCGCGCCGGGGACACCTCCGTACGCTTACGTGTCGATTTGTCATTAGACCACGCGTCACGATCATGA
CCATCTAAAAAATGAATTCTATAGTGACGCAGTAAAATCGACGGCAGATGATATGTTATATCATCGTC
TTTTTGA

```
+
FFFFFFFFFFFFFFFFFFFFFFFFFFFFFFFFFFFFFFFFFFFFFFFFFFFFFFFFFFFFFFFFFFFFFF
FFFFFFFFFFFF:FFFFFFFFFFFFFFF:FFFFFFFFFFFFFFFFFFFFFFFFFFFFFFFFFFFFFFFFF
FFFFFFF
@A00721:190:HGJW7DSXY:4:2533:5801:32017 1:N:0:TCTGCAAG+GCTGCACT
CCCGCGCCGGGGACACCTCCGTACGCTTACGTGTCGATTTGTCATTAGACCACGCGTCACGATCATGA
CCATCTAAAAAATGAATTCTATAGTGACGCAGTAAAATCGACGGCAGATGATATGTTATATCATCGTC
TTTTTGA
+
FFFFFFFFFFFFFFFFFFFFFFFFFFFFFFFFFFFFF,FFFFFFFFFFFFFFFFFFFFFFFFFFFFFFF
FFFFFFFFFFFFFFFFFFFFFFFFFFFFFFFFFFFFFFFFFFFFFFFFFFFFFFFFFFFFF:FFFFFFF
FFFFFFF
@A00721:190:HGJW7DSXY:4:2543:12491:31735 1:N:0:TCTGCAAG+GCTGCACT
CCCGCGCCGGGGACACCTCCGTACGCTTACGTGTCGATTTGTCATTAGACCACGCGTCACGATCATGA
CCATCTAAAAAATGAATTCTATAGTGACGCAGTAAAATCGACGGCAGATGATATGTTATATCATCGTC
TTTTTGA
+
FFFFFFFFFFFFFFFFFFFFFFFFFFFFFFFFFFFFFFFFFFFFFFFFFFFFFFFFFFFFFFFFFFF:FF
FFFFFFFFFFFFFFFFFFFF:::FFFFFFFFFFFFFFF:FFFF:FFFFFFFFFFFFFFFFFFFFFFFFFFF
FFFFFF:
@A00721:190:HGJW7DSXY:4:2544:4227:36495 1:N:0:TCTGCAAG+GCTGCACT
CCCGCGCCGGGGACACCTCCGTACGCTTACGTGTCGATTTGTCATTAGACCACGCGTCACGATCATGA
CCATCTAAAAAATGAATTCTATAGTGACGCAGTAAAATCGACGGCAGATGATATGTTATATCATCGTC
TTTTTGA
+
FF:F:FFFFF,FFFFFF:F:F:F:FF,FFF:F:FFFF:FFF,FF::F,FF,FFFFFF::FF:FFFF:F
FFFFFFFFFF:F:,:FFF::FF:FFFFF:FFF,F::FFFFFF:FF:,FFFFF,FFF,FFFF,FFFFFF
F,FFFF:
@A00721:190:HGJW7DSXY:4:2545:15881:17096 1:N:0:TCTGCAAG+GCTGCACT
CCCGCGCCGGGGACACCTCCGTACGCTTACGTGTCGATTTGTCATTAGACCACGCGTCACGATCATAA
CCATCTAAAAAATGAATTCTATAGTGACGCAGTAGAATCGACGGTCAATTTTGAAATTCAAAATTTAT
ATTTATTGTG
+
FFFFFFFFFFFFFFFFFFFFFFFFFFF:FFFFFFFFFFFFFFFFF,FFFFFFFFFFFFFFFFFFFFFFFF
FFFFFFFFFFFFFFFFFFFFFFFFFFFFFFFFFFFFFFFFFFFFFFFFFFFFFFF:FFFFFF:FFFFF
F,:,FFFF:F
@A00721:190:HGJW7DSXY:4:2614:5330:18584 1:N:0:TCTGCAAG+GCTGCACT
CCCGCGCCGGGGACACCTCCGTACGCTTACGTGTCGATTTGTCATTAGACCACGCGTCACGATCATGA
CCATCTAAAAAATGAATTCTATAGTGACGCAGTAAAATCGACGGCAGATGATATGTTATATCATCGTC
TTTTTGA
+
FFFFFFFFFFFFFFFFFFFFFFFFFFFF:FFFFFFFFFFFFFFFFFFFFFFFFFFFFFFFFFFFFFFFFF
FFFFFFFFFFFFFFFFFFFFFFFFFFFFFFFFFFFFFFFFFFFFFFFFFFFFFFFFFFFFFFFFFFFFFF
FFFFFFF
@A00721:190:HGJW7DSXY:4:2618:30897:21590 1:N:0:TCTGCAAG+GCTGCACT
CCCGCGCCGGGGACACCTCCGTACGCTTACGTGTCGATTTGTCATTAGACCACGCGTCACGATCATAA
CCATCTAAAAAATGAATTCTATAGTGACGCAGTAGAATCGACGGTCAATTTTGAAATTCAAAATTTAT
ATTTATTGTGT
+
FFFFFFFFFFFFFFFFFFFFFFFFFFFFFFFFFFFFFFFFFFFFFF:FFFFFFFFFFFFFFFFFFFFFFFF
FFFFFFFFFFFFFFFFFFFFFFFFFFFFFFFFFFF:FFFFFFFFFFFFFFFFFFFFFFFFFFFFFF,FF:
FFFFFFFFFFF
@A00721:190:HGJW7DSXY:4:2631:25626:1846 1:N:0:TCTGCAAG+GCTGCACT
CCCGCGCCGGGGACACCTCCGTACGCTTACGTGTCGATTTGTCATTAGACCACGCGTCACGATCATGA
```

```
CCATCTAAAAAATGAATTCTATAGTGACGCAGTAAAATCGACGGCAGATGATATGTTATATCATCGTC
TTTTTGA
+
FFFFFFFFFFFFFFFFFFFFFFFFFFFFFFFFFFFFFFFFFFFFFFFFFFFFFFFFFFFFFFFFFFFF
FFFFFFFFFFFFFFF:FFFFFFFFFFFFFFFFFFFFFFFFFFFFFFFFFFFFFFFFF,FFFFFFFFFF
FFFFFF:
@A00721:190:HGJW7DSXY:4:2677:8793:23265 1:N:0:TCTGCAAG+GCTGCACT
CCCGCGCCGGGGACACCTCCGTACGCTTACGTGTCGATTTGTCATTAGACCACGCGTCACGATCATAA
CCATCTAAAAAATGAATTCTATAGTGACGCAGTAGAATCGACGGTCAATTTTGAAATTCAAAATTTAT
ATTTATTGTGT
+
FFFFFFFFFFFFFFFFFFFFFFFFFFFFFFFFFFFFFFFFFFFFFFFF:FFFFFFFFFFFFFFFFFFF
FFFFFFFFFFFFFFFFFFFFFFFFFFF:FFFFFFFFFFFFFFFFFFFFFFF:FFFFFFFFFFFFFFFF
FFFFF:FFFF:
@A00721:190:HGJW7DSXY:4:2677:8730:24158 1:N:0:TCTGCAAG+GCTGCACT
CCCGCGCCGGGGACACCTCCGTACGCTTACGTGTCGATTTGTCATTAGACCACGCGTCACGATCATAA
CCATCTAAAAAATGAATTCTATAGTGACGCAGTAGAATCGACGGTCAATTTTGAAATTCAAAATTTAT
ATTTATTGTGT
+
FFFFFFFFFF:FFFFFFFFFFFFFFFFFFF:FFFFFFFFFFFFFFFFFFFFFFFFFFFFFFFFFFFFF
FFFF:FFFFFFFFFFFFFF:FFFFF:,FFFFFFFFFFFFFFFFFFFFFFFFFFFF,FFFFFFFFFFF
F:FFFFFFFFF
@A00721:190:HGJW7DSXY:4:1168:13883:7153 1:N:0:TCTGCAAG+GCTGCACT
ACCTCCGTACGCTTACGTGTCGATTTGTCATTAGACCACGCGTCACGATCATAACCATCTAAAAAATG
AAATCTATAGTGACGCAGTAGAATCGACGGTCAATTTTGAAATTCAAAATTTATATTTATTGTGTTGA
TGACTGCTTTAGCAG
+
F,FFFF,FFFFFF,FFFF,FF:FFF:F,,F:FF,F::F,,:FFFF:FFFFF,FFFFF,F:FFFFFFF:
FF,,,FFFF:,:FF::FF:FFFFF:FFF,:,FFF,FF,,FFFF::FFFFFF,F:F,:FF,F,,,FFFF
,,FFFF,,FFF,,FF
@A00721:190:HGJW7DSXY:4:1304:11912:1297 1:N:0:TCTGCAAG+GCTGCACT
ACCTCCGTACGCTTACGTGTCGATTTGTCATTAGACCACGCGTCACGATCATAACCATCTAAAAAATG
AATTCTATAGTGACGCAGTAGAATCGACGGTCAATTTTGAAATTCAAAATTTATATTTATTGTGTTGA
TGACTGCTTTAGCAG
+
FFFFFFFFFFFF:FFFFFFFFFFFFF:FFFFFFFFFFFFFFFFFFFFFFFF:FFFFFFFFFFF::FF
FF:FFFFFFFFF:FFFFFFFFFFFFFFFFFFFFFFFFFFFFFFFFFFFFFFFFFF:FFFF,,FFFFF
FFFFFFFFFFFFFFF
@A00721:190:HGJW7DSXY:4:1622:31665:13056 1:N:0:TCTGCAAG+GCTGCACT
ACCTCCGTACGCTTACGTGTCGATTTGTCATTAGACCACGCGTCACGATCATAACCATCTAAAAAATG
AATTCTATAGTGACGCAGTAGAATCGACGGTCAATTTTGAAATTCAAAATTTATATTTATTGTGTTGA
TGACTGCTTTAGCAG
+
FFFFFFFFFFFFFFFFFFFFFFFFFFFFFFFFFFFFFFFFFFFFFFFFFFFFFFFFFFFFFFFF:FFF
:FFFFFFFFFFF:FFFFFFFFFFFFFFFFFFFFFFFFFFFFFFFFFFF:FF:FFFFFFFFFFFFFFFFFF
FFFFFFFFFF:F:FF
@A00721:190:HGJW7DSXY:4:2168:15510:14512 1:N:0:TCTGCAAG+GCTGCACT
ACCTCCGTACGCTTACGTGTCGATTTGTCATTAGACCACGCGTCACGATCATAACCATCTAAAAAATG
AATTCTATAGTGACGCAGTAGAATCGACGGTCAATTTTGAAATTCAAAATTTATATTTATTGTGTTGA
TGACTGCTTTAGCAG
+
FFFFFFFFFFFFFFFFFF:FFFFFF::FFFFFFFFF:FFFFFFFFFFFFFFFFFFFFFFFFFFF,FFFF
FFFFFFF:FFF:FFFFFFFFFFF:FFFFFFFFFFF:FFFFFFFFFFFFFFFFF:FFFFF:FFFF:FFF
FFF:FFFFFFF:F,F
```

```
@A00721:190:HGJW7DSXY:4:2611:14977:25520 1:N:0:TCTGCAAG+GCTGCACT
ACCTCCGTACGCTTACGTGTCGATTTGTCATTAGACCACGCGTCACGATCATAACCATCTAAAAAATG
AATTCTATAGTGACGCAGTAGAATCGACGGTCAATTTTGAAATTCAAAATTTATATTTATTGTGTTGA
TGACTGCTTTAGCAG
+
FFFFFFFFFFFF:FFFFFFFFFFFFFFFFFFFFFFFFFFFFFFFFFFFFFFFFFFFFFFFFFFFFFFFFF:FF
FFFFFFFFFFFFFFFFFFFFFFFFFFF:FFFFFFFFFFFFFFF:FFFFFFFFFF:F,FFFFFFFFFFFF:
F::FFFFFFFFFFFF
@A00721:190:HGJW7DSXY:4:2340:11767:31172 1:N:0:TCTGCAAG+GCTGCACT
CCTCTGTACGCTTACGTGTCGATTTGTCATTAGACCACGCGTCACGATCATGACCATCTAAAAAATGA
ATTCTATAGTGACGCAGTAAAATCGACGGCAGATGATATGTTATATCATCGTCTTTTTGATAACCAAT
AGACTGTTAAAAGGT
+
FFFFFFFFFFFFFFFFFFFFFFFFFFFFFFFFFFFFFFFFFFFFFFFFFFFFFFFFFFFFFFFFF:FFFF
FFFF:F:FFFF:FFFFFFFFFFFFFFFF,FFFFFFF:FFFFFFFFFFFFFF::FFFFFFFFFFF:FFFFF
F,FFFFFFFFFFFFF
@A00721:190:HGJW7DSXY:4:1370:4544:5729 1:N:0:TCTGCAAG+GCTGCACT
CTCCGTACGCTTACGTGTCGATTTGTCATTAGACCACGCGTCACGATCATGACCATCTAAAAAATGAA
TTCTATAGTGACGCAGTAAAATCGACGGCAGATGATATGTTATATCATCGTCTTTTTGATAACCAATA
GACTGTTAAAAGGTC
+
FFFFFFFFFFFFFFFFFFFFFFFFFFFFFFFFFFFFFFFFFFFFFFFFFFFFFFFFFFFFFFFFFFFFFF
:FF::FFFFFFFFFFFFFFF:FFFFFFFFFFFFFFF:FFFFFFFFFFFFFFFFFF::FFFFF:FFFFFFF
FFFFFFFF::FFFFF
@A00721:190:HGJW7DSXY:4:1436:24496:15890 1:N:0:TCTGCAAG+GCTGCACT
CTCCGTACGCTTACGTGTCGATTTGTCATTAGACCACGCGTCACGATCATGACCATCTAAAAAATGAA
TTCTATAGTGACGCAGTAAAATCGACGGCAGATGATATGTTATATCATCGTCTTTTTGATAACCAATA
GACTGTTAAAAGGTC
+
FFFFFFFFFFFFFFFFFFFFFFFFFFFFFFFFFFFFFFFFFFFFFFFFFFFFFFFFFFFFFFFFFFFFFF
FFFFFFFFFFFFFF:FFFFFFFFFFFFFFFFFFFFFFFFFFFFFFFFFFFFFFFFFFFFFFFFFFFFFFF
FFFFFFFFFF:FFFF
@A00721:190:HGJW7DSXY:4:1445:1597:29810 1:N:0:TCTGCAAG+GCTGCACT
CTCCGTACGCTTACGTGTCGATTTGTCATTAGACCACGCGTCACGATCATGACCATCTAAAAAATGAA
TTCTATAGTGACGCAGTAAAATCGACGGCAGATGATATGTTATATCATCGTCTTTTTGATAACCAATA
GACTGTTAAAAGGTC
+
FFFF:FFFFFFFFFFFFFFFFFFFFFFFFFFF:FFFFFFFFF,F:FFF:F:FFFFFF:F:FFFF:FFFFF
,FFF,FFFFFFFFFFFFF:FFFFF:FFFFFFF:F,:F,F:FFFFFFFFFFFFFFF:FFFFFF::FFFFF:
:F:FFFFFF:,:FFF
@A00721:190:HGJW7DSXY:4:1478:26503:16423 1:N:0:TCTGCAAG+GCTGCACT
CTCCGTACGCTTACGTGTCGATTTGTCATTAGACCACGCGTCACGATCATGACCATCTAAAAAATGAA
TTCTATAGTGACGCAGTAAAATCGACGGCAGATGATATGTTATATCATCGTCTTTTTGATAACCAATA
GACTGTTAAAAGGTC
+
FFFFFFFFFFFFFFFFFFFFFFFF,FFFFFFFFFFFFFFFFFFFFFFFFFFFFFFFFFFFFFFFFFF:FF
FFFFF:FF:FFFFFFFFFFFFFF,FFF:FFFF:F:FFFFFF:FFFFF:FFFFFFFFFFFF:FF:FFFFF
FFFF,F:FFFFF:FF
@A00721:190:HGJW7DSXY:4:1627:11379:36229 1:N:0:TCTGCAAG+GCTGCACT
CTCCGTACGCTTACGTGTCGATTTGTCATTAGACCACGCGTCACGATCATGACCATCTAAAAAATGAA
TTCTATAGTGACGCAGTAAAATCGACGGCAGATGATATGTTATATCATCGTCTTTTTGATAACCAATA
GACTGTTAAAAGGTC
+
FFFFFFFFFFFFFFFFFFFFFFFFFFFFFFFFFFFFFFFFFFFFFFFFFFFFFFFFFFFFFFFFFFFFF:
```

FF:FFFFFFFFFFFFFFFFFFFFFFFFFFFFFFFFFF:FFFFFFFFFFFFF:FF:FFFFFFFFFFFF:
FFFFFFFFFFFFFFF
@A00721:190:HGJW7DSXY:4:2125:6325:8907 1:N:0:TCTGCAAG+GCTGCACT
CTCCGTACGCTTACGTGTCGATTTGTCATTAGACCACGCGTCACGATCATGACCATCTAAAAAATGAA
TTCTATAGTGACGCAGTAAAATCGACGGCAGATGATATGTTATATCATCGTCTTTTTGATAACCAATA
GACTGTTAAAAGGTC
+
FFFFFFFFFFFFFFFFFFFFF:FFFFFFFFFFFFFFFFFFFFFFFFFFFFFFFFFFFFFFFFF:FFFF
FFF:,FFFFFFFFF:FFF:FFFFFFFFFFFFFFFFFFFFFFFFFFFFFFFFFFF:FFFFFFFFFFFFF
FFFFFFFFFFFFFFF
@A00721:190:HGJW7DSXY:4:2160:17815:16313 1:N:0:TCTGCAAG+GCTGCACT
CTCCGTACGCTTACGTGTCGATTTGTCATTAGACCACGCGTCACGATCATGACCATCTAAAAAATGAA
TTCTATAGTGACGCAGTAAAATCGACGGCAGATGATATGTTATATCATCGTCTTTTTGATAACCAATA
GACTGTTAAAAGGTC
+
FFFFFFFFFFFFFFFFFFFFFFFFFFFFFFFFFFFFFFFFFFFFFFFFFFFFFFFFFFFFFFFFFFFF
FFFFFFFFFFFFFFFFFFFFFFFFFFFFFFFFFFFFFFFFFFFFFFFFFFFFFFFFFFFFFFFFFFFF
FFFFFFFFFFFFFFF
@A00721:190:HGJW7DSXY:4:2547:23692:10645 1:N:0:TCTGCAAG+GCTGCACT
CTCCGTACGCTTACGTGTCGATTTGTCATTAGACCACGCGTCACGATCATGACCATCTAAAAAATGAA
TTCTATAGTGACGCAGTAAAATCGACGGCAGATGATATGTTATATCATCGTCTTTTTGATAACCAATA
GACTGTTAAAAGGTC
+
FFFFFFFFFFFFFFFFFFFFFFFFFFF:FFFFFFFFFFFFFFFFFFFFFFFFFFFFFFFFFFFFFFFF
:FFFF:FFFFFFFFFFF:FFFFFFFFFFFFFFFFFFFFFFFFFFFFFFFFFFFFFFFFFFFFFFFFFF
FFFFFFFF:F:FFFF
@A00721:190:HGJW7DSXY:4:1153:20030:5838 1:N:0:TCTGCAAG+GCTGCACT
ACGCTTACGTGTCGATTTGTCATTAGACCACGCGTCACGATCATAACCATCTAAAAAATGAATTCTAT
AGTGACGCAGTAGAATCGACGGTCAATTTTGAAATTCAAAATTTATTGTGTTGATGACTGCTTTAGCA
GTTGATTGATGGATT
+
FFFFFFFFFFFFFFFFFFFFFFFFFFFFFFFFFFFFFFFFFFFFFFFFFFFF:FFFFFFFFFFFFFF,F
FFFFFFFFFFFFFFFFFFFFFFFFFFFFFFFFFFFFFFFFFFFFFFFFF:FFFF,FFFFFFFFFFFFFF
FFFFFFFFFFFFFFF
@A00721:190:HGJW7DSXY:4:1309:18295:35462 1:N:0:TCTGCAAG+GCTGCACT
ACGCTTACGTGTCGATTTGTCATTAGACCACGCGTCACGATCATAACCATCTAAAAAATGAATTCTAT
AGTGACGCAGTAGAATCGACGGTCAATTTTGAAATTCAAAATTTATTGTGTTGATGACTGCTTTAGCA
GTTGATTGATGGATT
+
FFFFFFFFFFFFFFFFFFFFFFFFFFFFFFFFFFFFFFFFFFFFFFFFFFFFFFFFFF,FFFFFFFFFF
FFFFFFFFFFFFFFFFFFFFFFFFFFFFFFFFFFFFFFFFFFFFFFFFFFFFFFFFFFFFFFFFFFFF
FF::FFFFFFF:FFF
@A00721:190:HGJW7DSXY:4:1331:21667:36417 1:N:0:TCTGCAAG+GCTGCACT
ACGCTTACGTGTCGATTTGTCATTAGACCACGCGTCACGATCATAACCATCTAAAAAATGAATTCTAT
AGTGACGCAGTAGAATCGACGGTCAATTTTGAAATTCAAAATTTATTGTGTTGATGACTGCTTTAGCA
GTTGATTGATGGATT
+
FFFFFFFFFFFFFFFFFFFFFFFFFFFFFFFFFFFFFFFFFFFFFFFFFFFFFFFFFFFFFFFFFFFF
FFFFFFFFFFFFFFFFFFFFFFFFFFFFFFFFFFFFFFF:FFFFFFFFFFFFFFFFFFFFFFFFFFFF
F:FFFFFFFFFFFFF
@A00721:190:HGJW7DSXY:4:1332:32678:4789 1:N:0:TCTGCAAG+GCTGCACT
ACGCTTACGTGTCGATTTGTCATTAGACCACGCGTCACGATCATAACCATCTAAAAAATGAATTCTAT
AGTGACGCAGTAGAATCGACGGTCAATTTTGAAATTCAAAATTTATTGTGTTGATGACTGCTTTAGCA
GTTGATTGATGGATT

```
+
FFFFFFFFFF:FFFFFFFFFFFFFFFFFFFFFFFFFFFF,FFF:FFFF:F:,FFFFFF,FFFF:FFFF:
FFFFFFFFFFFFFFFFFFFF,FFFFFFFFFFF:FF,FF::FFF:,F:FFFF:FFFFF,FFFFFFF:FF
FFFF:FFFFFFFFFF
@A00721:190:HGJW7DSXY:4:2271:13015:4210 1:N:0:TCTGCAAG+GCTGCACT
ACGCTTACGTGTCGATTTGTCATTAGACCACGCGTCACGATCATAACCATCTAAAAAATGAATTCTAT
AGTGACGCAGTAGAATCGACGGTCAATTTTGAAATTCAAAATTTATTGTGTTGATGACTGCTTTAGCA
GTTGATTGATGGATT
+
FFFFFFFFFFFFFFFFFFFFFFFFFFFFFFFFFFFFFFFFFFFFFFFFFFFFFFFFFFFFFFFFFFF:
FFFFFFFFFFFFFFFFFFFFFFFFFFFFFFFFFFFFFFFF:FFFFFFFFFFFFFFFFFFFFFFFFFFF
F:FFFFFF:FFFFFF
@A00721:190:HGJW7DSXY:4:2642:29559:11475 1:N:0:TCTGCAAG+GCTGCACT
ACGCTTACGTGTCGATTTGTCATTAGACCACGCGTCACGATCATAACCATCTAAAAAATGAATTCTAT
AGTGACGCAGTAGAATCGACGGTCAATTTTGAAATTCAAAATTTATTGTGTTGATGACTGCTTTAGCA
GTTGATTGATGGATT
+
FFFFFFFFFFFFFFFFFFFFFFFFFFFFFFFFFFFFFFFFFFFFFFFFFFFFFFFFFFFFFFFFFFFF
FFFFFFFFFFFFFFFFFFFFFFFFFFFFFFFFFFFFFFFFFFFFFFFFFFFFFFFFFFFFFFFFFFFF
FFFFFFFFFF:FFFF
@A00721:190:HGJW7DSXY:4:1249:23041:12587 1:N:0:TCTGCAAG+GCTGCACT
GCTTACGTGTCGATTTGTCATTAGACCACGCGTCACGATCATAACCATCTAAAAAATGAATTCTATAG
TGACGCAGTAGAATCGACGGTCAATTTTGAAATTCAAAATTTATATTTATTGTGTTGATGACTGCTTT
AGCAGTTGATTGATG
+
:FFFFFFFFFFFFFFFFFFFFFFFFFFFFFFFFFFFFFFFFFFFFFFFFFFFFFFFFFFFFFFFFFFF
FFFFFFFFFFFFFFFFFFFFFFFFFFFFFFFFFFFFFFFFFFFFFFFFF:FFFFFFFFFFFF:FFFFFFFF
F:FFFFF:FFFFFFF
@A00721:190:HGJW7DSXY:4:1253:8983:29042 1:N:0:TCTGCAAG+GCTGCACT
GCTTACGTGTCGATTTGTCATTAGACCACGCGTCACGATCATAACCATCTAAAAAATGAATTCTATAG
TGACGCAGTAGAATCGACGGTCAATTTTGAAATTCAAAATTTATATTTATTGTGTTGATGACTGCTTT
AGCAGTTGATTGATG
+
FFFFFFFFFFFFFFFFFFFFFFFFFFFFFFFFFFFFFFFFFFFFFFFFFFFFFFFFFFFFFFFFFFFF
FF:FFFFFFFFFFFF:FFFFFFFFFFFFFFFFFFFFFFFFFFFFF:FFFFFFFFFFFF:FF:FFFFFF
FFFFF:FFFFFFFFF
@A00721:190:HGJW7DSXY:4:1253:8802:29074 1:N:0:TCTGCAAG+GCTGCACT
GCTTACGTGTCGATTTGTCATTAGACCACGCGTCACGATCATAACCATCTAAAAAATGAATTCTATAG
TGACGCAGTAGAATCGACGGTCAATTTTGAAATTCAAAATTTATATTTATTGTGTTGATGACTGCTTT
AGCAGTTGATTGATG
+
FFFFFFFFFFFFFFFFFFFFFFFFFFFFFFFFFFFFFFFFFFFFFFFFFFFFFFFFFFFF:FFFFFFFF
FFFF:FFFFFFFFFFFFFFFFFFFFFFFFFFFFFFFFFFFFFFFFF:F:FFFFF:FFFFFFFFFFFFFFF
FFFFFFFFFFFFFFF
@A00721:190:HGJW7DSXY:4:1430:27127:3192 1:N:0:TCTGCAAG+GCTGCACT
GCTTACGTGTCGATTTGTCATTAGACCACGCGTCACGATCATAACCATCTAAAAAATGAATTCTATAG
TGACGCAGTAGAATCGACGGTCAATTTTGAAATTCAAAATTTATATTTATTGTGTTGATGACTGCTTT
AGCAGTTGATTGATG
+
FFFFFFFFFFFFFFFFFFFFFFFFFFFFFFFFFFFFFFFFFFFFFFFFFFFFFFFFFFFF::FFFFFFF
FFFFFFFFFFFFFFFFFFFFFFFFFFFFFFF:FFFFF:FFF:FFFFFFFFF:F:FF:FFFFFFFFFFF
FFFFFF:FF:FFFFF
@A00721:190:HGJW7DSXY:4:1457:4101:3427 1:N:0:TCTGCAAG+GCTGCACT
GCTTACGTGTCGATTTGTCATTAGACCACGCGTCACGATCATAACCATCTAAAAAATGAATTCTATAG
```

TGACGCAGTAGAATCGACGGTCAATTTTGAAATTCAAAATTTATATTTATTGTGTTGATGACTGCTTT
AGCAGTTGATTGATG
+
FFFFFFFFFFFFFFFFFFFFFFFFFFFFFFFFFFFFFFFFFFFFFFFFFFFFFFFFFFFFFF:FFFFFFF
FFFFFFFFFFFFFFFFFFFFFFFFFFFFFFFFFFFFFFFFFFFF,F,F:F:FFFFFFFFFFFFFFFFFFF
FFFFFFFFFFFFFFF
@A00721:190:HGJW7DSXY:4:1619:11785:6151 1:N:0:TCTGCAAG+GCTGCACT
GCTTACGTGTCGATTTGTCATTAGACCACGCGTCACGATCATAACCATCTAAAAAATGAATTCTATAG
TGACGCAGTAGAATCGACGGTCAATTTTGAAATTCAAAATTTATATTTATTGTGTTGATGACTGCTTT
AGCAGTTGATTGATG
+
FFFFFFFFFFFFFFFFFFFFFFFFFFFFFFFFFFFFFFFFFFFFFFFFFFFFFFFFFFFFFF:FFFFFFF
FFFFFFFFFFFFFFFFFFFFFFFFFFFFFFFFFFFFFFFFFFFFFFF,FFFFF:FFFFFFFFFFFF:FFF
FF:FFFFFFFFFFFF
@A00721:190:HGJW7DSXY:4:2108:15917:19946 1:N:0:TCTGCAAG+GCTGCACT
GCTTACGTGTCGATTTGTCATTAGACCACGCGTCACGATCATAACCATCTAAAAAATGAATTCTATAG
TGACGCAGTAGAATCGACGGTCAATTTTGAAATTCAAAATTTATATTTATTGTGTTGATGACTGCTTT
AGCAGTTGATTGATG
+
FFFFFFFFFFFFFFFFFFFFFFFFFFFFFFF:FFFFFFFFFFFFFFFFFFF:FFFFFFFFFFFFFFFFFF
FFFFFFFFFFFFFFFFFFFFFFFFFFFFFFFFFFFF,FFFF,FFFFFFFFF,FF,FFFFFFFF:FFFFFF
FFF:FF,F:FFF:FF
@A00721:190:HGJW7DSXY:4:2132:10755:12164 1:N:0:TCTGCAAG+GCTGCACT
GCTTACGTGTCGATTTGTCATTAGACCACGCGTCACGATCATAACCATCTAAAAAATGAATTCTATAG
TGACGCAGTAGAATCGACGGTCAATTTTGAAATTCAAAATTTATATTTATTGTGTTGATGACTGCTTT
AGCAGTTGATTGATG
+
FFFFFFFFFFFFFFFFFFFFFFFFFFFFFFFFFFFFFFFFFFFFFFFFFFFFFFFFFFFFFFFFFFFFFF
FFFFFFFFFFFFFFFFFFFFFFFFFFFFFFFFFFFFFFFFFFFFFFFFFFFFFF:FFFF:FFFFFFFFFF
FF:FFFFFFFFFFFF
@A00721:190:HGJW7DSXY:4:2138:16523:35243 1:N:0:TCTGCAAG+GCTGCACT
GCTTACGTGTCGATTTGTCATTAGACCACGCGTCACGATCATAACCATCTAAAAAATGAATTCTATAG
TGACGCAGTAGAATCGACGGTCAATTTTGAAATTCAAAATTTATATTTATTGTGTTGATGACTGCTTT
AGCAGTTGATTGATG
+
FFF,FFFF::FFF:FF:FFFF:FFFF:FFFFFFFFFFFFFF:FFFFFFFFFFFFFFF:F:FF,FFF:F:F
FFFFFFFFFFFFFFFFFF,FFFFFFFFFFFFFFFFFFFFF:FFFFF,FFFFFFFFF:FFFFFF,F,FFFF
FF:FFF,::,FFFFF
@A00721:190:HGJW7DSXY:4:1315:15691:1924 1:N:0:TCTGCAAG+GCTGCACT
CTTACGTGTCGATTTGTCATTAGACCACGCGTCACGATCATAACCATCTAAAAAATGAATTCTATAGT
GACGCAGTAGAATCGACGGTCAATTTTGAAATTCAAAATTTATATTTATTGTGTTGATGACTGCTTTA
GCAGTTGATTGATGG
+
FFFFFFFFFFFFFFFFFFFFFFFFFFFFFFFFFFFFFFFFFFFFFFFFFFFFFFFFFF:FF:FFFFFFFF
F:F:FFFFFFFFFFFFFFF:FFFFFFFFFFFFFFFFFFFFFFFFFFFFFFFFFFFFFFFFFFFFFFFFFF
FF:FFFFFFFFFFFF
@A00721:190:HGJW7DSXY:4:1350:24749:14544 1:N:0:TCTGCAAG+GCTGCACT
CTTACGTGTCGATTTGTCATTAGACCACGCGTCACGATCATAACCATCTAAAAAATGAATTCTATAGT
GACGCAGTAGAATCGACGGTCAATTTTGAAATTCAAAATTTATATTTATTGTGTTGATGACTGCTTTA
GCAGTTGATTGATGG
+
FFFFFFFFFFFFFFFFFFFFFFFFFFFFFFFFFFFFFFFFFFFFFFFFFFFFFFFFFFFFFFFFFFFFFF
FFFFFFFFFFFFFFFFFFFFFFFFFFFFFFFFFFFFFFFFFFFFFFFFFFFFFFFFFFFFFFFFFFFFFF
FFFFFFFFFFFFFFF

```
@A00721:190:HGJW7DSXY:4:1370:28058:17801 1:N:0:TCTGCAAG+GCTGCACT
CTTACGTGTCGATTTGTCATTAGACCACGCGTCACGATCATAACCATCTAAAAAATGAATTCTATAGT
GACGCAGTAGAATCGACGGTCAATTTTGAAATTCAAAATTTATATTTATTGTGTTGATGACTGCTTTA
GCAGTTGATTGATGG
+
FFFFFFFFFFFFFFFFFFFFFFFFFFFFFFFFFFFFFFFF:FFFF:FFFFFFFF:FFFFFFFFFF:FF
FFFFFFFF:FFFFFFFFFFFFFFFFFFFF:FFFFFFFF:FF:FF:,FFFFFFFFFFFFFFFFFFFFFF
FFFFFFFFFFF:FFF
@A00721:190:HGJW7DSXY:4:1531:6090:35931 1:N:0:TCTGCAAG+GCTGCACT
CTTACGTGTCGATTTGTCATTAGACCACGCGTCACGATCATAACCATCTAAAAAATGAATTCTATAGT
GACGCAGTAGAATCGACGGTCAATTTTGAAATTCAAAATTTATATTTATTGTGTTGATGACTGCTTTA
GCAGTTGATTGATGG
+
FFFFFFFFFFFFFFFFFFFFFFFFFFFFFFFFFFFFFFFFFFFFFFFFFFFFFFFFFFFFFFF:FFFF
FFF:FFFFFFFFFFFFFFFFFFFFFFFFFFFFFFFFFFFFFFFF:FFFFFF:F:FFFFFFFFFFFFFF
FFFFFFFFFFFFFFF
@A00721:190:HGJW7DSXY:4:2322:10881:19366 1:N:0:TCTGCAAG+GCTGCACT
CTTACGTGTCGATTTGTCATTAGACCACGCGTCACGATCATAACCATCTAAAAAATGAATTCTATAGT
GACGCAGTAGAATCGACGGTCAATTTTGAAATTCAAAATTTATATTTATTGTGTTGATGACTGCTTTA
GCAGTTGATTGATGG
+
FFFFFFFFFFFFFFFFFFFFFFFFFFFFFFFFFFFFFFFFFFFFFFFFFFFFFFFFFFFFFFFFFFFF
F:FFFFFFFFFFFFFFFFFFFFFFFFFFFFFFFFFFFFFFFFFFFFFFFF:FFFFFFFFFFFFFFFFF
F:FFFFFF:FFFFFF
@A00721:190:HGJW7DSXY:4:2475:30544:13651 1:N:0:TCTGCAAG+GCTGCACT
CTTACGTGTCGATTTGTCATTAGACCACGCGTCACGATCATAACCATCTAAAAAATGAATTCTATAGT
GACGCAGTAGAATCGACGGTCAATTTTGAAATTCAAAATTTATATTTATTGTGTTGATGACTGCTTTA
GCAGTTGATTGATGG
+
FFFFFFFFFFFFFFFFFFFFFFFFFFFFFFFFFFFFFFFFFFFFFFFFFFFFFFFFFFFFFFFFFFFF
FFFFFFFFFFFFFFFFFF:FFFFFFFFFF:FFFFFFFFFFFFFF,FFFFFFFFFFFFFF:FFFFFFFF
FFFFFFFFFFFFFFF
@A00721:190:HGJW7DSXY:4:2615:25337:20729 1:N:0:TCTGCAAG+GCTGCACT
CTTACGTGTCGATTTGTCATTAGACCACGCGTCACGATCATAACCATCTAAAAAATGAATTCTATAGT
GACGCAGTAGAATCGACGGTCAATTTTGAAATTCAAAATTTATATTTATTGTGTTGATGACTGCTTTA
GCAGTTGATTGATGG
+
FFFFFFFFFFFFFFFFFFFFFFFFFFFFFFFFFFFFF:FFFFFFFFFFFFFFFFFFFFFFFFFF:FFF
FFFFFFFFF:FFFFFFFFFFFFFFFFFFF,FFFFFFFFFFF,FFF:FFFFFFFFFFFFFFFFFF,FFF
::FF:FF,FFFFFFF
@A00721:190:HGJW7DSXY:4:1146:27317:25567 1:N:0:TCTGCAAG+GCTGCACT
TCTATAGTGACGCAGTAAAATCGACGGCAGATGATATGTTATATCATCGTCTTTTTGATAACCAATAG
ACTGTTAAAAGGTCTATATAAATGGCTATGCCATTTATCTTTTGAATGTTATTTCGTAATTTATCTAT
GAATTACGCGTTAAT
+
FFFFFFFFF:FFFFFFFFFFFFFFFFFFFF:FFFF:FFFFFFFFFFFFFFFFFFFFFFFFFFFFFFFF
FFFFFFFFFFFFFFFFFFFFFFF:FFF:FFFFFFFFFFFFFFFFFF:FFFFFFFF,FFF,FFFFFFFF
FFFFFF,FF:FFFFF
@A00721:190:HGJW7DSXY:4:1245:21106:12493 1:N:0:TCTGCAAG+GCTGCACT
TCTATAGTGACGCAGTAAAATCGACGGCAGATGATATGTTATATCATCGTCTTTTTGATAACCAATAG
ACTGTTAAAAGGTCTATATAAATGGCTATGCCATTTATCTTTTGAATGTTATTTCGTAATTTATCTAT
GAATTACGCGTTAAT
+
FFFFFFFFFFFFFFFFFFFFFFFFFFFFFFFFFFFFFFFFFFFFFFFFFFFFFFFFFFFFFFFFFFFF
```

```
FFFFFFFFFFFFFFFFFFFFFFFFFFF,FFFFF:FFFFFFFFFFFFFFFFFFFFFFFFFFFFFFFFFF
FFFFFFFFF:FFFFF
@A00721:190:HGJW7DSXY:4:1265:26829:21151 1:N:0:TCTGCAAG+GCTGCACT
TCTATAGTGACGCAGTAAAATCGACGGCAGATGATATGTTATATCATCGTCTTTTTGATAACCAATAG
ACTGTTAAAAGGTCTATATAAATGGCTATGCCATTTATCTTTTGAATGTTATTTCGTAATTTATCTAT
GAATTACGCGTTAAT
+
FFFFFFFFF:FFFFFFFFFFFFFFFFFFFFFFFFFFFFFFFFFFFFFFFFFFFFFFFFFFFFFFFFFF
FFFFFFFF::FFFFFFFFFFFFFFFFFF:FFF:FFFFFFF:FFFFFFFF:FFFFFFFFFFFFFFFFFF
FFF:FFF:FFFFF:F
@A00721:190:HGJW7DSXY:4:1424:8097:25473 1:N:0:TCTGCAAG+GCTGCACT
TCTATAGTGACGCAGTAAAATCGACGGCAGATGATATGTTATATCATCGTCTTTTTGATAACCAATAG
ACTGTTAAAAGGTCTATATAAATGGCTATGCCATTTATCTTTTGAATGTTATTTCGTAATTTATCTAT
GAATTACGCGTTAAT
+
FFFFFFFFFFFFFFFFFFFFFFF:FFFFFFFFFFFFFFFFFFFFFFFFFFFFFFFFFFFFFFFFFFFF
FFFFFFFFFFFFFFFFFFFFFFFFFFFFFFFFFFFFFFFF:FFFFFFFFFFFFFF:FFFFFFFFFFFF
FFFFFFFFFFFFFFF
@A00721:190:HGJW7DSXY:4:1443:12915:18944 1:N:0:TCTGCAAG+GCTGCACT
TCTATAGTGACGCAGTAAAATCGACGGCAGATGATATGTTATATCATCGTCTTTTTGATAACCAATAG
ACTGTTAAAAGGTCTATATAAATGGCTATGCCATTTATCTTTTGAATGTTATTTCGTAATTTATCTAT
GAATTACGCGTTAA
+
:FFFFFF:FFFFFFFFFFFFFFFFFFFFFFFFFFFFFFFFFFFFFFFFFFF:FFFFFFFFFFFFFFFF
FFFFF:FFFFFFFFFFFFFFFFFFFFFFFF,FF:FFFF:FFFFFFFF:FFFFFFFFFFFFFF:F:FFF
FFF:FFFF,F::FF
@A00721:190:HGJW7DSXY:4:1618:30879:28072 1:N:0:TCTGCAAG+GCTGCACT
TCTATAGTGACGCAGTAAAATCGACGGCAGATGATATGTTATATCATCGTCTTTTTGATAACCAATAG
ACTGTTAAAAGGTCTATATAAATGGCTATGCCATTTATCTTTTGAATGTTATTTCGTAATTTATCTAT
GAATTACGCGTTAAT
+
FFFFFFFFFFFFFFFFFFFFFFFFFFFFFFFFFFFFFFFFFFFFFFFFFFF:F:FFFFFFFFFFFFFF
FFFFFFFFFFFFFFFFFFFFFF:FFFFFFFFFF,FFFFFFFFFFFFFFFFFFFFFFFFFFFFFFFFFF
FFF:FFFFFFFFFFF
@A00721:190:HGJW7DSXY:4:1622:16179:32957 1:N:0:TCTGCAAG+GCTGCACT
TCTATAGTGACGCAGTAAAATCGACGGCAGATGATATGTTATATCATCGTCTTTTTGATAACCAATAG
ACTGTTAAAAGGTCTATATAAATGGCTATGCCATTTATCTTTTGAATGTTATTTCGTAATTTATCTAT
GACTTACGCGTTAAT
+
FF,FFFF:FFFFFFF,:FFFFFFFFFFFFFFF:FFFFF:,FF::FF:FFFFFFFF:FF:FFFFFF,FF
FFFFFFFFFFFFFFFFFF:FFFF:FFFFFFFFFFFF:F:FFFFFFFFF,FF:FFFFFFFF,FFFF:F,
:F,F,F:F:FFF:FF
@A00721:190:HGJW7DSXY:4:2363:23728:20447 1:N:0:TCTGCAAG+GCTGCACT
TCTATAGTGACGCAGTAAAATCGACGGCAGATGATATGTTATATCATCGTCTTTTTGATAACCAATAG
ACTGTTAAAAGGTCTATATAAATGGCTATGCCATTTATCTTTTGAATGTTATTTCGTAATTTATCTAT
GAATTACGCGTTAAT
+
FF:FFFFFFFFFFFFFFFFFFFFFFFFFFFFFFFFFFFFFFFFFFFFFFFFF:FFFFFFFFFFFFFFF
FFFFFFFFFFFFFFFFFFFFFF:FFFFFFFFFF:FFFFFFFFFFFFFFFFFFFFFFFFFFFFFF:FFF
FFFFF:FFFFFFFFF
@A00721:190:HGJW7DSXY:4:2376:20473:17378 1:N:0:TCTGCAAG+GCTGCACT
TCTATAGTGACGCAGTAAAATCGACGGCAGATGATATGTTATATCATCGTCTTTTTGATAACCAATAG
ACTGTTAAAAGGTCTATATAAATGGCTATGCCATTTATCTTTTGAATGTTATTTCGTAATTTATCTAT
GAATTACGCGTTAAT
```

+
FFFFFFFFF:FFFFFFFFFFFFFFFFFFFFFFFFFFF,FFFFFFFF:FFFFFFFFFFFFFFFFFFFFFF
F,FFFFF:FFFFFFFFFFFFFF:FFFFFFFFFF,F:FFFFFFFFFFFFFFFFFFFFFFFFFFFFFFFF
FFFFFFFFFFFFFFF
@A00721:190:HGJW7DSXY:4:2442:11388:10535 1:N:0:TCTGCAAG+GCTGCACT
TCTATAGTGACGCAGTAAAATCGACGGCAGATGATATGTTATATCATCGTCTTTTGATAACCAATAG
ACTGTTAAAAGGTCTATATAAATGGCTATGCCATTTATCTTTTGAATGTTATTTCGTAATTTATCTAT
GAATTACGCGTTAAT
+
FFFFFFFFFFFFFFFFFFFFFFFFFFFFFFFFFFFFFFFFFFFFFFFFFFFFFFFFFFFFFFFFFFFF
FFFFFFFFF,FFFFFFFFFFFFFFFFFFFFFFF:FFFFFFF:FFF:FFFFFFFFFFFFFFFFFFFFFF
FFF:FF,FFFFFFFF
@A00721:190:HGJW7DSXY:4:2267:32642:31375 1:N:0:TCTGCAAG+GCTGCACT
TGACGCAGTAAAATCGACGGCAGATGATATGTTATATCATCGTCTTTTGATAACCAATAGACTGTTA
AAAGGTCTATATAAATGGCTATGCCATTTATCTTTTGAATGTTATTTCGTAATTTATCTATGAATTAC
GCGTTAATAATTATA
+
FF:FFFFFFFFFFFFFFFFFFFFFFFFFFFFFFFFFFFFFFFF::F:F:FFFFFFFFFFFFFFFFFFF
F:FFFFFFFFFFFFFFFFFFF:F::FFFFFFFFFFFFFFFFFF:FFF:FFFFFFF:FFFFFFFFFFFF
FFF,FF:FF::FFF:
@A00721:190:HGJW7DSXY:4:1220:26232:35524 1:N:0:TCTGCAAG+GCTGCACT
GTAAAATCGACGGCAGATGATATGTTATATCATCGTCTTTTGATAACCAATAGACTGTTAAAAGGTC
TATATAAATGGCTATGCCATTTATCTTTTGAATGTTATTTCGTAATTTATCTATGAATTACGCGTTAA
TAATTATAGAGATCG
+
FFFFFFFFFFFFFFFF:FFFFFFFFFFFFFFFFFFFFFFFFFFFFFFFFFFFFFFFFFFFFFF:FFFF
FFFFFFFFFFFFFFFFFFF,FFFF:FFFFFFFFFFFFFFFFFFFFFFFFFFFFFFFFFFFFFFFF:FFF
FFFFFFFFFFFFFFF
@A00721:190:HGJW7DSXY:4:1261:1416:18630 1:N:0:TCTGCAAG+GCTGCACT
GTAAAATCGACGGCAGATGATATGTTATATCATCGTCTTTTGATAACCAATAGACTGTTAAAAGGTC
TATATAAATGGCTATGCCATTTATCTTTTGAATGTTATTTCGTAATTTATCTATGAATTACGCGTTAA
TAATTATAGAGATCG
+
FFFFFFFFFFFFFFFFFFFFFFFFFFFFFFFFFFFFFFFFFFFFFFFFFFFFFFFFFFFFFFF:FFFFF
FFFFFFFFFFFF:FFFFFFFFFFFFF:FFFFF:FFFFFFFFFFFFFFFFFFFFFFFFFFFFFFF,:F:
FFFFFFF:FFFFFF:
@A00721:190:HGJW7DSXY:4:1635:26865:31203 1:N:0:TCTGCAAG+GCTGCACT
GTAAAATCGACGGCAGATGATATGTTATATCATCGTCTTTTGATAACCAATAGACTGTTAAAAGGTC
TATATAAATGGCTATGCCATTTATCTTTTGAATGTTATTTCGTAATTTATCTATGAATTACGCGTTAA
TAATTATAGAGATCG
+
FFFFFFFFFFFFFFFF:FFFFFFFFFFFFFFFFFFFFFFFFFFFFFFFFFFFFFFFFFFFFFF,FFFF
FFFFFFF,FFFFFFFFFFFFFFFF,FFFFFFFFFFFFFFFFFF:FFFFFFFFFFFF:FFFFFFFFFFF
FFFFFFFFFFFFFFF
@A00721:190:HGJW7DSXY:4:2171:11803:3051 1:N:0:TCTGCAAG+GCTGCACT
GTAAAATCGACGGCAGATGATATGTTATATCATCGTCTTTTGATAACCAATAGACTGTTAAAAGGTC
TATATAAATGGCTATGCCATTTATCTTTTGAATGTTATTTCGTAATTTATCTATGAATTACGCGTTAA
TAATTATAGAGATCG
+
FFFFFFFFFFFFFFFFFFFFFFFFFFFF:FFFFFFFFFFFFFFFFFFFFFFFFFFFFFFFFFFFFFFFF
FFFFFFFFFFFFFFFFFFFFFFFFFFFF:FFFFFFFF:FFFFFFFFFFFFFFFFFFFF:FFFFFF:FFF
F:FFFFFFFFFFFFF
@A00721:190:HGJW7DSXY:4:2473:10610:6621 1:N:0:TCTGCAAG+GCTGCACT
GTAAAATCGACGGCAGATGATATGTTATATCATCGTCTTTTGATAACCAATAGACTGTTAAAAGGTC

```
TATATAAATGGCTATGCCATTTATCTTTTGAATGTTATTTCGTAATTTATCTATGAATTACGCGTTAA
TAATTATAGAGATCG
+
FFFFFFFFFFFFFFFFFFFFF:FFFFFFFFFFFFFFFFFFFFFFFFFFFFFFFFFFFFFFFFFF:FFFF
FFFFFFFFFFFFFFFFFFF,FFFFFFFFFFFFFFFFFFFFF:FFFFFFFFFFFFFFFFFFFFF:FFF
FFFFF:FFFFFFFFF
@A00721:190:HGJW7DSXY:4:2624:17309:32440 1:N:0:TCTGCAAG+GCTGCACT
GTAAAATCGACGGCAGATGATATGTTATATCATCGTCTTTTTGATAACCAATAGACTGTTAAAAGGTC
TATATAAATGGCTATGCCATTTATCTTTTGAATGTTATTTCGTAATTTATCTATGAATTACGCGTTAA
TAATTATAGAGATCG
+
FFFFFFFFFFFFFFFFFFFFFFFFFFFFFFFFFFFFFFFF:FFFFFFFFFFFFFFFFFFFFFFFF,FFFF
FFFFFFFFFFFFFFFFFFFFFFFFFFFFFFF:FFFF:F:FFFFFFFFFFFFF:FFFFFFFFFFFFFFFF
,FF:FF:FFFFFFFF
@A00721:190:HGJW7DSXY:4:1223:9317:25081 1:N:0:TCTGCAAG+GCTGCACT
ATCGTCTTTTTGATAACCAATAGACTGTTAAAAGGTCTATATAAATGGCTATGCCATTTATCTTTTGA
ATGTTATTTCGTAATTTATCTATGAATTACGCGTTAATAATTATAGTTTTCGTCTTGATTATTTGTAA
GATTTGTTTTGAACA
+
FFFFFFFFF:FFFFFFFFFFFFFFFFFFFFFFFFFFFFFFFFFFFFFFFFFFFFFFFFFFFFFFFFFF
F:FFFFFFFFFFFFFFFFFFFFFFFFF:FFFFFFFFFFFFFFFFFFFFFFFF:F,FF:,FFFFFFFFFF
FFFFFF:F,F,,FFF
@A00721:190:HGJW7DSXY:4:1277:14886:7138 1:N:0:TCTGCAAG+GCTGCACT
ATCGTCTTTTTGATAACCAATAGACTGTTAAAAGGTCTATATAAATGGCTATGCCATTTATCTTTTGA
ATGTTATTTCGTAATTTATCTATGAATTACGCGTTAATAATTATAGTTTTCGTCTTGATTATTTGTAA
GATTTGTTTTGAACA
+
FFFFFFFFFFFFFFFFFFFFFFFFFFFFFF,F:FFFFFFFFFFF,FFFFFFFFFFFFFFFFF:FFFFF
FFFFF:F:FFFFFF:F:FFFFFFFF::FFFFFFF:FFFFFFFFFFFFFF:FFF:::FF:FFFFFFFF:
FFFFFF:FFFFFFFF
@A00721:190:HGJW7DSXY:4:1334:2908:18396 1:N:0:TCTGCAAG+GCTGCACT
ATCGTCTTTTTGATAACCAATAGACTGTTAAAAGGTCTATATAAATGGCTATGCCATTTATCTTTTGA
ATGTTATTTCGTAATTTATCTATGAATTACGCGTTAATAATTATAGTTTTCGTCTTGATTATTTGTAA
GATTTGTTTTGAACA
+
FFFFFFFFFFFFFFFFFFFFFFFFFFFFFFFFF,FFFFFFFFFFFFFFFFFFFFFFFFFFFFFF:FFFFF
FFFFFFFFFFFFFFFFFFFFFF:FFFFFFFFFFFFFFFFFFFFFFFFFFFF:FFFFFFFFFFFFFFFFFFF
FFFFF:FFFF,:FFF
@A00721:190:HGJW7DSXY:4:1370:7916:2080 1:N:0:TCTGCAAG+GCTGCACT
ATCGTCTTTTTGATAACCAATAGACTGTTAAAAGGTCTATATAAATGGCTATGCCATTTATCTTTTGA
ATGTTATTTCGTAATTTATCTATGAATTACGCGTTAATAATTATAGTTTTCGTCTTGATTATTTGTAA
GATTTGTTTTGAACA
+
FFFFFFFFFFFFFFFFFFFFFFFFFFFFFFFFF,FFFFFFFFFFFFFFFFFFFFFFFFFFFFFFFFFFF
FFFFFFFFFFFFFFFFFFFFFFFFFFFFFFFFFFFF:FFFFFFFFFFFFFFFFFF:FFFFFFFFFFFFF
FFFFFFFFFFF,FFF
@A00721:190:HGJW7DSXY:4:1510:12599:13009 1:N:0:TCTGCAAG+GCTGCACT
ATCGTCTTTTTGATAACCAATAGACTGTTAAAAGGTCTATATAAATGGCTATGCCATTTATCTTTTGA
ATGTTATTTCGTAATTTATCTATGAATTACGCGTTAATAATTATAGTTTTCGTCTTGATTATTTGTAA
GATTTGTTTTGAACA
+
FFFFFFFFFFFFFFFFFFFFFFFFFFFFFFFFFFFFFFFFFFFFFF:FFFFFFFFFFFFFFFFFFFFFFF
FFF:FFFFFFFFFFFFF:FFFFF:FFFFFFFFFFFF:FFFFFFFFFFFFFFF::F,,FFFFFFFFFF
FFFFFFFFFFF:FFF
```

```
@A00721:190:HGJW7DSXY:4:1638:1163:10363 1:N:0:TCTGCAAG+GCTGCACT
ATCGTCTTTTTGATAACCAATAGACTGTTAAAAGGTCTATATAAATGGCTATGCCATTTATCTTTTGA
ATGTTATTTCGTAATTTATCTATGAATTACGCGTTAATAATTATAGTTTTCGTCTTGATTATTTGTAA
GATTTGTTTTGAACA
+
FFFFFF:FFFFFFFFFFFFFFFFFFFFFFFFFFFFFFFFFFFFFFFFFFFFFFFFFFFFFFFFFFFFF
FFFFFFFFFFFFF:FFFFFFFFFFFFFFFFFFFFFFFFFFFFFFFFFFFFFFF:F,FF,FFFFFFFFF
:FFFF:F:FFFFFFF
@A00721:190:HGJW7DSXY:4:2510:14452:18192 1:N:0:TCTGCAAG+GCTGCACT
ATCGTCTTTTTGATAACCAATAGACTGTTAAAAGGTCTATATAAATGGCTATGCCATTTATCTTTTGA
ATGTTATTTCGTAATTTATCTATGAATTACGCGTTAATAATTATAGTTTTCGTCTTGATTATTTGTAA
GATTTGTTTTGAACA
+
FFFFFFFFFFFF:FFFFFFFFFFFFFFFFFFFFFFFFFFFFFFFFFFFFFFFFFFFFFFFFFFFFFFF
FFFFFFFFFFFF:FFFFFFFFFFFFFFFF::FFF:FFFFFFFFFFFFFFF,FFFFFF:FFFFFFFFFF
F:FFF:FFFFFF,FF
@A00721:190:HGJW7DSXY:4:2536:9815:10254 1:N:0:TCTGCAAG+GCTGCACT
ATCGTCTTTTTGATAACCAATAGACTGTTAAAAGGTCTATATAAATGGCTATGCCATTTATCTTTTGA
ATGTTATTTCGTAATTTATCTATGAATTACGCGTTAATAATTATAGTTTTCGTCTTGATTATTTGTAA
GATTTGTTTTGAACA
+
FFFFFFFFFFFFFFFFFFFFFFFFFFFFFFFFF,FFFFFFFFFFFFF:FFFFFFFFFFFFFF,FFFFFF
FFFFFFFFFFFFFFFFFFF:FFFFFFFFF::FFFFFFFFFFFFFF,FFFF,FF,:FFFFFFFFF,FFF
:FFFFFFFFF,:FF:
@A00721:190:HGJW7DSXY:4:2123:10601:31000 1:N:0:TCTGCAAG+GCTGCACT
CGACGGACATTGAAAATGTATCTCGATTTGTTCAAAACAAATCTTACAAATAATCAAGACGAAAACTA
TAATTATTAACGCGTAATTCATAGATAAATTACGAAATAACATTCAAAAGATAAATGGCATAGCCATT
TATATAGACCTTTTA
+
FFFFFFFFFFFFFFFFFFFFFFFFFFFFFFFFFFFF:FFFFFFFFFFFFFFFFFFFFFFFFFF:FF:FF
FF:FFFFFFFFFFFFFFFFFFFFFFFFFFF:FF,FFFFFFFFFFFFFF,FFFFFFF:FFFFFFFFFFF
F:FFFF:F:FF:F:F
@A00721:190:HGJW7DSXY:4:2123:11333:32518 1:N:0:TCTGCAAG+GCTGCACT
CGACGGACATTGAAAATGTATCTCGATTTGTTCAAAACAAATCTTACAAATAATCAAGACGAAAACTA
TAATTATTAACGCGTAATTCATAGATAAATTACGAAATAACATTCAAAAGATAAATGGCATAGCCATT
TATATAGACCTTTTA
+
FFFFFFFFFFFFFFFFFFFFFFFFFFFFFFFFFFFFFFFFFFFFFFFFFFFFFFFFFFFFFFFFF:FFF
FFFFFFFFFFFFFFF:FFFFFFFFFFFFFFFFFFFFFFFFFFFFFFFFFFFFFFFFF,FFFFFFFFFF
FFFFFFF,FF,FFFF
@A00721:190:HGJW7DSXY:4:2145:7690:19914 1:N:0:TCTGCAAG+GCTGCACT
CGACGGACATTGAAAATGTATCTCGATTTGTTCAAAACAAATCTTACAAATAATCAAGACGAAAACTA
TAATTATTAACGCGTAATTCATAGATAAATTACGAAATAACATTCAAAAGATAAATGGCATAGCCATT
TATATAGACCTTTTA
+
FFFFFFFFFFFFFFFFFFFFFFFFFFFFFFFFFFFF:FFFFFFFFFFFFFFFFFFFFFFFFFFFFFFFF
FFFFFFFFFFFFFFFFFFFFFFFFFFFFFFFFFFFFFFFFFFF:FFFFFFFFFFFFFFFFFFFF:FF:F
FFFFFFFFFF,FFFF
@A00721:190:HGJW7DSXY:4:2242:28782:13698 1:N:0:TCTGCAAG+GCTGCACT
CGACGGACATTGAAAATGTATCTCGATTTGTTCAAAACAAATCTTACAAATAATCAAGACGAAAACTA
TAATTATTAACGCGTAATTCATAGATAAATTACGAAATAACATTCAAAAGATAAATGGCATAGCCATT
TATATAGACCTTTTA
+
FFFFFFFFFFFFFFFFFF:FFFFFFFFFFFFFFF,FFFFFFF,F::FFFFFFFFFFF:FFFF:FFFFF
```

FFFFFF,FFFFFFF,FFFF:FFFFFFFFFFFF,FFFFFFFFFFFF:::FFFFFFFFF:FFFF,F,F:F
:,FFFF,F:,FF,::
@A00721:190:HGJW7DSXY:4:2344:2356:11240 1:N:0:TCTGCAAG+GCTGCACT
CGACGGACATTGAAAATGTATCTCGATTTGTTCAAAACAAATCTTACAAATAATCAAGACGAAAACTA
TAATTATTAACGCGTAATTCATAGATAAATTACGAAATAACATTCAAAAGATAAATGGCATAGCCATT
TATATAGACCTTTTA
+
FFFFFFFFFFFFFFFF:FFFFFFFFFFFFFFFFFFFFFFFFFFFFFFFFFFFFFFFFFFFFFFFFFFF
FF:FFFFFFFFFFFFFFFFFFFFFFFFFFFFFFFFFFFFFFFFFFFFFFFFFFFF:FFFFFFF:F
FFFFFF:FFF::FFF
@A00721:190:HGJW7DSXY:4:2355:29559:26788 1:N:0:TCTGCAAG+GCTGCACT
CGACGGACATTGAAAATGTATCTCGATTTGTTCAAAACAAATCTTACAAATAATCAAGACGAAAACTA
TAATTATTAACGCGTAATTCATAGATAAATTACGAAATAACATTCAAAAGATAAATGGCATAGCCATT
TATATAGACCTTTTA
+
FFFFFFFFFFFFFFFFFFFFFFFFFFFFFFFFFFFFFFFFFFFFFFFFFFFFFFFFFFFFFFFFFFF:
F:FFFFFFFFFFFFFFFFFFFFFFFFFFFFFFFFFFFFFFF:FFFF:FFFFFFFFFFFFFFFFFF,F
FFFFFFFF:,:FFFF
@A00721:190:HGJW7DSXY:4:2124:31882:13964 1:N:0:TCTGCAAG+GCTGCACT
CGACGGACATTGAAAATGTATCTCGATTTGTTCAAAACAAATCTTACAAATAATCAAGACGAAAACTA
TAATTATTAACGCGTAATTCATAGATAAATTACGAAATAACATTCAAAAGATAAATGGCATAGCCAGT
TATATAGACCTTTT
+
FFFFF:FFFFFFFFFFFFFFFFFFFFFFFFFFFF,FFFFFFFFFFFFFFFFFFFFFFFFFFFFFF:FF:
FFFFFFFFF,FFFFFFFFFFFFF:FFF:F,FFFFFF:FF::FFFFF,FFF:F:FFFFF::FFFFF:,F
,,FFFFFF:,FF,:
@A00721:190:HGJW7DSXY:4:1201:21594:22576 1:N:0:TCTGCAAG+GCTGCACT
GTTATTTCGTAATTTATCTATGAATTACGCGTTAATAATTATAGTTTTCGTCTTGATTATTTGTAAGA
TTTGTTTTGAACAAATCGAGATACATTTTCAATATCCGTCGAAGGAGATCTGTTTGATCGAGATTTTA
AGACGAAAACGATCC
+
FFFFFFFFFFFFFFFFFFFFFFFFFFFFFFFFFFFFFFFFFFFFFFFFFFFFFF:FF:FFFFFFFFFF:
FFFFFFFFFFFFFFFFFFFFFFFFFFFFFFFFFFFFFFFFFFFFFFFFFFFF:FFFFFFFFFF:FFFF
F:FF:FFF:FFFFF:
@A00721:190:HGJW7DSXY:4:1204:15989:32377 1:N:0:TCTGCAAG+GCTGCACT
GTTATTTCGTAATTTATCTATGAATTACGCGTTAATAATTATAGTTTTCGTCTTGATTATTTGTAAGA
TTTGTTTTGAACAAATCGAGATACATTTTCAATATCCGTCGAAGGAGATCTGTTTGATCGAGATTTTA
AGACGAAAACGATCC
+
FFFFFFFFFFFFFFFFFFFFFFFF:FFFFFFFFFFFFFFFFFFFFFFFFFFFFFFFFFFFFFFFFFFF:
FFFFFFFFFFFFFFFFFFFFFFFFFFFFFFFFFFFFFFFFFFFFFFFFFFF,FFFFFFFFFFFFFFFF
F,:F:FFFFFFFFFF
@A00721:190:HGJW7DSXY:4:1319:30779:11303 1:N:0:TCTGCAAG+GCTGCACT
GTTATTTCGTAATTTATCTATGAATTACGCGTTAATAATTATAGTTTTCGTCTTGATTATTTGTAAGA
TTTGTTTTGAACAAATCGAGATACATTTTCAATGTCCGTCGAAGGAGATCTGTTTGATCGAGATTTTA
AGACGAAAACGATCC
+
FFFFFFFFFFFFFFFFFFFFFFFFFFFFFFFFFFFFFFFFFFFFFFFFFFFFFFFFFFFFFFFFFFFF
FFFFFFFFF,FFFFFFFFFFFFFFFFFFFFFFFFFFFFFFFFFFFFFFFFF:FFFFFFFFFFFFFF
FF:FFFFFF,FFF:F
@A00721:190:HGJW7DSXY:4:1446:18900:31814 1:N:0:TCTGCAAG+GCTGCACT
GTTATTTCGTAATTTATCTATGAATTACGCGTTAATAATTATAGTTTTCGTCTTGATTATTTGTAAGA
TTTGTTTTGAACAAATCGAGATACATTTTCAATGTCCGTCGAAGGAGATCTGTTTGATCGAGATTTTA
AGACGAAAACGATCC

```
+
FFFFFFFFFFFFFFFFFFFFFFFFFFFFFFFFFFFFFFFFFFFFFFFFFFFFFFFFFFFFFFFFFFFFFFFF:
FFFFFFFFFFFFFFFFFFFFFFFFFFF:FFFFFFFFFFFFFFFFFFFFF:FFF:FFF:FFF:FFF:FFFF
::FFFFFF,FFFF:F
@A00721:190:HGJW7DSXY:4:1513:9453:28980 1:N:0:TCTGCAAG+GCTGCACT
GTTATTTCGTAATTTATCTATGAATTACGCGTTAATAATTATAGTTTTCGTCTTGATTATTTGTAAGA
TTTGTTTTGAACAAATCGAGATACATTTTCAATATCCGTCGAAGGAGATCTGTTTGATCGAGATTTTA
AGACGAAAACGATCC
+
FFFFFFFFFFFFFFFFFFFFFFFF:FFFFFFFFFFFFFFFFFFFFFFFFFFFFFF:FFFFFFFFFFFFFF
FFFFFFFFFFFFFFFFFFFFFFFFFFFFFFFFFFFFFFFFFF:FFFFFFFFFFFFFF:FFFFFFFFFFFF
FF:FFFFFFFFFFF:F
@A00721:190:HGJW7DSXY:4:1567:19379:25254 1:N:0:TCTGCAAG+GCTGCACT
GTTATTTCGTAATTTATCTATGAATTACGCGTTAATAATTATAGTTTTCGTCTTGATTATTTGTAAGA
TTTGTTTTGAACAAATCGAGATACATTTTCAATATCCGTCGAAGGAGATCTGTTTGATCGAGATTTTA
AGACGAAAACGATCC
+
FFFFFFFFFFFFFFFFFFFFF:FFFFFFFFFFFFFFFFF:FFFFFFFFFFFF:FFFFFFFFFF::FFFFFF
:FFFFFFFFFFFFFFFFFFFFFFFFFFF:FFFFFFFFFFFF:FFF:FFFFFFFFFFFFFFFFF:FFFFF,FF
F:FFFFFFFF,FFFFF
@A00721:190:HGJW7DSXY:4:1614:1958:17660 1:N:0:TCTGCAAG+GCTGCACT
GTTATTTCGTAATTTATCTATGAATTACGCGTTAATAATTATAGTTTTCGTCTTGATTATTTGTAAGA
TTTGTTTTGAACAAATCGAGATACATTTTCAATATCCGTCGAAGGAGATCTGTTTGATCGAGATTTTA
AGACGAAAACGATCC
+
FFFFF:FFFFFFFFFFF:FF:FFFFFFFFFF:FFFFFFFFFFFFFFFFFFFFF:,FF:FFFFFFFFFFF
FFF:FFFFFFFFFFFFFFFFFFFFFFFFFFFFFFFFFF:FFFFFFFFFFFFFF:FF:FFFFFFFFFFFFFFF
FFFFFFFFFFF,FFFF
@A00721:190:HGJW7DSXY:4:2119:24740:21574 1:N:0:TCTGCAAG+GCTGCACT
GTTATTTCGTAATTTATCTATGAATTACGCGTTAATAATTATAGTTTTCGTCTTGATTATTTGTAAGA
TTTGTTTTGAACAAATCGAGATACATTTTCAATATCCGTCGAAGGAGATCTGTTTGATCGAGATTTTA
AGACTAAAACGATC
+
:FFF:FFFFFF:,FFFFFFFFFFFFFFF,::FF,FFFFFFFFF:FFFFFFF:FFFFFFFF,F,FFFF:
:F,,F,FF:FF:FFF:F,F:FFFFF,:FF:FFFFF:::F:FFF,,FFFFF:,FF::FF,FFFFF:FFF
F:::,FFFFF,FFF
@A00721:190:HGJW7DSXY:4:2361:24044:25160 1:N:0:TCTGCAAG+GCTGCACT
GTTATTTCGTAATTTATCTATGAATTACGCGTTAATAATTATAGTTTTCGTCTTGATTATTTGTAAGA
TTTGTTTTGAACAAATCGAGATACATTTTCAATATCCGTCGAAGGAGATCTGTTTGATCGAGATTTTA
AGACGAAAACGATCC
+
FFFFFFFFFFFF:FFFFFFFFFFFFFFFFFFFFFFFFFFFFFFFFFFFFFFFFFFFFFFFFFFFFFFFFF
FFFFFFFFFFFFFFFFFFFFFFFFFFFFFFFFFFFFFFFFFFFFFFFFFFFFFFFFFFFFFFFFFFFFFF
FFFFFFFFFFFFFFF
@A00721:190:HGJW7DSXY:4:2412:14570:9565 1:N:0:TCTGCAAG+GCTGCACT
GTTATTTCGTAATTTATCTATGAATTACGCGTTAATAATTATAGTTTTCGTCTTGATTATTTGTAAGA
TTTTTTTTGAACAAATAGAGATACAATTTCAATTTACGTCGAAGGAGATCAGTTTGATCGAGATTTTA
AGACGAAAACGATCC
+
FFFF:FFFFFFF:FFFFFFFFFF,FFFFFFFFFFFFFFFFFFFFFFFFFFFFFFFFFFFFFFFFFFFFFF
FFF,:FFF,,F,FFF,,,F,,,F:F,FFF,FF,,F,F,F,F:F,FF:::,,,FFF,,F,,FFFFFFFF
F:,F::FFF,:F,:F
@A00721:190:HGJW7DSXY:4:2422:20184:5822 1:N:0:TCTGCAAG+GCTGCACT
GTTATTTCGTAATTTATCTATGACTTACGCGTTAATAATTATAGTTTTCGTCTTGATTATTTGTAAGA
```

TTTGTTTTGAACAAATCGAGATACATTTTCAATGTCCGTCGAAGGAGATCTGTTTGATCGAGATTTTA
AGACGAAAACGATCC
+
FFFFFFFFFFFFFFFFFFFFFFF,FFFFFFFFFFFFFFFFFFFFFFFFFFFFFFFFFFFFFFFFFFFF
FFFFFFFFFFFFFFFFFFF:FFFFFFFFFFFFF:FFFFFFFFFFFFFFFFFFFFF::FFFFFFFFFFF
FF:FFF:FFFFFFF:
@A00721:190:HGJW7DSXY:4:2650:2935:3161 1:N:0:TCTGCAAG+GCTGCACT
GTTATTTCGTAATTTATCTATGAATTACGCGTTAATAATTATAGTTTTCGTCTTGATTATTTGTAAGA
TTTGTTTTGAACAAATCGAGATACATTTTCAATATCCGTCGAAGGAGATCTGTTTGATCGAGATTTTA
AGACGAAAACGATCC
+
FFFFFFFFFFFFFFFFFFFFFFF:FFFFFFFFFFFFFFFFFFFFFFFFFFFFFFFFFFFFFFFFFFFF
FFFFFFFFFFFFFFFFFFFFFFFFFFFFFFFFFFFFFFFFFFFFFFFFFFFF:FFFFFFFFFFFFFFF
FFFF:FFFFFFFFFF
@A00721:190:HGJW7DSXY:4:2658:20464:2801 1:N:0:TCTGCAAG+GCTGCACT
GTTATTTCGTAATTTATCTATGAATTACGCGTTAATAATTATAGTTTTCGTCTTGATTATTTGTAAGA
TTTGTTTTGAACAAATCGAGATACATTTTCAATGTCCGTCGAAGGAGATCTGTTTGATCGAGATTTTA
AGACGAAAACGATCC
+
FFFF:FFFFFFFFFFFFFFFFFFFFFFFFFFFFFFFFFFFFFFFFFFF:FFFF,FF,FFF:FFFFFFF
FFFFFFFFFFFFFFFFFFFFFFFFFFFFFFFFFFFFFFFFFFFFFFFFFFFFFFFFFFFFFFFFFFFF
FFFFFFFF,FFFFFF
@A00721:190:HGJW7DSXY:4:1606:26087:35524 1:N:0:TCTGCAAG+GCTGCACT
CCTGGATCGTTTTCGTCTTAAAATCTCGATCAAACAGATCTCCTTCGACGGACATTGAAAATGTATCT
CGATTTGTTCAAAACAAATCTTACAAATAATCAAGACGAAAACTATAATTATTAACGCGTAATTCATA
GATAAATTACGAAAT
+
FFFFFFFFFFFFFFFFFFFFFFFFFFFFFFFFFFFFFFFFFFFFFFFFFFFFFFFFFFFFFFFFFFFF
FFFFFFFFFFFFFFFFFFFFFFFFFFFFFFFFFFFFFFFFF:FFFFFFFFFFFF:FFFFFFFFFFFFF
FFFFFFFFFFFFFFF
@A00721:190:HGJW7DSXY:4:1610:2917:28588 1:N:0:TCTGCAAG+GCTGCACT
CCTGGATCGTTTTCGTCTTAAAATCTCGATCAAACAGATCTCCTTCGACGGACATTGAAAATGTATCT
CGATTTGTTCAAAACAAATCTTACAAATAATCAAGACGAAAACTATAATTATTAACGCGTAATTCATA
GATAAATTACGAAAT
+
FFFFFFFFFFFFFFFFFFFFFFFFFFFFFFFFFFFFFFFFFFFFFFFFFFFFFFFFFFFFFFFFFFFF
FFFFFFFFFFFFFFFFFFFFFFFFFFFFFFF:FFFFFFFFFFFFFFFFFFFFFFFFFFFFFFFFFFFF
FFFFFFFFFFF:FFF
@A00721:190:HGJW7DSXY:4:1657:31593:31000 1:N:0:TCTGCAAG+GCTGCACT
CCTGGATCGTTTTCGTCTTAAAATCTCGATCAAACAGATCTCCTTCGACGGACATTGAAAATGTATCT
CGATTTGTTCAAAACAAATCTTACAAATAATCAAGACGAAAACTATAATTATTAACGCGTAATTCATA
GATAAATTACGAAAT
+
FFFFFFFFFFFFFFFFFFFFFFFFFFFFFFFFFFFFFFFFFFFFFFFFFFFFFFFFFFFFFFFFFFFF
FFFFFFFFFFFF:FFFFFFFFFFFFFFFFFFFF::FFFFFFFFFFFFFFFFFFF:FFFFFFFFFFFFF
FFFFFFFFFFFFFFF
@A00721:190:HGJW7DSXY:4:1657:31756:31156 1:N:0:TCTGCAAG+GCTGCACT
CCTGGATCGTTTTCGTCTTAAAATCTCGATCAAACAGATCTCCTTCGACGGACATTGAAAATGTATCT
CGATTTGTTCAAAACAAATCTTACAAATAATCAAGACGAAAACTATAATTATTAACGCGTAATTCATA
GATAAATTACGAAAT
+
FFFFFFFFFFFFFFFFFFFFFFFFFFFFFFFFFFFFFFFFFFFFFFFFFFFFFFFFFFFFFFFFFFFF
FFFFFFFFFFFFFFFFFFFFFFFFFFFFFFFFFFFFFFFFFFFFFFFFF:FFFF:F,FFFFFFF:FFF
,FFFFFFFFF:FFFF

```
@A00721:190:HGJW7DSXY:4:2146:21504:32189 1:N:0:TCTGCAAG+GCTGCACT
CCTGGATCGTTTTCGTCTTAAAATCTCGATCAAACAGATCTCCTTCGACGGACATTGAAAATGTATCT
CGATTTGTTCAAAACAAATCTTACAAATAATCAAGACGAAAACTATAATTATTAACGCGTAATTCATA
GATAAATTACGAAAT
+
FFFFFFFFFFFFFFFFFFFFFFFFFFFFFFFFFFFFFFFFFFFFFFFFFFFFFFFFFFFFFFFFFFFF
FFFFFFFFFFFFFFFFFF:FF:FFFFFFFFFFFFF:FFFFFFFFFFFFFFFFFFF,FFFFFFFFFFFF
FFFFFF:FFF,FFFF
@A00721:190:HGJW7DSXY:4:1336:23809:10441 1:N:0:TCTGCAAG+GCTGCACT
CCCCAATTTCCATTAGCAGCAGCCTGGATCGTTTTCGTCTTAAAATCTCGATCAAACAGATCTCCTTC
GACGGACATTGAAAATGTATCTCGATTTGTTCAAAACAAATCTTACAAATAATCAAGACGAAAACTAT
AATTATTAACGCGTA
+
FFFFFFFFFFFFFFFFFFFFFFFF:FFFFFFFFFFFFFFFFFFFFFFFFFFFFFFFFFFFFFFFFFFF
FFFFFFFFFFFFF:FFFFFFFFFFFFFFFFFFFFF,FFFFFFFFFFFFFFFFFFFFFFFFFFFFFFFF
FFFFFFFFFFFFFFF
@A00721:190:HGJW7DSXY:4:1471:4444:22467 1:N:0:TCTGCAAG+GCTGCACT
CCCCAATTTCCATTAGCAGCAGCCTGGATCGTTTTCGTCTTAAAATCTCGATCAAACAGATCTCCTTC
GACGGACATTGAAAATGTATCTCGATTTGTTCAAAACAAATCTTACAAATAATCAAGACGAAAACTAT
AATTATTAACGCGTA
+
FFFFFFFFFFFFFFFFFFFFFFFFFFFFFFFFFFFFFFFFFFFFFFFFFFFFFFFFFFFFFFFFFFFF
F:FFFFFFFFFFFFFFFFFFFFFFFFFFFFFFFF:FFFFFFFFFFFFFFFFFFFFFF:FFFFFFFFFF
FFFFFFFF:FFFFFF
@A00721:190:HGJW7DSXY:4:1531:6298:6825 1:N:0:TCTGCAAG+GCTGCACT
CCCCAATTTCCATTAGCAGCAGCCTGGATCGTTTTCGTCTTAAAATCTCGATCAAACAGATCTCCTTC
GACGGACATTGAAAATGTATCTCGATTTGTTCAAAACAAATCTTACAAATAATCAAGACGAAAACTAT
AATTATTAACGCGTA
+
FFFFFF:FFFF::FFFFF:FF:FF,FFFFFFFF,,FFFFFFFFF::F:FFFFFFFFFFFFF:FFF,FF
F:FFFFFFF:FFFFF:FFFFFFF:FFFFF,F:FFFFFFF,,F,,:FFFFFF,,FF::FF::F,FFFF,
FF:FFF,FFF:FFFF
@A00721:190:HGJW7DSXY:4:2158:18584:30733 1:N:0:TCTGCAAG+GCTGCACT
CCCCAATTTCCATTAGCAGCAGCCTGGATCGTTTTCGTCTTAAAATCTCGATCAAACAGATCTCCTTC
GACGGACATTGAAAATGTATCTCGATTTGTTCAAAACAAATCTTACAAATAATCAAGACGAAAACTAT
AATTATTAACGCGTA
+
FFFFFFFFFFFFFFFFFFFFFFFFFFFFFFFFFFFFFFFFFFFFFFFFFFFFFFFFFFFFFFFFFFFF
FFFFFFFFFFFFFFFFFFFFFFFFFFFFF:FFFFFFFFFFFFFFFFFFFFFFFFFFFFFFFF:FFFFF
FFFFFFFFFFF:FFF
@A00721:190:HGJW7DSXY:4:2162:14624:35086 1:N:0:TCTGCAAG+GCTGCACT
CCCCAATTTCCATTAGCAGCAGCCTGGATCGTTTTCGTCTTAAAATCTCGATCAAACAGATCTCCTTC
GACGGACATTGAAAATGTATCTCGATTTGTTCAAAACAAATCTTACAAATAATCAAGACGAAAACTAT
AATTATTAACGCGTA
+
FFFFFFFFFFFFFFFFFFFFFFFFFFFFFFFFFFFFFFFFFFFFFFFFFFFFFFFFFFFFFFFFFFFF
FFFFFFFFFFFFFFFFFFFFFFFFFFFFFFFFFFF,FFFFFFFFFFFFFFFFFFFF:FFFFFFFFFFF
FFFFFFFFFF:FFFF
@A00721:190:HGJW7DSXY:4:2335:25726:17895 1:N:0:TCTGCAAG+GCTGCACT
CCCCAATTTCCATTAGCAGCAGCCTGGATCGTTTTCGTCTTAAAATCTCGATCAAACAGATCTCCTTC
GACGGACATTGAAAATGTATCTCGATTTGTTCAAAACAAATCTTACAAATAATCAAGACGAAAACTAT
AATTATTAACGCGTA
+
FFFFFFFFFFFFFFFFFFFFFFFFFFFFFFFFFFFFFFFFFFFFFFFFFFFFFFFFFFFFFFFFFFFF
```

```
FFFFFFFFFFFFFFFFFFFFFFFFFFFFFFFFFFFFF:FFFFFFFFFFFFFFFFFFFFFFF:FFFFFFFF
FFFFFFFF,F:FFFF
@A00721:190:HGJW7DSXY:4:2670:8793:29027 1:N:0:TCTGCAAG+GCTGCACT
CCCCAATTTCCATTAGCAGCAGCCTGGATCGTTTTCGTCTTAAAATCTCGATCAAACAGATCTCCTTC
GACGGACATTGAAAATGTATCTCGATTTGTTCAAAACAAATCTTACAAATAATCAAGACGAAAACTAT
AATTATTAACGCGTA
+
FFFFFFFFFFFFFFFFFFFFFFFFFFFFFFFFFFFFFFFFFFFFFFFFFFFFFFFFFFFFFFFFFFFFFF
FFFFFFF:FFFFFFFFFFFFFFFFFFFFFFFFFFFFFFFFFFFFFFFFFFFFF:FFFFFFFF
FFFFFFFFFFFFFFF
@A00721:190:HGJW7DSXY:4:1114:21269:5447 1:N:0:TCTGCAAG+GCTGCACT
ACGCGTTAATAATTATAGTTTTCGTCTTGATTATTTGTAAGATTTGTTTTGAACAAATCGAGATACAT
TTTCAATGTCCGTCGAAGGAGATCTGTTTGATCGAGATTTTAAGACGAAAACGATCCAGGCTGCTGCT
AATGGAAATTGGGGC
+
FFFFFF,FFFFFFFFFFFFFFFFFFFFFFFFFFFFFFFFFFFFFFFFFFFFFFFFFFFFFFFFFFFFFFF
FFFFFFFFFFFFFFFFFFFFFFFFFFFFFFF:FFFFFFFFFFF:FFF,FFFFFFFFFFFFFFFFFFFFFF
F,F,FF:FFFFFFFF
@A00721:190:HGJW7DSXY:4:1402:9634:12633 1:N:0:TCTGCAAG+GCTGCACT
ACGCGTTAATAATTATAGTTTTCGTCTTGATTATTTGTAAGATTTGTTTTGAACAAATCGAGATACAT
TTTCAATGTCCGTCGAAGGAGATCTGTTTGATCGAGATTTTAAGACGAAAACGATCCAGGCTGCTGCT
AATGGAAATTGGGGC
+
FFFFFFFFFFFFFFFFFFFFFFFFFFFFFFFFFFFFFFFFFFFFFFFFF:FFFFFFFFFFFFFFFFFFFF
FFFFFFFFFFFFFFFFFFFFFFFFFFFFFFFFFFFFFFFFFFF:FFFFFFFFFFFFFFFFFFFFF:FFFF
FFFFFF,FFFFFFFF
@A00721:190:HGJW7DSXY:4:2101:20419:16438 1:N:0:TCTGCAAG+GCTGCACT
ACGCGTTAATAATTATAGTTTTCGTCTTGATTATTTGTAAGATTTGTTTTGAACAAATCGAGATACAT
TTTCAATGTCCGTCGAAGGAGATCTGTTTGATCGAGATTTTAAGACGAAAACGATCCAGGCTGCTGCT
AATGGAAATTGGGGC
+
FFFFFFFFFFFFFFFFFFFFFFFFFFF:FFFFFFFFFFFFFFFFFFFFFFFFFFFFFFFFFFFFFFFFFF
FFFFFFFFFFFFFFFFFFFFFFFFFFFF:FFFFFFFFFFFFFFFFFFFFFFFFFFFFFFFFFFFFFFFFF
FFFFFF,FFFFFFFF
@A00721:190:HGJW7DSXY:4:1504:15772:15937 1:N:0:TCTGCAAG+GCTGCACT
CGCGTTAATAATTATAGTTTTCGTCTTGATTATTTGTAAGATTTGTTTTGAACAAATCGAGATACATT
TTCAATGTCCGTCGAAGGAGATCTGTTTGATCGAGATTTTAAGACGAAAACGATCCAGGCTGCTGCTA
ATGGAAATTGGGGCA
+
FFFFFFFFFFFFFFFFFFFFFFFFFFFFFF,FFFFFFFFFFFFFFFFFFFFFFFFFFFFFFFFFFFFFFF
FFFFFFFFFFFFFFFFFFFFFFFFFFFFFFFFFFFFFFFFFFFFFFFFFFFFFFFFFFFFFFFFFFFFFF
FFFFFFFFFFFFFFF
@A00721:190:HGJW7DSXY:4:2201:12527:23218 1:N:0:TCTGCAAG+GCTGCACT
CGCGTTAATAATTATAGTTTTCGTCTTGATTATTTGTAAGATTTGTTTTGAACAAATCGAGATACATT
TTCAATGTCCGTCGAAGGAGATCTGTTTGATCGAGATTTTAAGACGAAAACGATCCAGGCTGCTGCTA
ATGGAAATTGGGGCA
+
FFFFFFFFFFFFFFFFFFF:FFFFFF,FF:FFFFFFFFFFFFFFFFFFFFFFFFFFFF:F:FF:F,FF:F
FFFFFFFFFF:FF:FFFFF:FFF:FF:FFFFFFFFFFFFFFFFFFFF:FFFFFFFFFFF:FFFFFFFFFF
F,:FFFFFFFFFFFF
@A00721:190:HGJW7DSXY:4:2244:9136:8390 1:N:0:TCTGCAAG+GCTGCACT
CGCGTTAATAATTATAGTTTTCGTCTTGATTATTTGTAAGATTTGTTTTGAACAAATCGAGATACATT
TTCAATGTCCGTCGAAGGAGATCTGTTTGATCGAGATTTTAAGACGAAAAAGATCCAGGCTGCTGCTA
ATGGAAATTGGGGCA
```

```
+
FFFFFFFFFFFFFFFFFFFFFFFFFFFFFFFFFFFFFFFFFFFFFFFFFFFFFFFFFFFFFFFFFFFF
FFFFFFFFFF:FFFFFFFFFFFFFFFFFFFFFFFFFFFFFFFF:FFFFFFF,:FFF:FFFFFFFFFFFF
FFFFFFFFFFFFFFF
@A00721:190:HGJW7DSXY:4:2255:8811:17190 1:N:0:TCTGCAAG+GCTGCACT
CGCGTTAATAATTATAGTTTTCGTCTTGATTATTTGTAAGATTTGTTTTGAACAAATCGAGATACATT
TTCAATGTCCGTCGAAGGAGATCTGTTTGATCGAGATTTTAAGACGAAAACGATCCAGGCTGCTGCTA
ATGGAAATTGGGGCA
+
FFFFFFFFFFFFFFFFFFFFFFFFFFFFFFFF:FFFFFFFFFFFFFF:FFFFFFFFFFFFFFFFFFFFF
FFFFFFFFFFFFFFFFFFFFFFFF:F:FFFFFFFFFFFFFFFFFF:FFFFFFFFFFFFFFFFFFFFFFFF
FFFFFFFF:FFFFFF
@A00721:190:HGJW7DSXY:4:2318:20772:18490 1:N:0:TCTGCAAG+GCTGCACT
CGCGTTAATAATTATAGTTTTCGTCTTGATTATTTGTAAGATTTGTTTTGAACAAATCGAGATACATT
TTCAATGTCCGTCGAAGGAGATCTGTTTGATCGAGATTTTAAGACGAAAACGATCCAGGCTGCTGCTA
ATGGAAATTGGGGCA
+
FFFFFFFFFFFFFFFFFFFFFFFFFFFFFF:FFFFFFFFFFFFFFFFFFFFFFFFFFFFFFFFFFFFF
FFFFFFFFFFFFFFFFFFFFFFFFFFFFFFFFFFFFFFFFFFFFFFFFFFFFFFFFFFFFFFF:FFFFF
FFFFFFFFFFFFFFF
@A00721:190:HGJW7DSXY:4:2504:17472:20040 1:N:0:TCTGCAAG+GCTGCACT
CGCGTTAATAATTATAGTTTTCGTCTTGATTATTTGTAAGATTTGTTTTGAACAAATCGAGATACATT
TTCAATGTCCGTCGAAGGAGATCTGTTTGATCGAGATTTTAAGACGAAAACGATCCAGGCTGCTGCTA
ATGGAAATTGGGGCA
+
FFFFFFFFFFFFFFFFFFFFFFFFFFFFF:FFFFFFFFFFFFFFFFFFFFFFFFFFFFFFFFFFFFFF
FFFFFFFFFFFFFFFFFFFFFFFFFFFFF:FFF:FFFFFFFFF:FFFFFFFFFFF:FFFFFFFFFFF:F
FFFFF,FFFFFF:FF
@A00721:190:HGJW7DSXY:4:2609:7862:26224 1:N:0:TCTGCAAG+GCTGCACT
CGCGTTAATAATTATAGTTTTCGTCTTGATTATTTGTAAGATTTGTTTTGAACAAATCGAGATACATT
TTCAATGTCCGTCGAAGGAGATCTGTTTGATCGAGATTTTAAGACGAAAACGATCCAGGCTGCTGCTA
ATGGAAATTGGGGCA
+
FFFFFFFFFFFFFFFFFFFFFFFFFFFFFFFFFFFFFFFFFFFFFFFFFFFFFFFFFFFFFFFFFFFF
FFFFFFFFFFFFFFFFFFFFFFFFFFFFFFFFFFFFFFFFFFFF:FFFFFFFFFFFFFFFFFFFFFFFF
FFFFFFFFFFFFFF:
@A00721:190:HGJW7DSXY:4:1244:7509:6918 1:N:0:TCTGCAAG+GCTGCACT
GCGTTAATAATTATAGTTTTCGTCTTGATTATTTGTAAGATTTGTTTTGAACAAATCGAGATACATTT
TCAATGTCCGTCGAAGGAGATCTGTTTGATCGAGATTTTAAGACGAAAACGATCCAGGCTGCTGCTAA
TGGAAATTGGGGCAA
+
FFFFFFFFFF:FFFFFFFFFFFFF:FFFFFFF:FFFFFFFFFFFFFFFFFFFFFFFFFFFFFFFFFFFF
FFFFFFFFFFFFFFFFFFFFF:FFFFFFFFFFFFFFFFFFFFFFFFFFFFFFFFFFFFFFFFFFFFFFF
FFFFFFFFFFFFFFF
@A00721:190:HGJW7DSXY:4:1339:4119:9283 1:N:0:TCTGCAAG+GCTGCACT
GCGTTAATAATTATAGTTTTCGTCTTGATTATTTGTAAGATTTGTTTTGAACAAATCGAGATACATTT
TCAATGTCCGTCGAAGGAGATCTGTTTGATCGAGATTTTAAGACGAAAACGATCCAGGCTGCTGCTAA
TGGAAATTGGGGCAA
+
FFFFFFFFFFF:FFFFFFFFFFFFFFFFFFFFFFFFFFFFFFFFFFFFFFFFFFF:FFFFFFFFFFF
FFFFFFFFFFFFFFFF:FFF:FFFFFFFFFF:FFFFFFFFF,FFFFFFFFFFFFFFFFFFFFFFFFFF
FFF:FFFFFFFFFFF
@A00721:190:HGJW7DSXY:4:1616:4372:16203 1:N:0:TCTGCAAG+GCTGCACT
GCGTTAATAATTATAGTTTTCGTCTTGATTATTTGTAAGATTTGTTTTGAACAAATCGAGATACATTT
```

```
TCAATGTCCGTCGAAGGAGATCTGTTTGATCGAGATTTTAAGACGAAAACGATCCAGGCTGCTGCTAA
TGGAAATTGGGGCAA
+
FFFFFFFFFFFFFFFFFFFFFFFFFFFFFFFFFFFFFFFFFFFFFFFFFFFFFFFFFFFFFFFFFFFF
FFFFFFFFFFFFF:FFFFFFFFFFFFFFFFFFFFFFFFFFF:FFFFFFFFFFFFFFFFFFFFFFFFFF
FFFFFFF:FFFFFFF
@A00721:190:HGJW7DSXY:4:2102:28664:28902 1:N:0:TCTGCAAG+GCTGCACT
GCGTTAATAATTATAGTTTTCGTCTTGATTATTTGTAAGATTTGTTTTGAACAAATCGAGATACATTT
TCAATGTCCGTCGAAGGAGATCTGTTTGATCGAGATTTTAAGACGAAAACGATCCAGGCTGCTGCTAA
TGGAAATTGGGGCA
+
FFFFFFFFFFFFFFFFFFFFFFFF::FFFFFFFFFFFFFFFFFFFFFFF:FFFFFFFFFFFFFFFFFF
FFFFFFF:FFFFFFFFFFFFFFFFFFFFFFFFFFFFFFFFF,FFFF:FFF:FFFF:FF:FF,FFF:FF:
F:FFFFFF,:FFF:
@A00721:190:HGJW7DSXY:4:2257:24026:10974 1:N:0:TCTGCAAG+GCTGCACT
GCGTTAATAATTATAGTTTTCGTCTTGATTATTTGTAAGATTTGTTTTGAACAAATCGAGATACATTT
TCAATGTCCGTCGAAGGAGATCTGTTTGATCGAGATTTTAAGACGAAAACGATCCAGGCTGCTGCTAA
TGGAAATTGGGGCA
+
FFF,:FFFFFFF:FF,,FFFFF,,,FFFFFFFFFFFFFFFF:,FF:FFFFFFFFF:F,F:FFFFFF:F
FFFFFFF,F,F,FFF:FFFFFFFFFF:FF:FFF:FF,FFFFFFF:FFF:FF::F,F:,,:FFFFF:F,
,FFFF,F,:FFF,F
@A00721:190:HGJW7DSXY:4:2673:10520:34021 1:N:0:TCTGCAAG+GCTGCACT
GCGTTAATAATTATAGTTTTCGTCTTGATTATTTGTAAGATTTGTTTTGAACAAATCGAGATACATTT
TCAATGTCCGTCGAAGGAGATCTGTTTGATCGAGATTTTAAGACGAAAACGATCCAGGCTGCTGCTAA
TGGAAATTGGGGCAA
+
FFFFFFFFFFFFFFFFFFFFFFFF,FF:FFFFFFFFFFFFFFFFFFFFFFFFFFFFFFFFFFFFFFFF
FFFFFFFFFFFFFFFFFFFFFFFFFFFFFFFFFFFFFFFFFFFFFFFFF:FFFFFFFFFFFFFFFFFF
FFFFF,FFFFFFFFF
@A00721:190:HGJW7DSXY:4:1524:21513:20682 1:N:0:TCTGCAAG+GCTGCACT
ATCGTCTTGATTATTTGTAAGATTTGTTTTGAACAAATCGAGATACATTTTCAATGTCCGTCGAAGGA
GATCTGTTTGATCGAGATTTTAAGACGAAAACGATCCAGGCTGCTGCTAATGGAAATTGGGGCAATGT
AAAGCTCCAAATGCC
+
FFFFFFFFFFFFF,FFFFFF:FFFFFFFFF,FF:FFFF,FF:FFFFFFF::FFF,FF,::FF:FFFFF
FFF:FFFFFFFFFFFFFF,FFFF:FFFFFFFFFFFF:FFFFFF,FFFF:FFFFFFFF:::FFFFFF:F
FFF,FFFFFFFFF:F
@A00721:190:HGJW7DSXY:4:2153:18692:13980 1:N:0:TCTGCAAG+GCTGCACT
ATCGTCTTGATTATTTGTAAGATTTGTTTTGAACAAATCGAGATACATTTTCAATGTCCGTCGAAGGA
GATCTGTTTGATCGAGATTTTAAGACGAAAACGATCCAGGCTGCTGCTAATGGAAATTGGGGCAATGT
AAAGCTCCAAATGCC
+
FFFFFFFFFFFFF:FFFFFFFFFFFFFFFFFFFFFFFFFFFFFFFFFFFFFFFFFFFFFFFFFFFFFF
FFFFFFFFFFFF:FFFFFFFFFFFFFFFFFFFFFFFFFFFFFFFFFFFFFFFFFFFFFFFFFFFFFFF
FFFFFFFFFFFFFFF
@A00721:190:HGJW7DSXY:4:2252:21106:2409 1:N:0:TCTGCAAG+GCTGCACT
ATCGTCTTGATTATTTGTAAGATTTGTTTTGAACAAATCGAGATACATTTTCAATGTCCGTCGAAGGA
GATCTGTTTGATCGAGATTTTAATACGAAAACGATCCAGGCTGCTGCTAATGGAAATTGGGGCAATGT
AAAGCTCCAAATGC
+
FFFFF:,F:F:FFFFF::FFFFFFFFFFFFF:FFFFF,:,,FFFF,FFF:F::FFFFFF:F,FFFF:F
FF,FF,F,FFFFF,F:FFF,FFF:FF:FFFFF:FFF:F,FF,:FFF:FF:FF,FFFFFF::FFFFFFF
FFFFFFFFFFFFFF
```

```
@A00721:190:HGJW7DSXY:4:2306:2510:23124 1:N:0:TCTGCAAG+GCTGCACT
ATCGTCTTGATTATTTGTAAGATTTGTTTTGAACAAATCGAGATACATTTTCAATGTCCGTCGAAGGA
GATCTGTTTGATCGAGATTTTAAGACGAAAACGATCCAGGCTGCTGCTAATGGAAATTGGGGCAATGT
AAAGCTCCAAATGCC
+
FFFFFFFFFFFFFFFFFFFFFFFFFFF:FFFFFFFFFFFFFFF:FFFFFFFFFFFFFFFFFFFFFFFFF
FFFFFFFFFFFFFFFFFFFFFFFFFFFFFFFF:FFFFFFFF:FFFFFFFFFFFFFFFFF,FF:FFFFF
FFFFFFFFFFFFF:F
@A00721:190:HGJW7DSXY:4:2306:2528:23124 1:N:0:TCTGCAAG+GCTGCACT
ATCGTCTTGATTATTTGTAAGATTTGTTTTGAACAAATCGAGATACATTTTCAATGTCCGTCGAAGGA
GATCTGTTTGATCGAGATTTTAAGACGAAAACGATCCAGGCTGCTGCTAATGGAAATTGGGGCAATGT
AAAGCTCCAAATGCC
+
F:FFFFFFFFFFFF,FFFFFFFFFFFFFFFFFFFFFFFFF,FFFFFFF:FFFFFFFFFFFFFFFFFFFF
FF:FFFF:FFFFFFFFFF:FFFFFFFFFFF:FFF:FFFFFF:FFF:FFFFFFFF:FF:FFFFFFFFFF
FFFFFFFFFFFF:FF
@A00721:190:HGJW7DSXY:4:2322:15872:31767 1:N:0:TCTGCAAG+GCTGCACT
ATCGTCTTGATTATTTGTAAGATTTGTTTTGAACAAATCGAGATACATTTTCAATGTCCGTCGAAGGA
GATCTGTTTGATCGAGATTTTAAGACGAAAACGATCCAGGCTGCTGCTAATGGAAATTGGGGCAATGT
AAAGCTCCAAATGCC
+
FFFFFF:FFFFFFFFFFFFFFFFFFFFFFFFFFFFFFF:FFFFFFFFFFFFFFFFFFFFFFFFFFFFF
FFFFFFFFFFFFFFFFFFFFFFFFFFFFFFFFFFFFFFFFFFFF:FFFFFFFFFFFFFFFFFFFFFF,FFF
FFFFFFFFFFFF:FF
@A00721:190:HGJW7DSXY:4:2645:10257:18724 1:N:0:TCTGCAAG+GCTGCACT
ATCGTCTTGATTATTTGTAAGATTTGTTTTGAACAAATCGAGATACATTTTCAATGTCCGTCGAAGGA
GATCTGTTTGATCGAGATTTTAAGACGAAAACGATCCAGGCTGCTGCTAATGGAAATTGGGGCAATGT
AAAGCTCCAAATGCC
+
FFFFFFFFFFFFFFFFFFFFFFFFFFFFFFFFFFFFFFFFFFFFFFFFFFFFFFFFFFFFFFFFFFFFF
FFFFFFFFFFFFFFFFFFFFFFFFFFFFFFFFFFFFFFFFFFFFFFFFFFFFFFFFFFFF::FFFFFFF
FFFFFFFFFFFFFFF
@A00721:190:HGJW7DSXY:4:1527:4942:29841 1:N:0:TCTGCAAG+GCTGCACT
CGTCTTGATTATTTGTAAGATTTGTTTTGAACAAATCGAGATACATTTTCAATGTCCGTCGAAGGAGA
TCTGTTTGATCGAGATTTTAAGACGAAAACGATCCAGGCTGCTGCTAATGGAAATTGGGGCAATGTAA
AGCTCCAAATGCCCA
+
FFFFFFFFFFFFFFFFFFFFFFFFFFFFFFFFFFFFFFFFFFFFFFFFFFFFFFFFFFFFFFFFFFFFF
FFFFFFFFFFFFFFFFFFFFFFF:FFFFFFFFFFFFFFFFFFFFFFFFFFFFFFFFFFFFFFFFFFFFF
FFFFFFFFFFFFFFF
@A00721:190:HGJW7DSXY:4:1609:8269:6230 1:N:0:TCTGCAAG+GCTGCACT
CGTCTTGATTATTTGTAAGATTTGTTTTGAACAAATCGAGATACATTTTCAATGTCCGTCGAAGGAGA
TCTGTTTGATCGAGATTTTAAGACGAAAACGATCCAGGCTGCTGCTAATGGAAATTGGGGCAATGTAA
AGCTCCAAATGCCCA
+
FF,FFFFFFFFFFFFFFFFFFFFFFFFFFFFFFFFFFFFFFFFFFFFFFFFFFFFFFFFFFFFFFFFFF
FFFFFFFFFFFFFFFFFFFFFFFFFFFFFFFFFFFFFFFFFFFFFFFFFFFF:FFFFFFFFFFFFFFF
FFFFFFFFFFFFFFF
@A00721:190:HGJW7DSXY:4:1155:31882:22263 1:N:0:TCTGCAAG+GCTGCACT
GTCTTGATTATTTGTAAGATTTGTTTTGAACAAATCGAGATACATTTTCAATGTCCGTCGAAGGAGAT
CTGTTTGATCGAGATTTTAAGACGAAAACGATCCAGGCTGCTGCTAATGGAAATTGGGGCAATGTAAA
GCTCCAAATGCCCAG
+
FFFFFFFFFFFFFFFFFFFFFFFFFFFFFFFFFFFFFFFFFFFFFFFFFFFFFFFFFFFFFFFFFFFFF
```

FFFFFFFFFFFFFFFFFFFFFFFFFFFFFFFFFFFFFFFFFFFFFFFFFFFFFFFFFFFFFFFFFFFFFF
FFFFFFFFFFFFFFF
@A00721:190:HGJW7DSXY:4:1157:12762:10066 1:N:0:TCTGCAAG+GCTGCACT
GTCTTGATTATTTGTAAGATTTGTTTTGAACAAATCGAGATACATTTTCAATGTCCGTCGAAGGAGAT
CTGTTTGATCGAGATTTTAAGACGAAAACGATCCAGGCTGCTGCTAATGGAAATTGGGGCAATGTAAA
GCTCCAAATGCCCAG
+
FFFFFFFFFFFFFFFFFFFFFFFFFFFFFFFFFFFFFFFFFFFFFFFFFFFFFFFFFFFFFFFFFFFFFF
FFFFFFFFFFFFFFFFFFFFFFFFFFFFFFFFFFFFFFFFFFFFFFFFFFFFFFFFFF,FFFFFFFFFFF
FFFFFFFFFFFFFFF
@A00721:190:HGJW7DSXY:4:1302:21233:28494 1:N:0:TCTGCAAG+GCTGCACT
GTCTTGATTATTTGTAAGATTTGTTTTGAACAAATCGAGATACATTTTCAATGTCCGTCGAAGGAGAT
CTGTTTGATCGAGATTTTAAGACGAAAACGATCCAGGCTGCTGCTAATGGAAATTGGGGCAATGTAAA
GCTCCAAATGCCCAG
+
FFFFFFFFFF,F::FFF:F:FFFFFFFFFFFFFFF:FFFFFFFFFFFF:FFFFFFFFFFFFFF:FFFFFF
FFFFFFFFFFFFFFFFF:FFFFFFFFFFFFFFFFFFFFFFFFFFFFFFFFFFF:FFFFFFFFFFFFFFFF
FFFF:FFF::FF:FF
@A00721:190:HGJW7DSXY:4:1412:24252:33191 1:N:0:TCTGCAAG+GCTGCACT
GTCTTGATTATTTGTAAGATTTGTTTTGAACAAATCGAGATACATTTTCAATGTCCGTCGAAGGAGAT
CTGTTTGATCGAGATTTTAAGACGAAAACGATCCAGGCTGCTGCTAATGGAAATTGGGGCAATGTAAA
GCTCCAAATGCCCAG
+
FFFFFFFFFFFFFFFFFFFFFFFFFFFFFFFFFFFFFFFFFFFFFFFFFFFFFFFFFFFFFFF,FFFFFF
FFFFFFFFFFFFFFFFFFFFFFFFFFFFFFFFFFFFFFFFFFFFFFFFFFFFFFFFFFFFFFFFFFFFFF
FFFFFFFFFFFFFFF
@A00721:190:HGJW7DSXY:4:1412:24885:34256 1:N:0:TCTGCAAG+GCTGCACT
GTCTTGATTATTTGTAAGATTTGTTTTGAACAAATCGAGATACATTTTCAATGTCCGTCGAAGGAGAT
CTGTTTGATCGAGATTTTAAGACGAAAACGATCCAGGCTGCTGCTAATGGAAATTGGGGCAATGTAAA
GCTCCAAATGCCCAG
+
FFFF:FFFFFFFFFFFFFF:FFFFFFFFFFFFFFFFFFFFFFFFFFFFFFFFFFFFFFFFFFFFFFFFFF
FFFFFFFFFFFFFFFFFFFFFFFFFFFFFFFFFFFFFFFFFFFFFFFFFFFFFFFFFFFFFFFFFFFFFF
FFFFFFFFFFFFFFF
@A00721:190:HGJW7DSXY:4:2332:15854:17989 1:N:0:TCTGCAAG+GCTGCACT
GTCTTGATTATTTGTAAGATTTGTTTTGAACAAATCGAGATACATTTTCAATGTCCGTCGAAGGAGAT
CTGTTTGATCGAGATTTTAAGACGAAAACGATCCAGGCTGCTGCTAATGGAAATTGGGGCAATGTAAA
GCTCCAAATGCCCAG
+
F:FFFFFFFFFFFFFFFFFFFFFFFFFFFFFFFFFFFFFFFFFFFFFFFFFFFFFFFFFFFFFFFFFFFF
FFFFFFFFFFFF:FFFFFFFFFFFFFFFFFFFFF,FFFFFFFFFFFFFFFFFFFFFFFFFFFFFFFFFFF
FFFFFFFFFFFFFFF
@A00721:190:HGJW7DSXY:4:2539:1371:16110 1:N:0:TCTGCAAG+GCTGCACT
GTCTTGATTATTTGTAAGATTTGTTTTGAACAAATCGAGATACATTTTCAATGTCCGTCGAAGGAGAT
CTGTTTGATCGAGATTTTAAGACGAAAACGATCCAGGCTGCTGCTAATGGAAATTGGGGCAATGTAAA
GCTCCAAATGCCCAG
+
FFF:FFFFFFFFFFFFFFF:FFFFFFFFFFFFFFFFFFFFFFFFFFFFFFFFFFFFFFFFFFFFFFFFFF
FFFFFFFFFFFFFFFFFFFF:FFFFFFFFFFFFFFFFFFFFFFFFFFFFFFFFFFFFFFFFFFFFFFFFF
FFFFFFFFFFFFFFF
@A00721:190:HGJW7DSXY:4:2168:15799:20776 1:N:0:TCTGCAAG+GCTGCACT
CGAGATACATTTTCAATGTCCGTCGAAGGAGATCTGTTTGATCGAGATTTTAAGACGAAAACGATCCA
GGCTGCTGCTAATGGAAATTGGGGCAATGTAAAGCTCCAAATGCCCAGTATGGCCGATGTGATGTACA
ATAAGTTGAAGAAAC

+
FFFFFFFFFFFFFFFFFFFFFFFFFFFFFFFFFFFFFFFFFFFFFFFFFFFFFFFFFFFFFFFFFFFF
FFFFFFFFFFFFFFFFFFFFFFFFFFFFFFFFFFFFFFFFFFFFFFFFFFFFFFFFFFFFFFFFFFFF
FFFFFFFFFFF:FFF
@A00721:190:HGJW7DSXY:4:2336:7916:2206 1:N:0:TCTGCAAG+GCTGCACT
CGAGATACATTTTCAATGTCCGTCGAAGGAGATCTGTTTGATCGAGATTTTAAGACGAAAACGATCCA
GGCTGCTGCTAATGGAAATTGGGGCAATGTAAAGCTCCAAATGCCCAGTATGGCCGATGTGATGTACA
ATAAGTTGAAGAAAC
+
FFFFFFFFFFFFFFFFFFFFFFFFFFFFFFFFFFFFFFFFFFFFFFFFFFFFFFFFFFFFFFFFFFFF
FFFFFFFFFFFFFFFFFFFFFFFFFFFFFFFFFFFFFFFFFFFFFF:FF:FFFFFFF,FFFFFFFFFF
FFFFF:FFF:FFFFF
@A00721:190:HGJW7DSXY:4:2543:29640:24361 1:N:0:TCTGCAAG+GCTGCACT
CGAGATACATTTTCAATGTCCGTCGAAGGAGATCTGTTTGATCGAGATTTTAAGACGAAAACGATCCA
GGCTGCTGCTAATGGAAATTGGGGCAATGTAAAGCTCCAAATGCCCAGTATGGCCGATGTGATGTACA
ATAAGTTGAAGAAAC
+
FFFFFFFFFFFFFFFFFFFFFFFFFFFFFFFFFFFFFFFFFFFFFFFFFFFFFFFFFFFFFFFFFFFF
FFFFFFFFFFFFFFFFFFFFFFFFFFFFFFFF:FFFFFFFFFFFFFFFF,FFFFFFFFF:F,FFFF::
FFFFF:FFFFF,F:F
@A00721:190:HGJW7DSXY:4:2566:17852:23923 1:N:0:TCTGCAAG+GCTGCACT
CGAGATACATTTTCAATGTCCGTCGAAGGAGATCTGTTTGATCGAGATTTTAAGACGAAAACGATCCA
GGCTGCTGCTAATGGAAATTGGGGCAATGTAAAGCTCCAAATGCCCAGTATGGCCGATGTGATGTACA
ATAAGTTGAAGAAAC
+
FFFFFFFFFFFFFFFFFFFFFFFFFFFFFFFFFFFFFFFFFFFFFFFFFFFFFFFFFFFFFFFFFFFF
FFFFFFFFFFFFFFFFFFFFFFFFFFFFFFFF:FFFFFFFFFFFFFFFFFFFFFFFFFF,FFFFFFFF
FFF,F,FFFFFFFFF
@A00721:190:HGJW7DSXY:4:2602:24451:1031 1:N:0:TCTGCAAG+GCTGCACT
CGAGATACATTTTCAATGTCCGTCGAAGGAGATCTGTTTGATCGAGATTTTAAGACGAAAACGATCCA
GGCTGCTGCTAATGGAAATTGGGGCAATGTAAAGCTCCAAATGCCCAGTATGGCCGATGTGATGTACA
ATAAGTTGAAGAAAC
+
FFFFFFFFFFFFFFFFFFFFFFFFFFFF:FFFFFFFFFFFFFFFFFFFFFFFFFFFFFFFFFFFFFFF
FFFFFFFFFFFFFFFFFFFFFFF:FFFFFFFF:FFFFFFFF,FFFFFFFF::FF:FFFFFFFFFFF,F
:FFFFFFF,FFFFFF
@A00721:190:HGJW7DSXY:4:2602:23421:2409 1:N:0:TCTGCAAG+GCTGCACT
CGAGATACATTTTCAATGTCCGTCGAAGGAGATCTGTTTGATCGAGATTTTAAGACGAAAACGATCCA
GGCTGCTGCTAATGGAAATTGGGGCAATGTAAAGCTCCAAATGCCCAGTATGGCCGATGTGATGTACA
ATAAGTTGAAGAAAC
+
FFFFFFFFFFFFFFFFFFFFFFFFFFFFFFFFFFFFFFFFFFFFFFFFFFFFFFFFFFFFFFFFFFFF
FFFFFFFFFFFFFFFFFFFFFFFFFFFFFFFFFFFFFFFFFFF:FFFFF,FFFFFFFFFFFFFFFF:F
F:FFFFFFFFF:FFF
@A00721:190:HGJW7DSXY:4:1175:5475:3302 1:N:0:TCTGCAAG+GCTGCACT
GCGTGTTTCTTCAACTTATTGTACATCACATCGGCCATACTGGGCATTTGGAGCTTTACATTGCCCCA
ATTTCCATTAGCAGCAGCCTGGATCGTTTTCGTCTTAAAATCTCGATCAAACAGATCTCCTTCGACGG
ACATTGAAAATGTAT
+
FFFFFFFFFFFFFFFFFFFFFFFFFFFFFFFFF:FFFFFFFFFFFFFFFFFFFFFFFFFFFFFFFFFF
FFFFFFFFFFFFFFFFFFFFFFFFFFFFFFFFFFFFFFFFFFFFFFFFFFFFF:FFFFFFFFF:FFFF
FFFFFFFFFFFFFFF
@A00721:190:HGJW7DSXY:4:1178:24089:9173 1:N:0:TCTGCAAG+GCTGCACT
GCGTGTTTCTTCAACTTATTGTACATCACATCGGCCATACTGGGCATTTGGAGCTTTACAAAGCCCCA

```
ATTTCCATTAGCAGCAGCCTGGATCGTTTTCGTCTTAAAATCTCGATCAAACAGATCTCCTTCGACGG
ACATTGAAAATGTAT
+
FFFFFFFFFFFFFFFFFFFFFFFFFFFFFFFFFFFFFFFFFFFFFFFFF,FFFFFFFFFFF:,::FFFFF
FFFFFFFFFFFFFFFF:FFFFFFFFFFFFFFFFFFFFFFFFFFFFFFFFFFF,F:FFF:FFFFFFFFFFF
FFFFFFFFFFFFFFF
@A00721:190:HGJW7DSXY:4:1178:23185:10019 1:N:0:TCTGCAAG+GCTGCACT
GCGTGTTTCTTCAACTTATTGTACATCACATCGGCCATACTGGGCATTTGGAGCTTTACATTGCCCCA
ATTTCCATTAGCAGCAGCCTGGATCGTTTTCGTCTTAAAATCTCGATCAAACAGATCTCCTTCGACGG
ACATTGAAAATGTAT
+
FFFFFFFFFFFFFFFFFFFFFFFFFFFFFFFFFFFFFFFFFFFFFFFFFFFFFFFFFFFFFFFFFFFFFF
FFFFFFFFFFFFFFFFFFFFFFFFFFFFFFFFFFFFFFFFFFFFFFFFFFFFFFFFFFFFFFFFFFFFFF
FF:FFFFFFFFFFFF
@A00721:190:HGJW7DSXY:4:1256:2220:12727 1:N:0:TCTGCAAG+GCTGCACT
GCGTGTTTCTTCAACTTATTGTACATCACATCGGCCATACTGGGCATTTGGAGCTTTACATTGCCCCA
ATTTCCATTAGCAGCAGCCTGGATCGTTTTCGTCTTAAAATCTCGATCAAACAGATCTCCTTCGACGG
ACATTGAAAATGTAT
+
FFFFFFFFFFFFFFFFFFFFFFFFFFFFF:FFFFFFFFFFFFFFF:FFFFFFF:FFFFFFFFFFFFFFFF
FFFFFF:FFFFFFFFFFF:FFFFFFFFFFFFFFFFFFFFFFFFFFFFF:FFFFFFFFFFFFFFFFFFFFF
FFFFFFFFFFFFFFF
@A00721:190:HGJW7DSXY:4:1364:28718:28902 1:N:0:TCTGCAAG+GCTGCACT
GCGTGTTTCTTCAACTTATTGTACATCACATCGGCCATACTGGGCATTTGGAGCTTTACATTGCCCCA
ATTTCCATTAGCAGCAGCCTGGATCGTTTTCGTCTTAAAATCTCGATCAAACAGATCTCCTTCGACGG
ACATTGAAAATGTAT
+
FFFFFFFFFFFFFFFFFFFFFFFFFFFFFFFFFFFFFFFFFFFFFFFFFFFFFFFFFFFFFFFFFFFFFF
FFFFFFFFFFFFFFFFFFFFFFFFFFFFFFFFFFFFFFFFFFFFFFFFF:FFFFFFF:FFFFFFFFFF,F
FFFFFFFFFFFFFFF
@A00721:190:HGJW7DSXY:4:1475:29930:28494 1:N:0:TCTGCAAG+GCTGCACT
GCGTGTTTCTTCAACTTATTGTACATCACATCGGCCATACTGGGCATTTGGAGCTTTACATTGCCCCA
ATTTCCATTAGCAGCAGCCTGGATCGTTTTCGTCTTAAAATCTCGATCAAACAGATCTCCTTCGACGG
ACATTGAAAATGTAT
+
FFFFFFFFFFFFFFFFFFFFFFFFFFFFFFFFFFFFFFFFFFFFFFFFFFFFFFFFFFFFFFFFFFFFFF
FFFFFFFFFFFFFFFFFFFFFFFFFFFFFFFFFFFFFFFFFFFFFFFFFFFFFFFFFFFFFFFFFFFFFF
FFFFFFFFFFFFFFF
@A00721:190:HGJW7DSXY:4:1516:1506:34006 1:N:0:TCTGCAAG+GCTGCACT
GCGTGTTTCTTCAACTTATTGTACATCACATCGGCCATACTGGGCATTTGGAGCTTTACATTGCCCCA
ATTTCCATTAGCAGCAGCCTGGATCGTTTTCGTCTTAAAATCTCGATCAAACAGATCTCCTTCGACGG
ACATTGAAAATGTAT
+
FFFFFFFFFFFFFFFFFFFFFF:FFFFFFFFFFFFFF:FFFFFFFFFFFFFFFFFFFFFFFFFFFFFFFF
FFFFFFFFFFFFFFFFFFFFFF:FFFFFFFFFFFFFFFFFFFFFFFFFFFF:FFFFFFFFFFFFFFFFFF
FFFFFFFFFFFFFFF
@A00721:190:HGJW7DSXY:4:1560:27624:30232 1:N:0:TCTGCAAG+GCTGCACT
GCGTGTTTCTTCAACTTATTGTACATCACATCGGCCATACTGGGCATTTGGAGCTTTACATTGCCCCA
ATTTCCATTAGCAGCAGCCTGGATCGTTTTCGTCTTAAAATCTCGATCAAACAGATCTCCTTCGACGG
ACATTGAAAATGTAT
+
FFFFFFFFFFFFFFFFFFFFFFFFFFFFFFFFFFFFFFFFFFFFFF:FFFFFFFFFFFFFFF:FFFFFFF
FFFFFFFFFFFFFFFFFFFFFFFFFFFFFFFFFFFFFFFFFFFFFFFFFFFFFFFFFFFFFFFFFFFFFF
FF:FF:F:FFFFFFF
```

```
@A00721:190:HGJW7DSXY:4:1622:8630:29872 1:N:0:TCTGCAAG+GCTGCACT
GCGTGTTTCTTCAACTTATTGTACATCACATCGGCCATACTGGGCATTTGGAGCTTTACATTGCCCCA
ATTTCCATTAGCAGCAGCCTGGATCGTTTTCGTCTTAAAATCTCGATCAAACAGATCTCCTTCGACGG
ACATTGAAAATGTAT
+
FFFFFFFFFFFFFFFFFFFFFFFFFFFFFFFFFFFFFFFFFFFFFFFFFFFFFFFFFFFFFFFFFFFF
FFFFFFFFFFFFFFFFFFFFFFFFFFFFFFFFFFFFFFFFFFFFFFFFFF:FFFFFFFFFFFFFFFFF
FFFFFFFFFFFFFFF
@A00721:190:HGJW7DSXY:4:1640:9905:31297 1:N:0:TCTGCAAG+GCTGCACT
GCGTGTTTCTTCAACTTATTGTACATCACATCGGCCATACTGGGCATTTGGAGCTTTACATTGCCCCA
ATTTCCATTAGCAGCAGCCTGGATCGTTTTCGTCTTAAAATCTCGATCAAACAGATCTCCTTCGACGG
ACATTGAAAATGTAT
+
FFFFFFFFFFFFFFFFFFFFFFFFFFFFFFFFFFFFFFFFFFFFFFFFFFFFFFFFFFFFFFFFFFFF
FFFFFFFFFFF:FFFFFFFFFFFFFFFFFFFFFFFFFFFFFFFFFFFFFF:FFFFFFFFFFFFFFFFF
FFF:FFFFFFF:FFF
@A00721:190:HGJW7DSXY:4:2238:32814:5744 1:N:0:TCTGCAAG+GCTGCACT
GCGTGTTTCTTCAACTTATTGTACATCACATCGGCCATACTGGGCATTTGGAGCTTTACATTGCCCCA
ATTTCCATTAGCAGCAGCCTGGATCGTTTTCGTCTTAAAATCTCGATCAAACAGATCTCCTTCGACGG
ACATTGAAAATGTAT
+
FFFFFFFFFFFFFFFFFFFFFFFFFFFFFFFFFFFFFFFFFFF:FFFFFFFFFFFFFFFFFFFFFFFF
FFFFFFFFFFFFFFFFFFFF:FFFFFFFFFFFFFFFFF::FFFFFFFFFFFFFFFFFFF:FFFFFFFF
FFFFFFFFFFFFFFF
@A00721:190:HGJW7DSXY:4:2318:25807:4539 1:N:0:TCTGCAAG+GCTGCACT
GCGTGTTTCTTCAACTTATTGTACATCACATCGGCCATACTGGGCAATTGGAGCTTTACATTGCCCCA
ATAACCATTATCAGCAGCATGGATCGTTTTCGTCTTAAAAACTAGATCAAACAGATCACCTTCGACGT
ACATTGAAAAAGTAT
+
FFF:F:FFF,FF,FFFFF:F:,FFF,FF:FFFFF:FFFFFF,FFFF,,,:,F,F,,FFFFFF,FFF,F
F,,,FFF,FF,FFF,F,F,FF:F,:FFF:F:F,F,,FFFF,FF,FFFFFFFFFFF,F,,FF,FFF:,,
F,F:,,FFFF,,FFF
@A00721:190:HGJW7DSXY:4:2503:28926:7905 1:N:0:TCTGCAAG+GCTGCACT
GCGTGTTTCTTCAACTTATTGTACATCACATCGGCCATACTGGGCATTTGGAGCTTTACATTGCCCCA
ATTTCCATTAGCAGCAGCCTGGATCGTTTTCGTCTTAAAATCTCGATCAAACAGATCTCCTTCGACGG
ACATTGAAAATGTAT
+
FFFFFFFFFFFFFFFFFFFFFFFFFFFFFFFFFFFFFFFFFFFFFFFFFFFFFFFFFFFFFFFFFFFF
FFFFFFFF:FFFFFFFFFFFFFFFFFFFFFF:FFFFFFFFF,FFF:FF:FF:FF,F:FFF:::F:FFFF
FFF:F,FFFFF:FF:
@A00721:190:HGJW7DSXY:4:2525:25319:12868 1:N:0:TCTGCAAG+GCTGCACT
GCGTGTTTCTTCAACTTATTGTACATCACATCGGCCATACTGGGCATTTGGAGCTTTACATTGCCCCA
ATTTCCATTAGCAGCAGCCTGGATCGTTTTCGTCTTAAAATCTCGATCAAACAGATCTCCTTCGACGG
ACATTGAAAATGTAT
+
FFF:FFFFFFFFFFFFFFFFFFFFFFFFFFF,FFFFFFFFF:FFF:FFFFFFFFFF:FFFFF:FFFFF
FFFFFFFFFFF,FF,FF,FFFFFF:FFFFFF,F:FFFFFFF,FF,FF:FFFFF,F:,F,FFFFFFFFF
:FFFFFFFFFFFF:F
@A00721:190:HGJW7DSXY:4:2604:29613:3991 1:N:0:TCTGCAAG+GCTGCACT
GCGTGTTTCTTCAACTTATTGTACATCACATCGGCCATACTGGGCATTTGGAGCTTTACATTGCCCCA
ATTTCCATTAGCAGCAGCCTGGATCGTTTTCGTCTTAAAATCTCGATCAAACAGATCTCCTTCGACGG
ACATTGAAAATGTAT
+
FFFFFFFFFFFFFFFFFFFFFFF:FFFFFFFFFFFFFFFFFFFFFFFF:FFFFFFFFFFFFFFFFFFF
```

FFFFFFFF:FFFFFFFFFFFFFFF:FFFFFFFFFFFFFFFF:FFFFFFFFF:FFFF:F:,F,::FFFF
:,F:F:FFFF:FFFF
@A00721:190:HGJW7DSXY:4:1117:21802:4899 1:N:0:TCTGCAAG+GCTGCACT
GTCCGTCGAAGGAGATCTGTTTGATCGAGATTTTAAGACGAAAACGATCCAGGCTGCTGCTAATGGAA
ATTGGGGCAATGTAAAGCTCCAAATGCCCAGTATGGCCGATGTGATGTACAATAAGTTGAAGAAACAC
GCGATCATGATTGAT
+
FFFFFFFFFFFFFFFFFFFFFFFFFFFFFFFFFFFFFFFFFFFFFFFFF:FFFFFFFFFFFFFFFFFFF
FFFFFFFFFFFFFFFFFFFFFFFFFFFFFFFFF:FFFFFFFFFFF:F:FF:FF:FFFFFFFFFFFFFFF
FFFFFFFFFFFFFFF
@A00721:190:HGJW7DSXY:4:1367:28366:26537 1:N:0:TCTGCAAG+GCTGCACT
GTCCGTCGAAGGAGATCTGTTTGATCGAGATTTTAAGACGAAAACGATCCAGGCTGCTGCTAATGGAA
ATTGGGGCAATGTAAAGCTCCAAATGCCCAGTATGGCCGATGTGATGTACAATAAGTTGAAGAAACAC
GCGATCATGATTGAT
+
FFFFFFFFFFFFFFFFFFFFFFFFFFFFFFFFFFFFFFFFFFFFFFFFFFFFFFFFFFFFFFFFFFFF
FFFFFFFFFFFFFFFFFFFFFFFFFFFFFFFFFFFFFFFFFFFFFFFFFFFFFFFFFFFFFF:FFF:F
:FFFFFFFFFFFFFF
@A00721:190:HGJW7DSXY:4:1549:19831:31798 1:N:0:TCTGCAAG+GCTGCACT
GTCCGTCGAAGGAGATCTGTTTGATCGAGATTTTAAGACGAAAACGATCCAGGCTGCTGCTAATGGAA
ATTGGGGCAATGTAAAGCTCCAAATGCCCAGTATGGCCGATGTGATGTACAATAAGTTGAAGAAACAC
GCGATCATGATTGAT
+
FFFFFFFFFFFFFFFFFFFFFFFFFFFFFFFFFFFFFFFFFFFFFFFFFFFFFFFFFFFFFFFFFFFF
FFFFFFFFF:FFFFFFFFFFFFFFFFFFFFFFFFFFFFFFFFFFFFFFFFFFFFFFFFF:FFFFFFFF
FFFFFFFFFFFFFFF
@A00721:190:HGJW7DSXY:4:1644:6515:24768 1:N:0:TCTGCAAG+GCTGCACT
GTCCGTCGAAGGAGATCTGTTTGATCGAGATTTTAAGACGAAAACGATCCAGGCTGCTGCTAATGGAA
ATTGGGGCAATGTAAAGCTCCAAATGCCCAGTATGGCCGATGTGATGTACAATAAGTTGAAGAAACAC
GCGATCATGATTGAT
+
FFFFFFFFFFFFFFFFFFFFFFFFFFFFFFFFFFFFFFFFFFFFFFFFFFFFFFFFFFFFFFFFFFF:
FFFFFFFFFFFFFFFFFFFFFFFFFFFFFFFFF,FFFFFFFFFFFFFFFFFFFFFFFFFFFFFFFFFF
FFFFFFFFFFFFFFF
@A00721:190:HGJW7DSXY:4:2201:1714:33364 1:N:0:TCTGCAAG+GCTGCACT
GTCCGTCGAAGGAGATCTGTTTGATCGAGATTTTAAGACGAAAACGATCCAGGCTGCTGCTAATGGAA
ATTGGGGCAATGTAAAGCTCCAAATGCCCAGTATGGCCGATGTGATGTACAATAAGTTGAAGAAACAC
GCGATCATGATTGAT
+
:FFFFF::FFFFFFFFFFFFFF:FFF:FFFFFFFFFFFFFFFFFF:FFFFFF:FFF:FFFFFFFFFFF
FFFF,:FFFFFFFFFFFFFF:FFFF::F:FFFFFFFFFFF,:FFF:,FFFFF:FFF::FFF:F:FFFF
F:FFFFFF:FFFFFF
@A00721:190:HGJW7DSXY:4:2208:32443:27492 1:N:0:TCTGCAAG+GCTGCACT
GTCCGTCGAAGGAGATCTGTTTGATCGAGATTTTAAGACGAAAACGATCCAGGCTGCTGCTAATGGAA
ATTGGGGCAATGTAAAGCTCCAAATGCCCAGTATGGCCGATGTGATGTACAATAAGTTGAAGAAACAC
GCGATCATGATTGAT
+
FFFFFFFFFFFFFFFFFFFFFFFFFFFFFFFFFFFFF:FFFFFFFF:FFFFFFFFFFFFFFFFFFFFF:
FFFFFFFFFFFF:FFF:FFFFFFFFFFFFFFFFFFFF:FFFFFFFFFFFFFFFFFFFFFFFFFF:FFFF
,FFF:FFFFFFFF:F
@A00721:190:HGJW7DSXY:4:2344:12084:3443 1:N:0:TCTGCAAG+GCTGCACT
GTCCGTCGAAGGAGATCTGTTTGATCGAGATTTTAAGACGAAAACGATCCAGGCTGCTGCTAATGGAA
ATTGGGGCAATGTAAAGCTCCAAATGCCCAGTATGGCCGATGTGATGTACAATAAGTTGAAGAAACAC
GCGATCATGATTGAT

```
+
FFFFFFFFFFFFFFFFFFFFFFFFFFFFFFFFFFFFFFFFFFFFFFFFFFFFFFFFFFFFFFFFFFFF
FFFFFFFFFFFFFFFFFFFFFFFFFFFFFFFFFFFFFFFFFFFFFFFFFFFFFFFFFFFFFFFFFFFF
FFFFFFFFFFFFFFF
@A00721:190:HGJW7DSXY:4:2529:32009:6825 1:N:0:TCTGCAAG+GCTGCACT
GTCCGTCGAAGGAGATCTGTTTGATCGAGATTTTAAGACGAAAACGATCCAGGCTGCTGCTAATGGAA
ATTGGGGCAATGTAAAGCTCCAAATGCCCAGTATGGCCGATGTGATGTACAATAAGTTGAAGAAACAC
GCGATCATGATTGAT
+
FFFFFFFFFFFFFFFFFFFFFFFFFFFFFFFFFFFFF:FFFFFFFFFFFFFFFFFFFFFFFFFFFFFF
FFFFFFFFFFFFFFFFFFFFFFFFFFFFFFFFFFFFFFFFFFFFF:FFFFFFFFFFFFF:FFFFFF:F
FFFFFFFFFFFFF:F
@A00721:190:HGJW7DSXY:4:2649:8757:4319 1:N:0:TCTGCAAG+GCTGCACT
GTCCGTCGAAGGAGATCTGTTTGATCGAGATTTTAAGACGAAAACGATCCAGGCTGCTGCTAATGGAA
ATTGGGGCAATGTAAAGCTCCAAATGCCCAGTATGGCCGATGTGATGTACAATAAGTTGAAGAAACAC
GCGATCATGATTGAT
+
FFFFFFFFFFFFFFFFFFFFFFFFFFFFFFFFFFFFFFFFFFFFFFFFFFFFFFFFFFFFFFFFFFFF
FFFFFFFFFFFFFFFFFFFFFFFFFFFFFFFFFFFFFFFFFFFFFFFFFFFFFFFFFFFFFFFFFFFF
FFFFFFFFFFFFFFF
@A00721:190:HGJW7DSXY:4:1149:29044:19727 1:N:0:TCTGCAAG+GCTGCACT
CGGCCGTCGAAGGAGATCTGTTTGATCGAGATTTTAAGACGAAAACGATCCAGGCTGCTGCTAATGGA
AATTGGGGCAATGTAAAGCTCCAAATGCCCAGTATGGCCGATGTGATGTACAATAAGTTGAAGAAACA
CGCGATCATGATTGA
+
FFFFFFFFFFFFFFFFFFFFFFFFFFFFFFFFFFFFFFFFFFFFFFFFFFFFFFFFFFFFF:FFFFFF
FFFFFFFFFFFFFFFFFFFFFFFFFFFFFFFFFFFFFFFFFFFFFFFFFFFFFFFFFFFFFFFFFFFF
FFFF,FFFFFFFFFF
@A00721:190:HGJW7DSXY:4:1249:1841:9878 1:N:0:TCTGCAAG+GCTGCACT
CGGCCGTCGAAGGAGATCTGTTTGATCGAGATTTTAAGACGAAAACGATCCAGGCTGCTGCTAATGGA
AATTGGGGCAATGTAAAGCTCCAAATGCCCAGTATGGCCGATGTGATGTACAATAAGTTGAAGAAACA
CGCGATCATGATTGA
+
FFFFFFFFFFFFFFFFFFFFFFFFFFFFFFFFFFFFFFFFFFFFFFFFFFFFFFFFFFFFFFFFFFFF
FFFFFFFFFFFFFFFFFFFFFFFFFFFFFFFFFFFFFFFFFFFFF,FFFFF::FFFFFFFFFFFFFFFF
F:FFFFFFF:FF,FF
@A00721:190:HGJW7DSXY:4:1530:3812:31548 1:N:0:TCTGCAAG+GCTGCACT
CGGCCGTCGAAGGAGATCTGTTTGATCGAGATTTTAAGACGAAAACGATCCAGGCTGCTGCTAATGGA
AATTGGGGCAATGTAAAGCTCCAAATGCCCAGTATGGCCGATGTGATGTACAATAAGTTGAAGAAACA
CGCGATCATGATTGA
+
FFFFFFFFFFFFFFFFFFFFFFFFFFFFFFFFFFFFFFFFFFFFFFFFFFFFFFFFFFFFFFFFFFFF
:FFFFFFFFFFFFFFFFFFFFFFFFFFFFFFFFFFFFFFFFFFFFFFFFFFFF,FFFFF:FFFFFFF:
FFFFFFFFFFFFFF:
@A00721:190:HGJW7DSXY:4:1530:3775:31610 1:N:0:TCTGCAAG+GCTGCACT
CGGCCGTCGAAGGAGATCTGTTTGATCGAGATTTTAAGACGAAAACGATCCAGGCTGCTGCTAATGGA
AATTGGGGCAATGTAAAGCTCCAAATGCCCAGTATGGCCGATGTGATGTACAATAAGTTGAAGAAACA
CGCGATCATGATTGA
+
FFFFFFFFFFFFFFFFFFFFFFFFFFFFFFFFFFFFFFFFFFFFFFFFFFFFFFFFFFFFFFFFFFFF
FFFFFFFFFFFFFFFFFFFFFFFFFFFFFFFFFFFFF:FFFFFFFFFFFFFF:FFFFFFF:FF:FFFF
FFFFFFFFFFFFFFF
@A00721:190:HGJW7DSXY:4:1574:8404:17926 1:N:0:TCTGCAAG+GCTGCACT
CGGCCGTCGAAGGAGATCTGTTTGATCGAGATTTTAAGACGAAAACGATCCAGGCTGCTGCTAATGGA
```

AATTGGGGCAATGTAAAGCTCCAAATGCCCAGTATGGCCGATGTGATGTACAATAAGTTGAAGAAACA
CGCGATCATGATTGA
+
FFFFFFFFFFFFFFFFFFFFFFFFFFFFFFFFFFFFFFFF:FFFFFFFFFFFFFFFFF:FFFFF:FFFF
FFF:FFFFFFFFFFFFFFFFF:FFF:F:FF:FFFFFFFFFFFFFFF:FFFFFFFFFFFFFFFF:FFFF
FFFFFFFFFFFFFFF
@A00721:190:HGJW7DSXY:4:1656:10981:27962 1:N:0:TCTGCAAG+GCTGCACT
CGGCCGTCGAAGGAGATCTGTTTGATCGAGATTTTAAGACGAAAACGATCCAGGCTGCTGCTAATGGA
AATTGGGGCAATGTAAAGCTCCAAATGCCCAGTATGGCCGATGTGATGTACAATAAGTTGAAGAAACA
CGCGATCATGATTGA
+
FFFFFFFFFFFFFFFFFFFFFFFFFFFFFFFFFFFFFFFFFFFFF:FFFFFFFFFFFFFFFFFFFFFFF
FFFFFFFFFF:FF:FFFFFFFFFFFFFFFFFFFFFFFFFFFFFFF:FFFFF,F:F:FFFFFFFFFFFF
FFF:::FF:FFFFFF
@A00721:190:HGJW7DSXY:4:2144:10185:18161 1:N:0:TCTGCAAG+GCTGCACT
CGGCCGTCGAAGGAGATCTGTTTGATCGAGATTTTAAGACGAAAACGATCCAGGCTGCTGCTAATGGA
AATTGGGGCAATGTAAAGCTCCAAATGCCCAGTATGGCCGATGTGATGTACAATAAGTTGAAGAAACA
CGCGATCATGATTGA
+
FFFFFFFFFFFFFFFFFFFFFFFFFFFFFFFFFFFFFFFFFFFFFFFFFFFFFFFFFFFFFFFFFFFF
FFFFFFFFFFFFFFFFFFFFFFFFFFFFFFFFFFFFFFFFFFFFFFFFFFFFFFFFF:FFFFFFFFFFF
FFFFF,FFFFFFFFF
@A00721:190:HGJW7DSXY:4:2323:16495:26897 1:N:0:TCTGCAAG+GCTGCACT
CGGCCGTCGAAGGAGATCTGTTTGATCGAGATTTTAAGACGAAAACGATCCAGGCTGCTGCTAATGGA
AATTGGGGCAATGTAAAGCTCCAAATGCCCAGTATGGCCGATGTGATGTACAATAAGTTGAAGAAACA
CGCGATCATGATTGA
+
FFFFFFFFFFFFFFFFFFFFFFFFFFFFFFFFF:FFFFFFFFFFFFFFFFFFFFFFFFFFFFFF:FFFFF
FF:FFFFFFFFFFFFFFFFFFFFFFFFFFFFFFFFFF:FFFFFFFFFFFFFFFF,FFFF:FFFFFFFFFF
FFFF,FFFFFFFFF:
@A00721:190:HGJW7DSXY:4:1146:10719:34240 1:N:0:TCTGCAAG+GCTGCACT
CGTCGAAGGAGATCTGTTTGATCGAGATTTTAAGACGAAAACGATCCAGGCTGCTGCTAATGGAAATT
GGGGCAATGTAAAGCTCCAAATGCCCAGTATGGCCGATGTGATGTACAATAAGTTGAAGAAACACGCG
ATCATGATTGATCGC
+
FFFFFFFFFFFFFFFFFFFFFFFFFFFFFFF:FFFFFFFFFFFFFFFFFFFFFFFFFFFFFFFFFFFFFF
FFFFFF,FFFF:FFFFFFFF:FFFFFFFFFFFFFFF:FFFFFFFFFFFFFFFFFFFF:FFFFFFFFF
FFFFFFFF:FFFF:F
@A00721:190:HGJW7DSXY:4:1217:26078:9768 1:N:0:TCTGCAAG+GCTGCACT
CGTCGAAGGAGATCTGTTTGATCGAGATTTTAAGACGAAAACGATCCAGGCTGCTGCTAATGGAAATT
GGGGCAATGTAAAGCTCCAAATGCCCAGTATGGCCGATGTGATGTACAATAAGTTGAAGAAACACGCG
ATCATGATTGATCGC
+
FFFFFFFFFFFFFFFFFFFFFFFFFFFFFFFFFFFFFFFFFFFFFFFFFFFFFFFFFFFFFFFFFFFFF
FFFFFFFFFF:FFFFFFFFFFFFFFFFFFFFFFFFFFFFFFFFFF:FFFFFFFFFFFFF:FFFF:F:F
FFFFFFFFFF:FFFF
@A00721:190:HGJW7DSXY:4:1217:28311:13823 1:N:0:TCTGCAAG+GCTGCACT
CGTCGAAGGAGATCTGTTTGATCGAGATTTTAAGACGAAAACGATCCAGGCTGCTGCTAATGGAAATT
GGGGCAATGTAAAGCTCCAAATGCCCAGTATGGCCGATGTGATGTACAATAAGTTGAAGAAACACGCG
ATCATGATTGATCGC
+
FFFFFFFFFFFFFFFFFFFFFFFFFFFFFFFFFFFF:FFFFFFFFFF:FFFFFFFFFFFFFFFFFFFFFF
FFFFFFFFFFFFFFFFFFFFFFFFFFFFFFFFFFFFFFFFFFFFFFFFFFFFFFFFF:FF:FFFFFFFF
FFFF:FFFFF:FFFF

@A00721:190:HGJW7DSXY:4:1417:18982:23030 1:N:0:TCTGCAAG+GCTGCACT
CGTCGAAGGAGATCTGTTTGATCGAGATTTTAAGACGAAAACGATCCAGGCTGCTGCTAATGGAAATT
GGGGCAATGTAAAGCTCCAAATGCCCAGTATGGCCGATGTGATGTACAATAAGTTGAAGAAACACGCG
ATCATGATTGATCGC
+
FFFFFFFFFFFFFF:FFF:FFFFFFFFFFFFFFFFFFFFFF:FFFFFFF:FFFF:FFFFFFFFFFFFF
FFFFFFFFFFFFFFFFFFFFFFFFFF:FFFFFFFFFFFFFFFFFFFFFFFFFFFFFFFF:FFFFFFFF
FFFFFFFFFFFFFFF
@A00721:190:HGJW7DSXY:4:2269:25355:24862 1:N:0:TCTGCAAG+GCTGCACT
CGTCGAAGGAGATCTGTTTGATCGAGATTTTAAGACGAAAACGATCCAGGCTGCTGCTAATGGAAATT
GGGGCAATGTAAAGCTCCAAATGCCCAGTATGGCCGATGTGATGTACAATAAGTTGAAGAAACACGCG
ATCATGATTGATCGC
+
FFFFFFFFFFFFFFFFFFFFFFFFFFFFFFFFFF,FFFFFFFFFFFFFFFFFFFFFFFFFFFFFFFFF
FFFFFFFFFFFFF:FFFFFFFFF:FFFFFFFFFFFFFFFFFFFFFFFFF:FFFFFFFFF:FFFFFFFF
FFFFFFFFFFFF:FF
@A00721:190:HGJW7DSXY:4:2573:1696:11663 1:N:0:TCTGCAAG+GCTGCACT
CGTCGAAGGAGATCTGTTTGATCGAGATTTTAAGACGAAAACGATCCAGGCTGCTGCTAATGGAAATT
GGGGCAATGTAAAGCTCCAAATGCCCAGTATGGCCGATGTGATGTACAATAAGTTGAAGAAACACGCG
ATCATGATTGATCGC
+
FFFFFFFFFFFFFFFFFFFFFFFFFFF:FFFFFFFFFFFFFFFFFFFFFFFFFFFFFFFFFFFFFFFF
FFFFFFFFFFFFFFFFFFFFFFFFFFFFFFFFFFFFFFFFFFFFFFFFFFFFFFF:FFF:FFFFFFFF
FFFFFFFFFFFFF:F
@A00721:190:HGJW7DSXY:4:1311:5864:24956 1:N:0:TCTGCAAG+GCTGCACT
GTCGAAGGAGATCTGTTTGATCGAGATTTTAAGACGAAAACGATCCAGGCTGCTGCTAATGGAAATTG
GGGCAATGTAAAGCTCCAAATGCCCAGTATGGCCGATGTGATGTACAATAAGTTGAAGAAACACGCGA
TCATGATTGAGATCG
+
FFFFFFFFFFFFFFFFFFFFFFFFFFFFFFFFFFFFFFFFFFFFFFFFFFFFFFFFFFFFFFFFF:FF
FFFFF:FFFFFFFFFFFFFFFFFFFFFFFFFFFFFFFFFFFFFFFFFFFFFFFFFFFFFFFFFFFFFF
FFFFFFFFFFFFFF:
@A00721:190:HGJW7DSXY:4:1662:17671:7263 1:N:0:TCTGCAAG+GCTGCACT
GTCGAAGGAGATCTGTTTGATCGAGATTTTAAGACGAAAACGATCCAGGCTGCTGCTAATGGAAATTG
GGGCAATGTAAAGCTCCAAATGCCCAGTATGGCCGATGTGATGTACAATAAGTTGAAGAAACACGCGA
TCATGATTGAGATCG
+
FFFFFFFFFFFFFFFFFFFFFFFFFFFFFFFFFFFFFFFFFFFFFFFFFFFFFFFFFFFFFFFFFFFF
FFFFFFFFFFFFFFFFFFFFFFFFFFFF:FFFFFFFFFFFFFFFFFFFFFFFFFFFFFFFFFFF:FFF
FFFFFFF,FFFFFFF
@A00721:190:HGJW7DSXY:4:2246:23891:30123 1:N:0:TCTGCAAG+GCTGCACT
GTCGAAGGAGATCTGTTTGATCGAGATTTTAAGACGAAAACGATCCAGGCTGCTGCTAATGGAAATTG
GGGCAATGTAAAGCTCCAAATGCCCAGTATGGCCGATGTGATGTACAATAAGTTGAAGAAACACGCGA
TCATGATTGAGATCG
+
FFFFFFFFFFFFFFFFFFFFFFFFFFFFFFFFFFFFFFF:FFFFFFFFFFFFFFFFFFFFFFFFFFFF
FFFFFFFFFF,FFFFFFFFFFFFFFFFF,FFFFFFFFFFFF:FFFFFFFFFFFFFFFFFFFFFFFFFF
FFFFFF:FFFFFFFF
@A00721:190:HGJW7DSXY:4:2276:3513:1814 1:N:0:TCTGCAAG+GCTGCACT
GTCGAAGGAGATCTGTTTGATCGAGATTTTAAGACGAAAACGATCCAGGCTGCTGCTAATGGAAATTG
GGGCAATGTAAAGCTCCAAATGCCCAGTATGGCCGATGTGATGTACAATAAGTTGAAGAAACACGCGA
TCATGATTGAGATCG
+
FFFFFFFFFFFFFFFFFFFFFFFFFFFFFFFFFFFFFFFFFFFFFFFFFFFFFFFFFFFFFFFFFFFF

FFFFFFFFFFFFFFFFFFFFFFFFFFFFFFFFFFFFFFFFFFFFFFFFFFFFFFFF:FFFFFFFFF
FFFFFFFFF:FFFFF
@A00721:190:HGJW7DSXY:4:2375:15365:9690 1:N:0:TCTGCAAG+GCTGCACT
GTCGAAGGAGATCTGTTTGATCGAGATTTTAAGACGAAAACGATCCAGGCTGCTGCTAATGGAAATTG
GGGCAATGTAAAGCTCCAAATGCCCAGTATGGCCGATGTGATGTACAATAAGTTGAAGAAACACGCGA
TCATGATTGAGATCG
+
FFFFFFFFFFFFFFFFFFFFFFFFFFFFFFFFFFFFFFFFFFFFFFFFFFFFFFFFFFFFFFFFFFFF
FFFFFFFFFFFFFFFFFFFFFFFFFFFFFFFFFFFFFFFFFFFFFFFFFFFFFFFFFFFFFFFFFFFF
FFFFFF,:FFFFFFF
@A00721:190:HGJW7DSXY:4:2418:9588:18286 1:N:0:TCTGCAAG+GCTGCACT
GTCGAAGGAGATCTGTTTGATCGAGATTTTAAGACGAAAACGATCCAGGCTGCTGCTAATGGAAATTG
GGGCAATGTAAAGCTCCAAATGCCCAGTATGGCCGATGTGATGTACAATAAGTTGAAGAAACACGCGA
TCATGATTGAGATCG
+
FFFFFFFFFFFFFFFFFFFFFFFFFFFFFFFFFFFFFFFFFFFFFFFFFFFFFFFFFFFFFFF:FFFF
FFFFFFFFFFFFFFFFFFFFFFFFFFFFFFFFFFFFFFFFFFFFFFFFFFFFFFF:FFFFFFFFFFFF
FFFFFFF,FFFFFFF
@A00721:190:HGJW7DSXY:4:2525:31919:31688 1:N:0:TCTGCAAG+GCTGCACT
GTCGAAGGAGATCTGTTTGATCGAGATTTTAAGACGAAAACGATCCAGGCTGCTGCTAATGGAAATTG
GGGCAATGTAAAGCTCCAAATGCCCAGTATGGCCGATGTGATGTACAATAAGTTGAAGAAACACGCGA
TCATGATTGAGATCG
+
FFFFFFFFFFFFFFFFFFFFFFFFFFFFFFFFFFFFFFFFFFFFFFFFFFFFFFFFFFFFFFFFFFFF
FFFFFFFFFFFFFFFFFFFFFFFFFFFFFFFFFFFFFFFFFFFFFFFFFFFFFFFFFFFFFFFFFFFF
FFFFFFFFFFFFFFF
@A00721:190:HGJW7DSXY:4:1111:11885:31594 1:N:0:TCTGCAAG+GCTGCACT
CGAGATTTTAAGACGAAAACGATCCAGGCTGCTGCTAATGGAAATTGGGGCAATGTAAAGCTCCAAAT
GCCCAGTATGGCCGATGTGATGTACAATAAGTTGAAGAAACACGCGATCATGATTGATCGCTGTGTTA
TCGAGTATCGACACC
+
FFFFFFFFFFFFFFFFFFFFFFFFFFFFFFFFFFFF,FFFFFFFFFFFFFFFFFFFFFFFFFFFFFFF
FFFFFFFFFFFFFFFFFFFFF:FFFFFFFFFFFFFFFFFFFFFFFFFFFFFFFFFFFFFF:FFFFFFFF
FF:FFFFFFFFFFFF
@A00721:190:HGJW7DSXY:4:1375:30843:28009 1:N:0:TCTGCAAG+GCTGCACT
CGAGATTTTAAGACGAAAACGATCCAGGCTGCTGCTAATGGAAATTGGGGCAATGTAAAGCTCCAAAT
GCCCAGTATGGCCGATGTGATGTACAATAAGTTGAAGAAACACGCGATCATGATTGATCGCTGTGTTA
TCGAGTATCGACACC
+
FFFFFFFFFFFF:FFFFFFFFFFFFFFFFFFFFFFFFFFFFFFFFFFFFFFFFFFFFFFFFFFFFFFF
FFFFFFFFFFFFFFFFFFFFFFFFFFFFFFFFFFFFFFFFFFFFFFFFFFFFFFFFFFFFFFFFFFFF
FFFFFFFFFFFFFFF
@A00721:190:HGJW7DSXY:4:1654:14516:2300 1:N:0:TCTGCAAG+GCTGCACT
CGAGATTTTAAGACGAAAACGATCCAGGCTGCTGCTAATGGAAATTGGGGCAATGTAAAGCTCCAAAT
GCCCAGTATGGCCGATGTGATGTACAATAAGTTGAAGAAACACGCGATCATGATTGATCGCTGTGTTA
TCGAGTATCGACACC
+
FFF:FFF,FFFFFFFFFFFFFFFFFF,F:FF,FF:FFFFFFFFFFFFFFFFFFFFFFFFFFFFFFFFF:
FFF,F::FFFF:F,FF:F:FFFFFFFFF:FFFFFFF:FFFFFFFFFFF::,,FFFFFF:FFF:FFFFF
F,FF,FFFFFF,F:F
@A00721:190:HGJW7DSXY:4:2325:31114:32957 1:N:0:TCTGCAAG+GCTGCACT
CGAGATTTTAAGACGAAAACGATCCAGGCTGCTGCTAATGGAAATTGGGGCAATGTAAAGCTCCAAAT
GCCCAGTATGGCCGATGTGATGTACAATAAGTTGAAGAAACACGCGATCATGATTGATCGCTGTGTTA
TCGAGTATCGACACC

```
+
FFFFFFFFFFFFFFFFFFFFFFFFFFFFFFFFFFFFFFFFFFFFFFFFFFFFFFFFFFFFFFFFFFFFFF
FFFFFFFFFFFFFFFFFFFFFFFFFFFFFFFFFFFFFFFFFFFFFFFFFFFFFFFFFFFFFFFFFFFFFF
FFFFFFFFFFFFFFF
@A00721:190:HGJW7DSXY:4:1122:20582:2910 1:N:0:TCTGCAAG+GCTGCACT
CTGGTGTCGATACTCGATAACACAGCGATCAATCATGATCGCGTGTTTCTTCAACTTATTGTACATCA
CATCGGCCATACTGGGCATTTGGAGCTTTACATTGCCCCAATTTCCATTAGCAGCAGCCTGGATCGTT
TTCGTCTTAAAATCT
+
FFFFFFFFFFFFFFFFFFFFFFFFFFFFFFFFFFFFFFFFFFFFFFFFFFFFFFFFFFFFFFFFFFF,FF
FF,FFF:FF:FFFFFFFFFFFFFFFFFFFFFFF:FFFFFFFFFFFFFFFFFFFFFFFFFFFFFFFFFFF:
FFFFFFF:FFFFFFF
@A00721:190:HGJW7DSXY:4:1160:16387:13964 1:N:0:TCTGCAAG+GCTGCACT
CTGGTGTCGATACTCGATAACACAGCGATCAATCATGATCGCGTGTTTCTTCAACTTATTGTACATCA
CATCGGCCATACTGGGCATTTGGAGCTTTACATTGCCCCAATTTCCATTAGCAGCAGCCTGGATCGTT
TTCGTCTTAAAATCT
+
FFFFFFFFFFFFFFFFFFFFFFFFFFFFFFFF:FFFFFFFFFFFFFFFFFFFFFFFFFFFFFFF:FFFFF
FFFFFFFFFFFFFFFFFFFFFFFFFFF:FFFFFFFFFFFFFFFFFFFFFFFFFFFFFFFFFFFFFFFFFF
FFFFFFFFFFFFFFF
@A00721:190:HGJW7DSXY:4:1336:17264:23938 1:N:0:TCTGCAAG+GCTGCACT
CTGGTGTCGATACTCGATAACACAGCGATCAATCATGATCGCGTGTTTCTTCAACTTATTGTACATCA
CATCGGCCATACTGGGCATTTGGAGCTTTACAGTGCCCCAATTTCCATTAGCAGCAGCCTGGATCGTT
TTCGTCTTAAAATCT
+
:F,FFFFFFFFFFFFFFFFFFFFFFFFFF:FFFFFFFFFFFFFF,FFFFFFFFFF:F,FFFFFF:FFFFF
FFFFFFFFFFF:FFFFFF,FFFFFFFFF:FFFF,:FFFFFFFF:FFFFFFFFFFFFFF:FF,FFF:FF,F
FFFFFF,:FFF:FFF
@A00721:190:HGJW7DSXY:4:1552:8703:16783 1:N:0:TCTGCAAG+GCTGCACT
CTGGTGTCGATACTCGATAACACAGCGATCAATCATGATCGCGTGTTTCTTCAACTTATTGTACATCA
CATCGGCCATACTGGGCATTTGGAGCTTTACATTGCCCCAATTTCCATTAGCAGCAGCCTGGATCGTT
TTCGTCTTAAAATCT
+
FFFFFFFFFFFFFFFFFFFFFFFFFFFFFFFFFFFFFFFFFFFFFFFFFFFFFFFFFFFFFFFFFFFFFF
FFFFFFFFFFFFFFFFFFFFFFFFFFFFFFFFFFFFFFFFFFFFFFFFFFFFFFFFFFFFFFFFFFFFFF
FFFFFFFFFFFFFFF
@A00721:190:HGJW7DSXY:4:1608:19741:8093 1:N:0:TCTGCAAG+GCTGCACT
CTGGTGTCGATACTCGATAACACAGCGATCAATCATGATCGCGTGTTTCTTCAACTTATTGTACATCA
CATCGGCCATACTGGGCATTTGGAGCTTTACATTGCCCCAATTTCCATTAGCAGCAGCCTGGATCGTT
TTCGTCTTAAAATCT
+
FFFFFFFFFFFFFFFFFFFFFFFFFFFFFFF:FFFFFFFFFFFFFFFFFFFFFFFFFFFFFFFFFFFFFF
FFFFFFFFFFFFFFFFFFFFFFFFFFFFFFFF:FFFFFFFFFFFFFFFFFFFFFFFFFFFFFFFFFFFFF
FFFFFFFFFFFFFFF
@A00721:190:HGJW7DSXY:4:1677:27498:11819 1:N:0:TCTGCAAG+GCTGCACT
CTGGTGTCGATACTCGATAACACAGCGATCAATCATGATCGCGTGTTTCTTCAACTTATTGTACATCA
CATCGGCCATACTGGGCATTTGGAGCTTTACATTGCCCCAATTTCCATTAGCAGCAGCCTGGATCGTT
TTCGTCTTAAAATCT
+
FFFFFFFFFFFFFFFFFFFFFFFFFFFFFFFFFFFFFFFFFFFFF:FFFFFFF:FFFFFF::FFFFFFFF
FFFFFFFFFFFFFFFFFFFFFFFFFF::FFFFF,FF:FFFFFFFFFFFFFFF:FFFFFFFFFFFFFFFFF
FFFFFFFFFFFFFFF
@A00721:190:HGJW7DSXY:4:2258:32244:9361 1:N:0:TCTGCAAG+GCTGCACT
CTGGTGTCGATACTCGATAACACAGCGATCAATCATGATCGCGTGTTTCTTCAACTTATTGTACATCA
```

```
CATCGGCCATACTGGGCATTTGGAGCTTTACATTGCCCCAATTTCCATTAGCAGCAGCCTGGATCGTT
TTCGTCTTAAAATCT
+
FFFFFFFFFFFFFFFFFFFFFFFFFFFFFFFFFFFFFFFFFFFFFFFFFFFFFFFF:FFFFFFFFFFF
FFFFF:FFFFF:FF:FFFFFFFFFFFFFFF:FFFFFFFFFFFFFFFFFFFFFFFFFFFFFFF:FFFFF
FFFFFFFFFFFFF:F
@A00721:190:HGJW7DSXY:4:2258:30463:19241 1:N:0:TCTGCAAG+GCTGCACT
CTGGTGTCGATACTCGATAACACAGCGATCAATCATGATCGCGTGTTTCTTCAACTTATTGTACATCA
CATCGGCCATACTGGGCATTTGGAGCTTTACATTGCCCCAATTTCCATTAGCAGCAGCCTGGATCGTT
TTCGTCTTAAAATCT
+
FFFFFFFFFFFFFFFFFFFFFFFFFFFFFFFFFFFFFFFFFFFFFFFFFFFFFFFFFFFFFFFFFFFF
FFFFFFFFFFFFFFFFFFFFFFFFFFFFFFFFFFFFFFFFFFFFFFFFFFFFFFFFFFFFFFFFFFFF
FFFFFFFFFFFFFFF
@A00721:190:HGJW7DSXY:4:2409:8856:35117 1:N:0:TCTGCAAG+GCTGCACT
CTGGTGTCGATACTCGATAACACAGCGATCAATCATGATCGCGTGTTTCTTCAACTTATTGTACATCA
CATCGGCCATACTGGGCATTTGGAGCTTTACATTGCCCCAATTTCCATTAGCAGCAGCCTGGATCGTT
TTCGTCTTAAAATCT
+
FFFFFFFFFFFFFFFFFFFFFFFFFFFFFFFFFFFFFFFFFFFFFFFFFFFFFFFFFFFFFFFFFFFF
FFFFFFFFFFFFFFFFFFFFFFFFFFFFFFFFFFFFFFFFFFFFFFFFFFFFFFFFFFFFFFFFFFFF
FFFFFF:FFFFFFFF
@A00721:190:HGJW7DSXY:4:2533:27037:6511 1:N:0:TCTGCAAG+GCTGCACT
CTGGTGTCGATACTCGATAACACAGCGATCAATCATGATCGCGTGTTTCTTCAACTTATTGTACATCA
CATCGGCCATACTGGGCATTTGGAGCTTTACATTGCCCCAATTTCCATTAGCAGCAGCCTGGATCGTT
TTCGTCTTAAAATCT
+
FFFFFFFFFFFFFFFFFFFFFFFFFFFFFFFFFFFFFFFFFFFFFFFFFFFFFFFFFFFFFFFFFFFF
FFFFFFFFFFFFFFFFFFFFFFFFFFFF:FFFFFFFFFFFFFFFFFFFFF:FFF:FFFFFFFFFFFFF
FFFFFFFFFFFFFFF
@A00721:190:HGJW7DSXY:4:2408:4508:29716 1:N:0:TCTGCAAG+GCTGCACT
CTGGAGTCGATACTCGATAACACAGCGATCAATCATGATCGCGTGTTTCTTCAAATTATTGTAAATCA
CATCGGCCATACGGGGCAATTGGAGCTATACATTGCCCAAATTTCCATTAGCAGCAGCCTGGATCGTT
GTCGTCTTAAA
+
F:,::F::,F:FF:FFF:FF:F,FFF:FFF:FFFFF,FF,:,::,,FF:::FFF,FFF:F:FF,F::F
FFF:,FFFF:FF,,,,FF,FFFFFFF,,:FFF:,,,FF,FF,:,,FF,:F,F,,FF,FFF::F,,F,F
,:::FFF,F:F
@A00721:190:HGJW7DSXY:4:1559:8115:13291 1:N:0:TCTGCAAG+GCTGCACT
CAGGCTGCTGCTAATGGAAATTGGGGCAATGTAAAGCTCCAAATGCCCAGTATGGCCGATGTGATGTA
CAATAAGTTGAAGAAACACGCGATCATGATTGATCGCTGTGTTATCGAGTATCGACACCAGTGTCCGA
TTAATAGTGAGGGAT
+
FFFFFFFFFFFFFFFFFFFFFFFFFFFFFFFFFFFFFFFFFFFFFFFFFFF,FFFFFFFFFFFFFFFF
FFF,FFFFFFFFFFFFFFFFFFFFFFFFFFFFFFFFFFFFFFFFFFFFFFFFFFFFFFFFFFFFFFFF
FFFFFFFFFFFFFFF
@A00721:190:HGJW7DSXY:4:1671:29686:31955 1:N:0:TCTGCAAG+GCTGCACT
CAGGCTGCTGCTAATGGAAATTGGGGCAATGTAAAGCTCCAAATGCCCAGTATGGCCGATGTGATGTA
CAATAAGTTGAAGAAACACGCGATCATGATTGATCGCTGTGTTATCGAGTATCGACACCAGTGTCCGA
TTAATAGTGAGGGAT
+
FFFFFFFFFFFFFFFFFFFFFFFFFFFFFFFFFFFFFFFFFFFFFFFFFFFFFFFFFFFFFFFFFFFF
FFFFFFFFFFFFFFFFFFFFFFFFFFFFFFFFFFFFFFFFFFFFFFFFFFFFFFFFFFFFFFFFFFFF
FFFFFFFFFFFFFFF
```

```
@A00721:190:HGJW7DSXY:4:2202:25229:15718 1:N:0:TCTGCAAG+GCTGCACT
CAGGCTGCTGCTAATGGAAATTGGGGCAATGTAAAGCTCCAAATGCCCAGTATGGCCGATGTGATGTA
CAATAAGTTGAAGAAACACGCGATCATGATTGATCGCTGTGTTATCGAGTATCGACACCAGTGTCCGA
TTAATAGTGAGGGAT
+
FFFFFFFFFFFFFFFFFFFFFFFFFFFFFFFFFFFFFFFFFFFFFFFFFFFFFFFFFFFFF,FFFFFF
FFFFFFFFFFFFFFFFFFFFFFFFFFFFFFFFFFFFFFFFFFFFFFFFFFFFFFFFFFFFFFFFFFFF
FFFFFFFFFFFFFFF
@A00721:190:HGJW7DSXY:4:2428:20039:20979 1:N:0:TCTGCAAG+GCTGCACT
CAGGCTGCTGCTAATGGAAATTGGGGCAATGTAAAGCTCCAAATGCCCAGTATGGCCGATGTGATGTA
CAATAAGTTGAAGAAACACGCGATCATGATTGATCGCTGTGTTATCGAGTATCGACACCAGTGTCCGA
TTAATAGTGAGGGAT
+
FFFFFFFFFFFFFFFFFFFFFFFFFFFFFFFFFFFFFFFFFFFFFFFFFFF,FFFFFFFFF:FFFFFF
FFFFFFFFFFFFFFFFFFFFFFF:FFFFFFFFF:FF:FFFFFFFFFFFFFFFFFFFFFFFFFFFFFFF
FFFFFFFFFFFFFFF
@A00721:190:HGJW7DSXY:4:2428:19524:22529 1:N:0:TCTGCAAG+GCTGCACT
CAGGCTGCTGCTAATGGAAATTGGGGCAATGTAAAGCTCCAAATGCCCAGTATGGCCGATGTGATGTA
CAATAAGTTGAAGAAACACGCGATCATGATTGATCGCTGTGTTATCGAGTATCGACACCAGTGTCCGA
TTAATAGTGAGGGAT
+
FFFFFFFFFFFFFFFFFFFFFFFFFFFFFFFFFFFFFFFFFFFFFFFFFFFFFFFFFFFFFFFF:FFF
FFFFFFFFFFFFFFFFFFFFFFFFFF,FFFFFFFFFFFFFFFFFFFFFFFFFFFFFFFFFF:FFFFFF
FFFFFFFFFFFFFFF
@A00721:190:HGJW7DSXY:4:2563:14687:3505 1:N:0:TCTGCAAG+GCTGCACT
CAGGCTGCTGCTAATGGAAATTGGGGCAATGTAAAGCTCCAAATGCCCAGTATGGCCGATGTGATGTA
CAATAAGTTGAAGAAACACGCGATCATGATTGATCGCTGTGTTATCGAGTATCGACACCAGTGTCCGA
TTAATAGTGAGGGAT
+
FFFFFFFFFFFFFFFFFFFFFFFFFFFFFFFFFFFFFFFFFFFFFFFFFFFFFFFFFFFFFFFFFFFF
FFFFFFFFFFFFFFFFFFFFFFFFFFFFFFFFFFFFF:FFFFFFFFFFFFFFFFFFFFFFFFFFFFFF
FFFFFFFFFFFFFFF
@A00721:190:HGJW7DSXY:4:1306:24731:5368 1:N:0:TCTGCAAG+GCTGCACT
CTCCAAATGCCCAGTATGGCCGATGTGATGTACAATAAGTTGAAGAAACACGCGATCATGATTGATCG
CTGTGTTATCGAGTATCGACACCAGTGTCCGATTAATAGTGAGGGATCCGTTAGGATCCAGGTGCACG
ATAGTCGAATGATGG
+
FFFFFFFFFFFFFF:FFFFFFFFFF,FFFFFFFFF,FFFFFFFFFFFFFFFFFFFFF:FFFFFFFFFF
FFFFFFFFFFFFFFFFFFFFFFFFF,:FFFFFF:FFFFFFF:FFFFFFFFFF:FFFFFF:F:FFFFFF
FFFFFFFFFFFF:FF
@A00721:190:HGJW7DSXY:4:1326:9507:10379 1:N:0:TCTGCAAG+GCTGCACT
CTCCAAATGCCCAGTATGGCCGATGTGATGTACAATAAGTTGAAGAAACACGCGATCATGATTGATCG
CTGTGTTATCGAGTATCGACACCAGTGTCCGATTAATAGTGAGGGATCCGTTAGGATCCAGGTGCACG
ATAGTCGAATGATGG
+
FFFFFFFFFFFFFFFFFFFFFFFFFFFF:FFFFFFFFFFFFFFFFFFFFFFFFFFFFFFFFFFFFFFF
FFFFFFFFFFFFFFFFFFFFFFFFFFFFFFFFFFFFFFFFFFFFFFFFFFF,:FF:FFFFFF:FFFFF
FFFFFFFFFFFFFFF
@A00721:190:HGJW7DSXY:4:1344:24939:10739 1:N:0:TCTGCAAG+GCTGCACT
CTCCAAATGCCCAGTATGGCCGATGTGATGTACAATAAGTTGAAGAAACACGCGATCATGATTGATCG
CTGTGTTATCGAGTATCGACACCAGTGTCCGATTAATAGTGAGGGATCCGTTAGGATCCAGGTGCACG
ATAGTCGAATGATGG
+
:FFFFF:FFFFF,,,FFFFFFFFFF,:FFFFF,FFFFFFFF:FFFFFFFFFF:FFF:FFFFFFFFFFF
```

```
,FFFFF:FFF:FFFFFFFFFF,FFFF,:FF:F,FFFFFFFFF:,FFFF,,:FFFFFFFFF,:F,FFFF
FFF:FFFFFFFFFFF
@A00721:190:HGJW7DSXY:4:1417:5737:32283 1:N:0:TCTGCAAG+GCTGCACT
CTCCAAATGCCCAGTATGGCCGATGTGATGTACAATAAGTTGAAGAAACACGCGATCATGATTGATCG
CTGTGTTATCGAGTATCGACACCAGTGTCCGATTAATAGTGAGGGATCCGTTAGGATCCAGGTGCACG
ATAGTCGAATGATGG
+
FFFFFFFFFFFFFFFFFFFFFFFFFFF:FFFFFFFFFFFFFFFFFFFFFFFFFFFFFFFFFFFFFFFF
FFFFFFFFFFFFFFFFFFFFFFFFFFFFFFFFFFFFFFFFFFFFFFFFFFFFFFFFFFF:FFFFFFFF
FFFFFFFF,FFFFFF
@A00721:190:HGJW7DSXY:4:1442:31024:36808 1:N:0:TCTGCAAG+GCTGCACT
CTCCAAATGCCCAGTATGGCCGATGTGATGTACAATAAGTTGAAGAAACACGCGATCATGATTGATCG
CTGTGTTATCGAGTATCGACACCAGTGTCCGATTAATAGTGAGGGATCCGTTAGGATCCAGGTGCACG
ATAGTCGAATGATGG
+
FFFFFFFFFFFFFFFFFFFFFFFFFFFFFFFFFFFFFFFFFFFFFFFFFFFFFFFFFFFFFFFFFFFF
FFFFFFFFFFFFFFFFFFFFFFFFFFFFFFFFFFFFFFFFFFFFFFFFFFF::FF:FFFFFFFFFFFF
FFFFFFFFFFFFFFF
@A00721:190:HGJW7DSXY:4:1624:9968:25332 1:N:0:TCTGCAAG+GCTGCACT
CTCCAAATGCCCAGTATGGCCGATGTGATGTACAATAAGTTGAAGAAACACGCGATCATGATTGATCG
CTGTGTTATCGAGTATCGACACCAGTGTCCGATTAATAGTGAGGGATCCGTTAGGATCCAGGTGCACG
ATAGTCGAATGATGG
+
FFFFFFFFFFFFFFFFFFFFFFFFFFFFFFFFFFFFFFFFFFFFFFFFFFFFFFFFFFFFFFFFFFFF
FFFFFFFFFFFFFFFFFFFFFFFFFFFFFFFFFFFFFFFFFFFFFFFFFF,F:FFFFFFFFFFFFFFF
F::FFFFFFFFFFFF
@A00721:190:HGJW7DSXY:4:2119:30834:30968 1:N:0:TCTGCAAG+GCTGCACT
CTCCAAATGCCCAGTATGGCCGATGTGATGTACAATAAGTTGAAGAAACACGCGATCATGATTGATCG
CTGTGTTATCGAGTATCGACACCAGTGTCCGATTAATAGTGAGGGATCCGTTAGGATCCAGGTGCACG
ATAGTCGAATGATGG
+
FFFFFFFFFFFFFFFFFFFFFFFFFFFFFFFFFFFFFFFFFFFFFFFFFFFFFFFFFFFFFFFFFFFF
FFFFFFFFFFFFFFFFFFFFFFFFFFFFFFFF:FFFFFFFFFFFFFFFFF:F,FFFFFF:FFFFFFFF
FFFFFFFFFFFFFFF
@A00721:190:HGJW7DSXY:4:2549:18530:31798 1:N:0:TCTGCAAG+GCTGCACT
CTCCAAATGCCCAGTATGGCCGATGTGATGTACAAAAAGTTGAAGAAACACGCGATCATGATTGATCG
CTGTGTTATCGAGTATCGACACCAGTGTCCGATTAATAGTGAGGGATCCGTTAGGATCCAGGTGCACG
ATAGTCGAATGATGG
+
FF:FFFFFFF,FF,FFFFFF:,F:F:FF:,FFFFF,FFFFFFFFF:FFFFFF,,,FFF:FFFFFFFFF
FF,FFF::F,FF,,F:FFFFFFFFF:FFFF,FFFFFFFFFFF:,FFFFF:F,,,:FFF:F:,F:F,FF
FF:,FFFF,F,,F,F
@A00721:190:HGJW7DSXY:4:2608:22941:11882 1:N:0:TCTGCAAG+GCTGCACT
CTCCAAATGCCCAGTATGGCCGATGTGATGTACAATAAGTTGAAGAAACACGCGATCATGATTGATCG
CTGTGTTATCGAGTATCGACACCAGTGTCCGATTAATAGTGAGGGATCCGTTAGGATCCAGGTGCACG
ATAGTCGAATGATGG
+
FFFFFFFFFFFFFFFFFFFFFFFFFFFFFFF:FFFFFFFFFFFFFFFFFFFFFFF:FF:FFFFFFFFF
FFFFFFFFFFFF:FFFFFFFFFF,FFFFFFFFFFFFFFFFFFFFFFFFFF:,:FFFFFF,FFFFFFFF
F:FFFFFFFFFFFFF
@A00721:190:HGJW7DSXY:4:2644:25129:17550 1:N:0:TCTGCAAG+GCTGCACT
CTCCAAATGCCCAGTATGGCCGATGTGATGTACAATAAGTTGAAGAAACACGCGATCATGATTGATCG
CTGTGTTATCGAGTATCGACACCAGTGTCCGATTAATAGTGAGGGATCCGTTAGGATCCAGGTGCACG
ATAGTCGAATGATGG
```

```
+
FFFFFFFFFFFFFFFFFFFFFFFFFFFFFFFFFFFFFFFFFFFFFFFFFFFFFFFFF,FFFFFFFFFFFF
FFFFFFFFFFFFFFFFFFFFFFFFFFFFFFFFFFFFFFFFFFFFFFFFFFFF,FFFFFFFFFFFFFF:FF
FFFFFFFFFFFFFFF
@A00721:190:HGJW7DSXY:4:2122:27272:7952 1:N:0:TCTGCAAG+GCTGCACT
CCCAGTATGGCCGATGTGATGTACAATAAGTTGAAGAAACACGCGATCATGATTGATCGCTGTGTTAT
CGAGTATCGACACCAGTGTCCGATTAATAGTGAGGGATCCGTTAGGATCCAGGTGCACGATAGTCGAA
TGATGGGAACAGCGT
+
:FFFFF,FFFFFFFFFFFFFFFFF:FFFFFFFFFFFFFFFFFFFFF:FFFFFFFF:FFFFFFFFFFFF:F
FFFFFFFF:FFFFFFFFFFFFFFFFFFFFFFFFFFFFFFFFFFFF,FFFFFFFFFFFFFFFFFFFFFFFF
FFFFFFF:,FFFFFF
@A00721:190:HGJW7DSXY:4:2122:30273:8265 1:N:0:TCTGCAAG+GCTGCACT
CCCAGTATGGCCGATGTGATGTACAATAAGTTGAAGAAACACGCGATCATGATTGATCGCTGTGTTAT
CGAGTATCGACACCAGTGTCCGATTAATAGTGAGGGATCCGTTAGGATCCAGGTGCACGATAGTCGAA
TGATGGGAACAGCGT
+
FFFFFFFFFFFFFFFFFFFFFFFFFFFFFFFFFFFFFFFFFFFFFFFFFFFFFFFFFFFFFFFFFFFFFF
FFFFFFFFFFFFFFFFFFFFFFFFFFFFFFFFFFFFFFFFFFFF:FFFFFFFFFFFFFFFFFFFFFFFFF
FFFFFFFFFFFFFFF
@A00721:190:HGJW7DSXY:4:2161:1877:36683 1:N:0:TCTGCAAG+GCTGCACT
CCCAGTATGGCCGATGTGATGTACAATAAGTTGAAGAAACACGCGATCATGATTGATCGCTGTGTTAT
CGAGTATCGACACCAGTGTCCGATTAATAGTGAGGGATCCGTTAGGATCCAGGTGCACGATAGTCGAA
TGATGGGAACAGCGT
+
FFFFFF:FFFFFFFFFFFFFFFFFFFFFFFFFFFFFFFFFFFFFFFFFFFF:FFFFFFFFFFFFFFFFFF
FFFFFFFFFFFFFFFFFFFFFFFFFFFFFFFFFFFFFFFFFFF:F,FFF:FFFFFFFFFFFFFFFFFFFF
FFFFFFFF,FFFFFF
@A00721:190:HGJW7DSXY:4:1317:32027:23797 1:N:0:TCTGCAAG+GCTGCACT
AGTATGGCCGATGTGATGTACAATAAGTTGAAGAAACACGCGATCATGATTGATCGCTGTGTTATCGA
GTATCGACACCAGTGTCCGATTAATAGTGAGGGATCCGTTAGGATCCAGGTGCACGATAGTCGAATGA
TGGGAACAGCGAGAT
+
FFFFFFFFFFFFFFFFFFFFFFFFFFFFFFFFFFFFFFFFFFFFFFFFFFFFFFFFFFFFFFFFFFFFFF
FFFFFFFFFFFFFFFFFFFFFFFFFFFFFFFFFFFFFFFFF:FFFFFFFFFFFFFFFFFFFFFFFFFFFF
FFFFF:FFFFFFFFF
@A00721:190:HGJW7DSXY:4:1425:6153:31532 1:N:0:TCTGCAAG+GCTGCACT
AGTATGGCCGATGTGATGTACAATAAGTTGAAGAAACACGCGATCATGATTGATCGCTGTGTTATCGA
GTATCGACACCAGTGTCCGATTAATAGTGAGGGATCCGTTAGGATCCAGGTGCACGATAGTCGAATGA
TGGGAACAGCGAGAT
+
FFFFFFFFFFFFFFFFFFFFFFF,FFFFFFFFFFFFFFFFFFFFFFFFFFFFFFFFFFFFFFFFFFFFFF
FFFFFFFFFFFFFFFFFFFFFFFFFFFFFFFFFFFFFFFFF,FFFFFFFFFFFFFFFFFFFF:FFFFFFF
FFFFFFFFFFFFFFF
@A00721:190:HGJW7DSXY:4:1536:28619:15608 1:N:0:TCTGCAAG+GCTGCACT
AGTATGGCCGATGTGATGTACAATAAGTTGAAGAAACACGCGATCATGATTGATCGCTGTGTTATCGA
GTATCGACACCAGTGTCCGATTAATAGTGAGGGATCCGTTAGGATCCAGGTGCACGATAGTCGAATGA
TGGGAACAGCGAGAT
+
FFFFFFFFFFFFFFFFFFFFFFF:FFFFFFFFFFFFFFFFFFFFFFFFFFFFFFFFFFFFFFFFFFFFFF
FFFFFFFFFFFFFFFFFFFFFFFFFFFFFFFFFFFFFFF:FFFFFFFFFFFFFFFFFFFFFFFFFFFFFF
FFFFF:FFF:FFFFF
@A00721:190:HGJW7DSXY:4:1669:19352:20102 1:N:0:TCTGCAAG+GCTGCACT
AGTATGGCCGATGTGATGTACAATAAGTTGAAGAAACACGCGATCATGATTGATCGCTGTGTTATCGA
```

GTATCGACACCAGTGTCCGATTAATAGTGAGGGATCCGTTAGGATCCAGGTGCACGATAGTCGAATGA
TGGGAACAGCGAGAT
+
FFFFFFFFFFFFFFFFFFFFFFFFFFFFFFFFFFFFFFFFFFFFFFFFFFFFFFFFFFFFFFFFFFFFFF
FFFFFFFFFFFFFFFFFFFFFFFFFFFFFFFFFF:FFFF:FFFFFFFFFFFFFFFFFFFFFFFFFFFFFF
FFF:FF:FFFFFFFF
@A00721:190:HGJW7DSXY:4:2611:13331:11365 1:N:0:TCTGCAAG+GCTGCACT
AGTATGGCCGATGTGATGTACAATAAGTTGAAGAAACACGCGATCATGATTGATCGCTGTGTTATCGA
GTATCGACACCAGTGTCCGATTAATAGTGAGGGATCCGTTAGGATCCAGGTGCACGATAGTCGAATGA
TGGGAACAGCGAGAT
+
FFFFFFFFFFFFFFFFFFFFFFFFFFFFFFFFFFFFFFFFFFFFFFFFFFFFFFFFFFFFFFFFFFFFFF
FFFFFFFFFFFFFFFFFFFFFFFFFFFFFFFFFFFFFFFFFFFFFFFFFFFFFFFFFFFFFFFFFFFFFF
FFFFF:FFFFFFFFF
@A00721:190:HGJW7DSXY:4:1239:21856:16955 1:N:0:TCTGCAAG+GCTGCACT
GGCCGATGTGATGTACAATAAGTTGAAGAAACACGCGATCATGATTGATCGCTGTGTTATCGAGTATC
GACACCAGTGTCCGATTAATAGTGAGGGATCCGTTAGGATCCAGGTGCACGATAGTCGAATGATGGGA
ACAGCGTCTAAACAA
+
FFFFFFFFFFFFFFFFFFFFFFFFFFFFFFFFFFFFFFFFFFFFFFFFFFFFFFFFFFFFFFFFFFFFFF
FFFFFFFFFFFFFFFFFFFFFFFFFFFFFFFFFFFFFFFFFFFF:FFFFFFFFFFFFFFFFFFFFFFFFF
:FFFFFFFFFFFFFF
@A00721:190:HGJW7DSXY:4:1246:32832:21308 1:N:0:TCTGCAAG+GCTGCACT
GGCCGATGTGATGTACAATAAGTTGAAGAAACACGCGATCATGATTGATCGCTGTGTTATCGAGTATC
GACACCAGTGTCCGATTAATAGTGAGGGATCCGTTAGGATCCAGGTGCACGATAGTCGAATGATGGGA
ACAGCGTCTAAACAA
+
FFFFFFFFFFFFFFFFFFFFFFFFFFFFFFFFFFFFFFFFFFFFFFFFF:FFFFFFFFFFFFFFFFFFFF
FFFFFFFFFFFFFFFFFFFFFFFFFFFFFFFFFFFF,FFFFFFFFFFFFFFFFFFFFFFFFFFF,FFFFF
,FFFFFFFFFF,FFF
@A00721:190:HGJW7DSXY:4:1262:19651:31234 1:N:0:TCTGCAAG+GCTGCACT
GGCCGATGTGATGTACAATAAGTTGAAGAAACACGCGATCATGATTGATCGCTGTGTTATCGAGTATC
GACACCAGTGTCCGATTAATAGTGAGGGATCCGTTAGGATCCAGGTGCACGATAGTCGAATGATGGGA
ACAGCGTCTAAACAA
+
FFFFFFFFFFFFFFFFFFFFFFFFFFFFFFFFFFFFFFFFFFFFFFFFFFFFFFFFFFFFFFFFFFFFFF
FFFFFFFFFFFFFFFFFFFFFFFFFFFFF:FFFFFFFFFFFFFFFFFFFFFFFFFFFFFFFFFFFFFFFF
:FFFFFFFFFFFFFF
@A00721:190:HGJW7DSXY:4:1278:32669:27289 1:N:0:TCTGCAAG+GCTGCACT
GGCCGATGTGATGTACAATAAGTTGAAGAAACACGCGATCATGATTGATCGCTGTGTTATCGAGTATC
GACACCAGTGTCCGATTAATAGTGAGGGATCCGTTAGGATCCAGGTGCACGATAGTCGAATGATGGGA
ACAGCGTCTAAACAA
+
FFFFFFFF:FFFFFFFFFFFFFFFFFFFF:FFFFFFFFFFFFFFFF:FFFFFFFFFFFFFFFFFFFFFFF
FFFFFFFFFFFFFFFFFFFFFFFFFFFFFFFFFFFFFFFFFFFFFFFFFFFFFFFFFFFFFFFFFFFFFF
FFFFFFFFFFFFFFF
@A00721:190:HGJW7DSXY:4:1341:28185:25473 1:N:0:TCTGCAAG+GCTGCACT
GGCCGATGTGATGTACAATAAGTTGAAGAAACACGCGATCATGATTGATCGCTGTGTTATCGAGTATC
GACACCAGTGTCCGATTAATAGTGAGGGATCCGTTAGGATCCAGGTGCACGATAGTCGAATGATGGGA
ACAGCGTCTAAACAA
+
FFFFFFFFFFFFFFFFFFFFFFFFFFFFFFFFFFFFFFFFFFFFFFFFFFFFFFFFFFFFFFFFFFFFFF
FFFFFFFFFFFFFFFFFFFFFFFFFFFFF:FFFFFF,FFFFFFFFFFFFFFFFFFFFFFFFFFFFFFFFF
:FFFFFFFFFFFFFF

```
@A00721:190:HGJW7DSXY:4:1351:3965:28087 1:N:0:TCTGCAAG+GCTGCACT
GGCCGATGTGATGTACAATAAGTTGAAGAAACACGCGATCATGATTGATCGCTGTGTTATCGAGTATC
GACACCAGTGTCCGATTAATAGTGAGGGATCCGTTAGGATCCAGGTGCACGATAGTCGAATGATGGGA
ACAGCGTCTAAACAA
+
FFFFFFFFFFFFFFFFFFFFFFFFFFFFFFFFFFFFFFFFFFFFFFFFFFFFFFFFFFFFFFFFFFFFFF
FFFFFFFFFFFFFFFFFFFFFFFFFFFFF:FFFFFF:FFFFFF:FFFFFFFFFFFFFFFFF:FFFFFFFF
:FFFFFFFFFFFFFF
@A00721:190:HGJW7DSXY:4:1412:8151:36307 1:N:0:TCTGCAAG+GCTGCACT
GGCCGATGTGATGTACAATAAGTTGAAGAAACACGCGATCATGATTGATCGCTGTGTTATCGAGTATC
GACACCAGTGTCCGATTAATAGTGAGGGATCCGTTAGGATCCAGGTGCACGATAGTCGAATGATGGGA
ACAGCGTCTAAACAA
+
FFFFFFFFFFFFFFFFFF:FFFFFFFFF:FFFFFFFFFFFF:FFFFFFFFFFFFFFFFFFFFFFFFFFFF
FFFFFFFFFFFFFFFFFFFFFFFFFFFFFFFFFFFFFFFFFFFFFFFFFFF:FFFFFFFFFFFFFFFFFF
FFFFFFFFFFFFFFF
@A00721:190:HGJW7DSXY:4:1464:15058:28917 1:N:0:TCTGCAAG+GCTGCACT
GGCCGATGTGATGTACAATAAGTTGAAGAAACACGCGATCATGATTGATCGCTGTGTTATCGAGTATC
GACACCAGTGTCCGATTAATAGTGAGGGATCCGTTAGGATCCAGGTGCACGATAGTCGAATGATGGGA
ACAGCGTCTAAACAA
+
FFFFFFFFFFFFFFFFFFFFFFFFFFFFFFFFFFFFFFFFFFFFFFFFFFFFFFFFFFFFFFFFFFFFFF
FFFFFFFFFFFFFFFFFFFFFFFFFFFFFFFFFFFF:FFFFFFF:FFFFFFFFFFFFFFFFFFFFFFFFF
FFFFFFFFFFFFFFF
@A00721:190:HGJW7DSXY:4:1473:30309:14340 1:N:0:TCTGCAAG+GCTGCACT
GGCCGATGTGATGTACAATAAGTTGAAGAAACACGCGATCATGATTGATCGCTGTGTTATCGAGTATC
GACACCAGTGTCCGATTAATAGTGAGGGATCCGTTAGGATCCAGGTGCACGATAGTCGAATGATGGGA
ACAGCGTCTAAACAA
+
FFFFFFFFFFFFFFFFFFFFFFFFFFFFFFFFFFFFFFFFFFFFFFFFFFFFFFFFFFFFFFFFFFFFFF
FFFFFFFFFFFFFFFFFFFFFFFFFFFFFFFFFFFFFFFFFFFFFFFFFFFFFFFFFFFFFFFFFFFFFF
FFFFFFFFFFFFFFF
@A00721:190:HGJW7DSXY:4:1527:11469:27774 1:N:0:TCTGCAAG+GCTGCACT
GGCCGATGTGATGTACAATAAGTTGAAGAAACACGCGATCATGATTGATCGCTGTGTTATCGAGTATC
GACACCAGTGTCCGATTAATAGTGAGGGATCCGTTAGGATCCAGGTGCACGATAGTCGAATGATGGGA
ACAGCGTCTAAACAA
+
FFFFFFFF:FFFFFFFFF:FFFFFFFFFFFFFFFFFFFF:FFFFFFFFFFFFFFFFFFFFFFFFFFFFFF
FFFFFFFFFFFFFFFFFFFFFFFFFFF:FFFFF:,FFFFFFFFFFFFFFFFFFFFFFFFFFFFFFFFFFF
,FFFFFFFFFFFFFF
@A00721:190:HGJW7DSXY:4:1606:26087:15107 1:N:0:TCTGCAAG+GCTGCACT
GGCCGATGTGATGTACAATAAGTTGAAGAAACACGCGATCATGATTGATCGCTGTGTTATCGAGTATC
GACACCAGTGTCCGATTAATAGTGAGGGATCCGTTAGGATCCAGGTGCACGATAGTCGAATGATGGGA
ACAGCGTCTAAACAA
+
FFFFFFFFFFFFFFFFFFFFFFFFFFFFFFFFFFFFFFFFFFFFFFFFFFFFFFFFFFFFFFFFFFFFFF
FFFFFFFFFFFFFFFFFFFFFFFFFFFFF:FFFFFFFFFFFFFFFFFFFFFFFFFFFFFFFFFFFFFFFF
:FFFFFFFFFFFFFF
@A00721:190:HGJW7DSXY:4:2108:25509:8594 1:N:0:TCTGCAAG+GCTGCACT
GGCCGATGTGATGTACAATAAGTTGAAGAAACACGCGATCATGATTGATCGCTGTGTTATCGAGTATC
GACACCAGTGTCCGATTAATAGTGAGGGATCCGTTAGGATCCAGGTGCACGATAGTCGAATGATGGGA
ACAGCGTCTAAACAA
+
FF:FFFFFFFFFFF:FFFFFF:FFFFFFFFFFFFFFFFFFFFFFFFFF:FFFFFFFFFFFFFFFFFFFFF
```

```
FFFFFFFFFFFFFFFF:FFF,:FFFFFFFFFFFFFFFFFFFFFFFF:FFFFFFFFFFFF:FFFFFFFF
FFFFFFFFFFFFFFF
@A00721:190:HGJW7DSXY:4:2108:25120:9048 1:N:0:TCTGCAAG+GCTGCACT
GGCCGATGTGATGTACAATAAGTTGAAGAAACACGCGATCATGATTGATCGCTGTGTTATCGAGTATC
GACACCAGTGTCCGATTAATAGTGAGGGATCCGTTAGGATCCAGGTGCACGATAGTCGAATGATGGGA
ACAGCGTCTAAACAA
+
FFFFFFFFFFFFFFFFFFFFFFFFFFFFFFFFFFFFFFFFFFFFF:FFFFFFFFFFFFFFFFFFFFFF
FFFFFFFFFFFFFFFFFFFFFFFFFFFFFFFFFF:FFFFFFFFFFFFFFFFFFFFFFFFFFFFFFFFF
FFFFFFFFFFFFFFF
@A00721:190:HGJW7DSXY:4:2156:21712:5212 1:N:0:TCTGCAAG+GCTGCACT
GGCCGATGTGATGTACAATAAGTTGAAGAAACACGCGATCATGATTGATCGCTGTGTTATCGAGTATC
GACACCAGTGTCCGATTAATAGTGAGGGATCCGTTAGGATCCAGGTGCACGATAGTCGAATGATGGGA
ACAGCGTCTAAACAA
+
FFFFFFFF:FFFFFFFFFFFFFFFFFFFFFFFFFFFFFFFFFFFFFFFFFFFFFFFFFFFFFFFFFFF
FFFFFFFFFFFFFFFFFFFFFFFFFFFFFFFFFFF:FFFFFFFFFFFFFFFFFFFFFFFFFFFFFFFF
,FFFFFFFFFFFFFF
@A00721:190:HGJW7DSXY:4:2177:31864:12837 1:N:0:TCTGCAAG+GCTGCACT
GGCCGATGTGATGTACAATAAGTTGAAGAAACACGCGATCATGATTGATCGCTGTGTTATCGAGTATC
GACACCAGTGTCCGATTAATAGTGAGGGATCCGTTCGGATCCAGGTGCACGATAGTCGAATGATGGGA
ACAGCGTCTAAACAA
+
FFFFFFFFFFFFFFFFFFFFFFFFFFFF:FFFFFFFFFFFFFFFFFFFFFFFFFFFFFFFFFFFFFFF
FFFFFFFFFFFFFFFFFFFFFFFFFFFFFFFFFF:F,FFFFFFFFFFFFFFFFFFFFFFFFFFF,FFF
FFFFFFFFFFFFFFF
@A00721:190:HGJW7DSXY:4:2322:3540:23594 1:N:0:TCTGCAAG+GCTGCACT
GGCCGATGTGATGTACAATAAGTTGAAGAAACACGCGATCATGATTGATCGCTGTGTTATCGAGTATC
GACACCAGTGTCCGATTAATAGTGAGGGATCCGTTAGGATCCAGGTGCACGATAGTCGAATGATGGGA
ACAGCGTCTAAACAA
+
FF,,:F,FFFFF:,FFFF,FFFFF,FF,FFFFFFF,FFFFFFFFFF,FF::FF,F,,FFFFFF,:F:F
FFFFFF:FF,FFFFFF,FFFFFF:FFFF:F,,::FFFFFF,FF,FFF,FF:FFFF::FFF:,FFF,FF
,FF:F,F:FFFF,FF
@A00721:190:HGJW7DSXY:4:2371:15420:11788 1:N:0:TCTGCAAG+GCTGCACT
GGCCGATGTGATGTACAATAAGTTGAAGAAACACGCGATCATGATTGATCGCTGTGTTATCGAGTATC
GACACCAGTGTCCGATTAATAGTGAGGGATCCGTTAGGATCCAGGTGCACGATAGTCGAATGATGGGA
ACAGCGTCTAAACAA
+
FFFFFFFF:FFFFFFF:FFFFFFFFFFFFFFFFFFFFFFFFFFFFFFFFFFFFFFFFFFFFFFFFFFF
FFFFFFFFFFFFFFFFFFFFFFFFFFFFFFFFFF:FFFFFFFFFFFFFFF:FFFFFFFFFFFFFFFFF
FFFFFFFFFFFFFFF
@A00721:190:HGJW7DSXY:4:2375:26368:14716 1:N:0:TCTGCAAG+GCTGCACT
GGCCGATGTGATGTACAATAAGTTGAAGAAACACGCGATCATGATTGATCGCTGTGTTATCGAGTATC
GACACCAGTGTCCGATTAATAGTGAGGGATCCGTTAGGATCCAGGTGCACGATAGTCGAATGATGGGA
ACAGCGTCTAAACAA
+
FFFFFFFFFFFFFFFFFFFFFFFFF:FF:FFFFFFFFFFFFFFFFFFFFFFFFF:FFFFFFFFFFFFF
FFFFFF,FFFFFFFFFFFFFFFFFFFFFFFFFFFFFFFFFFFFFFFFFFFFFFFFFFFFFFF:FFFFF
FFFFFFFFFFFFFFF
@A00721:190:HGJW7DSXY:4:2411:23439:2347 1:N:0:TCTGCAAG+GCTGCACT
GGCCGATGTGATGTACAATAAGTTGAAGAAACACGCGATCATGATTGATCGCTGTGTTATCGAGTATC
GACACCAGTGTCCGATTAATAGTGAGGGATCCGTTAGGATCCAGGTGCACGATAGTCGAATGATGGGA
ACAGCGTCTAAACAA
```

+
FFFFFFFFFFFFFFFFF:FFFFFFFFFF:FFFFFFFFFFFFFFFFFFFFFFFFFFFFFFFFFFFFFFF
FFFFFFFFFFFFFFFFFFFFFFFFFFFFFFFF:FF,FFFFFF:FFFFFFFFFFFFFFFFFFFFFFFFF
FFFFFFFFFFFFFFF
@A00721:190:HGJW7DSXY:4:2436:7003:29622 1:N:0:TCTGCAAG+GCTGCACT
GGCCGATGTGATGTACAATAAGTTGAAGAAACACGCGATCATGATTGATCGCTGTGTTATCGAGTATC
GACACCAGTGTCCGATTAATAGTGAGGGATCCGTTAGGATCCAGGTGCACGATAGTCGAATGATGGGA
ACAGCGTCTAAACAA
+
FFFFFFFFFFFFFFFFFFFFFFFFFFFFFFFFFFFFFFFFFFFFFFFFFFFFFFFFFFFFFFFFFFFF
FFFFFFFFFFFFFFFFFFFFFFFFF:FFFFFFFFFFFFFFFFFFFFFFFFFFFFFFFFFFFFFFFFFF
FFFFFFFFFFFFFFF
@A00721:190:HGJW7DSXY:4:1122:2998:11130 1:N:0:TCTGCAAG+GCTGCACT
GCCGATGTGATGTACAATAAGTTGAAGAAACACGCGATCATGATTGATCGCTGTGTTATCGAGTATCG
ACACCAGTGTCCGATTAATAGTGAGGGATCCGTTAGGATCCAGGTGCACGATAGTCGAATGATGGGAA
CAGCGTCTAAACAAG
+
:FFFFFFFF:FFFFFFFF,FFFFFFFFF:FFFFFFFFFFFFFFF,FFFFFF:F:FFFFFFFF:F:FFF
FFFFFFFFFFFF:FFFFFFFFFFFFFF:FFFF:F,:FFFFFFFFFFFFFFFFFFFFFFFFFFFFF:F,
FFF:FF:FFF:FF:F
@A00721:190:HGJW7DSXY:4:1152:21640:20776 1:N:0:TCTGCAAG+GCTGCACT
GCCGATGTGATGTACAATAAGTTGAAGAAACACGCGATCATGATTGATCGCTGTGTTATCGAGTATCG
ACACCAGTGTCCGATTAATAGTGAGGGATCCGTTAGGATCCAGGTGCACGATAGTCGAATGATGGGAA
CAGCGTCTAAACAAG
+
FFFFFFFFFFFFFFFFFFFFFFFFFFFFFFFFFFFFFFFFFFFFFFFFFFFFFFFFFFFFFFFFFFFF
FFFFFFFFFFFFFFFFFFFFFFFFFFFFFFFFFFFFFFFFFFFFFFFFFFFFFFFFFFFFFFFFFFF,
FFFFFFFFFFFFF:F
@A00721:190:HGJW7DSXY:4:1351:29704:11005 1:N:0:TCTGCAAG+GCTGCACT
GCCGATGTGATGTACAATAAGTTGAAGAAACACGCGATCATGATTGATCGCTGTGTTATCGAGTATCG
ACACCAGTGTCCGATTAATAGTGAGGGATCCGTTAGGATCCAGGTGCACGATAGTCGAATGATGGGAA
CAGCGTCTAAACAAG
+
FFFFFFFFFFFFFFFFFFFFFFFFFFFFFFFFFFFFFFFFFFFFFFFFFFFFFFFFFFFFFFFFFFFF
FFFFFFFFFFFFFFFFFFFFFFFFFFFFFFFFFFFFFFFFFFFFFFFFFFFFFFFFFFFFFFFFFFF:
FFFFFFFFFFFFFFF
@A00721:190:HGJW7DSXY:4:1439:14434:8954 1:N:0:TCTGCAAG+GCTGCACT
GCCGATGTGATGTACAATAAGTTGAAGAAACACGCGATCATGATTGATCGCTGTGTTATCGAGTATCG
ACACCAGTGTCCGATTAATAGTGAGGGATCCGTTAGGATCCAGGTGCACGATAGTCGAATGATGGGAA
CAGCGTCTAAACAAG
+
FFFFFFFFFFFFFFFFFFFFFFFFFFFFFFFFFFFFFFFFFFFFFFFFFFFFFFFFFFFFFFFFFFFF
F:FFFFFFFFFFFFFFFFFFFFFFFFFFFFFF:F:FFFFFFFFF:FFFFFFFFFFFFFFFFFFFFFFF
FFFFFFFFFFFFFFF
@A00721:190:HGJW7DSXY:4:1474:31819:13730 1:N:0:TCTGCAAG+GCTGCACT
GCCGATGTGATGTACAATAAGTTGAAGAAACACGCGATCATGATTGATCGCTGTGTTATCGAGTATCG
ACACCAGTGTCCGATTAATAGTGAGGGATCCGTTAGGATCCAGGTGCACGATAGTCGAATGATGGGAA
CAGCGTCTAAACAAG
+
FFFFFFFFFFFFFFFFFFFFFFFFFFFFFFF:FFFFFFFFFFFFFF:FFFFFFFFFFFFFFFFFFFFF
FFFFFFFFFFFFFFFFFFFFFFFFFFFFFFFFF:FFFFFFFFFFFFFFFFFFFFFFFFFFFFF:FFF:
FFFFFFFFFFFFFFF
@A00721:190:HGJW7DSXY:4:1508:14823:3740 1:N:0:TCTGCAAG+GCTGCACT
GCCGATGTGATGTACAATAAGTTGAAGAAACACGCGATCATGATTGATCGCTGTGTTATCGAGTATCG

ACACCAGTGTCCGATTAATAGTGAGGGATCCGTTAGGATCCAGGTGCACGATAGTCGAATGATGGGAA
CAGCGTCTAAACAAG
+
FFFFFFFFFFFFFFFFFFFFFFFFFFFFFFF:FFFFFFFFFFFFFFFFFFFFFFFFFFFFFFFFFFFF
FFFFFFFFFFFFFFFFFFFFFFFFFFFFFF:FFF:FFFFFFFFFFFFFFFFFFFFFFFFFFFFFF:F:
FFFFFFFFFFFFFFF
@A00721:190:HGJW7DSXY:4:1538:20021:19038 1:N:0:TCTGCAAG+GCTGCACT
GCCGATGTGATGTACAATAAGTTGAAGAAACACGCGATCATGATTGATCGCTGTGTTATCGAGTATCG
ACACCAGTGTCCGATTAATAGTGAGGGATCCGTTAGGATCCAGGTGCACGATAGTCGAATGATGGGAA
CAGCGTCTAAACAAG
+
FFFFFFFFFFFFFFFF:FFFFFFFFFFFFFFFFFFFFFFFFFFFFFFFFFFFFFFFFFFFFFFFFFF
FFFFFFFFFFFFFFFFFFFFFFFFFFFFFFFFFFFF:FFFFFFFFFFFFFFFFFFFFFFFFFFFFFF:
FFFFFFFFFF:FFFF
@A00721:190:HGJW7DSXY:4:1559:11369:5588 1:N:0:TCTGCAAG+GCTGCACT
GCCGATGTGATGTACAATAAGTTGAAGAAACACGCGATCATGATTGATCGCTGTGTTATCGAGTATCG
ACACCAGTGTCCGATTAATAGTGAGGGATCCGTTAGGATCCAGGTGCACGATAGTCGAATGATGGGAA
CAGCGTCTAAACAAG
+
FFFFFFFFFFFFFFFFFFFFFFFFFFFFFFFFFFFFFFFFFFFFF,FFFFFFFFFFFFFFFFFFFFFF
FFFFF:FFFFFFFFFFFFFFFFFFFFFFFFFFF::FFFFFFFFFFFFFFFFFFFFFFFFFFFFFFFFF
FFFFFFFFFFFFFFF
@A00721:190:HGJW7DSXY:4:2116:25536:36855 1:N:0:TCTGCAAG+GCTGCACT
GCCGATGTGATGTACAATAAGTTGAAGAAACACGCGATCATGATTGATCGCTGTGTTATCGAGTATCG
ACACCAGTGTCCGATTAATAGTGAGGGATCCGTTAGGATCCAGGTGCACGATAGTCGAATGATGGGAA
CAGCGTCTAAACAA
+
FFFFFFFFFF,FFFFFF:FFF:FFFFFFFFFFFFF::FF,FFFFFFF,FFF,F:,FFFFFFFF:F:FF
FFFFFFFFFFFFFFF,FFFFF::FFFFF,FFF::,FFFFFFFF,FFFFFFFFF,F,FFF,FFFF,FF,
,FF:FFFFFFFFFF
@A00721:190:HGJW7DSXY:4:2168:29957:3051 1:N:0:TCTGCAAG+GCTGCACT
GCCGATGTGATGTACAATAAGTTGAAGAAACACGCGATCATGATTGATCGCTGTGTTATCGAGTATCG
ACACCAGTGTCCGATTAATAGTGAGGGATCCGTTAGGATCCAGGTGCACGATAGTCGAATGATGGGAA
CAGCGTCTAAACAAG
+
FFFFFFFFFFFFFFFFF:FFFFFF:FFFFFFFFFFFFFFFFFFFFFFFFFFFFFFFFFFFFFFFFFFF
FFFFFFFFFFFFFFFFFFFFFFFFFFFFFFFFFFFFFFFFFFFFFFFFFFFFFFFFFFFFFFFFFFF:
FFFFF:FFFFFFFFF
@A00721:190:HGJW7DSXY:4:2178:12689:17957 1:N:0:TCTGCAAG+GCTGCACT
GCCGATGTGATGTACAATAAGTTGAAGAAACACGCGATCATGATTGATCGCTGTGTTATCGAGTATCG
ACACCAGTGTCCGATTAATAGTGAGGGATCCGTTAGGATCCAGGTGCACGATAGTCGAATGATGGGAA
CAGCGTCTAAACAAG
+
FFFFFFFFFFFFFFFFFFFFFFFFFFF:FFFFFFFFFFFFFF:F:F:FFFFFFFFFFFFFFFFFFFFF
FFFFFFFFFFFFFFFFFFFFFFFFFFFFFFFF:F:FFFFFFFFFFFFFFFFFFFFFFF,FFFFFFFF:
FFFFFFFFFFFFFFF
@A00721:190:HGJW7DSXY:4:2178:12183:18239 1:N:0:TCTGCAAG+GCTGCACT
GCCGATGTGATGTACAATAAGTTGAAGAAACACGCGATCATGATTGATCGCTGTGTTATCGAGTATCG
ACACCAGTGTCCGATTAATAGTGAGGGATCCGTTAGGATCCAGGTGCACGATAGTCGAATGATGGGAA
CAGCGTCTAAACAAG
+
FFFFFFFFFFFFFFFFFFFFFFFFFFFF:FFFFFFFFFFFFFFFFFFFFFFFFFFFFFFFFFFFFFFF
FFFFFFFFFFFFFFFFFFFFFFFFFFFFFFFFFF,FFFFFFFFFFFFFFFFFFFFFFFFFFFFFFFFF
FFFFFFFFFFFFFFF

```
@A00721:190:HGJW7DSXY:4:2178:10791:22874 1:N:0:TCTGCAAG+GCTGCACT
GCCGATGTGATGTACAATAAGTTGAAGAAACACGCGATCATGATTGATCGCTGTGTTATCGAGTATCG
ACACCAGTGTCCGATTAATAGTGAGGGATCCGTTAGGATCCAGGTGCACGATAGTCGAATGATGGGAA
CAGCGTCTAAACAAG
+
FFFFFFFFFFFFFFFFFFFFFFFF:FF:FFF:FFFFFFFFFFFFFFFFFFFFFFFFFFFFFFFFFFFF
FFFFFFFFFFFFFFFFFFFFFFFFFFFFFFFFFFFFFFFFFFFFFFFFFFFFFFFFFF:FFFFFFFF:
FFFFFFFFFFFFFFF
@A00721:190:HGJW7DSXY:4:2178:10484:23312 1:N:0:TCTGCAAG+GCTGCACT
GCCGATGTGATGTACAATAAGTTGAAGAAACACGCGATCATGATTGATCGCTGTGTTATCGAGTATCG
ACACCAGTGTCCGATTAATAGTGAGGGATCCGTTAGGATCCAGGTGCACGATAGTCGAATGATGGGAA
CAGCGTCTAAACAAG
+
FFFFFFFFFFFFFFFFFFFFFFFFFFFFFFFFFFFFFFFFFFFFFFFFFFFFFFFFFFFFFFFFFFFF
FFFFFFFFFFFFFFFFFFFFFFFFFFFFFFFFFFFFFFFFFFFFFFFFFFFFFFFFFFFFFFFFFFFF
FFFFFFFFFFFFFFF
@A00721:190:HGJW7DSXY:4:2248:20356:31297 1:N:0:TCTGCAAG+GCTGCACT
GCCGATGTGATGTACAATAAGTTGAAGAAACACGCGATCATGATTGATCGCTGTGTTATCGAGTATCG
ACACCAGTGTCCGATTAATAGTGAGGGATCCGTTAGGATCCAGGTGCACGATAGTCGAATGATGGGAA
CAGCGTCTAAACAAG
+
FFFFFFFFFF,F:FFFF,FFFFFFFFFFFFFF,:FFFFFFFFFFFFFFFF,FFFFFFFFFFFFF:FFF
FFFFFFF:FFFFFFFFFFFFFFFFFFFFFFFFF:FFFFFFFFFFFFFFFFFFFFFF:FFFFFFF:FFF
FFFFFFFFFFFFFFF
@A00721:190:HGJW7DSXY:4:2428:16649:2487 1:N:0:TCTGCAAG+GCTGCACT
GCCGATGTGATGTACAATAAGTTGAAGAAACACGCGATCATGATTGATCGCTGTGTTATCGAGTATCG
ACACCAGTGTCCGATTAATAGTGAGGGATCCGTTAGGATCCAGGTGCACGATAGTCGAATGATGGGAA
CAGCGTCTAAACAAG
+
FFFFFFFFFFFFFFFFFFFFFFFFFFFFFFFFFFFFFFFFFFFFFFFFFFFFFFFFFFFFFFFFFFFF
FFFFFFFFFFFFFFFFFFFFFFFFFFFFFFFF,FFFFFFFFFFFFFFFFFFFFFFFFFFFFFFFFFF:
FFFFFFFFFFFFFFF
@A00721:190:HGJW7DSXY:4:2450:24071:20791 1:N:0:TCTGCAAG+GCTGCACT
GCCGATGTGATGTACAATAAGTTGAAGAAACACGCGATCATGATTGATCGCTGTGTTATCGAGTATCG
ACACCAGTGTCCGATTAATAGTGAGGGATCCGTTAGGATCCAGGTGCACGATAGTCGAATGATGGGAA
CAGCGTCTAAACAAG
+
FFFFFFFFFFFFFFFFFFFFFFFFFFFFFFFFFFFFFFFFFFFFFFFFFFFFFFFFFFFFFFFFFFFF
FFFFFFFFFFF:FFFFFFFFFFFFFFFFFFFF:F:FFFFFFFFFFFFFFFFFFFFFFFFFFFFFFFF:
FFFFFFFFFF:FFF:
@A00721:190:HGJW7DSXY:4:2631:30535:12790 1:N:0:TCTGCAAG+GCTGCACT
GCCGATGTGATGTACAATAAGTTGAAGAAACACGCGATCATGATTGATCGCTGTGTTATCGAGTATCG
ACACCAGTGTCCGATTAATAGTGAGGGATCCGTTAGGATCCAGGTGCACGATAGTCGAATGATGGGAA
CAGCGTCTAAACAAG
+
FFFFFFFFFFFFFFFFFFFFFFFFFFFFFFFFFFFFFFFFFFFFFFFFFFFFFFFFFFFFFFFFFFFF
FFFFFFFFFFFFFFFFFFFFFFFFFFFFFFFFFF,FFFFFFFFFFFFFFFFFFFFFFFFFFFFFFFFF
FFFFFFFFFFFFFFF
@A00721:190:HGJW7DSXY:4:1251:21260:35775 1:N:0:TCTGCAAG+GCTGCACT
TTTAGACGCTGTTCCCATCATTCGACTATCGTGCACCTGGATCCTAACGGATCCCTCACTATTAATCG
GACACTGGTGTCGATACTCGATAACACAGCGATCAATCATGATCGCGTGTTTCTTCAACTTATTGTAC
ATCACATCGG
+
FFFFFFFFFFFFFFFFFFFFFFFFFFFFFFFFFFFFFFFFFFFFFFFFFFFFFFFFFFFFFFFFFFFF
```

FFFFFFFFFFFFFFFFFFFFFFFFFFFFF:FFFFFFFFFFFFFFFFFFFFFFFFFFFFFFFFFFFFFFFF
FFFFFFFF:F
@A00721:190:HGJW7DSXY:4:1524:1380:7388 1:N:0:TCTGCAAG+GCTGCACT
TTTAGACGCTGTTCCCATCATTCGACTATCGTGCACCTGGATCCTAACGGATCCCTCACTATTAATCG
GACACTGGTGTCGATACTCGATAACACAGCGATCAATCATGATCGCGTGTTTCTTCAACTTATTGTAC
ATCACATCGG
+
FFFFFFFFFFFFFFFFFFFFFFFFFFFFFFFFFFFFFFFFFFFFFFFFFFF:FFFFFFFFFFFFFFFFFF
FFFFFFFFFFFFFFFFFFFFFFFFFFFFFFFFFFFFFFFFFFFFFFFFFFFFFFFFFFFFFFFFFFFFFF
FFFFFFFFFF
@A00721:190:HGJW7DSXY:4:2170:29677:3944 1:N:0:TCTGCAAG+GCTGCACT
TTTAGACGCTGTTCCCATCATTCGACTATCGTGCACCTGGATCCTAACGGATCCCTCACTATTAATCG
GACACTGGTGTCGATACTCGATAACACAGCGATCAATCATGATCGCGTGTTTCTTCAACTTATTGTAC
ATCACATCGG
+
FFFFFFFFFFFFFFFFFFFFFFFFFFFFFFFFFFFFFFFFFFFFFFFFFFFFFFFFFFFFFFFFFFFFFF
FFFFFFFFFFFFFFFFFFFFFFFFFFFFFFFFFFFF:FFFFFFFFFFFFFFFFFFFFFFFFFF:FFFFFF
FFFFFFFFFF
@A00721:190:HGJW7DSXY:4:2265:20781:12493 1:N:0:TCTGCAAG+GCTGCACT
TTTAGACGCTGTTCCCATCATTCGACTATCGTGCACCTGGATCCTAACGGATCCCTCACTATTAATCG
GACACTGGTGTCGATACTCGATAACACAGCGATCAATCATGATCGCGTGTTTCTTCAACTTATTGTAC
ATCACATCGG
+
FFFFFFFFFFFFFFFFFFFFFFFFFFFFFFFFFFFFFFFFFFFFFFFFFFFFFFFFFFFFFFFFFFFFFF
FFFFFFFFFFFFFFFFFFFFFFFFFFFFFFFFFFFFFFFFFFFFFFFFFFFFFFFFFFFFFFFFFFFFFF
FFFFFFFFFF
@A00721:190:HGJW7DSXY:4:2343:2311:15671 1:N:0:TCTGCAAG+GCTGCACT
TTTAGACGCTGTTCCCATCATTCGACTATCGTGCACCTGGATCCTAACGGATCCCTCACTATTAATCG
GACACTGGTGTCGATACTCGATAACACAGCGATCAATCATGATCGCGTGTTTCTTCAACTTATTGTAC
ATCACATCGG
+
FFFFFFFFFFFFFFFFFFFFFFFFFFFFFFFFFFFFFFFFF:FFFFFFFF,FFFFFFFFFF:FFFFFF:F
FFF:FFFFFFFFFFFFFFFFFF,FFFFFF:FFFFFFFFFFFFFFFFFFFFFF,FF:FF:F::,F,FFF,F
:FFFFFFFFF
@A00721:190:HGJW7DSXY:4:1402:20482:12696 1:N:0:TCTGCAAG+GCTGCACT
ATTACGAATTCAGCTTGTTTAGACGCTGTTCCCATCATTCGACTATCGTGCACCTGGATCCTAACGGA
TCCCTCACTATTAATCGGACACTGGTGTCGATACTCGATAACACAGCGATCAATCATGATCGCGTGTT
TCTTCAACTTATTGT
+
FFFFFFFFFFFFFFFFFFFFFFFFFFFFFFFFFFFFFFFFFFFFFFFFFFFFFFFFFFFFFFFFFFFFFF
FFFFFFFFFFFFFFFFFFFFFFFFFFFFFFFFFFFFFFFFFFFFFFFFFFFFFFFFFFFFFFFFFFFFFF
FFFFFFFFFFFFFFF
@A00721:190:HGJW7DSXY:4:1648:31403:1485 1:N:0:TCTGCAAG+GCTGCACT
ATTACGAATTCAGCTTGTTTAGACGCTGTTCCCATCATTCGACTATCGTGCACCTGGATCCTAACGGA
TCCCTCACTATTAATCGGACACTGGTGTCGATACTCGATAACACAGCGATCAATCATGATCGCGTGTT
TCTTCAACTTATTGT
+
FFFFFFFFFFFFFFFFFFFFFFFFFFFFFFFFFFFFFFFFFFFFFFFFFFFFFFFFFFFFFFFFFFFFF:
FF:FFFFFFFFFFFFFFFFFFFFFFFFFFFFFFFFFFFFFFFFFFFFFFFFFF:FFFFFFFFFFFFFFFF
FFFFFFFFFFFFFFF
@A00721:190:HGJW7DSXY:4:2133:8006:28291 1:N:0:TCTGCAAG+GCTGCACT
ATTACGAATTCAGCTTGTTTAGACGCTGTTCCCATCATTCGACTATCGTGCACCTGGATCCTAACGGA
TCCCTCACTATTAATCGGACACTGGTGTCGATACTCGATAACACAGCGATCAATCATGATCGCGTGTT
TCTTCAACTTATTGT

```
+
FFFFFFFFFFFFFFFFFF:FFFFFFFFFFFFFFFFFFFFFFFFFFFFFFFFFFFFFFFFFFFFF:FFFF,
FFFFFFFFFFFFFFFFFFFF:FFFFFFFFFFFFFFFFFFFFFFFFFFFFFFF:FFFFFFFFFFFFFFFF
FFFFFFFFFFFFFFF
@A00721:190:HGJW7DSXY:4:2362:11894:14450 1:N:0:TCTGCAAG+GCTGCACT
ATTACGAATTCAGCTTGTTTAGACGCTGTTCCCATCATTCGACTATCGTGCACCTGGATCCTAACGGA
TCCCTCACTATTAATCGGACACTGGTGTCGATACTCGATAACACAGCGATCAATCATGATCGCGTGTT
TCTTCAACTTATTGT
+
FFFFFFFFFFFFFFFFFFFFFFFFFFFFFFFFFFFFFFFFFFFFFFFFFFFFFFFFFFFFFFF:FFFFF
FFFFFFFFFFFFFFFFFFFFFFFFFFFFFFFFFFFFFFFFFFFFFFFFFFF:FFFFFFFFFFFFFFFF
FFFFFFFFFFFFFFF
@A00721:190:HGJW7DSXY:4:1523:24316:13291 1:N:0:TCTGCAAG+GCTGCACT
CTGGCATTACGAATTCAGCTTGTTTAGACGCTGTTCCCATCATTCGACTATCGTGCACCTGGATCCTA
ACGGATCCCTCACTATTAATCGGACACTGGTGTCGATACTCGATAACACAGCGATCAATCATGATCGC
GTGTTTCTTCAACTT
+
FFFFFFFFFFFFFFFFFFFFFFFFFFFFFFFFFFFFFFFFFFFFFFFFFFFFFFFFFFFFFFFFFFFF
FFFF::FFFFFFFFFFFFFFFFFFFFFFFFFFFFFFFFFFFFFFFFFFFFFFFFFFFFFFFFF:FFFF
FFFFFFFFFFFFFFF
@A00721:190:HGJW7DSXY:4:2304:2600:23469 1:N:0:TCTGCAAG+GCTGCACT
CTGGCATTACGAATTCAGCTTGTTTAGACGCTGTTCCCATCATTCGACTATCGTGCACCTGGATCCTA
ACGGATCCCTCACTATTAATCGGACACTGGTGTCGATACTCGATAACACAGCGATCAATCATGATCGC
GTGTTTCTTCAACTT
+
FFFFFFFFFFFFFFFFFFFFFFFFFFFFFFFFFFFFFFFFFFFFFFFFFFFFFFFFFFFFFFFFFF,F
FFFFFFFFFFFFFFFFFFFFFFFFFFFFFFFFFFFFFFFFFFFFFFFFFFF:FFFF,FFFF:FFFFFF
FFFFFFFFFFFFFFF
@A00721:190:HGJW7DSXY:4:2523:20961:17848 1:N:0:TCTGCAAG+GCTGCACT
CTGGCATTACGAATTCAGCTTGTTTAGACGCTGTTCCCATCATTCGACTATCGTGCACCTGGATCCTA
ACGGATCCCTCACTATTAATCGGACACTGGTGTCGATACTCGATAACACAGCGATCAATCATGATCGC
GTGTTTCTTCAACTT
+
FFFFFFFFFFFFFFFFFFFFFFFFFFFFFFFFFFFFFFFF:FFFFFFFFFFFFFFFFFFFFFFFFFFF
FFFFFFFFFFFFFFFFFFFFF:FFF:FFFFFFFFFFFFFFFFFFFFFFFFFFFFFFF:FFFFFFFFFF
FFFFFFFFFFFFFF:
@A00721:190:HGJW7DSXY:4:1129:1796:30906 1:N:0:TCTGCAAG+GCTGCACT
GCTGTGTTATCGAGTATCGACACCAGTGTCCGATTAATAGTGAGGGATCCGTTAGGATCCAGGTGCAC
GATAGTCGAATGATGGGAACAGCGTCTAAACAAGCTGAATTCGTAATGCCAGTTACGTGTAACTGCGA
TCTGACGTTTTACGG
+
FFFFFFFFFFFFFFFFFFFFFFFFFFFFFFFFFF,FFFFFFFFFFFFFFFFFF,:FFFFFFFFFFFFF
FFFFFFFFFFFFFFFFFFFFFFFFFFFFFFFFFFFFFFFFFFFFFFFFFFFFFFFFFFFFFFFFFFFF
:FFFFFFFFFFFFFF
@A00721:190:HGJW7DSXY:4:1434:28094:29481 1:N:0:TCTGCAAG+GCTGCACT
GCTGTGTTATCGAGTATCGACACCAGTGTCCGATTAATAGTGAGGGATCCGTTAGGATCCAGGTGCAC
GATAGTCGAATGATGGGAACAGCGTCTAAACAAGCTGAATTCGTAATGCCAGTTACGTGTAACTGCGA
TCTGACGTTTTACGG
+
FFFF:FFFFFFFFFFF:FFFF:FF:FFFFFFFFFFFFFF:FFFFFFFFFFF,:,FFF,FFFFFFFFFF
FFFFFFFFFFFFFFFFF::FFFFFFFFFFFFFFFFFFFFFFFFFF::FFFFFFFFFFFFFFFFFFFFFF
FFFFFFFFFFFFFFF
@A00721:190:HGJW7DSXY:4:2277:24695:27414 1:N:0:TCTGCAAG+GCTGCACT
GCTGTGTTATCGAGTATCGACACCAGTGTCCGATTAATAGTGAGGGATCCGTTAGGATCCAGGTGCAC
```

GATAGTCGAATGATGGGAACAGCGTCTAAACAAGCTGAATTCGTAATGCCAGTTACGTGTAACTGCGA
TCTGGCGTTTTACGG
+
FFFFFFFFFFFFFFFFFFFFFFFFFFFFFFFFFFFFFFFFFFFFFFFFFFF:F,FFFFFFFFFFFFFF
FFFFFFFFFFFFFFFFFF:FFFFFFFFFFFFFFFFFFFF:FFFFFFFFFFFFFFFFFFFFFFFFFFFF
FFFF,FFFFFFFFFF
@A00721:190:HGJW7DSXY:4:2555:3378:19022 1:N:0:TCTGCAAG+GCTGCACT
GCTGTGTTATCGAGTATCGACACCAGTGTCCGATTAATAGTGAGGGATCCGTTAGGATCCAGGTGCAC
GATAGTCGAATGATGGGAACAGCGTCTAAACAAGCTGAATTCGTAATGCCAGTTACGTGTAACTGCGA
TCTGACGTTTTACGG
+
FFFFFFFFFFFFFFFFFFFFFFFFFFFFFFFFFFFFFFFFFFFFFFFFFFF:F:FFFFFFFFFFFFFF
FFFFFFFFFFFFFFFFFFFFFFFFFFFFFFFFFFFFFFFFFFFF:FFFFFFFFFFFFFFFFFFFFFFF
FFFFFF:F:FFFFFF
@A00721:190:HGJW7DSXY:4:1141:30798:15843 1:N:0:TCTGCAAG+GCTGCACT
CTTCCGTAAAACGTCAGATCGCAGTTACACGTAACTGGCATTACGAATTCAGCTTGTTTAGACGCTGT
TCCCATCATTCGACTATCGTGCACCTGGATCCTAACGGATCCCTCACTATTAATCGGACACTGGTGTC
GATACTCGATAACAC
+
FFFFFFFFFFFFFFFFFFFFFFFFFFFFFFFFFFFFFFFFFFFFFFFFFFFFFFFFFFFFFFFFFFFF
FFFFFFFFFFFFFFFFFFFFFFFFFFFFFFFFFFFFFFFFFFFFFFFFFFFFFFFFFFFFFFFFFFFF
FFFFFFFFFFFFFFF
@A00721:190:HGJW7DSXY:4:1162:26359:12727 1:N:0:TCTGCAAG+GCTGCACT
CTTCCGTAAAACGTCAGATCGCAGTTACACGTAACTGGCATTACGAATTCAGCTTGTTTAGACGCTGT
TCCCATCATTCGACTATCGTGCACCTGGATCCTAACGGATCCCTCACTATTAATCGGACACTGGTGTC
GATACTCGATAACAC
+
FFFFFFFFFFFFFFFFFFFFFFFFFFFFFFFFFFFFFFFFFFFFFFFFFFFFFFFFFFFFFFFFFFFF
FFFFFFFFFFFFFFFFFFFFFFFFFFFFFFFFFFFFFF,FFFFFFFFFFFFFFFFFFFFFFFFFFFFF
FFFFFFFFFFFFFFF
@A00721:190:HGJW7DSXY:4:1413:22309:1454 1:N:0:TCTGCAAG+GCTGCACT
CTTCCGTAAAACGTCAGATCGCAGTTACACGTAACTGGCATTACGAATTCAGCTTGTTTAGACGCTGT
TCCCATCATTCGACTATCGTGCACCTGGATCCTAACGGATCCCTCACTATTAATCGGACACTGGTGTC
GATACTCGATAACAC
+
FFFFFFFFFFFFFFFFFFFFFFFFFFFFFFFFFFFFFFFFFFFFFFFFFFFFFFFFFFFFFFFFFFFF
FFFFFFFFFFFFFFFFFFFFFFFFFFFFFFFF:F:FFFF:FFFFFFFFFFFFFFF:FFFFFFF:FFFF
:FFFFFFFFFFFFFF
@A00721:190:HGJW7DSXY:4:1459:1759:19664 1:N:0:TCTGCAAG+GCTGCACT
CTTCCGTAAAACGTCAGATCGCAGTTACACGTAACTGGCATTACGAATTCAGCTTGTTTAGACGCTGT
TCCCATCATTCGACTATCGTGCACCTGGATCCTAACGGATCCCTCACTATTAATCGGACACTGGTGTC
GATACTCGATAACAC
+
FFFFFFFFFFFFF:FFFFFFFFFFFFFFFFFFFFFFFFFFFFFFFFFFFFFFFFFFFFFFFFFFFFFF
FFFFFFFFFFFFFFFFFFFFFFFFFFFFFFFFF:FFFF::FFFFFFFFFFFFFFFFFFFFFFFFFFFF
FFFFFFFFFFFFFFF
@A00721:190:HGJW7DSXY:4:1666:11867:12837 1:N:0:TCTGCAAG+GCTGCACT
CTTCCGTAAAACGTCAGATCGCAGTTACACGTAACTGGCATTACGAATTCAGCTTGTTTAGACGCTGT
TCCCATCATTCGACTATCGTGCACCTGGATCCTAACGGATCCCTCACTATTAATCGGACACTGGTGTC
GATACTCGATAACAC
+
FFFFFFFFFFFFFFFFFFFFFFFFFFFFFFFFFFFFFFFFFFFFFFFFFFFFF:FFFFFFFFFFFFFF
FFFFF:FFFFFFFFFFFFFFFFFFFFFFFFFFFFF:FF:FFFFFF:FFFFFFFFFFFFFF:FFFFFFF
FFFFFFFFFFFFFFF

@A00721:190:HGJW7DSXY:4:2325:24505:35540 1:N:0:TCTGCAAG+GCTGCACT
CTTCCGTAAAACGTCAGATCGCAGTTACACGTAACTGGCATTACGAATTCAGCTTGTTTAGACGCTGT
TCCCATCATTCGACTATCGTGCACCTGGATCCTAACGGATCCCTCACTATTAATCGGACACTGGTGTC
GATACTCGATAACAC
+
FFFFFFFFFFFFFFFFFFFFFFFFFFFFFFFFFFFFFFFFFFFFFFFFFFFFFFFFFFFFFFFFFFFF,
FFFFFFF:FFFFFFF:FFFFFFFFFFFFFFFFFFFFFF,,FFFFFFF:FF:FFFFFFFFFF,FFFFFF
FF:FFFFFFFFFFFF
@A00721:190:HGJW7DSXY:4:2362:5150:26725 1:N:0:TCTGCAAG+GCTGCACT
CTTCCGTAAAACGTCAGATCGCAGTTACACGTAACTGGCATTACGAATTCAGCTTGTTTAGACGCTGT
TCCCATCATTCGACTATCGTGCACCTGGATCCTAACGGATCCCTCACTATTAATCGGACACTGGTGTC
GATACTCGATAACAC
+
FFFFFFFFFFFFFFFFFFFFFFFFFFFFFFFFFFFFFFFFFFFFFFFFFFFFFFFFFFFFFFFFFFFF:
FFFFFFFFFFFFFFFFFFFFFFFFFFFFFFFFFFFFFF,FFFFFFFFFFFFFFFFFFFFFFFFFFFFF
FFFFFFFFFFFFFFF
@A00721:190:HGJW7DSXY:4:2563:21531:21715 1:N:0:TCTGCAAG+GCTGCACT
CTTCCGTAAAACGTCAGATCGCAGTTACACGTAACTGGCATTACGAATTCAGCTTGTTTAGACGCTGT
TCCCATCATTCGCCTATCGTGCACCTGGATCCTAACGGATCCCTCACTATTAATCGGACACTGGTGTC
GATACTCGATAACAC
+
FF:FFFFFFFFFF:FFFFFFFFFFFFFFFFFFFFFFFFF,FFFF:FFFFFF:F:FFFF:F,FFFFFFF
,FFFFFFFFFFF,FFFFFFFFF:FFFFFF:FF,:FFFFF,FFF,FFF,FFFFFFFF:FFF:FFF,FFF
FF:FF:FFFFFF:FF
@A00721:190:HGJW7DSXY:4:2641:31331:10504 1:N:0:TCTGCAAG+GCTGCACT
CTTCCGTAAAACGTCAGATCGCAGTTACACGTAACTGGCATTACGAATTCAGCTTGTTTAGACGCTGT
TCCCATCATTCGACTATCGTGCACCTGGATCCTAACGGATCCCTCACTATTAATCGGACACTGGTGTC
GATACTCGATAACAC
+
FFFFFFFFFFFFFFFFFFFFFFFFFFFFFFFFFFFFFFFFFFFFFFFFFFFFFFFFFFFFFFFFFFFF
FFFFFFFFFFFFFFFFFFFFFFFFFFFFFFFFFFFFFFFFFFFFFFFFFFFFFFFFFFFFFFFFFFFF
FFFFFFFFFFFFF,F
@A00721:190:HGJW7DSXY:4:1109:6533:12211 1:N:0:TCTGCAAG+GCTGCACT
CGACGCCAGTGTCCGATTAATAGTGAGGGATCCGTTAGGATCCAGGTGCACGATAGTCGAATGATGGG
AACAGCGTCTAAACAAGCTGAATTCGTAATGCCAGTTACGTGTAACTGCGATCTGACGTTTTACGGAA
GTTGTTGGAGTTCTG
+
FFFFFFFFFFFFFFFFFFFFFFFFFFFFFFFFFF::FFFFFFFFFFFFFFFFFFFFFFFFFFFFFFFF
FFFFFFFFFFFFFFFFFF:FFFFFFFFFFFFFFFFFFFFFFFFFFFFFFFFFFFFFFFFFFFFFFF:F
FFFFFFFFFFFFFFF
@A00721:190:HGJW7DSXY:4:1126:31991:13683 1:N:0:TCTGCAAG+GCTGCACT
CGACGCCAGTGTCCGATTAATAGTGAGGGATCCGTTAGGATCCAGGTGCACGATAGTCGAATGATGGG
AACAGCGTCTAAACAAGCTGAATTCGTAATGCCAGTTACGTGTAACTGCGATCTGACGTTTTACGGAA
GTTGTTGGAGTTCTG
+
FFFFFFFFFFFFFFFFFFFFFFFFFFFFFFFFFFF,,:FFFFFFFFFFFFFFFFFFFFFFFFFFFFFF
:,FFFFFFFFF:FFFFFFFFFFFFFFFFFFFFFFFFFFFFFFFFFFFFFFFFFFFFFF:FFFFFFFF
F:FFF:FFFFFFF:F
@A00721:190:HGJW7DSXY:4:1228:21540:13275 1:N:0:TCTGCAAG+GCTGCACT
CGACGCCAGTGTCCGATTAATAGTGAGGGATCCGTTAGGATCCAGGTGCACGATAGTCGAATGATGGG
AACAGCGTCTAAACAAGCTGAATTCGTAATGCCAGTTACGTGTAACTGCGATCTGACGTTTTACGGAA
GTTGTTGGAGTTCTG
+
FFFFFFFFFFFFFFFFFFFFFFFFFFFFFFFFFFFFFFFFFFFF:FFFFFFFFFFFFFFFFFFFFFFF

```
FFF,FFFFFFFFFFFFFFFFFF:FFFFFFFFFF:FFFFFFFFFFFFFFFFFFFFFFFFFFFFFFFFFFF
FFFFFFFFFFFFFFF
@A00721:190:HGJW7DSXY:4:1315:8730:7498 1:N:0:TCTGCAAG+GCTGCACT
CGACGCCAGTGTCCGATTAATAGTGAGGGATCCGTTAGGATCCAGGTGCACGATAGTCGAATGATGGG
AACAGCGTCTAAACAAGCTGAATTCGTAATGCCAGTTACGTGTAACTGCGATCTGACGTTTTACGGAA
GTTGTTGGAGTTCTG
+
FFFFFFFFF:FFFFFFFFFFFFFFFFFFFFFFFFF::,FFFFFFFFFFFFFFFFFFFFFFFFFFFFFFF
FFFFFFFFFFFFFFFF:FFFFFFFFFFFFFFFFFFFFFFFFFFFFFFFFFFFFFFFFFFFFFFF:FFFF
FFFFFFFFFFFFFF:
@A00721:190:HGJW7DSXY:4:2310:3513:4695 1:N:0:TCTGCAAG+GCTGCACT
CGACGCCAGTGTCCGATTAATAGTGAGGGATCCGTTAGGATCCAGGTGCACGATAGTCGAATGATGGG
AACAGCGTCTAAACAAGCTGAATTCGTAATGCCAGTTACGTGTAACTGCGATCTGACGTTTTACGGAA
GTTGTTGGAGTTCTG
+
FFFFFFFFFFFFFFFFFFFFFFFFFFFFFFFFFFFFFFFFFFFFFFFFFFFFFFFFFFFFFFFFFFFF
F:FFFFFFFFFFFFFFFFFF:FFFFFFFFFFFFFFFFFFFFFFFFFFFFFFFFFFFFFFFFFFFFFFFF
FFFFFFFFFFFFFFF
@A00721:190:HGJW7DSXY:4:2355:30219:20134 1:N:0:TCTGCAAG+GCTGCACT
CGACGCCAGTGTCCGATTAATAGTGAGGGATCCGTTCGGATCCAGGTGCACGATAGTCGAATGATGGG
AACAGCGTCTAAACAAGCTGAATTCGTAATGCCAGTTACGTGTAACTGCGATCTGACGTTTTACGGAA
GTTGTTGGAGTTCTG
+
FFFFFFFFFFFFFFFFFFFFFFFFFFFFFFFFFFF,FFFFFFFFFFFFFFFFFFFFFFFFFFF:FFFF
FFFFFFFFFFFFFFFFFFFFFFFFFFFFFFFFFFFFFFFF:FFFFFFFFFFFFFFFFFFFFFFFFF:F:
FF,FFFFFFFFFFFF
@A00721:190:HGJW7DSXY:4:2456:32759:26913 1:N:0:TCTGCAAG+GCTGCACT
CGACGCCAGTGTCCGATTAATAGTGAGGGATCCGTTAGGATCCAGGTGCACGATAGTCGAATGATGGG
AACAGCGTCTAAACAAGCTGAATTCGTAATGCCAGTTACGTGTAACTGCGATCTGACGTTTTACGGAA
GTTGTTGGAGTTCTG
+
FFFFFFFFFFFFFFFFFFFFFFFFFFFFFFFFFFFFFFFFFFFFFFFFFFFFFFFFFFFFFFFFFFFF
F:FFFFFFFFFFFFFFFFFFFFFFFFFFFFFFFFFFFFFFFFFFFFFFFFFF:FFFF:FFFFF,FFFF
FFFFFFFFFFFF,FF
@A00721:190:HGJW7DSXY:4:1125:15944:21402 1:N:0:TCTGCAAG+GCTGCACT
GTCCGATTAATAGTGAGGGATCCGTTAGGATCCAGGTGCACGATAGTCGAATGATGGGAACAGCGTCT
AAACAAGCTGAATTCGTAATGCCAGTTACGTGTAACTGCGATCTGACGTTTTACGGAAGTTGTTGGAG
TTCTGCATCGTCTGA
+
FFFFFFFFFFFFFFFFFFFFFFFFFFFFFFFFFFFFFFFFFFFFFFFFFFFFFFFFFFF,FFFFFFFF
FFFFFFFFFFFFFFF:FFFFF:FFFFFFFFFFFFFF:FFFFFFFFFFFFFFFFFFFF:FFFFF:FFFF
FFFFFFFFFFFFFFF
@A00721:190:HGJW7DSXY:4:1402:16152:14215 1:N:0:TCTGCAAG+GCTGCACT
GTCCGATTAATAGTGAGGGATCCGTTAGGATCCAGGTGCACGATAGTCGAATGATGGGAACAGCGTCT
AAACAAGCTGAATTCGTAATGCCAGTTACGTGTAACTGCGATCTGACGTTTTACGGAAGTTGTTGGAG
TTCTGCATCGTCTGA
+
FFFFFFFFFFFFFFFFFFFFFFFFFFFFFFFFFFFFFFFFFFFFFFFFFFF:FFFFFFFFFFFFFFFF
FFFFFFFFFFFFFFFFFFFFFFFFFFFFFFFFFFFFFFFFFFFFFFFFFFFFFFFFF:FFFFFFFFFF
FFFFFFFFFFFFFFF
@A00721:190:HGJW7DSXY:4:1451:30816:7576 1:N:0:TCTGCAAG+GCTGCACT
GTCCGATTAATAGTGAGGGATCCGTTAGGATCCAGGTGCACGATAGTCGAATGATGGGAACAGCGTCT
AAACAAGCTGAATTCGTAATGCCAGTTACGTGTAACTGCGATCTGACGTTTTACGGAAGTTGTTGGAG
TTCTGCATCGTCTGA
```

+
FFFFFFFFFFFFFFFFFFFFFFFFFFFFFFFFFFFFFFFFFFFFFFFFFFFFFFFFFFFFFFFFFFFFFF
FFFFFFFFFFFFFFFFFFFFFFFFFFFFFFFFFFFFFFFFFFFFFFFFFFFFFFFFFFFFFFFFFFFFFF
FFFFFFFFFFFFFFF
@A00721:190:HGJW7DSXY:4:1453:31060:17487 1:N:0:TCTGCAAG+GCTGCACT
GTCCGATTAATAGTGAGGGATCCGTTAGGATCCAGGTGCACGATAGTCGAATGATGGGAACAGCGTCT
AAACAAGCTGAATTCGTAATGCCAGTTACGTGTAACTGCGATCTGACGTTTTACGGAAGTTGTTGGAG
TTCTGCATCGTCTGA
+
FFFFFFFFFFFFFFFFFFFFFFFF::,FFFFFFFFFFFFFFFFFFFFFFFFFFFFFFFFFF:FFFFFFFF
FFFFFFFFFFFFFFFFFFFFFFFFFFFFFFFFFFFFFFFFFFFFFFFFFFFFFFFFFFFFF,FFFFFFFF
FFFFFFFFFFFFFFF
@A00721:190:HGJW7DSXY:4:1534:24560:35383 1:N:0:TCTGCAAG+GCTGCACT
GTCCGATTAATAGTGAGGGATCCGTTAGGATCCAGGTGCACGATAGTCGAATGATGGGAACAGCGTCT
AAACAAGCTGAATTCGTAATGCCAGTTACGTGTAACTGCGATCTGACGTTTTACGGAAGTTGTTGGAG
TTCTGCATCGTCTGA
+
FFFFFFFFFFFFFFFFFFFFFFFFFF:FFFFFFFFFFFFFFFFFFFFFFFFFFFFFFFFFF:FFFFFFFF
FFFFFFFFFFFFFFFFFF:FFFFFFFFFFFFFFFFFFFFFFFFFFFFFFFFFFFFF:FFFFFFFFFFFFF
FFF:FFFFFFFFFFF
@A00721:190:HGJW7DSXY:4:1611:2844:17754 1:N:0:TCTGCAAG+GCTGCACT
GTCCGATTAATAGTGAGGGATCCGTTAGGATCCAGGTGCACGATAGTCGAATGATGGGAACAGCGTCT
AAACAAGCTGAATTCGTAATGCCAGTTACGTGTAACTGCGATCTGACGTTTTACGGAAGTTGTTGGAG
TTCTGCATCGTCTGA
+
FFFFFFFFFFFFFFFFFFFFFFFFFFFFF:FFFFFFFFFFFFFFFFFFFFFFFFFFFFFFFFFFFFFFFF
FFFFFFFFFFFFFFFFFFFFFFFFFFFFF,:FFF:F:FFFFFFFFFFFFFFFFFFFFFFFFF:FFFFFFF
FFFFF:FFFFFF::F
@A00721:190:HGJW7DSXY:4:2115:14968:5462 1:N:0:TCTGCAAG+GCTGCACT
GTCCGATTAATAGTGAGGGATCCGTTAGGATCCAGGTGCACGATAGTCGAATGATGGGAACAGCGTCT
AAACAAGCTGAATTCGTAATGCCAGTTACGTGTAACTGCGATCTGACGTTTTACGGAAGTTGTTGGAG
TTCTGCATCGTCTGA
+
FFFFFFFFFFFFFFFFFFFFFFFF,F:FFFFFFFFFFFFFFFFFFFFFFFFFFFFFFFFFFFFFFFFFFF
FFFFFFFFFFFFFFFFFFFFFFFFFFFFFFFFFFFFFFFFFFFFFFFFFFFFFFFFFFFFFFFFFFFFFF
FFFFFFFFFFFFFFF
@A00721:190:HGJW7DSXY:4:2135:5547:3302 1:N:0:TCTGCAAG+GCTGCACT
GTCCGATTAATAGTGAGGGATCCGTTAGGATCCAGGTGCACGATAGTCGAATGATGGGAACAGCGTCT
AAACAAGCTGAATTCGTAATGCCAGTTACGTGTAACTGCGATCTGACGTTTTACGGAAGTTGTTGGAG
TTCTGCATCGTCTGA
+
FFFFFFFFFFFFFFFFFFFFFFFF:FFFFFFFFFFFFFFFFFFFFFFFFFFFFFF:FFFFF:FFFFFFFF
FFFFFFFFFFFFFFFFFFFFFFFFFFFFFFFFFFFFFFFFFFFFFFFFFFFFFFFFFFFFFFFFFFFFFF
FFFFFFFFFFFFFFF
@A00721:190:HGJW7DSXY:4:2372:15591:8296 1:N:0:TCTGCAAG+GCTGCACT
GTCCGATTAATAGTGAGGGATCCGTTAGGATCCAGGTGCACGATAGTCGAATGATGGGAACAGCGTCT
AAACAAGCTGAATTCGTAATGCCAGTTACGTGTAACTGCGATCTGACGTTTTACGGAAGTTGTTGGAG
TTCTGCATCGTCTGA
+
FFFFFFFFFFFFFFFFFFFFFFFF:F,FFFFFFFFFFFFFFFFFFFFFFFFFFFFFFFFFF:FFFFFFFF
FFFFFFFFFFF:FFFFFFFFFFFFFFFFFFFFFFFFFFFFFFFFFFFFFFFFFFFFFFFFFFFFFFFFFF
FFFFFFFFFFFFFFF
@A00721:190:HGJW7DSXY:4:2518:9317:11115 1:N:0:TCTGCAAG+GCTGCACT
GTCCGATTAATAGTGAGGGATCCGTTAGGATCCAGGTGCACGATAGTCGAATGATGGGAACAGCGTCT

AAACAAGCTGAATTCGTAATGCCAGTTACGTGTAACTGCGATCTGACGTTTTACGGAAGTTGTTGGAG
TTCTGCATCGTCTGA
+
FFFFFFFFFFFFFFFFFFFFFFFFFF:FFFFFFFFFFFFFFFFFFFFFFFFFFF:FFFF:FFFFFFFF
FFFFFFFFFFFF:FFFFFFFFFFFFFFFFFFFFFFFFFFFFFFFFFFFFFFFFFFFFFF,FFFFFFF
FFFFFFFFFFFFFFF
@A00721:190:HGJW7DSXY:4:2564:30463:35524 1:N:0:TCTGCAAG+GCTGCACT
CGCACGATAGTCGAATGATGGGAACAGCGTCTAAACAAGCTGAATTCGTAATGCCAGTTACGTGTAAC
TGCGATCTGACGTTTTACGGAAGTTGTTGGAGTTCTGCATCGTCTGATGAGATGCCTTGGAAGATGTT
GTATAGGTTGGAAAA
+
FFFFFFFFFFFFFFFFFFFFFFFFFFFFFFFFFFFFFFFFFFFFFFFFFFFFFFFFFFFFFFFFFFFF
FFFFFFFFFFFFFFFFFFFFFFF:FF:FFFFFFFFFFFFFFFFFFFFFFFFFFFFFFFFFFFFFFFFF
FFFFFFFFFFFFFFF
@A00721:190:HGJW7DSXY:4:1406:7057:10989 1:N:0:TCTGCAAG+GCTGCACT
GCACGATAGTCGAATGATGGGAACAGCGTCTAAACAAGCTGAATTCGTAATGCCAGTTACGTGTAACT
GCGATCTGACGTTTTACGGAAGTTGTTGGAGTTCTGCATCGTCTGATGAGATGCCTTGGAAGATGTTG
TATAGGTTGGAAAAT
+
FFFFFFFFFFFFFFFFF:FFFFFFFFFFFFFFFFFFFFFFFFFFFFFFFFFFFFFFFFFFFFFFFFFF
FF:FFFFFFFFFFFFFFFFF:FFFFFFFFFFFFFFFFFFFFFFFFFFFFFFFFFFFFFFFFFFFFFFF
FFFFFFFFFFFFFFF
@A00721:190:HGJW7DSXY:4:1116:9100:34225 1:N:0:TCTGCAAG+GCTGCACT
CCTGTTTTGAGGTTGGAATTTTCCAACCTATACAACATCTTCCAAGGCATCTCATCAGACGATGCAGA
ACTCCAACAACTTCCGTAAAACGTCAGATCGCAGTTACACGTAACTGGCATTACGAATTCAGCTTGTT
TAGACGCTGTTCCCA
+
FFFFFFFFFFFFFFFFFFFFFFFFFFFFFFFFFFFFFFFFFFFFFFFFFFFFFFFFFFFFFFFFFFFF
FFFFFFFFFFFFFFFFFFFFFFFFFFFFFFFFFFFFFFFFFFFFFFFFF:FFFFFFFFFFFFFFFFFF
FFFFFFFFFFFFFFF
@A00721:190:HGJW7DSXY:4:1163:8097:4742 1:N:0:TCTGCAAG+GCTGCACT
CCTGTTTTGAGGTTGGAATTTTCCAACCTATACAACATCTTCCAAGGCATCTCATCAGACGATGCAGA
ACTCCAACAACTTCCGTAAAACGTCAGATCGCAGTTACACGTAACTGGCATTACGAATTCAGCTTGTT
TAGACGCTGTTCCCA
+
FFFFFFFFFFFF:FFFFFFFFFFFFFFFFFFFFFFFFFFFFFFFFFFFFFFFFFFFFFFFFFFFFFFF
FFFFFFFFFFFFFFFFFFFFFFFFFFFFFFFFFFFFFFFFFFFFFFFFFFFFFFFFFFFFFFFFFFFF
FFFFFFFFFFFFFFF
@A00721:190:HGJW7DSXY:4:1501:12192:34976 1:N:0:TCTGCAAG+GCTGCACT
CCTGTTTTGAGGTTGGAATTTTCCAACCTATACAACATCTTCCAAGGCATCTCATCAGACGATGCAGA
ACTCCAACAACTTCCGTAAAACGTCAGATCGCAGTTACACGTAACTGGCATTACGAATTCAGCTTGTT
TAGACGCTGTTCCCA
+
F,FFF,FFFFFFFFFFFFFFFFFFFF::FF:FFFFFFFFFFFFFFF:FFF::FFFFFFFFFFFFFFFF
FFFFFFF:FFFF,,F:FFFFFF,FFFFF:FFFFFFFFFFFFFFFF,FFFFFFFF,FFF,:F,FFFFFF
FF,FFFFFFFFF:,F
@A00721:190:HGJW7DSXY:4:2129:14335:33489 1:N:0:TCTGCAAG+GCTGCACT
CCTGTTTTGAGGTTGGAATTTTCCAACCTATACAACATCTTCCAAGGCATCTCATCAGACGATGCAGA
ACTCCAACAACTTCCGTAAAACGTCAGATCGCAGTTACACGTAACTGGCATTACGAATTCAGCTTGTT
TAGACGCTGTTCCCA
+
FFFFFFFFFFFFFFFFFFFFFFFFFFFFFFFFFFFFFFFFFFFFFFFFFFFFFFFFFFFFFFFFFFFF
FFFFFFFFFFFFFFFFFFFF:FFFFFFFFFFFFFFFFFFFFFFFFFFFFFFFFFFFFFFFFFFFFFF:FFFFF
FFFFFFFFFFFFFFF

@A00721:190:HGJW7DSXY:4:2323:10131:33379 1:N:0:TCTGCAAG+GCTGCACT
CCTGTTTTGAGGTTGGAATTTTCCAACCTATACAACATCTTCCAAGGCATCTCATCAGACGATGCAGA
ACTCCAACAACTTCCGTAAAACGTCAGATCGCAGTTACACGTAACTGGCATTACGAATTCAGCTTGTT
TAGACGCTGTTCCCA
+
FFFFFFFFFFFFFFFFFFFFFFFFFFFFFFFFFFFFFFFFFFFFFFFFFFFFFFFFFFFFFFFFFFFF
FFFFFFFFFFFFFFFFFFFFFFFFFFFFFFFFFFFFFFFFFFFFFFFFFFFFFFFFFFFFFFFFFFFF
FF:FFFFFFFFFFFF
@A00721:190:HGJW7DSXY:4:2537:6361:8093 1:N:0:TCTGCAAG+GCTGCACT
CCTGTTTTGAGGTTGGAATTTTCCAACCTATACAACATCTTCCAAGGCATCTCATCAGACGATGCAGA
ACTCCAACAACTTCCGTAAAACGTCAGATCGCAGTTACACGTAACTGGCATTACGAATTCAGCTTGTT
TAGACGCTGTTCCCA
+
FFFFFFFFFFFFFFFFFFFFFFFFFFFFFFFFFFFFFFFFFFFFFFFFFFFFFFFFFFFFFFFFFFFF
FFFFFFFFFFFFFFFFFFFFFFFFFFFFFFFFFFFFFFFFFFFFFFFFFFFFFFFFFFFFFFFFFFFF
FF:FFFFFFFFFFFF
@A00721:190:HGJW7DSXY:4:1118:3667:12696 1:N:0:TCTGCAAG+GCTGCACT
GTCTAAACAAGCTGAATTCGTAATGCCAGTTACGTGTAACTGCGATCTGACGTTTTACGGAAGTTGTT
GGAGTTCTGCATCGTCTGATGAGATGCCTTGGAAGATGTTGTATAGGTTGGAAAATTCCAACCTCAAA
ACAGGTACTCTGTTT
+
:FFFFFFFFFFFFFFFFFFFFFFFFFFFFFFFFFFFFFFFFFFFFFFFFFFFFFFFFFFFF:FFFFFF
FFFFFFFFFF:FFFFFFFFFFFFF:FF:F,FF:FFFFFFFFFFFFF:FFFFFFFFFFFFFFFFFFFFF
FFFFFFFF::FFFFF
@A00721:190:HGJW7DSXY:4:2105:19217:28479 1:N:0:TCTGCAAG+GCTGCACT
GTCTAAACAAGCTGAATTCGTAATGCCAGTTACGTGTAACTGCGATCTGACGTTTTACGGAAGTTGTT
GGAGTTCTGCATCGTCTGATGAGATGCCTTGGAAGATGTTGTATAGGTTGGAAAATTCCAACCTCAAA
ACAGGTACTCTGTTT
+
FFFFFFFFFFFFFFFFFFFFFFFFFFFFFFFFFFFFFFFFFFFFFFFFFFFFFFFFFFFFFFFFFFFF
FFFFFFFFFFFFFFFFFFFFFFFFFFFFFFFFFFFFFFFFFFFFFFFFFFFFFFFFF:FFFFFFFFFF
FFFF:FFFFFFFFFF
@A00721:190:HGJW7DSXY:4:2350:13910:18724 1:N:0:TCTGCAAG+GCTGCACT
GTCTAAACAAGCTGAATTCGTAATGCCAGTTACGTGTAACTGCGATCTGACGTTTTACGGAAGTTGTT
GGAGTTCTGCATCGTCTGATGAGATGCCTTGGAAGATGTTGTATAGGTTGGAAAATTCCAACCTCAAA
ACAGGTACTCTGTTT
+
FFFFFFFFFFFFFFFFFFFFFFFFFFFFFFFFFFFFFFFFFFFFFFFFFFFFFFFFFFFFF:FFFFFF
FFFFFFFFFFFFFFFFFFFFFFFFFFFFFFFFFFFFFFFFFFFFFFFFFFFFFFFFFFFFFFFFFFFF
FFFFFFFFFFFFFFF
@A00721:190:HGJW7DSXY:4:2576:32850:21903 1:N:0:TCTGCAAG+GCTGCACT
GTCTAAACAAGCTGAATTCGTAATGCCAGTTACGTGTAACTGCGATCTGACGTTTTACGGAAGTTGTT
GGAGTTCTGCATCGTCTGATGAGATGCCTTGGAAGATGTTGTATAGGTTGGAAAATTCCAACCTCAAA
ACAGGTACTCTGTTT
+
FFFFFFFFFFFFFFFFFFFFFFFFFFFFFFFFFFFFFFFFFFFFFFFFF:FFFFFFFFFFFFFFFFFF
FFFFFFFFFFFFFFFFFFFFFFFFFFFFFFFFFFFFFFFFFFFFFFFFFFFFFFFFFFFFFFFFFFFF
FFFFFFFFFFFFFFF
@A00721:190:HGJW7DSXY:4:1261:30400:33974 1:N:0:TCTGCAAG+GCTGCACT
CTGCGATCTGACGTTTTACGGAAGTTGTTGGAGTTCTGCATCGTCTGATGAGATGCCTTGGAAGATGT
TGTATAGGTTGGAAAATTCCAACCTCAAAACAGGTACTCTGTTTTGTAGAATCAAAGGACGTCTGAAG
CTATGTGCTTCGAAG
+
FFFFFFFFFFFFFFFFFFFFFFFFFFFFFFFFFFFFFFFFFFFFFFFFFFFFFFFFFFFFFFFFF,FF

```
FFFFFFFFFFFFFFFFFFFFFFFFFFFFFFFFFFFFFFFFFFFFFFFF,FFFFFFFFFFFFFFFFF:F
FFFFFFFFFFFFFFF
@A00721:190:HGJW7DSXY:4:1452:12653:23876 1:N:0:TCTGCAAG+GCTGCACT
CTGCGATCTGACGTTTTACGGAAGTTGTTGGAGTTCTGCATCGTCTGATGAGATGCCTTGGAAGATGT
TGTATAGGTTGGAAAATTCCAACCTCAAAACAGGTACTCTGTTTTGTAGAATCAAAGGACGTCTGAAG
CTATGTGCTTCGAAG
+
FFFFFFFFFFFFFFFFFFFFFFFFFFFFFFFFFFFFFFFFFFFFFFFFFFFFFFFFFFFFFFFFF,FF
FFFFFFF:FFFFFFFFFFFFFFFFFFFFFFFFFFFFFFFFFFFFFFFFF:FFFFFFFFFFFFFFFFFF
FFFFFFFFFFFFFFF
@A00721:190:HGJW7DSXY:4:2273:1796:4914 1:N:0:TCTGCAAG+GCTGCACT
CTGCGATCTGACGTTTTACGGAAGTTGTTGGAGTTCTGCATCGTCTGATGAGATGCCTTGGAAGATGT
TGTATAGGTTGGAAAATTCCAACCTCAAAACAGGTACTCTGTTTTGTAGAATCAAAGGACGTCTGAAG
CTATGTGCTTCGAAG
+
FFFFFFFFFFFFFFFFFFFFFFFFFFF:FFFFFFFFFFFFFFFFFFFFFFFFFFFFFFFFFFFF:FFF
FFFFFFFFFFFFFFFFFFFFFFFFFFFFFFFFFFFFFFFFFFFFFFFFFF,FFFFFFFFFFFFFFFFF
FFFFFFFFFFFFFFF
@A00721:190:HGJW7DSXY:4:2273:1524:5008 1:N:0:TCTGCAAG+GCTGCACT
CTGCGATCTGACGTTTTACGGAAGTTGTTGGAGTTCTGCATCGTCTGATGAGATGCCTTGGAAGATGT
TGTATAGGTTGGAAAATTCCAACCTCAAAACAGGTACTCTGTTTTGTAGAATCAAAGGACGTCTGAAG
CTATGTGCTTCGAAG
+
FFFFFFFFFFFFFFFFFFFFFFFFFFF:FFFFFFFFFFFFFFFFFFFFFFFFFFFF:FFFFFFFFFFF
FFFFFFFFFFFFFFFFFFFFF:FFFFFFFFFFFFFFFFFFFFFFFFFF:FFFFFFFFFFFFFFFFFFF
FFFFFFFFFFFFFFF
@A00721:190:HGJW7DSXY:4:2273:1362:5071 1:N:0:TCTGCAAG+GCTGCACT
CTGCGATCTGACGTTTTACGGAAGTTGTTGGAGTTCTGCATCGTCTGATGAGATGCCTTGGAAGATGT
TGTATAGGTTGGAAAATTCCAACCTCAAAACAGGTACTCTGTTTTGTAGAATCAAAGGACGTCTGAAG
CTATGTGCTTCGAAG
+
FFFFFFFFFFFFFFFFFFFFFFFFFFFFFFFFFFFFFFFFFFFFFFFFFFFFFFFFFFFFFFFFFFFF
FFFFFFF:FFFFFFFFFFFFFFFFFFFFFFFFFFFFFFFFFFFFFFFFFFFFFFFFFFFFFFFFFFFF
FFFFFFFFFFF:FFF
@A00721:190:HGJW7DSXY:4:2665:29161:32174 1:N:0:TCTGCAAG+GCTGCACT
CTGCGATCTGACGTTTTACGGAAGTTGTTGGAGTTCTGCATCGTCTGATGAGATGCCTTGGAAGATGT
TGTATAGGTTGGAAAATTCCAACCTCAAAACAGGTACTCTGTTTTGTAGAATCAAAGGACGTCTGAAG
CTATGTGCTTCGAAG
+
FFFFFFFFFFFFFFFFFFFFFFFFF,FFFFFFFFFFFFFFFFFFFFFFFFFFFFFFFFFFFFFFFFFF
FFFFFFFFFFFFF:FFFFFFFFFFFFFFFFFFFFFFFFFFFFFFFFFFFFFFFFFFFFFFFFFFFFFF
FFFFFFFFFFFFFFF
@A00721:190:HGJW7DSXY:4:1138:18014:32221 1:N:0:TCTGCAAG+GCTGCACT
CTGGCGTTTTACGGAAGTTGTTGGAGTTCTGCATCGTCTGATGAGATGCCTTGGAAGATGTTGTATAG
GTTGGAAAATTCCAACCTCAAAACAGGTACTCTGTTTTGTAGAATCAAAGGACGTCTGAAGCTATGTG
CTTCGAAGCGCGTT
+
FFFFFFF:FFFFFFFFFF,FFFFFFFFFFFF:FF::FF,FFF:F,FFFFFFFFFFFFF,FFFFFFFF:
FF:FF:FFFFFFFFFFF:FFFFF:F::FFFFF,FFFFFFFFFFFF:FFFFFF::F,FFFFFFFFFFF:
FF,FFFF,FFFFFF
@A00721:190:HGJW7DSXY:4:1153:28447:29371 1:N:0:TCTGCAAG+GCTGCACT
CTGACGTTTTACGGAAGTTGTTGGAGTTCTGCATCGTCTGATGAGATGCCTTGGAAGATGTTGTATAG
GTTGGAAAATTCCAACCTCAAAACAGGTACTCTGTTTTGTAGAATCAAAGGACGTCTGAAGCTATGTG
CTTCGAAGCGCGTTG
```

```
+
FFFFFFFFFFFFFFFFFFFFFFFFFFFFFFFFFFFFFFFFFFFFFFFFFFFFFFFFFFFFFFFFFFFF
FFFFFFFFFFFFFFFFFFFFFFFFFFFFFFFFFFFFFFFFFFFFFFFF:FFFFFFFFFFFFFFFF:FF
FFFFFFFF:FFFFFF
@A00721:190:HGJW7DSXY:4:1166:1633:15248 1:N:0:TCTGCAAG+GCTGCACT
CTGACGTTTTACGGAAGTTGTTGGAGTTCTGCATCGTCTGATGAGATGCCTTGGAAGATGTTGTATAG
GTTGGAAAATTCCAACCTCAAAACAGGTACTCTGTTTTGTAGAATCAAAGGACGTCTGAAGCTATGTG
CTTCGAAGCGCGTTG
+
FFFFF:FFFFFFFFFFFFFFFFFFFFFFFFFFFFFFFFFFFFFFFFFFFFFFFFFFFF:FFFF:FFFF
FFFFFFFFFFFFFFFFFFFFF:FFFF:FFFFFFFFFFFFFFFFFFFFFFFFFFFFFFFFF:FFFFFF:
FFFFFFFFFFFF:FF
@A00721:190:HGJW7DSXY:4:1263:6759:15421 1:N:0:TCTGCAAG+GCTGCACT
CTGACGTTTTACGGAAGTTGTTGGAGTTCTGCATCGTCTGATGAGATGCCTTGGAAGATGTTGTATAG
GTTGGAAAATTCCAACCTCAAAACAGGTACTCTGTTTTGTAGAATCAAAGGACGTCTGAAGCTATGTG
CTTCGAAGCGCGTTG
+
FFFFFFFFFFFFFFF:FFFFFFFFFFFFFFFFFFFFFF:FFFFFFFFFFFFFFFF:FFFFFFFFFFFF
FFFFFF:FFFFFFFFFFFFFFFFFFFFFFFFFFFFFFFFF:FFFFFFFFFFFFFFFFFFFFFFFFFFF
FFFFFFFFFFFFFFF
@A00721:190:HGJW7DSXY:4:2203:3079:9580 1:N:0:TCTGCAAG+GCTGCACT
CTGGCGTTTTACGGAAGTTGTTGGAGTTCTGCATCGTCTGATGAGATGCCTTGGAAGATGTTGTATAG
GTTGGAAAATTCCAACCTCAAAACAGGTACTCTGTTTTGTAGAATCAAAGGACGTCTGAAGCTATGTG
CTTCGAAGCGCGTTG
+
FFFFFFFFFFFFFFFFFFFFFFFFF:FF:FFFF::FFFFFFFFFFFFFFFFFFFFFFFF::FFFFFF:
FFFF:FFFFFFFFFF:FFFFFFF:F::FFFFFFFF,FFFFF,FFF,FFFFFFF:FFF,FF,:FFFFFF
FFF,FFF:FFFFF,F
@A00721:190:HGJW7DSXY:4:2368:25771:33661 1:N:0:TCTGCAAG+GCTGCACT
CTGACGTTTTACGGAAGTTGTTGGAGTTCTGCATCGTCTGATGAGATGCCTTGGAAGATGTTGTATAG
GTTGGAAAATTCCAACCTCAAAACAGGTACTCTGTTTTGTAGAATCAAAGGACGTCTGAAGCTATGTG
CTTCGAAGCGCGTTG
+
FFFFFFFFFFFFFFFFFFFFFFFFFFFFFFFFFFFFFFFFFFFFFFFFFFFFFFFFFFFFFFFFFFFF
FFFFFFFFFFFFFFFFFFFFFFFFFFFFFFFFFFFFFFFFFFFFFFFFF:FFF:FFFFFFFFFFFFFF
FFFFFFFFFFFFFFF
@A00721:190:HGJW7DSXY:4:2378:12364:12477 1:N:0:TCTGCAAG+GCTGCACT
CTGGCGTTTTACGGAAGTTGTTGGAGTTCTGCATCGTCTGATGAGATGCCTTGGAAGATGTTGTATAG
GTTGGAAAATTCCAACCTCAAAACAGGTACTCTGTTTTGTAGAATCAAAGGACGTCTGAAGCTATGTG
CTTCGAAGCGCGTTG
+
FFFFFFFFFFFFFFFFF,FFFFFFFFFFFFFFFFFFFFFFFFFFFFFFFF:FFFFFFFFFFFFFFFFF
FFFFFFFFFFFFFFFFFFFFFFFFFFFFFFFFFFFFFFFFFFFFFFFFFFFFFFFFFF:FFFFFFFFF
FFFFFFFFFFFFFFF
@A00721:190:HGJW7DSXY:4:2467:7509:6355 1:N:0:TCTGCAAG+GCTGCACT
CTGACGTTTTACGGAAGTTGTTGGAGTTCTGCATCGTCTGATGAGATGCCTTGGAAGATGTTGTATAG
GTTGGAAAATTCCAACCTCAAAACAGGTACTCTGTTTTGTAGAATCAAAGGACGTCTGAAGCTATGTG
CTTCGAAGCGCGTTG
+
FFFFFFF:FFFFFFFFFFFFF:FFFFFFF:FFFFFFFFFFFFFFFFFFFFFFFFFFFF:FFFFFFFFF
FFFFFFFFFFFFFFFFFFFFFFFFFFFFFFFFFFFFFFFFF:FFFFFFFFFFF:FF:FFFFFFFFFFF
FFFFFFFFFFFFFFF
@A00721:190:HGJW7DSXY:4:2633:2736:17409 1:N:0:TCTGCAAG+GCTGCACT
CTGACGTTTTACGGAAGTTGTTGGAGTTCTGCATCGTCTGATGAGATGCCTTGGAAGATGTTGTATAG
```

GTTGGAAAATTCCAACCTCAAAACAGGTACTCTGTTTTGTAGAATCAAAGGACGTCTGAAGCGATGTG
CTTCGAAGCGCGTTG
+
FFFFFFFFFFFFFFFFFFFFF:FFFFFFFFFFFFFFFFFFFFFFFFFFFFFFFF:FFFFFF:FFFFFFFFF
FFFFFF:FFFFFFFFFFFFFFFFFFFFFFFFFFFFFFFFFFFFFFFF:FFFFFFFFFFF:FF,::FFF
FFFFF,:FFFFFFFF
@A00721:190:HGJW7DSXY:4:2678:12382:33332 1:N:0:TCTGCAAG+GCTGCACT
CTGACGTTTTACGGAAGTTGTTGGAGTTCTGCATCGTCTGATGAGATGCCTTGGAAGATGTTGTATAG
GTTGGAAAATTCCAACCTCAAAACAGGTACTCTGTTTTGTAGAATCAAAGGACGTCTGAAGCTATGTG
CTTCGAAGCGCGTTG
+
FFFFFFFFFFFFFFFFFFFFFFFFFFFFFFFFFFFFFFFFFFFFFFFFFFFFFFFFFFFFFFFFFFFFF
FFFFFFFFFFFFFFFFFFFFFFFFFFFFFFFFFFFFFFFFFFFFFFFFFFFFFF:F:FFFFFFFFFFF
FFFF:FFFFFFFFFF
@A00721:190:HGJW7DSXY:4:1317:20202:26240 1:N:0:TCTGCAAG+GCTGCACT
GACGTTTTACGGAAGTTGTTGGAGTTCTGCATCGTCTGATGAGATGCCTTGGAAGATGTTGTATAGGT
TGGAAAATTCCAACCTCAAAACAGGTACTCTGTTTTGTAGAATCAAAGGACGTCTGAAGCTATGTGCT
TCGAAGCGCG
+
FFFFFFFFFFFFFFFFFFFFFFFFFFFFFFFFFFFFFFFFFFFFFFFFFFFFFFFFFFFFFFFFFFFFF
FFFFFFFFFFFFFFFFFFFFFFFFFFFFFFFFFFFFFFFFFFFFFFFFFFFFFFFFFFFFF:FF:FFFF
FFFFFFFFFF
@A00721:190:HGJW7DSXY:4:1375:32488:6621 1:N:0:TCTGCAAG+GCTGCACT
GACGTTTTACGGAAGTTGTTGGAGTTCTGCATCGTCTGATGAGATGCCTTGGAAGATGTTGTATAGGT
TGGAAAATTCCAACCTCAAAACAGGTACTCTGTTTTGTAGAATCAAAGGACGTCTGAAGCTATGTGCT
TCGAAGCGCG
+
FFFFFFFFFFFFFFFFFFFF:FFFFFFFFFFFFFFFFFFFFFFFFFFFFFFFFFFFFFFFFFFFFFFFF
FFFFFFFFFFFFFFFFFFFFFFFFFFFFFFFFFFFFFFFFFFFFFFFF:FFFFFFFFFFFFFFFFFFFF
:FFFFFFFFF
@A00721:190:HGJW7DSXY:4:1376:17671:7232 1:N:0:TCTGCAAG+GCTGCACT
GACGTTTTACGGAAGTTGTTGGAGTTCTGCATCGTCTGATGAGATGCCTTGGAAGATGTTGTATAGGT
TGGAAAATTCCAACCTCAAAACAGGTACTCTGTTTTGTAGAATCAAAGGACGTCTGAAGCTATGTGCT
TCGAAGCGCG
+
FFFFFFFFFFFFFFFF:FFFFFFFFFFFFFFFFFFFFFFFFFFFFFFFFFFFFFFF:FFFFFF:FFFFFFFF
FFFFFFFFFFFFFFFFFFFFFFFFFFFFFFFFFFFFFFF,:FFFFFFFFFFFFFFFFFFFFFFFFFFFF
FFFFFFFFFF
@A00721:190:HGJW7DSXY:4:1619:27145:24236 1:N:0:TCTGCAAG+GCTGCACT
GACGTTTTACGGAAGTTGTTGGAGTTCTGCATCGTCTGATGAGATGCCTTGGAAGATGTTGTATAGGT
TGGAAAATTCCAACCTCAAAACAGGTACTCTGTTTTGTAGAATCAAAGGACGTCTGAAGCTATGTGCT
TCGAAGCGCG
+
FFFFFFFFFFFFF,FF:FF,:FFFFFFFFFFFFFFFFF::F:FF:FFF:FFFFFFFFFFFFFFFFFFFF
FFFFFFFFFFFFFFFFFFFFFFFFFFFFFFFFFFFFFFF:FFFFFFFFFFFFFFFFFFFFF,FFFFFFF
F:F:F:FFFF
@A00721:190:HGJW7DSXY:4:1105:6578:8625 1:N:0:TCTGCAAG+GCTGCACT
CCTGGGACTCCTGAACTCAATCTCCTCAACGCGCTTCGAAGCACATAGCTTCAGACGTCCTTTGATTC
TACAAAACAGAGTACCTGTTTTGAGGTTGGAATTTTCCAACCTATACAACATCTTCCAAGGCATCTCA
TCAGACGATGCAGAA
+
FFFFFFFFFFFFFFFFFFFFFFFFFFFFFFFFFFFFFFFFFFFFFFFFFFFFFFFFFFFFFFFFFFFFF
FFFF:FFFFFFFFFFFFFFFFFFFFFFFFFFFFFFFFFFFFFFFFF:FFFFFFFFFFFFFFFFFFFFFFFFF
FFFF:FFF:FFFFFF

```
@A00721:190:HGJW7DSXY:4:1251:7093:19727 1:N:0:TCTGCAAG+GCTGCACT
CCTGGGACTCCTGAACTCAATCTCCTCAACGCGCTTCGAAGCACATAGCTTCAGACGTCCTTTGATTC
TACAAAACAGAGTACCTGTTTTGAGGTTGGAATTTTCCAACCTATACAACATCTTCCAAGGCATCTCA
TCAGACGATGCAGAA
+
FFFFFFFFFFFFF:,FFFFFFFFFFFFFFFFFFFFFFFFFFFFFFFFFFFFFFFFFFFFFFFFFFFFFFF
FFFFFFFFFFFF:FFFFFFFFFFFFFFFF:FFFFFFFFFFFFFFFFFFFFFFFFFFFFFFF,FFFF:FFF
FFF:FFFFFFFFFFF
@A00721:190:HGJW7DSXY:4:1269:23782:27117 1:N:0:TCTGCAAG+GCTGCACT
CCTGGGACTCCTGAACTCAATCTCCTCAACGCGCTTCGAAGCACATAGCTTCAGACGTCCTTTGATTC
TACAAAACAGAGTACCTGTTTTGAGGTTGGAATTTTCCAACCTATACAACATCTTCCAAGGCATCTCA
TCAGACGATGCAGAA
+
::FFFFFF:FFFFFFFFFFFFFFFFFFFFFFFFFFFFFFFFFFFFFFFF:FF:FFFFFF:FFFFFFFFF:
FFFFF:FFFFFFFFFFFFFF:F:FFFFFFF,F:FFFFFFFFFFFFFFFFFFFFFF:FFFFFFFFFFFFFF
FFFF:FFFFFFFFFF
@A00721:190:HGJW7DSXY:4:1342:23818:34256 1:N:0:TCTGCAAG+GCTGCACT
CCTGGGACTCCTGAACTCAATCTCCTCAACGCGCTTCGGAGCACATAGCTTCAGACGTCCTTTGATTC
TACAAAACAGAGTACCTGTTTTGAGGTTGGAATTTTCCAACCTATACAACATCTTCCAAGGCATCTCA
TCAGACGATGCAGAA
+
:F:FF:FF,FF,:FFF,FFFFFFFFFFFFFFFFFF:FFFFFFFFFFFFFFFFFFFFF:FFFFF:FFFFFF
FFFFFFFFFFFF:FFFFFFFFFFFFFFFFFFFFFFFFFFFFFFFFFFFFFFFFFFFFF:FFFFFFFFFF
FFFFFFFFFFFFFFF
@A00721:190:HGJW7DSXY:4:2410:24876:21057 1:N:0:TCTGCAAG+GCTGCACT
CCTGGGACTCCTGAACTCAATCTCCTCAACGCGCTTCGAAGCACATAGCTTCAGACGTCCTTTGATTC
TACAAAACAGAGTACCTGTTTTGAGGTTGGAATTTTCCAACCTATACAACATCTTCCAAGGCATCTCA
TCAGACGATGCAGAA
+
FFFFFFFFFFFFFFFFFFFFFFFFFFFFFFFFFFFFFFFFFFFFFFFFFFFFFFFFFFFFFFFFFFFFFF
FFFFFFFFFFFFFFFFFFFFFFFFFFFFFFFFFFFFFFFFFFFFFFFFFFFFF:FFFF:FFFFFFFFFFF
FFFFFFFFFFF:FFF
@A00721:190:HGJW7DSXY:4:2511:18701:25614 1:N:0:TCTGCAAG+GCTGCACT
CCTGGGACTCCTGAACTCAATCTCCTCAACGCGCTTCGAAGCACATAGCTTCAGACGTCCTTTGATTC
TACAAAACAGAGTACCTGTTTTGAGGTTGGAATTTTCCAACCTATACAACATCTTCCAAGGCATCTCA
TCAGACGATGCAGAA
+
FFFFFFFFFFFFFFFFFFFFFFFFFFFFFFFFFFFFFFFFFFFFFFFFFFFFFFFFFFFFFFFFFFFFFF
FFFF:FFFFFFFFFFFFFFFFFFFFFFFFFFF:FFFFFF:FFFFFFFFFFFFFFFFFFFFFFFFFFFFFF
FFFFFFFFFFFFFFF
@A00721:190:HGJW7DSXY:4:2615:26078:32816 1:N:0:TCTGCAAG+GCTGCACT
CCTGGGACTCCTGAACTCAATCTCCTCAACGCGCTTCGAAGCACATAGCTTCAGACGTCCTTTGATTC
TACAAAACAGAGTACCTGTTTTGAGGTTGGAATTTTCCAACCTATACAACATCTTCCAAGGCATCTCA
TCAGACGATGCAGAA
+
FFFFFFFFFFFFF:FFFFFFFFFFFFFFFFFFFFFFFFFFFFFFFFFFFFFFFFFFFFFFFFFFFFFFFF
FFFFFFFFFFFFFFFFFFFFFFFFFFFFFFFFFFFFFFFFFFFFFFFFFFFFFFFFFFFFFFFFFF:FFF
FFFFFFFFFF,F,FF
@A00721:190:HGJW7DSXY:4:2676:22092:5932 1:N:0:TCTGCAAG+GCTGCACT
CCTGGGACTCCTGAACTCAATCTCCTCAACGCGCTTCGGAGCACATAGCTTCAGACGTCCTTTGATTC
TACAAAACAGAGTACCTGTTTTGAGGTTGGAATTTTCCAACCTATACAACATCTTCCAAGGCATCTCA
TCAGACGATGCAGAA
+
FFFFFFFFFFFFFFFFFFFFFFFFFFFFFFFFFFFFFFFFFFFFFFFFFFFFFFFFFFFFFFFFFFFFFF
```

```
FFFFFFFFFFFFFFFFFFFFFFFFFFFFFFFFFFFFFFFFFFFFFFFFFFFFFFFFFFFFFFFFFFFF
FFFFFFFFFFFFFFF
@A00721:190:HGJW7DSXY:4:1408:15826:23610 1:N:0:TCTGCAAG+GCTGCACT
TGATGAGATGCCTTGGAAGATGTTGTATAGGTTGGAAAATTCCAACCTCAAAACAGGTACTCTGTTTT
GTAGAATCAAAGGACGTCTGAAGCTATGTGCTTCGAAGCGCGTTGAGGAGATTGAGTTCAGGAGTCCC
AGGATCAACATCCTG
+
FFFFFFFFFFFFFFFFFFFFFFFFFFFFFFFFFFFF:FFFFFFFFFFFFFFFFFFFFFFFFFFFFFFF
FFFFFFFFFFFFFFF:FFFFFFFFFFFFFFFFFFFFFFFFFFFFFFFFFFFFFFFFFFFF:FFFFFFF
FFFFFFFFFFFFFFF
@A00721:190:HGJW7DSXY:4:1451:31159:16564 1:N:0:TCTGCAAG+GCTGCACT
TGATGAGATGCCTTGGAAGATGTTGTATAGGTTGGAAAATTCCAACCTCAAAACAGGTACTCTGTTTT
GTAGAATCAAAGGACGTCTGAAGCTATGTGCTTCGAAGCGCGTTGAGGAGATTGAGTTCAGGAGTCCC
AGGATCAACATCCTG
+
FFFFFFFFFFFFFFFFFFFFFFFFFFFFFFFFFFFFFFFFFFFFFFFFFFFFFFFFFFFFFFFFFFFF
FFFFFFFFFFFFFFFFFF:FFFFFFFFFFFFFFFFFFFFFFFFFFFFFFFFFFFFFFFFFFFFFFFFF
FFFFFFFFFFFFFFF
@A00721:190:HGJW7DSXY:4:1645:32398:15765 1:N:0:TCTGCAAG+GCTGCACT
TGATGAGATGCCTTGGAAGATGTTGTATAGGTTGGAAAATTCCAACCTCAAAACAGGTACTCTGTTTT
GTAGAATCAAAGGACGTCTGAAGCTATGTGCTTCGAAGCGCGTTGAGGAGATTGAGTTCAGGAGTCCC
AGGATCAACATCCTG
+
FFFFFFFFFFFFFFFFFFFFFFFFFFFFFFFFFFFFFFF:FFFFFFFFFFF:FFF:FFFFFFFFFFFFF
FFF,FFFFFF:FFFFF:FFFFFFFFFFFFFFFFFFFF,FFFFFFFFFFFFFFFFF:FF:FFFF:FF::
FF:FFFFF:FFFFFF
@A00721:190:HGJW7DSXY:4:2271:21233:21512 1:N:0:TCTGCAAG+GCTGCACT
TGATGAGATGCCTTGGAAGATGTTGTATAGGTTGGAAAATTCCAACCTCAAAACAGGTACTCTGTTTT
GTAGAATCAAAGGACGTCTGAAGCTATGTGCTTCGAAGCGCGTTGAGGAGATTGAGTTCAGGAGTCCC
AGGATCAACATCCTG
+
FFFFFFFFFFFFFFFFFFFFFFFFFFFFFFFFFFFFFFFFFFFFFFFFFFFFFFFFFFFFFFFFFFFF
FFFFFFFFFFFFFFFFFFFFFFFFFFFFFFFFFFFFFFFFFFFFFFFFFFFFFFFFFFFFFFFFFF:F
FFFFFFFFFFFFFFF
@A00721:190:HGJW7DSXY:4:2271:23023:22138 1:N:0:TCTGCAAG+GCTGCACT
TGATGAGATGCCTTGGAAGATGTTGTATAGGTTGGAAAATTCCAACCTCAAAACAGGTACTCTGTTTT
GTAGAATCAAAGGACGTCTGAAGCTATGTGCTTCGAAGCGCGTTGAGGAGATTGAGTTCAGGAGTCCC
AGGATCAACATCCTG
+
FFFFFFFFFFFFFFFFFFFFFFFFFFFFFFFFFFFF:FFFFFFFFFFFFFFFFFFFFFFFFFFFFFFF
FFFFFFFFFFFFFFFFFFFFFFFFFFFFFFFFFFFFFFFFFFFFFFFFFFFFFFFFFFFFFFFFFFFF
FFFFFFFFFFFFFFF
@A00721:190:HGJW7DSXY:4:2608:6072:2268 1:N:0:TCTGCAAG+GCTGCACT
TGATGAGATGCCTTGGAAGATGTTGTATAGGTTGGAAAATTCCAACCTCAAAACAGGTACTCTGTTTT
GTAGAATCAAAGGACGTCTGAAGCTATGTGCTTCGAAGCGCGTTGAGGAGATTGAGTTCAGGAGTCCC
AGGATCAACATCCTG
+
FFFFFFFFFFFFF:FFFFFFFF,FFFFFFFFFFFFFFFFFFFFFFFFFFFFFFFFFFFFFFFFFFFFF
FFFFFFFFFF,FFFFFFFFFFFFFF:FFFFFFFFFFFFFFFFFFFFFFFFFFFFFFFFF:FFFFFFFF
FFFFFFFFFFFFFFF
@A00721:190:HGJW7DSXY:4:2625:19117:10833 1:N:0:TCTGCAAG+GCTGCACT
TGATGAGATGCCTTGGAAGATGTTGTATAGGTTGGAAAATTCCAACCTCAAAACAGGTACTCTGTTTT
GTAGAATCAAAGGACGTCTGAAGCTATGTGCTTCGAAGCGCGTTGAGGAGATTGAGTTCAGGAGTCCC
AGGATCAACATCCTG
```

```
+
FFFFFFFFFFFFFFFFFFFFFFFFFFFFFFFFFFFFF:FFFFFFFFFFFFFFFFFFFFFFFFFFFFFFFF
FFFFFFFFFFFFFFFFFFFFFFFFFFFFFFFFFFFFFFFFFFFFFFFFFFFFFFFFFFFFFFFFFFFFFF
FFFFFFFFFFFFFFF
@A00721:190:HGJW7DSXY:4:1328:3233:23813 1:N:0:TCTGCAAG+GCTGCACT
TGGAAGATGTTGTATAGGTTGGAAAATTCCAACCTCAAAACAGGTACTCTGTTTTGTAGAATCAAAGG
ACGTCTGAAGCTATGTGCTTCGAAGCGCGTTGAGGAGATTGAGTTCAGGAGTCCCAGGATCAACATCC
TGTCGAAGAGGCATG
+
FFFFFFFFFFFFFFFFFFFFFFFFFFFFFFFFFFFFFFFFFFFFFFFFFFFFFFFFFFFFFFFFFFFFFF
FFFFFFFFFFFFFFFFFFFFFFFFFFFFFFFFFFFFFFFFFFFFFFFFFFFFFFFFFFFFFFFFFFFFFF
FFFFFFFFFFFFFFF
@A00721:190:HGJW7DSXY:4:2126:11207:34773 1:N:0:TCTGCAAG+GCTGCACT
TGGAAGATGTTGTATAGGTTGGAAAATTCCAACCTCAAAACAGGTACTCTGTTTTGTAGAATCAAAGG
ACGTCTGAAGCTATGTGCTTCGAAGCGCGTTGAGGAGATTGAGTTCAGGAGTCCCAGGATCAACATCC
TGTCGAAGAGGCATG
+
FFFFFFFFFFFFFFFFFFFFFFFFFFFFFFFF:FFFFFF:FFFFFFFFFFFFFFFFFFFFFFFFFFFFFF
FFFFFFFFFFFF:FFFFFFFFFFFFFFFFFFFFFFFFFFFFFFFFFFFFFFFFFFFFFFFFFFFFFF:FF
FFFFFFFFFFFFFFF
@A00721:190:HGJW7DSXY:4:2427:27516:28948 1:N:0:TCTGCAAG+GCTGCACT
TGGAAGATGTTGTATAGGTTGGAAAATTCCAACCTCAAAACAGGTACTCTGTTTTGTAGAATCAAAGG
ACGTCTGAAGCTATGTGCTTCGAAGCGCGTTGAGGAGATTGAGTTCAGGAGTCCCAGGATCAACATCC
TGTCGAAGAGGCATG
+
:FFFFFF:FFFFFFFFFFFFFFFF,FFFFFFFFFFFFFFFFFFFFF,FFFFFFFFFFFFFFFFFFFFFFF
FFFFFFFFFFF:FFFFFFFFFFFFFFFFFFFFFFFFFFFFFFFFFFF:FFFFFFFFFFFFFFFFFF:FFF
FFFFFFFFFFFFFFF
@A00721:190:HGJW7DSXY:4:1236:9652:22185 1:N:0:TCTGCAAG+GCTGCACT
GGAAGATGTTGTATAGGTTGGAAAATTCCAACCTCAAAACAGGTACTCTGTTTTGTAGAATCAAAGGA
CGTCTGAAGCTATGTGCTTCGAAGCGCGTTGAGGAGATTGAGTTCAGGAGTCCCAGGATCAACATCCT
GTCGAAGAGGCATGA
+
FFFFFFFFFFFFFFFFFFFFFFFFFFFFFFFFFFFFFFFFFFFFFFFFFFFFFFFFFFFFFFFFFFFFFF
FFFFFFFFFF:FFFFFFFFFFFFFFFFFF:FFFFFFFFFFFFFFFFFFFFFFFFFFFFFFFFFFFFFFFF
FFFFFFFFFFFFFFF
@A00721:190:HGJW7DSXY:4:2139:1624:26882 1:N:0:TCTGCAAG+GCTGCACT
GGAAGATGTTGTATAGGTTGGAAAATTCCAACCTCAAAACAGGTACTCTGTTTTGTAGAATCAAAGGA
CGTCTGAAGCTATGTGCTTCGAAGCGCGTTGAGGAGATTGAGTTCAGGAGTCCCAGGATCAACATCCT
GTCGAAGAGGCATGA
+
FFFFFFFFFFFFFFFFFFFFFFFFFFFFFFFFFFFFFFFFFFFFFFFFFFFFFFFFFFFFFFFFFFFFFF
FFFFFFFFFF,FFFFFFFFFFFFFFFFFFFFFFFFFFFFFFFFFFFFFFFFFFFFFFFFFFFFFFFFFFF
F:FFFFFFFFFFFF:
@A00721:190:HGJW7DSXY:4:2361:22218:32111 1:N:0:TCTGCAAG+GCTGCACT
GGAAGATGTTGTATAGGTTGGAAAATTCCAACCTCAAAACAGGTACTCTGTTTTGTAGAATCAAAGGA
CGTCTGAAGCTATGTGCTTCGAAGCGCGTTGAGGAGATTGAGTTCAGGAGTCCCAGGATCAACATCCT
GTCGAAGAGGCATGA
+
FFFFFFFFFFFFFFFFFFFFFFFFFFFFFFFFFFFFFF:FFFFFFFFFFFFFFFFFFFFFFFFFFFFF:F
FFFFFFFFFFFFFFFFFFFFFFFF,FFFFFFFFFFFFFFFFFFFFFFFFFFFFFFFFFFFFFFFFFFFFF
FFFFFFFFFFFFFFF
@A00721:190:HGJW7DSXY:4:2610:12337:1282 1:N:0:TCTGCAAG+GCTGCACT
GGAAGATGTTGTATAGGTTGGAAAATTCCAACCTCAAAACAGGTACTCTGTTTTGTAGAATCAAAGGA
```

```
CGTCTGAAGCTATGTGCTTCGAAGCGCGTTGAGGAGATTGAGTTCAGGAGTCCCAGGATCAACATCCT
GTCGAAGAGGCATGA
+
FFFFFFFFFFFFFFFFFFFFFFFFFFFFFFFFFFFFFFFFFFFFFFFFFFFFFFFFFFFFFFFFF:FF
FFFFFFFFFFF:FFFFFFFFFFFFFFFFFFFFFFFFFFFFFFFFFFFFFFFFFFFFFFFF
FFFFFFFFFFFFFFF
@A00721:190:HGJW7DSXY:4:2652:4906:12430 1:N:0:TCTGCAAG+GCTGCACT
GGAAGATGTTGTATAGGTTGGAAAATTCCAACCTCAAAACAGGTACTCTGTTTTGTAGAATCAAAGGA
CGTCTGAAGCTATGTGCTTCGAAGCGCGTTGAGGAGATTGAGTTCAGGAGTCCCAGGATCAACATCCT
GTCGAAGAGGCATGA
+
FFFFFFFFFFFFFFFFFFFFFFFFFFFFFFFFFFFFFFFFFFFFFFFFFFFFFFFFFFFFFFFFFFFF
FFFFFFFFFFFFFFFFFFFFFFFFFFFFFFFFFFFFFFFFFFFFFFFFFFFFFFFFFFFFFFFFFFFF
FFFFFFFFFFFFFFF
@A00721:190:HGJW7DSXY:4:1353:4634:22858 1:N:0:TCTGCAAG+GCTGCACT
CCTTGGCTTCATGCCTCTTCGACAGGATGTTGATCCTGGGACTCCTGAACTCAATCTCCTCAACGCGC
TTCGAAGCACATAGCTTCAGACGTCCTTTGATTCTACAAAACAGAGTACCTGTTTTGAGGTTGGAATT
TTCCAACCTATACAA
+
FFFFFFFFFFFFFFFFFFFFFFFFFFFFFFFFFFFFFFFF,FFFFFFF:FFFFFFFFFFFFFFFFFFF
FFFFFFFFFFFFFFFFFFFFFFFFFFFFFFF,FFFFFF,FFFFFFFFFFFFFFFFFFFFFFFFFFFFF
FFFFFFFFFFFFFFF
@A00721:190:HGJW7DSXY:4:1632:24089:29747 1:N:0:TCTGCAAG+GCTGCACT
CCTTGGCTTCATGCCTCTTCGACAGGATGTTGATCCTGGGACTCCTGAACTCAATCTCCTCAACGCGC
TTCGAAGCACATAGCTTCAGACGTCCTTTGATTCTACAAAACAGAGTACCTGTTTTGAGGTTGGAATT
TTCCAACCTATACAA
+
FFFFFFFFFFFFFFFFFFFFFFFFFFFFFFFFFFFFFFFFFFFFFFFF:FFFFFFFFFFFFFFFFFFF
FFFFFFFFFFFFFFFFFFFFFFFFFFFFFFFFFFFFFFFFFFFFFFFFFFFFFFFFFFFFFFFFFFFF
FFFFFFFFFFFFFFF
@A00721:190:HGJW7DSXY:4:2430:23222:14810 1:N:0:TCTGCAAG+GCTGCACT
CCTTGGCTTCATGCCTCTTCGACAGGATGTTGATCCTGGGACTCCTGAACTCAATCTCCTCAACGCGC
TTCGAAGCACATAGCTTCAGACGTCCTTTGATTCTACAAAACAGAGTACCTGTTTTGAGGTTGGAATT
TTCCAACCTATACAA
+
FFFFFFFFFFFFFFFFFFFFFFFFFFFFFFFFFFFFFFFFFFFFFFF:FFFFFFFFFFFFFFFFFFFF
FFFFFFFFFFFFFFFFFFFFFFFFFFFFFFFFFF:FFFFFFFFFFFFFFFFFFFFFFFFFFFFFF:FF
FFFFFFFFFFFFFFF
@A00721:190:HGJW7DSXY:4:2543:11469:28870 1:N:0:TCTGCAAG+GCTGCACT
CCTTGGCTTCATGCCTCTTCGACAGGATGTTGATCCTGGGACTCCTGAACTCAATCTCCTCAACGCGC
TTCGAAGCACATAGCTTCAGACGTCCTTTGATTCTACAAAACAGAGTACCTGTTTTGAGGTTGGAATT
TTCCAACCTATACAA
+
FFFFFFFFFFFFFFFFFFFFFFFFFFFFFFFFFFFFFFFF:FFFFFFFFFFFFFFFFFFFFFFFFFFF
FFFFFFFFFFFFFFFFFFFFFFFFFFFFFFFFFFFFFFFFFFFFFFFFFFFFF:FFF:FFFFFFFFFF
FFFFFFF::FFF:FF
@A00721:190:HGJW7DSXY:4:1629:12310:16673 1:N:0:TCTGCAAG+GCTGCACT
CTCTGTTTTGTAGAATCAAAGGACGTCTGAAGCTATGTGCTTCGAAGCGCGTTGAGGAGATTGAGTTC
AGGAGTCCCAGGATCAACATCCTGTCGAAGAGGCATGAAGCCAAGGATATAGATATGTGGCATGTTGA
ATATGCCACTAGTCA
+
FFFFFFFFFFFFFFFFFFFFFFFFFFFFFFFFFFFFFFFFFFFFFFFFFFF:FFFFFFFF:FFFFFFF
FFFFFFFFFFFFFFFFFFFFFFFFFFFFFFFFFFFFFFFFFFFFFFFFFFFFFFFFF:FFFFFFFFFF
FFFFFFFFFFFFFFF
```

```
@A00721:190:HGJW7DSXY:4:2221:12771:9142 1:N:0:TCTGCAAG+GCTGCACT
CTCTGTTTTGTAGAATCAAAGGACGTCTGAAGCTATGTGCTTCGAAGCGCGTTGAGGAGATTGAGTTC
AGGAGTCCCAGGATCAACATCCTGTCGAAGAGGCATGAAGCCAAGGATATAGATATGTGGCATGTTGA
ATATGCCACTAGTCA
+
FFFFFFFFFFFFFFFFFFF:FFFFFFFFFFFFFFFFFFFFFFFFFFFFFF:FFFFFFFFFFFFFFFFF
FFFFFFFFFFFFFFFFFFFFFFFFFFFFFFFFFFFFFFFFFFFFFFFFFFFFFFFFFFFFFFFFFFFF
FFFFF:FFFFFFFFF
@A00721:190:HGJW7DSXY:4:1238:1443:20181 1:N:0:TCTGCAAG+GCTGCACT
TGTAGAATCAAAGGACGTCTGAAGCTATGTGCTTCGAAGCGCGTTGAGGAGATTGAGTTCAGGAGTCC
CAGGATCAACATCCTGTCGAAGAGGCATGAAGCCAAGGATATAGATATGTGGCATGTTGAATATGCCA
CTAGTCAACCCACGT
+
FFFFFFFFFFF:FFFFFFFFFFFFFFFFFFFFFFFFFFFFFFF:FFFFFFFFFFFFFFFFFFFFFFFF
FFFFFFFFFFFFFFFFFFFFFF,FFFFFFFFFFFFFFFFFFFFFFFFFFFFFFFFFFFFFFFFFFFFF
FFFFFFFFFFFFFFF
@A00721:190:HGJW7DSXY:4:1472:9055:4429 1:N:0:TCTGCAAG+GCTGCACT
TGTAGAATCAAAGGACGTCTGAAGCTATGTGCTTCGAAGCGCGTTGAGGAGATTGAGTTCAGGAGTCC
CAGGATCAACATCCTGTCGAAGAGGCATGAAGCCAAGGATATAGATATGTGGCATGTTGAATATGCCA
CTAGTCAACCCACGT
+
FFFFFFFFFFFFFFFFFFFFFFFFFFFFFFFFFFFFFFFFFFFFFF:FFFFFFFFFFFFFFFFFFFFF
FFFFFFFFFFFFFFFFFFFFFFFFFFFFFFFFFFFFFF:FFFFFFFFFFFFFF:FFFFFF:FFFFFFF
FFFFFFFFFFFFFFF
@A00721:190:HGJW7DSXY:4:1578:25654:33708 1:N:0:TCTGCAAG+GCTGCACT
TGTAGAATCAAAGGACGTCTGAAGCTATGTGCTTCGAAGCGCGTTGAGGAGATTGAGTTCAGGAGTCC
CAGGATCAACATCCTGTCGAAGAGGCATGAAGCCAAGGATATAGATATGTGGCATGTTGAATATGCCA
CTAGTCAACCCACGT
+
FFFFFFFFFFFFFFFFFFFFFFFFFFFFFFFFFFFFFFFFFFFFFFFFFFFFFFFFFFFFFFFFFFFF
FFFFFFFFFFFFFFFFFFFFFF,FFFFFFFFFFFFFFFF:FFFFFFFFFFFFFFFFFFFFFFFFFFFF
FFFFFFFFFFFFFFF
@A00721:190:HGJW7DSXY:4:1678:5195:33943 1:N:0:TCTGCAAG+GCTGCACT
TGTAGAATCAAAGGACGTCTGAAGCTATGTGCTTCGAAGCGCGTTGAGGAGATTGAGTTCAGGAGTCC
CAGGATCAACATCCTGTCGAAGAGGCATGAAGCCAAGGATATAGATATGTGGCATGTTGAATATGCCA
CTAGTCAACCCACGT
+
FFFF:FFFFFFFFFFFFFFFFFFFFFFFFFFFFFFFFFFFFFFF:FFFFFFFFFFFF:FFFFFFFFFF
FFFFFFFFFFFFFFFFFFFFFFFFFFFFFFFFFFFFFFFFFFFFFFFFFFF:FFFFFFFFFFFFFFFF
FFFFFFFFFFFFFFF
@A00721:190:HGJW7DSXY:4:2107:29008:23954 1:N:0:TCTGCAAG+GCTGCACT
TGTAGAATCAAAGGACGTCTGAAGCTATGTGCTTCGAAGCGCGTTGAGGAGATTGAGTTCAGGAGTCC
CAGGATCAACATCCTGTCGAAGAGGCATGAAGCCAAGGATATAGATATGTGGCATGTTGAATATGCCA
CTAGTCAACCCACGT
+
FFFFFFFFFFFFFFFFFFFFFFFFFFFFFFFFFFFFFFFFFFFFFFFFFFFFFFFFFFFFFFFFFFFF
FFFFFFFFFFFFFFFFFFFFFFFFFFFFFFFFFFFFFFFFFFFFFFFFFFFFFFFFFFFFFFFF:FFF
FFFFFFFFFFFFFFF
@A00721:190:HGJW7DSXY:4:2206:2230:22482 1:N:0:TCTGCAAG+GCTGCACT
TGTAGAATCAAAGGACGTCTGAAGCTATGTGCTTCGAAGCGCGTTGAGGAGATTGAGTTCAGGAGTCC
CAGGATCAACATCCTGTCGAAGAGGCATGAAGCCAAGGATATAGATATGTGGCATGTTGAATATGCCA
CTAGTCAACCCACGT
+
FFFFFFFFFFFFFFFFFFFFFFFFFFFFFFFFFFFFFFFFFFFFFFFFFFFFFFFFFFFFFFFFFFFF
```

```
FFFFFFFFFFF:FFFFFFFFFFFFFFFFFFFFFFFFFFFFFFFFFFFFFFFF:FFFFFFFFFFFFFFFF
FFFFFFFFFFFFFFF
@A00721:190:HGJW7DSXY:4:2260:12066:8797 1:N:0:TCTGCAAG+GCTGCACT
TGTAGAATCAAAGGACGTCTGAAGCTATGTGCTTCGAAGCGCGTTGAGGAGATTGAGTTCAGGAGTCC
CAGGATCAACATCCTGTCGAAGAGGCATGAAGCCAAGGATATAGATATGTGGCATGTTGAATATGCCA
CTAGTCAACCCACGT
+
FFFFFFFFFFFFFFFFFFFFFFFFFFFFFFFFFFFFFFFFFFFFFFFFFFFFFFFFFFFFFFFFFFFFF
FFFFFFFFFFFFFFFFFFFFFFFFFFFFFFFFFFFFFFFFFFFFFFFFFFFFFFFFFFF:FFFFFFFFF
FFFFFFFFFFFFFFF
@A00721:190:HGJW7DSXY:4:2427:18078:1141 1:N:0:TCTGCAAG+GCTGCACT
TGTAGAATCAAAGGACGTCTGAAGCTATGTGCTTCGAAGCGCGTTGAGGAGATTGAGTTCAGGAGTCC
CAGGATCAACATCCTGTCGAAGAGGCATGAAGCCAAGGATATAGATATGTGGCATGTTGAATATGCCA
CTAGTCAACCCACGT
+
FFFFFFFFFFFFFFFFFFFFF:FFFFFFFFFFFFFFFFFFFFFFFFFFFFFFFFFFFFFFFFFFFFFFF
FFFFFFFFFFFFFFFFFFFFFFFFFFFFFFFFFFFFFFFFFFFFFFFFFFFFFFFFFFFFF:FFFFFFF
FFFFFFFFFFFFFFF
@A00721:190:HGJW7DSXY:4:2431:31638:11350 1:N:0:TCTGCAAG+GCTGCACT
TGTAGAATCAAAGGACGTCTGAAGCTATGTGCTTCGAAGCGCGTTGAGGAGATTGAGTTCAGGAGTCC
CAGGATCAACATCCTGTCGAAGAGGCATGAAGCCAAGGATATAGATATGTGGCATGTTGAATATGCCA
CTAGTCAACCCACGT
+
FFFFFFFFFFFFFFFFFFFFFFFFFFFFFFFFFFFFFFFFFFFFFFF:FFFFFFFFFFFFFFFFFFFFF
FFFFFFFFFFFFFFFFFFFFFFFFFFFFFFFFFFFFFFFFFFFFFFFFFFFFFFFFFFFFFFFFFFFFF
FFFFFFFFFFFFFFF
@A00721:190:HGJW7DSXY:4:1213:6867:36902 1:N:0:TCTGCAAG+GCTGCACT
CGGACGTCTGAAGCTATGTGCTTCGAAGCGCGTTGAGGAGATTGAGTTCAGGAGTCCCAGGATCAACA
TCCTGTCGAAGAGGCATGAAGCCAAGGATATAGATATGTGGCATGTTGAATATGCCACTAGTCAACCC
ACGTTGAACCGTGTG
+
FFFFFFFFFFFFFFF::FFFFFFFFF:FFFFFFFFFFFFFFF:FFFF:FFFFFFFFFFFFFFFFFFFFF
FFFFFFFFFFFFFFFFFFFFFFF:FFFF:FFFFFFFFFFFFFF:FFFFFFFFFFFFFFFFFFFFFFFFF
FFFFFFFFFFFFFFF
@A00721:190:HGJW7DSXY:4:1308:22932:24893 1:N:0:TCTGCAAG+GCTGCACT
CGGACGTCTGAAGCTATGTGCTTCGAAGCGCGTTGAGGAGATTGAGTTCAGGAGTCCCAGGATCAACA
TCCTGTCGAAGAGGCATGAAGCCAAGGATATAGATATGTGGCATGTTGAATATGCCACTAGTCAACCC
ACGTTGAACCGTGTG
+
FFFFFF,:F:FFFF,FF:F,FFFF:FF,FFFFFF,FFFF,FF:FF,:F:FF,F,F:F:F,:FF,FFFF
FF:FFF,FFF:F,F:FFFFF,FFFF,:FFF:FFFFF::FFF:FFF:::FFFFFF:FFFFFFFFFFFF:F
FFFFF:FF,F:FFFF
@A00721:190:HGJW7DSXY:4:1669:11487:24079 1:N:0:TCTGCAAG+GCTGCACT
CGGACGTCTGAAGCTATGTGCTTCGAAGCGCGTTGAGGAGATTGAGTTCAGGAGTCCCAGGATCAACA
TCCTGTCGAAGAGGCATGAAGCCAAGGATATAGATATGTGGCATGTTGAATATGCCACTAGTCAACCC
ACGTTGAACCGTGTG
+
FFFFFFFFFF:,FFFFFFFFFFFFFFFFFFFFFFFFFFFFFFFFFFFFFFFFFFFFFFFFFFFFFFFFF
FFFFFFFFFFFFFFFFFFFFFFFFFFFFFFFFFFFFFFFFF:FFFFFFFFFFFFFFFFFFFFFFFFFFF
FFFFFFFFFFFFFFF
@A00721:190:HGJW7DSXY:4:2228:25780:34240 1:N:0:TCTGCAAG+GCTGCACT
CGGACGTCTGAAGCTATGTGCTTCGAAGCGCGTTGAGGAGATTGAGTTCAGGAGTCCCAGGATCAACA
TCCTGTCGAAGAGGCATGAAGCCAAGGATATAGATATGTGGCATGTTGAATATGCCACTAGTCAACCC
ACGTTGAACCGTGTG
```

+
FFFFFFFFFFFFFFFFFFFFFFFFFFFFFFFFFFFFFFFFFFFFFFFFFFFFFFFFFFFFFFFFFFFFFF
FFFFFFFFFFFFFFFFFFFFFFFFFFFFFFFFFFFFFFFFFFFFFFFFFFFFFFFFFFFFFFFFFFFFFF
FFFFFFF:FFFFFFF
@A00721:190:HGJW7DSXY:4:2237:22110:19867 1:N:0:TCTGCAAG+GCTGCACT
CGGACGTCTGAAGCTATGTGCTTCGAAGCGCGTTGAGGAGATTGAGTTCAGGAGTCCCAGGATCAACA
TCCTGTCGAAGAGGCATGAAGCCAAGGATATAGATATGTGGCATGTTGAATATGCCACTAGTCAACCC
ACGTTGAACCGT
+
FF,FFFFFFFFFFFFF:FFFFFF:FFF,FFFFFFFFFFFFFFFFFFFFFF:FFFFFFFF,,FFFFFFF
,:F:FFF:FFFFF,FFF:FF,:FFF,:FFFFF:FFF,:F,FFF:,FFFFFFFFF,FF:FF:FFFFFFF
F:F:F:FFF,F:
@A00721:190:HGJW7DSXY:4:2261:4634:36354 1:N:0:TCTGCAAG+GCTGCACT
CGGACGTCTGAAGCTATGTGCTTCGAAGCGCGTTGAGGAGATTGAGTTCAGGAGTCCCAGGATCAACA
TCCTGTCGAAGAGGCATGAAGCCAAGGATATAGATATGTGGCATGTTGAATATGCCACTAGTCAACCC
ACGTTGAACCGTGTG
+
FFFFFFFFFFFFFF,FFFFFFFFFFFFFFFFFFFFFFFFFFFFFFFFFFFFFFFFFFFFFFFFFFFFFF
FFFFFFFFFFFFFFFFFFFFFFFFFFFFFFFFFFFFFFFFFFFFFFFFFFFFFFFFFFFFFFFFFFFFFF
FFFF:FFFFFFFFFF
@A00721:190:HGJW7DSXY:4:2337:27037:25739 1:N:0:TCTGCAAG+GCTGCACT
CGGACGTCTGAAGCTATGTGCTTCGAAGCGCGTTGAGGAGATTGAGTTCAGGAGTCCCAGGATCAACA
TCCTGTCGAAGAGGCATGAAGCCAAGGATATAGATATGTGGCATGTTGAATATGCCACTAGTCAACCC
ACGTTGAACCGTGTG
+
FF:FFFFFFFFFFFFFFFFFFFFF:FF:F:FFFFFF:FFFF:FFFFFFFFF:FFFFFFFFFFFFFFF
FF:FFFFFFFFF,FFFFFFFFF,FF:FFFFFFFFFFF,FFFFFFFF::F:FFF:FFFFFFFFFFFFFF
FFFFFFFFFF:::FF
@A00721:190:HGJW7DSXY:4:2525:28492:12759 1:N:0:TCTGCAAG+GCTGCACT
CGGACGTCTGAAGCTATGTGCTTCGAAGCGCGTTGAGGAGATTGAGTTCAGGAGTCCCAGGATCAACA
TCCTGTCGAAGAGGCATGAAGCCAAGGATATAGATATGTGGCATGTTGAATATGCCACTAGTCAACCC
ACGTTGAACCGTGTG
+
FFFFFFFFFFFFFF:FFFFFFFFFFFFFFFFFFFFFFFFFFFFFFFFFFFFFFFFFFFFFFFFFFFFFF
FFFFFFFFFFFFFFFFFFFFFFFFFFFFFFFFFFFFFFFFFFFFFFFFFFFFFFFFFFFFFFFFFFFFFF
FFF:FFFFFFFFFFF
@A00721:190:HGJW7DSXY:4:1311:7292:27743 1:N:0:TCTGCAAG+GCTGCACT
CTGAAGCTATGTGCTTCGAAGCGCGTTGAGGAGATTGAGTTCAGGAGTCCCAGGATCAACATCCTGTC
GAAGAGGCATGAAGCCAAGGATATAGATATGTGGCATGTTGAATATGCCACTAGTCAACCCACGTTGA
ACCGTGTGCTTTCGT
+
FFFFFFFFFFFFFFFFFFFFFFFFFFFFFFFFFFFFFFFFFFFFFFFFFFFFFFFFFFFFFFFFFFFFFF
FFFFFFFFFFFFFFFFFFFFFFFFFFFFFFFFFFFFFFFFFFFFFFFFFFFFFFFFFFFFFFFFFFFFFF
FFFFFFFFFFFFFFF
@A00721:190:HGJW7DSXY:4:1431:6017:3082 1:N:0:TCTGCAAG+GCTGCACT
CTGAAGCTATGTGCTTCGAAGCGCGTTGAGGAGATTGAGTTCAGGAGTCCCAGGATCAACATCCTGTC
GAAGAGGCATGAAGCCAAGGATATAGATATGTGGCATGTTGAATATGCCACTAGTCAACCCACGTTGA
ACCGTGTGCTTTCGT
+
FFFF:FF:FFFFFFFFFFFFFFFFFFFFFFFFFFFFFFFFFFFFFFFFFFFFFFFFFFFFFFFFFFFFFF
FFFFFFFFFFFFFFFFFFFFFFFFFFFF:FFFFFFFFFFFFFFFFFFFFFFFFFFFFFFFFFFFFFFFFF
FFFFFFFFFFFFFFF
@A00721:190:HGJW7DSXY:4:2136:1226:11663 1:N:0:TCTGCAAG+GCTGCACT
CTGAAGCTATGTGCTTCGAAGCGCGTTGAGGAGATTGAGTTCAGGAGTCCCAGGATCAACATCCTGTC

GAAGAGGCATGAAGCCAAGGATATAGATATGTGGCATGTTGAATATGCCACTAGTCAACCCACGTTGA
ACCGTGTGCTTTCGT
+
FFFFFFFFFFFFFFFFFFFFFFFFFFFFFFFFFFFFFFFFFFFFFFFFFFFFFFFFFFFFFFFFFFFFFF
FFFFFFFFFFFFFFFFFFFFFFFFFFFFFFFFFFFFFFFFFFFFFFFFFFFFFFFFFFFFFFFFFFFFFF
FFFFFFFFFFFFFFF
@A00721:190:HGJW7DSXY:4:2318:30825:15796 1:N:0:TCTGCAAG+GCTGCACT
CTGAAGCTATGTGCTTCGAAGCGCGTTGAGGAGATTGAGTTCAGGAGTCCCAGGATCAACATCCTGTC
GAAGAGGCATGAAGCCAAGGATATAGATATGTGGCATGTTGAATATGCCACTAGTCAACCCACGTTGA
ACCGTGTGCTTTCGT
+
FFFFFFFFFFFFFFFFFFFFFFFFFFFFFFFFFFFFFFFFFFFFFFFFFFFFFFFFFFFFFFFFFFFFFF
FFFFFFFFFFFFFFFFFFFFFFFFFFFFFFFFFFFFFFFFFFFFFFFFFFFFFFFFFFFFFFFFFFFFFF
FFFFFFFFFFFFFFF
@A00721:190:HGJW7DSXY:4:2410:8775:17942 1:N:0:TCTGCAAG+GCTGCACT
CTGAAGCTATGTGCTTCGAAGCGCGTTGAGGAGATTGAGTTCAGGAGTCCCAGGATCAACATCCTGTC
GAAGAGGCATGAAGCCAAGGATATAGATATGTGGCATGTTGAATATGCCACTAGTCAACCCACGTTGA
ACCGTGTGCTTTCGT
+
FFFFFFFFFFFFFFFFFFFFFFFFFFFFFFFFFFFFFFFFFFFFFFFFFFFFFFFFFFFFFFFFFFFFFF
FFFFFFFFFFFFFFFFFFFFFFFFFFFFFFFFFFFFFFFFFFFFFFFFFFFFFFFFFFFFFFFFFFFFFF
FFFFFFFFFFFFFFF
@A00721:190:HGJW7DSXY:4:2414:3739:33082 1:N:0:TCTGCAAG+GCTGCACT
CTGAAGCTATGTGCTTCGAAGCGCGTTGAGGAGATTGAGTTCAGGAGTCCCAGGATCAACATCCTGTC
GAAGAGGCATGAAGCCAAGGATATAGATATGTGGCATGTTGAATATGCCACTAGTCAACCCACGTTGA
ACCGTGTGCTTTCGT
+
FFFF:FFFFFFFFFFFFFFFFFFFFFFFFFFFFFFFFFFFFFFFFFFFFFFFFFFFFFFFFFFFFFFFF
FFFFFFFFFFFFFFFFFFFFFFFFFFFFFFFFFFFFFFFFFFFFFFFFFFFFFFFFFFFFFFFFFFFFFF
FFFFFFFFFFFFFFF
@A00721:190:HGJW7DSXY:4:2616:32353:34413 1:N:0:TCTGCAAG+GCTGCACT
CTGAAGCTATGTGCTTCGAAGCGCGTTGAGGAGATTGAGTTCAGGAGTCCCAGGATCAACATCCTGTC
GAAGAGGCATGAAGCCAAGGATATAGATATGTGGCATGTTGAATATGCCACTAGTCAACCCACGTTGA
ACCGTGTGCTTTCGT
+
FFFFFFF:FFFFFFFFFFFFFFFFFFFFFFFFFFFFFFFFFFFFFFFFFFFFFFFFFFFFFFFFFFFFFF
FFFFFFFFFFFFFFFFFFFFFFFFFFFFFFF:FFFFFFFFFFFFFFFFFFFFFFFFFFFFFFFFFFFFFF
FFFFFFFFFFFFFFF
@A00721:190:HGJW7DSXY:4:1268:28131:6370 1:N:0:TCTGCAAG+GCTGCACT
ACGCACGAAAGCACACGGTTCAACGTGGGTTGACTAGTGGCATATTCAACATGCCACATATCTATATC
CTTGGCTTCATGCCTCTTCGACAGGATGTTGATCCTGGGACTCCTGAACTCAATCTCCTCAACGCGCT
TCGAAG
+
FFFFFFFFFFFFFFFFFFFFFFFFFFFFFFFFFFFFFFFFFFFFFFFFFFFFFFFFFFFFFFFFFFFFFF
FFFFFFFFFFFFFFFF:FFFFFFFFFFFFFFFFFFFFFFFFFFFFFFF:FFFFFFFF:FFFFFFFFFFFF
FFFFFF
@A00721:190:HGJW7DSXY:4:1350:9091:1705 1:N:0:TCTGCAAG+GCTGCACT
ACGCACGAAAGCACACGGTTCAACGTGGGTTGACTAGTGGCATATTCAACATGCCACATATCTATATC
CTTGGCTTCATGCCTCTTCGACAGGATGTTGATCCTGGGACTCCTGAACTCAATCTCCTCAACGCGCT
TCGAAG
+
FFFFFFFFFFFFFFFFFFFFFFFFFFFFFFFFFFFFFFFFFFFFFFFFFFFFFFFFFFFFFFFFFFFFFF
FFFFFFFFFFFFFFFF,FFFFFFFFFFFFFFF:FFFFFFFFFFF,FF,:FFFFFFFFFFFFFFFFFFFF,
FF:FFF

@A00721:190:HGJW7DSXY:4:1628:16676:3443 1:N:0:TCTGCAAG+GCTGCACT
ACGCACGAAAGCACACGGTTCAACGTGGGTTGACTAGTGGCATATTCAACATGCCACATATCTATATC
CTTGGCTTCATGCCTCTTCGACAGGATGTTGATCCTGGGACTCCTGAACTCAATCTCCTCAACGCGCT
TCGAAG
+
FF:FFF:FFFFFFF:FFF:FFFFFFFFFFFFFFF:FF,F:FF:F,:FFFFFFFFFFFF:FFFFF,FFF
FFFFFFFF:FF:FFFFFFFFFFFFFFFFFFFFFFFFFFF,F:FFF:F:FFFFF:F:FFFFFFF:F::,
:FFFFF
@A00721:190:HGJW7DSXY:4:2277:27046:21934 1:N:0:TCTGCAAG+GCTGCACT
ACGCACGAAAGCACACGGTTCAACGTGGGTTGACTAGTGGCATATTCAACATGCCACATATCTATATC
CTTGGCTTCATGCCTCTTCGACAGGATGTTGATCCTGGGACTCCTGAACTCAATCTCCTCAACGCGCT
TCGAAG
+
FFFFFF:F:FFFFFFFFFFFFFFF::FFFFFFFF:FF,FFFFFF:,F:FFFFFF,FFF:,FF,FFFFF
:FFFFFFFFFF::FFFFFFFFFFFFFFFF,,F:FFFF::F:FFFFFFFFFFFFFF,FFFFFF:FF:F,
FFFFFF
@A00721:190:HGJW7DSXY:4:2451:30391:6778 1:N:0:TCTGCAAG+GCTGCACT
ACGCACGAAAGCACACGGTTCAACGTGGGTTGACTAGTGGCATATTCAACATGCCACATATCTATATC
CTTGGCTTCATGCCTCTTCGACAGGATGTTGATCCTGGGACTCCTGAACTCAATCTCCTCAACGCGCT
TCGAAG
+
FFFFFFFFFFFFFFFFFFFFFFFFFFFFFFFFFFFFFFFFFFFFFFFFFFFFFFFFFFFFFFFFFFFF
FFFFFFFFFFFFFFFFFFF:FFFFFFFFFFFFFFFFFFFFFFFFFFFFFFFFFFFFFFFFFFFFFFFF
FFFFFF
@A00721:190:HGJW7DSXY:4:2520:10484:1235 1:N:0:TCTGCAAG+GCTGCACT
ACGCACGAAAGCACACGGTTCAACGTGGGTTGACTAGTGGCATATTCAACATGCCACATATCTATATC
CTTGGCTTCATGCCTCTTCGACAGGATGTTGATCCTGGGACTCCTGAACTCAATCTCCTCAACGCGCT
TCGAAG
+
FFFFFFFFFFFFFFFFFFFFFFFFF,FFFFFFFFFFFFFFFFFFFFFF,FFFF:FFFFFFFFFFFFFF
FFFFFFFFFFFF:FFFFFFFFFFFFFFFFFFF,FFFFFFFF,F:FF,:FFFFFFFFFFFFFF:FFFF
FFFFFF
@A00721:190:HGJW7DSXY:4:2520:10529:1344 1:N:0:TCTGCAAG+GCTGCACT
ACGCACGAAAGCACACGGTTCAACGTGGGTTGACTAGTGGCATATTCAACATGCCACATATCTATATC
CTTGGCTTCATGCCTCTTCGACAGGATGTTGATCCTGGGACTCCTGAACTCAATCTCCTCAACGCGCT
TCGAAG
+
FFFFFFFFFFFFFFFFFFFFFFFFFFFFFFFFFFFFFFFFFFFFFFFFFFFFFFFFFFFFFFFFFFFF
FFFFFFFFFFFFFFFFFFFFFFFFFFFFFFFFFFFFFFFFFFFFFFF:FFFFF:FFFFFFFFFFFFFF
FFFFFF
@A00721:190:HGJW7DSXY:4:1223:30626:30264 1:N:0:TCTGCAAG+GCTGCACT
ACGCACGAAAGCACACGGGTCAACGTGGGTTGACTAGTGGCATATTAAACATGCCACATATCTATATC
CTTGGCTTCATGCCTCTTCGACAGGATGTTGATCCTGGGACTCCTGACCTCAATCTACTCAACGCGCT
TCGAA
+
FF,FFF:F,:FFF,:F:F,F,,FFFFFF,,,F,:,FFFF::FFFFF,FFF,FFFF,FF,,,F,:,:,,
FF:F:FFFFFFFF,,F,FFFFFF,FF,,:,F,FF,F,FFF:,,:F,F,F:FFF,,,,FFF,FFFF,,,
F,FF:
@A00721:190:HGJW7DSXY:4:1474:5475:11005 1:N:0:TCTGCAAG+GCTGCACT
CGTTGAGGAGATTGAGTTCAGGAGTCCCAGGATCAACATCCTGTCGAAGAGGCATGAAGCCAAGGATA
TAGATATGTGGCATGTTGAATATGCCACTAGTCAACCCACGTTGAACCGTGTGCTTTCGTGCGTAGAT
CGGAGAAGACTTAGT
+
FFFFFFFFFFFFFFFFFFFFFFFFFFFFFFFFFFFFFFFFFFFFFFFFFFFFFFFFFFFFFFFFFFFF

FFFFFFFFFFFFFFFFFFFFFFFFFFFFFFFFFFFFFFFFFFFFFFFFFFFFFFFFFFFFFFFFFFFFFF
FFFFFFFFFFFFFFF
@A00721:190:HGJW7DSXY:4:2201:28546:21308 1:N:0:TCTGCAAG+GCTGCACT
CGTTGAGGAGATTGAGTTCAGGAGTCCCAGGATCAACATCCTGTCGAAGAGGCATGAAGCCAAGGATA
TAGATATGTGGCATGTTGAATATGCCACTAGTCAACCCACGTTGAACCGTGTGCTTTCGTGCGTAGAT
CGGAGAAGACTTAGT
+
:FFF,FFFFFFFFFFF:F:FF:FFFFFFFFFFFFFFFF::FFFFF:FFFFF:FFFFFF:FFFFFFFFF
FF:FFFFF,FFFFFFFFFFFF:FFFFFFFF:FFF:FFFF:FFFFFFFF:FFFFFF:F,FFFFFF:FF
FFFFFFFFFF:FF:F
@A00721:190:HGJW7DSXY:4:2264:25292:19304 1:N:0:TCTGCAAG+GCTGCACT
CGTTGAGGAGATTGAGTTCAGGAGTCCCAGGATCAACATCCTGTCGAAGAGGCATGAAGCCAAGGATA
TAGATATGTGGCATGTTGAATATGCCACTAGTCAACCCACGTTGAACCGTGTGCTTTCGTGCGTAGAT
CGGAGAAGACTTAGT
+
FFFFFFFFFFFFFFFFFFFFFFFFFFFFFFFFFFFFFFFFFFFFFFFFFFFFFFFFFFFFFFFFFFFFFF
FFFFFFFFFFFFFFFFFFFFFFFFFFFFFFFFFFFFFFFFFFFFFFFFFFFFFFFFFFFFFFFFFFFFFF
FFFFFFFFFFFFFFF
@A00721:190:HGJW7DSXY:4:2505:29324:26193 1:N:0:TCTGCAAG+GCTGCACT
CGTTGAGGAGATTGAGTTCAGGAGTCCCAGGATCAACATCCTGTCGAAGAGGCATGAAGCCAAGGATA
TAGATATGTGGCATGTTGAATATGCCACTAGTCAACCCACGTTGAACCGTGTGCTTTCGTGCGTAGAT
CGGAGAAGACTTAGT
+
FFFFFFFFFFFFFFFFFFFFFFFFFFF:FFFFFFFFFFFFFFFFFFFFFFFFFFFFFFFFFFFFFFFF:FFFFFF
FFFFFFFFFFFFFFFFFFFFFFFFFFFFFFFFFFFFFFFFFFFFFFFFFFFFFFFFFFFFFFFFFFFFFF
FFFFFF:FFFFFFFF
@A00721:190:HGJW7DSXY:4:2561:27263:28479 1:N:0:TCTGCAAG+GCTGCACT
CGTTGAGGAGATTGAGTTCAGGAGTCCCAGGATCAACATCCTGTCGAAGAGGCATGAAGCCAAGGATA
TAGATATGTGGCATGTTGAATATGCCACTAGTCAACCCACGTTGAACCGTGTGCTTTCGTGCGTAGAT
CGGAGAAGACTTAGT
+
FFFFFFFFFFFFFFFFFFFFFFFFFFFFFFFFFFFFFFFFFFFFFFFFFFFFFFFFFFFFFFFFFFFFFF
FFFFFFFFFFFFFFFFFFFFFFFFFFFFFFFFFFFFFFFFFFFFFFFFFFFFFFFFFFFFFFFFFFFFFF
FFFFFFFFFFFFFFF
@A00721:190:HGJW7DSXY:4:2623:32235:21684 1:N:0:TCTGCAAG+GCTGCACT
CGTTGAGGAGATTGAGTTCAGGAGTCCCAGGATCAACATCCTGTCGAAGAGGCATGAAGCCAAGGATA
TAGATATGTGGCATGTTGAATATGCCACTAGTCAACCCACGTTGAACCGTGTGCTTTCGTGCGTAGAT
CGGAGAAGACTTAGT
+
FFFFFFFFFFFFFFFFFFFFFFFFFFFFFFFFFFFFFFFFFFFFFFFFFFFFFFFFFFFFFFFFFFFFFF
FFFFFFFFFFFFFFFFFFFFFFFFFFFFFFFFFFFFFFFFFFFFFFFFFFFFFFFFFFFFFFFFFF:FFF
FFFFFFFFFFFFFFF
@A00721:190:HGJW7DSXY:4:2666:8983:35806 1:N:0:TCTGCAAG+GCTGCACT
CGTTGAGGAGATTGAGTTCAGGAGTCCCAGGATCAACATCCTGTCGAAGAGGCATGAAGCCAAGGATA
TAGATATGTGGCATGTTGAATATGCCACTAGTCAACCCACGTTGAACCGTGTGCTTTCGTGCGTAGAT
CGGAGAAGACTTAGT
+
FFFFFFFFFFF:FFFFFFFFFFFFFFFFFFFFFFFFFFFFFFFFFFFFFFFFFFFFFFFFFFFFFFFFFFFF
FFFFFFFFFFFFFFFFFFFFFFFFFFFFFFFFFFFFFFFFFFFFFFFFFFFFFFFFFFFFFFFFFFFFFF
FFFFFFFFFFFFFFF
@A00721:190:HGJW7DSXY:4:1251:8612:3850 1:N:0:TCTGCAAG+GCTGCACT
GAGGAGATTGAGTTCAGGAGTCCCAGGATCAACATCCTGTCGAAGAGGCATGAAGCCAAGGATATAGA
TATGTGGCATGTTGAATATGCCACTAGTCAACCCACGTTGAACCGTGTGCTTTCGTGCGTAGATCGGA
GAAGACTTAGTTCTG

+
FFFFFFFFFFFFFFFFFFFFFFFFFFFFFFFFFFFFFFFFFFFFFFFFFFFFFFFFFFFFFFFFFFFF
FFFFFFFFF:FFFFFFFFFFFFFFFFFFFFFFFFFFFFFFFFFFFFFFFFFFFFFFFFFFFFFFFFFF
FFFFFF:FFFFFFFF
@A00721:190:HGJW7DSXY:4:1537:10755:35806 1:N:0:TCTGCAAG+GCTGCACT
GAGGAGATTGAGTTCAGGAGTCCCAGGATCAACATCCTGTCGAAGAGGCATGAAGCCAAGGATATAGA
TATGTGGCATGTTGAATATGCCACTAGTCAACCCACGTTGAACCGTGTGCTTTCGTGCGTAGATCGGA
GAAGACTTAGTTCTG
+
FFFFFFFFFFFFFFFFFFFFFFFFFFFFFFFFFFFFFFFFFFFFFFFFFFFFFFFFFFFFFFFFFFFF
FFFFFFFFFFFFFFFFFFFFFFFFFFFFFFF:FFFFFFFFFFFFFFFFFFFFFFFFFFFFFFFFFFFF
FFFFFFFFFFFFFFF
@A00721:190:HGJW7DSXY:4:1545:20347:27712 1:N:0:TCTGCAAG+GCTGCACT
GAGGAGATTGAGTTCAGGAGTCCCAGGATCAACATCCTGTCGAAGAGGCATGAAGCCAAGGATATAGA
TATGTGGCATGTTGAATATGCCACTAGTCAACCCACGTTGAACCGTGTGCTTTCGTGCGTAGATCGGA
GAAGACTTAGTTCTG
+
FFFFFFFFFFFFFFFFFFFFFFFFFFFFFFFFFFFFFFFFFFFFFFFFFFFFFFFFFFFFFFFFFFFF
FFFFFFFFFFFFFFFFFFFFFFFFFFFFFFFFFFFFFFFFFFFFFFFFFFFFFFFFFFFFFFFFFFFF
FFFFFFFFFFFFFFF
@A00721:190:HGJW7DSXY:4:2321:31295:26882 1:N:0:TCTGCAAG+GCTGCACT
GAGGAGATTGAGTTCAGGAGTCCCAGGATCAACATCCTGTCGAAGAGGCATGAAGCCAAGGATATAGA
TATGTGGCATGTTGAATATGCCACTAGTCAACCCACGTTGAACCGTGTGCTTTCGTGCGTAGATCGGA
GAAGACTTAGTTCTG
+
FFF:FFF,F:FFFFFFF,FF:F:FFFFFFFFFFFFFFFFF:FFFFFF::FFFFFFFFFF:FFFFFFFF
FFFFFFFF::FFFF,::FFF,FF:FFFFFFFFFF,:F:FFFFFFF,F,FFFFFFFF:FFFFFFFF,FF
FFF,,F::FFFFFFF
@A00721:190:HGJW7DSXY:4:2430:4137:2049 1:N:0:TCTGCAAG+GCTGCACT
GAGGAGATTGAGTTCAGGAGTCCCAGGATCAACATCCTGTCGAAGAGGCATGAAGCCAAGGATATAGA
TATGTGGCATGTTGAATATGCCACTAGTCAACCCACGTTGAACCGTGTGCTTTCGTGCGTAGATCGGA
GAAGACTTAGTTCTG
+
FFFFFFFFFFFFFFFFFFFFFFFFFFFFFFFFFFFFFFFFFFFFFFFFFFFFFFFFFFFFFFFFFFFF
FFFFFFFFFFFFFFFFFFFF:FFFFFFFFFFFFFFF:F:FFFFFFFFFFFFFFFFFFFFFFFFFFFFF
FFFFF:FFFFFFFFF
@A00721:190:HGJW7DSXY:4:2535:27272:34507 1:N:0:TCTGCAAG+GCTGCACT
GAGGAGATTGAGTTCAGGAGTCCCAGGATCAACATCCTGTCGAAGAGGCATGAAGCCAAGGATATAGA
TATGTGGCATGTTGAATATGCCACTAGTCAACCCACGTTGAACCGTGTGCTTTCGTGCGTAGATCGGA
GAAGACTTAGTTCTG
+
FFFFFFFFFFFFFFFFFFFFFFFFFFFFFFFFFFFFFFFFFFFFFFFFFFFFFFF:FFFFFFFFFF
FFFFFFFFF:FFFFFFFFFFFFFFFFFFFFFFFFFFFFFFF:FFFFFFFFFFFFFFFFF:FFFFFFFF
FFFFFFFFFFFFFFF
@A00721:190:HGJW7DSXY:4:2546:6262:12493 1:N:0:TCTGCAAG+GCTGCACT
GAGGAGATTGAGTTCAGGAGTCCCAGGATCAACATCCTGTCGAAGAGGCATGAAGCCAAGGATATAGA
TATGTGGCATGTTGAATATGCCACTAGTCAACCCACGTTGAACCGTGTGCTTTCGTGCGTAGATCGGA
GAAGACTTAGTTCTG
+
FFFFF:F:F:FF:FFFF:FFFF:FF:FFFFF::FFFF,,FF:FFFFF,FFF:F:FF,FFFFF:FFFFF
FFFFFFFFF,FFFFFFFFFFFFFFFFFFFFF:FFFFFFFFFF:F:FFFF:FFFF:FF:FFFFFF:F:F
:FFF:,:FFFFFFF:
@A00721:190:HGJW7DSXY:4:2546:7591:12947 1:N:0:TCTGCAAG+GCTGCACT
GAGGAGATTGAGTTCAGGAGTCCCAGGATCAACATCCTGTCGAAGAGGCATGAAGCCAAGGATATAGA

TATGTGGCATGTTGAATATGCCACTAGTCAACCCACGTTGAACCGTGTGCTTTCGTGCGTAGATCGGA
GAAGACTTAGTTCTG
+
FFFFFFF:FFFFFFFFFFFFFFFFFFFFFFFFFFFFFFFFFFFFFFFFFFFFFFFFFFFFFFF,FFFF
FFFFFFFFFFFFFFFFFFFFFFFFFFFFFFFFFFFFFFFF:FFFFFFFFFFFFFFFFFFF:FFFFFF
FFFFFF:FFFFFFFF
@A00721:190:HGJW7DSXY:4:1228:2428:1470 1:N:0:TCTGCAAG+GCTGCACT
GGAGATTGAGTTCAGGAGTCCCAGGATCAACATCCTGTCGAAGAGGCATGAAGCCAAGGATATAGATA
TGTGGCATGTTGAATATGCCACTAGTCAACCCACGTTGAACCGTGTGCTTTCGTGCGTAGATCGGAGA
AGACTTAGTTCTGAT
+
FFFFFFFFFFFFFFFFFFFFFFFFFFFFFFFFFFFFFFFFFFFFFFFFFFFFFFFFFFFFFFFFFFFF
FFFFFFFFFFFFF:FFFFFFFFFFFFFFFFFFFFFFFFFFFFFFFFFFFFFFFFFFFFFFFFFFFFFF
FFFFFFFFFFFFFFF
@A00721:190:HGJW7DSXY:4:1267:23755:11381 1:N:0:TCTGCAAG+GCTGCACT
GGAGATTGAGTTCAGGAGTCCCAGGATCAACATCCTGTCGAAGAGGCATGAAGCCAAGGATATAGATA
TGTGGCATGTTGAATATGCCACTAGTCAACCCACGTTGAACCGTGTGCTTTCGTGCGTAGATCGGAGA
AGACTTAGTTCTGAT
+
FFFFFFFFFFFFFFFFFFFFFFFFFFFFFFFFFFFFFFFFFFFFFFFFFFFFFFFFFFFFFFFFFFFF
FFFFFFFFFFFFFFFFFFFFFFFFFFFFFFFFFFFFFFFFFFFFFFFFFFFFFFFFFFFFFFFFFFFF
FFFFFFFFFFFFFFF
@A00721:190:HGJW7DSXY:4:1460:26946:6010 1:N:0:TCTGCAAG+GCTGCACT
GGAGATTGAGTTCAGGAGTCCCAGGATCAACATCCTGTCGAAGAGGCATGAAGCCAAGGATATAGATA
TGTGGCATGTTGAATATGCCACTAGTCAACCCACGTTGAACCGTGTGCTTTCGTGCGTAGATCGGAGA
AGACTTAGTTCTGAT
+
FFFFFFFFFFFFFFFFFFFFFFFFFFFFFFFFFFFFFFFFFFFFFFFFFFFFFFFFFFFFF,FFFFFF
FFFFFFFFFFFFFFFFFF:FFFFFFFFFFFFFFFFFFFFFFFFFFFFFFFFFFFFFFFFFFFFFFFFF
,F::FFFFFFFFF:F
@A00721:190:HGJW7DSXY:4:2210:29957:24126 1:N:0:TCTGCAAG+GCTGCACT
GGAGATTGAGTTCAGGAGTCCCAGGATCAACATCCTGTCGAAGAGGCATGAAGCCAAGGATATAGATA
TGTGGCATGTTGAATATGCCACTAGTCAACCCACGTTGAACCGTGTGCTTTCGTGCGTAGATCGGAGA
AGACTTAGTTCTGAT
+
FFFFFFFFFFFFFFFFFFFFFFFFFFFFFFFFFFFFFFFFFFFFFFFFFFFFFFFFFFFFFFFFFF:F
FFFFFFFFFFFFFFFFFFFFFFFFFFFFFFFFFFFFFFFFFFFFFFFFFFFFFFFFFFFFFFFFFFFF
FFFFFFFFFFFFFFF
@A00721:190:HGJW7DSXY:4:2457:21007:36057 1:N:0:TCTGCAAG+GCTGCACT
GGAGATTGAGTTCAGGAGTCCCAGGATCAACATCCTGTCGAAGAGGCATGAAGCCAAGGATATAGATA
TGTGGCATGTTGAATATGCCACTAGTCAACCCACGTTGAACCGTGTGCTTTCGTGCGTAGATCGGAGA
AGACTTAGTTCTGAT
+
FFFFFFFFFFFFFFFFFFFFFFFFFFFFFFFFFFFFFFFFFFFFFFFFFFFFFFFFFFFFFFFFFFFF
FFFFFFFFFFFFFFFFFFFFFFFFFFFFFFFFFFFFFFFFFFFFFFFFFFFFFFFFFFFFFFFFFFFF
FFFFFFFFFFFFFFF
@A00721:190:HGJW7DSXY:4:2558:21404:21057 1:N:0:TCTGCAAG+GCTGCACT
GGAGATTGAGTTCAGGAGTCCCAGGATCAACATCCTGTCGAAGAGGCATGAAGCCAAGGATATAGATA
TGTGGCATGTTGAATATGCCACTAGTCAACCCACGTTGAACCGTGTGCTTTCGTGCGTAGATCGGAGA
AGACTTAGTTCTGAT
+
FFFFFFFFFFFFFFFFFFFFFFFFFFFFFFFFFFFFFFFFFFFFFFFFFFFFFFFFFFFFFFFFFFFF
FFFFFFFFFFFFFFFFFFFFFFFFFFFFFFFFFFFFFFFFFFFF:FFFFFFFFFFFFFFFFFFFFFFF
FFFFFFFFFFFFFFF

```
@A00721:190:HGJW7DSXY:4:1109:2962:30170 1:N:0:TCTGCAAG+GCTGCACT
ATTGAGTTCAGGAGTCCCAGGATCAACATCCTGTCGAAGAGGCATGAAGCCAAGGATATAGATAAGTG
GCATGTAGAATATGCCAATAGTCAACCCACGGTGAACCGTGTGCTTTCGTGCGTAGATCGGAGAAGAC
TTAGTTCTGATTCCA
+
FFFFF,FFFF,,F,F:FFF:,FFFFFFF,:::,,F,FFFF,,FFF:FF,,FFFFF,:F:FFF:F,,F:
,FF,FF,:FF:FF:,:F,FF,FF:FF:FFF:,,FFFFF,:,,FFF,,,,,:,F,FFFFFFFFFF,,F,
F:FF,F:FFFFF:,F
@A00721:190:HGJW7DSXY:4:1174:9851:4742 1:N:0:TCTGCAAG+GCTGCACT
ATTGAGTTCAGGAGTCCCAGGATCAACATCCTGTCGAAGAGGCATGAAGCCAAGGATATAGATATGTG
GCATGTTGAATATGCCACTAGTCAACCCACGTTGAACCGTGTGCTTTCGTGCGTAGATCGGAGAAGAC
TTAGTTCTGATTCCA
+
FFFFFFFFFFFFFFFFFFFFFFFFFFFFFFFFFFFFFFFFFFFFFFFFFFFFFFFF:FFFFFFFFFFF
FF:FFFFFFFFFFFFFFFFFFFFFFFFFFFFFFFFFFFFFFFFFFFFFFFFFFFFFF:FFFFFFFFFF
FFFFFFFFFFFFFFF
@A00721:190:HGJW7DSXY:4:1438:17644:12759 1:N:0:TCTGCAAG+GCTGCACT
ATTGAGTTCAGGAGTCCCAGGATCAACATCCTGTCGAAGAGGCATGAAGCCAAGGATATAGATATGTG
GCATGTTGAATATGCCACTAGTCAACCCACGTTGAACCGTGTGCTTTCGTGCGTAGATCGGAGAAGAC
TTAGTTCTGATTCCA
+
FFFFFFFFFFFFFFFFFFFFFFFFFFFFFFFFFFFFFFFFFFFFFFFFFFFFFFFFFFFFFFFFFFFF
FFFFFFFFFFFFFFFFFFFFFFFFFFFFFFFFFFFFFFFFFFFFFFFFFFFFF:FF:FFFFFFFFFFF
FFFFFFFFFFFFFFF
@A00721:190:HGJW7DSXY:4:1478:9950:11741 1:N:0:TCTGCAAG+GCTGCACT
ATTGAGTTCAGGAGTCCCAGGATCAACATCCTGTCGAAGAGGCATGAAGCCAAGGATATAGATATGTG
GCATGTTGAATATGCCACTAGTCAACCCACGTTGAACCGTGTGCTTTCGTGCGTAGATCGGAGAAGAC
TTAGTTCTGATTCCA
+
F:FFFFFFFFFFFFFFFFFFFFFFFFFFFFF:FFFFFFFFFFFFFFFFFFF,FFFF,FFFFFFFFFFF
FFF:FFFFFFFFFFFFFFFFFFFFFFFFFFFFFFFFFFFFFF:FFFFFFFFFFFFFFFFFFFFF,FFF
FFFFFFFFFFFFFF:
@A00721:190:HGJW7DSXY:4:2104:5900:29121 1:N:0:TCTGCAAG+GCTGCACT
ATTGAGTTCAGGAGTCCCAGGATCAACATCCTGTCGAAGAGGCATGAAGCCAAGGATATAGATATGTG
GCATGTTGAATATGCCACTAGTCAACCCACGTTGAACCGTGTGCTTTCGTGCGTAGATCGGAGAAGAC
TTAGTTCTGATTCCA
+
FFFFFFFFFFFFFFFFFFFFFFFFFFFFFFFFFFFFFFFFFFFFFFFFFFFFFFFF,FFFFFFFFFFF
FFFFF:FFFFFFFFFFFFFFFFFFFFFFFFFFFFFFFFFFFFFF:FFFFFFFFFFFFFFFFFF:FFFF
FFFFFFFFFFFFFFF
@A00721:190:HGJW7DSXY:4:2109:3558:34929 1:N:0:TCTGCAAG+GCTGCACT
ATTGAGTTCAGGAGTCCCAGGATCAACATCCTGTCGAAGAGGCATGAAGCCAAGGATATAGATATGTG
GCATGTTGAATATGCCACTAGTCAACCCACGTTGAACCGTGTGCTTTCGTGCGTAGATCGGAGAAGAC
TTAGTTCTGATTCCA
+
FFFFFFFFFFFFFFFFFFFFFFFFFFFFFFFFFFFFFFFFFFFFFFFFFFFFFFFFFFFFFFFFFFFF
FFFFFFFFFFFFFFFFFFFFFFFFFFFFFFFFFFFFFFFFFFFFFFFFFFFFFFFFFFFFFFFFFFFF
FFFFFFFFFFFFFFF
@A00721:190:HGJW7DSXY:4:2531:31494:28197 1:N:0:TCTGCAAG+GCTGCACT
ATTGAGTTCAGGAGTCCCAGGATCAACATCCTGTCGAAGAGGCATGAAGCCAAGGATATAGATATGTG
GCATGTTGAATATGCCACTAGTCAACCCACGTTGAACCGTGTGCTTTCGTGCGTAGATCGGAGAAGAC
TTAGTTCTGATTCCA
+
FFFFFFFFFFFFFFFFFFFFFFFFFFFFFFFFFFFFFFFFFFFFFFFFFFFFFFFFFFFFFFFFFFFF
```

FFFFFFFFFFFFFFFFFFFFFFFFFFFFFFFFFFFFFFFFFFFFFFFFFFFFFFFFFFFFFFFFFFFFFF
FFFFFFFFFFFFFFF
@A00721:190:HGJW7DSXY:4:1274:19180:2832 1:N:0:TCTGCAAG+GCTGCACT
CCCAGGATCAACATCCTGTCGAAGAGGCATGAAGCCAAGGATATAGATATGTGGCATGTTGAATATGC
CACTAGTCAACCCACGTTGAACCGTGTGCTTTCGTGCGTAGATCGGAGAAGACTTAGTTCTGATTCCA
TTACGGTCTTGCCCG
+
FFFFFFFFFFFFFFFFFFFFFFFFFFFFFFFFFFFFFFFFFFFFFFFFFFFFFFFFFFFFFFFFFFFFFF
FFFFFFFFFFFFFFFFFFFFFFFFFFFFFFFFFFFFFFFFFFFFFFFFFFFFFFFF:FFFFFFFFFFFFF
FFFFFFFFFFFFFFF
@A00721:190:HGJW7DSXY:4:1601:27543:28839 1:N:0:TCTGCAAG+GCTGCACT
CCCAGGATCAACATCCTGTCGAAGAGGCATGAAGCCAAGGATATAGATATGTGGCATGTTGAATATGC
CACTAGTCAACCCACGTTGAACCGTGTGCTTTCGTGCGTAGATCGGAGAAGACTTAGTTCTGATTCCA
TTACGGTCTTGCCCG
+
FFFFFFFFFFFFFFFF:FFFFFFFFFFFFFFFFFFFFFFFFFFFFFFFFFFFFFFFFFFFFFFFFFFFFF
FFFFFFFFFFFFFFFFFFFFFFFFFFFFFFFFFFFFFFFFFFFFFFFFFFFFFFFFFFFFFFFFFFFFFF
FFFFFFFFFFFFFFF
@A00721:190:HGJW7DSXY:4:1157:12400:30765 1:N:0:TCTGCAAG+GCTGCACT
CAAGGATATAGATATGTAGCATGTTGAATATGCCACTAGTCAACCCACGTTGAACCGTGTGCTTTCGT
GCGTAGATCGGAGAAGACTTAGTTCTGATTCCATTACGGTCTTGCCCGGAATGTGTTACAGTGAGATA
TCCAGAAGATCGATA
+
FFFFFFFFFFFFFFFFFFFFFFFFFFFFFFFFFFFFFFFFFFFFFFFFFFFFFFFFFFFFFFFFFFFFFF
FFFFFFFFFFFFFFFFFFFFFFFFFFFFFFFFFFFFFFFFFFFFFFFFFFFFFFFFFF:FFFFFFFFFFF
FFFFFFF:FFFFFFF
@A00721:190:HGJW7DSXY:4:1329:2971:23359 1:N:0:TCTGCAAG+GCTGCACT
CAAGGATATAGATATGTAGCATGTTGAATATGCCACTAGTCAACCCACGTTGAACCGTGTGCTTTCGT
GCGTAGATCGGAGAAGACTTAGTTCTGATTCCATTACGGTCTTGCCCGGAATGTGTTACAGTGAGATA
TCCAGAAGATCGATA
+
FFFFFFFFFFFFFFFFFFFFFF,FFFFFFFFFFFFFFFFFFFFFFFFFFFFFFFFFFFFFFFFFFFFFFF
FFFFFFFFFFFFFFFFFFF::FFFFFFFFFFFFFFFFFFFFFFFFFFFFFFFFFFFFFFF:FFF:FFFF
FFFFFFFFFFFFFFF
@A00721:190:HGJW7DSXY:4:1359:23258:21574 1:N:0:TCTGCAAG+GCTGCACT
CAAGGATATAGATATGTAGCATGTTGAATATGCCACTAGTCAACCCACGTTGAACCGTGTGCTTTCGT
GCGTAGATCGGAGAAGACTTAGTTCTGATTCCATTACGGTCTTGCCCGGAATGTGTTACAGTGAGATA
TCCAGAAGATCGATA
+
FFFFFFFFFFFFFFFFFFFFFFFFFFFFFFFFFFFFFFFFFFFFFFFFFFFFFFFFFFFFFFFFFFFFFF
FFFFFFFFFFFFFFFFFFFFFFFFFFFFFFFFFFFFFFFFFFFFFFFFFFFFFFFFFF:FFFFFFFFFFF
FFFFFFFFFFFFFFF
@A00721:190:HGJW7DSXY:4:1469:24325:26115 1:N:0:TCTGCAAG+GCTGCACT
CAAGGATATAGATATGTAGCATGTTGAATATGCCACTAGTCAACCCACGTTGAACCGTGTGCTTTCGT
GCGTAGATCGGAGAAGACTTAGTTCTGATTCCATTACGGTCTTGCCCGGAATGTGTTACAGTGAGATA
TCCAGAAGATCGATA
+
FFFFFFFFFFFFFFFFFFFFFFFFFFFFFFFFFFFFFFFFFFFFFFFFFFFFFFFFFFFFFFFFFFFFFF
FFF:FFFFFFFFFFFFFFFFFFFFFFFFFFFFFFFFFFFFFFFFFFFFFFFFFFFF:FFFFFFFFFFFFF
FFFFFFFFFFFFFFF
@A00721:190:HGJW7DSXY:4:2131:27380:9862 1:N:0:TCTGCAAG+GCTGCACT
CAAGGATATAGATATGTAGCATGTTGAATATGCCACTAGTCAACCCACGTTGAACCGTGTGCTTTCGT
GCGTAGATCGGAGAAGACTTAGTTCTGATTCCATTACGGTCTTGCCCGGAATGTGTTACAGTGAGATA
TCCAGAAGATCGATA

```
+
FFFFFFFFFFFFFFFFFFFFFFFFFFFFFFFFFFFFFFFFFFFFFFFFFFFFFFFFFFFFFFFFFFFFFF
FFFFFFFFFFFFFFFFFFFFFFFFFFFFFFFFFFFFFFFFFFFFFFFFFFFFFFFFFFFFFFFFFFFFFF
FFFFFF:FFFFFFF:
@A00721:190:HGJW7DSXY:4:2176:4924:32628 1:N:0:TCTGCAAG+GCTGCACT
CAAGGATATAGATATGTAGCATGTTGAATATGCCACTAGTCAACCCACGTTGAACCGTGTGCTTTCGT
GCGTAGATCGGAGAAGACTTAGTTCTGATTCCATTACGGTCTTGCCCGGAATGTGTTACAGTGAGATA
TCCAGAAGATCGATA
+
FFFFFFFFFFFFFFFFFFFFFFFFFFFFFFFFFFFFFFFFFFFFFFFFFFFFFF,FFFFFFFFFFFFFFF
FFFFFFFFFFFFFFFFFFFFFFFFFFFFFFFFFFFFFFFFFFFFFFFFFFFF:FFFFFFFFFFFFFFFFF
FFFFFF,FFFFFFFF
@A00721:190:HGJW7DSXY:4:2423:13621:9173 1:N:0:TCTGCAAG+GCTGCACT
CAAGGATATAGATATGTAGCATGTTGAATATGCCACTAGTCAACCCACGTTGAACCGTGTGCTTTCGT
GCGTAGATCGGAGAAGACTTAGTTCTGATTCCATTACGGTCTTGCCCGGAATGTGTTACAGTGAGATA
TCCAGAAGATCGATA
+
FFFFF:,FFFFF:FFFFFFFFFF::F:FF:FF:,FFFFF:FFFFFF,FFF:,FFFF,F,F,FF:FFFFFF
FFF,::FFFFFF,FF,::F:F,:FFF:F:,:FFFFFF,:,FF:FFFFF,F::F,,,,::F:FFFFFFFF
F:FFFFF,F,FFFFF
@A00721:190:HGJW7DSXY:4:2550:23375:12571 1:N:0:TCTGCAAG+GCTGCACT
CAAGGATATAGATATGTAGCATGTTGAATATGCCACTAGTCAACCCACGTTGAACCGTGTGCTTTCGT
GCGTAGATCGGAGAAGACTTAGTTCTGATTCCATTACGGTCTTGCCCGGAATGTGTTACAGTGAGATA
TCCAGAAGATCGATA
+
FFFFFFFFFFFFFFFFFFFFFFFFFFFFFFFFFFFFFFFFFFFFFFFFFFFFFFFFFFFFFFFFFFFFFF
FFFFFFFFFFFFFFFFFFFFFFFFFFFFFFFFFFFFFFFFFFFFFFFFFFFFFFFFFFFFFFFFFFFFFF
FFFFFFFFFFFFFFF
@A00721:190:HGJW7DSXY:4:2669:2537:34788 1:N:0:TCTGCAAG+GCTGCACT
CAAGGATATAGATATGTAGCATGTTGAATATGCCACTAGTCAACCCACGTTGAACCGTGTGCTTTCGT
GCGTAGATCGGAGAAGACTTAGTTCTGATTCCATTACGGTCTTGCCCGGAATGTGTTACAGTGAGATA
TCCAGAAGATCGATA
+
FFFFFFFFFFFFFFFFFFFFFFFFF,FFFFFFFFFFFFFFFFFFFFFFF:F::FFFFFFFFFFFFFFFFF
FFF,FFFFF:FFFFFFFFFFFFF:FFFFFFFF:FFFFFFFFFFFFFFFFFFFFFFFF:F:FFFFFFFFFF
FFFFFFFFFF:FFFF
@A00721:190:HGJW7DSXY:4:1120:25310:26600 1:N:0:TCTGCAAG+GCTGCACT
GGATATAGATATGTGGCATGTTGAATATGCCACTAGTCAACCCACGTTGAACCGTGTGCTTTCGTGCG
TAGATCGGAGAAGACTTAGTTCTGATTCCATTACGGTCTTGCCCGGAATGTGTTACAGTGAGATATCC
AGAAGATCGATAGCT
+
:FFFFFFFFFFFFFFFFFFFFFFFFFFFFFFFFFFFFFFFFFFFFFFFFFFFFFFFFFFFFFFFFFFFFF
FFFFFFFFFFFFFFFF:FFFFFFFFFFFFFFFFFFFFFFFFFFFFFFFFFFFFFFFFFFFFFFFFFFFF
FFFFFFFFFFFFFFF
@A00721:190:HGJW7DSXY:4:1203:7572:31046 1:N:0:TCTGCAAG+GCTGCACT
GGATATAGATATGTGGCATGTTGAATATGCCACTAGTCAACCCACGTTGAACCGTGTGCTTTCGTGCG
TAGATCGGAGAAGACTTAGTTCTGATTCCATTACGGTCTTGCCCGGAATGTGTTACAGTGAGATATCC
AGAAGATCGATAGCT
+
FFFFFFFFFFFFFFFFFFFFFFF:FFFFFFFF:FFF:FFFFFFFFFFF,FFFFFFF,FFFF:FFFFFFF
FFFFFFFF:FFF:F,FFF:FFFFFFFFF,F:FF,FF:FF,,F,F:FFFFFFFFFFFFFFFFF:FFF:F
FFFFFFFF,FFFFFF
@A00721:190:HGJW7DSXY:4:1478:31873:29700 1:N:0:TCTGCAAG+GCTGCACT
GGATATAGATATGTGGCATGTTGAATATGCCACTAGTCAACCCACGTTGAACCGTGTGCTTTCGTGCG
```

```
TAGATCGGAGAAGACTTAGTTCTGATTCCATTACGGTCTTGCCCGGAATGTGTTACAGTGAGATATCC
AGAAGATCGATAGCT
+
FFFFFFFFFFFFFFFFFFFFFFFFFFFFFFFFFFFFFFFFFFFFFFFFFFFFFFFFFFFFFFFFFFFF
FFFFFFFFFFFFFFFFFFFFFFFFFFFFFFFFFFFFFFFFFFFFFFFFFFFFFFFFFFFFFFFFFFFF
FFFFFFFFFFFFFF:
@A00721:190:HGJW7DSXY:4:2270:1470:6668 1:N:0:TCTGCAAG+GCTGCACT
GGATATAGATATGTGGCATGTTGAATATGCCACTAGTCAACCCACGTTGAACCGTGTGCTTTCGTGCG
TAGATCGGAGAAGACTTAGTTCTGATTCCATTACGGTCTTGCCCGGAATGTGTTACAGTGAGATATCC
AGAAGATCGATAGCT
+
FFFFFFFFFFFFFFFFFFFFFFFFFFFFFFFFFFFFFFFFFFFFFFFFFFFFFFFFFFFFFFFFFFFF
FFFFFFFFFFFFFFFFFFFFFFFFFFFFFFFFFFFFFFFFFFFFFFFFFFFFFFFFFFFFFFFFFFFF
FFF::FFFFFFFFF:
@A00721:190:HGJW7DSXY:4:2346:19859:31062 1:N:0:TCTGCAAG+GCTGCACT
GGATATAGATATGTGGCATGTTGAATATGCCACTAGTCAACCCACGTTGAACCGTGTGCTTTCGTGCG
TAGATCGGAGAAGACTTAGTTCTGATTCCATTACGGTCTTGCCCGGAATGTGTTACAGTGAGATATCC
AGAAGATCGATAGCT
+
FFFFFFFFFFFFFFFFFFFFFFFFFFFFFFFFFFFFFFFFFFFFFFFFFFFFFFFFFFFFFFFFFFFF
FFFFFFFFFFFFFFFFFFFFFFFFFFFFFFFFFFFFFFFFFFFFFFFFFFFFFFFFFFFFFFFFFFFF
FFFFFFFFFFFFFFF
@A00721:190:HGJW7DSXY:4:2414:29460:28338 1:N:0:TCTGCAAG+GCTGCACT
GGATATAGATATGTGGCATGTTGAATATGCCACTAGTCAACCCACGTTGAACCGTGTGCTTTCGTGCG
TAGATCGGAGAAGACTTAGTTCTGATTCCATTACGGTCTTGCCCGGAATGTGTTACAGTGAGATATCC
AGAAGATCGATAGCT
+
FFFFFFFFFFFFFFFFFFFFFFFFFFFFFFFFFFFFFFFFFFFFFFFFFFFFFFFFFFFFFFFFFFFF
FFFFFFFFFFFFFFFFFFFFFFFFFFFFFFFFFFFFFFFFFFFFFFFFFFFFFFF:FFFFFFFFFFFF
FFFFFFFFFFFFFFF
@A00721:190:HGJW7DSXY:4:1329:2112:8218 1:N:0:TCTGCAAG+GCTGCACT
CACTAGTCAACCCACGTTGAACCGTGTGCTTTCGTGCGTAGATCGGAGAAGACTTAGTTCTGATTCCA
TTACGGTCTTGCCCGGAATGTGTTACAGTGAGATATCCAGAAGATCGATAGCTGAATGTGATAGAAGA
CATAGTTTTGAGATA
+
FFFFFFFFFFFFFFFFFFFFFFFFFFFFFFFFFFFFFFFFFFFFFFFFFFFFFFFFFFFFFFFFFFFF
FFFFFFFFFFFFFFFFFFFFFFFFFFFFFFFFFFFFFFFFFFFFFFFFFFFFF:FFFFFFFFFFFFFF
F:FFFF:F::F,FFF
@A00721:190:HGJW7DSXY:4:1631:5855:22999 1:N:0:TCTGCAAG+GCTGCACT
CACTAGTCAACCCACGTTGAACCGTGTGCTTTCGTGCGTAGATCGGAGAAGACTTAGTTCTGATTCCA
TTACGGTCTTGCCCGGAATGTGTTACAGTGAGATATCCAGAAGATCGATAGCTGAATGTGATAGAAGA
CATAGTTTTGAGATA
+
FFFFFFFFFFFFFFFFFFFFFFFFFFFFFFFFFFFFFFFFFFFFFFFFFFFFFFFFFFFFFFFFFFFF
FFFFFFFFFFFFFFFFFFFFFFFFFFFFFFFFFFFFFFFFFFFFFFFFFFFFFFFFFFFFFFFFFFFF
FFFFFFFFFFFFFFF
@A00721:190:HGJW7DSXY:4:1663:5701:34413 1:N:0:TCTGCAAG+GCTGCACT
CACTAGTCAACCCACGTTGAACCGTGTGCTTTCGTGCGTAGATCGGAGAAGACTTAGTTCTGATTCCA
TTACGGTCTTGCCCGGAATGTGTTACAGTGAGATATCCAGAAGATCGATAGCTGAATGTGATAGAAGA
CATAGTTTTGAGATA
+
FFFFFFFFFFFFFFFF:FFFFFFFFFFFFFFFFFFFFFF:FFFFFFFFFFFFFFFFFFFFFFF:FFFFFF
FFFFFFFFFFFFFFFFFFFFFFF:FF:FFFFFFFFFFFFFFFFFFFFFFFFFFFFFFFFFFFFFFFFF
FFFFFFFFFFFFFFF
```

```
@A00721:190:HGJW7DSXY:4:2156:10050:13542 1:N:0:TCTGCAAG+GCTGCACT
CACTAGTCAACCCACGTTGAACCGTGTGCTTTCGTGCGTAGATCGGAGAAGACTTAGTTCTGATTCCA
TTACGGTCTTGCCCGGAATGTGTTACAGTGAGATATCCAGAAGATCGATAGCTGAATGTGATAGAAGA
CATAGTTTTGAGATA
+
FFFFFFFFFFFFFFFFFFFFFFFFFFFFFFFFFFFFFFFFFFFFFFFFFFFFF,F:FFFFFFFFFFFF
FFFFFFFFFFFFFFFFFFFFFFFFFFFFFFFFFFFFFFFFFFFFFFFFFFFFFFFFFFFFFFFFFFFF
FFFFFFFFFFFFFFF
@A00721:190:HGJW7DSXY:4:2350:20591:4648 1:N:0:TCTGCAAG+GCTGCACT
CACTAGTCAACCCACGTTGAACCGTGTGCTTTCGTGCGTAGATCGGAGAAGACTTAGTTCTGATTCCA
TTACGGTCTTGCCCGGAATGTGTTACAGTGAGATATCCAGAAGATCGATAGCTGAATGTGATAGAAGA
CATAGTTTTGAGATA
+
FFFFFFFFF:FFFFFF::FFFFFFFF:FFFFFFFFFFFFFFFFFFF:FFFFFFF::FFFFF:FF,FFF
FFFFFFFFFFFFFFFFFFFF:F:F,F,F,FFFFFFFFFFFF:FFFFFFFFF:FFFFFFFFFFFF:FFF
FFFFF,FFFFFFFFF
@A00721:190:HGJW7DSXY:4:2355:2935:33442 1:N:0:TCTGCAAG+GCTGCACT
CACTAGTCAACCCACGTTGAACCGTGTGCTTTCGTGCGTAGATCGGAGAAGACTTAGTTCTGATTCCA
TTACGGTCTTGCCCGGAATGTGTTACAGTGAGATATCCAGAAGATCGATAGCTGAATGTGATAGAAGA
CATAGTTTTGAGATA
+
FFFFFFFFFFFFFFFFFFFFFFFFFFFFFFFFFFFFFFFFFFFFFFFFFFFFF:FFFFFFFFF:FFFF
:FFFFFFFFFFFFFFFFFFFFFFFFFFFFFFFFFFFFFFFFFFFFFFFFFFFFFFFFFFFFFFFF:FF
FF:FFFFFFFFFFFF
@A00721:190:HGJW7DSXY:4:2419:30346:16689 1:N:0:TCTGCAAG+GCTGCACT
CACTAGTCAACCCACGTTGAACCGTGTGCTTTCGTGCGTAGATCGGAGAAGACTTAGTTCTGATTCCA
TTACGGTCTTGCCCGGAATGTGTTACAGTGAGATATCCAGAAGATCGATAGCTGAATGTGATAGAAGA
CATAGTTTTGAGATA
+
FFFFFFFFFFFFFFFFFFFFFFFFFFFFFFFFFFFFFFFFFFFFFFFFFFFFFFFFFFFFFFFFFFFF
FFFFFFFFFFFFFFFFFFFFFFF:FFFFFFFFFFFFFFFFFFFFFFFFFFFFFFFFFFFFFFFFFFFF
FFF,:FFFFFFFFFF
@A00721:190:HGJW7DSXY:4:2464:1994:15092 1:N:0:TCTGCAAG+GCTGCACT
CACTAGTCAACCCACGTTGAACCGTGTGCTTTCGTGCGTAGATCGGAGAAGACTTAGTTCTGATTCCA
TTACGGTCTTGCCCGGAATGTGTTACAGTGAGATATCCAGAAGATCGATAGCTGAATGTGATAGAAGA
CATAGTTTTGAGATA
+
FFFFFFFFFFFFFFFFFFFFFFFFFFFFFFFFFFFFFFFFFFFFFFFFFFFFFFFFFFFFFFFFFFFF
FFFFFFFFFFFFFFFFFFFFFFFFFFFFFFFFF:FFFFFFFFFFFFFFFFFFFFFFFFFFFFFFFFFF
FFFFFFFFFFFFFFF
@A00721:190:HGJW7DSXY:4:2513:11803:9001 1:N:0:TCTGCAAG+GCTGCACT
CACTAGTCAACCCACGTTGAACCGTGTGCTTTCGTGCGTAGATCGGAGAAGACTTAGTTCTGATTCCA
TTACGGTCTTGCCCGGAATGTGTTACAGTGAGATATCCAGAAGATCGATAGCTGAATGTGATAGAAGA
CATAGTTTTGAGATA
+
FFFFFFFFFFFFFFFFFFFFFFFFFFFFFFFFFFFFFFFFFFFFFFFFFFFF:FFFFFFFFFFFFFFF
FFFFFFFFFFFFFFFFFFFFFFFFFFFFFFFFFFFFFFFFFFFFFFFFF:FFFFF:FF:FFFFFFFFF
FF,FFFFFFFFFFFF
@A00721:190:HGJW7DSXY:4:1538:30110:20322 1:N:0:TCTGCAAG+GCTGCACT
ACGTTGAACCGTGTGCTTTCGTGCGTAGATCGGAGAAGACTTAGTTCTGATTCCATTACGGTCTTGCC
CGGAATGTGTTACAGTGAGATATCCAGAAGATCGATAGCTGAATGTGATAGAAGACATAGTTTTGAGA
TATCGAGCAACGTCA
+
FFFFFFFFFFFFFFFFFFFFFFFFFF:FFFFFFFFFFFFFFFFFFFFFFFFFFFFFFFFFFFFFFFFF
```

FFFFFFFFF:FFF:FFFFFFFFFFFFFFFFFFFFFFFFFFFFFFFFFFFFFFFFF:,FFFFFFFFFFF
FFFFFF:FF,FFFFF
@A00721:190:HGJW7DSXY:4:1653:12662:3004 1:N:0:TCTGCAAG+GCTGCACT
ACGTTGAACCGTGTGCTTTCGTGCGTAGATCGGAGAAGACTTAGTTCTGATTCCATTACGGTCTTGCC
CGGAATGTGTTACAGTTAGATATCCAGAAGATCGAAAGCTGACAGTGATAGAAGACATAGTTTTGAGA
TATCGAGCAACGACA
+
F:FFFFFFFF:F::,,FFFF:,F:F,F:F,:FFFFFF,FFFFFF,,,FF:FFF,FF,F:,,F,F,::F
F::FF,F,:,FFFF,,,F,FFF,FFF,FFFFFF,F,F:FFFF,,,F:FFF,FF:FFF,F,,,FF,F:F
F,FF:FFFFFF:,FF
@A00721:190:HGJW7DSXY:4:2350:11532:8030 1:N:0:TCTGCAAG+GCTGCACT
ACGTTGAACCGTGTGCTTTCGTGCGTAGATCGGAGAAGACTTAGTTCTGATTCCATTACGGTCTTGCC
CGGAATGTGTTACAGTGAGATATCCAGAAGATCGATAGCTGAATGTGATAGAAGACATAGTTTTGAGA
TATCGAGCAACGTCA
+
FFFFFFFFFFFFFFFFFFFFFFFFFF:FFFFFFFFFFFFFFFFFFFFFFFFFFFFFFFFFFFFFFFFF
FFFFFFFFFFFFFFFFFFFFFFFFFFFFFFFFFFFFFFFFFFFFFFFFFFFFFFFF:FFFFFFFFFFF
FFFFFFFFF:FFFFF
@A00721:190:HGJW7DSXY:4:2607:28845:18756 1:N:0:TCTGCAAG+GCTGCACT
ACGTTGAACCGTGTGCTTTCGTGCGTAGATCGGAGAAGACTTAGTTCCGATTCCATTACGGTCTTGCC
CGGACTGTGTTACAGTGAGATATCCAGAAGATCGATAGCTGAATGTGATAGAAGACATAGTTTTGAGA
TATCTAGCAACGTCA
+
:FFFF:FF,FF,:,F:,FFF:FF:F:F:F,FFFFF:,:FF,,FFFFF,FFFFF:FFFFFF,FF,F,FF
F,FF,FFFFF,F,FFF:FFFF::FFFFFF,FF,:F:FFF,FF:FFF,,FF,,FFF:FFFFFF,F,FFF
FFFF,F,FFFF,:FF
@A00721:190:HGJW7DSXY:4:1116:18475:31328 1:N:0:TCTGCAAG+GCTGCACT
GCTTTCGTGCGTAGATCGGAGAAGACTTAGTTCTGATTCCATTACGGTCTTGCCCGGAATGTGTTACA
GTGAGATATCCAGAAGATCGATAGCTGAATGTGATAGAAGACATAGTTTTGAGATATCGAGCAACGTC
ATTGCTGATGC
+
FFFFFFFFFFFFFFFFFFFFFFFFFFFFFFF:F,,FFFFFFFFFFF:F:FFF:FFFFFFFF,F,FFFF
FFFFFFFFFFFFFFFF:FFFFF:F::FF:FF:FFFFFF:F:::FFF:F:F,FFFF,FFF:FFFFFF,F
FFF:FFFFFFF
@A00721:190:HGJW7DSXY:4:1136:13602:5071 1:N:0:TCTGCAAG+GCTGCACT
GCTTTCGTGCGTAGATCGGAGAAGACTTAGTTCTGATTCCATTACGGTCTTGCCCGGAATGTGTTACA
GTGAGATATCCAGAAGATCGATAGCTGAATGTGATAGAAGACATAGTTTTGAGATATCGAGCAACGTC
ATTGCTGATGC
+
FFFFFFFFFFFFFFFFFFFFFFFFFFFFFFFFFFFFFFFFF:FFFFFFFF:FFFFFFFFFF:FFFFFF
FFFFFFFFFFFFFFFFFFFFFFFFF:,FFFFFFFFFFFFFFFF:FFFFFFFF:FFFFFFFFFFFFFFF
FFFFFFFFFFF
@A00721:190:HGJW7DSXY:4:1167:31168:30044 1:N:0:TCTGCAAG+GCTGCACT
GCTTTCGTGCGTAGATCGGAGAAGACTTAGTTCTGATTCCATTACGGTCTTGCCCGGAATGTGTTACA
GTGAGATATCCAGAAGATCGATAGCTGAATGTGATAGAAGACATAGTTTTGAGATATCGAGCAACGTC
ATTGCTGATGC
+
FFFFFFFFFFFFFFFFFF:FFFFFFFFFFFFFFFFFFFFFFFFFFFFFFFFFFFFFFFFFFFFFFFFF
FFFFFFFFFFFFFFFFFFFFFFF:FFFFFFFFFFFFFFFFFFFFFFFFFFFFFFFFF:FFFFFFFFFFFF
FFFFFFFFFFF
@A00721:190:HGJW7DSXY:4:2161:18041:7592 1:N:0:TCTGCAAG+GCTGCACT
GCTTTCGTGCGTAGATCGGAGAAGACTTAGTTCTGATTCCATTACGGTCTTGCCCGGAATGTGTTACA
GTGAGATATCCAGAAGATCGATAGCTGAATGTGATAGAAGACATAGTTTTGAGATATCGAGCAACGTC
ATTGCTGATGC

+
FFFFFFFFFFFFFFFFFFFFFFFFFFFFFFFFFFFFFFFFFFFFFFFFFFFFFFFFFFFFFFFFFFFFFF
FFFFFFFFFFFFFFFFFFFFFFFFFFFFFFFFFFFFFFFFFFFFFFFFFFFFFFFFFFFFFFFFFFFFFF
FFFFFFFFFFF
@A00721:190:HGJW7DSXY:4:2241:23511:9612 1:N:0:TCTGCAAG+GCTGCACT
GCTTTCGTGCGTAGATCGGAGAAGACTTAGTTCTGATTCCATTACGGTCTTGCCCGGAATGTGTTACA
GTGAGATATCCAGAAGATCGATAGCTGAATGTGATAGAAGACATAGTTTTGAGATATCGAGCAACGTC
ATTGCTGATGC
+
FFFFFFFFFFFFFFFFFFFFFFFFFFFFFFFFFFFFFFFFFFFFFFFFFFFFFFFFFFFFFFFFFFFFFF
FFFFFFFFFFFFFF,FFFFFFFFFFFFFF:FFFFFFFFFFFFFFFFFFFFFFFFFFFFFFFFFFFFFFFF
FFFFFFFFFFF
@A00721:190:HGJW7DSXY:4:2242:14389:18991 1:N:0:TCTGCAAG+GCTGCACT
GCTTTCGTGCGTAGATCGGAGAAGACTTAGTTCTGATTCCATTACGGTCTTGCCCGGAATGTGTTACA
GTGAGATATCCAGAAGATCGATAGCTGAATGTGATAGAAGACATAGTTTTGAGATATCGAGCAACGTC
ATTGCTGATGC
+
FFFFFFFFFFFFFFFFFFFFFFFFFFFFFFFFFFFFFFFFFFFFFFFFFFFFFFFFFFFFFFFFFFFFFF
FFFFFFFFFFFFFF:FFFFFFFFFFFFFFFFFFFFFFFFFFFFFFFFFFFFFFFFFFFFFFFFFFFFFFFF
FFFFFFF:FFF
@A00721:190:HGJW7DSXY:4:2367:32533:31876 1:N:0:TCTGCAAG+GCTGCACT
GCTTTCGTGCGTAGATCGGAGAAGACTTAGTTCTGATTCCATTACGGTCTTGACCGGAATGTGTTACA
GTGAGATATCAAGAAGATCGATAGCTGAATGTGATAGAAGACATAGTTTTGAGATATCGAGCAACGTC
ATTGCTGATGC
+
FFFF:F:FFFFFFFFFFFFFF::::,FF:F::FFF,F::,:FFFFF,,FF,,:,FFFFFF:F,,FFFFF
:FFFFF,F,F,F,FFFFF,,FFFF,FF::FFFFFFFFFF,F:FFF,,FFFF,FFF,,,FFF,FFFFFF
F::,:FF:FFF
@A00721:190:HGJW7DSXY:4:2558:18331:34084 1:N:0:TCTGCAAG+GCTGCACT
GCTTTCGTGCGTAGATCGGAGAAGACTTAGTTCTGATTCCATTACGGTCTTGCCCGGAATGTGTTACA
GTGAGATATCCAGAAGATCGATAGCTGAATGTGATAGAAGACATAGTTTTGAGATATCGAGCAACGTC
ATTGCTGATGC
+
FFFFFFFFFFFFFFFFFFFFFFFFFFFFFFFFFFFFFFFFFFFFFFFFFFFFFFFFFFFFFFFFFFFFFF
FFFFFFFFFFFFFFFFFFFFFFFFFFFFFFFFFFFFFFFFFFFFFFFFFFFFFFFFFFFFFFFFFFFFFF
FFFFFFFFFFF
@A00721:190:HGJW7DSXY:4:2560:4707:13620 1:N:0:TCTGCAAG+GCTGCACT
GCTTTCGTGCGTAGATCGGAGAAGACTTAGTTCTGATTCCATTACGGTCTTGCCCGGAATGTGTTACA
GTGAGATATCCAGAAGATCGATAGCTGAATGTGATAGAAGACATAGTTTTGAGATATCGAGCAACGTC
ATTGCTGATGC
+
FFFFFFFFFFFFFFFFFFFFFFFFF:FFFFFFFFFFFFFFFFFFFFFFFFFFFFFFFFFFFFFFFFFFFF
FFFFFFFFFFFFFFFFFFFFFFFFFFFFFFFFFFFFFFFFFFFFFFFFFFFFFFFFFFFFFFFFFFFFFF
FFFFFFFFFFF
@A00721:190:HGJW7DSXY:4:2576:10719:25880 1:N:0:TCTGCAAG+GCTGCACT
GCTTTCGTGCGTAGATCGGAGAAGACTTAGTTCTGATTCCATTACGGTCTTGCCCGGAATGTGTTACA
GTGAGATATCCAGAAGATCGATAGCTGAATGTGATAGAAGACATAGTTTTGAGATATCGAGCAACGTC
ATTGCTGATGC
+
FFFFFFFFFFFFFFFFFFFFFFF:FFFF:FFFFFFFFFFFFFFFFFFFFFFFFFFFFFFFFFFFFFFFFF
FFFFFFFFFFFFFFF:F:FFFFFFFFFFF:FFFFFFFFFFFFF,FFFFFFFFFFFFFFFFFFFFFFFFF
FFFFFFFFFFF
@A00721:190:HGJW7DSXY:4:2670:18014:14591 1:N:0:TCTGCAAG+GCTGCACT
GCTTTCGTGCGTAGATCGGAGAAGACTTAGTTCTGATTCCATTACGGTCTTGCCCGGAATGTGTTACA

GTGAGATATCCAGAAGATCGATAGCTGAATGTGATAGAAGACATAGTTTTGAGATATCGAGCAACGTC
ATTGCTGATGC
+
FFFFFFFFFFFFFFFFFFFFFFFFFFFFFFFFFFFFFFFFFFFFFFFFFFFFFFFFFFFFFFFFFF
F:FFFFFFFFFFFFFFFFFFFFFFFFFFFFFFFFFFFFFFFFFFFFFFFFFFFFFFFFFFFFFFFF
FFFFFFFFFFF
@A00721:190:HGJW7DSXY:4:1117:31946:14575 1:N:0:TCTGCAAG+GCTGCACT
CGTGCGTAGATCGGAGAAGACTTAGTTCTGATTCCATTACGGTCTTGCCCGGAATGTGTTACAGTGAG
ATATCCAGAAGATCGATAGCTGAATGTGATAGAAGACATAGTTTTGAGATATCGAGCAACGTCATTGC
TGATGCCAAAGTTGA
+
FFFFFFFFFFFFFFFFFFFFFFFFFFFFFFFFFFFFFFFFFFFFFFFFFFFFFFFFFFFFFFFFFF
FFFFFFFFFFFFFFFFFFFFFFFFFFFFFFFFFFF:FFFFFFFFFFFFFFFFF:FFFFFFFFFFFFF
FFFF,FFF,FFFFFF
@A00721:190:HGJW7DSXY:4:1424:19018:33395 1:N:0:TCTGCAAG+GCTGCACT
CGTGCGTAGATCGGAGAAGACTTAGTTCTGATTCCATTACGGTCTTGCCCTGAAAGTGTTACAGTGAG
ATATACAGAAGATCTATATCAGAATGTGATATAAGACATAGTTTTGAGATATCGAGCAACGTCATTGC
AGATGCCAAAGATGA
+
FFFFFF:FFFFF,,F:FFF:F,:F:FFFFFFF,FFF:FF,F,::FF,F,F,FFF,:F,,,F::,:FF,
FFF:,FFFFFFFF:,,:,,:,,F:FFF,,F:,FF,F,FFF,:,,,:,FF,F,F,FFFFF,FFFF:F,F
,:FFF:FF:::,:,F
@A00721:190:HGJW7DSXY:4:1531:2148:31892 1:N:0:TCTGCAAG+GCTGCACT
CGTGCGTAGATCGGAGAAGACTTAGTTCTGATTCCATTACGGTCTTGCCCGGAATGTGTTACAGTGAG
ATATCCAGAAGATCGATAGCTGAATGTGATAGAAGACATAGTTTTGAGATATCGAGCAACGTCATTGC
TGATGCCAAAGTTGA
+
F,FFFFFFFFFFFFFFFFFF:FFFFFF:FFFFF:FFFFFFFFFFFF:FFFFFFF:FFFFFFFF:FF
FFFFFFFFF,,FFFFFFFF:FFFFFF::FFFFFFFFF:::FFFF,FFFFFF:FFFFFF,FFFFF:FF
FFFFFFFFFFF,FFF
@A00721:190:HGJW7DSXY:4:2276:32904:15765 1:N:0:TCTGCAAG+GCTGCACT
CGTGCGTAGATCGGAGAAGACTTAGTTCTGATTCCATTACGGTCTTGCCCGGAATGTGTTACAGTGAG
ATATCCAGAAGATCGATAGCTGAATGTGATAGAAGACATAGTTTTGAGATATCGAGCAACGTCATTGC
TGATGCCAAAGTTGA
+
FFFFFFFFFFFFFFFFFF:FFFFFFFFFFFFFFFFFFFFFFFFFFFFFFFFFFFFFFFFFFFFFFF
FFFFFFFFFFFFFFFFFFFFFFFFFFFFFFFFFFFFFFFFFFFFFFFFFFFF:FFFFFFFFFFFFF
FFFFFFFFFF:FFFF
@A00721:190:HGJW7DSXY:4:2642:5068:7795 1:N:0:TCTGCAAG+GCTGCACT
CGTGCGTAGATCGGAGAAGACTTAGTTCTGATTCCATTACGGTCTTGCCCGGAATGTGTTACAGTGAG
ATATCCAGAAGATCGATAGCTGAATGTGATAGAAGACATAGTTTTGAGATATCGAGCAACGTCATTGC
TGATGCCAAAGTTGA
+
FFFFFFFFFFFFFFFFFFFFFFFFFFFFFFFFFFFFFFFFFFFF:FFFFFFFFFFFFFF:FFFFFFF
FFFFFFFFFFFFFFFFFFFFFFFFFFFFFFFFFFFFF:FFFFFFFFFFFFFFFFFFFF::FFFFFFFF
FFFFFFFFFFFFFFF
@A00721:190:HGJW7DSXY:4:1228:7392:24189 1:N:0:TCTGCAAG+GCTGCACT
TGCGTAGATCGGAGAAGACTTAGTTCTGATTCCATTACGGTCTTGCCCGGAATGTGTTACAGTGAGAT
ATCCAGAAGATCGATAGCTGAATGTGATAGAAGACATAGTTTTGAGATATCGAGCAACGTCATTGCTG
ATGCCAAAGTTGAAG
+
FFFFFFFFFFFFFFFFFFFFFFFFFFFFFFFFFFFFFFFFFFFFFFFFFFFFFFFFFFFFFFFFFF
FFFFFFFFFFFFFFFFFFFFFFFFFFFFFFFFF,FFFFFFFFFFFFFFFFFFFFFFFFFFFFFFFFFF
FFFF:FF:FFFFFFF

```
@A00721:190:HGJW7DSXY:4:1350:2745:7153 1:N:0:TCTGCAAG+GCTGCACT
TGCGTAGATCGGAGAAGACTTAGTTCTGATTCCATTACGGTCTTGCCCGGAATGTGTTACAGTGAGAT
ATCCAGAAGATCGATAGCTGAATGTGATAGAAGACATAGTTTTGAGATATCGAGCAACGTCATTGCTG
ATGCCAAAGTTGAAG
+
FFFFFFFFFFFFFFFFFFFFFFFFFFFFFFFFFFFFFFFFFFFFFFFFFFFFFFFFFFFFFFFFFFFF
FFFFFFFF:FFFFFFFFFFFFFFFFFFFFFFFFFFFFFFFFFFFFFFFFFFFFFFFFFFFFFFFFFFF
FFFFFFFFFFFFFFF
@A00721:190:HGJW7DSXY:4:1350:2284:7169 1:N:0:TCTGCAAG+GCTGCACT
TGCGTAGATCGGAGAAGACTTAGTTCTGATTCCATTACGGTCTTGCCCGGAATGTGTTACAGTGAGAT
ATCCAGAAGATCGATAGCTGAATGTGATAGAAGACATAGTTTTGAGATATCGAGCAACGTCATTGCTG
ATGCCAAAGTTGAAG
+
FFFFFFFFFFF:FFFFFFFFFFFFFFFFFFFFFFFFFFFFFFFFFFFFFFFFFFFFFFFFFFFFFFFF
FFFFFFFF:FFFFFFFFFFFFFFFFFFFFFFFFFFFFFFFFFFFFFFFFFFFFFFFFFFFFFFFFFFF
FFFFFFFFFFFFFF:
@A00721:190:HGJW7DSXY:4:1362:2727:36057 1:N:0:TCTGCAAG+GCTGCACT
TGCGTAGATCGGAGAAGACTTAGTTCTGATTCCATTACGGTCTTGCCCGGAATGTGTTACAGTGAGAT
ATCCAGAAGATCGATAGCTGAATGTGATAGAAGACATAGTTTTGAGATATCGAGCAACGTCATTGCTG
ATGCCAAAGTTGAAG
+
FFFFFFFFFFFFFFFFFFFF:FFFFFFFF:FFFFFFFFFFFFFFFFFFFFFFFFFFFF:FFFFFFFFF
FFFF:FFFFFFFFFFFFFFFFF,FFFFFFFFFFFFFFFFFFFFFFFFFFFFFFFFFFFFFFFFFF:F::
FFFFFFFFFFFFFF:
@A00721:190:HGJW7DSXY:4:1366:11867:3255 1:N:0:TCTGCAAG+GCTGCACT
TGCGTAGATCGGAGAAGACTTAGTTCTGATTCCATTACGGTCTTGCCCGGAATGTGTTACAGTGAGAT
ATCCAGAAGATCGATAGCTGAATGTGATAGAAGACATAGTTTTGAGATATCGAGCAACGTCATTGCTG
ATGCCAAAGTTGAAG
+
FFFFFFFFFFFFFFFFFFFFFFFFFFFFFFFFFFFFFFFFFFFFFFFFFFFFFFFF:F:FFFFFFFFF
FFFFFFFFFFFFFFFFFFFFFF:FFFFFFFFFFFFFFFFFFFFFFFFFFFFFFFFFFFFFFFFFFFFF
FFFFFFFFFFFFFFF
@A00721:190:HGJW7DSXY:4:1368:29649:12164 1:N:0:TCTGCAAG+GCTGCACT
TGCGTAGATCGGAGAAGACTTAGTTCTGATTCCATTACGGTCTTGCCCGGAATGTGTTACAGTGAGAT
ATCCAGAAGATCGATAGCTGAATGTGATAGAAGACATAGTTTTGAGATATCGAGCAACGTCATTGCTG
ATGCCAAAGTTGAAG
+
FFFFFFFFFFFFFFFFFFFFFFFFFFFFFFFFFFFFFFFFFFFFFFFFFFF:FFFFFFFFFFFFF:FF
FFFFFFFFFFFFFFFFFFFFFFFFFFFFFFFFFFFFFFFFFFFFFFFF:FFFFFFFFFFFFFFFFFFF
FFFFFFFFFFFFFFF
@A00721:190:HGJW7DSXY:4:2220:16812:31641 1:N:0:TCTGCAAG+GCTGCACT
TGCGTAGATCGGAGAAGACTTAGTTCTGATTCCATTACGGTCTTGCCCGGAATGTGTTACAGTGAGAT
ATCCAGAAGATCGATAGCTGAATGTGATAGAAGACATAGTTTTGAGATATCGAGCAACGTCATTGCTG
ATGCCAAAGTTGAAG
+
FFFFFFFFFFFFFFFFFFFFFFFFFFFFFFFFFFFFFFFFFFFFFFFFFFFFFFFFFFFFFFFFFFFF
FFFFFFF,FFFF:FF:FFFFFFFFFFFFFFFFFFFFFFFFFFFFFFFFFFFFFFFFFFFFFFFFFFFF
FFF:F:FFFFFFFFF
@A00721:190:HGJW7DSXY:4:2220:16333:32002 1:N:0:TCTGCAAG+GCTGCACT
TGCGTAGATCGGAGAAGACTTAGTTCTGATTCCATTACGGTCTTGCCCGGAATGTGTTACAGTGAGAT
ATCCAGAAGATCGATAGCTGAATGTGATAGAAGACATAGTTTTGAGATATCGAGCAACGTCATTGCTG
ATGCCAAAGTTGAAG
+
FFFFF:FFFFFFFFFFFFFFFFFFFFFFFFFFFFFFFFFFFFFFFFFFFFFFFFFFFFFFFFFFFFFF
```

```
FFFFFFFFFFFFFFFFFFFFFFFFFFFFFFFFFFFFFFFFFFFFFFFFFFFFFFFFF:FFFFFFFFFF
FFFFFFFFFFFFFFF
@A00721:190:HGJW7DSXY:4:2302:1145:2440 1:N:0:TCTGCAAG+GCTGCACT
TGCGTAGATCGGAGAAGACTTAGTTCTGATTCCATTACGGTCTTGCCCGGAATGTGTTACAGTGAGAT
ATCCAGAAGATCGATAGCTGAATGTGATAGAAGACATAGTTTTGAGATATCGAGCAACGTCATTGCTG
ATGCCAAAGTTGAAG
+
FFFFFFFFFFFFFFFFFF:FFFFFFFFFFFFFFFFFFFFFFFFFFFFFFFFF:FFFFFFFFFFFFFFF
FFFFFFFF:FF:FFFFFFFFFFFFFFFFFFFFFFFFFFFFFFFFFFFFFFFFFFFFFFFFFFFFFFFF
FFFFFFF:FFFF:FF
@A00721:190:HGJW7DSXY:4:2345:32678:36699 1:N:0:TCTGCAAG+GCTGCACT
TGCGTAGATCGGAGAAGACTTAGTTCTGATTCCATTACGGTCTTGCCCGGAATGTGTTACAGTGAGAT
ATCCAGAAGATCGATAGCTGAATGTGATAGAAGACATAGTTTTGAGATATCGAGCAACGTCATTGCTG
ATGCCAAAGTTGAA
+
FFFFFFFFFFFFFFFFFFFFFFFFFFFFFFFFFFFFFFFFFFFFFFFFFFFFFFFFFFFFFFFFFFFF
FFFFFFF:FFFFFFFFFFFFFFFFFFFFFFFFFFFFFFFFFFFFFFFFFFFFFFFFFFFFFFFFFFFF
F:FFFFFFFFFFFFF
@A00721:190:HGJW7DSXY:4:2348:26982:24424 1:N:0:TCTGCAAG+GCTGCACT
TGCGTAGATCGGAGAAGACTTAGTTCTGATTCCATTACGGTCTTGCCCGGAATGTGTTACAGTGAGAT
ATCCAGAAGATCGATAGCTGAATGTGATAGAAGACATAGTTTTGAGATATCGAGCAACGTCATTGCTG
ATGCCAAAGTTGAAG
+
FFFFFFFFFFFFFFFFFFFFFFFFFFFFFFFFFFFFFFFFFFFFFFFFFFF,FFFFFFFFFFFFFFFF
FFFFFFFFFFFFFFFFFFFFFFFFFFFFFFFFFFFFFFFFFFFFFFFFFFFFFFFF,FFFFFFFFFFF
FFFFFFFFFFFFFFF
@A00721:190:HGJW7DSXY:4:2350:28980:21151 1:N:0:TCTGCAAG+GCTGCACT
TGCGTAGATCGGAGAAGACTTAGTTCTGATTCCATTACGGTCTTGCCCGGAATGTGTTACAGTGAGAT
ATCCAGAAGATCGATAGCTGAATGTGATAGAAGACATAGTTTTGAGATATCGAGCAACGTCATTGCTG
ATGCCAAAGTTGAAG
+
F:FF,FFF,FFFFFFFFF:FFFFFFFFFFFFF,FFFFFFFFF::FFFFF:FFF:FFFF:FFFF:FFFF
FF:F:FF,FFFFFFFF:::FFFFFF,FFFFFFFFFFFF,,:F:FFFF:FFFFFFFFF:FFFFF:F:FF
:FFFFFFF,,FFFF:
@A00721:190:HGJW7DSXY:4:2445:2266:29904 1:N:0:TCTGCAAG+GCTGCACT
TGCGTAGATCGGAGAAGACATAGTTCTGATTCCATTACGGTCTTGCCCGGAATGTGTTACAGTGAGAT
ATCCAGAAGATCGATAGCTGAATGTGATAGAAGACATAGTTTTGAGATATCGAGCAACGTCATTGCTG
ATGCCAAAGTTGAAG
+
FF:FFF:F:FFFFFFF:F,,FFFFFFF:F::FFFFF:FFFFFF,F:FF,FFFF::FFF:FFFFFFFF:
F,:FFF,F:F:F:FFF::::FF,FFFFFFFFFFFFFFFF:F,FFFFFFFF:FFFFF,FF,FF:FFFFF
F,FFFFFFFFFFFF:
@A00721:190:HGJW7DSXY:4:2476:23990:29606 1:N:0:TCTGCAAG+GCTGCACT
TGCGTAGATCGGAGAAGACTTAGTTCTGATTCCATTACGGTCTTGCCCGGAATGTGTTACAGTGAGAT
ATCCAGAAGATCGATAGCTGAATGTGATAGAAGACATAGTTTTGAGATATCGAGCAACGTCATTGCTG
ATGCCAAAGTTGAAG
+
FFFFFFFFFFFFFFFFFFFFFFFFFFFFFFFFFFFFFFFFFFF:FFFFFFF:FFFFFFFFFFFFFFFF
FFFFFFFF:FFFFFFFF:FFFFFFFFFFFFFFFFFFFFFFFFFFFFFFFFFFFFFFF::FFFFFFFFF
FF:F:FFFFF:FFFF
@A00721:190:HGJW7DSXY:4:2510:15980:26725 1:N:0:TCTGCAAG+GCTGCACT
TGCGTAGATCGGAGAAGACTTAGTTCTGATTCCATTACGGTCTTGCCCGGAATGTGTTACAGTGAGAT
ATCCAGAAGATCGATAGCTGAATGTGATAGAAGACATAGTTTTGAGATATCGAGCAACGTCATTGCTG
ATGCCAAAGTTGAAG
```

+
FFFFFFFFFFFFFFFFFFFFFFFFFFFFFFFFFFFFFFFFFFFF:FFFFFFFFFFFFFFFFFFFFFFFF
FFFFFFFFFFFFFFFFFFFFFFFFFFFFFFFFFFFFFFFFFFFFFFFFFFFFFFFFFFFFFFF:FFFFF
FFFFFFFFFF:FFFF
@A00721:190:HGJW7DSXY:4:2670:28601:30358 1:N:0:TCTGCAAG+GCTGCACT
TGCGTAGATCGGAGAAGACTTAGTTCTGATTCCATTACGGTCTTGCCCGGAATGTGTTACAGTGAGAT
ATCCAGAAGATCGATAGCTGAATGTGATAGAAGACATAGTTTTGAGATATCGAGCAACGTCATTGCTG
ATGCCAAAGTTGAAG
+
FFFFFFFFFFFFFFFFFFFFFFFFFFFFFFFFFFFFFFFFFFFFFFFFFFFFFFFFFFFFFFFFFFFF
FFFFFFFFFFFFFFFFFFFFFFFFFFFFFFF:FFFFFFFFFFFFFFFFFFFFFFFFFFFFFFFFFFFF
FFFFFFFFFFFFFFF
@A00721:190:HGJW7DSXY:4:1414:32154:24925 1:N:0:TCTGCAAG+GCTGCACT
CGTAGATCGGAGAAGACTTAGTTCTGATTCCATTACGGTCTTGCCCGGAATGTGTTACAGTGAGATAT
CCAGAAGATCGATAGCTGAATGTGATAGAAGACATAGTTTTGAGATATCGAGCAACGTCATTGCTGAT
GCCAAAGTTGAAGAT
+
FFFFFFFFFFFFFFFFFFFFFFFFFFFFFFFFFFFFFFFFFFFFFFFFFFFFFFFFFFFFFFFFFFFF
FFFFFFFFFFFFFFFFFFFFFFFFFFFFFFFFFFFFFFFFFFFFFFFFFFFFFFFFFFFFFFFFFFFF
F,:FFFFFFFFFFFF
@A00721:190:HGJW7DSXY:4:1442:28565:30326 1:N:0:TCTGCAAG+GCTGCACT
CGTAGATCGGAGAAGACTTAGTTCTGATTCCATTACGGTCTTGCCCGGAATGTGTTACAGTGAGATAT
CCAGAAGATCGATAGCTGAATGTGATAGAAGACATAGTTTTGAGATATCGAGCAACGTCATTGCTGAT
GCCAAAGTTGAAGAT
+
FFFFFFFFFFFFFFFFFF:FFFFFFFFFFFFFFFFFFFFFFFFFFFFFFFFFFF:FFFFFFFFFFFFFF
FFFFFFFFFFFFFFFFFFFFFFFFFFFFFFFFFFFFFFFFFFFFFFFFFFFFFFFFFFFFFFFFFFFF
FFFFFFFFFFFFFFF
@A00721:190:HGJW7DSXY:4:1464:19153:13338 1:N:0:TCTGCAAG+GCTGCACT
CGTAGATCGGAGAAGACTTAGTTCTGATTCCATTACGGTCTTGCCCGGAATGTGTTACAGTGAGATAT
CCAGAAGATCGATAGCTGAATGTGATAGAAGACATAGTTTTGAGATATCGAGCAACGTCATTGCTGAT
GCCAAAGTTGAAGAT
+
FFFFFFFFFFFFFFFFFFFFFFFFFFFFFFFFFFFFFFFFFFFFFFFFFFFFFFFFFFFFFFFFFFFF
FFF:FF,FFFFFFFFFFFFFFFFFFFFFFFF:FF:FFFFFFFFFFFFFFFFFFFFFFFFFFFFFFFFFF
FFFFFFFFFFFFFFF
@A00721:190:HGJW7DSXY:4:1651:30454:3693 1:N:0:TCTGCAAG+GCTGCACT
CGTAGATCGGAGAAGACTTAGTTCTGATTCCATTACGGTCTTGCCCGGAATGTGTTACAGTGAGATAT
CCAGAAGATCGATAGCTGAATGTGATAGAAGACATAGTTTTGAGATATCGAGCAACGTCATTGCTGAT
GCCAAAGTTGAAGAT
+
FFFFFFFFFFFFFFFFFFFFFFFFFFFFFFFFFFFFFFFFFFFFFFFFFFFFFFFFFFFFFFFFFFFF
:FFFFFFFFFFFFFFFFFFFFFFFFFFFFFFFFFFFFFFFFFFFFFFFFFFFFF:FFFFFFFF,FFFF
FFFFFFFFFFFFFFF
@A00721:190:HGJW7DSXY:4:2229:2799:17738 1:N:0:TCTGCAAG+GCTGCACT
CGTAGATCGGAGAAGACTTAGTTCTGATTCCATTACGGTCTTGCCCGGAATGTGTTACAGTGAGATAT
CCAGAAGATCGATAGCTGAATGTGATAGAAGACATAGTTTTGAGATATCGAGCAACGTCATTGCTGAT
GCCAAAGTTGAAGAT
+
FFFFFFFFFFFFFFFFFFFFFFFFFFFFFFFFFFFFFFFFFFFFFFFFFFFFFFFFFFF:FFFFFFFFF
FFFFFFFF:FFFFFFFFFFFFFFFFFFFFFFFFFFFFFFFF:FFFFFFFFFFFFFF:FFFFFFFFFFFFF
FFFFFFFFFFFFFFF
@A00721:190:HGJW7DSXY:4:2241:31204:28948 1:N:0:TCTGCAAG+GCTGCACT
CGTAGATCGGAGAAGACTTAGTTCTGATTCCATTACGGTCTTGCCCGGAATGTGTTACAGTGAGATAT

CCAGAAGATCGATAGCTGAATGTGATAGAAGACATAGTTTTGAGATATCGAGCAACGTCATTGCTGAT
GCCAAAGTTGAAGAT
+
FFFFFFFFFFFF:,FFFFFFFF:FFFFFF:FFFFFFF,FFFFFFFF,FF::FFFFFFFFFFFFFFFF
FFFFF:FFFFFFFF:FFFFFFFFFFFFF:FFFFFFFFF:FF,FFFFFFF:FF,FFF::FFF:FFFF:F
,,FFFFFFFF:F,FF
@A00721:190:HGJW7DSXY:4:1237:29243:20322 1:N:0:TCTGCAAG+GCTGCACT
TCGGAGAAGACTTAGTTCTGATTCCATTACGGTCTTGCCCAGAATGTGTTACAGTGAGATATCCAGAA
GATCGATAGCTGAATGTGATAGAAGACATAGTTTTGAGATATCGAGCAACGTCATTGCTGATGCCAAA
GTTGAAGATATTAGG
+
FFFFFFFFFFFFFFFFFFFFFFFFFFFFFFFFFFFFFFFFFFFFFFFFFFFFFFFFFFFFFFFFFFFF
FFFFFFFFFFFFFFFFFFFFFFFFFFFFFFFFFFFFFFFFFFFFFFFF:FFFFFFFFFFFFFFFFFFF
FFFF:FFFFFFFFFF
@A00721:190:HGJW7DSXY:4:1240:25780:14231 1:N:0:TCTGCAAG+GCTGCACT
TCGGAGAAGACTTAGTTCTGATTCCATTACGGTCTTGCCCGGAATGTGTTACAGTGAGATATCCAGAA
GATCGATAGCTGAATGTGATAGAAGACATAGTTTTGAGATATCGAGCAACGTCATTGCTGATGCCAAA
GTTGAAGATATTAGG
+
FFFFFFFFFFFFFFFFFFFFFFFFFFFFFFFF:FFFFFFFFFFFFFFFFFFFFFFFFFFFFFFFFFFF
FFFFFFFFFFFFFFFFFFFFFFFFFFFFF:FFFFFFFFFFFFFFFFFFFFFFFFFFFFFFFFFFFFFF
FFFFFFFFFFFFFFF
@A00721:190:HGJW7DSXY:4:1313:7129:32597 1:N:0:TCTGCAAG+GCTGCACT
TCGGAGAAGACTTAGTTCTGATTCCATTACGGTCTTGCCCGGAATGTGTTACAGTGAGATATCCAGAA
GATCGATAGCTGAATGTGATAGAAGACATAGTTTTGAGATATCGAGCAACGTCATTGCTGATGCCAAA
GTTGAAGATATTAGG
+
FFFFFFFFFFFFFF:FFFFFFFF,FFFFFFFFF:FFFFFFFFFFFFFFFFFFFFFFFFFFFF:FFFFF
FFFFFFFFFFFFFFFFFFFFFFFF:FFFFF,FFFFFFFFFFFFFFFFFFFFFFFFFFF:FFFF,FFFF
FFFFFFFFF:FFFFF
@A00721:190:HGJW7DSXY:4:1475:5267:30185 1:N:0:TCTGCAAG+GCTGCACT
TCGGAGAAGACTTAGTTCTGATTCCATTACGGTCTTGCCCGGAATGTGTTACAGTGAGATATCCAGAA
GATCGATAGCTGAATGTGATAGAAGACATAGTTTTGAGATATCGAGCAACGTCATTGCTGATGCCAAA
GTTGAAGATATTAGG
+
FFFFFFFFFFFFFFFFFFFFFFFFFFFFFFFFFFFFFFFFFFFFFFF:FFFFFFFFFFFFFFFFFFFF
FFFFFFFFFFFFFFFFFFFFFFFFFFFFFFFFFFFFFFFFFFFFFFFFFFFFFFFFFFFFFFFFFFFF
FFFFFFFFFFFFFFF
@A00721:190:HGJW7DSXY:4:1657:4092:18161 1:N:0:TCTGCAAG+GCTGCACT
TCGGAGAAGACTTAGTTCTGATTCCATTACGGTCTTGCCCGGAATGTGTTACAGTGAGATATCCAGAA
GATCGATAGCTGAATGTGATAGAAGACATAGTTTTGAGATATCGAGCAACGTCATTGCTGATGCCAAA
GTTGAAGATATTAGG
+
FFFFFFFFFFFF:FFFFFFFFFFFFFFFFFFFFFFFFFFFFFFFFFFFFFFFFFFFFFFFFFFFFFF
FFFFFFFFFFFFFFFFFFFFFFFFFFFFFF:FFFFFFFFFFFFFFFFFFFFFFFFF:FFFFFFFFFFFF
FFFFFFFFFFFFFFF
@A00721:190:HGJW7DSXY:4:1657:4806:19586 1:N:0:TCTGCAAG+GCTGCACT
TCGGAGAAGACTTAGTTCTGATTCCATTACGGTCTTGCCCGGAATGTGTTACAGTGAGATATCCAGAA
GATCGATAGCTGAATGTGATAGAAGACATAGTTTTGAGATATCGAGCAACGTCATTGCTGATGCCAAA
GTTGAAGATATTAGG
+
FFFFFFFFFFF:FFFFFFFFFFFFFFFFFFFFFFFF:FFFFFFFF:FFFF:FFFFFFFFFFFFFFFFFF
FFFFFFFFFFFFFFFFFFFFFFFFFFFFFFFFFFFFFFFFFFFFFF:FFFFFFFFFFFFFFFFFFFFFFF
FFFFFFFFFFFFFFF

```
@A00721:190:HGJW7DSXY:4:2363:2528:2895 1:N:0:TCTGCAAG+GCTGCACT
TCGGAGAAGACTTAGTTCTGATTCCATTACGGTCTTGCCCGGAATGTGTTACAGTGAGATATCCAGAA
GATCGATAGCTGAATGTGATAGAAGACATAGTTTTGAGATATCGAGCAACGTCATTGCTGATGCCAAA
GTTGAAGATATTAGG
+
FFFFFFFFFFFFFFFFFFFFFFFFFFFFFFFFFFFFFFFFFFF:FFFFFFFFFFFFFFFFFFFFFFF:
FFFFFFFFFFFFF:FFFFFFFFFFFFFF,FFFFFFFFFFFFFFFFFF:FFFFFFFFFFFFFFFFFFFF
FFFFFFFFF:FFFFF
@A00721:190:HGJW7DSXY:4:1117:10041:28933 1:N:0:TCTGCAAG+GCTGCACT
CCCGGAATGTGTTACAGTGAGATATCCAGAAGATCGATAGCTGAATGTGATAGAAGACATAGTTTTGA
GATATCGAGCAACGTCATTGCTGATGCCAAAGTTGAAGATATTAGGCCCGAAGATAGTGTTAGTCAGA
CGTCGAGTGTTAGAC
+
FFFFFFFFFFFFFFFFFFFFFFFFFFFFFFFFFFFFFFFFFFFFFFFFFFFFFFFFFFFFFFFFFFFF
FFFFFFFFFFFFFFFFFFFFFFFFFFFFFFFFFFFFFFFFFFFFFFFFFFFFFFFFFFFFFFFFFFF:
FFFFFFFFFFFFFFF
@A00721:190:HGJW7DSXY:4:1275:3351:22169 1:N:0:TCTGCAAG+GCTGCACT
CCCGGAATGTGTTACAGTGAGATATCCAGAAGATCGATAGCTGAATGTGATAGAAGACATAGTTTTGA
GATATCGAGCAACGTCATTGCTGATGCCAAAGTTGAAGATATTAGGCCCGAAGATAGTGTTAGTCAGA
CGTCGAGTGTTAGAC
+
FFFFFF:FFFFFFFFFFFFFFFFFFFFFFFFFFFFFFFFFFFFFFFFFFFFFFFFFFFFFFFFFFFFF
FFFFFFFFFFFFFFFFFFFFFFFFFFFFFFFFFFFFFFFF:FFFFFFFFFFFFFFFFFFFFFFFFFFF
FFFFFFFFFFFFFFF
@A00721:190:HGJW7DSXY:4:1407:23836:24267 1:N:0:TCTGCAAG+GCTGCACT
CCCGGAATGTGTTACAGTGAGATATCCAGAAGATCGATAGCTGAATGTGATAGAAGACATAGTTTTGA
GATATCGAGCAACGTCATTGCTGATGCCAAAGTTGAAGATATTAGGCCCGAAGATAGTGTTAGTCAGA
CGTCGAGTGTTAGAC
+
FFFFFFFFFFFFFFFFFFFFFFFFFFFFFFFFFFFFFFFFFFFFFFFFFFFFFFFFFFFFFFFFFFFF
FFFFFFFFFFFFFFFFFFFFFFFFFFFFFFFFFFFFFFFFFFFFFFFFFFFFFFFFFFFFFFFFFFFF
FFFFFFFFFFFFFFF
@A00721:190:HGJW7DSXY:4:1545:20871:35916 1:N:0:TCTGCAAG+GCTGCACT
CCCGGAATGTGTTACAGTGAGATATCCAGAAGATCGATAGCTGAATGTGATAGAAGACATAGTTTTGA
GATATCGAGCAACGTCATTGCTGATGCCAAAGTTGAAGATATTAGGCCCGAAGATAGTGTTAGTCAGA
CGTCGAGTGTTAGAC
+
FFFFFFFFFFFFFFFFFFFFFFFFFFFFFFFFFFFFFFFFFFFFFFFFFFFFFFFFFFFFFFFFFFFF
FFFFFFFFFFFFFFFFFFFFFFFFFFFFFFFFFFFFFFFFFFFFFFFFFFFFFFFFFFFFFFFFFFFF
FFFFFFFFFFFFFFF
@A00721:190:HGJW7DSXY:4:2141:27190:2644 1:N:0:TCTGCAAG+GCTGCACT
CCCGGAATGTGTTACAGTGAGATATCCAGAAGATCGATAGCTGAATGTGATAGAAGACATAGTTTTGA
GATATCGAGCAACGTCATTGCTGATGCCAAAGTTGAAGATATTAGGCCCGAAGATAGTGTTAGTCAGA
CGTCGAGTGTTAGAC
+
FFFFFFFFF:FFFFFFFFFFFFFFFFFFFFFFFFFFFF,FFFFFFFFFFFFFFFFFFFFFFFFFFFFF
FFFFFFFFFFFFFFFFFFFFFFFFFFFFFFFFFFFFFFFFFFFFFFFFFFFFFF,FF,FFFFFFFFFF
FFFFFFFFFFFFFFF
@A00721:190:HGJW7DSXY:4:2334:28782:13228 1:N:0:TCTGCAAG+GCTGCACT
CCCGGAATGTGTTACAGTGAGATATCCAGAAGATCGATAGCTGAATGTGATAGAAGACATAGTTTTGA
GATATCGAGCAACGTCATTGCTGATGCCAAAGTTGAAGATATTAGGCCCGAAGATAGTGTTAGTCAGA
CGTCGAGTGTTAGAC
+
FFFFFFFFFFFFFFFFFFFFFFFFFFFFFFFFFFFFFFFFFFFF:FFFFFFFFFF:FFFFFFFFFFFF
```

```
FFFFFFFFFFFFFFFFFFFFFFFFFFFFFFFFFFFFFFFFFFFFFFFFFFFFFFFFFFFFFFFFFF:F
FFFFFFFFFFFFFFF
@A00721:190:HGJW7DSXY:4:2407:25880:24987 1:N:0:TCTGCAAG+GCTGCACT
CCCGGAATGTGTTACAGTGAGATATCCAGAAGATCGATAGCTGAATGTGATAGAAGACATAGTTTTGA
GATATCGAGCAACGTCATTGCTGATGCCAAAGTTGAAGATATTAGGCCCGAAGATAGTGTTAGTCAGA
CGTCGAGTGTTAGAC
+
FFFFFF:FFFFFFFFFFFFFFFFFFFFFFFFFFFFFFFFFFFFFFFFFFFFFFFFFFFFFFFFFFFFF
FFFFFFFFFFFFFFFFFFFFFFFFFFFFFFFFFFF:FFFFFFFFFFFFFFFFFF:FFFFFFFFFFFFF
FFFFFFFFFFFFFFF
@A00721:190:HGJW7DSXY:4:1152:10375:34397 1:N:0:TCTGCAAG+GCTGCACT
GTGAGATATCCAGAAGATCGATAGCTGAATGTGATAGAAGACATAGTTTTGAGATATCGAGCAACGTC
ATTGCTGATGCCAAAGTTGAAGATATTAGGCCCGAAGATAGTGTTAGTCAGACGTCGAGTGTTAGACG
AGACCAGTGTCTGGC
+
F:FFFFFFFFFFFF,FFFFFFFFFFFFFFFFFFFFFFFFFFFFFFFFFFFFFFFFFFFFFFFFFFFFF
FFFFFFFFFFFFFFFFFFFFFFFF:FFFFFFFFFFFFFFFFFFFFFFFFFFFFFFFFFFFFFFFFFF:
FFFFFFFFFFFFF:F
@A00721:190:HGJW7DSXY:4:1259:1542:30029 1:N:0:TCTGCAAG+GCTGCACT
GTGAGATATCCAGAAGATCGATAGCTGAATGTGATAGAAGACATAGTTTTGAGATATCGAGCAACGTC
ATTGCTGATGCCAAAGTTGAAGATATTAGGCCCGAAGATAGTGTTAGTCAGACGTCGAGTGTTAGACG
AGACCAGTGTCTGGC
+
FFFFFFFFFFFFFFFFFFFFFFFFFFFFFFFFFFFFFFFFFFF:FFFFFFFFFFFFFFFFFFFFFFFF
FFFFFFFFFFFFFFFFFFFFFFFFFFFFFFFFFFFFFFFFFFFFFFFFFFFFFFFFFFFFFFFFFFFF
FFFFFFFFFFFFFFF
@A00721:190:HGJW7DSXY:4:2117:23529:36010 1:N:0:TCTGCAAG+GCTGCACT
GTGAGATATCCAGAAGATCGATAGCTGAATGTGATAGAAGACATAGTTTTGAGATATCGAGCAACGTC
ATTGCTGATGCCAAAGTTGAAGATATTAGGCCCGAAGATAGTGTTAGTCAGACGTCGAGTGTTAGACG
AGACCAGTGTCTGGC
+
FFFFFFFFFFFFFFFFFFFFFFFFFFFFFFFFFFFFFFFFFFFFFFFFFFFFFFFFFFFFFFFFFFFF
FFFFFFFFFFFFFFFFFFFFFFFFFFFFFFFFFFFFFFFFFFFFFFFFFFFFFFFFFFFFFFFFFFFF
FFFFFFFFFFFFFFF
@A00721:190:HGJW7DSXY:4:2145:20735:2112 1:N:0:TCTGCAAG+GCTGCACT
GTGAGATATCCAGAAGATCGATAGCTGAATGTGATAGAAGACATAGTTTTGAGATATCGAGCAACGTC
ATTGCTGATGCCAAAGTTGAAGATATTAGGCCCGAAGATAGTGTTAGTCAGACGTCGAGTGTTAGACG
AGACCAGTGTCTGGC
+
FFFFFFFFFFFFFFFFFFFFFFFFFFFFFFFFFFFFFFFFFFFFFFFFFFFFFFFFFFFFFFF:FFFF
FFFFFFFFFFFFFFFFFFFFFFFF,FFFFFFFFFFFFFF,FFFFFFFFFFFFFFFFFFFFFFFFFFFF
FFFFFFFFFFFFFFF
@A00721:190:HGJW7DSXY:4:1156:12527:9815 1:N:0:TCTGCAAG+GCTGCACT
ATCCAGAAGATCGATAGCTGAATGTGATAGAAGACATAGTTTTGAGATATCGAGCAACGTCATTGCTG
ATGCCAAAGTTGAAGATATTAGGCCCGAAGATAGTGTTAGTCAGACGTCGAGTGTTAGACGAGACCAG
TGTCTGGCTGGATCG
+
FFFFFFFFFFFFFFFFFFFFFFFFFFFFFFFFFFFFFFFFFFFFFFFFFFFFFFFFFFFFFFFFFFFF
FFFFFFFFFFFFFFFFFFFFFFFFFFFFFFFFFFFFFFFFFFFFFF:FFFFFFFFFFFFF:FFFFFFF
FFFFFFFFFFFFFF:
@A00721:190:HGJW7DSXY:4:1336:31882:6918 1:N:0:TCTGCAAG+GCTGCACT
ATCCAGAAGATCGATAGCTGAATGTGATAGAAGACATAGTTTTGAGATATCGAGCAACGTCATTGCTG
ATGCCAAAGTTGAAGATATTAGGCCCGAAGATAGTGTTAGTCAGACGTCGAGTGTTAGACGAGACCAG
TGTCTGGCTGGATCG
```

```
+
FFFFFFFFFFFFFFFFFFFFFFFFFFFFFFFFFFFFFFFFFFFFFFFFFFFFFFFFFFFFFFFFFFFF
FFFFFFFFFFFFFFFFFFFFFFFFFFFFFFFFFFFFFFFFFFFFFFFFFFFFFFFFFFFFFFFFFFFF
FFFFFFFFFFFFFF:
@A00721:190:HGJW7DSXY:4:1344:31295:19460 1:N:0:TCTGCAAG+GCTGCACT
ATCCAGAAGATCGATAGCTGAATGTGATAGAAGACATAGTTTTGAGATATCGAGCAACGTCATTGCTG
ATGCCAAAGTTGAAGATATTAGGCCCGAAGATAGTGTTAGTCAGACGTCGAGTGTTAGACGAGACCAG
TGTCTGGCTGGATCG
+
FFFFFFFF,FFFFFFFFFFFFFFFFFFFFFFFFFFFFFFFFFFFFFFFFFFFFFFFFFFFFFFFFFFF
FFFFFFFFFFFFFFFFFFFFFFFFFFFFFFFFFFFFFFFFFFFF:FF:FFFFFFFFFFFFFFFFFFFF
FFFFFFFFFFFFFFF
@A00721:190:HGJW7DSXY:4:2241:26684:12226 1:N:0:TCTGCAAG+GCTGCACT
ATCCAGAAGATCGATAGCTGAATGTGATAGAAGACATAGTTTTGAGATATCGAGCAACGTCATTGCTG
ATGCCAAAGTTGAAGATATTAGGCCCGAAGATAGTGTTAGTCAGACGTCGAGTGTTAGACGAGACCAG
TGTCTGGCTGGATCG
+
FFFFFFFFFFFFFFFFFFFFFFFFFFFFFFFFFFFFFFFFFFFFFFFFFFFFFFFFFFFFFFFFFFFF
FFFFF:FFFFFFFFFFFFFFFFFFFFFFFFFFFFFFFFFFFFFFFFFFFFFFFFFFFFFFFFFFFFFF
FFFFFFFFFFFFFFF
@A00721:190:HGJW7DSXY:4:2643:12536:36354 1:N:0:TCTGCAAG+GCTGCACT
ATCCAGAAGATCGATAGCTGAATGTGATAGAAGACATAGTTTTGAGATATCGAGCAACGTCATTGCTG
ATGCCAAAGTTGAAGATATTAGGCCCGAAGATAGTGTTAGTCAGACGTCGAGTGTTAGACGAGACCAG
TGTCTGGCTGGATCG
+
FFFFFFFFFFFFFFFFFFFFFFFFFFFFFFFFFFFFFFFFFFFFFFFFFFFFFFFFFFFFFFFFFFFF
FFFFFFFFFFFFFFFFFFFFFFFFFFFFFFFF:FFFFFFFFFFFFFFFFFFFFFFFFFFFFFFFFFFF
FFFFFFFFFFFFFFF
@A00721:190:HGJW7DSXY:4:1160:12391:30843 1:N:0:TCTGCAAG+GCTGCACT
TGCTGGTCGATCCAGCCAGACACTGGTCTCGTCTAACACTCGACGTCTGACTAACACTATCTTCGGGC
CTAATATCTTCAACTTTGGCATCAGCAATGACGTTGCTCGATATCTCAAAACTATGTCTTCTATCACA
TTCAGCTATCGATCT
+
FFFFFFFFFFFFFFFFFFFFFFFFFFFFFFFFFFFFFFFFFFFFFFFFFFFFFFFFFFFFFFFFFFFF
FFFFFFFFFFFFFFFFFFFFFFFFFFFFFFFFFFFFFFFFFFFFFFFFFFFFFFFFFFFFFFFFFFFF
FFFFFFFFFFFFFFF
@A00721:190:HGJW7DSXY:4:2437:15691:26412 1:N:0:TCTGCAAG+GCTGCACT
TGCTGGTCGATCCAGCCAGACACTGGTCTCGTCTAACACTCGACGTCTGACTAACACTATCTTCGGGC
CTAATATCTTCAACTTTGGCATCAGCAATGACGTTGCTCGATATCTCAAAACTATGTCTTCTATCACA
TTCAGCTATCGATCT
+
FFFFFFFFFFFFFFFFFFFFFFFFFFFFFFFFFFFFFFFFFFFFFFFFFFFFFFFFFFFFFFFFFFFF
FFFFFFFFF:FFFFFFFFFFFFFFFFFFFFFFFFFFFFFFFFF:FFFFFFFFF:FFFFFFFFFFFFFF
FFFFFFFFFFFFFFF
@A00721:190:HGJW7DSXY:4:2437:16170:26678 1:N:0:TCTGCAAG+GCTGCACT
TGCTGGTCGATCCAGCCAGACACTGGTCTCGTCTAACACTCGACGTCTGACTAACACTATCTTCGGGC
CTAATATCTTCAACTTTGGCATCAGCAATGACGTTGCTCGATATCTCAAAACTATGTCTTCTATCACA
TTCAGCTATCGATCT
+
FFFFFFFFFFFFFFFFFFFFFFFFFFFFFFFFFFFFFFFFFFFFFFFFF:FFFFFFFFFFFFFFFFFF
FFFFFFFFFFFFFFFFFFFFFFFFFFFFFFFFFFFFFFFFFFFFFFFFFFFFFFFFFFFFFFFFFFFF
FFFFFFFFFFFFFFF
@A00721:190:HGJW7DSXY:4:1251:8531:16517 1:N:0:TCTGCAAG+GCTGCACT
CGATAGCTGAATGTGATAGAAGACATAGTTTTGAGATATCGAGCAACGTCATTGCTGATGCCAAAGTT
```

GAAGATATTAGGCCCGAAGATAGTGTTAGTCAGACGTCGAGTGTTAGACGAGACCAGTGTCTGGCTGG
ATCGACCAGCATTAT
+
FFFFFFFFFFFFFFFFFFFFFFFFFFFFFFFFFFFFFFFFFFFFFFFFFFFFFFFFFFFFFFFFFFFFFF
FF:FFFFFF:FFFFFFFFFF:FFFFFFFFFFFFFFFFFFFFFFFFFFFFFFFFFFFFFFFFFFFFFFFF
FFFFFFFFFFFFFFF
@A00721:190:HGJW7DSXY:4:1270:25409:24643 1:N:0:TCTGCAAG+GCTGCACT
CGATAGCTGAATGTGATAGAAGACATAGTTTTGAGATATCGAGCAACGTCATTGCTGATGCCAAAGTT
GAAGATATTAGGCCCGAAGATAGTGTTAGTCAGACGTCGAGTGTTAGACGAGACCAGTGTCTGGCTGG
ATCGACCAGCATTAT
+
FFFFFFFFFFFFFFFFFFFFFFFFFFFFFFFFFFFFFFFFFFFFFFFFFFFFFF:FFFFFFFFFFFFF
FFFFFFFFF:FFFFFFFFFFFFFFFFFFFFFFFFFFFFFFFFFFFFFFFFFFFFFFFFFFFFFFFFFFFF
FFFFFFFFFFFFFFF
@A00721:190:HGJW7DSXY:4:2316:3667:28197 1:N:0:TCTGCAAG+GCTGCACT
CGATAGCTGAATGTGATAGAAGACATAGTTTTGAGATATCGAGCAACGTCATTGCTGATGCCAAAGTT
GAAGATATTAGGCCCGAAGATAGTGTTAGTCAGACGTCGAGTGTTAGACGAGACCAGTGTCTGGCTGG
ATCGACCAGCATTAT
+
FFFFFFFFFFFFFFFFFFFFFFFFFFFFFFFFFFFFFFFFFFFFFFFFFFFFFFFFFFFFFFFFFFFFFF
FFFFFFFFFFFFFFFFFFFFFFFFFFFFFFFFFFFFFFFFFFFFFFFFFFFFFFFFFFFFFFFFFFF:FF
FFFFFFFFFFFFFFF
@A00721:190:HGJW7DSXY:4:2423:1913:35055 1:N:0:TCTGCAAG+GCTGCACT
CGATAGCTGAATGTGATAGAAGACATAGTTTTGAGATATCGAGCAACGTCATTGCTGATGCCAAAGTT
GAAGATATTAGGCCCGAAGATAGTGTTAGTCAGACGTCGAGTGTTAGACGAGACCAGTGTCTGGCTGG
ATCGACCAGCATTAT
+
FFFFFFFFFFFFFFFFFFFFFFFFFFFFFFFFFFFFFFFFFFFFFFFFFFFFFFFFFFFFFFFFFFFFFF
FFFFFFFFFFFFFFFFFF:FFFFFFFFFFFFFFFFFFFFFFFFFFFFFFFFFFFFFFFFFFFFFFFFFFF
FFFFFFFFFFFFFFF
@A00721:190:HGJW7DSXY:4:2613:8124:12774 1:N:0:TCTGCAAG+GCTGCACT
CGATAGCTGAATGTGATAGAAGACATAGTTTTGAGATATCGAGCAACGTCATTGCTGATGCCAAAGTT
GAAGATATTAGGCCCGAAGATAGTGTTAGTCAGACGTCGAGTGTTAGACGAGACCAGTGTCTGGCTGG
ATCGACCAGCATTAT
+
FFFF:FFFFFFFFFFFFFFFFFFFFFFFFFF:FFFFFFFFFFFFFFFFFFFFFFFFFFFFFFFFFFFFFF
FFFFFFFFFFFFFFFFFFFFF:FFFFFFFFFFFFFFFFFFFFFFFFFFFFFFFFFFFFFFFFFFFFFFFF
FFFFFFFFFFFFFFF
@A00721:190:HGJW7DSXY:4:2173:1931:6965 1:N:0:TCTGCAAG+GCTGCACT
TGCTGGTCGATCCAGCCAGACACTGGTCTCGTCTAACACTCGACGTCTGACTAACACTATCTTCGGGC
CTAATATCTTCAACTTTGGCATCAGCAATGACGTTGCTCGAAATCTCAAAACTATGTCTACAATCACA
T
+
F,F:FF,F,F,,FFFFFFFF,:F,:,FF,F,,:F,:FFF::FFF,,F,:F,FFF,FFF:F:,FFF:::
:FFF,,,F,:F:::F,F,,FFFFF,F,FF,FF,FFFFFFFF,FFF,F:,F,F,:FFF,F,:,FFFF,F
F
@A00721:190:HGJW7DSXY:4:1222:24903:6668 1:N:0:TCTGCAAG+GCTGCACT
CCTATCCATAGCAATCTGCAATATATCGACCAGATCAGCATTCATAATGCTGGTCGATCCAGCCAGAC
ACTGGTCTCGTCTAACACTCGACGTCTGACTAACACTATCTTCGGGCCTAATATCTTCAACTTTGGCA
TCAGCAATGACGTTG
+
FFFFFFFFFFFFF,FFFFFFFFFFFFFFFFFFFFFFFFFFFFFFFFFFFFF:FFFFFFFFFFFFFFFFF
FFFFFFFFFFFFF:FFFFFFFFFFFFFFFFFFFFFFF,F:F:FFF:FFFFFFFFFFFFFFFFFFF:FFFFF
FF,FFFFFFFFFFFF

```
@A00721:190:HGJW7DSXY:4:2163:24162:5635 1:N:0:TCTGCAAG+GCTGCACT
CCTATCCATAGCAATCTGCAATATATCGACCAGATCAGCATTCATAATGCTGGTCGATCCAGCCAGAC
ACTGGTCTCGTCTAACACTCGACGTCTGACTAACACTATCTTCGGGCCTAATATCTTCAACTTTGGCA
TCAGCAATGACGTTG
+
FFFFFFFFFFFFFFFFFFFFFFFFFFFFFFFFFFFFFFFFFFFFFFFFFFFFFFFFFFFFFFFFFFFFFF
FFFFFFFFFFFFFFFFFFFFFFFFFFFFFFF:FFFFFFFFFFFFFFFFFFFFFFF,FFFFFFFF,FFFFF
FFFFFFFFFFFFFFF
@A00721:190:HGJW7DSXY:4:2252:24822:17519 1:N:0:TCTGCAAG+GCTGCACT
CCTATCCATAGCAATCTGCAATATATCGACCAGATCAGCATTCATAATGCTGGTCGATCCAGCCAGAC
ACTGGTCTCGTCTAACACTCGACGTCTGACTAACACTATCTTCGGGCCTAATATCTTCAACTTTGGCA
TCAGCAATGACGTTG
+
FFFFFFFFFFFFFFFFFFFFFFFFFFFFFFFFFFFFFFFFFFFFFFFFFFFFFFFFFFFFFFFFFFFFFF
FFFFFFFFFFFFFFFFFFFFFFFFFFFFFFFFFFFFFFFFFFFFFFFFFFFFFFFFF:FFFFF:FFFFFF
FFFFFFFFFF:FFFF
@A00721:190:HGJW7DSXY:4:2437:8269:5228 1:N:0:TCTGCAAG+GCTGCACT
CCTATCCATAGCAATCTGCAATATATCGACCAGATCAGCATTCATAATGCTGGTCGATCCAGCCAGAC
ACTGGTCTCGTCTAACACTCGACGTCTGACTAACACTATCTTCGGGCCTAATATCTTCAACTTTGGCA
TCAGCAATGACGTTG
+
FFFFFFFFFFFFFFFFFFFFFFFFFFFFFFFFFFFFFFFFFFFFFFFFFFFFFFFFFFFFFFFFFFFFFF
FFFFFFFFFFFFFFFFFFFFFFFFFFFFFFFF,F:FFFFFF:FFFFFFFFFFFF:FFFFFFFFFFFFFFF
FFFFFFFFFFFFFFF
@A00721:190:HGJW7DSXY:4:2611:8856:35243 1:N:0:TCTGCAAG+GCTGCACT
CCTATCCATAGCAATCTGCAATATATCGACCAGATCAGCATTCATAATGCTGGTCGATCCAGCCAGAC
ACTGGTCTCGTCTAACACTCGACGTCTGACTAACACTATCTTCGGGCCTAATATCTTCAACTTTGGCA
TCAGCAATGACGTTG
+
FFFFFFFFFFFFFFFFFFFFFFFFFFFFFFFFFFFFFFFFFFFFFFFFFFFFFFFFFFFFFFFFFFFFFF
FFFFFFFFFFFF:FFFFFFFFFFFFFFFFFFFFFFF:F:FFFFFFFFFFFFFFFFFFFFFFFFF:FFFFF
FFFFFFFFFFFFFFF
@A00721:190:HGJW7DSXY:4:2654:5430:31187 1:N:0:TCTGCAAG+GCTGCACT
CCTATCCATAGCAATCTGCAATATATCGACCAGATCAGCATTCATAATGCTGGTCGATCCAGCCAGAC
ACTGGTCTCGTCTAACACTCGACGTCTGACTAACACTATCTTCGGGCCTAATATCTTCAACTTTGGCA
TCAGCAATGACGTTG
+
FFFFFFFFFFFFFFFFFFFFFFFFFFFFFFFFFFFFFFFFFFFFFFFFFFFFFFFFF:FFFFFFFFFFFF
FFFFFFFFFFFFFFFFFFFFFFFFFFFFFFFFFFFFFFFFFFFFFFFFFFFFFFFFFFFFFFFFFFFFFF
FFFFFFFFFFF:FFF
@A00721:190:HGJW7DSXY:4:1262:10800:32377 1:N:0:TCTGCAAG+GCTGCACT
GCCAAAGTTGAAGATATTAGGCCCGAAGATAGTGTTAGTCAGACGTCGAGTGTTAGACGAGACCAGTG
TCTGGCTGGATCGACCAGCATTATGAATGCTGATCTGGTCGATATATTGCAGATTGCTATGGATAGGG
GAAAAAAATCAATAT
+
FFFFFFFFFFFFFFFFFFFFFFFFFFFFFFFFFFFFFFFFFFFFFFFFFFFFFFFFFFFFFFFFFFFFFF
FFFFFFFFFFFFFFFFFFF:FFFFFFFFFFFFFFFFFFFFFFFFFFFFFFFFFFFFFFFFFFFFFFFFFF
FFFFFFF:F::FFFF
@A00721:190:HGJW7DSXY:4:1315:12192:12148 1:N:0:TCTGCAAG+GCTGCACT
GCCAAAGTTGAAGATATTAGGCCCGAAGATAGTGTTAGTCAGACGTCGAGTGTTAGACGAGACCAGTG
TCTGGCTGGATCGACCAGCATTATGAATGCTGATCTGGTCGATATATTGCAGATTGCTATGGATAGGG
GAAAAAAATCAATAT
+
FFFFFFFFFFFFFFF:FF,FFFFFFFFFFFFFFFFFFFFFFFFFFFFFFFFFFFFFFFFFFFFFFFFFFF
```

```
FFFFFFFFFFFFFFFFFFFFFFFFFFFFFFFFFFFFFFFFFFFFF:FFFFFFFFFFFFFFFFFFFFFF
FF:FFFFFFFFFFFF
@A00721:190:HGJW7DSXY:4:1406:17671:17503 1:N:0:TCTGCAAG+GCTGCACT
GCCAAAGTTGAAGATATTAGGCCCGAAGATAGTGTTAGTCAGACGTCGAGTGTTAGACGAGACCAGTG
TCTGGCTGGATCGACCAGCATTATGAATGCTGATCTGGTCGATATATTGCAGATTGCTATGGATAGGG
GAAAAAAATCAATAT
+
FFFFFFFFFFFFFFFFFFFFFFFFFFFFFFFFFFFFFFFFFFFFFFFFFFFFFFFFFFFFFFFFFFFF
FFFFFF,FFFFFFFFFFFFFFFFFFFFFFFFFFFFFFFFFFFFFFFFF:FF:FFFF:FFFFFFFFFFF
FFFFFFF:FFFFFFF
@A00721:190:HGJW7DSXY:4:1471:3269:1767 1:N:0:TCTGCAAG+GCTGCACT
GCCAAAGTTGAAGATATTAGGCCCGAAGATAGTGTTAGTCAGACGTCGAGTGTTAGACGAGACCAGTG
TCTGGCTGGATCGACCAGCATTATGAATGCTGATCTGGTCGATATATTGCAGATTGCTATGGATAGGG
GAAAAAAATCAATAT
+
FFFF,FF::FFFFFFFFF,F:FFF:FFFFFFFFF::FFF:F:FFFFFFFF:FFFF:F:FFFFFFFFFF
FFF::FFFFFFF,FFFFFFFF:FF:FFFFFFFFFF:FF:FFFFF:FFFFFFFFFFFFF,FFFF:FFFF
FF,FFFF:FFFFFFF
@A00721:190:HGJW7DSXY:4:1668:4291:2472 1:N:0:TCTGCAAG+GCTGCACT
GCCAAAGTTGAAGATATTAGGCCCGAAGATAGTGTTAGTCAGACGTCGAGTGTTAGACGAGACCAGTG
TCTGGCTGGATCGACCAGCATTATGAATGCTGATCTGGTCGATATATTGCAGATTGCTATGGATAGGG
GAAAAAAATCAATAT
+
FFFFFFFFFFFFFFF::FFFFFFFFFFFFFFFFFFFFFFFFFFFFFFFFFFFFFFFFFFFFFFFFFFF
FFFFFFFFFFFFFFFFFFFFFFFFFFFFFFFFFFFFFFFFFFFFFFF:FFFFFFFFFFFFFFFFFFFF
FFFFFFFFFFFFFFF
@A00721:190:HGJW7DSXY:4:2243:16721:36996 1:N:0:TCTGCAAG+GCTGCACT
GCCAAAGTTGAAGATATTAGGCCCGAAGATAGTGTTAGTCAGACGTCGAGTGTTAGACGAGACCAGTG
TCTGGCTGGATCGACCAGCATTATGAATGCTGATCTGGTCGATATATTGCAGATTGCTATGGATAGGG
GAAAAAAATCAATAT
+
FFFFFFFFFFFFFFFFFFFFFFFFFFFFFFFFFFFFFFFFFFFFFFFFFFFFFFFFFFFFFFFFFFFF
FFFFFFFFFFFFFFF::FFFFFFFFFFFFFFFFFFFFFFFFFFFF:FFFFFFFFFFFFFFFFF:FFFF
FF:FFFFFFFFFFFF
@A00721:190:HGJW7DSXY:4:2446:16495:18067 1:N:0:TCTGCAAG+GCTGCACT
GCCAAAGTTGAAGATATTAGGCCCGAAGATAGTGTTAGTCAGACGTCGAGTGTTAGACGAGACCAGTG
TCTGGCTGGATCGACCAGCATTATGAATGCTGATCTGGTCGATATATTGCAGATTGCTATGGATAGGG
GAAAAAAATCAATAT
+
FFFFFFFFFFFFFFFFFFFFFFFFFFFFFFFFFFFFFFFFFFFFFFFFFFFFFFFFFFFFFFFFFFFF
FFFFFFFFFFFFFFFF:FFFFFFFFFFFFFFFFFFFFFFFFFFFFFFFFFFFFFFFFFFFFFFFFFFF
FFFFFFFFFFFFFFF
@A00721:190:HGJW7DSXY:4:2461:23484:23062 1:N:0:TCTGCAAG+GCTGCACT
GCCAAAGTTGAAGATATTAGGCCCGAAGATAGTGTTAGTCAGACGTCGAGTGTTAGACGAGACCAGTG
TCTGGCTGGATCGACCAGCATTATGAATGCTGATCTGGTCGATATATTGCAGATTGCTATGGATAGGG
GAAAAAAATCAATAT
+
FFFFFFFFFFFFFFFFFF:FFFFFFFFFF:FFFFFFFFFFFFFFFFFFFFFFFFFFFFFFFFFFFFFF
FFFFFFFFFFFFFFFFFFFFFFFFFFFFFFFFFFFFFFFFFFFFFFFFFFFFFFFFFFFFFFFFFFFF
FFFFFFFFFFFFFFF
@A00721:190:HGJW7DSXY:4:2528:6876:7513 1:N:0:TCTGCAAG+GCTGCACT
GCCAAAGTTGAAGATATTAGGCCCGAAGATAGTGTTAGTCAGACGTCGAGTGTTAGACGAGACCAGTG
TCTGGCTGGATCGACCAGCATTATGAATGCTGATCTGGTCGATATATTGCAGATTGCTATGGATAGGG
GAAAAAAATCAATAT
```

+
FFFFFFFFFFFFFFFFFFFFFFFFFFFFFFFFFFFFFFFFFF:FFFFFFFFFFFFFFFFFFFFFFFFF
FFFFFFFFFFFFFFFF:FFFFFFFFFFFFFFFFFFFFFFFFFFFFFFFFFFFFFFFFFFFFFFFFFFF
FF,FFFFFFFFFFFF
@A00721:190:HGJW7DSXY:4:2528:6596:7780 1:N:0:TCTGCAAG+GCTGCACT
GCCAAAGTTGAAGATATTAGGCCCGAAGATAGTGTTAGTCAGACGTCGAGTGTTAGACGAGACCAGTG
TCTGGCTGGATCGACCAGCATTATGAATGCTGATCTGGTCGATATATTGCAGATTGCTATGGATAGGG
GAAAAAAATCAATAT
+
FFFFFFFFFFFFFFFFFFFFFFFFFFFFFFFFFFFFFFFFFFFFFFFFFFFFFFFFFFFFFFFFFFFF
FFFFFFFFFFFFFF:FFFFFFFFFFFFFFFFFFFFF:FFFFFFFFFFFFFFFFFFFFFFFFFFFFFFF
FFFFFFFFFFFFFFF
@A00721:190:HGJW7DSXY:4:2545:25997:27038 1:N:0:TCTGCAAG+GCTGCACT
GCCAAAGTTGAAGATATTAGGCCCGAAGATAGTGTTAGTCAGACGTCGAGTGTTAGACGAGACCAGTG
TCTGGCTGGATCGACCAGCATTATGAATGCTGATCTGGTCGATATATTGCAGATTGCTATGGATAGGG
GAAAAAAATCAATAT
+
FFFFFFFFFFFFFFFFFFFFFFFFFFFFF:FFFFFFFFFFFFFFFFFFFFFFFFFFFFF:FFFFFFFF
FFFFFFFFFFFFFFFFFFFFFFFFFFFFFFFFFFFFFFFFFFFFFFFF:FFFFFFFFF:F:FFFF:FF
FFFFFFFFFFFFFFF
@A00721:190:HGJW7DSXY:4:2646:22209:9737 1:N:0:TCTGCAAG+GCTGCACT
GCCAAAGTTGAAGATATTAGGCCCGAAGATAGTGTTAGTCAGACGTCGAGTGTTAGACGAGACCAGTG
TCTGGCTGGATCGACCAGCATTATGAATGCTGATCTGGTCGATATATTGCAGATTGCTATGGATAGGG
GAAAAAAATCAATAT
+
FFFFFFFFFFFFFFFFFFFFFFFFFFFFFFFFFFFFFFFFFFFFFFFFFFFFFFFFFFFFFFFFFFFF
FFFFFFFFFFFFFFFFFFFFFFFFFFFFFFFFFFFFFFFFFFFFFFFFFFFFFFF:FFFFFFFFF:FF
FFFFFFFFFFFFFFF
@A00721:190:HGJW7DSXY:4:2676:26115:7513 1:N:0:TCTGCAAG+GCTGCACT
GCCAAAGTTGAAGATATTAGGCCCGAAGATAGTGTTAGTCAGACGTCGAGTGTTAGACGAGACCAGTG
TCTGGCTGGATCGACCAGCATTATGAATGCTGATCTGGTCGATATATTGCAGATTGCTATGGATAGGG
GAAAAAAATCAATAT
+
FFFFFFFFFFFFFFFFFF:FFFFFFFFFFFFFFFFFFFFFFFFFFFFFFFFFFFFFFFFFFFFFFFFF
FFFFFFFFFFFFFFFFFFFFFFFFFFFFFFFFFFFF:FFFFFFFFFF:FFFFFFFFFFFFFFFFFFFF
FFFFFFFFFFFFFFF
@A00721:190:HGJW7DSXY:4:1262:9426:31563 1:N:0:TCTGCAAG+GCTGCACT
CCAAAGTTGAAGATATTAGGCCCGAAGATAGTGTTAGTCAGACGTCGAGTGTTAGACGAGACCAGTGT
CTGGCTGGATCGACCAGCATTATGAATGCTGATCTGGTCGATATATTGCAGATTGCTATGGATAGGGG
AAAAAAATCAATAT
+
F,FFFFFFFFF:F:F:FF:FFFF:FFFFFFFFFFFFFF,FFF:F:F,FFFFFFFFFFFFFF:FFFFFF
,F::F:FFFFFFFF:FFFFF:FF,FFF,FF:FFFFFFFFFFFFFF:FFFF,FFFFF:,:FFFFFF,:F
FFFF:FF,FFF,FF
@A00721:190:HGJW7DSXY:4:1233:30409:21496 1:N:0:TCTGCAAG+GCTGCACT
GTATTAGGCCCGAAGATAGTGTTAGTCAGACGTCGAGTGTTAGACGAGACCAGTGTCTGGCTGGATCG
ACCAGCATTATGAATGCTGATCTGGTCGATATATTGCAGATTGCTATGGATAGGGGAAAAAAATCAAT
ATTTGACAGTCATAT
+
FFFFFFFFFFFFFFFFFFFFFFFFFFFFFFFFFFFFFFFFFFFFFFFFFFFFFFFFFFFFFFFFFFFF
FFFFFFFFFFFFFFFFF:FFFFFFFFFFFFFFFFFFFFFFFFFFFFFFFFFFFFFFFFFFFFFFFFFF
FFFF:FFFFFFFF:F
@A00721:190:HGJW7DSXY:4:1448:3513:36793 1:N:0:TCTGCAAG+GCTGCACT
GTATTAGGCCCGAAGATAGTGTTAGTCAGACGTCGAGTGTTAGACGAGACCAGTGTCTGGCTGGATCG

ACCAGCATTATGAATGCTGATCTGGTCGATATATTGCAGATTGCTATGGATAGGGGAAAAAAATCAAT
ATTTGACAGTCATAT
+
FFFFFFFFFFFFFFFFFFFFFFFFFFFFFFFFFFFFFFFFFFFFFFFFFFFFFFFFFF:FFFFFFFFF:F
FFFFFFFFFFFFFFFFFFFFFFFFFFFFFFFFFFFFFFFFFFFFFFFFFFFFFFFFF:FFFFFFFFFFFF
FFFFFFFFFFFFFFF
@A00721:190:HGJW7DSXY:4:2118:12852:15295 1:N:0:TCTGCAAG+GCTGCACT
GTATTAGGCCCGAAGATAGTGTTAGTCAGACGTCGAGTGTTAGACGAGACCAGTGTCTGGCTGGATCG
ACCAGCATTATGAATGCTGATCTGGTCGATATATTGCAGATTGCTATGGATAGGGGAAAAAAATCAAT
ATTTGACAGTCATAT
+
FFFFFFFFFFFFFFFFFFFFFFFFFFFFFFFFFFFFFFFFFFFFFFFFFFFFFFFFFFFFFFFFFFFFFF
FFFFFFFFFFFFFFFFFFFFFFFFFFFFFFFFFFFFFFFFFFFFFFFFFFFFFF:FFFFFFFFFFFFFFF
FFFFFFFFFFFFFFF
@A00721:190:HGJW7DSXY:4:1119:19208:12336 1:N:0:TCTGCAAG+GCTGCACT
CCCGAAGATAGTGTTAGTCAGACGTCGAGTGTTAGACGAGACCAGTGTCTGGCTGGATCGACCAGCAT
TATGAATGCTGATCTGGTCGATATATTGCAGATTGCTATGGATAGGGGAAAAAAATCAATATTTGACA
GTCATATTACTAAAG
+
FFFFFFFFFFFFFFFFFFF,FFFFFFFFFFFFFFFFFFFFFFFFFFFFFFFFFFFFFFFFFFFFFFFFFF
FFFFFFFFFFFFFFFFFFFFFFFFFFFFFFFFFFFFFFFFFFFFFFFFFF:FFFFFFFFFFFFFFFFFFF
FFF:FFFFFFFFFF:
@A00721:190:HGJW7DSXY:4:1260:14281:21559 1:N:0:TCTGCAAG+GCTGCACT
CCTGAAGATAGTGTTAGTCAGACGTCGAGTGTTAGACGAGACCAGTGTCTGGCTGGATCGACCAGCAT
TATGAATGCTGATCTGGTCGATATATTGCAGATTGCTATGGATAGGGGAAAAAAATCAATATTTGACA
GTCATATTACTAAA
+
FFFFFFFFFFFFFFFFFFFFFFFFFFFFFFFFFFFFFFFFFFFFFFFFFFFFFFFFFFFFFFFFFFFFFF
FFFFFFFFFFFFFF:FFFFFFFFFFFFFFFFFFFFFFFFFFFFFFFFFFFFFFFF:FFFFFFFFFFFFFF
FFFFFFFFFFFFFF
@A00721:190:HGJW7DSXY:4:1365:3396:3396 1:N:0:TCTGCAAG+GCTGCACT
CCCGAAGATAGTGTTAGTCAGACGTCGAGTGTTAGACGAGACCAGTGTCTGGCTGGATCGACCAGCAT
TATGAATGCTGATCTGGTCGATATATTGCAGATTGCTATGGATAGGGGAAAAAAATCAATATTTGACA
GTCATATTACTAAA
+
FFFFFFFFFFFFFFFFFFFFFFF:FFFFFFFFFFFFFFFF:FFFFFFFFFFFFFFFFFFFFFFFFFFFFF
FFFFFFFFFFFFFFFFFFFFFFFFFFFFFFF:FFFFFFFFFFFFFFFFFFFFFFFFFFFFFFFFFFFFFF
FFFFFFFFFFFFFF
@A00721:190:HGJW7DSXY:4:1436:10493:5822 1:N:0:TCTGCAAG+GCTGCACT
CCCGAAGATAGTGTTAGTCAGACGTCGAGTGTTAGACGAGACCAGTGTCTGGCTGGATCGACCAGCAT
TATGAATGCTGATCTGGTCGATATATTGCAGATTGCTATGGATAGGGGAAAAAAATCAATATTTGACA
GTCATATTACTAAAG
+
FFFFFFFFFFFFFFFFFFFFFFFFFFFFFFFFFFFFFFFFFFFFFFFFFFFFFFFFFFFFFFFF:FFFFF
FFFFFFFFFFFFFFFFFFFFFFFFFFFFFFFFFFFFFFFFFFFFFFFFFFFFF:FFFFFFFFFFFF:FFF
FFFFFFFFFFFFFFF
@A00721:190:HGJW7DSXY:4:1465:30544:5447 1:N:0:TCTGCAAG+GCTGCACT
CCCGAAGATAGTGTTAGTCAGACGTCGAGTGTTAGACGAGACCAGTGTCTGGCTGGATCGACCAGCAT
TATGAATGCTGATCTGGTCGATATATTGCAGATTGCTATGGATAGGGGAAAAAAATCAATATTTGACA
GTCATATTACTAAAG
+
FFFFFFFFFFFFFFFFFFFFFFFFFFFFFFFFFFFFFFFFFFFFFFFFFFFFFFFFFFFFFFFFFFFFFF
FFFFFFFFF:FFFFFFFFFFFFFFFFFFFFFFFFFFFFFFFFFFFFFFFFFFFFFFFFFFFFFFFFFFFF
FFFFFFFFFFFFFF:

```
@A00721:190:HGJW7DSXY:4:1613:29143:13354 1:N:0:TCTGCAAG+GCTGCACT
CCTGAAGATAGTGTTAGTCAGACGTCGAGTGTTAGACGAGACCAGTGTCTGGCTGGATCGACCAGCAT
TATGAATGCTGATCTGGTCGATATATTGCAGATTGCTATGGATAGGGGAAAAAAATCAATATTTGACA
GTCATATTACTAAA
+
FFFFFFFF:FFFFFFFFFFFFFFFFFFFFFFFFFFFFFFFFFFFFFFFFFFFFFF:FFFFFFFFFFF
FFFFFFFFFFFFFFFFFFFFFFFFFFFFFFFFFFFFFFFFF:FFFFFFFFFFFFFFFFFFFFFFFFF:
FFFFFFFFFFFFFF
@A00721:190:HGJW7DSXY:4:2175:19407:14716 1:N:0:TCTGCAAG+GCTGCACT
CCCGAAGATAGTGTTAGTCAGACGTCGAGTGTTAGACGAGACCAGTGTCTGGCTGGATCGACCAGCAT
TATGAATGCTGATCTGGTCGATATATTGCAGATTGCTATGGATAGGGGAAAAAAATCAATATTTGACA
GTCATATTACTAAAG
+
FFFFFFFFFFFFFFFFFFFFFFFFFFFFFFFFFFFFFFFFFFFFFFFFFFFFFFFFFFFF:FFFFFFF
FFFF:FFFFFFFFFFFFFFFFFFFFFFFFFFFFFFFFFFFFFFFFFFFFFFFFFFFFFFFFFFFFFFF
FFFFFFFFFFFFFFF
@A00721:190:HGJW7DSXY:4:2209:18701:31720 1:N:0:TCTGCAAG+GCTGCACT
CCTGAAGATAGTGTTAGTCAGACGTCGAGTGTTAGACGAGACCAGTGTCTGGCTGGATCGACCAGCAT
TATGAATGCTGATCTGGTCGATATATTGCAGATTGCTATGGATAGGGGAAAAAAATCAATATTTGACA
GTCATATTACTAAAG
+
FF:FFFFFFFFFFFFFFFFFFFFF,F:FF:,F,F:FFFFFFFFF:FFF:FFFFFFFFFF:F::FFFF:
FFFFFFF,F:FFFFFFF:FFFFFFF:F:,F:F,F,FFFFFFFFFFF:FFFFFFF,FFFFFFFFF:F::
:FFFFFFFFFFFFF:
@A00721:190:HGJW7DSXY:4:2226:15745:33144 1:N:0:TCTGCAAG+GCTGCACT
CCCGAAGATAGTGTTAGTCAGACGTCGAGTGTTAGACGAGACCAGTGTCTGGCTGGATCGACCAGCAT
TATGAATGCTGATCTGGTCGATATATTGCAGATTGCTATGGATAGGGGAAAAAAATCAATATTTGACA
GTCATATTACTAAAG
+
FFFFFFFFFFFFFFFFFFFFFFFFFFFFFFFFFFFFFFFFFFFFFFFFFFFFFFFFFFFFFFFFFFFF
FFFFFFFFF:FFFFFFFFFFFFFFFFFFFFFFFFFFFFFFFFFFFFFFFFFFFF:FFFFFFFFFF
FFFFFFFFFFFFFF:
@A00721:190:HGJW7DSXY:4:2233:20907:32033 1:N:0:TCTGCAAG+GCTGCACT
CCTGAAGATAGTGTTAGTCAGACGTCGAGTGTTAGACGAGACCAGTGTCTGGCTGGATCGACCAGCAT
TATGAATGCTGATCTGGTCGATATATTGCAGATTGCTATGGATAGGGGAAAAAAATCAATATTTGACA
GTCATATTACTAAAG
+
FFFFFFFFFFFFFFFFFFFFFFFFFFFFFFFFFFFFFFFFFFFFFFFFFFFFFFFFFFFFFFFFFFF:
FFFFFFFFFFFFFFFFFFFFFFFFFFFFFFFFFFFFFFFFFFFFFFFFFFFFFFFFFFFFFFFFFFFF
FFFFFFFFFFFFFFF
@A00721:190:HGJW7DSXY:4:2250:30364:6104 1:N:0:TCTGCAAG+GCTGCACT
CCCGAAGATAGTGTTAGTCAGACGTCGAGTGTTAGACGAGACCAGTGTCTGGCTGGATCGACCAGCAT
TATGAATGCTGATCTGGTCGATATATTGCAGATTGCTATGGATAGGGGAAAAAAATCAATATTTGACA
GTCATATTACTAAAG
+
FFFFFFFFFFFFFFFFFFFFFFFFFFFFFFFFFFFFFFFFFFFFFFFFFFFFFFFFFFFFFFFFFFFF
FFFFFFFFFFFFFFFFFFFFFFFFFFF:FFFFFFFF::FFFF:FFFFFFFFFFFFFFFFFFFFFFFFF
FFFFFFFFFFFFFFF
@A00721:190:HGJW7DSXY:4:2253:24840:24815 1:N:0:TCTGCAAG+GCTGCACT
CCCGAAGATAGTGTTAGTCAGACGTCGAGTGTTAGACGAGACCAGTGTCTGGCTGGATCGACCAGCAT
TATGAATGCTGATCTGGTCGATATATTGCAGATTTCTATGGATAGGGGAAAAAAATCAATATTTGACA
GTCATATTACTAAAG
+
FF:FFFFFF:,FF,:FFFFFFFFFFF,FFF,FFFFFF,FFFFFF,FFFFFFFFF:FFFFFFF:FFFFF
```

FFFFFFFFFFFFFFFFFFFFFFFFFFFFFFF:F,FFFFFFFFFF,FFFFFF:FFFFFF:FF,::FFF
:FFFFFF:F:FFFFF
@A00721:190:HGJW7DSXY:4:2261:23077:10864 1:N:0:TCTGCAAG+GCTGCACT
CCCGAAGATAGTGTTAGTCAGACGTCGAGTGTTAGACGAGACCAGTGTCTGGCTGGATCGACCAGCAT
TATGAATGCTGATCTGGTCGATATATTGCAGATTGCTATGGATAGGGGAAAAAAATCAATATTTGACA
GTCATATTACTAAAG
+
FFFFFFFFFFFF:::FFFFFFFFFFFFFFFFFFFFFFFFFFFFFFFFF,F:,FFF:FFFFFFF,FFFF
FFFFFF::FFFFFFFFFFFFF:FFFFFFFFFFFFFFFFFFFFFFFFFFFF,FFFFFFFFFFFF::FFFF
F::F:F:FFF,FFF:
@A00721:190:HGJW7DSXY:4:2261:23140:11819 1:N:0:TCTGCAAG+GCTGCACT
CCCGAAGATAGTGTTAGTCAGACGTCGAGTGTTAGACGAGACCAGTGTCTGGCTGGATCGACCAGCAT
TATGAATGCTGATCTGGTCGATATATTGCAGATTGCTATGGATAGGGGAAAAAAATCAATATTTGACA
GTCATATTACTAAAG
+
FFFFFFFFFFFF:FFFFFFFFFFFFFFFFFFFFFFFFFFFFFFFFFFFFFFFFFFFFFFFFFF:FFFFF
FFFFFFFFFFFFF:FFFFFFFFFFFFFFFFFFFFFFFFFF:FFFF:FFFFFFFFFFFFFFFFFFFFFFF
FFFFFFFFFFFFFFF
@A00721:190:HGJW7DSXY:4:2502:7383:6136 1:N:0:TCTGCAAG+GCTGCACT
CCCGAAGATAGTGTTAGTCAGACGTCGAGTGTTAGACGAGACCAGTGTCTGGCTGGATCGACCAGCAT
TATGAATGCTGATCTGGTCGATATATTGCAGATTGCTATGGATAGGGGAAAAAAATCAATATTTGACA
GTCATATTACTAAA
+
FFFFFFFFFFFFFFFFFFFFFFFFFFFFFFFFFFFFFFFFFFFFFFFFFFFFF:FFFFFFFFFFFFFF
FFFFFFFFFFFFFFFFFFFFFFFFFFFF,FFFFFFFFFFFFFFFFFFFFFFFFFFFFFFFFFFFFFFF
FF:FFFFFFFFFFF
@A00721:190:HGJW7DSXY:4:2531:19217:19930 1:N:0:TCTGCAAG+GCTGCACT
CCCGAAGATAGTGTTAGTCAGACGTCGAGTGTTAGACGAGACCAGTGTCTGGCTGGATCGACCAGCAT
TATGAATGCTGATCTGGTCGATATATTGCAGATTGCTATGGATAGGGGAAAAAAATCAATATTTGACA
GTCATATTACTAAAG
+
FFFFFFFFFFFFFFFFFFFFFFFFFFFFFFFFFFFFFFFFFFFFFFFFFFFFFFFFFFFFFFFFFFFF
FFFFFFFFFFFFFFFFFFFFFFFFFFFFF:FFFFFFFFF,FFFFFFFFFFFFFFFFFFFFFFFFFFFFF
FFFFFFFFFFFFFFF
@A00721:190:HGJW7DSXY:4:2541:15203:6590 1:N:0:TCTGCAAG+GCTGCACT
CCCGAAGATAGTGTTAGTCAGACGTCGAGTGTTAGACGAGACCAGTGTCTGGCTGGATCGACCAGCAT
TATGAATGCTGATCTGGTCGATATATTGCAGATTGCTATGGATAGGGGAAAAAAATCAATATTTGACA
GTCATATTACTAAAG
+
FFFFFFFFFFFFFFFFFFFFFFFFFFFFFFFFFFFFFFFFFFFFFFFFFFFFFFFFFFFFFFFFFFFF
FFFFFFFFFFFFFFFFFFFFFFFFFFFFFFFFFFFFFFFFFFFFFFFFFFFFFFFFFFFFFFFFFFFF
FFFFFFFFFFFFFFF
@A00721:190:HGJW7DSXY:4:1135:20817:17879 1:N:0:TCTGCAAG+GCTGCACT
CGAAGATAGTGTTAGTCAGACGTCGAGTGTTAGACGAGACCAGTGTCTGGCTGGATCGACCAGCATTA
TGAATGCTGATCTGGTCGATATATTGCAGATTGCTATGGATAGGGGAAAAAAATCAATATTTGACAGT
CATATTACTAAAGAA
+
FFFFFFFFFFFFFF:FFFFFFFFFFFFFFFFFFFFFFFFFFFFFFFFFFFFFFFFFFFFFFFFFFFFF
FFFFFFFFFFFFFFFFFFFFFFFFFF:FFFFFF:FFFFFFFFFFFFFFFFFFFFFFFFFFFFFFFFFF
FFFFFFFFFFFFFFF
@A00721:190:HGJW7DSXY:4:1154:14778:25676 1:N:0:TCTGCAAG+GCTGCACT
CGAAGATAGTGTTAGTCAGACGTCGAGTGTTAGACGAGACCAGTGTCTGGCTGGATCGACCAGCATTA
TGAATGCTGATCTGGTCGATATATTGCAGATTGCTATGGATAGGGGAAAAAAATCAATATTTGACAGT
CATATTACTAAAGAA

```
+
FFFFFFFFFFFFFFFFFFFFFFFFFFFFFFFFFFFFFFFFFFFFFFFFFFFFFFFFFFFFFFFFFFFFFF
FFFFFFFFFFFFFFFFFFFFFFFFFFFFFFFFFFFFFFFFFFFFFFFFFFFFFFFFFFFFFFFFFFFFFF
FFFFFFFFFFFFF:F
@A00721:190:HGJW7DSXY:4:1301:15628:24831 1:N:0:TCTGCAAG+GCTGCACT
CGAAGATAGTGTTAGTCAGACGTCGAGTGTTAGACGAGACCAGTGTCTGGCTGGATCGACCAGCATTA
TGAATGCTGATCTGGTCGATATATTGCAGATTGCTATGGATAGGGGAAAAAAATCAATATTTGACAGT
CATATTACTAAAGAA
+
FFFFFFFFFFFFFFFFFFFFFFFFFFFFFFFFFFFFFFFFFFFFFFFFFFFFFFFFFFFFFFFFFFFFFF
FFFFFFFFFFFFFFFFFFFFFFFFFF:FFFFFFF:FFFFFFFFFFFFFFF:FFFFFF:FFFFFFFFF:F
FFFFFFFFFFFFFFF
@A00721:190:HGJW7DSXY:4:1301:15682:24893 1:N:0:TCTGCAAG+GCTGCACT
CGAAGATAGTGTTAGTCAGACGTCGAGTGTTAGACGAGACCAGTGTCTGGCTGGATCGACCAGCATTA
TGAATGCTGATCTGGTCGATATATTGCAGATTGCTATGGATAGGGGAAAAAAATCAATATTTGACAGT
CATATTACTAAAGAA
+
FFFFFFFFFFFFFFFFFFFFFFFFFFFFFFFFFFFFFFFFFFFFFFFFFFFF:FFFFFFFFFFFFFFF
FFFFFFFFFFFFFFFFFFFFFFFFFF:FFFFFFFFF:FFFFF:FFFFFFFFFFFFFFFFFFFFFFFFFF
FFFFFFF:,FFFFFF
@A00721:190:HGJW7DSXY:4:1428:9833:15139 1:N:0:TCTGCAAG+GCTGCACT
CGAAGATAGTGTTAGTCAGACGTCGAGTGTTAGACGAGACCAGTGTCTGGCTGGATCGACCAGCATTA
TGAATGCTGATCTGGTCGATATATTGCAGATTGCTATGGATAGGGGAAAAAAATCAATATTTGACAGT
CATATTACTAAAGAA
+
FFFFFFFFFFFFFFFFFFFFFFFFFFFFFFFFFFFFFFFFFFFFFFFFFFFFFFFFFFFFFFFFFFFFFF
FFFFFFFFFFFFFFFFFFFFFFFFFFFFFFFFFFFFFFFFFFFFFFFFFFFFFFFFFFFFFFFFFFFFFF
FFFFFFFFFFFFFFF
@A00721:190:HGJW7DSXY:4:1572:26277:30655 1:N:0:TCTGCAAG+GCTGCACT
CGAAGATAGTGTTAGTCAGACGTCGAGTGTTAGACGAGACCAGTGTCTGGCTGGATCGACCAGCATTA
TGAATGCTGATCTGGTCGATATATTGCAGATTGCTATGGATAGGGGAAAAAAATCAATATTTGACAGT
CATATTACTAAAGAA
+
FFFFFFFFF:FFFFFFFFFFFFFFFFFFFFFFFFFFFFFFFFFFFFFFFFFF:FFFFFFFFFFFFFFF
FFFFFFFFFFFFFFFFFFFFFFFFFFFFFFFFFFFF,FFFFFFFFFFFFFFFFFFFFFFFFFFFFFFFFF
FFFFFFFFFFFFFFF
@A00721:190:HGJW7DSXY:4:1576:22173:4382 1:N:0:TCTGCAAG+GCTGCACT
CGAAGATAGTGTTAGTCAGACGTCGAGTGTTAGACGAGACCAGTGTCTGGCTGGATCGACCAGCATTA
TGAATGCTGATCTGGTCGATATATTGCAGATTGCTATGGATAGGGGAAAAAAATCAATATTTGACAGT
CATATTACTAAAGAA
+
FFFFFFFFFFFFFFF:FFFFFFFFFFFFFFFFFFFFFFFFFFFFFFFFF:FFFFFFFFFFFFFFFFFFFF
FFFFFFFFFFFFFFFFFFFFFFFFFFFFFFFFFFFFFFFFFFFFFFFFFFFFFFFFFFFFFFFFFFFFFF
FFFFFFFFFF:FFFF
@A00721:190:HGJW7DSXY:4:2319:13277:26334 1:N:0:TCTGCAAG+GCTGCACT
CGAAGATAGTGTTAGTCAGACGTCGAGTGTTAGACGAGACCAGTGTCTGGCTGGATCGACCAGCATTA
TGAATGCTGATCTGGTCGATATATTGCAGATTGCTATGGATAGGGGAAAAAAATCAATATTTGACAGT
CATATTACTAAAGAA
+
FFFFFFFFFFFFFFFFFFFFFFFFFFFFFFFFFFFFFFFFFFFFFFFFFFFFFFFFFFFFFFFFFFFFFF
FFFFFFFFFFFFFFFFFFFFFFFFFFFFFFFFFFFFFFFFFFFFFFFFFFFFFFFFFFFFFFFFFFFFFF
FFFFFFFFFFFFFFF
@A00721:190:HGJW7DSXY:4:2361:31530:26694 1:N:0:TCTGCAAG+GCTGCACT
CGAAGATAGTGTTAGTCAGACGTCGAGTGTTAGACGAGACCAGTGTCTGGCTGGATCGACCAGCATTA
```

TGAATGCTGATCTGGTCGATATATTGCAGATTGCTATGGATAGGGGAAAAAAATCAATATTTGACAGT
CATATTACTAAAGAA
+
FFFFFFFFFFFFFFFFFFFFFFFFFFFFFFFFFFFFFFFFFFFFFFFFFFFFFFFFFFFFFFFFFFFFFF
FFFFFFFFF:FFFFFFFFFFFFFFFFFFFFFFFFFFFFFFFFFFFFF:FFFFFFFFFFFFFF:FFFF
FFFFFFF:F:FFFFF
@A00721:190:HGJW7DSXY:4:2458:26386:5134 1:N:0:TCTGCAAG+GCTGCACT
CGAAGATAGTGTTAGTCAGACGTCGAGTGTTAGACGAGACCAGTGTCTGGCTGGATCGACCAGCATTA
TGAATGCTGATCTGGTCGATATATTGCAGATTGCTATGGATAGGGGAAAAAAATCAATATTTGACAGT
CATATTACTAAAGAA
+
FFFFFFFFFFFFFFFFFFFFFFFFFFFFFFFFFFFFFFFFFFFFFFFFFFFFFFFFFFFFFFFFFFFFFF
FFFFFFFFFFFFFFFFFFFFFFFFFFFFFFFFFFFF:FFFF,FFFFFFFFFFFFFFFFFFFFFFFFFFF
FFFFFFFFF:FFFFF
@A00721:190:HGJW7DSXY:4:2471:14290:9580 1:N:0:TCTGCAAG+GCTGCACT
CGAAGATAGTGTTAGTCAGACGTCGAGTGTTAGACGAGACCAGTGTCTGGCTGGATCGACCAGCATTA
TGAATGCTGATCTGGTCGATATATTGCAGATTGCTATGGATAGGGGAAAAAAATCAATATTTGACAGT
CATATTACTAAAGAA
+
FFFFFFFFFFFFFFFFFFFFFFFFFFFFFFFFFFFFFFFFFFFFFFFFFFFFFFFFFFFFFFFFFFFFFF
FF:FFFFFFFFFFFFFFFFFFFFF:FFFFFFFFFFFFFFFFFFFFFFFFFFFFFFFFFFFFFFFFFFFF
FFFFFFFFFFF:FFF
@A00721:190:HGJW7DSXY:4:2542:12988:22764 1:N:0:TCTGCAAG+GCTGCACT
CGAAGATAGTGTTAGTCAGACGTCGAGTGTTAGACGAGACCAGTGTCTGGCTGGATCGACCAGCATTA
TGAATGCTGATCTGGTCGATATATTGCAGATTGCTATGGATAGGGGAAAAAAATCAATATTTGACAGT
CATATTACTAAAGAA
+
FFFFFFFFFFF::FFFFFFFFFFFFFFFFFFFFFFFFFFFFFFFFFFFFFFFFFFFFFFFFFFFFFFFFF
FFFFFFFFFFFFFFFFFFFFFFFFFFFFFFFFFFFFFFFF:FFFFFFFFFFFFFFFFFFFFFFFFFFFF
FFFFFFFFFFFFFFF
@A00721:190:HGJW7DSXY:4:2572:29568:34820 1:N:0:TCTGCAAG+GCTGCACT
CGAAGATAGTGTTAGTCAGACGTCGAGTGTTAGACGAGACCAGTGTCTGGCTGGATCGACCAGCATTA
TGAATGCTGATCTGGTCGATATATTGCAGATTGCTATGGATAGGGGAAAAAAATCAATATTTGACAGT
CATATTACTAAAGAA
+
FFFFF:F:FFFFFFFFFFFFFFFFFFFFFFFFFFFFFFFFFFFFFFFFFFFFFFFFFFFFFFFFFF:FFF
FFFFFFFFFFFFFFFFFFFFFFFFFFFFFFFFFFFFFFFF:FF:FFFFFFFFFFFFFFFFFF:FFF:F
FFFFFFFF:FFFFFF
@A00721:190:HGJW7DSXY:4:2666:19298:20885 1:N:0:TCTGCAAG+GCTGCACT
CGAAGATAGTGTTAGTCAGACGTCGAGTGTTAGACGAGACCAGTGTCTGGCTGGATCGACCAGCATTA
TGAATGCTGATCTGGTCGATATATTGCAGATTGCTATGGATAGGGGAAAAAAATCAATATTTGACAGT
CATATTACTAAAGAA
+
FFF,FFFFFFFFFFFFFFFFFFFFFFFFFFFFFFFFFFFFFFFFFFFFFFFFFFFFFFFFFFFFFFFFFF
FFFFFFFFFFFFFFFFFFFFFFFFFFFFFFFFFFFFFFFFFFFFFFFFFFFFFFFFFFFFFFFFFFFFFF
FFFFFFFFFFFFFF:
@A00721:190:HGJW7DSXY:4:2168:10312:4852 1:N:0:TCTGCAAG+GCTGCACT
GAAGATAGTGTTAGTCAGACGTCGAGTGTTAGACGAGACCAGTGTCTGGCTGGATCGACCAGCATTAT
GAATGCTGATCTGGTCGATATATTGCAGATTGCTATGGATAGGGGAAAAAAATCAATATTTGACAGTC
ATATTACTAAAGAA
+
FFFFFFFFF:F:FFF:FF:FFFF:FFFFF:FFFFFFFFFFFF,FFF:F,FFFFFFFFFFF:FFFFFF
:FF:FFFFFFFF,F:FFF:FFFFFF,F,FFFFFFFF:FF:F,:FFFFF,FFFF,FFFFF,FFFFFF:F
FFFFFFFFFFF,FF

@A00721:190:HGJW7DSXY:4:1378:7265:3709 1:N:0:TCTGCAAG+GCTGCACT
CTTATTTCTTTCTTTAGTAATATGACTGTCAAATATTGATTTTTTTCCCCTATCCATAGCAATCTGCA
ATATATCGACCAGATCAGCATTCATAATGCTGGTCGATCCAGCCAGACACTGGTCTCGTCTAACACTC
GACGTCTGACTAACA
+
FFFFFFFFFFFFFFFFFFFFFFFFFFFFFFFFFFFFFFFF::FFFFFFFFFFFFFFFFFFFFFFFFFFFF
FFFFFFFFFFFFFFFFFFFFFFFFF:FFFFFFFFFFFFFF:FFFF:FFFFFFFFFFFFFFFFFFFFFFFF
FFFFFFFFFFFFFFF
@A00721:190:HGJW7DSXY:4:1450:30074:4288 1:N:0:TCTGCAAG+GCTGCACT
CTTATTTCTTTCTTTAGTAATATGACTGTCAAATATTGATTTTTTTCCCCTATCCATAGCAATCTGCA
ATATATCGACCAGATCAGCATTCATAATGCTGGTCGATCCAGCCAGACACTGGTCTCGTCTAACACTC
GACGTCTGACTAACA
+
FFFFFFFFFFFFFFFFFFFFFFFFFFFFFFFFFFFFFFFFFF:F:FFFFFF:FFFFFFFFF:FFFFFFFF
F:F:FFF,FF:FFFFF:FFFFFFFFFFFFFFFF:FFFFFF:FFFFFFFFFFFFFFFFFF,FFFFFFFFFF
FFFFFFFFFFFFF:F
@A00721:190:HGJW7DSXY:4:2238:20889:19445 1:N:0:TCTGCAAG+GCTGCACT
CTTATTTCTTTCTTTAGTAATATGACTGTCAAATATTGATTTTTTTCCCCTATCCATAGCAATCTGCA
ATATATCGACCAGATCAGCATTCATAATGCTGGTCGATCCAGCCAGACACTGGTCTCGTCTAACACTC
GACGTCTGACTAACA
+
FFFFFFFFFFFFFFFFFFFFFFFFFFFFFFFFFFFFFFFFFFFFFFFFFFFFFFFFFFFFFFFFFFFFFF
F:FFFFFFFFFFFFFFFFFFFFFFFFFFFFFFFFFFFFFFFFFFFFFFFFF:FFFFFFFFF:FFFFFFFF
FFFF:FFFFFFFFFF
@A00721:190:HGJW7DSXY:4:2245:12689:12696 1:N:0:TCTGCAAG+GCTGCACT
CTTATTTCTTTCTTTAGTAATATGACTGTCAAATATTGATTTTTTTCCCCTATCCATAGCAATCTGCA
ATATATCGACCAGATCAGCATTCATAATGCTGGTCGATCCAGCCAGACACTGGTCTCGTCTAACACTC
GACGTCTGACTAACA
+
FFFFFFFFF:FFFFFFFF::FFFFFFFFFFFFFFFFFFFFFFFFFFFFFFFFFFFFFFFFF:FFFFFFFF
:FFFFFFFFFFFFFFFFFFFFFFFFFFFFFFFFFFFFFFFFFFFFFFFFFFFFFFFFFFFFFFFFFFFFF
FFFFFFFFFFFFFFF
@A00721:190:HGJW7DSXY:4:2308:15700:31657 1:N:0:TCTGCAAG+GCTGCACT
CTTATTTCTTTCTTTAGTAATATGACTGTCAAATATTGATTTTTTTCCCCTATCCATAGCAATCTGCA
ATATATCGACCAGATCAGCATTCATAATGCTGGTCGATCCAGCCAGACACTGGTCTCGTCTAACACTC
GACGTCTGACTAACA
+
FFFFFFFFFFFFFFFFFF:FFFFFFFFFFFFFFFFFFFFFF:F:FFFFFFFFFFFFFFFFFFFFFFFFFF
FFFF:FF:FFFFFFFFFFFFFFFFFFFFFFFFFFFF:FFFFFF:FFFFFFFFFFFFFFFFFFFFFFFFFF
,FFFFFFFFFF:FFF
@A00721:190:HGJW7DSXY:4:2378:27877:13322 1:N:0:TCTGCAAG+GCTGCACT
CTTATTTCTTTCTTTAGTAATATGACTGTCAAATATTGATTTTTTTCCCCTATCCATAGCAATCTGCA
ATATATCGACCAGATCAGCATTCATAATGCTGGTCGATCCAGCCAGACACTGGTCTCGTCTAACACTC
GACGTCTGACTAACA
+
FFFFFFFFFFFFFFFFFF:FFFFFFFFFFFFF:FFFFFFFFFFFFFFFFFFFFFFFFFFFFFFFFFFFFF
FFFFFFFFFFFFFFFFFFFFFFFFFFFFFFFFFFFFFFFFFFFFFFFFFFFFFFFFFFFFFFFFFFFFFF
FFFFFFFFFFFFFFF
@A00721:190:HGJW7DSXY:4:1147:28049:2503 1:N:0:TCTGCAAG+GCTGCACT
TAATAAAATTACAAATTTTATTTCATTTCACGTCTTATTTCTTTCTTTAGTAATATGACTGTCAAATA
TTGATTTTTTTCCCCTATCCATAGCAATCTGCAATATATCGACCAGATCAGCATTCATAATGCTGGTC
GATCCAGCCAGACAC
+
FFFFFFFFFFFFFFFF:FFFFFFFFFFFFFFFFFFFF,FFFFFFFF,FFFFFF:FFFFFFFFFFFFFFFF

:FFFFFFFFFFFFFFFFFFFFFFF:F:FFF:FFFFFFFFFFFFFFFFFFFFF:FFFFFFFFFFFFFFFFF
FFFFFFFFFFFFFFF
@A00721:190:HGJW7DSXY:4:1344:14850:8265 1:N:0:TCTGCAAG+GCTGCACT
TAATAAAATTACAAATTTTATTTCATTTCACGTCTTATTTCTTTCTTTAGTAATATGACTGTCAAATA
TTGATTTTTTTCCCCTATCCATAGCAATCTGCAATATATCGACCAGATCAGCATTCATAATGCTGGTC
GATCCAGCCAGACAC
+
FFFFFFFFFFFFFFFFFFFFFFFFFFFFFFFFFFFFFFF:FFFFFFF::FFFFFFFFFFFFFFFF:FFFF
FFFFFFFFFFFFFFFFFFFFFFFFFFFFFFFFFFFFFFF:FFFFFFFFFFFFFFFFFFFFFFFFFFFFFF
FFFFFFFFFFFFFFF
@A00721:190:HGJW7DSXY:4:1410:19777:30859 1:N:0:TCTGCAAG+GCTGCACT
TAATAAAATTACAAATTTTATTTCATTTCACGTCTTATTTCTTTCTTTAGTAATATGACTGTCAAATA
TTGATTTTTTTCCCCTATCCATAGCAATCTGCAATATATCGACCAGATCAGCATTCATAATGCTGGTC
GATCCAGCCAGACAC
+
FFFFFFFFFFFFFFFFFFFFFFFFFFFFFFFFFFFF:FFF:FFFFFFFFFFFFFFFFFFFFFFFFFFFFF
FFFFFFFFFFFFFFFFFFFFFFFFFFFFFFFFFFFFFFFFFFFFFFFFFFFFFFFFFFFFFFFFFFFFFF
FFFFFFFFFFFFFFF
@A00721:190:HGJW7DSXY:4:1502:6289:22811 1:N:0:TCTGCAAG+GCTGCACT
TAATAAAATTACAAATTTTATTTCATTACACGTCTTATTTCTTTCTTTAGTAATATGACTGTCAAATA
TAGATTTTTTTCCCCTATCCATAGCAATCTGCAATATATCTACCAGATCAGCATTCATAATGCTGGTC
GATCCAGCCAGACAC
+
FFFFFF:F,F:FFF:FFFF:FFF:FFF,FFFFFF:FFFFF:,F:FFF:,,,,FFF::FF:,,F,FFF:,,
F,FFFFF:F,:::,FFF,:FFF:FFFFFFF:,:FFFFFF,F,FF,:FFFFFFFFFF:,:FFFF::F,:FF
FF:F:F:F:F,FFFF
@A00721:190:HGJW7DSXY:4:2102:25518:15374 1:N:0:TCTGCAAG+GCTGCACT
TAATAAAATTACAAATTTTATTTCATTTCACGTCTTATTTCTTTCTTTAGTAATATGACTGTCAAATA
TTGATTTTTTTCCCCTATCCATAGCAATCTGCAATATATCGACCAGATCAGCATTCATAATGCTGGTC
GATCCAGCCAGACAC
+
FFFFFFFFFFFFFFF:F:FFFFFFFFFFFFFFFFF:FFFFFFFFFFFFFFFFFFFFFFFFFFFFFFFFFF
FFFFFFFFFFFFF:FFFFFFFFFFFFFFFFFFFFFFFFFFFFFFFFFFFFFFFFF::FFFFFFFFFFFFF
FFFF:FFFFFFFFFF
@A00721:190:HGJW7DSXY:4:2251:32072:28886 1:N:0:TCTGCAAG+GCTGCACT
TAATAAAATTACAAATTTTATTTCATTTCACGTCTTATTTCTTTCTTTAGTAATATGACTGTCAAATA
TTGATTTTTTTCCCCTATCCATAGCAATCTGCAATATATCGACCAGATCAGCATTCATAATGCTGGTC
GATCCAGCCAGACAC
+
FFFFFFFFFFFFF:F:FFFFFFFFFFFFFFFFFFFFF:::F:FFFFFFFFFFFFFFFFF:FFFFFF::F:
FFFFFFFFF:FFFFFFFFFFFFFFFFFFF:FFFFFFFFFFFFFF,FFFFFFFFFFFFFFFFFF:,FFFFF
,:FFFFFFFFFFFFF
@A00721:190:HGJW7DSXY:4:2251:32443:28933 1:N:0:TCTGCAAG+GCTGCACT
TAATAAAATTACAAATTTTATTTCATTTCACGTCTTATTTCTTTCTTTAGTAATATGACTGTCAAATA
TTGATTTTTTTCCCCTATCCATAGCAATCTGCAATATATCGACCAGATCAGCATTCATAATGCTGGTC
GATCCAGCCAGACAC
+
FFFFFFFFFFFFFFFFFFFFFFFFFFFFFFFFFF:FFFFFF,FFFFFFFFFFFFFFFFF:FFFFFFFFFF
FFF:FFFFFFF:FFFFFFFFFFF:FFFF:FFFFFFFFFFF:FFFFFFFFFFFF:FFFFFFFFFFFFFF:F
FFF:FFFFFFFFFFF
@A00721:190:HGJW7DSXY:4:2262:27977:20603 1:N:0:TCTGCAAG+GCTGCACT
TAATAAAATTACAAATTTTATTTCATTTCACGTCTTATTTCTTTCTTTAGTAATATGACTGTCAAATA
TTGATTTTTTTCCCCTATCCATAGCAATCTGCAATATATCGACCAGATCAGCATTCATAATGCTGGTC
GATCCAGCCAGACAC

```
+
FFFFFFFFFFFFFFFFFFFFFFFFFFFFFFFFFFFFFFFFFFFFFFFFFFFFFFFFFFFFFFFFFFFFFFF
FFFFFFFFFFFFF:FFFFFFFFFFFFFFFFFFFFFFFFFF:FFFFFFFFFFFFFFFFFFFFFFFFFFFFFF
FFFFFFFFFFFFFFF
@A00721:190:HGJW7DSXY:4:2372:24641:10567 1:N:0:TCTGCAAG+GCTGCACT
TAATAAAATTACAAATTTTATTTCATTTCACGTCTTATTTCTTTCTTTAGTAATATGACTGTCAAATA
TTGATTTTTTTCCCCTATCCATAGCAATCTGCAATATATCGACCAGATCAGCATTCATAATGCTGGTC
GATCCAGCCAGACAC
+
FFFFFFF:FFFFFFF:FFFFFFFFFFFFFFFFFFFFFFFF:FFFFFFFFFFFFFF:FF:FFFFFFFFF
FF:FFFFFFFFFFFFFF:FFFFFFFFFF:FFFFFFFFFFFFFFFFFFFFFFFFFFFFFFF:FFFFFFFF
FFFFFFFFFFFFFF:
@A00721:190:HGJW7DSXY:4:1156:4716:25222 1:N:0:TCTGCAAG+GCTGCACT
CGATGTATTAGATGATTAATAAAATTACAAATTTTATTTCATTTCACGTCTTATTTCTTTCTTTAGTA
ATATGACTGTCAAATATTGATTTTTTTCCCCTATCCATAGCAATCTGCAATATATCGACCAGATCAGC
ATTCATAATGCTGGT
+
FFFFFFF:FFFFFFFFFFFFFFFFFFFFFFFF,FFFFFFFFFFFFFFFF:FF:F,FFFFFFFFFFFF
:F:FFFFFFFF:FF,FFFFFFFFFFFFFFFFFFFFFFFFFFFFFFFFFFFFFFFFFFFFFFFFFFFFF
FFFFFFFFFFFFFFF
@A00721:190:HGJW7DSXY:4:1555:32696:35916 1:N:0:TCTGCAAG+GCTGCACT
CGATGTATTAGATGATTAATAAAATTACAAATTTTATTTCATTTCACGTCTTATTTCTTTCTTTAGTA
ATATGACTGTCAAATATTGATTTTTTTCCCCTATCCATAGCAATCTGCAATATATCGACCAGATCAGC
ATTCATAATGCTGGT
+
FFFFFFFFFFFFF:FFFFFFFFFFFFFFFFFFFFFFFFFFFFFFF:FF::FFFF,:F::FFFF,FFFFF
FFF,,,FFFFFFFFFFFFFFFFFFFFFFF:FFFFFFFFFF,:FFFFFFFFFFFFFFFFFFFFFFFF,FF,
FF:FF:FFF::::,F
@A00721:190:HGJW7DSXY:4:2546:29496:10895 1:N:0:TCTGCAAG+GCTGCACT
CGATGTATTAGATGATTAATAAAATTACAAATTTTATTTCATTTCACGTCTTATTTCTTTCTTTAGTA
ATATGACTGTCAAATATTGATTTTTTTCCCCTATCCATAGCAATCTGCAATATATCGACCAGATCAGC
ATTCATAATGCTGGT
+
FFFFFFFFFFFFFFFFFFFFFFFFFFFFFFFFFFFFFFFFFFFFFFFFFFFF,FFFFFFFFFFFFFF
FFFFFFFFFFFFFFFFFFFFFFFFFFFF,FFFFFFFFFFFFFFFFFFFFFFFFFFFFFFFFFFFFFFFF
FFFF:FFFFFFFF::
@A00721:190:HGJW7DSXY:4:1357:31611:30248 1:N:0:TCTGCAAG+GCTGCACT
CGATGTATTAGATGATTAATAAAATTACAAATTTTATTTCATTTCAAGTCTTATTTCTTTCTTTAGTA
ATATGACTGTCAAATATTGATTTTTTTCCCCTATCCATAGCAATATTCAATATATCGAACAGATCAGC
AATCATAATTCTGT
+
FFFF,FF:,FFFF::,,F:FFFFF:,FFFFF,FFFFF,FFFFFFFF,F,:,,F:,FFF,FF,,:F,FF
:FF,,F,:F:,F:FFFF,FFF,:F,FFFF,F::,,FFFF,FFF,,,,,FFFFFFFF,F,FFFFF,F,:
:,:,F,FFF:F,::
@A00721:190:HGJW7DSXY:4:1269:22706:22247 1:N:0:TCTGCAAG+GCTGCACT
GGTCGATATATTGCAGATTGCTATGGATAGGGGAAAAAAATCAATATTTGACAGTCATATTACTAAAG
AAAGAAATAAGACGTGAAATGAAATAAAATTTGTAATTTTATTAATCATCTAATACATCGAACGCTAT
TGGCTGGTGTATTGG
+
FFFFFFFFFFFFFFFFFFFFFFFFFFFFFFFFFFFFFFFFFFFFFFFFF:FFFFFFFFFFFFFFFFFF
FFFFFFFFFFFFFFFFFFFFFFFFFFFFFFFFFFFFFFFFFFFFFFFFFFFFFFFFFFFFFFFFFFFF
F:FFFFFFFFFFFFF
@A00721:190:HGJW7DSXY:4:1530:18123:29246 1:N:0:TCTGCAAG+GCTGCACT
GGTCGATATATTGCAGATTGCTATGGATAGGGGAAAAAAATCAATATTTGACAGTCATATTACTAAAG
```

AAAGAAATAAGACGTGAAATGAAATAAAATTTGTAATTTTATTAATCATCTAATACATCGAACGCTAT
TGGCTGGTGTATTGG
+
FFFFFFFFFFFFFFFFFFFFFFFFFF,FFFFFFFFFFFFFFFFFFFFFFFFFFFFFFFFFFFFFFFFF
FFFF,FFFFFFFFFFFFFFFFFFF:FFFFFFFFFFF,FFFFFFFFFFFFFFFFFFFFFFFFFFFF:F:
FFFFFFFFFFFFFFF
@A00721:190:HGJW7DSXY:4:1618:3586:16532 1:N:0:TCTGCAAG+GCTGCACT
GGTCGATATATTGCAGATTGCTATGGATAGGGGAAAAAAATCAATATTTGACAGTCATATTACTAAAG
AAAGAAATAAGACGTGAAATGAAATAAAATTTGTAATTTTATTAATCATCTAATACATCGAACGCTAT
TGGCTGGTGTATTGG
+
FFFFF::F:FFFFFFFFFFFFFF,:FFFFFFFFFFFFFFFFFFFFFFFFFFFFFFFFFF:FFFFFFFF
FFF:FFFFFFFFF,F,FFFFFFFFFFFFFFF,FFFF:FFFFFFFFFFFFFFFFFF,FF,:FF,FFFF:
:FF:FFF,FFFFF,F
@A00721:190:HGJW7DSXY:4:2621:10791:21997 1:N:0:TCTGCAAG+GCTGCACT
GGTCGATATATTGCAGATTGCTATGGATAGGGGAAAAAAATCAATATTTGACAGTCATATTACTAAAG
AAAGAAATAAGACGTGAAATGAAATAAAATTTGTAATTTTATTAATCATCTAATACATCGAACGCTCT
TGGCTGGTGTATTGG
+
FFFFFFFFFFFFFFFF:FFFFF:F,FFFFFFFFFFFFFFF,,FF:F,F,FFFF:FFF:FFFFF:FFF:
FFFFF:F:FF,FFF,FFFFF,FFF:FFFFF:FFFF::,,FFFFFF:FFFFFFFFFFFF:F,FF:,F,F
,FF:FFFFFF:FFFF
@A00721:190:HGJW7DSXY:4:1269:8486:25833 1:N:0:TCTGCAAG+GCTGCACT
GCCTCCACCAAATCCTGATCTGAATCACTTTCCGTATTTAATAGGTCACTATGACCAGATGTCAGATA
CATTAATTCCAATACACCAGCCAATAGCGTTCGATGTATTAGATGATTAATAAAATTACAAATTTTAT
TTCATTTCACGTCTT
+
FFFFFFFFFFFFFFFFFFFFFFFFFFFFFFFFFFFF:FFFFFFFFFFFFFFFFFFFFFFFFFFFFFFF
FFFFFFFFFFFFFFFFFFFFFFFFFFFFFFFFFFFFFFF:FFFFFFFFFFFFFFF:FFFFFFFFFFFF
FFFFFFFFFFFFFFF
@A00721:190:HGJW7DSXY:4:1611:6298:24987 1:N:0:TCTGCAAG+GCTGCACT
GCCTCCACCAAATCCTGATCTGAATCACTTTCCGTATTTAATAGGTCACTATGACCAGATGTCAGATA
CATTAATTCCAATACACCAGCCAATAGCGTTCGATGTATTAGATGATTAATAAAATTACAAATTTTAT
TTCATTTCACGTCTT
+
FFF:FFFFFFFFFF:FFFFFFFFFFFFFFFFFFFFF::FFF:FFFFFF:FFFFFFFFFFFFF:FFFFF
FFFFFFFFF:FFFFFFFFFFFFFFFFFFFFFFFF:FF:FFFFFFFFFFF:FFFFFFFFFFFFF:FFFF
FFFFFFF,F,FFFFF
@A00721:190:HGJW7DSXY:4:2621:3432:10942 1:N:0:TCTGCAAG+GCTGCACT
GCCTCCACCAAATCCTGATCTGAATCACTTTCCGTATTTAATAGGTCACTATGACCAGATGTCAGATA
CATTAATTCCAATACACCAGCCAATAGCGTTCGATGTATTAGATGATTAATAAAATTACAAATTTTAT
TTCATTTCACGTCTT
+
:FFFFFFFFFFFFF:FFFFFFFFFFFFFFFFFFFFFFF,FFFFFF:FFFFFFFF:FFFFFFFFFFFFF
FF:FFFF::,FF:FFFF,FFFF:FFFFFF:FFF::FFF:FF,FF:FFFFFFFFFFFFF:F,::FFFFF
FF:FFFFFFFFFFFF
@A00721:190:HGJW7DSXY:4:1135:24768:11725 1:N:0:TCTGCAAG+GCTGCACT
GGCTGGTGTATTGGAATTAATGTATCTGACATCTGGTCATAGTGACCTATTAAATACGGAAAGTGATT
CAGATCAGGATTTGGTGGAGGATATCTAAAGTAGTCGTACATTAGAACGATTTCGTCTATTCCAACGA
AATCCATGCATCTGC
+
FFFFFFFFFFFFFFFFFFFFFFFFFFFFFFFFFFFFFFFFFFFFFFFFFFFFFFFFFFFFFFFFFFFF
FFFFFFFFFFFFFFFFFFFFFFFFFFFFFFFFFFFFFFFFFFFFFFFFFFFFFFFFFFFFFFFFFFFF
FFFFFFFFFFFFFFF

```
@A00721:190:HGJW7DSXY:4:1315:31557:23860 1:N:0:TCTGCAAG+GCTGCACT
GGCTGGTGTATTGGAATTAATGTATCTGACATCTGGTCATAGTGACCTATTAAATACGGAAAGTGATT
CAGATCAGGATTTGGTGGAGGATATCTAAAGTAGTCGTACATTAGAACGATTTCGTCTATTCCAACGA
AATCCATGCATCTGC
+
FFFFFFFFFFFFFFF,FFFFFFFFFFFFFFFFFFFFFFFFFFFFFFFFFFFFFFFFFFFFFFFFFFFFFF
FFFFFFFFFFFFFF:FFFFFFFFF:FFFFFFFFFFFFFFFFFFFFF:FFF:FFFFFFFF:FFFFFFFFFF
FFFFFFFF:FFFFFF
@A00721:190:HGJW7DSXY:4:1466:12554:36479 1:N:0:TCTGCAAG+GCTGCACT
GGCTGGTGTATTGGAATTAATGTATCTGACATCTGGTCATAGTGACCTATTAAATACGGAAAGTGATT
CAGATCAGGATTTGGTGGAGGATATCTAAAGTAGTCGTACATTAGAACGATTTCGTCTATTCCAACGA
AATCCATGCATCTGC
+
FFF,::::FFFFFFFFF,FFFFFFF:FFFFFFFFF:FF:FFFFFFFFFFFFFFFFFFFFFFFFFFFFF::
FFFFFFF,FF,FFF:FF:F,FFFF:FFFFFF,FFFFF::FFF,FFFFFFFFFF,FFF:FFF,FF:FFFFF
FF:F:FFFFFF:F,F
@A00721:190:HGJW7DSXY:4:2111:12093:28698 1:N:0:TCTGCAAG+GCTGCACT
GGCTGGTGTATTGGAATTAATGTATCTGACATCTGGTCATAGTGACCTATTAAATACGGAAAGTGATT
CAGATCAGGATTTGGTGGAGGATATCTAAAGTAGTCGTACATTAGAACGATTTCGTCTATTCCAACGA
AATCCATGCATCTGC
+
FFFFFFFFFFFFFFFFFFFFFFFFFFFFFFFFFFFFFFFFFFFFFFFFFFFFFFFFFFFFFFFFFFFFFF
FFFFFFFFFFFFFFFFFFFFFFFFFFFFFFFFFFFFFFFFFFFFFFFFFFFFFFFF:FFFFFFFFFF:FF
FFFFFFFFFFFFFF:
@A00721:190:HGJW7DSXY:4:2145:5647:6699 1:N:0:TCTGCAAG+GCTGCACT
GGCTGGTGTATTGGAATTAATGTATCTGACATCTGGTCATAGTGACCTATTAAATACGGAAAGTGATT
CAGATCAGGATTTGGTGGAGGATATCTAAAGTAGTCGTACATTAGAACGATTTCGTCTATTCCAACGA
AATCCATGCATCTGC
+
FFFFFFFFFFFFFFFFFFFFFFFFFFFFFFFFFFFFFFFFFFFFFFFFFFFFFFFFFFFFFFFFFFFFFF
FFFFFFFFFFFFFFFFFFFFFFFFFFFFFFFFFFFFFFFFFFFFFFFFFFFFFFFFFFFFFFFFFFFFFF
FFFFFFFFFFFFFFF
@A00721:190:HGJW7DSXY:4:2239:31656:34272 1:N:0:TCTGCAAG+GCTGCACT
GGCTGGTGTATTGGAATTAATGTATCTGACATCTGGTCATAGTGACCTATTAAATACGGAAAGTGATT
CAGATCAGGATTTGGTGGAGGATATCTAAAGTAGTCGTACATTAGAACGATTTCGTCTATTCCAACGA
AATCCATGCATCTGC
+
FFFFFFFFFFFFFFFFFFFFFFFFFFFFFFFFFFFFFFFFFFFFFFFFFFFFFFFFFFFFFFFFFFFFFF
FFFFFFFFFFFFFFFFFFFFFFFFFFFFFFFFFFFFFFFFFFFFFFFFFF,FFFFFFFFFFFFF:FFFFF
FFF::FFFFFFFFFF
@A00721:190:HGJW7DSXY:4:2274:24044:5650 1:N:0:TCTGCAAG+GCTGCACT
GGCTGGTGTATTGGAATTAATGTATCTGACATCTGGTCATAGTGACCTATTAAATACGGAAAGTGATT
CAGATCAGGATTTGGTGGAGGATATCTAAAGTAGTCGTACATTAGAACGATTTCGTCTATTCCAACGA
AATCCATGCATCTGC
+
FFFFFFFFFFFFFFFFFFFFFFFFFFF:FFFFFFFFFFFFFFFFFFFFFFFFFFFFFFFFFFFFFFFFFF
FFFFFFFFFFFFFFFFFFFFFFFFFFFFFFFFFFFFFFFFFFFFFFFFFFFFFFFFFFFFFFFF:FFFFF
FFFFFFFFFFFFFFF
@A00721:190:HGJW7DSXY:4:2420:1642:12477 1:N:0:TCTGCAAG+GCTGCACT
GGCTGGTGTATTGGAATTAATGTATCTGACATCTGGTCATAGTGACCTATTAAATACGGAAAGTGATT
CAGATCAGGATTTGGTGGAGGATATCTAAAGTAGTCGTACATTAGAACGATTTCGTCTATTCCAACGA
AATCCATGCATCTGC
+
FFFFFFFFFFFFFFFFFFFFFFFFFFFFFFFFFFFFFFFFFFFFFFFFFFFFFFFFFFFFFFFFFFFFFF
```

```
FFFFFFFFFF:FFFFFFFFFFFFFFFFFFFFFFFFFFFFFFFFFFFFFFFFFFFFFFFF:FFFFF
FFFF:FFF:FFFFFF
@A00721:190:HGJW7DSXY:4:2661:21612:16908 1:N:0:TCTGCAAG+GCTGCACT
GGCTGGTGTATTGGAATTAATGTATCTGACATCTGGTCATAGTGACCTATTAAATACGGAAAGTGATT
CAGATCAGGATTTGGTGGAGGATATCTAAAGTAGTCGTACATTAGAACGATTTCGTCTATTCCAACGA
AATCCATGCATCTGC
+
FFFFFFFFFFFFFFFFFFFFFFFFFFFFFFFFFFFFFFFFFFFFFFFFFFFFFFFFFFFFFFFFFFFF
FFFFFFFFFFFFFFFFFFFFFFFFFFFFFFFFFFFFFFFFFFFFFFFFFFFFFFFFFFFFFFFFFFFF
FFFFFFFFFFFFFFF
@A00721:190:HGJW7DSXY:4:1315:23231:2143 1:N:0:TCTGCAAG+GCTGCACT
CTGACATCTGGTCATAGTGACCTATTAAATACGGAAAGTGATTCAGATCAGGATTTGGTGGAGGATAT
CTAAAGTAGTCGTACATTAGAACGATTTCGTCTATTCCAACGAAATCCATGCATCTGCATTCCAGTTC
TTCGTAATAGTTATA
+
FFFFFFFFFFFFFFFFFFFFFFFFFFFFFFFFFFFFFFFFFFFFFFFFFFFFFFF:FFFFFFFFFFFF
FFFFFFFFFFFFFFFFFFFFFFFFFFFFFFFFFFFFFFFFFFFFFFFFFFFFFFFFFFFFFFFFFFFF
FFFFFFFFFFFFFFF
@A00721:190:HGJW7DSXY:4:1407:8793:35884 1:N:0:TCTGCAAG+GCTGCACT
CTGACATCTGGTCATAGTGACCTATTAAATACGGAAAGTGATTCAGATCAGGATTTGGTGGAGGATAT
CTAAAGTAGTCGTACATTAGAACGATTTCGTCTATTCCAACGAAATCCATGCATCTGCATTCCAGTTC
TTCGTAATAGTTATA
+
FFFFFFFFFFFFF:FFFFFFFFFFFFFFFFFFFFFFFFFFFFFFFFFFFFFFFFFFFFFFFFFFFFFF
FFFFFFFFFFFFFFFFFFFFFFFFFF:FFFFFFFFFFFFFFFFFFFFFFFFFFFFFFFFF:FFFFFFFFF
FFFFFFFFFFFFFFF
@A00721:190:HGJW7DSXY:4:1424:26576:24126 1:N:0:TCTGCAAG+GCTGCACT
CTGACATCTGGTCATAGTGACCTATTAAATACGGAAAGTGATTCAGATCAGGATTTGGTGGAGGATAT
CTAAAGTAGTCGTACATTAGAACGATTTCGTCTATTCCAACGAAATCCATGCATCTGCATTCCAGTTC
TTCGTAATAGTTATA
+
FFFFFFFFFFFFFFFFFFFFFFFFFFFFFFFFFFFFFFFFFFFFFFFFFFFFFFFFFFFFFFFFFFFF
FFFF:FFFFFFFFFFFFFFFFFFFFFFFFFFFFFFFFFFFFFFFFFFFFFFFFFFFFFFFFFFFFFFFF
:FFFFFFFFFFFFFF
@A00721:190:HGJW7DSXY:4:1441:29315:25050 1:N:0:TCTGCAAG+GCTGCACT
CTGACATCTGGTCATAGTGACCTATTAAATACGGAAAGTGATTCAGATCAGGATTTGGTGGAGGATAT
CTAAAGTAGTCGTACATTAGAACGATTTCGTCTATTCCAACGAAATCCATGCATCTGCATTCCAGTTC
TTCGTAATAGTTATA
+
FFFFFFFFFFFFFFFFFFFFFFFFFFFFFFFFFFFFFFFFFFFFFFFFFFFFFFFFFFFFFFFFFFFF
FFFFFFFFFFFFFFFFF:FFFFFFF,FFFFFFFF:FFFFFFFFFFFFFFFFFFFFFFFFFFFFFFFFFF
FFFFFFFFFFFF:FF
@A00721:190:HGJW7DSXY:4:1558:29134:8046 1:N:0:TCTGCAAG+GCTGCACT
CTGACATCTGGTCATAGTGACCTATTAAATACGGAAAGTGATTCAGATCAGGATTTGGTGGAGGATAT
CTAAAGTAGTCGTACATTAGAACGATTTCGTCTATTCCAACGAAATCCATGCATCTGCATTCCAGTTC
TTCGTAATAGTTATA
+
FFF:FFFFFFFFFFFFFFFFFFFFFFFFFFFFFFFFFFFFFFFFFFFFFFFFFFF,FFFFFFFFFFFF
FFFFFFFFFFFFFFFFFFFFFFFFFFFFFFFFFFFFFFFFFFFFFFFFFFFFFFFFFFFFFFFFFFFF
FFFFFF:FFFFFFFF
@A00721:190:HGJW7DSXY:4:2139:30192:31015 1:N:0:TCTGCAAG+GCTGCACT
CTGACATCTGGTCATAGTGACCTATTAAATACGGAAAGTGATTCAGATCAGGATTTGGTGGAGGATAT
CTAAAGTAGTCGTACATTAGAACGATTTCGTCTATTCCAACGAAATCCATGCATCTGCATTCCAGTTC
TTCGTAATAGTTATA
```

```
+
FFFFFFFFFFFFFFFFFFFFFFFFFFFFFFFFFFFFFFFFFFFFFFFFFFFFFFFFFFFFFFFFFFFFFF
FFFFFFFFFFFFFFFFFFFFFFFFFFFFFFFFFFFFFFFFFFFFFFFFFFFFFFFFFFFFFFFFFFFFFF
FFFFFFFFFFFFFFF
@A00721:190:HGJW7DSXY:4:2169:12038:7091 1:N:0:TCTGCAAG+GCTGCACT
CTGACATCTGGTCATAGTGACCTATTAAATACGGAAAGTGATTCAGATCAGGATTTGGTGGAGGATAT
CTAAAGTAGTCGTACATTAGAACGATTTCGTCTATTCCAACGAAATCCATGCATCTGCATTCCAGTTC
TTCGTAATAGTTATA
+
FFFFFFFFFFFFFFFFFFFFFFFFFFFFFFFFFFFFFFFFFFFFFFFFFFFFFFFFFFFFFFFFFFFFFF
FFFFFFFFFFFFFFFFFFFFFFFFFFFFFFFFFFFFFFFFFFFFFFFFFFFFFFFFFFFFFFFFFFFFFF
FFFFFFFFFFFFFFF
@A00721:190:HGJW7DSXY:4:2212:17806:1141 1:N:0:TCTGCAAG+GCTGCACT
CTGACATCTGGTCATAGTGACCTATTAAATACGGAAAGTGATTCAGATCAGGATTTGGTGGAGGATAT
CTAAAGTAGTCGTACATTAGAACGATTTCGTCTATTCCAACGAAATCCATGCATCTGCATTCCAGTTC
TTCGTAATAGTTATA
+
FFFFFFFFFFFFFFFFFFFFFFFFFFFFFFFFFFFFFFFFFFFFFFFFFFFFFFF:FFFFFFFFFFFFFF
FFFFFFFFFFFFFFFFFFFFFFFFFFFFFFFFFFFFFFFFFFFFFFFFFFFFFFFFFFFFFFFFFFFFFF
FFFFFFFFFFFFFFF
@A00721:190:HGJW7DSXY:4:2318:17779:15405 1:N:0:TCTGCAAG+GCTGCACT
CTGACATCTGGTCATAGTGACCTATTAAATACGGAAAGTGATTCAGATCAGGATTTGGTGGAGGATAT
CTAAAGTAGTCGTACATTAGAACGATTTCGTCTATTCCAACGAAATCCATGCATCTGCATTCCAGTTC
TTCGTAATAGTTATA
+
FFF:FFFFFFFFFFFFFFFFFFFFFFFFFFFFFFFFFFFFFFFFFFFFFFFFFFFFFFFFFFFFFFFFFF
FFFFFFFFFFFFFFFFFFFFFFFFFFFFFFFFFFFFFFFFFFFFFFFFFFFFFFF::FFFFFFFFFFFFF
FFFFFFFFFFFFFFF
@A00721:190:HGJW7DSXY:4:2424:26693:17284 1:N:0:TCTGCAAG+GCTGCACT
CTGACATCTGGTCATAGTGACCTATTAAATACGGAAAGTGATTCAGATCAGGATTTGGTGGAGGATAT
CTAAAGTAGTCGTACATTAGAACGATTTCGTCTATTCCAACGAAATCCATGCATCTGCATTCCAGTTC
TTCGTAATAGTTATA
+
F:FFFFFFFFFFFFFFFFFFFFFFFFFFFFFFFFFFFFFFFFFFFFFFFFFFFFFFFFFFFFFFFFFFFF
FFFFFFFFFFFFFFFFFFFFFFFFFFFFFFFFFFFFFFFFFFFFFFFFFFFFFFFFFFFFFFFFFFFFFF
FFFFFFFF:FFFFFF
@A00721:190:HGJW7DSXY:4:2571:3649:5494 1:N:0:TCTGCAAG+GCTGCACT
CTGACATCTGGTCATAGTGACCTATTAAATACGGAAAGTGATTCAGATCAGGATTTGGTGGAGGATAT
CTAAAGTAGTCGTACATTAGAACGATTTCGTCTATTCCAACGAAATCCATGCATCTGCATTCCAGTTC
TTCGTAATAGTTATA
+
FFFFFFFFFFFFFFFFFFFFFFFFFFFFFFFFFFFFFFFFFFFFFFFFFFFFFFFF:FFFFFFFFFFFFF
FFFFFFFFFFFFFFFFFFFFFFFFFFFFFFFFFFF:FFFFFFFFFFFFFFFFFFFFFFFFFFFFFFFFFF
FFFFFFFFFFFFFFF
@A00721:190:HGJW7DSXY:4:2578:7618:1157 1:N:0:TCTGCAAG+GCTGCACT
CTGACATCTGGTCATAGTGACCTATTAAATACGGAAAGTGATTCAGATCAGGATTTGGTGGAGGATAT
CTAAAGTAGTCGTACATTAGAACGATTTCGTCTATTCCAACGAAATCCATGCATCTGCATTCCAGTTC
TTCGTAATAGTTATA
+
F:FFFF:FFFFFFFFFFFFFFFFFFFFFFFFFFFFFFFFFFFFFFFFFFFFFFFFFFFFFFFFFFFFFFF
FFFFFFFFFFFFFFFFFFFFFFFFFFFFFFFFFFFFFFFFFFFFFFFFFFFFF:FFF,FFFFFFFFFFFF
FFFFFFFFFFFFFFF
@A00721:190:HGJW7DSXY:4:2603:2889:23469 1:N:0:TCTGCAAG+GCTGCACT
CTGACATCTGGTCATAGTGACCTATTAAATACGGAAAGTGATTCAGATCAGGATTTGGTGGAGGATAT
```

```
CTAAAGTAGTCGTACATTAGAACGATTTCGTCTATTCCAACGAAATCCATGCATCTGCATTCCAGTTC
TTCGTAATAGTTATA
+
FFFFFFFFFFFFFFFFF:FFFFFFFFFFF:FFFFFFFFFFFFFFFFFFFFFFFFFFFFFFFFFFFFFF
FFFFFFFFFFFFFFFFFFFFFFFFFFFFFFFFFFFFFFFFFFFFFFFFFFFFFFFFFFF:FFFFFF:F
FFFFFFFFFF:FFFF
@A00721:190:HGJW7DSXY:4:2613:6551:1282 1:N:0:TCTGCAAG+GCTGCACT
CTGACATCTGGTCATAGTGACCTATTAAATACGGAAAGTGATTCAGATCAGGATTTGGTGGAGGATAT
CTAAAGTAGTCGTACATTAGAACGATTTCGTCTATTCCAACGAAATCCATGCATCTGCATTCCAGTTC
TTCGTAATAGTTATA
+
FFFFFFFFFFFFFFFFFFFFFFFFFFFFFFFFFFFFFFFFFFFFFFFFFFFFFFFFFFFFFFFFFFFF
FFFFFFFFFFFFFFFFFFFFFFFFFFFFFFFFFFFFFFFFFFFFFFFFFFFFFFFFFFFFFFFFFFFF
FFFFFFFFFFFFFFF
@A00721:190:HGJW7DSXY:4:1603:2193:3881 1:N:0:TCTGCAAG+GCTGCACT
CGAAAACGAGTATAACTATTACGAAGAACTGGAATGCAGATGCATGGATTTCGTTGGAATAGACGAAA
TCGTTCTAATGTACGACTACTTTAGATATCCTCCACCAAATCCTGATCTGAATCACTTTCCGTATTTA
ATAGGTCACTATGAC
+
FFFFFFFFFFFFFFFFFFFFFFFFF:FFFFFFFFFFFFFFFFFFFFFFFFFFFFFFFFFFFFFFFFFF
FFFFFFFFFFFFFFFFFFFFFFFFFFFFFFFFFFFFFFFFFFFFFFFFFFFFFFFFFFFFFFFFFFFF
:FFF:FFFFFFFFFF
@A00721:190:HGJW7DSXY:4:2155:10917:13604 1:N:0:TCTGCAAG+GCTGCACT
CGAAAACGAGTATAACTATTACGAAGAACTGGAATGCAGATGCATGGATTTCGTTGGAATAGACGAAA
TCGTTCTAATGTACGACTACTTTAGATATCCTCCACCAAATCCTGATCTGAATCACTTTCCGTATTTA
ATAGGTCACTATGAC
+
FFFFF,FFFFFFFFFFFFFFFFFFFFFFFFFFFFFFFFFF:FFFFFFFFFFFFFFFFFFFFFFFFFFF
FFFFFFFFFFFFFFFFFFFFFFFFFFFFFFFFFFFFFFFFFFFFFFFFFFFFFFFFFFFFFFFFFFFF
FFFF:FFFFFFFFFF
@A00721:190:HGJW7DSXY:4:1277:29821:25895 1:N:0:TCTGCAAG+GCTGCACT
GTGGAGGATATCTAAAGTAGTCGTACATTAGAACGATTTCGTCTATTCCAACGAAATCCATGCATCTG
CATTCCAGTTCTTCGTAATAGTTATACTCGTTTTCGATATCATTAATTATATATTTCATCGAAACGAT
CACACTGTTGGGAAT
+
FFFFFFFFFFFFFFFFFFFFFFFFFFFFFFFFFFFFFFFFFFFFFFFFFF:FFFFFFFFFFFFFFFFF
FFFFFFFFFFFFFFFFFFFFFFFFFFFFFFFFFFFFFFFFFFFFFFFFFFFFFFFFFFFFFFFFFFFF
FFFFFFF:FFFFFFF
@A00721:190:HGJW7DSXY:4:1277:29857:25895 1:N:0:TCTGCAAG+GCTGCACT
GTGGAGGATATCTAAAGTAGTCGTACATTAGAACGATTTCGTCTATTCCAACGAAATCCATGCATCTG
CATTCCAGTTCTTCGTAATAGTTATACTCGTTTTCGATATCATTAATTATATATTTCATCGAAACGAT
CACACTGTTGGGAAT
+
FFFFFFFFFFFFFFFFFFFFFFFFFFFFFFFFFFFFFFFFFFFFFFFFFFFFFFFFFFFFFFFFFFFF
FFFFFFFFFFFFFFFFFFFFFFFFFFFFFFFFFFFFFFFFFFFFFFFFFFFFFFFFFFFFFFFFFFFF
FFFFFFFFFF:FFFF
@A00721:190:HGJW7DSXY:4:1339:14859:15828 1:N:0:TCTGCAAG+GCTGCACT
GTGGAGGATATCTAAAGTAGTCGTACATTAGAACGATTTCGTCTATTCCAACGAAATCCATGCATCTG
CATTCCAGTTCTTCGTAATAGTTATACTCGTTTTCGATATCATTAATTATATATTTCATCGAAACGAT
CACACTGTTGGGAAT
+
FFFFFFFF:FFFFFFFFFFFFFFFF:FFFFFFFFFFFFF:FFFFFFFFFFFFFFFFFF:FFFFFFFFF
FF,F:FFFFFFF:FFFFFFFFFFFFFFFFFFF:FFFF:,FFFFFFFFFFFFFFFFFFFFFFF::FFFFFF
,FFF,FFFFF:F:FF
```

@A00721:190:HGJW7DSXY:4:1460:2953:1125 1:N:0:TCTGCAAG+GCTGCACT
GTGGAGGATATCTAAAGTAGTCGTACATTAGAACGATTTCGTCTATTCCAACGAAATCCATGCATCTG
CATTCCAGTTCTTCGTAATAGTTATACTCGTTTTCGATATCATTAATTATATATTTCATCGAAACGAT
CACACTGTTGGGAAT
+
FFFFFFFFFFFFFFFFFFFFFFFFFFFFFFFFFFFFFFFFFFFFFFFFFFFFFFFFFFFFFFFFFFFF
FFFFFFFFFFFFFFFFFFFFFFFFFFFFFFFFFFFFFF:FFFFFFFFFFFFFFFFFFFFFFFFFFFFF
FFFFFFFFFFFFF:F
@A00721:190:HGJW7DSXY:4:2176:5358:2691 1:N:0:TCTGCAAG+GCTGCACT
GTGGAGGATATCTAAAGTAGTCGTACATTAGAACGATTTCGTCTATTCCAACGAAATCCATGCATCTG
CATTCCAGTTCTTCGTAATAGTTATACTCGTTTTCGATATCATTAATTATATATTTCATCGAAACGAT
CACACTGTTGGGAAT
+
F,F,F:FFFFFFFFF:F,F,,FFFFFFF:F:FFFFFF::,,FFFFFF:,F:F:FF:FFFFFF:FFFFF
FFF:FFF:FF,FFFF,FF::F:FFFF:F:,F,,FF:F,,:FF,,,FFFFFFFF,,FFFFFFFFFFFF,
F:,FFFF,F:FF::F
@A00721:190:HGJW7DSXY:4:2249:23149:32690 1:N:0:TCTGCAAG+GCTGCACT
GTGGAGGATATCTAAAGTAGTCGTACATTAGAACGATTTCGTCTATTCCAACGAAATCCATGCATCTG
CATTCCAGTTCTTCGTAATAGTTATACTCGTTTTCGATATCATTAATTATATATTTCATCGAAACGAT
CACACTGTTGGGAAT
+
FFFFFFFFFFFFFFFFFFFFFFFFFFFFFFFFFFFFFFFFFFFFFFFFFFFFFFFFFFFFFFFFFFFF
FFFFFFFFFFFFFFFFFFFFFFFFFFFFFFFFFFFFFFFFFFFFFFFFFFFFFFFFFFFFFFFFFFFF
FFFFF:FFFFFFFFF
@A00721:190:HGJW7DSXY:4:2442:15338:16250 1:N:0:TCTGCAAG+GCTGCACT
GTGGAGGATATCTAAAGTAGTCGTACATTAGAACGATTTCGTCTATTCCAACGAAATCCATGCATCTG
CATTCCAGTTCTTCGTAATAGTTATACTCGTTTTCGATATCATTAATTATATATTTCATCGAAACGAT
CACACTGTTGGGAAT
+
FFFFFFFFFFFFFFFFFFFFFFFFFFFFFFFFFFFFFFFFFFFFFFFFFFFFFFFFFFFFFFFFFFFF
FFFFFFFFFFFFFFFFFFFFFFFFFFFFFFFFFFFFFFFFFFFFFFFFFFFFFFFFFFFFFFFFFFFF
FFFFFFFFFFFFFFF
@A00721:190:HGJW7DSXY:4:2570:1931:18771 1:N:0:TCTGCAAG+GCTGCACT
GTGGAGGATATCTAAAGTAGTCGTACATTAGAACGATTTCGTCTATTCCAACGAAATCCATGCATCTG
CATTCCAGTTCTTCGTAATAGTTATACTCGTTTTCGATATCATTAATTATATATTTCATCGAAACGAT
CACACTGTTGGGAAT
+
FFFFFFFFFFFFFFFFFFFFFFFFFFFFFFFFFFFFFFFFFFFFFFFFFFFFFFFFFFFFFFFFFFFF
FFFFFFFFFFFFFFFFFFFFFFFFFFFFFFFFFFFFFF:FFF:FFFFFFFFFFFFFFFFFFFFFFFFF
FFFFFFFFFFFFFFF
@A00721:190:HGJW7DSXY:4:1146:7744:17284 1:N:0:TCTGCAAG+GCTGCACT
GGAGGATATCTAAAGTAGTCGTACATTAGAACGATTTCGTCTATTCCAACGAAATCCATGCATCTGCA
TTCCAGTTCTTCGTAATAGTTATACTCGTTTTCGATATCATTAATTATATATTTCATCGAAACGATCA
CACTGTTGGGAATGC
+
FFFFFFFFFFFFFFFFFFFFFFFFFFFFFFFFFF,FFFFFFFFFFFFFFFFFFFFFFFFFFFFFFFFF
FFFFFFFFFFFFFFFFFFFFFFFFFFFFFFFFFFFF:FFFFFFFFFFFFFFFFFFFFFFFFFFFFFFF
FFFFFFFFFFFFFF:
@A00721:190:HGJW7DSXY:4:1150:28320:16219 1:N:0:TCTGCAAG+GCTGCACT
GGAGGATATCTAAAGTAGTCGTACATTAGAACGATTTCGTCTATTCCAACGAAATCCATGCATCTGCA
TTCCAGTTCTTCGTAATAGTTATACTCGTTTTCGATATCATTAATTATATATTTCATCGAAACGATCA
CACTGTTGGGAATGC
+
FFFFFFFFFFFFFFFFFFFFFFFFFFFFFFFFFFFFFFFFFFFFFFFFFFF:FFFFFFFFFFFFFFFF

```
FFFFFFFFF:FFFFFFFFFFFFFFFFFFFFFFFFFFFFFFFFFFFFFFFFFFFFFFF:FFFFFFFFFF
FFFFFFFFFFFFFFF
@A00721:190:HGJW7DSXY:4:1165:26494:27461 1:N:0:TCTGCAAG+GCTGCACT
GGAGGATATCTAAAGTAGTCGTACATTAGAACGATTTCGTCTATTCCAACGAAATCCATGCATCTGCA
TTCCAGTTCTTCGTAATAGTTATACTCGTTTTCGATATCATTAATTATATATTTCATCGAAACGATCA
CACTGTTGGGAATGC
+
FFFFFFFFFFFFFFFFFFFFFFFFFFFFFFFFFFFFFFFFFFFFFFFFFFFFFFFFFFFFFFFFFFFF
FFFFFFFFF:FFFFFFFFFFFFFFFFFFFFFFFFFFFFFFFFFFFFFFFFFFFFFFFFFFFFFFFFFF
FFFFFFFFFFFFFFF
@A00721:190:HGJW7DSXY:4:1304:15709:15045 1:N:0:TCTGCAAG+GCTGCACT
GGAGGATATCTAAAGTAGTCGTACATTAGAACGATTTCGTCTATTCCAACGAAATCCATGCATCTGCA
TTCCAGTTCTTCGTAATAGTTATACTCGTTTTCGATATCATTAATTATATATTTCATCGAAACGATCA
CACTGTTGGGAATGC
+
FFFFFFFFFFFFFFFFFFFFFFFFFFFFFFFFFFFFFFFFFFFFFFFFFFFFFFFFFFFFFFFFFFFF
FFFFFFFFFFFFFFFFFFFFFFFFFFFFFFFFFFFFFFFFFFFFFFFFFFFFFFFFFFFFFFF:FFFF
FFFFFFFFFFF,FFF
@A00721:190:HGJW7DSXY:4:1643:32443:10363 1:N:0:TCTGCAAG+GCTGCACT
GGAGGATATCTAAAGTAGTCGTACATTAGAACGATTTCGTCTATTCCAACGAAATCCATGCATCTGCA
TTCCAGTTCTTCGTAATAGTTATACTCGTTTTCGATATCATTAATTATATATTTCATCGAAACGATCA
CACTGTTGGGAATGC
+
FFFFFFFFFFFFFFFFFFFFFFFFFFFFFFFFFFFFFFFFFFFFFFFFFFFFFFFFFFFFFFFFFFFF
FFFFFFFFF:FFFFFFFFFF:FFFFFFFFFFFFFFFFFFF:FFFFFFFFFFFFFFFFFFFFFFFF:FF
FFFFFFFFFFF,FFF
@A00721:190:HGJW7DSXY:4:1472:28565:33238 1:N:0:TCTGCAAG+GCTGCACT
AAAGTAGTCGTACATTAGAACGATTTCGTCTATTCCAACGAAATCCATGCATCTGCATTCCAGTTCTT
CGTAATAGTTATACTCGTTTTCGATATCATTAATTATATATTTCATCGAAACGATCACACTGTTGGGA
ATGCTGATCTG
+
FFFFFFFFFFFFFFFFFFFFFFFFFFFFFFFFFFFFFFFFFFFFFFFFFFFFFFFFFFFFFFFFFFFF
FFFFFFFFFFFFFFFFFFFFFFFFFFFFF:FFFFFFFFFFFFFFFFFFFFFFFFFFFFFFFFFFFFFF
:FFFFFFFFFF
@A00721:190:HGJW7DSXY:4:1474:15826:31657 1:N:0:TCTGCAAG+GCTGCACT
AAAGTAGTCGTACATTAGAACGATTTCGTCTATTCCAACGAAATCCATGCATCTGCATTCCAGTTCTT
CGTAATAGTTATACTCGTTTTCGATATCATTAATTATATATTTCATCGAAACGATCACACTGTTGGGA
ATGCTGATCTG
+
FFFFFFFFFFFFFFFFFFFFFFFFFFFFFFFFFFFFFFFFFFFFFFFFFFFFFFFFFFFFFFFFFFFF
FFFFFFFFFFFFFFFFFFFFFFFFFFFFF,FFFFFFFFFFFFFFFFFFFFFFFFFFFFFFFFFFFFFF
FFFFFFFFFFF
@A00721:190:HGJW7DSXY:4:1521:30716:24064 1:N:0:TCTGCAAG+GCTGCACT
AAAGTAGTCGTACATTAGAACGATTTCGTCTATTCCAACGAAATCCATGCATCTGCATTCCAGTTCTT
CGTAATAGTTATACTCGTTTTCGATATCATTAATTATATATTTCATCGAAACGATCACACTGTTGGGA
ATGCTGATCTG
+
FFFFFFFFFFFFFFFFFFFFFFFFFFFFFFFFFFFFFFFFFFFFFFFFFFFFFFFFFFFFFFFFF:FF
FFFFFFFFFFFFFFFFFFFFFFFFFFFFF:FFFFFFFFFFFFFFFFFFFFFF:FFFFFFFFFFFF:FF
FFFFFF:F:FF
@A00721:190:HGJW7DSXY:4:2132:26765:27305 1:N:0:TCTGCAAG+GCTGCACT
AAAGTAGTCGTACATTAGAACGATTTCGTCTATTCCAACGAAATCCATGCATCTGCATTCCAGTTCTT
CGTAATAGTTATACTCGTTTTCGATATCATTAATTATATATTTCATCGAAACGATCACACTGTTGGGA
ATGCTGATCTG
```

```
+
FFFFFFFFFFFFFFFFFFFFFFFFFFFFFFFFFFFFFFFFFFFFFFFFFFFFFFFFFFFFFFFFFFFF,F
FFFFFFFFFFFFFFFFFFFFFFF::FFFF:FFFFFFFFFFFFFFFFFFFFFFFFFFFFFFFFFFFFFFFF
FFFFFFFFFFF
@A00721:190:HGJW7DSXY:4:2135:9833:15577 1:N:0:TCTGCAAG+GCTGCACT
AAAGTAGTCGTACATTAGAACGATTTCGTCTATTCCAACGAAATCCATGCATCTGCATTCCAGTTCTT
CGTAATAGTTATACTCGTTTTCGATATCATTAATTATATATTTCATCGAAACGATCACACTGTTGGGA
ATGCTGATCTG
+
FFFFFFFFFFFFFFFFFFFFFFFFFFFFFFFFFFFFFFFFFFFFFFFFFFFFFFFFFFFFFFFFFFFFFF
FFFFFFFFFFFFFFFFFFFFF:FFFFF:F,F::,FFFFFFFFFFF,FFFFFFFFFFFFFFFFFFFFFFFF
FFFFFFFFFFF
@A00721:190:HGJW7DSXY:4:2135:12093:17425 1:N:0:TCTGCAAG+GCTGCACT
AAAGTAGTCGTACATTAGAACGATTTCGTCTATTCCAACGAAATCCATGCATCTGCATTCCAGTTCTT
CGTAATAGTTATACTCGTTTTCGATATCATTAATTATATATTTCATCGAAACGATCACACTGTTGGGA
ATGCTGATCTG
+
FFFFFFFFFFFFFFFFFFFFFFFFFFFFFFFFFFFFFFFFFFFFFFFFFFFFFFFFF:FFFFFFFFFFFF
FFFFFFFFFFFFFFFF:FFFFFFFFFFFFFFFFFFFFFFFFFFFFFFFFFFFFFFFFFFFFFF:FFFFF
:FFFFFFFFFF
@A00721:190:HGJW7DSXY:4:2249:11098:35744 1:N:0:TCTGCAAG+GCTGCACT
AAAGTAGTCGTACATTAGAACGATTTCGTCTATTCCAACGAAATCCATGCATCTGCATTCCAGTTCTT
CGTAATAGTTATACTCGTTTTCGATATCATTAATTATATATTTCATCGAAACGATCACACTGTTGGGA
ATGCTGATCTG
+
FFFFFFFFFFFFFFFFFFFFFFFFFFFFFFFFFFFFFFFFFFFFFFFFFFFFFFFFFFFFFFFFFFFFFF
FFFFFFFFFFFFFFFFFFFFFFFF,FFFFFFFFFFFFFFFFFFFFFFFFFFFFFFFFFFFFFFFFFFFFF
FFFFFFFFFFF
@A00721:190:HGJW7DSXY:4:2628:14344:9048 1:N:0:TCTGCAAG+GCTGCACT
AAAGTAGTCGTACATTAGAACGATTTCGTCTATTCCAACGAAATCCATGCATCTGCATTCCAGTTCTT
CGTAATAGTTATACTCGTTTTCGATATCATTAATTATATATTTCATCGAAACGATCACACTGTTGGGA
ATGCTGATCTG
+
FFFFFFFFFFFFFFFFFFFFFFF:FFFFFFFFFFFFFFFFFFFFFFFFFFFFFFFFFFFFFFFFFFFFFF
FFFFFFFFFFFFFFFFFFFFFFFFF:FF:FFFFFF:FFFFFFFFFFFFFFFFFFFFFFFFFFFFFFFFFF
FF:FFFFFFFF
@A00721:190:HGJW7DSXY:4:1405:13015:24878 1:N:0:TCTGCAAG+GCTGCACT
CTTCGTAATAGTTATACTCGTTTTCGATATCATTAATTATATATTTCATCGAAACGATCACACTGTTG
GGAATGCTGATCTGGTTAATTGTATAGACGACTTCGTCTATTACTTCCTTTACACGGATGTGCATGTC
TGAAAGTATGTCGTC
+
:FFFFFFFFFFFFFFFFFFFFFFFFFFFFFFFFFFFFFFFFFFFFFFFFFFFFFFFFFFFFFFFFFFFFF
FFFFFFFFFF:FFFFFFFFFFFFFFF::FFFFFFFFFFFFFFFFFFFFFFFFFFFFFFFFFFFFFFFFFF
FFFFFFFFFFFF:FF
@A00721:190:HGJW7DSXY:4:1409:11822:12821 1:N:0:TCTGCAAG+GCTGCACT
CTTCGTAATAGTTATACTCGTTTTCGATATCATTAATTATATATTTCATCGAAACGATCACACTGTCG
GGAATGCTGATCTGGTTAATTGTATAGACGACTTCGTCTATTACTTCCTTTACACGGATGTGCATGTC
TGAAAGTATGTCGTC
+
FFFFFFFFF:FFFFFFFFFFFFFFFFFFFFFFFFFFF:FFFFFFFFFFFFFFFFFFFFFFFFFFF,FF,F
FFFFFFFFFF:FFF:FFFFFFFFFFFF:FFFFFFFFFFFFFFFFFFFFFFFFFFFFFFFFFFFFFFFFFF
FFFFFFFFF:FFFFF
@A00721:190:HGJW7DSXY:4:1561:29369:22858 1:N:0:TCTGCAAG+GCTGCACT
CTTCGTAATAGTTATACTCGTTTTCGATATCATTAATTATATATTTCATCGAAACGATCACACTGTTG
```

```
GGAATGCTGATCTGGTTAATTGTATAGACGACTTCGTCTATTACTTCCTTTACACGGATGTGCATGTC
TGAAAGTATGTCGTC
+
FFFFFFFFFFFFFFFFFFFFFFFFFFF:FFFFFFFFFFFFFFFFFFFFFFFFFFFFFFFFFFFFFFFF:F
FFFFFFFFFFFFFFFFFFFFFFFFFFFF,FFFFFFFFFFFFFFFFFFFFFFFFFFFFFFFFFFFFFFFFF
FF:FFFFFFFFFFFF
@A00721:190:HGJW7DSXY:4:2202:10013:18959 1:N:0:TCTGCAAG+GCTGCACT
CTTCGTAATAGTTATACTCGTTTTCGATATCATTAATTATATATTTCATCGAAACGATCACACTGTTG
GGAATGCTGATCTGGTTAATTGTATAGACGACTTCGTCTATTACTTCCTTTACACGGATGTGCATGTC
TGAAAGTATGTCGTC
+
FFFFFFFFFFFFFFFFFFFFFFFFFFFFFFFFFFFFFFFFFFFFFFFFFFFFFFFFFFFFFFFFFFFFFF
FFF:FFFFFFFFFFFFF:FFFFFFFFFFFFFFFFFFFFFFFFFFFFFFFFFFFF:FF:FFFFFFFFFFFF
FFFFFFFFFFFF:FF
@A00721:190:HGJW7DSXY:4:2407:26711:3004 1:N:0:TCTGCAAG+GCTGCACT
CTTCGTAATAGTTATACTCGTTTTCGATATCATTAATTATATATTTCATCGAAACGATCACACTGTTG
GGAATGCTGATCTGGTTAATTGTATAGACGACTTCGTCTATTACTTCCTTTACACGGATGTGCATGTC
TGAAAGTATGTCGTC
+
FFFFFFFFFFFFFFFFFFFFFFFFFFFFFFFFFFFFFF,FFFFFFFFFFFFFFFFFFFFFFFFFFFFFFF
FFF:FFFFFFFFFFFFFFFFFFFFFFF:FFFFFFFFFFFFFFFFFFFFFFFFFFFFFFFFFFFFFFFFFF
FFFFFFFFFFFFFFF
@A00721:190:HGJW7DSXY:4:2427:28782:4272 1:N:0:TCTGCAAG+GCTGCACT
CTTCGTAATAGTTATACTCGTTTTCGATATCATTAATTATATATTTCATCGAAACGATCACACTGTTG
GGAATGCTGATCTGGTTAATTGTATAGACGACTTCGTCTATTACTTCCTTTACACGGATGTGCATGTC
TGAAAGTATGTCGTC
+
FFFFFFFFFFFFFFFFFFFFFFFFFFFF,FFFFFFFFFFFFFFFFFFFFFFFFFFFFFFFFFFFFFFFFF
FFFFFFFFFFFFFFFFFFFFFFFFFFF:FFFFFFFFFFFFFFF:FFFFFFF:FF:FF:FFFFFF:FFFFF
FFFFFFFFFFFFFFF
@A00721:190:HGJW7DSXY:4:2432:18502:7733 1:N:0:TCTGCAAG+GCTGCACT
CTTCGTAATAGTTATACTCGTTTTCGATATCATTAATTATATATTTCATCGAAACGATCACACTGTTG
GGAATGCTGATCTGGTTAATTGTATAGACGACTTCGTCTATTACTTCCTTTACACGGATGTGCATGTC
TGAAAGTATGTCGTC
+
FFFFFFFFFFFFFFFFFFFFFFFFFF:FFFFFFFF:FFFFFFFFFFFFFFFFFFFFFFFFFFFFFFFFFF
FFFFFFFFFFFFFFF,FFFFFFFFFFFFFFFFFFFFFFFFFFFFFFFFFFFFFFFFFFFFFFFFFFFFFF
FF:FFFFFFFFFFFF
@A00721:190:HGJW7DSXY:4:2518:21133:16642 1:N:0:TCTGCAAG+GCTGCACT
CTTCGTAATAGTTATACTCGTTTTCGATATCATTAATTATATATTTCATCGAAACGATCACACTGTTG
GGAATGCTGATCTGGTTAATTGTATAGACGACTTCGTCTATTACTTCCTTTACACGGATGTGCATGTC
TGAAAGTATGTCGTC
+
FFFFFFFFFFFFFFFFFFFFFFFFFF:F,FFFFF,FFFFFFFFFFFFFFFFFFFFFFFFFFFFFFFF:FF
FFFFFFFFFFFFFFFF:FFFFFFFFFFFFFFFFFFFFFFFFFFFFFFFF:FFFFFFFFFFFFFFFFFFFF
FFFFFFFFFFFFFFF
@A00721:190:HGJW7DSXY:4:2609:9923:19711 1:N:0:TCTGCAAG+GCTGCACT
CTTCGTAATAGTTATACTCGTTTTCGATATCATTAATTATATATTTCATCGAAACGATCACACTGTTG
GGAATGCTGATCTGGTTAATTGTATAGACGACTTCGTCTATTACTTCCTTTACACGGATGTGCATGTC
TGAAAGTATGTCGTC
+
FFFFFFFFFFFFFFFFFFFFFFFFFFFF:FFFFF:FFFFFFFFFFFFFFFFFFFFFFFFFFFFFFFF:FF
FFF:FFFFFFFFFFFFFFFFFFFFFFF:FFFFFFFFFFFFFFFFFFFFFFFFFFFFFFFFFFFFFF,FFF
FFFFFFFFFFFFFFF
```

```
@A00721:190:HGJW7DSXY:4:2364:13069:33614 1:N:0:TCTGCAAG+GCTGCACT
TTCGTAATAGTTATACTCGTTTTCGATATCATTAATTATATATTTCATCGAAACGATCACACTGTTGG
GAATGCTGATCTGGTTAATTGTATAGACGACTTCGTCTATTACTTCCTTTACACGGATGTGCATGTCT
GAAAGTATGTCGTC
+
FFFFFFFFFFFFFFFF:FFF,FFFFF:F,FFF:F::FFFF:FFFFFFFFFFFFFFFFFFFF:FFF,:F
FFFFF:FFFF:F:FFF:FFF:FFFF,FFFFFFFFFFFFFFFFFFFF:FFFFFF:FF:F:FF:F,,FFF
FFFFFFF:FFFF:F
@A00721:190:HGJW7DSXY:4:1410:30210:30859 1:N:0:TCTGCAAG+GCTGCACT
CTCGTTTTCGATATCATTAATTATATATTTCATCGAAACGATCACACTGTTGGGAATGCTGATCTGGT
TAATTGTATAGACGACTTCGTCTATTACTTCCTTTACACGGATGTGCATGTCTGAAAGTATGTCGTCT
CTGAAGAATGCGAAG
+
FFFFFFFFFFFFFFFFFFFFFFFFFFFFFFFFFFFFFFFFFFFFFFFFFFFFFFFFFFFFFFFFFFFF
FFFFFFFFFFF:FFF:FFFFFFFFFFFFFFFFFFFFFFFFFFFFFFFFFFFFFFFFFFFFFFFFFFFF
FFFFFFFFFFFFFFF
@A00721:190:HGJW7DSXY:4:1462:15835:2174 1:N:0:TCTGCAAG+GCTGCACT
CTCGTTTTCGATATCATTAATTATATATTTCATCGAAACGATCACACTGTTGGGAATGCTGATCTGGT
TAATTGTATAGACGACTTCGTCTATTACTTCCTTTACACGGATGTGCATGTCTGAAAGTATGTCGTCT
CTGAAGAATGCGAAG
+
FFFFFFFFFFFFF:::,FFFFFFFFFFFFFFFFFFFFFFFFFFFFFFFFFFFFFF:FFFFFFFFFFFF
FFFFFFFFFFF:FFFFFFFFFFFFFFFFFFFF:FFFFFFFFFFFFFFFFFFFFFFFFFFFFFFF:FFF
FFFFFFFFFFFFFFF
@A00721:190:HGJW7DSXY:4:2446:22254:25786 1:N:0:TCTGCAAG+GCTGCACT
CTCGTTTTCGATATCATTAATTATATATTTCATCGAAACGATCACACTGTTGGGAATGCTGATCTGGT
TAATTGTATAGACGACTTCGTCTATTACTTCCTTTACACGGATGTGCATGTCTGAAAGTATGTCGTCT
CTGAAGAATGCGAAG
+
FFFFFFFFFFF::FFFFFFFFFFFFFFFFFFFFFFFFFFFFFFFFFFFFFFFFFF:FFFFFFFFFFFF
FFFFFFFFFFF:FFFFFFFFFFFFFFFFFFFFFFFFFFFFFFFFFFFFFFFFFFFFFFFFFFFFFFFF
FFFF::FFFFF:FFF
@A00721:190:HGJW7DSXY:4:2503:31349:21339 1:N:0:TCTGCAAG+GCTGCACT
CTCGTTTTCGATATCATTAATTATATATTTCATCGAAACGATCACACTGTTGGGAATGCTGATCTGGT
TAATTGTATAGACGACTTCGTCTATTACTTCCTTTACACGGATGTGCATGTCTGAAAGTATGTCGTCT
CTGAAGAATGCGAAG
+
FFFFFFFFFFFFFFFFFFFFFFFFFFFFFFFFFFFFFFFFFFFFFFFFFFFFFFF:FFFFFFFFFFFF
FFFFFFFFFFF:FFFFFFFFFFFFFFFFFFFFFFFFFFFFFFFFFFFFFFFFFFFFFFFFFFFF:FFF
FFFFFFFFFFFFFFF
@A00721:190:HGJW7DSXY:4:2671:28610:9455 1:N:0:TCTGCAAG+GCTGCACT
CTCGTTTTCGATATCATTAATTATATATTTCATCGAAACGATCACACTGTTGGGAATGCTGATCTGGT
TAATTGTATAGACGACTTCGTCTATTACTTCCTTTACACGGATGTGCATGTCTGAAAGTATGTCGTCT
CTGAAGAATGCGAAG
+
FFFFFFFFFFFFFFFFFFFFFFFFFFFFFFFFFFFFFFFFFFFFFFFFFFFF:FFFFFFFFFFFFFFF
FFFFFFFFFFFFFFFFFFFFFFFFFFFFFFFF,FFFFFFFFFFFFFFF:FFFFFFFF:FFFFFFFFFFF
FFFFFFFF:FFFFFF
@A00721:190:HGJW7DSXY:4:2252:18502:16908 1:N:0:TCTGCAAG+GCTGCACT
GTCCGGATTACAATTGCTTCGCATTCTTCAGAGACGACATACTTTCAGACATGCACATCCGTGTAAAG
GAAGTAATAGACGAAGTCGTCTATACAATTAACCAGATCAGCATTCCCAACAGTGTGATCGTTTCGAT
GAAATATATAATTAA
+
F,FFFFFFFFFFFFFFF,F:FFFFFFFFFFFFFFFFFFFFFFFFFFFFFFFFFFFFFFFFF:FFFFFF
```

FFFFFFFFFFFF:FFFFFFFFFFFFFFFFFFFFFFFFFFFFFFFFFFFFFFFFFFFFFFFFFFFFF
FFF,FFFFFFFFFFF
@A00721:190:HGJW7DSXY:4:2568:3613:31203 1:N:0:TCTGCAAG+GCTGCACT
GTCCGGATTACAATTGCTTCGCATTCTTCAGAGACGACATACTTTCAGACATGCACATCCGTGTAAAG
GAAGTAATAGACGAAGTCGTCTATACAATTAACCAGATCAGCATTCCCAACAGTGTGATCGTTTCGAT
GAAATATATAATTAA
+
F:FFF:FF:FFFFF:FF,FFFFFFFFFFFF:FFFFFFFFFFFFF:FF:FFF:FFFFFFFFFFF:F:F:
FFFFFFFFFFFFFFFFFFFFFFFFFFFFFFFF:FFFFFFFFFFFFFFFFFF,FFFFFFFF:FFFFFF
FF:FFFFF:FFF,FF
@A00721:190:HGJW7DSXY:4:2568:3794:31516 1:N:0:TCTGCAAG+GCTGCACT
GTCCGGATTACAATTGCTTCGCATTCTTCAGAGACGACATACTTTCAGACATGCACATCCGTGTAAAG
GAAGTAATAGACGAAGTCGTCTATACAATTAACCAGATCAGCATTCCCAACAGTGTGATCGTTTCGAT
GAAATATATAATTAA
+
FFFFFFFFFFFFFFFFFFFFFFFFFFFFFFFFFFFFFFFFFFFFFFFFFFFFFFFFFFFFFFFFF:F
FF:FFFFFFFFFFFFFFFFFFFFFFFFFFFFFFFFFFFFFFFFFFFFFFFFFFFFFFFFFFFFFFFF
FFFFFFFFFFFFFFF
@A00721:190:HGJW7DSXY:4:1569:26982:34131 1:N:0:TCTGCAAG+GCTGCACT
GTCCGGATTACAATTGCTTCGCATTCTTCAGAGACGACATACTTTCAGACATGCACATCCGTGTAAAG
GAAGTAATAGACGAAGTCGTCTATACAATTAACCAGATCAGCATTCCCAACAGTGTGATCGTTTCGAT
GAAATATATAATTA
+
FFFFFFFFFFFFFFFFFFFFFFFFFFFFFFFFFFFFFFFFFFFFFFFFFFFFFFFFF:FFFF,F,FF
FFFFFFFFFFFF:F:FFFFFFFFFFFFFFFFFFFFFFFF,FFFFFFFFFFFF:FFFFFF:FFFF,FF
FFFFFFFFFFFFFF
@A00721:190:HGJW7DSXY:4:1438:3730:31093 1:N:0:TCTGCAAG+GCTGCACT
ATCGGAACGATCACACTGTTGGGAATGCTGATCTGGTTAATTGTATAGACGACTTCGTCTATTACTTC
CTTTACACGGATGTGCATGTCTGAAAGTATGTCGTCTCTGAAGAATGCGAAGCAATTGTAATCCGGAC
ACCAGAATGGATATA
+
FFFFFFFFFFFFFFFF:FFFFFFFFFFFFFFFFFFFFFFFFFFFFF:FFFFFFFFFFFFFFFFFFFF
FFFFFFFFFFFFFFFFFFFFFFFFFFFFFFFFFFFFFFFFFFFFFFFFFFFFFFFFFFFFFFFFFFF
FFFFFFF:FFFFFFF
@A00721:190:HGJW7DSXY:4:1517:19253:19210 1:N:0:TCTGCAAG+GCTGCACT
ATCGGAACGATCACACTGTTGGGAATGCTGATCTGGTTAATTGTATAGACGACTTCGTCTATTACTTC
CTTTACACGGATGTGCATGTCTGAAAGTATGTCGTCTCTGAAGAATGCGAAGCAATTGTAATCCGGAC
ACCAGAATGGATATA
+
FFFFFFFFFFFFFFFFFFFFFFFFF:FFFFFFFFFFFFFFFFFFFFF:FFFFFFFFFFFFFFFFFFF
FFFFFFFFFFFFFFFFFFFFFFFFFFFFFFFFFFFFFFFFFFFFFFFFFFFFFFFFFFFFFFFFFFF
FFFFFFFFFFFF:FF
@A00721:190:HGJW7DSXY:4:1524:3396:24439 1:N:0:TCTGCAAG+GCTGCACT
ATCGGAACGATCACACTGTTGGGAATGCTGATCTGGTTAATTGTATAGACGACTTCGTCTATTACTTC
CTTTACACGGATGTGCATGTCTGAAAGTATGTCGTCTCTGAAGAATGCGAAGCAATTGTAATCCGGAC
ACCAGAATGGATATA
+
FFFFFFFFFFFFFFFFFFFFFFFF,F:F:FFFFF:FFFFFFFFFFFFF:FF,FFFF,FFFFFF,FF,
:FFFFFFF:,FFFFF:FFFFFFFFFFF::FFFF:FFFF::FF:FF,:FFFFFFFF:FFFFFFFFFFFF
::FFFFFFFFFFFFF
@A00721:190:HGJW7DSXY:4:1536:24451:21512 1:N:0:TCTGCAAG+GCTGCACT
ATCGGAACGATCACACTGTTGGGAATGCTGATCTGGTTAATTGTATAGACGACTTCGTCTATTACTTC
CTTTACACGGATGTGCATGTCTGAAAGTATGTCGTCTCTGAAGAATGCGAAGCAATTGTAATCCGGAC
ACCAGAATGGATATA

```
+
FFFFFFFFFFFFFFFFFFFFFFFFFFFFFFFFFFFFFFFFF:FFFFFFF:FFFFFFFFFFFFFFFFFFF
FFFFFFFFFFFFFFFFFFFFFFFFFFFFFFFFFFFFFFFFFFFFFFFFFFFFFFFFFFFFFFFFF:FFFF
FFFFFFFFFFFFFFF
@A00721:190:HGJW7DSXY:4:2406:9254:16297 1:N:0:TCTGCAAG+GCTGCACT
ATCGGAACGATCACACTGTTGGGAATGCTGATCTGGTTAATTGTATAGACGACTTCGTCTATTACTTC
CTTTACACGGATGTGCATGTCTGAAAGTATGTCGTCTCTGAAGAATGCGAAGCAATTGTAATCCGGAC
ACCAGAATGGATATA
+
FFFFFFFFFFFFFFFFFFFFFFFFFFFFFFFFFFFFFFFFFFFFFFFFF:FFFFFFFFFFFFFFFFFFF
FFFFFFFFFFFFFFFFFFFFFFFFFFFFFFFFFFFFFFFFFFFFFFFFFFFFFFFFFFFFFFFFFF:FFF
FFFFFF,FFFFFFFF
@A00721:190:HGJW7DSXY:4:2632:2880:13088 1:N:0:TCTGCAAG+GCTGCACT
ATCGGAACGATCACACTGTTGGGAATGCTGATCTGGTTAATTGTATAGACGACTTCGTCTATTACTTC
CTTTACACGGATGTGCATGTCTGAAAGTATGTCGTCTCTGAAGAATGCGAAGCAATTGTAATCCGGAC
ACCAGAATGGATATA
+
FFFFFFFFFFFFFFF,FFFFFFF:FFFFFFFFFFFFFFFFFFFFFFFFFFFFFFFFFFFFF:FFFFFFF
FFFFFFFFFFFFFFFFF,FFFFFF:FFFFFFFFFFFFFFFFFFFFFFFFFFFFFFFFFFFFFFFFFFFFF
FFFFFFFFFFFFFFF
@A00721:190:HGJW7DSXY:4:1417:5367:27915 1:N:0:TCTGCAAG+GCTGCACT
GGTTAATTGTATAGACGACTTCGTCTATTACTTCCTTTACACGGATGTGCATGTCTGAAAGTATGTCG
TCTCTGAAGAATGCGAAGCAATTGTAATCCGGACACCAGAATGGATATATGATCTTCGTCATTTTCAG
AGCAGCTCTGAATCG
+
FFFFFFFFFFFFFF:FFFFFFFFFFFFFFFFFFFFFFFFFFFFFFFFFFFFFFFFFFFFFFFFFFFFFF
FFFFFFFFFFFFFFFFFFFFFFFFFFFFFFFFFFFFFFFFFFFFFFFFFFFFFFFFFFFFFFFFFFFFFF
FFFFFFFFFFFFFFF
@A00721:190:HGJW7DSXY:4:1467:18982:12884 1:N:0:TCTGCAAG+GCTGCACT
GGTTAATTGTATAGACGACTTCGTCTATTACTTCCTTTACACGGATGTGCATGTCTGAAAGTATGTCG
TCTCTGAAGAATGCGAAGCAATTGTAATCCGGACACCAGAATGGATATATGATCTTCGTCATTTTCAG
AGCAGCTCTGAATCG
+
FFFFFF:FFFFFFF:FFFFFFFFFFFFFFFFFFFFFFFFFFFFFFFFFFFFFFFFFFFFFFFFFFFFFF
FFFFFFFFFF:FFFFF,FFFF,FFFF:FFFFFFFFFFFFF:FFFFFFFFFFFFFFFFFFFFFFFFFFFF
FFFFFFFFFF:FFFF
@A00721:190:HGJW7DSXY:4:1576:26503:33301 1:N:0:TCTGCAAG+GCTGCACT
GGTTAATTGTATAGACGACTTCGTCTATTACTTCCTTTACACGGATGTGCATGTCTGAAAGTATGTCG
TCTCTGAAGAATGCGAAGCAATTGTAATCCGGACACCAGAATGGATATATGATCTTCGTCATTTTCAG
AGCAGCTCTGAATCG
+
FF:FFFFFFFFFFF:FFFFFFFFFFFFFFFFFFFFFFFFFFFFFFFFFFFFFFFFFFFFFFFFFFFFFF
FFFFFFFFFFFFFFFFFFFFFFFFFFFFFFFFFFFFFFFFFFFFFFFFFFFFFFFFFFFFFFFFFFFFFF
FFFFFFFFFFFFFFF
@A00721:190:HGJW7DSXY:4:1603:5484:23140 1:N:0:TCTGCAAG+GCTGCACT
GGTTAATTGTATAGACGACTTCGTCTATTACTTCCTTTACACGGATGTGCATGTCTGAAAGTATGTCG
TCTCTGAAGAATGCGAAGCAATTGTAATCCGGACACCAGAATGGATATATGATCTTCGTCATTTTCAG
AGCAGCTCTGAATCG
+
FFFFFFFFFFFFFF:FFFFFFFFFFFFFFFFFFFFFFFFFFFFFFFFFFFFFFFFFFFFFFFFFFFFFF
FFFFFFFFFFFFFFFFFFFF,FFFFFFFFFFFFFFFFFFFFFFFFFFFFFFFFFFFFFFFFFFFFFFFFF
FFFFFFFFFFFFFFF
@A00721:190:HGJW7DSXY:4:2202:30092:3787 1:N:0:TCTGCAAG+GCTGCACT
GGTTAATTGTATAGACGACTTCGTCTATTACTTCCTTTACACGGATGTGCATGTCTGAAAGTATGTCG
```

```
TCTCTGAAGAATGCGAAGCAATTGTAATCCGGACACCAGAATGGATATATGATCTTCGTCATTTTCAG
AGCAGCTCTGAATCG
+
FFFFFFFFFFFFFF:FFFFFFFFFFFFFFFFFFFFFFFFFFFFFFFFFFFFFFFFFFFFFFFFFFFFF
FFFFFFFFFFFFFFFFFFFFFFFFFFFFFFFFFFFFFFFFFFFFFFFFFFFFFFFFFFFFFFFFFFFF
FFFFFFFFFFF:FFF
@A00721:190:HGJW7DSXY:4:2212:3134:26303 1:N:0:TCTGCAAG+GCTGCACT
GGTTAATTGTATAGACGACTTCGTCTATTACTTCCTTTACACGGATGTGCATGTCTGAAAGTATGTCG
TCTCTGAAGAATGCGAAGCAATTGTAATCCGGACACCAGAATGGATATATGATCTTCGTCATTTTCAG
AGCAGCTCTGAATCG
+
FFFFFFFFFFFFFF:FFFFFFFFFFFFFFFFFFFFFFFFFFFFFFFFFFFFFFFFFFFFFFFFFFFFF
FFFFFFFFFFFFFFFFFFFF::FFFFFFFFFFFFFFFFFFFFFFFFFFFFFFFFFFFFFFFFFFFFFF
F:FFFFFFFFFFFF:
@A00721:190:HGJW7DSXY:4:2367:10746:5603 1:N:0:TCTGCAAG+GCTGCACT
GGTTAATTGTATAGACGACTTCGTCTATTACTTCCTTTACACGGATGTGCATGTCTGAAAGTATGTCG
TCTCTGAAGAATGCGAAGCAATTGTAATCCGGACACCAGAATGGATATATGATCTTCGTCATTTTCAG
AGCAGCTCTGAATCG
+
FFFFFFFFFFFFFFFFFFFFFFFFFFFFFFFFFFFFFFFFFFFFFFFFFFFFFFFFFFFFFFFFFFFF
FFFFFFFFFFFFFFFFFFFFFFFFFF:FFFFFFFFFFFFFFFFFFFFFFFFFFFFFFFFFFFFFFFFF
FFFFFFFFFFFFFFF
@A00721:190:HGJW7DSXY:4:2367:11912:5744 1:N:0:TCTGCAAG+GCTGCACT
GGTTAATTGTATAGACGACTTCGTCTATTACTTCCTTTACACGGATGTGCATGTCTGAAAGTATGTCG
TCTCTGAAGAATGCGAAGCAATTGTAATCCGGACACCAGAATGGATATATGATCTTCGTCATTTTCAG
AGCAGCTCTGAATCG
+
FF::FFFFFFFFFFFFFFFFFFFFFFFFFFFFFFFFFFFFFFFFFFFFFFFFFFFFFFFFFFFFFFFF
FFFFFFFFFFFFFFFFFFFFFFFFFFFF:FFFFFFFFFFF,FFFFFFFFFF:FFFFFFFFFFFFFFFF
FFFFFFFFFFFFFFF
@A00721:190:HGJW7DSXY:4:1147:32434:3771 1:N:0:TCTGCAAG+GCTGCACT
GTCTGAAAGTATGTCGTCTCTGAAGAATGCGAAGCAATTGTAATCCGGACACCAGAATGGATATATGA
TCTTCGTCATTTTCAGAGCAGCTCTGAATCGTGCTTCGTTTTCTACGTATCTGGACCAGTATATCTGC
TTGACTTGATGCTTC
+
FFFFFFFFFFFFFFFFFFFFFFFFFFFFFFFFFFFFFFFFFFFFFFFFFFFFFFFFFFFFFFFFFFFF
FFFFFFFFFFFFFFFFFFFFFFFFFFFFFFFFFFFFF:FFFFFFFFFFFFFFFFFFFFFF:FFF:FFF
FFF,FFFFFFFFFF:
@A00721:190:HGJW7DSXY:4:1168:1199:25488 1:N:0:TCTGCAAG+GCTGCACT
GTCTGAAAGTATGTCGTCTCTGAAGAATGCGAAGCAATTGTAATCCGGACACCAGAATGGATATATGA
TCTTCGTCATTTTCAGAGCAGCTCTGAATCGTGCTTCGTTTTCTACGTATCTGGACCAGTATATCTGC
TTGACTTGATGCTTC
+
FFFFFFFFFFFFFFFFFFFFFFFFFFFFFFFFFFFFFFFFFFFFFFFFFFFFFFFFFFFFFFFFFFFF
FFFFFFFFFFFFFFFFFFFFFFFFFFFFFFFFFFFFFFFFFFFFFFFFFFFFFFFFFFFFFFFFFFFF
FFFFFFFFFFFFFFF
@A00721:190:HGJW7DSXY:4:1209:16929:16532 1:N:0:TCTGCAAG+GCTGCACT
GTCTGAAAGTATGTCGTCTCTGAAGAATGCGAAGCAATTGTAATCCGGACACCAGAATGGATATATGA
TCTTCGTCATTTTCAGAGCAGCTCTGAATCGTGCTTCGTTTTCTACGTATCTGGACCAGTATATCTGC
TTGACTTGATGCTTC
+
FFFFFFFFFFFFFFFFFFFFFFFFFFFFFFFFFFFFFFFFFFFFFFFFFFFFFFFFFFFFFFFFFFFF
FFFFFFFFFFFFFFFFFFFFFFFFFFFFFFFFFFFFFFFFFFFFFFFFFFFFFFFFFFFFFFFFFFFF
FFFFFFFFFFFFFFF
```

```
@A00721:190:HGJW7DSXY:4:1269:2419:26068 1:N:0:TCTGCAAG+GCTGCACT
GTCTGAAAGTATGTCGTCTCTGAAGAATGCGAAGCAATTGTAATCCGGACACCAGAATGGATATATGA
TCTTCGTCATTTTCAGAGCAGCTCTGAATCGTGCTTCGTTTTCTACGTATCTGGACCAGTATATCTGC
TTGACTTGATGCTTC
+
FFFFFFFFFFFFFFFFFFFFFFFFFFFFFFFFFFFFFFFFFFFFFFFFFFFFFFFF,FFFFFFFFFFF
FFFFFFFFFFFFFFFFFFFFFFFFFFFFFFFFFFFFFFFFFFFFFFFFFFFFF:FFFFFFFFFFFFFF
FFFFFFFFFFFFFFF
@A00721:190:HGJW7DSXY:4:1269:2510:26287 1:N:0:TCTGCAAG+GCTGCACT
GTCTGAAAGTATGTCGTCTCTGAAGAATGCGAAGCAATTGTAATCCGGACACCAGAATGGATATATGA
TCTTCGTCATTTTCAGAGCAGCTCTGAATCGTGCTTCGTTTTCTACGTATCTGGACCAGTATATCTGC
TTGACTTGATGCTTC
+
FFFFFFFFFFFFFFFFFFFFFFFFFFFFFFFFFFFF,FFFFFFFFFFFFFFFFFF:FFFFFFFFF,FF
FFFFFFFFFFFFFFFFFFFFFFFFFFFFFFFFF:FFFFFFFFFFFFFFFFFFF:FFFFFFFFFFFFFF
FFFFFFFFFFFFFFF
@A00721:190:HGJW7DSXY:4:1310:15989:11397 1:N:0:TCTGCAAG+GCTGCACT
GTCTGAAAGTATGTCGTCTCTGAAGAATGCGAAGCAATTGTAATCCGGACACCAGAATGGATATATGA
TCTTCGTCATTTTCAGAGCAGCTCTGAATCGTGCTTCGTTTTCTACGTATCTGGACCAGTATATCTGC
TTGACTTGATGCTTC
+
FFFFFFFFFFFFFFFFFFFFFFFFFFFFF:FFFFFFFFFFFFFFFFFFFFFFFF:FFFFFFFFFFFFF
FFFFFFFFFFFFFFFFFFFFFFFFFFFFFFFFFFFFFFFF:FFFFFFFFFFFFFFFFFFFFFFFFFFF
FFFFFFFFFF:FFFF
@A00721:190:HGJW7DSXY:4:1313:19407:2002 1:N:0:TCTGCAAG+GCTGCACT
GTCTGAAAGTATGTCGTCTCTGAAGAATGCGAAGCAATTGTAATCCGGACACCAGAATGGATATATGA
TCTTCGTCATTTTCAGAGCAGCTCTGAATCGTGCTTCGTTTTCTACGTATCTGGACCAGTATATCTGC
TTGACTTGATGCTTC
+
FFFFFFFFFFFFFFFFFFFFFFFFFFFFFFFFFFFFFFFFFFFFFFFFFFFFFFFFFFFFFFFFFFFF
FFFFFFFFFFFFFFFFFFFFFFFFFFFFFFFFFFFFFFFFFFFFFFFFFFFFFFFFFFFFFFFFFFFF
FFFFFFFFFFFFFFF
@A00721:190:HGJW7DSXY:4:1327:8305:36323 1:N:0:TCTGCAAG+GCTGCACT
GTCTGAAAGTATGTCGTCTCTGAAGAATGCGAAGCAATTGTAATCCGGACACCAGAATGGATATATGA
TCTTCGTCATTTTCAGAGCAGCTCTGAATCGTGCTTCGTTTTCTACGTATCTGGACCAGTATATCTGC
TTGACTTGATGCTTC
+
FFFFFFFFFFFFFFFFFFFFFFFFFFFFFFFFFFFFFFFFFFFFFFFFFFFFFFFFFFFFFFFFFFFF
FFFFFFFFFFFFFFFFFFFFFFFFFFFFFFFFFFFFFFFFFF:FFFFFFFFFFFFFFFFFFFFFFFFF
FFFFFFFFFFFFFFF
@A00721:190:HGJW7DSXY:4:1339:32642:29027 1:N:0:TCTGCAAG+GCTGCACT
GTCTGAAAGTATGTCGTCTCTGAAGAATGCGAAGCAATTGTAATCCGGACACCAGAATGGATATATGA
TCTTCGTCATTTTCAGAGCAGCTCTGAATCGTGCTTCGTTTTCTACGTATCTGGACCAGTATATCTGC
TTGACTTGATGCTTC
+
FFFFFFFFFFFFFFFFFFFFFFFFFFFFFFFFFFFFFFFFFFFFFFFFFFFFFFFFFFFFFFFFFFFF
FFFFFFFFFFFFFFFFFFFFFFFFFFFFFFFFFFFFFFFFFFFFFFFFFFFFFFFFFFFFFFFFFFFF
FFFFFFFFFFFFFFF
@A00721:190:HGJW7DSXY:4:1362:9471:7122 1:N:0:TCTGCAAG+GCTGCACT
GTCTGAAAGTATGTCGTCTCTGAAGAATGCGAAGCAATTGTAATCCGGACACCAGAATGGATATATGA
TCTTCGTCATTTTCAGAGCAGCTCTGAATCGTGCTTCGTTTTCTACGTATCTGGACCAGTATATCTGC
TTGACTTGATGCTTC
+
FFFFFFFFFFFFFFFFFFFFFFFFFFFFFF:FFFFFF:FFFFFFFFFFFFFF,FFFFFFFFFFFFFFF
```

```
FFF:FFFFFFFFFFFFFFFFFFF:FFFFFFFFFFFFFFFFFFFFFFFFFFFFFFFFFFFFFFFFFFFF:
FF:FFFFFFFFFFFF
@A00721:190:HGJW7DSXY:4:1436:10456:5916 1:N:0:TCTGCAAG+GCTGCACT
GTCTGAAAGTATGTCGTCTCTGAAGAATGCGAAGCAATTGTAATCCGGACACCAGAATGGATATATGA
TCTTCGTCATTTTCAGAGCAGCTCTGAATCGTGCTTCGTTTTCTACGTATCTGGACCAGTATATCTGC
TTGACTTGATGCTTC
+
FFFFFFFFFFFFFFFFFFFFFFFFFFFFFFFFFFFFFFFFFFFFFFFFFFFFFFFFFFFFFFFFFFFF
FFFFFFFFFFFFFFFFFFFFFFFFFFFFFFFFFFFFFFFF:FFF:FF::FFFFFFFFFFFF:FFFFF
FFFFFFFFFFFFFFF
@A00721:190:HGJW7DSXY:4:1502:19271:32737 1:N:0:TCTGCAAG+GCTGCACT
GTCTGAAAGTATGTCGTCTCTGAAGAATGCGAAGCAATTGTAATCCGGACACCAGAATGGATATATGA
TCTTCGTCATTTTCAGAGCAGCTCTGAATCGTGCTTCGTTTTCTACGTATCTGGACCAGTATATCTGC
TTGACTTGATGCTTC
+
FFFFFFFFFFFFFFFFFFFFFFFFFFFFFFFFFFFFFFFFFFFFFFFFFFFFFFFFFFFFFFFFFFFF
FFFFFFFFFFFFFFFFFFFFFFFFFFFFFFFFFFFFFFFFFFFFFFFFFFFFFFFFFFFFFFFFFFF:
FFFFFFFFFFF:FFF
@A00721:190:HGJW7DSXY:4:1502:18475:32831 1:N:0:TCTGCAAG+GCTGCACT
GTCTGAAAGTATGTCGTCTCTGAAGAATGCGAAGCAATTGTAATCCGGACACCAGAATGGATATATGA
TCTTCGTCATTTTCAGAGCAGCTCTGAATCGTGCTTCGTTTTCTACGTATCTGGACCAGTATATCTGC
TTGACTTGATGCTTC
+
FFFFFFFFFFFFFFFFFFFFFFFFFFFFFFFFFFFFFFFFFFFFFFFFFFFFFFFFFFFFFFFFFFFF
FFFFFFFFFFFFFFFFFFFFFFFFFFFFFFFFFFFFFFFFFFFFFFFFFFFFFFFFFFFFFFFFFFFF
FFFFFFFFFFFFFFF
@A00721:190:HGJW7DSXY:4:1503:3387:14841 1:N:0:TCTGCAAG+GCTGCACT
GTCTGAAAGTATGTCGTCTCTGAAGAATGCGAAGCAATTGTAATCCGGACACCAGAATGGATATATGA
TCTTCGTCATTTTCAGAGCAGCTCTGAATCGTGCTTCGTTTTCTACGTATCTGGACCAGTATATCTGC
TTGACTTGATGCTTC
+
FFFFFFFFFFFFFFFFFFFFFFFFFFFFFFFFFFFFFFFFFFFFFFFFFFFFFFFFF:FFFFFFFFFF
FFFFFFFFFFFFFFFFFFFFFFFFFFFFFFFFFFFFFFFFFFFFFFFFFFFFFFFFFFFFFFFFFFFF
FFFFFFFFFFFFFFF
@A00721:190:HGJW7DSXY:4:1515:31774:20447 1:N:0:TCTGCAAG+GCTGCACT
GTCTGAAAGTATGTCGTCTCTGAAGAATGCGAAGCAATTGTAATCCGGACACCAGAATGGATATATGA
TCTTCGTCATTTTCAGAGCAGCTCTGAATCGTGCTTCGTTTTCTACGTATCTGGACCAGTATATCTGC
TTGACTTGATGCTTC
+
FFFFFFFFFFFFFFFFFFFFFFFFFFFFFFFFFFFFFFFFFFFFFFFFFFFFFFFF,FFFFFFFFFFF
FFFFFFFFFFFFFFFFFFFFFFFFFFFFFFFFFFFFFFFFFFFFFFFFFFFFFFFFFFFFFFFFFFFF
FFFFFFFFFFFFFFF
@A00721:190:HGJW7DSXY:4:1558:22507:21527 1:N:0:TCTGCAAG+GCTGCACT
GTCTGAAAGTATGTCGTCTCTGAAGAATGCGAAGCAATTGTAATCCGGACACCAGAATGGATATATGA
TCTTCGTCATTTTCAGAGCAGCTCTGAATCGTGCTTCGTTTTCTACGTATCTGGACCAGTATATCTGC
TTGACTTGATGCTTC
+
FFFFFFFFFFFFFFFFFFFFFFFFFFFFFFFFFFFFFFFFFFFFFFFFFFFFFFFFF:FFFF:FFFFFF
FFFFFFFFFFFFFFFFFFFFFFFFFFFFFFF:FFFFFFFFFFFFFFFFFFFFFFFFFFFFFFFFFFFF
FFFFFFFFFFFFFFF
@A00721:190:HGJW7DSXY:4:1614:5249:23610 1:N:0:TCTGCAAG+GCTGCACT
GTCTGAAAGTATGTCGTCTCTGAAGAATGCGAAGCAATTGTAATCCGGACACCAGAATGGATATATGA
TCTTCGTCATTTTCAGAGCAGCTCTGAATCGTGCTTCGTTTTCTACGTATCTGGACCAGTATATCTGC
TTGACTTGATGCTTC
```

+
FFFFFFFFFFFFFFFFFFFFFFFFFFFFFFFFFFFFFFFFFFF:FFFFFFFFFFFFFFFFFFFFFFFF
FFFFFFFFFFFFFFFFFFFFFFFFFFFFFFFFFFFFFFFFFFFFFFFF:FFFFFFFFFFFFFFFFFFF
FFFFFFFFFFFFFFF
@A00721:190:HGJW7DSXY:4:1624:7256:15280 1:N:0:TCTGCAAG+GCTGCACT
GTCTGAAAGTATGTCGTCTCTGAAGAATGCGAAGCAATTGTAATCCGGACACCAGAATGGATATATGA
TCTTCGTCATTTTCAGAGCAGCTCTGAATCGTGCTTCGTTTTCTACGTATCTGGACCAGTATATCTGC
TTGACTTGATGCTTC
+
FFFFFFFFFFFFFFFFFFFFFFFFFFFFFFFFFFFFFFFFFFFFFFFFFFFFFFFF:FFFFFFFFFFF
FFFFFFFFFFFFFFFFFFFFFFFFFFFFFFFFFFFFFFFFFFFFFFFFFFFFFFFFFFFFFFFFFFFF
FFFFFFFFFFFFFFF
@A00721:190:HGJW7DSXY:4:1624:8079:15452 1:N:0:TCTGCAAG+GCTGCACT
GTCTGAAAGTATGTCGTCTCTGAAGAATGCGAAGCAATTGTAATCCGGACACCAGAATGGATATATGA
TCTTCGTCATTTTCAGAGCAGCTCTGAATCGTGCTTCGTTTTCTACGTATCTGGACCAGTATATCTGC
TTGACTTGATGCTTC
+
FFFFFFFFFFFFFFFFFFFFFFFFFFFFFFFFFFFFFFFFFFFFFFFFFFFFFFFFFFFFFFFFFFFF
FFFFFFFFFFFFFFFFFFFFFFFFFFFFFFFFFFFFFFFFFFFFFFFFFFFFFFFFFFFFFFFFFFFF
FFFFFFFFFFFFFFF
@A00721:190:HGJW7DSXY:4:1665:32000:16485 1:N:0:TCTGCAAG+GCTGCACT
GTCTGAAAGTATGTCGTCTCTGAAGAATGCGAAGCAATTGTAATCCGGACACCAGAATGGATATATGA
TCTTCGTCATTTTCAGAGCAGCTCTGAATCGTGCTTCGTTTTCTACGTATCTGGACCAGTATATCTGC
TTGACTTGATGCTTC
+
FFFFFFFFFFFFFFFFFFFFFFFFFFFFFFFFFFFFFFFFFFFFFFFFFFFFFFFFFFFFFFFFFFFF
FFFFFFFFFFFFFFFFFFFFFFFFFFFFFFFFFFFFFFFFFFFFFFFF,FFFFFFFFFFFFFFFFFFF
FFF:FFFFFFFFFFF
@A00721:190:HGJW7DSXY:4:2153:23411:8750 1:N:0:TCTGCAAG+GCTGCACT
GTCTGAAAGTATGTCGTCTCTGAAGAATGCGAAGCAATTGTAATCCGGACACCAGAATGGATATATGA
TCTTCGTCATTTTCAGAGCAGCTCTGAATCGTGCTTCGTTTTCTACGTATCTGGACCAGTATATCTGC
TTGACTTGATGCTTC
+
FFFFFFFFFFFFFFFFFFFFFFFFFFFFFFFFFFFFFFFFFFFFFFFFFFFFFFFFFFFFFFFFFFFF
FFFFFFFFFFFFFFFFFFFFFFFFFFFFFFFFFFFFFFFFFFFFFFFFFFFFFFFFFFFFFFFFFFFF
FFFFFFFFFFFFFFF
@A00721:190:HGJW7DSXY:4:2225:5556:22200 1:N:0:TCTGCAAG+GCTGCACT
GTCTGAAAGTATGTCGTCTCTGAAGAATGCGAAGCAATTGTAATCCGGACACCAGAATGGATATATGA
TCTTCGTCATTTTCAGAGCAGCTCTGAATCGTGCTTCGTTTTCTACGTATCTGGACCAGTATATCTGC
TTGACTTGATGCTTC
+
FFFFFFFFFFFFFFFFFFFFFFFFFFFFFFFFFFFFFFFFFFFFFFFFFFFFFFFFFFFFFFFFFFFF
FFFFFFFFFFFFFFFFFFFFFFFFFFFFFFFFFFFFFFFFFFFFFFFFFFFFFFF:FFFFFFFF:FFF
FFFFFFFFFF::FFF
@A00721:190:HGJW7DSXY:4:2244:17743:25113 1:N:0:TCTGCAAG+GCTGCACT
GTCTGAAAGTATGTCGTCTCTGAAGAATGCGAAGCAATTGTAATCCGGACACCAGAATGGATATATGA
TCTTCGTCATTTTCAGAGCAGCTCTGAATCGTGCTTCGTTTTCTACGTATCTGGACCAGTATATCTGC
TTGACTTGATGCTTC
+
FFFFFFFFFFFFFFFFFFFFFFFFFFFFFFFFFFFFFFFFFFFFFFFFFFFFFFFF:FFFFFFFFFFF
FFFFFFFFFFFFFFFFFFFFFFFFFFFFFFFFFFFFFFFFFFFFFFFFFFFFFFFFFFFFFFFFFFFF
FFFFFFFFFFFFFFF
@A00721:190:HGJW7DSXY:4:2253:6108:17362 1:N:0:TCTGCAAG+GCTGCACT
GTCTGAAAGTATGTCGTCTCTGAAGAATGCGAAGCAATTGTAATCCGGACACCAGAATGGATATATGA

```
TCTTCGTCATTTTCAGAGCAGCTCTGAATCGTGCTTCGTTTTCTACGTATCTGGACCAGTATATCTGC
TTGACTTGATGCTTC
+
FFFFFFFFFFFFFFFFFFFFFFFFFFFFFFFFFFFFF:FFFFFFFFFFFFFFFFFFFFFFFFFFFFFFFF
FFFFFFFFFFFFFFFFFFFFFFFFFFFFFFFFFFFFFFFFFFFFFFFFFF:FFFFFFFFFFFFF:FFFFF
:FFFFFFFFFFFFF:
@A00721:190:HGJW7DSXY:4:2307:19967:4633 1:N:0:TCTGCAAG+GCTGCACT
GTCTGAAAGTATGTCGTCTCTGAAGAATGCGAAGCAATTGTAATCCGGACACCAGAATGGATATATGA
TCTTCGTCATTTTCAGAGCAGCTCTGAATCGTGCTTCGTTTTCTACGTATCTGGACCAGTATATCTGC
TTGACTTGATGCTTC
+
FFFFFFFFFFFFFFFFFFFFFFFFFFFFFFFFFFFFFFFFFFFFFFFFFFFFFFFFFFFFFFFFFFFFFF
:FFFFFFFFFFFFFFFFFFFFFFFFFFFFFFFFFFFFFFFFFFFFFFFFF:FFFFFFFFFFFFFFFFFFF
FFFFFFFFFFFFFFF
@A00721:190:HGJW7DSXY:4:2311:11288:17785 1:N:0:TCTGCAAG+GCTGCACT
GTCTGAAAGTATGTCGTCTCTGAAGAATGCGAAGCAATTGTAATCCGGACACCAGAATGGATATATGA
TATTCGTCATTTTCAGAGCAGCTCTGAATCGTGCTTCGTTTTCTACGTATCTGGACCAGTATATCTGC
TTGACTTGATGCTTC
+
FFFFFFFFF,FFFF:F,:F:F:FFFFFFFFFFFF:FFFFFFFFFF,FF:F::F:F:FFF:FFFFFFFFFF
F,FFF:FFF,FFFFF:F,FF,FFFFFFFFFFFF:FFFF,FFFFFFFF:F,FFFF,FFF,F:FF:FFFF:F
FFFF,,F,FF:FFFF
@A00721:190:HGJW7DSXY:4:2334:25590:33661 1:N:0:TCTGCAAG+GCTGCACT
GTCTGAAAGTATGTCGTCTCTGAAGAATGCGAAGCAATTGTAATCCGGACACCAGAATGGATATATGA
TCTTCGTCATTTTCAGAGCAGCTCTGAATCGTGCTTCGTTTTCTACGTATCTGGACCAGTATATCTGC
TTGACTTGATGCTTC
+
FFFFFFFFFFFFFFFFFFFFFFFFFFFFFFFFFFFFFFFFFFFFFFFFFFFFFFFFFFFFFFFFFFFFFF
FFFFFFFFFFFFFFFFFFFFFFFFFFFFFFFFFFFFFFFFFFFFFFFFFFFFFFFFFFFFFFFFFFFFFF
FFFFFFFFFFFFFFF
@A00721:190:HGJW7DSXY:4:2352:5602:31923 1:N:0:TCTGCAAG+GCTGCACT
GTCTGAAAGTATGTCGTCTCTGAAGAATGCGAAGCAATTGTAATCCGGACACCAGAATGGATATATGA
TCTTCGTCATTTTCAGAGCAGCTCTGAATCGTGCTTCGTTTTCTACGTATCTGGACCAGTATATCTGC
TTGACTTGATGCTTC
+
:FF:FFFFFFFF:FFFFF:FFFFFFFF,FFFFFFFFFFFFFFFF:FF:FFFFFFFFFFFFFFFFFFFFFF
FFFFFF:FF:FFFFFFFFFFFFFFFFFFFFFFF::FFFFF:FFFFFFFF:FFFFFFFFFFFFFFFFFF:F
F,FFFFF:FFFFFFF
@A00721:190:HGJW7DSXY:4:2377:32072:12915 1:N:0:TCTGCAAG+GCTGCACT
GTCTGAAAGTATGTCGTCTCTGAAGAATGCGAAGCAATTGTAATCCGGACACCAGAATGGATATATGA
TCTTCGTCATTTTCAGAGCAGCTCTGAATCGTGCTTCGTTTTCTACGTATCTGGACCAGTATATCTGC
TTGACTTGATGCTTC
+
FFFFFFFFFFFFFFFFFFFFFFFFFFFFFFFFFFFFFFFFFFFFFFFFFFFFFFFFFFFFFFFFFFFFFF
FFFFFFFFFFFFFFFFFFFFFFFFFFFFFFFFFF:FFFFFFFFFFF::FFFFFFFFFFFFFFFFFFFFFF
FFFF:FFFFFFFFFF
@A00721:190:HGJW7DSXY:4:2427:18213:30060 1:N:0:TCTGCAAG+GCTGCACT
GTCTGAAAGTATGTCGTCTCTGAAGAATGCGAAGCAATTGTAATCCGGACACCAGAATGGATATATGA
TCTTCGTCATTTTCAGAGCAGCTCTGAATCGTGCTTCGTTTTCTACGTATCTGGACCAGTATATCTGC
TTGACTTGATGCTTC
+
FFFFFFFFFFFFFFFFFFFFFFFFFFFFFFFFFFFFFFFFFFFFFFFFFFFFFFFFFFFFFFFFFFFFFF
FFFFFFFFFFFFFFFFFFFFFFFFFFFFFFFF:FFFFFFFFFFFFFFFFFFFFFFFFFFFFFFFFFFFFF
FFFFFFFFFFFFFFF
```

@A00721:190:HGJW7DSXY:4:2443:8585:23469 1:N:0:TCTGCAAG+GCTGCACT
GTCTGAAAGTATGTCGTCTCTGAAGAATGCGAAGCAATTGTAATCCGGACACCAGAATGGATATATGA
TCTTCGTCATTTTCAGAGCAGCTCTGAATCGTGCTTCGTTTTCTACGTATCTGGACCAGTATATCTGC
TTGACTTGATGCTTC
+
FFFFFFFFFFFFFFFFFFFFFFFFFFFFFFFFFFFFFF:FFFFFFF:FFFFFFFFFFFFFFFFFFFFFFF
FFFFFFFFF:F:FFFFFFFFFFF:FFFFFFFFFFF:FFFFFF:FFFFFF,FF,FF::FFF,,FFFFFFFF
FFFF,F,F,FFFFFF
@A00721:190:HGJW7DSXY:4:2443:8612:24205 1:N:0:TCTGCAAG+GCTGCACT
GTCTGAAAGTATGTCGTCTCTGAAGAATGCGAAGCAATTGTAATCCGGACACCAGAATGGATATATGA
TCTTCGTCATTTTCAGAGCAGCTCTGAATCGTGCTTCGTTTTCTTCGTATCTGGACCAGTATATCTGC
TTGACTTGATGCTTC
+
FFFFFFFFFFFFFFFFFFFFFFFFFFFFFFFFFFFFFFFFFFFFFFFFFFFFFFFFFFFFFFFFFFFFFF
FFFFFFFFFFFFFFFFFFFFFFFFFFFFFFFFFFFFFFFFFFFFFFFFFFFFFFFFFFFFFFFFFFFFFF
FFFFFFFFFF,FFFF
@A00721:190:HGJW7DSXY:4:2522:29803:32189 1:N:0:TCTGCAAG+GCTGCACT
GTCTGAAAGTATGTCGTCTCTGAAGAATGCGAAGCAATTGTAATCCGGACACCAGAATGGATATATGA
TCTTCGTCATTTTCAGAGCAGCTCTGAATCGTGCTTCGTTTTCTACGTATCTGGACCAGTATATCTGC
TTGACTTGATGCTTC
+
FFFFFFFFFFFFFFFFFFFFFFFFFFFFFFFFFFFFFFFFFFFFFFFFFFFFFFFFFFFFFFFFFFFFFF
FFFFFFFFFFFFFFFFFFFFFFFFFFFFFFFFFFFFFFFFFFFFFFFFFFFFFFFFFFFFFFFFFFFFFF
FFF:FFFFFFFFFFF
@A00721:190:HGJW7DSXY:4:2551:23213:15264 1:N:0:TCTGCAAG+GCTGCACT
GTCTGAAAGTATGTCGTCTCTGAAGAATGCGAAGCAATTGTAATCCGGACACCAGAATGGATATATGA
TCTTCGTCATTTTCAGAGCAGCTCTGAATCGTGCTTCGTTTTCTACGTATCTGGACCAGTATATCTGC
TTGACTTGATGCTTC
+
FFFFFFFFFFFFFFFFFFFFFFFFFFFFFFFFFFFFFFFFFFFFFFFFFFFFFFFFFFFFFFFFFFFFFF
FFFFFFFFFFFFFFFFFFFFFFFFFFFFFFFFFFFFFFFFFFFFFFFFFFFFFFFFFFFFFFFFFFFFFF
FFFFFFFFFFFFFFF
@A00721:190:HGJW7DSXY:4:2552:9607:19601 1:N:0:TCTGCAAG+GCTGCACT
GTCTGAAAGTATGTCGTCTCTGAAGAATGCGAAGCAATTGTAATCCGGACACCAGAATGGATATATGA
TCTTCGTCATTTTCAGAGCAGCTCTGAATCGTGCTTCGTTTTCTACGTATCTGGACCAGTATATCTGC
TTGACTTGATGCTTC
+
FFFFFFFFFFFFFFFFFFFFFFFFFFFFFFFFFFFFFFFFFFFFFFFFFFFFFFFFFFFFFFFFFFFFFF
FFFFFFFFFFFFFFFFFFFFFFFFFFFFFFFFFFFFFFFFFFFFFFFFFFFFFFFFFFFFFFFFFFFFFF
FFFFFFFFFFFFFFF
@A00721:190:HGJW7DSXY:4:2603:1145:22639 1:N:0:TCTGCAAG+GCTGCACT
GTCTGAAAGTATGTCGTCTCTGAAGAATGCGAAGCAATTGTAATCCGGACACCAGAATGGATATATGA
TCTTCGTCATTTTCAGAGCAGCTCTGAATCGTGCTTCGTTTTCTACGTATCTGGACCAGTATATCTGC
TTGACTTGATGCTTC
+
FFFFFFFFFFFFFFFFFFFFFFFFFFFF:FFFFFFFFFF:FFFFFFFFFFFFFFFFFFFFFFFFFFFFFF
FFFFFFFFFFFFFFFFFFFFFFFFFFFFFFFF:FFFFFFFFFFFFFFFFFFFF:FFFFFFFFFFFFFFFF
F:FFFFFFFFFFFFF
@A00721:190:HGJW7DSXY:4:1150:28718:16908 1:N:0:TCTGCAAG+GCTGCACT
GTCGTCTCTGAAGAATGCGAAGCAATTGTAATCCGGACACCAGAATGGATATATGATCTTCGTCATTT
TCAGAGCAGCTCTGAATCGTGCTTCGTTTTCTACGTATCTGGACCAGTATATCTGCTTGACTTGATGC
TTCAAGTCATCTGGG
+
FFFFFFFFFFFFFFFFFFFF:FFFFFFFFFFFFFFFFFFFFFFFFFFFFFFFFFFFFFFFF:FFFFFFFF

FFFFFFFFFFFFFFFFFFFFFFFFFFFFFFFFFFFFFFFFFFFFFFFFFF:FFFFFFFFFFFFFFFFFF
FFFFFFFFFFFFFFF
@A00721:190:HGJW7DSXY:4:1472:12057:14826 1:N:0:TCTGCAAG+GCTGCACT
GTCGTCTCTGAAGAATGCGAAGCAATTGTAATCCGGACACCAGAATGGATATATGATCTTCGTCATTT
TCAGAGCAGCTCTGAATCGTGCTTCGTTTTCTACGTATCTGGACCAGTATATCTGCTTGACTTGATGC
TTCAAGTCATCTGGG
+
FFFFFFFFFFFFFFFFFFFFFFFFF:FFFFFFFFFFFFFFFFFFFFFFFFFFFFFFFFFFFFFFFFFFFF
FFFFFFFFFFFFFFFFFFFFFFFFFFFFFFFFFFFFFFFFFFFFFFFFFFFFFFFFFFFFFFFFFFFF
FFFFFFFFFFFFFFF
@A00721:190:HGJW7DSXY:4:1510:31268:4476 1:N:0:TCTGCAAG+GCTGCACT
GTCGTCTCTGAAGAATGCGAAGCAATTGTAATCCGGACACCAGAATGGATATATGATCTTCGTCATTT
TCAGAGCAGCTCTGAATCGTGCTTCGTTTTCTACGTATCTGGACCAGTATATCTGCTTGACTTGATGC
TTCAAGTCATCTGGG
+
FFFFFFFFFFFFFFFFFFFFFFFFF:FFFFFFFFFFFFFFFFFFFFFFFFF,FFFFFFFFFFFFFFFFFF
FFFFFFFFFFFFFFFFFFFFFFFFFFFFFFFFFFFFFFFFFFFF:FFFFFFFFFFFFFFFFFFFFFFFF
FFFFF:FFFFFFFFF
@A00721:190:HGJW7DSXY:4:1603:26458:17002 1:N:0:TCTGCAAG+GCTGCACT
GTCGTCTCTGAAGAATGCGAAGCAATTGTAATCCGGACACCAGAATGGATATATGATCTTCGTCATTT
TCAGAGCAGCTCTGAATCGTGCTTCGTTTTCTACGTATCTGGACCAGTATATCTGCTTGACTTGATGC
TTCAAGTCATCTGGG
+
FFFFFFFFFFFFFFFFFFFFFFFFFFFFFFFFFFFFFFFFFFFFFFFFFFFFFFFFFFFFFFFFFFFF
FFFFFFFFFFFFFFFFFFFFFFFFFFFFFFFFFFFFFFFFFFFFFFFFFFFFFFFFFFFFFFFFFFFF
FFFFFFFFFFFFFFF
@A00721:190:HGJW7DSXY:4:1662:8314:13792 1:N:0:TCTGCAAG+GCTGCACT
GTCGTCTCTGAAGAATGCGAAGCAATTGTAATCCGGACACCAGAATGGATATATGATCTTCGTCATTT
TCAGAGCAGCTCTGAATCGTGCTTCGTTTTCTACGTATCTGGACCAGTATATCTGCTTGACTTGATGC
TTCAAGTCATCTGGG
+
:FFFFFF:F:FFF:FFFFFFFFFFFFFFFFF:FFFFFFFFFFFFF,FFFFFFFFFFF:F:FFFFFFFF
FFFFFFFFFFFFFFFFF:F:FFF:FFFF:FFFFFFFFFFFFFFFFFFFFFFFFF,FFFFFFFF:F:FFF
:FFFFFFFF:FF:F:
@A00721:190:HGJW7DSXY:4:1664:4517:27727 1:N:0:TCTGCAAG+GCTGCACT
GTCGTCTCTGAAGAATGCGAAGCAATTGTAATCCGGACACCAGAATGGATATATGATCTTCGTCATTT
TCAGAGCAGCTCTGAATCGTGCTTCGTTTTCTACGTATCTGGACCAGTATATCTGCTTGACTTGATGC
TTAAAGTCATCTGG
+
:FF::F,FFFFF,F:F::,FF,F:F:F,,FFFF:FFFFF:,FFF:F,::FFFFFFF,F:FF,F,FFF:
FF::F,,F,F:FFFFFF,F:FFF,:,,,F,FF:FF,:F:F,,FFFFF,FFFF,:,FF,:FFFF:FF::
F,,FF:F,,FFFFF
@A00721:190:HGJW7DSXY:4:2576:4951:26256 1:N:0:TCTGCAAG+GCTGCACT
GTCGTCTCTGAAGAATGCGAAGCAATTGTAATCCGGACACCAGAATGGATATATGATCTTCGTCATTT
TCAGAGCAGCTCTGAATCGTGCTTCGTTTTCTACGTATCTGGACCAGTATATCTGCTTGACTTGATGC
TTCAAGTCATCTGGG
+
FFFFFFFFFFFFFFFFFFFFFFFFFFFFFFFFFFFFFFFFFFFFFFFFFFFFFFFFFFFFFFFFFFFF
FFFFFFFFFFFFFFFFFFFFFFFFFFFFFFFFFFFFFFFFFFFFFFFFFFFFFFFFFFFFFFFFFFFF
FFFFFFFFFFFFFFF
@A00721:190:HGJW7DSXY:4:2617:24306:17910 1:N:0:TCTGCAAG+GCTGCACT
GTCGTCTCTGAAGAATGCGAAGCAATTGTAATCCGGACACCAGAATGGATATATGATCTTCGTCATTT
TCAGAGCAGCTCTGAATCGTGCTTCGTTTTCTACGTATCTGGACCAGTATATCTGCTTGACTTGATGC
TTCAAGTCATCTGGG

+
FFFFFFFFFFFFFFFFFFFFFFFFFFFFFFFFFFFFFFFFFFFFFFFFF:FFFFFFFFFFFFFFFF
FFFFFFFFFFFFFFFFFFFFFFFFFFFFFFFFFF:FFFFFFFFFFFFFFFFFFFFFFFFFFFFFFF
FFFFFFFFFFFFFFF
@A00721:190:HGJW7DSXY:4:1172:12111:35368 1:N:0:TCTGCAAG+GCTGCACT
GTCTCTGAAGAATGCGAAGCAATTGTAATCCGGACACCAGAATGGATATATGATCTTCGTCATTTTCA
GAGCAGCTCTGAATCGTGCTTCGTTTTCTACGTATCTGGACCAGTATATCTGCTTGACTTGATGCTTC
AAGTCATCTGGGAGA
+
FFFFFFFFFFFFFFFFFFFF:,FFFFF:FFFFFFFFFFFFF,FFF:FFFFFFFFFFFFFFF:FFFFFF
FFFFFFFFFFFFFFFFFFFFFFFFFFFFFFFFFF:F:FFFFFFFFFFFFFFFF:FFFF:FFFFFFFFF
:FFFFFFF:FFFFFF
@A00721:190:HGJW7DSXY:4:1648:12608:28025 1:N:0:TCTGCAAG+GCTGCACT
GTCTCTGAAGAATGCGAAGCAATTGTAATCCGGACACCAGAATGGATATATGATCTTCGTCATTTTCA
GAGCAGCTCTGAATCGTGCTTCGTTTTCTACGTATCTGGACCAGTATATCTGCTTGACTTGATGCTTC
AAGTCATCTGGGAGA
+
FFFFFFFFFFFFFFFFFFFFFFFFFFFFFFFFFFFFFFFFFFFFFFFFFFFFFFFFFFFFFFFFFFFF
FFFFFFFFFFFFFFFFFFFFFFFFFFFFFFFFFFFFFFFFFFFFFFFFFFFFFFFFFFFFFFFFFFFF
FFFFFFFFFFFFFFF
@A00721:190:HGJW7DSXY:4:2146:25672:16611 1:N:0:TCTGCAAG+GCTGCACT
GTCTCTGAAGAATGCGAAGCAATTGTAATCCGGACACCAGAATGGATATATGATCTTCGTCATTTTCA
GAGCAGCTCTGAATCGTGCTTCGTTTTCTACGTATCTGGACCAGTATATCTGCTTGACTTGATGCTTC
AAGTCATCTGGGAGA
+
FFFFFFFFFFFFFFFFFFFFFFFFFFF,FFFFFFFFFFFFF:FFFFFFFFFFFFFFFFFFFFFFFFFF
FF:FFFFFFFFFFFFFFFFFFFFFFFFFFFFFFFFFFFFFFFFFFFFF:FFFFFFF:FFFFFF::FFFF
FFFFFF,F,F:F,FF
@A00721:190:HGJW7DSXY:4:2178:11975:11240 1:N:0:TCTGCAAG+GCTGCACT
GTCTCTGAAGAATGCGAAGCAATTGTAATCCGGACACCAGAATGGATATATGATCTTCGTCATTTTCA
GAGCAGCTCTGAATCGTGCTTCGTTTTCTACGTATCTGGACCAGTATATCTGCTTGACTTGATGCTTC
AAGTCATCTGGGAGA
+
FFFFFFFFFFFFFFFFFFFFFFFFFFFFFFFFFFFFFFFFFFFFFFFFFFFFFFFFFFFFFFFFFFFF
FFFFFFFFFFFFFFFFFFFFFFFFFFFFFFFFFFFFFFFFFFFFFFFFFFFFFFFFFFFFFFFFFFFF
FFFFFF::FFFFFFF
@A00721:190:HGJW7DSXY:4:2178:12038:11256 1:N:0:TCTGCAAG+GCTGCACT
GTCTCTGAAGAATGCGAAGCAATTGTAATCCGGACACCAGAATGGATATATGATCTTCGTCATTTTCA
GAGCAGCTCTGAATCGTGCTTCGTTTTCTACGTATCTGGACCAGTATATCTGCTTGACTTGATGCTTC
AAGTCATCTGGGAGA
+
FFFFFFFFFFFFFFFFFFFFF,:FFFFFFFFFFFFFFF:FFFFFFFFFFFFFFFFFFFFFFFFFFFFFF
FFFFFFFFFFFFFFFFFFFFFFFFFFFFFFFFFFFFFFFFFFFFFFFFFFFFFFFFFFFFFFFFFFFF
FFFFFFFF,FFFFFF
@A00721:190:HGJW7DSXY:4:2601:7500:17206 1:N:0:TCTGCAAG+GCTGCACT
GTCTCTGAAGAATGCGAAGCAATTGTAATCCGGACACCAGAATGGATATATGATCTTCGTCATTTTCA
GAGCAGCTCTGAATCGTGCTTCGTTTTCTACGTATCTGGACCAGTATATCTGCTTGACTTGATGCTTC
AAGTCATCTGGGAGA
+
FFFFFFFFFFFFFFFFFFFFFFFFFFFFFFFFFFFFFFFFFF:FFFFFFFFFFFF:FFFFFFFFFFFF
FFFFFFFFFFFFFFFFFFFFFFFFFFFFFFFFFFFFFFF:FFFFFFFFFFFFFFFFFFFFFFFFFFFF
FFFFFFFFFFFFFFF
@A00721:190:HGJW7DSXY:4:2676:7274:28338 1:N:0:TCTGCAAG+GCTGCACT
GTCTCTGAAGAATGCGAAGCAATTGTAATCCGGACACCAGAATGGATATATGATCTTCGTCATTTTCA

```
GAGCAGCTCTGAATCGTGCTTCGTTTTCTACGTATCTGGACCAGTATATCTGCTTGACTTGATGCTTC
AAGTCATCTGGGAGA
+
FFFFFFFFFFFFFFFFFFFFFFFFFFFFFFFFFFFFFFFFFFFFFFFFFFFFFFFFFFFFFFFFFFFFFF
FFFFFFFFFFFFFFFFFFFFFFFFFFFFFFFFFFFFFFFFFFFFFFFFFFFFFFFFFFFFFFFFFFFFFF
FFFFFFFFFFFFFFF
@A00721:190:HGJW7DSXY:4:2538:23258:21543 1:N:0:TCTGCAAG+GCTGCACT
TCTGAAGAATGCGAAGCAATTGTAATCCGGACACCAGAATGGATATATGATCTTCGTCATTTTCAGAG
CAGCTCTGAATCGTGCTTCGTTTTCTACGTATCTGGACCAGTATATCTGCTTGACTTGATGCTTCAAG
TCATCTGGGAGA
+
:FFFFF,FFFF,:FF:FFFF,,FFF,:,::F:F,FFFF,F,:F,F,FFFFF,F,::F,F,FFF,F:FF
FFF,F:F,FFF,,F,:F:,:FFF:FFF,FFFFFF:,F,:F::FFFF,F:,FF,F,::FFF,::F,FF,
F,F:,F:,:FFF
@A00721:190:HGJW7DSXY:4:1336:22498:26960 1:N:0:TCTGCAAG+GCTGCACT
AATCCGGACACCAGAATGGATATATGATCTTCGTCATTTTCAGAGCAGCTCTGAATCGTGCTTCGTTT
TCTACGTATCTGGACCAGTATATCTGCTTGACTTGATGCTTCAAGTCATCTGGGAGAATGTTGTAGAT
ATCGTCAAATTTGAT
+
FFFFFFFFFFFFFFFFFFFFFFFFFFFFFFFFFFFFFFFFFFFFFFFFFFFFFFFFFFFFFFFFFFF:FF
FFFFFFFFFFFFFFFFFFFFFFFFFFFFFFFFFFFFFFFFFFFFFFFFFFFFFFFFFFFFFFFFFFFFFF
FFFFFFFFFFFFFFF
@A00721:190:HGJW7DSXY:4:2369:26033:15013 1:N:0:TCTGCAAG+GCTGCACT
AATCCGGACACCAGAATGGATATATGATCTTCGTCATTTTCAGAGCAGCTCTGAATCGTGCTTCGTTT
TCTACGTATCTGGACCAGTATATCTGCTTGACTTGATGCTTCAAGTCATCTGGGAGAATGTTGTAGAT
ATCGTCAAATTTGAT
+
FFFFFFFFFFFFFFFFFFFFFFFFFFFFFFFFFFFFFFFFFFFFFFFFFFFFFFFFFFFFFFFFFFFFFF
FFFFFFFFFFFFFFFFFFFFFFFFFFFFFFFFFFFFFFFFFFFFFFFFFFFFFFFFFFFFFFFFFFFFFF
FFFFFFFFFFFFFFF
@A00721:190:HGJW7DSXY:4:2438:28619:26725 1:N:0:TCTGCAAG+GCTGCACT
AATCCGGACACCAGAATGGATATATGATCTTCGTCATTTTCAGAGCAGCTCTGAATCGTGCTTCGTTT
TCTACGTATCTGGACCAGTATATCTGCTTGACTTGATGCTTCAAGTCATCTGGGAGAATGTTGTAGAT
ATCGTCAAATTTGAT
+
FFFFFFFFFFFFFFFFFFFFFFFFFFFFFFFFFFFFFFFFFFFFFFFFFFFFFFFFFFFFFFFFFFFFFF
FFFFFFFFFFFFFFFFFFFFFFFFFFFFFFFFFFFF:FFFFFFFFFFFFFFFFFFFFFFFFFFFFFFFFF
FFFFFF:FFFFFF,F
@A00721:190:HGJW7DSXY:4:2603:20708:19820 1:N:0:TCTGCAAG+GCTGCACT
AATCCGGACACCAGAATGGATATATGATCTTCGTCATTTTCAGAGCAGCTCTGAATCGTGCTTCGTTT
TCTACGTATCTGGACCAGTATATCTGCTTGACTTGATGCTTCAAGTCATCTGGGAGAATGTTGTAGAT
ATCGTCAAATTTGAT
+
FFFFFFFFFFFFFFF,:FFFFFFFFFFFFF:FFFFFFFFFFFFFFFFFFFFFF:FFFFFFFF,FFFFFFF
FFFFF::FFFFFFFFFFFFF:FFFFFFFFF,FFFFFF::FF:,F::FFFFFFFFFF:,FFF:F:,FFFFF
FF:FFF:FFFFFFFF
@A00721:190:HGJW7DSXY:4:2211:13078:5040 1:N:0:TCTGCAAG+GCTGCACT
ATCCGGACACCAGAATGGATATATGATCTTCGTCATTTTCAGAGCAGCTCTGAATCGTGCTTCGTTTT
CTACGTATCTGGACCAGTATATCTGCTTGACTTGATGCTTCAAGTCATCTGGGAGAATGTTGTAGATA
TCGTCAAATTTGATA
+
FFFFFFFF:FFFFFFFFFFFFFFFFFFFFFFFFFFFFFFFFFFFFFFFFFFFFFFFFFFFFFFFFFFFFF
FFFFFFFFFFFFFFFFFFFFFFFFFFFFFFFFFFFFFFFFFFFFFFFFFFFFFFFFFFFFFFFFFFFFFF
FFFFFFFFFFFFFFF
```

```
@A00721:190:HGJW7DSXY:4:2421:3025:3975 1:N:0:TCTGCAAG+GCTGCACT
ATCCGGACACCAGAATGGATATATGATCTTCGTCATTTTCAGAGCAGCTCTGAATCGTGCTTCGTTTT
CTACGTATCTGGACCAGTATATCTGCTTGACTTGATGCTTCAAGTCATCTGGGAGAATGTTGTAGATA
TCGTCAAATTTGATA
+
FF::FFFFFFF:FFFF:FFFFFFFFFFFFFF:FFFF:F,FFFFFFFF,F:FFFF::FFFFFFFF,,,:
:FF:,FFFF:::FFFF:F::FFF:FFFFFFFFF,F:,:FFFFFFFFF,F::F:FFF,FF:FFFFFFFF
FF::,FFF:,F,FFF
@A00721:190:HGJW7DSXY:4:2426:3685:13761 1:N:0:TCTGCAAG+GCTGCACT
ATCCGGACACCAGAATGGATATATGATCTTCGTCATTTTCAGAGCAGCTCTGAATCGTGCTTCGTTTT
CTACGTATCTGGACCAGTATATCTGCTTGACTTGATGCTTCAAGTCATCTGGGAGAATGTTGTAGATA
TCGTCAAATTTGATA
+
FFFFFFFFFFFFFFFFFFFFFFFFFFFFFFFFFFFFFFF:FFFFFF:FFFFFFFFFFF:FFFFFFFFF
FFF:FFFFFFF:FFFFF:FFFFFFFFFFFFFFFFFFFFFFFFFFFFFFFFFFFFFFFFFFF::FFFFF
F:FFFFFFFFFFFFF
@A00721:190:HGJW7DSXY:4:2528:12364:23657 1:N:0:TCTGCAAG+GCTGCACT
ATCCGGACACCAGAATGGATATATGATCTTCGTCATTTTCAGAGCAGCTCTGAATCGTGCTTCGTTTT
CTACGTATCTGGACCAGTAAATCTGCTTGACTTGATGCTTCAAGTCATCTGGGAGAATGTTGTAGATA
TCGTCAAATTTGATA
+
:FF:FFFFFFFFFFFF,:FFFFFF:F,FFFFF,FFFFF:FFFFFFFFFF:FFFFFF,,:F,FFFFF,F
FFFF:,FFF,F:F:FFF,F,FFFF,FFF:FFFFF:FFFFFFFF:,FFFF,,F:F::FF:F:FFF:FFF
FF,F:FFFF:,:FFF
@A00721:190:HGJW7DSXY:4:2571:28935:14403 1:N:0:TCTGCAAG+GCTGCACT
ATCCGGACACCAGAATGGATATATGATCTTCGTCATTTTCAGAGCAGCTCTGAATCGTGCTTCGTTTT
CTACGTATCTGGACCAGTATATCTGCTTGACTTGATGCTTCAAGTCATCTGGGAGAATGTTGTAGATA
TCGTCAAATTTGATA
+
FFFFFFFFFFFFFFFFFFFFFFFFFFFFFFFFFFFFFFFFFFFFFFFFFFFFFFFFFFFFFFFFFFFF
FFFFFFFFFFFFFFFFFFFFFFFFFFFFFFFFFFFFFFFFFFFFFFFFFFFFFFFFFFF:FFFFFFFF
FFF:FFFFFFFFFFF
@A00721:190:HGJW7DSXY:4:1110:24261:6120 1:N:0:TCTGCAAG+GCTGCACT
CCGGACACCAGAATGGATATATGATCTTCGTCATTTTCAGAGCAGCTCTGAATCGTGCTTCGTTTTCT
ACGTATCTGGACCAGTATATCTGCTTGACTTGATGCTTCAAGTCATCTGGGAGAATGTTGTAGATATC
GTCAAATTTGATAGT
+
FFFFFFFFFFFFFFFFFFFFFFFFFFFFFFFFFFFFFFFFFFFFFFFFFFFFFFFFFFFFFFF:FFFF
FFFFFFFFFFFFFFFFFFFFFFFFFFFFFFFFFFFFFFFFFFFFFFFFFFFFFFFFFFFFFFFF:FFF
F:FFF:FFFFFFFFF
@A00721:190:HGJW7DSXY:4:1202:4978:22514 1:N:0:TCTGCAAG+GCTGCACT
CCGGACACCAGAATGGATATATGATCTTCGTCATTTTCAGAGCAGCTCTGAATCGTGCTTCGTTTTCT
ACGTATCTGGACCAGTATATCTGCTTGACTTGATGCTTCAATTCATCTGGGAGAATGTTGTAGATATC
GTCAAATTTGATAGT
+
FF,FFFFFFFFFFF:F:FFF::F:FFFFFFFFFFFFFFFFF,FFFFFFF:FFFFFF:FFF:FFFFFFF
FFFFFFFFFFFFFF:FFFF:F,F:FFFFFF,,F,FFFFFFF,:FFF,:FFFFFFFF,:F::FFFF,F:
FFFFFF,FF:F:FFF
@A00721:190:HGJW7DSXY:4:1274:16242:4131 1:N:0:TCTGCAAG+GCTGCACT
CCGGACACCAGAATGGATATATGATCTTCGTCATTTTCAGAGCAGCTCTGAATCGTGCTTCGTTTTCT
ACGTATCTGGACCAGTATATCTGCTTGACTTGATGCTTCAAGTCATCTGGGAGAATGTTGTAGATATC
GTCAAATTTGATAGT
+
FFFFFFFFFFFFFFFFFFFFFFFFFFFFFFFFFFFFFFFFFFFFFFFFFFFFFFFFFFFFFFFFFFFF
```

```
FFFFFFFFFFFFFFFFFFFFFFFFFFFFFFFFFFFFFFFFFFFFFFFFFFFFFFFFFFFFFFFF:FFFFF
FFFFFFFFFFFFFFF
@A00721:190:HGJW7DSXY:4:1417:17146:30373 1:N:0:TCTGCAAG+GCTGCACT
CCGGACACCAGAATGGATATATGATCTTCGTCATTTTCAGAGCAGCTCTGAATCGTGCTTCGTTTTCT
ACGTATCTGGACCAGTATATCTGCTTGACTTGATGCTTCAAGTCATCTGGGAGAATGTTGTAGATATC
GTCAAATTTGATAGT
+
FFFFFFFFFFFFFFFFFFFFFFFFFFFFFFFFFFFFFFFFFFFFFFFFFFFFFFFFFFFFFFFFFFFFFF
FFFFFFFFFFFFFFFFFFFFFFFFFFFFFFFFFFFFFFFFFFFFFFFFFFFFFFFFFFFFFFFFFFFFFF
:FF:FFFFFFFFFFF
@A00721:190:HGJW7DSXY:4:1471:31458:32424 1:N:0:TCTGCAAG+GCTGCACT
CCGGACACCAGAATGGATATATGATCTTCGTCATTTTCAGAGCAGCTCTGAATCGTGCTTCGTTTTCT
ACGTATCTGGACCAGTATATCTGCTTGACTTGATGCTTCAAGTCATCTGGGAGAATGTTGTAGATATC
GTCAAATTTGATAGT
+
FFFFFFFFFFFFFFFFFFFFFFFFFFFFFFFFFFFFFFFFFFFFFFFFFFFFFFFFFFFFFFFFFFFFFF
FFFFFFFFFFFFFFFFFFFFFFFFFFFFFFFFFFFFFFFFFFFFFFFFFFFFFF:FFFFFFFFFFFFFFF
FFFFFFFFFFFFFFF
@A00721:190:HGJW7DSXY:4:1649:23529:5353 1:N:0:TCTGCAAG+GCTGCACT
CCGGACACCAGAATGGATATATGATCTTCGTCATTTTCAGAGCAGCTCTGAATCGTGCTTCGTTTTCT
ACGTATCTGGACCAGTATATCTGCTTGACTTGATGCTTCAAGTCATCTGGGAGAATGTTGTAGATATC
GTCAAATTTGATAGT
+
FFFFFF:FFFFFFFFFFFFFFFFFFFFFFFFFFFFFFFFFFFFFFFFFFFFFFFFFFFFFFFFFFFFFFF
FFFFFFFFFFFFFFFFFFFFFFFFFFFFFFFFFFFFFFFFFFFFFFFFFFFFFFFFFFFFFFFFFFFFFF
FFFFF:FFFFFFFFF
@A00721:190:HGJW7DSXY:4:2304:3061:34820 1:N:0:TCTGCAAG+GCTGCACT
CCGGACACCAGAATGGATATATGATCTTCGTCATTTTCAGAGCAGCTCTGAATCGTGCTTCGTTTTCT
ACGTATCTGGACCAGTATATCTGCTTGACTTGATGCTTCAAGTCATCTGGGAGAATGTTGTAGATATC
GTCAAATTTGATAGT
+
FFFFFFFFFFFFFFFFFFFFFFFFFFFFFFFFFFFFFFFFFFFFFFFFFFFFFFFFFFFFFFFFFFFFFF
FFFFFFFFFFFFFFFFFFFFFFFFFFFFFFFFFFFFFFFFFFFFFFFFFFFFFFFFFFFFFFFFFFFFF:
FFFFFFFFFFFFFFF
@A00721:190:HGJW7DSXY:4:2332:17418:13777 1:N:0:TCTGCAAG+GCTGCACT
CCGGACACCAGAATGGATATATGATCTTCGTCATTTTCAGAGCAGCTCTGAATCGTGCTTCGTTTTCT
ACGTATCTGGACCAGTATATCTGCTTGACTTGATGCTTCAAGTCATCTGGGAGAATGTTGTAGATATC
GTCAAATTTGATAGT
+
FFFFFFFFFFFFFFFFFFFFFFFFFFFFFFFFFFFFFFFFFFFFFFFFFFFFFFFFFFFFFFFFFFFFFF
FFFFF:FFFFFFFFFFFFFFFFFFFFFFFFFFFFFFFFFFFFFFFFFFFFFFFFFFFFFFFFFFFFFFFF
FFFFFFF,FFFFFFF
@A00721:190:HGJW7DSXY:4:2648:4472:33254 1:N:0:TCTGCAAG+GCTGCACT
CCGGACACCAGAATGGATATATGATCTTCGTCATTTTCAGAGCAGCTCTGAATCGTGCTTCGTTTTCT
ACGTATCTGGACCAGTATATCTGCTTGACTTGATGCTTCAAGTCATCTGGGAGAATGTTGTAGATATC
GTCAAATTTGATAGT
+
FFFFFFFFFFFFFFFFFFFFFFFFFFFFFFFFFFFFFFFFFFFFFFFFFFFFFFFFFFF:FFFFFFFFFF
:FFFFFFFFFFFFFFFFFFFFFFFFFFFFFFFFFFFFFFFFFFFFFFF:FFFFFFF:FFF:FFFFF:FFF
FF:FFFFFFFFFFFF
@A00721:190:HGJW7DSXY:4:1275:18213:25426 1:N:0:TCTGCAAG+GCTGCACT
CAGAATGGATATATGATCTTCGTCATTTTCAGAGCAGCTCTGAATCGTGCTTCGTTTTCTACGTATCT
GGACCAGTATATCTGCTTGACTTGATGCTTCAAGTCATCTGGGAGAATGTTGTAGATATCGTCAAATT
TGATAGTACCATAGT
```

+
FFFF:FFFFFFFFFFFFFFFFFFFFFFFFFFFFFFFFFFFFFFFFFFFFFFFFFFFFFFFFFFFFF
FFFFFFFFFFFFFFFFFFFFFFFFFFFFFFFFFFFFFFFFFFFFFFF,FFFFFFFFFFFFFF:FFFFFFF
FFFFFFFFFFFFFFF
@A00721:190:HGJW7DSXY:4:1275:18141:25457 1:N:0:TCTGCAAG+GCTGCACT
CAGAATGGATATATGATCTTCGTCATTTTCAGAGCAGCTCTGAATCGTGCTTCGTTTTCTACGTATCT
GGACCAGTATATCTGCTTGACTTGATGCTTCAAGTCATCTGGGAGAATGTTGTAGATATCGTCAAATT
TGATAGTACCATAGT
+
FFFFFFFFFFFFFFFFFFFFFFFFFFFFFFFFFFFFFFFFFFFFFFF:FFFFFFFFFFFFFFF,FFFFFF
FFFFFFFFFFFFFFFF:F:FFF:FFFFFFFFFFF:FFFF,FF,FF:FFFFFFFFFFFF,FFFFFF:FFFF
FFFFFFFF::FFFFF
@A00721:190:HGJW7DSXY:4:1329:1660:12477 1:N:0:TCTGCAAG+GCTGCACT
CAGAATGGATATATGATCTTCGTCATTTTCAGAGCAGCTCTGAATCGTGCTTCGTTTTCTACGTATCT
GGACCAGTATATCTGCTTGACTTGATGCTTCAAGTCATCTGGGAGAATGTTGTAGATATCGTCAAATT
TGATAGTACCATAGT
+
FFFF:FFFFFFFFFFFFFFFFFFFFFFFFFFFFFFFFFFFFFFFFF:FFFFFF:FFFFFFFFFFFFFFFF
FFFFFFFFFFFFFFFFFFFFFFFF:FFFFFFFFFFFFFFFFFFFFFF:FFFFFFFFFFFFFFFFFFFF:F
FFFFFFFFFFFFFFF
@A00721:190:HGJW7DSXY:4:1625:29668:11412 1:N:0:TCTGCAAG+GCTGCACT
CAGAATGGATATATGATCTTCGTCATTTTCAGAGCAGCTCTGAATCGTGCTTCGTTTTCTACGTATCT
GGACCAGTATATCTGCTTGACTTGATGCTTCAAGTCATCTGGGAGAATGTTGTAGATATCGTCAAATT
TGATAGTACCATAGT
+
FFFFFFFFFFFFFFFFFFFFFFFFFFFFFFFFFFFFFFFFFFFFFFFFFFFFFFFFFFFFFFFFFFFFFF
FFFFFFFFFFFFFFFFFFFFFFFFFFFFFFFFFFFFFFFFFFFFFFFFFFFFFFFFFFFFFFFFFFFFFF
FFFFFFFFFFFFFFF
@A00721:190:HGJW7DSXY:4:2129:14895:5368 1:N:0:TCTGCAAG+GCTGCACT
CAGAATGGATATATGATCTTCGTCATTTTCAGAGCAGCTCTGAATCGTGCTTCGTTTTCTACGTATCT
GGACCAGTATATCTGCTTGACTTGATGCTTCAAGTCATCTGGGAGAATGTTGTAGATATCGTCAAATT
TGATAGTACCATAGT
+
FFFFFFFFFFFFFFFFFFFFFFFFFFFFFFFFFFFFFFFFFFFFFFFFFFFFFFFFFFFFFFFFFFFFFF
FFFFFFFFFFFFFFF:FFFFFFFFFFFFFFFFFFFFFFFFFFFFFFFFFFFFFFFFFFFFFFFFFFFFFF
FFFFFFFF:FFFFFF
@A00721:190:HGJW7DSXY:4:2343:6045:13338 1:N:0:TCTGCAAG+GCTGCACT
CAGAATGGATATATGATCTTCGTCATTTTCAGAGCAGCTCTGAATCGTGCTTCGTTTTCTACGTATCT
GGACCAGTATATCTGCTTGACTTGATGCTTCAAGTCATCTGGGAGAATGTTGTAGATATCGTCAAATT
TGATAGTACCATAGT
+
FFFFFFFFFFFFFFFFFFFFFFFFFFFFFFFFFFFFFFFFFFFFFFFFFFFFFFFFFFFFFFFFFFFFFF
FFFFFFFFFFFFFFFFFFFFFFFFFFFFFFFFFFFFFFFFFFFFFFFFFFFFFFFFFFFFFFFFFFFFFF
FFFFFFFF:FFFFFF
@A00721:190:HGJW7DSXY:4:2406:1678:22153 1:N:0:TCTGCAAG+GCTGCACT
CAGAATGGATATATGATCTTCGTCATTTTCAGAGCAGCTCTGAATCGTGCTTCGTTTTCTACGTATCT
GGACCAGTATATCTGCTTGACTTGATGCTTCAAGTCATCTGGGAGAATGTTGTAGATATCGTCAAATT
TGATAGTACCATAGT
+
FFFFFFFFFFFFFFFFFFFFF:FFFFFFFFFFFFFFFFFFFFFFFFFFFFFFFFFFFFFFFFFFFFF:FF
FFFFFFFFFFFFFFFFFFFFFFFFFFFFFFFFFFFFFFFFFF:FFFFF:FFFFFFFFFFFFFFFFF:FFF
FFFFFFFFFFFFFFF
@A00721:190:HGJW7DSXY:4:2435:16007:35070 1:N:0:TCTGCAAG+GCTGCACT
CAGAATGGATATATGATCTTCGTCATTTTCAGAGCAGCTCTGAATCGTGCTTCGTTTTCTACGTATCT

```
GGACCAGTATATCTGCTTGACTTGATGCTTCAAGTCATCTGGGAGAATGTTGTAGATATCGTCAAATT
TGATAGTACCATAGT
+
FFFFFFFFFFFFFFFFFFFFFFFFFFFFFFFFFFFFFFFFFFFFFFFFFFFFFFFFFFFFFFFFFFFF
FFFFFFFFFFFFFFFFFFFFFFFFFFFFFFFFFFFFFFFFFFFFFFFFFFFFFFFFF,FFFFFFFFFF
F:FFFFFFFFFFFFF
@A00721:190:HGJW7DSXY:4:2435:16794:36808 1:N:0:TCTGCAAG+GCTGCACT
CAGAATGGATATATGATCTTCGTCATTTTCAGAGCAGCTCTGAATCGTGCTTCGTTTTCTACGTATCT
GGACCAGTATATCTGCTTGACTTGATGCTTCAAGTCATCTGGGAGAATGTTGTAGATATCGTCAAATT
TGATAGTACCATAGT
+
FFFFFFFFFFFFFFFFFFFFFFFFFFFFFFFFFFFFFFFFFFFFFFFFFFFFFFFFFFFFFFFFFFFF
FFFFFFFFFFFFFFFFFFFFFFFFFFFFFFFFFFFFFFFFFFFFFFFFFFFFFFFFFFFFFFFFFFF:
FFFFFFFF:FFFFFF
@A00721:190:HGJW7DSXY:4:2461:4264:31485 1:N:0:TCTGCAAG+GCTGCACT
CAGAATGGATATATGATCTTCGTCATTTTCAGAGCAGCTCTGAATCGTGCTTCGTTTTCTACGTATCT
GGACCAGTATATCTGCTTGACTTGATGCTTCAAGTCATCTGGGAGAATGTTGTAGATATCGTCAAATT
TGATAGTACCATAGT
+
FFFFFFFFFFFFFFFFFFFFFFFFFFFFFFFFFFFFFFFFFFFFFFFFFFFFFFFFFFFFFFFFFFFF
FFFFFFFFFFFFFFFFFFFFFFFFFFFFFFFFFFFFFFFFFFFFFFFFFFFFFFFFFFFFFFFFFFFF
FFFFFFFFFFFFFFF
@A00721:190:HGJW7DSXY:4:1259:24903:19695 1:N:0:TCTGCAAG+GCTGCACT
ATATGATCTTCGTCATTTTCAGAGCAGCTCTGAATCGTGCTTCGTTTTCTACGTATCTGGACCAGTAT
ATCTGCTTGACTTGATGCTTCAAGTCATCTGGGAGAATGTTGTAGATATCGTCAAATTTGATAGTACC
ATAGTTCGACGCCAT
+
FFFFFFFFFFFFFFFFFFFFFFFFFFFFFFFFFFFFFFFFFFFFFFFFFFFFFFFFFFFFFFFFFFFF
FFFFFFFFFFFFFFFFFFFFFFFFFFFFFFFFFFFFFFFFFFFFFFF:FFFF:FFFFFFFFFFFFFF
FFFFFFFFFFFFFFF
@A00721:190:HGJW7DSXY:4:2209:17544:36573 1:N:0:TCTGCAAG+GCTGCACT
ATATGATCTTCGTCATTTTCAGAGCAGCTCTGAATCGTGCTTCGTTTTCTACGTATCTGGACCAGTAT
ATCTGCTTGACTTGATGCTTCAAGTCATCTGGGAGAATGTTGTAGATATCGTCAAATTTGATAGTACC
ATAGTTCGACGCCAT
+
FFFFFFFFFFFFFFFFFFFFFFFFFFFFFFFFFFFFFFFFFFFFFFFFFFF:FFFFFFFFFFFFFFFF
FFFFFFFFFFFFFFFFFFFFFFFFFFFFFFFFFFFFFFFFFFFFFF:,FFFFFFFFFFFFF,FFFFFF
FFFFFF:FFFFFFFF
@A00721:190:HGJW7DSXY:4:1339:18032:27743 1:N:0:TCTGCAAG+GCTGCACT
CGTCATTTTCAGAGCAGCTCTGAATCGTGCTTCGTTTTCTACGTATCTGGACCAGTATATCTGCTTGA
CTTGATGCTTCAAGTCATCTGGGAGAATGTTGTAGATATCGTCAAATTTGATAGTACCATAGTTCGAC
GCCATTTGACACACT
+
FFFFFFFFFFFFFFFFFFFFFFFFFFFFFFFFFFFFFFFFFFFFFFFFFFFFFFFFFFFFFFFFFFFF
FFFFFFFFFFFFFFFFFFFFFFFFFFFFFFFFFF,:FFFFFFFFFFFFFFFFFFFFFFFFFFFFFF:FF
FFFFFFFFFFFFFFF
@A00721:190:HGJW7DSXY:4:1342:7835:35196 1:N:0:TCTGCAAG+GCTGCACT
CGTCATTTTCAGAGCAGCTCTGAATCGTGCTTCGTTTTCTACGTATCTGGACCAGTATATCTGCTTGA
CTTGATGCTTCAAGTCATCTGGGAGAATGTTGTAGATATCGTCAAATTTGATAGTACCATAGTTCGAC
GCCATTTGACACACT
+
FFFFFFFFFFFFFFFFFFFFFFFFFFFF:FFF:FFFFFFFF:FFFFFFFFFFFFFFFFFFFFFFFFFF
FFFFFFFFFFFFFFFFFFFFFFFFFFF,FFFFFFFFF:FFFFFFFF,FFFFFFFFFFFFFFFFFFFFF
FFFF,FFFFFFFFF:
```

@A00721:190:HGJW7DSXY:4:1449:12518:21198 1:N:0:TCTGCAAG+GCTGCACT
CGTCATTTTCAGAGCAGCTCTGAATCGTGCTTCGTTTTCTACGTATCTGGACCAGTATATCTGCTTGA
CTTGATGCTTCAAGTCATCTGGGAGAATGTTGTAGATATCGTCAAATTTGATAGTACCATAGTTCGAC
GCCATTTGACACACT
+
FFFFFFFFFFFFFFFFFFFFFFFFFFFFFFFFFFFFFFFFFFFFFFFFFFFFFFFFFFFFFFFFFFFF
:FFFFFFFFFFFFFFFFFFFF:FFFFFFFFFFF:FFFFFFFFFFFFFFFFFFFFFFF:FFFFFFFFFF
FFFFFFFFFFFFFFF
@A00721:190:HGJW7DSXY:4:1526:16667:29763 1:N:0:TCTGCAAG+GCTGCACT
CGTCATTTTCAGAGCAGCTCTGAATCGTGCTTCGTTTTCTACGTATCTGGACCAGTATATCTGCTTGA
CTTGATGCTTCAAGTCATCTGGGAGAATGTTGTAGATATCGTCAAATTTGATAGTACCATAGTTCGAC
GCCATTTGACACAC
+
FFFFFFFFFFFFFFFFFFFFFFFFFFFFFFFFFFFFFFFFFFFFFFFFFFFFFFFFFFFFFFFFFFFF
FFFFFFFFFFFFFFFFFFFF:FFFFF,FF:FFFFFFFFFFFFFF:FFFFFFFFFFFFFFFFFFFFFFF
:FFFFFFFFFFFFF
@A00721:190:HGJW7DSXY:4:2242:26549:15687 1:N:0:TCTGCAAG+GCTGCACT
CGTCATTTTCAGAGCAGCTCTGAATCGTGCTTCGTTTTCTACGTATCTGGACCAGTATATCTGCTTGA
CTTGATGCTTCAAGTCATCTGGGAGAATGTTGTAGATATCGTCAAATTTGATAGTACCATAGTTCGAC
GCCATTTGACACACT
+
FFFFFFFFFFFFFFFFFFFFFFFFFFFFFFFFFFFFFFFFFFFFFFFFFFFFFFFFFFFFFFFFFFFF
FFFFFFFFFFFFFFFFFFFFFFFFFFFFFFFFFFFFFFFFFFFFFF:FFFF,FFFFFFFFFFFF:FFF
FFFFFFFFFFFFFF:
@A00721:190:HGJW7DSXY:4:2318:15456:12665 1:N:0:TCTGCAAG+GCTGCACT
CGTCATTTTCAGAGCAGCTCTGAATCGTGCTTCGTTTTCTACGTATCTGGACCAGTATATCTGCTTGA
CTTGATGCTTCAAGTCATCTGGGAGAATGTTGTAGATATCGTCAAATTTGATAGTACCATAGTTCGAC
GCCATTTGACACACT
+
FFFFFFFFFFFFFFFFFFFFFFFFFFFFFFFFFFFFFFFFFFFFFFFFFFFFFFFFFFFFFFFFFFFF
FFFFFFFFFFFFFFFFFFFFFFFFFFFFF,F:FFFFFF,FFFFFFFFFF:FFFFFFFFFFFFFFFFFF
FFF,:FFFFFFFF:F
@A00721:190:HGJW7DSXY:4:2318:15474:13416 1:N:0:TCTGCAAG+GCTGCACT
CGTCATTTTCAGAGCAGCTCTGAATCGTGCTTCGTTTTCTACGTATCTGGACCAGTATATCTGCTTGA
CTTGATGCTTCAAGTCATCTGGGAGAATGTTGTAGATATCGTCAAATTTGATAGTACCATAGTTCGAC
GCCATTTGACACACT
+
FFFFFFFFFFFFFFFFFFFFFFFFFFF:FFFFFFFFFFFFFFFFF:FFFFFFFFFFFFFFFFFFFFFF
FFFFFFFFFFFFFFFFFF:FFFFFFF,FFFFFFFFFFFFFFFFFFF:FFFFFFFFFFFFFFFFFFFFF
:FFFFFFFFFFFFFF
@A00721:190:HGJW7DSXY:4:2360:24569:26412 1:N:0:TCTGCAAG+GCTGCACT
CGTCATTTTCAGAGCAGCTCTGAATCGTGCTTCGTTTTCTACGTATCTGGACCAGTATATCTGCTTGA
CTTGATGCTTCAAGTCATCTGGGAGAATGTTGTAGATATCGTCAAATTTGATAGTACCATAGTTCGAC
GCCATTTGACACACT
+
FFFFFFFFFFFFFFFFFFFFFFFFFFFFFFFFFFFFFFFFFFFFFFFFFFFFFFFFFFFFFFFFFFFF
F:FFFFFFFFFFFFFFFFFFFFFFF:FFFFFFFFFFFFFFFFFFFFF:FFFFFFFFFFFFFFFFFFFF
FFFF,FFFFFFFFFF
@A00721:190:HGJW7DSXY:4:2432:23149:36573 1:N:0:TCTGCAAG+GCTGCACT
CGTCATTTTCAGAGCAGCTCTGAATCGTGCTTCGTTTTCTACGTATCTGGACCAGTATATCTGCTTGA
CTTGATGCTTCAAGTCATCTGGGAGAATGTTGTAGATATCGTCAAATTTGATAGTACCATAGTTCGAC
GCCATTTGACACACT
+
FFFFFFFFFFFFFFFFFFFFFFFFFFFFFFFFFFFFFFFFFFFFFFFFFFFFFFFFFFFFFFFFFFFF

FFFFFFFFFFFFFFFFFFFFFFFFFFFFFFFFFFFFFFFFFFFFFFFFFFFFFFFFFFFFFFFFFFFFFF
FFFFFFFFFFFFFFF
@A00721:190:HGJW7DSXY:4:2464:5981:35493 1:N:0:TCTGCAAG+GCTGCACT
CGTCATTTTCAGAGCAGCTCTGAATCGTGCTTCGTTTTCTACGTATCTGGACCAGTATATCTGCTTGA
CTTGATGCTTCAAGTCATCTGGGAGAATGTTGTAGATATCGTCAAATTTGATAGTACCATAGTTCGAC
GCCATTTGACACACT
+
FFFFFFFFFFFFFFFFFFFFFFFFFFFFFFFFFFFFFFFFFFFFFFFFFFFFFFFFFFFFFFFFFFFFFF
FFFFFFFFFFFFFFFFFFFFFFFFFFF:FFFFF:FFFFFFFFFFFFFFFFFFFFFFFFFFFFFFFFFFFF
FFFFFFFFFFFFFFF
@A00721:190:HGJW7DSXY:4:2533:13973:25755 1:N:0:TCTGCAAG+GCTGCACT
CGTCATTTTCAGAGCAGCTCTGAATCGTGCTTCGTTTTCTACGTATCTGGACCAGTATATCTGCTTGA
CTTGATGCTTCAAGTCATCTGGGAGAATGTTGTAGATATCGTCAAATTTGATAGTACCATAGTTCGAC
GCCATTTGACACACT
+
FFFFFFFFFFFFFFFFFFFFFFFFFFFFFFFFFFFFFFFFFFFFFFFFFFFFFFFFFFFFFFFF:FFFFF
FFFFFFFFFFFFFFFFFFFFFFFFFFFFFFFFFF,FFFFFFFFFFFFFFFF:FFFFFFFFFFFFFFFFFF
FFFFF:FFFFFFFFF
@A00721:190:HGJW7DSXY:4:2629:23303:1485 1:N:0:TCTGCAAG+GCTGCACT
CGTCATTTTCAGAGCAGCTCTGAATCGTGCTTCGTTTTCTACGTATCTGGACCAGTATATCTGCTTGA
CTTGATGCTTCAAGTCATCTGGGAGAATGTTGTAGATATCGTCAAATTTGATAGTACCATAGTTCGAC
GCCATTTGACACACT
+
FFFFFFFFFFFFFFFFFFFFFFFFFFFFFFF:FFFFFFFFFFFFFFFFFFFFFFFFFFFFFFFFFFFFFF
FFFFFFFFFFFFFFFFFFFFFFFFFFFFFFFFFFFFFFFFFFFFFFFFFFFFFFFFFFFFFFFFFFFFFF
:FFFFFFFFFFFFFF
@A00721:190:HGJW7DSXY:4:1127:16016:24659 1:N:0:TCTGCAAG+GCTGCACT
CGACAGTGTGTCAAATGGCGTCGAACTATGGTACTATCAAATTTGACGATATCTACAACATTCTCCCA
GATGACTTGAAGCATCAAGTCAAGCAGATATACTGGTCCAGATACGTAGAAAACGAAGCACGATTCAG
AGCTGCTCTGAAAAT
+
FFFFFFFFFFFFFFFFFFFFFFFFFFFFFFFFF:FFFFF:FFFFFFFFFFFFFFFFFFFFFFFFFFFFFF
FFFFFFFFFFFFFFFFFFFFFFFFFFFFFFFFFFFFFFFFFFFFFFFFFFFFFFFF:FFFFFFFFFFFFF
FFFFFFFFFFFFFFF
@A00721:190:HGJW7DSXY:4:1171:12337:5822 1:N:0:TCTGCAAG+GCTGCACT
CGACAGTGTGTCAAATGGCGTCGAACTATGGTACTATCAAATTTGACGATATCTACAACATTCTCCCA
GATGACTTGAAGCATCAAGTCAAGCAGATATACTGGTCCAGATACGTAGAAAACGAAGCACGATTCAG
AGCTGCTCTGAAAAT
+
FFFFFFFFFFFFFFFFFFFFFFFFFFFFFFFFFFFFFFFFFFFFFFFFFFFFFFFFFFFFFFFFFFFFFF
FFFFFFFFFFFFFFFFFFFFFFFFFFFFFFFFFFFFFFFFFFFFFFFFFFFFFFFFFFFFFFFFFFFFFF
FFFFFFFFFFFFFFF
@A00721:190:HGJW7DSXY:4:1330:26630:6621 1:N:0:TCTGCAAG+GCTGCACT
CGACAGTGTGTCAAATGGCGTCGAACTATGGTACTATCAAATTTGACGATATCTACAACATTCTCCCA
GATGACTTGAAGCATCAAGTCAAGCAGATATACTGGTCCAGATACGTAGAAAACGAAGCACGATTCAG
AGCTGCTCTGAAAAT
+
FFFFFFFFFFFFFFFFFFFFFFFFFFFFFFFFFFFFF:FFFFFFFFFFFFFFFFFFFFFFFFFFFFFFFF
FFFFFFFFF:FFFFFFFFFFFFFFFFFFFFFFFFFFFFFFFFFFFFFFFFFFFFFFFFFFFFFFFFFFFF
FFFFFFFFFFFFFFF
@A00721:190:HGJW7DSXY:4:1454:24740:27398 1:N:0:TCTGCAAG+GCTGCACT
CGACAGTGTGTCAAATGGCGTCGAACTATGGTACTATCAAATTTGACGATATCTACAACATTCTCCCA
GATGACTTGAAGCATCAAGTCAAGCAGATATACTGGTCCAGATACGTAGAAAACGAAGCACGATTCAG
AGCTGCTCTGAAAAT

+
FFFFFFFFFFFFFFFFFFFFFFFFFFFFFFFFFFFFFFFFFFFFFFFFFFFFFFFFFFFFFFFFFFFF
FFFFFFFFFFFFFFFFFFFFFFFFFFFFFFFFFFFFFFF:FFFFFFFFFFFFFFFFFFFFFFFFFFFF
FFFFFF:FFFFFFFF
@A00721:190:HGJW7DSXY:4:1454:24180:27743 1:N:0:TCTGCAAG+GCTGCACT
CGACAGTGTGTCAAATGGCGTCGAACTATGGTACTATCAAATTTGACGATATCTACAACATTCTCCCA
GATGACTTGAAGCATCAAGTCAAGCAGATATACTGGTCCAGATACGTAGAAAACGAAGCACGATTCAG
AGCTGCTCTGAAAAT
+
FFFFFFFFFFFFFF:FFFFFFFFFFFFFFFFFFFFFFFFFFFFFFFFFFFFFFFFFFFFFFFFFFFFF
FFFFFFFFFFFFFFFFFFFFFFFFFFFFFFFFFFFFFFFFFFFFFFFFFFFFFFFFFFFFFFFFFFFF
FFFFFFFFFFFFFFF
@A00721:190:HGJW7DSXY:4:2269:5412:16219 1:N:0:TCTGCAAG+GCTGCACT
CGACAGTGTGTCAAATGGCGTCGAACTATGGTACTATCAAATTTGACGATATCTACAACATTCTCCCA
GATGACTTGAAGCATCAAGTCAAGCAGATATACTGGTCCAGATACGTAGAAAACGAAGCACGATTCAG
AGCTGCTCTGAAAAT
+
FFFFFFFFFFFFFFFFFFFFFFFFFFFFFFFFFFF,FFFFFFFFFFFFFFFFFFFFFFFFFFFFFFFF
FFFFFFFFFFFFFFFFFFFFFFFFFFFFFF::FFFFFFFFFFFFFFFFFFF:FFFFFFFFFFFFFFFF
FFFFFFFFFFFFFFF
@A00721:190:HGJW7DSXY:4:2536:1434:33473 1:N:0:TCTGCAAG+GCTGCACT
CGACAGTGTGTCAAATGGCGTCGAACTATGGTACTATCAAATTTGACGATATCTACAACATTCTCCCA
GATGACTTGAAGCATCAAGTCAAGCAGATATACTGGTCCAGATACGTAGAAAACGAAGCACGATTCAG
AGCTGCTCTGAAAAT
+
FFFFFFFFFFFFF:FFFFFFFFFFFFFFFFFFFFFFFFFFFFFFFFFFFFFFFFFFFFFFFFFFFFFF
FFFFFFFFFFFFFFFFFFFFFFFFFFFFFFF:FFFFFFFFFFFFFFFF,FFFFFFFFFFFFFFFFFFF
:FFFFFFFFFFFFFF
@A00721:190:HGJW7DSXY:4:1103:3757:7905 1:N:0:TCTGCAAG+GCTGCACT
TTTTCAGAGCAGCTCTGAATCGTGCTTCGTTTTCTACGTATCTGGACCAGTATATCTGCTTGACTTGA
TGCTTCAAGTCATCTGGGAGAATGTTGTAGATATCGTCAAATTTGATAGTACCATAGTTCGACGCCAT
TTAGATCGGAA
+
FFFFFFFFFFFFFFFFFFFFFFFFFFFFFF:FFFFFFFFFFFFFFFFFFFFFFFFFFFFFFFFFFFFF
FFFFFFF:FFFFFFFFFF:FF,:FFFFFFFFFFFFFFFFFF:FFFFFFFF:FFFFFFFFF:FFFFFFF
FFFFFFFFFFF
@A00721:190:HGJW7DSXY:4:1366:15356:27211 1:N:0:TCTGCAAG+GCTGCACT
TTTTCAGAGCAGCTCTGAATCGTGCTTCGTTTTCTACGTATCTGGACCAGTATATCTGCTTGACTTGA
TGCTTCAAGTCATCTGGGAGAATGTTGTAGATATCGTCAAATTTGATAGTACCATAGTTCGACGCCAT
TTAGATCGGAA
+
FFFFFFFFFFFFFFFFFFFFFFFFFFFFFFFFFFFFFFFFFFFFFFFFFFFFFFFFFFFFFFFFFFFF
FFFFFFFFFFFFFFFFFFFFF,FFFFFFFFFFF,FFFFFFFFFFFFFFFFFFFFFFFFFFFFFFFFFF
FFFFFFFFFFF
@A00721:190:HGJW7DSXY:4:1654:27661:33489 1:N:0:TCTGCAAG+GCTGCACT
TTTTCAGAGCAGCTCTGAATCGTGCTTCGTTTTCTACGTATCTGGACCAGTATATCTGCTTGACTTGA
TGCTTCAAGTCATCTGGGAGAATGTTGTAGATATCGTCAAATTTGATAGTACCATAGTTCGACGCCAT
TTAGATCGGAA
+
FFFFFFFFFFFFFFFFFFFFFFFFFFFFF:FFFFFFFFFFFFFFFFFFFFFFFFFFFFFFFFFFFFFF
FFFFFFFFFFFFFFFFFFFFF:FFFFF:FFFFFFFFFFF:FFFFFFFFFFFFFFFFFFFFFFFFFFF:
FFFFFFFFFFF
@A00721:190:HGJW7DSXY:4:2225:19732:19100 1:N:0:TCTGCAAG+GCTGCACT
TTTTCAGAGCAGCTCTGAATCGTGATTCGTTTTCTACGTATCTGGACCAGTATATCTGCTTTACTTGA

```
TGCTTCAAGTCAAATTGGAGAAAGTTTTAGATATCGTCAAATTTGATAGTACCATAGTTCGACGCCAT
TTAGATCGGA
+
F:FF:FFF,,FF:,FF,F:F:,,,,F:F,FFFF,:FFF,,FFF:,F,:F,,F,FFFF,:F:,F,:F:F
F:F,F,,FFFF:,,F,:,FFFF,FF,,FF,,F:FFFFF:FFF,FFFF:FFFFFF:F,F,F,FF,,FFF
FFF:FF:F,F
@A00721:190:HGJW7DSXY:4:2276:11478:33865 1:N:0:TCTGCAAG+GCTGCACT
TTTTCAGAGCAGCTCTGAATCGTGCTTCGTTTTCTACGTATCTGGACCAGTATATCTGCTTGACTTGA
TGCTTCAAGTCATCTGGGAGAATGTTGTAGATATCGTCAAATTTGATAGTACCATAGTTCGACGCCAT
TTAGATCGGAA
+
FFFFFFFFFFFFFFFFFFFFFFFFFFFFFFFFFFFFFFFFF:FFFFFFFFFFFFFFFFFFFFFFFFFFFF
FFFFFFFFFFFFFFFFFFFFF::FF:FFF:FFFFFFFFFFFF,FFFFFFFFFFFFFFFFFFFFFFFFFFF
FFFFFFFFFFF
@A00721:190:HGJW7DSXY:4:2323:1235:17065 1:N:0:TCTGCAAG+GCTGCACT
TTTTCAGAGCAGCTCTGAATCGTGCTTCGTTTTCTACGTATCTGGACCAGTATATCTGCTTGACTTGA
TGCTTCAAGTCATCTGGGAGAATGTTGTAGATATCGTCAAATTTGATAGTACCATAGTTCGACGCCAT
TTAGATCGGAA
+
FFFFFFFFFFFFFFFFFFFFFFFFFF:FFFFFFFFFFFFFFFFFFFFFFFFFFFFFFFFFFFFFFFFFFF
FFFFFFFFFFFFFFFFFFFFFFFFFFFFFFFF,FFFFFFFFFFFFFFFFF:FFFFFFF:FFFFFFFFFFF
FFFFFFFFFFF
@A00721:190:HGJW7DSXY:4:2468:13015:27289 1:N:0:TCTGCAAG+GCTGCACT
TTTTCAGAGCAGCTCTGAATCGTGCTTCGTTTTCTACGTATCTGGACCAGTATATCTGCTTGACTTGA
TGCTTCAAGTCATCTGGGAGAATGTTGTAGATATCGTCAAATTTGATAGTACCATAGTTCGACGCCAT
TTAGATCGGAA
+
FFFFFFFFFFFFFFFFFFFFFFFFFFFFFFFFFFFFFFFFFFFFFFFFFFFFFFFFFFFFFFFFFFFFFF
FFFF:FFFFFFFFFFFFFFFF:FFFFFFFFF:::FFFFFFFFFFFFFFFFFFFFFFFFFFFFFFFFFFF:
FFFFFFFFFFF
@A00721:190:HGJW7DSXY:4:1152:19208:22952 1:N:0:TCTGCAAG+GCTGCACT
GGAGAATGTTGTAGATATCGTCAAATTTGATAGTACCATAGTTCGACGCCATTTGACACACTGTTGAT
ATGATGAATTTGTAATATAATGTAAAATGTGTTGTGAGTTTTGTTATGTTCGAACAACACTACATTTA
TACAATGTTTAAGAT
+
FFFFFFFFFFFFFFFFFFFFFFFFFFFFFFFFFFFFFFFFFFFFFFFFFFFFFFFFFFFFFFFFFFFFFF
FFFF:FFFFFFFF:FFFFFFFFFFFFFFFFFFFFFFFFFFFFFFFFFFFF:FFFFFFFFFFFFFFFFFFF
FFFFFFFFFFFFFFF
@A00721:190:HGJW7DSXY:4:1323:30472:21104 1:N:0:TCTGCAAG+GCTGCACT
GGAGAATGTTGTAGATATCGTCAAATTTGATAGTACCATAGTTCGACGCCATTTGACACACTGTTGAT
ATGATGAATTTGTAATATAATGTAAAATGTGTTGTGAGTTTTGTTATGTTCGAACAACACTACATTTA
TACAATGTTTAAGAT
+
FFFFF:FFFFFFFFFFFFFFFFF:FFFFFFFFFFFFFFFFFFFFFFFFFFFFFFFFFFFFFFFFFFFFFF
FFFFFFFFFFFFFFFFFFFFFFFFFFFFFFFFFFFFFFFFFFFFFFFFFFFF:FFFFFFFFFFFFFFFFF
FFFFFFFFFFFFFFF
@A00721:190:HGJW7DSXY:4:1511:1832:25676 1:N:0:TCTGCAAG+GCTGCACT
GGAGAATGTTGTAGATATCGTCAAATTTGATAGTACCATAGTTCGACGCCATTTGACACACTGTTGAT
ATGATGAATTTGTAATATAATGTAAAATGTGTTGTGAGTTTTGTTATGTTCGAACAACACTACATTTA
TACAATGTTTAAGAT
+
FFFFFFFFFFFFFFFFFFFFFFFFFFFFFFFFFFFFFFFFFFFFFFFFFFF:FFFFFFFFFFFFFFFFFF
:FFF:FFFFFFFFFFFFFFFFFFFFFFFFFFFFFFFFFFFFFFFFFFF:FFF:FFFFFFFFFFFFFFFFF
FFFFFFFFFFFF:FF
```

```
@A00721:190:HGJW7DSXY:4:1672:27335:11725 1:N:0:TCTGCAAG+GCTGCACT
GGAGAATGTTGTAGATATCGTCAAATTTGATAGTACCATAGTTCGACGCCATTTGACACACTGTTGAT
ATGATGAATTTGTAATATAATGTAAAATGTGTTGTGAGTTTTGTTATGTTCGAACAACACTACATTTA
TACAATGTTTAAGAT
+
FFFFFFFFFFFFFFFFFFFFFFFFFFFFFFFFFFFFFFFFFFFFFFFFFFFFFFFFFFFFFFFFFFFFFF
FFFFFFF:FFFFFFFFFFFFFFFFFFFFFFFFFFFFFFFFFFFFFFFFFFFFFFFF:FFFFFFFFF
FFFFFFFFFFFFFFF
@A00721:190:HGJW7DSXY:4:2430:15239:22529 1:N:0:TCTGCAAG+GCTGCACT
GGAGAATGTTGTAGATATCGTCAAATTTGATAGTACCATAGTTCGACGCCATTTGACACACTGTTGAT
ATGATGAATTTGTAATATAATGTAAAATGTGTTGTGAGTTTTGTTATGTTCGAACAACACTACATTTA
TACAATGTTTAAGAT
+
FFFFFFFFFFFFFFFFFFFFFFFFFFFFFFFFFFFFFFFFFFFFFFFFFFFFFFFFFFFFFFFFFFFFF,
FFFFFFFFFFFFFFFFFFFFFFFFFF:FFFFFF:F:FFFFFFFFFFFFFFFFFFFFFFFFFFFFFFFFFF
FFFFFFFFFFFFFFF
@A00721:190:HGJW7DSXY:4:1318:16016:34679 1:N:0:TCTGCAAG+GCTGCACT
CATAGTTCGACGCCATTTGACACACTGTTGATATGATGAATTTGTAATATAATGTAAAATGTGTTGTG
AGTTTTGTTATGTTCGAACAACACTACATTTATACAATGTTTAAGATTCGATATGTATATAAGAGAAT
CTTGTTGCTTTTAAC
+
FFFFFFFFFFFFFFFFFFFFFFFFFFFFFFFFF:FFFFFFFFFFFFFFFFFFFFFFF:FFFFFFFFFFFF
FFFFFFFFFFFFFFFFFFFFFFFFFFFFFFFFFFFFFFFFFFFFFFFFFFFFFFFFFFFFFFFFFFFFFF
FFF:FFFFF:FFFFF
@A00721:190:HGJW7DSXY:4:1336:31385:2832 1:N:0:TCTGCAAG+GCTGCACT
CATAGTTCGACGCCATTTGACACACTGTTGATATGATGAATTTGTAATATAATGTAAAATGTGTTGTG
AGTTTTGTTATGTTCGAACAACACTACATTTATACAATGTTTAAGATTCGATATGTATATAAGAGAAT
CTTGTTGCTTTTAAC
+
FFFFFFFFFFFFFFFFFFFFFFFFFFFFFFFFFFFFFFFFFFFFFFFFFFFFFFFFFFFFFFFFFFFFFF
FFFFFFFFFFFFFFFFFFFFFFFFFFFFF:FFFFFFFFFFFFFFFFFFFFFFFFFFFFFFFFFFFFFFFF
FFFFFFFFFFFFF,F
@A00721:190:HGJW7DSXY:4:1352:1841:14450 1:N:0:TCTGCAAG+GCTGCACT
CATAGTTCGACGCCATTTGACACACTGTTGATATGATGAATTTGTAATATAATGTAAAATGTGTTGTG
AGTTTTGTTATGTTCGAACAACACTACATTTATACAATGTTTAAGATTCGATATGTATATAAGAGAAT
CTTGTTGCTTTTAAC
+
FFFFFFFFFFFFFFFFFFFFFFFFFFFFF:FFFFFFFFFFFFFFFF:FFFFFFFFFFFFFFFF:FFFF,F
FFFFFFFFFFFFFFFFFFFFFFFFFFFFFFFFFFFFF:FFFFFFF:FFFFFFFFFFFFFFFFFFFFFFF:
FFFFFFFFFFFFFFF
@A00721:190:HGJW7DSXY:4:1508:20338:16705 1:N:0:TCTGCAAG+GCTGCACT
CATAGTTCGACGCCATTTGACACACTGTTGATATGATGAATTTGTAATATAATGTAAAATGTGTTGTG
AGTTTTGTTATGTTCGAACAACACTACATTTATACAATGTTTAAGATTCGATATGTATATAAGAGAAT
CTTGTTGCTTTTAAC
+
FFFFFFFFFFFFFFFFFFFFFFFFFFFFFFFFFFFFFFFFFFFFFFFFFFFFFFFFFFFFFFFFFFFFFF
FFFFFFFFFFFFFFFFFFFFFFFFFFFFFFFFFFFFFFFFFFFFFFFFFFFFFFFFFFFFFFFFFFFFFF
,FFFFFFFFFFFFFF
@A00721:190:HGJW7DSXY:4:2117:2555:10551 1:N:0:TCTGCAAG+GCTGCACT
CATAGTTCGACGCCATTTGACACACTGTTGATATGATGAATTTGTAATATAATGTAAAATGTGTTGTG
AGTTTTGTTATGTTCGAACAACACTACATTTATACAATGTTTAAGATTCGATATGTATATAAGAGAAT
CTTGTTGCTTTTAAC
+
FFFFFFFFFFFFFFFFFFFFFFFFFF:F:FFFFFFFF:FFFFFFFFFFFFFFFFFFFFFFFFFFFFFF,F
```

FFFFFFFFFFFFF:FFFFFFFFFFFFFFFFFFFFFFFFFFFFFFFFFFFFFFF,FF,FFFFFFFFFFF
:FFF:FFF,FFFF:F
@A00721:190:HGJW7DSXY:4:2242:18313:7404 1:N:0:TCTGCAAG+GCTGCACT
CATAGTTCGACGCCATTTGACACACTGTTGATATGATGAATTTGTAATATAATGTAAAATGTGTTGTG
AGTTTTGTTATGTTCGAACAACACTACATTTATACAATGTTTAAGATTCGATATGTATATAAGAGAAT
CTTGTTGCTTTTAAC
+
FFFFFFFFFFFFFFFFFFFFFFFFFFFFFFFFFFFFFFFFFFFFFFFFFFFFF:FFFFFFFFFFF,F
FFFFFFFFFFFFFFFFFFFFFFFFFFFFFFFFFFF:F,:FF:FFFF:FFFF:FFFFFFFFFFFFFFF,
FFFFFFFFFFF:FFF
@A00721:190:HGJW7DSXY:4:2267:29369:15154 1:N:0:TCTGCAAG+GCTGCACT
CATAGTTCGACGCCATTTGACACACTGTTGATATGATGAATTTGTAATATAATGTAAAATGTGTTGTG
AGTTTTGTTATGTTCGAACAACACTACATTTATACAATGTTTAAGATTCGATATGTATATAAGAGAAT
CTTGTTGCTTTTAAC
+
FFFFFFFFFFFFFFFFFFFFFFFFFFFFFFFFFFFFFFFFFFFFFFFFFFFFFFFFFFFFFFFFFFFF
FFFFFFFFFFFFFFFFFFFFFFFFFFFFFFFFFFFFFFFFFFFFFFFFFF,FFFFFFFFF:FFFFF
FFFFFFFFFFFFFFF
@A00721:190:HGJW7DSXY:4:2352:3052:19085 1:N:0:TCTGCAAG+GCTGCACT
CATAGTTCGACGCCATTTGACACACTGTTGATATGATGAATTTGTAATATAATGTAAAATGTGTTGTG
AGTTTTGTTATGTTCGAACAACACTACATTTATACAATGTTTAAGATTCGATATGTATATAAGAGAAT
CTTGTTGCTTTTAAC
+
FFFFFFFFFFFFFFFFFFFFFFFFFFFFFFFFFFFFFFFFFFFFFFFFFFFFFFFFFFFFF:FFFFFF
FFFFFFFFFFFFFFFFFFFFFFFFFFFFFFFFFFFF:FFFFFFFFFFFFFFFFFFFFFFFFFFFFFFF
FFFFFFF:FFFFFF:
@A00721:190:HGJW7DSXY:4:2417:4318:23124 1:N:0:TCTGCAAG+GCTGCACT
CATAGTTCGACGCCATTTGACACACTGTTGATATGATGAATTTGTAATATAATGTAAAATGTGTTGTG
AGTTTTGTTATGTTCGAACAACACTACATTTATACAATGTTTAAGATTCGATATGTATATAAGAGAAT
CTTGTTGCTTTTAAC
+
FFFFFFFFFFFFFFFFFFFFFFFFF:F:FFF:FFFFFFFFFFFFFFFFFFFFFFFFFFFFFFFFFFFF
FFFFFFFFFFFFFFFFFFFFFFFFFFFFFFFFFFFFFFFFFFFF:FFFFFF:F:FFFFFFFFFFF:
FFFFFFFFF:FFFFF
@A00721:190:HGJW7DSXY:4:2509:17210:14826 1:N:0:TCTGCAAG+GCTGCACT
CATAGTTCGACGCCATTTGACACACTGTTGATATGATGAATTTGTAATATAATGTAAAATGTGTTGTG
AGTTTTGTTATGTTCGAACAACACTACATTTATACAATGTTTAAGATTCGATATGTATATAAGAGAAT
CTTGTTGCTTTTAAC
+
FFFFFFFFFFFFFF:F:FFFFFFFFFFFFFFFFFFF:FFFFFFFFFFFFFFFFFFFFF:FFFFFFFFF
FFFFFFFFFFFFFFFFFFFFFFFFFFFFFFFFFFFFFFFFFFFFFFFFF:FFFFFFFFFFFFFFF:
FFFFFFFFFFFFFFF
@A00721:190:HGJW7DSXY:4:2662:6885:1705 1:N:0:TCTGCAAG+GCTGCACT
CATAGTTCGACGCCATTTGACACACTGTTGATATGATGAATTTGTAATATAATGTAAAATGTGTTGTG
AGTTTTGTTATGTTCGAACAACACTACATTTATACAATGTTTAAGATTCGATATGTATATAAGAGAAT
CTTGTTGCTTTTAAC
+
FFFFFFFFFFFFFFFFFFFFFFFFFFFFFFFFFFFFFFFFFFFFFFFFFFFFFFFFFFFFFFFFFFFF
FFFFFFFFFFFFFFF:FFFFFFFFFFFFFFFFFFFFFFFFFFFFFFFFFFFFFFFFFFFFFFFFFF,F
FFFFFFFFFFFFFFF
@A00721:190:HGJW7DSXY:4:2671:20862:3333 1:N:0:TCTGCAAG+GCTGCACT
CATAGTTCGACGCCATTTGACACACTGTTGATATGATGAATTTGTAATATAATGTAAAATGTGTTGTG
AGTTTTGTTATGTTCGAACAACACTACATTTATACAATGTTTAAGATTCGATATGTATATAAGAGAAT
CTTGTTGCTTTTAAC

```
+
FFFFFFFFFFFFFFFFFFFFFFFFF:FFFFFFFFFFFFFFFFFFFFFFFFFFFFFFFFFFFFFFFFFF
FFFFFFFFFFFFFFFFFFFFFFFFFFFFFFFFFFFFFFFFFFFFFFFFFFFFFFFFFFFFFFFFFFFF
FF:FFFFFFFFFFFF
@A00721:190:HGJW7DSXY:4:1310:27570:28009 1:N:0:TCTGCAAG+GCTGCACT
AAATTCAAAAAAAGGTTAAAAGCAACAAGATTCTCTTATATACATATCGAATCTTAAACATTGTATAA
ATGTAGTGTTGTTCGAACATAACAAAACTCACAACACATTTTACATTATATTACAAATTCATCATATC
AACAGTGTGTC
+
FFFFFFFFFFFFFFFFF:FFFFFFFFFFFFFFFFFFFFFFFFFFFFFFFFFFFFFFFFFFFFFFFFFF
FFFFFFFFFFFFFFFFFFFFFFFFFFFFFFFF:FFFFFFFFFFFFFFFF:FFFFF,FFFFFFFFFFFF
FFF:F:FFFFF
@A00721:190:HGJW7DSXY:4:1611:8621:13385 1:N:0:TCTGCAAG+GCTGCACT
AAATTCAAAAAAAGGTTAAAAGCAACAAGATTCTCTTATATACATATCGAATCTTAAACATTGTATAA
ATGTAGTGTTGTTCGAACATAACAAAACTCACAACACATTTTACATTATATTACAAATTCATCATATC
AACAGTGTGTC
+
FFFFFFFFFFFFFFFFFFFFFFFFFFFFFFFFFFFFFFFFFFFFFFFFFFFFFFFFFFFFFFFFFFFF
FFFFFFFFFFFFFFFFFFFFFFFFFFFFFFFFFFFFFFFFFFFFFFFFFFFFFFFFFFFFFFFFFFFF
FFFFFFFFFFF
@A00721:190:HGJW7DSXY:4:2109:25672:7341 1:N:0:TCTGCAAG+GCTGCACT
AAATTCAAAAAAAGGTTAAAAGCAACAAGATTCTCTTATATACATATCGAATCTTAAACATTGTATAA
ATGTAGTGTTGTTCGAACATAACAAAACTCACAACACATTTTACATTATATTACAAATTCATCATATC
AACAGTGTGTC
+
F:FFFFFFFFFF:FFFFFFFFFFFFFFFF:FFFFFFFFFFFFFFFFFFFFFFFFFFFFFFFFFFFFFF
FFFFFFFFFFFFFFFFFFFFFFFFFFFFFFFFFFFFFFFFFFF:FFFFFFFFFFFFFFFFFFF:FFFF
FFFFFFFFFFF
@A00721:190:HGJW7DSXY:4:2423:5556:18223 1:N:0:TCTGCAAG+GCTGCACT
AAATTCAAAAAAAGGTTAAAAGCAACAAGATTCTCTTATATACATATCGAATCTTAAACATTGTATAA
ATGTAGTGTTGTTCGAACATAACAAAACTCACAACACATTTTACATTATATTACAAATTCATCATATC
AACAGTGTGTC
+
FFFFFFFFFFFFFFFFFFFFFFFFFFFFF:FFFFFFFFFF:FFFFFFFFFFFFFFFFFFFFFFFFFFF
FFFFFFFFFFFFFFFFFFFFFFFFFFFFFFFFFFFFFFFFFFFFFFFFFFFFFFFFFFFFFFFFFFFF
FFFFFFFFFFF
@A00721:190:HGJW7DSXY:4:1101:21965:30671 1:N:0:TCTGCAAG+GCTGCACT
TGTCAAAATTCAAAAAAAGGTTAAAAGCAACAAGATTCTCTTATATACATATCGAATCTTAAACATTG
TATAAATGTAGTGTTGTTCGAACATAACAAAACTCACAACACATTTTACATTATATTACAAATTCATC
ATATCAACAGTGTGT
+
FFFFFFFFFFFFFFFFFFFFFFFFFFFFFFFFFFFFFFFFFFFFFFFFFFFFFFFFFFFFFFFFFFFF
FFFFFFFFFFFFFFFFFFFFFFFFFFFFFF:FFFFFFFF:FFF:FFFF:FFFFFFFFFFFFFFFFFF:
FFFFFFFFFFFFFFF
@A00721:190:HGJW7DSXY:4:1170:2519:33661 1:N:0:TCTGCAAG+GCTGCACT
TGTCAAAATTCAAAAAAAGGTTAAAAGCAACAAGATTCTCTTATATACATATCGAATCTTAAACATTG
TATAAATGTAGTGTTGTTCGAACATAACAAAACTCACAACACATTTTACATTATATTACAAATTCATC
ATATCAACAGTGTGT
+
FFFFFFFFFFFFFFFFF:FFFFFFFFFFFFFFFFFFFFFFFFFFFFFFFFF:FFFFFFFFFFFFFFFF
:FFFFFFFFFF:FFFFFFFFFFFFFFFFFFFFFFFFFFFFFFF,FFFFFFFFFFFFFFFFFFFFFFFF
FFFFFFFFFFFFFFF
@A00721:190:HGJW7DSXY:4:1351:27787:7279 1:N:0:TCTGCAAG+GCTGCACT
TGTCAAAATTCAAAAAAAGGTTAAAAGCAACAAGATTCTCTTATATACATATCGAATCTTAAACATTG
```

TATAAATGTAGTGTTGTTCGAACATAACAAAACTCACAACACATTTTACATTATATTACAAATTCATC
ATATCAACAGTGTGT
+
FFFFFFFFFFFFFFFFFFFFFFFFFFFFFFFFFFFFFFFF:FFFFFFFF,FFFFFFFFFFFFFFF:FF
FFFFFFFFFFFFFFFFFFFFFFF,FFFFFFFFFFFFFFFFFF:FF:FFFFFFFFFFFFFFFFFFFFF,
,FFFFFFFFFFFFFF
@A00721:190:HGJW7DSXY:4:2109:8766:11162 1:N:0:TCTGCAAG+GCTGCACT
TGTCAAAATTCAAAAAAAGGTTAAAAGCAACAAGATTCTCTTATATACATATCGAATCTTAAACATTG
TATAAATGTAGTGTTGTTCGAACATAACAAAACTCACAACACATTTTACATTATATTACAAATTCATC
ATATCAACAGTGTGT
+
FFFFFFFFFFFFFFFFFFFFFFFFFFFFFFFFFFFFFFFFFFFFFFFFFFFFFFFFFFFFFFFFFFFF
FFFFFFFFFFF:FFFFFFFFFFFFFFFFFFFFFFFFFFFFFFFFFFFFFFFFFFFFFFFFFFFFFFFF
FFFFFFFFFFFFFFF
@A00721:190:HGJW7DSXY:4:2138:18810:22858 1:N:0:TCTGCAAG+GCTGCACT
TGTCAAAATTCAAAAAAAGGTTAAAAGCAACAAGATTCTCTTATATACATATCGAATCTTAAACATTG
TATAAATGTAGTGTTGTTCGAACATAACAAAACTCACAACACATTTTACATTATATTACAAATTCATC
ATATCAACAGTGTGT
+
FFFFFFFFFFFFFFFFFFFFFFFFFFFFFFFFFFFFFFFFFFFFF:FFFFFFFFFFFFFFFFFFFFFF
FFFFFFFFFF:FFFFFFFF:FFFFFFFFFFFFFFFFFFFFFFFFF:F:FFFFFFFFFFFFFFFFFFFF
FFFF:FF:F::FFFF
@A00721:190:HGJW7DSXY:4:2526:10520:22717 1:N:0:TCTGCAAG+GCTGCACT
TGTCAAAATTCAAAAAAAGGTTAAAAGCAACAAGATTCTCTTATATACATATCGAATCTTAAACATTG
TATAAATGTAGTGTTGTTCGAACATAACAAAACTCACAACACATTTTACATTATATTACAAATTCATC
ATATCAACAGTGTGT
+
FFFFFFFFFFFFFFFFF,FFFFFFFFFFFFFFFFFFFFFFFFFFFFFFFFFFFFFFFFFFFFFFFFFF
FFFFFFFFFFFFFFF:FFFFFFFFFFFFFFFFFFFFFFFFFFF:FFFFFFFFFFFFFF:FFFFFFFFF
FFFFFFFFFFFF:FF
@A00721:190:HGJW7DSXY:4:1514:17255:12900 1:N:0:TCTGCAAG+GCTGCACT
CATCTTTATGTCAAAATTCAAAAAAAGGTTAAAAGCAACAAGATTCTCTTATATACATATCGAATCTT
AAACATTGTATAAATGTAGTGTTGTTCGAACATAACAAAACTCACAACACATTTTACATTATATTACA
AATTCATCATATCAA
+
FFFFFFFFFFFFFFFFFFFFFFFFFFFFFFFFFFFFFFFFFFFFFFF:F:FFFFFFFFFFFFFFF,FF
FFFFFFFFFFFFF:F,FFF:FFFFFF,FF:FFFFFFFFFFFFF:FFFFFFF:,FFF:FF:FFFFFFFF
FFFFFFF:F:FFFFF
@A00721:190:HGJW7DSXY:4:2122:2727:36777 1:N:0:TCTGCAAG+GCTGCACT
CATCTTTATGTCAAAATTCAAAAAAAGGTTAAAAGCAACAAGATTCTCTTATATACATATCGAATCTT
AAACATTGTATAAATGTAGTGTTGTTCGAACATAACAAAACTCACAACACATTTTACATTATATTACA
AATTCATCATATCAA
+
FFFFFFFFFFFFFFFFFFFFFFFFFFFFFFFFFFFFFFFFFFFFFFFFFFFFFFFFFFFFFFFFFFFF
FFFFFFFFFFFFFFFFFF:FFFFFFFFFFFFFFFF:FFFFFFFFFFF:FFF,,FFFFFFFFFF:FFFF
FFFF:FFFFFFF:FF
@A00721:190:HGJW7DSXY:4:2625:8024:27007 1:N:0:TCTGCAAG+GCTGCACT
ATCTTTATGTCAAAATTCAAAAAAAGGTTAAAAGCAACAAGATTCTCTTATATACATATCGAATCTTA
AACATTGTATAAATGTAGTGTTGTTCGAACATAACAAAACTCACAACACATTTTACATTATATTACAA
ATTCATCATATCAA
+
FFFFF,FFFFFFFFF:FF:FFFFFFFFF:FF:FFFFFFFF,FF,:F,F:,FFFFFFF,FFFFFFFFFF
FFFFFF,FFFFFFFF:FF:FF:FFF,FFF:F:FFFFFFFFFFFFFFFFFF,FF:F:F,F:F:F:FFFF
FFFFFFFFFF:FFF

```
@A00721:190:HGJW7DSXY:4:1325:15338:8265 1:N:0:TCTGCAAG+GCTGCACT
CTGACTTAGATCATAAGTAATGTATACAGTAAATGTTTGTACAGTTGAAATAGATTTGAAATTCAAAT
TTCAAATTTGAAAATATGAGCCGTACAAACTTTGACAGTCGATTTCATCTTTATGTCAAAATTCAAAA
AAAGGTTAAAAGCAA
+
FFF:FFFFFFFFFFFFFFFFFFF:FFFFFFFFFFFFF:FFFFFFFFFFFFFFFFFFFFFFFFFFFF:F
FFFFFFFFFFFFFFFFFFF:FFFFFFFFFFFFFFFFFFFF:FFFFFFF,FFFFF,FFFFFFFF:FFFF
FFF::FFFFFF,:FF
@A00721:190:HGJW7DSXY:4:2422:11776:30749 1:N:0:TCTGCAAG+GCTGCACT
CTGACTTAGATCATAAGTAATGTATACAGTAAATGTTTGTACAGTTGAAATAGATTTGAAATTCAAAT
TTCAAATTTGAAAATATGAGCCGTACAAACTTTGACAGTCGAATTAATATTTATGTCAAAAATCAAAA
AAATTTTAAAAGAAA
+
F,:FFFFF,FFFF,FFFFFFF,FFFFFFFFFFFF,FFF,FFF::FF:FFFFFF:FFFFFFFFFFFFFF
FFFFFF:FF:FFFF:FFFFFF,F,FFFFF,:F,FF,FFFF:F,:,,F,:F,FFF,,,FFFF,FFFFF:
FF:,,,,:FFF,,:F
@A00721:190:HGJW7DSXY:4:1122:3613:15796 1:N:0:TCTGCAAG+GCTGCACT
CGCTTTTAACCTTTTTTTGAATTTTGACATAAAGATGAAATCGACTGTCAAAGTTTGTACGGCTCATA
TTTTCAAATTTGAAATTTGAATTTCAAATCTATTTCAACTGTACAAACATTTACTGTATACATTACTT
ATGATCTAAGTCAGC
+
FFFFFFFFFFFFFFFFFFFFFFFFFFFFFFFFFFFFFFFFFFFFFFFFFFFFFFFFFFFFFFFFFFFF
FFFFFFFFFFF:FFFFFFFFFFFFFFFFFFFFFFFFFFFFFFFFFFFFFFFFFFFFFFFFFFFFF,FF
FFFFFFFFFFFFF,F
@A00721:190:HGJW7DSXY:4:1122:4643:18771 1:N:0:TCTGCAAG+GCTGCACT
CGCTTTTAACCTTTTTTTGAATTTTGACATAAAGATGAAATCGACTGTCAAAGTTTGTACGGCTCATA
TTTTCAAATTTGAAATTTGAATTTCAAATCTATTTCAACTGTACAAACATTTACTGTATACATTACTT
ATGATCTAAGTCAGC
+
FFFFFFFFFFFFF:,FFF:FFFFFFF:FFFFF:FFFFFFFFFFFFFFFFFFFFFFFFFFFFFFFFFFF
FFFFFFFFFFFFFFFFFFFFFFF:FFFFFFFFFFFFFFFFFFFFFFFFF,FFFFFFFFFFFFFFF:FF
FF:FFFFFFFFFFFF
@A00721:190:HGJW7DSXY:4:1153:22173:3223 1:N:0:TCTGCAAG+GCTGCACT
CGCTTTTAACCTTTTTTTGAATTTTGACATAAAGATGAAATCGACTGTCAAAGTTTGTACGGCTCATA
TTTTCAAATTTGAAATTTGAATTTCAAATATATTTCAACTGTACAAACATATACTGTATACATTACTT
ATGATCTAAGTCAGC
+
FFF::FFFFF:FFFF:FF:FFFFF,:FFFFFFFFFFFFFFFF:FF::FFFFF,:F::FFF::,FFFFF
FFFFFFFFFF,:FFFF:F,:F::,,FFF:,,FFFF:FF,F,FFFFFFFFF,FF:FF:F:F,F,,FFF:
FFFFF,FFFF,FF,:
@A00721:190:HGJW7DSXY:4:1547:16161:26162 1:N:0:TCTGCAAG+GCTGCACT
CGCTTTTAACCTTTTTTTGAATTTTGACATAAAGATGAAATCGACTGTCAAAGTTTGTACGGCTCATA
TTTTCAAATTTGAAATTTGAATTTCAAATCTATTTCAACTGTACAAACATTTACTGTATACATTACTT
ATGATCTAAGTCAGC
+
FFFFFFFFFFFFFFFFFFFFFFFFFFFFFFFFFFFFFFFFFFFFFFFFFFFFFFFFFFFFFFFFFFFF
FFFFFFFFFFFFFFFFFFFFFFFFFFFFFFFFFFFFFFFFFFFFFFFFFFFFFFFFFFFFFFFFFFFF
FFFFFFFFFFFFFFF
@A00721:190:HGJW7DSXY:4:1630:18394:8484 1:N:0:TCTGCAAG+GCTGCACT
CGCTTTTAACCTTTTTTTGAATTTTGACATAAAGATGAAATCGACTGTCAAAGTTTGTACGGCTCATA
TTTTCAAATTTGAAATTTGAATTTCAAATCTATTTCAACTGTACAAACATTTACTGTATACATTACTT
ATGATCTAAGTCAGC
+
FFFFFFFFFFFFFFFFFFFFFFFFFF:FFFFFFFFFFFFFFFFFFFFFFFFFFFFFFFFFFFFFFFFF
```

FFFFFFFFFFFFFFFFFFFFFFFFFFFFFFFFFFFFFFFF:FF:FF:FFFFFFFFFFFFFFFFFFFFF
FF:FFFFFFFFFF:F
@A00721:190:HGJW7DSXY:4:1668:20772:21026 1:N:0:TCTGCAAG+GCTGCACT
CGCTTTTAACCTTTTTTTGAATTTTGACATAAAGATGAAATCGACTGTCAAAGTTTGTACGGCTCATA
TTTTCAAATTTGAAATTTGAATTTCAAATCTATTTCAACTGTACAAACATTTACTGTATACATTACTT
ATGATCTAAGTCAGC
+
FFFFFFFFFFFFFF:FFFFFFFFFFFFFFFFFFFFFFFFFFFFFFFFFFFFFFFFFFFFFFFFFFFFF
FFFFFFFFFFFFFFFFFFFFFFFFFFFFFFFFFFFFFFFFFFFFFF::F,FFFFFFFFFFFFFFFFFF
FFFFFF:F:FFFFFF
@A00721:190:HGJW7DSXY:4:2174:2365:7592 1:N:0:TCTGCAAG+GCTGCACT
CGCTTTTAACCTTTCTTTGAATTTTGACATAAAGATGAAATCGACTGTCAAAGTTTGTACGGCTCATA
TTTTCAAATTTGAAATTTGAATTTCAAATCTATTTCAACTGTACAAACATTTACTGTATACATTACTT
ATGATCTAAGTCAGC
+
FFFF:FFFFFFFFFFFFFFFFFFFFFFFFFFFFFFFFFFFFFFFFFFFFFFFFFFFFFFFFFFFFFFF
FFFFFFFFFFFFFFFFFFFFFFFF:FFFFFFFFFFFFFFFFFFFFFFFFFFFFFFFFFFFFFFFFFFF
FFFFFFFFFF,FFFF
@A00721:190:HGJW7DSXY:4:2525:18882:26177 1:N:0:TCTGCAAG+GCTGCACT
CGCTTTTAACCTTTTTTTGAATTTTGACATAAAGATGAAATCGACTGTCAAAGTTTGTACGGCTCATA
TTTTCAAATTTGAAATTTGAATTTCAAATCTATTTCAACTGTACAAACATTTACTGTATACATTACTT
ATGATCTAAGTCAGC
+
FF:F:FFFFFFFFFFF:FFFFFFFF,F:FFFFFFFFFFFFFFFFFFFFFFF:F:FFFFF:FFFFFFFF
FFFFFFFFFFFFFFFFFFFFFFFF:FFFFFFFFFF,FFFFFFFFFFFFFFF:F,FFFFFF,F:FFFF,
FFFFFFFFFFF,F:F
@A00721:190:HGJW7DSXY:4:2547:13087:32330 1:N:0:TCTGCAAG+GCTGCACT
CGCTTTTAACCTTTTTTTGAATTTTGACATAAAGATGAAATCGACTGTCAAAGTTTGTACGGCTCATA
TTTTCAAATTTGAAATTTGAATTTCAAATCTATTTCAACTGTACAAACATTTACTGTATACATTACTT
ATGATCTAAGTCAGC
+
FFFFFFFFFFFFFFFF,FFFFFFFFFFFFFFFFFFFFFFFF:FFFFFFFFFFFFFF,FFFF,FFFFFF
FFFFFFF:::F:FFFFFFFFF:F:FFFFFFFFFFFFFF:FFFFFFFFFFF:FFFFFFFFFFFF:FFFF
FF,FFFFFF:F,FFF
@A00721:190:HGJW7DSXY:4:2654:18493:3646 1:N:0:TCTGCAAG+GCTGCACT
CGCTTTTAACCTTTTTTTGAATTTTGACATAAAGATGAAATCGACTGTCAAAGTTTGTACGGCTCATA
TTTTCAAATTTGAAATTTGAATTTCAAATCTATTTCAACTGTACAAACATTTACTGTATACATTACTT
ATGATCTAAGTCAGC
+
FFFFFFFFFFFFF:FFFFFFFFFFFFF:FFFFFFFFFFFFFF:FFFFF:FFFF:FFFFFFFFFFFFFF
F:FF,FFFFF:FFFFFFF,FFFFFFFFFF:FFFFFFFFFFFFFFFFFFFFFFFFFFFFFF,FFFFFFF
FF,FF,FFF:FFFF:
@A00721:190:HGJW7DSXY:4:1112:16459:3317 1:N:0:TCTGCAAG+GCTGCACT
CTTTTTTTGAATTTTGACATAAAGATGAAATCGACTGTCAAAGTTTGTACGGCTCATATTTTCAAATT
TGAAATTTGAATTTCAAATCTATTTCAACTGTACAAACATTTACTGTATACATTACTTATGATCTAAG
TCAGCCACTTACATA
+
FFFFFFFFFFFFFFFFFFFFFFFFFFFFFFFFFFFFFFFFFFFFFFFFFFFFFFFFFFFFFFFFFFFF
FFFFFFFFFFFFFFFFFFFFFFFFFFFFFFFFFFFFFFFFFFFFFFFFFFFFFFFFFFFFFFFFFFFF
FFFFFFFFFFFFFFF
@A00721:190:HGJW7DSXY:4:1112:16405:3787 1:N:0:TCTGCAAG+GCTGCACT
CTTTTTTTGAATTTTGACATAAAGATGAAATCGACTGTCAAAGTTTGTACGGCTCATATTTTCAAATT
TGAAATTTGAATTTCAAATCTATTTCAACTGTACAAACATTTACTGTATACATTACTTATGATCTAAG
TCAGCCACTTACATA

+
FFFFFFFFFFFFFFFFFFFFFFFFFFFFFFFFFFFFFFFFFFFFFFFFFFFFFFFFFFFFFFFFFFFF
FFFFFFFFFFFFFFFFFFFFFFFFFFFFFFFFFFFFFFFFFFFFFFFFFFFFFFFFFFFFFFFFFFFF
FFF:FFFFF:FFFFF
@A00721:190:HGJW7DSXY:4:1117:1533:23249 1:N:0:TCTGCAAG+GCTGCACT
CTTTTTTTGAATTTTGACATAAAGATGAAATCGACTGTCAAAGTTTGTACGGCTCATATTTTCAAATT
TGAAATTTGAATTTCAAATCTATTTCAACTGTACAAACATTTACTGTATACATTACTTATGATCTAAG
TCAGCCACTTACATA
+
FFFF,FFFFFFFFFFFFFFFFFFFFFFFFFFFFFFFFFFFFFF:FFFFFFFFFFFFFFFFFFFFFFFF
FFFFFFFFFF:FFFFF::FFFFFFFFF,FFFFFFFF,F:FFFFFFFFFFFFFFFFFFF,FFFFFFFFF
FFFFFFFF:FFFFFF
@A00721:190:HGJW7DSXY:4:1228:6659:31720 1:N:0:TCTGCAAG+GCTGCACT
CTTTTTTTGAATTTTGACATAAAGATGAAATCGACTGTCAAAGTTTGTACGGCTCATATTTTCAAATT
TGAAATTTGAATTTCAAATCTATTTCAACTGTACAAACATTTACTGTATACATTACTTATGATCTAAG
TCAGCCACTTACATA
+
FFFFFFFFFFFFFFFFFF:FFFFFFFFFFFFFFFFFFFFFFFFFFFFFFFFFFFFFFFFFFF:FFFFF
:FFFFFFFFFFFFFFFFFFFFFFFFFFFFFFFFFFFFFFFFFFFFFFFFFFFFFFFFFFF:FFFFFFF
FFFF:FFFFFFFFFF
@A00721:190:HGJW7DSXY:4:1316:15917:25457 1:N:0:TCTGCAAG+GCTGCACT
CTTTTTTTGAATTTTGACATAAAGATGAAATCGACTGTCAAAGTTTGTACGGCTCATATTTTCAAATT
TGAAATTTGAATTTCAAATCTATTTCAACTGTACAAACATTTACTGTATACATTACTTATGATCTAAG
TCAGCCACTTACATA
+
FFFFFFFFFFFFFFFFFFFFFFFFFFFFFFFFFFFFFFFFFFFFFFFFFFFFFFFFFFFFFFFFFFFF
FFFFFFFFFFFFFFFFFFFFFFFFFFFFFFFFFFFFFFFFFFFFFFFFFFFFFFFFFFFFFFFFFFF:
FFFFFFFFFFFFFFF
@A00721:190:HGJW7DSXY:4:1341:7075:34945 1:N:0:TCTGCAAG+GCTGCACT
CTTTTTTTGAATTTTGACATAAAGATGAAATCGACTGTCAAAGTTTGTACGGCTCATATTTTCAAATT
TGAAATTTGAATTTCAAATCTATTTCAACTGTACAAACATTTACTGTATACATTACTTATGATCTAAG
TCAGCCACTTACATA
+
FFFFFFFFFFFFFFFFFFFFFFFFFFFFFFFFFFFFFFFFFFFFFFFFFFFFFFFFFFFFFFFFFFFF
FFFFFFFFFFFFFFFFFFFFFFFFFFFFFFFFFFFFFFFFFFF:FFFFFFFFFFFFFFFFFFFFFFFF
FFF:FFFFFFFFFFF
@A00721:190:HGJW7DSXY:4:1539:32145:31391 1:N:0:TCTGCAAG+GCTGCACT
CTTTTTTTGAATTTTGACATAAAGATGAAATCGACTGTCAAAGTTTGTACGGCTCATATTTTCAAATT
TGAAATTTGAATTTCAAATCTATTTCAACTGTACAAACATTTACTGTATACATTACTTATGATCTAAG
TCAGCCACTTACATA
+
FFFFFFFFFFFFFFFFFFFFFF,FFFFFFFFFFFFFFFFFFFFFFFFFFFFFFFFFFFFFFFFFFFFF
FFFFFFFFFFFFFFFFFFFFFFFFFFFFFFFFFFFF::FFFFFFFFFFFFFFFFFFFFFFF:FFFFFF
FFFFFFFFFFFFFFF
@A00721:190:HGJW7DSXY:4:1549:1678:5337 1:N:0:TCTGCAAG+GCTGCACT
CTTTTTTTGAATTTTGACATAAAGATGAAATCGACTGTCAAAGTTTGTACGGCTCATATTTTCAAATT
TGAAATTTGAATTTCAAATCTATTTCAACTGTACAAACATTTACTGTATACATTACTTATGATCTAAG
TCAGCCACTTACATA
+
FFFFFFFFFFFFFFFFFFFFFFFFFFFFFFFFFFFFFFFFFFFFFFFFFFFFFFFFFFFFFFFFFFFF
FFFFFFFFFFFFFFFFFFFFFFFFFFFFFFFFFFFFFFFFFFFFFFFFFFFFFFFFFFFFFFFFFFFF
FFFFFFFFFFFFFFF
@A00721:190:HGJW7DSXY:4:1620:13783:15906 1:N:0:TCTGCAAG+GCTGCACT
CTTTTTTTGAATTTTGACATAAAGATGAAATCGACTGTCAAAGTTTGTACGGCTCATATTTTCAAATT

```
TGAAATTTGAATTTCAAATCTATTTCAACTGTACAAACATTTACTGTATACATTACTTATGATCTAAG
TCAGCCACTTACATA
+
FFFFFFFFFFFFFFFFFFFFFFFFFFFFFFFFFFFFFFFFFFFFFFFFFFFFFFFFFFFFFFFFFFFF
FFFFFFFFFFFFFFFFFFFFFFFFFFFFFFFFFFFFFFFFFFFFFFFFFFFFFFFFFFF:FFFFFFFF
FFFFFFFFFFFFFFF
@A00721:190:HGJW7DSXY:4:1661:27136:5494 1:N:0:TCTGCAAG+GCTGCACT
CTTTTTTTGAATTTTGACATAAAGATGAAATCGACTGTCAAAGTTTGTACGGCTCATATTTTCAAATT
TGAAATTTGAATTTCAAATCTATTTCAACTGTACAAACATTTACTGTATACATTACTTATGATCTAAG
TCAGCCACTTACATA
+
FFFFFFFFFFFFFFFFFFFFFFFFFFFFFFFFFFFFFFFFFFFFFFFFFFFFFFFFFFFFFFFFFFFF
FF,FFFFFFFFFFFFFFFFFFFFFFFFFFFFFFFFFFFFF:FFFFFFFFFFFFFFFFFFFFFFFFFF,
FFFF,FFFFFFFFFF
@A00721:190:HGJW7DSXY:4:2113:17933:9220 1:N:0:TCTGCAAG+GCTGCACT
CTTTTTTTGAATTTTGACATAAAGATGAAATCGACTGTCAAAGTTTGTACGGCTCATATTTTCAAATT
TGAAATTTGAATTTCAAATCTATTTCAACTGTACAAACATTTACTGTATACATTACTTATGATCTAAG
TCAGCCACTTACATA
+
FFFFFFFFFFFFFFFFFFFFFFFFFFFFFFFFFFFFFFFFFFFFFFFFFFFFFFFFFFFFFFFFF:FFF
FFFFFFFFFFFFFFFFFFFFFFFFFFFFFFFFFFFFFFFFFFFFFFFFFFFFFFFFFFFFFFFFFFF,
FFFFFFFFFFFFFFF
@A00721:190:HGJW7DSXY:4:2122:1994:10269 1:N:0:TCTGCAAG+GCTGCACT
CTTTTTTTGAATTTTGACATAAAGATGAAATCGACTGTCAAAGTTTGTACGGCTCATATTTTCAAATT
TGAAATTTGAATTTCAAATCTATTTCAACTGTACAAACATTTACTGTATACATTACTTATGATCTAAG
TCAGCCACTTACATA
+
FFFFFFFFFFFFFFFFFFFFFFFFFFFFFFFFFFFFFFFFFFFFFFFFFFFFFFFFFFFFFFFFFFFF
FFFFFFFFFFFFFFFFFFFFFFFFFFFFFFFFFFFFFFFFFFFFFFFFFFFFFFFFFFFFFFFFFFFF
FFFF:FFFFFFFFFF
@A00721:190:HGJW7DSXY:4:2224:20039:5228 1:N:0:TCTGCAAG+GCTGCACT
CTTTTTTTGAATTTTGACATAAAGATGAAATCGACTGTCAAAGTTTGTACGGCTCATATTTTCAAATT
TGAAATTTGAATTTCAAATCTATTTCAACTGTACAAACATTTACTGTATACATTACTTATGATCTAAG
TCAGCCACTTACATA
+
FFFFFFFFFFFFFFFFFFFFFFFFFFFFFFFFFFFFFFFFFFFFFFFFFFFFFFFFFFFFFFFFFFFF
FFFFFFFFFFFFFFFFFFFFFFFFFFFFFFFFFFFFFFFFFFFFFFFFFFFFFFFFFFFF:FFFFFFF
F:F:FFFFFFFFFFF
@A00721:190:HGJW7DSXY:4:2463:20998:31093 1:N:0:TCTGCAAG+GCTGCACT
CTTTTTTTGAATTTTGACATAAAGATGAAATCGACTGTCAAAGTTTGTACGGCTCATATTTTCAAATT
TGAAATTTGAATTTCAAATCTATTTCAACTGTACAAACATTTACTGTATACATTACTTATGATCTAAG
TCAGCCACTTACATA
+
FFFFFFFFFFFFFFFFFFFFFFFFFFFFFFFFFFFFFFFFFFFFFFFFFFFFFFFFFFFFFFFFFFFF
FFFFFFFFFFFFFFFFFFFFFFFFFFFF:FFFFFFFFFFFFFFFFFFFFFFFFFFFFFFFFFFFFFFF
FFFFFFFFFFFFFFF
@A00721:190:HGJW7DSXY:4:2463:21278:32205 1:N:0:TCTGCAAG+GCTGCACT
CTTTTTTTGAATTTTGACATAAAGATGAAATCGACTGTCAAAGTTTGTACGGCTCATATTTTCAAATT
TGAAATTTGAATTTCAAATCTATTTCAACTGTACAAACATTTACTGTATACATTACTTATGATCTAAG
TCAGCCACTTACATA
+
FFFFFFFFFFFFFFFFFFFFFFFFFFFFFFFFFFFFFFFFFFFFFFFFFFFFFFFFFFFFFFFFFFFF
FFFFFFFFFFFFFFFFFFFFFFFFFFFF:FFFFFFF:FFF:FFFFFFFFFFFFFFFFFFFFFFFFFFF
FFFFFFFFFFFFFFF
```

```
@A00721:190:HGJW7DSXY:4:1259:17318:29293 1:N:0:TCTGCAAG+GCTGCACT
CAGTCCTCGAATCAGTAGATTCGAAATATATGACAAGTATATGTAAGTGGCTGACTTAGATCATAAGT
AATGTATACAGTAAATGTTTGTACAGTTGAAATAGATTTGAAATTCAAATTTCAAATTTTAAAATATG
AGCCGTACAAACTTT
+
:FFFFFFF:FF::FF:F:F:FF:FFFFFFFF,FFFFFFF,FF:FFF,,,FF:F:FF:F::FFFFFFFF
:::FF,FFFFFFFFF:FFFF::FFFFFFFFFFFFFFFFFF:FF:FFFFFFFFFFFFFF:FFFFFFFFF
FFFFFFF,FFFFFFF
@A00721:190:HGJW7DSXY:4:1459:20726:2597 1:N:0:TCTGCAAG+GCTGCACT
CAGTCCTCGAATCAGTAGATTCGAAATATATGACAAGTATATGTAAGTGGCTGACTTAGATCATAAGT
AATGTATACAGTAAATGTTTGTACAGTTGAAATAGATTTGAAATTCAAATTTCAAATTTTAAAATATG
AGCCGTACAAACTTT
+
FFFFFFFFFFFFFFFFFFFFFFFFFFFFFFFFFFFFFFFFFFFFFFFFFFFFFFFFFFFFFFFFFFFF
FFFFFFFFFFFFFFFFFFF:FFFFFFFFFFFFFFFFFFFFFFFFFFFFFFFFFFFFFFFFFFFFFFFF
FFFFFFFFFFFFFFF
@A00721:190:HGJW7DSXY:4:2169:21965:36025 1:N:0:TCTGCAAG+GCTGCACT
CAGTCCTCGAATCAGTAGATTCGAAATATATGACAAGTATATGTAAGTGGCTGACTTAGATCATAAGT
AATGTATACAGTAAATGTTTGTACAGTTGAAATAGATTTGAAATTCAAATTTCAAATTTTAAAATATG
AGCCGTACAAACTTT
+
FFFFFFFFFFFFFFFFFFFFFFFFFFFFFFFFFFFFFFFFFFFFFFFFFFFFFFFFFFFFFFFFFFFF
FFFFFFFFFFFFFFFFFFFFFFFFFFFFFFFFFFFFFFFFFFFFFFFFFFFFFFFFFFFFFFFFFFFF
FFFFFFFFFFFFFFF
@A00721:190:HGJW7DSXY:4:2277:20636:16188 1:N:0:TCTGCAAG+GCTGCACT
CAGTCCTCGAATCAGTAGATTCGAAATATATGACAAGTATATGTAAGTGGCTGACTTAGATCATAAGT
AATGTATACAGTAAATGTTTGTACAGTTGAAATAGATTTGAAATTCAAATTTCAAATTTTAAAATATG
AGCCGTACAAACTTT
+
FFFFFFFFFFFFFFFF:FFFFFFFFFFFFFFFFFFFFFFFFFFFFFFFFFFFFFFFFFFFFFFFFFF
:FFFFFFFFFFFFFFFFFFFFFFFFFFFFFFFFFFFFFFFFFFFFFFFFFFFFFFFFFFFFFFFFFFF
F:FFFFFFFFFFFFF
@A00721:190:HGJW7DSXY:4:1241:11568:20650 1:N:0:TCTGCAAG+GCTGCACT
CTCATATTTTCAAATTTGAAATTTGAATTTCAAATCTATTTCAACTGTACAAACATTTACTGTATACA
TTACTTATGATCTAAGTCAGCCACTTACATATACTTGTCATATATTTCGAATCTACTGATTCGAGGAC
TGTCATTTAATTTTG
+
FFFFFFFFFFFFFFFFFFFFFFFFFFFFFFFFFFFFFFFFFFFFFFFFFFFFFFFFFFFFFFFFFFFF
FFFFFFFFFFFFFFFFFFFFFFFFFFFFFFFFFFFFFFFFFFFFFFFFFFFFFFFFFFFFFFFFFFFF
FFFFFFFFFFF:FFF
@A00721:190:HGJW7DSXY:4:1241:11794:20729 1:N:0:TCTGCAAG+GCTGCACT
CTCATATTTTCAAATTTGAAATTTGAATTTCAAATCTATTTCAACTGTACAAACATTTACTGTATACA
TTACTTATGATCTAAGTCAGCCACTTACATATACTTGTCATATATTTCGAATCTACTGATTCGAGGAC
TGTCATTTAATTTTG
+
FFFFFFFFFFFFFFFFFFFFFF:FFFFFFFFFFFFFFFFFFFFFFFFFFFFFFF:FFFFFFFFFFFFFF:
FFFFFFFFFFFFFFFFFFFFFFFFFFFFFFFFFFFFFFFFF,FFFFFFFFFFFFFFFFFFFFFFFFFFF
FFFFF:FFFFFFFFF
@A00721:190:HGJW7DSXY:4:1427:24985:35618 1:N:0:TCTGCAAG+GCTGCACT
CTCATATTTTCAAATTTGAAATTTGAATTTCAAATCTATTTCAACTGTACAAACATTTACTGTATACA
TTACTTATGATCTAAGTCAGCCACTTACATATACTTGTCATATATTTCGAATCTACTGATTCGAGGAC
TGTCATTTAATTTTG
+
FFFFFFFFFFFFFFFFFFFFFFFFFFFFFFFFFF,FFFFFFFFFFFFFFFFFF:FFFFFFFFFFFFFFF
```

```
FFFFFFFFFFFFFFFFFFFFFF:FFFFFFFFFFFFFFFFFFFFFFFFFFFFFFF:FF:FF:FFF,FFFFF
FFFFF,FFFFFFFFF
@A00721:190:HGJW7DSXY:4:1624:23556:19022 1:N:0:TCTGCAAG+GCTGCACT
CTCATATTTTCAAATTTGAAATTTGAATTTCAAATCTATTTCAACTGTACAAACATTTACTGTATACA
TTACTTATGATCTAAGTCAGCCACTTACATATACTTGTCATATATTTCGAATCTACTGATTCGAGGAC
TGTCATTTAATTTTG
+
FFFFFFFFFFFFFFFFFFFFFFFFFFFFFFFFFFFFFFFFFFFFFFFFFFFFF:F:F:FFFFFFFFFFF
FFFFFFFFFFFFFFFFFFFFFFFFFFFFFFFFFFFFFFFFFFFFFFFFFFFFFFFFFFFFFFFFFFFFF:
FFFFFFFFF:F:FFF
@A00721:190:HGJW7DSXY:4:1626:30346:7451 1:N:0:TCTGCAAG+GCTGCACT
CTCATATTTTCAAATTTGAAATTTGAATTTCAAATCTATTTCAACTGTACAAACATTTACTGTATACA
TTACTTATGATCTAAGTCAGCCACTTACATATACTTGTCATATATTTCGAATCTACTGATTCGAGGAC
TGTCATTTAATTTTG
+
FFFFFFFFFFFFFFFFFFFFFFFFFFFFFFFFF,:FFFFFFFFFFFFFFFFFFF:FFFFFFFFFFFFFFF
FFFFF:FFFFFFFFFFFFFFFFFFFFFFFFFFFF:FFFFFFFFFFFFFFFFFFFFFFFFFFFFFFFFFFF
FFFFFFFFFFFFFFF
@A00721:190:HGJW7DSXY:4:2536:1859:14512 1:N:0:TCTGCAAG+GCTGCACT
CTCATATTTTCAAATTTGAAATTTGAATTTCAAATCTATTTCAACTGTACAAACATTTACTGTATACA
TTACTTATGATCTAAGTCAGCCACTTACATATACTTGTCATATATTTCGAATCTACTGATTCGAGGAC
TGTCAGTTAATTTTG
+
FFFFFFFFFFFFFFFFFFFFFFFFFFFF:FFFFF:FFFFFFFFFFFFFFFFFFF:FFFFFFFFFFFFFFF
FFFFFFFFFFFFFFFFFFFFFFFFFFFFFFFFFFFFFFFFFFFFFFFFFFFFFFFFFFFFFFFFFFFFFF
FFF:F,FFFF:FFFF
@A00721:190:HGJW7DSXY:4:1660:13593:14920 1:N:0:TCTGCAAG+GCTGCACT
TTTCATTATTCAAAATTAAATGACAGTCCTCGAATCAGTAGATTCGAAATATATGACAAGTATATGTA
AGTGGCTGACTTAGATCATAAGTAATGTATACAGTAAATGTTTGTACAGTTGAAATAGATTTGAAATT
CAAATTTCAAATTTG
+
:FFFFFFFFFFFFFFFFFFFFFFFFFFFFFFFFFFFFFFFFFFFFFFFFFFFFFFFFFFFFFFFFFFFFF
F:FFFFFFFFFFFFFFFFFFFFFFFFFFFFFFFFFFFFFF:FFFFFFF:FFFFFFFFFFFFFFFFFFFFF
FFFFFFFFFFFFFF:
@A00721:190:HGJW7DSXY:4:1660:13015:15421 1:N:0:TCTGCAAG+GCTGCACT
TTTCATTATTCAAAATTAAATGACAGTCCTCGAATCAGTAGATTCGAAATATATGACAAGTATATGTA
AGTGGCTGACTTAGATCATAAGTAATGTATACAGTAAATGTTTGTACAGTTGAAATAGATTTGAAATT
CAAATTTCAAATTTG
+
FFFFFFFFFFFFFFFFFFFFFFFFFFFFFFFFFFFFFFFFFFFFFFFFFFFFFFFFFFFFFFFFFFFFFF
,FFFFFFFFFFFFFFFFFFFFFF:FFFFFFFFFFFFFFFFFFFFFFFFFFFFFFFFFFFFFFFFFFFFFF
FFFFFFFFFFFFFFF
@A00721:190:HGJW7DSXY:4:2240:12816:8437 1:N:0:TCTGCAAG+GCTGCACT
TTTCATTATTCAAAATTAAATGACAGTCCTCGAATCAGTAGATTCGAAATATATGACAAGTATATGTA
AGTGGCTGACTTAGATCATAAGTAATGTATACAGTAAATGTTTGTACAGTTGAAATAGATTTGAAATT
CAAATTTCAAATTTG
+
FFFFFFFFFFFFFFFFFFFFFFFFFFFFFFFFFFFFFFFFFFFFFFFFFFFFFFFFFFFFFFFFFFFFFF
FFFFFFFFFFFFFFFFFFFFFFFFFFFFFFFFFFFFFFFFFFFFFFFFFFFFFFFFFFFFFFFF,FFFFF
FFFFFFFFFFFFFFF
@A00721:190:HGJW7DSXY:4:2438:11234:36229 1:N:0:TCTGCAAG+GCTGCACT
TTTCATTATTCAAAATTAAATGACAGTCCTCGAATCAGTAGATTCGAAATATATGACAAGTATATGTA
AGTGGCTGACTTAGATCATAAGTAATGTATACAGTAAATGTTTGTACAGTTGAAATAGATTTGAAATT
CAAATTTCAAATTTG
```

```
+
FFFFFFFFFFFFFFFFFFFFFFFFFFFFFFFFFFFFFFFFFFFFFFFFFFFFFFFFFFFFFFFFFFFFFF
:FFFFFFFFFFFFFFFFFFFFFFFFFFFF:FFFFFFFFFFFFFFFFFFFFFFFFFFFFFFFFFFFFFFFF
FFFFFFFFFFFFFFF
@A00721:190:HGJW7DSXY:4:1214:21856:8907 1:N:0:TCTGCAAG+GCTGCACT
CAACTGTACAAACATTTACTGTATACATTACTTATGATCTAAGTCAGCCACTTACATATACTTGTCAT
ATATTTCGAATCTACTGATTCGAGGACTGTCATTTAATTTTGAATAATGAAAGATCTGATATCATCTT
TCAAACGACGTGTTA
+
FFFFFFFFFFFFFFFFFFFFFFFFFFFFFFFFFFFFFFFFFFFFFFFFFFFFFFFFFFFFFFFFFFFFFF
FFFFFFFFFFFFFFFFFFFFFFFFFFFFFFFFFF:FFFFFFFFFFFFFFFFFFFFFFFFFFFFFFFFFFF
FFFFFF:FFFFFFFF
@A00721:190:HGJW7DSXY:4:1331:25500:17597 1:N:0:TCTGCAAG+GCTGCACT
CAACTGTACAAACATTTACTGTATACATTACTTATGATCTAAGTCAGCCACTTACATATACTTGTCAT
ATATTTCGAATCTACTGATTCGAGGACTGTCATTTAATTTTGAATAATGAAAGATCTGATATCATCTT
TCAAACGACGTGTTA
+
FFF,F:FFFFFFFFFFFFFFFFFFFFFFFFFFFFFFFFF::FF,::FFFFFFFFFFFFFFFF:FF:FFF,
F,FF:FFFFFFFFFFFFFFFF:FFFFFF:FFFFFFFF:FF:FFFFFFFF::F:FFF,FFFFF:FFFFFFF
,F:FFFFF:F:F:FF
@A00721:190:HGJW7DSXY:4:1506:18602:21183 1:N:0:TCTGCAAG+GCTGCACT
CAACTGTACAAACATTTACTGTATACATTACTTATGATCTAAGTCAGCCACTTACATATACTTGTCAT
ATATTTCGAATCTACTGATTCGAGGACTGTCATTTAATTTTGAATAATGAAAGATCTGATATCATCTT
TCAAACGACGTGTTA
+
FFFFFFFFFFFFFFFFFFFFFFFFFFFFFFFFFFFFFFFFFFFFFFFFFFFFFFFFFFFFFFFFFFFFFF
FFFFFFFFFFFFFFFFFFFFFFFFFFFFFFFFFFFFFFFFFFFFFFFFFFFFFFFFFFFFFFFFFFFFFF
FFFFFFFFFFFFFFF
@A00721:190:HGJW7DSXY:4:1558:21829:14779 1:N:0:TCTGCAAG+GCTGCACT
CAACTGTACAAACATTTACTGTATACATTACTTATGATCTAAGTCAGCCACTTACATATACTTGTCAT
ATATTTCGAATCTACTGATTCGAGGACTGTCATTTAATTTTGAATAATGAAAGATCTGATATCATCTT
TCAAACGACGTGTTA
+
FFFFFFFFFFF:FFFFFFFFFFFFFFFFFFFFFFFFFFFFFFFFFFFFFFFFFFFFFFFFFFFFFFFFF:
F:FFFFFFFFFFFFFFFFFFFFFFFFFFFFFFFFFFFFFFFFFFFFFFFFFFFFF:FFFFFFFFFFFFFF
FFFF:FF:FF:FFFF
@A00721:190:HGJW7DSXY:4:1617:5376:32503 1:N:0:TCTGCAAG+GCTGCACT
CAACTGTACAAACATTTACTGTATACATTACTTATGATCTAAGTCAGCCACTTACATATACTTGTCAT
ATATTTCGAATCTACTGATTCGAGGACTGTCATTTAATTTTGAATAATGAAAGATCTGATATCATCTT
TCAAACGACGTGTTA
+
FFFFFFFFFFFFFFFFFFFFFF:FFFFFFFFFFFFFFFFFFFFFFFFFFFFFFFFFFFFFFFFFFFFFF:
FFFFFFFFFFFFFFFFFFFFFFFFFFFFF:FFFFFFFFFFFFFFFFFFFFFFFFFFFFFFFFFFFFFFFF
FFFF:FFFFFFFFFF
@A00721:190:HGJW7DSXY:4:2258:9561:9846 1:N:0:TCTGCAAG+GCTGCACT
CAACTGTACAAACATTTACTGTATACATTACTTATGATCTAAGTCAGCCACTTACATATACTTGTCAT
ATATTTCGAATCTACTGATTCGAGGACTGTCATTTAATTTTGAATAATGAAAGATCTGATATCATCTT
TCAAACGACGTGTTA
+
FFFFFFFFFFF:FFFFFFFFFFFFFFFFFFFFFFFFFFFFFFFFFFFFFFFFFFFFFFFFFFFFFFFFF:
FFFFFFFFFFFFFFFFFFFFFFFFFFFFFFFFFFFFFFFFFFFFFFFFFFFFFFFFFFFFFFFFFFFFFF
FFFFFFFF:FFFFFF
@A00721:190:HGJW7DSXY:4:2261:24153:11913 1:N:0:TCTGCAAG+GCTGCACT
CAACTGTACAAACATTTACTGTATACATTACTTATGATCTAAGTCAGCCACTTACATATACTTGTCAT
```

ATATTTCGAATCTACTGATTCGAGGACTGTCATTTAATTTTGAATAATGAAAGATCTGATATCATCTT
TCAAACGACGTGTTA
+
FFFFFFFFFFFFFFFFFFFFFFFFFFFFFFFFFFFFFFFFFFFFFFFFFFFFFFFFFFFFFFFFFFFFFF
F:FFFFFFFFFFFFFFFFFFFFFFFFFFFFFFFFFFFFFFFFFFFFFFFFFFFFF:FFFFFFFFFFFFFF
FF:FFFFFFFFFFFF
@A00721:190:HGJW7DSXY:4:2333:4020:20509 1:N:0:TCTGCAAG+GCTGCACT
CAACTGTACAAACATTTACTGTATACATTACTTATGATCTAAGTCAGCCACTTACATATACTTGTCAT
ATATTTCGAATCTACTGATTCGAGGACTGTCATTTAATTTTGAATAATGAAAGATCTGATATCATCTT
TCAAACGACGTGTTA
+
FFFFFFFFFFFFFFFFFFFFFFFFFFFFFFFFFFFFFFFFFFFFFFFFFFFFFFFFFFFFFFFFFFFFFF
FFFFFFFFFFFFFFFFFFFFFFFFFFFFFFFFF:FFFFFFFFFFFFFFFF:FFFFFFFFFFFFFFFFFFF
FFFFFFFFFFFFFFF
@A00721:190:HGJW7DSXY:4:2514:6388:13150 1:N:0:TCTGCAAG+GCTGCACT
CAACTGTACAAACATTTACTGTATACATTACTTATGATCTAAGTCAGCCACTTACATATACTTGTCAT
ATATTTCGAATCTACTGATTCGAGGACTGTCATTTAATTTTGAATAATGAAAGATCTGATATCATCTT
TCAAACGACGTGTTA
+
FFFFFFFFFFFFF:FFFFFFFFFFFFFFFFFFFFFFFFFFFFFFFFFFFFFFFFFFFFFFFFFFFFFFFF
FFFFFFFFFFFFFFFFFFFFFFFFFFFFFFFFFFFFFFFFFFFFFFFFFFFFFFFFFFFFFFFFFFFFFF
FFFFFFFFFFFFFFF
@A00721:190:HGJW7DSXY:4:2533:15790:32033 1:N:0:TCTGCAAG+GCTGCACT
CAACTGTACAAACATTTACTGTATACATTACTTATGATCTAAGTCAGCCACTTACATATACTTGTCAT
ATATTTCGAATCTACTGATTCGAGGACTGTCATTTAATTTTGAATAATGAAAGATCTGATATCATCTT
TCAAACGACGTGTTA
+
FFFFFFFFFFFFFFFFFFFFFFFFFFFFFFFFFFFFFFFFFFFFFFFFFFFFFFFFFFFFFFFFFFFFF:
FFFFFFFFFFFFFFFFFFFFFFFFFFFFFFFFFFFFFFFFFFFFFFFFFFFFFFFFFFFFFFFFFFFFFF
FFFFFFFFF:FFFFF
@A00721:190:HGJW7DSXY:4:2546:19804:25614 1:N:0:TCTGCAAG+GCTGCACT
CAACTGTACAAACATTTACTGTATACATTACTTATGATCTAAGACAGCCACTTACATATACTTGTCAT
ATATTACGAATCTACTGATTCGAGGACTGTCATTTAATATAGAATAATGAAAGATCTGATATCATCTA
TCAAACGACGTGTTA
+
F:FFFFFFFFF,FFFFFFF,FFF,FFFFFFFFFFFFFFFF:FF,,FF,FFFFFFFFFFF,FF:F:FF:,
FF:FF,,,FFFFFF:F:F:F:,FFFFF:,FFF,F:FF,,F,FFF,FF,F:,F,:FF,FFFFFFFFF,,
,:F:F,FF,:,,FFF
@A00721:190:HGJW7DSXY:4:2629:14959:1251 1:N:0:TCTGCAAG+GCTGCACT
CAACTGTACAAACATTTACTGTATACATTACTTATGATCTAAGTCAGCCACTTACATATACTTGTCAT
ATATTTCGAATCTACTGATTCGAGGACTGTCATTTAATTTTGAATAATGAAAGATCTGATATCATCTT
TCAAACGACGTGTTA
+
FFFFFFFFFFF:FFF:FFFFFFFFFFFFFFFFFFFFFFFFFFFFFFFFFFFFFFFFFFFFFFFFFFFFFF
FFFFFFFFFFFFFFFFFFFFFFFFFFFFFFFFF::FFFFFFFFF:FFFFFFFFFFFFFFFFFFFFFFFFF
FFFFFFFFF:FFFFF
@A00721:190:HGJW7DSXY:4:1563:9860:14215 1:N:0:TCTGCAAG+GCTGCACT
CAGATAACCAGTTCTAACACGTCGTTTGAAAGATGATATCAGATCTTTCATTATTCAAAATTAAATGA
CAGTCCTCGAATCAGTAGATTCGAAATATATGACAAGTATATGTAAGTGGCTGACTTAGATCATAAGT
AATGTATACAG
+
FFFFFFFFFFFFFFFFFFFFFFFFFFFF:FFFFFFFF:FFFFFFFFFFFFFFFFFFFFFFFFFFFFFFFF
FFFFFFFFFFFFFFFFFFFFFFFFFFFFFFFFFFFFFFFFFFFFFFFFFFFFFFFFFFFFFFFFFFFFFF
FFFFFFFFFFF

@A00721:190:HGJW7DSXY:4:2401:15221:27070 1:N:0:TCTGCAAG+GCTGCACT
CAGATAACCAGTTCTAACACGTCGTTTGAAAGATGATATCAGATCTTTCATTATTCAAAATTAAATGA
CAGTCCTCGAATCAGTAGATTCGAAATATATGACAAGTATATGTAATTGGCTGACTTAGATCATAAGT
AATGTATACAG
+
FFFFFFFFFFFFFFFFFFFFFFFFFF::FF,:FFFFF,FF,F::FFF,FFFFFF,FFFFFFFFF,,
FF,F:FFF:FFFFFF,FFFF:FFFF:FFFF,F:FF:FFFFF,F:F,F,F:FFFFFF:FF,:FFF:F
:FFFFFFFFFF
@A00721:190:HGJW7DSXY:4:1119:11550:8938 1:N:0:TCTGCAAG+GCTGCACT
CGCCACTTACATATACTTGTCATATATTTCGAATCTACTGATTCGAGGACTGTCATTTAATTTTGAAT
AATGAAAGATCTGATATCATCTTTCAAACGACGTGTTAGAACTGGTTATCTGTGACGTATATCCATTT
CGGTGTAGAAACCAG
+
FFFFFFFFFFFFFFFFFFFFFFFFFFFFFFFFFFFFFFFFFFFFFFFFFFFFFFFFFF:FFFFFFF
FFFFFFFFFFFFFFFFFFFFFFFFFFFFFFFFFFFFFFFFFFFFFFFFFFFFFFFF:FFFFFFFFF
FFFFFFF:FFFFFFF
@A00721:190:HGJW7DSXY:4:1237:4499:34992 1:N:0:TCTGCAAG+GCTGCACT
CGCCACTTACATATACTTGTCATATATTTCGAATCTACTGATTCGAGGACTGTCATTTAATTTTGAAT
AATGAAAGATCTGATATCATCTTTCAAACGACGTGTTAGAACTGGTTATCTGTGACGTATATCCATTT
CGGTGTAGAAACCAG
+
FFFFFFFFFFFFFFFFFFFFFFFFFFFFFFFFFFFFFFFFFFFFFFFFFFFFFFFFFFFFFFFFFF
FFFFFFFFFFFFFFFFFFFFFFFFFFFFFFFFFFFFFF:FFFFFFFFFFFFFFFFFFFFFFFFFFF
FFFFFFFFFFFFFFF
@A00721:190:HGJW7DSXY:4:1306:10402:36949 1:N:0:TCTGCAAG+GCTGCACT
CGCCACTTACATATACTTGTCATATATTTCGAATCTACTGATTCGAGGACTGTCATTTAATTTTGAAT
AATGAAAGATCTGATATCATCTTTCAAACGACGTGTTAGAACTGGTTATCTGTGACGTATATCCATTT
CGGTGTAGAAACCAG
+
FFFFFFFFFFFFFFFFFFFFFFFFFFFFFFFFFFFFFFFFFFFFFFFFFFFFFFFF:FFFFFFFFF
FFFFF:FFFFFFFFFFFFFFFFFFFFFFFFF:FFFFFFFFFFF:FFFFFFFFFFFFFFFFFFFFFF
FFFFFFFFFF:FFFF
@A00721:190:HGJW7DSXY:4:1311:4860:31360 1:N:0:TCTGCAAG+GCTGCACT
CGCCACTTACATATACTTGTCATATATTTCGAATCTACTGATTCGAGGACTGTCATTTAATTTTGAAT
AATGAAAGATCTGATATCATCTTTCAAACGACGTGTTAGAACTGGTTATCTGTGACGTATATCCATTT
CGGTGTAGAAACCAG
+
FFFFFFFFFFFF:FFFFFFFFFF,FFFFFFFFFFFFFFFF:FFFFFFFF:FFFFFFFFFF:FFFFF
FFFFFF:FFFFFFFFFFFFFFFFFFFFFFFFF,FFFFFFFFFF:FFFFFFFFFFFFF,FFFFFFFF
FF,FFFFFFFFFFFF
@A00721:190:HGJW7DSXY:4:1442:24903:20102 1:N:0:TCTGCAAG+GCTGCACT
CGCCACTTACATATACTTGTCATATATTTCGAATCTACTGATTCGAGGACTGTCATTTAATTTTGAAT
AATGAAAGATCTGATATCATCTTTCAAACGACGTGTTAGAACTGGTTATCTGTGACGTATATCCATTT
CGGTGTAGAAACCAG
+
FFFFFFFFFFFFFFFFFFFFFFFFFFFFFFFFFFF:FFFFFFFFFFFFFFFFFFFFFFFFFFFFFF
FFFF:FFFFFFFFFFFFFFFFFFFFFFFFFFFFFFFFFFFFFFFFFFFFFFFFFFFFFFFFFFFFF
FFFFFFFFFFFFFFF
@A00721:190:HGJW7DSXY:4:2254:3586:35415 1:N:0:TCTGCAAG+GCTGCACT
CGCCACTTACATATACTTGTCATATATTTCGAATCTACTGATTCGAGGACTGTCATTTAATTTTGAAT
AATGAAAGATCTGATATCATCTTTCAAACGACGTGTTAGAACTGGTTATCTGTGACGTATATCCATTT
CGGTGTAGAAACCAG
+
FFFFFFFFFFFFFFFFFFFFF:FFFFFFFFFFFFFFFFFFFFFFFFFFFFFFFFFFFFFFFFFFFF

```
FFFFFFFFFFFFFFFFFFFFFFFFFFFFFFFFFFFFFFFFFFFFFFFF:FFFFFFFFFFFFFFFFF:FF
FFFFFFFFFFFFFFF
@A00721:190:HGJW7DSXY:4:2264:2157:33567 1:N:0:TCTGCAAG+GCTGCACT
CGCCACTTACATATACTTGTCATATATTTCGAATCTACTGATTCGAGGACTGTCATTTAATTTTGAAT
AATGAAAGATCTGATATCATCTTTCAAACGACGTGTTAGAACTGGTTATCTGTGACGTATATCCATTT
CGGTGTAGAAACCAG
+
FFFFFFFFFFFFFFFFFFFFF:,FFFF,FFFFFFFFFFFFFFFFFFFFFFFFFFFFFFFFFFFFFFFFF
FFFFFFFFFFFFFFFFFFFFF:FFFFFF,FFFFFFF:FFFFFFFFFFFFFFFFFFFFFF:FFFFF,FF
F:F:FFFFFFFF:FF
@A00721:190:HGJW7DSXY:4:1339:22625:19570 1:N:0:TCTGCAAG+GCTGCACT
GCCACTTACATATACTTGTCATATATTTCGAATCTACTGATTCGAGGACTGTCATTTAATTTTGAATA
ATGAAAGATCTGATATCATCTTTCAAACGACGTGTTAGAACTGGTTATCTGTGACGTATATCCATTTC
GGTGTAGAAACCAGA
+
FFFFFFFFFFFFFFFFFFFFFFFFFFFFFFFFFFFFFFFFFFFFFFFF:FF::FFFF:FFFFFFF
FFFFFFFFFFFFF:FFFFFFFFFFFFFFFFFFFFFFFFFFFFFFFFFFFFFFFFFFFFFFFFFFFFF
FFFFFFFFFFFFFFF
@A00721:190:HGJW7DSXY:4:1452:3052:24095 1:N:0:TCTGCAAG+GCTGCACT
GCCACTTACATATACTTGTCATATATTTCGAATCTACTGATTCGAGGACTGTCATTTAATTTTGAATA
ATGAAAGATCTGATATCATCTTTCAAACGACGTGTTAGAACTGGTTATCTGTGACGTATATCCATTTC
GGTGTAGAAACCAGA
+
FFFFFFFFFFFFFFFFFFFFFFFFFFFFFFFFFFFFFFFFFFFFFFFFFFFFFFFFFFFFFFFFFFFFF
FFFFFFFFFFFFFFFFFFFFFFFFFFFFFFFFF:FFFFFFFFFFF:FFFFFFFFFFFFFFFFFFFFFFFF
FFFFFFFFFFFFFFF
@A00721:190:HGJW7DSXY:4:1462:12961:10347 1:N:0:TCTGCAAG+GCTGCACT
GCCACTTACATATACTTGGCATATATTTCGAATCTACTGATTCGAGGACTGTCATTTAATTTTGAATA
ATGAAAGATCTGATATCATCTTTCAAACGACGTGTTAGAACTGGGTATCTGTGACGTATATCCATTTC
GGTGTAGAAACCAGA
+
FFF:FFFFFFFFFFFFFF,FFFF:F,FFFFFFFFFFFFFF:FFFFFFFFFFFFFFFFFFFFFFF:F,FF
FFFFFFFFFFFFFFFFFFFFFFFFFFFFFF,FF:FFFFFFFFFFF,FFFFFFFFFF:FFFFFFF,FFFF
FFFFFFFFFFFFFFF
@A00721:190:HGJW7DSXY:4:1555:14986:27070 1:N:0:TCTGCAAG+GCTGCACT
GCCACTTACATATACTTGTCATATATTTCGAATCTACTGATTCGAGGACTGTCATTTAATTTTGAATA
ATGAAAGATCTGATATCATCTTTCAAACGACGTGTTAGAACTGGTTATCTGTGACGTATATCCATTTC
GGTGTAGAAACAAGA
+
FFFFF,FF,FFF:FFF:FF,FFFFF:FF:,:FF:FF,F:,F::FF:FFFFFF,F:F:FF:,FF:FF,F
F:FFFFFF,F,FFFFFFF::FFF:FFF::F:FF:FF:F:FF:,:F,FF:F,FFF:FFFFF:,:FFFF,
FFFFFFFFFFF,F,F
@A00721:190:HGJW7DSXY:4:1669:21594:4413 1:N:0:TCTGCAAG+GCTGCACT
GCCACTTACATATACTTGTCATATATTTCGAATCTACTGATTCGAGGACTGTCATTTAATTTTGAATA
ATGAAAGATCTGATATCATCTTTCAAACGACGTGTTAGAACTGGTTATCTGTGACGTATATCCATTTC
GGTGTAGAAACCAGA
+
FFFFFFFFFFFFFFFFFFFFFFFFFFFFFFFFFFFFFFFFFFFFFFFFFFFFFFFFFFFFFFFFFFFFF
FFFFFFFFFFFFFFFFFFFFFFFFFFFFFFFFFFFFFFFFFFFFFFFFFFFFFFFFFFFFFFFFFFFFF
FFFFFFFFFFFFFFF
@A00721:190:HGJW7DSXY:4:2123:2275:27164 1:N:0:TCTGCAAG+GCTGCACT
GCCACTTACATATACTTGTCATATATTTCGAATCTACTGATTCGAGGACTGTCATTTAATTTTGAATA
ATGAAAGATCTGATATCATCTTTCAAACGACGTGTTAGAACTGGTTATCTGTGACGTATATCCATTTC
GGTGTAGAAACCAGA
```

```
+
FFFFFFFFFFFFFFFFFFFFF,FFFFFFFFFFFFFFFFFFFFFFFFFFFFFFFFFFFFFFFFFFFFFF
FFFFFFFFFFFFFFFFFFFFFFFFFFFFFFFFFFFFFFFFFFFF:FFFFFFFFFFFF:FFFFFFFFFF
FFFFFFFFFFFFFFF
@A00721:190:HGJW7DSXY:4:2153:22354:22670 1:N:0:TCTGCAAG+GCTGCACT
GCCACTTACATATACTTGTCATATATTTCGAATCTACTGATTCGAGGACTGTCATTTAATTTTGAATA
ATGAAAGATCTGATATCATCTTTCAAACGACGTGTTAGAACTGGTTATCTGTGACGTATATCCATTTC
GGTGTAGAAACCAGA
+
FFFFFFFFFFFFFFFFFFFFFFFF:FFFFFFFFFFFFFFFFFFFFFFFFFFFFFF:F:FFFFFFFFFF
FFFFFFFFFFFFFFFFFFFFFFFFFFFFFFFF:FFFFFFFFFFFFFFFFFFFFFFFFFFFFF,:FFFF
FF:FFF,:FFFFFFF
@A00721:190:HGJW7DSXY:4:2250:4499:29293 1:N:0:TCTGCAAG+GCTGCACT
GCCACTTACATATACTTGTCATATATTTCGAATCTACTGATTCGAGGACTGTCATTTAATTTTGAATA
ATGAAAGATCTGATATCATCTTTCAAACGACGTGTTAGAACTGGTTATCTGTGACGTATATCCATTTC
GGTGTAGAAACCAGA
+
FFFFFFFFFFFFFFFFFFFFFFFFFFFFFFFFFFFFFFFFFFFFFFFFFFFFFFFFFFFFFFFFFFFF
FFFFFFFFFFFFFFFFFFFFFFFFFFFFFFFFFFFFFFFFFFFFFFFFFFFFFFFFFFFFFFFFFFFF
FFFFFFFFFFFFFFF
@A00721:190:HGJW7DSXY:4:2353:32018:29011 1:N:0:TCTGCAAG+GCTGCACT
GCCACTTACATATACTTGTCATATATTTCGAATCTACTGATTCGAGGACTGTCATTTAATTTTGAATA
ATGAAAGATCTGATATCATCTTTCAAACGACGTGTTAGAACTGGTTATCTGTGACGTATATCCATTTC
GGTGTAGAAACCAGA
+
FFFFFFFFFFFFFFFFFFFFFFFFFFFFFFFFFFFFFFFFFFFFFFFFFFFFFFFF:FFFFFFFFFFF
FFFFFFFFFFFFFFFFFFFFFFFF:F,FFFFFFFFFFFFFFFFFFFFFFFFFFFFFFFFFFF:FFFFF
FFFFFFFFFFFFFFF
@A00721:190:HGJW7DSXY:4:2513:5258:18114 1:N:0:TCTGCAAG+GCTGCACT
GCCACTTACATATACTTGTCATATATTTCGAATCTACTGATTCGAGGACTGTCATTTAATTTTGAATA
ATGAAAGATCTGATATCATCTTTCAAACGACGTGTTAGAACTGGTTATCTGTGACGTATATCCATTTC
GGTGTAGAAACCAGA
+
FFFFFFFFFFFFFFFFFFFFFFFFFFFFFFFFFFFFFFFFFFFFFFFFFFFFFFFFFFFFFFFFFFFF
FFFFFFFFFFFFFFFFFFFFFFFFFFFFFFFFFFFFFFFFFFFFFFFFFFFFFFFFFFFFFFFFFFFF
FFFFFFFFFFFFFFF
@A00721:190:HGJW7DSXY:4:2629:13865:4648 1:N:0:TCTGCAAG+GCTGCACT
GCCACTTACATATACTTGTCATATATTTCGAATCTACTGATTCGAGGACTGTCATTTAATTTTGAATA
ATGAAAGATCTGATATCATCTTTCAAACGACGTGTTAGAACTGGTTATCTGTGACGTATATCCATTTC
GGTGTAGAAACCAGA
+
FFFFFFFFFFFFFFFFFFFFFFFFFFFFFFFFFFFFFFFFFFFFFFFFFFFFFF:FFFFFFFFFFFFF
FFFFFFFFFFFFFFFFFFFFFFFFFFFFFFFFFFFFFFFFFFF:FFFFFFFFFFFFFFFFFFFFFFFF
FFFFFF:FFFFFFFF
@A00721:190:HGJW7DSXY:4:2657:15628:1157 1:N:0:TCTGCAAG+GCTGCACT
GCCACTTACATATACTTGTCATATATTTCGAATCTACTGATTCGAGGACTGTCATTTAATTTTGAATA
ATGAAAGATCTGATATCATCTTTCAAACGACGTGTTAGAACTGGTTATCTGTGACGTATATCCATTTC
GGTGTAGAAACCAGA
+
FFFFFFFFFFFFFFFFFF:FFFFFFFFFFFFFFFFFFFFFFFFFFFFFFFFFFFFFFFFFFFFFFFFF
FFFFFFFFFFFFFFFFFFFFFFFFFFFFFFFFFFFFFFFFFFFFFFFFFFFFFFFFFFFFFFF:FFFF
FFFFFFFFFFFFFFF
@A00721:190:HGJW7DSXY:4:1134:23936:3270 1:N:0:TCTGCAAG+GCTGCACT
CACTTACATATACTTGTCATATATTTCGAATCTACTGATTCGAGGACTGTCATTTAATTTTGAATAAT
```

GAAAGATCTGATATCATCTTTCAAACGACGTGTTAGAACTGGTTATCTGTGACGTATATCCATTTCGG
TGTAGAAACCAGAGA
+
FFFFFFFFFFFFFFFFFFFFFFFFFFFFFFFFFFFFFFFFFFFFFFFFFFFFFFFFFFFFFFFFFFFF
FFFFFFFFFFFFFFFFFFFFFFFFFFFFFFFFFFFFFFFFFFFFFFFFFFFFFFFFFFFFFFFFFFFF
FFFFFFFFFFFFFFF
@A00721:190:HGJW7DSXY:4:1436:9570:3693 1:N:0:TCTGCAAG+GCTGCACT
CACTTACATATACTTGTCATATATTTCGAATCTACTGATTCGAGGACTGTCATTTAATTTTGAATAAT
GAAAGATCTGATATCATCTTTCAAACGACGTGTTAGAACTGGTTATCTGTGACGTATATCCATTTCGG
TGTAGAAACCAGAGA
+
FFFFFFFFFFFFFFFF::F:FFFFFFFFFFFFFFFFFFFFFFFFFFFFFFFFFFFFFFFFFFFFFFFF
FFFFFFFFFFFFFFFFFFFFFFFF:FFFFFFFFFFFF:FFFFFFFFFFFFFFFFFFFFFFFFFFFFFF
FFFF:FFFFF:FFFF
@A00721:190:HGJW7DSXY:4:1436:9634:3771 1:N:0:TCTGCAAG+GCTGCACT
CACTTACATATACTTGTCATATATTTCGAATCTACTGATTCGAGGACTGTCATTTAATTTTGAATAAT
GAAAGATCTGATATCATCTTTCAAACGACGTGTTAGAACTGGTTATCTGTGACGTATATCCATTTCGG
TGTAGAAACCAGAGA
+
FFFFFFFFFFFFFFFFFFFFFFFFFFFFFFFFFFFFFFFFFFFFFFFFFFFFFFFFFFFFFF:FFFFF
FFFFFFFFFFFFFFFFFFFFFFFFFFFFFFFFFFFFFFFFFFFFFFFFFFFFFFFFFFFFFFFFFFFF
FFFF,FFFFFFFFFF
@A00721:190:HGJW7DSXY:4:1456:26756:3458 1:N:0:TCTGCAAG+GCTGCACT
CACTTACATATACTTGTCATATATTTCGAATCTACTGATTCGAGGACTGTCATTTAATTTTGAATAAT
GAAAGATCTGATATCATCTTTCAAACGACGTGTTAGAACTGGTTATCTGTGACGTATATCCATTTCGG
TGTAGAAACCAGAGA
+
FFFFFFFFFFFFFFFFFFFFFFFFFFFFFFFFFFFFFFFFFFFFFFFFFFFFFFFFFFFFFFFFFFFF
FFFFFFFFFFFFFFFFFFFFFFFFFFFFFFFFFFFFFFFFFFFFFFFFFFFFFFF:FFF:FFFFFFFF
FF:FFFFFFFFFFFF
@A00721:190:HGJW7DSXY:4:1540:6849:4366 1:N:0:TCTGCAAG+GCTGCACT
CACTTACATATACTTGTCATATATTTCGAATCTACTGATTCGAGGACTGTCATTTAATTTTGAATAAT
GAAAGATCTGATATCATCTTTCAAACGACGTGTTAGAACTGGTTATCTGTGACGTATATCCATTTCGG
TGTAGAAACCAGAGA
+
FFFFFFFFFFFFFFFFFFFFFFFFFFFFFFFFFFFFFFFFFFFFFFFFFFFFFFFFFFFFFFFFFFFF
FFFFFFFFFFFFFFFFFFFFFFFF:FFFFFFFFFFFFFFFFFFFFFFFFFFFFFFFFFFFFFFFFFFF
FFFFFFFFFFFFFFF
@A00721:190:HGJW7DSXY:4:2512:25346:29105 1:N:0:TCTGCAAG+GCTGCACT
CACTTACATATACTTGTCATATATTTCGAATCTACTGATTCGAGGACTGTCATTTAATTTTGAATAAT
GAAAGATCTGATATCATCTTTCAAACGACGTGTTAGAACTGGTTATCTGTGACGTATATCCATTTCGG
TGTAGAAACCAGAGA
+
FFFFFFFFFFFFFFFFFFFFFFFFFFFFFFFFFFFFFFFFFFFFFFFFFFFFFFFFFFFFFFFFFFFF
FFFFFFFFFFFFFFFFFFFFFFFFFFFFFFFFFFFFFFFFFFFFFFFFFFFFFFFFFFFFFFFFFFFF
FFFFFFFFFFFFFFF
@A00721:190:HGJW7DSXY:4:1114:32579:9220 1:N:0:TCTGCAAG+GCTGCACT
TCATATATTTCGAATCTACTGATTCGAGGACTGTCATTTAATTTTGAATAATGAAAGATCTGATATCA
TCTTTCAAACGACGTGTTAGAACTGGTTATCTGTGACGTATATCCATTTCGGTGTAGAAACCAGAATC
CTTCAGATCGG
+
FFFFFFFFFFFFFFFFFFFFFFFFFFFFFFFFFFFF:FFFFFFFFFFFFFFFFFFFFFFFFFFFFFFF
FFFFFFFFFFFFFFFFFFFF:FFFFFFFFFFFFFFFFFFFFFFFFFFFFFFFFFFFFFFFFFFFFFFF
FFFFFFFFFFF

@A00721:190:HGJW7DSXY:4:1129:5755:3850 1:N:0:TCTGCAAG+GCTGCACT
TCATATATTTCGAATCTACTGATTCGAGGACTGTCATTTAATTTTGAATAATGAAAGATCTGATATCA
TCTTTCAAACGACGTGTTAGAACTGGTTATCTGTGACGTATATCCATTTCGGTGTAGAAACCAGAATC
CTTCAGATCGG
+
FFF,FFF:FFFFFFFFFFFFFFFFFFF:FFFFFFFFFFFFFFFFFFFFFFFFF:FFFFFFFFFFFFFFFF
FFFFFFFFFFFFFFFFFFFFFFFFFFFFFFFFFFFFFFFF:F:FFFFFFFFFFFFFFFF:FFFFFFFFFF
FFFFFFFFFFF
@A00721:190:HGJW7DSXY:4:1349:24433:7545 1:N:0:TCTGCAAG+GCTGCACT
TCATATATTTCGAATCTACTGATTCGAGGACTGTCATTTAATTTTGAATAATGAAAGATCTGATATCA
TCTTTCAAACGACGTGTTAGAACTGGTTATCTGTGACGTATATCCATTTCGGTGTAGAAACCAGAATC
CTTCAGATCGG
+
FFFFFFFFFFFFFFFFFFFFFFFFFFFFFFFFFFFFFFFFFFFFFFFFFFFFFFFFFFFFFFFFFFFFFF
FFFFFFFFFFFFFFFFFFFFFFFFFFFFF:FFFFFFFFFFFFFFFFFFFFFFFFFFFFFFFF:FFFFFFF
FFFFFFFFFFF
@A00721:190:HGJW7DSXY:4:1607:21206:24220 1:N:0:TCTGCAAG+GCTGCACT
TCATATATTTCGAATCTACTGATTCGAGGACTGTCATTTAATTTTGAATAATGAAAGATCTGATATCA
TCTTTCAAACGACGTGTTAGAACTGGTTATCTGTGACGTATATCCATTTCGGTGTAGAAACCAGAATC
CTTCAGATCGG
+
FFFFFFFFFFFFFFFFFFFFFFFFFFFFFFFFFFFFFFFFFFFFFFFFFFFFFFFFFFFFFFFFFFFFFF
FFF:FFFFFFFFFFFFFFFF:FFFFF:FFFFFFFF:FFFFFFFFFFFFFFFFFFFFFFFFFFFFFFFFFF
FFFFFFFF,FF
@A00721:190:HGJW7DSXY:4:1670:1253:15248 1:N:0:TCTGCAAG+GCTGCACT
TCATATATTTCGAATCTACTGATTCGAGGACTGTCATTTAATTTTGAATAATGAAAGATCTGATATCA
TCTTTCAAACGACGTGTTAGAACTGGTTATCTGTGACGTATATCCATTTCGGTGTAGAAACCAGAATC
CTTCAGATCGG
+
FFFFFFFFFFFFFFFFFFFFFFFFFFFFFFFFFFFFFFFFFFFFFFFFFFFFFFFFFFFFFFFFFF:FFFF
FFFFFFFFFFFFFFFFFFFFFFFFFFFFFFFFFFFFFFFFFFFFFFFFFFFFFFFFFFFFFFFFFFFFFF
FFFFFFFFF:F
@A00721:190:HGJW7DSXY:4:2529:7536:20400 1:N:0:TCTGCAAG+GCTGCACT
TCATATATTTCGAATCTACTGATTCGAGGACTGTCATTTAATTTTGAATAATGAAAGATCTGATATCA
TCTTTCAAACGACGTGTTAGAACTGGTTATCTGTGACGTATATCCATTTCGGTGTAGAAACCAGAATC
CTTCAGATCGG
+
FFFFFFFFFFFFFFFFFFFFFFFFFFFFFFFFFFFFFFFFFFFFFFFFFFFFFFFFFFFFFFFFFFFFFF
FFFFFFFFFFFFFFFFFFFFFFFFFFFFFFFFFFFFFFFFFFFFFFFFFFFFFFFFFFFFFFFFFFFFFF
FFFFFFFFF,F
@A00721:190:HGJW7DSXY:4:1654:20374:36933 1:N:0:TCTGCAAG+GCTGCACT
CTCGAGGACTGTCATTTAATTTTGAATAATGAAAGATCTGATATCATCTTTCAAACGACGTGTTAGAA
CTGGTTATCTGTGACGTATATCCATTTCGGTGTAGAAACCAGAATCCTTCAGATCGGCAATGAATTCC
GATGAGGATCTATCG
+
FFFFFFFFFFFFFFFFFFFFFFFFFFFFFFFFFFFFFFFFFFFFFFFFFFFFFFFF:FFFFFFFFFFFFF
FFFFFFFFFFFFFFFFFFFFFFFFFFFFFFFFFFFFFFFFFFFFFFFFFFFFFFFFFFFFF:FFFFFFFF
FFFFFFFFFF:FFFF
@A00721:190:HGJW7DSXY:4:2229:5746:34053 1:N:0:TCTGCAAG+GCTGCACT
CTCGAGGACTGTCATTTAATTTTGAATAATGAAAGATCTGATATCATCTTTCAAACGACGTGTTAGAA
CTGGTTATCTGTGACGTATATCCATTTCGGTGTAGAAACCAGAATCCTTCAGATCGGCAATGAATTCC
GATGAGGATCTATCG
+
FFFFFFFFFFFFFFFFFFFFFFFFFFFFFFFFFFFFFFFFFFFFFFFFFFFFFFFFFFFFFFFFFFFFFF

```
FFFFFFFFFFFFFFFFFFFFFFFFFFFFFFFFFFFFFFFF:FFFFFFFFFFFFFFFFFFFFFFFFFFFF:
FFFFFFFFFFFFFFF
@A00721:190:HGJW7DSXY:4:2325:10999:13025 1:N:0:TCTGCAAG+GCTGCACT
CTCGAGGACTGTCATTTAATTTTGAATAATGAAAGATCTGATATCATCTTTCAAACGACGTGTTAGAA
CTGGTTATCTGTGACGTATATCCATTTCGGTGTAGAAACCAGAATCCTTCAGATCGGCAATGAATTCC
GATGAGGATCTATCG
+
FFFFFFFFFFFFFF:FFFFFFFFFFFFFFFFFFFFFFFFFFFFFFFFFFFFFFFFFFFFFFFFFFFFFFF
FFFFFFFFFFFFFFFFFFFFFFFFFFFFFFFFFFFFFFFFFFFFFFFFFFFFFFFFFFFFFFFFFFFFF:
FFFF:FFFFFFFFF:
@A00721:190:HGJW7DSXY:4:2615:4264:10974 1:N:0:TCTGCAAG+GCTGCACT
CTCGAGGACTGTCATTTAATTTTGAATAATGAAAGATCTGATATCATCTTTCAAACGACGTGTTAGAA
CTGGTTATCTGTGACGTATATCCATTTCGGTGTAGAAACCAGAATCCTTCAGATCGGCAATGAATTCC
GATGAGGATCTATCG
+
FFFFFFFFFFFFFF:FFFFFFFFFFFFFFFFFFFFFFFFFFFFFFFFFFFFFFFFFFFFFFFFFFFFFFF
FFFFFFFFFFFFFFFFF,FFFFFF:FFFFFFFFFFFFFFFFFFFFFFFFFFFFFFFFFFF:FFFFFFFFF
F:FFFFFFFFFFFFF
@A00721:190:HGJW7DSXY:4:1248:18783:10598 1:N:0:TCTGCAAG+GCTGCACT
AGGACTGTCATTTAATTTTGAATAATGAAAGATCTGATATCATCTTTCAAACGACGTGTTAGAACTGG
TTATCTGTGACGTATATCCATTTCGGTGTAGAAACCAGAATCCTTCAGATCGGCAATGAATTCCGATG
AGGATCTATCGATCT
+
FFFFFFFFFFFFFFFFFFFFFFFFFFFFFFFFFFFFFFFFFFFFFFFFFFFFFFFFFFFFFFFFFFFFFF
FFFFFF:FFFFFFFFFFFFFFFFFFFFFFFFFFFFFFFFFFFFFFFFFFFFFFFFFFFFFFFFFFFFFFF
FFFFFFFFFFFFFFF
@A00721:190:HGJW7DSXY:4:2636:6433:27195 1:N:0:TCTGCAAG+GCTGCACT
AGGACTGTCATTTAATTTTGAATAATGAAAGATCTGATATCATCTTTCAAACGACGTGTTAGAACTGG
TTATCTGTGACGTATATCCATTTCGGTGTAGAAACCAGAATCCTTCAGATCGGCAATGAATTCCGATG
AGGATCTATCGATCT
+
FFFFFFFFFFFFFFFFFFFFFFFFFFFFFFFFFFFFFFFFFFFFFFFFFFFFFFFFFFFFFFFFFFFFFF
FFFFFFFFFFFFFFFFFF:FFFFFFFFFFFFFFFFFFFFFFFFFFFF:FFFFFFFFFFFFF:FF::FFFF
FFFFFFFFF:FFFFF
@A00721:190:HGJW7DSXY:4:1432:28791:2910 1:N:0:TCTGCAAG+GCTGCACT
CTGTCATTTAATTTTGAATAATGAAAGATCTGATATCATCTTTCAAACGACGTGTTAGAACTGGTTAT
CTGTGACGTATATCCATTTCGGTGTAGAAACCAGAATCCTTCAGATCGGCAATGAATTCCGATGAGGA
TCTATCGATCTTCAC
+
FFFFFFFFFFFFFFFFFFFFFFFFFFFFFFFFFFFFFFFFFFFFFFFFFFFFFFFFFFFFFFFFFFFFFF
FFFFFFFFFFFFFFFFFFFFFFFFFFFFFFFFFFFFFFFFFFFFFFFFFFFFFFFFFFFFFFFFFFFFFF
FFFFFFFFFFFFFFF
@A00721:190:HGJW7DSXY:4:1625:23466:24940 1:N:0:TCTGCAAG+GCTGCACT
CTGTCATTTAATTTTGAATAATGAAAGATCTGATATCATCTTTCAAACGACGTGTTAGAACTGGTTAT
CTGTGACGTATATCCATTTCGGTGTAGAAACCAGAATCCTTCAGATCGGCAATGAATTCCGATGAGGA
TCTATCGATCTTCAC
+
FFFFFFFFFFFFFFFFFFFFFFFFFFFFFFFFFFFFFFFFFFFFFFFFFFFFFFFFFFFFFFFFFFFFFF
FFFFFFFFFFFFFFFFFFFFFFFFFFFFFFFFFFFFFFFFFFFFFFFFFFFFFFFFFFFFFFFFFFFFFF
FF:FFFFF:FFFFFF
@A00721:190:HGJW7DSXY:4:1646:27001:15875 1:N:0:TCTGCAAG+GCTGCACT
CTGTCATTTAATTTTGAATAATGAAAGATCTGATATCATCTTTCAAACGACGTGTTAGAACTGGTTAT
CTGTGACGTATATCCATTTCGGTGTAGAAACCAGAATCCTTCAGATCGGCAATGAATTCCGATGAGGA
TCTATCGATCTTCAC
```

+
FFFFFFFFFFFFFFFFFFFFFFF:FFFFFFFFFFFFFFFFFFFFFFFFFFFFFFFFFFFFFFFFFFFF
FFFFFFFFFFFFFFFFFFFFFFFFFFFFFFFFFFFFFFFFFFFFFFFFFFFFFFFFFFFFFFFFFFFF
FFFFFFFFF:FFF:F
@A00721:190:HGJW7DSXY:4:1157:19488:8061 1:N:0:TCTGCAAG+GCTGCACT
AGCGCATATCGTCGACAATAGACAGAAGTGAAGATCGATAGATCCTCATCGGAATTCATTGCCGATCT
GAAGGATTCTGGTTTCTACACCGAAATGGATATACGTCACAGATAACCAGTTCTAACACGTCGTTTGA
AAGATGATATCAGAT
+
FFFFFFFFFFFFFFFFFFFFFFFFFFFFFFFFFFFFFFFFFFFFFFFFFFFFFFFFFFFFFFFFFFFF
FFFFFFFFFFFFFF:FFFFFFFFFFFFFFFFFFFFFFFFFFFFFFFFFFFFFFFFFFFFFFFFFFFFF
FFFFFFFFFFFFFFF
@A00721:190:HGJW7DSXY:4:1263:31295:6433 1:N:0:TCTGCAAG+GCTGCACT
AGCGCATATCGTCGACAATAGACAGAAGTGAAGATCGATAGATCCTCATCGGAATTCATTGCCGATCT
GAAGGATTCTGGTTTCTACACCGAAATGGATATACGTCACAGATAACCAGTTCTAACACGTCGTTTGA
AAGATGATATCAGAT
+
FFFFFFFFFFFFFFFFFFFFFFFFFFFFFFFFFFFFFFFFFFFFFFFFFFFFFFFFFFFFFFFFFFFF
FFFFFFFFFFFFFFFFFFFFFFFFFFFFFFFFFFFFFFFF:FFFFFFFFFFFFFFFFFFFFFFFFFFF
FFFFFFFFFFFFFFF
@A00721:190:HGJW7DSXY:4:1632:12301:13808 1:N:0:TCTGCAAG+GCTGCACT
AGCGCATATCGTCGACAATAGACAGAAGTGAAGATCGATAGATCCTCATCGGAATTCATTGCCGATCT
GAAGGATTCTGGTTTCTACACCGAAATGGATATACGTCACAGATAACCAGTTCTAACACGTCGTTTGA
AAGATGATATCAGAT
+
FFFFFFFFFFFFFFFFFFFFFFFFFFFFFFFFFFFFFFFFFFFFFFFFFFFFFFFFFFFFFFFFFFFF
FFFFFFFFFFFFFFFFFFFFFFFFFFFFFFFFFFFFFFFFFFFFFFFFFFFFFFFFFFFFFFFF:FFF
FFFFFFFFFFFFFFF
@A00721:190:HGJW7DSXY:4:2173:10402:28244 1:N:0:TCTGCAAG+GCTGCACT
AGCGCATATCGTCGACAATAGACAGAAGTGAAGATCGATAGATCCTCATCGGAATTCATTGCCGATCT
GAAGGATTCTGGTTTCTACACCGAAATGGATATACGTCACAGATAACCAGTTCTAACACGTCGTTTGA
AAGATGATATCAGAT
+
FFFFFFFFFFFFFFFFFFFFFFFFFFFFFFFFFFFFFFFFFFFFFFFFFFFFFFFFFFFFFFFFFFFF
FFFFFFFFFFFFFFFFFFFFFFFFFFFFFFFFFFFFFFFFFFFFFFFFFFFFFFFFFFFFFFFFFFFF
FFFFFFFFFFFFFFF
@A00721:190:HGJW7DSXY:4:1219:16468:28980 1:N:0:TCTGCAAG+GCTGCACT
GCGACGTGTTAGAACTGGTTATCTGTGACGTATATCCATTTCGGTGTAGAAACCAGAATCCTTCAGAT
CGGCAATGAATTCCGATGAGGATCTATCGATCTTCACTTCTGTCTATTGTCGACGATATGCGCTGCTG
GGAATGTTATGTATT
+
FFFFFFFFFFFFFFFFFFFFFFFFFFFFFFFFFFFFFFFFFFFFFFFFFFFFFFFFFFFFFFFFFFFF
FFFFFFFFFFFFFFFFFFFFFFFFFFFFFFFFFFFFFFFFFFFFFFFFFFFFFFFFFFFFFFFFFFFF
FFF,FFFFFFFFFFF
@A00721:190:HGJW7DSXY:4:1303:28076:29951 1:N:0:TCTGCAAG+GCTGCACT
GCGACGTGTTAGAACTGGTTATCTGTGACGTATATCCATTTCGGTGTAGAAACCAGAATCCTTCAGAT
CGGCAATGAATTCCGATGAGGATCTATCGATCTTCACTTCTGTCTATTGTCGACGATATGCGCTGCTG
GGAATGTTATGTATT
+
FFFFFFFFFFFFFFFFFFFFFFFFFFFFFFFFFFFFFFFFFFFFFFFFFFFFFFFFFFFFFFFFFFFF
FFFFFFFFFFFFF:FFFFFFFFFFFFFFFFFFFFFFFFFFFFFFFF:FFFFFFFFFFFFFFFF:FF:F
FFF,FFFFFFFFFFF
@A00721:190:HGJW7DSXY:4:1678:10954:35305 1:N:0:TCTGCAAG+GCTGCACT
GCGACGTGTTAGAACTGGTTATCTGTGACGTATATCCATTTCGGTGTAGAAACCAGAATCCTTCAGAT

```
CGGCAATGAATTCCGATGAGGATCTATCGATCTTCACTTCTGTCTATTGTCGACGATATGCGCTGCTG
GGAATGTTATGTATT
+
FFFFFFFFFFFF:FFFFFFFFFFFFFFFFFFFFFFFFFFFFFFFFFFFFFFFFFFFFFFFFFFFFFFFF
FFFFFFFFFFFFFFFFFFFFFFFFFFFFFFFFFFFFFFFFFFFFFFFFFFFFFFFFFFFFFFFFFFFFFF
FFFFFFFFFFFFFFF
@A00721:190:HGJW7DSXY:4:2116:25075:19554 1:N:0:TCTGCAAG+GCTGCACT
GCGACGTGTTAGAACTGGTTATCTGTGACGTATATCCATTTCGGTGTAGAAACCAGAATCCTTCAGAT
CGGCAATGAATTCCGATGAGGATCTATCGATCTTCACTTCTGTCTATTGTCGACGATATGCGCTGCTG
GGAATGTTATGTATT
+
FFFFFFFFFFFFFFFFFFFFFFFFFFFFFFFFFFFFFFFFFFFFFFFFFFFFFFFFFFFFFFFFFFFFFF
FFFFFFFFFFFFFFFFFF:FFFFFFFFFFFFFFFFFFFFFFFFFFFFFFFFFFFFFF:FFFFFFFFFFFF
FFFF:F:FFFFFFFF
@A00721:190:HGJW7DSXY:4:2203:28031:25394 1:N:0:TCTGCAAG+GCTGCACT
GCGACGTGTTAGAACTGGTTATCTGTGACGTATATCCATTTCGGTGTAGAAACCAGAATCCTTCAGAT
CGGCAATGAATTCCGATGAGGATCTATCGATCTTCACTTCTGTCTATTGTCGACGATATGCGCTGCTG
GGAATGTTATGTATT
+
FFFFFFFFFFFFFFFFFFFFFFFFFFFFFFFFFFFFFFFFFFFFFFFFFFFFFFFFFFFFFFFFFFFFFF
FFFFFFFFFFFFFFFFFFFFFFFFFFFFFFFFFFFFFFFFFFFFFF:FFFFFFFFFFFFFFFFFFFFFFF
FFF:FFFFFFFFFFF
@A00721:190:HGJW7DSXY:4:2269:6885:31454 1:N:0:TCTGCAAG+GCTGCACT
GCGACGTGTTAGAACTGGTTATCTGTGACGTATATCCATTTCGGTGTAGAAACCAGAATCCTTCAGAT
CGGCAATGAATTCCGATGAGGATCTATCGATCTTCACTTCTGTCTATTGTCGACGATATGCGCTGCTG
GGAATGTTATGTATT
+
FFFFFFFFFFFFFFFFFFFFFFFFFFFFFFFFFFFFFFFFFFFFFFFFFFFFFFFFFFFFFFFFFFFFFF
FFFFFFFFFFFFFFFFFFFFFFFFFFFFFFF:FFFFFFFFFFFFFFF:FFFFFFFFFFFFFFFFFFFFFF
FFF,FFFFFFFFFFF
@A00721:190:HGJW7DSXY:4:1345:10438:16470 1:N:0:TCTGCAAG+GCTGCACT
GACGTGTTAGAACTGGTTATCTGTGACGTATATCCATTTCGGTGTAGAAACCAGAATCCTTCAGATCG
GCAATGAATTCCGATGAGGATCTATCGATCTTCACTTCTGTCTATTGTCGACGATATGCGCTGCTGGG
AATGTTATGTATTCG
+
FFFFFFFFFFFFFFFFFFFFFFFFFFFFFFFFFFFFFFFFFFFFFFFFFFFFFFFFFFFFFFFFFFFFFF
FFFFFFFFFFFFFFFFFFFFFFFFFFFFFFFFFFFFFFFFFFFF:FFFFFFFFFFFFFFFFFFFFFFFFF
FFFFFFFFFFFFFFF
@A00721:190:HGJW7DSXY:4:1569:10854:33442 1:N:0:TCTGCAAG+GCTGCACT
GACGTGTTAGAACTGGTTATCTGTGACGTATATCCATTTCGGTGTAGAAACCAGAATCCTTCAGATCG
GCAATGAATTCCGATGAGGATCTATCGATCTTCACTTCTGTCTATTGTCGACGATATGCGCTGCTGGG
AATGTTATGTATTCG
+
FFFFFFFFFFFFFFFFFFFFFFFFFFFFFFFFFFFFFFFFFFFFFFFFFFFFFFFFFFFFFFFFFFFFFF
FFFFFFFFFFFFFF:FFFFFFFFFFFFFF:FFFFFFFFFFFFFFFFFFFFFFFFFFFFFFFFFFFFFFFF
FFFFFFF:FFFFFFF
@A00721:190:HGJW7DSXY:4:2158:13991:36808 1:N:0:TCTGCAAG+GCTGCACT
GACGTGTTAGAACTGGTTATCTGTGACGTATATCCATTTCGGTGTAGAAACCAGAATCCTTCAGATCG
GCAATGAATTCCGATGAGGATCTATCGATCTTCACTTCTGTCTATTGTCGACGATATGCGCTGCTGGG
AATGTTATGTATTCG
+
FFFFFFFFFFFFFFFFFFFFFFFFFFFFFFFFFFFFFFFFFFFFFFFFFFFFFFFFF:FFFFFFFFFFFF
FFFFFFFFFFFFFF:FFFFFFFFFFFFFFFFFFFFFFFFFFFFF,FFFFFFFFFFFFFFFF:FFFFFFFF
FFFFFFFFFFFFFFF
```

```
@A00721:190:HGJW7DSXY:4:2369:26304:18114 1:N:0:TCTGCAAG+GCTGCACT
GACGTGTTAGAACTGGTTATCTGTGACGTATATCCATTTCGGTGTAGAAACCAGAATCCTTCAGATCG
GCAATGAATTCCGATGAGGATCTATCGATCTTCACTTCTGTCTATTGTCGACGATATGCGCTGCTGGG
AATGTTATGTATTCG
+
FFFFFFFFFFFFFFFFFFFFFFFFFFFFFFFFFFFFFFFFFFFFFFF:FFFFFFFFFFFFFFFFFFFF
FFFFFFFFFFFFFFFFFFFFFFFFFFFFFFFFFFFFFFFFFFFF,FFFFFFFFFFFFFFFFFFFFFFF
FFFFFFFFFFFFFF:
@A00721:190:HGJW7DSXY:4:2578:15673:28635 1:N:0:TCTGCAAG+GCTGCACT
GACGTGTTAGAACTGGTTATCTGTGACGTATATCCATTTCGGTGTAGAAACCAGAATCCTTCAGATCG
GCAATGAATTCCGATGAGGATCTATCGATCTTCACTTCTGTCTATTGTCGACGATATGCGCTGCTGGG
AATGTTATGTATTCG
+
FFFFFFFFFFFFFFFFFFFFFFFFFFFFFFFFFFFFFFFFFFFFFFFFFFFFFFFFFFFFFFFFFFFF
FFFFFFFFFFFFFFFFFFFFFFFFFFFFFFFFFFFFFFFFFF:,:FFFFFFFFFFFFFFFF:FFFFFF
FFFFF:FFFFFFFFF
@A00721:190:HGJW7DSXY:4:2661:24758:27993 1:N:0:TCTGCAAG+GCTGCACT
GACGTGTTAGAACTGGTTATCTGTGACGTATATCCATTTCGGTGTAGAAACCAGAATCCTTCAGATCG
GCAATGAATTCCGATGAGGATCTATCGATCTTCACTTCTGTCTATTGTCGACGATATGCGCTGCTGGG
AATGTTATGTATTCG
+
FFFFFFFFFFFFFFFFFFFFFF:FFFFFFFFFFFFFFFFFFFFFFFFFFFFFFFFFFFFFFFFFFFFF
FFFFFFFFFFFFFFFFFFFFFFFFFFFFF,FFFFFFFFFFFFFFFFFFFFFFFFFFFFF:FFFF:FFF
F,FFFFFFFFFFFFF
@A00721:190:HGJW7DSXY:4:1123:27100:26005 1:N:0:TCTGCAAG+GCTGCACT
CGTGTTAGAACTGGTTATCTGTGACGTATATCCATTTCGGTGTAGAAACCAGAATCCTTCAGATCGGC
AATGAATTCCGATGAGGATCTATCGATCTTCACTTCTGTCTATTGTCGACGATATGCGCTGCTGGGAA
TGTTATGTATTCGAC
+
FFFF:FFFFFFFFFFF,FFFFFFFFFFFFFFFFFF:FFFFFFFFFFFFFFF:FFFFFF,FFFFFFFFF
FFFFFFFF:,FFFFFFFFFFFFFFFF,FFFFFFFF:FFFFFFFFFFFFFFF:FFFF,FFFF,,F:FF,
FF:FF:FFFFFF:FF
@A00721:190:HGJW7DSXY:4:1155:16947:24173 1:N:0:TCTGCAAG+GCTGCACT
CGTGTTAGAACTGGTTATCTGTGACGTATATCCATTTCGGTGTAGAAACCAGAATCCTTCAGATCGGC
AATGAATTCCGATGAGGATCTATCGATCTTCACTTCTGTCTATTGTCGACGATATGCGCTGCTGGGAA
TGTTATGTATTCGAC
+
:FFFFFFFFFFFF,F:FFF:FFFFFFFFFFFFFFFFFFFFFFFFFFFFFFFFFF:F::F:FFFFF:FF
F:FFFFFFF,FFF:FFFFFFFF:FFFFFF:FFFFFF:FFFFF:FFFFFFFFFFFF:FFF:FFFFFFF:
FF:FFFF:FFFFFFF
@A00721:190:HGJW7DSXY:4:1157:26476:18944 1:N:0:TCTGCAAG+GCTGCACT
CGTGTTAGAACTGGTTATCTGTGACGTATATCCATTTCGGTGTAGAAACCAGAATCCTTCAGATCGGC
AATGAATTCCGATGAGGATCTATCGATCTTCACTTCTGTCTATTGTCGACGATATGCGCTGCTGGGAA
TGTTATGTATTCGAC
+
FFFFFFFFFFFFFFFFFFFFFFFFFFFFFFFFFFFFFFFFFFFFFFFFFFFFFFFFFFFFFFFFFFFF
FFFFFFFFFFFFFFFFFFFFFFFFFFFFFFFFFFFFFFFFF:FFFFFFFFFFFFFFFFFFFFFFFFF:
FFFFFFFFFFFFFFF
@A00721:190:HGJW7DSXY:4:1157:26883:19648 1:N:0:TCTGCAAG+GCTGCACT
CGTGTTAGAACTGGTTATCTGTGACGTATATCCATTTCGGTGTAGAAACCAGAATCCTTCAGATCGGC
AATGAATTCCGATGAGGATCTATCGATCTTCACTTCTGTCTATTGTCGACGATATGCGCTGCTGGGAA
TGTTATGTATTCGAC
+
FFFFFFFFFFFFFFF:FFFFFFFFFFFFFFFFFFFFFFFFFFFFFFFFFFFFFFFFFFFFFFFFFFFF
```

FFFFFFFFFFFFFFFFFFFFFFFFFFFFFFFFFFFFFFFFFFFF,FFFFFFFFFFFFFFFFFFFFFFFFF
:FFFFFFFFFFFFFF
@A00721:190:HGJW7DSXY:4:1165:26711:8891 1:N:0:TCTGCAAG+GCTGCACT
CGTGTTAGAACTGGTTATCTGTGACGTATATCCATTTCGGTGTAGAAACCAGAATCCTTCAGATCGGC
AATGAATTCCGATGAGGATCTATCGATCTTCACTTCTGTCTATTGTCGACGATATGCGCTGCTGGGAA
TGTTATGTATTCGAC
+
FFFFFFFFFFFFFFFFFFFFFFFFFFFFFFFFFFFFFFFFFFFFFFFFFFFFFFFFFFFFFFFFFFFFFF
FFFFFF:FFFFFFFFFFFFFFFFFFFFFFFFFFFFFFFFFFFFF,FFFFFFFFFFFFFFFFFFFFFFFFF
FFFFFFFFFFFFFFF
@A00721:190:HGJW7DSXY:4:1205:13141:30608 1:N:0:TCTGCAAG+GCTGCACT
CGTGTTAGAACTGGTTATCTGTGACGTATATCCATTTCGGTGTAGAAACCAGAATCCTTCAGATCGGC
AATGAATTCCGATGAGGATCTATCGATCTTCACTTCTGTCTATTGTCGACGATATGCGCTGCTGGGAA
TGTTATGTATTCGAC
+
FFFFFFFFFFFFFFFFFFFFFFFFFFFF:FFFFFFFFFFFFFFFFFFFFFFFFFFFFFFFFFFFFFFFFF
FFFFFFFFFFFFFFFFFFFFFFFFFFFFFFFFFFFFFFFFFFF:FFFFFFFFF,FFFFFFFFFFFFFFF,
FFFFFFFFFFFFFFF
@A00721:190:HGJW7DSXY:4:1325:31458:9752 1:N:0:TCTGCAAG+GCTGCACT
CGTGTTAGAACTGGTTATCTGTGACGTATATCCATTTCGGTGTAGAAACCAGAATCCTTCAGATCGGC
AATGAATTCCGATGAGGATCTATCGATCTTCACTTCTGTCTATTGTCGACGATATGCGCTGCTGGGAA
TGTTATGTATTCGAC
+
FFFFFFFFFFFFFFFFFFFFFFFFFFFFFFFFFFFFFFFFFFFFFFFFFFFFFFFFFFFFFFFFFFFFFF
FFFFFFFFFFFFFFFFFFFFFFFFFFFFFFFFFFFFFFFFFFFFFFFFFFFFFFFFFFFFFFFFFFFFF,
FFFFFFFFFFFFFF:
@A00721:190:HGJW7DSXY:4:1360:29152:3255 1:N:0:TCTGCAAG+GCTGCACT
CGTGTTAGAACTGGTTATCTGTGACGTATATCCATTTCGGTGTAGAAACCAGAATCCTTCAGATCGGC
AATGAATTCCGATGAGGATCTATCGATCTTCACTTCTGTCTATTGTCGACGATATGCGCTGCTGGGAA
TGTTATGTATTCGAC
+
FFFFFFFFFFFFFFFFFFFFFFFFFFFFFFFFFFFFFFFFFFFFFFFFFFFFFFFFFFFFFFFFFFFFFF
FFFFFFFFFFFFFFFFFFFFFFFFFFFFFFFFFFFFFFFFFFF::FFFFFFFFFFFFFFFFFFFFFFFF:
FFFFFFFFFFFFFFF
@A00721:190:HGJW7DSXY:4:1557:13304:5556 1:N:0:TCTGCAAG+GCTGCACT
CGTGTTAGAACTGGTTATCTGTGACGTATATCCATTTCGGTGTAGAAACCAGAATCCTTCAGATCGGC
AATGAATTCCGATGAGGATCTATCGATCTTCACTTCTGTCTATTGTCGACGATATGCGCTGCTGGGAA
TGTTATGTATTCGAC
+
FF:FFFFFFFFFFFFFFFFF:FFFFFFFFFFFFFFFFFF:FFFFFFFFFFFFFFFFFFFFFFFFFFFFFF
FF:FFFFFFFFFFFFFFFFFFFFFFFFFFFFFFFFF:FFFFFFFFFFFFFFFFFFFFFFFFFFF:FFFFF
F,:FFFFFFF:FFFF
@A00721:190:HGJW7DSXY:4:2232:17861:15139 1:N:0:TCTGCAAG+GCTGCACT
CGTGTTAGAACTGGTTATCTGTGACGTATATCCATTTCGGTGTAGAAACCAGAATCCTTCAGATCGGC
AATGAATTCCGATGAGGATCTATCGATCTTCACTTCTGTCTATTGTCGACGATATGCGCTGCTGGGAA
TGTTATGTATTCGAC
+
FFFFFFFFFFFFFFFFFFFFFFFFFFFFFFFFFFFFFFFFFFFFFFFFFFFFFFFFFFFFFFFFFFFFFF
FFFFFFFFFFFFFFFFFFFFFFFFFFF:FFFFFFFFFFFFFFFF:FFFFFFFFF:FFFFFFFFFFFFFF,
FFFFFFFFFFFFFFF
@A00721:190:HGJW7DSXY:4:2306:13431:19179 1:N:0:TCTGCAAG+GCTGCACT
CGTGTTAGAACTGGTTATCTGTGACGTATATCCATTTCGGTGTAGAAACCAGAATCCTTCAGATCGGC
AATGAATTCCGATGAGGATCTATCGATCTTCACTTCTGTCTATTGTCGACGATATGCGCTGCTGGGAC
TGTTATGTATTCGAC

```
+
FFF:FFFFFFFFFFFFFFFFFFFFFFFFFFFFF,FFFFF:FF:FFFFFFF:FFFFFFFFFFFFFF:F::
FFFFFFFFF,:FFFFFFFFFFFFFFFFFFFFFFFFFFFFFFF:F:FFFFF,FFFF:F:FFFFFFFFF,
FFFFFF:FFFFF,FF
@A00721:190:HGJW7DSXY:4:2344:16215:28009 1:N:0:TCTGCAAG+GCTGCACT
CGTGTTAGAACTGGTTATCTGTGACGTATATCCATTTCGGTGTAGAAACCAGAATCCTTCAGATCGGC
AATGAATTCCGATGAGGATCTATCGATCTTCACTTCTGTCTATTGTCGACGATATGCGCTGCTGGGAA
TGTTATGTATTCGAC
+
FFFFFFFF:FFFFFFFFFFFFFFFFFFFFFFFFFFFFFFFFFFFFFFFFF:FFFFFFFFFFFFFFF,FF
FFFFFFF::FFFFFF:FFFF:FFF,F:FFFFF:FFFFFFF:FFFFFF:FFFFFFFFFFFFFFFFFFF,
F:FFFFFFFFFFFFF
@A00721:190:HGJW7DSXY:4:2369:11369:4805 1:N:0:TCTGCAAG+GCTGCACT
CGTGTTAGAACTGGTTATCTGTGACGTATATCCATTTCGGTGTAGAAACCAGAATCCTTCAGATCGGC
AATGAATTCCGATGAGGATCTATCGATCTTCACTTCTGTCTATTGTCGACGATATGCGCTGCTGGGAA
TGTTATGTATTCGAC
+
FFFFFFFFFFFFFFFFFFFFFFFFFFFFFFFFFFFFFFFFFFFFFFFFFFFFFFFFFFFFFFFFFFFF
FFFFFFFFFFFFFFFFFFFFFFFFFFFFFFFFFFFFFFFFFF,FFFFFFFFFFFFFFFFFFFFFFFFFF
FFFFFFFFFFFFFFF
@A00721:190:HGJW7DSXY:4:2469:12328:6339 1:N:0:TCTGCAAG+GCTGCACT
CGTGTTAGAACTGGTTATCTGTGACGTATATCCATTTCGGTGTAGAAACCAGAATCCTTCAGATCGGC
AATGAATTCCGATGAGGATCTATCGATCTTCACTTCTGTCTATTGTCGACGATATGCGCTGCTGGGAA
TGTTATGTATTCGAC
+
FFFFFFFFFFFFFFF:FFFFFFFFFFFFFFFFFFFFFFFFFFFFFFFFFFFFFFFFFFFFFFFFFFFF
FFFFFFFFFFFFFFFFFFFFFFFFFFFFFFFFFFFFFFFFFFFF,FFFFFFFFFFFFFFFFFFFFFFFF,
FFFFFFFFFFFFFFF
@A00721:190:HGJW7DSXY:4:2666:20157:22874 1:N:0:TCTGCAAG+GCTGCACT
CGTGTTAGAACTGGTTATCTGTGACGTATATCCATTTCGGTGTAGAAACCAGAATCCTTCAGATCGGC
AATGAATTCCGATGAGGATCTATCGATCTTCACTTCTGTCTATTGTCGACGATATGCGCTGCTGGGAA
TGTTATGTATTCGAC
+
FFFFFFFFFFFFFFFFFFFFFFFFFFFFFFFFFFFFFFFFFFFFFFFFFFFFFFFFFFFFFFFFFFFF
FFFFFFFFFFFFFFFFFFFFFFFFFFFFFFFFFFFFFFFFFFFF:FFFFFFFFFFFFFFFFFFFFFFFF
FFFFFFFFFFFFFFF
@A00721:190:HGJW7DSXY:4:1216:18973:11115 1:N:0:TCTGCAAG+GCTGCACT
TGTTAGAACTGGTTATCTGTGACGTATATCCATTTCGGTGTAGAAACCAGAATCCTTCAGATCGGCAA
TGAATTCCGATGAGGATCTATCGATCTTCACTTCTGTCTATTGTCGACGATATGCGCTGCTGGGAATG
TTATGTATTCGACGA
+
FFFFFFFFFFFFFFFFFFFFFFFFFFFFFFFFFFFFFFFFFFFFFFFFFFFFFFFFFFFFFFFFFFF:
FFFFFFFFFFFFFFFFFFFFFFFFFFFFFFFFFFFFFFFFFFFFFFFFFFFFFFFFFFFFFFFFF:FF
FFFFFFFFFFFFFFF
@A00721:190:HGJW7DSXY:4:1219:25473:32863 1:N:0:TCTGCAAG+GCTGCACT
TGTTAGAACTGGTTATCTGTGACGTATATCCATTTCGGTGTAGAAACCAGAATCCTTCAGATCGGCAA
TGAATTCCGATGAGGATCTATCGATCTTCACTTCTGTCTATTGTCGACGATATGCGCTGCTGGGAATG
TTATGTATTCGACGA
+
FFFFFFFFFFFFFFFFFFFFFFFFFFFFFFFFFFFFFFFFFFFFFFFFFFFFFFFFFFFFFFFFFFFF
FFFFFFFFFFFFFFFFFFFFFFFFFFFFFFFFFFFFFFFFF:FFFFFFFFFFFFFFFF:FFFFFFFF,F
:FFFFFFFFFFFFFF
@A00721:190:HGJW7DSXY:4:1445:19416:1861 1:N:0:TCTGCAAG+GCTGCACT
TGTTAGAACTGGTTATCTGTGACGTATATCCATTTCGGTGTAGAAACCAGAATCCTTCAGATCGGCAA
```

TGAATTCCGATGAGGATCTATCGATCTTCACTTCTGTCTATTGTCGACGATATGCGCTGCTGGGACTG
TTATGTATTCGACGA
+
FFFFFFFFFF:::FF:FFFFFFFF:FFFFF:FFFFF,FFF:F:FFFFF:FFFF:FF:FFFFFF:F::F
FFFFFFFF:F:FFFFFFFF:FF,FFFFFFFFFF:FF:FF::F:FFFFF:FF:,FFFF:FF:FFFF,,F
FFFFFFFFFF,FFFF
@A00721:190:HGJW7DSXY:4:2326:3477:12148 1:N:0:TCTGCAAG+GCTGCACT
TGTTAGAACTGGTTATCTGTGACGTATATCCATTTCGGTGTAGAAACCAGAATCCTTCAGATCGGCAA
TGAATTCCGATGAGGATCTATCGATCTTCACTTCTGTCTATTGTCGACGATATGCGCTGCTGGGAATG
TTATGTATTCGACGA
+
FFFFFFFFFFFFFFFFFFFFFFFFFFFFFFFFFFFFFFFFFFFFFFFFFFFFFFFFFFFFFFFFFFFF
FFFFFFFFFFFFFFFFFFFFFFFFFFFFFFFFFFFFFFFFFFFFFFFFFFFFFFFFFFFFFFFFF:FF
FFFFFFFFFFFFFFF
@A00721:190:HGJW7DSXY:4:1220:3504:22780 1:N:0:TCTGCAAG+GCTGCACT
CCAGAATCCTTCAGATCAGCAATGAATTCCGATGAGGATCTATCGATCTTCACTTCTGTCTATTGTCG
ACGATATGCGCTGCTGGGAATGTTATGTATTCGACGATTCATGTTCGCTTTCGTTATTGTAGAACAAT
AAAGTTCTGAATTC
+
FFFFFFFFFFFFFFFFFFFFFFFFFFFFFFFFFF:FFFFFFFFFFFFFFFFFFFFFFFFFFFFFFFFF
FFFFFFFFFFFFF:FFFFFF:FFFFFFFFFFFFFFFF:FFF:FFFFFFFFFFFFFFFFFFFFFFFFFF
FFFFFFFFFFFFFF
@A00721:190:HGJW7DSXY:4:1238:4056:32878 1:N:0:TCTGCAAG+GCTGCACT
CCAGAATCCTTCAGATCAGCAATGAATTCCGATGAGGATCTATCGATCTTCACTTCTGTCTATTGTCG
ACGATATGCGCTGCTGGGAATGTTATGTATTCGACGATTCATGTTCGCTTTCGTTATTGTAGAACAAT
AAAGTTCTGAATTCG
+
FFFFFFFFFFFFFFFFFFFFFFFFFFFFFFFFFFFFFFFFFFFFFFFFFFFFFFFFFFFF::FFFFFF
FFFFFFFFFFFFFFFFFFFFFFFFFFFFFFFFFFFFFFFFFFFFFFFFFFFFFFFFFFFFFFF:FFFFF
FFFFFFFFFFFFFFF
@A00721:190:HGJW7DSXY:4:1365:6316:2221 1:N:0:TCTGCAAG+GCTGCACT
CCAGAATCCTTCAGATCAGCAATGAATTCCGATGAGGATCTATCGATCTTCACTTCTGTCTATTGTCG
ACGATATGCGCTGCTGGGAATGTTATGTATTCGACGATTCATGTTCGCTTTCGTTATTGTAGAACAAT
AAAGTTCTGAATTCG
+
FFFFFFFFFFFFFFFFFFFFFFFFFFFFFFFFFFFFFFFFFFFFFFFFF:FFFFFFFFFFF,,FFFFF
FFFFFFFFFFFFFFFFFFFF,:FFFFFFFFFFFFFFFFFFFFFFFFFFFFFFFF:FFFFFFFFFFFFFF
FFFFFFFFFFFFF:F
@A00721:190:HGJW7DSXY:4:1453:18683:12023 1:N:0:TCTGCAAG+GCTGCACT
CCAGAATCCTTCAGATCAGCAATGAATTCCGATGAGGATCTATCGATCTTCACTTCTGTCTATTGTCG
ACGATATGCGCTGCTGGGAATGTTATGTATTCGACGATTCATGTTCGCTTTCGTTATTGTAGAACAAT
AAAGTTCTGAATTCG
+
FFFFFFFFFFFFFFFFFFFFFFFFFFFFFFFFFFFFFFFFFFFFFFFFF:FFF:FFFFFFFFFF,FFFFF
FFFFFFFFFFFFFFFFFFF:FFFFFFFFFFFFFFFFFFFFFFFFFFFFFFFFFFFFFFFFFFFFFFFF
FFFFFFFFFFFFFFF
@A00721:190:HGJW7DSXY:4:1656:29206:27868 1:N:0:TCTGCAAG+GCTGCACT
CCAGAATCCTTCAGATCAGCAATGAATTCCGATGAGGATCTATCGATCTTCACTTCTGTCTATTGTCG
ACGATATGCGCTGCTGGGAATGTTATGTATTCGACGATTCATGTTCGCTTTCGTTATTGTAGAACAAT
AAAGTTCTGAATTCG
+
FFFFFFFFFFFFFFFFFFFFFFFFFFFFFFFFFFFFFFFFFFFFFFFFFFFFFFFFFFFFFF,FFFFFF
FFFFFFFFFFFFFFFFFFFFFFFFFFFFFFFFFFFFFFFFFFFFFFFFFFFFFFFFFFFFFFFFFFFF
F:FF:FFFFFFFFFF

@A00721:190:HGJW7DSXY:4:2549:5348:22968 1:N:0:TCTGCAAG+GCTGCACT
CAGAATCCTTCAGATCAGCAATGAATTCCGATGAGGATCTATCGATCTTCACTTCTGTCTATTGTCGA
CGATATGCGCTGCTGGGAATGTTATGTATTCGACGATTCATGTTCGCTTTCGTTATTGTAGAACAATA
AAGTTCTGAATTC
+
FF:FF:FFFFFFFFFFF,FFFFFFFFF:FFFFFFFFFFFF::FF,FF:FFFFFFFFFFFFFF:FFF:F
F:FFF:FF:FF::F:,FFFFFF:FFF:FFFFFF,,FFFFFF:,FF,F:FFFFFFFFFFFFFFF:FFFF
F:FFFF:FFF::F
@A00721:190:HGJW7DSXY:4:1447:6244:14372 1:N:0:TCTGCAAG+GCTGCACT
GACGAATTCAGAACTTTATTGTTCTACAATAACGAAAGCGAACATGAATCGTCGAATACATAACATTC
CCAGCAGCGCATATCGTCGACAATAGACAGAAGTGAAGATCGATAGATCCTCATCGGAATTCATTGCC
GATCTGAAGGATTCT
+
FFFFFFFFFFFFFFFFFFFFFFFFFFFFFFFFFFFFFFFFFFFFFFFFFFFFF:FFFFFFFFFFFFFF
FFFFFFFFFFFFFFFFFFFFFFFFFFFFFFFFFFFFFFFFFFFFFFFFFFFFFFFFFFFFFFFFFFFF
FFFFFFFFFFFFFFF
@A00721:190:HGJW7DSXY:4:2522:7645:5588 1:N:0:TCTGCAAG+GCTGCACT
GACGAATTCAGAACTTTATTGTTCTACAATAACGAAAGCGAACATGAATCGTCGAATACATAACATTC
CCAGCAGCGCATATCGTCGACAATAGACAGAAGTGAAGATCGATAGATCCTCATCGGAATTCATTGCC
GATCTGAAGGATTCT
+
FFFFFFFFFFFFFFFFFFFFFFFFFFFFFFFFFFFFFFFFFFFFFFFFFFFFFFFFFFF:FFFFFFFF
FFFFFFFFFFFFFFFFFFFFFFFFFFFF:FFFFFFFFF,FFFFFFFFFFFFFFFFFFFFFFFFFFFFF
F:FFFFF:FFFF:FF
@A00721:190:HGJW7DSXY:4:1614:31729:16799 1:N:0:TCTGCAAG+GCTGCACT
GAATCCTTCAGATCGGCAATGAATTCCGATGAGGATCTATCGATCTTCACTTCTGTCTATTGTCGACG
ATATGCGCTGCTGGGAATGTTATGTATTCGACGATTCATGTTCGCTTTCGTTATTGTAGAACAATAAA
GTTCTGAATTCGTCA
+
FFFFFFFFFFFFFFFFFFFFFFFFFFFFFFFFFFFFFFFFFFFFFFFFFFFFFFFFFFFFFFFFFFFF
FFFFFFFFFFFFFFFFFFFFFFFFFFFFFFFFFFFFFFFFFFFFFFFFFFFFFFFFFFF:FFFFFFFF
FFFFFFFFFFF:FFF
@A00721:190:HGJW7DSXY:4:2506:18710:10723 1:N:0:TCTGCAAG+GCTGCACT
GAATCCTTCAGATCGGCAATGAATTCCGATGAGGATCTATCGATCTTCACTTCTGTCTATTGTCGACG
ATATGCGCTGCTGGGAATGTTATGTATTCGACGATTCATGTTCGCTTTCGTTATTGTAGAACAATAAA
GTTCTGAATTCGTCA
+
FFFFFFFFFFFFFFFFFFFFFFFFFFFFFFFFFFFFFFFFFFFFFFFFFFFFFFFFF:FFFFFFFFFF
FFFFFFFFFFFFFFFFFFFFFFFFFFFFFFFFFF,FFFFFFFFFFFFFFFFFFFFFFF:FFFFFFFFF
FFFFFFFFFFFFFFF
@A00721:190:HGJW7DSXY:4:2513:26359:28354 1:N:0:TCTGCAAG+GCTGCACT
GAATCCTTCAGATCGGCAATGAATTCCGATGAGGATCTATCGATCTTCACTTCTGTCTATTGTCGACG
ATATGCGCTGCTGGGAATGTTATGTATTCGACGATTCATGTTCGCTTTCGTTATTGTAGAACAATAAA
GTTCTGAATTCGTCA
+
FFFFFFFFFFFFFFFFFFFFFFFFFFFFFFFFFFFFFFFFFFFFFFFFFFFFFFFFFF:FFFFFFFFF
FFFFFFFFFFFFFFFFFFFFFFFFFFFFFFFFFFFFFFFFFFFFFFFFFFFFFFFFFFFFFFFFFFFF
FFFFFFFFFFF:FFF
@A00721:190:HGJW7DSXY:4:1116:22869:30859 1:N:0:TCTGCAAG+GCTGCACT
GCAATGAATTCCGATGAGGATCTATCGATCTTCACTGCTGTCTATTGTCGACGATATGCGCTGCTGGG
AATGTTATGTATTCGACGATTCATGTTCGCTTTCGTTATTGTAGAACAAAAAAGTTCTGAATTCGTCA
CACCA
+
FFFFFFFF,FFFFFFFFF:FFFFFFFFFF,:F:FFF,:::FFFFF,FF:FFFFF:FFFF:F:F::FF,

```
FFFFFF:F,F,FFFFF:FFFF:FFF,FFF:,FFFF,:FFFFFFFFF,::,FFF:F,FFFFF:,FFFFF
,FF,F
@A00721:190:HGJW7DSXY:4:1317:19226:31908 1:N:0:TCTGCAAG+GCTGCACT
GCAATGAATTCCGATGAGGATCTATCGATCTTCACTTCTGTCTATTGTCGACGATATGCGCTGCTGGG
AATGTTATGTATTCGACGATTCATGTTCGCTTTCGTTATTGTAGAACAATAAAGTTCTGAATTCGTCA
CACCA
+
FFFFFFFFFFFFFFFFFFFFFFFFFFFFF:FFFFFFFFFF:FFFF:FFFFFFFFFFFFFFFFFFFFFF
FFFFFFFFFFFFFFFFFFFFFFFFFFFFFFFFFF:FFFFFFFFFFFFFFFFFFFFFFFFFFFFFFFFF
FF,FF
@A00721:190:HGJW7DSXY:4:1412:23023:33943 1:N:0:TCTGCAAG+GCTGCACT
GCAATGAATTCCGATGAGGATCTATCGATCTTCACTTCTGTCTATTGTCGACGATATGCGCTGCTGGG
AATGTTATGTATTCGACGATTCATGTTCGCTTTCGTTATTGTAGAACAATAAAGTTCTGAATTCGTCA
CACCA
+
FFFFFFFFFFFFFFFFFFFFFFFFFFFFF:FFF:F:FFFFFFFF:FFFFFFFFFFFFFFFFFFF:FFF
FFFFFFFFFFFFFFFFFFFFFFFFFFFFFFFFFFFFFF,FFFFFFFFFFFFFFFFFFFFFFFFFFFFF
FFFFF
@A00721:190:HGJW7DSXY:4:1521:6677:10833 1:N:0:TCTGCAAG+GCTGCACT
GCAATGAATTCCGATGAGGATCTATCGATCTTCACTTCTGTCTATTGTCGACGATATGCGCTGCTGGG
AATGTTATGTATTCGACGATTCATGTTCGCTTTCGTTATTGTAGAACAATAAAGTTCTGAATTCGTCA
CACCA
+
FFFFFFFFFFFFFFFFFFFFFFFFFFFFF,:FFFFFFFFFFFFFFFFFFFFFFFFFFFFFFFFFFFFF
FFFFFFFFFFFFFFFFFFFFFFFFFFFFFFFFFFFFFFFFFFFF:FFFFFFFFFFFFFFF:FFFFFFFF
FFFFF
@A00721:190:HGJW7DSXY:4:1554:25491:5274 1:N:0:TCTGCAAG+GCTGCACT
GCAATGAATTCCGATGAGGATCTATCGATCTTCACTTCTGTCTATTGTCGACGATATGCGCTGCTGGG
AATGTTATGTATTCGACGATTCATGTTCGCTTTCGTTATTGTAGAACAATAAAGTTCTGAATTCGTCA
CACCA
+
FFFFFFFFFFFFFFFFFFFFFFFFFFFFF:FFFFFFFFFFFFF,FFFFFFFFFFFFFFFFF:FFFFFF
FFFFFFFFFFFFFFFFFFFFFFFFFFFFFFFFFFFFFFFFFFFFFFFFFFFFFFFFFFFFFFFFFFFF
FFFFF
@A00721:190:HGJW7DSXY:4:1603:32705:11788 1:N:0:TCTGCAAG+GCTGCACT
GCAATGAATTCCGATGAGGATCTATCGATATTCACTTCTGTCTATTGTCGACGATATGCGCTGCTGGG
AATGTTATGTATTCGACGATTCATGTTCGCTTTCGTTATTGTAGAACAATAAAGTTCTGAATTCGTCA
CACCA
+
FFFF:FFFFFFFF:FFFFFFFFFFFFFF,,,FFFFFF,FF:FFFFFFFFFFFFFFF,FFFF::FFFFF
FFFFFFFFF:FFFFFFF,F:FFF,FF,FFF:FFFFFF:FFFFF:FFFFFFFF:FFFFFFFFFFF:FFF
FFF,F
@A00721:190:HGJW7DSXY:4:2268:30472:1282 1:N:0:TCTGCAAG+GCTGCACT
GCAATGAATTCCGATGAGGATCTATCGATCTTCACTTCTGTCTATTGTCGACGATATGCGCTGCTGGG
AATGTTATGTATTCGACGATTCATGTTCGCTTTCGTTATTGTAGAACAATAAAGTTCTGAATTCGTCA
CACCA
+
FFFFFFFFFFFFFFFFFFFFFFFFFFFFFF,FFFFFFFFFFFFF:FFFFFFFFFFFFFFFFFFFFFFFF
FFFFFFFFFFFFFFFFFF,FFFFFFFFFFFFFFFFFFFFFFFFFFFFFFFFFF,FFFFFFFFFFFFFFF
FFFFF
@A00721:190:HGJW7DSXY:4:2373:19551:20885 1:N:0:TCTGCAAG+GCTGCACT
GCAATGAATTCCGATGAGGATCTATCGATCTTCACTTCTGTCTATTGTCGACGATATGCGCTGCTGGG
AATGTTATGTATTCGACGATTCATGTTCGCTTTCGTTATTGTAGAACAATAAAGTTCTGAATTCGTCA
CACCA
```

+
FFFFFFFFFFF::FF:FFFFFFFF:FFFF:FF:FFFFF:FFF::FFFFFFFFFF:FFFFFFFFFFFF:
:,FFFFFFFFFFFFFFFFF::FFFFFF,FFF:FFFFFFF:FFFFFFFFFFFFFFFFFF,FFFFFFFFF
FFFFF
@A00721:190:HGJW7DSXY:4:2377:17336:18082 1:N:0:TCTGCAAG+GCTGCACT
GCAATGAATTCCGATGAGGATCTATCGATCTTCACTTCTGTCTATTGTCGACGATATGCGCTGCTGGG
AATGTTATGTATTCGACGATTCATGTTCGCTTTCGTTATTGTAGAACAATAAAGTTCTGAATTCGTCA
CACCA
+
FFFFFFFFFFFFFFFF:FFFFFFFFFFFF:FFFFF:FFFFFFFFFFFFFFFFFFFFFFFFFFFFFFFFF
F,FFFFF:FFFFFFFFFF:FFFFFFFFFFFFFFFFFFFFFFFFF:FFFFFFFFFFFFFFF:FFFFFFFF
FFFFF
@A00721:190:HGJW7DSXY:4:2541:32190:22357 1:N:0:TCTGCAAG+GCTGCACT
GCAATGAATTCCGATGAGGATCTATCGATCTTCACTTCTGTCTATTGTCGACGATATGCGCTGCTGGG
AATGTTATGTATTCGACGAGTCATGTTCGCTTTCGTTATTGTAGAAAAATAAAGTTCTGAAATCGTCA
CACCA
+
FFFF,::FFFF:FF:,FFFFFF:FFFFFF,F,FF:FFFF:,F:FFF,:FFF:,FFFF,F:,FF:FFF,
FFFF:FFFFFF:,FFFF:F,,FFFFFF:,FF:FFFFFF:FFFF,FF,:F,FFF:F::F,F,,F:,:,F
FFF,F
@A00721:190:HGJW7DSXY:4:1342:26241:2848 1:N:0:TCTGCAAG+GCTGCACT
TCACTTCTGTCTATTGTCGACGATATGCGCTGCTGGGAATGTTATGTATTCGACGATTCATGTTCGCT
TTCGTTATTGTAGAACAATAAAGTTCTGAATTCGTCACACCAGAATGTTATTAGAACCTCTGACGTCA
TTCTG
+
FFFFFFFFFFFFFFFFFFFFFFFFFFFFFFFFFFFFFFFFFFFFFFFFFFFFFFFFFFFFFFFFFFFF
FFFFFFFFFFFFFFFFFFFFFFFFFFFFFFFFFFFFFFFFFFFFFFFFFFFFFFFFFFFFFFFFFFFF
FFFFF
@A00721:190:HGJW7DSXY:4:1645:12870:29324 1:N:0:TCTGCAAG+GCTGCACT
TCACTTCTGTCTATTGTCGACGATATGCGCTGCTGGGAATGTTATGTATTCGACGATTCATGTTCGCT
TTCGTTATTGTAGAACAATAAAGTTCTGAATTCGTCACACCAGAATGTTATTAGAACCTCTGACGTCA
TTCTG
+
FFFFFFFFFFFFFFFFFFFFFFFFFFFFFFFFFFFFFFFFFFFFFFFFFFFFFFFFFFFFFFFFFFFF
FFFFFFFFFFFFFFFFFFFFFFFF:FFFFFFFFFFFFFFFFFFFFFFFFFFFFFFFFFFFFFFFFFFF
FFFFF
@A00721:190:HGJW7DSXY:4:2249:24026:5400 1:N:0:TCTGCAAG+GCTGCACT
TCACTTCTGTCTATTGTCGACGATATGCGCTGCTGGGAATGTTATGTATTCGACGATTCATGTTCGCT
TTCGTTATTGTAGAACAATAAAGTTCTGAATTCGTCACACCAGAATGTTATTAGAACCTCTGACGTCA
TTCTG
+
FFFF::,FFFFFFFF:FFFF,FFFFFFF:FFF::FFFFFFFFFFFFFFFFFFFFFFFFFFF:FFFFF,F
FFFFFFFF:F:FF:FFFFFFFFFFFFFFFFFFFFFFFFFFFFFFFFFFFFFFFFFFF,FF:FFFFFFFF
F,FFF
@A00721:190:HGJW7DSXY:4:2508:18059:7373 1:N:0:TCTGCAAG+GCTGCACT
TCACTTCTGTCTATTGTCGACGATATGCGCTGCTGGGAATGTTATGTATTCGACGATTCATGTTCGCT
TTCGTTATTGTAGAACAATAAAGTTCTGAATTCGTCACACCAGAATGTTATTAGAACCTCTGACGTCA
TTCTG
+
FFFFFFFFFFFFFFFFFFFFFFFFFFFFFFFFFFFFFFFFFFFFFFFFFFFFFFFFFFFFFFFFFFFF
FFFFFFFFFFFFFFFFFFFFFFFFFFFFFFFFFFFFFFFFFFFFFFFFFFFFF:FFFFFFFFF:FFFF
FFFFF
@A00721:190:HGJW7DSXY:4:2539:24632:15029 1:N:0:TCTGCAAG+GCTGCACT
TCACTTCTGTCTATTGTCGACGATATGCGCTGCTGGGAATGTTATGTATTCGACGATTCATGTTCGCT

TTCGTTATTGTAGAACAATAAAGTTCTGAATTCGTCACACCAGAATGTTATTAGAACCTCTGACGTCA
TTCTG
+
FFFFFFFFFFFFFFFFFFFFFFFFFFFFFFFFFFFFFFFFFFFFF:FFFFFFFFFFFFFFFFFFFFFF
FFFFFFFFFFFFFFFFFFFFFFFFFFFFFFFF:FFFFFFFFFFFFFFFFFFFFFFFF:FFFFFFFFFF
FF:FF
@A00721:190:HGJW7DSXY:4:1129:20817:7827 1:N:0:TCTGCAAG+GCTGCACT
CTTCTGTCTATTGTCGACGATATGCGCTGCTGGGAATGTTATGTATTCGACGATTCATGTTCGCTTTC
GTTATTGTAGAACAATAAAGTTCTGAATTCGTCACACCAGAATGTTATTAGAACCTCTGACGTCATTC
TGGTGGTCCGCATCA
+
FFFFFFFFF:FFFFFFFFFFFFFFFFFFFF:FFFF,FFFFFFFFFFFFFFFFFF:FFFFFFFFFFFFF
FFFFFFFFFFFFFFFFFFFFFFFFFFFFFFFFFFFFFFFFF,FFFFFFF:FFFFFFFFFFFFFFFFFF
FFF,FFFFFFFF:FF
@A00721:190:HGJW7DSXY:4:2174:21703:11459 1:N:0:TCTGCAAG+GCTGCACT
CTTCTGTCTATTGTCGACGATATGCGCTGCTGGGAATGTTATGTATTCGACGATTCATGTTCGCTTTC
GTTATTGTAGAACAATAAAGTTCTGAATTCGTCACACCAGAATGTTATTAGAACCTCTGACGTCATTC
TGGTGGTCCGCATCA
+
FFF:FFFFFFFF,,,FFFFFFFFF:,FFFFFFFFF,FFFFFFFFF,FFFFF:FFFFF:FFFF,FF,FF
,,FFFFFFFFFFFFF,FF,FFFFFFFFFFFFFFF:FFFFFF,F:,,F:FF,FFFFFFFFFFFFFFFF:
FF:,:FFFFFFFFFF
@A00721:190:HGJW7DSXY:4:2329:1362:14121 1:N:0:TCTGCAAG+GCTGCACT
CTTCTGTCTATTGTCGACGATATGCGCTGCTGGGAATGTTATGTATTCGACGATTCATGTTCGCTTTC
GTTATTGTAGAACAATAAAGTTCTGAATTCGTCACACCAGAATGTTATTAGAACCTCTGACGTCATTC
TGGTGGTCCGCATCA
+
FFFFFFFFFFFFF:FFFFFFFFFFFFFFFFFFFFF:FFFFFFFFFFFFFFFFFFFFFFFFFFFFFFFF
FFFFFFFFFFFFFFFFFFFFFFFFFFFFFFFFFFFFFFFFFFFFFFFFFFFFFFFFF:FFFFFFFFFF
FFFFFFFF:FFFFFF
@A00721:190:HGJW7DSXY:4:2404:30897:10598 1:N:0:TCTGCAAG+GCTGCACT
CTTCTGTCTATTGTCGACGATATGCGCTGCTGGGAATGTTATGTATTCGACGATTCATGTTCGCTTTC
GTTATTGTAGAACAATAAAGTTCTGAATTCGTCACACCAGAATGTTATTAGAACCTCTGACGTCATTC
TGGTGGTCCGCATCA
+
FFFFFFFFFFFFFFFFFFFFFFFFFFFFFFFFFFFFFFFFFFFFFFFFFFFFFFFFFFFFFFFFFFFF
FFFFFFFFFFFFFFFFFFFFFFFFFFFFF:FFFFFFFFFFFFFFFFFFFF:FFFFFFFFFFFFFFFFF
FFFFF,FFFFFFFFF
@A00721:190:HGJW7DSXY:4:2413:3568:17347 1:N:0:TCTGCAAG+GCTGCACT
CTTCTGTCTATTGTCGACGATATGCGCTGCTGGGAATGTTATGTATTCGACGATTCATGTTCGCTTTC
GTTATTGTAGAACAATAAAGTTCTGAATTCGTCACACCAGAATGTTATTAGAACCTCTGACGTCATTC
TGGTGGTCCGCATCA
+
FFFFFFFFFFFFFFFFFFFFFFFFFFFFFFFFFFFFFFFFFFFFFFFFFFFFFFFFFFFFFFFFFFFF
FFFFFFFFFFFFFFFFFFFFFFFFFFFFF::FFFFFFFFFF:FFFFFFFFFFFFFFFFFFFFFFFFFF
FFFFFFFF:FFFFFF
@A00721:190:HGJW7DSXY:4:2469:31385:5588 1:N:0:TCTGCAAG+GCTGCACT
CTTCTGTCTATTGTCGACGATATGCGCTGCTGGGAATGTTATGTATTCGACGATTCATGTTCGCTTTC
GTTATTGTAGAACAATAAAGTTCTGAATTCGTCACACCAGAATGTTATTAGAACCTCTGACGTCATTC
TGGTGGTCCGCATCA
+
FFFFFFFFFFFFFFFFFFFFFFFFFFFFFFFFFFF:FFFFFFFFFFFFFFFFFFFFFFFFFFFFFFFF
FFFFFFFFFFFFFFF:FFFFFFFFFFFFFFF:FFFFFFFFFFFFFFFFFFFFFFFFF:F:FFFFFFF:
FFFFFFFFF:FFFFF

```
@A00721:190:HGJW7DSXY:4:2477:3007:11898 1:N:0:TCTGCAAG+GCTGCACT
CTTCTGTCTATTGTCGACGATATGCGCTGCTGGGAATGTTATGTATTCGACGATTCATGTTCGCTTTC
GTTATTGTAGAACAATAAAGTTCTGAATTCGTCACACCAGAATGTTATTAGAACCTCTGACGTCATTC
TGGTGGTCCGCATCA
+
FFFFFFFFF:FFFFFFFFFFFFFFFFFFFFFFFFFF,FFFFFFFFFFFFFFFFFFFFFFFFFFFFFFFFF
FFFFFFFFFFFFFFFFFFFFFFFFFFFFFFFFFFFFFFFFFFFFFFFFFFFFFFFFFFFFFFFFFFFFFF
FFFFF:FFFFFFFFF
@A00721:190:HGJW7DSXY:4:2535:19452:26162 1:N:0:TCTGCAAG+GCTGCACT
CTTCTGTCTATTGTCGACGATATGCGCTGCTGGGAATGTTATGTATTCGACGATTCATGTTCGCTTTC
GTTATTGTAGAACAATAAAGTTCTGAATTCGTCACACCAGAATGTTATTAGAACCTCTGACGTCATTC
TGGTGGTCCGCATC
+
FFFFFFFF:F:FFFFFFFFFFFFFFFFFFFFFFFFFFFFFF:FFFFFFF:FFFF:FFFFFFFFFFFFFFF
:FFFF:FFFFFFFFFFFF:FFFFFF:FFFFFFFFFFF:FFFFFFFFFFFFFFFFF:FFFFFFFF:FFFFF
F:FFFFFFFFFFFF
@A00721:190:HGJW7DSXY:4:2560:17797:32628 1:N:0:TCTGCAAG+GCTGCACT
CTTCTGTCTATTGTCGACGATATGCGCTGCTGGGAATGTTATGTATTCGACGATTCATGTTCGCTTTC
GTTATTGTAGAACAATAAAGTTCTGAATTCGTCACACCAGAATGTTATTAGAACCTCTGACGTCATTC
TGGTGGTCCGCATCA
+
FFFFFFFFFFFFFFFFFFFFFFFFFFFFFFFFFFFFFFFFFFFFFFFFFFFFFFFFFFFFFFFFFFFFFF
FFFFFFFFFFFFFFFFFFFFFFFFFFFFFFFFFFFFFFFFFFFFFFFFFFFFFFF:FFFFFFFFFFFFFF
FFFFFFFFFFFFFFF
@A00721:190:HGJW7DSXY:4:2641:7889:29747 1:N:0:TCTGCAAG+GCTGCACT
CTTCTGTCTATTGTCGACGATATGCGCTGCTGGGAATGTTATGTATTCGACGATTCATGTTCGCTTTC
GTTATTGTAGAACAATAAAGTTCTGAATTCGTCACACCAGAATGTTATTAGAACCTCTGACGTCATTC
TGGTGGTCCGCATCA
+
FFFFFFFFFFFFFFFFFFFFFFFFFFFFFFFFFFFFFFFFFFFFFFFFFFFFFFFFFFFFFFFFFFFFFF
FFFFFFFFFFFFFFFFFFFFFFFFFFFFFFFFFFFFFFFFFFFFFFFFFFFFFFFFFFFFFFFFFFFFFF
FFFFFFFFFFFFFFF
@A00721:190:HGJW7DSXY:4:1111:26521:30138 1:N:0:TCTGCAAG+GCTGCACT
AGTCTATTGTCGACGATATGCGCTGCTGGGAATGTTATGTATTCGACGATTCATGTTCGCTTTCGTTA
TTGTAGAACAATAAAGTTCTGAATTCGTCACACCAGAATGTTATTAGAACCTCTGACGTCATTCTGGT
GGTCCGCATCAATTG
+
FFFFFFFFFFFFFFFFFFFFFFFFFFFFFFFFFFFFFFFFFFFFFFFFFFFFFFFFFFFFFFFFFFFFFF
FFFFFFFFFFFFFFFFFFFFFFFFFFFFFFFFFFFFFFFFFFFFFFFFFFFFFFFFFFF:FFFFFFFFFF
FFFFFFFFFFFFFFF
@A00721:190:HGJW7DSXY:4:1346:31720:24017 1:N:0:TCTGCAAG+GCTGCACT
AGTCTATTGTCGACGATATGCGCTGCTGGGAATGTTATGTATTCGACGATTCATGTTCGCTTTCGTTA
TTGTAGAACAATAAAGTTCTGAATTCGTCACACCAGAATGTTATTAGAACCTCTGACGTCATTCTGGT
GGTCCGCATCAATTG
+
:F:FF,FFF:FFFF:F,F,F,FF,FFFF,FFF:FFFFF:FFFFFF,FFF::F,FFFFFF,FFFFFF,FFF
::FFFFFFF:FFFF,FFFFFFF:FFF:FFF:FFFFFFFFFFFF,FFFFFFFFFFFFFFFFFFF:FF:,FF
FFFFFFFFF:,FF::
@A00721:190:HGJW7DSXY:4:1452:32163:29982 1:N:0:TCTGCAAG+GCTGCACT
AGTCTATTGTCGACGATATGCGCTGCTGGGAATGTTATGTATTCGACGATTCATGTTCGCTTTCGTTA
TTGTAGAACAATAAAGTTCTGAATTCGTCACACCAGAATGTTATTAGAACCTCTGACGTCATTCTGGT
GGTCCGCATCAATTG
+
FFFFFFFFFFFFFFFFFFFFFFFFFFFFFFFFFFFFFFFFF:FFFFF:FFFFFFFFF:FFFFFFFFFFFF
```

FFFFFFFFFFFFFF:FFFFF:FFFFFFFFFFFFFFFFFFFFFFFFF,FFF,FFF,FFF:FFFFFFFFF
FFFFFFFFFFFFFFF
@A00721:190:HGJW7DSXY:4:1666:18304:4539 1:N:0:TCTGCAAG+GCTGCACT
AGTCTATTGTCGACGATATGCGCTGCTGGGAATGTTATGTATTCGACGATTCATGTTCGCTTTCGTTA
TTGTAGAACAATAAAGTTCTGAATTCGTCACACCAGAATGTTATTAGAACCTCTGACGTCATTCTGGT
GGTCCGCATCAATTG
+
FF,F:FFFFFFFFFFFFFFFFFFFFFFFFFFFFFFFFFFFFFFFFFFFFFFFFFFFFFFFFFFFFFFF
FFFFFFFFFFFFFFFFFFFFFFFFFFFFFFFFFFFFFF,FFFF,FFFFFFFFFFFFFFFFFFFFFFFFF
FFFFFFFFFFFFFFF
@A00721:190:HGJW7DSXY:4:2663:16776:12101 1:N:0:TCTGCAAG+GCTGCACT
AGTCTATTGTCGACGATATGCGCTGCTGGGAATGTTATGTATTCGACGATTCATGTTCGCTTTCGTTA
TTGTAGAACAATAAAGTTCTGAATTCGTCACACCAGAATGTTATTAGAACCTCTGACGTCATTCTGGT
GGTCCGCATCAATTG
+
FFFFFFFFFFFFFFFFFFFFFFFFFF,FFFFFFFFFFFFFFFFFFFFFFFFFFFFFFFFFFFFFFFFF
:FFFF:FFFFFFFF:FFFFFFFFFFFFFFFFFFFFFFFFFFFFFFFFFFFFFFFFFFFFFFFFFFFFF
FFFFFFFFFF:FFFF
@A00721:190:HGJW7DSXY:4:1127:4978:36511 1:N:0:TCTGCAAG+GCTGCACT
GTCTATTGTCGACGATATGCGCTGCTGGGAATGTTATGTATTCGACGATTCATGTTCGCTTTCGTTAT
TGTAGAACAATAAAGTTCTGAATTCGTCACACCAGAATGTTATTAGAACCTCTGACGTCATTCTGGTG
GTCCGCATCAATTGT
+
FFFF:FFFFFFFFFFFFFFFFFFFFFFFFFFFFFFFFFFFFFFFFFFFFFFFFFFFFFFFFFFFFFFF
FFFFFFFFFFFFFFFFFFFFFFFFFFFFFFFFFFFFFFFFF:FFFFFFFFFFFFFFFFFFFFFFFFFFF
FFFFFFFFFFFFFFF
@A00721:190:HGJW7DSXY:4:1206:15890:12226 1:N:0:TCTGCAAG+GCTGCACT
GTCTATTGTCGACGATATGCGCTGCTGGGAATGTTATGTATTCGACGATTCATGTTCGCTTTCGTTAT
TGTAGAACAATAAAGTTCTGAATTCGTCACACCAGAATGTTATTAGAACCTCTGACGTCATTCTGGTG
GTCCGCATCAATTGT
+
FFFF:FFFFFFFFFFFFFFFFFFFFFFFFFFFFFFFFFFFFFFFFFFFFFFFFFFFFFFFFFFFFFFF
FFFFFFFFFFFFFFFFFFFFFFFFFFFFFFFFFFFFFFFFFFFFFFFFFFFFFFFFFFFFFFFFFFFF
FFFFFFFFFFFFFFF
@A00721:190:HGJW7DSXY:4:1206:15420:15702 1:N:0:TCTGCAAG+GCTGCACT
GTCTATTGTCGACGATATGCGCTGCTGGGAATGTTATGTATTCGACGATTCATGTTCGCTTTCGTTAT
TGTAGAACAATAAAGTTCTGAATTCGTCACACCAGAATGTTATTAGAACCTCTGACGTCATTCTGGTG
GTCCGCATCAATTGT
+
::F:FFFFFFFFFFFFFFFFFFFFFFFFFFF::FFFFFF:FFFFFFFFFF:FFFFFF:FFFFFFFFFFF
F,FFFFFFFF:FFFFFFFFFFFFFFFFFFFFFFFF:FF:FFFFFFF::FFF:FFFFF,:FFF,FFF:FF
FFFFFF:FFFF:FFF
@A00721:190:HGJW7DSXY:4:1313:5095:12195 1:N:0:TCTGCAAG+GCTGCACT
GTCTATTGTCGACGATATGCGCTGCTGGGAATGTTATGTATTCGACGATTCATGTTCGCTTTCGTTAT
TGTAGAACAATAAAGTTCTGAATTCGTCACACCAGAATGTTATTAGAACCTCTGACGTCATTCTGGTG
GTCCGCATCAATTGT
+
FFFFFFFFFFFFFFFFFFFFFFFFFFFFFFFFFFFFFFFFFFFFFFFFFFFFFFFFFFFFFFFFFFFF
FFFFFFFFFFFFFFFFFFFFFFFFFFFFFFFFFFFFFFFFFFFFFFFF:FFFFFFFFFFFFFFFFFFF
FFF:FFFFFFFFFFF
@A00721:190:HGJW7DSXY:4:1538:29324:8500 1:N:0:TCTGCAAG+GCTGCACT
GTCTATTGTCGACGATATGCGCTGCTGGGAATGTTATGTATTCGACGATTCATGTTCGCTTTCGTTAT
TGTAGAACAATAAAGTTCTGAATTCGTCACACCAGAATGTTATTAGAACCTCTGACGTCATTCTGGTG
GTCCGCATCAATTGT

```
+
FFFFFFFFFFFFFFFFFFFFFFFFFFFFFF:FFFFFFFFFFFFFFFFFFFFFFFFFFFFFFFFFFFFFFFF
FFFFFFFFFFFFFFFFFFFFFFFFFFFFFFFFFFFFFFFFFFFFFFFFFFFFFFFFFFFFFFFFFFFFFFF
FFFFFFFFFFFFFFF
@A00721:190:HGJW7DSXY:4:2167:18078:17394 1:N:0:TCTGCAAG+GCTGCACT
GTCTATTGTCGACGATATGCGCTGCTGGGAATGTTATGTATTCGACGATTCATGTTCGCTTTCGTTAT
TGTAGAACAATAAAGTTCTGAATTCGTCACACCAGAATGTTATTAGAACCTCTGACGTCATTCTGGTG
GTCCGCATCAATTG
+
F,FFFF:,FFFFFFFFFFFFFFFFF,:F:F:FF:,FFFFFFFFFFF:FFFFF,FFF:FFFFF:FFFFF
F:FFFFFFFFFFFFFFFFFFFFFFFFF:FFFFFFFFFFFFFF,FF:FF,FFFFF:FFFFFFFF:FFFF:FF
FFFFFFFFFFFF,F
@A00721:190:HGJW7DSXY:4:2167:20772:19272 1:N:0:TCTGCAAG+GCTGCACT
GTCTATTGTCGACGATATGCGCTGCTGGGAATGTTATGTATTCGACGATTCATGTTCGCTTTCGTTAT
TGTAGAACAATAAAGTTCTGAATTCGTCACACCAGAATGTTATTAGAACCTCTGACGTCATTCTGGTG
GTCCGCATCAATTGT
+
FFFFFFFFFFFFFFFFFFFFFFFFFFFFFF:FFFFFFFFFFFFFFFFFFFFFFFFFFFFFFFFFFFFFFFF
FFFFFFFFFFFFFFFFFFFFFFFFFFFFFFFFFFFFFFFFFFFFFFFFFFFFF:FFFFFFFFFFFFFFF
FFFFFFFFFFFFFFF
@A00721:190:HGJW7DSXY:4:2356:13819:21856 1:N:0:TCTGCAAG+GCTGCACT
GTCTATTGTCGACGATATGCGCTGCTGGGAATGTTATGTATTCGACGATTCATGTTCGCTTTCGTTAT
TGTAGAACAATAAAGTTCTGAATTCGTCACACCAGAATGTTATTAGAACCTCTGACGTCATTCTGGTG
GTCCGCATCAATTGT
+
FFFF,FFFFFFFFFFFFFFFFFFFFFFFFFFFFFFFFFFFFFFFFFFFFFFFFFFFFFFFFFFFFFFFF
FFFFFFFFFFFFFFFFFFFFFFFFFFFFFFFFFFFFFFFFFFFFFFFFFFFFFFFFFFFFFFFFFFFFFFF
FFFFFFFFFFFFFFF
@A00721:190:HGJW7DSXY:4:2418:27335:33395 1:N:0:TCTGCAAG+GCTGCACT
GTCTATTGTCGACGATATGCGCTGCTGGGAATGTTATGTATTCGACGATTCATGTTCGCTTTCGTTAT
TGTAGAACAATAAAGTTCTGAATTCGTCACACCAGAATGTTATTAGAACCTCTGACGTCATTCTGGTG
GTCCGCATCAATTGT
+
FFFFF:FFFFFFFFFFFFFFFFFFFFFFFFF:FFFFFFFFFFFFFFFFFFFFFFFFFFFFFFFFFFFF:
FFFF:FFFFFFFFFFFFFFFFFFFFFFFFFFFFFFFFFFFFFFFFF,FFF:FF:FFFFFFFFFFFFFFF
FFFFFFFFFFFFFF:
@A00721:190:HGJW7DSXY:4:1147:19723:33833 1:N:0:TCTGCAAG+GCTGCACT
CTCTATTGTCGACGATATGCGCTGCTGGGAATGTTATGTATTCGACGATTCATGTTCGCTTTCGTTAT
TGTAGAACAATAAAGTTCTGAATTCGTCACACCAGAATGTTATTAGAACCTCTGACGTCATTCTGGTG
GTCCGCATCAATTGT
+
FFFFFFFFFFFFFFFFFFFFFFF:FFFFFFFFFFFFFFFFFFFFFFFFFFFFFFFFFFFFFFFFFFFFFFF
FFFFFFFFFFFFFFFFFFFFFFFFFFFFFFFFFFFFFFFFFFFFFFF:FFFFFFFFFFFFFFFFFFFFFFF
FFFFF:FFFFFFFFF
@A00721:190:HGJW7DSXY:4:1449:10257:32064 1:N:0:TCTGCAAG+GCTGCACT
CTCTATTGTCGACGATATGCGCTGCTGGGAATGTTATGTATTCGACGATTCATGTTCGCTTTCGTTAT
TGTAGAACAATAAAGTTCTGAATTCGTCACACCAGAATGTTATTATAACCTCTGACGTCATTCTGGTG
GTCCGCATCAATTGT
+
FFFF:FF:::FFFFFFFF,,F,FFFF:,,FFF:F,FFFFFFF,FFFFFFF,FF:FFFF,FFF,FFFFF
F,FF::F:FFFFFF::FFFFFFF:,FFFFF:FFFFF:FFF:FFFF,FFFFFF:FFFFF,FFFFF:FFF
,FF::,:,,FFFFFF
@A00721:190:HGJW7DSXY:4:1502:4472:33442 1:N:0:TCTGCAAG+GCTGCACT
CTCTATTGTCGACGATATGCGCTGCTGGGAATGTTATGTATTCGACGATTCATGTTCGCTTTCGTTAT
```

TGTAGAACAATAAAGTTCTGAATTCGTCACACCAGAATGTTATTAGAACCTCTGACGTCATTCTGGTG
GTCCGCATCAATTGT
+
FFFF:FFFFFFFFFFFFFFFFFFFFFFFFFFFFFFFFFFFFFFFFFFFFFFFFFFFFFFFFFFFFFFF
FFFFFFFFFFFFFFFFFFFFFFFF:FFFFFFFFFFFFF:FFFFFFFFFFFFF:FFFFFFFFFFFFFFF
FFFFFFFFFFFFFFF
@A00721:190:HGJW7DSXY:4:1526:4273:16626 1:N:0:TCTGCAAG+GCTGCACT
CTCTATTGTCGACGATATGCGCTGCTGGGAATGTTATGTATTCGACGATTCATGTTCGCTTTCGTTAT
TGTAGAACAATAAAGTTCTGAATTCGTCACACCAGAATGTTATTAGAACCTCTGACGTCATTCTGGTG
GTCCGCATCAATTGT
+
FFFF:FFFFFFFFFFFFFFFFF:FFFFFFFFFFFFFFFFFFFFFFFFFFFFFFFFFFFFFFFFFFFFF
FFFFFFFFFFFFFFFFFFFFFFFFFFFFFFFFFFFFFFFFFFFFFFFFFFFFFFFFFFFFFFFFFFFF
FFFFFFFFFFFFFFF
@A00721:190:HGJW7DSXY:4:1604:31033:23735 1:N:0:TCTGCAAG+GCTGCACT
CTCTATTGTCGACGATATGCGCTGCTGGGAATGTTATGTATTCGACGATTCATGTTCGCTTTCGTTAT
TGTAGAACAATAAAGTTCTGAATTCGTCACACCAGAATGTTATTAGAACCTCTGACGTCATTCTGGTG
GTCCGCATCAATTGT
+
FFFFFFFFFFFFFFFFFFFFFFFFFFFFFFFFFFFFFFFFFFFFFFFFFFFFFFFFFFFFFFFFFFFF
FFFFFFFFFFFFFFFFFFFFFFFFFFFFFFFFFFFFFFFFFFFFFFFFFFFF:FFFFFFFFFFFFFFF
FFFFFFFFFFFFFFF
@A00721:190:HGJW7DSXY:4:2106:16441:30686 1:N:0:TCTGCAAG+GCTGCACT
CTCTATTGTCGACGATATGCGCTGCTGGGAATGTTATGTATTCGACGATTCATGTTCGCTTTCGTTAT
TGTAGAACAATAAAGTTCTGAATTCGTCACACCAGAATGTTATTAGAACCTCTGACGTCATTCTGGTG
GTCCGCATCAATTGT
+
FFFF:FFFFFFFFFFFFFFFFFFFFFFFFF,FFFF:FFFFF:FFFFFFFFFFFFFFFFFFFFFFFFFF
FFFF:FFFFFFFFFFFFFFFFFFFFFFFFFFFFFFFFFFFFFFFFFFF:FFF:FFFFFFFFFFFFFFF
FFFFFFFFFFFF:F:
@A00721:190:HGJW7DSXY:4:2453:22137:29872 1:N:0:TCTGCAAG+GCTGCACT
CTCTATTGTCGACGATATGCGCTGCTGGGAATGTTATGTATTCGACGATTCATGTTCGCTTTCGTTAT
TGTAGAACAATAAAGTTCTGAATTCGTCACACCAGAATGTTATTAGAACCTCTGACGTCATTCTGGTG
GTCCGCATCAATTGT
+
FFFFFFFFFFFFFFFFFFFFFFFFFFFFFF:FFFFFFFFFFFFFFFFFFFFFFFFFFFFFFFFFFFFF
FFFFFFFFFFFFFFFFFFFFFFFFFFFFFFFFFFFFFFFFFFFFFFFFFFFFFFFFFFFFFF:FFFFF
FFFFFFFFFFFF:FF
@A00721:190:HGJW7DSXY:4:1564:9534:15123 1:N:0:TCTGCAAG+GCTGCACT
CTGTCGACGATATGCGCTGCTGGGAATGTTATGTATTCGACGATTCATGTTCGCTTTCGTTATTGTAG
AACAATAAAGTTCTGAATTCGTCACACCAGAATGTTATTAGAACCTCTGACGTCATTCTGGTGGTCCG
CATCAATTGTCTCAT
+
FFFFFFFFFFFFFFFFFFFFFFFFFFFFFFFFFFFFFFFFFFF:FFFFFFFFFFFFFFFFFFFFFFFF
FFFFFFFFFFFFFFFFFFFFFFFFFFFFFFFFFFFFFFFFFFFFFFFFFFFFFFFFFF:FFFFFFFFF
FFFFFFFFFFFFFFF
@A00721:190:HGJW7DSXY:4:1505:14235:10300 1:N:0:TCTGCAAG+GCTGCACT
CGACGATATGCGCTGCTGGGAATGTTATGTATTCGACGATTCATGTTCGCTTTCGTTATTGTAGAACA
ATAAAGTTCTGAATTCGTCACACCAGAATGTTATTAGAACCTCTGACGTCATTCTGGTGGTCCGCATC
AATTGTCTCATATCA
+
FFFFFFFFFFFFFFFFFFFFFFFFFFFFFFFFFFFFFFFFFFFFFFFFFFFFFFFFFFFFFFFFFFFF
FFFFFFFFFFFFFFFF:FFFFFFFFFFFFFFFFFFF,FFFFFFFFF:FFFFFFFFFFFFFFFFFFFFF
FFFFFFFFFFFFFFF

```
@A00721:190:HGJW7DSXY:4:2263:5791:18944 1:N:0:TCTGCAAG+GCTGCACT
CGACGATATGCGCTGCTGGGAATGTTATGTATTCGACGATTCATGTTCGCTTTCGTTATTGTAGAACA
ATAAAGTTCTGAATTCGTCACACCAGAATGTTATTAGAACCTCTGACGTCATTCTGGTGGTCCGCATC
AATTGTCTCATATCA
+
FFFFFFFFFFFFFFFFFFFFFFFFFFFFFFFFFFFFFFFFFFFFFFFFFFFFFFFFFFFFFFFFFFFF
FFFFFFFFFFFFFFFFFFFFFFFFFFFFFFFFFFFFFFFFFFFFFFFFFFFFFFFFFFFFFFFFFFFF
FFFFFFFFFFFFFFF
@A00721:190:HGJW7DSXY:4:2313:3866:30765 1:N:0:TCTGCAAG+GCTGCACT
CGACGATATGCGCTGCTGGGAATGTTATGTATTCGACGATTCATGTTCGCTTTCGTTATTGTAGAACA
ATAAAGTTCTGAATTCGTCACACCAGAATGTTATTAGAACCTCTGACGTCATTCTGGTGGTCCGCATC
AATTGTCTCATATCA
+
FFFFFFFFFFFFFFFFFFFFFFFFFFFFFFFFFFFFFFFFFFFFFFFFFFFFFFFFFFFFFFFFFFFF
FFFFFFFFFFFFFFFFFFFFFFFFFFFFFFFFFFFFFFFFFFFFFFFFFFFFFFFFFFFFFFFFFFFF
F:FFFFFFFFFFFFF
@A00721:190:HGJW7DSXY:4:2505:8558:8985 1:N:0:TCTGCAAG+GCTGCACT
CGACGATATGCGCTGCTGGGAATGTTATGTATTCGACGATTCATGTTCGCTTTCGTTATTGTAGAACA
ATAAAGTTCTGAATTCGTCACACCAGAATGTTATTAGAACCTCTGACGTCATTCTGGTGGTCCGCATC
AAATGTCTCATA
+
FFFFFF:FFFFFFFFFFF:FFFFF,FFFF,FFF:FFFFFFFFF:FFFF:FF,FF:FFFF:FFF,FF:F
FFFFFFF,FFFFF:F,FFFFFFFFFFFFF:FFFFFF,FFF,F:FFFFFF,F:,FF:FF,FFFFFFF,F
,F,,FFFF,F,F
@A00721:190:HGJW7DSXY:4:2602:11315:6590 1:N:0:TCTGCAAG+GCTGCACT
CGACGATATGCGCTGCTGGGAATGTTATGTATTCGACGATTCATGTTCGCTTTCGTTATTGTAGAACA
ATAAAGTTCTGAATTCGTCACACCAGAATGTTATTAGAACCTCTGACGTCATTCTGGTGGTCCGCATC
AATTGTCTCATATCA
+
FFFFFFFFFFFFFFFF:FFFFFFFFFFFFFFFFF:F:FFFFFFFF:FFFFFFFFFFFFFFFFFFFFFF
FFFFF:FFFFFFFFF:FFFFFFFFFFFFF:FFFFFFFFFFF,F:FFFFFFF:FFFFFFFFFFF:FFFF
FFFFFFFFFFFFFFF
@A00721:190:HGJW7DSXY:4:1247:15899:34131 1:N:0:TCTGCAAG+GCTGCACT
GACGATATGCGCTGCTGGGAATGTTATGTATTCGACGATTCATGTTCGCTTTCGTTATTGTAGAACAA
TAAAGTTCTGAATTCGTCACACCAGAATGTTATTAGAACCTCTGACGTCATTCTGGTGGTCCGCATCA
ATTGTCTCATATCAT
+
FFFFFFFFFFFFFFFFFFFFFFFFFFFFFFFFFFFFFF:FFFFFFFFFFFFFFFFFFFFFFFFFFFFF
FFFFFFFFFFFFFFFFFFFFFFFFFFFFFFFFFFFFFFFFFFFFFFFFFFFF:FFFFFFFFFFFFFFF
FFFFFFFFFFFFFFF
@A00721:190:HGJW7DSXY:4:1423:7048:22811 1:N:0:TCTGCAAG+GCTGCACT
GACGATATGCGCTGCTGGGAATGTTATGTATTCGACGATTCATGTTCGCTTTCGTTATTGTAGAACAA
TAAAGTTCTGAATTCGTCACACCAGAATGTTATTAGAACCTCTGACGTCATTCTGGTGGTCCGCATCA
ATTGTCTCATATCAT
+
FFFFFFFFFFFFFFFFFFFF:FFFFFFFFFFFFFFFFFFFFFFFFFFFFFFFFFFFFFFFFFFFFFFF
FFFFFFFFFFFFFFFFFFFFFFFFFFFFFFFFFFFFFFFFFFFFFFFFFFFFFFFFFFFFFFFFFFFF
FFFFFFFFFFFFFFF
@A00721:190:HGJW7DSXY:4:1510:28339:33755 1:N:0:TCTGCAAG+GCTGCACT
GACGATATGCGCTGCTGGGAATGTTATGTATTCGACGATTCATGTTCGCTTTCGTTATTGTAGAACAA
TAAAGTTCTGAATTCGTCACACCAGAATGTTATTAGAACCTCTGACGTCATTCTGGTGGTCCGCATCA
ATTGTCTCATATCAT
+
FFFFFFFFFFFFFFFFFFFFFFFFFFFFFFFFFFFFFFFFFFFFFFFF:FFFFFFFFFFFFFFFFFFF
```

:FFFFFFFFFFFFFFFFFFFFFFFFFFFFFFFFFF:FFFFFFFFFFFFFFFFFFFFFFFFFFFFFFFF
FFFFFFFFF::F:FF
@A00721:190:HGJW7DSXY:4:1662:19569:29215 1:N:0:TCTGCAAG+GCTGCACT
GACGATATGCGCTGCTGGGAATGTTATGTATTCGACGATTCATGTTCGCTTTCGTTATTGTAGAACAA
TAAAGTTCTGAATTCGTCACACCAGAATGTTATTAGAACCTCTGACGTCATTCTGGTGGTCCGCATCA
ATTGTCTCATATCAT
+
FFFFFFFFFFFFFFFFFFFFFFFFFFFFFFFFFFFFFFFFFFFFFFFFFFFFFFFFFFFFFFFFFFFF
FFFFFFFFFFFFFFFFFFFFFFFFFFFFFFFFFFFFFFFFFFFFFFFFFFFFFFFFFFFFFFFFFFFF
FFFFFFFFF::FFFF
@A00721:190:HGJW7DSXY:4:2344:6506:8343 1:N:0:TCTGCAAG+GCTGCACT
GACGATATGCGCTGCTGGGAATGTTATGTATTCGACGATTCATGTTCGCTTTCGTTATTGTAGAACAA
TAAAGTTCTGAATTCGTCACACCAGAATGTTATTAGAACCTCTGACGTCATTCTGGTGGTCCGCATCA
ATTGTCTCATATCAT
+
FFFFFFFFFFFFFFF,FFFFFFFFFFFFFFFFFFFFFF:FFFFFFFFFFFFFFFFFFFFFFFFFFFFF
FFFFFFFFFFFFFFFFFFFFFFFFFFFFFFFFFFFFFFFFFFFFFFFFFFFFFFFFFFFFFFFFFFF:
FFFFFFFFFFFFFFF
@A00721:190:HGJW7DSXY:4:2476:16179:18740 1:N:0:TCTGCAAG+GCTGCACT
GACGATATGCGCTGCTGGGAATGTTATGTATTCGACGATTCATGTTCGCTTTCGTTATTGTAGAACAA
TAAAGTTCTGAATTCGTCACACCAGAATGTTATTAGAACCTCTGACGTCATTCTGGTGGTCCGCATCA
ATTGTCTCATATCAT
+
FFF::FFFFF:FFFF,FFFFFFF:FFFFFFFFFFFFFF,FFFFFFFFFFFFFFFFF:FF:FF:FF:FF
FFF,::FF,FFFFFF:FFFFFF,FFFFFFFFFFFFFFFFFFF,FFFFFFFFFFFFFFFFFFFFFFFFF
:FFFF,FFFFFF,FF
@A00721:190:HGJW7DSXY:4:1106:17653:10802 1:N:0:TCTGCAAG+GCTGCACT
CGCGCTGCTGGGAATGTTATGTATTCGACGATTCATGTTCGCTTTCGTTATTGTAGAACAATAAAGTT
CTGAATTCGTCACACCAGAATGTTATTAGAACCTCTGACGTCATTCTGGTGGTCCGCATCAATTGTCT
CATATCATTTAGTTA
+
FFFFFFFFFFFFF:FFFFFFFFFFFFFFFFFFFFFFFFFFFFFFFFFFFFFFFFFFFFFFFFFFFFFFFF
FFFFFFFFFFFFFFFFFFFFFFFFFFFFFFFFFFFFFFFFFFFFFFFFFFFFFFFFFFFF:F,FFFFF
FFFFFF::FFFFFFF
@A00721:190:HGJW7DSXY:4:1132:21450:16094 1:N:0:TCTGCAAG+GCTGCACT
CGCGCTGCTGGGAATGTTATGTATTCGACGATTCATGTTCGCTTTCGTTATTGTAGAACAATAAAGTT
CTGAATTCGTCACACCAGAATGTTATTAGAACCTCTGACGTCATTCTGGTGGTCCGCATCAATTGTCT
CATATCATTTAGTTA
+
FFFFFFFFFFFFF:FFFFFFFFFFFFFFFFFFFFFFFFFFFFFFFFFFFFFFFFFFFFFFFFFFFFFFFF
F:FFFFFFFFFFFFFFFFF,FFFFFFFFFFFFFFFFFFFFFFFFFFF:FFFFFFFFFFFFFFFFFFFF
FF:FFFFFFFFFFFF
@A00721:190:HGJW7DSXY:4:1173:11523:1251 1:N:0:TCTGCAAG+GCTGCACT
CGCGCTGCTGGGAATGTTATGTATTCGACGATTCATGTTCGCTTTCGTTATTGTAGAACAATAAAGTT
CTGAATTCGTCACACCAGAATGTTATTAGAACCTCTGACGTCATTCTGGTGGTCCGCATCAATTGTCT
CATATCATTTAGTTA
+
FFFFFFFFFFFFF,FFFFFFFFFFFFFFFFFFFFFFFFFFFFFFFFFFFFFFFFFFFFFFFFFFFFFFFF
FFFFFFFFFFFFFFFFFFFFFFFFFFFFFFFFFFFFFFFFFFFFFFFFFFFFFFFFFFFFFFFFFFF:
FFFFFF:FFFFFFFF
@A00721:190:HGJW7DSXY:4:1310:32000:20556 1:N:0:TCTGCAAG+GCTGCACT
CGCGCTGCTGGGAATGTTATGTATTCGACGATTCATGTTCGCTTTCGTTATTGTAGAACAATAAAGTT
CTGAATTCGTCACACCAGAATGTTATTAGAACCTCTGACGTCATTCTGGTGGTCCGCATCAATTGTCT
CATATCATTTAGTTA

```
+
FFFFFFFFFFFFF,FFFFFFFFFFFFFFFFFFFFFFF:FFFFFFFFFFFFFFFFFFFFFFFFFFFFFF
FFF:FFFFFFFFFFFFFFFFFFFFFFFFFFF:FFFFFFFFFFFFFFFFFFFFFFFFFFFFFFFFFFFF
FFFFF:FFFFFFFFF
@A00721:190:HGJW7DSXY:4:1317:5755:26960 1:N:0:TCTGCAAG+GCTGCACT
CGCGCTGCTGGGAATGTTATGTATTCGACGATTCATGTTCGCTTTCGTTATTGTAGAACAATAAAGTT
CTGAATTCGTCACACCAGAATGTTATTAGAACCTCTGACGTCATTCTGGTGGTCCGCATCAATTGTCT
CATATCATTTAGTTA
+
FFFFFFFFFFFFFFFFFFFFFFFFFFFFFFFFFFFFFFFFFFFFFFFFFFFFFFFFFFFFFFFFFFFF
FFFFFFFFFFFFFFFFFFFFFFFF:FFFFFFFFFFFFFFFFFFFFFFFFFF:FFFFFFFFFFFFFFFF
FFFFFFFFFFFFFFF
@A00721:190:HGJW7DSXY:4:2257:7563:21386 1:N:0:TCTGCAAG+GCTGCACT
CGCGCTGCTGGGAATGTTATGTATTCGACGATTCATGTTCGCTTTCGTTATTGTAGAACAATAAAGTT
CTGAATTCGTCACACCAGAATGTTATTAGAACCTCTGACGTCATTCTGGTGGTCAGCATCAATTGTCT
CATATCATTTAGTTA
+
FF:FFFFFFF,FFFF:F,FF,FFF,,FFFFFF,FFFFFF::FF:FFFFFFFF::F,FFFFF:FFFFF:
FFFFFFF,F:FFFFFFF,F::FFFF,:F,:F,F::,FF,:FFF:FF,:F::FFF,F:F,FFFF:FFFF
FF,FFFF::FFFFFF
@A00721:190:HGJW7DSXY:4:2404:8404:16078 1:N:0:TCTGCAAG+GCTGCACT
CGCGCTGCTGGGAATGTTATGTATTCGACGATTCATGTTCGCTTTCGTTATTGTAGAACAATAAAGTT
CTGAATTCGTCACACCAGAATGTTATTAGAACCTCTGACGTCATTCTGGTGGTCCGCATCAATTGTCT
CATATCATTTAGTTA
+
FFFFFFFFFFFFFFFFFFFFFFFFFFFFFFFFFFFFFFFFFFFFFFFFFFFFFFFFFF:FFFFFFFFF
FFFFFFFF:FFFFFFFFFFFFFFFFFFFF,FFFFFFFFFFFFFFFFFFFFFFFFFFFFFFFFFFFFFF
FFFFFFF,FFFFFFF
@A00721:190:HGJW7DSXY:4:2661:1163:12774 1:N:0:TCTGCAAG+GCTGCACT
CGCGCTGCTGGGAATGTTATGTATTCGACGATTCATGTTCGCTTTCGTTATTGTAGAACAATAAAGTT
CTGAATTCGTCACACCAGAATGTTATTAGAACCTCTGACGTCATTCTGGTGGTCCGCATCAATTGTCT
CATATCATTTAGTTA
+
FFFFFFFFFFFFF:FFFFFFFFFFFFFFFFFFFFFFFFFFFFFFFFFFFFFFFFFFFFFFFFFFFFFF
FFFFFFFFFFFFFFFFFFFFFFFFFFFFFFFFFFFFFFFFFFFFFFFFFFFFF:FFFFFFFFFFF:FF
FFFFFFFFFFFFFFF
@A00721:190:HGJW7DSXY:4:1119:20817:2660 1:N:0:TCTGCAAG+GCTGCACT
TGCTGCTGGGAATGTTATGTATTCGACGATTCATGTTCGCTTTCGTTATTGTAGAACAATAAAGTTCT
GAATTCGTCACACCAGAATGTTATTAGAACCTCTGACGTCATTCTGGTGGTCCGCATCAATTGTCTCA
TATCATAGATCGGAA
+
FF:FFFFFFFFFFFFFFFFFFFFFFFFFFFFFFFFFFFFFFFFFFFFFFFFFFFFFFFFFFFFFFFFF
FFFFFFFFFFFFFFFFFFFF:FFFFFFFFFFFFFFFFFFFFFFFFFFFFFFFFFFFFFFFFFFFFFFF
FFFF,FFFFFFFFFF
@A00721:190:HGJW7DSXY:4:1173:22318:20259 1:N:0:TCTGCAAG+GCTGCACT
TGCTGCTGGGAATGTTATGTATTCGACGATTCATGTTCGCTTTCGTTATTGTAGAACAATAAAGTTCT
GAATTCGTCACACCAGAATGTTATTAGAACCTCTGACGTCATTCTGGTGGTCCGCATCAATTGTCTCA
TATCATAGATCGGAA
+
FFFFFFFFFFFFFFFFFFFFFFFFFFFFFFFFFFFFFFFFFFFFFFFFFFFFFFFFFFFFFFFFFFFF
FFFFFFFFFFFFFFFFFFFFFFFFFFFFFFFFFFFFFFFFFFFFFFFFFFFFFFFFFFFFFFFFFFFF
FFFFFFFFFFFFFFF
@A00721:190:HGJW7DSXY:4:1415:4689:35477 1:N:0:TCTGCAAG+GCTGCACT
TGCTGCTGGGAATGTTATGTATTCGACGATTCATGTTCGCTTTCGTTATTGTAGAACAATAAAGTTCT
```

GAATTCGTCACACCAGAATGTTATTAGAACCTCTGACGTCATTCTGGTGGTCCGCATCAATTGTCTCA
TATCATAGATCGGAA
+
:FFFFFFFFFF:FFF:FFFFFFFFF:FFFFFFFFFFFFFFFFFFFFFFF:FFFFFFFFFFFFF:FF:F
FFF:FFFFFFFFFFFFFFFFFFFFFFFFFFFFFFFFFFFFF,FFFFFFFFFFFFFFFFFFFFFFFFFF
FFFFFFFFFFF:FFF
@A00721:190:HGJW7DSXY:4:1445:21395:27618 1:N:0:TCTGCAAG+GCTGCACT
TGCTGCTGGGAATGTTATGTATTCGACGATTCATGTTCGCTTTCGTTATTGTAGAACAATAAAGTTCT
GAATTCGTCACACCAGAATGTTATTAGAACCTCTGACGTCATTCTGGTGGTCCGCATCAATTGTCTCA
TATCATAGATCGGAA
+
FFFFFF:FFFFFFFFFFFFFFFFFFFFFFFFFFFFFFFFFFFFFFFFFFFFFFFFFFFFFFFFFFFFF
FFFFFFFFFFFFFFFFFFFFFFFFFFF:FFFFFFFFFFFFFFFFFFFFFFFFFFFF:FF:FFFFFFFF
FFFFFFF:FFFFFFF
@A00721:190:HGJW7DSXY:4:1503:19605:5885 1:N:0:TCTGCAAG+GCTGCACT
TGCTGCTGGGAATGTTATGTATTCGACGATTCATGTTCGCTTTCGTTATTGTAGAACAATAAAGTTCT
GAATTCGTCACACCAGAATGTTATTAGAACCTCTGACGTCATTCTGGTGGTCCGCATCAATTGTCTCA
TATCATAGATCGGAA
+
FFFFFFFFFFFFFFF:FFFFF:FFFFFFFFFFFFFFFFFFFFFFFFFFFFFFFFFFFFFFFFFFFFFF
FFFFFFFFFFFFFFFFFFFFFFFFFFFFFFFFFFFFFFFFFFFFFFFFFFFFFFFFFFFFFFFFFFFF
FFFFFFF:FFFFFFF
@A00721:190:HGJW7DSXY:4:1649:6831:35117 1:N:0:TCTGCAAG+GCTGCACT
TGCTGCTGGGAATGTTATGTATTCGACGATTCATGTTCGCTTTCGTTATTGTAGAACAATAAAGTTCT
GAATTCGTCACACCAGAATGTTATTAGAACCTCTGACGTCATTCTGGTGGTCCGCATCAATTGTCTCA
TATCATAGATCGGAA
+
FFFFFFFFFFFFFFFFFFFFFFFFFFFFFFFFFFFFFFFFFFFFFFFFFFFFFFFFFFFFFFFFFFFF
FFFFFFFFFFFFFFFFFFFFFFFFFFFFFFFFFFFFFFFFFFFFFFFFFFFFFFFFFFFFFFFFFFFF
FFFFFFFFFFFFFFF
@A00721:190:HGJW7DSXY:4:2133:32832:11443 1:N:0:TCTGCAAG+GCTGCACT
TGCTGCTGGGAATGTTATGTATTCGACGATTCATGTTCGCTTTCGTTATTGTAGAACAATAAAGTTCT
GAATTCGTCACACCAGAATGTTATTAGAACCTCTGACGTCATTCTGGTGGTCCGCATCAATTGTCTCA
TATCATAGATCGGAA
+
FFFFFFFFFFFFFFFFFFFFFFFFFFFFFFFFFFFF:FFFFFFFFFFFFFFFFFFFFFFFF:FFFFFF
:FFFFFFFFFFFFFFFF,FFFFFFFFFFF,FFFF:FFFF:FFFFFFFFFFFFFF,FFFFFFFFF,F::
FFF:FFFFFFFF,F:
@A00721:190:HGJW7DSXY:4:2137:8558:30373 1:N:0:TCTGCAAG+GCTGCACT
TGCTGCTGGGAATGTTATGTATTCGACGATTCATGTTCGCTTTCGTTATTGTAGAACAATAAAGTTCT
GAATTCGTCACACCAGAATGTTATTAGAACCTCTGACGTCATTCTGGTGGTCCGCATCAATTGTCTCA
TATCATAGATCGGAA
+
FFFFFFFFFFF:FFFFFFFFFFFFFFFFFFFFFFFFFFFFFFFFFFFFFFFFFFFFFFFFFFFFFFFF
FFFFFFFFFFFFFFFFFFFFFFFFFFFFFFFFFFFFFFFFF::F::FFFFFFFFFFFFFFFFF:FFFF
FFF,FFF:FFFFFFF
@A00721:190:HGJW7DSXY:4:2223:22544:20118 1:N:0:TCTGCAAG+GCTGCACT
TGCTGCTGGGAATGTTATGTATTCGACGATTCATGTTCGCTTTCGTTATTGTAGAACAATAAAGTTCT
GAATTCGTCACACCAGAATGTTATTAGAACCTCTGACGTCATTCTGGTGGTCCGCATCAATTGTCTCA
TATCATAGATCGGAA
+
FFFFFFFFFFF:FFFFFFFFFFFFFFFFFFFFFFFFFFFFFFFFFFFFFFFFF:FFFFFFFFFFFFFF
:FFFFFFFFFFFFFFFFFFFFFFFFFFFFFFFFFFFFFFFFFFFFFFFFFFFFFFFFFFFFFFFFFFF
FFFFFFFFFFF:FFF

@A00721:190:HGJW7DSXY:4:2269:3341:34491 1:N:0:TCTGCAAG+GCTGCACT
TGCTGCTGGGAATGTTATGTATTCGACGATTCATGTTCGCTTTCGTTATTGTAGAACAATAAAGTTCT
GAATTCGTCACACCAGAATGTTATTAGAACCTCTGACGTCATTCTGGTGGTCCGCATCAATTGTCTCA
TATCATAGATCGGAA
+
FFFFFFFFFFF,FFF:FFFFFFFFFFFFFFFFFFFFFFFFFFFFFFFFFFFFFFFFFFFFFFFFFFFF
FFFFFFFFFFFFFFFFFFFFFFFFFFFFFFFFFFFFFFFFFFFFFFFFFFFFFFFFFFFFFFFFFFFF
FFFFFFFFFFFFFFF
@A00721:190:HGJW7DSXY:4:2378:10059:3975 1:N:0:TCTGCAAG+GCTGCACT
TGCTGCTGGGAATGTTATGTATTCGACGATTCATGTTCGCTTTCGTTATTGTAGAACAATAAAGTTCT
GAATTCGTCACACCAGAATGTTATTAGAACCTCTGACGTCATTCTGGTGGTCCGCATCAATTGTCTCA
TATCATAGATCGGAA
+
FFFFFFFFFFF:FFFFFFFFFFFFFFFFFFFFFFFFFFFFFFFFFFFFFFFF:FFFFFFFFFFFFFF
FFFF:F:FFFFFFFFFFFFFFFFFFFF:FFFFFFFFFFFFFFFFFFFFFFF:FFFFFFFFFFFFFFFFFF
FFFFFFF:FFFFFFF
@A00721:190:HGJW7DSXY:4:2378:9968:7482 1:N:0:TCTGCAAG+GCTGCACT
TGCTGCTGGGAATGTTATGTATTCGACGATTCATGTTCGCTTTCGTTATTGTAGAACAATAAAGTTCT
GAATTCGTCACACCAGAATGTTATTAGAACCTCTGACGTCATTCTGGTGGTCCGCATCAATTGTCTCA
TATCATAGATCGGAA
+
FFFFFFFFFFF,FFFFFFFFFFFFFFFFFFFFFFFFFFFFFF::FFFFFFFFFF:FFFFFFFF::FFF
:FFFFFFFFF:FFFFFFFFFFFFFFF,FF:FFF:FFFFFFF:F:FFFFFFFF:,FFFFFFFF,FFFFF
FFFFFFF,FFFFFFF
@A00721:190:HGJW7DSXY:4:2563:19416:34585 1:N:0:TCTGCAAG+GCTGCACT
TGCTGCTGGGAATGTTATGTATTCGACGATTCATGTTCGCTTTCGTTATTGTAGAACAATAAAGTTCT
GAATTCGTCACACCAGAATGTTATTAGAACCTCTGACGTCATTCTGGTGGTCCGCATCAATTGTCTCA
TATCATAGATCGGAA
+
FFFFFFFFFFFFFFFFFFFFFFFFFFFFFFFFFFFFFFFFFFFFFFFFFFFF:FFFFFFFFFFFFFF
FFFFFFFFFFFFFFFFFFFFFFFFFFFFFFFFFFFFFFFFFFFFFFFFFFFFFFFFFFFFFFFFFFFF
FFFFFFFFFFFFFFF
@A00721:190:HGJW7DSXY:4:2618:10140:14011 1:N:0:TCTGCAAG+GCTGCACT
TGCTGCTGGGAATGTTATGTATTCGACGATTCATGTTCGCTTTCGTTATTGTAGAACAATAAAGTTCT
GAATTCGTCACACCAGAATGTTATTAGAACCTCTGACGTCATTCTGGTGGTCCGCATCAATTGTCTCA
TATCATAGATCGGAA
+
FFFFFFFFFFF:FFFFFFFFFFFFFFFFFFFFFFFFFFFFFFFFFFFFFFFFFFFFFFFFFFFFFFFF
FFFFFFFFFFFFFFFFFFFFFFFFFFFFFFFFFFFFFFFFFFFFFFFFFFFFFFFFFFFFFFFFFFFF
FFFFFFFFFFFFFFF
@A00721:190:HGJW7DSXY:4:1149:12029:30718 1:N:0:TCTGCAAG+GCTGCACT
CATTAACTAAATGATATGAGACAATTGATGCGGACCACCAGAATGACGTCAGAGGTTCTAATAACATT
CTGGTGTGACGAATTCAGAACTTTATTGTTCTACAATAACGAAAGCGAACATGAATCGTCGAATACAT
AACATTCCCAGCAGC
+
FFFFFFFFFFFFFFFFFFFFFFFFFFFFFFFFFFFFFFFFFFFFFFFFFFFFFFFFFFF,FFFFFFFF
FFFFFFFFFFFFFFFFFFF:FFF:FFFFFFFFFFFFFFFFFFFFFFFFFFFFFFFFFFFFFFFF:FFF
FFFFFF:FFFFFFFF
@A00721:190:HGJW7DSXY:4:1320:8901:9925 1:N:0:TCTGCAAG+GCTGCACT
GCTGCTGGGAATGTTATGTATTCGACGATTCATGTTCGCTTTCGTTATTGTAGAACAATAAAGTTCTG
AATTCGTCACACCAGAATGTTATTAGAACCTCTGACGTCATTCTGGTGGTCCGCATCAATTGTCTCAT
ATCATTTAGTTAATG
+
FFFFFFFFFF,FFFFFFFFFFFFFFFFFFFFFFFFFFFFFFFFFFFFFFFFFFFFFFFFFFFFFF:FF

```
F::FFFFFFFFFFFFFFFFFFFFFFFFF:FFFFFFFFFFFFFFFFFFFFFFFFFFFFF:FFFFFFFFF
FFFFFFFFFFFFFF:
@A00721:190:HGJW7DSXY:4:1471:26793:35336 1:N:0:TCTGCAAG+GCTGCACT
GCTGCTGGGAATGTTATGTATTCGACGATTCATGTTCGCTTTCGTTATTGTAGAACAATAAAGTTCTG
AATTCGTCACACCAGAATGTTATTAGAACCTCTGACGTCATTCTGGTGGTCCGCATCAATTGTCTCAT
ATCATTTAGTTAATG
+
FFFFFFFFFFFFFFFFFFFFFFFFFFFFFFFFFFFFFFFFFFFF,FFFFFFFFFFFFFFFFFFFFFFF
FFFFFFFFFFFFFFFFFFFFFFFFFFFFFFFFFFFFFFFFFFFFFFFFFFFFFFFFFFFFFFFFFFFF
FFFFFFFFFFFFFFF
@A00721:190:HGJW7DSXY:4:1570:12536:33724 1:N:0:TCTGCAAG+GCTGCACT
CATTAACTAAATGATATGAGACAATTGATGCGGACCACCAGAATGACGTCAGAGGTTCTAATAACATT
CTGGTGTGACGAATTCAGAACTTTATTGTTCTACAATAACGAAAGCGAACATGAATCGTCGAATACAT
AACATTCCCAGCAGC
+
FFFFFFFFFFFFFFFFFFFFFFF:FFFFFFFFFFFF:FFFFFFFFFFFFFFFFFFFFFFFFFF:FFFF
FFFFFFFFFFFFFFFFFFFF:FFFFFFFFF:FFFFFFFFFFFFFFFFFFFFFFFFFFFFFFFFFFFFF
FFFFFFFFFFFFFFF
@A00721:190:HGJW7DSXY:4:1611:18611:9987 1:N:0:TCTGCAAG+GCTGCACT
GCTGCTGGGAATGTTATGTATTCGACGATTCATGTTCGCTTTCGTTATTGTAGAACAATAAAGTTCTG
AATTCGTCACACCAGAATGTTATTAGAACCTCTGACGTCATTCTGGTGGTCCGCATCAATTGTCTCAT
ATCATTTAGTTAATG
+
FFFFFFFFFFFFFFFFFFFFFFFFFFFFFFFFFFFFFFFFFFFFFFFFFFFFFFFFFFFFFFFFFFFF
FFFFFFFFFFFFFFFFFFFFFFFFF:FFFFFFFFFFFFFFFFFFFFFFFFFFFFFFFFFFFFFFFFF:
FFFFFFFFFF:FFFF
@A00721:190:HGJW7DSXY:4:1611:20012:10504 1:N:0:TCTGCAAG+GCTGCACT
GCTGCTGGGAATGTTATGTATTCGACGATTCATGTTCGCTTTCGTTATTGTAGAACAATAAAGTTCTG
AATTCGTCACACCAGAATGTTATTAGAACCTCTGACGTCATTCTGGTGGTCCGCATCAATTGTCTCAT
ATCATTTAGTTAATG
+
FFFFFFFFFFFFFFFFFFFFFFFFFFFFFFFFFFFFFFFFFFFFFFFFFFFFFFFFFFFFFFFFFFFF
FFFFFFFFFFFFFFFFFFFFFFFFFFFFFFFFFFFFFFFF,FFFFFFFFFFFFFFFFFFFFFFFFFFF
FFFFFFFFFFFFFFF
@A00721:190:HGJW7DSXY:4:1647:15338:33943 1:N:0:TCTGCAAG+GCTGCACT
CATTAACTAAATGATATGAGACAATTGATGCGGACCACCAGAATGACGTCAGAGGTTCTAATAACATT
CTGGTGTGACGAATTCAGAACTTTATTGTTCTACAATAACGAAAGCGAACATGAATCGTCGAATACAT
AACATTCCCAGCAGC
+
FFFFFFFFFFFFFFFF:FFFFFFFFFFFFFFFFFFFFFFFFFFFFFFFFFFFFFFFFFFFFFFFFFFF
FFFFFFFFFFFFFFFFFFFFFFFFFFFFFFFFFFFFFFFF:FFFFFFFFFFFFFFFFFFFFFFFFFFF
FFFFFFFFFFFFFFF
@A00721:190:HGJW7DSXY:4:2143:6732:33223 1:N:0:TCTGCAAG+GCTGCACT
GCTGCTGGGAATGTTATGTATTCGACGATTCATGTTCGCTTTCGTTATTGTAGAACAATAAAGTTCTG
AATTCGTCACACCAGAATGTTATTAGAACCTCTGACGTCATTCTGGTGGTCCGCATCAATTGTCTCAT
ATCATTTAGTTAATG
+
FFFFFFFFFFFFFFFFFFFFFFFFFFFFFFFFFFFFFFFFFFFFFFFFFFFFFFFFFFFFFFFFFFFF
FFFFFFFFFFFFFFFFFFFFFFFFFFFFFFFFFFFFFFFFFFFFFFFFFFFFFFFFFFFFFFFFFFFF
FFFFFFFFFFFFFF:
@A00721:190:HGJW7DSXY:4:2153:8196:27868 1:N:0:TCTGCAAG+GCTGCACT
GCTGCTGGGAATGTTATGTATTCGACGATTCATGTTCGCTTTCGTTATTGTAGAACAATAAAGTTCTG
AATTCGTCACACCAGAATGTTATTAGAACCTCTGACGTCATTCTGGTGGTCCGCATCAATTGTCTCAT
ATCATTTAGTTAAT
```

```
+
FFFFFF:FFF:FFFFFFFFFFFFFFFFFFFFFFFFFFFFFFFFFFFFFFFFFFF:FFFFFFFFFFFFFF:
FFFFFFFFFFFFFF:FFFFFFFFFF:FFFFFFFFFFFFFFFFFFF:FFFFFFF:FFFFFFFFF,FFFF
FFFF:FFFFFFFFF
@A00721:190:HGJW7DSXY:4:2162:28583:10473 1:N:0:TCTGCAAG+GCTGCACT
CATTAACTAAATGATATGAGACAATTGATGCGGACCACCAGAATGACGTCAGAGGTTCTAATAACATT
CTGGTGTGACGAATTCAGAACTTTATTGTTCTACAATAACGAAAGCGAACATGAATCGTCGAATACAT
AACATTCCCAGCAGC
+
FFFFFFFFFFFFFFFFFFFFFFFFFFFFFFFFFFFFFFFFFFFFFFFFFFFFFFFFFFFFFFFFFFFF
FFFFFFFF,FFFFFFF,FFFFFFFFFFFF:FFFFFFFFFFFFFFFFF,FFFFFFFFFFFFFFFFFFFF
FFFFFFFFFFFFFFF
@A00721:190:HGJW7DSXY:4:2523:27661:35775 1:N:0:TCTGCAAG+GCTGCACT
GCTGCTGGGAATGTTATGTATTCGACGATTCATGTTCGCTTTCGTTATTGTAGAACAATAAAGTTCTG
AATTCGTCACACCAGAATGTTATTAGAACCTCTGACGTCATTCTGGTGGTCCGCATCAATTGTCTCAT
ATCATTTAGTTAATG
+
FFFFFFFFFFFFFFFFFFFFFFFFFFFFFFFFFFFFFFFFFFFFFFFFFFFFFFFFFFFFFFFFFFFF
FFFFFFFFFFFFFFFF:FFFFFFFFFFFFFFFFFFFFFFFFFFFFFFFFFFFFFFFFFFFFFFFFFFF
FFFFFFFFFFFFFFF
@A00721:190:HGJW7DSXY:4:2548:17481:1329 1:N:0:TCTGCAAG+GCTGCACT
GCTGCTGGGAATGTTATGTATTCGACGATTCATGTTCGCTTTCGTTATTGTAGAACAATAAAGTTCTG
AATTCGTCACACCAGAATGTTATTAGAACCTCTGACGTCATTCTGGTGGTCCGCATCAATTGTCTCAT
ATCATTTAGTTAAT
+
FFFFFFFFFFFFFFFFFFFFFFFFFFFFFFFFFFFFFFFFFFFFFFFFFFFFFFFFFFFFFFFFFFFF
FFFFFFFFFFFFFF:FFFFFFFFFFFFFFFFFFFFFFFFFFFFFFFFFFFFFFF:FFFFFFFFFFFFFF
:FFF:FFFFFFFFF
@A00721:190:HGJW7DSXY:4:2652:16839:32910 1:N:0:TCTGCAAG+GCTGCACT
GCTGCTGGGAATGTTATGTATTCGACGATTCATGTTCGCTTTCGTTATTGTAGAACAATAAAGTTCTG
AATTCGTCACACCAGAATGTTATTAGAACCTCTGACGTCATTCTGGTGGTCCGCATCAATTGTCTCAT
ATCATTTAGTTAATG
+
FFFFFFFFFF:FFFFFFFFFFFFFFFFFFFFFFFFFFFFFFFFFFFFFFFFFFFFFFFFFFFFFFFFF
FFFFFFFFFFFFFFFFFFFFFFFFFFFFFFFFFFFFFFFFFFFFFFFFFFFFFFFFFFFFFFFFFFFF
FFFFFFFFFFFFFFF
@A00721:190:HGJW7DSXY:4:2676:31385:26318 1:N:0:TCTGCAAG+GCTGCACT
GCTGCTGGGAATGTTATGTATTCGACGATTCATGTTCGCTTTCGTTATTGTAGAACAATAAAGTTCTG
AATTCGTCACACCAGAATGTTATTAGAACCTCTGACGTCATTCTGGTGGTCCGCATCAATTGTCTCAT
ATCATTTAGTTAAT
+
FFFFFFFFFF:FFFFFFFFFFFFFFFFFFFFFFFFFFFFFFFFFFFFFFFFFFFFFFFFFFF:FFFFFF
FFFFFFFFFFFFFFFFFFFFFFFFFFFFFFFFFFFFFFFFFFFFFFFFFFFFFFFFFFFFFFFFFFFF
FFFFFFFFFFFFFF
@A00721:190:HGJW7DSXY:4:1535:31548:6057 1:N:0:TCTGCAAG+GCTGCACT
CGCTGGGAATGTTATGTATTCGACGATTCATGTTCGCTTTCGTTATTGTAGAACAATAAAGTTCTGAA
TTCGTCACACCAGAATGTTATTAGAACCTCTGACGTCATTCTGGTGGTCCGCATCAATTGTCTCATAT
CATTTAGTTAATGAG
+
FFFFFFFFFFFFFFFFFFFFFFFFFFFFFFFFFFFFFFFFFFFFFFFFFFFFFFFFFFFFFFFFFFFF
FFFFFFFFFFFFFFFFFFFFFFFFF,FFFFFFFFFFFFFFFFFFFFFFFFFFFFFFFFFFFFFFFFFF
FFFFFFFFFFFFF,F
@A00721:190:HGJW7DSXY:4:2208:24795:7357 1:N:0:TCTGCAAG+GCTGCACT
CGCTGGGAATGTTATGTATTCGACGATTCATGTTCGCTTTCGTTATTGTAGAACAATAAAGTTCTGAA
```

```
TTCGTCACACCAGAATGTTATTAGAACCTCTGACGTCATTCTGGTGGTCCGCATCAATTGTCTCATAT
CATTTAGTTAATGAG
+
FFF::F:F,FFFFFFFFFFFF:FFFFFFFFF:FFFFFFFFFFFFFFFFFFFFFFFF:FFFFFFFFFFF
FF:FFFFFFFFF:FFF::FFFFFFFF,FFF,FF:FFFFF::FFFFFFFF:FFFFFFFFFFF:::FFF:
FFFFFF:FFFFFFFF
@A00721:190:HGJW7DSXY:4:2278:4842:29512 1:N:0:TCTGCAAG+GCTGCACT
CGCTGGGAATGTTATGTATTCGACGATTCATGTTCGCTTTCGTTATTGTAGAACAATAAAGTTCTGAA
TTCGTCACACCAGAATGTTATTAGAACCTCTGACGTCATTCTGGTGGTCCGCATCAATTGTCTCATAT
CATTTAGTTAATGAG
+
FFFFFFFFFFFFFFFFFFFFFFFFFFFFFFFFFFFFFFFFFFFFFFFFFFFFFFFFFFFFFFFFFFFF
FFFFFFFFFFFFFFFFFFFFFFFFFFFFFFFFFFFFFFFFFFFFFFFFFFFFFFFFFFFFFFFFFFFF
FFFFFFFFFFFFFFF
@A00721:190:HGJW7DSXY:4:1153:20509:6668 1:N:0:TCTGCAAG+GCTGCACT
GCTGGGAATGTTATGTATTCGACGATTCATGTTCGCTTTCGTTATTGTAGAACAATAAAGTTCTGAAT
TCGTCACACCAGAATGTTATTAGAACCTCTGACGTCATTCTGGTGGTCCGCATCAATTGTCTCATATC
ATTTAGTTAATGAGG
+
FFFFFFFFFFFFFFFFFFFFFFFFFFFFFFFFFFFFFFFFFFFFFFFFFFFFFFF,FFFFFFFFFFFFF
FFFFFFFFFFFFFFFFFFFFFFFFFFFFFFFFFFFFFFFFFFFFFFFFFFFFFFFFFFFFFFFFFFFF
FFFFFFFFFFFFFFF
@A00721:190:HGJW7DSXY:4:1266:23032:30608 1:N:0:TCTGCAAG+GCTGCACT
GCTGGGAATGTTATGTATTCGACGATTCATGTTCGCTTTCGTTATTGTAGAACAATAAAGTTCTGAAT
TCGTCACACCAGAATGTTATTAGAACCTCTGACGTCATTCTGGTGGTCCGCATCAATTGTCTCATATC
ATTTAGTTAATGAGG
+
FFFFFFFFFFFFFFFFFFFFFFFFFFFFFFFFFFFFFFFFFFFFFFFFFFFFFFFFFFFFFFFFFFFF
FFFFFFFFFFFFFFFFFFFFFFFFFFFFFFFFFFFFFFFFFFFFFFFFFFFFFFFFFFFFFFFFFFFF
F:FFFFFFFFFFFFF
@A00721:190:HGJW7DSXY:4:1409:26811:9095 1:N:0:TCTGCAAG+GCTGCACT
GCTGGGAATGTTATGTATTCGACGATTCATGTTCGCTTTCGTTATTGTAGAACAATAAAGTTCTGAAT
TCGTCACACCAGAATGTTATTAGAACCTCTGACGTCATTCTGGTGGTCCGCATCAATTGTCTCATATC
ATTTAGTTAATGAGG
+
FFFFFFFFFFFFFFFFFFFFFFFFFFFFFFFFFFFFFFFFFFFFFFFFFFFFFFFFFFFFFFFFFFFF
FFFFFFFFFFFFFFFFFFFFFFFFFFFFFFFFFFFFFFFFFFFFFFFFFFFFFFFFFFFFFFFFFFFF
FFFFFFFFFFFFFFF
@A00721:190:HGJW7DSXY:4:1418:25970:21167 1:N:0:TCTGCAAG+GCTGCACT
GCTGGGAATGTTATGTATTCGACGATTCATGTTCGCTTTCGTTATTGTAGAACAATAAAGTTCTGAAT
TCGTCACACCAGAATGTTATTAGAACCTCTGACGTCATTCTGGTGGTCCGCATCAATTGTCTCATATC
ATTTAGTTAATGAGG
+
FFFFFFFFFFFFFFFFFFFFFFFFFFFFFFFFFFFF:FFFFFFFFFFFFFFFFFFFFFFFFFFFFFFF
FFFFFFFFFFFFFFFFFFFFFFFFFFFFFFFFFFFFFFFFFFFFFFFFFFFFFFFFFFFFFFFFF:FF
FFFFF:FFFFFFFFF
@A00721:190:HGJW7DSXY:4:1445:17246:34585 1:N:0:TCTGCAAG+GCTGCACT
GCTGGGAATGTTATGTATTCGACGATTCATGTTCGCTTTCGTTATTGTAGAACAATAAAGTTCTGAAT
TCGTCACACCAGAATGTTATTAGAACCTCTGACGTCATTCTGGTGGTCCGCATCAATTGTCTCATATC
ATTTAGTTAATGAGG
+
FFFFFFFFFFFFFFFFFFFFFFFFFFFFFFFFFFFFFFFFFFFF:FFFFFFFF:FFFFFFFFFFFFFF
FFFFFFFFFFFFFFFFFFFFFFFFFFFFFFFFFFFFFFFFFFFFFFFFFFFFFFFFFFFFFFFFFFFF
FFFFFFFFFFFFFFF
```

```
@A00721:190:HGJW7DSXY:4:2310:9525:27946 1:N:0:TCTGCAAG+GCTGCACT
GCTGGGAATGTTATGTATTCGACGATTCATGTTCGCTTTCGTTATTGTAGAACAATAAAGTTCTGAAT
TCGTCACACCAGAATGTTATTAGAACCTCTGACGTCATTCTGGTGGTCCGCATCAATTGTCTCATATC
ATTTAGTTAATGAGG
+
FFFFFFFFFFFFFFFFFFFFFFFFFFFFFFFFFFFFFFFFFFFFFFFFFFFFFFFFFFFFFFFFFFFF
FFFFFFFFFF:FFFFFFFFFFFFFFFFFFFFFFFFFFFFFFFFFFFFFFFFFFFFFFFFFFF:FFFFF
FFFFFFFFFFFFFF:
@A00721:190:HGJW7DSXY:4:2365:5475:33458 1:N:0:TCTGCAAG+GCTGCACT
GCTGGGAATGTTATGTATTCGACGATTCATGTTCGCTTTCGTTATTGTAGAACAATAAAGTTCTGAAT
TCGTCACACCAGAATGTTATTAGAACCTCTGACGTCATTCTGGTGGTCCGCATCAATTGTCTCATATC
ATTTAGTTAATGAGG
+
FFFFFFF:FFFFFFFFFFFFFFFFFFFFFFFFFFFFFFFFFFFFFFFFFFFFFFFFFFFFFFFFFFFF
FFFFFFFFFFFFFFFFFFFFFFFFFFFFFFFFFFFFFFFFFFFFFFFFFFFFFFFFFFFFFFFFFFFF
F:FFF:FFFFFFFFF
@A00721:190:HGJW7DSXY:4:2372:19868:26506 1:N:0:TCTGCAAG+GCTGCACT
GCTGGGAATGTTATGTATTCGACGATTCATGTTCGCTTTCGTTATTGTAGAACAATAAAGTTCTGAAT
TCGTCACACCAGAATGTTATTAGAACCTCTGACGTCATTCTGGTGGTCCGCATCAATTGTCTCATATC
ATTTAGTTAATGAGG
+
FFFFFFFFFFFFFFFFFFFFFFFFFFFFFFFFFF:FFFFFFFFFFF:FFFFFFFFFFFFFFFFFFFFF
FFFFFFFFFFFFFFFFFFFFFFFFFFF:FFFFFFFFFFF:FFFFFFFFFFFFF:FFFF:FFFFFFFFF
FFFFFF:FFFFFFF:
@A00721:190:HGJW7DSXY:4:2411:29903:8876 1:N:0:TCTGCAAG+GCTGCACT
GCTGGGAATGTTATGTATTCGACGATTCATGTTCGCTTTCGTTATTGTAGAACAATAAAGTTCTGAAT
TCGTCACACCAGAATGTTATTAGAACCTCTGACGTCATTCTGGTGGTCCGCATCAATTGTCTCATATC
ATTTAGTTAATGAGG
+
FFFFFFF:FFFFFFFFFFFFFFFFFFFFFFFFFFFFFFFFFFFFFFFFF:FFFFFFFFFFFFF:FFFF
FFFFFFFFFF,FFFFFFFFFFFFFF:FFFFFFFFFFFFFFF:FF,FFFFFFFFFFFFFFFFFFF,FFF
FF:FF:FFFFFFFF:
@A00721:190:HGJW7DSXY:4:2674:29966:13213 1:N:0:TCTGCAAG+GCTGCACT
GCTGGGAATGTTATGTATTCGACGATTCATGTTCGCTTTCGTTATTGTAGAACAATAAAGTTCTGAAT
TCGTCACACCAGAATGTTATTAGAACCTCTGACGTCATTCTGGTGGTCCGCATCAATTGTCTCATATC
ATTTAGTTAATGAG
+
FF:FFFFFF:FFFFFFFFF:FFFFFFFFF:F:FFFFF:,FF:FFF,FF:FFFFFF,FF::FFF,FFF:
F:,FFFFFFFF,F,F:F:F:FF,FF:::F:FFFF,FFFF,::FF:,F:F:FF,FFF:FFF,FFF:FFF
FFFFFFFFFFFFFFF
@A00721:190:HGJW7DSXY:4:1135:28989:13808 1:N:0:TCTGCAAG+GCTGCACT
CTGGGAATGTTATGTATTCGACGATTCATGTTCGCTTTCGTTATTGTAGAACAATAAAGTTCTGAATT
CGTCACACCAGAATGTTATTAGAACCTCTGACGTCATTCTGGTGGTCCGCATCAATTGTATCATATAA
ATTAGTTAATTAGGA
+
FFFFFF:FFFFFFFFFFFFFFFFFFFFFFFFFFFFFFFFFFFFFFFFFFFFFFFFFFFFFFFFFFFFF
FFFFFFFFFFFFFFFFFFFFFFFFFFFFFF:FFFFFFFFFFFFFFFFFFFF,,FF:F,F,::F:F,,F
,FF,:FFF,,:,,::
@A00721:190:HGJW7DSXY:4:1271:2682:27054 1:N:0:TCTGCAAG+GCTGCACT
CTGGGAATGTTATGTATTCGACGATTCATGTTCGCTTTCGTTATTGTAGAACAATAAAGTTCTGAATT
CGTCACACCAGAATGTTATTAGAACCTCTGACGTCATTCTGGTGGTCCGCATCAATTGTCTCATATCA
TTTAGTTAATGAGGA
+
FFFFFFFFFFFFFFFFFFFFFFFFFFFFFFFFFFFFFFFFFFFFFFF,FFFFFFFFFFFFFFFFFFFF
```

FFFFFFFFFFFFFFFFFFFFFFFFFFFFFFFFFFFFFFFFFFFFFFFFFF:FFF:FFFF,FFFFF,F,,F
,FFF,FFFF,FF,FF
@A00721:190:HGJW7DSXY:4:1639:31087:14027 1:N:0:TCTGCAAG+GCTGCACT
CTGGGAATGTTATGTATTCGACGATTCATGTTCGCTTTCGTTATTGTAGAACAATAAAGTTCTGAATT
CGTCACACCAGAATGTTATTAGAACCTCTGACGTCATTCTGGTGGTCCGCATCAATTGTCTAATATAA
TTTAGTTAATTATG
+
FFFFFFFFFFFFFFFFFFFFFFFFFFFFFFFFFFFFFFFFFFFFFFFFFFFFFFFFFFFFFFFFFFFF
FFFFFFFFFFFFFFFFFFFFFFFFFFFFFFFFFFFFFFFFFFFFFFFF:FFF,,,FF,F,F,F,F,F:,:
FFF,FFF,FF:F,F
@A00721:190:HGJW7DSXY:4:2150:25943:25034 1:N:0:TCTGCAAG+GCTGCACT
CTGGGAATGTTATGTATTCGACGATTCATGTTCGCTTTCGTTATTGTAGAACAATAAAGTTCTGAATT
CGTCACACCAGAATGTTATTAGAACCTCTGACGTCATTCTGGTGGTCCGCAACAATTGTATCAAATAA
ATTATTTAATT
+
FFFFF:FFFFFFFFFFFFFFFFFFFFFFFFFFFFFFFFFFFFFFFFFFFFFFFFFFFFFFFFFFFFFF
FFFFFFFFFF:FFFFFFFFFF:FFFFFFFFFFFFFFFF::FFFFFFF:FF,,FF,F,F,,,F,F:,F
:,F:,FFFF,F
@A00721:190:HGJW7DSXY:4:2466:11550:8187 1:N:0:TCTGCAAG+GCTGCACT
CTGGGAATGTTATGTATTCGACGATTCATGTTCGCTTTCGTTATTGTAGAACAATAAAGTTCTGAATT
CGACACACCAGAATGTTATTAGAACCTCTGACGTCATTCTGGTGGTCCGCATCAATTGTCTCATATAA
TTTAGTTAATGAGG
+
FFFFFFFFFFFFFFFFFFFFFFFFFFFFFFFFFFFFFFFFFF:FFFFFFFFFFFFFFFFFFFFFFFFF
FFFFFFFFFFFFFFFFFFFFFFFFF:FFFFFFFFFFFFFFFFFFFFFFFFFFF,:FF,F:FF,FFFF:,F
,FFF,FF:F:,F,F
@A00721:190:HGJW7DSXY:4:1261:28031:26146 1:N:0:TCTGCAAG+GCTGCACT
TGGGAATGTTATGTATTCGACGATTCATGTTCGCTTTCGTTATTGTAGAACAATAAAGTTCTGAATTC
GTCACACCAGAATGTTATTAGAACCTCTGACGTCATTCTGGTGGTCCGCATCAATTGTCTCATATCAT
TTAGTTAATGAGGAT
+
FFFFF:FFFF:FFFFFFFFFFFFFFFFFF,FFFFFFFFFFFFFFFFFFFFFFFFFF:FFFFFFFFFFF
FFF:FFFFFFFFFFFFFFFFFFFF:FFFFFF:FFFFFFFFFFFFFFFFFFFF:FFFFFFFFFFFFFFF
FFFFFFFFFFFFFFF
@A00721:190:HGJW7DSXY:4:1362:11523:35509 1:N:0:TCTGCAAG+GCTGCACT
TGGGAATGTTATGTATTCGACGATTCATGTTCGCTTTCGTTATTGTAGAACAATAAAGTTCTGAATTC
GTCACACCAGAATGTTATTAGAACCTCTGACGTCATTCTGGTGGTCCGCATCAATTGTCTCATATCAT
TTAGTTAATGAGGAT
+
FF,FFFFFFFFFFFFFFFFFFFFFFFFFFFFFFFFFFFFFFFFFFFF:FFFFFFFFFFFFFFFFFFFF
FFFFFFFFFFFFFFFF:FFFFFFFFFFFFFFFFFF:FFFFFFFFFFFFFF:FFF,FFFFFFFFFFFFF
::FFF:FFFFFFFFF
@A00721:190:HGJW7DSXY:4:1367:29776:35775 1:N:0:TCTGCAAG+GCTGCACT
TGGGACTGTTATGTATTCGACGATTCATGTTCGCTTTCGTTATTGTAGAACAATAAAGTTCTGAATTC
GTCACACCAGAATGTTATTAGAACCTCTGACGTCATTCTGGTGGTCCGCATCAATTGTCTCATATCAT
TTAGTTAATGAGGAT
+
FFFFF,FFFFFFFFFFFFFFFFFFFFFFFF:,FFFFFFFFFFFFFFFF:FFFFF,FF:FF:FFFFFFFF
FFFFFFFF:FFFF:FFF:FF,:FFFFFFFFF:FFFF:FF:F,F:FFFFFFFFFFF::FFFFFFFFFF:
F,F,,FFFF:,:,:F
@A00721:190:HGJW7DSXY:4:1440:3604:6981 1:N:0:TCTGCAAG+GCTGCACT
TGGGAATGTTATGTATTCGACGATTCATGTTCGCTTTCGTTATTGTAGAACAATAAAGTTCTGAATTC
GTCACACCAGAATGTTATTAGAACCTCTGACGTCATTCTGGTGGTCCGCATCAATTGTCTCATATCAT
TTAGTTAATGAGGAT

+
FFFFF,FFFFFFFFFFFFFFFFFFFFFFFFFFFFFFFFFFFFFFFFFFFFFFFFFFFFFFFFFF
FFFFFFFFFFFFFFFFFFFFFFFFFFFFFFFFFFFFFFFFFFFFFFFFFFFFFFFFFFFFFFFFFFF:
FFFFFFFFFFFFFFF
@A00721:190:HGJW7DSXY:4:1510:10791:26506 1:N:0:TCTGCAAG+GCTGCACT
TGGGAATGTTATGTATTCGACGATTCATGTTCGCTTTCGTTATTGTAGAACAATAAAGTTCTGAATTC
GTCACACCAGAATGTTATTAGAACCTCTGACGTCATTCTGGTGGTCCGCATCAATTGTCTCATATCAT
TTAGTTAATGAGGAT
+
FFFFFFFFFFFFFFFFFFFFFFFFFFFFFFFFFFFFFFFFFFFFFFFFFFFFFFFFFFFFFFFFFFFF
FFFFFFFFFFFFFFFFF:FFFFFFFFFFFFFFFFFFFFFFF:FFFFFFFFFFFF,FFFFFFFFFFFF:
FFF:FFFFFFF:FFF
@A00721:190:HGJW7DSXY:4:1511:21034:24424 1:N:0:TCTGCAAG+GCTGCACT
TGGGAATGTTATGTATTCGACGATTCATGTTCGCTTTCGTTATTGTAGAACAATAAAGTTCTGAATTC
GTCACACCAGAATGTTATTAGAACCTCTGACGTCATTCTGGTGGTCCGCATCAATTGTCTCATATCAT
TTAGTTAATGAGGAT
+
FFFFFFFFFFFFFFFFFFFFFFFFFFFFFFFFFFFFFFFFFFFFFFFFFFFFFFFFFF:FFF:FFFFF
FFFFFFFFFFFFFFFFFFFFFFFFFFFFFFFFFFFFFFFFFFFFFFFFFFFFFFFFFFFFFFFFFFFF
FFFFFFFFFF:FFFF
@A00721:190:HGJW7DSXY:4:1529:30147:9706 1:N:0:TCTGCAAG+GCTGCACT
TGGGAATGTTATGTATTCGACGATTCATGTTCGCTTTCGTTATTGTAGAACAATAAAGTTCTGAATTC
GTCACACCAGAATGTTATTAGAACCTCTGACGTCATTCTGGTGGTCCGCATCAATTGTCTCATATCAT
TTAGTTAATGAGGAT
+
FFFFFFFFFFFFFFFFFFFFFFFFFFFFFFFFFFFFFFFFFFFFFFFFFFFFFFFF:FFFFF:FFFFF
FFFFFFFFF:FFFFFFFFFFFFF,FFFFFFFFFFFFFFFFFFF:FFFFFFFFFFFFFFFFFFFFFFFF
FFFFFFFFFFFFFFF
@A00721:190:HGJW7DSXY:4:1630:1931:22435 1:N:0:TCTGCAAG+GCTGCACT
TGGGAATGTTATGTATTCGACGATTCATGTTCGCTTTCGTTATTGTAGAACAATAAAGTTCTGAATTC
GTCACACCAGAATGTTATTAGAACCTCTGACGTCATTCTGGTGGTCCGCATCAATTGTCTCATATCAT
TTAGTTAATGAGGAT
+
FFFFFFFFFFFFFFFFFFFFFFFFFFFFFFFFFFFFFFFFFFFFFFFFFFFFFFFFFFFFFFFFFFFF
FFFFFFFFFFFFFFFFFFFF:FFFFFFF:FFFF:FFF:FFFFFFFF,FFFFFFFFFFFFFFFFF:FFF
FFF,FFFFFFFFFFF
@A00721:190:HGJW7DSXY:4:1661:16532:15342 1:N:0:TCTGCAAG+GCTGCACT
TGGGAATGTTATGTATTCGACGATTCATGTTCGCTTTCGTTATTGTAGAACAATAAAGTTCTGAATTC
GTCACACCAGAATGTTATTAGAACCTCTGACGTCATTCTGGTGGTCCGCATCAATTGTCTCATATCAT
TTAGTTAATGAGGAT
+
FFFFF:FFFFFFFFFFFFFFFFFFFFFFFFFFFFFFFFFFFF,FFFFFFFFFFFFFFFFFFFFFFFFF
FFFFFFFFFFFFFFFFFFF:FFFFFFFFFFFFFFFFFFFFFFFFFFFFFFFFFFFFFFFFFFFFFFFF
FFFFF:FFFFF:FFF
@A00721:190:HGJW7DSXY:4:2106:1768:19523 1:N:0:TCTGCAAG+GCTGCACT
TGGGAATGTTATGTATTCGACGATTCATGTTCGCTTTCGTTATTGTAGAACAATAAAGTTCTGAATTC
GTCACACCAGAATGTTATTAGAACCTCTGACGTCATTCTGGTGGTCCGCATCAATTGTCTCATATCAT
TTAGTTAATGAGGAT
+
FFFFFFFFFFFFFFFFFFFFFFFFFFFFFFFFFFFFF:FFFFFFFFF,FFFFFFFFF:FFFFFFFFF:
FFFF:FFFF:FFFFFFFFFFFFFFFFFFFFFFFFFFFFFFFFFFFFFFF:,FF,FFFFFFFF:FF:F
FFFFFFFFFFFFFFF
@A00721:190:HGJW7DSXY:4:2242:28167:14450 1:N:0:TCTGCAAG+GCTGCACT
TGGGAATGTTATGTATTCGACGATTCATGTTCGCTTTCGTTATTGTAGAACAATAAAGTTCTGAATTC

```
GTCACACCAGAATGTTATTAGAACCTCTGACGTCATTCTGGTGGTCCGCATCAATTGTCTCATATCAT
TTAGTTAATGAGGAT
+
FFFFFFFFFFFFFFFFFFFFFFFFFFFFFFFFFFFFFFFF:FFFFFFFFFFF:FFFFFFFFFFFFFFFFF
FFFFFFFFFFFFFFFFFFFFFF:FFFFFFFFFFFFF:FFFFF:FFFFFFFFFFFF:FFFFFFFFFFFFFF
FFFFFFFFFF:F:FF
@A00721:190:HGJW7DSXY:4:2342:28330:16266 1:N:0:TCTGCAAG+GCTGCACT
TGGGAATGTTATGTATTCGACGATTCATGTTCGCTTTCGTTATTGTAGAACAATAAAGTTCTGAATTC
GTCACACCAGAATGTTATTAGAACCTCTGACGTCATTCTGGTGGTCCGCATCAATTGTCTCATATCAT
TTAGTTAATGAGGAT
+
FFFFFFFFFFFFFFFFFFFFFFFFFFFFFFFFFFFFFFFFFFFFFFFFFFFFFFFFFFFFFFFFFFFFFF
FFFFFFFFFFFFFFFFFFFFFFFFFFFFFFFFFFFFFFFFFFFFFFFFFFFFFFFFFFFFFFFFFFFFFF
FFFFFFFFFFFFFFF
@A00721:190:HGJW7DSXY:4:2420:31629:15718 1:N:0:TCTGCAAG+GCTGCACT
TGGGAATGTTATGTATTCGACGATTCATGTTCGCTTTCGTTATTGTAGAACAATAAAGTTCTGAATTC
GTCACACCAGAATGTTATTAGAACCTCTGACGTCATTCTGGTGGTCCGCATCAATTGTCTCATATCAT
TTAGTTAATGAGGAT
+
FFFFFFFFFFFFFFFFFFFFFFFFFFFFFFFFFFFFFFFFFFFFFFFFFFFFFFFFFFFFFFFFFFFFFF
FFFFFFFFFFFFFFFFFFFFFFFF:FFFFFFFFFFFFFFFFFFFFFFFFFFFFFFFFFFFFFFFFFFFF
FFFFFFFFF,FFFFF
@A00721:190:HGJW7DSXY:4:2420:30626:17206 1:N:0:TCTGCAAG+GCTGCACT
TGGGAATGTTATGTATTCGACGATTCATGTTCGCTTTCGTTATTGTAGAACAATAAAGTTCTGAATTC
GTCACACCAGAATGTTATTAGAACCTCTGACGTCATTCTGGTGGTCCGCATCAATTGTCTCATATCAT
TTAGTTAATGAGGAT
+
FFFFFFFFFFFFFFFFFFFFFFFFFF:FF:FFFFFFFFFFFFFFFFFFF:FFFFFFFFFFFFFFF:FFFF
FFFFFFFFFF:FFFFFFFFFFFFFFFFFFFFFFF,FFFFFFFFFFFFFFFFFFFFFFFFFFFFFFFFFFF
FF:FFFFFFFFFFFF
@A00721:190:HGJW7DSXY:4:2551:4553:8406 1:N:0:TCTGCAAG+GCTGCACT
TGGGAATGTTATGTATTCGACGATTCATGTTCGCTTTCGTTATTGTAGAACAATAAAGTTCTGAATTC
GTCACACCAGAATGTTATTAGAACCTCTGACGTCATTCTGGTGGTCCGCATCAATTGTCTCATATCAT
TTAGTTAATGAGGAT
+
FFFFFFFFFFFFFFFFFFFFFFFFFFFFFFFFFFFFFFFFFFFFFFFFFFFFFFFFFFFFFFFFFFFFFF
F,FFFFFFFFFFFF:FFFFFFFFFFFFFFFFFFFFFFFFFFFFFFFFFFFFFFFFFFFFFFFFFFFFFF
FFFFFFFFFFFFFFF
@A00721:190:HGJW7DSXY:4:2609:25400:25723 1:N:0:TCTGCAAG+GCTGCACT
TGGGAATGTTATGTATTCGACGATTCATGTTCGCTTTCGTTATTGTAGAACAATAAAGTTCTGAATTC
GTCACACCAGAATGTTATTAGAACCTCTGACGTCATTCTGGTGGTCCGCATCAATTGTCTCATATCAT
TTAGTTAATGAGGAT
+
FFFFFFFFFFFFFFFFFFFFFFFFFFFFFFFFFFFFFFFFFFFFFFFFF:FFFFFFFFFFFFFFFFFFF:
FFFFFFFFFFFFFFFFFFFFFFFFF:F::FFFFFFFFFF:FFFFFFFFFFFFFFFFFFFFFFFFFFFFFF
FFFFFFFFF:FFFFF
@A00721:190:HGJW7DSXY:4:2642:4571:28761 1:N:0:TCTGCAAG+GCTGCACT
TGGGAATGTTATGTATTCGACGATTCATGTTCGCTTTCGTTATTGTAGAACAATAAAGTTCTGAATTC
GTCACACCAGAATGTTATTAGAACCTCTGACGTCATTCTGGTGGTCCGCATCAATTGTCTCATATCAT
TTAGTTAATGAGGAT
+
FFFFFF,FFFFFFFFFFFFFFFFFFFFFFFFFFFFFFFFFFFFFFFFFFFFFFFFFFFFFFFFFFFFFFF
FFFFFFFFFFFFFFFFFFFFFFFFFFFFFFFFFFFFFFFFFFFFFFFFFFFFFFFFFFFFFFFFFFFFFF
F:FFFFFFFFFFFFF
```

@A00721:190:HGJW7DSXY:4:2675:26232:30452 1:N:0:TCTGCAAG+GCTGCACT
TGGGAATGTTATGTATTCGACGATTCATGTTCGCTTTCGTTATTGTAGAACAATAAAGTTCTGAATTC
GTCACACCAGAATGTTATTAGAACCTCTGACGTCATTCTGGTGGTCCGCATCAATTGTCTCATATCAT
TTAGTTAATGAGGAT
+
FFFFFFFFFFFFFFFFFFFFFFFFFFFFFFFFFFFFFFFFFFFFFFFFFFFFFFFFFFFFFFFFFFFFFF
FFFFFFFFFFFFFFFFFFFFFFFFFFFFFFFFFFFFFFFFFFFFFFFFFFFFFFFFFF:FFFFFFFFFFF
FFFFFFFFFFF,FFF
@A00721:190:HGJW7DSXY:4:1412:8413:1720 1:N:0:TCTGCAAG+GCTGCACT
ATGTATTCGACGATTCATGTTCGCTTTCGTTATTGTAGAACAATAAAGTTCTGAATTCGTCACACCAG
AATGTTATTAGAACCTCTGACGTCATTCTGGTGGTCCGCATCAATTGTCTCATATCATTTAGTTAATG
AGGATAATATTTATC
+
FFFFFFFFFFFFFFFFFFFFFFFFFFFFFFFFFFFFFFFFFFFFFFFFFFFFFFFFFFFFFFFFFFFFFF
FFFFFFFFFFFFFFFFFFFFFFFFFFFFFFFFFFFFFFFFFFFFFFFFFFFFFFFFFFFFFFFFFFFFFF
FFFFFFFFFFFFFFF
@A00721:190:HGJW7DSXY:4:2452:17598:7576 1:N:0:TCTGCAAG+GCTGCACT
ATGTATTCGACGATTCATGTTCGCTTTCGTTATTGTAGAACAATAAAGTTCTGAATTCGTCACACCAG
AATGTTATTAGAACCTCTGACGTCATTCTGGTGGTCCGCATCAATTGTCTCATATCATTTAGTTAATG
AGGATAATATTTATC
+
FFFFFFFFFFFFFFFFFFFFFFFFFFFFFFFFFFFFFFFFFFFFFFFFFFFFF:FFFFFFFFFFFFFFFF
FFFFFFFFFFFFFFFFFF:FFFFFFFFFFFFFFFFF:FFFFFFFFFFFFFFFFFFFF:FFFFFFFFFFFF
FF:FFFFFFFF:FFF
@A00721:190:HGJW7DSXY:4:1336:14733:8876 1:N:0:TCTGCAAG+GCTGCACT
CGACGATTCATGTTCGCTTTCGTTATTGTAGAACAATAAAGTTCTGAATTCGTCACACCAGAATGTTA
TTAGAACCTCTGACGTCATTCTGGTGGTCCGCATCAATTGTCTCATATCATTTAGTTAATGAGGATAA
TATTTATCCTTGATG
+
FFFFFFF:FFFFFFFFFFFFFFFFFFFFFFFFFFFFFFFFFFFFFFFFFFFFFFFFFFFFFFFFFFFFFF
FFFFFFFFFFFFFFFFFFFFFFFFFFFFFFFFFFFFFFFFFFFFFFFFFFFFFFFFFFFFFFFFFFFFFF
FFFF:FFFFFFFFF:
@A00721:190:HGJW7DSXY:4:1512:13313:27054 1:N:0:TCTGCAAG+GCTGCACT
CGACGATTCATGTTCGCTTTCGTTATTGTAGAACAATAAAGTTCTGAATTCGTCACACCAGAAATTTA
TTAGAACCTCTGACGTCATTCTGGTGGTCCGCATCAAATGTCTCATATCATTTAGTTAATGAGGATAA
TATTTATCCTTGATG
+
FFFFF:,F:F,FFFF:FF:FF,,:F,,,:F,FFFFFFFFFFFFF:,FF,,,,F:,F:F,FFFF,,FFF
FFF,FF,FF:F,FF,FFFF,FFF,F,,,F,FFF,:FF:F,,,:FF:F:,F,:,F,,,,F,,F,FF,:F
F:F,,FFFF,F,F:F
@A00721:190:HGJW7DSXY:4:2331:28221:3333 1:N:0:TCTGCAAG+GCTGCACT
CGACGATTCATGTTCGCTTTCGTTATTGTAGAACAATAAAGTTCTGAATTCGTCACACCAGAATGTTA
TTAGAACCTCTGACGTCATTCTGGTGGTCCGCATCAATTGTCTCATATCATTTAGTTAATGAGGATAA
TATTTATCCTTGATG
+
FFFFFFFFFFFFFFF:FFFF:FFFFFFFFFFFFFFFFFFFFFFFFFFFFFFFF:FFFFFF,FFFF::FFF
,FFFFFFFFFFFFFFFFFF:FFF:FFFFF:F:FF:FFFFFFFFFF:F:FF,FF:FF,FFFFF,FFFFFFF
FFF,:F:F,FFFFFF
@A00721:190:HGJW7DSXY:4:2549:2094:11443 1:N:0:TCTGCAAG+GCTGCACT
CGACGATTCATGTTCGCTTTCGTTATTGTAGAACAATAAAGTTCTGAATTCGTCACACCAGAATGTTA
TTAGAACCTCTGACGTCATTCTGGTGGTCCGCATCAATTGTCTCATATCATTTAGTTAATGAGGATAA
TATTTATCCTTGATG
+
FFFFFFFFFFFFFFFFFFFFFFFFFFFFFFFFFFFFFFFFFFFFFFFFFFFFFFFFFFFFFFFFFFFFFF

FFFFFFFFFFFFFFFFFFFFFFFFFFFFFFFFFFFFFFFFFFFFFFFFFFFFFFF:FFFFFFFFFFFF
FFFFFFFFFFFFFFF
@A00721:190:HGJW7DSXY:4:1254:12002:24377 1:N:0:TCTGCAAG+GCTGCACT
GATTCATGTTCGCTTTCGTTATTGTAGAACAATAAAGTTCTGAATTCGTCACACCAGAATGTTATTAG
AACCTCTGACGTCATTCTGGTGGTCCGCATCAATTGTCTCATATCATTTAGTTAATGAGGATAATATT
TATCCTTGATGATAT
+
FFFFFFFFFFFFFFFFFFFFFFFFFFFFFFFFFFFFFFFFFFFFFFFFFFFFFFFFFFFFFFFFFFFF
FFFFFF:FFFFFF:FFFFFFFFFFFFFFFFFFFFFFFFFFFFFFFF:FFFFFFFFFFFFFFFFFFFFF
:FFFFFFFF:FFFFF
@A00721:190:HGJW7DSXY:4:1261:19307:15170 1:N:0:TCTGCAAG+GCTGCACT
GATTCATGTTCGCTTTCGTTATTGTAGAACAATAAAGTTCTGAATTCGTCACACCAGAATGTTATTAG
AACCTCTGACGTCATTCTGGTGGTCCGCATCAATTGTCTCATATCATTTAGTTAATGAGGATAATATT
TATCCTTGATGATAT
+
FFFFFFFFFFFFFFFFFFFFFFFFFFFFFFFFFFFFFFFFFFFFFFFFFFFFFFFFFFFFFFFFFFF:
FFFFFFFFFFFFFFFFFFFFFFFFF:FFFFFFFFFFFFFFFFFFFFFFFFFFFFFFFF:FFFFFFFFF
FFFF,FFFFFFFFFF
@A00721:190:HGJW7DSXY:4:1434:8612:5572 1:N:0:TCTGCAAG+GCTGCACT
GATTCATGTTCGCTTTCGTTATTGTAGAACAATAAAGTTCTGAATTCGTCACACCAGAATGTTATTAG
AACCTCTGACGTCATTCTGGTGGTCCGCATCAATTGTCTCATATCATTTAGTTAATGAGGATAATATT
TATCCTTGATGATAT
+
FFFFFFFFFFFFFFFFFFFFFFFFFFFFFFFFFFFFFFFFFFFFFFFFFFFFFFFFFFFFFFFFFFFF
FFFFFFFFFFFFFFFFFFFFFFFFFFFFFFFFFFFFFFFFFFFFFFFFFFFFFFFF:F:FFFFFFFFF
FFFF:FFFF:FFFFF
@A00721:190:HGJW7DSXY:4:2319:3468:18552 1:N:0:TCTGCAAG+GCTGCACT
GATTCATGTTCGCTTTCGTTATTGTAGAACAATAAAGTTCTGAATTCGTCACACCAGAATGTTATTAG
AACCTCTGACGTCATTCTGGTGGTCCGCATCAATTGTCTCATATCATTTAGTTAATGAGGATAATATT
TATCCTTGATGATAT
+
FFFFFFFFFFFFFFFFFFFFFFFFFFFFFFFFFFFFFFFFFFFFFF:FFFFFFFFFFFFFFFFFFFF:
FFFFFFF:FFFFFFFFFFFFFFFFFFFFFFFFFFFFFFFFFFFFFFFFFFFFFFFFFFF:FFFFFFFF
FFFF,FFFFFFFFF:
@A00721:190:HGJW7DSXY:4:2418:14326:12179 1:N:0:TCTGCAAG+GCTGCACT
GATTCATGTTCGCTTTCGTTATTGTAGAACAATAAAGTTCTGAATTCGTCACACCAGAATGTTATTAG
AACCTCTGACGTCATTCTGGTGGTCCGCATCAATTGTCTCATATCATTTAGTTAATGAGGATAATATT
TATCCTTGATGATAT
+
FF:FFFFFFFFFFFFFFFFFFFF:FFFFFFFFFFFF:FFFFFFFFFFFFFFFFF:F:FFFFFFFFFFF
FFFFFFFFFFFF::FFFFFFFF:FFFFFFFFFFFFFFFFFFFFFFF:F,FFFFFFFFFFFFFFFFFFFFF
FFF,FFF:FFFFFFF
@A00721:190:HGJW7DSXY:4:2450:17626:33520 1:N:0:TCTGCAAG+GCTGCACT
GATTCATGTTCGCTTTCGTTATTGTAGAACAATAAAGTTCTGAATTCGTCACACCAGAATGTTATTAT
AACCTCTGACGTCATTCTTGTGGTCCGCATCAATTGTCTCATATCATTTAGTTAATGAGGATAATATT
TATCCTTGATGATAT
+
FFFFFFF,:FFFFFF:F,F:FFF,FF,FFFFF:F:FF:,FF,FFFF,::FFFF,,F:FFF,FFFFFF,
FFFFFF:F:FF:FFF,FF,,:F:FFF::FFFF::F,,:F:::FF,FFFFFFFFFFF:FF:FFFF,FFF
FFF,FFF:FF:F,FF
@A00721:190:HGJW7DSXY:4:1146:19651:22153 1:N:0:TCTGCAAG+GCTGCACT
GTTCGCTTTCGTTATTGTAGAACAATAAAGTTCTGAATTCGTCACACCAGAATGTTATTAGAACCTCT
GACGTCATTCTGGTGGTCCGCATCAATTGTCTCATATCATTTAGTTAATGAGGATAATATTTATCCTT
GATGATATGTTTGAT

```
+
FFFFFFFFFFFFFFFFFFFFFFFFFFFFFFFFFFFFFFFFFFFFFFFFFFFFFFFFFFFFFFFFFFFF
FFFFFFFF:FFFFFFFFFFFFFFFFFFFFFFFFFFFFFFFFFFFFFFFFFFFFFFFFFFFFFFFF:FF
FFFFFFFF:FFFFFF
@A00721:190:HGJW7DSXY:4:1152:26449:18552 1:N:0:TCTGCAAG+GCTGCACT
GTTCGCTTTCGTTATTGTAGAACAATAAAGTTCTGAATTCGTCACACCAGAATGTTATTAGAACCTCT
GACGTCAGTCTGGTGGTCCGCATCAATTGTCTCATATCATTTAGTTAATGAGGATAATATTTATCCTT
GATGATATGTTTGAT
+
FFFFFFFFFFFFFFFFFFFFFFFFFFFFFFFFFFFFFFFFFFFFFFFFFFFFFFFFFFFFFFFFFFFF
FFFFFFFF,FFFFFFFFFFFFFFFFFFFFFFFFFFFFFFFFFFFFFFFFFFFFFFFFFFFFFFFFFFF
FFFFFF:FFFFFFFF
@A00721:190:HGJW7DSXY:4:1159:28926:7905 1:N:0:TCTGCAAG+GCTGCACT
GTTCGCTTTCGTTATTGTAGAACAATAAAGTTCTGAATTCGTCACACCAGAATGTTATTAGAACCTCT
GACGTCATTCTGGTGGTCCGCATCAATTGTCTCATATCATTTAGTTAATGAGGATAATATTTATCCTT
GATGATATGTTTAGA
+
FFFFFFFFFFFFFFFFFFFFFFFFFFFFFFFFFFFFFFFFFFFFFFFFFFFFFFFFFFFFFFFFFFFF
FFFFFFFFFFFFFFFFFFFFFFFFFFFFFFFFFFFFFFFFFFFFFFFFFFFFFFFFFFFFFFFFFFFF
FFFFFFFFFFFFFF:
@A00721:190:HGJW7DSXY:4:1268:15176:1344 1:N:0:TCTGCAAG+GCTGCACT
GTTCGCTTTCGTTATTGTAGAACAATAAAGTTCTGAATTCGTCACACCAGAATGTTATTAGAACCTCT
GACGTCATTCTGGTGGTCCGCATCAATTGTCTCATATCATTTAGTTAATGAGGATAATATTTATCCTT
GATGATATGTTTGAT
+
FFFFFFFFFFFFFFFFFFFFFFFFFFFFFFFFFFFFFFFFFFFFFFFFFFFFFF:FFFFFFFFFFFFF
FFFFFFFFFFFFFFFFFFFFFFFF:FFFFFFFFFFFFFFFFFFFFF,FFFFFFFFFFFFFFFFFF:,F
FFFFFFFFFFFFFFF
@A00721:190:HGJW7DSXY:4:1328:15709:6903 1:N:0:TCTGCAAG+GCTGCACT
GTTCGCTTTCGTTATTGTAGAACAATAAAGTTCTGAATTCGTCACACCAGAATGTTATTAGAACCTCT
GACGTCATTCTGGTGGTCCGCATCAATTGTCTCATATCATTTAGTTAATGAGGATAATATTTATCCTT
GATGATATGTTTGAT
+
FFFFFFFFFFFFFFFFFFFFFFFFFFFFFFFFFFFFFFFFFFFFFFFFFFFFFFFFFFFFFFFFFFFF
FFFFFFFFFFFFFFFFFFFFFFFFFFFFFFFFFFFFFFF,FFFFFFFFFFFFFFFFFFFFFFFF:FF:
FF,FFFFFFFFFFFF
@A00721:190:HGJW7DSXY:4:1401:25355:8891 1:N:0:TCTGCAAG+GCTGCACT
GTTCGCTTTCGTTATTGTAGAACAATAAAGTTCTGAATTCGTCACACCAGAATGTTATTAGAACCTCT
GACGTCATTCTGGTGGTCCGCATCAATTGTCTCATATCATTTAGTTAATGAGGATAATATTTATCCTT
GATGATATGTTTAG
+
FFFFFFFFFFFFFFFFFFFFFFFFFFFFFFFFFFFFFFFF:FFFFFFF:FFFFFFFFFFFFFFFFFFF
FF:FFFFFFFFFFFFFF:FFFFFFFF:F:FFFFFFFFFFFFFF,FFFFFFFFFFFFFFFFF:FFF,FF
FFFFFFFFFF:FFF
@A00721:190:HGJW7DSXY:4:1435:26458:32002 1:N:0:TCTGCAAG+GCTGCACT
GTTCGCTTTCGTTATTGTAGAACAATAAAGTTCTGAATTCGTCACACCAGAATGTTATTAGAACCTCT
GACGTCATTCTGGTGGTCCGCATCAATTGTCTCATATCATTTAGTTAATGAGGATAATATTTATCCTT
GATGATATGTTTAGA
+
FFFFFFFFFFFFFFFFFFFFF:FFFFFFFFFFFFFFFFFFFFFFFFFFFFFFFFFFFFFFFFFFFFFF
FFFFFFFFFFFFFFFFFFFFFFFFFFFFFFFFFFFFFFFFFFFFFFFFFFFF:FFFFFFFFFFF,:FF
:FF:FFFF,FFFFFF
@A00721:190:HGJW7DSXY:4:1660:3305:18959 1:N:0:TCTGCAAG+GCTGCACT
GTTCGCTTTCGTTATTGTAGAACAATAAAGTTCTGAATTCGTCACACCAGAATGTTATTAGAACCTCT
```

GACGTCATTCTGGTGGTCCGCATCAATTGTCTCATATCATTTAGTTAATGAGGATAATATTTATCCTT
GATGATATGTTTGAT
+
FFFFFFFFFFFFFFFFFFFFFFFFFFFFFFFFFFFFFFFFFFFFFFFFFFFFFFFFFFFFFFFFFFFFFFF
FFFFFFFFFFFFFFFFFFFFFFFFFFFFFFFFFFFFFFFFFFFFFFFFFFFFFFFFFFFFFFFFFFFFFFF
FFFFFFFFFFFFFFF
@A00721:190:HGJW7DSXY:4:2233:11234:29590 1:N:0:TCTGCAAG+GCTGCACT
GTTCGCTTTCGTTATTGTAGAACAATAAAGTTCTGAATTCGTCACACCAGAATGTTATTAGAACCTCT
GACGTCATTCTGGTGGTCCGCATCAATTGTCTCATATCATTTAGTTAATGAGGATAATATTTATCCTT
GATGATATGTTTGAT
+
FFFFFFFFFFFFFFFFFFFFFFFFFFFFFFFFFFFFFFFFFFFFFFFFFFFFFFFFFFFFFFFFFFFFFFF
FFFFFFFFFFFFFFFFFFFFFFFFFFFFFFFFFFF:FFF:FFFFFFFFFFFFFFFFFFFFFFFFFFFFFFF
FFFFFFFFFFFFF,F
@A00721:190:HGJW7DSXY:4:2260:4453:23829 1:N:0:TCTGCAAG+GCTGCACT
GTTCGCTTTCGTTATTGTAGAACAATAAAGTTCTGAATTCGTCACACCAGAATGTTATTAGAACCTCT
GACGTCATTCTGGTGGTCCGCATCAATTGTCTCATATCATTTAGTTAATGAGGATAATATTTATCCTT
GATGATATGTTTAGA
+
FFFFFFFFFFFFFFFFFFFFFFFFFFFFFFFFFFFFFFFFFFFFFFFFFFFFFFFFFFFFFFFFFFFFFFF
FFFFFFFFFFFFFFFFFFFFFF:FFFFFFFFFFFFFFFF:FFFF:FFFFFFFFFFFFFFFFFFFFFFFFFF
FFFFFFFFFFFFFF:
@A00721:190:HGJW7DSXY:4:2528:14154:21026 1:N:0:TCTGCAAG+GCTGCACT
GTTCGCTTTCGTTATTGTAGAACAATAAAGTTCTGAATTCGTCACACCAGAATGTTATTAGAACCTCT
GACGTCATTCTGGTGGTCCGCATCAATTGTCTCATATCATTTAGTTAATGAGGATAATATTTATCCTT
GATGATATGTTTGAT
+
FFFFFFFFFFFFFFFFFFFFFFFFFFFFFFFFFFFFFFFFFFFFFFFFFFF:FFFFFFFFFFFFFFFFFFF
FFFFFF:FFFFFFFFFFFFFFFFFFFFFFFFFFFFFFFFFF:FFFFFFFFFFFFFFFFFFFFFFFFFFFFF
FFFFFFFF,FFFFFF
@A00721:190:HGJW7DSXY:4:1125:27651:35164 1:N:0:TCTGCAAG+GCTGCACT
GCTTTCGTTATTGTAGAACAATAAAGTTCTGAATTCGTCACACCAGAATGTTATTAGAACCTCTGACG
TCATTCTGGTGGTCCGCATCAATTGTCTCATATCATTTAGTTAATGAGGATAATATTTATCCTTGATG
ATATGTTTGATTTCA
+
FFFFFFFFFFFFFFFFFFFFFFFFF:FFFFFFFFFFFFFFFFFFFFFFFFFFFFFFFFFFFFFFFFF:F:F
FFFFFFFFFFFFFFFFFF:FFFFFFFFFFFFFFFFFF:F::FFFF,FFFFFFFFFFFFFFFFFFFFFFFFF
FFFFFFFFFFFFFFF
@A00721:190:HGJW7DSXY:4:2474:13919:24283 1:N:0:TCTGCAAG+GCTGCACT
GCTTTCGTTATTGTAGAACAATAAAGTTCTGAATTCGTCACACCAGAATGTTATTAGAACCTCTGACG
TCATTCTGGTGGTCCGCATCAATTGTCTCATATCATTTAGTTAATGAGGATAATATTTATCCTTGATG
ATATGTTTGATTTCA
+
FFFFFFFFFFFFFFFFFFFFFFFFFFFFFFFFFFFFFFFFFFFFFFFFFFFFFFFFFFFFFFFFFFFFFFF
FFFFFFFFFFFFFFFFFFFFFFFFFFFFFFFFFFFFFFFFFFFFFFFFFFFFFFFFFFFFFFFF:FFFFFF
F:FFFFFFFFFFFFF
@A00721:190:HGJW7DSXY:4:2545:25880:10269 1:N:0:TCTGCAAG+GCTGCACT
GCTTTCGTTATTGTAGAACAATAAAGTTCTGAATTCGTCACACCAGAATGTTATTAGAACCTCTGACG
TCATTCTGGTGGTCCGCATCAATTGTCTCATATCATTTAGTTAATGAGGATAATATTTATCCTTGATG
ATATGTTTGATTTCA
+
FFFFFFFFFFFFFFFFFFFFFFFFFFFFFFFFFFFFFFFFFFFFFFFFFFFFFFFFFFFFFFFFFFF:FFF
FFFFFFFFFFFFFFFFFFFFFFFFFFFFFFFFFF:FFFFFFFFFFFFFFFFFFFFFFFFFFFFF:FFF:FF
FFFFFFFFFFFFFFF

```
@A00721:190:HGJW7DSXY:4:2227:15564:20181 1:N:0:TCTGCAAG+GCTGCACT
CGTTATTGTAGAACAATAAAGTTCTGAATTCGTCACACCAGAATGTTATTAGAACCTCTGACGTCATT
CTGGTGGTCCGCATCAATTGTCTCATATCATTTAGTTAATGAGGATAATATTTATCCTTGATGATATG
TTTGATAGATCGGAA
+
FFFFFFFFFFFFFFFFFFFFFFFFFFFFFFFFFFFFFFFFFFFFFFFFFFFFFFFFFFF:FFFFFFFF
FFFFFFFFFFFFFFFFFFFFFFFFFFFFFFF:FFFFFFFFFFFFFFFFFFFFFFFFFFFFFFFFFFFF
FFFFFFFFFFFFFFF
@A00721:190:HGJW7DSXY:4:2530:12789:7169 1:N:0:TCTGCAAG+GCTGCACT
CGTTATTGTAGAACAATAAAGTTCTGAATTCGTCACACCAGAATGTTATTAGAACCTCTGACGTCATT
CTGGTGGTCCGCATCAATTGTCTCATATCATTTAGTTAATGAGGATAATATTTATCCTTGATGATATG
TTTGATAGATCGGAA
+
FFFFFFFFFFFFFFFFFFFFFFFFFFFFFFFFFFFFFFFFFFFFFFFFFFFFFFFFFFFFFFFFFFFF
FFFFFFFFFFFFFFFFFFFFFFFFFFFFFFFFFFFFFFFFFFFFFFFFFFFFFFF:FFFFFFFFFFF
FFFFFFFFFFFFFFF
@A00721:190:HGJW7DSXY:4:1517:25382:25598 1:N:0:TCTGCAAG+GCTGCACT
CACCAGAATGTTATTAGAACCTCTGACGTCATTCTGGTGGTCCGCATCAATTGTCTCATATCATTTAG
TTAATGAGGATAATATTTATCCTTGATGATATGTTTGATTTCATCGGGAAGAGATGAAAGTAAATTTC
TGAATATGATGTTAG
+
FFFFFFFFFFFFFFFFFFFFFFFFFFFFFFFFFFFFFFFFFFFFFFFFFFFFFFFFFFFFFFFFFFFF
FFFFFFFFFFFFFFFFFFFFFFFFFFFFFFFFFFFFFFFFFFFFF:FFFFFFFFFFFFFFFFFFFFFF
FFFFFFFFFFFFFFF
@A00721:190:HGJW7DSXY:4:1616:16559:14512 1:N:0:TCTGCAAG+GCTGCACT
CACCAGAATGTTATTAGAACCTCTGACGTCATTCTGGTGGTCCGCATCAATTGTCTCATATCATTTAG
TTAATGAGGATAATATTTATCCTTGATGATATGTTTGATTTCATCGGGAAGAGATGAAAGTAAATTTC
TGAATATGATGTTAG
+
FFFFFFFFFFFFFFFFFFFFFFFFFFFFFFFFFFFFFFFFFFFFFFFF,F:FFFFFFFFFFFFFF,FF
:FFFFFFFFFFFFFFFFFFFFFFFFFFFFFFFFFFFFFFFFFFFFFFFFFFFFFFFFFFFFFFFFFFF
FFFFFFFFFFFFFFF
@A00721:190:HGJW7DSXY:4:2147:28384:7780 1:N:0:TCTGCAAG+GCTGCACT
CACCAGAATGTTATTAGAACCTCTGACGTCATTCTGGTGGTCCGCATCAATTGTCTCATATCATTTAG
TTAATGAGGATAATATTTATCCTTGATGATATGTTTGATTTCATCGGGAAGAGATGAAAGTAAATTTC
TGAATATGATGTTAG
+
FFFFFFFFFF:FFFFF:FF::FFFFFF:FFFFFFFFF,FFFFFFF:FF:FFF:FFFFFFFFFFFFFFF
FFFFF:FF:FFFFFFFFFFF,FFF,FFFF:FFFFF:FFF,FFFF::FFFFFFFF:FFF:,FFFF,FFF
F::FFFF,FFFFFFF
@A00721:190:HGJW7DSXY:4:2360:4255:23703 1:N:0:TCTGCAAG+GCTGCACT
CACCAGAATGTTATTAGAACCTCTGACGTCATTCTGGTGGTCCGCATCAATTGTCTCATATCATTTAG
TTAATGAGGATAATATTTATCCTTGATGATATGTTTGATTTCATCGGGAAGAGATGAAAGTAAATTTC
TGAATATGATGTTAG
+
FFFFFFFFFFFFFFFFFFFFFFFFFFFFFFFFFFFFFFFFFFFFFFFFFFFFFFFFFFFFFFF:FFFF
FFFFFFFFFFFFFFFFFFFFFFFFFFFFFFFFFFFFFFFFFFFFFFFFFFFFFFFFFFFFFFFFFFFF
FFFFFFFFFFFFFFF
@A00721:190:HGJW7DSXY:4:1366:26449:36245 1:N:0:TCTGCAAG+GCTGCACT
ATATCATTTAGTTAATGAGGATAATATTTATCCTTGACGATATGTTTGATTTCATCGGGAAGAGATGA
AAGTAAATTTCTGAATATGATGTTAGCGTTAATGGAGAAACGCGATGAAGAAGATGAAGGAGTCCCCA
TTACTGTTAAATCAG
+
FFFFFFFFFFFFFFFFFFFFFFFFFFFFFFFFFFFFFFFFFFFF:FFFFFFFFFFFFFFFFFFFFFFF
```

```
FFFFFFFFFFFFFFFFFFFFFFFFFFFFFFFFFFFFFFFFFFFFFFFFFFF:FFFFFFFFFFFFFFFF
FFFFFFFFFFFFFFF
@A00721:190:HGJW7DSXY:4:1561:1732:17206 1:N:0:TCTGCAAG+GCTGCACT
ATATCATTTAGTTAATGAGGATAATATTTATCCTTGATGATATGTTTGATTTCATCGGGAAGAGATGA
AAGTAAATTTCTGAATATGATGTTAGCGTTAATGGAGAAACGCGATGAAGAAGATGAAGGAGTCCCCA
TTACTGTTAAATCAG
+
FFFFFFFFFFFFFFFFFFFFFFFFFFFFFFFFFFFFFFFFFFFFFFFFFFFFFFFF,FFF:FFFFFFF
FFF,FFFF:FFFFFFFFFFFFFFFFFFFFFFFFFFFFFFFFFFFFFFFFFFFFFFFFF,FFFFFFFF
FFFFFFFFFFFFFFF
@A00721:190:HGJW7DSXY:4:1565:16080:31814 1:N:0:TCTGCAAG+GCTGCACT
ATATCATTTAGTTAATGAGGATAATATTTATCCTTGATGATATGTTTGATTTCATCGGGAAGAGATGA
AAGTAAATTTCTGAATATGATGTTAGCGTTAATGGAGAAACGCGATGAAGAAGATGAAGGAGTCCCCA
TTACTGTTAAATCAG
+
FFFFFF:FFFFFFFFFFFFFFFFFFFFFFFFFFFFFFFFFFFFFFFFFFFFFFFFFFFFFFFFFFFFF
FFFFFFFFFFFFFFFFFFFFFFFFFF:FFF:FFFFFFFFFFFFFFFFFFF:FFFFFFFFFFFFFFFFF
FFFFFFFFFFFFFFF
@A00721:190:HGJW7DSXY:4:1648:15094:20932 1:N:0:TCTGCAAG+GCTGCACT
ATATCATTTAGTTAATGAGGATAATATTTATCCTTGATGATATGTTTGATTTCATCGGGAAGAGATGA
AAGTAAATTTCTGAATATGATGTTAGCGTTAATGGAGAAACGCGATGAAGAAGATGAAGGAGTCCCCA
TTACTGTTAAATCAG
+
FFFFFFFFFFFFFFFFFFFFFFFFFFFFFFFFFFFFFFFFFF:FFFFFFFFFFFFFFFFFFFFFFFFF
FFFFFFFFFFFFFFFFFFFF:FFFFFFFFFFF:FFFFFFFF:F:FFFFFFFFFFF:FFFFF:FFFFFF
,FFFFFFFFFFFFFF
@A00721:190:HGJW7DSXY:4:1670:14335:34648 1:N:0:TCTGCAAG+GCTGCACT
ATATCATTTAGTTAATGAGGATAATATTTATCCTTGATGATATGTTTGATTTCATCGGGAAGAGATGA
AAGTAAATTTCTGAATATGATGTTAGCGTTAATGGAGAAACGCGATGAAGAAGATGAAGGAGTCCCCA
TTACTGTTAAATCAG
+
FFFFFFFFFFFFFFFFFFFFFFFFFFFFFFFFFFFFFFFFFFF:FFFF:FFFFFFFFFFFFFFFFFFF
FFFFFFFFFFFFFFFFFFFFFFFFFFFFFFFFFF:FFFFFFFFFFFFFFFFFFFFFFFFFFFFFFFFF
FFFFFFFFFFFFFFF
@A00721:190:HGJW7DSXY:4:2167:32226:35289 1:N:0:TCTGCAAG+GCTGCACT
ATATCATTTAGTTAATGAGGATAATATTTATCCTTGATGATATGTTTGATTTCATCGGGAAGAGATGA
AAGTAAATTTCTGAATATGATGTTAGCGTTAATGGAGAAACGCGATGAAGAAGATGAAGGAGTCCCCA
TTACTGTTAAATCAG
+
FFFFFFFFFFFFFFFFFFFFFFFFFFFFFFFFFFFFFFFFFFFFFFFFFFFFFFFFFFFFFFFFFFFF
FFFFFFFFFFFFFFFFFFFFFFFFFFFFFFFFFFFFFFFFF:FFFFFF:FFFFFFFFFF:FFFF:FFF
FFFFFFFFFFFFFFF
@A00721:190:HGJW7DSXY:4:2173:32786:22858 1:N:0:TCTGCAAG+GCTGCACT
ATATCATTTAGTTAATGAGGATAATATTTATCCTTGATGATATGTTTGATTTCATCGGGAAGAGATGA
AAGTAAATTTCTGAATATGATGTTAGCGTTAATGGAGAAACGCGATGAAGAAGATGAAGGAGTCCCCA
TTACTGTTAAATCAG
+
FFFFFFFF:FFFFFFFFFFFFFFFFFFFFFFFFFFFFFFFFFFFFFFFFFFFFFFFFFFFFFFFFFFF
FFFFFFFFFFFFFFFFFFFFF:FFFF:FFFFFF:FFFFFFFFFFFFFFFFFFFFFFFFF:FFFF:FFF
FFFFFFFFFFFF:FF
@A00721:190:HGJW7DSXY:4:2460:1633:3067 1:N:0:TCTGCAAG+GCTGCACT
ATATCATTTAGTTAATGAGGATAATATTTATCCTTGATGATATGTTTGATTTCATCGGGAAGAGATGA
AAGTAAATTTCTGAATATGATGTTAGCGTTAATGGAGAAACGCGATGAAGAAGATGAAGGAGTCCCCA
TTACTGTTAAATCAG
```

```
+
FFFFFF:FFFFFFFFFFFFFFFFFFFFFFFFFFFFFFFFFFFFFFFFFFFFFFFFFFFFFFFFFFFFF
FFFFFFFFFFFFFFFFFFFFFFFFFFFFFFFFFFFFFFFFFFFFFFF:FFFFFFFFFFFFFFFFFFFF
FFFFFFFFFFFFFFF
@A00721:190:HGJW7DSXY:4:1216:30273:30091 1:N:0:TCTGCAAG+GCTGCACT
CTGATATGTTTGATTTCATCGGGAAGAGATGAAAGTAAATTTCTGAATATGATGTTAGCGTTAATGGA
GAAACGCGATGAAGAAGATGAAGGAGTCCCCATTACTGTTAAATCAGGGACATCCGCATATTAAGCAT
TATATTGGGGACTGG
+
FFFFFFFFFFFFFFFFFFFFFFFFFFFFFFFFFFFFFFFFFFFFFFFFFFFFFFFFFFFFFFFFFFFF
FFFFFFFFFFFFFFFFFFFFFFFFFFFFFFFFFFFFFFFFFFFFFFFFFFFFFFFFFFFFFFFFFFFF
FFF:FFFFFFFFFFF
@A00721:190:HGJW7DSXY:4:1329:12509:31172 1:N:0:TCTGCAAG+GCTGCACT
CTGATATGTTTGATTTCATCGGGAAGAGATGAAAGTAAATTTCTGAATATGATGTTAGCGTTAATGGA
GAAACGCGATGAAGAAGATGAAGGAGTCCCCATTACTGTTAAATCAGGGACATCCGCATATTAAGCAT
TATATTGGGGACTGG
+
FFFFFFFFFFFFFFFFFFFFFFFFFFFFFFFFFFFFFFFFFFFFFFFFFFFFFFFFFFFFFFFFFFFF
FFFFFFFFFFFFFFFFFFFFFFFF:FFFFFFFFFFFFFFFFFFFFFFFF:FFFFFFFFFFF:FFFFFF
FFFFF:FFFFFFFFF
@A00721:190:HGJW7DSXY:4:1455:17372:5306 1:N:0:TCTGCAAG+GCTGCACT
CTGATATGTTTGATTTCATCGGGAAGAGATGAAAGTAAATTTCTGAATATGATGTTAGCGTTAATGGA
GAAACGCGATGAAGAAGATGAAGGAGTCCCCATTACTGTTAAATCAGGGACATCCGCATATTAAGCAT
TATATTGGGGACTGG
+
FFFF:FFFFFFFFFFFFFFF:FFFFFFFFFFFFFFFFFFFFFFFFFFFFFFFFFFFFFFFFFFFFFFF
FFFFFFFFFFFFFFFFFFFFFFFFFFFFFFFFFFFFFFFFFFFFFFF:FFFFFFFFFFFFFFFFFFFF
FFF:FFFFF:FFFFF
@A00721:190:HGJW7DSXY:4:1455:16342:8719 1:N:0:TCTGCAAG+GCTGCACT
CTGATATGTTTGATTTCATCGGGAAGAGATGAAAGTAAATTTCTGAATATGATGTTAGCGTTAATGGA
GAAACGCGATGAAGAAGATGAAGGAGTCCCCATTACTGTTAAATCAGGGACATCCGCATATTAAGCAT
TATATTGGGGACTGG
+
FFFFFFFFFFFFFFFFFFFFFFFFFFFFFFFFFFFFFFFFFFFFFFF:FFFFFFFFFFFFFFFFFFFF
FFFFFFFFFFFFFFFFFFFFFFFFFFFFFFFFFFFFFFFFFFFFFFFFFFFFFFFFFFFFFFFFFFFF
FFFFFFFFFFFFFFF
@A00721:190:HGJW7DSXY:4:1542:17336:4460 1:N:0:TCTGCAAG+GCTGCACT
CTGATATGTTTGATTTCATCGGGAAGAGATGAAAGTAAATTTCTGAATATGATGTTAGCGTTAATGGA
GAAACGCGATGAAGAAGATGAAGGAGTCCCCATTACTGTTAAATCAGGGACATCCGCATATTAAGCAT
TATATTGGGGACTGG
+
FFFFF:FFFFFFFFFFFFFFFFFFFFFFFFFFFFFFFFFFFFFFFFFFFFFFFFFFFFFF:FFFFFFF
FFFFFFFFFFFFFFFFFFFFFFFFFFFFFFFFFFFFFFFFFFFFFFFFFFFFFFFFFFFFFFF:FFFF
FFFFFFFFFFFFFFF
@A00721:190:HGJW7DSXY:4:2361:11397:18411 1:N:0:TCTGCAAG+GCTGCACT
CTGATATGTTTGATTTCATCGGGAAGAGATGAAAGTAAATTTCTGAATATGATGTTAGCGTTAATGGA
GAAACGCGATGAAGAAGATGAAGGAGTCCCCATTACTGTTAAATCAGGGACATCCGCATATTAAGCAT
TATATTGGGGACTGG
+
FFFFFFFFFFFFFFFFFFFFFFFFFFFFFFFFFFFFFFFF:FFFFFFFFFFFFFFFFFFFFFFFFFFF
FFFFFFFFFFFFFFFFFFFFFFFFFFFFFFFFFFFFFFFFFFFFFFFFFFFFFFFFFFFFFFFFFFFF
FFFFFFF:FFFFFFF
@A00721:190:HGJW7DSXY:4:2404:12165:34460 1:N:0:TCTGCAAG+GCTGCACT
CTGATATGTTTGATTTCATCGGGAAGAGATGAAAGTAAATTTCTGAATATGATGTTAGCGTTAATGGA
```

GAAACGCGATGAAGAAGATGAAGGAGTCCCCATTACTGTTAAATCAGGGACATCCGCATATTAAGCAT
TATATTGGGGACTGG
+
:FFFFFFFFFFFFFFFFFFFFFFFFFFFFFFFFFFFFFFFFFFFFFFFFFFFFFFFFFFFFFFFFFFF
FFFFFFFFFFFFFFFFFFFFFFFFFFFFFFFFFFFFFFFFFFFFFFFF:FFFFFFFFFFFFFFFF:FF
FFFFFFFFFFFFFFF
@A00721:190:HGJW7DSXY:4:1656:14814:8672 1:N:0:TCTGCAAG+GCTGCACT
GAAGAGATGAAAGTAAATTTCTGAATATGATGTTAGCGTTAATGGAGAAACGCGATGAAGAAGATGAA
GGAGTCCCCATTACTGTTAAATCAGGGACATCCGCATATTAAGCATTATATTGGGGACTGGGGACCTA
TATATATGTATAGAG
+
FFFFFFFFFFFFFFFFFFFFFFFFFFFFFFFFFFFFFFFFFFFFFFFFFFFFFFFFFFFFFFFFFFFF
FFFFFFFFFFFFFFFFFFFFFFFFFFFFFFFFFFFFFFFFFFFFFFFFFFFFFFFFFFFFFFFFFFFF
FFFFFFFFFFFFFFF
@A00721:190:HGJW7DSXY:4:1656:15311:9627 1:N:0:TCTGCAAG+GCTGCACT
GAAGAGATGAAAGTAAATTTCTGAATATGATGTTAGCGTTAATGGAGAAACGCGATGAAGAAGATGAA
GGAGTCCCCATTACTGTTAAATCAGGGACATCCGCATATTAAGCATTATATTGGGGACTGGGGACCTA
TATATATGTATAGAG
+
FFFFFFFFFFFFFFFFFFFFFFFFFFFFFFFFF:FFFFFF:FFFFFFFFFFFFFFFFFFFFFFFFFFF
F,FF:FFFFFFFFFFF:FFFFFFFF,FFFFFFFFFFFFF,FFFFF:FFFFFFFFFFFFFFFFFFFFFF
FF:FFF:F,FFFF:F
@A00721:190:HGJW7DSXY:4:2265:17445:18208 1:N:0:TCTGCAAG+GCTGCACT
GAAGAGATGAAAGTAAATTTCTGAATATGATGTTAGCGTTAATGGAGAAACGCGATGAAGAAGATGAA
GGAGTCCCCATTACTGTTAAATCAGGGACATCCGCATATTAAGCATTATATTGGGGACTGGGGACCTA
TATATATGTATAGAG
+
FFFFFFFFFFFFFFFFFFFFFFFFFFFFFFFFFFFFFFFF:FFFFFFFFFFFFFFFFFFFFFFFFFFF
FFFFFFFFFFFFFFFFFFFFFFFFFFFFFFFFFFFFFFFFFFFFFFFFFFFFFFFFFFFFFFFFFFFF
FFFFFFFFFFFFFFF
@A00721:190:HGJW7DSXY:4:2601:1353:36401 1:N:0:TCTGCAAG+GCTGCACT
GAAGAGATGAAAGTAAATTTCTGAATATGATGTTAGCGTTAATGGAGAAACGCGATGAAGAAGATGAA
GGAGTCCCCATTACTGTTAAATCAGGGACATCCGCATATTAAGCATTATATTGGGGACTGGGGACCTA
TATATATGTATAGAG
+
FFFFFFFFFFFFF::FFF,FF::FF:::FFFFF:FFFFFFFFFFFFFFFFFFFFFFFFFFFFFFFFFF
FFFFFFFFFFF:FFFFFFFFFFFFFFFFFFFFF,FFFFF:FFFFFFFFFFF:FFFFFFFFFFFFFFFF
FFFFFFFFFFFFFFF
@A00721:190:HGJW7DSXY:4:1163:32407:35697 1:N:0:TCTGCAAG+GCTGCACT
CCGGATTACTCTATACATATATATAGGTCCCCAGTCCCCAATATAATGCTTAATATGCGGATGTCCCT
GATTTAACAGTAATGGGGACTCCTTCATCTTCTTCATCGCGTTTCTCCATTAACGCTAACATCATATT
CAGAAATTTACTTTC
+
FFFFFFFFFFFFFFFFFFFFFFFFFFFFFFFFFFFFFFFFFFFFFFFFFFFFFFFFFFFFFFFFFFFF
FFFFFFFFFFFFFFFFFFFFFFFFFFFFFFFFFFFFFFFFFFFF,FFFF,FF:FFFF,,FFFF:FFFF
FFFFF,FFFFFFFFF
@A00721:190:HGJW7DSXY:4:1325:12861:10958 1:N:0:TCTGCAAG+GCTGCACT
CCGGATTACTCTATACATATATATAGGTCCCCAGTCCCCAATATAATGCTTAATATGCGGATGTCCCT
GATTTAACAGTAATGGGGACTCCTTCATCTTCTTCATCGCGTTTCTCCATTAACGCTAACATCATATT
CAGAAATTTACTTTC
+
FFFFFFFFFFFFFFFFF:FFFFFFFFFFFFFFFFFFFFFFFFFFFFFFFFFFFFFFFFFFFFFFFFFF
FFFFFFFFFFFFFFFFFFFFFFFFFFFFFFFF:FFFFFFFFF:FFFFFFFFF:FFFFFF:FFFFFFFF
FFFFFFFFFFFFFFF

```
@A00721:190:HGJW7DSXY:4:1331:2239:14982 1:N:0:TCTGCAAG+GCTGCACT
CCGGATTACTCTATACATATATATAGGTCCCCAGTCCCCAATATAATGCTTAATATGCGGATGTCCCT
GATTTAACAGTAATGGGGACTCCTTCATCTTCTTCATCGCGTTTCTCCATTAACGCTAACGTCATATT
CAGAAATTTACTTTC
+
FFFFFFFFFFFFFFFFFFFF::FFFFFFFFFFFFFFFFFFFFFFFFFFFFFFFFFFFFFFFFFFFFFF
FFFFFFFFFFFFFFFFFFFFFFFFFFFFFFFFFFFFFFF:FFFFFFFFFFFFFFFFFFFFFFFFFFFF
FFFFFFFFFFFFFFF
@A00721:190:HGJW7DSXY:4:1433:22914:19132 1:N:0:TCTGCAAG+GCTGCACT
CCGGATTACTCTATACATATATATAGGTCCCCAGTCCCCAATATAATGCTTAATATGCGGATGTCCCT
GATTTAACAGTAATGGGGACTCCTTCATCTTCTTCATCGCGTTTCTCCATTAACGCTAACATCATATT
CAGAAATTTACTTTC
+
FFFFFFFFFFFFFFFFFFFFFFFFFFFFFFFFFFFFFFFFFFFFFFFFFFFFFFFFFFFFFFFFFFFF
FFFFFFFFFFFFFFFFFFFFFFFFFFFFFFFFFFFFFFFFFFFFFFFF:FF,FF,FFFFFFFFFFFFFFF
FFFFFFFFFFFFFFF
@A00721:190:HGJW7DSXY:4:2365:24017:26115 1:N:0:TCTGCAAG+GCTGCACT
CCGGATTACTCTATACATATATATAGGTCCCCAGTCCCCAATATAATGCTTAATATGCGGATGTCCCT
GATTTAACAGTAATGGGGACTCCTTCATCTTCTTCATCGCGTTTCTCCATTAACGCTAACATCATATT
CAGAAATTTACTTTC
+
FFFFFFFFFFFFFFFFFFFFF:FFFFFFFFFFFFFFFFFFFFFFFFFFFFFFFFFFFFFFFFFFFFFF
FFFFFFFFFFFFFFFFFFFFFFFFFFFFFFFFFFFFFFFFFFFFFFFFFFFFFFFFF:FFF,F:FFFF
FFFFFFFFFFFFFFF
@A00721:190:HGJW7DSXY:4:2528:16098:19351 1:N:0:TCTGCAAG+GCTGCACT
CCGGATTACTCTATACATATATATAGGTCCCCAGTCCCCAATATAATGCTTAATATGCGGATGTCCCT
GATTTAACAGTAATGGGGACTCCTTCATCTTCTTCATCGCGTTTCTCCATTAACGCTAACATCATATT
CAGAAATTTACTTTC
+
FFFFFFFFFFFFFFFFFFFFFFFFFFFFFFFFFFFFFFFFFFFFFFFFFFFFFFFFFFFFFFFFFFFF
FFFFFFFFFFFFFFFFFFFFFFFFFFFFFFFFFFFF:FFFFFFFFFFFF,FFFF:FFFFFFFFFFFFF
FFFFFFFFFFFFFFF
@A00721:190:HGJW7DSXY:4:1326:30165:25958 1:N:0:TCTGCAAG+GCTGCACT
CTCCGGATTACTCTATACATATATATAGGTCCCCAGTCCCCAATATAATGCTTAATATGCGGATGTCC
CTGATTTAACAGTAATGGGGACTCCTTCATCTTCTTCATCGCGTTTCTCCATTAACGCTAACATCATA
TTCAGAAATTTACTT
+
FFFFFFFFFFFFFFFFFFFFFFFFFFFFFFFFFFFFFFFFFFFFFFFFFFFFFFFFFFFFFFFFFFFF
FFFFFFFFFFFFFFFFFFFFFFFFFFFFFFFFFFFFFFFFFFFFFFFFFFF:FFFFFFFF:FFFFFFFF
FFFFFFFFFFFFFFF
@A00721:190:HGJW7DSXY:4:1464:22010:3630 1:N:0:TCTGCAAG+GCTGCACT
CTCCGGATTACTCTATACATATATATAGGTCCCCAGTCCCCAATATAATGCTTAATATGCGGATGTCC
CTGATTTAACAGTAATGGGGACTCCTTCATCTTCTTCATCGCGTTTCTCCATTAACGCTAACATCATA
TTCAGAAATTTACTT
+
FFFFFFFFFFFFFFFFFFFFFFFFFFFFFFFFFFFFFFFFFFFFFFFFFFFFFFFFFFFFFFFFFFFF
FFFFFFFFFFFF:FFFFFFFFFFFF:FFFFFFFFFFFF:FFFFFF:FFFF,F,FFF:FFFFF,FFFFF
FFFFFFFFFF:FFFF
@A00721:190:HGJW7DSXY:4:1521:31250:26209 1:N:0:TCTGCAAG+GCTGCACT
CTCCGGATTACTCTATACATATATATAGGTCCCCAGTCCCCAATATAATGCTTAATATGCGGATGTCC
CTGATTTAACAGTAATGGGGACTCCTTCATCTTCTTCATCGCGTTTCTCCATTAACGCTAACATCATA
TTCAGAAATTTACTT
+
FFFFFFFFFFFFFFFFFF:FFFFFFFFFFFFFFFFFFFFFFFFFFFFFFFFFFFFFFFFFFFFFFFFF
```

FFFF,FFFFFFFFFFFFFFFFFFFFFFFFFFFFFFFFFFFFFFF:FFFFF:F,:FFFFF,F,FF:FF
FFFFFFFFFFFFFFF
@A00721:190:HGJW7DSXY:4:1542:5520:18756 1:N:0:TCTGCAAG+GCTGCACT
TCCGGATTACTCTATACATATATATAGGTCCCCAGTCCCCAATATAATGCTTAATATGCGGATGTCCC
TGATTTAACAGTAATGGGGACTCCTTCATCTTCTTCATCGCGTTTCTCCAGTAACGCTAACATCATAT
TCAGAAATTTACTT
+
FFFFFFFFFFFFF,FFFFFFF:FFFFF:FFFFFFFFFFFFFFFFFF:FFFFFFF:FFF:FFFFFFFFF
FFFF:F:FF,FFFFFFFFFFFFFFFFFFFFFFFFFF,FFFFFF,F:FFF:,F::FF:F,:FFFFFFFF
F:FFFFF:FFFFFF
@A00721:190:HGJW7DSXY:4:2253:1072:20791 1:N:0:TCTGCAAG+GCTGCACT
CTCCGGATTACTCTATACATATATATAGGTCCCCAGTCCCCAATATAATGCTTAATATGCGGATGTCC
CTGATTTAACAGTAATGGGGACTCCTTCATCTTCTTCATCGCGTTTCTCCATTAACGCTAACATCATA
TTCAGAAATTTACTT
+
FFFFFFFFFFFFFFFFFFFFFFFFFFFFFFFFFFFFFFFFFFFFFFFFFFFFF:FFFFFFFFFFFFF:F
FFFFFFFFFFFFFFFFFFFFFFFFFFFFFFFFFFFFFFFF:FFFFFFFFF:F:FFFFFFF:FFFFFFF
FFFFFFFFFF:FFFF
@A00721:190:HGJW7DSXY:4:2565:21838:17237 1:N:0:TCTGCAAG+GCTGCACT
CTCCGGATTACTCTATACATATATATAGGTCCCCAGTCCCCAATATAATGCTTAATATGCGGATGTCC
CTGATTTAACAGTAATGGGGACTCCTTCATCTTCTTCATCGCGTTTCTCCATTAACGCTAACATCATA
TTCAGAAATTTACTT
+
FFFFFFFFFFFFFFFFFFFFFFFFFFFFFFFFFFFFFF:FFFFFFFFFFFFFFFFFFFFF:FFFFFFF
FFFFFFFFFFFFFFFFFF,FFFFFFFFFFFFFFFFFFFFFFFFFFFFFFFFF,FFFF:FFF:FFF:FF,F
FFFFFFFFFF:F::F
@A00721:190:HGJW7DSXY:4:2575:21495:25160 1:N:0:TCTGCAAG+GCTGCACT
CTCCGGATTACTCTATACATATATATAGGTCCCCAGTCCCCAATATAATGCTTAATATGCGGATGTCC
CTGATTTAACAGTAATGGGGACTCCTTCATCTTCTTCATCGCGTTTCTCCATTAACGCTAACATCATA
TTCAGAAATTTACTT
+
FFFFFFFFFFFFFFFFFFFFF,FFFFFFFFFFFFFFFFFFFFFFFFFFFFFFFFFFFFFF:FFFFFFFF
FFFF:FFFFFFFF:FFFFFFFFFFFF:F:FFFFF:FFFFFFFFF,FF:FFFFFF,FFF:F,F,:FF:F
FFFFFFFFF::FFFF
@A00721:190:HGJW7DSXY:4:2674:21169:16799 1:N:0:TCTGCAAG+GCTGCACT
CTCCGGATTACTCTATACATATATATAGGTCCCCAGTCCCCAATATAATGCTTAATATGCGGATGTCC
CTGATTTAACAGTAATGGGGACTCCTTCATCTTCTTCATCGCGTTTCTCCATTAACGCTAACAGCATA
TTCAGAAATTTACTT
+
FFFFFFFFFFFFFFFFFFFFFFFFFFFFFFFFFFFFFFFFFFFFFFFFFFFFFFFFFFFFFFFFFFFF
FFFFFFFFFFFFFFFFFFFFFFFFFFFFFFFFFFFFFFFFFFF:FFFF,F:FFFFFFF:FF,F:FF
FFFFFFFFFFFFFFF
@A00721:190:HGJW7DSXY:4:1341:2266:1626 1:N:0:TCTGCAAG+GCTGCACT
CCGGATTACTCTATACATATATATAGGTCCCCAGTCCCCAATATAATGCTTAATATGCGGATGTCCCT
GATTTAACAGTAATGGGGACTCCTTCATCTTCTTCATCGCGTTTCTCCATTAACGCTAACATCATATT
CAGAAATTTAGATCG
+
FFFFFFFFFFFFFFFFFFFFF:FFFFFFFFFFFFFFFFFFFFFFF:FFFFFFFFFFFFFFFFFFFFFFF
FFFFFFFFFFFFFFFFFFFFFFFFFFFFFFFFFFFFFFFFFFF:FFFFFFFFFFFFFFFFFFFFFFFFF
FFFFFFFFFFFFFF:
@A00721:190:HGJW7DSXY:4:2443:9381:3834 1:N:0:TCTGCAAG+GCTGCACT
CCGGATTACTCTATACATATATATAGGTCCCCAGTCCCCAATATAATGCTTAATATGCGGATGTCCCT
GATTTAACAGTAATGGGGACTCCTTCATCTTCTTCATCGCGTTTCTCCATTAACGCTAACATCATATT
CAGAAATTTAGATCG

+
FFFFFFFFFFFFFFFFFFFFFFFFFFFFFFFFFFFFFFFFFFFFFFFFFFFFFFFFFFFFFFFFFFFFFF
FFFFFFFFFFFFFFFFFFFFFFFFFFFFFFFFFFFFFFFFFFF:FFFFFFFFFFFFFFFFFFFFFFFFFF
FFFFFFFFFFFFFFF
@A00721:190:HGJW7DSXY:4:2445:11505:5415 1:N:0:TCTGCAAG+GCTGCACT
CCGGATTACTCTATACATATATATAGGTCCCCAGTCCCCAATATAATGCTTAATATGCGGATGTCCCT
GATTTAACAGTAATGGGGACTCCTTCATCTTCTTCATCGCGTTTCTCCATTAACGCTAACATCATATT
CAGAAATTTAGATCG
+
FFFFFFFFFFFFFFFFFFFFFFFFFFFFFFFFFFFFFFFFFFFFFFFFFFFFFFFFFFFFFFFFFFFFFF
FFFFFFFFFFFFFFFFFFFFFFFFFFFFFFFFFFFFFFFFFFFFFFFFF::FFFF:FFFFFFFFFFFFFF
FFFFFFFFFFFFFFF
@A00721:190:HGJW7DSXY:4:2466:11098:25003 1:N:0:TCTGCAAG+GCTGCACT
CCGGATTACTCTATACATATATATAGGTCCCCAGTCCCCAATATAATGCTTAATATGCGGATGTCCCT
GATTTAACAGTAATGGGGACTCCTTCATCTTCTTCATCGCGTTTCTCCATTAACGCTAACATCATATT
CAGAAATTTAGATCG
+
FFFFFFFFFFFFFFFFFFFFFFFFFFFFFFFFFFFFFFFFFFFFFFFFFFFFFFFFFFFFFFFFFFFFFF
FFFFFFFFFFFFFFFFFFFFFFFFFFFFFFFFFFFFFFFFFFFFFFFFFFFFFFFFFFFFFFFFFFFFFF
FFFFFFFFFFFFFFF
@A00721:190:HGJW7DSXY:4:2417:14163:36542 1:N:0:TCTGCAAG+GCTGCACT
CCGGATTACTCTGTACATATATATAGGTCCCCAGTCCCCAATATAATGCTTAATATGCGGATGTCCCT
GATTTAACAGTAATGGGGACTCCTTCATCTTCTTCATCGCGTTTCTCCATTAACGCTAACATCATATT
CAGAAATTTAGATC
+
FFFFFF:FF:F,:,FFFFFF:FFFFFF:FFFFFFFFFFFFFFFF:FF:F:F,FFFFFFFFFFF:FFFFFF
FFFFFFFFFFFFFFFFFFFFFFFFFFFFFFFFFFFFF::FFF:FFFFFFFFFFFF::FFFFFFFFFFFFF
FFF,:FFFF:,FFF
@A00721:190:HGJW7DSXY:4:2368:5358:34006 1:N:0:TCTGCAAG+GCTGCACT
CTGATGTTAGCGTTAATGGAGAAACGCGATGAAGAAGATGAAGGAGTCCCCATTACTGTTAAATCAGG
GACATCCGCATATTAAGCATTATATTGGGGACTGGGGACCTATATATATGTATAGAGTAATCCGGAGG
AATCTATCCCCAAGA
+
FFFFFFFFFFFFFFFFFFFFFFFFFFFFFFFFFFFFFFFFFFFFFFFFFFFFFFFFFFFFFFFFFFFFFF
FFFFFFFFFFFFFFFFFFFFFFFFFFFFFFFFFFFFFFFFFFFFFFFFFFFFFFFFFFFFFFFFFFFFFF
FFFFFFFFFFFFFFF
@A00721:190:HGJW7DSXY:4:2370:17770:30358 1:N:0:TCTGCAAG+GCTGCACT
CTGATGTTAGCGTTAATGGAGAAACGCGATGAAGAAGATGAAGGAGTCCCCATTACTGTTAAATCAGG
GACATCCGCATATTAAGCATTATATTGGGGACTGGGGACCTATATATATGTATAGAGTAATCCGGAGG
AATCTATCCCCAAGA
+
FFFFFFFFFFFFFFFFFFFFFFFFFFFFFFFFFFFFFFFFFFFFFFFFFFFFFFFFFFFFFFFFFFFFFF
FFFFFFFFFFFFFFFFFFFFFFFFFFFFFFFFFFFFFFFFFFFFFFFFFFFFFFFFFFFFFFFFFFFFFF
FFFFFFFFFFFFFFF
@A00721:190:HGJW7DSXY:4:2475:24804:8844 1:N:0:TCTGCAAG+GCTGCACT
CTGATGTTAGCGTTAATGGAGAAACGCGATGAAGAAGATGAAGGAGTCCCCATTACTGTTAAATCAGG
GACATCCGCATATTAAGCATTATATTGGGGACTGGGGACCTATATATATGTATAGAGTAATCCGGAGG
AATCTATCCCCAAGA
+
FFFFFFFFFFFFFFFFFFFFFFFFFFFFFFFFFFFFFFFFFFFFFFFFFFFFFFFFFFFFFFFFFFFFFF
FFFFFFFFFFFFFFFFFFFFFFFFFFFFFFFFFFFFFFFFFFFFFFFFFFFFFFFFFFFFFFFFFFFFFF
FFFFFFFFFFFFFFF
@A00721:190:HGJW7DSXY:4:2535:9715:22608 1:N:0:TCTGCAAG+GCTGCACT
CTGATGTTAGCGTTAATGGAGAAACGCGATGAAGAAGATGAAGGAGTCCCCATTACTGTTAAATCAGG

```
GACATCCGCATATTAAGCATTATATTGGGGACTGGGGACCTATATATATGTATAGAGTAATCCGGAGG
AATCTATCCCCAAGA
+
:FFFFFFFFFFFFFFFFFFFFFFFFFFFFFFFFFFFFFFFFFFFFFFFFFFFFFFFFFFFFFFFFFFFFF
FFFFFFFFFFFFFFFFFFFFFFFFFFFFFFFFFFFFFFFFFFFFFFFFFFFFFFFFFFFF:FFFFFFFFF
F:FFFFFFFFFFFFF
@A00721:190:HGJW7DSXY:4:2613:1461:23469 1:N:0:TCTGCAAG+GCTGCACT
CTGATGTTAGCGTTAATGGAGAAACGCGATGAAGAAGATGAAGGAGTCCCCATTACTGTTAAATCAGG
GACATCCGCATATTAAGCATTATATTGGGGACTGGGGACCTATATATATGTATAGAGTAATCCGGAGG
AATCTATCCCCAAGA
+
FFFFFFFFFFFFFFFFFFFFFFFFFFFFFFFFFFFFFFFFFFFFFFFFFFFFFFFFFFFFFFFFFFFFFF
FFFFFFFFFFFFFFFFFFFFFFFFFFFFFFFFFFFFFFFFFFFFFFFFFFFFF:FFFFFF:FFFFFFFFF
F:FFFFFFFFFFFFF
@A00721:190:HGJW7DSXY:4:2654:5891:16172 1:N:0:TCTGCAAG+GCTGCACT
CTGATGTTAGCGTTAATGGAGAAACGCGATGAAGAAGATGAAGGAGTCCCCATTACTGTTAAATCAGG
GACATCCGCATATTAAGCATTATATTGGGGACTGGGGACCTATATATATGTATAGAGTAATCCGGAGG
AATCTATCCCCAAGA
+
FFFFFFFFFFFFFFFF::FF:FFFFF:FF::FFFFFFFFFFFFF:FFFFF:FFFFFFFFFFFFFFFFFFF
FFFFFFFFFFFFFFFFFFFF:FFFFFF:FFFFFFFFFFFFFFFFFFFFFFFFFFF:FFFFFFFFFFFFFF
FFFFFFFFFFFFF:F
@A00721:190:HGJW7DSXY:4:1263:8567:28667 1:N:0:TCTGCAAG+GCTGCACT
CCTCAATATAATGCTTAATATGCGAATGTCCCTGATTTAACAGTAATGGGGACTCCTTCATCTTCTTC
ATCGCGTTTCTGCATTAACGCGAAGAATTATTTCTTAACTTATTCAAGATGTCCAATTTCCAAAGAAA
TTGTTCTCGACCAAA
+
FFFFFFFFFFFFFFFFFFFFFFFFFFFFFFFFFFFFFFFFFFFFFFFFFFFFF,FFFFFFFFFFFFFFFF
FFFFFFFFFFFFFFFFFFFFFFFFFFFFFFFFFFFFFFFFFFFFFFFFFFFFFFFFFFFFFFFFFFFFFF
FFFFFFFFFFFFFFF
@A00721:190:HGJW7DSXY:4:1309:12310:13135 1:N:0:TCTGCAAG+GCTGCACT
CCTCAATATAATGCTTAATATGCGAATGTCCCTGATTTAACAGTAATGGGGACTCCTTCATCTTCTTC
ATCGCGTTTCTGCATTAACGCGAAGAATTATTTCTTAACTTATTCAAGATGTCCAATTTCCAAAGAAA
TTGTTCTCGACCAAA
+
FFFFFFFFFFFFFFFFFFFFFFFFFFFFFFFFFFFFFFFFFFFFFFFFFFFFFFFFFFFFFFFFFFFFFF
FFFFFFFFFFFFFFFFFFFFFFFFFFFFFFFFFFFFFFF:FFFFFFF:FF:FFFF:FFFFFF,FFFF:FF
FFFFFFFFF:FFFFF
@A00721:190:HGJW7DSXY:4:1338:10592:23437 1:N:0:TCTGCAAG+GCTGCACT
CCTCAATATAATGCTTAATATGCGAATGTCCCTGATTTAACAGTAATGGGGACTCCTTCATCTTCTTC
ATCGCGTTTCTGCATTAACGCGAAGAATTATTTCTTAACTTATTCAAGATGTCCAATTTCCAAAGAAA
TTGTTCTCGACCAAA
+
FFFFFFFFFFFFFFFFFFFFFFFFFFFFFFFFFFFFFFFFFFFFFFFFFFFFFFFFFFFFFFFFFFFFFF
FFFFFFFFFFFFFFFFFFFFFFFFFFFFFFFFFFFFFFFFFFFFFFFFFFFFFFFFFFFFFFFFFFF:,F
FFFFFFFF:FFFFFF
@A00721:190:HGJW7DSXY:4:1457:30761:1470 1:N:0:TCTGCAAG+GCTGCACT
CCTCAATATAATGCTTAATATGCGAATGTCCCTGATTTAACAGTAATGGGGACTCCTTCATCTTCTTC
ATCGCGTTTCTGCATTAACGCGAAGAATTATTTCTTAACTTATTCAAGATGTCCAATTTCCAAAGAAA
TTGTTCTCGACCAAA
+
FFFFFFFFF:FFFFFFFFFFFFFFFFFFFFFFFFFFFFFFFFFFFFFFFFFFF:FFFFFFFFFFFFFFFF
FFFFFFFFFFFFFFFF:FFFFFFFFFFFFFFFFFFFFFFFFFFFFFFFFFFFFFFFFFFFFFFFFFFFFF
FFFFFFFFFFFFFFF
```

@A00721:190:HGJW7DSXY:4:1648:28763:29356 1:N:0:TCTGCAAG+GCTGCACT
CCTCAATATAATGCTTAATATGCGAATGTCCCTGATTTAACAGTAATGGGGACTCCTTCATCTTCTTC
ATCGCGTTTCTGCATTAACGCGAAGAATTATTTCTTAACTTATTCAAGATGTCCAATTTCCAAAGAAA
TTGTTCTCGACCAAA
+
FFFFFFFFFFFFFFFFFFFFFFFFFFFFFFFFFFFFFFFFFFFFFFFFFFF:FFFFFFFFFFFFFFFF
FFFFFFFFFFFFFFFFFFF:FFFFFFFFFFFFFFFFFFFFFFFFF:FFFFFFFFFFFFFFFFFFFFFF
FFFFFFFFFFFFFFF
@A00721:190:HGJW7DSXY:4:2226:18231:35039 1:N:0:TCTGCAAG+GCTGCACT
CCTCAATATAATGCTTAATATGCGAATGTCCCTGATTTAACAGTAATGGGGACTCCTTCATCTTCTTC
ATCGCGTTTCTGCATTAACGCGAAGAATTATTTCTTAACTTATTCAAGATGTCCAATTTCCAAAGAAA
TTGTTCTCGACCAAA
+
FFFFFFFFFFFFFFFFFFFFFFFFFFFFFFFFFFFFFFFFFFFFFFFFFFFFFFFFFFFFFFFFFFFF
FFFFFFFFFFFFFFFFF:FFFFFFFFFFFFFFF:FFFFFFFFFFFFFFFFFFFFFFFFFFFFFF:FFF
FFFFFFFFFFFFFFF
@A00721:190:HGJW7DSXY:4:2451:14733:32737 1:N:0:TCTGCAAG+GCTGCACT
CCTCAATATAATGCTTAATATGCGAATGTCCCTGATTTAACAGTAATGGGGACTCCTTCATCTTCTTC
ATCGCGTTTCTGCATTAACGCGAAGAATTATTTCTTAACTTATTCAAGATGTCCAATTTCCAAAGAAA
TTGTTCTCGACCAAA
+
FFFFFFFFFFFFFFFFFFFFFFFFFFFFFFFFFFFFFFFFFFFFFFFFFFFFFFFFFFFFFFFFFFFF
FFFFFFFFFFFFFFFFF:FFFFFFFFFFFFFFFFFFFFFFFFFFFFFFFFFFFFFFFFFFFFFFFFFF
FFFFFFFF:FFFFFF
@A00721:190:HGJW7DSXY:4:2452:6696:8484 1:N:0:TCTGCAAG+GCTGCACT
CCTCAATATAATGCTTAATATGCGAATGTCCCTGATTTAACAGTAATGGGGACTCCTTCATCTTCTTC
ATCGCGTTTCTGCATTAACGCGAAGAATTATTTCTTAACTTATTCAAGATGTCCAATTTCCAAAGAAA
TTGTTCTCGACCAAA
+
FFFFFFFFFFFFFFFFFFFFFFFFFFFFFFFFFFFFFFFFFFFFFFFFFFF:FFFFFFFFFFFFFFFF
FFFFFFFFFFFFFFFFFFFFFFFFFFFFFFFFFFFFFFFFFFFFFFFFFFFFFFFFFFFFFFFFFFFF
F:FFFFFFFFFFFFF
@A00721:190:HGJW7DSXY:4:2540:25229:6104 1:N:0:TCTGCAAG+GCTGCACT
CCTCAATATAATGCTTAATATGCGAATGTCCCTGATTTAACAGTAATGGGGACTCCTTCATCTTCTTC
ATCGCGTTTCTGCATTAACGCGAAGAATTATTTCTTAACTTATTCAAGATGTCCAATTTCCAAAGAAA
TTGTTCTCGACCAAA
+
FFFFFFFFFFFFFFFFFFFFFFFFFFFFFFFFFFFFFFFFFFFFFFFFFFFFFFFFFFFFFFFFFFFF
FFFFFFFFFFFFFFFFFFFFFFFFFFFFFFFFFFFFFF:FFFFFFFFF:F,FF,FFFFFF,FFF:FFF
FFFFFFFFFFFFFF:
@A00721:190:HGJW7DSXY:4:2572:25129:18928 1:N:0:TCTGCAAG+GCTGCACT
CCTCAATATAATGCTTAATATGCGAATGTCCCTGATTTAACAGTAATGGGGACTCCTTCATCTTCTTC
ATCGCGTTTCTGCATTAACGCGAAGAATTATTTCTTAACTTATTCAAGATGTCCAATTTCCAAAGAAA
TTGTTCTCGACCAAA
+
FFFFFFFFFFFFFFFFFFFFFFFFFFFFFFFFFFFFFFFFFFFFFFFFFFFFFFFFFFFFFFFFFFFF
FFFFFFFFFFFFFFFFFFFFFFFFF:FFFFFFFFFFFFFFFFFFFFFFFFFFFFFFFFFFFFFFFFFF
FF:FFFFFFFFFFFF
@A00721:190:HGJW7DSXY:4:1221:11767:18364 1:N:0:TCTGCAAG+GCTGCACT
GTCGAGAACAATTTCTTTGGAAATTGGACATCTTGAATAAGTTAAGAAATAATTCTTCGCGTTAATGC
AGAAACGCGATGAAGAAGATGAAGGAGTCCCCATTACTGTTAAATCAGGGACATTCGCATATTAAGCA
TTATATTGGGGACTG
+
FFFFFFFFFFFFFFFFFFFFFFFFFFFFFFFFFFFFFFFFFFFFFFFFFFFFFFFFFFFFFFFFFFFF

```
FFFFFFFFFFFFFFFFFFFFFFFFFFFFFF:FFFFFFFFFFFFFFFFFFFFFFFFFFFFFFFFFFFFFF
FFFFFFFFFFFFFFF
@A00721:190:HGJW7DSXY:4:2149:19199:32800 1:N:0:TCTGCAAG+GCTGCACT
GTCGAGAACAATTTCTTTGGAAATTGGACATCTTGAATAAGTTAAGAAATAATTCTTCGCGTTAATGC
AGAAACGCGATGAAGAAGATGAAGGAGTCCCCATTACTGTTAAATCAGGGACATTCGCATATTAAGCA
TTATATTGGGGACTG
+
FFFFFFFFFFFFFFFFFFFFFFFFFFFFFFFFFFFFFFFFFFFFFFFFFFFFFFFFFFFFFFFFFFFF
FFFFFFFFFFFFFFFFFFFFFFFFFFFFFFFFFFFFFFFFFFFFFFFFFFFFFFFFFFFFFFFFFFFF
FFFFFFFFF:FFFFF
@A00721:190:HGJW7DSXY:4:2356:3929:9549 1:N:0:TCTGCAAG+GCTGCACT
GTCGAGAACAATTTCTTTGGAAATTGGACATCTTGAATAAGTTAAGAAATAATTCTTCGCGTTAATGC
AGAAACGCGATGAAGAAGATGAAGGAGTCCCCATTACTGTTAAATCAGGGACATTCGCATATTAAGCA
TTATATTGGGGACTG
+
FFFFFFFFFFFFFFFFFFFFFFFFFFFFFFFFFFFFFFFFFFFFFFFFFFFFFFFFFFFFFFFFFFFF
FFFFFFFFFFFFFFFFFFF:FFFFFFFFFFFFFFFFFFFFFFFFFFFFFFFFFFFFFFFFFFFFFFFF
FFFFFFFFFFFFFFF
@A00721:190:HGJW7DSXY:4:2618:5773:23390 1:N:0:TCTGCAAG+GCTGCACT
GTCGAGAACAATTTCTTTGGAAATTGGACATCTTGAATAAGTTAAGAAATAATTCTTCGCGTTAATGC
AGAAACGCGATGAAGAAGATGAAGGAGTCCCCATTACTGTTAAATCAGGGACATTCGCATATTAAGCA
TTATATTGGGGACTG
+
FFFFFFFFFFFFFFFFFFFFFFFFFFFFFFFFFFFFFFFFFFFFFFFFFFFFFFFFFFFFFFFF:FFF
FFFFFFFFFFFFFFFFFFF:FFFFFFFFFFFFFFFFFFFFFFFFFFFFFFFFFFFFFFFFFFFFFFFF
FFFFFFFFFFFFFFF
@A00721:190:HGJW7DSXY:4:1129:23610:36714 1:N:0:TCTGCAAG+GCTGCACT
TCGAGAACAATTTCTTTGGAAATTGGACATCTTGAATAAGTTAAGAAATAATTCTTCGCGTTAATGCA
GAAACGCGATGAAGAAGATGAAGGAGTCCCCATTACTGTTAAATCAGGGACATTCGCATATTAAGCAT
TATATTGGGGACTGG
+
FFFFFFFFFFFFFFFFFFFFFF:FFFFFFFFFFFFFFFFFFFFFFFFFFFFFFFFFFFFFFFFFFFFF
FFFFFFFFFFFFFFFFFFFFFFFFFFFFFFFFFFFFFFFFFFFFFFFFFFFFFFFFFFFFFFFFFFFF
FFFFFFF:FFFFFFF
@A00721:190:HGJW7DSXY:4:1202:25880:25739 1:N:0:TCTGCAAG+GCTGCACT
TCGAGAACAATTTCTTTGGAAATTGGACATCTTGAATAAGTTAAGAAATAATTCTTCGCGTTAATGCA
GAAATGCGATGAAGAAGATGAAGGAGTCCCCATTACTGTTAAATCAGGGACATTCGCATATTAAGCAT
TATATTGGGGACTGG
+
FFFFFFFFFFFFFFFFFFFFFFFFFF:FFFFFFFFFFFFFFFFFFFFFFFFFFFFFFFFFFFFFFFFF
FFFFFFFFFFFFF:FFFFFFFFFF:FFFFFFF:FFFFFFFFFFFFFFFFFFFFFFFFFFFFFFFFFFF
:FFFFFFFFFFFFFF
@A00721:190:HGJW7DSXY:4:2527:21703:13777 1:N:0:TCTGCAAG+GCTGCACT
TCGAGAACAATTTCTTTGGAAATTGGACATATTGAATAAGTTAAGAAATAATTCTTCGCGTTAATGCA
GAAACGCGATGAAGAAGATGAAGGAGTCCCCATAACTGTTAAATCAGGGACATTCGCATATTAAGCAT
TATATTGGGGACTG
+
FF::FFF,FFFFF:F:FF,FF:F::,F,FF,F::F,:FF,F,FFFFFF:FF,FFF::,,::,FFFF:F
:FFFF:,:FFFFF:FFFFFFFFFFF:F:F,,FF,FF:,F,FFFF:F,,:F,FF,F:FF,F,F:F:,F,
FFFFF:F,,:F:,:
@A00721:190:HGJW7DSXY:4:2606:11333:23437 1:N:0:TCTGCAAG+GCTGCACT
TCGAGAACAATTTCTTTGGAAATTGGACATCTTGAATAAGTTAAGAAATAATTCTTCGCGTTAATGCA
GAAATGCGATGAAGAAGATGAAGGAGTCCCCATTACTGTTAAATCAGGGACATTCGCATATTAATCAT
TATATTGGGGACTG
```

```
+
FF:F,FFFFFFFFFF,FF:F:FFFFFFFFFF:FFFFFFFFFFFFFFFFFFFFF,F,FFFF,FFFFF:F
FFFFFFFFFFFFFFFFFFF:FFFFFF::FF:F,FFFFF,:FFFFFFF:FFFFF:FFFFFFFFFF,FFF
,FFF:F,F:FFFFF
@A00721:190:HGJW7DSXY:4:1512:10556:28573 1:N:0:TCTGCAAG+GCTGCACT
GGACATCTTGAATAAGTTAAGAAATAATTCTTCGCGTTAATGCAGAAACGCGATGAAGAAGATGAAGG
AGTCCCCATTACTGTTAAATCAGGGACATTCGCATATTAAGCATTATATTGGGGACTGGGGACCTATA
TATATGTATAGAGTA
+
FFFFFFFFFFF:FFFFFFFFFFFFFFFFFFFFFFFFFFFFFFFFFFFFFFFFFFFFFFFFFF:FFFFF
FFFFFFFFFFFFFFFFFFFFFFFFFFFFFFFFFFFFFFFFFFFFFFFFFFFFFFFFFFFFFFFFFFFF
FFFFFFFFFFFFFFF
@A00721:190:HGJW7DSXY:4:1607:25265:16438 1:N:0:TCTGCAAG+GCTGCACT
GGACATCTTGAATAAGTTAAGAAATAATTCTTCGCGTTAATGCAGAAACGCGATGAAGAAGATGAAGG
AGTCCCCATTACTGTTAAATCAGGGACATTCGCATATTAAGCATTATATTGGGGACTGGGGACCTATA
TATATGTATAGAGTA
+
::FFFFFFF::F:FFFFFFFFFFFFFFFFFFFFFFFFFFFFFFF:FFFFF:FFFFFF,FFFF::FFFF
FFFFFFFF,FFFFFF:FFF:FFFFFFFFFFFFFFFF:FFFFFFFFFF,F:F:FFFFFFF:FFFFFFFF
FFFFF:FFFFFFF,F
@A00721:190:HGJW7DSXY:4:2302:14416:26021 1:N:0:TCTGCAAG+GCTGCACT
GGACATCTTGAATAAGTTAAGAAATAATTCTTCGCGTTAATGCAGAAACGCGATGAAGAAGATGAAGG
AGTCCCCATTACTGTTAAATCAGGGACATTCGCATATTAAGCATTATATTGGGGACTGGGGACCTATA
TATATGTATAGAGTA
+
FFFFFFFFFFFFFFFFFFFFFFFFFFFFFFFFFFFFFFFFFFFFFFFFFFFFFFFFFFFFFFFFFFFF
FFFFFFFFFFFFFFFFFFFFFFFFFFFFFFFFFFFFFFFFFFFFFFFFFFFFFFFFFFFFFFFFFFFF
FFFFFFFFFFFFFFF
@A00721:190:HGJW7DSXY:4:2549:18295:28635 1:N:0:TCTGCAAG+GCTGCACT
GGACATCTTGAATAAGTTAAGAAATAATTCTTCGCGTTAATGCAGAAACGCGATGAAGAAGATGAAGG
AGTCCCCATTACTGTTAAATCAGGGACATTCGCATATTAAGCATTATATTGGGGACTGGGGACCTATA
TATATGTATAGAGTA
+
FFFFFFFFFFFFFFFFFFFFFFFFFFFFFFFFFFFFFFFFFFFFFFFFFFFFFFFFFFFFFFFFFFFF
FFFFFFFFFFFFFFFFFFFFFFFFFFFFFFFFFFFFFFFFFFFFFFFFFFFFFFFFFFFFFFFFFFFF
FFFFFFFFFFFFF:F
@A00721:190:HGJW7DSXY:4:1133:12066:21574 1:N:0:TCTGCAAG+GCTGCACT
CTCCGGATTACTCTATACATATATATAGGTCCCCAGTCCCCAATATAATGCTTAATATGCGAATGTCC
CTGATTTAACAGTAATGGGGACTCCTTCATCTTCTTCATCGCGTTTCTGCATTAACGCGAAGAATTAT
TTCTTAACTTATTCA
+
FFFFFFFFFFFFFFFFFFFFFFFFFFFFFFFFFFFFFFFFFFFFFFFFFFFFFFFFFFFFFFFFFFFF
FFFFFFFFFFFFF:FFFF:F::FFF:FFFFFFFFFFFFFFFFFFFFFFFFFF,:FFFFFFFFFFFFFF
FFFFFFFFFFFFFFF
@A00721:190:HGJW7DSXY:4:1136:24505:32659 1:N:0:TCTGCAAG+GCTGCACT
CTCCGGATTACTCTATACATATATATAGGTCCCCAGTCCCCAATATAATGCTTAATATGCGAATGTCC
CTGATTTAACAGTAATGGGGACTCCTTCATCTTCTTCATCGCGTTTCTGCATTAACGCGAAGAATTAT
TTCTTAACTTATTCA
+
FFFFFFFFFF:FFFF:FFFFFFFFFFF:FF:FF:F:F:FFFFFFFFFFF:FFFFFFFFFF:FFF:F:,
FFFFFFFF:FFFFFF,FFFF:FF,FFFFFFFFFFF:FFF,FFFFFFFF:F:FFFF,F:FF:FFFFF:
FFFFFFFFFFFFF,F
@A00721:190:HGJW7DSXY:4:1213:15076:32612 1:N:0:TCTGCAAG+GCTGCACT
CTCCGGATTACTCTATACATATATATAGGTCCCCAGTCCCCAATATAATGCTTAATATGCGAATGTCC
```

CTGATTTAACAGTAATGGGGACTCCTTCATCTTCTTCATCGCGTTTCTGCATTAACGCGAAGAATTAT
TTCTTAACTTATTCA
+
FFFFFFFFFFFFFFFFFFFFFFFFFFFFFFFFFFFFFFFFFFFFFFFFFFFFFFFFFFFFFFFFFFFFFF
FFFFFFF:FFFFFFFFFFFFFFFFFFFFFFFFFFFF:FFFFFFFFFF:FFFF,FFFFFFFFFFFF:,FFF
::FFFFFFFFFFF:F
@A00721:190:HGJW7DSXY:4:1264:29234:26475 1:N:0:TCTGCAAG+GCTGCACT
CTCCGGATTACTCTATACATATATATAGGTCCCCAGTCCCCAATATAATGCTTAATATGCGAATGTCC
CTGATTTAACAGTAATGGGGACTCCTTCATCTTCTTCATCGCGTTTCTGCATTAACGCGAAGAATTAT
TTCTTAACTTATTCA
+
FFFFFFFFFFFFFFFFFFFFFFFFFFFFFFFFFFFFFFFFFFFFFFFFFFFFFFFFFFFFFFFFFFFFFF
FFFFFFF:FFFFFFFFFFFFF::FFFFFFFFFFFFFFFFFFFFFFFFFFFFFFFFFFFFFFFFFF::FFF
F:FFFFFFFFFFFFF
@A00721:190:HGJW7DSXY:4:1277:23122:32800 1:N:0:TCTGCAAG+GCTGCACT
CTCCGGATTACTCTATACATATATATAGGTCCCCAGTCCCCAATATAATGCTTAATATGCGAATGTCC
CTGATTTAACAGTAATGGGGACTCCTTCATCTTCTTCATCGCGTTTCTGCATTAACGCGAAGAATTAT
TTCTTAACTTATTCA
+
FFFFFFFFFFFFFFFFFFFFFFF:FFFFFFFFFFFFFFFFFFFFFFFFFFFFFFFFFFFFFFFFFFFFFF
FFFFF:FFFFFFFFFFFFFF:F:FF::FFFFFFFFFFFFFFFFFFFFFFFFFFFFFFFFFFFFFFFFFFF
FFFFFFFFFFFFFFF
@A00721:190:HGJW7DSXY:4:1342:13557:26944 1:N:0:TCTGCAAG+GCTGCACT
CTCCGGATTACTCTATACATATATATAGGTCCCCAGTCCCCAATATAATGCTTAATATGCGAATGTCC
CTGATTTAACAGTAATGGGGACTCCTTCATCTTCTTCATCGCGTTTCTGCATTAACGCGAAGAATTAT
TTCTTAACTTATTCA
+
FFFFFFFFFFFFFFFFFFFFFFFFFFFFFFFFFFFFFFFFFFFFFFFFFFFFFFFFFFFFFFFFFFFFFF
FFFFFFFFFFFFFFFFFFFF:FFFFFFFFFFFFFFFFFFFFFFFFFFFFFFFF:FFFFFFFFFFFFFFFF
FFFFFFFFFFFFFFF
@A00721:190:HGJW7DSXY:4:1347:23421:11397 1:N:0:TCTGCAAG+GCTGCACT
CTCCGGATTACTCTATACATATATATAGGTCCCCAGTCCCCAATATAATGCTTAATATGCGAATGTCC
CTGATTTAACAGTAATGGGGACTCCTTCATCTTCTTCATCGCGTTTCTGCATTAACGCGAAGAATTAT
TTCTTAACTTATTCA
+
FFFFFFFFFFFFFFFFFFFFFFFFFFFFFFFFFFFFFFFFFFFFFFFFFFFFFFFFFFFFFFFFFFFFFF
FFFFFFFFFFFFFFFFFFFF:FFFFFFFFFFFFFFFFFFFFFFFFFFFFFFFFFFFFFFFFFFFFFFFFF
FFFFFFFFFFFFFFF
@A00721:190:HGJW7DSXY:4:1374:15908:28447 1:N:0:TCTGCAAG+GCTGCACT
CTCCGGATTACTCTATACATATATATAGGTCCCCAGTCCCCAATATAATGCTTAATATGCGAATGTCC
CTGATTTAACAGTAATGGGGACTCCTTCATCTTCTTCATCGCGTTTCTGCATTAACGCGAAGAATTAT
TTCTTAACTTATTCA
+
FFFFFFFFFFFFFFFFFFFFFFFFFFFFFFFFFFFFFFFFFFFFFFFFFFFFFFFFFF:FFFFFFFFFFF
FFFFFFFFFFFFFFFFFFFF,F:F:FFFFFFFFFFFFFFFFFFFFFFFFFFFF,FFFFFFFFFFFFFFFF
FFFFFFFFFFFFFFF
@A00721:190:HGJW7DSXY:4:1435:19199:32643 1:N:0:TCTGCAAG+GCTGCACT
CTCCGGATTACTCTATACATATATATAGGTCCCCAGTCCCCAATATAATGCTTAATATGCGAATGTCC
CTGATTTAACAGTAATGGGGACTCCTTCATCTTCTTCATCGCGTTTCTGCATTAACGCGAAGAATTAT
TTCTTAACTTATTCA
+
FFFFFFFFFFFFFFFFFFFFFFFFFFFFFFFFFFFFFFFFFFFFFFFFFFFFFFFFFFFFFFFFFFFFFF
FFFFFFFFFFFFFFFFFFFFFFFFFFFFFFFFFFFFFFFFFFFFFFFFFFFFFFFFFFFFFFFFFFF:FF
FF:FFFFFFFFFFFF

```
@A00721:190:HGJW7DSXY:4:1539:19488:29074 1:N:0:TCTGCAAG+GCTGCACT
CTCCGGATTACTCTATACATATATATAGGTCCCCAGTCCCCAATATAATGCTTAATATGCGAATGTCC
CTGATTTAACAGTAATGGGGACTCCTTCATCTTCTTCATCGCGTTTCTGCATTAACGCGAAGAATTAT
TTCTTAACTTATTCA
+
FFFFFFFFFFFFFFFFFFFFFFFFFFFFFFFFFFFFFFFFFFFFF:FFFFFFFFFFF:FFFFFFFF
FFFF,:FFFFFFF:FFFFFF::,FFFFFFFFFFFFFFFFFFFFFFFFFFFFFFFFFFFFFFFFFFFFF
FFFFFFFFFFFFFFF
@A00721:190:HGJW7DSXY:4:1545:10601:21543 1:N:0:TCTGCAAG+GCTGCACT
CTCCGGATTACTCTATACATATATATAGGTCCCCAGTCCCCAATATAATGCTTAATATGCGAATGTCC
CTGATTTAACAGTAATGGGGACTCCTTCATCTTCTTCATCGCGTTTCTGCATTAACGCGAAGAATTAT
TTCTTAACTTATTCA
+
FFFFFFFFFFFFFFFFFFFFFFFFFFFFFFFFFFFFFFFFFFFFFFF:FFFFFFFFFFFFFFFFFFF
FFFFFFFFFFFFFFFFFFFFFFFFFF,FFFFFFFFFFFFFFFFFFFFF:FFFFFFFFFFFF:F,FF::
FFFFFFFFFFFFF,F
@A00721:190:HGJW7DSXY:4:2519:29821:5979 1:N:0:TCTGCAAG+GCTGCACT
CTCCGGATTACTCTATACATATATATAGGTCCCCAGTCCCCAATATAATGCTTAATATGCGAATGTCC
CTGATTTAACAGTAATGGGGACTCCTTCATCTTCTTCATCGCGTTTCTGCATTAACGCGAAGAATTAT
TTCTTAACTTATTCA
+
FFFFFFFFFFFFFFFFFFFFFFFFFFFFF,FFFFFFFFFFFFFFFFFFFFFFFFFFFFFF:FFFFFFF:
FFFFFFFFFFFF:FF:F,,FFF::FFFFFFFFFFFFFFFF::FFFFFF:FFFFFFFFFFFFFF::F:F
FFFFFF,FFFFFF,F
@A00721:190:HGJW7DSXY:4:2666:21730:26318 1:N:0:TCTGCAAG+GCTGCACT
CTCCGGATTACTCTATACATATATATAGGTCCCCAGTCCCCAATATAATGCTTAATATGCGAATGTCC
CTGATTTAACAGTAATGGGGACTCCTTCATCTTCTTCATCGCGTTTCTGCATTAACGCGAAGAATTAT
TTCTTAACTTATTCA
+
FFFFFFFFFFFFFFFFFFFFFFFFFFFFFFFFFFFFFFFFFFFFFFFFFFFFFFFFFFFFFFFFFFFF
FFFF,:FFFFFFF:FFFFFFF::FF,FFFFFFFFFFFFFFFFF:FFFFFFFFFFFFFFFFFFFFFFFF
FFFFFFFFFFFFFFF
@A00721:190:HGJW7DSXY:4:1510:26395:35117 1:N:0:TCTGCAAG+GCTGCACT
GATAGATTCCTCCGGATTACTCTATACATATATATAGGTCCCCAGTCCCCAATATAATGCTTAATATG
CGAATGTCCCTGATTTAACAGTAATGGGGACTCCTTCATCTTCTTCATCGCGTTTCTGCATTAACGCG
AAGAATTATTTCTTA
+
FFFFFFFFFFFFFFFFFFFFFFFFFFFFFFFFFFFFFFFFFFFFFFFFFFFFFFFFFFFFFFFFFFFF
FFFFFFFFFFFFFFFFFFFFF:FFFFFFF:FFFFFFFFFFFFFFFFFFFFFFFFFFFFFFFFFF,FFF
FFFFFFFFFFF,FFF
@A00721:190:HGJW7DSXY:4:1556:12500:6386 1:N:0:TCTGCAAG+GCTGCACT
ATAGATTCCTCCGGATTACTCTATACATATATATAGGTCCCCAGTCCCCAATATAATGCTTAATATGC
GAATGTCCCTGATTTAACAGTAATGGGGACTCCTTCATCTTCTTCATCGCGTTTCTGCATTAACGCGA
AGAATTATTTCTTA
+
FFFFFFFFFFFFFFFF:F:FFFFFFFFFFFFFFFFF:FFFFFFFFFFFFFFFFFFFFFFFFFFFFFFF
FFFF::FFFF,FFFFFF:FF,FF:FFFF:FFFFFFFFFFFFFFF:FFFFFFFF:FFFFFF:FFFFFFF
FFFFFFFF:FFFFF
@A00721:190:HGJW7DSXY:4:2660:9715:10050 1:N:0:TCTGCAAG+GCTGCACT
GATAGATTCCTCCGGATTACTCTATACATATATATAGGTCCCCAGTCCCCAATATAATGCTTAATATG
CGAATGTCCCTGATTTAACAGTAATGGGGACTCCTTCATCTTCTTCATCGCGTTTCTGCATTAACGCG
AAGAATTATTTCTTA
+
FFFFFFFFFFFFFFFFFFFFFFFFFFFFFFFFFFFFFFFFFFFFFFFFFFFFFFFFFFFFFFFFFFFF
```

FFFFFFFFFFFFFFFFFFFFFFFFFFFFFFFFFFFFFFFFFFFFFFFFFFFFFFFFFFFFFFFFFFFFFF
FFFF:FFFFFFFFFF
@A00721:190:HGJW7DSXY:4:1212:23936:33364 1:N:0:TCTGCAAG+GCTGCACT
GACTATTCCAATTGGGGATAGATTCCTCCGGATTACTCTATACATATATATAGGTCCCCAGTCCCCAA
TATAATGCTTAATATGCGAATGTCCCTGATTTAACAGTAATGGGGACTCCTTCATCTTCTTCATCGCG
TTTCTGCATTAACGC
+
FFFFFF::FFFFFFFFFFFFFFFFFFFFFFFFFFFFFFFFFFFFFFFFFFFFFFFFFFFFFFFFFFFF
FFFFFFF:FFFFFFFFFFFFFFFFFFFFFFFFF:FFFF:FFFFFFF:FFFFFFFFFFFFFFFFFFFF:
FFFFFFFFFFFFFFF
@A00721:190:HGJW7DSXY:4:1351:12825:11490 1:N:0:TCTGCAAG+GCTGCACT
GACTATTCCAATTGGGGATAGATTCCTCCGGATTACTCTATACATATATATAGGTCCCCAGTCCCCAA
TATAATGCTTAATATGCGAATGTCCCTGATTTAACAGTAATGGGGACTCCTTCATCTTCTTCATCGCG
TTTCTGCATTAACGC
+
FFFF,FFFFFFFFFFFFFFFFFFFFFFFFFFFFFFFFFFFFFFFFFFFFFFFFFFFFFFFFFFFFFFF
FFFFFFFFFFFFFFFFFFFFFFFFFFFFF:FFF:FFFF:F:FFFFFFFFF:FFFFFFFFFFFFFFFF
FF:FFFFFFF:FFFF
@A00721:190:HGJW7DSXY:4:2136:30382:23641 1:N:0:TCTGCAAG+GCTGCACT
GACTATTCCAATTGGGGATAGATTCCTCCGGATTACTCTATACATATATATAGGTCCCCAGTCCCCAA
TATAATGCTTAATATGCGAATGTCCCTGATTTAACAGTAATGGGGACTCCTTCATCTTCTTCATCGCG
TTTCTGCATTAACGC
+
FFFFFFFFFFFFFFFFFFFFFFFFFFFFFFFFFFFFFFFFFFFFFFFFFFFFFFFFFFFFFFFFFFFF
FFFFFFFFFFFFFFFFFFFFFFFFFFFFFFFFFFFFFFF:FFFFFF:F:FFFFFFFFFFFFFFFFFFF
FFFFFFFFFFFFFFF
@A00721:190:HGJW7DSXY:4:2262:31964:24815 1:N:0:TCTGCAAG+GCTGCACT
GACTATTCCAATTGGGGATAGATTCCTCCGGATTACTCTATACATATATATAGGTCCCCAGTCCCCAA
TATAATGCTTAATATGCGAATGTCCCTGATTTAACAGTAATGGGGACTCCTTCATCTTCTTCATCGCG
TTTCTGCATTAACGC
+
FFFFFFFFFFFFFFFFFFFFFFFFFFFFFFFFFFFFFFFFFFFFFFFF:FFFFFFFFFFFFFFFFFFF
FFFFFFFFFFFFFFFFFFFFFFFFF:FFFFFFFFFFFFFFFFFFFFFF:FFFFFFFFFFFFFFFFFFFFF
FFF:FF::FFFFF::
@A00721:190:HGJW7DSXY:4:2536:8784:4022 1:N:0:TCTGCAAG+GCTGCACT
GACTATTCCAATTGGGGATAGATTCCTCCGGATTACTCTATACATATATATAGGTCCCCAGTCCCCAA
TATAATGCTTAATATGCGAATGTCCCTGATTTAACAGTAATGGGGACTCCTTCATCTTCTTCATCGCG
TTTCTGCATTAACGC
+
FFFFFFFFFFFFFFFFFFFFFFFFFFFFFFFFFFFFFFFFFFFFFFFFFFFFFFFFFFFFFFFFFFFF
FFFFFFFFFFFFFFFFFFFFFFFFFFFFFFFFFFFFFFF,FFFFFFF:FFFFFFFFFFFFFFFFFFFFF
FFFFFFFFFFFFF:F
@A00721:190:HGJW7DSXY:4:1111:14986:12853 1:N:0:TCTGCAAG+GCTGCACT
GCGGGATATTTTCAAATTTGAAATTTGAAATCATTACTATTCCAATTGGGGATAGATTCCTCCGGATT
ACTCTATACATATATATAGGTCCCCAGTCCCCAATATAATGCTTAATATGCGAATGTCCCTGATTTAA
CAGTAATGGGGACTC
+
FFFFFFFFFFFFFFFFFFFFFFFFFFFFFFFFFFFFFFFFFFFFFFFFFFFFFFFFFFFFFFFFFFFF
FFFFFFFFFFFFFFFFFFFFFFFFFFFFFFFFFF:FFFFFFFFFFFFFFFFFFFFFFFFFFFFFFFFF
FFFF:FFFFFFFFFF
@A00721:190:HGJW7DSXY:4:2242:18837:25285 1:N:0:TCTGCAAG+GCTGCACT
GCGGGATATTTTCAAATTTGAAATTTGAAATCATTACTATTCCAATTGGGGATAGATTCCTCCGGATT
ACTCTATACATATATATAGGTCCCCAGTCCCCAATATAATGCTTAATATGCGAATGTCCCTGATTTAA
CAGTAATGGGGACTC

+
FFFFFFFFFFFFFFFFFFFFFFFFFFFFFFFFFFFFFFFFFFFFFFFFFFFFFFFFFFFFFFFFFFFF
FFFFFFFFFFFFFFFFFFFFFFF:FFFFFFFFFFF,FFFFFFFFFFFFFFFFF:FFFFFFFFFFFFFF
FFFFFFFFFFFFFFF
@A00721:190:HGJW7DSXY:4:2457:1253:6355 1:N:0:TCTGCAAG+GCTGCACT
GCGGGATATTTTCAAATTTGAAATTTGAAATCATTACTATTCCAATTGGGGATAGATTCCTCCGGATT
ACTCTATACATATATATAGGTCCCCAGTCCCCAATATAATGCTTAATATGCGAATGTCCCTGATTTAA
CAGTAATGGGGACTC
+
FFFFFFFFFFFFFFFFFFFFFFFFFFFFFFFFFFFFFFF:FFFFFFFFFFFFFFFFFFFFFFFFFFFF
FFFFFFFFFFFFFFFFFFFFFFFFFFFFFFFFFFFFFFFFFFFFFFFFFFFFFFFFFFFFFFFFFFFF
FFFFFFFFFFFFFFF
@A00721:190:HGJW7DSXY:4:2548:14787:14262 1:N:0:TCTGCAAG+GCTGCACT
GCGGGATATTTTCAAATTTGAAATTTGAAATCATTACTATTCCAATTGGGGATAGATTCCTCCGGATT
ACTCTATACATATATATAGGTCCCCAGTCCCCAATATAATGCTTAATATGCGAATGTCCCTGATTTAA
CAGTAATGGGGACTC
+
FFFFFFFFFFFFFFFFFFFFFFFFFFFFFFFFFFFFFFF,FFFFFFFFFFFFFFFFFFFFFFFFFFFF
FFFFFFFFFFFFFFFFFFFFFFFFFFFFFFFFFFFFFFFFFFFFFFFFFFFFFFFFFFFFFFFFFFFF
FFFFFFFFFFFFFFF
@A00721:190:HGJW7DSXY:4:1212:28212:23140 1:N:0:TCTGCAAG+GCTGCACT
GGGCGGGATATTTTCAAATTTGAAATTTGAAATCATTACTATTCCAATTGGGGATAGATTCCTCCGGA
TTACTCTATACATATATATAGGTCCCCAGTCCCCAATATAATGCTTAATATGCGAATGTCCCTGATTT
AACAGTAATGGGGAC
+
FFFFFFFFFFFFFFFFFFF,,:FFFFFFFF:FFFF,FFFFFFFFFFFFFFFFFFFFFF,FF:FFFF:F
FFFFFFFFFF:FF:F:FFFFFFFFFFF,FFFFFFF:FFFFFFFF,FFF:FF,F:F,:FFFFFFFF,FF
F,F:F,FFFFFFFFF
@A00721:190:HGJW7DSXY:4:1240:8983:33301 1:N:0:TCTGCAAG+GCTGCACT
GGGCGGGATATTTTCAAATTTGAAATTTGAAATCATTACTATTCCAATTGGGGATAGATTCCTCCGGA
TTACTCTATACATATATATAGGTCCCCAGTCCCCAATATAATGCTTAATATGCGAATGTCCCTGATTT
AACAGTAATGGGGAC
+
FFFFFFFFFFFFFFFFFFFFFFFFFFFFFFFFFFFFFFFFFFFFFFFFFFFFFFFFFFFFFFFFFFFF
FFFFFFFFFFFFFFFFFFFFFFFFFFFFFFFFFFFFFFFFFFFFFFFFFFFFFFFFFFFFFFFFFFFF
FFFFFFFFFFFFFFF
@A00721:190:HGJW7DSXY:4:1304:14443:22467 1:N:0:TCTGCAAG+GCTGCACT
GGGCGGGATATTTTCAAATTTGAAATTTGAAATCATTACTATTCCAATTGGGGATAGATTCCTCCGGA
TTACTCTATACATATATATAGGTCCCCAGTCCCCAATATAATGCTTAATATGCGAATGTCCCTGATTT
AACAGTAATGGGGAC
+
FFFFFFFFFFFFFFFFFFFFFFFFFFFFFFFFFFFFFFFFFFFFFFFFFFFFFFFFFFFFFFFFFFFF
FFFFFFFFFFFFFFFFFFFFFFFFFFFFFFFFFFFFFFFFFFFFFFFFFFFFFFFFF:FFFFFFFFFF
FFFFFFFFFFFFFFF
@A00721:190:HGJW7DSXY:4:1415:14543:33160 1:N:0:TCTGCAAG+GCTGCACT
GGGCGGGATATTTTCAAATTTGAAATTTGAAATCATTACTATTCCAATTGGGGATAGATTCCTCCGGA
TTACTCTATACATATATATAGGTCCCCAGTCCCCAATATAATGCTTAATATGCGAATGTCCCTGATTT
AACAGTAATGGGGAC
+
FFFFFFFFFFFFFFFFFFFFFFFFFFFFFFFFFFFFFFFFFFFFFFFFFFFFFFFFFFFFFFFFFFFF
FFFFFFFFFFFFFFFFFFFFFFFFFFFFFFFFFFFFFFFFFFFFFFFFFFFFFFFFFFFFFFFFFFFF
FFFFFFFFFF:FFFF
@A00721:190:HGJW7DSXY:4:1673:19199:11099 1:N:0:TCTGCAAG+GCTGCACT
GGGCGGGATATTTTCAAATTTGAAATTTGAAATCATTACTATTCCAATTGGGGATAGATTCCTCCGGA

```
TTACTCTATACATATATATAGGTCCCCAGTCCCCAATATAATGCTTAATATGCGAATGTCCCTGATTT
AACAGTAATGGGGAC
+
FFFFFFFFFFFFFFFFFFFFFFFFFFFFFFFFFFFFFFFFFFFFFFFFFFFFFFFFFFFFFFFFFFFF
FFFFFFFFFFFFFFFFFFFFFFFFFFFFFFFFFFFFFFFF,FFFFFFFFFFFFFFFFFFFFFFFFFFF
FFFFFFFFFFFFFFF
@A00721:190:HGJW7DSXY:4:2443:14009:31735 1:N:0:TCTGCAAG+GCTGCACT
GGGCGGGATATTTTCAAATTTGAAATTTGAAATCATTACTATTCCAATTGGGGATAGATTCCTCCGGA
TTACTCTATACATATATATAGGTCCCCAGTCCCCAATATAATGCTTAATATGCGAATGTCCCTGATTT
AACAGTAATGGGGAC
+
FFFFFFFFFFFFFFFFFFFFFF:FFFFFFFFFFFFFFFF:FFFFFFFFFF,FFFFFFFFF,FFFFFFF
FFFFFFFFFFFFFFFFFFFF,:FF:FFFFFFFFFFF:FFFFF:FFFFFFFF::FFFFFFF:FFFFFFF
FFFFFFFFFFFF,FF
@A00721:190:HGJW7DSXY:4:2671:9896:20854 1:N:0:TCTGCAAG+GCTGCACT
GGGCGGGATATTTTCAAATTTGAAATTTGAAATCATTACTATTCCAATTGGGGATAGATTCCTCCGGA
TTACTCTATACATATATATAGGTCCCCAGTCCCCAATATAATGCTTAATATGCGAATGTCCCTGATTT
AACAGTAATGGGGAC
+
FFFFFFFFFFFFFFFFFFFFFFFFFFFFFFFFFFFFFFFFFFFFFFFFFFFFFFFFFFFFFFFFFFFF
FFFFFFFFFFFFFFFFFFFFFFFFFFFFFFFFFFFFFFFFFFFFFFFFFFFFFFFFFFFFFFFFFFFF
FFFFFFFFFFFFFFF
@A00721:190:HGJW7DSXY:4:2671:9905:21183 1:N:0:TCTGCAAG+GCTGCACT
GGGCGGGATATTTTCAAATTTGAAATTTGAAATCATTACTATTCCAATTGGGGATAGATTCCTCCGGA
TTACTCTATACATATATATAGGTCCCCAGTCCCCAATATAATGCTTAATATGCGAATGTCCCTGATTT
AACAGTAATGGGGAC
+
FFFFFFFFFFFFFFFFFFFFFFFFFFFFFFFFFFFFFFFFFFFFFFFFFFFFFFFFFFFFFFFFFFFF
FFFFFFFFFFFFFFFFFFFFFFFFFFFFFFFFFFFFFFFFFFFFFFFFFFFFFFFFFFFFFFFFFFFF
FFFFFFFFFFFFFFF
@A00721:190:HGJW7DSXY:4:1272:10520:25347 1:N:0:TCTGCAAG+GCTGCACT
CCCCATTACTGTTAAATCAGGGACATTCGCATATTAAGCATTATATTGGGGACTGGGGACCTATATAT
ATGTATAGAGTAATCCGGAGGAATCTATCCCCAATTGGAATAGTAATGATTTCAAATTTCAAATTTGA
AAATATCCCGCCCTA
+
FFFFFFFFFFFFFFFFFFF:FFFFFFFFFFFFFFFFFFFF:FFFF,FFFFFFFFFFFFFFFFF:FFFF
FFFFFFFFFFFFFFFFFFFFFFFFFFFFFFFFFFFFFFF:FFFFFFFFFFFFFFFFFFFFFFFFFFFF
FFFFFFFFFFF,FFF
@A00721:190:HGJW7DSXY:4:1343:26675:1971 1:N:0:TCTGCAAG+GCTGCACT
CCCCATTACTGTTAAATCAGGGACATTCGCATATTAAGCATTATATTGGGGACTGGGGACCTATATAT
ATGTATAGAGTAATCCGGAGGAATCTATCCCCAATTGGAATAGTAATGATTTCAAATTTCAAATTTGA
AAATATCCCGCCCTA
+
FFFFFFFFFFFFFFFFFFFFFFFFFFFFFFFFFFFFFFFFFFFFFFFFFFFFFFFFFFFFFFFFFFFF
FFFFFFFFFFFFFFFFF:FFFFFFFFFFFFFFFFFFFFFFFFFFFFFFFFFFFFFFFFFFFFFFFFFF
FFFFFFFFFFFFFFF
@A00721:190:HGJW7DSXY:4:1343:26096:2253 1:N:0:TCTGCAAG+GCTGCACT
CCCCATTACTGTTAAATCAGGGACATTCGCATATTAAGCATTATATTGGGGACTGGGGACCTATATAT
ATGTATAGAGTAATCCGGAGGAATCTATCCCCAATTGGAATAGTAATGATTTCAAATTTCAAATTTGA
AAATATCCCGCCCTA
+
FFFFFFFFFFFFFFFFF,,F,FFFF:FFFFFFFFFFFFFFFFF:F:FFFFFFFFFF:,FFFFFFFFFF
FFFFFFF,FFFFFF::F,FFFFFFFFFFF:FFFFF:FF:,FF::FF:,FFFFFFFFFFFFFF:FFFFF
FFFFFF,F:,,F:F:
```

@A00721:190:HGJW7DSXY:4:1406:24831:26209 1:N:0:TCTGCAAG+GCTGCACT
CCCCATTACTGTTAAATCAGGGACATTCGCATATTAAGCATTATATTGGGGACTGGGGACCTATATAT
ATGTATAGAGTAATCCGGAGGAATCTATCCCCAATTGGAATAGTAATGATTTCAAATTTCAAATTTGA
AAATATCCCGCCCTA
+
FFFFFFFFFFFFFFFFFFFFFFFFFFFFFFFFFFFFFFFFFFFFFFFFFFFFFFFFFFFFFFFFFFFF
FFFFFFFFFFFFFFFFFFFFFFFFFFFFFFFFFFFFFFF:FFFFFFFFFFFFFFFFFFFFFFFFFFFF
FFFFFFFFFFFFF:F
@A00721:190:HGJW7DSXY:4:2349:24713:12446 1:N:0:TCTGCAAG+GCTGCACT
CCCCATTACTGTTAAATCAGGGACATTCGCATATTAAGCATTATATTGGGGACTGGGGACCTATATAT
ATGTATAGAGTAATCCGGAGGAATCTATCCCCAATTGGAATAGTAATGATTTCAAATTTCAAATTTGA
AAATATCCCGCCCTA
+
FFFFFFFFFFFFFFFFFFF:FFFFFFFFFFFFFFFFFFFFFFFFFFFFFFFFFFFFFFFFF:F:FFFF
FFFFFFFFFFFFFFFFFFFFFFFFFFFFFFFFFFFFFFFF:FFFFFFFFFFFFFF:F:FFFFFFFF,,
FFFFFFFF:FF:FFF
@A00721:190:HGJW7DSXY:4:2636:18765:28072 1:N:0:TCTGCAAG+GCTGCACT
CCCCATTACTGTTAAATCAGGGACATTCGCATATTAAGCATTATATTGGGGACTGGGGACCTATATAT
ATGTATAGAGTAATCCGGAGGAATCTATCCCCAATTGGAATAGTAATGATTTCAAATTTCAAATTTGA
AAATATCCCGCCCT
+
FFFFFFFFFFF:FFFFFFFFFFFFFFFFFFFFFFFFFFFFFFFFF,FF,FFFFFF,FFF:FFFFFFFF
FFFFFFFFFFFFFFF,FFFFFF:FFFFFFFFFFF:,:FF:FFFFFFFFFFFFFFFFFFF:FFFFF:FF
:FFFFFFF,FF:FF
@A00721:190:HGJW7DSXY:4:1418:10004:3568 1:N:0:TCTGCAAG+GCTGCACT
CGGACATTCGCATATTAAGCATTATATTGGGGACTGGGGACCTATATATATGTATAGAGTAATCCGGA
GGAATCTATCCCCAATTGGAATAGTAATGATTTCAAATTTCAAATTTGAAAATATCCCGCCCTACAGT
ATACCAATTCAAATT
+
FFFFFFFFFFFFFFFFFFFFFFFFFFFFFFFFFFFFFF:F:FFFFFFFFFFFFFFFFFFFFFFFFFFF
FFFFFF:FFFFFFFFFFFFFFFFFFFFFFFFFF:FFFF,FFFFFFFFFFFF:FFFFF:::FFFFFFFF
FFF:FFFFFFFFFFF
@A00721:190:HGJW7DSXY:4:1525:7880:20525 1:N:0:TCTGCAAG+GCTGCACT
CGGACATTCGCATATTAAGCATTATATTGGGGACTGGGGACCTATATATATGTATAGAGTAATCCGGA
GGAATCTATCCCCAATTGGAATAGTAATGATTTCAAATTTCAAATTTGAAAATATCCCGCCCTACAGT
ATACCAATTCAAATT
+
FFFFFFFFFFFFFFFFFFFFFFFFFF:FFFFFFFFFFFFFFF:FFFFFFFFFFFFFFF:FFFFFFFFF
FFFFFFFFFFFFFFFFFFFFFFFFFFFFFFFFFFFFFFFFFFFFFFFFFFFFFFFFFFFFFFFFFFFF
FFFF:FFFFFFFFFF
@A00721:190:HGJW7DSXY:4:1526:9317:11960 1:N:0:TCTGCAAG+GCTGCACT
CGGACATTCGCATATTAAGCATTATATTGGGGACTGGGGACCTATATATATGTATAGAGTAATCCGGA
GGAATCTATCCCCAATTGGAATAGTAATGATTTCAAATTTCAAATTTGAAAATATCCCGCCCTACAGT
ATACCAATTCAAATT
+
FFFFFFFFFFFFFFFFFFFFFFFFFFFFFFFFFFFFFFFFFFFFFFFFFFFFFFFFFFFFFFFFFFFF
FFFFFFFFFFFFFFFFFFFFFFFFFFFFFFFFFFFFFFFFFFFFFFFFFFFFFFFFFFFFFFFFFFF,
FFFFFFFFFFFFFFF
@A00721:190:HGJW7DSXY:4:1529:9091:8594 1:N:0:TCTGCAAG+GCTGCACT
CGGACATTCGCATATTAAGCATTATATTGGGGACTGGGGACCTATATATATGTATAGAGTAATCCGGA
GGAATCTATCCCCAATTGGAATAGTAATGATTTCAAATTTCAAATTTGAAAATATCCCGCCCTACAGT
ATACCAATTCAAATT
+
FFFFFFFFFFFFFFFFFFFFFFFFFF:FFFFFFFFFFFFFFFFFFFFFFFFFFFFFFFFFFFFFFFFF

FFFFFFFFFFFFFFFFFFFFFFFFFFFFFFFFFFFFFFFFFFFFFFFFFFFFFFFFFFFFFFFFF,F:
FFFFFFFFFFFFFF:
@A00721:190:HGJW7DSXY:4:1547:18900:14309 1:N:0:TCTGCAAG+GCTGCACT
CGGACATTCGCATATTAAGCATTATATTGGGGACTGGGGACCTATATATATGTATAGAGTAATCCGGA
GGAATCTATCCCCAATTGGAATAGTAATGATTTCAAATTTCAAATTTGAAAATATCCCGCCCTACAGT
ATACCAATTCAAATT
+
FFFFFFFFFFFFFFFFFFFFFFFFFF:FFFFFFFFFFFFFFFFFFFFFFFFFFFFF:FFFFFFFFFFF
FFF:FFFFFFFFFFF:FFFFFFFFFFFFFFFFFFFFFFFFFFFFFFFFFFFFFFFFFFFFFFFFFFFF
FFFFFFF,FFFF:FF
@A00721:190:HGJW7DSXY:4:1565:11686:23109 1:N:0:TCTGCAAG+GCTGCACT
CGGACATTCGCATATTAAGCATTATATTGGGGACTGGGGACCTATATATATGTATAGAGTAATCCGGA
GGAATCTATCCCCAATTGGAATAGTAATGATTTCAAATTTCAAATTTGAAAATATCCCGCCCTACAGT
ATACCAATTCAAATT
+
FFFFFFFFFFFFFFFFFFFFFFFFFFFFFFFFFFFFFFFFFFFFFFFFFFFFFFFFFFFFFFFFF:FF
FFF:FFFFFFFFFFFFFFFFFFFFFFFFFFFFFFFFFF,FFFFFFFFFFFFFFFFFFFFFFFFFF:,F
FFFF:FFFFFFFFFF
@A00721:190:HGJW7DSXY:4:2239:9869:26976 1:N:0:TCTGCAAG+GCTGCACT
GTCCGCATATTAAGCATTATATTGGGGACTGGGGACCTATATATATGTATAGAGTAATCCGGAGGAAT
CTATCCCCAATTGGAATAGTAATGATTTCAAATTTCAAATTTGAAAATATCCCGCCCTACAGTATACC
AATTCAAATTCAAAT
+
FFFFF,FFFFFFFFFFFFFFFFFFFFFFFFFFFFFFFFFFFFFFFFFFFFFFFFFFFFFFFFFFFFFF
FFFFFFFFFFFFFFFFFFFFFFF:FFFFFFFFFFFFFFFFFFFFFFFFFFF:FFFFFFFFFFFFFFFF
FFFF,FF:FFFFFFF
@A00721:190:HGJW7DSXY:4:2639:6433:6214 1:N:0:TCTGCAAG+GCTGCACT
GTCCGCATATTAAGCATTATATTGGGGACTGGGGACCTATATATATGTATAGAGTAATCCGGAGGAAT
CTATCCCCAATTGGAATAGTAATGATTTCAAATTTCAAATTTGAAAATATCCCGCCCTACAGTATACC
AATTCAAATTCAAAT
+
FFFFFFFFFFFFFFFFFFFFFFFFFFFFFFFFFFFFFFFFFFFFFFFFFFFFFFFFFFFFFFFFFFFF
FFFFFFFFFFFFFFFFFFFFFFFFFFFFFFFFFFFFFFFFFFFFFFFFFFFFFFFFFFFFFFFFFFFF
FFFFFFFFFFFFFFF
@A00721:190:HGJW7DSXY:4:1140:6280:1783 1:N:0:TCTGCAAG+GCTGCACT
CCGCATATTAAGCATTATATTGGGGACTGGGGACCTATATATATGTATAGAGTAATCCGGAGGAATCT
ATCCCCAATTGGAATAGTAATGATTTCAAATTTCAAATTTGAAAATATCCCGCCCTACAGTATACCAA
TTCAAATTCAAATTC
+
FFFFFFFFFFFFFFFFFFF:FFFFFFFFFFFFFFFFFFF:FFFFFFFFFFFFFFFFFFFFFFFFFFFF
FFFFFFFFFFFFFFFFFFFFFFFFFFFF:FFFFFFFFFFFFFFFFFFFFFFFFFFFFFFFFFFFFFFF:
FFFFFFF:FF,FFFF
@A00721:190:HGJW7DSXY:4:1239:2926:9815 1:N:0:TCTGCAAG+GCTGCACT
CCGCATATTAAGCATTATATTGGGGACTGGGGACCTATATATATGTATAGAGTAATCCGGAGGAATCT
ATCCCCAATTGGAATAGTAATGATTTCAAATTTCAAATTTGAAAATATCCCGCCCTACAGTATACCAA
TTCAAATTCAAATTC
+
FFFFFFFFFFFFFFFFFFFFFFFFFFFFFFFFFFFFFFFFFFFFFFFFFFFFFF:FFFFFFFFF,FFF
FFFFFFFFFFFFFFFFFFF:FFFFFFFFFFFFFFFFFFFFFFFFFFFFFFFFFFFFFFFFFFFFFFF:
FFFFFFFFFF:FFFF
@A00721:190:HGJW7DSXY:4:1301:1389:25441 1:N:0:TCTGCAAG+GCTGCACT
CCGCATATTAAGCATTATATTGGGGACTGGGGACCTATATATATGTATAGAGTAATCCGGAGGAATCT
ATCCCCAATTGGAATAGTAATGATTTCAAATTTCAAATTTGAAAATATCCCGCCCTACAGTATACCAA
TTCAAATTCAAATTC

```
+
FFFFFFFFFFFFFFFFFFFFFFFFFFFFFFFFFFFFFFFFFFFFFFFFFFFFFFFFFFFFFFFFFFFF
FFFFFFFFFFFFFFFFFFFFFFFFFFFFFFFFFFFFFFFFFFFFFFFFFFFFFFFFFFFFFFFFFFFF
FFFFF:FFF:FFFFF
@A00721:190:HGJW7DSXY:4:1301:16278:35790 1:N:0:TCTGCAAG+GCTGCACT
CCGCATATTAAGCATTATATTGGGGACTGGGGACCTATATATATGTATAGAGTAATCCGGAGGAATCT
ATCCCCAATTGGAATAGTAATGATTTCAAATTTCAAATTTGAAAATATCCCGCCCTACAGTATACCAA
TTCAAATTCAAATTC
+
FFFFFFFFFFFFFFFFFF:FFFFFFFFFFFFFFFFFFFFFFFFFFFFFFFFFFF:FFFFFFFFFFFFF
FFFFFFFFFFFFFFFFFFFFFFFFFFFFFFFFFFFFFFFFFFFFFFFFFFFFFFFFFFFFFFFFFFFF
:FFFFFFFFFFFFFF
@A00721:190:HGJW7DSXY:4:1310:21206:18270 1:N:0:TCTGCAAG+GCTGCACT
CCGCATATTAAGCATTATATTGGGGACTGGGGACCTATATATATGTATAGAGTAATCCGGAGGAATCT
ATCCCCAATTGGAATAGTAATGATTTCAAATTTCAAATTTGAAAATATCCCGCCCTACAGTATACCAA
TTCAAATTCAAATTC
+
FFFFFFFFFFFFFFFFFFFFFFFFFFFFFFFFFFFFFFFFFFFFFFFFFFFFFF:FFFFFFFFF:FFF
FFFFFFFFFFFFFFFFFFFFFFFFFFFFFFFFFFFFFFFFFFFFFFFFFFFFFFFFFFFFFFFFFFFF
FFFFFFFFFFFFFFF
@A00721:190:HGJW7DSXY:4:1612:22869:5963 1:N:0:TCTGCAAG+GCTGCACT
CCGCATATTAAGCATTATATTGGGGACTGGGGACCTATATATATGTATAGAGTAATCCGGAGGAATCT
ATCCCCAATTGGAATAGTAATGATTTCAAATTTCAAATTTGAAAATATCCCGCCCTACAGTATACCAA
TTCAAATTCAAATTC
+
FFFFFFFFFFFFFFFFFFFFFFFFFFFFFFFFFFFFFFFFFFFFFFFFFFFFFFFFFFFFFFFFFFFF
FFFFFFFFFFFFFFFFFFFFFFFFFFFFFFFFFFFFFFFFFFFFFFFFF,FFFFF:FFFFFFFFFFFF
:FFFFFFFFFFFFFF
@A00721:190:HGJW7DSXY:4:2145:16947:17065 1:N:0:TCTGCAAG+GCTGCACT
CCGCATATTAAGCATTATATTGGGGACTGGGGACCTATATATATGTATAGAGTAATCCGGAGGAATCT
ATCCCCAATTGGAATAGTAATGATTTCAAATTTCAAATTTGAAAATATCCCGCCCTACAGTATACCAA
TTCAAATTCAAATTC
+
FFFFFFFFFFFFFFFFFFF:FFFFFFFFFFFFFFFFFFFFFFFFFFFFFFFFFFFFFFFFFFFFFFFF
FFFFFFFFFFFFFFFFFFFFFFFFFFFFFFFFFFFFFFFFFF:FFFFFFFFFFFFFFFFFFFFFFFFF
FFFFFFFFFFF:FFF
@A00721:190:HGJW7DSXY:4:2169:19307:31955 1:N:0:TCTGCAAG+GCTGCACT
CCGCATATTAAGCATTATATTGGGGACTGGGGACCTATATATATGTATAGAGTAATCCGGAGGAATCT
ATCCCCAATTGGAATAGTAATGATTTCAAATTTCAAATTTGAAAATATCCCGCCCTACAGTATACCAA
TTCAAATTCAAATTC
+
FFFFFFFFFFFFFFFFFFFFFFFFFFFFFFFFFFFFFFFFFFFFFFFFFFFFFFFFFFFFFFFFFFFF
FFFFFFFFFFFFFFFFFFFFFFFFFFFFFFFFFFFFFFFFFFFFFFFF:,FFFFFFFFFFFFFFFFFF
FFFFFFFFFFFFFFF
@A00721:190:HGJW7DSXY:4:2336:22968:27023 1:N:0:TCTGCAAG+GCTGCACT
CCGCATATTAAGCATTATATTGGGGACTGGGGACCTATATATATGTATAGAGTAATCCGGAGGAATCT
ATCCCCAATTGGAATAGTAATGATTTCAAATTTCAAATTTGAAAATATCCCGCCCTACAGTATACCAA
TTCAAATTCAAATTC
+
F:FFFFFFFFFFFFFFFFFFFFFFFFFFFFFFFFFFFFFFFFFFFFFFFFFFFFFF:FFFFFFFFFFFF
FFF:FFFFF:FFFFFFFFFFFFFFFFFFFFFFFFFFFFFFFFFFFFFFF:F:FF::FFFFFFFFFFFF
FFFFFFFF:FF:FF:
@A00721:190:HGJW7DSXY:4:2544:24153:5494 1:N:0:TCTGCAAG+GCTGCACT
CCGCATATTAAGCATTATATTGGGGACTGGGGACCTATATATATGTATAGAGTAATCCGGAGGAATCT
```

```
ATCCCCAATTGGAATAGTAATGATTTCAAATTTCAAATTTGAAAATATCCCGCCCTACAGTATACCAA
TTCAAATTCAAATTC
+
FFFFFFFFFFFFFFFFFFFFFFFFFFFFFFFFFFFFFFFFFFFFFFFFFFFFFFFF:FFFFFFFFF,FFF
FFFFFFFFFFFFFFFFFFFFFFFFFFFFFFFFFFFFFFFFFFFFFFFFFFFFFFFFFFFFFFFFFFFFFF
FFFFFFFFFFFFFFF
@A00721:190:HGJW7DSXY:4:2544:27697:5682 1:N:0:TCTGCAAG+GCTGCACT
CCGCATATTAAGCATTATATTGGGGACTGGGGACCTATATATATGTATAGAGTAATCCGGAGGAATCT
ATCCCCAATTGGAATAGTAATGATTTCAAATTTCAAATTTGAAAATATCCCGCCCTACAGTATACCAA
TTCAAATTCAAATTC
+
FFFFFFFFFFFFFFFFFFFFFFFFFFFFFFFFFFFFFFFFFFFFFFFFFFFFFFFFFFFFFFFFFF:FFF
FFFFFFFFFFFFFFFFFFFFFFFFFFFFFFFFFFFFFFFFFFFFFFFFFFFFFFFFFFFFFFFFFFFFFF
FFFFFFFFFFFFFFF
@A00721:190:HGJW7DSXY:4:1347:18954:30123 1:N:0:TCTGCAAG+GCTGCACT
ATCGCATATTAAGCATTATATTGGGGACTGGGGACCTATATATATGTATAGAGTAATCCGGAGGAATC
TATCCCCAATTGGAATAGTAATGATTTCAAATTTCAAATTTGAAAATCATTACTATTCCAATTGGGGA
TAGATTCCTCCGAGA
+
FFFFFFFFFFFFFFFFFFFFF,FFFFFFFFFFFFFFFFFFFFFFFFFFFFFFFFFFFFFFFFFFFFFFFF
FFFFFFFFFFFFFFF:FFFFFFFFFFFFFFFFFFFFFFFFFFFFFFF,FFFFF:FFF:FFFF:FF:FFF:
FFFFFFFFFFFFFFF
@A00721:190:HGJW7DSXY:4:1431:18358:3693 1:N:0:TCTGCAAG+GCTGCACT
ATCGCATATTAAGCATTATATTGGGGACTGGGGACCTATATATATGTATAGAGTAATCCGGAGGAATC
TATCCCCAATTGGAATAGTAATGATTTCAAATTTCAAATTTGAAAATCATTACTATTCCAATTGGGGA
TAGATTCCTCCGAGA
+
FFFFFFFFFFFFFFFFFFFFFFFFFFFFFFFFFFFFFFFFF:FFFFFFFFFFFFFFFFFFFFFFFFFFFF
FFFFFFFFFFFFFFFFFFFFFFFFFFFFFFFFFFFFF,FFFFFFFFFFFFFFFFFFFFF:F,F:FF:FFF
FFFFFFFFFFFFFFF
@A00721:190:HGJW7DSXY:4:2446:1787:6746 1:N:0:TCTGCAAG+GCTGCACT
ATCGCATATTAAGCATTATATTGGGGACTGGGGACCTATATATATGTATAGAGTAATCCGGAGGAATC
AATCCCCAATTGGAATAGTAATGATTTCAAATTTCAAATTTGAAAATCATTACTATTCCAATTGGGGA
TAGATTCCACCGAGA
+
FFFFFFFFF:FFFFFFFFFFFFF:FFFFFF,F,FFFFFFFF:FFF,F,FFF:::,FFFFFFFFFF:F,FF
,FFFFF:FFFFF:F,FFFFFFFFFFFF:FFFFFF,:F:FFF,:FFF:FFF:FFFFFFFFF:FF:F,F,F:
FFFFFFFF,FF,FF:
@A00721:190:HGJW7DSXY:4:1357:30698:19617 1:N:0:TCTGCAAG+GCTGCACT
CGCATATTAAGCATTATATTGGGGACTGGGGACCTATATATATGTATAGAGTAATCCGGAGGAATCTA
TCCCCAATTGGAATAGTAATGATTTCAAATTTCAAATTTGAAAATATCCCGCCCTACAGTATACCAAT
TCAAATTCAAATTCA
+
FFFFFFFFFFFFFFFFFFFFFFFFFFFFFFFFFFFFFFFFFFFFFFFFFFFFFFFFFFFFFFFFFFFFFF
FFFFFFFFFFFFFFFFFFFFFFFFFFFFFFFFFFFFFFFFFFFFFFFFFFFFFFFFFFFFFFFFFFFFFF
FFFFFFFFFFFFFFF
@A00721:190:HGJW7DSXY:4:1471:23294:3411 1:N:0:TCTGCAAG+GCTGCACT
CGCATATTAAGCATTATATTGGGGACTGGGGACCTATATATATGTATAGAGTAATCCGGAGGAATCTA
TCCCCAATTGGAATAGTAATGATTTCAAATTTCAAATTTGAAAATATCCCGCCCTACAGTATACCAAT
TCAAATTCAAATTCA
+
FF:F:FFFFFFFFFFFFFFFFFFFFFF:FFFFFFFFF:FFFFFFFFFFFFFFFFF:,:FFFFFFFFFFFF
:FFF,FFFF,FFFFF:FFFFFF:FFFFFF:FF::F:FF,F,FFFFFFF,FF:FF,FF,FFFF:FF:FF:
F,FFFF:::F,FFFF
```

```
@A00721:190:HGJW7DSXY:4:2177:32045:21136 1:N:0:TCTGCAAG+GCTGCACT
CGCATATTAAGCATTATATTGGGGACTGGGGACCTATATATATGTATAGAGTAATCCGGAGGAATCTA
TCCCCAATTGGAATAGTAATGATTTCAAATTTCAAATTTGAAAATATCCCGCCCTACAGTATACCAAT
TCAAATTCAAATTCA
+
FFFFFFFFFFFFFFFFFFFFFFFFFFFFFFFFFFFFFFFFFFFFFFFFFFFFFFFFFFFFFFFFFFFF
FFFFFFFFFFFFFFFFFFFFFFFFFFFFFFFFFFFFFFFFFFFFFFFFFF::FFFFFFFFFFFFFFFF
FFFFFFFFFFFFFFF
@A00721:190:HGJW7DSXY:4:2201:10809:17143 1:N:0:TCTGCAAG+GCTGCACT
CGCATATTAAGCATTATATTGGGGACTGGGGACCTATATATATGTATAGAGTAATCCGGAGGAATCTA
TCCCCAATTGGAATAGTAATGATTTCAAATTTCAAATTTGAAAATATCCCGCCCTACAGTATACCAAT
TCAAATTCAAATTCA
+
FFFFFFFFFFFFFFFFFFFFFFFFFFFFFFFFFFFFFFFFFFFFFFFFFFFFFFFFFFFFFFFFFFFF
FFFFFFFF:FFFFFFFFFFFFFFFFFFFFFFFFF:FFFFFFFFFFFFFFFFFFFFFFFFFFFFFFFFF
FFFF:FFFFFFFFFF
@A00721:190:HGJW7DSXY:4:1222:2492:22811 1:N:0:TCTGCAAG+GCTGCACT
GCATATTAAGCATTATATTGGGGACTGGGGACCTATATATATGTATAGAGTAATCCGGAGGAATCTAT
CCCCAATTGGAATAGTAATGATTTCAAATTTCAAATTTGAAAATATCCCGCCCTACAGTATACCAATT
CAAATTCAAATTCAA
+
FFFFFFFFFFFFFFFFFFFFFFFFFFFFFFFFFFF,FFFFFFFFFFFFFFFFFFFFFFFFFFFFFFFF
FFFFFFFFFFFFFFFF:FFFFFFFFFFFFFFFFFFFFFFFFFFFFFFFFFFFFFF:FFFFFFFFFFFF
FFFFFFFFFFFFFFF
@A00721:190:HGJW7DSXY:4:2216:32280:35477 1:N:0:TCTGCAAG+GCTGCACT
GCATATTAAGCATTATATTGGGGACTGGGGACCTATATATATGTATAGAGTAATCCGGAGGAATCTAT
CCCCAATTGGAATAGTAATGATTTCAAATTTCAAATTTGAAAATATCCCGCCCTACAGTATACCAATT
CAAATTCAAATTCAA
+
FFFFFFFFFFFFFFFFFFFFFFFFFFFFFFFFFFFFFFFFFFFFFFFFFFFFF:FFFFFFFFFFFFFF
FFFFFFFFFFFFFFFFFFFFFFFFFFFFFFFFFFFFFFFFFFF:FFFFFFFFFF,FFFFFFFFFFFFF
FFFFFFFFFFFFFFF
@A00721:190:HGJW7DSXY:4:1528:31403:36432 1:N:0:TCTGCAAG+GCTGCACT
GGGGACTGGGGACCTATATATATGTATAGAGTAATCCGGAGGAATCTATCCCCAATTGGAATAGTAAT
GATTTCAAATTTCAAATTTGAAAATATCCCGCCCTACAGTATACCAATTCAAATTCAAATTCAAATAG
AATTCCATAGGCCCC
+
FFFFFFFFFFFFFFFFFFFFFFFFFFFFFFFFFFFFFFFFFFFFFFFFFFFFFFFFFFFFFFFFFFFF
FFFFFFFFFFFFFFFFFFFF:FFFFFFFFFFFFFFFFFFF:FFFFFFFFFFFFFFFFFFFFFFFFFFF
FFFFFFFFFFFFFFF
@A00721:190:HGJW7DSXY:4:1650:16269:12414 1:N:0:TCTGCAAG+GCTGCACT
GGGGACTGGGGACCTATATATATGTATAGAGTAATCCGGAGGAATCTATCCCCAATTGGAATAGTAAT
GATTTCAAATTTCAAATTTGAAAATATCCCGCCCTACAGTATACCAATTCAAATTCAAATTCAAATAG
AATTCCATAGGCCCC
+
FFFFFFFFFFFFFFFFFFFFFFFFFFFFFFFFFFFFFFFFFFFFFFFFFFFFFFFFFFFFFFFFFFFF
FFFFFFFFFFFFFFFFFFFFFFFFFFFFFFFFFFFFFFFFFFFFFFFFFFFFFFFFFFFFFFFFFF:F
FFFFFFFFFFFFFFF
@A00721:190:HGJW7DSXY:4:2465:22146:8938 1:N:0:TCTGCAAG+GCTGCACT
GGGGACTGGGGACCTATATATATGTATAGAGTAATCCGGAGGAATCTATCCCCAATTGGAATAGTAAT
GATTTCAAATTTCAAATTTGAAAATATCCCGCCCTACAGTATACCAATTCAAATTCAAATTCAAATAG
AATTCCATAGGCCCC
+
FFFFFFFFFFFFFFFFFFFFFFFFFFFFFFFFFFFFFFFFFFFFFFFFFFFFFFFFFFFFFFFFFFFF
```

FFFFFFFFFFFFFFFFFFFFFFFFFFFFFFFFFFFFFFFFFFFFFFFFFFFFFFFFFFFFFFFFFFFF
FFFF:FFFF:FFFFF
@A00721:190:HGJW7DSXY:4:1201:6406:36824 1:N:0:TCTGCAAG+GCTGCACT
GGCTGGGGACCTATATATATGTATAGAGTAATCCGGAGGAATCTATCCCCAATTGGAATAGTAATGAT
TTCAAATTTCAAATTTGAAAATATCCCGCCCTACAGTATACCAATTCAAATTCAAATTCAAATAGAAT
TCCATAGGCCCCGCA
+
:FFFFFFFFFFFFFFFFFFFFFFFFFFFFFFFFFFFFFFF,FFFFFFFFFFF:FFFFFFFFFFFFF:F
FFFFFFFFFFFFFFFFFFFFFFFFF:FFFFFFFF::FFFFFFFFFFFFFFFFFFFFFFFFFFFFFFFF
:F:FFFF:FFFFFFF
@A00721:190:HGJW7DSXY:4:1228:23647:17989 1:N:0:TCTGCAAG+GCTGCACT
GGCTGGGGACCTATATATATGTATAGAGTAATCCGGAGGAATCTATCCCCAATTGGAATAGTAATGAT
TTCAAATTTCAAATTTGAAAATATCCCGCCCTACAGTATACCAATTCAAATTCAAATTCAAATAGAAT
TCCATAGGCCCCGCA
+
::F,FFFFF:FFFFFFFFFFFFFFFFF,FFFF,F::F,:FFFFFF:FFF:FF:FF,FF:F:FFF,:F:
F,:F::FFFF:FFF,FF:FFFFF:,,FFFFFFFFF::FFF,FFF:FFFF:FF:FFF::FFFFFF,FFF
:,:FFF:F,F,FF:F
@A00721:190:HGJW7DSXY:4:1305:31286:30655 1:N:0:TCTGCAAG+GCTGCACT
GGCTGGGGACCTATATATATGTATAGAGTAATCCGGAGGAATCTATCCCCAATTGGAATAGTAATGAT
TTCAAATTTCAAATTTGAAAATATCCCGCCCTACAGTATACCAATTCAAATTCAAATTCAAATAGAAT
TCCATAGGCCCCGCA
+
::F:FFFFFFFFFFFFFFFFFFFFFFFF:FFFFFFFFFFFF:FFFFFFFFFFFFFFFFFFFF:FFFFF
FFFFF:F:FFFFFF:FFFFF::FFFFFFFFF,FF:F:F:F:FFFFF,:F:FF,FFFFF:FF:FF,FFF
FF,FFF,,F:F,FFF
@A00721:190:HGJW7DSXY:4:1430:28971:2816 1:N:0:TCTGCAAG+GCTGCACT
GGCTGGGGACCTATATATATGTATAGAGTAATCCGGAGGAATCTATCCCCAATTGGAATAGTAATGAT
TTCAAATTTCAAATTTGAAAATATCCCGCCCTACAGTATACCAATTCAAATTCAAATTCAAATAGAAT
TCCATAGGCCCCGCA
+
FFFFFFFFFFFFFFFFFFFFFFFFFFFFFFFFFFFFFFFFFFFFFFFF:FFFFFFFFFFFFFFFFFFF
FFFFFFFFFFFF:FFFFFFFFFFFFFFFFFFFFFFFFFFFFFF:FFFFFFFFFFFFFFFFFFFF:FFF
FF:FFFFFFFFFFFF
@A00721:190:HGJW7DSXY:4:1472:9751:28401 1:N:0:TCTGCAAG+GCTGCACT
GGCTGGGGACCTATATATATGTATAGAGTAATCCGGAGGAATCTATCCCCAATTGGAATAGTAATGAT
TTCAAATTTCAAATTTGAAAATATCCCGCCCTACAGTATACCAATTCAAATTCAAATTCAAATAGAAT
TCCATAGGCCCCGCA
+
FFFFFFFFFFFFFFFFFFFFFFFFFFFFFFFFFFFFFFFF:FFFFFFFFFFFFFFFFFFFFFFFFFFF
FFFFFFFFFFFFFFFFFFFFFFFFFFFFFFFFFFFFFFFFFFFFFFFFFFFFFFFFFFFFFFFFFFFF
FFFFFFFFFFFFFFF
@A00721:190:HGJW7DSXY:4:1475:29469:6684 1:N:0:TCTGCAAG+GCTGCACT
GGCTGGGGACCTATATATATGTATAGAGTAATCCGGAGGAATCTATCCCCAATTGGAATAGTAATGAT
TTCAAATTTCAAATTTGAAAATATCCCGCCCTACAGTATACCAATTCAAATTCAAATTCAAATAGAAT
TCCATAGGCCCCGCA
+
FFFFFFFFFFFFFFFFFFFFFFFFFFFFFFFFFFFFFFFF:FFFFFFFFFFFFFFFFFFFFFFFFFFF
FFFFFFFFFFF,FFFFFFFFFFFF:FFFFFFFFFFFFFFFFFFFFFFFFFFFFFFFFFFFFFF,FFF
FFFFFFFFFFFFFFF
@A00721:190:HGJW7DSXY:4:1577:21169:5431 1:N:0:TCTGCAAG+GCTGCACT
GGCTGGGGACCTATATATATGTATAGAGTAATCCGGAGGAATCTATCCCCAATTGGAATAGTAATGAT
TTCAAATTTCAAATTTGAAAATATCCCGCCCTACAGTATACCAATTCAAATTCAAATTCAAATAGAAT
TCCATAGGCCCCGCA

```
+
FFFFFFFFFFF,FFFFFFFFFFFFFFFFFFFFFFFFFFFFFFFFFFFFFFFFFFFFFFFFFFFFFFFFF
FFFFFFFFFFFFFFFFFFFFFFFFFFFFFFFFFFFFFFFFFFFFFFFFFFFFFFFFFFFFFFFFF:FFF
FFFFFFFFFF:FFFF
@A00721:190:HGJW7DSXY:4:1635:8205:22874 1:N:0:TCTGCAAG+GCTGCACT
GGCTGGGGACCTATATATATGTATAGAGTAATCCGGAGGAATCTATCCCCAATTGGAATAGTAATGAT
TTCAAATTTCAAATTTGAAAATATCCCGCCCTACAGTATACCAATTCAAATTCAAATTCAAATAGAAT
TCCATAGGCCCCGCA
+
FFFFFFFFFFFFFFFFFFFFFFF:FFFFFFFFFFFFFFFFFFFFFFFFFFFFFFFFFFFFFFFFFFFFF
FFFFFFFFFFFFFFFFFFFFFFFFFFFFFFFFFFFFFFFFFFFFFFFFFFFFFFFFFFFFFFFFF,FFF
FF:F:FFFFFFFFFF
@A00721:190:HGJW7DSXY:4:2108:14425:34554 1:N:0:TCTGCAAG+GCTGCACT
GGCTGGGGACCTATATATATGTATAGAGTAATCCGGAGGAATCTATCCCCAATTGGAATAGTAATGAT
TTCAAATTTCAAATTTGAAAATATCCCGCCCTACAGTATACCAATTCAAATTCAAATTCAAATAGAAT
TCCATAGGCCCCGCA
+
FFFFFFFFFFFFFFFFFFFFFFFFFFFFFF:FFFFFFFFFFFFFFFFFFFFFFFFFFFFFFFFFFFFFF
FFFFFFFFFFFFFFFFFFFFFFFFFFFFFFFFFFFFFFFFFFFFFFFFFFFFFFFFFFFFFFFFFFFFF
FFFFFFF:FFFFFFF
@A00721:190:HGJW7DSXY:4:2211:19777:13135 1:N:0:TCTGCAAG+GCTGCACT
GGCTGGGGACCTATATATATGTATAGAGTAATCCGGAGGAATCTATCCCCAATTGGAATAGTAATGAT
TTCAAATTTCAAATTTGAAAATATCCCGCCCTACAGTATACCAATTCAAATTCAAATTCAAATAGAAT
TCCATAGGCCCCGCA
+
FFFFFF:FFFFFFFFFFFFFFFFFFFF:FFFFFFFFFFFFFFFF:FF:FFFFFFFFFFFFFFFFFFFFF
FFF:FFF:FFFFF:FFFFFFFFFFFFFFFFFFFFFFFFFFF,FFFFFFFFF:FFFFFFFFFF::F:FFF
FFFFFFFFFF:FFFF
@A00721:190:HGJW7DSXY:4:2222:9281:5760 1:N:0:TCTGCAAG+GCTGCACT
GGCTGGGGACCTATATATATGTATAGAGTAATCCGGAGGAATCTATCCCCAATTGGAATAGTAATGAT
TTCAAATTTCAAATTTGAAAATATCCCGCCCTACAGTATACCAATTCAAATTCAAATTCAAATAGAAT
TCCATAGGCCCCGCA
+
FFFFFFFFFFFFF:FFFFFFFFFFFFFFFFFFFFFFFFFFF:FFFFFFFFFFFFFFFFFFFFFFFFFFF
FFFFFFFFFFFFFFFFFFFFFFFFFFFFFFFFFFFFFFFFFFFF:FFFFFFFFFFFFFFFFFFFF:FFF
F,FFFFFFFFFFFFF
@A00721:190:HGJW7DSXY:4:2236:6207:29277 1:N:0:TCTGCAAG+GCTGCACT
GGCTGGGGACCTATATATATGTATAGAGTAATCCGGAGGAATCTATCCCCAATTGGAATAGTAATGAT
TTCAAATTTCAAATTTGAAAATATCCCGCCCTACAGTATACAAATTCAAATTCAAATTCAAATAGAAT
TACATATGCCCCGCA
+
FFFFFFFFFF::FFFFF:FFFFFFFFFFFFFFFF,FFFFFFFF,FF,FFFFFFF:FFFFFFFFFFFFFF
FFFFFFFFF,FFFFFF:FFFFFFF,:,F:FFFFFF:FFFFF,FFF,FFFF::FFFFFFFFFFFFFFFFF
,,,FFF,:::F:F:F
@A00721:190:HGJW7DSXY:4:2655:29053:6339 1:N:0:TCTGCAAG+GCTGCACT
GGCTGGGGACCTATATATATGTATAGAGTAATCCGGAGGAATCTATCCCCAATTGGAATAGTAATGAT
TTCAAATTTCAAATTTGAAAATATCCCGCCCTACAGTATACCAATTCAAATTCAAATTCAAATAGAAT
TCCATAGGCCCCGCA
+
FFFFFFFFFFFFFFFFFFFFFFFFFFFFFFFFFFFFFFFFFFFFFFFFFFFFF:FFFFFFFFFFFFFFF
FFFFFFFFFFFFFFFFFFFF:FFFFFFFFFFFFF:FFFFFFFFFFFFFFF:F:FFFFFFFFF,FFFFFF
F:FFFFFFFFFFFFF
@A00721:190:HGJW7DSXY:4:2226:21025:23030 1:N:0:TCTGCAAG+GCTGCACT
GCTGGGGACCTATATATATGTATAGAGTAATCCTGAGGAATCTATCCCCAATTGGAATAGTAATGATT
```

```
TCAAATTTCAAATTTGAAAATATCACGCACTACAGTATACCAATTCAAATTCAAATTCAAATAGAATT
CCATAGGCCCCGCA
+
FFF,F:FFFF:FFFFFFFF:FFFFFFFFF,,F,,:F,:FFF:FFFFF,FFFFFF:FFFF:,FFF:FFF
F,FF:,FFFFFFF,FFFFFF,F::,:,:,FF:FF,F,FF:FFF:,FFFFFF,FFF,,,FFFFF:FFFF
F:F,F:FFFFFFFF
@A00721:190:HGJW7DSXY:4:1214:22996:14606 1:N:0:TCTGCAAG+GCTGCACT
GGGCGTATGGAATTCTATTTGAATTTGAATTTGAATTTGAATTGGTATACTGTAGGGCGGGATATTTT
CAAATTTGAAATTTGAAATCATTACTATTCCAATTGGGGATAGATTCCTCCGGATTACTCTATACATA
TATATAGGTCCCCAG
+
FFFFFFFFFFFFFFFF:FFFFFFFFFFFFFFFFFFFFFFFFFFFFFFFFFFFFFFFFFFFFFFFFFFF
FFFFFFFFFFFFFFFFFFFFFFFFFFF,FFFF:FFFFFFFFFFFFFFFFFFFFFFFFFFFFFFFFFFF
FFFFFFFFFFFFFFF
@A00721:190:HGJW7DSXY:4:1244:17463:10347 1:N:0:TCTGCAAG+GCTGCACT
GGGCGTATGGAATTCTATTTGAATTTGAATTTGAATTTGAATTGGTATACTGTAGGGCGGGATATTTT
CAAATTTGAAATTTGAAATCATTACTATTCCAATTGGGGATAGATTCCTCCGGATTACTCTATACATA
TATATAGGTCCCCAG
+
FFFFFFFFFFFFFFFFFFFFFFFFFFFFFFFFFFFFFFFFFFFF:FFFFFFFFFFFFFFFFFFFFFFF
FFFFFFFFFFFFFFFFFFFFFFFFFFFFFFFFFFFFFFFFFFFFFFFFFFFFFFFFFFFFFFFFFFFF
FF:FFF:FFFFFFFF
@A00721:190:HGJW7DSXY:4:1436:6017:29481 1:N:0:TCTGCAAG+GCTGCACT
CTGGGGACCTATATATATGTATAGAGTAATCCGGAGGAATCTATCCCCAATTGGAATAGTAATGATTT
CAAATTTCAAATTTGAAAATATCCCGCCCTACAGTATACCAATTCAAATTCAAATTCAAATAGAATTC
CATAGGCCCCGCAGG
+
FFFFFFFFFFFFFFFFFFFFFFFFFFFFFFFFFFFFFFFFFFFFFFFFFFFFFFFFFFFFFFFFFFFF
FFFFFFFFFFFFFFFFFFFFFFFFFFFFFFFF:FFFFFFFFFFFFFFFFFFFFFFFFFFFFFFFFFFF
FFFFFFFFFFFFFFF
@A00721:190:HGJW7DSXY:4:1606:21043:24408 1:N:0:TCTGCAAG+GCTGCACT
CTGGGGACCTATATATATGTATAGAGTAATCCGGAGGAATCTATCCCCAATTGGAATAGTAATGATTT
CAAATTTCAAATTTGAAAATATCCCGCCCTACAGTATACCAATTCAAATTCAAATTCAAATAGAATTC
CATAGGCCCCGCAGG
+
FFFFFFFFFFFFFFFFFFFFFFFFFFFFFFFFFFFFFFFFFFFFFFFFFFFFFFFFFFFFFFFFFFFF
FFFFFFFFFFFFFFF:FFFFFFFFFFFFFFFFFFFFFFFFFFFFFFFFFFFFFFFFFFFFFF:FFFFF
FFFFFFFFFFFFFFF
@A00721:190:HGJW7DSXY:4:1651:23927:18004 1:N:0:TCTGCAAG+GCTGCACT
CTGGGGACCTATATATATGTATAGAGTAATCCGGAGGAATCTATCCCCAATTGGAATAGTAATGATTT
CAAATTTCAAATTTGAAAATATCCCGCCCTACAGTATACCAATTCAAATTCAAATTCAAATAGAATTC
CATAGGCCCCGCAGG
+
FFFFFFFFFFFFFFFFFFFFFFFFFFFFFFFFFFFFFFFFFFFFFFFFFFFFFFFFFFFFFFFFFFFF
FFFFFFFFFFFFFFFFFFFFFFFFFFFFFFFF,F:FFFFFFFFFFFFFFFFFF:FFFFFFFFFFFFFF
FFFFFFFFFFFFFFF
@A00721:190:HGJW7DSXY:4:1674:29116:17472 1:N:0:TCTGCAAG+GCTGCACT
GGGCGTATGGAATTCTATTTGAATTTGAATTTGAATTTGAATTGGTATACTGTAGGGCGGGATATTTT
CAAATTTGAAATTTGAAATCATTACTATTCCAATTGGGGATAGATTCCTCCGGATTACTCTATACATA
TATATAGGTCCCCAG
+
FFFFFFFFFFFFFFFFFFFFFFFFFFFFFFFFFFFFFFFFFFFFFFFFFFFFFFFFFFFFFFFFFFFF
FFFFFFFFFFFFFFFFFFFFFFFFFFFFFFFFFFFFFFFFFFFFFFFFFFFFFF:FFFFFFFFFFFFF
FFFFFFFFFFFFFFF
```

```
@A00721:190:HGJW7DSXY:4:2525:7708:18192 1:N:0:TCTGCAAG+GCTGCACT
CTGGGGACCTATATATATGTATAGAGTAATCCGGAGGAATCTATCCCCAATTGGAATAGTAATGATTT
CAAATTTCAAATTTGAAAATATCCCGCCCTACAGTATACCAATTCAAATTCAAATTCAAATAGAATTC
CATAGGCCCCGCAGG
+
FFFFFFFFFFFFFFFFFFFFFFFFFFFFFFFFFFFFFFFFFFFFFFFFFFFFFFFFFFFFFFFFFFFF
FFFFFFFFFFFFFFFFFFFFFFFFFFFFFFFFFFFFFFFFFFFFFFFFFFFFFFFFFFFFFFFFFFF,
FFFFFFFFFFFFFFF
@A00721:190:HGJW7DSXY:4:2536:3794:34898 1:N:0:TCTGCAAG+GCTGCACT
GGGCGTATGGAATTCTATTTGAATTTGAATTTGAATTTGAATTGGTATACTGTAGGGCGGGATATTTT
CAAATTTGAAATTTGAAATCATTACTATTCCAATTGGGGATAGATTCCTCCGGATTACTCTATACATA
TATATAGGTCCCCAG
+
FFFFFFFFFFFFFFFFFFFFFFFFFFFFFFFFFFFFFFFFFFFFFFFFFFFFFFFFFFFFFFFFFFFF
FFFFFFFFFFFFFFFFFFFFFFFFFFFFFFFFFFFFFFFFFFFFFFF,FFFFFFFFFFFFFFFF:FFF
FFFFFFFF,FFFFFF
@A00721:190:HGJW7DSXY:4:1460:9109:2300 1:N:0:TCTGCAAG+GCTGCACT
GGGACCTATATATATGTATAGAGTAATCCGGAGGAATCTATCCCCAATTGGAATAGTAATGATTTCAA
ATTTCAAATTTGAAAATATCCCGCCCTACAGTATACCAATTCAAATTCAAATTCAAATAGAATTCCAT
AGGCCCCGCAGGGGC
+
FFFFFFFFFFFFFFFFFFFFFFFFFFFFFFFFFFFFFFFFFFFFFFFFFFFFFFFFFFFFFFFFFFFF
FFFFFFFFFFFFFFFFFFFF:FFFFFFFFFFFFFFFFFFFFFFFFFFFFF:FFFFFFFF:FFFFF:FF
FFFFFFFFFFFFFFF
@A00721:190:HGJW7DSXY:4:2158:27769:23124 1:N:0:TCTGCAAG+GCTGCACT
GGGACCTATATATATGTATAGAGTAATCCGGAGGAATCTATCCCCAATTGGAATAGTAATGATTTCAA
ATTTCAAATTTGAAAATATCCCGCCCTACAGTATACCAATTCAAATTCAAATTCAAATAGAATTCCAT
AGGCCCCGCAGGGGC
+
FFFFFFFFFFFFFFFFFFFFFFFFFFFFFFFFFFFFFFFFFFFFFFFFFFFFFFFFFFFFFFFFFFFF
FFFFFFFFFFFFFFFFFFFFFFFFFFFFFFFFFFFFFFFFFFFFFFFFFFFFFFFFFFFFFFFFFFFF
FFFFF:FFFFFFFFF
@A00721:190:HGJW7DSXY:4:2326:7093:34976 1:N:0:TCTGCAAG+GCTGCACT
GGGACCTATATATATGTATAGAGTAATCCGGAGGAATCTATCCCCAATTGGAATAGTAATGATTTCAA
ATTTCAAATTTGAAAATATCCCGCCCTACAGTATACCAATTCAAATTCAAATTCAAATAGAATTCCAT
AGGCCCCGCAGGGGC
+
FFFFFFFFFFFFFFFFFFFFFFFFFFFFFFFFFFF:FFFFFFFFFFFFFFFFFFFFFFFFFFFFFFFF
FFFFFFFFFFFFFFFFFFFFFFFFFFFFFFFFFFFFFFFFFFFFFFFFFFFFFFFFFFFFFFFFFFFF
FFFFFFFFFFFFFFF
@A00721:190:HGJW7DSXY:4:2358:25238:16141 1:N:0:TCTGCAAG+GCTGCACT
GGGACCTATATATATGTATAGAGTAATCCGGAGGAATCTATCCCCAATTGGAATAGTAATGATTTCAA
ATTTCAAATTTGAAAATATCCCGCCCTACAGTATACCAATTCAAATTCAAATTCAAATAGAATTCCAT
AGGCCCCGCAGGGGC
+
FFFFFFFFFFFFFFFFFFFF:FFFFFFFFFFFFFFFFFFFFFFFFFFFFFFFFFFFFFFFF:FFFFFF
FFFFFFFFFFFFFFFFFFF,FFFFFFFFFFFFFFFF,FFFFFFFFFFFFFFFFFFFFFF:FFFF,FFF
F,F,FFFFFFFFFFF
@A00721:190:HGJW7DSXY:4:2461:3812:17362 1:N:0:TCTGCAAG+GCTGCACT
GGGACCTATATATATGTATAGAGTAATCCGGAGGAATCTATCCCCAATTGGAATAGTAATGATTTCAA
ATTTCAAATTTGAAAATATCCCGCCCTACAGTATACCAATTCAAATTCAAATTCAAATAGAATTCCAT
AGGCCCCGCAGGGGC
+
FFFFFFFFFF:FFFF::FFFFF:F:F::F::FFFFFFFFFFFF:FFFFFFFFFFFFFFFFFFFFFFFF
```

FFFFFFFFFFFFFFFFFFFF,:FFFFFFF:FFFFFFFFFFFF::FFFFF,:FFFFFFFF,F,FF::FF:F
FF:FFF:FFFF:FFF
@A00721:190:HGJW7DSXY:4:2555:13810:25285 1:N:0:TCTGCAAG+GCTGCACT
GGGACCTATATATATGTATAGAGTAATCCGGAGGAATCTATCCCCAATTGGAATAGTAATGATTTCAA
ATTTCAAATTTGAAAATATCCCGCCCTACAGTATACCAATTCAAATTCAAATTCAAATAGAATTCCAT
AGGCCCCGCAGGGGC
+
FFFFFFFFFFFFFFFFFFFFFFFFFFFFFFFFFFFF:FFFFFFFFFFFFFFFFFFFFFFFFFFFFFFFFF
FFFFFFFFFFFFFFFFFFFFFFFFFFFFFFFFFFFFFFFFFFFFFFFFFFFFF:FFFFF:FFFFFF::FF
FFFFFFFFFFFFFFF
@A00721:190:HGJW7DSXY:4:2651:2257:27508 1:N:0:TCTGCAAG+GCTGCACT
GGGACCTATATATATGTATAGAGTAATCCGGAGGAATCTATCCCCAATTGGAATAGTAATGATTTCAA
ATTTCAAATTTGAAAATATCCCGCCCTACAGTATACCAATTCAAATTCAAATTCAAATAGAATTCCAT
AGGCCCCGCAGGGGC
+
FFFFFFFFFFFFFFFFFFFFFFFFFFFFFFFFFFFFFFFF,FFFFFFFFFFFFFFFFFFFFFFFFFFFFF
FFFFFFFFFFFFFFFFFFFFFFFFFFFFFFFFFFFFFFFFFFFFFFFFFFFFFFFFFFFFF:FFFFFFFF
FFFFFFFFFFFFFFF
@A00721:190:HGJW7DSXY:4:1226:10963:36104 1:N:0:TCTGCAAG+GCTGCACT
GTATAGAGTAATCCGGAGGAATCTATCCCCAATTGGAATAGTAATGATTTCAAATTTCAAATTTGAAA
ATATCCCGCCCTACAGTATACCAATTCAAATTCAAATTCAAATAGAATTCCATAGGCCCCGCAGGGGC
ATCTTACCGCAGACC
+
FFFFFFFFFFFFFFFFFFFFFFFFFFFFFFFFFFFFFFFFFFFFFFFFFFFFFFFFFFFFFFFFFFFFFF
FFFFFFFFFFFFFFFF:FFFFFFFFFFFFFFFFFFFFFFFFFFFFFFFFFFFFFFFFFFFFFFF:FFFFF
FFFFFFFFFFFFFFF
@A00721:190:HGJW7DSXY:4:1234:20184:12712 1:N:0:TCTGCAAG+GCTGCACT
GTATAGAGTAATCCGGAGGAATCTATCCCCAATTGGAATAGTAATGATTTCAAATTTCAAATTTGAAA
ATATCCCGCCCTACAGTATACCAATTCAAATTCAAATTCAAATAGAATTCCATAGGCCCCGCAGGGGC
ATCTTACCGCAGACC
+
FFFFFFFFFFFFFFFFFFFFFFFFFFFFFFFFFFFFFFFFFFFFFFFFFFFFFFFFFFFFFFFFFFFFFF
FFFFFFFFFFFFFFFFFFFFFFFFFFFFFFFFFFFFFFFFFFFFFFFF:FFFFFFFF:FFFFFFFFFFFF
,FFFFFFFFFFFFFF
@A00721:190:HGJW7DSXY:4:1265:32108:30013 1:N:0:TCTGCAAG+GCTGCACT
GTATAGAGTAATCCGGAGGAATCTATCCCCAATTGGAATAGTAATGATTTCAAATTTCAAATTTGATA
ATATCCCGCCATACAGTATACCAATTCAAATTCAAATTCAAATAGAATTCCATAGGCCCCGCAGGGGC
ATCTTACCGCAGA
+
FFFFF:FFFFFF,FFFF:,F:FFFFFF,FFFFFFFFFFFFFFFF:FF:FFFFFFF::FF:,,:FFFFF,F
:FF:F:FFFF,:FFFF:FF::FFFF,FFFFFF,FFF:::FFFFF,:,:F:,,FF,F,,:,FFFF,F:,
FF,FFF,,:F:FF
@A00721:190:HGJW7DSXY:4:1519:3143:27383 1:N:0:TCTGCAAG+GCTGCACT
GTATAGAGTAATCCGGAGGAATCTATCCCCAATTGGAATAGTAATGATTTCAAATTTCAAATTTGAAA
ATATCCCGCCCTACAGTATACCAATTCAAATTCAAATTCAAATAGAATTCCATAGGCCCCGCAGGGGC
ATCTTACCGCAGACC
+
FFFFFFFFFFFFFFFFFFFFFFFFFFFFFFFFFFFFFFFFFFFFFFFFFFFFFFF:FFFFFFFFFFFFFF
FFFFFFFFFFFFFFFF:FFFFFFFFFFFFFFFFFFFFFFFFFFFFFFFFFFFFFFFFFFFFFFFFFFFFF
FFF:FFFFFFFFFFF
@A00721:190:HGJW7DSXY:4:2110:4779:14058 1:N:0:TCTGCAAG+GCTGCACT
GTATAGAGTAATCCGGAGGAATCTATCCCCAATTGGAATAGTAATGATTTCAAATTTCAAATTTGAAA
ATATCCCGCCCTACAGTATACCAATTCAAATTCAAATTCAAATAGAATTCCATAGGCCCCGCAGGGGC
ATCTTACCGCAGACC

```
+
FFFFFFFFFFFFFFFFFFFFFFFFFFFFFFFFFFFFFFFFFFFFFFFFFFFFF:FFFFFFFFFFFFFF
FFFFFFFFFFFFFFFFFFFFFFFFFFFFFFFFFFF:FFFFFFFFFFFFFFFFFFFFFFFFFFFFFFFF
FFFFFFFFFFFFFFF
@A00721:190:HGJW7DSXY:4:2110:2700:16157 1:N:0:TCTGCAAG+GCTGCACT
GTATAGAGTAATCCGGAGGAATCTATCCCCAATTGGAATAGTAATGATTTCAAATTTCAAATTTGAAA
ATATCCCGCCCTACAGTATACCAATTCAAATTCAAATTCAAATAGAATTCCATAGGCCCCGCAGGGGC
ATCTTACCGCAGACC
+
FFFFFFFFFFFFFFFFFFFF:FFFFFFFFFFFFFFFFFFFFFFFFFFFFFFFFFFFFFFFFFFFFFFF
FFFFFFFFFFFFFF:FFFFFFFFFFFFFFFFFFFFFFFFFFFF::FFFF:FFFFFFFFFFFFFFFFFF
:FFFFFF:FFFFFFF
@A00721:190:HGJW7DSXY:4:2241:10773:23531 1:N:0:TCTGCAAG+GCTGCACT
GTATAGAGTAATCCGGAGGAATCTATCCCCAATTGGAATAGTAATGATTTCAAATTTCAAATTTGAAA
ATATCCCGCCCTACAGTATACCAATTCAAATTCAAATTCAAATAGAATTCCATAGGCCCCGCAGGGGC
ATCTTACCGCAGACC
+
FFFFFFFFFFFFFFFFFFFFFFFFFFFFFFFFFFFFF:FFFF:FFFFFFFFFFFFFFFFFFFFFFFFF
FFFFFFFFFFF,FFFFFFFFFFFFFFFFFFFFFFFFFFFFFFFFFFFFFFFFFFFFFFFFFFFFFFFF
,FFFFFFFFFFFFFF
@A00721:190:HGJW7DSXY:4:2334:23665:19774 1:N:0:TCTGCAAG+GCTGCACT
GTATAGAGTAATCCGGAGGAATCTATCCCCAATTGGAATAGTAATGATTTCAAATTTCAAATTTGAAA
ATATCCCGCCCTACAGTATACCAATTCAAATTCAAATTCAAATAGAATTCCATAGGCCCCGCAGGGGC
ATCTTACCGCAGACC
+
FFFFFFFFFFFFFFFFFFFFFFFFFFFFFFFFFFFFFFFFFFFFFFFFFFFFFFFFFFFFFFFFFFFF
FFFFFFFFFFFFFFFFFFFFFFFFFFFFFFFFFFFFFFFFFFFFFF:FFFFFFF:FFFFFFFFFFFFF
:FFFFFFFFFFFFFF
@A00721:190:HGJW7DSXY:4:2406:28076:8155 1:N:0:TCTGCAAG+GCTGCACT
GTATAGAGTAATCCGGAGGAATCTATCCCCAATTGGAATAGTAATGATTTCAAATTTCAAATTTGAAA
ATATCCCGCCCTACAGTATACCAATTCAAATTCAAATTCAAATAGAATTCCATAGGCCCCGCAGGGGC
ATCTTACCGCAGACC
+
FFFFFFFFFFFFFFFFFFFFFFFFFFFFFFFFFFFFFFFFFFFFFFFFFFFFFFFFFFFFFFFFFFFF
FFFFFFFFFFFF:FFFFFFFFFFFFFFFFFFF:FFFFFFFF:FFFF,FFFFFFFFFFFFFFFFFFFFF
,FF,FF,FFF,FFFF
@A00721:190:HGJW7DSXY:4:2418:31159:20791 1:N:0:TCTGCAAG+GCTGCACT
GTATAGAGTAATCCGGAGGAATCTATCCCCAATTGGAATAGTAATGATTTCAAATTTCAAATTTGAAA
ATATCCCGCCCTACAGTATACCAATTCAAATTCAAATTCAAATAGAATTCCATAGGCCCCGCAGGGGC
ATCTTACCGCAGACC
+
FFFFFFFFFF,FFFFFFFFFFFFFFFFFFFFFFFFFFFFFFFFFFFFFFFFFFFFFFFFFFFFFFFFF
FFFFFFFFFFFFFFFFFFFFFFFFFFFFFFFFFFFFFFFFFFFFFFFFFFFFFFFFFFFFFFFFFFFF
FFFFFFFFFFFFFFF
@A00721:190:HGJW7DSXY:4:2437:23448:23813 1:N:0:TCTGCAAG+GCTGCACT
GTATAGAGTAATCCGGAGGAATCTATCCCCAATTGGAATAGTAATGATTTCAAATTTCAAATTTGAAA
ATATCCCGCCCTACAGTATACCAATTCAAATTCAAATTCAAATAGAATTCCATAGGCCCCGCAGGGGC
ATCTTACCGCAGACC
+
FF:FFFFFFFFFFFFFFFFFFFFFFFFFFFFFFFFFFFFFFFFFFFFFFFFFFFFFFFFFFFFFFFFF
FFFFFFFFFFFFFFFFFFFFFFFFFFFFFFFFFFFFFFFFF::FFFFFFFFFFFFFFFFFFFFFFFFF
FFFFFFFFFFFFFFF
@A00721:190:HGJW7DSXY:4:2441:19379:23657 1:N:0:TCTGCAAG+GCTGCACT
GTATAGAGTAATCCGGAGGAATCTATCCCCAATTGGAATAGTAATGATTTCAAATTTCAAATTTGAAA
```

```
ATATCCCGCCCTACAGTATACCAATTCAAATTCAAATTCAAATAGAATTCCATAGGCCCCGCAGGGGC
ATCTTACCGCAGACC
+
FFFFFFFFFF:FFFFFFFFFFFFFFFFFFFFFFFFFFFFFFFFFFFFFFFFFFFFFFFFFFFFFFFFFF
FFFFFFFFFFFFFFFFFFFFFFFFFFFFFFFFFFFFFFFFFFFFFFFFFFFFFFFFFFFFFFFFFFFFF
FFFFFFFFFFFFFFF
@A00721:190:HGJW7DSXY:4:2647:29288:9251 1:N:0:TCTGCAAG+GCTGCACT
GTATAGAGTAATCCGGAGGAATCTATCCCCAATTGGAATAGTAATGATTTCAAATTTCAAATTTGAAA
ATATCCCGCCCTACAGTATACCAATTCAAATTCAAATTCAAATAGAATTCCATAGGCCCCGCAGGGGC
ATCTTACCGCAGACC
+
FFFFF:FFFF:FFFFFFFFF:FFFFFFFFFFFFFFFFFFFFFFFFFFFFFFFFFFFFFFFFFFFFFFF
FFFFFFFFFFFFFFFFFFFFFFFFF:FFFF:F:FFFFFFFFFFF,FFFF,FFFFFFF:FF:FFFFFFF
FFFFFFFFFFF:FFF
@A00721:190:HGJW7DSXY:4:1148:19994:35994 1:N:0:TCTGCAAG+GCTGCACT
ATAGAGTAATCCGGAGGAATCTATCCCCAATTGGAATAGTAATGATTTCAAATTTCAAATTTGAAAAT
ATCCCGCCCTACAGTATACCAATTCAAATTCAAATTCAAATAGAATTCCATAGGCCCCGCAGGGGCAT
CTTACCGCAGACCGT
+
FFFFFFFFFFFFFFFFFFFFFF:FFFFFFFFFFFFFFFFFFFFFFFFFFFFFFFFFFFFFFFFFFFFF
FFFFFFFFFFFFFFFFFFFFFFFFFFFFFFFFFFFFFFFFFFFFFFFFFFFFFFFFFFFFFFFFFFFF
FFFFFFFFFFFFFFF
@A00721:190:HGJW7DSXY:4:1168:24306:15060 1:N:0:TCTGCAAG+GCTGCACT
ATAGAGTAATCCGGAGGAATCTATCCCCAATTGGAATAGTAATGATTTCAAATTTCAAATTTGAAAAT
ATCCCGCCCTACAGTATACCAATTCAAATTCAAATTCAAATAGAATTCCATAGGCCCCGCAGGGGCAT
CTTACCGCAGACCGT
+
FFFFFFFFFFFFFFFFFFFFFFFFFFFFFFFFFFFFFFFFFFFFFFFFFFFFFFFFFFFFFFFFFFFF
FFFFFFFFFFFFFFFFFFFFFFFFFFFFFFFFFFFFFFFFFFFFFFFFFFFFFF:FFFFFFFFFFF,F
FFFFFFFFFFFFFFF
@A00721:190:HGJW7DSXY:4:1178:23294:28494 1:N:0:TCTGCAAG+GCTGCACT
ATAGAGTAATCCGGAGGAATCTATCCCCAATTGGAATAGTAATGATTTCAAATTTCAAATTTGAAAAT
ATCCCGCCCTACAGTATACCAATTCAAATTCAAATTCAAATAGAATTCCATAGGCCCCGCAGGGGCAT
CTTACCGCAGACCGT
+
FFFFFFFF:FFFFFFFFFFFFFFFFFFFFFFFFFFFFFFFFFFFFFFFFFFFFFFFFFFFFFFFFFFF
FFFFFFFFF,FFFFFFF,FFFFFFFFFFFFFFFFFFFFFFFFF:FF:FFFFFF:FFFFFFFFFFFFF:F
FFF:FFFFFFFFFFF
@A00721:190:HGJW7DSXY:4:1228:6632:16861 1:N:0:TCTGCAAG+GCTGCACT
ATAGAGTAATCCGGAGGAATCTATCCCCAATTGGAATAGTAATGATTTCAAATTTCAAATTTGAAAAT
ATCCCGCCCTACAGTATACCAATTCAAATTCAAATTCAAATAGAATTCCATAGGCCCCGCAGGGGCAT
CTTACCGCAGACCGT
+
FFFFFFF:FFFFFFF::::FFF:F,FF:FFF,FFFFFFFFFFFF:F:FFFFFF:FFFFFFFFFFFFFF
FF:FFF:F::FFF:FFFFFFFFFFFFFFFFFFFFFFFFFFFF:FF:F:F:FFFF,FFFFFFFFF:FFFF
FFFFFFFFF:FFFFF
@A00721:190:HGJW7DSXY:4:1233:26304:13416 1:N:0:TCTGCAAG+GCTGCACT
ATAGAGTAATCCGGAGGAATCTATCCCCAATTGGAATAGTAATGATTTCAAATTTCAAATTTGAAAAT
ATCCCGCCCTACAGTATACCAATTCAAATTCAAATTAAAATAGAATTCCATAGGCCCCGCAGGGGCAT
CTTACAGCAGACCGT
+
FF,,FFFFFFFF:FF,:FFFFFFFFFF,FFF:FFFFFF:FFFFFFF:,FFFF,:FFFFFF,F:FFFF:
F:F:,:F,F,FFF,FF,FF,FFFFFFFF,FFFF,FF,FFF,::FF,F::FFF,,F,FF,,FF,F,::F
FF,FF,FFFFF:F,F
```

```
@A00721:190:HGJW7DSXY:4:1252:21323:29559 1:N:0:TCTGCAAG+GCTGCACT
ATAGAGTAATCCGGAGGAATCTATCCCCAATTGGAATAGTAATGATTTCAAATTTCAAATTTGAAAAT
ATCCCGCCCTACAGTATACCAATTCAAATTCAAATTCAAATAGAATTCCATAGGCCCCGCAGGGGCAT
CTTACCGCAGACCGT
+
FFFFFFFFFFFFFFFFFFFFFFFFFFFFFFFFFFFFFFFFFFFFFFFFFFF:FFFFFFFFFFFFFFFF
FFFFFFFFFFFFFFFFFFFFFFFFFFFFFFFFFFFFFFFFFFFFFFFF:FFFFFFFFFFFFFFFFFFF
FFFFFFFFFFFFFFF
@A00721:190:HGJW7DSXY:4:1312:31069:14152 1:N:0:TCTGCAAG+GCTGCACT
ATAGAGTAATCCGGAGGAATCTATCCCCAATTGGAATAGTAATGATTTCAAATTTCAAATTTGAAAAT
ATCCCGCCCTACAGTATACCAATTCAAATTCAAATTCAAATAGAATTCCATAGGCCCCGCAGGGGCAT
CTTACCGCAGACCGT
+
FFFFFFFFFFFFFFFFFFFFFFFFFFFFFFFFFFFFFFFFFFFFFFFFFFFFFFFFFFFFFFFFFFFF
FFFFFFFFFFFFFFFFFFFFFFFFFFF:FFFFFFFFFFFFFFFFFFFFFFFFFFFFFFFFFFFFFFFF
FFFFFFFFFFFFFFF
@A00721:190:HGJW7DSXY:4:1424:7093:20196 1:N:0:TCTGCAAG+GCTGCACT
ATAGAGTAATCCGGAGGAATCTATCCCCAATTGGAATAGTAATGATTTCAAATTTCAAATTTGAAAAT
ATCCCGCCCTACAGTATACCAATTCAAATTCAAATTCAAATAGAATTCCATAGGCCCCGCAGGGGCAT
CTTACCGCAGACCGT
+
FFFFFFFFFFFFFFFFFFFFFFFFFFFFFFFFFFFFFFFFFFFFFFFFFFFFFFFFFFFFFFFFFFFF
FFFFFFFFFFFFFFFFFFFFFFFFFFFFFFFFFFFFFFF:F,FFFFFFFFFFFFFFFFFFFFFFFFFF
FFFFFFFFFFFFFFF
@A00721:190:HGJW7DSXY:4:1574:10619:4413 1:N:0:TCTGCAAG+GCTGCACT
ATAGAGTAATCCGGAGGAATCTATCCCCAATTGGAATAGTAATGATTTCAAATTTCAAATTTGAAAAT
ATCCCGCCCTACAGTATACCAATTCAAATTCAAATTCAAATAGAATTCCATAGGCCCCGCAGGGGCAT
CTTACCGCAGACCGT
+
FFFFFFFFFFFFFFFFFFFFFFFFF:FFFFFFFFFFFFFFFFFFFFFFFFFFFFF:FFFFFFFFFFFF
FFFFFFFFFFFF:FFFFF:FFF,FFFFF:FFFFFFFFFFFFFFFFFFFFFFF:FFF:FFFFFFFFF:F
FFFFFFFFFFFFFFF
@A00721:190:HGJW7DSXY:4:1574:10619:4633 1:N:0:TCTGCAAG+GCTGCACT
ATAGAGTAATCCGGAGGAATCTATCCCCAATTGGAATAGTAATGATTTCAAATTTCAAATTTGAAAAT
ATCCCGCCCTACAGTATACCAATTCAAATTCAAATTCAAATAGAATTCCATAGGCCCCGCAGGGGCAT
CTTACCGCAGACCGT
+
FFFFFFFFFFFFFFFFFFFFFF:FFFFFFFFFFFFFFFFFFFFFFFFFFFFFFFFFFFFFFFFFFFFF
FFFFFFFFFFFF:FFFFFFFFFFFFFF:FFFFFFFFFFFFFFFFFFFFFF::FFFFFFFFFFFFFF,F
FFFFFFFFFFFFFFF
@A00721:190:HGJW7DSXY:4:1668:32307:27007 1:N:0:TCTGCAAG+GCTGCACT
ATAGAGTAATCCGGAGGAATCTATCCCCAATTGGAATAGTAATGATTTCAAATTTCAAATTTGAAAAT
ATCCCGCCCTACAGTATACCAATTCAAATTCAAATTCAAATAGAATTCCATAGGCCCCGCAGGGGCAT
CTTACCGCAGACCGT
+
FFFFFFFFFFFFFFFFFFFFFFFFFFFFFFFFFFFFFFFFFFFFFFFFFFFFFFFFFFFFFFFFFFFF
FFFFFFFFFFFFFFFFFFFFFFFFFFFFFFFFFFFFFFFFFF:FFFFFFFFFFFFFFFFFFFFFFFFF
FFFFFFFFFFFFFFF
@A00721:190:HGJW7DSXY:4:2209:1081:11475 1:N:0:TCTGCAAG+GCTGCACT
ATAGAGTAATCCGGAGGAATCTATCCCCAATTGGAATAGTAATGATTTCAAATTTCAAATTTGAAAAT
ATCCCGCCCTACAGTATACCAATTCAAATTCAAATTCAAATAGAATTCCATAGGCCCCGCAGGGGCAT
CTTACCGCAGACCGT
+
FFFFFFFFFFFFFFFFFFFFFFFFFFFFFFFFFFFFFFFFFFFFFFFFFFFFFFFFFFFFFFFFFFFF
```

FFFFFFFFFFFFFFFFFFFFFFFFFFFFFFFFF:FFFFFFFFFFFFFF:FFFFFFFFFFFFFFFFFFF
FFFFFFFFFFFFFFF
@A00721:190:HGJW7DSXY:4:2223:9001:1705 1:N:0:TCTGCAAG+GCTGCACT
ATAGAGTAATCCGGAGGAATCTATCCCCAATTGGAATAGTAATGATTTCAAATTTCAAATTTGAAAAT
ATCCCGCCCTACAGTATGCCAATTCAAATTCAAATTCAAATAGAATTCCATAGGCCCCGCAGGGGCAT
CTTACCGCAGACCGT
+
FFFFFFFFFFFFFFFFFFFFF:FF:FFFFFFFFFFFFFFFFFFFFFFFFFFFF:FFFFFFFFFFFFFF
FFFFFFFFFFFFFFFFF,FF:FFFFFFF,FFFFFFFFFFFFF:FFFF:,F,FF:FFF:F:FF:F:FFF
:FFFF,FFFFFF:FF
@A00721:190:HGJW7DSXY:4:2223:10167:2597 1:N:0:TCTGCAAG+GCTGCACT
ATAGAGTAATCCGGAGGAATCTATCCCCAATTGGAATAGTAATGATTTCAAATTTCAAATTTGAAAAT
ATCCCGCCCTACAGTATACCAATTCAAATTCAAATTCAAATAGAATTCCATAGGCCCCGCAGGGGCAT
CTTACCGCAGACCGT
+
FFFFFFFFFFFFFFFFFFFFFFFFFFFFFFFFFFFFFFFFFFFFFFFFFFFFFFFFFFFFFFFFFFFF
FFFFFFFFFFFFFFFFFFFFFFFFFFFFFFFFFFFFFFFF:FFFFFFFFFFFFFFFFFFFFFFFFFFF
FFFFFFFFFFFFFFF
@A00721:190:HGJW7DSXY:4:2226:6298:14559 1:N:0:TCTGCAAG+GCTGCACT
ATAGAGTAATCCGGAGGAATCTATCCCCAATTGGAATAGTAATGATTTCAAATTTCAAATTTGAAAAT
ATCCCGCCCTACAGTATACCAATTCAAATTCAAATTCAAATAGAATTCCATAGGCCCCGCAGGGGCAT
CTTACCGCAGACCGT
+
FFFFFFFFFFFFFFFFFFFFFFFFFFFFFFFFFFFFFFFFFFFFFFFFFFFFFFFFFFFFFFFFFFFF
FFFF:FFFFFFFFFFFFFFFFF:FFFF:FFFFFFFFFFF:FFFFFFFFFFFFFFFFFFFFFFFFFF:F
FFFFFFFFFFFFFFF
@A00721:190:HGJW7DSXY:4:2314:20509:12900 1:N:0:TCTGCAAG+GCTGCACT
ATAGAGTAATCCGGAGGAATCTATCCCCAATTGGAATAGTAATGATTTCAAATTTCAAATTTGAAAAT
ATCCCGCCCTACAGTATACCAATTCAAATTCAAATTCAAATAGAATTCCATAGGCCCCGCAGGGGCAT
CTTACCGCAGACCGT
+
FFFFFFFFFFFFFFFFFFFFFFFFFFFFFFFFFFFFFFFFFFFFFFFFFFF:FFFFFFFFFFFFFFFF
FFFFFFFFFFFFFFFFFFFFFFFFFFFFFFFFFFFFFFFFFFFFFFFFFFFFFFFFFFFFFFFFFFFF
FFFFFFFFFFFFFFF
@A00721:190:HGJW7DSXY:4:1128:5855:25598 1:N:0:TCTGCAAG+GCTGCACT
CCGGAGGAATCTATCCCCAATTGGAATAGTAATGATTTCAAATTTCAAATTTGAAAATATCCCGCCCT
ACAGTATACCAATTCAAATTCAAATTCAAATAGAATTCCATAGGCCCCGCAGGGGCATCTTACCGCAG
ACCGTGGGGCAAGCC
+
FFFFFFFF:FFFFFFFFFFFFFFFFFFFFFFFFFFFFFFFFFFFFFFFFFFFFFFFFFFFFFFF:FF:F
FFFFFFFFFFFF,FFFF:FFFFFFFFFFFFFFFFFFFF:FFFFFFFFFFFFFFFFFFFFFFFFFF,FF,
FFFFFFFFFFFFFFF
@A00721:190:HGJW7DSXY:4:1128:3540:35931 1:N:0:TCTGCAAG+GCTGCACT
CCGGAGGAATCTATCCCCAATTGGAATAGTAATGATTTCAAATTTCAAATTTGAAAATATCCCGCCCT
ACAGTATACCAATTCAAATTCAAATTCAAATAGAATTCCATAGGCCCCGCAGGGGCATCTTACCGCAG
ACCGTGGGGCAAGCC
+
FFFFFFFFFFFFFFFFFFFFFFFFFFFFFFFFFFFFFFFFFFFFFFFFFFFFFFFFFFFFFFFFFFFF
FFFFFFFFFFFFF:FFFFFFFFFFFFFFFFFF:FFFFFFFFFFFFFFFFFFFFFFFFFFFFFFFFFFF
FFFFFFFFFFF:FFF
@A00721:190:HGJW7DSXY:4:1172:20961:16344 1:N:0:TCTGCAAG+GCTGCACT
CCGGAGGAATCTATCCCCAATTGGAATAGTAATGATTTCAAATTTCAAATTTGAAAATATCCCGCCCT
ACAGTATACCAATTCAAATTCAAATTCAAATAGAATTCCATAGGCCCCGCAGGGGCATCTTACCGCAG
ACCGTGGGGCAAGCC

```
+
FFFFFFFFFFFFFFFFFFFFFFFFFFFFFFFFFFFFFFFFFFFFFFFFFFFFFFFFFFFFFFFFFFFFFFFFFFF
FFFFFFF:FFFFFFFFFFFFFFFFFFFFFFFFFFFFF:FFFFFFFFFFFFFFFFFFFFFFFFFFFFFFFF
FFFFFFFFFF:F:FF
@A00721:190:HGJW7DSXY:4:1263:30581:8766 1:N:0:TCTGCAAG+GCTGCACT
CCGGAGGAATCTATCCCCAATTGGAATAGTAATGATTTCAAATTTCAAATTTGAAAATATCCCGCCCT
ACAGTATACCAATTCAAATTCAAATTCAAATAGAATTCCATAGGCCCCGCAGGGGCATCTTACCGCAG
ACCGTGGGGCAAGCC
+
FFFFFFFFFFFFFFFFFFFFFFFFFFFFFFFFFFFFFFFFFFFFFFFFFFFFFFFFFFFF:FFFFFFF
FFFFF:FFFFFFFFFFFFFFFFFFFFFFFFFFFFFFFFFFFFFFFFFFFFFFFFFFFFFFFFFFFFFF
FFFFFFFFFFFFFFF
@A00721:190:HGJW7DSXY:4:1317:17535:17738 1:N:0:TCTGCAAG+GCTGCACT
CCGGAGGAATCTATCCCCAATTGGAATAGTAATGATTTCAAATTTCAAATTTGAAAATATCCCGCCCT
ACAGTATACCAATTCAAATTCAAATTCAAATAGAATTCCATAGGCCCCGCAGGGGCATCTTACCGCAG
ACCGTGGGGCAAGCC
+
FFFFFFFFFFFFFFFFFFFFFFFFFFFFFFFFFFFFFFFFFFFFFFFFF:FFFFFFF:FFFFFFFF,
FFFFFF:FFFF:FFFFFFFFFFFFFFFFF:FF:FFFFFFFFFFFFFFFF,FFFFFFFFFFFFFFFF
FFFFFFFFFFFFFFF
@A00721:190:HGJW7DSXY:4:2655:7229:20838 1:N:0:TCTGCAAG+GCTGCACT
CCGGAGGAATCTATCCCCAATTGGAATAGTAATGATTTCAAATTTCAAATTTGAAAATATCCCGCCCT
ACAGTATACCAATTCAAATTCAAATTCAAATAGAATTCCATAGGCCCCGCAGGGGCATCTTACCGCAG
ACCGTGGGGCAAGCC
+
FFFFFFFFFFFFFFFFFFFFFFFFFFFFFFFFFFFFFFFFFFFFFFFFFFFF:FFFFFFFFFFFFFF
FFFFFFF:,FFFFFFFFFFFFFFFFFFFF:FFFFFFFF:FFFF,FFFFFFFFFFFFFFFFFFFFFFFF
FFFFFFFFFFFFFFF
@A00721:190:HGJW7DSXY:4:1504:2862:4946 1:N:0:TCTGCAAG+GCTGCACT
CGGAGGAATCTATCCCCAATTGGAATAGTAATGATTTCAAATTTCAAATTTGAAAATATCCCGCCCTA
CAGTATACCAATTCAAATTCGAATTCAAATAGAATTCCATAGGCCCCGCAGGGGCATCTTACCGCAGA
CCGTGGGGCAAGCCA
+
FFFFFFFFFFFFFFFFFFFFFFFFFFFFFFFFFFFF:FFFFFFFFFFFFFFFFFFFFFFFFFFFFFFF
FFFFFFFFFFFFFFFFFFFFFFFF:FFFF,FFFFFFFFFFFFFFFFFFFFFFFFFFFFFFFFFFFFFFF
FFFFFFFFFFFFFFF
@A00721:190:HGJW7DSXY:4:2120:31611:18693 1:N:0:TCTGCAAG+GCTGCACT
CGGAGGAATCTATCCCCAATTGGAATAGTAATGATTTCAAATTTCAAATTTGAAAATATCCCGCCCTA
CAGTATACCAATTCAAATTCGAATTCAAATAGAATTCCATAGGCCCCGCAGGGGCATCTTACCGCAGA
CCGTGGGGCAAGCC
+
FFFFFFFFFFFFFFFFFFFFFFFFFFFFFFFFFFFFFFFFFFFFFFFFFFFFFFFFFFFFFFFFFFFF
FFF:FFFFFFFFFFFFFFFFFFFFFFFFFFFFFFFFFFFFFFFFFFFFFFFFF:FFFFFFFFFFFF
FFFFFFFFFFFFFF
@A00721:190:HGJW7DSXY:4:2242:18105:7482 1:N:0:TCTGCAAG+GCTGCACT
CGGAGGAATCTATCCCCAATTGGAATAGTAATGATTTCAAATTTCAAATTTGAAAATATCCCGCCCTA
CAGTATACCAATTCAAATTCGAATTCAAATAGAATTCCATAGGCCCCGCAGGGGCATCTTACCGCAGA
CCGTGGGGCAAGCC
+
FFFFFFFFFFFFFFFFFFFF:FFFFFFFFFFF:FFFFFFFFFFFFFFFFFFFFFFFFFFF:FFFFFFFFF,
FFF:,FFF:FFFFFFFFFFFFFFFFFF,FFFFFFFFFF:FFF:F:FFFFFFFFFFFFFFFFFFFFFFFF
F:FFFFFF:FFFFF
@A00721:190:HGJW7DSXY:4:2316:30120:26694 1:N:0:TCTGCAAG+GCTGCACT
CGGAGGAATCTATCCCCAATTGGAATAGTAATGATTTCAAATTTCAAATTTGAAAATATCCCGCCCTA
```

CAGTATACCAATTCAAATTCGAATTCAAATAGAATTCCATAGGCCCCGCAGGGGCATCTTACCGCAGA
CCGTGGGGCAAGCCA
+
FFFFFFF:FFFFFFFFFFFFFFFFFFFFFFFFFFFFFFFFFFFFFFFFFFFFFF:FFFFFFFFFFFFFFF
FFFFFF,FFFFFFFFFFFFFFFFFFFFFF,FFFFFFFFFFFFFFFFFFFFFFFFFFFFFFFFFFFFFFFF
FFFFFFFFFFFFFFF
@A00721:190:HGJW7DSXY:4:2641:6515:33818 1:N:0:TCTGCAAG+GCTGCACT
CGGAGGAATCTATCCCCAATTGGAATAGTAATGATTTCAAATTTCAAATTTGAAAATATCCCGCCCTA
CAGTATACCAATTCAAATTCGAATTCAAATAGAATTCCATAGGCCCCGCAGGGGCATCTTACCGCAGA
CCGTGGGGCAAGCCA
+
FFFFFFFFFFFFFFFFFFFFFFFFFFFFFFFFFFFFFFFFFFFFFFFFFFFFFFFFFFFFF:FFFFFFFF
FFFFFFFFFFFFFFFFFFFFFFFFFFFFFFFFFFFFFFFFFFF:FFFFFFFFFFFFFF:FFFFFFFFFFF
FFFFFFFFFFFFFFF
@A00721:190:HGJW7DSXY:4:2677:17815:23954 1:N:0:TCTGCAAG+GCTGCACT
CGGAGGAATCTATCCCCAATTGGAATAGTAATGATTTCAAATTTCAAATTTGAAAATATCCCGCCCTA
CAGTATACCAATTCAAATTCGAATTCAAATAGAATTCCATAGGCCCCGCAGGGGCATCTTACCGCAGA
CCGTGGGGCAAGCCA
+
FFFFFFFFFFFFFFFFFFFFFFFFFFFFFFFFFFFFFFFFFFFFFFFFFFFFFFFFFFFFFFFFFFFFFF
FFFFFFFFFFFFF:FFFFFFFFF:FF:FFFF:FFFFFFFFFFFFFFFFFFFFFFFFF:F:FFF:FFFFFF
FFFFFFFFFFFFFFF
@A00721:190:HGJW7DSXY:4:1110:21540:10332 1:N:0:TCTGCAAG+GCTGCACT
GGAGGAATCTATCCCCAATTGGAATAGTAATGATTTCAAATTTCAAATTTGAAAATATCCCGCCCTAC
AGTATACCAATTCAAATTCAAATTCAAATAGAATTCCATAGGCCCCGCAGGGGCATCTTACCGCAGAC
CGTGGGGCAAGCCAA
+
FFFFFFFFFFFFFFFFFFFFFFFFFFFFFFFFFFFFFFFFFFFFFFFFFFFFFFFFFFFFFFFFFFFF:F
FFFFFFFFFFFFFFFFFFFFFFFFFFFF:FFFFFFFFFFFFFFFFFFFFFFFFFFFFFFFFFFFFFFFFF
FFFFFFFFFFFFFFF
@A00721:190:HGJW7DSXY:4:1120:28022:24815 1:N:0:TCTGCAAG+GCTGCACT
GGAGGAATCTATCCCCAATTGGAATAGTAATGATTTCAAATTTCAAATTTGAAAATATCCCGCCCTAC
AGTATACCAATTCAAATTCAAATTCAAATAGAATTCCATAGGCCCCGCAGGGGCATCTTACCGCAGAC
CGTGGGGCAAGCCAA
+
FFFFFFFFFFFFFFFFFFFFFFFFFFFFFFFFFFFFFFFFFFFFFFFFFFFFFFFFFFFFFFFFFFFFFF
FFFFFFFFFFFFFFFFFFFFFFFFFFFFFFFFFFFFFFFFFFFFFFFFFFFFFFFFFFFFFFFFFFFFFF
FFFFFFFFFFFFFFF
@A00721:190:HGJW7DSXY:4:1301:8196:9048 1:N:0:TCTGCAAG+GCTGCACT
GGAGGAATCTATCCCCAATTGGAATAGTAATGATTTCAAATTTCAAATTTGAAAATATCCCGCCCTAC
AGTATACCAATTCAAATTCAAATTCAAATAGAATTCCATAGGCCCCGCAGGGGCATCTTACCGCAGAC
CGTGGGGCAAGCCAA
+
FFF:FFFFFFFFFF:FFF:,FF:FFFFFFFF,FFFFFFFFF:,FFFFFFFFFFFF:FFFFF,FFFF:,FF
FFFFFFF:F,::::FF:FFF,FFFFFF:FFFFFFFFFFF,F:F,F:FFFFFF:FF::,FFFF:FF:FF,F
:FF::FF:FF,FF,F
@A00721:190:HGJW7DSXY:4:1531:17472:19570 1:N:0:TCTGCAAG+GCTGCACT
GGAGGAATCTATCCCCAATTGGAATAGTAATGATTTCAAATTTCAAATTTGAAAATATCCCGCCCTAC
AGTATACCAATTCAAATTCAAATTCAAATAGAATTCCATAGGCCCCGCAGGGGCATCTTACCGCAGAC
CGTGGGGCAAGCCAA
+
FFFFFFFFFFFFFFFFFFFFFFFFFFFFFFFFFFFFFFFFFFFFFFFFFFFFFFFFFFFFFFFFFFFF:FF
FFFFFFFFFFFFFFFFFFFFFFFFFFFFFFFFFFFFFFFFFFFFFFFFFFFFFFFFFFFFFFFFFFFFFF
FFFFFFFFF:FFFFF

```
@A00721:190:HGJW7DSXY:4:1541:22037:30514 1:N:0:TCTGCAAG+GCTGCACT
GGAGGAATCTATCCCCAATTGGAATAGTAATGATTTCAAATTTCAAATTTGAAAATATCCCGCCCTAC
AGTATACCAATTCAAATTCAAATTCAAATAGAATTCCATAGGCCCCGCAGGGGCATCTTACCGCAGAC
CGTGGGGCAAGCCAA
+
FFFFFFFFFFFFFFFFFFFFFFFFFFFFFFFFFFFFFFFFFFFFFFFFFFFFFFFFFFFFFFFFFFFF
FFFFFFFFFFFFFFFFFFFFFF:FFF:FFFFFFFFFFFFFFFFFFFFFFFFFFFFFFFFFFFFFFFFF
FFFFFFFFFFFFFF:
@A00721:190:HGJW7DSXY:4:1641:27218:24205 1:N:0:TCTGCAAG+GCTGCACT
GGAGGAATCTATCCCCAATTGGAATAGTAATGATTTCAAATTTCAAATTTGAAAATATCCCGCCCTAC
AGTATACCAATTCAAATTCAAATTCAAATAGAATTCCATAGGCCCCGCAGGGGCATCTTACCGCAGAC
CGTGGGGCAAGCCAA
+
FFFFFFFFFFFFFFFFFFFFFFFFFFFFFFFFFFFFFFFFFFFFFFFFFFFFFFFFFFFFFFFFFFFF
FFFFFFFFFFFFFFFFFFFFFFFFFFFFF:FFFFFFFFFFFFFFFFFFFFFFFFFFFFFFFFFFFFFF
FFFFFFFFFFFFF:F
@A00721:190:HGJW7DSXY:4:2259:26449:7279 1:N:0:TCTGCAAG+GCTGCACT
GGAGGAATCTATCCCCAATTGGAATATTAATGATTTCAAATTTCAAATTTGAAAATATCCCGCCCTAC
AGTATACCAATTCAAATTCAAATTCAAATAGAATTCCATAGTCCCCGCAGGGGCCTCTTACCGCAGAA
CGTGGGGCAAGCCAA
+
FFFF,FFFFFFF,,:FFFFF:FFFFF:FFFF::FFFFFF:FFFFFF:FFF:FFF:FFFFFFF,F,FF,
F,F,F::FFFFFFFF,,FFFFFFF,:F,:F:FFFF:FF:F,,FF,FFF:,:,F,,FF,FF::,,F,F,
F,,,,F:::F::,FF
@A00721:190:HGJW7DSXY:4:2677:17833:23797 1:N:0:TCTGCAAG+GCTGCACT
GGAGGAATCTATCCCCAATTGGAATAGTAATGATTTCAAATTTCAAATTTGAAAATATCCCGCCCTAC
AGTATACCAATTCAAATTCGAATTCAAATAGAATTCCAAAGGCCCCGCAGGTGCATCTTACCGCATAC
CGTGGGGCAAGCC
+
FFF:FFFFFF:F:FFFFFFF:FFF,F,FFF,FF,,FFFFFFFF:F:FFFF:FFFFFFFFFF,F:FFF:
F,,:F,FFFFFF,FFF:F::::F,,FFF:,:FFFF,FF,FF:FF:FF:F,,,F,F::F:FFF:,:,,,
,FFFF:,FFF:,F
@A00721:190:HGJW7DSXY:4:1344:30373:13917 1:N:0:TCTGCAAG+GCTGCACT
ATTGGAATAGTAATGATTTCAAATTTCAAATTTGAAAATATCCCGCCCTACAGTATACCAATTCAAAT
TCAAATTCAAATAGAATTCCATAGGCCCCGCAGGGGCATCTTACCGCAGACCGTGGGGCAAGCCAATA
TAATATTACTGGCTT
+
FFFFFFFFFFFFFFFFFFFFFFFFFFFFFFFFFFFFFFFFFFFFFFFFFF:FFFFFFFFFFFFFFFFFF
FFFFFFFFFF:FFF:FFFFFFFFFFFFFFFFFFFFFFFFFFFFFFFFFFFFFFFFFFFFFFFFFFF,F
FFFFFFFFFFFFFFF
@A00721:190:HGJW7DSXY:4:1362:16559:8155 1:N:0:TCTGCAAG+GCTGCACT
ATTGGAATAGTAATGATTTCAAATTTCAAATTTGAAAATATCCCGCCCTACAGTATACCAATTCAAAT
TCAAATTCAAATAGAATTCCATAGGCCCCGCAGGGGCATCTTACCGCAGACCGTGGGGCAAGCCAATC
TAATATTACTGGCTT
+
F:F:FFFFF:FFFFFFFFFFFFFFFFFFFFFFFF:FFFFF:FFFFFFFF:FFFFFF:FFFFFFF:FFFFF
FFFFFFFFFFFFF:FFFFF:FFF:FFFFF:FFFFFFFFFFFFFFFFFFFFFFFFFF,F:FFFFFFFF:F,
FF:FFFFFFFFFFFF
@A00721:190:HGJW7DSXY:4:1432:15374:8359 1:N:0:TCTGCAAG+GCTGCACT
ATTGGAATAGTAATGATTTCAAATTTCAAATTTGAAAATATCACGCCCTACAGTATACCAATTCAAAT
TCAAATTCAAATAGAATTCCATAGGCCCCGCAGGGGCATCTTACCGCAGACCGTGGGGCAAGCCAATA
TAATATTACTGGCTT
+
FFF:FFFFFFFFF:FF:FFFFFFF,FFFFFFFFFFFFFFF:,,F,F,FFFFFFFFFFFF,:,FFFFFF
```

,FFFFF,:FF,FFFFF:F:FFFF,,FFFF,FF,FFFFFF:F,FFFF:FFFFF:F:FF,FFF,F,FF,F
,FFFFF:FFF,:F,F
@A00721:190:HGJW7DSXY:4:1668:7374:34240 1:N:0:TCTGCAAG+GCTGCACT
ATTGGAATAGTAATGATTTCAAATTTCAAATTTGAAAATATCCCGCCCTACAGTATACCAATTCAAAT
TCAAATTCAAATAGAATTCCATAGGCCCCGCAGGGGCATCTTACCGCAGACCGTGGGGCAAGCCAATA
TAATATTACTGGCTT
+
FFFFFFFFFFFFFFFFFFFFFFFFFFFFFFFFFFFFFFFFFFFFFFFFFFFFFFFFFFFFFFFFFFFF
FFFFFFFFFFFFFFFFFFFFFFFFFFFFFFFFFFFFFFFFFFFFFFFFFFFFFFFFFFFFFFFF:FFF
FFFFFFFFFFFFFFF
@A00721:190:HGJW7DSXY:4:1677:32208:4758 1:N:0:TCTGCAAG+GCTGCACT
ATTGGAATAGTAATGATTTCAAATTTCAAATTTGAAAATATCCCGCCCTACAGTATACCAATTCAAAT
TCAAATTCAAATAGAATTCCATAGGCCCCGCAGGGGCATCTTACCGCAGACCGTGGGGCAAGCCAATA
TAATATTACTGGCTT
+
FFFFFFFFFFFFFFFFFFFFFFFFFFFFFFFFFFFFFFFFFFFFFFF,FF,F:FFFFF:FFFFFFFF
FFFFFFFFFFFFF:FFFFFFFFFFFFFFFFFFFFFFFFFFFFFF:FFFFFFFFFFFFFFFFFFF:FFFFF
F:FFFFFFFFFFFFF
@A00721:190:HGJW7DSXY:4:2174:5267:34601 1:N:0:TCTGCAAG+GCTGCACT
ATTGGAATAGTAATGATTTCAAATTTCAAATTTGAAAATATCCCGCCCTACAGTATACCAATTCAAAT
TCAAATTCAAATAGAATTCCATAGGCCCCGCAGGGGCATCTTACCGCAGACCGTGGGGCAAGCCAATA
TAATATTACTGGCTT
+
FFFFFFFFFFFFFFFFFFFFFFFFFFFFFFFFFFFFFFFFFFFFFFFFFFFFFFFFFFFFFFFFFFFF
FFFFFFFFFFFFFFFFFFFFFFFFFFFFFFFFFFFFFF:FFFFFFFFFFFFFFFFFFFFFFF,FFF,FFF
F,FFFFFFFFFFFFF
@A00721:190:HGJW7DSXY:4:2267:5575:17534 1:N:0:TCTGCAAG+GCTGCACT
ATTGGAATAGTAATGATTTCAAATTTCAAATTTGAAAATATCCCGCCCTACAGTATACCAATTCAAAT
TCAAATTCAAATAGAATTCCATAGGCCCCGCAGGGGCATCTTACCGCAGACCGTGGGGCAAGCCAATA
TAATATTACTGGCTT
+
FFFFFFFFFFFFFFFFFFFFFFFFFFFFFFFFFFFFFFFFFFFFFFFFFFFFFFFFFFFFFFFFFFFF
FFFFFFFFFFFFFFFFFF:FFFFFFFFFFFFFFFFFFFFFFFFFFFFFFFFFFFFFFFFFFFFFFFFFF
FFFFFFFFFFFFFFF
@A00721:190:HGJW7DSXY:4:2327:10077:6324 1:N:0:TCTGCAAG+GCTGCACT
ATTGGAATAGTAATGATTTCAAATTTCAAATTTGAAAATATCCCGCCCTACAGTATACCAATTCAAAT
TCAAATTCAAATAGAATTCCATAGGCCCCGCAGGGGCATCTTACCGCAGACCGTGGGGCAAGCCAATA
TAATATTACTGGCTT
+
FFFFFFFFFFFFFFFFFFFFFFFFFFFFFFFFFFFFFFFFFFFFFFFFFFFFFFFFFFFFFFFFFFFF
FFFFFFFFFFFFFFFFFFFFFFFFFFFFFFFFFFFFFF,FFFFFFFFFFFFFFFFFFFFFFF:FFFFFF,
F:FFFFFFFFFFFFF
@A00721:190:HGJW7DSXY:4:2533:6506:11600 1:N:0:TCTGCAAG+GCTGCACT
ATTGGAATAGTAATGATTTCAAATTTCAAATTTGAAAATATCCCGCCCTACAGTATACCAATTCAAAT
TCAAATTCAAATAGAATTCCATAGGCCCCGCAGGGGCATCTTACCGCAGACCGTGGGGCAAGCCAATA
TAATATTACTGGCTT
+
FFFFFFFFFFFFFFFFFFFFFFFFFFFFFFFFFFFFFFFFFFFFFFFFFFFFFFFFFFFFFFFFFFFF
FFFFFFFFFF:FFFF,FFFFFFFFFFFFFFFFFFFFFFFFFFFFFFFFFFFFFFFFFFFFFFFFFFFF
FFF,FFFFFFFFFFF
@A00721:190:HGJW7DSXY:4:2670:10131:26647 1:N:0:TCTGCAAG+GCTGCACT
ATTGGAATAGTAATGATTTCAAATTTCAAATTTGAAAATATCCCGCCCTACAGTATACCAATTCAAAT
TCAAATTCAAATAGAATTCCATAGGCCCCGCAGGGGCATCTTACCGCAGACCGTGGGGCAAGCCAATA
TAATATTACTGGCTT

```
+
FFFFFFFFFFFFFFFFFFFFFFFFFFFFFFFFFFF:FFFFFFFFFFFFFFFF:FFFFF:FFFFFFFFFF
FFFFFFFFFFFFFFFFFFFF:FFF::FFFFFFFFFFFFFFFFFFFFFFFFFFFFFFFFFFFFFFFF:FF
FFFFFFFFFFFFFFF
@A00721:190:HGJW7DSXY:4:1536:32786:27806 1:N:0:TCTGCAAG+GCTGCACT
CCCGGCGCGGGCAAGCCAGTAATATTATATTGGCTTGCCCCACGGTCTGCGGTAAGATGCCCCTGCGG
GGCCTATGGAATTCTATTTGAATTTGAATTGGTATACTGTAGGGCGGGATATTTTCAAATTTGAAATT
TGAAATCATTACTAT
+
FFFFFFFFFFFF:F:FFFF:FFFFFFFFFFFFFFFFFFFFFFFFFFFF,FF:FFFFF:::F:FFF,FFFF
FFFF:F:FF:FFF:FFFFFF:FFFFFFFFF:FFFFFFFFF:FFFFFFFFFFFFFF,:FFFFF:FFFFF
F:,,FFFF:FF,FFF
@A00721:190:HGJW7DSXY:4:1537:22625:19914 1:N:0:TCTGCAAG+GCTGCACT
CCCGGCGCGGGCAAGCCAGTAATATTATATTGGCTTGCCCCACGGTCTGCGGTAAGATGCCCCTGCGG
GGCCTATGGAATTCTATTTGAATTTGAATTGGTATACTTTAGGGCGGGATATTTTCAAATTTGAAATT
TGAAATCATTACTAT
+
FFFFFFFFFFFFFF,FFF:FFFFFFFFFFFF,FFFF,FFFFFF:FF:::FFF:FF,FFFFFFFF:FFF
FF:F,F:,FFFFF,:FF:F,,FFFF,FFFF:FFFFFF,,FFF,FFFFFFFF:FFF,FFFFFF:FFF,F
F:FFFF:F:FFF,F:
@A00721:190:HGJW7DSXY:4:2278:23873:2848 1:N:0:TCTGCAAG+GCTGCACT
CCCGGCGCGGGCAAGCCAGTAATATTATATTGGCTTGCCCCACGGTCTGCGGTAAGATGCCCCTGCGG
GGCCTATGGAATTCTATTTGAATTTGAATTGGTATACTGTAGGGCGGGATATTTTCAAATTTGAAATT
TGAAATCATTACTAT
+
FFFFFFFFFFFFFFFFF:FFFFFFFFFFFFFFFFFFFFFFFFFFFFFFFFFFFFFFFFF:FFFFFFFFF
FFFFFFFFFFFFFF:FFFFFFFFFFFFFFF:FFFFFFFFFFFFFFFFFFFFFFFFFFFFFFFFFFFFF
FFFFFFFFFFFFFFF
@A00721:190:HGJW7DSXY:4:2665:7862:7185 1:N:0:TCTGCAAG+GCTGCACT
CCCGGCGCGGGCAAGCCAGTAATATTATATTGGCTTGCCCCACGGTCGGCGGTAAGATGCCCCTGCGG
GGCCTATGGAATTCTATTTGAATTTGAATTGGTATACTGTAGGGCGGGATATTTTCAAATTTGAAATT
TGAAATCATTACTAT
+
FFFFFFFFFFFF,:FFF:F:,,,F,FFFF:FFFF:FFFFFF:F:F:F,FFFF,FFFFFFFFFF:FFFF
FFFF,:,FFFF::FFFF:FF,F:FFFFF:FFFF,F:F,F:,FFFFFFFFFFFF:F:FFFFFFFF:FFF
F:,FF::FFFFF,FF
@A00721:190:HGJW7DSXY:4:1162:21649:31970 1:N:0:TCTGCAAG+GCTGCACT
CCCGGCGCGGGCAAGCCAGTAATATTATATTGGCTTGCCCCACGGTCTGCGGTAAGATGCCCCTGCGG
GGCCTATGGAATTCTATTTGAATTTGAATTTGAATTGGTATACTGTAGGGCGGGATATTTTCAAATTT
GAAATTTGAAATCAT
+
FFFFFFFFFFFFFFFFFFFF:FFF,:FFFFF:FFFFFFFFFFFFFFFFFFFFFFFFFFFFFFFFFFFFF
FFFFFFFFFFFFFFFFFFFF:FFFFFFFFFFFFFF:FFFFFFF:FFFFFFFFFFFFFFFFFFFFFFFF
FFFFFFFF:FFFFFF
@A00721:190:HGJW7DSXY:4:1223:7346:19038 1:N:0:TCTGCAAG+GCTGCACT
CCCGGCGCGGGCAAGCCAGTAATATTATATTGGCTTGCCCCACGGTCTGCGGTAAGATGCCCCTGCGG
GGCCTATGGAATTCTATTTGAATTTGAATTTGAATTGGTATACTGTAGGGCGGGATATTTTAAAATTT
GAAATTTGAAATAAT
+
FFFFFFFFFFFF:F::FF,FFF,FFFFFF,F:F:FFFFFFFFF:FF::::FFFFF,::::FFF,,FFF
FFFF,FF::FF,F,FFFFF,:F:FF,:F:FF,FF:F:FFFFF,:::::FFFFFFFFF,FFF,FFFFFF
,,FF:F::FFFF,FF
@A00721:190:HGJW7DSXY:4:1257:2745:13761 1:N:0:TCTGCAAG+GCTGCACT
CCCGGCGCGGGCAAGCCAGTAATATTATATTGGCTTGCCCCACGGTCTGCGGTAAGATGCCCCTGCGG
```

GGCCTATGGAATTCTATTTGAATTTGAATTTGAATTGGTATACTGTAGGGCGGGATATTTTCAAATTT
GAAATTTGAAATCAT
+
FFFFFFFFFFFFFF:FFFFFFFFF:FFFFFFFFFFFFFFFFFFFFFFFFFFFFFF:FFFFFFFFFFFF
FFFF,FFFFFFFF::F:F:F:FFFFFFF:FFFFFFFFFFFFFFFFFFFFFFFFFFFFFFFF:FFFFFF
FFFFFFF,:FF,FFF
@A00721:190:HGJW7DSXY:4:1346:30129:22326 1:N:0:TCTGCAAG+GCTGCACT
CCTGGCGCGGGCAAGCCAGTAATATTATATTGGCTTGCCCCACGGTCTGCGGTAAGATGCCCCTGCGG
GGCCTATGGAATTCTATTTGAATTTGAATTTGAATTGGTATACTGTAGGGCGGGATATTTTCAAATTT
GAAATTTGAAATCAT
+
FFFFFFFFFFFFFF:FFF:FFFF:FFFFF:FFFFFFFFFFFFFFFFF:F,FFFFF,F,FFFFF,FFFF
FFFF,FFFFFF:FF:FF:FF:FFFF:FF:FF::FFFFFFFFF:FFFFFFFFFFF:FFFFFF,FFF:FF
,FFFFFF,FFFFFF:
@A00721:190:HGJW7DSXY:4:1348:27850:2597 1:N:0:TCTGCAAG+GCTGCACT
CCCGGCGCGGGCAAGCCAGTAATATTATATTGGCTTGCCCCACGGTCTGCGGTAAGATGCCCCTGCGG
GGCCTATGGAAATCTATTTGAATTTGAATTTGAATTGGTATACTGTAGGGCGGGATATTTTCAAATTT
GAAATTTGAAATCAT
+
FFFFFFFFFFFF::FFF,FF,::FFF:FFFFFFFFFFFFFFFFFFFF:FFFF:F::FFFFFFF:FFFF
FFFF,FFFFF,,FF:::FFFF::FFF:F,FF:FFFFFFFFFF:FF:FFFFFFFF:FFFFFF,:FF:FF
FFFFFFFF:FFFFFF
@A00721:190:HGJW7DSXY:4:1351:19560:3677 1:N:0:TCTGCAAG+GCTGCACT
CCTGGCGCGGGCAAGCCAGTAATATTATATTGGCTTGCCCCACGGTCTGCGGTAAGATGCCCCTGCGG
GGCCTATGGAATTCTATTTGAATTTGAATTTGAATTGGTATACTGTAGGGCGGGATATTTTCAAATTT
GAAATTTGAAATCAT
+
FFFFFFFFFFFFFFFFF:FFF:FFF:FFFFFFFFFF,FFFFFF:FFFFFFFF:FFFFFFFFFF,FFFF
FFFFFFF:FFFFF,FFFFFFFFFFFFFFFFFFFFFFFFFF:FFFFFFFFFFFFFFFFFFFFFFFFFF:FF
FFFFFFF:FFFF,F:
@A00721:190:HGJW7DSXY:4:1451:3414:23312 1:N:0:TCTGCAAG+GCTGCACT
CCCGGCGCGGGCAAGCCAGTAATATTATATTGGCTTGCCCCACGGTCTGCGGTAAGATGCCCCTGCGG
GGCCTATGGAATTCTATTTGAATTTGAATTTGAATTGGTATACTGTAGGGCGGGATATTTTCAAATTT
GAAATTTGAAATCAT
+
FFFFFFFFFFFFFFFFFFFFFFFFFFFFFFFF,FFFF:FFFFFFFFFFF:FFFFFFF,F:FFFF::FFF
FFFFFFFFFFFFF,FFFFF::FF:FFFFFFF:FFFF:FFFFFFFFFF:FFFFFFFFFFFFFFFFF:FF
,FFFFFFFFFFFFFF
@A00721:190:HGJW7DSXY:4:1564:11478:14700 1:N:0:TCTGCAAG+GCTGCACT
CCTGGCGCGGGCAAGCCAGTAATATTATATTGGCTTGCCCCACGGTCTGCGGTAAGATGCCCCTGCGG
GGCCTATGGAATTCTATTTGAATTTGAATTTGAATTGGTATACTGTAGGGCGGGATATTTTCAAATTT
GAAATTTGAAATCAT
+
FFFFFFFFFFFF:FFFF:FFFFF:F:FFFFF:FFFFFFFFFFFFFFFFFFFFFFFFFFFFFFFF:FFFF
FFFF:FFFFF:FFFFFFFFFFFFFFFFFFFFFFFFFFFFFFFFF:FFFFFFFFFFFFFFFFFFFFFFFF
:FFFFFFFFFFFFFF
@A00721:190:HGJW7DSXY:4:2130:29035:30953 1:N:0:TCTGCAAG+GCTGCACT
CCTGGCGCGGGCAAGCCAGTAATATTATATTGGCTTGCCCCACGGTCTGCGGTAAGATGCCCCTGCGG
GGCCTATGGAATTCTATTTGAATTTGAATTTGAATTGGTATACTGTAGGGCGGGATATTTTCAAATTT
GAAATTTGAAATCAT
+
FFFFFFFFFFFFFF,FF,FFFFFFFFFFFFF:FFFFFFFFFF::FFFFFFFFFFFFF::FFFF:FFFF
FFFFFFFFFF::F,FFFFF,FFFFF:FFFFF,FF:F,FFFFFF,FFFFFFFFFFFFFFFFFFFF::FF:
,:FF:FF,,FFFFFF

```
@A00721:190:HGJW7DSXY:4:2571:32931:26772 1:N:0:TCTGCAAG+GCTGCACT
CCCGGCGCGGGCAAGCCAGTAATATTATATTGGCTTGCCCCACGGTCTGCGGTAAGATGCCCCTGCGG
GGCCTATGGAATTCTATTTGAATTTGAATTTGAATTGGTATACTGTAGGGCGGGATATTTTCAAATTT
GAAATTTGAAATCAT
+
FFFFFFFFFFFFFFFFFF:FFFFFFFFFFF:FFFFFFFFFFFFFFFFFF:FFFFFFFFFFFFFFFFFFFF
FFFFFF:FFFFFFFFFFFFFFFFFFF:FFFFFFFFFF,FFFFF:F:FF:FFFFFFFFFFFFFFFFFFFFF
:FFFFFF,:FF:,FF
@A00721:190:HGJW7DSXY:4:2656:3821:23265 1:N:0:TCTGCAAG+GCTGCACT
CCTGGCGCGGGCAAGCCAGTAATATTATATTGGCTTGCCCCACGGTCTGCGGTAAGATGCCCCTGCGG
GGCCTATGGAATTCTATTTGAATTTGAATTTGAATTGGTATACTGTAGGGCGGGATATTTTCAAATTT
GAAATTTGAAATCAT
+
FFFFFFFFFFFFFFFFFFFFFFFFFFFFFF:F:FFFFFFFFFFFFFFFFFFFFFFFFFFF:FFFFFFFFF
FFFF:FFFFFFF:F:FFFFFFFFFFFFFFFFFFFFFFFFFFFFF:F,:FFFFFFFFFFFFFFFFFFFFFF
F,FFFFFFFFFF:FF
@A00721:190:HGJW7DSXY:4:2142:13304:34240 1:N:0:TCTGCAAG+GCTGCACT
CCCGGCGCGGGCAAGCCCGTAATATTATATTGGCTTTCCCCACGGTCTGCGGTAAGATGCCCCTGCGG
GGCCTATGGAATTCTATTTGAATTTGAATTTGAATTGGTATACTGTAGGGCGGGATATTTTCAAATTT
GTAATTTGAAATCA
+
FFFFFFFFFFFF,F:,F,,:FFFFFF,,F,F::FFF,FFFFFFF,FF:F,FFF,FFFFFF:,FFF,,FFF
FFFF,F:FFFF:F,F,,FF:,F,FF,,F:FF,:FFFFF,:FF,:FFFFFF:FFFF,FF:FFF,FFFFFF
:,,FF,F,F:FFFF
@A00721:190:HGJW7DSXY:4:1259:26449:18646 1:N:0:TCTGCAAG+GCTGCACT
GTCCCCGGCGCGGGCAAGCCAGTAATATTATATTGGCTTGCCCCACGGTCTGCGGTAAGATGCCCCTG
CGGGGCCTATGGAATTCTATTTGAATTTGAATTTGAATTGGTATACTGTAGGGCGGGATATTTTCAAA
TTTGAAATTTGAAAT
+
FFFFFFFFFFFFFFFFFFF:F,FFFF::FFFFFFFFFFFFFFFFFFFFFFFFFF:FFFFFF:FFFFFFFF
FFFFFFF:F:FFFFFF,:FFFFF:FFFFFFFFFFFFFFFFFF::FFF,FF:FFFFFFFFFFFFFFF:FFF
FFFF,FFFFFFFFFF
@A00721:190:HGJW7DSXY:4:1413:26621:28463 1:N:0:TCTGCAAG+GCTGCACT
GTCCCCGGCGCGGGCAAGCCAGTAATATTATATTGGCTTGCCCCACGGTCTGCGGTAAGATGCCCCTG
CGGGGCCTATGGAATTCTATTTGAATTTGAATTTTAATTGGTATACTGTAGGGCGGGATATTTTCAAA
TTTGAAATTTGAAAT
+
FFFFFFFFFFFFFFFFF,F,FFF,::F,:FF::F,F,F:,F:FFFFFF,FF,:FFFF::F,F:,:FFF,:
F,FFF:F:F,::FFF:,:FFFF,:FFFF,:F,FF,:F,F,FF:FF,F:F:,:FF:FF,FF:FFF,FFF
,FFF:FF,FF,:FF:
@A00721:190:HGJW7DSXY:4:2366:25256:34961 1:N:0:TCTGCAAG+GCTGCACT
GTCCCCGGCGCGGGCAAGCCAGTAATATTATATTGGCTTGCCCCACGGTCTGCGGTAAGATGCCCCTG
CGGGGCCTATGGAATTCTATTTGAATTTGAATTTGAATTGGTATACTGTAGGGCGGGATATTTTCAAA
TTTGAAATTTGAAAT
+
FFFFFFFFFFFFFFFFFFFFFFFFF:FFFFFFFFFFFF:F:FFFFF:FFF:FFFFFFFFFFFF:FFFFFF
:FFFFFF::FFFFFF::FFFFFF:FFFFF:FFFFFFFFFFFFFF::FFFFFFFFFFFFFFFFFFFFFFFF
FFF:FFFFFFFFFFF
@A00721:190:HGJW7DSXY:4:2366:24587:35994 1:N:0:TCTGCAAG+GCTGCACT
GTCCCCGGCGCGGGCAAGCCAGTAATATTATATTGGCTTGCCCCACGGTCTGCGGTAAGATGCCCCTG
CGGGGCCTATGGAATTCTATTTGAATTTGAATTTGAATTGGTATACTGTAGGGCGGGATATTTTCAAA
TTTGAAATTTGAAAT
+
FFFFFFFFFFFFFFFFFFFFF:FFFFFF:FFFFFFFFFFFFFFFFFFFFFF:FFFFFFFFFFFFFFFFFF
```

FFFFFFFF:FFFFFFFFFF,FFF::FFFF,FFFFFFFFFFF:FFFFFFFFFFFFFFFFFFFFFFFFFFF
FFFF,FFFFFF:FFF
@A00721:190:HGJW7DSXY:4:2221:32117:31876 1:N:0:TCTGCAAG+GCTGCACT
CCTGGCGCGGGCAAGCCCGTAATATTAAATTGGCTTGCCCCAAGGTCTGCGGTAATATTCCCCTGCGG
GGCCTATGGAATTCTATTTGAATTTGAATTTTAATTGGTATACATTATGGCGGGATATTTTCAAATTT
TAAATTTTTAATAAT
+
FFFFFFFFFFFFF,,FF,FFFF:FFF:,F,F,:,::,F:FFF,,:,,,,,:F:FF,,F,F:,F,,F:F
FFFF,FFF:FFFF,F::FFF:FFFF,FFFF,,:FF,,FFFFF,,,FF,:::FFF:F,FFFF,FFFFFF
,,FFFFF,,,FF:FF
@A00721:190:HGJW7DSXY:4:2225:18376:12273 1:N:0:TCTGCAAG+GCTGCACT
ACGGAGGTGTCCCCGGCGCGGGCAAGCCAGTAATATTATATTGGCTTGCCCCACGGTCGGCGGTAAGA
TGCCCCAGCGGGGCCTATGGAATTCTATTTGAATTTGTATTTGAATTTGTATACTGTAGGGCGGGATA
TTTTCAAATTTGAAA
+
FFFFFFFFFFFFFFFFFFFFFF:,F,,F,,,,F,F,FF:F:F,F,,F:FFFF:F:FF:,F,FF::::F
:F,FFF,FFFFFF,F,,:FF:FFF,,F,FF,,F:FF,,:F:F,,FFF,FF:FF,,:,,,FF,FFF,:F
,:FF,:F::FF,,FF
@A00721:190:HGJW7DSXY:4:2264:32832:25974 1:N:0:TCTGCAAG+GCTGCACT
ACGGAGGTGTCCCCGGCGCGGGCAAGCCAGTAATATTATATTGGCTTGCCCCACGGTCTGCGGTAAGA
TGCCCCTGCGGGGCCTATGGAATTCTATTTGAATTTGAATTTGAATTGGTATACTGTAGGGCGGGATA
TTTTCAAATTTGAAA
+
FFFFFFFF:FFFF:FFFFFFFFFFFF:F:,FFFF:,F:FFFFFFF:FFFFFFF:FFF,,,,FFFFF,:
FF:FFFFFFFFFFFF,F:FFFF:F,F,F:FFFFFFFF,,FFF:FFFF:FFFF:FFFF,,FFFFFFFFF
F:FFFF,:FFFF,FF
@A00721:190:HGJW7DSXY:4:2664:30346:14372 1:N:0:TCTGCAAG+GCTGCACT
ACGGAGGTGTCCCCGGCGCGGGCAAGCCAGTAATATTATATTGGCTTGCCCCACGGTCTGCGGTAAGA
TGCCCCTGCGGGGCCTATGGAATTCTATTTGAATTTGAATTTGAATTGGTATACTGTAGGGCGGGATA
TTTTCAAATTTGAAA
+
FFFFFFFFFFFFFFFFFFFFFFFFFFFFF,FF:F,FF:F::F,FF,F:FFFFFF:FF:FF:FF:FFFF
FFFFFF,FFFFFFFFFF,,FFF,F,,:FFF:FFFFF:,F:FF,FFFF,FFFFFFF,F:FF:FFFFFFF
FFFF,F,FFFF:F:F
@A00721:190:HGJW7DSXY:4:1503:30056:17253 1:N:0:TCTGCAAG+GCTGCACT
ACGGAGGTGTCCCCGGCGCGGGCAAGCCAGTAATATTATATTTGGTTGCCCCACGGTCTGCGGTAAGA
TGGCCCGGCGGGGCCTATGGAATTATATTTGTATTTTTATTTTAATTTGTATAATGTAGGGGGGGGTA
TTTTAAAATTT
+
FFFFFFFFFFFFFFFFFFFFFF,,F,:F,,,,:,,,F,:,:F,,,,:,::FF::,F,,,F,FF,,F:,
,,,:FF,,::FFF,F,,,,,,F:F,:FFFF,,,FFF,,,,FF,,::F,,:,F,,,,:,:,F,FFF,::
:FFF,:F:,FF
@A00721:190:HGJW7DSXY:4:1128:6958:6026 1:N:0:TCTGCAAG+GCTGCACT
ATCCCGCCCTACAGTATACCAATTCAAATTCAAATTCAAATAGAATTCCATAGGCCCCGCAGGGGCAT
CTTACCGCAGACCGTGGGGCAAGCCAATATAATATTACTGGCTTGCCCGCGCCGGGGACACCTCCGTA
CGCTTACGTGTCGAT
+
FFFFFFFFFFFFFFFFFFFFFFFFFFFFFFFFFFFFFFFFFFFFFFFFFFFFFFFFFFFFFFFFFFFF
FFFFFFFFFFFFFFFFFFFFFFFFFFFFFFFFFFFFFFFFFFFFFFFFFFFFFFFFFFFFFFFFFFFF
FFFFFFFFFFFFFFF
@A00721:190:HGJW7DSXY:4:1435:2220:11475 1:N:0:TCTGCAAG+GCTGCACT
ATCCCGCCCTACAGTATACCAATTCAAATTCAAATTCAAATAGAATTCCATAGGCCCCGCAGGGGCAT
CTTACCGCAGACCGTGGGGCAAGCCAATATAATATTACTGGCTTGCCCGCGCCGGGGACACCTCCGTA
CGCTTACGTGTCGAT

+
FFFFFFFFFFFFFFFFFFFFFFFFFFFFFFFFFFFFFFFFFFFFFFFFFFFFFFFFFFFFFFFFFFFFFF
FFFFFFFFFFFFFF:FFFFFFFFFFFFF,FFFFFFFFFFFFFFFFFFFFFFFFFFFFFFFFFFFFFFFFF
FFFFFFFFFFFFFF:
@A00721:190:HGJW7DSXY:4:1471:23493:29684 1:N:0:TCTGCAAG+GCTGCACT
ATCCCGCCCTACAGTATACCAATTCAAATTCAAATTCAAATAGAATTCCATAGGCCCCGCAGGGGCAT
CTTACCGCAGACCGTGGGGCAAGCCAATATAATATTACTGGCTTGCCCGCGCCGGGGACACCTCCGTA
CGCTTACGTGTCGAT
+
FFFFFFFFFFFFFFFFFFFFFFFFFFFFFFFFFFFFFFFFFFFFFFFFFFFFFF:FFFFFFFFFFFFFFF
FFFFFFFFFFFFFFFFFFFFF::FFFFFFFFFFFFFFFFFFFFFFFFFFFFFFFFFFFFFFFFFFFF:FF
FFF,FF:FFFFFFFF
@A00721:190:HGJW7DSXY:4:1641:5231:35790 1:N:0:TCTGCAAG+GCTGCACT
ATCCCGCCCTACAGTATACCAATTCAAATTCAAATTCAAATAGAATTCCATAGGCCCCGCAGGGGCAT
CTTACCGCAGACCGTGGGGCAAGCCAATATAATATTACTGGCTTGCCCGCGCCGGGGACACCTCCGTA
CGCTTACGTGTCGAT
+
FFFFFFFFFFFFFFFFF:FFFFFFFFFFFFFFFFFFFFFFFFFFFFFFFFFFFFFFFFFFFFFFFFFFFF
FFFFFFFFFFFFFFFFFFFFFFFFFFFFFFFFFFFFFFFFFFFFFFFFFFFFFFFFFFFFFFFFFFFFFF
FFFFFFFFFFFFFFF
@A00721:190:HGJW7DSXY:4:2133:1542:32409 1:N:0:TCTGCAAG+GCTGCACT
ATCCCGCCCTACAGTATACCAATTCAAATTCAAATTCAAATAGAATTCCATAGGCCCCGCAGGGGCAT
CTTACCGCAGACCGTGGGGCAAGCCAATATAATATTACTGGCTTGCCCGCGCCGGGGACACCTCCGTA
CGCTTACGTGTCGAT
+
FFFFFFFFFFFFFFFFFFFFFFFFFFFFFFFFFFFFFFFFFFFFFFFFFFFFFFFFFFFFFFFFFFFFFF
FFFFFFFFFFFFFFFFFFFFF:FFFFFFFFFFFFFFFFFFFFFFFFFFFFFFFFFFFFFFFFFFFFFFFF
FFFFFFFFFFFFFFF
@A00721:190:HGJW7DSXY:4:2455:26775:12477 1:N:0:TCTGCAAG+GCTGCACT
ATCCCGCCCTACAGTATACCAATTCAAATTCAAATTCAAATAGAATTCCATAGGCCCCGCAGGGGCAT
CTTACCGCAGACCGTGGGGCAAGCCAATATAATATTACTGGCTTGCCCGCGCCGGGGACACCTCCGTA
CGCTTACGTGTCGAT
+
FFFFFFFFFFFF:FFFFFFF:FFFFFFFFFFFFFFFFFFFFFFFFFFFFFFFFFFFFFFFFFFFFFFFFF
FFFFFFFFFFFFFFFFFFFFFFFFFF:FFFFFFFFFFFFFFFFFFFFFFFFFFFFFFFFFFFFFFFFFFF
FFFF:FFFFFFFFFF
@A00721:190:HGJW7DSXY:4:1611:23149:18004 1:N:0:TCTGCAAG+GCTGCACT
CCCGCCCTACAGTATACCAATTCAAATTCAAATTCAAATAGAATTCCATAGGCCCCGCAGGGGCATCT
TACCGCAGACCGTGGGGCAAGCCAATATAATATTACTGGCTTGCCCGCGCCGGGGACACCTCCGTACG
CTTACGTGTCGATTT
+
FFFFFFFFFF:FFFFFFFFFFFFFFFFFFFFFFFFF:FFFFFFFFFFFFFFFFFFFFFFFFFFFFFFFFF
FFFFFFFFFFFFFFFFFFFFFFFFFF,FFFFFFFFFFFFFFFFFFFFFFFFFFFFF,FFFFFFFFFFFFF
FFFFFF:FFFFFFFF
@A00721:190:HGJW7DSXY:4:2216:14642:25629 1:N:0:TCTGCAAG+GCTGCACT
CCCGCCCTACAGTATACCAATTCAAATTCAAATTCAAATAGAATTCCATAGGCCCCGCAGGGGCATCT
TACCGCAGACCGTGGGGCAAGCCAATATAATATTACTGGCTTGCCCGCGCCGGGGACACCTCCGTACG
CTTACGTGTCGATTT
+
FFFFFFFFFFFFFFFFFFF:FFFFFFFFFFFFFFFFFFFFFFFFFFF:FFFF:FF:FFFFFFFFFFFFFF
FFFFFFFFFFFF:FFFFFFFFFFFF:FFFFFFFFFFFFFFFFFFFFFFFFFFFFFFFFFFFFFFFFFFFF
FFFFFFFFFFFFFF:
@A00721:190:HGJW7DSXY:4:1240:24876:2769 1:N:0:TCTGCAAG+GCTGCACT
CCGCCCTACAGTATACCAATTCAAATTCAGATTCAAATAGAATTCCATAGGCCCCGCAGGGGCATCTT

ACCGCAGACCGTGGGGCAAGCCAATATAATATTACTGGCTTGCCCGCGCCGGGGACACCTCCGTACGC
TTACGTGTCGATTTG
+
FFFFFFFFFFFFFFFFFFFFFFFFFFFFFFFFFFFFFFFFFFFFFFF:FFFFFFFFFFFFFFFFFFFFFF
FFFFFFFFFFFFFFFFFFFFFFFFFFFFFFFFFFFFFFFFFFFFFFFFFFFFFFFFFFFFFFFFFFFFFF
FFFFFFFFFFFFFFF
@A00721:190:HGJW7DSXY:4:1325:24822:5431 1:N:0:TCTGCAAG+GCTGCACT
CCGCCCTACAGTATACCAATTCAAATTCAAATTCAAATAGAATTCCATAGGCCCCGCAGGGGCATCTT
ACCGCAGACCGTGGGGCAAGCCAATATAATATTACTGGCTTGCCCGCGCCGGGGACACCTCCGTACGC
TTACGTGTCGATTTG
+
FFFFFFFFFFFFFFFFFFF,FFFFFFFFF:FFFFFFFFFFFFFFFF:FFFFFFFFFFFFFFFFFFFFFFF
FFFFFFFFFFFFFFFFFFFFFFFFFFFFFFFFFFFFFFFFFFFFFFFFFFFFFFFFFFFFFFFFFFFFFF
FFFFFFFFFFFFFFF
@A00721:190:HGJW7DSXY:4:1341:7229:5274 1:N:0:TCTGCAAG+GCTGCACT
CCGCCCTACAGTATACCAATTCAAATTCAAATTCAAATAGAATTCCATAGGCCCCGCAGGGGCATCTT
ACCGCAGACCGTGGGGCAAGCCAATATAATATTACTGGCTTGCCCGCGCCGGGGACACCTCCGTACGC
TTACGTGTCGATTTG
+
FFFFFFFFFFFFFFFFFFFFFFFFFFFFFFFFFFFFFFFFFFFFFFFFFFFFFFFFFFFFFFFFFFFFFF
FFFFFFFFFFFFFFFFFFF:FFFFFFFFFFFFFFFFFFFFFFFFFFFFFFFFFFFFFFFFFFFFFFFFFF
,FFFF:FFFFFFFFF
@A00721:190:HGJW7DSXY:4:1341:18484:7294 1:N:0:TCTGCAAG+GCTGCACT
CCGCCCTACAGTATACCAATTCAAATTCAAATTCAAATAGAATTCCATAGGCCCCGCAGGGGCATCTT
ACCGCAGACCGTGGGGCAAGCCAATATAATATTACTGGCTTGCCCGCGCCGGGGACACCTCCGTACGC
TTACGTGTCGATTTG
+
FFFFFFFFFFFFFFFFFFFFFFFFFFFFFFFFFFFFFFFFFFFFFFFFFFFFFFFFFFFFFFFFFFFFFF
FFFFFFFFFFFFFFFFFFFFFFFFFFFFFFFFFFFFFFFFFFFFFFFFFFFFFFFFFFFFFFFFFFFFFF
FFFFFFFFFFFFFFF
@A00721:190:HGJW7DSXY:4:1403:10529:8547 1:N:0:TCTGCAAG+GCTGCACT
CCGCCCTACAGTATACCAATTCAAATTCAAATTCAAATAGAATTCCATAGGCCCCGCAGGGGCATCTT
ACCGCAGACCGTGGGGCAAGCCAATATAATATTACTGGCTTGCCCGCGCCGGGGACACCTCCGTACGC
TTACGTGTCGATTTG
+
FFFFFFFFFFFFFFFFFFFFFFFFFFFFFFFFFFFFFFFF:FFFFFFFFFFFFFFFFFFFF:FFFFFFFF
FFFFFFFFFFFFFFFFFFFFFFF:FFFFFFFFFFF:FFFFFFFFFF:FFFFFFFFFFFFFFFFFFFFFFF
::FFFFFFFFFFFFF
@A00721:190:HGJW7DSXY:4:1418:16685:14168 1:N:0:TCTGCAAG+GCTGCACT
CCGCCCTACAGTATACCAATTCAAATTCAAATTCAAATAGAATTCCATAGGCCCCGCAGGGGCATCTT
ACCGCAGACCGTGGGGCAAGCCAATATAATATTACTGGCTTGCCCGCGCCGGGGACACCTCCGTACGC
TTACGTGTCGATTTG
+
FFFFFFFFFFFFFFFFFFFFFFFFFFFFFFFFFFFFFFFFFFFFFFFFFFFFFFFFFFFFFFFFFFFFFF
FFFFFFFFFFFFFFFFFFFFFFFFFFFFFFFFFFFFFFFFFFFFFFFFFFFFFFFFFFFFFFFFFF:FFF
FFFFFFFFFFFFFFF
@A00721:190:HGJW7DSXY:4:1516:32452:16423 1:N:0:TCTGCAAG+GCTGCACT
CCGCCCTACAGTATACCAATTCAAATTCAAATTCAAATAGAATTCCATAGGCCCCGCAGGGGCATCTT
ACCGCAGACCGTGGGGCAAGCCAATATAATATTACTGGCTTGCCCGCGCCGGGGACACCTCCGTACGC
TTACGTGTCGATTTG
+
FFFFFFF:FFFFFFFFFFFFFFFFFFFFFFFFFFFFFFFFFFFFFFFFF:FFFFFFFFFFFF
FFFFFFFFFFFFFFFFFFFFFFFFFFFFFFFFFFFFFFFFFFFFFFFFFFFFFFFFFFFFFFFFFFFFFF
FFFFF:FFFFFFFFF

```
@A00721:190:HGJW7DSXY:4:1532:10737:6715 1:N:0:TCTGCAAG+GCTGCACT
CCGCCCTACAGTATACCAATTCAAATTCAAATTCAAATAGAATTCCATAGGCCCCGCAGGGGCATCTT
ACCGCAGACCGTGGGGCAAGCCAATATAATATTACTGGCTTGCCCGCGCCGGGGACACCTCCGTACGC
TTACGTGTCGATTTG
+
FFFFFFFFFFFFFFFFFFFFFFFFFFFFFFFFFFFFFFFFFFFFF:FFF:FFFFFFFFFFFFFFFFFF
FFFFFFFFFFFFFFFFFFFFFFFFFFFFFFFFFFFFFFFFFFFFFFFFFFFFFFFFFFFFFFFFFFFF
:FFFFFFFFFFFFFF
@A00721:190:HGJW7DSXY:4:1545:20302:6402 1:N:0:TCTGCAAG+GCTGCACT
CCGCCCTACAGTATACCAATTCAAATTCAAATTCAAATAGAATTCCATAGGCCCCGCAGGGGCATCTT
ACCGCAGACCGTGGGGCAAGCCAATATAATATTACTGGCTTGCCCGCGCCGGGGACACCTCCGTACGC
TTACGTGTCGATTTG
+
FFFFFFFFFFFFFFFFFFFFFFFF:FFFFFFFFFFFFFFFFFFFFFFFFFFFFFFFFFFFFFF:FFFF
FFFFFFFFFFFFFFFFFFFFFF:FFFFFFFFFFFFFFFFFFFFFFFFFFFFFFFFF:FFFFFF:FFFF
FFFFF:FFFFFFFFF
@A00721:190:HGJW7DSXY:4:1613:24795:2879 1:N:0:TCTGCAAG+GCTGCACT
CCGCCCTACAGTATACCAATTCAAATTCAAATTCAAATAGAATTCCATAGGCCCCGCAGGGGCATCTT
ACCGCAGACCGTGGGGCAAGCCAATATAATATTACTGGCTTGCCCGCGCCGGGGACACCTCCGTACGC
TTACGTGTCGATTTG
+
FFFFFF:,FFFFFFFFFFF:FFFFF:FFFFFF:FFFFFFFFFFFF:F:FF:FFFFFFFFF,FFFFF:F
FFFFFF:FFFFF:FF:FFFFFFFF:FF:FFFFFFFF:FFFFFFFF,FFFFFFFFFFFFFFFFF,FF,F
FFFF:,F,FFFFFFF
@A00721:190:HGJW7DSXY:4:1671:13946:2973 1:N:0:TCTGCAAG+GCTGCACT
CCGCCCTACAGTATACCAATTCAAATTCAAATTCAAATAGAATTCCATAGGCCCCGCAGGGGCATCTT
ACCGCAGACCGTGGGGCAAGCCAATATAATATTACTGGCTTGCCCGCGCCGGGGACACCTCCGTACGC
TTACGTGTCGATTTG
+
FFFFFFFFFFFFFFFFFFFFFFFFFFFFFF:FFFFFFFF:FFFFF:FFFFFFFFFFFFFFFFFFFFFF
FFFFFFFFFFFFFFFFFFFFFFFFFFFFFFFFFFFFFFFFFFFFFFFFFFFFFFFFFFFFFFFFFFFF
:FFFFFFFFFFFF:F
@A00721:190:HGJW7DSXY:4:2131:7319:21746 1:N:0:TCTGCAAG+GCTGCACT
CCGCCCTACAGTATACCAATTCAAATTCAAATTCAAATAGAATTCCATAGGCCCCGCAGGGGCATCTT
ACCGCAGACCGTGGGGCAAGCCAATATAATATTACTGGCTTGCCCGCGCCGGGGACACCTCCGTACGC
TTACGTGTCGATTT
+
FFFFFFFFFFFFFFFFFFFFFFFFFFFFFFFFFFFFFFF:FFFF:FFFFFFFFFFFFFFFFFFFFFFF
FFFFFFFFFFFFFFFFFFFFFFFFFFFFFFFFFFFFFFFFFFFFFFFFFFFFFFFFFFFFFFFFFFFF
FFFFFFFFFFFFFF
@A00721:190:HGJW7DSXY:4:2148:22905:16611 1:N:0:TCTGCAAG+GCTGCACT
CCGCCCTACAGTATACCAATTCAAATTCAAATTCAAATAGAATTCCATAGGCCCCGCAGGGGCATCTT
ACCGCAGACCGTGGGGCAAGCCAATATAATATTACTGGCTTGCCCGCGCCGGGGACACCTCCGTACGC
TTACGTGTCGATTTG
+
FFFFFFFFF:FFFFFFFFFFFFFFFFFFFFFFFFFFFFFFFFFFFFFFFFFFFFFFFFFFFFFFFFFF
FFFFFFFFFFFFFFFFFFFFFFFFFF:FFFFFFFFFFFFFFFFFFFFFFFFFFFFFFFFFFFFFFFFF
FFFFF,FFFFFFFFF
@A00721:190:HGJW7DSXY:4:2163:26856:11647 1:N:0:TCTGCAAG+GCTGCACT
CCGCCCTACAGTATACCAATTCAAATTCAAATTCAAATAGAATTCCATAGGCCCCGCAGGGGCATCTT
ACCGCAGACCGTGGGGCAAGCCAATATAATATTACTGGCTTGCCCGCGCCGGGGACACCTCCGTACGC
TTACGTGTCGATTTG
+
FFFFFFFFFFFFFFFFFFFFFFFFFFFFFFFFFFFFFFFFFFFFFFFFFFFFFFFFFFFFFFFFFFFF
```

```
FFFFFFFFFFFFFFFFFFF:FFFFFFFFFFFFFFFFFFFFFFFFFFFFFFFFFFFFFFFFFFFFFFFFFF
FFFFFFFFFFFFFFF
@A00721:190:HGJW7DSXY:4:2205:16224:6574 1:N:0:TCTGCAAG+GCTGCACT
CCGCCCTACAGTATACCAATTCAAATTCAAATTCAAATAGAATTCCATAGGCCCCGCAGGGGCATCTT
ACCGCAGACCGTGGGGCAAGCCAATATAATATTACTGGCTTGCCCGCGCCGGGGACACCTCCGTACGC
TTACGTGTCGATTTG
+
FFFFFFFFFFFFFFFFFFFFFFFFFFFFFFFFFFFFFFFFFFFFFFFFFFFFFFFFFFFFFFFFFFFFFF
FFFFFFFFFFFFFFFFFFF:FFFFFFFFFFFFFFFFFFFFFFFFFFFFFFFFFFFFFFFFFFFFFFFFFF
FFFFFFFFFFFFFFF
@A00721:190:HGJW7DSXY:4:2210:15917:11240 1:N:0:TCTGCAAG+GCTGCACT
CCGCCCTACAGTATACCAATTCAAATTCAAATTCAAATAGAATTCCATAGGCCCCGCAGGGGCATCTT
ACCGCAGACCGTGGGGCAAGCCAATATAATATTACTGGCTTGCCCGCGCCGGGGACACCTCCGTACGC
TTACGTGTCGATTTG
+
FFFFFFFFFFFFFFFFFFFFFFFF:FFFFFFFFFFFFFFFFFFFFFF:FFFFFFFFFFFFFFFFFFFFFF
FFFFFFFFFFFFFFFFFFFFFFFFFFFFFFFFFFFFFFFFFFFFFFFFFFFFFFFFFFFFFFFFFFFFFF
F:FFFFFFFFFFFF:
@A00721:190:HGJW7DSXY:4:2240:17481:27759 1:N:0:TCTGCAAG+GCTGCACT
CCGCCCTACAGTATACCAATTCAAATTCAAATTCAAATAGAATTCCATAGGCCCCGCAGGGGCATCTT
ACCGCAGACCGTGGGGCAAGCCAATATAATATTACTGGCTTGCCCGCGCCGGGGACACCTCCGTACGC
TTACGTGTCGATTTG
+
FFFFFFFFFFFFFFFFFFFFFFFFFFFFFFFFFFFFFFFFFFFFFFFFFFFFFFFFFFFFFFFFFFFFFF
FFFFFFFFFFFFFFFFFFFFFFFFFFFFFFFFFFFFFFFFFFFFFFFFFFFFFFFFFFFFFFFFFFFFFF
FFFFFFFFFFFFFFF
@A00721:190:HGJW7DSXY:4:2240:14769:29074 1:N:0:TCTGCAAG+GCTGCACT
CCGCCCTACAGTATACCAATTCAAATTCAAATTCAAATAGAATTCCATAGGCCCCGCAGGGGCATCTT
ACCGCAGACCGTGGGGCAAGCCAATATAATATTACTGGCTTGCCCGCGCCGGGGACACCTCCGTACGC
TTACGTGTCGATTTG
+
FFFFFFFFFFFFFFFFFFFFFFFFFFFFFFFFFFFFFFFFFFFFFFFFFFFFFFFFFFFFFFFFFFFFFF
FFFFFFFFFFFFFFFFFFFFFFF:FFFFFFFFFFFFFFFFFFFFFFFFFFFFFFFFFFFFFFFFFFFFFF
FFFFFFFFFFFFFFF
@A00721:190:HGJW7DSXY:4:2307:20925:27492 1:N:0:TCTGCAAG+GCTGCACT
CCGCCCTACAGTATACCAATTCAAATTCAAATTCAAATAGAATTCCATAGGCCCCGCAGGGGCATCTT
ACCGCAGACCGTGGGGCAAGCCAATATAATATTACTGGCTTGCCCGCGCCGGGGACACCTCCGTACGC
TTACGTGTCGATTTG
+
FFFFFFFFFFFFFFFFFFFFFFFFFFFFFFFFFFFFF:FFFFFFFF:FF:FFFFFFFFFFFFFFFFFFFF
FFFFFFFFFFFFFFFFFFFFFFFFFF:FFFFFFFFFFFFFFFFFFFFFFFFFFFF:FFFFFFFFFFFFFF
FFFFFFFFFFFFFFF
@A00721:190:HGJW7DSXY:4:2324:28085:34757 1:N:0:TCTGCAAG+GCTGCACT
CCGCCCTACAGTATACCAATTCAAATTCAAATTCAAATAGAATTCCATAGGCCCCGCAGGGGCATCTT
ACCGCAGACCGTGGGGCAAGCCAATATAATATTACTGGCTTGCCCGCGCCGGGGACACCTCCGTACGC
TTACGTGTCGATTTG
+
FFFFFFF,FFFFFFFFFFFFFFFFFFFFFFFFFFFFFFFFFFFFFF:FFFFFF,FFFFFFFFF,FFFFFF
FFFFFFFFFFF,FFFFFFF:FFFFFFFFFFFFFFFF:F:FFFFF:FFFFFFFFFFF:F,FFFFFFFFFFF
FFFFFFFFFFFF:FF
@A00721:190:HGJW7DSXY:4:2362:15917:7670 1:N:0:TCTGCAAG+GCTGCACT
CCGCCCTACAGTATACCAATTCAAATTCAAATTCAAATAGAATTCCATAGGCCCCGCAGGGGCATCTT
ACCGCAGACCGTGGGGCAAGCCAATATAATATTACTGGCTTGCCCGCGCCGGGGACACCTCCGTACGC
TTACGTGTCGATTTG
```

+
FFFFFFFFFFFFFFFFFFFFFFFFFFFFFFFFFFFFFFFFFFFFFFFFFFFFFFFFFFFFFFFFFFFFFF
FFFFFFFFFFFFFFFFFFFFFFFFFFFFFFFFFFFFFFFFFFFFFFFFFFFFFFFFFFFFFFFFFFFFFF
FFFFFFFFFFFFFFF
@A00721:190:HGJW7DSXY:4:2369:7148:3223 1:N:0:TCTGCAAG+GCTGCACT
CCGCCCTACAGTATACCAATTCAAATTCAAATTCAAATAGAATTCCATAGGCCCCGCAGGGGCATCTT
ACCGCAGACCGTGGGGCAAGCCAATATAATATTACTGGCTTGCCCGCGCCGGGGACACCTCCGTACGC
TTACGTGTCGATTTG
+
FFFFFFFFFFFFFFFFFFFFFFFFFFFFFFFFFFFFFFFFFFFFFFFFFFFFFFFFFFFFFFFFFFFFFF
FFFFFFFFFFFFFFFFFFFFFFFFFFFFFFFFFFFFFFFFFFFFFFFFFFFFFFFFFFFFFFFFFFFFFF
FFFFFFFFFFFFFFF
@A00721:190:HGJW7DSXY:4:2435:25780:27539 1:N:0:TCTGCAAG+GCTGCACT
CCGCCCTACAGTATACCAATTCAAATTCAAATTCAAATAGAATTCCATAGGCCCCGCAGGGGCATCTT
ACCGCAGACCGTGGGGCAAGCCAATATAATATTACTGGCTTGCCCGCGCCGGGGACACCTCCGTACGC
TTACGTGTCGATTTG
+
FFFFFFFFFFFFFFFFFFFFFFFFFFFFFFFFFFFFF:FFFFFFFF:FFFFFFFFFFFFFF:FFFFFFFF
FFFFFFFFFFFFFFFFFFFFFFFFFFFFFFFFFFFFFF:FFFFF:FFFFFFFFFFFFFFFFFFFFFFFFF
FFFFFFFFFFFFFFF
@A00721:190:HGJW7DSXY:4:2445:4291:14591 1:N:0:TCTGCAAG+GCTGCACT
CCGCCCTACAGTATACCAATTCAAATTCAAATTCAAATAGAATTCCATAGGCCCCGCAGGGGCATCTT
ACCGCAGACCGTGGGGCAAGCCAATATAATATTACTGGCTTGCCCGCGCCGGGGACACCTCCGTACGC
TTACGTGTCGATTTG
+
FFFFFFFFF:FFFFFFFFFFFFFFFFFFFFFFFFFFFFFFFFFFFF:FFFFFFFFFFFFFFFFFFFFFFF
FFFFFFFFFFFFFFFFFFFFFFFFFFFFFFFFFFFFFFFFFFFFFFFFFFFFFFFFFFFFFFFFFF:FFF
FFFF:FFFFFFFFFF
@A00721:190:HGJW7DSXY:4:2445:4734:17362 1:N:0:TCTGCAAG+GCTGCACT
CCGCCCTACAGTATACCAATTCAAATTCAAATTCAAATAGAATTCCATAGGCCCCGCAGGGGCATCTT
ACCGCAGACCGTGGGGCAAGCCAATATAATATTACTGGCTTGCCCGCGCCGGGGACACCTCCGTACGC
TTACGTGTCGATTTG
+
FFFFFFFFFFFFFFFFFFFFFFFFFFFFFFFFFFFFFFFFFFFFFFFFFFFFFFFFFFFFFFFFFFFFFF
FFFFFFFFFFFFFFFFFFFFFFFFFFFFFFFFFFFFFFFFFFFFFFFFFFFFFFFFFFFFFFFFFFFFFF
:FFFFFFFFFFFFFF
@A00721:190:HGJW7DSXY:4:2446:10321:9220 1:N:0:TCTGCAAG+GCTGCACT
CCGCCCTACAGTATACCAATTCAAATTCAAATTCAAATAGAATTCCATAGGCCCCGCAGGGGCATCTT
ACCGCAGACCGTGGGGCAAGCCAATATAATATTACTGGCTTGCCCGCGCCGGGGACACCTCCGTACGC
TTACGTGTCGATTTG
+
FFFFFFFFFFFFFFFFFFFFFFFFFFFFFFFFFFFFFFFFFFFFFF:FFFFFFFFFFFFFFFFFFFFFFF
FFFFFFFFFFFFFFFFFF,FFFFFF,FF,FFFFFFFFFFFFFFFFFFFFFFFFFFFFFFFFFFFFFFFFF
FF:FFFFFFFFF:FF
@A00721:190:HGJW7DSXY:4:2471:28176:12868 1:N:0:TCTGCAAG+GCTGCACT
CCGCCCTACAGTATACCAATTCAAATTCAAATTCAAATAGAATTCCATAGGCCCCGCAGGGGCATCTT
ACCGCAGACCGTGGGGCAAGCCAATATAATATTACTGGCTTGCCCGCGCCGGGGACACCTCCGTACGC
TTACGTGTCGATTTG
+
FFFFFFFFFFFFFFFFFFF:FFFFFFFFFFFFFFFFFFFFFFFFFFFFFFFFFFFFFFFFFFFFFFFFFF
FFFFFFFFFFFFFFFFFFFFFFF:FFFFFFFFFFFFFFFFFFFFFFFFFFFFFFFFFFFFFFFFFFFFFF
FFFFFFFFFFF:FFF
@A00721:190:HGJW7DSXY:4:2506:8377:16219 1:N:0:TCTGCAAG+GCTGCACT
CCGCCCTACAGTATACCAATTCAAATTCAAATTCAAATAGAATTCCATAGGCCCCGCAGGGGCATCTT

ACCGCAGACCGTGGGGCAAGCCAATATAATATTACTGGCTTGCCCGCGCCGGGGACACCTCCGTACGC
TTACGTGTCGATTTG
+
FFFFFFFFFFFFFFFFFFFFFFFFFFFFFFFFFFFFFFFFFFFF:FFFFFFFFFFFFFFFFFF:FFFF
FFFFFFFFFFFFFFFFFFFFFFFFFFFFFFFFFFFFFFFFFFFFFFFFFFFFFFFFFFFFFFF:FFFF
FFFFFFFFFFFFFFF
@A00721:190:HGJW7DSXY:4:2519:28230:22044 1:N:0:TCTGCAAG+GCTGCACT
CCGCCCTACAGTATACCAATTCAAATTCAAATTCAAATAGAATTCCATAGGCCCCGCAGGGGCATCTT
ACCGCAGACCGTGGGGCAAGCCAATATAATATTACTGGCTTGCCCGCGCCGGGGACACCTCCGTACGC
TTACGTGTCGATTTG
+
FFFFFFFFFFFFFFFFFFFFFFFFFFFFFFFFFFFFFFFFFFFFF:FFFFFFFFFFFFFFFFFFFFFF
FFFFFFFFFFFFFFFFFFFFFFFFFFFFFFFFFFFFFFFFFFFFFFFFFFFFFFFFFFFFFFFFFFFF
FFFFFFFFFFFFFFF
@A00721:190:HGJW7DSXY:4:2521:8865:25488 1:N:0:TCTGCAAG+GCTGCACT
CCGCCCTACAGTATACCAATTCAAATTCAAATTCAAATAGAATTCCATAGGCCCCGCAGGGGCATCTT
ACCGCAGACCGTGGGGCAAGCCAATATAATATTACTGGCTTGCCCGCGCCGGGGACACCTCCGTACGC
TTACGTGTCGATTTG
+
FFFFFFFFFFFFFFFFFFFFFFFF:FFFFFFFFFFFFFFFFFFFFFFFFFFFFFFFFFFFFFFFFFFF
FFFFFFFFFFFFFFFFFFFFFFFFFFFFFFFFFFFFFFFFFFFFFFFFFFFFFFFFFFFFFFFFFFFF
FFFFFFFFFFFFFFF
@A00721:190:HGJW7DSXY:4:2525:27525:32659 1:N:0:TCTGCAAG+GCTGCACT
CCGCCCTACAGTATACCAATTCAAATTCAAATTCAAATAGAATTCCATAGGCCCCGCAGGGGCATCTT
ACCGCAGACCGTGGGGCAAGCCAATATAATATTACTGGCTTGCCCGCGCCGGGGACACCTCCGTACGC
TTACGTGTCGATTTG
+
FFFFFFFFF:FFFFFFFFFFFFFFFFFFFFFFFF:FFF:FFFFFF:FFFFFFFFFFFFFFFFF::FFF
FFFFFFFFFFFFFFFFFFFFFFFFFFFFFFFFFFFFFFFFFFFFFFFFFFFFFFFFFFFFFFFFFFFF
FFFFF,FFFFFFFFF
@A00721:190:HGJW7DSXY:4:2577:16333:29058 1:N:0:TCTGCAAG+GCTGCACT
CCGCCCTACAGTATACCAATTCAAATTCAAATTCAAATAGAATTCCATAGGCCCCGCAGGGGCATCTT
ACCGCAGACCGTGGGGCAAGCCAATATAATATTACTGGCTTGCCCGCGCCGGGGACACCTCCGTACGC
TTACGTGTCGATTTG
+
FFFFFFFFFFFFFFFFFFFFFFFFFFFFFFFFFFFFFFFFFFFFF:FFFFFFFFFFFFFFFFFFFFFF
FFFFFF:FFFFFFFFFFFFFFFFFFFFFFFFFFFFFFFFFFFFFFFFFFFFFF:FFFFFFFFFFFFFF
FFFFFFFFFFFFFFF
@A00721:190:HGJW7DSXY:4:2647:30680:3239 1:N:0:TCTGCAAG+GCTGCACT
CCGCCCTACAGTATACCAATTCAAATTCAAATTCAAATAGAATTCCATAGGCCCCGCAGGGGCATCTT
ACCGCAGACCGTGGGGCAAGCCAATATAATATTACTGGCTTGCCCGCGCCGGGGACACCTCCGTACGC
TTACGTGTCGATTTG
+
FFFFFFFFF:FFFFFFFFFFFFFFFFFFFFFFFFFFFFFFFFFF::FFFF,FFF:FFFFFFFFFFFFF
FFFFFF:FF:FFFFFFFFFF,FFFFFFFFFFFFFFFF::FFF:FFFFFFFFFFFFFFFFFFFF:FFFF
FFFFFFFFFFFFFFF
@A00721:190:HGJW7DSXY:4:2653:17544:34194 1:N:0:TCTGCAAG+GCTGCACT
CCGCCCTACAGTATACCAATTCAAATTCAAATTCAAATAGAATTCCATAGGCCCCGCAGGGGCATCTT
ACCGCAGACCGTGGGGCAAGCCAATATAATATTACTGGCTTGCCCGCGCCGGGGACACCTCCGTACGC
TTACGTGTCGATTTG
+
FFFFFFFFFFFFFFFFFFFFFFFFFFFFFFFFFFFFFFFFFFFFF:FFFFFFFFFFFFFFFFFFFFFF
FFFFFFFFFFFFFFFFFFFFFFFFFFFFFFFFFFFFFFFFFFFFFFFFFFFFFFFFFFFFFFFFFFFF
FFFFFFFFFFFFFFF

```
@A00721:190:HGJW7DSXY:4:2653:17852:34225 1:N:0:TCTGCAAG+GCTGCACT
CCGCCCTACAGTATACCAATTCAAATTCAAATTCAAATAGAATTCCATAGGCCCCGCAGGGGCATCTT
ACCGCAGACCGTGGGGCAAGCCAATATAATATTACTGGCTTGCCCGCGCCGGGGACACCTCCGTACGC
TTACGTGTCGATTTG
+
FFFFFFFFFFFFFFFFFFFFFFFFFFFFFFFFFFFFFFFFFFFFFFFFFFFFFFFFFFFFFFFFFFFF
FFFFFFFFFFFFFFFFFFFFFFFFFFFFFFFFFFFFFF:FFFFFFFFFFFFFFFFFFFFFFFFFFFFF
FFFFF:FFFFFFFFF
@A00721:190:HGJW7DSXY:4:1103:9064:11866 1:N:0:TCTGCAAG+GCTGCACT
CGCCCTACAGTATACCAATTCAAATTCAAATTCAAATAGAATTCCATAGGCCCCGCAGGGGCATCTTA
CCGCAGACCGTGGGGCAAGCCAATATAATATTACTGGCTTGCCCGCGCCGGGGACACCTCCGTACGCT
TACGTGTCGATTTGT
+
FFFFFF:FFFFFFFFFF:FFFFFFFFFFFFFFFFFFFFFFFFFFFFFFFFFFFFFFFFFFFFFFFFFF
FFFFFFFFFFFFFFFFFFFFFFFFFFFFFFF:FFFFFFFFFFFFFFFFFFFFFFFFFFFFFFFFFFFF
FFFFFFFFFFFFFFF
@A00721:190:HGJW7DSXY:4:1166:12554:2284 1:N:0:TCTGCAAG+GCTGCACT
CGCCCTACAGTATACCAATTCAAATTCAAATTCAAATAGAATTCCATAGGCCCCGCAGGGGCATCTTA
CCGCAGACCGTGGGGCAAGCCAATATAATATTACTGGCTTGCCCGCGCCGGGGACACCTCCGTACGCT
TACGTGTCGATTTGT
+
FFFFFFFFFFFFFFFFFFFFFFFFFFFFFFFFFFFFFFFFFFFFFFFFFFFFFFFFFFFFFFFFFFFF
FFFFFFFFFFFFFFFFFFFFFFFFFFFFF:FFFFFFFFFFFFFFFFFFFFFFFFFFFFFFFFFFFFFF
FFFFFFFFFFFFFFF
@A00721:190:HGJW7DSXY:4:1331:22110:18020 1:N:0:TCTGCAAG+GCTGCACT
CGCCCTACAGTATACCAATTCAAATTCAAATTCAAATAGAATTCCATAGGCCCCGCAGGGGCATCTTA
CCGCAGACCGTGGGGCAAGCCAATATAATATTACTGGCTTGCCCGCGCCGGGGACACCTCCGTACGCT
TACGTGTCGATTTGT
+
FFFFFFFFFFFFFFFFFFFFFFFFFFFFF:FFFFFFFFFFFFFFFFFFFFFFFFFFFFFFFFFFFFFF
FFFFFFFFFFFFFFFFFFFFFFFFFFFFFFFFFFFFFFFFFFFFFFFFFFFFFFFFFFFFFFFFFFFF
F:FFFFFFFFFFFFF
@A00721:190:HGJW7DSXY:4:1622:28718:24893 1:N:0:TCTGCAAG+GCTGCACT
CGCCCTACAGTATACCAATTCAAATTCAAATTCAAATAGAATTCCATAGGCCCCGCAGGGGCATCTTA
CCGCAGACCGTGGGGCAAGCCAATATAATATTACTGGCTTGCCCGCGCCGGGGACACCTCCGTACGCT
TACGTGTCGATTTGT
+
FFFFFFFFFFFFFFFFFFFFFFFFFFFFFFFFFFFFFFFFFFFFFFFFFFFFFFFFFFFFFFFFFFFF
FFFFFFFFFFFFFFFFFFFFFFFFFFFFFFFFFFFFFFFFFFFFFFFFFFFFFFFFFFFFFFFFFFFF
FFFFFFFFFFFFFFF
@A00721:190:HGJW7DSXY:4:2456:4625:10191 1:N:0:TCTGCAAG+GCTGCACT
CGCCCTACAGTATACCAATTCAAATTCAAATTCAAATAGAGTTCCATAGGCCCCGCAGGGGCATCTTA
CCGCAGACCGTGGGGCAAGCCAATATAATATTACTGGCTTGCCCGCGCCGGGGACACCTCCGTACGCT
TACGTGTCGATTTGT
+
FFFFFFFFFFFFFFFFFFFFFFFFFFFFFFFFFFFFFFFFFFFFF:FFFFFFFFFFFFFFFFFFFFFF
FFFFFFFFFFFFFFFFFFFFFF:FFFFFFFFFFFFFFFFFFFFFFFFFFFFFFFFFFFFFFFFFFFFF
FFFFFFFFFFFFFFF
@A00721:190:HGJW7DSXY:4:2510:11116:9001 1:N:0:TCTGCAAG+GCTGCACT
CGCCCTACCGTATACCAATTCAAATTCAAATTCAAATAGAATTCCATAGGCCCCGCAGGGGCATCTTA
CCGCAGACCGTGGGGCAAGCCAATATAATATTACTGGCTTGCCCGCGCCGGGGACACCTCCGTACGCT
TACGTGTCGATTTGT
+
FFFFFFFF,::FFFFFFFFFFFFF:,FFFFFFFFFFFFFF:FFF,FFF,FFFFFFFFFF:FF,,:FF
```

```
:FFFFFFFFFFF,FFFFF:FFFF:FFFF,,F,FFFFFF:FFF:FFFF:F,F:F,F:FF,FFF,F:FFF
,FFFFF:FFFF:FFF
@A00721:190:HGJW7DSXY:4:2673:9715:5447 1:N:0:TCTGCAAG+GCTGCACT
CGCCCTACAGTATACCAATTCAAATTCAAATTCAAATAGAATTCCATAGGCCCCGCAGGGGCATCTTA
CCGCAGACCGTGGGGCAAGCCAATATAATATTACTGGCTTGCCCGCGCCGGGGACACCTCCGTACGCT
TACGTGTCGATTTGT
+
FFFFFFFFFFFFFFFFFFFFFFFFFFFFFFFFFFFFFFFF:FFFFFFFFFFFFFF:FFFFFFFF
FFFFFFFFFF:FFFFFFFFFF:FFFFFFFFFFFFFFFFFF:FFFFFFFFFFFFFFFFFFF:FFFFFFF
FFFF:FFFFFF:FFF
@A00721:190:HGJW7DSXY:4:1253:25129:27007 1:N:0:TCTGCAAG+GCTGCACT
GCCCTACAGTATACCAATTCAAATTCAAATTCAAATAGAATTCCATAGGCCCCGCAGGGGCATCTTAC
CGCAGACCGTGGGGCAAGCCAATATAATATTACTGGCTTGCCCGCGCCGGGGACACCTCCGTACGCTT
ACGTGTCGATTTGTC
+
FFFFFFFFFFFFFFFFFFFFFFFFFFFFFFFFFFFFFFFFFFFFFFFFFFFFFFFFFFFFFFFFFFFF
FFFFFFFFFFFFFFFFFFFFFFFFFFFFFFFFFFFFFFFFFFFFFFFFFFFFFFFFFFFFFFFFFFFF
FFFFFFFFFFFFFFF
@A00721:190:HGJW7DSXY:4:1255:23628:12633 1:N:0:TCTGCAAG+GCTGCACT
GCCCTACAGTATACCAATTCAAATTCAAATTCAAATAGAATTCCATAGGCCCCGCAGGGGCATCTTAC
CGCAGACCGTGGGGCAAGCCAATATAATATTACTGGCTTGCCCGCGCCGGGGACACCTCCGTACGCTT
ACGTGTCGATTTGTC
+
FFFFFFFFFFFFFFFFFFFFFFFFFFFFFFFFFFFFFFFFFFFFFFFFFFFFFFFFFFFFFFFFFFFF
FFFFFFFFFFFFFFFFFFFFFFFFFFFFFFFFFFFFFFFFFFFFFFFFFFFF,FFFFFFFFFFFFFFF
FFFFFFFFFFFFFFF
@A00721:190:HGJW7DSXY:4:1460:12391:12680 1:N:0:TCTGCAAG+GCTGCACT
GCCCTACAGTATACCAATTCAAATTCAAATTCAAATAGAATTCCATAGGCCCCGCAGGGGCATCTTAC
CGCAGACCGTGGGGCAAGCCAATATAATATTACTGGCTTGCCCGCGCCGGGGACACCTCCGTACGCTT
ACGTGTCGATTTGTC
+
FFFFFFFFFFFFFFFFFFFFFFFFFFFFFFFFFFFFFFFFFFFFFFFFFFFFFFFFFFFFFFFFFFFF
FFFFFFFFFFFFFFFFFFFFFFFFFFFFFFFFFFFFFFFFFFFFFFFFFFF:FFFF:FFFFFFFFFF
FFFFFFFFFFFFFFF
@A00721:190:HGJW7DSXY:4:2673:31810:15374 1:N:0:TCTGCAAG+GCTGCACT
GCCCTACAGTATACCAATTCAAATTCAAATTCAAATAGAATTCCATAGGCCCCGCAGGGGCATCTTAC
CGCAGACCGTGGGGCAAGCCAATATAATATTACTGGCTTGCCCGCGCCGGGGACACCTCCGTACGCTT
ACGTGTCGATTTGTC
+
FFFFFFFFFFFFFFFFFFFFFFFFFFFFFFFFFFFFFFFFFFFFFFFFFFFFFFFFFFFFFFFFFFFF
FFFFFFFFFFFFFFFFFFFFFFFFFFFFFFFFFFFFFFFFFFFFFFFFFFFFFFFFFFFFFFFFFFFF
FFFFFFFFFFFFFFF
@A00721:190:HGJW7DSXY:4:1237:4661:7310 1:N:0:TCTGCAAG+GCTGCACT
CCTACAGTATACCAATTCAAATTCAAATTCAAATAGAATTCCATAGGCCCCGCAGGGGCATCTTACCG
CAGACCGTGGGGCAAGCCAATATAATATTACTGGCTTGCCCGCGCCGGGGACACCTCCGTACGCTTAC
GTGTCGATTTGTCAG
+
FFFFFFF,FFFFFF:::FFFFFF:FFF,FFFFF,FFFFFF:FF,F,FFFFF:FFFF,FFFF:FFF:FF
:FFF::FF:FFFFFFF,FFFFFFFFFFFF:FFF:FF:FF:FFF:F,FFFFFFFF:FF:FFFF:FF:FF
FFFFF:FFFF,FFFF
@A00721:190:HGJW7DSXY:4:1336:25464:1720 1:N:0:TCTGCAAG+GCTGCACT
CCTACAGTATACCAATTCAAATTCAAATTCAAATAGAATTCCATAGGCCCCGCAGGGGCATCTTACCG
CAGACCGTGGGGCAAGCCAATATAATATTACTGGCTTGCCCGCGCCGGGGACACCTCCGTACGCTTAC
GTGTCGATTTGTCAG
```

```
+
FFFFFFFFFFFF:FFFFFFFFFFFFFFFFFFFFFFFFFFFFFFFFFF:FFFFFFFFFFFFFFFFFFFF
FFFFFFFFFFFFFFFFFFF,FFFFFFFFFFFF:FF:FFFFFFFFFFFFFFFFFFFFFFFFFFFFFF:FF
FFFFFFF:FFFFFFF
@A00721:190:HGJW7DSXY:4:1371:16676:26678 1:N:0:TCTGCAAG+GCTGCACT
CCTACAGTATACCAATTCAAATTCAAATTCAAATAGAATTCCATAGGCCCCGCAGGGGCATCTTACCG
CAGACCGTGGGGCAAGCCAATATAATATTACTGGCTTGCCCGCGCCGGGGACACCTCCGTACGCTTAC
GTGTCGATTTGTCAG
+
FFFFFFFFFFFFFFFFFFFF:FFFFFFFFFFFFFFFFFFFFFFFFFFFFFFFFFFFFFFFFFFFFFFF
FFFFFFFFFFFFFFFFFFFFFFFFFFFFFFFFFFFFFFFFFFFFFFFFFFFFFFFFFFFFFFFFFFFF
FFFFFFFFFFFFFFF
@A00721:190:HGJW7DSXY:4:1430:18927:10567 1:N:0:TCTGCAAG+GCTGCACT
CCTACAGTATACCAATTCAAATTCAAATTCAAATAGAATTCCATAGGCCCCGCAGGGGCATCTTACCG
CAGACCGTGGGGCAAGCCAATATAATATTACTGGCTTGCCCGCGCCGGGGACACCTCCGTACGCTTAC
GTGTCGATTTGTCAG
+
FFFFFFFFFFFFFFFFFFFFFFFFFFFFFFFFFFFFFFFFFFFFFFFFFFFFFFFFFFF:FFFFFFFF
FFFFFFFFFFFFFFFFFFFFFFFFFFFFFFFFFFFFFFFFFFFFFFFFFFFFFFFFFFFFFFFFFFFF
FFFFFFFFFFFFFFF
@A00721:190:HGJW7DSXY:4:1530:3938:2487 1:N:0:TCTGCAAG+GCTGCACT
CCTACAGTATACCAATTCAAATTCAAATTCAAATAGAATTCCATAGGCCCCGCAGGGGCATCTTACCG
CAGACCGTGGGGCAAGCCAATATAATATTACTGGCTTGCCCGCGCCGGGGACACCTCCGTACGCTTAC
GTGTCGATTTGTCAG
+
FFFFF:FFFFFFFFFFFFFFFFFFFFFFFFFFFFFFFFFFFFFFFFFFFFFFFFFFFFFFFFFFFFFF
FFFFFFFFFFFFFFFFFF:FFFFF:FFFFFFFFFFFFFFFFFFFFFFFFFFFFFFFFFFFFFFFFFFF
FFFFFFFFFFFFFFF
@A00721:190:HGJW7DSXY:4:1677:20799:11522 1:N:0:TCTGCAAG+GCTGCACT
CCTACAGTATACCAATTCAAATTCAAATTCAAATAGAATTCCATAGGCCCCGCAGGGGCATCTTACCG
CAGACCGTGGGGCAAGCCAATATAATATTACTGGCTTGCCCGCGCCGGGGACACCTCCGTACGCTTAC
GTGTCGATTTGTCAG
+
FFFFFFFFFFFFFFFFFFFFFFFFFFFFFFFFFFFFFFFFFFFFFFFFFFFFFFFFFFFFFFFFFFFF
FFFFFFFFFFFFFFFFFF:FFFFFFFFFFFFFFFFFFFFFFFFFFFFFFFFFFFFFFFFFFFFFFFFF
FFFFFFFFFFFFFFF
@A00721:190:HGJW7DSXY:4:1128:23402:8923 1:N:0:TCTGCAAG+GCTGCACT
CTACAGTATACCAATTCAAATTCAAATTCAAATAGAATTCCATAGGCCCCGCAGGGGCATCTTACCGC
AGACCGTGGGGCAAGCCAATATAATATTACTGGCTTGCCCGCGCCGGGGACACCTCCGTACGCTTACG
TGTCGATTTGTCATT
+
F:FFFFFFFFFFFFFFFFFF::FFFFFFFFFFFFFFFFFF:FFFFFFFFFFFF:FFFFFFFFFFFFFF
FFFFFFFFFFFFFFFFFFFFFFFFFFFFFF:FF:FFFFFFFFFFFFFFFFFFFFFFFFFFFFFFFFFF:
FFFFFFFFFFFFFF:
@A00721:190:HGJW7DSXY:4:1231:6415:31391 1:N:0:TCTGCAAG+GCTGCACT
CTACAGTATACCAATTCAAATTCAAATTCAAATAGAATTCCATAGGCCCCGCAGGGGCATCTTACCGC
AGACCGTGGGGCAAGCCAATATAATATTACTGGCTTGCCCGCGCCGGGGACACCTCCGTACGCTTACG
TGTCGATTTGTCATT
+
FFFFFFFFFFFFFFFFFFFFFFFFFFFFFFFFFFFFFFFFFFFFFFFFFFFFFFFFFFFFFFFFFFFF
FFFFFFFFFFFFFFFFFFFFFFFFFFFFFFFFFFFFFFFFFFFFFFFFFFFFFFFFFFFFFFFFFFFF
FFFFFFFFFFFFFFF
@A00721:190:HGJW7DSXY:4:1268:16324:5431 1:N:0:TCTGCAAG+GCTGCACT
CTACAGTATACCAATTCAAATTCAAATTCAAATAGAATTCCATAGGCCCCGCAGGGGCATCTTACCGC
```

AGACCGTGGGGCAAGCCAATATAATATTACTGGCTTGCCCGCGCCGGGGACACCTCCGTACGCTTACG
TGTCGATTTGTCATT
+
FFFFFFFFFFFFFFFFFFFFFFFFFFFFFFFFFFFFFFFFFFFFFFFFFFFFFFFFFFFFFFFFFFFF
FFFFFFFFFFFFFFFFFFFFFFFFFFFFFFFFFFFFFFFFFFFFFFFFFFFFFFFFFFFFFFFFFFFF
FFFF:FFFFFFFFFF
@A00721:190:HGJW7DSXY:4:1433:1036:3380 1:N:0:TCTGCAAG+GCTGCACT
CTACAGTATACCAATTCAAATTCAAATTCAAATAGAATTCCATAGGCCCCGCAGGGGCATCTTACCGC
AGACCGTGGGGCAAGCCAATATAATATTACTGGCTTGCCCGCGCCGGGGACACCTCCGTACGCTTACG
TGTCGATTTGTCATT
+
FFFFFFFFFFFFFFFFFFFFFFFFFFFFFFF:FFF:FFFF:FFFFFFFFFFFFFFFFFFFFFFFFFFF
FFFFFFFFFFFFFFFFFFFFFFFFFFFFFFFFFFFFF:FFFFFFFFFFF:FFFFFFFFFFFFF:FFFF
FFFFFFFFFFFFFFF
@A00721:190:HGJW7DSXY:4:1457:6470:2832 1:N:0:TCTGCAAG+GCTGCACT
CTACAGTATACCAATTCAAATTCAAATTCAAATAGAATTCCATAGGCCCCGCAGGGGCATCTTACCGC
AGACCGTGGGGCAAGCCAATATAATATTACTGGCTTGCCCGCGCCGGGGACACCTCCGTACGCTTACG
TGTCGATTTGTCATT
+
FFFFFFFFFFFFFFFFFFF:FFFFFFFFFFFFFFFFFFFFFFFFFFFFFFFFFFFFFFFFFFFFFFFF
FFFFFFFFFFFFFFFFFFFFFFFFFFFFFFFFFFFFFFFFFFFFFFFFFFFFFFFFFFFFFFFFFFFF
FFFFFFFFFFFFFFF
@A00721:190:HGJW7DSXY:4:1464:1280:7498 1:N:0:TCTGCAAG+GCTGCACT
CTACAGTATACCAATTCAAATTCAAATTCAAATAGAATTCCATAGGCCCCGCAGGGGCATCTTACCGC
AGACCGTGGGGCAAGCCAATATAATATTACTGGCTTGCCCGCGCCGGGGACACCTCCGTACGCTTACG
TGTCGATTTGTCATT
+
FFFFFFFFFFFFFFFFFFFFFFFFFFFFFFFFFFFFFFFF:FFFFFFFFFFFFFFFFFFFFFFFFFFF
FFFFFFFFFFFFFFFFFFFFFFFFFFFFFFFFFFFFFFFFFFFFFFFFFFFFFFFFFFFFFFFFFFFF
FFFFFFFFFFFFFFF
@A00721:190:HGJW7DSXY:4:1535:17942:12868 1:N:0:TCTGCAAG+GCTGCACT
CTACAGTATACCAATTCAAATTCAAATTCAAATAGAATTCCATAGGCCCCGCAGGGGCATCTTACCGC
AGACCGTGGGGCAAGCCAATATAATATTACTGGCTTGCCCGCGCCGGGGACACCTCCGTACGCTTACG
TGTCGATTTGTCATT
+
FFFFFFFFFFFFFF:F:FFFFFFFFFFF::FFFFFFFFFF:F:FF:FFFFFFFFFFFFFFFFFFFFFF
FFFFFFFFFFFFFFFFFFFFF:FFFFFFFFFFFFFF,FFFFFFFFFFFFFFF:FFFFFFFFFFF:FF,
FFFFFFFFF:FFFFF
@A00721:190:HGJW7DSXY:4:1644:11451:13088 1:N:0:TCTGCAAG+GCTGCACT
CTACAGTATACCAATTCAAATTCAAATTCAAATAGAATTCCATAGGCCCCGCAGGGGCATCTTACCGC
AGACCGTGGGGCAAGCCAATATAATATTACTGGCTTGCCCGCGCCGGGGACACCTCCGTACGCTTACG
TGTCGATTTGTCATT
+
F:FFFFFFFFFFFFFFFFFFFFFFFFFFFFFFFFFFFFFF:FFFFFFFFFFFFFFFFFFFFFFFFFFF
FFFFFFFFFFFFFFFFFFFFFFFF:FFFFFFFFFFFFFFFFFFFFFFFFFFFFFFFFFFFFFFFFFF
:FFFFFFFFFFFFFF
@A00721:190:HGJW7DSXY:4:2202:15863:18975 1:N:0:TCTGCAAG+GCTGCACT
CTACAGTATACCAATTCAAATTCAAATTCAAATAGAATTCCATAGGCCCCGCAGGGGCATCTTACCGC
AGACCGTGGGGCAAGCCAATATAATATTACTGGCTTGCCCGCGCCGGGGACACCTCCGTACGCTTACG
TGTCGATTTGTCATT
+
FFFFFFFFFFFFFFFFFFFFFFFFFFFFFFFFFFFFFFF::FFFFFFFFFFFFFFFFFFFFFFFFFFF
FFFFFFFFFFFFFFFFFFF:FFFFFFFFFFFF,FFFFFFFFFFFFFFFFFFFFFFFFFFFFFFFFFFF
FFFFFF:FFFFFFFF

```
@A00721:190:HGJW7DSXY:4:2230:26250:3176 1:N:0:TCTGCAAG+GCTGCACT
CTACAGTATACCAATTCAAATTCAAATTCAAATAGAATTCCATAGGCCCCGCAGGGGCATCTTACCGC
AGACCGTGGGGCAAGCCAATATAATATTACTGGCTTGCCCGCGCCGGGGACACCTCCGTACGCTTACG
TGTCGATTTGTCATT
+
FFFFFFFFFFFFFFFFFFFFFFFFFFFFFFFFFFFFFFFFFFFFFFFFFFFFFFFFFFFFFFFFFFFF
FFFFFFFFFFFFFFFFFFFF:FFFFFFFFFFFFFFFFFFFFFFFFFFFFFFFFFFFFFFFFFFFFFFF
FFFFFFFFFFFFF:F
@A00721:190:HGJW7DSXY:4:2458:3224:24142 1:N:0:TCTGCAAG+GCTGCACT
CTACAGTATACCAATTCAAATTCAAATTCAAATAGAATTCCATAGGCCCCGCAGGGGCATCTTACCGC
AGACCGTGGGGCAAGCCAATATAATATTACTGGCTTGCCCGCGCCGGGGACACCTCCGTACGCTTACG
TGTCGATTTGTCA
+
:FFFFF:FFFFF:FFFFFFFF,FFFFF,,FFFFF,FF,FF:FFFFFFFFFFFF:F:FFF:FFF:FFFF
F:FFFFFFFF:FFF,F:FF,F,FFFF:FF:::,:,FFFFFFFFFF,FFFFFFFF,FF:FF:FF,:FFF
FFFF,FF:FFFFF
@A00721:190:HGJW7DSXY:4:2658:18132:9502 1:N:0:TCTGCAAG+GCTGCACT
CTACAGTATACCAATTCAAATTCAAATTCAAATAGAATTCCATAGGCCCCGCAGGGGCATCTTACCGC
AGACCGTGGGGCAAGCCAATATAATATTACTGGCTTGCCCGCGCCGGGGACACCTCCGTACGCTTACG
TGTCGATTTGTCATT
+
FFFFFFFFFFFFFFFFFFFFFFFFFFFFFFFFFFFFFFFFFFFFFFFFFFFFFFFFFFFFFFFFFFFF
FFFFFFFFFFFFFFFFFFFFFFFFFFFFFFFFFFFFFFFFFFFFFFFFFFFFFFFFFFFFFFFFFFFF
FFFF:FFFFFFFFFF
@A00721:190:HGJW7DSXY:4:1119:8558:22200 1:N:0:TCTGCAAG+GCTGCACT
TACCAATTCAAATTCAAATTCAAATAGAATTCCATAGGCCCCGCAGGGGCATCTTACCGCAGACCGTG
GGGCAAGCCAATATAATATTACTGGCTTGCCCGCGCCGGGGACACCTCCGTACGCTTACGTGTCGATT
TGTCATTAGACCACG
+
FFFFFFFFFFFFFFFFFFFFFFFFFFFFFFFFFFFFFFFFFFFFFFFFFFFFFFFFFFFFFFFFFFFF
FFFFFFFFFFFFFFFFFFFFFFFFFFFFFFFFFFFFFFFFFFFFFFFFFFFFFFFFFFF:FFFFFF,F
FFFFFFFF:FFFFFF
@A00721:190:HGJW7DSXY:4:1344:4562:35133 1:N:0:TCTGCAAG+GCTGCACT
TACCAATTCAAATTCAAATTCAAATAGAATTCCATAGGCCCCGCAGGGGCATCTTACCGCAGACCGTG
GGGCAAGCCAATATAATATTACTGGCTTGCCCGCGCCGGGGACACCTCCGTACGCTTACGTGTCGATT
TGTCATTAGACAGAT
+
FFFFFFFFFFFFFFFFFFFFFFF:FFFFFFFFFFFFFFFFFFFFFFFFFFFFFFFFFFFFFFFFFFFF
FFFFFFFFFFFFFFFFFFFFFFFFFFFFFFFFFFFFFFFFFFFFFFFFFF:FFFFFFFFFFFFFFFFF
FFFFF:FFFFFFFFF
@A00721:190:HGJW7DSXY:4:1647:11939:23703 1:N:0:TCTGCAAG+GCTGCACT
TACCAATTCAAATTCAAATTCAAATAGAATTCCATAGGCCCCGCAGGGGCATCTTACCGCAGACCGTG
GGGCAAGCCAATATAATATTACTGGCTTGCCCGCGCCGGGGACACCTCCGTACGCTTACGTGTCGATT
TGTCATTAGACCACG
+
:FF:F::F:FFFF:FFFFF:FFF,FFFFFFFFFFF,F,FFF:FFFFF:FF::F,:FFFF:FFFFFF,F
F:F:FF,FFFF:FFFFFFFFFF:FFFF,F,FFFFFFF:FFF:FF,FFFFF,FFFF:,FFF:FFF,FFF
FFFFFFFFFFF:FF:
@A00721:190:HGJW7DSXY:4:1648:5692:33144 1:N:0:TCTGCAAG+GCTGCACT
TACCAATTCAAATTCAAATTCAAATAGAATTCCATAGGCCCCGCAGGGGCATCTTACCGCAGACCGTG
GGGCAAGCCAATATAATATTACTGGCTTGCCCGCGCCGGGGACACCTCCGTACGCTTACGTGTCGATT
TGTCATTAGACAGAT
+
FFFFFFFFFFFFFFFFFFFFFFFFFFFFFFFFFFFFFFFFFFFFFFFFFFFFFFFFFFFFFFFFFFFF
```

```
FFFFFFFFFFFFFFFFFFFFFFFFFFFFFFFFFFFFFFFFFFFFFFFFFFFFFF:FFFFFFFFFF:F
FFFFFFFFFFFFFFF
@A00721:190:HGJW7DSXY:4:2648:7780:27336 1:N:0:TCTGCAAG+GCTGCACT
TACCAATTCAAATTCAAATTCAAATAGAATTCCATAGGCCCCGCAGGGGCATCTTACCGCAGACCGTG
GGGCAAGCCAATATAATATTACTGGCTTGCCCGCGCCGGGGACACCTCCGTACGCTTACGTGTCGATT
TGTCATTAGACAGAT
+
FFFFFFFFFFFFFFFFFFFFFFFFFFFFFFFFFFFFFFFFFFFFFFFFFFFFFFFFFFFFFFFFFFF
FFFFFFFFF,F:FFFFFFFFFFFFFFFFFFFFFFFFFFFFFFFFFFFFFFFFFFFF:FFFFFFFFFF
FFFFFFFFFFFFFFF
@A00721:190:HGJW7DSXY:4:2664:18177:3317 1:N:0:TCTGCAAG+GCTGCACT
TACCAATTCAAATTCAAATTCAAATAGAATTCCATAGGCCCCGCAGGGGCATCTTACCGCAGACCGTG
GGGCAAGCCAATATAATATTACTGGCTTGCCCGCGCCGGGGACACCTCCGTACGCTTACGTGTCGATT
TGTCATTAGACCACG
+
FFFFF,FFFFFFFFFFFFFFFFFFFFFFFFFFFFFFFFFFFFFFFFFFFFFFFFFFFFFFFFFFFFF
FFFFFFFFFFFFFFFFFFFFFFFFFFFFFFFFFFFFFFFFFFFFFFFFFFFFFFFFFFFFFFFFFFF
FFFFFFFFFFFFFFF
@A00721:190:HGJW7DSXY:4:1123:2618:33896 1:N:0:TCTGCAAG+GCTGCACT
GGTCATGATCGTGACGCGTGGTCTAATGACAAATCGACACGTAAGCGTACGGAGGTGTCCCCGGCGCG
GGCAAGCCAGTAATATTATATTGGCTTGCCCCACGGTCTGCGGTAAGATTCCCCTGCGGGGCCTATGG
AATTCTATTTGAATT
+
FFFFFFFFFFFFFFFFFFFFFFFFFFFFFFFFFFFFFFFFFFFFFFFFFFFFFFFFFFFFFFFFFFF
FFFFF:FF,,:FF,F,FFFF:F:F::F,FFFF:FFFF,:F:FFFFF:FF,:FFF:FF:FFF:F:F,,F
FF:F:FF,FF,,F:F
@A00721:190:HGJW7DSXY:4:1142:27263:14387 1:N:0:TCTGCAAG+GCTGCACT
GGTCATGATCGTGACGCGTGGTCTAATGACAAATCGACACGTAAGCGTACGGAGGTGTCCCCGGCGCG
GGCAAGCCAGTAATATTATATTGGCTTGCCCCACGGTCTGCGGTAAGATGCCCCTGCGGGGCCTATGG
AATTCTATTTGAATT
+
FFFFFF:FFFFF:FFFFFF:FFFFFFFFFFFFFF:FFFFFFFFFFFFFFFFFFFFFFFFFFFFFFFF
FFFFFFFF,,FFF,FFF:,:,F,FF,F,FF::,,F::F,FF,:FF::FF:FFFFFFFFF:FFF,F:::
FF:F:FF,FF:FF,F
@A00721:190:HGJW7DSXY:4:2362:28357:34757 1:N:0:TCTGCAAG+GCTGCACT
GGTCATGATCGTGACGCGTGGTCTAATGACAAATCGACACGTAAGCGTACGGAGGTGTCCCCGGCGCG
GGCAAGCCAGTAATATTATATTGGCTTGCCCCACGGTCTGCGGTAAGATGCCCCTGCGGGGCCTATGG
AATTCTATTTGAATT
+
FFFFFFFFFFFFFFFFFFFFFFFFFFFFFFFFFFFFFFFFFFFFFFFFFFFFFFFFFFFFFFFFFFF
FFFFFFFFFFFFF:FFFFFFFFFFFFFFFFFFFFFFFFFFFFFFFFFFFFFFFFFFFFFFFFFFFFF
F:FFFF:FFFFFFFF
@A00721:190:HGJW7DSXY:4:2412:30689:25332 1:N:0:TCTGCAAG+GCTGCACT
GGTCATGATCGTGACGCGTGGTCTAATGACAAATCGACACGTAAGCGTACGGAGGTGTCCCCGGCGCG
GGCAAGCCAGTAATATTATATTTGCTTGCCCCACGGTCTGCGGTAAGATGCCCCTGCGGGGCCTATGG
AATTCTATTTGAATT
+
FFFFFFFFFFFFFFFFFFFFFFFFFFFFFFFFFFFFFFFFFFFFFFFFFFFFFFFFFFFFFFFFFFF
FF:FF,FF::,FF:F,::,:FF,FF:F,FFFF::,::,,::FF::FFF:,:FFF,FFFFFF,F,FFFF
F:,FFF,,F:,::,F
@A00721:190:HGJW7DSXY:4:2441:26666:24251 1:N:0:TCTGCAAG+GCTGCACT
GGTCATGATCGTGACGCGTGGTCTAATGACAAATCGACACGTAAGCGTACGGAGGTGTCCCCGGCGCG
GGCAAGCCAGTAATATTATATTGGCTTGCCCCACGGTCTGCGGTAAGATGCCCCTGCGGGGCCTATGG
AATTCTATTTGAATT
```

```
+
FFFFFFFFFFFFFFFFFFFFFFFFFFFFFFFFFFFFFFFFFFFFFFFFFFFFFFFFFFFFFFFFFFFFFF
FFFFF,FF:FFFFF:FFF:FFF:,::FFFFFFFF:FF:F:,FFFFF:FF,,FFF,:FFFFFFF,FFFF
FF,FFF:::FFFF:F
@A00721:190:HGJW7DSXY:4:1214:3179:35180 1:N:0:TCTGCAAG+GCTGCACT
TACGATCATAACCATCTAAAAAATGAATTCTATAGTCCCCGCAGGGGCATCTTACCGCAGACCGTGGG
GCAAGCCAATATAATATTACTGGCTTGCCCGCGCCGGGGACACCTCCGTACGCTTACGTGTCGATTTG
TCATTAGACCACGCG
+
FFFFFFFFFFFFFFFFFFFFFFFFFFFF:FFFFFFFFFFFFFFFFFFFFFFFFFFFFFFFFFFFFFFFF
FFF:FFFFFFFFFFFFFFFFFFFFFFFFFFFFFFFFFFFFFFFFFFFFFFFFFFFFFFFFFFFF:FFF
FFFFFFFFFFFFFFF
@A00721:190:HGJW7DSXY:4:1417:10936:12884 1:N:0:TCTGCAAG+GCTGCACT
TACGATCATAACCATCTAAAAAATGAATTCTATAGTCCCCGCAGGGGCATCTTACCGCAGACCGTGGG
GCAAGCCAATATAATATTACTGGCTTGCCCGCGCCGGGGACACCTCCGTACGCTTACGTGTCGATTTG
TCATTAGACCACGCG
+
FFFFFFFFFFFFFFFFFFFFFF:FFFFFFFFFFFFFFFFFFFFFFFFF:FFFFFFFFFFFFFFFFFFF
FFFFFFFFFFFFFFFFFFFFFFFFFFFFFFFFFFFFFFFFFFFFFFFFF:FFFFFFFFFFFFFFFFFFF
FFFFFFFFFFFFFFF
@A00721:190:HGJW7DSXY:4:1621:25852:28729 1:N:0:TCTGCAAG+GCTGCACT
TCCATAGGCCCCGCAGGGGCATCTTACCGCAGACCGTGGGGCAAGCCAATATAATATTACTGGCTTGC
CCGCGCCGGGGACACCTCCGTACGCTTACGTGTCGATTTGTCATTAGACCACGCGTCACGATCATAAC
CATCTAAAAAATGAA
+
FFFFFFFFFFFFFFFFFFFFFFFFFFFFFFFFFFFFFFFFFFFFFFFFFFFFFFFFFFFFFFFFFFFFF
FFFFFFFFFFFFFFFFFFFFFFFFFFFFFFFFFFFFFFFFFFFFFFFFFFFFFFFFFFFFFFFFFFFFF
FFFFFFFF:FFFFFF
@A00721:190:HGJW7DSXY:4:1656:13892:10770 1:N:0:TCTGCAAG+GCTGCACT
TCCATAGGCCCCGCAGGGGCATCTTACCGCAGACCGTGGGGCAAGCCAATATAATATTACTGGCTTGC
CCGCGCCGGGGACACCTCCGTACGCTTACGTGTCGATTTGTCATTAGACCACGCGTCACGATCATAAC
CATCTAAAAAATGAA
+
FFFFFFFFFFFFFFFFFFFFFFFFFFFFFFFFFFFFFFFFFFFFFFFFFFFFFFFFFFFFFFFFFFFFF
FFFFFFFFFFFFFFFFFFFFFFFFFFFFFFFFFFFFFFFFFFFFFFFFFFFFFFFFFFFFFF:FFFFFF
FFFFF:FFFFFFFFF
@A00721:190:HGJW7DSXY:4:2253:9100:6104 1:N:0:TCTGCAAG+GCTGCACT
TCCATAGGCCCCGCAGGGGCATCTTACCGCAGACCGTGGGGCAAGCCAATATAATATTACTGGCTTGC
CCGCGCCGGGGACACCTCCGTACGCTTACGTGTCGATTTGTCCTTAGACCACGCGTCACGATCATAAC
CATCTAAACAATGAA
+
F:FFFFFFFFFFFFFFFFFFFFFFFFFFFF::FFFF:FFFF,FFFF:,F::,F:FFFFF:FFFF:FFF
FFFFF,,F:F:FF:F:FFF::,F:FF,FF,F::,FF:FFFF,,F:F:FFFFFFFFFFFFFFFF,FF:F
FFF::FFF,FFFFFF
@A00721:190:HGJW7DSXY:4:1103:9561:34867 1:N:0:TCTGCAAG+GCTGCACT
CCATAGGCCCCGCAGGGGCATCTTACCGCAGACCGTGGGGCAAGCCAATATAATATTACTGGCTTGCC
CGCGCCGGGGACACCTCCGTACGCTTACGTGTCGATTTGTCATTAGACCACGCGTCACGATCATAACC
ATCTAAAAAATGAAT
+
FFFFFFFFFFFFFFFFFFFFFFFFFFFFFFFFFFFFFFFFFFFFFFFFFFFF:FFF:FFFFFFFFFFF
FFFFFFFFFFFFFFFFFFFFFFFFFFFFFFFFFFFFFFFFFFFFF:FF:FFFFFFFFFFFFFFFFFFFFFF
FFFFFFFFFFFFFFF
@A00721:190:HGJW7DSXY:4:1121:22996:18646 1:N:0:TCTGCAAG+GCTGCACT
CCATAGGCCCCGCAGGGGCATCTTACCGCAGACCGTGGGGCAAGCCAATATAATATTACTGGCTTGCC
```

CGCGCCGGGGACACCTCCGTACGCTTACGTGTCGATTTGTCATTAGACCACGCGTCACGATCATAACC
ATCTAAAAAATGAAT
+
FFFFFFFFFFFFFFFFFFFFFFFFFFFFFFFFFFFFFFFFFFFFFFFFFFFFFFFFFFFFFFFFFFFF
FFFFFFFFFFFFFFFFFFFFFFFFFFFFFFFFFFFFFFFFFFFFFFFFFFFFFFFFFFFFFFFFFFFF
FFFFFFFFFFFFFFF
@A00721:190:HGJW7DSXY:4:1210:2862:33442 1:N:0:TCTGCAAG+GCTGCACT
CCATAGGCCCCGCAGGGGCATCTTACCGCAGACCGTGGGGCAAGCCAATATAATATTACTGGCTTGCC
CGCGCCGGGGACACCTCCGTACGCTTACGTGTCGATTTGTCATTAGACCACGCGTCACGATCATAACC
ATCTAAAAAATGAA
+
FFFFFFFFFFFFFFFFFFF:FFFFFFFFFFFFFFFFFFFFFFFFFFFFFFFFFFFFFFFFFFFFFFFF
FFFFFFFFFFFFFFFFFFFFFFFF:FFFFFFFFFFFFFFFFF:F:FFFFFFFFFFFFFFFFFFFFFFF
FFFFFFFFFFFFFF
@A00721:190:HGJW7DSXY:4:1223:10917:35900 1:N:0:TCTGCAAG+GCTGCACT
CCATAGGCCCCGCAGGGGCATCTTACCGCAGACCGTGGGGCAAGCCAATATAATATTACTGGCTTGCC
CGCGCCGGGGACACCTCCGTACGCTTACGTGTCGATTTGTCATTAGACCACGCGTCACGATCATAACC
ATCTAAAAAATGAAT
+
FFFFFFFFFFFFFFFFFFFFFFFFFFFFFFFFFFFFFFFFFFFFFFFFFFFFFFFFFFFFFFFFF:FF
FFFFFFFFFFFFFFFFFFFFFFFFFFFFFFFFFFFFFFFFFFFFFF:FFFFFFFFFFFFFFFFFFFFF
FFFFFFFFFFFFFFF
@A00721:190:HGJW7DSXY:4:1234:28827:29277 1:N:0:TCTGCAAG+GCTGCACT
CCATAGGCCCCGCAGGGGCATCTTACCGCAGACCGTGGGGCAAGCCAATATAATATTACTGGCTTGCC
CGCGCCGGGGACACCTCCGTACGCTTACGTGTCGATTTGTCATTAGACCACGCGTCACGATCATAACC
ATCTAAAAAATGAAT
+
FFFFFFFFFFFFFFFFFFFFFFFFFFFFFFFFFFFFFFFFFFFFFFFFFFFFFFFFFFFFFFFFFFFF
FFFFFFFFFFFFFFFFFFFFFFFF,FFFFFFFFFFFFFFFFF::FF:FFFFFFFFFFFFFFFFFFFFF
FFFFFFFFFFFFFFF
@A00721:190:HGJW7DSXY:4:1240:2790:33505 1:N:0:TCTGCAAG+GCTGCACT
CCATAGGCCCCGCAGGGGCATCTTACCGCAGACCGTGGGGCAAGCCAATATAATATTACTGGCTTGCC
CGCGCCGGGGACACCTCCGTACGCTTACGTGTCGATTTGTCATTAGACCACGCGTCACGATCATAACC
ATCTAAAAAATGAAT
+
FFFFFFFFFFFFFFFFFFFFF:FFFFFFFFFFFFFFFFFFFFFFFFFFFFFFFFFFFFFFFFFFFFFF
FFFFFFFFFFFFFFFFFFFFFFFFFFFFFFFFFFF:FFFF::FFFFFFFFFF:FFFFFFF,FFFF:FF
FFFFFFFFFF:FFF:
@A00721:190:HGJW7DSXY:4:1246:3739:17049 1:N:0:TCTGCAAG+GCTGCACT
CCATAGGCCCCGCAGGGGCATCTTACCGCAGACCGTGGGGCAAGCCAATATAATATTACTGGCTTGCC
CGCGCCGGGGACACCTCCGTACGCTTACGTGTCGATTTGTCATTAGACCACGCGTCACGATCATAACC
ATCTAAAAAATGAAT
+
FF:FFFFFFFFFFFFFFFF:FFFFFFFFFFFFFFFFFFFFFFFFFFFFFFFF:FFFFFFFFFF:FFFF
FFFFFFFFFFFFFFFFFFFFFFF:F:FFFFFFFFFFFFFFFFFFFFFFFFFFFFFFFFFF:FFFFFFF
FFFFFFFFFFFFFFF
@A00721:190:HGJW7DSXY:4:1530:25048:7670 1:N:0:TCTGCAAG+GCTGCACT
CCATAGGCCCCGCAGGGGCATCTTACCGCAGACCGTGGGGCAAGCCAATATAATATTACTGGCTTGCC
CGCGCCGGGGACACCTCCGTACGCTTACGTGTCGATTTGTCATTAGACCACGCGTCACGATCATAACC
ATCTAAAAAATGAAT
+
FFFFFFFFFFFFFFFFFFFFFFFFFFFFFF:FFFFFFFFFFFFFFFFFFFFFFFFFFFFFFFFFFFFF
FFFFFFFFFFFFFFFFFFFFFFFF:FFFFFFFFFFFFFFFFFFFFFFFFFFFFFFFFFFFFFFFFFF
FFFFFFFFFFFFFFF

```
@A00721:190:HGJW7DSXY:4:1634:13801:1783 1:N:0:TCTGCAAG+GCTGCACT
CCATAGGCCCCGCAGGGGCATCTTACCGCAGACCGTGGGGCAAGCCAATATAATATTACTGGCTTGCC
CGCGCCGGGGACACCTCCGTACGCTTACGTGTCGATTTGTCATTAGACCACGCGTCACGATCATAACC
ATCTAAAAAATGAAT
+
FFFFFFFFFFFFFFFFFFFFFFFFFFFFFFFFFFFFFFFFFFFFFFFFFFFFFFFFFFFFFFFFFFFF
FFFFFFFFFFFFFFFFFFFFFFFFFFFFFFFFFFFFFFFFFFFFFFFFFFFFFFFFFFFFFFFFFFFF
FFFFFFFFFFFFFFF
@A00721:190:HGJW7DSXY:4:1674:17924:7200 1:N:0:TCTGCAAG+GCTGCACT
CCATAGGCCCCGCAGGGGCATCTTACCGCAGACCGTGGGGCAAGCCAATATAATATTACTGGCTTGCC
CGCGCCGGGGACACCTCCGTACGCTTACGTGTCGATTTGTCATTAGACCACGCGTCACGATCATAACC
ATCTAAAAAATGAAT
+
FFFFFFFFFFFFFFFFFFFFFFFFFFFFFFFFFFFFFFFFFFFFFFFFFFFFFFFFFFFFFFFFFFFF
FFFFFFFFFFFFFFFFFFFFFFFFFFFFFFFFFFFFFFFFFFFFFFFFFFFFFFFFFFFFFFFFFFFF
FFFFFFFFFFFFFFF
@A00721:190:HGJW7DSXY:4:2124:28809:9298 1:N:0:TCTGCAAG+GCTGCACT
CCATAGGCCCCGCAGGGGCATCTTACCGCAGACCGTGGGGCAAGCCAATATAATATTACTGGCTTGCC
CGCGCCGGGGACACCTCCGTACGCTTACGTGTCGATTTGTCATTAGACCACGCGTCACGATCATAACC
ATCTAAAAAATGAAT
+
FFFFFFFFFFFFFFFFFFFFFFFFFFFFFFFFFFFFFFFFFFFFFFFFFFFFFFFFFFFFFFFFFFFF
FFFFFFFFFFFFFFFFFFFFFFFFFFFFFFFFFFFFFFFFFFFFFFFFFFFFFFFFFFFFFFFFFFFF
FFFFFFFFFFFFFFF
@A00721:190:HGJW7DSXY:4:2210:6081:26584 1:N:0:TCTGCAAG+GCTGCACT
CCATAGGCCCCGCAGGGGCATCTTACCGCAGACCGTGGGGCAAGCCAATATAATATTACTGGCTTGCC
CGCGCCGGGGACACCTCCGTACGCTTACGTGTCGATTTGTCATTAGACCACGCGTCACGATCATAACC
ATCTAAAAAATGAAT
+
FFFFFFFFFFFFFFFFFFFFFFFFFFFFFFFFFFFFFFFFFFFFFFFFFFFFFFFFFFFFFFFFFFFF
FFFFFFFFFFFFFFFFFFFFFFFFFFFFFFFFFFFFFFFFFFFFFFFFFFFF:FFFFFFFFFFFFFFF
FFFFFFFFFFFFFFF
@A00721:190:HGJW7DSXY:4:2238:20311:11616 1:N:0:TCTGCAAG+GCTGCACT
CCATAGGCCCCGCAGGGGCATCTTACCGCAGACCGTGGGGCAAGCCAATATAATATTACTGGCTTGCC
CGCGCCGGGGACACCTCCGTACGCTTACGTGTCGATTTGTCATTAGACCACGCGTCACGATCATAACC
ATCTAAAAAATGAAT
+
FFFF:FFFFFFFFFFFFFFFFFFFFFFFFFFFFFFFFFFFFFFFFFFFFFFFFFFFF:FFFFFFFFFF
FFFFFFFFFFFFFFFFFFFFFFFFFFFFFFFFFFFFFFFFFFFFFFFFFFFFFFFFFFFFFFFFFFFF
FFFFFFFFFFFFFFF
@A00721:190:HGJW7DSXY:4:2257:4589:26005 1:N:0:TCTGCAAG+GCTGCACT
CCATAGGCCCCGCAGGGGCATCTTACCGCAGACCGTGGGGCAAGCCAATATAATATTACTGGCTTGCC
CGCGCCGGGGACACCTCCGTACGCTTACGTGTCGATTTGTCATTAGACCACGCGTCACGATCATAACC
ATCTAAAAAATGAAT
+
FFFFFFF:FFFFFFFFFFFFFFFFFFFFFFFFFFFFFFFFFFFFFFFFFFFFFFFFFFFFFFFFFFFF
FFFFFFFFFFFFFFFFFFFFFFFFFFFFFFFFFFF,FFFFFFFFFFFFFFFFFFFFFFFF:FFFFFFF
FFFF:FFFFFFFFFF
@A00721:190:HGJW7DSXY:4:2257:4833:27179 1:N:0:TCTGCAAG+GCTGCACT
CCATAGGCCCCGCAGGGGCATCTTACCGCAGACCGTGGGGCAAGCCAATATAATATTACTGGCTTGCC
CGCGCCGGGGACACCTCCGTACGCTTACGTGTCGATTTGTCATTAGACCACGCGTCACGATCATAACC
ATCTAAAAAATGAAT
+
FFFFFFFFFFFFFFFFFFFFFFFFFFFFFFFFFFFFFFFFFFFFFFFFFFFFFFFFFFFFFFFFFFFF
```

```
FFFFFFFFFFFFFFFFFFFFFFFFFFFFFFFFFFFFFFFFFFFFFFFFFFFFFFFFFFFFFFFFFFFF
FFFFFFFFF:FFFFF
@A00721:190:HGJW7DSXY:4:2348:22073:16235 1:N:0:TCTGCAAG+GCTGCACT
CCATAGGCCCCGCAGGGGCATCTTACCGCAGACCGTGGGGCAAGCCAATATAATATTACTGGCTTGCC
CGCGCCGGGGACACCTCCGTACGCTTACGTGTCGATTTGTCATTAGACCACGCGTCACGATCATAACC
ATCTAAAAAATGAAT
+
FFFFFFFFFFFFFFFFFFFFFFFFFFFFFFFFFFFFFFFFFFFFFFFFFFFFFFFFFFFFFFFFFFFF
FFFFFFFFFFFFFFFFFFFFFFFF:FFFFFFFFFFFFFFFFFFFFFFFFFFFFFFFFFFF,F:FFFFF
FFFFFFFFFFFFFFF
@A00721:190:HGJW7DSXY:4:2630:6560:23657 1:N:0:TCTGCAAG+GCTGCACT
CCATAGGCCCCGCAGGGGCATCTTACCGCAGACCGTGGGGCAAGCCAATATAATATTACTGGCTTGCC
CGCGCCGGGGACACCTCCGTACGCTTACGTGTCGATTTGTCATTAGACCACGCGTCACGATCATAACC
ATCTAAAAAATGAAT
+
FFFFFFFFFFFFFFFFFFFFFFFFFFFFFFFFFFFFFFFFFFFFFFFFFFFFFFFFFFFFFFFFFFFF
FFFFFFFFFFFFFFFFFFFFFFFFF,FFFFFFFFFFFFFFFFFFFFFFFFFFFFFFFFFFFFFFFFFF
FFFFFFFFFFFFFFF
@A00721:190:HGJW7DSXY:4:1308:4553:21715 1:N:0:TCTGCAAG+GCTGCACT
CCTAGGCCCCGCAGGGGCATCTTACCGCAGACCGTGGGGCAAGCCAATATAATATTACTGGCTTGCCC
GCGCCGGGGACACCTCCGTACGCTTACGTGTCGATTTGTCATTAGACCACGCGTCACGATCATAACCA
TCTAAAAAATGAATT
+
FFFFFFFFFFFFFFFFFFFFFFFFFFFFFFFFFFFFFFFFFFFFFFFFFFFFFFFFFFFFFFFFFFFF
FFFFFFFFFFFFFFFFFFFFFFFF:FFFFFFFFFFFFFFFFFFFFFFFFFFFFFFFFFFFF,FFFFF
FFFFFFFFF:FFFF:
@A00721:190:HGJW7DSXY:4:2612:5168:33207 1:N:0:TCTGCAAG+GCTGCACT
CCTAGGCCCCGCAGGGGCATCTTACCGCAGACCGTGGGGCAAGCCAATATAATATTACTGGCTTGCCC
GCGCCGGGGACACCTCCGTACGCTTACGTGTCGATTTGTCATTAGACCACGCGTCACGATCATAACCA
TCTAAAAAATGAATT
+
FFFFFFFFFFFFFFFFFFFFFFFFFFFFFFFFFFFFFFFFFFFFFFFFFFFFFFFFFFFFFFFFFFFF
FFFFFFFFFFFFFFFFFFFFFFFFFFFFFFFFFFFFFFFFFFFFFFFFFFFFFFFFFFFFFFFFFFFF
FFFFFFFFFFFF,FF
@A00721:190:HGJW7DSXY:4:1176:29514:28432 1:N:0:TCTGCAAG+GCTGCACT
GGCCCTGCAGGGGCATCTTACCGCAGACCGTGGGGCAAGCCAATATAATATTACTGGCTTGCCCGCGC
CGGGGACACCTCCGTACGCTTACGTGTCGATTTGTCATTAGACCACGCGTCACGATCATAACCATCTA
AAAAATGAATAGATC
+
FFFFFFFFFFFFFF:FFFFFFFFFFFFFFFFFFFFFFFFFFFFFFFFF:FFFFF:FFFFFFFFFFFFF
FFFFFFFFFFFFFFFFFFFFFFFFFFFFFFFF,FFFFFFFFFFFFFFFFFFFFFFFFFFFFFFFFFFF
FFFFFFFFFFF:FFF
@A00721:190:HGJW7DSXY:4:1509:27407:7247 1:N:0:TCTGCAAG+GCTGCACT
GGCCCTGCAGGGGCATCTTACCGCAGACCGTGGGGCAAGCCAATATAATATTACTGGCTTGCCCGCGC
CGGGGACACCTCCGTACGCTTACGTGTCGATTTGTCATTAGACCACGCGTCACGATCATAACCATCTA
AAAAATGAATAGATC
+
FFFFFFFFFFFFFFFFFFFFFFFFFFFFFF:FFFFFFFFFF:FF,FFFFFFFFFFFFFFFFFFFFFFF
FFFFF:FFFFFFFF:FFFF:FFFFFFFFFFFFFFFFFFFFFFFFFFFFFFFFFFFFFFFFFFFFFFFF
FFFFFFFF:FFFFFF
@A00721:190:HGJW7DSXY:4:1525:28519:27430 1:N:0:TCTGCAAG+GCTGCACT
GGCCCCGCAGGGGCATCTTACCGCAGACCGTGGGGCAAGCCAATATAATATTACTGGCTTGCCCGCGC
CGGGGACACCTCCGTACGCTTACGTGTCGATTTGTCATTAGACCACGCGTCACGATCATGACCATCTA
AAAAATGAATTCTAT
```

+
FFFFFFFFFFFFFFFFFFFFFFFFFFFFFFFFFFFFFFFF:FFFFFFFFFFFFFFFFFFFFFFFFFFFFF
FFFFFFFFFFFFFFFFFFFFFFFFFFFFFFFFFFFFFF:FFFFFFFFFFFFFFFFFFFFFFFFFFFFFFF
FFFFFFFFFFFFFFF
@A00721:190:HGJW7DSXY:4:1551:13467:17143 1:N:0:TCTGCAAG+GCTGCACT
GGCCCTGCAGGGGCATCTTACCGCAGACCGTGGGGCAAGCCAATATAATATTACTGGCTTGCCCGCGC
CGGGGACACCTCCGTACGCTTACGTGTCGATTTGTCATTAGACCACGCGTCACGATCATAACCATCTA
AAAAATGAATAGATC
+
FFFFFFFFFFFFFFF:FFFFFFFFFFFFFFFFFFFFFFFFFFFFF:FFFFFFFFFFFFFFFFFFFFFFFF
FFFFFFFFFFFFFFFFFFFFFFFFFFFFFFFFFFFFFFFFFFFFFFFFFFFFFFFFFFFFFFFFFFFFFF
FFFFFFFFFFFFFFF
@A00721:190:HGJW7DSXY:4:1636:10791:3677 1:N:0:TCTGCAAG+GCTGCACT
GGCCCTGCAGGGGCATCTTACCGCAGACCGTGGGGCAAGCCAATATAATATTACTGGCTTGCCCGCGC
CGGGGACACCTCCGTACGCTTACGTGTCGATTTGTCATTAGACCACGCGTCACGATCATAACCATCTA
AAAAATGAATAGATC
+
FFFFFFFFFFFFFFFFFFFFFFFFFFFFFFFFFFFFFFFFFFFF:FFFFFFFFFFFFFFFFFFFFFFFFF
FFFFFFFFFFFFFFFFFFFFFFFFFFFFFFFFFFFFFFFFFFFFFFFFFFFFFFFFFFFFFFFFFFFFFF
FFFFFFFFFFFFFFF
@A00721:190:HGJW7DSXY:4:2123:15637:25535 1:N:0:TCTGCAAG+GCTGCACT
GGCCCTGCAGGGGCATCTTACCGCAGACCGTGGGGCAAGCCAATATAATATTACTGGCTTGCCCGCGC
CGGGGACACCTCCGTACGCTTACGTGTCGATTTGTCATTAGACCACGCGTCACGATCATAACCATCTA
AAAAATGAATAGATC
+
FFFFFFFFFFFFFFFFFFFFFFFFFFFFFFFFFFFFFFFFFFFFFFFFFFFFFFFFFFFFFFFFFFFFFF
FFFFFFFFFFFFFFFFFFFFFFFFFFFFFFFFFFFFFF:FFFFFFFFFFFFFFFFFFFF:FFFFFFFFFF
FFFFFFFFFFFFFFF
@A00721:190:HGJW7DSXY:4:2123:14362:26209 1:N:0:TCTGCAAG+GCTGCACT
GGCCCTGCAGGGGCATCTTACCGCAGACCGTGGGGCAAGCCAATATAATATTACTGGCTTGCCCGCGC
CGGGGACACCTCCGTACGCTTACGTGTCGATTTGTCATTAGACCACGCGTCACGATCATAACCATCTA
AAAAATGAATAGATC
+
FFFFFFFFFFFFFFFFFFFFFFFFFFFFFFFFFFFFFFFFFFFFFFFFFFFFFFFFFFFFFFFFFFFFFF
FFFFFFF:FFFFFFFFFFFFFFFFFFFFFFFFFFFFFFFFFFFFFFFFFFFFFFFFFFFFFFFFFFFFFF
FFFFFFFFFFFFFFF
@A00721:190:HGJW7DSXY:4:2150:27154:17519 1:N:0:TCTGCAAG+GCTGCACT
GGCCCTGCAGGGGCATCTTACCGCAGACCGTGGGGCAAGCCAATATAATATTACTGGCTTGCCCGCGC
CGGGGACACCTCCGTACGCTTACGTGTCGATTTGTCATTAGACCACGCGTCACGATCATAACCATCTA
AAAAATGAATAGATC
+
FFFFFFFFFFFFFFFFFFFFFFFFFFFFFFFFFFFFFFFFFFFFFFFFFFFFFF:FFFFFFFFFFFFFFF
FFFFFFFFFFFFFFFFFFFFFFFFFFFFFFFFFFFFFFFFF:FFFFFFFFFFFFFFFFF,FFFFFFFFFF
:FFFFFFFFFFFFFF
@A00721:190:HGJW7DSXY:4:2355:14805:9283 1:N:0:TCTGCAAG+GCTGCACT
GGCCCTGCAGGGGCATCTTACCGCAGACCGTGGGGCAAGCCAATATAATATTACTGGCTTGCCCGCGC
CGGGGACACCTCCGTACGCTTACGTGTCGATTTGTCATTAGACCACGCGTCACGATCATAACCATCTA
AAAAATGAATAGATC
+
FFFFFFFFFFFFFFF:FFFFFFFFFFFFFFFFFFFFFFFFFFFFFF:FFFFFFFFFFFFFFFFFFFFFFF
FFFFFFFFFFFFFFFFFFFFFFFFFFFFFFFFFFFFFFFFFFFFFFFFFFFFFFFFFFFFFFFFFFFFFF
FFFFFF:FFFFFFFF
@A00721:190:HGJW7DSXY:4:2374:19334:20979 1:N:0:TCTGCAAG+GCTGCACT
GGCCCTGCAGGGGCATCTTACCGCAGACCGTGGGGCAAGCCAATATAATATTACTGGCTTGCCCGCGC

CGGGGACACCTCCGTACGCTTACGTGTCGATTTGTCATTAGACCACGCGTCACGATCATAACCATCTA
AAAAATGAATAGATC
+
FFFFFFFFFFFFFFFFFFFFFFFFFFFFFFFFFFFFFFFFFFFFFFFFFFFFFFFFFFFFFFFFFFFFFF
FFFFFFFFFFFFFFFFFFFFFFFFFFFFFFFFFFFFFFFFFFFFFFFFFFFFFFFFFFFFFFFFFFFFFF
F:FFFFFFFFFFFFF
@A00721:190:HGJW7DSXY:4:2511:30644:30984 1:N:0:TCTGCAAG+GCTGCACT
GGCCCCGCAGGGGCATCTTACCGCAGACCGTGGGGCAAGCCAATATAATATTACTGGCTTGCCCGCGC
CGGGGACACCTCCGTACGCTTACGTGTCGATTTGTCATTAGACCACGCGTCACGATCATGACCATCTA
AAAAATGAATTCTAT
+
FFFFFFFFFFFFFFFFFFFFFFFFFFFFFFFFFFFFFFFFFFFFFFFFFFFFFFFFFFFFFFFFFFFFFF
FFFFFFFFFFFFFFFFFFFFF:FFFFFFFFFFFFFFFFFFFFFF:FFFFFFFFFFFFFFFFFFFFFFFFF
FFFFFFF:FFFFFFF
@A00721:190:HGJW7DSXY:4:2569:12997:13448 1:N:0:TCTGCAAG+GCTGCACT
GGCCCCGCAGGGGCATCTTACCGCAGACCGTGGGGCAAGCCAATATAATATTACTGGCTTGCCCGCGC
CGGGGACACCTCCGTACGCTTACGTGTCGATTTGTCATTAGACCACGCGTCACGATCATGACCATCTA
AAAAATGAATTCTAT
+
FFFFFFFFFFFFFFFFFFFFFFFFFFFFFFFFFFFFFFFFFFFFFFFFFFFFFFFFFFFFFFFFFFFFFF
FFFFFFFFFF:FFFFFFFFFFFFFFFFFFFFFFFFFFFFFFFFFFFFFFFFFFFFFFFFFFFFFFFFFFF
FFFFFFFFFFFFFFF
@A00721:190:HGJW7DSXY:4:2673:13584:25989 1:N:0:TCTGCAAG+GCTGCACT
GGCCCTGCAGGGGCATCTTACCGCAGACCGTGGGGCAAGCCAATATAATATTACTGGCTTGCCCGCGC
CGGGGACACCTCCGTACGCTTACGTGTCGATTTGTCATTAGACCACGCGTCACGATCATAACCATCTA
AAAAATGAATAGATC
+
FFFFFFFFFFFFFFFFFFFFFFFFFFFFFFFFFFFFFFFFFFFFFFFFFFFFFFFFFFFFFFFFFFFFFF
FFFFFFFFFFFFFFFFFFFFFFFFFFFFFFFFFFFFFFFFFFFFFFFFFFFFFFFFFFFFFFFFFFFFFF
FFFFFFFFFFFFFFF
@A00721:190:HGJW7DSXY:4:1176:19732:36855 1:N:0:TCTGCAAG+GCTGCACT
GCTCCGCAGGGGCATCTTACCGCAGACCGTGGGGCAAGCCAATATAATATTACTGGCTTGCCCGCGCC
GGGGACACCTCCGTACGCTTACGTGTCGATTTGTCATTAGACCACGCGTCACGATCATAACCATCTAA
AAAATGAATTCTATA
+
FFFFFFFFFFFFFFFFFFFFFFFFFFFFFFFFFFFFFFFFFFFFFFFFFFFFFFFFFFFFFFFFFFFFFF
FFFFFFFFFFFFFFFFFFFFFFFFFFFFFFFFFFFFFFFFFFFFFFFFFFFFFFFFFFFFFFFFFFFFFF
FFFFFFFF:FFFF:F
@A00721:190:HGJW7DSXY:4:1427:21414:12994 1:N:0:TCTGCAAG+GCTGCACT
GCTCCGCAGGGGCATCTTACCGCAGACCGTGGGGCAAGCCAATATAATATTACTGGCTTGCCCGCGCC
GGGGACACCTCCGTACGCTTACGTGTCGATTTGTCATTAGACCACGCGTCACGATCATAACCATCTAA
AAAATGAATTCTATA
+
FFFFFFFFFFF:FFFFFFFFFFFFFFFFFFFFFFFFFFFFFFFFFFFFFFFFFFFFFFFFFFFFFFFFFF
FFFFFFFFFFFFFFFFFFFFFFFFFFFFFFFFFFFFFFFFFFFFFFFFFFFFFFFFFFFFFFFFFFFFFF
FFFF,FF,F:FFFFF
@A00721:190:HGJW7DSXY:4:1464:12906:5682 1:N:0:TCTGCAAG+GCTGCACT
GCTCCGCAGGGGCATCTTACCGCAGACCGTGGGGCAAGCCAATATAATATTACTGGCTTGCCCGCGCC
GGGGACACCTCCGTACGCTTACGTGTCGATTTGTCATTAGACCACGCGTCACGATCATAACCATCTAA
AAAATGAATTCTATA
+
FFFFFFFFFFFFFFFFFFFFFFFFFFF:FFFFFFFFFFFFFFFFFFFFFFFFFF:FFFFFFFFFFFFFFF
FFFFFFFFFFFFFFFFFFFFFFFFFFFFFFFF:FFFF,FFFFFFFFFFF:FFFFFFFFFFFFFFF,FFFF
FFFF,FFFFFFFF::

```
@A00721:190:HGJW7DSXY:4:2254:4363:28745 1:N:0:TCTGCAAG+GCTGCACT
GCTCCGCAGGGGCATCTTACCGCAGACCGTGGGGCAAGCCAATATAATATTACTGGCTTGCCCGCGCC
GGGGACACCTCCGTACGCTTACGTGTCGATTTGTCATTAGACCACGCGTCACGATCATAACCATCTAA
AAAATGAATTCTATA
+
FFFFFFFFFFFFFFFFFFFFFFFFFFFFFFFFFFFFFFFFFFFFFFFFFFFFFF:FFFFFFFFFFFFFFF
FFFFFFFFFFFFFFFFFFFFFFFFFFFFFF:FFFFFFFFFFFFFFFFFFFFFFFFFFFFFFFFFFFFFFF
FFFFFFFFF:FFFFF
@A00721:190:HGJW7DSXY:4:2319:23854:34569 1:N:0:TCTGCAAG+GCTGCACT
GCCCCGCAGGGGCATCTTACCGCAGACCGTGGGGCAAGCCAATATAATATTACTGGCTTGCCCGCGCC
GGGGACACCTCCGTACGCTTACGTGTCGATTTGTCATTAGACCACGCGTCACGATCATAACCATCTAA
AAAATGAATTCTATA
+
FFFFFFFFFFFFFFFFFFFFFFFFFFFFFFFFFFFFFFFFFFFFFFFFFFFFFFFFFFFFFFFFFFFFFF
FFFFFFFFFFFFFFFFFFFFFFFFFFFFFFFFFFFFFFFFFFFFFFFFFFFFFFFFFFFFFFFFFFFFFF
FFFFFFFFFFFFFFF
@A00721:190:HGJW7DSXY:4:2605:23701:12226 1:N:0:TCTGCAAG+GCTGCACT
GCTCCGCAGGGGCATCTTACCGCAGACCGTGGGGCAAGCCAATATAATATTACTGGCTTGCCCGCGCC
GGGGACACCTCCGTACGCTTACGTGTCGATTTGTCATTAGACCACGCGTCACGATCATAACCATCTAA
AAAATGAATTCTATA
+
FFFFFFFFFFFFFFFFFFFFFFFFFFFFFFFFFFFFFFFFFFFFFFFFFFFFFFFFFFFFFFFFFFFFFF
FFFFFFFFFFFFFFFFFFFFFFFFFFFFFFFFFFFFFFFFFFFFFFFFFFFFFFFFFFFFFFFFFFFFFF
FFFFFFFFFFFFFFF
@A00721:190:HGJW7DSXY:4:2678:15383:9157 1:N:0:TCTGCAAG+GCTGCACT
GCTCCGCAGGGGCATCTTACCGCAGACCGTGGGGCAAGCCAATATAATATTACTGGCTTGCCCGCGCC
GGGGACACCTCCGTACGCTTACGTGTCGATTTGTCATTAGACCACGCGTCACGATCATAACCATCTAA
AAAATGAATTCTATA
+
FFFFFFFFFFFFFFFFFFFFFFFFFFFFFFFFFFFFFFFFFFFFFFFFFFFFFFFFFFFFFFFFFFFFFF
FFFFFFFFFFFFFFFFFFFFFFFFFFFFFFFFFFFFF:FFFFFFFFFFFFFFFFFFFFFFFFFFFFFFFF
:FF,FFFFFFFFFFF
@A00721:190:HGJW7DSXY:4:1114:8775:7294 1:N:0:TCTGCAAG+GCTGCACT
CCCCGCAGGGGCATCTTACCGCAGACCGTGGGGCAAGCCAATATAATATTACTGGCTTGCCCGCGCCG
GGGACACCTCCGTACGCTTACGTGTCGATTTGTCATTAGACCACGCGTCACGATCATGACCATCTAAA
AAATGAATTCTATAG
+
FFFFFFFFFFFFFFFFFFFFFFFFFFFFFFFFFFFFFFFFFFFFFFFFFFFFFFFFFFFFFFFFFFFFFF
FFFFFFFFFFFFFFFFFFF,FFFFFFFFFFFFFFFFFFF:FFFFFFFFFFFFFF:FFFFFFFFFFFFFFF
FFFFFFFF:FF:F:F
@A00721:190:HGJW7DSXY:4:1262:31051:32565 1:N:0:TCTGCAAG+GCTGCACT
CCCCGCAGGGGCATCTTACCGCAGACCGTGGGGCAAGCCAATATAATATTACTGGCTTGCCCGCGCCG
GGGACACCTCCGTACGCTTACGTGTCGATTTGTCATTAGACCACGCGTCACGATCATGACCATCTAAA
AAATGAATTCTATAG
+
FFFFFFFFFFFFFFFFFFFFFFFFFFFFFFFFFFFFFFFFFFFFFFFFFFFFFFFFFFFFFFFFFFFFFF
FFFFFFFFFFFFFFFFF,FFFFFFFFFFFFFFFFFFFFFFFFFFFFFFFFFFFFFFFFFFFFFFFFFFFF
FFFFFFFFFF::FFF
@A00721:190:HGJW7DSXY:4:1262:31575:33661 1:N:0:TCTGCAAG+GCTGCACT
CCCCGCAGGGGCATCTTACCGCAGACCGTGGGGCAAGCCAATATAATATTACTGGCTTGCCCGCGCCG
GGGACACCTCCGTACGCTTACGTGTCGATTTGTCATTAGACCACGCGTCACGATCATGACCATCTAAA
AAATGAATTCTATAG
+
FFFFFFFFFFFFFFFFFFFFFFFFFFFFFFFFFFFFFFFFFFFFFFFFFFFFFFFFFFFFFFFFFFFFFF
```

FFFFFFFFFFFFFFFFFFFFFFFFFFFFFFFFFFFFFFFFFFFFFFFFFFFFFFF:FFFFFFFFF:FF
FFFFFFFFFFFFFFF
@A00721:190:HGJW7DSXY:4:1311:4851:26396 1:N:0:TCTGCAAG+GCTGCACT
CCCCGCAGGGGCATCTTACCGCAGACCGTGGGGCAAGCCAATATAATATTACTGGCTTGCCCGCGCCG
GGGACACCTCCGTACGCTTACGTGTCGATTTGTCATTAGACCACGCGTCACGATCATAACCATCTAAA
AAATGAATTCTATAG
+
FFFFFFFFFFFFFFFFFFFFFFFFFFFFFFFFFFFFFFFFFFF:FFFFFFFFFFFFFFFFFFFFFFFFF
FFFFFFFFFFFFFFFFFFFFFFFFFFFFFFFFFFFFFFFFFFFFFFFFFFFFFFFFFFFFFFFFFFFFF
FFFFFFFFFFFF,FF
@A00721:190:HGJW7DSXY:4:1522:2410:36793 1:N:0:TCTGCAAG+GCTGCACT
CCCCGCAGGGGCATCTTACCGCAGACCGTGGGGCAAGCCAATATAATATTACTGGCTTGCCCGCGCCG
GGGACACCTCCGTACGCTTACGTGTCGATTTGTCATTAGACCACGCGTCACGATCATGACCATCTAAA
AAATGAATTCTATAG
+
FFFFFFFFFFFFFFFFFFFFFFFFFFFFFFFFFFFFFFFFFFFFFFFFFFFFFFFFFFFFFFFFFFFFF
FFFFFFFFFFFFFFFFFFFFFFFFFFFFFFFFFFFFFFFFFFFFFFFFFFFFFFFFFFFFFFFFFFFFF
FFFFFF,FF:F::FF
@A00721:190:HGJW7DSXY:4:1614:4833:35790 1:N:0:TCTGCAAG+GCTGCACT
CCCCGCAGGGGCATCTTACCGCAGACCGTGGGGCAAGCCAATATAATATTACTGGCTTGCCCGCGCCG
GGGACACCTCCGTACGCTTACGTGTCGATTTGTCATTAGACCACGCGTCACGATCATGACCATCTAAA
AAATGAATTCTATAG
+
FFFFFFFFFFFFFFFFFFFFFFFFFFFFFFFF:FFFFFFF::FFFFFFFFFFFFFFFFFFFFFFFFFFF
FFFFFFFFFFFFFF:FF:FFFFFFF:FFFFFFFFF,FFFF::FFFFFFFFFFFFFF:FFFFFFFFFFF
FFFFFFFFF:F:,FF
@A00721:190:HGJW7DSXY:4:1616:6894:26710 1:N:0:TCTGCAAG+GCTGCACT
CCCCGCAGGGGCATCTTACCGCAGACCGTGGGGCAAGCCAATATAATATTACTGGCTTGCCCGCGCCG
GGGACACCTCCGTACGCTTACGTGTCGATTTGTCATTAGACCACGCGTCACGATCATAACCATCTAAA
AAATGAATTCTATAG
+
FFFFFFFFFFFFFFFFFFFFFFFFFFFFFFFFFFFFFFFFFFFFFFFFFFFFFFFFFFFFFFFFFFFFF
FFFFFFFFFFFFFFFFFFFFFFFFFFFFFFFFFFFFFFFFFFFFFFFFFFFFFFFFFFFFFFFFFFFFF
FFFFFFFFFFF,FFF
@A00721:190:HGJW7DSXY:4:2123:21151:29168 1:N:0:TCTGCAAG+GCTGCACT
CCCCGCAGGGGCATCTTACCGCAGACCGTGGGGCAAGCCGATATAATATTACTGGCTTGCCCGCGCCG
GGGACACCTCCGTACGCTTACGTGTCGATTTGTCATTAGACCACGCGTCACGATCATGACCATCTAAA
AAATGAATTCTATAG
+
FFFFFFFFFFFFFFFFFFFFFFFFFFFFFFFFFFFFFFFF,FFFFFFFFFFFFFFFFFFFFFFFFFFFF
FFFFFFFFFFFFFFFFFFFFFFFFFFFFFFFFFFFFFFFFFFFFFFFFFFFFFFFFFFFFFFFFFF,FF
FFFFFFFFFFFFFFF
@A00721:190:HGJW7DSXY:4:2206:16794:32675 1:N:0:TCTGCAAG+GCTGCACT
CCCCGCAGGGGCATCTTACCGCAGACCGTGGGGCAAGCCAATATAATATTACTGGCTTGCCCGCGCCG
GGGACACCTCCGTACGCTTACGTGTCGATTTGTCATTAGACCACGCGTCACGATCATAACCATCTAAA
AAATGAATTCTATAG
+
FFFFFFFFFFFFFFFFFFFFFFFFFFFFFFFFFFFFFFFFFFFFFFFFFFFFFFFFFFFFFFFFFFFFF
FFFFFFFFFFFFFFFFFFFFFFFFFFFFFFFFFFFFFFFFFFFFFFFFFFF:FFF:FFFFFFFFFFF
FFFF:FFFFF:FFFF
@A00721:190:HGJW7DSXY:4:2209:17635:1438 1:N:0:TCTGCAAG+GCTGCACT
CCCCGCAGGGGCATCTTACCGCAGACCGTGGGGCAAGCCAATATAATATTACTGGCTTGCCCGCGCCG
GGGACACCTCCGTACGCTTACGTGTCGATTTGTCATTAGACCACGCGTCACGATCATGACCATCTAAA
AAATGAATTCTAT

+
FFFFFFFFFFFFFFFFFFFFFFFFFFFFFFFFFFFFFFFFFFFFFFFFFFFFFFFFFFFFFFFFFFFF
FFFFFFFFFFFFFFFFFFFFFFFFFFFFFFFFFFFFFFFFFFFFFFFFFFFFF:F:FFFFFFFFFFFF
FF:FFFFFFFFF:
@A00721:190:HGJW7DSXY:4:2209:16911:1783 1:N:0:TCTGCAAG+GCTGCACT
CCCCGCAGGGGCATCTTACCGCAGACCGTGGGGCAAGCCAATATAATATTACTGGCTTGCCCGCGCCG
GGGACACCTCCGTACGCTTACGTGTCGATTTGTCATTAGACCACGCGTCACGATCATGACCATCTAAA
AAATGAATTCTATAG
+
FFFFFFFFFFFFFFFFFFFFFFFFFFFFFFFFFFFFFFFFFFFFFFFFFFFFFFFFFFFFFFFFFFFF
FFFFFFFFFFFFFFFFFF,FFFFFFFFFFFFFFFFFFFFFFFFFFFFFFFFFFFFFFFFFFFFFFFFF
FFFFFFFFFFFFFFF
@A00721:190:HGJW7DSXY:4:2304:12084:33254 1:N:0:TCTGCAAG+GCTGCACT
CCCCGCAGGGGCATCTTACCGCAGACCGTGGGGCAAGCCAATATAATATTACTGGCTTGCCCGCGCCG
GGGACACCTCCGTACGCTTACGTGTCGATTTGTCATTAGACCACGCGTCACGATCATAACCATCTAAA
AAATGAATTCTATAG
+
FFFFFFFFFFFFFFFFFFFFFFFFFFFFFFFFFFFF:FFFFFFFFFFFFFFFFFFFFFFFFFFFFFFF
FFFFFFFFFFFFFFFFFFFFFFFFFFFFFFFFFFFFFFFFFFFFF:FFFFFFFFFFFFFFFFFFFFFF
FFFFFFFFFFFFFF:
@A00721:190:HGJW7DSXY:4:2509:10384:30686 1:N:0:TCTGCAAG+GCTGCACT
CCCCGCAGGGGCATCTTACCGCAGACCGTGGGGCAAGCCAATATAATATTACTGGCTTGCCCGCGCCG
GGGACACCTCCGTACGCTTACGTGTCGATTTGTCATTAGACCACGCGTCACGATCATAACCATCTAAA
AAATGAATTCTATAG
+
FFFFFFFFFFFFFFFFFFFFFFFFFFFFFFFFFFFFFFFF,FFFFFFFFFFFFFFFFFFFFFFFFFFF
FFFFFFFFFFFFFFFFFFFFFFFFFFFFFFFFFFFFFFFFFFFFFFFFFFFFFFFFFFFFFFFFF:FF
FFFFFF:FFF:FFFF
@A00721:190:HGJW7DSXY:4:2608:5484:6887 1:N:0:TCTGCAAG+GCTGCACT
CCCCGCAGGGGCATCTTACCGCAGACCGTGGGGCAAGCCAATATAATATTACTGGCTTGCCCGCGCCG
GGGACACCTCCGTACGCTTACGTGTCGATTTGTCATTAGACCACGCGTCACGATCATGACCATCTAAA
AAATGAATTCTATA
+
FFFFFFFFFFFFFFFFFFFFFFFFFFFFFFFFFFFFFFFFFFFFFFFFFFFFFFFFFFFFFFFFFFFF
FFFFFFFFFFFFFFFFF,FFFFFFFFFFFFFFFFFFFFFFFFFFFFFFFFFFFFFFFFFFFFFFFFFF
FFFFFFFFFFFF:F
@A00721:190:HGJW7DSXY:4:2647:25852:7028 1:N:0:TCTGCAAG+GCTGCACT
CCCCGCAGGGGCATCTTACCGCAGACCGTGGGGCAAGCCAATATAATATTACTGGCTTGCCCGCGCCG
GGGACACCTCCGTACGCTTACGTGTCGATTTGTCATTAGACCACGCGTCACGATCATGACCATCTAAA
AAATGAATTCTAGAG
+
FFFFFFFFFFFFFFFFFFFFFFFFFFFFFFFFFFFFFFFFFFFFFFFFFFFFFFFFFFFFFFFFFFFF
FFFFFFFFFFFFFFFFFFFFFFFFFFFFFFFFFFF,FFFFFFFFFFFFFFFF,FFFFFFFFFFFFFFF
FF:FFF,:FFFF,FF
@A00721:190:HGJW7DSXY:4:1123:28791:14653 1:N:0:TCTGCAAG+GCTGCACT
CCCGCAGGGGCATCTTACCGCAGACCGTGGGGCAAGCCAATATAATATTACTGGCTTGCCCGCGCCGG
GGACACCTCCGTACGCTTACGTGTCGATTTGTCATTAGACCACGCGTCACGATCATAACCATCTAAAA
AATGAATTCTATAGT
+
FFFFFFFFFFFFFFFFFFFFFFFFFFFFFFFFFFFFFFFFFFFFFFFFFFFFFFFFFFFFFFFFFFFF
FFFFFFFFFFFFFFFFFFFFFFFFFFFFFFFFFFFFFFFFFFFFFFFFFFFFFFFFFFFFFFFFFFFF
FFFFFFFFFFFF::F
@A00721:190:HGJW7DSXY:4:1367:31656:30514 1:N:0:TCTGCAAG+GCTGCACT
CCCGCAGGGGCATCTTACCGCAGACCGTGGGGCAAGCCAATATAATATTACTGGCTTGCCCGCGCCGG

GGACACCTCCGTACGCTTACGTGTCGATTTGTCATTAGACCACGCGTCACGATCATAACCATCTAAAA
AATGAATTCTATAGT
+
FFFFFFFFFFFFFFFFFFFFFFFFFFFFFFFFFFFFFFFFFFFFFFFFFFFFFFFFFFFFFFFFFFFFFF
FFFFFFFFFFFFFFFFFFFFFFFFFFFFFFFFFFFFFFFFFFFFFFFFFFFFFFFFF:FFFFFFFFFFF
FFFFFFFFFFF::FF
@A00721:190:HGJW7DSXY:4:1402:4146:21793 1:N:0:TCTGCAAG+GCTGCACT
CCCGCAGGGGCATCTTACCGCAGACCGTGGGGCAAGCCAATATAATATTACTGGCTTGCCCGCGCCGG
GGACACCTCCGTACGCTTACGTGTCGATTTGTCATTAGACCACGCGTCACGATCATAACCATCTAAAA
AATGAATTCTATAGT
+
FFFFFFFFFFFFFFFFFFFFFFFFFFFFFFFFFFFFFFFFFFFFFFFFFFFFFFFFFFFFFFFFFFFFFF
FFFFFFFFFFFFFFFFFFFFFFFFFFFFFFFFFFFFFFFFFFFFFFFFFFFFFFF,FFFFFFFFFFFFF
FFFFFF,FF,FFFFF
@A00721:190:HGJW7DSXY:4:1464:24804:36276 1:N:0:TCTGCAAG+GCTGCACT
CCCGCAGGGGCATCTTACCGCAGACCGTGGGGCAAGCCAATATAATATTACTGGCTTGCCCGCGCCGG
GGACACCTCCGTACGCTTACGTGTCGATTTGTCATTAGACCACGCGTCACGATCATAACCATCTAAAA
AATGAATTCTATAGT
+
FFFFFFFFFFFFFFFFFFFFFFFFFFFFFFFFFFFFFFFFFFFFFFFFFFFFFFFFFFFFFFFFFFFFFF
FFFFFFFFFFFFFFFFFFFFFFFFFFFFFFFFFFFFFFFFFFFFFFFFFFFFFFFFFFFFFFFFFF,FFF
FFFFFFFFFFFFFFF
@A00721:190:HGJW7DSXY:4:1470:32072:7811 1:N:0:TCTGCAAG+GCTGCACT
CCCGCAGGGGCATCTTACCGCAGACCGTGGGGCAAGCCAATATAATATTACTGGCTTGCCCGCGCCGG
GGACACCTCCGTACGCTTACGTGTCGATTTGTCATTAGACCACGCGTCACGATCATAACCATCTAAAA
AATGAATTCTATAGT
+
FFFFFFFFFFFFFFFFFFFFFFFFFFFFFFFFFFFFFFFFFFFFFFFFFFFFFFF:FFFFFFFFFFFF
FFFFFFFFFFFFFFFFFFFFFFFFFFFFFFFFFFF:FFFFFFFFFFFFFFFFFFFFFFFFFFFFFFFFFF
FFFFF,FF:FFFFFF
@A00721:190:HGJW7DSXY:4:1566:24053:9893 1:N:0:TCTGCAAG+GCTGCACT
CCCGCAGGGGCATCTTACCGCAGACCGTGGGGCAAGCCAATATAATATTACTGGCTTGCCCGCGCCGG
GGACACCTCCGTACGCTTACGTGTCGATTTGTCATTAGACCACGCGTCACGATCATAACCATCTAAAA
AATGAATTCTATAGT
+
FFFFFFFFFFFFFFFFFFFFFFFFFFFFFFFFFFFFFFFFFFFFFFFFFFFFFFFFFFFFFFFFFFFFFF
FFFFFFFFFFFFFFFFFFFFFFFFFFFFFFFFFFFFFFFFFFFFFFFFFFFFFFFFFFFFFFFFFFFFFF
FFFFFFFFFFFF:FF
@A00721:190:HGJW7DSXY:4:1667:12301:20948 1:N:0:TCTGCAAG+GCTGCACT
CCCGCAGGGGCATCTTACCGCAGACCGTGGGGCAAGCCAATATAATATTACTGGCTTGCCCGCGCCGG
GGACACCTCCGTACGCTTACGTGTCGATTTGTCATTAGACCACGCGTCACGATCATGACCATCTAAAA
AATGAATTCTATAG
+
FFFFFFFFFFFFFFFFFFFF,FFFFFF,,FFFFFFF:FF:,FFFFFFF,FFFFFF:,FFFF:FFFFFF
:FFFF:FFFFF,F:FF,F:FF:F:FFF:,FFFFF:FF,FFFFFFFFFFFFFFFFFFFF,FF:FFFFFF
FF:FFFF:FF:,F:
@A00721:190:HGJW7DSXY:4:2611:17237:2315 1:N:0:TCTGCAAG+GCTGCACT
CCCGCAGGGGCATCTTACCGCAGACCGTGGGGCAAGCCAATATAATATTACTGGCTTGCCCGCGCCGG
GGACACCTCCGTACGCTTACGTGTCGATTTGTCATTAGACCACGCGTCACGATCATAACCATCTAAAA
AATGAATTCTATAGT
+
FFFFFFFFFFFFFFFFFFFFFFFFFFFFFFFFFFFFFFFFFFFFFFFFFFFFFFFFFFFFFFFFFFFFFF
FFFFFFFFFFFFFFFFFFFFFFFFFFFFFFFFFFFFFFFFFFFFFFFFFFFFFFFFFFFFF:FFFFFFF
FFFFFFFFF:FF:FF

```
@A00721:190:HGJW7DSXY:4:1528:12816:22467 1:N:0:TCTGCAAG+GCTGCACT
CCGCAGGGGCATCTTACCGCAGACCGTGGGGCAAGCCAATATAATATTACTGGCTTGCCCGCGCCGGG
GACACCTCCGTACGCTTACGTGTCGATTTGTCATTAGACCACGCGTCACGATCATAACCATCTAAAAA
ATGAATTCTATAGTG
+
FFFFFFFFFFFFFFFFFFFFFFFFFFFFFFFFF:FFFFFFFFFFFFFFFFFFFFFFFFFFFFFFFFFF
FFFFFFFFFFFFFFFFFFFFFFFFFFFFFFFFFFFFFFFFFFFFFFFFFFFFFFFFFFFFFFFFFFFF
FFFF:FFFFFFFFFF
@A00721:190:HGJW7DSXY:4:2123:22923:15327 1:N:0:TCTGCAAG+GCTGCACT
CCGCAGGGGCATCTTACCGCAGACCGTGGGGCAAGCCAATATAATATTACTGGCTTGCCCGCGCCGGG
GACACCTCCGTACGCTTACGTGTCGATTTGTCATTAGACCACGCGTCACGATCATAACCATCTAAAAA
ATGAATTCTATAGTG
+
FFFFFFFFFFFFFFFFFFFFFFFFFFFFFFFFFFFFFFF:FFFFFFFFFFFFFFFFFFFFFFFFFFFF
FFFFFFFFFFFFFFFFFFFFFFFFF:F:FFFFFFFFFFFFFFFFFFFFFFFFFFFFFFFFFFFFFFFF
FFFFFFFFFF,FFFF
@A00721:190:HGJW7DSXY:4:2167:3106:31579 1:N:0:TCTGCAAG+GCTGCACT
CCGCAGGGGCATCTTACCGCAGACCGTGGGGCAAGCCAATATAATATTACTGGCTTGCCCGCGCCGGG
GACACCTCCGTACGCTTACGTGTCGATTTGTCATTAGACCACGCGTCACGATCATAACCATCTAAAAA
ATGAATTCTATAGTG
+
FFFFFFFFFFFFFFFFFFFFFFFFFFFFFFFFFFFFFFFFFFFFFFFFFFFFFFFFFFFFFFFFFFFF
FFFFFFFFFFFFFFFFFFFFFFFFFFFFFFFFFFFFFFFFFFFFFFFFFFFFFFFFFFFFFFFFFFFF
FFFFFFFFFF,FFFF
@A00721:190:HGJW7DSXY:4:1313:29785:22639 1:N:0:TCTGCAAG+GCTGCACT
CGTCACTATAGAATTCATTTTTTAGATGGTCATGATCGTGACGCGTGGTCTAATGACAAATCGACACG
TAAGCGTACGGAGGTGTCCCCGGCGCGGGCAAGCCAGTAATATTATATTGGCTTGCCCCACGGTCTGC
GGTAAGATGCCCCTG
+
FFFFFFFFFFFFFFFFFFFFFFFFFFFFFFFFFFFFFFFFFFFFFFFFFFFFFFFFFFFFFFFFFFFF
FFFFFFFFFFFFFFFFFFFFFFFFFFFFFFFFFFF,FFFF:FFFFFFFFFFFFF:FFFFFFFFFFFFF
FFFFFFFFFFFFFFF
@A00721:190:HGJW7DSXY:4:1424:21793:12085 1:N:0:TCTGCAAG+GCTGCACT
CGTCACTATAGAATTCATTTTTTAGATGGTCATGATCGTGACGCGTGGTCTAATGACAAATCGACACG
TAAGCGTACGGAGGTGTCCCCGGCGCGGGCAAGCCAGTAATATTATATTGGCTTGCCCCACGGTCTGC
GGTAAGATGCCCCTG
+
FFFFFFFFFFFFFFFFFFFFFFFFFFFFFFFFFFFFFFFFFFFFFFFFFFFFFFFFFFF:FFFFFFFF
FFFFFFFFFFFFF:FFFFFFFFFFFFFFFFFF,FF,FFFFFFFFFFFFFFFFFF:FFFFFFFFF,FF:
FFFFFF:FF,FFFFF
@A00721:190:HGJW7DSXY:4:1565:12780:26068 1:N:0:TCTGCAAG+GCTGCACT
CGTCACTATAGAATTCATTTTTCAGATGGTCATGATCGTGACGCGTGGTCTAATGACAAATCGACACG
TAAGCGTACGGAGGTGTCCCCGGCGCGGGCAAGCCAGTAATATTATATTGGCTTGCCCCACGGTCTGC
GGTAAGATGCCCCTG
+
FFFFFFFFFFFFFFFFFFFFFFFFFFFFFFFFFFFFFFFFFFFFFFFFFFFFFFFFFFFFFFFFFFFF
FFFFFF:FFFFFFFFFFFFFFFFFFFFFFFF:FFFF:FFFF,FFFFF:FFFFFFFFFFFF,FFFFFFFF
FFFFFF,F:FFFFFF
@A00721:190:HGJW7DSXY:4:2344:31873:27007 1:N:0:TCTGCAAG+GCTGCACT
CGTCACTATAGAATTCATTTTTTAGATGGTCATGATCGTGACGCGTGGTCTAATGACAAATCGACACG
TAAGCGTACGGAGGTGTCCCCGGCGCGGGCAAGCCAGTAATATTATATTGGCTTGCCCCACGGTCTGC
GGTAAGATGCCCCTG
+
FFFFFFFFFFFFFFFFFFFFFFFFFFFFFFFFFFFFFFFFFFFFFFFFFFFFF:FFFFFFFFFFFFFF
```

F:FFFFFFFFFFFFFFFFFFFFFFFFFFFFFFFFFFFFF:FFFFFFFFFF,:FFFF,FFFFFFFF:FF:
FFFFFFFFF:FFFFF
@A00721:190:HGJW7DSXY:4:2355:20971:13479 1:N:0:TCTGCAAG+GCTGCACT
CGTCACTATAGAATTCATTTTTTAGATGGTCATGATCGTGACGCGTGGTCTAATGACAAATCGACACG
TAAGCGTACGGAGGTGTCCCCGGCGCGGGCAAGCCAGTAATATTATATTGGCTTGCCCCACGGTCTGC
GGTAAGATGCCCCTG
+
FFFFFFFFFFFFFFFFFFFFFFFFFFFFFFFFFFFFFFFFFFFFFFFFFFFFFFFFFFFFFFFFFFFF
FFFFFFFFFFFFFFFFFFFFFFFFFFFFFFFFFFFFFF:FFFFFFFFFF:FFFFFFFFFFFFFFFFFF
FFFFF:FFFFFFFFF
@A00721:190:HGJW7DSXY:4:2167:4200:32847 1:N:0:TCTGCAAG+GCTGCACT
AGGGGCATCTTACCGCAGACCGTGGGGCAAGCCAATATAATATTACTGGCTTGCCCGCGCCGGGGACA
CCTCCGTACGCTTACGTGTCGATTTGTCATTAGACCACGCGTCACGATCATAACCATCTAAAAAATGA
ATTCTATAGTG
+
FF:F,:F,FFFF:FFFF,FFF:,FFFF::,,F:FFFFFFF,FF,FF:FFFFFF:FF:FF,F:,FFF:F
FFFF:FFFFFFF,FFFFFFFFF,F,FFFFFFF:FFFF:FF::FFFFFFFFFFFFFF,:FFFFFFFFF:
F,FF,FFF:FF
@A00721:190:HGJW7DSXY:4:1147:31837:24972 1:N:0:TCTGCAAG+GCTGCACT
ACCGCAGACCGTGGGGCAAGCCAATATAATATTACTGGCTTGCCCGCGCCGGGGACACCTCCGTACGC
TTACGTGTCGATTTGTCATTAGACCACGCGTCACGATCATAACCATCTAAAAAATGAATTCTATAGTG
ACGCAGTAGAATCGA
+
FFFFFF:FFFFF::FFFFF:FFFF:FFFF:FFFFFFF:FFF,FFFFFFFFF::FFFFFFFFFFFFFFF
:FFFFFFFFFFFFFFFFF:FF:FFFFFFFFFFFFFFFF:FFFFFFFFFFFFFFFFFFF,FF,,:FFFF
:::FF:FFF:FFFFF
@A00721:190:HGJW7DSXY:4:1147:31783:25160 1:N:0:TCTGCAAG+GCTGCACT
ACCGCAGACCGTGGGGCAAGCCAATATAATATTACTGGCTTGCCCGCGCCGGGGACACCTCCGTACGC
TTACGTGTCGATTTGTCATTAGACCACGCGTCACGATCATAACCATCTAAAAAATGAATTCTATAGTG
ACGCAGTAGAATCGA
+
FFFFFFFFFFFFFFFFFFFFFFFFFFFFFFFFFFFFFFFFFFFFFFFFFFFFFFFFFFFFFFFFFFFF
FFFFFFFFFFFFFFFFFFFFFFFFFFFFFFFFFFFFFFFFFFFFFFFFFFFFFFFFFFFFFFFFFFFF
FFFFFFFFFFFFFFF
@A00721:190:HGJW7DSXY:4:1526:22272:35462 1:N:0:TCTGCAAG+GCTGCACT
ACCGCAGACCGTGGGGCAAGCCAATATAATATTACTGGCTTGCCCGCGCCGGGGACACCTCCGTACGC
TTACGTGTCGATTTGTCATTAGACCACGCGTCACGATCATAACCATCTAAAAAATGAATTCTATAGTG
ACGCAGTAGAATCGA
+
FFFFFFFFFFFFFFFFFFFFFFFFFFFFFFFFFFFFFFFFFFFFFFFFFFFFFFFFFFFFFFFFFFFF
FFFFFFFFF:FFFFFFFFFFFFFFFFFFFFFFFFFFFFFFFFFFFFFFFFFFFFFFFFFFFFF:FFFF
FFFFFFFFFFFFFFF
@A00721:190:HGJW7DSXY:4:1620:30427:20337 1:N:0:TCTGCAAG+GCTGCACT
ACCGCAGACCGTGGGGCAAGCCAATATAATATTACTGGCTTGCCCGCGCCGGGGACACCTCCGTACGC
TTACGTGTCGATTTGTCATTAGACCACGCGTCACGATCATAACCATCTAAAAAATGAATTCTATAGTG
ACGCAGTAGAATCGA
+
FFFFFFFFFFFFFFFFFFFFFFFFFFFFFFFFFFFFFFFFFFFFFFFFFFFFFFFFFFFFFFFFFFFF
FFFFFFFFFFFFFFFFFF:FFFFFFFFFFFFFFFFFFFFFFFFFFFFFFFFFFFFFFFFFFFFFFFFF
FFFFFFFFFFFFFFF
@A00721:190:HGJW7DSXY:4:1110:23321:23406 1:N:0:TCTGCAAG+GCTGCACT
GCCGCAGACCGTGGGGCAAGCCAATATAATATTACTGGCTTGCCCGCGCCGGGGACACCTCCGTACGC
TTACGTGTCGATTTGTCATTAGACCACGCGTCACGATCATAACCATCTAAAAAATGAATTCTATAGTG
ACGCAGTAGAATCGA

```
+
FFFFFFFFFFFFFFFFFFFFFFFFFFFFFFFFFFFFFFFFFFFFFFFFFFFFFFFFFFFFFFFFFFFFFF
FFFFFFFFFFF:FFFFF:FFFFFFFFFFFFFFFFFFFFFF:FFFFF:FFFFFFFFFFFFFFFFF:FFFFF
FFFF,FFFFFFFFFF
@A00721:190:HGJW7DSXY:4:2320:20745:14027 1:N:0:TCTGCAAG+GCTGCACT
GCCGCAGACCGTGGGGCAAGCCAATATAATATTACTGGCTTGCCCGCGCCGGGACACCTCCGTACGC
TTACGTGTCGATTTGTCATTAGACCACGCGTCACGATCATAACCATCTAAAAAATGAATTCTATAGTG
ACGCAGTAGAATCGA
+
FFFFFFFFFFFFFFFFFFFFFFF:FFFFFFFFFFFFFFFFFFFFFFFFFFFFFFFFFFFFFFFFFFFFFF
FFFFFFFFFFFFFFFFFFFFFFFFFFFFFFFFFFFFFFFFFFFFFFFFFFFFFFFFFFFFFFFFF:FFFF
FFFFFFFFFFFFFFF
@A00721:190:HGJW7DSXY:4:2331:11668:20447 1:N:0:TCTGCAAG+GCTGCACT
GCCGCAGACCGTGGGGCAAGCCAATATAATATTACTGGCTTGCCCGCGCCGGGACACCTCCGTACGC
TTACGTGTCGATTTGTCATTAGACCACGCGTCACGATCATAACCATCTAAAAAATGAATTCTATAGTG
ACGCAGTAGAATCGA
+
FFFFFFFFFFFFFFFFFFFFFFFF:FFFFFFFFFFFFFFFFFFFFFFFFFFFFFFFFFFFFFFFFFFFFF
FFFFFFFFFFFFFFFFFFFFFFFFFFFFFFFFFFFFFFFFFFFFFFFFFFFFFF:FFFFFFFFFF:FFFF
FFFFFFFFFFFFFFF
@A00721:190:HGJW7DSXY:4:2472:18331:9377 1:N:0:TCTGCAAG+GCTGCACT
GCCGCAGACCGTGGGGCAAGCCAATATAATATTACTGGCTTGCCCGCGCCGGGACACCTCCGTACGC
TTACGTGTCGATTTGTCATTAGACCACGCGTCACGATCATAACCATCTAAAAAATGAATTCTATAGTG
ACGCAGTAGAATCGA
+
:FFFFFFFFFFFFFFFFFFFFFFFFFFFFF:FFFFFFFFFFFFFFFFFFFFFFFFFFFFFFFFFFFFFFF
FFFFFFFFFFFFFFFFFFFFFFFFFFFFFFFFFFFFFFFFFFFFFFFFFFFFFFFFFFFFFFFFF:FFFF
FF:FFFFFFFFFFFF
@A00721:190:HGJW7DSXY:4:2472:18285:9424 1:N:0:TCTGCAAG+GCTGCACT
GCCGCAGACCGTGGGGCAAGCCAATATAATATTACTGGCTTGCCCGCGCCGGGACACCTCCGTACGC
TTACGTGTCGATTTGTCATTAGACCACGCGTCACGATCATAACCATCTAAAAAATGAATTCTATAGTG
ACGCAGTAGAATCGA
+
FFFFFFFFFFFFFFFFFFFFFFF::FFFFFFFFFFFFFFFFFFFFFFFFFFFFFFFFFFF:FFFF:FFFF
FFFFFFFFFFFFFFFFFFFFFFFFFFFFFFFFFFFFFFFFFFFFFFFFFFFFFFFFFFFFFFFFFFFFFF
FFFFFFFFFFFFFFF
@A00721:190:HGJW7DSXY:4:1338:16685:19899 1:N:0:TCTGCAAG+GCTGCACT
CCGCAGACCGTGGGGCAAGCCAATATAATATTACTGGCTTGCCCGCGCCGGGACACCTCCGTACGCT
TACGTGTCGATTTGTCATTAGACCACGCGTCACGATCATGACCATCTAAAAAATGAATTCTATAGTGA
CGCAGTAAAATAGAT
+
FFFFFFFFFFFFFFFFFFFFFFFFFFFFFFFFFFFFFFFFFFFFFFFFFFFFFFFFFFFFFFFFFFFFFF
FFFFFFFFFFFFFFFFFFFFFFFFFFFFFFFFFFFFFFF:FFFFFFFFFFFFFF:FFFFFFFFFFFFFFF
FFFFFFFFFFFFFFF
@A00721:190:HGJW7DSXY:4:2424:28022:34679 1:N:0:TCTGCAAG+GCTGCACT
CCGCAGACCGTGGGGCAAGCCAATATAATATTACTGGCTTGCCCGCGCCGGGACACCTCCGTACGCT
TACGTGTCGATTTGTCATTAGACCACGCGTCACGATCATGACCATCTAAAAAATGAATTCTATAGTGA
CGCAGTAAAATAGAT
+
FFFFFFFFFFFFFFFFFFFFF:FFFFFFFFFFFFFFFFFFFFFFFFFFFFFFFFFFFFFFFFFFFFFFFF
FFFFFFFFFFFFFFFFFFFFFFFFFFFFFFFFFFFFFFFFFFFFFFFF,FFFFFFFFFFFFFFFFFFFFF
FFFFFFFFFFFFFFF
@A00721:190:HGJW7DSXY:4:2504:9290:10128 1:N:0:TCTGCAAG+GCTGCACT
CCGCAGACCGTGGGGCAAGCCAATATAATATTACTGGCTTGCCCGCGCCGGGACACCTCCGTACGCT
```

TACGTGTCGATTTGTCATTAGACCACGCGTCACGATCATGACCATCTAAAAAATGAATTCTATAGTGA
CGCAGTAAAATAGAT
+
FFFFFFFFFFFFFFFFFFFFFFFFFFFFFFFFFFFFFFFFFFFFFFFFFFFFFFFFFFFFFFFFFFFF
FFFFFFFFFFFFFFFFFFFFFFFFFFFFFFFFFFFFFFFFFFFFFFFFFFFFFFFFFFFFFFFFFFFF
FFFFFFFFFFFFFFF
@A00721:190:HGJW7DSXY:4:2522:31864:13088 1:N:0:TCTGCAAG+GCTGCACT
CCGCAGACCGTGGGGCAAGCCAATATAATATTACTGGCTTGCCCGCGCCGGGGACACCTCCGTACGCT
TACGTGTCGATTTGTCATTAGACCACGCGTCACGATCATGACCATCTAAAAAATGAATTCTATAGTGA
CGCAGTAAAATAGAT
+
FFFFFFFFFFFFFFFFFFFFFFFFFFFFFFFFFFFFFFFFFFFFFFFFFFFFFFFFFFFFFFFFFFFF
FFFFFFFFFFFFFFFFFFFFFFFFFFFFFFFFFFFFFFFFFFFFFFFFFFFFFFFFFFFFFFFFFFFF
FFFFFFFFFFFFFFF
@A00721:190:HGJW7DSXY:4:2573:15275:27414 1:N:0:TCTGCAAG+GCTGCACT
TATAAATTTTGAATTTCAAAATTGACCGTCGATTCTACTGCGTCACTATAGAATTCATTTTTTAGATG
GTTATGATCGTGACGCGTGGTCTAATGACAAATCGACACGTAAGCGTACGGAGGTGTCCCCGGCGCGG
GCAAGCCAGTAATAT
+
FFFFFFFFFFFFFF:FFFFFFFFFFFFFFFFF,FF:FF:FFFFFFFFFF:FF:FFFF:FFFFF:F:FF
FFFFFF:F:FFFFFFFFFFFFFFF,FFFF:FFFFFFFF:F:FFFF:FFFFFFFFF:FFFFFFFFFFFF
FFFFFFFF,FFFFFF
@A00721:190:HGJW7DSXY:4:2660:18973:4633 1:N:0:TCTGCAAG+GCTGCACT
TATAAATTTTGAATTTCAAAATTGACCGTCGATTCTACTGCGTCACTATAGAATTCATTTTTTAGATG
GTTATGATCGTGACGCGTGGTCTAATGACAAATCGACACGTAAGCGTACGGAGGTGTCCCCGGCGCGG
GCAAGCCAGTAATAT
+
FFFFFFFFFFFFFFFFFFFFFFFFFFFFFFFFFFFFFFFFFFFFFFFFF:F:FFFFFFFFFFFFF,FF
FFFFFF:FFFFFFFFFFFFFFFFFFFF:FFFFFFFFFFFFFFFFFFFFF:FFFF:FFFFFFFFFFFFF
F,,FFFF:FFFFFFF
