# Supplementary material for: Characterization of Spanish Olive Virome by High Throughput Sequencing Opens New Insights and Uncertainties
Source: Viruses. 2021 Nov 6;13(11):2233. doi: 10.3390/v13112233 (PMC8622421; doi:10.3390/v13112233)
Supplement: Supplementary file 1 [file reads OLyAV V64.pdf › reads OLyAV V64.pdf]

```
@A00721:190:HGJW7DSXY:4:1102:7916:34773_1:N:0:TCTGCAAG+GCTGCACT
CAGACGTGTGCTCTTCCGATCTCGCTAACTAGGTGAATTCCTGGCGAACCGCAAATCAATTGAAGAAA
ACCACTCCCTTCAATTCAATAACATAACATACCATACACTCCCCAAACATAACATACCATAACCTACC
ATG
+
FF::,:FFFFFF,FFFFFFF:FF:F,FFFF,FFF,F,FFF,FFFFFFFF:F:FFFF,F,FF,FF:FFF
FFFFFFFFFFFFFFFFFFFF:FFFFFFFFFFFFFFFFFFFFFFFFFFFFFFFFFFFFFFFFFFFFFFF
FFF
@A00721:190:HGJW7DSXY:4:1449:27751:18865_2:N:0:TCTGCAAG+GCTGCACT
GCTCCAACTGGCACGCCAGACGTAGGGTCAAACCTACTACCGCCGGTCGGCAGATCGGCACCACTGGT
CTCTACGTTCAGGGACCCGCTGTGCAGAATAACCTTCCCAAATCTAGA
+
FFFFFFFFFFFFFFFFFFFFFFFFFFFFFFFFFFFFFFFFFFFFFFFF,FFFFFFFFFFFFFFFFFFF
FFFFFFFFFFFFFFFFFFFFFFFFFFFFFFFFFFFFFFFFFFFFFFFF
@A00721:190:HGJW7DSXY:4:1239:2175:22420_2:N:0:TCTGCAAG+GCTGCACT
CTGAAGGTCGTTACCGGACGACCACTCAAAGGAAACGGGCTGTTGCCCAGAGGAAAGCAGCGACCACC
GAGGTCGCTCGTAACATTGACGGCAGTCCCGTCATCTCTGTGGGAACT
+
FFFFFFFFFFFFFFFFFFFFFFFFFFFFFFFFFFFFFFFFFFFFFFFFFFFFFFFFFFFFFFFFFFFF
FFFFFFFFFFFFFFFFFFFFFFFFFFFFFFFFFFFFFFFFFFFFF:FF
@A00721:190:HGJW7DSXY:4:1107:27489:25864_1:N:0:TCTGCAAG+GCTGCACT
GCTCTTCCGATCTATTCACCACTCCCATACCAATCAATGATGTGACTATACAGTTCGGCCATTTCAAG
AGTGGTCAATACGTCGTGGGAGGTACTGAAGCCTTCCCCTCCGCTGGCACGAAGAACACTCCCAAGCC
AAT
+
FFFFFFFFFFFFFF:FFFFFFFFFFFFFFFFFFFFFFFFFFFFFFFFFFFFFFFFFFFFFFFFFFFFF
FFFFFFFFF:FFFFFFFFFFFFFFFFFFFFFFFFFFFFFFFFFFFFFFFFFFFFFFFFFFFFFFFFFF
FFF
@A00721:190:HGJW7DSXY:4:1414:14751:34648_2:N:0:TCTGCAAG+GCTGCACT
TCTGACCAGAATGAAAAGGGGTTTACCAGGGTAGTCTCTAAGTCTACCAGGAAGAGGGGAAATGTCCA
CAAAACTGAGAAGAATAAAGGGAAAGGTCCATCAGGACCTAAGAAGGG
+
FFFFF:FF,FF,FFFFFFF,,F:FFF:F,,F,,:F:::FF:FF:FFF,F,,FFFFF:FFFFF,FFFF,
FFFFFFF:FFF::FFFF::FF:FFF::FF:FFFFF:FFFFFF::FF:F
@A00721:190:HGJW7DSXY:4:1332:10312:14873_1:N:0:TCTGCAAG+GCTGCACT
CTAATTCTGAAGGAAGATCTCCCACCATCTGGGGTTGGGAATAAGAAATCTGCACGGGCCAAAGAGGC
CGCTGCTACGAAGTCGCTGAAGGCGGCTTGGGTCATTAAGGGTAGCTGTAAAGTTGCGCCTGCCGACC
CCA
+
FFFF,FFFFFFFFFFFFFFFFFFFFFFFFFFFFFFFFFFFFFFFFFFF,FFFFFFFFFFFFFFFFFFF
FFFFFFFFFFFFFFFFFFFFFFFFFFFFFFFFFFFFFFFFFFFFFFFFFFFFFFFFFFFFFFFFFFFF
FFF
@A00721:190:HGJW7DSXY:4:1316:22137:7138_2:N:0:TCTGCAAG+GCTGCACT
GACTCTCTACAGTAGGTTCGTGCCTACTATGAGCAAATCTGTTGAATTCGTCACTGAGGACGAAGGTG
AGAGGACTTTTGGCTACTGGCGACAAACAGATCGGAAGAGCGTCGTGT
+
FFFFFFFFFFFFFFFFFFFFFFFFFFFFFFFF:FFFFFFFFFFFFFFFF:FFFFFFFFFFFF:FFFFFF
FFFFFFFFFFFFFFFFFFFFFFFFFFFFFFFFFFFFFFFFFFFFFFF:
@A00721:190:HGJW7DSXY:4:1158:6723:13980_1:N:0:TCTGCAAG+GCTGCACT
ATCTTGCAGAGTGACTGGAGTTCAGACGTGTGCTCTTCCGATCTTGTCAACGGCGTTTGGAGGATTTT
ATCCGCGCTTAACTATGGGGCGAAGATCCCCTATGGAGTCTTGAAGGACTTAGGTTGGCATATTTATG
CCG
+
FFF,FFFFFFFFFFFFFFFFFFFFFFFFFFFFFFFFFFFFFFFFFFFFFFFFFFFFFFFFFFFFFFFF
```

FFFFFFFFFFFFFFFFFFFFFFFFFFFFFFFFFFFFFFFFFFFFFFFFFFFFFFFFFFFFFFFFFFFF
FFF
@A00721:190:HGJW7DSXY:4:2309:2013:15499_1:N:0:TCTGCAAG+GCTGCACT
GCATGTCACCCTTAAGCACCGAGGTAAGCTCGGGACTTACAAGAGAGAGTGTGAGAGAGTCGCGGTCC
CAATATTTTCCCAGAAAGCGGGCGTTCTGACTGATGAGGTTAAAAC
+
FFFFFFFFF:F,FFFFFFFF:F,:FFFFF:F:FFFFFFFFF,FFFFFFFFFF:F:FFFFFFF,,,,F,
F,:,,::F,,FF:,,::,F,FF,,,:,F:,F:FFF,,FF:F:FFF:
@A00721:190:HGJW7DSXY:4:1166:32199:32612_2:N:0:TCTGCAAG+GCTGCACT
ATTCACTGGTTCTGGGAGGCTGCTTTCCTCCATAATTACAACCTCTCTTTAGAGAGCATTAGAAGGAA
GTTGGGCAAATTCCCACATATGGTAGACGTTGTCACACTTTTCTTGAA
+
FFFF,:FFFFFFFFFFFFFFFFFFFFFFFFFFFFFFFFFFFFFFFFFFFFFFFFFFFF:FFFFFFFF
FFFFF,FFFFF:FFFFFFFFFFFFFF:FFFFFFFFFF:FFFFFFFFFF
@A00721:190:HGJW7DSXY:4:1360:31675:18208_1:N:0:TCTGCAAG+GCTGCACT
TTCCTCCGTCATTGGGGATTTCGATGTGAGAAGGTGGAAGGTGTCAAGGGTCTTCTTCATGCTGATCT
TTCAGGACCTAATCACATCATGATTGATAGTTACGGCTCGTATCGAGTTGGAGGGAGGAAGTTCTCCG
AA
+
FFF,FFF,FFFFF:FFF::FFFF:FFF,FFFFFFF:FF:FFFFF,FFFFFF,FFFFF:FFF,FF:FFF
FFFFFFF,FFFFFFFF:FFFFFFFF::,F,:FF,F:,F:FFFFFFFFFFFFFFFFFFFFFFFF,FFFFF
F:
@A00721:190:HGJW7DSXY:4:1215:9236:30452_1:N:0:TCTGCAAG+GCTGCACT
GCTGTAACTGTCATTTCAGAGAGGGGAGCTCTGAAAAGGAAACTTCGTCAGAAGATGAGGAAGTCTAA
GAGGAGGAGTTTGAGGAAGGCTCTAGAGAAGATCTCACCACCTGGAGAAGGCAAGTGGAGAGTCCAGA
AGT
+
FFFFFFFFFFFFFFFFFFFFFFFFFFFFFFFFF:FFFFFFFFFFFFFFFFFFFFFFFFFFFFFFFFFF
FFFF,FFFFFFFFFFFFFFFFFFFFFFFFFFFFFFF,FFFFFFFFFFFFFFFFFFFFFFFFFFFFFFF
FFF
@A00721:190:HGJW7DSXY:4:1264:30418:17096_2:N:0:TCTGCAAG+GCTGCACT
GGATTATTTGAAGCAGGGGTTACCACTTCCTAAGGTGGATGAGGGAGGTTGTTTCTTTGGAGTTTTAG
ATAATCTTTTGAGAAGGGGTCATAGAGCCAGCAAGAGAAGCTTATATG
+
FFFFFFFFFFFFFFFFFFFFFFFFFFFFFFFFFFFFFFFFFFFFFFFFFFFFFFFFFFFFFFFFFFFF
FFFFFFFFFFFFFFFFFFFFFFFFFFFFFFFFFFFFFFFFFFFFFFFF
@A00721:190:HGJW7DSXY:4:1160:14778:14998_2:N:0:TCTGCAAG+GCTGCACT
CCTTGAGAAGACAAGCCACATTAAGATATAATTTAGGTCAACTTGGGGTTTCGAATGTCATTAGTGCC
TTACGATTGAAAGAGGTCCTGTTGAGTATCGACAGATCTTTCGATCTT
+
FFFFFFFFFFFFFFFFFFFFFFFFFFFFFFFFFFFFFFFFF,FFFFFFFFFFFFFFFFFFFFFFFFFF,
FFFFFFFFFFFFFFFFFFFF,:FFFFFFFFFFFFFF:FFFFFFFFFF
@A00721:190:HGJW7DSXY:4:1211:25003:32393_2:N:0:TCTGCAAG+GCTGCACT
TTAACGTGGCAGAATTCGGTAACTTACCATATTGTTCCTTCCGGGAAGTTGTGGGATTTAACTTTGAC
TCAACTACTCCGATCAGATCCGGATGGTTTGATTGGTATGGACTACTC
+
FFFFFFFFFFFFFFFFFFFFFFFFFFFFFFFFFFFFFFFFFFF,FF:FFFFFFFFFFFFFFFFFFF
FFFFFFFFFFFFFFFFFFFFFFFFF:FFF:FFFFFFFFFFFFFFFFFFF
@A00721:190:HGJW7DSXY:4:1249:16767:17628_1:N:0:TCTGCAAG+GCTGCACT
TAGTTGTCTAGGGTCATGGCAGATACTACCGATAAGGTGAAAAATATGCAGGTCAAAGATTCTGTAGC
TCTGAGGGCGGTTGAGGGAGCCGTTTTGGCCGACTTTAACTATTCTAAGAGTCTTTATGATTCAAAAC
CTA
+
FFFFFFFFFFFF:FFFFFFFFFFFFFFFFFFFFFFFFFFFFFFFFFFFFFFFFFFFFFFFFFFFFFFF

FFFFFFFFFFFFFFFFFFFFFFFFFFFFFFFFFFFFFFFFFFFFFFFFFFFFFFFFFFFFFFFFFFFF
FFF
@A00721:190:HGJW7DSXY:4:1152:18873:9690_2:N:0:TCTGCAAG+GCTGCACT
ACCTTACAAGGTTGAGGAGACTTTACAGCGGCTTATTGTCGCGCAGTTTCCAGAATTGCGGTTGAAGT
TCACCCATTCTATCTTGAATGCTCACGCCGTCGCAGCCATATCGAGAC
+
FFFFFFFFFFFFFFFFFFFFFFFFFFFFFFFFFFFFFFFFFFFFFFFFFFFFFF,FFFFFFFFFFFFF
FFFFFFFFFFFFFFFFFFFFFFFFFFFFFFFFFFFFFFF,FFFFF:FF
@A00721:190:HGJW7DSXY:4:1314:3947:36824_2:N:0:TCTGCAAG+GCTGCACT
AAAGTACAGATTTAGTGATTTAGATATTATTGATATTGGAGGCGATATTCGTTTTCATATCCAAAACT
CCTTGAATGGAGTACATATTTGCAACCCTATTCTTGATGTGAAAGATG
+
FFFFFFFFFFFFFFFFFFFFFFFFFFFFFFFFFFFFFFFFFFFFFFFFFFFFFFFFFFFFFFFFFFFF
FFFFFFFFFFFFFFFFFFFFFFFF:FFF:FFFFFFFFFFFFFFF:FF:
@A00721:190:HGJW7DSXY:4:1564:20808:3396_2:N:0:TCTGCAAG+GCTGCACT
AACATTGATTGGGAACGTACCTACTCACGAGACGTCATGAAAGCTATCGGAGGGAAGCAAAATGTTTC
GTTCTGTTACAAGAACGCTTTGGATTGTGACCATAAAGCCTCAGTTGC
+
FFFFFFF:FFFFFFFFFFFFFFFFFFFFFFFFFFFFFFFFFFFFFFFFFFFFFFFFFFFFFFFFFFFF
F:FFFFFFFFFFFFFFFFFFFFFFFFFFFFFFFFFFFFFFFFFFFFFF
@A00721:190:HGJW7DSXY:4:1369:16984:36793_2:N:0:TCTGCAAG+GCTGCACT
ACGATATCTCTCTTGATGATGTAGCCACTATAATGCTTAAGAGAGAGATTGATGTATGTCATTTCGTC
TTAGTTTGTCCCGGCGAATTATTGGACACGATGAAAAATGACATACCC
+
F:FF:FFFFFFFFFF:FFF:FFFFFFFFFF,FFFFF:FFFFFFFFFFF:FFFFF,FFFFFFFF:FFFF
FFFFF:FFFFFFF:FFF:F,FFFFFFF:FFFFFFFFF::FFFFFFF,F
@A00721:190:HGJW7DSXY:4:1209:19877:26772_1:N:0:TCTGCAAG+GCTGCACT
TCGTTATCCGTCATGGAGATGATGGCGCATCACTTGATTACTATATGCCCGAAGGATTGGGTTATTCT
CACAACAAAGATAAGATTTTATCTTTCTTCAAACACCCTGTTTTCCTTAAGGGCGGACAATTGTTCCA
TTG
+
FFFFFFFFFFFFFFFFFFFFFFFFFFFF:FFFFFFFFFFFFFFFF,FFFFFFFFFFFFFFFFFFFFFF
FFFFFFF,FFFFFFFFFF:FFFFFFFFFFFFFFFFFFFFFFFFFFFFFFFFFFFFFFFFFFFFF:FFF
FFF
@A00721:190:HGJW7DSXY:4:1234:6777:22028_2:N:0:TCTGCAAG+GCTGCACT
ATTGAGCATTAATGAGTTCCGAATAACTCGTAATAAATCTTTCTCTAGTGGACAATTCAGAATACCTA
TTTCCGTACCAAGAGCCACTGCCAATCTCATCGCTATACGCATACCTG
+
FFFFFFFFFFFFFFFFFFFFFFFFFFFFFFFFFFFFFFFFFFFFFFFFFFFFFFFFFFFFFFFFFFFF
FFFFFFFFFFFFFFFFFFFFFFFFFFFFFFFFFFFFFFFFF,FFFFFF
@A00721:190:HGJW7DSXY:4:1141:14660:10222_1:N:0:TCTGCAAG+GCTGCACT
GAGGTTCGTTCCTCAATAAAAATTTTTCTCAAGAGATTATGGTTGATAAAGTTTTCTTCATAAGGGCT
ATTCAGTACGTCATGGGGATCACTTCTTCGGTCAATGACAAAACCTTCGAATATGTTACGACATGGCT
TCG
+
FFFFFFFFFFFFFFFFFFFFFFFFFFF:FFFFFFFFFFFFFFFFFFFFFFFFFFFFFFFFFFFFFFFF
FFFFFFFFFFFFFFFFFFFFFFFFFFFFFFFFFFFFFFFFFFFFFFFFFFFFFFFFFFFFFFFFFFFF
FFF
@A00721:190:HGJW7DSXY:4:1258:2474:25191_2:N:0:TCTGCAAG+GCTGCACT
AGGATTGTCATCTCGGGGAAAGTGATTCACCAAGATGTTCATATAGATACGAGATACCTAGAGAAGGT
CGCAGCGATCTTACTCGTTATCGGAGCTAAGAAGAAGACTTTGGGTAC
+
FFFFFFFFFFFFFFFFFFFFFFFFF:FFFFFFFFFFFFFFFFFFFFFFFFFFFFFFFFFFFFFFFFFF
FFFFFFFFFFFFFFFFFFFFFFFFFFFFFFFFFFFFFFFF:FFFFFFF

@A00721:190:HGJW7DSXY:4:1111:27344:29684_2:N:0:TCTGCAAG+GCTGCACT
AAACTTAACGTTAGTACCGGAGGTGATCTCTCTGCTTCTGCGTTACTGAGAGAAAAGATTCAGGACGC
CTACGGTGACTTTATGGACAAGATGCAAGTTCTTGGGAAATACTTGTT
+
FF:FFFFFFFFFFFFFFFFFFFFFFFFFFFFFFFFFFFFFFFFFFFFFFFFFFFF:FFFFFFFFFFFF
FFFFFFFFFFFFF:FF:FFFFFFFFFFFFFFFFFFFFFFFFFFFFFFF
@A00721:190:HGJW7DSXY:4:1223:18403:30420_2:N:0:TCTGCAAG+GCTGCACT
TCGATTCTTTGTATAGGGAGTGTGAGAAGACAACGAATCTTGAAACTACGGTCCTTATCAAAGAAAAT
GGTGAACCGGTCTCTAAATCTTTTGAGATTGAGAAGATTGAAGAACAC
+
FFFFFFF:FFFFFFFFFFFFFF:FFFFFFFFFFFFFFFFFFF:FFFFFFFFFFFFFFFFFFFFFFFFF
FFFFFFFFFFFFFFFFFFFFFFFFFFFFF:FFFFFFFFFFFFF:FFFFF
@A00721:190:HGJW7DSXY:4:1232:14714:22091_2:N:0:TCTGCAAG+GCTGCACT
GCTCGGCGTTGTTCTTGAAAACCAAGTTAAGAAGGAGGCTGATCATGGAGTCTTTGTACCTTCTGTCA
AACTTCGAGGTTCAGCTACGAGAGGTGATCCCGGATGTGGATTGAAGG
+
FFFFFFF:FFFFF:FFFFFF:FFFFFFFFF:FFFFFFFFFFFFFF:FFFFFFFFFFFFFF:FFFFFF
FFFF:FFFFFFFFFFFFFFFFFFF:F:FFFFFFFFFFFFFFFF:FFFFF
@A00721:190:HGJW7DSXY:4:1316:14036:26584_2:N:0:TCTGCAAG+GCTGCACT
GGAGACTCCTTCTTTTTGAATGTTGTAAAGAATTGGGTTGAGAAGTGTGCTTTACAAGGAGTTAGACT
CTTCTTCAAGTTTTTAGATCTTCTTGATAATCTTAAAGAAAAATTTTC
+
FFFFFFFFFFFFFFFFFFFFFFFFFFFFFFFFFFFFFFFFFFFFFFFFFFFFFFFFFFFFFFFFFFFF
FFFFFFFF:FFFFFFFFFFFFFFFFFFFFFFFFFFFFFFFFFFFFFFF
@A00721:190:HGJW7DSXY:4:1339:10782:19194_1:N:0:TCTGCAAG+GCTGCACT
AGTCTGTACTTACATTCTTTACGCCAGATTCTTCATGGGGCGACGCTGTTTCTCTTGGTATTTCTGAA
ATAGTTTATCAGAGCGTCATTTTCCTTTGGGATCTTTCCTCTGGTAAGGGAATTATTAAATCATTCTT
CTC
+
FFFFFFFFFFFFFFFFFFFFFFFFFFFFFFFFFFFFFFFFFFFFFFFFFFFFFFFFFFFFFFFFFFFF
FFFFFFFFFFFFFFFFFFFFFFFFFFFFFFFFFFFFFFFFFFFFFFFFFFFFFFFFFFFFFFFFFFFF
FFF
@A00721:190:HGJW7DSXY:4:1220:7943:10895_2:N:0:TCTGCAAG+GCTGCACT
ATTATTACTGTTTTCTCTACCCATAACTTCTTTTTCAAAAATGCGGATGGGTCTACTGATGTTTTCCT
ACAGAGTGCAGTCGTTTCAACACTTATCACCACTTTTTAAAGGAAAAT
+
FFFFFFFFFFFFFFFFFFFFFFFFFFFFFFFFFFFFFFFFFFFFFFFFFFF:FFFFFFFFFFFFFFFF
FFFFFFFFFFFFFFFFF,FF,FF,FFF:FFFF:,FF:,:F::F:FFFF
@A00721:190:HGJW7DSXY:4:1238:2311:31046_1:N:0:TCTGCAAG+GCTGCACT
AAATACGTATGTCGGTGGGGTTCTCAAGGCTCTCATCGCCACTGGGATTCTAAGGGGTTCCATAAGAT
ATTATCTGACATTGTGGTTCAATGATCAGGATTCTTCTTTTGGTCCTTACGTCTTAAGTAATTTGGAT
GTT
+
FFFFFFFFFFFFFFFFFFFFFFFFFFFFFFFFFFFFFFFFFFFFFFFFFFFFFFFFFFFFFFFFFFFF
FFFFFFFFFFFFFFFFFFFFFFFFFFFFFFFFFFFFFFFFFFFFFFFFFFFFFFFFFFFFFFFFFFFF
FFF
@A00721:190:HGJW7DSXY:4:1138:5791:25770_2:N:0:TCTGCAAG+GCTGCACT
GTTCTTGTCACGATTCTTAAAAGAAGTTTATTAAAACTTCAGAGTCTCTTGGAAGTCCAGGTAGCTAA
TGTTCTTGCAATAGCCACAGACTTTCTGGGAAAAGAGACCTCTGTTGG
+
FFFFFFFFFFFFFFFFFFFFFFFFFFFFFFFFFFFFFFFFFFFFFFFFFFFFFFFFFFFFFFFFFFFF
FFFFFFFFFFFFFFFFFFFFFFFFFFFFFFFFFFFFFFFFFFFFFFFF
@A00721:190:HGJW7DSXY:4:1162:23963:11397_1:N:0:TCTGCAAG+GCTGCACT
GAGAATTACTCAGATTCAATCTGGCATCACTGAACGTCTACAACGAGTTAGGGAGTGGCTAGGTTTTA

```
AAAGATCCTTTGCACAAGCAATACCTTCCACTGAGTTCATCACCCAGAGTTTCGACGTCGCGGAAAAC
GAT
+
FFFFFFFFFFFFFFFFFF,FFFFFFFFFFFFFFFFFFFFFFFFFFFFFFFFFFFFFFFFFFFFFFFFF
FFFFFFFFFFFFFFFF:FF:FFFFFFFFFFFFFFFFFFFFFFFFFFFFFFFFFFFFFFFFFFFFFFFF
FFF
@A00721:190:HGJW7DSXY:4:1257:15908:18145_2:N:0:TCTGCAAG+GCTGCACT
CACTGTTCGAGAATCTTCTGATGCTGAATTTGAGGAAAATTTTTCGGAAGTTTTTGGTATAAATCCTG
ATGGCTCAGTACAGGGAGGAGATCGGAAGAGCGTCGTGTAGGGAAAGA
+
FFFFFFFFFFFFFFFFFFFFFFFFFFFFF:FFFFFFFFFFFFFFFFFFFFFFFFFFFFFFFFFFFFFF
FFFFFFFFFFFFFFFFFFFFFFFFFFFFFFFFFFFFFFF:FFFF,:FF:
@A00721:190:HGJW7DSXY:4:1107:17490:33348_1:N:0:TCTGCAAG+GCTGCACT
ATGGACAAAATCTGGGATCATAGGTATGTGTCACGCTACACTCGAAATATGGGCCCTCATAAGGAAGT
ATAGTTGGCGTCTTGTCGTTGACGTCATTACAGTCTGGAAGTTAGATCATTTGTTCAGGAAGATCTCT
ACA
+
FFFFFFFFFFFFFFFFFFFFFFFFFFFFFFFFFFFFFFFFFFFFFFFFFFFFFFFFFFFFFFFFFFFF
FFFFFFFFFF:FFFFFFFFFFFFFFFFFFFFFFFFFFFFFFFFFFFFFFFFFFFFFFFFFFFFFFFFF
FFF
@A00721:190:HGJW7DSXY:4:1267:28989:7232_1:N:0:TCTGCAAG+GCTGCACT
CTTTAGCAACTTTAGATAAATTCTTTCCGGGCTCAATGACCTTTGCTATGTACGTGGATCTAATCCTT
GGTGATCTGAATCTGACCATCGCAAAGAAGACTTTATTAGTTCATGAAGTTCTTACACTTCTCGCGAC
TAA
+
FFFFFFFFFFFFFFFFFFFFFFFFFFFFFFFFFFFFFFFFFFFFFFFFFFFFFFFFFFFFFFFFFFFF
FFFFFFFFFFFFFFFFFFFFFFFFFFFFFFFFFFFFFFFFFFFFFFFFF:FFFFFFFFFFFFFFFFFF
FFF
@A00721:190:HGJW7DSXY:4:1435:10276:35133_1:N:0:TCTGCAAG+GCTGCACT
GCTTTATCTTAAAGCATTACCACCAAATTTAGCACTTTTTGAATATGACAGAAGCTCGCCATATCAGA
TTAGAGACATGCCAGTTAGAGTTCTTCTAACCAGGACTGATGGTGAGATTCTTTTACCTTTTGGAGGT
CCA
+
FFFFFFFF:FFFFFFFFFFFF,FFFFFFFFFFFFFFFFFFFFFFFFFFFFFFFFFFFFFFFFFFFFFF
FFFFFFFFFFFFFFFFFFFFFFFFFFFFFFFFFFFFFFFFFFFFFFFFFFFFFFFFFFFFFFFFFFFF
FFF
@A00721:190:HGJW7DSXY:4:1261:22281:28369_2:N:0:TCTGCAAG+GCTGCACT
CCTAGTGTCTTTTCTAGAGTCCAAGGACGTTTCCGTCGGAGGATGACAGGTGGGAGACCGCTCAATAA
CACTCTATTGAATCCCTTTTCCACGATGAAAGCTCTTTATGAGGTGCT
+
FF:FFFFF:FFFFFFFFFFFFFFFFFFFFFFFFFFFFFFFFFFFFFFFFFFFFFFFFFFFFFFFFFFF
FFFFFFFFFFFFFFFFFFFFFFFFFFFFFFFFFFFF:FFFFF:FFF:F
@A00721:190:HGJW7DSXY:4:1447:29161:32424_1:N:0:TCTGCAAG+GCTGCACT
TCTTTGGTGATATCGCCTCTGTCCTTTCTCCTTTAATCGTGAGGTTACGAGAGTTTCGTACTTGGGTT
TATCGGTTGTATTTTGATAAATTTGTTGGAATCGATAATGATACTGACGAAGGTTCTATTACCACCCT
TGT
+
FFFFFFFFFFFFFFFFFFFFFFFFFFFFFFFFFFFFFFFFFFFFFFFFFFFFFFFFFFFFFFFFFFFF
FFFFFFFFFFFFFFFFFFFFFFFFFFFFFFFFFFFFFFFFFF:FFFFFFFFFFFFFFFFFFFFFFFFF
,FF
@A00721:190:HGJW7DSXY:4:1159:17680:14418_2:N:0:TCTGCAAG+GCTGCACT
GTGATGCTTTCTTAGAGGCTCGTCTAGCCGCTGAGATTCGTCTTACCTATTTACAATCTGTAGAGAGA
GAGGTTAATCAGAATTTGAGTGATAACGATTTAGTACTGGGAAATGGT
+
```

FFFFFFFFFFFFFFFFFFFFFFFFFFFFFFFFFFFFFFFFFFF:F:F:FFFFFFFFFFF:FFFFFFFF
FFFF:FFFFFFFF:FFFFFFFFFFFFFFFFFFFFFFFFFFFF:F,:FF
@A00721:190:HGJW7DSXY:4:1156:17264:36839_1:N:0:TCTGCAAG+GCTGCACT
TTTTCTTTCCCTCCCCTTTTTCGATTGGATTCGACGGGGTCTTCGTCTCACGACTAATTTGATAAGGT
TTTTCTTTTATGGGACTCTTGTTTGTACTTATGAAGGAGGTATTACTTCGGAAGTTCTTAATGTAGGT
CTC
+
FFFFFF,FFFFFF:F:FFF:FFFFFFFFFFFFFFFFFFFFFFF:FFFFFFFFFFFFFFFFFFFFFFFF
FFFFFFFFFF:FFFFFFFFFFFFFFFFFFFFFFFFFFFFF:FFFFFFFFFFFFFFFF:FFFFFF:FFF
FFF
@A00721:190:HGJW7DSXY:4:1401:3631:9784_2:N:0:TCTGCAAG+GCTGCACT
GGTTTATGTTTCGGTTTCAGCTGGATGGATCTTTTGATGGTATGTTTCACATTGCGTGATCACTTCCC
GTGGACTTGGGAAGAGATTAAACATAAAATCCAGCGATTCTATTATGT
+
FF:FFFFFFFFFFFFFFFFFFFFFFFFFFFFFFFFFFFFFFFFFFFFFFFFFFFFFFFFFFFFF:FFF
FFFFFFFFFFFFFFFFFFFFFFFFFFF:F:FFFFFFFFFFFFFFFFF:
@A00721:190:HGJW7DSXY:4:1150:16821:35102_2:N:0:TCTGCAAG+GCTGCACT
TCGATGATGTTCGGGCTGTGGGGATTAACTTCCCTCGTGAAACTTACGACGATGTTCTCGCTCGCATT
TCACAAGGATTACTAAGTCTGAGATCTGCCACTTCTAATTTTAGGAAA
+
FFFFFFFFFFFFFFFFFFFFFFFFFFFFFFFFFFFFFFFFFFFFFFFFFFFFFFFFFFFFFFFFFFFF
FFFFFFFFFFFFFFFFFFFFFFFFFFFFFFFFFF:FFFFFFFFFFFFF
@A00721:190:HGJW7DSXY:4:1127:11153:24001_2:N:0:TCTGCAAG+GCTGCACT
AGAAAGTTCATGAGGATGTCTTGGTCGAAAAATATCGCCGGGAACTTCGAATAGAGGATCCTGATGAC
ATTATTTCAACGGATACCGATCTCAGTTCTACCACAGAAACCGTCTCA
+
FFF:FFFFFFFFF:FF,FFFFF:FFFFFFFF:FFFFFF,FFFF::FFF,FFF,:F:FFFF:F:::FFF
FFFFFFFFFFF:FFFFFF:FFFFFFFFFFFFFFFFFF:FFF:FFFFFF
@A00721:190:HGJW7DSXY:4:1446:11758:30843_2:N:0:TCTGCAAG+GCTGCACT
ACTGATTCGGAATTAGAGAAAGACGACCTTCTTGTGGGGACTGAGACCTCTGAATTTTCAGGGTGCCC
TGATGAGGACGTTGAAAGGGATAATTTCAACTCTGAGACTTCTAAGAG
+
FFFFF,FFFFFFFFFFFFFFFFFFFFFFFFFFFFFFFFFFFFFFFFFFFFFFFFFFFFFFFFFFFFFF
FFF:FFFFFFFFFF:FFFFFFFFFFFFFFFFFFFFFFFFFFFFFFFFF
@A00721:190:HGJW7DSXY:4:1125:9073:17519_2:N:0:TCTGCAAG+GCTGCACT
ACCAGAAACGTAACAAGAAGAATAAAGGTAGGAAGGGGAGAAAACAGAACAAGCGAGCGGTTGAAGAG
AAAACTGATATGGTTGCGGCTGATCGTACCCCAATTCCTGCTAGAAAG
+
FFFFFFFFFFFFFFFFFFFFFFFFFFFFFFFFFFFFFFFFFFFFFFFFFFFFFFFFFFFFFFFFFFFF
FFFFFFFFFFFFFFFFFFFFFFFFFFFFFFFFFFFFFFFFF,FFFFFF
@A00721:190:HGJW7DSXY:4:1103:23231:6308_1:N:0:TCTGCAAG+GCTGCACT
ATACCACTGATCTTCCAGAGTTACGTAGTGGTCGTTCTTTTGGTGATTTTATCAAAGAGAAAGTTTCT
GCTAGTCGCGACGATAGTATTTATCGGAGTGATATTAACAAGTTAAACTTCGAACTGACCACTTACAA
GAG
+
F:FFFFFFFFFFFFFFFFFFFFFFFFFFF:FFFFFF:FFFFFFFF:FFFFFFFFFFFFFFFFFFFF:F
FFFFFFFFFFFFFF:FFFFFFFFFFFFFFFFFFFFFFFFFFFFFFFFFFFFFFFFFFFFFFFFFFFFFF
FFF
@A00721:190:HGJW7DSXY:4:1117:16957:34992_1:N:0:TCTGCAAG+GCTGCACT
GCAACATGGATCAGTTTTCCAAAAAGCTATTAATAGCAGTTGCGGGGCTATAATACCGCCTCCAGATT
ACTCTAAACTGAATCCACTCTCTTGGGATATAAATGAGTACATCTATAATAGAGTTTTGGAACTGTGT
AAT
+
FFFFFFFFFFFFFF,FFFFFFFFFFFFFFFFFFFFFFFF:FFFFFFF:FFFFFFFFFFFFFFFFFFFF

FFFFFFFFFF:FFFFFFFFFFFFFFFFFFFFFFFFFFFFFFFFFFFFFFFFF:FFFFFFFFFFFFFFF
FFF
@A00721:190:HGJW7DSXY:4:1240:8404:22435_1:N:0:TCTGCAAG+GCTGCACT
GGCTTCTCTAAAGAACAGCAACTTTCAACATGAATTAGTTATTGAAAGTCTTGACCCTCACGTCTCAC
TTTATCACCTTGGAGATGGTAAAGTCATTAACAAAGGAAATGTTAAGGCTGATGACTTAAATTTCGCA
TTC
+
FFFFFFFFFFFFFFFFFFFFFFFFFFFFFFFFFFFFFFFFFFFFFFFFFFFFFFFFFFFFFFFFFFFF
FFFFFFFFFFFFFFFFFFFFFFFFFFFFFFFFFFF:FFFFFFFFFFFFFFFFFFFFFFFFFFFFFFFF
FFF
@A00721:190:HGJW7DSXY:4:1244:15745:8375_2:N:0:TCTGCAAG+GCTGCACT
AGACCTTTCTTGGGGAGTAAAGAGGGTTTGAAAATTACGGAACCTTTCCTCGTTCTTCATGAAGATCT
TCGAGTGTTTTATAGTGAGTTATTCTTGCGAAGGTTCAAGTCGATTGA
+
FFFFF:FF:FFFFFFFFFF,FFFFFFFFFF:FFFFFFFFFFFFFFFFFFFFFFFF:FFFFFFFFFFFF
FFFFFFFFFFFFF:FFFFFFFFFFFFFFFFFFF:FFFFFFFFFFFFFF
@A00721:190:HGJW7DSXY:4:1221:1768:12696_1:N:0:TCTGCAAG+GCTGCACT
ACGAGATCTTGCAGAGTGACTGGAGTTCAGACGTGTGCTCTTCCGATCTGTGGAGGGAAGACCACTGA
ACTTATAGCGACTTTTGTCGAATTTTTCGCAAAAGGTCTTGAAGTACGTATTGTCACTGCTAATCGTA
ATT
+
FF,,FFFFFFFFFFFFFFFFFFFFFFFFFFFFFFFFFFFFFFFFFFFFFFFFFFFFFFFFFFFFFFFF
FFFFFFFF:FFFFFFFFFFFFF:FFFFFFFFFFFFFFFFFFFFFFFFFFFFFFFFFFFFFFFFFFFFF
F:F
@A00721:190:HGJW7DSXY:4:1247:6460:25332_1:N:0:TCTGCAAG+GCTGCACT
GTGACTAAAGTTTTGGCACTTCTAGTTCATCGCGGTATTTGTGAAATTAAGACAGACGTGAAAGTTAT
GATGAGTGATCGTATTAGAACAGCTGATTCTTATTGCATGAACGTCGCTGAGGTCAAACCTGTGGACG
TTC
+
FFFFFFFFFFFFFFFFFFFFFFFFFFFFFFFFF:FFFFFFFFFFFFFFFFFFFFFF:FFFFFFFFF:F
FFFFFFFFFFFFFFFFFFFFFFFFFFF:FFFFFFFFFFFFFFFFFFFFFFFFFFFFFFFFF:FFFFFF
F:F
@A00721:190:HGJW7DSXY:4:1151:13313:23453_2:N:0:TCTGCAAG+GCTGCACT
CTGGTCAGACCCTTTTTCTTACACATCTACTTAAGGCGGCTTATACCATAGGTTACGGTGATTCCAAT
CAAATCGGATTCATCAATCGTATTGATCTCCAAGTCGCAAACTACTTC
+
FFFFFFFFFFFFFFFFFFFFFFFFFFFFFFFFFFFFFFFFFFFFFFFFFFFFFFFFFFFFFFFFFFFF
FFFFFFFFFFFFFFFFFFFFFFFFFFFFFFFFFFFFFFFF:FFFFFFF
@A00721:190:HGJW7DSXY:4:1139:31503:9048_1:N:0:TCTGCAAG+GCTGCACT
GTGTGCTCTTCCGATCTTTCTTATCGAAGTCTCAGTTATCGCTGCCCGGCCGACGTTTGTCGCGAACT
CTCAGAGGTTTACGGCAGAAAAATTTCTCCTGCTTGTAATTCGAGAGAGGACACTATGGAAATTAGAC
CTA
+
FFFFFFFFFFFFFFFFFFFFFFFFFFFFFFFFFFFFFFFFFFFFFFFFFFFFFFFFFFFFFFFFFFFF
FFFFFFFFFFFFF:FFFFFFFFFFFFFFFFFFFFFFFFFFFFFFFFFFFFFFFFFFFFFFFFFFFFFF
FFF
@A00721:190:HGJW7DSXY:4:1104:5014:13777_2:N:0:TCTGCAAG+GCTGCACT
ATATTACTTTCACTCAAGGTGAAAAGTTTGAAATTAACAAAGTCCTTGGTAAGAGTAAGGTTAAATGT
GAAGATGCTCAGACTATTCATGAAGTTCAAGGTAATACTTACCAGCAC
+
FFFFFFFFFFFFFFFFFFFFFFFFFFF:FFFFFFFFFFFFFFF,FFFFFFFFFFFFFFFFFFFFFFFF
FFFFFFF:FFFFFFFFFFFFFFFFFFFFFFF:FFFFFFFFF,FFF:FF
@A00721:190:HGJW7DSXY:4:1538:22218:24158_2:N:0:TCTGCAAG+GCTGCACT
AAACCTCAAGATGACCCTGTTTTTGCATCACAAACACATATTACCGTTGCTCTATCACGACACGTTGA

```
ATCTTTACGATACTTTGTTATCAATGTAAAGTTAAACGATGCTACTTG
+
FFFFFFFFFFFFFFFFFFFFFFFFFFFFFFFFFFFFFFFFFFFFFFFFFFFFFFFFFFFFFFFFFFFFFF
FFFFFFFFFFFFFFFFFFFFFFFFFFFFFFFFFFFFFFFFFFFFFFFF
@A00721:190:HGJW7DSXY:4:1547:9263:14998_2:N:0:TCTGCAAG+GCTGCACT
TAAGGAACTGTCACGTTTAGCACGGGAGGTTGCAGATTCAACCCCTTATATCGAGGCTATCGAGGACG
ATGAGTATCTTGAAGTTGTTGACGACAAGCCTCAAGAGGGTAGAGGAC
+
FFFFFFFFFFFFFFFFFFFFFFFFFFFFFFFFFFFFFFFFFFFFFF:FFFFFFFFFFFFFFFFFFFFFFF
FFFFFFFFFFFFFFFFFFFFFFFFFFFFFFFFFFFFFFFFFFFFFFFF
@A00721:190:HGJW7DSXY:4:1205:13277:14528_2:N:0:TCTGCAAG+GCTGCACT
GGTTATTCCTGGAACCACCTTAACGTCACTCGGTAATCTTTCAGCCGAATTAAGTACTAATGAGTTCA
AGTCGGTTGTCGATCGAATTAAGATCAAGGCTAATACCTTTATAGGTA
+
FFFFFFFFFFFFFFFFFFFFFFFFFFFFFFFFFF:FFFFFFFFFFFFFFFFFFFFFFFFFFFFFFFFFFF
FFFFFFFFFFFFFFFFFFFFFFFFFFFFFFFFFFFFFFF::FFFFFFFF
@A00721:190:HGJW7DSXY:4:1108:26530:14967_2:N:0:TCTGCAAG+GCTGCACT
GCCGCGTAGTCAGCGCGTTTAGCTGTATCATCAGTCAGAGTATACCCGATAGGGTACCGAGTCTCCAA
GAGAATATTTATTCTTATGAGGCTCGAAACTTCAACGTTTTCACAACG
+
FFFFFFFFFFFFFFFFFF,FFFFFFFFFFFFFFFFFFF:FFFFFFFFFFFFFFFFFFFFFFFFFFFFFFF
FFFFFFFFFFFFFFFFFFFFFFFFFFFFFFFFFFFFFFFFFFFFFFFF
@A00721:190:HGJW7DSXY:4:1110:27100:14575_2:N:0:TCTGCAAG+GCTGCACT
TCGCACCCGAAATTTTTGGGCAGAATGCTCTTAACGCGGTTCTTGAACGGTGTTTTGATCAGTCTAAG
TTGGATAAAGTTATAGGAGACTCAGTTAACCTGAGAGCTCCTCAATTG
+
FFFFFFFFFF:FFFFFFFFFFFFFFFFFFFFFFFFFFFFFFFFFFFFFFFFFFFFFFFFFFFFFFFFFFF
FFFFFFFFFFFFFFFFFFFFFFFFFFFFFFFFFFFFFFFFFFFFFFFF
@A00721:190:HGJW7DSXY:4:1456:3513:30906_1:N:0:TCTGCAAG+GCTGCACT
AACAATAGGGACGCTAGTATACTTCGTAAAGTCGACAGCGAAATGGTCAATATTGATAATTGGACCGA
CTATATGACCTATTTTAAGCTCATGGTGAAAAGGGAAGCTAAGGTTAAACTTGATGCTTCAGCCTTTT
CGA
+
FFFFFFFFFFFFFFFFFFFFFFFFFFFFFFFF:FFFFFFFFFFFFFFFFFFFFFFFFFFFFFFFFFFFFF
FFFFFFFFFFFFFFFFFFFFF,FFFFFFFFFFFFFFFFFFFFFFFFFFFFFFFFFFFFFFFFFFFFFFFF
FFF
@A00721:190:HGJW7DSXY:4:1246:15754:6981_1:N:0:TCTGCAAG+GCTGCACT
AGAATATCGTTTATCACTCAAAATTTATTAATGCAATTTTCGGACCTATCTTTAAGGTTCTTGTGAGG
AGAGTCAAACAGTGTCTTTTACCCAACATTTTTATGTATACAGAGATGACCAGTGACGATTTCTCCCG
GAA
+
FFFFFFF,F,FFFFF:F,::FFFFFFFFFFFFFFF:FFFFFF:F:F:FFFFF:FFFFFF,FFFFFFFF
FFFFFFFFFFFFFFFFF:FF:FFFFFFFFFFFFF,FFFFFFFFFFFFFFFF:FFFF:FFF:FFFF:F:
FFF
@A00721:190:HGJW7DSXY:4:2333:14832:10081_1:N:0:TCTGCAAG+GCTGCACT
CTCTTAGGAGGTCATGATGACTACTATAAGGGTGAAATCGATTTTTCGAAGTTTGACAAATCTCAAGA
TTTCTTTTGTAAAGCCTATGAGATAAAGATGTATTCTTATTTAGGCTTCGATGCTGAATTTCTTGAAC
TTT
+
FFFFFFFFFFFF,FFFFFFFFFFFF:FFFFFFFFFFFFFFFFFFFFFFFFFF::FFFFFF:FFFFFFFFF
FFFF:FFFFFFFFFFFFFFFFFFFFFFFFFFFFFFFFFFFFFFFFFFFFFFFFFFFFFFFFF:FFFFFF
FFF
@A00721:190:HGJW7DSXY:4:1232:26060:6762_2:N:0:TCTGCAAG+GCTGCACT
TGTCACGCCAGGAGTATCCATAACGATTTGTCATTTACCACCTTTGCTCAACGACGAAGTGGAACGGC
```

CAACACTTTCTTTGGTAATTGTATGATCACTTTGATGATTTTATCACT
+
FFFFFFFFFFFFFFFFFFFFFFFFFFFFFFFFFFFFFFFFFFFFFFFFFFFFFFFFFFFFFFFFFFFFF
FFFFFFFFFFFFFFFFFFFFFFFFFFFFFFFFFFFFFFFFFFFFFFFF
@A00721:190:HGJW7DSXY:4:1136:17137:35556_1:N:0:TCTGCAAG+GCTGCACT
CTTGGAAAGTTTTCGGCTGTGGTTGTCGCTGGGGACGATTCCATTCTGTTTTCAAAGACTCCAATAAC
TGATCATTCTGATCATATGATTTTGGACAATGGTTTCGAAGCGAAATTTTTGAGGGATAAACCAGCGT
ACT
+
FFFFFFFFFFFFFFFFFFFFFFFFFFFFFFFFF:FFFFFFFFFFFFFFFFFFFFFFFFFFFFFFFFFFF
FFFFFF:FFFFFFFFFFFFFFFFFFFFFFFFFFFFFFFFFFFFFFFFFFFFFFFFFFFFFFFFFFFFFF
FFF
@A00721:190:HGJW7DSXY:4:1439:16532:23109_2:N:0:TCTGCAAG+GCTGCACT
CCTGATCCTTACAAGATCATCGTTAAGTTAGGTAAACCCCTAACCGATGTTACTCTTGTAGGACTTTG
GGAGAGATTCGTTAGTTTTAAAGACTATACTAAATATCTCCACTACGA
+
FFFFFFFFFFFFFFFFFFF,FF,FFFFFFFFFF:F:FFFFFFFFFF:FFFFFFFFFFFFFFFFFFFFF
FFFFFFF:FFFFFFFFFFFF:FFFFFFFFFFF:FFFFFFFFFFFFFFF
@A00721:190:HGJW7DSXY:4:1562:6017:21997_2:N:0:TCTGCAAG+GCTGCACT
CGATTAGCTCTTGCGACTAATATTCGATATTCGATTGAGGGTCGTTATACCTATTCGGCTATCTGTGC
TTTGAATTGTATAAGATCAAATTTTAAACGATTCCGAGAGGTTTATCC
+
FFFFFFFFFFFFFFFFFFFFFF:FFFFFFFFF:FFFFFFFFFFF,FFFFF:FFFFFFFFFFFFFFFF:F
FF:FFFFFFFFFFFFFFFFFFFFFFFF,F,F,FFFFFFFFFFF:FFFF:F
@A00721:190:HGJW7DSXY:4:1215:30807:8343_1:N:0:TCTGCAAG+GCTGCACT
GGAGTTCTTGAAGCATCCCTACCTTTCCTTGTGAAAATTTTGATGCGGTTTCATAAGATTATCAGTAA
TCCTGATTCTACTACGGATTCATGGACCTTCCATCCTGTACCTCTTCTGGAGAGACGTATCTCAAAGA
TCG
+
FFFFFFF:FFFFFFFFFFFFFFFF:FFFFFFFFFFFFFFFFFFFFFFFFFFFFFFFFFFFFFFFFFFF:
FFFFFFFFFFFFFFFFFFFFFFFFFFFFFFFFFFFFFFFFFFFFFFFFFFFFFFFFFFFFFFFFFFFFF
FFF
@A00721:190:HGJW7DSXY:4:1444:11966:28855_1:N:0:TCTGCAAG+GCTGCACT
GGTGATATTGTTGAGCGAGAGCGACCAAGATCTAAATTCGGTAACGGTGCGTGGCGCTTTATTTTAAA
AAGCTCCCTCCTCTCGCAAGTTTGATCTGATACTTATCACGAAAGGACTTGAGTCTTTTGATCAGTCT
CTG
+
FFFFFFF:FFFFFFFFFFFFF:FFFFFFFFFFFFFFFFFFFFFFFFFFFFFFFFFFFFFFFFFFFFFFF
FFFFFFFFFFFFFFFFFFFFFFFFFFFFFFFFFFFFFFFFFFFFFFFFFFFFFFFFFFFFFFFFFFFFF
FFF
@A00721:190:HGJW7DSXY:4:1335:4616:6355_2:N:0:TCTGCAAG+GCTGCACT
TAAATGACGATCATTAAGACTCTCTTTCTTCTTTGTTTCCTTCTTTGCACTGATTTTTCCTCATCAGC
TTGTGTTTTACCTAATCTGTGTTTTTACGATAACAACTATCAGTTGAT
+
FFFFFFFFFFFFFFFFFFFFFFF:FFFFF::F:FFFF:FFFFFF:FFFFFFFFFFFFFFF:FFFFFFFF
FFFFFFFFFFFFFFFFFFFFFFFFFFFFFFFFFFFFFFFFFFFFFFFF
@A00721:190:HGJW7DSXY:4:2249:9498:33035_1:N:0:TCTGCAAG+GCTGCACT
TTCCGATCTGAACTTTGGGAATTTGAAGCTCAGGGTGAAGCACAACGTTTGACCGTGACGAACTGTGG
TGTTCTTGATCTACGTTACGGATATACTCTCCTTGAGTTCACTCGTAAGGACACTACCACTTATTACG
AT
+
FF,FFFFFF:FF,FFFF,FFFFFFFFFFFFFFFFFFFFFFFF:FFF:FFFFFFFFFFF,FFFFFFFFFF
F:FFFFFFFFFFFFFFFFFFFFFFFFFFFFF:FFFFFFFFFFFFFFFFFFFFFFF:FFFFFFFFFFFFF:
FF

```
@A00721:190:HGJW7DSXY:4:1304:14642:16423_2:N:0:TCTGCAAG+GCTGCACT
CCTTTAAGCGTCTTCTGTTCATCGCAACCAGATGATATCTTCTATTCGAACTTATGTTTCGATCAGTG
TTTCTTCTATCAGGAGGAAAACACTTGTTCTAGTCCTTGTAAGATTAA
+
FFF:FFFF:FFFFFFF:FFFFFFFFFFFFFFFFFFFFFF:FFFFFF,:FFFFFFFFFFFF:FFFFFFF
FFFFFFFFF:,FF:FF,FFFF,F,F:FFF,FFFF::FFFFFFFFFFF:
@A00721:190:HGJW7DSXY:4:1329:6451:27727_2:N:0:TCTGCAAG+GCTGCACT
TGTTTACCACCGAAATATCAAGGTTCTTGTCCTTCAAACGATTGGAAGGTTAGTTATCTTAATAGTAC
TAAAGGGGTTTACTTACAACCCTATGACGATAGTTTCTCTTTACACAC
+
FFFFFFFFFFFFFFFFFFFFFFFFFFFFFFFFFFFF:FFFFFFFFFF:FFFFFFFFFFFFFFF:FFF
FFFFFFF:FFFFFFFFFFFF:FFFFFFFFFFFFFFFFFFFFFFFFFFFF
@A00721:190:HGJW7DSXY:4:1421:7581:33692_2:N:0:TCTGCAAG+GCTGCACT
TGTTTTCTTCTAATGTGGCCTGAACTACGTTTCACTTTGTGTGTACTTGTCTCTATTTTAAGTTTTGT
CTTCCTTGTTTGTCTTATTCTATTAATCACTCATTTTAGTGAGATTAT
+
FF:FF:FFFFFFFFFFF:FFFFFFFFFF:FFFFFFFFFFFFF:FFF:FFFFFFFF:FFFFFFFFFFFF
FFFF:F::FFFFFFFFFFFFFFFFFFFFFFFFFFFF:FF:F:FFFFFFF
@A00721:190:HGJW7DSXY:4:1116:25048:20729_2:N:0:TCTGCAAG+GCTGCACT
CTAAAACGTCTTCGACAACAGCAGGGGTTTTCGAATAGTGATAGCCACCCGCCTTACAGAGGGGCAGC
AGCATGACCATAATTGGTATTGACTTTGGTACCACCTTTTCTACTGTC
+
FFFFFFFFFFFFFFFFFFF:FFFFFFF:FFFFFFF:FFFFFFFFFFFFFFFFFFFFFFFFFFFFFFFF
FFFFFFFFFFFFFFFFFFFFFFFFFFFFFFFFFFFFF:FFFFF:FFFFF
@A00721:190:HGJW7DSXY:4:1123:31765:16391_1:N:0:TCTGCAAG+GCTGCACT
TTCGAATGAAGTTTTAATGCTTAACAACAATGATAGTGAATTTATACCTACAATCATTGGTTTTTCTA
ATAATGATGACACTATTGTTTATGGATATGATGCTATTACTCATGATGGTTACGGTCAGGGAGGATTC
TCA
+
FFFFFFFFFF,FFFFFFFFF:FFFFFFFFFF,FFFFFFFFFFFFFF:FFFFFFFFFF:FFFFFFFFFF
FFFFFFFF:FFFFFFFFF:FFFFFFFFFFFFFFFFFFFFFFFFFFFFF:FFFFF::FFFFFFFFFFFFF
FFF
@A00721:190:HGJW7DSXY:4:1443:25545:20776_1:N:0:TCTGCAAG+GCTGCACT
CTATGGATCGGGGTTAATTCTAAAACATTACCCGAAAGACGTGCTAAATTAAAACCATTGTACGATGT
CGTCTGTCTCGGCGATCACTTTTCACTAAGAATTGGTCCTACTTTTGGACAAGGCCAACTTCGTAATC
TTC
+
F:FFFFFFFFFFFFFFFFFFFFFFFFFFFFFFF:FFFFFFFFFFFFFFFFFFFFFFFFFFFFFFFFFF
FFFFFFFFFFFFFFFFFFFFFFFFFFFFFFFF:FFFFFFFFFFFFF:F:FFFFFFFFFFFF:FFFFFFF
FFF
@A00721:190:HGJW7DSXY:4:1435:20763:4069_2:N:0:TCTGCAAG+GCTGCACT
TCTTTATCTCTTTGGTTGTTAGGGATTTTGAGAGAGTGAAAGGCTTCAATTGTAGTGGTTTGGTCGTT
TCAGTACCCAGTCAGTATACCTCTACGCAAAGATATTTCATGAAAACT
+
FFFFFFFFFFFFFFFFFF:FFFFFFFFFFF:FFFFFFFFFFFFFFFFFFFFF:FFFFFFFFFFFFFFF
:FFFFFFFFFF:FFFFFFFFFFFFFF:FFFFFF:FFFFFFFFFFFFFF
@A00721:190:HGJW7DSXY:4:1111:15013:34569_1:N:0:TCTGCAAG+GCTGCACT
CCGGATTACCTGTCTTTCATATTATGAATGAACCTTCGGCCGCTCTCTTTGCTTCGATGTTGGATGTC
AAGAAGGGTTCAGAATGGGATTCCTACGTAGTTTATGACTTTGGTGGTGGTACTTTCGATACTTCTAT
CGT
+
F:FFFFFFFFFFFFFFFFFFFFFFFFFFFFFFFFFFFFFFFFFFFFFFF:FFFFFFFFFFFFFFFFFFF
FFFFFFFFFFFFFFFFFFFFFFFFFFFFFFFFFFFFFFFFFFF:FFFFFFFFFFFFFFFFFFFFFFFFFF
FFF
```

@A00721:190:HGJW7DSXY:4:1125:31358:13307_2:N:0:TCTGCAAG+GCTGCACT
TACTATAGTGTTATTTATTCTGGTGGTGATGATAATCTTGGAGGTCGTGACGTTGATGAAGCTATAAG
AGCGTTCTTATTACGGCGCACTTCTTTACCCGCCACCACACAACTTTC
+
:F,FFFFF:F:FFFFFF:FFFFF:FFFFFFFFFFFFFFFFFFFFFFFFFF:FFFFFFFFFFF:FFFFF
FFFFFFFFFFFF,F,,FFF,F,,FFF:FFFFF:FFFFFFFF:FFF:FF
@A00721:190:HGJW7DSXY:4:1152:24858:17394_1:N:0:TCTGCAAG+GCTGCACT
TGAAGGAGGAGGTTTCTGTTACTGGAAAACCCTCGTCAATACTTTTCAATAATATACGTCTTGAACTC
ACTTATGACGATCTAGTGAAATTAACGCGTCCTTTTCTGGAACGTAGTTTTACTATTATGAACAAAGT
TAT
+
FFFFFFFFFFFFFFFFFFFFFFFFFFFFFFFFFFFFFFFFFFFF:FFF:FFFF,FFFFFFFFFFFFFFF
FFFFFFFFFFFFFFFFFFFFFFFFFFFFFFFFFFFFFFFFFFFFFFFFFFFFFFFFFFFFFF:FFFFFF
FFF
@A00721:190:HGJW7DSXY:4:1353:27679:11036_2:N:0:TCTGCAAG+GCTGCACT
GCAAACTTACCCTGATTCCGGCCGGTGGTAGCTCATTTCTACCAGGGGCTATTACTCTTGCTGAAACA
GCTATTAAAGCTGTTGAGCGGGTAATTACACCTAAACGAGCTCGATCT
+
FFFFFFFFFFFF:FFF:FFFFF:FF:FFFFFFFFFFFFFFFFFFFFFFFFFFFF:F:FFFFF:FFFFFF
FFFFFFFFFFFFFFFFFFFFFFFFF:F:FFFFF:FFFFFFFFFFFFFFFF
@A00721:190:HGJW7DSXY:4:1139:20726:29403_1:N:0:TCTGCAAG+GCTGCACT
AAGGATGTGCACTCTTTTCTGCTACTTTAACGGAGCGTAGTGTACTCTTCGTCGATTGTATCAACAGT
AACATTACTTCTAATAAAGGTCTCTATCAAGCTCGTATTATCGTCGCTGCTGGATCTGCGTTACCGTT
GAA
+
FFFFFFFFFFFFFFFFFFFFFFFFFFFFFFFFFFFFFFFFFFFFFFFFFFFFFFFFFFFFFFFFFFFF
FFFFFFFFFFFFFFFFFFFFFFFFFFFFFFFFFFFFFFFFFFFFFFFFFFFFFFFFFFFFFFFFFFFF
FFF
@A00721:190:HGJW7DSXY:4:1241:7708:7639_2:N:0:TCTGCAAG+GCTGCACT
AGGTAATTATGAAGACTGTGATTTATCTTTCACTCTGGCCTTGTATGAGGGTGTTTCTAATCGTAACA
TTAATAATTTCAATCTTCTTAAAAAGAAGATAGAGAGTCATACTTTGG
+
FFFFFFFFFFFFFFFFFFFFFFFFFFFFFFFFFFFFF:FFFFFF,FFFFFFFFFFFFFFF:FFFFFFFF
FFFFFFFFFFFFFF:FFFFFFFFFFFFFFFFFFFFFFFFFFFF:FFFF:
@A00721:190:HGJW7DSXY:4:1136:28013:24612_2:N:0:TCTGCAAG+GCTGCACT
GATAGCCAATATTGAAGTGGACACCTCTGGTAGTATCGAAGTAAAGATAACCAATAAGGAAGGAGCTT
ATACTATGGTTGAATCAACTAAGCATACTACTTTTGCTTTGATTACTC
+
FFF:FFFFFFFFFFFFFFFFFFFFFFFFFFFFFFFFFFFFFFFFFFFFFFFFFFFFFFFFFFFFFFFF
FFFFFFFFFFFFF:F:FFFFFFF:FFFFF:FFFFFFFFFFFFFFFFF:
@A00721:190:HGJW7DSXY:4:1339:18385:29262_1:N:0:TCTGCAAG+GCTGCACT
CTTCCGATCTGTTAAAGAGTCGATTAAACAAGTTGTACTTATCAATGATTTTATCGCTGGTTGTTATC
TCGAAAAGTCTCTTCTAAAAGGAACGTCAGTTCTAACCCAAAGAGAATATCTAAAATATTTACAAAAT
CTT
+
FFFFFFFFFFFFFFFFFF,FFFFFFFFFFFFFFFFFFFFFFFFFFFF:FFFFFFFF:FF:FFFFFFFF:
FFFFFFFFFFF:FFFFFFFFFFFFFFFFFFFF:FFFFFFFFFFFFFFFFFFFFFFFFFFFFFFFFFFF
FFF
@A00721:190:HGJW7DSXY:4:2247:17119:9909_1:N:0:TCTGCAAG+GCTGCACT
ACGAGATCTTGCAGAGTGACTGGAGTTCAGACGTGTGCTCTTCCGATCTGTACAATCTTACTTGGAAC
GAAGGCGACCTGTTCCGGAGTTTTTACGGGATCCGGAATGTTCTGTCTATATTAGGTGAAGCGAAATA
CGA
+
FFF,FFFFF,FFFFFFFFFFFFFF:FFFFFFFFFFFFFFFFFFFFFFFFFFFFFFFFF,FFFFFFFFFF

```
FFFFFFFFFFFF:FFFFFFFFFFFFFFFFFFFFFFFFFFFFFFFFFFFFFFFFFFFF:FFFFFFFFF
FFF
@A00721:190:HGJW7DSXY:4:1257:7220:35039_1:N:0:TCTGCAAG+GCTGCACT
TCGTACCTTGATGCTGACTTTCGTCAATATGATTTGATCTGGTTGCTCAATTCTAAGCGTGTTCCGCT
TAGTGCGAGGCCTCTCTTCTACGAGTATTGCTATTTATACGGTTACGAGAAGAAGGTTCGAAAGTTTG
AGA
+
FFFFFF,FFFFFFFFF,FFFFFFFFFFFFFFFFFF:FFFFFF:FFFFFFFFFFFFFFFFFFFFFFFFF
FFFFFFFF:FFFFFF:FFFFFFFFFFFFFFFFFFFFFFFFFFFF:FFFFFFFFFFFFFFFF:FFFFF
FFF
@A00721:190:HGJW7DSXY:4:1147:15908:13667_2:N:0:TCTGCAAG+GCTGCACT
CTATACGTGACATACTAAATACTAGATATTTGGATGATCTTGATAAAGAACTTCATGAATTTTCAGAG
GATGAAAAATTTTCTTCCTTGGATTTTAATATAAAGAATCTTTACCAG
+
FFFFFFFFFFFFFFFFFFFFFFFFFFFFFFFFFFFFFFFFFFFFFFFFFFFFFFFFFFFFFFFFFFFF
FFFFFFFFFFFFFFFFFFFFFFFFFFFFFFFFFFFFFFFF:FFFFFF:FF
@A00721:190:HGJW7DSXY:4:1120:13548:22983_1:N:0:TCTGCAAG+GCTGCACT
AGGAGGCAGTCTTCTGATCGTAAGAAGTTTATAAGGAACATATCCATTTGTTGTAATAGGGTCATCAA
CTTAGAACTCGAAACTCTAAGTGGTAATACTGTCTTTCCAAGCTTACCTGTATCTTCAATGAAATTGG
ACT
+
:FFFFFFFFFF:FFFFFFFFFFFFFF:FFF:FFFFF,FFF:FFFFFFFFFFFFFFFFFFFFFFFFFFF
FFFFFFFFFFFFFFFFFFFFFFFFFFFFFFFFFFFFFFFFFFFFFFFFFFFFFFFFFFFFFFFFFFFF
FFF
@A00721:190:HGJW7DSXY:4:1155:4038:20071_2:N:0:TCTGCAAG+GCTGCACT
GTGGGCTTTCCGTAAAGGAAGATCTGTTATCGACGGTTTATCGTTTACGAAGTATCACTCCACCGGTG
AATGGTTTAAGAATACTATAAATAGAATAGCTATTACTACTAATGGCG
+
FFFFFFFFFFFFFFFFFFFFFFFFFFFFFFFFFFFFFFFFFFFFFF:FFFFFFFFFFFFFFFFFFFFF
FFFFFFFFFFFFFFFFFFFFFFFFFFFFFFFFFFFFFFFFFFFFFFFF
@A00721:190:HGJW7DSXY:4:1176:20410:6339_2:N:0:TCTGCAAG+GCTGCACT
TAGTGACATCTGGTCAATTTCGTTTTTAGGTGGTCTTTATTCTATTATTGATTTTTATGAGATTGATA
CGTTAACAGAAGATGATGTTAGAGTAGAATTCCTGAAGAGTCCTTCCG
+
FFFFFFFFFFFFFFFFFFFFFFFFFFFFFFFFFFFFFFFFFFFFFFFF:FFFF:FFFFFFFFFFFFFF
FFFFFFFFFFFF:FFFFFFFFFFFFF:FFFFFFF:FFFFFFFFF:FFF
@A00721:190:HGJW7DSXY:4:1638:27082:16767_1:N:0:TCTGCAAG+GCTGCACT
TAATAGTTAAATCTAGGATTATGTCTGTGTTGAAAGACTTTCTACCGATAAGGGTCACTAGTCTTACA
CAATTAAGAAATAATCCTACATTGACTGATTATCTAAAGATTGAAGGAAAATTTTATCCTCGTTTAAC
TTA
+
FFFFFFFFFFFFFFFFFFFFFFFFFFFFFFFFFFFFFFFFFFFFFFFFFFFFFFFFFFFFFFFFFFFF
FFFFFFFFFFFFFFFFFFFFFFFFFFFFFFFFFFFFFFFFFFFFFFFFFFFFFFFFFFFFFFFFFFFF
:FF
@A00721:190:HGJW7DSXY:4:1136:10800:22576_1:N:0:TCTGCAAG+GCTGCACT
GTGCTCTTCCGATCTATAACTTCTAATTTACGTTTGCTACTCAGAGCTGGTCCTCTCAGCAAAGGGAT
GCATGGTGTTATCTCCTCAGGAGTCCCGGCATTTGATAATAATGACGAGACTTTCGTCATAATCGCTG
CAT
+
FFFFFFFFFFFFF:FFFFFFFFFFFFFFFFFFFF:FFFFFFFFFFFFFFFFFFFFFFFFFFFFFFFFFF
FFFFFFFFFFFFFFFFFFFFFFFFFFFFFFFFFFFFFFFFFFFFFFFFFFFFFFFFFFFFFFFFFFFF
FFF
@A00721:190:HGJW7DSXY:4:1144:22770:15186_1:N:0:TCTGCAAG+GCTGCACT
GGTACCAGCGCATCTCGTGTCGAGCCTCGACCCGATTATATGGTATTAACTTATAAAGGTGAGGAGAC
```

```
CATTTTCTTGCTGAAATTTATAGAAGATTATTTTGACAGAATCAATAAGAATCATCGATTCTCGATTC
GTA
+
FFFFFFFFFFFFFFFFFFFFFFFFFFF:FFFF:FFFFFFFFFFFFFFFFFFFFFFFFFFFFFFFFFFF
FFFFFFFFFFFFFFFFFFFFFFFFFFFFFFFFFFFFFFFFFFFFFFFFFFFFFFFFFFFFFFFFFFFF
FFF
@A00721:190:HGJW7DSXY:4:2261:30942:7921_1:N:0:TCTGCAAG+GCTGCACT
TACATTTATGCTGATCTAAAATTTCGATCTAGATGGATCCACTCCTCGAGTTATCATGATGTTAGTAT
GTTATATACTGATTTACAACGATATAGGAGTAATCTACGAGAAGATTCTAGAATTCTTAATTATAATA
TT
+
FFFFFFFFFFFFFFFFFFFFFFFFFFFFFFFFFFFFFFFFFFFFFFFFFFFFFFFFFFFFFFFFFFFF
FFFFFFFFFFFFFFFFFFFFFFFFFFFFFFFFFFFFFFFFF::FFFFFFFFFFFFFFFFFFFFFFFFF
FF
@A00721:190:HGJW7DSXY:4:1145:21522:18161_2:N:0:TCTGCAAG+GCTGCACT
GAGATCACACATTTTGAGAACTAACTATGTTATGTTCTCTGATCATTTTGACAATCTCTTCTTATAAT
AAATAAATAAATAATATTACTAATGGCAAATAGTGGAAAGGCTCCAGT
+
FFFFFFFFFFF:FFFFFFFFFFFFFFFFFFFFFFFFFFFFFFFFFFFFFFFFFFFFFFFFFFFFFFFF
FFFFFFFFFFFFFFFFFFFFFFFFFFFFFFFFFFFFFFFFFFFFFFFF
@A00721:190:HGJW7DSXY:4:1253:14714:28980_2:N:0:TCTGCAAG+GCTGCACT
CTGCTTTGGATTACGATGACGTGAGAATAGGTGAAGGGATAGTTTTTACCTCGACCAACATTAAGGCG
GCGGCGACTAATCTTAAAGCTTCACCCAAAATGAAGGACACTTCGAAA
+
FFFFFFFFFFFFFFFFFFFFFFFFFFFFFFFFF:FFFFFFFFFF:FFFFFFFFFFFFFFFFFFFFFFF
FFFFFFFFFFFFFFFFFFFFFFFFFFFFFFFFFFFFFFFFFFFFFFFF
@A00721:190:HGJW7DSXY:4:1169:13169:23672_2:N:0:TCTGCAAG+GCTGCACT
AAAAGGCTCTAAAGGACACTATACAATATCCAGACGACGACGAAGCTTATACTACTTTAATTTTGGGT
ATTATGTGGAGATATCGTGATATCAGTACGTCCAGATCCTCTGCTTTT
+
FFF:FFFFFFFFFFFFFFFFFFFFFFFFFFFFFFFFFFFFFFFFFFFFFFFFFFFFFFFFFFF:FFFF
FFFFFFFFFFFFFFFFFFFFFFFFFFFFFFFFFFFFFFFFFFFFFFFF
@A00721:190:HGJW7DSXY:4:1155:25644:27586_2:N:0:TCTGCAAG+GCTGCACT
ATCAAATATAAAGAAAAGGACTATGAAATAAAAGATTTGGTGCTTAGGGATATAATGAATTCAGCACC
ACATTTTGGTCAAGTCAATCCGTTGAGAAAATTTCTTAGAGGATATTC
+
FFFFFFFFFFFFFFFFFFFFFFFFFFFFFFFFFFFFFFFFFFFFFFFFFFFFFFFFFFFFFFFFFFFF
FFFFFFFFFFFFFFFFFFFF:FFFFFFFFFFFFFFFFFFFFFFFFFFF
@A00721:190:HGJW7DSXY:4:1136:4237:11397_1:N:0:TCTGCAAG+GCTGCACT
CCAACATTTACCACCAAATTCTTTTGATACAACTCTAGCCTTCAAGTGGGGTATGCCTGCGGAATACG
CTTGCTATTCACCAGATTGGTTCGAACCCGTTGATGCTATGTCAGAAGAAGCTATTCAAGCTTGTCGT
TTA
+
F:F:FFFFF,,FFFFF:FFFFFF:FFFFF,FFFFFFFFFF:,F:FFFFFFFFFFFFFFFFF,FFFFFF
:FFFFFFFFFFFFFFFFFF:FFFFF:::F,,:FFFFFF:FFFFFFF:FFFFFFFFF:FF,,FFFF::F
FFF
@A00721:190:HGJW7DSXY:4:1130:11315:14418_2:N:0:TCTGCAAG+GCTGCACT
TCAAACAAGCCCAGACTCAAGGAGTCCAATCAAAGGTTTTGAATTTAGCTCAACTGGGGAGACGTGCT
TAAATGGGTGATAAAAATTATTTAGAGATCTTTTCTACTTACAAAGGA
+
FFFF:FFFFFFFFFFFFFFFFFFFFFFFFFFFF:FFFFFFFFFFFFFFFFFFFFFFFFFFFFFFFFFF
FFF:FFFFFFFFFFFFFFFFFFFFFFFFFFFFFFFFFFFFFFFFFFFF:FFF
@A00721:190:HGJW7DSXY:4:1437:12753:20415_2:N:0:TCTGCAAG+GCTGCACT
AATTCTACGGAAAAGTCCGGAGTATATTGTATGTTAATAGATAGTGGGATAATAGTTCTACCGATACG
```

```
TAGTGAACGCGTTCGTAGAACCAAGTACTTTCCGTCTGAAGAGTTTAG
+
FFFFFFFFFFFFFFFFFFFFFFFFFFFFFFFFF:FFFFFFF,,FFFFFFFFFFFFFFFFFFFFFFFFF
:FFFFFFFFFFFFFFFFFFFFFFFFFFFFFFFFFFFFFF:FFFF,FFF
@A00721:190:HGJW7DSXY:4:1352:21703:4601_1:N:0:TCTGCAAG+GCTGCACT
TAACCGATAGGAGTTGTATAGTTGAAGATGTGGCTTTAGACGATAATCCTTTAAGGACTATGTTGACT
GACGAAGATTTTCCCACAGATTTGTCTACGTTGCAGATCGCCACTCCAAACACTATATACATTAAAAT
ATA
+
FFFFFFFFFFFF:FF,FFFFFFF:FFFFFFFFF:FFFFFFFFFFFFFF:FFFFFFFF,FFFFFFFFFF
FFFFFFFFFFFFFFFFFFFFFFFFFFFFFFFFFFFFFFFFFFFFFFFFFFFFFFFFFFFFFFFFFFFF
FFF
@A00721:190:HGJW7DSXY:4:1566:12301:1564_2:N:0:TCTGCAAG+GCTGCACT
AGTCATAACTTTTGTTACCAAATTCTTGGCTTTCAACGAAGTCTCTGATTTTATTAATCATTACTTTA
ACGATCTCTCGGTCATTTACTCTGTACTTGATCTACGTCGTTTAACTG
+
FFFFFFFFFFFFFFFFFFFFFFFFFFFFFFFFFFFF:FFFFFFFFFFFFFFFFFFFFFFFFFFFFFFF
FFFFFFFFFF:FFFFFFFFFFFFFFFFFFFFFFFFFF:FFFF:FFFFFF
@A00721:190:HGJW7DSXY:4:1147:31430:22670_2:N:0:TCTGCAAG+GCTGCACT
CTTAGAATGACTCTATCTTTTCAGACACTTCTTATATTATTTACAATAGTTAATATCATATTAGTAAT
ACTCACTATCGTTTTCTGCTTTTTCTTAGCTAATAGATTAATAAAGGA
+
FFFFFFFFFFFFFFFFFFFFFFFFFFFFFFFFFFFFFFFFFFFF:FFFFFFFF:FFFFFFFFFFFFFF
FFFFFFFFFFFFFFFFFFFFFFFFF:FFFFFFFFFFFFFFFFFFFF:FF
@A00721:190:HGJW7DSXY:4:1325:4408:23750_2:N:0:TCTGCAAG+GCTGCACT
ACCAACTCTTCGTTACGAGACCTGATGGTAGAATAGATTTAGTCTCTACACCCATCTCAACGAGGATA
AAGCGAGTTTTCCGTCCTGAACGTAATACACGAAGAGATCATCCGCAA
+
FFFFFFFFFFFFFFFFFFFFFFFFFFFFFFFFFFFFFFFFFFFFFFFFFFFFFFFFFFFFFFFFFFFF
FFFFFFFFFFFFFFFFFFFFFFFFFFFFFFFFFFFFFFFFFFFFFFFF
@A00721:190:HGJW7DSXY:4:2344:31928:32863_2:N:0:TCTGCAAG+GCTGCACT
ATAAAAGTCCCTTAAAAATTTAGATTATTAATACTTCCTTTAATCAATTTATAGAAGTTATTTATTTT
GTACTTTTTATAATTTATAAACATAATATAAATTTTGGTATTTTTTTT
+
::,FFF,F:F::F,,:F,,F:,,FFFFF,:F,,:,,F:,FF:,,:F,FFF:FF,,F,,:::FF,,,FF
,F,:FFFFF,FFF:FF,F:FFF,F,,F:F,,,FFFF,:FFF:FFFF,F
@A00721:190:HGJW7DSXY:4:1212:27896:17049_2:N:0:TCTGCAAG+GCTGCACT
GAAGACATTATCTCTTATGTACTTATTGTAACGATCATTCTTTCGAGTTTTGTCATTCTTATTCTTTT
ATTCCTTTAACTGTGTATGAGGAGGATTTATAATGCTTTTCGATAGTG
+
FFFFFFF,FF:FFFFFFFFFFFFFFFFFFFFFFFFFFFFFFFFFFFFFFFFFFFFFFFFFFFFFFFFF
FFFFF,FFFFFFFFF:F:F,FFFFFFFFFFFFFFFFFFFFF:FFFFFF
@A00721:190:HGJW7DSXY:4:1565:19244:24862_2:N:0:TCTGCAAG+GCTGCACT
ATTACTGTTTCAAATCATCTTTACTCACAGACTCCGAAAATGGTTTCAAATGAGGTCAAAGCTCTATT
TATTAAACGAACTATTATCCGATTCTTCGAGGGTATCACTACCACTGA
+
FFFFFFF::FFFFFFFFFFFF,FF:FFFF:FFFFFFFFFFF::FFF:FFFFFFFF,,FFFF:F,FF:F
FFF:FFFFFFF:FF::F:F::FFFFF,FFFF,FFF,FFFFF:,FFFFF
@A00721:190:HGJW7DSXY:4:1302:10682:14481_2:N:0:TCTGCAAG+GCTGCACT
CAAGGAACTTCAATTCCTTCGATGTTTATATTTTTCTATAAGGGAAAATAGCAATAATCAAATTTCGA
CAAATTTTAGAACTTTATTTCAGAACTCTCTCCGATCACCTTGGTGTG
+
FFFFF,F:FFF:,:FF,FFFFFFFFFFFFFFFFF,FFFFF:F::FFFFF,FFFFFFFF:FFFFF:F,F
::FF:FF:F:FFFF:,FF,F,F:FFFFFFFF,FFFF,FF,:FF,FFFF
```

```
@A00721:190:HGJW7DSXY:4:1178:16993:33739_1:N:0:TCTGCAAG+GCTGCACT
TCTTTTCAATAAGATGTATGATGATAATCTACTATTTTTAAATAGTGATATTCAGCGTATACTTGAAT
ACTTTATCAAAGATTTGGCTTGGTCTATTTACCGCAAAAAGTTGGAAGAGGTCTATGATATTTATTTT
ACT
+
:FFFFFFF:FFFFFFFFFFFFFFFFFFFFFFFFFFFFFFFFFFFFFFFFFFFFFFFFFFFFFFFFFFF
FFFFFFFFFFFFFFFFFFFFFFFFFFFFFFFFFFFFFFFFFFFFFFFFFF:FFFFFFFFFFFFFFF
FFF
@A00721:190:HGJW7DSXY:4:1418:21386:26944_2:N:0:TCTGCAAG+GCTGCACT
CACCGATTATAACTTGACTGTTGATAATATTACAATAGCTCTTTCTCAGAAGATTAGAGAAGTAACCG
GTGAACACGTCGGTAGCATTGATTTACTTTTACCGTTAGAACTGAAAA
+
FFFFFFFFFF:FFFFFF:FFFF,FFFFFFFF:,FFFFFFFFFF:FF:FFF::FFF:FFFFFFF,:F:
FFFFFFFFFF:FFFFFFFFFFFFFFFFFFFFFF:F:FFFFF:FFFFFF
@A00721:190:HGJW7DSXY:4:1260:3468:24878_1:N:0:TCTGCAAG+GCTGCACT
ACCTATAAGTTACAAACGATTCTTCGACTATATTAAAGAAATAAAAATACTTCAAGTACCTGAACTTT
ACATTTTTAATGGTATGGTATAATGTGTCTTACAAATAGAACCTTCACTCTCGGTAATGAGAGTGTAT
TCC
+
FF:FFFFFFFFFFFFFFFFFFFFFFFFFFFFFFFFFFFFFFFFFFFFFFFFFFFFFFFFFFFFFFFFF
FFFFFFFFFFFFFFFFFFFFFFFFFFFFF:FFFFF:FFFF:FFFFFFFFFFFFFFFFFFFFFFFFFFF
FFF
@A00721:190:HGJW7DSXY:4:1251:32072:32142_1:N:0:TCTGCAAG+GCTGCACT
GGCATTACTAGGATTTATCACACTCCTAAATTTGATAGCAATTTATGTTTTAAATGCTAGGAAAATTT
TAGAAAAACTCCATTGTATAGTTCAAGGAGTACTTAATTCAATAGCTTTAGTATTATTTACTATCGCC
TTT
+
:FFFFFFFF:FFFFFFFFFFFFFF:FFFFF,:FFFFFFFFFFFFFFFFFFFFFFFFFFFFFFFFFFFF
FFFFFFFFFFFFFFFFFF,F:FFFFFFFFFFFFFFFFF:FFFF:,FF:FF:FFFFFFFFFFFFFFFFF
FFF
@A00721:190:HGJW7DSXY:4:1234:24623:27915_2:N:0:TCTGCAAG+GCTGCACT
AGTTAATTGCTTACACAAAAGTGACGCGCGAAGGAATCTTAATTGATTGAAGCTAGGCGTTGGGGAGA
ATGAACTGCCGCTTCCCGGAAGAAACGATCTATTTACAGAGTATTTAG
+
FFFFFFFFFFFFFFFFFFFFFFFFFFFFFFFFFFFFFFFFFFFF:FFFF,FFFFFFFFFFFFFFFFFF
FFFFFFFFFFFFFFFFFFFFFFFFF:FFFFFF,FFFFFFFFFFFFFFF
@A00721:190:HGJW7DSXY:4:1234:9706:14920_1:N:0:TCTGCAAG+GCTGCACT
CAGACGTGTGCTCTTCCGATCTCGCTAACTAGGTGAATTCCTGGCGAACCGCAAATCAATTGAAGAAA
ACCACTCCCTTCAATTCAATAACATAACATACCATACACTCCCCAAACATAACATACCATAACCTACC
ATG
+
FFFF:FFFFFFFFFFF:FFFF:F:,FFFFF::FFFF:FFFFFFFF:FFF,F:FFFFFFFF:,FFF:FF
FFFFFFFFFFFFFFF:FFFFFFFFFFFFFFFFFFFFFFFFFFFFFFFFFFFFFFFFFFFFFFFFFFFF
:FF
@A00721:190:HGJW7DSXY:4:1453:22724:9189_2:N:0:TCTGCAAG+GCTGCACT
GCTCCAACTGGCACGCCAGACGTAGGGTCAAACCTACTACCGCCGGTCGGCAGATCGGCACCACTGGT
CTCTACGTTCAGGGACCCGCTGTGCAGAATAACCTTCCCAAATCTAGA
+
FFFFFFFFFFFFFFFFFFFFFFFFFFFFFFFFFFFFFFFFFFFFFFFFFFFFFFFFFFFFFFFFFFFF
FFFFFFFFFFFFFFFFFFFFFFFFFFFFFFFFFFFFFFFFF:FFFFFFF
@A00721:190:HGJW7DSXY:4:1322:19388:27712_2:N:0:TCTGCAAG+GCTGCACT
CTGAAGGTCGTTACCGGACGACCACTCAAAGGAAACGGGCTGTTGCCCAGAGGAAAGCAGCGACCACC
GAGGTCGCTCGTAACATATACGGCAGTCACGTCATCTCTGTGGGAACT
+
```

```
:F,FF,::,FFFF,,,,FF,FF:FFFFFFF,:FFF:,F:,FFFF:,:::,F:,FFF,FFF::FF,FF:
,F,FFF,FFF:FFF,FF,,FF,,FF,FF,:,,:F,:FF,:F:,,F:,,
@A00721:190:HGJW7DSXY:4:1167:31430:24048_1:N:0:TCTGCAAG+GCTGCACT
GCTCTTCCGATCTATTCACCACTCCCATACCAATCAATGATGTGACTATACAGTTCGGCCATTTCAAG
AGTGGTCAATACGTCGTGGGAGGTACTGAAGCCTTCCCCTCCGCTGGCACGAAGAACACTCCCAAGCC
AAT
+
FFFFFFF:FFFFFFFFFFFFFFFFFFFFFFFFFFFF:FFF:FFFFFFFFFFFFFFFFFFFFFFFFFFF
FFFFFFFFFFFFFFFFFFFFFFFFFFFFFFFFFFFFFFFFFFFFFFFFFFFFFFFFFFFFFFFFFFFF
FFF
@A00721:190:HGJW7DSXY:4:1414:15257:36839_2:N:0:TCTGCAAG+GCTGCACT
TCTGACCAGAATGAAAAGGGGTTTACCAGGGTAGTCTCTAAGTCTACCAGGAAGAGGGGAAATGTCCC
CAAAACTGAGAAGAATAAAGGGAAAGGTCCATCAGGACCTAAGAAGGG
+
FFFFFFFFFFFFFFFFFFFFFFFFFFFFFFFFFFFFFFFFFFFFFFFFFFF:FFFFFFFFFFFFFFFF
FFFF,FFFFFFFFFFFFFFFFFFFFFFFFFFFFFFFFFFFFFFFFFFF
@A00721:190:HGJW7DSXY:4:1422:28375:29340_1:N:0:TCTGCAAG+GCTGCACT
CTAATTCTGAAGGAAGATCTCCCACCATCTGGGGTTGGGAATAAGAAATCTGCACGGGCCAAAGAGGC
CGCTGCTACGAAGTCGCTGAAGGCGGCTTGGGTCATTAAGGGTAGCTGTAAAGTTGCGCCTGCCGACC
CCA
+
FFFFFFFFFFFFFFFFFFFFFFFFFFFFFFFFFFFFFFFFFFFFFFFFFFFFFFFFFFFFFFFFFFFF
FFFFFFFFFFFFFFFFFFFFFFFFFFFFFFFFFFFFFFFFFFFFFFFFFFFFFFFFFFFFFFFFFFFF
FFF
@A00721:190:HGJW7DSXY:4:1316:24261:8688_2:N:0:TCTGCAAG+GCTGCACT
GACTCTCTACAGTAGGTTCGTGCCTACTATGAGCAAATCTGTTGAATTCGTCACTGAGGACGAAGGTG
AGAGGACTTTTGGCTACTGGCGACAAACAGATCGGAAGAGCGTCGTGT
+
FFFFFF:FFFFFFFFFFFFFFFFFFFFFFF:FFFFFFFFFFFFF:FFFFFFFFFFF,FF:F::FFFFFF
F:FFFF:FF:FF:,:FFFF:F:FFFFF:FFFFF:FFF:FFF:FFF:FF
@A00721:190:HGJW7DSXY:4:2647:23583:15718_1:N:0:TCTGCAAG+GCTGCACT
ATCTTGCAGAGTGACTGGAGTTCAGACGTGTGCTCTTCCGATCTTGTCAACGGCGTTTGGAGGATTTT
ATCCGCGCTTAACTATGGGGCGAAGATCCCCTATGGAGTCTTGAAGGACTTAGGTTGGCATATTTATG
CCG
+
FFFFFFFFFFFFFFFFFFFFFFFFFFFFFFFFFFFF:FFFFFFFFFFFFFFFFFFFFFFFFFFFFFFF
FFFFFFFFFFFFFFFFFFFFFFFFFFFFFFFFFFFFFFFFFFFFFFFFFFFFFFFFFFFFFFFFFFFF
FFF
@A00721:190:HGJW7DSXY:4:1514:32841:2722_1:N:0:TCTGCAAG+GCTGCACT
GCATGTCACCCTTAAGCACCGAGGTAAGCTCGGGACTTACAAGAGAGAGTGTGAGAGAGTCGCTCTCA
CTATTTTTTCCCAGAATGCGGGCTTTCTGACTGATGAGGTTAAAACTCTTGCTGCTTCGGTGGGTGAG
GG
+
FFFFFFFFFFFFFFFFFFFFFFFFFFFFFFFFFFFFFFFFFF:FFFFFFFFFFFFFFFFFFFFFF,F:F
F:FFFFFF,FFFF:FFFF:FFF,,FF,FFF::F,F,,,FFF:FFF,,,,FF,F,F:F,,,:FFF:F,F
FF
@A00721:190:HGJW7DSXY:4:2259:22833:30232_2:N:0:TCTGCAAG+GCTGCACT
ATTCACTGGTTCTGGGAGGCTGCTTTCCTCCATAATTACAACCTCTCTTTAGAGAGCATTAGAAGGAA
GTTGGGCAAATTCCCACATATGGTAGACGTTGTCACACTTTTCTTGAA
+
FFFFFFFFFFFFFFFFFFFFFFFFFFFFFFFFFFFFFFFFFFFFFFFFFF:F:FFFFFFFFFFFFFF:
FFFFFFFFFFFFFFFFFF:FFFFFFFFFFFFFFFFFFFFFFFFFFFFFF
@A00721:190:HGJW7DSXY:4:1528:25889:1423_1:N:0:TCTGCAAG+GCTGCACT
TTCCTCCGTCTTTGGGGATTTCGATGTGAGAAGGTGGTAGGTGTCAAGGGTGTTATTCATGCTGATCT
```

TTAAGGACCTAATCACATCATGATTGATAGTTACGGCTCGTATCGAGTTGGAGGGAGGAAGTTCTCCG
AA
+
F:F,F:,FF:,:,,F,FFFF:,F,::FF::F,FFFF:,,FF,FFFFFF::F,FF,FF,F::FFF:F:F
,F,FFFF:FFFFF,F,FF,FF:FFFF:F,,:FFFF:FFFFFFFFFFFF,FFF:FF,F::FFFFFFF:F
:F
@A00721:190:HGJW7DSXY:4:1217:22155:3286_1:N:0:TCTGCAAG+GCTGCACT
GCTGTAACTGTCATTTCAGAGAGGGGAGCTCTGAAAAGGAAACTTCGTCAGAAGATGAGGAAGTCTAA
GAGGAGGAGTTTGAGGAAGGCTCTAGAGAAGATCTCACCACCTGGAGAAGGCAAGTGGAGAGTCCAGA
AGT
+
:,FFFF,,F,FF:FFFFF:F:F:F:FFFFFFFFFFFFF,:FFFFF:FFFFFFFFFFFFFFFFFFFFFF
FFFFFFFFFFF:,F:FFFF:FF:FFFFFF:FFF:F:,:F:FFFFFF:FF:FFFFFFFFFF,:F:FFF,
F:F
@A00721:190:HGJW7DSXY:4:1456:29243:24737_2:N:0:TCTGCAAG+GCTGCACT
GGATTATTTGAAGCAGGGGTTACCACTTCCTAAGGTGGATGAGGGAGGTTGTTTCTTTGGAGTTTTAG
ATAATCTTTTGAGAAGGGGTCATAGAGCCAGCAAGAGAAGCTTATATG
+
FFFFFFFFFFFFFFFFFFFFFFFFFFFFFFFFFFFFFFFFFFFFFFFFFFFFFFFFFFFFFFFFFFFF
FFF:FFFFFFFFFFFFFFFFFFFFFFFFFFFFFFFFFFFFFFFFFFFF
@A00721:190:HGJW7DSXY:4:1165:26178:25880_2:N:0:TCTGCAAG+GCTGCACT
CCTTGAGAAGACAAGCCACATTAAGATATAATTTAGGTCAACTTGGGGTTTCGAATGTCATTAGTGCC
TTACGATTGAAAGAGGTCCTTTTGAGTATCGACAGATCTTTCGATCTT
+
FFFFFFFFFFFFF:FFFFFFFF::FF:FFFFFFF:F:FFFFFFFFF:FF:FFFF:FFFFFF:FFFFF:
FFF:FFFFFFFFFFF:FFFF:FFFFFFFFFF:FFFFFFFFFFFF,FFF
@A00721:190:HGJW7DSXY:4:1211:25021:32424_2:N:0:TCTGCAAG+GCTGCACT
TTAACGTGGCAGAATTCGGTAACTTACCATATTGTTCCTTCCGGGAAGTTGTGGGATTTAACTTTGAC
TCAACTACTCCGATCAGATCCGGATGGTTTGATTGGTATGGACTACTC
+
FFFFFFFFFFFFFFFFFFFFFFFFFFFFFFFFFFFFFFFFFFFFFFFFFFFFFFFFFFFFFFFFFFFF
FFFFFFFFFFFFFFFFFFFFFFFFFFFFFFFFFFFFFFFFFFFFFFFF
@A00721:190:HGJW7DSXY:4:1368:7048:23500_1:N:0:TCTGCAAG+GCTGCACT
TAGTTGTCTAGGGTCATGGCAGATACTACCGATAAGGTGAAAAATATGCAGGTCAAAGATTCTGTAGC
TCTGAGGGCGGTTGAGGGAGCCGTTTTGGCCGACTTTAACTATTCTAAGAGTCTTTATGATTCAAAAC
CTA
+
FFFFFFFFFFFFFFFFFFFFFFFFFFFFFFFFFFFFFFFFFFFFFFFFFFFFFFFFFFFFFFFFFFFF
FFFFFFFFFFFFFFFFFFFFFFFFFFFFFFFFFFFFFFFFFFFFFFFFFFFFFFFFFFFFFFFFF:FF
FFF
@A00721:190:HGJW7DSXY:4:1212:3016:13385_2:N:0:TCTGCAAG+GCTGCACT
ACCTTACAAGGTTGAGGAGACTTTACAGCGGCTTATTGTCGCGCAGTTTCCAGAATTGCGGTTGAAGT
TCACCCATTCTATCTTGAATGCTCACGCCGTCGCAGCCATATCGAGAC
+
FFFFFFFFFFFFFFFFFFFFFFFFFFFFFFFFF:F:FFFFFFFFFFFFFFFFFFFFFFFFFFFFFFFF
FFFFFFFFFFFFFFFFFFFFFFFFFFFFFFFFFFFFFFFFFFFFFFFF
@A00721:190:HGJW7DSXY:4:1445:5801:21214_2:N:0:TCTGCAAG+GCTGCACT
AAAGTACAGATTTAGTGATTTAGATATTATTGATATTGGAGGCGATATTCGTTTTCATATCCAAAACT
CCTTGAATGGAGTACATATTTGCAACCCTATTCTTGATGTGAAAGATG
+
FFFFF::FFFFFFFFFFFFFFFFFFFFFFFFFFFFFFFFFFFFFFFFFFFFFFFFF:FFFFFFFF:FF
FFFFFFFFFFFFFFFFFFFFFFFFFFFFFFFF:FFFFF,F::FFFFFF
@A00721:190:HGJW7DSXY:4:2336:30553:35305_2:N:0:TCTGCAAG+GCTGCACT
AACATTGATTGGGAACGTACCTACTCACGAGACGTCATGAAAGCTATCGGAGGGAAGCAAAATGTTTC

GTTCTGTTACAAGAACGCTTTGGATTGTGACCATAAAGCCTCAGTTGC
+
FFFFFFF:FFFFFFFFFFFFFFFFFFFFFFFFFFFFFFFFFFFFFFFFFFFFFFFFFFFFFFFFFFFF
FFFFFFFFFFFFFFFFFFFFFFFFFFFFF:FFFFFFFFFFFFFFFFFF
@A00721:190:HGJW7DSXY:4:1371:29514:24768_2:N:0:TCTGCAAG+GCTGCACT
ACGATATCTCTCTTGATGATGTAGCCACTATAATGCTTAAGAGAGAGATTGATGTATGTCATTTCGTC
TTAGTTTGTCCCGGCGAATTATTGGACACGATGAAAAATGACATACCC
+
FFFFFFFFFFFF:FFFFFFFFFFFFF:FFFF:FFFFFF:FF,FFFFFFFFFFFFFFFFFFFF,FFFFF
FFFFFFFFFFFFFFFFFFF:FFFFFFFFFFFF,FFFFFFFF:FFFFFFF
@A00721:190:HGJW7DSXY:4:1416:8558:13839_1:N:0:TCTGCAAG+GCTGCACT
TCGTTATCCGTCATGGAGATGATGGCGCATCACTTGATTACTATATGCCCGAAGGATTGGGTTATTCT
CACAACAAAGATAAGATTTTATCTTTCTTCAAACACCCTGTTTTCCTTAAGGGCGGACAATTGTTCCA
TTG
+
F,FFFFFF::FFF:F:F:FFF:FFF,FFFF,FFFFFFFFF:FFFFFFFFFFFFF:FFFF:FFFFFFF:
:FFFFFF,:FFFFF:FFFFFFF:FFFFFF:FFFFF:F:FFFFFF,FFF:F:FFFFFFFFFFFF:FFFF
FFF
@A00721:190:HGJW7DSXY:4:1621:16477:16282_2:N:0:TCTGCAAG+GCTGCACT
ATTGAGCATTAATGAGTTCCGAATAACTCGTAATAAATCTTTCTCTAGTGGACAATTCAGAATACCTA
TTTCCGTACCAAGAGCCACTGCCAATCTCATCGCTATACGCATACCTG
+
FFFFFFFFFFFFFFFFFFFFFFFFFFFFFFFFFFFFFFFFFFFFFFFFFFFFFFFFFFFFFFFFFFFF
FFFFFFFFFFFFFFFFFFFFFFFFFFFFFFFFFFFFFFFFFFFFFFFF
@A00721:190:HGJW7DSXY:4:1348:2392:36793_1:N:0:TCTGCAAG+GCTGCACT
GAGGTTCGTTCCTCAATAAAAATTTTTCTCAAGAGATTATGGTTGATAAAGTTTTCTTCATAAGGGCT
ATTCAGTACGTCATGGGGATCACTTCTTCGGTCAATGACAAAACCTTCGAATATGTTACGACATGGCT
TCG
+
FFFFFFFF:FFFFFFFFFFFFFFFFFFFFFFF:FFFFFFFFFFFFFFFF:FFFFFFFFFFFF:FFFFFF
FFFFFFFFFFFFFFFFFFFFFFFFFFFFFFFFFFFFFFFFFFFFFFFFFFFFFFFFFFFFFFFFFFFF
FFF
@A00721:190:HGJW7DSXY:4:1519:23032:23343_2:N:0:TCTGCAAG+GCTGCACT
AGGATTGTCATCTCGGGGAAAGTGATTCACCAAGATGTTCATATAGATACGAGATACCTAGAGAAGGT
CGCAGCGATCTTACTCGTTATCGGAGCTAAGAAGAAGACTTTGGGTAC
+
FFFFFFFFFFFFFFFFFFFFFFFFFFFFFFFFFFFFFFFFFFFFFFFFFFFFFFFFFFFFFFFFFFFF
FFFFFFFFFFFFFFFFFFFFFFFFFF:FFFFFFFF:FFFFFFFFFFFF
@A00721:190:HGJW7DSXY:4:1231:12111:25535_2:N:0:TCTGCAAG+GCTGCACT
AAACTTAACGTTAGTACCGGAGGTGATCTCTCTGCTTCTGCGTTACTGAGAGAAAAGATTCAGGACGC
CTACGGTGACTTTATGGACAAGATGCAAGTTCTTGGGAAATACTTGTT
+
FF:FFFFFFF:FFFFFFFFFFFFFFFFFFFFFFFFFFFFFFFFFFFFFFFFFFFFFFFFFFFFFFFFF
FFFFFFFFFFFFFFFFFFFFFFFFFFFFFFFFFFFFFFFFFFFFFFFF
@A00721:190:HGJW7DSXY:4:1223:18783:30358_2:N:0:TCTGCAAG+GCTGCACT
TCGATTCTTTGTATAGGGAGTGTGAGAAGACAACGAATCTTGAAACTACGGTCCTTATCAAAGAAAAT
GGTGAACCGGTCTCTAAATCTTTTGAGATTGAGAAGATTGAAGAACAC
+
FFFFFFF,FFFFFFFFFFFFFFFFFFFFFFFFFFFFFFFFFFFFFFFFFFFFFFFFFFFFF,FFFFFF
FFFFFFFFFFFFFFFFFFFFFFFFFFFFFFFFFFFFFFFFFFFFFFFF
@A00721:190:HGJW7DSXY:4:1476:14109:31532_2:N:0:TCTGCAAG+GCTGCACT
GCTCGGCGTTGTTCTTGAAAACCAAGTTAAGAAGGAGGCTGATCATGGAGTCTTTGTACCTTCTGTCA
AACTTCGAGGTTCAGCTACGAGAGGTGATCCCGGATGTGGATTGAAGG
+

FFFFFFFFFFFFFFFFFFFFFFFFFFFFFFFFFFFFFFFFFFFFFFFFFFFFFFFFFFFFFFFFFFFFFFFF
FFFFFFFFFFFFFFFFFFFFFFFFFFFFFFFFFFFFFFFFFFFFFFFFFFF
@A00721:190:HGJW7DSXY:4:1339:16640:5478_2:N:0:TCTGCAAG+GCTGCACT
GGAGACTCCTTCTTTTTGAATGTTGTAAAGAATTGGGTTGAGAAGTGTGCTTTACAAGGAGTTAGACT
CTTCTTCAAGTTTTTAGATCTTCTTGATAATCTTAAAGAAAAATTTTC
+
FFFFFFFFFFFFFFFFFFFFFFFFFFFFFFFFF:FFFFFFFFFFF,FFFFFFFFFFFFF::FFFFFFFFF
FFFFFFFFFFFFFFFF:FFFFF:FFFFFFFFFFFFFFFFFFFFFFFFFFF
@A00721:190:HGJW7DSXY:4:1653:10737:21057_1:N:0:TCTGCAAG+GCTGCACT
AGTCTGTACTTACATTCTTTACGCCAGATTCTTCATGGGGCGACGCTGTTTCTCTTGGTATTTCTGAA
ATAGTTTATCAGAGCGTCATTTTCCTTTGGGATCTTTCCTCTGGTAAGGGAATTATTAAATCATTCTT
CTC
+
FFFFFFFFFFFFFFFF,FFFFFFFFFFFFFFFFFFFFFFFFFFFFFFFFFFFFFFFFFFFFFFF:FFFF
FFFFFFFFFFFFFFFF:FF,FFFFFFFFFFFFFFFFFFFFFFFFFFFFFFFFFFFFFFFFFFFFFFFF
FFF
@A00721:190:HGJW7DSXY:4:1315:31693:33896_2:N:0:TCTGCAAG+GCTGCACT
ATTATTACTGTTTTCTCTACCCATAACTTCTTTTTCAAAAATGCGGATGGGTCTACTGATGTTTTCCT
ACAGAGTGCAGTCGTTTCAACACTTATCACCACTTTTGAAAGGAAAAT
+
FFFFFFFFFFFFFFFFFFFFFFFFFFFFFFFFFFFF:F:FFFFFFFFFFFFFFFFFFFFFFFFFFFFFFF
F:FFFFFFFFFFFFF:F,FFFFFFFFF,F,FF:,FFFF:F,,F,FFF:
@A00721:190:HGJW7DSXY:4:1509:10285:8030_1:N:0:TCTGCAAG+GCTGCACT
AAATACGTATGTCGGTGGGGTTCTCAAGGCTCTCATCGCCACTGGGATTCTAAGGGGTTCCATAAGAT
ATTATCTGACATTGTGGTTCAATGATCAGGATTCTTCTTTTGGTCCTTACGTCTTAAGTAATTTGGAT
GTT
+
FFFFFFFFF:FFFFFFFFFFFFFFFFFFFFFFFFFFFFF:FFFFFFFFFFFFFFFFFFFFFFFFFFFFFF
FFFFFFFFFFFFFFFFFFFFFFFFFFFFFFFFFFFFFFFFFFFFFFFFFFFFFFFFFFFFFFFFFFFF:F
FFF
@A00721:190:HGJW7DSXY:4:1267:19000:36777_2:N:0:TCTGCAAG+GCTGCACT
GTTCTTGTCACGATTCTTAAAAGAAGTTTATTAAAACTTCAGAGTCTCTTGGAAGTCCAGGTAGCTAA
TGTTCTTGCAATAGCCACAGACTTTCTGGGAAAAGAGACCTCTGTTGG
+
FFFFFFFFFFFFFFFFFFFFFFFF:FFFFFFFFFFFFFFFFFFFFFFFFFFFFFFFFFFFFFFFFFFF:F
FFFFFFFFFFFFFFFFFFFFFFFFFFFFFF,FFFF:FFFFFFFFFFFF
@A00721:190:HGJW7DSXY:4:1405:10872:2722_1:N:0:TCTGCAAG+GCTGCACT
GAGAATTACTCAGATTCAATCTGGCATCACTGAACGTCTACAACGAGTTAGGGAGTGGCTAGGTTTTA
AAAGATCCTTTGCACAAGCAATACCTTCCACTGAGTTCATCACCCAGAGTTTCGACGTCGCGGAAAAC
GAT
+
FFFFFFFFFFFFFFFFFFFFFFFFFFFFFFFFFFFFFFFFFFFFFFFFFFFFFFFFFFFFFFFFFFFFFF
FFFFFFFFFFFFFFFFFFFFFF:FFFFFFFFFFFFFFFFFFFFFFFFFFFFFFFFFFFFFFFFFFFFFFF
FFF
@A00721:190:HGJW7DSXY:4:1314:3685:14920_2:N:0:TCTGCAAG+GCTGCACT
CACTGTTCGAGAATCTTCTGATGCTGAATTTGAGGAAAATTTTTCGGAAGTTTTTGGTATAAATCCTG
ATGGCTCAGTACAGGGAGGAGATCGGAAGAGCGTCGTGTAGGGAAAGA
+
FFFFFFFFFFFFFFFFFFFFFFFFFFFFFF,FFFFFFFFFFFFFFFFFFFFFFFFFFFFFFFFFFFFFFF
FFFFFFFFFFF:FFFFFFFFFFFFFF:FFFFFFFFFFF:FFFF:FFFF
@A00721:190:HGJW7DSXY:4:1139:12726:20556_1:N:0:TCTGCAAG+GCTGCACT
ATGGACAAAATCTGGGATCATAGGTATGTGTCACGCTACACTCGAAATATGGGCCCTCATAAGGAAGT
ATAGTTGGCGTCTTGTCGTTGACGTCATTACAGTCTGGAAGTTAGATCATTTGTTCAGGAAGATCTCT
ACA

+
FFFFFFFFFFFFFFFFFFFFFFFFFFFFFFFFFFFFFFFFFFFFFF:FFFFFFFFFFFFFFFF:FFFFFF
FFFFFFFFFFFFFFFFFFFFFFFFFFFFFFFFFFFFFFFFFFFFFFFFFFFFFFFFFFFFFFFFFFFFFF
FFF
@A00721:190:HGJW7DSXY:4:1267:30852:4131_1:N:0:TCTGCAAG+GCTGCACT
CTTTAGCAACTTTAGATAAATTCTTTCCGGGCTCAATGACCTTTGCTATGTACGTGGATCTAATCCTT
GGTGATCTGAATCTGACCATCGCAAAGAAGACTTTATTAGTTCATGAAGTTCTTACACTTCTCGCGAC
TAA
+
FFFFFFFFFFFFFFFFFFFFFFFFFFFFFFFFFFFFFFFFFFFFFFFFFFFFFFFFFFFFFFFFFFFFFF
FFFFFFFFFFFFFFFFFFFFFFFFFFFFFFFFFFFFFFFFFFFFFFFFFFFFFFFFFFFFFFFFFFFFFF
FFF
@A00721:190:HGJW7DSXY:4:2208:22245:26271_1:N:0:TCTGCAAG+GCTGCACT
GCTTTATCTTAAAGCATTACCACCAAATTTAGCACTTTTTGAATATGACAGAAGCTCGCCATATCAGA
TTAGAGACATGCCAGTTAGAGTTCTTCTAACCAGGACTGATGGTGAGATTCTTTTACCTTTTGGAGGT
CCA
+
FFFFFFFFFFFFFFFFFFFFFFFFFFFFFFFFFFFFFFFFFFFFFFFFFFFFFFFFFFFFFFFFFFFFFF
FFFFFFFFFFFFFFFFFFFFFFFFFFFFFFFFFFFFFFFFFFFFFFFFFFFFFFFFFFFFFFFFFFFFFF
FFF
@A00721:190:HGJW7DSXY:4:1403:31937:20791_2:N:0:TCTGCAAG+GCTGCACT
CCTAGTGTCTTTTCTAGAGTCCAAGGACGTTTCCGTCGGAGGATGACAGGTGGGAGACCGCTCAATAA
CACTCTATTGAATCCCTTTTCCACGATGAAAGCTCTTTATGAGGTGCT
+
FFFFFFFFFFFFFFFFFFF:FFFFFFFFFFFF:FFFFFFFFFFFFFFFFFFFFFFFFFFFFFFFFFFFFF
FFFFFFFF,FFFFF,F:FFFF,F:FFFFFFFF,FFFFFFF:FFFF:FF
@A00721:190:HGJW7DSXY:4:2110:17463:10755_1:N:0:TCTGCAAG+GCTGCACT
TCTTTGGTGATATCGCCTCTGTCCTTTCTCCTTTAATCGTGAGGTTACGAGAGTTTCGTACTTGGGTT
TATCGGTTGTATTTTGATAAATTTGTTGGAATCGATAATGATACTGACGAAGGTTCTATTACCACCCT
TGT
+
:F:FFFFFFFF:,FFFFFFFF:,FFFFFFFF,,:,:F:FFFFFFFFFFFFFFFFFFFFFF:FFFFFFF:F
F:FFFFF:FFFF:FFFFFFFFFF:FFFFFFFF:FFFFFFFFF,FFFFFFFFFFFFFFFFF:FFFFFFFFF
FFF
@A00721:190:HGJW7DSXY:4:1562:27814:14810_2:N:0:TCTGCAAG+GCTGCACT
GTGATGCTTTCTTAGAGGCTCGTCTAGCCGCTGAGATTCGTCTTACCTATTTACAATCTGTAGAGAGA
GAGGTTAATCAGAATTTGAGTGATAACGATTTAGTACTGGGAAATGGT
+
FFFFFFFFFFFFFFFFFFFFFFFFFFFFFFFFFFFFFFFFFFFFFFFFFFFFFFFFFFFFFFFFFFFFFF
FFFFFFFFFFFFF:FFFFFFFFFFF:FFFFFFFFFFFFFFFFF,FFFF
@A00721:190:HGJW7DSXY:4:1253:12970:19351_1:N:0:TCTGCAAG+GCTGCACT
TTTTCTTTCCCTCCCCTTTTTCGATTGGATTCGACGGGGTCTTCGTCTCACGACTAATTTGATAAGGT
TTTTCTTTTATGGGACTCTTGTTTGTACTTATGAAGGAGGTATTACTTCGGAAGTTCTTAATGTAGGT
CTC
+
FFFFFFFFFFFFFFFFFFFFFFFFFFFFFFFFF:FFFFFFFFFFFFFFFFFFFFFFFFFFFFFFFFFFFF
FFFFFFFFFFFFFFFFFFFFFFFFFFFFFFFFFFFFFFFFFFF,FFFF:FFFFFFFFFFFFFFFFFFFFF
FFF
@A00721:190:HGJW7DSXY:4:1478:11930:28197_2:N:0:TCTGCAAG+GCTGCACT
GGTTTATGTTTCGGTTTCAGCTGGATGGATCTTTTGATGGTATGTTTCACATTGCGTGATCACTTCCC
GTGGACTTGGGAAGAGATTAAACATAAAATCCAGCGATTCTATTATGT
+
FFFFFFFFFFFFFFFFFFFFFFFFFFFFFFFFFFFFFFFFFFFFFFFFFFFFFFFFFFFFFFFFFFFFFF
FFFFFFFFFFFF:FFFFFFFFFFFFFF:FFFFFFFFFFFFFFFFF,FF

```
@A00721:190:HGJW7DSXY:4:2168:23701:7373_2:N:0:TCTGCAAG+GCTGCACT
TCGATGATGTTCGGGCTGTGGGGATTAACTTCCCTCGTGAAACTTACGACGATGTTCTCGCTCGCATT
TCACAAGGATTACTAAGTCTGAGATCTGCCACTTCTAATTTTAGGAAA
+
FFFFFFFFFFFFFFFFFFFFFFFFFFFFFFFFFFFFFFFFFFFFFFFFFFFFFFFFFFFFFFFFFFFF
FFFFFFFFFFFFFFFFFFF:FFFFFFFFFFFFFFFFFFFFFFFFFFFF
@A00721:190:HGJW7DSXY:4:1349:14696:4586_2:N:0:TCTGCAAG+GCTGCACT
AGAAAGTTCATGAGGATGTCTTGGTCGAAAAATATCGCCGGGAACTTCTAATAGAGGATCCTGATGAC
ATTATTTCAACGGATACCGATCTCAGTTCTACCACAGAAACCGTCTCA
+
:FFFFFFFFFFFFFFFFFFFFFFFFF:FFFFFFFFFFFFFF:FFF:FFF:,:FFFFFFFFFFFFFFF
FFFFFFFFF:FFFFFFFFFFFFFFFFFFFFFFFFFFFFFFFFFFFFFF
@A00721:190:HGJW7DSXY:4:1627:6876:7639_2:N:0:TCTGCAAG+GCTGCACT
ACTGATTCGGAATTAGAGAAAGACGACCTTCTTGTGGGGACTGAGACCTCTGAATTTTCAGGGTGCCC
TGATGAGGACGTTGAAAGGGATAATTTCAACTCTGAGACTTCTAAGAG
+
FFFFF,FFFFFFFFFFFFFFFFFFFFFFFFFFFFFFFFFFFFFFFFFFF:FFFFFFFFFFFFFFFFFF
FFFFFFFFFFFFFFFFFFFFFFFFFFFFFFFFFFFFFFFFFFFFFFFF
@A00721:190:HGJW7DSXY:4:1168:3486:36777_2:N:0:TCTGCAAG+GCTGCACT
ACCAGAAACGTAACAAGAAGAATAAAGGTAGGAAGGGGAGAAAACAGAACAAGCGAGCGGTTGAAGAG
AAAACTGATATGGTTGCGGCTGATCGTACCCCAATTCCTGCTAGAAAG
+
FFFFFFFFFFFF:FFFFFFFFFFF:FFFFFFFFFFFFFFFFFFFFFFFFFFFFFFFFFFFFFFFFFFF
FFFFFFFFFFFFFFFFF:FFFFFFFFFFFFFFFFFFFFFFFFFFFFFF
@A00721:190:HGJW7DSXY:4:1129:11785:8437_1:N:0:TCTGCAAG+GCTGCACT
ATACCACTGATCTTCCAGAGTTACGTAGTGGTCGTTCTTTTGGTGATTTTATCAAAGAGAAAGTTTCT
GCTAGTCGCGACGATAGTATTTATCGGAGTGATATTAACAAGTTAAACTTCGAACTGACCACTTACAA
GAG
+
FFFFFFFFFFFFFFFFFFFFFFFF:FFFFFFFFFFFFFFFFFFFFFFFFFFFFFFFFFFFFFFFFFFF
FFFFFFFFFFFFFF:FFFFFFFFFFFFFFFFFFFFFFFFFFFFFFFFFFFFFFFFFFFFFFFFFFFFF
FFF
@A00721:190:HGJW7DSXY:4:1554:25708:34710_1:N:0:TCTGCAAG+GCTGCACT
GCAACATGGATCAGTTTTCCAAAAAGCTATTAATAGCAGTTGCGGGGCTATAATACCGCCTCCAGATT
ACTCTAAACTGAATCCACTCTCTTGGGATATAAATGAGTACATCTATAATAGAGTTTTGGAACTGTGT
AAT
+
FFFFFFFFF:FFFFFFFFFFFFFFFFFFFFFF:FFFFFFFFFFFFFFFFFFFFFFFFFFFFFFFFFFFF
FFFFFFFFFFFFFFFFFFFFFFFFFFFFFFFFFFFFFFFFFFFFFFFFFFFFFFFFFFFFFFFFFFFF
FFF
@A00721:190:HGJW7DSXY:4:1240:9787:18098_1:N:0:TCTGCAAG+GCTGCACT
GGCTTCTCTAAAGAACAGCAACTTTCAACATGAATTAGTTATTGAAAGTCTTGACCCTCACGTCTCAC
TTTATCACCTTGGAGATGGTAAAGTCATTAACAAAGGAAATGTTAAGGCTGATGACTTAAATTTCGCA
TTC
+
F:FFFFFFFFF:FF:FFFFFFFFFFFFFFFFFFF:FFFFFFFFFFFFFFFFF:FFFFFFFFFFFFFFF
FFFFFFFFFFFFFFFFFFFFFFFFFFFFFF,FFFF:FFFFFFFFFFFFFFFFFFFFFFFFFFFFFFFF
FFF
@A00721:190:HGJW7DSXY:4:1459:20094:26553_2:N:0:TCTGCAAG+GCTGCACT
AGACCTTTCTGGGGGAGTAAAGAGGGTTTGAAAATTACGGAACCTTTCCTCGTTCTTCATGAAGATCT
TCGAGTGTTTTATAGTGAGTTATTCTTGCGAAGGTTCAAGTCGATTGA
+
FFFFFFF:F,,FFFF:FFFF,F:FF:FFFFFFFFFFFFFFFFFFFFFFF,FFFFFF,F::FF:F:FFF:
F:FFF,FFF,FFFFFFFFFFFFFFFFFFFFF,,FFFFF,FF:FF,:F,
```

```
@A00721:190:HGJW7DSXY:4:1657:32325:21590_1:N:0:TCTGCAAG+GCTGCACT
ACGAGATCTTGCAGAGTGACTGGAGTTCAGACGTGTGCTCTTCCGATCTGTGGAGGGAAGACCACTGA
ACTTATAGCGACTTTTGTCGAATTTTTCGCAAAAGGTCTTGAAGTACGTATTGTCACTGCTAATCGTA
ATT
+
FFF:FFFFF:FFFFFFFFFFFFFFFFFFFFFFFFFFFFFFFFFFFFFFFFFFFFFFFFF:FFFFFFF
FFFFFFFFFFFFFFFFFFFFFFFFFFFFFFFFFFFFFFFFFFFFFFFFFFFFFFFFFFFFFFFFFFFFF
FFF
@A00721:190:HGJW7DSXY:4:1371:26178:15076_1:N:0:TCTGCAAG+GCTGCACT
GTGACTAAAGTTTTGGCACTTCTAGTTCATCGCGGTATTTGTGAAATTAAGACAGACGTGAAAGTTAT
GATGAGTGATCGTATTAGAACAGCTGATTCTTATTGCATGAACGTCGCTGAGGTCAAACCTGTGGACG
TTC
+
FFFFFFFFFFFFFFFFFFFFFFF:FFFFFFFFFFFFF:FFFFFFFFFFFFFFFFFFFFFFFFFFFFFF
FFFFFFFFFFFFFFFFFFFFFFFFFFFFFFFFFFFFFFFFFFFFFFFFFFFFFFFFFFFFFFFFFFFFF
FFF
@A00721:190:HGJW7DSXY:4:1476:14488:24643_2:N:0:TCTGCAAG+GCTGCACT
CTGGTCAGACCCTTTTTCTTACACATCTACTTAAGGCGGCTTATACCATAGGTTACGGTGATTCCAAT
CAAATCGGATTCATCAATCGTATTGATCTCCAAGTCGCAAACTACTTC
+
FFFFFFFFFFFFFFFFFFFFFFFFFFFFFFFFFFFFFFFFFFFFFFFFFFFFFFFFFFFFFFFFFFFFF
FFFFFFFFFFFFFFFFFFFFFFFFFFFFFFFFFFFFFFFFFFFFFFFF
@A00721:190:HGJW7DSXY:4:1322:19171:2347_1:N:0:TCTGCAAG+GCTGCACT
GTGTGCTCTTCCGATCTTTCTTATCGAAGTCTCAGTTATCGCTGCCCGGCCGACGTTTGTCGCGAACT
CTCAGAGGTTTACGGCAGAAAAATTTCTCCTGCTTGTAATTCGAGAGAGGACACTATGGAAATTAGAC
CTA
+
FFFFFFFFFFFFFFFFFFFFFFFFFFFFFFFFFFFFFFFFFFFFFFFFFFFFFFFFFFFFFFFFFFFFF
FFFFFFFFFFFFFFFFFFFFFFFF:FFFFFFFFFFFFFFFFFFFFFFFFFFFFFFFFFFFFFFFFFFF
FFF
@A00721:190:HGJW7DSXY:4:1303:23294:29183_2:N:0:TCTGCAAG+GCTGCACT
ATATTACTTTCACTCAAGGTGAAAAGTTTGAAATTAACAAAGTCCTTGGTAAGAGTAAGGTTAAATGT
GAAGATGCTCAGACTATTCATGAAGTTCAAGGTAATACTTACCAGCAC
+
FFFFFFFFFFFF:FFFFF:FFFFFFF:,FFFFFFFFFFFFFF:FFFFFFFFFFFFFF:FFFFFFFF
FFFFFFFFFFFFFFFFFFFFFFFFFFFFFFF::FFFFFFFFF:FFFFF
@A00721:190:HGJW7DSXY:4:1676:22001:31673_2:N:0:TCTGCAAG+GCTGCACT
AAACCTCAAGATGACCCTGTTTTTGCATCACAAACACATATTACCGTTGCTCTATCACGACACGTTGA
ATCTTTACGATACTTTGTTATCAATGTAAAGTTAAACGATGCTACTTG
+
FFFFFFFFFF:FFFFFFFFFFFFFFFFFFFFFFFFFFFFFFFFFFFFFFFFFFFFFFFFFFFFFFFFFF
FFFFFFFFFFFFFFFFFFFFFFFFFFFFFFFFFFFF,FFFFFFFFFFF
@A00721:190:HGJW7DSXY:4:1653:17264:35023_2:N:0:TCTGCAAG+GCTGCACT
TAAGGAACTGTCACGTTTAGCACGGGAGGTTGCAGATTCAACCCCTTATATCGAGGCTATCGAGGACG
ATGAGTATCTTGAAGTTGTTGACGACAAGCCTCAAGAGGGTAGAGGAC
+
FFFFFF:FFFFFFFFFFFFFFFFFFFFFFFFFFFFFFFFFFFFFFFFFFFFFFFFFFFFFFFFFFFFFF
FFFFFFFFFFFFFFFFFFFFFFFFFFFFFFFFFFFFFFFFFFFFFFFF
@A00721:190:HGJW7DSXY:4:1322:26937:8061_2:N:0:TCTGCAAG+GCTGCACT
GGTTATTCCTGGAACCACCTTAACGTCACTCGGTAATCTTTCAGCCGAATTAAGTACTAATGAGTTCA
AGTCGGTTGTCGATCGAATTAAGATCAAGGCTAATACCTTTATAGGTA
+
FFFFFFFFFFFFFFFFFFFFFFFFFFFFFFFFFFF:FFFFFFFFFFFFFFFFFFFFFFF:FFFFFFFFF
FFFFFFFFFFFFFFFFFFFFFFFFFFFFFFFFFFF:FFFFFFFFFFFF
```

```
@A00721:190:HGJW7DSXY:4:1430:19623:7420_2:N:0:TCTGCAAG+GCTGCACT
GCCGCGTAGTCAGCGCGTTTAGCTGTATCATCAGTCAGAGTATACCCGATAGGGTACCGAGTCTCCAA
GAGAATATTTATTCTTATGAGGCTCGAAACTTCAACGTTTTCACAACG
+
FFFFFFFFFFFFFFFFFFFFFFFFFFFFFFFFFFFFFFFFFFFFFFFFFFFFFFFFFFFFFFFFFFFF
FFFFFFFFFFFFFFFFFFFFFFFFFFFFFFFFFFFFFFFFFFFFFFFF
@A00721:190:HGJW7DSXY:4:1268:18728:36182_2:N:0:TCTGCAAG+GCTGCACT
TCGCACCCGAAATTTTTGGGCAGAATGCTCTTAACGCGGTTCTTGAACGGTGTTTTGATCAGTCTAAG
TTGGATAAAGTTATAGGAGACTCAGTTAACCTGAGAGCTCCTCAATTG
+
:FFFFFFFFFFFFFFFFFFFFFFFFFFFFFFFFFFFFFFFFFF:FFFFFF:FFFFFFFFFFFFFFFFF
FFFFFFFFFFFFFFFFFFFFFFFFFFFFFFFFFFFFFFFFFFFFFFFF
@A00721:190:HGJW7DSXY:4:2150:1181:33254_1:N:0:TCTGCAAG+GCTGCACT
AACAATAGGGACGCTAGTATACTTCGTAAAGTCGACAGCGAAATGGTCAATATTGATAATTGGACCGA
CTATATGACCTATTTTAAGCTCATGGTGAAAAGGGAAGCTAAGGTTAAACTTGATGCTTCAGCCTTTT
CGA
+
FFFF,FFFFFFFFFFFFFFFFFFF:FFFFFFFFFFF::FFFFFFFFFFFFFFFFFFFFFFFFFFFFFF
FFFFFFFFFFFFFFFFFFFFFFFFFFFFFFFFFFFFFFFFFFFF:FFFFFFFFFFFFFFFFFFFFFFF
FFF
@A00721:190:HGJW7DSXY:4:1368:6596:22842_1:N:0:TCTGCAAG+GCTGCACT
AGAATATCGTTTATCACGCAAAATTTATTAATGCAATTTTCGGACCTATCTTTAAGGTTCTTGTGAGG
AGAGTCAAACAGTGTCTTTTACCCAACATTTTTATGTATACAGAGATGACCAGTGACGATTTCTCCCG
GAA
+
FFFFF:FFFFFFFFFFFFFFFF:FFFFFFFFFFFFFFFFFFFFFFFFFFFFFFFFFFFFFFFFFFFFF
FFFFFFFFFFFFFFFFFFFFFFFFFFFFFFFFFFFFFFFFFFFFFFFFFFFFFFFFFFFFFFFFFFFF
FFF
@A00721:190:HGJW7DSXY:4:2363:18340:35728_1:N:0:TCTGCAAG+GCTGCACT
CTCTTAGGAGGTCATGATGACTACTATAAGGGTGAAATCGATTTTTCGAAGTTTGACAAATCTCAAGA
TTTCTTTTGTAAAGCCTATGAGATAAAGATGTATTCTTATTTAGGCTTCGATGCTGAATTTCTTGAAC
TTT
+
FFFFFFFFFFFFFFFFFFFFFFFFFFFFFFFFFFFFFFFFF:FFFFFFFFFFFFFFFFFFFFFFFFFF
FF:F:FFFFFFFFFFFFFFFFFFFFFFFFFFFFFFFFFFFFFFFFFFFFFFFFFFFFFFFFFFFFFFF
FFF
@A00721:190:HGJW7DSXY:4:1336:30147:11898_2:N:0:TCTGCAAG+GCTGCACT
TGTCACGCCAGGAGTATCCATAACGATTTGTCATTTACCACCTTTGCTCAACGACGAAGTGGAACGGC
CAACACTTTCTTTGGTAATTGTATGATCACTTTGATGATTTTATCACT
+
FFFFFFFFFFFFFFFFFFFFFFFFFFFFFFFFFFFFFFFFFFFFFFFFFFFFFFFFF:FFFFFFFFFF
FFFFFFFFFFFFFFFFFFFFFFFFFFFFFFFFFFFFFFFFFFFFFFFF
@A00721:190:HGJW7DSXY:4:1170:18991:30217_1:N:0:TCTGCAAG+GCTGCACT
CTTGGAAAGTTTTCGGCTGTGGTTGTCGCTGGGGACGATTCCATTCTGTTTTCAAAGACTCCAATAAC
TGATCATTCTGATCATATGATTTTGGACAATGGTTTCGAAGCGAAATTTTTGAGGGATAAACCAGCGT
ACT
+
FFFFFFFFFFFFFFFFFFFFFFFFFFFFFFFFFFFFFFFFFFFFFFFFFFFFFFFFFFFFFFFFFFFF
FFFFFFFFFFFFFFFFFFFFFFFFFFFFFFFFFFFFFFFFFFFFFFFFFFFFFFFFFFF:FFFFFFFF
FFF
@A00721:190:HGJW7DSXY:4:1446:18466:3599_2:N:0:TCTGCAAG+GCTGCACT
CCTGATCCTTACAAGATCATCGTTAAGTTAGGTAAACCCCTAACCGATGTTACTCTTGTAGGACTTTG
GGAGAGATTCGTTAGTTTTAAAGACTATACTAAATATCTCCACTACGA
+
```

FFFFFFFFFFFFFFFFFFFFFFFFFFFFFFFFF:FFFFFFF:FFFFFFFFFFFFF:FF:FFFFFFFFF
:FFFFFFFFFFFFFFFFF:F:FFFFFFFF:FFFFFFFFFFFFFFF:FF
@A00721:190:HGJW7DSXY:4:1272:25518:22608_2:N:0:TCTGCAAG+GCTGCACT
TTGCGACTAATATTCGATATTCGATTGAGGGTCGTTATACCTATTCGGCTATCTGTGCTTTGAATTGT
ATAAGATCAAATTTTAAACGATTCCGAGAGGTTTATCCTGATGTGGAG
+
FFFFFFFFFFFFFFFFFFFFFFFFF:FFFFFFFFFFFFFFFFFFFFFFFFFF:FFFFFFFFFFFFFFF
FFFFFFFFFFFFFFFFFFFF:FFFF:FF:FFFFFFFFFFFFFFFF,FFF
@A00721:190:HGJW7DSXY:4:1311:28474:26850_1:N:0:TCTGCAAG+GCTGCACT
ATCCCGACCTTTCCTTGTGAAAATTTTGATGCGGTTTCATAAGATTATCAGTAATCCTGATTCTACTA
CGGATTCATGGACCTTCCATCCTGTACCTCTTCTGGAGAGACGTATCTCAAAGATCGACTTCGAGACA
GGT
+
FFFFFFFFFF:FFFFFFFFFFFFFFFFFF:FFFFFFFFFFFFFFFFFFFFFFFFFFFFFFFFFFFFFF
FFFFFFFFFFFFFFFFFFFFFFFFFFFFFFFFFFFFFFFFFFFFFFFFFFFFFFFFFFFFFFFFFFFF
FFF
@A00721:190:HGJW7DSXY:4:2659:22842:34757_1:N:0:TCTGCAAG+GCTGCACT
AGCGTTAGCGACCAAGATCTAAATTCGTTAACGGTGCGTGGCGCTTTATTTTAAAAAGCTCCCTCCTC
TCGCAAGTTTGATCTTATACTTATCACGATAGGACTTGAGTCTTTTGATCAGTCTCTGACTTGACCAT
+
FF,:,,FFF:F,F,F,:FFF,F,,FFF,FFF,F:F::FF,,,,:FFF:F:FF,F:F:F,F:,FFFFF,
FFF,F,FFFFFF:,F,FF,:FF,F,F,,:,:FFF,FFFF,FFFFFFFFF,:FFFF:FF,:FFF::,,F
@A00721:190:HGJW7DSXY:4:1178:21323:34068_1:N:0:TCTGCAAG+GCTGCACT
TGACGATCATTAAGACTCTCTTTCTTCTTTGTTTCCTTCTTTGCACTGATTTTTCCTCATCAGCTTGT
GTTTTACCTAATCTGTGTTTTTACGATAACAACTATCAGTTGATTAAGACTCTTCCGAAAGGCGAACT
TTG
+
FFFFFFFFFFFFFFFFFFFFFFFFFFFFFFFFFFFFFFFFFFFFFFFFFFFFFFFFFFFFFFFFFFFF
FFFFFFFFFFFFFFFFFFFFFFFFFFFFFFFFFFFFFFFFFFFFFFFFFFFFFFFFFFFFFFFFFFFF
FFF
@A00721:190:HGJW7DSXY:4:1357:2835:4679_2:N:0:TCTGCAAG+GCTGCACT
GGGTGAAGCACAACGTTTGACCGTGACGAACTGTGGTGTTCTTGATCTACGTTACGGATATACTCTCC
TTGAGTTCACTCGTAAGGACACTACCACTTATTACGATATTTCTTTAG
+
FFFFFFFFFFFFFFFFFFFFFFFFFFFFFFFFFFFFFFFFFFFFFFFFFFFFFFFFFFFFFFFFFFFF
FFFFFFFFFFFFFFF:FFFFFFFFFFFFFFFFFFFFFF,FFF:FFFFFF
@A00721:190:HGJW7DSXY:4:2224:12002:28166_2:N:0:TCTGCAAG+GCTGCACT
CCTTTAAGCGTCTTCTGTTCATCGCAACCAGATGATATCTTCTATTCGAACTTATGTTTCGATCAGTG
TTTCTTCTATCAGGAGGAAAACACTTGTTCTAGTCCTTGTAAGATTAA
+
FFFFFFFFFFFFFFFFFFFFFFFFFFFFFFFFFFFFFFFFFFFFFFFFFFFFFFFFFFFFFFF:FFFF
FFFFFFFFFFFFFFFFFFFFFFFFFFFFF:FFFFFFFFFFFFFF:F:FF
@A00721:190:HGJW7DSXY:4:1550:24216:3286_2:N:0:TCTGCAAG+GCTGCACT
TGTTTACCACCGAAATATCAAGGTTCTTGTCCTTCAAACGATTGGAAGGTTAGTTATCTTAATAGTAC
TAAAGGGGTTTACTTACAACCCTATGACGATAGTTTCTCTTTACACAC
+
FFFFFF:,FFFFFFFFFFFFF::F:FFFFFFF,FFFFFFFFFFFFFFF::F:FF:FF:FFFFFF,FF:
FFFFFF:FFFFFF,FFFFFF:::FF,FFFF,FF:FFFF,FFFF:FF:F
@A00721:190:HGJW7DSXY:4:1603:22046:5321_2:N:0:TCTGCAAG+GCTGCACT
TGTTTTCTTCTAATGTGGCCTGAACTACGTTTCACTTTGTGTGTACTTGTCTCTATTTTAAGTTTTGT
CTTCCTTGTTTGTCTTATTCTATTAATCACTCATTTTAGTGAGATTAT
+
FF:FFFFFFFFFFFF:FFFFFFFFFFFFFFFFFFFFFFF,FFFFFF:FFFFFFFFFFFFFFFFFFFFF
FFFF:FFFFFFFFFFFFFF:FFF:FF:FFF:FFFFFFFFF,:FFF:FF

@A00721:190:HGJW7DSXY:4:1151:16929:32690_2:N:0:TCTGCAAG+GCTGCACT
CTAAAACGTCTTCGACAACAGCAGGGGTTTTCGAATAGTGATAGCCACCCGCCTTACAGAGGGGCAGC
AGCATGACCATAATTGGTATTGACTTTGGTACCACCTTTTCTACTGTC
+
FFFFFFFFFFFFFFFFFFFFFFFFFFFF::FFFFFFFFFFFFFFFFFFFFFFFFFFFFFFFFFFFFFF
FFFFFFFFFFFFFFFFFFFFFFFF:FFFFFFFFFFFFFFFFFFFFFFFF
@A00721:190:HGJW7DSXY:4:1363:17155:7654_1:N:0:TCTGCAAG+GCTGCACT
TTCGAATGAAGTTTTAATGCTTAACAACAATGATAGTGAATTTATACCTACAATCATTGGTTTTTCTA
ATAATGATGACACTATTGTTTATGGATATGATGCTATTACTCATGATGGTTACGGTCAGGGAGGATTC
TCA
+
FFFFFFFFFFFFFFFFFFFFFFFFFFFFFFFFFFFFFFFFFFFFFFFFFFFFFFFFFFFFFFFFFFFF
FFFFFFFFFFFFFFFFFFFFFFFFFFFFFFFFFFFFFFFFFFFFFFFFFFFFFFFFFFFFFFFFFFFF
FFF
@A00721:190:HGJW7DSXY:4:1310:5909:5776_2:N:0:TCTGCAAG+GCTGCACT
ACATTACCCGAAAGACGTGCTAAATTAAAACCATTGTACGATGTCGTCTGTCTCGGCGATCACTTTTC
ACTAAGAATTGGTCCTACTTTTGGACAAGGCCAACTTCGTAATCTTCA
+
:FFFFFFFFFFFFFFFFFFFFFFFFFFFF:FFF:FFFFFFFFFFFFFFFFFFFFFFFFFFFFFFFFFF
FFFFFFFFFFFFFFFFFFFFFF:FFFFFFFFFFFFFFFFFFFFFFFFFF
@A00721:190:HGJW7DSXY:4:1435:22598:5776_2:N:0:TCTGCAAG+GCTGCACT
TCTTTATCTCTTTGGTTGTTAGGGATTTTGAGAGAGTGAAAGGCTTCAATTGTAGTGGTTTGGTCGTT
TCAGTACCCAGTCAGTATACCTCTACGCAAAGATATTTCATGAAAACT
+
FFFFFFFFFFFFFFF:FFFFFFFFFFFFFFFFFFFFFFFFFFFFFFFFF:FFFFFF::FFFFFFFFFFF
FFF,FFFFF:FFFFFFFFFF:FFFFFFFFFFFFFFFFFFFFFFFFFFF
@A00721:190:HGJW7DSXY:4:1212:25970:26866_1:N:0:TCTGCAAG+GCTGCACT
CCGGATTACCTGTCTTTCATATTATGAATGAACCTTCGGCCGCTCTCTTTGCTTCGATGTTGGATGTC
AAGAAGGGTTCAGAATGGGATTCCTACGTAGTTTATGACTTTGGTGGTGGTACTTTCGATACTTCTAT
CGT
+
FFFFFFFFFFFFFFFFFFFFFFFFFFFFFFFFFFFFFFFFFFFFFFFFFFFFFFFFFFFFFFFFFFFF
FFFFFFFFFFFFFFFFFFFFFFFFFFFFFFFFFFFFFFFFFFFFFFFFFFFFFFFFFFFFFFFFFFFF
FFF
@A00721:190:HGJW7DSXY:4:1238:1994:11209_2:N:0:TCTGCAAG+GCTGCACT
TACTATAGTGTTATTTATTCTGGTGGTGATGATAATCTTGGAGGTCGTGACGTTGATGAAGCTATAAG
AGCGTTCTTATTACGGCGCACTTCTTTACCCGCCACCACACAACTTTC
+
FFFF:FFFFFFFFFFFFFFFFFFFFFFFFFFFF:FFFFFFFFFFFFFFFFFFFFFFFFFF:FFFFFFFF
FFFFFFFFFFFFFFFFFFFFFFFFFFF:FFF:F:F:FFFFFFF:FF:FF
@A00721:190:HGJW7DSXY:4:1177:25997:32737_1:N:0:TCTGCAAG+GCTGCACT
TGAAGGAGGAGGTTTCTGTTACTGGAAAACCCTCGTCAATACTTTTCAATAATATACGTCTTGAACTC
ACTTATGACGATCTAGTGAAATTAACGCGTCCTTTTCTGGAACGTAGTTTTACTATTATGAACAAAGT
TAT
+
FFFFFFFFFFFFFFFFFFFFFFFFFFFFFFFFFFFFFFFFFFFFFFFFFFFFF:FFFFFFFFFFFFFFF
FFFFFFFFFFFFFFFFFFFFFFFFFFFFFFFFFFFFFFFFFFFFFFFFFFFFFFFFFFFFFFFFFFFF
FFF
@A00721:190:HGJW7DSXY:4:1474:5837:11694_2:N:0:TCTGCAAG+GCTGCACT
GCAAACTTACCCTGATTCCGGCCGGTGGTAGCTCATTTCTACCAGGGGCTATTACTCTTGCTGAAACA
GCTATTAAAGCTGTTGAGCGGGTAATTACACCTAAACGAGCTCGATCT
+
FFFFFFFFFFFFFFFFFFFFFFFFFFFFFFFFFFFFFFFFFFFFFFFFFFFFFFFFFFF:FFFFFFFFF
FFFFFFFFFFFFFFFFFFFFFFFFFFFFFFFFFFFFFFFFFFFFFFFF

```
@A00721:190:HGJW7DSXY:4:1139:20808:27853_1:N:0:TCTGCAAG+GCTGCACT
AAGGATGTGCACTCTTTTCTGCTACTTTAACGGAGCGTAGTGTACTCTTCGTCGATTGTATCAACAGT
AACATTACTTCTAATAAAGGTCTCTATCAAGCTCGTATTATCGTCGCTGCTGGATCTGCGTTACCGTT
GAA
+
FFFFFFFFFFFFFFFFFFFFFFFFFFFFF:FFFFFFFFFFFFFFFFFFFFFFFFFFFFFFFFFFFFFF
FFFFFFFFFFFFFFFFFFFFFFFFF:FFFFFFFFFFFFFFFFFFFFFFFFFFFFFFFFFFFFFFFFFF
FFF
@A00721:190:HGJW7DSXY:4:1355:21676:20713_2:N:0:TCTGCAAG+GCTGCACT
AGGTAATTATGAAGACTGTGATTTATCTTTCACTCTGGCCTTGTATGAGGGTGTTTCTAATCGTAACA
TTAATAATTTCAATCTTCTTAAAAAGAAGATAGAGAGTCATACTTTGG
+
FFFFFFFFFFFFFFFFFFFFFFFFFFFFFFFFFFFFFFFFFFFFF:FFFFFFFFFFFFFFFFFFFFFF
FFFFFFFFFFFFFFFFFFFFFFFFFFF:FFFFF:FFFFFFFFFFFFFF
@A00721:190:HGJW7DSXY:4:1154:8241:27884_2:N:0:TCTGCAAG+GCTGCACT
GATAGCCAATATTGAAGTGGACACCTCTGGTAGTATCGAAGTAAAGATAACCAATAAGGAAGGAGCTT
ATACTATGGTTGAATCAACTAAGCATACTACTTTTGCTTTGATTACTC
+
FFFFFFFFFFFFFFFFF:FFFFFFFFFFFFFFFFFFFFFFFFFFF:FFFFFFFFFFFFFFF:FFFFFFF
FFFFFFFFFFFFFFFFFFFFFFFF:F:FFFFFF,FFFFFFFFFFFFFF
@A00721:190:HGJW7DSXY:4:1562:28664:36980_1:N:0:TCTGCAAG+GCTGCACT
CTTCCGATCTGTTAAAGAGTCGATTAAACAAGTTGTACTTATCAATGATTTTATCGCTGGTTGTTATC
TCGAAAAGTCTCTTCTAAAAGGAACGTCAGTTCTAACCCAAAGAGAATATCTAAAATATTTACAAAAT
CTT
+
FFFFFFFFFFFFFFFFFFFFFFFFFFFFFFFFFFFFFFFFFFFFFFFFFFFFFFFFFFFFFFFFFFFF
FFFFFFFFFFFFFFFFFFFFFFFFFFFFFFFFFFFFFFFFFFFFF:FFFFFFFFFFFFFFFFFFFFFF
FFF
@A00721:190:HGJW7DSXY:4:2540:23692:31250_1:N:0:TCTGCAAG+GCTGCACT
ACGAGATCTTGCAGAGTGACTGGAGTTCAGACGTGTGCTCTTCCGATCTGTACAATCTTACTTGGAAC
GAAGGCGACCTGTTCCGGAGTTTTTACGGGATCCGGAATGTTCTGTCTATATTAGGTGAAGCGAAATA
CGA
+
FF,,FFFFFFFFFFFFFFFFFFFFFFFFFFFFFFFFFFFFFFFFFFFFFFFFFFFFFFFFFFFFFFFF
FFFFFFFFFFFFFFFFFFFFFFFFFFFFFFFFFFFFFFFFFFFFFFFFFFFFFFFFFFFFFFFFFFFF
FFF
@A00721:190:HGJW7DSXY:4:1257:7355:33865_1:N:0:TCTGCAAG+GCTGCACT
TCGTACCTTGATGCTGACTTTCGTCAATATGATTTGATCTGGTTGCTCAATTCTAAGCGTGTTCCGCT
TAGTGCGAGGCCTCTCTTCTACGAGTATTGCTATTTATACGGTTACGAGAAGAAGGTTCGAAAGTTTG
AGA
+
FFFFFFFFFFFFFF::FFFFFFFFFFFFFFFFFFFFFFFFFFF:FFFFFFFFFFFFFFFFFFFFFFFF
FFFFFFFFFFFFFFFFFFFFFFFFFFFFFFFFFFFFFFFFFFFFFFFFFFFFFFFFFFFFFFFFFFFF
FFF
@A00721:190:HGJW7DSXY:4:1409:10050:1579_2:N:0:TCTGCAAG+GCTGCACT
CTATACGTGACATACTAAATACTAGATATTTGGATGATCTTGATAAAGAACTTCATGAATTTTCAGAG
GATGAAAAATTTTCTTCCTTGGATTTTAATATAAAGAATCTTTACCAG
+
FFFFFFFFFFFFFFFFFFFFFFFFFFFFFFFFFFFFFFFFFFFFFFFFFFFFFFFFFFFFFFFFFFFF
FFFFFFFFFFFFFFFFFFFFFFFFFFFFFFFFFFFFFFFFFFFFFFFF
@A00721:190:HGJW7DSXY:4:2238:28953:6762_1:N:0:TCTGCAAG+GCTGCACT
AGGAGGCAGTCTTCTGATCGTAAGAAGTTTATAAGGAACATATCCATTTGTTGTAATAGGGTCATCAA
CTTAGAACTCGAAACTCTAAGTGGTAATACTGTCTTTCCAAGCTTACCTGTATCTTCAATGAAATTGG
ACT
```

+
FFFFFFFFFF:FFFFFFFFFFFFFFFFFFFFFFFFFFFFFFFFFFFFFFFFFFFF:FFFFFFFFFFF
FFFFFFFFFFFFFFFFFFFFFFFFFFFFFFFFFFFFFFFFFFFFFFFFFFFFFFFFFFFFFFFFFFFFFFFF
FF:
@A00721:190:HGJW7DSXY:4:1170:24930:4993_2:N:0:TCTGCAAG+GCTGCACT
GTGGGCTTTCCGTAAAGGAAGATCTGTTATCGACGGTTTATCGTTTACGAAGTATCACTCCACCGGTG
AATGGTTTAAGAATACTATAAATAGAATAGCTATTACTACTAATGGCG
+
FFFFFFFFFFFFFFFFF:FFFFFFFFFFF:FFFFFFFFFFFFF:FFFF:FFFFFFFFF:FFFFF:FFF
F:FF:FFFFFF:F:FFFFFFFFFFFF:,F,FF:FFFFFFFFFFFF:F,
@A00721:190:HGJW7DSXY:4:1217:9579:16861_2:N:0:TCTGCAAG+GCTGCACT
TAGTGACATCTGGTCAATTTCGTTTTTAGGTGGTCTTTATTCTATTATTGATTTTTATGAGATTGATA
CGTTAACAGAAGATGATGTTAGAGTAGAATTCCTGAAGAGTCCTTCCG
+
FFFFFFFFFFFFFFFFF:FFFF:FFFFFFF:FFFFFFF:FFFFFF:FFFFFFFFFFFFFF,FF:FFFF
FF:FFFFFFFFFFFFFFFFF,FFFFF:FFFFFFF:FFFFFFFFFFFF,F
@A00721:190:HGJW7DSXY:4:2565:3992:12853_1:N:0:TCTGCAAG+GCTGCACT
TAATAGTTAAATCTAGGATTATGTCTGTGTTGAAAGACTTTCTACCGATAAGGGTCACTAGTCTTACA
CAATTAAGAAATAATCCTACATTGACTGATTATCTAAAGATTGAAGGAAAATTTTATCCTCGTTTAAC
TTA
+
FFFFFFFFFFFFFFFFFFFFFFFFFFFFFFFFFFFFFFFFFF:FFFFFFFFFFFFFFFFFFFFFFFFFF
FFFFFFFF:FFFFFFFFFFFFFFFFFFFFFFFFFFFFFFFFFFFFFFFFFFFFFFFFFFFFFFFFFFFF
FFF
@A00721:190:HGJW7DSXY:4:1254:32217:5118_1:N:0:TCTGCAAG+GCTGCACT
GTGCTCTTCCGATCTATAACTTCTAATTTACGTTTGCTACTCAGAGCTGGTCCTCTCAGCAAAGGGAT
GCATGGTGTTATCTCCTCAGGAGTCCCGGCATTTGATAATAATGACGAGACTTTCGTCATAATCGCTG
CAT
+
FFFFFFFFFFFFFFFFFFFFFFFFFFFFFFFFFFFFFFFFFFFFFFFFFFFFFFFFFFFFFFFFFFFFFFFF
FFFFFFFFFFFFFFFFFFFFFFFFFFFFFFFFFFFFF:FFFFFFFFFFFFFFFFFFFFFFFFFFFFFFFF
FFF
@A00721:190:HGJW7DSXY:4:1250:17183:35728_1:N:0:TCTGCAAG+GCTGCACT
GGTACCAGCGCATCTCGTGTCGAGCCTCGACCCGATTATATGGTATTAACTTATAAAGGTGAGGAGAC
CATTTTCTTGCTGAAATTTATAGAAGATTATTTTGACAGAATCAATAAGAATCATCGATTCTCGATTC
GTA
+
FFFFFFFFFFFFFFFFFFFFFFFFFFFFFFFFFFFFFFFFFFFFFFFFFFFFFFFFFFFFFFFFFFFFFFFF
FFFFFFFFFFFFFFFFFFFFFFF:FFF,FF:FFFF:F:FF,FFFFFFFFFFFFFFFFFFFFFFFFFFF
FFF
@A00721:190:HGJW7DSXY:4:1222:28320:25739_1:N:0:TCTGCAAG+GCTGCACT
TACATTTATGCTGATCTAAAATTTCGATCTAGATGGATCCACTCCTCGAGTTATCATGATGTTAGTAT
GTTATATACTGATTTACAACGATATAGGAGTAATCTACGAGAAGATTCTAGAATTCTTAATTATAATA
TTC
+
FFFFFFFFFFFFFFFFFFFFFFF:F,F,FFF,FFFFFFFFFFF:FFFFFF:FFFFFF:FF:FFFFFFF
FFFFFFFF,FFFFFFFFFFF:FF:FF,FFFFFFFFFFF:FFFFFFF:FF:F:FFFF:FFFFFFFFF:F
:,F
@A00721:190:HGJW7DSXY:4:1156:16206:7294_2:N:0:TCTGCAAG+GCTGCACT
GAGATCACACATTTTGAGAACTAACTATGTTATGTTCTCTGATCATTTTGACAATCTCTTCTTATAAT
AAATAAATAAATAATATTACTAATGGCAAATAGTGGAAAGGCTCCAGT
+
FFFFFFFFFFFFFFFFFFFFFFFFFFFFFFFFFFFFFFFFFFFFFFFFFFFFFFFFFFFFFFFFFFFFFFFF
FFFFFFFFFFFFFFFFFFFFFFFFFFFFFFFFFFFFFFFFFFFFFFFF

```
@A00721:190:HGJW7DSXY:4:1325:11207:5525_2:N:0:TCTGCAAG+GCTGCACT
CTGCTTTGGATTACGATGACGTGAGAATAGGTGAAGGGATAGTTTTTACCTCGACCAACATTAAGGCG
GCGGCGACTAATCTTAAAGCTTCACCCAAAATGAAGGACACTTCGAAA
+
:FFFFFFFFFFFFFFFFFFFFFFFFFFFFFFFFFFFFFFFFFFFFFFFFFFF:FFFFFFFFFFFFFFF
FFF::FFFFFFFF:FFFF:FFFFFFFFFFFFF:FFF,FFFFFFFFFFF
@A00721:190:HGJW7DSXY:4:1172:31358:17033_2:N:0:TCTGCAAG+GCTGCACT
AAAAGGCTCTAAAGGACACTATACAATATCCAGACGACGACGAAGCTTATACTACTTTAATTTTGGGT
ATTATGTGGAGATATCGTGATATCAGTACGTCCAGATCCTCTGCTTTT
+
FFFFFFFFFFFFFFFFFFFFFFFFFFFFFFFFF:FFFFFFFFFFFFFFFFFFFFFFFFFFFFFFFFFF
FFFFFFF::FFFFFFFFFFFFFFFFFFFFFFFFFFFFFFFFFFFFFFF
@A00721:190:HGJW7DSXY:4:1155:25690:27602_2:N:0:TCTGCAAG+GCTGCACT
ATCAAATATAAAGAAAAGGACTATGAAATAAAAGATTTGGTGCTTAGGGATATAATGAATTCAGCACC
ACATTTTGGTCAAGTCAATCCGTTGAGAAAATTTCTTAGAGGATATTC
+
FFFFFFFFFFFFFFFFFFFFFFFFFFFFFFFFFFFFFFFFFFFFFFFFFFFFFFFFFFFFFFFFFFFF
FFFFFFFFFFFFF:FF:FFFFFFFFFFFFFFFFF:FFFFFFFFFFFFF
@A00721:190:HGJW7DSXY:4:1156:18891:36213_1:N:0:TCTGCAAG+GCTGCACT
CCAACATTTACCACCAAATTCTTTTGATACAACTCTAGCCTTCAAGTGGGGTATGCCTGCGGAATACG
CTTGCTATTCACCAGATTGGTTCGAACCCGTTGATGCTATGTCAGAAGAAGCTATTCAAGCTTGTCGT
TTA
+
FFFFFFFFFFFFFFFFFFFFFF:FFFFFFFFFFFFFFFFFFFFFFFFFFFFFFFFFFFFFFFFFFFFF
F,FFFFFFFFFFFFFFFFFFFFFFFFFFFFFFFFFFFFFFFFFFFFFFFFFFFFFFFFFFFFFFFFFF
FFF
@A00721:190:HGJW7DSXY:4:2309:11306:3662_2:N:0:TCTGCAAG+GCTGCACT
TCAAACAAGCCCAGACTCAAGGAGTCCAATCAAAGGTTTTGAATTTAGCTCAACTGGGGAGACGTGCT
TAAATGGGTGATAAAAATTATTTAGAGATCTTTTCTACTTACAAAGGA
+
FFFFFFFFFFFFFFFFFFFFFFFFFFFFFFFFFFF:FFFFFFFFFFFFFFFFFFF:FFFFFFFFFF:FFF
FFFFFFFFFFFFFFF:FFFFFFFF:FFFFFFFFFFFFFFFFFFF,FFF
@A00721:190:HGJW7DSXY:4:1462:28628:11209_2:N:0:TCTGCAAG+GCTGCACT
AATTCTACGGAAAAGTCCGGAGTATATTGTATGTTAATAGATAGTGGGATAATAGTTCTACCGATACG
TAGTGAACGCGTTCGTAGAACCAAGTACTTTCCGTCTGAAGAGTTTAG
+
FFFFFFFFFFFFFFFFFFFFFFFFFFFFFFFFFFFFFFFFFFFFFFFFFFFFFFFFFFFFFFFFFFFF
FFFFFFFFFFFFFFFFFFFFFFFFFFFFFFFFFFFFFFFFFFFFFFFF
@A00721:190:HGJW7DSXY:4:1423:23710:34695_1:N:0:TCTGCAAG+GCTGCACT
TAACCGATAGGAGTTGTATAGTTGAAGATGTGGCTTTAGACGATAATCCTTTAAGGACTATGTTGACT
GACGAAGATTTTCCCACAGATTTGTCTACGTTGCAGATCGCCACTCCAAACACTATATACATTAAAAT
ATA
+
FFFFFFFFFFFFFFFFFFFFFFFFFFFFFFFFFFFFFFFFFFFFFFFFFFFFFFFFFFFFFFFFFFFF
FFFFFFFFFFFFFFFFFFFFFFFFFFFFFFFFFFFFFFFFFFFFFFFFFFFFFFFFFFFFFFFFFFFF
FFF
@A00721:190:HGJW7DSXY:4:2129:29423:2440_2:N:0:TCTGCAAG+GCTGCACT
AGTCATAACTTTTGTTACCAAATTCTTGGCTTTCAACGAAGTCTCTGATTTTATTAATCATTACTTTA
ACGATCTCTCGGTCATTTACTCTGTACTTGATCTACGTCGTTTAACTG
+
FFFFFFFFFFFFFFFFFFFFFFFFFFFFFFFFFFFFFFFFFFFFFFFFFFFFFFFFFFFFFFFFFFFF
FFFFF:F:FFFFFFFFFFFFFFFFFFFFFFFFFFFFFFFFFFFFFFF:
@A00721:190:HGJW7DSXY:4:1265:26241:27398_2:N:0:TCTGCAAG+GCTGCACT
CTTAGAATGACTCTATCTTTTCAGACACTTCTTATATTATTTACAATAGTTAATATCATATTAGTAAT
```

ACTCACTATCGTTTTCTGCTTTTTCTTAGCTAATAGATTAATAAAGGA
+
FFFF:FFFF:FFFFFFFFFFFFFFFFFFFF:,FFFFFFFFFFFFFFFFFFFFFFFFFFFFF,FFFFFF
F:FFFFFFFFFFFFFFFFFFFFFF:FFFF,FFFF,,FFFFFFFFFFF
@A00721:190:HGJW7DSXY:4:1407:27670:14058_2:N:0:TCTGCAAG+GCTGCACT
ACCAACTCTTCGTTACGAGACCTGATGGTAGAATAGATTTAGTCTCTACACCCATCTCAACGAGGATA
AAGCGAGTTTTCCGTCCTGAACGTAATACACGAAGAGATCATCCGCAA
+
FFFFFFFFFFFFFFFFFFFFFFFFFFFFFFFFFFFFFFFFFFFFFFFFFFFFFFFFFFFFFFFFFFFF
FFFFFFFFFFFFFFFFFFFFFFFFF:FFFFFFFFFFFFFFFFFFFFFF
@A00721:190:HGJW7DSXY:4:1135:11080:31203_2:N:0:TCTGCAAG+GCTGCACT
AAAGTCCCTTCTAACTTTAGATTATGAATCCTGCCTTTACTCATTTTATAGCAGTTATTTATGTTGTA
CTTTTTATAATTTATAACCTTCTTATACTTTTGGGTATGTTTTGTGAA
+
FFFFFFFFFFFFFFFFFFFFFFFFF:FFFFFFFFFFFFFFFFFFFFFFFFFFFFFFF,FFFFFFFFFF
FFFFFFFFFFFFFF:FFFFFFFFFFFF::FFF:FFF:FFFFFFFFFFF
@A00721:190:HGJW7DSXY:4:1275:11198:18505_2:N:0:TCTGCAAG+GCTGCACT
GAAGACATTATCTCTTATGTACTTATTGTAACGATCATTCTTTCGAGTTTTGTCATTCTTATTCTTTT
ATTCCTTTAACTGTGTATGAGGAGGATTTATAATGCTTTTCGATAGTG
+
FFFFFFFFFFFFFFFFFFFFFFFFFFFFFFFFFFFFFFFFFFFFFFFFF:FFFFF,FF:F:FFFFFFF
FFFFFFFFFFFFFFFF:FFFFFF:FFFFFFFFFF:FFF:FFFFFFFFF
@A00721:190:HGJW7DSXY:4:1612:10592:24909_2:N:0:TCTGCAAG+GCTGCACT
ATTACTGTTTCAAATCATCTTTACTCACAGACTCCGAAAATGGTTTCAAATGAGGTCAAAGCTCTATT
TATTAAACGAACTATTATCCGATTCTTCGAGGGTATCACTACCACTGA
+
FFFFFFFFFFFFFFFFFFFFFFFFFFFFFFFFFFFFFFFFFF:FFFFFFFFFFFFFFF:F:F:FFFFFF
FFFFFFFFFFFFFFFFFFFFFFFFF:FFFFFFFFFFFFFFFFFFFFFF
@A00721:190:HGJW7DSXY:4:1377:1208:2362_2:N:0:TCTGCAAG+GCTGCACT
CAAGGAACTTCAATTCCTTCGATGTTTATATTTTTCTATAAGGGAAAATAGCAATAATCAAATTTCGA
CAAATTTTAGAACTTTATTTCAGAACTCTCTCCGATCACCTTGGTGTG
+
FFFFFFFFFFFFFFFFFFFFFFFFFFFFF:FFFFFFFFFFFFFFFFFFFFFFFFFFFFFFFFFFFFFF
FFFFFFFFFFFF:FFFFFFFFFFFFFFFFFFFFFFFFFFFFF:FFFFFF
@A00721:190:HGJW7DSXY:4:1504:5141:8641_1:N:0:TCTGCAAG+GCTGCACT
TCTTTTCAATAAGATGTATGATGATAATCTACTATTTTTAAATAGTGATATTCAGCGTATACTTGAAT
ACTTTATCAAAGATTTGGCTTGGTCTATTTACCGCAAAAAGTTGGAAGAGGTCTATGATATTTATTTT
ACT
+
FFFFFFFFFFFFFFFFFFFFFFFFFFFFFFFFFFF:FFFFFFFFFFFFFFFFFFFFFFFFFFFFFFFF
FFFFFFFFFFFFFFFFFFFFFFFFFFFFFFFFFFFFFFFFFFFFFFFFFFFFFFFFFFFFFFFFFFFF
FFF
@A00721:190:HGJW7DSXY:4:2120:28727:22842_2:N:0:TCTGCAAG+GCTGCACT
CACCGATTATAACTTGACTGTTGATAATATTACAATAGCTCTTTCTCAGAAGATTAGAGAAGTAACCG
GTGAACACGTCGGTAGCATTGATTTACTTTTACCGTTAGAACTGAAAA
+
FF:F,FFFFFFFFFFFFFFFFFFFFFFFFFFFFFFFFFFFFFFFFFFFFFFFFFFFFFFFFFFFFFFF
FFFFFFFFFFFFFFFFFFFFFFFFFFFF::FFFFFFFFFFFFFFFFFF
@A00721:190:HGJW7DSXY:4:1429:27254:1720_1:N:0:TCTGCAAG+GCTGCACT
ACCTATAAGTTACAAACGATTCTTCGACTATATTAAAGAAATAAAAATACTTCAAGTACCTGAACTTT
ACATTTTTAATGGTATGGTATAATGTGTCTTACAAATAGAACCTTCACTCTCGGTAATGAGAGTGTAT
TCC
+
FFFFFF:FFF:,F:,FF,F,FFFFFFF:FFFFFFFFF:FFFFFFFFFFFFFFF:FFFFFFFFFFFFFF

FFFFFFFFFFFFFFFF,FFFFFFFFFFFFFFFFFF:FFFFF,FFFFFFFFFF:FFFFFFFFFFFFFFF
FFF
@A00721:190:HGJW7DSXY:4:1370:7916:24721_1:N:0:TCTGCAAG+GCTGCACT
GGCATTACTAGGATTTATCACACTCCTAAATTTGATAGCAATTTATGTTTTAAATGCTAGGAAAATTT
TAGAAAAACTCCATTGTATAGTTCAAGGAGTACTTAATTCAATAGCTTTAGTATTATTTACTATCGCC
TTT
+
FFFF:FFFFFF:FFFFFFFFFFFFFFFFFFFFFFFFFFFFFFFFFFFFFFF:FFFFFFFFFFFFFFFF
FFFFFFFFFFFFFFFFFFFFFFFFFFFFFFFFFFFFFFFF:FFFFFFFFFFFFFFFFFFFFFFFFFFF
FFF
@A00721:190:HGJW7DSXY:4:1302:32542:8218_2:N:0:TCTGCAAG+GCTGCACT
AGTTAATTGCTTACACAAAAGTGACGCGCGAAGGAATCTTAATTGATTGAAGCTAGGCGTTGGGGAGA
ATGAACTGCCGCTTCCCGGAAGAAACGATCTATTTACAGAGTATTTAG
+
FFFFFFF:FFFFFFFFFFFFFFFFFFFFFFFFFFFFFFFFFFFFFFFFFFFFFFFFF:FFFFFFFFFF
FFFFFFFFFFFFFFFFFFFFFFFFFFFFFFFFFFFFF:FFFFFFFFFFF
@A00721:190:HGJW7DSXY:4:1102:7916:34773_2:N:0:TCTGCAAG+GCTGCACT
GAATTCCTGGCGAACCGCAAATCAATTGAAGAAAACCACTCCCTTCAATTCAATAACATAACATACCA
TACACTCCCCAAACATAACATACCATAACCTACCATGACCAATCATCG
+
FFFFFFFFFFFFFFFFFFFFFFFFFFFFFFFFFFFFFFFFFFFFFFFFFFFFFFFFFFFFFFFFFFF:
FFFFFFFFFFFFFFFFFFFFFFFF:FFFFFFFFFFFFFFFFFFFFFFF
@A00721:190:HGJW7DSXY:4:1132:17436:26835_1:N:0:TCTGCAAG+GCTGCACT
ACGCCAGACGTAGGGTCAAACCTACTACCGCCGGTCGGCAGATCGGCACCACTGGTCTCTACGTTCAG
GGACCCGCTGTGCAGAATAACCTTCCCAAATCTAGATCTGCACAGAAGAAGGCTGAAGGTCGTTACCG
GAC
+
:FFFFFFFFFF,:FFFFFFFFFF:FF,FFFFFFFFFFF,FFF,FFFFF:FFF,FFFFFFFFFF:FFFF
FFFFFF,FFFFFFFFF,FF:,FFFFFF:FFFFFF:F:F:FFFFFFFFFFFFFFFFFFFFFFFF:F:FFF
FFF
@A00721:190:HGJW7DSXY:4:1203:3685:17112_1:N:0:TCTGCAAG+GCTGCACT
GGCTGTTGCCCAGAGGAAAGCAGCGACCACCGAGGTCGCTCGTAACATTGACGGCAGTCCCGTCATCT
CTGTGGGAACTGAGGCAGTACCTCAGTCAAAAGTCACATTCACCACTCCCATGCCAATCAATGATGTG
ACT
+
FFF:FF:FFFFFFFFFFFFFFFFFFFFFFFFFFFFFFFFFFFFFFF:FFFFFFFFFFFFFFFFFFFFF
FFFFFFFFFFFFFFFFFF:FFFFFFFFFFFFFFFFFFFF:FFFFF,FFFFFFFFFFFFFFF:FFFFFFF
FFF
@A00721:190:HGJW7DSXY:4:2357:9191:32628_2:N:0:TCTGCAAG+GCTGCACT
GTGGTCAATACGTCGTGGGAGGTACTGAAGCCTTCCCCTCCGCTGGCACGAAGAACACTCCCAAGCCA
ATCAATAGTTCTTCTGACCAGAATGAAAAGGGGTTTACCAGGGAGATC
+
FFFFFFFFFFFFFFFFFFFFFFFFFFFFFFFFFFFFFFFFFFFFFFFFFFFFFFFFFFFFFFFFFFFF
FFFFFFFFFFFFFFFFFFFFFFFFFFFFFFFFFFF:FFFFFFFFFFFFF
@A00721:190:HGJW7DSXY:4:1217:10348:29121_2:N:0:TCTGCAAG+GCTGCACT
CAGGAAGAGGGGAAATGTCCCCAAAACTGAGAAGAATAAAGGGAAAGGTCCATCAGGACCTAAGAAGG
GGAAATTACCCCTAATTCTGAAGGAAGATCTCCCACCATCTGGGGTTG
+
FFFFFFFFFFFFFFFFFFFFFFFFFFFFFFFFFFFFFFFFFFFFFFFFFFFFFFFFFFFFFFFFFFFF
FFFFFFFFFFFFFFFFFFFFFFFFFFFFFFFFFFFFFFFFFFFFFFFFF
@A00721:190:HGJW7DSXY:4:1259:28212:14810_2:N:0:TCTGCAAG+GCTGCACT
ATCTGCACGGGCCAAAGAGGCCGCTGCTACGAAGTCGCTGAAGGCGGCTTGGGTCATTAAGGGTAGCT
GTAAAGTTGCGCCTGCCGACCCCATCTTTGAGACCAAGACTCTCTACA
+

FFFFFFFFFFFFFF:FFFFFFFFFFFFFFFFFFFFFFFFFFFFFFFFFFFFFFFFFFFFFFFFFFFFF
FF:FFFFFFFFFFFFFFFFFFFFFFFFFFFFFFFFFFFFFFFFFFFFF
@A00721:190:HGJW7DSXY:4:1608:14488:24330_1:N:0:TCTGCAAG+GCTGCACT
TACTATGAGCAAATCTGTTGAATTCGTCACTGAGGACGAAGGTGAGAGGACTTTTGGCTACTGGCGAC
AAACTGAGAGCAGTCGCTGGGGAAAGAGAATTGCGTGGGTTGAAGGCCGACGCTTCGAGCTTGAGTGT
GAC
+
FFFFFFFFFFFFFFFFFF:,FFFFFFFFFFFFFFFFFF:FFFFFFFFFFFFFFFFFFFFFFFFFFFFF
FFFFFFFFFFFFFFFFFFFFFFFFFFFFFF,FFFFFFFFFFFFFFFFFFFFFFFFFFFFFFFFFFFFF
FFF
@A00721:190:HGJW7DSXY:4:1148:29441:1814_2:N:0:TCTGCAAG+GCTGCACT
GTTTTTGTCAACGGCGTTTGGAGGATTTTATCCGCGCTTAACTATGGGGCGAAGATCCCCTATGGAGT
CTTGAAGGACTTAGGTTGGCATATTTATGCCGACGATAATAAGCATGT
+
FFFF:FFFFFFFFFFFF,FFF:FFFFFFFFFFFFFFFFFFFFFFFFFFFFFFFFFFFFFFFFFFFFFF
FFFFF,FFFFFFFFFFFFFFFFFFFFFFFFFFFFFFFFFFFFFFFFFF
@A00721:190:HGJW7DSXY:4:1242:25825:17347_1:N:0:TCTGCAAG+GCTGCACT
CCGAGGTAAGCTCGGGACTTACAAGAGAGAGTGTGAGAGAGTCGCTCTCACTATTTTTGCCCAGAATG
CGGGCTTTCTGACTGATGAGGTTAAAACTCTTGCTCCTTCTGTGGGTGAGGGTTTTTGTTATATTCAC
TGG
+
FFFFFFFFFFFFFFFFFFFFFFFFFFFFFFFFFFFFFFFFFFFFFFFFFFFFFFF:FFFFFFFFFFFF
FFFFFFFFFFFFFFFFFFFFFFFFFFFFFFFFFFFFFFFFFFFFFFFFFFFFFFFFFFFFFFFFFFFF
FFF
@A00721:190:HGJW7DSXY:4:1129:7880:26130_2:N:0:TCTGCAAG+GCTGCACT
TCCTCCATAATTACAACCTCTCTTTAGAGAGCATTAGAAGGAAGTTGGGCAAATTCCCACATATGGTA
GACGTTGTCACACTTTTCTTGAAAATTTTTCCTTTCCTCCGTCATTGG
+
FFFFFFFFFFFFFFFFFFFFFFF:FFFFFFFFFFFFFFFFFFF,FFFFFFFFFFFFFFFFF:FFFFFF
FFFFFFFFFFFFFF:FFFFFFF:FFFFFFFFFFFFFFFFFFFF:F,FF
@A00721:190:HGJW7DSXY:4:1530:10402:27430_2:N:0:TCTGCAAG+GCTGCACT
GTGAGAAGGTGGAAGGTGTCAAGGGTCTACTTCATGCTGATCTTTCAGGACCTAATCACATCATGATT
GATAGTTACGGCTCGTATCGAGTTGGAGGGAGGAAGTTCTCCGAAATT
+
FFFF:FFFF,F:FFFFFFFF:FFFF,F,,FF,FFF:FFFFFFF,,FFFFFFFFFF:FFFF,F,FFFFF
FFFFF,FFFFFFFFFFFFFFFFFFFFFFFF,FFF,FFFFFFFF,F:FF
@A00721:190:HGJW7DSXY:4:1269:2745:22404_1:N:0:TCTGCAAG+GCTGCACT
GCTGTAACTGTCATTTCAGAGAGGGGAGCTCTGAAAAGGAAACTTCGTCAGAAGATGAGGAAGTCTAA
GAGGAGGAGTTTGAGGAAGGCTCTAGAGAAGATCTCACCACCTGGAGAAGGCAAGTGGAGAGTCCAGA
AGT
+
FFFFFFFFFFFFFF:FFFFFFFFFFFFFFFFFFFFFFFFFFFFFFFF:FFFFFFFFFFFFFFFFFFFF
FFFFFFFFFFFFFFFFFFFFFFFFFFFFFFFFFFFF,FFFFFFFFFFFFFFFFFFFFFFFFFFFFFFF
FFF
@A00721:190:HGJW7DSXY:4:1511:16034:36307_2:N:0:TCTGCAAG+GCTGCACT
GGATTATTTGAAGCAGGGGTTACCACTTCCTAAGGTGGATGAGGGAGGTTGTTTCTTTGGAGTTTTAG
ATAATCTTTTGAGAAGGGGTCATAGAGCCAGCAAGAGAAGCTTATATG
+
FFFFFFFFFFFFFFFFFFFFFFFFFF:FFFFFFFFFFFFFFFFFFFFFFFFFFFFFFFFFFF,FFFFF
FFFFFFF,FFFFFFFFFFFFFFFFFFFFFFFFFFFFFFFFFFFFFFFF
@A00721:190:HGJW7DSXY:4:1212:4490:27461_2:N:0:TCTGCAAG+GCTGCACT
CCTTGAGAAGACAAGCCACATTAAGATATAATTTAGGTCAACTTGGGGTTTCGAATGTCATTAGTGCC
TTACGATTGAAAGAGGTCCTTTTGAGTATCGACAGATCTTTCGATCTT
+

```
FFFFFFFFFFFFFFFFFFFFFFFFFFFFFFFFFFFFFFFFFFFFFFFF:FFFFFFFFFFFFFFFFFFF
FFFFFFFFFF:FFFF,FF:FFFFFFFFFFF:FFFFFFFFFFF:FF:FF
@A00721:190:HGJW7DSXY:4:1308:18204:12696_2:N:0:TCTGCAAG+GCTGCACT
TTAACGTGGCAGAATTCGGTAACTTACCATATTGTTCCTTCCGGGAAGTTGTGGGATTTAACTTTGAC
TCAACTACTCCGATCAGATCCGGATGGTTTGATTGGTTTGGACTACTC
+
F,FFFFFFFFFFFFFFF,FFF:FFFFFFFF:FF:FF,FFFF:FFFFFFFFF,FFFFFFFFFFFFFFF:
F:FFFFFFFFFFF,FFFF:,FFFFFFFFF:FF:,FF:,FFFFFFFFF:
@A00721:190:HGJW7DSXY:4:1426:4616:28087_1:N:0:TCTGCAAG+GCTGCACT
TAGTTGTCTAGGGTCATGGCAGATACTACCGATAAGGTGAAAAATATGCAGGTCAAAGATTCTGTAGC
TCTGAGGGCGGTTGAGGGAGCCGTTTTGGCCGACTTTAACTATTCTAAGAGTCTTTATGATTCAAAAC
CTA
+
FFFFFFFFFFFFFFFF:FFFFFFFFFFFFFFFFFFFFFFFFFFFFFFFFFFFFFFFFFFFFFFF,FFF
FFFFFFFFFFFFFFFFFFFFFFFFFFFFFFFFFFFFFFFFFFFFFFFFFFFFFFFFFFFFFFFFFFFF
FFF
@A00721:190:HGJW7DSXY:4:1429:11767:30232_2:N:0:TCTGCAAG+GCTGCACT
ACCTTACAAGGTTGAGGAGACTTTACAGCGGCTTATTGTCGCGCAGTTTCCAGAATTGCGGTTGAAGT
TCACCCATTCTATCTTGAATGCTCACGCCGTCGCAGCCATATCGAGAC
+
FFFFFFFFFFFFFFFFFFFFFFFFFFFFFFFF:FFFFFFFFFFFFFFFFFFFFFFFFFFFFFFFFFFF
FFFFFFFFFFFFFFFFFFFFFFFFFFFFFFFFFFFFFFFFFFFFFFFF
@A00721:190:HGJW7DSXY:4:1452:9308:6840_2:N:0:TCTGCAAG+GCTGCACT
AAAGTACAGATTTAGTGATTTAGATATTATTGATATTGGAGGCGATATTCGTTTTCATATCCAAAACT
CCTTGAATGGAGTACATATTTGCAACCCTATTCTTGATGTGAAAGATG
+
FFFFF:FFFFFFFFFFFFFFFFFFFFFFFFFFFFFFFFFFFFFFFF:FFFFFFFFF:FFF:FFFFFFF
:FFFFFFFFFFFFFFFFFFFFFFFFFFFFFFF:FFFFFFFFFF,FFFF
@A00721:190:HGJW7DSXY:4:2365:12536:7138_2:N:0:TCTGCAAG+GCTGCACT
AACATTGATTGGGAACGTACCTACTCACGAGACGTCATGAAAGCTATCGGAGGGAAGCAAAATGTTTC
GTTCTGTTACAAGAACGCTTTGGATTGTGACCATAAAGCCTCAGTTGC
+
FFFFFFFFFFFFFFFFFFFFFFFFFF:FFFF,FFFFFFFFFFFFFFFF:FFFFFFFFFFFFFFFFFFF
FFFFFFFFFFFFFFFF:FFFFFFFFFFFFFFFFFFFFFFFFFFFFFFF
@A00721:190:HGJW7DSXY:4:1648:27697:25786_2:N:0:TCTGCAAG+GCTGCACT
ACGATATCTCTCTTGATGATGTAGCCACTATAATGCTTAAGAGAGAGATTGATGTATGTCATTTCGTC
TTAGTTTGTCCCGGCGAATTATTGGACACGATGAAAAATGACATACCC
+
FFFFFFFFFFFFFFFFFFFFFFFFFF:FFFFFFFFFFFFFFFFFFFFFFFFFFFFFFFFFFFFFFFFF
FFFFFFFFFFFFFFFFFF,FF,FFFFFFFF:FFF:FFFFFFFFFFFFF
@A00721:190:HGJW7DSXY:4:1434:4273:13808_1:N:0:TCTGCAAG+GCTGCACT
TCGTTATCCGTCATGGAGATGATGGCGCATCACTTGATTACTATATGCCCGAAGGATTGGGTTATTCT
CACAACAAAGATAAGATTTTATCTTTCTTCAAACACCCTGTTTTCCTTAAGGGCGGACAATTGTTCCA
TTG
+
FFF:FFFFFFFFFFFFFFFFFFFFFFFFFFFFFFFFFFFFFFFFFFFFFFFFFFFFFFFFFFFFFFFF
FFFFFFFFFFFFFFFFFFFFFFFFFFF:FFFFFF:FFFFFFFFFFFFFFFFFFFFFFFFFFFF,FFFF
FFF
@A00721:190:HGJW7DSXY:4:2330:19271:5118_2:N:0:TCTGCAAG+GCTGCACT
ATTGAGCATTAATGAGTTCCGAATAACTCGTAATAAATCTTTCTCTAGTGGACAATTCAGAATACCTA
TTTCCGTACCAAGAGCCACTGCCAATCTCATCGCTATACGCATACCTG
+
FFF:F:FFFFFFF:FFFFFFFF:FFFFFFFFFFFFFFFFF,FF:FFFF,:FFFFFFFFFFFF,FFFFF
FF:F:F:FF:FFFFFFFFF,:FFFF:FFFFFF,FFF:F,,FF:FF,,F
```

```
@A00721:190:HGJW7DSXY:4:1643:7970:13479_1:N:0:TCTGCAAG+GCTGCACT
GAGGTTCGTTCCTCAATAAAAATTTTTCTCAAGAGATTATGGTTGATAAAGTTTTCTTCATAAGGGCT
ATTCAGTACGTCATGGGGATCACTTCTTCGGTCAATGACAAAACCTTCGAATATGTTACGACATGGCT
TCG
+
FFFFFFFFFFFFFFFFFF,FFFFFF:FFFFF:FFFFFFFFFFFFFFFFFFFFFFFFFFFFFF:FFFFF
FFFFFFFFFFFFFFFFFFFFFFFFFFFFFFFFFFFFFFFFFFFFFFFFFFFFFFFFFFFFFFFFFFFF
FFF
@A00721:190:HGJW7DSXY:4:1628:19958:17957_2:N:0:TCTGCAAG+GCTGCACT
AGGATTGTCATCTCGGGGAAAGTGATTCACCAAGATGTTCATATAGATACGAGATACCTAGAGAAGGT
CGCAGCGATCTTACTCGTTATCGGAGCTAAGAAGAAGACTTTGGGTAC
+
FFFFFFFFFFFFFFFFFFFFFFFFFFFFFFFFFFFFFFFFFFFFF:FFFFFFFFFFFFFFFFFFFFFF
FFF:FFFFFFFFFFFFFFFFFFFFFFFFFFFFFFFFFFFFFFFFFF:F
@A00721:190:HGJW7DSXY:4:1460:18945:2268_2:N:0:TCTGCAAG+GCTGCACT
AAACTTAACGTTAGTACCGGAGGTGATCTCTCTGCTTCTGCGTTACTGAGAGAAAAGATTCAGGACGC
CTACGGTGACTTTATGGACAAGATGCAAGTTCTTGGGAAATACTTGTT
+
FFFFFFFFFFFFFFFFFFFFFFFFFFFFFFFFFFFFFFFFFFFFF:FFFFFFFFFFFFFFFFFFFFFF
FFFFFFFFFFFFFF:FFFFFFFFFFFFFFFFFFFFFFFFFFF:FFFFFF
@A00721:190:HGJW7DSXY:4:1432:2917:9643_2:N:0:TCTGCAAG+GCTGCACT
TCGATTCTTTGTATAGGGAGTGTGAGAAGACAACGAATCTTGAAACTACGGTCCTTATCAAAGAAAAT
GGTGAACCGGTCTCTAAATCTTTTGAGATTGAGAAGATTGAAGAACAC
+
FFFFFFFF:FF:FF,FFFFFFFFFFFFFFFFFFFFF:FFFFFFFFFFFFFFFFFFFFFFFFFFFFFFF
FFFFFFFFFFFFFFFFFFFF:FFFFFFFFFFFFFFFFFFFFFFFFFFF
@A00721:190:HGJW7DSXY:4:1538:32949:33285_2:N:0:TCTGCAAG+GCTGCACT
GCTCGGCGTTGTTCTTGAAAACCAAGTTAAGGAGGAGGCTGATCATGGAGTCTTTGTACCTTCTGTCA
AACTTCGAGGTTCAGCTACGAGAGGTGATCCCGGATGTGGATTGAAGG
+
FFFFFFFFFFFFFF:FFFFF:FF,FFFFFFFFFFFFFFFFFFFFFFFFFFFFFFFFFFFF,FFFFFFF
FFFFFFFFFFFFFFFFFFFFFFFFFFFFFFFFFFFF:FFFFFFFF::FF
@A00721:190:HGJW7DSXY:4:1454:27823:26913_2:N:0:TCTGCAAG+GCTGCACT
GGAGACTCCTTCTTTTTGAATGTTGTAAAGAATTGGGTTGAGAAGTGTGCTTTACAAGGAGTTAGACT
CTTCTTCAAGTTTTTAGATCTTCTTGATAATCTTAAAGAAAAATTTTC
+
FFFFFFFFFFFFFFFF:FFFFFFFFFFF:FFF,FFFFFFFFFFFFFFFFFFFFFFFFFFFFFFFFFFF
FFFFFFFFFFF,FFFFFFFFFFFFFFFFFFFFFFFFFFFFFFFFFFFF
@A00721:190:HGJW7DSXY:4:2436:15275:10535_1:N:0:TCTGCAAG+GCTGCACT
AGTCTGTACTTACATTCTTTACGCCAGATTCTTCATGGGGCGACGCTGTTTCTCTTGGTATTTCTGAA
ATAGTTTATCAGAGCGTCATTTTCCTTTGGGATCTTTCCTCTGGTAAGGGAATTATTAAATCATTCTT
CTC
+
FFFFFFFFFFFFFFFFFFFFFFFFFFFFFFFFFFFFFFFFFFFFFFFFFFFFFFFFFFFFFFFFFFFF
FFFFFFFFFFFFFFFFFFFFFFFFFFFFFFFFFFFFFFFFFFFFFFFFFFFFFFFFFFFFFFFFFFFF
FFF
@A00721:190:HGJW7DSXY:4:1526:11442:23281_2:N:0:TCTGCAAG+GCTGCACT
ATTATTACTGTTTTCTCTACCCATAACTTCTTTTTCAAAAATGCGGATGGGTCTACTGATGTTTTCCT
ACAGAGTGCAGTCGTTTCAACACTTATCACCACTTTTGAAAGGAAAAT
+
FFFFFFFFFFFFFFFFFFFFFFFFFFFFF:FFFFFFFFFFFFFFFFFFFFFFFFFFFFFFFFFFFFFF
FFFFFFF:FFFFFFFFFFFFFF,,FFF,FF:FF,FFF,FFFFFFFFFF
@A00721:190:HGJW7DSXY:4:1624:5475:14043_1:N:0:TCTGCAAG+GCTGCACT
AAATACGTATGTCGGTGGGGTTCTCAAGGCTCTCATCGCCACTGGGATTCTAAGGGGTTCCATAAGAT
```

```
ATTATCTGACATTGTGGTTCAATGATCAGGATTCTTCTTTTGGTCCTTACGTCTTAAGTAATTTGGAT
GTT
+
FFFFFFFFFFFFFFFFFFFFFFFFFFFF:FFFFFFFFFFFFFFFFFFFF:FFFFFFFFFFFFFFFFFF
FFFFFFFFFFFFFFFFFFFFFFFFFFFFFFFFFFFFFFFFFFFFFFFFFFFFFFFFFFFFFFFFFFFF
FFF
@A00721:190:HGJW7DSXY:4:1301:16758:31767_2:N:0:TCTGCAAG+GCTGCACT
GTTCTTGTCACGATTCTTAAAAGAAGTTTATTAAAACTTCAGAGTCTCTTGGAAGTCCAGGTAGCTAA
TGTTCTTGCAATAGCCACAGACTTTCTGGGAAAAGAGACCTCTGTTGG
+
FFFFFFFFFFFFFFFFFFFFFFFFFFFF:FFFFFFFFFFFFFFFFFFFFFFFFFFFFFFFFFFFFFFF
FFFFFFFFFFFFFFFFFFFFFFFFFFFFFFFFFFF:FFFFFFFFFFFF
@A00721:190:HGJW7DSXY:4:1439:23366:31876_1:N:0:TCTGCAAG+GCTGCACT
GAGAATTACTCAGATTCAATCTGGCATCACTGAACGTCTACAACGAGTTAGGGAGTGGCTAGGTTTTA
AAAGATCCTTTGCACAAGCAATACCTTCCACTGAGTTCATCACCCAGAGTTTCGACGTCGCGGAAAAC
GAT
+
FFFFFFFFFFFFFFFFFFFFFFFFFFFFFFFFFFFFFFFFFFFFFFFFFFFFFFFFFFFFFFFFFFFF
FFFFFFFFFFFFFFFFFFFFFFFFFFFFFFFFFFFFFFFFFFFFFFFFFFFFFFFFFFFFFFFFFFFF
FFF
@A00721:190:HGJW7DSXY:4:1649:10393:6903_2:N:0:TCTGCAAG+GCTGCACT
CACTGTTCGAGAATCTTCTGATGCTGAATTTGAGGAAAATTTTTCGGAAGTTTTTGGTATAAATCCTG
ATGGCTCAGTACAGGGAGGAGATCGGAAGAGCGTCGTGTAGGGAAAGA
+
FFFFFFFFFFFFFFFFFFFFFF:FFFFFFFFFFFFFF:FFFFFF:FFFFFFFFF:FFFFFFFFFFFFFF
FFFFF,FFFFF::FFFFFFFFFFFFFFFFFFFFFFFF:F:FFFFFFFFF
@A00721:190:HGJW7DSXY:4:1142:29839:24017_1:N:0:TCTGCAAG+GCTGCACT
ATGGACAAAATCTGGGATCATAGGTATGTGTCACGCTACACTCGAAATATGGGCCCTCATAAGGAAGT
ATAGTTGGCGTCTTGTCGTTGACGTCATTACAGTCTGGAAGTTAGATCATTTGTTCAGGAAGATCTCT
ACA
+
FFFFFFFFFFFFFFFFFFFFFFFFFFF:FFFFFFFFFFFFFFFFFFFFFFFFFFFFFFFFFFFFFFFF
FFFFFFFFFFFFFFFFFFFFFFFFFFFFFFFFFFFFFFFFFFFFFFFFFFFFFFFFFFFFFFFFFFFF
FFF
@A00721:190:HGJW7DSXY:4:1430:26142:35243_1:N:0:TCTGCAAG+GCTGCACT
CTTTAGCAACTTTAGATAAATTCTTTCCGGGCTCAATGACCTTTGCTATGTACGTGGATCTAATCCTT
GGTGATCTGAATCTGACCATCGCAAAGAAGACTTTATTAGTTCATGAAGTTCTTACACTTCTCGCGAC
TAA
+
FFFFFFFFFFFFFFFFFFFFFFFFFFFFFFFFFFFF:FFFFFFFFFFFFFFFFFFFFFFFFFFFFFFF
FFFFFFFFFFFFFFFFFFFFFFFFFFFFFFFFFFFFFFFFFFFFFFFFFFFFFFFFFFFFFFFFFFFF
FFF
@A00721:190:HGJW7DSXY:4:2470:15980:19617_1:N:0:TCTGCAAG+GCTGCACT
GCTTTATCTTAAAGCATTACCACCAAATTTAGCACTTTTTGAATATGACAGAAGCTCGCCATATCAGA
TTAGAGACATGCCAGTTAGAGTTCTTCTAACCAGGACTGATGGTGAGATTCTTTTACCTTTTGGAGGT
CCA
+
FFFFFFFFFFFFFFFFFFFFFFFFFF:,FFFFFFFFF:FFFFFFFFFFFFFFFFFFFFFFFFFFFFFF
FFFFFFFFFFFFFFFFFFFFFFFFFFFFFFFFFFFFFFFFFFFFFFFFFFFFFFFFFFFFFFFFFFFF
FFF
@A00721:190:HGJW7DSXY:4:1623:18376:32878_2:N:0:TCTGCAAG+GCTGCACT
CCTAGTGTCTTTTCTAGAGTCCAAGGACGTTTCCGTCGGAGGATGACAGGTGGGAGACCGCTCAATAA
CACTCTATTGAATCCCTTTTCCACGATGAAAGCTCTTTATGAGGTGCT
+
```

```
FFFFFFFFFFFFFFFFFFFFFFFFFFFFFFFFFFFFFFFFFFFFFFFFFFFFFFFFFFFFFFFFFFFF
FFFFFFFF:FFFFFFFFFFFFFFFFFFFFFFFFFFFFFFFFFFFFFFF
@A00721:190:HGJW7DSXY:4:2538:31946:32111_1:N:0:TCTGCAAG+GCTGCACT
TCTTTGGTGATATCGCCTCTGTCCTTTCTCCTTTAATCGTGAGGTTACGAGAGTTTCGTACTTGGGTT
TATCGGTTGTATTTTGATAAATTTGTTGGAATCGATAATGATACTGACGAAGGTTCTATTACCACCCT
TGT
+
FF:FFFFFFFFFFFFFFFFFFFFFFFFFFFFF:FFFFFFFFFFFFFFFFFFFFFFFFFFFFFFFFFFFF
FFFFFFFFFFFFFFFFFFFFFFFFFFFFFFFFFFFFFFFFFFFFFFFFFFFFFFFFFFFFFFFFFFFF
:FF
@A00721:190:HGJW7DSXY:4:2136:27525:17127_2:N:0:TCTGCAAG+GCTGCACT
GTGATGCTTTCTTAGAGGCTCGTCTAGCCGCTGAGATTCGTCTTACCTATTTACAATCTGTAGAGAGA
GAGGTTAATCAGAATTTGAGTGATAACGATTTAGTACTGGGAAATGGT
+
FFFFFFFFFFFFFFFFFFFFFFFFFF:FFFFFFFFFFFFFFFFFFFFFF:FFFFFFF:FFFFFFFFFF
FFFFFFFFFFFFFFF:FFF:FFFFFFFFFF:FFFFFFFFFFFF,FF::
@A00721:190:HGJW7DSXY:4:1326:8974:8766_1:N:0:TCTGCAAG+GCTGCACT
TTTTCTTTCCCTCCCCTTTTTCGATTGGATTCGACGGGGTCTTCGTCTCACGACTAATTTGATAAGGT
TTTTCTTTTATGGGACTCTTGTTTGTACTTATGAAGGAGGTATTACTTCGGAAGTTCTTAATGTAGGT
CTC
+
FFFFFF:FFFFFFFFF,FFFFFFFF:FF:FFFFFFFF:FFFFFFFFFFFFFFFFFFFFFFFFFFFFFF
FFFFFFFFFFFFFFFFFFFFFFFFFFFFFFFFFFFFFFFF:FFFFFFFFFFFFFFFFFFFFFFFFFFF
FFF
@A00721:190:HGJW7DSXY:4:2153:27588:33708_2:N:0:TCTGCAAG+GCTGCACT
GGTTTATGTTTCGGTTTCAGCTGGATGGATCTTTTGATGGTATGTTTCACATTGCGTGATCACTTCCC
GTGGACTTGGGAAGAGATTAAACATAAAATCCAGCGATTCTATTATGT
+
FFFFFFFFFFFFFFFFFFFFFFFFFFFFFFFFFFFFFFF:FFFFFFFFFFFFFFFFFFFFFFFFFFF:
FFFFFFFFFFFFFFFFFFFFFFFFFFFF,FFF:FFFF:FFFFFFFFFFF
@A00721:190:HGJW7DSXY:4:2417:32407:6292_2:N:0:TCTGCAAG+GCTGCACT
TCGATGATGTTCGGGCTGTGGGGATTAACTTCCCTCGTGAAACTTACGACGATGTTCTCGCTCGCATT
TCACAAGGATTACTAAGTCTGAGATCTGCCACTTCTAATTTTAGGAAA
+
FFFFFFFFFFFFFFFFFFFFFFFFFFFFFFFFFFFFF:FFFFFFFFFFFFF:FFFFFFFFFFFFF,FF
FFFFFFFFFFFFFFFFFFFFFFFFFFFFFFFFFFFFF:FFFFFFFFFF
@A00721:190:HGJW7DSXY:4:1476:22164:16203_2:N:0:TCTGCAAG+GCTGCACT
AGAAAGTTCATGAGGATGTCTTGGTCGAAAAATATCGCCGGGAACTTCTAATAGAGGATCCTGATGAC
ATTATTTCAACGGATACCGATCTCAGTTCTACCACAGAAACCGTCTCA
+
FFFFF:FFFFFFFFFF:FFFFF:::FFFFFFF:FFFFFFF::F,,F,FFFFFF:FF:FFFFFFFFFFF
FFFFF,FFFF:FFFFFFF:FFFFFF,,FFFFFFF:F:FFFF,,:FFFF
@A00721:190:HGJW7DSXY:4:1676:1398:20666_2:N:0:TCTGCAAG+GCTGCACT
ACTGATTCGGAATTAGAGAAAGACGACCTTCTTGTGGGGACTGAGACCTCTGAATTTTCAGGGTGCCC
TGATGAGGACGTTGAAAGGGATAATTTCAACTCTGAGACTTCTAAGAG
+
FFFFFFFFFFFFFFFFFFFFFFFFFFFFFFFFFFFFFFFFFFF:FFFFFFFFFFFFFFFFFFFFFFFF
FFFFFFFFFFFFFFFFFFFFFFFFFFFFFFFFFFFFFFF:FFFFFFFFF
@A00721:190:HGJW7DSXY:4:1169:2410:1532_2:N:0:TCTGCAAG+GCTGCACT
ACCAGAAACGTAACAAGAAGAATAAAGGTAGGAAGGGGAGAAAACAGAACAAGCGAGCGGTTGAAGAG
AAAACTGATATGGTTGCGGCTGATCGTACCCCAATTCCTGCTAGAAAG
+
FFFFFF,FFFFFFF:F:FFFFFFFFFFFF,,F:FFFFFFFFFFFFFFF,FFF,:FF:FF:FFF,FFFF
FFFFFFFFFFFFFFFFFFFFFF:FFFF:FFFFFFFFFF,FFFFF,FFF
```

```
@A00721:190:HGJW7DSXY:4:1129:4526:12085_1:N:0:TCTGCAAG+GCTGCACT
ATACCACTGATCTTCCAGAGTTACGTAGTGGTCGTTCTTTTGGTGATTTTATCAAAGAGAAAGTTTCT
GCTAGTCGCGACGATAGTATTTATCGGAGTGATATTAACAAGTTAAACTTCGAACTGACCACTTACAA
GAG
+
FFFFFFFFFFFFFFFFFFFFFFFFFFFF:FFFFFFF:FFFFFFFFFFFFFFFFFFFFFFFFFFFFFFF
FFFFFFFFFF:FFFFFFFFFFFFFFFFFFFFFFFFFFFFFFFFFFFFFFFFFFF:F:FFFFFFFFFFFF
FFF
@A00721:190:HGJW7DSXY:4:2108:32624:20635_1:N:0:TCTGCAAG+GCTGCACT
GCAACATGGATCAGTTTTCCAAAAAGCTATTAATAGCAGTTGCGGGGCTATAATACCGCCTCCAGATT
ACTCTAAACTGAATCCACTCTCTTGGGATATAAATGAGTACATCTATAATAGAGTTTTGGAACTGTGT
AAT
+
F:::FFFF:FFFFFFFF:FFFF:FFFFFFFF:FFFF:FF:FFFFF:FFFFFFFFFFFFFFFFFFFFFFF
FFFFFFFFFFFFFF::FFFFFFFFFFFFFFFFFFFFFFFFFF:F:F:FF:FFFFFFFFFFFFFFFFFFF
FF,
@A00721:190:HGJW7DSXY:4:1263:31611:7420_1:N:0:TCTGCAAG+GCTGCACT
GGCTTCTCTAAAGAACAGCAACTTTCAACATGAATTAGTTATTGAAAGTCTTGACCCTCACGTCTCAC
TTTATCACCTTGGAGATGGTAAAGTCATTAACAAAGGAAATGTTAAGGCTGATGACTTAAATTTCGCA
TTC
+
FFFFFFFFFFFFFFFFFFFFFFFFFFFFFFFFFFFFFFFFFF,FFFFFFFFFFFFFFFFFFFFFFFFFF
FFFFFFFFFFFFFFFFFFFFFFFFFFFFF:FFFF:FFFFFFFFFFFFFFFFFFFFF:FFFFFFFFFFFF
FFF
@A00721:190:HGJW7DSXY:4:1633:12581:32894_2:N:0:TCTGCAAG+GCTGCACT
AGACCTTTCTTGGGGAGTAAAGAGGGTTTGAAAATTACGGAACCTTTCCTCGTTCTTCATGAAGATCT
TCGAGTGTTTTATAGTGAGTTATTCTTGCGAAGGTTCAAGTCGATTGA
+
FFFFFFFFFFFFFFFFFFFFFFFFFFFFFFFFFFFFFFFFFFFFFFFFFFFFFFFFFFFFFFFFFFFF
FFFFFFFFFFFFFFFFFFFFFFFFFFFFFFFFFFFFFFFFFFFFFFFF
@A00721:190:HGJW7DSXY:4:2312:8820:4805_1:N:0:TCTGCAAG+GCTGCACT
ACGAGATCTTGCAGAGTGACTGGAGTTCAGACGTGTGCTCTTCCGATCTGTGGAGGGAAGACCACTGA
ACTTATAGCGACTTTTGTCGAATTTTTCGCAAAAGGTCTTGAAGTACGTATTGTCACTGCTAATCGTA
ATT
+
:FF,FFFFFFFFFFFFFFFFFFFFFFFFFFFFFFFFFFFFFFFFFFFFFFFFFFFFFFFFFFFFFF:F
FFFFFFFFFFFFFFFFFFFFFFFFFFFFFFFFFFFFFFFFFFFFFFFFFFFFFFFFFFFFFFFFFFFF
FFF
@A00721:190:HGJW7DSXY:4:1463:11957:9612_1:N:0:TCTGCAAG+GCTGCACT
GTGACTAAAGTTTTGGCACTTCTAGTTCATCGCGGTATTTGTGAAATTAAGACAGACGTGAAAGTTAT
GATGAGTGATCGTATTAGAACAGCTGATTCTTATTGCATGAACGTCGCTGAGGTCAAACCTGTGGACG
TTC
+
FFFFFFFFFFFFFFFFFFFFFFFFFFFFFFFFFFFFFFFFFFFFFFFFFFFFFFFFFFFFFFFFFFFF
FFFFFFFFFFFFFFFFFFFFFFFFFFFFF,FFFFFFFFFFFFFFFFFFFFFFFFFFFFFFFFFFFFFF
F:F
@A00721:190:HGJW7DSXY:4:1672:10041:23735_2:N:0:TCTGCAAG+GCTGCACT
CTGGTCAGACCCTTTTTCTTACACATCTACTTAAGGCGGCTTATACCATAGGTTACGGTGATTCCAAT
CAAATCGGATTCATCAATCGTATTGATCTCCAAGTCGCAAACTACTTC
+
FFFFFFFFFFFFFFFFFFFFFFFFFFFFFFFFFFFFFFFFFFFFFFFFFFFFFFFFFFFFFFFFFFFF
FFFFFFFFFFFFFFFFF:FFFFFFFFFFFFFFFFFFFFFFFFFF,FFFF
@A00721:190:HGJW7DSXY:4:1402:8395:26459_1:N:0:TCTGCAAG+GCTGCACT
GTGTGCTCTTCCGATCTTTCTTATCGAAGTCTCAGTTATCGCTGCCCGGCCGACGTTTGTCGCGAACT
```

CTCAGAGGTTTACGGCAGAAAAATTTCTCCTGCTTGTAATTCGAGAGAGGACACTATGGAAATTAGAC
CTA
+
FFFFFFFFFFFFFFFFFFFFFFFFFFFFFFFFFFFFFFFFFFFFFFFFFFFFFFFFFFFFFFFFFFFF
FFFFFFFFFFFFFFFFFFFFFFFFFFFFFFFFFFFFFFFFFFFFFFFFFFFFFFFFFFFFFFFFFFFF
FFF
@A00721:190:HGJW7DSXY:4:1629:27407:22341_2:N:0:TCTGCAAG+GCTGCACT
ATATTACTTTCACTCAAGGTGAAAAGTTTGAAATTAACAAAGTCCTTGGTAAGAGTAAGGTTAAATGT
GAAGATGCTCAGACTATTCATGAAGTTCAAGGTAATACTTACCAGCAC
+
FFFFFFFFFFFFFFF:FFFFFFFFFFFFFFFFFFFFFFFFFFFFF:FFFFFFFFFFFFFFFFFFFFFF
FFFFFFFFFFFFFFFFFFFFFFFFFFFFFFFFFFFFFFFFFFFFFFFF
@A00721:190:HGJW7DSXY:4:1677:27064:29136_2:N:0:TCTGCAAG+GCTGCACT
AAACCTCAAGATGACCCTGTTTTTGCATCACAAACACATATTACCGTTGCTCTATCACGACACGTTGA
ATCTTTACGATACTTTGTTATCAATGTAAAGTTAAACGATGCTACTTG
+
FFFFFFFFFFFFFFFFFFFFFFFFFFFFFFFFFFFFFFFFFFFFFFFFFFFFFFFFFFFFFFFFFFFF
FFFFFFFFFFFFFFFFFFFFFFFFFFFFFFFFFFFFFFFFFFFFFFFF
@A00721:190:HGJW7DSXY:4:2214:18738:35415_2:N:0:TCTGCAAG+GCTGCACT
TAAGGAACTGTCACGTTTAGCACGGGAGGTTGCAGATTCAACCCCTTATATCGAGGCTATCGAGGACG
ATGAGTATCTTGAAGTTGTTGACGACAAGCCTCAAGAGGGTAGAGGAC
+
FFFFFFFFFFFFFFFFFFFFFFFFFFFFFFFFFFFFFFFFFFFFFFFFFFFFFFFFFFFFFFFFF:F:F
F,FFFFFFFFFFFFF:FFFFFFFFFFFFFFFFFFFFFFFFFFFFFFFF
@A00721:190:HGJW7DSXY:4:1504:12138:18411_2:N:0:TCTGCAAG+GCTGCACT
GGTTATTCCTGGAACCACCTTAACGTCACTCGGTAATCTTTCAGCCGAATTAAGTACTAATGAGTTCA
AGTCGGTTGTCGATCGAATTAAGATCAAGGCTAATACCTTTATAGGTA
+
FFFFFFFFFFFFFFFFFFFFFFFFFF:FFFFFFFFFFFFFFFFFFFFFFFFFFF:FFFFFFFFFFFFFF
FFFFFFFFFFFFFFF:FFFFFFFFFFFF:FFFFFFF:FFFFFFFFFFF
@A00721:190:HGJW7DSXY:4:1547:2383:3646_2:N:0:TCTGCAAG+GCTGCACT
GCCGCGTAGTCAGCGCGTTTAGCTGTATCATCAGTCAGAGTATACCCGATAGGGTACCGAGTCTCCAA
GAGAATATTTATTCTTATGAGGCTCGAAACTTCAACGTTTTCACAACG
+
FFFFFFFFFFFFFFFFFFFFFFFFFFFFFFFFFFFFFFFFF:F:FFFFFFFFFFFFFFFFFFFFFFFF
FFFF:FFFFFFFFFFFFFFFFFFFFFFFFFFFFFFFFFFFFFFFF:FF
@A00721:190:HGJW7DSXY:4:1277:30436:7827_2:N:0:TCTGCAAG+GCTGCACT
TCGCACCCGAAATTTTTGGGCAGAATGCTCTTAACGCGGTTCTTGAACGGTGTTTTGATCAGTCTAAG
TTGGATAAAGTTATAGGAGACTCAGTTAACCTGAGAGCTCCTCAATTG
+
FFFFFFFFFFFFFFFFFFFFFFFFFFFFFFFFFFFFFFFFFFFFFFFFFFFFFFFFFFFFFFFF:FFF
F,FFFFFFFFFFFFFFFFFFFFFFFFFFFFFFFF:FFFFFFFFFFFFF
@A00721:190:HGJW7DSXY:4:2174:25943:3020_1:N:0:TCTGCAAG+GCTGCACT
AACAATAGGGACGCTAGTATACTTCGTAAAGTCGACAGCGAAATGGTCAATATTGATAATTGGACCGA
CTATATGACCTATTTTAAGCTCATGGTGAAAAGGGAAGCTAAGGTTAAACTTGATGCTTCAGCCTTTT
CGA
+
FF,FFFFF,FFF:FFFFFFFFFFFF:FFFF:FFFFFFF::FFFFF:FFFFFFFFFFFFFFFFFF::FF
FFFFFFFFFFFFFFFF:FFFFFFFFFFFFFFFFFFFFFFFFFFFFFFFFFFFFFFFFFFFFFFF:FFF
FFF
@A00721:190:HGJW7DSXY:4:1373:3079:1720_1:N:0:TCTGCAAG+GCTGCACT
AGAATATCGTTTATCACGCAAAATTTATTAATGCAATTTTCGGACCTATCTTTAAGGTTCTTGTGAGG
AGAGTCAAACAGTGTCTTTTACCCAACATTTTTATGTATACAGAGATGACCAGTGACGATTTCTCCCG
GAA

```
+
FFFFFFFFFFFF:FFFFFFFFFFFFFFFFF:FFFFFFFFFFFFFFFF:FF:FFFFFFFFFFFFFF:FF
FFFFFFFFFFFFFFFFFFFFFFFFFFFFFF,FFFFFFFFFFFFF:FFFFFFFFFFFFFFFFFFFFFFFFF
FFF
@A00721:190:HGJW7DSXY:4:1106:12292:25441_1:N:0:TCTGCAAG+GCTGCACT
CGAGATCTTGCAGAGTGACTGGAGTTCAGACGTGTGCTCTTCCGATCTTTTCTTTTGTAAAGCCTATG
AGATAAAGATGTATTCTTATTTAGGCTTCGATGCTGAATTTCTTGAACTTTGGAGTGCCAGTGAATAT
TTT
+
:F:FFFFFFF:FFFFFFFFFFFFFFFFFFFFFFFFF:FFFFFF,FFFFFFFFFFFFFFFFFFFFFFFFF
F:FFFFFFFFFFFFFFFFFFFFFFFFFFFFFFFFFFFFFFFFFFFFFFFFFFFFFFFFFFF:FFFFFF
FFF
@A00721:190:HGJW7DSXY:4:1139:11116:13228_1:N:0:TCTGCAAG+GCTGCACT
GAGTATCCATAACGATTTGTCATTTACCACCTTTGCTCAACGACGAAGTGGAACGGCCAACACTTTCT
TTGGTAATTGTATGATCACTTTGATGATTTTATCACTTTATTATGACCTTGGAAAGTTTTCGGCTGTG
GTT
+
FFFFFFFFFFFFFFFFFFFFFFFFFFFFFF:FFFFFFFFFFFFFFFFFFFFFFFFFFFFFFFFFFFFF
FFFFFFFFFFFFFFFFFFFFFFFFFFFFFFFFFFFFFFFFFFFFFFFFFFFFFFFFFFFFFFFFFFFF
FFF
@A00721:190:HGJW7DSXY:4:1234:23873:23703_1:N:0:TCTGCAAG+GCTGCACT
CCATTCTGTTTTCAAAGACTCCAATAACTGATCATTCTGATCATATGATTTTGGACAATGGTTTCGAA
GCGAAATTTTTGAGGGATAAACCAGCGTACTTCTGCTCGCGATTTATTCTTTTTGTCAATGATTACAT
CTA
+
:FFF,:F:FFFFF:FFF,FF,:FFFF,,:,,F:FFF:FFFFFF,FF:FFFFF:FF:FFF,FFFF:FFF
F:F:,FFFFFFFFF,FFF:,FF:F:F:FF:FF:FFFFFFFFF:FFF,F,:FFFF::,F,FFFFFFFFF
FFF
@A00721:190:HGJW7DSXY:4:1231:10484:29982_1:N:0:TCTGCAAG+GCTGCACT
CCTTACAAGATCATCGTTAAGTTAGGTAAACCCCTAACCGATGTTACTCTTGTAGGACTTTGGGAGAG
ATTCGTTAGTTTTAAAGACTATACTAAATATCTCCACTACGAGGTTGTTGTTTGGCGATTAGCTCTTG
CGA
+
FFFFFFFFFFFFFFFFFFFFFFFFFFFFFFFFFFFFFFFFFFFFFFFFFFFFFFFFFFFFFFFFFFFF
FFFFFFFFFFFFFFFFFFFFFFFFFFFFFFFFFFFFFFFFFFFFFFFFFFFFFFFFFFFFFFFFFFFF
FFF
@A00721:190:HGJW7DSXY:4:1224:11225:15045_1:N:0:TCTGCAAG+GCTGCACT
TATTCGATTGAGGGTCTTTATACCTTTTCGGCTTTCTGGGCTTTGAATTGTATAAGATCTAATTTTTA
ACGATTCCGAGAGGTTTATCCTGATGTGGAGGGAGTTCTTGAAGCATCACGACATTTCCTTGTGAAAA
+
F,FF,FFFF:,:,:F,,FFFF:,::,F:,FFFF,F:FF,,::FF,FFFF:F,,FF,,FF,,FFFFF,,
F,F:FF,FFF:F:,F,FFFFFFFFFF::FFFFF:FF,F,FFFF,F,F,,F:,F,:FF,,,F:FFFF,:
@A00721:190:HGJW7DSXY:4:1134:4065:32299_1:N:0:TCTGCAAG+GCTGCACT
TTTCATAAGATTATCAGTAATCCTGATTCTACTACGGATTCATGGACCTTCCATCCTGTACCTCTTCT
GGAGAGACGTATCTCAAAGATCGACTTCGAGACAGGTGATATTGTTGAGCGAGAGCGACCAAGATCTA
AAT
+
FF:FFFFFFFFFFFFFFFFFFFFFFFFFFFFFFFFFFFFFFFFFFFFFFFFFFFFFFFFFFFFFFFFF
FFFFFFFFFFFFFFFFFFFFFFFFFFFFFFFFFFFFFFFFFFFFFFFFFFFFFFFFFFFFFFFFFFFF
FFF
@A00721:190:HGJW7DSXY:4:1127:18511:25128_1:N:0:TCTGCAAG+GCTGCACT
CGTGGCGCTTTATTTTAAAAAGCTCCCTCCTCTCGCAAGTTTGATCTGATACTTATCACGAAAGGACT
TGAGTCTTTTGATCAGTCTCTGACTTGACCATCTTTCATTTTAAATGACGATCATTAAGACTCTCTTT
CTT
```

```
+
FFFFFFFFFFFFFFFFFFFFFFFFFFFFFFFFFFFFFFFFFFFFFFFFFFFFFFFFFFFFFFFFFFFFFF:FFFF
FFFFFFFFFFFFFFFFFFFFFFFFFFFFFFFFFFFF:FFFFFFFFFFFFFFFFFFFFFFFFFFFFFFFFFFFFF
FFF
@A00721:190:HGJW7DSXY:4:1217:32651:5055_2:N:0:TCTGCAAG+GCTGCACT
CTTTGCACTGATTTTTCCTCATCAGCTTGTGTTTTACCTAATCTGTGTTTTTACGATAACAACTATCA
GTTGATTAAGACTCTTCCGAAAGGCGAACTTTGGGAATTTGAAGCTCA
+
FFFFFFFFFFFFFFFFFFFFFFFFFFFFFFFF:FFFFFFFF:FFFFFFFF:FFFFFFFFFFFFFFFFFFFFF
FFFFFFFFF:FFFFFFFFFFFF:,:FFF:FFFFFF:FFFFFFFFFFFF
@A00721:190:HGJW7DSXY:4:1232:17065:15139_1:N:0:TCTGCAAG+GCTGCACT
CGTTTGACCGTGACGAACTGTGGTGTTCTTGATCTACGTTACGGATATACTCTCCTTGAGTTCACTCG
TAAGGACACTACCACTTATTACGATATTTCTTTAGTCGATGGGTTTTCGACTCCTTTAAGCGTCTTCT
GTT
+
FFFFFFFFFFFFFFFFFFFFFFFFFFFFFFFFFFFFFFFFFFFFFFFFFFFFFFFFFFFFFFFFFFFFFFFFFF
FFFFFFFFFFFFFFFFFFFFFFFFFFFFFFFFFFFFFFFFFFFFFFFFFFFFFFFFFFFFFFFFFFFFFFFFFF
FFF
@A00721:190:HGJW7DSXY:4:1557:8919:21042_2:N:0:TCTGCAAG+GCTGCACT
AATCCTAACGTAGAAGATTGTTGTTTACCACCGAAATATCAAGGTTCTTGTCCTTCAAACGATTGGAA
GGTTACTTATCTTAATAGTACTAAAGGGGTTTACTTACAACCCTATGA
+
FFFFFFFFFFFFFFFFFFFFFFFFFFFFFFFFFFFFFFFFFFFFFFFFFFFFFFFFFFFFFFFFFFFFFFFFFF
FFFFFFFFFFFFFFFFFFFFFFFFFFFFFFFFFFFFFFFFFFFFFFFFFF
@A00721:190:HGJW7DSXY:4:1631:15058:11256_2:N:0:TCTGCAAG+GCTGCACT
TGTTTTCTTCTAATGTGGCCTGAACTACGTTTCACTTTGTGTGTACTTGTCTCTATTTTAAGTTTTGT
CTTCCTTGTTTGTCTTATTCTATTAATCACTCATTTTAGTGAGATTAT
+
FFFFFFFFFFFFFFFFFFFFFFFFFFFFFFFFFFFFFFFFFFFFFFFFFFFFFFFFFFFFFFFFFFFFFFFFFF
:FFFFFFFFFFFFFF:FFFFFFFFFFFFFFFFFFFFFFFFFFFF:FFFFF
@A00721:190:HGJW7DSXY:4:1214:22607:28056_2:N:0:TCTGCAAG+GCTGCACT
CTAAAACGTCTTCGACAACAGCAGGGGTTTTCGAATAGTGATAGCCACCCGCCTTACAGAGGGGCAGC
AGCATGACCATAATTGGTATTGACTTTGGTACCACCTTTTCTACTGTC
+
FFFFFFFFFFFFFFFFFFFFFFFFFFFFFFFFFFFFFFFFFFFFFFFFFFFFFFFFFFFFFFF:FFFFFFFFFF
FFFFFFFFFFF,FFFFFFFFFFFFFFFFFF::FFFFFFFFFFFFFFFF
@A00721:190:HGJW7DSXY:4:1453:13666:21277_1:N:0:TCTGCAAG+GCTGCACT
TTCGAATGAAGTTTTAATGCTTAACAACAATGATAGTGAATTTATACCTACAATCATTGGTTTTTCTA
ATAATGATGACACTATTGTTTATGGATATGATGCTATTACTCATGATGGTTACGGTCAGGGAGGATTC
TCA
+
FF:FFFFFFFFFFFFFFFFFFFFFFFFFFFFFFFFFFFFFFFFF,FFFFFFFFFFFFFFFFFFFFFFFFFFFFF
F,F:FFFFFFFFFFFFFFFFFFFFFFFFFFFFFFFFFFFFFFFFFFFFFFFFFFFFFFFFFFFFFFFFFFFFFF
FFF
@A00721:190:HGJW7DSXY:4:1510:12753:10645_2:N:0:TCTGCAAG+GCTGCACT
ACATTACCCGAAAGACGTGCTAAATTAAAACCATTGTACGATGTCGTCTGTCTCGGCGATCACTTTTC
ACTAAGAATTGGTCCTACTTTTGGACAAGGCCAACTTCGTAATCTTCA
+
FFFF:F:FF,FFFFFFFF:F:FFFFFFFFFF:F::FFFFFFFF,FFFFFFFFFF:F:FFFFFF:FF,F
FFFFFFFFFFFFFFFFFFFFFFFFFFFF,F:FFFFFFF::FFFFFFFFFF
@A00721:190:HGJW7DSXY:4:2604:14461:23657_2:N:0:TCTGCAAG+GCTGCACT
TCTTTATCTCTTTGGTTGTTAGGGATTTTGAGAGAGTGAAAGGCTTCAATTGTAGTGGTTTGGTCGTT
TCAGTACCCAGTCAGTATACCTCTACGCAAAGATATTTCATGAAAACT
+
```

FFFFFFFFFF:FFFFFFFFFFFFFFFFFFFFFFFFFFFFFFFFFFFFFFFFFFFFFFFFFFFFFFFFF
FFFFFFFFFFFFFFFFFFFFFFFFFFFFFFFFFFFFFFFFFFF:FFFF
@A00721:190:HGJW7DSXY:4:1403:5891:11475_1:N:0:TCTGCAAG+GCTGCACT
CCGGATTACCTGTCTTTCATATTATGAATGAACCTTCGGCCGCTCTCTTTGCTTCGATGTTGGATGTC
AAGAAGGGTTCAGAATGGGATTCCTACGTAGTTTATGACTTTGGTGGTGGTACTTTCGATACTTCTAT
CGT
+
FFFFFFFFFFFFFFFFFFFFFFFFFFFFFFFFFFFFFFFFFFFFFFFFFFFFFFFFFFFFFFFF:FFF
FFFFFFFFFFFFFFFFFFFFFFFFFFFFFFFFFFFFFFFFFFFFFFFFFFFFFFFFFFFFFFFFFFFF
FFF
@A00721:190:HGJW7DSXY:4:1467:19759:7404_2:N:0:TCTGCAAG+GCTGCACT
TACTATAGTGTTATTTATTCTGGTGGTGATGATAATCTTGGAGGTCGTGACGTTGATGAAGCTATAAG
AGCGTTCTTATTACGGCGCACTTCTTTACCCGCCACCACACAACTTTC
+
FFFF:FFFFFFFFFFFFFFFFFFFFFFFFFFFFFFFFFFFFFFFFFFFFFFFFFFFFFFFFFFFFFFF
FFFFFFFFFFFFFFFFFFF:FFFFFF:FFFFFFFFF:FFFFFFFFFFF
@A00721:190:HGJW7DSXY:4:1201:32606:36104_1:N:0:TCTGCAAG+GCTGCACT
TGAAGGAGGAGGTTTCTGTTACTGGAAAACCCTCGTCAATACTTTTCAATAATATACGTCTTGAACTC
ACTTATGACGATCTAGTGAAATTAACGCGTCCTTTTCTGGAACGTAGTTTTACTATTATGAACAAAGT
TAT
+
FFFFFFFFFFFFFFFFFFFF:FFF:FFFFFFFFFF,FFFFFF,F:FFFFFFFFFFFFFFFFFFFFFFF
FFFF:FFFFFFFFFFFFFFFFFFFFFFFFFFF:FFFFFFFFFFFFFFFFFFFFFFFFFFFFFFFFFFF
FFF
@A00721:190:HGJW7DSXY:4:1649:10276:18286_2:N:0:TCTGCAAG+GCTGCACT
GCAAACTTACCCTGATTCCGGCCGGTGGTAGCTCATGTCTACCAGGGGCTATTACTCTTGCTGAAACA
GCTATTAAAGCTGTTGAGCGGGTAATTACACCTAAACGAGCTCGATCT
+
FF,FFF:FFFFFFFFFFF,F:FF,:F::FF:F:FFF,FF:,F,F,:FFF,,F,F,,FFFF,FFFFF,F
:FFFFFFFF,:FFF,F:,FF:FFFFFFF:F::FFF,F,F,,,:FF,:F
@A00721:190:HGJW7DSXY:4:1255:8793:36949_1:N:0:TCTGCAAG+GCTGCACT
AAGGATGTGCACTCTTTTCTGCTACTTTAACGGAGCGTAGTGTACTCTTCGTCGATTGTATCAACAGT
AACATTACTTCTAATAAAGGTCTCTATCAAGCTCGTATTATCGTCGCTGCTGGATCTGCGTTACCGTT
GAA
+
FFFFFFFFFFFFFFFFFFFFFFFFFFFFFFFFFFFFFFFFFFFFFFFFFFFFFFFFFFFFFFFFFFFF
FFFFFFFFFFFFFFFFFFFFFFFFFFFFFFFFFFFFFFFFFFFFFFFFFFFFFFFFFFFFFFFFFF,F
FFF
@A00721:190:HGJW7DSXY:4:2109:13295:17347_2:N:0:TCTGCAAG+GCTGCACT
AGGTAATTATGAAGACTGTGATTTATCTTTCACTCTGGCCTTGTATGAGGGTGTTTCTAATCGTAACA
TTAATAATTTCAATCTTCTTAAAAAGAAGATAGAGAGTCATACTTTGG
+
FFFFFFFFFFFFFFFFFFFFFFFFFFFFFFFFFFFFFFFFFFFFFFFFFFFFFFFFFFFFFFFFFFFF
FFFFFFFFFFFFFFFFFFFFFFFF:FFFFFFFFFFFFFFFFFFFFFFF
@A00721:190:HGJW7DSXY:4:1223:32524:28103_2:N:0:TCTGCAAG+GCTGCACT
GATAGCCAATATTGAAGTGGACACCTCTGGTAGTATCGAAGTAAAGATAACCAATAAGGAAGGAGCTT
ATACTATGGTTGAATCAACTAAGCATACTACTTTTGCTTTGATTACTC
+
FFFFFFFFFFFFFFFFFFFFFFFFFFFFFFFFFFFFFFFFFFFFFFFFFFFFFFFFFFFFFFFFFFFF
FFFFFFFFFFFF:FFFFFFFFFFFFFFFFFFFFFFFFFFFFFFFFFFF
@A00721:190:HGJW7DSXY:4:2177:5032:4758_1:N:0:TCTGCAAG+GCTGCACT
CTTCCGATCTGTTAAAGAGTCGATTAAACAAGTTGTACTTATCAATGATTTTATCGCTGGTTGTTATC
TCGAAAAGTCTCTTCTAAAAGGAACGTCAGTTCTAACCCAAAGAGAATATCTAAAATATTTACAAAAT
CTT

```
+
FFFFFFFFFFFFFFFFFFFFFFFFFFFFFFFFFFFFFFFFFFFFFFFFFFFFFFFFFFFFFFFFFFFFFF
FFFFFFFFFFFFFFFFFFFFFFFFFFFFFFFFFFFFFFFFFFFFFFFFFFFFFFFFFFFFFFFFFFFFFF
FFF
@A00721:190:HGJW7DSXY:4:2675:13105:26788_1:N:0:TCTGCAAG+GCTGCACT
ACGCGATCTTGCAGAGTGACTGGAGTTCAGACGTGTGCTCTTCCGATCTGTACAATCTTACTTGGAAC
GAAGGCGACCTGTTCCGGAGTTTTTACGGGATCCGGAATGTTCTGTCTATATTAGGTGAAGCGAAATA
CGA
+
FF:,FFFFFFFFFFFFFFFFFFFFFFFFFFFFFFFFFFFFFFFFFFF,FFFFFFFFFFFFFFFFFFFFF
FFFFFFFFFFFFFFFFFFFFFFFFFFFFFFFFFFFFFFFFFFFFFFFFFFFFFFFFFFFFFFFFFFFFFF
FFF
@A00721:190:HGJW7DSXY:4:1261:2510:3834_1:N:0:TCTGCAAG+GCTGCACT
TCGTACCTTGATGCTGACTTTCGTCAATATGATTTGATCTGGTTGCTCAATTCTAAGCGTGTTCCGCT
TAGTGCGAGGCCTCTCTTCTACGAGTATTGCTATTTATACGGTTACGAGAAGAAGGTTCGAAAGTTTG
AGA
+
FFFFFFFFFFFFFFFFFFFFFFFF,FFFFFFFFFFFFFFFF:FFFFFFFFFFFFFFFFFFFFFFFFFF
FFFFFFFFFFFFFFFFFFFFFFFFFFFF,FFFFFFFFFFFFFFFFFFFFFFFFFFFFFFFFFFFFFFFFF
FFF
@A00721:190:HGJW7DSXY:4:1575:3821:19351_2:N:0:TCTGCAAG+GCTGCACT
CTATACGTGACATACTAAATACTAGATATTTGGATGATCTTGATAAAGAACTTCATGAATTTTCAGAG
GATGAAAAATTTTCTTCCTTGGATTTTAATATAAAGAATCTTTACCAG
+
FFFFFFFFFFFFFFFFFFFFFFFFFFFFFFFFFFFFFFFFFFFFFFFFFFFFFFFFFFFFFFFFFFFFFF
FFFFFFF:FFFFFFFFFFFFFFFFFFFFFFFFFFFFFFFFFFFFFFF:F
@A00721:190:HGJW7DSXY:4:2434:8070:8609_1:N:0:TCTGCAAG+GCTGCACT
AGGAGGCAGTCTTCTGATCGTAAGAAGTTTATAAGGAACATATCCATTTGTTGTAATAGGGTCATCAA
CTTAGAACTCGAAACTCTAAGTGGTAATACTGTCTTTCCAAGCTTACCTGTATCTTCAATGAAATTGG
ACT
+
:FFFFFFFFFFFFFFFFFFFFFFFFFFF:FF:FF:FF:FFFFFFFFFFFFFFFF:FFFFFFFFFFFFF:
FFFFFFFFFFFFFFFFFFFFFFFFFFFFFFFFF:FFFFFFFFFFFFFFFFFFFFFFFFFFFFFFFFFFFF
FFF
@A00721:190:HGJW7DSXY:4:1205:16848:4022_2:N:0:TCTGCAAG+GCTGCACT
GTGGGCTTTCCGTAAAGGAAGATCTGTTATCGACGGTTTATCGTTTACGAAGTATCACTCCACCGGTG
AATGGTTTAAGAATACTATAAATAGAATAGCTATTACTACTAATGGCG
+
FFFFFFFFFFFFFFFFFFFFFFFFFFFFFFFFFFFFFFFFFFFFFFFFFFFFFFFFF:FFFFFFFFFFF
:FFFFFFFFFFFFFFFF:FFFFFFFFFFF:FFFFFFFFFFFFFFFFF:
@A00721:190:HGJW7DSXY:4:1335:21133:15577_2:N:0:TCTGCAAG+GCTGCACT
TAGTGACATCTGGTCAATTTCGTTTTTAGGTGGTCTTTATTCTATTATTGATTTTTATGAGATTGATA
CGTTAACAGAAGATGATGTTAGAGTAGAATTCCTGAAGAGTCCTTCCG
+
FFFFFFF:FFFFFFFFFFFFFFFFFFFFFFFFFFFFF,FFFFFF:FFFFFFFFFFFFFFFFFFFFFFFF
FFFFFFFFFFFFFFFFFFFFFFFFFFFFFFFFF:FFFFF:FFFFFFFFFF
@A00721:190:HGJW7DSXY:4:2565:4110:12649_1:N:0:TCTGCAAG+GCTGCACT
TAATAGTTAAATCTAGGATTATGTCTGTGTTGAAAGACTTTCTACCGATAAGGGTCACTAGTCTTACA
CAATTAAGAAATAATCCTACATTGACTGATTATCTAAAGATTGAAGGAAAATTTTATCCTCGTTTAAC
TTA
+
FFFFFFFFFFFFFFFFFFFFFFFFFFFF:FFFFFFFFFFFFFFFFFFFFFFFFFFFFFFFFFFFFFFFFF
FFFFFFFFFFFFFFFFFFFFFFFFFFFFFFFFFFFFFFFFFFFFFFFFFFFFFFFFFFFFFFFF:FFFFF
FFF
```

@A00721:190:HGJW7DSXY:4:1274:11116:23218_1:N:0:TCTGCAAG+GCTGCACT
GTGCTCTTCCGATCTATAACTTCTAATTTACGTTTGCTACTCAGAGCTGGTCCTCTCAGCAAAGGGAT
GCATGGTGTTATCTCCTCAGGAGTCCCGGCATTTGATAATAATGACGAGACTTTCGTCATAATCGCTG
CAT
+
FFFFFFFFFFFFFFFFF,FFFFFFFFFFFFFF:FFFFFFFFFFFFFFFFFFFFFFFFFFFFFFFFFFF
FFFFFFFFFFFFFFFFFFFFFFFFFFFFFFFFFFFFFFFFFFFFFFFFFFFFFFFFFFFFFFFFFFFF
FFF
@A00721:190:HGJW7DSXY:4:1270:12662:24893_1:N:0:TCTGCAAG+GCTGCACT
GGTACCAGCGCATCTCGTGTCGAGCCTCGACCCGATTATATGGTATTAACTTATAAAGGTGAGGAGAC
CATTTTCTTGCTGAAATTTATAGAAGATTATTTTGACAGAATCAATAAGAATCATCGATTCTCGATTC
GTA
+
FFFFF:FFF:FFF:FFFFFFFFF,FFFFFFFFF,,FFFFFF:FFFFFFF:FFFFFFFFFFF,:FF,FF
,FFFFFFFF:FFFFFFFFFFFFFF::FFFFFFFFFF:FFFFFFFFFFF:FFFF,F:FFFFFFFFFFF:
FF:
@A00721:190:HGJW7DSXY:4:1166:10022:16219_1:N:0:TCTGCAAG+GCTGCACT
TATGCTGATCTAAAATTTCGATCTAGATGGATCCACTCCTCGAGTTATCATGATGTTAGTATGTTATA
TACTGATTTACAACGATATAGGAGTAATCTACGAGAAGATTCTAGAATTCTTAATTATAATATTCAAC
CTC
+
FFFFFFFFFFFFFFFFFFFFFFFFFFFFFFFFFFFFFFFFFFFFFFFFFFFFFFFFFFFFFFFFFFFF
FFFFFFF:FFFFFFFFFFFFFFFFFFFFFFFFFFFFFFFFFFFFFFFFFFFFFFFFFFFFFFFFFFFF
FFF
@A00721:190:HGJW7DSXY:4:1233:17888:1251_2:N:0:TCTGCAAG+GCTGCACT
GAGATCACACATTTTGAGAACTAACTATGTTATGTTCTCTGATCATTTTGACAATCTCTTCTTATAAT
AAATAAATAAATAATATTACTAATGGCAAATAGTGGAAAGGCTCCAGT
+
FFFFFFFFFF:FFFFFFFFFFFFFFFFFFFFFFFFFFFFFFFFFFFFFFFFFFFFFF:FFF:FFFFF
FFFFFF:FFFFFFFFFFFFFFFFFFFFFFFFFFFFFFFFFFFFFFFFF
@A00721:190:HGJW7DSXY:4:1449:8901:25676_2:N:0:TCTGCAAG+GCTGCACT
CTGCTTTGGATTACGATGACGTGAGAATAGGTGAAGGGATAGTTTTTACCTCGACCAACATTAAGGCG
GCGGCGACTAATCTTAAAGCTTCACCCAAAATGAAGGACACTTCGAAA
+
FFFFFFFFFFFFFFFFFFFFFFFFFFF:FFFFFFFFFFFFFFFFFFFFFFFFFFFFFFFFFFFFFFFF
FFFFFFFFFFFFFFFFFFFFFFFFFFFFFFFFFFFFFFFFFFFFFFFF
@A00721:190:HGJW7DSXY:4:1413:24795:4351_2:N:0:TCTGCAAG+GCTGCACT
AAAAGGCTCTAAAGGACACTATACAATATCCAGACGACGACGAAGCTTATACTACTTTAATTTTGGGT
ATTATGTGGAGATATCGTGATATCAGTACGTCCAGATCCTCTGCTTTT
+
FFFFFFFFFFFFFFFFFFFFFFFFFFFFFFFFFFFFFFFFFFFFFFFFFFFFFFFFFF,FFFFFFFFF
:FFFFFFFFFFFFFFFFFFFFFFFFFFFFFFFFFFFFFFFFFFFFFFF
@A00721:190:HGJW7DSXY:4:1516:19542:4930_2:N:0:TCTGCAAG+GCTGCACT
ATCAAATATAAAGAAAAGGACTATGAAATAAAAGATTTGGTGCTTAGGGATATAATGAATTCAGCACC
ACATTTTGGTCAAGTCAATCCGTTGAGAAAATTTCTTAGAGGATATTC
+
FFFFFFFFF:FFFFFFFFFFFFFFFFFFFFFFFFFFFFFFFF:F,FFFFFFFF:FFFFFFFFFFFFFF:
FFFFFFFFFFFFFFFFFFFFF:FFFFFFFFFFFFFFFFFFFFFFFFFF
@A00721:190:HGJW7DSXY:4:1662:22345:23938_1:N:0:TCTGCAAG+GCTGCACT
CCAACATTTACCACCAAATTCTTTTGATACAACTCTAGCCTTCAAGTGGGGTATGCCTGCGGAATACG
CTTGCTATTCACCAGATTGGTTCGAACCCGTTGATGCTATGTCAGAAGAAGCTATTCAAGCTTGTCGT
TTA
+
:FFFFFFFFFFFFFFFFFFFFFFFFFFFFFFFFFF:F:FFFFFFFFFFFFFF:FFFFFFFFFFFFFF

FFFFFFFFFFFFFFFFFFFFFFFFFFFFFFFFFFFFFFFFFFFFFFFFFFFFFFFFFFFFFFFFFFFF
FFF
@A00721:190:HGJW7DSXY:4:2413:4752:35963_2:N:0:TCTGCAAG+GCTGCACT
TCAAACAAGCCCAGACTCAAGGAGTCCAATCAAAGGTTTTGAATTTAGCTCAACTGGGGAGACGTGCT
TAAATGGGTGATAAAAATTATTTAGAGATCTTTTCTACTTACAAAGGA
+
FFFF:FFFFFFFFFFFFFF:FFFFFFFFFF:FF,FFFFFFFFFFFFFFFFFFFFFFFFFFFFFFFFFF
FF:FFFFFFFFFF,FF:FF:FFFFFFFFFFFFFFFFFFFFFFFFFFFF
@A00721:190:HGJW7DSXY:4:1572:30346:35383_2:N:0:TCTGCAAG+GCTGCACT
AATTCTACGGAAAAGTCCGGAGTATATTGTATGTTAATAGATAGTGGGATAATAGTTCTACCGATACG
TAGTGAACGCGTTCGTAGAACCAAGTACTTTCCGTCTGAAGAGTTTAG
+
F:FFFFFFFFFFFFFFFFFFFFFFFFFFFFFFFFFFFFFFFFFF:FFFFFFFFFFFFFFFFFFFFFFF
:FFFFF:FFFFFFFFFFFFFFFFFFFFFFFFFFFFFFFFFF:F:FFFF
@A00721:190:HGJW7DSXY:4:1434:25418:25379_1:N:0:TCTGCAAG+GCTGCACT
TAACCGATAGGAGTTGTATAGTTGAAGATGTGGCTTTAGACGATAATCCTTTAAGGACTATGTTGACT
GACGAAGATTTTCCCACAGATTTGTCTACGTTGCAGATCGCCACTCCAAACACTATATACATTAAAAT
ATA
+
FFFFFFFFFFFFFFFFFFFFFFFFFFFFFFFFFFFFFFFFFFFFFFFFFFFFFFFFFFFFFFFFFFFF
FFFFFFFFFFFFFFFFFFFFFFFFFFFFFFFFFFFFFFFFFFFFFFFFFFFFFFFFFFFFFFFFFFFF
FFF
@A00721:190:HGJW7DSXY:4:2129:30047:2080_2:N:0:TCTGCAAG+GCTGCACT
AGTCATAACTTTTGTTACCAAATTCTTGGCTTTCAACGAAGTCTCTGATTTTATTAATCATTACTTTA
ACGATCTCTCGGTCATTTACTCTGTACTTGATCTACGTCGTTTAACTG
+
FFFFFFFFFFFFFFFFFFFFFFFFFFFFFFFFFFFFFFFFFFFFFFFFFFFFFFFFFFFFFFFFFFFF
F:FFFFF:FF:FFFFFFFFFFFFFFFFFFF:FFFFF,FFFFFFFF,FF
@A00721:190:HGJW7DSXY:4:1344:21052:24079_2:N:0:TCTGCAAG+GCTGCACT
CTTAGAATGACTCTATCTTTTCAGACACTTCTTATATTATTTACAATAGTTAATATCATATTAGTAAT
ACTCACTATCGTTTTCTGCTTTTTCTTAGCTAATAGATTAATAAAGGA
+
FFFFFFFFFFFFFFFFFFFFFFFFFFFFFFFFFFFFFFFFFFFFFFFFFFFFF:FFFFFFFFFFFFFF
FFFFFFFFFFFFFFFFFFFFFFFFFFFFFFFFF:FFFFFFFFFFFFFF
@A00721:190:HGJW7DSXY:4:2173:26639:21699_2:N:0:TCTGCAAG+GCTGCACT
ACCAACTCTTCGTTACGAGACCTGATGGTAGAATAGATTTAGTCTCTACACCCATCTCAACGAGGATA
AAGCGAGTTTTCCGTCCTGAACGTAATACACGAAGAGATCATCCGCAA
+
FFFFFFFFFFFFFFFFFFFFFFFFF:FFFFFFFFFFFFFFFFFFFFFFFFFFFFFFFFFFFFFFFFFF
FFFFFFFFFFFFFFFFFFFFFFFFFFFFFFFFFFFFFFFFFFFFFFFF
@A00721:190:HGJW7DSXY:4:1364:11234:17785_2:N:0:TCTGCAAG+GCTGCACT
AAAGTCCCTTCTAACTTTAGATTATGAATCCTGCCTTTACTCATTTTATAGCAGTTATTTATGTTGTA
CTTTTTATAATTTATAACCTTCTTATACTTTTGGGTATGTTTTGTGAA
+
FFFFFFFFFFFFFFF:FFFFFFFFFFFFFFFFFFFFFF:FFFFFFFFFFFFFFFF:FF:FFFFFFFFF
FFFFFFFFFFFFFFFFFFFFFFFFFFFFFFFFFFFFFFFFFFF:F:FF
@A00721:190:HGJW7DSXY:4:1446:28782:23782_2:N:0:TCTGCAAG+GCTGCACT
GAAGACATTATCTCTTATGTACTTATTGTAACGATCATTCTTTCGAGTTTTGTCATTCTTATTCTTTT
ATTCCTTTAACTGTGTATGAGGAGGATTTATAATGCTTTTCGATAGTG
+
FFFFFFF:FFFF:FFFFFFFFFFFFFFFFFFFFFFFFFFFFFFFFFFFFFFFFFFF:FFFFFFFFFFF
FFFFFFFFF,:FFFFFFFF::FF:FFFFFFFFFFFFFFFFFFFFFFFF
@A00721:190:HGJW7DSXY:4:2450:19253:2206_2:N:0:TCTGCAAG+GCTGCACT
ATTACTGTTTCAAATCATCTTTACTCACAGACTCCGAAAATGGTTTCAAATGAGGTCAAAGCTCTATT

TATTAAACGAACTATTATCCGATTCTTCGAGGGTATCACTACCACTGA
+
FFFFFFFFFFFFFFFFFFFFFFFFFFFFFFFFFFFFF:FFFFFFFF:FFFFFFFFFFFFF:FFFFFFF
FFFFFFFFFFFFFFFFFFF,FFFFFFFFFFFFFFFFFFFFFFFFFFFFF
@A00721:190:HGJW7DSXY:4:1412:2401:25817_2:N:0:TCTGCAAG+GCTGCACT
CAAGGAACTTCAATTCCTTCGATGTTTATATTTTTCTATAAGGGAAAATAGCAATAATCAAATTTCGA
CAAATTTTAGAACTTTATTTCAGAACTCTCTCCGATCACCTTGGTGTG
+
FFFFFFFFFFFFFFFFFFFFFFFFFFFFFFFFFFFFFFFFFFFFFFFFFFFFFFFFFFFFFFFFFFFF
FF:FFFFFFFFFFFFFFFFFFFFFFFFFFFFFFFFFFFFFFFFFFFFF
@A00721:190:HGJW7DSXY:4:2148:12753:3129_1:N:0:TCTGCAAG+GCTGCACT
TCTTTTCAATAAGATGTATGATGATAATCTACTATTTTTAAATAGTGATATTCAGCGTATACTTGAAT
ACTTTATCAAAGATTTGGCTTGGTCTATTTACCGCAAAAAGTTGGAAGAGGTCTATGATATTTATTTT
ACT
+
FFFFFFFFFFFFFFFFFFFFF:FFFFFFFFFFFFFFFFFFFFFFFFFFFFFFFFFFFFFFFFFFFFFF
FFFFFFFFFFFFFFFFFFFFFFFFFFFFFFFFFFFFFFFFFFFFFFFFFFFF:FFFFFFFFFFFFFFF
FFF
@A00721:190:HGJW7DSXY:4:2156:26178:14544_2:N:0:TCTGCAAG+GCTGCACT
CACCGATTATAACTTGACTGTTGATAATATTACAATAGCTCTTTCTCAGAAGATTAGAGAAGTAACCG
GTGAACACGTCGGTAGCATTGATTTACTTTTACCGTTAGAACTGAAAA
+
FFFFFFFFFFFFFFFFFFFFFFFFFFFFFFFFFFFFFFFFFFFFFFFFFFFFFFFFFFFFFFFFFFFF
FFFFFFFFFFFFFFFFFFFFFFFFFFFFFFFFFFFFFFFFFFFFFFFF
@A00721:190:HGJW7DSXY:4:1471:20446:32737_1:N:0:TCTGCAAG+GCTGCACT
ACCTATAAGTTACAAACGATTCTTCGACTATATTAAAGAAATAAAAATACTTCAAGTACCTGAACTTT
ACATTTTTAATGGTATGGTATAATGTGTCTTACAAATAGAACCTTCACTCTCGGTAATGAGAGTGTAT
TCC
+
FF:FFFFFFFFFFFFFFFFFFFFFFFFFFFFFFFFF:FFFFFFFFFFFFFFFFFFFFFFFFFFFFFFF
FFFFFFFFFFFFFFFFFFFFFFFFFFFFFFFFFFFFFFFFFFFFFFFFFFFFFFFFFFFFFFFFFFFF
FFF
@A00721:190:HGJW7DSXY:4:1372:30526:27774_1:N:0:TCTGCAAG+GCTGCACT
GGCATTACTAGGATTTATCACACTCCTAAATTTGATAGCAATTTATGTTTTAAATGCTAGGAAAATTT
TAGAAAAACTCCATTGTATAGTTCAAGGAGTACTTAATTCAATAGCTTTAGTATTATTTACTATCGCC
TTT
+
FFFFFF:FFFF:FFFFFFFFFFFFF,FFFFFFFFFFFFFFFFFFFFFF:FFFFFFFFFFFFFFFFFF:
FFFFFF:FFFFFFFFFFFFFFFFFFFFFFFFFFFFFFFFFFFFFFFFFFFFFFFFFFFFFFFFFFFFF
FFF
@A00721:190:HGJW7DSXY:4:1309:17788:1235_2:N:0:TCTGCAAG+GCTGCACT
AGTTAATTGCTTACACAAAAGTGACGCGCGAAGGAATCTTAATTGATTGAAGCTAGGCGTTGGGGAGA
ATGAACTGCCGCTTCCCGGAAGAAACGATCTATTTACAGAGTATTTAG
+
FFFFFFFFFFFFFFFFFFFFFFFFFFFFFFFFFFFFFFFFFFFFFFFFFFFFFFFFFFF:FFFFFFFF
FFFFFFFFFFFFFFFFFFFFFFFFFFFFFFFFFFFFFFF:FFFFFFFF
@A00721:190:HGJW7DSXY:4:1234:9706:14920_2:N:0:TCTGCAAG+GCTGCACT
GAATTCCTGGCGAACCGCAAATCAATTGAAGAAAACCACTCCCTTCAATTCAATAACATAACATACCA
TACACTCCCCAAACATAACATACCATAACCTACCATGACCAATCATCG
+
FFFFFFFFFFFFFFFFFFFFFFFFFFFFFFFFFFFFFFFFFFFFFFFFFFFFFFFFFFFFFFFFFFFF
FFFFF:FFFFFF:FFFFFFFFFFFFFFFFFFFFFFFFFFFFFFFFFFF
@A00721:190:HGJW7DSXY:4:1266:7970:11945_1:N:0:TCTGCAAG+GCTGCACT
ACGCCAGACGTAGGGTCAAACCTACTACCGCCGGTCGGCAGATCGGCACCACTGGTCTCTACGTTCAG

GGACCCGCTGTGCAGAATAACCTTCCCAAATCTAGATCTGCACAGAAGAAGGCTGAAGGTCGTTACCG
GAC
+
FFFFFFFFFFFFFFFFFFF,FFFFFFFFFFFFFFFFFFFFFFFFFFFFFFF:FFFFFFFFFFFFFFFF
FFFFFFFFFFFFFFFFFFFFFFFFFFFFFFFFFFFFFFFFFFFFFFFFFFFFFFFFFFFFFFFFFFFF
FFF
@A00721:190:HGJW7DSXY:4:1519:13548:29496_1:N:0:TCTGCAAG+GCTGCACT
GGCTGTTGCCCAGAGGAAAGCAGCGACCACCGAGGTCGCTCGTAACATTGACGGCAGTCCCGTCATCT
CTGTGGGAACTGAGGCAGTACCTCAGTCAAAAGTCACATTCACCACTCCCATGCCAATCAATGATGTG
ACT
+
FFFFFFFFFFFFFFFFFFFFFFFFFFFFFFFFFFFFFFFFFFFFFFFFFFFFFFFFFFFFFFFFFFFF
FFFFFFFFFFFFFFFFFFFFFFFFFFFFFFFFFFFFFFFFFFFFFFFFFFFFFFFFFFFFFFFFFFFF
FFF
@A00721:190:HGJW7DSXY:4:2377:7455:23703_2:N:0:TCTGCAAG+GCTGCACT
GTGGTCAATACGTCGTGGGAGGTACTGAAGCCTTCCCCTCCGCTGGCACGAAGAACACTCCCAAGCCA
ATCAATAGTTCTTCTGACCAGAATGAAAAGGGGTTTACCAGGGAGATC
+
FFFFFFFFFFFFFFFFFFFFFFFFFFFFFFFFFFFFFFFFFFFFFFFFFFFFF:FFFFFFFFFFFFFF
FFFFFFFFFFFFFFFFFFFFFFFFFF:FFFFFFFFFFFFFFFFFFFFFF
@A00721:190:HGJW7DSXY:4:1252:30092:7764_2:N:0:TCTGCAAG+GCTGCACT
CAGGAAGAGGGGAAATGTCCCCAAAACTGAGAAGAATAAAGGGAAAGGTCCATCAGGACCTAAGAAGG
GGAAATTACCCCTAATTCTGAAGGAAGATCTCCCACCATCTGGGGTTG
+
FFFFFFFFFFFFFFFFFFFFFFFFFFFFFFFFFFFFFFFFFFFFF:FFFFFFFFFFFFFFFFFFFFFF
FFFF:FFFFFFFFFFFFFFFFFFFF:FFFFFFFFFFFFFFFFFFFFFF
@A00721:190:HGJW7DSXY:4:1432:27914:9189_2:N:0:TCTGCAAG+GCTGCACT
ATCTGCACGGGCCAAAGAGGCCGCTGCTACGAAGTCGCTGAAGGCGGCTTGGGTCATTAAGGGTAGCT
GTAAAGTTGCGCCTGCCGACCCCATCTTTGAGACCAAGACTCTCTACA
+
FFFFFFFFFFFFF:FFFFFFFFFFFFFFFFFFFFFFFFFFFFFFFFFFF:FFFFFFFF,FFFFFF:FFF
FFF:FFFFFFFFFFFFFFFFFFFFFFFF:FFFFFFFFFFFFFFFF:FFF
@A00721:190:HGJW7DSXY:4:2363:17119:30389_1:N:0:TCTGCAAG+GCTGCACT
TACTATGAGCAAATCTGTTGAATTCGTCACTGAGGACGAAGGTGAGAGGACTTTTGGCTACTGGCGAC
AAACTGAGAGCAGTCGCTGGGGAAAGAGAATTGCGTGGGTTGTAGGCCGACGCTTCGAGCTTGAGTGT
GAC
+
FFFFFFFFFFFFFFFFFFFFFFFFFFFFFFFFFFFFFFFFFFFFFFFFFFFFFFFFFFFFFFFFFFFF
F,FFFFFFFFFFFFFFFFFFFFFFFFFFFFFFFFFFFFFFFF,FFFFFFFFFFFFFFFFFFFFFFFFF
FFF
@A00721:190:HGJW7DSXY:4:1233:2175:2691_2:N:0:TCTGCAAG+GCTGCACT
GTTTTTGTCAACGGCGTTTGGAGGATTTTATCCGCGCTTAACTATGGGGCGAAGATCCCCTATGGAGT
CTTGAAGGACTTAGGTTGGCATATTTATGCCGACGATAATAAGCATGT
+
FFF:FFF:FFFFFFFFFFF:FFFFFFFFFFFFFFFFFFFFFF:FFFFFFFFFFFFFFFF,F:FFFFF
F,:FFFFFFF:FFFFFFFFFFFFFFFFFFFFFFFFFFFFFFFFFFFFFF
@A00721:190:HGJW7DSXY:4:1354:4996:14935_1:N:0:TCTGCAAG+GCTGCACT
CCGAGGTAAGCTCGGGACTTACAAGAGAGAGTGTGAGAGAGTCGCTCTCACTATTTTGCCCAGAATG
CGGGCTTTCTGACTGATGAGGTTAAAACTCTTGCTCCTTCTGTGGGTGAGGGTTTTTGTTATATTCAC
TGG
+
FFFFFFFFFFFFFFFFFFFFFFFFFFFFFFFFFFFFFFFFFFFFFFFFFFFFFFFFFFFFFFFFFFFF
FFFFFFFFFFFFFFFFFFFFFFFFFFFFFFFFFFFFFFFFFFFFFFFFFFFFFFFFFFFFFFFFFFFF
FFF

```
@A00721:190:HGJW7DSXY:4:1129:8052:26146_2:N:0:TCTGCAAG+GCTGCACT
TCCTCCATAATTACAACCTCTCTTTAGAGAGCATTAGAAGGAAGTTGGGCAAATTCCCACATATGGTA
GACGTTGTCACACTTTTCTTGAAAATTTTTCCTTTCCTCCGTCATTGG
+
FFFFFFF,FFFFFFFFFFFFFFF:FFFFFFFFF:FFFFFFFFFFFFFFFFFFFFFFFFF:F,FFFFFF
FFFFFFFFFFFFFFFFFFFFFFFFFFFFFFFFFFFFFFFFFFFF:FFFF
@A00721:190:HGJW7DSXY:4:2649:15157:11021_2:N:0:TCTGCAAG+GCTGCACT
GTGAGAAGGTGGAAGGTGTCAAGGGTCTTCTTCATGCTGATCTTTCAGGACCTAATCACATCATGATT
GATAGTTACGGCTCGTATCGAGTTGGAGGGAGGAAGTTCTCCGAAATT
+
FFFFFFFFFFFFFFFFFFFFFFFFFFFFFFFFFFFFFFFFFFFFFFFFFFFFFFFFFFFFFFFFFFFF
FFFFFFFFFFFFFFFFFFFFFFFFFFFFFFFFFFFFFFFFFFFFFFFF
@A00721:190:HGJW7DSXY:4:1656:2582:4586_1:N:0:TCTGCAAG+GCTGCACT
GCTGTAACTGTCATTTCAGAGAGGGGAGCTCTGAAAAGGAAACTTCGTCAGAAGATGAGGAAGTCTAA
GAGGAGGAGTTTGAGGAAGGCTCTAGAGAAGATCTCACCACCTGGAGAAGGCAAGTGGAGAGTCCAGA
AGT
+
FFF:FFFFFFFFFF:FFFFFFFFFFFFFFFFFFFFFFFFFFFFFFFFFFFFFFFFFFFFFFF:FFFFFF
FFFFFFFFFF:FFFFFFFFFFFFFFFFFFFFFFFFFF,FFFFFFFFFFFFFFFFFFFFFFFFFFFFFFF
FFF
@A00721:190:HGJW7DSXY:4:2266:9353:15562_2:N:0:TCTGCAAG+GCTGCACT
GGATTATTTGAAGCAGGGGTTACCACTTCCTAAGGTGGATGAGGGAGGTTGTTTCTTTGGAGTTTTAG
ATAATCTTTTGAGAAGGGGTCATAGAGCCAGCAAGAGAAGCTTATATG
+
FFFFFFFFFFFFFFFFFFFFFFFFFFFFFFFFFFFFFFF,FFFFFFFFFFFFFFFFFFFFFFFF:FFFF
FFFFFFF:FFFFFFFFFFFFFF:FFFFFFFFFFFFFFFFFFFFFFFFFF
@A00721:190:HGJW7DSXY:4:1306:6831:34835_2:N:0:TCTGCAAG+GCTGCACT
CCTTGAGAAGACAAGCCACATTAAGATATAATTTAGGTCAACTTGGGGTTTCGAATGTCATTAGTGCC
TTACGATTGAAAGAGGTCCTTTTGAGTATCGACAGATCTTTCGATCTT
+
F:FFFFFFF,FFFF:FFFFFFFFFFFFFFFFFFFFFFFFFFFFFFFFFFFFFFFFFFFFFFFFFFFF:F
FF::F::FFFFFF:FFFFF:FFFFFFFFFFFFFFFFFFFFFFFFFFFFF
@A00721:190:HGJW7DSXY:4:1342:12626:29653_2:N:0:TCTGCAAG+GCTGCACT
TTAACGTGGCAGAATTCGGTAACTTACCATATTGTTCCTTCCGGGAAGTTGTGGGATTTAACTTTGAC
TCAACTACTCCGATCAGATCCGGATGGTTTGATTGGTATGGACTACTC
+
:FFFFFFFFFFFFFFFF:FFFFFFFFFFFFFFFFFFFFFFFFFFFFFF,FFFFFFFFFFFFFFFFFFFF
FFFFFFFFFFFFFFFFFFFFFFFF:FFFFFFFFFFFFFFFFFFFFFFF
@A00721:190:HGJW7DSXY:4:1439:19587:11898_1:N:0:TCTGCAAG+GCTGCACT
TAGTTGTCTAGGGTCATGGCAGATACTACCGATAAGGTGAAAAATATGCAGGTCAAAGATTCTGTAGC
TCTGAGGGCGGTTGAGGGAGCCGTTTTGGCCGACTTTAACTATTCTAAGAGTCTTTATGATTCAAAAC
CTA
+
FFFFFFFFFFFFFFFFFFFFFFFFFFFFFFFFFFFFFFFFFFFFFFFFFFFFFFFFFFFFFFFFFFFF
FFFFFFFFFFFFFFFFFFFFFFFFFFFFFFFFFFFFFFFFFFFFFFFFFFFFFFFFFFFFFFFF:FFF
FFF
@A00721:190:HGJW7DSXY:4:1447:24189:19523_2:N:0:TCTGCAAG+GCTGCACT
ACCTTACAAGGTTGAGGAGACTTTACAGCGGCTTATTGTCGCGCAGTTTCCAGAATTGCGGTTGAAGT
TCACCCATTCTATCTTGAATGCTCACGCCGTCGCAGCCATATCGAGAC
+
FFFFFFFFFFFFFFFFFFFFFFFFFFFFFFFFFFFFFFFFFFFFFFFFFFFFFFFFFFFFFFFFFFFF
FFFFFFFFFFFFF:FFFFFFF:FF:FFFFFFFFFFFFFFFFFFFFFFFF
@A00721:190:HGJW7DSXY:4:1552:2962:30295_2:N:0:TCTGCAAG+GCTGCACT
AAAGTACAGATTTAGTGATTTAGATATTATTGATATTGGAGGCGATATTCGTTTTCATATCCAAAACT
```

CCTTGAATGGAGTACATATTTGCAACCCTATTCTTGATGTGAAAGATG
+
FFFFFFFFFFFFFFF:FFFFFFFFFFFFFFFFFFFFFFFFFFFFFFFFFFFFFFFFFFFFFFFFFFFFFF
FFFFFFFFF:FFFFFFFFFFFFFFFFFFFFFFFFFFFFFFFFFFFFFF
@A00721:190:HGJW7DSXY:4:2368:31024:9126_2:N:0:TCTGCAAG+GCTGCACT
AACATTGATTGGGAACGTACCTACTCACGAGACGTCATGAAAGCTATCGGAGGGAAGCAAAATGTTTC
GTTCTGTTACAAGAACGCTTTGGATTGTGACCATAAAGCCTCAGTTGC
+
FFFFFFFFFFFFFFFFFFFFFFFFFFFFFFFFFFFFFFFFFFFFFFFFFFFFFFF,FFFF:FFFFFFF
FFFFFFFFFFFFFFF:FFFFFFFFFFFFFFFFFFFFFFFFFF:FFFFFF
@A00721:190:HGJW7DSXY:4:2358:27037:9987_2:N:0:TCTGCAAG+GCTGCACT
ACGATATCTCTCTTGATGATGTAGCCACTATAATGCTTAAGAGAGAGATTGATGTATGTCATTTCGTC
TTAGTTTGTCCCGGCGAATTATTGGACACGATGAAAAATGACATACCC
+
FFFFFFFFFFFFFFFF:FFFFF:FFFFFF:FFFFFFF:FFFFFFF,FFFFFFFFFFFFF:F:FFFFF
FFFFFFFFFFFFFFFFFFFFFF:FFFFF:FFFFFFFFFFFFFFFFFFFF
@A00721:190:HGJW7DSXY:4:1564:17291:36605_1:N:0:TCTGCAAG+GCTGCACT
TCGTTATCCGTCATGGAGATGATGGCGCATCACTTGATTACTATATGCCCGAAGGATTGGGTTATTCT
CACAACAAAGATAAGATTTTATCTTTCTTCAAACACCCTGTTTTCCTTAAGGGCGGACAATTGTTCCA
TTG
+
FFFFFFFFFFFFFFFFFFFFFFFFFFFFFFFFFFFFFFFFFFFFFFFFFFFFFFFFFFFFFFFFFFFF
FFFFFFFF:FFFFFFFFFFFFFFFFFFFFFFFFFFFFFFFFFFFFFFFFFFFFFFFFFFFFFFFFFFF
FFF
@A00721:190:HGJW7DSXY:4:2627:1642:19993_2:N:0:TCTGCAAG+GCTGCACT
ATTGAGCATTAATGAGTTCCGAATAACTCGTAATAAATCTTTCTCTAGTGGACAATTCAGAATACCTA
TTTCCGTACCAAGAGCCACTGCCAATCTCATCGCTATACGCATACCTG
+
FFFFFFFFFFFFFFFFFFFFFFFFFFFFFFFFFFFFFFFFFFFFFFFFFFFFFFFFFFFFFFFFFFFF
FFFF:FFFFFFFFFFFFFFFF:FFFFFFFFFF,F:FFFFFFFFFFFFF
@A00721:190:HGJW7DSXY:4:2450:3821:28839_1:N:0:TCTGCAAG+GCTGCACT
GAGGTTCGTTCCTCAATAAAAATTTTTCTCAAGAGATTATGGTTGATAAAGTTTTCTTCATAAGGGCT
ATTCAGTACGTCATGGGGATCACTTCTTCGGTCAATGACAAAACCTTCGAATATGTTACGACATGGCT
TCG
+
FFFF:FFFFFFFFFFFFF::FFFFFFFFFFFFFFFFF,FFFFFFFFFFFFFFFFFFFFF,FFFFFFFF
FFFFFFFFFFFFFFFFFFFFFFFFFFFFFFFFFFFFFFFFFFFFFFFFFFF:FFFFFFFFFFFFFFFF
FFF
@A00721:190:HGJW7DSXY:4:1664:27859:33927_2:N:0:TCTGCAAG+GCTGCACT
AGGATTGTCATCTCGGGGAAAGTGATTCACCAAGATGTTCATATAGATACGAGATACCTAGAGAAGGT
CGCAGCGATCTTACTCGTTATCGGAGCTAAGAAGAAGACTTTGGGTAC
+
FFFFFFFFFFFFFFFFFF:FFFFFFFFFFFFFFFF,FFFFFFFFFFFFFFFFFFFFFFFFFFFFFFFF
FFFFFFFFFFFFFFFFF:FFFFFFFFFF:FFFFFFFFFFF:FFFFFFF
@A00721:190:HGJW7DSXY:4:1615:6298:21731_2:N:0:TCTGCAAG+GCTGCACT
AAACTTAACGTTAGTACCGGAGGTGATCTCTCTGCTTCTGCGTTACTGAGAGAAAAGATTCAGGACGC
CTACGGTGACTTTATGGACAAGATGCAAGTTCTTGGGAAATACTTGTT
+
FFFFFFFFFFFFFFFFFFFFFFFFFFFFFFFFFFFFFFFFFFFFFFFFFFFFFFFFFFFFFFFFFFFF
FFFFFFFFFFFFFFFFFFFFFFFFFFFF:FFFFFFFFFF:FFFFFFFFF
@A00721:190:HGJW7DSXY:4:2353:8865:11490_2:N:0:TCTGCAAG+GCTGCACT
TCGATTCTTTGTATAGGGAGTGTGAGAAGACAACGAATCTTGAAACTACGGTCCTTATCAAAGAAAAT
GGTGAACCGGTCTCTAAATCTTTTGAGATTGAGAAGATTGAAGAACAC
+

```
FFFFFFFF,FF:F,FFFFFFFFFFFFFFFFFFFFFFFFFFFFFFFFFFFFFFFFFF:FFFF,FFFFF:
FFFFFFFFFFFFFFFFF:FFF::FFFF,FFF,FFFFFFFFFFFFFFFF
@A00721:190:HGJW7DSXY:4:1570:3477:12117_2:N:0:TCTGCAAG+GCTGCACT
GCTCGGCGTTGTTCTTGAAAACCAAGTTAAGAAGGAGGCTGATCATGGAGTCTTTGTACCTTCTGTCA
AACTTCGAGGTTCAGCTACGAGAGGTGATCCCGGATGTGGATTGAAGG
+
FFFFFFFFFFFFFFFFFFFFFFFFFFFFFFFFFFFFFFFFFFFFFFFFFFFFFFFFFFFFFFFFFFFF
FFFFFFFFFFFFFFFFFFFFFFFFFFFFFFFFFFFF,FFFFFFFFFFFF
@A00721:190:HGJW7DSXY:4:1568:13386:30279_2:N:0:TCTGCAAG+GCTGCACT
GGAGACTCCTTCTTTTTGAATGTTGTAAAGAATTGGGTTGAGAAGTGTGCTTTACAAGGAGTTAGACT
CTTCTTCAAGTTTTTAGATCTTCTTGATAATCTTAAAGAAAAATTTTC
+
FFFFFFFFFFFFFFFFFFFFFFFFFFFFFFFFFFFFFFFFFFFFFFFFFFFFFFFFFFF:FFFFFFFFFF
FFFFFFFFFFFFFFFF,FFFFFFFFFFFFFFFFFFFF:FFFFFFFFF:
@A00721:190:HGJW7DSXY:4:2456:28791:27618_1:N:0:TCTGCAAG+GCTGCACT
AGTCTGTACTTACATTCTTTACGCCAGATTCTTCATGGGGCGACGCTGTTTCTCTTGGTATTTCTGAA
ATAGTTTATCAGAGCGTCATTTTCCTTTGGGATCTTTCCTCTGGTAAGGGAATTATTAAATCATTCTT
CTC
+
FFF:FFFFFFFFFFFFFFFFFFFFFFFFFFFFFFFFFFFFFFFFFFFFFFFFFFFFFFFFFFFFFFFF
FFFFF:FFFFFFFFFFFFFFFFFFFFFFFFFFFFFFFFFFFFFFFFFFFFFFFFFFFFFFFFFFFFFF
FFF
@A00721:190:HGJW7DSXY:4:1535:6903:5995_2:N:0:TCTGCAAG+GCTGCACT
ATTATTACTGTTTTCTCTACCCATAACTTCTTTTTCAAAAATGCGGATGGGTCTACTGATGTTTTCCT
ACAGAGTGCAGTCGTTTCAACACTTATCACCACTTTTGAAAGGAAAAT
+
FFFFFFFFFFFFFFFFFFFFFFFFFFFFFFFFFFFFFFFFFFFFFFFFFFFFFFFFFFFFFFFFFFFF
FFFFFFFFFFFFFFFFF:FFFF:::FF:FFFFFFF:FFFFFFFF:FFF
@A00721:190:HGJW7DSXY:4:2438:7509:18443_1:N:0:TCTGCAAG+GCTGCACT
AAATACGTATGTCGGTGGGGTTCTCAAGGCTCTCATCGCCACTGGGATTCTAAGGGGTTCCATAAGAT
ATTATCTGACATTGTGGTTCAATGATCAGGATTCTTCTTTTGGTCCTTACGTCTTAAGTAATTTGGAT
GTT
+
FFFFFFFFF:FFFFFFFFFFFFFFFFFFFFFFFFFFFFFFFFFFFFFFFFFFFFFFFFFFFFFFFFFF
FFFFFFFFFFFFFFFFFFFFFFFFFFFFFFFFFFFFFFFFFFFFFFFFFFFFFFFFFFFFFFFFFFFF
FFF
@A00721:190:HGJW7DSXY:4:2423:4689:22451_2:N:0:TCTGCAAG+GCTGCACT
GTTCTTGTCACGATTCTTAAAAGAAGTTTATTAAAACTTCAGAGTCTCTTGGAAGTCCAGGTAGCTAA
TGTTCTTGCAATAGCCACAGACTTTCTGGGAAAAGAGACCTCTGTTGG
+
FFFFFFFFFFFFFFFFFFFFFFFFFFFFFFFFFFFFFFFFFFFFFFFFFFFFFFFFFFFFFFFFFFFF
:FFFFFFFFF:FFFFF,FFFFFFFFFFFFFFFFFFFFFFFFFFFFFFF
@A00721:190:HGJW7DSXY:4:1502:24071:7044_1:N:0:TCTGCAAG+GCTGCACT
GAGAATTACTCAGATTCAATCTGGCATCACTGAACGTCTACAACGAGTTAGGGAGTGGCTAGGTTTTA
AAAGATCCTTTGCACAAGCAATACCTTCCACTGAGTTCATCACCCAGAGTTTCGACGTCGCGGAAAAC
GAT
+
FFFFFFFFFFFFFF,FFFFFF:FFFFFFFFFFFFFFFFFFFFFFFFFFFFFFFFFFFFFFFFFFFFFF
FFFFFFFFFFFFFFFFFFFFFFFFFFFFFFFFFFFFFFFFFFFFFFFFFFFFFFFFFFFFFFFFFFFF
FFF
@A00721:190:HGJW7DSXY:4:1672:6931:17440_2:N:0:TCTGCAAG+GCTGCACT
CACTGTTCGAGAATCTTCTGATGCTGAATTTGAGGAAAATTTTTCGGAAGTTTTTGGTATAAATCCTG
ATGGCTCAGTACAGGGAGGAGATCGGAAGAGCGTCGTGTAGGGAAAGA
+
```

FFFFFFFFFFFFFFFFFFFFFFFFFFFFFFFFFFFFFFFFFFF:FFFFFFFFFFFFFFFFFFFFFFFFFFF
FFFFFFFFFFFFFFFFFFFFFFFFFFFFFFFFFFFFF,FFFFFF:FFF:
@A00721:190:HGJW7DSXY:4:1232:28311:5744_1:N:0:TCTGCAAG+GCTGCACT
ATGGACAAAATCTGGGATCATAGGTATGTGTCACGCTACACTCGAAATATGGGCCCTCATAAGGAAGT
ATAGTTGGCGTCTTGTCGTTGACGTCATTACAGTCTGGAAGTTAGATCATTTGTTCAGGAAGATCTCT
ACA
+
FFFFFFFFFFFFFFFFFFFFFFFFFFFFFFFFFFFFFFFFFFFFFFFFFFFFFFFFFFFFFFFFFFFFFF
FFFFFFFFFFFFFFFFFFFFFFFFFFFFFFFFFFFFFFFFFFFFFFFFFFFFFFFFFFFFFFFFFFFFFF
FFF
@A00721:190:HGJW7DSXY:4:1565:25292:14982_1:N:0:TCTGCAAG+GCTGCACT
CTTTAGCAACTTTAGATAAATTCTTTCCGGGCTCAATGACCTTTGCTATGTACGTGGATCTAATCCTT
GGTGATCTGAATCTGACCATCGCAAAGAAGACTTTATTAGTTCATGAAGTTCTTACACTTCTCGCGAC
TAA
+
:FFFF,FF,,FFFFFFFFFFFF,,F::::::,:FFF:FFF:FFF:FFFF,:F:,FFFFFFF,FF:,FF
:FFFFF:FF,FFFFFFFFF:,FFF:FF,FFFFFF::F::FFFF,F:,FFFFFF:FF:FFFFFFFFFFF
FF:
@A00721:190:HGJW7DSXY:4:2614:30581:10394_1:N:0:TCTGCAAG+GCTGCACT
GCTTTATCTTAAAGCATTACCACCAAATTTAGCACTTTTTGAATATGACAGAAGCTCGCCATATCAGA
TTAGAGACATGCCAGTTAGAGTTCTTCTAACCAGGACTGATGGTGAGATTCTTTTACCTTTTGGAGGT
CCA
+
FFFFFF:,FFFFF,FFFFFFFFFFFF:FFFF:FFF::FFFFFFFFFFFFFF:FFFFFFFFFFFFFFFF
FFFFFFFFFFFFFFF:FFFFFF:FFFFFFFFFFFFFFFFFFFFFFFFFFFFFFF:FFFFFF:FFFFFFF
FFF
@A00721:190:HGJW7DSXY:4:1623:7780:36292_2:N:0:TCTGCAAG+GCTGCACT
CCTAGTGTCTTTTCTAGAGTCCAAGGACGTTTCCGTCGGAGGATGACAGGTGGGAGACCGCTCAATAA
CACTCTATTGAATCCCTTTTCCACGATGAAAGCTCTTTATGAGGTGCT
+
FFFFFFFFFFFFFFFFFFFFFFFFFFFFFFFFFFFFFFFFFFF,FFFFFFFFFFFFFFFFFFFFFFFFF
FFFFFFFFFFFFFFFFFFFFFFFFFFFFFFFFFFFFFFFFFFFFFFFF
@A00721:190:HGJW7DSXY:4:2556:15727:11005_1:N:0:TCTGCAAG+GCTGCACT
TCTTTGGTGATATCGCCTCTGTCCTTTCTCCTTTAATCGTGAGGTTACGAGAGTTTCGTACTTGGGTT
TATCGGTTGTATTTTGATAAATTTGTTGGAATCGATAATGATACTGACGAAGGTTCTATTACCACCCT
TGT
+
FFFFFFFFFFFFFFFFFFF:FFFFFFFFFFFFFFFFFFFFFFFFFFFFFFFFFFFFFFFFFFFFFFFFFF
FFFFFFFFFFFFFFFFFFFFFFFFFFFFFFFFFFFFFFFFFFFFFFFFFFFFFFFFFFFFFFFFFFFFFF
FFF
@A00721:190:HGJW7DSXY:4:2443:15501:5102_2:N:0:TCTGCAAG+GCTGCACT
GTGATGCTTTCTTAGAGGCTCGTCTAGCCGCTGAGATTCGTCTTACCTATTTACAATCTGTAGAGAGA
GAGGTTAATCAGAATTTGAGTGATAACGAGTTAGTACTGGGAAATGGT
+
FFFFFFFFFFFFFFFFFFFFFFFFFFFFFFFFFFFFFFFFFFFFFFFFFFFFFFFFFFFFFFFFFFFFFF
FFFFFFFFFFFFF:F:FFFFFFFFFFFFF,FFFFF:F:FFFF:FFFFF
@A00721:190:HGJW7DSXY:4:1573:1931:34992_1:N:0:TCTGCAAG+GCTGCACT
TTTTCTTTCCCTCCCCTTTTTCGATTGGATTCGACGGGGTCTTCGTCTCACGACTAATTTGATAAGGT
TTTTCTTTTATGGGACTCTTGTTTGTACTTATGAAGGAGGTATTACTTCGGAAGTTCTTAATGTAGGT
CTC
+
FFFFFFFFFFFFFFFFFFF:FFFFFFFFFFFFFFFFFFFFFFFFFFFFFFFFFFFFFFFFFFFFFFFFFF
FFFFFFFFFFFFFFFFFFFFFFFFFFFFFFFFFFF:FFFFF:F:,:FFFFFFFFFFFFFFFFFFFFFFFF
FFF

```
@A00721:190:HGJW7DSXY:4:2268:7808:8437_2:N:0:TCTGCAAG+GCTGCACT
GGTTTATGTTTCGGTTTCAGCTGGATGGATCTTTTGATGGTATGTTTCACATTGCGTGATCACTTCCC
GTGGACTTGGGAAGAGATTAAACATAAAATCCAGCGATTCTATTATGT
+
FFFFFFFFFFFFFFFFFFFFFFFFFFFFFFFFFFFFFFFFFFFFFFFFFFFFFFFFFFFFFFFFFFFF
FFFFFFFFFFFFFFFFFFFFFFFFFFFFFFFFFFFFFFFFFFFFFFFF
@A00721:190:HGJW7DSXY:4:2432:24948:28980_2:N:0:TCTGCAAG+GCTGCACT
TCGATGATGTTCGGGCTGTGGGGATTAACTTCCCTCGTGAAACTTACGACGATGTTCTCGCTCGCATT
TCACAAGGATTACTAAGTCTGAGATCTGCCACTTCTAATTTTAGGAAA
+
FFFFFFFFFFFFFFFFFFFFFFFFFFFFFFFFFFFFFFFFFFFFFFFFFFFFFFFFFFFFFFF:FFFF
FFFFFFFFFFFFFFF:FFFFFFFFFFFFFFFFFFFFFFFFFFFFFFFF
@A00721:190:HGJW7DSXY:4:1507:8061:8656_2:N:0:TCTGCAAG+GCTGCACT
AGAAAGTTCATGAGGATGTCTTGGTCGAAAAATATCGCCGGGAACTTCTAATAGAGGATCCTGATGAC
ATTATTTCAACGGATACCGATCTCAGTTCTACCACAGAAACCGTCTCA
+
FFFFFFFFFFFFFFFFFF,FFFFFFFFFFFFFFFFFFFFFFFF,,FF,FFFFF:F:FFFFFFFFFFFF
,FFFFFFFFFFFFFFFFFFFFFFFFFFFFFFFFFFFFFFFFFFFFFFF
@A00721:190:HGJW7DSXY:4:2101:24433:13620_2:N:0:TCTGCAAG+GCTGCACT
ACTGATTCGGAATTAGAGAAAGACGACCTTCTTGTGGGGACTGAGACCTCTGAATTTTCAGGGTGCCC
TGATGAGGACGTTGAAAGGGATAATTTCAACTCTGAGACTTCTAAGAG
+
FFFFFFFFFFFFFFFFFFFFFFFFFFFFFFFFFFFFFFFFFFFFFFFFFFFFFFFFFFFFFFFFFFFF
FFFFFFFFF:FFFFFFFFFFFFFFFFFFFFFFFFFFFFFFFFFFFFF:
@A00721:190:HGJW7DSXY:4:1230:25925:4993_2:N:0:TCTGCAAG+GCTGCACT
ACCAGAAACGTAACAAGAAGAATAAAGGTAGGAAGGGGAGAAAACAGAACAAGCGAGCGGTTGAAGAG
AAAACTGATATGGTTGCGGCTGATCGTACCCCAATTCCTGCTAGAAAG
+
:FFFFFFFFFFFFFFFFFFFFFFFFF:FFFFFFFFFFFFFFFFFFFFFFFFFFFFFFFFFFF:FF:FFFF
FFFFFFFFFFFFF:FFFFFFFFFFFFFFFFFFFFFFFFFFFFFFFFFF
@A00721:190:HGJW7DSXY:4:1378:13865:10285_1:N:0:TCTGCAAG+GCTGCACT
ATACCACTGATCTTCCAGAGTTACGTAGTGGTCGTTCTTTTGGTGATTTTATCAAAGAGAAAGTTTCT
GCTAGTCGCGACGATAGTATTTATCGGAGTGATATTAACAAGTTAAACTTCGAACTGACCACTTACAA
GAG
+
FFFFFFFFFFFFFFFFFFFFFFFFFFFFFFFFFFFFFFFFFFFFFFFF:FFFFFFFFFFFFFFFFFFF
FFFFFFFFFFFFFFF:FFF:FFFFFFFFFFFFFFFFFFFFFFFFFFFFFFFFFFFFFFFFFFFFFFFF
FFF
@A00721:190:HGJW7DSXY:4:2472:24279:9533_1:N:0:TCTGCAAG+GCTGCACT
GCAACATGGATCAGTTTTCCAAAAAGCTATTAATAGCAGTTGCGGGGCTATAATACCGCCTCCAGATT
ACTCTAAACTGAATCCACTCTCTTGGGATATAAATGAGTACATCTATAATAGAGTTTTGGAACTGTGT
AAT
+
FFFFFFFFFFFFFFFFFFF:FFFFFFFFFFFFFFF:FFFFFFFFFFFFFFFFFFFFFFFFFFFFFFFFF
FFFFFFFFFFFFFFFFFFFFFFFFFFFFFFFFFFFFFFFFFFFFFFFFFFFFFFFFFFFFFFFFFFFF
FFF
@A00721:190:HGJW7DSXY:4:1530:30092:3724_1:N:0:TCTGCAAG+GCTGCACT
GGCTTCTCTAAAGAACAGCAACTTTCAACATGAATTAGTTATTGAAAGTCTTGACCCTCACGTCTCAC
TTTATCACCTTGGAGATGGTAAAGTCATTAACAAAGGAAATGTTAAGGCTGATGACTTAAATTTCGCA
TTC
+
FFF:FFFFFFFFFFFFFFFFFFFFFFFFFFFFFFFFFFFFFFFFFFFFFFFFFFFFFFFFFFFFFFFF
FFFFFFFFFFFFFFFFFFFFFFFFFFFFFF:FFFFFFFFFFFFFFFFFFFFFFFFFFFFFFFFFFFFF
FFF
```

```
@A00721:190:HGJW7DSXY:4:2234:31168:8343_2:N:0:TCTGCAAG+GCTGCACT
AGACCTTTCTTGGGGAGTAAAGAGGGTTTGAAAATTACGGAACCTTTCCTCGTTCTTCATGAAGATCT
TCGAGTGTTTTATAGTGAGTTATTCTTGCGAAGGTTCAAGTCGATTGA
+
FFFFFFFFFFFFFFFFFFFFFF:FFFFFFFFFFFFFF:FF:FFFFFFFFFFFFFFFFFFFFFFFFFFF
:FFFFFFFFFFFFFFFFFFFFFFFF:FFFFFFFFFFFFFFFFFFFFFF::
@A00721:190:HGJW7DSXY:4:2622:30318:35931_1:N:0:TCTGCAAG+GCTGCACT
ACGAGATCTTGCAGAGTGACTGGAGTTCAGACGTGTGCTCTTCCGATCTGTGGAGGGAAGACCACTGA
ACTTATAGCGACTTTTGTCGAATTTTTCGCAAAAGGTCTTGAAGTACGTATTGTCACTGCTAATCGTA
ATT
+
FF::FFFF,FFFFFFFFFF:FFFFFFFFFFFFFFFFFFFFFFFFFFFFFFFFFFFFFFFFFFFFFF:F
FFFFFFFFFFFFFFFFFFFFFFFFFFFFFFFFFFFFFFFFFFFFFFFFFFFFFFFFFFFFFFFFFFFF
FFF
@A00721:190:HGJW7DSXY:4:2456:12237:21652_1:N:0:TCTGCAAG+GCTGCACT
GTGACTAAAGTTTTGGCACTTCTAGTTCATCGCGGTATTTGTGAAATTAAGACAGACGTGAAAGTTAT
GATGAGTGATCGTATTAGAACAGCTGATTCTTATTGCATGAACGTCGCTGAGGTCAAACCTGTGGACG
TTC
+
FFFFFFFFFFFFFFFFFFF:FFFFFFFFFFFFFFFFFFFFFFFFFFFFFFFFFFFFFFFFFFFFFFFF
FFFFFFFFFFFFFF:FFFFFFFFFFFFFFFFFFFFFFFFFFFFFFFFFFFFFFFFFFFFFFFFFFFFF
FFF
@A00721:190:HGJW7DSXY:4:1672:10050:23750_2:N:0:TCTGCAAG+GCTGCACT
CTGGTCAGACCCTTTTTCTTACACATCTACTTAAGGCGGCTTATACCATAGGTTACGGTGATTCCAAT
CAAATCGGATTCATCAATCGTATTGATCTCCAAGTCGCAAACTACTTC
+
FFFFFFFFFFFFFF:FFFFFFFFFFFFFFFFFFFFFFF:FFFFFFFFFFFF:FFFFFFFFFFFFFFFFFFF
FFFFFFFFFFFFFFFFFFFFFFFFFFFFFFFFFFFFFFFFFFFFFFFF
@A00721:190:HGJW7DSXY:4:1230:17029:17581_2:N:0:TCTGCAAG+GCTGCACT
TTGATGAGTGTCGTTCTTATCGAAGTCTCAGTTATCGCTGCCCGGCCGACGTTTGTCGCGAACTCTCA
GAGGTTTACGGCAGAAAAATTTCTCCTGCTTGTAATTCGAGAGAGGAC
+
FFFFFFFFFFFFFFFFFFFFFFFFFFFFFFFFFFFFFFFFFFFFFFFFFFFFFFFFFFFFFFFFFFFF
FFFF:FFFFFFFFFFFFFFFFFFFFFFFFFFFFFFFFFFFFFFFFFFF
@A00721:190:HGJW7DSXY:4:1237:28311:10598_1:N:0:TCTGCAAG+GCTGCACT
TTAGACCTATCAGATGTCTTGACGACTTCGAATTTGAGGACGGAGTTAAATATATTACTTTCACTCAA
GGTGAAAAGTTTGAAATTAACAAAGTCCTTGGTAAGAGTAAGGTTAAATGTGAAGATGCTCAGACTAT
TCA
+
FFFFFFFFFFFFFFFFFFFFFFFFFFFFFFFFFFFFFFFFFFFFFFFFFFFFFFFFFFFFFFFFFFFF
FFFFFFFFFFFFFFFFFFFFFFFFFFFFFFFFFFFFFFFFFFFFFFFFFFFFFFFFFFFFFFFFFFFF
FFF
@A00721:190:HGJW7DSXY:4:1214:29912:33661_2:N:0:TCTGCAAG+GCTGCACT
ACTTACCAGCACGTTCGGCTGGTTAGAATTAAACCTCAAGATGACCCTGTTTTTGCATCACAAACACA
TATTACCGTTGCTCTATCACGACACGTTGAATCTTTACGATACTTTGT
+
FFFFFFFFFFFFFFFFFFFFFFFFFFFFFFFFFFFFFFFFFFFFFFFFFFFFFFFF:FFFFFFFFFFF
:FFFFFFFFFFFFFFFFFFFFFFFFFFFFFF:FFFFFFFFFFFFFFFFF
@A00721:190:HGJW7DSXY:4:1302:15049:17002_1:N:0:TCTGCAAG+GCTGCACT
TTAAACGATGCTACTTGTGAGAGGATCAATCACAGTAAGTAACTGTCACGTTTAGCACGGGAGGTTGC
AGATTCAACCCCTTATATCGAGGCTATCGAGGACGATGAGTATCTTGAAGTTGTTGACGACAAGCCTC
AAG
+
:FFFFFFFFF,FFFFFF:FF:F,FFFF:F:FF::FFF:F:FF:FFFFFFFFFFFFFFFFFF:FFFFFF
```

```
,FFFFFF:FFF:FFFF,FFF:FFFFFFFF:FFFFFFFFFFF,FFFF:F::FFFFFF:FFFFFFFFFFF
FFF
@A00721:190:HGJW7DSXY:4:1527:19822:6449_2:N:0:TCTGCAAG+GCTGCACT
TCTTCTACTGTCTCTGCCTTCCAAGATTTCTTAGAAGAGGTTATTCCTGGAACCACCTTAACGTCACT
CGGTAATCTTTCAGCCGAATTAAGTACTAATGAGTTCAAGTCGGTTGT
+
FFFFFFFFFFFFFFFFFFFFFFFFFFFFFFFFFFFFFFFFFFFFFFFFFFFFFFFFFFFFFFFFFFFF
FFFFFFFF,,FFFFFFFF,F:FFFFFFFFFFFFFFFFFFF:FFF,FFF
@A00721:190:HGJW7DSXY:4:1119:9986:29684_1:N:0:TCTGCAAG+GCTGCACT
GATCAAGGCTAATACCTTTATAGGTAAGAAGCCCCTTGAGCCGCGTAGTCAGCGCGTTTAGCTGTATC
ATCAGTCAGAGTATACCCGATAGGGTACCGAGTCTCCAAGAGAATATTTATTCTTATGAGGCTCGAAA
CTT
+
FFF:FFFFFFFFFFFFFF:F:FFFFFFFFFFFFFFFFFFFFFFFFFFFFFFFFFFFFFFFFFFFFFFF
FFFFFFFFFFFFFFFFFF:FFFFFFFFFF:FFFFFFFFFFFFFFFFFFF:FFFFFFFFFFFFFFFFFF
FFF
@A00721:190:HGJW7DSXY:4:1343:27597:7670_1:N:0:TCTGCAAG+GCTGCACT
CAACGGAAAGACATATCGCACCCGAAATTTTTGGGCAGAATGCTCTTAACGCGGTTCTTGAACGGTGT
TTTGATCAGTCTAAGTTGGATAAAGTTATAGGAGACTCAGTTAACCTGAGAGCTCCTCAATTGATAGA
TTG
+
FFFF:FFF:FFFFF,FFFFFFFFFFFFFFF:FFFFFFFFFFFFFFF:FFFFFFFFFFFFFFFFFFFFF
FFFFFFFFFFFFFFFFFFFFFFFFFFFFFFFFFFFFFFFFFFFFFFFFFFFFFFFFF:FFFF,:FF
:FF
@A00721:190:HGJW7DSXY:4:1143:16631:28322_2:N:0:TCTGCAAG+GCTGCACT
CTAGTATACTTCGTAAAGTCGACAGCGAAATGGTCAATATTGATAATTGGACCGACTATATGACCTAT
TTTAAGCTCATGGTGAAAAGGGAAGCTAAGGTTAAACTTGATGCTTCA
+
F:FFFFFFFFFFFFFFFFFFFFFFFFFFFFFFFFFFFFFFFFFFFFFFFFFFFFFFFFFFFFFFFFFF
FFFFFFFFFF,FFFFFFFFFFFF:FFFFFFFFFFF:FFFFFFFF:FFF
@A00721:190:HGJW7DSXY:4:1316:6352:30624_2:N:0:TCTGCAAG+GCTGCACT
CATAACCCCGCACAGAATATCGTTTATCACGCAAAATTTATTAATGCAATTTTCGGACCTATCTTTAA
GGTTCTTGTGAGGAGAGTCAAACAGTGTCTTTTACCCAACATTTTTAT
+
FFFFFFFFFFFFFFFFFFFFFFFFFFFFFFFFFFFFFFFFFFFFFFFFFFFFFFFFFFFFFFFFFFFF
FFFFF:FFFFFFFFFFFFFFFFFFFFFFFFF,FFFF:FFFFFFFFFFF
@A00721:190:HGJW7DSXY:4:1209:8332:15013_1:N:0:TCTGCAAG+GCTGCACT
TGACCAGTGACGATTTCTCCCGGAAGCTCTTCTCTCTCTTAGGAGGTCATGATGACTACTATAAGGGT
GAAATCGATTTTTCGAAGTTTGACAAATCTCAAGATTTCTTTTGTAAAGCCTATGAGATAAAGATGTA
TT
+
FFFFFFFFFFFFFFFFFFFFFFFFFFFFFFFFFFFFFFFFFFFFFFFFFFFFFF:FFFFFFFFFFFFF
F:FFFFFFFFFFF:FFFFFFFFFFFFFFFFFFFFFFFFFFFFFFFFFFFFF,FF,FFFFFFFFFFFFFFF
FF
@A00721:190:HGJW7DSXY:4:1177:16568:21010_2:N:0:TCTGCAAG+GCTGCACT
TTCGATGCTGAATTTCTTGAACTTTGGAGTGCCAGTGAATATTTTTGTCACGCCAGGAGTATCCATAA
CGATTTGTCATTTACCACCTTTGCTCAACGACGAAGTGGAACGGCCAA
+
FFFFFFFFFFFFFFFFFFFFFFFFFFFFFFFFFFFFFFFFFFFFFFFFFFFFFFFFFFFFFFFFFFFF
FFFFFFFFFFFFFFFFF:FFFFFFFFFFFFFFFFFFFFFFFFFFFFFFF
@A00721:190:HGJW7DSXY:4:1309:21061:14638_2:N:0:TCTGCAAG+GCTGCACT
TAATTGTATGATCACTTTGATGATTTTATCACTTTATTATGACCTTGGAAAGTTTTCGGCTGTGGTTG
TCGCTGGGGACGATTCCATTCTGTTTTCAAAGACTCCAATAACTGATC
+
```

FFFFFFFFFFFFFFFFFFFFFFFFFFFFFFFFFFFFFFFFFFFFFFFFFFFFFFFFFFFFFFFFFFFFFFFFFFFFFFFFFFFFFFFF
FFFFFFFFFFFFFFFFFFFFFFFFFFFFFFFFFFFFFFFFFFFFFFFFFFFF
@A00721:190:HGJW7DSXY:4:1325:11469:11177_2:N:0:TCTGCAAG+GCTGCACT
CAGCGTACTTCTGCTCGCGATTTATTCTTTTTGTCAATGATTACATCTACTTCGTTCCTGATCCTTAC
AATATCATCGTTAAGTTAGGTAAACCCCTAACCGATGTTACTCTTGTA
+
FF:FF:FFF:,::F,F,FFF,:FF,,FFF,F,FFFFFF:F:FFFF:FFF::FF:F:::,:F:F,FFF,
FF,F:,FFFF,,FF::FF:,:FFFF,,FFFF:,FFF,F,F:F,FF:FF
@A00721:190:HGJW7DSXY:4:1378:10845:5181_1:N:0:TCTGCAAG+GCTGCACT
TTCGTTAGTTTTAAAGACTATACTAAATATCTCCACTACGAGGTTGTTGTTTGGCGATTAGCTCTTGC
GACTAATATTCGATATTCGATTGAGGGTCGTTATACCTATTCGGCTATCTGTGCTTTGAATTGTATAA
TAT
+
FFF:FF:FFFF:FFF,,F,FFFFFFFFFFF:FF:FF:FFFFFF,FFFFFFF:,:F,FFFFF:FFF:F:
:F:FFFF:FFFFFFFFFF:FFF,:FF,FF,FFFFFFF:F,:FF:FFFF:F,F:FFFFFFFFF,:FFFF
,FF
@A00721:190:HGJW7DSXY:4:1134:7717:36119_2:N:0:TCTGCAAG+GCTGCACT
GATTCCGAGAGGTTTATCCTGATGTGGAGGGAGTTCTTGAAGCATCCCGACCTTTCCTTGTGAAAATT
TTGATGCGGTTTCATAAGATTATCAGTAATCCTGATTCTACTACGGAT
+
FFFFFFFFFFFFFFFFFFFFFFFFFFFFFFFFFFFFFFFFFFFFFFFFFFFFFFFFF,FFFFFFFFFF
FFFFFFFFFFF,FFFFFFFFFFFFFFFFFFFFFFFFFFFFFFFFFFFF
@A00721:190:HGJW7DSXY:4:1562:6017:21997_1:N:0:TCTGCAAG+GCTGCACT
CCATCCTGTACCTCTTCTGGAGAGACGTATCTCAAAGATCGACTTCGAGACAGGTGATATTGTTGAGC
GAGAGCGACCAAGATCTAAATTCGGTAACGGTGCGTGGCGCTTTATTTTAAAAAGCTCCCTCCTCTCG
CAA
+
FFFFFFFFFFFFFFFF::FFFFFFFFF,FFFFFF:FFFFFFFFFFFFFFFFFFFFFFFFFFFFFFFFF
FFFFFFFFFFFFFFFFFFFFFFFFFFFF:FFFFFFFFFFFFFFFFFFFFFFFFFFFFFFFFFFFFFFF
FFF
@A00721:190:HGJW7DSXY:4:2202:22616:23938_1:N:0:TCTGCAAG+GCTGCACT
ATACTTATCACGAAAGGACTTGAGTCTTTTGATCAGTCTCTGACTTGACCATCTTTCATTTTAAATGA
AGATCATTAAGACTCTCTTTCTTCTTTGTTTCCTTCTTTGCACTGATTTTTCCTCATCAGCTTGTGTT
TT
+
FFFFFFFFFF:FFFF,F,:FFF:FF:F,FFFFFF:FFFFFFFFFFFFFF:FFFFFFFFFFFFF:FFF:
,FFFFFFFFFFF,F:FFFFFFFF,FFFFFFF:FFF:FFF::::FFFFFFFFFFFFFFFFFF:FFFFFF
FF
@A00721:190:HGJW7DSXY:4:1171:27751:9220_1:N:0:TCTGCAAG+GCTGCACT
GTTTTTACGATAACAACTATCAGTTGATTAAGACTCTTCCGAAAGGCGAACTTTGGGAATTTGAAGCT
CAGGGTGAAGCACAACGTTTGACCGTGACGAACTGTGGTGTTCTTGATCTACGTTACGGATATACTCT
CCT
+
FFFFFF:FFFFFFFFFFF,FFFFF:FFFFFFFFFFFFFFFFFFFFFFFFFFFFFFFFFFFFFFFFFFF
FFFFFFFFFFFFFFFFFFFFFFFFFFFFFFFFFFFFFFFFFFFFFFFFFFFFFFFFFFFFFFFFFFFF
FFF
@A00721:190:HGJW7DSXY:4:1113:6515:12493_1:N:0:TCTGCAAG+GCTGCACT
ACACTACCACTTATTACGATATTTCTTTAGTCGATGGGTTTTCGACGCCTTTAAGCGTCTTCTGTTCA
TCGCAACCAGATGATATCTTCTATTCGAACTTATGTTTCGATCAGTGTTTCTTCTATCAGGAGGAAAA
CAC
+
FFFFFFFFFFFFFFF:,FFFFFFFFFFFFFFFFFFFFFFFFFFFFF:FFFFFFFFFFFFFFFFFFFFF
FFFFFFFF:FFFFFFFFFFFFFFFFFFF,F:F:FF:FFFFFFFFFFFFFFFFFFFFFFFFFFFFFFFF
FFF

```
@A00721:190:HGJW7DSXY:4:2342:11849:17002_2:N:0:TCTGCAAG+GCTGCACT
AATCCTAACGTAGAAGATTGTTGTTTACCACCGAAATATCAAGGTTCTTGTCCTTCAAACGATTGGAA
GGTTACTTATCTTAATAGTACTAAAGGGGTTTACTTACAACCCTATGA
+
FFFFFFFFFFFFFFFF:FFFFFFFFFFFFFFFFFFFFFFFFFFF:FFFFFFFFFFFFFFFFFFFFFFFF
FFFFFFFFFFFFFFFFFFFFFFFFFFFFFFFFFFFFFFFFFFFFFFFF
@A00721:190:HGJW7DSXY:4:1643:4164:9267_2:N:0:TCTGCAAG+GCTGCACT
TGTTTTCTTCTAATGTGGCCTGAACTACGTTTCACTTTGTGTGTACTTGTCTCTATTTTAAGTTTTGT
CTTCCTTGTTTGTCTTATTCTATTAATCACTCATTTTAGTGAGATTAT
+
,FFFFFFFFF,FFFFFFFFFFFFFFFFFFF:FFFFFFFFFFFFFFFFFFFFFFFFFFFFFFFFFFFFF
F:FFF::FF:FFFFF:FF:FF,FFFFFFFF:F:FFF:FF::FFFFFF:
@A00721:190:HGJW7DSXY:4:2312:9290:5494_2:N:0:TCTGCAAG+GCTGCACT
CTAAAACGTCTTCGACAACAGCAGGGGTTTTCGAATAGTGATAGCCACCCGCCTTACAGAGGGGCAGC
AGCATGACCATAATTGGTATTGACTTTGGTACCACCTTTTCTACTGTC
+
FFF:FFFFFFFFFFFFFFF:FFFFFFFFFFFFFFFFFFFFFFFFFFFFFFFFFFFFFFFFFFFFFFFF
FFFFFFFFFFFF,FFFFFFFFFFF:FFFFFFFFFFFFFFFFFFFFFFF
@A00721:190:HGJW7DSXY:4:1472:8648:16908_1:N:0:TCTGCAAG+GCTGCACT
TTCGAATGAAGTTTTAATGCTTAACAACAATGATAGTGAATTTATACCTACAATCATTGGTTTTTCTA
ATAATGATGACACTATTGTTTATGGATATGATGCTATTACTCATGATGGTTACGGTCAGGGAGGATTC
TCA
+
FFFFFFFFFFFFFFFFFF:FFFF::FFFFFFFF::FFFFFFFFFFFFFFFFFFFFFFFFFFFFFFFFF
FFFFFFFFFFF:FFFFFF:FFFFFFFFFFFF,FFFFFF,FFFFFFFFFFFFFFFFF:FFFFFF:FFFF
FFF
@A00721:190:HGJW7DSXY:4:1601:27489:14716_2:N:0:TCTGCAAG+GCTGCACT
ACATTACCCGAAAGACGTGCTAAATTAAAACCATTGTACGATGTCGTCTGTCTCGGCGATCACTTTTC
ACTAAGAATTGGTCCTACTTTTGGACAAGGCCAACTTCGTAATCTTCA
+
FFFFFFFFFFFFFFFFF:FFFFFF:FFFFFFFFF,F:FFFFFFFFFFFFFFFFFFFFFFFFFFFFFFF
FF:FFFFF:FFFFFFF:FFFFFFFFFFFFFFFFFFFFFFFFFFFFFFF:
@A00721:190:HGJW7DSXY:4:1202:31259:20431_2:N:0:TCTGCAAG+GCTGCACT
TTTATCTCTTTGGTTGTTAGGGATTTTGAGAGAGTGAAAGGCTTCAATTGTAGTGGTTTGGTCGTTTC
AGTACCCAGTCAGTATACCTCTACGCAAAGATATTTCATGAAAACTAC
+
FFFFFFF,F:FFF,:FFFFFFFFFFFFF:FFFFFFFFFFFFFFFFFFFFFFFFFFFFFFFFFFFFFFF
FFFFFFF:FFFFFFFFF:FFFFFFFF::FFFFFFFFFFFF,FF,:FFF
@A00721:190:HGJW7DSXY:4:1572:22580:11694_1:N:0:TCTGCAAG+GCTGCACT
CCGGATTACCTGTCTTTCATATTATGAATGAACCTTCGGCCGCTCTCTTTGCTTCGATGTTGGATGTC
AAGAAGGGTTCAGAATGGGATTCCTACGTAGTTTATGACTTTGGTGGTGGTACTTTCGATACTTCTAT
CGT
+
:,FFFFFF:FFFF:FFFFFF,F,FF:FFFF:F,FFFFFFFFFFFFFFFFFFFFFFFFFFFFFFFFF:F
FF:FFFFFFFFFFFFFFFFF:FFFFFFFFFFF,FFFFFFFFFFFFFFFFFFFFFFFFFFFFFFFFFFF
FFF
@A00721:190:HGJW7DSXY:4:1554:17219:24267_2:N:0:TCTGCAAG+GCTGCACT
TACTATAGTGTTATTAATTCTGGTGGTGATGATAATCTTGGAGGTCGTGACGTTGATGAAGCTATAAG
AGCGTTCTTATTACGGCGCACTTCTTTACCCGCCACCACACAACTATC
+
:FFFFFFF:FF:F,:,FFFF,FFFF:F:F,:FFFFFF,:FFFFFFFF::FF:,,FFF:FFFFF:FFFF
F:F,FFFFFF:FFFFFF:FFF,F:,F:FFFF,:FFFF:FFFFF:,,:F
@A00721:190:HGJW7DSXY:4:1535:22589:27524_1:N:0:TCTGCAAG+GCTGCACT
TGAAGGAGGAGGTTTCTGTTACTGGAAAACCCTCGTCAATACTTTTCAATAATATACGTCTTGAACTC
```

ACTTATGACGATCTAGTGAAATTAACGCGTCCTTTTCTGGAACGTAGTTTTACTATTATGAACAAAGT
TAT
+
FF:,FFFF:FFFFFFFFFFFF:FFFFFFF:FFFFF::FF:FFFFFFFFFF:FFFFFFFFFFFF:F:F,
FFFFFFFFF,FFFFFFFFFFFFFFFFF:FFFF,FFFFFFFFFFFFFFFFFFFFFFFFFFFFF:FFFFF
FFF
@A00721:190:HGJW7DSXY:4:2158:28583:1266_2:N:0:TCTGCAAG+GCTGCACT
GCAAACTTACCCTGATTCCGGCCGGTGGTAGCTCATTTCTACCAGGGGCTATTACTCTTGCTGAAACA
GCTATTAAAGCTGTTGAGCGGGTAATTACACCTAAACGAGCTCGATCT
+
FFFFFFFFFFFFFFFFFFFFFFFFFFFFFFFFFFFFFFFFFFFFFFFFFFFFFFFFFFFFFFFFFFFF
FFFFFFFFFFFFFFFFFFFFFFFFFFFFFFFFFFFFFFFFF:FFFFFFF
@A00721:190:HGJW7DSXY:4:1348:17616:33379_1:N:0:TCTGCAAG+GCTGCACT
AAGGATGTGCACTCTTTTCTGCTACTTTAACGGAGCGTAGTGTACTCTTCGTCGATTGTATCAACAGT
AACATTACTTCTAATAAAGGTCTCTATCAAGCTCGTATTATCGTCGCTGCTGGATCTGCGTTACCGTT
GAA
+
FFFFFFFFFFFFFFFFFFFFFFFFFFFFFFFFFF:FFFFFFFFFFFFFFFFFFFFFFFFFFFFFFFFF
FFFFFFFFFFFFFFFFFFFFFFFFFFFFFFFFFFFFFFFFFFFFFFFFFFFFFFFFFFFFFFFFFFFF
FFF
@A00721:190:HGJW7DSXY:4:2203:28094:6026_2:N:0:TCTGCAAG+GCTGCACT
AGGTAATTATGAAGACTGTGATTTATCTTTCACTCTGGCCTTGTATGAGGGTGTTTCTAATCGTAACA
TTAATAATTTCAATCTTCTTAAAAAGAAGATAGAGAGTCATACTTTGG
+
FFFFFFFFFFFFFFFFFFFFFFFFFFFFFFFFFFFFFFFFFFFFFFFFFFFFFFFFFFFFFFFFFFFF
FFFFFFFFFFFFFFFFFFFFFFFFFFFFFFFFFFFFFFFFFFFFFFF:
@A00721:190:HGJW7DSXY:4:1324:23448:35023_2:N:0:TCTGCAAG+GCTGCACT
GATAGCCAATATTGAAGTGGACACCTCTGGTAGTATCGAAGTAAAGATAACCAATAAGGAAGGAGCTT
ATACTATGGTTGAATCAACTAAGCATACTACTTTTGCTTTGATTACTC
+
FFFFFFFFFFFFFFFFFFFFFFFFFFFFFFFFFFFFFFFFFFFFFFFFFFFFFFFFFFFFFFFFFFFF
FFFFFFFFFFFFFFFFFFFFFFFFFFFFFFFFFFFFFFFFFFFFFFFF
@A00721:190:HGJW7DSXY:4:2306:10782:27899_1:N:0:TCTGCAAG+GCTGCACT
CTTCCGATCTGTTAAAGAGTCGATTAAACAAGTTGTACTTATCAATGATTTTATCGCTGGTTGTTATC
TCGAAAAGTCTCTTCTAAAAGGAACGTCAGTTCTAACCCAAAGAGAATATCTAAAATATTTACAAAAT
CTT
+
:FFFFFFFFFFFFFFFFFFF:FFFFFFFF:FFFFFFFFFFFFFFFFFFFFFFFFF:FF:FFFFFFFFF
FF:FFFFFFFFF,FFFFFFFFFFFFFFFFFFFFFFFFFFFFFFFFFFFFFFFFFFFFFFFFFFFFFFF
FFF
@A00721:190:HGJW7DSXY:4:1259:4318:17707_1:N:0:TCTGCAAG+GCTGCACT
GATCTGTTGCTTGTCGCTTCCTCCTTTAATGGCACTACCTCGGTACAATCTTACTTGGAACGAAGGCG
ACCTGTTCCGGAGTTTTTACGGGATCCGGAATGTTCTGTCTATATTAGGTGAAGCGAAATACGATTTC
GAA
+
FFFFFFFFFFFFFFFFFFFFFFFFFFFFFFFFFFFFFFFFFFFFFFFFFFFFFFFFFFFFFFFFFFFF
FFFFFFFFFFFFF:FFFFFFFFFFFFFFFFFFFFFFFFFFFFFFFFFFFFFFFFFFFFFFFF,FFFF
FFF
@A00721:190:HGJW7DSXY:4:1261:2564:3646_1:N:0:TCTGCAAG+GCTGCACT
TCGTACCTTGATGCTGACTTTCGTCAATATGATTTGATCTGGTTGCTCAATTCTAAGCGTGTTCCGCT
TAGTGCGAGGCCTCTCTTCTACGAGTATTGCTATTTATACGGTTACGAGAAGAAGGTTCGAAAGTTTG
AGA
+
FFF:FFFFFFFFFFFFFFFFFFFFFFFFFFFFFFFFFFFFFFFFFFFFFFFFFFFFFFFFFFFFF

```
FFFFFFFFFFFFFFFF:FFFFFFFFFFFFFFFFFFFFFFFFFFFFFFFFFFFFFFFFFFFF:FFFFF
FFF
@A00721:190:HGJW7DSXY:4:2659:10610:33144_2:N:0:TCTGCAAG+GCTGCACT
CTATACGTGACATACTAAATACTAGATATTTGGATGATCTTGATAAAGAACTTCATGAATTTTCAGAG
GATGAAAAATTTTCTTCCTTGGATTTTAATATAAAGAATCTTTACCAG
+
FFFFFFFFFF:FFFFFF:FFFFFFFFFFFFFFFFFFFFFFFFFFFFFFFFFFFF:FF:FFFFFFFFF
FF:FFFFFFFFFFFFFF,FFFFFF,::FFFFFFFFFF:FF:FFFFFFF
@A00721:190:HGJW7DSXY:4:2441:1985:14669_1:N:0:TCTGCAAG+GCTGCACT
AGGAGGCAGTCTTCTGATCGTAAGAAGTTTATAAGGAACATATCCATTTGTTGTAATAGGGTCATCAA
CTTAGAACTCGAAACTCTAAGTGGTAATACTGTCTTTCCAAGCTTACCTGTATCTTCAATGAAATTGG
ACT
+
FFFFFFFFFF,FFFFFFFFFFFFFF:FFFFFFFFFFFFFFFFFFFFFFFFFFFFFFFFFFFFFFFFFF
FFFFFFFFFFFFFFFFFFFFFFFFFFFFFFFFFFFFFFFFFFFFFFFFFFFFFFFFFFFFFFFFFFFF
FFF
@A00721:190:HGJW7DSXY:4:1545:11433:20666_2:N:0:TCTGCAAG+GCTGCACT
GTGGGCTTTCCGTAAAGGAAGATCTGTTATCGACGGTTTATCGTTTACGAAGTATCACTCCACCGGTG
AATGGTTTAAGAATACTATAAATAGAATAGCTATTACTACTAATGGCG
+
FFF:FFFFFFFF:FF:FFFFFFFFFF:FFFFFFFFFFFFFFFFFFFFFFFFFFFFFFFFFFFFFFFFF
F:FFFFFFFFFFFFFFFFFFFFFFFFFFFFFFFFFFFFFFFFFFFFFF
@A00721:190:HGJW7DSXY:4:1645:17490:9048_2:N:0:TCTGCAAG+GCTGCACT
TAGTGACATCTGGTCAATTTCGTTTTTAGGTGGTCTTTATTCTATTATTGATTTTTATGAGATTGATA
CGTTAACAGAAGATGATGTTAGAGTAGAATTCCTGAAGAGTCCTTCCG
+
FFFFFFFFFFFFFFFFF,FFFFFFFFFF:FFFFFFFFFFFFFFFFFFFF,FFFFFFFFFFFFFFFFFF
FFFFFFFFF:FFFF,FFFFFFF:FF::FFFFF:FFFFFFFFFFFFFF
@A00721:190:HGJW7DSXY:4:1170:24930:4993_1:N:0:TCTGCAAG+GCTGCACT
TTAAATCTAGGATTATGTCTGTGTTGAAAGACTTTCTACCGATAAGGGTCACTAGTCTTACACAATTA
AGAAATAATCCTACATTGACTGATTATCTAAAGATTGAAGGAAAATTTTATCCTCGTTTAACTTACTT
AGA
+
FFFFFFFFFFFFFFFFFFFFFFFFFFFFFFFFFFFF,FFFFFFFFFFFFFFFFFFFFFFFFFFFFFFF
FFFFFFFFFFFF:FFFFFFFFFF:FF:FFFFFFFFFFFFFFFFFFFFFFFFFFFFFFFFFFFFFFFFF
FFF
@A00721:190:HGJW7DSXY:4:1146:26476:17409_1:N:0:TCTGCAAG+GCTGCACT
ATACAAATGATAACTTCTAATTTACGTTTGCTACTCAGAGCTGGTCCTCTCAGCAAAGGGATGCATGG
TGTTATCTCCTCAGGAGTCCCGGCATTTGATAATAATGACGAGACTTTCGTCATAATCGCTGCATATT
ACG
+
FFFFFFFFFFFFFFFFFFFFFFFFFFFFFFFFFFFFFFFFFFFFFFFF:FFFFFF:FFFFFFFFFFFF
FFFFFFFFFFFFFFFFFFFFFFFFFFFFFFFFFFFFFFFF:FFFFFFFFFFFFFFFFFFF:FFFFFFFF
FFF
@A00721:190:HGJW7DSXY:4:1310:13512:1877_2:N:0:TCTGCAAG+GCTGCACT
CCAGCGCATCTCGTGTCGAGCCTCGACCCGATTATATGGTATTAACTTATAAAGGTGAGGAGACCATT
TTCTTGCTGAAATTTATAGAAGATTATTTTGACAGAATCAATAAGAAT
+
FFFFFFFFFFFFFFFFFFFFFFFFFFFFFFFFFFFFFFFFFFFFF:FFFFFFFFFFFFFFFFFFFFFFF
FFFFFFFFFFFFFFFFFFFFFFFFFFFF:FFFFFFFFFFFFFFFFFFFF
@A00721:190:HGJW7DSXY:4:1178:13910:35509_1:N:0:TCTGCAAG+GCTGCACT
TATGCTGATCTAAAATTTCGATCTAGATGGATCCACTCCTCGAGTTATCATGATGTTAGTATGTTATA
TACTGATTTACAACGATATAGGAGTAATCTACGAGAAGATTCTAGAATTCTTAATTATAATATTCAAC
CTC
```

+
FFFFFFFFF:FFFFFFFFFFFFFFFFFFFFFFFFFFFFFFFFFFFFFFFFFFFFFFFFFFFFFFFF
FFFFFFFFFFFFFFFFFFFFFFFFFFFFFFFFFFFFFFFFFFFFFFFFFFFFFFFFFFFFFFFF:FFFFF
FFF
@A00721:190:HGJW7DSXY:4:1251:24713:6277_2:N:0:TCTGCAAG+GCTGCACT
GAGATCACACATTTTGAGAACTAACTATGTTATGTTCTCTGATCATTTTGACAATCTCTTCTTATAAT
AAATAAATAAATAATATTACTAATGGCAAATAGTGGAAAGGCTCCAGT
+
:FFFFFFF:FF,FFFFFFFFFFFFFFFFFFFFFFFFFF:FFFFFFF:FF:FFFFFFFFFFFFFFFFFFFF
FFFFFFF:FFFFFFFFFFFFFFFF,FF:FFF,FF:FFFFFFFFFFFFFF
@A00721:190:HGJW7DSXY:4:1665:27145:20415_2:N:0:TCTGCAAG+GCTGCACT
CTGCTTTGGATTACGATGACGTGAGAATAGGTGAAGGGATAGTTTTTACCTCGACCAACATTAAGGCG
GCGGCGACTAATCTTAAAGCTTCACCCAAAATGAAGGACACTTCGAAA
+
FFFFFFFFFFFFFFFFFFFFFFFFFFFFFFFFFFFFFFF:FFFFFFFFFFFFFFFFFFFFFFFFFFFFFF
FFFFFFFFF:FFFFFFFFFFFFFFFFFFFFFFFFFFFFFFFFFFFFFFF
@A00721:190:HGJW7DSXY:4:1449:21884:26522_2:N:0:TCTGCAAG+GCTGCACT
AAAAGGCTCTAAAGGACACTATACAATATCCAGACGACGACGAAGCTTATACTACTTTAATTTTGGGT
ATTATGTGGAGATATCGTGATATCAGTACGTCCAGATCCTCTGCTTTT
+
FFFFFFFFFFFFFFFFFFFFFFFFFFFFFFFFFFFFFFFFFFFFFFFFFFFFFFFFFFFFFFFFFFFFFF
FFFFFFFF:FFFFFFFFFFFFFFFFFFFFF:FFFFFFFFFFFFFFFFFF
@A00721:190:HGJW7DSXY:4:2105:1262:6277_2:N:0:TCTGCAAG+GCTGCACT
ATCAAATATAAAGAAAAGGACTATGAAATAAAAGATTTGGTGCTTAGGGATATAATGAATTCAGCACC
ACATTTTGGTCAAGTCAATCCGTTGAGAAAATTTCTTAGAGGATATTC
+
FFFFFFFFFFFFFFFFFFFFFFFFFFFFFFFFFFFFFFFFFFFFFFFFFFFFFFFFFFFFFFFFFFFFFF
FFFFFFFFFFFFFFFFFFFFFFFFFFFFFFFFFFFFFFFFFFFFFFFFF
@A00721:190:HGJW7DSXY:4:1662:22417:23907_1:N:0:TCTGCAAG+GCTGCACT
CCAACATTTACCACCAAATTCTTTTGATACAACTCTAGCCTTCAAGTGGGGTATGCCTGCGGAATACG
CTTGCTATTCACCAGATTGGTTCGAACCCGTTGATGCTATGTCAGAAGAAGCTATTCAAGCTTGTCGT
TTA
+
FFFFFFFFFFFFFFFFFFFFFFFFFFFFFFFFFFFFFFFFFFFFFFFFFFFFFFFFFFFFFFFFFFFFFF
FFFFFFFFFFFFFFFFFFFFFFFFFFFFFFFFFFFFFF:FFFFFFFFFFFFFFFFFFFFFFFFFFFFFFF
FFF
@A00721:190:HGJW7DSXY:4:2619:29107:15984_2:N:0:TCTGCAAG+GCTGCACT
TCAAACAAGCCCAGACTCAAGGAGTCCAATCAAAGGTTTTGAATTTAGCTCAACTGGGGAGACGTGCT
TAAATGGGTGATAAAAATTATTTAGAGATCTTTTCTACTTACAAAGGA
+
FFFFFFFFFFFFFFFFFFFFFFFFFFFFFFFFFFFFFFF:FFFFFFFFFFFFFFFFFFFFFFFFFFFFFF
FF:F:FFFFFFFFFF:FF:FFFFFFFFFFFFFFFFFF:FFFFFFFFFFF
@A00721:190:HGJW7DSXY:4:2105:20039:35164_2:N:0:TCTGCAAG+GCTGCACT
AATTCTACGGAAAAGTCCGGAGTATATTGTATGTTAATAGATAGTGGGATAATAGTTCTACCGATACG
TAGTGAACGCGTTCGTAGAACCAAGTACTTTCCGTCTGAAGAGTTTAG
+
:FFFFFFFFFFFFFFFFFFFFFFFFF,FFFFFFFFFFFFFFFFFFFFFFFFFFFFFFFFFFFFFFFFFFF
FFFFFF:FFFFFFFFFFFFFFFFFFFFFFFFFFFFFFFFFFFFF,FFFF
@A00721:190:HGJW7DSXY:4:1653:32660:16157_1:N:0:TCTGCAAG+GCTGCACT
TAACCGATAGGAGTTGTATAGTTGAAGATGTGGCTTTAGACGATAATCCTTTAAGGACTATGTTGACT
GACGAAGATTTTCCCACAGATTTGTCTACGTTGCAGATCGCCACTCCAAACACTATATACATTAAAAT
ATA
+
FFFF,FF:FFFFFFFFFFFFFFFF:FFFFFFFFFFFFFFFFFFFFFFFFFFFFFFFFFFFFFFFFFFFFF

FFFFFFFF:FFFFFFFFFFFFFFFFFFFFFFFFFFFFFFFFFFFFFFFFFFFFFFFFFFFFFFFFFFF
FFF
@A00721:190:HGJW7DSXY:4:2170:19840:19069_2:N:0:TCTGCAAG+GCTGCACT
AGTCATAACTTTTGTTACCAAATTCTTGGCTTTCAACGAAGTCTCTGATTTTATTAATCATTACTTTA
ACGATCTCTCGGTCATTTACTCTGTACTTGATCTACGTCGTTTAACTG
+
FFFFFFFFFFFFFFFFFFFFFFFFFFFFFFFFFFFFFFFFFFFFFFFFFFFFFFFFFFFFFFFFFFFF
FFFF:FFFFFFFFFFFFFFFFFFFFFFFFFFFFFFFFFFFFFFFFFFF
@A00721:190:HGJW7DSXY:4:1406:22941:26506_2:N:0:TCTGCAAG+GCTGCACT
CTTAGAATGACTCTATCTTTTCAGACACTTCTTATATTATTTACAATAGTTAATATCATATTAGTAAT
ACTCACTATCGTTTTCTGCTTTTTCTTAGCTAATAGATTAATAAAGGA
+
FFFFFFFFFFFFFFFFFFFFFFFFFFFFFFFFFFFFFFFFFFFFFFFFFFFFFFFFFFFFFFF:FFFF
FFFFFFFFFFFFFFFFFFFFFFFFFFFFFFF::FFFFFFFFFFFFFFF
@A00721:190:HGJW7DSXY:4:2274:31430:32064_2:N:0:TCTGCAAG+GCTGCACT
ACCAACTCTTCGTTACGAGACCTGATGGTAGAATAGATTTAGTCTCTACACCCATCTCAACGAGGATA
AAGCGAGTTTTCCGTCCTGAACGTAATACACGAAGAGATCATCCGCAA
+
FFFFFFFFFFFFFFFFFFFFFFFFFFFFFFFFFFFFFFFFFFFFFFFFFFFFFFFFFFFFFFFFFFFF
FFFFFFFFFFFFFFFFFFFFFFFFFFFFFFFFFFFFFFFFFFFFFFFF
@A00721:190:HGJW7DSXY:4:2136:11993:25989_2:N:0:TCTGCAAG+GCTGCACT
AAAGTCCCTTCTAACTTTAGATTATGAATCCTGCCTTTACTCATTTTATAGCAGTTATTTATGTTGTA
CTTTTTATAATTTATAACCTTCTTATACTTTTGGGTATGTTTTGTGAA
+
FFFFFFFFFFFFFFFFFFFFFFFFFFFFFFFFFFF:FFFFF::F,FFFFFFFFFFFF,FFFFFFF:FF
FFFFFFFFFF,FFFFFFFFF,FF:FFF:FFFF:,FFFFFFFFFFFFFF
@A00721:190:HGJW7DSXY:4:2269:4942:16971_2:N:0:TCTGCAAG+GCTGCACT
GAAGACATTATCTCTTATGTACTTATTGTAACGATCATTCTTTCGAGTTTTGTCATTCTTATTCTTTT
ATTCCTTTAACTGTGTATGAGGAGGATTTATAATGCTTTTCGATAGTG
+
FFFFFFFFFFFFFFFFFFF:FFFFFFFFFFFFFFFFFFFFFFFFFFFFF:FFFFFFFFFFF:FFFFFF
FFF:F:FFFFFFFFFFFFFFFFFFFFFFFFFFFFF,FFFFF,FFFFFF
@A00721:190:HGJW7DSXY:4:2623:28592:1125_2:N:0:TCTGCAAG+GCTGCACT
ATTACTGTTTCAAATCATCTTTACTCACAGACTCCGAAAATGGTTTCAAATGAGGTCAAAGCTCTATT
TATTAAACGAACTATTATCCGATTCTTCGAGGGTATCACTACCACTGA
+
FFFFFFFFFFFFFFFFFFFFFFFFFFFFFFFFFFFFFFFFFFFFFFFFF:FFFFFFFFF:FFFFFFFF
FFFFFFFFFFFFFFFFFFFFFFFFFFFFFFFF:FFFFFFFFFFFFFFF
@A00721:190:HGJW7DSXY:4:1427:26178:14450_2:N:0:TCTGCAAG+GCTGCACT
CAAGGAACTTCAATTCCTTCGATGTTTATATTTTTCTATAAGGGAAAATAGCAATAATCAAATTTCGA
CAAATTTTAGAACTTTATTTCAGAACTCTCTCCGATCACCTTGGTGTG
+
FFFFFFFFFFFFFFFFFFFFFFFFFFFFFFFFFFFFFFFFFFFFFFFFFFFFFFFFFFFFFFFFFFFF
FFFF:FFFFFFFF:FFFFFFFFFFFFFFFFFFFFFFFFFF,FF:FFFFF
@A00721:190:HGJW7DSXY:4:2415:14931:24909_1:N:0:TCTGCAAG+GCTGCACT
TCTTTTCAATAAGATGTATGATGATAATCTACTATTTTTAAATAGTGATATTCAGCGTATACTTGAAT
ACTTTATCAAAGATTTGGCTTGGTCTATTTACCGCAAAAAGTTGGAAGAGGTCTATGATATTTATTTT
ACT
+
::FFF:FFFFFF,FFFFFF,FF:FFFFFFFFFFFFFFFFFFFFFFF,:FFFFFFFFF:FFF:FFFFFF
FFFFFFFFFFFF,FFFFF:FFFFFF:FFFFFFFF,FFFFFFFFF:FFFF:FFFFFFFFFFFFFFFFFF
FFF
@A00721:190:HGJW7DSXY:4:2457:23809:18082_2:N:0:TCTGCAAG+GCTGCACT
CACCGATTATAACTTGACTGTTGATAATATTACAATAGCTCTTTCTCAGAAGATTAGAGAAGTAACCG

```
GTGAACACGTCGGTAGCATTGATTTACTTTTACCGTTAGAACTGAAAA
+
FFFFFFFFFFFFFFFFFFFFFFFFFFFFFFFFFFFFFFFFFFFFFFFFFFFFFFFFFFFFFFFFFFFFFFF
FFFFFFFFFFFFFFFFFFFFFFFFFFFFFFFFFFFFFFFFFFFFFFFF
@A00721:190:HGJW7DSXY:4:1511:24894:20400_1:N:0:TCTGCAAG+GCTGCACT
ACCTATAAGTTACAAACGATTCTTCGACTATATTAAAGAAATAAAAATACTTCAAGTACCTGAACTTT
ACATTTTTAATGGTATGGTATAATGTGTCTTACAAATAGAACCTTCACTCTCGGTAATGAGAGTGTAT
TCC
+
FFFFFFFFFF:FFF:FFFFFFFFFFFFFFFFFFFFFFFFFFFFFFFFFFFFFFFFFFFFFFFFFFFFF
FFFFFFFFFFFFFFFFFFFFFFFFFFFFF:FFFFFFFFFFFFFFFFFFFFFFFFFFFFFFFFFFFFFF
FFF
@A00721:190:HGJW7DSXY:4:1406:18747:3302_1:N:0:TCTGCAAG+GCTGCACT
GGCATTACTAGGATTTATCACACTCCTAAATTTGATAGCAATTTATGTTTTAAATGCTAGGAAAATTT
TAGAAAAAACTCCATTGTATAGTTCAAGGAGTACTTAATTCAATAGCTTTAGTATTATTTACTATCGCC
TTT
+
FFFFFFFFFFFFFF:FFFFFFFFFFFFFF,FFFFFFFFFFFFFFFFFFFFFFFFFFFFFFFFFFFFFF
FFFFFFFFFFFFFF:FFFFFFFFFFFFFFFFFFFFFFFFFFFFF:FFFFFFFFFFFFFFFFFFFFFFFF
FFF
@A00721:190:HGJW7DSXY:4:1352:28782:4523_2:N:0:TCTGCAAG+GCTGCACT
AGTTAATTGCTTACACAAAAGTGACGCGCGAAGGAATCTTAATTGATTGAAGCTAGGCGTTGGGGAGA
ATGAACTGCCGCTTCCCGGAAGAAACGATCTATTTACAGAGTATTTAG
+
FFFFFFFFFFFFFFFFFFFFFFFFFFFFFFFFFFFFFFFFFFFFFFFFFFFFFFFFFFFFFFFFFFFFF
FFFFFFFFFFFFFFFFFFFFFF,FFFFFFF:FFFFFFFFFFFF,FFFF
@A00721:190:HGJW7DSXY:4:1103:16712:20885_2:N:0:TCTGCAAG+GCTGCACT
CCGCAAATCAATTGAAGAAAACCACTCCCTTCAATTCAATAACATAACATACCATACACTCCCCAAAC
ATAACATACCATAACCTACCATGACCAATCATCGCTCCAACTGGCACG
+
FFFFFFFFFFFFFFFFFFFFFF:,:FF:FF,FFFFFFFFF,FFFFFFFFFFFFFFFFFFFFFFFFFFF
FFFFFFF,FFFFF:FFFFFFFFFF:FF::FFFFFFFFF,FFFFFFFFF
@A00721:190:HGJW7DSXY:4:1125:12337:27085_1:N:0:TCTGCAAG+GCTGCACT
CTACTACCGCCGGTCGGCAGATCGGCACCACTGGTCTCTACGTTCAGGGACCCGCTGTGCAGAATAAC
CTTCCCAAATCTAGATCTGCACAGAAGAAGGCTGAAGGTCGTTACCGGACGACCACTCAAAGGAAACG
GGC
+
F:FFFFFFFFFFFFFFFFF:FFFFFFFFFFFFFFFFFFFFFFFFFF:FFFFFFFFFFFFFFFFFFFFFF
FFFFFFF:FFFFFFFFFFFFFFFFFFFFFFFFFFFFFFFFFF:FFFFFFFFFFFFFFFFFFFFFFFFFF
FFF
@A00721:190:HGJW7DSXY:4:1239:2175:22420_1:N:0:TCTGCAAG+GCTGCACT
GAGGTCGCTCGTAACATTGACGGCAGTCCCGTCATCTCTGTGGGAACTGAGGCAGTACCTCAGTCAAA
AGTCACATTCACCACTCCCATACCAATCAATGATGTGACTATACAGTTCGGCCATTTCAAGAGTGGTC
AAT
+
FFFFFFFFFFFFFFFFFFFFFFFFFFF:FFFFFFFFFFFFFFFFFFFFFFFFFFFFFFFFFFFFFFFFF
FFFFFFF:FFFFFFFFFFFFFFFFFFFFFFFFFFFFFFFF:FFFFFFFFFFFFFFFFFFFFFFFFFFFF
FFF
@A00721:190:HGJW7DSXY:4:1119:29830:9533_1:N:0:TCTGCAAG+GCTGCACT
GTACTGAAGCCTTCCCCTCCGCTGGCACGAAGAACACTCCCAAGCCAATCAATAGTTCTTCTGACCAG
AATGAAAAGGGGTTTACCAGGGTAGTCTCTAAGTCTACCAGGAAGAGGGGAAATGTCCCCAAAACTGA
GAA
+
FFFFFFFFFFF:FFFFFFF:FFFF:FFFFFFFFFFFFFFFFFFFFFFFFFFFFFFFFFFF:FFFFFFFFF
```

FFFFFFFFFFFFFFFFFFFFFFFFFFFFFFFFFFFFFFFFFFFFFFFFFFFFFFFF,FFFFFFFFFFFFFF
FFF
@A00721:190:HGJW7DSXY:4:1101:28438:17080_2:N:0:TCTGCAAG+GCTGCACT
AGGTCCATCAGGACCTAAGAAGGGGAAATTACCCCTAATTCTGAAGGAAGATCTCCCACCATCTGGGG
TTGGGAATAAGAAATCTGCACGGGCCAAAGAGGCCGCTGCTACGAAGT
+
FFFFFFFFFFFFFFFFFFFFFFFFFFFFFFFFFFFFFFFFFFFFFFFFFFFFFFFFFFFFFFFFFFFFF
FFFFFFFFFFFFFFFFFFFFFFFFFFFFFFFFFFFFFFFFFFFFFFFFF
@A00721:190:HGJW7DSXY:4:1218:30996:32221_1:N:0:TCTGCAAG+GCTGCACT
GCTTGGGTCATTAAGGGTAGCTGTAAAGTTGCGCCTGCCGACCCCATCTTTGAGACCAAGACTCTCTA
CAGTAGGTTCGTGCCTACTATGAGCAAATCTGTTGAATTCGTCACTGAGGACGAAGGTGAGAGGACTT
TTG
+
FFFFFFFFFFFFFFFFFFFFFFFFFFFFFFFFFFFFFFFFFFFFFFFFF:FFFFFFFFFFFFFFFFFFFF
FFFFFFFFFFFFFFFFFFFFFFFFFFFFFFFFFFFFFFFFFFFFFFFFFFFFFFFFFFFFFFFFFFFFF
FFF
@A00721:190:HGJW7DSXY:4:1341:12572:10802_1:N:0:TCTGCAAG+GCTGCACT
CAAACTGAGAGCAGTCGCTGGGGAAAGAGAATTGCGTGGGTTGAAGGCCGACGCTTCGAGCTTGAGTG
TGACGAGGCCGGTGACGTTTTTGTCAACGGCGTTTGGAGGATTTTATCCGCGCTTAACTATGGGGCGA
AGA
+
FF:FFFFFFFFFFFFFFFFFFFFFFFFFFFFFFFFFFFFFFFFFFFFFFFFFF:FFFFFFFFFFFFFFFF
FFFFFFFFFFFFFFFFF:FFFFFFFFFFFFFF,FFFFFFFFFFF:FFFFFFFFFFFFFFFFFFFFFFFF
FFF
@A00721:190:HGJW7DSXY:4:1214:4001:14967_1:N:0:TCTGCAAG+GCTGCACT
AGTCTTGAAGGACTTAGGTTGGCATATTTATGCCGACGATAATAAGCATGTCACCCTTAAGCACCGAG
GTAAGCTCGGGACTTACAAGAGAGAGTGTGAGAGAGTCGCTCTCACTATTTTTGCCCAGAATGCGGGC
TTT
+
FFFFFFFFFFFFF:F:FF:FFFFFFFFFF,FFFFFFFFFFFFFFFFFF:FFFFFFFFF,FFFFFFFFF
FFF,FFFFFFFFFFFFFFFFFFFFFFFFFFFFFFFFFFFFFFFFFFFFFFFFFFFFFFFFFFFFFFFFF
FFF
@A00721:190:HGJW7DSXY:4:1178:28076:20149_2:N:0:TCTGCAAG+GCTGCACT
GCTCCTTCTGTGGGTGAGGGTTTTTGTTATATTCACTGGTTCTGGGAGGCTGCTTTCCTCCATAATTA
CAACCTCTCTTTAGAGAGCATTAGAAAGATCGGAAGAGCGTCGTGTAG
+
FFFFFFFFFFFFFFFFFFFFFFFFFFF:FFFFFFFFFFFFFFFFFFFFFFFFFFFFFFFFFFFFFFFFF
FFFFFFFFF,FF:FFFFFFFF:FFFFFFFFFFFF:FFFFFFFF,F,FF
@A00721:190:HGJW7DSXY:4:2112:13123:9439_2:N:0:TCTGCAAG+GCTGCACT
GTTGTCACACTTTTCTTGAAAATTTTTCCTTTCCTCCGTCATTGGGGATTTCGATGTGAGAAGGTGGA
AGGTGTCAAGGGTCTTCTTCATGCTGATCTTTCAGGACCTAATCACAT
+
FFFFFFFFFFFFFFFFFFFFFFFFFFFFFFFFFFFFFFFFFFFF:FFFFFFFFFFFFFFFF:FFFFFFFF
FFFFFFFFF,FFFFFFFFFFFF:FFFFFFFFFFFFFFFFFFFFFFFFFF
@A00721:190:HGJW7DSXY:4:1144:8323:12618_1:N:0:TCTGCAAG+GCTGCACT
ACGGCTCGTATCGAGTTGGAGGGAGGAAGTTCTCCGAAATTAAAGACGGGGGGATTAAGAGTCCAGCT
GTAACTGTCATTTCAGAGAGGGGAGCTCTGAAAAGGAAACTTCGTCAGAAGATGAGGAAGTCTAAGAG
GAG
+
:FFFFFFFFFFFFFFFFFFFFFFF:FFF:FFFFFFFFFFFFFFFFFFFFFFFFFFFFFFFFFF:FFFFFFF
FFFFFFFFFFFFFFFFFFFFFFFFFFFFFFFFFFFFFFFFFFFFF:FFFFFFFFFFFFFFFFFFFFFFFF
FFF
@A00721:190:HGJW7DSXY:4:2149:8278:27164_2:N:0:TCTGCAAG+GCTGCACT
AAGGCTCTAGAGAAGATATCACCACCTGGAGAAGGCAAGTGGAGAGTCCAGAAGTTGATGAAGAAGGA

TTATTTGAAGCAGGGGTTACCACTTCCTAAGGTGGATGAGGGAGGTTG
+
FFF,FFFF:FFFF:FF,,:FFF:FF:FFFFFFF::FFFF:FF:FFF:,FF,F::,,,,FFFF::FFFF
FFFF,FFF::F::FFFFFF:FF:FFFFF:FF,FF:F,,FFFFFFFFFF
@A00721:190:HGJW7DSXY:4:1378:16450:36808_1:N:0:TCTGCAAG+GCTGCACT
TTTTGAGAAGGGGTCATAGAGCCAGCAAGAGAAGCTTATATGCTGGTTTAAGTGATGATTATCACTCC
TTGAGAAGACAAGCCACATTAAGATATAATTTAGGTCAACTTGGGGTTTCGAATGTCATTAGTGCCTT
ACG
+
FFFFFFFFFFF:F:FF:FF:FFFFFFFFFFFFFFFFFFFFFFFFFFFFFFFFFFFFFFFFFFFFFFFF
FFFFFFFFFFFFFFFFFFFFF:FFFFFFFFFFFFFFFFFFFFFFFFFFFFFFFFFFFFFF:FFFFFFFFF
FFF
@A00721:190:HGJW7DSXY:4:1253:31810:17347_2:N:0:TCTGCAAG+GCTGCACT
TCCTTTTGAGTATCGACAGATCTTTCGATCTTGAGGACGTACATTTAACGTGGCAGAATTCGGTAACT
TACCATATTGTTCCTTCCGGGAAGTTGTGGGATTTAACTTTGACTCAA
+
FFFFFFFFFFFFFFFFFF,FFFFFFFFFFFFFFFFFFFFFFFFFFFFFFFFFFFFFFFFFFFFFFFFF
FFFF:FFFFFFFFFFFFFFFF::FFFFFFFFFFFFFFFFFFFFFFFFFF
@A00721:190:HGJW7DSXY:4:2372:17101:6558_2:N:0:TCTGCAAG+GCTGCACT
ATTGGTATGGACTACTCGACTTTTGATCTGGAGTTGTCTAGGGTCATGGCAGATACTACCGATAAGGT
GAAAAATATGCAGGTCAAAGATTCTGTAGCTCTGAGGGCGGTTGAGGA
+
FFFFFFFFFFFFFFFFFFFFFFFFFFFFFFFFFFFFFFFFFFFFFFFFFFFFFFFFFFFFFFFFFFFF
FFFFFFFFFFFFFFFFFFFFFFFFFFFFFFFFFFFFFFFFFFFFFFFF
@A00721:190:HGJW7DSXY:4:1261:7798:32033_1:N:0:TCTGCAAG+GCTGCACT
TTCTAAGAGTCTTTATGATTCAAAACCTATCGTTGTCGTACCTTACAAGGTTGAGGAGACTTTACAGC
GGCTTATTGTCGCGCAGTTTCCAGAATTGCGGTTGAAGTTCACCCATTCTATCTTGAATGCTCACGCC
GTC
+
FFFFFFFFFFFFFFFFFFFFFFFFFFFFFFFFFFFFFFFFFFFFFFFFFFFFFFFFFFFFFFFFFFFF
FFFFFFFFFFFFFFFFFFFFFFFFFFFFFFFFFFFFFFFFFFFFFFFFFFFFFFFFFFFFFFFFFFFF
FFF
@A00721:190:HGJW7DSXY:4:1127:26919:1329_2:N:0:TCTGCAAG+GCTGCACT
TTATAATCACTTGTTAAAGTACAGATTTAGTGATTTAGATATTATTGATATTGGAGGCGATATTCGTT
TTCATATCCAAAACTCCTTGAATGGAGTACATATTTGCAACCCTATTC
+
FFFFFFFFFFFFFFFFFFFFFFFFFFFFFFF,FFFFFFFFFFFFFFFF:FFFFF:FFFF:FFFFFFFFF
FFFFFFFFFFFFFFFFFFFFFFFFFFFFFFFFFF:FFFFFFFFFFFF,
@A00721:190:HGJW7DSXY:4:1106:21423:14043_1:N:0:TCTGCAAG+GCTGCACT
GATGCATCGAGGTATACGTCTAGGAACATTGATTGGGAACGTACCTACTCACGAGACGTCATGAAAGC
TATCGGAGGGAAGCAAAATGTTTCGTTCTGTTACAAGAACGCTTTGGATTGTGACCATAAAGCCTCAG
TTG
+
FFFFFFFFFFFFFFFFFFFF,FFFFFFFFFFFFFFFFFFFFFFFFFFFFFFFFFFFFFFFFFFFFFFF
FFFFFFFFFFFFFFFFFFFFFFFFFFFFFFFFFFFFFFFFFFFFFFFFFFFFFFFFFFFFFFFFFFFF
FFF
@A00721:190:HGJW7DSXY:4:2419:30490:33536_2:N:0:TCTGCAAG+GCTGCACT
ACGATATCTCTCTTGATGATGTAGCCACTATAATGCTTAAGAGAGAGATTGATGTATGTCATTTCGTC
TTAGTTTGTCCCGGCGAATTATTGGACACGATGAAAAATGACATACCC
+
FFFFFFFFFFFFFFFFFFFFFFFFFFFFFFFFFFFFFFFFFFFFFFFFFFFFFFFFFFFFFFFFFFFF
FFFFFFFFFFFFFFFF:F:F,FFFFFFFFFFFFFFFFFFFFFFFFFFF
@A00721:190:HGJW7DSXY:4:2214:12915:29966_1:N:0:TCTGCAAG+GCTGCACT
TCGTTATCCGTCATGGAGATGATGGCGCATCACTTGATTACTATATGCCCGAAGGATTGGGTTATTCT

CACAACAAAGATAAGATTTTATCTTTCTTCAAACACCCTGTTTTCCTTAAGGGCGGACAATTGTTCCA
TTG
+
F:FFFFFF:FFFFFFFFFF:FFFFFFFFFFFFFFFFFFFFFF:FFFFFFFFFFFFFFFFFFFFFFFFF
FFFFFFF:FFFFFFFFFFFFFFFFFFFFFFFFFFFFFFFFFFFFFFFFFFFFFFFFFFFFF,FFFF
FFF
@A00721:190:HGJW7DSXY:4:2631:21242:31861_2:N:0:TCTGCAAG+GCTGCACT
ATTGAGCATTAATGAGTTCCGAATAACTCGTAATAAATCTTTCTCTAGTGGACAATTCAGAATACCTA
TTTCCGTACCAAGAGCCACTGCCAATCTCATCGCTATACGCATACCTG
+
FFFFFFFF:FFF:FFFFFFFFFFFFFFFFFFFFFFFFFFFFFFFFFF:FF:FFFFFFFFFFFFFFFFF
FFF:FFFFFFFFFFFFFFFFFFFFFFF,F:FF:,FFFFFFFFFFFF:FF
@A00721:190:HGJW7DSXY:4:1275:15221:9345_2:N:0:TCTGCAAG+GCTGCACT
AGGTTCGTTCCTCAATAAAAATTTTTCTCAAGAGATTATGGTTGATAAAGTTTTCTTCATAAGGGCTA
TTCAGTACGTCATGGGGATCACTTCTTCGGTCAATGACAAAACCTTCG
+
FFFFFFFFFFFFFFFFFFFFFFFFFFFFFFFFFFF,F:FFFFFFFFFFFFFFFFFFFFFFFFFFFFFF
FFFFFFFFFFFFFFFFFFFFFFFFFFFFFFFFFFFFFFFFFFFFFFFF
@A00721:190:HGJW7DSXY:4:1119:20961:23703_2:N:0:TCTGCAAG+GCTGCACT
CTTCGTAACAACAGTGCGAGGATTGTCATCTCGGGGAAAGTGATTCACCAAGATGTTCATATAGATAC
GAGATACCTAGAGAAGGTCGCAGCGATCTTACTCGTTATCGGAGCTAA
+
FFFFFFFFFFFFFFFFFFFFFFFFFFFFFFFFFFFFFFFFFFFFFFFFFFFFFFFFFFFFFFFFFFFF
FFFFFF:FFFFFFFFFFFFFFFFFFFFFFFFFFFFFFFFFFFFFFFFF
@A00721:190:HGJW7DSXY:4:2318:7111:7420_2:N:0:TCTGCAAG+GCTGCACT
TACACGTTATGCTCGCAAACTTAACGTTAGTACCGGAGGTGATCTCTCTGCTTCTGCGTTACTGAGAG
AAAAGATTCAGGACGCCTACGGTGACTTTATGGACAAGATGCAAGTTC
+
FFFFFFFFFFFFFFFFFFFFFFFFFFFFFFFFFFFFFFFFFFFFFFFFFFFFFFFFFFFFFFFFFFFF
FFFFFFFFFFFFFFFFFFFFFFFFFFFFFFFFFFFFFFFFFFFFFFFF
@A00721:190:HGJW7DSXY:4:1113:21151:7654_2:N:0:TCTGCAAG+GCTGCACT
TTTAGGTTTAGTAGGTGATCTTGACGTCGATTCTTTGTATAGGGAGTGTGAGAAGACAACGAATCTTG
AAACTACGGTCCTTATCAAAGAAAATGGTGAACCGGTCTCTAAATCTT
+
FFFFFFFFFFFFFFFFFFFFFFFFFFFFFF:F:FFFFFFFFFFFFFFFFFFFFF,FFFFFFFFFFFFF
FFFFFFFFFFFFFFFF,:FFFFFFFFFFFFFFFFFFFFFFFFFFFF:FF
@A00721:190:HGJW7DSXY:4:1240:28556:29684_2:N:0:TCTGCAAG+GCTGCACT
GAAGATTGAAGAACACGTCAAGAAGTCGCTCGGCGTTGTTCTTGAAAACCAAGTTAAGAAGGAGGCTG
ATCATGGAGTCTTTGTACCTTCTGTCAAACTTCGAGGTTCAGCTACGA
+
FFFFFFFFFFFFFFFFF:FFFFFFFFFFFFFFFFFFFFFFFFFFFFFFFFFFFFFFFFFFFFFFFFFF
FFFFFFFFFFFFFFFFFFFFFFFFFFFFFFFFFFFFFFFFFFFFFFFF
@A00721:190:HGJW7DSXY:4:1265:6813:11099_1:N:0:TCTGCAAG+GCTGCACT
TGGATTGAAGGCTGGCTCTAAGGGAGACTCCTTCTTTTTGAATGTTGTAAAGAATTGGGTTGAGAAGT
GTGCTTTACAAGGAGTTAGACTCTTCTTCAAGTTTTTAGATCTTCTTGATAATCTTAAAGAAAAATTT
TCG
+
FFFFFFFFFFFF:FFFFFFFFFFFFFFFFFFFFFFFFFFFFFFFFFFFFFFFFFFFFFFFFFFFFFFF
FFFFFFFFFFFFFFFFFFFFFFFFFFFFFFFFFFFFFFFFFFFFFFFFFFFFFFFFFFFFFFFFFFFF
FFF
@A00721:190:HGJW7DSXY:4:1119:1750:24314_1:N:0:TCTGCAAG+GCTGCACT
GTCTGTACTTACATTCTTTACGCCAGATTCTTCATGGGGCGACGCTGTTTCTCTTGGTATTTCTGAAA
TAGTTTATCAGAGCGTCATTTTCCTTTGGGATCTTTCCTCTGGTAAGGGAATTATTAAATCATTCTTC
TCA

```
+
FFF:FFFFFFFFF,FFFFFFFFFFFF:FFFFFF:FFFFFFFFFFFFF:FFFFFFFFFFFFFFFFFFFF
FFFFFFFFFFFFFFFFFFFFFFFFFFFFFFFFFFFFFFFFFFFFFFFFFFFFFFFFFFFFFFFFFFFF
FFF
@A00721:190:HGJW7DSXY:4:1604:15383:10661_2:N:0:TCTGCAAG+GCTGCACT
ATTATTACTGTTTTCTCTACCCATAACTTCTTTTTCAAAAATGCGGATGGGTCTACTGATGTTTTCCT
ACAGAGTGCAGTCGTTTAAACACATATAACCACTTTTGAAAGGAAAAT
+
FFFFFFFFFFFFFFFFFFFFFFFFFFFFFFFFFFFFFFFFFFFFFFFFFFFFFFFFFFFFFFFFFFFF
FFFFFFFFFFFFFFF:F,FF,F,,FF:,F,:F,,FFF,FFF,:FFFF,
@A00721:190:HGJW7DSXY:4:2635:19190:18349_1:N:0:TCTGCAAG+GCTGCACT
AAATACGTATGTCGGTGGGGTTCTCAAGGCTCTCATCGCCACTGGGATTCTAAGGGGTTCCATAAGAT
ATTATCTGACATTGTGGTTCAATGATCAGGATTCTTCTTTTGGTCCTTACGTCTTAAGTAATTTGGAT
GTT
+
FFFFFFFF:FFFFFFFFFFFFFFFFFFFFFFFFFFFFFFFFFFFFFFFFFFFFFFFFFFFFFFFFFFF
FFFFFFFFFFFFFFFFFFFFFFFFFFFFFFFFFFFFFFFFFFFFFFFFFFFFFFFFFFFFFFFFFFFF
FFF
@A00721:190:HGJW7DSXY:4:2451:30029:6026_2:N:0:TCTGCAAG+GCTGCACT
GTTCTTGTCACGATTCTTAAAAGAAGTTTATTAAAACTTCAGAGTCTCTTGGAAGTCCAGGTAGCTAA
TGTTCTTGCAATAGCCACAGACTTTCTGGGAAAAGAGACCTCTGTTGG
+
FFFF:FFFFFFFFFFFFFFFFFFFFFF:FFFFFFFFFFFFFFFFFFFFFFFFFFFFFFFFFFFFFFFF
FFFFFFFFFFFFFFFFFFFFFFFFFFF,FFFFFFFFFFFF,FFFFFFF:
@A00721:190:HGJW7DSXY:4:1502:24542:6073_1:N:0:TCTGCAAG+GCTGCACT
GAGAATTACTCAGATTCAATCTGGCATCACTGAACGTCTACAACGAGTTAGGGAGTGGCTAGGTTTTA
AAAGATCCTTTGCACAAGCAATACCTTCCACTGAGTTCATCACCCAGAGTTTCGACGTCGCGGAAAAC
GAT
+
FFFFFFFFFFFFFFFFFFFFF:FFF:FFFFFFFFFFFFFFFFFFFFFFFFFFFFFFFFFFFFF:FFFF
FFFFFFFFFFFFFFFFFFFFFFFFFFFFFFFFFFFFF:FFFFFFFFFFFFFFFFFFFFFFFFFFFFFF
FFF
@A00721:190:HGJW7DSXY:4:1122:3007:30185_2:N:0:TCTGCAAG+GCTGCACT
ACTGTTCGAGAATCTTCTGATGCTGAATTTGAGGAAAATTTTTCGGAAGTTTTTGGTATAAATCCTGA
TGGCTCAGTACAGGGAGGAGGTAATAGTTATAAAACCTTCTTTTATCA
+
FFFFFFFFFFFFFFFFFFFF:FFFFFFFFFFFFFFFFFFFFFFFFFFFFFF:FFFFFFFFFFFFFFFF
FFFFFFFFFFFFFFFFFFFFF:FF,FFFFFFFFFFFFFFFFFFFFFFF
@A00721:190:HGJW7DSXY:4:1259:1741:12493_1:N:0:TCTGCAAG+GCTGCACT
ATGGACAAAATCTGGGATCATAGGTATGTGTCACGCTACACTCGAAATATGGGCCCTCATAAGGAAGT
ATAGTTGGCGTCTTGTCGTTGACGTCATTACAGTCTGGAAGTTAGATCATTTGTTCAGGAAGATCTCT
ACA
+
FFFFFFF:F:FFFFFFFFFFFFFFFFFFFFFFFFFFFFFFFFFFFFFFFFFFFFFFFFFFFFFFFFFF
FFFF,FFFFFFFFFFFFFFFFFFFFFFFFFFFFFFFFFFFFFFFFFFFFFFFFFFFFFFFFFFFFFFF
FFF
@A00721:190:HGJW7DSXY:4:2374:22327:15859_1:N:0:TCTGCAAG+GCTGCACT
CTTTAGCAACTTTAGATAAATTCTTTCCGGGCTCAATGACCTTTGCTATGTACGTGGATCTAATCCTT
GGTGATCTGAATCTGACCATCGCAAAGAAGACTTTATTAGTTCATGAAGTTCTTACACTTCTCGCGAC
TAA
+
FFFFFFFFFFFFFFFFFFFFFFFFFFFFFFFFFFFFFFFFFFFFFFFFFFFFFFFFFFFFFFFFFFFF
FFFFFFFFFFFFFFFFFFFFFFFFFFFFFFFFFFFFFFFFFFFFFFFFFFFFFFFFFFFFFFFFFFFF
FFF
```

@A00721:190:HGJW7DSXY:4:2664:18484:27336_1:N:0:TCTGCAAG+GCTGCACT
GCTTTATCATAAAGCATTACCACCAAATTTAGCACTTTTTGAATATGACAGAAGCTCGCCATATCAGA
TTAGAGACATGCCAGTTAGAGTTCTTCTAACCAGGACTGATGGTGAGATTCTTTTACCTTTTGGAGGT
CCA
+
FFF:FFF,,FFFFFF:FFFF:FF,F:,F:,F::,FFFFFF,FFFFFFFFF,,,FFFFFFF:FFFFFFFFF
FFFFFFF::FFF:FFFFFF::FF:FF,FFF:FF:F::FFFFFFF:FFF:FFFFFFFFFFFFFFFF::FF
F,F
@A00721:190:HGJW7DSXY:4:2209:5376:20697_2:N:0:TCTGCAAG+GCTGCACT
CCTAGTGTCTTTTCTAGAGTCCAAGGACGTTTCCGTCGGAGGATGACAGGTGGGAGACCGCTCAATAA
CACTCTATTGAATCCCTTTTCCACGATGAAAGCTCTTTATGAGGTGCT
+
FFFFFFFFFF:FFFFFFFFFFFFFFFFFFFFFFFFFFFFFFFFFFFFFFFFFFFFFFFFFFFFFFFFFFF
FFFFFFFFFFFFFFFFFFFFFFFFFFFFFFFFFFFFFFFFFFFFFFFF
@A00721:190:HGJW7DSXY:4:2633:19587:15718_1:N:0:TCTGCAAG+GCTGCACT
TCTTTGGTGATATCGCCTCTGTCCTTTCTCCTTTAATCGTGAGGTTACGAGAGTTTCGTACTTGGGTT
TATCGGTTGTATTTTGATAAATTTGTTGGAATCGATAATGATACTGACGAAGGTTCTATTACCACCCT
TGT
+
FFFFFFFFFFFFFFF:FFFFFFFFFF::FFFFFFF:FFFFFFFFFFFFFFFFFFFFFFFFFFFFFFFFFF
FFFFFFFFFFFFFFFFFFFFFFFFFFFFFFFFFFFFFFFFFFFFFFFFFFFFFFFFFFFFFFFFFFFFFF
FFF
@A00721:190:HGJW7DSXY:4:1156:17264:36839_2:N:0:TCTGCAAG+GCTGCACT
TGATGCTTTCTTAGAGGCTCGTCTAGCCGCTGAGATTCGTCTTACCTATTTACAATCTGTAGAGAGAG
AGGTTAATCAGAATTTGAGTGATAACGATTTAGTACTGGGAAATGGTG
+
FFFFFFFFFFFFFFFFFFFFFFFFFFFFFFFFFFFFFFFFFFFFFFFF:FFFFFFFFFFFFFFFFFFFFF
FFFFFFFFFFFF,FFFFFFFFFFFFFFFFFFFFFFF:FFFFFFFFFFF
@A00721:190:HGJW7DSXY:4:1610:3323:14011_1:N:0:TCTGCAAG+GCTGCACT
TTTTCTTTCCCTCCCCTTTTTCGATTGGATTCGACGGGGTCTTCGTCTCACGACTAATTTGATAAGGT
TTTTCTTTTATGGGACTCTTGTTTGTACTTATGAAGGAGGTATTACTTCGGAAGTTCTTAATGTAGGT
CTC
+
FFFFFFFFFFFFFFFFFFFFFFFFFFFFFFFFFFFFFFFFFFFFFFFFFFFFFFFFFFFFFFFFFFFFFF
FFFFFFFFFFFFFFFFFFFFFFFFFFF:FFF:FFFFFFFFFFF:FFFFFFFFFFFFFFFFFFFFFFFFFF
FFF
@A00721:190:HGJW7DSXY:4:1473:31684:4570_1:N:0:TCTGCAAG+GCTGCACT
GGTTTATGTTTCGGTTTCAGCTGGATGGATCTTTTGATGGTATGTTTCACATTGCGTGATCACTTCCC
GTGGACTTGGGAAGAGATTAAACATAAAATCCAGCGATTCTATTATGTCGCTAAAGCCTGTCCAGTTG
TCG
+
FFFFFFFFFFFFFFFFFFFFFFFFFFFFFFFFFFFFFFFFFFFFFFFF:FFFFFFFFFFFFFFFF,FFFF
FFFFFFFFFFFFFFFFFFFFFFFFFF:FFFFFFFFFFFF:FFFFFFFFFFFFFFFFFFFFFFFFFFFFFF
FFF
@A00721:190:HGJW7DSXY:4:1130:19750:18787_2:N:0:TCTGCAAG+GCTGCACT
GGCTGTGGGGATTAACTTCCCTCGTGAAACTTACGACGATGTTCTCGCTCGCATTTCACAAGGATTAC
TAAGTCTGAGATCTGCCACTTCTAATTTTAGGAAACGTCGCGAAAAGA
+
FFF:FFFFFFFFFFFFFFFFFFFFFFFFFFFFFFFFFFFFFFFFFFFFFFFFFFFFFFFFFFFFFFFFFF
FFFFFFFFFFFFFFFFFFFFFFFFFFFFFFFFF:FFFFFFFFF:FFFF
@A00721:190:HGJW7DSXY:4:1117:16016:11350_2:N:0:TCTGCAAG+GCTGCACT
GTCTTGGTCGAAAAATATCGCCGGGAACTTCTAATAGAGGATCCTGATGACATTATTTCAACGGATAC
CGATCTCAGTTCTACCACAGAAACCGTCTCAGGAACTGTCTCCACTGA
+

```
FFFFFFFFFFFFFFFFFFFFFFFFFF:FFFFFFFF,FFFFFFFFFFFFFFFFFFFFFFFF:FFFFFFF
FFFFFFFFFFFFFFFFFFFFFFFFFFFFFFFFFF:FFFFFFFFFFFFF
@A00721:190:HGJW7DSXY:4:1130:9037:28667_2:N:0:TCTGCAAG+GCTGCACT
AAAGACGACCTTCTTGTGGGGACTGAGACCTCTGAATTTTCAGGGTGCCCTGATGAGGACGTTGAAAG
GGATAATTTCAACTCTGAGACTTCTAAGAGTGTTTCAAAAGACCAGAA
+
FFFFFFFFFFFFFFFFFFFFFFFFFFFFFFFFFFFFFFFFFFFFFFFFFFFFFFFFFFFFFFFFFFFF
FFFFFFFFFFFFFFFFFFFFFFFFFFFFFFFFFFFFFFFFFFFFFFFF
@A00721:190:HGJW7DSXY:4:1121:26323:32299_1:N:0:TCTGCAAG+GCTGCACT
TAAAGGTAGGAAGGGGAGAAAACAGAACAAGCGAGCGGTTGAAGAGAAAACTGATATGGTTGCGGCTG
ATCGTACCCCAATTCCTGCTAGAAAGATCACTCTCGGCGATACCACTGATCTTCCAGAGTTACGTAGT
GGT
+
FFFFFFFFFFFFFFFFFFFFFFFFFFFFF:FFFFFFFFFFFFFFFFFFFFFFFFFFFFFFFFFFFFFF
FFFFFFFFFFFFFFFFFFFFFFFFFFFFFFFFFFFFFFFFFFFFFFFFFFFFFFFFFFFFFFFFFFFF
FFF
@A00721:190:HGJW7DSXY:4:1310:26793:23563_1:N:0:TCTGCAAG+GCTGCACT
TTTTATCAAAGAGAAAGTTTCTGCTAGTCGCGACGATAGTATTTATCGGAGTGATATTAACAAGTTAA
ACTTCGAACTGACCACTTACAAGAGTGTTGAAGTACCGCAACATGGATCAGTTTTCCAAAAAGCTATT
AAT
+
FFFFFFFFFFFFFFFFFFFFFFFFFFFFFFFFFFFFFFFFFFFFFFFFFFFFFFFFFFFFFFFFFFFF
FFFFFFFFFFFFFFFFFFFFFFFFFFFFFFFFFFFFFFFFFFFFFFFFFFFFFFFFFFFFFFFFFFFF
FFF
@A00721:190:HGJW7DSXY:4:1224:18593:29966_2:N:0:TCTGCAAG+GCTGCACT
AAACTGAATCCACTCTCTTGGGATATAAATGAGTACATCTATAATAGAGTTTTGGAACTGTGTAATGT
CCTACGTACTGTTCAAGGTGTTTGGGCTTCTCTAAAGAACAGCAACTT
+
FFFFFFF,FFFFFFFFFF:FFFFFFFFFFFFFFFFFFFFFFFFFFFFFFFFFFFFFFFFFFFFFFFFF
FFFFFFFFFFFFFFFFFFFFF:FFFFFFFFFFFFFFFFFFFFFFFFFF
@A00721:190:HGJW7DSXY:4:1126:25274:35775_2:N:0:TCTGCAAG+GCTGCACT
GGCTGATGACTTAAATTTCGCATTCTGCTTCGACGGTAATTCCATAAGACCTTTCTTGGGGAGTAAAG
AGGGTTTGAAAATTACGGAACCTTTCCTCGTTCTTCATGAAGATCTTC
+
FFFFFFFFFFFFFFFFFFFFFFFFFFFFFFFFFFFFFFFFFFFF:FFFFFFFFFFFFFFFFFFFFFFF
FFFFFFFFFFFFFFFFFFFFFFFFFFFFFFFFFFFFFFFFFF::F:FF
@A00721:190:HGJW7DSXY:4:1236:21748:32268_2:N:0:TCTGCAAG+GCTGCACT
GTTATTCTTGCGAAGGTTCAAGTCGATTGATTTGTTGACTACGATCTCTCTTGTAATAAGAAGTTCT
TCGCAGTTGAATCTCCACCAGGTGGAGGGAAGACCACTGAACTTATAG
+
FFFFFFFFFFFFFFFFFF:FFFFFFF,FFFFFFFFFF:FFFF:FFFFFFF::FFFFFFFFFFFFFFFF
FFFFFFFFFFFFFF:FFFFFFFFFFFFF::,FFFFF:FFFFFFFFFFF
@A00721:190:HGJW7DSXY:4:1237:18276:1078_1:N:0:TCTGCAAG+GCTGCACT
TTCGCAAAAGGTCTTGAAGTACGTATTGTCACTGCTAATCGTAATTCTCGAGAAGAGATAGTGACTAA
AGTTTTGGCACTTCTAGTTCATCGCGGTATTTGTGAAATTAAGACAGACGTGAAAGTTATGATGAGTG
ATC
+
FFFFFFFF,FFFFFFFFFFF,,FF:FFFFF::FF,F,FFFFFFFFF:,:F:FFFFF,FFFFFFFFFF,
FFFFFFFFF:FFFFFFFFF::F::F:FFFFFFFF:FFFFFF,:F,FF:FFFF,:FFFFFFF,FFFFF:
FF:
@A00721:190:HGJW7DSXY:4:1225:13657:24173_2:N:0:TCTGCAAG+GCTGCACT
AGCTGATTCTTATTGCATGAACGTCGCTGAGGTCAAACCTGTGGACGTTCTTTTCATCGACGAATTAT
TCATGATTCACGCTGGTCAGACCCTTTTTCTTACACATCTACTTAAGG
+
```

FFFFFFFFFFFFFFFFFFFFFFFFFFFFFFFFFFFF:FFFFFFFFFFFFFFFFFFFF:FFFFFFFFFF
FFFFFFFFFFFFFFFFFFFFFFFFFFFFFFFFFFFFFFFFFFFFFFFF
@A00721:190:HGJW7DSXY:4:1112:30644:9283_2:N:0:TCTGCAAG+GCTGCACT
GTTACGGTGATTCCAATCAAATCGGATTCATCAATCGTATTGATCTCCAAGTCGCAAACTACTTCAAC
ATCAACGACATCATTCTTGATGAGTGTCGTTCTTATCGAAGTCTCAGT
+
FFFFFFFFFFFFFFFFFFFFFFFFFFFFFFFFFFFFFFFFFFFFFFFFFFFFFFFFFFFFFFFFFFFF
FFFFFFFFFFFFFFFFFFFFFFFFFFFFFFFFFFFFFFFFFFFFFFFF
@A00721:190:HGJW7DSXY:4:1237:28311:10598_2:N:0:TCTGCAAG+GCTGCACT
CGGCCGACGTTTGTCGCGAACTCTCAGAGGTTTACGGCAGAAAAATTTCTCCTGCTTGTAATTCGAGA
GAGGACACTATGGAAATTAGACCTATCAGATGTCTTGACGACTTCGAA
+
FFFFFF:FFFFFFFFFFFFFFFFFFFFFFFFFFFFFFFFFFFFFFFFFFFFFFFFFFFFFFFFFFFFF
FFFFFFFFF:FFFFF:FFFFF:FFFFFFFFFFFFFFFFFFFFF,F:FF
@A00721:190:HGJW7DSXY:4:2320:3215:30326_2:N:0:TCTGCAAG+GCTGCACT
ATATTACTTTCACTCAAGGTGAAAAGTTTGAAATTAACAAAGTCCTTGGTAAGAGTAAGGTTAAATGT
GAAGATGCTCAGACTATTCATGAAGTTCAAGGTAATACTTACCAGCAC
+
FFFFFFFFFFFFFFFFFFFFFFF:FFFFFFFFFFFFFFFFFFFFFFFFFFFFFFFFFFFFFFFFFFFF
FFFFFFFFFFFFFFFFFFFFFFFFFFFFFF,FFF:FFFFFFFFFFFFF
@A00721:190:HGJW7DSXY:4:1326:3992:2049_1:N:0:TCTGCAAG+GCTGCACT
ATGACCCTGTTTTTGCATCACAAACACATATTACCGTTGCTCTATCACACACGTTGAATCTTTACGAT
ACTTTGTTATCAATGTAAAGTTAAACGATGCTACTTGTGAGAGGATCAATCACAGTAAGGAACTGTCA
CGT
+
FFFFFFFFFFFFFFFFFFFFFFFFFFFFFF:FFFFFFFFFFFFFFFFFFFFFFFFFFFFFFFFFFFFF
FFFFFFFFFFFFFFFFFFFFFFFFFFFFFFFFFFFFFFFFFFFFFFFF,FFFFFFFFFFFFFFFFFFF
FFF
@A00721:190:HGJW7DSXY:4:1252:21016:36730_1:N:0:TCTGCAAG+GCTGCACT
TGCAGATTCAACCCCTTATATCGAGGCTATCGAGGACGATGAGTATCTTGAAGTTGTTGACGACAAGC
CTCAAGAGGGTAGAGGACCACCTGGATCTTCTACTGTCTCTGCCTTCCAAGATTTCTTAGAAGAGGTT
ATT
+
FFFFFFFFFFFFFFFFFFFFFFFFFFFFFFFFFFFFFFFFFFFFFFFFFFFFFFFFFFFFFFFFFFFF
FFFFFFFFFFFFFFFFFFFFFFFFFFFFFFFFFFFFFFFFFFFFFFFFFFFFFFFFFFFFFFFFFFFF
FFF
@A00721:190:HGJW7DSXY:4:1263:16568:17284_1:N:0:TCTGCAAG+GCTGCACT
CTTAACGTCACTCGGTAATCTTTCAGCCGAATTAAGTACTAATGAGTTCAAGTCGGTTGTCGATCGAA
TTAAGATCAAGGCTAATACCTTTATAGGTAAGAAGCCCCTTGAGCCGCGTAGTCAGCGCGTTTAGCTG
TAT
+
FFFF:FFFFFFFFFFFFFFFFFFFFFFFFFFFFFFFFFFFF,FFFFFF:FFFFFFFFFFFFFFF:FFF
FFFFFFFFFFFFFFFF,F:FFFFFF:FFFFFFFFFFFFFFFFFFFFFFFFFFFFFFFFFFFFFFFFFF
FFF
@A00721:190:HGJW7DSXY:4:1148:26404:34976_1:N:0:TCTGCAAG+GCTGCACT
TATACCCGATAGGGTACCGAGTCTCCAAGAGAATATTTATTCTTATGAGGCTCGAAACTTCAACGTTT
TCACAACGGAAAGACATATCGCACCCGAAATTTTTGGGCAGAATGCTCTTAACGCGGTTCTTGAACGG
TGT
+
FFFFFFFFFFFFFFFFFFFFFFFFFFFFFFFFFFFFFFFFFFFFFFFFFFFFFFFFFFFFFFFFFFFF
FFFFFFFFFFFFFFFFFFFFFFFFFFFFFF:FFFFFFFFFFFFFFFFFFFFFFFFFFFFFFFFFFFFF
FFF
@A00721:190:HGJW7DSXY:4:1338:8467:20259_2:N:0:TCTGCAAG+GCTGCACT
CTAAGTTGGATAAAGTTATAGGAGACTCAGTTAACCTGAGAGCTCCTCAATTGATAGATTGGGTCAAC

AATAGGGACGCTAGTATACTTCGTAAAGTCGACAGCGAAATGGTCAAT
+
FFFFFFF:FFFFFFFFFFFFFF:FFFFFFFFFFFFFFFFFFFFFFFFFFFFFFFFFFFFFFFFFFFFFFF
FFFFFFFFFFFFFFFFFFFFFFFFFFFFFFFFFFFFFFFFFFFFFFFF
@A00721:190:HGJW7DSXY:4:1314:7997:1877_1:N:0:TCTGCAAG+GCTGCACT
ATATGACCTATTTTAAGCTCATGGTGAAAAGGGAAGCTAAGGTTAAACTTGATGCTTCAGCCTTTTCG
AAGCATAACCCCGCACAGAATATCGTTTATCACGCAAAATTTATTAATGCAATTTTCGGACCTATCTT
TAA
+
FFFFFFFFFFFFFFFFFFFFFFFFFFFFFFFFFFFFFFFFFFFFFFFFFFFFFFFFFFFFFFFFFFFF
FFFFFFFFFFFFFFFFFFFFFFFFFFFFFFFFFFFFFFFFFFFFFFFFFFFFFFFFFFFFFFFFFFFF
FFF
@A00721:190:HGJW7DSXY:4:1150:26612:13385_1:N:0:TCTGCAAG+GCTGCACT
TGTCTTTTACCCAACATTTTTATGTATACAGAGATGACCAGTGACGATTTCTCCCGGAAGCTCTTCTC
TCTCTTAGGAGGTCATGATGACTACTATAAGGGTGAAATCGATTTTTCGAAGTTTGACAAATCTCAAG
ATT
+
FF:FFFFFFFFFFFFFFFF:FFFFFFFFFFFFFFFFFFFFFFFFFFFFFFFFFFFFFFFFFFFF,FFF
FFFFFFFFFFFFFFFFFFFFFFFFFFFFFFFFFFFFFFFFFF:FFFFFFFFFFFFFFFFFFFFFFFFF
FFF
@A00721:190:HGJW7DSXY:4:1106:12292:25441_2:N:0:TCTGCAAG+GCTGCACT
AGCCTATGAGATAAAGATGTATTCTTATTTAGGCTTCGATGCTGAATTTCTTGAACTTTGGAGTGCCA
GTGAATATTTTTGTCACGCCAGGAGATCGGAAGAGCGTCGTGTAGGGA
+
FFFFFFFFFFFFFFFFFFFFFFFFFFFFFFFF:FFFFFFFFFFFFFFFFFFFFFFFFFFFFFF,FFFF
FFFFFFFFFFFFFFFFF,FFFFFFFFFFFFFFFFFFFFFF,F,FFFFF
@A00721:190:HGJW7DSXY:4:1268:10890:8265_2:N:0:TCTGCAAG+GCTGCACT
ACGGCCAACACTTTCTTTGGTAATTGTATGATCACTTTGATGATTTTATCACTTTATTATGACCTTGG
AAAGTTTTCGGCTGTGGTTGTCGCTGGGGACGATTCCATTCTGTTTTC
+
FFFFFFFFFFFFFFFFFFFFFFFF:FFFFFFFFFFFFFFFFFFFFFFFFFFFFFFFF,FFFFFFFF,FFF
F,FFFFFFFFFFFFFFFFFFFFFF,FFFFFFFFFFFFF,FFFFFFFFF
@A00721:190:HGJW7DSXY:4:1378:26910:1063_2:N:0:TCTGCAAG+GCTGCACT
CAGCGTACTTCTGCTCGCGATTTATTCTTTTTGTCAATGATTACATCTACTTCGTTCCTGATCCTTAC
AAGATCATCGTTAAGTTAGGTAAACCCCTAACCGATGTTACTCTTGTA
+
FFFFFFFFFFFFFFFFFFFFFFFFFFFFFFFFFFFFFFFFFFFFFFFFFFFFFFFFFFFF:FFFFFFF
FFF:FFFFFFFFFFFFFFFFFFFFFFFFFFFFFFFFFFFFFFFFFFFF
@A00721:190:HGJW7DSXY:4:1524:30101:16110_1:N:0:TCTGCAAG+GCTGCACT
TTCGTTAGTTTTAAAGACTATACTAAATATCTCCACTACGAGGTTGTTGTTTGGCGATTAGCTCTTGC
GACTAATATTCGATATTCGATTGAGGGTCGTTATACCTATTCGGCTATCTGTGCTTTGAATTGTATAA
GAT
+
FFFFFFFFFFFFFFFFFFFFFFFFFFFFFFFFFFFFFFFFFFFFFFFFFFFFFFFFFFFFFFFFFFFF
FFFFFFFFFFFFFFFFFFFFFFFFFFFFFFFFFFFFFFFFFFFFFFFFFFFF:FFFFFFFFFFFFFFF
FFF
@A00721:190:HGJW7DSXY:4:1166:18159:5415_2:N:0:TCTGCAAG+GCTGCACT
GATTCCGAGAGGTTTATCCTGATGTGGAGGGAGTTCTTGAAGCATCCCGACCTTTCCTTGTGAAAATT
TTGATGCGGTTTCATAAGATTATCAGTAATCCTGATTCTACTACGGAT
+
FFFFFFFFF:FFFFF:FFFFFFFFFFFFFFFFFFFFFFFFFFFFFFFFFFFFFFFFFFFF,F:FFFFFFF
FFFFFFFFFFFFFFFFFFFFFFFFFFFFFFFFFFFFFFFFFFFFFFFF
@A00721:190:HGJW7DSXY:4:1116:27651:27398_1:N:0:TCTGCAAG+GCTGCACT
GACTGGAGTTCAGACGTGTGCTCTTCCGATCTCAAAGATCGACTTCGAGACAGGTGATATTGTTGAGC

```
GAGAGCGACCAAGATCTAAATTCGGTAACGGTGCGTGGCGCTTTATTTTAAAAAGCTCCCTCCTCTCG
CAA
+
F::FFFFFF:F,FFFFFFFFFFFF:F,FF::::,F::FFFFFFFFFF:FFF:FFFFFF:F:FFFFFFF
FFFFFFFFFFFFF:FFFFFFFFFF:F:FFFFFFFFFFFFFFFFFFFFFFFFF:FFFFF:FFFFFFFFFF
FFF
@A00721:190:HGJW7DSXY:4:1178:21323:34068_2:N:0:TCTGCAAG+GCTGCACT
ACTTGAGTCTTTTGATCAGTCTCTGACTTGACCATCTTTCATTTTAAATGACGATCATTAAGACTCTC
TTTCTTCTTTGTTTCCTTCTTTGCACTGATTTTTCCTCATCAGCTTGT
+
FFFFFFFFFFFFFFFFFFFFFFFFFFFFFFFFFFFFFFFFFFFFFFFFFFFFFFFFFFFFFFFFFFFFF
F,:F,FFFFFFFFFFFFFFFFFFFFFFFFFFFFFFFFFFFFFFFFFFF
@A00721:190:HGJW7DSXY:4:1373:28953:8390_1:N:0:TCTGCAAG+GCTGCACT
GTTTTTACGATAACAACTATCAGTTGATTAAGACTCTTCCGAAAGGCGAACTTTGGGAATTTGAAGCT
CAGGGTGAAGCACAACGTTTGACCGTGACGAACTGTGGTGTTCTTGATCTACGTTACGGATATACTCT
CCT
+
FFF:FFFFFFFFFFFFFFFFFFFFFFFFFFFFFFFFFFFFFFFFFFFFFFFFFFFFFFFFFFFFFFFFF
FFFFFFFFFFFFF,FFF:FFFFFFFFFFFFFFFFFFFFFFFFFFFFFFFFFFFFFFFFFFFFFFFFFFF
FFF
@A00721:190:HGJW7DSXY:4:1155:29306:35305_1:N:0:TCTGCAAG+GCTGCACT
ACACTACCACTTATTACGATATTTCTTTAGTCGATGGGTTTTCGACGCCTTTAAGCGTCTTCTGTTCA
TCGCAACCAGATGATATCTTCTATTCGAACTTATGTTTCGATCAGTGTTTCTTCTATCAGGAGGAAAA
CAC
+
FFFFFFFFFFFFFFFFFFFFFFFFFFFFFFFFFFFFFFFFFFFFFFFFFFFFFFFFFFFFFFFFFFFFF
FFFFFFFFFFFFFFFFFFFFFFFFFFFFFFFFF,FFFFFFFFFFFFFFFFFFFFFFFFFFFFFFFFFFF
FFF
@A00721:190:HGJW7DSXY:4:2111:18231:27586_2:N:0:TCTGCAAG+GCTGCACT
TGTTTACCACCGAAATATCAAGGTTCTTGTCCTTCAAACGATTGGAAGGTTAGTTATCTTAATAGTAC
TAAAGGGGTTTACTTACAACCCTATGACGATAGTTTCTCTTTACACAC
+
FFFFFFFFFFFFFFFFFFFFFFFFFFFFFFFFFFFFFFFFFFFFFFFFFFFFFFFFFFFFFFFFFFFFF
FFFFF:FFFFFFFFFFFFFFFFFFFFFFFFFFFFFFFF:FF,FFFFFFFF
@A00721:190:HGJW7DSXY:4:2401:31403:12947_2:N:0:TCTGCAAG+GCTGCACT
TGTTTTCTTCTAATGTGGCCTGAACTACGTTTCACTTTGTGTGTACTTGTCTCTATTTTAAGTTTTGT
CTTCCTTGTTTGTCTTATTCTATTAATCACTCATTTTAGTGAGATTAT
+
FFFFFFFFFFFFFFFFFFFF:FFFFFFFFFFFFFFFFF:FFFFFFFFFFFFFFFFFFFFFFFFFFFFFF
FFF:,FFFFFFFF:FFFFF:FFFFFFFFF:FFFFFFFF,FFF,FF:FF
@A00721:190:HGJW7DSXY:4:2441:14669:26396_2:N:0:TCTGCAAG+GCTGCACT
CTAAAACGTCTTCGACAACAGCAGGGGTTTTCGAATAGTGATAGCCACCCGCCTTACAGAGGGGCAGC
AGCATGACCATAATTGGTATTGACTTTGGTACCACCTTTTCTACTGTC
+
FFFFFFFFFFFFFFFFFFF:FF:FFFFFFFFFFFFFFFFFFFFFFFFFFFFFFFFFFFFFFFFFFFFFF
FFFFFFFFFFFFF:FFFF:FFFFFFFFFFFFFFFFFF,FFFFFFFFFF
@A00721:190:HGJW7DSXY:4:1570:10357:3114_1:N:0:TCTGCAAG+GCTGCACT
TTCGAATGAAGTTTTAATGCTTAACAACAATGATAGTGAATTTATACCTACAATCATTGGTTTTTCTA
ATAATGATGACACTATTGTTTATGGATATGATGCTATTACTCATGATGGTTACGGTCAGGGAGGATTC
TCA
+
FFFFF:FFFFFFFFFFFFFFFFFFFFF:F::FFFFFFFFF,FFFF:FFFFFFFF:FFFFFFFFFFFFFF
FFFFFFFFFFFFF,:FFFFFFFF,FFFFFFFFFFFFFFFFFFFFFFFFFFFFFFFFFFFFFFFFFFFFF
FFF
```

@A00721:190:HGJW7DSXY:4:1601:27751:14763_2:N:0:TCTGCAAG+GCTGCACT
ACATTACCCGAAAGACGTGCTAAATTAAAACCATTGTACGATGTCGTCTGTCTCGGCGATCACTTTTC
ACTAAGAATTGGTCCTACTTTTGGACAAGGCCAACTTCGTAATCTTCA
+
FFFFFFFFFFFFFFFFFFFFF,FFFFFFFFFFF:FFFFFFF:F:FFFFFFFFFFFFFFFFFFFF:FFF
FFFFFFFFFFFFFFFFFFFFFFFFFFFFFFFFFFFFFFFFFFFFFFFF
@A00721:190:HGJW7DSXY:4:1325:1325:27305_2:N:0:TCTGCAAG+GCTGCACT
TTTATCTCTTTGGTTGTTAGGGATTTTGAGAGAGTGAAAGGCTTCAATTGTAGTGGTTTGGTCGTTTC
AGTACCCAGTCAGTATACCTCTACGCAAAGATATTTCATGAAAACTAC
+
FFFFFFFFFFFFFFF:,FFF:FFFF:FFFFFFFFFFFFF,FFF:FFF:FFFFFFFFFFFFFFFFFFFF
F:FFFFFFFFFFFFFFFFFFFFFFFFFFFFFFFFFFFFFFFFFFFFFF
@A00721:190:HGJW7DSXY:4:1636:19533:28494_1:N:0:TCTGCAAG+GCTGCACT
CCGGATTACCTGTCTTTCATATTATGAATGAACCTTCGGCCGCTCTCTTTGCTTCGATGTTGGATGTC
AAGAAGGGTTCAGAATGGGATTCCTACGTAGTTTATGACTTTGGTGGTGGTACTTTCGATACTTCTAT
CGT
+
FFFFFFFFFFFFFFFFFFFFFFFFFFFFFFFFFFFFFFFFFFFFFFFFFFFFFFFFFFFFFFF:FFFF
FFFFFFFFFFFFFFFFFFFFF:FFFFFFFFFFFFFFFFFFFFFFFFFFFFFFFFFFFFFFFFFFFFFF
FFF
@A00721:190:HGJW7DSXY:4:1562:11379:10332_2:N:0:TCTGCAAG+GCTGCACT
TACTATAGTGTTATTTATTCTGGTGGTGATGATAATCTTGGAGGTCGTGACGTTGATGAAGCTATAAG
AGCGTTCTTATTACGGCGCACTTCTTTACCCGCCACCACACAACTTTC
+
FFFFFFFFFFFFFFFFFFFFFFFFFFFFFFFFFFFFFFFFFFFFFFFFFFFFFFFFFFFFFF:F:FFF:
FFFFFFFFFFFFFFFFFFFFFFFFFFFFFFFFFFFFFFFFFFFFFFFF
@A00721:190:HGJW7DSXY:4:1569:6533:28181_1:N:0:TCTGCAAG+GCTGCACT
TGAAGGAGGAGGTTTCTGTTACTGGAAAACCCTCGTCAATACTTTTCAATAATATACGTCTTGAACTC
ACTTATGACGATCTAGTGAAATTAACGCGTCCTTTTCTGGAACGTAGTTTTACTATTATGAACAAAGT
TAT
+
FFFFFFFFFFFFFFFFFFFFFFFFFFFFFFFFFFFFFFFFFFFFFFFFFFFFFFFFFFF:FFFFFFFFF
FFFFFFFFFFF:FFFFFFFFFFFFFFFFFFFFFFFFFFFFFFFFFFFFFFFFFFFFFFFFFFFF:FFFF
FFF
@A00721:190:HGJW7DSXY:4:1353:27679:11036_1:N:0:TCTGCAAG+GCTGCACT
CCCTGATTCCGGCCGGTGGTAGCTCATTTCTACCAGGGGCTATTACTCTTGCTGAAACAGCTATTAAA
GCTGTTGAGCGGGTAATTACACCTAAACGAGCTCGATCTGCCATCGCTGAAGGATGTGCACTCTTTTC
TGC
+
FFFFFFFFFFFFFFFF:FFFFFFFFF::FFFFFF:FFFFFFFFFFFFFFFFFFFFFFFFFF:FFF:FF,
FFFFF::FFFFFFFFFFFFFFFFFFFFFFFFFFFFF,FFFFFFFFFFFFFFFFFFFFFFFF:FFFFFFFF
FFF
@A00721:190:HGJW7DSXY:4:1358:24279:2268_1:N:0:TCTGCAAG+GCTGCACT
CTTCGTCGATTGTATCAACAGTAACATTACTTCTAATAAAGGTCTCTATCAAGCTCGTATTATCGTCG
CTGCTGGATCTGCGTTACCGTTGAAGGCTAAACTCCAAGGTAATTATGAAGACTGTGATTTATCTTTC
ACT
+
FFFFFFFFFFFFFFFFFFFFFF::FFFFFFFFFFFFFFFFFFFFFFFFFFFFFFFFFFFFFFFFFFFF
FFFFFFFFFFFFFFFFFFFFFFFFFFFFFFFFFFFFFFFFFFFFFFFFFFFFFFFFFFFFFFFFFFFF
FFF
@A00721:190:HGJW7DSXY:4:1252:30689:37012_1:N:0:TCTGCAAG+GCTGCACT
ACGTGTGCTCTTCCGATCTATAATTTCAATCTTCTTAAAAAGAAGATAGAGAGTCATACTTTGGGCTA
TCGTTCAGGTGCATGGGGTTGGATAGCCAATATTGAAGTGGACACCTNTGGTAGTATCGAAGTAAAGA
TAA

+
::FFFFFFF:FFF:FFFFFFFFFFFFFFFF:FFFFFFFFFFFFFFFF:FFFFFFFFFFFFFFFFFFFF
F:FFFFFFFFFFFFFFFFFFFFFFFFFFFFFFFFFFFFFFFFFF#FFFFFFFFFFFFFFFFFFFF
FFF
@A00721:190:HGJW7DSXY:4:1136:28013:24612_1:N:0:TCTGCAAG+GCTGCACT
ATACTATGGTTGAATCAACTAAGCATACTACTTTTGCTTTGATTACTCTACCGCCTTACAAGTCTAAG
GTTAAAGAGTCGATTAAACAAGTTGTACTTATCAATGATTTTATCGCTGGTTGTTATCTCGAAAAGTC
TCT
+
FFFFF:FFFFFFFFFFFFFFF,FFFFFFFFFFFFFFFFFFFFF:FFFF:FFFFFFFFFFFFFFFFFFF
FFFFFFFFFFFF:FFFFFFFFFFFFFFFFFFFFFFFFFFFFFFFFFFFFFFFFFFFFFFFFFFFFFF:
FFF
@A00721:190:HGJW7DSXY:4:1204:6325:4085_2:N:0:TCTGCAAG+GCTGCACT
TCAGTTCTAACCCAAAGAGAATATCTAAAATATTTACAAAATCTTACCACTATCTACGAACCTAAAGT
TCTGAAGACGTTGCTTGTCGCTTCCTCCTTTAATGGCACTACCTCGGT
+
FFFFFFFFFFFFFFFFFFFFFFFFFFFFF:FFFFFFFFFFFFFFFFFFFFFFFFFFFFFFFFFFFFFF
FFFFFFFFFFFFFFFFFFFFFFFFFFFFFFFFFFFFFFFFFFFFFFFF
@A00721:190:HGJW7DSXY:4:2247:17119:9909_2:N:0:TCTGCAAG+GCTGCACT
TTGGAACGAAGGCGACCTGTTCCGGAGTTTTTACGGGATCCGGAATGTTCTGTCTATATTAGGTGAAG
CGAAATACGATTTCGAACGTCAAGATCGGAAGAGCGTCGTGTAGGGAA
+
FFFFFFFFFFFFFFFFFFFFFFFFFFFFFFFFFFFFFFFFFFFFFFFFFFFFFFFFFFFFFFFFFFFF
FFF:FFFFFFFFFFFFFFFFFFFFFFFFFFFFFFFFFFF:FFFFFFFF:
@A00721:190:HGJW7DSXY:4:1163:11840:12853_2:N:0:TCTGCAAG+GCTGCACT
ATTTGATCTGGTTGCTCAATTCTAAGCGTGTTCCGCTTAGTGCGAGGCCTCTCTTCTACGAGTATTGC
TATTTATACGGTTACGAGAAGAAGGTTCGAAAGTTTGAGAACTTAGTT
+
FF:FFFFFFFFFFFFFFFFFFFFFFFFFFFFFFFFFFFFFFFFFFFFFFFFFFFFFFFFFFFFFFFFF
FFFFFF:FFFFFFFFFFFFFFFFFFFFFFFFFFFFFFFFFFFFFFFFF
@A00721:190:HGJW7DSXY:4:1262:15293:5400_2:N:0:TCTGCAAG+GCTGCACT
ACATACTAAATACTAGATATTTGGATGATCTTGATAAAGAACTTCATGAATTTTCAGAGGATGAAAAA
TTTTCTTCCTTGGATTTTAATATAAAGAATCTTTACCAGGACTCCTAC
+
FFFFFFFFFFFFFFFFFFFFFFFFFFFFFFFFFFFFFF:FF:FFFFFFFFFFFFFFFFFFFFFF:FF:
FFFFFFFFF:FFFFFFFFF:FFFFFFFFFFFFFFFFFFFFFFFFFFFF
@A00721:190:HGJW7DSXY:4:1170:1561:36761_2:N:0:TCTGCAAG+GCTGCACT
TCTGATCGTAAGAAGTTTATAAGGAACATATCCATTTGTTGTAATAGGGTCATCAACTTAGAACTCGA
AACTCTAAGTGGTAATACTGTCTTTCCAAGCTTACCTGTATCTTCAAT
+
FFFFFFFFFFFFFFFFFFFFFFF:FFFFFFF:FFFFFFFFFF:FFFFFFFFFFFFFFFFFFFFFFFFF
FFFFFFFFFFFFFFFFFFFFFFFFFFFFFFFFFFFFFFFFFFFFFFF:
@A00721:190:HGJW7DSXY:4:1458:31638:1611_1:N:0:TCTGCAAG+GCTGCACT
TTCGATAGGGGACATTTTATCGTGGGCTTTCCGTAAAGGAAGATCTGTTATCGACGGTTTATCGTTTA
CGAAGTATCACTCCACCGGTGAATGGTTTAAGAATACTATAAATAGAATAGCTATTACTACTAATGGC
GTT
+
FFFFFFFFFFFFFFFFFFFFFFFFFFFFFFFFFFFFFFFFFFFFFFFFFFFFFFFFFFFFFFFFFFFF
FFFFFFFFFFFFFFFFFFFFFFFFFFFFFFFFFFFFFFFFFFFFFFFFFFFFFFFFFFFFFFFFFFFF
FFF
@A00721:190:HGJW7DSXY:4:1650:11749:12226_2:N:0:TCTGCAAG+GCTGCACT
TAGTGACATCTGGTCAATTTCGTTTTTAGGTGGTCTTTATTCTATTATTGATTTTTATGAGATTGATA
CGTTAACAGAAGATGATGTTAGAGTAGAATTCCTGAAGAGTCCTTCCG
+

FFFF:FFFFFFFFFFFFFFFFFFFFFFFFFFFFFFFF:FFFFFFFFFFFFFFFFFF:FFFFFFFFFFF
F:FFF,FFFFFFFFFFFFFFF:FFFFFFFFFFFFFFFFFFFF:F:FFF
@A00721:190:HGJW7DSXY:4:1205:16848:4022_1:N:0:TCTGCAAG+GCTGCACT
TTAAATCTAGGATTATGTCTGTGTTGAAAGACTTTCTACCGATAAGGGTCACTAGTCTTACACAATTA
AGAAATAATCCTACATTGACTGATTATCTAAAGATTGAAGGAAAATTTTATCCTCGTTTAACTTACTT
AGA
+
FFFFFFFFFFFFFFFFFFFFFFFFFFFFFFFFFFFFFFFFFFFFFFFFFFFFFFFFFFFFFFFFFFFF
FFFFFFFFFFFFFFFFFFFFFFFFFFF:FFFFFFFFFFFFFFFFFFFFFFFFFFFFFFFFFFFF:FFFFF
FFF
@A00721:190:HGJW7DSXY:4:1414:3676:18630_1:N:0:TCTGCAAG+GCTGCACT
ATACAAATGATAACTTCTAATTTACGTTTGCTACTCAGAGCTGGTCCTCTCAGCAAAGGGATGCATGG
TGTTATCTCCTCAGGAGTCCCGGCATTTGATAATAATGACGAGACTTTCGTCATAATCGCTGCATATT
ACG
+
FFFFFFFFFFFFFFFFFFFFFFFFFFFFFFFF,FFFFFFFFFFFFFFFFFFFFFFFFFFF:FFF:FFFF
FFFFFFFFFFFFF:FFFFFFFF:FFFFFFFFFFFFFFFFFFFFFFFFFFFFFFFFFFFFFFFFFFFFF
:FF
@A00721:190:HGJW7DSXY:4:1342:12979:20494_2:N:0:TCTGCAAG+GCTGCACT
CCAGCGCATCTCGTGTCGAGCCTCGACCCGATTATATGGTATTAACTTATAAAGGTGAGGAGACCATT
TTCTTGCTGAAATTTATAGAAGATTATTTTGACAGAATCAATAAGAAT
+
FFFFFFFFFFFFFFFF:FFFFFFFFFFFFFFFFFFFFFFFFFFFFFFFFFFF:F:FFFFFFFFFFFFFF
FFFFFFFFFFFFFFFFFFFFFFFFFFFFFFFFFFFFFFFFFFFFFFFF
@A00721:190:HGJW7DSXY:4:1538:11243:24721_1:N:0:TCTGCAAG+GCTGCACT
TATGCTGATCTAAAATTTCGATCTAGATGGATCCACTCCTCGAGTTATCATGATGTTAGTATGTTATA
TACTGATTTACAACGATATAGGAGTAATCTACGAGAAGATTCTAGAATTCTTAATTATAATATTCAAC
CTC
+
FFFF:FFFFFFFFFFFFFFFFFFFFFFFFFFFFFFFFF,F::FFFFFFFFF:FFFFFFFFFFFFFFF
FFFFFFF:FFFFFFFFFFFFFFFFFFFFF,FFFFF:FFFFFFFFFFFFFFFFFFFFFFFFFFFFFFFFF
FFF
@A00721:190:HGJW7DSXY:4:1363:4345:4914_2:N:0:TCTGCAAG+GCTGCACT
GAGATCACACATTTTGAGAACTAACTATGTTATGTTCTCTGATCATTTTGACAATCTCTTCTTATAAT
AAATAAATAAATAATATTACTAATGGCAAATAGTGGAAAGGCTCCAGT
+
FFFFFFFFFFFFFFFFFF:FF:FFFFFFFFFFFFFFFFFFFFFFFFFFFF:FFFFFFFFFFFFFFFFFF
FFFFFFFFFFFFFFFFF:FFFFFFFFFFF:FFFFFFFF:FFFFFFFFF
@A00721:190:HGJW7DSXY:4:2407:8052:17910_2:N:0:TCTGCAAG+GCTGCACT
CTGCTTTGGATTACGATGACGTGAGAATAGGTGAAGGGATAGTTTTTACCTCGACCAACATTAAGGCG
GCGGCGACTAATCTTAAAGCTTCACCCAAAATGAAGGACACTTCGAAA
+
FFFFFFFFFFFFFFFFFFFFFFFFFFFFFFFFFFFFFFFFFFFFFFFFFFFFFFFFFFFFFFFFFFFF
FFFFFFFFFFFFFFFFFFFFFFFFFFFFFFFFFFFFFFFFFFFFFFFF
@A00721:190:HGJW7DSXY:4:2146:15872:25755_2:N:0:TCTGCAAG+GCTGCACT
AAAAGGCTCTAAAGGACACTATACAATATCCAGACGACGACGAAGCTTATACTACTTTAATTTTGGGT
ATTATGTGGAGATATCGTGATATCAGTACGTCCAGATCCTCTGCTTTT
+
FFFFFFFFFFFFFFFFF:FFFFFFFFFFFFFFFFFFFFFFFFFFFFFFFFFFFFFFFFFFFF::FFFF:
FF:FFFFFFFFFF:FFFFFFFFFFFFFFFFFFFFFFFFFFFFFFFFFFFF
@A00721:190:HGJW7DSXY:4:2430:2139:10833_2:N:0:TCTGCAAG+GCTGCACT
ATCAAATATAAAGAAAAGGACTATGAAATAAAAGATTTGGTGCTTAGGGATATAATGAATTCAGCACC
ACATTTTGGTCAAGTCAATCCGTTGAGAAAATTTCTTAGAGGATATTC
+

F:FFFFFFFFFFFFFFFFFFFFFFFFFFFFFFFFFFFFFFFFFFFFFFFFFFFFFFFFFFFFFFFFFF
FFFFFFFFFFFFFFFFF:FFFF:FFFFFFFFFFFFFFFFFFFFFFFFFF
@A00721:190:HGJW7DSXY:4:2118:32922:11162_1:N:0:TCTGCAAG+GCTGCACT
CCAACATTTACCACCAAATTCTTTTGATACAACTCTAGCCTTCAAGTGGGGTATGCCTGCGGAATACG
CTTGCTATTCACCAGATTGGTTCGAACCCGTTGATGCTATGTCAGAAGAAGCTATTCAAGCTTGTCGT
TTA
+
FFFFFFFFFFFFFFFFFFFFFFFFFFFFFFFFFFFFFFFFFFFFFFFFFFFFFFFFFFFFFFFFFFFF
FFFFFFFFFFFFFFFFFFFFFFFFFFFFFFF:FFFFFFFFFFFFFFFFFFFFFFFFFFFFFFFFFFF:
FFF
@A00721:190:HGJW7DSXY:4:1162:29469:16266_2:N:0:TCTGCAAG+GCTGCACT
AAACAAGCCCAGACTCAAGGAGTCCAATCAAAGGTTTTGAATTTAGCTCAACTGGGGAGACGTGCTTA
AATGGGTGATAAAAATTATTTAGAGATCTTTTCTACTTACAAAGGACA
+
FF:FFFFFFFFF:FFFFFFFFFFFFFFFF:F:FF:FFFFFFF:FFFFFFF:FFFFFFFFFFFFFFFFF
F,FFFFFFFFFFFF,FFFFFFFFFFFF:FFFFFFFFFFFFFF:FFFFF
@A00721:190:HGJW7DSXY:4:2131:23321:11162_2:N:0:TCTGCAAG+GCTGCACT
AATTCTACGGAAAAGTCCGGAGTATATTGTATGTTAATAGATAGTGGGATAATAGTTCTACCGATACG
TAGTGAACGCGTTCGTAGAACCAAGTACTTTCCGTCTGAAGAGTTTAG
+
FFFFFFFFFFFFFFFFFFFFFFFFFFFFFFFFFFFFFFFFFFFFFFFFFFFFFFFFFFFFFFFFFFFF
FFFFFFFFFFFFFFFFFFFFFFFFFFFFFFFFFFFFFFFFFFFFFFFF
@A00721:190:HGJW7DSXY:4:2161:18394:1063_1:N:0:TCTGCAAG+GCTGCACT
TAACCGATAGGAGTTGTATAGTTGAAGATGTGGCTTTAGACGATAATCCTTTAAGGACTATGTTGACT
GACGAAGATTTTCCCACAGATTTGTCTACGTTGCAGATCGCCACTCCAAACACTATATACATTAAAAT
ATA
+
FFFFFFFFFFFFFFFFFFFFFFFFFFFFFFFFFFFFFFFFFFFFFFFFFFFFFFFFFFFFFFFFFFFF
FFFFFFFFFFFFFFFFFFFFFFFFFFFFFFFFFFFFFFFFFFFFFFFFFFFFFFFFFFFFF:FFFFFF
FFF
@A00721:190:HGJW7DSXY:4:2278:19144:6496_2:N:0:TCTGCAAG+GCTGCACT
AGTCATAACTTTTGTTACCAAATTCTTGGCTTTCAACGAAGTCTCTGATTTTATTAATCATTACTTTA
ACGATCTCTCGGTCATTTACTCTGTACTTGATCTACGTCGTTTAACTG
+
FFFFFFFFFFFFFFFFFFFFFFFFFFFFFFFFFFFFFFFFFFFFFFFFFFFFFFFFFFFFFFFFFFFF
FFFFF:FFFFFFFFFFFF:FFFFFFFFF:FFF:FFFFFFFFFFFFFF
@A00721:190:HGJW7DSXY:4:2103:1181:6136_2:N:0:TCTGCAAG+GCTGCACT
CTTAGAATGACTCTATCTTTTCAGACACTTCTTATATTATTTACAATAGTTAATATCATATTAGTAAT
ACTCACTATCGTTTTCTGCTTTTTCTTAGCTAATAGATTAATAAAGGA
+
F:FFFFFFFFFFFFFFF:F::FFFFFFFF,F:FFFFFFFFF:FFFFFFF:FFF:FFFFFFFFFFFFFF
FF,FF,FFFFFF:FFFF:F,FFFFFFFF,,FFFFF,F:FFFFFFFF,F
@A00721:190:HGJW7DSXY:4:2314:29252:8187_2:N:0:TCTGCAAG+GCTGCACT
ACCAACTCTTCGTTACGAGACCTGATGGTAGAATAGATTTAGTCTCTACACCCATCTCAACGAGGATA
AAGCGAGTTTTCCGTCCTGAACGTAATACACGAAGAGATCATCCGCAA
+
FFFFFFFFFFFFFFFFFFFFFFFFFFFFFFFFFFFFFFFFFFFFFFFFFFFFFFFFFFFFFFFFFFFF
FFFFFFFFFFFFFFFFFFFFFFFFFF:FFFFFFF:FFFFFFFFFFFFFF
@A00721:190:HGJW7DSXY:4:2136:13856:26083_2:N:0:TCTGCAAG+GCTGCACT
AAAGTCCCTTCTAACTTTAGATTATGAATCCTGCCTTTACTCATTTTATAGCAGTTATTTATGTTGTA
CTTTTTATAATTTATAACCTTCTTATACTTTTGGGTATGTTTTGTGAA
+
FFFFFFFFFFFFFFF,FFFFFFFFFFFFFFFFFFFFFFFFFFFFFFFFFFFFFFFFFFF:FFFFFFFF
FFFFFFFFFFFFFFFFFFFFFFFFFFFFFFF:FFFFFFFFFF:F,FFFF

```
@A00721:190:HGJW7DSXY:4:2511:31485:29966_2:N:0:TCTGCAAG+GCTGCACT
GAAGACATTATCTCTTATGTACTTATTGTAACGATCATTCTTTCGAGTTTTGTCATTCTTATTCTTTT
ATTCCTTTAACTGTGTATGAGGAGGATTTATAATGCTTTTCGATAGTG
+
FFFFFFFFFFFFFFFFFFFFFFFFFFFFFFF:FFFFFFFFFFFFFFFFFFFFFFFF:FFFFFFFFFF
FFFF,FFFF::FFFFFFFFF,FFFFFFFFFFFFF,:FFFF,FFFFFFF
@A00721:190:HGJW7DSXY:4:1323:18728:18364_2:N:0:TCTGCAAG+GCTGCACT
CTTTACTCACAGACTCCGAAAATGGTTTCAAATGAGGTCAAAGCTCTATTTATTAAACGAACTATTAT
CCGATTCTTCGAGGGTATTACTACCACTGATCTGCCGACTTTAGTCAA
+
FFFFFFFFFFFFFFFFFFFFFFFFFFFFFFFFFFFFFFFFFFFFFFFFFFFFFFFFFFFFFFFFFFFF
FFFFFFFFFFFFFFFFFFFFFFFFFFFFFFFFFFFFFFFFFFFF:FFF
@A00721:190:HGJW7DSXY:4:1342:20844:29105_2:N:0:TCTGCAAG+GCTGCACT
TTCCTTCGATGTTTATATTTTTCTATAAGGGAAAATAGCAATAATCAAATTTCGACAAATTTTAGAAC
TTTATTTCAGAACTCTCTCCGATCACCTTGGTGTGATCTCCCGGAGGA
+
FFFFFFFFFFFFFFFFFFFFFFFFFFFFFFFFFFFFFFFFFFFFFFFFFFFFFF:FFFFFFFFFFFFF
FFF:FFFFFFFFFFFFFFFFFFFFFFFFFFFFFFFFFFFFFFFFFFFF
@A00721:190:HGJW7DSXY:4:1146:26323:30859_2:N:0:TCTGCAAG+GCTGCACT
ATGTATGATGATAATCTACTATTTTTAAATAGTGATATTCAGCGTATACTTGAATACTTTATCAAAGA
TTTGGCTTGGTCTATTTACCGCAAAAAGTTGGAAGAGGTCTATGATAT
+
FFFFFFFFFFFFFFFFFFFFFFFFFFFFFFFFFFFFFFFFFFFFFFFFFFFFFFFFFFFFFFFFFFFF
FFFF:FFFFFFFFFFFFFFFFFFFFFFFFFFFFFFFFFFFFFFFFFFF
@A00721:190:HGJW7DSXY:4:1254:19560:14794_2:N:0:TCTGCAAG+GCTGCACT
TATACTTTAAATAGCACCGATTATAACTTGACTGTTGATAATATTACAATAGCTCTTTCTCAGAAGAT
TAGAGAAGTAACCGGTGAACACGTCGGTAGCATTGATTTACTTTTACC
+
FFFFFFFFFFFFFFFFFFFFFFFFFFFFFFFFFFFFFFFFFFFFFFFFFFFFFFFFFFFFFFFFFF:F
FFFFFFFFFFFFFFFFFFFFFFFFFFFF:FFFFFFFFFFFFFFFFFFF
@A00721:190:HGJW7DSXY:4:1220:15293:9690_2:N:0:TCTGCAAG+GCTGCACT
TTTCGAAAAGAAACCTATAAGTTACAAACGATTCTTCGACTATATTAAAGAAATAAAAATACTTCAAG
TACCTGAACTTTACATTTTTAATGGTATGGTATAATGTGTCTTACAAA
+
FF,FFFFFFFFFF:FFFFFFFFFFFFFFFFFFFFFFFFFFFFFFFFFF:FFFFFFFFFFFFFFF:FFF
FFFFFFF:FFFFFFFFFFFFFFFFFF,FFFFFFFFFFFFFFFFFFFFF
@A00721:190:HGJW7DSXY:4:1148:20880:22529_2:N:0:TCTGCAAG+GCTGCACT
CTCTCGGTAATGAGAGTGTATTCCTTAATATTCTAGTAACCGCTATGTGGGCATTACTAGGATTTATC
ACACTCCTAAATTTGATAGCAATTTATGTTTTAAATGCTAGGAAAATT
+
FFFFFFFFFFFFFFFFFFFFFFFFFFFFFFFFFFFFFFFFFFFFFFFFFFFFFFFFFFFFFFFFFFFF
FFFFFFFFFFF:FFFFFFFFFFFFFFFFF:FFFFFFFFFFFFFFFFFF
@A00721:190:HGJW7DSXY:4:1501:11315:27414_2:N:0:TCTGCAAG+GCTGCACT
TTGTATAGTTCAAGGAGTACTTAATTCAATAGCTTTAGTATTATTTACTATCGCCTTTATTGAAAATT
GTATAAGAGTTAATTGCTTACACAAAAGTGACGCGCGAAGGAATCTTA
+
FFFFFFFFFFFFFFFFFFFFFFFFFFFFFFFFFFFFFFFFFFF:FFFFFFFFFF:,FF,FFFFFFFFF
FFFFFFFFFFFFFFFFFFFFFFFFFFFFFFFFFFFFFFFFFF,FFFFF
@A00721:190:HGJW7DSXY:4:1406:30770:35931_1:N:0:TCTGCAAG+GCTGCACT
GGGGAGAATGAACTGCCGCTTCCCGGAAGAAACGATCTATTTACAGAGTATTTAGAGTAGTCCAAAGT
TTGTATAAATTTATATGATTTTCTATATATATACCACATATATATATATATAAAATCCTATAAATTTG
TAC
+
FF,:F:FFFFFFFFFFFFFFFFFFFFFF,FFFFFFFFFFFFFFFFFFFFFFFFFFFF:FFFFFFFFFFF
```

FF::FFFFF:FFFFFFFFFF::,FFFFFFFFFFFFFFF:FFFFFFFFFFFFFFFFFFFFFFFFFFFFF
FF:
@A00721:190:HGJW7DSXY:4:1612:5240:11694_2:N:0:TCTGCAAG+GCTGCACT
CCGCAAATCAATTGAAGAAAACCACTCCCTTCAATTCAATAACATAACATACCATACACTCCCCAAAC
ATAACATACCATAACCTACCATGACCAATCATCGCTCCAACTGGCACG
+
FFFFFFFFFFFFFFFFF:FF:FF,FFFFFFFFF:FFFFFFFFFFFFFFFFFFFFFFFFFFFFFFFFFF
FFFFFFFFFFF:FFFFF:FFFFFFFFFFFFFFFFFFFFFFFFFFFFFF
@A00721:190:HGJW7DSXY:4:1149:13838:9392_1:N:0:TCTGCAAG+GCTGCACT
CTACTACCGCCGGTCGGCAGATCGGCACCACTGGTCTCTACGTTCAGGGACCCGCTGTGCAGAATAAC
CTTCCCAAATCTAGATCTGCACAGAAGAAGGCTGAAGGTCGTTACCGGACGACCACTCAAAGGAAACG
GGC
+
FFF,F:F:FFFF:F:FFF,FFF:FF,F,F:FF:FFFF,FFFFFFFFFFFFF,FFFFFFFFF,FFFF,F
FF:FF,F,FFF,FFFF:FFFF,,FFF:F,FFFFF,FF,F:FFFFFFFFFFFF:::FF,:FF:FFFFFF
,FF
@A00721:190:HGJW7DSXY:4:1322:19388:27712_1:N:0:TCTGCAAG+GCTGCACT
GAGGTCGCTCGTAACATTGACGTCAGTCCCGTCATCTCTGTGGGAACTGAGGCAGTACCACAGTCAAA
AGTCACATTCACCACTACCATACCAATCAATGATGTGACTATACAGTTCGGCCATTTCAAGAGTGGTC
AAT
+
F::,F,FFFF,F,F,FFF,FF:,:,:F:,FFF:F,,F,FFF,F,F,,:FF:F:,,F,F,,,FFFFF,,
FFFFFFFF::FFF,FF,,:FFF,:,FF:::F,FF,F:F:FFF::,FFF,,:,:,FFFFFF:F:FFFFF
FFF
@A00721:190:HGJW7DSXY:4:1633:31819:1110_1:N:0:TCTGCAAG+GCTGCACT
GTACTGAAGCCTTCCCCTCCGCTGGCACGAAGAACACTCCCAAGCCAATCAATAGTTCTTCTGACCAG
AATGAAAAGGGGTTTACCAGGGTAGTCTCTAAGTCTACCAGGAAGAGGGGAAATGTCCCCAAAACTGA
GAA
+
FFFFFFFFFFFFFFFFFFFFFFFFFFFFF:FFFFFFFFFFFFFFF:FFFFFFFFFFFFFFFFFFFFFFF
FFFFFFFFFFFFFFFFFFFFF:FFFFFFFFF:FFFFFFFF:FFFFFFFFFFFFFFFFFFFFFFFFFFFF
FFF
@A00721:190:HGJW7DSXY:4:1326:20537:2675_2:N:0:TCTGCAAG+GCTGCACT
AGGTCCATCAGGACCTAAGAAGGGGAAATTACCCCTAATTCTGAAGGAAGATCTCCCACCATCTGGGG
TTGGGAATAAGAAATCTGCACGGGCCAAAGAGGCCGCTGCTACGAAGT
+
FFFFFFFFFFFFFFFFFFFFFFFFFFFFFFFFFFFFFFFFFFFFFFFFFFFFFFFFFFFFFFF:FFFF
,FFFFFFFFFFFFFFFFFFFFFFFFFFFFFFFFFFFFFFFFFFFFFFF
@A00721:190:HGJW7DSXY:4:1343:30129:25113_1:N:0:TCTGCAAG+GCTGCACT
GCTTGGGTCATTAAGGGTAGCTGTAAAGTTGCGCCTGCCGACCCCATCTTTGAGACCAAGACTCTCTA
CAGTAGGTTCGTGCCTACTATGAGCAAATCTGTTGAATTCGTCACTGAGGACGAAGGTGAGAGGACTT
TTG
+
FFFFFFFFFFFFFFFFFFFFFFFFFFFFFFFFFFFFFFFFFFFFFF:FFFFFFFFFFFFFFFFFFFFF
FFFFFFFFFFFFFFFFFFFFFFFFFFFFFFFFFFFFFFFFFFFFFFFFFFFFFFFFFFFFFFFFFFFF
FFF
@A00721:190:HGJW7DSXY:4:1408:30987:9533_1:N:0:TCTGCAAG+GCTGCACT
CAAACTGAGAGCAGTCGCTGGGGAAAGAGAATTGCGTGGGTTGAAGGCCGACGCTTCGAGCTTGAGTG
TGACGAGGCCGGTGACGTTTTTGTCAACGGCGTTTGGAGGATTTTATCCGCGCTTAACTATGGGGCGA
AGA
+
F:,FFFFFFFFFFF:FFFF,FFFFFFFFFF,F:FFFFFFFFFFFFFFFFF:FFFFFFFFFFFFFFFF
FFFFFFFF:FFFFFFFFFFFFFFFFFFFFFFFFFFFFFFFFFFFFFFF:FF:FFFFFFFFFFFFFFFFFFF
FFF

@A00721:190:HGJW7DSXY:4:1410:2953:11898_1:N:0:TCTGCAAG+GCTGCACT
AGTCTTGAAGGACTTAGGTTGGCATATTTATGCCGACGATAATAAGCATGTCACCCTTAAGCACCGAG
GTAAGCTCGGGACTTACAAGAGAGAGTGTGAGAGAGTCGCTCTCACTATTTTTGCCCAGAATGCGGGC
TTT
+
FFFFFFFFFFFFFFF:FFFFFFFFFFF,F,FFFFFFFFFFFFFF::FFFFFFFFFFFFFF,FFFFFFFFF
FFF:FFFFFFFFFFFFFFFFFFFFFFFFFFFFFFFFFFFFFFFFFFFFFFFFFFFFFFFFFFFFFFFFFF
FFF
@A00721:190:HGJW7DSXY:4:1314:23972:25473_2:N:0:TCTGCAAG+GCTGCACT
GCTCCTTCTGTGGGTGAGGGTTTTTGTTATATTCACTGGTTCTGGGAGGCTGCTTTCCTCCATAATTA
CAACCTCTCTTTAGAGAGCATTAGAAAGATCGGAAGAGCGTCGTGTAG
+
FFFFFFFFFFFFFFFFFFFFFFFFFFFFFFFFFFFFFFFF:FF:FFFFFFFFFFFFFFF:FFFFFFFFFF
FFFFFFF:FFFFFFF:FFFFFFFFFFFFFFFFFFFFF,FF:FFFFFF:
@A00721:190:HGJW7DSXY:4:2466:5620:33082_2:N:0:TCTGCAAG+GCTGCACT
GTTGTCACACTTTTCTTGAAAATTTTTCCTTTCCTCCGTCATTGGGGATTTCGATGTGAGAAGGTGGA
AGGTGTCAAGGGTCTTCTTCATGCTGATCTTTCAGGACCTAATCACAT
+
FFFFFFF:FFFFFFFFF,FFFFFFFFF:,FFFFFFFFF,FFFFFF,F,:FFFFFFFF:F:FFF:F:FF:F
:FFF:FFFF::FFFFFFFF:FFFFFFFF,FFFFFF,F::FFFFFF:FF
@A00721:190:HGJW7DSXY:4:1244:14461:29324_1:N:0:TCTGCAAG+GCTGCACT
ACGGCTCGTATCGAGTTGGAGGGAGGAAGTTCTCCGAAATTAAAGACGGGGGGATTAAGAGTCCAGCT
GTAACTGTCATTTCAGAGAGGGGAGCTCTGAAAAGGAAACTTCGTCAGAAGATGAGGAAGTCTAAGAG
GAG
+
:FFFFFFFFFFFFFFFFFFFFFFFFFFFFFFFFFFFFFFFFFFFFFFFFFFFFFFFFFFFFFFFFFFFFF
FFFFFFFFFFFFFFFFFFFFFFFFFFFFFFFFFFFFFFFFFFFFFFFFFFFFFFFFFFFFFFFFFFFFFF
FFF
@A00721:190:HGJW7DSXY:4:1316:17861:34084_1:N:0:TCTGCAAG+GCTGCACT
AAGGCTCTAGAGAAGATCTCCCCACCTGGAGAAGGCAAGTGGAGAGTCCAGAAGTTGATGAAGAAGGA
TTATTTGAAGCAGGGGTTACCACTTCCTAAGGTGGATGAGGGAGGTTGTTTCTTTGGAGTTTTAGATA
ATC
+
FFFFFFFFFFFFFFFFFFFF,FFFFFFFFFFFFFFFFFFFFFFFFFFFFFFFF:FFFFFFFFFFFFFFFF
FFFFFFFFFFFFFFFFF:FFFFFFFFFFFFFFFFFFFFFFFFFFFFFF:FFFFFFFFFFFFFFFFFFF:F
FFF
@A00721:190:HGJW7DSXY:4:1671:23005:3317_2:N:0:TCTGCAAG+GCTGCACT
GGTCATAGAGCCAGCAAGAGAAGCTTATATGCTGGTTTAAGTGATGATTATCACTCCTTGAGAAGACA
AGCCACATTAAGATATAATTTAGGTCAACTTGGGGTTTCGAATGTCAT
+
FFFFFFFF:FFFFFFFFFFFFFFFFFFFFFFFFFFFFFFFFFFF:FFFFFFFFFFFFFFFFFFFFFFFFF
FFFFFFFFFFFFFFFFFFFFFFFFFFFFFFFFFFFFFFFFFFFFFFFF
@A00721:190:HGJW7DSXY:4:1106:7771:5024_2:N:0:TCTGCAAG+GCTGCACT
AGGTCCTTTTGAGTATCGACAGATCTTTCGATCTTGAGGACGTACATTTAACGTGGCAGAATTCGGTA
ACTTACCATATTGTTCCTTCCGGGAAGTTGTGGGATTTAACTTTGACT
+
FFFFFFFFFFFFFFFFFFFFFFFFFFFFFFFFFFFFFFFFFFFFFFFFFFFFFFFFFFFFFFFFFFFFFF
FFFFFFFFFFFFFFFFFFFFFFFFF,FFFFFFFFFFFFFFFFFFFFFF
@A00721:190:HGJW7DSXY:4:1338:19859:1564_2:N:0:TCTGCAAG+GCTGCACT
GGTATGGACTACTCGACTTTTGATCTGGAGTTGTCTAGGGTCATGGCAGATACTACCGATAAGGTGAA
AAATATGCAGGTCAAAGATTCTGTAGCTCTGAGGGCGGTTGAGGGAGC
+
FFFFFFFFF:FFFFFFFFFFFFFF:FFFFFFFFFFF,FFFFFFFFFFFF:FFFFFFFFFFFF,FFFFFFF
FFF:FFFFFFFF::FFFFFFFFFFFF:FFFFFFFF,FFFFFFFFFFFF

@A00721:190:HGJW7DSXY:4:1407:11704:21136_1:N:0:TCTGCAAG+GCTGCACT
TTCTAAGAGTCTTTATGATTCAAAACCTATCGTTGTCGTACCTTACAAGGTTGAGGAGACTTTACAGC
GGCTTATTGTCGCGCAGTTTCCAGAATTGCGGTTGAAGTTCACCCATTCTATCTTGAATGCTCACGCC
GTC
+
FFFFFFFFFFFFFFFFFFFFFFFFFFFFFFFFFFFFFF:FFFFFFFFFFFFFFFFFFFFFFFFFFFFF
FFFFFFFFFFFFFFFFFFFFFFFFFFFFFFFFFFFFFFFFFFFFFFFFFFFFFFFFFFFFFFFFFFFF
FFF
@A00721:190:HGJW7DSXY:4:1420:15121:13182_2:N:0:TCTGCAAG+GCTGCACT
TTATAATCACTTGTTAAAGTACAGATTTAGTGATTTAGATATTATTGATATTGGAGGCGATATTCGTT
TTCATATCCAAAACTCCTTGAATGGAGTACATATTTGCAACCCTATTC
+
FFFFFFFFFFFFFFFFFFFFFFFFFFFF:FFFF:FFFFFFFFFFFFFFFFFFFFFFF:FFFFFF:FFF
FFFFFFFFFFFFFFFFFFFFFFFFFFFFFFFFFFFFFFFFFF:FFFFF
@A00721:190:HGJW7DSXY:4:1343:8223:12320_1:N:0:TCTGCAAG+GCTGCACT
GATGCATCGAGGTATACGTCTAGGAACATTGATTGGGAACGTACCTACTCACGAGACGTCATGAAAGC
TATCGGAGGGAAGCAAAATGTTTCGTTCTGTTACAAGAACGCTTTGGATTGTGACCATAAAGCCTCAG
TTG
+
FFFFFFFFFFFFFFFFFFFFFFFFFFFFFFFFFFFFFFFFFFFFFF:FFFFFFFFFFFFFFFFFFFFF
:FFFFFFFFFFF:FFFFFFFFFFFFFFFFFFFFFFFFFFFFFFFFFFFFFFFFFFFFF::FFFFFFFF
FFF
@A00721:190:HGJW7DSXY:4:2524:19361:35837_2:N:0:TCTGCAAG+GCTGCACT
ACGATATCTCTCTTGATGATGTAGCCACTATAATGCTTAAGAGAGAGATTGATGTATGTCATTTCGTC
TTAGTTTGTCCCGGCGAATTATTGGACACGATGAAAAATGACATACCC
+
FFFFFFFFFFFFFFFFFFFFFFFFFFFFFFFFFFFFFFFFFFFFFFFFFFFFFFFFFFFFFFFFFFFF
FFFFFFFFFFFFFFFFFFFFFFFFFFFFFFFFFFFFFFFFFFFFFFFF
@A00721:190:HGJW7DSXY:4:2467:16776:32863_1:N:0:TCTGCAAG+GCTGCACT
TCGTTATCCGTCATGGAGATGATGGCGCATCACTTGATTACTATATGCCCGAAGGATTGGGTTATTCT
CACAACAAAGATAAGATTTTATCTTTCTTCAAACACCCTGTTTTCCTTAAGGGCGGACAATTGTTCCA
TTG
+
FFFFFFFFFFFFFF:FFF:FFFFFFFFFFFFFFFFFFFFFFF,FFFFFFF:FFFF:FFFFFFF:FFFF
FF:F:FFFFFFFFFFFFFFFFFFFFFFFFFF:FF,FFFFFFFFFFFFFFFFFFFFFFFFFFFFFFFFF
FFF
@A00721:190:HGJW7DSXY:4:1140:11433:12242_2:N:0:TCTGCAAG+GCTGCACT
GCATTAATGAGTTCCGAATAACTCGTAATAAATCTTTCTCTAGTGGACAATTCAGAATACCTATTTCC
GTACCAAGAGCCACTGCCAATCTCATCGCTATACGCATACCTGATTGG
+
FFFFFFFFFFFFFF:FFFFFFFFFFFFFFFFFFFFFF:FFFF:F:FF,:FFFFF:F:FFF,FFF:F:F
F:FFFFF,FF:FFFF::FFF:F:FF,F,FF:FFFF:FFF:FF,F::,F
@A00721:190:HGJW7DSXY:4:1115:10872:33473_2:N:0:TCTGCAAG+GCTGCACT
AAATTTTTCTCAAGAGATTATGGTTGATAAAGTTTTCTTCATAAGGGCTATTCAGTACGTCATGGGGA
TCACTTCTTCGGTCAATGACAAAACCTTCGAATATGTTACGACATGGC
+
FFFFFFFFFFFFFFFFFFFFFFFF:FFFFFFFFFFFFFFFF:FFFFFFFFFFFFFFFFFFFFFFFFFF
:FFFFFFFFF:FFFFFFFFFFFFFFFF:FFFFFFFFFFFFFFFFFF,F
@A00721:190:HGJW7DSXY:4:1672:25021:11663_2:N:0:TCTGCAAG+GCTGCACT
AGGATTGTCATCTCGGGGAAAGTGATTCACCAAGATGTTCATATAGATACGAGATACCTAGAGAAGGT
CGCAGCGATCTTACTCGTTATCGGAGCTAAGAAGAAGACTTTGGGTAC
+
FFFFFFFFFFFFFFFFFFFFFFFFF:FFFFF:FFFFFF:FFFFFFFFFFFFFFFFFFFFFFFFFFFFF
FFFFFFFFFFFFFFFFFFFFFFFFFFFFFFFFFFFFFFFFF:FFFFFF

```
@A00721:190:HGJW7DSXY:4:1252:2121:5854_2:N:0:TCTGCAAG+GCTGCACT
TAACGTTAGTACCGGAGGTGATCTCTCTGCTTCTGCGTTACTGAGAGAAAAGATTCAGGACGCCTACG
GTGACTTTATGGACAAGATGCAAGTTCTTGGGAAATACTTGTTTCCCT
+
FFFFFFFFFFFFFFFFFFFFFFFFFFFFFFFFFFFFFFFFFFFFFFFFFFFFFFFFFFFFFFFFFFFF
FFFFFFFFFFFFFFFFFFFFFFFFFFFFFFFFFFFFFFFFFFFFFFFF
@A00721:190:HGJW7DSXY:4:2474:14705:4351_2:N:0:TCTGCAAG+GCTGCACT
TCGATTCTTTGTATAGGGAGTGTGAGAAGACAACGAATCTTGAAACTACGGTCCTTATCAAAGAAAAT
GGTGAACCGGTCTCTAAATCTTTTGAGATTGAGAAGATTGAAGAACAC
+
FFFFFFFFFFF,:FFFFFFFFFFFFFFFFFFFFFFFFFFFFFFFFFFFFFFFFFFFFFFFFFFFFFFF
FFFFFFFFFFFFFFFFFFFFFFFFFFFFFFFFFFFFFFFFFFFFFFFF
@A00721:190:HGJW7DSXY:4:1638:32994:8469_2:N:0:TCTGCAAG+GCTGCACT
GCTCGGCGTTGTTCTTGAAAACCAAGTTAAGAAGGAGGCTGATCATGGAGTCTTTGTACCTTCTGTCA
AACTTCGAGGTTCAGCTACGAGAGGTGATCCCGGATGTGGATTGAAGG
+
FFFFFFFFFFFFFFFFFFFFFFFFFFFFF,FFFFFFFFFFFFFFFFFFFF:FFFFFFFFFFFFFFFFF
:FFFFFFFFFFFFFFFFFFFFFFFFFFFFFFFFFFFFFFFFFFFF:FFF
@A00721:190:HGJW7DSXY:4:2465:28402:21715_2:N:0:TCTGCAAG+GCTGCACT
GGAGACTCCTTCTTTTTGAATGTTGTAAAGAATTGGGTTGAGAAGTGTGCTTTACAAGGAGTTAGACT
CTTCTTCAAGTTTTTAGATCTTCTTGATAATCTTAAAGAAAAATTTTC
+
FFFFFFFFFFFFFFFFFFFFFFFFFFFFFFFFFFFFFFFFFFFFFFFFFFFFFFFFF:FFFFFFFFFF
FFFFFFFFFFFFFFFFFFFF:FFFFFFFFFFFFFFFFF,FFFFFFFFF,
@A00721:190:HGJW7DSXY:4:1162:29921:13197_1:N:0:TCTGCAAG+GCTGCACT
GTCTGTACTTACATTCTTTACGCCAGATTCTTCATGGGGCGACGCTGTTTCTCTTGGTATTTCTGAAA
TAGTTTATCAGAGCGTCATTTTCCTTTGGGATCTTTCCTCTGGTAAGGGAATTATTAAATCATTCTTC
TCA
+
FFFFFFFFFFFFFFF:FFFFFFFFFFFFFFFFFFFFFFFFFFFFFFFFFFFFFFFFFFFFFFFFFFFF
FFFFFFFFFFFFFFFFFFFFFFFFFFFFFFFFFFFFFFFFFFFFFFFFFFFFFFFFF:FFFFFFFFFF
FFF
@A00721:190:HGJW7DSXY:4:1647:19669:33896_2:N:0:TCTGCAAG+GCTGCACT
ATTATTACTGTTTTCTCTACCCATAACTTCTTTTTCAAAAATGCGGATGGGTCTACTGATGTTTTCCT
ACAGAGTGCAGTCGTTTCAACACTTATCACCACTTTTGAAAGGAAAAT
+
FFFFFFFFFFFFFFFFFFFFFFFFFFFFFFFFFFFFFFFFFFFFFFFFFFFFFFFFFFFFFFFFFFFF
FFF:FFFFFFFFFFF:F,FFFFF,FFFFFFFFFF:FFF:FF,FFFFFF
@A00721:190:HGJW7DSXY:4:1326:16821:10989_2:N:0:TCTGCAAG+GCTGCACT
AATACGTATGTCGGTGGGGTTCTCAAGGCTCTCATCGCCACTGGGATTCTAAGGGGTTCCATAAGATA
TTATCTGACATTGTGGTTCAATGATCAGGATTCTTCTTTTGGTCCTTA
+
FFFFFFFFFFFFFFFFFFFFFFFFFFFFFFFFFFFFFFFFFFFFFFFFFFFFFFFFFFFFFFFFFFFF
FFFFFFFFFFFFFFFFFFFFF:FFFFFFFFFFFFFFFFFFFFFFFFFF
@A00721:190:HGJW7DSXY:4:1162:22535:32095_1:N:0:TCTGCAAG+GCTGCACT
ATTTGGATGTTGTCGAAAACTATCAGGTTCTTGTCACGATTCTTAAAAGAAGTTTATTAAAACTTCAG
AGTCTCTTGGAAGTCCAGGTAGCTAATGTTCTTGCAATAGCCACAGACTTTCTGGGAAAAGAGACCTC
TGT
+
FFFFFFFFFFFFFFF:FFFFFFFFFFFFFFFFF:FFFFFFFFFFFFFFFFFFFFFFFFFFFFF:FFFFFF
FFFFFFFFFFFFFFFFFFFFFFFFFFFFFFFFFFFFFFFFFF:FFFFFFFFFFFFFFFFFFFFFFFFF:
FFF
@A00721:190:HGJW7DSXY:4:1504:29098:17566_1:N:0:TCTGCAAG+GCTGCACT
GAGAATTACTCAGATTCAATCTGGCATCACTGAACGTCTACAACGAGTTAGGGAGTGGCTAGGTTTTA
```

AAAGATCCTTTGCACAAGCAATACCTTCCACTGAGTTCATCACCCAGAGTTTCGACGTCGCGGAAAAC
GAT
+
FFFFFFFFFFFFFFFFFFFFFFFFFFFFFFFFFFFFFFFFFFFFFFFFFFFFFFFFFFFFFFFFFFFFF
FFFFFFFFFFFFFFFFFFFFF:FFFFFFFFFFFFFFFFFFFFFFFFFFFFFFFFFFFFFFFFFFFF:FFF
FFF
@A00721:190:HGJW7DSXY:4:1159:29803:19132_2:N:0:TCTGCAAG+GCTGCACT
ACTGTTCGAGAATCTTCTGATGCTGAATTTGAGGAAAATTTTTCGGAAGTTTTTGGTATAAATCCTGA
TGGCTCAGTACAGGGAGGAGGTAATAGTTATAAAACCTTCTTTTATCA
+
FFFFFFFFFFFFFFFFFF:::,,F:FFFF,FFF:FFFFFFFFFFFFFFFFFFFFF:FFFFFFFFFF:F
FFFFFFFFFFFFFFFFF,:F,FFFFFFFFF:FFFFFFFF,FFFFFF,F
@A00721:190:HGJW7DSXY:4:1619:5267:27806_1:N:0:TCTGCAAG+GCTGCACT
ATGGACAAAATCTGGGATCATAGGTATGTGTCACGCTACACTCGAAATATGGGCCCTCATAAGGAAGT
ATAGTTGGCGTCTTGTCGTTGACGTCATTACAGTCTGGAAGTTAGATCATTTGTTCAGGAAGATCTCT
ACA
+
FFFFFFFFFFFFFFFFFFFFFFFFFFFFFFFF:FFF:FFFFFF,FFFFFFFFFFFFFFFFFFF:F:FFF
FFFFFFFFFF::FFFFFFF:FFFFFFFFFFFFFFFFFFFFFFFFFFFFFFFFFFFFFFFFFFFFFFFF
FFF
@A00721:190:HGJW7DSXY:4:2575:23773:13260_1:N:0:TCTGCAAG+GCTGCACT
CTTTAGCAACTTTAGATAAATTCTTTCCGGGCTCAATGACCTTTGCTATGTACGTGGATCTAATCCTT
GGTGATCTGAATCTGACCATCGCAAAGAAGACTTTATTAGTTCATGAAGTTCTTACACTTCTCGCGAC
TAA
+
:FFFFFFFFFFFFFFFFFFFFFFFFFFFFFFFF:::FFFFFFFFFFFFFFFFFFFFFFFF,FFFFFFFF
FFFFFFFFFFFFFFFFFFFFFFFFFFFFFFFFFFFFFFFFFFFFFFFFFFFFFFFFFFFFFFFFFFFFF
FFF
@A00721:190:HGJW7DSXY:4:1534:17047:24251_2:N:0:TCTGCAAG+GCTGCACT
CTTTATCTTAAAGCATTACCACCAAATTTAGCACTTTTTGAATATGACAGAAGCTCGCCATATCAGAT
TAGAGACATGCCAGTTAGAGTTCTTCTAACCAGGACTGATGGTGAGAT
+
FFFFFFFFFFFFFFFFFFFFFFFFFFFFFFFFFFFFFFFFFFFFFFFFFFFFFFFFFFFFFFFFFFFFF
FFFFFFFFFFFFFFFFFFFFFFFFFFFFFFFFFFFFFFFFFFFFFFFF
@A00721:190:HGJW7DSXY:4:1177:26413:29450_2:N:0:TCTGCAAG+GCTGCACT
GGAGGTCCAGGAACCTTTACTCCTAGTGTCTTTTCTAGAGTCCAAGGACGTTTCCGTCGGAGGATGAC
AGGTGGGAGACCGCTCAATAACACTCTATTGAATCCCTTTTCCACGAT
+
FFFFFFFFFFFFFFFFFFFFFFFFFFFFFFFFFFFFFFFFFFFFFFFFFFFFFFFFFFFFFFFFFFFFF
FFFFFFFFFFFFFFFFFFFFFFFFFFFFFFFFFFFFFFFF:FFFFFFF
@A00721:190:HGJW7DSXY:4:1105:20437:36949_1:N:0:TCTGCAAG+GCTGCACT
GATATCGCCTCTGTCCTTTCTCCTTTAATCGTGAGGTTACGAGAGTTTCGTACTTGGGTTTATCGGTT
GTATTTTGATAAATTTGTTGGAATCGATAATGATACTGACGAAGGTTCTATTACCACCCTTGTCATTG
ATA
+
FFFFFFFFFFF:FFFFFFFFFFFFFFFFFFFFFFFFFFFFFFFFFFFFFFFFF:FFFFFFFFFFFFFFF
FFFFFFFFFFFFFFFFFFFFFFFFFFFFFFFFFFFFFFFFFFFFFFFFFFFFFFFFFFFFFFFFFFFFF
FFF
@A00721:190:HGJW7DSXY:4:1253:12970:19351_2:N:0:TCTGCAAG+GCTGCACT
TGATGCTTTCTTAGAGGCTCGTCTAGCCGCTGAGATTCGTCTTACCTATTTACAATCTGTAGAGAGAG
AGGTTAATCAGAATTTGAGTGATAACGATTTAGTACTGGGAAATGGTG
+
FFFFFFFFFFFFFFFFFFFFFFFFFFFFFFFFFFFFFFFFFFFFFFFFF:FF:FFFFFFFFFFFFFFFF
FFFFFFFFFFFFFFFFFFFF:FFF:FFFFFFFFFFFFFFFFFFFFFFFF

@A00721:190:HGJW7DSXY:4:1675:12436:35806_1:N:0:TCTGCAAG+GCTGCACT
TTTTCTTTCCCTCCCCTTTTTCGATTGGATTCGACGGGGTCTTCGTCTCACGACTAATTTGATAAGGT
TTTTCTTTTATGGGACTCTTGTTTGTACTTATGAAGGAGGTATTACTTCGGAAGTTCTTAATGTAGGT
CTC
+
FFFFFFFFFFFFFFFFFFFFFFFFFFFFFFFFFFFFFFFFFFFFFFFFFFFFFFFFFFFFFFFFFFFF
FFFFFFFFFFFFFFFFFFFFFFFFFFF:FFFFFFFFFFFFFFFFFFFFFFFFFFFFFFFFFFFFFFFF
FFF
@A00721:190:HGJW7DSXY:4:1610:16929:26209_1:N:0:TCTGCAAG+GCTGCACT
GGTTTATGTTTCGGTTTCAGCTGGATGGATCTTTTGATGGTATGTTTCACATTGCGTGATCACTTCCC
GTGGACTTGGGAAGAGATTAAACATAAAATCCAGCGATTCTATTATGTCGCTAAAGCCTGTCCAGTTG
TCG
+
FFFFF:FFFFFFFFFFFFFFFFFFFFFFFFFFFFF::FFFF:FFFFFFFF:FFFFFF:FFFFF:FFFF
FFFFFF:FFFFFFFFFFFFFFFFFFFFFFFFFFFFFFFFFFFFFFFFFFFFFFFFFFFFFFFFFFFFF
FFF
@A00721:190:HGJW7DSXY:4:1229:14199:16282_2:N:0:TCTGCAAG+GCTGCACT
GGCTGTGGGGATTAACTTCCCTCGTGAAACTTACGACGATGTTCTCGCTCGCATTTCACAAGGATTAC
TAAGTCTGAGATCTGCCACTTCTAATTTTAGGAAACGTCGCGAAAAGA
+
FFFFFFFFFFFFFFFFFFFFFFFFFFFFFFF,FFFFFFFFFFFFFFFFFFFFFFFFFFFFFFFFFFFF
FFFFFFF:FFFFFFFFFF:FFFFFFFFFFFF:FFFFFFFFFFFFFFFF
@A00721:190:HGJW7DSXY:4:1362:19940:12790_2:N:0:TCTGCAAG+GCTGCACT
GTCTTGGTCGAAAAATATCGCCGGGAACTTCTAATAGAGGATCCTGATGACATTATTTCAACGGATAC
CGATCTCAGTTCTACCACAGAAACCGTCTCAGGAACTGTCTCCACTGA
+
FFFFFFFFFFFFFFFFFFFFFFFFFF,FFFFFFFFFFFFFFFFFFFFFFFFFFF:FFFFFFFFFFFFF
F:FFFFFFFFFFFFFFFFFFFFFFFFFFFFFFFF:FFFFFFFF:FFFF
@A00721:190:HGJW7DSXY:4:1321:23647:5932_2:N:0:TCTGCAAG+GCTGCACT
AAAGACGACCTTCTTGTGGGGACTGAGACCTCTGAATTTTCAGGGTGCCCTGATGAGGACGTTGAAAG
GGATAATTTCAACTCTGAGACTTCTAAGAGTGTTTCAAAAGACCAGAA
+
FFFFFFFFFFFFFFFFFFFFFFFFFFFFFFFFFFFFFFFFFFFFFFFFFFFFFFFFFFFFFFFFFFFF
FFFFFFFFFFFFFFFFFFFFFFFFFFFFFFFFFFFFFFFFFFFFFFFF
@A00721:190:HGJW7DSXY:4:1314:26530:35008_1:N:0:TCTGCAAG+GCTGCACT
TAAAGGTAGGAAGGGGAGAAAACAGAACAAGCGAGCGGTTGAAGAGAAAACTGATATGGTTGCGGCTG
ATCGTACCCCAATTCCTGCTAGAAAGATCACTCTCGGCGATACCACTGATCTTCCAGAGTTACGTAGT
GGT
+
FFF,FFFFFFFFFFFFFFFFFFFFFFFFFFFFFFFFFFFFFFFFFFFF:FFFFFFFFFFFFFFFFFFF
FFFFFFFFFFFFFFFFFFFFFFFFFFFFFFFFF:FFFFFFFFFFFFFFFFFFFFFFFFFFFFFFFFFF
FFF
@A00721:190:HGJW7DSXY:4:1316:29288:22654_1:N:0:TCTGCAAG+GCTGCACT
TTTTATCAAAGAGAAAGTTTCTGCTAGTCGCGACGATAGTATTTATCGGAGTGATATTAACAAGTTAA
ACTTCGAACTGACCACTTACAAGAGTGTTGAAGTACCGCAACATGGATCAGTTTTCCAAAAAGCTATT
AAT
+
FFFFFFFFFFFFFFFFFFFFFFFFFFFFFFFFFFFFFFFFFFFF:FFFFFFFFFFFFFFFFFFFFFFF
FFFFFFFFFFFFFFFFFFFFFFFFFFFFFFFFFFFFFFFFFFFFFFFFFFFFFFFFFFFFFFFFFFF:
FFF
@A00721:190:HGJW7DSXY:4:1460:8061:27070_2:N:0:TCTGCAAG+GCTGCACT
AAACTGAATCCACTCTCTTGGGATATAAATGAGTACATCTATAATAGAGTTTTGGAACTGTGTAATGT
CCTACGTACTGTTCAAGGTGTTTGGGCTTCTCTAAAGAACAGCAACTT
+

```
FFFFFFFFFFFFFFFFF:FFFFFFFFFFFFFFFFFFFFFFFFFFFFFFFFFFFFFFFF:FFFFFFFFFFF
FFFFFFFFFFFFFFFFFFFFFFFFFFFFFFFFFFFFFF:FFFFFFFFFF
@A00721:190:HGJW7DSXY:4:1614:5990:23766_2:N:0:TCTGCAAG+GCTGCACT
GGCTGATGACTTAAATTTCGCATTCTGCTTCGACGGTCATTCCATAAGACCTTTCTTGGGGAGTACAG
AGGGTTTGAAAATTACGGAACCTTTCCTCGTTCTTCCTGAAGATCTTC
+
,FF,FFF:F:F,FFFFFFFFFFFFF,:F,::FFF,FF,F,FFFFFF,FFF,,,:FFFFF,F,F:F,F,
F:,,FF:FFFFFF,F:::FF::F,F:,FFF,FFFFF,FF:FFF:F,,:
@A00721:190:HGJW7DSXY:4:1423:13919:15013_2:N:0:TCTGCAAG+GCTGCACT
GTTATTCTTGCGAAGGTTCAAGTCGATTGATTTGTTGACTACGATCTCTCTTGTAATAAGAAGTTCT
TCGCAGTTGAATCTCCACCAGGTGGAGGGAAGACCACTGAACTTATAG
+
FFFFFFFFFFFFFFFFFFFFFFFFFFFFFFFFFFFFFFF:FFFFFFFFFFFFFFFFFFFFFFFFFFFFF
F:FFFFFFFFFFFFFFFFFFFFFFFFFFFFFF:F:FFF:FFFFFFFFFF
@A00721:190:HGJW7DSXY:4:1239:12879:1564_1:N:0:TCTGCAAG+GCTGCACT
TTCGCAAAAGGTCTTGAAGTACGTATTGTCACTGCTAATCGTAATTCGCGAGAAGAGATAGTGACTAA
AGTTTTGGCACTTCTAGTTCATCGCGGTATTTGTGAAATTAAGACAGACGTGAAAGTTATGATGAGTG
ATC
+
FFFFFFFF:F:F,FFFFFFFFFFF:FFFFFFFFFFFFFFF:FFFFFFF:FFFFFFFFFFFFFFFFFFF
FFFFFFFFFFFFFFFFF:FFFFFFFFFF:FFFFFFFFFFFFFFFFFFFFFFFFFFFFFFFFFFFFF:F
FFF
@A00721:190:HGJW7DSXY:4:1226:25744:26631_2:N:0:TCTGCAAG+GCTGCACT
AGCTGATTCTTATTGCATGAACGTCGCTGAGGTCAAACCTGTGGACGTTCTTTTCATCGACGAATTAT
TCATGATTCACGCTGGTCAGACCCTTTTTCTTACACATCTACTTAAGG
+
FFFFFFFFFFFFFFFFFFFFFFFFFFFFFFFFFFFFFFFFFFFFFFFFFFFFFFFFFFFFFFFFFFFF
FFFFFFFFFFFFFFFFFFFFFFFFFFFFFFFFFFFFFFFFFFFFFFFF
@A00721:190:HGJW7DSXY:4:1261:7437:3286_2:N:0:TCTGCAAG+GCTGCACT
GTTACGGTGATTCCAATCAAATCGGATTCATCAATCGTATTGATCTCCAAGTCGCAAACTACTTCAAC
ATCAACGACATCATTCTTGATGAGTGTCGTTCGTATCGAAGTCTCAGT
+
F:FFFFFFFF:FFFFFFFFFFF:,FF:FFFFFFFFFFFF,:FFFFFFFFFFFF:FFFFFFFFFFFFFF
FFFFF::FFFF:FFFF:,FFFFFFFF:FFFFF,FFFFFF:FF:FFFF,
@A00721:190:HGJW7DSXY:4:1266:5240:8782_2:N:0:TCTGCAAG+GCTGCACT
CGGCCGACGTTTGTCGCGAACTCTCAGAGGTTTACGGCAGAAAAATTTCTCCTGCTTGTAATTCGAGA
GAGGACACTATGGAAATTAGACCTATCAGATGTCTTGACGACTTCGAA
+
FFFFFFFFFFFFFFFFFFFFFFFFFFFFFFFFFFFFFFFFFF:FFFFFFFFFFFFFF:FFFFFFF
FFFFFFFFF:FFFFFFFFFFFFFFFFFFFFFFFFFF:FFFFFFF,FFFF
@A00721:190:HGJW7DSXY:4:2456:15356:15718_2:N:0:TCTGCAAG+GCTGCACT
ATATTACTTTCACTCAAGGTGAAAAGTTTGAAATTAACAAAGTCCTTGGTAAGAGTAAGGTTAAATGT
GAAGATGCTCAGACTATTCATGAAGTTCAAGGTAATACTTACCAGCAC
+
FF:FFFFFFFF:FFFFFFF:FF,FFFFFFFF:FFFFFFF:FFFFF:FFFFFFFFFFFFFFFFFFFFFF
FFFFFFFF:FFFFFF:FFFFFF,FFFFFFFFFFFFFFFFFFFFFFFFFF
@A00721:190:HGJW7DSXY:4:2615:13358:22060_1:N:0:TCTGCAAG+GCTGCACT
ATGACCCTGTTTTTGCATCACAAACACATATTACCGTTGCTCTATCACACACGTTGAATCTTTACGAT
ACTTTGTTATCAATGTAAAGTTAAACGATGCTACTTGTGAGAGGATCAATCACAGTAAGGAACTGTCA
CGT
+
FFFFFFFFFFFFFFFFFFFFFF,FFFFFFFFFFFFFFFFFFFFFFFFFFFFFFFFFFFFFF:FFFFFFF
FFF:FFFFFFFFFFF:FFFFFFFF:FFFFFFFFF:FFFFFFFFFFFFFFFFFFFFFFFFFFFFFFFFF
FFF
```

```
@A00721:190:HGJW7DSXY:4:1351:32036:20870_1:N:0:TCTGCAAG+GCTGCACT
TGCAGATTCAACCCCTTATATCGAGGCTATCGAGGACGATGAGTATCTTGAAGTTGTTGACGACAAGC
CTCAAGAGGGTAGAGGACCACCTGGATCTTCTACTGTCTCTGCCTTCCAAGATTTCTTAGAAGAGGTT
ATT
+
FFFFFF:FFFFFFFFFFFFFFFFFFFFFFFFFFFFFFFFFFFFFFFFFFFFFFFFFFFFFFFFFFFFF
FFFFFFFFFFFFFFFFFFFFFFFFFFFFFFFFFFFFFFFFFFFFFFFFFFFFFFFFFFFFFFFFFFFF
FFF
@A00721:190:HGJW7DSXY:4:1454:32334:32189_1:N:0:TCTGCAAG+GCTGCACT
CTTAACGTCACTCGGTAATCTTTCAGCCGAATTAAGTACTAATGAGTTCAAGTCGGTTGTCGATCGAA
TTAAGATCAAGGCTAATACCTTTATAGGTAAGAAGCCCCTTGAGCCGCGTAGTCAGCGCGTTTAGCTG
TAT
+
FFFFFFFFFFFFFFFFFFFFFFFFFFFFFFFFFFFFFFFFFFFFF:FFFFFFFFFFFFFFFF:FFFFF
FFFFFFFFFFFFFFFFFFFFFFFFFFFFFFFFFFFFFFFFFFFFFFFFFFFFFFFFFFFF:FFFFFFF
FFF
@A00721:190:HGJW7DSXY:4:1374:21938:11647_1:N:0:TCTGCAAG+GCTGCACT
TATACCCGATAGGGTACCGAGTCTCCAAGAGAATATTTATTCTTATGAGGCTCGAAACTTCAACGTTT
TCACAACGGAAAGACATATCGCACCCGAAATTTTTGGGCAGAATGCTCTTAACGCGGTTCTTGAACGG
TGT
+
FFFFFFFFF:FFFFFFFFFFFFFFFFFFFFFFFFFFFFFFFFFFFFFFFFFFFFFFFFFFFFFFFFFF
FFFFFFFFFFFFFFFFFFFFFFFFFFFFFFFFFFFFFFFFFFFFFFFFFFFFFFFFFF:FFFFFFFFF
FFF
@A00721:190:HGJW7DSXY:4:1533:12283:15468_2:N:0:TCTGCAAG+GCTGCACT
CTAAGTTGGATAAAGTTATAGGAGACTCAGTTAACCTGAGAGCTCCTCAATTGATAGATTGGGTCAAC
AATAGGGACGCTAGTATACTTCGTAAAGTCGACAGCGAAATGGTCAAT
+
FFFFFFFFFFFF:FFFFFFFFFFFFFFFFFFFFFFFFFFFFFFFFFFFFFFFFFFFFFFFFFFFFFFF
FFFFFFFFFFFFFFFFFFFFFFFFFFFFFFFFFFFFFFFFFFFFFFFF
@A00721:190:HGJW7DSXY:4:2242:14651:16689_1:N:0:TCTGCAAG+GCTGCACT
ATATGACCTATTTTAAGCTCATGGTGAAAAGGGAAGCTAAGGTTAAACTTGATGCTTCAGCCTTTTCG
AAGCATAACCCCGCACAGAATATCGTTTATCACGCAAAATTTATTAATGCAATTTTCGGACCTATCTT
TAA
+
FFFFFFF,F:FFFFFFFFFFFF:F:FFFF:FFFFFFFFFFF:F:FFF:FFF,FFFFFFFFFFFFFFFF
FF,FFFFF::F,FFFFFFFFFFFFF:FFFF::F,FFFFFFFFFFFFFFFFFF:FFFFFFFF,FFFFFF
FFF
@A00721:190:HGJW7DSXY:4:1150:27561:14559_1:N:0:TCTGCAAG+GCTGCACT
TGTCTTTTACCCAACATTTTTATGTATACAGAGATGACCAGTGACGATTTCTCCCGGAAGCTCTTCTC
TCTCTTAGGAGGTCATGATGACTACTATAAGGGTGAAATCGATTTTTCGAAGTTTGACAAATCTCAAG
ATT
+
FFFFFFF:FFFFFFFFFFFFFF:FFFFFFFFFFF:FFFFFFFFFFFFFFFFFFFFFFFFFFFFFFFFF
FFFFFFFFFFFFFFFFFFFFFFFFFFFFFFFFFFFFFFFFFFFFFFFFFFFFFFFFFFFF:FFFFFFF
FFF
@A00721:190:HGJW7DSXY:4:1138:31204:10003_2:N:0:TCTGCAAG+GCTGCACT
AGCCTATGAGATAAAGATGTATTCTTATTTAGGCTTCGATGCTGAATTTCTTGAACTTTGGAGTGCCA
GTGAATATTTTTGTCACGCCAGGAGATCGGAAGAGCGTCGTGTAGGGA
+
FFFFFFFFFFFFFFFFFFFFFFFFFFFFFFFFFFFFFFFFFFFFFFFFFFFFFFFFFFFFFFFFFFFF
FFFFFFFFFFFFFFFFFFFFFFFFFFFF:FF,FFF,FFFFFFFFFFFF
@A00721:190:HGJW7DSXY:4:2278:26666:18114_2:N:0:TCTGCAAG+GCTGCACT
ACGGCCAACACTTTCTTTGGTAATTGTATGATCACTTTGATGATTTTATCACTTTATTATGACCTTGG
```

```
AAAGTTTTCGGCTGTGGTTGTCGCTGGGGACGATTCCATTCTGTTTTC
+
FFFFFFFFFFFFFFFFFFFFFFFFFFFFFFFFFFFFFFFFFFFFFFFFFFFFFFFFFFFFFFFFFFFF
FFFFFFFFFFFFFFFFFFFFFFFFFFFFFFFFFFFFFFFFFFFFFFFF
@A00721:190:HGJW7DSXY:4:1428:32949:15217_2:N:0:TCTGCAAG+GCTGCACT
CAGCGTACTTCTGCTCGCGATTTATTCTTTTTGTCAATGATTACATCTACTTCGTTCCTGATCCTTAC
AAGATCATCGTTAAGTTAGGTAAACCCCTAACCGATGTTACTCTTGTA
+
FFFFF:FFFFFFFFFFFFFFFFFFF:FFFFFFFFFFFFFFFFFFFFFFFFFFFFFFFFFFFFFFFFFF
FFFFFFFFFFFFFFFFFFFFFFFFFFFFFFF,FFFFFFFFFFFFFFFF
@A00721:190:HGJW7DSXY:4:1229:31792:36793_1:N:0:TCTGCAAG+GCTGCACT
TTTAAAGACTATACTAAATATCTCCACTACGAGGTTGTTGTTTGGCGATTAGCTCTTGCGACTAATAT
TCGATATTCGATTGAGGGTCGTTATACCTATTCGGCTATCTGTGCTTTGAATTGTATAAGATCAAATT
TTA
+
FFFFF::F,FFFF:FFFFFF::::FF:FFFF,F,FF:FFFFF:FFF:FFFFFFFFFFFF,F,FF,FFF
FF::FFFFFF:FFF,FFFF:FFF,FFF:F,F:FFF:F,F:FFFF:FFFFF,FF:FFFF:,F::FF,FF
FFF
@A00721:190:HGJW7DSXY:4:1322:4869:36886_2:N:0:TCTGCAAG+GCTGCACT
CCTGATGTGGAGGGAGTTCTTGAAGCATCCCGACCTTTCCTTGTGAAAATTTTGATGCGGTTTCATAA
GATTATCAGTAATCCTGATTCTACTACGGATTCATGGACCTTCCATCC
+
FFFFFFFFFFFFFFFFFFFFFFFFFFFFFFFFFFFFFFFFFFFFFFFFFFFFFFFFFFFFFFFFFFFF
FFFFFFFFFFFFFFFFFFFFFFFFFFFFFFFFFFFFFFFFFFFFFFFF
@A00721:190:HGJW7DSXY:4:1444:11966:28855_2:N:0:TCTGCAAG+GCTGCACT
GAGAGACGTATCTCAAAGATCGACTTCGAGACAGGTGATATTGTTGAGCGAGAGCGACCAAGATCTAA
ATTCGGTAACGGTGCGTGGCGCTTTATTTTAAAAAGCTCCCTCCTCTC
+
FFFFFFFFFFFFFFFFFFFFFFFF:FFFFFF:FFFFFFFFFFFFFFFFFFFFFFFFF:FFFFFFFFFFFF
FFFFFFFFFFFFFFFFFFFFFFFFFFFFFFFFFFFFFFFFFFFFFFFF
@A00721:190:HGJW7DSXY:4:1171:27751:9220_2:N:0:TCTGCAAG+GCTGCACT
CTGATACTTATCACGAAAGGACTTGAGTCTTTTGATCAGTCTCTGACTTGACCATCTTTCATTTTAAA
TGACGATCATTAAGACTCTCTTTCTTCTTTGTTTCCTTCTTTGCACTG
+
FFFFFFFFFFFFFFFFFFFFFFFFFFFFFFFFFFFFFFFFFFFFFFFFFFFFFFFFFFFFFFFFFFFF
FF:FFFFFFFFFFFFFFFFFFFFFFFFFFFF:FFFFFFFFFFF:FFFFF
@A00721:190:HGJW7DSXY:4:2310:5620:26287_2:N:0:TCTGCAAG+GCTGCACT
CAGCTTGTGTTTTACCTAATCTGTGTTTTTACGATAACAACTATCAGTTGATTAAGACTCTTCCGAAA
GGCGAACTTTGGGAATTTGAAGCTCAGGGTGAAGCACAACGTTTGACC
+
FFFFFFFFFFFFFFF,FFF:FFFFFFFFF:FFFFF:FFFFFFFFFFFFF:FFFFFFFFFFFFFFFFFF
FFFFFFFF,:FFFFFFFFFFFFFFFFFFFF:FFFFFFFFF:,FFFF,F
@A00721:190:HGJW7DSXY:4:1477:23276:5635_1:N:0:TCTGCAAG+GCTGCACT
GTTACGGATATACTCTCCTTGAGTTCACTCGTAAGGACACTACCACTTATTACGATATTTCTTTAGTC
GATGGGTTTTCGACGCCTTTAAGCGTCTTCTGTTCATCGCAACCAGATGATATCTTCTATTCGAACTT
ATG
+
FFFFFFFFFFFFFFFFFFFFFFFFFFFFFFFFFFFFFFFFFFFFFFFFFFFFFFFFFFFFFFFFFFFF
FFFFFF:FFFFFFFFFFFFFFFFFFF:FFFFFFFFFFFFFFFFFFFFFFFFFFFFFFFFFFFFFF:FFF
FFF
@A00721:190:HGJW7DSXY:4:2250:23131:20666_2:N:0:TCTGCAAG+GCTGCACT
TGTTTACCACCGAAATATCAAGGTTCTTGTCCTTCAAACGATTGGAAGGTTAGTTATCTTAATAGTAC
TAAAGGGGTTTACTTACAACCCTATGACGATAGTTTCTCTTTACACAC
+
```

```
FFFFFFFFFFF:FFFFFFFFFFFFFF,FFFFFFFFFF:FFFFFFFFFF:FFFFFF:FFFFFFFF,FFF
FFFFFF:FFFFFFFFFFFFFFFFFF:FF,FFFFFFFFFFFFFF:FFF:F
@A00721:190:HGJW7DSXY:4:2642:5321:36573_2:N:0:TCTGCAAG+GCTGCACT
TGTTTTCTTCTAATGTGGCCTGAACTACGTTTCACTTTGTGTGTACTTGTCTCTATTTTAAGTTTTGT
CTTCCTTGTTTGTCTTATTCTATTAATCACTCATTTTAGTGAGATTAT
+
FFFFFFFFFFF:FFFFFFFFFFFFFF:FFFFFFFFFFFFFFFFFFFFFFFFFFFFFFFF:FFFFFFFF
FFFFFFFFFFFFFFFFFFFFFFFFFFFFFFFFFFFFFFFFFFFFFFFF
@A00721:190:HGJW7DSXY:4:2551:15600:10629_2:N:0:TCTGCAAG+GCTGCACT
CTAAAACGTCTTCGACAACAGCAGGGGTTTTCGAATAGTGATAGCCACCCGCCTTACAGAGGGGCAGC
AGCATGACCATAATTGGTATTGACTTTGGTACCACCTTTTCTACTGTC
+
FFFFFFFFFFFFFFFFFFFFFFFFFFFFF:FFFFFFFFFFFFFFFFFFFFFFFF:FFFFFFFFFFFFFF
FFFFFFFFFFFFFF:FFFFFFFFFFFFFFFFFFFFFFFFFFFFFFFFF
@A00721:190:HGJW7DSXY:4:1627:18692:21089_1:N:0:TCTGCAAG+GCTGCACT
TTCGAATGAAGTTTTAATGCTTAACAACAATGATAGTGAATTTATACCTACAATCATTGGTTTTTCTA
ATAATGATGACACTATTGTTTATGGATATGATGCTATTACTCATGATGGTTACGGTCAGGGAGGATTC
TCA
+
FFF:FFFFFFFFFFFFFFFFFFFFFFFFFFFFFFFFFFFFFFFFFFFFFFFFFFFFFFFF,FFFFFFF
FFFFFF:FFFFFFFFFFFFFFFFFFFFFFFFFFFFFFFFFFFFFFFFFFFFFFFFFFFFFFFFFFFFF
FFF
@A00721:190:HGJW7DSXY:4:2115:19877:29215_2:N:0:TCTGCAAG+GCTGCACT
ACATTACCCGAAAGACGTGCTAAATTAAAACCATTGTACGATGTCGTCTGTCTCGGCGATCACTTTTC
ACTAAGAATTGGTCCTACTTTTGGACAAGGCCAACTTCGTAATCTTCA
+
FFFFFFFFFFFFFFFFFFFFFFFFFFFFFFFFFFFFFFFFFFFFFFFFFFFFFFFFFFFFFFFFFFFF
FFF::FFFFFFFFFFFFFFFF:FFFFFFFFFFFFFFFFFFFFFFFFFF
@A00721:190:HGJW7DSXY:4:1467:23204:23766_2:N:0:TCTGCAAG+GCTGCACT
TTTATCTCTTTGGTTGTTAGGGATTTTGAGAGAGTGAAAGGCTTCAATTGTAGTGGTTTGGTCGTTTC
AGTACCCAGTCAGTATACCTCTACGCAAAGATATTTCATGAAAACTAC
+
FFFFFFFFF:FFFFFFFFFFFFFFFFFFFFFFFFFFFFFFFFFFFF:FFFFFFFFFFFFFFFFFFFFF
FFFFFFFFFFFFFF:FFFFFFFFFFFFFFFFFFFFFFFFFFFFFFFFF
@A00721:190:HGJW7DSXY:4:2305:10945:27367_1:N:0:TCTGCAAG+GCTGCACT
CCGGATTACCTGTCTTTCATATTATGAATGAACCTTCGGCCGCTCTCTTTGCTTCGATGTTGGATGTC
AAGAAGGGTTCAGAATGGGATTCCTACGTAGTTTATGACTTTGGTGGTGGTACTTTCGATACTTCTAT
CGT
+
FFFFFFFFFFFFFFFFFFFFFFFFFFFFFFFFFFFFFFFFFFFFFFFFFFFFFFFFFFFFFFF:FFFF
FFFFFFFFFFFFFFFFFFFFFFFFFFFFFFFFFFFFFFFFFFFFFFFFFFFFFFFFFFFFFFFFFFFF
FFF
@A00721:190:HGJW7DSXY:4:2314:5728:15107_2:N:0:TCTGCAAG+GCTGCACT
TACTATAGTGTTATTTATTCTGGTGGTGATGATAATCTTGGAGGTCGTGACGTTGATGAAGCTATAAG
AGCGTTCTTATTACGGCGCACTTCTTTACCCGCCACCACACAACTTTC
+
FFFFFFFFFFFFFFFFFFFFFFFFFFFFFFFFFFFFFFFFFFFFFFFFFFFFFFFFFFFF::FFFFFF
FFFFFFFFFFFFFFF:F:FFFFFFFFFF:F::F:FFFFFFFFFFFFFF
@A00721:190:HGJW7DSXY:4:1618:5701:30718_1:N:0:TCTGCAAG+GCTGCACT
TGAAGGAGGAGGTTTCTGTTACTGGAAAACCCTCGTCAATACTTTTCAATAATATACGTCTTGAACTC
ACTTATGACGATCTAGTGAAATTAACGCGTCCTTTTCTGGAACGTAGTTTTACTATTATGAACAAAGT
TAT
+
FFFFFFFFFFFFFFFFFFFFFFFFFFFFFFFFFFFFFFFFFFFFFFFFF:FFFFFFFFFFFFFFFFFF
```

```
FFFFFFFFFFFFFFFFFFFFFFFFFFFFFFFFFFFFFFFFFFFFFFFFFFFFFFFFFFFFFFFFFFFFFF
FFF
@A00721:190:HGJW7DSXY:4:1474:5837:11694_1:N:0:TCTGCAAG+GCTGCACT
CCCTGATTCCGGCCGGTGGTAGCTCATTTCTACCAGGGGCTATTACTCTTGCTGAAACAGCTATTAAA
GCTGTTGAGCGGGTAATTACACCTAAACGAGCTCGATCTGCCATCGCTGAAGGATGTGCACTCTTTTC
TGC
+
FFFFFF,:FFFFFFFFFFFFFFFFFFF,FFFFFFFFFFFFFFFFFFFFFFFFFFFFFFFFFFFFFFFFFF
FFFFFFFFFFFFFFFFFFFFFFFFFFFFFFFFFFFFFFFFFFFFFFFFFFFFFFFFFFFFFFFFFFFFFF
FFF
@A00721:190:HGJW7DSXY:4:1437:21395:16564_1:N:0:TCTGCAAG+GCTGCACT
CTTCGTCGATTGTATCAACAGTAACATTACTTCTAATAAAGGTCTCTATCAAGCTCGTATTATCGTCG
CTGCTGGATCTGCGTTACCGTTGAAGGCTAAACTCCAAGGTAATTATGAAGACTGTGATTTATCTTTC
ACT
+
FFFFFFFFFFFFF:FFFFFFFF:FFFFFFFFFFFFFFFFFFFFFFFFFFFFFFFFFFFFFFFFFFFFFFF
FFFFFFFFFFFFFFF,FFFFFFFFFFFFFFFFFFFFFFFFFFFFFFFFFFFFFFFFFFFFFFFFFFFFFF
FFF
@A00721:190:HGJW7DSXY:4:1266:23710:11334_1:N:0:TCTGCAAG+GCTGCACT
ACGTGTGCTCTTCCGATCTATAATTTCAATCTTCTTAAAAAGAAGATAGAGAGTCATACTTTGGGCTA
TCGTTCAGGTGCATGGGGTTGGATAGCCAATATTGAAGTGGACACCTCTGGTAGTATCGAAGTAAAGA
TAA
+
FFFFFFFFFFFF:FF:F,FFFFFFFFFFFF,FFF:FFFF:FFF:FFF:FFFFFFFFFFFFFFFFFFFF
FFFFFFFFFFFFFFFFFFFFFFFFFFFFFFFFFFFFFFFFFFFFFFFFFF:FFFFFFFFFFFFFFFFFF
FFF
@A00721:190:HGJW7DSXY:4:1154:8241:27884_1:N:0:TCTGCAAG+GCTGCACT
ATACTATGGTTGAATCAACTAAGCATACTACTTTTGCTTTGATTACTCTACCGCCTTACAAGTCTAAG
GTTAAAGAGTCGATTAAACAAGTTGTACTTATCAATGATTTTATCGCTGGTTGTTATCTCGAAAAGTC
TCT
+
FFFFFF:FFFFFFFFFFFFF:FFFFFFFFFFFFFFFFFFFFFFFFFFFFFFFFFFFFFFFFFFFFFFFFF
FFFFFFFFFFFFFFFFFFFFFFFFFFFFFFFFFFFFFFFFFFFFFFFFFFFFFFFFFFFFFFFFFFFFFF
FFF
@A00721:190:HGJW7DSXY:4:1270:23773:24659_2:N:0:TCTGCAAG+GCTGCACT
TCAGTTCTAACCCAAAGAGAATATCTAAAATATTTACAAAATCTTACCACTATCTACGAACCTAAAGT
TCTGAAGACGTTGCTTGTCGCTTCCTCCTTTAATGGCACTACCTCGGT
+
FFFFFFFFFFFFFFFFFFFFFFFFFFFFFFFFFFFFFFFFFFFFFFFFFFFFFFFFFFFFFFFFFFFFFF
FFFFFFFFFFFFFFFFFFFFFFFFF:FFFFFFFFFFFFFFFFFFFFFFF
@A00721:190:HGJW7DSXY:4:2540:23692:31250_2:N:0:TCTGCAAG+GCTGCACT
TTGGAACGAAGGCGACCTGTTCCGGAGTTTTTACGGGATCCGGAATGTTCTGTCTATATTAGGTGAAG
CGAAATACGATTTCGAACGTCAAGATCGGAAGAGCGTCGTGTAGGGAA
+
FFFFFFFFFFFFFFFFFFFFFFFFFFFFFFFFFFFFFFFFFFFFFFFFFFFFFFFFFFFFFFFFFFFFFF
FFFFFFFFFFFFFFFFFF:FFFFFFFFFFFFFFFFFFFFFFF,FFFFFF
@A00721:190:HGJW7DSXY:4:1227:19334:31626_2:N:0:TCTGCAAG+GCTGCACT
ATTTGATCTGGTTGCTCAATTCTAAGCGTGTTCCGCTTAGTGCGAGGCCTCTCTTCTACGAGTATTGC
TATTTATACGGTTACGAGAAGAAGGTTCGAAAGTTTGAGAACTTAGTT
+
FFFFFFFFFFFFFFFFFFFFFFFFFFFFFFFFFFFFFFFFFFFFFFFFFFFFFFFFFFFFFFFFFF:FFF
FFFFFFFFFFFFFFFFFFFFFFFFFFFFFFFFFFFFFFFFFFFFFFFF
@A00721:190:HGJW7DSXY:4:2204:8034:33786_2:N:0:TCTGCAAG+GCTGCACT
ACATACTAAATACTAGATATTTGGATGATCTTGATAAAGAACTTCATGAATTTTCAGAGGATGAAAAA
```

TTTTCTTCCTTGGATTTTAATATAAAGAATCTTTACCAGGACTCCTAC
+
FFFFFFFFFFFFFFFFFF,FFFFFFFFFFFFFFFFFFF:FFFFFFF:FFF:::FFFFFFFFFFFFFFFF
:FFFFFFFF:FFFFFFFFF,F:FF,F:FFFFFFFFFFFFFF:FF:FFF
@A00721:190:HGJW7DSXY:4:1447:2799:28260_2:N:0:TCTGCAAG+GCTGCACT
TCTGATCGTAAGAAGTTTATAAGGAACATATCCATTTGTTGTAATCGGGTCATCAACTTAGAACTCGA
AACTCTAAGTGGTAATACTGTCTTTCCAAGCTTACCTGTATCTTCAAT
+
FFFFFFFFFFFFFFFFFFFFF,FFFFFFFFFFFFF,F,FF:F,FFF,FFFFFFFFFFFFFFFFFFFFFF
FFFFF::FFFFFFF:FFFFFFFF,FFFF:FF:FFFFFF::FFFFFFFF
@A00721:190:HGJW7DSXY:4:1478:6298:32315_1:N:0:TCTGCAAG+GCTGCACT
TTCGATAGGGGACATTTTATCGTGGGCTTTCCGTAAAGGAAGATCTGTTATCGACGGTTTATCGTTTA
CGAAGTATCACTCCACCGGTGAATGGTTTAAGAATACTATAAATAGAATAGCTATTACTACTAATGGC
GTT
+
FFFFFFFFFFFFF:FFFFFFFFFFFF:FFFFFFFFFFFFFFFFFFFFFFFFFFFFFFFFFFFFFFFFFF
FFFFFFFFFFFFFFFFFFFFFFFFFF:FFFFFFFFFFFFFFFFFFFFF:FFFFFFFFFFFFFFFFFFFF
FFF
@A00721:190:HGJW7DSXY:4:1653:24740:6042_2:N:0:TCTGCAAG+GCTGCACT
TAGTGACATCTGGTCAATTTCGTTTTTAGGTGGTCTTTATTCTATTATTGATTTTTATGAGATTGATA
CGTTAACAGAAGATGATGTTAGAGTAGAATTCCTGAAGAGTCCTTCCG
+
FFFFFFFFFFFFFFFFFFFFFFFF:FFFFFFFFFFFFFFFF:FFFFFFFFFFFFFFFFFF:FFFFFFFF
F:FFFFFFFFFFFFFFFFFFFFFFFF:FFFFFFFFFFF,FFFFFFFFF:
@A00721:190:HGJW7DSXY:4:1545:11433:20666_1:N:0:TCTGCAAG+GCTGCACT
TTAAATCTAGGATTATGTCTGTGTTGAAAGACTTTCTACCGATAAGGGTCACTAGTCTTACACAATTA
AGAAATAATCCTACATTGACTGATTATCTAAAGATTGAAGGAAAATTTTATCCTCGTTTAACTTACTT
AGA
+
FFFFFF:FFFFFFFFFFF:FFFFFFFFFFFFFFFFFFFFFFFFFFFFFFFFFFFFFFFFFFFFFFFFFF
FFFFFFFFFFFFFFFFFFFFFFFFFFFFFFFFFFFFFFFFFFFFFFFFFFFFFFFFFFFFFFF,FFFFF
FFF
@A00721:190:HGJW7DSXY:4:1523:30987:25254_1:N:0:TCTGCAAG+GCTGCACT
ATACAAATGATAACTTCTAATTTACGTTTGCTACTCAGAGCTGGTCCTCTCAGCAAAGGGATGCATGG
TGTTATCTCCTCAGGAGTCCCGGCATTTGATAATAATGACGAGACTTTCGTCATAATCGCTGCATATT
ACG
+
F:FFFFFFFFFFFFFFFFFFFFFFFFFFFFFFFFFFFFFFFFFFFFFFFFFFFFFFFFFFFFFFFFFF
FFFFFFFFFFFF,FFFFFFFFFFFFFFFFFFFFFFFFFFFFFFFFFFFFFFFFFFFFFFFFFFFFFFF
FFF
@A00721:190:HGJW7DSXY:4:1342:12997:20556_2:N:0:TCTGCAAG+GCTGCACT
CCAGCGCATCTCGTGTCGAGCCTCGACCCGATTATATGGTATTAACTTATAAAGGTGAGGAGACCATT
TTCTTGCTGAAATTTATAGAAGATTATTTTGACAGAATCAATAAGAAT
+
FFFFF:FFFFFFF:FFFFFFFFFFFFFFFFFFFFFFFFF:FFF:FFFFFFFFFFFFFFFFFFFFFFFFF
FFFFFFFFFFFFFFFFFFFF:FFFFFFFFFFFFFFFFFFFFFFFFFFFFF
@A00721:190:HGJW7DSXY:4:1620:3396:20932_1:N:0:TCTGCAAG+GCTGCACT
TATGCTGATCTAAAATTTCGATCTAGATGGATCCACTCCTCGAGTTATCATGATGTTAGTATGTTATA
TACTGATTTACAACGATATAGGAGTAATCTACGAGAAGATTCTAGAATTCTTAATTATAATATTCAAC
CTC
+
FFF,FFFFF:FF:FFFFFFFFFFFFFFFFFFFFF:F:F:FF:F:FFFFF:FFF:FFFFFFF:F,F:FFF
F:FFFFFFFF:F,FFFFFFFFFF:::FFFFFF:F:FFFFFFFFFFFFFFFFFFFFFFFFFFFFFFFFFF
FFF

```
@A00721:190:HGJW7DSXY:4:1367:27190:14606_2:N:0:TCTGCAAG+GCTGCACT
GAGATCACACATTTTGAGAACTAACTATGTTATGTTCTCTGATCATTTTGACAATCTCTTCTTATAAT
AAATAAATAAATAATATTACTAATGGCAAATAGTGGAAAGGCTCCAGT
+
F:FFFFFFFFFFFFFFFFFFFFFFFFFFFFFFFFFFFFFFFFFFFFFFFFFFFFFFFFFFFFFFFFFF
FFFFFFFFFFFFFFFFFF,FFFFFFF,:FFFFFFFFFFFFFFFFFFFF
@A00721:190:HGJW7DSXY:4:2569:22263:6324_2:N:0:TCTGCAAG+GCTGCACT
CTGCTTTGGATTACGATGACGTGAGAATAGGTGAAGGGATAGTTTTTACCTCGACCAACATTAAGGCG
GCGGCGACTAATCTTAAAGCTTCACCCAAAATGAAGGACACTTCGAAA
+
FFFFFFFFFFFFFFFFFFFFFFFFFFFFFFFFFFFFFFFFFFFFFFFFFFFFFFFFFFFFFFFFFFFF
FFFFFFFFFFFFFFFFFFFFFFFFFFFFFFFFFFFFFFFFFFFFFFFF
@A00721:190:HGJW7DSXY:4:2208:23122:26819_2:N:0:TCTGCAAG+GCTGCACT
AAAAGGCTCTAAAGGACACTATACAATATCCAGACGACGACGAAGCTTATACTACTTTAATTTTGGGT
ATTATGTGGAGATATCGTGATATCAGTACGTCCAGATCCTCTGCTTTT
+
FFFFFFFFFFFFFFFFFFFFFFFFFFFFFFFFFFFFFFFFFFFFFFFFFFFFFFFFFFFFFFFFFFFF
,FFFF:FFFFFFFFFFFFFFFFFFFFFFFFFFFFF:FFFFFFFFFFFFF
@A00721:190:HGJW7DSXY:4:2449:14181:11146_2:N:0:TCTGCAAG+GCTGCACT
ATCAAATATAAAGAAAAGGACTATGAAATAAAAGATTTGGTGCTTAGGGATATAATGAATTCAGCACC
ACATTTTGGTCAAGTCAATCCGTTGAGAAAATTTCTTAGAGGATATTC
+
FFFFFFFFFFFFFFFFFFFFFFFFFFFFFFFFF:FFFFFFFFFFFFFFFFFFFFFFFFFFFFFFFFFF
FFFFFFFFFFFFFFFFFFFFFFF:FFFFFFFFFFFFFFFFFFFFFFFF
@A00721:190:HGJW7DSXY:4:2167:25554:2848_1:N:0:TCTGCAAG+GCTGCACT
CCAACATTTACCACCAAATTCTTTTGATACAACTCTAGCCTTCAAGTGGGGTATGCCTGCGGAATACG
CTTGCTATTCACCAGATTGGTTCGAACCCGTTGATGCTATGTCAGAAGAAGCTATTCAAGCTTGTCGT
TTA
+
FFFFFFFF:FFFFFFFFFFFFFFFFFFFFFFFFFFFF:FFFFFFFFFFFFFFFFFFFFFFFFFFFFFF
FFFFFFFFFFFFFFFFFFFFFFFFFFFFFFFFFFFFFFFFFFFFFFFFFFFFFFFFFFFFFFFFFFFF
FFF
@A00721:190:HGJW7DSXY:4:1275:25581:11381_2:N:0:TCTGCAAG+GCTGCACT
AAACAAGCCCAGACTCAAGGAGTCCAATCAAAGGTTTTGAATTTAGCTCAACTGGGGAGACGTGCTTA
AATGGGTGATAAAAATTATTTAGAGATCTTTTCTACTTACAAAGGACA
+
FFFFFFFFFFFFFFFFFFFFFFFFFFFFFFFF:FFFFFFFFFFFFFFFFFFFFFFFFFFFFFFFFFFF
FF:FFFFFFFFFFFFFFFFFFFFFFFFFFFFFFFFFFFFFFFFFFFFF
@A00721:190:HGJW7DSXY:4:2211:16450:14481_2:N:0:TCTGCAAG+GCTGCACT
AATTCTACGGAAAAGTCCGGAGTATATTGTATGTTAATAGATAGTGGGATAATAGTTCTACCGATACG
TAGTGAACGCGTTCGTAGAACCAAGTACTTTCCGTCTGAAGAGTTTAT
+
F:FFF,:,:FFFFF,::F:,F,:FFFF::FF:FF,F,:,,F,FFF,FFFFFFFFFFF:FF,F,FFFFF
FF:F:FFF:FFFFF:FFFFFFFFFF,F:FF,F,:F,F,F,:F,FFFF,
@A00721:190:HGJW7DSXY:4:2161:19533:1626_1:N:0:TCTGCAAG+GCTGCACT
TAACCGATAGGAGTTGTATAGTTGAAGATGTGGCTTTAGACGATAATCCTTTAAGGACTATGTTGACT
GACGAAGATTTTCCCACAGATTTGTCTACGTTGCAGATCGCCACTCCAAACACTATATACATTAAAAT
ATA
+
FFFFFFFFFFFFFFFFFFFFFFFFFFFFFFFFFFFFFFFFFFFFFFFFFFFFFFFFFFFFFFFFFFF:
FFFFFFFFFFFFFFFFFFFFFFFFFFFFFFFFFFFFFFFFFFFFFFFFFFFFFFFFFFFFFFFFFFFF
FFF
@A00721:190:HGJW7DSXY:4:2533:9028:23265_2:N:0:TCTGCAAG+GCTGCACT
AGTCATAACTTTTGTTACCAAATTCTTGGCTTTCAACGAAGTCTCTGATTTTATTAATCATTACTTTA
```

ACGATCTCTCGGTCATTTACTCTGTACTTGATCTACGTCGTTTAACTG
+
FFFFFFFFFFFFFFFFFFFFFFFFFFFFFFFFFFFFFFFFFFFFFFFFFFFFFFFFFFFFFFFFFFFFFF
F:F:FFFFFFFFFFFFFFFFFFFFFFFFFFFFFFFF:FF:FFFFFF:FF
@A00721:190:HGJW7DSXY:4:2112:17047:33739_2:N:0:TCTGCAAG+GCTGCACT
CTTAGAATGACTCTATCTTTTCAGACACTTCTTATATTATTTACAATAGTTAATATCATATTAGTAAT
ACTCACTATCGTTTTCTGCTTTTTCTTAGCTAATAGATTAATAAAGGA
+
FFFFFFFFFFFFFFFFFFFFFFFFFFFFFFFFFFFFFFFFFFFFFFFFFFFFFFFFFFFFFFFFFFF:,F
FFFFFFFFFFFF:FFFFFFFFFFFF,FFFFF:FFFFFFFF:FFFF,FF
@A00721:190:HGJW7DSXY:4:1139:30436:20854_1:N:0:TCTGCAAG+GCTGCACT
CCAACTCTTCGTTACGATACTTGTTGGTATAATATATTTAGTCTCTACACCCATCTCAACGAGTAAAA
AGCGAGTTTTCCGTCCTTAACGTAATACACGAAGAGAACATCCGCAAACGACTCGAAACCGAGACGCC
GTA
+
F,FF:F:F,,,FFFF,F,:,,F,,,,,FF,F,F,,,FFF:,,F:,,FFFFF,F,,,FF,F,,:,,,F:
,,:,,,FFFFF:,,F,,,F:::FFFF:FFF,:,,:,F,::,FF,,F,FFFFFFFFFFFFFFFFFFFF
FFF
@A00721:190:HGJW7DSXY:4:1544:19027:24518_2:N:0:TCTGCAAG+GCTGCACT
TTTAGATTATGAATCCTGCCTTTACTCATTTTATAGCAGTTATTTATGTTGTACTTTTTATAATTTAT
AACCTTCTTATACTTTTGGGTATGTTTGTGAAAAACATGAGATACCC
+
FFFFFFFFFFFFFFFFFFFFFFFFFFFFFFFFFFFFFFFFFFFFFFFFFFFFFFFFFFFFFFFFFFFFFF
FFFFFFFFFFFFFFFFFFFFFFFFFFFFFFFFFFFFFFFFFFFFFF,F
@A00721:190:HGJW7DSXY:4:1208:26702:21621_1:N:0:TCTGCAAG+GCTGCACT
TATGTACTTATTGTAACGATCATTCTTTCGAGTTTTGTCATTCTTATTCTTTTATTCCTTTAACTGTG
TATGAGGAGGATTTATAATGCTTTTCGATAGTGATTGTTATCAATTTAAATACATTACTGTTTCAAAT
CAT
+
FFFFFFFFFFFFFFFFFFFFFFFFFFFFFFFFFFFFFFFFFFFFFFFFFFFFFFFFFFFFFFFFFFFFFF
FFFFFFFFFFFFFFFFFFFFFFFFFFFFFFFFFFFFFFFFFFFFFFFFFFFFFFFFFFFFFFFFFFFFFF
FFF
@A00721:190:HGJW7DSXY:4:1318:4634:25708_2:N:0:TCTGCAAG+GCTGCACT
GACTCCGAAAATGGTTTCAAATGAGGTCAAAGCTCTATTTATTAAACGAACTATTATCCGATTCTTCG
AGGGTATCACTACCACTGATCTGCCGACTTTAGTCAAGGAACTTCAAT
+
FFFFFFFFFFFFFFFFFFFFFFFFFFFFFFFFFFFFFFFFFFFFFFFFFFFFFFFFFFFFFFFFFFFFFF
FFFFFFFFFFFFFFFFFFFFFFFFFFFFFFFFFFFFFFFFF,:FFFFFF
@A00721:190:HGJW7DSXY:4:1117:10673:20854_2:N:0:TCTGCAAG+GCTGCACT
TATTTTTCTATAAGGGAAAATAGCAATAATCAAATTTCGACAAATTTTAGAACTTTATTTCAGAACTC
TCTCCGATCACCTTGGTGTGATCTCCCGGAGGATCTTTTCAATAAGAT
+
FFFFFFFFFFFFFFFFFFFFFFFFFFFFFFFFFFFFFFFFFFFFFFFFFFFFFFFFFFFFFFFFFFFFFF
FFFFFFFFFFFFFFFFFFFFFFFFFFFFFFFFFFFFFFFFFFFFFFFF
@A00721:190:HGJW7DSXY:4:1124:8386:34053_2:N:0:TCTGCAAG+GCTGCACT
ATTTTTAAATAGTGATATTCAGCGTATACTTGAATACTTTATCAAAGATTTGGCTTGGTCTATTTACC
GCAAAAAGTTGGAAGAGGTCTATGATATTTATTTTACTTATACTTTAA
+
FFF:FFFFFFFFFFFFFFFFFFFFFFFFFFFFFFFFFFFFFFFFFFF,FFFFF:FFFFFFFFFFFFFFFF
FFFFFFFFFFFFFFFFFFFFFFFFFFFFFFFFFFFFFFFFFF:FFFFF
@A00721:190:HGJW7DSXY:4:1266:13801:12148_1:N:0:TCTGCAAG+GCTGCACT
AACTTGACTGTTTATAATATTACAATAGCTCTTTCTCAGAAGATTAGAGAAGTAACCGGTGAACACGT
CGGTAGCATTGATTTACTTTTACCGTTAGAACTGAAAAAGATTCTTTTCGAAAAGAAACCTATAAGTT
ACA

+
F:,:FFFFFFFF,FFFFFFFFF:FFFFF:FFFFFFFFFFFF,FFFFFFFFF::FFF:FF:FFFFFFF,FF
FFFFFFFFFFF:FFFFFFF:F:,FFFFFFFFFF,FFFFFFFFFFFFFFFFFFFFFFFFF:FFFFFFFFFF
FFF
@A00721:190:HGJW7DSXY:4:1148:20880:22529_1:N:0:TCTGCAAG+GCTGCACT
TATATTAAAGAAATAAAAATACTTCAAGTACCTGAACTTTACATTTTTAATGGTATGGTATAATGTGT
CTTACAAATAGAACCTTCACTCTCGGTAATGAGAGTGTATTCCTTAATATTCTAGTAACCGCTATGTG
GGC
+
FFFFFFFFFFFFFFFFFFFFFFFFFFFFFFFFFFFFFFFFFFFFFFFFFFFFFFFFFFFFFFFFFFFFFF
FFFFFFFFFFFFFFFFFFFFFFFFFFFFFFFFFFFFFFFFFFFFFFFFFFFFFFFFFFFFFFFFFFFFFF
FFF
@A00721:190:HGJW7DSXY:4:1435:27480:10629_2:N:0:TCTGCAAG+GCTGCACT
TCCTAAATTTGATAGCAATTTATGTTTTAAATGCTAGGAAAATTTTAGAAAAACTCCATTGTATAGTT
CAAGGAGTACTTAATTCAATAGCTTTAGTATTATTTACTATCGCCTTT
+
FFFFFFF,:FFFFFFFF,:::FFFFFF,FFFFF,:FFFFFFFFFFFFFFFFFFFFFFFFF,FF:FFFFFF
FFFFFFFFFFFFFFFFFFFFFFFFFFFFFFFF:FFFFFF:FFFFF:,F
@A00721:190:HGJW7DSXY:4:1361:28827:4194_2:N:0:TCTGCAAG+GCTGCACT
AGTTAATTGCTTACACAAAAGTGACGCGCGAAGGAATCTTAATTGATTGAAGCTAGGCGTTGGGGAGA
ATGAACTGCCGCTTCCCGGAAGAAACGATCTATTTACAGAGTATTTAG
+
FFFFFFFFFFFFFFFFFF:FFFF:FFFFFFFFFFF:FFFFFFFFFFFFFFFFFFFFFFFFFFFFFFFFFF
FFFFFFFFFFFFFFFFFFFFFFFFFFFFFFFFFFFFFFFFFFFFF:FF
@A00721:190:HGJW7DSXY:4:2676:27507:17190_2:N:0:TCTGCAAG+GCTGCACT
CCGCAAATCAATTGAAGAAAACCACTCCCTTCAATTCAATAACATAACATACCATACACTCCCCAAAC
ATAACATACCATAACCTACCATGACCAATCATCGCTCCAACTGGCACG
+
FFFFFFFFFFFFFFF:FFFFFFF,F:FFFFFFFF:FFFFFFFFFFF,FFFFFFFFFFFF:FFFFFFFFFF
F:FFFFFFFFFF,FFFFFFFFFFFFFFFFFFFFFFFFFFFFFFFF,FF
@A00721:190:HGJW7DSXY:4:1149:13856:9455_1:N:0:TCTGCAAG+GCTGCACT
CTACTACCGCCGGTCGGCAGATCGGCACCACTGGTCTCTACGTTCAGGGACCCGCTGTGCAGAATAAC
CTTCCCAAATCTAGATCTGCACAGAAGAAGGCTGAAGGTCGTTACCGGACGACCACTCAAAGGAAACG
GGC
+
FFFFFFFFFFFFFFFFFFFFFFFFFFFFFFFFFFFFFFFFFFFFFFFFFFFFFFFFFFFFFFFFFFFFFF
FFFFFFFFFFFFF::FFFFFFFFFFFFF,FFFFFFFFFFFFFFFFFFFFFFFFFFFFFFFFFFFFFFFFF
FFF
@A00721:190:HGJW7DSXY:4:2632:3070:31923_1:N:0:TCTGCAAG+GCTGCACT
GAGGTCGCTCGTAACATTGACGACAGTCCCGTCATCTCTGTGGGAACTGAGGCAGTACCTCAGTCAAA
AGTCACATTCACCACTCCCATACCAATCAATGATGTGACTATACAGTTCGGCCATTTCAAGAGTGGTC
AAT
+
FFFFFFFFFFFFFFFFFFFFFFFFFFFFFFFFFFFFFFFFFFFFFFFFFFFFFFFFFFFFFFFFFFFFFF
FFFFFFFFFFFFFFFFFFFFFFFFFFFF:FFFFFFFFFFFFFFFFFFFFFFFFFFFFFFFFFFFFFFFFF
FF:
@A00721:190:HGJW7DSXY:4:2517:30192:14356_1:N:0:TCTGCAAG+GCTGCACT
GTACTGAAGCCTTCCCCTCCGCTGGCACGAAGAACACTCCCAAGCCAATCAATAGTTCTTCTGACCAG
AATGAAAAGGGGTTTACCAGGGTAGTCTCTAAGTCTACCAGGAAGAGGGGAAATGTCCCCAAAACTGA
GAA
+
FFFFFFFFF:F:FFFFFFFFFFFFFFFFFFFFFFFFFFFFFFFFFFFFFFFFFFFFFFFFFFFFFFFFFF
FFFFFFFFFFFFFFFFFF:FFFFFFFF:FFFFFFFFFFFFFFFFFFFFFFFFFFFFFFFFFFFFFFFFFF
FFF

@A00721:190:HGJW7DSXY:4:1459:17327:19539_2:N:0:TCTGCAAG+GCTGCACT
AGGTCCATCAGGACCTAAGAAGGGGAAATTACCCCTAATTCTGAAGGAAGATCTCCCACCATCTGGGG
TTGGGAATAAGAAATCTGCACGGGCCAAAGAGGCCGCTGCTACGAAGT
+
FFFFFFFFFFFFFFFFFFFFFFFFFFFFFFFFFFFFFFFFFFFFFF:FFFFFFFFFFFFFF:FFFF
FFFFFFFFFFFFFF:FFFFFFFFFFFFFFFFFFFFFFFFFFFFFFFFFFF
@A00721:190:HGJW7DSXY:4:1343:31467:23610_1:N:0:TCTGCAAG+GCTGCACT
GCTTGGGTCATTAAGGGTAGCTGTAAAGTTGCGCCTGCCGACCCCATCTTTGAGACCAAGACTCTCTA
CAGTAGGTTCGTGCCTACTATGAGCAAATCTGTTGAATTCGTCACTGAGGACGAAGGTGAGAGGACTT
TTG
+
F,FFFF,FFFFF:FFFFFFFFFFFFFFFFFFFFFFF:FFFFFF:FFFFFFFFFFFFFFFFFFFFFF,FFF
FFFFFFFFFFFFFFFFFF:FFFFFFFF:FFFFFFFFFFFFFFFFFFFFFFFFFFFFFFFFFFFFFFFFF
FFF
@A00721:190:HGJW7DSXY:4:1622:10908:1971_1:N:0:TCTGCAAG+GCTGCACT
CAAACTGAGAGCAGTCGCTGGGGAAAGAGAATTGCGTGGGTTGAAGGCCGACGCTTCGAGCTTGAGTG
TGACGAGGCCGGTGACGTTTTTGTCAACGGCGTTTGGAGGATTTTATCCGCGCTTAACTATGGGGCGA
AGA
+
FFFFFFFFFFFFFFFFFFFFFFFFFFFFFFFFFFFFFFFF:FFFFFFFFFFFFFFFFFFFFFFFFFFF
FFFFFFFFFFFFFFFFFFFFFFFF,FFFFFFFF:FFFFFFFFFFFF:FFFFFFFFFFFFFFFFFFFFFF
FFF
@A00721:190:HGJW7DSXY:4:1414:32533:21355_1:N:0:TCTGCAAG+GCTGCACT
AGTCTTGAAGGACTTAGGTTGGCATATTTATGCCGACGATAATAAGCATGTCACCCTTAAGCACCGAG
GTAAGCTCGGGACTTACAAGAGAGAGTGTGAGAGAGTCGCTCTCACTATTTTTGCCCAGAATGCGGGC
TTT
+
FFFFFFFFFFFFFFFF:FFFFFFFFFFFFFFFFFFFFFFFFFFFF,FFFFFFFFFFFFFFFFFFFFFFF
FFFFFFFFFFFFFFFFFFFFFFFFFFFFFFFFFFFF:FFFFFFFFFFFFFF:FFFFFFFFFFFFFFFFF
FFF
@A00721:190:HGJW7DSXY:4:2124:32506:25661_2:N:0:TCTGCAAG+GCTGCACT
GCTCCTTCTGTGGGTGAGGGTTTTTGTTATATTCACTGGTTCTGGGAGGCTGCTTTCCTCCATAATTA
CAACCTCTCTTTAGAGAGCATTAGAAAGATCGGAAGAGCGTCGTGTAG
+
FFF:FF:FFFFFFFFFFFFFFFFFFFF:FFFFFFFFFFFFFFFFFFFFFFFFFFFFFFFFFFFFFFFFF
FFFFFFFFFFFFFFFFFF:FF:FFFFFFFFFFFFFFFFFFFFFF:F,,F
@A00721:190:HGJW7DSXY:4:2466:5954:33473_2:N:0:TCTGCAAG+GCTGCACT
GTTGTCACACATTTCTTTAAAATTTTACCTTTCATCCTACATTGGGGATTTCGATGTTAGAAGGTGGA
ATGTGTCAAGGGTCTTCTTCATTATGATCTTTAATGACCTAATCACAT
+
,::,FFF::F,,FF,:F,FFFFFFF,,,,::FF:,FF,,FF::,F,,F,,FF,F,FF,F,,F,:,,,F
F,,,,FFFFFF,FF:FFFFFFF,,F,F:FFFF,F,FFFF,F:,,FFFF
@A00721:190:HGJW7DSXY:4:1447:29541:17394_1:N:0:TCTGCAAG+GCTGCACT
ACGGCTCGTATCGAGTTGGAGGGAGGAAGTTCTCCGAAATTAAAGACGGGGGGATTAAGAGTCCAGCT
GTAACTGTCATTTCAGAGAGGGGAGCTCTGAAAAGGAAACTTCGTCAGAAGATGAGGAAGTCTAAGAG
GAG
+
FFFFFFFFFFFFFFFFFFFFFFFFFFFFFF:FFFFFFFFFFFFFFFFFFFFFFFFFFFFFFFFFFFFF
FFFFFFFFFFFFFFFFFFFFFFFFFFFFFFFFFFFFFFFFFFFFFFFFFFFFFFFFFFFFFFFFFFFF
FFF
@A00721:190:HGJW7DSXY:4:1366:3097:27774_1:N:0:TCTGCAAG+GCTGCACT
AAGGCTCTAGAGAAGATCTCACCACCTGGAGAAGGCAAGTGGAGAGTCCAGAAGTTGATGAAGAAGGA
TTATTTGAAGCAGGGGTTACCACTTCCTAAGGTGGATGAGGGAGGTTGTTTCTTTGGAGTTTTAGATA
ATC

+
FFFFFFFFFFFF,FFFFFFF,FFFFF:FFFFFFFFFFFFFFFFFFFFFFFFFFFFFFFFFFFF,:F:FF
FFF:FFF:FFFFFFFFFFFFFFFFFFFFFF:FFFFFFFFFFFFFFFFFFFFFFFFFFFFFF:FFFFFFFF
FFF
@A00721:190:HGJW7DSXY:4:2477:20546:10676_2:N:0:TCTGCAAG+GCTGCACT
GGTCATAGAGCCAGCAAGAGAAGCTTATATGCTGGTTTAAGTGATGATTATCACTCCTTGAGAAGACA
AGCCACATTAAGATATAATTTAGGTCAACTTGGGGTTTCGAATGTCAT
+
FFFFFFFFFFFFFFFFFFFFFFFFFFFFFFFFFFFFFFFFFFFFFFFFFFFFFFFFFFFFFFFFFFFFF
FFFFFFFFFFFFFFFFFFFFFFFFFFFFFFFFFFFFFFFFFFFFFFFF
@A00721:190:HGJW7DSXY:4:1501:20799:24612_2:N:0:TCTGCAAG+GCTGCACT
AGGTCCTTTTGAGTATCGACAGATCTTTCGATCTTGAGGACGTACATTTAACGTGGCAGAATTCGGTA
ACTTACCATATTGTTCCTTCCGGGAAGTTGTGGGATTTAACTTTGACT
+
FFFFFFFFFFFFFFFFFFFFFFFFFFFF::FFFFFFFFFFFFFFFFFFFFFFFFFFFFFFFFFFF:FFF
FFFFFF:FFFFFFFFFFFFFFFFFFFFFFFFFFFFF:FFFFFFFFFFFF
@A00721:190:HGJW7DSXY:4:2146:6271:32299_2:N:0:TCTGCAAG+GCTGCACT
GGTATGGACTACTCGACTTTTGATCTGGAGTTGTCTAGGGTCATGGCAGATACTACCGATAAGGTGAA
AAATATGCAGGTCAAAGATTCTGTAGCTCTGAGGGCGGTTGAGGGAGC
+
FFFFFFFFF:FFF:FFFFFFFFFFFFFFF:FFFFFFFF:FFFFFFFFFFFFFFFFFFFFFFFF:FF:FF
FFFFFFFFFFFFFFF:FFFFFFFFFFFF:FFF:FF::FFFFFFFFFFF
@A00721:190:HGJW7DSXY:4:1535:18032:19288_1:N:0:TCTGCAAG+GCTGCACT
TTCTAAGAGTCTTTATGATTCAAAACCTATCGTTGTCGTACCTTACAAGGTTGAGGAGACTTTACAGC
GGCTTATTGTCGCGCAGTTTCCAGAATTGCGGTTGAAGTTCACCCATTCTATCTTGAATGCTCACGCC
GTC
+
FFFFFFFFFFF:FFFFFFFFFFFFFFFFFFFFFFFFFFFFFFFFFFFFFFFFFFFFFFFFFFFFFFFFF
FFFFFF:FFFFF:FFFFFFFFFFFFFFFFFFFFFFFFFFFFFFFFFFFFFFFFFFFFFFFFFFFFFFFF
FFF
@A00721:190:HGJW7DSXY:4:1575:3088:7968_2:N:0:TCTGCAAG+GCTGCACT
TTATAATCACTTGTTAAAGTACAGATTTAGTGATTTAGATATTATTGATATTGGAGGCGATATTCGTT
TTCATATCCAAAACTCCTTGAATGGAGTACATATTTGCAACCCTATTC
+
FFFFFFFFFFFFFFFFFFFFFFFFFFFFFFFFFFFFFFFFFFFFFFFFFFFFFFFFFFFFFFFFFFFFF
FFFFFFFFFFFFFFFFFFFFF:FFFFFFFFFFFFFFFFFFFFFF,FFF
@A00721:190:HGJW7DSXY:4:2456:9968:13714_1:N:0:TCTGCAAG+GCTGCACT
GATGCATCGAGGTATACGTCTAGGAACATTGATTGGGAACGTACCTACTCACGAGACGTCATGAAAGC
TATCGGAGGGAAGCAAAATGTTTCGTTCTGTTACAAGAACGCTTTGGATTGTGACCATAAAGCCTCAG
TTG
+
FFFFFFFFFFFFFFFFFFFFFFFFFFFFFFFFFFFFFFFFFFFFFFFFFFFFFFFFFFFFFFFFFFFFF
FFFFFFFFFFFFFFFFFFFFFFFFFFFFFFFFFFFFFFFFFFFFFFFFFFFFFFFFFFFFFFFFFFFFF
FFF
@A00721:190:HGJW7DSXY:4:1314:1027:16110_2:N:0:TCTGCAAG+GCTGCACT
ATATCTCTCTTGATGATGTAGCCACTATAATGCTTAAGAGAGAGAGATTGATGTATGTCATTTCGTCTTA
GTTTGTCCCGGCGAATTATTGGACACGATGAAAAATGACATACCCGTT
+
FFFFFFFFFFFFFFFFFFFFFFFFFFFFFFFFFFFFFFFFFFFFFFFFFFFFFFFFFFFFFFFFFFFFF
FFFFFFFFFFFFFFFFFFFFFFFFFFFFFFFFFFFFFFFFFFF:FFFF
@A00721:190:HGJW7DSXY:4:1364:24026:4022_2:N:0:TCTGCAAG+GCTGCACT
TCATGGAGATGATGGCGCATCACTTGATTACTATATGCCCGAAGGATTGGGTTATTCTCACAACAAAG
ATAAGATTTTATCTTTCTTCAAACACCCTGTTTTCCTTAAGGGCGGAC
+

```
FFFFFFFFFFFFFFFFFFFFFF:FFF:FF:FFFFFFFFFFFF,FFFFFFFFFFF,FFFFFFFFFFFFF
FFFFFFFF:FFFFFFFFFFFFF:FFFFFFFFF:FFFFFF:FFFFFFFF
@A00721:190:HGJW7DSXY:4:1604:31015:23202_2:N:0:TCTGCAAG+GCTGCACT
TGTGAACTCTTGGGGACTAGATTGAGCATTAATGAGTTCCGAATAACTCGTAATAAATCTTTCTCTAG
TGGACAATTCAGAATACCTATTTCCGTACCAAGAGCCACTGCCAATCT
+
FFFFFFFF:FFFFFF,FFFFFFFFFFFFFFF:FF:FFFFFFFFFFFFFFFFFFFFFFFFFFFFFFFFF
FFFFFFFFFFFFFFFFFFFFFFFFFFFFFFFFFFFFFFFFFFFFFFFF
@A00721:190:HGJW7DSXY:4:1312:13639:36573_2:N:0:TCTGCAAG+GCTGCACT
CGCATACCTGATTGGAAGAAGCGAGGTTCGTTCCTCAATAAAAATTTTTCTCAAGAGATTATGGTTGA
TAAAGTTTTCTTCATAAGGGCTATTCAGTACGTCATGGGGATCACTTC
+
FFFFFFFFFFFFFFFFFF:,FFFFFFFFFFFFFFFFFFFFFFFFFFFFFFFF:FFFFFFFF,FFFFFF
FFFFFFFFFFFFFFFFFFF:FFFFFFFFFFFFFFFFFFFFFFFFFFFF
@A00721:190:HGJW7DSXY:4:1256:29785:16282_2:N:0:TCTGCAAG+GCTGCACT
ACAAAACCTTCGAATATGTTACGACATGGCTTCGTAACAACAGTGCGAGGATTGTCATCTCGGGGAAA
GTGATTCACCAAGATGTTCATATAGATACGAGATACCTAGAGAAGGTC
+
FFFFF:FFFFFFFFFFFFFFFFFFFFFFFFFFFFFFFFFFFFFFFFFFFFFFFFFFFFFFFFFFFF,F
FFFFFFFFFFFFFF,FFFFFFFFF:FFFFFFFFFFF:FFFFFFFFF:F
@A00721:190:HGJW7DSXY:4:1357:31358:27806_1:N:0:TCTGCAAG+GCTGCACT
CTCGTTATCGGAGCTAAGAAGAAGACTTTGGGTACACGTTATGCTCGCAAACTTAACGTTAGTACCGG
AGGTGATCTCTCTGCTTCTGCGTTACTGAGAGAAAAGATTCAGGACGCCTACGGTGACTTTATGGACA
AGA
+
FF:FFFFFFFFFFFFFFFFFFFFFFFFFFFFFFFFFFFFFFFFFFFFFFFFFFFFFFFFFFFFFFFFF
FFFFFFFFFFFFFFFFFFFFFFFFFFFFFFFFFFFFFFFFFFFFFFFFFFFFFFFFFFFFFFFFFFFF
FFF
@A00721:190:HGJW7DSXY:4:1337:30120:28447_2:N:0:TCTGCAAG+GCTGCACT
TTTAGGTTTAGTAGGTGATCTTGACGTCGATTCTTTGTATAGGGAGTGTGAGAAGACAACGAATCTTG
AAACTACGGTCCTTATCAAAGAAAATGGTGAACCGGTCTCTAAATCTT
+
FFFFFFFFFFFFFFFFFFFFFFFFFFFFFFFFFFFFFFFFFFFFFFFFFFFFFFFFFFFFFFFFFFFF
FFFFFFFFFFFFFFFFFFF:F:FFFFF,FFFFFFFFFFFFFFFFF:FF
@A00721:190:HGJW7DSXY:4:1616:7979:22639_2:N:0:TCTGCAAG+GCTGCACT
GAAGATTGAAGAACACGTCAAGAAGTCGCTCGGCGTTGTTCTTGAAAACCAAGTTAAGAAGGAGGCTG
ATCATGGAGTCTTTGTACCTTCTGTCAAACTTCGAGGTTCAGCTACGA
+
FFFFFFFFFFFFFFFFFFFFFFFFFFF:FFFFFFFFFFFFFFFFFFFFFFFFF:FFFFFFFFFFFFFF
FFFF:FFFF:FFFFFFFFFFFFFFFFFFFFFFFFFFFFFFFFF:FFFF
@A00721:190:HGJW7DSXY:4:1347:18059:20306_1:N:0:TCTGCAAG+GCTGCACT
TGGATTGAAGGCTGGCTCTAAGGGAGACTCCTTCTTTTTGAATGTTGTAAAGAATTGGGTTGAGAAGT
GTGCTTTACAAGGAGTTAGACTCTTCTTCAAGTTTTTAGATCTTCTTGATAATCTTAAAGAAAAATTT
TCG
+
FFFFFFFFFFFFFFFFFFFFFFFFFFFFFF:FFFFFFFFFFFFFFFFFFFFFFFFFFFFFFFFFFFFF
FFFFFFFFFFFFFFFFFFFFFFFF:FFFFFFF:FFFFFFFFFFFFFFFFFFFFFFFFFFFFFFFFFFF
FFF
@A00721:190:HGJW7DSXY:4:1311:31647:8328_1:N:0:TCTGCAAG+GCTGCACT
GTCTGTACTTACATTCTTTACGCCAGATTCTTCATGGGGCGACGCTGTTTCTCTTGGTATTTCTGAAA
TAGTTTATCAGAGCGTCATTTTCCTTTGGGATCTTTCCTCTGGTAAGGGAATTATTAAATCATTCTTC
TCA
+
FFFFFFFFFFFFFFFFFFFFFFFFFFFFFFFFFFFFFFFFFFFFFFFFFFFFFFFFFFFFFFFFFFFF
```

```
FFFFFFFFFFFFFFFFFFFFFFFFFFFFFFFFFFFFFFFFFFFFFFFFFFFFFFFFFFFFFFFFFFFFFFF
FFF
@A00721:190:HGJW7DSXY:4:2375:8106:7795_2:N:0:TCTGCAAG+GCTGCACT
ATTATTACTGTTTTCTCTACCCATAACTTCTTTTTCAAAAATGCGGATGGGTCTACTGATGTTTTCCT
ACAGAGTGCAGTCGTTTCAACACTTATCACCACATTTGAAAGGAAAAT
+
FFFFFFFFFFFFFFFFFFFFFFFFFFFFFFFFFFFFFFFFFFFFFFFFFFFFFFFFFFFFFFFFFFFFFFF
FFFFFFFFFFFFFFFFF:FFFFF:FFF,F:FF,,FFFFFFF:FFFFF:
@A00721:190:HGJW7DSXY:4:1343:10728:25864_2:N:0:TCTGCAAG+GCTGCACT
AATACGTATGTCGGTGGGGTTCTCAAGGCTCTCATCGCCACTGGGATTCTAAGGGGTTCCATAAGATA
TTATCTGACATTGTGGTTCAATGATCAGGATTCTTCTTTTGGTCCTTA
+
FFFFFFFFFFFFFFFFFFFFFFFFFFFFFFFFFFFFFFFFFFFFFF,FFFFFFFFFFFFFFFFFFF:F
FFFFFFFFFFFFFFFFFFFFFFFFFFFFFFFFFFFFFFFFFF,FFFFFF
@A00721:190:HGJW7DSXY:4:2372:2474:29293_1:N:0:TCTGCAAG+GCTGCACT
ATTTGGATGTTGTCGAAAACTATCAGGTTCTTGTCACGATTCTTAAAAGAAGTTTATTAAAACTTCAG
AGTCTCTTGGAAGTCCAGGTAGCTAATGTTCTTGCAATAGCCACAGACTTTCTGGGAAAAGAGACCTC
TGT
+
F:FFFFFF:FFFF,FFFFFFFFFFFFFFF,FFFFFFFF,FFFFFF:F,,,FFFFFFFFF,,FFFFFFF
FFFFFFFFF,FFFF,F:F:F:F:F:FFFFFFFFFFFFFFFF:FFFF,FFFFFFFFFFFF:FFFFFFFF
FFF
@A00721:190:HGJW7DSXY:4:1527:24704:12085_1:N:0:TCTGCAAG+GCTGCACT
GAGAATTACTCAGATTCAATCTGGCATCACTGAACGTCTACAACGAGTTAGGGAGTGGCTAGGTTTTA
AAAGATCCTTTGCACAAGCAATACCTTCCACTGAGTTCATCACCCAGAGTTTCGACGTCGCGGAAAAC
GAT
+
FFFFFFFFFFFFFFFFFFFFFFFFFFFFFFFFFFFFFFFFFFFFFFFFFFFFFFFFFFFFFFFFFFFFFFF
FFFFFFFFFFFFFFFFFFFFFFFFFFFFFFFFFFFFFFFFFFFFFFFFFFFFFFFFFFFFFFFFFFFFFFF
FFF
@A00721:190:HGJW7DSXY:4:1269:4029:24596_2:N:0:TCTGCAAG+GCTGCACT
ACTGTTCGAGAATCTTCTGATGCTGAATTTGAGGAAAATTTTTCGGAAGTTTTTGGTATAAATCCTGA
TGGCTCAGTACAGGGAGGAGGTAATAGTTATAAAACCTTCTTTTATCA
+
FFFFFFFFFFFFFFFFFFFFFFFFFFFFFFF:FFFFFF:FFFFFFFFFFFFFFFFFFFFFFFFFFFFFF
FFFFFFFFFFFFFFFFFFFFFFFF:FFFFF:FFFFFFFFFFFFFFFFFF
@A00721:190:HGJW7DSXY:4:2116:4716:30765_1:N:0:TCTGCAAG+GCTGCACT
ATGGACAAAATCTGGGATCATAGGTATGTGTCACGCTACACTCGAAATATGGGCCCTCATAAGGAAGT
ATAGTTGGCGTCTTGTCGTTGACGTCATTACAGTCTGGAAGTTAGATCATTTGTTCAGGAAGATCTCT
ACA
+
FFFFFFFFFFFF:FFFFFFFFFFFFFFFFFFFFFFFFFFFFFFFFFFFFFFFFFFFFFFFFFFFFFFFFF
FFFFFFFFFFFFFFFFFFFFFFFFFFFFFFFFFFFFFFFFFFFFFFFFFFFFFFFFFFFFFFFFFFFFFFF
FFF
@A00721:190:HGJW7DSXY:4:2254:32922:22091_1:N:0:TCTGCAAG+GCTGCACT
TTTAGCAACTTTAGATAAATTCTTTCCGGGCTCAATGACCTTTGCTATGTACGTGGATCTAATCCTTG
GTGATCTGAATCTGACCATCGCAAAGAAGACTTTATTAGTTCATGAAGTTCTTACACTTCTCGCGACT
AA
+
FFFFFFFFFFFFFFFFFFFF,FFFFFFFFFFFFFFFFFFFFFFFFFFFFFFFFFFF,FFFF,FFFFFFFFF
FFFFFFFFFFFFFFFFFFFFFFFF:FFFFFFFFFFFFFFFFFFFFFFFFFFFFFFFFFFFFFFFFFFFFF
FF
@A00721:190:HGJW7DSXY:4:2217:3866:28761_2:N:0:TCTGCAAG+GCTGCACT
CTTTATCTTAAAGCATTACCACCAAATTTAGCACTTTTTGAATATGACAGAAGCTCGCCATATCAGAT
```

TAGAGACATGCCAGTTAGAGTTCTTCTAACCAGGACTGATGGTGAGAT
+
F::FFFFFFFFFFFFFFFFFFFFFFFFFFFFFF:F:FFFFFFFFFFFFFFFFFFFFFFFFFFFFFF:
FFFFFFFFFFFFFFFFFFFFFFFFFFFFFFFFFFFFFFFFFFFFFFFF
@A00721:190:HGJW7DSXY:4:1605:28320:10488_2:N:0:TCTGCAAG+GCTGCACT
GGAGGTCCAGGAACCTTTACTCCTAGTGTCTTTTCTAGAGTCCAAGGACGTTTCCGTCGGAGGATGAC
AGGTGGGAGACCGCTCAATAACACTCTATTGAATCCCTTTTCCACGAT
+
FFFFFFFFFFFFFFFFFFFFFFFFFFFFFFFFFFFFFFFFFFFFF:FFFFFFFFFFFFFFFFFFFFFFFF
FFFFFFFFFFFFFFFFFFFFFFFFFFFFFFFFFF:FF:FFFFFFFFFFFF
@A00721:190:HGJW7DSXY:4:1476:20916:19429_1:N:0:TCTGCAAG+GCTGCACT
GATATCGCCTCTGTCCTTTCTCCTTTAATCGTGAGGTTACGAGAGTTTCGTACTTGGGTTTATCGGTT
GTATTTTGATAAATTTGTTGGAATCGATAATGATACTGACGAAGGTTCTATTACCACCCTTGTCATTG
ATA
+
FFFFFFFFFFFFFFFFFF:FFFFFFFFFFFFFFFFFFFFFFFFFFFF:FFFFFFFFFFFFFFFFFFFFFFF
FFFFFFFFFF:FFFFFFFFFFFFFFFFFFFFFFFFFFFFFFFFFFFFFFFFFFFFFFFFFFFFFFFFFFF
FFF
@A00721:190:HGJW7DSXY:4:1326:8974:8766_2:N:0:TCTGCAAG+GCTGCACT
TGATGCTTTCTTAGAGGCTCGTCTAGCCGCTGAGATTCGTCTTACCTATTTACAATCTGTAGAGAGAG
AGGTTAATCAGAATTTGAGTGATAACGATTTAGTACTGGGAAATGGTG
+
FFFFFFFFFFFFFFFFFFFFFFFFFFFFFFFFFFFFFFFFFFFFFFFFFFFFFFFFFFFFFFFFFFFF
FFFFFFFFFFFFFFFFFFFFFFFFFFFFFFFFFFFFFFFFFFFFFFFF
@A00721:190:HGJW7DSXY:4:2660:26476:36699_1:N:0:TCTGCAAG+GCTGCACT
TTTTCTTTCCCTCCCCTTTTTCGATTGGATTCGACGGGGTCTTCGTCTCACGACTAATTTGATAAGGT
TTTTCTTTTATGGGACTCTTGTTTGTACTTATGAAGGAGGTATTACTTCGGAAGTTCTTAATGTAGGT
CTC
+
FFFFFFFFFFFFFFFFFFFFFFFFFFFFFFFFFFFFFFFFFFFFFFFFFFFFFFFFFFFFFFFFFFFF:
FFFFFFFFFFFFFFFFFFFFFFFFFFFFFFFFFFFFFFFFFFFFFFFFFFFFFFFFFFFFFFFFFFFFF
FFF
@A00721:190:HGJW7DSXY:4:1610:17906:28056_1:N:0:TCTGCAAG+GCTGCACT
GGTTTATGTTTCGGTTTCAGCTGGATGGATCTTTTGATGGTATGTTTCACATTGCGTGATCACTTCCC
GTGGACTTGGGAAGAGATTAAACATAAAATCCAGCGATTCTATTATGTCGCTAAAGCCTGTCCAGTTG
TCG
+
FFFFF:,FF:FFFFFFFFFFFFFFFFFFFFFFFFFFFFFFFFFFFF:FFFFFFFFFFFFFFFFFFFFFF
FFF:FFFFFFFFFFFFFFFFFFFFFFFFFFFFFFFFFFFFFFFFFFFFFFFFFFFFFFFFFFFFFFFFF
FFF
@A00721:190:HGJW7DSXY:4:1455:7780:5321_2:N:0:TCTGCAAG+GCTGCACT
GGCTGTGGGGATTAACTTCCCTCGTGAAACTTACGACGATGTTCTCGCTCGCATTTCACAAGGATTAC
TAAGTCTGAGATCTGCCACTTCTAATTTTAGGAAACGTCGCGAAAAGA
+
FFFFFFFFFFFFFFFFFFFFFFFFFF:FFFFFFFFFFF:F:FFFFFFFFFFFFFFFFFFFFFFFFFFFFFF
F:FF:FFF:FFFFFFFFFFFFFF,FF:FFF:FFFFFFFF:FFFF:FFF
@A00721:190:HGJW7DSXY:4:2109:28718:36135_2:N:0:TCTGCAAG+GCTGCACT
GTCTTGGTCGAAAAATATCGCCGGGAACTTCTAATAGAGGATCCTGATGACATTATTTCAACGGATAC
CGATCTCAGTTCTACCACAGAAACCGTCTCAGGAACTGTCTCCACTGA
+
FFFFFFFFFFFFFFFFFFFFFFFFFFFFFFFFF,:F:FFFFFFFFFFFFFFFFFFFFFFFFFFFF:FFFFF
FFFFFFFFFFFFFFFFFFFFFFFFFFFFFFFFFFFFFFFFFFFFFFFF
@A00721:190:HGJW7DSXY:4:1462:12102:35321_2:N:0:TCTGCAAG+GCTGCACT
AAAGACGACCTTCTTGTGGGGACTGAGACCTCTGAATTTTCAGGGTGCCCTGATGAGGACGTTGAAAG

GGATAATTTCAACTCTGAGACTTCTAAGAGTGTTTCAAAAGACCAGAA
+
FFFFFFFFFFFFFFFFFFFFFFFFFFFFFFFFFFFFFFFFFFFFFFFFFFF:FFFFFFF:FFFFFF
:FFFFFF:FFFFFFFFFFFFFFFFFFFF:FFFFFFFFFFFFFFFFFF:
@A00721:190:HGJW7DSXY:4:1662:21974:20071_1:N:0:TCTGCAAG+GCTGCACT
TAAAGGTAGGAAGGGGAGAAAACAGAACAAGCGAGCGGTTGAAGAGAAAACTGATATGGTTGCGGCTG
ATCGTACCCCAATTCCTGCTAGAAAGATCACTCTCGGCGATACCACTGATCTTCCAGAGTTACGTAGT
GGT
+
FF:FFFFFFF:,FFFFF:F:F,FFFFFFF,:FF:FFFFFFF:FFFFF:,FF:FFF,F::FFFFFF:FF
,FF::F:FFFF:FF::FFFFFFF:FFFF:FFFFFFF::FFFFF:F:FFFFFFFFFF,:FFF:F:FFFF
,FF
@A00721:190:HGJW7DSXY:4:1373:13819:2033_1:N:0:TCTGCAAG+GCTGCACT
TTTTATCAAAGAGAAAGTTTCTGCTAGTCGCGACGATAGTATTTATCGGAGTGATATTAACAAGTTAA
ACTTCGAACTGACCACTTACAAGAGTGTTGAAGTACCGCAACATGGATCAGTTTTCCAAAAAGCTATT
AAT
+
FFFFFFFFFFFFFFF:FFFFFFFFFFFFFFFFFFFFFFFFFF::FFFFFFFFFFFFFFFFFFFFFFFF
FFFFFFFFFFFFFFFFFFFF:FFFFFFFFFFFFFFFFFFFFFFFFFFFFFFFFFFFFFFFFFFFFFFF
FF:
@A00721:190:HGJW7DSXY:4:1648:7988:9220_2:N:0:TCTGCAAG+GCTGCACT
AAACTGAATCCACTCTCTTGGGATATAAATGAGTACATCTATAATAGAGTTTTGGAACTGTGTAATGT
CCTACGTACTGTCCAAGGTGTTTGGGCTTCTCTAAAGAACAGCAACTT
+
FFFFFFFFFFFFFFFFFFFFFFFFFFFFFFFFFFFFFFFFFFFFFFFFFFFFFFFFFFFFFFFFFFFF
FFFFFFFFF:FF,FF:FFFFF:FFFFFFFFFFFFFFFFFFFFFFFFFFF
@A00721:190:HGJW7DSXY:4:1630:22752:11616_2:N:0:TCTGCAAG+GCTGCACT
GGCTGATGACTTAAATTTCGCATTCTGCTTCGACGGTAATTCCATAAGACCTTTCTTGGGGAGTAAAG
AGGGTTTGAAAATTACGGAACCTTTCCTCGTTCTTCATGAAGATCTTC
+
FFFFFFFFFFFFFFFFFFFFFFFFFFFFF,FFFFFFFFFFFFFF,FFFFFFFFF,F,FFFFF:FFF:FF
FFFFFFFFFFFFFFFFFFFFFFFFFFFFFFFFFFFFFFFFFFFFF:,FF
@A00721:190:HGJW7DSXY:4:1423:14009:14638_2:N:0:TCTGCAAG+GCTGCACT
GTTATTCTTGCGAAGGTTCAAGTCGATTGATTTGTTGACTACGATCTCTCTTGTAATAAGAAGTTCT
TCGCAGTTGAATCTCCACCAGGTGGAGGGAAGACCACTGAACTTATAG
+
FFFFFFFFFFFFFFFFFFFFFFFFFFFF::FFFFFFFFFFFF:FFF:FFF,FFFFFFFFFF:F:FFFF
FFFFFF:FFFFFFFFFFFFFFF:FFFFFFFFFFFFFFFFFFFFFFFFFF
@A00721:190:HGJW7DSXY:4:1433:24207:5306_1:N:0:TCTGCAAG+GCTGCACT
TTCGCAAAAGGTCTTGAAGTACGTATTGTCACTGCTAATCGTAATTCGCGAGAAGAGATAGTGACTAA
AGTTTTGGCACTTCTAGTTCATCGCGGTATTTGTGAAATTAAGACAGACGTGAAAGTTATGATGAGTG
ATC
+
FFFFFFFFFFFFFFFFFFF:FFFFFFFFFFFFF:FFFF:F::FFFFFFFFFFFFFFFFFFFFFFFFFF
FF:FFFFFFFFFFFFFFFFFFFFFFFFFFFFFFFFFFFFFFFFFFFFFFFFFFFFFFFFFFFFFFFFF
FFF
@A00721:190:HGJW7DSXY:4:1262:9155:28087_2:N:0:TCTGCAAG+GCTGCACT
AGCTGATTCTTATTGCATGAACGTCGCTGAGGTCAAACCTGTGGACGTTCTTTTCATCGACGAATTAT
TCATGATTCACGCTGGTCAGACCCTTTTTCTTACACATCTACTTAAGG
+
FFFF,F::FFFF:FFFFF:FFFFF,FFFFFFFFFFF,FF:,F:FFFFFF:FF,FFFFFF:F,FFFFFF
F,FFFFFFFFFF:F,F:FFFFFFFFF,FF:FFF,,FF:FFFFFFFF,F
@A00721:190:HGJW7DSXY:4:1361:10782:23578_2:N:0:TCTGCAAG+GCTGCACT
GTTACGGTGATTCCAATCAAATCGGATTCATCAATCGTATTGATCTCCAAGTCGCAAACTACTTCAAC

```
ATCAACGACATCATTCTTGATGAGTGTCGTTCTTATCGAAGTCTCAGT
+
FFFFFFFFFFFFFFFFFFFFFFFFFFFFFFFFFFFFFFFFFFFFFFFFFFFFFFFFFFFFFFFFFFFFFF
FFFFFFFFFFFFFFFFFFFFFFFFFFFFFFFFFFFFFFFFFFFFFFFF
@A00721:190:HGJW7DSXY:4:1266:5882:7200_2:N:0:TCTGCAAG+GCTGCACT
CGGCCGACGTTTGTCGCGAACTCTCAGAGGTTTACGGCAGAAAAATTTCTCCTGCTTGTAATTCGAGA
GAGGACACTATGGAAATTAGACCTATCAGATGTCTTGACGACTTCGAA
+
FFFFFFFFFFFFFFFFFFFFFFFFFFFFFFFFFFFFFFFF:FFFFFFFFFFFFFFFFFFF:FFFFFF
FFFFFF,FFFFFFFFFFFFFFFFFFFFFFFFFFFFFFFF:FFFFFFFF
@A00721:190:HGJW7DSXY:4:2605:1994:32659_2:N:0:TCTGCAAG+GCTGCACT
ATATTACTTTCACTCAAGGTGAAAAGTTTGAAATTAACAAAGTCCTTGGTAAGAGTAAGGTTAAATGT
GAAGATGCTCAGACTATTCATGAAGTTCAAGGTAATACTTACCAGCAC
+
FFFFFFFFFFFFFFFFFFFFFF:F:FFFFFFFFFFFFFFFFFFFFFFFFFFFFF:FFFFFFFFFFFF:
FFFF:FFFFFFFFFFF:FF:FFFFFFFFFFFFF:FFFFFFFF:FFFFF
@A00721:190:HGJW7DSXY:4:2631:9968:21042_1:N:0:TCTGCAAG+GCTGCACT
ATGACCCTGTTTTTGCATCACAAACACATATTACCGTTGCTCTATCACACACGTTGAATCTTTACGAT
ACTTTGTTATCAATGTAAAGTTAAACGATGCTACTTGTGAGAGGATCAATCACAGTAAGGAACTGTCA
CGT
+
FFF:FFFFFFF:FFFFFFFFFFFFFFFFFFFFFFFFFFFFFFFFFFFFFFFFFFFFFF::FFFFFFF
FFFFFFFFFFFFFFFFFFFFFFFFFFFFFFFFFF:FFFFFFFFFFFFFFFFFFFFFFFFFFFFFFFFF
FFF
@A00721:190:HGJW7DSXY:4:1555:11822:20212_1:N:0:TCTGCAAG+GCTGCACT
TGCAGATTCAACCCCTTATATCGAGGCTATCGAGGACGATGAGTATCTTGAAGTTGTTGACGACAAGC
CTCAAGAGGGTAGAGGACCACCTGGATCTTCTACTGTCTCTGCCTTCCAAGATTTCTTAGAAGAGGTT
ATT
+
FFFFFFFFFFFFFFFFFFFFFFFFFFFFFFFFFFFFFFFFFFFFFFFFFFFFFFFFFFFFFFFFFFFF
FFFFFFFFFFFFFFFFFFFFFFFFFFFFFFFFFFFFFFFFFFFFFFFFFF:FFFFFFFFFFFFFFFFF
FFF
@A00721:190:HGJW7DSXY:4:1641:13901:10222_1:N:0:TCTGCAAG+GCTGCACT
CTTAACGTCACTCGGTAATCTTTCAGCCGAATTAAGTACTAATGAGTTCAAGTCGGTTGTCGATCGAA
TTAAGATCAAGGCTAATACCTTTATAGGTAAGAAGCCCCTTGAGCCGCGTAGTCAGCGCGTTTAGCTG
TAT
+
FFFFFFFFFFFFFFFFFFFFFFFFF:FFFFFFFFFFFFFFFFFFFFFFFFFFFFFFFFFFFFFFFFFF
FFFFFFFFFFFFFFFFFFF:FFFFFFFFFFFFFFFFFFFFFFFFFFFFFFFFFFFFFFFFFFFFFFFF
FFF
@A00721:190:HGJW7DSXY:4:1446:4101:18302_1:N:0:TCTGCAAG+GCTGCACT
TATACCCGATAGGGTACCGAGTCTCCAAGAGAATATTTATTCTTATGAGGCTCGAAACTTCAACGTTT
TCACAACGGAAAGACATATCGCACCCGAAATTTTTGGGCAGAATGCTCTTAACGCGGTTCTTGAACGG
TGT
+
F:FFFFFFFFFFFFFFFFFFFFFFFFFFFFFFFFFFFFFFFFFFFFFFFFFFFFFFFFFFFF:FFFFF
FFFFFFFFFFFFFFFFFFFFFFFFFFFFFFFFFFFFFFFFFFFFFFFFFFFFFFFFFFFFFFFFFFFF
FFF
@A00721:190:HGJW7DSXY:4:1621:29441:3004_2:N:0:TCTGCAAG+GCTGCACT
CTAAGTTGGATAAAGTTATAGGAGACTCAGTTAACCTGAGAGCTCCTCAATTGATAGATTGGGTCAAC
AATAGGGACGCTAGTATACTTCGTAAAGTCGACAGCGAAATGGTCAAT
+
FFFFFFFFFFFFFFFFFFFFFFFFFFFFFFFFFFFFFFFFFFFFFFFFFFFFFFFFFFFFFFFFFFFF
FFFFFFFFF:FFF:FFFFFFFFFFFFFFFFFFFFFFFF,FFFFFF,FF
```

```
@A00721:190:HGJW7DSXY:4:2450:27579:31187_1:N:0:TCTGCAAG+GCTGCACT
ATATGACCTATTTTAAGCTCATGGTGAAAAGGGAAGCTAAGGTTAAACTTGATGCTTCAGCCTTTTCG
AAGCATAACCCCGCACAGAATATCGTTTATCACGCAAAATTTATTAATGCAATTTTCGGACCTATCTT
TAA
+
FFFFFFFFFFFFFFFFFFFFFFFFFFFFFFFFFFFFFFFFFFFFFFFFFFFFFFFFFFFFFFFFFFFF
FFFFFFFFFFFFFFFFFFFFFFFFFFFFFFFFFFFFFFFFFFFFFFFFFFFFFFFFFFFFFFFFFFFF
FFF
@A00721:190:HGJW7DSXY:4:1240:3884:3521_1:N:0:TCTGCAAG+GCTGCACT
TGTCTTTTACCCAACATTTTTATGTATACAGAGATGACCAGTGACGATTTCTCCCGGAAGCTCTTCTC
TCTCTTAGGAGGTCATGATGACTACTATAAGGGTGAAATCGATTTTTCGAAGTTTGACAAATCTCAAG
ATT
+
FFFFFFFFFFFFFFFFFFFFFFFFFFFFF:FFFFFFFFFFFFFFFF:FFFFFFFFFFFFFFFFFFFFF
FFFFFFFFFFFFFFFFFFFFFFFFFFFFFFFFFFFFFFFFFFFFFFFFFFFFFFFFFFFFFFFFFFFF
FFF
@A00721:190:HGJW7DSXY:4:1152:18665:30311_2:N:0:TCTGCAAG+GCTGCACT
AGCCTATGAGATAAAGATGTATTCTTATTTAGGCTTCGATGCTGAATTTCTTGAACTTTGGAGTGCCA
GTGAATATTTTTGTCACGCCAGGAGATCGGAAGAGCGTCGTGTAGGGA
+
FFFFFFFFFFFFFFFFFFFFFFFF:FFFFFFFFFFFFFFFFFFFFFFFFFFFF:FFFF:FFFFFFFFF
FFFF:FFFFFFFFFFFFFFFFFFFFFFFFFFFFFFFFFFFFF:FFFFF
@A00721:190:HGJW7DSXY:4:1108:2736:3380_1:N:0:TCTGCAAG+GCTGCACT
TAACACATTCTTTGGTAATTGTATGATCACTCTTATGATGCTCTCTCTTTATTACGACCTTCGGAAGT
TTAGAGCCGTCGCTGTCGCCGGAGACGATTCGATCCTCTTTTCGGACGTACCGGTCCAGGATTATTCA
GAT
+
FFFFFFFFFFFFFFFFFFFFFFFFFFFFFFFFFFFFFFFFFFFFFFFFFFFFFFFFFFFFFFFFFFFF
FFFFFFFFFFFFFFFFFFFFFFFFFFFFFFFFFFFFFFFFFFFFFFFFFFFFFFFFFFFFFFFFFFFF
FFF
@A00721:190:HGJW7DSXY:4:1553:21450:14810_2:N:0:TCTGCAAG+GCTGCACT
CAGCGTACTTCTGCTCGCGATTTATTCTTTTTGTCAATGATTACATCTACTTCGTTCCTGATCCTTAC
AAGATCATCGTTAAGTTAGGTAAACCCCTAACCGATGTTACTCTTGTA
+
FFFFFFFFFFFFF,FF::FFFFFFFF:FFFFFFFFFFFFFFFFFFF:FFFFFFFFFFFFFFFFFFFFF
FFFFFFFFFFFFFF:FFFFFF:FFFFFFFFFFFFFFFFFF:FFFFFFF
@A00721:190:HGJW7DSXY:4:1265:15167:7435_1:N:0:TCTGCAAG+GCTGCACT
TTTAAAGACTATACTAAATATCTCCACTACGAGGTTGTTGTTTGGCGATTAGCTCTTGCGACTAATAT
TCGATATTCGATTGAGGGTCGTTATACCTATTCGGCTATCTGTGCTTTGAATTGTATAAGATCAAATT
TTA
+
FFFFFF,FFFFFFFFFFFF:FFFFFF:FF:F,FFFF,FF,FF:FFF,FFF::FF,FFFFFF,FFFFFF
F:FFFFFF:FFFF:FF:FF:FFFFFFF:FFFFFFF:FFFFFFFFFFFF,F,FFFFFFFFFFFFFFFFF
FFF
@A00721:190:HGJW7DSXY:4:1633:22833:30733_2:N:0:TCTGCAAG+GCTGCACT
CCTGATGTGGAGGGAGTTCTTGAAGCATCCCGACCTTTCCTTGTGAAAATTTTGATGCGGTTTCATAA
GATTATCAGTAATCCTGATTCTACTACGGATTCATGGACCTTCCATCC
+
FFFFFFFFFFFFFFFFFFFFFFFFFFFFFFFFFFFFFFFFFFFFFFFFFFFFFFFFFFFFFFFFFFFF
FFFF:FFFFFFFFFFFFFFFFFFFFFFFFFFFFFFFFFFFFFFFFFF
@A00721:190:HGJW7DSXY:4:1444:12048:28870_2:N:0:TCTGCAAG+GCTGCACT
GAGAGACGTATCTCAAAGATCGACTTCGAGACAGGTGATATTGTTGAGCGAGAGCGACCAAGATCTAA
ATTCGGTAACGGTGCGTGGCGCTTTATTTTAAAAAGCTCCCTCCTCTC
+
```

FFFFFFFFFFFFFFFFFFFFFFFFFFFFFFFFFFFFFFFFFFFFFFFFFFFFFFFFFFFFFFFFFFFFFFFF
FFFFFFFFFFFFFFFFFFFFFFFFFFFFFFFFFFFFFFFFFFFFFFFFFFF
@A00721:190:HGJW7DSXY:4:1373:28953:8390_2:N:0:TCTGCAAG+GCTGCACT
CTGATACTTATCACGAAAGGACTTGAGTCTTTTGATCAGTCTCTGACTTGACCATCTTTCATTTTAAA
TGACGATCATTAAGACTCTCTTTCTTCTTTGTTTCCTTCTTTGCACTG
+
FFFFFFF:FFFFFFFFFFFFFFFFFFFFFFFFFFFFFFFFFFFFFFFFFFFFF:FFFFFFFFFFFFFF
FF:FFFFFFFFF:FFFFFFFFF:FFFFFFFF:FFFFFFFFFFFFFFFF
@A00721:190:HGJW7DSXY:4:2524:26160:24189_2:N:0:TCTGCAAG+GCTGCACT
CAGCTTGTGTTTTACCTAATCTGTGTTTTTACGATAACAACTATCAGTTGATTAAGACTCTTCCGAAA
GGCGAACTTTGGGAATTTGAAGCTCAGGGTGAAGCACAACGTTTGACC
+
FFFFFFFFFFFFFFFFFFFF:FFFFFFFFFFFFFFFFFFFFFFFFFFFFFFFFFFFFF:FFFFFFFFFF
FFFFFFFFFFFFFFFFFFFFFFFFFFFFFFFFFFFFFFFFFFFFFFFF
@A00721:190:HGJW7DSXY:4:1558:21603:27445_1:N:0:TCTGCAAG+GCTGCACT
GTTACGGATATACTCTCCTTGAGTTCACTCGTAAGGACACTACCACTTATTACGATATTTCTTTAGTC
GATGGGTTTTCGACGCCTTTAAGCGTCTTCTGTTCATCGCAACCAGATGATATCTTCTATTCGAACTT
ATG
+
FFFFFFFFFFFFFFFFFFFFFFFFFFFFFFFFFFFFFFFF:FFFFFFFFFFFFFFFFFFFFFFFFFFF
FFFFFFFFFFFFFFFFFFFFFFFFFFFFFFFFFFFFFFFFFFFFFFFFFFFFFFFFFFFFFFFFFFFF
FFF
@A00721:190:HGJW7DSXY:4:1557:8919:21042_1:N:0:TCTGCAAG+GCTGCACT
GTTTACCACCGAAATATCAAGGTTCTTGTCCTTCAAACGATTGGAAGGTTACTTATCTTAATAGTACT
AAAGGGGTTTACTTACAACCCTATGACGATAGTTTCTCTTTACACACTTGTGAAGCCAATTTAGTTGT
TTT
+
FFFFFFFFF:FFFFF:FFFFFFFFFFFFFFFFFFFFFFFFFFFFFFFFFFFFFFFFFFFFFFFFFFFF
FFFFFFFFFFFFFFFFFFFFFFFFFFFFFFFFFFFFFFFFFFFFFFFF:FFFFFFFFFFFFFFFFFFF
FFF
@A00721:190:HGJW7DSXY:4:1142:32235:13072_2:N:0:TCTGCAAG+GCTGCACT
TCACTTTGTGTGTACTTGTCTCTATTTTAAGTTTTGTCTTCCTTGTTTGTCTTATTCTATTAATCACT
CATTTTAGTGAGATTATTAAAAACGCACTAAAACGTCTTCGACAACAG
+
FFFFFFFFFFFFFF,:FFFFFFFFFFFFF:FFF:FFFFFFFFFFFFFFFFFFFFFFFFFFFFFFFFFF
FFFFFFFFFFFFFFFFFFFFFF,,FFFFFFFFFFFFFFFFFFFFFFFFF
@A00721:190:HGJW7DSXY:4:1411:19425:33191_2:N:0:TCTGCAAG+GCTGCACT
TGATAGCCACCCGCCTTACAGAGGGGCAGCAGCATGACCATAATTGGTATTGACTTTGGTACCACCTT
TTCTACTGTCTGTCTCGCCTCTTCGAATGAAGTTTTAATGCTTAACAA
+
FFFFFFFFFFFFFFFFFFFFFFFFFFFFFFFFFFFFFFFFFFFFFFFFFFFFFFFFFFFFFFFFFFFF
FFFFFFFFFFFFFFFFFFFFFFFFFFFFFFF,FFFFFFFFFFFFFFFF
@A00721:190:HGJW7DSXY:4:1405:22236:2644_2:N:0:TCTGCAAG+GCTGCACT
CTACAATCATTGGTTTTTCTAATAATGATGACACTATTGTTTATGGATATGATGCTATTACTCATGAT
GGTTACGGTCAGGGAGGATTCTCAGTTTACAAGGACCTTAAGCGATGG
+
FFFFFFFFFFFFFFFFFFFFFFFFFFFFFFFFFFFFFFFFFFFFFFFFFFFFFFFFFFFFFFFFFFFF
FFFFFFFFFFFFFF:FFFFFFFFFFFFFFFFFFFFFFFFFFFFFFFFF:F
@A00721:190:HGJW7DSXY:4:2273:12617:35900_2:N:0:TCTGCAAG+GCTGCACT
ACATTACCCGAAAGACGTGCTAAATTAAAACCATTGTACGATGTCGTCTGTCTCGGCGATCACTTTTC
ACTAAGAATTGGTCCTACTTTTGGACAAGGCCAACTTCGTAATCTTCA
+
:FFFFFFFFFFFFFFFFFFFFFFFFFFFFF:FFFFFFFFFFFFFFFFFFFFFFFFFFFFFFFFF:FFFF
FFFF:FFFFF:FFFFFFFFFF:FFFFFFFFFFFFFFFF:FFFFFFFFF

```
@A00721:190:HGJW7DSXY:4:1575:31485:1971_2:N:0:TCTGCAAG+GCTGCACT
TTTATCTCTTTGGTTGTTAGGGATTTTGAGAGAGTGAAAGGCTTCAATTGTAGTGGTTTGGTCGTTTC
AGTACCCAGTCAGTATACCTCTACGCAAAGATATTTCATGAAAACTAC
+
FFFFFFFFFF,FF:FFFFFFFFF:FFFFFFFFFFFFFF:FFFFFFFF:FFFFFFFFFFFFFFFFFFFF
FFFF:FFFFFFFFFFF:FFFFFF,FFFFFFFFFFFF:FFFFFFFFFFF
@A00721:190:HGJW7DSXY:4:2369:31114:14951_1:N:0:TCTGCAAG+GCTGCACT
CCGGATTACCTGTCTTTCATATTATGAATGAACCTTCGGCCGCTCTCTTTGCTTCGATGTTGGATGTC
AAGAAGGGTTCAGAATGGGATTCCTACGTAGTTTATGACTTTGGTGGTGGTACTTTCGATACTTCTAT
CGT
+
FFFFFFFFFFFFF,FF:FFFFFFFFFFFFFFFFFFFFFFFFFFFFFFFFFF:FFFFFFFFFFFFFFFF
FFFFFFFFFFF:FFFFFFFFFFFFFFFFFFFFFFFFFFFFFFFFFFFFFFFFFFFFFFFFFFFFFF:F
FFF
@A00721:190:HGJW7DSXY:4:2416:1624:28291_2:N:0:TCTGCAAG+GCTGCACT
TACTATAGTGTTATTTATTCTGGTGGTGATGATAATCTTGGAGGTCGTGACGTTGATGAAGCTATAAG
AGCGTTCTTATTACGGCGCACTTCTTTACCCGCCACCACACAACTTTC
+
FFFFFFFFFFFFFFFFFFFFFFFFFFFFFFFFFFFFFFFFFFFFFFFFFFFFFFFFFFFFFFFFFFFF
FFFFFFFFFFFFFFFFF:FFFFFFFFFFFF:FFFFFFFFFFFFFFFFF
@A00721:190:HGJW7DSXY:4:2333:32371:9017_1:N:0:TCTGCAAG+GCTGCACT
TGAAGGAGGAGGTTTCTGTTACTGGAAAACCCTCGTCAATACTTTTCAATAATATACGTCTTGAACTC
ACTTATGACGATCTAGTGAAATTAACGCGTCCTTTTCTGGAACGTAGTTTTACTATTATGAACAAAGT
TAT
+
:FFFFFFFFFFFFFFFFFF,FFFFFFFFFFFFFFFFFFFFFFFFFF:FFFFFFFFFFFFFFFFFFFFFF
FFFFFFFFFFFFFFFFFFFFFFFFFF:FFFFFFFFFFFFFFFFFFFFFFFFFFFFFFFFFFFFFFFFF
FFF
@A00721:190:HGJW7DSXY:4:2158:28583:1266_1:N:0:TCTGCAAG+GCTGCACT
CCCTGATTCCGGCCGGTGGTAGCTCATTTCTACCAGGGGCTATTACTCTTGCTGAAACAGCTATTAAA
GCTGTTGAGCGGGTAATTACACCTAAACGAGCTCGATCTGCCATCGCTGAAGGATGTGCACTCTTTTC
TGC
+
FFFFFFFFFFFFFFFF:FFFFFFFFFFFFFFFFFFFFFFFFFFFFFFFFFFFFF:FFFFFFFFFFFFFF
FFF:FFFFFFFFFFFFFFFFFFFFFFFFFFFFFFFFFF:FFFFFFFFFFFFFFFFFFFFFFFFFFFFF
FFF
@A00721:190:HGJW7DSXY:4:1476:11252:8703_1:N:0:TCTGCAAG+GCTGCACT
CTTCGTCGATTGTATCAACAGTAACATTACTTCTAATAAAGGTCTCTATCAAGCTCGTATTATCGTCG
CTGCTGGATCTGCGTTACCGTTGAAGGCTAAACTCCAAGGTAATTATGAAGACTGTGATTTATCTTTC
ACT
+
FFFFFFFFFFFFFFFFFFFFFFFFFFFFFFFFFFFFFFF:F:FFFFFFFFFFFFFFFFFFFFFFFFFF
FFFFFFFFFFFFFFFFFFFFFFFFFFFFFFFFFFFFFFFFFFFFFFFFFFFFFFFFFFFFFFFFFFFF
FF:
@A00721:190:HGJW7DSXY:4:1308:14561:15092_1:N:0:TCTGCAAG+GCTGCACT
ACGTGTGCTCTTCCGATCTATAATTTCAATCTTCTTAAAAAGAAGATAGAGAGTCATACTTTGGGCTA
TCGTTCAGGTGCATGGGGTTGGATAGCCAATATTGAAGTGGACACCTCTGGTAGTATCGAAGTAAAGA
TAA
+
FFFFFFFFFFFFF:FFF:FFFFFFFFFFFF:FFFFFFFFFFFFFFFF:FFFFFFFFFFFFFFFF:FFF
F:FFFFFFFFFFFFFFFFFFFFFFFFFFFFFFFFFFFFFFFFFFFFFFFFFFFFFFFFFFFFFFFFFF
FFF
@A00721:190:HGJW7DSXY:4:1223:32524:28103_1:N:0:TCTGCAAG+GCTGCACT
ATACTATGGTTGAATCAACTAAGCATACTACTTTTGCTTTGATTACTCTACCGCCTTACAAGTCTAAG
```

```
GTTAAAGAGTCGATTAAACAAGTTGTACTTATCAATGATTTTATCGCTGGTTGTTATCTCGAAAAGTC
TCT
+
FFFFFFFFFFFFFFFFFFFFFFFFFFF:FFFFFFFFFFFFFFFFFFFFFFFFFFFFFFFFFFFFFFFFF
FFFFFFFFFFFFFFFFFFFFFFFFFFFFFFFFFFFFFFFFFFFFFFFFFFFFFFFFFFFFFFFFFFFF
FFF
@A00721:190:HGJW7DSXY:4:2156:29107:29512_2:N:0:TCTGCAAG+GCTGCACT
TCAGTTCTAACCCAAAGAGAATATCTAAAATATTTACAAAATCTTACCACTATCTACGAACCTAAAGT
TCTGAAGACGTTGCTTGTCGCTTCCTCCTTTAATGGCACTACCTCGGT
+
FFFFFFFFFFFFFFFFFFFF:FFFFFFFFFFFFFFFFFFFFFFFFFFFFFFFFFFFFFFFFFFFFFFF
FFFFFFFFFF:FFFFFFFFF:FFFFFFFFFFFFFFFFFFFFFFFFFFF
@A00721:190:HGJW7DSXY:4:2675:13105:26788_2:N:0:TCTGCAAG+GCTGCACT
TTGGAACGAAGGCGACCTGTTCCGGAGTTTTTACGGGATCCGGAATGTTCTGTCTATATTAGGTGAAG
CGAAATACGATTTCGAACGTCAAGATCGGAAGAGCGTCGTGTAGGGAA
+
FFFFFFF:FFFFFF:FFFFFFFFFFFFFFFFFFFFFFFFFFFFFFFFFFFF:FFFFFFFFFFFFFFFF
FFFFFFFFFFFFFFFFFFFFFFFF:FFFFFFFFFFFFFFFFF:FFFFF:
@A00721:190:HGJW7DSXY:4:1237:22064:13432_2:N:0:TCTGCAAG+GCTGCACT
ATTTGATCTGGTTGCTCAATTCTAAGCGTGTTCCGCTTAGTGCGAGGCCTCTCTTCTACGAGTATTGC
TATTTATACGGTTACGAGAAGAAGGTTCGAAAGTTTGAGAACTTAGTT
+
FFFFFFFFFFFFFFFFFFF:FFFFFFFFFFFFFFFFFFFFFFFFFFFFFFFFFFF:FFFFFFFFFFFFF
FFFFFFFFFFFFFFFFFFFFFFFFFFFFFFFFF:FFFFFFFFFFFFFFF
@A00721:190:HGJW7DSXY:4:2208:5014:33348_2:N:0:TCTGCAAG+GCTGCACT
ACATACTAAATACTAGATATTTGGATGATCTTGATAAAGAACTTCATGAATTTTCAGAGGATGAAAAA
TTTTCTTCCTTGGATTTTAATATAAAGAATCTTTACCAGGACTCCTAC
+
FFFFFFFFFFFFFFFFFFFFFFFFFFFFFFFFFFFFFFFFFFFFFFFFFFF:FFFFFFFFFFFFFFF:
FFFFFFFFFFFFFFFFFFFFFFFFFF:FFFFF:FFFFFFFFFFFFFFFF
@A00721:190:HGJW7DSXY:4:1455:7862:13917_2:N:0:TCTGCAAG+GCTGCACT
TCTGATCGTAAGAAGTTTATAAGGAACATATCCATTTGTTGTAATAGGGTCATCAACTTAGAACTCGA
AACTCTAAGTGGTAATACTGTCTTTCCAAGCTTACCTGTATCTTCAAT
+
FFFFFFFFFFFFFFFFFFFFFFFFFFFFFFFF:FFFFFFFFFFFF:FFFFFFF:FFFFFFFFFFFFF:F
FFFF:FFFFFFFFFFFFFF:FFFFFFFFFFFFFFFFFFFFFFFFF:FFFF
@A00721:190:HGJW7DSXY:4:2167:17273:20353_1:N:0:TCTGCAAG+GCTGCACT
TTCGATAGGGGACATTTTATCGTGGGCTTTCCGTAAAGGAAGATCTGTTATCGACGGTTTATCGTTTA
CGAAGTATCACTCCACCGGTGAATGGTTTAAGAATACTATAAATAGAATAGCTATTACTACTAATGGC
GTT
+
FFFFFFFFFFFFFFFFFFFFFFFFFFFFFFFFFFFFFFFFFFFFF:FFFFFFFFFFFFFFFFFFFFFFF
FFFFFFFFFFFFFFFFFFFFFFFFFFFFFFFFFFFFFFFFFFFFF:FFFFFFFFFFFFFFFFFFFFFFFFF
FFF
@A00721:190:HGJW7DSXY:4:2141:9842:26365_2:N:0:TCTGCAAG+GCTGCACT
TAGTGACATCTGGTCAATTTCGTTTTTAGGTGGTCTTTATTCTATTATTGATTTTTATGAGATTGATA
CGTTAACAGAAGATGATGTTAGAGTAGAATTCCTGAAGAGTCCTTCCG
+
FFFFFFFFFFFFFFFFFFFFFFFFF:FFFFF:FFFFFFFFFFF,FFFFFF:FFFFFFFFFFFFFFFFFF
FFFFFFFFFFFFFFFFFFFFFFFFFFFFFFFF:FFFFFFFFF:FFF,FF
@A00721:190:HGJW7DSXY:4:2341:28980:10191_1:N:0:TCTGCAAG+GCTGCACT
TTAAATCTAGGATTATGTCTGTGTTGAAAGACTTTCTACCGATAAGGGTCACTAGTCTTACACAATTA
AGAAATAATCCTACATTGACTGATTATCTAAAGATTGAAGGAAAATTTTATCCTCGTTTAACTTACTT
AGA
```

+
FFFFFFFFFF,FFFFFFFFFFFFFFFFFF:FFFFFFFFFFFFFFFFFFFFFFFFFFF:FFFFFFFFF
FFFFFFFFFFFFFFFFFFFFFFFFFFFFFFFFFFFFFFFFFFFFFFFFFFFFFFFFFFFFFFFFFFF
FFF
@A00721:190:HGJW7DSXY:4:1570:29170:10207_1:N:0:TCTGCAAG+GCTGCACT
ATACAAATGATAACTTCTAATTTACGTTTGCTACTCAGAGCTGGTCCTCTCAGCAAAGGGATGCATGG
TGTTATCTCCTCAGGAGTCCCGGCATTTGATAATAATGACGAGACTTTCGTCATAATCGCTGCATATT
ACG
+
FFFFFFFFFFFFFFFFFFFFFFFFFFFFFFFFFFFFFFFFFFFFFFFFFFFFFFFFFFFFFFFFFFF
FFFFFFFFFFFF:FFFFFFFFFFFFFFFFFFFFFFFFFFFFFFFFFFFFFFFFFFFFFFFFFFFFFF
FFF
@A00721:190:HGJW7DSXY:4:1522:29297:25019_2:N:0:TCTGCAAG+GCTGCACT
CCAGCGCATCTCGTGTCGAGCCTCGACCCGATTATATGGTATTAACTTATAAAGGTGAGGAGACCATT
TTCTTGCTGAAATTTATAGAAGATTATTTTGACAGAATCAATAAGAAT
+
FF,FFFFFFFFFFFFFFFFFFFFFFFFFFFFFFFFFFFFFFFFFFFFFFFFFFFFFFFFFFFFFFFF
FFFF:FFFFFFFFFFFFFFF:FFFF:FFFFFFFFFFFFFFFFFFFFFF
@A00721:190:HGJW7DSXY:4:2609:8901:32127_1:N:0:TCTGCAAG+GCTGCACT
TATGCTGATCTAAAATTTCGATCTAGATGGATCCACTCCTCGAGTTATCATGATGTTAGTATGTTATA
TACTGATTTACAACGATATAGGAGTAATCTACGAGAAGATTCTAGAATTCTTAATTATAATATTCAAC
CTC
+
FFF:FFFFFFFFFFFFFFFFFFFFFFFFFFFFFFFFFFFFFFFFFFFFFFFFFFFFFFFFFFFFFFF
FFFFFFFFFFFFFFFFFFFFFFFFFFFFFFFFFFFFFFFF:FFFFFFFFFFFFFFFFFFFFFFFFFF
FFF
@A00721:190:HGJW7DSXY:4:1371:27299:28541_2:N:0:TCTGCAAG+GCTGCACT
GAGATCACACATTTTGAGAACTAACTATGTTATGTTCTCTGATCATTTTGACAATCTCTTCTTATAAT
AAATAAATAAATAATATTACTAATGGCAAATAGTGGAAAGGCTCCAGT
+
FFFFFFFFFFFFFFFF:FFFFFFFFFFFFFFFFFFFFFFFFFFFFFFFFFFFFFFFFFFFFFFFFFF
FFFFFFFFFFFFFFFFFFFFFFFFFFFFFFFFFFFFFFFFFFFFFFFF
@A00721:190:HGJW7DSXY:4:1215:28221:18458_2:N:0:TCTGCAAG+GCTGCACT
TGCTTTGGATTACGATGACGTGAGAATAGGTGAAGGGATAGTTTTTACCTCGACCAACATTAAGGCGG
CGGCGACTAATCTTAAAGCTTCACCCAAAATGAAGGACACTTCGAAAC
+
FFFFFFFFFFFFFFFFFFFFFFFFFFFFFFFFFFFFFFFFFFFFFFFFFFFFFFFFFFFFFFFFFFF
FFFFFFFFFFFFFFFFFFFFFFFFFFFFFFFFFFFFFFFFFFFFFFFF
@A00721:190:HGJW7DSXY:4:2219:22363:19680_2:N:0:TCTGCAAG+GCTGCACT
AAAAGGCTCTAAAGGACACTATACAATATCCAGACGACGACGAAGCTTATACTACTTTAATTTTGGGT
ATTATGTGGAGATATCGTGATATCAGTACGTCCAGATCCTCTGCTTTT
+
FFFFF:FFFFFFFFFFFFFFFFFFFFFFFFFFFFFFFFFFFFFFFFFFFF:FFFFFFFFFFF:FFFF
FFFFFFFFFFFFFFFF:FFFFFFFFFFFFFFFFFFFFFF:FFFFFFFF
@A00721:190:HGJW7DSXY:4:2475:11849:18599_2:N:0:TCTGCAAG+GCTGCACT
ATCAAATATAAAGAAAAGGACTATGAAATAAAAGATTTGGTGCTTAGGGATATAATGAATTCAGCACC
ACATTTTGGTCAAGTCAATCCGTTGAGAAAATTTCTTAGAGGATATTC
+
FFFF:FFFFFFFFFFFFFFFFFFFFFFFFFFFFFFFFFFFFFFFFFFFFFFFFFFFFFFFFFFFFFF
FFFFFFFFFFFFFFFFFFFFFFFFFFFFFFFFFFFFFFFFFFFFFFFF
@A00721:190:HGJW7DSXY:4:2167:25717:2597_1:N:0:TCTGCAAG+GCTGCACT
CCAACATTTACCACCAAATTCTTTTGATACAACTCTAGCCTTCAAGTGGGGTATGCCTGCGGAATACG
CTTGCTATTCACCAGATTGGTTCGAACCCGTTGATGCTATGTCAGAAGAAGCTATTCAAGCTTGTCGT
TTA

+
FFFFFFFFFFFFFFFFFFFFFFFFFFFFFFFFFFFFF:FFFFFF:FFFFFFFFFFFFFFF:FFFFFFFF
FFFFFFFFFFFFFFFFFFFFFFFFFFFFFFFFFFFFFFFFFFFFFFFFFFFFFFFFFFFFFFFFFFFFF
FFF
@A00721:190:HGJW7DSXY:4:1331:24279:15765_2:N:0:TCTGCAAG+GCTGCACT
AAACAAGCCCAGACTCAAGGAGTCCAATCAAAGGTTTTGAATTTAGCTCAACTGGGGAGACGTGCTTA
AATGGGTGATAAAAATTATTTAGAGATCTTTTCTACTTACAAAGGACA
+
FF:FFF,FFFFFFFFFFFFFFFFFFFFFFFFFFFFFFFFFFFFFF:FFFFFFF,FFFFFFFFFFFFFF
F,FFFFFFFFFFFF:FFFFFFF,FFFFFFFFFFFFFFFFFFFF,FF:F
@A00721:190:HGJW7DSXY:4:2309:16387:35383_2:N:0:TCTGCAAG+GCTGCACT
AATTCTACGGAAAAGTCCGGAGTATATTGTATGTTAATAGATAGTGGGATAATAGTTCTACCGATACG
TAGTGAACGCGTTCGTAGAACCAAGTACTTTCCGTCTGAAGAGTTTAG
+
:FFFFFFFFFFFFFFFFFFFFFFFF,::FFFFFFFF,FFFFFFFFFFF:,FFFFFFFFFFFFFFF:FF
FFFFFFFFFFFFFFFFFFFFFFFFFFFFFFFFFFFFF:FFFF,,FFF
@A00721:190:HGJW7DSXY:4:2222:10267:24972_1:N:0:TCTGCAAG+GCTGCACT
TAACCGATAGGAGTTGTATAGTTGAAGATGTGGCTTTAGACGATAATCCTTTAAGGACTATGTTGACT
GACGAAGATTTTCCCACAGATTTGTCTACGTTGCAGATCGCCACTCCAAACACTATATACATTAAAAT
ATA
+
F:F:FFFFFFFF:FFFFFFFFFFFFFFFFFFF:FFFFFFFFFFFFFFFFFFFFFFFFFFFFFFFFFFFF
FFFFFFFFFFFFFFFFFFFFFFFFFFFFFFFFFFFFFFFFFFFFFFFFFFFFFFFFFFFFFFFFFFFFF
FFF
@A00721:190:HGJW7DSXY:4:1102:5249:19820_1:N:0:TCTGCAAG+GCTGCACT
CATAACTTTTGTTACCAAATTCTTGGCTTTCAACGAAGTCTCTGATTTTATTAATCATTACTTTAACG
ATCTCTCGGTCATTTACTCTGTACTTGATCTACGTCGTTTAACTGTCGGAACAACCTTTACTTAGAAT
GAC
+
FFFFF:FFFFFFFFFFFF,FF:FFF:,FFFFFFFFF,FFFFFF,:FFFFFFF:FFFFFFFFFFFFFF
FFFFFFFF:FF,FFFFFFFFFFFFFFFFFFFFFFFF:FFFFFFFFFFFFFFFFFFFFFFFFFFFFFFFF
FFF
@A00721:190:HGJW7DSXY:4:1323:14642:29575_2:N:0:TCTGCAAG+GCTGCACT
TTCTTATATTATTTACAATAGTTAATATCATATTAGTAATACTCACTATCGTTTTCTGCTTTTTCTTA
GCTAATAGATTAATAAAGGATGAGTACGAAAACCAACTCTTCGTTACG
+
FFFFFF:FFFFFFFFFFFFFFFFFFFFFFFFF:FFFFFFFFFFFFFFFFFFFFFFFFFFFFFFFFF:FF
FFFFFFFFF,FFFFFFFFFFFFFFFFFFFFFFFFFFFFFFFFFFFFFF
@A00721:190:HGJW7DSXY:4:1139:3748:34068_1:N:0:TCTGCAAG+GCTGCACT
GTAGAATAGTTTTAGTCTCTACACCCATCTCAACGCGGATAACGCGTGTTTTTCGTCCTGAACGTAAT
ACACGAAGAGATCAGCCGCAAACGACTCGAAACCGAGACGCCGTAGATCAAAGTCCCTTCTAACTTTA
GAT
+
F:,,F,F:,,FF:,:,,,:,,F:FFFF,,,:F,,,,:,FF:F,,,F,,FFFF,::,F:F,F,F,:F:,
:F,F,:,:,,:,FF,FF,,F,:FF,FFFFFFFFFFFFFFFFFFFFFFFFFFF:FFFFF:FFFFFFFFFF
FFF
@A00721:190:HGJW7DSXY:4:1219:8730:33489_1:N:0:TCTGCAAG+GCTGCACT
CTTTACTCATTTTATAGCAGTTATTTATGTTGTACTTTTTATAATTTATAACCTTCTTATACTTTTGG
GTATGTTTTGTGAAAAACATGAGATACCCAAGTCTATCGAAGACATTATCTCTTATGTACTTATTGTA
ACG
+
F:FFFFF:FFFFFFFF:FFFFFFFFFFFFFFFFFFFFFFFFFFFFFFFFFFFFFFFFFFFFFFF:FFFF
FFFFFF:FFFFFFFFFFFFFFFFFFFFFFFFFFFFFFFFFFFFFFFFFFFFFFFFFFFFFFFFFFFFFF
FFF

```
@A00721:190:HGJW7DSXY:4:1307:30002:25927_1:N:0:TCTGCAAG+GCTGCACT
TTCTTATTCTTTTATTCCTTTAACTGTGTATGAGGAGGATTTATAATGCTTTTCGATAGTGATTGTTA
TCAATTTAAATACATTACTGTTTCAAATCATCTTTACTCACAGACTCCGAAAATGGTTTCAAATGAGG
TCA
+
FFFFFFFFFFFFFFFFFFFFFFFFFFFFFFFFFFFFFF:FFFFFFFFFFFFFFF:FFFFFFFFFFFFF
FFFFFFFFFFFFFFFFFFFFFFFFFFFFFFFFFFFFFFFFFFFFFFFFFF:FFFFFFFFFFF:FFFF:
FFF
@A00721:190:HGJW7DSXY:4:1307:30002:25927_2:N:0:TCTGCAAG+GCTGCACT
TATTAAACGAACTATTATCCGATTCTTCGAGGGTATCACTACCACTGATCTGCCGACTTTAGTCAAGG
AACTTCAATTCCTTCGATGTTTATATTTTTCTATAAGGGAAAATAGCA
+
:FFFF:FFFFFFFFFFF:FFFF:FFFFFFFF:FFFFFF:FFFFFFFFFFFFFFFFFF:FFFFFFFFFF
FFFF::FFFFFFFFFFFFF:FFFFFFFFF:FFFFFFFFFFFFFF:FFF
@A00721:190:HGJW7DSXY:4:1162:3405:26459_2:N:0:TCTGCAAG+GCTGCACT
TTCGACAAATTTTAGAACTTTATTTCAGAACTCTCTCCGATCACCTTGGTGTGATCTCCCGGAGGATC
TTTTCAATAAGATGTATGATGATAATCTACTATTTTTAAATAGTGATA
+
FFFF:FFFFFFFFFFFFFFFFFF:F:FFFFF:FFFFFFFFFFFF::FFFFF:FFFFFFFFFFFFFFFF
FFFFFFFFFFFFFF:FFFFFFFFFFFFFFFFFFFFFFFFFFFFFFFFFF
@A00721:190:HGJW7DSXY:4:1418:21386:26944_1:N:0:TCTGCAAG+GCTGCACT
ACTTTATCAAAGATTTGGCTTGGTCTATTTACCGCAAAAAGTTGGAAGAGGTCTATGATATTTATTTT
ACTTATACTTTAAATAGCACCGATTATAACTTGACTGTTGATAATATTACAATAGCTCTATCTCAGAA
GA
+
FF:FFFFFFF,:FFFFFF,FF:,,F:FFFFFF,FFFFF:,F:FFFFFFFF,FFFFFFFFFFFFFF,,F
F,F,FFFFFFFFFFFFFF:FF:FFFFF:F,::FFFFF:F:FFF,:FFFFFFF,FF,FFF,:FFFFFFF
,F
@A00721:190:HGJW7DSXY:4:1133:14064:32706_2:N:0:TCTGCAAG+GCTGCACT
AACCGGTGAACACGTCGGTAGCATTGATTTACTTTTACCGTTAGAACTGAAAAAGATTCTTTTCGAAA
AGAAACCTATAAGTTACAAACGATTCTTCGACTATATTAAAGAAATAA
+
FF:FF,FFFF:FFFF:,FFFFFFFFFFFFFFFFFFFFFFFF:F,:FFFFFFF:FFFFFFFFFFFFFFF
FFFFFFFFFFFFFFFFFFFFFFFFFFFFFFFFFFFFFFFFFFFFFFFF
@A00721:190:HGJW7DSXY:4:1130:30391:12195_2:N:0:TCTGCAAG+GCTGCACT
ACCTGAACTTTACATTTTTAATGGTATGGTATAATGTGTCTTACAAATAGAACCTTCACTCTCGGTAA
TGAGAGTGTATTCCTTAATATTCTAGTAACCGCTATGTGGGCATTACT
+
FFFFFFFFFFFFFFFFFFFFFFFFFFFFFFFFFFFFFFFFFFFFFFFFFFFFFFFFFFFFFFFFFFFF
FFFFFFFFFFFF:FFFFFFFFFFFFFFFFFFFFFFFF,FFFF,FFFF:F
@A00721:190:HGJW7DSXY:4:1325:22562:27445_1:N:0:TCTGCAAG+GCTGCACT
GATAGCAATTTATGTTTTAAATGCTAGGAAAATTTTAGAAAAACTCCATTGTATAGTTCAAGGAGTAC
TTAATTCAATAGCTTTAGTATTATTTACTATCGCCTTTATTGAAAATTGTATAAGAGTTAATTGCTTA
CAC
+
FFFFFFFFFFFFF:FFFFFFFFFFFFFFFFFFFFFFFFFFFFFFFFFFFFFFFFFF,FFFFFFFFFFF
FFFFFFFFFFFFFFFFFFFFFFFFFFFFFFFFFFFFFFFFFFFFFFFFFFFFFFFFFFFFFFFFFFFF
FFF
@A00721:190:HGJW7DSXY:4:1406:30770:35931_2:N:0:TCTGCAAG+GCTGCACT
TTAATTGATTGAAGCTAGGCGTTGGGGAGAATGAACTGCCGCTTCCCGGAAGAAACGATCTATTTACA
GAGTATTTAGAGTAGTCCAAAGTTTGTATAAATTTATATGATTTTCTA
+
FFFFFFFFFFFFFFFFFFFFFFFFFFFFFFFFFFFFFFFFFFFFFFFFFFFFFFFFFFFFFFFFFFFF
FFFFFFFFFFFFFFFFFFFFFFFFFFFFFFFFFFFFFFFFFFFFFFFF
```

```
@A00721:190:HGJW7DSXY:4:1320:23683:15421_2:N:0:TCTGCAAG+GCTGCACT
GAAGAAAACCACTCCCTTCAATTCAATAACATAACATACCATACACTCCCCAAACATAACATACCATA
ACCTACCATGACCAATCATCGCTCCAACTGGCACGCCAGACGTAGGGT
+
FFFFFFFFFF,FFFFF,FFFF,FFFFFFFFFFFFFFFFFFFFFFFFFFFFFF:FFFFFFFFFFFFFF
FFFFFFFFFFFFFFFFFFFFFFFFFFFFFFFFFFFFFFF:FFFFFFFF
@A00721:190:HGJW7DSXY:4:1115:17083:35587_2:N:0:TCTGCAAG+GCTGCACT
ACCGCCGGTCGGCAGATCGGCACCACTGGTCTCTACGTTCAGGGACCCGCTGTGCAGAATAACCTTCC
CAAATCTAGATCTGCACAGAAGAAGGCTGAAGGTCGTTACCGGACGAC
+
:FF:FFF::F:F:FFF:F,FFFFFFFF::,FFFFFFFF,FFFFFFFF,FFFFFFFFFFF:FFFFF:FF
:F,F:FFFFFFFFF:FF:FFF:FF:::FF:FFF,FFFFFFFFF:FFFF
@A00721:190:HGJW7DSXY:4:2164:14353:29168_1:N:0:TCTGCAAG+GCTGCACT
GGCTGTTGCCCAGAGGAAAGCAGCGACCACCGAGGTCGCTCGTAACATTGACGGCAGTCCCGTCATCT
CTGTGGGAACTGAGGCAGTACCTCAGTCAAAAGTCACATTCACCACTCCCATGCCAATCAATGATGTG
ACT
+
FFFFFF:FFFFFFFFFFFFFFFFFFFFFFFFFFFFFFFFFFFFFF:FFFFFFFFFFFFFFFFFFFFF
FFFFFFFFFFFFFFFFFFFFFFFFFFFFFFFFFFFFFFFFFFFFFFFFFFFFFFFFFFFFFFFFFFFF
FFF
@A00721:190:HGJW7DSXY:4:1630:28357:9173_2:N:0:TCTGCAAG+GCTGCACT
TCAATACGTCGTGGGAGGTACTGAAGCCTTCCCCTCCGCTGGCACGAAGAACACTCCCAAGCCAATCA
ATAGTTCTTCTGACCAGAATGAAAAGGGGTTTACCAGGGTAGTCTCTA
+
FFFFFFFFFFFFFFFFFFFFFFFFFFFFFFFFFFFFFFFFFFFFFFFFFFFFFFFFFFFFFFFFFFFF
FFFFFFFFFFFFFFFFFFFFFFFF:FFFFFFFFFFFFFFFFFFFFFFF
@A00721:190:HGJW7DSXY:4:1414:15257:36839_1:N:0:TCTGCAAG+GCTGCACT
GAAGAGGGGAAATGTCCCCAAAACTGAGAAGAATAAAGGGAAAGGTCCATCAGGACCTAAGAAGGGGA
AATTACCCCTAATTCTGAAGGAAGATCTCCCACCATCTGGGGTTGGGAATAAGAAATCTGCACGGGCC
AAA
+
FFFFFFFFFF:FFF:FFFFFFFFFFFFFF::FF,:,:FFFFF:FFFFFFFFFFFFFFFFFFFFFFFFF
:F,FFFFFFFFFFFFFF:FFFFFFFFFFFFFFFFFFFFFFFFFFFFFFFFFFFFFFFFFFFFFFFFFF
FF:
@A00721:190:HGJW7DSXY:4:1142:12798:10410_2:N:0:TCTGCAAG+GCTGCACT
CTACGAAGTCGCTGAAGGCGGCTTGGGTCATTAAGGGTAGCTGTAAAGTTGCGCCTGCCGACCCCATC
TTTGAGACCAAGACTCTCTACAGTAGGTTCGTGCCTACTATGAGCAAT
+
FFFFFFFFFFFFFFFFFFFFFF:FFFFFFFF,FFFFFF,FFFFFFFFFFFFFFFFFFFFFFFFFFFFF
FFFFFFFFFFFFFFFFFFFFFFFF:FFFFFFFFFFFFFFFFFFFFFFF
@A00721:190:HGJW7DSXY:4:1275:11650:27555_2:N:0:TCTGCAAG+GCTGCACT
TCGTCACTGAGGACGAAGGTGAGAGGACTTTTGGCTACTGGCGACAAACTGAGAGCAGTCGCTGGGGA
AAGAGAATTGCGTGGGTTGAAGGCCGACGCTTCGAGCTTGAGTGTGAC
+
F:FFFFFFF:FFFFFFFFFFFFFFFFFFFFFFFFFFFFFFFFFFFFFFFFFFFFFFFFFFFFFFFFFF
,FFFFFFFFFFFFFFFFFFFFFFFFFFFFFFFFFFFFFFFFFFFFFFF
@A00721:190:HGJW7DSXY:4:1574:21305:13870_2:N:0:TCTGCAAG+GCTGCACT
GTTTTTGTCAACGGCGTTTGGAGGATTTTATCCGCGCTTAACTATGGGGCGAAGATCCCCTATGGAGT
CTTGAAGGACTTAGGTTGGCATATTTATGCCGACGATAATAAGCATGT
+
FFFFFFFFFFFFFFFF:FFFFFFFFFFFFFFFFFFFFFFFFFFFFFFFFFFFFFFFFFFFF:F:FFFFF
FFFFFFFFFFFFFFFFFF:FFFFFFFFFFFFFFFFFFFFFFFFFFFFF
@A00721:190:HGJW7DSXY:4:1529:4345:4758_1:N:0:TCTGCAAG+GCTGCACT
CCGAGGTAAGCTCGGGACTTACAAGAGAGAGTGTGAGAGAGTCGCTCTCACTATTTTTGCCCAGAATG
```

```
CGGGCTTTCTGACTGATGAGGTTAAAACTCTTGCTCCTTCTGTGGGTGAGGGTTTTTGTTATATTCAC
TGG
+
FFFFFFF,FFFFFFFFF:FF:FFFFFFF:FFFFFFFFFFFFF:FFFFFFF,FFFFFFFFFFFFFFFFF
,FFFFFFFFFFFF:FFFFFFFFFF,:FFFF:FFFFFFFFFF:FFFFFFF:FF:FFFFFFFFFFFFFFF
FFF
@A00721:190:HGJW7DSXY:4:1234:29839:20165_2:N:0:TCTGCAAG+GCTGCACT
TCCTCCATAATTACAACCTCTCTTTAGAGAGCATTAGAAGGAAGTTGGGCAAATTCCCACATATGGTA
GACGTTGTCACACTTTTCTTGAAAATTTTTCCTTTCCTCCGTCATTGG
+
FFFFFFFF:FFFFFFFFFFFFFF:FFFFFFFFFFFFFFFFFFF,FFFFFFFFFFFFFFFFFFFFFFFFF
FFFFFFFFFFFFFF,FFF:FFFFFFFFFFFFFFF:FFF:FFF:F,FFF
@A00721:190:HGJW7DSXY:4:2649:15447:11083_2:N:0:TCTGCAAG+GCTGCACT
GTGAGAAGGTGGAAGGTGTCAAGGGTCTTCTTCATGCTGATCTTTCAGGACCTAATCACATCATGATT
GATAGTTACGGCTCGTATCGAGTTGGAGGGAGGAAGTTCTCCGAAATT
+
FFFFFFFFFFFFFFFFFFFFFFFFFFFFFFFFFFFFFFFFFFFFFFFFFFFFFFFFFFFFFFFFFFFF
FFFFFFFFFFFFFFFFFFFFFFFFF:FFF:FFFFFFFFFFFFFFFFFFF
@A00721:190:HGJW7DSXY:4:2315:15022:30765_1:N:0:TCTGCAAG+GCTGCACT
GCTGTAACTGTCATTTCAGAGAGGGGAGCTCTGAAAAGGAAACTTCGTCAGAAGATGAGGAAGTCTAA
GAGGAGGAGTTTGAGGAAGGCTCTAGAGAAGATCTCACCACCTGGAGAAGGCAAGTGGAGAGTCCAGA
AGT
+
FFFFFF:FFF:FFFFFF:FF:FFFFFFF:FFFFFFFFFFFFF:FFFFFFFFFFFFFFFF::FFFFFF:
FFFFFFFFFFFFFFFFFFFFF:FFFFFFFFFFFFFF,FFFFFFFFFFFFFFFFFFFFFFFFFFF:FFF:
:FF
@A00721:190:HGJW7DSXY:4:2418:32515:17409_2:N:0:TCTGCAAG+GCTGCACT
GGATTATTTGAAGCAGGGGTTACCACTTCCTAAGGTGGATGAGGGAGGTTGTTTCTTTGGAGTTTTAG
ATAATCTTTTGAGAAGGGGTCATAGAGCCAGCAAGAGAAGCTTATATG
+
FFFFFFFFFFFFFFFFFFFFFFFFFFFFFFFF:FFFFFFFFFFFFFFFFFFFFFF:FFFFFFFF,FFFF
F:FFFFFFFF:FFFFFFF:FFFFFFF:FFFFFF,FFFFFFFFFFFFFF
@A00721:190:HGJW7DSXY:4:1319:29568:7576_2:N:0:TCTGCAAG+GCTGCACT
CCTTGAGAAGACAAGCCACATTAAGATATAATTTAGGTCAACTTGGGGTTTCGAATGTCATTAGTGCC
TTACGATTGAAAGAGGTCCTTTTGAGTATCGACAGATCTTTCGATCTT
+
FFFFFFFFFFFFFFFFFFFFFFFFFFFFFFFFFFFFFFFFFFFFFFFFFFFFFFFFFFFFFFFFFFFF
FFFFFFFFFFFFFFFFFFF:F,FFFFFFFFFFFFFFFFFFFF:FFFFF
@A00721:190:HGJW7DSXY:4:1455:7789:27633_2:N:0:TCTGCAAG+GCTGCACT
TTAACGTGGCAGAATTCGGTAACTTACCATATTGTTCCTTCCGGGAAGTTGTGGGATTTAACTTTGAC
TCAACTACTCCGATCAGATCCGGATGGTTTGATTGGTATGGACTACTC
+
FFFFFFFFFFFFFFFFFFFFFFFFFFFFFFFFFFFFFFFFFFFFF:FFFFFFFFFFFFFF:FFFFFF
FFFFFFFFFFFFFFFFFFFFFFFFFFFF,:FFFFFFFFFFFFFFFFFFF
@A00721:190:HGJW7DSXY:4:1439:19623:12242_1:N:0:TCTGCAAG+GCTGCACT
TAGTTGTCTAGGGTCATGGCAGATACTACCGATAAGGTGAAAAATATGCAGGTCAAAGATTCTGTAGC
TCTGAGGGCGGTTGAGGGAGCCGTTTTGGCCGACTTTAACTATTCTAAGAGTCTTTATGATTCAAAAC
CTA
+
FFF:FFFFFFFFFFFFFF:FFFFFFFFFFFFFFF:FFFFFFFFFFFFFFFFFFFFFFFFFF:FFFFFFFF
FFFFFFFFFFFFFFFFFFFFFFFFFFFFFFFFFFFFFFFFFFFFFFFFFFFFFFFFFFFFFF:FFFFFFF
FFF
@A00721:190:HGJW7DSXY:4:1553:25943:8719_2:N:0:TCTGCAAG+GCTGCACT
ACCTTACAAGGTTGAGGAGACTTTACAGCGGCTTATTGTCGCGCAGTTTCCAGAATTGCGGTTGAAGT
```

```
TCACCCATTCTATCTTGAATGCTCACGCCGTCGCAGCCATATCGAGAC
+
FFFFFFF:FFFFFFFFFFFFFFFFFFFFFFFFFFFFFFFFFFFFFFFFFFFFFFFFFFFFFFFFFFFF
FFFFFF,FFFFFFFFFFFFFFFFFFFFFFFFFFFFFFF,FFFFFFFFF
@A00721:190:HGJW7DSXY:4:1609:15320:13714_2:N:0:TCTGCAAG+GCTGCACT
AAAGTACAGATTTAGTGATTTAGATATTATTGATATTGGAGGCGATATTCGTTTTCATATCCAAAACT
CCTTGAATGGAGTACATATTTGCAACCCTATTCTTGATGTGAAAGATG
+
FFFFFFFFFFFFFFFFFFFFFFFFFFFFFFFFFFFFFFFF:FFFFFFFF:FFFFFFFFFFF:FFFFFF
FFFFFFFFFFFFFFFFFFFFFFFFFFFFFFFFFFFFFFF,FFFFFFFFF
@A00721:190:HGJW7DSXY:4:2670:20401:13056_2:N:0:TCTGCAAG+GCTGCACT
AACATTGATTGGGAACGTACCTACTCACGAGACGTCATGAAAGCTATCGGAGGGAAGCAAAATGTTTC
GTTCTGTTACAAGAACGCTTTGGATTGTGACCATAAAGCCTCAGTTGC
+
F:FFFFF:F:FFFFFFFFFFFFFFFFFFFFFFFFFFFFFFFFFFFFFFF,F:FFFF:FFF:,,,FFFFF
F,FF:FFFFFFFFFFFFF,FFFF:FFFFFFFFFFFFFFFFFFF:,FFF
@A00721:190:HGJW7DSXY:4:1102:12255:33176_1:N:0:TCTGCAAG+GCTGCACT
CTCTCTTGATGATGTAGCCACTATAATGCTTAAGAGAGAGATTGATGTATGTCATTTCGTCTTAGTTT
GTCCCGGCGAATTATTGGACACGATGAAAAATGACATACCCGTTCCTGACCTTGGTATCGTTATCCGT
CAT
+
FFFF:FFFFFFFFFFFFFFFFFF:FFFFFFFFFFFFFFFFFFFFFFFFFFFFFFFFFFFFFFFFFFFFF
FFFFFFFFFFF:FFFFFFFFFFFFFFFFFFFFFFFFF:FFFFFFFFFFFFFFFFFFFFFFFFFFFFFFF
FFF
@A00721:190:HGJW7DSXY:4:1119:4716:32706_2:N:0:TCTGCAAG+GCTGCACT
CGAAGGATTGGGTTATTCTCACAACAAAGATAAGATTTTATCTTTCTTCAAACACCCTGTTTTCCTTA
AGGGCGGACAATTGTTCCATTGTGAACTCTTGGGGACTAGATTGAGCA
+
FFFFFFFFFFFFFFFFFFFFFFFFFFFFF:FFFFFFFFF:FFFFFFFFFFFFFFFFFFFFFFFFFFFFF
FFFFFFFFFFFFFFFFFFFFFFFFFFFFFFFFFFFFFFFFFFF:FFFFF
@A00721:190:HGJW7DSXY:4:1147:6451:31485_1:N:0:TCTGCAAG+GCTGCACT
AGTGACTGGAGTTCAGACGTGTGCTCTTCCGATCTATTCAGAATACCTATTTCCGTACCAAGAGCCAC
TGCCAATCTCATCGCTATACGCATACCTGATTGGAAGAAGCGAGGTTCGTTCCTCAATAAAAATTTTT
CTC
+
F:FFFFFFFFFFFFFFFFFFFFFFFF:FFFFFFFFFFFF:FFFFFFFFFFFFFFFFFFFFFFFFFFFF:
FFFFFFFFFFFFF:FFFFFFFFFFFFFFFFFFFFFFFFFFFFFFFFFFFFFFFFFFFFFFFFFFFFFF
FFF
@A00721:190:HGJW7DSXY:4:1535:17951:27821_1:N:0:TCTGCAAG+GCTGCACT
TAAAGTTTTCTTCATAAGGGCTATTCAGTACGTCATGGGGATCACTTCTTCGGTCAATGACAAAACCT
TCGAATATGTTACGACATGGCTTCGTAACAACAGTGCGAGGATTGTCATCTCGGGGAAAGTGATTCAC
CAA
+
FFFFFFFFFFFFFFFFFFFFFFFFFFFFFFFFF,FFFFFFFFFFFFFFFFFFFFFFFFFFFFFFFFFFF
FFFF:FFFFFFFFFFFFFFFFFFFFFFFFFFFFFFFFFFFFFFFFFFFFFFFFFFFFFFFFFFFFFFF
FFF
@A00721:190:HGJW7DSXY:4:1323:27633:6261_1:N:0:TCTGCAAG+GCTGCACT
GAGATACCTAGAGAAGGTCGCAGCGATCTTACTCGTTATCGGAGCTAAGAAGAAGACTTTGGGTACAC
GTTATGCTCGCAAACTTAACGTTAGTACCGGAGGTGATCTCTCTGCTTCTGCGTTACTGAGAGAAAAG
ATT
+
FFFF:FFFFFFFFFFFFF:FFFFFFFFFFFFFFFFFFFFFFFFFFFFFFFFFFFFFFFF:FFFFFFFFFF
FFFFFFFFFFFFFFFFFFFFFFFFFFFFFFFFFFFFF:FFFFFFFFFFFFFFFFFFFFFFFFFFFFFFF
FFF
```

```
@A00721:190:HGJW7DSXY:4:1228:18014:7858_2:N:0:TCTGCAAG+GCTGCACT
ACGGTGACTTTATGGACAAGATGCAAGTTCTTGGGAAATACTTGTTTCCCTTTTTAGGTTTAGTAGGT
GATCTTGACGTCGATTCTTTGTATAGGGAGTGTGAGAAGACAACGAAT
+
FFFFFFFFFFFFFFFFFFFFFFFFFF:FFFFFFFFFFFFFFFFFFFFFFF:FFFFFF,FFFFF:FFFFF
FFFFFFFFF:FFFFFF,:FF:FFFFFFFFF:FFFFFFFFFFFFFFFFF
@A00721:190:HGJW7DSXY:4:2251:29270:1955_2:N:0:TCTGCAAG+GCTGCACT
TATCAAAGAAAATGGTGAACCGGTCTCTAAATCTTTTGAGATTGAGAAGATTGAAGAACACGTCAAGA
AGTCGCTCGGCGTTGTTCTTGAAAACCAAGTTAAGAAGGAGGCTGATC
+
FFFFFFFFFFFFFFFFFFFFFFFFFFFFFFFFFFFFFFFFFFFFFFFFFFFFFFFFFFFFFFFFFFF:F
FFFFFFFFFFFFFFFFFFFFFFFFF:FFFFFFFFFFFFFFFFFFFFFFF
@A00721:190:HGJW7DSXY:4:1266:26106:9846_2:N:0:TCTGCAAG+GCTGCACT
GTACCTTCTGTCAAACTTCGAGGTTCAGCTACGAGAGGTGATCCCGGATGTGGATTGAAGGCTGGCTC
TAAGGGAGACTCCTTCTTTTTGAATGTTGTAAAGAATTGGGAGATCGG
+
FFFFFFFFFFFFFFFFFFFFFFFFFFFFFFFFFFFFFFFFFFFFFFFFFF,FFFFFFFFFFFFFFFFFF
FF:FFFFFFFFFFFFFFF::FF:::FFFF,FFFFFFF:FFFFFFFFFF
@A00721:190:HGJW7DSXY:4:2226:11930:17989_1:N:0:TCTGCAAG+GCTGCACT
AGGAGTTAGACTCTTCTTCAAGTTTTTAGATCTTCTTGATAATCTTAAAGAAAAATTTTCGAATAAAC
TAATGAGATGTATAGGCTTAGTCAAGTCTGTACTTACATTCTTTACGCCAGATTCTTCATGGGGCGAC
+
FFFFFFFF,F:F:,,:FF:F:FFFFFFFFFFFFFF:FFFFFFFFFFFF:FF:FF,:FFF:,FF,:FFF
FFFF:F:,,,FFFFFF,FFF:FFFFFF,:FF:FFF:FFFFF:F:FFFFFFFFFF,FFFFF:F:FFF,F
@A00721:190:HGJW7DSXY:4:2559:9191:16282_1:N:0:TCTGCAAG+GCTGCACT
TTGGTATTTCTGAAATAGTTTATCAGAGCGTCATTTTCCTTTGGGATCTTTCCTCTGGTAAGGGAATT
ATTAAATCATTCTTCTCACTCTTGACGAGAATTATTACTGTTTTCTCTACCCATAACTTCTTTTTCAA
AA
+
:F:FFFFFF:FFFFFFFFFFF:FFF:FFFFF:F:FFFFFFFFFF,FFFFFF:FFFFFFFFFFFFFFFF
FFFFFFFFFFFFFFFFFFFF:FF,FFFFFFFFFFFFFFFFF::FFFFFFFFF,FF:FFF:FFFFFF,F
FF
@A00721:190:HGJW7DSXY:4:1360:14678:31234_1:N:0:TCTGCAAG+GCTGCACT
ACTGGAGTTCAGACGTGTGCTCTTCCGATCTGTTTCAACACTTATCACCACTTTTGAAAGGAAAATAA
TTGGTGATGAAAATACGTATGTCGGTGGGGTTCTCAAGGCTCTCATCGCCACTGGGATTCTAAGGGGT
TCC
+
FFFFFFFFFFFFFFFFFFFFFFFFFFFFFF:FFFFFFFFFF:FFFFFFFFFF:FFFFFFFFFFFFFFFF
FFFFFFFFFFFFFFFFFFFFFFFFFFFFFFFFFFFFFFFFFFFFFFFFFFFFFFFFFFFFFFFFFFFF
FFF
@A00721:190:HGJW7DSXY:4:1162:22535:32095_2:N:0:TCTGCAAG+GCTGCACT
TTCAATGATCAGGATTCTTCTTTTGGTCCTTACGTCTTAAGTAATTTGGATGTTGTCGAAAACTATCA
GGTTCTTGTCACGATTCTTAAAAGAAGTTTATTAAAACTTCAGAGTCT
+
FFFFFFFFFFFFFFFFFFFFFFFFFFFFFFFFFFFFFFFFFFFFF:FFFFFFFFFFFFFFFFFFFFFFF
FFFFFF:FFFFFFFFFFFFFFFFFFFFFFFFFFFFFFFFF:FFFFFFF,
@A00721:190:HGJW7DSXY:4:1111:26919:13855_1:N:0:TCTGCAAG+GCTGCACT
GGTAGCTAATGTTCTTGCAATAGCCACAGACTTTCTGGGAAAAGAGACCTCTGTTGTAGAGGAGTTGA
GAGAATTACTCAGATTCAATCTGGCATCACTGAACGTCTACAACGAGTTAGGGAGTGGCTAGGTTTTA
AAA
+
:FFFFFFFFFFFFFFFFFFFFFFFFFFFFFFFFFFFFFFFFFFFFFFFFFFFFFFFFFFFFFFFFFFFF
FFFFFFFFFFFFFFFFFFFFFFFFFFFFFFFFFFFFFFFFFFFFFFFFFFFFFFFFFFFFFFFF:FFF
FFF
```

```
@A00721:190:HGJW7DSXY:4:1221:1280:33677_1:N:0:TCTGCAAG+GCTGCACT
AGCAATACCTTCCACTGAGTTCATCACCCAGAGTTTCGACGTCGCGGAAAACGATGAAGATTTCCACA
CTGTTCGAGAATCTTCTGATGCTGAATTTGAGGAAAATTTTTCGGAAGTTTTTGGTATAAATCCTGAT
GGC
+
:FFFFFFFFFFFFFFFFFFFFFFFFFFFFFFF:FFFFFFFFFFFFFFFFFFFFFFFFFFFF,FFFFFFF
FFFFFFFFFFFFFFFFFFFFFFFFFFFFFFFFFFFFFFFFFFFFFFFFFFFFFFFFFFFFFFFFFFFF
FFF
@A00721:190:HGJW7DSXY:4:2270:27010:25191_2:N:0:TCTGCAAG+GCTGCACT
AGTTATAAAACCTTCTTTTATCATTGCAACAGGACTTTGAGAAGACTGTTGAAATGGACAAAATCTGG
GATCATAGGTATGTGTCACGCTACACTCGAAATATGGGCCCTCATAAG
+
FFFFFFFFFFFFFFFFFFFFF:FFFFFFFFFFFFFFFFFFFFF:FFFFFFFFFFF:FF:FFFFFFFFFF
FFFFF:::FFFFFFFFFFFFFFFFFFF:FFFFFFFFFFFFFFFFFFFFF:
@A00721:190:HGJW7DSXY:4:1411:30463:10848_1:N:0:TCTGCAAG+GCTGCACT
CGTAATTACAGTCTGGAAGTTAGATCATTTGTTCAGGAAGATCTCTACAGAGATTATCGAGAGTTGTG
ATCTAGTTCTAAATTTAGGTCGACGGACTTTAGCAACTTTAGATAAATTCTTTCCGGGCTCAATGACC
TTT
+
FFF,:FF,:FFFFFFF:FFFF:FFFF,FFFFFFFFFF:FFFFFFFFFFF,F,FFFFF,FF:FFFFFFF
FFFF,FFFFFFF:FFFFFFFFFFF,F:FFFFFF:FF:FFF,FFF:FFFFFFFF,F:FFFFFFFFFFFF
FFF
@A00721:190:HGJW7DSXY:4:1112:30689:32189_1:N:0:TCTGCAAG+GCTGCACT
TCTGACCATCGCAAAGAAGACTTTATTAGTTCATGAAGTTCTTACACTTCTCGCGACTAATGTCAGCT
TCACTACAAAGGCGTTACGTAAAGTAGTTTTCACGCTTTATCTTAAAGCATTACCACCAAATTTAGCA
CTT
+
FF:FFFFFFFFFFFFFFFFFFFFFFFFFFFFFFFFFFFFFFFFFFFFFFFFFFF:F:FFFFFFFFFFF
FFFFFFFFFFFFFFFFFFFFFFFFFFFFFFFFFFFFFFFFFFFFFFFFFFFFFFFFFFFFFFFFFFFF
FFF
@A00721:190:HGJW7DSXY:4:1114:9715:26647_1:N:0:TCTGCAAG+GCTGCACT
GAAGCTCGCCATATCAGATTAGAGACATGCCAGTTAGAGTTCTTCTAACCAGGACTGATGGTGAGATT
CTTTTACCTTTTGGAGGTCCAGGAACCTTTACTCCTAGTGTCTTTTCTAGAGTCCAAGGACGTTTCCG
TCG
+
FF:FFFFFFFFFFFFFFF:FFFFFFFFFFFFFFFFFFFFFFFFF::FFF:FFFFF,FFFFFFFFFFFFFF
FFFFFFFFFFFFFFFFFFFFFFFFFFFFFFFFFFFFF:FFFFFFFFFFFFFFFFFFFFFFFFFF:FFFF
FFF
@A00721:190:HGJW7DSXY:4:1111:19343:12665_1:N:0:TCTGCAAG+GCTGCACT
GTGGGAGACCGCTCAATAACACTCTATTGAATCCCTTTTCCACGATGAAAGCTCTTTATGAGGTGCTC
CATGCAAATTTACAGTTCCTCTTTGGTGATATCGCCTCTGTCCTTTCTCCTTTAATCGTGAGGTTACG
AGA
+
FFFFFFFFFFFFFFFFFFF:FFFFFFFFFFFFFFFFFFFFFFFFFFFFFFFFFFFFFFFFFFFFFFFFF
FFFFFFFFFFFFFFFFFFFFFFFFFFFFFFFFFFFFFFFFFFFFFFFFFFFFFFFFFFFFFFFFFFFF
FFF
@A00721:190:HGJW7DSXY:4:1105:27055:32033_2:N:0:TCTGCAAG+GCTGCACT
GGTTTATCGGTTGTATTTTGATAAATTTGTTGGAATCGATAATGATACTGACGAAGGTTCTATTACCA
CCCTTGTCATTGATACAAACATTCTAGGTGATGCTTTCTTAGAGGCTC
+
FFFFFFFFFFFFFFFFFFFFFFFFFFFFFFFFFFFFFFFFF,FFFF:FFFFFFFFFFFFFFFFFFFFFFF
FFFFFFFFFFFFFFFFFFFFFFFFFFFFFFFFFFFFFFFFFFFFFFFF
@A00721:190:HGJW7DSXY:4:1161:6388:35947_2:N:0:TCTGCAAG+GCTGCACT
TATTTACAATCTGTAGAGAGAGAGGTTAATCAGAATTTGAGTGATAACGATTTAGTACTGGGAAATGG
```

```
TGGTAGTCAGTTTACTACTTTTCTTTCCCTCCCCTTTTTCGATTGGAT
+
FFFFFFFFFFFFFF:FFFFFFFFFFFFFFFFFFFFFFFFFFFFFFFFFFF,FFFFFFFFFFFFFFFFF
FFFFFFFFFFFFFFFFFFFFFFFFFFFFFFFFFFFFFFFFFFFFFFFF
@A00721:190:HGJW7DSXY:4:1349:18150:15922_2:N:0:TCTGCAAG+GCTGCACT
CGTCTCACGACTAATTTGATAAGGTTTTTCTTTTATGGGACTCTTGTTTGTACTTATGAAGGAGGTAT
TACTTCGGAAGTTCTTAATGTAGGTCTCCTAGTACTTAAGGGTTTATG
+
FFFFFFFFFFFFFFFFFFFFFFFFF,,FFFF:FFFFFFFFF:F:FFF:FFFFFF:FFFFFF:FFFFFF
FFFFFFFFFFFFFFFFFFFFF:FFF:FFFFFFFFFFFFFFFFFFFFFF
@A00721:190:HGJW7DSXY:4:1101:19289:36213_2:N:0:TCTGCAAG+GCTGCACT
TTTTGATGGTATGTTTCACATTGCGTGATCACTTCCCGTGGACTTGGGAAGAGATTAAACATAAAATC
CAGCGATTCTATTATGTCGCTAAAGCCTGTCCAGTTGTCGATGATGTT
+
FFFFFFFFFFFFFFFFFFFFFFFFFFFFFFFFFFFFFFFFF,F:FFFFFFFFFFF:FF:FFFFFFFFF
FFFFF:,FF:FFFFFFFFFFF:FFFFFFFFFFFFFFFF:FFFFFFFFF
@A00721:190:HGJW7DSXY:4:1253:19099:23171_2:N:0:TCTGCAAG+GCTGCACT
TCGTGAAACTTACGACGATGTTCACTCTCGCATTTCACAAGGATTACTAAGTCTGAGATCTGCCACTT
CTAATTTTAGGAAACGTCTCGAAAAGAAAGTTCATGAGGATGTCTTGG
+
FF::FFFF,FFFFF,F:F:FF:F,:,F:F::FF,,,FFFFFFFF,FFFF::FF,,,FFFFF:,::FFF
F::F:FF:F,:FFFFFFF,FFFF:FFFFF,FF,::,FF,FF:,:F,F,
@A00721:190:HGJW7DSXY:4:1603:30671:20415_2:N:0:TCTGCAAG+GCTGCACT
GGGAACTTCTAATAGAGGATCCTGATGACATTATTTCAACGGATACCGATCTCAGTTCTACCACAGAA
ACCGTCTCAGGAACTGTCTCCACTGATTCGGAATTAGAGAAAGACGAC
+
FFFF:FFFFF:FFFFFFFFFFFFFFFFFFFFFFFFFFFFFFFFFFFFFFFFFFFFFFFFFFFFFFFFF
FFFFFFFF:FFF,FFFFFFFFFFFFF:FFFFFFFFFF:FFFFF:FFFF
@A00721:190:HGJW7DSXY:4:1250:10782:6574_1:N:0:TCTGCAAG+GCTGCACT
TCTCTGAGACCTCTGAATTTTCAGGGTGCCCTGATGAGGACGTTGAAAGGGATAATTTCAACTCTGAG
ACTTCTAAGAGTGTTTCAAAAGACCAGAAACGTAACAAGAAGAATAAAGGTAGGAAGGGGAGAAAACA
GAA
+
FFFFFFFFFFFFFFFFFFFFFFFFFFFFFFFFFFFFFFFFFFFFFFFFFFFFFFFFFFFFFFFFFFFF
FFFFFFFFFFFFFFFFFFFFFFFFFFFFFFFFFFFFFFFFFFFFFFFFFFFFFFFFFFFFFFFFFFFF
FFF
@A00721:190:HGJW7DSXY:4:1108:24569:2049_2:N:0:TCTGCAAG+GCTGCACT
ACTGATATGGTTGCGGCTGATCGTACCCCAATTCCTGCTAGAAAGATCACTCTCGGCGATACCACTGA
TCTTCCAGAGTTACGTAGTGGTCGTTCTTTTGTTGATTTTATCAAAGA
+
FFFFFFFFFFFFFFFFFFFFFFFFFFFFFFFFFFFFFFFFFFFFFFFFFFFFFFFFFFFFFFFFFFFF
FFFFFFFFFFFFFFFFFFFFFFFFFFFFFFFFFFFFFFFFFF:FFFFF
@A00721:190:HGJW7DSXY:4:1654:18936:23516_2:N:0:TCTGCAAG+GCTGCACT
GATATTAACAAGTTAAACTTCGAACTGACCACTTACAAGAGTGTTGAAGTACCGCAACATGGATCAGT
TTTCCAAAAAGCTATTAATAGCAGTTGCGGGGCTATAATACCGCCTCC
+
FFFFFFFFFF:FFFFFFFFFFFFFFFFFFFFFFFFFFFFFF:FFFFFFFFFFFFFFFFFFFFFFFFFF
FFFFFFFFFFFFFFFFFFFFFF:FFFFFFFFFFFFFFFFFFFFFFFFF
@A00721:190:HGJW7DSXY:4:2139:1823:14043_2:N:0:TCTGCAAG+GCTGCACT
AAACTGAATCCACTCTCTTGGGATATAAATGAGTACATCTATAATAGAGTTTTGGAACTGTGTAATGT
CCTACGTACTGTTCAAGGTGTTTGGGCTTCTCTAAAGAACAGCAACTT
+
FFFFFFFFFFFFFFFFFFFFFFFFFFFFFFFFFFFFFF:FFFFFFFFFFFFFFFFFFFFFFFFFFFFF
FFFFFFFFFFFFFFFFFF,FF:FFFFFFFFFFFFFFFFFFFFFFFFFF
```

@A00721:190:HGJW7DSXY:4:2164:7491:25207_2:N:0:TCTGCAAG+GCTGCACT
GGCTGATGACTTAAATTTCGCATTCTGCTTCGACGGTAATTCCATAAGACCTTTCTTGGGGAGTAAAG
AGGGTTTGAAAATTACGGAACCTTTCCTCGTTCTTCATGAAGATCTTC
+
FFFFFFFFFFFFFFFFFFFFFFFFFFFFFFFFFFFFF:FFFFFFFFFFFFFFFF,FFFFFFFFFFFFF
FFFFFFFFFFFFFFFF:FFFFFFFFFFFFFFFFFFFFFFFFF:FFFFF
@A00721:190:HGJW7DSXY:4:1611:18059:7341_2:N:0:TCTGCAAG+GCTGCACT
GTTATTCTTGCGAAGGTTCAAGTCGATTGATTTTGTTGACTACGATCTCTCTTGTAATAAGAAGTTCT
TCGCAGTTGAATCTCCACCAGGTGGAGGGAAGACCACTGAACTTATAG
+
FFFFFFFFFFFFFFFFFFFFFFFFFFFFFFFFFFFFFFFFFFFFFF:F:FF:FFFFFFFFFFF:FF:F
FFFF:F:FF:FF:FFFFFFFFFFFFF:FFFFFFFFFF:FFFF::FFFF
@A00721:190:HGJW7DSXY:4:2125:23104:21277_1:N:0:TCTGCAAG+GCTGCACT
TTCGCAAAAGGTCTTGAAGTACGTATTGTCACTGCTAATCGTAATTCGCGAGAAGAGATAGTGACTAA
AGTTTTGGCACTTCTAGTTCATCGCGGTATTTGTGAAATTAAGACAGACGTGAAAGTTATGATGAGTG
ATC
+
FFF:FFFFFFFFFFFFFFFFFFFFFFFFFFFFFFFFFFFFFFFFFFFFFFFFFFFFFF:FFFFFFFFF
FFFFFFFFFFF:FFFFF:FFFFFFFFFFFFFFFFFFFFFFFFFFFFFFFFFFFFFFFFFFFFFFFFFF
FFF
@A00721:190:HGJW7DSXY:4:1404:5448:12054_2:N:0:TCTGCAAG+GCTGCACT
AGCTGATTCTTATTGCATGAACGTCGCTGAGGTCAAACCTGTGGACGTTCTTTTCATCGACGAATTAT
TCATGATTCACGCTGGTCAGACCCTTTTTCTTACACATCTACTTAAGG
+
FFFFFFFFFFFFF,FFFFFFFFFFFFFFF:FFFFFFFFFFFFFFFFFFFFFFFFFF:FFFFFFFFFFF
:FFF:FFFF:FFFFFFFFFFFFFF:FFFF,FFFFFFFFFFFFFFFFFF
@A00721:190:HGJW7DSXY:4:1565:21712:23030_2:N:0:TCTGCAAG+GCTGCACT
GTTACGGTGATTCCAATCAAATCGGATTCATCAATCGTATTGATCTCCAAGTCGCAAACTACTTCAAC
ATCAACGACATCATTCTTGATGAGTGTCGTTCTTATCGAAGTCTCAGT
+
FFFFFFFFFFFFFFFFFFFFFFFFFFFFFFFFFFFFFFFFFFFFFFFFFFFFFFFFFFFFFFFFFFFF
FFFFFFFFFFFFFFFFFFFFFFFFFFFFFFFFFFFFFFFFFFFFFFFF
@A00721:190:HGJW7DSXY:4:1470:26955:22216_2:N:0:TCTGCAAG+GCTGCACT
CGGCCGACGTTTGTCGCGAACTCTCAGAGGTTTACGGCAGAAAAATTTCTCCTGCTTGTAATTCGAGA
GAGGACACTATGGAAATTAGACCTATCAGATGTCTTGACGACTTCGAA
+
FFFFFFFFFFFFFFFFFFFFFFFFFFFFFFFFFFFFFFFFFFFFFFFFFFFFFFFFFFFFFFFFFFFF
FFFFFFFFFFFFFFFFFFFFFFFFFFFFFFFFFFFFFFFFF:FFFFFF
@A00721:190:HGJW7DSXY:4:2670:16089:25755_2:N:0:TCTGCAAG+GCTGCACT
ATATTACTTTCACTCAAGGTGAAAAGTTTGAAATTAACAAAGTCCTTGGTAAGAGTAAGGTTAAATGT
GAAGATGCTCAGACTATTCATGAAGTTCAAGGTAATACTTACCAGCAC
+
FFFFFFFFFFFFFFFFFFFFFFFF:FFFFF:FFFFFFFFFFFFFFFFFFFFFFFFFFFFFFFFFFFFF
FFFFFFFFFFFFFFFFFFFFFFFFFFFFFFFFFFFFFFFFFFFFFFFF
@A00721:190:HGJW7DSXY:4:2665:31864:18662_1:N:0:TCTGCAAG+GCTGCACT
ATGACCCTGTTTTTGCATCACAAACACATATTACCGTTGCTCTATCACACACGTTGAATCTTTACGAT
ACTTTGTTATCAATGTAAAGTTAAACGATGCTACTTGTGAGAGGATCAATCACAGTAAGGAACTGTCA
CGT
+
FFFFFFFFF,FFFFFFFFFFFFFFFFFFFFFFFFFFFFFFFFFFFFFFFFFFFFFFFFFFFFFFFFF:
FFFFFFFFFFFFFFFFFFFFFFFFFFFFFFFFFF:FFFFFFFFFFFFFFFFFFFFFFFFFFFFFFFFF
FFF
@A00721:190:HGJW7DSXY:4:2365:10655:5447_1:N:0:TCTGCAAG+GCTGCACT
TGCAGATTCAACCCCTTATATCGAGGCTATCGAGGACGATGAGTATCTTGAAGTTGTTGACGACAAGC

CTCAAGAGGGTAGAGGACCACCTGGATCTTCTACTGTCTCTGCCTTCCAAGATTTCTTAGAAGAGGTT
ATT
+
FFFFFFFFFFFFFF,FFFFFFFFFFF:FFFFFFFFFFFFFFFFFFFFFFFFFFFFFFFFFFFFFFFFF
FFFF:FFFFFFFFFFFFFFFFFF:FFFFFFFFFFFFFFFFFFFFFFFF:FFFFF:FFFFFFFFFFFF
FFF
@A00721:190:HGJW7DSXY:4:2523:16712:16532_1:N:0:TCTGCAAG+GCTGCACT
CTTAACGTCACTCGGTAATCTTTCAGCCGAATTAAGTACTAATGAGTTCAAGTCGGTTGTCGATCGAA
TTAAGATCAAGGCTAATACCTTTATAGGTAAGAAGCCCCTTGAGCCGCGTAGTCAGCGCGTTTAGCTG
TAT
+
FFFFFFFFFFFFFFFFFFFFFFFFFFF:FFFFFFFFFFFFFFFFF:FFFFFFFFFFFFFFFFFFFFFF
FFFFFFFFFFFFFFFFFFFFFFFFFFFFFFFFFFFFFFFFFFFFFFFFFFFFFFFFFFFFFFFFFFFF
FFF
@A00721:190:HGJW7DSXY:4:2549:29197:14826_1:N:0:TCTGCAAG+GCTGCACT
TATACCCGATAGGGTACCGAGTCTCCAAGAGAATATTTATTCTTATGAGGCTCGAAACTTCAACGTTT
TCACAACGGAAAGACATATCGCACCCGAAATTTTTGGGCAGAATGCTCTTAACGCGGTTCTTGAACGG
TGT
+
FFFFFFFFFFFFFF:FFFFFFFFFFFFFFFFFFFFFF:FFFFFFFFFFFFFFFFFFFFFFFFFFFFFF
FFFFF,FFFFFFFFFFFFFFFFFFFFFFFFFFFFFFFFFFFFFFFFFFFFFFFFFFFFFFFFFFFFFF
FFF
@A00721:190:HGJW7DSXY:4:1654:28248:11334_2:N:0:TCTGCAAG+GCTGCACT
CTAAGTTGGATAAAGTTATAGGAGACTCAGTTAACCTGAGAGCTCCTCAATTGATAGATTGGGTCAAC
AATAGGGACGCTAGTATACTTCGTAAAGTCGACAGCGAAATGGTCAAT
+
FFFFFF:FFFFFFFFFFFFFFFFFFFFFFFFFFFFFFFFFFFFFFFFFFFFFFFFFFFF:FFFFFFFF
FFFFFFFFFFFFFFFFFFFFFFFF:FFFFFFFFFFFFFFFFFF,FF:FF
@A00721:190:HGJW7DSXY:4:2563:21703:25770_1:N:0:TCTGCAAG+GCTGCACT
ATATGACCTATTTTAAGCTCATGGTGAAAAGGGAAGCTAAGGTTAAACTTGATGCTTCAGCCTTTTCG
AAGCATAACCCCGCACAGAATATCGTTTATCACGCAAAATTTATTAATGCAATTTTCGGACCTATCTT
TAA
+
FFFFFF:FFF:FFFFFFFFFFF:FFFF::FFFFFFFFFFFFFFFFFFFFFFFFFFFFFFFFFFFFFFF
FFFFFFFFFFFFFFFFFFFFFFFFFFFFFFFFFFFFFFFFFFFFFFFFFFFFFFFFFFFFFFFFFFFF
FFF
@A00721:190:HGJW7DSXY:4:1546:18448:30499_1:N:0:TCTGCAAG+GCTGCACT
TGTCTTTTACCCAACATTTTTATGTATACAGAGATGACCAGTGACGATTTCTCCCGGAAGCTCTTCTC
TCTCTTAGGAGGTCATGATGACTACTATAAGGGTGAAATCGATTTTTCGAAGTTTGACAAATCTCAAG
ATT
+
FFFFFFFFFFFFFFFFFFFFFFFFFFFFFFFFFFFFFFFFFFFFFFFFFFFFFFFFFFFFFFFFFFFF
FFFFFFFFFFFFFFFFFFFFFFFFFFFFFFFFFFFFFFFFFFFFFFFFFFFFFFFFFFFFFFFFFFFF
FFF
@A00721:190:HGJW7DSXY:4:1477:26720:24659_2:N:0:TCTGCAAG+GCTGCACT
AGCCTATGAGATAAAGATGTATTCTTATTTAGGCTTCGATGCTGAATTTCTTGAACTTTGGAGTGCCA
GTGAATATTTTTGTCACGCCAGGAGATCGGAAGAGCGTCGTGTAGGGA
+
FFFFFFFFFFFFFFFFFFFFFFFFFFF:FFFFFFFFFFFFFFFF:FFFFFFFF:FFFF:FFFFFFFFF
FFFFFFFFFFFFFFFFFFFFFFFFFFFFFFFFFFFFFFFFFFF,FFFFF
@A00721:190:HGJW7DSXY:4:1108:8160:7420_1:N:0:TCTGCAAG+GCTGCACT
TAACACATTCTTTGGTAATTGTATGATCACTCTTATGATGCTCTCTCTTTATTACGACCTTCGGAAGT
TTAGAGCCGTCGCTGTCGCCGGAGACGATTCGATCCTCTTTTCGGACGTACCGGTCCAGGATTATTCA
GAT

```
+
FFFFFFFFFFFFFFFFFFFFFFFFFFFFFFFFFFFFFFFFFFFFFFFFFFFFFFFFFFFFFFFFFFFFFFF
FFFFFFF:FFFFFFFFFFFFFFFFFFFFF:FFFFFFFFFFFFFFFFFFFFFFFFFFFFFFFFFFFFFFFFF
FFF
@A00721:190:HGJW7DSXY:4:1577:32172:24361_2:N:0:TCTGCAAG+GCTGCACT
CAGCGTACTTCTGCTCGCGATTTATTCTTTTTGTCAATGATTACATCTACTTCGTTCCTGATCCTTAC
AAGATCATCGTTAAGTTAGGTAAACCCCTAACCGATGTTACTCTTGTA
+
FFFFFF:FFFFFF,:F:FFFFFFFF::FFFFFFFFFFFFFF:FFFFFFFFFFF:FFF:FFFFFF::FF
FFFFFF:FFFFFFFFFFFFFFFFF:FFFFFFFF,FFFFFFF:F,FFFFF
@A00721:190:HGJW7DSXY:4:1274:8097:19085_1:N:0:TCTGCAAG+GCTGCACT
TTTAAAGACTATACTAAATATCTCCACTACGAGGTTGTTGTTTGGCGATTAGCTCTTGCGACTAATAT
TCGATATTCGATTGAGGGTCGTTATACCTATTCGGCTATCTGTGCTTTGAATTGTATAAGATCAAATT
TTA
+
FFFFFFFFFFFFFFFFFFFFF:FFFFFFFFFFFFFFFFFFFFFFFFFFFFFFFFFFFFFFFFF:FFFFFF
FFFFFFFFFFFFFFFFFFFFFFFFFFFFFFFFFFFFFFFFFFFFFFFFFFFFFFFFFFFFFFFFFFFFFFF
FFF
@A00721:190:HGJW7DSXY:4:1645:7129:14873_2:N:0:TCTGCAAG+GCTGCACT
CCTGATGTGGAGGGAGTTCTTGAAGCATCCCGACCTTTCCTTGTGAAAATTTTGATGCGGTTTCATAA
GATTATCAGTAATCCTGATTCTACTACGGATTCATGGACCTTCCATCC
+
FFFFFFFFFFFFFFFFFFFFFFFFFFFFFFFFFFFFFFFFFFFFFFFFFFF:FFFF:FFFFFFFFFF
FFFF:FFFFFFFFFFFFFFFFFFFFFFFFFFFFFFFFFFFFFFFFFFF
@A00721:190:HGJW7DSXY:4:1230:30960:1219_2:N:0:TCTGCAAG+GCTGCACT
GACGTATCTCAAAGATCGACTTCGAGACAGGTGATATTGTTGAGCGAGAGCGACCAAGATCTAAATTC
GGTAACGGTGCGTGGCGCTTTATTTTAAAAAGCTCCCTCCTCTCCCAA
+
FFFFFFFFFFFFF:FFFFFFFFFFFFFFFFFFFFFFFFF:FFFFF:FFFFF:FFFF:FF:FFFFFF:F:F
FFFF:FF:F:FFFFFFFFFFFFFFFFFF,FF:FFFFFFFFFFFFFFFF
@A00721:190:HGJW7DSXY:4:1207:12129:3709_2:N:0:TCTGCAAG+GCTGCACT
ACTTGAGTCTTTTGATCAGTCTCTGACTTGACCATCTTTCATTTTAAATGACGATCATTAAGACTCTC
TTTCTTCTTTGTTTCCTTCTTTGCACTGATTTTTCCTCATCAGCTTGT
+
FFFFFFFFFFFFFFFFFFFFFFFFFFFFFFFFFFFFFFFFFFFFFFFFFFFFFFFFFFFFFFFFFFFFFFF
FFFFFFFFFFFFFFFFFFFFFFFFFFFFFFFFFFFFFFFFFFFFFFFF
@A00721:190:HGJW7DSXY:4:1546:5710:34898_1:N:0:TCTGCAAG+GCTGCACT
GTTTTTACGATAACAACTATCAGTTGATTAAGACTCTTCCGAAAGGCGAACTTTGGGAATTTGAAGCT
CAGGGTGAAGCACAACGTTTGACCGTGACGAACTGTGGTGTTCTTGATCTACGTTACGGATATACTCT
CCT
+
FFFFFFFFFFFFFFFFFF,FFFFFFFFFFFFFFFFFFFFFFFF,FFFFFFFFFFFFFFFFFFFFFFFFFFF
FFFFFFFFFFFFFFFFFFFFFFFFFFFFFFFFFFFFFFFFFFF:FFFFFFFFFFFFFFFFFFFFFFFFFFF
FFF
@A00721:190:HGJW7DSXY:4:1315:1597:21104_1:N:0:TCTGCAAG+GCTGCACT
ACACTACCACTTATTACGATATTTCTTTAGTCGATGGGTTTTCGACGCCTTTAAGCGTCTTCTGTTCA
TCGCAACCAGATGATATCTTCTATTCGAACTTATGTTTCGATCAGTGTTTCTTCTATCAGGAGGAAAA
CAC
+
FFFFFFFFFFFFFFFFFFFF:FFFFFFFFFFFFFFFFFFFFFFFFFFFFFFFFFFFFFFFFFFFFFFFFFF
FFFFFFFFFFFFFFFFFFFFFFFFFFFFFFFFFFFFFFFFFFFFFFFFFFFF:FFFFFFFFFFFFFFFFFF
FFF
@A00721:190:HGJW7DSXY:4:2342:11849:17002_1:N:0:TCTGCAAG+GCTGCACT
GTTTACCACCGAAATATCAAGGTTCTTGTCCTTCAAACGATTGGAAGGTTACTTATCTTAATAGTACT
```

AAAGGGGTTTACTTACAACCCTATGACGATAGTTTCTCTTTACACACTTGTGAAGCCAATTTAGTTGT
TTT
+
FFFFFFFFFFFFFFFFFFFFFFFFFFFFFFFFFFFFFFFFFFFFFF:FFFFFFFFFFFFFFFFFFFFF
FFFFFFFFFFFFFFFFFFFFFFFFFFFFFFFFFFFFFFFFFFFFFFFFF:FFFFFFFFFFFFFFFFFF
FFF
@A00721:190:HGJW7DSXY:4:1309:29179:29324_2:N:0:TCTGCAAG+GCTGCACT
TCACTTTGTGTGTACTTGTCTCTATTTTAAGTTTTGTCTTCCTTGTTTGTCTTATTCTATTAATCACT
CATTTTAGTGAGATTATTAAAAACGCACTAAAACGTCTTCGACAACAG
+
FFFFFFFFFFFFFF:FFFFFFFF:FFFFFFF:F:FFFFFFFFF:FFFFFFFFFFFFFFFFFFFFFFFF
FFFFFFFFFFFFFFFFFF:FFF:FFFFFFFFF:FFFFFFF:FFFFFFF
@A00721:190:HGJW7DSXY:4:1367:30563:23484_1:N:0:TCTGCAAG+GCTGCACT
GCCTTACAGAGGGGCAGCAGCATGACCATAATTGGTATTGACTTTGGTACCACCTTTTCTACTGTCTG
TCTCGCCTCTTCGAATGAAGTTTTAATGCTTAACAACAATGATAGTGAATTTATACCTACAATCATTG
GTT
+
FFFFFFFFFFFFFFFFFFFFFFFFFFFFFFFFFFFFFFFFFFFFFFFFFFFFFFFFFFFFFFFFFFFF
FFFFFFFFFFFFFFFFFFFFFFFFFFFFFFFFFFFFFFFFFFFFFFFFF:FFFFFFFFFFFFFFFFFF
FFF
@A00721:190:HGJW7DSXY:4:1128:24189:24439_2:N:0:TCTGCAAG+GCTGCACT
TGTTTATGGATATGATGCTATTACTCATGATGGTTACGGTCAGGGAGGATTCTCAGTTTACAAGGACC
TTAAGCGATGGATCGGGGTTAATTCTAAAACATTACCCGAAAGACGTG
+
FFFFFFFFFFFFFFFFFFFFFFFFFFFFFFFFFFFFFFFFFFFFFFFFFFFFFFFFFFFFFFFFFFFF
FFFF:FFFFFFFFFFFFFFFFFFFFFFFFFFFFFFFFFFFFFFFFFF
@A00721:190:HGJW7DSXY:4:1121:17273:20666_2:N:0:TCTGCAAG+GCTGCACT
GTACGATGTCGTCTGTCTCGGCGATCACTTTTCACTAAGAATTGGTCCTACTTTTGGACAAGGCCAAC
TTCGTAATCTTCATGAGATCGTTTCTTTCTTTATCTCTTTGGTTGTTA
+
FFFFFFFFFFFFFFFFFFFFFFFFFFFFFFFFFFFFFFFFFFFFFFFFFFFFFF:FFFFFFFFFFFFF
FFFFFFFFFFFFFFFFFFFFFFFFFFFFFFFFFFFFFFFFFFFFFFFF
@A00721:190:HGJW7DSXY:4:1105:10511:30373_2:N:0:TCTGCAAG+GCTGCACT
GAGTGAAAGGCTTCAATTGTAGTGGTTTGGTCGTTTCAGTACCCAGTCAGTATACCTCTACGCAAAGA
TATTTCATGAAAACTACAGAGGAACGTACCGGATTACCTGTCTTTCAT
+
FFFFFFFFFFFF:FFFFFFFFFFFFF,FFFFFFFFFFFFFFFFFFFFF,FFFFFFFFFFFFFFFFFFF
FFFFFFFFFFFFFFFFFFFFFFF:FFFFFFFFFFFFFFFFFFFFFFFF
@A00721:190:HGJW7DSXY:4:1562:11207:26663_1:N:0:TCTGCAAG+GCTGCACT
CTTCGGCCGCTCTCTTTGCTTCGATGTTGGATGTCAAGAAGGGTTCAGAATGGGATTCCTACGTAGTT
TATGACTTTGGTGGTGGTACTTTCGATACTTCTATCGTCTTACGTTACGGTAATTACTATAGTGTTAT
TTA
+
FFFFFFFFFFFFFF,FFFFFFFFFFFFFFFFFFFFFFFFFFFFFFFFFFFFFFFFFFFFFFFFFFFFFF
FFFFFFFFFFFFFFFFFFFFFFFFFFFFFFFFFFFFFFFFFFFFFFFFFFFFFFFFFFFFFFFFFFFF
FFF
@A00721:190:HGJW7DSXY:4:1133:13169:28557_1:N:0:TCTGCAAG+GCTGCACT
ATAATCTTGGAGGTCGTGACGTTGATGAAGCTATAAGAGCGTTCTTATTTCGGCGCACTTCTTTACCC
GCCACCACACAACTTTCAGTTTCACAACTGAAGGAGGAGGTTTCTGTTACTGGAAAACCCTCGTCAAT
ACT
+
FFFFFFFFFFFFFFFFFFFFF:FFFFFFFFFFFFFFFFFFFFFFFFFF:FFFFFFFFFFFFFFFFFFF
FFFFFFFFFFFFFFFFFFFFFFFFFFFFFFFFFFFFFFFFFFFFFFFFFFFFFFFFFFFFFFFFFFFF
FFF

```
@A00721:190:HGJW7DSXY:4:2315:28330:22968_1:N:0:TCTGCAAG+GCTGCACT
TGGATTTCAGACGTGTTCTCTTCCGATCTGTGAAATTAACGCGTCCTTTTCTGGAACGTAGTTTTACT
ATTATGAACAAAGTTATCAAAGATAGTGGCATTCAAGGCTATCTTACCCTGATTCCGGCCGGTGGTAG
CT
+
F:,,:,:FF:FFFF,F,,FFFFF:FFF,F,:FFF,F:,F,F,FF,F,FF,FF,FFF,FF:FFFFF,,:
F:F,F,:F,F:F,F:,FFFF,FFFFFFFF,:FFF:,FF,,F,,FF,,,,FF,FF:F::F,FFFFFFFF
:F
@A00721:190:HGJW7DSXY:4:1225:23348:8359_2:N:0:TCTGCAAG+GCTGCACT
AGGGGCTATTACTCTTGCTGAAACAGCTATTAAAGCTGTTGAGCGGGTAATTACACCTAAACGAGCTC
GATCTGCCATCGCTGAAGGATGTGCACTCTTTTCTGCTACTTTAACGG
+
FFFFFFFFFFFFFFF:FFFFFFFFFFFFFFFFFFFFFFFFFFFFFFFFFFFFFFFFFFF:FFFFFFFFF
FFFFFFFFF:FFFFFFF:FFFFFFFFFFF:FFFFFFFFFFFFFF:FFF
@A00721:190:HGJW7DSXY:4:2110:31774:10081_1:N:0:TCTGCAAG+GCTGCACT
CTTCGTCGATTGTATCAACAGTAACATTACTTCTAATAAAGGTCTCTATCAAGCTCGTATTATCGTCG
CTGCTGGATCTGCGTTACCGTTGAAGGCTAAACTCCAAGGTAATTATGAAGACTGTGATTTATCTTTC
ACT
+
FFFFF:FFFFFFFFFFFFFFF:FFFFFFFFFFFFFFFFFFFFFFFFFFFFFFFFFFFFFFFFFFFFFF
FFFFFFFFFFFFFFFFFFFFFFFFFFFFFFFFFFFFFFFFFFFFFFFFFFFFFFFFFFFFFFFFFFFF
FFF
@A00721:190:HGJW7DSXY:4:1470:29821:29121_1:N:0:TCTGCAAG+GCTGCACT
ACGTGTGCTCTTCCGATCTATAATTTCAATCTTCTTAAAAAGAAGATAGAGAGTCATACTTTGGGCTA
TCGTTCAGGTGCATGGGGTTGGATAGCCAATATTGAAGTGGACACCTCTGGTAGTATCGAAGTAAAGA
TAA
+
FFFFFFFF:FFFFFFFFFFFFFFFFFFFFFFFFFFFFFFFFFFFFF:FFFFFFFFFFFFFFFFFFFFFF
FFFFFFFFFFFFFFFFFFFFFFFFFFFFFFFFFFFFFFFFFFFFFFFFFFFFFFFFFFFFFFFFFFFF
FFF
@A00721:190:HGJW7DSXY:4:1324:23448:35023_1:N:0:TCTGCAAG+GCTGCACT
ATACTATGGTTGAATCAACTAAGCATACTACTTTTGCTTTGATTACTCTACCGCCTTACAAGTCTAAG
GTTAAAGAGTCGATTAAACAAGTTGTACTTATCAATGATTTTATCGCTGGTTGTTATCTCGAAAAGTC
TCT
+
:FFFFFFFFFFFFFFFFF:FF:FFFFFFFFFFFFFFFFFFFFFFFFFFFFFFFFFFFFFFFFFFFFFF
FFFFFFFFFFFFFFFFFFFFFFFFFFFFFFFF:FFFFFFFFFFFFFFFFFFFFFFFFFFFFFFFFFFF
FFF
@A00721:190:HGJW7DSXY:4:2431:31195:8923_2:N:0:TCTGCAAG+GCTGCACT
TCAGTTCTAACCCAAAGAGAATATCTAAAATATTTACAAAATCTTACCACTATCTACGAACCTAAAGT
TCTGAAGACGTTGCTTGTCGCTTCCTCCTTTAATGGCACTACCTCGGT
+
FFFFFFFFFFFFFFFFFFFFFFFFFFFFFFFFFFFFFFFFFFFFFFFFFFFFFFFFFFFFFFFFFFFF
FFFF:FF:FFFFFFFFFFFFFFFFFFFFFFFFFFFFFFFFFFFFFFFF
@A00721:190:HGJW7DSXY:4:1531:10954:35806_1:N:0:TCTGCAAG+GCTGCACT
TTGGAACGAAGGCGACCTGTTCCGGAGTTTTTACGGGATCCGGAATGTTCTGTCTATATTAGGTGAAG
CGAAATACGATTTCGAACGTCAACGTTCACCGAGTTTCGTTTCGTACCTTGATGCTGACTTTCGTCAA
TAT
+
F:FFF:FFFFFFFFFFFFFFFFFFFFFF:FFFFFFFFFFFFFFFFFFFFFFFFFFFFFFFFFFFFFFF
FFFFFFFFFFFFFFFFFFFFFFF:FFFFFFFFFFFFFFFFFFFFFFFFFFFFFFFFFFFFFFFFFFFF
FFF
@A00721:190:HGJW7DSXY:4:1122:17842:28635_1:N:0:TCTGCAAG+GCTGCACT
TAGTGCGAGGCCTCTCTTCTACGAGTATTGCTATTTATACGGTTACGAGAAGAAGGTTCGAAAGTTTG
```

AGAACTTAGTTTACGAACTTGACTCTACTATACGTGACATACTAAATACTAGATATTTGGATGATCTT
GAT
+
FFFFFFFFFFFFFFFFFFFFFFFFFFFFFFFFFFFFFFFFFFFFFFFFFFFFFFFFFFFFFFFFFFFF
FFFFFFFFFFFFFFFFFFFFFFFFFFFFFFFFFFFFFFFFFFFFFF,FFFFFFFFFFFFFFFFFFFFF
FFF
@A00721:190:HGJW7DSXY:4:1121:29342:19492_2:N:0:TCTGCAAG+GCTGCACT
TTCAGAGGATGAAAAATTTTCTTCCTTGGATTTTAATATAAAGAATCTTTACCAGGACTCCTACTACA
GGAGGCAGTCTTCTGATCGTAAGAAGTTTATAAGGAACATATCCATTT
+
FFF,:FF:FFFFFF:FFFFFFFFFFFFFFFFF:F:FFFFFFFFFFFF:FFFF,F:FFFFFFFF:FFFF
FFFFFFFFFFFFFFFFFFFFFFFFFFFFFFFFF,FFFFFFFFF:FFFF
@A00721:190:HGJW7DSXY:4:1113:29912:11271_1:N:0:TCTGCAAG+GCTGCACT
ACTCGAAACTCTAAGTGGTAATACTGTCTTTCCAAGCTTACCTGTATCTTCAATGAAATTGGACTCTT
CGATAGGGGACATTTTATCGTGGGCTTTCCGTAAAGGAAGATCTGTTATCGACGGTTTATCGTTTACG
AAG
+
F:F::FFFFF::F:FFFFFFFF,FFFFFFFF::FF,FFFFFFF::FFFFF,FFFFFFFFFFFFFF:FF
:F:FF:F:FFFFFFFFFFFFFFFFFFFFFFFFFFFF,FFF,FFFFFFFFF::::FFFFFF:FFFFF,F
:FF
@A00721:190:HGJW7DSXY:4:1647:16640:15624_2:N:0:TCTGCAAG+GCTGCACT
GTTTAAGAATACTATAAATAGAATAGCTATTACTACTAATGGCGTTGGCCTAGTGGATGTTATTAGTG
ACATCTGGTCAATTTCGTTTTTAGGTGGTCTTTATTCTATTATTGATT
+
FFFFFFFFFFFFFFFFFFFFFFFFFFFFFFFFFFFFFFFFFFFFFFFFFFFFFFFFFFFFFFFFFFFF
FFFFFFFFFFFFFFFFFFFFFFFFFFFFFFFFFFFFFFFFFFFFFFFF
@A00721:190:HGJW7DSXY:4:1110:29939:8124_1:N:0:TCTGCAAG+GCTGCACT
ACAGAAGATGATGTTAGAGTAGAATTCCTGAAGAGTCCTTCCGTAACCAACTTCTTGACAATTTTCTT
CAATCCTTCGATAATAGTTAAATCTAGGATTATGTCTGTGTTGAAAGACTTTCTACCGATAAGTGTCA
CTA
+
FFFFFFFFFFFFFFFFFFFFFFFFFFFFFFFFFFFFFFFFFFFFFFFFFFFFFFFFFFFFFFFFFFFF
FFFF:FFFFFFFFFFFFFFFFF:FFFFFFFFFF:F:FFFFFFFFF::F,FFFFFFFFFF,FFF,FFFF
FFF
@A00721:190:HGJW7DSXY:4:1101:22290:27665_2:N:0:TCTGCAAG+GCTGCACT
TAAGAAATAATCCTACATTGACTGATTATCTAAAGATTGAAGGAAAATTTTATCCTCGTTTAACTTAC
TTAGACTTTCCTACGATACAAATGATAACTTCTAATTTACGTTTGCTA
+
FFFFFFFFFFFFFFFFFFFFFFFFFFFFFFFFFFFFFFFFFFFFFFFFFFFFFFFFFFFFFFFFFFFF
FFFFFFFFFFFFFFFFFFFFFFFFFFFFFFFFFFFFFFFFFFFFFFFF
@A00721:190:HGJW7DSXY:4:1135:11369:12070_2:N:0:TCTGCAAG+GCTGCACT
CAAAGGGATGCATGGTGTTATCTCCTCAGGAGTCCCGGCATTTGATAATAATGACGAGACTTTCGTCA
TAATCGCTGCATATTACGGATTACACGGTACCAGCGCATCTCGTGTCG
+
FFFFFFFFFFFFFFFFFF:FFFFFFFFFFFFFFFFFFFFFFFFFFFF:FFFFFFFFFFFFFFFFFFFF
FFFFFFFFFFFFFFFFFFFFFFFFFFFFF:FFFFFFFFFFFFFFFFFF
@A00721:190:HGJW7DSXY:4:1347:20871:16971_1:N:0:TCTGCAAG+GCTGCACT
GTATTAACTTATAAAGGTGAGGAGACCATTTTCTTGCTGAAATTTATAGAAGATTATTTTGACAGAAT
CAATAAGAATCATCGATTCTCGATTCGTAGAGCTTATCTAGGTACTCTCATTTACTATGTTGATTTAG
TCT
+
F,FFFFFFFFFFFFFFFFFFFFFFFFFFFFFFFFF:FFFFFFFFFFFFFFFFFFFFFFFFFFFFF:FF
FFFFFFFFFFFFFFFFFFFFFFFFFFFFFFFF:FFFFFFFFFFFFFFFFFFFFFFFFFFFFFFFFFFF
FFF

@A00721:190:HGJW7DSXY:4:1351:26332:12994_2:N:0:TCTGCAAG+GCTGCACT
TGATCTAAAATTTCGATCTAGATGGATCCACTCCTCGAGTTATCATGATGTTAGTATGTTATATACTG
ATTTACAACGATATAGGAGTAATCTACGAGAAGATTCTAGAATTCTTA
+
FFFFFFFFFFFFFFF:FFFFFFFFFFFFFFFFFFFF:FFFFFFFF:FFFFFFFFFFFFFFFFFFFF
FFFFFFF,FFFFFF,FFFFFFFFFFFFFFFFFF:FFFFFFFFFFFFFF
@A00721:190:HGJW7DSXY:4:1440:27037:22138_2:N:0:TCTGCAAG+GCTGCACT
GAGATCACACATTTTGAGAACTAACTATGTTATGTTCTCTGATCATTTTGACAATCTCTTCTTATAAT
AAATAAATAAATAATATTACTAATGGCAAATAGTGGAAAGGCTCCAGT
+
FFFFFFFFFFFFFFFFFFFFFFFFFFFFFFFFFFFFFFFFFFFFFFFFFFFFFFFFFFFFFFFFFFFF
FFFFFFFFFFFFFFFFFFFFFFFFFFFFFFFFFFFFFFFFFFFFFFFF
@A00721:190:HGJW7DSXY:4:1301:13467:21433_2:N:0:TCTGCAAG+GCTGCACT
TGCTTTGGATTACGATGACGTGAGAATAGGTGAAGGGATAGTTTTTACCTCGACCAACATTAAGGCGG
CGGCGACTAATCTTAAAGCTTCACCCAAAATGAAGGACACTTCGAAAC
+
FFFFFFFFFFFFFFFFFFFFFFFFFFFFFFFFFFFFFFFFFFFFFFFFFFFFFFFFFFFFFFFFFFFF
FFFFFFFF:FFFF:FFFFFFFFFFFFFFF:FFFFFFFFFFFFFFF:FF
@A00721:190:HGJW7DSXY:4:2244:30083:12258_2:N:0:TCTGCAAG+GCTGCACT
AAAAGGCTCTAAAGGACACTATACAATATCCAGACGACGACGAAGCTTATACTACTTTAATTTTGGGT
ATTATGTGGAGATATCGTGATATCAGTACGTCCAGATCCTCTGCTTTT
+
FFFFFFFFFFFFFFFFFFFFFFFFFFFFFFFFFFFFFFFFFFFFFFFFFFFFFFFFFFFFFFFFFFFF
,FFFFFFFFFFFFFFFFFFFFFFFFFFFFF:FFFFFFFFFFFFFFFFFF
@A00721:190:HGJW7DSXY:4:2672:10791:17863_2:N:0:TCTGCAAG+GCTGCACT
ATCAAATATAAAGAAAAGGACTATGAAATAAAAGATTTGGTGCTTAGGGATATAATGAATTCAGCACC
ACATTTTGGTCAAGTCAATCCGTTGAGAAAATTTCTTAGAGGATATTC
+
FFFF:FFFFFFFFFFFFFFFFFFFFFFFF:FF:FFFFFFFFFFFFFFF:FFFFFFFFF:FFFFFF::FF
FFFFFFF:FFFFFFF:FFFFFFFFFFFFFFFFFFFFFFF,FFFFFFFFF
@A00721:190:HGJW7DSXY:4:2445:9579:28228_1:N:0:TCTGCAAG+GCTGCACT
CCAACATTTACCACCAAATTCTTTTGATACAACTCTAGCCTTCAAGTGGGGTATGCCTGCGGAATACG
CTTGCTATTCACCAGATTGGTTCGAACCCGTTGATGCTATGTCAGAAGAAGCTATTCAAGCTTGTCGT
TTA
+
FFFFFFFFF::FF:F,F:FFFFFFFFFFFFFFFFFFFF,FFFFFF:FFFFFF:FFFFFF,:FFFFFFF
FFFFFFFFFFFFFFFFFFFFFFFF,F:FFFFF,FFFF:FFFFFF:F:FFFFFF:FFFFFFFF:FFFFFF
FF:
@A00721:190:HGJW7DSXY:4:1644:15826:16971_2:N:0:TCTGCAAG+GCTGCACT
AAACAAGCCCAGACTCAAGGAGTCCAATCAAAGGTTTTGAATTTAGCTCAACTGGGGAGACGTGCTTA
AATGGGTGATAAAAATTATTTAGAGATCTTTTCTACTTACAAAGGACA
+
FF:FFFFFFF:FFFFFF:FFFFFFFFFFFFF,FFFFFFFFFFFFFFFFFFFFFFFFFFFFFFFFFFFFF
FFFFFFFFFFFFFFF,FF:FF:FFFFFFFFFFFFFFFFFFFFFFFFFF,
@A00721:190:HGJW7DSXY:4:2422:6225:17691_2:N:0:TCTGCAAG+GCTGCACT
AATTCTACGGAAAAGTCCGGAGTATATTGTATGTTAATAGATAGTGGGATAATAGTTCTACCGATACG
TAGTGAACGCGTTCGTAGAACCAAGTACTTTCCGTCTGAAGAGTTTAG
+
FFFFFFFFFFFFFFFFFFFFFFF:FFFF:FF:FFFFFFFFFFFFFFFFFFFFFFFFFFFFFFFFFFFFF
:FFFFFFFFFFFFF:FFFFFFFFFFFFFFFFFF:FFFFFFFFFFF:FFF
@A00721:190:HGJW7DSXY:4:2320:13792:6151_1:N:0:TCTGCAAG+GCTGCACT
TAACCGATAGGAGTTGTATAGTTGAAGATGTGGCTTTAGACGATAATCCTTTAAGGACTATGTTGACT
GACGAAGATTTTCCCACAGATTTGTCTACGTTGCAGATCGCCACTCCAAACACTATATACATTAAAAT
ATA

```
+
FFFFFFFFFFFFFFFFFFFFFFFFFFFFFFFFFFFFFFFFFFFFFFFFFFFFFFFFFFFFFFFFFFFFFF
FFFFFFFF:FFFFFFFFFFFFFFFFFFFFFFFFFFFFFFFFFFFFFFFFFFFFFFFFFFFFFFFFFFFFF
FFF
@A00721:190:HGJW7DSXY:4:1203:27796:27868_1:N:0:TCTGCAAG+GCTGCACT
CATAACTTTTGTTACCAAATTCTTGGCTTTCAACGAAGTCTCTGATTTTATTAATCATTACTTTAACG
ATCTCTCGGTCATTTACTCTGTACTTGATCTACGTCGTTTAACTGTCGGAACAACCTTTACTTAGAAT
GAC
+
FFFFFFFFFFFFFFFFFFFFFFFFFFFFFFFFFFFFFFFFFFFFFFFFFFFFFFFFF:FFFFFFFFFF
FFFFFFFFFFFFFFFFFFFFFFFFFFFFFFFFFFFFFFFFFFFFFFFFFFFFFFFFFFFFFFFFFFFF:
FFF
@A00721:190:HGJW7DSXY:4:1323:21187:6872_2:N:0:TCTGCAAG+GCTGCACT
TTCTTATATTATTTACAATAGTTAATATCATATTAGTAATACTCACTATCGTTTTCTGCTTTTTCTTA
GCTAATAGATTAATAAAGGATGAGTACGAAAACCAACTCTTCGTTACG
+
FFFFFFFFFFFFFFFFFFFFFFFFFFFFFFFFFFFFFFFFFFFFFFFFFFFFFFFFFFFFFFFFFFFF
FFFFF:FFFFFFFFFFFFFFFFFFFFFFFFFFFFFFFFFFFFFFFFFF
@A00721:190:HGJW7DSXY:4:1269:22480:2033_1:N:0:TCTGCAAG+GCTGCACT
GTATAATAGATTTAGTCTCTACACCCATCTCAACGAGGATAAAGCGAGTTTTCCGTCCTGAACTTAAT
ACACGAAGAGATCATCCGCAAACGACTCGAAACCGAGACGCCGTAGATCAAAGTCCCTTCTAACTTTA
GAT
+
FFF,FFF,:FFFF::F,FF:FFF:FFF:::FF::,:,:F:FF,:,:,:FFFFF:,F,:F:F:F,FFFF
F,::,FF,F:F,,F,F:FFF:FFFFFFFFFF,FFFFFFFFFFFFFFFFFFFFFFFFFFFFFF:FF:FFF
FFF
@A00721:190:HGJW7DSXY:4:2123:22679:22764_1:N:0:TCTGCAAG+GCTGCACT
CTTTACTCATTTTATAGCAGTTATTTATGTTGTACTTTTTATAATTTATAACCTTCTTATACTTTTGG
GTATGTTTTGTGAAAAACATGAGATACCCAAGTCTATCGAAGACATTATCTCTTATGTACTTATTGTA
ACG
+
FFF::FFFFFFFFFFFF:FFFFFFFFFFFFFFFFFFFFFFFFFFFFFFFFFFFFFFFFFFFFFFFFFF:
FFFFFFFFFFFFFFFFFFFFFFFFFFFFFFFFFFFFFFFFFFFFFFFFFFFFFFFF:FFFFFFFFFFFF
FFF
@A00721:190:HGJW7DSXY:4:1530:26567:28870_1:N:0:TCTGCAAG+GCTGCACT
TTCTTATTCTTTTATTCCTTTAACTGTGTATGAGGAGGATTTATAATGCTTTTCGATAGTGATTGTTA
TCAATTTAAATACATTACTGTTTCAAATCATCTTTACTCACAGACTCCGAAAATGGTTTCAAATGAGG
TCA
+
FFFFFFFFFFFFFFF:FFFFFFFFFFFF:FFFFFFFFFFFFFFFFFFFFFFFFFFFFFFFFFFFFFFFF
FFFFFFFFFFFFFFFFFFFFFFFFFFFFFFFFFFFFFFF:F:FFFFFFFFFFFFFF:FFFFFF:FFFF,
FFF
@A00721:190:HGJW7DSXY:4:1530:26567:28870_2:N:0:TCTGCAAG+GCTGCACT
TATTAAACGAACTATTATCCGATTCTTCGAGGGTATCACTACCACTGATCTGCCGACTTTAGTCAAGG
AACTTCAATTCCTTCGATGTTTATATTTTTCTATAAGGGAAAATAGCA
+
FF:FFFFFFFFFFFFFFFFFFF:FF,FFFFF:FFF:FFFFFFFFFFFF:FFFF:FFFFFFFFFFFFFF
FFFF:FFFFFFFFFFFFFF:FFFFFFFFF:FFFFFFFFFFFFFF:FFF
@A00721:190:HGJW7DSXY:4:1317:18231:24173_2:N:0:TCTGCAAG+GCTGCACT
TTCGACAAATTTTAGAACTTTATTTCAGAACTCTCTCCGATCACCTTGGTGTGATCTCCCGGAGGATC
TTTTCAATAAGATGTATGATGATAATCTACTATTTTTAAATAGTGATA
+
FFFFFFFFFFFFFFFFFFFFFFFFFFFFFFFFFFFFFFFFFFFFFFFFFFFFFFFFFFFFFFFFFFFF
FFFFFFFFFFFFFFFFFFFFFFFFFFFFFFFFFFFFFFFFFFFFFFFF
```

@A00721:190:HGJW7DSXY:4:2120:28727:22842_1:N:0:TCTGCAAG+GCTGCACT
ACTTTATCAAAGATTTGGCTTGGTCTATTTACCGCAAAAAGTTGGAAGAGGTCTATGATATTTATTTT
ACTTATACTTTAAATAGCACCGATTATAACTTGACTGTTGATAATATTACAATAGCTCTTTCTCAGAA
GAT
+
FFFFFFFFFFFFFFFFFFFFFFFFFFFFFFFFFFFFFF:FFFFFFFFFFFFFFF:FFFFFFFFFFFF
FFFFFFFFFFFFFFFFFFFFFFFFFFFFFFFFFFFFFFFFFFFFFFFFFFFFFFFFFFFFFFFFFFFF
FFF
@A00721:190:HGJW7DSXY:4:1304:5303:6793_1:N:0:TCTGCAAG+GCTGCACT
ACCGGTGAACACGTCGGTAGCATTGATTTACTTTTACCGTTAGAACTGAAAAAGATTCTTTTCGAAAA
GAAACCTATAAGTTACAAACGATTCTTCGACTATATTAAAGAAATAAAAATACTTCAAGTACCTGAAC
TTT
+
FFFFFFFFFFF::FF:FFFF,:FF:FFFFF:FFFFFFFFFFF:FFFF:FFFFFFFFFFFF:FF,FFFF
FFFFF:FFFF::F:,FF,FFFFFFFFFFFFFFFFFFFFFFFFFFFFFFFFFFFFFFFFF:FFFFFFFF
FFF
@A00721:190:HGJW7DSXY:4:1342:32913:27336_1:N:0:TCTGCAAG+GCTGCACT
ATGGTATAATGTGTCTTACAAATAGAACCTTCACTCTCGGTAATGAGAGTGTATTCCTTAATATTCTA
GTAACCGCTATGTGGGCATTACTAGGATTTATCACACTCCTAAATTTGATAGCAATTTATGTTTTAAA
TGC
+
FFFFFFFFF::FFFFFFFFFFFFFFFFFFFFFFFFFFFFF:FFFFFFFFFFFFFFFFFFFFFFFFFFF
FFFFFFFFFFFFFFFFFFFFFFFFFFFFFFFFFFFFFFFFFFFFF:FFFFFFFFFFFFFFFFFFFFFFF
FFF
@A00721:190:HGJW7DSXY:4:1170:26449:11099_2:N:0:TCTGCAAG+GCTGCACT
AAAAACTCCATTGTATAGTTCAAGGAGTACTTAATTCAATAGCTTTAGTATTATTTACTATCGCCTTT
ATTGAAAATTGTATAAGAGTTAATTGCTTACACAAAAGTGACGCGCGA
+
FFFFFFFFFFFFFFFFFFFFFFFFFFFFFFFFFFFFFFFFFFFFF:FFFFFFFFFFFFFFFFFFFFFFF
FFFFFFFFFFFFFFFFFFFFFFFFFFFFFFFFFFFFFFFFFFFFFFFF
@A00721:190:HGJW7DSXY:4:1542:23185:28839_1:N:0:TCTGCAAG+GCTGCACT
GGGGAGAATGAACTGCCGCTTCCCGGAAGAAACGATCTATTTACAGAGTATTTAGAGTAGTCCAAAGT
TTGTATAAATTTATATGATTTTCTATATATATACCACATATATATATATATAAAATCCTATAAATTTG
TAC
+
FFFFFFFFFFFFFFFFFFFFFFFFFF:FFFFFF:FF:FFFFFFFFFF:FFFFF:FFFFFFFFFF:F:,
FFFFFFFFFFFFFFFFFFFF:FFFFFFFFFFFFFFFFFF:FFFFFFFFFFFFFFFFFFFFFFFFFFFFF
FFF
@A00721:190:HGJW7DSXY:4:1551:32741:26287_2:N:0:TCTGCAAG+GCTGCACT
GAAGAAAACCACTCCCTTCAATTCAATAACATAACATACCATACACTCCCCAAACATAACATACCATA
ACCTACCATGACCAATCATCGCTCCAACTGGCACGCCAGACGTAGGGT
+
FFFFFFF::FF:FFFFFFFFFFFFFFFF,FFFFFFFFFFFFFFFFFFFFFFF:FFFF:FFF:FFF:FF
:FFFFFFFFF:FF::FFFFFFFFFFFFFFFFFFFFFFFFFFFFFFFFFF
@A00721:190:HGJW7DSXY:4:1323:21685:2127_2:N:0:TCTGCAAG+GCTGCACT
ACCGCCGGTCGGCAGATCGGCACCACTGGTCTCTACGTTCAGGGACCCGCTGTGCAGAATAACCTTCC
CAAATCTAGATCTGCACAGAAGAAGGCTGAAGGTCGTTACCGGACGAC
+
FFFFFFFFFFFFFFFFFFFFFFFFFFFFFFFFFFFFFFFFFFFFFFFFFFFFFFFFFFFFFFFFFFFF
F:FF:FFFFFFFFFFFFFFFFFFFFFFFFFFFFFFFFFFFFFFFFFFF
@A00721:190:HGJW7DSXY:4:2456:22336:31219_1:N:0:TCTGCAAG+GCTGCACT
GGCTGTTGCCCAGAGGAAAGCAGCGACCACCGAGGTCGCTCGTAACATTGACGGCAGTCCCGTCATCT
CTGTGGGAACTGAGGCAGTACCTCAGTCAAAAGTCACATTCACCACTCCCATGCCAATCAATGATGTG
ACT

+
FFFFFFFFFFFFFFFFFF:FFFFFFFFFF,F:FFFFFFF,FFFFFFFF:FFF:FFFF::FFFFFFFFFF
,FFFFFFFFFFFFFFFFFFFFFFFFFFFFFFFFFFFFFF:FFF:FFFFFF:FFFFFFFFF,FF:FFF
FFF
@A00721:190:HGJW7DSXY:4:2134:28610:5415_2:N:0:TCTGCAAG+GCTGCACT
TCAATACGTCGTGGGAGGTACTGAAGCCTTCCCCTCCGCTGGCACGAAGAACACTCCCAAGCCAATCA
ATAGTTCTTCTGACCAGAATGAAAAGGGGTTTACCAGGGTAGTCTCTA
+
FFFFFFFFFFFFFFFFFFFFFFFFFFFFFFFFFFFFFFFFFFFFFFFFFFFFFFFFFFFFFFFF,FFF
FFFFFFFFFFFFFFFFFFFFFFFFFFFFFFFFFFFFFFFFF:FFFFFFF
@A00721:190:HGJW7DSXY:4:1414:14751:34648_1:N:0:TCTGCAAG+GCTGCACT
AGAGGGGAAATGTACCCAAAACTGAGAAGAATAAAGGGAAAGGTCCATCAGGACCTAAGAAGGGGAAA
TTACCCCTAATTCTGAAGGAAGATCTCCCACCATCTGGGGTTGGGAATAAGAAATCTGCACGGGCCAA
A
+
FFFF,FFFFFF:F,FFFFFFFFFFF,FFFFFFFF:FFFFFFFFF:,FFFF::FFFFF:FF::F:F:FF
FFFFF::F:FFFFFFF:F,FFFFFFFFFFFF::FFFFFFFFFFFF,FF:FFFFFFFFFFFFFFF:FFF
F
@A00721:190:HGJW7DSXY:4:1369:20636:3129_2:N:0:TCTGCAAG+GCTGCACT
CTACGAAGTCGCTGAAGGCGGCTTGGGTCATTAAGGGTAGCTGTAAAGTTGCGCCTGCCGACCCCATC
TTTGAGACCAAGACTCTCTACAGTAGGTTCGTGCCTACTATGAGCAAT
+
FFFFFFFFFFFFFFFFFFFFFFFFFFFF:FFFF:FFFFFFFFFFFF:F:FFFFFFFFFFFFFFFFFFF
FFFFFFFFFFFFFFFFFFFFFFFFF:FFFFFFFFFFFFFF:FFFFF:F:
@A00721:190:HGJW7DSXY:4:1416:29008:17315_2:N:0:TCTGCAAG+GCTGCACT
TCGTCACTGAGGACGAAGGTGAGAGGACTTTTGGCTACTGGCGACAAACTGAGAGCAGTCGCTGGGGA
AAGAGAATTGCGTGGGTTGAAGGCCGACGCTTCGAGCTTGAGTGTGAC
+
FFFFFFFFFFFFFFFFFFFFFFFFFFFFFFFFFFFFFFFFFFFFFFFFFFFFFFFFFFFFFFF:FFFFF
FFFFFFFFFFFFFFFFFFFFFFFFFFFFFFFFFFFFFFFFFFFFFFFFF
@A00721:190:HGJW7DSXY:4:2229:24686:8985_2:N:0:TCTGCAAG+GCTGCACT
GTTTTTGTCAACGGCGTTTGGAGGATTTTATCCGCGCTTAACTATGGGGCGAAGATCCCCTATGGAGT
CTTGAAGGACTTAGGTTGGCATATTTATGCCGACGATAATAAGCATGT
+
FFFFFFFFFFFFFFFF::FFFFFFFFFFFFFFFFFFFFFFFFFFFFFFFFFFFFFFFFFFFFFFFFFF
FFFFFFFFFFFFFFFF:F:FFFFFFFFFFFFFFFFFFFFFFFFF:FFFF
@A00721:190:HGJW7DSXY:4:2272:26558:8782_1:N:0:TCTGCAAG+GCTGCACT
CCGAGGTAAGCTCGGGACTTACAAGAGAGAGTGTGAGAGAGTCGCTCTCACTATTTTTGCCCAGAATG
CGGGCTTTCTGACTGATGAGGTTAAAACTCTTGCTCCTTCTGTGGGTGAGGGTTTTTGTTATATTCAC
TGG
+
FFFFFFFFFFFFFFFFFFFFFFFFFFFFFFFFFFFFFFFFFFFFFFFFFFFFFFF:FFFFFFFFFFFF
FFFFFFFFFFFFFFFFFFFFFFFFFFFFFFFFFFFFFFFFFFFFFFFFFFFFFFFFFFFFFFFFFFFF
FFF
@A00721:190:HGJW7DSXY:4:1320:25843:27899_2:N:0:TCTGCAAG+GCTGCACT
TCCTCCATAATTACAACCTCTCTTTAGAGAGCATTAGAAGGAAGTTGGGCAAATTCCCACATATGGTA
GACGTTGTCACACTTTTCTTGAAAATTTTTCCTTTCCTCCGTCATTGG
+
FFFFFFFF,FFFFFFFFFFFFFFFFFFFFFFF:FFFFFFFFF:F:FFFFFF:FFFFFFFFFFFFFFFF
F:FFFFFFFFFFFF,FFFFFF:FFFFF:FFFFFFFFFFFFFFFF,FFF
@A00721:190:HGJW7DSXY:4:2669:14543:26710_2:N:0:TCTGCAAG+GCTGCACT
GTGAGAAGGTGGAAGGTGTCAAGGGTCTTCTTCATGCTGATCTTTCAGGACCTAATCACATCATGATT
GATAGTTACGGCTCGTATCGAGTTGGAGGGAGGAAGTTCTCCGAAATT
+

```
FFFFFF::FFFFFFFFFFFFF,FFFFFFFFFFFFFFFFFFFFFFFF:FFFFFFFFFFFFFFFFFFFFF
FFFFFFFFFFFFFFF:FFFFFFFFFFFFFFFFFFFFFFFFF:FFFFFF
@A00721:190:HGJW7DSXY:4:1103:31421:11318_2:N:0:TCTGCAAG+GCTGCACT
TCAGAGAGGGGAGCTCTGAAAAGGAAACTTCGTCAGAAGATGGGGAAGTCTAAGAGGAGGAGTTTGAG
GAAGGCTCTAGAGAAGATCTCACCACCTGGAGAAGGCAAGTGGAGAGT
+
FFFFFFFFFFFFFFFFFFFFFFFFFFFFFFFFFFFFFFFFFFFFFFFFFFFFFFFFFFF:FFFFFFFF
FFF,FFFFFFFFFFFF:FFFFFFFF:FFFFFFFFF,FFFFF,F,FFFF
@A00721:190:HGJW7DSXY:4:1113:9426:25081_2:N:0:TCTGCAAG+GCTGCACT
AAGAAGGATTATTTGAAGCAGGGGTTACCACTTCCTAAGGTGGATGAGGGAGGTTGTTTCTTTGGAGT
TTTAGATAATCTTTTGAGAAGGGGTCATAGAGCCAGCAAGAGAAGCTT
+
FFFFFFFFFFFFF:FFFFFFFFFFFFFFFFFFFFFFFFFFF:FFFFF:FFFFFFFFFFFFFFFFFFFF
FFFFFFFFFFF:FFFFFFFFFFFFFFFFFFFFFFFFFFFFFFFFFFFF
@A00721:190:HGJW7DSXY:4:1323:32805:3098_2:N:0:TCTGCAAG+GCTGCACT
CCTTGAGAAGACAAGCCACATTAAGATATAATTTAGGTCAACTTGGGGTTTCGAATGTCATTAGTGCC
TTACGATTGAAAGAGGTCCTTTTGAGTATCGACAGATCTTTCGATCTT
+
FFFFFFF:FFFFFFFFFFFFFFFFF:FFFFFFFFFFFF:FFFFFFFFFFFFFFFFFFFFFFFFFFFFF
FFFF:FFFFF:F,F:FFF:FFFFFFFFFFFFFFFFFFFFFFFFFFFFF
@A00721:190:HGJW7DSXY:4:1570:26106:27226_2:N:0:TCTGCAAG+GCTGCACT
TTAACGTGGCAGAATTCGGTAACTTACCATATTGTTCCTTCCGGGAAGTTGTGGGATTTAACTTTGAC
TCAACTACTCCGATCAGATCCGGATGGTTTGATTGGTATGGACTACTC
+
FFFFFFFFFFFFFFFFFFFFFFFFFFFFFFFFFFFFFFFFFFFFFF:FFFFFFFFF:FFFFFFFFFFF
FF:FFFFFFFFFFFFFFFFFFFFF:FF,:FFFFFFFFFFFFFFFFFFF
@A00721:190:HGJW7DSXY:4:1463:30120:4679_1:N:0:TCTGCAAG+GCTGCACT
TAGTTGTCTAGGGTCATGGCAGATACTACCGATAAGGTGAAAAATATGCAGGTCAAAGATTCTGTAGC
TCTGAGGGCGGTTGAGGGAGCCGTTTTGGCCGACTTTAACTATTCTAAGAGTCTTTATGATTCAAAAC
CTA
+
FFF:FFFFFFFFFFFFFFFFFFFFFFFFFFFFFFFFFFFFFFFFFFFFFFFFFFFFFFFFFFFFFFFF
FFFFFFFFFFFFFFFFFFFFFFFFFFFFFFFFFFFFFFFFFFFFFFFFFFFFFFFFFFFFFFFFFFFF
FFF
@A00721:190:HGJW7DSXY:4:1664:15682:26866_2:N:0:TCTGCAAG+GCTGCACT
ACCTTACAAGGTTGAGGAGACTTTACAGCGGCTTATTGTCGCGCAGTTTCCAGAATTGCGGTTGAAGT
TCACCCATTCTATCTTGAATGCTCACGCCGTCGCAGCCATATCGAGAC
+
FFFFFFFFFFFFFFFFFFFFFFFFFFFFFFFFFFFFFFFFFFFFFFFFFFFFFFFFFFFFFFFFFFFF
FFFFFFFFFFFFFFFFFFFFFFFFFFFFFFFFFFFFFFFFFFFFFFFF
@A00721:190:HGJW7DSXY:4:1665:32461:19852_2:N:0:TCTGCAAG+GCTGCACT
AAAGTACAGATTTAGTGATTTAGATATTATTGATATTGGAGGCGATATTCGTTTTCATATCCAAAACT
CCTTGAATGGAGTACATATTTGCAACCCTATTCTTGATGTGAAAGATG
+
F:FFFFFFFFFFFFFFFFFFFFFFFFFFFFFFFFFFFFFFFFFFFF,FFFFFFFFFFFFF:FFF,FFF
FFFF:FFFFFFFF:FFFF,FFFFF:FF:FFFF,FF,FFFFF:FFFFFF
@A00721:190:HGJW7DSXY:4:2670:20907:11835_2:N:0:TCTGCAAG+GCTGCACT
AACATTGATTGGGAACGTACCTACTCACGAGACGTCATGAAAGCTATCGGAGGGAAGCAAAATGTTTC
GTTCTGTTACAAGAACGCTTTGGATTGTGACCATAAAGCCTCAGTTGC
+
FFFFFFFFFFFFFF:FFFFFFFFFFFFFFFFFFFFFFFFFFFFFFFFFFFFFFFF,FFFFFFFFFFFF
FFFFFFFFFFFFFFFFFFFFFFFFFFFFFFFFFFFFFFFFFFFFFFFF
@A00721:190:HGJW7DSXY:4:1134:15429:27931_1:N:0:TCTGCAAG+GCTGCACT
CTCTCTTGATGATGTAGCCACTATAATGCTTAAGAGAGAGATTGATGTATGTCATTTCGTCTTAGTTT
```

```
GTCCCGGCGAATTATTGGACACGATGAAAAATGACATACCCGTTCCTGACCTTGGTATCGTTATCCGT
CAT
+
FF,FFFFFFFFFFF:FF,FFFFFFF:FF:FF:F::FFFFFFFFFF:FFFFFFFFFF:FFFFFFFFFFF
:FF:FF,:FFF::FF:FFFFFFFFFFFFFFFFF:F:,F:::FFFF:,FFF:FFF:F:F:FFF:F:F,F
FF,
@A00721:190:HGJW7DSXY:4:2529:13829:20744_2:N:0:TCTGCAAG+GCTGCACT
CGAAGGATTGGGTTATTCTCACAACAAAGATAAGATTTTATCTTTCTTCAAACACCCTGTTTTCCTTA
AGGGCGGACAATTGTTCCATTGTGAACTCTTGGGGACTAGATTGAGCA
+
FFFFFFFFFFFFFFFFFFFFFFFFFFFFFFFFFFFF:FFFFFFFFFFFFFFFFFF:FFFFFFFFFFFF
FFFFF:FFFFFFFFFFF:FFFFFFFFFFFFFFFFFFFFFFFFFFFFFFF
@A00721:190:HGJW7DSXY:4:1331:13973:4586_1:N:0:TCTGCAAG+GCTGCACT
AGTGACTGGAGTTCAGACGTGTGCTCTTCCGATCTATTCAGAATACCTATTTCCGTACCAAGAGCCAC
TGCCAATCTCATCGCTATACGCATACCTGATTGGAAGAAGCGAGGTTCGTTCCTCAATAAAAATTTTT
CTC
+
FFFFFFFFFFFFFFFFFFFFFF:FF:FF:FFFFFFFFFFFFFFFFFFFFFFFFFFFFF:FFFFFF:FF
FFFFFFFFFFFFF:FFFFFFFFFFFFFFFFFFFFFFFFFFFFFFFFFFFF,FFFFFFFFFFFFFFFFFF
FFF
@A00721:190:HGJW7DSXY:4:2519:10574:36025_1:N:0:TCTGCAAG+GCTGCACT
TAAAGTTTTCTTCATAAGGGCTATTCAGTACGTCATGGGGATCACTTCTTCGGTCAATGACAAAACCT
TCGAATATGTTACGACATGGCTTCGTAACAACAGTGCGAGGATTGTCATCTCGGGGAAAGTGATTCAC
CAA
+
FFFFFFFFFFFFFFFFFFFFFFFFFFFFFFFFFFFFFFFFFFFFFFFFFFFFFFFFFFFFFFFFFFFF
FFFFFFFFFFFFFFFFFFFFFFFFFFFFFFFFFFFFFFFFFFFFFFFFFFFFFFFFFFFFFFFFFFFF
FFF
@A00721:190:HGJW7DSXY:4:1324:13964:2816_1:N:0:TCTGCAAG+GCTGCACT
GAGATACCTAGAGAAGGTCGCAGCGATCTTACTCGTTATCGGAGCTAAGAAGAAGACTTTGGGTACAC
GTTATGCTCGCAAACTTAACGTTAGTACCGGAGGTGATCTCTCTGCTTCTGCGTTACTGAGAGAAAAG
ATT
+
FFFFFFFFFFFFFFFFFFFFFFFFFFFFFFFFFFFFFFFFFFFFFFFFFFFFFFFFFFFFF:FFFFFF
FFFFFFFFFFFFFFFFFFFFFFFFFFFFFFFFFFFFFFFFFFFFFFFFFFFFFFFFFFFFFFFFFFFF
FFF
@A00721:190:HGJW7DSXY:4:1335:10700:8218_2:N:0:TCTGCAAG+GCTGCACT
ACGGTGACTTTATGGACAAGATGCAAGTTCTTGGGAAATACTTGTTTCCCTTTTTAGGGTTAGTAGGT
GATCTTGACGTCGATTCTTTGTATAGGGAGTGTGAGAAGACAACGAAT
+
FFFFFFFFFFFFFFFFFFFFFFFFFFFF:FFFFFFFFFFFFFFFFFFF:FF::FF,FF,FFFFFFFFF
F,F:FFFFFFFFFFFFFFFFF:FFFFFFFFFFFFFFFF,FFF:FFFF
@A00721:190:HGJW7DSXY:4:2444:23529:27993_2:N:0:TCTGCAAG+GCTGCACT
TATCAAAGAAAATGGTGAACCGGTCTCTAAATCTTTTGAGATTGAGAAGATTGAAGAACACGTCAAGA
AGTCGCTCGGCGTTGTTCTTGAAAACCAAGTTAAGAAGGAGGCTGATC
+
FFFFFFFFFFFFFFFFFFFFFFFFFFFFFFFFFFFFFFFFFFFFFFFFFFFFFFFFFFFFFFFFFFFF
FFFFFFFFFFFFFFFFFFFFFFFFFFFFFFFFFFFFFFFFFFFFFFFF
@A00721:190:HGJW7DSXY:4:1575:8730:27821_2:N:0:TCTGCAAG+GCTGCACT
GTACCTTCTGTCAAACTTCGAGGTTCAGCTACGAGAGGTGATCCCGGATGTGGATTGAAGGCTGGCTC
TAAGGGAGACTCCTTCTTTTTGAATGTTGTAAAGAATTGGGAGATCGG
+
FFFFFFFFFFFFFFFFFFFF:FFFFFFFFFFFFFFF:FF:FF:FFFF:,FFFFFFFFFF:FFFFFFFF
FF,FF:FFFFFFFFFFFFF,FFFFFFFFFFFF:FFFFF:FFFFFFFFF
```

```
@A00721:190:HGJW7DSXY:4:1175:1759:31375_1:N:0:TCTGCAAG+GCTGCACT
AGTTTTTAGATCTTCTTGATAATCTTAAAGAAAAATTTTCGAATAAACTAATGAGAGGTATAGGCTTA
GTCAAGTCTGTACTTACATTCTTTACGCCAGATTCTTCATGGGGCGACGCTGTTTCTCTTGGTATTTC
TGA
+
FFFFFFFFFFFFFFFFFFFFFFFFFFFFFFFFFFFFFFFFFFFFFFFFFFFFFF:FFFFFFFFFFF:FF
FFFFFFFFFFFFFFFFFFFFFFFFFFFFFFFFFFFFFFFFFFFFFFFFFFFFFFFFFFFFFFFFFFFFF
FFF
@A00721:190:HGJW7DSXY:4:1151:3369:31250_2:N:0:TCTGCAAG+GCTGCACT
CAGAGCGTCATTTTCCTTTGGGATCTTTCCTCTGGTAAGGGAATTATTAAATCATTCTTCTCACTCTT
GACGAGAATTATTACTGTTTTCTCTACCCATAACTTCTTTTTCAAAAA
+
FFFFFFFFFFFFFFFFFFFFFFFFFFFFFFFFFFF,F:FFFFFFFFFFFFFFFFFFFFFFFFFFFFFF
FFFFFFF:FFFFFFFFFFFFFFFFFFFFFFFFFFFFFFFFFFFFF,FFF
@A00721:190:HGJW7DSXY:4:2635:20943:33630_1:N:0:TCTGCAAG+GCTGCACT
TGGAGTTCAGACGTGTGCTCTTCCGATCTGTTTCAACACTTATCACCACTTTTGAAAGGAAAATAATT
GGTGATGAAAATACGTATGTCGGTGGGGTTCTCAAGGCTCTCATCGCCACTGGGATTCTAAGGGGTTC
C
+
FFFFFFF,FF:,FF:F,,FFFF,FF:FFF:FFFFF::,,FFFFFFFFF,FFFFFFFF,F:FF:F:,F:
FFFF:FFFF,,F,:FF:FFF:FFFFFFFFFFF,F:FFFFFFFFFFFFFFFFFFF,FFFFFFFFF::FF
F
@A00721:190:HGJW7DSXY:4:2372:2474:29293_2:N:0:TCTGCAAG+GCTGCACT
TTCAATGATCAGGATTCTTCTTTTGGTCCTTACGTCTTAAGTAATTTGGATGTTGTCGAAAACTATCA
GGTTCTTGTCACGATTCTTAAAAGAAGTTTATTAAAACTTCAGAGTCT
+
,F:FFFFFFF:,FFFFFFF,::FF:FFFFFFFFFFF:F:FFFFFFFFFFFF::FFFFFFFFFF:FFFF
FFFFFFFFFFFFFF,FF:FFFFFFFFF,FFFFFF::F:::FFFF:FFF
@A00721:190:HGJW7DSXY:4:1111:27769:13166_1:N:0:TCTGCAAG+GCTGCACT
GGTAGCTAATGTTCTTGCAATAGCCACAGACTTTCTGGGAAAAGAGACCTCTGTTGTAGAGGAGTTGA
GAGAATTACTCAGATTCAATCTGGCATCACTGAACGTCTACAACGAGTTAGGGAGTGGCTAGGTTTTA
AAA
+
FFFFFFFFFFFFFFFFFFFF,FFFFFFFFFFFFFFFFFFFFFFFFFFFFFFFFFFFFFFFFFFFFFFF
FFFFFFFFFFFFFFFFFFFFFFFFFFFFFFFFFFFFFFFFFFFFFFFFFFFFFFFFFFFFFFFFFFFFF
FFF
@A00721:190:HGJW7DSXY:4:1258:17535:1642_1:N:0:TCTGCAAG+GCTGCACT
AGCAATACCTTCCACTGAGTTCATCACCCAGAGTTTCGACGTCGCGGAAAACGATGAAGATTTCCACA
CTGTTCGAGAATCTTCTGATGCTGAATTTGAGGAAAATTTTTCGGAAGTTTTTGGTATAAATCCTGAT
GGC
+
FFFFFFFFFFFFFFFFFFFFFFFFFFFFFFFFFFFFFFFFFFFFFFFFFFFFFFFFFFF:FFFFFFFF
FFFFFFFFFFFFFFFFFFFFFFFFFFFFFFFFFFFFFFFFFFFFFFFFFFFFFFFFFFFFFFFFFFFFF
FFF
@A00721:190:HGJW7DSXY:4:2462:9715:16564_2:N:0:TCTGCAAG+GCTGCACT
AGTTATAAAACCTTCTTTTATCATTGCAACAGGACTTTGAGAAGACTGTTGAAATGGACAAAATCTGG
GATCATAGGTATGTGTCACGCTACACTCGAAATATGGGCCCTCATAAG
+
FFFFFFFFFFFFFFF:FFFFFFFFFFFFFFFFFFFFFFFFFFFFFFFFFFFFFFFFFFFFFFFFFFFFF
FFFFFFFFFFFFFFFFFFFFFF:FFFFFFFFFFFFFFFFFFFFFFFFFF
@A00721:190:HGJW7DSXY:4:1501:10547:19163_1:N:0:TCTGCAAG+GCTGCACT
CGTCATTACAGTCTGGAAGTTAGATCATTTGTTCAGGAAGATCTCTACAGAGATTATCGAGAGTTGTG
ATCTAGTTCTAAATTTAGGTCGACGGACTTTAGCAACTTTAGATAAATTCTTTCCGGGCTCAATGACC
TTT
```

```
+
FFFFFF,FFFFFFFFFFFFFFFFFFFF:FFFFFFFFFFFFFFFFFFFFFFFFFFFFFFFFFFFFFFFFF
FFFFFFFFFFFFFFFFFFFFFFFFFFFFFFFFFFFFFFFFFFFFFFFFFFFFFFFFFFFFFFFFFFFFF
FFF
@A00721:190:HGJW7DSXY:4:1168:19804:2534_1:N:0:TCTGCAAG+GCTGCACT
TCTGACCATCGCAAAGAAGACTTTATTAGTTCATGAAGTTCTTACACTTCTCGCGACTAATGTCAGCT
TCACTACAAAGGCGTTACGTAAAGTAGTTTTCACGCTTTATCTTAAAGCATTACCACCAAATTTAGCA
CTT
+
FFFFFFFFFFFFFFFFFFFFFFFFFFFFFFFFFFFFFFFFFFFFFFFFFFFFFFFFFFFFFFFFFFF::
:FFFFFFFFFFFFFFFFFFFF:FFFFFFFFFFFFFFFFFFFFFF:FFFFFFFFFFFFFFFFFFFFFFFF
FFF
@A00721:190:HGJW7DSXY:4:1266:5403:12602_1:N:0:TCTGCAAG+GCTGCACT
GAAGCTCGCCATATCAGATTAGAGACATGCCAGTTAGAGTTCTTCTAACCAGGACTGATGGTGAGATT
CTTTTACCTTTTGGAGGTCCAGGAACCTTTACTCCTAGTGTCTTTTCTAGAGTCCAAGGACGTTTCCG
TCG
+
FFFFFFFFFFFFFFFFFFFFFFFFFFFFFFFFFF:FFFFFFFFFFFFFFFFFFFFFFFFFFFFFFFFFF
FFFFFFFFFFFFFFFFFFFFFFFFFFFF:FFFFFFFFFFFFFFFFFFFFFFFFFFFFFFFFFFFFFFFF
FFF
@A00721:190:HGJW7DSXY:4:1173:2537:34006_1:N:0:TCTGCAAG+GCTGCACT
GTGGGAGACCGCTCAATAACACTCTATTGAATCCCTTTTCCACGATGAAAGCTCTTTATGAGGTGCTC
CATGCAAATTTACAGTTCCTCTTTGGTGATATCGCCTCTGTCCTTTCTCCTTTAATCGTGAGGTTACG
AGA
+
FFFFFFFFFFFFFFFFFFFFFFFFFFFFFFFFFFFFFFFFFFFFFFFFFFFFFFFF:,FFFFFFFFFF
F::FFFFFFFFFF,F,FFFFFFFFFFFFFFFFFFFFFFFFFFFFF,FFFFFFFF:FFFFFFFFFFFFFF
FFF
@A00721:190:HGJW7DSXY:4:1157:12943:6527_2:N:0:TCTGCAAG+GCTGCACT
GGTTTATCGGTTGTATTTTGATAAATTTGTTGGAATCGATAATGATACTGACGAAGGTTCTATTACCA
CCCTTGTCATTGATACAAACATTCTAGGTGATGCTTTCTTAGAGGCTC
+
FFFFFFFFFFFFFFFF:FFFFFFFFFFFFFFFFFFFFFFF:FFFFFFFFFFFFFFF:FFFFFFFFFFFF
FFFFFFFFFFFFFFFFFFFFFFFFFFFFFFFFFFFFFFFFFFFFF::FF:
@A00721:190:HGJW7DSXY:4:1221:21757:14372_2:N:0:TCTGCAAG+GCTGCACT
TATTTACAATCTGTAGAGAGAGAGGTTAATCAGAATTTGAGTGATAACGATTTAGTACTGGGAAATGG
TGGTAGTCAGTTTACTACTTTTCTTTCCCTCCCCTTTTTCGATTGGAT
+
FFFFFFFFFFF:FFFFFFFFFF,FFFFFFFFFFFFFFFF:FFFFFFFFFFF:FFFFFFFFFFFFFFFF
,FF:FFFFFFFFFFFFFFFFFFFFFFFFFFFFFFFFFFFFFFFFFFFFF
@A00721:190:HGJW7DSXY:4:1473:31684:4570_2:N:0:TCTGCAAG+GCTGCACT
CGTCTCACGACTAATTTGATAAGGTTTTTCTTTTATGGGACTCTTGTTTGTACTTATGAAGGAGGTAT
TACTTCGGAAGTTCTTAATGTAGGTCTCCTAGTACTTAAGGGTTTATG
+
FFFFFFFFFFF,FFFFFFFFFFFFFFFFFFFF:FFFFFFFFFFFFFFFFFFFFFFFFFFFFFFFFFFFF
FFFFFF:FFFFFFFFFFFFFFFFFFFFFFFFFFFFFFFFFFFFFFFFFF
@A00721:190:HGJW7DSXY:4:1127:19271:31109_2:N:0:TCTGCAAG+GCTGCACT
TTTTGATGGTATGTTTCACATTGCGTGATCACTTCCCGTGGACTTGGGAAGAGATTAAACATAAAATC
CAGCGATTCTATTATGTCGCTAAAGCCTGTCCAGTTGTCGATGATGTT
+
:FFFFFFFFF:FFFFFFFFFFFFFFFFFFFFFFFFFFFFFFFFFFFFFFFFFFFFFFFFFFFFFFFFFF
:FFFF,,FF:FFFFFFFFFFFFFFFFFFFFFFFFFFFFFFFFFFFFFFF
@A00721:190:HGJW7DSXY:4:1468:6180:26256_2:N:0:TCTGCAAG+GCTGCACT
TCGTGAAACTTACGACGATGTTCTCGCTCGCATTTCACAAGGATTACTAAGTCTGAGATCTGCCACTT
```

```
CTAATTTTAGGAAACGTCGCGAAAAGAAAGTTCATGAGGATGTCTTGG
+
FFFFFFFFFFFFFFFFFFFFFFFFFFFFFFFFFFF:FFFFFFFFFFFFFFFFFFFFFFFFFFFFFF:FFF
FFFF:FFFFFFFFFFFFFFFFFFFFFFFFFF,FFFFFFFFFF:F:FFF
@A00721:190:HGJW7DSXY:4:1178:22535:17347_2:N:0:TCTGCAAG+GCTGCACT
GAACTTCTAATAGAGGATCCTGATGACATTATTTCAACGGATACCGATCTCAGTTCTACCACAGAAAC
CGTCTCAGGAACTGTCTCCACTGATTCGGAATTAGAGAAAGACGACCT
+
F:FFF:FF:FFFFFFFFFFFFFFFFFFFFFFFFFFFFFFFFFFFFFFFFFFFFFFFFFFFFFFFFFFFFF
FFFFFFFFFFFFFFFFFFFFFFFFFFFFFFFFFFFFFFFFFFFFFFFF
@A00721:190:HGJW7DSXY:4:1248:5258:29763_1:N:0:TCTGCAAG+GCTGCACT
GAGACCTCTGAATTTTCAGGGTGCCCTGATGAGGACGTTGAAAGGGATAATTTCAACTCTGAGACTTC
TAAGAGTGTTTCAAAAGACCAGAAACGTAACAAGAAGAATAAAGGTAGGAAGGGGAGAAAACAGAACA
AGC
+
FFFFFFFFFFFFFFFFFFFFFFFFFFFFFFFFFFFFFFFFFFFFFFFFFFFFFFFFFFFFFFFFFFFFFF
FFFFFFFFFFFFFFFFFFFFFFFFFFFFFFFFFFFFFFFFFFFFFFFFFFFFFFFFFFFFFFFFFFFFFF
FFF
@A00721:190:HGJW7DSXY:4:1231:23285:24752_2:N:0:TCTGCAAG+GCTGCACT
ACTGATATGGTTGCGGCTGATCGTACCCCAATTCCTGCTAGAAAGATCACTCTCGGCGATACCACTGA
TCTTCCAGAGTTACGTAGTGGTCGTTCTTTTGTTGATTTTATCAAAGA
+
FFFFFFFFFFFFFFFFFFFFFFFFFFFFFFFFFFFFFFFFFFFFFFFFFFFFFFFFFFFFFFFFFFFFFF
FFFFF:FFFFFFFFFFFFFFFFFFFFF:FFFFFFFFFFFFFFFF::FF
@A00721:190:HGJW7DSXY:4:1317:11496:11224_2:N:0:TCTGCAAG+GCTGCACT
CAAGTTAAACTTCGAACTGACCACTTACAAGAGTGTTGAAGTACCGCAACATGGATCAGTTTTCCAAA
AAGCTATTAATAGCAGTTGCGGGGCTATAATACCGCCTCCAGATTACT
+
FFFFFFFFFFFFFFFFFFFFFFFFFFFFFFFFFFFFFFFFFFFFFFFFFFFFFFFFFFFFFFFFFFFFFF
FFFFF:FFFFFFFFFFFFFFFFFFFFFFFFFFFFFFFFFFFFF:FFFF
@A00721:190:HGJW7DSXY:4:1224:18593:29966_1:N:0:TCTGCAAG+GCTGCACT
AGAGTTTTGGAACTGTGTAATGTCCTACGTACTGTTCAAGGTGTTTGGGCTTCTCTAAAGAACAGCAA
CTTTCAACATGAATTAGTTATTGAAAGTCTTGACCCTCACGTCTCACTTTATCACCTTGGAGATGGTA
AAG
+
FFFFFFFFFFFFFFFFFFFFFFFFFFFFFFFFFFFFFFFFFFFFFFFFFFFFFFFFFFFFFFFFFFFFFF
FFFFFFFFFFFFFFFFFFFFFFFFFFFFFFFFFFFFFFFFFFFFFFFFFFFFFFFFFFFFFFFFFFFFFF
FFF
@A00721:190:HGJW7DSXY:4:2513:6623:33223_2:N:0:TCTGCAAG+GCTGCACT
GGCTGATGACTTAAATTTCGCATTCTGCTTCGACGGTAATTCCATAAGACCTTTCTTGGGGAGTAAAG
AGGGTTTGAAAATTACGGAACCTTTCCTCGTTCTTCATGAAGATCTTC
+
FFFFFFFFFFFFFFF,FFFFFFFFFFFFFFFFFFFFFFFFF:FFFFFFFFFFFFFFFFFFFFFFFFFFFF
F:FFFFFF:FFFFFFFFFFFFFFFFFFFFFFFFFFF:FFFF:::FF,F
@A00721:190:HGJW7DSXY:4:1271:29966:15655_1:N:0:TCTGCAAG+GCTGCACT
TCTTGCGAAGGTTCAAGTCGATTGATTTTGTTGACTACGATCTCTCTTGTAATAAGAAGTTCTTCGCA
GTTGAATCTCCACCAGGTGGAGGGAAGACCACTGAACTTATAGCGACTTTTGTCGAATTTTTCGCAAA
AGG
+
FFFFFFFFFFFFFFFFFFFFFFFFFFF:FFFFFF:FFFFFFFFFFFFFFFFFFFFFFFFFFFFFFFFFFF
FFFFFFFFFFFFFFFFFFFFFFFFFFFFFFFFFFFF:FFFFFFFFFFFFFFFFFFFFFFFFFFFFFFFFF
FFF
@A00721:190:HGJW7DSXY:4:1165:24460:7310_2:N:0:TCTGCAAG+GCTGCACT
ATTGTCACTGCTAATCGTAATTCGCGAGAAGAGATAGTGACTAAAGTTTTGGCACTTCTAGTTCATCG
```

```
CGGTATTTGTGAAATTAAGACAGACGTGAAAGTTATGATGAGTGATCG
+
FFFFFFFFFFFFFFFFFFFFFFFFFFFFFFFFFFFFFFFFFFFFFFFFFFFFFFFFFFFFFFFFFFFFF
FFFFFFFFFFFFFFFFFFFFFFFFFFFFFFFFFFFFFFFFFFFFFFFF
@A00721:190:HGJW7DSXY:4:1342:7374:1861_2:N:0:TCTGCAAG+GCTGCACT
ACGTCGCTGAGGTCAAACCTGTGGACGTTCTTTTCATCGACGAATTATTCATGATTCACGCTGGTCAG
ACCCTTTTTCTTACACATCTACTTAAGGCGGCTTATACCATAGGTTAC
+
FFFFFFFFFFFFFFFFFFFF:FF:FFFFFFFFFFFFFFFFFFFFFFFFFFFF:FFFFFFF:FFFFFF:
FF::FFFFFFFF:FFF:FFFF,FFFF:FFF:FFFFFFF:FFFFFFFFF
@A00721:190:HGJW7DSXY:4:1315:13313:32941_2:N:0:TCTGCAAG+GCTGCACT
CAAATCGGATTCATCAATCGTATTGATCTCCAAGTCGCAAACTACTTCAACATCAACGACATCATTCT
TGATGAGTGTCGTTCTTATCGAAGTCTCAGTTATCGCTGCCCGGCCGA
+
FFFFFFFFFFFFFFF:FF::FFFFFFFFFF:FFF:FF:FFF:,FFFFFFFFFF:FFFFFFFF:FFFFF
FFF:F:FFFFFFFF:FFFF:FFFF::,F:FFFFFFFFFF:FFFFFFFF
@A00721:190:HGJW7DSXY:4:1363:12328:10128_2:N:0:TCTGCAAG+GCTGCACT
GAACTCTCAGAGGTTTACGGCAGAAAAATTTCTCCTGCTTGTAATTCGAGAGAGGACACTATGGAAAT
TAGACCTATCAGATGTCTTGACGACTTCGAATTTGAGGACGGAGTTAA
+
FFFFFFFFFFFFF:FFFFFFFFFFFFFFF:FFFFFFFFFFFFFFFFFFFFFFFFFF:FFFFFFFFFFFF
FFFFFFFFFFFFFFFFFFFFFFFFFFFFFFFFFFFFFFFFFFFFFFFF
@A00721:190:HGJW7DSXY:4:1326:3992:2049_2:N:0:TCTGCAAG+GCTGCACT
AATTAACAAAGTCCTTGGTAAGAGTAAGGTTAAATGTGAAGATGCTCAGACTATTCATGAAGTTCAAG
GTAATACTTACCAGCACGTTCGGCTGGTTAGAATTAAACCTCAAGATG
+
FFFFFFFFFFFFFFFFFFFFFFFFFFF:FFFFFFFFFFFFFFFFFFFFFFFFFFFFFFFFFFFFFFFFF
FFF:FFFFFFFFFFFFFFFFFFFFFFFFFFFFFFFFFFFFFFFFFFFF
@A00721:190:HGJW7DSXY:4:1305:29396:9251_2:N:0:TCTGCAAG+GCTGCACT
CACAAACACATATTACCGTTGCTCTATCACGACACGTTGAATCTTTACGATACTTTGTTATCAATGTA
AAGTTAAACGATGCTACTTGTGAGAGGATCAATCACAGTAAGGAACTG
+
FFFFFFFFFFFFFFFFFFFFFFFFFFFFFFFFFFFFFFFFFFFFFFFFFFFFFFF,FFFFFFFFFFFFF
FFFFFFF:FFFFFFFFFFFFFFFFFFFFFFFFFFFFFFFFFFFFFFFF
@A00721:190:HGJW7DSXY:4:1156:10890:23985_2:N:0:TCTGCAAG+GCTGCACT
ACGGGAGGTTGCAGATTCAACCCCTTATATCGAGGCTATCGAGGACGATGAGTATCTTGAAGTTGTTG
ACGACAAGCCTCAAGAGGGTAGAGGACCACCTGGATCTTCTACTGTCT
+
FFFFFFFFFFFFFFFFFFFFFFFFFFFFFFFFFFFFFFFFFFFFFFFFFFFFFFFFFFFFFFFFFFFFF
FFFFFFFFFFFFFFFFFFFF:FFFFFFFFFFFFFFFFFFFFFFFFFFF
@A00721:190:HGJW7DSXY:4:1525:2365:1767_2:N:0:TCTGCAAG+GCTGCACT
GGTTATTCCTGGACCCACCTTAACGTCACTCGGTAATCTTTCAGCCGAATTAAGTACTAATGAGTTCA
AGTCGGTTGTCGATCGAATTAAGATCAAGGCTAATACCTGTATAGGTA
+
:FFFFFF:FFFFF,FFFFFFF:FFFFFFFFFFFF,:FFF,,FFFFFFF:FFFFFFFFFFFFFFFFFFF
FFFFF::,FFFFFFFFFF,FFFFFFFFFFFFFF:,FFF:,FF:FF:,F
@A00721:190:HGJW7DSXY:4:2166:28167:4742_2:N:0:TCTGCAAG+GCTGCACT
GCCGCGTAGTCAGCGCGTTTAGCTGTATCATCAGTCAGAGTATACCCGATAGGGTACCGAGTCTCCAA
GAGAATATTTATTCTTATGAGGCTCGAAACTTCAACGTTTTCACAACG
+
FFFFFFFFFFFFFFFFFFFFFFFFFFFFFFFFFFFFFFFFFFFFFFFFFFFFFFFFFFFFFFFFFFFFF
FFFFFFFFFFFFFFFFFFFFFFFFFFFFFFFFFFFFFFFFFFFFFFFF
@A00721:190:HGJW7DSXY:4:1569:18041:16830_2:N:0:TCTGCAAG+GCTGCACT
TCGCACCCGAAATTTTTGGGCAGAATGCTCTTAACGCGGTTCTTGAACGGTGTTTTGATCAGTCTAAG
```

TTGGATAAAGTTGTAGGAGACTCAGTTAACCTGAGAGCTCCTCAATTG
+
FFFFFFFFFFFFFFFFFFFFFFFF:FFFFFFFFF:FFFFFFFFFFFFFFFFFFF,FFFFFF:FFFF:FF
F,FFFFFFFFFF:FFFFFF:FFFFFFFFFFFFFFFFFFFFFFFFFF:FF
@A00721:190:HGJW7DSXY:4:2335:11695:9502_1:N:0:TCTGCAAG+GCTGCACT
AACAATAGGGACGCTAGTATACTTCGTAAAGTCGACAGCGAAATGGTCAATATTGATAATTGGACCGA
CTATATGACCTATTTTAAGCTCATGGTGAAAAGGGAAGCTAAGGTTAAACTTGATGCTTCAGCCTTTT
CGA
+
FF:FFFFFF:FF:FF:FFFFF:FF:,FFFFFFFFFFFF::FFFFFFFFFFFFFFFFFFFFFFFFFFFFF
FFFFFFFFFFFFF:FFFFFFFFFFFFFFFFFFFFFFFFFFFFFFFFFFFFFFFFFFFFFFFFFFFFFFF
FFF
@A00721:190:HGJW7DSXY:4:1544:3540:15013_1:N:0:TCTGCAAG+GCTGCACT
AGAATATCGTTTATCACGCAAAATTTATTAATGCAATTTTCGGACCTATCTTTAAGGTTCTTGTGAGG
AGAGTCAAACAGTGTCTTTTACCCAACATTTTTATGTATACAGAGATGACCAGTGACGATTTCTCCCG
GAA
+
FFFFFFFFFFFFFFFFFFFFFFFFFFFFFFFFFFFFFFFFFFFFF:FFFFFFFFFFFFFF:FFFFFFFF
FFFFFFFFFFFFFFFFFFFFFFFFFFFFFFFFFFFFFFFFFFFFFFFFFFFFFFFFFFFFFFFFFFFFF
FFF
@A00721:190:HGJW7DSXY:4:1138:31204:10003_1:N:0:TCTGCAAG+GCTGCACT
CGAGATCTTGCAGAGTGACTGGAGTTCAGACGTGTGCTCTTCCGATCTTTTCTTTTGTAAAGCCTATG
AGATAAAGATGTATTCTTATTTAGGCTTCGATGCTGAATTTCTTGAACTTTGGAGTGCCAGTGAATAT
TTT
+
F:,FFFFFFFFFFFFFFFFFFFFFFFFFFFFFFFFFFFFFFFFFFFFFFFFFFFFFFFFFFFFFFFFFF
FFFFFFFFFFFFFFFFFFFFFFFFFFFFFFFFFFFFFFFFFFFFFFFFFFFFFFFFFFFFFFFFFFFFF
FFF
@A00721:190:HGJW7DSXY:4:1139:11342:13526_1:N:0:TCTGCAAG+GCTGCACT
GAGTATCCATAACGATTTGTCATTTACCACCTTTGCTCAACGACGAAGTGGAACGGCCAACACTTTCT
TTGGTAATTGTATGATCACTTTGATGATTTTATCACTTTATTATGACCTTGGAAAGTTTTCGGCTGTG
GTT
+
F:FFFFFFF,FFFFFFFFFFFFF::FFFFFFFFFFFFFFFFFFFFF::FFFFFFFFFFFFFFFFFFF:F
FFFFFF:FFFFFFFFFFFFFFFFFFFFFFFFFFFFFFFFFFFFFFFFFFFFFFFFFFFFFFFFFFFFFF
FFF
@A00721:190:HGJW7DSXY:4:1420:27498:29888_1:N:0:TCTGCAAG+GCTGCACT
CCATTCTGTTTTCAAAGACTCCAATAACTGATCATTCTGATCATATGATTTTGGACAATGGTTTCGAA
GCGAAATTTTTGAGGGATAAACCAGCGTACTTCTGCTCGCGATTTATTCTTTTTGTCAATGATTACAT
CTA
+
F:FFFFFFF:FFFFFFFFFFFFF:F:,FFFFF:FFFFFFFFFFFFFFFFFFFFFFFFFFFFFF:FFFFF
FFFFFFFFFFFFFFFFFFFFFFFFFFFFFFFFFFFFFFFFFFFFFFFFFFF:FFFFFFFFF:F:FFFF
FFF
@A00721:190:HGJW7DSXY:4:1311:2392:31814_1:N:0:TCTGCAAG+GCTGCACT
CCTTACAAGATCATCGTTAAGTTAGGTAAACCCCTAACCGATGTTACTCTTGTAGGACTTTGGGAGAG
ATTCGTTAGTTTTAAAGACTATACTAAATATCTCCACTACGAGGTTGTTGTTTGGCGATTAGCTCTTG
CGA
+
FFFFFFFFFFFFFFFFFF:FFFFFFFFFFFFFFFFFFFFFFFFFFFFFFFFFFFFFFFFFFFFFFFFFF
FFFFFFFFFFFFFFFFFFFFFFFFFFFFFFFFFFFFFFFFFFFFFFFFFFFFFFFFFFFFFFFFFFFFF
FFF
@A00721:190:HGJW7DSXY:4:2213:15619:18020_1:N:0:TCTGCAAG+GCTGCACT
TATTCGATTGAGGGTCGTTATACCTATTCGGCTATCTGTGCTTTGAATTGTATAAGATCAAATTTTAA

```
ACGATTCCGAGAGGTTTATCCTGATGTGGAGGGAGTTCTTGAAGCATCCCGACCTTTCCTTGTGAAAA
+
F,:F:::F::,FF,::,F:FFFF:F:FFFF::FFF:F,FFFFFF,F,FFFFFFFF,FFFFFFFFFFF:
FFFFFFFFFFFFFFFFFFFFFFFFFFFFFFF:FFFFFFFFFFFFFFFFFFFFFFFFFFFFFFFFFFFF
@A00721:190:HGJW7DSXY:4:1155:23068:15013_1:N:0:TCTGCAAG+GCTGCACT
TTTCATAAGATTATCAGTAATCCTGATTCTACTACGGATTCATGGACCTTCCATCCTGTACCTCTTCT
GGAGAGACGTATCTCAAAGATCGACTTCGAGACAGGTGATATTGTTGAGCGAGAGCGACCAAGATCTA
AAT
+
:FFFFFFFFFFFFFFFFFFFFFFFF::FFFFFFFFFFFFFFFFFFFFFFFFFFFFFFFFFFFFFFFFF
FFFFFFFFFFFFFFFFF:F:FFFFFFFFFFFFFFF:FFFFFFFFFFFFFFFFFFFFFFFFFFFFFFFF
F:F
@A00721:190:HGJW7DSXY:4:1170:18096:25598_1:N:0:TCTGCAAG+GCTGCACT
CGTGGCGCTTTATTTTAAAAAGCTCCCTCCTCTCGCAAGTTTGATCTGATACTTATCACGAAAGGACT
TGAGTCTTTTGATCAGTCTCTGACTTGACCATCTTTCATTTTAAATGACGATCATTAAGACTCTCTTT
CTT
+
FFFFFFFFFFFFFFFFFFFFFFFFFFFFFFFFFFFFFFFFFFFFFFFFFFFFFFFFFFFFFFFFFFFF
FFFFFFF:FFFFFFFFFFFFFFFFFFFFFFFFFFFFFFFFFFFFF:F:FFFFFFFFFFFFFFFFFFFFFF
FFF
@A00721:190:HGJW7DSXY:4:1242:26919:33708_2:N:0:TCTGCAAG+GCTGCACT
CTTTGCACTGATTTTTCCTCATCAGCTTGTGTTTTACCTAATCTGTGTTTTTACGATAACAACTATCA
GTTGATTAAGACTCTTCCGAAAGGCGAACTTTGGGAATTTGAAGCTCA
+
FFFFFFFFFFFFFFFFFFFFFFFFFFFFFFFFFFFFFFFFF:F:FFFFFFFFF::FFFFFFFFFFFF
:FFFFFFF:F:FFFFFFF:FFFFFFFFFFFFFFFFFFFFFFF:F:F:F
@A00721:190:HGJW7DSXY:4:1275:28935:34131_1:N:0:TCTGCAAG+GCTGCACT
CGTTTGACCGTGACGAACTGTGGTGTTCTTGATCTACGTTACGGATATACTCTCCTTGAGTTCACTCG
TAAGGACACTACCACTTATTACGATATTTCTTTAGTCGATGGGTTTTCGACTCCTTTAAGCGTCTTCT
GTT
+
FFFFFFFFFFFFFFFFFFFFFFFFFFF:FFFFFFFFFFFFFFFFFFFFFFFFFFFFFFFFFFFFFFFFF
FFFFFFFFFFFFFFFFFFFFFFFFFFFFFFFFFFFFFFFFFFFFFFFFFFFFFFFFFFFFFFFF:FFF
FFF
@A00721:190:HGJW7DSXY:4:2652:32470:32831_2:N:0:TCTGCAAG+GCTGCACT
TGTTTTCTTCTAATGTGGCCTGAACTACGTTTCACTTTGTGTGTACTTGTCTCTATTTTAAGTTTTGT
CTTCCTTGTTTGTCTTATTCTATTAATCACTCATTTTAGTGAGATTAT
+
FFFFF:FFFFFFFFF:FFFFFFFFFFFFFFFFFFFFFFFFFFFFFFFFFFFFFFFFFFFFF:FFFFFF
FFF,FFF:FFFFFFFFFFFFFFFFFFFFFF,FFFFFFFFFFFF:FFFFF
@A00721:190:HGJW7DSXY:4:2633:27028:12477_2:N:0:TCTGCAAG+GCTGCACT
CTAAAACGTCTTCGACAACAGCAGGGGTTTTCGAATAGTGATAGCCACCCGCCTTACAGAGGGGCAGC
AGCATGACCATAATTGGTATTGACTTTGGTACCACCTTTTCTACTGTC
+
FFFFFFFFFFFFFFFFFFFFFFFFFFFF:FFFFFF,FFFFFFFFFFFFFFFFFFF:FFFFFFFFFFFF
:FFFFFFFFFFFFFFFFF::FFFF:FFFFF:FFFFFF:FFFFFFFFFF
@A00721:190:HGJW7DSXY:4:2464:14778:34601_1:N:0:TCTGCAAG+GCTGCACT
TTCGAATGAAGTTTTAATGCTTAACAACAATGATAGTGAATTTATACCTACAATCATTGGTTTTTCTA
ATAATGATGACACTATTGTTTATGGATATGATGCTATTACTCATGATGGTTACGGTCAGGGAGGATTC
TCA
+
FF:FFFFF:FFFFFFFFFFFFFFFFFFFFFFFFFFFFFFFFFFFFFFFFFFFFFFFFFFFF:FFFFFFF
FFFFFFFFFFFFFFFFFFFFFFFFFFFFFFFFFFFFFFFFFFFFFFFFFFFFFFFFFFFFFFFFFFFF
FFF
```

```
@A00721:190:HGJW7DSXY:4:2273:12626:35916_2:N:0:TCTGCAAG+GCTGCACT
ACATTACCCGAAAGACGTGCTAAATTAAAACCATTGTACGATGTCGTCTGTCTCGGCGATCACTTTTC
ACTAAGAATTGGTCCTACTTTTGGACAAGGCCAACTTCGTAATCTTCA
+
FFFFFFFFFFFFFFFFFFFFFFFFF:FFFFF:FFFFFFFFFFFFFFFFFFFFFFFFFFFFFFFFFFFF
:FFFFFFFFFFFFFFFFFFFFFFFFFFFFFFFFFFFFFFFFFFFF:FFF
@A00721:190:HGJW7DSXY:4:1640:24035:16063_2:N:0:TCTGCAAG+GCTGCACT
TTTATCTCTTTGGTTGTTAGGGATTTTGAGAGAGTGAAAGGCTTCAATTGTAGTGGTTTGGTCGTTTC
AGTACCCAGTCAGTATACCTCTACGCAAAGATATTTCATGAAAACTAC
+
FFFFFFFFFFFFFF:FFFFFFFFFFFFFFFFFFFFFFFFFFFFFFFFFFFFFFFFFFFFFFFFFFFFF
FFFFFFFFFFFFFFFFFFFFFFFFFFFFFFFFFFFFFFFFFFFF:FFFF
@A00721:190:HGJW7DSXY:4:2628:27570:15264_1:N:0:TCTGCAAG+GCTGCACT
CGGATTACCTGTCTTTCATATTATGAATGAACCTTCGGCCGCTCTCTTTGCTTCGATGTTGGATGTCA
AGAAGGGTTCAGAATGGGATTCCTACGTAGTTTATGACTTTGGTGGTGGTACTTTCGATACTTCTATC
GT
+
:F:FFFFFFFFFFFFF:FFFFFFFFFFFFF:F:FFFFF,FFFFFF:FFFFF,FFF,FFFFFFFFFF:,
FF,FFF:,FFFFFFFFFFFFF:FFF:FFF,F:F:FFF:FFFFFFFFF,FFFFFFFFFFFFFFFFFFF:
FF
@A00721:190:HGJW7DSXY:4:2607:15935:10332_2:N:0:TCTGCAAG+GCTGCACT
TACTATAGTGTTATTTATTCTGGTGGTGATGATAATCTTGGAGGTCGTGACGTTGATGAAGCTATAAG
AGCGTTCTTATTACGGCGCACTTCTTTACCCGCCACCACACAACTTTC
+
FFFFFFFFFF,FFFFFFFFFFFFFFFFFFFFFFFFFFFFFFFFFFFFFFFFFFFFFFFFFFFF:FFFF
FFFFFFFFFFFFFFFFFFFFFFFFFFFFFFFFFFFFF:FFFFFFF:FFF
@A00721:190:HGJW7DSXY:4:2410:25852:17080_1:N:0:TCTGCAAG+GCTGCACT
TGAAGGAGGAGGTTTCTGTTACTGGAAAACCCTCGTCAATACTTTTCAATAATATACGTCTTGAACTC
ACTTATGACGATCTAGTGAAATTAACGCGTCCTTTTCTGGAACGTAGTTTTACTATTATGAACAAAGT
TAT
+
FFFFFFFFFFFFFFFFFFFFFFFFFFFFFFFFFFFFFFFFFFFFFFFFF:FFFFFFFF:F:FFFFFFF
FFFFFFFFFFFFFFFFFFFFFFFFFFFFFFFFFFFFFFFFFFFFFFFFFFFFFFFFFFFFFFFFFFFF
FFF
@A00721:190:HGJW7DSXY:4:1260:27796:26929_1:N:0:TCTGCAAG+GCTGCACT
CCTGATTCCGGCCGGTGGTAGCTCATTTCTACCAGGGGCTATTACTCTTGCTGAAACAGCTATTAAAG
CTGTTGAGCGGGTAATTACACCTAAACGAGCTCGATCTGCCATCGCTGAAGGATGTGCACTCTTTTCT
GCT
+
FFFFF:FFFFFFFFF:FF,FFFFFFF:FFFFFFFFFFFFFFF:FFFFFFFFFFFFFFFFFFFFFFFFF
FFFFFFFFFFFFFFFF:FFFFFFFFFFFFFFFFFFFFFFFFFFFFFFFFFFFFFFFFFFF:FFFFFFF
FFF
@A00721:190:HGJW7DSXY:4:2137:26549:17785_1:N:0:TCTGCAAG+GCTGCACT
CTTCGTCGATTGTATCAACAGTAACATTACTTCTAATAAAGGTCTCTATCAAGCTCGTATTATCGTCG
CTGCTGGATCTGCGTTACCGTTGAAGGCTAAACTCCAAGGTAATTATGAAGACTGTGATTTATCTTTC
ACT
+
FFFFFFFFFFFFFFFF:FFFFFFFFF,FFFFFFFFFFFFFFFFFFFFFFFFFFFFFFFFFFFFFFFFF
FFFFFFFFFFF:FF:FFFFFFFFFFFF:FFFFFFFF:FFFFFFFFFFFFFFFFFFFFFFF:FFFFFFFF
FFF
@A00721:190:HGJW7DSXY:4:1506:15582:5963_1:N:0:TCTGCAAG+GCTGCACT
ACGTGTGCTCTTCCGATCTATAATTTCAATCTTCTTAAAAAGAAGATAGAGAGTCATACTTTGGGCTA
TCGTTCAGGTGCATGGGGTTGGATAGCCAATATTGAAGTGGACACCTCTGGTAGTATCGAAGTAAAGA
TAA
```

+
FFFFFFFFFFFFFFFFFFFFFFFFFFFFFFFFFFFFFFFFFFFFFFFFFF:FFFFFFF:FFFFFFFFFF
FFFFFFFFFFFFFFFFFFFFFFFFFFFFFFFFFFFFFFFFFFFFFFFFFFFFFFFFFFFFFFFFFFFFF
FFF
@A00721:190:HGJW7DSXY:4:1342:24921:26240_1:N:0:TCTGCAAG+GCTGCACT
ATACTATGGTTGAATCAACTAAGCATACTACTTTTGCTTTGATTACTCTACCGCCTTACAAGTCTAAG
GTTAAAGAGTCGATTAAACAAGTTGTACTTATCAATGATTTTATCGCTGGTTGTTATCTCGAAAAGTC
TCT
+
FFFFFF:FFFFFFFFFFFFFFFFFFFFFFFFFFFFFFFFFFFFFFFFFFFFFFFFFFFFFFFFFFFFF
FFFFFFFFFFFFFFFFFFFFFFFFFFFFFFFFFFFFFFFFFFFFFFFFFFFFFFFFFFFFFFFFFFFFF
FFF
@A00721:190:HGJW7DSXY:4:1140:9444:18975_2:N:0:TCTGCAAG+GCTGCACT
TTCTAACCCAAAGAGAATATCTAAAATATTTACAAAATCTTACCACTATCTACGAACCTAAAGTTCTG
AAGACGTTGCTTGTCGCTTCCTCCTTTAATGGCACTACCTCGGTACAA
+
FFFFF:FFFFFFFFFFFF:FFFFFFFFFFFFFF:FFFFFFFFFFFFF:FFFFFF:FFFFFFFFFFFFF
FFFFFFFFFFFFFFFFFFFFFFFF:FFFFFFFFFFFF,FFFFFFF:FFF
@A00721:190:HGJW7DSXY:4:1563:13286:25567_2:N:0:TCTGCAAG+GCTGCACT
GAAGGCGACCTGTTCCGGAGTTTTTACGGGATCCGGAATGTTCTGTCTATATTAGGTGAAGCGAAATA
CGATTTCGAACGTCAACGTTCACCGAGTTTCGTTTCGTACCTTGATGA
+
FFFFFFFFFFFFFFFFFFFFFFFFFFFFFF:FFFFFFFFFFFFFFFFFFFFFFFFFFFFFFFFFFFFF
FFFFFFFFFFFFFFFFFFFFFFFFFFFFFFFFFFFFFFFFFFFFFFFF
@A00721:190:HGJW7DSXY:4:1453:13720:29418_2:N:0:TCTGCAAG+GCTGCACT
ATTTGATCTGGTTGCTCAATTCTAAGCGTGTTCCGCTTAGTGCGAGGCCTCTCTTCTACGAGTATTGC
TATTTATACGGTTACGAGAAGAAGGTTCGAAAGTTTGAGAACTTAGTT
+
FFFFFFFFFFFFFFFFFFFFFFFFFFFFFFFFFFFFFFFFFFFFFFFFFFFFFFFFFFFFFFFFFFFF
FFFFFFFFFFFFFFFFFFFFFFFFFFFFFFFFFFFFFFFFFFFFFFFF
@A00721:190:HGJW7DSXY:4:2515:10357:1485_2:N:0:TCTGCAAG+GCTGCACT
ACATACTAAATACTAGATATTTGGATGATCTTGATAAAGAACTTCATGAATTTTCAGAGGATGAAAAA
TTTTCTTCCTTGGATTTTAATATAAAGAATCTTTACCAGGACTCCTAC
+
FFFFFFFFFFFFFFFFFFFFFFFFFFFFFFFFFFFFFFFFFFFFFFFFFFFFFFFFFFFFFFFF:F:FF
FFFFFFFFFFFFFFFFFFFFFF,FFFFFFFFFFFFFFFFFFFFFFFFFF
@A00721:190:HGJW7DSXY:4:2122:15863:11397_2:N:0:TCTGCAAG+GCTGCACT
TCTGATCGTAAGAAGTTTATAAGGAACATATCCATTTGTTGTAATAGGGTCATCAACTTAGAACTCGA
AACTCTAAGTGGTAATACTGTCTTTCCAAGCTTACCTGTATCTTCAAT
+
FFFFFFFFFFFFFFFFFFFFFFFFFF,FFFFF:FFFFFFFFFFF:FFFFFFFFFFF:FFFFFFFFFF:F
FF:FFFFFFFFFFFFFFFFFFFFFFFFFFFFFFFFFFFFFFFFFFFFF
@A00721:190:HGJW7DSXY:4:2236:7491:30561_1:N:0:TCTGCAAG+GCTGCACT
TTCGATAGGGGACATTTTATCGTGGGCTTTCCGTAAAGGAAGATCTGTTATCGACGGTTTATCGTTTA
CGAAGTATCACTCCACCGGTGAATGGTTTAAGAATACTATAAATAGAATAGCTATTACTACTAATGGC
GTT
+
F:FFFFFFFFFFFFFFFFFFFFFFFFFFFFFFFFFFFFFFFFFFFFFFFFFFFFFFFFFFFFFFF:FFF
FFFFFFFFFFFFFFFFFFFFFFFFFFFFFFFFFFFFFFFFFFF:FFFFFFFFFFFF:FFFFFFFFFFFFF
FFF
@A00721:190:HGJW7DSXY:4:2254:20509:28119_2:N:0:TCTGCAAG+GCTGCACT
TAGTGACATCTGGTCAATTTCGTTTTTAGGTGGTCTTTATTCTATTATTGATTTTTATGAGATTGATA
CGTTAACAGAAGATGATGTTAGAGTAGAATTCCTGAAGAGTCCTTCCG
+

```
FFFFFFFFFFFFFFFFFFFFFFFFFFFFFFFFFFFFFFFFFFFFFFFFFFFFFFFFFFFFFFFFFFFFFF
FFFFFFFFFFFFFFFFFFFFFFFFFFFFFFFFFFFFFFFFFFFFFFFFFF
@A00721:190:HGJW7DSXY:4:1136:30156:32362_2:N:0:TCTGCAAG+GCTGCACT
CTAGGATTATGTCTGTGTTGAAAGACTTTCTACCGATAAGGGTCACTAGTCTTACACAATTAAGAAAT
AATCCTACATTGACTGATTATCTAAAGATTGAAGGAAAATTTTATCCT
+
FFFFFFFFFFFFFFFFFFFFFFFFFFFFFFFFFFFFFFFFFF:FFFFFFFFFFFFFFFFFFFFFFFFF
FFFFFFFF,FFFFFFFF:FFFFFFFFFFFFFFFFFFFFFFFFFFFFFF
@A00721:190:HGJW7DSXY:4:1101:22290:27665_1:N:0:TCTGCAAG+GCTGCACT
GACTTTCCTACGATACAAATGATAACTTCTAATTTACGTTTGCTACTCAGAGCTGGTCCTCTCAGCAA
AGGGATGCATGGTGTTATCTCCTCAGGAGTCCCGGCATTTGATAATAATGACGAGACTTTCGTCATAA
TCG
+
FFFFFFFFFFFFFFFFFFFFFFFFFFFFFFFFFFFFFFFFFFFFFFFFFFFFFFFFFFFFFFFFFFFF
FFFFFFFFFFFFFFFFFFFFFFFFFFFFFFFFFFFFFFFFFFFFFFFFFFFFFFFFFFFFFFFFFFFF
FFF
@A00721:190:HGJW7DSXY:4:2301:11460:20275_1:N:0:TCTGCAAG+GCTGCACT
GATTACACGGTACCAGCGCATCTCGTGTCGAGCCTCGACCCGATTATATGGTATTAACTTATAAAGGT
GAGGAGACCATTTTCTTGCTGAAATTTATAGAAGATTATTTTGACAGAATCAATAAGAATCATCGATT
CT
+
FFFFFFFFFFFFFFFFFFFFFFFFFFFFFFFFFFFFFFFFFFFFFFFFFFFF:FFF:FFFFFFFFFFFFFF
FFFFFFFFFF:FFF,FFF:FFFFFFFFFFF,:FFFFFFFFFF,FFF,FF,FFF,FF,FF,,F,,:F,F
:F
@A00721:190:HGJW7DSXY:4:2630:5249:35008_1:N:0:TCTGCAAG+GCTGCACT
TATGCTGATCTAAAATTTCGATCTAGATGGATCCACTCCTCGAGTTATCATGATGTTAGTATGTTATA
TACTGATTTACAACGATATAGGAGTAATCTACGAGAAGATTCTAGAATTCTTAATTATAATATTCAAC
CTC
+
FFFF,F,F:,FFFFFFFF,FFFFFFFFFFFFFF:FFFFF:FF::FFFFFFFFFFFFFF:FFFFFFF
FFFFFFFFFFFFFFFFFFFFFFFFFFFFFFFFFFFFFFFFFFFFFFFF:FFFFFFFFFFFFFFFFFFF
FFF
@A00721:190:HGJW7DSXY:4:1528:15492:25692_2:N:0:TCTGCAAG+GCTGCACT
GAGATCACACATTTTGAGAACTAACTATGTTATGTTCTCTGATCATTTTGACAATCTCTTCTTATAAT
AAATAAATAAATAATATTACTAATGGCAAATAGTGGAAAGGCTCCAGT
+
FFFFFFFFFFF:FFFFFFFFFFFFFFFFFFFFFFFFFFFFFFFFFFFFFFF:FFFFFFFFFFFFFFFFF
FFFFFFFF:FFFFFFFFFFFFFFFFFFFFFFFFF:FFFFFFFFFFFFFF
@A00721:190:HGJW7DSXY:4:2669:9516:2816_2:N:0:TCTGCAAG+GCTGCACT
TGCTTTGGATTACGATGACGTGAGAATAGGTGAAGGGATAGTTTTTACCTCGACCAACATTAAGGCGG
CGGCGACTAATCTTAAAGCTTCACCCAAAATGAAGGACACTTCGAAAC
+
FFFFFFFFFFFFFFFFFFFFFFFFFFFFFFFFFFFFFFFFFFFFFFFFFFFFFFFFFFFFFFFFFFFF
FFFFFFFFFFFFFFFFFFFFFFFFFFF:FFFFFFFFFFFFFFFFFFFF
@A00721:190:HGJW7DSXY:4:1169:13169:23672_1:N:0:TCTGCAAG+GCTGCACT
GGCTCTAAAGGACACTATACAATATCCAGACGACGACGAAGCTTATACTACTTTAATTTTGGGTATTA
TGTGGAGATATCGTGATATCAGTACGTCCAGATCCTCTGCTTTTACCGATAACGATAAATTCGTCATC
AAA
+
FFFFFFFFFFFFFFFFFFFFFFFFFFFFFFFFFFFFFFFFFFF:FFFFFFFFFFFFFFFFFFFFFFFFF
FFFFFFFFFFFFFFFFFFFFFFFFFFFFFFFFFFFFFFFFFFFFFFFFFFFFFFFFFFFFFFFFFFFF
FFF
@A00721:190:HGJW7DSXY:4:1155:25644:27586_1:N:0:TCTGCAAG+GCTGCACT
TTGGTGCTTAGGGATATAATGAATTCAGCACCACATTTTGGTCAAGTCAATCCGTTGAGAAAATTTCT
```

TAGAGGATATTCGCCATTGTGGTACGAATTGGTCCAACATTTACCACCAAATTCTTTTGATACAACTC
TAG
+
FFFFFFFFFFFFFFFFFFFFFFFFFFFFFFFF:FFFFFFFFFFFFFFFFFFFFFFFFFFFFFFFFFFF
FFFFFFFFFFFFFFFFFFFFFFFFFFFFFFFFFFFFFFFFFFFFFFFFFFFFFFFFFFFFFFFFFFFF
FFF
@A00721:190:HGJW7DSXY:4:1457:14470:3630_1:N:0:TCTGCAAG+GCTGCACT
TGCCTGCGGAATACGCTTGCTATTCACCAGATTGGTTCGAACCCGTTGATGCTATGTCAGAAGAAGCT
ATTCAAGCTTGTCGTTTAAAGACTGCAGAGGCTGTCAAACAAGCCCAGACTCAAGGAGTCCAATCAAA
GGT
+
FFFFFFFFFFFFFFFFFFFFFFFFFFFFFFFFFFFFFFFFFFFFFFFFFFFFFFFFFFFFFFFFFFFF
FFFFFFFFFFFFFF:FFFFFFFFFFFFFFFFFFFFFFFFFFFFFFFFFFFFFFFFFFFFFFFFFFFFF
:FF
@A00721:190:HGJW7DSXY:4:1352:21703:4601_2:N:0:TCTGCAAG+GCTGCACT
TGGGTGATAAAAATTATTTAGAGATCTTTTCTACTTACAAAGGACAATTCAGTTTCATTAATTCTACG
GAAAAGTCCGGAGTATATTGTATGTTAATAGATAGTGGGATAATAGTT
+
FFFFFFFFFFFF:FFFFFFFFFFFFFFFFFFFFFFFFFFFFFFFFFFFFFFFFF:FFFFFFFFFF:FFFF
FFFFFFFF:FFFFFFFFFFFFFFFFFFFFF,:FFFF::FFFFFFFFFF
@A00721:190:HGJW7DSXY:4:1437:12753:20415_1:N:0:TCTGCAAG+GCTGCACT
GTAGTGAACGCGTTCGTAGAACCAAGTACTTTCCGTCTGAAGAGTTTAGAGCTGCTATTGATTATGTA
ACCGATAGGAGTTGTATAGTTGAAGATGTGGCTTTAGACGATAATCCTTTAAGGACTATGTTGACTGA
CGA
+
FFFFFFF:FFFFFFFFFFFFFFFFFFFFFFFFFFFFFFFFFFFFFFFFFFFFFFFFFFFFFFFFFFFF
FFFFFFFFFFFFFFFFFFFFFFFFFFFFFFFFFFFFFFFFFFFFFFFFFFFFFFFFFFFFFFFFFFFF
FFF
@A00721:190:HGJW7DSXY:4:1313:3549:26146_2:N:0:TCTGCAAG+GCTGCACT
TTGTCTACGTTGCAGATCGCCACTCCAAACACTATATACATTATAAAATATAAAAATAAGGGAGTCAT
AACTTTTGTTACCAAATTCTTGGCTTTCAACGAAGTCTCTGATTTTAT
+
FFFFFFFFFFFFFFFFFFFFFFFFFFFFFFFFFFFFFFFFFFFFFFFFFFFFFFFFFFFFFFFFFFFF
FFFFFFFFFFFFFFFFFFFFFFFFFFFFFFFFFFFFFFFFFFFFFFFF
@A00721:190:HGJW7DSXY:4:1105:11849:30091_1:N:0:TCTGCAAG+GCTGCACT
TAACGATCTCTCGGTCATTTACTCTGTACTTGATCTACGTCGTTTAACTGTCGGAACAACCTTTACTT
AGAATGACTCTATCTTTTCAGACACTTCTTATATTATTTACAATAGTTAATATCATATTAGTAATACT
CAC
+
FFFFFFFFFFFFFFFFFFFFFFFF:FFFFFFFFFFFFF:FFFFFFFFFFFFFFFFFFFFFFFFFFFFFF
FFFFFFF::FFFFFFFFFFFFFFFFFFFFFFFFFFFFFFFFFFFFFFFFFFFFFFFFFFFFFFFFFFF
FFF
@A00721:190:HGJW7DSXY:4:1125:5385:7592_2:N:0:TCTGCAAG+GCTGCACT
GCTTTTTCTTAGCTAATAGATTAATAAAGGATGAGTACGAAAACCAACTCTTCGTTACGAGACCTGAT
GGTAGAATAGATTTAGTCTCTACACCCATCTCAACGAGGATAAAGCGA
+
FFFFFFFFFFFFFFFFFFFFFFFFFFFFFFFFFFFFFFFFFFFFFFFFFFFFFFFFFFFFFFFFFFFF
FFFFFFFFFFFFFFFFFFFFFFFFFFFFFFFFFFFFFFFFFFFFFFFF
@A00721:190:HGJW7DSXY:4:2130:23565:24518_1:N:0:TCTGCAAG+GCTGCACT
TTAACTTAATACACCAATAGAAAATCCGCAAACGACTCGAAACCGAGACGCCGTAGATCAAAGTCCCT
TCTAACTTTAGATTATGAATCCTGCCTTTACTCATTTT
+
:,::,,,::F::,,,:,,,,,,,F,F:,,F,FFFFFFF:FFFFFFFFFFFF:FFFFFFFFFFFFFFFF
FFFFFFFFF:FFFFFFFFFFFFFFFFFFFFFFFFFFFF

@A00721:190:HGJW7DSXY:4:1239:9498:26866_2:N:0:TCTGCAAG+GCTGCACT
TTTTTATAATTTATAACCTTCTTATACTTTTGGGTATGTTTTGTGAAAAACATGAGATACCCAAGTCT
ATCGAAGACATTATCTCTTATGTACTTATTGTAACGATCATTCTTTCG
+
FFFFFFFF:FFFFFFFFFFFFFFFFFFFFFFF:FF:F:FFFFFFFFFFFFFFFFFFFFFFFFFFFFFF
FFFFFFFFFFFFFF:F:FFF:FFFFFFFFFFFFFFFFFFFFFFFFFFFF
@A00721:190:HGJW7DSXY:4:1617:7211:4899_1:N:0:TCTGCAAG+GCTGCACT
TTCTTATTCTTTTATTCCTTTAACTGTGTATGAGGAGGATTTATAATGCTTTTCGATAGTGATTGTTA
TCAATTTAAATACATTACTGTTTCAAATCATCTTTACTCACAGACTCCGAAAATGGTTTCAAATGAGG
TCA
+
FFFFFFFFFFFFFFFFFFFFFFFFFFFF:FF:FFFFFFFFFF:FFFFFFFFFFF:FFFFFFFFFFFFF
FFFFFFFFFFFFFFFFFFFFFFFFFFFFFFFFFFFFFFFFFFFFFFFFFFFFFFFFFF::FFFFFFF:
FFF
@A00721:190:HGJW7DSXY:4:1617:7211:4899_2:N:0:TCTGCAAG+GCTGCACT
TATTAAACGAACTATTATCCGATTCTTCGAGGGTATCACTACCACTGATCTGCCGACTTTAGTCAAGG
AACTTCAATTCCTTCGATGTTTATATTTTTCTATAAGGGAAAATAGCA
+
FFFFFFFFFFFFFFFFFFFFFFFFFFFFFFFFFFFFFFF::FFFFFFFFFFFFFFF:FFFFFFFFFFF
F:F:FFF:FFFFFFFFFFFFFFFFFFFFFFFFFF:FFFFFFFFFFFFFF
@A00721:190:HGJW7DSXY:4:1318:1723:30812_2:N:0:TCTGCAAG+GCTGCACT
TTCGACAAATTTTAGAACTTTATTTCAGAACTCTCTCCGATCACCTTGGTGTGATCTCCCGGAGGATC
TTTTCAATAAGATGTATGATGATAATCTACTATTTTTAAATAGTGATA
+
FFFFFFFFFF:FFFFFFFFFFFFFFFFFFFFFFFFFFFFFFFFFFFFFFFFFFFFFFFFFFFFFFFFF
FFFFFFFFFFFFFFFFFFFFFFFFFFFFFFFFFFFFFFFFFFFFFFFF
@A00721:190:HGJW7DSXY:4:2156:26178:14544_1:N:0:TCTGCAAG+GCTGCACT
ACTTTATCAAAGATTTGGCTTGGTCTATTTACCGCAAAAAGTTGGAAGAGGTCTATGATATTTATTTT
ACTTATACTTTAAATAGCACCGATTATAACTTGACTGTTGATAATATTACAATAGCTCTTTCTCAGAA
GAT
+
FFFFFFFFFFFFF,FFFFFFFFFFFFFFFFFFFFFFFFFF,FFFFFFFFFFFFFF:FFFFFFFFFFFFF
F::FFFFFFFF,FFFFFFFFFFFFFFFFFFFFFFFFFFFFFFFFFFFFFF:FFFFFFFFFFFFFFFFFF
FFF
@A00721:190:HGJW7DSXY:4:1425:19958:25003_1:N:0:TCTGCAAG+GCTGCACT
ACCGGTGAACACGTCGGTAGCATTGATTTACTTTTACCGTTAGAACTGAAAAAGATTCTTTTCGAAAA
GAAACCTATAAGTTACAAACGATTCTTCGACTATATTAAAGAAATAAAAATACTTCAAGTACCTGAAC
TTT
+
F:FF,F:FFFFFF::FFFFF,FFF,FFFFF:FFFF:FF::F:FFF,FF,FFF:,FF,F:FFFFFFF:,
FFFF,F:FF,:FF:F,FFF,FFFFFFF:F::FFFFFFFFFFFFFFFFFFFFFFFFFF,FFFFFFFFFFF
FFF
@A00721:190:HGJW7DSXY:4:1403:22607:31375_1:N:0:TCTGCAAG+GCTGCACT
ATGGTATAATGTGTCTTACAAATAGAACCTTCACTCTCGGTAATGAGAGTGTATTCCTTAATATTCTA
GTAACCGCTATGTGGGCATTACTAGGATTTATCACACTCCTAAATTTGATAGCAATTTATGTTTTAAA
TGC
+
FF::FFFF,FFF:FFFFF,FFFFFFFFFF,F:FFFFFFF:FFFFFFFFFFFF:FFFFFFFFFFF:F:
FFFF:,FFF,FFFFFFFFFF:FFF:FFFFFFFFFF,FFFFFF,:FFFFFF::FFF:,FFFFFFFFFF,
FFF
@A00721:190:HGJW7DSXY:4:1426:7102:33207_2:N:0:TCTGCAAG+GCTGCACT
AAAAACTCCATTGTATAGTTCAAGGAGTACTTAATTCAATAGCTTTAGTATTATTTACTATCGCCTTT
ATTGAAAATTGTATAAGAGTTAATTGCTTACACAAAAGTGACGCGCGA
+

```
FFFF:FFFFF:FFFFFFFFF,FFFFFFFFFFFFFFFFFFFFFFF:FFFFFFFFFFFF:FF,FF:FFFF
FF:FFFFFF:FFFFFFFFFFFFFFFF,:FF:F:FF:FFFFFF:FFFFF
@A00721:190:HGJW7DSXY:4:2570:29333:28025_1:N:0:TCTGCAAG+GCTGCACT
GGGGAGAATGAACTGCCGCTTCCCGGAAGAAACGATCTATTTACAGAGTATTTAGAGTAGTCCAAAGT
TTGTATAAATTTATATGATTTTCTATATATATACCACATATATATATATATAAAATCCTATAAATTTG
TAC
+
FFFFFF:FFFFFF,FFFFFFFFFFFFF:FFFFFFF:FFFFFFFFFFFFFFFFFFFFFFFFFFFFFFFF
FFFFFFFFFFFFFFFFFFFFFFFFFFFFFFFFFFFFFFFFFFFFFFFFFFFFFFFFFFFFFFFFFFFF
FFF
@A00721:190:HGJW7DSXY:4:2274:12798:19085_2:N:0:TCTGCAAG+GCTGCACT
GAAGAAAACCACTCCCTTCAATTCAATAACATAACATACCATACACTCCCCAAACATAACATACCATA
ACCTACCATGACCAATCATCGCTCCAACTGGCACGCCAGACGTAGGGT
+
FFFFFFFFFF,:FFFF:,FFFFFFFFFFFFFFFFFFFFFFFFFFFFFFFFFFFFFFFFFFFFFFFFFF
FFFFFFFFFFFFFFFFFFFFFFFFFF:FFFFFFFFFFFFFFFF:FFFF
@A00721:190:HGJW7DSXY:4:2338:1099:33959_2:N:0:TCTGCAAG+GCTGCACT
ACCGCCGGTCGGCAGATCGGCACCACTGGTCTCTACGTTCAGGGACCCGCTGTGCAGAATAACCTTCC
CAAATCTAGATCTGCACAGAAGAAGGCTGAAGGTCGTTACCGGACGAC
+
FFFFFFFFFFFFFFFFFFFFFFFFFFFFFFFFFFFFFFFFFFFFFFFFFFFFFFFFFFFFFFFFFFFF
FFFFFFFFFFFFFFFFFFFFFFFFFFFFFFFFFFFFFFFFFFFFFFFF
@A00721:190:HGJW7DSXY:4:2472:11008:20212_1:N:0:TCTGCAAG+GCTGCACT
GGCTGTTGCCCAGAGGAAAGCAGCGACCACCGAGGTCGCTCGTAACATTGACGGCAGTCCCGTCATCT
CTGTGGGAACTGAGGCAGTACCTCAGTCAAAAGTCACATTCACCACTCCCATGCCAATCAATGATGTG
ACT
+
FFFFFFFFFFFFFFFFFFFFFFFFFFFFFFFFFFFFFFFFFFFFFFFFFFFFFFFFFFFFFFFFFFFF
FFFFFFFFFFFFFFFFFFFFFFFFFFFFFFFFFFFFFFFFFFFFFFFFFFFFFFFFFFFFFFFFFFFF
FFF
@A00721:190:HGJW7DSXY:4:2544:1732:9784_2:N:0:TCTGCAAG+GCTGCACT
TCAATACGTCGTGGGAGGTACTGAAGCCTTCCCCTCCGCTGGCACGAAGAACACTCCCAAGCCAATCA
ATAGTTCTTCTGACCAGAATGAAAAGGGGTTTACCAGGGTAGTCTCTA
+
FFFFFFFFFFFFFFFFFFFFFFFFFFFFFFFFFFFFFFF,FFFFFFFFFFFFFFFF:FFFFFFFFF:F
FFFF:FFFFFFFFFFFFFFFFF:FFF:,FF:F:FFFFFFF,FFFFFFF
@A00721:190:HGJW7DSXY:4:1318:22851:36683_1:N:0:TCTGCAAG+GCTGCACT
AGAGGGGAAATGTCCCCAAAACTGAGAAGAATAAAGGGAAAGGTCCATCAGGACCTAAGAAGGGGAAA
TTACCCCTAATTCTGAAGGAAGATCTCCCACCATCTGGGGTTGGGAATAAGAAATCTGCACGGGCCAA
AGA
+
FFFFFFFFFFFFFFFF,FFFFFFFFFFFFFFF,:FF:FFFFFF:FFFFFFFFFFFFFFFFFFFFFFFF
FFFFFFFFFFFFFFFFFFFFFFFFFFFFFFFFFFFFFFFFFFFFFFFFFFFFFFFFFFFFFFFFFFFF
FFF
@A00721:190:HGJW7DSXY:4:1154:21404:25222_1:N:0:TCTGCAAG+GCTGCACT
CGAAGTCGCTGAAGGCGGCTTGGGTCATTAAGGGTAGCTGTAAAGTTGCGCCTGCCGACCCCATCTTT
GAGACCAAGACTCTCTACAGTAGGTTCGTGCCTACTATGAGCAAATCTGTTGAATTCGTCACTGAGGA
CGA
+
FFFFFFFFFFFFFFFFFFFFFFFFFFF:F:FFFFFFFFFFFFFFFFFFFFFFFFFFFFFFFFFFF:FF
FFFFFFFFFFFFFFF:FFFFFFFFFFFFFFFFFFFFFFFFFFFFFFFFFFFFFFFFFFFFFFFFFFFF
FFF
@A00721:190:HGJW7DSXY:4:1350:2410:5822_1:N:0:TCTGCAAG+GCTGCACT
ACTTTTGGCTACTGGCGACAAACTGAGAGCAGTCGCTGGGGAAAGAGTATTGCGTGGGTTGTAGGCCG
```

ACGCTTCGAGCTTGAGTGTGACGAGGCCGGTGACGTTTTTGTCAACGGCGTTTGGAGGATTTTATCCG
C
+
:FFFFFFFFFFF:FFFF,FF:FFFFF::F,,FFFF::FFF:FF,FF,,F,FF:FF,FFFFF,:FFFFF
,FFFFF,FFFFFF,FFFFFF,,F,FFF:FF:FF:FFFFFFFF:FF:FFFFFFF,F,:FFFFFFFF:,F
F
@A00721:190:HGJW7DSXY:4:1235:18765:3615_2:N:0:TCTGCAAG+GCTGCACT
GCGAAGATCCCCTATGGAGTCTTGAAGGACTTAGGTTGGCATATTTATGCCGACGATAATAAGCATGT
CACCCTTAAGCACCGAGGTAAGCTCGGGACTTACAAGAGAGAGTGTGA
+
FFFFFFFFFFFFFFFFFFFFFFFFFFFFFFFFFFFFFFFFF:FFFFFFFFFFFFFF,F:FFFF:FF
FFFFFFFFFFFFFFFFFFFFFFFFFFFFFFFFF:FFFFFFFFFFFFFFF
@A00721:190:HGJW7DSXY:4:1167:19741:16172_1:N:0:TCTGCAAG+GCTGCACT
CACTATTTTTGCCCAGAATGCGGGCTTTCTGACTGATGAGGTTAAAACTCTTGCTCCTTCTGTGGGTG
AGGGTTTTTGTTATATTCACTGGTTCTGGGAGGCTGCTTTCCTCCATAATTACAACCTCTCTTTAGAG
AGC
+
FFFFF:FFFFFFFFFFFFFFFFFFFFFFFFFFFFFFFFFFFFFFFFFFFFFFFFFFFFFFFFFFFFFF
FFFFFFFFFFFFFFFFFFFFFFFFFFFFFFFFFFFFFFFFFFFFFFFFFFFFFFFFFFFFFFFFFFFF
FFF
@A00721:190:HGJW7DSXY:4:1109:8477:7560_2:N:0:TCTGCAAG+GCTGCACT
AGTTGGGCAAATTCCCACATATGGTAGACGTTGTCACACTTTTCTTGAAAATTTTTCCTTTCCTCCGT
CATTGGGGATTTCGATGTGAGAAGGTGGAAGGTGTCAAGGGTCTTCTT
+
FFFFFFFFFFFFFFFFFFFFFFFFFF,FFFFFFFFFFFFFFFFFFFFFFFFFFFFFFFFFFFFFFFFF
FFF:FFFFFFFFFFFFF,FFFF,FFFFFFFFFFFFFFFFFFFFFFFFFF
@A00721:190:HGJW7DSXY:4:1136:23158:9157_2:N:0:TCTGCAAG+GCTGCACT
TTTCAGGACCTAATCACATCATGATTGATAGTTACGGCTCGTATCGAGTTGGAGGGAGGAAGTTCTCC
GAAATTAAAGACGGGGGGATTAAGAGTCCAGCTGTAACTGTCATTTCA
+
:FFFFFFFFFFFFFFFFFFFFFFFFFFFFFFFFFFFFFF:FF:FFFFFFFFFFFFFFFFFFFFFFFFFF
FFFFFFFF:FFFFFFFFFFFFFFFFFFFFFFFFFFFFFFFFF:FFFFFFF
@A00721:190:HGJW7DSXY:4:1226:11993:13307_2:N:0:TCTGCAAG+GCTGCACT
AAGAAAACTTCGTCAGAAGATGAGGAAGTCTAAGAGGAGGAGTTTGAGGAAGGCTCTAGAGAAGATCT
CACCACCTGGAGAAGGCAAGTGGAGAGTCCAGAAGTTGATGAAGAAGG
+
FFFFFF:FFFFFFFFFFFFFFFFFFFFFFFFF:FFFFFFFFFFF:FFFFFFFFFFFFFFFFFFFFFFFF
FFFFFFFFFFFFFFFFFFFFFF:FF:FFFFFFFFFFFFFFFFFFF:F,
@A00721:190:HGJW7DSXY:4:1226:8404:18396_2:N:0:TCTGCAAG+GCTGCACT
CAGGGGTTACCACTTCCTAAGGTGGATGAGGGAGGTTGTTTCTTTGGAGTTTTAGATAATCTTTTGAG
AAGGGGTCATAGAGCCAGCAAGAGAAGCTTATATGCTGGTTTAAGTGA
+
FFFFFFFFFFFFFFFFFFFFFFFFFFFFFFFFFFFFFFFFFFFFFFFFFFFFFFFFFFFFFFFFFFFF
FFFFFFFFFFFFFFFFFFFFFFFFFFFFFFFFFFFFFFFFF:FFFFFFF
@A00721:190:HGJW7DSXY:4:1414:14986:35180_2:N:0:TCTGCAAG+GCTGCACT
CCTTGAGAAGACAAGCCACATTAAGATATAATTTAGGTCAACTTGGGGTTTCGAATGTCATTAGTGCC
TTACGATTGAAAGAGGTCCTTTTGAGTATCGACAGATCTTTCGATCTT
+
FFFFFFFFFFF:FFFFFFFFFFFFFFFFFFFFFFFFFFFFFFFFFFFFFFFFFFFFFFFFFFF:FFFF
FFFFFFFFFFFF::FFFFFF:FFFFFFFFFFFFFFFFFFFFFFFFFFFFF
@A00721:190:HGJW7DSXY:4:2321:25870:1454_2:N:0:TCTGCAAG+GCTGCACT
TTAACGTGGCAGAATTCGGTAACTTACCATATTGTTCCTTCCGGGAAGTTGTGGGATTTAACTTTGAC
TCAACTACTCCGATCAGATCCGGATGGTTTGATTGGTATGGACTACTC
+

```
FFFFFFFFFFFFFFFFFFFFFFFFFFFFFFFFFF:FFFFFFFFFFFF:FFFFFF:FFFFFFFFFFFFFF
FFFFFFFFFFFFFFFFFFFFFFFFF:FFFFFFF,FFFFF:FFFFFFFFF
@A00721:190:HGJW7DSXY:4:2437:3947:35884_1:N:0:TCTGCAAG+GCTGCACT
TAGTTGTCTAGGGTCATGGCAGATACTACCGATAAGGTGAAAAATATGCAGGTCAAAGATTCTGTAGC
TCTGAGGGCGGTTGAGGGAGCCGTTTTGGCCGACTTTAACTATTCTAAGAGTCTTTATGATTCAAAAC
CTA
+
F:FFFFFFFFFFFFFFFFFF:FFFFFFFFFFFFFFFFFFFFFFFFFFFFFFFF:FFF:FFFFFFF:FFF
FF:FFFFFFFFFFFFFFFFFFFFFFFFFFFFFFFFFFFFFFFFFF:FFFFFFFFFFFFFFFFFFFF
FFF
@A00721:190:HGJW7DSXY:4:2657:23963:14184_2:N:0:TCTGCAAG+GCTGCACT
ACCTTACAAGGTTGAGGAGACTTTACAGCGGCTTATTGTCGCGCAGTTTCCAGAATTGCGGTTGAAGT
TCACCCATTCTATCTTGAATGCTCACGCCGTCGCAGCCATATCGAGAC
+
FFFFFFFFFFFFFFFFFFFFFFFFFFFFFFFFFFFFFFFFFFFFFFFFFFFFFFFFFFFFFFFFFFFF
FFFFFF:FFFFFFFFFFFFFFFF:FFFFFFFFFFFFFFFFFFFFFFFF
@A00721:190:HGJW7DSXY:4:2314:23547:31751_2:N:0:TCTGCAAG+GCTGCACT
AAAGTACAGATTTAGTGATTTAGATATTATTGATATTGGAGGCGATATTCGTTTTCATATCCAAAACT
CCTTGAATGGAGTACATATTTGCAACCCTATTCTTGATGTGAAAGATG
+
FFFFFFFFFFFFFFFFFFFFFFFFFFFFF:FFFFFFFFFFFFFFFFFFFFFFFFFFFFFFFFFFFFFFF
FFFFFFFFFFFFFFFFFFFFFF:FFFFFFFFFFFFFFFFFFFFFFFFF:
@A00721:190:HGJW7DSXY:4:1340:32479:30185_1:N:0:TCTGCAAG+GCTGCACT
ATTGATTGGGAACGTACCTACTCACGAGACGTCATGAAAGCTATCGGAGGGAAGCAAAATGTTTCGTT
CTGTTACAAGAACGCTTTGGATTGTGACCATAAAGCCTCAGTTGCGATAGCTGTAGAGGTATACGATA
TCT
+
FFFFFFFFFFFFFFFFFFF:FFFFFFFFFFFFFFFFFFFFFFFFFFFFFFFFFFFFFFFFFFFFFFFF
FFFFFFFFFFFFFFFFFFFFFFFFFFFFFFFFFFFFFFFFFFFFFFFFFFFFFFFFFFFF:FFFFFFF
FFF
@A00721:190:HGJW7DSXY:4:1333:27905:27367_2:N:0:TCTGCAAG+GCTGCACT
GCCACTATAATGCTTAAGAGAGAGATTGATGTATGTCATTTCGTCTTAGTTTGTCCCGGCGAATTATT
GGACACGATGAAAAATGACATACCCGTTCCTGACCTTGGTATCGTTAT
+
FFFFFFFFFFFFFFFF:FFFFFFFFFFFFFFF:FFFFFFFFFFFFFF:FFFFFF:FFFFFFF::F:FF
FFFFFFFFFFFFFFFFFFF,FFFFFFFFFFFFFFFFFFFFFFFFFFFF
@A00721:190:HGJW7DSXY:4:1223:26024:29215_2:N:0:TCTGCAAG+GCTGCACT
GGTTATTCTCACAACAAAGATAAGATTTTATCTTTCTTCAAACACCCTGTTTTCCTTAAGGGCGGACA
ATTGTTCCATTGTGAACTCTTGGGGACTAGATTGAGCATTAATGAGTT
+
FFFFFFFFFFFFFFFFFFFFFFFFFFFFFFFFFFFFFFFFFFFFFFFFFFFFFFFFFFFFFFFFFFFF
FFFFFFFFFFFFFFFFFFFFFF:FFFFFFFFFFFFFFFFFFFFFFFFF
@A00721:190:HGJW7DSXY:4:1125:13702:10692_2:N:0:TCTGCAAG+GCTGCACT
CTTTCTCTAGTGGACAATTCAGAATACCTATTTCCGTACCAAGAGCCACTGCCAATCTCATCGCTATA
CGCATACCTGATTGGAATAAGCGAGGTTCGTTCCTCAATAAAAATTTT
+
FFFFFFFFFFFFFFFFFFFFFFFFFFFFFFFFFFFFFFFFFFFFFFFF:FFFFFFFFFFFFFFFFFFF
FFFFFFFFFFFFFFFFFFFFFFFFFFFFFFFFFFFFFFFFFFFFFFFF
@A00721:190:HGJW7DSXY:4:1214:25301:14340_1:N:0:TCTGCAAG+GCTGCACT
AAGTTTTCTTCATAAGGGCTATTCAGTACGTCATGGGGATCACTTCTTCGGTCAATGACAAAACCTTC
GAATATGTTACGACATGGCTTCGTAACAACAGTGCGAGGATTGTCATCTCGGGGAAAGTGATTCACCA
AGA
+
FFFFFFFFFFF:FFFFFFFFFFFFFF:FFFFFFFFFFFFFFFFFFFFFFFFFFFFFFFFFFFFFFFFF
```

```
FFFFFFFFFFFFFFFFFFFFFFFFFFFFFFFFFFFFFFFFFFFFFFFFFFFFFFFFFFFFFFFFFFFF
FFF
@A00721:190:HGJW7DSXY:4:1365:6750:25739_1:N:0:TCTGCAAG+GCTGCACT
GAGATACCTAGAGAAGGTCGCAGCGATCTTACTCGTTATCGGAGCTAAGAAGAAGACTTTGGGTACAC
GTTATGCTCGCAAACTTAACGTTAGTACCGGAGGTGATCTCTCTGCTTCTGCGTTACTGAGAGAAAAG
ATT
+
FFFFFFFFFFFFFFFFFFFFFFFFFFFFFFFFFFFFFFFFFFFFFFFFFFFFFFFFFFFFFFFFFFFF
FFFFFFFFFFFFFFFFFFFFFFFFF:FFFFFFFFFFFFFFFFFFFFFFFFFFFFFFFFFFFFFFFFFF
FFF
@A00721:190:HGJW7DSXY:4:1553:1841:2581_2:N:0:TCTGCAAG+GCTGCACT
ACGGTGACTTTATGGACAAGATGCAAGTTCTTGGGAAATACTTGTTTCCCTTTTTAGGTTTAGTAGGT
GATCTTGACGTCGATTCTTTGTATAGGGAGTGTGAGAAGACAACGAAT
+
FFFFFFFFFFFFFFFFFFFFFFFFFFFFFFFFFFFFFFFFF:FFFFFFFFFFF:FFFFF:FFFFFFFF
:F:FFFFFFFFFFFFF::F:FFFF:FFFFF,FFF:FFFFFFFFFFFFF
@A00721:190:HGJW7DSXY:4:2444:28999:12508_2:N:0:TCTGCAAG+GCTGCACT
TATCAAAGAAAATGGTGAACCGGTCTCTAAATCTTTTGAGATTGAGAAGATTGAAGAACACGTCAAGA
AGTCGCTCGGCGTTGTTCTTGAAAACCAAGTTAAGAAGGAGGCTGATC
+
FFFFF::FFFFFFFFFFFFFFFFFFFFFFFFFFFFFFFFFFF,FFFFFFF:FFFFFF:FFFFFFFFFF:
FFFFFFFFFFFFFFFFFFFFFFFFF:FFFFFFFFFFFFFFFFFFFFFFF
@A00721:190:HGJW7DSXY:4:1639:16197:15765_2:N:0:TCTGCAAG+GCTGCACT
GTACCTTCTGTCAAACTTCGAGGTTCAGCTACGAGAGGTGATCCCGGATGTGGATTGAAGGCTGGCTC
TAAGGGAGACTCCTTCTTTTTGAATGTTGTAAAGAATTGGGAGATCGG
+
FFFFF:::FFFFFFFFFFFFF,FF,FFFFFFFFF:FFFFFFFFFFFFF:FFFFFFFFF,FFFFF:FFF
:FFF,FF:FFFFFFFF:,F:FFFFFFFFFFFFFFFFFF,FFFFFFF:F
@A00721:190:HGJW7DSXY:4:1564:25807:13526_1:N:0:TCTGCAAG+GCTGCACT
AGTTTTTAGATCTTCTTGATAATCTTAAAGAAAAATTTTCGAATAAACTAATGAGAGGTATAGGCTTA
GTCAAGTCTGTACTTACATTCTTTACGCCAGATTCTTCATGGGGCGACGCTGTTTCTCTTGGTATTTC
TGA
+
FFFFFFFFFFFFFFFFFFFFFF:FFFFFFFFFFFFFFFFFFFFFFFFFFFFFFFF:FFFFFFFFFFFFF
FFFFFFFFFFFFFFFFFFFFFFFFFFFFFFFFFFFFFFFFFFFFFFFFFFFFFFFFFFFFFFFFFF:FF
FFF
@A00721:190:HGJW7DSXY:4:1456:25771:32158_2:N:0:TCTGCAAG+GCTGCACT
CAGAGCGTCATTTTCCTTTGGGATCTTTCCTCTGGTAAGGGAATTATTAAATCATTCTTCTCACTCTT
GACGAGAATTATTACTGTTTTCTCTACCCATAACTTCTTTTTCAAAAA
+
FFFFFFFFFFFFFFFFFFFFFFFFFFFFFFFFFFFFFFFF:FFFFFFFFFFFFFFFFFFFFFFFFFF:F
:FFFFFFFFFFFFFFFFFFFFFFFFFFFFFFFFFFF:F:FFFFFFFFFFFF
@A00721:190:HGJW7DSXY:4:1126:18150:20243_1:N:0:TCTGCAAG+GCTGCACT
CTGATGTTTTCCTACAGAGTGCAGTCGTTTCAACACTTATCACCACTTTTGAAAGGAAAATAATTGGT
GATGAAAATACGTATGTCGGTGGGGTTCTCAAGGCTCTCATCGCCACTGGGATTCTAAGGGGTTCCAT
AAG
+
FFFFFFFFFFFFF:FFFFFFFFFFFFFFFFFFFFFFFFFFFFFFFFFFFFFFFFFFFFFFFFFFFFFF
FFFFFFFFFFFFFFFFFFFFFFFFFFFFFFFFFFFFFFFFFFFFFFFFFFFFFFFFFFFFFFFFFFFF
FFF
@A00721:190:HGJW7DSXY:4:2574:2899:22545_2:N:0:TCTGCAAG+GCTGCACT
TTCAATGATCAGGATTCTTCTTTTGGTCCTTACGTCTTAAGTAATTTGGATGTTGTCGAAAACTATCA
GGTTCTTGTCACGATTCTTAAAAGAAGTTTATTAAAACTTCAGAGTCT
+
```

```
FFFFFFFFFFFFFFFFFFFFFFFFFFFFFFFFFFFFFFFFFFFFFFFFFFFFFFFFFFFFFFFFFFFFFFF
FFFFFFFFFFFFFFFFFFFFFFFFFFFFFFFFFFFFFFFFFFFFFFFFFF
@A00721:190:HGJW7DSXY:4:1158:11379:29716_1:N:0:TCTGCAAG+GCTGCACT
GGTAGCTAATGTTCTTGCAATAGCCACAGACTTTCTGGGAAAAGAGACCTCTGTTGTAGAGGAGTTGA
GAGAATTACTCAGATTCAATCTGGCATCACTGAACGTCTACAACGAGTTAGGGAGTGGCTAGGTTTTA
AAA
+
FFFFF:FFFFFFFFFFFFFFFFFFFFFFFFFFFFFFFFFFFFFFFFFFFFFFFFFFFFFFFFFFFFFFFF
FFFFFFFFFFFFFFFFFF,FFFFFFFFFFFFFFFFFFFFFFFFFFFFFFFFFFFFFFFFFFFFFFFFFFF
FFF
@A00721:190:HGJW7DSXY:4:1267:6858:8046_1:N:0:TCTGCAAG+GCTGCACT
AGCAATACCTTCCACTGAGTTCATCACCCAGAGTTTCGACGTCGCGGAAAACGATGAAGATTTCCACA
CTGTTCGAGAATCTTCTGATGCTGAATTTGAGGAAAATTTTTCGGAAGTTTTTGGTATAAATCCTGAT
GGC
+
FFFFFFFFFFFFFFFFFFFFFFFFFFF,FFFFFFFFFF:FFFFFFFFF:F:FFFFFFFFF::FFFFFF
FFFFFFFFFFFFFFFFFF::FFFFFFFFFF:FFFFFFFFFFFFFFFFFFFFFFFFFFFFFFFF:FFFFF
FFF
@A00721:190:HGJW7DSXY:4:1150:2510:36151_1:N:0:TCTGCAAG+GCTGCACT
TAAAACCTTCTTTTATCATTGCAACAGGACTTTGAGAAGACTGTTGAAATGGACAAAATCTGGGATCA
TAGGTATGTGTCACGCTACACTCGAAATATGGGCCCTCATAAGGAAGTATAGTTGGCGTCTTGTCGTT
GAC
+
FFFFFFFFFFFFFFFFFFFFFFFFFFFFFFFFFFFFFFFFFFFFFFFFFFFFFFFFFFFFFFFFFFFF
FFFFFFFFFFFFFFFFFFFFFFFFFFFFFFFFFFFFFFFFFFFFFFFFFFFFFFFFFFFFFFFFFFFF
FFF
@A00721:190:HGJW7DSXY:4:1218:19208:33035_2:N:0:TCTGCAAG+GCTGCACT
AGTTAGATCATTTGTTCAGGAAGATCTCTACAGAGATTATCGAGAGTTGTGATCTAGTTCTAAATTTA
GGTCGACGGACTTTAGCAACTTTAGATAAATTCTTTCCGGGCTCAATG
+
FFFFFFFFFFFFFFFFFFFFFFFFFFFFFFFFFFFFFFFFFFFFFFFFFFFFFFFFFFFFFFFFFFFF
FFFFFFFFFFFFFFFFFFFFFFFFFFF:FFFFFFFFFFFFFFFFFFFF
@A00721:190:HGJW7DSXY:4:1227:11514:1423_1:N:0:TCTGCAAG+GCTGCACT
TCTGACCATCGCAAAGAAGACTTTATTAGTTCATGAAGTTCTTACACTTCTCGCGACTAATGTCAGCT
TCACTACAAAGGCGTTACGTAAAGTAGTTTTCACGCTTTATCTTAAAGCATTACCACCAAATTTAGCA
CTT
+
FFFFF:FFFFFFFFFFFFFFFFFFFFFFFFFFFFFFFFFF:FFFFFFF:FFFFFFFFFFFFFFFFFFFF
FFFFFFFFFFFFFFFFFFFFFFFFFFFFFFFFFFFFFFFFFFFFFFFFFFFFFFFFFFFFFFFFFFFF
FFF
@A00721:190:HGJW7DSXY:4:2246:7554:19586_1:N:0:TCTGCAAG+GCTGCACT
GAAGCTCGCCATATCAGATTAGAGACATGCCAGTTAGAGTTCTTCTAACCAGGACTGATGGTGAGATT
CTTTTACCTTTTGGAGGTCCAGGAACCTTTACTCCTAGTGTCTTTTCTAGAGTCCAAGGACGTTTCCG
TCG
+
FF:FFFFFFFFF:FFFFFFFFFFFFFFFFFFFFFFFFFFF,FFFFF:FFFFFFFFFFFFFFFFFFFFFFF
FFFFFFFF:FFFFFFFFFFFFFFFFFFFFFFFFFFFFFFFFFFFFFFFFFFFFFFFFFFFFFFFFFFFFFF
FFF
@A00721:190:HGJW7DSXY:4:1362:16152:7200_1:N:0:TCTGCAAG+GCTGCACT
GTGGGAGACCGCTCAATAACACTCTATTGAATCCCTTTTCCACGATGAAAGCTCTTTATGAGGTGCTC
CATGCAAATTTACAGTTCCTCTTTGGTGATATCGCCTCTGTCCTTTCTCCTTTAATCGTGAGGTTACG
AGA
+
FFFFFFFFFFFF:FFFFF,:FFFFFFFFFFFFFFFFFFFFFFFFFFFFFFFFFFFFFFFFFFFFF:FFF
```

FFFFFFFFFFFFFFFFFFFFFFFFFFFFFFFFFFFFFFFFFFFFFFFFFFFFF:FFFFFFFFFFFFFF
FFF
@A00721:190:HGJW7DSXY:4:1432:23610:22686_2:N:0:TCTGCAAG+GCTGCACT
GGTTTATCGGTTGTATTTTGATAAATTTGTTGGAATCGATAATGATACTGACGAAGGTTCTATTACCA
CCCTTGTCATTGATACAAACATTCTAGGTGATGCTTTCTTAGAGGCTC
+
FFFFFFFFFFFFFFFFFFFFFFFFFFFFFFFFFFFFFFFFFFFF,FFFFFFFFFFFFFFFFFFFFFFF
FFFFFFFFFFFFFFFFFFFFFFFFFFFFFFFFFFFFFFFFFFFFFFFF
@A00721:190:HGJW7DSXY:4:1235:10013:3364_2:N:0:TCTGCAAG+GCTGCACT
TATTTACAATCTGTAGAGAGAGAGGTTAATCAGAATTTGAGTGATAACGATTTAGTACTGGGAAATGG
TGGTAGTCAGTTTACTACTTTTCTTTCCCTCCCCTTTTTCGATTGGAT
+
FFFFFFFFFF,FFFFFFFFFFFFFFFFFFFFFFFFFFFFFFFFFFFFFFFFFFFFFFFFFFFFFFFFF
FFFFFFFFFFFFFFFFFFF:FFFFFFFFFFFFFFFFF:FFF,FFFFFF
@A00721:190:HGJW7DSXY:4:1610:16929:26209_2:N:0:TCTGCAAG+GCTGCACT
CGTCTCACGACTAATTTGATAAGGTTTTTCTTTTATGGGACTCTTGTTTGTACTTATGAAGGAGGTAT
TACTTCGGAAGTTCTTAATGTAGGTCTCCTAGTACTTAAGGGTTTATG
+
FFFFFFFFFFFFFFFFFFFFFFFFFF:FFFFFFFFFFFFFFFFFFFFFFFFFFFFFFFFFFFFFFF:F
FF:FFFFFFFFFFFFFFFFFFFFFFFFF:FFFFFFFFFF:FFFFFFFF
@A00721:190:HGJW7DSXY:4:1259:3097:10895_2:N:0:TCTGCAAG+GCTGCACT
TTTTGATGGTATGTTTCACATTGCGTGATCACTTCCCGTGGACTTGGGAAGAGATTAAACATAAAATC
CAGCGATTCTATTATGTCGCTAAAGCCTGTCCAGTTGTCGATGATGTT
+
FFFFFFFFFF:FFFFFFFFFFFFFFFFFFFFFFFFFFFFFFFFFFFFFFFFFFFFFFFFF:FFF:FFF
FFFFFF,FFFFF:FFFFFFFF:FFFFFFFFFFFFFFFFFFFFFFFFFF
@A00721:190:HGJW7DSXY:4:2124:13648:10441_2:N:0:TCTGCAAG+GCTGCACT
TCGTGAAACTTACGACGATGTTCTCGCTCGCATTTCACAAGGATTACTAAGTCTGAGATCTGCCACTT
CTAATTTTAGGAAACGTCGCGAAAAGAAAGTTCATGAGGATGTCTTGG
+
FFFFFFFFFFFFFFFFFFFFFFFFFFFFFFFFFFFFFFFFFFFFFFFFFFFFFFFFFFFFFFFFFFFF
FFFFFFFFFFFFFFFFFFFFFFFFFFFFFFFFFFFFFFFFFFFFFFFF
@A00721:190:HGJW7DSXY:4:1315:14995:27931_2:N:0:TCTGCAAG+GCTGCACT
GAACTTCTAATAGAGGATCCTGATGACATTATTTCAACGGATACCGATCTCAGTTCTACCACAGAAAC
CGTCTCAGGAACTGTCTCCACTGATTCGGAATTAGAGAAAGACGACCT
+
FF,FFFFF:FFFFFFFFFFFFFFFFFFFFFFFFFFFFF:FFFFFFFFFFFFFFFFFFFFFFFFFFFFF
FFFFFFFFFFFFFFFFFFFFFFFFFFFFFFFFFFFFFFFFFFFFFFFF
@A00721:190:HGJW7DSXY:4:1340:28592:7983_1:N:0:TCTGCAAG+GCTGCACT
GAGACCTCTGAATTTTCAGGGTGCCCTGATGAGGACGTTGAAAGGGATAATTTCAACTCTGAGACTTC
TAAGAGTGTTTCAAAAGACCAGAAACGTAACAAGAAGAATAAAGGTAGGAAGGGGAGAAAACAGAACA
AGC
+
FFFFFFFFF:F:FFFF:FFFFFF:F:FFFFFFF:FFFFF:FFFFFFFFFFFFFFFFFFFFFFFF,FFF
FFFFFFFFFFFFFFFFFFFF:FFFFFFFFFFFFFFFFFFFFFFFFFFFFFFFFFFFFFFFFFFFFFF
FFF
@A00721:190:HGJW7DSXY:4:1421:25129:11381_2:N:0:TCTGCAAG+GCTGCACT
ACTGATATGGTTGCGGCTGATCGTACCCCAATTCCTGCTAGAAAGATCACTCTCGGCGATACCACTGA
TCTTCCAGAGTTACGTAGTGGTCGTTCTTTTGTTGATTTTATCAAAGA
+
FFFFFFFFFFF:FFFFFFFFFFFFFFFFFFFFFFFFFFFFFFFFFFFFFFFFFFFFFFFFFFFFFFFF
FFFFFFFFFFFFFFFFFFFFFFFFFFFFFFFFFFFFFFFFFFFFFFFF
@A00721:190:HGJW7DSXY:4:2170:3106:24408_2:N:0:TCTGCAAG+GCTGCACT
CAAGTTAAACTTCGAACTGACCACTTACAAGAGTGTTGAAGTACCGCAACATGGATCAGTTTTCCAAA

AAGCTATTAATAGCAGTTGCGGGGCTATAATACCGCCTCCAGATTACT
+
FFFFFFFF:FFFFFFFFFFFFFFFFFFFF:FFFFFFFFFFFFFFFFFF,FFFFFFFFFFFFFFFFFFF
FFFFFFFFFFFFFFFFFFFFFFFFFF:FFFFFFFFFFFFFFFFFFFFF
@A00721:190:HGJW7DSXY:4:1460:8061:27070_1:N:0:TCTGCAAG+GCTGCACT
AGAGTTTTGGAACTGTGTAATGTCCTACGTACTGTTCAAGGTGTTTGGGCTTCTCTAAAGAACAGCAA
CTTTCAACATGAATTAGTTATTGAAAGTCTTGACCCTCACGTCTCACTTTATCACCTTGGAGATGGTA
AAG
+
FFFFFFFFFFFFFFFFFFFFFFFFFFFFFFFFFFFFFFFFFFF:FFFFFFFFFFFFFFFFFFFFFFFF
FFFFFFFFFFFFFFFFFFFFFFFFFFFFFFFFFFFFFFFFFFFFFFFFFFFFFFFFFFFFFFFFFFFF
FFF
@A00721:190:HGJW7DSXY:4:1264:13738:11663_2:N:0:TCTGCAAG+GCTGCACT
TAAATTTCGCATTCTGCTTCGACGGTAATTCCATAAGACCTTTCTTGGGGAGTAAAGAGGGTTTGAAA
ATTACGGAACCTTTCCTCGTTCTTCATGAAGATCTTCGAGTGTTTTAT
+
FFFFFFFFFFFFFFFFFFFFFFFFFFFFFFFFFFFFFFFFFFF:FFFFFFFFFFF:FFFFFFFFF:FF
FFFFF:FFFFFFFFFFFFFFFFFFFFFFFFFF:FFFFFFFFFFF,FFFF
@A00721:190:HGJW7DSXY:4:1523:26865:23406_1:N:0:TCTGCAAG+GCTGCACT
TCTTGCGAAGGTTCAAGTCGATTGATTTTGTTGACTACGATCTCTCTTGTAATAAGAAGTTCTTCGCA
GTTGAATCTCCACCAGGTGGAGGGAAGACCACTGAACTTATAGCGACTTTTGTCGAATTTTTCGCAAA
AGG
+
FFFFF:FFFFFFFFFFFFFFFFFFFFFFFFFFFFFFFFFFFFFFFFFFFFFFFFFFFFFFFFFFFFFF
FFFFFFFFFFFFFFFFFFFFFFFFFFFFFFFFFFFFFFFFFFFFFFFFFFFFFFFFFFFFFFFFFFFF
FFF
@A00721:190:HGJW7DSXY:4:1168:2736:17190_2:N:0:TCTGCAAG+GCTGCACT
ATTGTCACTGCTAATCGTAATTCGCGAGAAGAGATAGTGACTAAAGTTTTGGCACTTCTAGTTCATCG
CGGTATTTGTGAAATTAAGACAGACGTGAAAGTTATGATGAGTGATCG
+
FFFFFFFFFFFFFFFFFFFFFFFFFFFFFFFFFFFFFFFFFFFFFFFFFFFFFFFFFFFFFFFFFFFF
FFFFFFFFFFFFFFFFFFFFFFFFFFFFFFFFFFFFFFFFFFFFFFFF
@A00721:190:HGJW7DSXY:4:1450:15266:24079_2:N:0:TCTGCAAG+GCTGCACT
ACGTCGCTGAGGTCAAACCTGTGGACGTTCTTTTCATCGACGAATTATTCATGATTCACGCTGGTCAG
ACCCTTTTTCTTACACATCTACTTAAGGCGGCTTATACCATAGGTTAC
+
::FFF:FFFFFFFFFFFFFFFFFFFFFFFFFF:FFFFFFFFFFFFF:FFFF,FF:FFFFFF::FFFFFFF
FFF,,,FF,F:F:,FFF::FFFFFFFF:FFFFF,F,FF:F:FF,F:FF
@A00721:190:HGJW7DSXY:4:1370:2935:31000_2:N:0:TCTGCAAG+GCTGCACT
CAAATCGGATTCATCAATCGTATTGATCTCCAAGTCGCAAACTACTTCAACATCAACGACATCATTCT
TGATGAGTGTCGTTCTTATCGAAGTCTCAGTTATCGCTGCCCGGCCGA
+
FFFFFFFFFFFFFFFFFFFFFFFFFFFFFFFFFFFFFFFFFFFFFFFFFFFFFFFFFFFFFFFFFFFF
FFFFFFFFFFF::FFFFFFFFFFFFFFFFFFFFFFFFFFFFFFFFFFF
@A00721:190:HGJW7DSXY:4:1378:13783:19601_2:N:0:TCTGCAAG+GCTGCACT
GAACTCTCAGAGGTTTACGGCAGAAAAATTTCTCCTGCTTGTAATTCGAGAGAGGACACTATGGAAAT
TAGACCTATCAGATGTCTTGACGACTTCGAATTTGAGGACGGAGTTAA
+
FFFFFFFFFFFFFFFFFFFFFFFFFFFFFFFFFFFFFFFFFFFFFFFFFFFFFFFFFFFFFFFFFFFF
FFFFFFFFFFFFFFFFFFFFFFFFFFFFFFFFFFFFFFFFFFFFFFFF
@A00721:190:HGJW7DSXY:4:1417:8323:33974_2:N:0:TCTGCAAG+GCTGCACT
AATTAACAAAGTCCTTGGTAAGAGTAAGGTTAAATGTGAAGATGCTCAGACTATTCATGAAGTTCAAG
GTAATACTTACCAGCACGTTCGGCTGGTTAGAATTAAACCTCAAGATG
+

FFFFFFFFFFFFFFFFFFFFFFFFFFFFFFFFFFF:FFFFFFFFFFFFFFFFFFFFFFFFFFFFFFFFFF
FFFFFFFFFFFFFFFFFFFFFFFFFFFF,FFFFFFFFFF,FFFFFFFFFF
@A00721:190:HGJW7DSXY:4:1474:1597:18912_2:N:0:TCTGCAAG+GCTGCACT
CACAAACACATATTACCGTTGCTCTATCACGACACGTTGAATCTTTACGATACTTTGTTATCAATGTA
AAGTTAAACGATGCTACTTGTGAGAGGATCAATCACAGTAAGGAACTG
+
FFFFFFFFFFFFFFFFFFFFFFFFFFFFFFFFFFFFFFFFFFFFFFFFFFFFFFFFFFFFFFFFFFFFF,
FFFFFFFFFFFFFFF:FFFFFFFFFFFFFFFFFFFFFFFFFFF:FF:FFF
@A00721:190:HGJW7DSXY:4:1465:19461:6073_2:N:0:TCTGCAAG+GCTGCACT
ACGGGAGGTTGCAGATTCAACCCCTTATATCGAGGCTATCGAGGACGATGAGTATCTTGAAGTTGTTG
ACGACAAGCCTCAAGAGGGTAGAGGACCACCTGGATCTTCTACTGTCT
+
FFFFFFFFFFFFFFFFFFFFFFFFFFFFFFFFFFFFFFFFFFFFFFFFF,FFFFFFFFFFFFFFFFFFF
FFFFFFF:FFFFFFFFFFFFFFFFFFFFFFFFFFF:FFFFF:FFFFFFFF
@A00721:190:HGJW7DSXY:4:1540:11840:20995_2:N:0:TCTGCAAG+GCTGCACT
GGTTATTCCTGGAACCACCTTAACGTCACTCGGTAATCTTTCAGCCGAATTAAGTACTAATGAGTTCA
AGTCGGTTGTCGATCGAATTAAGATCAAGGCTAATACCTTTATAGGTA
+
FFFFFFFFFFFFFFFFFFFFFFFFFFFFFFFFFFFFFFFFFFFFFFFFFF:FFFFFFFFFFFFFFFFFF
FFFFFFFFFFFFFFFFFFFFFFFFFFFFFFFFFFFFFFFFFFFFFFFFFF
@A00721:190:HGJW7DSXY:4:2245:25373:3035_2:N:0:TCTGCAAG+GCTGCACT
GCCGCGTAGTCAGCGCGTTTAGCTGTATCATCAGTCAGAGTATACCCGATAGGGTACCGAGTCTCCAA
GAGAATATTTATTCTTATGAGGCTCGAAACTTCAACGTTTTCACAACG
+
FFFFFFFFFFFFFFFFFFFFFFFFFFFFFFFFFFFFFFFFFFFF:FFFFFFFFFFFFFFFFFFFFFFFFF
FFFFFFFFFFFFFFFFFFFFFFFFFFFFFFFFFFFFFFFFFFFFFFFFFF
@A00721:190:HGJW7DSXY:4:1569:29695:14278_2:N:0:TCTGCAAG+GCTGCACT
TCGCACCCGAAATTTTTGGGCAGAATGCTCTTAACGCGGTTCTTGAACGGTGTTTTGATCAGTCTAAG
TTGGATAAAGTTATAGGAGACTCAGTTAACCTGAGAGCTCCTCAATTG
+
FFFFFFFFFFFFFFFFFFFFFFFFFFFFFFFFFFFFFFFFFFFFFFFFFFFFFFFFFFFFFFFFFFFFFF
FFFFFFFFFFFFFFFFFFFFFFFFFFFFFFFFFFFFFFFFFFFFFFFFFF
@A00721:190:HGJW7DSXY:4:1143:16631:28322_1:N:0:TCTGCAAG+GCTGCACT
CAATAGGGACGCTAGTATACTTCGTAAAGTCGACAGCGAAATGGTCAATATTGATAATTGGACCGACT
ATATGACCTATTTTAAGCTCATGGTGAAAAGGGAAGCTAAGGTTAAACTTGATGCTTCAGCCTTTTCG
AAG
+
FFFFFFFFFFFFFFFFFFFFFFFFFFFFFFFFFFFFFFFFFFFFFFFFFF:FFFFFFFFFFFFFFFFFF
FFFFFFFFFFFFFFFFFFFFFFFFFFFFFFFFFFFFFFFFFFFFFFFFFFFFFFFFFFFFFFFFFFFFF
FFF
@A00721:190:HGJW7DSXY:4:1577:10719:10066_1:N:0:TCTGCAAG+GCTGCACT
AGAATATCGTTTATCACGCAAAATTTATTAATGCAATTTTCGGACCTATCTTTAAGGTTCTTGTGAGG
AGAGTCAAACAGTGTCTTTTACCCAACATTTTTATGTATACAGAGATGACCAGTGACGATTTCTCCCG
GAA
+
FFFFFFFFFFFFFFFFFFFFFFFFFFF:FFFFFFFFFFFFFFFFFFFFFFFF,FFFF:FFF,FFFFFFFF
FFFFFFFFFFFF:FFFFFFFFFFFFFFFFFFFFFFFFFFFFFFFFFFFFFFFFFFFFFFFFFFFFFFFFF
FFF
@A00721:190:HGJW7DSXY:4:1152:18665:30311_1:N:0:TCTGCAAG+GCTGCACT
CGAGATCTTGCAGAGTGACTGGAGTTCAGACGTGTGCTCTTCCGATCTTTTCTTTTGTAAAGCCTATG
AGATAAAGATGTATTCTTATTTAGGCTTCGATGCTGAATTTCTTGAACTTTGGAGTGCCAGTGAATAT
TTT
+
F:,FFFFFFFFFFFFFFFFFFFFFFFFFFFFFFFFFFFFFFFFFFFFFFFFFFFFFFFFFFFFFFFFF

```
FFFFFFFFFFFFFFFFFFFFFFFFFFFFFFFFFFFFFFFFFFFFFFFFFFFFFFFFFFFFFFFFFFFFFFFF
FFF
@A00721:190:HGJW7DSXY:4:1267:16785:10958_1:N:0:TCTGCAAG+GCTGCACT
GAGTATCCATAACGATTTGTCATTTACCACCTTTGCTCAACGACGAAGTGGAACGGCCAACACTTTCT
TTGGTAATTGTATGATCACTTTGATGATTTTATCACTTTATTATGACCTTGGAAAGTTTTCGGCTGTG
GTT
+
FFFFFFFFFFFFFFFFFFFFFFF,FFFFFFFFF:FFFFFFFFFFFFFFFFFFFFFFFFFFFFFFFFFFFF
F:FFFFFFFFFFFFFFFFFFFFFFFFFFFFFFFFFFFFFFFFFFFFFFFFFFFFFFFFFFFFFFFFFFFFF
FFF
@A00721:190:HGJW7DSXY:4:1566:13856:23547_1:N:0:TCTGCAAG+GCTGCACT
CCATTCTGTTTTCAAAGACTCCAATAACTGATCATTCTGATCATATGATTTTGGACAATGGTTTCGAA
GCGAAATTTTTGAGGGATAAACCAGCGTACTTCTGCTCGCGATTTATTCTTTTTGTCAATGATTACAT
CTA
+
FFFFFFFFFFFFFFFFFFFFFF:F:FFFFFFFFFFFFFFFFFFFFFFFFFFFFFFFFF:FFFFFFFFFF
FFFFFFFFFFFFFFFFFFFF::FFFFFFFFFFFFFFFFFFFFFFFFFFFFFFFFFFFF:FFFFFFFFFF
FFF
@A00721:190:HGJW7DSXY:4:1311:4282:27946_1:N:0:TCTGCAAG+GCTGCACT
CCTTACAAGATCATCGTTAAGTTAGGTAAACCCCTAACCGATGTTACTCTTGTAGGACTTTGGGAGAG
ATTCGTTAGTTTTAAAGACTATACTAAATATCTCCACTACGAGGTTGTTGTTTGGCGATTAGCTCTTG
CGA
+
FFF:FFFFFFFFFFFFFFFFFFFFFFFFFFFFFFFFFFFFFFFFFFFFFFFFFFFFFFFFFFFFFFFFFF
FFFFFFFFFFFFFFFFFFFFFFFFFFFFFFFFFFFFFFFFFFFFFFFFFFFFFFFFFFFFFFFFFFFFFFF
FFF
@A00721:190:HGJW7DSXY:4:2304:4969:4398_1:N:0:TCTGCAAG+GCTGCACT
TATTCTATTGAGGGTCGTTATACCTATTCGGCTATCTGTGCTTTGAATTGTATAAGATCAAATTTTAA
ACGATTCCGAGAGGTTTATCCTGATGTGGAGGGAGTTCTTGAAGCATCCCGACCTTTCCTTGTGAAAA
+
::FFF,,FFFFFFFFFFFFFFFF:F,F,FFF,FFF,FFFF,FFF,FFFFFFFFFF,F:FFFFFFF:F,
:FFFFFFFFFFFFFFFFFFFFFFF:FFFFFFFFFFFFFFFFFFFFFFFFFFFFFFFF:FFFFFFFFFFF
@A00721:190:HGJW7DSXY:4:2163:9914:5415_1:N:0:TCTGCAAG+GCTGCACT
TTTCATAAGATTATCAGTAATCCTGATTCTACTACGGATTCATGGACCTTCCATCCTGTACCTCTTCT
GGAGAGACGTATCTCAAAGATCGACTTCGAGACAGGTGATATTGTTGAGCGAGAGCGACCAAGATCTA
AAT
+
FFFFFFFFFFFFFFFFFFFFFFFFFFFFFFFFFFFFFFFFFFFFFFFFFFFFFFFFFFFFFFFFFFFFFF
FFFFFFFFFFFFFFFFFFFFFFFFFFFFFFFFFFFFFFFFFFFFFFFFFFFFFFFFFFFFFFFFFFFFFFF
FFF
@A00721:190:HGJW7DSXY:4:1314:3803:1908_1:N:0:TCTGCAAG+GCTGCACT
CGTGGCGCTTTATTTTAAAAAGCTCCCTCCTCTCGCAAGTTTGATCTGATACTTATCACGAAAGGACT
TGAGTCTTTTGATCAGTCTCTGACTTGACCATCTTTCATTTTAAATGACGATCATTAAGACTCTCTTT
CTT
+
FFFFFFFFFFFFFFFFFFFFFFFFFFFFFFFFFFFFFFFFFFFFFFFFFFFFFFFFFFFFFFFFFFFFFF
FFFFFFFFFFFFFFFFFFFFFFFFFFFFFFFFFF:FFFFFFFFFFFFFFFFFFFFFFFF:FFFFFFFFF
FFF
@A00721:190:HGJW7DSXY:4:1274:28483:17315_2:N:0:TCTGCAAG+GCTGCACT
CTTTGCACTGATTTTTCCTCATCAGCTTGTGTTTTACCTAATCTGTGTTTTTACGATAACAACTATCA
GTTGATTAAGACTCTTCCGAAAGGCGAACTTTGGGAATTTGAAGCTCA
+
FFFFFFF:::FFFFFFFFFFFFFFFFFFF:FFFFFFFFFFFFFFFFFFFFFFFFF:FFFFFFFFFFFF
FFFFFF::FF:FFFFFFFF:FFFFFFFFFFFFF:FF,FFFFFFFFFF
```

@A00721:190:HGJW7DSXY:4:1471:2727:13291_1:N:0:TCTGCAAG+GCTGCACT
CGTTTGACCGTGACGAACTGTGGTGTTCTTGATCTACGTTACGGATATACTCTCCTTGAGTTCACTCG
TAAGGACACTACCACTTATTACGATATTTCTTTAGTCGATGGGTTTTCGACTCCTTTAAGCGTCTTCT
GTT
+
FFFFFFFFFFFFFFFFFFFFFFFFFFFF:FFFFFFFFFFFFFFFFFFFFFFFFFFFFFFFFFFFFFFF
FFFFFFFFFFFFFFFFFFFFFFFFFFFFFFFFFFFFFFFFFFFFFFFFFFFFFFFFFFFFFFFFFFFF
FFF
@A00721:190:HGJW7DSXY:4:1318:5159:12336_2:N:0:TCTGCAAG+GCTGCACT
TTTCTTCTAATGTGGCCTGAACTACGTTTCACTTTGTGTGTACTTGTCTCTATTTTAAGTTTTGTCTT
CCTTGTTTGTCTTATTCTATTAATCACTCATTTTAGTGAGATTATTAA
+
FFFFFFFFFFFFFFFFFFFFFFFFFFFFFFFFFFFFFFFFFFFFFFFFFFFFFFFFFFFFFFFFFFFF
FFFFFFFFFFFFFFFFFFFFFFFFFFFFFFFFFFFFFFFFFFF:FFFF
@A00721:190:HGJW7DSXY:4:1368:16315:25927_1:N:0:TCTGCAAG+GCTGCACT
GTCTTCGACAACAGCAGGGGTTTTCGAATAGTGATAGCCACCCGCCTTACAGAGGGGCAGCAGCATGA
CCATAATTGGTATTGACTTTGGTACCACCTTTTCTACTGTCTGTCTCGCCTCTTCGAATGAAGTTTTA
ATG
+
FFFFFFFFFFFFFFFFFFFF:FFFFFFFFFFFFFFFFFFFFFFFFFFFFFFFFFFFFFFFFFFFFFFF
FFFFF:FFFFFFFFFFFFFFFFFFFFFFFFFFFFFFFFFFFFFFFFFFFFFFFFFFFFFFFFFFFFFF
FFF
@A00721:190:HGJW7DSXY:4:1142:6216:10222_2:N:0:TCTGCAAG+GCTGCACT
TGATAGTGAATTTATACCTACAATCATTGGTTTTTCTAATAATGATGACACTATTGTTTATGGATATG
ATGCTATTACTCATGATGGTTACGGTCAGGGAGGATTCTCAGTTTACA
+
FFFFFFFFFFFFFFFFFFFFF:FFFFFFFFFFFFFFFFFFFFFFFFFFFFFFFFFFFFFFFFFFFFFF
:FFFF,FFFFFFFFFFFFFFFFFFFFFFFFFFFFFFFFFFFFFFFFFF
@A00721:190:HGJW7DSXY:4:2274:13150:1564_2:N:0:TCTGCAAG+GCTGCACT
ACATTACCCGAAAGACGTGCTAAATTAAAACCATTGTACGATGTCGTCTGTCTCGGCGATCACTTTTC
ACTAAGAATTGGTCCTACTTTTGGACAAGGCCAACTTCGTAATCTTCA
+
FFFFFFFFFFFFFFFFFFFFFFFFFFFFFFFFFFFFFFFFFFFFFFFFFFFFFFFFFFFFFFFFFFFF
FFFFFFFFFFFFFFFFFFFFFFFFFFFFFFFFFFFFFFFFFFFFFFFF
@A00721:190:HGJW7DSXY:4:2201:11478:36182_2:N:0:TCTGCAAG+GCTGCACT
TTTATCTCTTTGGTTGTTAGGGATTTTGAGAGAGTGAAAGGCTTCAATTGTAGTGGTTTGGTCGTTTC
AGTACCCAGTCAGTATACCTCTACGCAAAGATATTTCATGAAAACTAC
+
FFFFFFFFFFFFF:FFFFFFFFFFFFFFFFFFFFFFFFFFFFFFFFFFFFFFFFFFFFFFFFFFFFFF
FFFFFFFFFFFFFFFFFFFFFFFFFFFFFFFFFFFFFFFFFFFFFFFF
@A00721:190:HGJW7DSXY:4:1433:14018:31657_2:N:0:TCTGCAAG+GCTGCACT
ATTACCTGTCTTTCATATTATGAATGAACCTTCGGCCGCTCTCTTTGCTTCGATGTTGGATGTCAAGA
AGGGTTCAGAATGGGATTCCTACGTAGTTTATGACTTTGAGATCGGAA
+
FFFFFFFFFFFFFFFFFFFFFFFFFFFFFFFFFFFFFFFFFFFFFFFFFFFFF:,FFFFFFFFFFF:FF
FFFFFFFFFFFFFFFFFFFFF:FFFFFFFFFFFFFFFF:FFFFFFFFF
@A00721:190:HGJW7DSXY:4:1114:26250:26130_1:N:0:TCTGCAAG+GCTGCACT
TCTTACGTTACGGTAATTACTATAGTGTTATTTATTCTGGTGGTGATGATAATCTTGGAGGTCGTGAC
GTTGATGAAGCTATAAGAGCGTTCTTATTACGGCGCACTTCTTTACCCGCCACCACACAACTTTCAGT
TTC
+
FFFFFFFFFFFFFFFFFFFFFFFFFFFFFFFFFFFFFFFFFFFFFFFFFFFFFFFFFFFFFFFFFFFF
FFFFFFFFFFFFFFFFFFFFFFFFFFFFFFFFFFFFFFFFFFFFFFFFFFFFFFFFFFFFFFFFFFFF
FFF

@A00721:190:HGJW7DSXY:4:2123:20690:8516_2:N:0:TCTGCAAG+GCTGCACT
TGTTACTGGAAAACCCTCGTCAATACTTTTCAATAATATACGTCTTGAACTCACTTATGACGATCTAG
TGAAATTAACGCGTCCTTTTCTGGAACGTAGTTTTACTATTATGAACA
+
FFFFFFFFFFFFFFFFFFFFFFFFFFFFFFFFFFFFFFFFFFFFFFFFFFFFFFFFFFFFFFFFFFFF
FFFFFFFFFFFFFFFFFFFFFFFFFFFFFFFFFFFFFFFFFFFFFFFF
@A00721:190:HGJW7DSXY:4:1252:16776:12258_1:N:0:TCTGCAAG+GCTGCACT
ACGTGTGCTCTTCCGATCTAACTTACCCTGATTCCGGCCGGTGGTAGCTCATTTCTACCAGGGGCTAT
TACTCTTGCTGAAACAGCTATTAAAGCTGTTGAGCGGGTAATTACACCTAAACGAGCTCGATCTGCCA
TCG
+
FFFFFFFF:FFFFFFFFFFFFFF:FFFFFFFFFFFFFFFFF:FFFFFFFFFFFFFFFFFFFFFFFFFF:
FFFFF:FFFFFFFFFFFFFFFFFFF:FFFFFFFFFFFFFFFFFFFFFFFFFFFFFFFFFFFFFF:FFFF
FFF
@A00721:190:HGJW7DSXY:4:1114:23023:9298_2:N:0:TCTGCAAG+GCTGCACT
CACTCTTTTCTGCTACTTTAACGGAGCGTAGTGTACTCTTCGTCGATTGTATCAACAGTAACATTACT
TCTAATAAAGGTCTCTATCAAGCTCGTATTATCGTCGCTGCTGGATCT
+
FFFFFFFFFFFFFFFFFFFFFFFFFFFFFFFFFFFFFFFFFFF:FF:FFFFFFFFFFFFFFFFFFFFF
FFFFFFFFFFFFFFFFFFFFFFFFFFFFFFFFFFFFFFFFFF:FFFFFF
@A00721:190:HGJW7DSXY:4:1274:12789:28588_1:N:0:TCTGCAAG+GCTGCACT
AAGGCTAAACTCCAAGGTAATTATGAAGACTGTGATTTATCTTTCACTCTGGCCTTGTATGAGGGTGT
TTCTAATCGTAACATTAATAATTTCAATCTTCTTAAAAAGAAGATAGAGAGTCATACTTTGGGCTATC
GTT
+
FFFFFFFFF:F:FFF:FFFFFFFFFFFFFFFFFFFFFFFFFFFF:FFFFFFFFFFFFFFFFFFFFFFFFFF
FFFFFFFFFF::FFFFFFFFFFFFFFFFFF:FFFFF:FFFFFFFFFFFFFFFFFFFFFFFFFFFFFFFF
FFF
@A00721:190:HGJW7DSXY:4:1342:24921:26240_2:N:0:TCTGCAAG+GCTGCACT
GATAGCCAATATTGAAGTGGACACCTCTGGTAGTATCGAAGTAAAGATAACCAATAAGGAAGGAGCTT
ATACTATGGTTGAATCAACTAAGCATACTACTTTTGCTTTGATTACTC
+
FFFFFFFFFFFFFFFFFFFFFFFFFFFFFFFFFFFFFFFFFFFFFFFFFFFFFFFFFFFFFFFF:FFFF
FFFFFFFFFFFFFFFFFFFFFFF:FFFFFF::FFF:FFFFFFFFFFF:F
@A00721:190:HGJW7DSXY:4:2342:1624:36808_1:N:0:TCTGCAAG+GCTGCACT
CTTCCGATCTGTTAAAGAGTCGATTAAACAAGTTGTACTTATCAATGATTTTATCGCTGGTTGTTATC
TCGAAAAGTCTCTTCTAAAAGGAACGTCAGTTCTAACCCAAAGAGAATATCTAAAATATTTACAAAAT
CTT
+
:FFFFFFFFFFFFFFFFFFFFF:FFFFFFFFFFFFFFFFF:FFFFFFFFFFF:FFF:FFFFFFFFFFFF
FFFFFFFFFFFFFFFFFFFFFFFF:FFFFFFFFFFFFFFFFFFFFFFFFFFFFFFFFFFFFFFFFFFFFF
FFF
@A00721:190:HGJW7DSXY:4:1425:9444:6825_1:N:0:TCTGCAAG+GCTGCACT
GATCTGTTGCTTGTCGCTTCCTCCTTTAATGGCACTACCTCGGTACAATCTTACTTGGAACGAAGGCG
ACCTGTTCCGGAGTTTTTACGGGATCCGGAATGTTCTGTCTATATTAGGTGAAGCGAAATACGATTTC
GAA
+
FFFFFFFFFFFFFFFFF:FFFFFFFFFFFFFFFFFFFFFFFFFFFFFF:FFFFFFFFFFFFFFFFFFFFFF
FFFFFFFFFFFFFFFFFFFFFFFFFFFFFFFFFFFFFFFFFFFFFFFFFFFFFFFFFFFFFFFFFFFF
FFF
@A00721:190:HGJW7DSXY:4:1261:2582:3646_1:N:0:TCTGCAAG+GCTGCACT
TCGTACCTTGATGCTGACTTTCGTCAATATGATTTGATCTGGTTGCTCAATTCTAAGCGTGTTCCGCT
TAGTGCGAGGCCTCTCTTCTACGAGTATTGCTATTTATACGGTTACGAGAAGAAGGTTCGAAAGTTTG
AGA

+
FFFFFFFFFFFFFFFFFF:FFFFFFFFFFFFF:FFFFFFFFFFFFFFFFF:FFFFFFFFFF:FFFFFF
FFFFFFFFFFFFFFFFFFFFFFFFFFFFFFFFFFFFFFFFFFFFFFFFFFFFFFFFFFFFFFF,FFFFF
FFF
@A00721:190:HGJW7DSXY:4:1220:3070:36370_2:N:0:TCTGCAAG+GCTGCACT
TACTAAATACTAGATATTTGGATGATCTTGATAAAGAACTTCATGAATTTTCAGAGGATGAAAAATTT
TCTTCCTTGGATTTTAATATAAAGAATCTTTACCAGGACTCCTACTAC
+
FFFFFFFFFFFFFF:FFFFFFFFFFFFFFFFFFFFFFFFFFFFFFFFFFFFFFFFFFFFFFFFFFFFF
FFFFFFFFFFFFFFFFFFFFFFFFFFFFFFFFFFFFFFFFFFFFFFFF
@A00721:190:HGJW7DSXY:4:2213:29215:11475_2:N:0:TCTGCAAG+GCTGCACT
TCTGATCGTAAGAAGTTTATAAGGAACATATCCATTTGTTGTAATAGGGTCATCAACTTAGAACTCGA
AACTCTAAGTGGTAATACTGTCTTTCCAAGCTTACCTGTATCTTCAAT
+
FFFFFFFFFFFFF:FFFFFFFFFFFFFFFFFFFFFFFFFFFFFF:F:FFFFFFFFFFFFFFFFFFFFFF
FFFFFFFFFFFFFFFFFFFFFFFFFFFF:FFFFFFFFFFFFFFFFFFF
@A00721:190:HGJW7DSXY:4:2374:31891:32362_1:N:0:TCTGCAAG+GCTGCACT
TTCGATAGGGGACATTTTATCGTGGGCTTTCCGTAAAGGAAGATCTGTTATCGACGGTTTATCGTTTA
CGAAGTATCACTCCACCGGTGAATGGTTTAAGAATACTATAAATAGAATAGCTATTACTACTAATGGC
GTT
+
FF:FFF,FFF:F::FFFFFF:FF,:F:FFFF,,FFFF,FFFFFF:FFFFFF,FFF:FFFFFFFFFFF:
F::FFFFFF:FF:::::F,:FFFF::FFF:FFFFFFFFFFF:FFFFFFFFFFF:FFF:FF:FFFFFFF
FFF
@A00721:190:HGJW7DSXY:4:2319:11550:33426_2:N:0:TCTGCAAG+GCTGCACT
TAGTGACATCTGGTCAATTTCGTTTTTAGGTGGTCTTTATTCTATTATTGATTTTTATGAGATTGATA
CGTTAACAGAAGATGATGTTAGAGTAGAATTCCTGAAGAGTCCTTCCG
+
FFFFFFFFFFFFFFFFFFFFFFFFFFFFFFF:FFFFFFFFFFF:FFFFFFFFFFFFFFFFFFFFFFFFF
FFFFFF:FFFF:FF:FFFFFFFFFFFFFFFFFFFFFFFF:FFF:FFF,F
@A00721:190:HGJW7DSXY:4:1219:9896:22200_2:N:0:TCTGCAAG+GCTGCACT
CTAGGATTATGTCTGTGTTGAAAGACTTTCTACCGATAAGGGTCACTAGTCTTACACAATTAAGAAAT
AATCCTACATTGACTGATTATCTAAAGATTGAAGGAAAATTTTATCCT
+
FFFFFFFFFFF:FFFFFFFFF:FFFFFFF:FFFFFFFFFFFFFFFFFFFFFFFFFFFFFFFFFFFFFFF
FFFFFFFFFFFFFFFFFFFFFFFFFFFFFFFFFFFFFFFFFFFFFFFF
@A00721:190:HGJW7DSXY:4:1102:9796:11412_1:N:0:TCTGCAAG+GCTGCACT
GACTTTCCTACGATACAAATGATAACTTCTAATTTACGTTTGCTACTCAGAGCTGGTCCTCTCAGCAA
AGGGATGCATGGTGTTATCTCCTCAGGAGTCCCGGCATTTGATAATAATGACGAGACTTTCGTCATAA
TCG
+
FFFFFFFFFFFFFFFFFFFFFFFFFFFFF:FFF:F:FFFFFFFFFFFFFFFFFFFFFFFFFFFFFFFFF
FF:FFFFFFFFFFFFFFFFFFFFFFFFFFFFFFFFFFFFFFFFFFFFFFFFFFFFFFFFFFFFF:FFF:
FFF
@A00721:190:HGJW7DSXY:4:1310:13512:1877_1:N:0:TCTGCAAG+GCTGCACT
GATTACACGGTACCAGCGCATCTCGTGTCGAGCCTCGACCCGATTATATGGTATTAACTTATAAAGGT
GAGGAGACCATTTTCTTGCTGAAATTTATAGAAGATTATTTTGACAGAATCAATAAGAATCATCGATT
CTC
+
FFFFFFFFFFFFFF:FFFFFFFFFFFFFFFFFFFFFFFFFFFFFFFFFFFFFFFFFFFFFFFFFFFFFF
FF:FFFFFFF:FFFFFFFF:FFFFFFFFFFFFF,FFFFFFFF:F:F:FFFFFFFFF:FFF:FF,FFFF
,F:
@A00721:190:HGJW7DSXY:4:1659:17219:5321_1:N:0:TCTGCAAG+GCTGCACT
CTAGATGGATCCACTCCTCGAGTTATCATGATGTTAGTATGTTATATACTGATTTACAACGATATAGG

AGTAATCTACGAGAAGATTCTAGAATTCTTAATTATAATATTCAACCTCAAAAGTTACGTGTTTACAG
TAA
+
FFFFFFFFFFFFFFFFFFFFFFFFFFFFFFFFFFF:FFFFFFFFFFFFFFFFFFFFFFFFFFFFFFFFF
FFFFFFFFFFFFFFFFFFFFFFFFFFFFFFFFFFF,FFFFFFFFFFFFFFFFF:F:FFFFFFFFFFFF
FFF
@A00721:190:HGJW7DSXY:4:1158:4399:18724_2:N:0:TCTGCAAG+GCTGCACT
CATTTTGAGAACTAACTATGTTATGTTCTCTGATCATTTTGACAATCTCTTCTTATAATAAATAAATA
AATAATATTACTAATGGCAAATAGTGGAAAGGCTCCAGTCAACACTAG
+
FF,FFFFFFFFFFFFFFFFFFFFFFFFFFFFFFFFFFFFF:FFFFFFFFFFFFFFFFFFFFFFFFFFF
FFFFFFFFFFFFFFFFFFFFFFFFFFFFFFFFFFFFFFFFFFFFFFFF
@A00721:190:HGJW7DSXY:4:1226:22670:7780_2:N:0:TCTGCAAG+GCTGCACT
TACGATGACGTGAGAATAGGTGAAGGGATAGTTTTTACCTCGACCAACATTAAGGCGGCGGCGACTAA
TCTTAAAGCTTCACCCAAAATGAAGGACACTTCGAAACTGAATAATGA
+
FFFFFFFFFFFFFFFFFFFFFFF:FFFFFFFFFFFFFFFFFFFFFFFFFFFFFF:FFFFFFFFFFFFF
FFFFFFFFFFFFFFFFFFFFFFFFFFFFFFFFFFFFFFFFFFFFFFFF
@A00721:190:HGJW7DSXY:4:1413:24795:4351_1:N:0:TCTGCAAG+GCTGCACT
GGCTCTAAAGGACACTATACAATATCCAGACGACGACGAAGCTTATACTACTTTAATTTTGGGTATTA
TGTGGAGATATCGTGATATCAGTACGTCCAGATCCTCTGCTTTTACCGATAACGATAAATTCGTCATC
AAA
+
FFFFFFFFFF:FFFFFFFFFFFFFFFFFFFFFFFFFFFFFFFFFFFFFFFFF::F::FFFFFFFFFFF
FFFFFFFFFFFFFFFFFFFFFFFFFFFFFFFFFFFFFFFFFFFFFFFFFFFFFFFFFFFFFFFFFFFF
FFF
@A00721:190:HGJW7DSXY:4:1155:25690:27602_1:N:0:TCTGCAAG+GCTGCACT
TTGGTGCTTAGGGATATAATGAATTCAGCACCACATTTTGGTCAAGTCAATCCGTTGAGAAAATTTCT
TAGAGGATATTCGCCATTGTGGTACGAATTGGTCCAACATTTACCACCAAATTCTTTTGATACAACTC
TAG
+
F,FFFFFFFFFFFF:FFFFFFFFFFFFFFFFFFFFFFFFFFFFFFFFFFFFFFFFFFFFFFFFFFFFF
FFFFFFFFFFFFFFFFFFFFFFFFFFFFFFFFFFFFFFFFFFFFFFFFFFFFFFFFFFFFFFFFFFFF
FFF
@A00721:190:HGJW7DSXY:4:1525:17038:14904_1:N:0:TCTGCAAG+GCTGCACT
TGCCTGCGGAATACGCTTGCTATTCACCAGATTGGTTCGAACCCGTTGATGCTATGTCAGAAGAAGCT
ATTCAAGCTTGTCGTTTAAAGACTGCAGAGGCTGTCAAACAAGCCCAGACTCAAGGAGTCCAATCAAA
GGT
+
FFFFFFFFFFFFFFFFFFFFFFFFFFFFFFFFFFFFFFFFFFFFFFFFFFFFFFFFFFFFFFFF:FFF
FFFFFFFFF:FFF:FFFF:FFFFFF:FFFFFFFFFFFFFFFFF:FFFF:FFFFFFFFFFFFF:FFFF:
FFF
@A00721:190:HGJW7DSXY:4:1423:23710:34695_2:N:0:TCTGCAAG+GCTGCACT
TGGGTGATAAAAATTATTTAGAGATCTTTTCTACTTACAAAGGACAATTCAGTTTCATTAATTCTACG
GAAAAGTCCGGAGTATATTGTATGTTAATAGATAGTGGGATAATAGTT
+
FFFFFFFFFFFFFFFFFFFFFFFFFFFFFFFFFFFFFFFFFFFFFFFFFFFFFFFFFFFFFFFFFFFF
FFFFFFFFFFFFFFFFFFFFFFFFFFFFFFFFFFFFFFFFFFFFFFFF
@A00721:190:HGJW7DSXY:4:1462:28628:11209_1:N:0:TCTGCAAG+GCTGCACT
GTAGTGAACGCGTTCGTAGAACCAAGTACTTTCCGTCTGAAGAGTTTAGAGCTGCTATTGATTATGTA
ACCGATAGGAGTTGTATAGTTGAAGATGTGGCTTTAGACGATAATCCTTTAAGGACTATGTTGACTGA
CGA
+
FFFFFFFFFFFFFFFFFFFFFFFFFFFFFFF:FFFFFFFFFFFFFFFFFFFFFFFFFFFFFFFFFFFF

FFFFFFFFFFFFFFFFFFFFFFFFFFFFFFFFFFFFFFFFFFFFFFFFFFFFFFFFFFFFFFFFFFFF
FFF
@A00721:190:HGJW7DSXY:4:1378:12436:14857_2:N:0:TCTGCAAG+GCTGCACT
TTGTCTACGTTGCAGATCGCCACTCCAAACACTATATACATTATAAAATATAAAAATAAGGGAGTCAT
AACTTTTGTTACCAAATTCTTGGCTTTCAACGAAGTCTCTGATTTTAT
+
FFFFFFFFFFFFFFFFFFFFFFFFFFFF:F:FFFFFFFFF::FFF:FFFFFFFFF:FFFFFF:F,FFF
FFFF:,FFFFFFFFFFFFFFFFFFFFFFFFFFFFFFFFFFFFF:FFFFF
@A00721:190:HGJW7DSXY:4:1315:32325:17644_1:N:0:TCTGCAAG+GCTGCACT
TAACGATCTCTCGGTCATTTACTCTGTACTTGATCTACGTCGTTTAACTGTCGGAACAACCTTTACTT
AGAATGACTCTATCTTTTCAGACACTTCTTATATTATTTACAATAGTTAATATCATATTAGTAATACT
CAC
+
FFFFFFFFFFFFFFFFFFFFFFFFFFFFFFFFFFFFFFFFFFFFFFFFFFFFFFFFFFFFFFFFFFFF
FFFFFFFFFFFFFFFFFFFFFFFFFFFFFFFFFFFFFFFFFFFFFFFFFFFFFFFFFFFFFFFFFFFF
FFF
@A00721:190:HGJW7DSXY:4:1202:23547:33630_2:N:0:TCTGCAAG+GCTGCACT
GCTTTTTCTTAGCTAATAGATTAATAAAGGATGAGTACGAAAACCAACTCTTCGTTACGAGACCTGAT
GGTAGAATAGATTTAGTCTCTACACCCATCTCAACGAGGATAAAGCGA
+
FFFFFFFFFFFFFFFFFFFFFFFFFFFFFFFFFFFFFFFFFFFFFFFFFFFFFFFFFFFF:FFFFFFF
FFFF:FFFFFFFFFFFFFFFFFFFFFFFFFFFFFFFFFFFFFFFFFFF
@A00721:190:HGJW7DSXY:4:2221:30427:7686_1:N:0:TCTGCAAG+GCTGCACT
AATACACGAAGAGATAACCCGAAAACGACTCGAAACCGAGACGCCGTAGATCAAAGTCCCTTCTAACT
TTAGATTATGAATCCTGCCTTTACTCA
+
FF,::,:,F,,:,F,,,,F:,,F,FFFFFFFFFFFFFFFFFFFFFFFFFFFFFFFFFFFFFFFFFFFF
FFFFFFFFFFFFFFFFFFF:FFFFFFF
@A00721:190:HGJW7DSXY:4:1239:9833:26725_2:N:0:TCTGCAAG+GCTGCACT
TTTTTATAATTTATAACCTTCTTATACTTTTGGGTATGTTTTGTGAAAAACATGAGATACCCAAGTCT
ATCGAAGACATTATCTCTTATGTACTTATTGTAACGATCATTCTTTCG
+
FFFFFFFFFFFFFFFFFF:FFFFFFFFFFFFFFFFFFFFFFFFFFFFFFFFF,FFFFFFFFFFFFFFFFFF
FFFFFF::FFFFFFFFFFFFFFF:FFFFFFFFFFFFFFFFFFFFFFFF
@A00721:190:HGJW7DSXY:4:2102:29243:36699_1:N:0:TCTGCAAG+GCTGCACT
TTCTTATTCTTTTATTCCTTTAACTGTGTATGAGGAGGATTTATAATGCTTTTCGATAGTGATTGTTA
TCAATTTAAATACATTACTGTTTCAAATCATCTTTACTCACAGACTCCGAAAATGGTTTCAAATGAGG
TCA
+
FF:FFFFFFFFFFFFF,FFF:FFFF,FFFFFFFFFFFFFFF:FFFFF::FFF:FFFFF::FFFF:FFF
FFFFFFFFFFFF,FFFF:FF:FFFFFFFFFFFFFFFFFFF,F:FF:F,FFFF:,:FFFF:FFFF:F,:
FFF
@A00721:190:HGJW7DSXY:4:2102:29243:36699_2:N:0:TCTGCAAG+GCTGCACT
TATTAAACGAACTATTATCCGATTCTTCGAGGGTATCACTACCACTGATCTGCCGACTTTAGTCAAGG
AACTTCAATTCCTTCGATGTTTATATTTTTCTATAAGGGAAAATAGCA
+
FFFFFF:,FFFFFF,F,FFFFFFFFFFFFFFFFFFFFFFFFFFFFFFFFFFFFFFFFFFFFFFFFF:F
FFFFFFFFFFFF:FFFFFF,FFF:FFFFFFFFFF,FFFFFFFFF:FF:
@A00721:190:HGJW7DSXY:4:1318:1750:31078_2:N:0:TCTGCAAG+GCTGCACT
TTCGACAAATTTTAGAACTTTATTTCAGAACTCTCTCCGATCACCTTGGTGTGATCTCCCGGAGGATC
TTTTCAATAAGATGTATGATGATAATCTACTATTTTTAAATAGTGATA
+
FFFFF:FF:FFFFFFFFFFFFFFFFFFFFFFFFFFFF:FF,FFFF,F,F,FFFFFFFFFFFFFFFFFF
FF,FFFFFFFFFFFFFFF:FF:FFFF:FFFFFFFF:FFFFFFFF:FF

```
@A00721:190:HGJW7DSXY:4:2457:23809:18082_1:N:0:TCTGCAAG+GCTGCACT
ACTTTATCAAAGATTTGGCTTGGTCTATTTACCGCAAAAAGTTGGAAGAGGTCTATGATATTTATTTT
ACTTATACTTTAAATAGCACCGATTATAACTTGACTGTTGATAATATTACAATAGCTCTTTCTCAGAA
GAT
+
FFFFFFFFFFFFFFFFFFFFFFFFFFFFFFFFFFFFFFFFFFFFFFFFFFFFFFFFFFFFFFFFFFFF
FFFFFFFFFFFFFFFFFFF:FFFFFFFFFFFFFFFFFFFFFFFFFFFFFFFFFFFFFFFFFFFFFFFF
FFF
@A00721:190:HGJW7DSXY:4:1431:5999:22686_1:N:0:TCTGCAAG+GCTGCACT
ACCGGTGAACACGTCGGTAGCATTGATTTACTTTTACCGTTAGAACTGAAAAAGATTCTTTTCGAAAA
GAAACCTATAAGTTACAAACGATTCTTCGACTATATTAAAGAAATAAAAATACTTCAAGTACCTGAAC
TTT
+
FFFFFFFFFFFFFFFFFFFFFFFFFFFFFFFFFFF::FF,FFFFFFFFFFFFFFFFFFFFFFFFFF:FF
FFFFFFF:FF:F:F:F:F:FFFFFFFFFFFFFFFFFFFF:FFFFFFFFFFFFFFFFFFFFFFFFFFFFF
FFF
@A00721:190:HGJW7DSXY:4:1418:2239:35837_1:N:0:TCTGCAAG+GCTGCACT
ATGGTATAATGTGTCTTACAAATAGAACCTTCACTCTCGGTAATGAGAGTGTATTCCTTAATATTCTA
GTAACCGCTATGTGGGCATTACTAGGATTTATCACACTCCTAAATTTGATAGCAATTTATGTTTTAAA
TGC
+
FFFFFFF,:FFFFFFFFFFFFF,FFFFFFFFF:FFFFFFFFFFFFFFFFFFFFFFFFFFF:FFFFFFF
FFFFFFFFFFFFFFFFFFFFFFFFFFFFFFFFFFFFFFFFFFFFFFFFFFFFFFFFFFFFFFFFFFF:F
FFF
@A00721:190:HGJW7DSXY:4:1602:32551:2722_2:N:0:TCTGCAAG+GCTGCACT
AAAAACTCCATTGTATAGTTCAAGGAGTACTTAATTCAATAGCTTTAGTATTATTTACTATCGCCTTT
ATTGAAAATTGTATAAGAGTTAATTGCTTACACAAAAGTGACGCGCGA
+
FFFFFFFFFFFFFFFFFFFFFF:,FF:FFFFFFFFFFFFFFFFFFFFFFFFFFFFFFFFFFFFFF:FFF
FFFFFFFFFFFFFFFFFFFFFF:FFFFFFF:FF:F::FFFFFFFFFF
@A00721:190:HGJW7DSXY:4:2656:29930:7451_1:N:0:TCTGCAAG+GCTGCACT
GGGGAGAATGAACTGCCGCTTCCCGGAAGAAACGATCTATTTACAGAGTATTTAGAGTAGTCCAAAGT
TTGTATAAATTTATATGATTTTCTATATATATACCACATATATATATATATAAAATCCTATAAATTTG
TAC
+
FF:FF:FF::,,F::FFF:FFFFF:,FF,FFFFFF:FFFFFFFFFFF:FFFFFFFF:FFFFFFFF:FF
FF::FFFFFFFFFFFFFFFFFFFFFFFFFFFFFFFFFFFFFFFFFFFFFFFFFFFFFFFFFFFFFFFF
FFF
@A00721:190:HGJW7DSXY:4:2335:2582:20212_2:N:0:TCTGCAAG+GCTGCACT
GAAGAAAACCACTCCCTTCAATTCAATAACATAACATACCATACACTCCCCAAACATAACATACCATA
ACCTACCATGACCAATCATCGCTCCAACTGGCACGCCAGACGTAGGGT
+
FFFFFFFFFFFF,FFFFFFF::FFF:,FFFF:FFFFFFFF:F:FFFF:FFFFFFFFFFFFFFFFFFFF
FFFF,F:F:FFFFFFFFFFFFFFFFFFFFFFFFFF:FF:FF:FFFFF:F
@A00721:190:HGJW7DSXY:4:2338:2257:35556_2:N:0:TCTGCAAG+GCTGCACT
ACCGCCGGTCGGCAGATCGGCACCACTGGTCTCTACGTTCAGGGACCCGCTGTGCAGAATAACCTTCC
CAAATCTAGATCTGCACAGAAGAAGGCTGAAGGTCGTTACCGGACGAC
+
FFFFFFFFFFFFFFFFFFFFFFFFFFFFFFFFFFFFFFFFFFFFFFFFFFFFFFFFFFFFFFFFFFFF
FFFFFFFFFF:FFFFFFFFF:FFFFFFFFFFFFFFFFFFFFFFF:FFFF
@A00721:190:HGJW7DSXY:4:1136:6099:13714_1:N:0:TCTGCAAG+GCTGCACT
GACGTGTGCTCTTCCGATCTCCGCGACCACCGAGGTCGCTCGTAACATTGACGGCAGTCCCGTCATCT
CTGTGGGAACTGAGGCAGTACCTCAGTCAAAAGTCACATTCACCACTCCCATGCCAATCAATGATGTG
ACT
```

```
+
FFFFFFFFFF:FFFFFFFFFF,FFFFFFFFFFFFFFFFFFFFFFFFFFFFFFFFFFFFFFFFFFFF
FFFFFFFFFFFFFFFFFFFFFFFFFFFFFFFFFFFFFFFFFFFFFFFFFFFFFFFFFFFFFFFFFFFFFF
FFF
@A00721:190:HGJW7DSXY:4:2545:11731:31955_2:N:0:TCTGCAAG+GCTGCACT
TCAATACGTCGTGGGAGGTACTGAAGCCTTCCCCTCCGCTGGCACGAAGAACACTCCCAAGCCAATCA
ATAGTTCTTCTGACCAGAATGAAAAGGGGTTTACCAGGGTAGTCTCTA
+
FFFFFFFFFFFFFFFFFFFFFFFFFFFFFFFFFFFFFFFFFFFFFFFFFFFFFFFFFFFFFFFFFFFFFF
FFFFFFFFFFFFFFFFFFF:FFFF:FFFFFFFFFFFFFFFFFFFFFFFF
@A00721:190:HGJW7DSXY:4:1334:15664:23891_1:N:0:TCTGCAAG+GCTGCACT
AGAGGGGAAATGTCCCCAAAACTGAGAAGAATAAAGGGAAAGGTCCATCAGGACCTAAGAAGGGGAAA
TTACCCCTAATTCTGAAGGAAGATCTCCCACCATCTGGGGTTGGGAATAAGAAATCTGCACGGGCCAA
AGA
+
FFFFF:FFFFFFFFFFFFFFFFFFFF:FFFFFFFFFFFFFF:FFFFFFFFFFFFFFFFFFFFF:FFF,
FFFFFF:FFFFFFFFFFFFFFFFFFFFFFFFFFFFFFFFFFFFFFFFFFFF:FFFFFFFFFFFFFFF
FF:
@A00721:190:HGJW7DSXY:4:1246:2618:18928_1:N:0:TCTGCAAG+GCTGCACT
CGAAGTCGCTGAAGGCGGCTTGGGTCATTAAGGGTAGCTGTAAAGTTGCGCCTGCCGACCCCATCTTT
GAGACCAAGACTCTCTACAGTAGGTTCGTGCCTACTATGAGCAAATCTGTTGAATTCGTCACTGAGGA
CGA
+
FFFFFFFFFFFFFFFFFFFFFFFFFFFFFFFFFFF:FFFFFFFFFFFFFFFFFFFFFFFFFFFFFFFFFF
FFFFFFFFFFFFFFF:FFFFFFFF:FFFFFFFFFFFFFFFFFFFFFFFFFFFFFFFFFFFFFFFFFFFFF
FFF
@A00721:190:HGJW7DSXY:4:1124:3549:14121_1:N:0:TCTGCAAG+GCTGCACT
ACTTTTGGCTACTGGCGACAAACTGAGAGCAGTCGCTGGGGAAAGAGAATTGCGTGGGTTGAAGGCCG
ACGCTTCGAGCTTGAGTGTGACGAGGCCGGTGACGTTTTTGTCAACGGCGTTTGGAGGATTTTATCCG
CGC
+
FF:F,FFFFFFFFFFFFFFFFFFFFFFFFFFFFFFFFFFFFFFFFFFFF:FFFFFFFFFFFFFFFFFF
FFFFFFFFFFFFFFFFFF:FFFFFFFFFFFFFFFFF:FFFFFFFFFFFFFF:FFFFFFFFFFFFFFFFFF
FFF
@A00721:190:HGJW7DSXY:4:1304:20889:31782_2:N:0:TCTGCAAG+GCTGCACT
GCGAAGATCCCCTATGGAGTCTTGAAGGACTTAGGTTGGCATATTTATGCCGACGATAATAAGCATGT
CACCCTTAAGCACCGAGGTAAGCTCGGGACTTACAAGAGAGAGTGTGA
+
FFFFFFFFFFFFFFFFFFFFFFFFFFFFFFFFFFFFFFFFFFFFFFFFFFFFFFFFFFFFFFFFFFFFFF
FFFFFFFFFFFFFFFFFFFFFFFFFFFFFFFFFF,FF:FFFFFFFFFFFF
@A00721:190:HGJW7DSXY:4:1216:28266:17409_1:N:0:TCTGCAAG+GCTGCACT
CACTATTTTTGCCCAGAATGCGGGCTTTCTGACTGATGAGGTTAAAACTCTTGCTCCTTCTGTGGGTG
AGGGTTTTTGTTATATTCACTGGTTCTGGGAGGCTGCTTTCCTCCATAATTACAACCTCTCTTTAGAG
AGC
+
FFFFFFFF,FFFFFFFFFFFFFFFFFFFFFFFFFFFFFFFFFFFFFFFFFFFFFFFFFFFFFFFFFFFF
FFFFFFFFFFFFFFFFFFFFFFFFFFFFFFFFFFFFFFFFFFFFFFFFFFFFFFFFFFFFFFFFFFFFFF
FFF
@A00721:190:HGJW7DSXY:4:1275:4029:37027_2:N:0:TCTGCAAG+GCTGCACT
AGTTGGGCAAATTCCCACATATGGTAGACGTTGTCACACTTTTCTTGAAAATTTTTCCTTTCCTCCGT
CATTGGGGATTTCGATGTGAGAAGGTGGAAGGTGTCAAGGGTCTTCTT
+
F::FFF:FF:FF:F:FF,FFFFFFFFF:FFF:FFFFFFFF,:FFFFFFFFFF:,FF:FFFFFFFFFFF
,F,,FFF:FF:::FFFFFFFFF,:F,FFF,FF,FFF:FFF,FFFFFFF
```

```
@A00721:190:HGJW7DSXY:4:1269:18005:1360_2:N:0:TCTGCAAG+GCTGCACT
TTTCAGGACCTAATCACATCATGATTGATAGTTACGGCTCGTATCGAGTTGGAGGGAGGAAGTTCTCC
GAAATTAAAGACGGGGGGATTAAGAGTCCAGCTGTAACTGTCATTTCA
+
FFFFFFFFFFFFFFFFFFFFFFFFFFFFFFFFFFFFFFFFF,FFFFFFFFFFFFFFFFF:FFFFFFF
FFFFFFFFFF:FFFFFFFFFFFFFFFFFFFFFFFFFFFFFFFFFFFFF
@A00721:190:HGJW7DSXY:4:1226:13241:8296_2:N:0:TCTGCAAG+GCTGCACT
AAGAAAACTTCGTCAGAAGATGAGGAAGTCTAAGAGGAGGAGTTTGAGGAAGGCTCTAGAGAAGATCT
CACCACCTGGAGAAGGCAAGTGGAGAGTCCAGAAGTTGATGAAGAAGG
+
FFFFFFFFFFFFFFFFFFFFFF:FFFFFFFFF,FFFFFFFFF:FFFFFFFFFFFFFFFFFFFFFFFFF
FFFFFFF:FFFFFFFFFF:FFFFFFFFFFFFFFFFFFFFFFFF:FFFFF
@A00721:190:HGJW7DSXY:4:1363:22553:34225_2:N:0:TCTGCAAG+GCTGCACT
CAGGGGTTACCACTTCCTAAGGTGGATGAGGGAGGTTGTTTCTTTGGAGTTTTAGATAATCTTTTGAG
AAGGGGTCATAGAGCCAGCAAGAGAAGCTTATATGCTGGTTTAAGTGA
+
FFFFFFFFFFFFFFFFF:FFFFFFFFFFFFFFFFFFFFFFFFFFFFFFF:FFFFFFFFFFFFFF:FFFF
FFFFFFFFFFFFFFFFFFFFFFFFF:FFFFFFFFF:FFF:FFFF:FFFF
@A00721:190:HGJW7DSXY:4:1414:25834:25254_2:N:0:TCTGCAAG+GCTGCACT
CCTTGAGAAGACAAGCCACATTAAGATATAATTTAGGTCAACTTGGGGTTTCGAATGTCATTAGTGCC
TTACGATTGAAAGAGGTCCTTTTGAGTATCGACAGATCTTTCGATCTT
+
FFFFFFFFFFFFFFFFFFFFFFFFFFFFFFFFFFFFF:FFFFFFFFFFFFFFFF:FFFFFFFFFFFFF
FFFFFFFFFFFFFFFFFFFFFFFFFFFFFFFFFF,FFFFFFFFFFFFFF
@A00721:190:HGJW7DSXY:4:2366:17788:3834_2:N:0:TCTGCAAG+GCTGCACT
TTAACGTGGCAGAATTCGGTAACTTACCATATTGTTCCTTCCGGGAAGTTGTGGGATTTAACTTTGAC
TCAACTACTCCGATCAGATCCGGATGGTTTGATTGGTATGGACTACTC
+
FFF,FFFFFFFFFFFFFFFFFFFFFFFFFFFFFFFFFFFFFFFFFFF,FFFFFFFFFFFFFFFFFFFF
FFFFFFFFFFFFF:FFFFFFFFFF,FF:,FFFFFFF:FFFFFFFFFFF
@A00721:190:HGJW7DSXY:4:2510:19560:14920_1:N:0:TCTGCAAG+GCTGCACT
TAGTTGTCTAGGGTCATGGCAGATACTACCGATAAGGTGAAAAATATGCAGGTCAAAGATTCTGTAGC
TCTGAGGGCGGTTGAGGGAGCCGTTTTGGCCGACTTTAACTATTCTAAGAGTCTTTATGATTCAAAAC
CTA
+
FFFFFFFFFFFFFFFFFFFFFFFFFFFFFFFFFFFFFFFFFFFFFFFFFFFF:FFFFFFFFFFFFFFF
FFFFFFFFFFFFFFFFFFFFFFFFFFFFFFFFFFFFFFFFFFFFFFFFFFFFFFFFFFFFFFFFFFFF
FFF
@A00721:190:HGJW7DSXY:4:1115:1850:32534_1:N:0:TCTGCAAG+GCTGCACT
TACAGCGGCTTATTGTCGCGCAGTTTCCAGAATTGCGGTTGAAGTTCACCCATTCTATCTTGAATGCT
CACGCCGTCGCAGCCATATCGAGACTATGTTATAATCACTTGTTAAAATACAGATTTAGTGATTTAGA
TAT
+
FFFFFFFFFFFFFFFFFFFFFFFF:FFFFFFF:FFFFFFFFFFFFFFFFFFFFFFFFFFFFFFFFFFF
FFFFFFFFFFFFFFFFF:FFFFFFFFFFFFFFFF:FFFFFFFFFFFFFFFFFFFFFFFFFFFFFFFFF
:FF
@A00721:190:HGJW7DSXY:4:1275:31385:24408_1:N:0:TCTGCAAG+GCTGCACT
GGAGGCGATATTCGTTTTCATATCCAAAACTCCTTGAATGGAGTACATATTTGCAACCCTATTCTTGA
TGTGAAAGATGCATCGAGGTATACGTCTAGGAACATTGATTGGGAACGTACCTACTCACGAGACGTCA
TGA
+
FFF:FFF:FFFF,F:FFFFFFFFFFFFFFFFFFFFFFFFFFFFFFFFFFFFFFFFFFFFFFFFFFFFF
FFFFFFFFFFFFFFFFFFFFFFFFFFFFFF:FFFF:FFFFFFFFFFFFFFFFFFFFFFFFFFFFFFFF
FFF
```

```
@A00721:190:HGJW7DSXY:4:1239:32425:6856_1:N:0:TCTGCAAG+GCTGCACT
ATGTTTCGTTCTGTTACAAGAACGCTTTGGATTGTGACCATAAAGCCTCAGTTGCGATAGCTGTAGAG
GTATACGATATCTCTCTTGATGATGTAGCCACTATAATGCTTAAGAGAGAGATTGATGTATGTCATTT
CGT
+
FF,F:F::FF:FFF,:FFFFFFFF:FFFF:FFFFFFFFFF,,F:::FF:FFFFFFFFFF:F:FFFFFF
,FF:FF:FFFFFFFFFFFF:FFF:F:FFFFF:FFF,FFF:FFFFFFFFFFFFFFFFFFFFFFFFFFFF
FF,
@A00721:190:HGJW7DSXY:4:1254:2112:16297_1:N:0:TCTGCAAG+GCTGCACT
GAATTATTGGACACGATGAAAAATGACATACCCGTTCCTGACCTTGGTATCGTTATCCGTCATGGAGA
TGATGCCGCATCACTTGATTACTATATGCCCGAAGGATTGGGTTATTCTCACAACAAAGATAAGATTT
TAT
+
FFFF:FFFFFFFFFFFFFFFFFFFFFFFF:FFFFFFFFFFFFFFFFFFFFFFFFF:FFFFFFFFFFFFF
FFFFFFFFFFFFFFFFFFFFFFFF,F:FFFFFFFFFFFFFFFFFFFFFFFFFFFFFFFFFFFFFFFFFF
FFF
@A00721:190:HGJW7DSXY:4:1233:17002:32565_1:N:0:TCTGCAAG+GCTGCACT
GTTTTCCTTAAGGGCGGACAATTGTTCCATTGTGAACTCTTGGGGACTAGATTGAGCATTAATGAGTT
CCGAATAACTCGTAATAAATCTTTCTCTAGTGGACAATTCAGAATACCTATTTCCGTACCAAGAGCCA
CTG
+
FF::FF:FFFFFF:F:FFFFFFFF,FFFF:FFFFFF,F:FFFF:FFFFF:FFF:F,FFFFF:FFFFFF
F:FFFFF,,FF:FF:FFFFFFF:FFF:F,FFFFF:FFFFF:FFFFFFFFFFFFFF:FFFFF,:FFF::
FFF
@A00721:190:HGJW7DSXY:4:1657:29776:10410_2:N:0:TCTGCAAG+GCTGCACT
CGCATACCTGATTGGAAGAAGCGAGGTTCGTTCCTCAATAAAAATTTTTCTCAAGAGATTATGGTTGA
TAAAGTTTTCTTCATAAGGGCTATTCAGTACGTCATGGGGATCACTTC
+
FFFFFFFFFFFFFFFFF:FFFFFFFFFFFFFFFFFFFFFFFFFFFFFFFFFFFFFFFFFFFFFFFFFF
FFFFFFFFFFFFFFFF:FFFFFFFFFFFFFFFFFFFFFFFFFFFFFFF
@A00721:190:HGJW7DSXY:4:1337:31919:34632_2:N:0:TCTGCAAG+GCTGCACT
ACAAAACCTTCGAATATGTTACGACATGGCTTCGTAACAACAGTGCGAGGATTGTCATCTCGGGGAAA
GTGATTCACCAAGATGTTCATATAGATACGAGATACCTAGAGAAGGTC
+
FFFFFFFFFFFFFFFFFFFFFFFFFFFFFFFF:FFFF:FFFFFFFFFFFFFFFFFFFFFFFFFFFFFF
FFFFFFF:FF,FFFFFFFFFFFF:FFFFFFFFFFFF:FFFFFFFFFFF
@A00721:190:HGJW7DSXY:4:1456:17897:15577_1:N:0:TCTGCAAG+GCTGCACT
CTCGTTATCGGAGCTAAGAAGAAGACTTTGGGTACACGTTATGCTCGCAAACTTAACGTTAGTACCGG
AGGTGATCTCTCTGCTTCTGCGTTACTGAGAGAAAAGATTCAGGACGCCTACGGTGACTTTATGGACA
AGA
+
FFFFFFFFFFFFFFFFFFFFFFFFFFFFFFFF:FFFFFFFFFFFFFFFFFFFFFFFFFFFFFFFFFFF
FFFFFFFFFFFFFFFFFFFFFFFFFFFFFFFFFFFFFFFFFFFFF:FFFFFFFFFFFFFFFFFFFFFF
FFF
@A00721:190:HGJW7DSXY:4:1338:4218:20008_2:N:0:TCTGCAAG+GCTGCACT
TTTAGGTTTAGTAGGTGATCTTGACGTCGATTCTTTGTATAGGGAGTGTGAGAAGACAACGAATCTTG
AAACTACGGTCCTTATCAAAGAAAATGGTGAACCGGTCTCTAAATCTT
+
FFFFFF,FFFFFFFFFFFFFFFFFFFFFFFFFFFFFFFFFF:FFFFFFFFFFFFFFFFFFFFFFFFFF
F:FFFFFFFF:F:FFFFFFF:FFFFFF,FFFFFFFFFFFFFFFFFFFF
@A00721:190:HGJW7DSXY:4:2154:26341:10974_2:N:0:TCTGCAAG+GCTGCACT
GAAGATTGAAGAACACGTCAAGAAGTCGCTCGGCGTTGTTCTTGAAAACCAAGTTAAGAAGGAGGCTG
ATCATGGAGTCTTTGTACCTTCTGTCAAACTTCGAGGTTCAGCTACGA
+
```

FFFFFFFFFFFFFFFFFFFFFFFFFFFFFFFFFFFFFFFFFFFFFFFFFFFFFFFFFFFFFFFFFFFF
FFFFFFFFFFFFFFFFFFFFFFFFFFFFFFFFFFFFFFFFFFFFFFFF
@A00721:190:HGJW7DSXY:4:1347:31078:34522_1:N:0:TCTGCAAG+GCTGCACT
TGGATTGAAGGCTGGCTCTAAGGGAGACTCCTTCTTTTTGAATGTTGTAAAGAATTGGGTTGAGAAGT
GTGCTTTACAAGGAGTTAGACTCTTCTTCAAGTTTTTAGATCTTCTTGATAATCTTAAAGAAAAATTT
TCG
+
FFFFFFFFFFFFFFFFFFFFFFFFFFFFF:FFFFFFFFFFFFFFFFFFFFFFFFFFFFF:FFFFF:FF
FFFF:FFFFFFFFFFFFFFFFFFFFFFFFFFF:FFFFFFFFFFFFFFFFFFFF:FFFFFFFFFFFFFF
FFF
@A00721:190:HGJW7DSXY:4:2277:17644:13730_1:N:0:TCTGCAAG+GCTGCACT
GTCTGTACTTACATTCTTTACGCCAGATTCTTCATGGGGCGACGCTGTTTCTCTTGGTATTTCTGAAA
TAGTTTATCAGAGCGTCATTTTCCTTTGGGATCTTTCCTCTGGTAAGGGAATTATTAAATCATTCTTC
TCA
+
FFFFFFFFFFFFFFFFFFFFFFFFFFF:FFFFFFFFFFFFFFFFFFFFFFFFFFFFFFFFFFFFFFFF
FFFFFFFFFFFFFFFFFFFFFFFFFFFFFFFFFFFFFFF:FFFFFFFFFFFFFFFFFFFFFFFFFFFF
FFF
@A00721:190:HGJW7DSXY:4:1328:9146:23375_2:N:0:TCTGCAAG+GCTGCACT
TTTTCTCTACCCATAACTTCTTTTTCAAAAATGCGGATGGGTCTACTGATGTTTTCCTACAGAGTGCA
GTCGTTTCAACACATATCACCACTTTTGAAAGGAAAATAATTGGTGAT
+
FFFFFFFFFFFFFFFFFFFFFFFFFFFFFFFFFFFFFFFFFFFFFFFFFFFFFFFFFFFFFFFFFFFF
FFFFF:F,FF:FF,FFF,FFF,::FFF,:FF:FFFFF,FFFF:FF:FF
@A00721:190:HGJW7DSXY:4:2124:9986:30874_1:N:0:TCTGCAAG+GCTGCACT
GTCGGTGGGGTTCTCAAGGCTCTCATCGCCACTGGGATTCTAAGGGGTTCCATAAGATATTATCTGAC
ATTGTGGTTCAATGATCAGGATTCTTCTTTTGGTCCTTACGTCTTAAGTAATTTGGATGTTGTCGAAA
ACT
+
FFFFFFFFFFFFF,FF,FFF:FFFFFFFFFFFFF:FFFFFFFFF:FFF:FFFFFFFFFFFFFFFFFFF
:FFFFFFFFFFFFFFFFFFFFFFFFFFFFFFFFFFFFFFFFFFFFFFFFFFFFFFFFFFFFFFFFFFF
FFF
@A00721:190:HGJW7DSXY:4:2520:24325:3693_2:N:0:TCTGCAAG+GCTGCACT
TGTCACGATTCTTAAAAGAAGTTTATTAAAACTTCAGAGTCTCTTGGAAGTCCAGGTAGCTAATGTTC
TTGCAATAGCCACAGACTTTCTGGGAAAAGAGACCTCTGTTGGTAGAG
+
FFFFFFFFFFFFFFFFFFFFFFFFFFFFFFFFFFFFFFFFFFFFFFFFFFFFFFFFFFFFFFFFFFFF
FFFFFFFFFFFFFFFFFFFFFFFFFFFFFFFFFFFFFFFFFF:FFFFF
@A00721:190:HGJW7DSXY:4:1173:7880:33238_2:N:0:TCTGCAAG+GCTGCACT
ATTACTCAGATTCAATCTGGCATCACTGAACGTCTACAACGAGTTAGGGAGTGGCTAGGTTTTAAAAG
ATCCTTTGCACAAGCAATACCTTCCACTGAGTTCATCACCCAGAGTTT
+
,FFFFFFFFF:FFFFFFFF:FFFFFFFFFFFFFF:FFF:FF:FFFFF:FFFFFFFFFFFFFF:FFFF:
FFFFFFFFFFFFFFFFFFFFFFFFF:FFFFFFFFFFFFFFFFFFF,FF
@A00721:190:HGJW7DSXY:4:1275:31295:32362_2:N:0:TCTGCAAG+GCTGCACT
ACTGTTCGAGAATCTTCTGATGCTGAATTTGAGGAAAATTTTTCGGAAGTTTTTGGTATAAATCCTGA
TGGCTCTGTACAGGGAGGAGGTAATAGTTATAAAACCTTCTTTTATCA
+
FFFF,FFFFFFFFF:F:FF,:F:FF:FF,F,:,FFF:FF,FF,FF:FFFFF,:,F:F:FFFF,FF:FF
:F:,,F,FFF:FF:FFF,FFFFFF:FFF:FFFFFF::,FFF,FFFFFF
@A00721:190:HGJW7DSXY:4:1107:17490:33348_2:N:0:TCTGCAAG+GCTGCACT
GGACAAAATCTGGGATCATAGGTATGTGTCACGCTACACTCGAAATATGGGCCCTCATAAGGAAGTAT
AGTTGGCGTCTTGTCGTTGACGTCATTACAGTCTGGAAGTTAGATCAT
+

```
FFFFFFFFFFFFFFFFFFFFFFFFFFFFFFFFFFFFFFFFFFFFFF:FFFFFFFFFFFFFFFFFF,FFFF
FFFFFFFFFFFFFFFFFFFFFFFFFFFFFFFFFFFFFFFFFFF:FFFFFF
@A00721:190:HGJW7DSXY:4:1267:28989:7232_2:N:0:TCTGCAAG+GCTGCACT
CGAGAGTTGTGATCTAGTTCTAAATTTAGGTCGACGGACTTTAGCAACTTTAGATAAATTCTTTCCGG
GCTCAATGACCTTTGCTATGTACGTGGATCTAATCCTTGGTGATCTGA
+
FFFFFFFFF:FFFFFFFFFFFFFFFFFFFFFFFFFFFFFFFFFFFFFFFFFFFFFFFFFFFFFFFFFFFF
FFFFFFFF:FFFFFFFFFFFFFFFFFFFFFFFFFFFFFFFFFFFFFFF
@A00721:190:HGJW7DSXY:4:1130:26187:34068_2:N:0:TCTGCAAG+GCTGCACT
TATTAGTTCATGAAGTTCTTACACTTCTCGCGACTAATGTCAGCTTCACTACAAAGGCGTTACGTAAA
GTAGTTTTCACGCTTTATCTTAAAGCATTACCACCAAATTTAGCACTT
+
FFFFFFFFFFFFFFFFFFFF:FFFFFFFFFFFFFFFFFFFFFFFFFFFFFFFFFFFFFFFFFFFFFFFFF
FFFFFFFFFFFFFFFFFFFFFFFFFFF,FFFFFFFFFFFFFFFFFFFFF
@A00721:190:HGJW7DSXY:4:2264:1371:30890_1:N:0:TCTGCAAG+GCTGCACT
GAAGCTCGCCATATCAGATTAGAGACATGCCAGTTAGAGTTCTTCTAACCAGGACTGATGGTGAGATT
CTTTTACCTTTTGGAGGTCCAGGAACCTTTACTCCTAGTGTCTTTTCTAGAGTCCAAGGACGTTTCCG
TCG
+
FFFFFFFFFFFFFFFFFFFFFFFFFFFFFFFFFFFFFFFFFFFFFF:FFFFFFFFFFFFFFFFFFFFF:
FFFFFFFFFFFFFFFFFFFFFFFFFFFFFFFFFFFFFFFFFFFFFFFFFFFFFFFFFFFFFFFFFFFFFF
FFF
@A00721:190:HGJW7DSXY:4:1477:17517:8531_1:N:0:TCTGCAAG+GCTGCACT
GTGGGAGACCGCTCAATAACACTCTATTGAATCCCTTTTCCACGATGAAAGCTCTTTATGAGGTGCTC
CATGCAAATTTACAGTTCCTCTTTGGTGATATCGCCTCTGTCCTTTCTCCTTTAATCGTGAGGTTACG
AGA
+
FFFFFFFFFFF:FFFFFF:FFFFFFFFFFFFFFFFFFFFFFF:FFFFFFFFFF:FF:::FFFFFFFFF:
FFFFFFFFF:FFFFFFFFFF::FFFFFFFFFFFFFFFFFFFFFFFFFFFFFFFFFFFFFFFFFFFFFFFF
FFF
@A00721:190:HGJW7DSXY:4:1432:24388:24314_2:N:0:TCTGCAAG+GCTGCACT
GGTTTATCGGTTGTATTTTGATAAATTTGTTGGAATCGATAATGATACTGACGAAGGTTCTATTACCA
CCCTTGTCATTGATACAAACATTCTAGGTGATGCTTTCTTAGAGGCTC
+
FFFFFFFFFFFFFFFFFFFFFFFFFFFFFFFFFFF,FFFFFFFFF:FFFFFFFFFFFFFFFFFFFFF:F
FFFFFFFFFFFFFFFFFFFFFFFFFFFFFFFFFFFFFFFFFF,:FFFFF
@A00721:190:HGJW7DSXY:4:1416:14317:35963_2:N:0:TCTGCAAG+GCTGCACT
TATTTACAATCTGTAGAGAGAGAGGTTAATCAGAATTTGAGTGATAACGATTTAGTACTGGGAAATGG
TGGTAGTCAGTTTACTACTTTTCTTTCCCTCCCCTTTTTCGATTGGAT
+
FFFFFFFFFFFFFFFFFFFFFFFFFFFFFFFFFFFFFFFFFFFFFFF:FFFFFFFFFFF:FFFFFFFFFF
FFFFFFFFFFFFFFFFFFFFFFFFF:FFFF:FFFFFFFFFFFFFFFFFF
@A00721:190:HGJW7DSXY:4:1610:17906:28056_2:N:0:TCTGCAAG+GCTGCACT
CGTCTCACGACTAATTTGATAAGGTTTTTCTTTTATGGGACTCTTGTTTGTACTTATGAAGGAGGTAT
TACTTCGGAAGTTCTTAATGTAGGTCTCCTAGTACTTAAGGGTTTATG
+
FFFFFFFFFFFFFFFFFFFFFFFFFFFFFFFFFFFFFFFFFFF:F,:FFFFFFFF::FFFF:FFFFFFFFF
FF:FFFFFFFFFFFFFFFFFFFFFFFFF:FFFFFFFFFFFFFFFFFFFF
@A00721:190:HGJW7DSXY:4:1313:23746:32002_2:N:0:TCTGCAAG+GCTGCACT
TTTTGATGGTATGTTTCACATTGCGTGATCACTTCCCGTGGACTTGGGAAGAGATTAAACATAAAATC
CAGCGATTCTATTATGTCGCTAAAGCCTGTCCAGTTGTCGATGATGTT
+
FFFFFFFFFFFFFFFFFFFFFFFFFFFFFFFFFFFFFFFFFFFFFFFFFFFFFFFFFFFFFFFFFFFFFF
:FFFFFFFFFFFFFFFFFFFFFFFFFFF:FFFFFFFFFFFFFFFFFFFF
```

```
@A00721:190:HGJW7DSXY:4:2267:11894:20275_2:N:0:TCTGCAAG+GCTGCACT
TCGTGAAACTTACGACGATGTTCTCGCTCGCATTTCACAAGGATTACTAAGTCTGAGATCTGCCACTT
CTAATTTTAGGAAACGTCGCGAAAAGAAAGTTCATGAGGATGTCTTGG
+
FFFFFFFFFFFFFFFFFFFFFFFFFFFFFFFFFFFFFFFFFFFFFFFFFFFFFFFFFF:FFFFFFFFF
FFFFFFFFFFFFFFFFFFFFFFFFFFFFFFFFFFFFFFFFFFFFFFFF
@A00721:190:HGJW7DSXY:4:1315:15176:28150_2:N:0:TCTGCAAG+GCTGCACT
GAACTTCTAATAGAGGATCCTGATGACATTATTTCAACGGATACCGATCTCAGTTCTACCACAGAAAC
CGTCTCAGGAACTGTCTCCACTGATTCGGAATTAGAGAAAGACGACCT
+
FFFFFFF:FFFFFFFFFFFFFFFFFFFFFFFFFFFFFFFFFFFFFFFFFFFFFFFFFFFFFFFFFFFF
FFFFFFFFFFFFFFFFFFFFFFFFFFFFFFFFFFFFFFFFF,FFFFFF
@A00721:190:HGJW7DSXY:4:1346:9887:32456_1:N:0:TCTGCAAG+GCTGCACT
GAGACCTCTGAATTTTCAGGGTGCCCTGATGAGGACGTTGAAAGGGATAATTTCAACTCTGAGACTTC
TAAGAGTGTTTCAAAAGACCAGAAACGTAACAAGAAGAATAAAGGTAGGAAGGGGAGAAAACAGAACA
AGC
+
FFFFFFFFF:FFFFFFFFFF:FFFFFF:,FFFFFFFFFFFFFFFFFF:FFFFFFFFFFFFFFFF:FFF
FFFFFFFFFFF:FFFFFFFFFF:FFFFFFFFFFFFFFFFFFFFFFFFFFFFFFFFFFFFFFFFFFFFF
FFF
@A00721:190:HGJW7DSXY:4:1422:2672:1329_2:N:0:TCTGCAAG+GCTGCACT
ACTGATATGGTTGCGGCTGATCGTACCCCAATTCCTGCTAGAAAGATCACTCTCGGCGATACCACTGA
TCTTCCAGAGTTACGTAGTGGTCGTTCTTTTGTTGATTTTATCAAAGA
+
FFFFFFFFFFFFFFFFFFFFFFFFFFFFFFFFFFF:FFFFFFFFFFFFFFFFFFFFFFFFFFFFFFFF
FFFFFFFFFFFFFFFFFFFFFFFFFFF:FFFFFFFFFFFFFFFFFFFF
@A00721:190:HGJW7DSXY:4:2217:26024:19288_2:N:0:TCTGCAAG+GCTGCACT
CAAGTTAAACTTCGAACTGACCACTTACAAGAGTGTTGAAGTACCGCAACATGGATCAGTTTTCCAAA
AAGCTATTAATAGCAGTTGCGGGGCTATAATACCGCCTCCAGATTACT
+
FFFFFFFFFFFFFFFFFFFFFFFFFFFFF,F:FFFFFFFFFFFFFFFFFFFFFFFFFFFFFFFFFFFF
FFFFFFFFFFFFF,FFFFFFFFFFFF:FFFFFF:,FFFFFFFFFFFFF
@A00721:190:HGJW7DSXY:4:1648:7988:9220_1:N:0:TCTGCAAG+GCTGCACT
AGAGTTTTGGAACTGTGTAATGTCCTACGTACTGTCCAAGGTGTTTGGGCTTCTCTAAAGAACAGCAA
CTTTCAACATGAATTAGTTATTGAAAGTCTTGACCCTCACGTCTCACTTTATCACCTTGGAGATGGTA
AAG
+
FFFFFFFFFFFFFFFFFFFFFFFFFFFFFFFFFFF,FFFFFFFFFFFFFFFFFFFFFFFFFFF:FFFF
FFFFFFFFFFFFFFFFFFFFFFFFFFFFFFFFFFFFFFFFFFFFFFFFFFFFFFFFFFFFFFFFFFFF
FFF
@A00721:190:HGJW7DSXY:4:1271:29966:15655_2:N:0:TCTGCAAG+GCTGCACT
ATTCTGCTTCGACGGTAATTCCATAAGACCTTTCTTGGGGAGTAAAGAGGGTTTGAAAATTACGGAAC
CTTTCCTCGTTCTTCATGAAGATCTTCGAGTGTTTTATAGTGATTTAT
+
FFFFFFFFFFFFFFFFFFFFFF:FFFFFFFFFFF,FFFFFFFFFFFFFFFFFFFFFFFFFFFFFFFFF
FFFFFFFFFFFFFFFF:::FFFFFFFFFFFFFFFFFFFFFFFFFFFFF
@A00721:190:HGJW7DSXY:4:1133:10773:7059_1:N:0:TCTGCAAG+GCTGCACT
CAAGTCGATTGATTTTGTTGACTACGATCTCTCTTGTAATAAGAAGTTCTTCGCAGTTGAATCTCCAC
CAGGTGGAGGGAAGACCACTGAACTTATAGCGACTTTTGTCGAATTTTTCGCAAAAGGTCTTGAAGTA
CGT
+
FFFFFFFFFFFFFFFFFFFFFFFFFFFFFFFFFFFFFFFFFFFFFFFFFFFFFFFFFFFFFFFFFFFF
FFFFFFFFFFFFFFFFFFFFFFFFFFFFFFFFFFFFFFFFFFFFFFFFFFFFFFFFFFFFFFFFFFFF
FFF
```

```
@A00721:190:HGJW7DSXY:4:1109:24912:13197_2:N:0:TCTGCAAG+GCTGCACT
TCGTAATTCGCGAGAAGAGATAGTGACTAAAGTTTTGGCACTTCTAGTTCATCGCGGTATTTGTGAAA
TTAAGACAGACGTGAAAGTTATGATGAGTGATCGTATTAGACCAGCTG
+
FFFFFFFFFFFFFFFFFFFFFFFFFFFFFFFFFFFFFFFFFFFFFFFFFFFFFFFFFFFFFFFFFFFF
FFFFFFFFFF:FFFFFFFFFFFFFFFFFFFFFFFFFFFFFFF,FFFFFF
@A00721:190:HGJW7DSXY:4:1550:18855:8061_2:N:0:TCTGCAAG+GCTGCACT
ACGTCGCTGAGGTCAAACCTGTGGACGTTCTTTTCATCGACGAATTATTCATGATTCACGCTGGTCAG
ACCCTTTTTCTTACACATCTACTTAAGGCGGCTTATACCATAGGTTAC
+
FFFFFFFFFFFFFFFFFFFFFFFFFFFFFFFFFFFFFFFFFFFFFFFFFFFFFFFFFFFFFFFFFFFF
FF:FF,FFFFFFFFFFFFF:FFFFFFFFFFFFFFFFFFFFFFF:FFFFF
@A00721:190:HGJW7DSXY:4:2265:12038:14356_2:N:0:TCTGCAAG+GCTGCACT
CAAATCGGATTCATCAATCGTATTGATCTCCAAGTCGCAAACTACTTCAACATCAACGACATCATTCT
TGATGAGTGTCGTTCTTATCGAAGTCTCAGTTATCGCTGCCCGGCCGA
+
FFFFFFFFFFFFFFFFFFFFFFFFFFFFFFFFFFFFFFFFFFFFFFFFFFFFFFFFFFFFFFFFFFFF
FFFFFFFFFFFFFFFFFFFFFFFFFFFFF,FFFFFF:FFFFFFFFFFFF
@A00721:190:HGJW7DSXY:4:1509:28655:9784_2:N:0:TCTGCAAG+GCTGCACT
GAACTCTCAGAGGTTTACGGCAGAAAAATTTCTCCTGCTTGTAATTCGAGAGAGGACACTATGGAAAT
TAGACCTATCAGATGTCTTGACGACTTCGAATTTGAGGACGGAGTTAA
+
FFFFFFFFFFFFFFFFFFFFFFFFFFFFFFFFFFFFFFFFFFFFFFFFFFFFFFFFFFFF:FFFF:FF
FFF:FFFFFFFFFFFFFFFFFFFF:FFFFFF::FFFFFFFFFFFFFFF
@A00721:190:HGJW7DSXY:4:2615:13358:22060_2:N:0:TCTGCAAG+GCTGCACT
AATTAACAAAGTCCTTGGTAAGAGTAAGGTTAAATGTGAAGATGCTCAGACTATTCATGAAGTTCAAG
GTAATACTTACCAGCACGTTCGGCTGGTTAGAATTAAACCTCAAGATG
+
FFFFFFFFFFFFFFFFFFFFFFFFFFFFFFFFFFFFFFFFFFFFFFFFFFFFFFFFFFFFFFFFFFFF
FFFFFFFFFFFFFFFFFFFFFFFFFFFFFFFFF,FFFF,FFFFFFFFFF
@A00721:190:HGJW7DSXY:4:2622:9109:12038_2:N:0:TCTGCAAG+GCTGCACT
CACAAACACATATTACCGTTGCTCTATCACGACACGTTGAATCTTTACGATACTTTGTTATCAATGTA
AAGTTAAACGATGCTACTTGTGAGAGGATCAATCACAGTAAGGAACTG
+
FFFFFFFFFFFFFFFFFFFFFFFFFFFFFFFFFFFFFFFFFFFFFFFFFFFFFFFFFFFFFFFFFFFF
FFFFFFFFFFFFFFFFFFFFFFFFFFFFFFFFFFFFFFFFF:FFF:FFF
@A00721:190:HGJW7DSXY:4:1508:8296:10347_2:N:0:TCTGCAAG+GCTGCACT
ACGGGAGGTTGCAGATTCAACCCCTTATATCGAGGCTATCGAGGACGATGAGTATCTTGAAGTTGTTG
ACGACAAGCCTCAAGAGGGTAGAGGACCACCTGGATCTTCTACTGTCT
+
FFFFFFFFFFFFFFFFFFFFFFFFFFFFFFFFFFFFFFFFFFF:FFFFFFFFFFFFFFFFFFFFF:FFF
FFFFFFFFFFFFFFFFFFF:F:FFFFFFFFFFFFFFFFFFFFFFFFFFF
@A00721:190:HGJW7DSXY:4:1565:2410:22294_2:N:0:TCTGCAAG+GCTGCACT
GGTTATTCCTGGAACCACCTTAACGTCACTCGGTAATCTTTCAGCCGAATTAAGTACTAATGAGTTCA
AGTCGGTTGTCGATCGAATTAAGATCAAGGCTAATACCTTTATAGGTA
+
FFFFFFFFFFFFFFFFFFFFFFFFFFFFFFFFFFFFFFFFFFFFFFFFFFFFFFFFFFFFFFFFFFFF
FFFFFFFFFFFFFFFFFFFFFFFFFFFFFFFFFFFFFFFFFFFFF:FFF
@A00721:190:HGJW7DSXY:4:2267:6949:20447_2:N:0:TCTGCAAG+GCTGCACT
GCCGCGTAGTCAGCGCGTTTAGCTGTATCATCAGTCAGAGTATACCCGATAGGGTACCGAGTCTCCAA
GAGAATATTTATTCTTATGAGGCTCGAAACTTCAACGTTTTCACAACG
+
FFFFFFFFFFF:FFFFFFFFFFFFFFFF:FFFFFFFFFFFFFFFFFFFFFFFFFFF:FFFFFFFFFFFF
FFFFFFFFFFFFFFFFFFFF:FFFFFFFFFFFFFFFFFFFFFFFFFFFF
```

@A00721:190:HGJW7DSXY:4:1620:26341:23249_2:N:0:TCTGCAAG+GCTGCACT
TCGCACCCGAAATTTTTGGGCAGAATGCTCTTAACGCGGTTCTTGAACGGTGTTTTGATCAGTCTAAG
TTGGATAAAGTTATAGGAGACTCAGTTAACCTGAGAGCTCCTCAATTG
+
FFFFFFFFFFFF:FFFFFFFFFFFFFFFFFFFFFFFFFFFFFFFFF:FFFFFFFFFFF:FFFFFFFFFF
FFFFFFFFFFFFFFFFFFFFFFFFFFFFFFFFFFFFFFFFFFFFFFFF
@A00721:190:HGJW7DSXY:4:1336:15438:9878_1:N:0:TCTGCAAG+GCTGCACT
CAATAGGGACGCTAGTATACTTCGTAAAGTCGACAGCGAAATGGTCAATATTGATAATTGGACCGACT
ATATGACCTATTTTAAGCTCATGGTGAAAAGGGAAGCTAAGGTTAAACTTGATGCTTCAGCCTTTTCG
AAG
+
FF,,:FFFFF:FFF:FFFFFFFF,FF,,,,FFFF,,FFF::F:FFFFF,,:FFFFF:,FFFF,,:F:F
FFFFFFFFFFFFFFF:FFF,FF,FFFF,F:F,:FFFFFFFFFF:FFFFFFFFFFFFF:FFFFFFFFFF
FFF
@A00721:190:HGJW7DSXY:4:2254:17309:4476_1:N:0:TCTGCAAG+GCTGCACT
AGAATATCGTTTATCACGCAAAATTTATTAATGCAATTTTCGGACCTATCTTTAAGGTTCTTGTGAGG
AGAGTCAAACAGTGTCTTTTACCCAACATTTTTATGTATACAGAGATGACCAGTGACGATTTCTCCCG
GAA
+
FFFFFFFFFFFFFFFFFFFFFFFFFFFFF:FF:FFFFFFFFFFFFFFFFFFFFFFFFFF:FFFFFFFFFF
FFFFFFFFFFFFFFFFFFFFFFFFFFFFFFFFFFFFFFFFFFFFFF:FFFFFFFFFFFFFFFFFFFFF
FFF
@A00721:190:HGJW7DSXY:4:1477:26720:24659_1:N:0:TCTGCAAG+GCTGCACT
CGAGATCTTGCAGAGTGACTGGAGTTCAGACGTGTGCTCTTCCGATCTTTTCTTTTGTAAAGCCTATG
AGATAAAGATGTATTCTTATTTAGGCTTCGATGCTGAATTTCTTGAACTTTGGAGTGCCAGTGAATAT
TTT
+
F:,FFFFFFFFFFFFFFFFFFFFFFFFFFFFFFFFFFFFFFFFFFFFFFFFFFFFFFFFFFFFFFFFF
FFFFFFFFFFFFFFFFFFFFFFFFFFFFFFFFFFFFFFFFFFFFFFFFFFFFFFFFFFFFFFFFFFFF
FFF
@A00721:190:HGJW7DSXY:4:1267:17363:10802_1:N:0:TCTGCAAG+GCTGCACT
GAGTATCCATAACGATTTGTCATTTACCACCTTTGCTCAACGACGAAGTGGAACGGCCAACACTTTCT
TTGGTAATTGTATGATCACTTTGATGATTTTATCACTTTATTATGACCTTGGAAAGTTTTCGGCTGTG
GTT
+
FFFFFFFFFFFFFFFFFFFFFFFFF,FFFFFF:FFFFFFF:FFFFFFFFFFFFFFFFFFFFFFFFFF,FF
FFFFFFFFFFFFFFFFFFFFFFFFFFFFFFFFFFFFFFF:FF:FFFFF:FFFFFFFFFFFFFFFFFFFF
FFF
@A00721:190:HGJW7DSXY:4:2330:23158:17550_1:N:0:TCTGCAAG+GCTGCACT
CCATTCTGTTTTCAAAGACTCCAATAACTGATCATTCTGATCATATGATTTTGGACAATGGTTTCGAA
GCGAAATTTTTGAGGGATAAACCAGCGTACTTCTGCTCGCGATTTATTCTTTTTGTCAATGATTACAT
CTA
+
FF:FFFFFFFFFFF,FFFFFFF:FFFFFFF,FFFFFFFFFFFFFFFFFFFFFFFFFFFF:FF:FFFFFF
FFFF:FFFFFFFFFFFFFFFFFFFFFFFFFFFFFFFFFFFFFFFFFF:FFFFFFFFFFFFFFFFFFF
FFF
@A00721:190:HGJW7DSXY:4:1353:24316:12571_1:N:0:TCTGCAAG+GCTGCACT
CCTTACAAGATCATCGTTAAGTTAGGTAAACCCCTAACCGATGTTACTCTTGTAGGACTTTGGGAGAG
ATTCGTTAGTTTTAAAGACTATACTAAATATCTCCACTACGAGGTTGTTGTTTGGCGATTAGCTCTTG
CGA
+
FFFFFFFFFFFFFFFFFFFFFFFFFFFFFFFFFFFFFFFFFFFFFFFFFFFFFFFFFFFFFFFFFFFF
FFFFFFFFFFFFFFFFFFFFFFFFFFFFFFFFFFFFFFFFFFFFFFFFFFF:FFFFFFFFFFFFFFFFF
FFF

```
@A00721:190:HGJW7DSXY:4:1250:4878:7122_2:N:0:TCTGCAAG+GCTGCACT
ATTGAGGGTCGTTATACCTATTCGGCTATCTGTGCTTTGAATTGTATAAGATCAAATTTTAAACGATT
CCGAGAGGTTTATCCTGATGTGGAGGGAGTTCTTGAAGCATCCCGACC
+
FFFFFFFFFFFFFFFFFFFFFFFFFFFFFFFFFFFFFFFFFFFF:FFFFFFFFFF:FFFFFFFFFFF
FFFFFFFFFFFFFFFFFF:FFFFFFFFFFFFFFFFFFFFFFFFFFFFF
@A00721:190:HGJW7DSXY:4:1167:23529:8766_1:N:0:TCTGCAAG+GCTGCACT
TGATGCGGTTTCATAAGATTATCAGTAATCCTGATTCTACTACGGATTCATGGACCTTCCATCCTGTA
CCTCTTCTGGAGAGACGTATCTCAAAGATCGACTTCGAGACAGGTGATATTGTTGAGCGAGAGCGACC
AAG
+
FF:F:FF,FFFFFFFFF:FFFF:FFFF:F::F,,FF:FFFF,:F:,FFFFFFF:FFFF:,FF::F:F:
,FF,FF:FFF:F:FF:FF:F,F,FFF,,FFF,:FF:FF:,,FFFF:,FFFFFFF,FF:F,FFF:F::,
FF:
@A00721:190:HGJW7DSXY:4:1506:9471:7623_1:N:0:TCTGCAAG+GCTGCACT
CGTGGCGCTTTATTTTAAAAAGCTCCCTCCTCTCGCAAGTTTGATCTGATACTTATCACGAAAGGACT
TGAGTCTTTTGATCAGTCTCTGACTTGACCATCTTTCATTTTAAATGACGATCATTAAGACTCTCTTT
CTT
+
FFFFFFFFFFFFFF:FFFFFFFFFFFFFFFFFFFFFFFFFFFFFFFFFFFFFFFFFFFFFFFFFFFFF
FFFFFFFFFFFFFFFFFFFFFFFFFFFFFFFFFFFFFFFFFFFFFFFFFFFFFFFF:FFFFFFFFFFF
FFF
@A00721:190:HGJW7DSXY:4:1430:10185:20008_2:N:0:TCTGCAAG+GCTGCACT
CTTTGCACTGATTTTTCCTCATCAGCTTGTGTTTTACCTAATCTGTGTTTTTACGATAACAACTATCA
GTTGATTAAGACTCTTCCGCAAGGCGAACTTTGGGAATTTGAAGCTCA
+
FFFFFFFFFFFFF,:FFFFFFFFFFFF,:FFFFFFFFFFFFFFFFF:F:FFFFFFFFF:FFFFFFFFF
FFFF:FFFFFFFF:FFFFF,FFF,FFFFFFFF:::FFFF::FFFFF:F
@A00721:190:HGJW7DSXY:4:2374:32009:27211_1:N:0:TCTGCAAG+GCTGCACT
CGTTTGACCGTGACGAACTGTGGTGTTCTTGATCTACGTTACGGATATACTCTCCTTGAGTTCACTCG
TAAGGACACTACCACTTATTACGATATTTCTTTAGTCGATGGGTTTTCGACTCCTTTAAGCGTCTTCT
GTT
+
FFFFFFFFFFFFFFFFFFFFFFFFFFFFFFFF:FFFFFFFFFFFFFFFFFFFFFFFFFFFFFFFFFFF
FFFFFFFFFFFFFFFFFFFFFFFFFFFFFFFFFFFFFFFFFFFFFFFFFFFFFFFFFFFFFFFFFF:FF
FFF
@A00721:190:HGJW7DSXY:4:1378:11749:28354_2:N:0:TCTGCAAG+GCTGCACT
TTTCTTCTAATGTGGCCTGAACTACGTTTCACTTTGTGTGTACTTGTCTCTATTTTAAGTTTTGTCTT
CCTTGTTTGTCTTATTCTATTAATCACTCATTTTAGTGAGATTATTAA
+
FFFFFFFFFFFFFFFFFFFFFFFF:FFFFFFFFFFFFFFFFFFFFFFFFFFFFFFFFF:FFFFFFFFFF
FFFFFFFFFFFFFFFFFFFFFFF:FFFFFFFFFFFFFFFFFFFFFFFFF
@A00721:190:HGJW7DSXY:4:1607:5502:26303_1:N:0:TCTGCAAG+GCTGCACT
GTCTTCGACAACAGCAGGGGTTTTCGAATAGTGATAGCCACCCGCCTTACAGAGGGGCAGCAGCATGA
CCATAATTGGTATTGACTTTGGTACCACCTTTTCTACTGTCTGTCTCGCCTCTTCGAATGAAGTTTTA
ATG
+
FFFFFFFFFFFFFFFFFF:FFFFFFFFFFFFFFFFFFFFFFFFFFFFFFFFFFFFFFFFFFFFFFFFFF
FFFFFFFFFFFFFFFFFFFFFFFFFFFFFFFFFFFFFFFFFFFFFFFFFFFFFFFFFFFFFFFFFFFFF
FFF
@A00721:190:HGJW7DSXY:4:1169:29550:31939_2:N:0:TCTGCAAG+GCTGCACT
TGATAGTGAATTTATACCTACAATCATTGGTTTTTCTAATAATGATGACACTATTGTTTATGGATATG
ATGCTATTACTCATGATGGTTACGGTCAGGGAGGATTCTCAGTTTACA
+
```

```
FFFF,FFFFFFFF:FFFFFFFFFFFFFFFFFFFFFFFFFFFFFFFFFF:FFFFFFFFF,FFFFFFFFFF
FFFFFFFFFFFFFFFFFFFFFFFFFFFFFFFFFFFFFFFFFFFFFFFFF
@A00721:190:HGJW7DSXY:4:2302:4562:32972_2:N:0:TCTGCAAG+GCTGCACT
ACATTACCCGAAAGACGTGCTAAATTAAAACCATTGTACGATGTCGTCTGTCTCGGCGATCACTTTTC
ACTAAGAATTGGTCCTACTTTTGGACAAGGCCAACTTCGTAATCTTCA
+
FFFFFFFFFFFFFFFFFFFFFFFFFFFFFFFFFFFFFFFFFFFFFFFFFFFFFFFFFFFFFFFFFFFFF
FFF:FFFFFFFFFFFFFFFFF:FFFFFFFFFFFFFFFFFFFFFFFFFFF
@A00721:190:HGJW7DSXY:4:2278:11153:4773_2:N:0:TCTGCAAG+GCTGCACT
TTTATCTCTTTGGTTGTTAGGGATTTTGAGAGAGTGAAAGGCTTCAATTGTAGTGGTTTGGTCGTTTC
AGTACCCAGTCAGTATACCTCTACGCAAAGATATTTCATGAAAACTAC
+
FFFFF:FFFFFFFFFFFFFFFFFFFFFFFFF:F:::FFF:::FFFFF:FFFFFFFFFFFFFFF,FFF:
F,FF,F,FFF:FFFFFF,,F:FF:,FFFF,FFF:FF,FF,FFFF,,F,
@A00721:190:HGJW7DSXY:4:1445:18647:20603_2:N:0:TCTGCAAG+GCTGCACT
ATTACCTGTCTTTCATATTATGAATGAACCTTCGGCCGCTCTCTTTGCTTCGATGTTGGATGTCAAGA
AGGGTTCAGAATGGGATTCCTACGTAGTTTATGACTTTGAGATCGGAA
+
FFFFFFFFFFFFFFFFFFFFFFFFFFFFFFFFFFFFFFFFFFFFFFFFFFFFFFFF:FFF:FF:FFFF:F
F:FFFF:F,FF:::FFFF:F,F:FFFFFFFFF,FF:FF,F,FF:,,FF
@A00721:190:HGJW7DSXY:4:1209:10818:17534_1:N:0:TCTGCAAG+GCTGCACT
TCTTACGTTACGGTAATTACTATAGTGTTATTTATTCTGGTGGTGATGATAATCTTGGAGGTCGTGAC
GTTGATGAAGCTATAAGAGCGTTCTTATTACGGCGCACTTCTTTACCCGCCACCACACAACTTTCAGT
TTC
+
F:FFF:FFFFFFFFFFFFFFF:FF:FF:FFFFFFFFF:FF,F:FF:FFFFFFFFFFFFFFFFFFFF:
FFFFF,FFFFFFFFFFFFFFFFFFFFFFFFFFF:FFFFFFFFF:FFFF:FFFFFFFFFFFFFFFFFFF
FFF
@A00721:190:HGJW7DSXY:4:2325:16839:10113_2:N:0:TCTGCAAG+GCTGCACT
TGTTACTGGAAAACCCTCGTCAATACTTTTCAATAATATACGTCTTGAACTCACTTATGACGATCTAG
TGAAATTAACGCGTCCTTTTCTGGAACGTAGTTTTACTATTATGAACA
+
FFFFFFFFFF:FFFFFFFFFFFFFFFFFFFFFFFFFFFFFFFFFFFFFFFFFFFFFFFFFFFFFFFFFF
FFFFFFFFFFFFFFFFFF,FFFFFFFF:FFFFFFFFFFFFFFFFFFFF
@A00721:190:HGJW7DSXY:4:1572:16667:33051_1:N:0:TCTGCAAG+GCTGCACT
ACGTGTGCTCTTCCGATCTAACTTACCCTGATTCCGGCCGGTGGTAGCTCATTTCTACCAGGGGCTAT
TACTCTTGCTGAAACAGCTATTAAAGCTGTTGAGCGGGTAATTACACCTAAACGAGCTCGATCTGCCA
TCG
+
FFFFFFFFFFFFFFFFFFFFFFFF,FFFFFFF:FFFFFFFFF,FF:FFFFFFFFFFFFFFFFFFFFFFF
FFFFFFFFFFFFFFFFFFFFFFFF:FFFFFFFFFFFFFFFFFFFFFFFFFFFFFFFFFFFFFFFFFFFF
FFF
@A00721:190:HGJW7DSXY:4:1155:32389:14309_2:N:0:TCTGCAAG+GCTGCACT
CACTCTTTTCTGCTACTTTAACGGAGCGTAGTGTACTCTTCGTCGATTGTATCAACAGTAACATTACT
TCTAATAAAGGTCTCTATCAAGCTCGTATTATCGTCGCTGCTGGATCT
+
FFFFFFFFFFFFFFFFFFFFFFFFFFFFFFFFFFFFFFFFFFFFFFFFFFFFFFFFFFFFFFFFFFFFF
FFFFFFFF,F:FFFFFFFFFFFFFFFFFFFFFFFFFFFFFFFFFFFF:F
@A00721:190:HGJW7DSXY:4:1611:27706:12211_1:N:0:TCTGCAAG+GCTGCACT
AAGGCTAAACTCCAAGGTAATCATGAAGACTGTGATTTATCTTTCACTCTGGCCTTGTATGAGGGTGT
TTCTAATCGTAACATTAATAATTTCAATCTTCTTAAAAAGAAGATAGAGAGTCATACTTTGGGCTATC
GTT
+
:FFFFFFFFFFFFFFFFFFFFFFFFFFFFFFFFFFFFFFFFFFFFFFFFFFFFFFFFFFFFFFFFFFFF
```

FFFFFFFFFFFFFFFFFFFF,FFFFFFFFFFFFFFFFFFFFFFFFFFFFFFFFFFFFFFFFFFFFFFFF
FFF
@A00721:190:HGJW7DSXY:4:1356:32090:36245_2:N:0:TCTGCAAG+GCTGCACT
GATAGCCAATATTGAAGTGGACACCTCTGGTAGTATCGAAGTAAAGATAACCAATAAGGAAGGAGCTT
ATACTATGGTTGAATCAACTAAGCATACTACTTTTGCTTTGATTACTC
+
FFF:FFFFFFFFFFFFFFFFFFFFFFFFFFFFFFFFFFFFFFFFFFFFFFFFFFFFFFFFFFFFFFFF
FF:FFFFFFFFFFFFFFFFFFFFFFFF:FFFFFFFFF,FFFFFFFFFFF
@A00721:190:HGJW7DSXY:4:2345:20500:12383_1:N:0:TCTGCAAG+GCTGCACT
CTTCCGATCTGTTAAAGAGTCGATTAAACAAGTTGTACTTATCAATGATTTTATCGCTGGTTGTTATC
TCGAAAAGTCTCTTCTAAAAGGAACGTCAGTTCTAACCCAAAGAGAATATCTAAAATATTTACAAAAT
CTT
+
FFFF:FFFFFFFFFFFFFFFFFFFFFFFFFFFFFFFFFFFFFFFFFFFFFFFFFFFFFFFFFFFFFFF
FFFFFFFFFFFFFFFFFFFFFFFFFFFFFFFFFFFFFFFFFFFFFFFFFFFFFFFFFFFFFFFFFFFF
FFF
@A00721:190:HGJW7DSXY:4:1553:5421:20024_1:N:0:TCTGCAAG+GCTGCACT
GATCTGTTGCTTGTCGCTTCCTCCTTTAATGGCACTACCTCGGTACAATCTTACTTGGAACGAAGGCG
ACCTGTTCCGGAGTTTTTACGGGATCCGGAATGTTCTGTCTATATTAGGTGAAGCGAAATACGATTTC
GAA
+
FFFFFFFFFFFFFFFFF:FFFFFFFFFFF:FFFFFFFFFFFFFFFFFFFFFFF:FFFFFFFFFFFFFFF
FFFFFFFFFFFFFFFFFFFFFFFFFFFFFFFF:FFFFFFFFFFFFFFFFFFFFFFFFFFFFFFFFFFF
FFF
@A00721:190:HGJW7DSXY:4:1350:5810:36229_1:N:0:TCTGCAAG+GCTGCACT
TCGTACATTGATGCTGACTTTCGTCAATATGATTTGATCTGGTTGCTCAATTCTAAGCGTGTTCCGCT
TAGTGCGAGGCCTCTCTTCTACGAGTATTGCTATTTATACGGTTACGAGAAGAAGGTTCGAAAGTTTG
AGA
+
FF,:FF,FF:FFFF:FFFFFFFFFFFFFFFF:FFFFFFFFFFFF:F::FFFFFFFFFFFFFFFFFFFF
FFFFFFFFFFFFF,FF,FFFFFFFFF::FFFFFFFFFFFFFFFF:FF:FFFFFFFFFFFFFFFFFFF:
:FF
@A00721:190:HGJW7DSXY:4:1455:22941:35556_2:N:0:TCTGCAAG+GCTGCACT
TACTAAATACTAGATATTTGGATGATCTTGATAAAGAACTTCATGAATTTTCAGAGGATGAAAAATTT
TCTTCCTTGGATTTTAATATAAAGAATCTTTACCAGGACTCCTACTAC
+
FFFFFFFFFFFFFFFFFFFFFFFFFFFFFFFFFFFFFFFFFFFFFFFFFFFFFFFFFFFFFFFFFFFF
FFFFF:FFFFFFFFFFFFFFFFFFFFFFFFF:FFFFFFFFFFFFFFFF
@A00721:190:HGJW7DSXY:4:2213:29234:11350_2:N:0:TCTGCAAG+GCTGCACT
TCTGATCGTAAGAAGTTTATAAGGAACATATCCATTTGTTGTAATAGGGTCATCAACTTAGAACTCGA
AACTCTAAGTGGTAATACTGTCTTTCCAAGCTTACCTGTATCTTCAAT
+
FFFFFFFFFFFFFFFFFFFFFFFFF,F,FFFFFF:FFFFFF,FFF:FFF:F:FFFFFFFF:FFFFFFF
FFFFFFFFF:FFF:FFFFFFFFFFFFFFFFF:FFFFFFF:FFFFFF,F
@A00721:190:HGJW7DSXY:4:2501:5168:4554_1:N:0:TCTGCAAG+GCTGCACT
TTCGATAGGGGACATTTTATCGTGGGCTTTCCGTAAAGGAAGATCTGTTATCGACGGTTTATCGTTTA
CGAAGTATCACTCCACCGGTGAATGGTTTAAGAATACTATAAATAGAATAGCTATTACTACTAATGGC
GTT
+
FFFFFFFFFFFF,F:FFFFFFFF:FFFF:FFFFFFFFFFFFFFF,FFFFFFFFFF:FFFFFF:FFFFF
FFFFFFFFFFFFFFFFFFFFFFFFFFFFFFFFFFFFFFFFFFF:,FFF:F,FFFFFFFFFFFFFFFFF
FFF
@A00721:190:HGJW7DSXY:4:2319:12301:33849_2:N:0:TCTGCAAG+GCTGCACT
TAGTGACATCTGGTCAATTTCGTTTTTAGGTGGTCTTTATTCTATTATTGATTTTTATGAGATTGATA

```
CGTTAACAGAAGATGATGTTAGAGTAGAATTCCTGAAGAGTCCTTCCG
+
FFFFFFFFFFFFFFFFFFFFFFFFFFFFFFFFFFFFFFFFFFFFFFFFFFFFFFFFFFFFFFFFFFFFFF
FFFFFFFFFFFFFFFFFFFFFFFFFFFFFFF:FFFFFFFFFFFFFFFF
@A00721:190:HGJW7DSXY:4:1374:21956:2033_2:N:0:TCTGCAAG+GCTGCACT
CTAGGATTATGTCTGTGTTGAAAGACTTTCTACCGATAAGGGTCACTAGTCTTACACAATTAAGAAAT
AATCCTACATTGACTGATTATCTAAAGATTGAAGGAAAATTTTATCCT
+
FFFFFFFFFFFFF:FFFFFFFFFFFFFFFFFFFFFFFFF,FFFFFFFFFFFFFFFFFFFFFFFFFFFFF
FFFFFFFFFFFFFFFFFFFFFFFFFFFFFFFFFFFFFFFFFFFFFFFF
@A00721:190:HGJW7DSXY:4:1221:30282:5337_1:N:0:TCTGCAAG+GCTGCACT
GACTTTCCTACGATACAAATGATAACTTCTAATTTACGTTTGCTACTCAGAGCTGGTCCTCTCAGCAA
AGGGATGCATGGTGTTATCTCCTCAGGAGTCCCGGCATTTGATAATAATGACGAGACTTTCGTCATAA
TCG
+
FFFFFFFFFFFFFFFFFF,FFFFFFFFFFFFFFFFFFFFFFFFFFFFFFFFFFFFFFFFFFFFFFFFFFF
FFFFFFFFFFFFFFFFFFFFFFFFFFFFFFFFFFFFFFFFFFFFFFFFFFFFFFFFFFFFFFFFFFFFFF
FFF
@A00721:190:HGJW7DSXY:4:1342:12979:20494_1:N:0:TCTGCAAG+GCTGCACT
GATTACACGGTACCAGCGCATCTCGTGTCGAGCCTCGACCCGATTATATGGTATTAACTTATAAAGGT
GAGGAGACCATTTTCTTGCTGAAATTTATAGAAGATTATTTTTACAGAATCAATAAGAATCATCGATT
CTC
+
FFFFFFFFFFF:FFFFFFFFFFFFFFFFFFFFFFFFFFFFFFFF:FFFFFFFF,F,FF,F:FFFFFF,F
FFFFF,FFFF,F,F,:FF,F,F,FFFFFF,,FF,FF,FFFFF,FFF,FFF:FF:FF:FF,,F:,,FFF
F,:
@A00721:190:HGJW7DSXY:4:2528:30572:34773_1:N:0:TCTGCAAG+GCTGCACT
CTAGATGGATCCACTCCTCGAGTTATCATGATGTTAGTATGTTATATACTGATTTACAACGATATAGG
AGTAATCTACGAGAAGATTCTAGAATTCTTAATTATAATATTCAACCTCAAAAGTTACGTGTTTACAG
TAA
+
FFFFF,FFFFFFFFFF:FFFFFFFFFFFFFFFFFFFFFFFFFFFFFFFFFFFFFFFFFFF:FFFFFFF
FFFFFFFFFFFFFFFFFFFFFFFFFFFFFFFFFFFFFFFFFFFFFFFFFFFF:FFFFFFFFFFFFFFFF
FFF
@A00721:190:HGJW7DSXY:4:1170:19162:9878_2:N:0:TCTGCAAG+GCTGCACT
CATTTTGAGAACTAACTATGTTATGTTCTCTGATCATTTTGACAATCTCTTCTTATAATAAATAAATA
AATAATATTACTAATGGCAAATAGTGGAAAGGCTCCAGTCAACACTAG
+
FFFFFFFFFFFFFFFFFFFFFFFFFFFFFFFFFFFFFFFFFFFFFFFFFFFFFFFFFFFFFFFFFFFFFF
FFFFFFFFF:FFFFFFFFFFFFFFFFFFFFFFFFFFFFFFFFFFFFFF
@A00721:190:HGJW7DSXY:4:1343:20518:18396_2:N:0:TCTGCAAG+GCTGCACT
TACGATGACGTGAGAATAGGTGAAGGGATAGTTTTTACCTCGACCAACATTAAGGCGGCGGCGACTAA
TCTTAAAGCTTCACCCAAAATGAAGGACACTTCGAAACTGAATAATGA
+
FF:FFFFFFFFFFFFFFFFFFFFFFFFFFF,FFFFFF,FFFFFFFFFFFFFFF,FFFFFFFFFFFFFFF
FF:FFFFFFFF:FFFFFFFFFFFFFFFFFFFFFFFFFFFFFFFF:FFFFF
@A00721:190:HGJW7DSXY:4:1449:21884:26522_1:N:0:TCTGCAAG+GCTGCACT
GGCTCTAAAGGACACTATACAATATCCAGACGACGACGAAGCTTATACTACTTTAATTTTGGGTATTA
TGTGGAGATATCGTGATATCAGTACGTCCAGATCCTCTGCTTTTACCGATAACGATAAATTCGTCATC
AAA
+
FFFFFFF:FFFFFFFFFFFFFFFFFFFFFFFFFFFFFFFFFFFFFFFFFFFFFFFFF:FFFFFFFFFF
FFFFFFFFFFFFFFFFFFFFFFFFFFFFFFFFFFFFFFFFF:FFFFFFFFFFFFFFFFFFFFFFFFFFFF
FFF
```

```
@A00721:190:HGJW7DSXY:4:1516:19542:4930_1:N:0:TCTGCAAG+GCTGCACT
TTGGTGCTTAGGGATATAATGAATTCAGCACCACATTTTGGTCAAGTCAATCCGTTGAGAAAATTTCT
TAGAGGATATTCGCCATTGTGGTACGAATTGGTCCAACATTTACCACCAAATTCTTTTGATACAACTC
TAG
+
FFFFFFFFFFFFFFFFFF:FFFFFFFFFFFFFFFFFFF:FFFFFFFFFFFFFFFFFFFFFFFFFFFFF
FFFFFFFFFFFFFFFFFFFFFFFFFFFFFFFFFFFFFFFFFFFFFFFFFFFFFFFFFFFFFFFFFFFF
FFF
@A00721:190:HGJW7DSXY:4:1531:32371:33098_1:N:0:TCTGCAAG+GCTGCACT
TGCCTGCGGAATACGCTTGCTATTCACCAGATTGGTTCGAACCCGTTGATGCTATGTCAGAAGAAGCT
ATTCAAGCTTGTCGTTTAAAGACTGCAGAGGCTGTCAAACAAGCCCAGACTCAAGGAGTCCAATCAAA
GGT
+
FFFFFFFFF:FFFFF:,FFFFFFFFFF:FFFFF:FFFFFFFFFFFFFFFFFFFFFFFFFFFFFFFFFF
F:FFFFFFFFFFFF:FFFFFFFFFFFFFFFFFFFFFFFFFFFFFFFFFFFFFFFFFFFFFFFFFFFFF
FFF
@A00721:190:HGJW7DSXY:4:1434:25418:25379_2:N:0:TCTGCAAG+GCTGCACT
TGGGTGATAAAAATTATTTAGAGATCTTTTCTACTTACAAAGGACAATTCAGTTTCATTAATTCTACG
GAAAAGTCCGGAGTATATTGTATGTTAATAGATAGTGGGATAATAGTT
+
FFFFFFFFFFFFFFFFFFFFFFFFFFFFFFFFFFFFFFFFFFFFFFFFFFFFFFFFFFFF:FFFFFFF
FFFFFFFFFFFFFFFFFFFFFFFFFFFFFF,FFFF:F,FFFFFFFFFF
@A00721:190:HGJW7DSXY:4:1572:30346:35383_1:N:0:TCTGCAAG+GCTGCACT
GTAGTGAACGCGTTCGTAGAACCAAGTACTTTCCGTCTGAAGAGTTTAGAGCTGCTATTGATTATGTA
ACCGATAGGAGTTGTATAGTTGAAGATGTGGCTTTAGACGATAATCCTTTAAGGACTATGTTGACTGA
CGA
+
FFFFFFF,FFFFFFFFFFFFFFFFFFFFFFFFFFFFFFFFFFFFFFFFFFFFFFFFFFFFFFFFFFFF
FFFFFFFFFFFFFFFFFFFFFFFFFFFFFFFFFFFFFFFFFFFFFFFF,FFFFFFFFFFFFFFFFFFFF
FFF
@A00721:190:HGJW7DSXY:4:1420:22941:24815_2:N:0:TCTGCAAG+GCTGCACT
TTGTCTACGTTGCAGATCGCCACTCCAAACACTATATACATTATAAAATATAAAAATAAGGGAGTCAT
AACTTTTGTTACCAAATTCTTGGCTTTCAACGAAGTCTCTGATTTTAT
+
F:FF:FF:FF:FFFFF,FFFFFFFFFFFFFFFFFFFFF:FFFFFFFFFFFFFF::FFFFFFFFFFFF
FFF,FFF,FFF:FFFFFFFFFF:FFFF:FFFFFFFFFFFFF:FFFFFF
@A00721:190:HGJW7DSXY:4:1354:7600:17378_1:N:0:TCTGCAAG+GCTGCACT
TAACGATCTCTCGGTCATTTACTCTGTACTTGATCTACGTCGTTTAACTGTCGGAACAACCTTTACTT
AGAATGACTCTATCTTTTCAGACACTTCTTATATTATTTACAATAGTTAATATCATATTAGTAATACT
CAC
+
FFFFFFFFFFFFFFFFFFFFFFFFFFFFFFFFFFFFFFFFFFFFFFFFFFFFFFFFFFFFFFFFFFFF
FFFFFFFFFFFFFFFFFFFFFFFFFFFFFFFFFFFFFFFFFFFFFFFFFFFFFFFFFFFFFFFFFFFF
FFF
@A00721:190:HGJW7DSXY:4:1460:14597:8547_2:N:0:TCTGCAAG+GCTGCACT
GCTTTTTCTTAGCTAATAGATTAATAAAGGATGAGTACGAAAACCAACTCTTCGTTACGAGACCTGAT
GGTAGAATAGATTTAGTCTCTACACCCATCTCAACGAGGATAAAGCGA
+
FFFFFFFFFFFFFFFFFFFF:FFFFFF:FFFFFFFFFFFFFFFFFFFFFFFFFFFFFFFF:FFFFFFF
FFFFFFFFFFFFFFFFFFFFFFFFFF,FFFFFFFFFFFF:FFFFFFFFF
@A00721:190:HGJW7DSXY:4:1165:22200:11819_1:N:0:TCTGCAAG+GCTGCACT
TACACGAAGAGATAATCCGCAAACGACTCGAAACCGAGACGCCGTAGATCAAAGTCCCTTCTAACTTT
AGATTATGAATCCTGCCTTTACTCATTTTATAGCAGTTATTTATGTTGTACTTTTTATAATTTATAAC
CTT
```

+
FFF:,FF,F::F,,F:FF:FF::FFFFFFFFFFFFFFFFFFF:FFFFFFFFFFFFFFFF:FFFFF:FF
FFFFFFFFFFFFFFFFFFFFFFFFFFFFFFFFFFFFFFFFFFFFFFFFFFFFFFFFFFFFFFFFFFFF
FFF
@A00721:190:HGJW7DSXY:4:1571:11306:26772_1:N:0:TCTGCAAG+GCTGCACT
TATGTACTTATTGTAACGATCATTCTTTCGAGTTTTGTCATTCTTATTCTTTTATTCCTTTAACTGTG
TATGAGGAGGATTTATAATGCTTTTCGATAGTGATTGTTATCAATTTAAATACATTACTGTTTCAAAT
CAT
+
FFFF:FFFFFFFFF,:FFFF,FFFFFFFFFFFF:FFFF:FFFFFFFFFFFFFFFFFF:FFF:FFFFFF
FFFFFFFFFFFFFF:FFFF,F:FFF,FFF:FFFFFF:FF:F:FF:FFFFFFF:FFFF:FFFFFFFFFF
FFF
@A00721:190:HGJW7DSXY:4:1318:5448:26897_2:N:0:TCTGCAAG+GCTGCACT
GACTCCGAAAATGGTTTCAAATGAGGTCAAAGCTCTATTTATTAAACGAACTATTATCCGATTCTTCG
AGGGTATCACTACCACTGATCTGCCGACTTTAGTCAAGGAACTTCAAT
+
FFFFFFFFFFFFFFFFFFFFFFFFFFFFFF:FFFFFFFFFFFFFFFFFF:FFFFFFF::FFF,FFF,
F:FFFFF,FFFFF:F,FFFF,F,FFFF:F:FFF:FFF:FFFFF,FFFF
@A00721:190:HGJW7DSXY:4:1247:16098:27524_2:N:0:TCTGCAAG+GCTGCACT
TATTTTTCTATAAGGGAAAATAGCAATAATCAAATTTCGACAAATTTTAGAACTTTATTTCAGAACTC
TCTCCGATCACCTTGGTGTGATCTCCCGGAGGATCTTTTCAATAAGAT
+
FFFFFFFFFFFFFFFFFFFFFFFFFFFFFFFFFFFFFFFFF:FFFFFFFFFFFFFFFFFFFFFFFFFF
FFFFFFFFFFFF:FFFFFFFFFFFFFFFFFFFFFFFFFFFFFFFFFFF
@A00721:190:HGJW7DSXY:4:1364:30101:20807_2:N:0:TCTGCAAG+GCTGCACT
ATTTTTAAATAGTGATATTCAGCGTATACTTGAATACTTTATCAAAGATTTGGCTTGGTCTATTTACC
GCAAAAAGTTGGAAGAGGTCTATGATATTTATTTTACTTATACTTTAA
+
FF:FFF:FFFFFFFFFF,FFFFFF:FFFFFFFFFFFFFFFFFFFFFFFFFFFFFFFFFFFFFFFFFFF
FFFFFF:FFFFFFFFFFFFFFF,FFFFFFFFFFFFFFFFFFFFFFFFF:
@A00721:190:HGJW7DSXY:4:1345:12283:13870_1:N:0:TCTGCAAG+GCTGCACT
AACTTGACTGTTGATAATATTACAATAGCTCTTTCTCAGAAGATTAGAGAAGTAACCGGTGAACACGT
CGGTAGCATTGATTTACTTTTACCGTTAGAACTGAAAAAGATTCTTTTCGAAAAGAAACCTATAAGTT
ACA
+
FF,FF:FFF:FF:FFFFFFFFF:FFFFF,F:FFFFFFFFFF:FFFFFFFFFFFFFFF:FF:FFFFFFF
FFFFFFFFFF:FFFFF:FFFFFFF:FFF,FFFFFFFFF::FFFFFFFFFFFFFFFFF:FFFFFFFFFF
FFF
@A00721:190:HGJW7DSXY:4:1166:28266:9455_1:N:0:TCTGCAAG+GCTGCACT
TATATTAAAGAAATAAAAATACTTCAAGTACCTGAACTTTACATTTTTAATGGTATGGTATAATGTGT
CTTACAAATAGAACCTTCACTCTCGGTAATGAGAGTGTATTCCTTAATATTCTAGTAACCGCTATGTG
GGC
+
FFFFFFFFFFFFFFFFFFFFFFFFFFFFFFFFFFFFFFFFFFFFFFFFFFFFFFFFFFFFFFFFFFFF
FFFFFFFFFFFFFFFFFFFFFFFFFF,FFFFFFFFFFFFFFFFFFFFFFFFFFFFFFFF:FFFFFFFFF
FFF
@A00721:190:HGJW7DSXY:4:1417:27525:35884_1:N:0:TCTGCAAG+GCTGCACT
GATAGCAATTTATGTTTTAAATGCTAGGAAAATTTTAGAAAAACTCCATTGTATAGTTCAAGGAGTAC
TTAATTCAATAGCTTTAGTATTATTTACTATCGCCTTTATTGAAAATTGTATAAGAGTTAATTGCTTA
CAC
+
FFFFFFFFF:FF:FFFFFFFFFFFFFFFFFFFFFFFFFFFFFFFF:FFFFFFFFFFFFFFFFFFFFFFF
FFFFFFFFFFFFFFFFFFFF:FFFFFFFFFFFFFFFFFFFFFFFFFFFFFF,FFFFFFF,FF:FFFFFF
FFF

@A00721:190:HGJW7DSXY:4:1542:23185:28839_2:N:0:TCTGCAAG+GCTGCACT
TTAATTGATTGAAGCTAGGCGTTGGGGAGAATGAACTGCCGCTTCCCGGAAGAAACGATCTATTTACA
GAGTATTTAGAGTAGTCCAAAGTTTGTATAAATTTATATGATTTTCTA
+
FFFF:FFFFFFFFFFF:FFFFFFFFFFFFFFFFFFFFFFFFFFFFFFFFFFFFFFFFFFFFFFFFFFFFF
FFFFFFFFFFFFFFFFF:FFFFF:FFFFFFFFFFFFFFFFFFFFFFFF
@A00721:190:HGJW7DSXY:4:1513:5864:34225_2:N:0:TCTGCAAG+GCTGCACT
TTCAATAACATAACATACCATACACTCCCCAAACATAACATACCATAACCTACCATGACCAATCATCG
CTCCAACTGGCACGCCAGACGTAGGGTCAAACCTACTACCGCCGGTCG
+
FFFFFFFFFFFFFFFFFF:FFFFFFFFFFFFFFFFFFFFFFFFFFFFFF:FFFFFFFFFFFFFFFFFFFF
FFFFFFFFFFFFFFFFFFFFFFFFFFFFFFFFFFFFFFFFFFFFFFFF
@A00721:190:HGJW7DSXY:4:1139:2465:22263_1:N:0:TCTGCAAG+GCTGCACT
TACGTTCAGGGACCCGCTGTGCAGAATAACCTTCCCAAATCTAGATCTGCACAGAAGAAGGCTGAAGG
TCGTTACCGGACGACCACTCAAAGGAAACGGGCTGTTGCCCAGAGGAAAGCAGCGACCACCGAGGTCG
CTC
+
FFFFFFFFFFFFFFFFFFFFF:FFFFFFFF:FFFFF::F,FFF,FFF::FFFFFFFF,FF:FFF:FFFFF
FF:FFFFFFFFFFFFFFFFFFFFFFFFFFFFFFFFFFFFFFFFFF:FFFFFFFFFFFFFFFFFFFFFFFF
FFF
@A00721:190:HGJW7DSXY:4:1166:4363:16219_2:N:0:TCTGCAAG+GCTGCACT
GCAGTCCCGTCATCTCTGTGGGAACTGAGGCAGTACCTCAGTCAAAAGTCACATTCACCACTCCCATG
CCAATCAATGATGTGACTATACAGTTCGGCCATTTCAAGAGTGGTCAA
+
FFFFFFFFFFFFFFFFFFFFFFFFFFFFFFFFFFFFFFFFFFFFFFFFFFFFFFFFFFFFFFFFFFFFFF
FFFFFFFFFFFFFFFFFFFFFFFFFFFFFFFFFFFFFFFFF:FFFFFF
@A00721:190:HGJW7DSXY:4:1313:24975:24674_1:N:0:TCTGCAAG+GCTGCACT
GAGGTACTGAAGCCTTCCCCTCCGCTGGCACGAAGAACACTCCCAAGCCAATCAATAGTTCTTCTGAC
CAGAATGAAAAGGGGTTTACCAGGGTAGTCTCTAAGTCTACCAGGAAGAGGGGAAATGTCCCCAAAAC
TGA
+
FFFFFFFFFFFFFFFFFFFFFFFFFFFFFF,FFFFFFFFFFFFFFFFFFFFFFFFFFFFFFFFFFFFFFF
FFFFFFFFFFFFFFFFFFFFFFFFFFFFFFFFFFFFFFFFFFFFFFFFFFFFFFFFFFFFFFFFFFFF:F
FFF
@A00721:190:HGJW7DSXY:4:1440:27190:19460_1:N:0:TCTGCAAG+GCTGCACT
TACGAGATCTTGCAGAGTGACTGGAGTTCAGACGTGTGCTCTTCCGATCTGGAAGATCTCCCACCATC
TGGGGTTGGGAATAAGAAATCTGCACGGGCCAAAGAGGCCGCTGCTACGAAGTCGCTGAAGGCGGCTT
+
::F,,F:FF:FFFFFFFFFFFFFFFFFFFFFFFFFFFFFFFFFFFFFFFFFFFFFFFFFFFFFFFFFFFF
FFFFFFFFFFFFFFFFFFFFFFFFFFFFFFFFFFFFFFFFFFFFFFFFFFFFFFFFFFFFFFFFFFFFFF
@A00721:190:HGJW7DSXY:4:1130:19316:4539_2:N:0:TCTGCAAG+GCTGCACT
TGTAAAGTTGCGCCTGCCGACCCCATCTTTGAGACCAAGACTCTCTACAGTAGGTTCGTGCCTACTAT
GAGCAAATCTGTTGAATTCGTCACTGAGGACGAAGGTGAGAGGACTTT
+
:FFFFFFFFFFFFFFFFFFFFFFFFFFFFFFFFF:FFFFFFFFFFFFFFFFFFFFFFFFFFFFFFFFFFF
FFFFF:FFFFFFFFFFFF:FFFFFFFFFFFFFFFFFFFFFFFFFFFFF
@A00721:190:HGJW7DSXY:4:1623:14407:33238_1:N:0:TCTGCAAG+GCTGCACT
CAAACTGAGAGCAGTCGCTGGGGAAAGAGAATTGCGTGGGTTGAAGGCCGACGCTTCGAGCTTGAGTG
TGACGAGGCCGGTGACGTTTTTGTCAACGGCGTTTGGAGGATTTTATCCGCGCTTAACTATGGGGCGA
AGA
+
FFFFFFFFFFFFFFFFFFFFFFFFFFFFFFFFFFFFFFFFFFFFFFFFFFFFFFFFFFFFFFFFFFFFFF
FFFFFFFFFFFFFFFFFFFFFFFFFFFFFFFFFFFFFFFFFFFFFFFFFFFFFFFFFFFFFFFFFFFFFF
FFF

```
@A00721:190:HGJW7DSXY:4:1421:13006:18098_1:N:0:TCTGCAAG+GCTGCACT
AGTCTTGAAGGACTTAGGTTGGCATATTTATGCCGACGATAATAAGCATGTCACCCTTAAGCACCGAG
GTAAGCTCGGGACTTACAAGAGAGAGTGTGAGAGAGTCGCTCTCACTATTTTTGCCCAGAATGCGGGC
TTT
+
FFFFFFFFFFFFFFFFFFFFFFFFFFFFFFFFFFFFFFFFFFFFFFFFFFFFFFFFFFF:FFFFFFFFF
FFF:FFFFFFFFFFFFFF,FFFFFFFFFFFFFFFFF:FFFFFFFFFFFFFFFFFFFFFFFFFFFFFFFF
FFF
@A00721:190:HGJW7DSXY:4:2124:32759:25911_2:N:0:TCTGCAAG+GCTGCACT
GCTCCTTCTGTGGGTGAGGGTTTTTGTTATATTCACTGGTTCTGGGAGGCTGCTTTCCTCCATAATTA
CAACCTCTCTTTAGAGAGCATTAGAAAGATCGGAAGAGCGTCGTGTAG
+
FFFFFFFFFFFFFFFFFFFFFFFFFFFFFFFFFFFF:FFFFFFFFFFFFFFFFFFFFFFFFF:FFFFFFF
FFFFFFFFF:FFFFFF:FFFFFFFFF:FFFFFFFFFFFFFFFFFFFFF
@A00721:190:HGJW7DSXY:4:2652:32877:17942_2:N:0:TCTGCAAG+GCTGCACT
GTTGTCACACTTTTCTTGAAAATTTTTCCTTTCCTCCGTCATTGGGGATTTCGATGTGAGAAGGTGGA
AGGTGTCAAGGGTCTTCTTCATGCTGATCTTTCAGGACCTAATCACAT
+
FFFF:FFFFFFFFFFFFFFFFFFFFFFFFF:FFF:,F:FF,FFFFFFFFFFFFFFFF,FFFFFF:FFFFF
FFFFFFFFFF,:F:FFFFFFFFF,FF:F,FFFFFFFFF,FFFFFF,FF
@A00721:190:HGJW7DSXY:4:2159:24478:21057_1:N:0:TCTGCAAG+GCTGCACT
ACGGCTCGTATCGAGTTGGAGGGAGGAAGTTCTCCGAAATTAAAGACGGGGGGATTAAGAGTCCAGCT
GTAACTGTCATTTCAGAGAGGGGAGCTCTGAAAAGGAAACTTCGTCAGAAGATGAGGAAGTCTAAGAG
GAG
+
:FFFFFFFFFFF:FFFFFFFFF:FF:F,:FFFFFFFFFFFFFFFFFFFFFFFFFFFFFFFFFFFFFFFF
FFFFFFFFFFFFF:FFFFF:FFFFFFFFFFFFFFFFFFF:FFFFFFFFFFFFFF:FFFFFFFFFFFFF
FFF
@A00721:190:HGJW7DSXY:4:1374:18882:30248_1:N:0:TCTGCAAG+GCTGCACT
AAGGCTCTAGAGAAGATCTCCCCACCTGGAGAAGGCAAGTGGAGAGTCCAGAAGTTGATGAAGAAGGA
TTATTTGAAGCAGGGGTTACCACTTCCTAAGGTGGATGAGGGAGGTTGTTTCTTTGGAGTTTTAGATA
ATC
+
FF:FFFFFFFFFFFFFFFFFF,,F:FFFFF::FF:FFFFFFFFFFFFFFFFFFFFFFF::FFFFF:FFFF
,F,FF:FFFFFFFFFFF,::FFFFF:FFFF:F:FFFFFFFFFFFFFFFFF,FFFFFFFFFFFFFFFF:
FF:
@A00721:190:HGJW7DSXY:4:1206:9869:26757_2:N:0:TCTGCAAG+GCTGCACT
AGCAAGAGAAGCTTATATGCTGGTTTAAGTGATGATTATCACTCCTTGAGAAGACAAGCCACATTAAG
ATATAATTTAGGTCAACTTGGGAGATCGGAAGAGCGTCGTGTAGGGAA
+
FFFFFFFFFFFFFFFFFFFFFFFFFFFFFFFFFFFFFFFFFFFFFFFFFFFFFFFFFFFFFFFFFFFFF
FFFFFFFFFFFFFFFFFFFFFFFFFFFFFFFFFFFF:FF,F::FFFF,
@A00721:190:HGJW7DSXY:4:1334:21748:36808_2:N:0:TCTGCAAG+GCTGCACT
TCCTTTTGAGTATCGACAGATCTTTCGATCTTGAGGACGTACATTTAACGTGGCAGAATTCGGTAACT
TACCATATTGTTCCTTCCGGGAAGTTGTGGGATTTAACTTTGACTCAA
+
FFFFFFFFFFFFFFFFFFFFF:FFFFFFFFFFFFFFFFFFFFFFFFF:F:FFFFFFFFFFFFFFFFFFF
FFF:FFFFFFFFFFFFFFF:FFFFFFFFF:FFFFFFFFFFFFFFFFFF
@A00721:190:HGJW7DSXY:4:2250:14000:9768_2:N:0:TCTGCAAG+GCTGCACT
GGTATGGACTACTCTACTTTTGATCTGGAGTTGTCTAGGGTCATTGCAGATACTACCGATAAGGTGAA
AAATATGCAGGTCAAAGATTCTGTAGCTCTGAGGGCGGTAGAGGGAGC
+
FF,F,:FFF,FFFF,FFFF,FFFFFFFFF,F,:,FFF,F,F,F:,F:FF:FF:,FF,F,:F:F:,,:F
F,F,F,,F:F,,FF:F,F,FF:,FF,:FFFF:F,FF:FF,FFFFFFF,
```

```
@A00721:190:HGJW7DSXY:4:1568:25870:31140_1:N:0:TCTGCAAG+GCTGCACT
TTCTAAGAGTCTTTATGATTCAAAACCTATCGTTGTCGTACCTTACAAGGTTGAGGAGACTTTACAGC
GGCTTATTGTCGCGCAGTTTCCAGAATTGCGGTTGAAGTTCACCCATTCTATCTTGAATGCTCACGCC
GTC
+
FFFF:FFFFFFFFFFFFFFFFFFFFFFFFFFFFFFFFFFFFFFFFF:FFFFFFFFFFFFFFFFFFFF
FFFFFFFFFFFFFFFFFFFFFFFFFFFFFFFFFFFFFFFFFFFFFFFFFFFFFFFFFFFFFFFFFFFF
FFF
@A00721:190:HGJW7DSXY:4:2141:20302:18208_2:N:0:TCTGCAAG+GCTGCACT
TTATAATCACTTGTTAAAGTACAGATTTAGTGATTTAGATATTATTGATATTGGAGGCGATATTCGTT
TTCATATCCAAAACTCCTTGAATGGAGTACATATTTGCAACCCTATTC
+
FFFFFFFFFFFFFFFF:FFFF:FFFFFFFFFFFFFFFFF:FFFFFFFFFFFFFFFF:FFFFFF:FFFF
FF:FFFFFFFFFFFFFF:FF:FFFFFFFFFFFFFFFFFFFFFFFF::FF
@A00721:190:HGJW7DSXY:4:2460:10755:35994_1:N:0:TCTGCAAG+GCTGCACT
GATGCATCGAGGTATACGTCTAGGAACATTGATTGGGAACGTACCTACTCACGAGACGTCATGAAAGC
TATCGGAGGGAAGCAAAATGTTTCGTTCTGTTACAAGAACGCTTTGGATTGTGACCATAAAGCCTCAG
TTG
+
FF:,:FFFFF:FFFFFFFFFFFFFF:FFFFFF:FFFF::FFF:FF:FFFF:FFFFFFFF:FFFFFFF,
FFFFFFFFFFFFFFFFFFF,FFFFFFFF:FFFFFFFFFFF:FFFFFFFFFFFFFFF:FFFFFFFFFFF
FFF
@A00721:190:HGJW7DSXY:4:1250:29116:29152_1:N:0:TCTGCAAG+GCTGCACT
CTCTCTTGATGATGTAGCCACTATAATGCTTAAGAGAGAGATTGATGTATGTCATTTCGTCTTAGTTT
GTCCCGGCGAATTATTGGACACGATGAAAAATGACATACCCGTTCCTGACCTTGGTATCGTTATCCGT
CAT
+
FFFFFFFFFFFFFFFFFFFFFFF:FF:FFFFFFFFFFFFFFFFFFFFFFFFFFFFFFFFFFFFFFFFF
FFFFFFFFFFFFFFFFFFFFFFFFFFFFFFFFFFFFF,FFFFFFFFFFFFFFFFFFFFFFFFFFFFFF
FFF
@A00721:190:HGJW7DSXY:4:2132:31539:17221_2:N:0:TCTGCAAG+GCTGCACT
GGTTATTCTCACAACAAAGATAAGATTTTATCTTTCTTCAAACACCCTGTTTTCCTTAAGGGCGGACA
ATTGTTCCATTGTGAACTCTTGGGGACTAGATTGAGCATTAATGAGTT
+
FFFFFFFFFFFFFFFFFFFFFF:FFFFF:FFFFFFFFFFFFFFFFFFFFFFFFFFFF:FFFFFFFFFF
FFFFFFFFFFFFFFFFFFFFFFFFFFFFFFFFFFFFFFF:F::F:FF,
@A00721:190:HGJW7DSXY:4:2101:2709:4836_2:N:0:TCTGCAAG+GCTGCACT
CTTTCTCTAGTGGACAATTCAGAATACCTATTTCCGTACCAAGAGCCACTGCCAATCTCATCGCTATA
CGCATACCTGATTGGAATAAGCGAGGTTCGTTCCTCAATAAAAATTTT
+
FFFFFFFFFFFFFFFFFFFFFFFFFFFFFFFFFFF:FFFFFFFFFFFF:FFF:FFFFFFFFF:FFF:FF
FFF:F:FFFFFFFFFF:FFFFFFFFFFFFFFFFFFFFFFF:FFFFFFFF
@A00721:190:HGJW7DSXY:4:1128:32624:10457_2:N:0:TCTGCAAG+GCTGCACT
TTTCTTCATAAGGGCTATTCAGTACGTCATGGGGATCACTTCTTCGGTCAATGACAAAACCTTCGAAT
ATGTTACGACATGGCTTCGTAACAACAGTGCGAGGATTGTCATCTCGG
+
FFFFFFFFFFFFFFFFFFFFFFFFFFFFFFFFFFFFFFFFFFFFFFFFFFFFFFFFFFFFFFFFFFFF
FFFFFFF:FFFFFFFFFFFFFFFFFFFFFFF:FFFFFFFFFFFFFFFF
@A00721:190:HGJW7DSXY:4:1119:20961:23703_1:N:0:TCTGCAAG+GCTGCACT
TCACCAAGATGTTCATATAGATACGAGATACCTAGAGAAGGTCGCAGCGATCTTACTCGTTATCGGAG
CTAAGAAGAAGACTTTGGGTACACGTTATGCTCGCAAACTTAACGTTAGTACCGGAGGTGATCTCTCT
GCT
+
FFF,F:FFFFFFF:FFFFFFFFF:FFFFFFFFFF:FFF,FFFFFFFFFFFFFFFFFFFFFFFFFFFFF
```

FFFFFFFF:FFFFFFFFFFFFFF:FFFFFFFFFFFF:FFFFFFFFFFFFFFFFFFFF:FFFFFFFFFFFF
FFF
@A00721:190:HGJW7DSXY:4:1404:24722:18662_1:N:0:TCTGCAAG+GCTGCACT
GAGAGAAAAGATTCAGGACGCCTACGGTGACTTTATGGACAAGATGCAAGTTCTTGGGAAATACTTGT
TTCCCTTTTTAGGTTTAGTAGGTGATCTTGACGTCGATTCTTTGTATAGGGAGTGTGAGAAGACAACG
AAT
+
FFFFFFFFFFFFFFFFFFFFFFFFFFFFFFFFFFFFFFFFFFFFFFFFFFFFFFFFFFFFFFFFFFFFFF
FFFFFFFFFFFFFFFFFFFFFFFFFF:FFFFFFFFFFFFFFFFFFFFFFFFFFFFFFFFFFFFFFFFFFF
FFF
@A00721:190:HGJW7DSXY:4:1111:30861:14074_1:N:0:TCTGCAAG+GCTGCACT
GAACCGGTCTCTAAATCTTTTGAGATTGAGAAGATTGAAGAACACGTCAAGAAGTCGCTCGGCGTTGT
TCTTGAAAACCAAGTTAAGAAGGAGGCTGATCATGGAGTCTTTGTACCTTCTGTCAAACTTCGAGGTT
CAG
+
F:FFF:FFFFFFFFFFFFFFFFFFFFFFFFFFFFFFFFFFFFFFFFFFFFFFFFFFFFFFFFFFFFFFFF
FFFFFFFFFFFFFFFFFFFFFFFFFFFFFFFFFFFFFFF:FFFFFFFFFFFFFFFFFFFFFFFFFFFFFF
FFF
@A00721:190:HGJW7DSXY:4:1442:9200:13448_2:N:0:TCTGCAAG+GCTGCACT
GATCCCGGATGTGGATTGAAGGCTGGCTCTAAGGGAGACTCCTTCTTTTTGAATGTTGTAAAGAATTG
GGTTGAGAAGTGTGCTTTACAAGGAGAGATCGGAAGAGCGTCGTGTAG
+
FFFFFFFFF:F,FFFFFFFFFFFFFFFFFFF,FFFFFFFFFFFFFFFFFFFFFFFFFFFFFFFFFFFFFF
FFFFFFFFFFFFFFFFFFFFFFFFFFFFFFFFFFFFFFFFFFFF,FFFF
@A00721:190:HGJW7DSXY:4:1168:20799:2910_2:N:0:TCTGCAAG+GCTGCACT
TAAAGAAAAATTTTCGAATAAACTAATGAGAGGTATAGGCTTAGTCAAGTCTGTACTTACATTCTTTA
CGCCAGATTCTTCATGGGGCGACGCTGTTTCTCTTGGTATTTCTGAAA
+
FFFFFFFFFFFFFF:FFFFFFFFFF:FFFFFFFFFFFFFFFFFFFFFFFFFFFFFFFFFFFFFFFFFFFF
F::FFFFFFFFFFFFFFFFFFFFFFFFF:F:FFFFFFFF:FFFFFFFFF
@A00721:190:HGJW7DSXY:4:1314:6479:2722_2:N:0:TCTGCAAG+GCTGCACT
CGTCATTTTCCTTTGGGATCTTTCCTCTGGTAAGGGAATTATTAAATCATTCTTCTCACTCTTGACGA
GAATTATTACTGTTTTCTCTACCCATAACTTCTTTTTAAAAAATGCGG
+
FFFFFFFFFF,FFFFFFFFFFFFF,FFFFF:FFFFFFFFFFFFFFFFFFFFF::FFFFFFF,FF:FFFFF
FFF:FFFFFFFFFFFFFFF:FFF,FFF:FFFFFFFFFF,FFFFFFFFFF
@A00721:190:HGJW7DSXY:4:1168:21594:5102_2:N:0:TCTGCAAG+GCTGCACT
TGATGTTTTCCTACAGAGTGCAGTCGTTTCAACACTTATCACCACTTTTGAAAGGAAAATAATTGGTG
ATGAAAATACGTATGTCGGTGGGGTTCTCAAGGCTCTCATCGCCACTG
+
FFFFFFFFFFFFFFFFFFFFFFFFFFFFFFFFFFF:F:FFFFFFF:FFFFF,:FFFFFF:FFFF:FFF,F
,FFFFFFF,F::FFF:FFFFFFFFFFFFFFFFF:FF,,:F:FF:,,F,F
@A00721:190:HGJW7DSXY:4:1130:11234:11835_2:N:0:TCTGCAAG+GCTGCACT
GGGTTCCATAAGATATTATCTGACATTGTGGTTCAATGATCAGGATTCTTCTTTTGGTCCTTACGTCT
TAAGTAATTTGGATGTTGTCGAAAACTATCAGGTTCTTGTCACGATTC
+
FFF:FFFFFFFFFFFFFFFFFFFFFFFFFFFFFFFFFFFFFFFFFFFFFFFFFFFFFFFFFFFFFFFFFF
FFFFFFFFFFFFFFFFFFFF:FFFFFFFFF:FFFFFFFFFFFFFFF:FF
@A00721:190:HGJW7DSXY:4:1111:26919:13855_2:N:0:TCTGCAAG+GCTGCACT
TTAAAACTTCAGAGTCTCTTGGAAGTCCAGGTAGCTAATGTTCTTGCAATAGCCACAGACTTTCTGGG
AAAAGAGACCTCTGTTGTAGAGGAGTTGAGAGAATTACTCAGATTCAA
+
FFFFFFFFFFFFFFFFFFFFFFFFFFFFFFFFFFFFFFFFFFFFFFFFFFFFFFFFFFFFFFFFFFFFFF
FFFFFFFFFFFFFFFFFFFFFFFFFFFFFFFFFFFFFFFFFFFFFFFFF

```
@A00721:190:HGJW7DSXY:4:1130:11234:11835_1:N:0:TCTGCAAG+GCTGCACT
ACGTCTACAACGAGTTAGGGAGTGGCTAGGTTTTAAAAGATCCTTTGCACAAGCAATACCTTCCACTG
AGTTCATCACCCAGAGTTTCGACGTCGCGGAAAACGATGAAGATTTCCACACTGTTCGAGAATCTTCT
GAT
+
FF:FFFFFFFFFFFF:FFFFFFFFFFFFFF:FFFFFFFFFFFFFF:FFFFFFFF:FFFFFFFFFFFFF
FFFFFFFFFFFF:FFFFFFFFFFFFFFFFFFFFFFFFFFFFFFFFFFFFFFFFFFFFFFFFFFFFFFF
FFF
@A00721:190:HGJW7DSXY:4:1325:24641:7028_1:N:0:TCTGCAAG+GCTGCACT
TGTGCTCTTCCGATCTCCTGATGGCTCAGTACAGGGAGGAGGTAATAGTTATAAAACCTTCTTTTATC
ATTGCAACAGGACTTTGAGAAGACTGTTGAAATGGACAAAATCTGGGATCATAGGTATGTGTCACGCT
ACA
+
FFFFFFFFFFFFFFFFFFFFFFFFFFFFFFFFFFFFFFFFFFFFFFFFFFF:F:FFFFFFFFFFFFF
FFFFFFFFFFFFFFFFFFFFFFFFFFFFFFFFFFFFFFFFFFFFFFFFFFFFFFFFFFFFFFFFFFFF
FFF
@A00721:190:HGJW7DSXY:4:1152:32850:36213_2:N:0:TCTGCAAG+GCTGCACT
GTTGGCGTCTTGTCGTTGACGTCATTACAGTCTGGAAGTTAGATCATTTGTTCAGGAAGATCTCTACA
GAGATTATCGAGAGTTGTGATCTAGTTCTAAATTTAGGTCGACGGACT
+
FFFFFFFFFFFFFFFFFFFFFFFFFF:FFFFFFFFFFFFFFF:FFFFFFFFFFFFFFF,FFFFFFFFFF
FFFFF:FFFFFFF,:F:::F:FFFFFFFFFFFFFFF:FFFFFFFFFFF
@A00721:190:HGJW7DSXY:4:1112:30689:32189_2:N:0:TCTGCAAG+GCTGCACT
ATGTACGTGGATCTAATGCTTGGTGATCTGAATCTGACCATCGCAAAGAAGACTTTATTAGTTCATGA
AGTTCTTACACTTCTCTCGACTAATGTCAGCTTCACTACAAAGGCGTT
+
FFFFFF,FFFFFFFFFFFFFFFFFFFFFFFF:FFFF:F,FFF:,FFFFFFF,FFFF:FFFF:FFFFF,F
F,FF:FFFFF:FFFFF,F,:FFFFF:FFF:FFFFF:FF:FFF,FF:FF
@A00721:190:HGJW7DSXY:4:2515:28718:31375_2:N:0:TCTGCAAG+GCTGCACT
CTTTATCTTAAAGCATTACCACCAAATTTAGCACTTTTTGAATATGACAGAAGCTCGCCATATCAGAT
TAGAGACATGCCAGTTAGAGTTCTTCTAACCAGGACTGATGGTGAGAT
+
FFFFFFFFFFFFFFFFFFFFFFFFFFFFFFFFFFFFFFFFFFFFFFFFFFFFFFFFFFFFFFFFFFFF
FFFFFFFFFFFFFFFFFFFFFFFFFFFFFFFFFFFF:FFFFFFFFFFF
@A00721:190:HGJW7DSXY:4:1677:1814:25833_2:N:0:TCTGCAAG+GCTGCACT
GGAGGTCCAGGAACCTTTACTCCTAGTGTCTTTTCTAGAGTCCAAGGACGTTTCCGTCGGAGGATGAC
AGGTGGGAGACCGCTCAATAACACTCTATTGAATCCCTTTTCCACGAT
+
FFFFFFFFFFFFFFFFFFFFFFFFFFFFFFFFFFFFFFFFFFFFFFFFFFFF:FFFFFFFFFFFFFF
FFFFFFFFFFFFFFFFF:FFFFFFFFFFFFFFFFFFFFFFFFFFFFFF:F
@A00721:190:HGJW7DSXY:4:1478:22905:7279_1:N:0:TCTGCAAG+GCTGCACT
GATATCGCCTCTGTCCTTTCTCCTTTAATCGTGAGGTTACGAGAGTTTCGTACTTGGGTTTATCGGTT
GTATTTTGATAAATTTGTTGGAATCGATAATGATACTGACGAAGGTTCTATTACCACCCTTGTCATTG
ATA
+
FFFFFFFFFFFFFFFFFFFFFFFFFFFFFFFFFFFFFFFFFFFFFFFF:FFFFFFFFFFFFFFFFFFF
FFFFFFFFFFFFFFFFFFFFFFFFFFFFFFFFFFFFFFFFFFFFFFF:FFFFFFFFFFFFFFFFFFFFF
FFF
@A00721:190:HGJW7DSXY:4:1573:1931:34992_2:N:0:TCTGCAAG+GCTGCACT
TGATGCTTTCTTAGAGGCTCGTCTAGCCGCTGAGATTCGTCTTACCTATTTACAATCTGTAGAGAGAG
AGGTTAATCAGAATTTGAGTGATAACGATTTAGTACTGGGAAATGGTG
+
FFFFFFFFFFFFFFFFFFFFFFFFFFFFFFFFFFFFFFFFFFFFFFFFFFFFFFFFFFFFFFFFFFFF
FFFFFFFFFFFFFFFFFFFFFFFFFFFFFFFFFFFFFFFFFF,FFFFF
```

```
@A00721:190:HGJW7DSXY:4:2666:32199:31767_1:N:0:TCTGCAAG+GCTGCACT
TTTTCTTTCCCTCCCCTTTTTCGATTGGATTCGACGGGGTCTTCGTCTCACGACTAATTTGATAAGGT
TTTTCTTTTATGGGACTCTTGTTTGTACTTATGAAGGAGGTATTACTTCGGAAGTTCTTAATGTAGGT
CTC
+
FFFF:F,FFFFFF:FFF:FFFFF:FFFFFFFFF:FFFFFFFFFFFFFFFFFF:FFFFFF:FFFFFFF
FFFFFFFFFFFFFFFFFFFFFFFFFFFFFFF:FFFFFFFFFF,:FF:FFFFF:FFFFFFFFFFFFFFF
FFF
@A00721:190:HGJW7DSXY:4:1617:27444:31579_1:N:0:TCTGCAAG+GCTGCACT
GGTTTATGTTTCGGTTTCAGCTGGATGGATCTTTTGATGGTATGTTTCACATTGCGTGATCACTTCCC
GTGGACTTGGGAAGAGATTAAACATAAAATCCAGCGATTCTATTATGTCGCTAAAGCCTGTCCAGTTG
TCG
+
FFFFFFFFFFFFFFFFFFFFFFFFFFFFFFFFFFFFFFFFFFFFF:FFFFFFFFFFFFFFFFFF:,FFF
FFFFFFFFFFFFFFFFFFFF,FFFFFFFFFFFFFFFFFFFFFFF:FFFFFFFFFFFFFFFFFFFFFFFF
FFF
@A00721:190:HGJW7DSXY:4:1463:10022:17785_2:N:0:TCTGCAAG+GCTGCACT
GGCTGTGGGGATTAACTTCCCTCGTGAAACTTACGACGATGTTCTCGCTCGCATTTCACAAGGATTAC
TAAGTCTGAGATCTGCCACTTCTAATTTTAGGAAACGTCGCGAAAAGA
+
FFFFFFFFFFFFFFFFFFFFFFFFFFFFFFFFFFFFFFFFFFFFFFF:FFFFFFF:FFFFFFFFFFFF
FFFFFFFFFFFFFFFFFFFFFFFFFFFF:FF:FFFFFFFFFFFFFFF:F
@A00721:190:HGJW7DSXY:4:1127:11153:24001_1:N:0:TCTGCAAG+GCTGCACT
TCTTGGTCGAAAAATATCGCCGGGAACTTCTAATAGAGGATCCTGATGACATTATTTCAACGGATACC
GATCTCAGTTCTACCACAGAAACCGTCTCAGGAACTGTCTCCACTGATTCGGAATTAGAGGAAGACGA
CCT
+
FFFF:FFF:FFFFFFFFFFFF::FFFFF:FFFFFFFFFFFFFFFFFFFFFFFFFFFFFFFFFF,FFFFF
FFFFFFFFFFFFFFFFFFFFFFFFF:::FFFF:FFFFFF:FFFFFFFFFFFFFFFFFFF:F,F:F:FFF
F:F
@A00721:190:HGJW7DSXY:4:1669:13250:19586_1:N:0:TCTGCAAG+GCTGCACT
GAGACCTCTGAATTTTCAGGGTGCCCTGATGAGGACGTTGAAAGGGATAATTTCAACTCTGAGACTTC
TAAGAGTGTTTCAAAAGACCAGAAACGTAACAAGAAGAATAAAGGTAGGAAGGGGAGAAAACAGAACA
AGC
+
FFFFFFFFFFFFFFFFFFFFFFFFFFFFFFFFFFFFFFFFFFFFFFFFFFFFFFFFFFFFFFFFFFFF
FF:FFFFFFFFFFFFFFFFFFFFFFFFFFFFFFFFFFFFFFFFFFFFFFFFFFFFFFFFFFFFFFFFFF
FFF
@A00721:190:HGJW7DSXY:4:1520:25970:5008_2:N:0:TCTGCAAG+GCTGCACT
ACTGATATGGTTGCGGCTGATCGTACCCCAATTCCTGCTAGAAAGATCACTCTCGGCGATACCACTGA
TCTTCCAGAGTTACGTAGTGGTCGTTCGTTTGTTGATTTTATCAAAGA
+
FF:FFFFFFFFF:FFFFFFFFFF,:FFFFFFFFFFFF:FFFFFFFFFFFF,FFFFFFFFFFFFFFFFF
FFFFF:FFFFFFFFFFF,FF:FFFFF:,,:FFFFFFFFFFFFFF,FFF
@A00721:190:HGJW7DSXY:4:2404:17933:5368_2:N:0:TCTGCAAG+GCTGCACT
CAAGTTAAACTTCGAACTGACCACTTACAAGAGTGTTGAAGTACCGCAACATGGATCAGTTTTCCAAA
AAGCTATTAATAGCAGTTGCGGGGCTATAATACCGCCTCCAGATTACT
+
FFFFFFFFFFFFFFFFFFFFFFFFFFFFFFFFFFFFFFFFFFFFFFFFFFFFFFFFFFFFFFFFFFFF
FFFFFFFFFFFFFFFFFFFFFFFFFFFFFFFFFFFFFFFFFFFFFFFF
@A00721:190:HGJW7DSXY:4:2139:1823:14043_1:N:0:TCTGCAAG+GCTGCACT
AGAGTTTTGGAACTGTGTAATGTCCTACGTACTGTTCAAGGTGTTTGGGCTTCTCTAAAGAACAGCAA
CTTTCAACATGAATTAGTTATTGAAAGTCTTGACCCTCACGTCTCACTTTATCACCTTGGAGATGGTA
AAG
```

```
+
FFFFFFFFFFFFFFFFFFFFFFFFFFFFFFFFFFFFFFFFFFFFFFFFFFFFFFFFFFFFFFFFFFFF
FFFFFFFFFFFFFFFFFFFFFFFFFFFFFFFFFFFFFFFFFFFFFFFFFFFFFFFFFFFFFFFFFFFF
FFF
@A00721:190:HGJW7DSXY:4:1523:26865:23406_2:N:0:TCTGCAAG+GCTGCACT
ATTCTGCTTCGACGGTAATTCCATAAGACCTTTCTTGGGGAGTAAAGAGGGTTTGAAAATTACGGAAC
CTTTCCTCGTTCTTCATGAAGATCTTCGAGTGTTTTATAGTGATTTAT
+
FFFFFFFFFFFFFFFFFFFFFFFFFFFFFFFFF:FFFFFFFFFFF,FFFFFFFFFFFFFFFFFFFFFF
FFFFFFFFFFFFFFF:FFFFFFFFFFFFFFFFFFFFFFFFFFFFFFFF
@A00721:190:HGJW7DSXY:4:1266:32633:34084_1:N:0:TCTGCAAG+GCTGCACT
CAAGTCGATTGATTTTGTTGACTACGATCTCTCTTGTAATAAGAAGTTCTTCGCAGTTGAATCTCCAC
CAGGTGGAGGGAAGACCACTGAACTTATAGCGACTTTTGTCGAATTTTTCGCAAAAGGTCTTGAAGTA
CGT
+
FFFFFFFFFFFF,FFFFFFFFFFFFFFFFFFFFFFFFFFFFFFFF:FFFFFFFFFF:FFFFFFFFFFF
FFFFFFFFFFFFFFFFFFFFFFFFFFFFFFFFFF:FFFFFFFFFFFFFFFFFFFFFFFFFFFFFFFFF
FFF
@A00721:190:HGJW7DSXY:4:1358:30092:25363_2:N:0:TCTGCAAG+GCTGCACT
TCGTAATTCGCGAGAAGAGATAGTGACTAAAGTTTTGGCACTTCTAGTTCATCGCGGTATTTGTGAAA
TTAAGACAGACGTGAAAGTTATGATGAGTGATCGTATTAGAACAGCTG
+
FFFFFFFFFFFFFFFFFFFFFFFFFFFFFFFFFFFFFFFFFFFFFFFFFFFFFFFFFFFFFFFFFFFF
FFFFFFFFFFFFFFFFFFFFFFFFFFFFFFFFFFFFFFFFFFFFFFFF
@A00721:190:HGJW7DSXY:4:1662:22525:9471_2:N:0:TCTGCAAG+GCTGCACT
ACGTCGCTGAGGTCAAACCTGTGGACGTTCTTTTCATCGACGAATTATTCATGATTCACGCTGGTCAG
ACCCTTTTTCTTACACATCTACTTAAGGCGGCTTATACCATAGGTTAC
+
FFFFFFFFFFFFFFFFFFFFF:FFFFFFFFFF:FFFFF:FFFFF:F:FFFFFFFFFFF:FFFFFFF,F
FFFFFFFFFFFFFFFFFFFFFFFFFFFFFFFFFFFFFFFFFFFFFFFF
@A00721:190:HGJW7DSXY:4:2353:13476:28463_2:N:0:TCTGCAAG+GCTGCACT
CAAATCGGATTCATCAATCGTATTGATCTCCAAGTCGCAAACTACTTCAACATCAACGACATCATTCT
TGATGAGTGTCGTTCTTATCGAAGTCTCAGTTATCGCTGCCCGGCCGA
+
FFFFFFFFFFFFF:FFFFFFFFFFFFFFFFFFFFFFFFFFFFFFFF::FFFFFFFFFFFFFFFFFFFF
FFFFFFFFFFFFFFF:FF:FFFFFFFFFFFFFFF:FFFFFFFFFFFFF
@A00721:190:HGJW7DSXY:4:2513:26169:20134_2:N:0:TCTGCAAG+GCTGCACT
GAACTCTCAGAGGTTTACGGCAGAAAAATTTCTCCTGCTTGTAATTCGAGAGAGGACACTATGGAAAT
TAGACCTATCAGATGTCTTGACGACTTCGAATTTGAGGACGGAGTTAA
+
FFFFFFFF:FFFFFFFFFFFFFFFFFFFFFFFFFFFFFFFFFFFFFFFFFFFFFFFFFFFFFFFFFFF
FFFFFFFFFFFFFFFFFFFFFFFFF,FF:FFF:FFFFFFFFFFFF:F:
@A00721:190:HGJW7DSXY:4:2631:9968:21042_2:N:0:TCTGCAAG+GCTGCACT
AATTAACAAAGTCCTTGGTAAGAGTAAGGTTAAATGTGAAGATGCTCAGACTATTCATGAAGTTCAAG
GTAATACTTACCAGCACGTTCGGCTGGTTAGAATTAAACCTCAAGATG
+
FFFFFFFFFFFFFFFFFFFF,F:FFFFFFFFFFFFF:FFFFFFF:FFFFFFFFFFFFF:FFFFFFFFF
FFFFFFFFFFFFFFFFFFFFFFFFFFFFFFFFFFFFFFFFFFFFFFFF
@A00721:190:HGJW7DSXY:4:2622:9498:13526_2:N:0:TCTGCAAG+GCTGCACT
CACAAACACATATTACCGTTGCTCTATCACGACACGTTGAATCTTTACGATACTTTGTTATCAATGTA
AAGTTAAACGATGCTACTTGTGAGAGGATCAATCACAGTAAGGAACTG
+
FFFFFFFFFFFFFFFFFFFFFFFFFFFFFFFFFFFFFFFFFFFFFFFFFFFFFFFFFFFFFFFFFFFF
FFFFFFFFFFFFFFFFFFFFFFFFFFFFFFFFFFFFFFFFFFFFFFFF
```

```
@A00721:190:HGJW7DSXY:4:1511:21640:26506_2:N:0:TCTGCAAG+GCTGCACT
ACGGGAGGTTGCAGATTCAACCCCTTATATCGAGGCTATCGAGGACGATGAGTATCTTGAAGTTGTTG
ACGACAAGCCTCAAGAGGGTAGAGGACCACCTGGATCTTCTACTGTCT
+
FFFFFFFF:FFFFFFFFFFFFFFFFFFFFFFFFFFF:FFFFFF,FFFFFFFFFFFFFFFFFFFFFFF
FFFFFFFFFFFFFFFFFFFFFFFFFFFFF:FFFFFF,FFFFFFFFFFFF
@A00721:190:HGJW7DSXY:4:1606:7075:27398_2:N:0:TCTGCAAG+GCTGCACT
GGTTATTCCTGGAACCACCTTAACGTCACTCGGTAATCTTTCAGCCGAATTAAGTACTAATGAGTTCA
AGTCGGTTGTCGATCGAATTAAGATCAAGGCTAATACCTGTATAGGTA
+
FFFFFFFFFFFFFFFFFFFFFFFFFFFFFFFFFFF:FFF:FF:FFFFFFFFFFFFFFFFFFFFFFFFF
FFFFFFFFFFFFFFFFFFFFFFFFFFFFFFFFFFFFFFFF,FFFFFFFF
@A00721:190:HGJW7DSXY:4:2310:26856:32346_2:N:0:TCTGCAAG+GCTGCACT
GCCGCGTAGTCAGCGCGTTTAGCTGTATCATCAGTCAGAGTATACCCGATAGGGTACCGAGTCTCCAA
GAGAATATTTATTCTTATGAGGCTCGAAACTTCAACGTTTTCACAACG
+
FFFFFFFFFFFFFFFFFFFFFFFFFFFFFFFFFFFFFFFFFFFFFFFFFFFFFFFFFFFFFFFFFFFF
FFFFFFFFFFFFFFFFFFFFFFFFFFFFFFFFFFFFFFFFFFFFFFFF
@A00721:190:HGJW7DSXY:4:2242:22589:29904_2:N:0:TCTGCAAG+GCTGCACT
TCGCACCCGAAATTTTTGGGCAGAATGCTCTTAACGCGGTTCTTGAACGGTGTTTTGATCAGTCTAAG
TTGGATAAAGTTATAGGAGACTCAGTTAACCTGAGAGCTCCTCAATTG
+
FFFFFFFFFFFFFFFFFFFFFFFFFFFFFFFFFFFFFFFFFFFFFFFFFFFFFFFFFFFFFFFFFFFF
FFFFFFFFFFFFFFFFFFFFFFFFFFFFF:FFFFFFFFFFFFFFFFFF
@A00721:190:HGJW7DSXY:4:2120:19443:12367_1:N:0:TCTGCAAG+GCTGCACT
CAATAGGGACGCTAGTATACTTCGTAAAGTCGACAGCGAAATGGTCAATATTGATAATTGGACCGACT
ATATGACCTATTTTAAGCTCATGGTGAAAAGGGAAGCTAAGGTTAAACTTGATGCTTCAGCCTTTTCG
AAG
+
FFF::FFFFFFFFFFFFFFFFFFFFFFFFFFFFFFFFFFFFFFFF:FFFFFFFFFFFFF:FFFFFFFFF
FFFFFFFFFFFFFFFFFFFFFFFFFFFFFFFFFFFFFFFFFFFFFFFFFFFFFFFFFFFFFFFFFFFF
FFF
@A00721:190:HGJW7DSXY:4:2366:13340:28886_1:N:0:TCTGCAAG+GCTGCACT
AGAATATCGTTTATCACGCAAAATTTATTAATGCAATTTTCGGACCTATCTTTAAGGTTCTTGTGAGG
AGAGTCAAACAGTGTCTTTTACCCAACATTTTTATGTATACAGAGATGACCAGTGACGATTTCTCCCG
GAA
+
FFFFFFFFFFFFFFFFFFFFFFFFFFFFFFFFFFFFFFFFFFFFFFFFFFFFFFFFFFFFFFFFFFFF
FFFFFFFFFFFFFFFFFFFFFFFFFFFFFFFFFFFFFFFFFFFFFFFFFFFFFFFFFFFFFFFFFFFF
FFF
@A00721:190:HGJW7DSXY:4:2346:15148:29356_1:N:0:TCTGCAAG+GCTGCACT
CGAGATCTTGCAGAGTGACTGGAGTTCAGACGTGTGCTCTTCCGATCTTTTCTTTTGTAAAGCCTATG
AGATAAAGATGTATTCTTATTTAGGCTTCGATGCTGAATTTCTTGAACTTTGGAGTGCCAGTGAATAT
TTT
+
FF:FFFFFFFFFFFFFFFFFFFFFFFFFFFFFFFFFFFFFFFFFFFFFFFFFFFFFFFFFFFFFFFFF
FFFFFFFFFFFFFFFFFFFFFFFFFFFFFFFFFFFFFFFFFFFFFFFFFFFFFFFFFFFFFFFFFFFF
FFF
@A00721:190:HGJW7DSXY:4:1273:26503:10817_1:N:0:TCTGCAAG+GCTGCACT
GAGTATCCATAACGATTTGTCATTTACCACCTTTGCTCAACGACGAAGTGGAACGGCCAACACTTTCT
TTGGTAATTGTATGATCACTTTGATGATTTTATCACTTTATTATGACCTTGGAAAGTTTTCGGCTGTG
GTT
+
FFFFFFFFFFF:FFFFFFFFFFFF:FFFFFFFFFFFFFFFFFFFFFFFFFFFFFFFFFFFFFFFFFF:
```

```
FFFFFFFFFFFFFFFFFFFFFFFFFFFFFFFFFFFFFFFFFFFFFFFFFFFFFFFFFFFFFFFFFFFF
FFF
@A00721:190:HGJW7DSXY:4:2335:3305:17895_1:N:0:TCTGCAAG+GCTGCACT
CCATTCTGTTTTCAAAGACTCCAATAACTGATCATTCTGATCATATGATTTTGGACAATGGTTTCGAA
GCGAAATTTTTGAGGGATAAACCAGCGTACTTCTGCTCGCGATTTATTCTTTTTGTCAATGATTACAT
CTA
+
:FFFFFFF:FFFFFFFFFFFFF,FFFFFFFFFFF,FFFFFFFFFFFFFFF:FFFFFFF:FFFFFFFFF
FFFFFFFFFFFFFFFFFFF,FFFFFFFFFFFFFFFFFFFFFF:FFFFFFFFFFFFFFFFFFFF:FFFF
FFF
@A00721:190:HGJW7DSXY:4:1377:23809:26256_1:N:0:TCTGCAAG+GCTGCACT
CCTTACAAGATCATCGTTAAGTTAGGTAAACCCCTAACCGATGTTACTCTTGTAGGACTTTGGGAGAG
ATTCGTTAGTTTTAAAGACTATACTAAATATCTCCACTACGAGGTTGTTGTTTGGCGATTAGCTCTTG
CGA
+
FFFFFFFFFFFFFFFFFFFF,FFFFFFF:FFFFFF,:FFFFFFFFFFFFFFFFFFFFFFFFFFFFFFF
FFFFFFFFFFFFFFFFFFFFFFFFFFFFFFFFFFFFFFFFFFFFFFFFFFFFFFFFFFFFFFFFFFFF
FFF
@A00721:190:HGJW7DSXY:4:1428:27118:12195_2:N:0:TCTGCAAG+GCTGCACT
ATTGAGGGTCGTTATACCTATTCGGCTATCTGTGCTTTGAATTGTATAAGATCAAATTTTAAACGATT
CCGAGAGGTTTATCCTGATGTGGAGGGAGTTCTTGAAGCATCCCGACC
+
F:FFFFFFFFFFFFFFFFFFFFFFFFFFFFFFFFFFFFF:FFFFF,FFFFFFF:FF:FFFFFFFFFFF
F:FFFFFFFFFFFFFFF:FFFFFFFF,FFFFFFFFF,,FFFFF:FFFF
@A00721:190:HGJW7DSXY:4:1542:15248:8578_1:N:0:TCTGCAAG+GCTGCACT
TGATGCGGTTTCATAAGATTATCAGTAATCCTGATTCTACTACGGATTCATGGACCTTCCATCCTGTA
CCTCTTCTGGAGAGACGTATCTCAAAGATCGACTTCGAGACAGGTGATATTGTTGAGCGAGAGCGACC
AAG
+
FFFFFF:FFFFFFFFFFFFF:FFFFFFFFFFFFFFFFFFFFFFFFFFFFFFFFFFFFFFFF:FFFFFFF
FFFFFFFFFFFFFFFFFFFFFFFFFFFFFFFFFFFFFFFFFFFFFFFFFFFFFFFFFFFFFFFFFFFF
FFF
@A00721:190:HGJW7DSXY:4:1508:20518:22968_1:N:0:TCTGCAAG+GCTGCACT
CGTGGCGCTTTATTTTAAAAAGCTCCCTCCTCTCGCAAGTTTGATCTGATACTTATCACGAAAGGACT
TGAGTCTTTTGATCAGTCTCTGACTTGACCATCTTTCATTTTAAATGACGATCATTAAGACTCTCTTT
CTT
+
FFFFFFFFFFFFFFFFFFFFFFFFFFFFFF:FFFFFFFFFFFFFFFFFFFFFFFFFFFFFFFFFFFFF
FFFFFFFFFFFFFFFFFFFFFFFFFFFFFFFFFFFFFFFFFFFFFFFFFFFFFFFFFFFFFFFFFFFF
FFF
@A00721:190:HGJW7DSXY:4:1448:4164:31939_2:N:0:TCTGCAAG+GCTGCACT
CTTTGCACTGATTTTTCCTCATCAGCTTGTGTTTTACCTAATCTGTGTTTTTACGATAACAACTATCA
GTTGATTAAGACTCTTCCGAAAGGCGAACTTTGGGAATTTGAAGCTCA
+
FFFFFFFFFFFFFFFFFFFFFFFFFFFFFFFFFFFFFFFFFFFFFFFFFFFFFFFFFFFFFFFFFFFF
FFFFFFFFFFFFFFFFF:FFFF:FFFFFFFFFFFFF,FFFFFFFFFFF
@A00721:190:HGJW7DSXY:4:2416:25789:3881_1:N:0:TCTGCAAG+GCTGCACT
CGTTTGACCGTGACTAACTGTGGTGTTCTTGATATAAGTTACGGATATACTCTCCTTGAGTTCACTCG
TAAGGACACTACCACTTATTACGATATTTCTTTAGTCGATGGGTTTTCGACTCCTTTTAGCGTCTTCT
GTT
+
FF,FFFFF:FFFF,,FFFFFF,:FFF:FFF,FF,F:,FFFF,,,FFF:FFF:FF,FF,FFFFF,,FFF
FFFF:,,:,F:,FFFFFF:FF:,FFFFFFFFFF,FF:FFFFFFFFFF:F,FF:FFFF,,::,F,FF,F
FFF
```

```
@A00721:190:HGJW7DSXY:4:1417:21884:7795_2:N:0:TCTGCAAG+GCTGCACT
TTTCTTCTAATGTGGCCTGAACTACGTTTCACTTTGTGTGTACTTGTCTCTATTTTAAGTTTTGTCTT
CCTTGTTTGTCTTATTCTATTAATCACTCATTTTAGTGAGATTATTAA
+
FFFFFFFFFFFFF:FFFFFFFFFFFFFFFFFFF:FFFFFFFFFFFFFFFFFFFFFFFFFFFFF:FFFF
FFFFFFFFFFFFFFFFFFFFFFF:FFFFFFF:FFFFFFFFFFFF:FFFF
@A00721:190:HGJW7DSXY:4:1607:5511:26287_1:N:0:TCTGCAAG+GCTGCACT
GTCTTCGACAACAGCAGGGGTTTTCGAATAGTGATAGCCACCCGCCTTACAGAGGGGCAGCAGCATGA
CCATAATTGGTATTGACTTTGGTACCACCTTTTCTACTGTCTGTCTCGCCTCTTCGAATGAAGTTTTA
ATG
+
FFFFFFFFFFFFFFFFFFFFFFFFFFFFFFFFFFFFFFFFFFFFFFFFFFFFFFFFFFFFFFFFFFFF
FFFFFFFFFFFFFFFFFFFFFFFFFFFFFFFFFFFFFFFFFFFFFFFFFFF:FFFFFFFFFFFFFFFF
FFF
@A00721:190:HGJW7DSXY:4:1412:9697:24674_2:N:0:TCTGCAAG+GCTGCACT
TGATAGTGAATTTATACCTACAATCATTGGTTTTTCTAATAATGATGACACTATTGTTTATGGATATG
ATGCTATTACTCATGATGGTTACGGTCAGGGAGGATTCTCAGTTTACA
+
FFFFFFFFFFFFFFFFFFFFFFFFFFFFFFFFFFFFFFFFFFFFFFFFFFFFFFFFFFFFFFFFFFFF
FFFFF:FFFFFFFFFFFFFFFFFFFFFFFFFFFFFFFFFFFFFFFFFF
@A00721:190:HGJW7DSXY:4:2323:19488:25034_2:N:0:TCTGCAAG+GCTGCACT
ACATTACCCGAAAGACGTGCTAAATTAAAACCATTGTACGATGTCGTCTGTCTCGGCGATCACTTTTC
ACTAAGAATTGGTCCTACTTTTGGACAAGGCCAACTTCGTAATCTTCA
+
FFFFFFFFFFFFFFFFFFFFFFFFFFFFFFFFFFFFFFFFFFFFFFFFFFFFFFFFFFFFFFFFFFFF
FFFFFFFFFFFFFFFFFFFFF:FFFFFFFFFFFFFFFFFFFFFFFFFF
@A00721:190:HGJW7DSXY:4:2325:7500:21496_2:N:0:TCTGCAAG+GCTGCACT
TTTATCTCTTTGGTTGTTAGGGATTTTGAGAGAGTGAAAGGCTTCAATTGTAGTGGTTTGGTCGTTTC
AGTACCCAGTCAGTATACCTCTACGCAAAGATATTTCATGAAAACTAC
+
FFFFFFF::FFFFF,F::FFFFFFFFFFFFFFFFFFFFFFFFF:FF,FFFFFFFFFFF:FFFFFFFFFF
FFFFFFFFFFFFFF,FFFFFFFFFFF:FFFFFFF:FFFFFFFFFFFF
@A00721:190:HGJW7DSXY:4:1637:15230:36417_2:N:0:TCTGCAAG+GCTGCACT
ATTACCTGTCTTTCATATTATGAATGAACCTTCGGCCGCTCTCTTTGCTTCGATGTTGGATGTCAAGA
AGGGTTCAGAATGGGATTCCTACGTAGTTTATGACTTTGGTGGTGGTA
+
FFFFFFFFFFFFFFFFFF:FFFFFFFFFFFFFFFFFFFFFFFFFF:FFFFFFFFF,FFFFFFFFFFFFF
FFFF:FFFFFFFFFFFFFFFF::FFFFFFFFFF:,:FFFFFFFFFFFF
@A00721:190:HGJW7DSXY:4:1250:24207:29136_1:N:0:TCTGCAAG+GCTGCACT
TCTTACGTTACGGTAATTACTATAGTGTTATTTATTCTGGTGGTGATGATAATCTTGGAGGTCGTGAC
GTTGATGAAGCTATAAGAGCGTTCTTATTACGGCGCACTTCTTTACCCGCCACCACACAACTTTCAGT
TTC
+
F:FF:FFFFFFF:FFFFFFFFFFFFFFFFFFFFFFFFFFFFF:FFFFFFFFFFFFFFFFFFFFFFFFFF
FFFFFFFFFFFFFFFFFFFFFFFFFFFFFFFFFFFFFFFFFFFFFFFFFFFFFFFFFFFFFFFFFFFF
FFF
@A00721:190:HGJW7DSXY:4:2325:17255:9549_2:N:0:TCTGCAAG+GCTGCACT
TGTTACTGGAAAACCCTCGTCAATACTTTTCAATAATATACGTCTTGAACTCACTTATGACGATCTAG
TGAAATTAACGCGTCCTTTTCTGGAACGTAGTTTTACTATTATGAACA
+
FFFFFFFFFFFFFFFFFFFFFFFFFFFFFFFFFFFFFFFFFFFFFFFFFFFFFFFFFFFFFFFFFFFF
FFFFFFFFFFFFFFFFFFFFFFFFFFFFFFFFFFFFFFFFFFFFFFFF
@A00721:190:HGJW7DSXY:4:1619:31882:33692_1:N:0:TCTGCAAG+GCTGCACT
ACGTGTGCTCTTCCGATCTAACTTACCCTGATTCCGGCCGGTGGTAGCTCATTTCTACCAGGGGCTAT
```

TACTCTTGCTGAAACAGCTATTAAAGCTGTTGAGCGGGTAATTACACCTAAACGAGCTCGATCTGCCA
TCG
+
FFFFFFFFFFFFFFFFFFFFFFFFFFFFFFF,FFFFFFFFF,FFFFFFFFFFFFFFFFFFFFFFFFFF
FFFFFFFFFFFFFFFFFFFFFFFFFFFFFFFFFFFFFFFFFFFFFFFFFFFFFFFFFFFFFFFFFFFF
FFF
@A00721:190:HGJW7DSXY:4:1456:27254:16438_2:N:0:TCTGCAAG+GCTGCACT
CACTCTTTTCTGCTACTTTAACGGAGCGTAGTGTACTCTTCGTCGATTGTATCAACAGTAACATTACT
TCTAATAAAGGTCTCTATCAAGCTCGTATTATCGTCGCTGCTGGATCT
+
FFFFFFFFFFFFFFFFFFFFFFFFFFFFFFFFFFFFFFFFFFFFFFFFFFFFFFFFFFFFFFFFFFFF
FFFFFFFFFFFFFFFFFFFFF:FFFFFFFFFFFFFFFFFFFFFFFFFF
@A00721:190:HGJW7DSXY:4:1629:20889:15843_1:N:0:TCTGCAAG+GCTGCACT
AAGGCTAAACTCCAAGGTAATTATGAAGACTGTGATTTATCTTTCACTCTGGCCTTGTATGAGGGTGT
TTCTAATCGTAACATTAATAATTTCAATCTTCTTAAAAAGAAGATAGAGAGTCATACTTTGGGCTATC
GTT
+
FFFFFFFFFFFFFFFFFFFFFFFFFFFFFFFFFFFFFFFFFFFFFFFFFFFFFFFFFFFFFFFFFFFF
FFFFFFFFFFFFFFFFFFFFFFFFFFFFFFFFFFFFFFFFFFFFFFFFFFFFFFFFFFFFFFFFFFFF
FFF
@A00721:190:HGJW7DSXY:4:1447:11234:23015_2:N:0:TCTGCAAG+GCTGCACT
GATAGCCAATATTGAAGTGGACACCTCTGGTAGTATCGAAGTAAAGATAACCAATAAGGAAGGAGCTT
ATACTATGGTTGAATCAACTAAGCATACTACTTTTGCTTTGATTACTC
+
FFFFFFFFFFFFF:FFFFFFFFFFFFFFFFFFFFFFFFFFFFFFFF,FFFFFFFFFFFFFFFFFFFFF
FFFFFFFFFFFFFFFFFFFFFFFFFFFFFFFFFFF:FFFFFFFFFFFF
@A00721:190:HGJW7DSXY:4:2540:20193:4930_1:N:0:TCTGCAAG+GCTGCACT
CTTCCGATCTGTTAAAGAGTCGATTAAACAAGTTGTACTTATCAATGATTTTATCGCTGGTTGTTATC
TCGAAAAGTCTCTTCTAAAAGGAACGTCAGTTCTAACCCAAAGAGAATATCTAAAATATTTACAAAAT
CTT
+
FFFF::FFFFF:FFFFFFFFFFFFFFFFFFF:FFFFFFFFFFFFFFFFFFFFFFFFFFFFFFFFFFFF
FFFFFFFFFFFFFFFFFFFFFFFFFFFFFFF:FFFFFFFFFFFFFFFFFFFFFFFFFFFFFFFFFFFF
FFF
@A00721:190:HGJW7DSXY:4:1624:24894:32612_1:N:0:TCTGCAAG+GCTGCACT
GATCTGTTGCTTGTCGCTTCCTCCTTTAATGGCACTACCTCGGTACAATCTTACTTGGAACGAAGGCG
ACCTGTTCCGGAGTTTTTACGGGATCCGGAATGTTCTGTCTATATTAGGTGAAGCGAAATACGATTTC
GAA
+
FFFFFF:FFFFFFFFFFFFFFFFFFFFFFFFFFFFFFFFFFFFFFFFFFFFFFFFFFFFFFFFFFFFF
FFFFFFFFFFFFFFFFFFFFFFFFFFFFFFFFFFFFFFFFFFFFFFFFFFFFFFFFFFFFFFFFFFFF
FFF
@A00721:190:HGJW7DSXY:4:1403:14579:23923_1:N:0:TCTGCAAG+GCTGCACT
TCGTACCTTGATGCTGACTTTCGTCAATATGATTTGATCTGGTTGCTCAATTCTAAGCGTGTTCCGCT
TAGTGCGAGGCCTCTCTTCTACGAGTATTGCTATTTATACGGTTACGAGAAGAAGGTTCGAAAGTTTG
AGA
+
FFFFF:FFFFFFFFFFFFFFFFFFFFFFFFFFFFFFFFFFFFFFFFFFFFFFFFFFFFFFFFFFFFFF
FFFFFFFFFFFFFFFFFFFFFFFFFFFFFFFFFFFFFFFFFFFFFFFFFFFFFFFFFFFFF,FFFFFF
FFF
@A00721:190:HGJW7DSXY:4:2401:26313:10802_2:N:0:TCTGCAAG+GCTGCACT
TACTAAATACTAGATATTTGGATGATCTTGATAAAGAACTTCATGAATTTTCAGAGGATGAAAAATTT
TCTTCCTTGGATTTTAATATAAAGAATCTTTACCAGGACTCCTACTAC
+

FFFFF::FFFFFFFFFFFFFFFFFFFFFFFFFFFFFFFFFFFFFFFFFFFFFFFFFFFFF:FFFFFFF
FFFFFFFFFFFFFFFFFFFFFFFFF:FFFFFFFFFFFFFFFFFFFFF,F
@A00721:190:HGJW7DSXY:4:2432:23366:9925_2:N:0:TCTGCAAG+GCTGCACT
TCTGATCGTAAGAAGTTTATAAGGAACATATCCATTTGTTGTAATAGGGTCATCAACTTAGAACTCGA
AACTCTAAGTGGTAATACTGTCTTTCCAAGCTTACCTGTATCTTCAAT
+
FFFFFFFFFFFFFFFFFFFFFFFFFFFFFFFFFFFFFFFFFFFFFFFFFFFFFFFFFFFFFFFFFFFF
FFFFFFFFFFFFFFFFFFFFFFFFFFF,FFFFFFF:FFFFFFFFFFFFF
@A00721:190:HGJW7DSXY:4:2567:10068:26663_1:N:0:TCTGCAAG+GCTGCACT
TTCGATAGGGGACATTTTATCGTGGGCTTTCCGTAAAGGAAGATCTGTTATCGACGGTTTATCGTTTA
CGAAGTATCACTCCACCGGTGAATGGTTTAAGAATACTATAAATAGAATAGCTATTACTACTAATGGC
GTT
+
:FFFFFFFFFFFFFFFFFFFFFFFFFFF:FFFFFFFFFFFFFFFFFFFFFFFFFFFFFFFFFFFFFFFF
FFFFFFFFFFFFFFFFFFFFFFFFFFFFFFFFFFFFFFFFFFFFFFFFFFFFFFFFFFFFFFFFFFFF
FFF
@A00721:190:HGJW7DSXY:4:1241:13042:19758_1:N:0:TCTGCAAG+GCTGCACT
TAGTGACATCTGGTCAATTTCGTTTTTAGGTGGTCTTTATTCTATTATTGATTTTTATGAGATTGATA
CGTTAACAGAAGATGATGTTAGAGTAGAATTCCTGAAGAGTCCTTCCGTAACCAACTTCTTGACAATT
TTC
+
FFFFFFFFFFFFFFFFFFFFFFFFFFFFFFFFFFFFFFFFFFFFFFFFFFFFFFFFFFFFFFFFFFFF
FFFFFFFFFFFFFFFFFFFFFFFFFFFFFFFFFFFFFFFFFFFFFFFFFF:FFFFFFFFFFFFFFFFFF
FFF
@A00721:190:HGJW7DSXY:4:2421:2257:27164_2:N:0:TCTGCAAG+GCTGCACT
CTAGGATTATGTCTGTGTTGAAAGACTTTCTACCGATAAGGGTCACTAGTCTTACACAATTAAGAAAT
AATCCTACATTGACTGATTATCTAAAGATTGAAGGAAAATTTTATCCT
+
FFFFFFF:FFFF:FFFFFFFFFFFFFFFFFFFFFFF:FFFFFFFFFFFFFFFFFFFFFF:FFFFFFFFFF
FFFFFFFFFFFFFFFFFFFFFFFFFFFFFFFFFFFFFFFFFFFFFFFFF
@A00721:190:HGJW7DSXY:4:1449:18747:15734_1:N:0:TCTGCAAG+GCTGCACT
GACTTTCCTACGATACAAATGATAACTTCTAATTTACGTTTGCTACTCAGAGCTGGTCCTCTCAGCAA
AGGGATGCATGGTGTTATCTCCTCAGGAGTCCCGGCATTTGATAATAATGACGAGACTTTCGTCATAA
TCG
+
FFFFFFFFFFFFFFFFFFFFFFFFFFFFFFFFFFFFFFFFFFFFFFFFFFFFFFFFFFFFFFFFFFFF
FFFFFFFFFFFFFFFFFFFFFFFFFFFFFFFFFFFFFFFFFFFFFFFFFFFFFFFFFFFFFFFFFFFF
FFF
@A00721:190:HGJW7DSXY:4:1342:12997:20556_1:N:0:TCTGCAAG+GCTGCACT
GATTACACGGTACCAGCGCATCTCGTGTCGAGCCTCGACCCGATTATATGGTATTAACTTATAAAGGT
GAGGAGACCATTTTCTTGCTGAAATTTATAGAAGATTATTTTGACAGAATCAATAAGAATCATCGATT
CTC
+
FFFFFFFFFFFFFFFFFFFFFFFFFFFFFFFFFFFFFFFFFFFFFFFFFFFFFFFFFFFFFFFFFFFF
FFFFFFFFFFFFFFFFFFFFFFFFFFFFFFFFFFF:FFFFFFFFFFFFFFFFFFFFFFFFFFFFFFFF
FFF
@A00721:190:HGJW7DSXY:4:1251:18096:15295_2:N:0:TCTGCAAG+GCTGCACT
GGATCCACTCCTCGAGTTATCATGATGTTAGTATGTTATATACTGATTTACAACGATATAGGAGTAAT
CTACGAGAAGATTCTAGAATTCTTAATTATAATATTCAACCTCAAAAG
+
FFFFFFFFFFFFFFFFFFFFFFFFFFFFFFFFFFFFFFFFFFFFFFFFFFFFFFFFFFFFFFFFFFFF
FFFFFFFFFF,FFFFFFF:FFFFFFFFF:FFF:FFFFFFFFFFFFFFF
@A00721:190:HGJW7DSXY:4:1531:14371:11725_2:N:0:TCTGCAAG+GCTGCACT
GAGATCACACATTTTGAGAACTAACTATGTTATGTTCTCTGATCATTTTGACAATCTCTTCTTATAAT

```
AAATAAATAAATAATATTACTAATGGCAAATAGTGGAAAGGCTCCAGT
+
FFFFFFFFFFFFFFFFF:FFFFFFFFFFFFFFFFFFF:FFF:FFFFFFFF:FFFFFFFFFFFFFFFFF
:FFFFFFFFFFFFFFFFFF:FFFFFFF:F,FFFFFFFFFFF::FFFFF
@A00721:190:HGJW7DSXY:4:1551:2067:25269_2:N:0:TCTGCAAG+GCTGCACT
TACGATGACGTGAGAATAGGTGAAGGGATAGTTTTTACCTCGACCAACATTAAGGCGGCGGCGACTAA
TCTTAAAGCTTCACCCAAAATGAAGGACACTTCGAAACTGAATAATGA
+
FFFFF:FFFFFFFFFFFF:FFFFFFFFFFFFFFFFFFFFFFFFFFFFFF:FFFFFF,FFFFF:FFFFF
FFFFFFFFFFFFFFFFFFFF:FFFFF:FFFFFFFFFFFFFFFFFFFFF
@A00721:190:HGJW7DSXY:4:2146:15872:25755_1:N:0:TCTGCAAG+GCTGCACT
GGCTCTAAAGGACACTATACAATATCCAGACGACGACGAAGCTTATACTACTTTAATTTTGGGTATTA
TGTGGAGATATCGTGATATCAGTACGTCCAGATCCTCTGCTTTTACCGATAACGATAAATTCGTCATC
AAA
+
:FFFFFFFF:FFFFFFFFFFFFF,FFFFFFFFFFFF:FFFFFFFFFFFFFFFFFFFFFFFFFFFFFFF
FFFFFFFFFFFFFFFFFFFFFFFFFFFFFFFFFFFFFFFFFFFFFFFF:FFFFFFFFFFFFFFFFFFF
FFF
@A00721:190:HGJW7DSXY:4:2105:1262:6277_1:N:0:TCTGCAAG+GCTGCACT
TTGGTGCTTAGGGATATAATGAATTCAGCACCACATTTTGGTCAAGTCAATCCGTTGAGAAAATTTCT
TAGAGGATATTCGCCATTGTGGTACGAATTGGTCCAACATTTACCACCAAATTCTTTTGATACAACTC
TAG
+
FF:FFFFFFFFFFFFFFFFFFF:FFFFFFFFFFF:FFFFFFFFFFFFFFFFFFFFFFFFFFFFFFFFFF
FFFFFFFFFFFFFFFFFFFFFFFFFFFFFFFFFFFF:FFFFFFFFFFFFFFFFFF:FFFFFFFFFFFF
FFF
@A00721:190:HGJW7DSXY:4:2473:23493:8797_1:N:0:TCTGCAAG+GCTGCACT
TGCCTGCGGAATACGCTTGCTATTCACCAGATTGGTTCGAACCCGTTGATGCTATGTCAGAAGAAGCT
ATTCAAGCTTGTCGTTTAAAGACTGCAGAGGCTGTCAAACAAGCCCAGACTCAAGGAGTCCAATCAAA
GGT
+
FFFFFFFFFFFFFFFFFFFFFFFF:FFFFFFFFFFFFFFFFFFFFFFFFFFFFFFFFFFFFFFFFFFF
FFFFFFFFFFFFFFFFFFFFFFFFFFFFFFFFFFFFFFFFFFFFFFFFFFFFFFFFFFFFFFFFFFFF
FFF
@A00721:190:HGJW7DSXY:4:1653:32660:16157_2:N:0:TCTGCAAG+GCTGCACT
TGGGTGATAAAAATTATTTAGAGATCTTTTCTACTTACAAAGGACAATTCAGTTTCATTAATTCTACG
GAAAAGTCCGGAGTATATTGTATGTTAATAGATAGTGGGATAATAGTT
+
FFFFFFFFFFFFFFFFFFFFFFFFFFFFFFFFFFFFFFFFFFFFFFFFFFFFFFFFFFFFFFFFFFFF
FFFFFFFFFFFFFFFFF:FFFFFFFFF:FFFFFFFFFFF:FF:FFFFF
@A00721:190:HGJW7DSXY:4:2105:20039:35164_1:N:0:TCTGCAAG+GCTGCACT
GTAGTGAACGCGTTCGTAGAACCAAGTACTTTCCGTCTGAAGAGTTTAGAGCTGCTATTGATTATGTA
ACCGATAGGAGTTGTATAGTTGAAGATGTGGCTTTAGACGATAATCCTTTAAGGACTATGTTGACTGA
CGA
+
FFFFFF:FFFFFFFFFFFFFFFFFFFFFFFFF:FFFFFFFFFFFFFFFFFFFFFFFFFFFFFFFFFFFF
FFFFFFFFFFFFFFFFFFFFFFFFFFFFFFFFFFFFFFFFFFFFFFFFFFFFFFFFFFFFFFFFFFFF
FFF
@A00721:190:HGJW7DSXY:4:2466:2790:8860_2:N:0:TCTGCAAG+GCTGCACT
TTGTCTACGTTGCAGATCGCCACTCCAAACACTATATACATTATAAAATATAAAAATAAGGGAGTCAT
AACTTTTGTTACCAAATTCTTGGCTTTCAACGAAGTCTCTGATTTTAT
+
FFFFFFFFFFFFFFFFFFFFFFFFFFFFFFFFFFFFFFFFFFFFF:FFFFFFFFFFFFFFFFFFFFFFF
FFFFFFFFFFFFFFFF:FFFFF:FFFFFFFF:FFFFFFFFFFFFFFFF
```

```
@A00721:190:HGJW7DSXY:4:1355:2781:31829_1:N:0:TCTGCAAG+GCTGCACT
TAACGATCTCTCGGTCATTTACTCTGTACTTGATCTACGTCGTTTAACTGTCGGAACAACCTTTACTT
AGAATGACTCTATCTTTTCAGACACTTCTTATATTATTTACAATAGTTAATATCATATTAGTAATACT
CAC
+
:FFFFFFFFFFFF:FFFFFFFFFFFFFFFFFFFFFFFFFFFFFFFFFFFFFFFFFFFFFFFFFFFFFFF
FFFFFFFFF:FFFFFFFFFFFFF:FFFFFFFFFFFFFFFFFFFFFFFFFFFFFFFFFFFFFFFFFFFFF
FFF
@A00721:190:HGJW7DSXY:4:2348:28989:21104_2:N:0:TCTGCAAG+GCTGCACT
GCTTTTTCTTAGCTAATAGATTAATAAAGGATGAGTACGAAAACCAACTCTTCGTTACGAGACCTGAT
GGTAGAATAGATTTAGTCTCTACACCCATCTCAACGAGGATAAAGCGA
+
FFFFFFFFFFFFFFFFFFFFFFFFFFFFFFFF:FFFFFFFFFFFFFFFFFFFFFFFFFFFFFFFFFFFFF
FFFFFFFFF:FFFFFFFFFFFFFFFFFFFFFFFFFFFFFFFFFFFFFF
@A00721:190:HGJW7DSXY:4:1325:16459:4382_1:N:0:TCTGCAAG+GCTGCACT
TACACTAATATATCATCCGCAAACGACTCGAAACCGAGACGCCGTAGATCAAAGTCCCTTCTAACTTT
AGATTATGAATCCTGCCTTTACTCATTTTATAGCAGTTATTTATGTTGTACTTTTTATAATTTATAAC
CTT
+
FF::F,F,,,,F:FF,FF,FF,FFFFFFFFFFFFFFFFFFFFFFFFFFFFFFFFFFFFFFFFFFFFFFFF
FFFFFFFFF:FFFFFFFFFFFFFFFFFFFFFFFFFFFFFFFFFFFFFFFFFFFFFFFFFFFFFFFFFFFF
FFF
@A00721:190:HGJW7DSXY:4:1674:27651:34945_1:N:0:TCTGCAAG+GCTGCACT
TATGTACTTATTGTAACGATCATTCTTTCGAGTTTTGTCATTCTTATTCTTTTATTCCTTTAACTGTG
TATGAGGAGGATTTATAATGCTTTTCGATAGTGATTGTTATCAATTTAAATACATTACTGTTTCAAAT
CAT
+
:FFFFFFFFFFFFFFFFFFFFFFFFFFFFFFFFFFFFFF:FFFFFFFFFFFFFFFFFFFFFF:FFFFFFF
FFFFFFFFFFFF:FFFFFFFFFFFFFFFFFFFFFFFFFFFFFFFFFFFFFFFFFFFFFFFFFFFFFFFF
FFF
@A00721:190:HGJW7DSXY:4:1409:3522:34115_2:N:0:TCTGCAAG+GCTGCACT
GACTCCGAAAATGGTTTCAAATGAGGTCAAAGCTCTATTTATTAAACGAACTATTATCCGATTCTTCG
AGGGTATCACTACCACTGATCTGCCGACTTTAGTCAAGGAACTTCAAT
+
FFFFFFFFFFFFFFFFFFFF:FF:FFFFFF:FFFFFFFFFFFFFFFFFFFFFFFFFFFFFFFFFFFFFFF
FFFFFFFFFFFFFFFFFFF:FFF:FFFFF:FFFFFFFFFFFFFFFFFF
@A00721:190:HGJW7DSXY:4:2564:12011:25175_2:N:0:TCTGCAAG+GCTGCACT
TATTTTTCTATAAGGGAAAATAGCAATAATCAAATTTCGACAAATTTTAGAACTTTATTTCAGAACTC
TCTCCGATCACCTTGGTGTGATCTCCCGGAGGATCTTTTCAATAAGAT
+
FFFFFFFFFFFFFFFFFFFFFFFFFFFFFFFFFFFFFFF:FFFFFFFFFFFFFFFFFFFFFFFFFFFFFF
FFFFFFFFFFFFFFFFFFFFFFFFFFFFFFFFFFFFFFFFFFFFFFFF
@A00721:190:HGJW7DSXY:4:1420:4562:6699_2:N:0:TCTGCAAG+GCTGCACT
ATTTTTAAATAGTGATATTCAGCGTATACTTGAATACTTTATCAAAGATTTGGCTTGGTCTATTTACC
GCAAAAAGTTGGAAGAGGTCTATGATATTTATTTTACTTATACTTTAA
+
FFFFFFFFFFFFFFFFFFFFFFFFFFFFFFFFFFFFFFFFFFFFFFFFFFFFFFFFFFFFFFFFFFFFFF
FFFFFFFFFFFFFFFFFFFFFFFFFFFFFFFFFFFFFFFFFFFFFFFF
@A00721:190:HGJW7DSXY:4:1425:27055:12743_1:N:0:TCTGCAAG+GCTGCACT
AACTTGACTGTTGATAATATTACAATAGCTCTTTCTCAGAAGATTAGAGAAGTAACCGGTGAACACGT
CGGTAGCATTGATTTACTTTTACCGTTAGAACTGAAAAAGATTCTTTTCGAAAAGAAACCTATAAGTT
ACA
+
FF:FF:F:FFFF:FFFFFFFFFF:FFFFF:FFFFFFFFFFFFFFFFFFFFFFFFFFFFFFFFFFFFFFFF
```

```
FF:FFFFFFFF:FFFFFFFFFFFFFFFFFFFFFFFFFFFFFFFFFFFFFFFFFFF:FFFFFFFFFFFF
FFF
@A00721:190:HGJW7DSXY:4:1202:21269:34319_1:N:0:TCTGCAAG+GCTGCACT
TATATTAAAGAAATAAAAATACTTCAAGTACCTGAACTTTACATTTTTAATGGTATGGTATAATGTGT
CTTACAAATAGAACCTTCACTCTCGGTAATGAGAGTGTATTCCTTAATATTCTAGTAACCTCTATGTG
GGC
+
FFFFFFFFFFFF::FF:FF:FFFFFFFFFFFFFFFFF:FFFF:F:FFFFFFF,F,FFFFFFFFFFFFF
F:FFFFFFFFFFFFFFFFFFF:FF:F,FFFFFFFFFFFFF:,FFFFFFFFFFFFFFFF:F,FFFF:FF
::F
@A00721:190:HGJW7DSXY:4:1452:32913:11678_1:N:0:TCTGCAAG+GCTGCACT
GATAGCAATTTATGTTTTAAATGCTAGGAAAATTTTAGAAAAACTCCATTGTATAGTTCAAGGAGTAC
TTAATTCAATAGCTTTAGTATTATTTACTATCGCCTTTATTGAAAATTGTATAAGAGTTAATTGCTTA
CAC
+
FFFFFFFF:FFFFFFFFFFFFFFFFFFFFFFFFFFFFFFFFFFFFFFFFFFFFFFFFFFFFFFFFFFF
FFFFFFFFFFFFFFFFFFFFFFFFFFFFFFFFFFFFFFFFFFFFFFFFFFFFFFFFFFFFFFFFFFFF
FFF
@A00721:190:HGJW7DSXY:4:2110:29170:9612_2:N:0:TCTGCAAG+GCTGCACT
TTAATTGATTGAAGCTAGGCGTTGGGGAGAATGAACTGCCGCTTCCCGGAAGAAACGATCTATTTACA
GAGTATTTAGAGTAGTCCAAAGTTTGTATAAATTTATATGATTTTCTA
+
FFFFFFFFFFFFFFFFFFFFFFFFFFFFFFFFFFFFFFFFFFFFFFFFFFFFFFFFFFFFFFFFFFFF
FFFF:FF:FFFFFFFFFFFFFFFFFFFFFFFFFFFFFFFFFFFFFF:FF
@A00721:190:HGJW7DSXY:4:2464:21007:9596_2:N:0:TCTGCAAG+GCTGCACT
TTCAATAACATAACATACCATACACTCCCCAAACATAACATACCATAACCTACCATGACCAATCATCG
CTCCAACTGGCACGCCAGACGTAGGGTCAAACCTACTACCGCCGGTCG
+
FFFFFFFFFFFFFFFFFFFF:FFFFFFFFFFFFFFFFFFFFFFFFFFFFFF:FFFFFFFFFFFFFFFF
FFF:FFF:F:FFFFFFFF,FFFFFFFFFFFFFFFFFF:F:FFFFFFFF
@A00721:190:HGJW7DSXY:4:1515:12436:34366_1:N:0:TCTGCAAG+GCTGCACT
TACGTTCAGGGACCCGCTGTGCAGAATAACCTTCCCAAATCTAGATCTGCACAGAAGAAGGCTGAAGG
TCGTTACCGGACGACCACTCAAAGGAAACGGGCTGTTGCCCAGAGGAAAGCAGCGACCACCGAGGTCG
CTC
+
F:FFFFFFFFFFFFFFFFFFFFFFFFFFFFFFFFFFFFFFFFFFFFFFFFFFFFFFFFFFFFFFFFFF
FFFFFFFFFFFFFFFFFFFFFFFFFFFFFFFFFFFFFFFFFFFFFFFFFFFFFFFFFFFFFFFFFFFF
FFF
@A00721:190:HGJW7DSXY:4:1311:30517:7373_2:N:0:TCTGCAAG+GCTGCACT
GCAGTCCCGTCATCTCTGTGGGAACTGAGGCAGTACCTCAGTCAAAAGTCACATTCACCACTCCCATG
CCAATCAATGATGTGACTATACAGTTCGGCCATTTCAAGAGTGGTCAA
+
FFFFFFFFFFFFFFFFFFFFFFFFFFFFFFFFFFFFFFFFFFFFFFFF:FFFFFFFFFFFFFFFFFFF
FFFFFFFFFFFFFFFFFFFFFFFFFFFFFFFFFFFFFFFFFFFFFFFF
@A00721:190:HGJW7DSXY:4:1551:6388:34851_1:N:0:TCTGCAAG+GCTGCACT
GAGGTACTGAAGCCTTCCCCTCCGCTGGCACGAAGAACACTCCCAAGCCAATCAATAGTTCTTCTGAC
CAGAATGAAAAGGGGTTTACCAGGGTAGTCTCTAAGTCTACCAGGAAGAGGGGAAATGTCCCCAAAAC
TGA
+
FFFFFFFFFFFFFFFFFFFFFFFFFFFFFFFFFFFFFFFFFFFFFFFFFFFFFFFFFFFFFFFFFFF:
FFFFFFFFFFFFFFFFFFFFFFFFFFFFFFFFFFFFFFFFFFFFFFFFFFFFFFFFFFFFFFFFFFFF
FFF
@A00721:190:HGJW7DSXY:4:1459:18530:19836_2:N:0:TCTGCAAG+GCTGCACT
AGGTCCATCAGGACCTAAGAAGGGGAAATTACCCCTAATTCTGAAGGAAGATCTCCCACCATCTGGGG
```

```
TTGGGAATAAGAAATCTGCACGGGCCAAAGAGGCCGCTGCTACGAAGT
+
FFFFFFFFFFFFFFFFFFFFFFFFFFFFFFFFFFFFFFFFFFFFFFFFFFFFFFFFFFFFFFF:FFFF
FFFFFFFFFFFFFFFFFFFFFFFFFFFFFFFFFFFFFFFFFFFFFFFF
@A00721:190:HGJW7DSXY:4:1633:6668:21527_1:N:0:TCTGCAAG+GCTGCACT
GCTTGGGTCATTAAGGGTAGCTGTAAAGTTGCGCCTGCCGACCCCATCTTTGAGACCAAGACTCTCTA
CAGTAGGTTCGTGCCTACTATGAGCAAATCTGTTGAATTCGTCACTGAGGACGAAGGTGAGAGGACTT
TTG
+
FFFFFFFFFF:FFFFFFFFFFFFFFFFFFFFFFFFFFFFFFFFFFFFFFF:FFFFFFFFFFFFFFFFF:
FFFFFFFFFFFFFFFFFFFFFFFFFFFFFFFFFFFFFFFFFFFFFFFFFFFFFFFFFFFFFFFFFFFF
FFF
@A00721:190:HGJW7DSXY:4:2154:15808:26804_1:N:0:TCTGCAAG+GCTGCACT
CAAACTGAGAGCAGTCGCTGGGGAAAGAGAATTGCGTGGGTTGAAGGCCGACGCTTCGAGCTTGAGTG
TGACGAGGCCGGTGACGTTTTTGTCAACGGCGTTTGGAGGATTTTATCCGCGCTTAACTATGGGGCGA
AGA
+
FFFFFFFFFFFFFFFFFFFFFFFFFFFFFFFFFFFFFFFFFFFFFFFFFFFFFFFFFFFFFFFFFFFF
FFFFFFFFFFFFFFFFFF:FFFFFFFFFFFFFFF:FFFFFFFFFFFFFFFFFFFFFFFFFFFFFFFFFF
FFF
@A00721:190:HGJW7DSXY:4:1470:5791:25113_1:N:0:TCTGCAAG+GCTGCACT
AGTCTTGAAGGACTTAGGTTGGCATATTTATGCCGACGATAATAAGCATGTCACCCTTAAGCACCGAG
GTAAGCTCGGGACTTACAAGAGAGAGTGTGAGAGAGTCGCTCTCACTATTTTTGCCCAGAATGCGGGC
TTT
+
FF:FFFFF:FF,FFF,FFFFFFFFFFFFF::FFF:F:F:FF,F,FFFFF::F:FF:FFFFFFFFF:FF
FFFFF:FFF:FFFFFFFF:F:FFFFFFFFFFFFFFFFFFFFFFFFFFFFFFFFFFFFFF:FFFF:FFFF
FF:
@A00721:190:HGJW7DSXY:4:2303:12048:17190_2:N:0:TCTGCAAG+GCTGCACT
GCTCCTTCTGTGGGTGAGGGTTTTTGTTATATTCACTGGTTCTGGGAGGCTGCTTTCCTCCATAATTA
CAACCTCTCTTTAGAGAGCATTAGAAAGATCGGAAGAGCGTCGTGTAG
+
FFFFFFFFFFFFFFFFFFFFFFFFFFFFFFFFFFFFFFFFFFFFFFFFFFFFFFFFFFFFFF:FFFFFF
FFFFFFFFFFFFFFFFFFFFFFFFFFFFFFFFFFFFFFFFFFFFFF:,
@A00721:190:HGJW7DSXY:4:1202:24605:4335_1:N:0:TCTGCAAG+GCTGCACT
CACACTTTTCTTGAAAATTTTTCCTTTCCTCCGTCATTGGGGATTTCGATGTGAGAAGGTGGAAGGTG
TCAAGGGTCTTCTTCATGCTGATCTTTCAGGACCTAATCACATCATGATTGATAGTTACGGCTCGTAT
CGA
+
FFFFFFFFFFFFFFFFFFFFFFFFFF:FFFFFFFFFFFFFFFFFFFFFFF:FFFFFFF:FFFFFFFFFFF
FFFFFFFFF:FFFFFFFFFFFFFF:FFFFFFFFFFFFFFFFFFFFFFFFFFFFFFFFFFFFFFFFFFF
FFF
@A00721:190:HGJW7DSXY:4:1207:32136:31375_2:N:0:TCTGCAAG+GCTGCACT
AAGTTCTCCGAAATTAAAGACGGGGGGATTAAGAGTCCAGCTGTAACTGTCATTTCAGAGAGGGGAGC
TCTGAAAAGGAAACTTCGTCAGAAGATGAGGAAGTCTAAGAGGAGAGA
+
FFFFFFFFFFFFFFFFFFFFFFFFFFFF:FFFFFFFFFFFFFFFFFFFFFFFFFFFFFFFFFFFFFFFF
FF:FFFFFFFFFFFFFFFFFFFFFFFFFFFFFFFFFFF:FFFFFFFFF
@A00721:190:HGJW7DSXY:4:1166:31331:6402_1:N:0:TCTGCAAG+GCTGCACT
CTCTAGAGAAGATCTCACCACCTGGAGAAGGCAAGTGGAGAGTCCAGAAGTTGATGAAGAAGGATTAT
TTGAAGCAGGGGTTACCACTTCCTAAGGTGGATGAGGGAGGTTGTTTCTTTGGAGTTTTAGATAATCT
TTT
+
FFFFFFFFFFFFFFFF,FF:FFF:FFFFFFFFF:FFFFFFFFFFFFFFFFF:FFFFFFFFFFFFFFFF
```

FFFFFFFFFFFFFFFFFFFFFFFFFFFFFFFFFFFFFFFFFFFFFFFFFFFFFFFFFFFFFFFFFFFF
FFF
@A00721:190:HGJW7DSXY:4:1429:20826:11318_2:N:0:TCTGCAAG+GCTGCACT
CCTTGAGAAGACAAGCCACATTAAGATATAATTTAGGTCAACTTGGGGTTTCGAATGTCATTAGTGCC
TTACGATTGAAAGAGGTCCTTTTGAGTATCGACAGATCTTTCGATCTT
+
FFFFFFFFFFFFFFFFFFFFFFFFFFFFFFFFFFFFFFFFFFFFFFFFFFFFFFFFFFFFFFFFFFFF
FFFFFFFFFFFFFFF,FFFFFFFFFF:FFFFFFFFFFFFFFFFFFFFF
@A00721:190:HGJW7DSXY:4:2601:31891:18897_2:N:0:TCTGCAAG+GCTGCACT
TTAACGTGGCAGAATTCGGTAACTTACCATATTGTTCCTTCCGGGAAGTTGTGGGATTTAACTTTGAC
TCAACTACTCCGATCAGATCCGGATGGTTTGATTGGTATGGACTACTC
+
FFFFFFFFFFFFFFFFFFFFFFFFFFFFFFF:FFFFFFFFFFFFFF:FFFFFFFFFFFFFFFFFFFFFF
FFFFFFFFFFFFFFFFFFFFFFFF:FFFFFFFFFFFFF,FFFFFFFFFF
@A00721:190:HGJW7DSXY:4:2341:4146:16250_2:N:0:TCTGCAAG+GCTGCACT
GTTGTCTAGGGTCATGGCAGATACTACCGATAAGGTGAAAAATATGCAGGTCAAAGATTTTGTAGCTC
TGAGGGCGGTTGAGGGAGCCGTTTTGGCCGACTTTAACTATTCTAAGA
+
FFFFFFFFFFFFFFFFFFFFFFFFFFFFFFFFFFFFFFFFFFFFFFFFFFFFFFFFFFFFFFFFFFFF
FFFFFFFFFFFFFFFFFFFFFFFFFFFFFFFFF,FFFFF:FFFFFFFFF
@A00721:190:HGJW7DSXY:4:1115:1850:32534_2:N:0:TCTGCAAG+GCTGCACT
CAAAACCTATCGTTGTCGTACCTTACAAGGTTGAGGAGACTTTACAGCGGCTTATTGTCGCGCAGTTT
CCAGAATTGCGGTTGAAGTTCACCCATTCTATCTTGAATGCTCACGCC
+
FFFFFFFFF:FFFFFFFFFFFFFFFF,FFFFFFFFFFF:FFFF,FFFFFFFFFFFFFFFFFFFFF::F
FFFFFFFFFFFF::FFF:FF:FF:FFFFF:FFFFFFFFFFFFFFFFFF
@A00721:190:HGJW7DSXY:4:2342:30481:15076_2:N:0:TCTGCAAG+GCTGCACT
TTATAATCACTTGTTAAAGTACAGATTTAGTGATTTAGATATTATTGATATTGGAGGCGATATTCGTT
TTCATATCCAAAACTCCTTGAATGGAGTACATATTTGCAACCCTATTC
+
FFFFFFFFFFFFFFFFFFFFFFFFFFFFFFFFFFFFFFFFFFFFFFF:FFFFFFFFFFFFFFFFFFFF
FFFFFFFFFFFF:FFFFFFFFFFF:FFFFFFFFFFFFFFFFFFFFFFFF
@A00721:190:HGJW7DSXY:4:2460:9697:36730_1:N:0:TCTGCAAG+GCTGCACT
GATGCATCGAGGTATACGTCTAGGAACATTGATTGGGAACGTACCTACTCACGAGACGTCATGAAAGC
TATCGGAGGGAAGCAAAATGTTTCGTTCTGTTACAAGAACGCTTTGGATTGTGACCATAAAGCCTCAG
TTG
+
FFFFFFF:FFFFFF:FFFFFFFFFFFFFFFFFFFFFFFFFFFFFFFFFFFFFFFFFFFFFFFFFFFFF
FFFFFFFFFFFFFFFFFFFFFFFFFFFFFFFFFFFFFFFFFFFFFFFFFFFFFFFFFFFFFFFFFFFF
FFF
@A00721:190:HGJW7DSXY:4:1459:6940:19617_1:N:0:TCTGCAAG+GCTGCACT
CTCTCTTGATGATGTAGCCACTATAATGCTTAAGAGAGAGATTGATGTATGTCATTTCGTCTTAGTTT
GTCCCGGCGAATTATTGGACACGATGAAAAATGACATACCCGTTCCTGACCTTGGTATCGTTATCCGT
CAT
+
FFFFFFFFF:FFFFFFFFFFFFFFFFFFFFFFFFFFFFFFFFFFFFFFFFFFFFFFFFFFFFFFFFFF
FFFFFFFF:FFFFFF::FFFFFFFFFFFFFFFFFFFFFFFFFFFFFFFFFFFFFFFFFFFFFFFFFFF
FFF
@A00721:190:HGJW7DSXY:4:2211:3721:5368_2:N:0:TCTGCAAG+GCTGCACT
GGTTATTCTCACAACAAAGATAAGATTTTATCTTTCTTCAAACACCCTGTTTTCCTTAAGGGCGGACA
ATTGTTCCATTGTGAACTCTTGGGGACTAGATTGAGCATTAATGAGTT
+
FFFFFFFFFFFFFFFFFFFFFFFFFFFFFFFFFFFFFFFFFFFFFFFFFFFFFFFFFFFFFFFFFFFF
FFFFFFFFFFFFFFFFFFFFFFFFFFFFFFFFFFFFFFFFFFFFFFFF

```
@A00721:190:HGJW7DSXY:4:1234:6777:22028_1:N:0:TCTGCAAG+GCTGCACT
TCTCTAGTGGACAATTCAGAATACCTATTTCCGTACCAAGAGCCACTGCCAATCTCATCGCTATACGC
ATACCTGATTGGAAGAAGCGAGGTTCGTTCCTCAATAAAAATTTTTCTCAAGAGATTATGGTTGATAA
AGT
+
FFFF:FFFFFFFFFFFFFFFF,FFFFFFFFFFFFFFFFFFFFFFFFFFFFFFFFFFF:FFFFFFFFFF
FFFFFFFFFFFFFFFFFFFFFFFFFFFFFFFFFFFFFFFFFFFFFFFF:F:FFFFFFFFFFFFFFF
FFF
@A00721:190:HGJW7DSXY:4:1115:11695:33927_1:N:0:TCTGCAAG+GCTGCACT
AGGGCTATTCAGTACGTCATGGGGATCACTTCTTCGGTCAATGACAAAACCTTCGAATATGTTACGAC
ATGGCTTCGTAACAACAGTGCGAGGATTGTCATCTCGGGGAAAGTGATTCACCAAGATGTTCATATAG
AT
+
::FFFFFFFFF:FF,FFFFFF,FFFF:FFFF:FFFFFFFFFFFFFFFFF:FFFFFF:F:F:FFFFFFF
FFF,FFF,FFFF,FFFFFFF:FFFFFFFFFFFFFFFFFFFFFFFFFFFFFFFFFFFFFFFFFFFFFFF
FF
@A00721:190:HGJW7DSXY:4:1258:5773:21010_1:N:0:TCTGCAAG+GCTGCACT
GAAGGTCGCAGCGATCTTACTCGTTATCGGAGCTAAGAAGAAGACTTTGGGTACACGTTATGCTCGCA
AACTTAACGTTAGTACCGGAGGTGATCTCTCTGCTTCTGCGTTACTGAGAGAAAAGATTCAGGACGCC
TAC
+
FFFFFFFFFFFFFFFFFFFFFFFFFFFFFFFFFFFFFFFFFFF:FFFFFFFFFFFFFFFFFFFFFFFF
FFFFFF,FFFFFF:FFFFFFFFFFFFFFFFFFFFFFFFFFFFFFFFFFFFFFFFFFFFFFFFFFFFFF
FFF
@A00721:190:HGJW7DSXY:4:1645:26892:25332_2:N:0:TCTGCAAG+GCTGCACT
TTTAGGTTTAGTAGGTGATCTTGACGTCGATTCTTTGTATAGGGAGTGTGAGAAGACAACGAATCTTG
AAACTACGGTCCTTATCAAAGAAAATGGTGAACCGGTCTCTAAATCTT
+
FFFFFFFFFFF:FFFFFFFFFFFFFFFFFFFFFFFFFFFFFFFFFFFFFFFFFFF,FFFFFFFFFFFF:
:FFFFFFFFFFFFFFFFFFFFFFFFFFFFF:FFFFFFFFFFFFFFFFFF
@A00721:190:HGJW7DSXY:4:2351:2953:26240_2:N:0:TCTGCAAG+GCTGCACT
GAAGATTGAAGAACACGTCAAGAAGTCGCTCGGCGTTGTTCTTGAAAACCAAGTTAAGAAGGAGGCTG
ATCATGGAGTCTTTGTACCTTCTGTCAAACTTCGAGGTTCAGCTACGA
+
FFFFFFFF:FFFFFFFFFFFFFFFFFFFFFFFFFFFFFFFFFFFFFFFFFFFFFFFFFFFFFFFFFFF
FFFFFFFFFFFFFFFFFFFFFFFFFF:FFFFFFFFFFFFFFFFFFFFF
@A00721:190:HGJW7DSXY:4:1430:14507:27461_1:N:0:TCTGCAAG+GCTGCACT
TGGATTGAAGGCTGGCTCTAAGGGAGACTCCTTCTTTTTGAATGTTGTAAAGAATTGGGTTGAGAAGT
GTGCTTTACAAGGAGTTAGACTCTTCTTCAAGTTTTTAGATCTTCTTGATAATCTTAAAGAAAAATTT
TCG
+
FFFFFFFFFFFFFFFFFFFFFFFFFFFFFFFFFFFFFFFFFFFFFFFFFFFFFFFFFFFFFFFFFFFF
FFFFFFFFFFFFFFFFFFFFFFFFFFFFFFFFF:FFFFFFFFFFFFFFFFFFFFFFFFFFFFFFFFFF
FFF
@A00721:190:HGJW7DSXY:4:2537:28556:28087_1:N:0:TCTGCAAG+GCTGCACT
GTCTGTACTTACATTCTTTACGCCAGATTCTTCATGGGGCGACGCTGTTTCTCTTGGTATTTCTGAAA
TAGTTTATCAGAGCGTCATTTTCCTTTGGGATCTTTCCTCTGGTAAGGGAATTATTAAATCATTCTTC
TCA
+
:FFFFFFFFFFFFFFFFFFFFFFFFFFF,FFFFFFFFFFFFFFFFFFFFFFFFFFFFFFFFFFFFFFFF
FFFFFFFFFFFFFFFFFFFFFFFFFFFFFFFFFFF:FFFFFFFFFFFFFFFFFFFFFFFFFFFFFFFF
FFF
@A00721:190:HGJW7DSXY:4:1336:19316:5666_2:N:0:TCTGCAAG+GCTGCACT
TTTTCTCTACCCATAACTTCTTTTTCAAAAATGCGGATGGGTCTACTGATGTTTTCCTACAGAGTGCA
```

```
GTCGTTTCAACACTTATCACCACTTTTGAAAGGAAAATAATTGGTGAT
+
FFFFFFFFFFFFFFFFFF:FFFFFFFFFFFFFFFFFFFF:FFFFFFFFFFFFFFFFFFFFFFFFFFFF
FFFFF,FFFF,FF,FFF:FFF::::FFFFFF,FFFFF:FFF:,,F:,F
@A00721:190:HGJW7DSXY:4:2242:25057:20995_1:N:0:TCTGCAAG+GCTGCACT
GTCGGTGGGGTTCTCAAGGCTCTCATCGCCACTGGGATTCTAAGGGGTTCCATAAGATATTATCTGAC
ATTGTGGTTCAATGATCAGGATTCTTCTTTTGGTCCTTACGTCTTAAGTAATTTGGATGTTGTCGAAA
ACT
+
FFFFFFFF:F,FFFFF,:FFFFFF:FFFFFFFFFFFFFFFFFFFFFFFF:FFFFF:FFFF:FFFFFFFF
FFF:FFFFFFFFFFFFFFFFFFFFFFFFFFFFFFFFFFF:FFFFFFFFFF:FFFFFFFFFFFFFFFFF:
FFF
@A00721:190:HGJW7DSXY:4:2620:21549:11162_2:N:0:TCTGCAAG+GCTGCACT
TGTCACGATTCTTAAAAGAAGTTTATTAAAACTTCAGAGTCTCTTGGAAGTCCAGGTAGCTAATGTTC
TTGCAATAGCCACAGACTTTCTGGGAAAAGAGACCTCTGTTGGTAGAG
+
FFFFFFFFFFFFFFFFFFFFFFFFFFFFFFFFFFFFFFFFFFFFFFFFFFFFFFFFFFFFFF:FFFFFF
FFFFFFFFFFFFFFFFFFFFFFFFFFFFFFFFFFFFFFFFFFFFFFFF
@A00721:190:HGJW7DSXY:4:1214:27651:17848_2:N:0:TCTGCAAG+GCTGCACT
ATTACTCAGATTCAATCTGGCATCACTGAACGTCTACAACGAGTTAGGGAGTGGCTAGGTTTTAAAAG
ATCCTTTGCACAAGCAATACCTTCCACTGAGTTCATCACCCAGAGTTT
+
FFFFFFFFFF,FFFF:FFFFFFFFFFFFFFFFFFFFFFFFFFFF:F,FFFFFFFFF,FFFFFFFFFFF
FFFFFFFFFFFF:FFFFFFFFFFFFFFFFFFFFFF:FFFFFFFFFFFFF
@A00721:190:HGJW7DSXY:4:1508:9299:28995_2:N:0:TCTGCAAG+GCTGCACT
ACTGTTCGAGAATCTTCTGATGCTGAATTTGAGGAAAATTTTTCGGAAGTTTTTGGTATAAATCCTGA
TGGCTCAGTACAGGGAGGAGGTAATAGTTATAAAACCTTCTTTTATCA
+
FFFFFFFFFFFFFFFFFFFFFFFFFF:FFFFFFFFFFFFFFFFFFFFFFFF:FFFFFFFFFFFFFFFFF
FFF:FF:FFFFFFFFFFFFFFFFF:FFFFFFFFFFFFFFFFFFFFFF:F
@A00721:190:HGJW7DSXY:4:1139:12726:20556_2:N:0:TCTGCAAG+GCTGCACT
GGACAAAATCTGGGATCATAGGTATGTGTCACGCTACACTCGAAATATGGGCCCTCATAAGGAAGTAT
AGTTGGCGTCTTGTCGTTGACGTCATTACAGTCTGGAAGTTAGATCAT
+
FFFFFFFFFFFFFFFFFFFFFFFFFFFFFFFFFFFFFFFFFFFFFFF:FFFFFFFFFF:FFFFFFFFFF
FFFFFFFFFFFFFFFFFFFFFFFFFFFFFFFFFFFFFFFFFFFFFFFF
@A00721:190:HGJW7DSXY:4:1267:30852:4131_2:N:0:TCTGCAAG+GCTGCACT
CGAGAGTTGTGATCTAGTTCTAAATTTAGGTCGACGGACTTTAGCAACTTTAGATAAATTCTTTCCGG
GCTCAATGACCTTTGCTATGTACGTGGATCTAATCCTTGGTGATCTGA
+
FFFFFFFFFFFFF:FFFFFFFFFFFFFFFFFFFFFFFFFFFFFFFFFFFFFFFFFFFFFFFFFFFFFF
FFFFFFFFFFFFFFFFFFFFFFFFFFFFFFFFFFFFFF,FFFFFFFFFF
@A00721:190:HGJW7DSXY:4:1355:25418:19867_2:N:0:TCTGCAAG+GCTGCACT
TATTAGTTCACGAAGTTCTTACACTTCTCGCGACTAATGTCAGCTTCACTACAAAGGCGTTACGTAAA
GTAGTTTTCACGCTTTATCTTAAAGCATTACCACCAAATTTAGCACTT
+
FFFFFFFFFF:FFFFFFFFFFFFFFFFFFFFFFFFFFFFFFFFFFFFFFFFFFFF:FFFFFFFFFFFFF
F:FFFFFFFFFFFFFF,FFFFFFFFFFFFFFFFFFFF:FF:FFFF:FF
@A00721:190:HGJW7DSXY:4:2264:1524:30405_1:N:0:TCTGCAAG+GCTGCACT
GAAGCTCGCCATATCAGATTAGAGACATGCCAGTTAGAGTTCTTCTAACCAGGACTGATGGTGAGATT
CTTTTACCTTTTGGAGGTCCAGGAACCTTTACTCCTAGTGTCTTTTCTAGAGTCCAAGGACGTTTCCG
TCG
+
FFFFFFFFFFFFFFFFFFFFFFFFFFFFFFFFFFFFFFFFFFFFFFFFFFFFFFFFFFFFFFFFFFFF
```

FFFFFFFFFFFFFFFFFFFFFFFFFFFFFFFFFFFFFFFFFFFFFFFFFFFFFFFFFFFFFFFFFFFF
FFF
@A00721:190:HGJW7DSXY:4:1621:22290:29543_1:N:0:TCTGCAAG+GCTGCACT
GTGGGAGACCGCTCAATAACACTCTATTGAATCCCTTTTCCACGATGAAAGCTCTTTATGAGGTGCTC
CATGCAAATTTACAGTTCCTCTTTGGTGATATCGCCTCTGTCCTTTCTCCTTTAATCGTGAGGTTACG
AGA
+
FFFFFFFFFFFFFFFFFFFFFFFFFFFFFFFFFFFFFFFFFFFFFFFFFFFFFFFFFFFFFFF,FFFF
FFFFFFFFFFFFFFFFFFFFFFFFFFFFFFFFFFFFFFF:FFFFFFFFFFF,FFFFFFFFFFFFFFFFF
FFF
@A00721:190:HGJW7DSXY:4:1471:2736:28307_2:N:0:TCTGCAAG+GCTGCACT
GGTTTATCGGTTGTATTTTGATAAATTTGTTGGAATCGATAATGATACTGACGAAGGTTCTATTACCA
CCCTTGTCATTGATACAAACATTCTAGGTGATGCTTTCTTAGAGGCTC
+
FFFFFFFFFF,FFFFFFFFFFFFFFFFFFFFF:FFFFFF::FFFFFFFFF:FFFFFFFFFFFFFFF:F
FFFFFFFFF:FFFFFFFF:FFFFFFFF:FFFFFFFFFFFFFFFFFFF:
@A00721:190:HGJW7DSXY:4:2130:15356:8954_2:N:0:TCTGCAAG+GCTGCACT
TATTTACAATCTGTAGAGAGAGAGGTTAATCAGAATTTGAGTGATAACGATTTAGTACTGGGAAATGG
TGGTAGTCAGTTTACTACGTTTCTTTCCCTCCCCTTTTTCGATTGGAT
+
,F::FFF,FFFFF:,::F:FFFFFFFFF:FF:FF:FFFFFFFFFF:,FFF:FF:FF,F:FFF:F,:FF
,FFFFF:FFFFFFFF,FF,,FFFFFFFFFFFFFFFFFF:F:,,FFFFF
@A00721:190:HGJW7DSXY:4:1617:27444:31579_2:N:0:TCTGCAAG+GCTGCACT
CGTCTCACGACTAATTTGATAAGGTTTTTCTTTTATGGGACTCTTGTTTGTACTTATGAAGGAGGTAT
TACTTCGGAAGTTCTTAATGTAGGTCTCCTAGTACTTAAGGGTTTATG
+
FFFFFFFFFFFF,:FFFFFFFFFFFFF:FF:,FFFFFFFFFFF,:F,FFFFF,:FFFFFFFF:FFF,F
FF:F:FFFFFFFFFFFF,:FFFFFFFFFFFFFFFFFFFFFFFFFFF,FFF
@A00721:190:HGJW7DSXY:4:1558:11577:16658_2:N:0:TCTGCAAG+GCTGCACT
TTTTGATGGTATGTTTCACATTGCGTGATCACTTCCCGTGGACTTGGGAAGAGATTAAACATAAAATC
CAGCGCTTCTATTATGTCGCTAAAGCCTGTCCAGTTGTCGATGATGTT
+
F,FFFFFFFFFFFFFFFFFFFFFFFFFFFFFFFFFFFFFFFFFFFFFFFFFFFFFFFFFFFFFFFFFF
FFFFF,:FFFFFFFFFFFFF:FFFFFFFFFFFFFFFFFFFFFFFFFFF
@A00721:190:HGJW7DSXY:4:1614:1307:10895_2:N:0:TCTGCAAG+GCTGCACT
AAACTTACGACGATGTTCTCGCTCGCATTTCACAAGGATTACTAAGTCTGAGATCTGCCACTTCTAAT
TTTAGGAAACGTCGCGAAAAGAAAGTTCATGAGGATGTCTTGGTCGAA
+
FFFFFFFFFFFFFFFFFF:FFFFFFFFFFFFFFFFFFFFFFFF:FFFFFFFFFFFFFFFFFFFFFFFFF
FFF:FF::FFFFFFFFFFFFF:FFFFFF::FFFFFFFFF:FFFFFFFF
@A00721:190:HGJW7DSXY:4:1368:7916:12414_2:N:0:TCTGCAAG+GCTGCACT
GAACTTCTAATAGAGGATCCTGATGACATTATTTCAACGGATACCGATCTCAGTTCTACCACAGAAAC
CGTCTCAGGAACTGTCTCCACTGATTCGGAATTAGAGAAAGACGACCT
+
FF:FF:FF,:FFFFFFFFFFFFFFFFFFFFFFFFFFFFFFFFFFFFFFFFFFFFFFFFFFFFFFFFFFF
FFFFFFFFFFFFFFFFFFFFFFFFFFFFFFFF:,FFFFFFFFFFFFFFF
@A00721:190:HGJW7DSXY:4:2211:15040:35274_1:N:0:TCTGCAAG+GCTGCACT
GAGACCTCTGAATTTTCAGGGTGCCCTGATGAGGACGTTGAAAGGGATAATTTCAACTCTGAGACTTC
TAAGAGTGTTTCAAAAGACCAGAAACGTAACAAGAAGAATAAAGGTAGGAAGGGGAGAAAACAGAACA
AGC
+
F:FFF,F:F,F:FFFFF,F:FFFF:FFF:FFFF:F::FFF:F:FF:FFFFFFFFFF,FFFFFFF,FFF
FF:FFFFFFFFFFFFF:FFFF:FFFFFFFFFFFFFFF:FFFFFFFFFFFFFFFFFFFFFFFFFFFFFFF
FFF

```
@A00721:190:HGJW7DSXY:4:2260:8196:30937_2:N:0:TCTGCAAG+GCTGCACT
ACTGATATGGTTGCGGCTGATCGTACCCCAATTCCTGCTAGAAAGATCACTCTCGGCGATACCACTGA
TCTTCCAGAGTTACGTAGTGGTCGTTCTTTTGTTGATTTTATCAAAGA
+
:FFFFFFFFFFFFFFFFFFFFFFFFFFFFFFFFFFFFFFFFFFF::FF:FFFFFFFFFFFFFFFFFFF
FFFFFFFFFFFFFFFFFF:FFFFFFFF:FFFFFFFFF,FFFFFFFFFF
@A00721:190:HGJW7DSXY:4:2448:23547:28494_2:N:0:TCTGCAAG+GCTGCACT
CAAGTTAAACTTCGAACTGACCACTTACAAGAGTGTTGAAGTACCGCAACATGGATCAGTTTTCCAAA
AAGCTATTAATAGCAGTTGCGGGGCTATAATACCGCCTCCAGATTACT
+
FFFFFFFFFFFFFFFFFFFFFFFFFFFFFFF:FFFFFFFFFFFFFFFFFFFFFFFFFFFFFFFFFFFF
FFFFFFFFF,FFFFFFFFFFFFFFFFFFF,FFFFFFFFFFFFFFFFFF
@A00721:190:HGJW7DSXY:4:2165:15962:33113_1:N:0:TCTGCAAG+GCTGCACT
AGAGTTTTGGAACTGTGTAATGTCCTACGTACTGTTCAAGGTGTTTGGTCTTCTCTAAAGAACAGCAA
CTTTCAACATGAATTAGTTATTGAAAGTCTTGACCCTCACGTCTCACTTTATCACCTTGGAGATGGTA
AAG
+
FF:,FFFFFFFFFFFF,FF,FFFFFFFFFFF,FFFF:,FFFFFFFF,F,FFFFFFF:FFFFFFFFFFF
FFF:FFF:FFFF:FF,FFFFFF:FFFFF,FF:FFF:F,FFFF,FF,FFFFFF,:F,FFF,:FFF,FFF
:,:
@A00721:190:HGJW7DSXY:4:2147:20003:15655_2:N:0:TCTGCAAG+GCTGCACT
ATTCTGCTTCGACGGTAATTCCATAAGACCTTTCTTGGGGAGTAAAGAGGGTTTGAAAATTACGGAAC
CTTTCCTCGTTCTTCATGAAGATCTTCGAGTGTTTTATAGTGATTTAT
+
FFFFFFFFFFFFFFFFFFFFFFFFFFFFFFFFFFFFFFFFFFFFFFFFFFFFFFFFFFFFFFFFFFFF
FFFFFFFFFFFFFF:FFFFFFFFFFFFFFFFFFFFFFFFFFFFFFFFF
@A00721:190:HGJW7DSXY:4:1417:3061:17973_1:N:0:TCTGCAAG+GCTGCACT
CAAGTCGATTGATTTTGTTGACTACGATCTCTCTTGTAATAAGAAGTTCTTCGCAGTTGAATCTCCAC
CAGGTGGAGGGAAGACCACTGAACTTATAGCGACTTTTGTCGAATTTTTCGCAAAAGGTCTTGAAGTA
CGT
+
FFFFFFFFFFFFFFFFFFFFFFFFFFFFFFFFFFFFFFFFFFFFFFFFFFFFFFFFFFFFFFFFFFFF
FFFFFFFFFFFFFFFFFFFFFFFFFFFFFFFFFFFFFFFFFFFFFFFFFFFFFFFFFFFFFFFFFFFF
FFF
@A00721:190:HGJW7DSXY:4:1358:30192:25473_2:N:0:TCTGCAAG+GCTGCACT
TCGTAATTCGCGAGAAGAGATAGTGACTAAAGTTTTGGCACTTCTAGTTCATCGCGGTATTTGTGAAA
TTAAGACAGACGTGAAAGTTATGATGAGTGATCGTATTAGAACAGCTG
+
FFFFFFFFFFFFFFFFFFFFF:FFFFFFFFFFFFFFFFFFFFFF:FFFFFFFFFFFFFFFFFFFFFFF
FFFFFFFFFFFFFFFFFFFFFFFFFFFFFFFFFFFFFFFFF:FFFFFF
@A00721:190:HGJW7DSXY:4:2455:3423:11146_1:N:0:TCTGCAAG+GCTGCACT
ACGTCGCTGAGGTCAAACCTGTGGACGTTCTTTTCATCGACGAATTATTCATGATTCACGCTGGTCAG
ACCCTTTTTCTTACACATCTACTTAAGGCGGCTTATACCATAGGTTACGGTGATTCCAATCAAATCGG
ATT
+
FFFFFFFFFFFFFFFFFFFFFFFFFFFFFFFFFFF,FFFFFFFFFFFFFFFFFFFFFFFFFFFFFFFF
FFFFFFFFFFFFFFFFFFFFFFFFFFFFFFFFFFFFFFFFFFFFFFFFFFFFFFFFFFFFFFF:FFFF
FFF
@A00721:190:HGJW7DSXY:4:2378:18566:24721_2:N:0:TCTGCAAG+GCTGCACT
TGATCTCCAAGTCGCAAACTACTTCAACATCAACGACATCATTCTTGATGAGTGTCGTTCTTATCGAA
GTCTCAGTTATCGCTGCCCGGCCGACGTTTGTCGCGAACTCTCAGAGG
+
:FFFFFFFFFFFFFFFFFFFFFFFFFFFFFFFFFFFFFFFFFFFFFFFFFFFFFFFFFFFFFFFFFFF
FFFFFFFFFFFFFFFFFFFFFFFFFFFFFFFFFFFFFFFFFFFFFFFF
```

@A00721:190:HGJW7DSXY:4:2353:13476:28463_1:N:0:TCTGCAAG+GCTGCACT
AATTTCTCCTGCTTGTAATTCGAGAGAGGACACTATGGAAATTAGACCTATCAGATGTCTTGACGACT
TCGAATTTGAGGACGGAGTTAAATATATTACTTTCACTCAAGGTGAAAAGTTTGAAATTAACAAAGTC
CTT
+
FFFFFFFFFFF,FFFFFFFFFFFFFFFFFFFFFFFFFFFFFFFFFFFFFFFFFFFFFFFFFFFFFFFF
FFFFFFFFFFFFFFFFFFFFFFFFFFFFFFFFFFFFFFFFFFFFFFFFFFF:FFFFFFFFFFFFFFFF
FFF
@A00721:190:HGJW7DSXY:4:1220:18511:19648_1:N:0:TCTGCAAG+GCTGCACT
GTTCAAGGTAATACTTACCAGCACGTTCGGCTGGTTAGAATTAAACCTCAAGATGACCCTGTTTTTGC
ATCACAAACACATATTACCGTTGCTCTATCACGACACGTTGAATCTTTACGATACTTTGTTATCAATG
TAA
+
FFFFFFFFFFFFFFFFFFFFFFFFFFFFFFFFFFFFFFFFFFFFFFFFFFFFFFFFFFFFFFFFFFFF
FFFFFFFFFFFFFFFFFFFFFFFFFFFFFFFFFFFFFFFFFFFFFFFFFFFFFFFFFFFFFFFFFFFF
FFF
@A00721:190:HGJW7DSXY:4:1252:21016:36730_2:N:0:TCTGCAAG+GCTGCACT
TGAGAGGATCAATCACAGTAAGGAACTGTCACGTTTAGCACGGGAGGTTGCAGATTCAACCCCTTATA
TCGAGGCTATCGAGGACGATGAGTATCTTGAAGTTGTTGACGACAAGC
+
:FFFFFFFFFFFFFFFFFFFFFFFFFFFFFFFFFFFFFFFFFFFFFFFFFFFFFFFFFFFFFFFFFFF
FFFFFFFFFFFFFFFFFFFFFFFFFFFFFFFFFFFFFFFFFFFFFFFF
@A00721:190:HGJW7DSXY:4:1210:6813:30420_1:N:0:TCTGCAAG+GCTGCACT
TAGAGGACCACCTGGATCTTCTACTGTCTCTGCCTTCCAAGATTTCTTAGAAGAGGTTATTCCTGGAA
CCACCTTAACGTCACTCGGTAATCTTTCAGCCGAATTAAGTACTAATGAGTTCAAGTCGGTTGTCGAT
CGA
+
:FFFFFFFFFFFFFFFFFFFFFFFFFFFFFFFFFFFFFFFFFFFFFFFFFFFFFFFFFFFFFFFFFFF
FFFFFFFFFFFFFFFFFFFFFFFFFFFFFFFFFFFFFFFFFFFFFFFFFFFFFFFFFFFFFFFFFFFF
FFF
@A00721:190:HGJW7DSXY:4:1364:32371:28494_2:N:0:TCTGCAAG+GCTGCACT
GGCTAATACCTTTATAGGTAAGAAGCCCCTTGAGCCGCGTAGTCAGCGCGTTTAGCTGTATCATCAGT
CAGAGTATACCCGATAGGGTACCGAGTCTCCAAGAGAATATTTATTCT
+
FFFFFFFFFFFFFFFFFFFFFFFFFFFFFFFFFFFFFFFFFFFFFFFFFFFFFFFFFFFFFFFFFFFF
FFFFFFFFFFFFFFFFFFFFFFFFFFFFFFFFF,FFFFFFFFFFFFFF
@A00721:190:HGJW7DSXY:4:1132:25003:28166_1:N:0:TCTGCAAG+GCTGCACT
ACGTTTTCACAACGGAAAGACATATCGCACCCGAAATTTTTGGGCAGAATGCTCTTAACGCGGTTCTT
GAACGGTGTTTTGATCAGTCTAAGTTGGATAAAGTTATAGGAGACTCAGTTAACCTGAGAGCTCCTCA
ATT
+
FFF:FF:FFFFFFFF:F:FFFFFFFFFFFFFFFF:FFFFFFFFFFFF:FFFF:FFF:FFFFFFF:FFF
FF:FFFFF,:FFFF,F:FFFF:FF:FFFFF:FFFFF:FFFFFFFFFFFFFFFFFFFFFFF:FFFFFFFF
FFF
@A00721:190:HGJW7DSXY:4:2204:23773:12289_1:N:0:TCTGCAAG+GCTGCACT
CAATAGGGACGCTAGTATACTTCGTAAAGTCGACAGCGAAATGGTCAATATTGATAATTGGACCGACT
ATATGACCTATTTTAAGCTCATGGTGAAAAGGGAAGCTAAGGTTAAACTTGATGCTTCAGCCTTTTCG
AAG
+
FFFF:F:FFFFFFFFFFFFFFFFFFFFFFFFFFFFFFFFFFFFFFFFFFFFFFFFFFF:FFFFFFFFF
FFFFFFFFFFFFFFFFFFFFFFFFFFFFFFFFFFFFFFFFFFFFFFFFFFFFFFFFFFFFFFFFFFFF
FFF
@A00721:190:HGJW7DSXY:4:1316:6352:30624_1:N:0:TCTGCAAG+GCTGCACT
GCAATTTTCGGACCTATCTTTAAGGTTCTTGTGAGGAGAGTCAAACAGTGTCTTTTACCCAACATTTT

```
TATGTATACAGAGATGACCAGTGACGATTTCTCCCGGAAGCTCTTCTCTCTCTTAGGAGGTCATGATG
ACT
+
FFFFFFFFF,FFFFFFFFFFFFFFFFFFFFFFFFFFFFFFFFFFFFFFFFFFFFFFFFFFFFFFFFFF
FFFFFFFFFFFFFFFFFFFFFFFFFFFFFFFFFFFFFFFFFFFFFFFFFFFFFFFFFFFFFFFFFFFF
FFF
@A00721:190:HGJW7DSXY:4:1263:16839:19194_2:N:0:TCTGCAAG+GCTGCACT
TTTCGAAGTTTGACAAATCTCAAGATTTCTTTTGTAAAGCCTATGAGATAAAGATGTATTCTTATTTA
GGCTTCGATGCTGAATTTCTTGAACTTTGGAGTGCCAGTGAATATTTT
+
FFFFFFFFFFFFFFFFFFFFFFFFFF:FFFFFFFFFFFFFFFFFFFFFFFFFFFFFFFFFFFFFFFFF
FFFFFFFFFFFFFFFFFFFFFFFFFFFFFFFFFFFFF:FFFFFF:FFF
@A00721:190:HGJW7DSXY:4:1302:21658:20650_1:N:0:TCTGCAAG+GCTGCACT
GAGTATCCATAACGATTTGTCATTTACCACCTTTGCTCAACGACGAAGTGGAACGGCCAACACTTTCT
TTGGTAATTGTATGATCACTTTGATGATTTTATCACTTTATTATGACCTTGGAAAGTTTTCGGCTGTG
GTT
+
FFFFFFFFFFFFFFFFFFFFFFFF:FFF,FF::FFFFF,FFFFFFFFFFFFFFFFFFFFFFFFFFFF:
FFFFFFF,FFFFFFFFFFFFFFFFFFFFFFFFFFFFFFFFFFFFFFFFFFFFFFFFFFFFFFFFFFFF
FFF
@A00721:190:HGJW7DSXY:4:2461:4444:11287_1:N:0:TCTGCAAG+GCTGCACT
CCATTCTGTTTTCAAAGACTCCAATAACTGATCATTCTGATCATATGATTTTGGACAATGGTTTCGAA
GCGAAATTTTTGAGGGATAAACCAGCGTACTTCTGCTCGCGATTTATTCTTTTTGTCAATGATTACAT
CTA
+
F:FFFFFFFFFFFFFFFFFFFFFFF,FFFFFFF:FFF:FFFFFFFFFFFFFFF,FFFF,FFFFFFFFFF
FFFFFFFFFFFFFFF:FFF:F:F:FFFFFFFFFF:FFFFFFFF:FFFFFFFFFFFFF:FFFFFFFFFF
FF:
@A00721:190:HGJW7DSXY:4:1459:19849:10410_1:N:0:TCTGCAAG+GCTGCACT
CCTTACAAGATCATCGTTAAGTTAGGTAAACCCCTAACCGATGTTACTCTTGTAGGACTTTGGGAGAG
ATTCGTTAGTTTTAAAGACTATACTAAATATCTCCACTACGAGGTTGTTGTTTGGCGATTAGCTCTTG
CGA
+
FFFF:FFFFFFFFFFFFFFFFFFFFFFFFFFFFFFFF,FFFFFFFFFFFFFFFFFFFFFFFFFFFFFF
FFFFFFFFFFFFFFFFFFFFFFFFFFFFFFFFFFFFFFFFFFFFFFFFFFFFFFFFFFFFFFFFFFFF
FFF
@A00721:190:HGJW7DSXY:4:1449:8974:11960_2:N:0:TCTGCAAG+GCTGCACT
ATTGAGGGTCGTTATACCTATTCGGCTATCTGTGCTTTGAATTGTATAAGATCAAATTTTAAACGATT
CCGAGAGGTTTATCCTGATGTGGAGGGAGTTCTTGAAGCATCCCGACC
+
FFFFFFFFFFFFFFFFFFFFFFFFFFFF:FFFFFFFFFFFFFFFFFFFFFFFFFFFFFFFFFFFFFFF
FFFFFFFFFFFFFFFFFF,FFFFFFFFFFFFFFFF,FFFFFFFFFFFF
@A00721:190:HGJW7DSXY:4:1624:19334:14403_1:N:0:TCTGCAAG+GCTGCACT
TGATGCGGTTTCATAAGATTATCAGTAATCCTGATTCTACTACGGATTCATGGACCTTCCATCCTGTA
CCTCTTCTGGAGAGACGTATCTCAAAGATCGACTTCGAGACAGGTGATATTGTTGAGCGAGAGCGACC
AAG
+
FFFFFFFFFFFFFFFFFFFFFFFFFFFFFFFFFFFFFFFFFFFFFFFFFFFFFFFFFFFFFFFFFFFF
FFF,FFFFFFFFFFFFFFFFFFFFFFFFFFFFFFFFFFFFFFFFF:FFFFFFFFFFFFFFFFFFFFFF
FFF
@A00721:190:HGJW7DSXY:4:1534:7744:36104_1:N:0:TCTGCAAG+GCTGCACT
CGTGGCGCTTTATTTTAAAAAGCTCCCTCCTCTCGCAAGTTTGATCTGATACTTATCACGAAAGGACT
TGAGTCTTTTGATCAGTCTCTGACTTGACCATCTTTCATTTTAAATGACGATCATTAAGACTCTCTTT
CTT
```

```
+
F:FFFFF:FFFFFFFFF:FFFFFFFFFFFFFFFFFFFFFFFFFFFFFFFFF,FFFFFFFFFFFFFFFF
FFFFFFFFFFFFFFFFF:FFFFFFFFFFFF,FFFFFFFFFF:FFFF:FFFFFFFFFFFFFFFFFFFFF
FFF
@A00721:190:HGJW7DSXY:4:1448:4345:31814_2:N:0:TCTGCAAG+GCTGCACT
CTTTGCACTGATTTTTCCTCATCAGCTTGTGTTTTACCTAATCTGTGTTTTTACGATAACAACTATCA
GTTGATTAAGACTCTTCCGAAAGGCGAACTTTGGGAATTTGAAGCTCA
+
FFFFFFFFFFFFFFFFFFFFFF:F::F:FFFFFFFFFF:FFFFFFF:FFFFFFF,FFFFFFFFFFF:F
FFF:FFF,FFFFFF:FFFFFFF:,:FFFFFFFFFFFFFFFFFFFFFFFF
@A00721:190:HGJW7DSXY:4:2531:5656:33896_1:N:0:TCTGCAAG+GCTGCACT
CGTTTGACCGTGACGAACTGTGGTGTTCTTGATCTACGTTACGGATATACTCTCCTTGAGTTCACTCG
TAAGGACACTACCACTTATTACGATATTTCTTTAGTCGATGGGTTTTCGACTCCTTTAAGCGTCTTCT
GTT
+
FFFFFFFFFFFFFFFFFFFFFFFFFFFFFFFFFFFFFFFFFFFFFFFFFFFFFFFFFFFFFFFFFFFF
FFFFFFFFFFFFFFFFFFFFFFFFFFFFFFFFFFFFFFFFFFFFFFFFFFFFFFFFFFFFFFFFFFFF
FFF
@A00721:190:HGJW7DSXY:4:1517:22996:15546_2:N:0:TCTGCAAG+GCTGCACT
TTTCTTCTAATGTGGCCTGAACTACGTTTCACTTTGTGTGTACTTGTCTCTATTTTAAGTTTTGTCTT
CCTTGTTTGTCTTATTCTATTAATCACTCATTTTAGTGAGATTATTAA
+
FFFFFFFFFFFFFFFFFFFFFFFFFFFFFFFFFFFFFFFFFFFFFFFFFFFFFFFFFFFFFFFFFFFF
FFF:FFFF,FFFFFFF:F:FFFFFFFFFFF:FF:FFFFFFFFFFFFFF
@A00721:190:HGJW7DSXY:4:1607:6045:30436_1:N:0:TCTGCAAG+GCTGCACT
GTCTTCGACAACAGCAGGGGTTTTCGAATAGTGATAGCCACCCGCCTTACAGAGGGGCAGCAGCATGA
CCATAATTGGTATTGACTTTGGTACCACCTTTTCTACTGTCTGTCTCGCCTCTTCGAATGAAGTTTTA
ATG
+
FFFFFFFFFFFF:FFFFFFFFFFFFFFF:FFFFFFFFFFFFFFFFFFFFFFFFFFFFFFFFFFFFFFF
FFFFFFFFFFFFFFFFFFFFFFFFFFFFFFFFFFFFFFFFFFFFFFFFFFF:FFFFFFFFFFFFFFFF
FFF
@A00721:190:HGJW7DSXY:4:1417:27742:4977_2:N:0:TCTGCAAG+GCTGCACT
TGATAGTGAATTTATACCTACAATCATTGGTTTTTCTAATAATGATGACACTATTGTTTATGGATATG
ATGCTATTACTCATGATGGTAACGGTCAGGGAGGATTCTCAGTTTACA
+
F:F:FF,FFF::F:FFFFFFFFFFFF,FFFFFFFFFFFFFFFFFFFFFFFFFF,,FFFFFFFFFFFF:
:FFFFF,FFFFFFFFFFFF,FFF,FFFFFFFFFFFFFFF::,FFFF
@A00721:190:HGJW7DSXY:4:1176:17137:6057_2:N:0:TCTGCAAG+GCTGCACT
CCGAAAGACGTGCTAAATTAAAACCATTGTACGATGTCGTCTGTCTCGGCGATCACTTTTCACTAAGA
ATTGGTCCTACTTTTGGACAAGGCCAACTTCGTAATCTTCATGAGATC
+
FFFFFFFFFFFFFFFFFFFFFFFF:FFFFFFFF,FFFFFFFFF:F:FFFFFFFFFFFFFF:FFFFF,
FFFFFFFFF:FF:FFFFFFFFFFFFFFFFF:FFFFF:FFFFFFFFFF
@A00721:190:HGJW7DSXY:4:1128:13593:26694_2:N:0:TCTGCAAG+GCTGCACT
TATCTCTTTGGTTGTTAGGGATTTTGAGAGAGTGAAAGGCTTCAATTGTAGTGGTTTGGTCGTTTCAG
TACCCAGTCAGTATACCTCTACGCAAAGATATTTCATGAAAACTACAG
+
FFFFFFFFFFFFFFFFFFFF:FFFFFFFFFFFFFFFFF:FFFF:FFFFFFFFFF:F:FFF:FFFFFF
FFFFFFFFFFFFFFF,FFFFFFFFFFFFFFF:FFFFFFFFFFFFFFFF
@A00721:190:HGJW7DSXY:4:2130:4698:17801_2:N:0:TCTGCAAG+GCTGCACT
ATTACCTGTCTTTCATATTATGAATGAACCTTCGGCCGCTCTCTTTGCTTCGATGTTGGATGTCAAGA
AGGGTTCAGAATGGGATTCCTACGTAGTTTATGACTTTGGTGGTGGTA
+
```

```
FFFFF:FFFFFFFF:FFFFFFFFFFFFFFFFFFFFFFFFFFFF:F:FFF,FF:FFFF:FFFFFFFFFF
FFF:FFFFFFFFFFFFFFFFFF,FFFF:FFFFFF,:FFFFFFFFFFF
@A00721:190:HGJW7DSXY:4:1519:14136:24972_1:N:0:TCTGCAAG+GCTGCACT
TCTTACGTTACGGTAATTACTATAGTGTTATTTATTCTGGTGGTGATGATAATCTTGGAGGTCGTGAC
GTTGATGAAGCTATAAGAGCGTTCTTATTACGGCGCACTTCTTTACCCGCCACCACACAACTTTCAGT
TTC
+
FFFFFFFFFFFFFFFFFFFFF:FFFFFFFFFFFFFFFFFFFFFFFFFFFF:FFFFFFFFFFFFFFFFFF
FFFFFFFFFFFFFFFFFFFFFFFFFFFFFFFFFFFFFFFFFFFFFFFFFFFFFFFFFFFFFFFFFFFF
FFF
@A00721:190:HGJW7DSXY:4:2333:31548:15045_2:N:0:TCTGCAAG+GCTGCACT
TGTTACTGGAAAACCCTCGTCAATACTTTTCAATAATATACGTCTTGAACTCACTTATGACGATCTAG
TGAAATTAACGCGTCCTTTTCTGGAACGTAGTTTTACTATTATGAACA
+
FFFFFFFFFFFF:FFFF:FFFF:FFFFFFFFFFFFFFFFFFFFFFFFFFFFFFFFFFFFFFFFFFFFF
F:FFFFFFF:F:FFFF,F:FFF:FFFFF:FFFFF:FFF,FFFFFFF:F
@A00721:190:HGJW7DSXY:4:1620:32099:9737_1:N:0:TCTGCAAG+GCTGCACT
ACGTGTGCTCTTCCGATCTAACTTACCCTGATTCCGGCCGGTGGTAGCTCATTTCTACCAGGGGCTAT
TACTCTTGCTGAAACAGCTATTAAAGCTGTTGAGCGGGTAATTACACCTAAACGAGCTCGATCTGCCA
TCG
+
FFFFFFFFFFFFFFFFFFFFFFFFFFFFFFFF:FFFFFFFFFFFFFFFFFFFFFFFFFFFFFFFFFFF
FFFFFFFFFFFFFFFFFFFFFFFFFFFFFFFFFFFFFFFFFFFFFFFFFFFFFFFFFFFFFFFFFFFF
FFF
@A00721:190:HGJW7DSXY:4:1477:26458:18223_2:N:0:TCTGCAAG+GCTGCACT
CACTCTTTTCTGCTACTTTAACGGAGCGTAGTGTACTCTTCGTCGATTGTATCAACAGTAACATTACT
TCTAATAAAGGTCTCTATCAAGCTCGTATTATCGTCGCTGCTGGATCT
+
FFFFFFFFFFFFFFFFFFFFFFFFFFFFFFFFFFFFFFFFFFFFF:FFFFFFFFFFFFFFFFFFFFFF
FFFFFFFFFFFFFFFFFFFFFFFFFFFFFFFFFFFFFFFFFFFFFFFF
@A00721:190:HGJW7DSXY:4:1638:7410:31923_1:N:0:TCTGCAAG+GCTGCACT
AAGGCTAAACTCCAAGGTAATTATGAAGACTGTGATTTATCTTTCACTCTGGCCTTGTATGAGGGTGT
TTCTAATCGTAACATTAATAATTTCAATCTTCTTAAAAAGAAGATAGAGAGTCATACTTTGGGCTATC
GTT
+
FFFFFFF:FFFFFFFFFFFFFFFFFFFFFFFFFFFFFFFFFFFFFFFFFFFFFFFFFFFFFFFFFFFF
FFFFFFFFFFFFFFFFFFFFFFFFFFFFFFFFFFFFFFFFFFFFFFFFFFFFFFFFFFFFFFFFFFFF
FFF
@A00721:190:HGJW7DSXY:4:1578:17815:3192_2:N:0:TCTGCAAG+GCTGCACT
GATAGCCAATATTGAAGTGGACACCTCTGGTAGTATCGAAGTAAAGATAACCAATAAGGAAGGAGCTT
ATACTATGGTTGAATCAACTAAGCATACTACTTTTGCTTTGATTACTC
+
FFFFFFFFFFFFFFFFFFFFFFFFFFFFFFFFFFFFFFFFFFFFFFFFFFFFFFFFFFFFFFFFFFFF
FFFFF:FF:FFFFFFFFFFFFFFFFFFFFFFFFFFFF:F:FFFFFFFF
@A00721:190:HGJW7DSXY:4:2638:21730:14356_1:N:0:TCTGCAAG+GCTGCACT
CTTCCGATCTGTTAAAGAGTCGATTAAACAAGTTGTACTTATCAATGATTTTATCGCTGGTTGTTATC
TCGAAAAGTCTCTTCTAAAAGGAACGTCAGTTCTAACCCAAAGAGAATATCTAAAATATTTACAAAAT
CTT
+
FFFF:FFFFFFFFFFFFFFFFFFFFFFFFFFFFFF,FF:FF:FFFFFFFFFFFFF:FFFFFFFFFFFF
FFFFFFFFFFFF:FFFFFFFFFFFFFFFFFFFFFFFFFFFFFFFFFFFFFFFFFFFFFFFFFFFFFFF
FFF
@A00721:190:HGJW7DSXY:4:1160:22797:12759_2:N:0:TCTGCAAG+GCTGCACT
AGACGTTGCTTGTCGCTTCCTCCTTTAATGGCACTACCTCGGTACAATCTTACTTGGAACGAAGGCGA
```

```
CCTGTTCCGGAGTTTTTACGGGATCCGGAATGTTCTGTCTATATTAGG
+
FFFFFFFFFFFFFFFFFFFFFFFFFFF:FFFFFFFFFFFFFFFFFFFFFFFFFFFFFFFFFFFFFFFFFF
FFFFFFFFFFFFFFFFFFFFFFF,FFFFFF,FFFFF,FFFFFFFFFFFF
@A00721:190:HGJW7DSXY:4:1122:17842:28635_2:N:0:TCTGCAAG+GCTGCACT
TCGAACGTCAACGTTCACCGAGTTTCGTTTCGTACCTTGATGCTGACTTTCGTCAATATGATTTGATC
TGGTTGCTCAATTCTAAGCGTGTTCCGCTTAGTGCGAGGCCTCTCTTC
+
FFFFFFFFFFFFFFFFFFFFFFFFFFFFFFFFFFFFFFFFFFFFFFFFFFFFFFFFFFFFFFFFFFFFFF
FFFFFFFFFFFFFFFFFFFFFFFFFFFFFFFFFFFFFFFFFFFFFFFF
@A00721:190:HGJW7DSXY:4:2221:24686:22639_2:N:0:TCTGCAAG+GCTGCACT
TATTTATACGGTTACGAGAAGAAGGTTCGAAAGTTTGAGAACTTAGTTTACGAACTTGACTCTACTAT
ACGTGACATACTAAATACTAGATATTTGGATGATCTTGATAAAGAACT
+
FFFFFFFFFFFFFFFFFFFFFFFFFFFFFFFFFFFFFFFFFFFFFFFFFFFFFFFFFFFFFFFFFFFFFF
FFFFFFFFFFFFFFFFFFFFFFFFFFFF:FFFFFFFFF:FFFFFFFFFF
@A00721:190:HGJW7DSXY:4:1113:29912:11271_2:N:0:TCTGCAAG+GCTGCACT
AAAATTTTCTTCCTTGGATTTTAATATAAAGAATCTTTACCAGGACTCCTACTACAGGAGGCAGTCTT
CTGATCGTAAGAAGTTTATAAGGAACATATCCATTTGTTGTAATAGGG
+
FF:FFFF:FFF:FFFFFFFFFFFFFFFFFFF,F:FFFFFFFFF,FFFFFFFFFFFFFFFFFFFFFFFFF
FFFFFF::FFF:FF,FFF:FF,FFF:FF:F,,FFFFFFFFFFFFFFF,
@A00721:190:HGJW7DSXY:4:1521:2465:18129_1:N:0:TCTGCAAG+GCTGCACT
ACTCGAAACTCTAAGTGGTAATACTGTCTTTCCAAGCTTACCTGTATCTTCAATGAAATTGGACTCTT
CGATAGGGGACATTTTATCGTGGGCTTTCCGTAAAGGAAGATCTGTTATCGACGGTTTATCGTTTACG
AAG
+
FFFFFFFFFFFFFFFFFFFFFFFFFFFFFFFFFFFFFFFFFFFFFFFFFFFFFFFFFFFFFFFFFFFFFF
FFFFFFFFFFFFFFFFFFFFFFFFFFF:FFFFFFFFFFFFFFFFFFFFFFFFF:FFFFFFFFFFFFFFFF
FFF
@A00721:190:HGJW7DSXY:4:2252:8269:12900_2:N:0:TCTGCAAG+GCTGCACT
GTTTAAGAATACTATAAATAGAATAGCTATTACTACTAATGGCGTTGGCCTAGTGGATGTTATTAGTG
ACATCTGGTCAATTTCGTTTTTAGGTGGTCTTTATTCTATTATTGATT
+
F:FFFFFFFFFFFFFF:FFFFFFFFFFFFFFFFFFFFFF:FFFFFFFFFFFFFFFFFFFFFFFFFFFFFF
FFFFFFFFFFFFFFFFFFFFFFFFFFFFFFFFFFFFFFFFFFFFFFFF
@A00721:190:HGJW7DSXY:4:1133:1922:34569_1:N:0:TCTGCAAG+GCTGCACT
ACAGAAGATGATGTTAGAGTAGAATTCCTGAAGAGTCCTTCCGTAACCAACTTCTTGACAATTTTCTT
CAATCCTTCGATAATAGTTAAATCTAGGATTATGTCTGTGTTGAAAGACTTTCTACCGATAAGGGTCA
CTA
+
FFFFFFFFFFFFFFF:FFFFFFFFFFFFFFF:FFFFFFFFFFFFFFFFFFFFFFFFFFFFFFFFFFFFFF
FFFFFFFFFFFFF:FFFFFFFFFFFFFFFFFFFFFFFFFFFFFFFFFFFFFFFFFFFFFFFFFFFFF:FF
FFF
@A00721:190:HGJW7DSXY:4:1102:9796:11412_2:N:0:TCTGCAAG+GCTGCACT
TAAGAAATAATCCTACATTGACTGATTATCTAAAGATTGAAGGAAAATTTTATCCTCGTTTAACTTAC
TTAGACTTTCCTACGATACAAATGATAACTTCTAATTTACGTTTGCTA
+
FFFFFFFFFFFFFFFFF,FFFFFF:FFFFFFFFFFF,FFFFFFF:,:FF::FFFF,FFF:FFFFFFFF
F:FFFF::FFFF,FFFFFFFF:FFFF,FFFF:FF,FFFFFFFFFFFFF
@A00721:190:HGJW7DSXY:4:1554:32551:32377_2:N:0:TCTGCAAG+GCTGCACT
CAAAGGGATGCATGGTGTTATCTCCTCAGGAGTCCCGGCATTTGATAATAATGACGAGACTTTCGTCA
TAATCGCTGCATATTACGGATTACACGGTACCAGCGCATCTCGTGTCG
+
```

FFFFFFFFFFFFFFFFFFFFFFFFFFFFFFFFFFFFFFFFFFFFFFFFFFFFFFF:FFFFFFFFFFFFFF
FFFFFFFFFFFFFFFFFFF:FFFFFFFFFFFFF:FFFFFFFFFFFFFFFF
@A00721:190:HGJW7DSXY:4:1532:9118:22451_1:N:0:TCTGCAAG+GCTGCACT
GTATTAACTTATAAAGGTGAGGAGACCATTTTCTTGCTGAAATTTATAGAAGATTATTTTGACAGAAT
CAATAAGAATCATCGATTCTCGATTCGTAGAGCTTATCTAGGTACTCTCATTTACTATGTTGATTTAG
TCT
+
FFFFFFFFFFFFFFFFFFFFFFFFFFFFFFFFFFFFFFFFFFFFFFFFFFFFFFFFF::FFFFFFFFFF
FFFFFFFFFFFFFFFFF:FFFFFFFFFFFFFFFFFFFFFFFFFFFFFFFFFFFFFFFFFFFFFFFFFFFF
FFF
@A00721:190:HGJW7DSXY:4:1404:26332:27962_2:N:0:TCTGCAAG+GCTGCACT
GGATCCACTCCTCGAGTTATCATGATGTTAGTATGTTATATACTGATTTACAACGATATAGGAGTAAT
CTACGAGAAGATTCTAGAATTCTTAATTATAATATTCAACCTCAAAAG
+
F:FF,FFFFFF:FFFFF,FFFFF:FFF:F,FFFFFFFFFF:FFF:FFFFFFFF::FFFFFFFFFFFFF
FFFFFF:FFFFFFFFFFFFFF::FFFFFFFFFFFFFFFFFFF:FF:FFF
@A00721:190:HGJW7DSXY:4:1643:13060:21856_2:N:0:TCTGCAAG+GCTGCACT
GAGATCACACATTTTGAGAACTAACTATGTTATGTTCTCTGATCATTTTGACAATCTCTTCTTATAAT
AAATAAATAAATAATATTACTAATGGCAAATAGTGGAAAGGCTCCAGT
+
FFFFFFFFFFFFF,FFFFFFFFFFFFFFFFFFFFFFF:FFFFFFFFFFFFFFFFFFF:FFFFFFFFFFF
FFFFFFFFFFFFFFFFFFFFFFFFFFF:FFFFFFFFFFFFF,FFFF:FF
@A00721:190:HGJW7DSXY:4:1652:13015:27414_2:N:0:TCTGCAAG+GCTGCACT
TACGATGACGTGAGAATAGGTGAAGGGATAGTTTTTACCTCGACCAACATTAAGGCGGCGGCGACTAA
TCTTAAAGCTTCACCCAAAATGAAGGACACTTCGAAACTGAATAATGA
+
FFFFFFFFFFFFFFFFFFFFFFFFFFFFFFFFFFFFFFFFFFFFFFFFFFFFFFFFFFFFFFFFFFFFF
FFFFFFFFFFFFFFFFFFFFF:FFFFFFFFFFFFFFFFFFFFFFFFFFF
@A00721:190:HGJW7DSXY:4:2208:23122:26819_1:N:0:TCTGCAAG+GCTGCACT
GGCTCTAAAGGACACTATACAATATCCAGACGACGACGAAGCTTATACTACTTTAATTTTGGGTATTA
TGTGGAGATATCGTGATATCAGTACGTCCAGATCCTCTGCTTTTACCGATAACGATAAATTCGTCATC
AAA
+
:FFFFFFFFFFF:FFFFFFFFFFFFFFFFFFFF:FFF:FFFFFFFFFF,FFFFFFFFFFFFF:FFFFFF
FFFFFFFFFFFFFFFFFFFFFFFFFFFFFFFFFFFFFFFFFFFFFFFFFFFFFFFFFFFFFFFFFFFFF
FFF
@A00721:190:HGJW7DSXY:4:2430:2139:10833_1:N:0:TCTGCAAG+GCTGCACT
TTGGTGCTTAGGGATATAATGAATTCAGCACCACATTTTGGTCAAGTCAATCCGTTGAGAAAATTTCT
TAGAGGATATTCGCCATTGTGGTACGAATTGGTCCAACATTTACCACCAAATTCTTTTGATACAACTC
TAG
+
FFFFFFFFFFFFFFFFFF:FFFFF,FFFFFFFFFFFFFFFFFFFFFFFFFFFFFFFFFFFFFFFFFFFF
:FFFFFFFFFFFFFFFFFFFFFFFFFFFFFFFFFFFFFFFFFFFFFFFFFFFFFFFFFFFFFFFFFFFF
FFF
@A00721:190:HGJW7DSXY:4:1155:30454:32127_2:N:0:TCTGCAAG+GCTGCACT
CGGAATACGCTTGCTATTCACCAGATTGGTTCGAACCCGTTGATGCTATGTCAGAAGAAGCTATTCAA
GCTTGTCGTTTAAAGACTGCAGAGGCTGTCAAACAAGCCCAGACTCAA
+
FFFFFFFFFFFFFFFFFFFFFFFFFFFFFFFFFFFFFFFFFFFFFFFFF:FFFFFFFFFFFFFFFFFFF
FFFFFFFFFFFFFFFFFFFFFFFFFFFFFFFFFF,FFFF:FFFFFFFFFF
@A00721:190:HGJW7DSXY:4:1316:24795:10770_1:N:0:TCTGCAAG+GCTGCACT
CAAAGGTTTTGAATTTAGCTCAACTGGGGAGACGTGCTTAAATGGGTGATAAAAATTATTTAGAGATC
TTTTCTACTTACAAAGGACAATTCAGTTTCATTAATTCTACGGAAAAGTCCGGAGTATATTGTATGTT
AAT

```
+
FFFFFFFFFFFFFFFFFFFFFFFFFFFFFFFFFFFFFFFFFFFFFFFFFFFFFFFFFFFFFFFFFFFFFF
FF:FFFFFFFFFF,:FFFFFF:FFFF:FFFFFFFFFFFFFFFFFFFFFFFFFFFFFFFFFFFFFFFFFFF
FFF
@A00721:190:HGJW7DSXY:4:1239:4381:14121_1:N:0:TCTGCAAG+GCTGCACT
AATAGTTCTACCGATACGTAGTGAACGCGTTCGTAGAACCAAGTACTTTCCGTCTGAAGAGTTTAGAG
CTGCTATTGATTATGTAACCGATAGGAGTTGTATAGTTGAAGATGTGGCTTTAGACGATAATCCTTTA
AGG
+
FFFFFFFFFFFFFFFFFFFFFFFFFFF:FFFFFFFFFFFFFFFFFFFFFFFFFFFFFFFFFFFFFFFFFF:
FFFFFFFFFFFFFFFFFFFFFFFFFFFFFFFFFFFFFFFFFFFFFFFFFFFFFFFFFFFFFFFFFFFF:F
FFF
@A00721:190:HGJW7DSXY:4:1125:15049:22200_1:N:0:TCTGCAAG+GCTGCACT
GAAGATTTTCCCACAGATTTGTCTACGTTGCAGATCGCCACTCCAAACACTATATACATTATAAAATA
TAAAAATAAGGGAGTCATAACTTTTGTTACCAAATTCTTGGCTTTCAACGAAGTCTCTGATTTTATTA
ATC
+
FFFFFFFFFFFFFFFFFFFFFFFFFFFFFFFFFFFFFFFFFFFFFFFFFFFFFFFFFFFFFFFFFFFFFF
FFFFFFFFFFFFFFFFFFFFFFFFFFFFFFFFFFFFFFFFFFFFFFFF:FFFFFFFFFFFFFFFFFFFF
FFF
@A00721:190:HGJW7DSXY:4:1566:12301:1564_1:N:0:TCTGCAAG+GCTGCACT
CGATCTCTCGGTCATTTACTCTGTACTTGATCTACGTCGTTTAACTGTCGGAACAACCTTTACTTAGA
ATGACTCTATCTTTTCAGACACTTCTTATATTATTTACAATAGTTAATATCATATTAGTAATACTCAC
TAT
+
FFFFFFFFFFFFFFF:FFFFFFFFFFFFFFFFFFFFFFFF:FFFFFFFFFFFFFFFFFFFFFFFFFF:FF
FFFFFFFFFFFFFFFFFFFFFFFFFFFFFFFFFFFFFFFFFFFFFFFFFFFFFFFFFFFFFFFFFFFFFF
FF:
@A00721:190:HGJW7DSXY:4:1457:32027:3286_2:N:0:TCTGCAAG+GCTGCACT
TTTTTCTTAGCTAATAGATTAATAAAGGATGAGTACGAAAACCAACTCTTCGTTACGAGACCTGATGG
TAGAATAGATTTAGTCTCTACACCCATCTCAACGAGGATAAAGCGAGT
+
FFFFF:FF:FFFFFFFFFFFFFFFFFFFFFFFFFFFFFFFF:FFFFFFFFFFFFFFFFFFFFFFFFFFFF
FF::FFFFF:FFFFFFFFFFFFFFFFFFFFFFFFFFFFFFFFFFF,FFF
@A00721:190:HGJW7DSXY:4:1369:2835:3176_1:N:0:TCTGCAAG+GCTGCACT
TACACGAAGAGATCATCCGCAAACGACTCGAAACCGAGACGCCGTAGATCAAAGTCCCTTCTAACTTT
AGATTATGAATCCTGCCTTTACTCATTTTATAGCAGTTATTTATGTTGTACTTTTTATAATTTATAAC
CTT
+
:F:,:,F,F,:F,,,,FF,,::FFFFFFFFFFFFFFFFFFFFFFFFFFFFFFFFFFFFFFFFFFFFFFFF
FFFFFFFFFFFFFFFFFFFFFFFFFFFFFFFFFFFFFFFFFFFFFFFFFFFFFFFFFFFFFFFFFFFFFF
FFF
@A00721:190:HGJW7DSXY:4:2534:3115:25488_1:N:0:TCTGCAAG+GCTGCACT
TATGTACTTATTGTAACGATCATTCTTTCGAGTTTTGTCATTCTTATTCTTTTATTCCTTTAACTGTG
TATGAGGAGGATTTATAATGCTTTTCGATAGTGATTGTTATCAATTTAAATACATTACTGTTTCAAAT
CAT
+
FFFFFFFFFFFFFFFFFFFFFFFFFFFFFFFFFFFFFFFFFFFFFFFFFFFFFFFFFFFFFFFFFFFFFF
FFFFFFFFFFFFFFFFFFFFFFFFFFFFFFFFFFFFFFFFFFFFFFFFFFFFFFFFFFFFFFFFFFFFFF
FFF
@A00721:190:HGJW7DSXY:4:2246:1904:1470_2:N:0:TCTGCAAG+GCTGCACT
GACTCCGAAAATGGTTTCAAATGAGGTCAAAGCTCTATTTATTAAACGAACTATTATCCGATTCTTCG
AGGGTATCACTACCACTGATCTGCCGACTTTAGTCAAGGAACTTCAAT
+
```

```
FFFFFFFFFFFFFFFFFFFFFFFFFFFFF:FFFFFFFFFFFFFFFFFFFFFFFFFFFFFFFFFFFFFFFF
FFFFFFFFFFFFFFFFFFFFFFFFFFFFFFFFFFFFFFFFFFF:FFFFFF
@A00721:190:HGJW7DSXY:4:2564:12943:26443_2:N:0:TCTGCAAG+GCTGCACT
TATTTTTCTATAAGGGAAAATAGCAATAATCAAATTTCGACAAATTTTAGAACTTTATTTCAGAACTC
TCTCCGATCACCTTGGTGTGATCTCCCGGAGGATCTTTTCAATAAGAT
+
FFFFFFFFFFFFFFFFFFFFFFFFFFFFFFFFFFFFFFFFFF:FF:FFFFFFFFF,FFFFFFFFFFFFF
FFFFFFFFFFFFFFFFFFFFFFFFFFFFFFFFFFFFFFFFFFFFFFFFFF
@A00721:190:HGJW7DSXY:4:1610:2266:14810_2:N:0:TCTGCAAG+GCTGCACT
ATTTTTAAATAGTGATATTCAGCGTATACTTGAATACTTTATCAAAGATTTGGCTTGGTCTATTTACC
GCAAAAAGTTGGAAGAGGTCTATGATATTTATTTTACTTATACTTTAA
+
FFFFFFFFFFFFFFFFFFFFFFFFFFFFFFFFFFFFFFFFFFFFFFFFFFFFFFFFFFFFFFFFFFFFFF
FFFFFFFFFFFFFFFFFFFFFFFF,FFFFFFFFFFFFFFFFFFFFFFFFFF
@A00721:190:HGJW7DSXY:4:1501:22327:28667_1:N:0:TCTGCAAG+GCTGCACT
AACTTGACTGTTGATAATATTACAATAGCTCTTTCTCAGAAGATTAGAGAAGTAACCGGTGAACACGT
CGGTAGCATTGATTTACTTTTACCGTTAGAACTGAAAAAGATTCTTTTCGAAAAGAAACCTATAAGTT
ACA
+
FF,FFF::F,FF,FFFFFFFFF::FFFF:F:FFFFF:FFFF:FFFFFF:F::FFFFFF:FF:F:F:FF
FF:F:F,:FF,:FFFF:FFFFFF:,FF::FF,F:FFFF,F,FF:FFFFFFFFF:,FFFF:FFF:::F:
FFF
@A00721:190:HGJW7DSXY:4:1424:6840:34194_1:N:0:TCTGCAAG+GCTGCACT
TATATTAAAGAAATAAAAATACTTCAAGTACCTGAACTTTACATTTTTAATGGTATGGTATAATGTGT
CTTACAAATAGAACCTTCACTCTCGGTAATGAGAGTGTATTCCTTAATATTCTAGTAACCGCTATGTG
GGC
+
FFFFFFFFFFFFFFFFFFFFFFFFFFFFFFFFFFFFFFFFFFFFFFFFFFFFFFFFFFFFFFFFFFFFFF
FFFFFFFFFFFFFFFFFFFFFFFFFFFFFFFFFFFFFFFFFFFFFFFFFFFFFFFFFFFFFFFFFFFFFF
FFF
@A00721:190:HGJW7DSXY:4:1460:7374:6464_1:N:0:TCTGCAAG+GCTGCACT
GATAGCAATTTATGTTTTAAATGCTAGGAAAATTTTAGAAAAACTCCATTGTATAGTTCAAGGAGTAC
TTAATTCAATAGCTTTAGTATTATTTACTATCGCCTTTATTGAAAATTGTATAAGAGTTAATTGCTTA
CAC
+
FFFFFFFFF:FFFFFFFFFFFFFFFFF::F,FFFFFFFFFFFFFFFFFFFF,F:F:FFFFFF:FFFFF
FFF:FFFF:F,FFFFFFFFFFFFFFFFFFFFFFFF:FFFFFFFFFFFFFFFFFFFFFFFFFFFFFFFFFF
FFF
@A00721:190:HGJW7DSXY:4:2570:29333:28025_2:N:0:TCTGCAAG+GCTGCACT
TTAATTGATTGAAGCTAGGCGTTGGGGAGAATGAACTGCCGCTTCCCGGAAGAAACGATCTATTTACA
GAGTATTTAGAGTAGTCCAAAGTTTGTATAAATTTATATGATTTTCTA
+
FFFFFFFFFFFFFFFFFFFFFF:FFFFFFFFFFFFFFFFFFFFFFFFFFFFFFFFFFFFFFFFFF:FFF
FFFF,FFFFFFFFFFFFFFFFFFFFFFFFFF:FFF:FFFFFFFFFFFF
@A00721:190:HGJW7DSXY:4:1255:1298:25379_2:N:0:TCTGCAAG+GCTGCACT
AACATACCATACACTCCCCAAACATAACATACCATAACCTACCATGACCAATCATCGCTCCAACTGGC
ACGCCAGACGTAGGGTCAAACCTACTACCGCCGGTCGGCAGATCGGCA
+
FFFFFFFFFFFFFFFFFFFFFFFFFFF,FFFFFFFFFFFFFFFFFFFFFFFFFFFFFFFFFFFFFFFFFF
FFFFFFF:FFFFFFFFFFFFFFFFFFFFFFFFFFFFFFFFFFFFFFFFFF
@A00721:190:HGJW7DSXY:4:2370:25861:35759_1:N:0:TCTGCAAG+GCTGCACT
TACGTTCAGGGACCCGCTGTGCAGAATAACCTTCCCAAATCTAGATCTGCACAGAAGAAGGCTGAAGG
TCGTTACCGGACGACCACTCAAAGGAAACGGGCTGTTGCCCAGAGGAAAGCAGCGACCACCGAGGTCG
CTC
```

+
FFFFFFFF:,FFF:,,FFFFFFFFFFFFFF,F,FF,FFFF,FF:,FFF::F:FFFFF:FFFFFFFF,,
F:FFF:FFFFFFFFFFFFFFF,::F:FFFFFF,FFFFFFF,FFFFFFFFFFFFFF::FFFFFFFFFFF
:FF
@A00721:190:HGJW7DSXY:4:1526:7048:1548_2:N:0:TCTGCAAG+GCTGCACT
GCAGTCCCGTCATCTCTGTGGGAACTGAGGCAGTACCTCAGTCAAAAGTCACATTCACCACTCCCATG
CCAATCAATGATGTGACTATACAGTTCGGCCATTTCAAGAGTGGTCAA
+
FFFFFFFFFFFFFFFF:FFFFFFFFFFFFFFFFFFFFFFFFFFFFFFFFFFFFFFFFFFFFFFFFFFF
FFFFFFFFFF:FFFFF:FFFFFFFFFFFFFFFFFFF:FFFFFFFFFFF
@A00721:190:HGJW7DSXY:4:1629:8241:19335_1:N:0:TCTGCAAG+GCTGCACT
GAGGTACTGAAGCCTTCCCCTCCGCTGGCACGAAGAACACTCCCAAGCCAATCAATAGTTCTTCTGAC
CAGAATGAAAAGGGGTTTACCAGGGTAGTCTCTAAGTCTACCAGGAAGAGGGGAAATGTCCCCAAAAC
TGA
+
FFFFFFFFFFFFFF:FFFFFFFFFFFFFFFFFFFFFFFFFFFFFFFFF:FFFFFFFFFFFFFFFFFFF
FFFFFFFFFF:FFFFFFFFFFFFFFFFFFFFFFFFFFFFFFFFFFFFFFFFFFFFFFFFFFFFFFFFF
FFF
@A00721:190:HGJW7DSXY:4:1503:10212:16924_2:N:0:TCTGCAAG+GCTGCACT
AGGTCCATCAGGACCTAAGAAGGGGAAATTACCCCTAATTCTGAAGGAAGATCTCCCACCATCTGGGG
TTGGGAATAAGAAATCTGCACGGGCCAAAGAGGCCGCTGCTACGAAGT
+
FFFFFFFFFFFFFFFFFFFFFFFFFFFFFFFFFFFFFFFFFFFFFFFFFFFFFFFFFFFFFFFFFFFF
FFFFFFF:FFFFFFFFFFFFFFFFFFFFFFFFFFFFFFFFFFFFFFF:FF
@A00721:190:HGJW7DSXY:4:2113:18900:9674_1:N:0:TCTGCAAG+GCTGCACT
GCTTGGGTCATTAAGGGTAGCTGTAAAGTTGCGCCTGCCGACCCCATCTTTGAGACCAAGACTCTCTA
CAGTAGGTTCGTGCCTACTATGAGCAAATCTGTTGAATTCGTCACTGAGGACGAAGGTGAGAGGACTT
TTG
+
FFFFFFF:FFFFFFFFFF:FFFFFFF,FFFFFFFFFFFFFFFFFFFFFFFFFFFFFFFFFFFFFFF,F
FFFFFFFFFFFFFFFFFFFFFFFFFFFFFFFFFFFFFFFFFFFFFFFFFFFFFFFFFFFFFFFFFFFF
FFF
@A00721:190:HGJW7DSXY:4:2278:9860:8923_1:N:0:TCTGCAAG+GCTGCACT
CAAACTGAGAGCAGTCGCTGGGGAAAGAGAATTGCGTGGGTTGAAGGCCGACGCTTCGAGCTTGAGTG
TGACGAGGCCGGTGACGTTTTTGTCAACGGCGTTTGGAGGATTTTATCCGCGCTTAACTATGGGGCGA
AGA
+
FFF:FFFFFFFFFFFFFFFFFFFFFFFFFFFFFFFFFFFFFFFFFFFFFFFFFFFFFFFFFFFFFFFF
FF,FFFFFFFFFFFFFFFFFFFFFFFFFFFF,FFFFFFFFFFF:FFFFFFFFFFFFFFFFFFFFFF
FFF
@A00721:190:HGJW7DSXY:4:1503:23402:16157_1:N:0:TCTGCAAG+GCTGCACT
AGTCTTGAAGGACTTAGGTTGGCATATTTATGCCGACGATAATAAGCATGTCACCCTTAAGCACCGAG
GTAAGCTCGGGACTTACAAGAGAGAGTGTGAGAGAGTCGCTCTCACTATTTTTGCCCAGAATGCGGGC
TTT
+
FFFFFFFFFFFFF:FFFF:FFFFFFFFFF:FFFFFFFFFFFFFFFFFFFFFFFFFFFFFFFF:FFFFF
FFF,FFFFFFFFFFFF::FF:FFFFFFFFFFFFFFFFFF:FF:FFFFFFFFFFFFFFFFFFFFFFFFF
FFF
@A00721:190:HGJW7DSXY:4:2369:3351:35884_2:N:0:TCTGCAAG+GCTGCACT
GCTCCTTCTGTGGGTGAGGGTTTTTGTTATATTCACTGGTTCTGGGAGGCTGCTTTCCTCCATAATTA
CAACCTCTCTTTAGAGAGCATTAGAAAGATCGGAAGAGCGTCGTGTAG
+
FFFFFFFFFFFFFFFFFFFFFFFFFFFFFFFFFFFFFFFFFFFFFFFFFFFFFFFFFFFFFFFFFFFF
FFFFFFFFFFFFFFFFFFFFFFFFFFFFFFFFFFFFFFFFFFFFFFF:FF

```
@A00721:190:HGJW7DSXY:4:1229:10212:11287_1:N:0:TCTGCAAG+GCTGCACT
CACACTTTTCTTGAAAATTTTTCCTTTCCTCCGTCATTGGGGATTTCGATGTGAGAAGGTGGAAGGTG
TCAAGGGTCTTCTTCATGCTGATCTTTCAGGACCTAATCACATCATGATTGATAGTTACGGCTCGTAT
CGA
+
FFFFFFFFFFFFFFFFFFFFFFFFFF,FFFFFFFFFFFFFFFFFFFFFFFFFFFFFFFFFFFFFFFFFF
FFFFFFFFFFFFF:FFFFFFFFFFF::FFFFFFF:FFFF:FFFFFFFFFFFF:FFFFFFFFFFFFFFF
FFF
@A00721:190:HGJW7DSXY:4:1351:24117:12258_2:N:0:TCTGCAAG+GCTGCACT
AAGTTCTCCGAAATTAAAGACGGGGGGATTAAGAGTCCAGCTGTAACTGTCATTTCAGAGAGGGGAGC
TCTGAAAAGGAAACTTCGTCAGAAGATGAGGAAGTCTAAGAGGAGAGA
+
FFFFFFFFFFFFFFFFFFFFFFFFFFFFFFFFFFFFFFFFFFFFFFF:FFFFFFFFFFFF:FFFFF
FFFFFFFFFFFFFFFFFFFFFFFFFF:FFFFFFFFFFFFFFFFFFFFFF
@A00721:190:HGJW7DSXY:4:1169:24831:17628_1:N:0:TCTGCAAG+GCTGCACT
CTCTAGAGAAGATCTCCCCACCTGGAGAAGGCAAGTGGAGAGTCCAGAAGTTGATGAAGAAGGATTAT
TTGAAGCAGGGGTTACCACTTCCTAAGGTGGATGAGGGAGGTTGTTTCTTTGGAGTTTTAGATAATCT
TTT
+
FFFFFFFFFFFFFFFF,FFFFFFFFFFFFFFF:FFFFFFFFFFFFFFFFFFFFFFFFFFFFFFF:FFFF
FFFFFFFFFFFFFFFFFFFFFFFFFFFFFFFFFFFFFFFFFFFFFFFFFFFFFFFFFFFFFFFFFFFF
FFF
@A00721:190:HGJW7DSXY:4:1429:21097:11569_2:N:0:TCTGCAAG+GCTGCACT
CCTTGAGAAGACAAGCCACATTAAGATATAATTTAGGTCAACTTGGGGTTTCGAATGTCATTAGTGCC
TTACGATTGAAAGAGGTCCTTTTGAGTATCGACAGATCTTTCGATCTT
+
FFFFFFFFFFFFFFFFFFFFFFFF,FFFFFFFFFFFFFFFFFFFFFFFFFFFFFFFFFFFFFFFFFFF
FFFFFFFFFFFFFFFFFFFFFFFFFFFFFF:FFFFFFFFFFFFFF:FF
@A00721:190:HGJW7DSXY:4:2667:5339:13870_2:N:0:TCTGCAAG+GCTGCACT
TTAACGTGGCAGAATTCGGTAACTTACCATATTGTTCCTTCCGGGAAGTTGTGGGATTTAACTTTGAC
TCAACTACTCCGATCAGATCCGGATGGTTTGATTGGTATGGACTACTC
+
FFFFFFFFFFFFFFFFFFFFFFFFFFFFFFFFFFFFFFFFFFFFFFFF:FFFFFFFFFFFFFFFFFFF
FFFFFFFFFFFFFFFFFFFFFFFFFFFFFFFFF:FFF:FFFFFFFFFFF
@A00721:190:HGJW7DSXY:4:1134:7591:8656_1:N:0:TCTGCAAG+GCTGCACT
TAGGGTCATGGCAGATACTACCGATAAGGTGAAAAATATGCAGGTCAAAGATTCTGTAGCTCTGAGGG
CGGTTGAGGGAGCCGTTTTGGCCGACTTTAACTATTCTAAGAGTCTTTATGATTCAAAACCTATCGTT
GTC
+
FFFFFFFFFFFFFFFF,FFFFFFFFFFFFFFFFFFFFFFFFFFFFFFFFFFFFFFFFFFFFFFFFFFF
FFFFFFFFFFFFFFFFFFFFFFFFFFFFFFFFFFFFFFFFFFFFFFFFFFFFFFFFFFFFFFFFFFFF
FFF
@A00721:190:HGJW7DSXY:4:1140:2049:9580_1:N:0:TCTGCAAG+GCTGCACT
TACAGCGGCTTATTGTCGCGCAGTTTCCAGAATTGCGGTTGAAGTTCACCCATTCTATCTTGAATGCT
CACGCCGTCGCAGCCATATCGAGACTATGTTATAATCACTTGTTAAAGTACAGATTTAGTGATTTAGA
TAT
+
:FFFFFFFFF:FFFFFFFFFFFFFFFFFFFFFFFFFFFFFFFFFFF:FFFFFFFFFF:FFFFFFFFFFF
FFFFFFFFFFFFFFFFFFFFFFFFFFF:FFFFFF:FFFFFFFFFFFFFFFFFFFFFFFFFFFFFFFFFF
FFF
@A00721:190:HGJW7DSXY:4:1320:13829:24220_1:N:0:TCTGCAAG+GCTGCACT
GGAGGCGATATTCGTTTTCATATCCAAAACTCCTTGAATGGAGTACATATTTGCAACCCTATTCTTGA
TGTGAAAGATGCATCGAGGTATACGTCTAGGAACATTGATTGGGAACGTACCTACTCACGAGACGTCA
TGA
```

+
FFFFFFFFFFFFFFFFFFFFFFFFFFFFFFFFFFFFFFFFFFFFFFFFFFFFFFFFFFFFFFFFFFFFFF
FFFFFFFFFFFFFFFFFFFFFFFFFFFFFFFFFFFFFFFFFFFFFFFFFFFFFFFFFFFFFFFFFFFFFF
FFF
@A00721:190:HGJW7DSXY:4:1406:7663:32456_1:N:0:TCTGCAAG+GCTGCACT
ATGTTTCGTTCTGTTACAAGAACGCTTTGGATTGTGACCATAAAGCCTCAGTTGCGATAGCTGTAGAG
GTATACGATATCTCTCTTGATGATGTAGCCACTATAATGCTTAAGAGAGAGATTGATGTATGTCATTT
CGT
+
FFFFFFFFFFFFFFFFFFFFFFFFFFFFFFFFFFFFFFFFFFFFFFFFFFFFFFFFFFFFFFFFFFFFFF
FFFFFFFFFFFFFFFFFFFFFFFFFFFFFFFFFFFFFFFFFFFFFFFFFFFFFFFFFFFFFFFFFFFFFF
FF:
@A00721:190:HGJW7DSXY:4:1351:6867:25191_1:N:0:TCTGCAAG+GCTGCACT
GAATTATTGGACACGATGAAAAATGACATACCCGTTCCTGACCTTGGTATCGTTATCCGTCATGGAGA
TGATGCCGCATCACTTGATTACTATATGCCCGAAGGATTGGGTTATTCTCACAACAAAGATAAGATTT
TAT
+
FFFFFFFFFFFFFFFFFFFFFFFFFFFFFFFFFFFFFFFFFFFFFFFFFFFFFFFFFFFFFFFFFFFFF:
FFFFFFFFFFFFFFFFFFFFFFFFFFFFFFFFFFFFFFFFFFFFFFFFFFFFFFFFFFF::FFFFFFFFF
FFF
@A00721:190:HGJW7DSXY:4:1528:13069:29794_1:N:0:TCTGCAAG+GCTGCACT
GTTTTCCTTAAGGGCGGACAATTGTTCCATTGTGAACTCTTGGGGACTAGATTGAGCATTAATGAGTT
CCGAATAACTCGTAATAAATCTTTCTCTAGTGGACAATTCAGAATACCTATTTCCGTACCAAGAGCCA
CTG
+
FFFFFF:FFFFFFFFFFFF:FFFFFFFFFFFFFF:FF:FFFFFFFFFFFFFFFFFFFFFFFFFFFFFFFF
FFFFFFFFFFFFFFFFFFF:FFFFFFFFFFFFFFF::FFF:FFF:FFFFFFFFFFFFFFFFFFFFFFFFF
FFF
@A00721:190:HGJW7DSXY:4:1132:1163:11490_2:N:0:TCTGCAAG+GCTGCACT
TGGAAGAAGCGAGGTTCGTTCCTCAATAAAAATTTTTCTCAAGAGATTATGGTTGATAAAGTTTTCTT
CATAAGGGCTATTCAGTACGTCATGGGGATCACTTCTTCGGTCAATGA
+
FFFFFFFFFFFFFFFFFFFFFF:FFFFFFFFF:FFFFFFFFFFFFFFFFF:FFFFFFFFFFFFFFFFFFF
FFFF:FFFFFFFFFFFFFFFFFFFFFFFF:FF:FFFFFFFFFFFFFFFFF
@A00721:190:HGJW7DSXY:4:1178:17933:7404_1:N:0:TCTGCAAG+GCTGCACT
AATATGTTACGACATGGCTTCGTAACAACAGTGCGAGGATTGTCATCTCGGGGAAAGTGATTCACCAA
GATGTTCATATAGATACGAGATACCTAGAGAAGGTCGCAGCGATCTTACTCGTTATCGGAGCTAAGAA
GAA
+
FFFFFFFFFFFFFFFFFFFFFFFFFFFFFFFFFFFFFFFFFFFFFFFFFFFFFFFFFFFFFFFFFFFFFF
FFFFFFFFFFFFFFFFFFFFFFFFFFFFFFFFFFFFFFFFFFFFFFFFFFFFFFFFFFFFFFFFFFFFFF
FFF
@A00721:190:HGJW7DSXY:4:1277:24749:19648_2:N:0:TCTGCAAG+GCTGCACT
GTTATGCTCGCAAACTTAACGTTAGTACCGGAGGTGATCTCTCTGCTTCTGCGTTACTGAGAGAAAAG
ATTCAGGACGCCTACGGTGACTTTATGGACAAGATGCAAGAGATCGGA
+
FFFFFFFFFFFFFFFFFFFFFFFFFFFFFFFFFFFFFFFFFFFFFFFFFFFFFFFFFFFFFFFFFFFFFF
FFFFFFFFFFFFFFFFFFFFFFFFFFFFFFFFFFFFFFFFFFFFFFFFFF
@A00721:190:HGJW7DSXY:4:2146:28185:15640_2:N:0:TCTGCAAG+GCTGCACT
TTTAGGTTTAGTAGGTGATCTTGACGTCGATTCTTTGTATAGGGAGTGTGAGAAGACAACGAATCTTG
AAACTACGGTCCTTATCAAAGAAAATGGTGAACCGGTCTCTAAATCTT
+
FFFFFFFFFFFFFFFFFFFFFFFFFFFFFFFFFFFFFFFFFFFFFFFFFFFFFFFFFFFFFFFFFFFFFF
FFFFFFFFFFFFFFFFFFFFFFFFF,FFFFFFFF:FFFFFFFFFFFFFFF

```
@A00721:190:HGJW7DSXY:4:2374:25229:16720_2:N:0:TCTGCAAG+GCTGCACT
GAAGATTGAAGAACACGTCAAGAAGTCGCTCGGCGTTGTTCTTGAAAACCAAGTTAAGAAGGAGGCTG
ATCATGGAGTCTTTGTACCTTCTGTCAAACTTCGAGGTTCAGCTACGA
+
FFFFFFFFFFFFFFFFFFFFFFFFFFFFFFFFFFFFFFFFFFFFFFFFFFFFFFFFFFFFFFFFFF:F
:FFFFFFFFFFFFFFFFFFFFFFFF:FFFFFFFFFFFFFFFFFFFFFF
@A00721:190:HGJW7DSXY:4:1506:17689:2096_1:N:0:TCTGCAAG+GCTGCACT
TGGATTGAAGGCTGGCTCTAAGGGAGACTCCTTCTTTTTGAATGTTGTAAAGAATTGGGTTGAGAAGT
GTGCTTTACAAGGAGTTAGACTCTTCTTCAAGTTTTTAGATCTTCTTGATAATCTTAAAGAAAAATTT
TCG
+
FFF,FFFFFFFFFFFFFFF,FF:FFFFFFFFFFFFFFFFFFFFFFFFFFFFF:FFFFFFFFFFFFFFF
FFFFFFFFF::FFFFFF:FFFFFFFFFFFFFFFFFFF,FFF:FFFFFFFFFFFFFFFFFFFFFFFFFF
FFF
@A00721:190:HGJW7DSXY:4:1224:1949:24659_2:N:0:TCTGCAAG+GCTGCACT
CTGTACTTACATTCTTTACGCCAGATTCTTCATGGGGCGACGCTGTTTCTCTTGGTATTTCTGAAATA
GTTTATCAGAGCGTCATTTTCCTTTGGGATCTTTCCTCTGGTAAGGGA
+
:FFFFFF:FFFFFFFFFFFFFFFFF:FFFFFF::FF:FFFFFFFFFFFFFFFFFFFFFFFFFFFFFFF
::FFFFFFFF:FF:FFF,FFF:F,:F:FFFFFFFFF:FFFFFFFFF::
@A00721:190:HGJW7DSXY:4:1107:18900:5478_2:N:0:TCTGCAAG+GCTGCACT
ATTCTTCTCACTCTTGACGAGAATTATTACTGTTTTCTCTACCCATAACTTCTTTTTCAAAAATGCGG
ATGGGTCTACTGATGTTTTCCTACAGAGTGCAGTCGTTTCAACACTTA
+
FFFFFFFFFFFFFFFFFFFFFFFFFFFFFFFFFFFFFFFFFFFFFFFFFFFFFFFFFFFFFFFFFFFF
FFFFFFFFF,FFFFFFFFFFFFFFFFFFFFFFFFFFFFFF,FF:,F,FF
@A00721:190:HGJW7DSXY:4:1238:2311:31046_2:N:0:TCTGCAAG+GCTGCACT
GGAAAATAATTGGTGATGAAAATACGTATGTCGGTGGGGTTCTCAAGGCTCTCATCGCCACTGGGATT
CTAAGGGGTTCCATAAGATATTATCTGACATTGTGGTTCAATGATCAG
+
FFFFFFFFFFFFFFFFFFFFFFFFFFFFFFFFFFFFFFFFFFFFFFFFFFFFFFFFFFFFFFFFFFFF
FFFFFFFFFFFFFFFFFFFFFFFFFFFFFFFFFFFFFFFFFFFFFFFF
@A00721:190:HGJW7DSXY:4:1303:27651:26396_2:N:0:TCTGCAAG+GCTGCACT
TGGTCCTTACGTCTTAAGTAATTTGGATGTTGTCGAAAACTATCAGGTTCTTGTCACGATTCTTAAAA
GAAGTTTATTAAAACTTCAGAGTCTCTTGGAAGTCCAGGTAGCTAATG
+
FFFFFFFFFFFFFFFFFF::FFFFFFFFFFFFFFFFFFFFFFFFFFFFFFFFFFFFFFFFFFFFFFFF
FFFFFFFFFFFFFFFFFFFFFFFFFFFFFFFFFFFFFFFF:FFFFFFF
@A00721:190:HGJW7DSXY:4:1231:25382:33395_2:N:0:TCTGCAAG+GCTGCACT
GCCACAGACTTTCTGGGAAAAGAGACCTCTGTTGGTAGAGGAGTTGAGAGAATTACTCAGATTCAATC
TGGCATCACTGAACGTCTACAACGAGTTAGGGAGTGGCTAGAGATCGG
+
FFFFFFFFFFFFF:FFFFFFFFFFFFFFFFFFFFFFFFFFFFFFFFFFFFFFF:FFFFFFFFFFFFFFF
FFFFFFFFFFFFFFFFFFFFFFFFFFFFFFFFFFFFFFFFFFFFFFFF
@A00721:190:HGJW7DSXY:4:1276:11098:18114_1:N:0:TCTGCAAG+GCTGCACT
AGCAATACCTTCCACTGAGTTCATCACCCAGAGTTTCGACGTCGCGGAAAACGATGAAGATTTCCACA
CTGTTCGAGAATCTTCTGATGCTGAATTTGAGGAAAATTTTTCGGAAGTTTTTGGTATAAATCCTGAT
GGC
+
FFFFF:FFFFFFFFFFFFFF:FFFFFF:FFFFFFFFFFFFFFFFFFFFFFFFFFFF:F:FFFFFF:FFF:
FFFFF:FFFFFFFFF:FFFFFFFFFFFFFFFFFFFFFFFFFFFFFFFFFF:FFFFFFFFFFFFFF:FFFF
FF:
@A00721:190:HGJW7DSXY:4:1150:2519:36198_1:N:0:TCTGCAAG+GCTGCACT
TAAAACCTTCTTTTATCATTGCAACAGGACTTTGAGAAGACTGTTGAAATGGACAAAATCTGGGATCA
```

TAGGTATGTGTCACGCTACACTCGAAATATGGGCCCTCATAAGGAAGTATAGTTGGCGTCTTGTCGTT
GAC
+
FFFFFFFFFFFFFFFFFFFFFF:FFFFFFFFFFFFFFFFFFFFFFFFFFFFFFFFFFFFFFFFFFFFFF
FFFFFFFFFFFFFFFFFFFFFFFFFFFFFFFFFFFFFFFFFFFFFFF:FFFFFFFFFFFFFFFFFFFF
FFF
@A00721:190:HGJW7DSXY:4:1258:15239:27383_2:N:0:TCTGCAAG+GCTGCACT
AGTTAGATCATTTGTTCAGGAAGATCTCTACAGAGATTATCGAGAGTTGTGATCTAGTTCTAAATTTA
GGTCGACGGACTTTAGCAACTTTAGATAAATTCTTTCCGGGCTCAATG
+
FFFFFFFFFFFFFFFFFFFFFFFFFFFFFFFF:FFFF:FFFFFFFFFFFFFFFFFFFFFFFFFFFFFF
FFFFFFFFFFFFFFF:FFFFFFFFFFFFFFFFFFFFFFFFFFFFFFFFF
@A00721:190:HGJW7DSXY:4:1273:11939:27524_1:N:0:TCTGCAAG+GCTGCACT
TCTGACCATCGCAAAGAAGACTTTATTAGTTCATGAAGTTCTTACACTTCTCGCGACTAATGTCAGCT
TCACTACAAAGGCGTTACGTAAAGTAGTTTTCACGCTTTATCTTAAAGCATTACCACCAAATTTAGCA
CTT
+
:FFFFFFFFFFFFFFFFFFFFFFFFFFFFFFFFFFFFFFFFFFFFFFFFFFFFFFFFFFFFFFFFFFF
FFFFFFFFFFFFFFFFFFFFFFFFFFFFFFFFFFFFFFFFFFFFFFFFFFFFFFF:FFFFFFFFFFFF
FFF
@A00721:190:HGJW7DSXY:4:1138:10682:36652_1:N:0:TCTGCAAG+GCTGCACT
CTCGCCATATAAGATTAGAGACATGCCAGTTAGAGTTCTTCTAACCAGGTCTGATGGTGAGATTCTTT
TTCCTTTTGGAGGTCCAGGAACCTTTACTCCTAGTGTCTTTTCTAGAGTCCAAGGACGTTTCCGTCG
+
FF,F,,,F,F,FFF,F,FFFF,FFF:FFFFFF:F,FF,FF,F:F,,,:,,,FFFFFF,:FFFFF,F:F
F,:F,,FFFFF:FFFFFF:FF,FFFFF::F,:FF,FF,FFF:,:FF:FF:,FF,F,FFFFFF,FF:F
@A00721:190:HGJW7DSXY:4:1644:14461:27633_1:N:0:TCTGCAAG+GCTGCACT
GTGGGAGACCGCTCAATAACACTCTATTGAATCCCTTTTCCACGATGAAAGCTCTTTATGAGGTGCTC
CATGCAAATTTACAGTTCCTCTTTGGTGATATCGCCTCTGTCCTTTCTCCTTTAATCGTGAGGTTACG
AGA
+
FFFFFFFFFFFF:FFFFFFFFFFFFFFFFFFFFFFFFFFFFFFFFFFFFFFFFFFF,FFFFFFFFFFF
FFFFFFFFFFFFFFFFFFFFFFFFFFFFFFFFFFFFFFFFFFFFF:FFFFFFFFFFFFFFFFFFFFFFFF
FFF
@A00721:190:HGJW7DSXY:4:2257:11803:19304_2:N:0:TCTGCAAG+GCTGCACT
GGTTTATCGGTTGTATTTTGATAAATTTGTTGGAATCGATAATGATACTGACGAAGGTTCTATTACCA
CCCTTGTCATTGATACAAACATTCTAGGTGATGCTTTCTTAGAGGCTC
+
FFFFFFFFFFFFFFFFFFFFFFFFF:FFFFFFFFFFFFFFFFFF:FFFFFFFFFFFFFFFFFFFFF:F
FFFFFFFFFFFFFFFFFFFFFFFFFFFFFFFFFFFFFFFFFFF,FFFFF
@A00721:190:HGJW7DSXY:4:2451:32289:36495_2:N:0:TCTGCAAG+GCTGCACT
TATTTACAATCTGTAGAGAGAGAGGTTAATCAGAATTTGAGTGATAACGATTTAGTACTGGGAAATGG
TGGTAGTCAGTTTACTACTTTTCTTTCCCTCCCCTTTTTCGATTGGAT
+
FFFFFFFFFFFFFFFFFFFFFFFFFFFFFFFFFFFFFFFFFFFFFFFFFFFFFFFF:FFFFFFFFFF
FFFFFFFFFFFFFFFFFFFFFFFFFFFFFFFFFFFFFFFFFFFFFFFFF
@A00721:190:HGJW7DSXY:4:2346:12943:2049_2:N:0:TCTGCAAG+GCTGCACT
CGTCTCACGACTAATTTGATAAGGTTTTTCTTTTATGGGACTCTTGTTTGTACTTATGAAGGAGGTAT
TACTTCGGAAGTTCTTAATGTAGGTCTCCTAGTACTTAAGGGTTTATG
+
FFFFFFF:FFFFFF:FFFFFFFFFFFFFFFFFFFFFFFFFFFF,F:FFFFFFF:FFFFFFFFFFFFFF
FFFFFFFFFFFFFFFFFFFFFFFFFFFFFFFFFFFFFFFF:FFFF,FFFF
@A00721:190:HGJW7DSXY:4:2177:21974:31876_2:N:0:TCTGCAAG+GCTGCACT
TTTTGATGGTATGTTTCACATTGCGTGATCACTTCCCGTGGACTTGGGAAGAGATTAAACATAAAATC

```
CAGCGATTCTATTATGTCGCTAAAGCCTGTCCAGTTGTCGATGATGTT
+
FFFFFFFFFFFFF:FFFFFFFFFFFFFFFFFFFFFFFFFFFFFFFFFFFFFFFFFFFFFFFFFFFFFF
FFFFFFFFFFFFFFFFFFFFFFFFFFFFFFFFFFFFFFFFFFFFFFFF
@A00721:190:HGJW7DSXY:4:1641:14262:31422_2:N:0:TCTGCAAG+GCTGCACT
AAACTTACGACGATGTTCTCGCTCGCATTTCACAAGGATTACTAAGTCTGAGATCTGCCACTTCTAAT
TTTAGGAAACGTCGCGAAAAGAAAGTTCATGAGGATGTCTTGGTCGAA
+
FFFFFFFFFFFFFFFFFFFFFFFFFFFFFFFFFFFFFFFFFFFFF:FFFFFFFFFFFFFFFFFFFFFF
FFFFFFFFFFFFFFFFFFFFFFFFFFFFF:FFFFFFFFFFFFFFFFFF
@A00721:190:HGJW7DSXY:4:1436:31096:10504_2:N:0:TCTGCAAG+GCTGCACT
GAACTTCTAATAGAGGATCCTGATGACATTATTTCAACGGATACCGATCTCAGTTCTACCACAGAAAC
CGTCTCAGGAACTGTCTCCACTGATTCGGAATTAGAGAAAGACGACCT
+
FF,FFFFFFFFFFFFFFFFFFFFFFFFFFFFFFFFFFFFFFFFFFFFFFFFFFFFFFFFFFFFFFFFF
FFFFFFFFFFFFFFFFFFFFFFFFFFFFFFFFF:FFFFFFFF:FFFFFF
@A00721:190:HGJW7DSXY:4:2371:19985:22545_1:N:0:TCTGCAAG+GCTGCACT
GAGACCTCTGAATTTTCAGGGTGCCCTGATGAGGACGTTGAAAGGGATAATTTCAACTCTGAGACTTC
TAAGAGTGTTTCAAAAGACCAGAAACGTAACAAGAAGAATAAAGGTAGGAAGGGGAGAAAACAGAACA
AGC
+
FFFFF:F:F:FFFFFFFFFFFFFFFFFFFFFFFFFFFFFFFFFFF:FFFFFFFFFFFFFFFFFFFFFF
FFFFFFFFFFFFFFFFFFFFFFFFFFFFFF,FFFFFFF:FFFFFFFFFFFFFFFFFFFFFFFFFFFFFF
FFF
@A00721:190:HGJW7DSXY:4:1329:17291:23202_2:N:0:TCTGCAAG+GCTGCACT
ATGGTTGCGGCTGATCGTACCCCAATTCCTGCTAGAAAGATCACTCTCGGCGATACCACTGATCTTCC
AGAGTTACGTAGTGGTCGTTCTTTTGGTGATTTTATCAAAGAGAAAGT
+
F:FFFFFF:FFF:FFFFFFFFFFFFFFFFFFFFFFFFFFFFFFFFFFFFF:FFFFFFFFFFFFFFFFFF
FFFFFFF:FFFFFFFFFFFF:FFFFFFFFF:FFFFFFFFFFFFFF:FF
@A00721:190:HGJW7DSXY:4:2549:23592:25254_2:N:0:TCTGCAAG+GCTGCACT
CAAGTTAAACTTCGAACTGACCACTTACAAGAGTGTTGAAGTACCGCAACATGGATCAGTTTTCCAAA
AAGCTATTAATAGCAGTTGCGGGGCTATAATACCGCCTCCAGATTACT
+
FFFFFFFFFFFFFFFFFFFFFFFF:FFFFFFFFFF,FFFFFFFFFFFFFFFFFFFFFFFFF:FFFF:F
FFFFFFFFFFFFFFFFFFFFFFFFFFFFFFFFFFFFFFFFFFFFFFFF
@A00721:190:HGJW7DSXY:4:2207:29306:29199_1:N:0:TCTGCAAG+GCTGCACT
AGAGTTTTGGAACTGTGTAATGTCCTACGTACTGTTCAAGGTGTTTGGGCTTCTCTAAAGAACAGCAA
CTTTCAACATGAATTAGTTATTGAAAGTCTTGACCCTCACGTCTCACTTTATCACCTTGGAGATGGTA
AAG
+
FFFFFFFFF,FFFFFFFFFFFFFFFFFFFFFFFFFFFFFFFFFFFFFFF:FFFFFFFFFFFFFFFFFF
FFFFFFFFFFFFFFFFFFFFFFFFFFFFFFFFFFFFFFFFFFFFFFFFFFFFFFFFFFFFFFFFFFFF
FFF
@A00721:190:HGJW7DSXY:4:2209:4887:29653_2:N:0:TCTGCAAG+GCTGCACT
ATTCTGCTTCGACGGTAATTCCATAAGACCTTTCTTGGGGAGTAAAGAGGGTTTGAAAATTACGGAAC
CTTTCCTCGTTCTTCATGAAGATCTTCGAGTGTTTTATAGTGATTTAT
+
FFFFFFFFFFFFFFFFFFFFFFFFFFFFFFFFFFF:FFFFFFFFFFFF:FFFFFFFFFFFFFFFFFF:F
FFFFFFFFFFFFFFFFFFFFFFFFFFFFFFF:FFFFFFFFF:FFFFFFF
@A00721:190:HGJW7DSXY:4:2150:29677:16000_1:N:0:TCTGCAAG+GCTGCACT
CAAGTCGATTGATTTTGTTGACTACGATCTCTCTTGTAATAAGAAGTTCTTCGCAGTTGAATCTCCAC
CAGGTGGAGGGAAGACCACTGAACTTATAGCGACTTTTGTCGAATTTTTCGCAAAAGGTCTTGAAGTA
CGT
```

```
+
FFFFFFFFFFFFFFFFFFFFFFFFFFFFFFFFFFFFFFFFFFFFFFFFFFFFFFFFFFFFFFFFFFFF
FFFFFFFFFFFFFFFFFFFFFFFFFFFFFFFFFFFFFFFFFFFFFFFFFFFFFFFFFFFFFFFFFFFF
FFF
@A00721:190:HGJW7DSXY:4:1567:29668:36464_2:N:0:TCTGCAAG+GCTGCACT
TCGTAATTCGCGAGAAGAGATAGTGACTAAAGTTTTGGCACTTCTAGTTCATCGCGGTATTTGTGAAA
TTAAGACAGACGTGAAAGTTATGATGAGTGATCGTATTAGAACAGCTG
+
FFFFFFFFFFFFFFFFFFFFFFFFFFFFFFFFFFFFFFFFFFFFFFFFFFFFFFFFFFFFFFFFFFFF
FFFFFFFFFFFFFFFFFFFFFFFFFFFFFFFFFFFFFFFFFFFFFFFF
@A00721:190:HGJW7DSXY:4:1307:14181:12305_2:N:0:TCTGCAAG+GCTGCACT
CTGAGGTCAAACCTGTGGACGTTCTTTTCATCGACGAATTATTCATGATTCACGCTGGTCAGACCCTT
TTTCTTACACATCTACTTAAGGCGGCTTATACCATAGGTTACGGTGAT
+
FFFFFFFFFFFFFFFFFFFFFFFFFFFFFFFFFFFFFFFFFFFFFFFFFFFFFFFFFFFFFFFFFFFF
FFFFF:F:F,F:FFFFFFF:FF:FFFFFFFF:,FFFFFFFFF:FFFFF
@A00721:190:HGJW7DSXY:4:1226:15121:14058_1:N:0:TCTGCAAG+GCTGCACT
GACGTGTGCTCTTCCGATCTCCAAGTCGCAAACTACTTCAACATCAACGACATCATTCTTGATGAGTG
TCGTTCTTATCGAAGTCTCAGTTATCGCTGCCCGGCCGACGTTTGTCGCGAACTCTCAGAGGTTTACG
GCA
+
FFFFFFFFFFFFFFFFFFFFFFF:FFFFFFFFFFFFFFFFFFF:FFFFFFFFFFFF,FFFFFFFFFFF
FFFFFFFFFFFFFFFFFFFFFFFFFFFFFFFFFFFFFFFFFFFFFFFFFFFFFFFFFFFFFFFFFFFF
FFF
@A00721:190:HGJW7DSXY:4:1519:3513:32440_1:N:0:TCTGCAAG+GCTGCACT
AATTCGAGAGAGGACACTATGGAAATTAGACCTATCAGATGTCTTGACGACTTCGAATTTGAGGACGG
AGTTAAATATATTACTTTCACTCAAGGTGAAAAGTTTGAAATTAACAAAGTCCTTGGTAAGAGTAAGG
TTA
+
:FFFFFFFFFFFFFFFFFFF:F:FFFFFFFFFFFFFFF:FFF,FFF:F:FFF:FF,FFFFFFFFF:F:
FFFFFFFFFFFFFFFFFFFFFFFFFFFFFFFFFFFFFFFFFFFFFFF,:FFFFFFFFFFFFFFFFFFF
FFF
@A00721:190:HGJW7DSXY:4:1241:31620:15640_1:N:0:TCTGCAAG+GCTGCACT
GTTCAAGGTAATACTTACCAGCACGTTCGGCTGGTTAGAATTAAACCTCAAGATGACCCTGTTTTTGC
ATCACAAACACATATTACCGTTGCTCTATCACGACACGTTGAATCTTTACGATACTTTGTTATCAATG
TAA
+
FFFFFFFFFF,F:FFFFF:F:FF:FFFFFFFFFFFFFFFFFFFFFFFFFFF:FFFFFFFFFFFFFFF:F
FFFFFF:FFF:FFFFFFFFFFFFFFFFFFFFFFFFFFF,FFFFFFFFFFF:FFFFFFFFFFFF:FFFFFF
FFF
@A00721:190:HGJW7DSXY:4:1351:32036:20870_2:N:0:TCTGCAAG+GCTGCACT
TGAGAGGATCAATCACAGTAAGGAACTGTCACGTTTAGCACGGGAGGTTGCAGATTCAACCCCTTATA
TCGAGGCTATCGAGGACGATGAGTATCTTGAAGTTGTTGACGACAAGC
+
FFFFFFFFFFFFFFFFFFFFFFFFF:FFFFFFFFFFFFFFFFFFFFFFFFFFFFFFFFFF:FFFFFFFFF
FFFFFFFFFFFFFFFFFFFFFFFFFFFFFFFFFFFFFFF:FFFFFFFF
@A00721:190:HGJW7DSXY:4:1506:10917:10066_1:N:0:TCTGCAAG+GCTGCACT
TAGAGGACCACCTGGATCTTCTACTGTCTCTGCCTTCCAAGATTTCTTAGAAGAGGTTATTCCTGGAA
CCACCTTAACGTCACTCGGTAATCTTTCAGCCGAATTAAGTACTAATGAGTTCAAGTCGGTTGTCGAT
CGA
+
FFFF:FFFFFFFFF:FFFFFFFFFFFFFFFFFF:FFFFFFFFFFFFFFF,FFFF:FFFFFFFFFFFFF
:FFFFFFFFFFFFFFF:FFFFFFFFFFFFFFFFFF:FFF:FFFF:FFF:FFFFFFFFF:FFFFFFFFFF
FFF
```

```
@A00721:190:HGJW7DSXY:4:1527:23511:11647_2:N:0:TCTGCAAG+GCTGCACT
GGCTAATACCTTTATAGGTAAGAAGCCCCTTGAGCCGCGTAGTCAGCGCGTTTAGCTGTATCATCAGT
CAGAGTATACCCGATAGGGTACCGAGTCTCCAAGAGAATATTTATTCT
+
FFFFFFFFFF:FFFFFFFFFFFFFFFFFFFFFFFFFFFFFFFFFFFFFFFFFFFFFFFFFFFFFFFFF
FFFFFFFFFFFFFFFFFFFFFFFFFFFFFFFFFFFFFFFFFFFFFFFF
@A00721:190:HGJW7DSXY:4:1570:21251:27524_1:N:0:TCTGCAAG+GCTGCACT
ACGTTTTCACAACGGAAAGACATATCGCACCCGAAATTTTTGGGCAGAATGCTCTTAACGCGGTTCTT
GAACGGTGTTTTGATCAGTCTAAGTTGGATAAAGTTATAGGAGACTCAGTTAACCTGAGAGCTCCTCA
ATT
+
FFFFFFFFFFFFFFFFFFFFFFFFFFFFFFFFFFFFFFFFFFFFFFFFFFFFFFFFFFFFFFFF:FFF
FFFFFFFF:FFFFFFFFFFFFFFFFFFFFFFFFFFFFFFFFFFFFFFFFFFFFFFFFFFFFFFFFFFF
FFF
@A00721:190:HGJW7DSXY:4:2529:27968:20525_1:N:0:TCTGCAAG+GCTGCACT
CAATAGGGACGCTAGTATACTTCGTAAAGTCGACAGCGAAATGGTCAATATTGATAATTGGACCGACT
ATATGACCTATTTTAAGCTCATGGTGAAAAGGGAAGCTAAGGTTAAACTTGATGCTTCAGCCTTTTCG
AAG
+
FFFFFFFFFFF,FFFFFFFFFFFFFFFFFFFFFFFFFFFFFFFFFFFFFFFFFFFFFFFFFFFFFFFF
FFFFFFFFFFFFFFFFFFFFFFFFFFFFFFFFFFFFFFFFFFFFFFFFFFFFFFFFFFFFFFFFFFFF
FFF
@A00721:190:HGJW7DSXY:4:1376:4481:5149_1:N:0:TCTGCAAG+GCTGCACT
GCAATTTTCGGACCTATCTTTAAGGTTCTTGTGAGGAGAGTCAAACAGTGTCTTTTACCCAACATTTT
TATGTATACAGAGATGACCAGTGACGATTTCTCCCGGAAGCTCTTCTCTCTCTTAGGAGGTCATGATG
ACT
+
FFFFFFFFFFFFFFFFFFFFFFFFFFFFFFFFFFFFFFFFFFFFFFFFFFFFFFFFFFFFFFFFFFFF
FFFFFFFFFFFFFFFFFFFFFFFFFFFFFF:FFFFFFFFFFFFFFFFFFFFFFFFFFFFFFFFFFFFF
FFF
@A00721:190:HGJW7DSXY:4:1355:32859:4038_2:N:0:TCTGCAAG+GCTGCACT
TTTCGAAGTTTGACAAATCTCAAGATTTCTTTTGTAAAGCCTATGAGATAAAGATGTATTCTTATTTA
GGCTTCGATGCTGAATTTCTTGAACTTTGGAGTGCCAGTGAATATTTT
+
FFFFFFFFFFFFFFFFFFFFFFFFFFFFFFFFFFFFFF:FFFFFFFFFFFFFFFFFFFFFFFFFFFFF
FFFFFFFFFFFFFFFFFFFFFFF:F:FFFFFFFFFFFFFFFFFFFFFF
@A00721:190:HGJW7DSXY:4:1348:31675:6621_1:N:0:TCTGCAAG+GCTGCACT
GAGTATCCATAACGATTTGTCATTTACCACCTTTGCTCAACGACGAAGTGGAACGGCCAACACTTTCT
TTGGTAATTGTATGATCACTTTGATGATTTTATCACTTTATTATGACCTTGGAAAGTTTTCGGCTGTG
GTT
+
FFF:FFFFFFFFFFFFF:FFFFFFFFFFFFF,FFFFFFFFFFFFFFFFFFFFFFFFFFFFFFFFFFFF
:FFFFFFFFFFFFFFFFFFFFFFFFFFFFFFFFFFFFFFFFFFFFFFFFFFFFFFFFFFFFFFFFFFF
FFF
@A00721:190:HGJW7DSXY:4:2517:32108:33176_1:N:0:TCTGCAAG+GCTGCACT
CCATTCTGTTTTCAAAGACTCCAATAACTGATCATTCTGATCATATGATTTTGGACAATGGTTTCGAA
GCGAAATTTTTGAGGGATAAACCAGCGTACTTCTGCTCGCGATTTATTCTTTTTGTCAATGATTACAT
CTA
+
FFF:FFFF,FFFFFFFFFFFFFF:FFFFFFFFFFFFFFFFFFFFFFFFFF:FFFFFFFFFFFF:FFFFF
FFFFFFFFFFFFFFFFFFFF:FFFFFFFFFFFFFFFFFFFFFFFFFFFFFFFFFFFFFFFFFFFFFFF
FFF
@A00721:190:HGJW7DSXY:4:1509:27661:4429_1:N:0:TCTGCAAG+GCTGCACT
CCTTACAAGATCATCGTTAAGTTAGGTAAACCCCTAACCGATGTTACTCTTGTAGGACTTTGGGAGAG
```

ATTCGTTAGTTTTAAAGACTATACTAAATATCTCCACTACGAGGTTGTTGTTTGGCGATTAGCTCTTG
CGA
+
FFFFFFFFFFFFFFFFFFFFFFFFFFFFFFFFFFFFFFFFFFFFFFFFFFFFFFFFFFFFFFFFFFFF
FFFFFFFFFFFFFFFFFFFFFFFFFFFFFFFFFFFFFF:FFFFFFFFFFFFFFFFFFFFFFFFFFFFF
FFF
@A00721:190:HGJW7DSXY:4:1508:4237:12774_2:N:0:TCTGCAAG+GCTGCACT
ATTGAGGGTCGTTATACCTATTCGGCTATCTGTGCTTTGAATTGTATAAGATCAAATTTTAAACGATT
CCGAGAGGTTTATCCTGATGTGGAGGGAGTTCTTGAAGCATCCCGACC
+
F:FFFFFFFFFFFFFFFFFFFFFFFFFFFFFFFFFFFFFFFFFF,FFFFFFFFFFFFFFFFFFFFFFF
FFFF:FFFFFFFFFFFFFFFFFFFFFFFFFFFFFF:FFFFFFFFFFFF
@A00721:190:HGJW7DSXY:4:1660:14389:36652_1:N:0:TCTGCAAG+GCTGCACT
TGATGCGGTTTCATAAGATTATCAGTAATCCTGATTCTACTACGGATTCATGGACCTTCCATCCTGTA
CCTCTTCTGGAGAGACGTATCTCAAAGATCGACTTCGAGACAGGTGATATTGTTGAGCGAGAGCGACC
AAG
+
FFFFFF:FFFFFFFFFFFFFFFFFFFFFFFFFFFFFFFFFF:FFFFFFFFFFFFFFFFFFFFFFFFFF
FFFFFFFFFFFFFFFFFFFFFF:FFFFFFFFFFFFFFFFFFFFFFFFFFFFFFFFFFFFFFFFFFFFF
FFF
@A00721:190:HGJW7DSXY:4:2127:17553:29982_1:N:0:TCTGCAAG+GCTGCACT
CGTGGCGCTTTATTTTAAAAAGCTCCCTCCTCTCGCAAGTTTGATCTGATACTTATCACGAAAGGACT
TGAGTCTTTTGATCAGTCTCTGACTTGACCATCTTTCATTTTAAATGACGATCATTAAGACTCTCTTT
CTT
+
FF:FFFF,FFFFFFFFFFFFFFFFFFFFFFFFFFFFFFFFFFFFFFFFFFFFFFFFFFFFFFFFFFFF
FFFFFFFFFFFFFFFFFFFFFFFFFFFFFFFFFFFFFFFFFFFFFFFFFFFFFFFFFFFFFFFFFFFF
FFF
@A00721:190:HGJW7DSXY:4:1458:7102:8343_2:N:0:TCTGCAAG+GCTGCACT
CTTTGCACTGATTTTTCCTCATCAGCTTGTGTTTTACCTAATCTGTGTTTTTACGATAACAACTATCA
GTTGATTAAGACTCTTCCGAAAGGCGAACTTTGGGAATTTGAAGCTCA
+
FFFFFFFFFFFFFFFFF:FFFFFFFFFFFFFFFFFFFFFFFFFFFFFFFFFFFF:FFFFFFFFFFFFF
FFFFFFFFFFFFFFFFF,FFFFFFFFF:FFFFFFFF:FFFFFFFFFF:
@A00721:190:HGJW7DSXY:4:1477:23276:5635_2:N:0:TCTGCAAG+GCTGCACT
ACCGTGACGAACTGTGGTGTTCTTGATCTACGTTACGGATATACTCTCCTTGAGTTCACTCGTAAGGA
CACTACCACTTATTACGATATTTCTTTAGTCGATGGGTTTTCGACGCC
+
FFFFFF:FFFFFFFFFFFFFFFFFFFFFFFFFFFFFFFFFFFFFFFFFFFFFFFFFFFFFFFFFFFFF
FFFFFFFFFFFFFFFFFFFFFFFFFFFFFFFFFFFFFFFFFFFFFFFF
@A00721:190:HGJW7DSXY:4:1553:18367:22435_2:N:0:TCTGCAAG+GCTGCACT
TTTCTTCTAATGTGGCCTGAACTACGTTTCACTTTGTGTGTACTTGTCTCTATTTTAAGTTTTGTCTT
CCTTGTTTGTCTTATTCTATTAATCACTCATTTTAGTGAGATTATTAA
+
FFFFFFFFFFF,FF:FFFFFFF:FF:FFFFF:FFFFFFFFFF:FFFFFFFFFFFFFFFFFFFF:FFFF
:FFF:FFFFFFFFFFF:FFFFFFF,FF:FFF:FFFF:FFFFFFFFFFF
@A00721:190:HGJW7DSXY:4:2171:29939:8437_1:N:0:TCTGCAAG+GCTGCACT
GTCTTCGACAACAGCAGGGGTTTTCGAATAGTGATAGCCACCCGCCTTACAGAGGGGCAGCAGCATGA
CCATAATTGGTATTGACTTTGGTACCACCTTTTCTACTGTCTGTCTCGCCTCTTCGAATGAAGTTTTA
ATG
+
F:FFFFFFFFFFFFFFFFFFFFFFFFFFFFFFFFFFFFFFFFFFFFFFFFFFF:FFFFFFFFFFFFFF
FFFFFFFFFFFFFFFFFFFFFFFFFFFFF:FFFFFFFFFFFFFFFFFFFFFFFFFFFFFFFFFFFFFF
FFF

```
@A00721:190:HGJW7DSXY:4:1417:28293:3208_2:N:0:TCTGCAAG+GCTGCACT
TGATAGTGAATTTATACCTACAATCATTGGTTTTTCTAATAATGATGACACTATTGTTTATGGATATG
ATGCTATTACTCATGATGGTTACGGTCAGGGAGGATTCTCAGTTTACA
+
FFFFFFFFFFFFFFFFFFFFFFFFFF:FFFFFFFFFFFFFFFFFFFFFFFFFF,FFFFFFFFFFFFFF
FFFFFFFFFFFFFFFFFFFFFFFFFFFFFFFFFFFFFFFFFFFFFFFF
@A00721:190:HGJW7DSXY:4:1245:21034:23860_2:N:0:TCTGCAAG+GCTGCACT
CCGAAAGACGTGCTAAATTAAAACCATTGTACGATGTCGTCTGTCTCGGCGATCACTTTTCACTAAGA
ATTGGTCCTACTTTTGGACAAGGCCAACTTCGTAATCTTCATGAGATC
+
FFFFFFFFFFFFFFFFFFFFFFFFFFFFFFFFFFFF:FFFFFFFFFFFFFFFFFFFFFFFFFFFFFFF
FFFFFFFFFFFFFFFFFFFFFFFFFFFFFFFFFFFFFFFFFFFFFFFF
@A00721:190:HGJW7DSXY:4:1360:15293:33051_2:N:0:TCTGCAAG+GCTGCACT
TATCTCTTTGGTTGTTAGGGATTTTGAGAGAGTGAAAGGCTTCAATTGTAGTGGTTTGGTCGTTTCAG
TACCCAGTCAGTATACCTCTACGCAAAGATATTTCATGAAAACTACAG
+
FFFFFF:FFFFFFFFFFFFFFFFFFFFFFFFFFFFFFFFFFFFFFFFFFFF:FFFFFFFFFFFFF:F
FFFFFFFFFFFFFFFFFFF:FFFFFFFFFFFFFFFFFFFFFFFFFFFF:
@A00721:190:HGJW7DSXY:4:2248:17282:2206_2:N:0:TCTGCAAG+GCTGCACT
ATTACCTGTCTTTCATATTATGAATGAACCTTCGGCCGCTCTCTTTGCTTCGATGTTGGATGTCAAGA
AGGGTTCAGAATGGGATTCCTACGTAGTTTATGACTTTGGTGGTGGTA
+
FFFFFFFFFFFFFF:FFFFFFFFFFFFFFFFFFFFFFFFFFFFFF:FFFFFFFFFF,:FFFFFFFFFF:
FFFFFFFFFFF:FFFFFFFFFFFFF:FFFFFFFF::FFFFFFFFFFFF
@A00721:190:HGJW7DSXY:4:2527:5954:11553_1:N:0:TCTGCAAG+GCTGCACT
TCTTACGTTACGGTAATTACTATAGTGTTATTTATTCTGGTGGTGATGATAATCTTGGAGGTCGTGAC
GTTGATGAAGCTATAAGAGCGTTCTTATTACGGCGCACTTCTTTACCCGCCACCACACAACTTTCAGT
TTC
+
FFFFFFFFFFFFFFFFFFFFFFF,FFFFFFFFFFFFFFFFFFFFFFFFFFFFFFFFFFFFFFFFFFFF
FFFFFFFFFFFFFFFFFFFFFFFFFFFFFFFFFFFFFFFFFFFFFFFFFFFFFFFFFFFFFFFFFFFF
FFF
@A00721:190:HGJW7DSXY:4:2468:16468:28886_2:N:0:TCTGCAAG+GCTGCACT
TGTTACTGGAAAACCCTCGTCAATACTTTTCAATAATATACGTCTTGAACTCACTTATGACGATCTAG
TGAAATTAACGCGTCCTTTTCTGGAACGTAGTTTTACTATTATGAACA
+
FF:FFFFFFFFFFFFFFFFFFFFFFFFFFFFFFFFFFFFFFFFFFFFFFFFFFFFFFFFFFFFFFFF
FFFFFFFFFFFFFFFFFFFFFFFFFFFFFFFFFFFFFFF,FFFFFFFFF
@A00721:190:HGJW7DSXY:4:1647:18692:6809_1:N:0:TCTGCAAG+GCTGCACT
ACGTGTGCTCTTCCGATCTAACTTACCCTGATTCCGGCCGGTGGTAGCTCATTTCTACCAGGGGCTAT
TACTCTTGCTGAAACAGCTATTAAAGCTGTTGAGCGGGTAATTACACCTAAACGAGCTCGATCTGCCA
TCG
+
FFFFFFFFFFFFFFFFFFFFFFFFFFFFFFFFFFFFFFFFFFFFF,FFFFFFFFFFFFFFFFFFFFFF
FFFFFFFFFFFFFFFFFFFFFFFFFFFFFFFFFFFFFFFFFFFFFFFFFFFFFFFFFFFFFFFFFFFF
FFF
@A00721:190:HGJW7DSXY:4:1522:12029:28432_2:N:0:TCTGCAAG+GCTGCACT
CACTCTTTTCTGCTACTTTAACGGAGCGTAGTGTACTCTTCGTCGATTGTATCAACAGTAACATTACT
TCTAATAAAGGTCTCTATCAATCTCGTATTATCGTCGCTGCTGGATCT
+
:FF:FFFF:FFFFFF:FFFFFFFFFFFFFFFFFFFFFFFFFF:FF:FFFFFFFFFFFFFFFFFF:FFFF
:,:FFFFFFFFFFFFFF::FF,FF,:F:FFFFFFF,F:F:FF,FFFFF
@A00721:190:HGJW7DSXY:4:2111:6686:35524_1:N:0:TCTGCAAG+GCTGCACT
AAGGCTAAACTCCAAGGTAATTATGAAGACTGTGATTTATCTTTCACTCTGGCCTTGTATGAGGGTGT
```

```
TTCTAATCGTAACATTAATAATTTCAATCTTCTTAAAAAGAAGATAGAGAGTCATACTTTGGGCTATC
GTT
+
FFFFFFFFFFFFFFFF:FFFFFFFFFFFFFFFFFFFFFFFFFFFFFFFFFFFFFFFFFFFFFFFFFFF
FFFFFFFFFFFFFFFFFFFFFFFFFFFFFFFFFFFFFFFFFFFFFFFFFFFFFFFFFFFFFFFFFFFF
FFF
@A00721:190:HGJW7DSXY:4:2104:5394:36385_2:N:0:TCTGCAAG+GCTGCACT
GATAGCCAATATTGAAGTGGACACCTCTGGTAGTATCGAAGTAAAGATAACCAATAAGGAAGGAGCTT
ATACTATGGTTGAATCAACTAAGCATACTACTTTTGCTTTGATTACTC
+
FFFFFFFFFFFFFFFFFFFFFFFFFFF:FFFFFFFFFFFFFFFFFFFFFFFFFFFFFFFF:FFFFF:FFF
FFFFFFFF,FFFFFFFFFFFFFFFF,FF,F,FFFFF,FFFFFFFF:F:
@A00721:190:HGJW7DSXY:4:2611:8838:6308_1:N:0:TCTGCAAG+GCTGCACT
TTCCGATCTGTTAAATAGTCTATTAAACAAGTTGTACTTATCAATGATTTTATCGCTGTTTGTTATCT
CGAAAAGTCTCTTCTAAAAGGAACGTCAGTTCTAACCCAAAGAGAATATCTAAAATATTTACAAAATC
TT
+
FF,,FFFFF,:FF:F,F,FF,FFFFFFFFF:FF,FF,FFFFF,FFF:FFFFFFF,FFF,FFFFFFF:F
FFFFFF,F,F,FFFFFF,FF,:F,FF,F,FFFFF::FFFFFFFFFFFFF:FFFFFFFFFFF:F:FFF,
FF
@A00721:190:HGJW7DSXY:4:1160:23158:13135_2:N:0:TCTGCAAG+GCTGCACT
AGACGTTGCTTGTCGCTTCCTCCTTTAATGGCACTACCTCGGTACAATCTTACTTGGAACGAAGGCGA
CCTGTTCCGGAGTTTTTAAGGGATCCGGAATGTTCTGTCTATATTAGG
+
F,FF:,:FF,FFFFF:FFFFFFF:,FFF,FFFFF,FFFFFFFFFF,FF:FFFFFF:FFF:FFFF:FF:
:F::FFF::FF,:,FFFF,,FF:,F,F:F,:F,FF:FF:FF:FF:FFF
@A00721:190:HGJW7DSXY:4:1255:18349:35399_2:N:0:TCTGCAAG+GCTGCACT
TCGAACGTCAACGTTCACCGAGTTTCGTTTCGTACCTTGATGCTGACTTTCGTCAATATGATTTGATC
TGGTTGCTCAATTCTAAGCGTGTTCCGCTTAGTGCGAGGCCTCTCTTC
+
FFFFFFFFFFFFFFFFFFFFFFFFFFFFFFFFFFFFFFFFFFFFFFF:FFFFFFFFFFFFFFFFFFFFF
FFFFFFFFFFFFFFFFFFFFFFFFFFFFFFFFFFFFFFFFFFFFFFFF
@A00721:190:HGJW7DSXY:4:1310:4137:16892_2:N:0:TCTGCAAG+GCTGCACT
AGTTTACGAACTTGACTCTACTATACGTGACATACTAAATACTAGATATTTGGATGATCTTGATAAAG
AACTTCATGAATTTTCAGAGGATGAAAAATTTTCTTCCTTGGATTTTA
+
FFFFF:FFFFFFFFFFFFFFFFFF:FFFFFFFFFFFFFFFFFFFFFFFFFFF:FFFFFFFF:F:FFF:FFF
FFF:FFFFFF:FFFFFFFFFFFFFFFF:FF,FFFF,FFFFFFFF:FFF
@A00721:190:HGJW7DSXY:4:1565:11686:35196_1:N:0:TCTGCAAG+GCTGCACT
TACAGGAGGCAGTCTTCTGATCGTAAGAAGTTTATAAGGAACATATCCATTTGTTGTAATAGGGTCAT
CAACTTAGAACTCGAAACTCTAAGTGGTAATACTGTCTTTCCAAGCTTACCTGTATCTTCAATGAAAT
TGG
+
FF:F:FFF:FF:FFFFFF:FF,F:,FFFFFFFFFF:F,FFFFFFFF:,FFFFFFFFFFFFF,,FFFF:
FF:FFFFFFFFFFFFFFFFFFF:FFFFFFFFFFFFF,FFFF,FFF:FFF:F:FFF:FFFFFFFFF:FF
FFF
@A00721:190:HGJW7DSXY:4:2139:31168:10285_2:N:0:TCTGCAAG+GCTGCACT
GTGGGCTTTCCGTAAAGGAAGATCTGTTATCGACGGTTTATCGTTTACGAAGTATCACTCCACCGGTG
AATGGTTTAAGAATACTATAAATAGAATAGCTATTACTACTAATGGCG
+
FFFFFFFFFFFFFFF:FFFFFFFFFFFFFFFFFFFFFFFFFFFFFFFFFFFFFFFFFFFFFFFFFFFF
FFFFFFFFFFFFFFF:FFFFFF:FFFFFF:FFFFFFFFFFFFFFF:FF
@A00721:190:HGJW7DSXY:4:1369:24614:5916_1:N:0:TCTGCAAG+GCTGCACT
TAGTGACATCTGGTCAATTTCGTTTTTAGGTGGTCTTTATTCTATTATTGATTTTTATGAGATTGATA
```

```
CGTTAACAGAAGATGATGTTAGAGTAGAATTCCTGAAGAGTCCTTCCGTAACCAACTTCTTGACAATT
TTC
+
FFFFFFFFFFFFFFFFFFFFFFF:FFFFFFFFFFFFFFFFFF:FFFFFFFFFFFFFFFFFF:FFFFFFF
FF:FFFFFFFFFFFFFFF:FFFFFFFFFFFFFFFFFFFFFFFFFFFFFFFFFFFFFFFFFFFFFFFFFF
FFF
@A00721:190:HGJW7DSXY:4:2565:23583:7607_2:N:0:TCTGCAAG+GCTGCACT
CTAGGATTATGTCTGTGTTGAAAGACTTTCTACCGATAAGGGTCACTAGTCTTACACAATTAAGAAAT
AATCCTACATTGACTGATTATCTAAAGATTGAAGGAAAATTTTATCCT
+
FFFFFFFFFFFFFFFFFFFFFFFFFFFFFF:FFFFFFFFFFFFFFFFFFFFFFFFFFFFFFFFFFFFFF
FFFFFFFFFFFFFFFFFFFFFFFFFFFFFFFFFFFFFFFFFFFFFFFF
@A00721:190:HGJW7DSXY:4:2359:14118:24001_1:N:0:TCTGCAAG+GCTGCACT
GACTTTCCTACGATACAAATGATAACTTCTAATTTACGTTTGCTACTCAGAGCTGGTCCTCTCAGCAA
AGGGATGCATGGTGTTATCTCCTCAGGAGTCCCGGCATTTGATAATAATGACGAGACTTTCGTCATAA
TCG
+
FFFFFFFFFFFFFFFFFFFFFFFFFFFFFFFFFFFFFFFFFFFFFFFFFFFFFFFFFFFFFFFFFFFF
FFFFFFFFFFFFFFFFFFFFFFFFFFFFFFFFFFFFFFFFFFFFFFFFFFFFFFFFFFFFFFFFFFFF
FFF
@A00721:190:HGJW7DSXY:4:1522:29297:25019_1:N:0:TCTGCAAG+GCTGCACT
GATTACACGGTACCAGCGCATCTCGTGTCGAGCCTCGACCCGATTATATGGTATTAACTTATAAAGGT
GAGGAGACCATTTTCTTGCTGAAATTTATAGAAGATTATTTTGACAGAATCAATAAGAATCATCGATT
CTC
+
FFFFFFFFFFFFFFFFFFFFFFFFFFF:FFFFFFFFFFFFFFF:FFFFFFFFFFFFFFFFFFFFFFFFF
FFFFFFFFFFFFFF:,FFFFFFF:FFFFFFFFF:FFFFFFFF,,,FFFFF:FFFFF,FF:FFF:FFFF
:FF
@A00721:190:HGJW7DSXY:4:2413:4065:32550_2:N:0:TCTGCAAG+GCTGCACT
GGATCCACTCCTCGAGTTATCATGATGTTAGTATGTTATATACTGATTTACAACGATATAGGAGTAAT
CTACGAGAAGATTCTAGAATTCTTAATTATAATATTCAACCTCAAAAG
+
FFFFFFFFFFFFFFFFFFFFFFFFFFFFFFFFFFFFFFFFFFFFFFFFFFFFFFFFFFFFFFFFFFFF
FFFFFFFFFFFFFFFFFFFFFFFFFFFFFFFFFFFFFFFFFFFFFFFF
@A00721:190:HGJW7DSXY:4:1669:23574:4366_2:N:0:TCTGCAAG+GCTGCACT
GAGATCACACATTTTGAGAACTAACTATGTTATGTTCTCTGATCATTTTGACAATCTCTTCTTATAAT
AAATAAATAAATAATATTACTAATGGCAAATAGTGGAAAGGCTCCAGT
+
FFFFFFFFFFFFFFFFFFFFFFFFFFFFFFFFFFFFFFFFFFFFFFFFFFFFFFFFFFFFFFFFFFFF
FFFFFFFFFFFFFFFFFFFFFFFFFFFFFFFFF:FFFFFFFFFFFFFF
@A00721:190:HGJW7DSXY:4:2161:20491:17065_2:N:0:TCTGCAAG+GCTGCACT
TACGATGACGTGAGAATAGGTGAAGGGATAGTTTTTACCTCGACCAACATTAAGGCGGCGGCGACTAA
TCTTAAAGCTTCACCCAAAATGAAGGACACTTCGAAACTGAATAATGA
+
FFFFFFFFFFFFFFFFFFFFFFFFFFFFFFFFFFFFFFFFFFFFFFFFFFFFFFFFFFFFFFFFFFFF
FFFFFFFFFFFFFFFFF:FFFFFFFFFFFFFFFFFFFFFFF:FFFFFFF
@A00721:190:HGJW7DSXY:4:2219:22363:19680_1:N:0:TCTGCAAG+GCTGCACT
GGCTCTAAAGGACACTATACAATATCCAGACGACGACGAAGCTTATACTACTTTAATTTTGGGTATTA
TGTGGAGATATCGTGATATCAGTACGTCCAGATCCTCTGCTTTTACCGATAACGATAAATTCGTCATC
AAA
+
FF:FFFFFFFFFFF:FFFFFFFFFFFFFFFFFFFFFFFFFFFFFFFFFFFFF:FFFFFFF:FFFFFFFFF
FFFFFFFFFFFFFFFFFFFFFFFFFFFFFFFFFFFFFFFFFFFFFFFFFFFFFFFFFFFFFFFFFFFF
FFF
```

```
@A00721:190:HGJW7DSXY:4:2449:14181:11146_1:N:0:TCTGCAAG+GCTGCACT
TTGGTGCTTAGGGATATAATGAATTCAGCACCACATTTTGGTCAAGTCAATCCGTTGAGAAAATTTCT
TAGAGGATATTCGCCATTGTGGTACGAATTGGTCCAACATTTACCACCAAATTCTTTTGATACAACTC
TAG
+
FFFFFFFFFFFFFF:FFFFFFFFFFFFFFFFFFFFFFFFFFFFFFFFFFFFFFFFFFFFFFFFFFFFF
FFFFFFFFFFFFFFFFFFFFFFFFFFFFFFFFFFFFFFFFFFFFFFFFFFFFFFFFFFFFFFFFFFFF
FFF
@A00721:190:HGJW7DSXY:4:1169:12744:30671_2:N:0:TCTGCAAG+GCTGCACT
CGGAATACGCTTGCTATTCACCAGATTGGTTCGAACCCGTTGATGCTATGTCAGAAGAAGCTATTCAA
GCTTGTCGTTTAAAGACTGCAGAGGCTGTCAAACAAGCCCAGACTCAA
+
FFFFFFFFFFFFFFFFFFFFFFF:FF:FFFFFFFFFFFFFFFFFFFFFF:FFFFFFFFFFFFFFFFFFF
FFFFFFFFFFFFFFFFFFFFFFFFFFFFFFFFFFFFFFFFFFFF:FFFFF
@A00721:190:HGJW7DSXY:4:1370:20528:22044_1:N:0:TCTGCAAG+GCTGCACT
CAAAGGTTTTGAATTTAGCTCAACTGGGGAGACGTGCTTAAATGGGTGATAAAAATTATTTAGAGATC
TTTTCTACTTACAAAGGACAATTCAGTTTCATTAATTCTACGGAAAAGTCCGGAGTATATTGTATGTT
AAT
+
FFFFFFFFFFFFFFFFFFFFFFFFFFFFFFFFFFFFFFFFFFFFFFFFFFFF:FFFFFFFFFFFFF:F
FFFFF,FFFFFFFFFFFFFFFFFFFFFFFFFFFFFFFFFFFFFFFFFFFFFFFFFFFFFFFFFFFFFF
FFF
@A00721:190:HGJW7DSXY:4:1308:13349:14841_1:N:0:TCTGCAAG+GCTGCACT
AATAGTTCTACCGATACGTAGTGAACGCGTTCGTAGAACCAAGTACTTTCCGTCTGAAGAGTTTAGAG
CTGCTATTGATTATGTAACCGATAGGAGTTGTATAGTTGAAGATGTGGCTTTAGACGATAATCCTTTA
AGG
+
FFFFFFFFFFFFFFFFFFFFFFFFFFFFFFFFFFFFFFFFFFFFF:FFFFFFFFFFFFFFFFFFFFFFF
FFFFFFFFFFFFFFFFFFFFFFFFFFFFFFFFFFFFFFFFFFFFFFFFFFFFFFFFFFFFFFFFFFFF
FFF
@A00721:190:HGJW7DSXY:4:1213:18466:21198_1:N:0:TCTGCAAG+GCTGCACT
GAAGATTTTCCCACAGATTTGTCTACGTTGCAGATCGCCACTCCAAACACTATATACATTATAAAATA
TAAAAATAAGGGAGTCATAACTTTTGTTACCAAATTCTTGGCTTTCAACGAAGTCTCTGATTTTATTA
ATC
+
FFFFFFFFFFFFFFFFFFFFFFFFFFFFFFFFFFFFFFFFFFFFFF,FFFFFFFFFFFFFFFFFFFFFF
FFFFFFFFFFFFFFFFFFFFFFFFFFFFFFFFFFFFFFFFFFFFFFFFFFFFFFFFFFFFFFFFFFFF
FFF
@A00721:190:HGJW7DSXY:4:2129:29423:2440_1:N:0:TCTGCAAG+GCTGCACT
CGATCTCTCGGTCATTTACTCTGTACTTGATCTACGTCGTTTAACTGTCGGAACAACCTTTACTTAGA
ATGACTCTATCTTTTCAGACACTTCTTATATTATTTACAATAGTTAATATCATATTAGTAATACTCAC
TAT
+
FFFFFFFFFFFFFFFFFFFFFFFFFFFFFFF:FFFFFFFFFFFFFFFFFFFFFFFFFFFF:FFFFFFFFF
FFFFFFFFFFFFFFFFFFFFFFFFFFFFFFFFFFFFFFF:FFFFFFFFFFFFFFFFFFFFFFFFFFFFF
FFF
@A00721:190:HGJW7DSXY:4:2272:3043:14027_2:N:0:TCTGCAAG+GCTGCACT
TTTTTCTTAGCTAATAGATTAATAAAGGATGAGTACGAAAACCAACTCTTCGTTACGAGACCTGATGG
TAGAATAGATTTAGTCTCTACACCCATCTCAACGAGGATAAAGCGAGT
+
FFFFFFFFFFFFFFFF,FFFFFFFFFFFFFFFFFFFFFFFFFFFFFFFFFFFFFFFFFFFFFFFFFFF
FFFF:FFFFFFFFFFFFFFFFFFFFFFFFFFFFFFFFFFFFFFFFFFF
@A00721:190:HGJW7DSXY:4:1659:8666:22639_1:N:0:TCTGCAAG+GCTGCACT
TACACGAAGAGATCATCCGCAAACGACTCGAAACCGAGACGCCGTAGATCAAAGTCCCTTCTAACTTT
```

AGATTATGAATCCTGCCTTTACTCATTTTATAGCAGTTATTTATGTTGTACTTTTTATAATTTATAAC
CTT
+
:FFF:,FF::FFFFF:FF:,F:FFFFFFFFFFFFFFFFFFFFFFFFFFFFF:FF:FFFFFFFFFFFF
FFFFFFFFFFFFFFFFFFFFFFFFFFFFFFFFFFFFFFFFFFFFFFFFFFFFFFFFFFFFFFFFFFFFF
FFF
@A00721:190:HGJW7DSXY:4:1135:11080:31203_1:N:0:TCTGCAAG+GCTGCACT
TACTTATTGTAACGATCATTCTTTCGAGTTTTGTCATTCTTATTCTTTTATTCCTTTAACTGTGTATG
AGGAGGATTTATAATGCTTTTCGATAGTGATTGTTATCAATTTAAATACATTACTGTTTCAAATCATC
TTT
+
FFFFFFFFFFFFFFFFFFFFFFFFFFFFFFFFFFFFFFFFFFFFFFFFFFFFFFFFFFFFFFFFFFFFF
FFFFFFFFFFFFFFFFFFFFFFFFFFFFFFFFFFFFFFFFFFFFFFFFFFFFFFFFFFFFFFFFFFFFF
FFF
@A00721:190:HGJW7DSXY:4:1470:21621:5400_1:N:0:TCTGCAAG+GCTGCACT
GAAAATGGTTTCAAATGAGGTCAAAGCTCTATTTATTAAACGAACTATTATCCGATTCTTCGAGGGTA
TCACTACCACTGATCTGCCGACTTTAGTCAAGGAACTTCAATTCCTTCGATGTTTATATTTTTCTATA
AGG
+
FFFFFFFFFFFFF:FFFFFFFFFF:FFFFFFFFFFFFFFFFFFFFF:FFFFFFFFFFFFFFFFFFFF
FFFFFFFFFFFFFFFFFFFFFFFFFFFFFFFFFFFFFFFFFFF,FFFFFFFFFFFFFFFF:FFFFFFFFF
:FF
@A00721:190:HGJW7DSXY:4:1105:1199:6511_2:N:0:TCTGCAAG+GCTGCACT
TAATCAAATTTCGACAAATTTTAGAACTTTATTTCAGAACTCTCTCCGATCACCTTGGTGTGATCTCC
CGGAGGATCTTTTCAATAAGATGTATGATGATAATCTACTATTTTTAA
+
FFFFFFFFFFFFFFFFFFFF,FFFFFFFFFFFFFFFFFFFFFFFFFFFFFFFF:FFFFFFFFFFFFFF
FFFFFFFFFFFFFFFFFFFFFFFFFFFFFFFFFFFFFFFFFFFFFFFF
@A00721:190:HGJW7DSXY:4:1227:25039:1955_1:N:0:TCTGCAAG+GCTGCACT
AGCGTATACTTGAATACTTTATCAAAGATTTGGCTTGGTCTATTTACCGCAAAAAGTTGGAAGAGGTC
TATGATATTTATTTTACTTATACTTTAAATAGCACCGATTATAACTTGACTGTTGATAATATTACAAT
AGC
+
FFFFFFFFFFFFFFFFFFFFF:FFFFFFFFFFFFFFFFFFFFFFFFFFFFFFFFFFFFFFFFFFFFFFF
FFFFFFFFFFFFFFFFFFFFFFFFFFFFFFFFFFFFFFFFFFFFFFFFFFFFFFFFFFFFFFFFFFFFF
FFF
@A00721:190:HGJW7DSXY:4:1147:14308:15687_2:N:0:TCTGCAAG+GCTGCACT
AAGATTAGAGAAGTAACTCGGTGAACACGTCGGTAGCATTGATTTACTTTTACCGTTAGAACTGAAAA
AGATTCTTTTCGAAAAGAAACCTATAAGTTACAAACGATTCTTCGACT
+
FFFFFFFFFFFFFFFFFFFFFF:FFFFFFFFFFFFFFFFFFFFFFFFFFFFFFFFFFFFFFFFFFFFFF
FFFFF:FFFFFFFFFFFFFFF:FFFFFFFFFFFFFFFFFFFFFFFFFFF
@A00721:190:HGJW7DSXY:4:1144:27254:19257_1:N:0:TCTGCAAG+GCTGCACT
AATAAAAATACTTCAAGTACCTGAACTTTACATTTTTAATGGTATGGTATAATGTGTCTTACAAATAG
AACCTTCACTCTCGGTAATGAGAGTGTATTCCTTAATATTCTAGTAACCGCTATGTGGGCATTACTAG
GAT
+
FFFFFFFFFFFFFFFFFFFFFFFFFFFFFFFFFFFFF:FFFFFFFFFFFFFFFFFFFFFFFFFFFFFFF
FFFFFFFFFFFFFFFFFFFFFFFFFFFFFFFFFFFFFFFFFFFFFFFFFFFFFFFFFFFFFFFFFFFFF
FFF
@A00721:190:HGJW7DSXY:4:1532:28176:7889_1:N:0:TCTGCAAG+GCTGCACT
GATAGCAATTTATGTTTTAAATGCTAGGAAAATTTTAGAAAAACTCCATTGTATAGTTCAAGGAGTAC
TTAATTCAATAGCTTTAGTATTATTTACTATCGCCTTTATTGAAAATTGTATAAGAGTTAATTGCTTA
CAC

```
+
FFFFFFFFFFFFFFFFFFFFFFFFFFFFFFFFFFFFFFFF::FFFFFFFFFFFFFFFFFFF:FFFFFFF
FFFFFFFFFF,FFFFFFFFFFFFFFFFFFFFFFFFFFFFFFFFFFFFFFFFFFFFFFFFFFFFFFFFFF
FFF
@A00721:190:HGJW7DSXY:4:2656:29930:7451_2:N:0:TCTGCAAG+GCTGCACT
TTAATTGATTGAAGCTAGGCGTTGGGGAGAATGAACTGCCGCTTCCCGGAAGAAACGATCTATTTACA
GAGTATTTAGAGTAGTCCAAAGTTTGTATAAATTTATATGATTTTCTA
+
FFFFFFFFFFFFFFFFFFFFFFF:FFFFFFFFFFFFFFFFFFFFFFFFFFFFFFFFFFFFFFFFFFFFF
FFFFFFFFFFFFFFFFFFFFFFFFFFFFFFFFFFFFFFFFFFFFFFFF
@A00721:190:HGJW7DSXY:4:1634:14100:26068_2:N:0:TCTGCAAG+GCTGCACT
AACATACCATACACTCCCCAAACATAACATACCATAACCTACCATGACCAATCATCGCTCCAACTGGC
ACGCCAGACGTAGGGTCAAACCTACTACCGCCGGTCGGCAGATCGGCA
+
FFFFFFFFFFFFFFFFFFFFFFFFFFFFFFFFFFFFFFFFFFFFFFFFFFFFFFFFFFFFFFFFFFFFF
FFFFFFFFFFFFFFFFFFFFFFFFFFFFFFFFFFFFFFFFFFFFFFFF
@A00721:190:HGJW7DSXY:4:2469:20094:10708_1:N:0:TCTGCAAG+GCTGCACT
TACGTTCAGGGACCCGCTGTGCAGAATAACCTTCCCAAATCTAGATCTGCACAGAAGAAGGCTGAAGG
TCGTTACCGGACGACCACTCAAAGGAAACGGGCTGTTGCCCAGAGGAAAGCAGCGACCACCGAGGTCG
CTC
+
FFFFFFFFFFFFFFFFFFFFFFFFFFFFFFFFFFFFFFFFFFFFFFFFFFFFFFFFFFFFFFFFFFFFF
FFFFFFFFFFFFFFFFFFFFFFFFFFFFFFFFFFFFFFFFFFFFFFFFFFFFFFFFFFFFFFFFFFFFF
FFF
@A00721:190:HGJW7DSXY:4:2369:6189:12900_2:N:0:TCTGCAAG+GCTGCACT
GCAGTCCCGTCATCTCTGTGGGAACTGAGGCAGTACCTCAGTCAAAAGTCACATTCACCACTCCCATG
CCAATCAATGATGTGACTATACAGTTCGGCCATTTCAAGAGTGGTCAA
+
FFFFFFFFFFFFFFFFFFFFFFFFFFFFFFFFFFFFFFFFFFFFFFFFFFFFFFFFFFFFFFFFFFFFF
FFFFFFFFFFFFFFFFFFFFFFFFFFFFFFFFFFFFFFFFFFFFFFFF
@A00721:190:HGJW7DSXY:4:1657:5855:12446_1:N:0:TCTGCAAG+GCTGCACT
GAGGTACTGAAGCCTTCCCCTCCGCTGGCACGAAGAACACTCCCAAGCCAATCAATAGTTCTTCTGAC
CAGAATGAAAAGGGGTTTACCAGGGTAGTCTCTAAGTCTACCAGGAAGAGGGGAAATGTCCCCAAAAC
TGA
+
FFFFFFFFFFFFFFFFFFFFFFFFFFFFFFFFFFFFFFFFFFFFFFFFFFFFFFFFFFFFFFFFFFFFF
FFFFFFFFFFFFFFFFFFFFFFFFFFFFFFFFFFFFFFFFFFFFFFFFFFFFFFFFFFFFFFFFFFFFF
FFF
@A00721:190:HGJW7DSXY:4:1656:13883:34773_2:N:0:TCTGCAAG+GCTGCACT
AGGTCCATCAGGACCTAAGAAGGGGAAATTACCCCTAATTCTGAAGGAAGATCTCCCACCATCTGGGG
TTGGGAATAAGAAATCTGCACGGGCCAAAGAGGCCGCTGCTACGAAGT
+
FFFFFFFFFFFFFFFFFF:FFFFFFFFFFFFFFFFFFFFFFFFFFFFFFFFFFFFF:FFFFFFFFFFFFFF
:FFFFF:FFFFFFFFFF:FFFFFFFFFFFFFFFFFFFFFFFFFFFFFF
@A00721:190:HGJW7DSXY:4:2321:11460:6339_1:N:0:TCTGCAAG+GCTGCACT
GCTTGGGTCATTAAGGGTAGCTGTAAAGTTGCGCCTGCCGACCCCATCTTTGAGACCAAGACTCTCTA
CAGTAGGTTCGTGCCTACTATGAGCAAATCTGTTGAATTCGTCACTGAGGACGAAGGTGAGAGGACTT
TTG
+
FFFFFFFFFFFF:FFFFFFFFFFFFFFFFFFFFFFFFFFFFFFFFFFFFFFFFFFFFFFFFFFFFFFFF
FFFFFFFFFFFFFFFFFFFFFFFFFFFFFFFFFFFFFFFFFFFFFFFFFFFFFFFFFFFFFFFFFFFFF
FFF
@A00721:190:HGJW7DSXY:4:2302:6831:28479_1:N:0:TCTGCAAG+GCTGCACT
CAAACTGAGAGCAGTCGCTGGGGAAAGAGAATTGCGTGGGTTGAAGGCCGACGCTTCGAGCTTGAGTG
```

TGACGAGGCCGGTGACGTTTTTGTCAACGGCGTTTGGAGGATTTTATCCGCGCTTAACTATGGGGCGA
AGA
+
FFFFFFFFFFFF,FFFFFFFFFFFFFFF:,,F:F:FF,F,FFF::FFFFFF:FFF:F::F:FFFF,F:
FFFFFFFFFFFFFFFFFFFFFFFFFFFFF::FFFFFF,::FFF:FFFFFFFFFFFF,FF:FFFF:FFF
FFF
@A00721:190:HGJW7DSXY:4:1516:27787:15295_1:N:0:TCTGCAAG+GCTGCACT
AGTCTTGAAGGACTTAGGTTGGCATATTTATGCCGACGATAATAAGCATGTCACCCTTAAGCACCGAG
GTAAGCTCGGGACTTACAAGAGAGAGTGTGAGAGAGTCGCTCTCACTATTTTTGCCCAGAATGCGGGC
TTT
+
FFFFFFFFFFFFFF:FFF:FFFFFF,FF:F,FFFFFFFFF:FFFFFFFFFFFFFFFFFFFFFFFFFFF
F:F:FFFFFFFFFF:FF:FFFFFFFFFFFF:FFFFFFFFFFFFFFFFF:FF:FFFFFFFF,FFFFFFF
FFF
@A00721:190:HGJW7DSXY:4:2440:24279:20431_2:N:0:TCTGCAAG+GCTGCACT
GCTCCTTCTGTGGGTGAGGGTTTTTGTTATATTCACTGGTTCTGGGAGGCTGCTTTCCTCCATAATTA
CAACCTCTCTTTAGAGAGCATTAGAAAGATCGGAAGAGCGTCGTGTAG
+
FFFFFFFFFFFFFFFFFFFFFFFFFFFFFFFFFFFFFFFFFFFFFFFFFFFFFFFFFFFFF:F:FFFF
F:FFFFFFFFFFFFFFFFFFFFFFFFFFFFFFFFFFFFFFFFF:F,:F
@A00721:190:HGJW7DSXY:4:1306:9634:29825_1:N:0:TCTGCAAG+GCTGCACT
CACACTTTTCTTGAAAATTTTTCCTTTCCTCCGTCATTGGGGATTTCGATGTGAGAAGGTGGAAGGTG
TCAAGGGTCTTCTTCATGCTGATCTTTCAGGACCTAATCACATCATGATTGATAGTTACGGCTCGTAT
CGA
+
FFFFFFFFFFFFFFFFFFFFFFFFF,FFFFFFF:FFFFFFFFFFFFFFF:FFFFFFFFFFFFFFFFFF
FFFFFFFFFFFFFFFFFFFFFFFFFFFFFFFFFFFFFFF:FFFFFFFFFFFFFFFFFFFFFFFFFFFF
FFF
@A00721:190:HGJW7DSXY:4:1436:32551:3474_2:N:0:TCTGCAAG+GCTGCACT
AAGTTCTCCGAAATTAAAGACGGGGGGATTAAGAGTCCAGCTGTAACTGTCATTTCAGAGAGGGGAGC
TCTGAAAAGGAAACTTCGTCAGAAGATGAGGAAGTCTATGAGGAGAGA
+
FFFFFFFFFFFFFFFFFFFFFFFFFFFFFFFFFFFFFFFFFFFFFFFFFFFFFFFFFFFFFFFFFFFF
FFFF:FF,FFFFFFFFFFFFFFFFFFFFFFFF,FFFF:,F:FFFFFFF
@A00721:190:HGJW7DSXY:4:1368:10131:22263_1:N:0:TCTGCAAG+GCTGCACT
CTCTAGAGAAGATCTCACCACCTGGAGAAGGCAAGTGGAGAGTCCAGAAGTTGATGAAGAAGGATTAT
TTGAAGCAGGGGTTACCACTTCCTAAGGTGGATGAGGGAGGTTGTTTCTTTGGAGTTTTAGATAATCT
TTT
+
FFFFFFFF:FFFFFFF,FFFFFFFFFFFFFFFFFFFFFFFFFFFFFFFFFFFFFFFFFFFFFFFFFFF
FFFFFFFFFFFFFFFFFFFFFFFFFFFFFFFFFFFFFFFFFFFFFFFFFFFFFFFFFFFFFFFF,FFF
FFF
@A00721:190:HGJW7DSXY:4:1441:12897:1470_2:N:0:TCTGCAAG+GCTGCACT
CCTTGAGAAGACAAGCCACATTAAGATATAATTTAGGTCAACTTGGGGTTTCGAATGTCATTAGTGCC
TTACGATTGAAAGAGGTCCTTTTGAGTATCGACAGATCTTTCGATCTT
+
FFFFFFFFFFFFFFFFFFFFFFFFFFFFFFFFFFFFFFFFFFFFFFFFFFFFFFFFFFFFFFFFFFFF
FFFFFFFFFFFFFFFFFFF:FFFFFFFFFFFFF:FFFFFFFFFFFFFF
@A00721:190:HGJW7DSXY:4:1340:16541:34679_2:N:0:TCTGCAAG+GCTGCACT
ACGTGGCAGAATTCGGTAACTTACCATATTGTTCCTTCCGGGAAGTTGTGGGATTTAACTTTGACTCA
ACTACTCCGATCAGATCCGGATGGTGTGATTGGTATGGACTACTCGAC
+
FFFFF:FFFFFFFFFFFFFFFFFFFFFFFF:FFFFFFFFFFFFFFFFFFFFFFFFFFFFFFFFFFFFF
FFFFFFFFFFFFFFFFFFFFFFFFF,FFFFFFFFF:FFFFFFFFF:FF

```
@A00721:190:HGJW7DSXY:4:1134:7636:8672_1:N:0:TCTGCAAG+GCTGCACT
TAGGGTCATGGCAGATACTACCGATAAGGTGAAAAATATGCAGGTCAAAGATTCTGTAGCTCTGAGGG
CGGTTGAGGGAGCCGTTTTGGCCGACTTTAACTATTCTAAGAGTCTTTATGATTCAAAACCTATCGTT
GTC
+
FFFFFFFFFFFFFFFFFFFFFFFFFFFFFFFFFFFFFFFFFFFFFFFFFFFFFFFFF:FFFFFFFFFF
FFFFFFFFFFFFFFFFFFFFFFFFFFFFFFFFFFFFFFFFFFFFFFFFFFFFFFFFFFFFFFFFFFFF
FFF
@A00721:190:HGJW7DSXY:4:1304:9570:19664_1:N:0:TCTGCAAG+GCTGCACT
TACAGCGGCTTATTGTCGCGCAGTTTCCAGAATTGCGGTTGAAGTTCACCCATTCTATCTTGAATGCT
CACGCCGTCGCAGCCATATCGAGACTATGTTATAATCACTTGTTAAAGTACAGATTTAGTGATTTAGA
TAT
+
FFFFFFFFFFFFFFFFFFFFFFFFFFFFFFFFFFFFFFFFFFFFFFFFFFFFFFFFFFFFF:FFFFFF
FFFFFFF:FFFFFFFFFFFFFFFF::FFFF:FFFFFFFFFFFFFFFFFFFFFFFFFFFFFFFFFFFFF
FFF
@A00721:190:HGJW7DSXY:4:1320:15628:24298_1:N:0:TCTGCAAG+GCTGCACT
GGAGGCGATATTCGTTTTCATATCCAAAACTCCTTGAATGGAGTACATATTTGCAACCCTATTCTTGA
TGTGAAAGATGCATCGAGGTATACGTCTAGGAACATTGATTGGGAACGTACCTACTCACGAGACGTCA
TGA
+
FFFFFFFFFFFFFFFFFFFFFFFFFFFFFFFFFFFFFFFFFFFFFFFFFFFFFFFFFFFFFFFFFFFF
FFFFFFFFFFFFFFFFFFFFFFFFFFFFFFFFFFFFFFFFFFFFFFFFFFFFFFFFFFFFFFFFFFFF
FFF
@A00721:190:HGJW7DSXY:4:1406:7717:31673_1:N:0:TCTGCAAG+GCTGCACT
ATGTTTCGTTCTGTTACAAGAACGCTTTGGATTGTGACCATAAAGCCTCAGTTGCGATAGCTGTAGAG
GTATACGATATCTCTCTTGATGATGTAGCCACTATAATGCTTAAGAGAGAGATTGATGTATGTCATTT
CGT
+
FFFFFFFFFFFFFFFFFFFFFFFFFFFFFFFFFFFFFFFFFFFFFFFFFFFFFFFFFFFFFFFFFFF:
FFFFFFFFFFFFFFFFFFFFFFFFFFFFFFFFFFFFFFFFFFFFFFFFFFFFFFFFFFFFFFFFFFFF
FFF
@A00721:190:HGJW7DSXY:4:1445:12825:7420_1:N:0:TCTGCAAG+GCTGCACT
GAATTATTGGACACGATGAAAAATGACATACCCGTTCCTGACCTTGGTATCGTTATCCGTCATGGAGA
TGATGCCGCATCACTTGATTACTATATGCCCGAAGGATTGGGTTATTCTCACAACAAAGATAAGATTT
TAT
+
FFFFFFFFFFFFFFFFFFFFFFFFFFFFFFFFFFFFFFFFFFFFFFFFFFFFFFFFFFFFFFFFFFFF
FFFFFFFFFFFFFFFFFFFFFFFFFFFFFFFFFFFFFFFFFFFFFFFFFFFFFFFFFFFFFFFFFFFF
FFF
@A00721:190:HGJW7DSXY:4:1620:30346:24706_1:N:0:TCTGCAAG+GCTGCACT
GTTTTCCTTAAGGGCGGACAATTGTTCCATTGTGAACTCTTGGGGACTAGATTGAGCATTAATGAGTT
CCGAATAACTCGTAATAAATCTTTCTCTAGTGGACAATTCAGAATACCTATTTCCGTACCAAGAGCCA
CTG
+
FFFFFFFFFFFFFFFFFFFFFFFFFFFFFFFFFFFFFF:FFFFFFF:FFFFFFFFFFFFFFFFFFFFF
FFFFFFFFFFFFFFFFFFFFFFFFFFFFFFFFFFFFF:FFFFFFFFFFFFFFFFFFFFFFFFFFFFFF
FFF
@A00721:190:HGJW7DSXY:4:1438:5755:11459_2:N:0:TCTGCAAG+GCTGCACT
TGGAAGAAGCGAGGTTCGTTCCTCAATAAAAATTTTTCTCAAGAGATTATGGTTGATAAAGTTTTCTT
CATAAGGGCTATTCAGTACGTCATGGGGATCACTTCTTCGGTCAATGA
+
FFFFFFFFFFFFFFFFFFFFFFFF,FFFFFFFFFFFFFFFFFFFFF:FFFFFFFFF:FF:FFFFFF:F
FFFFFFFFFFFFFFFFFFFFFFFFFFFFFFFFFFFFFFFFFFFFFFFF
```

```
@A00721:190:HGJW7DSXY:4:1258:25274:31266_1:N:0:TCTGCAAG+GCTGCACT
AATATGTTACGACATGGCTTCGTAACAACAGTGCGAGGATTGTCATCTCGGGGAAAGTGATTCACCAA
GATGTTCATATAGATACGAGATACCTAGAGAAGGTCGCAGCGATCTTACTCGTTATCGGAGCTAAGAA
GAA
+
FFFFFFFFFFFFFFFFFFFFFFFFFFFFFFFFF:FFFFFFFFFFFFFFFFFFFFFFFFFFFFFFFFFF
FFFFFFFFFFFFFFFFFFFFFFFFFFFFFFFFFFFFFFFFFFFFFFFFFFFFFFFFFFFFFFFFFFFF
FFF
@A00721:190:HGJW7DSXY:4:1468:17852:34663_2:N:0:TCTGCAAG+GCTGCACT
GTTATGCTCGCAAACTTAACGTTAGTACCGGAGGTGATCTCTCTGCTTCTGCGTTACTGAGAGAAAAG
ATTCAGGACGCCTACGGTGACTTTATGGACAAGATGCAAGAGATCGGA
+
FFFFFFFFFFFFFFFFFFFFFFFFFFFFFFFFFFFFFFFFFFFFFFFFFFFFFFFFFFFFFFFFFFFF
FFFFFFFFFFFFFFFFFFFFFFFFFFFFFFFFFFFFFFFFFFFFFFFF
@A00721:190:HGJW7DSXY:4:2151:11189:20181_2:N:0:TCTGCAAG+GCTGCACT
TTTAGGTTTAGTAGGTGATCTTGACGTCGATTCTTTGTATAGGGAGTGTGAGAAGACAACGAATCTTG
AAACTACGGTCCTTATCAAAGAAAATGGTGAACCGGTCTCTAAATCTT
+
FFFFFFF,FFFFFFFFFFFFFFFFFFFFFFFFFFFFFFFFFFFFFFFFFFFFFFFFFFFFFFFFFFFF
FFFFFFFFFFFFFFFF:FFFFFFFFFFFFFFF:FFFFFFFFFFFF:FF
@A00721:190:HGJW7DSXY:4:1161:20781:25582_2:N:0:TCTGCAAG+GCTGCACT
AGATTGAAGAACACGTCAAGAAGTCGCTCGGCGTTGTTCTTGAAAACCAAGTTAAGAAGGAGGCTGAT
CATGGAGTCTTTGTACCTTCTGTCAAACTTCGAGGTTCAGCTACGAGA
+
FFFFFFFFFFFFFFFFFFFFFFFFFFFFFFFFFFFFFFFFFFFFFFFFFFFFFFFFFFFFFFFFFFFF
FFFFFFFFFFFFFFFFFFFFFFFFFFFFFFFFFFFFFFFFFFFFFFFF
@A00721:190:HGJW7DSXY:4:1524:18313:22936_1:N:0:TCTGCAAG+GCTGCACT
TGGATTGAAGGCTGGCTCTAAGGGAGACTCCTTCTTTTTGAATGTTGTAAAGAATTGGGTTGAGAAGT
GTGCTTTACAAGGAGTTAGACTCTTCTTCAAGTTTTTAGATCTTCTTGATAATCTTAAAGAAAAATTT
TCG
+
:F:FFFFF:FF:,FFFFF,:,,FFFFFFFFFFFFFFFFFFFFFFFFFFFFFFF,FFFFFFFFFFFFFF
FFFFFFFFF:FFFFFFFFFFFFFFFFFFFFFFFFFFFFFFFFFFFFFFFFFFFFFFFFFFF:FFFFFF
FFF
@A00721:190:HGJW7DSXY:4:1260:3061:9204_2:N:0:TCTGCAAG+GCTGCACT
CTGTACTTACATTCTTTACGCCAGATTCTTCATGGGGCGACGCTGTTTCTCTTGGTATTTCTGAAATA
GTTTATCAGAGCGTCATTTTCCTTTGGGATCTTTCCTCTGGTAAGGGA
+
FFFFFFFFFFFFFFFFFFFFFFFFF:,FFFFFF:FFFFFFFFFFFFF:FFFFF:FFF:FFFFFFFFFFF
FFFFFFFFFF:FFFFFFFFF:FF:FFFFFFFFFFFFFFFFFF::FFFF
@A00721:190:HGJW7DSXY:4:1273:26422:5259_2:N:0:TCTGCAAG+GCTGCACT
ATTCTTCTCACTCTTGACGAGAATTATTACTGTTTTCTCTACCCATAACTTCTTTTTCAAAAATGCGG
ATGGGTCTACTGATGTTTTCCTACAGAGTGCAGTCGTTTCAACACTTA
+
FFFFFFFFFFFFFFFFFFFFFFFFFFFFFFFFFFFFFFFFFFFFFFFFFFFFFFFFFFFFFFFFFFFF
FFFFFFFFFFFFFFFFFFFF:FF:FFFFFFFFFFFFFFF:FF,FFFFF
@A00721:190:HGJW7DSXY:4:1509:10285:8030_2:N:0:TCTGCAAG+GCTGCACT
GGAAAATAATTGGTGATGAAAATACGTATGTCGGTGGGGTTCTCAAGGCTCTCATCGCCACTGGGATT
CTAAGGGGTTCCATAAGATATTATCTGACATTGTGGTTCAATGATCAG
+
FFFFFFFFFFFFFFFFFFFF,FFFFFFFFFFFFFFFFFFFFFFFFFFFFFFFFFFFFFFFFFFFFFFF
FF:FF:FFFFFFFFFFFFFFFFFFFFFFFFF:FFFFFFFFFFFFFFFFF
@A00721:190:HGJW7DSXY:4:1328:28429:21950_2:N:0:TCTGCAAG+GCTGCACT
TGGTCCTTACGTCTTAAGTAATTTGGATGTTGTCGAAAACTATCAGGTTCTTGTCACGATTCTTAAAA
```

```
GAAGTTTATTAAAACTTCAGAGTCTCTTGGAAGTCCAGGTAGCTAATG
+
FFFFFFFFFFFFFFFFFFFFFFFFFFFFFFFFFFFFFFFFFFFFFFFFFFFFFFFFFFFFFFFFFF::FF
F:::FFFFFFFF:FFFF:,FFFFFFFFFFFFFFFFFFF:FFFFFF:FF
@A00721:190:HGJW7DSXY:4:1401:12192:32628_2:N:0:TCTGCAAG+GCTGCACT
GCCACAGACTTTCTGGGAAAAGAGACCTCTGTTGGTAGAGGAGTTGAGAGAATTACTCAGATTCAATC
TGGCATCACTGAACGTCTACAACGAGTTAGGGAGTGGCTAGAGATCGG
+
FFFFFFFFFFFFFFFFFFFFFFFFFFFFFFFFFFFF:FFFFFFFFFFFFFFFFFFFFFFFFFFFFFFFF
FFFFFFFFFFFFFFFFFFFFFFFFFFFFFFFFFFFFFFFFFFFFFFFF
@A00721:190:HGJW7DSXY:4:2627:12147:31516_1:N:0:TCTGCAAG+GCTGCACT
AGCAATACCTTCCACTGAGTTCATCACCCAGAGTTTCGACGTCGCGGAAAACGATGAAGATTTCCACA
CTGTTCGAGAATCTTCTGATGCTGAATTTGAGGAAAATTTTTCGGAAGTTTTTGGTATAAATCCTGAT
GGC
+
FFFFFFFFFFFFFFFFFFFFFFFFFFFFFFFFFFFFFFFFFF:FFFFFFFFFFFFFF:FF,FFFFFFF
FFFFFFFFFFFFFFFFFFFFFFFFFFFFFFFFFFFFFFFFFFFFFFFFFFFFFFFFFFFFFFFFFFFF
FFF
@A00721:190:HGJW7DSXY:4:1150:3549:36354_1:N:0:TCTGCAAG+GCTGCACT
TAAAACCTTCTTTTATCATTGCAACAGGACTTTGAGAAGACTGTTGAAATGGACAAAATCTGGGATCA
TAGGTATGTGTCACGCTACACTCGAAATATGGGCCCTCATAAGGAAGTATAGTTGGCGTCTTGTCGTT
GAC
+
FFFFF,FFF,FFFFFFFFFFFFFFFF,FFFFFFFFFFFFFFFFFFFFFFFFFFFFFFFFFFFFFFFF:F
FFFFFFFFFFFFFFFFFFFFFFFFFFFFFFFFFFFFFFFFFFFFFFFFFFFFFFFFFFFFFFFFFFFF
FFF
@A00721:190:HGJW7DSXY:4:1317:11243:23782_2:N:0:TCTGCAAG+GCTGCACT
AGTTAGATCATTTGTTCAGGAAGATCTCTACAGAGATTATCGAGAGTTGTGATCTAGTTCTAAATTTA
GGTCGACGGACTTTAGCAACTTTAGATAAATTCTTTCCGGGCTCAATG
+
FFFFFFFFFFFFFFFFFFFFFF:FFFFFFFF:FFFFFFFFFFFFFFFFFFFFFFFFFFFFFFFFFFFFF
F:FFFFFFFFFFFFFFFFFFFFFFFFFFFFF:FFFFFF:FFFFFFFF:,
@A00721:190:HGJW7DSXY:4:1273:12120:29590_1:N:0:TCTGCAAG+GCTGCACT
TCTGACCATCGCAAAGAAGACTTTATTAGTTCATGAAGTTCTTACACTTCTCGCGACTAATGTCAGCT
TCACTACAAAGGCGTTACGTAAAGTAGTTTTCACGCTTTATCTTAAAGCATTACCACCAAATTTAGCA
CTT
+
FFFFFFFFFF:FFF,FFFFFFFFFFFFFFFFFFFFFFFFFFFFFFFFFFFFFFFFFFFFFFFFFFFFF
FFFFFF:F,FFFFFFFFFFFFFFFFFFFFFFFFF:FFFFFFFFFF:FFFFFFFFFFFFFFFF:FFFFF
FFF
@A00721:190:HGJW7DSXY:4:1220:25482:3630_1:N:0:TCTGCAAG+GCTGCACT
CTCGCCATATCAGATTAGAGACATGCCAGTTAGAGTTCTTCTAACCAGGACTGATGGTGAGATTCTTT
TACCTTTTGGAGGTCCAGGAACCTTTACTCCTAGTGTCTTTTCTAGAGTCCAAGGACGTTTCCGTCGG
AGG
+
FFFFFFFFFFFFFFFFFFFFFFFFFFFFFFF:FFFFFFFFFFFFFFFFFFFFFFFFFFFFFFFFFFFF
FFFFFF:FFFFFFFFFFFFFFFFFFFFFFFFFFFFFFFFFFFFFFFFFFFFFFFFFFFFFFFFFFFFF
FFF
@A00721:190:HGJW7DSXY:4:1616:20745:30780_1:N:0:TCTGCAAG+GCTGCACT
GAGACCGCTCAATAACACTCTATTGAATCCCTTTTCCACGATGAAAGCTCTTTATGAGGTGCTCCATG
CAAATTTACAGTTCCTCTTTGGTGATATCGCCTCTGTCCTTTCTCCTTTAATCGTGAGGTTACGAGAG
TTT
+
FF:FFF:F::FF:FFFF:F::F:FFFFF:FF:FFFFFF:F,FFFFFFFF::F:::FFFFFFFFFFFF
```

:FFFFFFFFFF,FFFFFFFF:FFFFFFFFFFFFFFFFFFFFFFFFFFFFFFFFFFFFFFFFFFFFFFF
FFF
@A00721:190:HGJW7DSXY:4:1372:31412:2472_1:N:0:TCTGCAAG+GCTGCACT
GTATTTTGATAAATTTGTTGGAATCGATAATGATACTGACGAAGGTTCTATTACCACCCTTGTCATTG
ATACAAACATTCTAGGTGATGCTTTCTTAGAGGCTCGTCTAGCCGCTGAGATTCGTCTTACCTATTTA
CAA
+
:FFFFFFFFFFFFFFFFFFFFFFFFFFFFFFFFFFFFF:FFFFFFFFFFFFFFFFFFFFFFFFFFFFF
FFFFFFFFFFFFFFFFFFFFFFFFFF:FFFFFF:FFFFFFFFFFFFFFFFFFFF,FFFFFFFFFFFFF
FFF
@A00721:190:HGJW7DSXY:4:1228:21115:16235_2:N:0:TCTGCAAG+GCTGCACT
TTTGAGTGATAACGATTTAGTACTGGGAAATGGTGGTAGTCAGTTTACTACTTTTCTTTCCCTCCCCT
TTTTCGATTGGATTCGACGGGGTCTTCGTCTCACGACTAATTTGATAA
+
FFFFFFFFFFF:FFFFFFFFF:FFFFFFFF:FF:FF,FFFFFFFFFFFFF:FFFFFFFFFFFFF:FFF
FFFFFFFFFFFFFFFFFFFFFFFFFFFFFFFFFFFF::F:FFFFFFFF
@A00721:190:HGJW7DSXY:4:1224:25599:18709_2:N:0:TCTGCAAG+GCTGCACT
TACTTATGAAGGAGGTATTACTTCGGAAGTTCTTAATGTAGGTCTCCTAGTACTTAAGGGTTTATGTT
TCGGTTTCAGCTGGATGGATCTTTTGATGGTATGTTTCACATTGCGTG
+
FFFFFFFFFFFFFFFFFFFFFFFFFFFFFFFFFFFFFFFFFFFFFFFFFFFFFFFFFF:FFFFFFFFF
FF:FFFFFFFFFFFFFFFFFFFFFFFFFFFFFFFFFFFFFFFFFFFFF
@A00721:190:HGJW7DSXY:4:1473:3323:34491_2:N:0:TCTGCAAG+GCTGCACT
TGGACTTGGGAAGAGATTAAACATAAAATCCAGCGATTCTATTATGTCGCTAAAGCCTGTCCAGTTGT
CGATGATGTTCGGGCTGTGGGGATTAACTTCCCTCGTGAAACTTACGA
+
FFFFFFFFFFFFFFF,FFFFFFFFFFFFFFFFFFF,FFFFFFFFFFFFFFFFFFFFFFFFFFFFFFFF
FFFFFF:FFFFFFFFFFFFF:FFFFFFFFFFFFFFFFF,FFFFFFFFF
@A00721:190:HGJW7DSXY:4:1208:7075:12242_1:N:0:TCTGCAAG+GCTGCACT
CACAAGGATTACTAAGTCTGAGATCTGCCACTTCTAATTTTAGGAAACGTCGCGAAAAGAAAGTTCAT
GAGGATGTCTTGGTCGAAAAATATCGCCGGGAACTTCTAATAGAGGATCCTGATGACATTATTTCAAC
GGA
+
FFFFFFFFFFFFFFFFFFFFFFFFFFFFFFFFFFFFFFFFFFFFFFFFFFFFFFFFFFFFFFFFFFFF
FFFFFFFFFFFFFFFFFFFFFFFFFFFFFFFFFFFFFFFFFFFFFFFFFFFFFFFFFFFFFFFFFFFF
FFF
@A00721:190:HGJW7DSXY:4:1105:3586:36761_2:N:0:TCTGCAAG+GCTGCACT
AGTTCTACCACAGAAACCGTCTCAGGAACTGTCTCCACTGATTCGGAATTAGAGAAAGACGACCTTCT
TGTGGGGACTGAGACCTCTGAATTTTCAGGGTGCCCTGATGAGGACGT
+
FFFFFFFFFFFFFFFFFFFFFFFFFFFFFFFFFFFFFFFFFF:FFFFFFFFFFFFFFFFFFFFFFF:F
FFFFFFFFFFFFFFFFFFFFFFFFFFFFFFFFFFFFFFF:FFFFFFFF
@A00721:190:HGJW7DSXY:4:1134:14091:13714_1:N:0:TCTGCAAG+GCTGCACT
ACTCTGAGACTTCTAAGAGTGTTTCAAAAGACCAGAAACGTAACAAGAAGAATAAAGGTAGGAAGGGG
AGAAAACAGAACAAGCGAGCGGTTGAAGAGAAAACTGATATGGTTGCGGCTGATCGTACCCCAATTCC
TGC
+
FFFFFFFFFFFF:FFFFFFFFFFFFFFFF:FFFFFFFFFFFFFFFFFFFFFFFFFF:FFFFFFFFFF:
FFFF:FFFFFFFFFFFFFFFFFFFFFFFFFFFFFFFFFFFFFFF:FFFFFFFFFFFFFFFFFFFFFFF
FFF
@A00721:190:HGJW7DSXY:4:1160:8495:30545_1:N:0:TCTGCAAG+GCTGCACT
TCTCGGCGATACCACTGATCTTCCAGAGTTACGTAGTGGTCGTTCTTTTGGTGATTTTATCAAAGAGA
AAGTTTCTGCTAGTCGCGACGATAGTATTTATCGGAGTGATATTAACAAGTTAAACTTCGAACTGACC
ACT

+
FFFFFFFFF,FFFFFFFFFFFFFFFFFFFFFFFFFFFFFFFFFFFFFFFFFFFFFFFFFFFFFFFFFFFFFF
FFFFFFFFFFFFFFFFFFFFFFF:FFFFFFFFFFFFFFFFFFFFFFFFFFFFFFFFFFF:FFFFFFFFFF
FFF
@A00721:190:HGJW7DSXY:4:1117:16957:34992_2:N:0:TCTGCAAG+GCTGCACT
TTGAAGTACCGCAACATGGATCAGTTTTCCAAAAAGCTATTAATAGCAGTTGCGGGGCTATAATACCG
CCTCCAGATTACTCTAAACTGAATCCACTCTCTTGGGATATAAATGAG
+
FFFFFFFFFFFFFFFFFFFFFFFFFFFFFFFFFFFFFFFFFFFFFFFFFFFFFFFFFFFFFFFFFFFFFF
FFFFFFFFFFFFFFFFFFFFFFFFFFFFFFFFFFFFFFFFFFFFFFFF
@A00721:190:HGJW7DSXY:4:2210:21748:28573_1:N:0:TCTGCAAG+GCTGCACT
AGAGTTTTGGAGCTGTGTAATGTCCTACGTACTGTTCAAGGTGTTTGGGCTTCTCTAAAGAACAGCAA
CTTTCAACATGAATTAGTTATTGAAAGTCTTGACCCTCACGTCTCACTTTATCACCTTGGAGATGGTA
AAG
+
FFFFFFFFFFFFFFFFFFFFFFFFFFFFFFFFFFFFFFFFFFFFFFFFFFFFFFFFFFFFFFFFFFFFFF
FFFFFFFFFFFFFFFFFFFFFFFFFFFFF:FFFFFFFFFFFFFFFFFFFFFFFFFFFFFFFFFFFFFFFF
FFF
@A00721:190:HGJW7DSXY:4:2209:4960:28870_2:N:0:TCTGCAAG+GCTGCACT
ATTCTGCTTCGACGGTAATTCCATAAGACCTTTCTTGGGGAGTAAAGAGGGTTTGAAAATTACGGAAC
CTTTCCTCGTTCTTCATGAAGATCTTCGAGTGTTTTATAGTGATTTAT
+
FFFFFFFFFFFFFFFFF:FFFFFFFFFFFFFFFFFFFFFFFFF:FFFFFFFFFFFFFFFFFFFFFFF::F
FFFFFFFFFFFFFFFFFFFFFFFFFFF:FFFFFFFFFFFF,FFFFFFFF
@A00721:190:HGJW7DSXY:4:2447:20799:24017_1:N:0:TCTGCAAG+GCTGCACT
CAAGTCGATTGATTTTGTTGACTACGATCTCTCTTGTAATAAGAAGTTCTTCGCAGTTGAATCTCCAC
CAGGTGGAGGGAAGACCACTGAACTTATAGCGACTTTTGTCGAATTTTTCGCAAAAGGTCTTGAAGTA
CGT
+
FFFFFFFFFFFFFFFFFFFFFF:FFFFFFFFF:FFFFFFFFFFFFFFFFF:FFFFFFFFFFFFFFFFFF
FFFFFFFFFFFFFFFFFFFFFFFFFFFFFFFFFFFFFFFFFFFFFFFFFFFFFFFFFFFFFFFFFFFFFF
FFF
@A00721:190:HGJW7DSXY:4:2555:19226:30561_2:N:0:TCTGCAAG+GCTGCACT
TCGTAATTCGCGAGAAGAGATAGTGACTAAAGTTTTGGCACTTCTAGTTCATCGCGGTATTTGTGAAA
TTAAGACAGACGTGAAAGTTATGATGAGTGATCGTATTAGAACAGCTG
+
FFFFFFFFFFFFFFFFFFFFFFFF:FFFFFFFFF:FFFFFFF:FFFFFFFFFFFFFFFF::FFFFFFFF
FFFFFFFFFFFF:FFFFFFFFFFFFFFFFFFFFFFFFF:FFFF:FFFFFF
@A00721:190:HGJW7DSXY:4:1361:4553:13761_2:N:0:TCTGCAAG+GCTGCACT
CTGAGGTCAAACCTGTGGACGTTCTTTTCATCGACGAATTATTCATGATTCACGCTGGTCAGACCCTT
TTTCTTACACATCTACTTAAGGCGGCTTATACCATAGGTTACGGTGAT
+
FFFFFFFFFFFFFFFFFFFFFFFFFFFFFFFFFFFFFFFFFFFFFFFFFFFFFFFFFFFFFFFFFFFFFF
FFFFFFFFFFF:FFFFFFFFFFFFFFF,FFFFF,FFFFFFFFFFFFFF
@A00721:190:HGJW7DSXY:4:1365:6451:23625_1:N:0:TCTGCAAG+GCTGCACT
GACGTGTGCTCTTCCGATCTCCAAGTCGCAAACTACTTCAACATCAACGACATCATTCTTGATGAGTG
TCGTTCTTATCGAAGTCTCAGTTATCGCTGCCCGGCCGACGTTTGTCGCGAACTCTCAGAGGTTTACG
GCA
+
FFFFFFFFFFFFFFFFFFFFFFF,:FFFFFFFFFFFFFFFFFFFF:FFFFFFFFFFFFFFFFFFFFFFFFF
FFFFFFFFFFFFFFFFFFFFFFFFFFFFFFFFFFFFFFFFFFFFFFFFFFFFFFFFFFF:FFFF:FFFFF
FFF
@A00721:190:HGJW7DSXY:4:1601:6406:2221_1:N:0:TCTGCAAG+GCTGCACT
AATTCGAGAGAGGACACTATGGAAATTAGACCTATCAGATGTCTTGACGACTTCGAATTTGAGGACGG

```
AGTTAAATATATTACTTTCACTCAAGGTGAAAAGTTTGAAATTAACAAAGTCCTTGGTAAGAGTAAGG
TTA
+
FFFF,FFFF,FFFFFF,FFFFFFFFFFFFF,FFFF:FFFFFF:FFFFFFFFFFFFFFFFFFFFFFFFF
FFFFFFFFFF:FFFFFFFFFFFFFFFFFFFFFFFFFFFFFFFFFFF:F:FF:FFF:FFFFFFFFFFFF
FFF
@A00721:190:HGJW7DSXY:4:1438:4399:27117_1:N:0:TCTGCAAG+GCTGCACT
GTTCAAGGTAATACTTACCAGCACGTTCGGCTGGTTAGAATTAAACCTCAAGATGACCCTGTTTTTGC
ATCACAAACACATATTACCGTTGCTCTATCACGACACGTTGAATCTTTACGATACTTTGTTATCAATG
TAA
+
FFFFFFFFFFFFFFFFFFFFFFFFFFFFFFFFFFFFFFFFFFFFFFFFFFFFFFFFFFFFFFFFFFFF
FFFFFFFFFFFFFFFFFFFFFFFF:FFFFFFFFFFFFFFFFFFFFFFFFFFFFFFFFFFFFFFFFFFF
FFF
@A00721:190:HGJW7DSXY:4:1555:11822:20212_2:N:0:TCTGCAAG+GCTGCACT
TGAGAGGATCAATCACAGTAAGGAACTGTCACGTTTAGCACGGGAGGTTGCAGATTCAACCCCTTATA
TCGAGGCTATCGAGGACGATGAGTATCTTGAAGTTGTTGACGACAAGC
+
FFFFFFFFFFFFFFFFFFFFFFFF,FFFFFFFFFFFF:FFFFFFFFFFFFFFFFFFFFFFFFFFFFFF
FFFFFFFFFFFFFFFFFFFFFFFFFFFFFFFFFFFFFFFFFFFFFFFF
@A00721:190:HGJW7DSXY:4:1552:26051:22999_1:N:0:TCTGCAAG+GCTGCACT
TAGAGGACCACCTGGATCTTCTACTGTCTCTGCCTTCCAAGATTTCTTAGAAGAGGTTATTCCTGGAA
CCACCTTAACGTCACTCGGTAATCTTTCAGCCGAATTAAGTACTACTGAGTTCAAGTCGGTTGTCGAT
CGA
+
FF:FFFFFFFFFFFFFFFF:FFFFFFFFFFFFFFFFFF:FFF:FFFFFFFFFFFFFFFFFF:FFFF:F
FFFFFFFFFFFFFFFFFFF,FFFFFFFFFFFFFFFFFFFFFFFFFF,FFFFFFFFFFFFFFF,FFFFFF
FFF
@A00721:190:HGJW7DSXY:4:2104:3685:35430_2:N:0:TCTGCAAG+GCTGCACT
GGCTAATACCTTTATAGGTAAGAAGCCCCTTGAGCCGCGTAGTCAGCGCGTTTAGCTGTATCATCAGT
CAGAGTATACCCGATAGGGTACCGAGTCTCCAAGAGAATATTTATTCT
+
:FFFFFFFFF::FFFFFFFFFFFFFFFFFFFFFFFFFFFFFFFFFFFFFFF:FFFFFFFFFFFFFFFF
FFFFFFFFFFFFFFFFFFFF:FFFFFFFFFFFFFFFF:F:FFFFFFFF
@A00721:190:HGJW7DSXY:4:1603:2510:28541_1:N:0:TCTGCAAG+GCTGCACT
ACGTTTTCACAACGGAAAGACATATCGCACCCGAAATTTTTGGGCAGAATGCTCTTAACGCGGTTCTT
GAACGGTGTTTTGATCAGTCTAAGTTGGATAAAGTTATAGGAGACTCAGTTAACCTGAGAGCTCCTCA
ATT
+
F:FFFFFFFFFF:FFFFF:FFFFFF:F::F,FF:,FFFFFF:FFFFFFFFF,FFFFF,FFF:FFF,FF
F:F:FFFFFFFFFFFFFFFFFFFFFFFFFFF:,FFFF,FFFFFF::FFFFFFFFF::FF:,FFFFFFFF
FFF
@A00721:190:HGJW7DSXY:4:1460:4797:22357_2:N:0:TCTGCAAG+GCTGCACT
AATAGGGACGCTAGTATACTTCGTAAAGTCGACAGCGAAATGGTCAATATTGATAATTGGACCGACTA
TATGACCTATTTTAAGCTCATGGTGAAAAGGGAAGCTAAGGTTAAACT
+
FFFFFFFFFFFFFFFFFFFFFFFFFFFFFFFFFFFFFFFFFFFFFFFFFFFFFFFFFFFFFFFFFFFF
FFFFFFFFFFFFFFFFFFFFFFFFFFFFF:FFFF:FFF:FFFFFFFFFF
@A00721:190:HGJW7DSXY:4:1376:4481:5149_2:N:0:TCTGCAAG+GCTGCACT
CATAACCCCGCACAGAATATCGTTTATCACGCAAAATTTATTAATGCAATTTTCGGACCTATCTTTAA
GGTTCTTGTGAGGAGAGTCAAACAGTGTCTTTTACCCAACATTTTTAT
+
FFFFFFFFFFFFFFF:FFFFFFFFFFFFFFFFFFFFFFFFFFFFFFFFFFFFFFFFFFFFFFFFFFF:
FFFFFFFFFFFFFFFFFFFFFFFFFFFFFFF,FFFFFFFFFFFFFFFFF
```

@A00721:190:HGJW7DSXY:4:1164:10728:5885_2:N:0:TCTGCAAG+GCTGCACT
CCAGTGACGATTTCTCCCGGAAGCTCTTCTCTCTCTTAGGAGGTCATGATGACTACTATAAGGGTGAA
ATCGATTTTTCGAAGTTTGACAAATCTCAAGATTTCTTTTGTAAAGCC
+
FFFFFFFFFFFFFFFFFFFFFFFFFFFFFFFFFFFFFFFFFFFFFFFFFFFFFFFFFFFFFFFFFFFFFF
FFFFFFFFFFFFFFFFFFFFFFFFFFFFFFFFFFFFFFFFFFFFFFFFF
@A00721:190:HGJW7DSXY:4:1209:8332:15013_2:N:0:TCTGCAAG+GCTGCACT
TATTCTTATTTAGGCTTCGATGCTGAATTTCTTGAACTTTGGAGTGCCAGTGAATATTTTTGTCACGC
CAGGAGTATCCATAACGATTTGTCATTTACCACCTTTGCTCAACGACG
+
FFFFFFFFFFFFFFFFFFFFFFFFFFFFFFFFFFFFFFFFFFFFFFFFFFFFFFFFFFFFFFFFFFFFFF
FFFFFFFFFFFFFFFFFFFFFFFFFFFFFFFFFFFFFFFFFFFFFFFFF
@A00721:190:HGJW7DSXY:4:1122:10230:4178_1:N:0:TCTGCAAG+GCTGCACT
TAACACATTCTTTGGTAATTGTATGATCACTCTTATGATGCTCTCTCTTTATTACGACCTTCGGAAGT
TTAGAGCCGTCGCTGTCGCCGGAGACGATTCGATCCTCTTTTCGGACGTACCGGTCCAGGATTATTCA
GAT
+
FFFFFFFFFFFFFFFFFFFFFFFFFFFFFFFFFFFFFFFFFFFFFFFFFFFFFFFFFFFFFFFFFFFFFF
FFFFFFFFFFFFFFFFFFFFFFFFFFFFFFFFFFFFFFFFFFFFFFFFFFFFFFFFFFFFFFFFFFFFFF
FFF
@A00721:190:HGJW7DSXY:4:1630:8648:16438_2:N:0:TCTGCAAG+GCTGCACT
CAGCGTACTTCTGCTCGCGATTTATTCTTTTTGTCAATGATTACATCTACTTCGTTCCTGATCCTTAC
AAGATCATCGTTAAGTTAGGTAAACCCCTAACCGATGTTACTCTTGTA
+
FFFFFFFFFFFFFFFFFFFF::FFFFFFFFFFFFFFFFFFFF:FFFFFFFFFFFFFFFFFFFFFFFFFFF
FF:FFFFFFFFFFFFFFFFFFFFFFFFFFFFFFFFFFFFFFFFFFFFFF
@A00721:190:HGJW7DSXY:4:1536:1380:26772_1:N:0:TCTGCAAG+GCTGCACT
TTTAAAGACTATAATAAATATCTCCACTACGAGGTTGTTGTTTGGCGATTAGCTCTTGCGACTAATAT
TCGATATTCGATTGAGGGTCGTTATACCTATTCGGCTATCTGTGCTTTGAATTGTATAAGATCAAATT
TTA
+
FFFFFF,F,FFFF,FFFFFFF:FF:FFF:FF:FFFFFFFFFFFFFFFF:,FF,F:F,FFFFFFFFFFF,F
FFFFFFFF:F,FFF,FF:FFFFF,FFFFFFFFFFFFFFFFF,FFFF:FFFFFFFFFF,FFF:,FFFFFFF
F:F
@A00721:190:HGJW7DSXY:4:2128:20175:7999_2:N:0:TCTGCAAG+GCTGCACT
CCTGATGTGGAGGGAGTTCTTGAAGCATCCCGACCTTTCCTTGTGAAAATTTTGATGCGGTTTCATAA
GATTATCAGTAATCCTGATTCTACTACGGATTCATGGACCTTCCATCC
+
FFFFFFFFFFFFFFFFFFFFFFFFFFFFFFFFFFFFFFFFFFFF:FFFFFFFFFFFF:FFFFFFFFFFFF
:FFFFFFFFFFFFFFFFFFFFFFFFFFF:FFFFFFFFFFF:,FFFFFFF
@A00721:190:HGJW7DSXY:4:1362:29731:1752_2:N:0:TCTGCAAG+GCTGCACT
GACGTATCTCAAAGATCGACTTCGAGACAGGTGATATTGTTGAGCGAGAGCGACCAAGATCTAAATTC
GGTAACGGTGCGTGGCGCTTTATTTTAAAAAGCTCCCTCCTCTCCCAA
+
FFFFFFFFFFFFFFFFFFFFFFFFFFFFFFFFFFFFFFFFFFFFFFFFFFFFFFFFFFFFFFFFFFFFFF
FFFFFFFFFFFFFFFFFFFFFFFFFFFFFFFFFFFFFFFFFFFFFFFFF
@A00721:190:HGJW7DSXY:4:1214:28212:2221_2:N:0:TCTGCAAG+GCTGCACT
ACTTGAGTCTTTTGATCAGTCTCTGACTTGACCATCTTTCATTTTAAATGACGATCATTAAGACTCTC
TTTCTTCTTTGTTTCCTTCTTTGCACTGATTTTTCCTCATCAGCTTGT
+
FFFFFFFFF:FFFFFFFFFFF,FFFFFFFFFFFFFFFFFFFFFFFFFFFFFFFFFF,FFFFFFF:FFFFF
F:,F:FF,,FF:FFFFFFFFFFFFFFFFF,FF:F,FFFFFFFFFF,FFF
@A00721:190:HGJW7DSXY:4:2143:18593:4507_1:N:0:TCTGCAAG+GCTGCACT
GTTTTTACGATAACAACTATCAGTTGATTAAGACTCTTCCGAAAGGCGAACTTTGGGAATTTGAAGCT

CAGGGTGAAGCACAACGTTTGACCGTGACGAACTGTGGTGTTCTTGATCTACGTTACGGATATACTCT
CCT
+
FFFFFFFFFF:FFFFFFFFFFFF:FFFFFFFFFFFFFFFFFFFFFFFFFFFFFFFFFFFFFFFFFFFFF
FFFFFFFFFFFFFFFFFFF,FFFFFFFFFFFFFFFFFFFFFFFFFFFFFFFFFFFFFFFFFFFFFFFFF
FFF
@A00721:190:HGJW7DSXY:4:1452:3378:11162_1:N:0:TCTGCAAG+GCTGCACT
ACACTACCACTTATTACGATATTTCTTTAGTCGATGGGTTTTCGACGCCTTTAAGCGTCTTCTGTTCA
TCGCAACCAGATGATATCTTCTATTCGAACTTATGTTTCGATCAGTGTTTCTTCTATCAGGAGGAAAA
CAC
+
FFFFFFFFFFFFFFFFFFFFFFFFFFFFFFFF:FFFFFFFFFFFFFFFFFFFFFFFFFFF:FFFFFFFFF
FFFFFFFFFFFFFFFFFFFFFFFFFFFFFFFFFFFFFFFFFFFFFFFFFFF:FFFFFFF:FFFFFFFFF
FFF
@A00721:190:HGJW7DSXY:4:2113:3893:7138_2:N:0:TCTGCAAG+GCTGCACT
TTTCTTCTAATGTGGCCTGAACTACGTTTCACTTTGTGTGTACTTGTCTCTATTTTAAGTTTTGTCTT
CCTTGTTTGTCTTATTCTATTAATCACTCATTTTAGTGAGATTATTAA
+
FFFFFFFFFFFFFFF:FFFFFFF:FFFFFFF,FFFFFFFFFFFFFFFFFFF:FFFFFFFFFFFFFFFF
FFFFFFFFFFFFFFFFFFFFFFFFF:FFFFFFFFFF:F:F:FFFFFFFF
@A00721:190:HGJW7DSXY:4:2249:19678:8797_1:N:0:TCTGCAAG+GCTGCACT
GTCTTCGACAACAGCAGGGGTTTTCGAATAGTGATAGCCACCCGCCTTACAGAGGGGCAGCAGCATGA
CCATAATTGGTATTGACTTTGGTACCACCTTTTCTACTGTCTGTCTCGCCTCTTCGAATGAAGTTTTA
ATG
+
F:,,FFFFFF:FFF:::,:,,FF:F:FF:,F,:FFFF,FFF,F:FFFFFFFFFF:F,:,:FFFFF:F:
FF:FFFF:FFFFFFFFFFFFFFFFF:FF,,FFFF:F,F,:FFFFFFFFFF:F,FFFF:FF:F,FFFFF:
FFF
@A00721:190:HGJW7DSXY:4:2105:15763:35462_2:N:0:TCTGCAAG+GCTGCACT
TGATAGTGAATTTATACCTACAATCATTGGTTTTTCTAATAATGATGACACTATTGTTTATGGATATG
ATGCTATTACTCATGATGGTTACGGTCAGGGAGGATTCTCAGTTTACA
+
FFF,FFFFFFFFFFFFFFFFFFF:FFFFFFFFF:FFF:FFFFFFFFFFFFFFF,FF:F,FFF:FFFFF
FFFFFFFFFFFFF:FFFFFFFFFFFFFFFFFFFFFF,FFFFFFFFFFFF
@A00721:190:HGJW7DSXY:4:1260:1199:35978_2:N:0:TCTGCAAG+GCTGCACT
CCGAAAGACGTGCTAAATTAAAACCATTGTACGATGTCGTCTGTCTCGGCGATCACTTTTCACTAAGA
ATTGGTCCTACTTTTGGACAAGGCCAACTTCGTAATCTTCATGAGATC
+
FFFFFFFFFFFFFFFFFFFFFFFFFFFFFFFFFFF:FFFFFFFFFFFFFFFFFFFFFFFFFFFFFFF:FF
FFFFFFFFFFFFFFFFFF:FFFFFF:FF:FFFFFFFF:FF,FFFFFFF
@A00721:190:HGJW7DSXY:4:1368:20003:31031_2:N:0:TCTGCAAG+GCTGCACT
TATCTCTTTGGTTGTTAGGGATTTTGAGAGAGTGAAAGGCTTCAATTGTAGTGGTTTGGTCGTTTCAG
TACCCAGTCAGTATACCTCTACGCAAAGATATTTCATGAAAACTACAG
+
FFFFFFFFFFFF:FFFFFFFFFFFFFFFFFFF:FFFFF:F:FFFFFFFFFFFFF:FFFFFFFFFFFF:
FFFFFFFFFFFFFFFF,FFFFFFFFFFFFFFFFFFFFFFFFFFFFFFFF
@A00721:190:HGJW7DSXY:4:2314:19759:6997_2:N:0:TCTGCAAG+GCTGCACT
ATTACCTGTCTTTCATATTATGAATGAACCTTCGGCCGCTCTCTTTGCTTCGATGTTGGATGTCAAGA
AGGGTTCAGAATGGGATTCCTACGTAGTTTATGACTTTGGTGGTGGTA
+
FFFFFFFFFFFFFF:FFFFFFFF:FFFFFFFFFFFFFFFFFFFFF:FFFFFF::FFFFFFFFFF,FFF
FFFFFFFFFF:F:FFFFFFFFFFFFFFF:FFFFF:::FFFFFFFFFF:
@A00721:190:HGJW7DSXY:4:2527:7636:12179_1:N:0:TCTGCAAG+GCTGCACT
TCTTACGTTACGGTAATTACTATAGTGTTATTTATTCTGGTGGTGATGATAATCTTGGAGGTCGTGAC

```
GTTGATGAAGCTATAAGAGCGTTCTTATTACGGCGCACTTCTTTACCCGCCACCACACAACTTTCAGT
TTC
+
FFFFFFFFFFFFFFFF:FFFFFFFFFFFFFFFFFFFFFFFFF:FFFFFFFFFFFFFFFFFFFFFFFFFF
FFFFFFFFFFFFFFFFFFFFFFFFFFFFFFFFFFFFFFFFFFFFFFFFFFFFFFFFFFFFFFFFFFFF
FFF
@A00721:190:HGJW7DSXY:4:2664:19497:34350_2:N:0:TCTGCAAG+GCTGCACT
TGTTACTGGAAAACCCTCGTCAATACTTTTCAATAATATACGTCTTGAACTCACTTATGACGATCTAG
TGAAATTAACGCGTCCTTTTCTGGAACGTAGTTTTACTATTATGAACA
+
FFFFFFFFFFFFFFFFFFFFFFFFFFFFFFFFFFFFFFFFFFFFFFFFFFFFFFFFFFFFFFFFF:FF
FFFFFFFF:FFFFFFFFFFFFFFFFFFFFFFFFFFFFFFFFFFFFFFF
@A00721:190:HGJW7DSXY:4:1647:19859:4695_1:N:0:TCTGCAAG+GCTGCACT
ACGTGTGCTCTTCCGATCTAACTTACCCTGATTCCGGCCGGTGGTAGCTCATTTCTACCAGGGGCTAT
TACTCTTGCTGAAACAGCTATTAAAGCTGTTGAGCGGGTAATTACACCTAAACGAGCTCGATCTGCCA
TCG
+
FFFFFFFFFFFFFFFFFFFFFFFFFFFFFFF,FFFFFFFFF,FF:FFFFFFFFFFFFFFFFFFFFFFF
FFFFFFFFFFFFFFFFFFFFFFFFFFFFFFFFFFFFFFFFFFFFFFFFFFFFFFFFFFFF:FFFFFFF
:FF
@A00721:190:HGJW7DSXY:4:1647:8296:27132_2:N:0:TCTGCAAG+GCTGCACT
CACTCTTTTCTGCTACTTTAACGGAGCGTAGTGTACTCTTCGTCGATTGTATCAACAGTAACATTACT
TCTAATAAAGGTCTCTATCAAGCTCGTATTATCGTCGCTGCTGGATCT
+
FFFFFFFFFFFFFFFFFFFFFFFFFFFFFFFFFFFFFFFFFFFFFFFFFFFFFFFFFFFFFFFFFFFF
FFFFFFFF,FFFFFFFFFFFFFFFFFFFFFFFFFFFFFFFFFFFFFF:FF
@A00721:190:HGJW7DSXY:4:2303:6858:35102_1:N:0:TCTGCAAG+GCTGCACT
AAGGCTAAACTCCATGGTAATTATGAAGACTGTGATTTATCTTTCACTCTGGCCTTGTATGCGGGTGT
TTCTAATCGTAACATTAATAATTTCAATCTTCTTAAAAAGAAGATAGAGAGTCATACTTTGGGCTATC
GTT
+
FF,FFF,FFFFFFF,,FFF,FFFFFF:F,FFFF,F:FFFF:FFFFFFF,F,F:,,FFFFFF,F,FFFF
FF:FF:F,FF,:,:FF,FFF,:FFF,FFFFF,FF,:F,::F:::FFFF::FF::FF,FFFF:,:FFF:
FFF
@A00721:190:HGJW7DSXY:4:2133:14100:25097_2:N:0:TCTGCAAG+GCTGCACT
GATAGCCAATATTGAAGTGGACACCTCTGGTAGTATCGAAGTAAAGATAACCAATAAGGAAGGAGCTT
ATACTATGGTTGAATCAACTAAGCATACTACTTTTGCTTTGATTACTC
+
FFFFFFFFFFFFFFFFFFFFFFFFFFFFFFFF:FFFFFFFFFFFFFFFFFFFFFFFFFFFFFFFFFFF
FFFFFFFFFFFFFFFFFFFFFFFFFFFFFFFFFFFFFFFFFFFFFFFF
@A00721:190:HGJW7DSXY:4:1508:18548:1391_1:N:0:TCTGCAAG+GCTGCACT
TCCGATCTGTTAAAGAGTCGATTAAACAAGTTGTACTTATCAATGATTTTATCGCTGGTTGTTATCTC
GAAAAGTCTCTTCTAAAAGGAACGTCAGTTCTAACCCAAAGAGAATATCTAAAATATTTACAAAATCT
T
+
FFFFFFFFF:FFFFFFFFFFFFFFFFFF:FFFFFFFFFFFFFFFFFFFFFFFFFF:FFFFFFFFFFFFFF
FFFFFFF:FFF:FFFFFFFFFFFFFFFFF:FFFFFFFFFFFFFFFFFFFFFFFFFFFFFFFFFFFFFFF
F
@A00721:190:HGJW7DSXY:4:1257:7220:35039_2:N:0:TCTGCAAG+GCTGCACT
AGACGTTGCTTGTCGCTTCCTCCTTTAATGGCACTACCTCGGTACAATCTTACTTGGAACGAAGGCGA
CCTGTTCCGGAGTTTTTACGGGATCCGGAATGTTCTGTCTATATTAGG
+
FFFFFFFFFFFFFFFFFFFFFFFFFFFFFFFFFFFFFFFFFFFFFFFFFFFFFFFFFFFFFFFFFFFF
FF,FFFFFFFFFF:FFFFFFFF,FFFF,FF:,FFFFFFFFFFFFFFFF
```

```
@A00721:190:HGJW7DSXY:4:1255:18629:35258_2:N:0:TCTGCAAG+GCTGCACT
TCGAACGTCAACGTTCACCGAGTTTCGTTTCGTACCTTGATGCTGACTTTCGTCAATATGATTTGATC
TGGTTGCTCAATTCTAAGCGTGTTCCGCTTAGTGCGAGGCCTCTCTTC
+
FFFFFFFFFF,FFFFFFFFFFFFFFFFFFFFFFFFFFF:FFFFFFFFFFFFFFFFFFFFFFFFFFFFF:F
FFFFFFFFFFFFFFFFFFFFFFFFFFFFFFF:FFFFFFFFFFFFFFFFFF
@A00721:190:HGJW7DSXY:4:1338:18367:6214_2:N:0:TCTGCAAG+GCTGCACT
AGTTTACGAACTTGACTCTACTATACGTGACATACTAAATACTAGATATTTGGATGATCTTGATAAAG
AACTTCATGAATTTTTCAGAGGATGAAAAAATTTTCTTCCTTGGATTTTA
+
FFFFFFFFFFFFFFFFFFFFFFFFFFFFFFFFFFFFFFFFFFFFFFFFFFFFF:FFFFFFFFFFFFFFFF
FFFFFFFFFFFFFFFF:FFFFFFFFFFFFFFFFFFFFFFFFFFFFFFFFF
@A00721:190:HGJW7DSXY:4:1662:32208:24518_1:N:0:TCTGCAAG+GCTGCACT
TACAGGAGGCAGTCTTCTGATCGTAAGAAGTTTATAAGGAACATATCCATTTGTTGTAATAGGGTCAT
CAACTTAGAACTCGAAACTCTAAGTGGTAATACTGTCTTTCCAAGCTTACCTGTATCTTCAATGAAAT
TGG
+
:FFFFFFFFFFFFFFFFFFFFFFFFFFFFFFFFFFFFFFFFFFFFFFFFFFFFFFFFFFFFFFFFFFFFF
FFFFFFFFFFFFFFFFFFFFFFFFFFFFFFFFFFFFFFFFFFFFFFFFFFFFFFFFFFFFFFFFFFFFFF
FFF
@A00721:190:HGJW7DSXY:4:2341:28980:10191_2:N:0:TCTGCAAG+GCTGCACT
GTGGGCTTTCCGTAAAGGAAGATCTGTTATCGACGGTTTATCGTTTACGAAGTATCACTCCACCGGTG
AATGGTTTAAGAATACTATAAATAGAATAGCTATTACTACTAATGGCG
+
FFFFFFFFFFFFFFFFFFFFFFFFFFFFFFFFFFFFFFFFFFFFFFFFFFFFFFFFFFFFFFFFFFFFFF
FFFFFFFFFFFFFFFFFFFFFFFFFFFFFFFFFFFFFFFFF:FFFFF:,F
@A00721:190:HGJW7DSXY:4:1447:15293:32393_1:N:0:TCTGCAAG+GCTGCACT
TAGTGACATCTGGTCAATTTCGTTTTTAGGTGGTCTTTATTCTATTATTGATTTTTATGAGATTGATA
CGTTAACAGAAGATGATGTTAGAGTAGAATTCCTGAAGAGTCCTTCCGTAACCAACTTCTTGACAATT
TTC
+
FFFFFFFFFFF:FFFFFFFFFFFFFFFFFFFF:FFFFFFFFFFFFFFFFFFFFFFFFFFFFFFFFFFF:F
FFFFFFFFFFFFFFFFFFFFFFFFFFFFFFFFFFFFFFFFFFFFFFFFFFFFFFFFFFFFFFFFFFFFFF
F:F
@A00721:190:HGJW7DSXY:4:2674:3125:23594_2:N:0:TCTGCAAG+GCTGCACT
CTAGGATTATGTCTGTGTTGAAAGACTTTCTACCGATAAGGGTCACTAGTCTTACACAATTAAGAAAT
AATCCTACATTGACTGATTATCTAAAGATTGAAGGAAAATTTTATCCT
+
FFFFFFFFFFFFFFFFFFFFFFFFFFFFFFFFFFFFFFFFFFFFFFFFFFFFFFF:FFFFFFFFFFFF:F
FFFFFFFFFFFFFFFFFFFFFFFFFFFFFFFFFFFFFFFFFFFFFFFFFF
@A00721:190:HGJW7DSXY:4:2546:14931:20932_1:N:0:TCTGCAAG+GCTGCACT
GACTTTCCTACGATACAAATGATAACTTCTAATTTACGTTTGCTACTCAGAGCTGGTCCTCTCAGCAA
AGGGATGCATGGTGTTATCTCCTCAGGAGTCCCGGCATTTGATAATAATGACGAGACTTTCGTCATAA
TCG
+
FFFFFFFFFFFFFFFFFFFFFFFFFFFFFFFFFFFFFFFFFFFFFFFFFFFFFFFFFFFFFFFFFFFFFF
FFFF,FFFFFFFFFFFFFFFFFFFFFFFFFFFFFFFFFFFFFFFFFFFFFFFFFFFFFFFFFFFFFFFFF
FFF
@A00721:190:HGJW7DSXY:4:1651:19072:23375_1:N:0:TCTGCAAG+GCTGCACT
GATTACACGGAACCAGCGCATCTCGTGTCGAGACTCGACCCGATTATATTGTATTAACTTATAAAAGT
GAGGAGACCATTATCTTGCTGAAATATATAGAAGATTATTTTGACAGAATCAATAAGAATCATCGATT
CTC
+
,:FF:FF,,,,F:FF,F,:F:FF,F,:F,:FF,FF:,F,F::FFF,,F,,FF,F,FF,,,FFF:F,,F
```

```
:FF,::F:FFFF,:FFFF,,,FFFF,FFFF,FFFFFFF,FFFF:,F,FF,:FF:FF,F:FFFFF,:F:
:,:
@A00721:190:HGJW7DSXY:4:2473:15167:24533_2:N:0:TCTGCAAG+GCTGCACT
GGATCCACTCCTCGAGTTATCATGATGTTAGTATGTTATATACTGATTTACAACGATATAGGAGTAAT
CTACGAGAAGATTCTAGAATTCTTAATTATAATATTCAACCTCAAAAG
+
FFFFFFFFFFFFFF:FFF:FFFFFFFFFFFFFFFFFFFFFFFFFFFFFFFFFFFFFFFFFFFFFFFFF
FFFFFFFFFF:FFFFFFFFFFFFFFFFFFFFFFFFFFFFFFFFFFF,FF
@A00721:190:HGJW7DSXY:4:2165:17725:9173_2:N:0:TCTGCAAG+GCTGCACT
GAGATCACACATTTTGAGAACTAACTATGTTATGTTCTCTGATCATTTTGACAATCTCTTCTTATAAT
AAATAAATAAATAATATTACTAATGGCAAATAGTGGAAAGGCTCCAGT
+
FFFFFFFFFFFFFFFFFFFFFFFFFFFFFFFFFFFFFFFFFFFFFFFFFFFFFFFFFFFFFFFFFFFF
FFFFFFFFFFFFFFFFFFFFFFFFFF:FFFF:FFFFFFFFFFFFFFFFF
@A00721:190:HGJW7DSXY:4:2654:18846:11240_2:N:0:TCTGCAAG+GCTGCACT
TACGATGACGTGAGAATAGGTGAAGGGATAGTTTTTACCTCGACCAACATTAAGGCGGCGGCGACTAA
TCTTAAAGCTTCACCCAAAATGAAGGACACTTCGAAACTGAATAATGA
+
FFFFFFFFFFFFFFFFFFFFFFFFFFFFFFFFFFFFFFFFFFFFFFFFFFFFFFFFFFFFFFFFFFFF
FFFFFFFFFFFFFFFFFFFFFFFFFFFFFFFFFFFFFFFFFFFFFFFF
@A00721:190:HGJW7DSXY:4:2244:30083:12258_1:N:0:TCTGCAAG+GCTGCACT
GGCTCTAAAGGACACTATACAATATCCAGACGACGACGAAGCTTATACTACTTTAATTTTGGGTATTA
TGTGGAGATATCGTGATATCAGTACGTCCAGATCCTCTGCTTTTACCGATAACGATAAATTCGTCATC
AAA
+
FFFFFFFFFFFFFFFFFFFFFFFFFFFFFFFFFFFFFFFFFFFF:FFF:FFFF:FFFFFFFFFFFFF
FFFFFFFFFFFFFFFFFFFFFFFFFFFFFFFFFFFFFFFFFFFFFFFFFFFFFFFFFFF:FFFFFFFF
FFF
@A00721:190:HGJW7DSXY:4:2475:11849:18599_1:N:0:TCTGCAAG+GCTGCACT
TTGGTGCTTAGGGATATAATGAATTCAGCACCACATTTTGGTCAAGTCAATCCGTTGAGAAAATTTCT
TAGAGGATATTCGCCATTGTGGTACGAATTGGTCCAACATTTACCACCAAATTCTTTTGATACAACTC
TAG
+
FFFFFFFFFFFFFFFFFFFF:FFFFFFFFF::FFFFF:FFFFFFFFFFFFFF:FFFFFFFFF,FFFFF
FFFFFFFFF:FFFFFFFFFFFF:FF:F:FFFFFFFFFFFFF:F:FF:FFFFFFFFFFFF:FFFFFFFF
FFF
@A00721:190:HGJW7DSXY:4:1340:4779:16877_2:N:0:TCTGCAAG+GCTGCACT
CGGAATACGCTTGCTATTCACCAGATTGGTTCGAACCCGTTGATGCTATGTCAGAAGAAGCTATTCAA
GCTTGTCGTTTAAAGACTGCAGAGGCTGTCAAACAAGCCCAGACTCAA
+
FFFFFFFFFFFFFFFFFFFFFFFFF,FFFFF:FFFFFFFFFFFFFFF,:FFFFFFFFFFFFFFF:FFFF
FFFFFFFFFFFFFFFFFFFFFFFFFFFFFFFFFFFFFFFFFFFFFFFF
@A00721:190:HGJW7DSXY:4:1544:7717:33677_1:N:0:TCTGCAAG+GCTGCACT
CAAAGGTTTTGAATTTAGCTCAACTGGGGAGACGTGCTTAAATGGGTGATAAAAATTATTTAGAGATC
TTTTCTACTTACAAAGGACAATTCAGTTTCATTAATTCTACGGAAAAGTCCGGAGTATATTGTATGTT
AAT
+
FFFFFFFFFFFFFFFFFFFFFFFFFFFFFFFFFFFFFFFFFFFFFFFFFFFFFFFFFFFFFFFFFFFF
FFFFFFFFFFFFFFFFFFFFFFFFFFF:FFFFFFFFFFFFFFFFFFFFFFFFFFFFFFF:FFFFFFFFFFF
FFF
@A00721:190:HGJW7DSXY:4:1308:13693:14967_1:N:0:TCTGCAAG+GCTGCACT
AATAGTTCTACCGATACGTAGTGAACGCGTTCGTAGAACCAAGTACTTTCCGTCTGAAGAGTTTAGAG
CTGCTATTGATTATGTAACCGATAGGAGTTGTATAGTTGAAGATGTGGCTTTAGACGATAATCCTTTA
AGG
```

```
+
FFFFFFFFFFFFFFFFFFFFFFFFFFFFFFFFFFFFFFFFFFFFFFFFFFFFFFFFFFFFFFFFFFFFFF
FFFF,FFFFFFFFFFFFFFFFFF:FFFFFFFFFFFFFFFFFFFFFFFFFFFFFFFFFFFFFFFFFFFFFF
FFF
@A00721:190:HGJW7DSXY:4:1242:22932:1720_1:N:0:TCTGCAAG+GCTGCACT
GAAGATTTTCCCACAGATTTGTCTACGTTGCAGATCGCCACTCCAAACACTATATACATTATAAAATA
TAAAAATAAGGGAGTCATAACTTTTGTTACCAAATTCTTGGCTTTCAACGAAGTCTCTGATTTTATTA
ATC
+
FFFFFFFFFFFFFFFFFFFFFFFFFFFFFFFFFFFFFFFFFFFFFFFFFFFFFFFFFFFFFFFFFFFFFF
FFFFFFFFFFFFFFFFFFFF:FFFFFFFFFFFFFFFFFFFFFFFFFFFFFFFFFFFFFFFFFFFFFFFFF
FFF
@A00721:190:HGJW7DSXY:4:2129:30047:2080_1:N:0:TCTGCAAG+GCTGCACT
CGATCTCTCGGTCATTTACTCTGTACTTGATCTACGTCGTTTAACTGTCGGAACAACCTTTACTTAGA
ATGACTCTATCTTTTCAGACACTTCTTATATTATTTACAATAGTTAATATCATATTAGTAATACTCAC
TAT
+
FFFFFFFFFFFFFFFFFFFFFFFFFFFFFFFFFFFFFF:FFFFFFFFFFFFFFFFFFFFFFFFFFFFFFF
FFFFFFFFFFFFFFFFFFFFFFFFFFFFFFFFFFFFFFFFFFFFFFFFFFFFFFFFFFFFFFFFFFFFFF
FFF
@A00721:190:HGJW7DSXY:4:2361:20030:10347_2:N:0:TCTGCAAG+GCTGCACT
TTTTTCTTAGCTAATAGATTAATAAAGGATGAGTACGAAAACCAACTCTTCGTTACGAGACCTGATGG
TAGAATAGATTTAGTCTCTACACCCATCTCAACGAGGATAAAGCGAGT
+
FFFFFFFFFFFFFF:FFFFFFFFFFFF:F:FFFFFFFFFFFFFFFFFFFFFFFFFFFFFFFFFFFFFFFF
FFFFFFF,FFFFFFFFFFFFFFFFFFFFFFFFFFF:FFFFFFFF:FFFF
@A00721:190:HGJW7DSXY:4:1121:32732:11804_2:N:0:TCTGCAAG+GCTGCACT
ACACGAAGAGATCATCCGCAAACGACTCGAAACCGAGACGCCGTAGATCAAAGTCCCTTCTAACTTTA
GATTATGAATCCTGCCTTTACTCATGTTATAGCAGTTATTTCTGTTGT
+
FFFFFFFFFFF:FFFFFFFFFFFFFFFFFFFFFFFFFFFFFFFFFFFFFFFFFFFFFFFFFFFFFFFF,F
FFFFFFFFFFFFFFFFF:FF:FFFF,,FFFFFFFFF,FFF,F,FFFF:F
@A00721:190:HGJW7DSXY:4:1228:17400:25332_1:N:0:TCTGCAAG+GCTGCACT
TTTATAACCTTCTTATACTTTTGGGTATGTTTTGTGAAAAACATGAGATACCCAAGTCTATCGAAGAC
ATTATCTCTTATGTACTTATTGTAACGATCATTCTTTCGAGTTTTGTCATTCTTATTCTTTTATTCCT
TTA
+
FFFFFFFFFFFFFFFFFFFFFFFFFFFFFFFFFFFFFFFFFFFFFFFFFFFF:FFFFFFFFFFFFFFFFFF
FFFFFFFFFFFFFFFFFFFFFFFFFFFFFFFFFFFFFFFFFFFFFFFFFFFFFFF:FFFFFFFFFFFFF
FFF
@A00721:190:HGJW7DSXY:4:1161:14127:12461_2:N:0:TCTGCAAG+GCTGCACT
TTTATAATGCTTTTCGATAGTGATTGTTATCAATTTAAATACATTACTGTTTCAAATCATCTTTACTC
ACAGACTCCGAAAATGGTTTCAAATGAGGTCAAAGCTCTATTTATTAA
+
FFFFFFFFFFFFFFFFFFFFFFFFFFFFFFFFFFFFFFFFFFFFFFFFFFFFFFF:F:FFFFFFFFFFFF
FFFFFFFFFFFFFFFFFFFFFFFF:FFF:FFFFFFFFFFFFFFFFFFFFF
@A00721:190:HGJW7DSXY:4:1251:16450:7185_1:N:0:TCTGCAAG+GCTGCACT
ATCCGATTCTTCGAGGGTATCACTACCACTGATCTGCCGACTTTAGTCAAGGAACTTCAATTCCTTCG
ATGTTTATATTTTTCTATAAGGGAAAATAGCAATAATCAAATTTCGACAAATTTTAGAACTTTATTTC
AGA
+
FFFFFFFFFFFFF:FFFFFFFFFF,FFF:FFFFFFFFFFFFFFFFFFFFFFFFFFFFFFFFFFF,FFFFFFF
FFF:FFFFFFFFFFFF::FFFFFFFFFFFFFFFFFFFFFFFFFFFFFFFFFFFFFFFFFFFFFFFFFFFF
FFF
```

```
@A00721:190:HGJW7DSXY:4:1316:16649:19304_2:N:0:TCTGCAAG+GCTGCACT
ATCACCTTGGTGTGATCTCCCGGAGGATCTTTTCAATAAGATGTATGATGATAATCTACTATTTTTAA
ATAGTGATATTCAGCGTATACTTGAATACTTTATCAAAGATTTGGCTT
+
FFFFFFFFFFFFFFFFFFFFFFFFFFFFFFFFFFF:FFFFFFFF:FFFFFFFFFFFFFFFFFFFFFF
FFFFFFFFFFFFFFFFFFFFFFFFFFFFFFFFFFFFFFF:FFFFFFFF
@A00721:190:HGJW7DSXY:4:1147:14308:15687_1:N:0:TCTGCAAG+GCTGCACT
CAAAAAGTTGGAAGAGGTCTATGATATTTATTTTACTTATACTTTAAATAGCACCGATTATAACTTGA
CTGTTGATAATATTACAATAGCTCTTTCTCAGAAGATTAGAGAAGTAACTCGGTGAACACGTCGGTAG
CAT
+
FFFFFFFFFFFFFFFFFFFFFFFF:FFFFFFF:F:FFFFFFFFFFFFFFFFFFFFFFFFFFFFFFFFF
F:FFFFFF:FFFFFFFFFFFFFF:FFFFFFFFFFFFFFFFFFFFFFFFFFFFFFFFF:FFFFFFFFFF
FFF
@A00721:190:HGJW7DSXY:4:1170:9064:21418_1:N:0:TCTGCAAG+GCTGCACT
AGAACTGAAAAAGATTCTTTTCGAAAAGAAACCTATAAGTTACAAACGATTCTTCGACTATATTAAAG
AAATAAAAATACTTCAAGTACCTGAACTTTACATTTTTAATGGTATGGTATAATGTGTCTTACAAATA
GAA
+
F:FFFF,FFF:FF,FF:FFFFFFFFFFFFFF,FF:FFFFFFFFFFFFFFFFFFFFFFFFFFFFF:FFF
FFFFFFFFFFF:FFFFFFFFFF:FF:F:FFFFFFFFFF,FFFFFFFFFFFFF:FFFFFFFFF:FFFFF
FFF
@A00721:190:HGJW7DSXY:4:1470:31123:7952_2:N:0:TCTGCAAG+GCTGCACT
TAATGAGAGTGTATTCCTTAATATTCTAGTAACCGCTATGTGGGCATTACTAGGATTTATCACACTCC
TAAATTTGATAGCAATTTATGTTTTAAATGCTAGGAAAATTTTAGAAA
+
FFFFFFFFFFFFFFFFFFFFFFFFFFFFFFFFFFFFFFFFFFFFFFFFFFFFFFFFFFFFFFFFFFFF
FFFFFFFFFFFFF:FFFFFFFFFFFF:,FFF,FFFFF,:F:FFFFFFF
@A00721:190:HGJW7DSXY:4:1670:18511:12477_1:N:0:TCTGCAAG+GCTGCACT
TATAGTTCAAGGAGTACTTAATTCAATAGCTTTAGTATTATTTACTATCGCCTTTATTGAAAATTGTA
TAAGAGTTAATTGCTTACACAAAAGTGACGCGCGAAGGAATCTTAATTGATTGAAGCTAGGCGTTGGG
GA
+
FFFFFFFFFFFFFFFFFFFFFFFFFFFFFFFFFFFFFFFFFFFFFFFFFFFFFFFFFFFFFFFFFFFF
FFFFF:FFFFFFFFFFFFFFFFFFFFFFFFFFFFFFFFFFFFFFFFFFF:FFFFFFFFFFFFFFFF
FF
@A00721:190:HGJW7DSXY:4:2110:29170:9612_1:N:0:TCTGCAAG+GCTGCACT
ATACAAGATATATTTACAGATTATTTAGTGTAGTCCAAAGTTTGTATAAATTTATTTGATTTTATATA
TATATCCCACATATATATATATATAAAATCCTATAAATTTGTAC
+
F,F:,,,FF,F,F,,F,F:,,FFF,:FF,,,,:,,,F,,,FFF,FFFF,,F,FFF,:,,F,,F,FFFF
FFFF,,:FF,:FFFFFFFFFFFFFFFFFFFFFFFFFFFFFFFFFF
@A00721:190:HGJW7DSXY:4:2448:20302:13886_2:N:0:TCTGCAAG+GCTGCACT
AACATACCATACACTCCCCAAACATAACATACCATAACCTACCATGACCAATCATCGCTCCAACTGGC
ACGCCAGACGTAGGGTCAAACCTACTACCGCCGGTCGGCAGATCGGCA
+
FFFFFFFFFFFFFFFFFFFFFFFFFFFFFFFFFFFFFFFFFFFFFFFFFFFFFFFFFFFFFFFFFFFF
FFFFFFFFFFFFFFFFFFFFFFFFFFFFFFFFFFFFFFFFFFFFFFFF
@A00721:190:HGJW7DSXY:4:1578:31015:27899_2:N:0:TCTGCAAG+GCTGCACT
ACCCGCTGTGCAGAATAACCTTCCCAAATCTAGATCTGCACAGAAGAAGGCTGAAGGTCGTTACCGGA
CGACCACTCAAAGGAAACGGGCTGTTGCCCAGAGGAAAGCAGCGACCA
+
FFFFFFFFFFFFFF:FFFFFFFFFFFFFFFFFF:FFFFFFFFFFFFFFFF:FFF::FFFFFFFFFFFF
FFFFFFFFFF:FFFFFFFFFFFFFFFFFFFFFFFFFFFFFFF:FF:FFFF
```

```
@A00721:190:HGJW7DSXY:4:1403:11433:12399_2:N:0:TCTGCAAG+GCTGCACT
GTAACATTGACGGCAGTCCCGTCATCTCTGTGGGAACTGAGGCAGTACCTCAGTCAAAAGTCACATTC
ACCACTCCCATGCCAATCAATGATGTGACTATACAGTTCGGCCATTTC
+
FFFFFFFFFFFFFFFFFFFFFFFFFFFFFFFFF:FFFFFFFFFFFFFFFFFFFFFFFFFFFFFFFF
FFFFFFFFFFFFFFFFFFFFFFFFFFFFFFFFFFFFFFFFFFFFFFFF
@A00721:190:HGJW7DSXY:4:1152:32570:29841_1:N:0:TCTGCAAG+GCTGCACT
ATACGTCGTGGGAGGTACTGAAGCCTTCCCCTCCGCTGGCACGAAGAACACTCCCAAGCCAATCAATA
GTTCTTCTGACCAGAATGAAAAGGGGTTTACCAGGGTAGTCTCTAAGTCTACCAGGAAGAGGGGAAAT
GTC
+
FFFFFFFFFFFFFFF:FFF:FFFFFFFFFFFFFFFFFFFFFFFFFFFFFFFFFFFFF:FFFFFF,FFF:
FFF,FFFFFFFFFFFFFFFFFFFFFFFFFF,FFFFFFFFFFFFFF:FFFFFFFFFF:FFFFFFFF:FFF
FFF
@A00721:190:HGJW7DSXY:4:1245:24162:7513_1:N:0:TCTGCAAG+GCTGCACT
AAAGGGAAAGGTCCATCAGGACCTAAGAAGGGGAAATTACCCCTAATTCTGAAGGAAGATCTCCCACC
ATCTGGGGTTGGGAATAAGAAATCTGCACGGGCCAAAGAGGCCGCTGCTACGAAGTCGCTGAAGGCGG
CTT
+
F:FF,FFFFF:,FFFFFFFFFFFFFFFFFFFF:FFFFFFFFFFFFFFFFFFFFFFFFFFFFFFFFFFF
FFFFFFFFFFFFFFFFFFFFFFFFFFFFFFFFFFFFFFFFFFFFFFFFFFFFFFFFFFFFFFFFFFFF
FFF
@A00721:190:HGJW7DSXY:4:1346:17128:28557_2:N:0:TCTGCAAG+GCTGCACT
TGTAAAGTTGCGCCTGCCGACCCCATCTTTGAGACCAAGACTCTCTACAGTAGGTTCGTGCCTACTAT
GAGCAAATCTGTTGAATTCGTCACTGAGGACGAAGGTGAGAGGACTTT
+
FFFFFFFFFF:FFFFFFFFFFFFFFFFFFFFFFFFFFFFFFFFFFFFFFFFFFFFFFFFFFFFFF:FF
FFFFFFFFFFFFFFFFFFFFFFFFFFFFFFFFFF:FFFFFFFFFFFFF
@A00721:190:HGJW7DSXY:4:2312:18367:7153_1:N:0:TCTGCAAG+GCTGCACT
CAAACTGAGAGCAGTCGCTGGGGAAAGAGAATTGCGTGGGTTGAAGGCCGACGCTTCGAGCTTGAGTG
TGACGAGGCCGGTGACGTTTTTGTCAACGGCGTTTGGAGGATTTTATCCGCGCTTAACTATGGGGCGA
AGA
+
FFFFFFFFFFFFFFFFFFFFFFFFFFFFFFFF:FFFFFFFFFFFFFFFFFFFFFFFFFFFFFFFFFFF
FFFFFFFFFFFFFFFFFF,FFFFFFFFFFFFFFFFFFFFFFFFFFFFFFFFFFFFFFFFFFFFFFFFF
FFF
@A00721:190:HGJW7DSXY:4:1550:22525:14951_1:N:0:TCTGCAAG+GCTGCACT
AGTCTTGAAGGACTTAGGTTGGCATATTTATGCCGACGATAATAAGCATGTCACCCTTAAGCACCGAG
GTAAGCTCGGGACTTACAAGAGAGAGTGTGAGAGAGTCGCTCTCACTATTTTTGCCCAGAATGCGGGC
TTT
+
FFFFFFFFFFFFFFFFFFFFFFFFFFFFFFFFFFFFFFFFFFFFFFFFFFFFFFFFFFFFFFFFFFFF
FFFFFFFFFFFFFFFFFFFFFFFFFFFFFFFFFFFFF:FFFFFFFFFFFFF:FFFFFFFFFFFFFFFFF
:FF
@A00721:190:HGJW7DSXY:4:2440:25355:21355_2:N:0:TCTGCAAG+GCTGCACT
GCTCCTTCTGTGGGTGAGGGTTTTTGTTATATTCACTGGTTCTGGGAGGCTGCTTTCCTCCATAATTA
CAACCTCTCTTTAGAGAGCATTAGAAAGATCGGAAGAGCGTCGTGTAG
+
FFFFFFFFFFFFFFFFFFFFFFFFFFFFFFFFFFFFFFFFFFFFFFFFFFFFFFFFFFFFFFFFFFFF
FFFFFFFFFF:FFFFFFFFFFFFFFFFFFFFFFFFF,FFFFFFFF:FFFF
@A00721:190:HGJW7DSXY:4:2167:17418:34663_1:N:0:TCTGCAAG+GCTGCACT
CACACTTTTCTTGAAAATTTTTCCTTTCCTCCGTCATTGGGGATTTCGATGTGAGAAGGTGGAAGGTG
TCAAGGGTCTTCTTCATGCTGATCTTTCAGGACCTAATCACATCATGATTGATAGTTACGGCTCGTAT
CGA
```

+
FFFFFFFFFFFFFFFFFFF:FFFFF,FFFFFFFFFFFFFFFFFFFFFFFFFFFFFFFFFFFFFFFFFF
FFFFFFFFFFFFFFFFFFFFFFFFFFFFFFFFFFFFFFFFFFFFFFFFFFF:FFFF:FFFFFFFFFF
FFF
@A00721:190:HGJW7DSXY:4:1642:30373:31485_2:N:0:TCTGCAAG+GCTGCACT
AAGTTCTCCGAAATTAAAGACGGGGGGATTAAGAGTCCAGCTGTAACTGTCATTTCAGAGAGGGGAGC
TCTGAAAAGGAAACTTCGTCAGAAGATGAGGAAGTCTAAGAGGAGAGA
+
FFFFFFFFFFFFFFFFF:FFFFFFFFFFFFFFFFFFFFFFFFFFFFFFFFFFFFFFFFFFFFFFFFFF
FFFFF:FFFFFFFFFFFFFFFFFFFFFFFFFFF::FFF::FFFFFFFFFF
@A00721:190:HGJW7DSXY:4:1368:11849:23046_1:N:0:TCTGCAAG+GCTGCACT
CTCTAGAGAAGATCTCCCCACCTGGAGAAGGCAAGTGGAGAGTCCAGAAGTTGATGAAGAAGGATTAT
TTGAAGCAGGGGTTACCACTTCCTAAGGTGGATGAGGGAGGTTGTTTCTTTGGAGTTTTAGATAATCT
TTT
+
FFFFFFFFFFFFFFFF,FFFFFFFFFFFFFFFFFFFFFFFFFFFFFFFFFFFFFFFFFFFFFFFFFFF
:FF,FFFFFFFFFFFFFFFFFFFFFFFFFFFFFFFFFFFFFFFFFFFFFFFFFFFFFFFFFFFFFFFF
FFF
@A00721:190:HGJW7DSXY:4:1519:10484:12665_2:N:0:TCTGCAAG+GCTGCACT
CCTTGAGAAGACAAGCCACATTAAGATATAATTTAGGTCAACTTGGGGTTTCGAATGTCATTAGTGCC
TTACGATTGAAAGAGGTCCTTTTGAGTATCGACAGATCTTTCGATCTT
+
FFFFFFFFFFFFFFFFFFFFFFFFFFFFFFFFFFFFFFFFFFFFFFFFFFFFFFFFFFFFFFFFFFFF
FFFFFFFFFFFFFFFFFFFFFFFFFFFFFFFFFFFFFFFFFFFF:FFFF
@A00721:190:HGJW7DSXY:4:2511:6045:1438_2:N:0:TCTGCAAG+GCTGCACT
ACGTGGCAGAATTCGGTAACTTACCATATTGTTCCTTCCGGGAAGTTGTGGGATTTAACTTTGACTCA
ACTACTCCGATCAGATCCGGATGGTTTGATTGGTATGGACTACTCGAC
+
FFFFFFFFFFFFFFFFFFFFFFFFFFF,FFFFFFFFFFFFFFFFFFFFFF::FFFFFFF:FFFFFFFFFF
FFFFFFFFFFFFFFFFFFFFFF,FF,,FFFFFFFFFFFF:FFFFFFFFF
@A00721:190:HGJW7DSXY:4:1325:18032:34569_1:N:0:TCTGCAAG+GCTGCACT
TAGGGTCATGGCAGATACTACCGATAAGGTGAAAAATATGCAGGTCAAAGATTCTGTAGCTCTGAGGG
CGGTTGAGGGAGCCGTTTTGGCCGACTTTAACTATTCTAAGAGTCTTTATGATTCAAAACCTATCGTT
GTC
+
FFFFFFFFFFFFFFFFFFFFFFFFFFFFFFFFFFFFFFFFFFFFFFFF:FFFFFFFFFFFFFFFFFFF
FFFFFFFFFFFFFFFFFFFFFFFFFFF:FFFFFFFFFFFFFFFFFFFFFFFFFFFFFFFFFFFFFFFF
FFF
@A00721:190:HGJW7DSXY:4:1512:3441:4413_1:N:0:TCTGCAAG+GCTGCACT
TACAGCGGCTTATTGTCGCGCAGTTTCCAGAATTGCGGTTGAAGTTCACCCATTCTATCTTGAATGCT
CACGCCGTCGCAGCCATATCGAGACTATGTTATAATCACTTGTTAAAGTACAGATTTAGTGATTTAGA
TAT
+
FFFFFFFFFFFFFFFFFFFFFFFFFFFFFFFFFFFFFFFFFFFFFFFFFFFFFFFFFFFFFFFFFFFF
FFFFFFFFFFFFFFFFFFFFFFFFFFFFFFFFFFFFFFFFFFFFFFFFFFFFFFFFFFFFFFFFFFFF
:FF
@A00721:190:HGJW7DSXY:4:1368:10402:27806_1:N:0:TCTGCAAG+GCTGCACT
GGAGGCGATATTCGTTTTCATATCCAAAACTCCTTGAATGGAGTACATATTTGCAACCCTATTCTTGA
TGTGAAAGATGCATCGAGGTATACGTCTAGGAACATTGATTGGGAACGTACCTACTCACGAGACGTCA
TGA
+
FFFFFFFFFFFF:FFFFFFFFFFFFFFFFFFFFFFFFFFFFFFFFFFFFFFFFFFFFFFFFFFFFFFF
FFFFFFFFFFFFFFFFFFFFFFFFFFF:,FFFFFFFFFFFFFFFFFFFFFFFFFFFFFFFFFFFFFFF
FFF

@A00721:190:HGJW7DSXY:4:1437:32642:22294_1:N:0:TCTGCAAG+GCTGCACT
ATGTTTCGTTCTGTTACAAGAACGCTTTGGATTGTGACCATAAAGCCTCAGTTGCGATAGCTGTAGAG
GTATACGATATCTCTCTTGATGATGTAGCCACTATAATGCTTAAGAGAGAGATTGATGTATGTCATTT
CGT
+
FFFFFFFFFFFFFFFFFF:FFFFFF:FFFF:FFFFFFFFFFF,FFFFFFFFFFFFFFFF:FFFFFFFF
FFFFF:F:FFFFFFFFFFFFFFFFFFFFFFFFFFFFFFFFFFFFFFFFFFFFFFFFFFFFFFFFFFFF
FFF
@A00721:190:HGJW7DSXY:4:1522:7401:4100_1:N:0:TCTGCAAG+GCTGCACT
GAATTATTGGACACGATGAAAAATGACATACCCGTTCCTGACCTTGGTATCGTTATCCGTCATGGAGA
TGATGCCGCATCACTTGATTACTATATGCCCGAAGGATTGGGTTATTCTCACAACAAAGATAAGATTT
TAT
+
:FFFFFFFFFFFFFFFFFFFFFFFFFFFFFFFFFFFFFFFFFFFFFFFFFFFFFFFFFFFFFFFFFFF
FFFFFFFFFFFFFFFFFFFF:FFFFFF:FFFFFFFFFFFFFFFFFFFFFFFFFFFFFFFFFFFFFFFF
FF:
@A00721:190:HGJW7DSXY:4:1659:12029:9862_1:N:0:TCTGCAAG+GCTGCACT
GTTTTCCTTAAGGGCGGACAATTGTTCCATTGTGAACTCTTGGGGACTAGATTGAGCATTAATGAGTT
CCGAATAACTCGTAATAAATCTTTCTCTAGTGGACAATTCAGAATACCTATTTCCGTACCAAGAGCCA
CTG
+
FFF:FFFFFFFFFFFF:FF,FFFFFF:FFFFFFFFFFFFFFFFFFFFFFFFFFFFFFFFFFFFFF,:F
FFFFFFFFFFFFFFFFFFFFFFFFFFFFFFFFFFFFFFFFFFFFF,FF:FFFFFFFFFFFFFFFFFFF
FFF
@A00721:190:HGJW7DSXY:4:1548:10890:8359_2:N:0:TCTGCAAG+GCTGCACT
TGGAAGAAGCGAGGTTCGTTCCTCAATAAAAATTTTTCTCAAGAGATTATGGTTGATAAAGTTTTCTT
CATAAGGGCTATTCAGTACGTCATGGGGATCACTTCTTCGGTCAATGA
+
FFFFFFFFFFFFFFFFFFFFFFFFFFFFFFFFFFFFFFFFFFFFFFFFFFFFFFFFFFFFFFFFFFFF
FF:FFFFFFFFFFFFFFFFFFFFFFFFFFFFFFFFFFFFFFFFFFFFF
@A00721:190:HGJW7DSXY:4:1262:17372:26256_1:N:0:TCTGCAAG+GCTGCACT
AATATGTTACGACATGGCTTCGTAACAACAGTGCGAGGATTGTCATCTCGGGGAAAGTGATTCACCAA
GATGTTCATATAGATACGAGATACCTAGAGAAGGTCGCAGCGATCTTACTCGTTATCGGAGCTAAGAA
GAA
+
FFFFFF,FFFFFFFFFFFFFFFFFFFFFFFFFFFFFFFFFFFFFFFFFFFFFFFFFFFFFFFFFFFFF
FFFFFFFFFF,FFFFFFFFFFFFFFF:FFFFFFFFFFFFFFFFFFFFFFFFFFFFFFFFFFFFFFFFF
FFF
@A00721:190:HGJW7DSXY:4:1668:24795:15969_2:N:0:TCTGCAAG+GCTGCACT
GTTATGCTCGCAAACTTAACGTTAGTACCGGAGGTGATCTCTCTGCTTCTGCGTTACTGAGAGAAAAG
ATTCAGGACGCCTACGGTGACTTTATGGACAAGATGCAAGAGATCGGA
+
FFFFFFFFFFFFFFFFFFFFFFFFFFFFFFFFFFFFFFFFFFFFFFFFFFFFFFFFF:FFFFFFFFFF
,FFFFFFFFFFFFFFFFFFFFFFFFFFFFFFFFF:FFFFFFFFFFFFFF
@A00721:190:HGJW7DSXY:4:2234:8621:3959_2:N:0:TCTGCAAG+GCTGCACT
TTTAGGTTTAGTAGGTGATCTTGACGTCGATTCTTTGTATAGGGAGTGTGAGAAGACAACGAATCTTG
AAACTACGGTCCTTATCAAAGAAAATGGTGAACCGGTCTCTAAATCTT
+
F:FFFFFFFFFFFFFFFFFFFFFFFFFFFFFFFFF:FFFFFFF:FFFFFFFFFFFFFFFFFFFFFFFF
FFFFFFFFFFFFFFFFFFFFFFFFFFFFFFFFFFFFFFFFFFFFF:FF
@A00721:190:HGJW7DSXY:4:2446:26431:24158_2:N:0:TCTGCAAG+GCTGCACT
AGATTGAAGAACACGTCAAGAAGTCGCTCGGCGTTGTTCTTGAAAACCAAGTTAAGAAGGAGGCTGAT
CATGGAGTCTTTGTACCTTCTGTCAAACTTCGAGGTTCAGCTACGAGA
+

```
FFFFFFFFF:FFFFFFFFFF:FFFFFFFFFFFFFFFFFFFFFFFFFFFFFFFFFFFFFFFFFFFFF
FFFFFFFFFFFFFFFFFFFFFFFFFF:FFFFFFFFFFFFFFFFFFFFF
@A00721:190:HGJW7DSXY:4:1533:16369:32409_1:N:0:TCTGCAAG+GCTGCACT
TGGATTGAAGGCTGGCTCTAAGGGAGACTCCTTCTTTTTGAATGTTGTAAAGAATTGGGTTGAGAAGT
GTGCTTTACAAGGAGTTAGACTCTTCTTCAAGTTTTTAGATCTTCTTGATAATCTTAAAGAAAAATTT
TCG
+
FFFFFFFFFFFFFFFFFFFFFFFFFFFFFFFFFFFFFFFFFFFFFFFFFFFFFFFFFFFFFFFFFFFF
FFFFFFFFFFFFFFFFFFFFFFFFFFFFFFFFFFFFFFFFFFFF:FFFF:FFFFFFFFFFFFFFFFFF
FFF
@A00721:190:HGJW7DSXY:4:1446:3640:32847_2:N:0:TCTGCAAG+GCTGCACT
CTGTACTTACATTCTTTACGCCAGATTCTTCATGGGGCGACGCTGTTTCTCTTGGTATTTCTGAAATA
GTTTATCAGAGCGTCATTTTCCTTTGGGATCTTTCCTCTGGTAAGGGA
+
FFFFFFFFF:FFFFFFFFFFFFFFFFFFFFFFFFFFFFFFFFFFFFFFFFFFFFFFFFFFFFFFFFFF
FFFFFFFFFFFFFFFFFFFFFFF:FFFFFFFFFFFFFFFFFFFFFFFF
@A00721:190:HGJW7DSXY:4:1520:8829:12712_2:N:0:TCTGCAAG+GCTGCACT
ATTCTTCTCACTCTTGACGAGAATTATTACTGTTTTCTCTACCCATAACTTCTTTTTCAAAAATGCGG
ATGGGTCTACTGATGTTTTCCTACAGAGTGCAGTCGTTTCAACACTTA
+
FFFFFFFFFFFFFFFF:FFFFFF:FFFFFFFFFFFFFFFFFFFFFFFFFFFFFFFFFF:FFFFFFFFF
FFF:FFFFFFFFFFFFFFFF,FFFFFFFFFFFFFFFF:FFFF:F:::F
@A00721:190:HGJW7DSXY:4:1624:5475:14043_2:N:0:TCTGCAAG+GCTGCACT
GGAAAATAATTGGTGATGAAAATACGTATGTCGGTGGGGTTCTCAAGGCTCTCATCGCCACTGGGATT
CTAAGGGGTTCCATAAGATATTATCTGACATTGTGGTTCAATGATCAG
+
FFFFFFFFFFFFFFFFFFFFFFFFFFFFFFFFFFFFFFFFFFFFFFFFF:FFFFF:FFFFFFFFFFFFFF
FFFFFFFFFFFFFFFFFFFFFFFFFFFFFFFFFFFFFFFFFFFFF:FF
@A00721:190:HGJW7DSXY:4:1534:8567:16423_2:N:0:TCTGCAAG+GCTGCACT
TGGTCCTTACGTCTTAAGTAATTTGGATGTTGTCGAAAACTATCAGGTTCTTGTCACGATTCTTAAAA
GAAGTTTATTAAAACTTCAGAGTCTCTTGGAAGTCCAGGTAGCTAATG
+
FFFFFFFFFFFFFFFFFFFFFFFFFFFFFFFFFFFFFFFFFFFFFFFFFFFFFFFFFFFFFFFFFFFF
FFFFFF,:FFFFFFFFFFF,FFFFFFFFFFFFFFFFFFFFFFFFFFFF
@A00721:190:HGJW7DSXY:4:1453:27444:27602_2:N:0:TCTGCAAG+GCTGCACT
GCCACAGACTTTCTGGGAAAAGAGACCTCTGTTGGTAGAGGAGTTGAGAGAATTACTCAGATTCAATC
TGGCATCACTGAACGTCTACAACGAGTTAGGGAGTGGCTAGAGATCGG
+
FFF:FFFFF:FFFFFFFF:FFFFFFFFFFFFFFFFFFFFFFFFFFFFFFF,F,FFFFFFFFFFFFFF:
FFFFFFFFF:FFFFFFFFFFFFFFFF,FFFFFFF:FFFFFFFFFFFF
@A00721:190:HGJW7DSXY:4:1119:30138:27164_1:N:0:TCTGCAAG+GCTGCACT
CAATACCTTCCACTGAGTTCATCACCCAGAGTTTCGACGTCGCGGAAAACGATGAAGATTTCCACACT
GTTCGAGAATCTTCTGATGCTGAATTTGAGGAAAATTTTTCGGAAGTTTTTGGTATAAATCCTGATGG
CTC
+
FFFFFFFFFFFFFFFFFFFFFFFFFFFFFFFFFFFFFFFF:FFFFFFFFFFFFFFFF:FFFFFFF:FF
FFFFFFFFFFFFFFFFFFFFFFFFFFFFFFFFFFFFFFFFFFFFFFFFFFFFFFFFF:FFFFFFFFFF
FFF
@A00721:190:HGJW7DSXY:4:1276:5231:1658_1:N:0:TCTGCAAG+GCTGCACT
TAAAACCTTCTTTTATCATTGCAACAGGACTTTGAGAAGACTGTTGAAATGGACAAAATCTGGGATCA
TAGGTATGTGTCACGCTACACTCGAAATATGGGCCCTCATAAGGAAGTATAGTTGGCGTCTTGTCGTT
GAC
+
FFFFFFFFFFFFFFFFFFFFFFFFFFFFFFFFFFFFFFFFFFFFFFFFFFFFFFFFFFFFFFFFFFFF
```

FFFFFFFFFFFFFFFFFFFFFFFFFFF:FFFFFFFFFFFFFF:FFFFFFFFFFFFFFFFFFFFFFFFFF
FFF
@A00721:190:HGJW7DSXY:4:1356:20076:5228_2:N:0:TCTGCAAG+GCTGCACT
AGTTAGATCATTTGTTCAGGAAGATCTCTACAGAGATTATCGAGAGTTGTGATCTAGTTCTAAATTTA
GGTCGACGGACTTTAGCAACTTTAGATAAATTCTTTCCGGGCTCAATG
+
FFFFFFFFFFFFFFFFFFFFFFFFFFFFFFFFFFFFFFFFFFFFFFFFFFFFFFFFFFFFFFFFFFF:
FFFFFFFFFFFF:FFFFFFFF:FFFFFFFFFFFFFFFFFFFFFFFFFF
@A00721:190:HGJW7DSXY:4:1308:29225:4977_1:N:0:TCTGCAAG+GCTGCACT
TCTGACCATCGCAAAGAAGACTTTATTAGTTCATGAAGTTCTTACACTTCTCGCGACTAATGTCAGCT
TCACTACAAAGGCGTTACGTAAAGTAGTTTTCACGCTTTATCTTAAAGCATTACCACCAAATTTAGCA
CTT
+
FFFFFFFFFF,FFFFFFFFFF:FFFFFFFFFFFFFFFFFFFFFFFFFFFFFFFFFFFFFFFFFFFFFF
FFFFFFFFFFFFFFFFFFFFFFFFFFFFFFFFFFFFFFFFFFFFFFFFFFFFFFFFFFFFFFFFFFFF
F:F
@A00721:190:HGJW7DSXY:4:1250:22218:8093_1:N:0:TCTGCAAG+GCTGCACT
CTCGCCATATCAGATTAGAGACATGCCAGTTAGAGTTCTTCTAACCAGGACTGATGGTGAGATTCTTT
TACCTTTTGGAGGTCCAGGAACCTTTACTCCTAGTGTCTTTTCTAGAGTCCAAGGACGTTTCCGTCGG
AGG
+
FFFFFFFFFFFFFFFFFFFFFFFFFFFFFFFFFFFFFFFFFFFFFFFFFFFFFFFFFFFFFFFFFFFF
FFFFFFFFFFFFFFFFFFFFFFFFFFFFFFFFFFFFFFFFFFFFFFFFFFFFFFFFFFFFFFFFFFFF
FFF
@A00721:190:HGJW7DSXY:4:2242:21368:9502_1:N:0:TCTGCAAG+GCTGCACT
GAGACCGCTCAATAACACTCTATTGAATCCCTTTTCCACGATGAAAGCTCTTTATGAGGTGCTCCATG
CAAATTTACAGTTCCTCTTTGGTGATATCGCCTCTGTCCTTTCTCCTTTAATCGTGAGGTTACGAGAG
TTT
+
FFFFFFFFFF,FFFFFFF:F:FFFFFFF:F:FFFF:F,:FFF:FF,,FFFF,F:FFFF:,:FF:F,FF
F,FFFF,,F,FFFFFFFFFFFF:FFFFF:FFFFFF:FFF,:FFF::::,::FFF,,:FF:::FFFF,F
FFF
@A00721:190:HGJW7DSXY:4:1408:26115:14653_1:N:0:TCTGCAAG+GCTGCACT
GTATTTTGATAAATTTGTTGGAATCGATAATGATACTGACGAAGGTTCTATTACCACCCTTGTCATTG
ATACAAACATTCTAGGTGATGCTTTCTTAGAGGCTCGTCTAGCCGCTGAGATTCGTCTTACCTATTTA
CAA
+
FFFFFFFFFFFFFFFFFFFFFFF:FFFFFFFFFFFFFFFFFFFFFFFFFFFFFFFFFFFFFFFFFFFF
FFFFFFFFFFFFFFFFFFFFFFFFFFFFFFFFFFFFFFFFFFFFFFFFFFFFFFFFFFFFFFFFFFFF
FFF
@A00721:190:HGJW7DSXY:4:1408:29297:2691_2:N:0:TCTGCAAG+GCTGCACT
TTTGAGTGATAACGATTTAGTACTGGGAAATGGTGGTAGTCAGTTTACTACTTTTCTTTCCCTCCCCT
TTTTCGATTGGATTCGACGGGGTCTTCGTCTCACGACTAATTTGATAA
+
FFFFFFFFFFFFFFFFFFFFFFFFFFFFFFFFFFFFFFFFFFFFFFFFFFFFFFFFFFFFFFFFF:FF
FFFFFFFFFFFFFFFFFFFFFFFFFFFFFFFFFFFFF:FFFFFFFFFFF
@A00721:190:HGJW7DSXY:4:1339:28908:24752_2:N:0:TCTGCAAG+GCTGCACT
TACTTATGAAGGAGGTATTACTTCGGAAGTTCTTAATGTAGGTCTCCTAGTACTTAAGGGTTTATGTT
TCGGTTTCAGCTGGATGGATCTTTTGATGGTATGTTTCACATTGCTTG
+
FFFFFFFFFFFFFFFFFFFFFFFFFFF:FFFF,FFFFFFFFFFFFFFFFFFFFFFFFFF:FFFFFFFFF
FFFFFFF:FFFFF:FF:FFF:FFFFFFF:FFFFFFFFFFFFFF,F,FF
@A00721:190:HGJW7DSXY:4:1606:1118:4617_2:N:0:TCTGCAAG+GCTGCACT
TGGACTTGGGAAGAGATTAAACATAAAATCCAGCGATTCTATTATGTCGCTAAAGCCTGTCCAGTTGT

CGATGATGTTCGGGCTGTGGGGATTAACTTCCCTCGTGAAACTTACGA
+
FFFFFFFFFFF:FFFFFFFFFFFFFFFFFFFFFFFF:F:FF:FFFFFFFFFFFFFFF:FFFFFFF::FF
FFFFFFFFFFFFFFFFF:FFFFFFFFFFFFFFFFFFFFFFFFF:FFFFF
@A00721:190:HGJW7DSXY:4:1272:13078:4820_1:N:0:TCTGCAAG+GCTGCACT
CACAAGGATTACTAAGTCTGAGATCTGCCACTTCTAATTTTAGGAAACGTCGCGAAAAGAAAGTTCAT
GAGGATGTCTTGGTCGAAAAATATCGCCGGGAACTTCTAATAGAGGATCCTGATGACATTATTTCAAC
GGA
+
FFFFFFFFFFFFFFFFFFFFFFFFFFFFFFFFFFFFFFFFFFFFFFFFFFFFFFFFFFFFFFFFFFFF
FFFFFFFFFFFFFFFFFFFFFFFFFFFFFFFFFFFFFFFFFFFFFFFF:FFFFFFFFFFFFFFFFFFF
FFF
@A00721:190:HGJW7DSXY:4:1146:22064:26396_2:N:0:TCTGCAAG+GCTGCACT
AGTTCTACCACAGAAACCGTCTCAGGAACTGTCTCCACTGATTCGGAATTAGAGAAAGACGACCTTCT
TGTGGGGACTGAGACCTCTGAATTTTCAGGGTGCCCTGATGAGGACGT
+
FFFFFFFFFFFFFFFFFFFFFFFFFFFFFFFFFFFFFFFFFFFFFFFFFFFFFFFFFF:FFFFFFFFF
FFFFFFFFFFFFFFFFFFFFFFFFFFFFFFFFFFFFFFFFF:FFFFFF
@A00721:190:HGJW7DSXY:4:1262:23637:13495_1:N:0:TCTGCAAG+GCTGCACT
ACTCTGAGACTTCTAAGAGTGTTTCAAAAGACCAGAAACGTAACAAGAAGAATAAAGGTAGGAAGGGG
AGAAAACAGAACAAGCGAGCGGTTGAAGAGAAAACTGATATGGTTGCGGCTGATCGTACCCCAATTCC
TGC
+
FFFFFFFFFFFFFFFFFFFFFFFFFFFFFFFFFFFFFFFFFFFFFFFFFFFFFFFFFFFFFFFFFFFF
FFFF:FFFFFFFFFFFFFFFFFFFFFFFFFFFFFFFFFFFFFFFFFFFFFFFFFFFFFFFFFFFFFFF
FFF
@A00721:190:HGJW7DSXY:4:1544:28103:6386_1:N:0:TCTGCAAG+GCTGCACT
TCTCGGCGATACCACTGATCTTCCAGAGTTACGTAGTGGTCGTTCTTTTGGTGATTTTATCAAAGAGA
AAGTTTCTGCTAGTCGCGACGATAGTATTTATCGGAGTGATATTAACAAGTTAAACTTCGAACTGACC
ACT
+
FFFFFF:FFFFFFFFFFFFFFFFFFFFFFFFFFFFFFFFFFFFFFFFFFFFFFFFFFFFFFFFFFFFF
FFFFFFFFFF:FFFFFFF:FFFFFFFFF::,FFFFFFFFFFFF:FFF:FFFFFFFFFFFF,FFFFFF
FFF
@A00721:190:HGJW7DSXY:4:1554:25708:34710_2:N:0:TCTGCAAG+GCTGCACT
TTGAAGTACCGCAACATGGATCAGTTTTCCAAAAAGCTATTAATAGCAGTTGCGGGGCTATAATACCG
CCTCCAGATTACTCTAAACTGAATCCACTCTCTTGGGATATAAATGAG
+
FFFFFFFFFFFFFFFFFFFFFFFFFFFFFFFFFFFFFFFFFFFFFFFFFFFFFFFFFFFFFFFFFFFF
FFFFFFFFFFFFFFFFFFFFFFFFFFFFFFFFFFFFFFFFFFFFFFFF
@A00721:190:HGJW7DSXY:4:2225:29993:16861_1:N:0:TCTGCAAG+GCTGCACT
AGAGTTTTGGAACTGTGTAATGTCCTACGTACTGTTCAAGGTGTTTGGGCTTCTCTAAAGAACAGCAA
CTTTCAACATGAATTAGTTATTGAAAGTCTTGACCCTCACGTCTCACTTTATCACCTTGGAGATGGTA
AAG
+
FFFFFFFFFFFFFFFFFFFFFFFFFFFFFFFFFFFFFFFFFFFFFFFFFFFFFFFFFFFFFFFFFFFF
FFFFFFFFFFFFFFFFFFFFFFFFFFFFFFFFFFFFFFFFFFFFFFFFFFFFFFFFFFFFFFFFFFFF
FFF
@A00721:190:HGJW7DSXY:4:1264:13738:11663_1:N:0:TCTGCAAG+GCTGCACT
CTTCGACGGTAATTCCATAAGACCTTTCTTGGGGAGTAAAGAGGGTTTGAAAATTACGGAACCTTTCC
TCGTTCTTCATGAAGATCTTCGAGTGTTTTATAGTGAGTTATTCTTGCGAAGGTTCAAGTCGATTGAT
TTT
+
FFF:FFFFFFF,FFFFFFFFFFFFFFFFFFFFFFFFFFFFFFFFFFFFFFFFFFFFFFFFFFFFFFFF

FFFFFFFFFFFFFFFFFFFFFFFFFFF:FFFFFFFFFFFFFF:FFFFFFFFFFFFFFFFFFFFFFFFFF
FF:
@A00721:190:HGJW7DSXY:4:1148:13358:17581_2:N:0:TCTGCAAG+GCTGCACT
AATAAGAAGTTCTTCGCAGTTGAATCTCCACCAGGTGGAGGGAAGACCACTGAACTTATAGCGACTTT
TGTCGAATTTTTCGCAAAAGGTCTTGAAGTACGTATTGTCACTGCTAA
+
FFFFFFF:FFFFFFFFFFFFFFF:FFFFFFFFFFFFFFFFFFFFFFFFFFFFFFF,FFFFFFFFFFFF
FFFFFFFFFFFFFFFFFFFFFFFFF:FFFFFFFFFFFFFFFFFFFFFFFF
@A00721:190:HGJW7DSXY:4:2435:9263:1939_2:N:0:TCTGCAAG+GCTGCACT
CGAGAAGAGATAGTGACTAAAGTTTTGGCACTTCTAGTTCATCGCGGTATTTGTGAAATTAAGACAGA
CGTGAAATTTATGATGAGTGATCGTATTAGAACAGCTGATTCTTATTG
+
FFFFFFFFFFFFFFFFFF:FFFFF:FFFFFFFFFFFFFFFFFFFFFFFFFFFFFFFFFFFFFFFFFFF
FFFFFFFFFFFFFFFFFFFFFFFFFFFFFFFFFFFFFFFFFFFFFFFFFF
@A00721:190:HGJW7DSXY:4:1402:23023:17190_2:N:0:TCTGCAAG+GCTGCACT
CTGAGGTCAAACCTGTGGACGTTCTTTTCATCGACGAATTATTCATGATTCACGCTGGTCCGACCCTT
TTTCTTACACATCTACTTAAGGCGGCTTATACCATAGGTTACGGTGAT
+
FFFFFFFFFFFFFFFFFFFFFFFFFFFFFFFFF:FFFFFFFFFFFFFFFFFFFFFFFF:FF,FFFFFFF
FFFFFFFF:FFFF:FFFFFFFFFFFFFFFFFFFFFFFFFFFFFFFFFFF:
@A00721:190:HGJW7DSXY:4:1414:29758:8500_1:N:0:TCTGCAAG+GCTGCACT
GACGTGTGCTCTTCCGATCTCCACGTCGCAAACTACTTCAACATCAACGACATCATTCTTGATGAGTG
TCGTTCTTATCGAAGTCTCAGTTATCGCTGCCCGGCCGACGTTTGTCGCGAACTCTCAGAGGTTTACG
GCA
+
FFFFFF:FFFFFFFFFFFFFFFF,FFFFFFFFFFFFFFFFFFF:FFFFFFFFFFFFFFFFFFFFFFFFF
FFFFFFFFFFFF,FFFFFFFFFFFFFFFFFFFFFFFFFFFFFFFFFFFFFFFFFFFFFFFFFFFFFFFF
FFF
@A00721:190:HGJW7DSXY:4:2468:32624:28119_1:N:0:TCTGCAAG+GCTGCACT
AATTCGAGAGAGGACACTATGGAAATTAGACCTATCAGATGTCTTGACGACTTCGAATTTGAGGACGG
AGTTAAATATATTACTTTCACTCAAGGTGAAAAGTTTGAAATTAACAAAGTCCTTGGTAAGAGTAAGG
TTA
+
FFFFFFFFFFFFFF:FFFFFFFFFFFFFFF:FFFFF:FFFFFFFFFFFFFFFFFFFFFFFFFF:,F:FF
FFFFF::FFFFFFFFFFFFFFFFFFFFFFFFFFF:FFFFFFFFFFFFFFFFFFFFFFFFFFFFFFFFFF
FFF
@A00721:190:HGJW7DSXY:4:1509:23529:33567_1:N:0:TCTGCAAG+GCTGCACT
GTTCAAGGTAATACTTACCAGCACGTTCGGCTGGTTAGAATTAAACCTCAAGATGACCCTGTTTTTGC
ATCACAAACACATATTACCGTTGCTCTATCACGACACGTTGAATCTTTACGATACTTTGTTATCAATG
TAA
+
FFFFFFFFFFFFFFF:FFFFFFF:FFFFFFFFFFFFFFFFFFFFFFFFFFFFFFFFFFFFFFFFFFFFF
FFFFFFFFFFFFFFFFFFFFFFFFFFFFFFFFFFFFFFFFFFFFFFFFFFFFFFFFFFFFFFFFFFFFF
FFF
@A00721:190:HGJW7DSXY:4:2365:10655:5447_2:N:0:TCTGCAAG+GCTGCACT
TGAGAGGATCAATCACAGTAAGGAACTGTCACGTTTAGCACGGGAGGTTGCAGATTCAACCCCTTATA
TCGAGGCTATCGAGGACGATGAGTATCTTGAAGTTGTTGACGACAAGC
+
FFFFFFFFFFFFFFFFFFFF:FFFFFFFFFFFFFFFFFFFFFFFFFFFFFFFF:FFFFFFFFFFFFFFF
FFFFFFFFFFFFF:FFFF:FFFFFFFFFFFFFFFFFFFFFFFFFFFFF:F
@A00721:190:HGJW7DSXY:4:1569:30273:5071_1:N:0:TCTGCAAG+GCTGCACT
TAGAGGACCACCTGGATCTTCTACTGTCTCTGCCTTCCAAGATTTCTTAGAAGAGGTTATTCCTGGAA
CCACCTTAACGTCACTCGGTAATCTTTCAGCCGAATTAAGTACTAATGAGTTCAAGTCGGTTGTCGAT
CGA

+
FFFFFFFFFFFFFFFFFFFFFFFFFFFFFFFFFFFFFFFF:FFFFF:FFFFFFF:FFFFFFFFFFFFFFFF
FFFFFFFFFFFFFFFFFFFFFFFFFFFFFFFFFFFFFFFFFFFFFFFFFFFFFFFFFFFFFFFFFFFFFFF
FFF
@A00721:190:HGJW7DSXY:4:2107:12147:31391_2:N:0:TCTGCAAG+GCTGCACT
GGCTAATACCTTTATAGGTAAGAAGCCCCTTGAGCCGCGTAGTCAGCGCGTTTAGCTGTATCATCAGT
CAGAGTATACCCGATAGGGTACCGAGTCTCCAAGAGAATATTTATTCT
+
FFFFFFFFFFFFFFFFFFFFFFFFFFFFFFFFFFFFFFFFFFFFFFFFFF:FFFFFFF,FFFFFFFFF
FFFFFFFFFFFFFFFFFFFFFFFFFFFFFFFFFFFFFFFFFFFFFFFF
@A00721:190:HGJW7DSXY:4:1674:31819:28416_1:N:0:TCTGCAAG+GCTGCACT
ACGTTTTCACAACGGAAAGACATATCGCACCCGAAATTTTTGGGCAGAATGCTCTTAACGCGGTTCTT
GAACGGTGTTTTGATCAGTCTAAGTTGGATAAAGTTATAGGAGACTCAGTTAACCTGAGAGCTCCTCA
ATT
+
FFFFFFFFFFFFF:FFFFFFFFFFFFFFFFFFFFFFFFFFFFFFFFFFFFFFFFFFFFFFF:FFFFFFF
FFFFFFF:FFFFFFFFFFF:FFFFFFFFFFFFFFFF:FFFFFFFFFFF:FFFFFFFFFFFFFF:FFFF
FFF
@A00721:190:HGJW7DSXY:4:1468:10456:15123_2:N:0:TCTGCAAG+GCTGCACT
AATAGGGACGCTAGTATACTTCGTAAAGTCGACAGCGAAATGGTCAATATTGATAATTGGACCGACTA
TATGACCTATTTTAAGCTCATGGTGAAAAGGGAAGCTAAGGTTAAACT
+
FFFFFFFFFFFFFFFFFFFFFFFFFFFFFFFFFFFFFFFFFFFFFFFF,FFFFFFFFFFFFFFFFFFF
FFFFFFFFFFFFFFFFFFFFFFFFFFFFFFFFFFF,FFFFFFFF:FFFFF
@A00721:190:HGJW7DSXY:4:2452:6985:35603_2:N:0:TCTGCAAG+GCTGCACT
CATAACCCCGCACAGAATATCGTTTATCACGCAAAATTTATTAATGCAATTTTCGGACCTATCTTTAA
GGTTCTTGTGAGGAGAGTCAAACAGTGTCTTTTACCCAACATTTTTAT
+
FFFFFFFFFFFFFFFFFFFFFFFFFFFFFFFFFFFFFFFFFFFFF:FFFF:FFFFFFFFFFFF:F:FFF,
FFFFFFFFFFFFFFFFFFFFFFFFFFFFFF,FFF:FFFFFFFFFFFFFF
@A00721:190:HGJW7DSXY:4:2160:7039:30279_2:N:0:TCTGCAAG+GCTGCACT
CCAGTGACGATTTCTCCCGGAAGCTCTTCTCTCTCTTAGGAGGTCATGATGACTACTATAAGGGTGAA
ATCGATTTTTCGAAGTTTGACAAATCTCAAGATTTCTTTTGTAAAGCC
+
FFFFFFFFFFFFFFFFFFFFFFFFFFFFFFFFFFFFFFFFFFFFFFFFFFFFFFFFFFFFFFFFFFFF
FFFFFFFFFFFFFFFFFFFFFFFFFFFFFFFFFFFFFFFFFFFFFFFF
@A00721:190:HGJW7DSXY:4:1177:16568:21010_1:N:0:TCTGCAAG+GCTGCACT
ATTTAGGCTTCGATGCTGAATTTCTTGAACTTTGGAGTGCCAGTGAATATTTTTGTCACGCCAGGAGT
ATCCATAACGATTTGTCATTTACCACCTTTGCTCAACGACGAAGTGGAACGGCCAACACTTTCTTTGG
TAA
+
FFFFFFFFFFFFFFFFFFFFFFFFFFFFFFFFFFFFFFFFFFFFFFFFFFFFF:FFFFFFFFFFFFFF
FFFFFFFFFFFFFFFFFFFFFFFFFFFFFFFFFFFFFFFFFFFFFFFFFFFFFFFFFFFFFFFFFFFF
FFF
@A00721:190:HGJW7DSXY:4:1655:10176:26005_1:N:0:TCTGCAAG+GCTGCACT
ACTTTGATGATTTTATCACTTTATTATGACCTTGGAAAGTTTTCGGCTGTGGTTGTCGCTGGGGACGA
TTCCATTCTGTTTTCAAAGACTCCAATAACTGATCATTCTGATCATATGATTTTGGACAATGGTTTCG
AA
+
FFFFFFFF,FFFFFFF::F:FF,FF:FFF,:F,F,F:FFFFFFFF:FFFFFFFFFFFFFFFFFF:FFF
FF:,FFFFF::FFFFFFF,F:FF,,FFF:FFFFF,F:FFFFFFFFFFF:::FFFF,FFF,FFFFFFFF
F,
@A00721:190:HGJW7DSXY:4:1630:8666:16689_2:N:0:TCTGCAAG+GCTGCACT
CAGCGTACTTCTGCTCGCGATTTATTCTTTTTGTCAATGATTACATCTACTTCGTTCCTGATCCTTAC

AAGATCATCGTTAAGTTAGGTAAACCCCTAACCGATGTTACTCTTGTA
+
:FFFFF,FFFF::F,F:FFF,FFFF,F:,FFF,FFFF,FFF,F:FFF,FF:F,FFFFFFFFFF:,,FF
FF:FFFF,FFFFFF:::::FFFFF,FFFFFFFFF,F,,FFFFF:FFF
@A00721:190:HGJW7DSXY:4:2133:20374:5494_1:N:0:TCTGCAAG+GCTGCACT
TTTAAAGACTATAATATATATGTCCACTACGAGGTTGTTGTTTGGGTATTAGCTCTTGCGACTAATAT
TCGTTATTCGATTGAGGGTCTTTATACCTATTCGGCTATCTGTGCTTTTAATTGTATAAGATCAAATT
TTA
+
F:FF:,,F,F,F,,FF,,FFF,FF,F,FFF,:::FF:FF:FF,FF,,,FF:F,F:FFF,FF,F,,F,F
F,:,F,FF,,,FFF,FF:F,,FFFFF:,FF:FFF:,FFFFF:FF,FFF,:,F,:F:FFF::F:FF,FF
FFF
@A00721:190:HGJW7DSXY:4:2454:22598:33364_2:N:0:TCTGCAAG+GCTGCACT
CCTGATGTGGAGGGAGTTCTTGAAGCATCCCGACCTTTCCTTGTGAAAATTTTGATGCGGTTTCATAA
GATTATCAGTAATCCTGATTCTACTACGGATTCATGGACCTTCCATCC
+
FFFFFFFFFFFFFFFFFFFFFFFFFFFFFFFFFFFFFFFFFFFFFFFFFFFFFFFFFFFFFFFFFFFF
FFFFFFFFFFFFFFFFFFFFFFFFFFFFFFFFFFFFFFFFFFFFFFFF
@A00721:190:HGJW7DSXY:4:1655:7600:33974_2:N:0:TCTGCAAG+GCTGCACT
GACGTATCTCAAAGATCGACTTCGAGACAGGTGATATTGTTGAGCGAGAGCGACCAAGATCTAAATTC
GGTAACGGTGCGTGGCGCTTTATTTTAAAAAGCTCCCTCCTCTCCCAA
+
FFFFFFFFFFFFFFFFFFFFFFFFFFFFFFFFFFFF:FFFFFFFFFFFFFFFFFF:FFFFFFFFFF:FF
:FFFFFFFFFFFFFFFFFFFFFFFFFFFF:FFFFFFFFFFFFFFFFFFF
@A00721:190:HGJW7DSXY:4:1257:3730:12555_2:N:0:TCTGCAAG+GCTGCACT
ACTTGAGTCTTTTGATCAGTCTCTGACTTGACCATCTTTCATTTTAAATGACGATCATTAAGACTCTC
TTTCTTCTTTGTTTCCTTCTTTGCACTGATTTTTCCTCATCAGCTTGT
+
FFFFFFFFFFFFFFFFFFFFFFFFFFFFFFFFFFFFFFFFFFFFFFFFFFFFFFFFFFFFFFFFFFFF
F:FFFFFFFFFFFFFFFFFFFFFFFFFFFFFFFFFFFFFFFFFFFFFF
@A00721:190:HGJW7DSXY:4:2369:12246:3411_1:N:0:TCTGCAAG+GCTGCACT
GTTTTTACGATCACAACTATCAGTTGATTAAGACTCTTCCGAAAGGCGAACTTTGGGAATTTGAAGCT
CAGGGTGAAGCACAACGTTTGACCGTGACGAACTGTGGTGTTCTTGATCTACGTTACGGATATACTCT
CCT
+
FFFFF:FFFF:,,FFFFF:F::FF:FFF:FFF,,,F:F,,FF,FF:,FFFFFFFF,FFF,FFF,FFFF
F,FF,:,FFFFFFFF,F:F,:,FF:F:::FFF:FFFFFFFF::FF:FF,F:FFFFFFF:FFFF,:FF:
,FF
@A00721:190:HGJW7DSXY:4:2412:27597:3349_1:N:0:TCTGCAAG+GCTGCACT
ACACTACCACTTATTACGATATTTCTTTAGTCGATGGGTTTTCGACGCCTTTAAGCGTCTTCTGTTCA
TCGCAACCAGATGATATCTTCTATTCGAACTTATGTTTCGATCAGTGTTTCTTCTATCAGGAGGAAAA
CAC
+
FFFFF:FFFFFF,:FFFFFFFFF:FFF:FFFFFF,FFFFFFFFF:FFFFF:FFFFFFFFFFFFFFFFF
FFFF:FFF:FFFFFFFFFFFFFFFFFFFFFFFFFFFFFFFFFFFFFFFFFFFFFFFFFFFFFFFFFFF
FFF
@A00721:190:HGJW7DSXY:4:2174:2645:2315_2:N:0:TCTGCAAG+GCTGCACT
TTTCTTCTAATGTGGCCTGAACTACGTTTCACTTTGTGTGTACTTGTCTCTATTTTAAGTTTTGTCTT
CCTTGTTTGTCTTATTCTATTAATCACTCATTTTAGTGAGATTATTAA
+
FFFFFFFFFFFFFFFFFFFFFFFFFFFFFFFFFFFFFFFFF:FFFFFFFFFFFFFFFFFFFFFFFFFF
FFFFFFFFFFFFF:FFFFFFFFFFFFFF:FFFFFFFFFFFFFFFFFFF
@A00721:190:HGJW7DSXY:4:2626:4246:5682_1:N:0:TCTGCAAG+GCTGCACT
GTCTTCGACAACAGCAGGGGTTTTCGAATAGTGATAGCCACCCGCCTTACAGAGGGGCAGCAGCATGA

CCATAATTGGTATTGACTTTGGTACCACCTTTTCTACTGTCTGTCTCGCCTCTTCGAATGAAGTTTTA
ATG
+
FFFFFFFFFFFFFFFFFFFFFFFF:FFF:FFFFFFFFFFFFFFFFFFFFFFFFFFFFFFFFFFFFFFFF
FFFFFFFFFFFFFFFFFFFFFF:FFFFFFFFFFFFFFFFFFFFFFFFFFFFFFFFFFFFFFFFFFFFFF
FFF
@A00721:190:HGJW7DSXY:4:2125:16947:34444_2:N:0:TCTGCAAG+GCTGCACT
TGATAGTGAATTTATACCTACAATCATTGGTTTTTCTAATAATGATGACACTATTGTTTATGGATATG
ATGCTATTACTCATGATGGTTACGGTCAGGGAGGATTCTCAGTTTACA
+
FFFFFFFFFFFFFFFFFFFFFFFFFFFFFFFFFFFFFFFFFFFFFFFFFFFFFFFFFFFFFFFFFFFF
FFFFFFFFF:FFFFFFFFFFFFFFFFFFFFFFFFF:FFFFF,FFFFFF
@A00721:190:HGJW7DSXY:4:1260:1416:35008_2:N:0:TCTGCAAG+GCTGCACT
CCGAAAGACGTGCTAAATTAAAACCATTGTACGATGTCGTCTGTCTCGGCGATCACTTTTCACTAAGA
ATTGGTCCTACTTTTGGACAAGGCCAACTTCGTAATCTTCATGAGATC
+
FFFFFFFFFFFFFFFFFFFFFFFFFFFFFFFFFFFFFFF:FFFFFFFFFFFFFFFFFFFFFFFFFFFFF
FFFF,FFFFFF:FFFFFFFFFFFFF:FFF:FFFFF:FFFFFFFFFF:F
@A00721:190:HGJW7DSXY:4:1368:27715:29731_2:N:0:TCTGCAAG+GCTGCACT
TATCTCTTTGGTTGTTAGGGATTTTGAGAGAGTGAAAGGCTTCAATTGTAGTGGTTTGGTCGTTTCAG
TACCCAGTCAGTATACCTCTACGCAAAGATATTTCATGAAAACTACAG
+
FFFFFFFFFFFFFF:FFFFFFFFFFFFFFFFFFFFFFFFFFFFFFF:FFFFFFFFF:FF:FFFFFFFFF
FFF:FFFFFFFFFFFFFFFFFFFFFFFFFFFFFF:F:FFFFFFFFFFFF
@A00721:190:HGJW7DSXY:4:2563:29767:32283_2:N:0:TCTGCAAG+GCTGCACT
ATTACCTGTCTTTCATATTATGAATGAACCTTCGGCCGCTCTCTTTGCTTCGATGTTGGATGTCAAGA
AGGGTTCAGAATGGGATTCCTACGTAGTTTATGACTTTGGTGGTGGTA
+
FFFFFFFFFFFFFFFFFFFFFFFFFFFFFFFFFFFFFFFFFFFFFFFFFFFFF:FFFFFFFFFFFFFF
FFFFFFFFFFFFFFFFFFFFFFFFFFFFFFFFFFFFFFFFFFFFFFFF
@A00721:190:HGJW7DSXY:4:1164:20600:32064_1:N:0:TCTGCAAG+GCTGCACT
ACGTGTGCTCTTCCGATCTTATAGTGTTATTTATTCTGGTGGTGATGATAATCTTGGAGGTCGTGACG
TTGATGAAGCTATAAGAGCGTTCTTATTACGGCGCACTTCTTTACCCGCCACCACACAACTTTCAGTT
TCA
+
FFFFFFFFFFFFFFFFFFFFFFFFFFFFFFFFFFFFFFFFFFFFFFFFFFFFFFFFFFFFFFFFFFFF
FFFFFFFFFFFFFFFFFFFFFFFFFFFFFFFFFFFFFFFFFFFFFFFFFFFFFFFFFFFFFFFFFFFF
FFF
@A00721:190:HGJW7DSXY:4:2249:16785:34569_1:N:0:TCTGCAAG+GCTGCACT
TACTGGAAAACCCTCGTCAATACTTTTCAATAATATACGTCTTGAACTCACTTATGACGATCTAGTGA
AATTAACGCGTCCTTTTCTGGAACGTAGTTTTACTATTATGAACAAAGTTATCAAAGATAGGGCATTC
AAG
+
FFFFF:FFFFFFFFFFFFFFFFFFFFFFFFFFFFFFFFFFFFFFFFFFFFFFFF:FFFFFFFFFFF
FFFFFFFFFFFFFFFFFFFFFFFFFFFFFFFFFFFFFFFFFFFFFFFFFFFFFFFFFFFFFFFFFFFF
FFF
@A00721:190:HGJW7DSXY:4:2158:32072:34240_1:N:0:TCTGCAAG+GCTGCACT
CCTGATTCCGGCCGGTGGTAGCTCATTTCTACCAGGGGCTATTACTCTTGCTGAAACAGCTATTAAAG
CTGTTGAGCGGGTAATTACACCTAAACGAGCTCGATCTGCCATCGCTGAAGGATGTGCACTCTTTTCT
GCT
+
FFFFFFFFFFFF:FF:FFFFFFFFFFFFFFFFFFFFFFFFFFFFFFFFFFFFFFFFFFFFFFFFFFFF
FFFFFFFFFFFFFFFFFFFFFFFFFFFFFFFFFFFFFFFFFFFFFFFFFFFFFFFFFFFFFF:FFFFF
FFF

```
@A00721:190:HGJW7DSXY:4:1274:12789:28588_2:N:0:TCTGCAAG+GCTGCACT
TCGATTGTATCAACAGTAACATTACTTCTAATAAAGGTCTCTATCAAGCTCGTATTATCGTCGCTGCT
GGATCTGCGTTACCGTTGAAGGCTAAACTCCAAGGTAATTATGAAGAC
+
FFFFFF:FFFFFFFFFFFFFFFFFFFFFFFFFFFFFFFFFFFFFFFFFFFFFFFFFFFFFFFFFFFFF
F:FFFF::FFFFFFFFFFFFFFFFFFFFFFFFFFFFF:F:FFFFF:FFF
@A00721:190:HGJW7DSXY:4:2118:19768:31657_2:N:0:TCTGCAAG+GCTGCACT
TTTCACTCTGGCCTTGTATGAGGGTGTTTCTAATCGTAACATTAATAATTTCAATCTTCTTAAAAAGA
AGATAGAGAGTCATACTTTGGGCTATCGTTCAGGTGCATGGGGATGGA
+
::FFFF:FFFFFFF:F:FF,FF:F,FF:F:FFFFFF:FFFF,,FF,FF,,FFFFFFFFF::FFF:F:F
FFF:F:F,FFFFFFF,F,FF,FFF,,F,:F,FF,,F,F:,FF,,,F,:
@A00721:190:HGJW7DSXY:4:1240:15383:24314_2:N:0:TCTGCAAG+GCTGCACT
TGAAGTGGACACCTCTGGTAGTATCGAAGTAAAGATAACCAATAAGGAAGGAGCTTATACTATGGTTG
AATCAACTAAGCATACTACTTTTGCTTTGATTACTCTACCGCCTTACA
+
FFFFFFFFFFFFFFFFFFFFFFFFFFFFFFFFFFFFFFFFFFFFFFFFFFFFFFFFFFFFFFFFFFFF
FFFFFFFFFFFFFFFFFFFFFFFFFFFFFFFFFFFFFFFFFFFFFFFF
@A00721:190:HGJW7DSXY:4:1122:12726:30984_2:N:0:TCTGCAAG+GCTGCACT
TAAAGAGTCGATTAAACAAGTTTTACTTATCAATTAATTTATCGCGGTTTGTTATCTCGAAAAGTCTA
ATATAAAATGAACGTAAGTTATAACCCAAAGAGAATATCTAAAATAAT
+
:FFF:F:FFFF,:FFF,FF,:F:F::,FFFFFF,:F:FFFF,,::,,,,F,:F::,F,,,FFF,,,,,
,F,FFFFF,,,FF,F,:,:F,,FFFFF:FF,F,FF,FF:,FFFF,F,F
@A00721:190:HGJW7DSXY:4:1122:12726:30984_1:N:0:TCTGCAAG+GCTGCACT
TACCACTATCTACGAACCTAAAGTTCTGAAGACGTTGCTTGTCGCTTCCTCCTTTAATGGCACTACCT
CGGTACAATCTTACTTGGAACGAAGGCGACCTGTTCCGGAGTTTTTACGGGATCCGGAATGTTCTGTC
TAT
+
:FF:F,F:FFF::FFFFF:FFFFFFFFFFFFF,FFFF:F,FFFFFFFF:FF,FFF,FF:F:FFFFFFF
F:FF:FFFF,F,FF:F:,FFF:FF:FFF:FFF:FF,FF,FFFF,:FF::FFFF::FF:FFFFFFF:F,
F:F
@A00721:190:HGJW7DSXY:4:1429:23014:17080_1:N:0:TCTGCAAG+GCTGCACT
AATACGATTTCGAACGTCAACGTTCACCGAGTTTCGTTTCGTACCTTGATGCTGACTTTCGTCAATAT
GATTTGATCTGGTTGCTCAATTCTAAGCGTGTTCCGCTTAGTGCGAGGCCTCTCTTCTACGAGTATTG
CTA
+
FFFFFFFFFFFFFFFFFFFFFFFFFFFFFFFFFFFFFFFFFFFFFFFFFFFFFFFFFFFFFFFFFFFF
FFFFFFFFFFFFFFFFFFFFFFFFFFFFFFFFFFFFFFFFFFFFFFFFFFFFFFFFFFFFFFFFFFFF
FFF
@A00721:190:HGJW7DSXY:4:2232:4571:7028_2:N:0:TCTGCAAG+GCTGCACT
AGTTTACGAACTTGACTCTACTATACGTGACATACTAAATACTAGATATTTGGATGATCTTGATAAAG
AACTTCATGAATTTTCAGAGGATGAAAAATTTTCTTCCTTGGATTTCA
+
FFFFFF:FFFFFFFFF:FFFFFFFFFFFFFFFFFFF:FFFFFFF:FFFFFFFFFFF:FFFFFFFFFFF
FFFFFFFFF:FFFFFFFFFFFFFFFFFFFFFFFFFFFFFFFFFFF:FFFF
@A00721:190:HGJW7DSXY:4:2114:5249:23578_1:N:0:TCTGCAAG+GCTGCACT
TACAGGAGGCAGTCTTCTGATCGTAAGAAGTTTATAAGGAACATATCCATTTGTTGTAATAGGGTCAT
CAACTTAGAACTCGAAACTCTAAGTGGTAATACTGTCTTTCCAAGCTTACCTGTATCTTCAATGAAAT
TGG
+
FFFFFFFFFFFFFFFFFFFFFFFFFFFFFFFFFFFFFFFFFFFFFFFFFFFFFFFFFFFFFFFFFFFF
FFFFFFFFFFFFFFFFFFFFFFFFFFFFFFFFFFFFFFFFFFFFFFFFFFFFFFFFFFFFFFFFFFFF
FFF
```

@A00721:190:HGJW7DSXY:4:2545:7419:7513_2:N:0:TCTGCAAG+GCTGCACT
GTGGGCTTTCCGTAAAGGAAGATCTGTTATCGACGGTTTATCGTTTACGAAGTATCACTCCACCGGTG
AATGGTTTAAGAATACTATAAATAGAATAGCTATTACTACTAATGGCG
+
FFFFFFFFFFFFFFFFFFFFFFFFFFFFFFFFFFF:FFFFFFFFFFFFFFFFFFFFFFFFFFFFFFFF
FF,FFFFFFFFFFFFFFFFFF:FFFFFFFFFFFFFFFFFFFFFFFFFFF
@A00721:190:HGJW7DSXY:4:1670:28140:35289_1:N:0:TCTGCAAG+GCTGCACT
TAGTGACATCTGGTCAATTTCGTTTTTAGGTGGTCTTTATTCTATTATTGATTTTTATGAGATTGATA
CGTTAACAGAAGATGATGTTAGAGTAGAATTCCTGAAGAGTCCTTCCGTAACCAACTTCTTGACAATT
TTC
+
FFFFFFFFFFFFFFFFF:FFF::FFFFFFFFFFFFFFFFFF:FFFFFFFFFFFFFFFFFFFFFFFFFF
FFFFFFFFFFFFFFFFFFFFFFFFFFFFFFFFFFFFFFFFFFFFFFFFFFFFFFF:FFFFFFFFFFFF
FFF
@A00721:190:HGJW7DSXY:4:2674:3450:24251_2:N:0:TCTGCAAG+GCTGCACT
CTAGGATTATGTCTGTGTTGAAAGACTTTCTACCGATAAGGGTCACTAGTCTTACACAATTAAGAAAT
AATCCTACATTGACTGATTATCTAAAGATTGAAGGAAAATTTTATCCT
+
FFFFFFFFFF:FFFFFFFFFFFFFFFFFFFFFFFFFFFFFFFFFFFFFFFFFFFFFFFFFFFFFFFFF
FFFFFFFFFFFFFFFFFFFFFFFFFFFFFFFFFFFFFFFFFFFFFFFF
@A00721:190:HGJW7DSXY:4:2674:27597:26271_1:N:0:TCTGCAAG+GCTGCACT
GACTTTCCTACGATACAAATGATAACTTCTAATTTACGTTTGCTACTCAGAGCTGGTCCTCTCAGCAA
AGGGATGCATGGTGTTATCTCCTCAGGAGTCCCGGCATTTGATAATAATGACGAGACTTTCGTCATAA
TCG
+
FFFFFFFFFFFFFFFFFFFFFFFFFFFFFFFFFFFFFFFFFFFFFFFFFFFFFFFFFFFFFFFFFFFF
FFFFFFFFFFFFFFFFFFFFFFFFFFFFFFFFFFFFFFFFFFFFFFFFFFFFFFFFFFFFFFFFFFFF
FFF
@A00721:190:HGJW7DSXY:4:1660:26449:2080_1:N:0:TCTGCAAG+GCTGCACT
GATTACACGGTACCAGCGCATCTCGTGTCGAGCCTCGACCCGATTATATGGTATTAACTTATAAAGGT
GAGGAGACCATTTTCTTGCTGAAATTTATAGAAGATTATTTTGACAGAATCAATAAGAATCATCGATT
CTC
+
FFFFFFFFFFFFFFFFFFFFFFFFFFFFFFFFFFFFFFFFFFFFFFFFFFFFFFFFFFFFFFFFFFFF
FFFFFFFFFFFFFFFFFFFFFFFFFFFFFF,:FFFFFFFFFFFFFFFFFF:FFFFFFFFFFF:F:FFF
FFF
@A00721:190:HGJW7DSXY:4:1337:5041:25567_2:N:0:TCTGCAAG+GCTGCACT
GATTTACAACGATATAGGAGTAATCTACGAGAAGATTCTAGAATTCTTAATTATAATATTCAACCTCA
AAAGTTACGTGTTTACAGTAACGAGATCACACATTTTGAGAACTAACT
+
FFFFFFFFFFFFFFFFFFFFFFFFFFFFFFF,FFFFFFFFFFFFF:FFFFFFFFFFFFFFFFFFFFFF
FF:F:FFFFFFFFFFFFFFFFFFFFFFFFFFFFFFFFF:FFFF:FFFFF:
@A00721:190:HGJW7DSXY:4:2310:14290:20384_1:N:0:TCTGCAAG+GCTGCACT
CTGATCATTTTGACAATCTCTTCTTATAATAAATAAATAAATAATATTACTAATGGCAAATAGTGGAA
AGGCTCCAGTCAACACTAGTTCGAGTGCTGCTTTGGATTACGATGACGTGAGAATAGGTGAAGGGATA
GTT
+
FFFFFFFFF:FFFFFFFF:FFFFFFFFFFFFFFFFFFFFFFFF:FF:F,FFFFFFFFFFFFFFFFFFF
FFFFFFFFFFFFFFFFFFFFFFFFFFFFFFFFFFFFFFFFFFFFFFFFFFFFFFFFFFFFFFFFFFFF
FFF
@A00721:190:HGJW7DSXY:4:1148:28384:32706_1:N:0:TCTGCAAG+GCTGCACT
GATCTATTAAGGCGGCGGCGACTAATCTTAAAGCTTCACCCAAAATGAAGTACACTTCGAAACTGAAT
AATGATGCACTACAAACAAGTATCGCTAATCATCTTAAAAAGGCTCTAAAGGACACTATACAATATCC
AGA

+
F,F,FFFFF::F,FFFF,FFF,FFFF::FF:FF:FFF:F,,F,:,FF,,:,:FFFFF,FF,F:FFFFF
FFFFF:,,:,FFF:F,F,F,FFFF,:FFFFFFFFF,FFFFF:F,F:FFFFF:,,,FFFF:FFFFFFF:
FFF
@A00721:190:HGJW7DSXY:4:1378:32967:35884_2:N:0:TCTGCAAG+GCTGCACT
TGCTTTTACCGATAACGATAAATTCGTCATCAAATATAAAGAAAAGGACTATGAAATAAAAGATTTGG
TGCTTAGGGATATAATGAATTCAGCACCACATTTTGGTCAAGTCAATC
+
FFFFFFFFFFFFFFFFFFFFFFFFFFFF:FF,FFFFFFFFFFFFFFFFFFFFFFFFFFFFFFFFFFFF
FFFFFFFFFFFFFFFFFFFFFFFFFFFFFFFFFFFFFFFFF:FFFFF:F
@A00721:190:HGJW7DSXY:4:1120:25843:19100_2:N:0:TCTGCAAG+GCTGCACT
TCTTAGAGGATATTCGCCATTGTGGTACGAATTGGTCCAACATTTACCACCAAATTCTTTTGATACAA
CTCTAGCCTTCAAGTGGGGTATGCCTGCGGAATACGCTTGCTATTCAC
+
FFFFFFFFFFFFFFFFFFFFFFFFFFFFFFFFFFFFFFFFFFFFFFFFFFF:FFFFFFFFFFFFFFFF
FFFFFF:FFFFFFFFFFFFFFFFFFFFFFFFFFFFFFFFFFFFFFFFFF
@A00721:190:HGJW7DSXY:4:1106:26142:24721_2:N:0:TCTGCAAG+GCTGCACT
CGAACCCGTTGATGCTATGTCAGAAGAAGCTATTCAAGCTTGTCGTTTAAAGACTGCAGAGGCTGTCA
AACAAGCCCAGACTCAAGGAGTCCAATCAAAGGTTTTGAATTTAGCTC
+
FFFFFFFFFFFFFFFFFFFFFFFFFFFFFFFFFFFFFFFFFFFFFFFFFFFFFFFF:FF:FFFFFFFFF
FFFFFFFFFFFFFFFFFFFFFFFFFFFF:FFFFFFFFF:FFFFFFFFFF
@A00721:190:HGJW7DSXY:4:2161:18394:1063_2:N:0:TCTGCAAG+GCTGCACT
TGGGTGATAAAAATTATTTAGAGATCTTTTCTACTTACAAAGGACAATTCAGTTTCATTAATTCTACG
GAAAAGTCCGGAGTATATTGTATGTTAATAGATAGTGGGATAATAGTT
+
FFFFFFFFFFFFFFFFFFFFFFFFFFFFFFFFFFFFFFFFFFFFFFFFFFFFFFFFFFFFFFFFFFFF
FFFF:FFFF:FFFFFFFFFFFFFFFFFFFFFFFFFFFF:FFFFFFF:FF
@A00721:190:HGJW7DSXY:4:2131:23321:11162_1:N:0:TCTGCAAG+GCTGCACT
GTAGTGAACGCGTTCGTAGAACCAAGTACTTTCCGTCTGAAGAGTTTAGAGCTGCTATTGATTATGTA
ACCGATAGGAGTTGTATAGTTGAAGATGTGGCTTTAGACGATAATCCTTTAAGGACTATGTTGACTGA
CGA
+
FFFFFFFFFFFFFFFFFFFFFFFFFFFFFFFFFFFFFFFFFFFFF:FFFFFFFFFFFFFFFFFFFFFFF
FFFFFFFFFFFFFFFFFFFFFFFFFFFFFFFFFFFFFFFFFFFFFFFFFFFFFFFFFFFFFFFFFFFFF
FFF
@A00721:190:HGJW7DSXY:4:1105:11849:30091_2:N:0:TCTGCAAG+GCTGCACT
GATCGCCACTCCAAACACTATATACATTATAAAATATAAAAATAAGGGAGTCATAACTTTTGTTACCA
AATTCTTGGCTTTCAACGAAGTCTCTGATTTTATTAATCATTACTTTA
+
FFFFFFFFFFFFFFFFFFFFFFFFFFFFFFFFFFFFFFFFFFFFFFFFFFFFFFFFFFFFFFFFFFFF
FFFFFFFFFFFFFFFFFFFFFFFFFFFFFFFFFFFFFFFFFFFFFFFFF
@A00721:190:HGJW7DSXY:4:1218:31033:26584_2:N:0:TCTGCAAG+GCTGCACT
GGTCATTTACTCTGTACTTGATCTACGTCGTTTAACTGTCGGAACAACCTTTACTTAGAATGACTCTA
TCTTTTCAGACACTTCTTATATTATTTACAATAGTTAATATCATATTA
+
FFFFFFFFFFFFFFFFFFFFFFFFFFFFFFFFFFFFFFFFFFFFFFFFF:FFFF:F:FFFFFFFFFFF
FFFFFFFFFFFFFF,FFFFFFFFFFFFFFFFFFFFFFFFFFFFFFFFFF
@A00721:190:HGJW7DSXY:4:1139:3748:34068_2:N:0:TCTGCAAG+GCTGCACT
CTATCGTTTTCTGCTTTTTCTTAGCTAATAGATTAATAAAGGATGAGTACGAAAACCAACTCTTCGTT
ACGAGACCTGATGGTAGAATAGATTTAGTCTCTACACCCATCTCAACG
+
FFFFFFFFFFFFFFFFFFFFFFFFFFFFF,FFF:FFFFFFFFFFFFFFFFFFFFFFFFFFFFFFFFFFF
FFFFFFFFFFF:FFFFFFFFFFFFFFFFFFFFFFFFFFFFFFFFFFFFF

```
@A00721:190:HGJW7DSXY:4:1127:15411:20134_1:N:0:TCTGCAAG+GCTGCACT
TTTTTTTCCGTCCTTAACTTAATACACTAAGTTATAATCCGCAAACGACTCGAAACCGAGACGCCGTA
GATCAAAGTCCCTTCTAACTTTAGA
+
F::FFF,F,,FF:F,F,,FFF,,,,,,,F,,,,FF,:,F,,,F:FFFFFFFFFFFF:FFFFFFFFFFF
FFFFFFFFFFFFFFFFFFFFFFFFF
@A00721:190:HGJW7DSXY:4:2157:24334:9690_1:N:0:TCTGCAAG+GCTGCACT
CTTTACTCATTTTATAGCAGTTATTTATGTTGTACTTTTTATAATTTATAACCTTCTTATACTTTTGG
GTATGTTTTGTGAAAAACATGAGATACCCAAGTCTATCGAAGACATTATCTCTTATGTACTTATTGTA
ACG
+
FFFFFFFFFFFFFFFFFFFFFFFFFFFFFFFFFFFFFFFFFFFFFFFFFFFFFFFFFFFFFFFFFFFF
FFFFFFFFFFFFFFFFFFFFFFFFFFFFFFFFFFFFFFFFFFFFFFFFFFFFFFFFFFFFFFFFFFFF
FFF
@A00721:190:HGJW7DSXY:4:2354:30680:23970_1:N:0:TCTGCAAG+GCTGCACT
TTCTTATTCTTTTATTCCTTTAACTGTGTATGAGGAGGATTTATAATGCTTTTCGATAGTGATTGTTA
TCAATTTAAATACATTACTGTTTCAAATCATCTTTACTCACAGACTCCGAAAATGGTTTCAAATGAGG
TCA
+
FFFFFFFFFFFFFFFFFFFFFFFFFFFFFFFFF:FFFFFFFFFFFFFFFFFFFFFFFFFFFFFFFFFF
FFFFFFFFFFFFFFFFFFFFFFFFFFFFFFFF:FFFFFFFFFFFFFFFFFF:FFFFFFFFFFF:FFFFF
FFF
@A00721:190:HGJW7DSXY:4:2354:30680:23970_2:N:0:TCTGCAAG+GCTGCACT
TATTAAACGAACTATTATCCGATTCTTCGAGGGTATCACTACCACTGATCTGCCGACTTTAGTCAAGG
AACTTCAATTCCTTCGATGTTTATATTTTTCTATAAGGGAAAATAGCA
+
FFFFFFFFFFFFFF:FFFFFFFFFFF:FFF:FFFFFFFFFFFFFFFFFFFFFFFFFFFFFFFFF:FFFF
FF:FFFFFFFFFFFFFFFFFFFFFFFFFFFFFFFFFFFFFFFFF:FFF
@A00721:190:HGJW7DSXY:4:1577:24234:25927_2:N:0:TCTGCAAG+GCTGCACT
TTCGACAAATTTTAGAACTTTATTTCAGAACTCTCTCCGATCACCTTGGTGTGATCTCCCGGAGGATC
TTTTCAATAAGATGTATGATGATAATCTACTATTTTTAAATAGTGATA
+
FFFFFF:FFFFFFFFFFFFFFFFFFFFFFFFFFFFFFFFFFFFFFFFFFFFFFFFFFFFFFFFFFFFF
FFFFFFFFFFFFFFFFFFFFFFFFFFFFFFFFFFFFFFFFFFFFFFFF
@A00721:190:HGJW7DSXY:4:1360:5231:7012_1:N:0:TCTGCAAG+GCTGCACT
CTTTATCAAAGATTTGGCTTGGTCTATTTACCGCAAAAAGTTGGAAGAGGTCTATGATATTTATTTTA
CTTATACTTTAAATAGCACCGATTATAACTTGACTGTTGATAATATTACAATAGCTCTTTCTCAGAAG
ATT
+
FFFFFFFFFFFFFFFFFFFFFFFFFFFFF:FFFFFFFFFFFFFFFFFFFFFFFFFFFFFFFFFFFFFF
FFFFFFFFFFFFFFFFFFFFFFFFFFFFFFF:FFFFFFFFFFFFFFFF:FFFFFFFFFFFFFFFFFFFF
FFF
@A00721:190:HGJW7DSXY:4:1156:7066:15013_2:N:0:TCTGCAAG+GCTGCACT
GGTGAACACGTCGGTAGCATTGATTTACTTTTACCGTTAGAACTGAAAAAGATTCTTTTCGAAAAGAA
ACCTATAAGTTACAAACGATTCTTCGACTATATTAAAGAAATAAAAAT
+
FFFFFFFFFFFFFFFFFFFFFFFFFFFFFFFFFFFFFFFFFFFFFFFFFFFFFFFFFFFFFFFFF:F:F
FF:FFFFF:FFFFFF:FFFFFFFFF:FFFFFFFFFFFFFFFFFFFFFF
@A00721:190:HGJW7DSXY:4:1325:22562:27445_2:N:0:TCTGCAAG+GCTGCACT
CTTTACATTTTTAATGGTATGGTATAATGTGTCTTACAAATAGAACCTTCACTCTCGGTAATGAGAGT
GTATTCCTTAATATTCTAGTAACCGCTATGTGGGCATTACTAGGATTT
+
FFFFFFFFFFFFF:FFFFFFFFFFFFFFFFFFFFFFFFFFFFFFFFFFFFFFFFFFFFFFFFFF:FFF
FFFFFFFFFFFFFFFFFFFF:FFFFFF:FFFFFFFFFFFFFF::FFFF
```

@A00721:190:HGJW7DSXY:4:1606:17011:3114_1:N:0:TCTGCAAG+GCTGCACT
GATAGCAATTTATGTTTTAAATGCTAGGAAAATTTTAGAAAAACTCCATTGTATAGTTCAAGGAGTAC
TTAATTCAATAGCTTTAGTATTATTTACTATCGCCTTTATTGAAAATTGTATAAGAGTTAATTGCTTA
CAC
+
FFFFFFFFFFFFFFFFFFFFFFFFFFFFFFFFFFFFFFFFFFFFFFFFFFFFFFFF:FFFFFFFFFFF
FFFFFFFFFF,FF:FFFFFFFFFFFFFFFFFFFFFFFFFFFFFFFFFFFFFFFFFFFFFFFFFFFFFF
FFF
@A00721:190:HGJW7DSXY:4:2656:31150:6277_2:N:0:TCTGCAAG+GCTGCACT
TTAATTGATTGAAGCTAGGCGTTGGGGAGAATGAACTGCCGCTTCCCGGAAGAAACGATCTATTTACA
GAGTATTTAGAGTAGTCCAAAGTTTGTATAAATTTATATGATTTTCTA
+
FFFFFF:FFFF:FFFFFF,FFFFFFFFFFFFFFFFFF:FF:FFFFFFFFFF::FFFFFFFFFFF:FFF
FFFFFFFFF:FFFFFFFFFFFFFFFFFFFFFFFFFFFFFFFFF:FFFF
@A00721:190:HGJW7DSXY:4:2462:29423:2785_2:N:0:TCTGCAAG+GCTGCACT
AACATACCATACACTCCCCAAACATAACATACCATAACCTACCATGACCAATCATCGCTCCAACTGGC
ACGCCAGACGTAGGGTCAAACCTACTACCGCCGGTCGGCAGATCGGCA
+
FFFFFFFFFFFFFFFFFFFFFFFFFFFFFFFFFFFFFFFFFFFFFFFFFFFFFFFFFFFFFFFFFFFF
FFFFFFFFFFFFFFFFFFFFFFFFFFFFFFFFFFFFFFFFFFFFFFFF
@A00721:190:HGJW7DSXY:4:1619:7319:3145_2:N:0:TCTGCAAG+GCTGCACT
ACCCGCTGTGCAGAATAACCTTCCCAAATCTAGATCTGCACAGAAGAAGGCTGAAGGTCGTTACCGGA
CGACCACTCAAAGGAAACGGGCTGTTGCCCAGAGGAAAGCAGCGACCA
+
FFF:FFFFFFFFFFFFFFFFFFFFFFFFFFFFFFFFFFFFFFFFFFFFFFFFFFFFFFFFFFFFFFFF
FFFFFFFFFF:FFFFFFFFFFFFFFFFFFFFFFFFF,FFFFFFFFFFF
@A00721:190:HGJW7DSXY:4:1509:19470:10222_2:N:0:TCTGCAAG+GCTGCACT
GTAACATTGACGGCAGTCCCGTCATCTCTGTGGGAACTGAGGCAGTACCTCAGTCAAAAGTCACATTC
ACCACTCCCATGCCAATCAATGATGTGACTATACAGTTCGGCCATTTC
+
FFFFFFFFFFFFFFFFFFFFFFFFFFFFFFFFFFFFFFFFFFFFFFFFFFFFFFFFFFFFFFFFFFFF
FFFFFFFFFFFFFFFFFFFFFFFFFFFFFFFFFFFFFFFFFFFFFFFF
@A00721:190:HGJW7DSXY:4:1168:7880:16360_1:N:0:TCTGCAAG+GCTGCACT
ATACGTCGTGGGAGGTACTGAAGCCTTCCCCTCCGCTGGCACGAAGAACACTCCCAAGCCAATCAATA
GTTCTTCTGACCAGAATGAAAAGGGGTTTACCAGGGTAGTCTCTAAGTCTACCAGGAAGAGGGGAAAT
GTC
+
FFFFFFFFFFFFFFFFFFFFFFFFF,FFFFFFFFFFFFFFFFFFFFFFFFFFFFFFFFFFFFFFFFFF
FFFFFFFFFFFFFFFFFFFFFFFFFFFFFFFFFFFFFFFFFFFFFFFFFFFFFFFFFFFFFF:FFFFF
FFF
@A00721:190:HGJW7DSXY:4:1371:25183:29763_1:N:0:TCTGCAAG+GCTGCACT
AAAGGGAAAGGTCCATCAGGACCTAAGAAGGGGAAATTACCCCTAATTCTGAAGGAAGATCTCCCACC
ATCTGGGGTTGGGAATAAGAAATCTGCACGGGCCAAAGAGGCCGCTGCTACGAAGTCGCTGAAGGCGG
CTT
+
FFFFFFFFFFFFFFFFFFFFFFFFFFFFFFFFFFFF:FFFFFFFFFFFFFFFFFFFFFFFFFFFFFFF
FFFFFFFFFFFFFFFFFFFFFFFFFFFFFFFFFFFFFFFFFFFFFFFFFFFFFFFFFFFFFFFFFFFF
FFF
@A00721:190:HGJW7DSXY:4:1403:2058:32925_2:N:0:TCTGCAAG+GCTGCACT
TGTAAAGTTGCGCCTGCCGACCCCATCTTTGAGACCAAGACTCTCTACAGTAGGTTCGTGCCTACTAT
GAGCAAATCTGTTGAATTCGTCACTGAGGACGAAGGTGAGAGGACTTT
+
FFFFFFFFFFFFFFFFFFFFFFFFFFFFFFFFFFFFFFFFFFFFFFFFFFFFFFFFFF:FFFFFFFFF
FFFFF:FFFFFF:FFFFF,FFFFFFFFFFFFFFFFFFFFFFFFFFFFF

```
@A00721:190:HGJW7DSXY:4:2354:26467:21934_1:N:0:TCTGCAAG+GCTGCACT
CAAACTGAGAGCAGTCGCTGGGGAAAGAGAATTGCGTGGGTTGAAGGCCGACGCTTCGAGCTTGAGTG
TGACGAGGCCGGTGACGTTTTTGTCAACGGCGTTTGGAGGATTTTATCCGCGCTTAACTATGGGGCGA
AGA
+
FFF,FFFFFFFFFFFFFFFFFFFFFFFFFFFFFFFFFFFFFFFFFFFFFFFFFFFFFFFF:FFFFFF
FFFFFFFFFFFFFFFFFFFFFFFFFFFFFFFF,FFFFFFFFFFFFFFFFFFFFFFFFFFFFFFFFFFF
FFF
@A00721:190:HGJW7DSXY:4:1551:24117:36088_1:N:0:TCTGCAAG+GCTGCACT
AGTCTTGAAGGACTTAGGTTGGCATATTTATGCCGACGATAATAAGCATGTCACCCTTAAGCACCGAG
GTAAGCTCGGGACTTACAAGAGAGAGTGTGAGAGAGTCGCTCTCACTATTTTTGCCCAGAATGCGGGC
TTT
+
FFFFFFFFFFFFFFFFFFFFFFFFFFF:FFFFFFFFFFFFFFFFFFFFFFFFFFFFFFFFFFFFFFFF
FFFFFFFFFFFFFFFFFFFFFFFFFFFFFFFFFFFFFFFFFFFFFFFFF:FFFFFFFFFFFFFFFFFFF
FFF
@A00721:190:HGJW7DSXY:4:2408:13413:8030_2:N:0:TCTGCAAG+GCTGCACT
ATTCACTGGTTCTGGGAGGCTGCTTTCCTCCATAATTACAACCTCTCTTTAGAGAGCATTAGAAGGAA
GTTGGGCAAATTCCCACATATGGTAGACGTTGTCACACTTTTCTTGAA
+
:F,:F:,F:::FF,FFF:F,FFFFF::FFFFF:FFFFFFFFFFFFFFF,FF:FFFFFFFFF,FFFFF:
FFF::FFFFFFFFFFFFFFF::FFFFFFFFFFFFFFFF:F:F:F:FFF
@A00721:190:HGJW7DSXY:4:2336:14479:14231_1:N:0:TCTGCAAG+GCTGCACT
TTCCTCCGTCATTGGGGATTTCGATGTGAGAAGGTGGAAGGTGTCAAGGGTCTTCTTCATGCTGATCT
TTCAGGACCTAATCACATCATGATTGATAGTTACGGCTCGTATCGAGTTGGAGGGAGGAAGTTCTCCG
AA
+
FFFF:,FFFFFFFFFFFFFFF:FFFFFFFFFF,FF,F:FFFFFFF::FFFFFFFFFF:FFFFF:FF:F
FFFF,FF:FFF,FFFFFF:FFFFFFFFFF,FFF:FF:F,:FFFFFFFFFFFFFFFF,FFF:FFFFF:F
FF
@A00721:190:HGJW7DSXY:4:1345:6126:18051_2:N:0:TCTGCAAG+GCTGCACT
TCAGAGAGGGGAGCTCTGAAAAGGAAACTTCGTCAGAAGATGAGGAAGTCTAAGAGGAGGAGTTTGAG
GAAGGCTCTAGAGAAGATCTCACCACCTGGAGTAGGCAAGTGGAGAGT
+
FFFFFFFFFFFFFFFFFFFF::FFF:FFFFFFFFFFF:FF:FFFF,FFFF,F,FFFFFFFFFFFFFFF
F,FFFFFF::F:F:,,FFFFFFFFF:F:FFFF,:FFF::FFFF,F:FF
@A00721:190:HGJW7DSXY:4:1327:26087:10254_2:N:0:TCTGCAAG+GCTGCACT
AAGAAGGATTATTTGAAGCAGGGGTTACCACTTCCTAAGGTGGATGAGGGAGGTTGTTTCTTTGGAGT
TTTAGATAATCTTTTGAGAAGGGGTCATAGAGCCAGCAAGAGAAGCTT
+
FFFFFF:FFFFFFFFFFFFFFFFFFFFFFFFFFFFFFFFF:FFFFFFFFFFFFFFFFFFFFFFFFFFF
FFFFFFFFFFFFFFFF:FFFFFFFFFFFFFF,FFFFFFFFFFFFFFFF
@A00721:190:HGJW7DSXY:4:1522:26060:8390_2:N:0:TCTGCAAG+GCTGCACT
CCTTGAGAAGACAAGCCACATTAAGATATAATTTAGGTCAACTTGGGGTTTCGAATGTCATTAGTGCC
TTACGATTGAAAGAGGTCCTTTTGAGTATCGACAGATCTTTCGATCTT
+
FFFFFFFFFFFFFFFFFFFFFFFFFFFFFFFFFFFFFFFFFFFFFFFFFFFFFFFFFFFFFFFFFFFF
FFFFFFFFFFFFFFFFFFFFFFFFFFFFFFFFFFFFFFFFFFF:FFFFF
@A00721:190:HGJW7DSXY:4:1217:22327:30232_2:N:0:TCTGCAAG+GCTGCACT
GTGGCAGAATTCGGTAACTTACCATATTGTTCCTTCCGGGAAGTTGTGGGATTTAACTTTGACTCAAC
TACTCCGATCAGATCCGGATGGTTTGATTGGTATGGACTACTCGACTT
+
FFFFFFFFFFFFFFFFFFFFFFFFFFFFFFFFFFFFFFFFFFFFFFFFFFFFFFFFFFFFFFFFFFFF
FFFFFFFFFFFFFFFFFFFFFFFFFFFFFFF:FFFFFFFFFFFF:FFFF
```

```
@A00721:190:HGJW7DSXY:4:1325:2230:22388_1:N:0:TCTGCAAG+GCTGCACT
TAGGGTCATGGCAGATACTACCGATAAGGTGAAAAATATGCAGGTCAAAGATTCTGTAGCTCTGAGGG
CGGTTGAGGGAGCCGTTTTGGCCGACTTTAACTATTCTAAGAGTCTTTATGATTCAAAACCTATCGTT
GTC
+
FFFFFFFFFFFFFFFFFFFFFFFFFFFFFFFFFFFFFFFFFFFFFFFFFFFFFFFFF:FFFFFFFFFFF
FFFFFFFFFFFFFFFFFFFFFFFFFFFFFFFFFFFFFFFFFFFFFFFFFFFFFFFFFFFFFFFFFFFFF
FFF
@A00721:190:HGJW7DSXY:4:1577:20699:22467_1:N:0:TCTGCAAG+GCTGCACT
TACAGCGGCTTATTGTCGCGCAGTTTCCAGAATTGCGGTTGAAGTTCACCCATTCTATCTTGAATGCT
CACGCCGTCGCAGCCATATCGAGACTATGTTATAATCACTTGTTAAAGTACAGATTTAGTGATTTAGA
TAT
+
FFFFFFFFFFFFFFFFFFFFFFFFFFFFFFFFFFFFFFFF:FFFFFF:FFFFFFFFFFF:FFFFFFFFFF
FFFFFFFFFFFFFFFFFFFFFFFFF:FFF:FFFF:FFFFFFFFFFFFFFFFFFFFFFFFFFFFFFFFFFF
FFF
@A00721:190:HGJW7DSXY:4:1368:11731:30389_1:N:0:TCTGCAAG+GCTGCACT
GGAGGCGATATTCGTTTTCATATCCAAAACTCCTTGAATGGAGTACATATTTGCAACCCTATTCTTGA
TGTGAAAGATGCATCGAGGTATACGTCTAGGAACATTGATTGGGAACGTACCTACTCACGAGACGTCA
TAA
+
FFFFF:FFFFFFFF:FFFFFFFFFFFFFFFFFFFFFFFFFFFFFFFFFFFFFFFFFFFFFF:FFFFFF
FFFFFFFFFFFFFFFFFFFFFFFFFFFFFFFFFFFFFFFFFFFFFFFFFFFFFFFFFFFFFFFFFFFFF
F,F
@A00721:190:HGJW7DSXY:4:1564:20808:3396_1:N:0:TCTGCAAG+GCTGCACT
ATGTTTCGTTCTGTTACAAGAACGCTTTGGATTGTGACCATAAAGCCTCAGTTGCGATAGCTGTAGAG
GTATACGATATCTCTCTTGATGATGTAGCCACTATAATGCTTAAGAGAGAGATTGATGTATGTCATTT
CGT
+
FFFFFFFFF::FFFFFFFFFFFFFFFFFFFFFFFFFFFFFFFFFFFFFFFFFF::FFFFFFFFFFFFFF
FFFFFFFFFFFFFFFFFFFFFFFFFFFFFFFFFFFFFFFFFFFFFFFFFFFFFFFFFFFFFFFFFFF:F
FFF
@A00721:190:HGJW7DSXY:4:1544:20220:6981_1:N:0:TCTGCAAG+GCTGCACT
GAATTATTGGACACGATGAAAAATGACATACCCGTTCCTGACCTTGGTATCGTTATCCGTCATGGAGA
TGATGGCGCATCACTTGATTACTATATGCCCGAAGGATTGGGTTATTCTCACAACAAAGATAAGATTT
TAT
+
FFFFFFF:FFFFFFFFFFFFFFFFFFFFFFFFFFFFFFFFFFFFFFFFFFFFFFFFFFFFFFFFFFFFF
FFFFFFFFFFFFFFFFFFFFFFFFFFFFFFFFFFFFFFFFFFFFFFFFFFFFFFFFFFFFFFFFFFFFF
FF:
@A00721:190:HGJW7DSXY:4:2620:31313:17801_1:N:0:TCTGCAAG+GCTGCACT
GTTTTCCTTAAGGGCGGACAATTGTTCCATTGTGAACTCTTGGGGACTAGATTGAGCATTAATGAGTT
CCGAATAACTCGTAATAAATCTTTCTCTAGTGGACAATTCAGAATACCTATTTCCGTACCAAGAGCCA
CTG
+
FFFFFFFFFFFFFFFFFFFFFFFFF:F:FFFFFFFFFFFFFFFFFFFFFFFFFF:FFFFFFFFFFFFFFF
FFFFFFFFFFFFFFFFFFFFFFFFFFFFFFFFFFFFFFFFFFFFFFFFFFFFFFFFFFFFFFFFFFFFF
FFF
@A00721:190:HGJW7DSXY:4:1548:11008:8688_2:N:0:TCTGCAAG+GCTGCACT
TGGAAGAAGCGAGGTTCGTTCCTCAATAAAAATTTTTCTCAAGAGATTATGGTTGATAAAGTTTTCTT
CATAAGGGCTATTCAGTACGTCATGGGGATCACTTCTTCGGTCAATGA
+
FFF:FFF,FFFFFF:FFFFFF:FFFFFFFFFFFFFFFFFFFFFFFFFFFF:FF,FFFFFFFFFFFFFFF
F,::FFFFF:FFFFFFFFFFFFFFFFFFFFFFFFFFF,FFFFFFF,FFF
```

```
@A00721:190:HGJW7DSXY:4:1562:15411:35790_1:N:0:TCTGCAAG+GCTGCACT
AATATGTTACGACATGGCTTCGTAACAACAGTGCGAGGATTGTCATCTCGGGGAAAGTGATTCACCAA
GATGTTCATATAGATACGAGATACCTAGAGAAGGTCGCAGCGATCTTACTCGTTATCGGAGCTAAGAA
GAA
+
FFFFFFF,FFFFFFFFFFFFFFFFFFFFFFFFFFFFFFFFFFFFFFFFF:FFFFFFFFFFFFFFFF
FFFFFFFFFFFFFFFFFFFFFFFFFFFFFFFFFFFFFFFFFFFFFFFFFFFFFFFFFFFFFFFFFFFF
FFF
@A00721:190:HGJW7DSXY:4:2376:3495:15186_2:N:0:TCTGCAAG+GCTGCACT
GTTATGCTCGCAAACTTAACGTTAGTACCGGAGGTGATCTCTCTGCTTCTGCGTTACTGAGAGAAAAG
ATTCAGGACGCCTACGGTGACTTTATGGACAAGATGCAAGAGATCGGA
+
FFFFFFFFFFFFFFFFFFFFFFFFFFFFFFFFFFFFFFFFFFFFFFFFFFFFFFFFFFFFFFFFFFFF
FFFFFFFFFFFFFFFFFFFFFFFFFFFFFFFFFF,FFFFFFFF:FFFF
@A00721:190:HGJW7DSXY:4:2240:4182:34882_2:N:0:TCTGCAAG+GCTGCACT
TTTAGGTTTAGTAGGTGATCTTGACGTCGATTCTTTGTATAGGGAGTGTGAGAAGACAACGAATCTTG
AAACTACGGTCCTTATCAAAGAAAATGGTGAACCGGTCTCTAAATCTT
+
F,FFFFF:FFF:FFFFFF:FFFFFF:FFF:F:FF:FFFFFFFFF:FFFFF:FFF::F:F:FF:FFFFF
F:F:FFF,FFFFF:FFFFF:FFFF:F:,F:FF,FFFFFFFF:FFFFFF
@A00721:190:HGJW7DSXY:4:2647:32027:5415_2:N:0:TCTGCAAG+GCTGCACT
AGATTGAAGAACACGTCAAGAAGTCGCTCGGCGTTGTTCTTGAAAACCAAGTTAAGAAGGAGGCTGAT
CATGGAGTCTTTGTACCTTCTGTCAAACTTCGAGGTTCAGCTACGAGA
+
FFFFFFFFFFFFFFFFFFFFFFFFFFFFFFFFFFFFFFFFFFFFFFFFFFFFFFFFFFFFFFFFFFFF
FFFFFFFFFFFFFFFFFFFFFFFFFFFFFFFF:FFFFFFFFFFFF:FF
@A00721:190:HGJW7DSXY:4:2364:2844:19382_1:N:0:TCTGCAAG+GCTGCACT
TGGATTGAAGGCTGGCTCTAAGGGAGACTCCTTCTTTTTGAATGTTGTAAAGAATTGGGTTGAGAAGT
GTGCTTTACAAGGAGTTAGACTCTTCTTCAAGTTTTTAGATCTTCTTGATAATCTTAAAGAAAAATTT
TCG
+
FFFFFFFFFFFFFFFFFFF:FFFFFFFFFFFFFFFFFFFFFFFFFFFFFFFFFFFFFFFFFFFFFFFF
FFFFFFFFFFFFFFFFFFFFFFFFFFFFFFFFFFFFFFFFFFFFFFFFFFFFFFFFFFFFFFFFFFFF
FFF
@A00721:190:HGJW7DSXY:4:1543:29803:31501_2:N:0:TCTGCAAG+GCTGCACT
CTGTACTTACATTCTTTACGCCAGATTCTTCATGGGGCGACGCTGTTTCTCTTGGTATTTCTGAAATA
GTTTATCAGAGCGTCATTTTCCTTTGGGATCTTTCCTCTGGTAAGGGA
+
FFFFFFFFFFFFFF,FFFFFFF,FFFFFFFFF:FFFFFFFFFF,FFFF:FF:FFFFFFFFFFFFFFFF
FFFFFFFFFFFFFFFFFFFFFFF,:FFFFFFFFFFFFFFFFFFFFFFFF
@A00721:190:HGJW7DSXY:4:2107:16577:10786_2:N:0:TCTGCAAG+GCTGCACT
ATTCTTCTCACTCTTGACGAGAATTATTACTGTTTTCTCTACCCATAACTTCTTTTTCAAAAATGCGG
ATGGGTCTACTGATGTTTTCCTACAGAGTGCAGTCGTTTAAACACTTA
+
FFFFFFFFFFFFFFFFFFFFF::FFFFFFFFFFFFFFFFFFFFFFFFFFFFFFFFFFFFF,FF:FFFFF
FFFFFFFFFFFFFFFFFFFFFFFFFF:FFFFFFFFFFF:F,,:,FF,FF
@A00721:190:HGJW7DSXY:4:2438:7509:18443_2:N:0:TCTGCAAG+GCTGCACT
GGAAAATAATTGGTGATGAAAATACGTATGTCGGTGGGGTTCTCAAGGCTCTCATCGCCACTGGGATT
CTAAGGGGTTCCATAAGATATTATCTGACATTGTGGTTCAATGATCAG
+
FFFFFFFFFFFFFFFFFFFFFFFFFFFFFFFFFFFFFFFFFFFFFFFFFFFFFFFFFFFFFFFFFFFF
FFFFFFFFFFFFFFFFFFFFFFFFFFFFFFFFFFFFFFFFFFFFFFFF
@A00721:190:HGJW7DSXY:4:2168:27037:36292_2:N:0:TCTGCAAG+GCTGCACT
TGGTCCTTACGTCTTAAGTAATTTGGATGTTGTCGAAAACTATCAGGTTCTTGTCACGATTCTTAAAA
```

GAAGTTTATTAAAACTTCAGAGTCTCTTGGAAGTCCAGGTAGCTAATG
+
FFFFFFFFFFFFFFFFFFFFFFFFFFFFFFFFFFFFFFFFFFFFFFFFFFFFFFFFFFFFFFFFFFFFFFF
FFFFFFF:FFFFFF:FFFFFFFFFFFFFFFFFFFFFFFFF:FFFF:FF
@A00721:190:HGJW7DSXY:4:1466:32868:9909_2:N:0:TCTGCAAG+GCTGCACT
GCCACAGACTTTCTGGGAAAAGAGACCTCTGTTGGTAGAGGAGTTGAGAGAATTACTCAGATTCAATC
TGGCATCACTGAACGTCTACAACGAGTTAGGGAGTGGCTAGAGATCGG
+
FFFFFFFFFFFFFFFFFFFFFFFFFFFFFFFFFFFFFF,FFFFFFFFFFFFFFFFFFFFFFFFFFFFF
FFFFFFFFFFF:FFFFFFFFFFFFFFFFFFFFFFFFFFFFFFFFFFFF
@A00721:190:HGJW7DSXY:4:1209:26096:3818_1:N:0:TCTGCAAG+GCTGCACT
CAATACCTTCCACTGAGTTCATCACCCAGAGTTTCGACGTCGCGGAAAACGATGAAGATTTCCACACT
GTTCGAGAATCTTCTGATGCTGAATTTGAGGAAAATTTTTCGGAAGTTTTTGGTATAAATCCTGATGG
CTC
+
FFFFFFFFFFFFFFFFFFFFFFFFFFFFFFFFFFFFFFFFFFFFFFFFFFFFFFFFFFFFFFFFFFFF
FFFFFFFFFFFFFFFFFFFFFFFFFFFFFFFFFFFFFFFFFFFFFFFFFFFFFFFFFFFFFFFFFFFF
FFF
@A00721:190:HGJW7DSXY:4:1357:8522:26209_1:N:0:TCTGCAAG+GCTGCACT
TAAAACCTTCTTTTATCATTGCAACAGGACTTTGAGAAGACTGTTGAAATGGACAAAATCTGGGATCA
TAGGTATGTGTCACGCTACACTCGAAATATGGGCCCTCATAAGGAAGTATAGTTGGCGTCTTGTCGTT
GAC
+
FF:FFFFFFFFFFFFFFFFFFFFFFFFFFFFFFFFFFFFFFFFFFFFFFFFFFFFFFFFFFFFFFFFF
FFFFFFFFFFFFFFFFFFFFFFFFFFFFFFFFFFFFFFFFFFFFFFFFFFFFFFFFFFFFFFFFFFFF
FFF
@A00721:190:HGJW7DSXY:4:2452:8594:33880_2:N:0:TCTGCAAG+GCTGCACT
AGTTAGATCATTTGTTCAGGAAGATCTCTACAGAGATTATCGAGAGTTGTGATCTAGTTCTAAATTTA
GGTCGACGGACTTTAGCAACTTTAGATAAATTCTTTCCGGGCTCAATG
+
FFFFFFFFFFFFFFFFFFFFFFFFFFFFFFFFFFFFFFFFFFFFFFFFFFFFFFFFFFFFFFFFFFFF
FFFFFFFFFFFFFFFFFFFFFFFFFFFFFFFFFFFFFFFFFFFFFFFF
@A00721:190:HGJW7DSXY:4:1570:24840:11162_1:N:0:TCTGCAAG+GCTGCACT
TCTGACCATCGCAAAGAAGACTTTATTAGTTCATGAAGTTCTTACACTTCTCGCGACTAATGTCAGCT
TCACTACAAAGGCGTTACGTAAAGTAGTTTTCACGCTTTATCTTAAAGCATTACCACCAAATTTAGCA
CTT
+
FFFFFFFFFFFFFFFFFFFFFFFFFFFFFFFFFFFFFFFFFFFFFFFFFFFFFFFFFFFFFFFFFFFF
FFFFFFFFFFFFFFFFFFFFFFFFFFFFFFFFFFFFFFFFFFFFFFFFFFFFFFFFFFFF:FFFFFFF
FFF
@A00721:190:HGJW7DSXY:4:1341:14208:34272_1:N:0:TCTGCAAG+GCTGCACT
CTCGCCATATCAGATTAGAGACATGCCAGTTAGAGTTCTTCTAACCAGGACTGATGGTGAGATTCTTT
TACCTTTTGGAGGTCCAGGAACCTTTACTCCTAGTGTCTTTTCTAGAGTCCAAGGACGTTTCCGTCGG
AGG
+
FFFFFFFFFFFFFFFFFFFFFFFFFFFFFFFFFFFFFFFFFFFFFFFFFFFFFFFFFFFFFFFFFFFF
FFFFFFFFFFFFFFFFFFFFFFFFFFFFFFFFFFFFFFFFFFFFFFFFFFFFFFFFFFFFFFFFFFFF
FFF
@A00721:190:HGJW7DSXY:4:2555:19868:26036_1:N:0:TCTGCAAG+GCTGCACT
GAGACCGCTCAATAACACTCTATTGAATCCCTTTTCCACGATGAAAGCTCTTTATGAGGTGCTCCATG
CAAATTTACAGTTCCTCTTTGGTGATATCGCCTCTGTCCTTTCTCCTTTAATCGTGAGGTTACGAGAG
TTT
+
FFFFFFFFFFFFFFFFFFFFFFFFFFFFFFFFFFFFFFFFFFFFFFFFFFFF,FFFFFFFFFFFFFFF

FFFFFFFFFFFFFFFFFFFFFFFFFFFFFFFFFFFFFFFFFFFFFFFFFFFFFFFFFFFFFFFFFFFFFF
FFF
@A00721:190:HGJW7DSXY:4:1421:8811:24298_1:N:0:TCTGCAAG+GCTGCACT
GTATTTTGATAAATTTGTTGGAATCGATAATGATACTGACGAAGGTTCTATTACCACCCTTGTCATTG
ATACAAACATTCTAGGTGATGCTTTCTTAGAGGCTCGTCTAGCCGCTGAGATTCGTCTTACCTATTTA
CAA
+
FFFFFFFFFFFFFF:FFFFFFF:F:FFFFFFFFFFFFF,FFFFFFFF,FFF::FFFFFFFFFFFFFFF
FFFFFFFFF:FFFFFFFFFFFFFFFFFFFFFFFFFFFFFFFFFFFFFFFFFFFFF:FFFFFFFFFFF
F:F
@A00721:190:HGJW7DSXY:4:1416:17101:33896_2:N:0:TCTGCAAG+GCTGCACT
TTTGAGTGATAACGATTTAGTACTGGGAAAGGGTGGTAGTCAGTTTACTACTTTTCTTTCCCTCCCCT
TTTTCGATTGGATTCGACGGGGTCTTCGTCTCACGACTAATTTGATAA
+
FFFFFFFFFFFFFFF:,FFFFFFFFFFFFF,FF:FFFFFFFFFFFFFFFFFF,F:FF:FFFFFFFFFF
FFFFFFFFFFFF:FFFFFFFFFFFFFFF:FFFFFFFFF:FFFFFFFFF
@A00721:190:HGJW7DSXY:4:1421:32054:2644_2:N:0:TCTGCAAG+GCTGCACT
TACTTATGAAGGAGGTATTACTTCGGAAGTTCTTAATGTAGGTCTCCTAGTACTTAAGGGTTTATGTT
TCGGTTTCAGCTGGATGGATCTTTTGATGGTATGTTTCACATTGCTTG
+
FF::FF:FFF:FFF:FFFFFF,FFFFF:::F,FFFFFF,F,::,FFF:,,FFFFF::,::FFFF,F:F
FF:F,FFF:F:F,:FFF:F:FFFFFFFF:,FF:,F:FFFF::F,F,::
@A00721:190:HGJW7DSXY:4:2121:27362:14841_2:N:0:TCTGCAAG+GCTGCACT
TGGACTTGGGAAGAGATTAAACATAAAATCCAGCGATTCTATTATGTCGCTAAAGCCTGTCCAGGTGT
CGATGATGTTCGGGCTGTGGGGATTAACTTCCCTCGTGAAACTTACGA
+
FFFFFFFFFFFFFFFFFFFFFFFFFFFFFFFFFFFF::FFFFFFFFFFFFFF:FFFFFFFFFFF,F:F
FFFFFF,FFFFFFFFFF:FFFFF:FFFFFFFFFFFFFFFF:FFFFFFF
@A00721:190:HGJW7DSXY:4:1313:28149:1830_1:N:0:TCTGCAAG+GCTGCACT
CACAAGGATTACTAAGTCTGAGATCTGCCACTTCTAATTTTAGGAAACGTCGCGAAAAGAAAGTTCAT
GAGGATGTCTTGGTCGAAAAATATCGCCGGGAACTTCTAATAGAGGATCCTGATGACATTATTTCAAC
GGA
+
:FFFFFF,,F::FF,FFFFFFFFFFFF,F:FFFFFFFFFFFFF:FFFFFF:F:FF,FFFFFFFFF:FF
FFF:F,,F:FFFFF:FFFFFFFF:FF:FF,FFFFFFFFFFFFFFF,F::FFFFFFF:FFFFFFF,:FF
F:F
@A00721:190:HGJW7DSXY:4:1465:15429:27837_2:N:0:TCTGCAAG+GCTGCACT
AGTTCTACCACAGAAACCGTCTCAGGAACTGTCTCCACTGATTCGGAATTAGAGAAAGACGACCTTCT
TGTGGGGACTGAGACCTCTGAATTTTCAGGGTGCCCTGATGAGGACGT
+
FFFFFFFFFFFFFFFFFFFFFFFFFFFFFFFFFFFFFFFFFFFFFFFF:FFFFFFFFFFFFFFFFFFF
:FFFFFFFFFFFFFFFFF,FFFF:FFFFFFFFFFFFFFF:FFFFFFFF
@A00721:190:HGJW7DSXY:4:1355:27082:23782_1:N:0:TCTGCAAG+GCTGCACT
ACTCTGAGACTTCTAAGAGTGTTTCAAAAGACCAGAAACGTAACAAGAAGAATAAAGGTAGGAAGGGG
AGAAAACAGAACAAGCGAGCGGTTGAAGAGAAAACTGATATGGTTGCGGCTGATCGTACCCCAATTCC
TGC
+
FFF:FFFFF:FF,FFFFFFF:FF:FFFF::FFFFFF,F:FFFF,:::FFF:FF,,,FFFFF,FFF,:F
FFFF:FF:F:FF,FFF:FFF:FFFFFFF:FFF,::FFFF:FFFF:F:FFFFF:FF,FFFF,FFF:FF,
F:F
@A00721:190:HGJW7DSXY:4:2319:12897:11522_1:N:0:TCTGCAAG+GCTGCACT
TCTCGGCGATACCACTGATCTTCCAGAGTTACGTAGTGGTCGTTCTTTTGGTGATTTTATCAAAGAGA
AAGTTTCTGCTAGTCGCGACGATAGTATTTATCGGAGTGATATTAACAAGTTAAACTTCGAACTGACC
ACT

```
+
FFF:F,FFF:F,::::FFF:FF,F::FFFFF:FF,:FF:F::FFF:FFFFFFFFFF:FFF,FFFF,F:
:FFFFF:F:F,FFFF:FFFFFF,,FFFFFFFFFFFFFFF:FFFFFFFFFFF:,F,,:FFF::FFF:,F
FFF
@A00721:190:HGJW7DSXY:4:2108:32624:20635_2:N:0:TCTGCAAG+GCTGCACT
TTGAAGTACCGCAACATGGATCAGTTTTCCAAAAAGCTATTAATAGCAGTTGCGGGGCTATAATACCG
CCTCCAGATTACTCTAAACTGAATCCACTCTCTTGGGATATAAATGAG
+
,F::FFFFFF:FFFFFFFFFFFFF:FFFFFFFFFFFFFFFFFFFFFFFFFFFFFFF:FFFFFFFFFFF
FFFFFFFFF:,FFFFFFFFF,F:FFFFFFFFFF:FFFFFFFFFFFFFF
@A00721:190:HGJW7DSXY:4:2256:32796:35399_1:N:0:TCTGCAAG+GCTGCACT
AGAGTTTTGGAACTGTGTAATGTCCTACGTACTGTTCAAGGTGTTTGGGCTTCTCTAAAGAACAGCAA
CTTTCAACATGAATTAGTTATTGAAAGTCTTGACCCTCACGTCTCACTTTATCACCTTGGAGATGGTA
AAG
+
FFFFFFFFFFFFFFFFFFFFFFFFFFFFFFFFFFFFFFFFFFFFFFFFFFFFFFFFFFFFFFFFFFFF
FFFFFFFFFFFFFFFFFFFFFFFFFFFFFFFFFFFFFFFFFFFFFFFFFFFFFFFFFFFFFFFFFFFF
FFF
@A00721:190:HGJW7DSXY:4:2354:17897:36432_2:N:0:TCTGCAAG+GCTGCACT
AGACCTTTCTTGGGGAGTAAAGAGGGTTTGAAAATTACGGAACCTTTCCTCGTTCTTCATGAAGATCT
TCGAGTGTTTTATAGTGAGTTATTCTTGCGAAGGTTCAAGTCGATTGA
+
FFFFFFFFF,FFFFFFFF::FFFFFFFFFFFFFFFFFFFFFFFFFFFFFFFFFFFFFFFFFFFFFFFF
FFFFFFFFFFFFFFFFFFFFFFFFFFFFFFFFFFF:FFFFFFFFF,FFF
@A00721:190:HGJW7DSXY:4:2143:12689:5807_1:N:0:TCTGCAAG+GCTGCACT
CGAGATCTTGCAGAGTGACTGGAGTTCAGACGTGTGCTCTTCCGATCTGTGGAGGGAAGACCACTGAA
CTTATAGCGACTTTTGTCGAATTTTTCGCAAAAGGTCTTGAAGTACGTATTGTCACTGCTAATCGTAA
TT
+
FF,F:F,FF,FFFF:FF,FFFFFFFFF:FFFFFFFF:F,FFFFFFFFFFFFFFF,FFFFFFFFFFFFF
FFFFFFFFF,:FFFFFFFFFFF:FFFFFFFFFF:FFFFF:F:FF:F,FFFFFFFFFFFFFFFFFFF,:
,F
@A00721:190:HGJW7DSXY:4:2527:29351:20760_1:N:0:TCTGCAAG+GCTGCACT
GTGACTAAAGTTTTGGCACTTCTAGTTCATCGCGGTATTTGTGAAATTAAGACAGACGTGAAAGTTAT
GATGAGTGATCGTATTAGAACAGCTGATTCTTATTGCATGAACGTCGCTGAGGTCAAACCTGTGGACG
TTC
+
FFFF,FF:FFFFFFFFFFFFFFFFFFFFFFFFFFFFFFFFFFFFFFFFFFFFFFFFFFFFFFFFFFFF
FFFFFFFFFFFFFFFFFFFFFFFFFFFFFFFFFF,FFFFFFFFFFFFFFFFFFFFFFFFFFFFFFFFF
FFF
@A00721:190:HGJW7DSXY:4:2151:12933:28244_2:N:0:TCTGCAAG+GCTGCACT
CTGGTCAGACCCTTTTTCTTACACATCTACTTAAGGCGGCTTATACCATAGGTTACGGTGATTCCAAT
CAAATCGGATTCATCAATCGTATTGATCTCCAAGTCGCAAACTACTTC
+
FFFFFFFFFFFFFFFFFFFFFF:FFFFFFF:F::FF:,:,F,,F:F,:F,FF:,FFFFF,FF,,FFF:F
FFFFFFF,F,FFFFFF:F:FFFFFFFFFFFFF::FFF,FFF,FF:FFF
@A00721:190:HGJW7DSXY:4:1230:18457:16297_2:N:0:TCTGCAAG+GCTGCACT
TTGATGAGTGTCGTTCTTATCGAAGTCTCAGTTATCGCTGCCCGGCCGACGTTTGTCGCGAACTCTCA
GAGGTTTACGGCAGAAAAATTTCTCCTGCTTGTAATTCGAGAGAGGAC
+
FFFFFFFFFFFFFFFFFFFFFFFFFFFFFFFFFFFFFFFFFFFFFFFFFFFFFFFF:FFFFFFFFFFF
FFFF:FFFFFFFFFFFFFFFFFFFFFFFF:FFFFFFFFFFFFFFFFF::
@A00721:190:HGJW7DSXY:4:1266:5240:8782_1:N:0:TCTGCAAG+GCTGCACT
TTAGACCTATCAGATGTCTTGACGACTTCGAATTTGAGGACGGAGTTAAATATATTACTTTCACTCAA
```

GGTGAAAAGTTTGAAATTAACAAAGTCCTTGGTAAGAGTAAGGTTAAATGTGAAGATGCTCAGACTAT
TCA
+
:FFFFFFFFFFFFFFFFFFFFFFFFFFFFFFFFFFFFFFFFFFFFFFFFFFFFFFFFFFFFFFFFFFF
FFFFFFFFFFFFFFFFFFFFFF:FFFFFFFFFFFFFFFFF:FFFFFFFFFFFF:FFFFFFFFFFFFFF
FFF
@A00721:190:HGJW7DSXY:4:1442:20347:22357_2:N:0:TCTGCAAG+GCTGCACT
ACTTACCAGCACGTTCGGCTGGTTAGAATTAAACCTCAAGATGACCCTGTTTTTGCATCACAAACACA
TATTACCGTTGCTCTATCACGACACGTTGAATCTTTACGATACTTTGT
+
FFFFFFFFFFFFFFFFFFFFFFFF:FFFFFFF:FFFFFFFFFFFFFFFFFFFFFFFFFFFFFFFFFFF
FFFFFFFFFFFFFFFFFFFFFFFFFFFFFFFFFFFFFFFFFFFFFFFF
@A00721:190:HGJW7DSXY:4:1507:8079:36182_1:N:0:TCTGCAAG+GCTGCACT
TTAAACGATGCTACTTGTGAGAGGATCAATCACAGTAAGTAACTGTCACGTTTAGCACGGGAGGTTGC
AGATTCAACCCCTTATATCGAGGCTATCGAGGACGATGAGTATCTTGAAGTTGTTGACGACAAGCCTC
AAG
+
FFFFFFFFFFFFFFFFFFFFFFFFFFFFFFFFFFFFFFFFFFFFFFFFFFFFFFFFFFFFFFFFFFFF
FFFFFFFFFFFFFFF:FFFFFFFFFFFFFFFFFFFFFFFFFFFFFFFFFFFFFFFFFFFFFFFFFFFF
FFF
@A00721:190:HGJW7DSXY:4:2367:5493:4836_2:N:0:TCTGCAAG+GCTGCACT
TCTTCTACTGTCTCTGCCTTCCAAGATTTCTTAGAAGAGGTTATTCCTGGAACCACCTTAACGTCACT
CGGTAATCTTTCAGCCGAATTAAGTACTAATGAGTTCAAGTCGGTTGT
+
FFFFFFFFFFFFFFFFFFFFFFFFFFFFFFFFFFFFFFFFFFFFFFFFFFFFFFFFFFFFFFFFFFFF
FFFFFFFFFFFFFFFFFFFFFFFFFFFFFFFFFFFFFFFFFFFFFFFF
@A00721:190:HGJW7DSXY:4:1418:5737:20008_1:N:0:TCTGCAAG+GCTGCACT
GATCAAGGCTAATACCTTTATAGGTAAGAAGCCCCTTGAGCCGCGTAGTCAGCGCGTTTAGCTGTATC
ATCAGTCAGAGTATACCCGATAGGGTACCGAGTCTCCAAGAGAATATTTATTCTTATGAGGCTCGAAA
CTT
+
FFFFFFFFFFFFFF:FFFFF,FFFFFFFFFFFFFFFFFFFFFFFFFFFFFFFFFFF:FFFFFFFFFFF
FFFFFFFFFFFFFFFFFFFFFFFFFFFFFFFFFFFFFFFFFFFFFFFFFFFFFFFFFFFFFFFFFF:F
FFF
@A00721:190:HGJW7DSXY:4:1508:24551:6214_1:N:0:TCTGCAAG+GCTGCACT
CAACGGAAAGACATATCGCACCCGAAATTTTTGGGCAGAATGCTCTTAACGCGGTTCTTGAACGGTGT
TTTGATCAGTCTAAGTTGGATAAAGTTATAGGAGACTCTGTTAACCTGAGAGCTCCTCAATTGATAGA
TTG
+
FF,F:FFFF:FFFFFF,FFFFFFFF:FF:FFFFF,F:FF:F:FF,FFFF:FF,FFFFFFF,:FFFFFF
FFFFFF::FFF,:F:FFF:FF:F,FFFFFFFFFFFFFF,FFFFFFF,FF:FF,F::FFFFFFFFFFFF
F,,
@A00721:190:HGJW7DSXY:4:1336:15438:9878_2:N:0:TCTGCAAG+GCTGCACT
CTAGTATACTTCGTAAAGTCGACAGCGAAATGGTCAATATTGATAATTGGACCGACTATATGACCTAT
TTTAAGCTCATGGTGAAAAGGGAAGCTAAGGTTAAACTTGATGAGTCA
+
FFF:FFFFF,,FFFFFFFFFFFFFFFF::F:,FFFFFFFFFFFFFFFFFFF:FFFF:FFFFFFF:FF:
FFF,FFFFFFFF,FFFFFFFFFFFFFFF:FFFFFFFFFFFFFF,,FFF
@A00721:190:HGJW7DSXY:4:2452:6985:35603_1:N:0:TCTGCAAG+GCTGCACT
GCAATTTTCGGACCTATCTTTAAGGTTCTTGTGAGGAGAGTCAAACAGTGTCTTTTACCCAACATTTT
TATGTATACAGAGATGACCAGTGACGATTTCTCCCGGAAGCTCTTCTCTCTCTTAGGAGGTCATGATG
ACT
+
FFFFFFFFFFFFFFFFFFFF:FFFFFFFFFFFFFFFFFFFFFFFFFFFFFFFFFFFFFFFFFFFFFFF

FFFFFFFFFFFFFFFFFFFFFFFFFFFFFFFFFFFFFFFFFFFFFFFFFFFFFFFFFFFFFFFFFFFF
FFF
@A00721:190:HGJW7DSXY:4:1456:30563:6887_2:N:0:TCTGCAAG+GCTGCACT
TTTCGAAGTTTGACAAATCTCAAGATTTCTTTTGTAAAGCCTATGAGATAAAGATGTATTCTTATTTA
GGCTTCGATGCTGAATTTCTTGAACTTTGGAGTGCCAGTGAATATTTT
+
F:FFFFFFFFFFFFFFFFFFFFFFFFFFFFFFFFFFFFFFFFFFFFFFFFFF,FFFFFFFFFFFFFFFF
FFFFFFFFFFFFFFFFFFFFFFFFFFFFFFFFFFFFFFFFFFFFFFFFF
@A00721:190:HGJW7DSXY:4:1445:14335:21934_1:N:0:TCTGCAAG+GCTGCACT
GAGTATCCATAACGATTTGTCATTTACCACCTTTGCTCAACGACGAAGTGGAACGGCCAACACTTTCT
TTGGTAATTGTATGATCACTTTGATGATTTTATCACTTTATTATGACCTTGGAAAGTTTTCGGCTGTG
GTT
+
FFFFFF:FFFFFFFFFFF:FFFFFFFF:FFFFF:FFFFFFF:FF:FFFFFFF:FFFFFFFFFFFFFFF
FFFFFFFFFFFFFFFFFFFF:FFFFFFFFFFFFFFFFFFFFFFFFF:FFFFFFFFFFFFFFFFFFFFFFF
FFF
@A00721:190:HGJW7DSXY:4:2526:6587:30968_1:N:0:TCTGCAAG+GCTGCACT
CCATTCTGTTTTCAAAGACTCCAATAACTGATCATTCTGATCATATGATTTTGGACAATGGTTTCGAA
GCGAAATTTTTGAGGGATAAACCAGCGTACTTCTGCTCGCGATTTATTCTTTTTGTCAATGATTACAT
CTA
+
FFFFFFFFFFFF:FFFFFFFFFF,FFFFFFFFF:FFFFFFFFFFFFFFFFFFFFFFFFFFFFFFFFFF
FFFFFFFFFFFFFFFFFFFFFFFFFFFFFFFFFFFFFFFFFFFFFFFFFFFFFFFFFFFFFFFFFFFF
FFF
@A00721:190:HGJW7DSXY:4:2112:29396:3583_1:N:0:TCTGCAAG+GCTGCACT
CCTTACAAGATCATCGTTAAGTTAGGTAAACCCCTAACCGATGTTACTCTTGTAGGACTTTGGGAGAG
ATTCGTTAGTTTTAAAGACTATACTAAATATCTCCACTACGAGGTTGTTGTTTGGCGATTAGCTCTTG
CGA
+
:FF:F:FFFFFFFFFFFFFF:FFFFFFFFFFFFFFF,:FFFFFFFFFFF:FFFF:FF:FFFFFFFFFF
FFFFFFFFFFFFFFFFFFFFFFFFFFFFF:FFFF:F:FFFFFFFFFFFFFFFFFFFFFFFFFFFFFFFF
FFF
@A00721:190:HGJW7DSXY:4:1644:31448:3411_2:N:0:TCTGCAAG+GCTGCACT
ATTTAGGGTCGTTATACCTATTCGGCTATCTGTGCTTTGAATTGTATAAGATCAAATTTTAAACGATT
CCGAGAGGTTTATCCTGATGTGGAGGGAGTTCTTGAAGCATCCCGACC
+
FFF,FF,FFFFFFFFF:FFF,FFFFFFFF,FFFFFFFFFFFFF:FFFFF:FFFFFFFFFFFFFFFFFF
,:FFFF:,:FFFF::F:F:FFFFFFF:FFFF:FFF:FF,F:F:::FFF
@A00721:190:HGJW7DSXY:4:1660:14425:36151_1:N:0:TCTGCAAG+GCTGCACT
TGATGCGGTTTCATAAGATTATCAGTAATCCTGATTCTACTACGGATTCATGGACCTTCCATCCTGTA
CCTCTTCTGGAGAGACGTATCTCAAAGATCGACTTCGAGACAGGTGATATTGTTGAGCGAGAGCGACC
AAG
+
FFFFFFFFFFFFFFFFFFFFFFFFFFFFFFFFFFFFFFFFF:FFFFFFFFFFFFFFFFFFFFFFFFFF
FFFFFFFFFFFFFFFFFFFFFFFFFFFFFFFFFFFFFFFFFFFFFFFFFFFFFFFFFFFFFFFFFFFF
FFF
@A00721:190:HGJW7DSXY:4:2364:32750:18380_1:N:0:TCTGCAAG+GCTGCACT
CGTGGCGCTTTATTTTAAAAAGCTCCCTCCTCTCGCAAGTTTGATCTGATACTTATCACGAAAGGACT
TGAGTCTTTTGATCAGTCTCTGACTTGACCATCTTTCATTTTAAATGACGATCATTAAGACTCTCTTT
CTT
+
FFFFFF::F::F:FFFFFFFFFFFFF:FFFFFFFFFFFFFFFFFFFFFFFFFFFFFFFFFFFFFFFFF
FFFF:F:FFFFFFFFFFFFFFFFFFFFFFFFFFFFFFFFFF:FFFFFFFFFFFFFFFFFFFFFFFFFFF
FFF

@A00721:190:HGJW7DSXY:4:1621:13874:6918_2:N:0:TCTGCAAG+GCTGCACT
CTTTGCACTGATTTTTCCTCATCAGCTTGTGTTTTACCTAATCTGTGTTTTTACGATAACAACTATCA
GTTGATTAAGACTCTTCCGAAAGGCGAACTTTGGGAATTTGAAGCTCA
+
FFFFFFFFFFFFFFFFFFFFFFFFFFFFFFFFFFF:FFFFFFFFFFFFFFFFFFFFFFFFFFFFFFFF
FFFFFFFFFFFFFFFFFFFFFFFF:FFFFFF,FFFFF:FFFFFFFFFFF
@A00721:190:HGJW7DSXY:4:1558:21603:27445_2:N:0:TCTGCAAG+GCTGCACT
ACCGTGACGAACTGTGGTGTTCTTGATCTACGTTACGGATATACTCTCCTTGAGTTCACTCGTAAGGA
CACTACCACTTATTACGATATTTCTTTAGTCGATGGGTTTTCGACGCC
+
FFFFFFFFFFFFFF:FFFFFFFFFFFFFFFFFFFFFFFFFFFFFFFFFFFFFFFFFFFFFFFFFFFFF
FFFFFFFFFFFFFFFFFFFFFFFFFFFFFFFFFFFFFFFFFFFFFFFF
@A00721:190:HGJW7DSXY:4:2517:27597:29215_2:N:0:TCTGCAAG+GCTGCACT
TTTCTTCTAATGTGGCCTGAACTACGTTTCACTTTGTGTGTACTTGTCTCTATTTTAAGTTTTGTCTT
CCTTGTTTGTCTTATTCTATTAATCACTCATTTTAGTGAGATTATTAA
+
FFFFFFFFFFFFFFFFFFFFFFFF:FFFFFFFF:FFFFFFFFFFFFFFFFFFFF:FFFFFFFFFFFFF
FFFFFFFFFFFFFFFFFFFFFFFFFFFFFFFFFFFFFFFFFFFFFFFF
@A00721:190:HGJW7DSXY:4:1411:19425:33191_1:N:0:TCTGCAAG+GCTGCACT
ACGTGTGCTCTTCCGATCTGTTTTCGAATAGTGATAGCCACCCGCCTTACAGAGGGGCAGCAGCATGA
CCATAATTGGTATTGACTTTGGTACCACCTTTTCTACTGTCTGTCTCGCCTCTTCGAATGATGTTTTA
ATG
+
FFFFFFFFFFFFFFFFFFFFFFFFFFFFFFFFFFFFFFFFFFFFFFFFFFFFFFFFFFFFFFFFFFFF
FFFFF:FFFFFFFFFFFFFFFFFFFFFFFFFFFFFFFFFFFFFFFFFFFFFFFFFFFFFFF,FFFFFF
FFF
@A00721:190:HGJW7DSXY:4:2323:2202:13291_2:N:0:TCTGCAAG+GCTGCACT
TGATAGTGAATTTATACCTACAATCATTGGTTTTTCTAATAATGATGACACTATTGTTTATGGATATG
ATGCTATTACTCATGATGGTTACGGTCAGGGAGGATTCTCAGTTTACA
+
FFFF:FFFFFFFFFFFFFFFFFFFFFFFFFFFFFFFFFFFFFFFFFFFFFFF::FFFFFFFFFFFFFF
FFFFFFFFFFFFFFFFFFFFFFFF:FFFFFFFFFFFFFFFFFFFFFFFF
@A00721:190:HGJW7DSXY:4:1271:7780:17002_2:N:0:TCTGCAAG+GCTGCACT
CCGAAAGACGTGCTAAATTAAAACCATTGTACGATGTCGTCTGTCTCGGCGATCACTTTTCACTAAGA
ATTGATCCTACTTTTGGACAAGGCCAACTTCGTAATCTTCATGAGATC
+
FFFFFFFFFFF,FFFFF,FFFFFFFFFF:FFFFFFF:FF:F,,F:FF:F:FFFFF,:FFFFF,FFF,F
FFF,,F:,FF:F::F:FFFFFF,F:FFF:FF::FFF:FFFFF,F,FF:
@A00721:190:HGJW7DSXY:4:1515:10601:26115_2:N:0:TCTGCAAG+GCTGCACT
TATCTCTTTGGTTGTTAGGGATTTTGAGAGAGTGAAAGGCTTCAATTGTAGTGGTTTGGTCGTTTCAG
TACCCAGTCAGTATACCTCTACGCAAAGATATTTCATGAAAACTACAG
+
FFFFFFFF,FFFFFF,FFFFF:FFFFFFFFFFFFFFFFFFFF:F,FFF:FFFFF,FFFFFFFFFFFFF
FFFFFFFFFFFFFFFFFFFF:FFFFFFFFFFF:FFFFFFFFFFFFFFF
@A00721:190:HGJW7DSXY:4:2612:5972:16344_2:N:0:TCTGCAAG+GCTGCACT
ATTACCTGTCTTTCATATTATGAATGAACCTTCGGCCGCTCTCTTTGCTTCGATGTTGGATGTCAAGA
AGGGTTCAGAATGGGATTCCTACGTAGTTTATGACTTTGAGATCGGAA
+
FFFFFFFFFFFFFF:FFFFFFFFFFFFFFFFFFFF:FFFFFFFFFFFFFFFFF,FFFFFFFFF:FFFF
FFFF,FFFFF,FFFFFFFFFFFFFFFFFFFFFFFFFFFFFFFFF:FFFF
@A00721:190:HGJW7DSXY:4:1305:13150:26897_1:N:0:TCTGCAAG+GCTGCACT
ACGTGTGCTCTTCCGATCTTATAGTGTTATTTATTCTGGTGGTGATGATAATCTTGGAGGTCGTGACG
TTGATGAAGCTATAAGAGCGTTCTTATTACGGCGCACTTCTTTACCCGCCACCACACAACTTTCAGTT
TCA

+
FFFFFFFF:FFFFFFFFFF:FFFFFFFFFFFFFFFFFFFFFFFFFFFFFFFFFFFFFFFFFFFFFFFFF
FFFFFFFFFFFFFFFFFFFFFFFFFFFFFFFFFFFFFFFFFFFFFFFFFFFFFFFFFFFFFFFFFF:F:F
FFF
@A00721:190:HGJW7DSXY:4:1113:16405:24987_1:N:0:TCTGCAAG+GCTGCACT
ACTGGAAAACCCTCGTCAATACTTTTCAATAATATACGTCTTGAACTCACTTATGACGATCTAGTGAA
ATTAACGCGTCCTTTTCTGGAACGTAGTTTTACTATTATGAACAAAGTTATCAAAGATAGTGGCATTC
AAG
+
FFFFFFFFFF:FF:FFFFFFFFFFFFFFFFFFFFFFFFFFFFFFFFFFFFFFFFFFFFFFFFFFFFFFFF
FFFFFFFFFFFFFF:FFFFFFFFFFFFFFFFFFFFFFFFFFFFFFFFFFFFFFFFFFFFFFFFFFFFFF
FFF
@A00721:190:HGJW7DSXY:4:2173:1967:4993_1:N:0:TCTGCAAG+GCTGCACT
CCTGATTCCGGCCGGTGGTAGCTCATTTCTACCAGGGGCTATTACTCTTGCTGAAACAGCTATTAAAG
CTGTTGAGCGGGTAATTACACCTAAACGAGCTCGATCTGCCATCGCTGAAGGATGTGCACTCTTTTCT
GCT
+
FFFFF:FFFFFFFFFFFFFFFFFFFF:FFFFFFFFFFFFFFFFFFFFFFFFFFFFFFFFFFFFFFFFFFF
FFFFFFFFFFFFFFFFFFFFFFFFFFFFFFFFFFFFFFFFFFFFFFFFFFFFFFFFFFFFFFFFFFFFF
FFF
@A00721:190:HGJW7DSXY:4:1310:20690:18724_2:N:0:TCTGCAAG+GCTGCACT
TCGATTGTATCAACAGTAACATTACTTCTAATAAAGGTCTCTATCAAGCTCGTATTATCGTCGCTGCT
GGATCTGCGTTACCGTTGAAGGCTAAACTCCAAGGTAATTATGAAGAC
+
FFFFFFFFFFFFFFFFFFFFFFFFFFFFFFFFFFFFFFFF:FFFFFFFFFFF,F:FFFFFFFFFFFF:FF
FFFFFFFFFFFFFFFFFFFFFFFFFFFFFFFFFFFFFFFFFF,FF,FF
@A00721:190:HGJW7DSXY:4:2110:8712:19022_1:N:0:TCTGCAAG+GCTGCACT
CGTGTGCTCTTCCGATCTGGTGTTTCTAATCGTAACATTAATAATTTCAATCTTCTTAAAAAGAAGAT
AGAGAGTCATACTTTGGGCTATCGTTCAGGTGCATGGGGTTGGATAGCCAATATTGAAGTGGACACCT
CTG
+
FFFFFFFF:F,:F,,FF:FFFFFFF,FFFFF:FFFFFFFFFFF:F,FFF,F::FFFFFFF,,F,FFFF
:FFFFFFFFF::FFFF::FFFFFFFFFFF:FF:FFFFFFFFFFFFFFF::FFFFFFF:FFFFFFFFFF
:FF
@A00721:190:HGJW7DSXY:4:1240:15383:24314_1:N:0:TCTGCAAG+GCTGCACT
AGATAACCAATAAGGAAGGAGCTTATACTATGGTTGAATCAACTAAGCATACTACTTTTGCTTTGATT
ACTCTACCGCCTTACAAGTCTAAGGTTAAAGAGTCGATTAAACAAGTTGTACTTATCAATGATTTTAT
CGC
+
FFFFFFFFFFFFFFFFFFFFFFFFFFFFFFFFFFFFFFFFFFFFF:FFFFFFFFFFFFFFFFFFFFFFF
FFFFF:FFFFFFFFFFFFFFFFFFFFFFFFFFFFFFFFFFFFFFFFFFFFFFFFFFFFFFFFFFFFFFF
FFF
@A00721:190:HGJW7DSXY:4:1352:31774:16219_2:N:0:TCTGCAAG+GCTGCACT
TTCTAACCCAAAGAGAATATCTAAATTATTTACAAAATCTTACCACTATCTACGAACCTAAAGTTCTG
AAGACGTTGCTTGTCGCTTCCTCCTTTAATGGCACTACCTCGGTACAA
+
FFFFFFFFFFFFFFFFFFFFFF:F:,FFFFFFFFFFFFFFFFFFFFFFFFFFFF:FFFF:FFFFFFFFF
FFF:FFFFFFFFFFFFFFFFFFFFFFFFFFF,FFFFFF:FFFFFFFFFF
@A00721:190:HGJW7DSXY:4:2146:3558:29700_2:N:0:TCTGCAAG+GCTGCACT
GAAGGCGACCTGTTCCGGAGTTTTTACGGGATCCGGAATGTTCTGTCTATATTAGGTGAAGCGAAATA
CGATTTCGAACGTCAACGTTCACCGAGTTTCGTTTCGTACCTTGATGA
+
FFFFFFFFFFFFFFFFFFFFFFFFFFFFFFFFFFFFFFFFFFFFFFFFFFFFFFFFFFFFFFFFFFFFF
FFFFFFFFFFFFFFFFFFFFFFFFFFFFFFFFFFFFFFFFFFFFFFFF

```
@A00721:190:HGJW7DSXY:4:1622:32931:35321_2:N:0:TCTGCAAG+GCTGCACT
ATTTGATCTGGTTGCTCAATTCTAAGCGTGTTCCGCTTAGTGCGAGGCCTCTCTTCTACGAGTATTGC
TATTTATACGGTTACGAGAAGAAGGTTCGAAAGTTTGAGAACTTAGTT
+
F,FFFFFFFFFFFFF:FFFFFFFFFFFFFFFFFFFFFFFFF:FFFFFFFFF,FFFFFFFFFFFFFFFFF
FFFFFF:FFFFFFFFFFFFFFFFFFFFFFFFFFFF:FFFFFFFFFFFFFF
@A00721:190:HGJW7DSXY:4:2523:20898:28103_2:N:0:TCTGCAAG+GCTGCACT
TACTAAATACTAGATATTTGGATGATCTTGATAAAGAACTTCATGAATTTTCAGAGGATGAAAAATTT
TCTTCCTTGGATTTTAATATAAAGAATCTTTACCAGGACTCCTACTAC
+
FFFFFFFFFFFFFF:F:FFFFFFFFFFFFFFFFF,FFFFFFFFFFFFFFFFFFFFFFFFFFFFFFFFF
FFFFFFF:FFFFFFFFFFFFF,FFFFFFFFFF:FFFFFFFFFFFFFFF
@A00721:190:HGJW7DSXY:4:2544:18539:29058_2:N:0:TCTGCAAG+GCTGCACT
TCTGATCGTAAGAAGTTTATAAGGAACATATCCATTTGTTGTAATAGGGTCATCAACTTAGAACTCGA
AACTCTAAGTGGTAATACTGTCTTTCCAAGCTTACCTGTATCTTCAAT
+
FFFFFFFFFFFFFFFFFFFFFFFFFFFFFFFFFFFFFFFFFFFFFFFFFFFFFFFFFFFFFFFFFFFF
FFFFFFF:FFFFFFFFFFFFFFFFFFFFFFFFFFFFFFFFFFFFFFFF
@A00721:190:HGJW7DSXY:4:2567:10520:26130_1:N:0:TCTGCAAG+GCTGCACT
TTCGATAGGGGACATTTTATCGTGGGCTTTCCGTAAAGGAAGATCTGTTATCGACGGTTTATCGTTTA
CGAAGTATCACTCCACCGGTGAATGGTTTAAGAATACTATAAATAGAATAGCTATTACTACTAATGGC
GTT
+
FFFF:F,FF,F:FFFFFFFF::FFFFFF:FFF:FFF:FFFFF,F:FFFFFF:FFF,F:FF:F:FFFFF
FFFFFFFFFFFFFFF:FF,FFFFFFFFFFFFFFFFFFFFFFFFFF:F,FFF::FFFFFFF:FFFFFFFF
FFF
@A00721:190:HGJW7DSXY:4:2157:28646:24205_1:N:0:TCTGCAAG+GCTGCACT
TAGTGACATCTGGTCAATTTCGTTTTTAGGTGGTCTTTATTCTATTATTGATTTTTATGAGATTGATA
CGTTAACAGAAGATGATGTTAGAGTAGAATTCCTGAAGAGTCCTTCCGTAACCAACTTCTTGACAATT
TTC
+
FFFFFF:FFFFFFFFFFFFFFF:FFFFFFFF:FFFFFFFFF,FFFFFFFFFFFFFFFFFFFFFFFFFF
FFFFFFFFFFFFFFFFFFFFFFFFFFFFFFFFFFFFFFFFFFFFFFFFFFFF:FFFFFFFFFFFFFF
FFF
@A00721:190:HGJW7DSXY:4:1110:29939:8124_2:N:0:TCTGCAAG+GCTGCACT
AGGATTATGTCTGTGTTGAAAGACTTTCTACCGATAAGGGTCACTAGTCTTACACAATTAAGAAATAA
TCCTACATTGACTGATTATCTAAAGATTGAAGGAAAATTTTATCCTCG
+
FFFFFFFFFFFFFFFFFFFFFFFFFFFFFFFFF:FFF,FFFFFFFFFFFFFFFFFF:FFF:FFFFFFFF
FFFFFFFFFF:FFFFFFFFFFFFFFFFFFFFFFFFFFFFFFFFFF:FFFF
@A00721:190:HGJW7DSXY:4:2674:8892:2988_1:N:0:TCTGCAAG+GCTGCACT
GACTTTCCTACGATACAAATGATAACTTCTAATTTACGTTTGCTACTCAGAGCTGGTCCTCTCAGCAA
AGGGATGCATGGTGTTATCTCCTCAGGAGTCCCGGCATTTGATAATAATGACGAGACTTTCGTCATAA
TCG
+
FFFFFFFFFFFFFFFFFFFFFFFFFFFFFFFFFFFFFFFFFFFFFFFFFFFFFFFFFFFFFFFFFFFF
FFFFFFFFFFFFFFFFFFFFFFFFFFFFFFFFFFFFFFFFFFFFFFFFFFFFFFFFFFFFFFFFFFFF
FFF
@A00721:190:HGJW7DSXY:4:1663:25816:36495_1:N:0:TCTGCAAG+GCTGCACT
GATTACACGGTACCAGCGCATCTCGTGTCGAGCCTCGACCCGATTATATGGTATTAACTTATAAAGGT
GAGGAGACCATTTTCTTGCTGAAATTTATAGAAGATTATTTTGACAGAATCAATAAGAATCATCGATT
CTC
+
FFFFFFFFFFFFFFFFFFFFFFFFFFFFFFFFFFFFFFFFFFFFFFFFFFFFFFFFFFFFFFFFFFFF
```

FFFFFFFFFFFFFFFFFFFFFFFFFFFFFFFFFFFFFFFFFFFFF,FFFFFFFFFFFFFFFFFFFFFFFFF
FF:
@A00721:190:HGJW7DSXY:4:1638:24261:30138_2:N:0:TCTGCAAG+GCTGCACT
GATTTACAACGATATAGGAGTAATCTACGAGAAGATTCTAGAATTCTTAATTATAATATTCAACCTCA
AAAGTTACGTGTTTACAGTAACGAGATCACACATTTTGAGAACTAACT
+
FFFFFFFFFFFFFFFFFFFFFFFFFFFFFFFFFFFFFFFFFFFFFFFFFFFF:FFFFFFFFFFFFFFF
F:FFFFFF:FFFFFF:FFFFFFFFFFFFFFFFFFFFFFFFFFFFFFFFF
@A00721:190:HGJW7DSXY:4:2557:6361:15201_1:N:0:TCTGCAAG+GCTGCACT
CTGATCATTTTGACAATCTCTTCTTATAATAAATAAATAAATAATATTACTAATGGCAAATAGTGGAA
AGGCTCCAGTCAACACTAGTTCGAGTGCTGCTTTGGATTACGATGACGTGAGAATAGGTGAAGGGATA
GTT
+
:FFFFF:FFFFFFF:FFFF,FFFFFFFFFFFFFFFFFFFFFF:FFFFFFFFFFFFFFFFFFFFFFFFF
FFFFFFFFFFFFFF:FFFFF:FFFFF:FFFFFFFFFFFFF:F:FFFFFFF:FFFFF:FFFFFFFFFFF
FFF
@A00721:190:HGJW7DSXY:4:1148:28899:29966_1:N:0:TCTGCAAG+GCTGCACT
GATCTATTAAGGCGGCGGCGACTAATCTTAAAGCTTCACCCAAAATGAAGGACACTTCGAAACTGAAT
AATGATGCACTACAAACAAGTATCGCTAATCATCTGAAAAAGGCTCTAAAGGACACTATACAATATCC
AGA
+
FFFFFFFFFFFFFFFFFF:FFFFFFFF:FFFFFFFFFFFF,FFFFFFFFFFFFFFFFFFFFFFFFFFF
FFFFF:FFF,FFFFFFFFF:FFFF:FFFFFFFFFFFFFFFFFFFFFFFFFF:FFFFFFFFFFFFFFFF
FFF
@A00721:190:HGJW7DSXY:4:2127:2853:32675_2:N:0:TCTGCAAG+GCTGCACT
TGCTTTTACCGATAACGATAAATTCGTCATCAAATATAAAGAAAAGGACTATGAAATAAAAGATTTGG
TGCTTAGGGATATAATGAATTCAGCACCACATTTTGGTCAAGTCAATC
+
:FF:FFFFF,FFFFFFFF::FF,FFF:FF,F:FFFF:FFF::F:FFFFF:F,FFFFFFFFFFF:F,:
F:FF,:FFF:FFFFFFFFFF,FFFFFFFFFF,:,,F,:FFFF:FF:FF
@A00721:190:HGJW7DSXY:4:1303:28393:1626_2:N:0:TCTGCAAG+GCTGCACT
TCTTAGAGGATATTCGCCATTGTGGTACGAATTGGTCCAACATTTACCACCAAATTCTTTTGATACAA
CTCTAGCCTTCAAGTGGGGTATGCCTGCGGAATACGCTTGCTATTCAC
+
FFFFFF:FFFFFFFFFFFFFF:FFFFFFFFFFFFFFFFFFFFFFFFFFFFFFFFFFF:FF:FFF:FFFF
FFFFF,FFFFFFF:FFFFFFFFFFFFFFFFFFFFFF,FFFFFFFFFFF
@A00721:190:HGJW7DSXY:4:1231:10881:17331_2:N:0:TCTGCAAG+GCTGCACT
CGAACCCGTTGATGCTATGTCAGAAGAAGCTATTCAAGCTTGTCGTTTAAAGACTGCAGAGGCTGTCA
AACAAGCCCAGACTCAAGGAGTCCAATCAAAGGTTTTGAATTTAGCTC
+
FFFFFFFFFFFFFFFFFFFFFFFFFFFFFFFFFFFFFFFF:FFFFFFFFFF::FFFFFFFFFFFFFFF
FFFFFFFFFFFFFFFFFFFFFFFFFFFFFFFFFFF,FFFFFFFFF:FFF
@A00721:190:HGJW7DSXY:4:2161:19533:1626_2:N:0:TCTGCAAG+GCTGCACT
TGGGTGATAAAAAATTATTTAGAGATCTTTTCTACTTACAAAGGACAATTCAGTTTCATTAATTCTACG
GAAAAGTCCGGAGTATATTGTATGTTAATAGATAGTGGGATAATAGTT
+
FFFFFFFFFFFFFFFFFFFFFFFFFFFFFFFFFFFFFFFFFFFFFFFFFFFFFFFFFFFFFFFFFFFF
FFFFFFFFFFFFFFFFFFFFFFFF:FFFFFF:FFF:FFFFF:FFFFFFF
@A00721:190:HGJW7DSXY:4:2211:16450:14481_1:N:0:TCTGCAAG+GCTGCACT
GTAGTGAACGCGTTCGTAGAACCAAGTACTTTCCGTCTGAAGAGTTTAGAGCTGCTATTGATTATGTA
ACCGATAGGAGTTGTATAGTTGAAGATGTGGCTTTAGTCGATAATCCTTTAAGGACTATGTTGACTGA
CGA
+
FFFFF:,,FFFFFFFFFF:,F,FFFFF,,:FF,:FF:FFF:,FFFFF::FF:FF,,,FF:FFFFF:F:

F:FF:F::FFFFF:FF:F::,,,FF,FFF,F,FFFF,,FFFFF:FFF,FFF:FF,FF,F:FFFFFFF:
FFF
@A00721:190:HGJW7DSXY:4:1641:14778:21104_2:N:0:TCTGCAAG+GCTGCACT
GATCGCCACTCCAAACACTATATACATTATAAAATATAAAAATAAGGGAGTCATAACTTTTGTTACCA
AATTCTTGGCTTTCAACTAAGTCTCTGATTTTATTAATCATTACTTTA
+
FFFFFFFFFFFFFF,FFFFFFFFFFFFFFFFFFFFFFFFFFF:FFFF:FFFFFFFFFFF:FFFFFFFF
FFFFFFFFFFFFFFFFF,FF:F:FFF:FFFFFFFFFFFFFFFF,FFFF
@A00721:190:HGJW7DSXY:4:1515:28709:22842_2:N:0:TCTGCAAG+GCTGCACT
GGTCATTTACTCTGTACTTGATCTACGTCGTTTAACTGTCGGAACAACCTTTACTTAGAATGACTCTA
TCTTTTCAGACACTTCTTATATTATTTACAATAGTTAATATCATATTA
+
FFFFFFFFF:FFFFFFFFFFFFFFFFFFFFFFFFFFFFFFFFFFFFFFFFFFFFFFFFFFFFFFFFFF
FFFFF:FFFFFFFFFFFFFFFFFFF:FFFFFFFFFFFFFFFFFFFFFF
@A00721:190:HGJW7DSXY:4:1212:30635:35352_2:N:0:TCTGCAAG+GCTGCACT
CTATCGTTTTCTGCTTTTTCTTAGCTAATAGATTAATAAAGGATGAGTACGAAAACCAACTCTTCGTT
ACGAGACCTGATGGTAGAATAGATTTAGTCTCTACACCCATCTCAACG
+
FF,FFF:FFFFFFFFFFFFFFFFFFF,FFFFFFFFFFFFFFFFFFFFFFFFFFFFFFFFFFFFFFFFF
FFFFFFFFFFFFFFFF:FFFFFFFFFFFFFFFFFFFFFFFFFFFFFFF
@A00721:190:HGJW7DSXY:4:1241:15591:1188_1:N:0:TCTGCAAG+GCTGCACT
GTGTGCTCTTCCGATCTCTTAATACACGAACACACAACCCCCACACGACTCGAAACCGAGACGCCGTA
GATCAAAGTCCCTTCTAACTTTAGATT
+
:F:FF,:,:,F,,F,,,,,,F:,::,:,F,,:,:,,F,F,,:F,FFFFFFFFFFFFFFFFFFFFFFFF
FF:FFFFFFFFFFFFFFFFFFFFFFFFF
@A00721:190:HGJW7DSXY:4:2216:31937:17816_1:N:0:TCTGCAAG+GCTGCACT
CTTTACTCATTTTATAGCAGTTATTTATGTTGTACTTTTTATAATTTATAACCTTCTTATACTTTTGG
GTATGTTTTGTGAAAAACATGAGATACCCAAGTCTATCGAAGACATTATCTCTTATGTACTTATTGTA
ACG
+
::FFFFFFF:FFFFFFFFFFFFFFFFFFFFFF,FFFFFFFFFFFFFFFFFFFFFFFFFFFFFFFFFFF,
FFFFFFFFFFFFFFFFFFFFFFFFFFFFFFFFFFFFFFFFFFFFFFFFFFFFFFFFFFFFFFFFFFFF
FFF
@A00721:190:HGJW7DSXY:4:2462:16938:2926_1:N:0:TCTGCAAG+GCTGCACT
TTCTTATTCTTTTATTCCTTTAACTGTGTATGAGGAGGATTTATAATGCTTTTCGATAGTGATTGTTA
TCAATTTAAATACATTACTGTTTCAAATCATCTTTACTCACAGACTCCGAAAATGGTTTCAAATGAGG
TCA
+
FFFF,FFFFFFFFFFF,FFFFFF:FFFFFFFFF:,FF:FF,,FFF:F:,FF:FFFFFFF:,FFFFFFF
F,FFFFFFFFFF,FFFFF,FFFFFFFFF:FFFF,F:F::FFF,FF,F,FFFFFF,:FFFFFF,FFFFF
FFF
@A00721:190:HGJW7DSXY:4:2462:16938:2926_2:N:0:TCTGCAAG+GCTGCACT
TATTAAACGAACTATGATCCGATTCTTCGAGGGTATCACTACCACTGATCTGCCGACTTTAGTCAAGG
AACTTCAATTCCTTCGATGTTTATATTTTTCTATAAGGGAAAATAGCA
+
FFFFFF,FFF:FF,F,FFFFFFFF:FFFFFFF:FFFFFFF:F::FF,FFFF:FFFF:FFFFFFFFFFF
F::FFF:,FF:,FFFFFFFFFFFFFFFFFFFFFFFFFF:FFFFFFFFFF
@A00721:190:HGJW7DSXY:4:1631:12319:11052_2:N:0:TCTGCAAG+GCTGCACT
TTCGACAAATTTTAGAACTTTATTTCAGAACTCTCTCCGATCACCTTGGTGTGATCTCCCGGAGGATC
TTTTCAATAAGATGTATGATGATAATCTACTATTTTTAAATAGTGATA
+
FFFFFF,FFF:,FF:F,FFFFFFFFFFFFFF,FFFFFFFFFFFF:FFF,FFFFFF:FFF,FFFF,F,F
FFFF,FFFFF:FFFFF:FFF:FFFFFFFF,FFFF,FFFF:FFFFFFFF

```
@A00721:190:HGJW7DSXY:4:2236:15691:34554_1:N:0:TCTGCAAG+GCTGCACT
CTTTATCAAAGATTTGGCTTGGTCTATTTACCGCAAAAAGTTGGAAGAGGTCTATGATATTTATTTTA
CTTATACTTTAAATAGCACCGATTATAACTTGACTGTTGATAATATTACAATAGCTCTTTCTCAGAAG
ATT
+
FFFFFF:F,FF:FFF:,FFFFFF:F:FFFF:FFFF:FFF,FFFFFFF,:F:FFFF,FFFFFFFFFFFF
FFFFF:FFFFFFF:FFFF:FFFFFFFF:FFFFFFFFFFFFFFFFF:FFFFF:FFF:FFFFFFFFFFFF
FFF
@A00721:190:HGJW7DSXY:4:1578:16586:31594_2:N:0:TCTGCAAG+GCTGCACT
GGTGAACACGTCGGTAGCATTGATTTACTTTTACCGTTAGAACTGAAAAAGATTCTTTTCGAAAAGAA
ACCTATAAGTTACAAACGATTCTTCGACTATATTAAAGAAATAAAAAT
+
FFFFFFFFFFFFFFFFFFFFFFFFFFFFFFFFFFFFFFFFFF:FFFFFFFFFFFFFF:FFFFFFFFFFF
FFFFFFFFFFFFFFFFFFFFFFFFFF:FFFFFFFFFFFFFFFFFFFF:F
@A00721:190:HGJW7DSXY:4:1417:27525:35884_2:N:0:TCTGCAAG+GCTGCACT
CTTTACATTTTTAATGGTATGGTATAATGTGTCTTACAAATAGAACCTTCACTCTCGGTAATGAGAGT
GTATTCCTTAATATTCTAGTAACCGCTATGTGGGCATTACTAGGATTT
+
FFFFFFFFFFFFFFFFFFFFFFFF:FFFFFFFFFFFFFFFFFFFFFFFFFFFFFFFFFFFFFFFFFFF
FFFFFFFFFFFFFFFFFFFFFFFFFFFFFFFFFFFF:FFFF:FFFFFFF
@A00721:190:HGJW7DSXY:4:2148:7157:14137_1:N:0:TCTGCAAG+GCTGCACT
GATAGCAATTTATGTTTTAAATGCTAGGAAAATTTTAGAAAAACTCCATTGTATAGTTCAAGGAGTAC
TTAATTCAATAGCTTTAGTATTATTTACTATCGCCTTTATTGAAAATTGTATAAGAGTTAATTGCTTA
CAC
+
FFFFF,FFFFFFFFFFFFFFF:FFFFFFFFFFFFFFFFFFFFFFFFFF:FFFFFFFFFFFFFFFFFFFF
FFFFFFFFF:FFF:FFFFFFFFFFFFFFFFFFFFFFFFFFFFFFFFFFFFFFFFFFFFFFFFFFFFFF
FFF
@A00721:190:HGJW7DSXY:4:2656:31150:6277_1:N:0:TCTGCAAG+GCTGCACT
GGGGAGAATGAACTGCCGCTTCCCGGAAGAAACGATCTATTTACAGAGTATTTAGAGTAGTCCAAAGT
TTGTATAAATTTATATGATTTTCTATATATATACCACATATATATATATATAAAATCCTATAAATTTG
TAC
+
::,FFFFFFFF,F:FFF,FFF:FF:FF,,FFF::FFF:F:FFFFF,F,FFFFFFFF:FF::,FFF:FF
,F:F,FFFF:FFFFFF,FFFFFFF,F:FFFFFFFFFFFFFF,FFF:FFF,FFF:FFFFFFFFFFFFFF
FFF
@A00721:190:HGJW7DSXY:4:2510:1407:33207_2:N:0:TCTGCAAG+GCTGCACT
AACATACCATACACTCCCCAAACATAACATACCATAACCTACCATGACCAATCATCGCTCCAACTGGC
ACGCCAGACGTAGGGTCAAACCTACTACCGCCGGTCGGCAGATCGGCA
+
FFFFFFFFFFFFFFFFFFFFFFFFFFFFFFFFFFFFFFFFFFFFFFFFFFFFFFFFFFFFFFFFFFFF
FFFFFFF:FFFFFFFFFFFFFFFFFFFFFFFFFFFFFFFFFF:FF:FFF
@A00721:190:HGJW7DSXY:4:2102:29523:35775_2:N:0:TCTGCAAG+GCTGCACT
ACCCGCTGTGCAGAATAACCTTCCCAAATCTAGATCTGCACAGAAGAAGGCTGAAGGTCGTTACCGGA
CGACCACTCAAAGGAAACGGGCTGTTGCCCAGAGGAAAGCAGCGACCA
+
FFFFFFFFFFFFFF:FFFFFFFFFFFFFFFFFFFFFFFFFFFF:FFFFFFFFFFFFFFFFFFFFFFFFF
FFFFFFFFFFF:FFFFFFFFFFFFFFFFFFFFFFFFFFF:FFFFFFFFFF
@A00721:190:HGJW7DSXY:4:2153:26539:36933_2:N:0:TCTGCAAG+GCTGCACT
GTAACATTGACGGCAGTCCCGTCATCTCTGTGGGAACTGAGGCAGTACCTCAGTCAAAAGTCACATTC
ACCACTCCCATACCAATCAATGATGTGACTATACAGTTCGGCCATTTC
+
FFFFFFFFFFF:FFFFFFFFFFFFFFFFF:FFFFFFFFFFFFF,FFFFFFFFFFFFFFFFFFFFFFFF
FFFFFFFFFFFFFFFFFFFFFFFF:FFFFFFFFFFFFFFFFFFFFFFF:F
```

```
@A00721:190:HGJW7DSXY:4:1402:5638:3740_1:N:0:TCTGCAAG+GCTGCACT
ATACGTCGTGGGAGGTACTGAAGCCTTCCCCTCCGCTGGCACGAAGAACACTCCCAAGCCAATCAATA
GTTCTTCTGACCAGAATGAAAAGGGGTTTACCAGGGTAGTCTCTAAGTCTACCAGGAAGAGGGGAAAT
GTC
+
FFFFFFFFFFFFFFFFFFFFFFFFF,FFFFFFFFFFFFFFFFFFFFFFFFFFFFFFFFFFFFFFFFFF
FFFFFFFFFFFFFFFFFFFFFFFFFFFFFFFFFFFFFFFFFFFFFFFFFFFFFFFFFFFFFFFFFFFF
FFF
@A00721:190:HGJW7DSXY:4:2505:25220:3114_1:N:0:TCTGCAAG+GCTGCACT
AAAGGGAAAGGTCCATCAGGACCTAAGAAGGGGAAATTACCCCTAATTCTGAAGGAAGATCTCCCACC
ATCTGGGGTTGGGAATAAGAAATCTGCACGGGCCAAAGAGGCCGCTGCTACGAAGTCGCTGAAGGCGG
CTT
+
F,FF:FFFF:FFFFF:FFFFFFFFFFFFFFFFFFFF:FFFFFFFFFF:FFFFFFFFFFFFFFFFFFFF
FFFFFFFFFFFFFFFFFFFFFFFFFFFFFFFFFFFFFFFFFFFFFFFFFFFFFFFFFFFFFFFFFFFF
FFF
@A00721:190:HGJW7DSXY:4:1426:8522:10520_2:N:0:TCTGCAAG+GCTGCACT
TGTAAAGTTGCGCCTGCCGACCCCATCTTTGAGACCAAGACTCTCTACAGTAGGTTCGTGCCTACTAT
GAGCAAATCTGTTGAATTCGTCACTGAGGACGAAGGTGAGAGGACTTT
+
FFFFFFFFFFFFFFFFFFFFFFFFFFFFFFFFFFFFFFFFFFFFFFFFFFFFFFFFFFFFFFFFFFFF
F:FFFFFFFFFFFFF:FF:FFFFFFFFFFFFFFFFFFFFFFFFFFF:FFF
@A00721:190:HGJW7DSXY:4:2509:22733:36667_1:N:0:TCTGCAAG+GCTGCACT
CAAACTGAGAGCAGTCGCTGGGGAAAGAGAATTGCGTGGGTTGAAGGCCGACGCTTCGAGCTTGAGTG
TGACGAGGCCGGTGACGTTTTTGTCAACGGCGTTTGGAGGATTTTATCCGCGCTTAACTATGGGGCGA
AGA
+
FF,FFFFFFFFFFFFFFFFFFFFFFFFFFFFFFFFFFFFFFFFFFFFFFFFFFFFFFFFFFFFFFFFF
FFFFFFFFFFFFFFFFF,FFFFFFFFFFFFFF:FFFFFFFFFFFFFFFFFFFFFFFFFFFFFFFFFFF
FFF
@A00721:190:HGJW7DSXY:4:2111:17219:28557_1:N:0:TCTGCAAG+GCTGCACT
AGTCTTGAAGGACTTAGGTTGGCATATTTATGCCGACGATAATAAGCATGTCACCCTTAAGCACCGAG
GTAAGCTCGGGACTTACAAGAGAGAGTGTGAGAGAGTCGCTCTCACTATTTTTGCCCAGAATGCGGGC
TTT
+
FFFFFFFFFFFFFFFFFFFFFFFFFFFFFFFFFFFFFFFFFFFFFFFFFFFFFFFFFFFFFFFFFFFF
F,FFFFFFFFFFF:FFFFFFFFFFFFFFFFFFFFFFFFFFFFFFFFFFF:FFFFFFFFFFFFFFFFFFF
FFF
@A00721:190:HGJW7DSXY:4:2456:14009:1454_2:N:0:TCTGCAAG+GCTGCACT
ATTCACTGGTTCTGGGAGGCTGCTTTCCTCCATAATTACAACCTCTCTTTAGAGAGCATTAGAAGGAA
GTTGGGCAAATTCCCACATATGGTAGACGTTGTCACACTTTTCTTGAA
+
FFF:FFFFFFFFFFFFFFFFFF::FFFFF::FFFFFFFF:FFFFFFF,FFFFFFFFFFFF:FFF:FF:
:FFFFFFFFF:FFFFFFFFFFF::FFFFFFFFFF:F::,FFFFFFFFF
@A00721:190:HGJW7DSXY:4:1244:7988:10848_1:N:0:TCTGCAAG+GCTGCACT
CTCTTCCGATCTTTGGGGATTTCGATGTGAGAAGGTGGAAGGTGTCAAGGGTCTTCTTCATGCTGATC
TTTCAGGACCTAATCACATCATGATTGATAGTTACGGCTCGTATCGAGTTGGAGGGAGGAAGTTCTCC
GAA
+
FFFFFFFFFFFFFFFFFFFFFFFFFFFFFFFFFFFFFFFFFFFFFFFFFFFFFFFFFFFFFFFFFFFF
FFFFFFFFFFFFFFFFFFFFFFFFFFFFFFFFFFFFFFFFFFFFFFFFFFFFFFFFFFFFFFFFFFFF
FFF
@A00721:190:HGJW7DSXY:4:1406:8974:6010_2:N:0:TCTGCAAG+GCTGCACT
TCAGAGAGGGGAGCTCTGAAAAGGAAACTTCGTCAGAAGATGAGGAAGTCTAAGAGGAGGAGTTTGAG
```

```
GAAGGCTCTAGAGAAGATCTCACCACCTGGAGAAGGCAAGTGGAGAGT
+
FFFF::,F:FFF:FF:,FFFFF:FFFFFF:FFFFFFFFFFFFFF:FFFFFFFFFFFFFFFFFFFFFFF
FF:F,FF::F:FFFFFFFFFFFFFFFF:::FFFFF:,FFFFF:FFFFF
@A00721:190:HGJW7DSXY:4:1327:26151:10426_2:N:0:TCTGCAAG+GCTGCACT
AAGAAGGATTATTTGAAGCAGGGGTTACCACTTCCTAAGTTGGATGAGGGAGGTTGTTTCTTTGGAGT
TTTAGATAATCTTTTGAGAAGGGGTCATAGAGCCAGCAAGAGAAGCTT
+
FFFFFF,FFFFF:FFFFFFFF,FF:,F,:FF:FF,F:FF,,FFFFF:F,FFF,F,F:F:F:F:FF:,F
F:FFFF:FFFF:FF,:F,:FF,:::FFFFFFF:,F:FFF::FF:,,FF
@A00721:190:HGJW7DSXY:4:1537:32054:36558_2:N:0:TCTGCAAG+GCTGCACT
CCTTGAGAAGACAAGCCACATTAAGATATAATTTAGGTCAACTTGGGGTTTCGAATGTCATTAGTGCC
TTACGATTGAAAGAGGTCCTTTTGAGTATCGACAGATCTTTCGATCTT
+
FFFF:FFFF:FFFFFFFFFFFFFFFFFFFFFFFFFFFFFFFFFFFF:FFFFFFFFFFFFFFFFFFFFF
FFFFFFFFFFFFFFFFF:FFFFFFFFFFFF::FFFFFFFFF::FF:FF
@A00721:190:HGJW7DSXY:4:1245:8693:34616_2:N:0:TCTGCAAG+GCTGCACT
GTGGCAGAATTCGGTAACTTACCATATTGTTCCTTCCGGGAAGTTGTGGGATTTAACTTTGACTCAAC
TACTCCGATCAGATCCGGATGGTTTGATTGGTATGGACTACTCGACTT
+
FFFFFFFFFFFFFFFFFFFFFFFFFFFFFFFFFFFFFFFFF:FFFFFFFFFFFFFFFFFFFFFFFFFF
FFFFFFFFFFF:FFF,FFFFFF:FFFFFFFF:FFFFFFFFFFFFFFFF
@A00721:190:HGJW7DSXY:4:1562:31123:4633_1:N:0:TCTGCAAG+GCTGCACT
TAGGGTCATGGCAGATACTACCGATAAGGTGAAAAATATGCAGGTCAAAGATTCTGTAGCTCTGAGGG
CGGTTGAGGGAGCCGTTTTGGCCGACTTTAACTATTCTAAGAGTCTTTATGATTCAAAACCTATCGTT
GTC
+
FFFFFFFFFF:FFFFFFFFFFFFFFFFFFFFFFFFFFFFFFFFFFFFFFFFFFFFFFFFFFFFFFFFF
FFFFFFFFFFFFFFF,FFFFFFFFFFFFFFFFFFFFFFFFFFFFFFF:,FFFFFFFFFFFFFFFFFFF
FFF
@A00721:190:HGJW7DSXY:4:2216:16233:18208_1:N:0:TCTGCAAG+GCTGCACT
TACAGCGGCTTATTGTCGCGCAGTTTCCAGAATTGCGGTTGAAGTTCACCCATTCTATCTTGAATGCT
CACGCCGTCGCAGCCATATCGAGACTATGTTATAATCACTTGTTAAAGTACAGATTTAGTGATTTAGA
TAT
+
FFFFFFFFFFFFFFFFFFFFFFFFFFFFFFFF:FFFFFFFFFFFFFFFFFFFFFFFFFFFFFFFFFFF
FFFFFFFFFFFFFFFFF:FFFFFFFF:FFFFFFFFFFFFFFFFFFFFFFFFFFFFFFFFFFFFFFFFF
FFF
@A00721:190:HGJW7DSXY:4:1415:19000:36965_1:N:0:TCTGCAAG+GCTGCACT
GGAGGCGATATTCGTTTTCATATCCAAAACTCCTTGAATGGAGTACATATTTGCAACCCTATTCTTGA
TGTGAAAGATGCATCGAGGTATACGTCTAGGAACATTGATTGGGAACGTACCTACTCACGAGACGTCA
TGA
+
FFFFFFFFFFFFFF:FFFFFF:FFFFFFFFFFFFFFFFFFFFFFFFFFFFFFFFFFFFFFFFFFFFFF
FF:FFFFFFFFFFFFFFFFFFFFFFFFFFFFFFFFFFFFFFFFFFFFFFFFFFFFFFFFFFFFFFFFF
FFF
@A00721:190:HGJW7DSXY:4:1644:14579:13745_1:N:0:TCTGCAAG+GCTGCACT
ATGTTTCGTTCTGTTACAAGAACGCTTTGGATTGTGACCATAAAGCCTCAGTTGCGATAGCTGTAGAG
GTATACGATATCTCTCTTGATGATGTAGCCACTATAATGCTTAAGAGAGAGATTGATGTATGTCATTT
CGT
+
FFFFFFFFFF:FFFFFFFFFFFFFFFFFFFFFFFFFFF:FFFFFFFFFFFFFFFF:FFFFFFFFFFFF
FFFFFFFFFFFFFFFFFFFFFFFFFFFFFFFFFFFFFFFFFFFFFFFFFFFFFFFFFFFFFFFFFFFF
FFF
```

```
@A00721:190:HGJW7DSXY:4:1560:18674:8907_1:N:0:TCTGCAAG+GCTGCACT
GAATTATTGGACACGGTGAAAAATGACATACCCGTTCCTGACCTTGGTATCGTTATCCGTCATGGAGA
TGATGGCGCATCACTTGATTACTATATGCCCGAAGGATTGGGTTATTCTCACAACAAAGATAAGATTT
TAT
+
FFFFFF:FFFFFFFFFFF:F:FFFFFFFFFFFFFFFFF,FFFFFFFFF:FFFFFFFFFFFFFFFFFFF
FFFFFFFF:FFFFFFFFFFFFFFFFFFFFFFFFFFFFFFFF:FFFFFFFFFFFFFFFFFFFFFFFFFF
FF:
@A00721:190:HGJW7DSXY:4:1604:15709:36526_1:N:0:TCTGCAAG+GCTGCACT
TTTTCATTAAGTGCGGACAATTGTTCCATTGTGAACTCTTGGGGACTAGATTGAGCATTAATGAGTTC
CGAATAACTCGTATTAAATCTTTCTCTAGTGGACAATTCAGATTACCTATTTCCGTACCAAGAGCCAC
TG
+
FF:F,,FFFFF,FF,:F,F,FF::FF,,FF,F:FFFF:FFF,FF:,FF::F,F,F,:FFFFFF:FFFF
,,,,FFF:F:FFF,FF,FFFFFFF:FFF,F,FF,F:FF:FFF,FF,FFFFF:FF:FF::,,F,FFF::
F,
@A00721:190:HGJW7DSXY:4:2160:17092:32503_2:N:0:TCTGCAAG+GCTGCACT
TGGAAGAAGCGAGGTTCGTTCCTCAATAAAAATTTTTCTCAAGAGATTATGGTTGATAAAGTTTTCTT
CATAAGGGCTATTCAGTACGTCATGGGGATCACTTCTTCGGTCAATGA
+
FFFFFFFFFFFFFFFFFFFFFFFFFFFFFFFFFFFFFFFFFFFFFFFFFFFFFFFFFF:FFFFFFFFF
FFFFFFFFFFFFFFFFFFFFFFFFFFFFFFFFFFFFFFF:FFFFFFFF
@A00721:190:HGJW7DSXY:4:1675:8097:20337_1:N:0:TCTGCAAG+GCTGCACT
AATATGTTACGACATGGCTTCGTAACAACAGTGCGAGGATTGTCATCTCGGGGAAAGTGATTCACCAA
GATGTTCATATAGATACGAGATACCTAGAGAAGGTCGCAGCGATCTTACTCGTTATCGGAGCTAAGAA
GAA
+
FFFFFFFFFFFFFFFFFFFFFFFFFFFFFFFFFFFFFFFFFFFFFFFFFFFFFFFFFFFFFFFFFFFF
FFFFFFFFFFFFFFFFFFFFFFFFFFFFFFFFFFFFFFFF:FFFFFFFFFFFFFFFFFFFFFFFFFFF
FFF
@A00721:190:HGJW7DSXY:4:2466:25644:9862_2:N:0:TCTGCAAG+GCTGCACT
GTTATGCTCGCAAACTTAACGTTAGTACCGGAGGTGATCTCTCTGCTTCTGCGTTACTGAGAGAAAAG
ATTCAGGACGCCTACGGTGACTTTATGGACAAGATGCAAGAGATCGGA
+
FFFFFFFFFFFFFFFFFFFFFFFFFFFFFFFFFFFFFFFFFFFFFFFFFFFFFFFFFFFFFFFFFFFF
FFFFFF:FFFFFFFFFFFFFFF:FFFFFFFFFFFFFF:FFFFFF:FFF
@A00721:190:HGJW7DSXY:4:2455:7690:14497_2:N:0:TCTGCAAG+GCTGCACT
TTTAGGTTTAGTAGGTGATCTTGACGTCGATTCTTTGTATAGGGAGTGTGAGAAGACAACGAATCTTG
AAACTACGGTCCTTATCAAAGAAAATGGTGAACCGGTCTCTAAATCTT
+
FFFFFFFFFFFFFFFFFFFFFFFFFFFFFFFFFFFFF,FFFF::FFFFF:FFFF,FFFFF,FFFFFFF
FFFFFFFFFFFFFFFFFFFF:FFF:F,FFFFFF::FFF:FFFFFFFFF
@A00721:190:HGJW7DSXY:4:2651:15619:13103_2:N:0:TCTGCAAG+GCTGCACT
AGATTGAAGAACACGTCAAGAAGTCGCTCGGCGTTGTTCTTGAAAACCAAGTTAAGAAGGAGGCTGAT
CATGGAGTCTTTGTACCTTCTGTCAAACTTCGAGGTTCAGCTACGAGA
+
FFFFFFFF:FFFFFFFFFFFFFFFFFFFFFFFFFFFFFFFFFFFFFFFFFFFFFFFFFFFFFFFFFFF
FFFFFFFFFFFFFFFFFFFFFFFFFFFFFFFFFFFFFFFF:F:FFFFF
@A00721:190:HGJW7DSXY:4:2404:6072:9095_1:N:0:TCTGCAAG+GCTGCACT
TGGATTGAAGGCTGGCTCTAAGGGAGACTCCTTCTTTTTGAATGTTGTAAAGAATTGGGTTGAGAAGT
GTGCTTTACAAGGAGTTAGACTCTTCTTCAAGTTTTTAGATCTTCTTGATAATCTTAAAGAAAAATTT
TCG
+
F,FFFFFFFFFFF:FFFFFFFFFFFFF:F:FFFFFFFFFFFFFFFFFFFFF:FFFFFFFFFFFFFF:F
```

FFFFFFFFF:FFFFF:FFFFFFFFFFFFFFFFFFFFFFFFFFFFFFFFFFFFFFFFFFFFFFFFFFF
FFF
@A00721:190:HGJW7DSXY:4:2101:18927:13886_2:N:0:TCTGCAAG+GCTGCACT
CTGTACTTACATTCTTTACGCCAGATTCTTCATGGGGCGACGCTGTTTCTCTTGGTATTTCTGAAATA
GTTTATCAGAGCGTCATTTTCCTTTGGGATCTTTCCTCTGGTAAGGGA
+
FFFFFFFFFFFFFFFFFFFFFFFFFFFFFFFFFFFFFFFFFFFFFFFFF:FFFF,FFFFFF:FFF
FFFFFFFFFFFFFFFFFFFFFF:,FFFFFFFFFFFFFFFFFF:FFFF
@A00721:190:HGJW7DSXY:4:2213:4074:13275_2:N:0:TCTGCAAG+GCTGCACT
ATTCTTCTCACTCTTGACGAGAATTATTACTGTTTTCTCTACCCATAACTTCTTTTTCAAAAATGCGG
ATGGGTCTACTGATGTTTTCCTACAGAGTGCAGTCGTTTCAACACTTA
+
FF,FFFFFFF:FFFFFFFFFFFF:FFFFFFFF:FFFFFFFFFFFFFFF:FFFFFF,FFFFFFFF:FF
F:FFFFFFFFFFF:FFFFFFFFFFFFFFFFFFFFFFFF::FFFF:F:,F
@A00721:190:HGJW7DSXY:4:2635:19190:18349_2:N:0:TCTGCAAG+GCTGCACT
GGAAAATAATTGGTGATGAAAATACGTATGTCGGTGGGGTTCTCAAGGCTCTCATCGCCACTGGGATT
CTAAGGGGTTCCATAAGATATTATCTGACATTGTGGTTCAATGATCAG
+
FFFFFFFFF,FFFFFFFFFFFFFFFFFFFFFFFFFFFFFFFFFFFFFFFFFFFFF:FFFFFFFFFF
FFFFFFFFFFFFFFFFFFF,:FFFFFFFFFFFFFFFFFFFFFFFFFFF
@A00721:190:HGJW7DSXY:4:2621:26250:21840_2:N:0:TCTGCAAG+GCTGCACT
TGGTCCTTACGTCTTAAGTAATTTGGATGTTGTCGAAAACTATCAGGTTCTTGTCACGATTCTTAAAA
GAAGTTTATTAAAACTTCAGAGTCTCTTGGAAGTCCAGGTAGCTAATG
+
FFFFFFFFFFFFFFFFFFFFFFFFFFFFFFFFFFFFFFFFFFFFFFFFFFFFFFFFFFFFFFFFFFFFF
F:FFFFFFFFFFFFFFFFFFFFFFFFFFFFFF:FFFFFFFFFFFFFFF
@A00721:190:HGJW7DSXY:4:1528:10068:20118_2:N:0:TCTGCAAG+GCTGCACT
GCCACAGACTTTCTGGGAAAAGAGACCTCTGTTGGTAGAGGAGTTGAGAGAATTACTCAGATTCAATC
TGGCATCACTGAACGTCTACAACGAGTTAGGGAGTGGCTAGAGATCGG
+
FFFFFFFFFFFFFFFFFFFFFFFFFFFFFFFFFFFFFFFFFFFFFFFFF,FFFFFFFFFFFFFFFFFFF
FFFFFFFFFFFFFFFFFFFF:FFFFFFFF:FFFFFFFFF:FFFFFFFF
@A00721:190:HGJW7DSXY:4:1209:26503:3897_1:N:0:TCTGCAAG+GCTGCACT
CAATACCTTCCACTGAGTTCATCACCCAGAGTTTCGACGTCGCGGAAAACGATGAAGATTTCCACACT
GTTCGAGAATCTTCTGATGCTGAATTTGAGGAAAATTTTTCGGAAGTTTTTGGTATAAATCCTGATGG
CTC
+
FFFFF:FFFFFF:FFFFFFFFFFFFF:FFF,FFFFFFF:FFFF,FFFFFFFFFFFFFFFFFFFFFFFFF
FFFFFF:F,FFFFFFFFFFFFFFFFFFFFFFFFFFFFFFFFFFFF:F:FFFFFFFFFFFFFFFFFFFFF
FFF
@A00721:190:HGJW7DSXY:4:2106:21450:5353_1:N:0:TCTGCAAG+GCTGCACT
TAAAACCTTCTTTTATCATTGCAACAGGACTTTGAGAAGACTGTTGAAATGGACAAAATCTGGGATCA
TAGGTATGTGTCACGCTACACTCGAAATATGGGCCCTCATAAGGAAGTATAGTTGGCGTCTTGTCGTT
GAC
+
FFFFFFFFFFFFFFFFFFFFFFFFFFF:FFFFFFFFFFFFFFFFFFFFFFFFFFFFFFFFFFFFFFFFF
FFFFFFFFFFFFFFFFFFFFFFFFFFFFFFFFFFFFFFFFFFFFFFFFFFFFFFFFFFFFFFFFFFFFF
FFF
@A00721:190:HGJW7DSXY:4:2523:17833:7607_2:N:0:TCTGCAAG+GCTGCACT
AGTTAGATCATTTGTTCAGGAAGATCTCTACAGAGATTATCGAGAGTTGTGATCTAGTTCTAAATTTA
GGTCGACGGACTTTAGCAACTTTAGATAAATTCTTTCCGGGCTCAATG
+
FFFFFFFFFFFFFFFFFFFFFFFFFFFFFFFFFFFF:FFFFFFFFFFFFFFFFFFFFFFFFFFFFFFFF
FFFFFFFFFFFFFFFFFFFF:FFFFFFFFFFFFFFFFFFFFFFFFFFFF

@A00721:190:HGJW7DSXY:4:2105:13530:3161_1:N:0:TCTGCAAG+GCTGCACT
TCTGACCATCGCAAAGAAGACTTTATTAGTTCATGAAGTTCTTACACTTCTCGCGACTAATGTCAGCT
TCACTACAAAGGCGTTACGTAAAGTAGTTTTCACGCTTTATCTTAAAGCATTACCACCAAATTTAGCA
CTT
+
FFFFF,FFFFFFFFFFFF:F:FFFFFFF:FFFFFFFFFFF,FFFFFFFFFFFF:FFFFFFFF:F:FFF
:F,FFFFFFFFFF,:FFFFFFFFFFFF:FFFFFF:FFFFFFFFFFFFFFFFFFFFF:F:FFFFFF:FF
FFF
@A00721:190:HGJW7DSXY:4:1552:15293:10034_1:N:0:TCTGCAAG+GCTGCACT
CTCGCCATATCAGATTAGAGACATGCCAGTTAGAGTTCTTCTAACCAGGACTGATGGTGAGATTCTTT
TACCTTTTGGAGGTCCAGGAACCTTTACTCCTAGTGTCTTTTCTAGAGTCCAAGGACGTTTCCGTCGG
AGG
+
FFFFFFFFFFFFFFFFFFFFFFFFFFFFFFFFFFFFFFFFFFFFFFFFFFFFFFFFFFFFFFFFFFFF
FFFFFFF:FFFFFFFFFFFFFFFFFFFFFFFFFFFFFFFFFFFFFFFFFFFFFFFFFFFFFFFFFFFF
FFF
@A00721:190:HGJW7DSXY:4:1105:20437:36949_2:N:0:TCTGCAAG+GCTGCACT
GACCGCTCAATAACACTCTATTGAATCCCTTTTCCACGATGACAGCTCTTTATGAGGTGCTCCATGCA
AATTTACAGTTCCTCTTTGGTGATATCGCCTCTGTCCTTTCTCCTTTA
+
FFFFFFFFFFFFFFFFFF,FFFFFFFFFFF:FFFFFFFFFFF,FFFFFFFFFFFFFFFFFFFFFFFFF
FFFFFFFFFFFFFFFF,FFFFFFFFFFFFFFFFFFFFFFFFFFFFFFF
@A00721:190:HGJW7DSXY:4:1372:31412:2472_2:N:0:TCTGCAAG+GCTGCACT
ACTTGGGTTTATCGGTTGTATTTTGATAAATTTGTTGGAATCGATAATGATACTGACGAAGGTTCTAT
TACCACCCTTGTCATTGATACAAACATTCTAGGTGATGCTTTCTTAGA
+
FFFFFFFFFFFFFFF:FFFFFFFFFFFFFFF:FFFFFFFFFFFFFFFFF::FFF:FFFFFFFFFFFFF
FFFFFFFFFFFFFFFFFFFFFFFFFFFFFFFFFFFFFFFFFFF:FFFF
@A00721:190:HGJW7DSXY:4:1120:22200:33458_2:N:0:TCTGCAAG+GCTGCACT
TTACAATCTGTAGAGAGAGAGAGGTTAATCAGAATTTGAGTGATAACGATTTAGTACTGGGAAATGGTGG
TAGTCAGTTTACTACTTTTCTTTCCCTCCCCATTTTCGATTGGATTCG
+
FFFFFFFFFF,FFFFF:FFF:FFFFFFFF:F:FFFFFFFFFFFFFFFFFFFF:FFF::FFFFFF,FFF
FFF:FF,:::FFFFFFF,:F,F,:::FF:F:,FF::,:F,FF,FFFF:
@A00721:190:HGJW7DSXY:4:1156:29785:10207_2:N:0:TCTGCAAG+GCTGCACT
TTGATAAGGTTTTTCTTTTATGGGACTCTTGTTTGTACTTATGAAGGAGGTATTACTTCGGAAGTTCT
TAATGTAGGTCTCCTAGTACTTAAGGGTTTATGTTTCGGTTTCAGCTG
+
FFFFFFFFFFFFFFFFFFFFFFFFFFFFFFFFFFFFFFFFFFFFFFFFFFFFFFFFFFFFFFFFFFFF
FFFFFFFF:FFFFFFFFFFF:FFFFFFFFFFFFF,FFFFF:FFFFFFF
@A00721:190:HGJW7DSXY:4:1129:32235:28698_2:N:0:TCTGCAAG+GCTGCACT
TGTTTCACATTGCGTGATCACTTCCCGTGGACTTGGGAAGAGATTAAACATAAAATCCAGCGATTCTA
TTATGTCGCTAAAGCCTGTCCAGTTGTCGATGATGTTCGGGCTGTGGG
+
FFFFFFFFFFFFFFFFFFFFFFFFFFFFFFFFFFFFFFFFFFFFFFFFFFFFF:FFFFFFFFFFFFFF
FFFFFFFFFFFFFFFFFFFFFFFFFFFFFFFFFFFFFFFFFFFFFFFF
@A00721:190:HGJW7DSXY:4:1641:14434:32002_2:N:0:TCTGCAAG+GCTGCACT
AAACTTACGACGATGTTCTCGCTCGCATTTCACAAGGATTACTAAGTCTGAGATCTGCCACTTCTAAT
TTTAGGAAACGTCGCGAAAAGAAAGTTCATGAGGATGTCTTGGTCGAA
+
FFF:FFFFFFF:FFFFFFFFFFFFFFFFFFFFFFFFFFFFFFFFFFFFFFFFFFFFFFFFFFFFFFFF
FFFFFFFFFFFFFFFFFFFFFFFFFFFFFFFFFFFFFFFFFF::FFFFF
@A00721:190:HGJW7DSXY:4:1469:10366:6668_2:N:0:TCTGCAAG+GCTGCACT
GAACTTCTAATAGAGGATCCTGATGACATTATTTCAACGGATACCGATCTCAGTTCTACCACAGAAAC

CGTCTCAGGAACTGTCTCCACTGATTCGGAATTAGAGAAAGACGACCT
+
FF,FF:FF,FFFFFFFFFFFFFF:FFFFFFFFFFFFFFFFFFFFFFFFFFFFFFFFFFFFFFFFFF
FFFFFFFFFFFFFFFFFFFFFFFFFFFFFF,FFFFFF:FFFFFFFFFF
@A00721:190:HGJW7DSXY:4:2547:32931:1438_1:N:0:TCTGCAAG+GCTGCACT
GAGACCTCTGAATTTTCAGGGTGCCCTGATGAGGACGTTGAAAGGGATAATTTCAACTCTGAGACTTC
TAAGAGTGTTTCAAAAGACCAGAAACGTAACAAGAAGAATAAAGGTAGGAAGGGGAGAAAACAGAACA
AGC
+
FFFFFFFFFFF:FFF:F,FFFFFFFFF,FFFFF:F,FFFFFFF:F,FFFFFFFFFFFFF:F:F:FFF
FFFFFFFFFFFFFFFFFFFFFFFFFF:FFFFFFFFFF:FFFFFFFFFFFFFFFFFFFFFFFFFFFFFF
:FF
@A00721:190:HGJW7DSXY:4:2403:28330:1548_2:N:0:TCTGCAAG+GCTGCACT
ATGGTTGCGGCTGATCGTACCCCAATTCCTGCTAGAAAGATCACTCTCGGCGATACCACTGATCTTCC
AGAGTTACGTAGTGGTCTTTCTTTTGGTGATTTTATCAAAGAGAAAGT
+
FF,F:FF,F,FFFFFFF:F::F,FFFF:F:,:FF,FFFFFF:F,FFF,F,:FF:F,FF::FFF:FFF:
F:FF:FF::,F,F:,FF,FFFF:FF::F,F:F,FFFFFFF:F:FF:,,
@A00721:190:HGJW7DSXY:4:1231:23285:24752_1:N:0:TCTGCAAG+GCTGCACT
CAAGTTAAACTTCGAACTGACCACTTACAAGAGTGTTGAAGTACCGCAACATGGATCAGTTTTCCAAA
AAGCTATTAATAGCAGTTGCGGGGCTATAATACCGCCTCCAGATTACTCTAAACTGAATCCACTCTCT
TGG
+
FFFFFFFFFFFFFFFFFFFFFFFFFFFFFFFFFFFFFFFFFFFFFFFFFFFFFFFFFFFFFFFFFFFF
FFFFFFFFFF:FFFFFFFFFFFFF:FFFFFFFFFFFFFFFFFFFFFFFFFFFFFFFFFFFFFFFFFFF
FFF
@A00721:190:HGJW7DSXY:4:2345:16089:13291_1:N:0:TCTGCAAG+GCTGCACT
AGAGTTTTGGAACTGTGTAATGTCCTACGTACTGTTCAAGGTGTTTGGGCTTCTCTAAAGAACAGCAA
CTTTCAACATGAATTAGTTATTGAAAGTCTTGACCCTCACGTCTCACTTTATCACCTTGGAGATGGTA
AAG
+
FFF:FFFFFFFFFFFFFFFFFFFFFFFFFFFFFFFFFFFFFFF:F:FFFFF:FFFFFFFFFFFFFFFF
FFFFFFFFFFFFFFFFFFFFFFFFFFFFFFFFFFFFFFFFFFFFFFFFFFFFFFFFFFFFFFFFFFFF
FFF
@A00721:190:HGJW7DSXY:4:2354:26087:13823_2:N:0:TCTGCAAG+GCTGCACT
AGACCTTTCTTGGGGAGTAAAGAGGGTTTGAAAATTACGGAACCTTTCCTCGTTCTTCATGAAGATCT
TCGAGTGTTTTATAGTGAGTTATTCTTGCGAAGGTTCAAGTCGATTGA
+
FFFFFFFF:FFFFFFFF:FFFFFFFFFFFFFFFFFFFFFFFFFFFFFFFFFFFFFFFFF:FFFFFFFF:
FF:FFFFFFFFFFFFFFFFFFFFFFFFFFFFFFFFFF,FFFFFFFFFF,
@A00721:190:HGJW7DSXY:4:2304:29894:15248_1:N:0:TCTGCAAG+GCTGCACT
CGCGATCTTGCAGAGTGACTGGAGTTCAGACGTGTGCTCTTCCGATCTGTGGAGGGAAGACCACTGAA
CTTATAGCGACTTTTGTCGAATTTTTCGCAAAAGGTCTTGAAGTACGTATTGTCACTGCTAATCGTAA
TT
+
FF,FFFFF,FFFFFFFFFFFFFFFFFFFFFFFFFFFFFFFFFFFFFFFFFFFFFFFFFFFFFFFFFFFF
FFFFFFFFFFFFFFFFFFFFFFFFFFFFFFFFFFFFFFFFFFFFFFFFFFFFFFFFFFFFFFFFFFFF
FF
@A00721:190:HGJW7DSXY:4:1151:28004:13698_2:N:0:TCTGCAAG+GCTGCACT
TTGGCACTTCTAGTTCATCGCGGTATTTGTGAAATTAAGACAGACGTGAAAGTTATGATGAGTGATCG
TCTTAGAACAGCTGATTCTTATTGCATGAACGTCGCTGAGGTCAAACC
+
FF:FFFFFFFFFFFFFFFFFFFFF,FFFFFFFFFFFFFFFFFFFFFFFFF:FFFFFFFF:FFFFFF:FF
F,FFFFFFFFFFFF:FFF,:FF:FFFFFFFFFFFFFFFFFFFFFFFFF

```
@A00721:190:HGJW7DSXY:4:1138:24306:25363_2:N:0:TCTGCAAG+GCTGCACT
TTTTCATCGACGAATTATTCATGATTCACGCTGGTCAGACCCTTTTTCTTACACATCTACTTAAGGCG
GCTTATACCATAGGTTACGGTGATTCCAATCAAATCGGATTCATCAAT
+
FFFFFFFFFFFFFFFFFFFFFFFFFFFFFFFFFFFFFFFFFFFFFFFFFFFFFFFFFFFFFFFFFFFF
FFFFFFFFFFFFFFFFFFFFFFFFFFFFFFFFFFFFFFFFFFFFFFFF
@A00721:190:HGJW7DSXY:4:1167:7880:22592_2:N:0:TCTGCAAG+GCTGCACT
TCCAAGTCGCAAACTACTTCAACATCAACGACATCATTCTTGATGAGTGTCGTTCTTATCGAAGTCTC
AGTTATCGCTGCCCGGCCGACGTTTGTCGCGAACTCTCAGAGGTTTAC
+
FFFFFFFFFFFFFFFFFFFFFFFFFFFFFFFFFFFFFFFFFFFFFFFFFFFFFFFFFFFFFFFFFFFF
FFFFFFFFFFFFFFFFFFFFFFFFFF:FFFFFFFFFFFFFFFFFFFFF
@A00721:190:HGJW7DSXY:4:1168:10800:23954_2:N:0:TCTGCAAG+GCTGCACT
CTATGGAAATTAGACCTATCAGATGTCTTGACGACTTCGAATTTGAGGACGGAGTTAAATATATTACT
TTCACTCAAGGTGAAAAGTTTGAAATTAACAAAGTCCTTGGTAAGAGT
+
FFFFFFFFFFFFFFFFFFFFFFFFFFFFFFFFFFFFFFFFFFFFFFFFFFFFFFFF:FFFFFFFFFFF
FFFFFFFFFFFFFFFF,FFFFFFFFFFFFFFFFFFFFFFFFFFFFFFF
@A00721:190:HGJW7DSXY:4:2261:29631:32957_1:N:0:TCTGCAAG+GCTGCACT
GTTCAAGGTAATACTTACCAGCACGTTCGGCTGGTTAGAATTAAACCTCAAGATGACCCTGTTTTTGC
ATCACAAACACATATTACCGTTGCTCTATCACGACACGTTGAATCTTTACGATACTTTGTTATCAATG
TAA
+
FFFFFFFFFFFFFFFFFFFFFFFFFFFFFFFFFFFFFFFFFFFFFFFFFFFFFFFFFFFFFFFFFFFF
FFFFFFFFFFFFFFFFFFFFFFFFFFFFFFFFFFFFFFFFFFFFF:FFFFFFFFFFFFFFFFFFFFFF
FFF
@A00721:190:HGJW7DSXY:4:2430:9136:15969_2:N:0:TCTGCAAG+GCTGCACT
TGAGAGGATCAATCACAGTAAGGAACTGTCACGTTTAGCACGGGAGGTTGCAGATTCAACCCCTTATA
TCGAGGCTATCGAGGACGATGAGTATCTTGAAGTTGTTGACGACAAGC
+
FFFFFFFFFFFFFFFFFFFF:FFFFFFFFFFFF,FFFFFFFFFFFFFFFFFFFFFFFFFFFFF:FFFF
FFFFFFFFFFFFFFFFFFFFFFFFFFFFFFFFFFFFFFFFFFFFFFFF
@A00721:190:HGJW7DSXY:4:1617:19551:19100_1:N:0:TCTGCAAG+GCTGCACT
TAGAGGACCACCTGGATCTTCTACTGTCTCTGCCTTCCAAGATTTCTTAGAAGAGGTTATTCCTGGAA
CCACCTTAACGTCACTCGGTAATCTTTCAGCCGAATTAAGTACTAATGAGTTCAAGTCGGTTGTCGAT
CGA
+
FFFFFFFFFF:FFFFFF:FFFFFFFFFFFFFF:FFFFFFFFFFFF,FFFFF:FFFFFFF:FFFFFFF:
:FFFFFFFFFFFFF:FFFFFFFFFFFFFFFFFFFFFFFF:FFFFF:FFFFFFFFFFFFFFFFFFFFFF
F:F
@A00721:190:HGJW7DSXY:4:2277:9914:27492_2:N:0:TCTGCAAG+GCTGCACT
GGCTAATACCTTTATAGGTAAGAAGCCCCTTGAGCCGCGTAGTCAGCGCGTTTAGCTGTATCATCAGT
CAGAGTATACCCGATAGGGTACCGAGTCTCCAAGAGAATATTTATTCT
+
FFFFFFFFFFFFFFFFFFFFFFFFFFFFFFFFFFFFFFFFFFFF:FFFFFFFFFFFFF:FFFFFFFFF
FFFFFFFFFFFFFFFFFFFFFFFFFFFFFFFFFFFFFF:FF,FFFFFFFF
@A00721:190:HGJW7DSXY:4:2171:9914:5979_1:N:0:TCTGCAAG+GCTGCACT
ACGTTTTCACAACGGAAAGACATATCGCACCCGAAATTTTTGGGCAGAATGCTCTTAACGCGGTTCTT
GAACGGTGTTTTGATCAGTCTAAGTTGGATAAAGTTATAGGAGACTCAGTTAACCTGAGAGCTCCTCA
ATT
+
FFFFFFFFFFFFFFFFFFFFFFFFFFFFFFFFFFFFFFFFFFFFFF:FFFFFFFFF:FFFFFFF:FF
FFFFFFFFFFFFFFFFFFFFFFFFFFFFFFFFF:FFFFFFFFFFFFFFFFFFFFFFFFFFFFFFFFFF
FFF
```

```
@A00721:190:HGJW7DSXY:4:2127:28854:8124_2:N:0:TCTGCAAG+GCTGCACT
AATAGGGACGCTAGTATACTTCGTAAAGTCGACAGCGAAATGGTCAATATTGATAATTGGACCGACTA
TATGACCTATTTTAAGCTCATGGTGAAAAGGGAAGCTAAGGTTAAACT
+
FFFFFFFFFFFFFFF:FFFFFFFFFFFFFFFFFFFFFFFFFFFFFFFFFFFFFFFFFFFFFFFFFFFF
FFFFFFFFFFFFFFFFFFFFFFFFFFFFFFFFF:FFFFFFFF:FFFFF
@A00721:190:HGJW7DSXY:4:1103:1768:6809_2:N:0:TCTGCAAG+GCTGCACT
CCTATCTTTAAGGTTCTTGTGAGGAGAGTCAAACAGTGTCTTTTACCCAACATTTTTATGTATACAGA
GATGACCAGTGACGATTTCTCCCGGAAGCTCTTCTCTCTCTTAGGAGG
+
FFFFFFF:FFFFFFFFFFFFFFFFFFFFFFFFFFFFFFFFFFFFFFFFFFFFFFFFFFFFFFFFFFFF
FFFFFFFFFFFFFFFFFFFFFFFFFFFF:FFFFFFFFFFFFFFFFFFF
@A00721:190:HGJW7DSXY:4:1511:29071:18928_2:N:0:TCTGCAAG+GCTGCACT
TTTCGAAGTTTGACAAATCTCAAGATTTCTTTTGTAAAGCCTATGAGATAAAGATGTATTCTTATTTA
GGCTTCGATGCTGAATTTCTTGAACTTTGGAGTGCCAGTGAATATTTT
+
FFFFFFFFFF:FFFFFFFFFFFFFFFFFFFFFFFF:FFFFFFFFFFFFFFFFF:FFFFFFFFFF:FFF
FFFFFFFFFFFFFFFFFFFFFF,FFFFFFFFFFFFFFFFFFFFFFFFF
@A00721:190:HGJW7DSXY:4:1510:12364:13448_1:N:0:TCTGCAAG+GCTGCACT
GAGTATCCATAACGATTTGTCATTTACCACCTTTGCTCAACGACGAAGTGGAACGGCCAACACTTTCT
TTGGTAATTGTATGATCACTTTGATGATTTTATCACTTTATTATGACCTTGGAAAGTTTTCGGCTGTG
GTT
+
FFFFFFFFFFFFFFF:FFFFFFFFFFFFFFF,FFFFFFFFFFFFFFFFFFFFFFFFFFFFFFFFF:F:
:FFFFFFFFFFFFFFFFFFFFFFFFFFFFFFFFFFFFFFFFFFFFFFFFFFFFFFFFFFFFFFFFFFF
FFF
@A00721:190:HGJW7DSXY:4:2546:17707:4163_1:N:0:TCTGCAAG+GCTGCACT
CCATTCTGTTTTCAAAGACTCCAATAACTGATCATTCTGATCATATGATTTTGGACAATGGTTTCGAA
GCGAAATTTTTGAGGGATAAACCAGCGTACTTCTGCTCGCGATTTATTCTTTTTGTCAATGATTACAT
CTA
+
FFFFFFFFFFFFFF:FFFF::FFFFFFFFFFFFFFFFFFFFFFFFFFFFFFFFFFFFFFFFFFFFFFF
FFFFFFFFFFFFFFFFFFFFFFFFFFFFFFFFFFFFFFFFFFFFFFFFFFFFFFFFFFFFFFFFFFFF
FFF
@A00721:190:HGJW7DSXY:4:2146:20347:8202_1:N:0:TCTGCAAG+GCTGCACT
CCTTACAAGATCATCGTTAAGTTAGGTAAACCCCTAACCGATGTTACTCTTGTAGGACTTTGGGAGAG
ATTCGTTAGTTTTAAAGACTATACTAAATATCTCCACTACGAGGTTGTTGTTTGGCGATTAGCTCTTG
CGA
+
FFFFFFFFFFFFFFFFFFFFFFFFFFFFFFFFFFF:FFFFFFFFFFFFFFFFFFFFFFFFFFFFFFFF
FFFFFFFFFFFFFFFFFFFFFFFFFFFFFFFFFFFFFFFFFFFFFFFFFFFFFFFFFFFFFFFFFFFF
FFF
@A00721:190:HGJW7DSXY:4:2209:29062:29246_2:N:0:TCTGCAAG+GCTGCACT
ATTGAGGGTCGTTATACCTATTCGGCTATCTGTGCTTTGAATTGTATAAGATCAAATTTTAAACGATT
CCGAGAGGTTTATCCTGATGTGGAGGGAGTTCTTGAAGCATCCCGACC
+
F:FFFFFFFFFFFFFFFFFFFFFFFFF,FFFFFFFFFFFFFFFFFFFFFFFFFFFFFFFFFFFFFFFF
::FFFFFFFFFFFFFFFFFFFFFFF:FFFFFFF:FFFFFFFFFFFFFF
@A00721:190:HGJW7DSXY:4:2120:18249:6198_1:N:0:TCTGCAAG+GCTGCACT
TGATGCGGTTTCATAAGATTATCAGTAATCCTGATTCTACTACGGATTCATGGACCTTCCATCCTGTA
CCTCTTCTGGAGAGACGTATCTCAAAGATCGACTTCGAGACAGGTGATATTGTTGAGCGAGAGCGACC
AAG
+
FFFFFFFFFFF:,F,,,FFFFFFFFF,:FFF:FFFFFFFFFFFFF:FFFFFFFFFFFF:,,FFFFFF,
```

```
FFFF:FFFFFFFFF,FFFFFFFFF:FFFFF:,,FF:FFFFFFFFFFFFFF:FFFFFFFFF:FFFFFF
FFF
@A00721:190:HGJW7DSXY:4:2376:23990:28009_1:N:0:TCTGCAAG+GCTGCACT
CGTGGCGCTTTATTTTAAAAAGCTCCCTCCTCTCGCAAGTTTGATCTGATACTTATCACGAAAGGACT
TGAGTCTTTTGATCAGTCTCTGACTTGACCATCTTTCATTTTAAATGACGATCATTAAGACTCTCTTT
CTT
+
FFFFFFF:FFFFFFFFFFFFFFFFFFFFFFFFFFFFFFFFFFFFFFFFFFFFFFFFFFFFFFFFFFFF
FFFFFFFFFFFFFFFFFFFFFFFFFFFFFF:FFFFFFFFFFFFFFFFFFFFFFFFFFFFFFFFFFFF:
FFF
@A00721:190:HGJW7DSXY:4:2103:12481:21261_2:N:0:TCTGCAAG+GCTGCACT
CTTTGCACTGATTTTTCCTCATCAGCTTGTGTTTTACCTAATCTGTGTTTTTACGATAACAACTATCA
GTTGATTAAGACTCTTCCGAAAGGCGAACTTTGGGAATTTGAAGCTCA
+
FFFFFFFFFFFFFF:FFFFFFFFFFFFFFFFFFFFFFFFFFFFFFFFFFFFFFFFFFFF:FFFFF,FFF
FFFFFFFFFFFFFFFFFFFFFFFF:FFF:FFFFFFFF:FFFF:FF:FFF
@A00721:190:HGJW7DSXY:4:1611:20989:11256_2:N:0:TCTGCAAG+GCTGCACT
ACCGTGACGAACTGTGGTGTTCTTGATCTACGTTACGGATATACTCTCCTTGAGTTCACTCGTAAGGA
CACTACCACTTATTACGATATTTCTTTAGTCGATGGGTTTTCGACGCC
+
FFFFFFFFFFFFFF:FFFFFFFFFFFFFFFFFFFFFFFFFFFFFFFFFFFFFFFFFFFFFFFFFFFFF
FFFFFFFFFFFFFFFFFFFFFFFFFFFFFFFFFF,FFFFFFFFFFFFF
@A00721:190:HGJW7DSXY:4:2519:29234:35775_2:N:0:TCTGCAAG+GCTGCACT
TTTCTTCTAATGTGGCCTGAACTACGTTTCACTTTGTGTGTACTTGTCTCTATTTTAAGTTTTGTCTT
CCTTGTTTGTCTTATTCTATTAATCACTCATTTAGTGAGATTATTAA
+
FFFFFFFFFFFFFFFFFFFFFFFFFFFFFFFFFFFFFFFFFFFFFFFFFFFFFFFFFFFFFFFFFFFF
FFFFFFFFFFFFFFFFFFFFFFFFFFFFFFF:FFFF:FFFFFFFFFFF
@A00721:190:HGJW7DSXY:4:1142:32235:13072_1:N:0:TCTGCAAG+GCTGCACT
TCTTCGACAACAGCAGGGGTTTTCGAATAGTGATAGCCACCCGCCTTACAGAGGGGCAGCAGCATGAC
CATAATTGGTATTGACTTTGGTACCACCTTTTCTACTGTCTGTCTCGCCTCTTCGAATGAAGTTTTAA
TGC
+
FFFFFFFFFFFFFFFFFFFFFFF:FFFFFFFFFFFFFFFFFFFFFFFFFFFFFFFFFFFFFFFFFFFFF
FFFFFFFFFFFFFFFFFFFFFFFFFFFFFFFFFFFFFFFFFFFFFFFFFFFFFFFFFFFFFF::FFFFF
FFF
@A00721:190:HGJW7DSXY:4:2435:23999:4069_2:N:0:TCTGCAAG+GCTGCACT
TGATAGTGAATTTATACCTACAATCATTGGTTTTTCTAATAATGATGACACTATTGTTTATGGATATG
ATGCTATTACTCATGATGGTTACGGTCAGGGAGGATTCTCAGTTTACA
+
FF:FFFF:FFFFFFFFFFFFFFFFFFFFFFFFFFFFFFFFFFFFFFFFFF:FF,FFFFFFFFFFFFFF
FFFFFFFFFFFFFFFFFFFFFFFFF:FFFFFFFFFFF:F:FFFFFFFFFF
@A00721:190:HGJW7DSXY:4:1363:6135:11616_2:N:0:TCTGCAAG+GCTGCACT
CCGAAAGACGTGCTAAATTAAAACCATTGTACGATGTCGTCTGTCTCGGCGATCACTTTTCACTAAGA
ATTGGTCCTACTTTTGGACAAGGCCAACTTCGTAATCTTCATGAGATC
+
FFFFFFFFFFFFFFFFFFFFFF,FFF,FFFFFFF:F:FFFF:FFFFFFFFFFFFFFFFFFFFFFFFFF
FFFFFFFFFFFF,FFFFFFFFFFFFFFFF:FFFFFFFF:FFFFFFFFF
@A00721:190:HGJW7DSXY:4:1527:22200:4366_2:N:0:TCTGCAAG+GCTGCACT
TATCTCTTTGGTTGTTAGGGATTTTGAGAGAGTGAAAGGCTTCAATTGTAGTGGTTTGGTCGTTTCAG
TACCCAGTCAGTATACCTCTACGCAAAGATATTTCATGAAAACTACAG
+
FFFFF:,,FFFFFF,FFFFF:FFFFFFFF:FFF:FFF:FFFF,FF:FFFFFFFFF:FFFFFFFFFFFF
:FFFFFF::FFFFF:FFFF:FFFFFFF:FFFFFFFFFF,FFF,FFFFF
```

```
@A00721:190:HGJW7DSXY:4:1419:22688:34867_1:N:0:TCTGCAAG+GCTGCACT
ACCTGTCTTTCATATTATGAATGAACCTTCGGCCGCTCTCTTTGCTTCGATGTTGGATGTCAAGAAGG
GTTCAGAATGGGATTCCTACGTAGTTTATGACTTTGGTGGTGGTACTTTCGATACTTCTATCGTCTTA
CGT
+
FFFFFFFFFFFFFFFFFFFFFFFFFFFFFFFFFFFFFFFFFFFFFFFFFFFFFFFFFFFFFFFFFFFF
FFFFFFFFFFFFF:FFFFFFFFFFFFFFFFFFFFFFFFFFFFFFFFFFFFFFFFFFFFFFFFFFFFFF
FFF
@A00721:190:HGJW7DSXY:4:1159:27308:4351_2:N:0:TCTGCAAG+GCTGCACT
AGTGTTATTTATTCTGGTGGTGATGATAATCTTGGAGGTCGTGACGTTGATGAAGCTATAAGAGCGTT
CTTATTACGGCGCACTTCTTTACCCGCCACCACACAACTTTCAGTTTC
+
FFFFFFFFFFFFFFFFFFFFFFFFFFFFFFFFFFFFFFFFFFFFFFFFFFFFFFFFFFFFFFFFFFFF
FFFFFFFFFFFFFFFFFFFFFFFFFFFFFFFFFFFFFFFFFFFFF:FFF
@A00721:190:HGJW7DSXY:4:1227:25545:30201_1:N:0:TCTGCAAG+GCTGCACT
ACTGGAAAACCCTCGTCAATACTTTTCAATAATATACGTCTTGAACTCACTTATGACGATCTAGTGAA
ATTAACGCGTCCTTTTCTGGAACGTAGTTTTACTATTATGAACAAAGTTATCAAAGATAGTGGCATTC
AAG
+
FFFFFFFFFFFFFFFFFFFFFFFFFFFFFFFFFFFFFFFFFFFFFFFFFFFFFFFFFFFFFFFFFFFF
FFFFFFFFFFFFFFFFFFFFF:FFFFF:FFF:FFFFFFFFFFFFFFFFFFFFFFFFFFFFFFFFFFFF
FFF
@A00721:190:HGJW7DSXY:4:2173:2003:5024_1:N:0:TCTGCAAG+GCTGCACT
CCTGATTCCGGCCGGTGGTAGCTCATTTCTACCAGGGGCTATTACTCTTGCTGAAACAGCTATTAAAG
CTGTTGAGCGGGTAATTACACCTAAACGAGCTCGATCTGCCATCGCTGAAGGATGTGCACTCTTTTCT
GCT
+
FFFFFFFFFFFFFFFFFFFFFFFFFFFFFFFFFFFFFFFFFFFFFFFF:FFFFFFFFFFFFFFFFFFF
FFFFFFFFFFFFFFFFFFFFFFFFFFFFFFFFFFFFFFFFFFFFFFFFFFFFFFFFFFFFFFFFFFFF
FFF
@A00721:190:HGJW7DSXY:4:1327:13774:18114_2:N:0:TCTGCAAG+GCTGCACT
TCGATTGTATCAACAGTAACATTACTTCTAATAAGGGTCTCTATCAAGCTCGTATTATCGTCGCTGCT
GGATCTGCGTTACCGTTGAAGGCTAAACTCCAAGGTAATTATGAAGAC
+
FFFF:FFFFFFFFFFF:FFFFFFFFFFFFFF:F,FFFFFFFFFFFFFFFFFFFFFFFFFFFFFFFFFF
FFFFFFFFFFFFFFFFFFFFFFFFFFF:FFFFFFFFFF:FFFFF::FFF
@A00721:190:HGJW7DSXY:4:2608:11677:29763_1:N:0:TCTGCAAG+GCTGCACT
CGTGTGCTCTTCCGATCTGGTGTTTCTAATCGTAACATTAATAATTTCAATCTTCTTAAAAAGAAGAT
AGAGAGTCATACTTTGGGCTATCGTTCAGGTGCATGGGGTTGGATAGCCAATATTGAAGTGGACACCT
CTG
+
FFFFFFFFFFFFFFFFF:FFFFFFFFFFFFFFFFFFFFFFFF:FFFFFFFFFFFFFFFFFFFFFFFFF
FFFFFFFF:FFFFFFFFFFFFFFFFFFFFFFFFFFFFFFFFFFFFFFFFFFFFFFFFFFFFFFFFFFF
FFF
@A00721:190:HGJW7DSXY:4:1361:32877:25426_1:N:0:TCTGCAAG+GCTGCACT
AGATAACCAATAAGGAAGGAGCTTATACTATGGTTGAATCATCTAAGCATACTACTTTTGCTTTGATT
ACTCTACCGCCTTACAAGTCTAAGGTTAAAGAGTCGATGAAACAAGTTGTACTTATCAATGATTTTAT
CGC
+
FFFFFFF:FFF::F:,F,FFF:FFFFFFFF:FFFF:F:FFF,FF:::FFFF:FFF:FFFFF:FFFFFF
:F::FFFF:F:F:FFFF,FFFFFF:FFF:FFFFFFFFF,FFFFFFFFFFFFFFFFFFFFFFFFFFFFF
FF:
@A00721:190:HGJW7DSXY:4:1426:8169:27727_2:N:0:TCTGCAAG+GCTGCACT
TTCTAACCCAAAGAGAATATCTAAAATATTTACAAAATCTTACCACTATCTACGAACCTAAAGTTCTG
```

```
AAGACGTTGCTTGTCGCTTCCTCCTTTAATGGCACTACCTCGGTACAA
+
FFFFFFFFFFFFFFFFFFFFFFFFFFFFFFFFFFFFFFFFFFFFFFFFFFFFFFFFFFFFFFFFFFFFFFFF
FFF:FFFFFFFFFFFFFFFFFFFFFFFFFFFFFFFFFFFFFFFFFFFF:
@A00721:190:HGJW7DSXY:4:2518:29378:7592_2:N:0:TCTGCAAG+GCTGCACT
GAAGGCGACCTGTTCCGGAGTTTTTACGGGATCCGGAATGTTCTGTCTATATTAGGTGAAGCGAAATA
CGATTTCGAACGTCAACGTTCACCGAGTTTCGTTTCGTACCTTGATGA
+
FFFFFFFFFFFFFFFFFFFFFFFFFFFFFF,FFFFFF,FFFFF:FFFFFFFFFFFFFFFFFFFF,F,F
FFFFFFFFFFFFFFFFFFFFFFFFFFFFFFFFFF:FFFFFFFFFF:FF:
@A00721:190:HGJW7DSXY:4:2333:16830:6903_2:N:0:TCTGCAAG+GCTGCACT
ATTTGATCTGGTTGCTCAATTCTAAGCGTGTTCCGCTTAGTGCGAGGCCTCTCTTCTACGAGTATTGC
TATTTATACGGTTACGAGAAGAAGGTTCGAAAGTTTGAGAACTTAGTT
+
,FFFFFFF,FF:FF:FFFFFFF:F:FFF:FFFFFFF:FFFFF:FF::FFFFFF:FFFFFFFFFFF,FF
FFF:F:FFFFF:F:FFFFFFFFFFFFFFFFFFFF:F:FFFFFFFFFFFF
@A00721:190:HGJW7DSXY:4:2647:2003:7686_2:N:0:TCTGCAAG+GCTGCACT
TACTAAATACTAGATATTTGGATGATCTTGATAAAGAACTTCATGAATTTTCAGAGGATGAAAAATTT
TCTTCCTTGGATTTTAATATAAAGAATCTTTACCAGGACTCCTACTAC
+
FFFFFFFFFFFFFFFFFFFFFFFFFFFFFFFFFFFFFFFFFFFFFFFFFFFFFFFFFFFFFFFFFFFF
FFFFFFFFFFFFFFFFFFFFFFFFFFFFFFFFFF:FFFFFFFFFFFFF
@A00721:190:HGJW7DSXY:4:1128:12762:2080_1:N:0:TCTGCAAG+GCTGCACT
TGATCGTAAGAAGTTTATAAGGAACATATCCATTTGTTGTAATAGGGTCATCAACTTAGAACTCGAAA
CTCTAAGTGGTAATACTGTCTTTCCAAGCTTACCTGTATCTTCAATGAAATTGGACTCTTCGATAGGG
GAC
+
FFFFFFFFFFFFFFFFFF:FFFF:FFFFFFFFFFFF:FFFFFFFFFF:FFFFFFFFFFFFFFFFFFFF
FFFFF,FFFFFFFFFFFFFFFFFFFFFFFFFFFFFFF,FFFFFFFFFFFFFFFFFF:FFFFFFFFFFF
FFF
@A00721:190:HGJW7DSXY:4:1553:16324:6621_2:N:0:TCTGCAAG+GCTGCACT
TAAAGGAAGATCTGTTATCGACGGTTTATCGTTTACGAAGTATCACTCCACCGGTGAATGGTTTAAGA
ATACTATAAATAGAATAGCTATTACTACTAATGGCGTTGGCCGAGTGG
+
FFFFFFFFFFFFFFFFFFFFFFFFFFFF:FFFFFFFFFFFFFFFFFFFFFFFFFF:FFFFFFFFFFFF
FF:FFFFF:FF::,FF:FF,FFFFFFFFFF:FFFFF:FFFF,,FFFFF
@A00721:190:HGJW7DSXY:4:1266:26648:31767_1:N:0:TCTGCAAG+GCTGCACT
TTTAGGTGGTCTTTATTCTATTATTGATTTTTATGAGATTGATACGTTAACAGAAGATGATGTTAGAG
TAGAATTCCTGAAGAGTCCTTCCGTAACCAACTTCTTGACAATTTTCTTCAATCCTTCGATAATAGTT
AAA
+
FFFFFFFFFFFFFFFFFFF,FFFFFFFFFFFFFFFFFFFFFFFFFFFFFFFFF:FFFFFFFFFFFFFF
FFFFFFFFFFFFFFFFFFFFFFFFFFFFFFFFFFFFFFFFF:F:FFFFFFFFFFFFFFFFFFFFFFFF
FFF
@A00721:190:HGJW7DSXY:4:1225:24849:22670_1:N:0:TCTGCAAG+GCTGCACT
CCGATAAGGGTCACTAGTCTTACACAATTAAGAAATAATCCTACATTGACTGATTATCTAAAGATTGA
AGGAAAATTTTATCCTCGTTTAACTTACTTAGACTTTCCTACGATACAAATGATAACTTCTAATTTAC
GTT
+
F,FFF:FFFFFF,FFFFFFFF:FFFFFFFFFFFFFFFFF,FFF,FFFF:FFFFFFFF:FFF,FFFFFF
FFF:FFF:FFFFFFFFFFFFFFFFFFFFFF,FFFFFFFFFFF:FFFFF:FFFFFFFFFFFFFFFFFFFF
FFF
@A00721:190:HGJW7DSXY:4:1644:20103:34773_2:N:0:TCTGCAAG+GCTGCACT
CAAAGGGATGCATGGTGTTATCTCCTCAGGAGTCCCGGCATTTGATAATAATGACGAGACTTTCGTCA
```

TAATCGCTGCATATTACGGATTACACGGTACCAGCGCATCTCGTGTCG
+
FFFFFFFFFFFFFFFFFFFFFFFFFFFFFFFFFFFFFFFFFFFFFFFFFFFFFFFFFFFFF:FFFFFF
FFFFFFFFFFFFFFFFFFFFFFFFFFFFFFFFFFFFFFFFFFFFFFFF
@A00721:190:HGJW7DSXY:4:1573:16288:10504_1:N:0:TCTGCAAG+GCTGCACT
GTATTAACTTATAAAGGTGAGGAGACCATTTTCTTGCTGAAATTTATAGAAGATTATTTTGACAGAAT
CAATAAGAATCATCGATTCTCGATTCGTAGAGCTTATCTAGGTACTCTCATTTACTATGTTGATTTAG
TCT
+
FFFFF:FFFF:FFFFFFFFFFFFFFFFFFFFFFFFF:FFFFFFFFFFFFFFFFFFFF:FFFFFFFFFF
FFFFFFFF,FFFFFFFFFFFFFFFFFFFFFFFFFFFFFFFFFFFFFFFFFFFFFFFFFFFFFFFFFF
FFF
@A00721:190:HGJW7DSXY:4:1678:28709:35055_2:N:0:TCTGCAAG+GCTGCACT
GATTTACAACGATATAGGAGTAATCTACGAGAAGATTCTAGAATTCTTAATTATAATATTCAACCTCA
AAAGTTACGTGTTTACAGTAACGAGATCACACATTTTGAGAACTAACT
+
FFFFFFFFFFFF:FFFFFFFFFFFFFFFFFFFFFFFFFFFF:FFFFFFFFFFFFFFFFFFFFFFFFFF
FFFFFFFFFFFFFFFFFFFFFFFFFFFFFFFFFFF,FFFF:FFFFFFF
@A00721:190:HGJW7DSXY:4:2645:4300:1642_1:N:0:TCTGCAAG+GCTGCACT
CTGATCATTTTGACAATCTCTTCTTATAATAAATAAATAAATAATATTACTAATGGCAAATAGTGGAA
AGGCTCCAGTCAACACTAGTTCGAGTGCTGCTTTGGATTACGATGACGTGAGAATAGGTGAAGGGATA
GTT
+
FF:FFFFFFFFFFFFFFFF:FFFF:FF,::FFFF::FFFF:FFF,,FFFF:F,:F,:FF::FFFFFFF
FFFFFFFFFFFFFFFFFFFF:FFFFFFFFFFFFFFFFF,FFFFFFFFFF:FFFFFFFFFFFFFFF,:F
FFF
@A00721:190:HGJW7DSXY:4:1240:15067:30843_1:N:0:TCTGCAAG+GCTGCACT
GATCTATTAAGGCGGCGGCGACTAATCTTAAAGCTTCACCCAAAATGAAGGACACTTCGAAACTGAAT
AATGATGCACTACAAACAAGTATCGCTAATCATCTGAAAAAGGCTCTAAAGGACACTATACAATATCC
AGA
+
:FF:FFFFFFFFFFFFFF,FF:FFFFFFFFFFFFFFFFFFFFFF,FFFFFFFFFFFFFFFF::FFFFF
FFFFFFFFFFFFFFFFFF,FFFFFFFFFFFFFFFFFFFFFFFFFFFFFFFFFFFFFFFFFFFFFFFFF
FFF
@A00721:190:HGJW7DSXY:4:2365:12472:28291_2:N:0:TCTGCAAG+GCTGCACT
TGCTTTTACCGATAACGATAAATTCGTCATCAAATATAAAGAAAAGGACTATGAAATAAAAGATTTGG
TGCTTAGGGATATAATGAATTCAGCACCACATTTTGGTCAAGTCAATC
+
FFFFFFFFFFFFFFFFFFFFFFFFFFFFFFFFFFFFFFFFFFFFFFFFFFFFFFFFFFFFFFFFFFFF
FFFFFFFFFFFFFFFFF:FFFFFFFFFFFFFFFFFFFFFFFFFFFFFF
@A00721:190:HGJW7DSXY:4:1507:28510:17080_2:N:0:TCTGCAAG+GCTGCACT
TCTTAGAGGATATTCGCCATTGTGGTACGAATTGGTCCAACATTTACCACCAAATTCTTTTGATACAA
CTCTAGCCTTCAAGTGGGGTATGCCTGCGGAATACGCTTGCTATTCAC
+
FFFFFFFFFFFFFFFFFFFFFFFFFFFFFFFFFFFFFFFFFFFFFFFFFFFFFFFFFFFFFFFFFFFF
FFFFFFFFFFFFF,FFFFFFFFFFFFFFFFFF:F:FFFFFFF:FFFFF
@A00721:190:HGJW7DSXY:4:1235:1072:1188_2:N:0:TCTGCAAG+GCTGCACT
CGAACCCGTTGATGCTATGTCAGAAGAAGCTATTCAAGCTTGTCGTTTAAAGACTGCAGAGGCTGTCA
AACAAGCCCAGACTCAAGGAGTCCAATCAAAGGTTTTTAATTTAGCTC
+
,FFFFFF:FF,FF:,F:FFF:F:FFFFFF,FFFFFFFFFFFFFFFF,FFF:FFF:FFFF:FF,FFFFF
F,F:F::,FFFF:FFFFF,F,F:FFF:FFFF,FFFFF,::F:FF:,,:
@A00721:190:HGJW7DSXY:4:2222:10267:24972_2:N:0:TCTGCAAG+GCTGCACT
TGGGTGATAAAAATTATTTAGAGATCTTTTCTACTTACAAAGGACAATTCAGTTTCATTAATTCTACG

```
GAAAAGTCCGGAGTATATTGTATGTTAATAGATAGTGGGATAATAGTT
+
FFFFFFFFFFFFFFFFFFFFFF:FFFFFFFFFFFFFFFFFFFFFFFFFFFFFFFFFFFFFFF:FFFFFFF
FFFFF::F:FFFFFFFFFFFFFFFF:FFFFFFFF::FFFFFFFFFFFF
@A00721:190:HGJW7DSXY:4:2309:16387:35383_1:N:0:TCTGCAAG+GCTGCACT
GTAGTGAACGCGTTCGTAGAACCAAGTACTTTCCGTCTGAAGAGTTTAGAGCTGCTATTGATTATGTA
ACCGATAGGAGTTGTATAGTTGAAGATGTGGCTTTAGACGATAATCCTTTAAGGACTATGTTGACTGA
CGA
+
FFFFFFFFFFFFFFFFFFFFFFFFFFFFFFFFFFFFFFFFFFFFFFFFFFFFFFFFFFFFFFFFFFFF
FFFFFFFFFFFFFFFFFFFFFFFFFFFFFFFFFFFFFFFFFFFFFFFFFFFFFFFFFFFFFFFFFFFF
FFF
@A00721:190:HGJW7DSXY:4:2353:19108:19366_2:N:0:TCTGCAAG+GCTGCACT
GATCGCCACTCCAAACACTATATACATTATAAAATATAAAAATAAGGGAGTCATAACTTTTGTTACCA
AATTCTTGGCTTTCAACGAAGTCTCTGATTTTATTAATCATTACTTTA
+
FFFFFFFFFFFFFFFFFFFFFFFFFFFF,F:FFFFFFFFFFFFFFFFFFFFFFFFFFFFFFFFFFFFF:
FFFFFFFFFFFFFFFFFFFF:FFFFFFFF:FFFFF,,FFFFFFFFFFF
@A00721:190:HGJW7DSXY:4:1140:26838:23390_2:N:0:TCTGCAAG+GCTGCACT
TCATTTACTCTGTACTTGATCTACGTCGTTTAACTGTCGGAACAACCTTTACTTAGAATGACTCTATC
TTTTCAGACACTTCTTATATTATTTACAATAGTTAATATCATATTAGT
+
:FFFFFFFFFFFFFFFFFFFF:FFFFFFFFFFFFFFFFFFF:FFFFF:FFFFFFFFFFFFFFFFFFFF
FFFFFFFFFFFFFFFFFFFFFFFFFFFFFFFFFFFFFFFFFFFFFFFF
@A00721:190:HGJW7DSXY:4:2422:30373:13479_2:N:0:TCTGCAAG+GCTGCACT
GCTTTTTCTTAGCTAATAGATTAATAAAGGATGAGTACGAAAACCAACTCTTCGTTACGATACCTGAT
GGTATAATAGATTTATTCTCTACACCCATCTCAACGAGGATAAAGCGA
+
FFFFFF,FF:F::FFFFF,FF:FFFFFF,:FF,F,FFF:FFFF,,FF,FFFFF,F:F,FF,FF,F,FF
:,FF,FF:F:FFFFF,F,F,FFFF:,FF:FFFFF,,FF,FFFFF:FFF
@A00721:190:HGJW7DSXY:4:1208:16080:21480_2:N:0:TCTGCAAG+GCTGCACT
ACACGAAGAGATCATCCGCAAACGACTCGAAACCGAGACGCCGTAGATCAAAGTCCCTTCTAACTTTA
GATTATGAATCCTGCCTTTACTCATTTTATAGCAGTTATTTATGTTGT
+
FFFFFFFFFFFFFFFFFFFFFF,FFFFFFFFFFFFFFFFFFFFFFFFFFFFFFFFFFFFFFFFFF:FFF
FF:FFFFFFFFFFFFF,FFFFFFFFFFFFFFFF:FFFFFF:FFF,FFF
@A00721:190:HGJW7DSXY:4:1470:28022:17331_1:N:0:TCTGCAAG+GCTGCACT
TTTATAACCTTCTTATACTTTTGGGTATGTTTTGTGAAAAACATGAGATACCCAAGTCTATCGAAGAC
ATTATCTCTTATGTACTTATTGTAACGATCATTCTTTCGAGTTTTGTCATTCTTATTCTTTTATTCCT
TTA
+
FFFFFFFFFFFFFFFFFFFFFFFFFFF:FFFFFFFFFFFFFFFFFFFFFFFFFFFFFFFFFFFFFFFFF
FFFFFFFFFFFFFFFFFFFFF:FFFFFFFFFFFFFFFFFFFFFFFFFFFFFFFFFFFFFFFFF,FFFF
FFF
@A00721:190:HGJW7DSXY:4:1219:32289:19022_2:N:0:TCTGCAAG+GCTGCACT
TTTATAATGATTTTCGATAGTGATTGTTATCAATTTAAATACATTACTGTTTCAAATCATCTTTACTC
ACAGACTCCGAAAATGGTTTCAAATGAGGTCAAAGCTCTATTTATTAA
+
F,:FFFFFF,FF:F,FFFF:FFFF:::FFF:FF:,FFFFFFFF:,F:FF,F:FFFFFFFFFFFFFFF,
F:F:FF,FF,FFFF,FFF,FFFF,FF,FFFFFF:F,FFFFFFF:,:FF
@A00721:190:HGJW7DSXY:4:1336:24722:18474_1:N:0:TCTGCAAG+GCTGCACT
ATCCGATTCTTCGAGGGTATCACTACCACTGATCTGCCGACTTTTGTCAAGGAACTTCAATTCCTTCG
ATGTTTATATTTTTCTATAAGGGAAAATAGCAATAATCAAATTTCGACTAATTTTAGATCTTTATTTC
AGA
```

```
+
:FF,FFFF,FF,F,F:FFFF,,FF:,:F,F:,FFF:,F,:FFFF,FF,,,FF:,FF:,,F:FFF,FF:
FF,,FFF:FFFFFF:FFF,F,F:FFFFF:FFFFF,:F,FF:F,:FF,:,,::FFFF,F,FFFFFFF::
FFF
@A00721:190:HGJW7DSXY:4:2201:2275:27320_2:N:0:TCTGCAAG+GCTGCACT
ATCACCTTGGTGTGATCTCCCGGAGGATCTTTTCAATAAGATGTATGATGATAATCTACTATTTTTAA
ATAGTGATATTCAGCGTATACTTGAATACTTTATCAAAGATTTGGCTT
+
FFFFFF,FFFFFFFFFFFFFFFFFFFFFFF,F:FFFFFFFFFFFFFFFFFFFFFFFFFFFFFF:FFFF
FFFFFFFFFFFF:FFFF::FFFFFF:FFFFFFFFFF:FFFFFFFFFFF
@A00721:190:HGJW7DSXY:4:1210:18765:1924_1:N:0:TCTGCAAG+GCTGCACT
CAAAAAGTTGGAAGAGGTCTATGATATTTATTTTACTTATACTTTAAATAGCACCGATTATAACTTGA
CTGTTGATAATATTACAATAGCTCTTTCTCAGAAGATTAGAGAAGTAACTCGGTGAACACGTCGGTAG
CAT
+
FFFFFFFFFFFFFFFFFFFFFFFFFFFFFFFFFFFFFFFFFFFFFFFFFFFFFFFFFFFFFFFFFFFF
FFFFFFFFFFFFFFFFFFFFFFFFFFFFFFFFFFF:FFF:FFFFFFFFFFFFFFFFFFFFFFFFFF:F
FFF
@A00721:190:HGJW7DSXY:4:1269:18439:7216_1:N:0:TCTGCAAG+GCTGCACT
AGAACTGAAAAAGATTCTTTTCGAAAAGAAACCTATAAGTTACAAACGATTCTTCGACTATATTAAAG
AAATAAAAATACTTCAAGTACCTGAACTTTACATTTTTAATGGTATGGTATAATGTGTCTTACAAATA
GAA
+
FFFFFFFFFFFFFFF:FFFFFFFFFFFFFFFFFFFFFFFFFFFFF:FFFFFFFFFFFFFFFFFFFFFF:F
FFFFFFFFFFFFFFFFFF:FFFFFFFFFFFFFFFFFFFFFFFFFFFFFFFFFFFFFFFFFFFFFFFFF
FFF
@A00721:190:HGJW7DSXY:4:1528:32904:25316_2:N:0:TCTGCAAG+GCTGCACT
TAATGAGAGTGTATTCCTTAATATTCTAGTAACCGCTATGTGGGCATTACTAGGATTTATCACACTCC
TAAATTTGATAGCAATTTATGTTTTAAATGCTAGGAAAATTTTAGAAA
+
FFFFFFFFFFFFFFFFFF:FFFFFFFFFFFFFFFFFFFFFFFFFFFFFFFFFFFFFFFFFFFFFFFFF
FFF:FFFFFFFFFF:FFFFFFFFFFFF:FFFFFFFF,FF,FFFFFFFF
@A00721:190:HGJW7DSXY:4:1435:27480:10629_1:N:0:TCTGCAAG+GCTGCACT
AAGGAGTACTTAATTCAATAGCTTTAGTATTATTTACTATCGCCTTTATTGAAAATTGTATAAGAGTT
AATTGCTTACACAAAAGTGACGCGCGAAGGAATCTTAATTGATTGAAGCTAGGCGTTGGGGAGAATGA
ACT
+
FF:FFFFFFFFFFF,FFF,FFFFFFFFFFFFFFFFFFFFFFFFFFFFFFFFFFFFFFFFFF:FFFFFFF
FFFFFFFFFFFFFFFFFFF:FF:FFFFFFF:FFFFFFFFFFFFFFFFFFFFFFFFFFFFFFFFFFFFF
FFF
@A00721:190:HGJW7DSXY:4:1255:1298:25379_1:N:0:TCTGCAAG+GCTGCACT
AACATACCATACACTCCCCAAACATAACATACCATAACCTACCATGACCAATCATCGCTCCAACTGGC
ACGCCAGACGTAGGGTCAAACCTACTACCGCCGGTCGGCAGATCGGCACCACTGGTCTCTACGTTCAG
GGA
+
F:F:FFFFFFFFFFFFFFFFF,F:F:,F,:FFFFFF:FFFFFFF:FFFFFFFFFFFFFFFFFFFFFFFF
FFFFFFFFFFFFFFFFFFFFFFFFFFFFFFFFFFFFFFFFFFFFFFFFFFFFFFFFFFFFFFFFFFFF
FFF
@A00721:190:HGJW7DSXY:4:1602:27832:31782_1:N:0:TCTGCAAG+GCTGCACT
CCTATCTCCTTCCCAAATCTAGATCTGCACAGAAGAAGGCTGAAGGTCGTTACCGGACGACCACTCAA
AGGAAACGGGCTGTTGCCCAGAGGAAAGCAGCGACCACCGAGGTCGCTCGTAACATTGACGGCAGTCC
CGT
+
FF,FFFF:,,FFFFF,FFFF:FFFFFFFFFFFFFFF:FF::F,FFF:FFFFFF:FFFFFFF,FFF:FF
```

FFFFF:FFFF:,:FFFF,FFFFFFF::FF:FFFFFFF:FFFFF,FF:FFF:FFFFFFFFFFF::FFFF
FFF
@A00721:190:HGJW7DSXY:4:1260:26268:8813_1:N:0:TCTGCAAG+GCTGCACT
AACTGAGGCAGTACCTCAGTCAAAAGTCACATTCACCACTCCCATACCAATCAATGATGTGACTATAC
AGTTCGGCCATTTCAAGAGTGGTCAATACGTCGTGGGAGGTACTGAAGCCTTCCCCTCCGCTGGCACG
AAG
+
FFFFFFFFFFFFFFFFFFFFFFFFFFFFFFFFFFFFFFFFFFFFF:FFFFFFFFFFFFFFFFFFFFFF
FFFFFFFFFFFFFFFFFFFFFFFFFFFFFFFFFFFFFFFFFFFFFFFFFFFFFFFFFFFFFFFFFFFF
FFF
@A00721:190:HGJW7DSXY:4:1120:24352:10473_2:N:0:TCTGCAAG+GCTGCACT
GCCAATCAATAGTTCTTCTGACCAGAATGAAAAGGGGTTTACCAGGGTAGTCTCTAAGTCTACCAGGA
AGAGGGGAAATGTCCCCAAAACTGAGAAGAATAAAGGGAAAGGTCCAT
+
FFFFFFFFFFFFFFFFFFFFFFFFFFFFFFFFFFFFFFFFFFFFFFFFFFFFFFFFFFFFFFFFFFFF
FFFFFFFFFFFFFFFFFFF,FFFFFFFFFFFFFFFFFFFFFFFFFFFF
@A00721:190:HGJW7DSXY:4:1559:23529:12399_2:N:0:TCTGCAAG+GCTGCACT
GAAGGGGAAATTATCCCTAATTCTGAAGGAAGATCTCCCACCATCTGGGGTTGGGAATAAGAAATCTG
CACGGGCCAAAGAGGCCGCTGCTACGAAGTCGCTGAAGGCGGCTTGGG
+
:FFFFFFFFFFFFFFFFFFFFFFFFFFFFFFF:FFFFFFFFFFFFFFF:F:FF:FF,FFFFFFFFFF:
FFFFFF:FFFFF:FFFFFFFFFFFFFFFFFFFFFFFFFFFFFFF:FFF
@A00721:190:HGJW7DSXY:4:1445:31015:26459_2:N:0:TCTGCAAG+GCTGCACT
TGTAAAGTTGCGCCTGCCGACCCCATCTTTGAGACCAAGACTCTCTACAGTAGGTTCGTGCCTACTAT
GAGCAAATCTGTTGAATTCGTCACTGAGGACGAAGGTGAGAGGACTTT
+
FFFFFFFFFFFFFFFFFFFFFFFFFFFFFFFF:FFFFFFFFFFFFFFFFFFFFFFFFFFFFFFFFFF:
FFFFFFFF:FFFFFFFFF:FFFFFFFFFFFFF:FFFFFF:FFFFFFFF
@A00721:190:HGJW7DSXY:4:2509:23357:36683_1:N:0:TCTGCAAG+GCTGCACT
CAAACTGAGAGCAGTCGCTGGGGAAAGAGAATTGCGTGGGTTGAAGGCCGACGCTTCGAGCTTGAGTG
TGACGAGGCCGGTGACGTTTTTGTCAACGGCGTTTGGAGGATTTTATCCGCGCTTAACTATGGGGCGA
AGA
+
FFF,FFFFFFFFFFFFFFFFFFFFFFFFFFFFFFFFFFFFFFFFFFFFFFFFFFFFFFFFFFFFFFFF
FFFFFFFFFFFFFFFFF:FFFFFFFFFFFFFF,FFFFFFFFFFFFFFFFFFFFFFFFFFFFFFFFFFF
FFF
@A00721:190:HGJW7DSXY:4:2511:22200:30358_1:N:0:TCTGCAAG+GCTGCACT
AGTCTTGAAGGACTTAGGTTGGCATATTTATGCCGACGATAATAAGCATGTCACCCTTAAGCACCGAG
GTAAGCTCGGGACTTACAAGAGAGAGTGTGAGAGAGTCGCTCTCACTATTTTTGCCCAGAATGCGGGC
TTT
+
FFFFFFFFFFFFFFFFFFF:FFFFFFFFF::FFFFFFFFFFFFFFFFFFFFFFFFFFFFFFFFFFFFF
FFFFFFFFFFFFFFFFFFFFFFFFFF:FFFFFFFFFFFFFFFFFFFFFFFFFFFFFFFFFF:FFFFFF
FFF
@A00721:190:HGJW7DSXY:4:2671:11496:25097_2:N:0:TCTGCAAG+GCTGCACT
ATTCACTGGTTCTGGGAGGCTGCTTTCCTCCATAATTACAACCTCTCTTTAGAGAGCATTAGAAGGAA
GTTGGGCAAATTCCCACATATGGTAGACGTTGTCACACTTTTCTTGAA
+
FFFFFFFFFFFFFFFFFFFFFFFFFFFFFFFFFFFFFFFFFFFFFFFFFFFFFFFFFFFFFFFFFFFF
FFFFFFFFFFFFFFFFF:FFFFFFFFFFFFFFFFFFFFFFFFFFFFFF
@A00721:190:HGJW7DSXY:4:1605:3450:28698_1:N:0:TCTGCAAG+GCTGCACT
CTCTTCCGATCTTTGGGGATTTCGATGTGAGAAGGTGGAAGGTGTCAAGGGTCTTCTTCATGCTGATC
TTTCAGGACCTAATCACATCATGATTGATAGTTACGGCTCGTATCGAGTTGGAGGGAGGAAGTTCTCC
GAA

+
FFFFFFFFFFFFFFFFFFFFFFFFFFFFFFFFFFFFFFFFFFFFFFFFFFFFFFFFFFFFFFFFFFFFFF
FFFFFFFFFFFFF:FFFFFFFFFFFFFFFFFFFFFFFFFFF:FFFFFFFFFFFFFFFFFFFFFFFFFFFFFF
FFF
@A00721:190:HGJW7DSXY:4:1467:16206:8359_2:N:0:TCTGCAAG+GCTGCACT
TCAGAGAGGGGAGCTCTGAAAAGGAAACTTCGTCAGAAGATGAGGAAGTCTAAGAGGAGGAGTTTGAG
GAAGGCTCTAGAGAAGATCTCACCACCTGGAGAAGGCAAGTGGAGAGT
+
:FFFFF:FFFFFFFFFFFFFF:FFFFFFFFFFFFFFFFFF::FFF:FFFFFF:FFF:FFFFFFFFFFF
FFFFFFFFFFFFFFFFFFFFF:FFFFFFFFFFFFFFFFFFFFFFFFFFFFF
@A00721:190:HGJW7DSXY:4:1353:31177:14058_2:N:0:TCTGCAAG+GCTGCACT
AAGAAGGATTATTTGAAGCAGGGGTTACCACTTCCTAAGGTGGATGAGGGAGGTTGTTTCTTTGGAGT
TTTAGATAATCTTTTGAGAAGGGGTCATAGAGCCAGCAAGAGAAGCTT
+
FFFFFFFFFFFFFFFFFFFFFFFFFFFFFFFFFFFFFFFFFFFFFFFFFFFFFFFFFFFFFFFFFFFFFF
FFFFFFFFFFFFFFFFFFFFFF,FFFFFFFFFFFFFFFFFFFFFFFFFFF
@A00721:190:HGJW7DSXY:4:1554:31828:6386_2:N:0:TCTGCAAG+GCTGCACT
CCTTGAGAAGACAAGCCACATTAAGATATAATTTAGGTCAACTTGGGGTTTCGAATGTCATTAGTGCC
TTACGATTGAAAGAGGTCCTTTTGAGTATCGACAGATCTTTCGATCTT
+
FFFFFFFFFFFFFFFFFFFFFFFF:FFFFFFFFFFFFFFFFFFFFFFFFFFFFFFFFFFFFFFFFFFFFF
FFFFFFFFFFFFFFFFFFFFF:FFFFFFFFFFFFFFFFFFFFFFFFFFFF
@A00721:190:HGJW7DSXY:4:1347:17137:5776_2:N:0:TCTGCAAG+GCTGCACT
GTGGCAGAATTCGGTAACTTACCATATAGTTCCTTCCGGGACGTTGTGGGATTTAACTTTGACTCAAC
TACTCCGATCAGATCCGGATGGTTTGATTGGTATGGACTACTCGACTT
+
:FFF:FFF:,FFF,FFFFFFFFFF,FF,FF:FFF,FF:,FF,:,,F,F::F,F:FF,::,FFF,FFFF
FF,FFFFFFFF:FFFF:FFFFFF:FFFFFFF:FF:,:F,FFF,FFFF:
@A00721:190:HGJW7DSXY:4:2142:27760:19006_1:N:0:TCTGCAAG+GCTGCACT
TAGGGTCATGGCAGATACTACCGATAAGGTGAAAAATATGCAGGTCAAAGATTCTGTAGCTCTGAGGG
CGGTTGAGGGAGCCGTTTTGGCCGACTTTAACTATTCTAAGAGTCTTTATGATTCAAAACCTATCGTT
GTC
+
FFFFFF:FFFFFFFFFFF:FFFFFF:FFFFFFFFFFFFFFFFFFFFFFFFFFFFFFFFFFFFFFFFFFF
FFFFFFFFFFFFFFFFFFFFFFFFFFFFFFFFFFFFFFFFFFFFFFFFFFFFFFFFFFFFFFFFFFFFFF
FFF
@A00721:190:HGJW7DSXY:4:2221:29559:13510_1:N:0:TCTGCAAG+GCTGCACT
TACAGCGGCTTATTGTCGCGCAGTTTCCAGAATTGCGGTTGAAGTTCACCCATTCTATCTTGAATGCT
CACGCCGTCGCAGCCATATCGAGACTATGTTATAATCACTTGTTAAAGTACAGATTTAGTGATTTAGA
TAT
+
FFFFFFFFFF,FFFFFFFFFFFFFFFFFFFFFF,FFFFFFFFFFFFFFFFFFFFFFFFFFFFFFFFFFFFF
FFFFFFFFFFFFFFFFFFFFFFFFFFFFFFFFFFFFFFFF:FFFFFFFFFFFFFFFFFFFFFFFFFFFFFF
FFF
@A00721:190:HGJW7DSXY:4:1515:19732:22733_1:N:0:TCTGCAAG+GCTGCACT
GGAGGCGATATTCGTTTTCATATCCAAAACTCCTTGAATGGAGTACATATTTGCAACCCTATTCTTGA
TGTGAAAGATGCATCGAGGTATACGTCTAGGAACATTGATTGGGAACGTACCTACTCACGAGACGTCA
TGA
+
FFFFFFFFFFFFFFF:FFFFFFFFFFFFFFFFFFFFFFFFFFFFFFFFFFFFFFFFFFFFFFFFFFFFFF
FFFFFFFFFFFFFFFFF:FFFFFFFFFFFFFFFFFFFFFFFFFFFFFFFFFFFFFFFFFFFFFFFFFFF
FFF
@A00721:190:HGJW7DSXY:4:2168:12988:2472_1:N:0:TCTGCAAG+GCTGCACT
ATGTTTCGTTCTGTTACAAGAACGCTTTGGATTGTGACCATAAAGCCTCAGTTGCGATAGCTGTAGAG

```
GTATACGATATCTCTCTTGATGATGTAGCCACTATAATGCTTAAGAGAGAGATTGATGTATGTCATTT
CGT
+
FFFFFFFFFFFFFFFFFFFFFFFFFFFFFFFFFFFFFFFFFFFFFFFFFF:FFFFFFFFFFFFFFFFFF
FFFFFFFFFFFFFFFFFFFFFFFFFFFFFFFFFFFFFFFFFFFFFFFFFFFFFFFFFFFFFFFFFFFFF
FFF
@A00721:190:HGJW7DSXY:4:1629:11623:25911_1:N:0:TCTGCAAG+GCTGCACT
GAATTATTGGACACGATGAAAAATGACATACCCGTTCCTGACCTTGGTATCGTTATCCGTCATGGAGA
TGATGCCGCATCACTTGATTACTATATGCCCGAAGGATTGGGTTATTCTCACAACAAAGATAAGATTT
TAT
+
FFFFFFFFFFFFFFFFFFFFFFFFFFFFF,FFFFFFFFFFFFFFFFFFFFFFFFFFFFFFFFFFFFFFF
FFFFFFFFFFFFFFFFFFFFFFFFFFFFFFFFFFFFFFFFFFFFFFFFFFF:FFFFFFFFFFFFFFFFF
FFF
@A00721:190:HGJW7DSXY:4:2309:31819:20118_1:N:0:TCTGCAAG+GCTGCACT
TTTTCCTTAAGGGCGGACAATTGTTCCATTGTGAACTCTTGGGGACTAGATTGAGCATTAATGAGTTC
CGAATAACTCGTAATAAATCTTTCTCTAGTGGACAATTCAGAATACCTATTTCCGTACCAAGAGCCAC
TG
+
FFFFFF::FFF:FFFFFFFFFFFFFF,FFFFF:FFFFFFFFFFFF:FFFFFFFFFFFFFFFFFFF:F:F
FFFFFFFF:FFFFFFFFFFFFFFFFFFFFFFFFFFFFFFFFFFFFFFFFFFFFFFFFFFFFFFFFFFFF
FF
@A00721:190:HGJW7DSXY:4:2308:11695:25222_2:N:0:TCTGCAAG+GCTGCACT
AGGTTCGTTCCTCAATAAAAATTTTTCTCAAGAGATTATGGTTGATAAAGTTTTCTTCATAAGGGCTA
TTCAGTACGTCATGGGGATCACTTCTTCGGTCAATGACAAAACCTTCG
+
FFFFFFFFFFFFFFFFFFFFFFFFFFFFFFFFFFFFF:,FFF,FFFFFFFFFFFFFFFF:FFFFFFF,
FFFFFFFFFFFFFFFFFFFFFFFFFFFF:FFFFFFFFFFFFFFFFFFF
@A00721:190:HGJW7DSXY:4:1366:1678:22780_2:N:0:TCTGCAAG+GCTGCACT
CTTCGTAACAACAGTGCGAGGATTGTCATCTCGGGGAAAGTGATTCACCAAGATGTTCATATAGATAC
GAGATACCTAGAGAAGGTCGCAGCGATCTTACTCGTTATCGGAGCTAA
+
FFFFFFFFFF:FFFFFFFFFFFFFFFFFFFFFFFFFFFFFFFFFFFFFFFFFFFFFFF:FFFFFFFFFFF
FFFFFFFFFFFFFFFFFFFFFFFFFFFFFFFFFFFFFF,FFFFFFFFFF
@A00721:190:HGJW7DSXY:4:2347:24722:12555_2:N:0:TCTGCAAG+GCTGCACT
TACACGTTATGCTCGCAAACTTAACGTTAGTACCGGAGGTGATCTCTCTGCTTCTGCGTTACTGAGAG
AAAAGATTCAGGACGCCTACGGTGACTTTATGGACAAGATGCAAGTTC
+
FFFFFFFFFFFFFFFFFFFFFFFFFFFFFFFFFFFFFFFFFFFFFFFFFFFFFFFFFFFFFFFFFFFFF
FFFFFFFFFFFFFFFFFFFFFFFFFF:FFFFFFFFFFFF:FFFFFFFFF
@A00721:190:HGJW7DSXY:4:2511:18430:21668_2:N:0:TCTGCAAG+GCTGCACT
TTTAGGTTTAGTAGGTGATCTTGACGTCGATTCTTTGTATAGGGAGTGTGAGAAGACAACGAATCTTG
AAACTACGGTCCTTATCAAGGAAAATGGTGAACCGGTCTCTAAATCTT
+
FFFFFFFFFFFFFFFFFFFFFFFFFFFFFFFFF:FFFFFFFFFFFFFFFFFFF:FFFFFFFFFFFFFFF
FFFFFFFFFFFFFFFFFFFFFFFFFFFFFFFFFFFFFFFFFFFFFFF:FF
@A00721:190:HGJW7DSXY:4:1158:21061:33583_1:N:0:TCTGCAAG+GCTGCACT
GATTGAAGAACACGTCAAGAAGTCGCTCGGCGTTGTTCTTGAAAACCAAGTTAAGAAGGAGGCTGATC
ATGGAGTCTTTGTACCTTCTGTCAAACTTCGAGGTTCAGCTACGAGAGGTGATCCCGGATGTGGATTG
AAG
+
FFFFFFFFFFFFFFFFFFFFFFFFFFFFFFFFFFFFFFFFFFFFFFFFFFF,FF::::F::FFFFF,F
:FFFFF:FFFFFFFFFFFFFFFFFFFFFFFFFFFFFFFFFFFFFFFFFFFFFFFFFFFFFFFFFFFFFF
FFF
```

```
@A00721:190:HGJW7DSXY:4:1339:10782:19194_2:N:0:TCTGCAAG+GCTGCACT
TCCTTCTTTTTGAATGTTGTAAAGAATTGGGTTGAGAAGTGTGCTTTACAAGGAGTTAGACTCTTCTT
CAAGTTTTTAGATCTTCTTGATAATCTTAAAGAAAAATTTTCGAATAA
+
FFFFFFFFFFFFFFFFFFFFFF:FFFFFFFFFFFFFFFFFFFFFF:FFFFFFFFFFFFFFFFFFFFFF
FFFFFFFFFFFFFFFFFFFFFFFFF:FFFFFFFFFFFFFFFFFFFFFF
@A00721:190:HGJW7DSXY:4:2265:26006:24424_2:N:0:TCTGCAAG+GCTGCACT
CTGTACTTACATTCTTTACGCCAGATTCTTCATGGGGCGACGCTGTTTCTCTTGGTATTTCTGAAATA
GTTTATCAGAGCGTCATTTTCCTTTGGGATCTTTCCTCTGGTAAGGGA
+
FFFFFFFFFFFFFFFFFFFFFFFFFFFFFFFFFFFFFFFFFF:FFF:F:FFFFFFFFFFFFFFF:FFF
FFFFFFFFFFFFFFFFFFFFFFFFFFFFFFFFFFFFFFFFFFFFFF:F
@A00721:190:HGJW7DSXY:4:2566:13160:35086_2:N:0:TCTGCAAG+GCTGCACT
TTCTCACTCTTGACGAGAATTATAACTGTTTTCTCTACCCATAACTTCTTTTTCAAAAATGCGGATGG
GTCTACTGAAGTTATCCTACAGAGTGCAGTCGTTTCAAAACTTATCAC
+
,FF,FFF,:FFFFFFFF:FFFF:,FF,,,FF:FFFFF:FFFFF:F,FFFF:F::FFFFFF,,,F,FF,
FFFFFFFF:,,FF,:,,FF,F:FFF:FF:FF,F:,,FF,FFFFF,,:F
@A00721:190:HGJW7DSXY:4:1151:3369:31250_1:N:0:TCTGCAAG+GCTGCACT
AATAATTGGTGATGAAAATACGTATGTCGGTGGGGTTCTCAAGGCTCTCATCGCCACTGGGATTCTAA
GGGGTTCCATAAGATATTATCTGACATTGTGGTTCAATGATCAGGATTCTTCTTTTGGTCCTTACGTC
TTA
+
FFFFFFFFFFFFFFFFFFFFFFFFFFFFFFFFFFFFFFFFFFFFFFFFFFFFFFFFFFFFFFFFFFFF
FFFFFFFFFFFFFFFFFFFFFFFFFFFFFFFFFFFFFFFFFFFFFFFFFFFFFFFFFFFFFFFFFFFF
FFF
@A00721:190:HGJW7DSXY:4:2472:22336:34820_2:N:0:TCTGCAAG+GCTGCACT
GTTCTTGTCACGATTCTTAAAAGAAGTTTATTAAAACTTCAGAGTCTCTTGGAAGTCCAGGTAGCTAA
TGTTCTTGCAATAGCCACAGACTTTCTGGGAAAAGAGACCTCTGTTGG
+
F,FFFFFFFFFFFFFFFFFFFFFFFFFFF:FFFFFFFFFFFFFFFFFF:FFFF:FFFF:FFFFFFFF:
FFFFFF:FFFFFFFFFFFFFFFFFFFFFFFF,F:FFFFFFFFFFFFFF
@A00721:190:HGJW7DSXY:4:1533:29613:29356_1:N:0:TCTGCAAG+GCTGCACT
GAGAATTACTCAGATTCAATCTGGCATCACTGAACGTCTACAACGAGTTAGGGAGTGGCTAGGTTTTA
AAAGATCCTTTGCACAAGCAATACCTTCCACTGAGTTCATCACCCAGAGTTTCGACGTCGCGGAAAAC
GAT
+
FFFFFFFFFFFFFF,FFFFFF,FFFFFFFFFFFFFFFFFFFFFFFFFFFFFFFFFFFFFFFFFFFFFF
F:FFFFFFFFFFFFFFFFFFF:FFFFFFF:FFFFFFFFFFFFFFFFFFFFFFFFFFFFFFFFFFFFFF
FFF
@A00721:190:HGJW7DSXY:4:2149:7383:4131_2:N:0:TCTGCAAG+GCTGCACT
ACTGTTCGAGAATCTTCTGATGCTGAATTTGAGGAAAATTTTTCGGAAGTTTTTGGTATAAATCCTGA
TGGCTCAGTACAGGGAGGAGGTAATAGTTATAAAACCTTCTTTTATCA
+
FFFFFFFFFFFFFFFFFFFFFFFFFFFFFFFFFFFFFFFFFFFFFFFFFFFFFFFFFFFFFFFFFFFF
FFFFFFFFF:FFFFFFFFFFFFFFFFFFFFFFFFFFFFFFFFFFFFFF
@A00721:190:HGJW7DSXY:4:1142:29839:24017_2:N:0:TCTGCAAG+GCTGCACT
GGACAAAATCTGGGATCATAGGTATGTGTCACGCTACACTCGAAATATGGGCCCTCATAAGGAAGTAT
AGTTGGCGTCTTGTCGTTGACGTCATTACAGTCTGGAAGTTAGATCAT
+
FFFFFFFFFFFFFFFFFFFFFFFFFFFFFFFFFFFFFFFFFFFFFFFFFFFFFFFFFFFFFFF:FFFF
FFFFFFFFFFFFFFFFFFFFFFFF:FFFFFFFFFFFF:FFFFFFFFFF
@A00721:190:HGJW7DSXY:4:1430:26142:35243_2:N:0:TCTGCAAG+GCTGCACT
CGAGAGTTGTGATCTAGTTCTAAATTTAGGTCGACGGACTTTAGCAACTTTAGATAAATTCTTTCCGG
```

```
GCTCAATGACCTTTGCTATGTACGTGGATCTAATCCTTGGTGATCTGA
+
FFFFFFFFFFFFFFFFFFFFFFFFFFFFFFFFFFFFFFFFFFFFFFFFFFFFFFFFFFFFFFFFFFFFFFF
FFFFFFFF:FFFFFFFFFFFFFFFFFFFFFFFFFF:FFFFF:F:F:FFF
@A00721:190:HGJW7DSXY:4:1534:15293:16454_2:N:0:TCTGCAAG+GCTGCACT
TATTAGTTCATGAAGTTCTTACACTTCTCGCGACTAATGTCAGCTTCACTACAAAGGCGTTACGTAAA
GTAGTTTTCACGCTTTATCTTAAAGCATTACCACCAAATTTAGCACTT
+
FFFFFFFFFFFFFFFFFFFFFFFFFFFFFFFFFFFFFFFFF:FFFFFFFFFFFFFFFFFFFFFFFFFFF:
FFFFFFFFFFFFFFFFFFFFFFFFFFFFFFFFFFFFFFFFFFFFFFFFF
@A00721:190:HGJW7DSXY:4:2103:28899:12524_1:N:0:TCTGCAAG+GCTGCACT
CTCGCCATATCAGATTAGAGACATGCCAGTTAGAGTTCTTCTAACCAGGACTGATGGTGAGATTCTTT
TACCTTTTGGAGGTCCAGGAACCTTTACTCCTAGTGTCTTTTCTAGAGTCCAAGGACGTTTCCGTCGG
AGG
+
:FFF,FFFFFF,,:F,FFFFFFFFFF::,FFFF:FFF,F::FFF::FFFFF:F:,FFFFFFFFFFFFF
F::FFFFFFFFFFFFFF:FFF::FFFFFF:FFF:FFF:FFFFF,FFFFFFFFFFFFFFFFFFFFFFFF
FFF
@A00721:190:HGJW7DSXY:4:1476:20916:19429_2:N:0:TCTGCAAG+GCTGCACT
GACCGCTCAATAACACTCTATTGAATCCCTTTTCCACGATGAAAGCTCTTTATGAGGTGCTCCATGCA
AATTTACAGTTCCTCTTTGGTGATATCGCCTCTGTCCTTTCTCCTTTA
+
FFFFFFFFFFFFFFFFFFFFFFFFFFFFFFFFFFFFFFFFFFFFFFFFFFFFFFFFFFFFFFFFFFFFFFF
FFFFFFFFFFFFFFFFFFFFFFFFFFFFFFFFFFFFFFFFFFFFFFFFF
@A00721:190:HGJW7DSXY:4:1408:26115:14653_2:N:0:TCTGCAAG+GCTGCACT
ACTTGGGTTTATCGGTTGTATTTTGATAAATTTGTTGGAATCGATAATGATACTGACGAAGGTTCTAT
TACCACCCTTGTCATTGATACAAACATTCTAGGTGATGCTTTCTTAGA
+
FFFFFFFFFFFFFFFFFFFFFFFFFFFFFFFFFFFFFFFFFFFFFFFFFFFFF:FFFFFFFFFFFFFF
FFFFFFFFFF:FFFFFFFFF:FFFFFFFFFFFFFFFFFFFFFFFFFFFF
@A00721:190:HGJW7DSXY:4:1214:4698:13260_2:N:0:TCTGCAAG+GCTGCACT
TTACAATCTGTAGAGAGAGAGAGGTTAATCAGAATTTGAGTGATAACGATTTAGTACTGGGAAATGGTGG
TAGTCAGTTTACTACTTTTCTTTCCCTCCCCTTTTTCGATTGGATTCG
+
FFFFFFFFFFF:FFFFFFFFFFFFFFFFFFFFFFFFFFFFFFFFFFFFF:FFFFFFFFFFFFFFFFFFFF
FFFFFFFFFFFFFFFFFFFFFFFFFFFFFFFFFFFFFFFFFFFFFFFF:
@A00721:190:HGJW7DSXY:4:1156:29794:10347_2:N:0:TCTGCAAG+GCTGCACT
TTGATAAGGTTTTTCTTTTATGGGACTCTTGTTTGTACTTATGAAGGAGGTATTACTTCGGAAGTTCT
TAATGTAGGTCTCCTAGTACTTAAGGGTTTATGTTTCGGTTTCAGCTG
+
FFFFFFFFFFFFFFFFFFFFFFFFFFFFFFFFFFFFFFFFFFFFFFFFFFFFFFFFFFFFFFFFFFFFFFF
FFFFFFFFFFFFFFFFFFFFFFFFFFFFFFFFFFFFFFFFFFFFFFFFF
@A00721:190:HGJW7DSXY:4:1129:32606:26584_2:N:0:TCTGCAAG+GCTGCACT
TGTTTCACATTGCGTGATCACTTCCCGTGGACTTGGGAAGAGATTAAACATAAAATCCAGCGATTCTA
TTATGTCGCTAAAGCCTGTCCAGTTGTCGATGATGTTCGGGCTGTGGG
+
FFFFFFFFFFFFFFFFFFFFFFFFFFFFFFFFFFFFFFFFF:FFFFFFFFFFFFFFFFFFFFFFFFFF,FFF:
FFFFFFFFFFFFFFFFFFFFFFFFFF,FFFFF:FFFFFFFFFF:FFFFFF
@A00721:190:HGJW7DSXY:4:2256:19452:9032_2:N:0:TCTGCAAG+GCTGCACT
AAACTTACGACGATGTTCTCGCTCGCATTTCACAAGGATTACTAAGTCTGAGATCTGCCACTTCTAAT
TTTAGGAAACGTCGCGAAAAGAAAGTTCATGAGGATGTCTTGGTCGAA
+
FFFFFFFFFFFFFFFFFFFFFFFFFFFFFFFFFFFFFFFFFFFFFFFFFFFFFFFFFFFFFFFFFFFFFFF
FFFFFFFFFFFFFFFFFFFFFFFFFFFFFFFFFFFFFFFFFFFFFFFFF
```

```
@A00721:190:HGJW7DSXY:4:1640:21251:29778_2:N:0:TCTGCAAG+GCTGCACT
GAACTTCTAATAGAGGATCCTGATGACATTATTTCAACGGATACCGATCTCAGTTCTACCACAGAAAC
CGTCTCAGGAACTGTCTCCACTGATTCGGAATTAGAGAAAGACGACCT
+
FFFFFFF:FFFFFFFFFF:FFFFFFFFFFFFFFFFFFFFFFFFF:FFFFFFFFFFFFFFFFFF:FFFF
FF:FFFFF:FFFFFFFFFFFFFFF:F:FFFFFFF,FFFFF:FFFFF:F
@A00721:190:HGJW7DSXY:4:1276:17571:19398_2:N:0:TCTGCAAG+GCTGCACT
AGACCTCTGAATTTTCAGGGTGCCCTGATGAGGACGTTGAAAGGGATAATTTCAACTCTGAGACTTCT
AAGAGTGTTTCAAAAGACCAGAAACGTAACAAGAAGAATAAAGGTAGG
+
FFFFFFFFFFFFFFFFFFFFFFFFFFFFFFFFFFFFFFFFFFFFFFFFFFFFFFFFFFFFFFFFFFFF
FFFFF:FFFFFFFFFFFFFFFFFFFFFFFFFFFFFFFFF:FFFFF:FFF
@A00721:190:HGJW7DSXY:4:1129:11785:8437_2:N:0:TCTGCAAG+GCTGCACT
AGCGAGCGGTTGAAGAGAAAACTGATATGGTTGCGGCTGATCGTACCCCAATTCCTGCTAGAAAGATC
ACTCTCGGCGATACCACTGATCTTCCAGAGTTACGTAGTGGTCGTTCT
+
FFFFFFFFFFFFFFFFFFFFFFFFFFFFFFFFFFFFFFFFFFFFFFFFFFFFFFFFFFFFFFFFFFFF
FFFFFFFFFFFFFFFFFFFFFFFFFFFFFFFFFFFFFFFFFFFFFFFF,
@A00721:190:HGJW7DSXY:4:1154:3821:36730_1:N:0:TCTGCAAG+GCTGCACT
AAGTTTCTGCTAGTCGCGACGATAGTATTTATCGGAGTGATATTAACAAGTTAAACTTCGAACTGACC
ACTTACAAGAGTGTTGAAGTACCGCAACATGGATCAGTTTTCCAAAAAGCTATTAATAGCAGTTGCGG
GGC
+
FFFFF,FFFFFFFFFFFFFFFFFFFFFFFFF,FFFFFFFFFFFFFFFFFF::FFFFFFFFFFFFFFFFFF
FFFFFFFFFFFFFFFFFFFFFFFFFFFFFFFFFFFFFFFFFFFFFFFFFFFFFFFFF:FFFFFFFFFFF
FFF
@A00721:190:HGJW7DSXY:4:2165:15962:33113_2:N:0:TCTGCAAG+GCTGCACT
AAACTGAATCCACTCTCTTGGGATATAAATGAGTACATCTATAATAGAGTTTTGGAACTGTGTAATGT
CCTACGTACTGTTCAAGGTGTTTGGGCTTCTCTAAAGAACAGCAACTT
+
FFFF:FFFFFFFF:FFF:FF:FFFFFFFFFFFFFFFFFFFFFFFFFFFFFFFFF:F:FFFF::FFFFFF:
FFFFF,FFF::FFFFF:FFFFFF,FFFFFFFFFFFF:FFFFFFF:FF:
@A00721:190:HGJW7DSXY:4:2359:14633:15468_2:N:0:TCTGCAAG+GCTGCACT
AGACCTTTCTTGGGGAGTAAAGAGGGTTTGAAAATTACGGAACCTTTCCTCGTTCTTCATGAAGATCT
TCGAGTGTTTTATAGTGAGTTATTCTTGCGAAGGTTCAAGTCGATTGA
+
FFFFF:FFF:FFFFFFFFF::FFFFFFFFFFFFFFFFFFFFF,FFFFFFFFFFFFFFFFFFFFFFFFFF:
FFFFFFFFFFFFFFFFFFFFFF:FFFFFFFFFFFFFFFFFFFFFFFF:FFF
@A00721:190:HGJW7DSXY:4:1148:14470:19132_2:N:0:TCTGCAAG+GCTGCACT
AATAAGAAGTTCTTCGCAGTTGAATCTCCACCAGGTGGAGGGAAGACCACTGAACTTATAGCGACTTT
TGTCGAATTTTTCGCAAAAGGTCTTGAAGTACGTATTGTCACTGCTAA
+
FF:FFFFFF:FFF,FFFFF:FFFFFFFFFF:FFFFFF,F:FFFFFFFFFF::FFFF,F:F,FFFF:FF
::F,FFFF:FFFF,FFFF:FFFFFFFFFFFFF:FFF,FFFFFFF:F:F
@A00721:190:HGJW7DSXY:4:2626:31439:26099_2:N:0:TCTGCAAG+GCTGCACT
CGAGAAGAGATAGTGACTAAAGTTTTGGCACTTCTAGTTCATCGCGGTATTTGTGAAATTAAGACAGA
CGTGAAATTTATGATGAGTGATCGTATTAGAACAGCTGATTCTTATTG
+
FFFFFFF:FFFFFFFFFFFFFFFFFFFFFFFFFFFFFFFFFFFFFFFFF:FFFFFFFFFFFFFFF:FFF
FFFFFFFFFFFF:FFF:FFFFFFFF:FFFFFFFFFFFFFFFFFFF::FF
@A00721:190:HGJW7DSXY:4:1527:17969:2206_2:N:0:TCTGCAAG+GCTGCACT
CTGAGGTCAAACCTGTGGACGTTCTTTTCATCGACGAATTATTCATGATTCACGCTGGTCAGACCCTT
TTTCTTACACATCTACTTAAGGCGGCTTATACCATAGGTTACGGTGAT
+
```

FFFFFFFFFFFFFFFFFFFFFFFFF::FFFFFFFFFFFFFF::FFFFFFFFFFFFFFFF:FFFFFFFF
F:FFFFFFFFFF:FFFFFFFFFFFFFFFFFFFFFFF:FFFFFFFFFFF
@A00721:190:HGJW7DSXY:4:1576:4679:27727_1:N:0:TCTGCAAG+GCTGCACT
GACGTGTGCTCTTCCGATCTCCAAGTCGCAAACTACTTCAACATCAACGACATCATTCTTGATGAGTG
TCGTTCTTATCGAAGTCTCAGTTATCGCTGCCCGGCCGACGTTTGTCGCGAACTCTCAGAGGTTTACG
GCA
+
FFFFFFFFFFFFFFFFFFFFFFF,FFFFFFFFFFFFFFFFFFFFFF:FFFFFFFFFFFFFFFFFFFFF
:FFFFFFFFFFFFFFFFFFFFFFFFFFFFFFFFFFFFFFFFFFFFFFFFFFFFFFFFFFFFFFFFFFF
FFF
@A00721:190:HGJW7DSXY:4:1201:9010:16219_2:N:0:TCTGCAAG+GCTGCACT
CTATGGAAATTAGACCTATCAGATGTCTTGACGACTTCGAATTTGAGGACGGAGTTAAATATATTACT
TTCACTCAAGGTGAAAAGTTTGAAATTAACAAAGTCCTTGGTAAGAGT
+
FFFFFFFFFFFFFFFFFFFFFFFFFFFFFFFFFFF:FFFFFFFFFFFFFFFFFFFFFFFFFFFFFFFF
FFFFFFFFFFF:FFFFFFFFFFFFFFFFFFFFFFFFFF:FFFFFFFFF
@A00721:190:HGJW7DSXY:4:2336:23032:29888_1:N:0:TCTGCAAG+GCTGCACT
GTTCAAGGTAATACTTACCAGCACGTTCGGCTGGTTAGAATTAAACCTCAAGATGACCCTGTTTTTGC
ATCACAAACACATATTACCGTTGCTCTATCACGACACGTTGAATCTTTACGATACTTTGTTATCAATG
TAA
+
FF:FFFFFFFFFFFFFFFFFFFFFFFFFFFF:FFFFFFFFFFFFFFFFFFFFFFFFFFFFF:FFFFFF
FFFFFFFFFFFFFFFFFFFFFFFFFFFFFFFFFFFFFFFFFFFFFFFFFFFFFFFFFFFFFFFFFFFF
FFF
@A00721:190:HGJW7DSXY:4:2631:1687:30154_2:N:0:TCTGCAAG+GCTGCACT
TAAGGAACTGTCACGTTTAGCACGGGAGGTTGCAGATTCAACCCCTTATATCGAGGCTATCGAGGACG
ATGAGTATCTTGAAGTTGTTGACGACAAGCCTCAAGAGGGTAGAGGAC
+
FFFFFFFFFFFFFFFFFFFFFFFFFFFFFFFFFFFFFFFFFFFFFFFFF:FFFFFFFFFFFFFFFFFF
FFFFFFFFFFFFFFFF:FFFFFFFFFFFF:FFFFFFFFFFF,FFFFFF
@A00721:190:HGJW7DSXY:4:2131:12807:18411_2:N:0:TCTGCAAG+GCTGCACT
GGTTATTCCTGGAACCACCTTAACGTCACTCGGTAATCTTTCAGCCGAATTAAGTACTAATGAGTTCA
AGTCGGTTGTCGATCGAATTAAGATCAAGGCTAATACATTTATAGGTA
+
FF,FF,FF:F,,F:FFF:FFFFF,:FFFF,:F:FFFF:,F:FFFF,FF,FFFFFF:F::F,,F,:FFF
FF,F,,,F,F:,F,:,F:FFFF,FF:FFF,F,FFFFF,,,FFFF,,FF
@A00721:190:HGJW7DSXY:4:2327:1805:1673_2:N:0:TCTGCAAG+GCTGCACT
GCCGCGTAGTCAGCGCGTTTAGCTGTATCATCAGTCAGAGTATACCCGATAGGGTACCGAGTCTCCAA
GAGAATATTTATTCTTATGAGGCTCGAAACTTCAACGTTTTCACAACG
+
FF,,FFFFFFFFFFFFF:FFF:,FFFFFFFFFFFF,F:F:FFFFF,FFF:FF:FFF,F,FF:F:FFFF
,:FFFFFFFFF,F:,FF:,F:::F,FFFFFFF::FF,FFFFFFFFF:F
@A00721:190:HGJW7DSXY:4:2276:10004:12461_2:N:0:TCTGCAAG+GCTGCACT
TCGCACCCGAAATTTTTGGGCAGAATGCTCTTAACGCGGTTCTTGAACGGTGTTTTGATCAGTCTAAG
TTGGATAAAGTTATAGGAGACTCAGTTAACCTGAGAGCTCCTCAATTG
+
FFFFFFFFFFFF:FFFFFFFFFFFFFFFFFFFFFFFFFF:FFFFFFFFFF:FFFFFFFFFFFFFFFFF
F:FFF:FFFFFFFFFFFFFFFFF,FFFFFFFFFFFFFFFFFFFFFFFF
@A00721:190:HGJW7DSXY:4:2304:10836:34256_2:N:0:TCTGCAAG+GCTGCACT
AATAGGGACGCTAGTATACTTCGTAAAGTCGACAGCGAAATGGTCAATATTGATAATTGGACCGACTA
TATGACCTATTTTAAGCTCATGGTGAAAAGGGAAGCTAAGGTTAAACT
+
FFFFFFFFFFFF,FFFFFFFFFFFFFFFFFFFFFFFFF:FFFF:FFFFF:FFFFFFFFFFFFFFF:FF
FFFFFFFFFFFFFFFFFFFFFFFFFFFF,F::F:FFFFFFFFFFFFFF

@A00721:190:HGJW7DSXY:4:1135:12997:9533_2:N:0:TCTGCAAG+GCTGCACT
CCTATCTTTAAGGTTCTTGTGAGGAGAGTCAAACAGTGTCTTTTACCCAACATTTTTATGTATACAGA
GATGACCAGTGACGATTTCTCCCGGAAGCTCTTCTCTCTCTTAGGAGG
+
FFFFFFFFFFFFFFFFFFFFFFFFFFFFFFFFFFFFFFFFFFFFFFFFFFFFFFFFFFFFFFFFFFFF
FFFFFFFFFFFFFFFFFFFFFFFFFFFFFFFFFFFFFFFFFFFFFFFF
@A00721:190:HGJW7DSXY:4:1566:4526:18662_2:N:0:TCTGCAAG+GCTGCACT
TTTCGAAGTTTGACAAATCTCAAGATTTCTTTTGTAAAGCCTATGAGATAAAGATGTATTCTTATTTA
GGCTTCGATGCTGAATTTCTTGAACTTTGGAGTGCCAGTGAATATTTT
+
FFFFFFFFFF:FFFFFFFFFFFFFFFFF:FFFFFFFFFFFFFFFFFFFFFFFFFFFFFFFFFFFFFFFF
FFF:FFFFFFFFFFFFFFFFFFFFFFFFFFFFFFFFFFFFFFFFFFFF
@A00721:190:HGJW7DSXY:4:1619:2049:27837_1:N:0:TCTGCAAG+GCTGCACT
GAGTATCCATAACGATTTGTCATTTACCACCTTTGCTCAACGACGAAGTGGAACGGCCAACACTTTCT
TTGGTAATTGTATGATCACTTTGATGATTTTATCACTTTATTATGACCTTGGAAAGTTTTCGGCTGTG
GTT
+
FF,FFF,FFFFF,,FFFFF:FFFFF:,FF:FFF::FFFFFF,FFFF:FFFFFFFF:FFF:FF:FFF,F
FFF:FF:FFFFFF:FFF::FFFFFFFFFFFFFFFFF:FFFFFFFFFFFFFFFF,FFFFFFFFF:FFFF
,FF
@A00721:190:HGJW7DSXY:4:2601:1280:28103_1:N:0:TCTGCAAG+GCTGCACT
CCATTCTGTTTTCAAAGACTCCAATAACTGATCATTCTGATCATATGATTTTGGACAATGGTTTCGAA
GCGAAATTTTTGAGGGATAAACCAGCGTACTTCTGCTCGCGATTTATTCTTTTTGTCAATGATTACAT
CTA
+
F:FFFFFFFFFFFFFFFFFF:FFFF:FFFFFFFFFFFFFFFFFFFFFFFFFFFFFF::F:FFFFFFFFF
F:FFFFFFFFFFFFFFFFFFFFFFFFFFFF,FFFFFFFFFFFFFFFFFFFFFFFFFFFFFFFFFFFFFF
FFF
@A00721:190:HGJW7DSXY:4:2313:5538:2096_1:N:0:TCTGCAAG+GCTGCACT
CCTTACAAGATCATCGTTAAGTTAGGTAAACCCCTAACCGATGTTACTCTTGTAGGACTTTGGGAGAG
ATTCGTTAGTTTTAAAGACTATACTAAATATCTCCACTACGAGGTTGTTGTTTGGCGATTAGCTCTTG
CGA
+
FFFFF::FFFFFFFFFFF:F,FFFFFFFFFFFFFFFFFFFFF:FFFFFFFFFFFFFFFFFFFFFFFFFF
FFFFFFFFFFFFFFFFFFFFFFFFFFFFFFFFFFFFFFFFFFFFFFFFFFFFFFFFFFFFFFFFFFFF
FFF
@A00721:190:HGJW7DSXY:4:2244:31132:36808_2:N:0:TCTGCAAG+GCTGCACT
ATTGAGGGTCGTTATACCTATTCGGCTATCTGTGCTTTGAATTGTATAAGATCAAATTTTAAACGATT
CCGAGAGGTTTATCCTGATGTGGAGGGAGTTCTTGAAGCATCCCGACC
+
FFFFFFFFFF:FFFFFFFFFFFFFFFFFFFFFFFFFFFFFFFFFFFFFFFFFFFFFFFFFFF,:,FFF
F,FFFFFFFFFFFFFF,:FFF,FFF:FFF:F:FFF:F:FFFF,FFFF,
@A00721:190:HGJW7DSXY:4:2365:28899:34945_1:N:0:TCTGCAAG+GCTGCACT
TGATGCGGTTTCATAAGATTATCAGTAATCCTGATTCTACTACGGATTCATGGACCTTCCATCCTGTA
CCTCTTCTGGAGAGACGTATCTCAAAGATCGACTTCGAGACAGGTGATATTGTTGAGCGAGAGCGACC
AAG
+
FFFFF,:FF:FFFFFFFFFFFF:F,FF:FF,FFFFFFFFFFFFFFFFFF:FFFFFFFFFFFF,FFFFF
:FF::FFFFFFFFF,:FFFF:F,:,F:FFFFF,FF:::FFF,FFFF:FFFFFFF:F::FFFFFF,F::
:F:
@A00721:190:HGJW7DSXY:4:2378:31656:34115_1:N:0:TCTGCAAG+GCTGCACT
CGTGGCGCTTTATTATAAAAAGCTCCCTCCTCTCGCAAGTTTGATCTGATACTTATCACGAAAGGACT
TGAGTCTTTTGATCAGTCTCTGACTTGACCATCTTTCATTTTAAATGACGATCATTAAGACTCTCTTT
CTT

+
FFFFFFFFFFFF:F,FFFFFFFFFFFFFFF,FFFFFFFF:FFFFFFFFFFFFFFFFFFFFFFFFFFFFF
FFFFFF:FFFFFFFFFFFFFFFFFFFFFFFF:FFFF,FF:FFFFFFFFFFFFFFFFFFFFFFFFFFFFF
FFF
@A00721:190:HGJW7DSXY:4:2103:14018:22983_2:N:0:TCTGCAAG+GCTGCACT
CTTTGCACTGATTTTTCCTCATCAGCTTGTGTTTTACCTAATCTGTGTTTTTACGATAACAACTATCA
GTTGATTAAGACTCTTCCGAAAGGCGAACTTTGGGAATTTGAAGCTCA
+
FFFFFFFFFFFFFFFFFFFFFFFFFFFFFFFFFFFFFFFFFFFFFFFFFFFFFFFFFFFFFFFFFFFF
FFFFFFFFFFFFFFFF:FFFFFFFFFFFFFFFFFFFFFFFFFFFFFFF
@A00721:190:HGJW7DSXY:4:1627:17870:11428_2:N:0:TCTGCAAG+GCTGCACT
ACCGTGACGAACTGTGGTGTTCTTGATCTACGTTACGGATATACTCTCCTTGAGTTCACTCGTAAGGA
CACTACCACTTATTACGATATTTCTTTAGTCGATGGGTTTTCGACGCC
+
FFFFFFFFFFFFFFFFFFFFFFFFFFFFFFFFFFFFFFFFFFFFFFFFFFFFFFFFFFFFFFFFFFFF
FFFFFFFFFFFFFFFFFFFFFFFFFFFFFFFFFFFFFFFFFFFFFFFF
@A00721:190:HGJW7DSXY:4:2320:11831:11334_2:N:0:TCTGCAAG+GCTGCACT
ATTCTTCTAATGTGGCCTGAACTACGATACACTTTGTGTGTACTTGTCTCTATTTTAAGTTTTGTCTT
CCTTTATTGTCTTATTCTATTAAACACTCATTTTAGTGAGATTATTAA
+
,F,,,,F,:F:,F,:FF,,FFFFFFF,,,:FF,F:F,F,:FF,FF,,,F,FF,F,FFF,FF:F:FF,F
,FF,,,,,:F,F,FF,:FFF:FF,,F:::F,:F,FFF:FFFFFFFFFF
@A00721:190:HGJW7DSXY:4:1163:12264:31438_1:N:0:TCTGCAAG+GCTGCACT
TCTTCGACAACAGCAGGGGTTTTCGAATAGTGATAGCCACCCGCCTTACAGAGGGGCAGCAGCATGAC
CATAATTGGTATTGACTTTGGTACCACCTTTTCTACTGTCTGTCTCGCCTCTTCGAATGAAGTTTTAA
TGC
+
FFFFFFFFFFFFFFFFFFFFFFFFFFFFFFFFFFFFFFFFFFFFFFFFFFFFFFFFFFFFFFFFFFFF
FFFFFFFFFFFFFFFFFFFFFFFFFFFFFFFFFFFFFFFFFFFFFFFFFFFFFFFFFFFFFFFFFFFF
FFF
@A00721:190:HGJW7DSXY:4:1438:22381:12258_2:N:0:TCTGCAAG+GCTGCACT
CTACAATCATTGGTTTTTCTAATAATGATGACACTATTGTTTATGGATATGATGCTATTACTCATGAT
GGTTACGGTCAGGGAGGATTCTCAGTTTACAAGGACCTTAAGCGATGG
+
FFFFFFFFFFFFFFFFFFFFFFFFFFFFFFFFFFFFFFFFFFFFFFFFFFFFFFFFFFFFFFFFFFFF
FFFFFFFFFFFFFFFFFFFF:FFFFFFFFFFFFFFFFFFFFF:FFFFF
@A00721:190:HGJW7DSXY:4:1371:15049:23610_2:N:0:TCTGCAAG+GCTGCACT
CCGAAAGACGTGCTAAATTAAAACCATTGTACGATGTCGTCTGTCTCGGCGATCACTTTTCACTAAGA
ATTGGTCCTACTTTTGGACAAGGCCAACTTCGTAATCTTCATGAGATC
+
FFFFFFFFFFFFFFFFFFFFFFFFFFFFFFFFFFFFFFFFFFFFFFFFFFFFFFFFFFFFFFFFFFFF
FFFFFFFFFFFFFFFFFFFFFFFFFFFFFFFFFFFFFFFFFFFFFFFF
@A00721:190:HGJW7DSXY:4:2124:19280:4006_2:N:0:TCTGCAAG+GCTGCACT
TATCTCTTTGGTTGTTAGGGATTTTGAGAGAGTGAAAGGCTTCAATTGTAGTGGTTTGGTCGTTTCAG
TACCCAGTCAGTATACCTCTACGCAAAGATATTTCATGAAAACTACAG
+
FF:F:F,FF:FFFFFF,FFFFFFFFFFFFFFF:FFFF:FFF,FFFFFFFFFFFF:FFFFFFF:FFFFF
FF,FFF:FFF::FFF:FFFFF:FF:FFFFFFF,FFFFFFF:FFFFFFF
@A00721:190:HGJW7DSXY:4:1501:4038:7858_1:N:0:TCTGCAAG+GCTGCACT
ACCTGTCTTTCATATTATGAATGAACCTTCGGCCGCTCTCTTTGCTTCGATGTTGGATGTCAAGAAGG
GTTCAGAATGGGATTCCTACGTAGTTTATGACTTTGGTGGTGGTACTTTCGATACTTCTATCGTCTTA
CGT
+
FFFFFFFFFFFFFFFFFFFFFFFFFFFFFFFFFFFFFFF:FFFFFFFFFFFFFFFFFFFFFFFFFFFF

FFFFFFFFFFFFFFFFFFFFFFFFFFFFFFFFFFFFFFFFFFFFFFFFFFFFFFFFFFFFF:FFFFFFF
FFF
@A00721:190:HGJW7DSXY:4:1257:23131:18724_2:N:0:TCTGCAAG+GCTGCACT
AGTGTTATTTATTCTGGTGGTGATGATAATCTTGGAGGTCGTGACGTTGATGAAGCTATAAGAGCGTT
CTCATTACGGCGCACTTCTTTACCCGCCACCACACAACTTTCAGTTTC
+
FFFFFFFFFF:FFFFFFFFFFFFFFFFFFFFFFFFFFFFFF:FFFFFFFFFFFFFFFF:FFFFFFFF
FFFFFFFFFF:FFFFFF:FFFFFFFFFFFFFFFFFF:FFF:FFFFFFF
@A00721:190:HGJW7DSXY:4:1247:2094:7373_1:N:0:TCTGCAAG+GCTGCACT
ACTGGAAAACCCTCGTCAATACTTTTCAATAATATACGTCTTGAACTCACTTATGACGATCTAGTGAA
ATTAACGCGTCCTTTTCTGGAACGTAGTTTTACTATTATGAACAAGGTTATCAAAGATAGTGGCATTC
AAG
+
FFFFFFFFFFFFF:FFFFFFFFFFFFFFFFFFFFFFFFFFFFFFFFFFFFFFFFFFFFFFFFFFFFFF
FFFFFFFFF:FF::FFFFFFFFFFFFFFFFFFFFFFFFFFFFFFFFFFF:FFFFF:FFFFFFFFFFFFF
FFF
@A00721:190:HGJW7DSXY:4:2476:15817:22936_1:N:0:TCTGCAAG+GCTGCACT
CCTGATTCCGGCCGGTGGTAGCTCATTTCTACCAGGGGCTATTACTCTTGCTGAAACAGCTATTAAAG
CTGTTGAGCGGGTAATTACACCTAAACGAGCTCGATCTGCCATCGCTGAAGGATGTGCACTCTTTTCT
GCT
+
FFFFF,FF:,F,FFFFFFFFFFF,FFFFFFF:FFFFFF,FFFF:FFFFFFFFF,FFFFFFFFFFFFFF
FFFFFFFFFFFFFFF:FFFF:FFF:,FFFF:FFF:F:FFF:FF:FFF:,:FFFFFFF:F:FFF,FFFF
F,F
@A00721:190:HGJW7DSXY:4:1358:24279:2268_2:N:0:TCTGCAAG+GCTGCACT
TCGATTGTATCAACAGTAACATTACTTCTAATAAAGGTCTCTATCAAGCTCGTATTATCGTCGCTGCT
GGATCTGCGTTACCGTTGAAGGCTAAACTCCAAGGTAATTATGAAGAC
+
FFFF:FFFFFFFFFFFFFFFFFFFFFFFFFFFFFFFFFFFFFFFFFFFFFFFFFFFFFFFFFFFFFFF
FFFFFFFFFFFFFFF:FFFFFFFFFFFFFFFFFFFFFFFFFFFFFFFF
@A00721:190:HGJW7DSXY:4:2625:30951:8719_1:N:0:TCTGCAAG+GCTGCACT
CGTGTGCTCTTCCGATCTGGTGTTTCTAATCGTAACATTAATAATTTCAATCTTCTTAAAAAGAAGAT
AGAGAGTCATACTTTGGGCTATCGTTCAGGTGCATGGGGTTGGATAGCCAATATTGAAGTGGACACCT
CTG
+
FFFFFFF:FFFF:FFFFFFFFFFFFFFFFFFFFFFFFF:FFFFFFFFFFFFFFFFFFFFFFFFFFFFF
FFFFFFFFFFFFFFFFFFFFFFFFFFFFFFFFFFFFFFFFFFFFFFFFFFFFFFFFFFFFFFFFFFFF
FFF
@A00721:190:HGJW7DSXY:4:1446:19849:12352_1:N:0:TCTGCAAG+GCTGCACT
AGATAACCAATAAGGAAGGAGCTTATACTATGGTTGAATCAACTAAGCATACTACTTTTGCTTTGATT
ACTCTACCGCCTTACAAGTCTAAGGTTAAAGAGTCGATTAAACAAGTTGTACTTATCAATGATTTTAT
CGC
+
FFFFF:FFFFF::FFFFFFFFFFFFFFFFFFFFFFFFFFFFFFFFFFFFFFFFFFF,FFFFFFFFFF
FFFFFFFFFFFFFFFFFFFFFFFFFFFFFFFFFFFFFFFFFFFFFFFFFFFFF:FFFFFFFFFFFFFF
FFF
@A00721:190:HGJW7DSXY:4:2364:12111:19304_2:N:0:TCTGCAAG+GCTGCACT
TTCTAACCCAAAGAGAATATCTAAAATATTTACAAAATCTTACCACTATCTACGAACCTAAAGTTCTG
AAGACGTTGCTTGTCGCTTCCTCCTTTAATGGCACTACCTCGGTACAA
+
FFFFFFFFFFFFFFFFF:FFFFF:FFFFFFFFFFFFFFFFFFFFFFFFFFFFFFFFFFF:FFFFFFFF
FFFFFFFFF::FFFFFFFFFFFFFFFFFFFF:FFFFFFFFFFFFFFFF
@A00721:190:HGJW7DSXY:4:2638:4264:8563_2:N:0:TCTGCAAG+GCTGCACT
GAAGGCGACCTGTTCCGGAGTTTTTACGGGATCCGGAATGTTCTGTCTATATTAGGTGAAGCGAAATA

CGATTTCGAACGTCAACGTTCACCGAGTTTCGTTTCGTACCTTGATGA
+
FFFFFFFFFFFFFFFFFFFFFFFFFFFFFFFFFFFFFFFFFFFFFFFFFF:FFFFFFFFFFFFFFFFFF
FFFFFFFFFFFFFFFFFFFFFFFFFFFFFFFFFFFFFFFFFFFFFFFFF
@A00721:190:HGJW7DSXY:4:2438:11713:30608_2:N:0:TCTGCAAG+GCTGCACT
ATTTGATCTGGTTGCTCAATTCTAAGCGTGTTCCGCTTAGTGCGAGGCCTCTCTTCTACGAGTATTGC
TATTTATACGGTTACGAGAAGAAGGTTCGAAAGTTTGAGAACTTAGTT
+
FFFFFFFFFFFFFFFFFFFFFFF:FFFFFFFFFFFFFFFFFFFFFFFFFFFFFFFFFF:FFFFFFFFFF
FFFFFFFFFFFFFFFFFFFFFFFFFFFFFFFFFFFFFFFFFFFFFFFFF
@A00721:190:HGJW7DSXY:4:1201:31557:7952_2:N:0:TCTGCAAG+GCTGCACT
AAATACTAGATATTTGGATGATCTTGATAAAGAACTTCATGAATTTTCAGAGGATGAAAAATTTTCTT
CCTTGGATTTTAATATAAAGAATCTTTACCAGGACTCCTACTACAGGA
+
F:FFFFFFFFFFFFFFFFFFFFFFFFFFFFFFFFFFFFFFFFFFFFFFFFFFFFFFFFFFFFFFFFFFF
::FFFFFFFFFFFFFFFFFFFFFFFFFFFFFFFFFFFFFFFFFFFFFFF
@A00721:190:HGJW7DSXY:4:1335:25183:11099_1:N:0:TCTGCAAG+GCTGCACT
TGATCGTAAGAAGTTTATAAGGAACATATCCATTTGTTGTAATAGGGTCATCAACTTAGAACTCGAAA
CTCTAAGTGGTAATACTGTCTTTCCAAGCTTACCTGTATCTTCAATGAAATTGGACTCTTCGATAGGG
GAC
+
FFFF:FFFFFFFFFFFFFFFFF:FFFFFFFFFFFFFFFFFFFFFFFFFFFFFFFFFFFFFFFFFFFFF:
FFFFFF:FFFFFFFFFFFFFFFFFFFFFFFFFFFFF:FFFFFFFFFFFFFFFFFFFFFFFFFFFFFFFF
FFF
@A00721:190:HGJW7DSXY:4:1643:20103:17049_2:N:0:TCTGCAAG+GCTGCACT
TAAAGGAAGATCTGTTATCGACGGTTTATCGTTTACGAAGTATCACTCCACCGGTGAATGGTTTAAGA
ATACTATAAATAGAATAGCTATTACTACTAATGGCGTTGGCCTAGTGG
+
FF,:FFFFF:FFFFFFFFFFFFFFFFFFFFFFFFFFFFFFFFFFFFFFFFFFFFFFFFFFFFFFFFFFF
F:FFFFFFFFFFFFFFFFFFF:FFFFFFFFF:FFFFFFFFFFFFFFFFF
@A00721:190:HGJW7DSXY:4:1427:24532:17080_1:N:0:TCTGCAAG+GCTGCACT
TTTAGGTGGTCTTTATTCTATTATTGATTTTTATGAGATTGATACGTTAACAGAAGATGATGTTAGAG
TAGAATTCCTGAAGAGTCCTTCCGTAACCAACTTCTTGACAATTTTCTTCAATCCTTCGATAATAGTT
AAA
+
FFFFFFFFFFFFFFFFFFFFFFFFFFFFFFFFFFFFFFFFFFFFFFFFFFFFFFFFFFFFFFFFFFFFF
FF:FFFF:F:FFFFFFF:FFFFFFFFFF,FFFFFFFFFFFFFFF:FF:FFFFF::FFFF,FFFFFFFFF
FFF
@A00721:190:HGJW7DSXY:4:2101:2953:11209_1:N:0:TCTGCAAG+GCTGCACT
CCGATAAGGGTCACTAGTCTTACACAATTAAGAAATAATCCTACATTGACTGATTATCTAAAGATTGA
AGGAAAATTTTATCCTCGTTTAACTTACTTAGACTTTCCTACGATACAAATGATAACTTCTAATTTAC
GTT
+
FFFFFFFFFFFFFFF:FFFFFFFFFFFFFFFFFFFFFFFFFFFFFF:FFFFFFFFFFFF:FFFFFFFFF
FFFFFFFFFFFFFFFFFFFFFFFFFFFFFFFFFFFFFFFFFFFF,FFFFFFFFFFFFFFFFFFFFFFFF
FFF
@A00721:190:HGJW7DSXY:4:1668:21576:22983_2:N:0:TCTGCAAG+GCTGCACT
CAAAGGGATGCATGGTGTTATCTCCTCAGGAGTCCCGGCATTTGATAATAATGACGAGACTTTCGTCA
TAATCGCTGCATATTACGGATTACACGGTACCAGCGCATCTCGTGTCG
+
FFFFFFFFFFFFFFFFFFFFFFFFFFFFFFFFFFFFFFFFFFFFFFFFFFFFFFFFFFFFFFFFFFFFF
FFFFFFFFFFFFFFFFFFFFFFFFFFFFFFFFFFFFFFFFFFFFFFFFF
@A00721:190:HGJW7DSXY:4:2208:1199:13275_1:N:0:TCTGCAAG+GCTGCACT
GTATTAACTTATAAAGGTGAGGAGACCATTTTCTTGCTGAAATTTATAGAAGATTATTTTGACAGAAT

```
CAATAAGAATCATCGATTCTCGATTCGTAGAGCTTATCTAGGTACTCTCATTTACTATGTTGATTTAG
TCT
+
FFFFFFFFFFFFFFFFFFFFFFFFFF:FFFFFFFFF:FFFFFFFFFFFFFFFFFFFF,FFFFFF,F:F
FFFF:FFFFFFFFFFFFFFFFFFFFFFFFFFFFFFFFFFFFFFFFFFFFFFFFFFFFFFFFFFFFFFF
FFF
@A00721:190:HGJW7DSXY:4:2355:24659:22686_2:N:0:TCTGCAAG+GCTGCACT
GATTTACAACGATATAGGAGTAATCTACGAGAAGATTCTAGAATTCTTAATTATAATATTCAACCTCA
AAAGTTACGTGTTTACAGTAACGAGATCACACATTTTGAGAACTAACT
+
FFFFFFFFFFFFFFFFFFFFFFFFFFFFFFFF:FFFFFFFFFFFFFFFFFFFFFFFFFFFFFFFFFFF
FFFFFFFFFFFFFFFFFFFFFFFFFFFFFFFFFFFF:F:FFFFFFFFFF
@A00721:190:HGJW7DSXY:4:1140:21160:4006_1:N:0:TCTGCAAG+GCTGCACT
TGATCATTTTGACAATCTCTTCTTATAATAAATAAATAAATAATATTACTAATGGCAAATAGTGGAAA
GGCTCCAGTCAACACTAGTTCGAGTGCTGCTTTGGATTACGATGACGTGAGAATAGGTGAAGGGATAG
TT
+
F,FF:FFFFFFFFFFF:FFFF:,FFFFFFFFFFFFFFFFF,:FFFF:FFFFFF:FFFFFFFFFFFFFF
F:FFFFFFFFFFFFFF:FFFFFFFFFFFFFFFFFFFFFFFFFFFFFFFFFFFFFFFFFF:FFFF,FF
FF
@A00721:190:HGJW7DSXY:4:1441:13024:13714_1:N:0:TCTGCAAG+GCTGCACT
GATCTATTAAGGCGGCGGCGACTAATCTTAAAGCTTCACCCAAAATGAAGGACACTTCGAAACTGAAT
AATGATGCACTACAAACAAGTATCGCTAATCATCTGAAAAAGGCTCTAAAGGACACTATACAATATCC
AGA
+
FFFFFFFFFFFFFFFFFFFFF:FFFFFF::FFFFFFF:FFFFFFFFFFFFFFFFFFFFFFFFFFFFFF
FFFFF:FFFFFFFFFFFFFFFFFFFFFFFFFFFFFFFFFFFFFFFFFFFFFFFFFFFFFFFFFFFFFF
FFF
@A00721:190:HGJW7DSXY:4:2472:25328:27946_2:N:0:TCTGCAAG+GCTGCACT
TGCTTTTACCGATAACGATAAATTCGTCATCAAATATAAAGAAAAGGACTATGAAATAAAAGATTTGG
TGCTTAGGGATATAATGAATTCAGCACCACATTTTGGTCAAGTCAATC
+
FFFFFFFFFFFFFFFFFFFFFFFFFFFFF:FFFFFFFFFFFFFFFFFFFFFFFFFFFFFFFFFFFFFF
FFFFFF,FFFFFFFFFFFFFFFFFFFFFFFFFFFFFFFFFFFFFFFFF
@A00721:190:HGJW7DSXY:4:2356:31693:20901_2:N:0:TCTGCAAG+GCTGCACT
TCTTAGAGGATATTCGCCATTGTGGTACGAATTGGTCCAACATTTACCACCAAATTCTTTTGATACAA
CTCTAGCCTTCAAGTGGGGTATGCCTGCGGAATACTCTTGCTATTCAC
+
FFFFFFF,FFFFFF:F:FFFFFFF,FFFFF:FFFFFFFFF,FFFFFFFF,FFFFFF::FFFFF,FF:F
,FFFFF:FFFFF,:FFFFFFFFFF,FFF,FFFFFF,FFFFFFFFFFFF
@A00721:190:HGJW7DSXY:4:1248:30183:28244_2:N:0:TCTGCAAG+GCTGCACT
CGAACCCGTTGATGCTATGTCAGAAGAAGCTATTCAAGCTTGTCGTTTAAAGACTGCAGAGGCTGTCA
AACAAGCCCAGACTCAAGGAGTCCAATCAAAGGTTTTGAATTTAGCTC
+
FFFFFFFFFFFFFFFFFFFFFFFFFFFFFFFFFFFFFFFFFFFFFFFFFFFFFFFFFFFFFFFFFFFF
FFFFFFFFFFFFFFFFFFFFFFFFFFFF,FF:FFFFFF,FFFFFFF:F:
@A00721:190:HGJW7DSXY:4:2320:13792:6151_2:N:0:TCTGCAAG+GCTGCACT
TGGGTGATAAAAATTATTTAGAGATCTTTTCTACTTACAAAGGACAATTCAGTTTCATTAATTCTACG
GAAAAGTCCGGAGTATATTGTATGTTAATAGATAGTGGGATAATAGTT
+
FFFFFFFFFFFFFFF:FFFFFFFFFFFFFFFFFFFFFFFFFFFFFFFFFFFFFF:FFFFFFFFFFFFFF,
FFFFF:FF:FFFFFFFFFFFFFFF,FFFFFF:FFF:FFFFFFFFFFF:F
@A00721:190:HGJW7DSXY:4:2422:6225:17691_1:N:0:TCTGCAAG+GCTGCACT
GTAGTGAACGCGTTCGTAGAACCAAGTACTTTCCGTCTGAAGAGTTTAGAGCTGCTATTGATTATGTA
```

```
ACCGATAGGAGTTGTATAGTTGAAGATGTGGCTTTAGACGATAATCCTTTAAGGACTATGTTGACTGA
CGA
+
FFFFFFFFF:FFFFFFFFFFFFFFFFFFFFFFFFFFFFFFFFFFFFFFFFFFFFFFFFFFFFFFFFFF
FFFFFFFFFFFFFFFFFFFFFFFFFFFFFFFFFFFFFFFFFFFFFFFFFFFFFFFFFFFFFFFFFFFF
FFF
@A00721:190:HGJW7DSXY:4:2453:30716:14544_2:N:0:TCTGCAAG+GCTGCACT
GATCGCCACTCCAAACACTATATACATTATAAAATATAAAAATAAGGGAGTCATAACTTTTGTTACCA
AATTCTTGGCTTTCAACGAAGTCTCTGATTTTATTAATCATTACTTTA
+
FFFFFFFFFFFFFFFFFFF:FFFFFFFFFFFF:FFFFFFFFFFFFFFFFFFFFFFFFFFFFFFFFFFF
FFFFFFFFFFFFFFF:FFFFFFFF:FF:FFFFFFFFFF,FFFFFFFFF
@A00721:190:HGJW7DSXY:4:1140:27118:22529_2:N:0:TCTGCAAG+GCTGCACT
TCATTTACTCTGTACTTGATCTACGTCGTTTAACTGTCGGAACAACCTTTACTTAGAATGACTCTATC
TTTTCAGACACTTCTTATATTATTTACAATAGTTAATATCATATTAGT
+
FFFFFFFFFFFF:FFFFFFFFFFFFFFF:FFFFFFFFFFFFFFFFFFFFFFFFFFFFFFFFFFFFFFF
FFFFFFFFFFFFFFFFFFFFFF:FFFFFFFFFFFFFFFFFFFFFFFFFF
@A00721:190:HGJW7DSXY:4:1177:10447:4053_2:N:0:TCTGCAAG+GCTGCACT
TTTTCTTAGCTAATAGATTAATAAAGGATGAGTACGAAAACCAACTCTTCGTTACGAGACCTGATGGT
AGAATAGATTTAGTCTCTACACCCATCTCAACGAGGATAAAGCGAGTT
+
FFFFFFFFFFFFFFFFFFFFFFFFFFFFFFFFFFFFFFFFFFFFFFFFFFFFFFFFFFFFFFFFFFFF
FFFFFFFFFFFFFFFFFFF:FFFFFFFFFFFFFFFFF:FFFFFFFFFF
@A00721:190:HGJW7DSXY:4:1221:1443:20901_2:N:0:TCTGCAAG+GCTGCACT
ACACGAAGAGATCATCCGCAAACGACTCGAAACCGAGACGCCGTAGATCAAAGTCCCTTCTAACTTTA
GATTATGAATCCTGCCTTTACTCATTTCATAGCAGTTATTGATGTTGT
+
FFFFFFFFFFFFFFFFFFFFFFFFFFFFFFFFFFFFFFFFFFFFFFFFFFF:FFFFF:FFFFFF::FF
FFF,FFFFFFFFFFFFFFFF:FFFFFFFFFFFFFFF:FFF,FFFFFFF
@A00721:190:HGJW7DSXY:4:1567:27163:19789_1:N:0:TCTGCAAG+GCTGCACT
TTTATAACCTTCTTATACTTTTGGGTATGTTTTGTGAAAAACATGAGATACCCAAGTCTATCGAAGAC
ATTATCTCTTATGTACTTATTGTAACGATCATTCTTTCGAGTTTTGTCATTCTTATTCTTTTATTCCT
TTA
+
F:FFF,:FFFFFFFF,,F,FFFFF:FF,FF:F::FFF,FFFF:FF:FFFFF,:FFFF,F,FF:F,,F,
:FFFFFFFFFF:FF:,FFFFFFFF:,FFFF,FFFFFF,F::F::FFFFFFFFF:,FF,FFFFFFFFFF
F:F
@A00721:190:HGJW7DSXY:4:1272:23249:36213_2:N:0:TCTGCAAG+GCTGCACT
TTTATAATGCTTTTCGATAGTGATTGTTATCAATTTAAATACATTACTGTTTCAAATCATCTTTACTC
ACAGACTCCGAAAATGGTTTCAAATGAGGTCAAAGCTCTATTTATTAA
+
FFFFFFFFFFFFFFFFFFFFFFFFFFFFFFFFFFFFFFFFFF:FFFFFFFFFFFFFFFFFF:FFFFFF
:FFFFFFFFFFFF,FFFFFFFFFFFFFFFFF:FFFFFFF:FFFF:FFF
@A00721:190:HGJW7DSXY:4:1318:4634:25708_1:N:0:TCTGCAAG+GCTGCACT
CACTACCACTGATCTGCCGACTTTAGTCAAGGAACTTCAATTCCTTCGATGTTTATATTTTTCTATAA
GGGAAAATATCAATAATCAAATTTCGACAAATTTTTGAACTTTATTTCAGAACTCTCTCCGATCACCT
TGG
+
FFFFFFFFFFFFFFFFFF:FFFFFFFFFFFFFFFFFF:FFFFFFFFFFFFFFFFFFFFFFFF:F::::F:FF
FFFFFFFFFFFFFFFFFFFFFFFFFFFFFFFFFFF:FFFFFFFFFFFFFFFFFFFFFFFFFFFFF:F:FFF
FFF
@A00721:190:HGJW7DSXY:4:1417:14000:29152_2:N:0:TCTGCAAG+GCTGCACT
GGAGGATCTTTTCAATAAGATGTATGATGATAATCTACTATTTTTAAATAGTGATATTCAGCGTATAC
```

TTGAATACTTTATCAAAGATTTGGCTTGGTCTATTTACCGCAAAAAGT
+
FFFFFFFFFFFFFFFFFFFFFFFFFFFFFFFFFFFFFFFFFFFFFFFFFFFFFFFFFFFFFFFFFFFF
FFFFFFFFFFFFFFFFFFFFFFFFFFFFFFFFFFFFFFFFFFFFFFFF
@A00721:190:HGJW7DSXY:4:2471:32461:33066_2:N:0:TCTGCAAG+GCTGCACT
CTATGATATTTATTTTACTTATACTTTAAATAGCACCGATTATAACTTGACTGTTGATAATATTACAA
TAGCTCTTTCTCAGAAGATTAGAGAAGTAACCGGTGAACACGTCGGTA
+
FFFFFFFFFF:FF:F:FFFF:FFFFFFF:FFFFFFFFFFF:FFFFFFFFFFFFFFFFFFFFFFF:FFF
:F:FFFFF:FFFF:FFFFFFFFFFFFF:FFFFFFFFFFFFF,FFFFFF
@A00721:190:HGJW7DSXY:4:1146:7265:1172_1:N:0:TCTGCAAG+GCTGCACT
ACTTTTACCGTTAGAACTGAAAAAGATTCTTTTCGAAAAGAAACCTATAAGTTACAAACGATTCTTCG
ACTATATTAAAGAAATAAAAATACTTCAAGTACCTGAACTTTACATTTTTAATGGTATGGTATAATGT
GTC
+
FFFFFF,F:::FFFF:F::F,FFFFFFF::FF::FFFFFFFFF:FFFF,F,FFFF:,FFFFFFFFFFF
,FFFFFFF:::FFFFFFFFFFFFFFF:FFFFFFFFFFFFFFFFFFFF:FFFFFFFFFFFFFFFFFFFF
FFF
@A00721:190:HGJW7DSXY:4:1130:30391:12195_1:N:0:TCTGCAAG+GCTGCACT
TTCACTCTCGGTAATGAGAGTGTATTCCTTAATATTCTAGTAACCGCTATGTGGGCATTACTAGGATT
TATCACACTCCTAAATTTGATAGCAATTTATGTTTTAAATGCTAGGAAAATTTTAGAAAAACTCCATT
GTA
+
FFFFFFFFFFFFFFFFFFFFFFFFFFFFFFFFFFFFFFFFFFFFFFFFFFFFFFFFFFFFFFFFFFFF
FFFFFFFFFFFFFFFFFFFFFFFFFFFFFFFFFFFFFFFFFFFFFFFFFFFFFFFFFFFFFFFFFFFF
FFF
@A00721:190:HGJW7DSXY:4:1438:11975:11929_2:N:0:TCTGCAAG+GCTGCACT
GTACTTAATTCAATAGCTTTAGTATTATTTACTATCGCCTTTATTGAAAATTGTATAAGAGTTAATTG
CTTACACAAAAGTGACGCGCGAAGGAATCTTAATTGATTGAAGCTAGG
+
FFFF:FFFFFFFFFFFFFFFFFFFFFFF::FFFFFFFF,FFFFFFFFFFFFFFFFFFF:FF,FFFFFF
:FFFFFFFFFF:FFFFFFFFFFFFFFFFFFFFFFFFFFFFFF:FFFFF
@A00721:190:HGJW7DSXY:4:1634:14100:26068_1:N:0:TCTGCAAG+GCTGCACT
AACATACCATACACTCCCCAAACATAACATACCATAACCTACCATGACCAATCATCGCTCCAACTGGC
ACGCCAGACGTAGGGTCAAACCTACTACCGCCGGTCGGCAGATCGGCACCACTGGTCTCTACGTTCAG
GGA
+
FFFF:FFFFFFFFFFFFFFFFFF,FF,FFFFFF:FFFFFFFFFFFFFFF:FFFFFFFF:FFF,FFFFF
FFFFFFFFFFFFFFFFFFFFFFF:FFFFFFFFFFFFFFFFFFFFFFFFFFFFFFFFFFFFFFFFFFFF
FFF
@A00721:190:HGJW7DSXY:4:1133:5095:17394_2:N:0:TCTGCAAG+GCTGCACT
TAACCTTCCCAAATCTAGATCTGCACAGAAGAAGGCTGAAGGTCGTTACCGGACGACCACTCAAAGGA
AACGGGCTGTTGCCCAGAGGAAAGCAGCGACCACCGAGGTCGCTCGTA
+
FFFFFFFFFFFFFFFFFFFFFFFFFFFFFFFF:FFFFFF:FFFFFFFFFFFFFFFFFF,FFFFFFFFF
FFFFFFFFFF:FFFFFFFFFFFFFFFFFFFFFFFFFFFFFFFFFFFFF
@A00721:190:HGJW7DSXY:4:1166:4363:16219_1:N:0:TCTGCAAG+GCTGCACT
AGTCCCGTCATCTCTGTGGGAACTGAGGCAGTACCTCAGTCAAAAGTCACATTCACCACTCCCATGCC
AATCAATGATGTGACTATACAGTTCGGCCATTTCAAGAGTGGTCAATACGTCGTGGGAGGTACTGAAG
CCT
+
FFFFFFFFFFFFFFFFFFFFFFFFFFFFFFFFFFFFFFFFFFFFFFFFFFFFFFFFFFFFFFFFFFFF
FFFFFFFFFFFFFFFFFFFFFFFFFFFFFFFFFFFFFFFFFFFFFFFFFFFFFFFFFFFFFFFFFFFF
FFF

```
@A00721:190:HGJW7DSXY:4:1361:11089:12680_1:N:0:TCTGCAAG+GCTGCACT
CACGAAGAACACTCCCAAGCCAATCAATAGTTCTTCTGACCAGAATGAAAAGGGGTTTACCAGGGTAG
TCTCTAAGTCTACCAGGAAGAGGGGAAATGTCCCCAAAACTGAGAAGAATAAAGGGAAAGGTCCATCA
GGA
+
FFFFFFFFFFFFFFFFFFFFFFFFFFFFFFFFFFFFFFFFFFFFFFFFFFFFFFFFFFFFFFFFFFFF
FFFFFFFFFFFFFFFFFFFFFFFFFFFFFFFFFFFFFFFFFFFFF:FFFFFFFFFFFFFFFFFFFFFF
FFF
@A00721:190:HGJW7DSXY:4:1154:21404:25222_2:N:0:TCTGCAAG+GCTGCACT
ATTACCCCTAATTCTGAAGGAAGATCTCCCACCATCTGGGGTTGGGAATAAGAAATCTGCACGGGCCA
AAGAGGCCGCTGCTACGAAGTCGCTGAAGGCGGCTTGGGTCATTAAGG
+
FFFFFFFFFFFFFFFFFFFFFFFFFFFFFFFFFFFFFFFFFFFFFFFFFFFFFFFFFFFFFFFFFFF:
FFFFFFFFFFFFFFFFFFFFFFFFFFFFFFFFFF,FFF,FF:FFF:FF
@A00721:190:HGJW7DSXY:4:1130:7808:27633_1:N:0:TCTGCAAG+GCTGCACT
AGTTGCGCCTGCCGACCCCATCTTTGAGACCAAGACTCTCTACAGTAGGTTCGTGCCTACTATGAGCA
AATCTGTTGAATTCGTCACTGAGGACGAAGGTGAGAGGACTTTTGGCTACTGGCGACAAACTGAGAGC
AGT
+
FFFFFFFFFFFFFFFF:F:F,FFFFFFF:F::FF,FFFF:F:FF,:FFFFF:FFFF:FFFF:FF:FFF
F,F:FFFFFFFFFFFFF:FFFFFFFFF:FFFFFFF:FF:,FFFFFFFFFFFFFFFFFFFFFFF:F:FF
FFF
@A00721:190:HGJW7DSXY:4:1272:4634:13275_2:N:0:TCTGCAAG+GCTGCACT
AGAATTGCGTGGGTTGAAGGCCGACGCTTCGAGCTTGAGTGTGACGAGGCCGGTGACGTTTTTGTCAA
CGGCGTTTGGAGGATTTTATCCGCGCTTAACTATGGGGCGAAGATCCC
+
FFFFFFFFFFFFFFFFFFFFFFFFFFFFFFFFFFFFFFFFFFFFFFFFFFFFFFFFFFFF:FFFFFF:F
FFFFFFFFFFFFFFFFFFFFFFFFFFFFFFFFF,FFFFFFFF:FFFFFF
@A00721:190:HGJW7DSXY:4:1367:8052:13589_2:N:0:TCTGCAAG+GCTGCACT
AAGGACTTAGGTTGGCATATTTATGCCGACGATAATAAGCATGTCACCCTTAAGCACCGAGGTAAGCT
CGGGACTTACAAGAGAGAGTGTGAGAGAGTCGCTCTCACTATTTTTGC
+
:FFFFFFFFFFFFFFFFFFFFFFFFFFFFFFFFFFFFFFFFFFFFFFFFFFFFFFFFFFFFFFFFFFF
FFFFFFFFFFFFFFFFFFFFFFFFFFFFFFFFFFFFFFFFFFFFFF,F
@A00721:190:HGJW7DSXY:4:1261:4996:23453_1:N:0:TCTGCAAG+GCTGCACT
TTTCTGACTGATGAGGTTAAAACTCTTGCTCCTTCTGTGGGTGAGGGTTTTTGTTATATTCACTGGTT
CTGGGAGGCTGCTTTCCTCCATAATTACAACCTCTCTTTAGAGAGCATTAGAAGGAAGTTGGGCAAAT
TCC
+
FFFFFFFFFFFFFFFF::FF,FFFFFFFFFFFFFFFFFFFFFFFFFFFF:FFFFFFFF,FFFFFFFFFF
FFFFFFFFFFFFFFFFFFFFFFFFFFFFFFFFFFFFFFFFFFFFFFFFFFFFFFFFFFFFFFFFFFFF
FFF
@A00721:190:HGJW7DSXY:4:2306:27588:18082_1:N:0:TCTGCAAG+GCTGCACT
CACACTTTTCTTGAAAATTTTTCCTTTCCTCCGTCATTGGGGATTTCGATGTGAGAAGGTGGAAGGTG
TCAAGGGTCTTCTTCATGCTGATCTTTCAGGACCTAATCACATCATGATTGATAGTTACGGCTCGTAT
CGA
+
FFFFFFFFFFFFFFFFFFFF:FFF:FFFFFF:FFFFFFFFFFFFFFFFFFFFFFFFFFFFFFFFFFFFF
FFFFFFFFFFFFFFFFFFFFFFFFFFFFFFFFFFFF:FFFFFFFFFFFFFFF:FFFF,FFFFFFFFFF
FFF
@A00721:190:HGJW7DSXY:4:2642:30499:24752_2:N:0:TCTGCAAG+GCTGCACT
AAGTTCTCCGAAATTAAAGACGGGGGGATTAAGAGTCCAGCTGTAACTGTCATTTCAGAGAGGGGAGC
TCTGAAAAGGAAACTTCGTCAGAAGATGAGGAAGTCTAAGAGGAGAGA
+
```

FFFFFFFFFFFFFFFFFFFFFFFFFFFFFFFFFFFFFFFFFFFFFFFFFFFFFFFFFFFFFFFFFFFFFF
FFFFFFF:FFFFFFFFFFFFFFFFFFFFFFFFFFFFF,FFFFFFFFFFF
@A00721:190:HGJW7DSXY:4:1504:12861:14027_1:N:0:TCTGCAAG+GCTGCACT
CTCTAGAGAAGATCTCACCACCTGGAGAAGGCAAGTGGAGAGTCCAGAAGTTGATGAAGAAGGATTAT
TTGAAGCAGGGGTTACCACTTCCTAAGGTGGATGAGGGAGGTTGTTTCTTTGGAGTTTTAGATAATCT
TTT
+
FFFFFFFFFFFFFFFF,FFFFFFFFFFFFFFFFFFFFFFFFFFFFFFFFFFFFFFFFFFFFFFFFFFFFF
FFFFFFFFFFFFFFFFFFFFFFFFFFFFFFFFFFFFFFFFFFFFFFFFFFFFFFFFFFFFFFFFFFFFFF
FFF
@A00721:190:HGJW7DSXY:4:1609:12301:15217_2:N:0:TCTGCAAG+GCTGCACT
CCTTGAGAAGACAAGCCACATTAAGATATAATTTAGGTCAACTTGGGGTTTCGAATGTCATTAGTGCC
TTACGATTGAAAGAGGTCCTTTTGAGTATCGACAGATCTTTCGATCTT
+
FFFFFFFFFFFFFFFFFFFFFFFFFFFFFFFFFFFFFFFFFFFFFFFFFFFFFFFFFFFFFFFFFFFFFF
FFFFFFFFFFFFFFFFFFFFFFFFFFFFFFF:FFFFFFFFFFFFFFFFF
@A00721:190:HGJW7DSXY:4:1351:7518:14293_2:N:0:TCTGCAAG+GCTGCACT
GTGGCAGAATTCGGTAACTTACCATATTGTTCCTTCCGGGAAGTTGTGGGATTTAACTTTGACTCAAC
TACTCCGATCAGATCCGGATGGTTTGATTGGTATGGACTACTCGACTT
+
FFFFFFFFFFFFFFFFFFFFFFFFFFFFFFFFFFFFFFFFFFFFFFFFFFFFFFFFFFFFFFFFFFFFFF
FFFFFFFFFFFFFFFFFFF:FFFFFFFFFFFFFFFFFFFFFFFFFFFFF
@A00721:190:HGJW7DSXY:4:2261:15781:22310_1:N:0:TCTGCAAG+GCTGCACT
TAGGGTCATGGCAGATACTACCGATAAGGTGAAAAATATGCAGGTCAAAGATTCTGTAGCTCTGAGGG
CGGTTGAGGGAGCCGTTTTGGCCGACTTTAACTATTCTAAGAGTCTTTATGATTCAAAACCTATCGTT
GTC
+
FFFFFFFF:FFFFFFFFFFFFFFFFFFFFFFFFFFFFFFFFFFFFFFFFFFFFFFFFFFFFFFFFFFFFF
FFFFFFFFFFFFFFFFFFFFFFFFFFFFFFFFFFFFFFF:FFFFFFFFFFFFFFFFFFFFFFFFFFFFFF
FFF
@A00721:190:HGJW7DSXY:4:2363:14850:5384_1:N:0:TCTGCAAG+GCTGCACT
TACAGCGGCTTATTGTCGCGCAGTTTCCAGAATTGCGGTTGAAGTTCACCCATTCTATCTTGAATGCT
CACGCCGTCGCAGCCATATCGAGACTATGTTATAATCACTTGTTAAAGTACAGATTTAGTGATTTAGA
TAT
+
FFFFFFFFFFFF:FFFFFFFFFFFFFFFFFFFFFFFFFFFFFFFFFFFFFFFFFFFFFFFFFFFFFFFF
FFFFFFFFFFFFFFFFFFFFFFFFFFFFFFFFFF,FFFFFFFFFFFFFFFFFFFFFFFFFFFFFFFFFFF
FFF
@A00721:190:HGJW7DSXY:4:1515:3495:27680_1:N:0:TCTGCAAG+GCTGCACT
GGAGGCGATATTCGTTTTCATATCCAAAACTCCTTGAATGGAGTACATATTTGCAACCCTATTCTTGA
TGTGAAAGATGCATCGAGGTATACGTCTAGGAACATTGATTGGGAACGTACCTACTCACGAGACGTCA
TGA
+
FFFFFFFFFFFFFFFFFFFFFFFFFFFFFFFFFFFFFFFFFFFFFFFFFFFFFFFFFFFFFFFFFFFFFF
FFFFFFFFFFFFFFFFFFFFFFFFFFFFFFFFFFFFFFFFFFFFFFFFFFF:FFFFFFFFFFFFFFFFFF
FFF
@A00721:190:HGJW7DSXY:4:2336:30553:35305_1:N:0:TCTGCAAG+GCTGCACT
ATGTTTCGTTCTGTTACAAGAACGCTTTGGATTGTGACCATAAAGCCTCAGTTGCGATAGCTGTAGAG
GTATACGATATCTCTCTTGATGATGTAGCCACTATAATGCTTAAGAGAGAGATTGATGTATGTCATTT
CGT
+
FFFFFFFFFFFFFFFFFF:FFFFFFFFFFFF:FFFFFFFFFFFFFFFFFFFFFFFFFFFFFFFFFFFFFF
FFFFFFFFFFFFFFFFFFFFFFFFFFFFFFFFFFFFFFFFFFFFFFFFFFFFFFFFFFFFFFFFFFFFFF
FFF

```
@A00721:190:HGJW7DSXY:4:2223:17219:14309_1:N:0:TCTGCAAG+GCTGCACT
GAATTATTGGACACGATGAAAAATGACATACCCGTTCCTGACCTTGGTATCGTTATCCGTCATGGAGA
TGATGCCGCATCACTTGATTACTATATGCCCGAAGGATTGGGTTATTCTCACAACAAAGATAAGATTT
TAT
+
FFFFFFFFFFFFFFFFFFFFFFFFFFFFFFFFFFFFFFFFFFFFFFFFFFFFFFFFFFFFFFFFFFFF
FFFFFFFFFFFFFFFFFFFFFFFFFFFFFFFFFFFFFFFFFFFFFFFFFFFFFFFFFFFFFFFFFFFF
FFF
@A00721:190:HGJW7DSXY:4:1223:26024:29215_1:N:0:TCTGCAAG+GCTGCACT
TAAGGGCGGACAATTGTTCCATTGTGAACTCTTGGGGACTAGATTGAGCATTAATGAGTTCCGAATAA
CTCGTAATAAATCTTTCTCTAGTGGACAATTCAGAATACCTATTTCCGTACCAAGAGCCACTGCCAAT
CTC
+
FFFFFFFFFFFFFFFFFFFFFFFFFFFFFFFFFFFFFFFFFFFFFFFFFFFFFFFFFFFFFFFFFFFF
FFFFFFFFFFFFFFFFFFFFFFFFFFFFFFFFFFFFFFFFFFFFFFFFFFFFFFFFFFFFFFFFFFFF
FFF
@A00721:190:HGJW7DSXY:4:2454:17644:5556_2:N:0:TCTGCAAG+GCTGCACT
AGGTTCGTTCCTCAATAAAAATTTTTCTCAAGAGATTATGGTTGATAAAGTTTTCTTCATAAGGGCTA
TTCAGTACGTCATGGGGATCACTTCTTCGGTCAATGACAAAACCTTCG
+
FFFFF:FFFFFFFFFFFFF:F:FFFF,FF:FF:FFF::FFF:FF:F,,FF,F:F:FF,,,F:F,:FFF
:FFFFFFFFF:F,,:F:FFFFFFF::F:FFFFFFFFF:FF:FFFFF:F
@A00721:190:HGJW7DSXY:4:1406:29143:18051_2:N:0:TCTGCAAG+GCTGCACT
CTTCGTAACAACAGTGCGAGGATTGTCATCTCGGGGAAAGTGATTCACCAAGATGTTCATATAGATAC
GAGATACCTAGAGAAGGTCGCAGCGATCTTACTCGTTATCGGAGCTAA
+
FFFFFFFFFFFFFFFFFFFFFFFFFFFFFFFFFFFFFFFFFFFFFFFFFF:FFFFFFFFFFFFFFFFF
FFFFFFFFFFFFFFFFFFFFFFFFFFFFFFFFFFFFFFFFFFFFFFFF
@A00721:190:HGJW7DSXY:4:2432:7166:5040_2:N:0:TCTGCAAG+GCTGCACT
TACACGTTATGCTCGCAAACTTAACGTTAGTACCGGAGGTGATCTCTCTGCTTCTGCGTTACTGAGAG
AAAAGATTCAGGACGCCTACGGTGACTTTATGGACAAGATGCAAGTTC
+
FFFFFFFFFFFFFFFFFFFFFFFFFFFFFFFFFFFFFFFFFFFFFFFFFFFFFFFFFFFFFFFFFFFF
FFFFFFFFFFFFFFFFFFF:FFFFFFFFFFFFFFFFFFFFFFFFFFFF
@A00721:190:HGJW7DSXY:4:2550:12924:6402_2:N:0:TCTGCAAG+GCTGCACT
TTTAGGTTTAGTAGGTGATCTTGACGTCGATTCTTTGTATAGGGAGTGTGAGAAGACAACGAATCTTG
AAACTACGGTCCTTATCAAAGAAAATGGTGAACCGGTCTCTAAATCTT
+
FFFFFFFFFFFFFFFFFF:FFFFFFFFFFFFFFFFFFFFFFFFFFFFFFFFFFFFFFFFFFFFFFFFF
FF:FFFFFFFFFFFFFFFFFFFFFFFFFFFFFF:FFFFFFF:FFFFFF
@A00721:190:HGJW7DSXY:4:1360:2564:11099_1:N:0:TCTGCAAG+GCTGCACT
GATTGAAGAACACGTCAAGAAGTCGCTCGGCGTTGTTCTTGAAAACCAAGTTAAGAAGGAGGCTGATC
ATGGAGTCTTTGTACCTTCTGTCAAACTTCGAGGTTCAGCTACGAGAGGTGATCCCGGATGTGGATTG
AAG
+
FFFFFFFFFFFFFFFFFFFFFFFFFFFFFFFFFFFFFFFFFFFFFFFFFFF:F:FF::FFFF:F,FF:F
FFFFFFFFFFFFFFFFFFFFFFFFFFFFFFFFFFFFFFFFFFFFFFFFFFFFFFFFFFFFFFFFFFFF
FFF
@A00721:190:HGJW7DSXY:4:1653:10737:21057_2:N:0:TCTGCAAG+GCTGCACT
TCCTTCTTTTTGAATGTTGTAAAGAATTGGGTTGAGAAGTGTGCTTTACAAGGAGTTAGACTCTTCTT
CAAGTTTTTAGATCTTCTTGATAATCTTAAAGAAAAATTTTCAAATAA
+
FFFFFFFFFFFF:FFFFFFFF,FFFFFFFFFFFFFFFFFFFFFFFFFFFFFFFFFFFFFFFFFFFFFF
FFFFFFFFFFFFFF:FFFFFFFFFFFFFFFFFF:FFFFFFFF,FFFFF
```

@A00721:190:HGJW7DSXY:4:2337:25491:16297_2:N:0:TCTGCAAG+GCTGCACT
CTGTACTTACATTCTTTACGCCAGATTCTTCATGGGGCGACGCTGTTTCTCTTGGTATTTCTGAAATA
GTTTATCAGAGCGTCATTTTCCTTTGGGATCTTTCCTCTGGTAAGGGA
+
F:,FFFFFFFFFFFFFFFFFFFFFFFFFFFFFFFFF:FFFFFF:FFFF,:FFFFFFF:FFFFFF:FFF
FFFFFFFFFF:FFFFFFFFF:FFF,,FFFFF:FFFFFFFFFFFFF:FF
@A00721:190:HGJW7DSXY:4:1242:3341:26036_1:N:0:TCTGCAAG+GCTGCACT
ACGAGAATTATTACTGTTTTCTCTACCCATAACTTCTTTTTCAAAAATGCGGATGGGTCTACTGATGT
TTTCCTACAGAGTGCAGTCGTTTCAACACTTATCACCACTTTTGAAAGGAAAATAATTGGTGATGAAA
ATA
+
FFFFFFFFFFFFFFFFFFFFFFFFFFFFFF:FFFFFFFFFFFFFFFFFFFFFFFFFFFFFFFFFFFFF
FFFFFF,FFFFFFFFFFFFFFFFFFFFFFF,FFFFFF:FFFFFFFFFFFFFFFFFFFFFFFFFFFFFF
FFF
@A00721:190:HGJW7DSXY:4:1175:15456:23970_2:N:0:TCTGCAAG+GCTGCACT
GGGTTCTCAAGGCTCTCATCGCCACTGGGATTCTAAGGGGTTCCATAAGATATTATCTGACATTGTGG
TTCAATGATCAGGATTCTTCTTTTGGTCCTTACGTCTTAAGTAATTTG
+
FFFFFFFFFFFFFFFFFFFFFFFFFFFFFFFFFFFFFFFFFFFFFFFFFFFFFFFFFFFFFFFFFFFF
FFFFFFFFFFFFFFFFFFFFFFFFFFFFFFFFFFFFFFF:FFFFFFFF
@A00721:190:HGJW7DSXY:4:1175:15456:23970_1:N:0:TCTGCAAG+GCTGCACT
TCTTGTCACGATTCTTAAAAGAAGTTTATTAAAACTTCAGAGTCTCTTGGAAGTCCAGGTAGCTAATG
TTCTTGCAATAGCCACAGACTTTCTGGGAAAAGAGACCTCTGTTGGTAGAGGAGTTGAGAGAATTACT
CAG
+
FFFFFFFFFFFFFFFFFFFFFFFFFFFFFFFFFFFFFFFFFFFFFFF:FFFFFFFFFFFFFFFFFFFF
FFFFFFFFFFFFFFFFFFFFFFFFFFFFFFFFFFFFFFFFFFFFFFFFFFFFFFFFFFFFFFFFFFFF
FFF
@A00721:190:HGJW7DSXY:4:1609:3369:8202_1:N:0:TCTGCAAG+GCTGCACT
ACGTCTACAACGAGTTAGGGAGTGGCTAGGTTTTAAAAGATCCTTTGCACAAGCAATACCTTCCACTG
AGTTCATCACCCAGAGTTTCGACGTCGCGGAAAACGATGAAGATTTCCACACTGTTCGAGAATCTTCT
GAT
+
FFFFFFFFFFFFFFFFFFFFFFFFFFFFFFFFF:FFFFFFFFFFFFFFFFFFFFFFFFFFFFFFFFFF
FFFFFFFFFFFF:FFFFFFFFFFFFFFFFFFFFFFFFFFFFFFFFFFFFFFFFFFFFFFFFFFFFFFF
FFF
@A00721:190:HGJW7DSXY:4:1618:19786:28526_1:N:0:TCTGCAAG+GCTGCACT
TGTGCTCTTCCGATCTCCTGATGGCTCAGTACAGGGAGGAGGTAATAGTTATAAAACCTTCTTTTATC
ATTGCAACAGGACTTTGAGAAGACTGTTGAAATGGACAAAATCTGGGATCATAGGTATGTGTCACGCT
ACA
+
FFFFFFFFFFFFFFFFFFFFFFFFFFFFFFFFFFFFFFFFFFFFF,FFFFFFFFFFFFFFFFFFFFFF
FFFFFFFFFFFFFFFFFFFFFFFFFFFFFFFFFFFFFFFFFFFFFFFFFFFFFFFFFFFFFFFFFF:F
FFF
@A00721:190:HGJW7DSXY:4:1211:11107:32377_2:N:0:TCTGCAAG+GCTGCACT
GTTGGCGTCTTGTCGTTGACGTCATTACAGTCTGGAAGTTAGATCATTTGTTCAGGAAGATCTCTACA
GAGATTATCGAGAGTTGTGATCTAGTTCTAAATTTAGGTCGACGGACT
+
FFFFFFFFFFFFFFFFFFFFFFFFFFFFFFFFFFFFFFFFFFFFFFFFFFFFFFFFFFFFFFFFFFFF
FFFFFFFFFFFFFFFFFFFFFFFFFFFFFFFFFFFFFF:FFFFFFFFFF
@A00721:190:HGJW7DSXY:4:1227:11514:1423_2:N:0:TCTGCAAG+GCTGCACT
ATGTACGTGGATCTAATGCTTGGTGATCTGAATCTGACCATCGCAAAGAAGACTTTATTAGTTCATGA
AGTTCTTACACTTCTCGCGACTAATGTCAGCTTCACTACAAAGGCGTT
+

```
:FFFFFFFFFFFFFFFFFFFFFFFFFFFFFFFFFFFFFFFFFFFFFFFFFFFFFFFFFFFFF:FFFFFFFF
FFFFFFFFFFFFFFFFFFFFFFFFFFFFFFFFFFFFFFFFFFFFFFFFFF
@A00721:190:HGJW7DSXY:4:2613:22824:18818_2:N:0:TCTGCAAG+GCTGCACT
CTTTATCTTAAAGCATTACCACCAAATTTAGCACTTTTTGAATATGACAGAAGCTCGCCATATCAGAT
TAGAGACATGCCAGTTAGAGTTCTTCTAACCAGGACTGATGGTGAGAT
+
:FFFFFFFFFFFFFFFFFFFFFFFFF:FFFFFFFFF:FFFFFFFFFF,FFFFFFFFFFFFFFFFFFFF:
FFFFFFFFFFFFFFFFFFFFFF:FFFFFFFFFFFFFF:FFFFFFFFFFFFFF
@A00721:190:HGJW7DSXY:4:2108:19714:3756_2:N:0:TCTGCAAG+GCTGCACT
GGAGGTCCAGGAACCTTTACTCCTAGTGTCTTTTCTAGAGTCCAAGGACGTTTCCGTCGGAGGATGAC
AGGTGGGAGACCGCTCAATAACACTCTATTGAATCCCTTTTCCACGAT
+
FFFFFFFFFFFFFFFFFFFFFFFFFFFFFFFFFFFFFFFFFFFFFFFFFFFFFFFFFFFFFFFFFFFFFF
FFFFFFFFFFFFFFFFFFFFFFFFFFFFFFFFFFFFFFFFFFFFFFFFFF
@A00721:190:HGJW7DSXY:4:1576:18891:34867_1:N:0:TCTGCAAG+GCTGCACT
GATATCGCCTCTGTCCTTTCTCCTTTAATCGTGAGGTTACGAGAGTTTCGTACTTGGGTTTATCGGTT
GTATTTTGATAAATTTGTTGGAATCGATAATGATACTGACGAAGGTTCTATTACCACCCTTGTCATTG
ATA
+
FFFFFFFFFFFFFFFFFFFFFFFFFFFFFFFFFFFFFFFFFFFFFFFFFFFFFFFFFFFFFFFFFFFFFF
FFFFFFFFFFFFFFFFFFFFFFFFFFFFFFFFFFFFFFFFFFFFFFFFFFFFFFFFFFFFFFFFFFFFFF
FFF
@A00721:190:HGJW7DSXY:4:1610:3323:14011_2:N:0:TCTGCAAG+GCTGCACT
TGATGCTTTCTTAGAGGCTCGTCTAGCCGCTGAGATTCGTCTTACCTATTTACAATCTGTAGAGAGAG
AGGTTAATCAGAATTTGAGTGATAACGATTTAGTACTGGGAAATGGTG
+
FFFFFFFFFFFFFFFFFFFFFFFFFFFFFFFFFFFFFFFFFFFFFFFFFFFFFFFFFFFFFFFFFF:FFF
FFFFFFFFFFFF,FFFFFFF:FFFFFFFF:FFFF,FFFFFF,F:FFFF
@A00721:190:HGJW7DSXY:4:1141:10429:20149_1:N:0:TCTGCAAG+GCTGCACT
CCTTTTTCGATTGGATTCGACGGGGTCTTCGTCTCACGACTAATTTGATAAGGTTTTTCTTTTATGGG
ACTCTTGTTTGTACTTATGAAGGAGGTATTACTTCGGAAGTTCTTAATGTAGGTCTCCTAGTACTTAA
GGG
+
FFFFFFFFFFFFFFFFFFFFFFFFFFFFFFFFFFFFFFFFFFFFFFFFFFFFFFFFFFFFFFFFFFFFFF
FFFFFFFFFFFFFFFFFFFFFFFFFFFFFFFFFFFFFFFFFFFFFFFFFFFFFFFFFFFFFFFFFFFFFF
FFF
@A00721:190:HGJW7DSXY:4:1370:1380:21668_2:N:0:TCTGCAAG+GCTGCACT
GTTTCAGCTGGATGGATCTTTTGATGGTATGTTTCACATTGCGTGATCACTTCCCGTGGACTTGGGAA
GAGATTAAACATAAAATCCAGCGATTCTATTATGTCGCTAAAGCCTGT
+
FFFFFFFFFFFFFFFFFFFFFFFFFFFFFFFFFFFFFFFFFFFFF:FFFFFFFFFFFFFFFFFFFFFFFFF
FFFFFFFFF:FFFFFFFFFFFFFF:FFFFFFFFFFFFFFFFFFFFFFFFF
@A00721:190:HGJW7DSXY:4:1167:28320:34444_2:N:0:TCTGCAAG+GCTGCACT
GTTCGGGCTGTGGGGATTAACTTCCCTCGTGAAACTTACGACGATGTTCTCGCTCGCATTTCACAAGG
ATTACTAAGTCTGAGATCTGCCACTTCTAATTTTAGGAAACGTCGCGA
+
FFFFFFFFFFFFFFFFFFFFFFFFFFFFFFFFF,FFFFFFFFFFFF:FFFFFFFFFFFFFFFFF:FFFFF
FFFFFFFFFFFFFF:FFFF:FFFFFFF:FFFFFFFFFFFFFFFFFFFF
@A00721:190:HGJW7DSXY:4:1459:24415:11271_2:N:0:TCTGCAAG+GCTGCACT
ATGAGGATGTCTTGGTCGAAAAATATCGCCGGGAACTTCTAATAGAGGATCCTGATGACATTATTTCA
ACGGATACCGATCTCAGTTCTACCACAGAAACCGTCTCAGGAACTGTC
+
FFFFFFFFFFFFFFFFFFFFFFFFFFFFFFFFFFFFFFFFF:FFFFFFFFFFFFFFFFFFFFFFFFFFFFF
FFFFFF:FFFFFFFFFFFFFFFFFFFFFFFFFFFFFFFFFFFFFFFFFFF
```

```
@A00721:190:HGJW7DSXY:4:1343:25690:22842_1:N:0:TCTGCAAG+GCTGCACT
GGAATTAGAGAAAGACGACCTTCTTGTGGGGACTGAGACCTCTGAATTTTCAGGGTGCCCTGATGAGG
ACGTTGAAACGGATAATTTCAACTCTGAGACTTCTAAGAGTGTTTCAAAAGACCAGAAACGTAACAAG
AAG
+
FFFFFFFFFFFFFFFFFFFFFFFFFFFFFFFFFFFFFFFFFFFFFFFFFFFFFFFFFFFFFFFFFFFF
FFFFFFFFFFFFFFFFFFFFFFFFFFFFFFFFFFFFFFFFFFFFFFFFFFFFFFFFFFFFFFFFFFFF
FFF
@A00721:190:HGJW7DSXY:4:1103:23231:6308_2:N:0:TCTGCAAG+GCTGCACT
GGAAGGGGAGAAAACAGAACAAGCGAGCGGTTGAAGAGAAAACTGATATGGTTGCGGCTGATCGTACC
CCAATTCCTGCTAGAAAGATCACTCTCGGCGATACCACTGATCTTCCA
+
FFFFFFFFFFFFFFFFFF:FFFFFFFFFFFFFFFFFF:F:FFFFFFFFFFFFFF:FFFFFFFFFFFFF
FFFFFF:FFFFFFFFFFFFFFFFFFFFFFFFFFFFFFFFFF:FFFFFFF
@A00721:190:HGJW7DSXY:4:1308:21242:22905_1:N:0:TCTGCAAG+GCTGCACT
GGTCGTTCTTTTGGTGATTTTATCAAAGAGAAAGTTTCTGCTAGTCGCGACGATAGTATTTATCGGAG
TGATATTAACAAGTTAAACTTCGAACTGACCACTTACAAGAGTGTTGAAGTACCGCAACATGGATCAG
TTT
+
FFFFFFFFFFFFFFFFFFFFFFFFFFFFFFFFFFFFFFFFFFFFFFFFFFFFFFFFFFFFFFFFFFFF
FFFFFFFFFFFFFFFFFFFFFFFFFFFFFFFFFFFFFFFFFFFFFFFFFFFFFFFFFFFFFFFFFFFF
FFF
@A00721:190:HGJW7DSXY:4:2207:29306:29199_2:N:0:TCTGCAAG+GCTGCACT
AAACTGAATCCACTCTCTTGGGATATAAATGAGTACATCTATAATAGAGTTTTGGAACTGTGTAATGT
CCTACGTACTGTTCAAGGTGTTTGGGCTTCTCTAAAGAACAGCAACTT
+
FFFFFFFFFFFFFFFFFFFFFFFFFFFFFFFFFFFFFFFFFF:FFFFFFFFFFFFFFFFFFFFFFFFF
FFFFFFFFFFFFFFFFFFFFFFFFFFFFFFFFFFF:FFFFFFFFFFFF
@A00721:190:HGJW7DSXY:4:2376:11812:22733_2:N:0:TCTGCAAG+GCTGCACT
AGACCTTTCTTGGGGAGTAAAGAGGGTTTGAAAATTACGGAACCTTTCCTCGTTCTTCATGAAGATCT
TCGAGTGTTTTATAGTGAGTTATTCTTGCGAAGGTTCAAGTCGATTGA
+
FFFFFFFFFFFFFFFFFFFFFFFFFFFFFFFFFFFFFFFFFFFFFFFFFFFFFFFFFFFFFFFFFFFF
FFFFF:FFFFFF:FFFFFFFFFFFFFFFFFFFFFFFFFFFFFFFFF::FFF
@A00721:190:HGJW7DSXY:4:1217:12879:26553_2:N:0:TCTGCAAG+GCTGCACT
AATAAGAAGTTCTTCGCAGTTGAATCTCCACCAGGTGGAGGGAAGACCACTGAACTTATAGCGACTTT
TGTCGAATTTTTCGCAAAAGGTCTTGAAGTACGTATTGTCACTGCTAA
+
FFFFFFFFFFFFFFFFFFFFFFFFFFFFFFFFFFFFFFFFFFFFFFFFFFFFFFFFFFFF,FFFFFFF
FFFFFFFFFFFFFF:FFFFFFFFFFFFFFFFFFFFFFF:FFFFFFFFFF
@A00721:190:HGJW7DSXY:4:1465:13214:24627_2:N:0:TCTGCAAG+GCTGCACT
TTGGCACTTCTAGTTCATCGCGGTATTTGTGAAATTAAGACAGACGTGAAAGTTATGATGAGTGATCG
TATTAGAACAGCTGATTCTTATTGCATGAACGTCGCTGAGGTCAAACC
+
FFFFFFFFFFFFFFFF:FFFFFFFFFFFFFFFFFFFFFFFFFFFFFF:FFFFFFFFFFFFFFFF:FFFF
FFFFFFFFFFFFFFFFFFFFFFFF:FFFFFFFFFFF:FFFFFFFFF,FF
@A00721:190:HGJW7DSXY:4:1634:6253:1767_2:N:0:TCTGCAAG+GCTGCACT
TTTTCATCGACGAATTATTCATGATTCACGCTGGTCAGACCCTTTTTCTTACACATCTACTTAAGGCG
GCTTATACCATAGGTTACGGTGATTCCAATCAAATCGGATTCATCAAT
+
FFFFFFFFFFFF:FFFFFFFFFFFFFFFFFFFFFFFFFFFFFFFFFFFFFFFFFFFFFFFFFFFFFFF
FFFFFFFFFFFFFFFFFFFFFFFFFFFFFFFFFFF:F:FFFFFFFFFFF
@A00721:190:HGJW7DSXY:4:1167:8323:22357_2:N:0:TCTGCAAG+GCTGCACT
TCCAAGTCGCAAACTACTTCAACATCAACGACATCATTCTTGATGAGTGTCGTTCTTATCGAAGTCTC
```

AGTTATCGCTGCCCGGCCGACGTTTGTCGCGAACTCTCAGAGGTTTAC
+
FFFFFFFFFFFFFFFFFFFFFFFFFFFFFFFFFFFFFFFFF:FFFFFF,FFFFFFFFFFFFFFFFFF
FFFFFFFFFFFFFFFFFFFFFFF,FFFFFFFFFFFFFFFFFFFFFFFFF
@A00721:190:HGJW7DSXY:4:1458:5285:9518_2:N:0:TCTGCAAG+GCTGCACT
CTATGGAAATTAGACCTATCAGATGTCTTGACGACTTCGAATTTGAGGACGGAGTTAAATATATTACT
TTCACTCAAGGTGAAAAGTTTGAAATTAACAAAGTCCTTGGTAAGAGT
+
FFFFFFF,FFFFFFFF:FFFFFF:FFF,F:FF:FF,FFFFFFFFFF:FFFFFFFF:FFFFFFF,FFFF
FFFFFFFFFFFFFFFFFFFFFFFFFFFFFFFFFF:FF:FF:FFFFFFF
@A00721:190:HGJW7DSXY:4:2373:2510:8437_1:N:0:TCTGCAAG+GCTGCACT
GTTCAAGGTAATACTTACCAGCACGTTCGGCTGGTTAGAATTAAACCTCAAGATGACCCTGTTTTTGC
ATCACAAACACATATTACCGTTGCTCTATCACGACACGTTGAATCTTTACGATACTTTGTTATCAATG
TAA
+
FFFFFFFFFFFFFFFFFFFFFFFFFFFFFFFFFFFFFFFFFFFFFFFFFFFFFFFFFFFFFFFFFFFF
FFFFFFFFFFFFFFFFFFFFFFFFFFFFFFFFFFFFFFFFFFFFFFFFFFFFFFFFFFFFFFFFFFFF
FFF
@A00721:190:HGJW7DSXY:4:2677:7319:5838_2:N:0:TCTGCAAG+GCTGCACT
TAAGGAACTGTCACGTTTAGCACGGGAGGTTGCAGATTCAACCCCTTATATCGAGGCTATCGAGGACG
ATGAGTATCTTGAAGTTGTTGACGACAAGCCTCAAGAGGGTAGAGGAC
+
FFFFFF:FFFFFFFFFFFFFFFFFFFFFFFFFFFFFFFFFFFFFFFFFFFFFFFFFFFFFFFFFFFFF
FFFFFFFFFFFFFFFFFFFFFFFFFFFFFFFFFFFFFFFFFFFFFFFF
@A00721:190:HGJW7DSXY:4:2358:3450:24126_2:N:0:TCTGCAAG+GCTGCACT
GGTTATTCCTGGAACCACCTTAACGTCACTCGGTAATCTTTCAGCCGAATTAAGTACTAATGAGTTCA
AGTCGGTTGTCGATCGAATTAAGATCAAGGCTAATACCTTTATAGGTA
+
FFFFFFFFFFFFFFFFFFFFFFFFFFFFFFFFFFFFFFF:FFFFFFFFFFFFFFFFFFFFFFFFFFFF
:FFFFFFFFFFFFFFFFFFFFFFFFFFFFFFFFFFFFFFF,FFFFFFFF
@A00721:190:HGJW7DSXY:4:2521:9625:14810_2:N:0:TCTGCAAG+GCTGCACT
GCCGCGTAGTCAGCGCGTTTAGCTGTATCATCAGTCAGAGTATACCCGATAGGGTACCGAGTCTCCAA
GAGAATATTTATTCTTATGAGGCTCGAAACTTCAACGTTTTCACAACG
+
FFFFFFFFFFFFFFFFFFFFFFFFFFFF:FFFFFFFFFFFFFFFFFFFFFFFFFFFFFFFFFFFFFFF
FFFFFFFFFFF:FFFFFFFFFFFFFFFFFFF:FFFFFFFFFFFFFFF:
@A00721:190:HGJW7DSXY:4:2563:5394:12837_2:N:0:TCTGCAAG+GCTGCACT
TCGCACCCGAAATTTTTGGGCAGAATGCTCTTAACGCGGTTCTTGAACGGTGTTTTGATCAGTCTAAG
TTGGATAAAGTTATAGGAGACTCAGTTAACCTGAGAGCTCCTCAATTG
+
FFFFFFFFFFFFFFFFFFFFFFFFFFFFFFFFF:FFFFFFFFFFFFFFFFFFFFFFFFFFFFFFFFFF
,FFFFFFFFFFFFFFFFFFFFFFFFFFFFFFFFFFFFFFFFFFFFFFF
@A00721:190:HGJW7DSXY:4:2470:22742:15013_2:N:0:TCTGCAAG+GCTGCACT
AATAGGGACGCTAGTATACTTCGTAAAGTCGACAGCGAAATGGTCAATATTGATAATTGGACCGACTA
TATGACCTATTTTAAGCTCATGGTGAAAAGGGAAGCTAAGGTTAAACT
+
FFFFFFFFFFFFFFFFFFFFFFFFFFFFFFFFFFFFFFFFFFFFFFFFFFFFFFFFFFFFFFFFFFFF
FFFFFFFFFFFFFFFFFFFFFFFFFFFFFFFFFF,FFFFFFFFFFFFF
@A00721:190:HGJW7DSXY:4:1236:14724:16125_2:N:0:TCTGCAAG+GCTGCACT
CCTATCTTTAAGGTTCTTGTGAGGAGAGTCAAACAGTGTCTTTTACCCAACATTTTTATGTATACAGA
GATGACCAGTGACGATTTCTCCCGGAAGCTCTTCTCTCTCTTAGGAGG
+
FFFFFF,FFFF:FFFF,F:::F,,FF::,:FFFF:::,FFF,:FFFFFFFFFF,F,FF,,,FFF:FFF
,F,FF,,,:F,FF:FF:::,FFFF,FF,FFFFF:FF:,FFFFF:,FFF

```
@A00721:190:HGJW7DSXY:4:2675:26494:20760_2:N:0:TCTGCAAG+GCTGCACT
TTTCGAAGTTTGACAAATCTCAAGATTTCTTTTGTAAAGCCTATGAGATAAAGATGTATTCTTATTTA
GGCTTCGATGCTGAATTTCTTGAACTTTGGAGTGCCAGTGAATATTTT
+
FFFFFFFFFFFFFFFFFFFFFFF:FFFFFFFFFFFFFFFFFFFFFFFFFFFFFFFFFFFFFFFFFFFF
FFFFFFFFFFFFFFFFFFFFF:FFFFFFFFFFFFF,:FFFFFFFFFFFF
@A00721:190:HGJW7DSXY:4:1662:31828:32189_1:N:0:TCTGCAAG+GCTGCACT
GAGTATCCATAACGATTTGTCATTTACCACCTTTGCTCAACGACGAAGTGGAACGGCCAACACTTTCT
TTGGTAATTGTATGATCACTTTGATGATTTTATCACTTTATTATGACCTTGGAAAGTTTTCGGCTGTG
GTT
+
FFFFFFFFFFF:FFFFFFFFFFFFFFFFFFFFFFFFF:FF,FFFFFFFFFFFFFFFFFFFFFFFFF:FF
F,FFFFFFFFFFFFFFFFFFFFFFFFFFFFFFFFFFFFFFFFFFFFFFFFFFFFFFFFFFFFFFFFFF
FFF
@A00721:190:HGJW7DSXY:4:2503:28610:22357_1:N:0:TCTGCAAG+GCTGCACT
CATTCTGTTTTCAAAGACTCCAATAACTGATCATTCTGATCATATGATTTTGGACAATGGTTTCGAAG
CGAAATTTTTGAGGGATAAACCAGCGTACTTCTGCTCGCGATTTATTCTTTTTGTCAATGATTACATC
TAC
+
FFFFFFFFFFFFFFFFFFF:FFFFFFFFFFFFFFFFFFFFFFFFFFFFFFFFFFFFFFFFFFFFFFFF
FFFFFFFFFFFFFFFFFFFFFFFFFFFFFFFFFFFFFFFFFFFFFFFFFFFFFFFFFFF:FFFFFFFFF
FFF
@A00721:190:HGJW7DSXY:4:2622:12454:19335_1:N:0:TCTGCAAG+GCTGCACT
CCTTACAAGATCATCGTTAAGTTAGGTAAACCCCTAACCGATGTTACTCTTGTAGGACTTTGGGAGAG
ATTCGTTAGTTTTAAAGACTATACTAAATATCTCCACTACGAGGTTGTTGTTTGGCGATTAGCTCTTG
CGA
+
FFFFFFFFFFFFFFFFFFFFFFFFFFFFFFFFFFF::FFFFFFFFFFFFFFFFFFFFFFFFFFFFFFFF
FFFFFFFFFFFFFFFFFFFFFFFFFFFFF:FFFFFFFFFFFFFFFFFFFFFFFFFFFFFFFFFFFFFF
FFF
@A00721:190:HGJW7DSXY:4:2474:31430:21042_2:N:0:TCTGCAAG+GCTGCACT
ATTGAGGGTCGTTATACCTATTCGGCTATCTGTGCTTTGAATTGTATAAGATCAAATTTTAAACGATT
CCGAGAGGTTTATCCTGATGTGGAGGGAGTTCTTGAAGCATCCCGACC
+
FFFFFF:FFFFFFFFFFFFFFFFF:FFFFFFFFFFFFFFFFFFFFFFFFFFFFFFFFFFFFF,FFFFF
FFFFFFFFFFFFFFFFFFFFFFF:FF:FFFFFFFFF,FFF:FFFFFFF:
@A00721:190:HGJW7DSXY:4:1218:22950:26804_2:N:0:TCTGCAAG+GCTGCACT
GGTTTCATAAGATTATCAGTAATCCTGATTCTACTACGGATTCATGGACCTTCCATCCTGTACCTCTT
CTGGAGAGACGTATCTCAAAGATCGACTTCGAGACAGGTGATATTGTT
+
FFFFFFFFFFFFFFFFFFFFFFFFFFFFFFFFFFFFFFFFFFFFFFFFFFFFFFFFFFFFFFFFFFFF
FFFF:FFFFFFFFFFFFFFFFFFFFFFFFFFFFFFFFFFFFFFFFFFF
@A00721:190:HGJW7DSXY:4:1139:28212:3129_1:N:0:TCTGCAAG+GCTGCACT
ACCAAGATCTAAATTCGGTAACGGTGCGTGGCGCTTTATTTTAAAAAGCTCCCTCCTCTCGCAAGTTT
GATCTGATACTTATCACGAAAGGACTTGAGTCTTTTGATCAGTCTCTGACTTGACCATCTTTCATTTT
AAA
+
::FFFFFF,FFFFFF:FFFF::FFFFFFFF,FFFFFFFFFFF:FFFFFFFF:FF:FFFFF:F:FFFFF
FFFFFFFFF:FFF:FFFFFF:FFFFFFF,FFFFFFFFFFFFFFFFFFFF:FFFF,FFFFFFFFFFFFF
FFF
@A00721:190:HGJW7DSXY:4:1274:4426:21371_2:N:0:TCTGCAAG+GCTGCACT
TTTCTTCTTTGTTTCCTTCTTTGCACTGATTTTTCCTCATCAGCTTGTGTTTTACCTAATCTGTGTTT
TTACGATAACAACTATCAGTTGATTAAGACTCTTCCGAAAGGCGAACT
+
```

FFFFFFFFFFFFFFFFF:FFFFFFFFFFFFFFFFFFF:FFFFFFFFFFFFFFFFFFFFFFFFFFFFFF
FFFFFFFFFFFFFFFFFFFFFFFFFFFFFFFFFFFFFFFFFFFFFFFFFF:
@A00721:190:HGJW7DSXY:4:1336:15628:24424_2:N:0:TCTGCAAG+GCTGCACT
GAAGCTCAGGGTGAAGCACAACGTTTGACCGTGACGAACTGTGGTGTTCTTGATCTACGTTACGGATA
TACTCTCCTTGAGTTCACTCGTAAGGACACTACCACTTATTACGATAT
+
FFFFFFFFFFFFFFFF:FFFFFFF:FFFFF:FFFF:FFF:FFFFFFFFF:FFFFFFFFFFFFF:F:FF
FFFFFFFFFFFFFFFFFFFFFFFFFFFFFFFFFFF,FFF:FFFFFFF:FF
@A00721:190:HGJW7DSXY:4:2371:4300:20462_2:N:0:TCTGCAAG+GCTGCACT
CCTTTAAGCGTCTTCTGTTCATCGCAACCAGATGATATCTTCTATTCGAACTTATGTTTCGATCAGTG
TTTCTTCTATCAGGAGGAAAACACTTGTTCTAGTCCTTGTAAGATTAA
+
FFFFFFFFFFFFFFFFFFFFFFFFFFF:FFFFFFFFFFFFFFFFFFFFFFFFFFFFFFFFFFFFFFFFF
FFFFFFFFFFFFFFFFFFFFFFFFFF:FFFFFFFFFFFFFFFFFF,FFF
@A00721:190:HGJW7DSXY:4:1105:19325:24846_2:N:0:TCTGCAAG+GCTGCACT
TCTTCTAATGTGGCCTGAACTACGTTTCACTTTGTGTGTACTTGTCTCTATTTTAAGTTTTGTCTTCC
TTGTTTGTCTTATTCTATTAATCACTCATTTTAGTGAGATTATTAAAA
+
FFFFFFFFFFFFFFFFFFFFFFFFFFFFFFFFFFFFFFFFFFFFFFFFFFFFFFFFFFFF:FFFFFFF
FFFFFFFFFFFFFFF:FFFFFFFFFFFFFFFFFFF,FFFFFF,FFFFFFF
@A00721:190:HGJW7DSXY:4:1309:29179:29324_1:N:0:TCTGCAAG+GCTGCACT
TCTTCGACAACAGCAGGGGTTTTCGAATAGTGATAGCCACCCGCCTTACAGAGGGGCAGCAGCATGAC
CATAATTGGTATTGACTTTGGTACCACCTTTTCTACTGTCTGTCTCGCCTCTTCGAATGAAGTTTTAA
TGC
+
FFFFFFFFFFFFFFFFFFFFFFFFFFFFFFFFFFFFFFFFFFFFFFFFFF:FFFFFFFFFFFFFFFFFF
FFFFFFFFFFFFFFFFFFFFFFFFFFFFFFFFFFFFFFFFFFFFFFFFFFFFFFFFFFFFFFFFFFFFF
FFF
@A00721:190:HGJW7DSXY:4:1449:32398:30702_2:N:0:TCTGCAAG+GCTGCACT
CTACAATCATTGGTTTTTCTAATAATGATGACACTATTGTTTATGGATATGATGCTATTACTCATGAT
GGTTACGGTCAGGGAGGATTCTCAGTTTACAAGGACCTTAAGCGATGG
+
FFFFFFFFFFFFFFFFFFFFFFFFFFFFFFFFFFFFFFFFFFFFFFFFFFFFFFFFFFFFFFFFFFFFF
FFFFFFFFFFFFFFFFFFFFFFFFFFFFFFFFFF,FFFFFFFFF:FFFF:
@A00721:190:HGJW7DSXY:4:1406:28908:15013_2:N:0:TCTGCAAG+GCTGCACT
CCGAAAGACGTGCTAAATTAAAACCATTGTACGATGTCGTCTGTCTCGGCGATCACTTTTCACTAAGA
ATTGGTCCTACTTTTGGACAAGGCCAACTTCGTAATCTTCATGAGATC
+
FFFFFFFFFFFFFFFFFFFFFFFFFFFFFFFFFFFFFFFFFFFFFFFFFFFFFFFFFFFFFFFFFFFFF
FFFFFFFFFFFFFFFFFFFFFFFFFFFFFFFFFFFFFFFFFFFFFFFFF
@A00721:190:HGJW7DSXY:4:2628:20193:29324_2:N:0:TCTGCAAG+GCTGCACT
TATCTCTTTGGTTGTTAGGGATTTTGAGAGAGTGAAAGGCTTCAATTGTAGTGGTTTGGTCGTTTCAG
TACCCAGTCAGTATACCTCTACGCAAAGATATTTCATGAAAACTACAG
+
FFFFFFFFFFFFFF,FFFFFFFFFFFFFFFFFFFFF:FFFFFFFFFFFFFFFFFFFFFFFFFFFFFFFF
FFFFFFFFFFFFFFFFFFFFFFFFFFFFFFFFFFFFFFFFFFFFFFFFF
@A00721:190:HGJW7DSXY:4:1533:10059:30154_1:N:0:TCTGCAAG+GCTGCACT
ACCTGTCTTTCATATTATGAATGAACCTTCGGCCGCTCTCTTTGCTTCGATGTTGGATGTCAAGAAGG
GTTCAGAATGGGATTCCTACGTAGTTTATGACTTTGGTGGTGGTACTTTCGATACTTCTATCGTCTTA
CGT
+
FFFFFFFFFFFFFFFFFFFFFFFFFFFFFFFFFFFFFFFFFFFFFFFFFFFFFFFFFFFFFFFFFFFFF
FFFFFFFFFFFFFFFFFFFFFFFFFFFFFFFFFFFFFFFFFFFFFFFFFFFFFFFFFFFFFFFFFFFFF
FFF

```
@A00721:190:HGJW7DSXY:4:1435:20835:28244_2:N:0:TCTGCAAG+GCTGCACT
AGTGTTATTTATTCTGGTGGTGATGATAATCTTGGAGGTCGTGACGTTGATGAAGCTATAAGAGCGTT
CTTATTACGGCGCACTTCTTTACCCGCCACCACACAACTTTCAGTTTC
+
FFFFF:FFFFFFFFFFFFFFFFFFFFFFFFFF:FFFFFF,FFFFFFF::FFFFFFFF:FFFFFFFFFF
FF:FFFF,FFFFFFFFFFFFFFF::FFFFFFF:FFFFFFFF:F:FFFF
@A00721:190:HGJW7DSXY:4:1323:29098:31031_1:N:0:TCTGCAAG+GCTGCACT
ACTGGAAAACCCTCGTCAATACTTTTCAATAATATACGTCTTGAACTCACTTATGACGATCTAGTGAA
ATTAACGCGTCCTTTTCTGGAACGTAGTTTTACTATTATGAACAAAGTTATCAAAGATAGTGGCATTC
AAG
+
FF:FFFFFFFFFFFFFFFFFFFFFFFFFFFFFFFFFFFFFFFFFFFFFFFFFFFFFFFFFFFFFFFFF
FFFFFFFFFFFFFFFFFFFFFFFFFFFFFFFFFFFFFFFFFFFFFFFFFFFFFFFFFFFFFFFFFFFF
FFF
@A00721:190:HGJW7DSXY:4:1625:6696:30874_1:N:0:TCTGCAAG+GCTGCACT
AGATCTTGCAGAGTGACTGGAGTTCAGACGTGTGCTCTTCCGATCTCTTGCTGAAACAGCTATTAAAG
CTGTTGAGCGGGTAATTACACCTAAACGAGCTCGATCTGCCATCGCTGAAGGATGTGCACTCTTTTCT
GCT
+
:FFFFFFFFFFFFFFFFFFFFFFFFFFFFFFFFFFFFFFFFFFFFFFF,FFFFFFFFFFFFFFFFFFFF
FFFFFFFFFFFFFFFFFFFFFFFFFFFFFFFFFFF:FFFFFFFFFFFFFFFFFFFFFFFFFFFFFFFF
FFF
@A00721:190:HGJW7DSXY:4:1437:21395:16564_2:N:0:TCTGCAAG+GCTGCACT
TCGATTGTATCAACAGTAACATTACTTCTAATAAAGGTCTCTATCAAGCTCGTATTATCGTCGCTGCT
GGATCTGCGTTACCGTTGAAGGCTAAACTCCAAGGTAATTATGAAGAC
+
FFFFFFFFFFFFFFFF:FFFFFFFFFFFFFFFFFFFFFFFFFFFFFFFFFFFFF,FFF,FFFFFFFFFF
FFFFFFFFFFFFFFF,FFFFFFFFFFFFFFFFFFFFFFFFFFFFFFFFF
@A00721:190:HGJW7DSXY:4:1209:15148:15890_1:N:0:TCTGCAAG+GCTGCACT
GTATGAGGGTGTTTCTAATCGTAACATTAATAATTTCAATCTTCTTAAAAAGAAGATAGAGAGTCATA
CTTTGGGCTATCGTTCAGGTGCATGGGGTTGGATAGCCAATATTGAAGTGGACACCTCTGGTAGTATC
GAA
+
FFFFFFFFFFFFFFFFFFFF:FFFFFFFFFFFFFFFFFFFFFFFFFFFFFFFFFFFFFFFFFFFFFFF
FFFFFFFFFFFFFFFFFFFFFFFFFFFFFFFFFFFFFFFFFFFFFFFFFFFFFFFFFFFFFFFFFFFF
FFF
@A00721:190:HGJW7DSXY:4:1356:32090:36245_1:N:0:TCTGCAAG+GCTGCACT
ATACTATGGTTGAATCAACTAAGCATACTACTTTTGCTTTGATTACTCTACCGCCTTACAAGTCTAAG
GTTAAAGAGTCGATTAAACAAGTTGTACTTATCAATGATTTTATCGCTGGTTGTTATCTCGAAAAGTC
TCT
+
FFFFFFFFFFFFFFFFFFFFFFFFFFFFFFFFFFF,FFFFFFFFFFFFFFFFFFFFFFFFFFFFFFFF
FFFFFFFFFFFFFFFFFFFFFFFFFFFFFFFFFFFFFFFFFFFFFFFFFFFFFFFFFFFFFFFFFFFF
FFF
@A00721:190:HGJW7DSXY:4:2406:9968:17033_2:N:0:TCTGCAAG+GCTGCACT
TTCTAACCCAAAGAGAATATCTAAAATATTTACAAAATCTTACCACTATCTACGAACCTAAAGTTCTG
AAGACGTTGCTTGTCGCTTCCTCCTTTAATGGCACTACCTCGGTACAA
+
FFFFFFFFFFFFFFFFFFFFFFFFFFFFFFFFFFFFFFFFFFFFFFFFFFFFFFFFFFFFFFFFF:FF
FFFFFFFFFFFFFFFFFFFFFFFFFFFFFFF:FFFFFF:FFFFFFFFFF
@A00721:190:HGJW7DSXY:4:2676:9362:19398_2:N:0:TCTGCAAG+GCTGCACT
GAAGGCGACCTGTTCCGGGGTTTTTACGGGATCCGGAATGTTCTGTCTATATTAGGTGAAGCGAAATA
CGATTTCGAACGTCAACGTTCACCGAGTTTCGTTTCGTACCTTGATGA
+
```

```
FFF,FFFFFFFFFFFFFF,FFFFFFFFFFFFFFFFFFFFFFFFFFFFFFFFFFFFFFFFFFFFFFFFF
FFFFFFFFFFFFFFFFFFFFFFFFFFFFFFFFFFFFFFFFFFFFFFFFF
@A00721:190:HGJW7DSXY:4:1255:18349:35399_1:N:0:TCTGCAAG+GCTGCACT
TAGTGCGAGGCCTCTCTTCTACGAGTATTGCTATTTATACGGTTACGAGAAGAAGGTTCGAAAGTTTG
AGAACTTAGTTTACGAACTTGACTCTACTATACGTGACATACTAAATACTAGATATTTGGATGATCTT
GAT
+
FFFFFFFFFF:FFFFFFFFFFFFFFFF:FFFFFFFFFFFFFFFFFFFFFFFFFFFFFFFFFFFFFFFFF
FFFFFFFFFFFFFFFFFFFFFFFFFFFFFFFFFFFFFFFFFFFFFFFFFFFFFFFFFFFFFFFFFF:FF
FFF
@A00721:190:HGJW7DSXY:4:2238:5602:23531_2:N:0:TCTGCAAG+GCTGCACT
TTCAGAGTATGAAAAATTTTCTTCCTTGGATTTTAATATAAAGAATCTTTACCAGGACTCCTACTACA
GGAGGCAGTCTTCTGATCGTAAGAAGTTTATAAGGAACATATCCATTT
+
:FFFFFF,FFFF:FFFFFFF:FF:FFFFFFFFFFFF:FFFFFFFFFF:FFF,FF:FFFFFFFFFFFFF
FFFFFFFF:FFFFFFFFFFFFF:FFF,FFFFFFFFFFFFFFFFF:FFF
@A00721:190:HGJW7DSXY:4:1569:2718:12837_1:N:0:TCTGCAAG+GCTGCACT
ACTCGAAACTCTAAGTGGTAATACTGTCTTTCCAAGCTTACCTGTATCTTCAATGAAATTGGACTCTT
CGATAGGGGACATTTTATCGTGGGCTTTCCGTAAAGGAAGATCTGTTATCGACGGTTTATCGTTTACG
AAG
+
FFFFFFFFFFFFFFFFFFFFFFFFFFFFFFFFFFFFF::FFFFFFFFFFFFFFFFFFFFFFFFFFFFF
FFFFFFFFFFFFFFFFFFFFFFFFFFFFFFFFFFFFFFFFFFFFFFFFFFFFFFFFFFFFFFFFFFFF
FFF
@A00721:190:HGJW7DSXY:4:2319:7889:26303_2:N:0:TCTGCAAG+GCTGCACT
GTTTAAGAATACTATAAATAGAATAGCTATTACTACTAATGGCGTTGGCCTAGTGGATGTTATTAGTG
ACATCTGGTCAATTTCGTTTTTAGGTGGTCTTTATTCTATTATTGATT
+
FFFFFFFFFFFFFFFFFFFFFFFF:FFFFFFFFFFFFF:FFFFFFFFFFFFFFFFFFFFFFFFFFFFF
FFFFFFFFFFFFFFFFF:FFFFFFFFFFFFFFFFFFFFFFFFFFFFFF
@A00721:190:HGJW7DSXY:4:1260:8938:31532_1:N:0:TCTGCAAG+GCTGCACT
ACAGAAGATGATGTTAGAGTAGAATTCCTGAAGAGTCCTTCCGTAACCAACTTCTTGACAATTTTCTT
CAATCCTTCGATAATAGTTAAATCTAGGATTATGTCTGTGTTGAAAGACTTTCTACCGATAAGGGTCA
CTA
+
FFFFFFFFFFFFFFFFFFFFFFFFFFFFFFFFFFFFFFFFFFFFFFFFFFFFFFFFFFFFFFFFFFFF
FFFFFFFFFFFFFFFFFFFFFFFFFFFFFFFFFFFFFFFFFFFFFFFF:FFFFFFFFFFFFFFFFFFF
FFF
@A00721:190:HGJW7DSXY:4:1221:30282:5337_2:N:0:TCTGCAAG+GCTGCACT
TAAGAAATAATCCTACATTGACTGATTATCTAAAGATTGAAGGAAAATTTTATCCTCGTTTAACTTAC
TTAGACTTTCCTACGATACAAATGATAACTTCTAATTTACGTTTGCTA
+
FFFFFFFFFFFFFFFFFFFFF:FF:FFFFFFFFFFFFFFFFFF:FFFFFFFFFFFFFFFFFFFFFFFF
FFFFFFFFFFFFFFFFFFFFFFFFFFFFFFFFFFFFFFFFFFFFFFFF
@A00721:190:HGJW7DSXY:4:2333:27453:25895_2:N:0:TCTGCAAG+GCTGCACT
CAAAGGGATGCATGGTGTTATCTCCTCAGGAGTCCCGGCATTTGATAATAATGACGAGACTTTCGTCA
TAATCGCTGCATATTACGGATTACACGGTACCAGCGCATCTCGTGTCG
+
FFFFFFFFFFFF:FFFF:FF:FFFFFFFFFFFFFFFFFFFFFFFFFFFFFFFFFFFFFFFFFFFF:FF
FFFFFFFFFFFF:FFFFFFFFFFFFFFFF:FFFFFFFFFFFFFFFFFF
@A00721:190:HGJW7DSXY:4:2250:6777:4116_1:N:0:TCTGCAAG+GCTGCACT
GTATTAACTTATAAAGGTGAGGAGACCATTTTCTTGCTGAAATTTATAGAAGATTATTTTGACAGAAT
CAATAAGAATCATCGATTCTCGATTCGTAGAGCTTATCTAGGTACTCTCATTTACTATGTTGATTTAG
TCT
```

+
FFFFFFFFFFFFFFFFFFFFFFFFFFFFFFFFFFFFFFFFFFFFFFFFF:FFFFFFFFFFFFFFFFFF
FFFFFFFFFFFFFFFFFFFFFFFFFFFFFFFFFFFFFFFFFFFFFFFFFFFFFFFFFFFFFFFFFFFF
FFF
@A00721:190:HGJW7DSXY:4:2456:29677:33849_2:N:0:TCTGCAAG+GCTGCACT
GATTTACAACGATATAGGAGTAATCTACGAGAAGATTCTAGAATTCTTAATTATAATATTCAACCTCA
AAAGTTACGTGTTTACAGTAACGAGATCACACATTTTGAGAACTAACT
+
FFFFFFFFFFFFFFFFFFFFFFFFFFFFFFFFFFFFFFFFFFFFFFFFFFFFFFFFFFFFFFFFFFFF
FFFFFFFFFFFFFFFFFFFFFFFFFFFFFFFFFFFFFFF:FFFFFFFF
@A00721:190:HGJW7DSXY:4:1306:12924:5525_1:N:0:TCTGCAAG+GCTGCACT
CAATCTCTTCTTATAATAAATAAATAAATAATATTACTAATGGCAAATAGTGGAAAGGCTCCAGTCAA
CACTAGTTCGAGTGCTGCTTTGGATTACGATGACGTGAGAATAGGTGAAGGGATAGTTTTTACCTCGA
CCA
+
FFFFFFFFFFFFFFFFFFFFFFFFFFFFFFFFFFFFFFFFFFFFFFFFFFFFFFFFFFFFFFFFFFFF
FFFFFFFFFFFFFFFFFFFFFFFFFFFFFFFFFFFFFFFFFFFFFFFFFFFFFFFFFFFFFFFFFFFF
FFF
@A00721:190:HGJW7DSXY:4:1148:28384:32706_2:N:0:TCTGCAAG+GCTGCACT
GCGACTAATCTTAAAGCTTCACCCAAAATGAAGGACACTTCGAAACTGAATAATGATGCACTACAAAC
AAGTATCGCTAATCATCTGAAAAAGGCTCTAAAGGACACTATACAATA
+
,F:FFFFFF:FFFFFF:FFFFFFFFFFFFFFFFFFFF:FFFFFFFFFF:FF:FFF:F:FFFFFFFFFF
:FFFFFFFFFFFFFFFFFFFFFFF:F,F,FFFFFFFFFFFFFFFFFFFF
@A00721:190:HGJW7DSXY:4:2641:32244:29309_2:N:0:TCTGCAAG+GCTGCACT
TGCTTTTACCGATAACGATAAATTCGTCATCAAATATAAAGAAAAGGACTATGAAATAAAAGATTTGG
TGCTTAGGGATATAATGAATTCAGCACCACATTTTGGTCAAGTCAATC
+
FFFFFFFFFFFFFFFFFFFFFFFFFFFFFFFFFFFFFFFFFFFFFFFFFFFFFFFFFFFFFFFFFFFF
FFF:F:FFFFFFFFFFFFFFFFFFFFFFFFFFFFFFFFFFFFFFFFFF
@A00721:190:HGJW7DSXY:4:1378:32967:35884_1:N:0:TCTGCAAG+GCTGCACT
TCTTAGAGGATATTCGCCATTGTGGTACGAATTGGTCCAACATTTACCACCAAATTCTTTTGATACAA
CTCTAGCCTTCAAGTGGGGTATGCCTGCGGAATACGCTTGCTATTCACCAGATTGGTTCGAACCCGTT
GAT
+
FFFFFFFFFFFFFFFFFFFFFFFFFFFFFFFFFFFFFFFFFFFFF,:FFFFFFFF:FFFFFFFFFFFF
FFFFFFFFFFFFFFFFFFFFFFFFFFFFFFFFFFFFFFFFFFFFFFFFFFFFFFFFFFFFFFFFFFFF
FFF
@A00721:190:HGJW7DSXY:4:1161:9344:7780_1:N:0:TCTGCAAG+GCTGCACT
AGCTATTCAAGCTTGTCGTTTAAAGACTGCAGAGGCTGTCAAACAAGCCCAGACTCAAGGAGTCCAAT
CAAAGGTTTTGAATTTAGCTCAACTGGGGAGACGTGCTTAAATGGGTGATAAAAATTATTTAGAGATC
TTT
+
FFFFFFFFFFFFFFFF,FFFFFFFFFFF,FFFFFFFFFFFFFFFFFFFFFFFFFFFFFFFFFFFFFFF
FFFFFFFFFFFFFFFFFFFFFFFFFFFFFFFFFFFFFF:FFFFFFFFFFFFFFFFF:FF:FFFFFFF
FFF
@A00721:190:HGJW7DSXY:4:1668:18656:29105_2:N:0:TCTGCAAG+GCTGCACT
CTACGGAAAAGTCCGGAGTATATTGTATGTTAATAGATAGTGGGATAATAGTTCTACCGATACGTAGT
GAACGCGTTCGTAGAACCAAGTACTTTCCGTCTGAAGAGTTTAGAGCT
+
FFFFFFFFFFFFF:FFFFFFFFFFFFFFFFFFFFFFFFFF:FFFFFFFFFFFFFFFFFFFFFFFFFFFF
FFFF:FFFFFFFFFFFFFFFFFFFFFFFFFFFFFF,FFFFFFFFFFFFF
@A00721:190:HGJW7DSXY:4:2326:31241:31798_1:N:0:TCTGCAAG+GCTGCACT
TAACCGATAGGAGTTGTATAGTTGAAGATGTGGCTTTAGACGATAATCCTTTAAGGACTATGTTGACT

```
GACGAAGATTTTCCCACAGATTTGTCTACGTTGCAGATCGCCACTCCAAACACTATATACATTAAAAT
ATA
+
FFFFFF:F::FF:FFFFF:FFFFFFFFFFFFFFFFFFFFFFFFFFFFFFFFFFFFFFFFFFFFFFFFF
FFFFFFFFFFFFFFFFFFFFFFFFFFFFFFFFFFFFFFFFFFFFFFFFFFFFFFFFFFFFFFFFFFF:
FFF
@A00721:190:HGJW7DSXY:4:1370:17788:8155_1:N:0:TCTGCAAG+GCTGCACT
CATAACTTTTGTTACCAAATTCTTGGCTTTCAACGAAGTCTCTGATTTTATTAATCATTACTTTAACG
ATCTCTCGGTCATTTACTCTGTACTTGATCTACGTCGTTTAACTGTCGGAACAACCTTTACTTAGAAT
GAC
+
:FFFFFFFFFFFFFFFFFF:FFFFFFFFFFFFFFF:FFFFFFFFFFFFFFFFFFFFFFFFFFFF:FFF
FFFFF:FFFFFFFFFFFFFFFFFFFFFFFFFFFFFFFFFFFFFFFFFFFFFFFFFFFFFFFFFFFFFF
FFF
@A00721:190:HGJW7DSXY:4:1323:24252:8453_2:N:0:TCTGCAAG+GCTGCACT
TTCTTATATTATTTACAATAGTTAATATCATATTAGTAATACTCACTATCGTTTTCTGCTTTTTCTTA
GCTAATAGATTAATAAAGGATGAGTACGAAAACCAACTCTTCGTTACG
+
FFFFFFFFFFFFFFFFFFFFFFFFFFFFFFFFFFFFFFFFFFFFFFFFFFFFFFFFFFFFFFFFFFFF
FFFFFFFFFFFFFFFFFFFFFFFFFFFFFFFFFFFFFFFFFFFFFFF:
@A00721:190:HGJW7DSXY:4:2452:5132:30452_1:N:0:TCTGCAAG+GCTGCACT
GTAGAATATATTTATTCTCTACACCCATCTCAACGTGGATAAAGCGATTTTTCCGTCCTGAACGTAAT
ACACTAAGAGATCATCCGAAAACGACTCGAAACCGAGACGCCGTAGATCAAAGTCCCTTCTAACTTTA
GAT
+
FF,,F,F,:,FFF,,,:,,,:FFFF,F::,,F:F,,:,F,FF,F:,,,FF:,F:F,F,F:F,:,FF,:
F,,:,F,,F,F,,:,F,:,:,FFFFFFFFFFFFFFFFFFFFFFFFFFFFFFFFFFFFFFFFFFFFFFF
FFF
@A00721:190:HGJW7DSXY:4:2467:25880:25269_1:N:0:TCTGCAAG+GCTGCACT
CTTTACTCATTTTATAGCAGTTATTTATGTTGTACTTTTTATAATTTATAACCTTCTTATACTTTTGG
GTATGTTTTGTGAAAAACATGAGATACCCAAGTCTATCGAAGACATTATCTCTTATGTACTTATTGTA
ACG
+
FFFFFFFFFFFFFFFFFFFFFFFFFFFFFFFFFFFFFFFFFFFFFFFFFFFFFFFFFFFFFFFFFFFF
FFFFFF:FFFFFFFFFFFFFFFFFFFFFFFFFFFFFFFFFFFFFFFFFFFFFFFFFFFFFFFFFFFFF
FFF
@A00721:190:HGJW7DSXY:4:2556:9191:2848_1:N:0:TCTGCAAG+GCTGCACT
TTCTTATTCTTTTATTCCTTTAACTGTGTATGAGGAGGATTTATAATGCTTTTCGATAGTGATTGTTA
TCAATTTAAATACATTACTGTTTCAAATCATCTTTACTCACAGACTCCGAAAATGGTTTCAAATGAGG
TCA
+
FFFFFFFFFFFFFFFFFFFFFFFFFFFFFFFFFFFFFFFFFFFFFFFFFFFFFFFFFFFFFFFFFFFF
FFFFFFFF:FFFFFFFFFFFFF:FF:FFFFFFFFFFFF:FFF:FFFFFFF,FFFFFFFFFFFFFFF:F
FFF
@A00721:190:HGJW7DSXY:4:2556:9191:2848_2:N:0:TCTGCAAG+GCTGCACT
TATTAAACGAACTATTATCCGATTCTTCGAGGGTATCACTACCACTGATCTGCCGACTTTAGTCAAGG
AACTTCAATTCCTTCGATGTTTATATTTTTCTATAAGGGAAAATAGCA
+
FF:FFFFFFFFFFFFFFFFFFFF:FFFFFFFFFFFFFFFFFFFFFFFFF:F:FFFFFF:FFF:FFFFFF
FFF:FFF,FFFFFFFF,FFFFFFFFF,::FFFFF:FFFFFFFFFFFFF
@A00721:190:HGJW7DSXY:4:2122:24279:15828_2:N:0:TCTGCAAG+GCTGCACT
TTCGACAAATTTTAGAACTTTATTTCAGAACTCTCTCCGATCACCTTGGTGTGATCTCCCGGAGGATC
TTTTCAATAAGATGTATGATGATAATCTACTATTTTTAAATAGTGATA
+
```

FFFFFFFFFFFFFFFFFFFFFFFFFFFFFFFFFFFFFFFFFFFFFFFFFFFFFFFFFFFFFFFFFFFFFF
FFFFFFFFFFFFFFFFFFFFFFFFFFFFFFFFF,FFFFF:FFFFFFFF
@A00721:190:HGJW7DSXY:4:2462:2320:2159_1:N:0:TCTGCAAG+GCTGCACT
CTTTATCAAAGATTTGGCTTGGTCTATTTACCGCAAAAAGTTGGAAGAGGTCTATGATATTTATTTTA
CTTATACTTTAAATAGCACCGATTATAACTTGACTGTTGATAATATTACAATAGCTCTTTCTCAGAAG
ATT
+
FFFFFFFFFFFFFFFFF,FF,FFFFFFFFFFFFFFFFF:FFFFFFF:F:FF:FFFFFFFFFFFFFFFF
:F:FFF:FFFFFFFFFFFFFFFFFFFFFF:FFF:FFFFFFFFFFFFFFFF:FFFFFFFFFFFFFFFF:
FFF
@A00721:190:HGJW7DSXY:4:1306:32163:13792_2:N:0:TCTGCAAG+GCTGCACT
GGTGAACACGTCGGTAGCATTGATTTACTTTTACCGTTAGAACTGAAAAAGATTCTTTTCGAAAAGAA
ACCTATAAGTTACAAACGATTCTTCGACTATATTAAAGAAATAAAAAT
+
F,F:FFFFF:FFFFFFFFF:FFFFFFFFFFFFFFF,:F,FFFFFFFFFFFFFFFFFFFFFFFFF:FF
:FFFFFFFFFFFFFFFFFFFFFFFF,FFFF:FFFFFFFFFFFFFFFFF
@A00721:190:HGJW7DSXY:4:1452:32913:11678_2:N:0:TCTGCAAG+GCTGCACT
CTTTACATTTTTAATGGTATGGTATAATGTGTCTTACAAATAGAACCTTCACTCTCGGTAATGAGAGT
GTATTCCTTAATATTCTAGTAACCGCTATGTGGGCATTACTAGGATTT
+
F:FFFFFFFFFFF:FFFFFFFFFFFFFFFFFFFFFFFFF:FFFFFFFFFFFF,FFFFFFFFFFFFFFF
FFFFFFFFFF:FFFFFFFFFFFFFFFFF:FFFFFFFFFFFFFF:FFFF
@A00721:190:HGJW7DSXY:4:2435:20564:21480_1:N:0:TCTGCAAG+GCTGCACT
GATAGCAATTTATTTTTTAAATGCTAGGAAAATTTTAGAAAAACTCCATTGTATAGTTCAAGGAGTAC
TTAATTCAATAGCTTTAGTATTATTTACTATCGCCTTTATTGAAAATTGTATAAGAGTTAATTGCTTA
CAC
+
FFFF,FFFFFFFF,F:FFFFFFF,FF,FFFF:FFFFFFFFFFFFFF,FFFFFFFFFFFFFF:FF:FFF
FFFFFFFFFFFF::FFFFFFFFFFFFFFFFFFFFF:FFFFFFFFFF:FF:FFFF,FFFFFFFFFFFFFF
FFF
@A00721:190:HGJW7DSXY:4:1302:32542:8218_1:N:0:TCTGCAAG+GCTGCACT
GGGAGAATGAACTGCCGCTTCCCGGAAGAAACTATCTATTTACAGAGTATTTAGAGTAGTCCAAAGTT
TGTATAAATTTATATGATTTTCTATATATATACCACATATATATATATATAAAATCCTATAAATTTGT
ACA
+
::FF:FFF,FFFF:FFF,F:F:F,:FF,FFF:,F:,FFF:FF:F,:,FFFFFF:FFFFF:F:F:F:FF
:FFFFFF:FFFFFFFFFFFFF:FF:FFFFF:FF:FFFFFFFFFFF:FFFFFFFFFFF:FFFFFFFF:F
F:F
@A00721:190:HGJW7DSXY:4:2448:20302:13886_1:N:0:TCTGCAAG+GCTGCACT
AACATACCATACACTCCCCAAACATAACATACCATAACCTACCATGACCAATCATCGCTCCAACTGGC
ACGCCAGACGTAGGGTCAAACCTACTACCGCCGGTCGGCAGATCGGCACCACTGGTCTCTACGTTCAG
GGA
+
F,FFF:FFFFFF:F:FFFFFF,FFFF,FF:FFF:FF,FF:FFF,,,FFFFFFF:FFFFFFFF:FFFFF
FFFFFFFFFFFFFFFFFFFFFFF:FFFFFFFFFFFFFFFFFF:FFFFFFFFFFFFFFFFF:FFFFFFF
FFF
@A00721:190:HGJW7DSXY:4:1633:10529:30311_2:N:0:TCTGCAAG+GCTGCACT
TAACCTTCCCAAATCTAGATCTGCACAGAAGAAGGCTGAAGGTCGTTACCGGACGACCACTCAAAGGA
AACGGGCTGTTGCCCAGAGGAAAGCAGCGACCACCGAGGTCGCTCGTA
+
FFFFFFFFFFFFFFFFFFFFFFFFFFF:FFFFFFFFFFFFFFFFFFFFFFFFFFFFFFFFFFFFFFFF
FFFFFFFFFFFFFFFFFFFFFFFFFFFFFFFFFFFFFFFFFFFFFFFF
@A00721:190:HGJW7DSXY:4:1311:30517:7373_1:N:0:TCTGCAAG+GCTGCACT
AGTCCCGTCATCTCTGTGGGAACTGAGGCAGTACCTCAGTCAAAAGTCACATTCACCACTCCCATGCC

```
AATCAATGATGTGACTATACAGTTCGGCCATTTCAAGAGTGGTCAATACGTCGTGGGAGGTACTGAAG
CCT
+
FFFFFFF:FFFFFFFFFFFFFFFFFFFFFFFFFFFFFFFFFFFFFFFFFFFFFFFFFFFFFFFFFFFF
FFFFFFFFFFFFFFFFFFFFFFFFFFFFFFFFFFFFFFFFFFFFFFFFFFFFFFFFFFFFFFFFFFFF
FF:
@A00721:190:HGJW7DSXY:4:1558:27670:14278_1:N:0:TCTGCAAG+GCTGCACT
ATCTAAGAACACTCCCAAGCCAATCAATAGTTCTTCTGACCAGAATGAAAAGGGGTTTACCAGGGTAG
TCTCTAAGTCTACCAGGAAGAGGGGAAATGTCCCCAAAACTGAGAAGACTAAAGGGAAAGGTCCATCA
GGA
+
FFFFFFFF::FFFFFFF,FFFF,FFF:FFFFFFFFFFFFFFFF:FFFFFFFF:FFFFFFFF:FFFF:F
FFFFFF,FF:FF:FFFFFFFFFFFFFFFFFFFFFFFFF:FFF:FFF:F,FF:,FFFF:FFFFFFFFFF
FFF
@A00721:190:HGJW7DSXY:4:1246:2618:18928_2:N:0:TCTGCAAG+GCTGCACT
ATTACCCCTAATTCTGAAGGAAGATCTCCCACCATCTGGGGTTGGGAATAAGAAATCTGCACGGGCCA
AAGAGGCCGCTGCTACGAAGTCGCTGAAGGCGGCTTGGGTCATTAAGG
+
F:FFFFFFFFFFFFFFFFFFF:FFFFFFFFFFFFFFFFFFFFFFFFFFFFFFFFFFFFFFFFFFFFFF
FFFFFFFFFFFFFFFFFFFFFFFFFFFFFFFFFF:FFFFFFFFFFFFFF
@A00721:190:HGJW7DSXY:4:1224:17598:33004_1:N:0:TCTGCAAG+GCTGCACT
AGTTGCGCCTGCCGACCCCATCTTTGAGACCAAGACTCTCTACAGTAGGTTCGTGCCTACTATGAGCA
AATCTGTTGAATTCGTCACTGAGGACGAAGGTGAGAGGACTTTTGGCTACTGGCGACAAACTGAGAGC
AGT
+
FFFFFFFFFFFFFFFFFFFFFFFFFFFFFFFFFFFFFFFFFFFFFFFFFFFFFFFFFFFFFFFFFFFF
FFFFFFFFFFFFFFFFFFFFFF:FF:FFFFFFFFFFFFFFFFFFFFFFFFFFFFFFFFFFFFFFFFFF
FFF
@A00721:190:HGJW7DSXY:4:1549:29152:13401_2:N:0:TCTGCAAG+GCTGCACT
AGAATTGCGTGGGTTGAAGGCCGACGCTTCGAGCTTGAGTGTGACGAGGCCGGTGACGTTTTTGTCAA
CGGCGTTTGGAGGATTTTATCCGCGCTTAACTATGGGGCGAAGATCCC
+
FFFFFFFFFFFFFFFFFFFFFFFFFFFFFFF:FFFFFFFFF:FFFFFFFFFFFFFFFFFFFFFFFFFF
FFFFFFFFFFFFFFFFFFFFFFFFFFFFFFFFFFFFFFFFFFFFFFFFF
@A00721:190:HGJW7DSXY:4:2253:28239:19930_2:N:0:TCTGCAAG+GCTGCACT
AAGGACTTAGGTTGGCATATTTATGCCGACGATAATAAGCATGTCACCCTTAAGCACCGAGGTAAGCT
CGGGACTTACAAGAGAGAGTGTGAGAGAGTCGCTCTCACTATTTTTGC
+
FFFFFFFFFFFFFFFFFFFFFFFFFFFFFFFFFFFFFFFFFFFFFFFFFFFFFFFFFFFFFFFF:FFF
FFFFFFFFFFFFFFFFFFFFFFFFFFFFFFFF:FFFF,FFFFFFFFFFF
@A00721:190:HGJW7DSXY:4:2515:5367:1141_1:N:0:TCTGCAAG+GCTGCACT
TTTCTGACTGATGAGGTTAAAACTCTTGCTCCTTCTGTGGGTGAGGGTTTTTGTTATATTCACTGGTT
CTGGGAGGCTGCTTTCCTCCATAATTACAACCTCTCTTTAGAGAGCATTAGAAGGAAGTTGGGCAAAT
TCC
+
FFFFFFFFFF:FF:FFF:FFFFFFFFFFFFFFFFFFFFFFFFFFFFFFFFFFFFFFFFFFFFFFFFFF
FFFFFFFFFFFFFFFFFFFFFFFFFFFFF:FFFFFFFFFFFFFFFF::FFFFFFFFFFFFFFFFFFFF
FFF
@A00721:190:HGJW7DSXY:4:2362:19886:26099_1:N:0:TCTGCAAG+GCTGCACT
CACACTTTTCTTGAAAATTTTTCCTGTCCTCCGTCATTGGGGATTTCGATGTGAGAAGGTGGAAGGTG
TCAAGGGTCTTCTTCATGCTGATCTTTCAGGACCTAATCACATCATGATTGATAGTTACGGCTCGTAT
CGA
+
FFFFFFFFFFF,FFFFFFFFFFFFF,FFFFFFFFFFF:FFFFFFFFFFFFFFFFFFFFFFFFFFFFFF
```

FF,FFFFFFFFFFFFFFFFFFFFFFFFFFFFFFFFFFFFFFFFFFFFFFFFFFFFFF:FFFFFFFFFF
FFF
@A00721:190:HGJW7DSXY:4:1127:24704:6793_2:N:0:TCTGCAAG+GCTGCACT
CGAAATTAAAGACGGGGGGATTAAGAGTCCAGCTGTAACTGTCATTTCAGAGAGGGGAGCTCTGAAAA
GGAAACTTCGTCAGAAGATGAGGAAGTCTAAGAGATCGGAAGAGCGTC
+
FFFFFFFFFFFF,FFFFFFFFFFFFFFFFFFFFFFFFFFFFFFFFFFFFFFFFFFFFFFFFFFFFFFF
FFF:FFFFFFFFFFFFFFFFFFFFFFFFFFF:FFFFFFFFF:FFFFFFF
@A00721:190:HGJW7DSXY:4:1132:18765:18302_2:N:0:TCTGCAAG+GCTGCACT
TCTCACCACCTGGAGAAGGCAAGTGGAGAGTCCAGAAGTTGATGAAGAAGGATTATTTGAAGCAGGGG
TTACCACTTCCTAAGGTGGATGAGGGAGGTTGTTTCTTTGGAGTTTTA
+
FFFFFFFFFFFF:FFFFFFFFFFFFFFFFFFFFFFFFFFFFFFFFFFFFFFFFFFFFFFFFFFFF:FF
FFFFFFFFFF:FFFFFFFFF:FFFFFFFFFFFFFFFFFFFFFFFF::FF
@A00721:190:HGJW7DSXY:4:1635:3378:3583_2:N:0:TCTGCAAG+GCTGCACT
CCTTGAGAAGACAAGCCACATTAAGATATAATTTAGGTCAACTTGGGGTTTCGAATGTCATTAGTGCC
TTACGATTGAAAGAGGTCCTTTTGAGTATCGACAGATCTTTCGATCTT
+
FFFFF:FFFFFFFFFFFFFFFFFFFFFFFF:FFFF,FFFFFFFFFFFFFFFFFFFFFFFFFFFFFFFF
FFF:FFFFFFFFFFFFFFF:FFFF:FFFFFFFFFFFFFFFF:FFFFFF
@A00721:190:HGJW7DSXY:4:1376:21160:34632_2:N:0:TCTGCAAG+GCTGCACT
GTGGCAGAATTCGGTAACTTACCATATTGTTCCTTCCGGGAAGTTGTGGGATTTAACTTTGACTCAAC
TACTCCGATCAGATCCGGATGGTTTGATTGGTATGGACTACTCGACTT
+
FFFFFFFFFFFFFFFFFFFFFFFFFFFFFFFFFFFFFFFFFFFFFFFFFFFFFFFFFFFFFFFFFFFF
FFFFFFFFFFFFFFFFFFFFFFFF,FFFFFFF:FFFFFFFFFFFFFFFF
@A00721:190:HGJW7DSXY:4:2278:7066:29262_1:N:0:TCTGCAAG+GCTGCACT
TAGGGTCATGGCAGATACTACCGATAAGGTGAAAAATATGCAGGTCAAAGATTCTGTAGCTCTGAGGG
CGGTTGAGGGAGCCGTTTTGGCCGACTTTAACTATTCTAAGAGTCTTTATGATTCAAAACCTATCGTT
GTC
+
:FFFFFFFFFFFFFFFFFFFFFFFFFFFFFFFFFFFFFFFFFFFFFFFFFFFFFFFFFFFFFFFFFFF
FFFFFFFFFFFFFFFFFFFFFFFFFFFFFFFFFFFFFFFFFFFFFFFFFFFFFFFFFFFFFFFFFFFF
FFF
@A00721:190:HGJW7DSXY:4:2363:17264:5118_1:N:0:TCTGCAAG+GCTGCACT
TACAGCGGCTTATTGTCGCGCAGTTTCCAGAATTGCGGTTGAAGTTCACCCATTCTATCTTGAATGCT
CACGCCGTCGCAGCCATATCGAGACTATGTTATAATCACTTGTTAAAGTACAGATTTAGTGATTTAGA
TAT
+
FFFFFFFFFFFFFFFFFFFFFFFFF:FFFFFFFF:FFFFFFFFFFFFFFFFFFFFFFFFFFFFFFFFF
FFFFFFFFFFFFFFFFFFFFFFFFFFFFFFF:FFFFFFFF:FFFFFFFFFFFFFFFFFFFFFFFFFFF
FFF
@A00721:190:HGJW7DSXY:4:1539:8395:27461_1:N:0:TCTGCAAG+GCTGCACT
GGAGGCGATATTCGTTTTCATATCCAAAACTCCTTGAATGGAGTACATATTTGCAACCCTATTCTTGA
TGTGAAAGATGCATCGAGGTATACGTCTAGGAACATTGATTGGGAACGTACCTACTCACGAGACGTCA
TGA
+
FFFFFFFFFFFFFFFFFFFFFFFFFFFFFFFFFFFFFFFFFFFFFFFFFF:FFFFFFFFFFFF:FFFFF
FFFFFFFFFFFFFFFFFFFFFFFFFFFFFFFFFFFFFFFFFFFFFFFFFFFFFFFFFFFFFFFFFFFF
FFF
@A00721:190:HGJW7DSXY:4:2365:12536:7138_1:N:0:TCTGCAAG+GCTGCACT
ATGTTTCGTTCTGTTACAAGAACGCTTTGGATTGTGACCATAAAGCCTCAGTTGCGATAGCTGTAGAG
GTATACGATATCTCTCTTGATGATGTAGCCACTATAATGCTTAAGAGAGAGATTGATGTATGTCATTT
CGT

+
FFFFFFFFFFFF,FFFFFF,:FF:FFFFFFF:FFFFF,FFFF:,FFFFF:F:FFFFFF:F:FFFFFFF
FFFFFF:FFFFFFFFFFFFFFFFFFFFFFFFFF:FFFFFFFFFF,FFFFF,F:FFFFFFFFFFFFFF,
FFF
@A00721:190:HGJW7DSXY:4:2252:12500:26365_1:N:0:TCTGCAAG+GCTGCACT
GAATTATTGGACACGATTAAAAATGACATACCCGTTCCTGACCTTGGTATCGTTATCCGTCATGGAGA
TGATGGCGCATCACTTGATTACTATATGCCCGAAGGATTGGGTTATTCTCACAACAAAGATAAGATTT
TAT
+
FFFFFFFFFF:FFFFF:,F,F,FFFFFFF,FFFFFFF,FF:F,FFFFF:FFFFFFFF:FFFFFFFFFF
FFFFFFFFF:FFFFFFF,FF:FF:FFFFFFFFFFFFFFFFFFFFFFFFFFFFFF,FF,FFFFFF:FFF:
FFF
@A00721:190:HGJW7DSXY:4:2132:31539:17221_1:N:0:TCTGCAAG+GCTGCACT
TAAGGGCGGACAATTGTTCCATTGTGAACTCTTGGGGACTAGATTGAGCATTAATGAGTTCCGAATAA
CTCGTAATAAATCTTTCTCTAGTGGACAATTCAGAATACCTATTTCCGTACCAAGAGCCACTGCCAAT
CTC
+
FFFFFFFFFFF:F:F:F:FFFFFFFFFFFFFFFFFFFFFFFFFFFFFFFF:FFFFFFFFFFFFFFFFF
FFFFFFFFFFFFFFFFFFFFFFFFFFFFFFFFFFFFFFFFFFFFFFFFFFFFFFFFFFFFFFFFFFFF
FFF
@A00721:190:HGJW7DSXY:4:1115:11695:33927_2:N:0:TCTGCAAG+GCTGCACT
AAATTTTTCTCAAGAGATTATGGTTGATAAAGTTTTCTTCATAAGGGCTATTCAGTACGTCATGGGGA
TCACTTCTTCGGTCAATGACAAAACCTTCGAATATGTTACGACATGGC
+
FFFFFFFFFFFF::FFFFFFFFFFFFF:FFFFFFF:FFFFFFFFFFFFFFFFFFFFFFFFFFFFF,FFF
FFFFFFFF:FFFFFFFFFFFFFFFFFFFFFFFFFF:FFFFFFFFFF:F
@A00721:190:HGJW7DSXY:4:2326:14796:28494_2:N:0:TCTGCAAG+GCTGCACT
AGGATTGTCATCTCGGGGAAAGTGATTCACCAAGATGTTCATATAGATACGAGATACCTAGAGAAGGT
CGCAGCGATCTTACTCGTTATCGGAGCTAAGAAGAAGACTTTGGGTAC
+
FFFFFFFFFFFFFFFFFFFFFFFFFFFFFFFFFFFFFFFFFFFFFFFFFFFFFFFFFFFFFFFFFFFF
FFFFFFFFFFFFFFFFFFFFFFFFFFFFFFFFFFFFFFFFFFFFFFFF
@A00721:190:HGJW7DSXY:4:1371:30671:10050_2:N:0:TCTGCAAG+GCTGCACT
TAACGTTAGTACCGGAGGTGATCTCTCTGCTTCTGCGTTACTGAGAGAAAAGATTCAGGACGCCTACG
GTGACTTTATGGACAAGATGCAAGTTCTTGGGAAATACTTGTTTCCCT
+
FF,FFFFFFFFFFFFFFFFFFFFFFFFFFFFFFFFFFFFFFFFFFFFFFFFFFFFFFFFFFFFFFFFF
FFFFFF:FFFFFFFFFFFFFFFFFFFFFFFFFFFFFFFFFFFFFFFFF
@A00721:190:HGJW7DSXY:4:1129:21404:6872_2:N:0:TCTGCAAG+GCTGCACT
TCTTTGTATAGGGAGTGTGAGAAGACAACGAATCTTGAAACTACGGTACTTATCAAAGAAAATGGTGA
ACCGGTCTCTAAATCTTTTGAGATTGAGAAGATTGAAGAAAACGTCAA
+
:F:FF:::FFFFFFFFF:FF:FFFFFFFF:FF:F,FFFFFFFFFF,F,,F:F,FFFF:FF,,F,F,::
FFF:,,:F:F::FF:F::,:FFF,,:FFF,::,FF:::FF,FF::FFF
@A00721:190:HGJW7DSXY:4:1116:6578:35149_2:N:0:TCTGCAAG+GCTGCACT
TTGTTCTTGAAAACCAAGTTAAGAAGGAGGCTGATCATGGAGTCTTTGTACCTTCTGTCAAACTTCGA
GGTTCAGCTACGAGAGGTGATCCCGGATGTGGATTGAAGGCTGGCTCT
+
:FFFF:FFFFFFFF:FFFFF:FFFFFFFFFFFFFFFFFFFFFFFFFFFFFF:FFF:FFFFFF:FFF:FF
FFFFFFFFFFF:FFFFFFFFFF:FFF:FFFFFFFFFFFFFFFFFFFFF
@A00721:190:HGJW7DSXY:4:1214:14769:16673_2:N:0:TCTGCAAG+GCTGCACT
CTTTTTGAATGTTGTAAAGAATTGGGTTGAGAAGTGTGCTTTACAAGGAGTTAGACTCTTCTTCAAGT
TTTTAGATCTTCTTGATAATCTTAAAGAAAAATTTTCGAATAAACTAA
+

```
FFFFFFF:FFFFFFFFFFFFFFFFFFFFFFFFFF,FFFFFFFFFFFFFFFFFFFFFFFFFFFFFF:FF
FFFFFFFFFFFFFFFFFFFFFFFFFFFFF,FFFFFFFFFFFFFFFFFFF
@A00721:190:HGJW7DSXY:4:2559:9191:16282_2:N:0:TCTGCAAG+GCTGCACT
CTGTACTTACATTCTTTACGCCAGATTCTTCATGGGGCGACGCTGTTTCTCTTGGTATTTCTGAAATA
GTTTATCAGAGCGTCATTTTCCTTTGGGATCTTTCCTCTGGTAAGGGA
+
FF:FFFFFFFFFF:FFFFFFFFFFFFFFFFFFFF:FFFFFFFFFFFFFF:FF:FFFFF:FFFFFFFFF
FFFFFFFFFFFFFFFFFFFF:,FFF,FFFFFFF:F::FFF:FFFF:FF
@A00721:190:HGJW7DSXY:4:1268:16839:9142_1:N:0:TCTGCAAG+GCTGCACT
ACGAGAATTATTACTGTTTTCTCTACCCATAACTTCTTTTTCAAAAATGCGGATGGGTCTACTGATGT
TTTCCTACAGAGTGCAGTCGTTTCAACACTTATCACCACTTTTGAAAGGAAAATAATTGGTGATGAAA
ATA
+
FFFFFFFFFFFFFFFFFFFFFFFFF,,FFF:FFFFFF:FFFFFFF:FFFFFFFFFFFFFFF:FFFFFF
FFFFFFFFFFFFFFFFFFFFFFFFFF:F:FFFFFFFFFFFFFFFFFFFFFFFFFFFFFFFFFFFFFFF
FFF
@A00721:190:HGJW7DSXY:4:1646:2844:3630_2:N:0:TCTGCAAG+GCTGCACT
GGGTTCTCAAGGCTCTCATCGCCACTGGGATTCTAAGGGGTTCCATAAGATATTATCTGACATTGTGG
TTCAATGATCAGGATTCTTCTTTTGGTCCTTACGTCTTAAGTAATTTG
+
FFFFFFFFFFFFFFFFFFFFFFFFFFFFFFFFFFF:FFFFFFFFFF:FFF:FFFFFFFF:FFFFFFF
FFFFFF:FFF:FFFFFFFFFF,FF:FFF,FF:F:FFFFFFF,FFFFF:
@A00721:190:HGJW7DSXY:4:1228:5801:7341_1:N:0:TCTGCAAG+GCTGCACT
TCTTGTCACGATTCTTAAAAGAAGTTTATTAAAACTTCAGAGTCTCTTGGAAGTCCAGGTAGCTAATG
TTCTTGCAATAGCCACAGACTTTCTGGGAAAAGAGACCTCTGTTGGTAGAGGAGTTGAGAGAATTACT
CAG
+
FFFFFFFFFFFFFFFFFFFFFFFFFFFFFFFFFFFFFFFFFFFFFFFFFFFFFFFFFFFFFFFFFFFF
FFFFFFFFFFFFFFFFFFFFFFFFFFFFFFFFFFFFFFFFFFFFFFFFFFFFFFFFFFFFFFFFFFFF
FFF
@A00721:190:HGJW7DSXY:4:2415:15474:35775_1:N:0:TCTGCAAG+GCTGCACT
ACGTCTACAACGAGTTAGGGAGTGGCTAGGTTTTAAAAGATCCTTTGCACAAGCAATACCTTCCACTG
AGTTCATCACCCAGAGTTTCGACGTCGCGGAAAACGATGAAGATTTCCACACTGTTCGAGAATCTTCT
GAT
+
FFFFFFFFFFFFFFFFFFFFFFFFFFFFFFFFFFFF:FFFFF:FFFFFFFFFFFFFFFFFFFFFFFFF
FFFFFFFFFFFFFFFFFFFFFFFFFFFFFFFFFFFFFFFFFFFFFFFFFFFF:FFFFFFFFFFFFFFF
FFF
@A00721:190:HGJW7DSXY:4:2274:16984:11271_1:N:0:TCTGCAAG+GCTGCACT
TGTGCTCTTCCGATCTCCTGATGGCTCAGTACAGGGAGGAGGTAATAGTTATAAAACCTTCTTTTATC
ATTGCAACAGGACTTTGAGAAGACTGTTGAAATGGACAAAATCTGGGATCATAGGTATGTGTCACGCT
ACA
+
FFFFFFFFFFFFFFFFFFFFFFFFF:FFFFFFFFFFFFFFFFFF:FFFFFFFFFFFFFFFFFFFFFFFF
FFFFFFFFFFFFFFFFFFFFFFFFFFFFFFFFFFFFFFFFFFFFFFFFFFFFFFFFFFFFFFFFFFFF
FFF
@A00721:190:HGJW7DSXY:4:1501:1380:10363_2:N:0:TCTGCAAG+GCTGCACT
GTTGGCGTCTTGTCGTTGACGTCATTACAGTCTGGAAGTTAGATCATTTGTTCAGGAAGATCTCTACA
GAGATTATCGAGAGTTGTGATCTAGTTCTAAATTTAGGTCGACGGACT
+
FFFFFFFFFFFFFFF:FFFFFFFFFFFF:FFFFFFFFFFFFFFFFFFFFFFFFFFFFFFFFFFFFFFF
FFFF:FFFFFFFFFFFFF:FFFFFFFFFFFFFFFFFFFF:FF,F,FFF
@A00721:190:HGJW7DSXY:4:1570:24840:11162_2:N:0:TCTGCAAG+GCTGCACT
ATGTACGTGGATCTAATGCTTGGTGATCTGAATCTGACCATCGCAAAGAAGACTTTATTAGTTCATGA
```

AGTTCTTACACTTCTCGCGACTAATGTCAGCTTCACTACAAAGGCGTT
+
FF:FFF,FFFFFFFFFFFFF,,FF:FFFF:FFFFFFF,:F,:FFFFF,:FFFFF:,FF:F,F::FF,F
FFFF:F:F,F:F::F,,:,FF,FF:,FFF,:,FFFF:F,:FF,,::F,
@A00721:190:HGJW7DSXY:4:2410:31964:14575_1:N:0:TCTGCAAG+GCTGCACT
GATCTTGCAGAGTGACTGGAGTTCAGACGTGTGCTCTTCCGATCTGAAGCTCGCCATATCAGATTAGA
GACATGCCAGTTAGAGTTCTTCTAACCAGGACTGATGGTGAGATTCTTTTACCTTTTGGAGGTCCAGG
AAC
+
:FF:FFFFFFF:FFFFFFFFFFFFFFFFFFFF::F:FFFFFFFF::FFFFF:::FFFFFFFFFFFFFF
:F:,FFF,:,FFFFFFFFFFF,FFFF,F:FFFFFFF:FFFFFFFF,FFFFFFFFF:FFFFF:FFFFFF
FFF
@A00721:190:HGJW7DSXY:4:1111:19343:12665_2:N:0:TCTGCAAG+GCTGCACT
CCAAGGACGTTTCCGTCGGAGGATGACAGGTGGGAGACCGCTCAATAACACTCTATTGAATCCCTTTT
CCACGATGAAAGCTCTTTATGAGGTGCTCCATGCAAATTTACAGTTCC
+
FFFFFFFFFFFFFFFFFFFFFFFFFFFFFFFFFFFFFFFFFFFFFFFFFFFFFFFFFFFFFFFFFFFF
FFFFFFFFFFFFFFFFFFFFFF:FFFFFFFFFFF:FFFFFFFFFFFF
@A00721:190:HGJW7DSXY:4:1221:13349:17065_2:N:0:TCTGCAAG+GCTGCACT
TTCTCCTTTAATCGTGAGGTTACGAGAGTTTCGTACTTGGGTTTATCGGTTGTATTTTGATAAATTTG
TTGGAATCGATAATGATACTGACGAAGGTTCTATTACCACCCTTGTCA
+
FFFFFFFFFFFFFF:FFFFF:FFFFFFFF:FFFFFFFFFFFFFFFFFFFFFFF:FFFFFFFFFFFFFF
FFF,FFF::F:FFFFFFF:FF,FFFFFFFFFFFFFF::FFF,FFFFFF
@A00721:190:HGJW7DSXY:4:1675:12436:35806_2:N:0:TCTGCAAG+GCTGCACT
TGATGCTTTCTTAGAGGCTCGTCTAGCCGCTGAGATTCGTCTTACCTATTTACAATCTGTAGAGAGAG
AGGTTAATCAGAATTTGAGTGATAACGATTTAGTACTGGGAAATGGTG
+
FFFFFFFFFFFFF:FFFFFFFFFFFFFFFFFFFFF:FFFFFFFFFF,FFFFFFFFFF:,FF:FFFFFF
:F:FFFFFFFFFFFFFFFFFFF:FFF,F,FFFFFFFFFFFFF:F,FF
@A00721:190:HGJW7DSXY:4:1156:24008:22874_1:N:0:TCTGCAAG+GCTGCACT
CCTTTTTCGATTGGATTCGACGGGGTCTTCGTCTCACGACTAATTTGATAAGGTTTTTCTTTTATGGG
ACTCTTGTTTGTACTTATGAAGGAGGTATTACTTCGGAAGTTCTTAATGTAGGTCTCCTAGTACTTAA
GGG
+
F:F:FFFFFFFFFFFFFFFFFFF:FFFFFFFFFFFFFFF:FFFFFFFFFFFFF:FFFFFFFFFFFFFF
FFFFFFFFFFFFFFFFFFFFFFFFFFFFFFFFFFFFFFFFFFFFFFFFFFFFFFFFFFFFFFFFFFFF
FFF
@A00721:190:HGJW7DSXY:4:1374:3721:4805_2:N:0:TCTGCAAG+GCTGCACT
GTTTCAGCTGGATGGATCTTTTGATGGTATGTTTCACATTGCGTGATCACTTCCCGTGGACTTGGGAA
GAGATTAAACATAAAATCCAGCGATTCTATTATGTCGCTAAAGCCTGT
+
FFFFFFFF:FFFFFFFFFFFFFFFFF:FFFFFFFFFFFF:FFFFFFFFFFFFFFF:FFFFFFFFFFF
FFFFFF:FFFFFFFFFF::FF:F:F,FFFFFFFF:F,FFFFF,:FFFF
@A00721:190:HGJW7DSXY:4:1306:4408:3521_2:N:0:TCTGCAAG+GCTGCACT
GTTCGGGCTGTGGGGATTAACTTCCCTCGTGAAACTTACGACGATGTTCTCGCTCGCATTTCACAAGG
ATTACTAAGTCTGAGATCTGCCACTTCTAATTTTAGGAAACGTCGCGA
+
FFFFFFFF:FFFFFFFFFFFFFFFFFF:FFFFFFFF:FFFFFFFFFFFFFFFFFFFFFFFFFFFFFFF
FFFFFFFFFFFFFFFF:FFFFFFFFFF:FFFFFFF:FFFFFFFFFFFF
@A00721:190:HGJW7DSXY:4:1542:4200:15530_2:N:0:TCTGCAAG+GCTGCACT
ATGAGGATGTCTTGGTCGAAAAATATCGCCGGGAACTTCTAATAGAGGATCCTGATGACATTATTTCA
ACGGATACCGATCTCAGTTCTACCACGGAAACCGTCTCAGGAACTGTC
+

```
FFFFFFFFFFFFFFFFFFFFFFFFFFFFFFFFFF,FFFFFFFF:FFFFFFFFFFFFFFFFFFFFFFFF
FFFFFFFFFFFFFFFFFFFFFFFFFF,FFFFFFFFFFFFFFFFFFFFF
@A00721:190:HGJW7DSXY:4:1343:26494:22232_1:N:0:TCTGCAAG+GCTGCACT
GGAATTAGAGAAAGACGACCTTCTTGTGGGGACTGAGACCTCTGAATTTTCAGGGTGCCCTGATGAGG
ACGTTGAAACGGATAATTTCAACTCTGAGACTTCTAAGAGTGTTTCAAAAGACCAGAAACGTAACAAG
AAG
+
FFFFFFFFFFFFFFFFFFFFFFFFFFFFFFFFFFFFFFFFFFFFFFFFFFFFFFFFFFFFFFFFFFFF
FFFFFFFFFFFFFFFFFFFFF:FFFFFFFFFFFFFFFFFFFFFFFFFFFFFFFFFFFFFFFFFFFFFF
FFF
@A00721:190:HGJW7DSXY:4:1378:13865:10285_2:N:0:TCTGCAAG+GCTGCACT
GGAAGGGGAGAAAACAGAACAAGCGAGCGGTTGAAGAGAAAACTGATATGGTTGCGGCTGATCGTACC
CCAATTCCTGCTAGAAAGATCACTCTCGGCGATACCACTGATCTTCCA
+
FFF:FFFFFFFFFFFFFFFFFFFFFFFFFFFFFFFF:FFFFFFFFFFFFFFFFFFF:FFFFFFFFFFF
FFFFFFFFFFFFFFFFFFFFFFFFFFFFFFFFFFFFFFFFFFFFFFFF
@A00721:190:HGJW7DSXY:4:1659:31991:17159_1:N:0:TCTGCAAG+GCTGCACT
GGTCGTTCTTTTGGTGATTTTATCAAAGAGAAAGTTTCTGCTAGTCGCGACGATAGTATTTATCGGAG
TGATATTAACAAGTTAAACTTCGAACTGACCACTTACAAGAGTGTTGAAGTACCGCAACATGGATCAG
TTT
+
FFFFFFFF,FFFFFFFFFFFFFFFFFFFFFFFFFFFFFFFFFFFFFFFFFFFFFFFFFFFFFFFFFFF
FFFFFFFFFFFF:FFFFFFFFFFFFFFFFFFFFFFFFFFFFFFFFFFFFFFFFFFFFFFFFFFFFFF
FFF
@A00721:190:HGJW7DSXY:4:2210:21748:28573_2:N:0:TCTGCAAG+GCTGCACT
AAACTGAATCCACTCTCTTGGGATATAAATGAGTACATCTATAATAGAGTTTTGGAGCTGTGTAATGT
CCTACGTACTGTTCAAGGTGTTTGGGCTTCTCTAAAGAACAGCAACTT
+
FFFFFFFFFFFFFFFFFFFFFFFFFFFFFFFFFFFFFFFFFFFFFFFFFFFFFFFFFFFFFFFFFFFF
FFFFFFFFFFFFFFFFFFFFFFFFFFFFFFFFFFFFFFFFFFFFFFFF
@A00721:190:HGJW7DSXY:4:2417:18584:4773_2:N:0:TCTGCAAG+GCTGCACT
AGACCTTTCTTGGGGAGTAAAGAGGGTTTGAAAATTACGGAACCTTTCCTCGTTCTTCATGAAGATCT
TCGAGTGTTTTATAGTGAGTTATTCTTGCGAAGGTTCAAGTCGATTGA
+
FFFFFFFFF:,FFFFFFFFF,FFFFFFFFFFFFFFFFFFFFFFFFFFFFFFFFFF,FFFFFFFFFFFF
FFFFFFFFFFFFFFFFF,FFFF:FFFFFFFFFFFFFFFFFFFFFFFFF
@A00721:190:HGJW7DSXY:4:1521:21630:10582_2:N:0:TCTGCAAG+GCTGCACT
AATAAGAAGTTCTTCGCAGTTGAATCTCCACCAGGTGGAGGGAAGACCACTGAACTTATAGCGACTTT
TGTCGAATTTTTCGCAAAAGGTCTTGAAGTACGTATTGTCACTGCTAA
+
F,FFFFFFFFFFFFFFFFFFFFFFFFFFFFFFFFFFFFFFFFFFFFFFFFFFFFFFFFFFFFFFFFFF
FFFFFFFFFFFFFFFFFFFFFFFFFFFFFFFF:FFFFFFFFFFFFFFF
@A00721:190:HGJW7DSXY:4:1526:25265:1814_2:N:0:TCTGCAAG+GCTGCACT
TTGGCACTTCTAGTTCATCGCGGTATTTGTGAAATTAAGACAGACGTGAAAGTTATGATGAGTGATCG
TATTAGAACAGCTGATTCTTATTGCATGAACGTCGCTGAGGTCAAACC
+
FFFFFFFFFFFFFFFFFFFFFFFFFFFFFFFFFFFFFFFFFFFFFFFFFFF:FFFFFFFFFFFFFF:F
FFFFFFFFFFFFFFF,FFFFFFF:FFFFFFFFFFFFFFFFFFFFFFFF
@A00721:190:HGJW7DSXY:4:2339:18756:23046_2:N:0:TCTGCAAG+GCTGCACT
TTTTCATCGACGAATTATTCATGATTCACGCTGGTCAGACCCTTTTTCTTACACATCTACTTAAGGCG
GCTTATACCATAGGTTACGGTGATTCCAATCAAATCGGATTCATCAAT
+
FFFFFFFFFFFFFFFFFFFFFFFFFFFFFFFFFFFFFFFFFFFFFFFFFFFFFFFFFFFFFFFFFFF:
FFFFFFFFFFFFFFFFFFFFFFFFFFFFFFFFFFFFFFFFFFFFFFFF
```

@A00721:190:HGJW7DSXY:4:1470:32832:11663_2:N:0:TCTGCAAG+GCTGCACT
TCCAAGTCGCAAACTACTTCAACATCAACGACATCATTCTTGATGAGTGTCGTTCTTATCGAAGTCTC
AGTTATCGCTGCCCGGCCGACGTTTGTCGCGAACTCTCAGAGGTTTAC
+
FFFFFFFFFFFFFFFFFFFFFFFFFFFFFFFFFFFFFFFFFFFFFFFFFFFFFFFFFFFFFFFF:FF:FF
FFFFFFFFFFFFFFFFFFFFFFFFFFFFFFF:FFFFFFFFFFFFFFFF
@A00721:190:HGJW7DSXY:4:1627:10818:11678_2:N:0:TCTGCAAG+GCTGCACT
CTATGGAAATTAGACCTATCAGATGTCTTGACGACTTCGAATTTGAGGACGGAGTTAAATATATTACT
TTCACTCAAGGTGAAAAGTTTGAAATTAACAAAGTCCTTGGTAAGAGT
+
FFFFFFFF:FFFFFFFFFFFFFFFFFFFFFFFFFFFFFFFFFFFFFFFFFFFFFFFFFFFFFFFFFFF
FFFFFFFFFFFFFFFFF:FFFFFFFFFFFFFFFFFFFFFFFFFFFFFF
@A00721:190:HGJW7DSXY:4:2373:3387:8234_1:N:0:TCTGCAAG+GCTGCACT
GTTCAAGGTAATACTTACCAGCACGTTCGGCTGGTTAGAATTAAACCTCAAGATGACCCTGTTTTTGC
ATCACAAACACATATTACCGTTGCTCTATCACGACACGTTGAATCTTTACGATACTTTGTTATCAATG
TAA
+
FFFFFFFFFFFFFFFFFFFFFFFFFFFFFFFFFFFFFFFFFFFFFFFFFFFFFFFFFFFFFFFFFFFF
FFFFFFFFFFFFFFFFFFFFFFFFFFFFFFFFFFFFFFFFFFFFFFFFFFFFFFFFFFFFFFFFFFFF
FFF
@A00721:190:HGJW7DSXY:4:1438:11984:22435_2:N:0:TCTGCAAG+GCTGCACT
AAGGAACTGTCACGTTTAGCACGGGAGGTTGCAGATTCAACCCCTTATATCGAGGCTATCGAGGACGA
TGAGTATCTTGAAGTTGTTGACGACAAGCCTCAAGAGGGTAGAGGACC
+
FFFFFFFFFFFFFFFFFFFFFFFFFFFFFFFFFFFFFFFFFFFFFFFFFFFFFFFFFF:FFFFFFFFF
FFFFF,FFFFFFFFFFFFFFFFFFFF,FFFFFFFFFFFFFFFFFFFFF
@A00721:190:HGJW7DSXY:4:2374:1090:10019_2:N:0:TCTGCAAG+GCTGCACT
GGTTATTCCTGGAACCACCTTAACGTCACTCGGTAATCTTTCAGCCGAATTAAGTACTAATGAGTTCA
AGTCGGTTGTCGATCGAATTAAGATCAAGGCTAATACCTTTATAGGTA
+
FFFFFFFFFFFFFFFFFFFFFFFFFFFFFFFFFFFFFFFFFFFFFFFFFFFFFFFFFFFFFFFFFFFF
FFFFFFFFFFFFFFFFFFFFFFFFFFFFFFFFFFF:FFFFFFFFFFFF
@A00721:190:HGJW7DSXY:4:1271:24433:18223_1:N:0:TCTGCAAG+GCTGCACT
TCATCAGTCAGAGTATACCCGATAGGGTACCGAGTCTCCAAGAGAATATTTATTCTTATGAGGCTCGA
AACTTCAACGTTTTCACAACGGAAAGACATATCGCACCCGAAATTTTTGGGCAGAATGCTCTTAACGC
GGT
+
FFFFFFFFFFFFFFFFFFFFFFFFFFFFFFFFFFFFFFFFFFFFFFFFF:FFFFFFFFFFFFFFFFFF
FFFFFFFFFFFFFFFFFF:FFFFFFFFFFF,FFFFFFFFFFFFFFFFFFFFFFFFFFFFFFFFFFFFF
FFF
@A00721:190:HGJW7DSXY:4:1213:1280:2174_2:N:0:TCTGCAAG+GCTGCACT
GATCAGTCTAAGTTGGATAAAGTTATAGGAGACTCAGTTAACCTGAGAGCTCCTCAATTGATAGATTG
GGTCAACAATAGGGACGCTAGTATACTTCGTAAAGTCGACAGCGAAAT
+
FFFFFFFFFFFF:FFFFF,FFFFFFFFFFFFFFFFFFFFFFFFFFFFFFFFFFFFFFFFFFFFFFFFF
FFFFFFFFFFFFFFFFFFFFFFFFFFFFFFFFFFFFFFFFF:FFFFFF
@A00721:190:HGJW7DSXY:4:1143:31684:10989_2:N:0:TCTGCAAG+GCTGCACT
TAATTGGACCGACTATATGACCTATTTTAAGCTCATGGTGAAAAGGGAAGCTAAGGTTAAACTTGATG
CTTCAGCCTTTTCGAAGCATAACCCCGCACAGAATATCGTTTATCACG
+
FFFFFFFFFFFFFFFFFFFFFFFFFFFF:FFFFFFFFFFFFFFFFFFFFFFFFFFFFFFFFFFFF:FFF
FF,FF:FFFFFFFFFFFFFFFFFFFFFFFFFFFFFFFFFFFFFFFFFF
@A00721:190:HGJW7DSXY:4:1259:5918:29027_2:N:0:TCTGCAAG+GCTGCACT
CCTATCTTTAAGGTTCTTGTGAGGAGAGTCAAACAGTGTCTTTTACCCAACATTTTTATGTATACAGA

GATGACCAGTGACGATTTCTCCCGGAAGCTCTTCTCTCTCTTAGGAGG
+
FFFFFFFFFFFFFFFFFFFFFFFFFFFFFFFFFFFFFFFFFFFFFFFFFFFFFFFFFFFFFFFF:FFFFF
FFFFFFFFFFFFFFFFFFFFFFFFFFFFFFFFFFFFFFFFFFFFFFFF
@A00721:190:HGJW7DSXY:4:2207:27263:35336_2:N:0:TCTGCAAG+GCTGCACT
TCAAGATTTCTTTTGTAAAGCCTATGAGATAAAGATGTATTCTTATTTAGGCTTCGATGCTGAATTTC
TTGAACTTTGGAGTGCCAGTGAATATTTTTGTCACGCCAGGAGTATCC
+
FFFFFFFFFFFFFFFFFFFFFFFFFFFFFFFFFFFFFFFFFFFFFFFFFFFFFFFFFFFFFFFFFFFFFF
FFFFFFFFFFFFFFFFFFFFFFFFFFFFFFFFFFFFFFFFFFFFFFFF
@A00721:190:HGJW7DSXY:4:1220:13602:33786_1:N:0:TCTGCAAG+GCTGCACT
ATTTACCACCTTTGCTCAACGACGAAGTGGAACGGCCAACACTTTCTTTGGTAATTGTATGATCACTT
TGATGATTTTATCACTTTATTATGACCTTGGAAAGTTTTCGGCTGTGGTTGTCGCTGGGGACGATTCC
ATT
+
FFFFFFFFFFFFFFFFFF,FFFFFFFFFFFFFFFFFFFFFFFFFFFFFF,FFFF:FFFFFFFFFFFFFF
FFFFFFFFFFFFFFFFFFFFFFFFFFFFFFFFFFFFFFFFFFFFFFFFFFFFFFFFFFFFFFFFFFFFFF
FFF
@A00721:190:HGJW7DSXY:4:2210:28646:35383_2:N:0:TCTGCAAG+GCTGCACT
AGACTCCAATAACTGATCATTCTGATCATATGATTTTGGACAATGGTTTCGAAGCGAAATTTTTGAGG
GATAAACCAGCGTACTTCTGCTCGCGATTTATTCTTTTTGTCAATGAT
+
FF,FF:FFFFFFFFFFFF,,FFFF,FFF:FFFFFFFFFFFFF:FFFFFFFFFFFFFF,F:FFFFFFFF
FFFFFF:F,FFFFF,F,FFF,FF:FFF:,F:FF:FFFFFFFFFFFFFF
@A00721:190:HGJW7DSXY:4:1646:26404:12680_2:N:0:TCTGCAAG+GCTGCACT
CCTGATCCTTACAAGATCATCGTTAAGTTAGGTAAACCCCTAACCGATGTTACTCTTGTAGGACTTTG
GGAGAGATTCGTTAGTTTTAAAGACTATACTAAATATCTCCACTACGA
+
FFFFFFFFFFFFFFFFFFFFFFFFFFFFFFFFFFFFFFFFFFFFFFFFFFFFFFFFF::FFFFFFFFFFF
FFFFFFFFFFFFFFFFFFFFFF:FFFFFFFFFFFFFFFFFFFFFFFF:FF
@A00721:190:HGJW7DSXY:4:1220:1208:34898_2:N:0:TCTGCAAG+GCTGCACT
GCGACTAATATTCGATATTCGATTGAGGGTCGTTATACCTATTCGGCTATCTGTGCTTTGAATTGTAT
AAGATCAAATTTTAAACGATTCCGAGAGGTTTATCCTGATGTGGAGGG
+
FFFFFFFFFFFFFFFFFFFFFFFF:FFFFFFFFFFFFFFFFF:FF:F:FFF:F::FFF:FFF:FFFFFF
FFFFFFFF,FF,F,F,FFFFFFFFFFFFFFFFFFF:FFFFFFFFFFFFF
@A00721:190:HGJW7DSXY:4:1467:22516:16814_1:N:0:TCTGCAAG+GCTGCACT
ATCCCGACCTTTCCTTGTGAAAATTTTGATGCGGTTTCATAAGATTATCAGTAATCCTGATTCTACTA
CGGATTCATGGACCTTCCATCCTGTACCTCTTCTGGAGAGACGTATCTCAAAGATCGACTTCGAGACA
GGT
+
FFFFFFFFFFFFFFFFFFFFFFFFFFFFFFFFFFFFFFFFFFFFFFFFFFFFFFFFFFFFFFFFFFFFFF
FFFFFFFFFFFFFFFFFFFFFFFFFFFFFFF:FFFFFFFFFFFFFF:FFFFFFFFFFFFFFFFFFFFFFF
FFF
@A00721:190:HGJW7DSXY:4:1141:14073:19194_2:N:0:TCTGCAAG+GCTGCACT
GCGAGAGCGACCAAGATCTAAATTCGGTAACGGTGCGTGGCGCTTTATTTTAAAAAGCTCCCTCCTCT
CGCAAGTTTGATCTGATACTTATCACGAAAGGACTTGAGTCTTTTGAT
+
FFFFFFFFFFFFFFFFFFFFFFFFFFFFFFFFFFFFFFFFFFFFFFFFFFFFFFFFFFFFFFFFFFFFFF
FFFFFFFFFF:FFFFFFFFFFFFF:FFFFFFFFFFFFFFFFFFFFFFFFF
@A00721:190:HGJW7DSXY:4:1146:4652:14904_2:N:0:TCTGCAAG+GCTGCACT
TCTTTCATTTTAAATGACGATCATTAAGACTCTCTTTCTTCTTTGTTTCCTTCTTTGCACTGATTTTT
TCCTCATCAGCTTGTGTTTTACCTAATCTGTGTTTTTACGATAACAAC
+

FFFFFFFFFFFFFFFFFFFFFFFFFFFFFFFFFF:FFFFFFFFFFFFFFFFFFFFFFFFFFFFFFFFF
FFFFFFFFFFFFFFFFFFFFFFF:FFFFFFFFFFFFFFFFFFFFFFFF
@A00721:190:HGJW7DSXY:4:1113:6515:12493_2:N:0:TCTGCAAG+GCTGCACT
TCTTCCGAAAGGCGAACTTTGGGAATTTGAAGCTCAGGGTGAAGCACAACGTTTGACCGTGACGAACT
GTGGTGTTCTTGATCTACGTTACGGATATACTCTCCTTGAGTTCACTC
+
FF:FFFFFFFFFFFFFFFFFFFFF:FFFFFFFFFFFFFFFFFFFFFFFFF:FF,FFFFFFFFFFFFFF
FFFFFFFFFFFFFFFFFFFFFFFFFFFFFFFFFFFFFFFFFFFFFFFF
@A00721:190:HGJW7DSXY:4:1144:23375:20713_1:N:0:TCTGCAAG+GCTGCACT
CACTTATTACGATATTTCTTTAGTCGATGGGTTTTCGACGCCTTTAAGCGTCTTCTGTTCATCGCAAC
CAGATGATATCTTCTATTCGAACTTATGTTTCGATCAGTGTTTCTTCTATCAGGAGGAAAACACTTGT
TCT
+
FFFFFF:FFFFFFFFFFFFFFFFFFFFFFFFFFFFFFFFFFFFFFFFFFFFFFFFFFFFFFFFFFFFF
FFFFFFFFFFFFF:FFFFFFFFFFFFFFFFFFFFFFFFFFFFFFFFFFFFFFFFFFFFFFFFFFFFFF
FFF
@A00721:190:HGJW7DSXY:4:1203:24044:32111_2:N:0:TCTGCAAG+GCTGCACT
TCTTCTAATGTGGCCTGAACTACGTTTCACTTTGTGTGTACTTGTCTCTATTTTAAGTTTTGTCTTCC
TTGTTTGTCTTATTCTATTAATCACTCATTTTAGTGAGATTATTAAAA
+
FFFFFFFFFFF:FFFFFFFFFFFFFFFFFFFFFFFFFFFFFFFFFFFFFFFFFFFFFFFFFFFFFFFF
FFFFFFFFFFFFFFFFFFFFFFFFFFFFFFFFFFFFFFFFFFFFFF:F
@A00721:190:HGJW7DSXY:4:1477:22860:6668_1:N:0:TCTGCAAG+GCTGCACT
TCTTCGACAACAGCAGGGGTTTTCGAATAGTGATAGCCACCCGCCTTACAGAGGGGCAGCAGCATGAC
CATAATTGGTATTGACTTTGGTACCACCTTTTCTACTGTCTGTCTCGCCTCTTCGAATGAAGTTTTAA
TGC
+
FFFFFFFFFFFFFFFFFFFFFFFFFFF:FFFFFFFFFFFFFFFFFFFFF,FFFFFFFFFFFFFFFFF:
FFFFFFFFFFFFFFFFFFFFFFFFFFFFFFFFFFFFFFFFFFFFFFFFFFFFF,FFFFFFFFFFFFFF
FFF
@A00721:190:HGJW7DSXY:4:1510:18114:8437_2:N:0:TCTGCAAG+GCTGCACT
CTACAATCATTGGTTTTTCTAATAATGATGACACTATTGTTTATGGATATGATGCTATTACTCATGAT
GGTTACGGTCAGGGAGGATTCTCAGTTTACAAGGACCTTAAGCGATGG
+
FFFFFFFFFFFFFFFFFFFFFFFFFFFFFFFFFFFF,FFFFFF:FFFFFFFF:FFFFFFFFFFFFFFF
F:FFFFFFFFFFFFFFFFFFFFFFFFFFFFFFFFFFFFFFFFFFFFFF
@A00721:190:HGJW7DSXY:4:1468:24930:6746_2:N:0:TCTGCAAG+GCTGCACT
CCGAAAGACGTGCTAAATTAAAACCATTGTACGATGTCGTCTGTCTCGGCGATCACTTTTCACTAAGA
ATTGGTCCTACTTTTGGACAAGGCCAACTTCGTAATCTTCATGAGATC
+
FFFFFFFFFF:FFFFFFFFFFFFFFFFFFFFFFFFFFFFFFFFFFFFFFFFFFFFFFFFFFFFFFFFF
FFFFFFFFFFFFFFFFFFFFFFFFFFFFFFFFFFFFFFFFFFFFFFFF
@A00721:190:HGJW7DSXY:4:2628:20365:30029_2:N:0:TCTGCAAG+GCTGCACT
TATCTCTTTGGTTGTTAGGGATTTTGAGAGAGTGAAAGGCTTCAATTGTAGTGGTTTGGTCGTTTCAG
TACCCAGTCAGTATACCTCTACTCAAAGATATTTCATGAAAACTACAG
+
FF:FFF,::FFFF:FFFF::F:F,FFF:FFF:FFFFFF,,,F:FFFFFFFFF:F:FFFFFFF,FFF:F
,FF:,FFFFF::F:F,F,FF,:,FFFF:FFFF,F:F:FFFFF,FF,FF
@A00721:190:HGJW7DSXY:4:2303:7166:23171_1:N:0:TCTGCAAG+GCTGCACT
ACCTGTCTTTCATATTATGAATGAACCTTCGGCCGCTCTCTTTGCTTCGATGTTGGATGTCAAGAAGG
GTTCAGAATGGGATTCCTACGTAGTTTATGACTTTGGTGGTGGTACTTTCGATACTTCTATCGTCTTA
CGT
+
:FFFFFFFFF:FFFFFFFFFFFFFFFFFFFFF:FFFFFF,F:FFFFFFFFFFFFFFFFFFFF:FFFFF

FFFFFF:FFFFFFFFFFF,FFFFFFFFFFFFFFFFFFFFFFFFFFFFFFFFFFFFF:FFFFFFFFF:
FFF
@A00721:190:HGJW7DSXY:4:1477:18909:16329_2:N:0:TCTGCAAG+GCTGCACT
AGTGTTATTTATTCTGGTGGTGATGATAATCTTGGAGGTCGTGACGTTGATGAAGCTATAAGAGCGTT
CTTATTACGGCGCACTTCTTTACCCGCCACCACACAACTTTCAGTTTC
+
:FFFFFFF:FFFFFFFFFFFFFFFFFFFFFFFFFFFF,FFFFF:FFF:FFFFF:FFFFFFFFF:FFFF
FFFFF:FFFFFFFFFFFFFFFFFFFFFFFFFFFFFFFFFFF:FFFFFF
@A00721:190:HGJW7DSXY:4:1427:7618:15436_1:N:0:TCTGCAAG+GCTGCACT
ACTGGAAAACCCTCGTCAATACTTTTCAATAATATACGTCTTGAACTCACTTATGACGATCTAGTGAA
ATTAACGCGTCCTTTTCTGGAACGTAGTTTTACTATTATGAACAAAGTTATCAAAGATAGTGGCATTC
AAG
+
FFFFFFFFFFFFFFFFFFFFFFFF:FFFFF:FFFFFFF:FFFFFFFFFFFFFFFFFFFFFFFFFFFFF
FFFFFFFFFFFFF:FFFFFFFFFFFFFFFFFFFFFFFFFFFFFFFFFFFFFFFFFFFFFFFFFFFFFF
FFF
@A00721:190:HGJW7DSXY:4:1655:1904:17347_1:N:0:TCTGCAAG+GCTGCACT
AGATCTTGCAGAGTGACTGGAGTTCAGACGTGTGCTCTTCCGATCTCTTGCTGAAACAGCTATTAAAG
CTGTTGAGCGGGTAATTACACCTAAACGAGCTCGATCTGCCATCGCTGAAGGATGTGCACTCTTTTCT
GCT
+
:FFFFFFFFFFFFFFFFFFFFFFFFFFFFFFFFFFFFFFFFFFFFFFF,FFFFFFFFFFFFFFFFFFFF
FFFFFFFFFFFFFFFFFFFFFFFFFFFFFFFFFFFFFFFFFFFFFFFFFFFFFFFFFFFFFFFFFFFF
FFF
@A00721:190:HGJW7DSXY:4:1472:30996:1063_2:N:0:TCTGCAAG+GCTGCACT
TCGATTGTATCAACAGTAACATTACTTCTAATAAAGGTCTCTATCAAGCTCGTATTATCGTCGCTGCT
GGATCTGCGTTACCGTTGAAGGCTAAACTCCAAGGTAATTATGAAGAC
+
FFFFFFFFFFFF:FFFFFFFFFFFFFFFFFFFFFFFFFFFFFFFFFFFFFFFFFFFFFFFFFFFF:FF
FFFFFFFFFF:FFFF,FF:FFFFFFFFFFFFF,FFFFFFFFFF:FFF:
@A00721:190:HGJW7DSXY:4:1304:7853:35164_1:N:0:TCTGCAAG+GCTGCACT
GTATGAGGGTGTTTCTAATCGTAACATTAATAATTTCAATCTTCTTAAAAAGAAGATAGAGAGTCATA
CTTTGGGCTATCGTTCAGGTGCATGGGGTTGGATAGCCAATATTGAAGTGGACACCTCTGGTAGTATC
GAA
+
FF:FFFFFFFFFFFFFFFFFFFFFFFFFFFFFFFFFFFFFFFFFFFFFFFFFFFFFFFFFFFFFFFFFF
FFFFFFFFFFFFFFFFFFFFFFFFFFFFFFFFFFFFFFFFFFFFFFFFFFFFFFFFFFFFFFFFFFF:
FFF
@A00721:190:HGJW7DSXY:4:1447:11234:23015_1:N:0:TCTGCAAG+GCTGCACT
ATACTATGGTTGAATCAACTAAGCATACTACTTTTGCTTTGATTACTCTACCGCCTTACAAGTCTAAG
GTTAAAGAGTCGATTAAACAAGTTGTACTTATCAATGATTTTATCGCTGGTTGTTATCTCGAAAAGTC
TCT
+
FFFFFFFFFFFFFFFFFFFF:F,FFFFFFFFFFFFFFFFFFFFFFFFFFFFFFFFFFFFFFFFFFFFFF
FFFFFFFFFFFFFFFFFFFFFFFFFFFFFFFFFFFFFFFFFFFFFFFFFFFFFFFFFFFFFFFFFFFF
FFF
@A00721:190:HGJW7DSXY:4:2555:15139:2409_2:N:0:TCTGCAAG+GCTGCACT
TTCTAACCCAAAGAGAATATCTAAAATATTTACAAAATCTTACCACTATCTACGAACCTAAAGTTCTG
AAGACGTTGCTTGTCGCTTCCTCCTTTAATGGCACTACCTCGGTACAA
+
FFFFFFFFFFFFFFFFFFFFFFFFFFFFFFFFFFFFFFFFFFFFFFFFFFFFFFFFFFFFFFFFFFFF
FFFFFFFFFFFFFFFFFF:FFFFFFFFFFFFFFFFF:FFFFFFFFFFF
@A00721:190:HGJW7DSXY:4:1429:23014:17080_2:N:0:TCTGCAAG+GCTGCACT
GCGACCTGTTCCGGAGTTTTTACGGGATCCGGAATGTTCTGTCTATATTAGGTGAAGCGAAATACGAT

```
TTCGAACGTCAACGTTCACCGAGTTTCGTTTCGTACCTTGATGCTGAC
+
FFFFFFFFFFFFFFFFFFFFFFFFFF:FFFFFFF:FFFFFFFFFF:FFFFFFFFFFFFFFFFFFFFFF
FFFFFFFFFFFFFFFFFFFFFFFFFFFFFFFFFFFFFFFF:FFFFFFF
@A00721:190:HGJW7DSXY:4:1255:18629:35258_1:N:0:TCTGCAAG+GCTGCACT
TAGTGCGAGGCCTCTCTTCTACGAGTATTGCTATTTATACGGTTACGAGAAGAAGGTTCGAAAGTTTG
AGGACTTAGTTTACGAACTTGACTCTACTATACGTGACATACTAAATACTAGATATTTGGATGATCTT
GAT
+
FFFFFFFFFFFFFFF:FFFFFFFF:FFFFFFFFFFFFFFFFFFFFFFFFFFFFFFFFFFFFFFFFFFF
FFFFFFFFFFFFFFFFFFFFFFFFFF:FFFFFFFFFFFFFFFFFFFFFFFFFFFFFFFFFFFFFFFFF
FFF
@A00721:190:HGJW7DSXY:4:2650:14516:3051_2:N:0:TCTGCAAG+GCTGCACT
TTCAGAGGATGAAAAATTTTCTTCCTTGGATTTTAATATAAAGAATCTTTACCAGGACTCCTACTACA
GGAGGCAGTCTTCTGATCGTAAGAAGTTTATAAGGAACATATCCATTT
+
FFFFFFFFFFFFFFFFFFFFFFFFFFF:FFF:FF,FFFFF,FFFFFFFFFFFFFFFFFFFFFFFFFFF
FFFFFFFFFFFFFFFFFFFFFFFFFFFFFFFFFFFFFFFFFFFFFFFF
@A00721:190:HGJW7DSXY:4:2146:25798:29857_1:N:0:TCTGCAAG+GCTGCACT
ACTCGAAACTCTAAGTGGTAATACTGTCTTTCCAAGCTTACCTGTATCTTCAATGAAATTGGACTCTT
CGATAGGGGACATTTTATCGTGGGCTTTCCGTAAAGGAAGATCTGTTATCGACGGTTTATCGTTTACG
AAG
+
FFFFFFFFFFFFFFFFFFFFFFFFFFFFFFFFFFFFFFFFFFFFFFF:FFFFFFFFFFFFFFFFFFF:
FFFFFFFFFFFFFFFFFFFFFFFFFFFFFFFF:FFFFFFFFFFFFFFFFFFFFFFFF:FFFFFFFFFFF
FFF
@A00721:190:HGJW7DSXY:4:2414:2600:4178_2:N:0:TCTGCAAG+GCTGCACT
GTTTAAGAATACTATAAATAGAATAGCTATTACTACTAATGGCGTTGGCCTAGTGGATGTTATTAGTG
ACATCTGGTCAATTTCGTTTTTAGGTGGTCTTTATTCTATTATTGATT
+
FFFFFFFFF::FFFFF:FFFFFF,FFFF:,,FFF:F:::FFFFFFFFFFF,:FF:FF,FFFFF:FFF:
FFFFF:FFFFFF,FFFFFFFFFFFFFFFFFFFFFF:FFFFFFFFFFFFF
@A00721:190:HGJW7DSXY:4:1419:14787:4523_1:N:0:TCTGCAAG+GCTGCACT
ACAGAAGATGATGTTAGAGTAGAATTCCTGAAGAGTCCTTCCGTAACCAACTTCTTGACAATTTTCTT
CAATCCTTCGATAATAGTTAAATCTAGGATTATGTCTGTGTTGAAAGACTTTCTACCGATAAGGGTCA
CTA
+
FFFFFFFFFFFFFFFFFFFFFFFFFFFFFFFFFFFFFFFFFFFFFFFFFFFFFFFFFFFFFFFFFFFF
FFFFFFFFFFFFFFFFFFFFFFFFFFFFFFFFFFFFFFFFFFFFFFFFFFFFFFFFFFFFFFFFFFFF
FFF
@A00721:190:HGJW7DSXY:4:1449:18747:15734_2:N:0:TCTGCAAG+GCTGCACT
TAAGAAATAATCCTACATTGACTGATTATCTAAAGATTGAAGGAAAATTTTATCCTCGTTTAACTTAC
TTAGACTTTCCTACGATACAAATGATAACTTCTAATTTACGTTTGCTA
+
FFFFFFFFFFFFFFFF,FFFFFFFFFFFFFFFFFFFFFFFFFFFF:F:FFFFF:FFFFFFFFFFFFF:F
FFFFFFFFFFFFFFFFFFFFFFFFFFFFFFFFFFFFFFFFFFFFFFFF
@A00721:190:HGJW7DSXY:4:2436:31928:11694_2:N:0:TCTGCAAG+GCTGCACT
CAAAGGGATGCATGGTGTTATCTCCTCAGGAGTCCCGGCATTTGATAATAATGACGAGACTTTCGTCA
TAATCGCTGCATATTACGGATTACACGGTACCAGCGCATCTCGTGTCG
+
FFFFFFFFFFFFFFFFFFFFFFFFFFFFFFFFFFFFFFFFFFFFFFFFFFFFF:FFFFFFFFFFFFFF
FFFFFFFFFFFFFFFFFFFFFFFFFFFFFF,FFFFFFFFFFFF:FFFFF
@A00721:190:HGJW7DSXY:4:2309:4345:10708_1:N:0:TCTGCAAG+GCTGCACT
GTATTAACTTATAAAGGTGAGGAGACCATTTTCTTGCTGAAATTTATAGAAGATTATTTTGACAGAAT
```

CAATAAGAATCATCGATTCTCGATTCGTAGAGCTTATCTAGGTACTCTCATTTACTATGTTGATTTAG
TCT
+
FFFFFFFFFF:F,FF:FFFFFFFF,,,FFFFFFFFF,F,FF:FFFFFFFFF:,FFFFFFF:FFF:,:F
:FFFFF:FFF,FFF:,FFFF:FFFFFFFFFFFFF:FFFFFFFFFFFFFFFFFFF:FFF:FFF,FFFFF
F:F
@A00721:190:HGJW7DSXY:4:2529:22399:2707_2:N:0:TCTGCAAG+GCTGCACT
GATTTACAACGATATAGGAGTAATCTACGAGAAGATTCTAGAATTCTTAATTATAATATTCAACCTCA
AAAGTTACGTGTTTACAGTAACGAGATCACACATTTTGAGAACTAACT
+
FFFFFFFFFFFFFFFFFFFFFFFFFFFFFFFFFFFFFFFFFFFFFFFFFFFFFFFFFFFFFFFFFFFF
FFFFFFFFFFFFFFFFFFFFFFFFFFFFFFFFFFFFFFFFFFFFFFFF
@A00721:190:HGJW7DSXY:4:1634:3341:21527_1:N:0:TCTGCAAG+GCTGCACT
CAATCTCTTCTTATAATAAATAAATAAATAATATTACTAATGGCAAATAGTGGAAAGGCTCCAGTCAA
CACTAGTTCGAGTGCTGCTTTGGATTACGATGACGTGAGAATAGGTGAAGGGATAGTTTTTACCTCGA
CCA
+
FFFFFFFFFF:FF:FFFFFFFFFFFFFFFFFFFFFFFFF::FFFFFFFFFFFFFFFFFFFFFFFFFFF
FFFFFFFFFFFFFFFFFFFFFFFFFFFFFFFFFFFFFFFFFFFFFFFFFFFFFF:FFFFFFFFFFFFFF
FFF
@A00721:190:HGJW7DSXY:4:1148:28899:29966_2:N:0:TCTGCAAG+GCTGCACT
GCGACTAATCTTAAAGCTTCACCCAAAATGAAGGACACTTCGAAACTGAATAATGATGCACTACAAAC
AAGTATCGCTAATCATCTGAAAAAGGCTCTAAAGGACACTATACAATA
+
FFFFFFFFFFFFFFFFFFFFFFFFFFFFFFFFFFFFFFFFFFFFFFFFFFFFFFFFFFFFFFFFFFFF
FFFFFFFFFFFFFFFFFFFFFFFFFFFFFFFFFFFFFFFFFFFFFFFF
@A00721:190:HGJW7DSXY:4:2675:10203:1846_2:N:0:TCTGCAAG+GCTGCACT
ATCAAATATAAAGAAAAGGACTATGAAATAAAAGATTTGGTGCTTAGGGATATAATGAATTCAGCACC
ACATTTTGGTCAAGTCAATCCGTTGAGAAAATTTCTTAGAGGATATTC
+
FFFFFFFFFFFFFFFFFFFFFFFFFFFFFFFF:FFFFFFFFFFFFFFFFFFFFFFFFFFFFFFFFFFF
FFFFFFF:FFFFFFFFFFFFFFFFFFFFFFFFFFFFFFFFFFF:FFFFFF
@A00721:190:HGJW7DSXY:4:1329:4761:20102_2:N:0:TCTGCAAG+GCTGCACT
CAACATTTACCACCAAATTCTTTTGATACAACTCTAGCCTTCAAGTGGGGTATGCCTGCGGAATACGC
TTGCTATTCACCAGATTGGTTCGAACCCGTTGATGCTATGTCAGAAGA
+
FFFFFFFFFFFFFFFFFFFFFFFFFFFFFFFFFFFFFFFFFFFFFFFFFFFFFFFFFFFFFFFFFFFF
FFFFFFFFFF:FFFFFF:FFFFFFFFFFF:FFFFFFFFFFFFFFFFFF
@A00721:190:HGJW7DSXY:4:1128:1570:21183_2:N:0:TCTGCAAG+GCTGCACT
TGTCGTTTAAAGACTGCAGAGGCTGTCAAACAAGCCCAGACTCAAGGAGTCCAATCAAAGGTTTTGAA
TTTAGCTCAACTGGGGAGACGTGCTTAAATGGGTGATAAAAATTATTT
+
FFFFF::FFFFFFFFFFFFFFFF:FFFFFFFFF:FFF,FFFFFFFFFFFFFFFFF:FFFFFFFFFFFF
FFFFFFFFFF:,FFFFFFFFFFFFFFFFF,FFFFFFFFFFFFF:FFFF
@A00721:190:HGJW7DSXY:4:1544:19488:21277_1:N:0:TCTGCAAG+GCTGCACT
TACTTACAAAGGACAATTCAGTTTCATTAATTCTACGGAAAAGTCCGGAGTATATTGTATGTTAATAG
ATAGTGGGATAATAGTTCTACCGATACGTAGTGAACGCGTTCGTAGAACCAAGTACTTTCCGTCTGAA
GAG
+
FFFFFFFFFFFFFFFFFFFFFFFFFFFFFFFFFFFFFFFFFFFF:FFFFFFFFFFFFFFFFFFFFFFF
FFFFFFFFFFFFFFFFFFFFFFFFFFFFFFFFFFFFFFFFFFFFFFFFFFFFFFFFFF:FFFFFFFFFF
FFF
@A00721:190:HGJW7DSXY:4:1144:30011:13041_2:N:0:TCTGCAAG+GCTGCACT
TATTGATTATGTAACCGATAGGAGTTGTATAGTTGAAGATGTGGCTTTAGACGATAATCCTTTAAGGA

CTATGTTGACTGACGAAGATTTTCCCACAGATTTGTCTACGTTGCAGA
+
FFFFFFFFFFFFFFFFFFFFFFFFFFF:FFFFFFFFFFFFFFFFFF:FFFFFFFFFFFFFFFFFFF:F
FFFFFFFFFFFFFFFFFFFFFFFFFFFFFFFFFFFFFFFF:FFFFFFF
@A00721:190:HGJW7DSXY:4:1163:16911:33473_2:N:0:TCTGCAAG+GCTGCACT
CACTATATACATTATAAAATATAAAAATAAGGGAGTCATAACTTTTGTTACCAAATTCTTGGCTTTCA
ACGAAGTCTCTGATTTTATTAATCATTACTTTAACGATCTCTCGGTCA
+
FFFFFFFFFFFFFF:FFFFF:FFFFFFFFFFFFFFFFFFFFFFFFFFFF:FFFFFFFFFFFFFFFFFF
FFFFFFFFFFFFFFFFFFFFFFFFFFFFFFFFFFFFFFFFFFFFFFFF
@A00721:190:HGJW7DSXY:4:1426:22580:14606_2:N:0:TCTGCAAG+GCTGCACT
TTGATCTACGTCGTTTAACTGTCGGAACAACCTTTACTTAGAATGACTCTATCTTTTCAGACACTTCT
TATATTATTTACAATAGTTAATATCATATTAGTAATACTCACTATCGT
+
FFFFFFFFFFFFFFFFFFFFFFFFFFFFFFFFFFFFFFFFFFFFFFFFF:FFFFFFFFFFFFFFFFFF
FFFFFFFFFFFFFFFFFFFFFFFFFFFFFFFFFFFFFFFFFFFFFFFF
@A00721:190:HGJW7DSXY:4:1333:32036:20337_1:N:0:TCTGCAAG+GCTGCACT
GCTCTTCCGATCTGATTAATAAAGGATGAGTACGAAAACCAACTCTTCGTTACGAGACCTGATGGTAG
AATAGATTTAGTCTCTACACCCATCTCAACGAGGATAAAGCGAGTTTTCCGTCCTGAACGTAATACAC
GAA
+
FFFFFFF:FFFFF:FFFFFFFFFFFFFFFFFFFFFFFFFFFFFFFFFFFFFFFFFFFFFFFFFFFFFF
FFFFFFFFFFFFFFFFFFFFFFFFFFFFFFFFFFFFFFFFFFFFFFFFFFFFFFFFFFFFFFFFFFFF
FFF
@A00721:190:HGJW7DSXY:4:1637:31033:12054_2:N:0:TCTGCAAG+GCTGCACT
CAAACGACTCGAAACCGAGACGCCGTAGATCAAAGTCCCTTCTAACTTTAGATTATGAATCCTGCCTT
TACTCATTTTATAGCAGTTATTTATGTTGTACTTTTTATAATTTATAA
+
FFF,FFFFFFFFFFFFF:FFFFFFFFFFFFFFFFFFFFFFFFFF:FFFFFFFFFFFFFFFF:F:FFFF
FFFFFFFFFFFF:FFFFFFFF:FFFFFFFF:FFFFFFFFFFFFFFFFF
@A00721:190:HGJW7DSXY:4:1323:18728:18364_1:N:0:TCTGCAAG+GCTGCACT
TTTGGGTATGTTTTGTGAAAAACATGAGATACCCAAGTCTATCGAAGACATTATCTCTTATGTACTTA
TTGTAACGATCATTCTTTCGAGTTTTGTCATTCTTATTCTTTTATTCCTTTAACTGTGTATGAGGAGG
ATT
+
FFFFFFFFFFFFFFFFFFFFFFFFFFFFFFFFFFF:FFFFFFFFFFFFFF:FFFFFFFFFFFFFFFFF
FFFFFFFFFFFFFFFFFFFFFFFFFFFFFFFF:FFFFFFFFFFFFFF:FFFFFFFFFFFFFFFFFFFF
FFF
@A00721:190:HGJW7DSXY:4:1239:9498:26866_1:N:0:TCTGCAAG+GCTGCACT
TCGATAGTGATTGTTATCAATTTAAATACATTACTGTTTCAAATCATCTTTACTCACAGACTCCGAAA
ATGGTTTCAAATGAGGTCAAAGCTCTATTTATTAAACGAACTATTATCCGATTCTTCGAGGGTATCAC
TAC
+
F:FFF:FFFFFFFFFFFF:FFFFFFFFFFFFFFFFFFFFFFFFFFFFFFFFFFFFFF:FFFFFFFFFF
FFFFFFFFFFFFFFFFFFFFFFFFFFFFFFFFFFFFFFF::FF:FFFFFFFFFFFFFFFFFFFFFFFF
FFF
@A00721:190:HGJW7DSXY:4:1302:10682:14481_1:N:0:TCTGCAAG+GCTGCACT
GACTTTATTCAAGGAACTTCAATTCCTTCGATGTTTATATTTTTCTATAAGGGAAAATAGCAATAATC
AAATTTAGACAAATTTTAGAACTTTATTTCAGAACTCTCTCCGATCACCTTGGTGTGATCTCCCGGAG
GAT
+
FF:FFFF,FFF:,FFF,FF,F:FF,FFF,FFFFF:FFFFFFFFFFF,FFFFF:FFFFFFFFFFF:FFF
FFFFFF,FF:FFFFFFFFF:F:FFFFFFFFF,::FF:FFFF:FF:F,F:FF,FFFFFFF::FFFFF,F
FFF

@A00721:190:HGJW7DSXY:4:1248:15483:30906_2:N:0:TCTGCAAG+GCTGCACT
ATGTATGATGATAATCTACTATTTTTAAATAGTGATATTCAGCGTATACTTGAATACTTTATCAAAGA
TTTGGCTTGGTCTATTTACCGCAAAAAGTTGGAAGAGGTCTATGATAT
+
FFFFFFFFFFFFFFFFFFFFFFFFFFFFFFFFFFFFFFFFFFFFFFFFFFF:FFFFFF:FFFFFFFFFF
F,FFFFFFFFFFFFFFFFFFFFFFF:F:FFFFFFFFFFFFFFFFFFFF:F
@A00721:190:HGJW7DSXY:4:1345:12283:13870_2:N:0:TCTGCAAG+GCTGCACT
TATACTTTAAATAGCACCGATTATAACTTGACTGTTGATAATATTACAATAGCTCTTTCTCAGAAGAT
TAGAGAAGTAACCGGTGAACACGTCGGTAGCATTGATTTACTTTTACC
+
FFFFFFFFFFFFFFFFFFFFFFFFFFFFFFFFFFFFFFFFFFFFFFFFFFFFFFFFFFFFFFFFFFFF:
FFFFFFFFFFFFFFFFFFFFFFFFFFFFFFFFFFFFFFFFFFFFFFFF:
@A00721:190:HGJW7DSXY:4:1221:18204:8907_2:N:0:TCTGCAAG+GCTGCACT
TTTCGAAAAGAAACCTATAAGTTACAAACGATTCTTCGACTATATTAAAGAAATAAAAATACTTCAAG
TACCTGAACTTTACATTTTTAATGGTATGGTATAATGTGTCTTACAAA
+
FFFFFFFFFFFFFFFFFFFFFFFFFFFFFFFFFFFFFFFFFFFFFFFFFFFFFFFFF:FFFFFFFFFF
FFFFFFFF:FFFFFFFFFFFFFFFFFFFFF:FFFFFFFFFFFFFFFFFF
@A00721:190:HGJW7DSXY:4:1166:28266:9455_2:N:0:TCTGCAAG+GCTGCACT
CTCTCGGTAATGAGAGTGTATTCCTTAATATTCTAGTAACCGCTATGTGGGCATTACTAGGATTTATC
ACACTCCTAAATTTGATAGCAATTTATGTTTTAAATGCTAGGAAAATT
+
FFFFFFFFFFFFFFFFFFFFFFFFFFFFFFFFFFFFFFFFFFFFFFFFFFFFFFFFFFFFFFFFFFFF
FFFFFFFFFFFFFFFFFFFFFFFFFFFFFFFFFFFF:FFFFFFFFFFFF
@A00721:190:HGJW7DSXY:4:1572:29713:19132_2:N:0:TCTGCAAG+GCTGCACT
TTGTATAGTTCAAGGAGTACTTAATTCAATAGCTTTAGTATTATTTACTATCGCCTTTATTGAAAATT
GTATAAGAGTTAATTGCTTACACAAAAGTGACGCGCGAAGGAATCTTA
+
FFFFFFFFFFFFFFFFFFFFFFFFFFFFFFFFFFFF:FFFF:FFFFFFFFFFFFFFFFFFFFFFFFFFF
FFFFFFFFFFFFFFFFF:FFFFFFFFF:FFFFFF:FFFFFFF:FFF,F
@A00721:190:HGJW7DSXY:4:1352:28782:4523_1:N:0:TCTGCAAG+GCTGCACT
GGGAGAATGAACTGCCGCTTCCCGGAAGAAACGATCTATTTACAGAGTATTTAGAGTAGTCCAAAGTT
TGTATAAATTTATATGATTTTCTATATATATACCACATATATATATATATAAAATCCTATAAATTTGT
ACA
+
FFFFFFFFFFFFFFFFFFFFFFFFF:FFFFFF:FFFFFFF:FFFFFFFF,FFFFFFFFFFFFFFFFFF:
F:FFFFFF,FFFFFFFFF:FF:F:FFFFFFFFFFF:F:FFFFFFFFFFFFFFFFFFFFFFFFFFF:FFFF
FFF
@A00721:190:HGJW7DSXY:4:2462:29423:2785_1:N:0:TCTGCAAG+GCTGCACT
AACATACCATACACTCCCCAAACATAACATACCATAACCTACCATGACCAATCATCGCTCCAACTGGC
ACGCCAGACGTAGGGTCAAACCTACTACCGCCGGTCGGCAGATCGGCACCACTGGTCTCTACGTTCAG
GGA
+
:FF,,FFFFFFFFFFFFFFFF,FFFFFFFF:FFFFFFFF,FFFFFFFFF:FFFFFFFFFFFF:FFFFF
FFFFFFFFFFFFFFF:FFFFFFFFFFFFFFFFFFFFFFFFFFFFFFFFFFFFFFFFFFFF,FFFFFFF
FFF
@A00721:190:HGJW7DSXY:4:1675:18385:5212_2:N:0:TCTGCAAG+GCTGCACT
TAACCTTCCCAAATCTAGATCTGCACAGAAGAAGGCTGAAGGTCGTTACCGGACGACCACTCAAAGGA
AACGGGCTGTTGCCCAGAGGAAAGCAGCGACCACCGAGGTCGCTCGTA
+
FFFFFFFFFFFFFFFFFFFFFFFFFFFFFFFFFFFFFFFFFFFFFFFFFFFFFFFFFFFFFFFFFFFF
FFFFFFFFFFFFFFFFFFFFFFFFFF:FFFFFFFFFFFFFFFFFFFFFF
@A00721:190:HGJW7DSXY:4:1526:7048:1548_1:N:0:TCTGCAAG+GCTGCACT
AGTCCCGTCATCTCTGTGGGAACTGAGGCAGTACCTCAGTCAAAAGTCACATTCACCACTCCCATGCC

```
AATCAATGATGTGACTATACAGTTCGGCCATTTCAAGAGTGGTCAATACGTCGTGGGAGGTACTGAAG
CCT
+
FFFFFFFFFFFFFFFFFFFFFFFFFFFFFFFFFFFFFFFFFFFFFFFFFFFFFFFFFFFFFFFFFFFFFF
FFFFFFFFFFFFFFFFFFFFFFFFFFFFFFFFFFFFFFFFFFFFFFFFFFFFFFFFFFFFFFFFFFFFFF
FFF
@A00721:190:HGJW7DSXY:4:1657:24523:33098_1:N:0:TCTGCAAG+GCTGCACT
ATCTAAGAACACTCCCAAGCCAATCAATAGTTCTTCTGACCAGAATGAAAAGGGGTTTACCAGGGTAG
TCTCTAAGTCTACCAGGAAGAGGGGAAATGTCCCCAAAACTGAGAAGAATAAAGGGAAAGGTCCATCA
GGA
+
FFFFF:FFFFFFFFFFFF:FFFFFFFFFFFFFFFFFFFFFFFFFFFF:FFFFFFFFFFFFFFFFFFFFFF
FFFFFFFFFFFFFFFFFFFFFFFFFFFFFFFFFFFFFFFFFFFFFFFFFFFFFFFFFFFFFF:FFFFFFF
FFF
@A00721:190:HGJW7DSXY:4:1319:13856:28557_2:N:0:TCTGCAAG+GCTGCACT
ATTACCCCTAATTCTGAAGGAAGATCTCCCACCATCTGGGGTTGGGAATAAGAAATCTGCACGGGCCA
AAGAGGCCGCTGCTACGAAGTCGCTGAAGGCGGCTTGGGTCATTAAGG
+
FFFFFFFFFFF:FF:FFFFFFFFFFFFFFFFFFFFFFFFFFFFFFFFFFFFFFFF:FFFFFFFFFFFFFF
FFF,FFFFFFFFFFFFFFFFFF:FFFFFFFFFFFFFFFFFFFFFF:FF
@A00721:190:HGJW7DSXY:4:2176:5493:9095_1:N:0:TCTGCAAG+GCTGCACT
AGTTGCGCCTGCCGACCCCATCTTTGAGACCAAGACTCTCTACAGTAGGTTCGTGCCTACTATGAGCA
AATCTGTTGAATTCGTCACTGAGGACGAAGGTGAGAGGACTTTTGGCTACTGGCGACAAACTGAGAGC
AGT
+
FFFFFFFFFFFFFFFFFFFFFFFFFFFFFFFFFFFFFFFFFFFFFFFFFFFFFFFFFFFFFFFFFFFFFF
FFFFFFFFFFFFFFFFFFFFFFFFFFFFFFFFFFFFFFFFFFFFFFFFFFFFFFFFFFFFFFFFFFFFFF
FFF
@A00721:190:HGJW7DSXY:4:2566:8712:20212_2:N:0:TCTGCAAG+GCTGCACT
AGAATTGCGTGGGTTGAAGGCCGACGCTTCGAGCTTGAGTGTGACGAGGCCGGTGACGTTTTTGTCAA
CGGCGTTTGGAGGATTTTATCCGCGCTTAACTATGGGGCGAAGATCCC
+
FFFFFFFFFFFFFFFFFFFFFFFFFFFFFFFFFFFFFFFFFFFFFF:FFFFFFFFFFFFFFFFFFFFFFF
FFFFFFFFFFFFFFFFFFF:FFFFFFFFFFFFFFFF:FFFFFFFFF:F
@A00721:190:HGJW7DSXY:4:2559:24026:7560_2:N:0:TCTGCAAG+GCTGCACT
AAGGACTTAGGTTGGCATATTTATGCCGACGATAATAAGCATGTCACCCTTAAGCACCGAGGTAAGCT
CGGGACTTACAAGAGAGAGTGTGAGAGAGTCGCTCTCACTATTTTTGC
+
FFFFFFFFFFFFFFFFFFFFFFFFFFFFFFFFFFFFFFFFFFFFFFFFFFFF:FFFFFFFFFFFFFFFFF
FFFFFFFFFFFFFFFFFFFFFFFFFFFFFFFFFFFFFFFFFFFFFF,F
@A00721:190:HGJW7DSXY:4:1166:25798:12790_2:N:0:TCTGCAAG+GCTGCACT
TTCTGACTGATGAGGTTAAAACTCTTGCTCCTTCTGTGGGTGAGGGTTTTTGTTATATTCACTGGTTC
TGGGAGGCTGCTTTCCTCCATAATTACAACCTCTCTTTAGAGAGCATT
+
FFFFFFFFFFFFFFFFFFFFFFFFFFFFFFFFFFFFFFFFFFFFFFFFFFFF:FFFFFFFFFFFFFFFFF
FFFFFFFFFFFFFFFFFFFFFFFFFFFFFFFFFFF:FFFFFFF:FF:F
@A00721:190:HGJW7DSXY:4:1207:27859:27164_2:N:0:TCTGCAAG+GCTGCACT
AAATTCCCACATATGGTAGACGTTGTCACACTTTTCTTGAAAATTTTTCCTTTCCTCCGTCATTGGGG
ATTTCGATGTGAGAAGGTGGAAGGTGTCAAGGGTCTTCTTCATGCTGA
+
F,FFFFFFFFFFF,FFFFFFFFFFFFFFFFFFF:F:FFFFFFFFFFFFFFFFFFFFFFFFFFFF,FFFF:
:FFFFFF,FFFFFFFFFFFFFF,FFFFFFFFFFFFFFFFFFFFFFFFF
@A00721:190:HGJW7DSXY:4:1152:30318:20149_1:N:0:TCTGCAAG+GCTGCACT
TTCAGACGTGTGCTCTTCCGATCTCGGCTCGTATCGAGTTGGAGGGAGGAAGTTCTCCGAAATTAAAG
```

ACGGGGGGATTAAGAGTCCAGCTGTAACTGTCATTTCAGAGAGGGGAGCTCTGAAAAGGAAACTTCGT
CAG
+
FFFFFFFFFFFFFFFFFFFFFFFFFFFFFFFFFFFFFFFFFFFFFFFFFFFFFFFFFFFFFFFFFFFFFF
FFFFFFFFFFFFFFFFFFFFFFFFFFFFFFFFFFFFFFFFFFFFFFFFFFFFFFFFFFFFFFFFFFFFFF
FFF
@A00721:190:HGJW7DSXY:4:1578:3929:14591_2:N:0:TCTGCAAG+GCTGCACT
AGAGGGGAGGAGTTTGAGGAAGGCTCTAGAGAAGATCTCACCACCTGGAGAAGGCAAGTGGAGAGTCC
AGAAGTTGATGAAGAAGGATTATTTGAAGCAGGGGTTACCACTTCCTA
+
FFFFFFFFFFFFFFFFFFFFF:FFFFFFFFFFFFFFFFFFF:FFFFFFFFFFFFFFFFFFFFFFFFFFFF
FFFFFFFFFFF:FFFFFFFFFFFFFFFFFFFFFFFFFFFFFFFFFFFFF
@A00721:190:HGJW7DSXY:4:1378:16450:36808_2:N:0:TCTGCAAG+GCTGCACT
TTCTTTGGAGTTTTAGATAATCTTTTGAGAAGGGGTCATAGAGCCAGCAAGAGAAGCTTATATGCTGG
TTTAAGTGATGATTATCACTCCTTGAGAAGACAAGCCACATTAAGATA
+
FFFFFFFFFFFFFFFFFFFFFFFFFFFFFFFFFFFFFFFFFFFFFFFFFFFFFFFFFFFFFFFFFFFFFF
FFFFFFFFFFFFFFFFFFFFFFFFFFFFFFFFFF::FFFFFFFFFFFFF
@A00721:190:HGJW7DSXY:4:1361:27208:7905_2:N:0:TCTGCAAG+GCTGCACT
CTTGGGGTTTCGAATGTCATTAGTGCCTTACGATTGAAAGAGGTCCTTTTGAGTATCGACAGATCTTT
CGATCTTGAGGACGTACATTTAACGTGGCAGAATTCGGTAACTTACCA
+
FFFFFFFFFFFFFFFFFFFFFFFFFFFFFFFFFFFFFFFFFFFFFFFFFFFFFFFFFFFFFFFFFFFFFF
FFFFFFFFFFFFFFFFFFFFFFFFFFFFFFFFFFFFFFFF:FFFFFFFF
@A00721:190:HGJW7DSXY:4:1206:25274:33865_2:N:0:TCTGCAAG+GCTGCACT
CGGGAAGTTGTGGGATTTAACTTTGACTCAACTACTCCGATCAGATCCGGATGGTTTGATTGGTATGG
ACTACTCGACTTTTGATCTGGAGTTGTCTAGGGTCATGGCAGATACTA
+
FFFFFFFFFFFFFFFFFFFFFFFFFFFFFFFFFFFFFFFFFFFFFFFFFFFFFFFFFFFFF:FFFFFFFF
FFFFFFFFFFFF:FFFFFFFFFFFFFFFFFFFFFFFFFFFFFFFFFFFF
@A00721:190:HGJW7DSXY:4:1535:8929:22654_2:N:0:TCTGCAAG+GCTGCACT
AAAATATGCAGGTCAAAGATTCTGTAGCTCTGAGGGCGGTTGAGGGAGCCGTTTTGGCCGACTTTAAC
TATTCTAAGAGTCTTTATGATTCAAAACCTATCGTTGTCGTACCTTAC
+
FFFFFFFFFFFFFFFFFFFFFFFFFFFFFFFFFFFFFFFFFFFFFFFFFFFFFFFFFFFFFFFFFFFFF:
FFFFF:FFFFFFFFFFFFFF:FFFFFFFFFFFFFFFFFFFFFFFFFFFF
@A00721:190:HGJW7DSXY:4:2408:17246:6621_1:N:0:TCTGCAAG+GCTGCACT
TACAGCGGCTTATTGTCGCGCAGTTTCCAGAATTGCGGTTGAAGTTCACCCATTCTATCTTGAATGCT
CACGCCGTCGCAGCCATATCGAGACTATGTTATAATCACTTGTTAAAGTACAGATTTAGTGATTTAGA
TAT
+
FFFFFFFFFFFFFFFFFFFFFFFFFFFFFFFFFFFFFFFFFFFFFFFFFFFFFFFFFFFFFFFFFFFFFF
FFFFFFFFFFFFFFFFFFFFFFFFFFFFFFFFFFFFFFFF:FFFFFFFFFFFFFFFFFFFFFFFFFFFFF
FFF
@A00721:190:HGJW7DSXY:4:2303:1434:14528_1:N:0:TCTGCAAG+GCTGCACT
GGAGGCGATATTCGTTTTCATATCCAAAACTCCTTGAATGGAGTACATATTTGCAACCCTATTCTTGA
TGTGAAAGATGCATCGAGGTATACGTCTAGGAACATTGATTGGGAACGTACCTACTCACGAGACGTCA
TGA
+
:F::,::FFFFF:FFFFF:FFFFF:FFFF,F:FFFF,FF:FFFFFFF:FFFFF,:F:FF:FFFFFF:FF
FFF:FFF:FFFFFFFF,F::F:FF,FFFFFFF:FFFFF,FFFF:::FFFFFFF:FFF,FFFFFFFFFF
FFF
@A00721:190:HGJW7DSXY:4:2368:31024:9126_1:N:0:TCTGCAAG+GCTGCACT
ATGTTTCGTTCTGTTACAAGAACGCTTTGGATTGTGACCATAAAGCCTCAGTTGCGATAGCTGTAGAG

GTATACGATATCTCTCTTGATGATGTAGCCACTATAATGCTTAAGAGAGAGATTGATGTATGTCATTT
CGT
+
FFFFFFFFFFFFFFFFFFFFFFFFF,F:FFFFFFFFFFFFFFFFFFF:FFFFFFFFFFFFFFFFFFFF
FFFFFFFFFFFFFFFFFFFFFFFFFFFFFFFFFFFFFFFFFFFFFFFFFFFFFFFFFFFFFFF:FFFFF
FFF
@A00721:190:HGJW7DSXY:4:2268:13376:20149_1:N:0:TCTGCAAG+GCTGCACT
GAATTATTGGACACGATGAAAAATGACATACCCGTTCCTGACCTTGGTATCGTTATCCGTCATGGAGA
TGATGGCGCATCACTTGATTACTATATGCCCGAAGGATTGGGTTATTCTCACAACAAAGATAAGATTT
TAT
+
FFFFFFFFFFFFFFFFF:FFF:FFFFFFFFFFFFFFFFFFFFFFFFFFFFFFFFFFFFFFFFFFFFFF
FFFFFFFFFFFFFFFFFFFFFFFFFFFFFFFFFFFFFFFFFFFFFFFFFFFFFFFFFFFFFFFFFFFF
FFF
@A00721:190:HGJW7DSXY:4:2211:3721:5368_1:N:0:TCTGCAAG+GCTGCACT
TAAGGGCGGACAATTGTTCCATTGTGAACTCTTGGGGACTAGATTGAGCATTAATGAGTTCCGAATAA
CTCGTAATAAATCTTTCTCTAGTGGACAATTCAGAATACCTATTTCCGTACCAAGAGCCACTGCCAAT
CTC
+
FFFFFFFF:FFFFF:F:FFFF,FFFFFFFFFFFFFFFFFFFFFFFFFFFFFFFFFFFFFFFFFFFFFF
FFFFFFFFFFFFFFFFFFFFFFFFFFFFFFFFFFFFFFFFFFFFF:FFFFFFFFFFFFFFFFFFFFFFF
FFF
@A00721:190:HGJW7DSXY:4:1156:2718:16877_2:N:0:TCTGCAAG+GCTGCACT
AAATTTTTCTCAAGAGATTATGGTTGATAAAGTTTTCTTCATAAGGGCTATTCAGTACGTCATGGGGA
TCACTTCTTCGGTCAATGACAAAACCTTCGAATATGTTACGACATGGC
+
FFFFFFFFFFFFFFFFFFF::FFFFFFF:FFFFFFFF:FFFFFFFFFFFFFFFFFFFFFFFFFFFFFF
FFFFFFFFFFFFFFFFFFFFFFFFFFFFFFFFFFFFFFFFFFFFFFFF
@A00721:190:HGJW7DSXY:4:2425:7581:30530_2:N:0:TCTGCAAG+GCTGCACT
AGGATTGTCATCTCGGGGAAAGTGATTCACCAAGATGTTCATATAGATACGAGATACCTAGAGAAGGT
CGCAGCGATCTTACTCGTTATCGGAGCTAAGAAGAAGACTTTGGGTAC
+
FFFFFFFFFFFFFFFFFFF,FFFFFFFFFFFFFFF,F:FFFFFFFFFFFFFFFFFFFFFFFFFFFFFF
FFFFFFFFFFFFFFFFFFFFFFFFFFFFFFFFFFFFFFFF:FFFFFFFF
@A00721:190:HGJW7DSXY:4:1405:2230:36761_2:N:0:TCTGCAAG+GCTGCACT
TAACGTTAGTACCGGAGGTGATCTCTCTGCTTCTGCGTTACTGAGAGAAAAGATTCAGGACGCCTACG
GTGACTTTATGGACAAGATGCAAGTTCTTGGGAAATACTTGTTTCCCT
+
FFFFFFFFFFFFFFFFFFFFFFFFFFFFFFFFFFFFFFFFFFFFFFFFFFFFFFFFFFFFFFFFFFFF
FFFFFF,FFFFFFFFFFFFFF:FFFFFFFFFFFFFFFFFFFFFFFFFFF
@A00721:190:HGJW7DSXY:4:1330:13494:29089_2:N:0:TCTGCAAG+GCTGCACT
TCTTTGTATAGGGAGTGTGAGAAGACAACGAATCTTGAAACTACGGTCCTTATCAAAGAAAATGGTGA
ACCGGTCTCTAAATCTTTTGAGATTGAGAAGATTGAAGAACACGTCAA
+
FF,FFF,FFFFFFFFFFFFFFFFFFFFFFFFFFFFFFFFFFFFFFFFFFFFFFFFF:FFFFFFFFFFF
F:FFFFFFFFFFFFFFFFFFFFFFFF:FFFFFFFFFFFF:FFFFFFFFFF
@A00721:190:HGJW7DSXY:4:1408:24334:9095_2:N:0:TCTGCAAG+GCTGCACT
TTGTTCTTGAAAACCAAGTTAAGAAGGAGGCTGATCATGGAGTCTTTGTACCTTCTGTCAAACTTCGA
GGTTCAGCTACGAGAGGTGATCCCGGATGTGGATTGAAGGCTGGCTCT
+
FFFFFFFFFFFFFFFFFFFFFFFFFFFFFFFFFFFFFFFFFFFFFFFFFFFFFFFFFFFFFF:FFFFFF
FFFFFFFFFFFFFFFFFFFFFFFFFFFFFFFFFFFFFFFFFFFFFFFF
@A00721:190:HGJW7DSXY:4:1362:17047:29825_2:N:0:TCTGCAAG+GCTGCACT
CTTTTTGAATGTTGTAAAGAATTGGGTTGAGAAGTGTGCTTTACAAGGAGTTAGACTCTTCTTCAAGT

TTTTAGATCTTCTTGATAATCTTAAAGAAAAATTTTCGAATAAACTAA
+
FFFF:FFFF,FF:FFFFFFF:F:F:FFFFFFFFFFFFFFFFFFFFFFFFFFFFFFFFFFFFFFF:F
FFFFF,FFFFFFFFFF:FFFFFFFFF,F,FFFFFFFFFFFFFFF:FFF
@A00721:190:HGJW7DSXY:4:2614:32497:19695_2:N:0:TCTGCAAG+GCTGCACT
CTGTACTTACATTCTTTACGCCAGATTCTTCATGGGGCGACGCTGTTTCTCTTGGTATTTCTGAAATA
GTTTATCAGAGCGTCATTTTCCTTTGGGATCTTTCCTCTGGTAAGGGA
+
FFFFFFFFFF:FFFFFFFFFFFFFFFFFFFFFFFFFFFFFFFFF:FFFF:F:FFFFFF:FFFFFFF:FF
FFFFFFFFFFFFFFFFFFFFFFF:FFFFFFFFFFFF:FFFFFFFFF:FF
@A00721:190:HGJW7DSXY:4:2566:13160:35086_1:N:0:TCTGCAAG+GCTGCACT
ACGAGAATTATTACTGTTTTCTCTACCCATAACTTCTTTTTCAAAAATGCGGATGGGTCTACTGATGT
TTTCCTACAGAGTGCAGTCGTTTCAACACTTATCACCACTTTTGAAAGGAAAATAATTGGTGATGAAA
ATA
+
:FF:,F,:FFFFF,FF,FFFF:FFF:F,F:,FFFFF,F,FFFFFF::FF:FF,FFFFFFF,FFFFFF,
FFF::F:FFFFFFF:FFFFFFFFFF:,FF::F,FFFF:,FF::F:,FFFF,FFFFFFFFFFF,FFFFF
FFF
@A00721:190:HGJW7DSXY:4:2177:23999:33505_2:N:0:TCTGCAAG+GCTGCACT
GGGTTCTCAAGGCTCTCATCGCCACTGGGATTCTAAGGGGTTCCATAAGATATTATCTGACATTGTGG
TTCAATGATCAGGATTCTTCTTTTGGTCCTTACGTCTTAAGTAATTTG
+
FFFFF,FFFF:FFFFFFFFFFFFFF,FFFFFFFFF:FFFFFFFFF:FFFFF:FFFFFFFFFF:FFF::
FF:FFFFFF:FFFFFF:FFFFF,F:FFFFFFFFFFFFF:F:FF:FFFF
@A00721:190:HGJW7DSXY:4:1248:16658:34820_1:N:0:TCTGCAAG+GCTGCACT
TCTTGTCACGATTCTTAAAAGAAGTTTATTAAAACTTCAGAGTCTCTTGGAAGTCCAGGTAGCTAATG
TTCTTGCAATAGCCACAGACTTTCTGGGAAAAGAGACCTCTGTTGGTAGAGGAGTTGAGAGAATTACT
CAG
+
FFFFFFFFFFFFFFFFFFFFFFFFFFFFFF,FFFFFFFFFFFFFFFFFFFFFFFFFFFFFFFFFFFFF
FFFFFFFFFFFFFFFFFFFFFFFFFFFFFFFFFFFFFFFFFFFFFFFFFFFFFFFFFFFFFFFFFFFF
FFF
@A00721:190:HGJW7DSXY:4:1233:11261:7216_2:N:0:TCTGCAAG+GCTGCACT
ACAACGAGTTAGGGAGTGGCTAGGTTTTAAAAGATCCTTTGCACAAGCAATACCTTCCACTGAGTTCA
TCACCCAGAGTTTCGACGTCGCGGAAAACGATGAAGATTTCCACACTG
+
FFFFFFFF:FFFFFFFFFFFFFFFFFFFFFFFFFFFFFFFFFFFFFFFFFFFFFFFFFFFFFFFFFFF
FFFFFFFFFFFFFFFFFFFFFFFFFFFFFFFFFFFFFFFFFFFFFFFF
@A00721:190:HGJW7DSXY:4:2246:9579:2268_1:N:0:TCTGCAAG+GCTGCACT
TTCTGATGCTGAATTTGAGGAAAATTTTTCTGATGTTTTTGGTATATATCCTGATGGCTCAGTTCAGG
GTGGTGGTTATAGTTATAAAACCTTCTTTTATCATTGCAACAGGACTTTGAGAAGTCTGTTGAAATGG
AC
+
FF,FF:FF,FF:FFFFF:,FFFF,FF,FF,,F,,FFFFFF:,FFF,,FF:,:F,,,F,FF:FF,:,FF
F,:F,F,F,:F,FFF,FFFF,,,FF:FF,F:FFFFFF,FFF:,FFFFFF,:,F:,,FFFFFFFFFFFF
FF
@A00721:190:HGJW7DSXY:4:1228:15429:4131_2:N:0:TCTGCAAG+GCTGCACT
TCATAGGTATGTGTCACGCTACACTCGAAATATGGGCCCTCATAAGGAAGTATAGTTGGCGTCTTGTC
GTTGACGTCATTACAGTCTGGAAGTTAGATCATTTGTTCAGGAAGATC
+
FFFFFFFFFFFFFFFFFFFFFFFFFFFFFF:FFFFFFFFFFFFFFFFFFFFFFFFFFFFFFFFFFFFF
FFFFFFFFFFFFFFFFFFFFFFFFFFFFFF:FFFFFFFFFFFFFFFFF
@A00721:190:HGJW7DSXY:4:1565:25292:14982_2:N:0:TCTGCAAG+GCTGCACT
CGAGAGTTGTGATCTAGTTCTAAATTTAGGTCGACGGACTTTAGCAACTTTAGATAAATTCTTTCCGG

GCTCAATGACCTTTGCTATGTACGTGGATCTAATCCTTGGTGATCTGA
+
FFFFFFFFFF:FFFFFFFFFFFFFFFFFF:FFFFFFFF:F::FF:FFFF:FFFFFFFFF:FFFFFFFF
FFFFFFFFF,F:::FFFFF:FFF:FF:FFFFFFFFFFF::FFFFFFFF
@A00721:190:HGJW7DSXY:4:1647:12500:29152_2:N:0:TCTGCAAG+GCTGCACT
TATTAGTTCATGAAGTTCTTACACTTCTCGCGACTAATGTCAGCTTCACTACAAAGGCGTTACGTAAA
GTAGTTTTCACGCTTTATCTTAAAGCATTACCACCAAATTTAGCACTT
+
FF:FFFFFFFFFFFFFFFFFFFFFFFFFFFFFFFFFFFFFFFFFFFFFFFFFFF:FFFFFFFFFFFF:
FFFFF:FFFFFFFFFFFFFFFFF:FFFFFFFFFFFFFFFFFFFFFFFF
@A00721:190:HGJW7DSXY:4:2407:12626:5071_1:N:0:TCTGCAAG+GCTGCACT
CTCGCCATATCAGATTAGAGACATGCCAGTTAGAGTTCTTCTAACCAGGACTGATGGTGAGATTCTTT
TACCTTTTGGAGGTCCAGGAACCTTTACTCCTAGTGTCTTTTCTAGAGTCCAAGGACGTTTCCGTCGG
AGG
+
FFFFFFFFFFFFFFFFFFFFFFFFFFFFFFFFFFFFFFFFFFFFFFFFFFFFFFFFFFFFFFFFFFFF
FFFFFFFFFFFFFFFFFFFFFFFFFFFFFFFFFFFFFFFFFFFFFFFFFFFFFFFFFFFFFFFFFFFF
FFF
@A00721:190:HGJW7DSXY:4:1478:22905:7279_2:N:0:TCTGCAAG+GCTGCACT
GACCGCTCAATAACACTCTATTGAATCCCTTTTCCACGATGAAAGCTCGTTATGAGGTGCTCCATGCA
AATTTACAGTTCCTCTTTGGTGATATCGCCTCTGTCCTTTCTCCTTTA
+
FFFFFFFFFFFFFFFFFFFFFFFFFFFFF,F,FFFFFFFFFFFFFFFF,FFFFFFFFFFFFFFFFFFF
:FFFFFFFFFFFFFFF:FFFFFFFFFFFFFFFFFFFFFFFFFFFF:FF
@A00721:190:HGJW7DSXY:4:1421:8811:24298_2:N:0:TCTGCAAG+GCTGCACT
ACTTGGGTTTATCGGTTGTATTTTGATAAATTTGTTGGAATCGATAATGATACTGACGAAGGTTCTAT
TACCACCCTTGTCATTGATACAAACATTCTAGGTGATGCTTTCTTAGA
+
FFFFFFFFFFFFFFFFFFFFFFFFFFFFFFFFFFFFFFFFFFFFFFF:FFFFFFFFFFFFFFFFFFFF
:FF:F:FFFFFF,FFFFFFFFFFFFFFFFFF:FFFF:FFFFFFFFFFF
@A00721:190:HGJW7DSXY:4:2170:10936:2581_2:N:0:TCTGCAAG+GCTGCACT
TTACAATCTGTAGAGAGAGAGGTTAATCAGAATTTGAGTGATAACGATTTAGTACTGGGAAATGGTGG
TAGTCAGTTTACTACTTTTCTTTCCCTCCCCTTTTTCGATTGGATTCG
+
FFFFFFFFFFFFFFFFFFFFFFFFFFFFFFFFFFFFFFFFFFFFFFFFFFFFFFFFFFFFFFFFFFFF
FFFFFFFFFFFFFFFFFFFFFFFFFFFFFFFFFFFFFFFFFFFFFFFF
@A00721:190:HGJW7DSXY:4:1163:28393:28745_2:N:0:TCTGCAAG+GCTGCACT
TTGATAAGGTTTTTCTTTTATGGGACTCTTGTTTGTACTTATGAAGGAGGTATTACTTCGGAAGTTCT
TAATGTAGGTCTCCTAGTACTTAAGGGTTTATGTTTCGGTTTCAGCTG
+
FFFFFFFF:FFFFFFFFF,FFF,FFFF:FFFFF:FFFFFFFFFFFFFF:FFFFF:FFFFFFFFFFFFF
FFFFFFFFFFFFF:FFFFFFFFFFFFFFFFFFFFFFFFFFFFFFFFFF
@A00721:190:HGJW7DSXY:4:1204:26078:3912_2:N:0:TCTGCAAG+GCTGCACT
TGTTTCACATTGCGTGATCACTTCCCGTGGACTTGGGAAGAGATTAAACATAAAATCCAGCGATTCTA
TTATGTCGCTAAAGCCTGTCCAGTTGTCGATGATGTTCGGGCTGTGGG
+
FFFFFFFFFFFFFFFFFFFFFFFFFFFFFFFFFFFFFFFFFFFFFFF:FFFFFFFFFFFFFF,:FFFF
:FF:FFFFFFFFFFFFFFFFFFFFFFFFFFFFFFFFFFFFF,FFFFF
@A00721:190:HGJW7DSXY:4:2266:16161:16172_2:N:0:TCTGCAAG+GCTGCACT
AAACTTACGACGATGTTCTCGCTCGCATTTCACAAGGATTACTAAGTCTGAGATCTGCCACTTCTAAT
TTTAGGAAACGTCGCGAAAAGAAAGTTCATGAGGATGTCTTGGTCGAA
+
FFFFFFFFFFFFFFFFFFFFFFFFFFFFFFFFFFFFFFFFFFFFFFFFFF:FFFFFFFFFFFFFFFFF
FFFFFFFFFFFFFFFFFFFFFFFFFFFFFFFFFFFFFFFFFFFFFFFF

```
@A00721:190:HGJW7DSXY:4:2264:14434:35446_2:N:0:TCTGCAAG+GCTGCACT
GAACTTCTAATAGAGGATCCTGATGACATTATTTCAACGGATACCGATCTCAGTTCTACCACAGAAAC
CGTCTCAGGAACTGTCTCCACTGATTCGGAATTAGAGAAAGACGACCT
+
FF,FFFFF:FFFFFFFFFFFFFFFFFFFFFFFFFFFFFFFFFFFFFFFFFFFFFFFFFFFFFFFFFFF
FFFFFFFFFFFFFFFFFFFFFFFFFFFFFFFFFF,FFFFFFFFFFFFFF
@A00721:190:HGJW7DSXY:4:1466:21929:12665_2:N:0:TCTGCAAG+GCTGCACT
AGACCTCTGAATTTTCAGGGTGCCCTGATGAGGACGTTGAAAGGGATAATTTCAACTCTGAGACTTCT
AAGAGTGTTTCAAAAGACCAGAAACGTAACAAGAAGAATAAAGGTAGG
+
FFFFFFFFFFFFFFFFFFFFFFFFFFFFFFFFFFFFF:FFFFFFFFFFFFFFFFFFFFFFFFFFFFFFF
FFFFFFFFFFFFFFFFFFFFFFFFFFFFFFFFFFFFFF:FFFFFFFFFF
@A00721:190:HGJW7DSXY:4:1129:4526:12085_2:N:0:TCTGCAAG+GCTGCACT
AGCGAGCGGTTGAAGAGAAAACTGATATGGTTGCGGCTGATCGTACCCCAATTCCTGCTAGAAAGATC
ACTCTCGGCGATACCACTGATCTTCCAGAGTTACGTAGTGGTCGTTCT
+
FFFFFFFFFFFFFFFFFFFFFFFFFFFFFFFFFFFFFFFFFFFFFFFFFFFFFFFFFFFFFFFFFFFF
FFFFFFFFFFFFFFFFFFFFFFFFFFFFFFFFFFFFFFFFFFFFFFFF
@A00721:190:HGJW7DSXY:4:1605:14959:9706_1:N:0:TCTGCAAG+GCTGCACT
AAGTTTCTGCTAGTCGCGACGATAGTATTTATCGGAGTGATATTAACAAGTTAAACTTCGAACTGACC
ACTTACAAGAGTGTTGAAGTACCGCAACATGGATCAGTTTTCCAAAAAGCTATTAATAGCAGTTGCGG
GGC
+
FFFFFFFFF:FFFFFFFF:FFFFFFFFFFFFFFFFFFFFFFFFF,FFFFFFFFFFFFFFFFFFFFFFFF
FFFFFFFFFFFFFFFFFFFFFFFFFFFFFFFFFFFFF:FFFFFFFFFFFFFFFFFFFFFFFFFFFFFF
FFF
@A00721:190:HGJW7DSXY:4:2225:29993:16861_2:N:0:TCTGCAAG+GCTGCACT
AAACTGAATCCACTCTCTTGGGATATAAATGAGTACATCTATAATAGAGTTTTGGAACTGTGTAATGT
CCTACGTACTGTTCAAGGTGTTTGGGCTTCTCTAAAGAACAGCAACTT
+
FFFFFFFFFFFFFFFFFF:FFFFFFFFFFFFFFFFFFFFFFFFFFFFFFFFFFFFFFFFFFFFFFFFF
FFFFFFFFFFFFFFFFFFFFFFFFFFFFFFFFFFFFFFFFFFFFFFFF
@A00721:190:HGJW7DSXY:4:2425:29577:23406_2:N:0:TCTGCAAG+GCTGCACT
AGACCTTTCTTGGGGAGTAAAGAGGGTTTGAAAATTACGGAACCTTTCCTCGTTCTTCATGAAGATCT
TCGAGTGTTTTATAGTGAGTTATTCTTGCGAAGGTTCAAGTCGATTGA
+
FFFFFFFFFFFFFFF:F:F:FF:FFFF:FFFFFFFFFFFFFF:FFFFFFFFFFFFFFFFFFFFFFFFFF:
FFFFFFFFF:FFFFFFFFFFFFFFFFFFFFFF,FFFFF:FFFFFFFFFF
@A00721:190:HGJW7DSXY:4:1611:29225:33505_2:N:0:TCTGCAAG+GCTGCACT
AATAAGAAGTTCTTCGCAGTTGAATCTCCACCAGGTGGAGGGAAGACCACTGAACTTATAGCGACTTT
TGTCGAATTTTTCGCAAAAGGTCTTGAAGTACGTATTGTCACTGCTAA
+
FFFFFFFFFFFFFFFFFFFFFFFFFFFFFFFFFFFFFFFFFFFFFFFFFFFFFFFFFFFFFF:FFFFF
FFFFFFFFFFFFFFFFFFFFF:FFFFFFFFFFFFFFFFFFFFFFFFFF
@A00721:190:HGJW7DSXY:4:1612:2808:35634_2:N:0:TCTGCAAG+GCTGCACT
TTGGCACTTCTAGTTCATCGCGGTATTTGTGAAATTAAGACAGACGTGAAAGTTATGATGAGTGATCG
TATTAGAACAGCTGATTCTTATTGCATGAACGTCGCTGAGGTCAAACC
+
FFFFFFFFFFFFFFFFFFFFFFFFFFFFFFFFFFFFFFFFFF,FFFFFFFFFFFFFFFFFFFFFFFFF
FFFFFFF,FFFFFFFFFFFFFFFFFFFFFFFFFFFFFFFFFFF:FFFF
@A00721:190:HGJW7DSXY:4:2543:6144:28917_2:N:0:TCTGCAAG+GCTGCACT
TTTTCATCGACGAATTATTCATGATTCACGCTGGTCAGACCCTTTTTCTTACACATCTACTTAAGGCG
GCTTATACCATAGGTTACGGTGATTCCAATCAAATCGGATTCATCAAT
+
```

FFFFFFFFFFFFFFFFFFFFFFFFFFFFFFFFFFFFFFFFFFFFFFFFFFFFFFFFFFFFFFFFFFFF
FFFFFFFFFFFFFFFFFFFFFFFFFFFFFFFFFFFFFFFFFFFFFFFF
@A00721:190:HGJW7DSXY:4:1477:1615:20290_2:N:0:TCTGCAAG+GCTGCACT
TCCAAGTCGCAAACTACTTCAACATCAACGACATCATTCTTGATGAGTGTCGTTCTTATCGAAGTCTC
AGTTATCGCTGCCCGGCCGACGTTTGTCGCGAACTCTCAGAGGTTTAC
+
FFFFFFFFFFFFFFFFFFFFFFFFFFFFFFFFFFFFFFFFFFFFFFFFFFFFFFF:FFFFFFFFFFFF
FFFFFFFFFFFFFFFFFFFFFF:,FFFFFFFFFFFFFFFFFFFFFFFF
@A00721:190:HGJW7DSXY:4:1635:30282:12540_2:N:0:TCTGCAAG+GCTGCACT
CTATGGAAATTAGACCTATCAGATGTCTTGACGACTTCGAATTTGAGGACGGAGTTAAATATATTACT
TTCACTCAAGGTGAAAAGTTTGAAATTAACAAAGTCCTTGGTAAGAGT
+
FFFFFFFFFFFFFFF:FFFFFFFFF:FFFFFFFFF:FFFFFFFFFFFFFFFFFFFFFF:FFFFFFFFF
FFF:FFFFFFFFFFFF:FFFFFFFFFFFFFFF:FFF:FFFFFFFFFFF
@A00721:190:HGJW7DSXY:4:2417:6153:20290_1:N:0:TCTGCAAG+GCTGCACT
GTTCAAGGTAATACTTACCAGCACGTTCGGCTGGTTAGAATTAAACCTCAAGATGACCCTGTTTTTGC
ATCACAAACACATATTACCGTTGCTCTATCACGACACGTTGAATCTTTACGATACTTTGTTATCAATG
TAA
+
FFFFFFFFFFFFFFFFFFFFFFFFFFFFFFFFF:FFFFFFFFFFFFFFFFFFFFFFFFFFFFFFFFFF
FFFFFFFFFFFFFFFFFFFFFFFFFFFFFFFFFFFFFFFFFFFFFFFFFFFFFFFFFFFFFFFFF:FF
FFF
@A00721:190:HGJW7DSXY:4:1606:32796:23625_2:N:0:TCTGCAAG+GCTGCACT
AAGGAACTGTCACGTTTAGCACGGGAGGTTGCAGATTCAACCCCTTATATCGAGGCTATCGAGGACGA
TGAGTATCTTGAAGTTGTTGACGACAAGCCTCAAGAGGGTAGAGGACC
+
FFFFFFFFFFFFFFF,FFFFFFFFFFFFF:F,FFFFFFFFFFFFFFFFFFFFFF,FFFFFFFFFFFFF
,FF,FFFFFFFFFFFFFFFF:FFFFFFFF:FFFFF:FF,FF:FF:FFF
@A00721:190:HGJW7DSXY:4:2442:28818:1799_2:N:0:TCTGCAAG+GCTGCACT
GGTTATTCCTGGAACCACCTTAACGTCACTCGGTAATCTTTCAGCCGAATTAAGTACTAATGAGTTCA
AGTCGGTTGTCGATCGAATTAAGATCAAGGCTAATACCTTTATAGGTA
+
FFFFFFFFFFFFFFFFFFFFFFFFFFFFFFFFFFFFFFFFFFFFFFFFFFFFFFFFFFFFFFFFFFF:
F:FFFFFFFFFFF:FFFFFFFFFFFFFFFFFFFFFFFFFFFFFF,FFF
@A00721:190:HGJW7DSXY:4:1344:32407:27837_1:N:0:TCTGCAAG+GCTGCACT
TCATCAGTCAGAGTATACCCGATAGGGTACCGAGTCTCCAAGAGAATATTTATTCTTATGAGGCTCGA
AACTTCAACGTTTTCACAACGGAAAGACATATCGCACCCGAAATTTTTGGGCAGAATGCTCTTAACGC
GGT
+
FFF:FFFFFFFF:FFFFFFFFF:FFF:FFFFFF:FFFFFFF,FFFFFFFF:FFFFFFFFFFFFFFFFF
FFFFFFFFFF,FFFFFFFFFFFFFFFFFFFFFFFFFFFFFFFF,FFFFFFFFFFFFFFF:FFFFFFFF
FFF
@A00721:190:HGJW7DSXY:4:1347:17363:7169_2:N:0:TCTGCAAG+GCTGCACT
GATCAGTCTAAGTTGGATAAAGTTATAGGAGACTCAGTTAACCTGAGAGCTCCTCAATTGATAGATTG
GGTCAACAATAGGGACGCTAGTATACTTCGTAAAGTCGACAGCGAAAT
+
FFFFF:FFFFFFF:FFFFFFFFFF:FFFFFFFFFFFFFFFFFFFFFFFFFFFFFFFFFFFFFFFFFFF
FFFFFFFFFFFFFFFFFF:FFFFFFFFFFFF:FFFFFFFFFFFFFFFF
@A00721:190:HGJW7DSXY:4:1143:32117:13338_2:N:0:TCTGCAAG+GCTGCACT
TAATTGGACCGACTATATGACCTATTTTAAGCTCATGGTGAAAAGGGAAGCTAAGGTTAAACTTGATG
CTTCAGCCTTTTCGAAGCATAACCCCGCACAGAATATCGTTTATCACG
+
FFFFFFFFF,FF,FFFFFF:FFFFF:FFFFFFFFFFFFFFFFF,FFFFFFFFFFFF:FF::FFFFFFF
FFFFFFFFFFFFFFFFFFF:FFFFFFFFFF,FFFFFFFFF,FFFFFFF

```
@A00721:190:HGJW7DSXY:4:1320:16288:5838_2:N:0:TCTGCAAG+GCTGCACT
CCTATCTTTAAGGTTCTTGTGAGGAGAGTCAAACAGTGTCTTTTACCCAACATTTTTATGTATACAGA
GATGACCAGTGACGATTTCTCCCGGAAGCTCTTCTCTCTCTTAGGAGG
+
FFFFFFFFFFFFFFFFFFFFFFFFFFFFFFFFFFFFFFFF:FFFFFFFFFFFFFFFFFFFFFFFFFFFFF
FFFFFFFFFFFFFFFFFFFFFFFFFFFFFFFFFFFFFFFFFFFFFFFFF
@A00721:190:HGJW7DSXY:4:1158:24560:13369_2:N:0:TCTGCAAG+GCTGCACT
TTTCTTTTGTAAAGCCTATGAGATAAAGATGTATTCTTATTTAGGCTTCGATGCTGAATTTCTTGAAC
TTTGGAGTGCCAGTGAATATTTTTGTCACGCCAGGAGTATCCATAACG
+
FFFFFFFFFFFFFFFFFFFFFFFFFFFFFFFFFFFFFFFFFFFFFFFFFFFFF,FF:FFFFFFFFFFFFF
:FFFFFFFFFFFFFFFFFFFFFFFFFFFFFFFFFFFFFFFFFFFFFFFF
@A00721:190:HGJW7DSXY:4:1373:12011:16407_2:N:0:TCTGCAAG+GCTGCACT
TACCACCTTTGCTCAACGACGAAGTGGAACGGCCAACACTTTCTTTGGTAATTGTATGATCACTTTGA
TGATTTTATCACTTTATTATGACCTTGGAAAGTTTTCGGCTGTGGTTG
+
FFFFFFFFFFFFFFFFFFFFFFFFFFFFFFFFFFFFFFFFFFFFFF:FFFFFFFFFFFFFFFFF:FFFFF
FFFFFFFFFFFFFFFFFFFFF:FFFF:FFFFFFFFFFFFFFFFFFFFFF
@A00721:190:HGJW7DSXY:4:2656:12689:10848_1:N:0:TCTGCAAG+GCTGCACT
CATTCTGTTTTCAAAGACTCCAATAACTGATCATTCTGATCATATGATTTTGGACAATGGTTTCGAAG
CGAAATTTTTGAGGGATAAACCAGCGTACTTCTGCTCGCGATTTATTCTTTTTGTCAATGATTACATC
TAC
+
FFFFFFF:FFFFFFFFFFFFFFFFFFFFFFFFFFFFFFFFFFFFFFFFFFFFFFFFFFFFFFFFFFFFFF
FFFFFFFFFFFFFFFFFFFFFFFFFFFFFFFFFFFFFFFFFFFFFFFFFFFFFFFFFFFFFFFFFFFFFF
FFF
@A00721:190:HGJW7DSXY:4:2638:7518:9377_1:N:0:TCTGCAAG+GCTGCACT
CCTTACAAGATCATCGTTAAGTTAGGTAAACCCCTAACCGATGTTACTCTTGTAGGACTTTGGGAGAG
ATTCGTTAGTTTTAAAGACTATACTAAATATCTCCACTACGAGGTTGTTGTTTGGCGATTAGCTCTTG
CGA
+
FFFF:FFFFFFFFFFFFFFFFFFFFFFFFFFFFFFF:FFFFFFFFFFFFFFFFFFFFFFFFFFFFFFFFF
FFFFFFFFFFFFFF,FFFFFFFFFFFFFFFFFFFFFFFFFFFFFFFFFFFFFFFFFFFFFFFFFFFFFFF
FFF
@A00721:190:HGJW7DSXY:4:2577:4191:32518_2:N:0:TCTGCAAG+GCTGCACT
ATTGAGGGTCGTTATACCTATTCGGCTATCTGTGCTTTGAATTGTATAAGATCAAATTTTAAACGATT
CCGAGAGGTTTATCCTGATGTGGAGGGAGTTCTTGAAGCATCCCGACC
+
F,FFFFFFFFFFFFFFFFFFFFFFFFFFFFFFFFFFFFFFFFFFFFFFFFFFFFFFFFFFFFFFFFFFFF
FFFFFFFFFFFFFFFFFFFFFFFFF:FFFFFFFFFFFFFFFFFFF:FFF
@A00721:190:HGJW7DSXY:4:1306:20021:21104_2:N:0:TCTGCAAG+GCTGCACT
GGTTTCATAAGATTATCAGTAATCCTGATTCTACTACGGATTCATGGACCTTCCATCCTGTACCTCTT
CTGGAGAGACGTATCTCAAAGATCGACTTCGAGACAGGTGATATTGTT
+
FFFFFFFFFFFFFFFFFFFFFFFFFFFFFFFFFFFFFFFFFFFFFFFFFFFFFFFFFFFFFFFFFFFFFF
F:FFFFFFFFFFFFFFFFFFFFFFFFFFFFFFFFFFFFFFFFFFFFFFF
@A00721:190:HGJW7DSXY:4:1310:18973:5979_1:N:0:TCTGCAAG+GCTGCACT
ACCAAGATCTAAATTCGGTAACGGTGCGTGGCGCTTTATTTTAAAAAGCTCCCTCCTCTCGCAAGTTT
GATCTGATACTTATCACGAAAGGACTTGAGTCTTTTGATCAGTCTCTGACTTGACCATCTTTCATTTT
AAA
+
FF:FFFFFFFFFFFFFFFFFFFFFFFFFFFFFFFFFFFFFFFFFFFFFFFFFFFFFFFFFFFFFFFFFFF
FFFFFFFFFFFFFFFFFFFFFFFFFFFFFFFFFFFFFFFFFFFFFFFFFFFFFFFFFFFFFFFFFFFFFF
FFF
```

@A00721:190:HGJW7DSXY:4:1334:25672:17926_2:N:0:TCTGCAAG+GCTGCACT
TTTCTTCTTTGTTTCCTTCTTTGCACTGATTTTTCCTCATCAGCTTGTGTTTTACCTAATCTGTGTTT
TTACGATAACAACTATCAGTTGATTAAGACTCTTCCGAAAGGCGAACT
+
FFFFFFFFFFFFFFFFFFFFFF::FFFFFFFFFFFFFFFFFFFFFFFFFFFFFFFFFFFFF,F:FFFFF
FF::FF,FFFFFFFFF:F,FFFFFFFFFF,FFFFFFFFFF:FFFFFFF
@A00721:190:HGJW7DSXY:4:1372:4544:21919_2:N:0:TCTGCAAG+GCTGCACT
GAAGCTCAGGGTGAAGCACAACGTTTGACCGTGACGAACTGTGGTGTTCTTGATCTACGTTACGGATA
TACTCTCCTTGAGTTCACTCGTAAGGACACTACCACTTATTACGATAT
+
FFFFFFFFFFFFFFFFFFFFFFFFFFFFFFFFFFFFFFFFF:FFFFFFFFFFFFFFFFFFFFFFFFFF
FFFFFFFFFFFFFFFFFFFFFFFF:FFFFFFFFFFFFFFFFFFFFFFFF
@A00721:190:HGJW7DSXY:4:2572:10691:23923_2:N:0:TCTGCAAG+GCTGCACT
CCTTTAAGCGTCTTCTGTTCATCGCAACCAGATGATATCTTCTATTCGAACTTATGTTTCGATCAGTG
TTTCTTCTATCAGGAGGAAAACACTTGTTCTAGTCCTTGTAAGATTAA
+
FFFFFF:FFFFFFFFFFFFFF:FFFFFFFFFFFFFFFFFFFFFFFFFFFFFFFFFFFFFFFFFFFFFF
FFFFFFFFFFFFFFFFFFFFFFFFFFFFFFFFFFFF:FFFF:FFFFFF
@A00721:190:HGJW7DSXY:4:1210:12500:16532_2:N:0:TCTGCAAG+GCTGCACT
TCTTCTAATGTGGCCTGAACTACGTTTCACTTTGTGTGTACTTGTCTCTATTTTAAGTTTTGTCTTCC
TTGTTTGTCTTATTCTATTAATCACTCATTTTAGTGAGATTATTAAAA
+
FFFFFFFFFFFFFFFFFFFFFFFFFFFFFFFFFFFFFFFFFFFFFFFFFFFFFFFFFFFFFFFFFFFF
FFFFFFFFFFFFFFFFFFFFFFF:F:FFFFFFFFFFFFFFFFFFFFFF
@A00721:190:HGJW7DSXY:4:1508:31937:30686_1:N:0:TCTGCAAG+GCTGCACT
TCTTCGACAACAGCAGGGGTTTTCGAATAGTGATAGCCACCCGCCTTACAGAGGGGCAGCAGCATGAC
CATAATTGGTATTGACTTTGGTACCACCTTTTCTACTGTCTGTCTCGCCTCTTCGAATGAAGTTTTAA
TGC
+
FFFFFFFFFFFFFFFFFFFFFFFFFFFFFFFFFFFFFFFFFFFFFFFFFFFFFFFFFFFFFFFFFFFF
FFFFFFFFFFFFFFFFFFFFFFFFFFFFFFFFFFFFFFFFFFFFFFFFFFFFFFFFFFFFFFFFFFFF
FFF
@A00721:190:HGJW7DSXY:4:1606:4652:34068_2:N:0:TCTGCAAG+GCTGCACT
CTACAATCATTGGTTTTTCTAATAATGATGACACTATTGTTTATGGATATGATGCTATTACTCATGAT
GGTTACGGTCAGGGAGGATTCTCAGTTTACAAGGACCTTAAGCGATGG
+
FFFFF:FFFFFFFFFFFFFFFFFFFFFFFFFFFFF:FFFF,FFFFFFFFFFFFFFFFFFFFFFFFFFF
FFFFFFFFFFFFFFFFFFFFFFFFFFFFFFFFFFFFFFFFFFFFFFFF
@A00721:190:HGJW7DSXY:4:1551:18069:4382_2:N:0:TCTGCAAG+GCTGCACT
CCGAAAGACGTGCTAAATTAAAACCATTGTACGATGTCGTCTGTCTCGGCGATCACTTTTCACTAAGA
ATTGGTCCTACTTTTGGACAAGGCCAACTTCGTAATCTTCATGAGATC
+
FFFFFFFFFFFFFFFFFFFFFFFFFFFFFFFFFFFFFFFFFFFFFFFFFFFFFFFFFFFFFFFFFFFF
FFFFFFFFFFFFFFF,FFFFFFFFFFFFFFFFFFFFFFFFFFFFFFFF
@A00721:190:HGJW7DSXY:4:1435:20763:4069_1:N:0:TCTGCAAG+GCTGCACT
TGTTAGGGATTTTGAGAGAGTGAAAGGCTTCAATTGTAGTGGTTTGGTCGTTTCAGTACCCAGTCAGT
ATACCTCTACGCAAAGATATTTCATGAAAACTACAGAGGAACGTACCGGATTACCTGTCTTTCATATT
ATG
+
FFFFFFFFFFFFFFFFFFFFFFFFFFFFFFFFFF:FFFFFFFFFFFFFFF:FFFFFFFFFFFFFFFFF
FFFFFFFFFFFFFFFFFFFFFFFFFFFFFFFFFFFFFFFFFFFFFFFFFFFFFFFFFFFFFFFFFFFF
FFF
@A00721:190:HGJW7DSXY:4:1168:23999:29465_1:N:0:TCTGCAAG+GCTGCACT
CTCTTTGCTTCGATGTTGGATGTCAAGAAGGGTTCAGAATGGGATTCCTACGTAGTTTATGACTTTGG

TGGTGGTACTTTCGATACTTCTATCGTCTTACGTTACGGTAATTACTATAGTGTTATTTATTCTGGTG
GTG
+
FFFFFFFFFFFFFFFFFFFFFFFFFFFFFFFFFFFFFFFFFFFFFFFFFFFFFFFFFFFFFFFFFFFF
FFFFFFFFFFFFFFFFFFFFFFFFFFFFFFFFFFFFFFFFFFFFFFFFFFFFFFFFFFFFFFFFFFFF
FFF
@A00721:190:HGJW7DSXY:4:1202:30101:32487_2:N:0:TCTGCAAG+GCTGCACT
AGGTCGTGACGTTGATGAAGCTATAAGAGCGTTCTTATTACGGCGCACTTCTTTACCCGCCACCACAC
AACTTTCAGTTTCACAACTGAAGGAGGAGGTTTCTGTTACTGGAAAAC
+
FFFFFFFFFFFFFFFFFFFFFFFFFFFFFFFFFFFFFFFFFFFFFFFFFFFFFFFFFFFFFFFFFFFF
FFFFFFFFFFFFFFFFFFFFFFFFFFFFFFFFFFFFFFFFFFFFFFFF
@A00721:190:HGJW7DSXY:4:1112:30400:33285_1:N:0:TCTGCAAG+GCTGCACT
ACTTTTCAATAATATACGTCTTGAACTCACTTATGACGATCTAGTGAAATTAACGCGTCCTTTTCTGG
AACGTAGTTTTACTATTATGAACAAAGTTATCAAAGATAGTGGCATTCAAGGCAAACTTACCCTGATT
CCG
+
F,FFFFFFFFFFFFFFFFFFFFFFFFFFFFFFFFFFFFFFFFFFFFFFFFFFFFFFFFFFFFFFFFFF
FFFFFFFFFFFFFFFFFFFFFFFFFFFFFFFFFFFFFFFFFFFFFFFFFFFFFFFFFFFFFFFFFFFF
FFF
@A00721:190:HGJW7DSXY:4:1137:30888:34475_1:N:0:TCTGCAAG+GCTGCACT
TTCTACCAGGGGCTATTACTCTTGCTGAAACAGCTATTAAAGCTGTTGAGCGGGTAATTACACCTAAA
CGAGCTTGATCTGCCATCGCTGAAGGATGTGCACTCTTTTCTGCTACTTTAACGGAGCGTAGTGTACT
CTT
+
FFFFFFFFFFFFFFFFFFFFFFFFFFFFFFFFFFFFFFFFFFFFFFFFFFFFFFFFFFFFFFFFFFFF
FFFFFFFFFFFFF:FFFFFFFFFFFFFFFFFFFFFFFFFFFFFFFFFFFFFFFFFFFFFFFFFFFFFFFF
FFF
@A00721:190:HGJW7DSXY:4:1114:23023:9298_1:N:0:TCTGCAAG+GCTGCACT
CAACAGTAACATTACTTCTAATAAAGGTCTCTATCAAGCTCGTATTATCGTCGCTGCTGGATCTGCGT
TACCGTTGAAGGCTAAACTCCAAGGTAATTATGAAGACTGTGATTTATCTTTCACTCTGGCCTTGTAT
GAG
+
FFFFFFFFFFFFFFFFFFFFFFF:FFFFFFFFFFFFFFFFFFFFFFFFFFFFFFFFFFFFFFFFFFFF
FFFFFFFFFFFFFFFFFFFFFFFFFFFFFFFFFFFFFFFFFFFFFFFFFFFFFFFFFFFFFFFFFFFF
FFF
@A00721:190:HGJW7DSXY:4:2111:18539:1877_2:N:0:TCTGCAAG+GCTGCACT
ATCGTAACATTAATAATTTCAATCTTCTTAAAAAGAAGATAGAGAGTCATACTTTGGGCTATCGTTCA
GGTGCATGGGGTTGGATAGCCAATATTGAAGTGGACACCTCTGGTAGT
+
FFFFFFFFFFFFFFFFFFFFFFFFFFFFFFFFFFFFFFFFFFFFFFFFFFFFFFFFFFFFFFFFFFFF
FFFFFFFFFFFFFFFFFFFFFFFFFFFFFFFFFFFFFFFFFFFFFFFF
@A00721:190:HGJW7DSXY:4:2602:27959:29496_1:N:0:TCTGCAAG+GCTGCACT
AGATAACCAATAAGGAAGGAGCTTATACTATGGTTGAATCAACTAAGCATACTACTTTTGCTTTGATT
ACTCTACCGCCTTACAAGTCTAAGGTTAAAGAGTCGATTAAACAAGTTGTACTTATCAATGATTTTAT
CGC
+
FFFFFFFFF:FFFFFFF:FFFFFFFFFFFFFFFFFFFFFFFFFFFFFFFFFFFFFFFFFFFFFFFFFF
FFFFFFFFFFFFFFFFFFFFFFFFFFFFFFFFFFFFFFFFFFFFFFFFFFFFFFFFFFFFFFFFFFFF
FFF
@A00721:190:HGJW7DSXY:4:1478:32533:3881_1:N:0:TCTGCAAG+GCTGCACT
ACCCAAAGAGAATATCTAAAATATTTACAAAATCTTACCACTATCTACGAACCTAAAGTTCTGAAGAC
GTTGCTTGTCGCTTCCTCCTTTAATGGCACTACCTCGGTACAATCTTACTTGGAACGAAGGCGACCTG
TTC

+
FFFFFF:FFFFFFFFFFFFFFFFFFFFFFFFFFFFFFFFF:FFF:FFFFFFFFFFFFFFFFFFFFFFFFF
FFFFFFFFFFFFFFFFFFFFFFFFFFFFFFFFFFFFFFFFFFFFFFFFFFFFFFFFFFFFFFFFFFFFFF
FFF
@A00721:190:HGJW7DSXY:4:1163:11840:12853_1:N:0:TCTGCAAG+GCTGCACT
TGTTCTGTCTATATTAGGTGAAGCGAAATACGATTTCGAACGTCAACGTTCACCGAGTTTCGTTTCGT
ACCTTGATGCTGACTTTCGTCAATATGATTTGATCTGGTTGCTCAATTCTAAGCGTGTTCCGCTTAGT
GCG
+
FFFFFFFFFFFFFF:FFFFFFFFFFFFFFFFFFFFFF:FFFFFFFFFFFFFFFFFFFFFFFFFFFFFFF
FFFFFFFFFFFFFFFFFFFFFFFFFFFFFFFF::FFFFFFFFFFFFFFFFFFFFFFFFFFFFFFFFFFFF
FF:
@A00721:190:HGJW7DSXY:4:1218:31331:11663_2:N:0:TCTGCAAG+GCTGCACT
GAGTATTGCTATTTATACGGTTACGAGAAGAAGGTTCGAAAGTTTGAGAACTTAGTTTACGAACTTGA
CTCTACTATACGTGACATACTAAATACTAGATATTTGGATGATCTTGA
+
FFFFFFFFFFFFFFFFFFFFFFFFFFFFFFFFFFFFFFFFFFFFFFFFFFFFFFFFFFFFFFFFFFFFFF
FFFFFFFFFFFFFFFFFFFFFFFFFFFFFFFFFFFFFF:FFFFFFFFFF
@A00721:190:HGJW7DSXY:4:1243:26060:18474_2:N:0:TCTGCAAG+GCTGCACT
CATGAATTTTCAGAGGATGAAAAATTTTCTTCCTTGGATTTTAATATAAAGAATCTTTACCAGGACTC
CTACTACAGGAGGCAGTCTTCTGATCGTAAGAAGTTTATAAGGAACAT
+
FFFFFFFFFFFFFFFF:FFFFFFFFF:FFFFFFFFFFFFFFFFFFFFFFFFFFFFFFFFFFFFFFFFF:F
FFFFFF:FFFFFFFFFFFFFFFFFFFFFFFFFFFFFFFFF:FFFFFFFFF
@A00721:190:HGJW7DSXY:4:2177:16152:19006_1:N:0:TCTGCAAG+GCTGCACT
ACTCGAAACTCTAAGTGGTAATACTGTCTTTCCAAGCTTACCTGTATCTTCAATGAAATTGGACTCTT
CGATAGGGGACATTTTATCGTGGGCTTTCCGTAAAGGAAGATCTGTTATCGACGGTTTATCGTTTACG
AAG
+
FFFFFFFFFFFFFFFFFFFFFFFFFFFFFFFFFFFFFFFFFFFFFF:FFF,FFFFFFFFFFFFFFFFFFF
FFFFFFFFFFFFFFFFFFFFFFFFFFFFFFFFFFFFF,FFFFFFF:FFFFFFFFFFFFFFFFFFFF:FFFFF
FFF
@A00721:190:HGJW7DSXY:4:2452:3314:11115_2:N:0:TCTGCAAG+GCTGCACT
GTTTAAGAATACTATAAATAGAATAGCTATTACTACTAATGGCGTTGGCCTAGTGGATGTTATTAGTG
ACATCTGGTCAATTTCGTTTTTAGGTGGTCTTTATTCTATTATTGATT
+
FFFFFFFFFFFFFFFFFFFFFFFFFFFFFFFFFFFFFFFFFFFFFFFFFFFFFFFFFFFFFFFFFFFFFF
FFFFFFFFFFFFFFFFFFFFFFFFFFFFFFFFFFFFFFFFFFFFFFFFF
@A00721:190:HGJW7DSXY:4:1457:6750:27774_1:N:0:TCTGCAAG+GCTGCACT
ACAGAAGATGATGTTAGAGTAGAATTCCTGAAGAGTCCTTCCGTAACCAACTTCTTGACAATTTTCTT
CAATCCTTCGATAATAGTTAAATCTAGGATTATGTCTGTGTTGAAAGACTTTCTACCGATAAGGGTCA
CTA
+
FFFFFFFFFFFFFFFFFFFFFF:FFFFFFFFFF:FFFFFFFFFFFFFFFFFFFF:FFFFFFFFFFFFFF
FFFFF:FFFFFFFFFFFFFFFFFFFFF:FFFFFFFFFFFFFFFFFFFFFFFFFFFFF:FFFFFFFFFFF
FFF
@A00721:190:HGJW7DSXY:4:2356:32009:3411_2:N:0:TCTGCAAG+GCTGCACT
TAAGAAATAATCCTACATTGACTGATTATCTAAAGATTGAAGGAAAATTTTATCCTCGTTTAACTTAC
TTAGACTTTCCTACGATACAAATGATAACTTCTAATTTACGTTTGCTA
+
FFFF:FFFFFFFFFFFFFFFFFFFFFFFFFFFFFFFFFFFFFFFFF::FFFF:FF:FFFFFFFFFFFFF
FFFFFFFFFFFFFFFFFFFFFFFFFFFFF:FFFFFFFFFFF:FFFF,FF
@A00721:190:HGJW7DSXY:4:2441:24695:27696_2:N:0:TCTGCAAG+GCTGCACT
CAAAGGGATGCATGGTGTTATCTCCTCAGGAGTCCCGGCATTTGATAATAATGACGAGACTTTCGTCA

```
TAATCGCTGCATATTACGGATTACACGGTACCAGCGCATCTCGTGTCG
+
FFFFFFFFFFFFFFFFFFFFFFFFFFFFFFFFFFFFFFFFF:FFFFFFFFFFFFFFFFFFFFFFFFFFFFF
FFFFFFFFFFF,FFFFFFFFFFFFFFFFFFFFFFFFFFFFFFFFFFFF
@A00721:190:HGJW7DSXY:4:1314:6207:14904_1:N:0:TCTGCAAG+GCTGCACT
ATTAACTTATAAAGGTGAGGAGACCATTTTCTTGCTGAAATTTATAGAAGATTATTTTGACAGAATCA
ATAAGAATCATCGATTCTCGATTCGTAGAGCTTATCTAGGTACTCTCATTTACTATGTTGATTTAGTC
TAC
+
FFFFFFFFFFFFFFFFFFFFFFFFFFFFFFFFFFFFFFFFFFFFFFFFFFFFFFFFF:FFF:FFFF:FFF
FFFFFFFFFFFFFFFFFFFFFFFFFFFFFFFFFFFFFFFFFFFFFFFFFFFFFFFFFFFFFFFFFFFFFF
FFF
@A00721:190:HGJW7DSXY:4:2532:2456:7623_2:N:0:TCTGCAAG+GCTGCACT
GATTTACAACGATATAGGAGTAATCTACGAGAAGATTCTAGAATTCTTAATTATAATATTCAACCTCA
AAAGTTACGTGTTTACAGTAACGAGATCACACATTTTGAGAACTAACT
+
FFFFFFFFFFFFF:FFFFFFFFFFFFFFFFFFFFFFFFFFFFFFFFFFFFFFFFFFFFFFFFFFFFFFFFF
FFFFFFFFFFFFFFFFFFFFFFFFFFFFFFFFFFF:F:FFFFFFFFFFFF
@A00721:190:HGJW7DSXY:4:2666:11912:30326_1:N:0:TCTGCAAG+GCTGCACT
CAATCTCTTCTTATAATAAATAAATAAATAATATTACTAATGGCAAATAGTGGAAAGGCTCCAGTCAA
CACTAGTTCGAGTGCTGCTTTGGATTACGATGACGTGAGAATAGGTGAAGGGATAGTTTTTACCTCGA
CCA
+
:FFFFFFFFFFFFFFFFFFFFFFFFFFFFFFFFFFFFFFFFFFFFFFFFFFFFFFFFFFFFFFFFFFFFFF
FFFFFFFFFFFFFFFFFFFFFFFFFFFFFFFFFFFFFFFFFFFFFFFFFFFFFFFFFFFFFFFFFFFFFF
FFF
@A00721:190:HGJW7DSXY:4:1240:15067:30843_2:N:0:TCTGCAAG+GCTGCACT
GCGACTAATCTTAAAGCTTCACCCAAAATGAAGGACACTTCGAAACTGAATAATGATGCACTACAAAC
AAGTATCGCTAATCATCTGAAAAAGGCTCTAAAGGACACTATACAATA
+
FFFFFFFFFFFFFFFFFFFFFFFFFFFFFFFFFFFFFFFFFFFFFFFFFFFFFFFFFFFFFFFFFFFFFF
FFFF:FFFFFFFFFFFFFFFFFFF,FFFFFFFFFFFFFFFFFFFFFFFFF
@A00721:190:HGJW7DSXY:4:2672:10791:17863_1:N:0:TCTGCAAG+GCTGCACT
TTGGTGCTTAGGGATATAATGAATTCAGCACCACATTTTGGTCAAGTCAATCCGTTGAGAAAATTTCT
TAGAGGATATTCGCCATTGTGGTACGAATTGGTCCAACATTTACCACCAAATTCTTTTGATACAACTC
TAG
+
FFFF:F,FFF,FFFFFF:FFFFFFFFFFFFF:FFFFFFFFFFFFFFFFFFFF:FFFFF,FFFFFFFFF
FFFFFFFFFFF:FFFFFFFFFFFFFFFFFFFFFFF:FFFFFFFFFFFFFFFFFFFF:FFFFFFFFFF:F
FFF
@A00721:190:HGJW7DSXY:4:1528:29722:23907_2:N:0:TCTGCAAG+GCTGCACT
CGGAATACGCTTGCTATTCACCAGATTGGTTCGAACCCGTTGATGCTATGTCAGAAGAAGCTATTCAA
GCTTGTCGTTTAAAGACTGCAGAGGCTGTCAAACAAGCCCAGACTCAA
+
FFFFFFFFFFFFFFFFFFFFFFF:FFFFFFFFFFFFFFFFFFFFFFFFF:FFFFFFFFFFFFFFFFFFFF
FFFFFFFFFFFFFFFFFFFFFFFFFFFFFFFFF:FFF:FFFFFFFFFFF
@A00721:190:HGJW7DSXY:4:1127:23068:6433_1:N:0:TCTGCAAG+GCTGCACT
AATTTAGCTCAACTGGGGAGACGTGCTTAAATGGGTGATAAAAATTATTTAGAGATCTTTTCTACTTA
CAAAGGACAATTCAGTTTCATTAATTCTACGGAAAAGTCCGGAGTATATTGTATGTTAATAGATAGTG
GGA
+
FFFFFFFFF:FFFFFFFFFFFFFFF:FFFFFFFFFFFFFFFFFFFFFFFFFFFFFFFFFFFFFFFFFFFFF
FFFFFFFFFFFFFFFFFFFFFFFFFFFFFFFFFFFFFFFFFFFFFFFFFFFFFFFFFFFFFFFFFFFFFF
FFF
```

@A00721:190:HGJW7DSXY:4:1110:5981:4398_2:N:0:TCTGCAAG+GCTGCACT
GAACGCGTTCGTATAACCAAGTACTTTCCGTCTGAAGAGTTTAGAGCTGCTATTGATTATGTAACCGA
TAGGAGTTGTATAGTTGAAGATGTGGCTTTAGACGATAATCCTTTAAG
+
FF:FFFFFFFFF:FFFFFFFFFFFFFFFFFFFFFFFFFFFFFFFFFFFFFFFFFFFFFFFFFFFFFF
,FFFFFFFF:FFFFFFFFFFFFFFFFFFFFFFFFFFFFFFFFFFFFFF
@A00721:190:HGJW7DSXY:4:1264:6027:13056_1:N:0:TCTGCAAG+GCTGCACT
GAAGATTTTCCCACAGATTTGTCTACGTTGCAGATCGCCACTCCAAACACTATATACATTATAAAATA
TAAAAATAAGGGAGTCATAACTTTTGTTACCAAATTCTTGGCTTTCAACGAAGTCTCTGATTTTATTA
ATC
+
FFFFFFFFFFFFFFFFFFFFFFFFFFFFFFFFFFFFFFFFFFFFF,FFFFFFFFFFFFFFFFFFFFFF
FFFFFFFFFFFFFFFFFFFFFFFFFFFFFFFFFFFFFFFFFFFFFFFFFFFFFFFFFFFFFFFFFFFF
FFF
@A00721:190:HGJW7DSXY:4:2278:19144:6496_1:N:0:TCTGCAAG+GCTGCACT
CGATCTCTCGGTCATTTACTCTGTACTTGATCTACGTCGTTTAACTGTCGGAACAACCTTTACTTAGA
ATGACTCTATCTTTTCAGACACTTCTTATATTATTTACAATAGTTAATATCATATTAGTAATACTCAC
TAT
+
FFFFFFFFFFFFFFFFFFFFFFFFFFFFFFFFFFFFFFFFFFFFFFFFFFFFFFFFFFFFFFF:FFFF
FFFFFFFFFFF:FFFFFFFFFFFFFFFFFFFFFFFFFFFFFFFFFFFFFFFFFFFFFFFF
FFF
@A00721:190:HGJW7DSXY:4:1208:17481:35837_2:N:0:TCTGCAAG+GCTGCACT
TTTTCTTAGCTAATAGATTAATAAAGGATGAGTACGAAAACCAACTCTTCGTTACGAGACCTGATGGT
AGAATAGATTTAGTCTCTACACCCATCTCAACGAGGATAAAGCGAGTT
+
FFFFFFFFFFFFFFFFF:FFFFFFFFFFFFFFFFFFFFFFFFFFFFFFFFFFFFFFFFFFFFFFFFFF
FFFFFFFFF:FFFFFFFFFFFFFFFFFFFFFFFFFFFFFFFFFFFFFF
@A00721:190:HGJW7DSXY:4:1517:28501:22952_2:N:0:TCTGCAAG+GCTGCACT
ACACGAAGAGATCATCCGCAAACGACTCGAAACCGAGACGCCGTAGATCAAAGTCCCTTCTAACTTTA
GATTATGAATCCTGCCTTTACTCATTTTATAGCAGTTATTTATGTTGT
+
FFFFFFFFFFFFFFFFFFFFFFFFFFFFF:FFFFFFFFFFFFFFFFFFFFFFFFFFFFFFFFF:FFFF
FFFFF:FFFFFFFFF:FFFFFFFFFFFFFFFFFFFFFFFF:FFFFFFF
@A00721:190:HGJW7DSXY:4:1673:15022:4460_1:N:0:TCTGCAAG+GCTGCACT
TTTATAACCTTCTTATACTTTTGGGTATGTTTTGTGAAAAACATGAGATACCCAAGTCTATCGAAGAC
ATTATCTCTTATGTACTTATTGTAACGATCATTCTTTCGAGTTTTGTCATTCTTATTCTTTTATTCCT
TTA
+
FFFFFFFFFFFFFFFFFFF:FFFFFFFFFFFFFFFFFFFFFFFFFFFFFFFFFFFFFFFFFFFFFFFF
FFFFFFFFFFFFFFFFFFFFFFFFFFFFFFFFFFFFFFFFFFFFFFFFFFFFFFFFFFFFFFFFFFFF
FFF
@A00721:190:HGJW7DSXY:4:1361:22037:18145_2:N:0:TCTGCAAG+GCTGCACT
TTTATAATGCTTTTCGATAGTGATTGTTATCAATTTAAATACATTACTGTTTCAAATCATCTTTACTC
ACAGACTCCGAAAATGGTTTCAAATGAGGTCAAAGCTCTATTTATTAA
+
FFFFFFFFFFFF:FFFFFFFFFFFFFFFFFFFFFFFFFFFFFFFFFFFFFFFFFF:FFFFFFFFFFFF
FFFFFFFFFFFFFFFFFFFF:FFFFFFFFFFFF,FFFFFFFFFFFF:F
@A00721:190:HGJW7DSXY:4:1409:3522:34115_1:N:0:TCTGCAAG+GCTGCACT
CACTACCACTGATCTGCCGACTTTAGTCAAGGAACTTCAATTCCTTCGATGTTTATATTTTTCTATAA
GGGAAAATATCAATAATCAAATTTCGACAAATTTTAGAACTTTATTTCAGAACTCTCTCCGATCACCT
TGG
+
FFFFFFFFFFFFFFFFFFFFFFFFFFFFFFFFFFFFFFFFF:FFFFFFFFF:FFFFFFFFFFFFFFFF

F:FFFFFFFFFFFFF:FFFFFFFFFFFFFFFFFFFFFFFFFFFFFFFFFFFFFFFFFFFFFFFFFFFF
FFF
@A00721:190:HGJW7DSXY:4:1513:21730:23500_2:N:0:TCTGCAAG+GCTGCACT
GGAGGATCTTTTCAATAAGATGTATGATGATAATCTACTATTTTTAAATAGTGATATTCAGCGTATAC
TTGAATACTTTATCAAAGATTTGGCTTGGTCTATTTACCGCAAAAAGT
+
FFFFFFFFFFFFFFFFFFFFFFFFFFFFFFFFFFFFFFFFFFFFFFFF:FFFFFFFFFFFFFFFFFFF
FFFFFFFFFFFFFFFFFFFFFFFFFF:FFFFFFFFFFFFF:F:FFFFFF
@A00721:190:HGJW7DSXY:4:1304:5303:6793_2:N:0:TCTGCAAG+GCTGCACT
TATGATATTTATTTTACTTATACTTTAAATAGCACCGATTATAACTTGACTGTTGATAATATTACAAT
AGCTCTTTCTCAGAAGATTAGAGAAGTAACCGGTGAACACGTCGGTAG
+
FFFFFF:FFFFFFFFFFFFFFFFFFF,FFFF:FFF:FFFFFFFFFFFFFFFFF:FFFFFFFFFFFF:F
FFFFFFFFFFFFFFFFFFFFFFFFFF:FFFFFFF,FFFFFFFFFF:FFFF
@A00721:190:HGJW7DSXY:4:1232:15908:27101_1:N:0:TCTGCAAG+GCTGCACT
CTTTTACCGTTAGAACTGAAAAAGATTCTTTTCGAAAAGAAACCTATAAGTTACAAACGATTCTTCGA
CTATATTAAAGAAATAAAAATACTTCAAGTACCTGAACTTTACATTTTTAATGGTATGGTATAATGTG
TCT
+
FFFFFFFFFFFFFFFFFFFFFFFFF:FF::FFFFFFFFFFFFFFFFFFF,,:FFFFFFFFFF,FFFFFF
FFFFFFFFFFFFFFFFFFFF:FFFFFFFFFFFFF:FFFFFFFFFFFFFFFFFFFFFFFFFFFFFFFFFF
FFF
@A00721:190:HGJW7DSXY:4:1232:4155:7654_1:N:0:TCTGCAAG+GCTGCACT
TTCACTCTCGGTAATGAGAGTGTATTCCTTAATATTCTAGTAACCGCTATGTGGGCATTACTAGGATT
TATCACACTCCTAAATTTGATAGCAATTTATGTTTTAAATGCTAGGAAAATTTTAGAAAAACTCCATT
GTA
+
FFFFFFFFFFFFFFFFFFFFFFFFF:FFFF:FFFFFFFFFFFFFFFFFFFFFFFFFFFFFFFFFFFFFF
FFFFFFFFFFFFFFFFFFFFFFFFFFFFFFFFFFFFFFFFFFFFFFFFFFFFFFFFFFFFFFFFFFF:F
FFF
@A00721:190:HGJW7DSXY:4:2219:9480:18035_2:N:0:TCTGCAAG+GCTGCACT
GTACTTAATTCAATAGCTTTAGTATTATTTACTATCGCCTTTATTGAAAATTGTATAAGAGTTAATTG
CTTACACAAAAGTGACGCGCGAAGGAATCTTAATTGATTGAAGCTAGG
+
FFFFFFFFFFFFFFFFFFFFFFFFFFFFFFFFFFFFFFF:FFFFFFFFFFFFFFFFFFFFFFFFFFFFF
FFFFFFFFFFFFFFFFFFFFFFFFFFFFFFFFFFFF:FFFFF:FFFFFFF
@A00721:190:HGJW7DSXY:4:2510:1407:33207_1:N:0:TCTGCAAG+GCTGCACT
AACATACCATACACTCCCCAAACATAACATACCATAACCTCCCATGACCAATCATCGCTCCAACTGGC
ACGCCAGACGTAGGGTCAAACCTACTACCGCCGGTCGGCAGATCGGCACCACTGGTCTCTACGTTCAG
GGA
+
FFFFFFFFFF:F:F:FF::,,FFFF:::,F,FFFF::FFF,FFFF,:FFF:,FF,FFF:FFF:F:F,F
FFFFFFFFFFF,FFFFFFFFF:FF:F,FFFFFFFF:FFFFF,::FFFFFF:FFFFFFFFFFFFFFFFF
FFF
@A00721:190:HGJW7DSXY:4:2175:7211:18114_2:N:0:TCTGCAAG+GCTGCACT
TAACCTTCCCAAATCTAGATCTGCACAGAAGAAGGCTGAAGGTCGTTACCGGACGACCACTCAAAGGA
AACGGGCTGTTGCCCAGAGGAAAGCAGCGACCACCGAGGTCGCTCGTA
+
FFFFFFFFFFFFFFFFFFFFFFFFFFFFFFFFFFFFFFFFFFFFFFFFFFFFFFFFFFFFFFFFFFFF
:FFFFFFFFFFFFFFFFFFFFFFFFFFFFFFFFFFFFFFFFFFFFFFF
@A00721:190:HGJW7DSXY:4:2369:6189:12900_1:N:0:TCTGCAAG+GCTGCACT
AGTCCCGTCATCTCTGTGGGAACTGAGGCAGTACCTCAGTCAAAAGTCACATTCACCACTCCCATGCC
AATCAATGATGTGACTATACAGTTCGGCCATTTCAAGAGTGGTCAATACGTCGTGGGAGGTACTGAAG
CCT

```
+
FFFFFFFFFFFFFFFFFFFFFFFFFFFFFFFFFFFFFFFFFFFFFFFFFFFFFFFFFFFFFFFFFFFF
FFFFFFFFFFFFFFFFFFFFFFFFFFFFFFFFFFFFFFFFFFFFFFFFFFFFFFFFFFFFFFFFFFFF
FFF
@A00721:190:HGJW7DSXY:4:1675:4327:10144_1:N:0:TCTGCAAG+GCTGCACT
ATCTAAGAACACTCCCAAGCCAATCAATAGTTCTTCTGACCAGAATGAAAAGGGGTTTACCAGGGTAG
TCTCTAAGTCTACCAGGAAGAGGGGAAATGTCCCCAAAACTGAGAAGAATAAAGGGAAAGGTCCATCA
GGA
+
FFFFFFFFFFFFFFFFFFFFFFFFFFFFFFFFF:FFFFFFFFFFFFFFFFFFFFFF:FFFF:FFFFFF
FFFFFFFFFFFFFFFFFFFFFFFFFFFFFFFFFFFFF:FFFFFFFFFFFFFFFFFFFFFFFFFFFFFF
FFF
@A00721:190:HGJW7DSXY:4:2133:16071:23281_2:N:0:TCTGCAAG+GCTGCACT
ATTACCCCTAATTCTGAAGGAAGATCTCCCACCATCTGGGGTTGGGAATAAGAAATCTGCACGGGCCA
AAGAGGCCGCTGCTACGAAGTCGCTGAAGGCGGCTTGGGTCATTAAGG
+
FFFFFFFFFFFFFFFFFFFFFFFFFFFFFFFFFFFFFFFFFFFFFFFF,FFFFFFFFFFFFFFFFFFF
FFFFFFFFFFFFFFFFFFFFFFFFFFFFFFFFFFFFFFFFFFFFFFFF
@A00721:190:HGJW7DSXY:4:2578:24270:6543_1:N:0:TCTGCAAG+GCTGCACT
AGTTGCGCCTGCCGACCCCATCTTTGAGACCAAGACTCTCTACAGTAGGTTCGTGCCTACTATGAGCA
AATCTGTTGAATTCGTCACTGAGGACGAAGGTGAGAGGACTTTTGGCTACTGGCGACAAACTGAGAGC
AGT
+
FFFFFFFFFFFFFFFFFFFFFFFFFFFFFFFF:FFFFFFFFFFFFFFFFFFFFFFFFFFFFFFFFFFF
FFFFFFFFFFFFFFFFFFFFFFFFFFFFFFFFFFFFFFFFFFFFFFFFFFFFFFFFFFFFFFFFFFFF
FFF
@A00721:190:HGJW7DSXY:4:2665:24361:8609_2:N:0:TCTGCAAG+GCTGCACT
AGAATTGCGTGGGTTGAAGGCCGACGCTTCGAGCTTGAGTGTGACGAGGCCGGTGACGTTTTTGTCAA
CGGCGTTTGGAGGATTTTATCCGCGCTTAACTATGGGGCGAAGATCCC
+
FFFFFFFFFFFFFFFFFFFFFFFFFFFFFFFFFFFFFFFFFFFFFFFFFFFFFFF:FFFF:FFFFFFF
FFFFF:FFFFFFFFFFFFFFFFFFFFFFFFFFFFFFFFFFFFFFFFFF
@A00721:190:HGJW7DSXY:4:1125:3839:16344_2:N:0:TCTGCAAG+GCTGCACT
AGGTTGGCATATTTATGCCGACGATAATAAGCATGTCACCCTTAAGCACCGAGGTAAGCTCGGGACTT
ACAAGAGAGAGTGTGAGAGAGTCGCTCTCACTATTTTTGCCCAGAATG
+
FFFFFFFFFFFFFFFFFFFFFFFFFFFFFFFFFFFFFFFFFFFFFFFFFFFFFFFFFFFFFFFFFFFF
FFFFFFFFFFFFFFFFFFFFFFFFFFFFFFFFFFFFFFF,FFFFFFFF:
@A00721:190:HGJW7DSXY:4:1619:16170:7639_1:N:0:TCTGCAAG+GCTGCACT
CTGATGAGGTTAAAACTCTTGCTCCTTCTGTGGGTGAGGGTTTTTGTTATATTCACTGGTTCTGGGAG
GCTGCTTTCCTCCATAATTACAACCTCTCTTTAGAGATCATTAGAAGGAAGTTGGGCAAATTCCCACA
TAT
+
:FFFFFFFF:FFFF:FFFFFFFF:FFF:FFFF:FF:FFF::FFFFFFFFFF:FFF:FFFFFFF,FFFF
F:FFFFFF,FF,FF:F:FFFFFFFFFFFFFFFFFFFFF:FFFFFF:FF:FFFFFFFFFFF,FFFFFF,
F:F
@A00721:190:HGJW7DSXY:4:2369:5294:1955_1:N:0:TCTGCAAG+GCTGCACT
CACACTTTTCTTGAAAATTTTTCCTTTCCTCCGTCATTGGGGATTTCGATGTGAGAAGGTGGAAGGTG
TCAAGGGTCTTCTTCATGCTGATCTTTCAGGACCTAATCACATCATGATTGATAGTTACGGCTCGTAT
CGA
+
FFFFFFFFFFFF:FFFFFFFFFFFFFFFFFFFFFFFFFFFF:FFFFFFFFFFFF:FFFFFFFFFFFFFF
FFFFFFFFFFFFFFFFFFFFFFFF,FFFFFFFFFFFFFFFFFFFFFFFFFFFFF:FFFFFFFF:FFFFFF
FFF
```

@A00721:190:HGJW7DSXY:4:1130:8232:24298_2:N:0:TCTGCAAG+GCTGCACT
CGAAATTAAAGACGGGGGGATTAAGAGTCCAGCTGTAACTGTCATTTCAGAGAGGGGAGCTCTGAAAA
GGAAACTTCGTCAGAAGATGAGGAAGTCTAAGAGGAGGAGTTTGAGGA
+
FFFFFFFFFFFFFFFFFFFFFFFFFFFFFFFFFFFFFFFFFFFFFFFFFFFFFFFFFFFFFFFFFFFF
FFFFFFFFFFFFFFFFFFFFFFFFFFFFFFFFFFFFFFFFFFFFFFFF
@A00721:190:HGJW7DSXY:4:1132:6361:8782_2:N:0:TCTGCAAG+GCTGCACT
GAAGTTGATGAAGAAGGATTATTTGAAGCAGGGGTTACCACTTCCTAAGGTGGATGAGGGAGGTTGTT
TCTTTGGAGTTTTAGATAATCTTTTGAGAAGTGGTCATAGAGCCAGCA
+
,,,FFF:,F,,,F,::,,F:,:F:F,,F::FF,F:F,:,::F:::F,:FFF,F,FF,FFF,F,F::FF
:,FF:FF,FF::F,,,F,::,FF:FF,F:,F,:,F::F,,,F,:::F:
@A00721:190:HGJW7DSXY:4:2112:16062:33630_2:N:0:TCTGCAAG+GCTGCACT
CCTTGAGAAGACAAGCCACATTAAGATATAATTTAGGTCAACTTGGGGTTTCGAATGTCATTAGTGCC
TTACGATTGAAAGAGGTCCTTTTGAGTATCGACAGATCTTTCGATCTT
+
FFFFFFFFFFFFFFFFFFFF:FFFFFFFFFFFFFFFFFFFFFFFFFFFFFFFFFFFFFFFFFFFFFFF
FFFFFFFFFFFFFFFFFFFFFFFFFFFFFFFFFFFFFFFFFFFFFFFF
@A00721:190:HGJW7DSXY:4:2171:28375:1376_2:N:0:TCTGCAAG+GCTGCACT
GTGGCAGAATTCGGTAACTTACCATATTGTTCCTTCCGGGAAGTTGTGGGATTTAACTTTGACTCAAC
TACTCCGATCAGATCCGGATGGTTTGATTGGTATGGACTACTCGACTT
+
FFFFFFFFFFFFFFFFFFFFFFFFFFFFFFFFFFFFFFFFFFFFFFFFFFFFFFFFFFFFFFFFFFFF
FFFFFFF:FFFFFFFFFFFFFF:FFFFFFFFFFFFFFFFFFFFFFFFF
@A00721:190:HGJW7DSXY:4:2301:7437:3912_1:N:0:TCTGCAAG+GCTGCACT
TAGGGTCATGGCAGATACTACCGATAAGGTGAAAAATATGCAGGTCAAAGATTCTGTAGCTCTGAGGG
CGGTTGAGGGAGCCGTTTTGGCCGACTTTAACTATTCTAAGAGTCTTTATGATTCAAAACCTATCGTT
GTC
+
FFFFFFFFFFFFFF:FFFFFFFFFFFFFFFFFFFFFFFFFFFFFFFFFFFFFFFFFFFFFFFFFFFFF
FFFFFFFFFFFFFFFFFFFFFF:FFFFFFFFFFFFFFFFFFFFFFFFFFFFFFFFFFFFFFFFFFFFF
FFF
@A00721:190:HGJW7DSXY:4:2534:7301:7122_1:N:0:TCTGCAAG+GCTGCACT
TACAGCGGCTTATTGTCGCGCAGTTTCCAGAATTGCGGTTGAAGTTCACCCATTCTATCTTGAATGCT
CACGCCGTCGCAGCCATATCGAGACTATGTTATAATCACTTGTTAAAGTACAGATTTAGTGATTTAGA
TAT
+
FFFFFFFFFFFFFFFFFFFFFFFFFFFFFFFF:FFFFFFFFFFFFFFFFFFFFFFFFFFFFFFFFFFF
FFFFFFFFFFFFFFFFFFFFFFFFFFFFFFFFFFFFFFFFFFFFFFFFFFFFFFFFFFFFFFFFFFFF
FFF
@A00721:190:HGJW7DSXY:4:1250:16947:4852_1:N:0:TCTGCAAG+GCTGCACT
GAGGCGATATTCGTTTTCATATCCAAAACTCCTTGAATGGAGTACATATTTGCAACCCTATTCTTGAT
GTGAAAGATGCATCGAGGTATACGTCTAGGAACATTGATTGGGAACGTACCTACTCACGAGACGTCAT
GAA
+
FFFFFFFFF:FFFFFFFFFFFFFFFFFFFFF:FFFFFFFFFFFFFFFFFFFFFF:FFFFFFFFFFFFF
FFFFFFFFFFFFFFFFFFFFFFFFFFFFFFFFFFFFFFFFFFFFFFFFFFFFFFFFFFFFFFFFFFF:
FFF
@A00721:190:HGJW7DSXY:4:2670:20401:13056_1:N:0:TCTGCAAG+GCTGCACT
ATGTTTCGTTCTGTTACAAGAACGCTTTGGATTGTGACCATAAAGCCTCAGTTGCGATAGCTGTAGAG
GTATACGATATCTCTCTTGATGATGTAGCCACTATAATGCTTAAGAGAGAGATTGATGTATGTCATTT
CGT
+
FFFFFFFFFF:FFFF::,F:,F,F,FFFFFFFFFF:FF,FF:FFFF:FFFFFFFFF:FFFFF:FFFF,

,F,FF,FFFFF:FFFFFFFFFFFFFFFFF:FFFFFFFFF,FFFFF:FFFF:FFF,FFFF:FF:FFFFF
FFF
@A00721:190:HGJW7DSXY:4:2364:10538:3145_1:N:0:TCTGCAAG+GCTGCACT
GAATTATTGGACACGATGAAAAATGACATACCCGTTCCTGACCTTGGTATCGTTATCCGTCATGGAGA
TGATGCCGCATCACTTGATTACTATATGCCCGAAGGATTGGGTTATTCTCACAACAAAGATAAGATTT
TAT
+
FFFFFFF:FFFFFFFF:FFFFFFFFFFFFFFF:FFFFFFFFF,FFFFFFFFFFFFFFF:FFFFFFFFF
FFFFFFFFF:FFFFFFFFFFFFFFFFF:FFFFFFFFFFFFFFFFFFFFFFFFFFFFFFFFFFFFFFFF
FFF
@A00721:190:HGJW7DSXY:4:2335:16197:30044_1:N:0:TCTGCAAG+GCTGCACT
TAAGGGCGGACAATTGTTCCATTGTGAACTCTTGGGGAATAGATTGAGCATTAATGAGTTCCGAATAA
CTCGTAATAAATCTTTCTCTAGTGGACAATTCAGAATACCTATTTCCGTACCAAGAGCCACTGCCAAT
CTC
+
FFFFFFFFFFFFFFFF:FFFF:FFFFFFFFFFFFFFFF,FFFFFFFFFFFFFFFFFFFFFFFFFFFFF
FFFFFFFFFFFFFFFFFFFFFFFFFFFFFFFFFFFFFFFFFFFFFFFFFFFFFFFFFFFFFFFFFFFF
FFF
@A00721:190:HGJW7DSXY:4:1323:18331:16799_2:N:0:TCTGCAAG+GCTGCACT
AAATTTTTCTCAAGAGATTATGGTTGATAAAGTTTTCTTCATAAGGGCTATTCAGTACGTCATGGGGA
TCACTTCTTCGGTCAATGACAAAACCTTCGAATATGTTACGACATGGC
+
FFFFFFFFFFFFFFFFF:FFFFF:FFFFFFFFFFFFFF:FF:FFFFFFFFFFFFFFFF:FFFFFFFFF
FFFFFFFFFFFFFFFFFFFFFFFFFFF:FFF:FFFFFFFFFFFFFFFFF
@A00721:190:HGJW7DSXY:4:2425:7925:30780_2:N:0:TCTGCAAG+GCTGCACT
AGGATTGTCATCTCGGGGAAAGTGATTCACCAAGATGTTCATATAGATACGAGATACCTAGAGAAGGT
CGCAGCGATCTTACTCGTTATCGGAGCTAAGAAGAAGACTTTGGGTAC
+
FFFFFFFFFFFFFFFFFFFFFFFFFFFFFFFFFFFFFFFFFFFFFFFF:FFFFFFF:FFFFFFFFFFFF
FFFFFFFFFFFFFFFFFFFF:FFFFFFF,:FFFFF:FFF:FFFFFFFF
@A00721:190:HGJW7DSXY:4:1458:18267:34350_2:N:0:TCTGCAAG+GCTGCACT
TAACGTTAGTACCGGAGGTGATCTCTCTGCTTCTGCGTTACTGAGAGAAAAGATTCAGGACGCCTACG
GTGACTTTATGGACAAGATGCAAGTTCTTGGGAAATACTTGTTTCCCT
+
FFFFFFFFFFFFFFFFFFFFFFFFFFFFFFFFFFFFFFFFFFFFFFFFFFFFFFFFFFFFFFFFFFFF
FFFFFFFFFFFFFFFFFFFFFFFFFFFFFFFFFFFFFFFFFFFFFFFF
@A00721:190:HGJW7DSXY:4:1369:3504:20149_2:N:0:TCTGCAAG+GCTGCACT
TCTTTGTATAGGGAGTGTGAGAAGACAACGAATCTTGAAACTACGGTCCTTATCAAAGAAAATGGTGA
ACCGGTCTCTAAATCTTTTGAGATTGAGAAGATTGAAGAACACGTCAA
+
F,F,FFFF:FFFFFFFFFFFFFFFFFFFFFFFFFFFFFFFFFFFFFFFFFFF,:FFFFF:F:FFFFF
,FFFF:FFFF:FFFFF::FFFFFFFFFFF,FFFF:FFFFFFFFF:F:F
@A00721:190:HGJW7DSXY:4:1558:29080:16125_2:N:0:TCTGCAAG+GCTGCACT
TTGTTCTTGAAAACCAAGTTAAGAAGGAGGCTGATCATGGAGTCTTTGTACCTTCTGTCAAACTTCGA
GGTTCAGCTACGAGAGGTGATCCCGGATGTGGATTGAAGGCTGGCTCT
+
FFFFFFFFFFFFFFFFFFFFFFFFFFFFFFFFFFFFFFFFFFFFFFFFFFFFFFFFFFFFFFFFFFFF
FFFFFFFFF:FFFFFFFFFFFFFFFFFFF,FFFFFFFFFFFFFFFFFF
@A00721:190:HGJW7DSXY:4:1636:17336:27101_2:N:0:TCTGCAAG+GCTGCACT
CTTTTTGAATGTTGTAAAGAATTGGGTTGAGAAGTGTGCTTTACAAGGAGTTAGACTCTTCTTCAAGT
TTTTAGATCTTCTTGATAATCTTAAAGAAAAATTTTCGAATAAACTAA
+
FF,FFFFFFFFFFFFFFFF:FFFFFFFFFFFFFFFFFFFFFFFFFFFFFF,FFFFFFFFFFFFFFFF:
FFFFFFFFFFFFFFFFFFFFFFF,FFFFFFF:FF:FF:FFFFFFFFFF

```
@A00721:190:HGJW7DSXY:4:2636:4065:17519_2:N:0:TCTGCAAG+GCTGCACT
CTGTACTTACATTCTTTACGCCAGATTCTTCATGGGGCGACGCTGTTTCTCTTGGTATTTCTGAAATA
GTTTATCAGAGCGTCATTTTCCTTTGGGATCTTTCCTCTGGTAAGGGA
+
F::FFFFFFFF::FF:FFFFFFFFFFFFFF:FFFFF,FFFFFFFFFFF::FF:F:F:FFFFFF:::FF
FFFFFFFFFF,FFFFFF,FFFFFFFFFFFFFFFFF,FFF,FFF,:FFF
@A00721:190:HGJW7DSXY:4:1126:18150:20243_2:N:0:TCTGCAAG+GCTGCACT
CGAGAATTATTACTGTTTTCTCTACCCATAACTTCTTTTTCAAAAATGCGGATGGGTCTACTGATGTT
TTCCTACAGAGTGCAGTCGTTTCAACACTTATCACCACTTTTGAAAGG
+
FFFFFFFFFFFFFFFFFFFFFFFFF:FFFFFFFFFFFFFFFFFFFFFFFFFFFFFFFFFFFFFFFFFF
FFFFFFFFFFFFFFFFFFFFFF:FFFFF,FF:,F,FFF,,FF,FFF,F
@A00721:190:HGJW7DSXY:4:1443:25355:9549_1:N:0:TCTGCAAG+GCTGCACT
GTGATGAAAATACGTATGTCGGTGGGGTTCTCAAGGCTCTCATCGACACTGGGATTCTAAGGGGTTCC
ATAAGATTTTATCTGACATTGTGGTTCAATGATCAGGATTCTTCTTTTGGTCCTTACGTCTTAAGTAA
TT
+
FF,FFFF,FFF,:FF,FFFF,,:::FFFFFF:::FF,FFF,FF:F,F,FFF:F,FFF::FFFF:,FFF
:F:FF:F,F:,FFFFF:F,FFF,FFF:,:FF:FFFFF:F:,FF,FFFFFFFFFFF,FFF:FFF:FFF:
FF
@A00721:190:HGJW7DSXY:4:1410:4725:25363_1:N:0:TCTGCAAG+GCTGCACT
TCTTGTCACGATTCTTAAAAGAAGTTTATTAAAACTTCAGAGTCTCTTGGAAGTCCAGGTAGCTAATG
TTCTTGCAATAGCCACAGACTTTCTGGGAAAAGAGACCTCTGTTGGTAGAGGAGTTGAGAGAATTACT
CAG
+
FFFFFFFF,FF:FFFFFFFFFFFFFFFFFFFFFFFFFFFFFFFFFFFFFFFFFFFFFFFFFFFFFFFF
FFFFFFFFFFFFFFFFFFFFFFFFFFFFFFFFFFFFFFFFFFFFFFFFFFFFFFFFFFFFFFFFFFFF
FFF
@A00721:190:HGJW7DSXY:4:1233:11550:7936_2:N:0:TCTGCAAG+GCTGCACT
ACAACGAGTTAGGGAGTGGCTAGGTTTTAAAAGATCCTTTGCACAAGCAATACCTTCCACTGAGTTCA
TCACCCAGAGTTTCGACGTCGCGGAAAACGATGAAGATTTCCACACTG
+
FFFF:FFF:FFFFFFFFFFFFFFFFFFFFFFFFF:FFFFFFFFFFFFFFFFFFFFFFFFFFFFFFFFFF
FFFFFFFFFFFFFFFFFF:FFFFFFFFFFFFFFFFFFFFFFFFFFFFF
@A00721:190:HGJW7DSXY:4:1204:22670:18803_2:N:0:TCTGCAAG+GCTGCACT
TCTGATGCTGAATTTGAGGAAAATTTTTCGGAAGTTTTTGGTATAAATCCTGATGGCTCAGTACAGGG
AGGAGGTAATAGTTATAAAACCTTCTTATATCATTGCAACAGGACTTT
+
FFFFFFFFFFFFF,FFFFFFFF:FFF,FFFFFFFFF:FF:F,FFFFF:FFF:FFFF::FFF::F::FF
FFFFFFFFFFFF:FFFFFF,FFFFFFF,FFF,FF,:FFF:FFFFFF::
@A00721:190:HGJW7DSXY:4:1232:28311:5744_2:N:0:TCTGCAAG+GCTGCACT
GGACAAAATCTGGGATCATAGGTATGTGTCACGCTACACTCGAAATATGGGCCCTCATAAGGAAGTAT
AGTTGGCGTCTTGTCGTTGACGTCATTACAGTCTGGAAGTTAGATCAT
+
FFFFFFFFFFFFFFFFFFFFFFFFFFFFFFFFFFFFFFFFFFFFFFFFFF:FFFFFF:FFFFFFFFFF
FFFFFFFFFFFFFFFFFFFFFFFFFF:FFFFFFFFFFFFFFFFF:FFF:
@A00721:190:HGJW7DSXY:4:2254:32922:22091_2:N:0:TCTGCAAG+GCTGCACT
CGAGAGTTGTGATCTAGTTCTAAATTTAGGTCGACGGACTTTAGCAACTTTAGATAAATTCTTTCCGG
GCTCAATGACCTTTGCTATGTACGTGGATCTAATCCTTGGTGATCTGA
+
FFFFFFFFFFFFFFFFFFFFFFFFFFFFFFFFFFFFFFFFFFFF:,FFFFFFFFFFFFFFFFFFFFFFF
FFFFFFFF:FF:FF:FFFFFFFFFFFFFFFFF:FFFFFFF:FFFFFFF
@A00721:190:HGJW7DSXY:4:1677:13702:2832_2:N:0:TCTGCAAG+GCTGCACT
TATTAGTTCATGAAGTTCTTACACTTCTCGCGACTAATGTCAGCTTCACTACAAAGGCGTTACGTAAA
```

```
GTAGTTTTCACGCTTTATCTTAAAGCATTACCACCAAATTTAGCACTT
+
FFFFFFFFFFFFF:FFFFFFFFFFFFFFFFFFFFFFFFFFFFFFFFFFFFFFFF,FFFFFFFFFFFFF
FF:FFFFFFFFFFFFFFFFFFFF:FFFFFFFFFFFF:FFFFFFFFFFF
@A00721:190:HGJW7DSXY:4:2436:22254:5462_1:N:0:TCTGCAAG+GCTGCACT
CTCGCCATATCAGATTAGAGACATGCCAGTTAGAGTTCTTCTAACCAGGACTGATGGTGAGATTCTTT
TACCTTTTGGAGGTCCAGGAACCTTTACTCCTAGTGTCTTTTCTAGAGTCCAAGGACGTTTCCGTCGG
AGG
+
FFFFFFFFFFFFFFFFFFFFFFFFFFFFFFFFFFFFFFFFFFFFFFFFFFFFFFFFFFFFFFFFFFFF
FFFFFFF:FFFFFFFFFFFFFFFFFFFFFFFFFFFFFFFFFFFFFFFFFFFFFFFFFFFFFFFFFFFF
FFF
@A00721:190:HGJW7DSXY:4:1576:18891:34867_2:N:0:TCTGCAAG+GCTGCACT
GACCGCTCAATAACACTCTATTGAATCCCTTTTCCACGATGAAAGCTCTTTATGAGGTGCTCCATGCA
AATTTACAGTTCCTCTTTGGTGATATCGCCTCTGTCCTTTCTCCTTTA
+
FFFFFFFFFFFFFFFFFFFFFFFFFFFFFFF:FFFFFFFFFFFFFFFFFFFFFFFFFFFFFFFFFFFF
FFFFFFFFFFFFFFFFF:,FFFFFFFF:FFFF:FFFFFFFFFFFFFFF
@A00721:190:HGJW7DSXY:4:1478:24252:28025_2:N:0:TCTGCAAG+GCTGCACT
ACTTGGGTTTATCGGTTGTATTTTGATAAATTTGTTGGAATCGATAATGATACTGACGAAGGTTCTAT
TACCACCCTTGTCATTGATACAAACATTCTAGGTGATGCTTTCTTAGA
+
FFFFFFFFFFFFFFFFFFFFFFFFFFFFFFFFFFFFFFFFFFFFFFFFFFFFFF:FFFFFF:FFFFF
FFFFFFFFFFFFFFFFFFFFFFFFFFFFFFFFFFFFFFFFFFFFFFF:
@A00721:190:HGJW7DSXY:4:2212:13819:32189_2:N:0:TCTGCAAG+GCTGCACT
TTACAATCTGTAGAGAGAGAGAGGTTAATCAGAATTTGAGTGATAACGATTTAGTACTGGGAAATGGTGG
TAGTCAGTTTACTACTTTTCTTTCCCTCCCCTTTTTCGATTGGATTCG
+
FFFFFFFFFFFFF:FFFFFFFFFFF:FFFFFFFFFFFFFFFFFFFFFFFFFFFFFFFFFFFFFFFFFF
FFFFFFFFFFFFFFFFFFFFFFFFFFFFFF:FFFF:FFFFFFFFFFFFFF
@A00721:190:HGJW7DSXY:4:1207:1877:30671_2:N:0:TCTGCAAG+GCTGCACT
TTGATAAGGTTTTTCTTTTATGGGACTCTTGTTTGTACTTATGAAGGAGGTATTACTTCGGAAGTTCT
TAATGTAGGTCTCCTAGTACTTAAGGGTTTATGTTTCGGTTTCAGCTG
+
FFFFFFFFFFFF:FFF:FFFFFFFFFFF::FFFFFFFF:FFFFFFFFFFFFFFFFFFFF:FFFFFFFF
FFFFFFFFFFFFFFFFFFFFFFFFFFFFFFFFFFFFFFFFFFFFF:FF,
@A00721:190:HGJW7DSXY:4:1250:5782:24251_2:N:0:TCTGCAAG+GCTGCACT
TGTTTCACAATGCGTGATCACTTCCCGTGGACTTGGGAAGAGATTAAACATAAAATCCAGCGATTCTA
TTATGTCGCTAAAGCCTGTCCAGTTGTCGATGATGTTAGGGCTGTGGG
+
F:,,F,FFF,:,,FF,FFFF,FFFFFF,,,FF:F,F,,:,FFFFFFFFFF,FF:FFFFF:,FFFFF,F
FFFF:F::FFFFF,FFFFFFFF::,FFFFFF,F,,FF,F,:FFF:F:F
@A00721:190:HGJW7DSXY:4:2420:22580:28541_2:N:0:TCTGCAAG+GCTGCACT
AAACTTACGACGATGTTCTCGCTCGCATTTCACAAGGATTACTAAGTCTGAGATCTGCCACTTCTAAT
TTTAGGAAACGTCGCGAAAAGAAAGTTCATGAGGATGTCTTGGTCGAA
+
FFFFFFFFFFFFFFFFFFFFFFFFFFFFFFFFFFFFFFFFFFFFFFFFFFFFFFFFFFFFFFFFFFFF
FFFFFFFFFFFFFFFFFFFFFF::FFFFFFFFFFFFFFFFFFFFFFFFFF
@A00721:190:HGJW7DSXY:4:2353:12599:8782_2:N:0:TCTGCAAG+GCTGCACT
GAACTTCTAATAGAGGATCCTGATGACATTATTTCAACGGATACCGATCTCAGTTCTACCACAGAAAC
CGTCTCAGGAACTGTCTCCACTGATTCGGAATTAGAGAAAGACGACCT
+
FF:FF:FF,FFFFFFFFFFFFFFFFFFFFFFFFFFFF:FFFFFFFFFFFFFFFFFFFFFFFFFFFFFFF
FFFFFFFFFF:FFFFFFFFFFFFFFFFFFFFF:FFFFFFFFFFFFFFFF
```

```
@A00721:190:HGJW7DSXY:4:2463:7066:33959_2:N:0:TCTGCAAG+GCTGCACT
AGACCTCTGAATTTTCAGGGTGCCCTGATGAGGACGTTGAAAGGGATAATTTCAACTCTGAGACTTCT
AAGAGTGTTTCAAAAGACCAGAAACGTAACAAGAAGAATAAAGGTAGG
+
FFFFFFFFFFFFFFFFFFFFFFFFFFFF:FFFFFFFFFFFFFFFFFFFFFFFFFFFFFFFFFFFFFFF
FFFFFFFFFFFFFFFFFFFFFFFFFFFFFFFFFFFFFFFFFFFFFFFF
@A00721:190:HGJW7DSXY:4:2103:22245:14372_2:N:0:TCTGCAAG+GCTGCACT
AGCGAGCGGTTGAAGAGAAAACTGATATGGTTGCGGCTGATCGTACCCCAATTCCTGCTAGAAAGATC
ACTCTCGGCGATACCACTGATCTTCCAGAGTTACGTAGTGGTCGTTCT
+
FFFFFFFFF,FFFFFFFFFFFFFFFFFFFFFFFFFFFFFFFFFFFFFFFFFFFFFFFFFFFFFFFFFF
FFFFFFFFFFFFFFFFFFFFFFFFFFFFFFFFFFFFFFFFFFFFFFFF
@A00721:190:HGJW7DSXY:4:2219:30834:22013_1:N:0:TCTGCAAG+GCTGCACT
AAGTTTCTGCTAGTCGCGACGATAGTATTTATCGGAGTGATATTAACAAGTTAAACTTCGAACTGACC
ACTTACAAGAGTGTTGAAGTACCGCAACATGGATCAGTTTTCCAAAAAGCTATTAATAGCAGTTGCGG
GGC
+
:FFFFFFFFFFFFFFF:FF:FFFFFFFFFF:FFFFFFFFFFFFFFFFF::FFFFFFFFFFFFFFFFFF
FFFFFFFFFFFFFFFFFFFFFFFFFFFFFFFFFFFFF:FFFFFFFFFFFFFFFFFFFF,FFFFFFFFF
FFF
@A00721:190:HGJW7DSXY:4:2256:32796:35399_2:N:0:TCTGCAAG+GCTGCACT
AAACTGAATCCACTCTCTTGGGATATAAATGAGTACATCTATAATAGAGTTTTGGAACTGTGTAATGT
CCTACGTACTGTTCATGGTGTTTGGGCTTCTCTAAAGAACAGCAACTT
+
FFFFFFFFFFFFFF:F:FFFFFFFFFFFFFFFFFFFFFFFFFFFFFFFFFFFFFFFFFFF:FFF:FFF
FFFFFFFFFFFFF:,,FFFF:FFFFFFFFFFFFF:,F,FFFFFFFFFF
@A00721:190:HGJW7DSXY:4:2436:21866:27398_2:N:0:TCTGCAAG+GCTGCACT
AGACCTTTCTTGGGGAGTAAAGAGGGTTTGAAAATTACGGAACCTTTCCTCGTTCTTCATGAAGATCT
TCGAGTGTTTTATAGTGAGTTATTCTTGCGAAGGTTCAAGTCGATTGA
+
FFFFFFFFFFFFFFFFFFFFFFFFFFFFFFFFFFFFFFFFFFFFFFFFFFFFFFFFFFFFFFFFFFFF
FFFFFFFFFFFFFFFFFFFFFFFFFFFFFFFFF:FFFFF:FFFFFFFF
@A00721:190:HGJW7DSXY:4:1639:25382:27696_2:N:0:TCTGCAAG+GCTGCACT
AATAAGAAGTTCTTCGCAGTTGAATCTCCACCAGGTGGAGGGAAGACCACTGAACTTATAGCGACTTT
TGTCGAATTTTTCGCAAAAGGTCTTGAAGTACGTATTGTCACTGCTCA
+
FFFFFFFFFFFFFFFFFFFFFFFFFFFFFFFFFFFFFFFFFFF:FFF:FFFFFFFFFFFFFFFFFFFF
FFFFFFFFFFFFFFFFFF,FFFFFFFFFFFFFFFFFFFFFFFFFFF,F
@A00721:190:HGJW7DSXY:4:2228:25382:34115_2:N:0:TCTGCAAG+GCTGCACT
TTGGCACTTCTAGTTCATCGCGGTATTTGTGAAATTAAGACAGACGTGAAAGTTATGATGAGTGATCG
TATTAGAACAGCTGATTCTTATTGCATGAACGTCGCTGAGGTCAAACC
+
FFFFF:F:FFFFFFFFFFFFFFFFFFFFFFFFFFFFFFFFFFFFFFFFFFFF:F:FFFFFFFFFFFFF
:FFFFFF,FFFFFFFF:FFF:FFFFFFFFFFFFFFFFFFFFFFFFFFF
@A00721:190:HGJW7DSXY:4:1328:18493:29731_2:N:0:TCTGCAAG+GCTGCACT
TTCATCGACGAATTATTCATGATTCACGCTGGTCAGACCCTTTTTCTTACACATCTACTTAAGGCGGC
TTATACCATAGGTTACGGTGATTCCAATCAAATCGGATTCATCAATCG
+
FFFFFFFFFFFFFFFFFFFFFFFFF:FFFFFFFFFFFFFFFFFFFFFFFFFFFFFFFFFFFFFFFFFF
FFFFFF:FFFFFFFFFFFFFFFFFFFFFFFFFFFFFFFFFFFFFFFFF
@A00721:190:HGJW7DSXY:4:1105:13720:12884_2:N:0:TCTGCAAG+GCTGCACT
CAAGTCGCAAACTACTTCAACATCAACGACATCATTCTTGATGAGTGTCGTTCTTATCGAAGTCTCAG
TTATCGCTGCCCGGCCGACGTTTGTCGCGAACTCTCAGAGGTTTACGG
+
```

F:FFFFFFFFFFFFFFFFFFFFFFFFFFFFFFFFFFFFFFFFFFFFFFFFFFFFFFFFFFFFFF:FFFF
FFFFFFFFFFFFFFFFFFFF,FFFFFFFFFFFFFFF:FFFFF:FFFFF
@A00721:190:HGJW7DSXY:4:1635:30960:10770_2:N:0:TCTGCAAG+GCTGCACT
CTATGGAAATTAGACCTATCAGATGTCTTGACGACTTCGAATTTGAGGACGGAGTTAAATATATTACT
TTCACTCAAGGTGAAAAGTTTGAAATTAACAAAGTCCTTGGTAAGAGT
+
:FFFFFFFFFFFFFFFFFFFFFFFFF:FF:,FFFF,FFFFFFFFFFFFFFFF:FFFFFFFFF,F:,FF,
F::FFFFFFF:FFFFFFFFFFFFFF:FFFFFFF:,:FFFF,FFF:FFF
@A00721:190:HGJW7DSXY:4:2451:19343:18020_1:N:0:TCTGCAAG+GCTGCACT
GTTCAAGGTAATACTTACCAGCACGTTCGGCTGGTTAGAATTAAACCTCAAGATGACCCTGTTTTTGC
ATCACAAACACATATTACCGTTGCTCTATCACGACACGTTGAATCTTTACGATACTTTGTTATCAATG
TAA
+
:FFFFFFFFFFFFFFFFFFFFFFFFFFFFFFFFFFFFFFFFFFFFFFFFFFFFFFFFFFFFFFFFFFF
FFFFFFFFFFFFFFFFFFFFFFFFFFFFFFFFFFFFFFFFFFFFFFFFF,FFFFFFFFFFFFFFFFFFF
FFF
@A00721:190:HGJW7DSXY:4:1678:9182:31548_2:N:0:TCTGCAAG+GCTGCACT
AAGGAACTGTCACGTTTAGCACGGGAGGTTGCAGATTCAACCCCTTATATCGAGGCTATCGAGGACGA
TGAGTATCTTGAAGTTGTTGACGACAAGCCTCAAGAGGGTAGAGGACC
+
FFFFFFFFFFFFFFFFFFFFFFFFFFFFFFFFFFFFFFFFFFFFFFFFFFFFFFFFFFFFFFFFFFFF:
FFFFFFFFFFFFFFFFFFFFFFFFFFFF:FFFFFFFFFFFF:FFFFFFF
@A00721:190:HGJW7DSXY:4:2472:2745:1485_2:N:0:TCTGCAAG+GCTGCACT
GGTTATTCCTGGAACCACCTTAACGTCACTCGGTAATCTTTCAGCCGAATTAAGTACTAATGAGTTCA
AGTCGGTTGTCGATCGAATTAAGATCAAGGCTAATACCTTTATAGGTA
+
FFFFF:F:FFF:FFFFFFFFFFFFF:FFFFFFFF,FFF:FFFFFFFFFF:F:FFFFFFFFFFFFFFFF
FF:FFFF,FF:FFFFFFFFFFFFFFFFFFFFFFFF,FF,F,FFFFF:FF
@A00721:190:HGJW7DSXY:4:2522:18656:4742_1:N:0:TCTGCAAG+GCTGCACT
TCATCAGTCAGAGTATACCCGATAGGGTACCGAGTCTCCAAGAGAATATTTATTCTTATGAGGCTCGA
AACTTCAACGTTTTCACAACGGAAAGACATATCGCACCCGAAATTTTTGGGCAGAATGCTCTTAACGC
GGT
+
FFFFFFFFFFFFFFFFFFFFFFFFFFFF:FFFFFFFFFFFFFFFFFFFFFF:FFFFFFFFFFFFFFFFFF
,FFFFF:FFFFFFFFFFFFFFFFFFFFFFFF:FFFFFFFFFFFFFFFFFFFFFFFFFFFFFFFFFFFFF
FFF
@A00721:190:HGJW7DSXY:4:1623:2049:36542_2:N:0:TCTGCAAG+GCTGCACT
GATCAGTCTAAGTTGGATAAAGTTATAGGAGACTCAGTTAACCTGAGAGCTCCTCAATTGATAGATTG
GGTCAACAATAGGGACGCTAGTATACTTCGTAAAGTCGACAGCGAAAT
+
FFFFFFFFFFFFFFFFFFFFFFFFFFFFFFFFFFFFFFFFFFFFFFFFFFFFFFFFFFFFFFFFFFFFF
FFFFFFFFF:FFFFFFFF:FFFFFFFFFFFFFFFFFFFFFFFFFFFFFF
@A00721:190:HGJW7DSXY:4:1640:6741:7654_2:N:0:TCTGCAAG+GCTGCACT
TAATTGGACCGACTATATGACCTATTTTAAGCTCATGGTGAAAAGGGAAGCTAAGGTTAAACTTGATG
CTTCAGCCTTTTCGAAGCATAACCCCGCACAGAATATCGTTTATCACG
+
F,FFFFFFFFFFFFFFFFFFFFFF:F:FFFFFFFFFF:FFFFFFFFFF:FFFFFFFFFFFFFFFFFFF
FFFFFFFFFF,FF:FFFFFFFFFFFFFFFFFFFFFFFFFFFFF:FFFF,
@A00721:190:HGJW7DSXY:4:1535:18891:12101_2:N:0:TCTGCAAG+GCTGCACT
CCTATCTTTAAGGTTCTTGTGAGGAGAGTCAAACAGTGTCTTTTACCCAACATTTTTATGTATACAGA
GATGACCAGTGACGATTTCTCCCGGAAGCTCTTCTCTCTCTTAGGAGG
+
FFFFFFFFFFFFFFFFFFFFFFFFFFFFFFFFF,FFFFFFFFFFFFFFFFFFFFFFFFFFFFFFFFFF:F
FFF:FFFFF:FFFFFFF:FFFFFFFFFFFFFFFFFFFFFFFFFFF:,FFF

```
@A00721:190:HGJW7DSXY:4:1511:2419:16736_2:N:0:TCTGCAAG+GCTGCACT
TTTCTTTTGTAAAGCCTATGAGATAAAGATGTATTCTTATTTAGGCTTCGATGCTGAATTTCTTGAAC
TTTGGAGTGCCAGTGAATATTTTTGTCACGCCAGGAGTATCCATAACG
+
FFFFFFFFFFFFFFFFFFFFFFFFFFFFFFFFFFFFFFFFFFFFFFFFFFFF,FFFFFFFFFFFFFFF
FFFFFFFFFFFFFFFFFFFFFFFFFFFFFFFFFFFFFFFFFFFFFFFF
@A00721:190:HGJW7DSXY:4:1564:22625:22388_2:N:0:TCTGCAAG+GCTGCACT
TACCACCTTTGCTCAACGACGAAGTGGAACGGCCAACACTTTCTTTGGTAATTGTATGATCACTTTGA
TGATTTTATCACTTTATTATGACCTTGGAAAGTTTTCGGCTGTGGTTG
+
FFFFFFFFFFFFFFFFFFFFFFFFFFFFFFFFFFFFFFFFFFFFFFFFFFFFFFFFFFFFFFFFFFFF
FFFFFFFFFFFFFFFFF,FFFF:FFFFFFFFFFFFFFF,FFFFFFFFF
@A00721:190:HGJW7DSXY:4:2129:7039:14152_1:N:0:TCTGCAAG+GCTGCACT
TCTGTTTTCAAAGACTCCAATAACTGATCATTCTGATAATTTGATTTTGGACAATGGTTTCGAAGCGA
AATTTTTGAGGGATAAACCAGCGTACTTCTGCTCGCGATTTATTCTTTTTGTCAATGATTACATCTA
+
F,FF:,FFFFFFFFF:F,,FFFFFFFFFF:FF,FFFF,FF,FF:FFFFFFFFF:FF:FFFF:,FF,FF
:FFFFFFF:FFFF:F:,FFFF:FFF,FF::F:F:F,FF:FFFFFFFFFF,F::FF,FFFF::FFF:F
@A00721:190:HGJW7DSXY:4:2643:4734:21433_1:N:0:TCTGCAAG+GCTGCACT
CCTTACAAGATCATCGTTAAGTTAGGTAAACCCCTAACCGATGTTACTCTTGTAGGACTTTGGGAGAG
ATTCGTTAGTTTTAAAGACTATACTAAATATCTCCACTACGAGGTTGTTGTTTGGCGATTAGCTCTTG
CGA
+
FFFFFFFFFFFFFFFFFFFFFFFFFFFFFFFFFFFF:FFFFFFFFFFFFFFFFFFFFFFFF:FFFFFFF
FFFFFFFFF:FFFFFFFFFFFFFFFFFFFFFFFFFFFFFFFFFFFFFFFFFFFFFFFFFFFFFFFFFF
FFF
@A00721:190:HGJW7DSXY:4:2619:30174:32957_2:N:0:TCTGCAAG+GCTGCACT
ATTGAGGGTCGTTATACCTATTCGGCTATCTGTGCTTTGAATTGTATAAGATCAAATTTTAAACGATT
CCGAGAGGTTTATCCTGATGTGGAGGGAGTTCTTGAAGCATCCCGACC
+
FFFFFFFFFFFFFFFFFFFFFFFFFFFFFFFFFFFFFFFFFFFFFFFFFFFFFFFFFFFFFF:FFFFF
FFFFFFFFFFFFFFFFFFFFFFFFFFFFFFFFFFFFFFFFFFFFFFFF
@A00721:190:HGJW7DSXY:4:2125:6677:35008_2:N:0:TCTGCAAG+GCTGCACT
GGTTTCATAAGATTATCAGTAATCCTGATTCTACTACGGATTCATGGACCTTCCATCCTGTACCTCTT
CTGGAGAGACGTATCTCAAAGATCGACTTCGAGACAGGTGATATTGTT
+
FFFFFFFFFFFFFFFFFFFFFFFFFFFFFFFFFFFFFFFFFFFFFFFFFFFFFFFFFFFFFFFFFFFF
FFF:FFFFFFFFFFFFFFFFFFFFFFF:FFFFFFFFFFFFFFFFFFFFF
@A00721:190:HGJW7DSXY:4:1310:19289:6026_1:N:0:TCTGCAAG+GCTGCACT
ACCAAGATCTAAATTCGGTAACGGTGCGTGGCGCTTTATTTTAAAAAGCTCCCTCCTCTCGCAAGTTT
GATCTGATACTTATCACGAAAGGACTTGAGTCTTTTGATCAGTCTCTGACTTGACCATCTTTCATTTT
AAA
+
FFFFFFFFFFFFFFFFFFFF::FFFFFFFFFFF,FFFFFFFF:FFFFFFFFFFFFFF:FFFFFFFFFF
FFFFFFFFFFFFFFFFFFFFFF,FFFFFFFFFFFFFFFFFFFFFFFFFFFFFFFFFFFFFF,FFFFFFF
FFF
@A00721:190:HGJW7DSXY:4:1403:11586:2018_2:N:0:TCTGCAAG+GCTGCACT
TTTCTTCTTTGTTTCCTTCTTTGCACTGATTTTTCCTCATCAGCTTGTGTTTTACCTAATCTGTGTTT
TTACGATAACAACTATCAGTTGATTAAGACTCTTCCGAAAGGCGAACT
+
F,FFFFFFFFFFFFFFFFFFFFF:FFFFFFFFFFFFFFFFFFFFFFFFFFF:FFFFFFFFFFFFFFFF
FFFF:FFFFFF,:FFFFFFFFFFFFFFFFFFFFFFFFFFFFF:FFF:FF
@A00721:190:HGJW7DSXY:4:1533:19922:5901_2:N:0:TCTGCAAG+GCTGCACT
GAAGCTCAGGGTGAAGCACAACGTTTGACCGTGACGAACTGTGGTGTTCTTGATCTACGTTACGGATA
```

TACTCTCCTTGAGTTCACTCGTAAGGACACTACCACTTATTACGATAT
+
FFFFFFFFFFFFFFFFFFFFFFFFFFFFFFFFF:FFFFFFFFFFFFFFFFFFFFFFFFFFFFFFFFF
FFFFFFFFFFFFFFFFFFFFFFFFFFFFFFFFFFFFFFFFF:FFFFFF
@A00721:190:HGJW7DSXY:4:2609:30318:21621_2:N:0:TCTGCAAG+GCTGCACT
CCTTTAAGCGTCTTCTGTTCATCGCAACCAGATGATATCTTCTATTCGAACTTATGTTTCGATCAGTG
TTTCTTCTATCAGGAGGAAAACACTTGTTGTAGTCCTTGTAAGATTAA
+
FFFFFFFFFFFF:FFFFFFFFFF:F:FFFFFFFFFFFFFFFFFFFF:FFF:FFFFFFFFFFFF:FFFF
FFF:FF,FFFF:FFFFFFFF:FFFFFFFF,F:FFFFFFFF:,:FFFF:
@A00721:190:HGJW7DSXY:4:1228:30436:3756_2:N:0:TCTGCAAG+GCTGCACT
TCTTCTAATGTGGCCTGAACTACGTTTCACTTTGTGTGTACTTGTCTCTATTTTAAGTTTTGTCTTCC
TTGTTTGTCTTATTCTATTAATCACTCATTTTAGTGAGATTATTAAAA
+
FFFFFFFFFFFF:FFFFFFFFFFFFFFFFFFFFFFFFFFFFFFF:FFFFFFFFFFFFFFFFFFFFFF::
FFFF:FFFFFFFFFFFFFFFFFFFFFFFFFFFFF:FFFFFFFFFFFFFF
@A00721:190:HGJW7DSXY:4:2255:18511:17049_1:N:0:TCTGCAAG+GCTGCACT
TCTTCGACAACAGCAGGGGTTTTCGAATAGTGATAGCCACCCGCCTTACAGAGGGGCAGCAGCATGAC
CATAATTGGTATTGACTTTGGTACCACCTTTTCTACTGTCTGTCTCGCCTCTTCGAATGAAGTTTTAA
TGC
+
FFFFFFFFFFF:FFFFFFFFFFFFFFFFFFFFFF:FFFFFFFFFFFFFFFFFFFFFFFFFFFFFFFFFF
FFFFFFFFFFFFFFFFFFFFFFFFFFFFFFFFFFFFFFFFFFFFFFFFFFFFFFFFFFFFFFFFFFFF
FFF
@A00721:190:HGJW7DSXY:4:2145:1488:23109_2:N:0:TCTGCAAG+GCTGCACT
CTACAATCATTGGTTTTTCTAATAATGATGACACTATTGTTTATGGATATGATGCTATTACTCATGAT
GGTTACGGTCAGGGAGGATTCTCAGTTTACAAGGACCTTAAGCGATGG
+
FFFFFFFFFFFFFFFFFFFFFFFFFFFFFFFFFF:FFFFFFFFFFFFFFFFFFFFFFF:FFFFFFFFFFF
FFFFFFFFFFFFFFFFFFFFFFFFF:FFFFFFF:FFFFFFF:F:FFFF
@A00721:190:HGJW7DSXY:4:1551:18891:4210_2:N:0:TCTGCAAG+GCTGCACT
CCGAAAGACGTGCTAAATTAAAACCATTGTACGATGTCGTCTGTCTCGGCGATCACTTTTCACTAAGA
ATTGGTCCTACTTTTGGACAAGGCCAACTTCGTAATCTTCATGAGATC
+
FFFFFFFFFF:FFFFFFFFFFFFFFF:FFFFFFFFF:FFFFFFFFFFFFFFFFFFFFFFFFFFFF:FFF
FFFFFFFFFFFFFFF:FFFFFFFFF:FFFFFFFF:FFFFFFFFFFFFF
@A00721:190:HGJW7DSXY:4:1435:22598:5776_1:N:0:TCTGCAAG+GCTGCACT
TGTTAGGGATTTTGAGAGAGTGAAAGGCTTCAATTGTAGTGGTTTGGTCGTTTCAGTACCCAGTCAGT
ATACCTCTACGCAAAGATATTTCATGAAAACTACAGAGGAACGTACCGGATTACCTGTCTTTCATATT
ATG
+
FFFFFFFFFFFFFFFFFFFFFFFFFFFFFFFFFFFFF:FFFFFFFFFFFFFFFFFFFFFFFFFFFFFFFF
FFFFFFFFFFFFF:FFFFFFFFFFFFFFFFFFFFFFFFFFFFFFFFFFFFFFFFFFFFFFFFFFFFFFFF
FFF
@A00721:190:HGJW7DSXY:4:1566:32515:35634_1:N:0:TCTGCAAG+GCTGCACT
CTCTTTGCTTCGATGTTGGATGTCAAGAAGGGTTCAGAATGGGATTCCTACGTAGTTTATGACTTTGG
TGGTGGTACTTTCGATACTTCTATCGTCTTACGTTACGGTAATTACTATAGTGTTATTTATTCTGGTG
GTG
+
FF:FFFFFFFFFFFFFFFFFFFFFFFFFFFFFF:FFFFFFFFFFFFFFFFFFFFFFFFFFFFFFFFFFFF
FFFFFFFFFFFFFFFFFFFFFFFFFFFFFFFFFFFFFFFFFFFFFFFFFF,FF:FFFFFFFFFFFFFFFF
FFF
@A00721:190:HGJW7DSXY:4:1669:16911:27023_2:N:0:TCTGCAAG+GCTGCACT
AGGTCGTGACGTTGATGAAGCTATAAGAGCGTTCTTATTACGGCGCACTTCTTTACCCGCCACCACAC

AACTTTCAGTTTCACAACTGAAGGAGGAGGTTTCTGTTACTGGAAAAC
+
FFFFFFFFFFFFFFF:FFFFFFFFFFFFFFFFFFFFFFFFFFFFFFFFFFFFFFFFFFFFFFFFFFFFF
FFFFFFFFFFFFFFFFFFFFFFFFFFFFFFFFFFFFFFF:FFFFFFFFF
@A00721:190:HGJW7DSXY:4:1419:25943:1924_1:N:0:TCTGCAAG+GCTGCACT
ACTTTTCAATAATATACGTCTTGAACTCACTTATGACGATCTAGTGAAATTAACGCGTCCTTTTCTGG
AACGTAGTTTTACTATTATGAACAAAGTTATCAAAGATAGTGGCATTCAAGGCAAACTTACCCTGATT
CCG
+
FFFFFFFFFFFFFFFFFFFFFFFFFFFFFFFFFFFFFFFFFFFFFFFFFFFFFFFFFFFFFFF:FFFF
FFFFFFFFFFFFFFFFFFFFFFFFFFFFFFFFFFFFFFFFFFFFFFFFFFFFFFFFFFFFFFFFFFF,F
FFF
@A00721:190:HGJW7DSXY:4:1212:30201:8735_1:N:0:TCTGCAAG+GCTGCACT
TTCTACCAGGGGCTATTACTCTTGCTGAAACAGCTATTAAAGCTGTTGAGCGGGTAATTACACCTAAA
CGAGCTTGATCTGCCATCGCTGAAGGATGTGCACTCTTTTCTGCTACTTTAACGGAGCGTAGTGTACT
CTT
+
FFFFFFFFFFFFFFFFFFFFFFFFFFFFFFF:FFF,FF:FFFFFFFFFFFFFFFFFFFFFFFFFFFFF
FFFFFFFFFFFFFFFFFFFFFFFFFF:FFFFFFFFFFFFFFFFFF,FFFFFFFFFFFFFFFFFFFFFFFF
F:F
@A00721:190:HGJW7DSXY:4:1155:32389:14309_1:N:0:TCTGCAAG+GCTGCACT
CAACAGTAACATTACTTCTAATAAAGGTCTCTATCAAGCTCGTATTATCGTCGCTGCTGGATCTGCGT
TACCGTTGAAGGCTAAACTCCAAGGTAATTATGAAGACTGTGATTTATCTTTCACTCTGGCCTTGTAT
GAG
+
FFFFFFFFFFFFFFFF:FFFFFFFFFFFFFFFFFFFFFFFFFFFFFFFFFFFFFFFFFFFFFFFFFFF
FFFFFFFFFFFFFFFFFFFFFFFFFFFFFFFFFFFFFFFFFFFFFFFFFFFFFFFFFFFFFFFFFFFFF
FFF
@A00721:190:HGJW7DSXY:4:2145:15510:12665_2:N:0:TCTGCAAG+GCTGCACT
ATCGTAACATTAATAATTTCAATCTTCTTAAAAAGAAGATAGAGAGTCATACTTTGGGCTATCGTTCA
GGTGCATGGGGTTGGATAGCCAATATTGAAGTGGACACCTCTGGTAGT
+
FFFFFFFFFFFFFFFFFFFFFFFFFFFFF:FFFFFFFFFFFFFFFFFFFFFFFFFFFFFFFFFFFFFFF
FFFFFFFFFFFFFFFFFFFFFFFFFFFFFFFFFFFFFFFFFFFFFFFF
@A00721:190:HGJW7DSXY:4:2659:22860:11334_1:N:0:TCTGCAAG+GCTGCACT
AGATAACCAATAAGGAAGGAGCTTATACTATGGTTGAATCAACTAAGCATACTACTTTTGCTTTGATT
ACTCTACCGCCTTACAAGTCTAAGGTTAAAGAGTCGATTAAACAAGTTGTACTTATCAATGATTTTAT
CGC
+
FFFFFFFFF:FFF:FFFFFFFFFFFFFFFFFF:FFFFFFFFFFFF:FFF:FFFFFFFFFFFFFFFFFF
FFFFFFFFFFFF:FFFFFFFFFFFFFFFFFFFFFFFFFFFFFFFFFFFFFFFFFFFFFFFFFFFFFFFF
FFF
@A00721:190:HGJW7DSXY:4:1510:16125:31704_1:N:0:TCTGCAAG+GCTGCACT
ACCCAAAGAGAATATCTAAAATATTTACAAAATCTTACCACTATCTACGAACCTAAAGTTCTGAAGAC
GTTGCTTGTCGCTTCCTCCTTTAATGGCACTACCTCGGTACAATCTTACTTGGAACGAAGGCGACCTG
TTC
+
FFFFFF:FFFFFFFFFFFFFFFFFFFFFFFFFFFFFFFFFFFFF,FFFFFFF:FFFFFFFFFFFFFFFFF
FFFFFFFFFFFFFFFFFFFFFFFFFFFFFFFFFFFFFFFFFFFFFFFFFFF:FFFFFFFFFFFFFFFFF
FFF
@A00721:190:HGJW7DSXY:4:1227:19334:31626_1:N:0:TCTGCAAG+GCTGCACT
TGTTCTGTCTATATTAGGTGAAGCGAAATACGATTTCGAACGTCAACGTTCACCGAGTTTCGTTTCGT
ACCTTGATGCTGACTTTCGTCAATATGATTTGATCTGGTTGCTCAATTCTAAGCGTGTTCCGCTTAGT
GCG

+
FFFFFFFFFFFFFFFFFFFFFFFF:FFFFFFFFFFFFFFFFFFFFFFFFFF:FFFFFFFFFFFFFFFF
FFFFFFFFFFFFFFFFFFFFF,FFFFFFFFFFFFFFFFFFFFFFFFFF:FFFFFFFFFFFF:FFFFFF
FF:
@A00721:190:HGJW7DSXY:4:1638:28953:18850_2:N:0:TCTGCAAG+GCTGCACT
GAGTATTGCTATTTATACGGTTACGAGAAGAAGGTTCGAAAGTTTGAGAACTTAGTTTACGAACTTGA
CTCTACTATACGTGACATACTAAATACTAGATATTTGGATGATCTTGA
+
FFFFFFFFFFFFFFFFFFFFFFFFFFFFFFFFFFFFFFFFFFFFFFFFFFFFFFFFFFFFFFFFFFFF
FFFFFFFFFFFFFFFFFFFFFFFFFFFFFFFFFFFFFFFFFFFFFFFF
@A00721:190:HGJW7DSXY:4:1533:10149:15843_2:N:0:TCTGCAAG+GCTGCACT
CATGAATTTTCAGAGGATGAAAAATTTTCTTCCTTGGATTTTAATATAAAGAATCTTTACCAGGACTC
CTACTACAGGAGGCAGTCTTCTGATCGTAAGAAGTTTATAAGGAACAT
+
FFFFFFFFFFFFFFFFFFFF:FFFFFFFFFFFFFFFFFFFFFFFFFFFF:FFFFFFFFFFFFFFFFFF
FFFFFFFFFFFFFFFFFFFFF:FFFFFFFFFFFFFFFFFFFF:FFFFF
@A00721:190:HGJW7DSXY:4:2327:6668:24846_1:N:0:TCTGCAAG+GCTGCACT
ACTCGAAACTCTAAGTGGTAATACTGTCTTTCCAAGCTTACCTGTATCTTCAATGAAATTGGACTCTT
CGATAGGGGACATTTTATCGTGGGCTTTCCGTAAAGGAAGATCTGTTATCGACGGTTTATCGTTTACG
AAG
+
FFFFFFFFFFFF::FFF:FFFFF:FF:FFFFF:F:FF:FF:FF,F:FF:FFF:FFFFFFFFFFFFF,F
FFFFFFF:FFFFFFFFFFFFFFFFFFFFF:FFFFFFFFFFFFFFFFFFFF:FFFFFFFFFFFFFFFFFFF
FFF
@A00721:190:HGJW7DSXY:4:2678:26838:6887_2:N:0:TCTGCAAG+GCTGCACT
GTTTAAGAATACTATAAATAGAATAGCTATTACTACTAATGGCGTTGGCCTAGTGGATGTTATTAGTG
ACATCTGGTCAATTTCGTTTTTAGGTGGTCTTTATTCTATTATTGATT
+
F,F,FFFFFFF:FFFFF:FFFF:FFFFFFFFFF,FFFFFFFFFFFFFFFFF:FFFFFFFFFFFFFFFF
FFFFFFFFFFFFFFFFFFFFFFFFFFFFFFFFFFFFFFFFFF:FFFFF
@A00721:190:HGJW7DSXY:4:1550:12879:32440_1:N:0:TCTGCAAG+GCTGCACT
ACAGAAGATGATGTTAGAGTAGAATTCCTGAAGAGTCCTTCCGTAACCAACTTCTTGACAATTTTCTT
CAATCCTTCGATAATAGTTAAATCTAGGATTATGTCTGTGTTGAAAGACTTTCTACCGATAAGGGTCA
CTA
+
FFFFFFFFFF:FFFFFFFFFFFFFFFFFFFFFFFFFFFFFFFFFFFFFFFFFFFFFFFFFFFFFFFFF
FFFFFFFFFFFFFFFFFFFFFFFFFFFFFFFFFFFFFFFFFFFFFFFFFFFFFFFFFFFFF:FFFFFFF
FF:
@A00721:190:HGJW7DSXY:4:2359:14118:24001_2:N:0:TCTGCAAG+GCTGCACT
TAAGAAATAATCCTACATTGACTGATTATCTAAAGATTGAAGGAAAATTTTATCCTCGTTTAACTTAC
TTAGACTTTCCTACGATACAAATGATAACTTCTAATTTACGTTTGCTA
+
FFFFFFFFFFFFFFFFFFFFFFFFFFFFFFFFFFFFFFFFFFFFFFFFFF,FFFFFFFFFFFFFFFFF
FFFFFFFFFFFFF:FF:FFFFFFFFFFFFFFFFFFFFFFF:FFFFFFF
@A00721:190:HGJW7DSXY:4:2556:30743:1063_2:N:0:TCTGCAAG+GCTGCACT
CAAAGGGATGCATGGTGTTATCTCCTCAGGAGTCCCGGCATTTGATAATAATGACGAGACTTTCGTCA
TAATCGCTGCATATTACGGATTACACGGTACCAGCGCATCTCGTGTCG
+
FFFFFFFFFFFFFFFFFFFFFFFFFFFFFFFFFFFFFFFFFFFFFFFFFFFFFFFFFFFFFFFFFFFF
FFFF:FFFFFFFFFFFFFFFFFFFFFFFFFFFFFFFFFFFFFFFFFFF
@A00721:190:HGJW7DSXY:4:1648:19895:4288_1:N:0:TCTGCAAG+GCTGCACT
ATTAACTTATAAAGGTGAGGAGACCATTTTCTTGCTGAAATTTATAGAAGATTATTTTGACAGAATCA
ATAAGAATCATCGATTCTCGATTCGTAGAGCTTATCTAGGTACTCTCATTTACTATGTTGATTTAGTC
TAC

```
+
FFFFFFFFF:::FFFFFFFFFFFFFFFFFFFFFF:FFFFFFFFFFFFFFFFF:FFFFFFFFFFFFFF
FFFFFFFFFFFFFFFFFFFFFFFFFFFFFFFFFFFFFFFFFFFFFFFFFFFFFFFFFFFFFFFFFFF
FFF
@A00721:190:HGJW7DSXY:4:2559:2248:20760_2:N:0:TCTGCAAG+GCTGCACT
GATTTACAACGATATAGGAGTAATCTACGAGAAGATTCTAGAATTCTTAATTATAATATTCAACCTCA
AAAGTTACGTGTTTACAGTAACGAGATCACACATTTTGAGAACTAACT
+
FFFFFFFFFFFFFFFFFFFFFFFFFFFFFFFFFFF,FFFFFFFFFFFFFFFFFFFFFFFFFFFFFFFF
FFFFFFFFFFFFFFFFFFFFFFFFFFFFFFFFFFFFFFFFFFFFFFFF
@A00721:190:HGJW7DSXY:4:1238:13819:3505_1:N:0:TCTGCAAG+GCTGCACT
TCTCTTCTTATAATAAATAAATAAATAATATTACTAATGGCAAATAGTGGAAAGGCTCCAGTCAACAC
TAGTTCGAGTGCTGCTTTGGATTACGATGACGTGAGAATAGGTGAAGGGATAGTTTTTACCTCGACCA
ACA
+
FFF:FFFFFFFFFFFFFFFFFFFFFFFFFFFFFFFFFFFFFFFFFFFFFFFFFFFFFFFFFFFFFFFF
FFFFFFFFFFFFFFFFFFFFFFF:FFFFFFFFFFFFFFFFFFFFFFFFFFFFFFFFFFFFFFFFFF:F
FFF
@A00721:190:HGJW7DSXY:4:1441:13024:13714_2:N:0:TCTGCAAG+GCTGCACT
GCGACTAATCTTAAAGCTTCACCCAAAATGAAGGACACTTCGAAACTGAATAATGATGCACTACAAAC
AAGTATCGCTAATCATCTGAAAAAGGCTCTAAAGGACACTATACAATA
+
FFFFFFFFFFFFFFFFFFFFFFFFFFFFFFFFFFFFFFFFFFFFFFFFFFFFFFFFFFFFFFFFFFFF
FFFFFFFFFFFFFFFFFFFFFFFFFFFFFFFFFFFFFFFFFFFFFFFF
@A00721:190:HGJW7DSXY:4:2675:10203:1846_1:N:0:TCTGCAAG+GCTGCACT
TTGGTGCTTAGGGATATAATGAATTCAGCACCACATTTTGGTCAAGTCAATCCGTTGAGAAAATTTCT
TAGAGGATATTCGCCATTGTGGTACGAATTGGTCCAACATTTACCACCAAATTCTTTTGATACAACTC
TAG
+
FFFFFFFFFFFFFFFFFFFFFFFFFFFFFFFFFFFFFFFFFFFFFFFFFFFFFFFFFFFFF:FFFFFFF
FFFFFFFFF:FFFFFFFFFFFFFFFFFFFFFFFFFFFFFFFF:FFFFFFFFFFFFFFFFFFFFFFFFFF
FFF
@A00721:190:HGJW7DSXY:4:1602:12319:15311_2:N:0:TCTGCAAG+GCTGCACT
CGGAATACGCTTGCTATTCACCAGATTGGTTCGAACCCGTTGATGCTATGTCAGAAGAAGCTATTCAA
GCTTGTCGTTTAAAGACTGCAGAGGCTGTCAAACAAGCCCAGACTCAA
+
FFFFFFFFFFFFFFFFFFFFF::FFFFFFFFFFFFFFFFFFFFFFFFFFFFFFFFFFFFFFFFFFFFF
FFFFFFFFFFFFFF,F,F,FFFF,FFF:F,FF,FFF:FFFFFFFFFFF
@A00721:190:HGJW7DSXY:4:2257:13928:3161_1:N:0:TCTGCAAG+GCTGCACT
AATTTAGCTCAACTGGGGAGACGTGCTTAAATGGGTGATAAAAATTATTTAGAGATCTTTTCTACTTA
CAAAGGACAATTCAGTTTCATTAATTCTACGGAAAAGTCCGGAGTATATTGTATGTTAATAGATAGTG
GGA
+
FFFFFFFFFF:FFFFFFFFFFFFFFFFFFFFFFFFFFFFFFFFFFFFFFFFFFFFFFFFFFFFFFFFF
FF:FFFFFFFFFFFFFFFFFFFFFFFFFFFFFFFFFFFFFFFFFFFFFFFFFFFFFFFFFFFFFFFFF
FFF
@A00721:190:HGJW7DSXY:4:1311:28999:29763_2:N:0:TCTGCAAG+GCTGCACT
GAACGCGTTCGTATAACCAAGTACTTTCCGTCTGAAGAGTTTAGAGCTGCTATTGATTATGTAACCGA
TAGGAGTTGTATAGTTGAAGATGTGGCTTTAGACGATAATCCTTTAAG
+
FFFFFFFFFFFFFFFFFFFFFFFFFFFFFFFFFFFFFFFFFFFFFFFFF:FFFFFFFFFFFFFFF:FF
FFFFFFFFFFFFFFFFFFFFFFFFFFFFFFFFFFFFFFFFFFFFFFFF
@A00721:190:HGJW7DSXY:4:1356:2175:23234_1:N:0:TCTGCAAG+GCTGCACT
GAAGATTTTCCCAAAGATTTGTCTACGTTGCAGATCGCCACTCCAAACACTATATACATTATAAAATA
```

```
TAAAAATAAGGGAGTCATAACTTTTGTTACCAAATTCTTGGCTTTCAACGAAGTCTCTGATTTTATTA
ATC
+
FFFFFFFFFFF,,,F::FFF,FFF,F:FFFFF::FFF,:FFFFFFFFF,:F:F:FF,,FFFF::FFFF
FFFF,FF,F,F,FFF:,:FFFF:FFFFFFF:F,:FFFF::F:FFFF:F,FFFFF:FFFF,FFFF,FFF
:FF
@A00721:190:HGJW7DSXY:4:2533:9028:23265_1:N:0:TCTGCAAG+GCTGCACT
CGATCTCTCGGTCATTTACTCTGTACTTGATCTACGTCGTTTAACTGTCGGAACAACCTTTACTTAGA
ATGACTCTATCTTTTCAGACACTTCTTATATTATTTACAATAGTTAATATCATATTAGTAATACTCAC
TAT
+
FFFFFFFFFFFFFFFFFFFFFFFFFFFFFFFFFFFFFFFFFFFFFFFFF:FFFFFFFFFFFFFFFFFF
FFFFFFFFFFFFFFFFFFFFFFFFFFFFFFFFFFFFFFFFFFFFFFFFFFFFFFFFFFFFFFFFFFFF
FFF
@A00721:190:HGJW7DSXY:4:1215:28203:23062_2:N:0:TCTGCAAG+GCTGCACT
TTTTCTTAGCTAATAGATTAATAAAGGATGAGTACGAAAACCAACTCTTCGTTACGAGACCTGATGGT
AGAATAGATTTAGTCTCTACACCCATCTCAACGAGGATAAAGCGAGTT
+
F:FFFFFFFFFFFFFFFFFFFFFFFFFFFFFFFFFFFFFFFFFFFFFFFFFFFFFFFFFFFFFFFFFF
FFFFFFFF:FFFFFFFFFFFFFFFFFFFFFFFFFFFFFFFFFFFFFFF
@A00721:190:HGJW7DSXY:4:2436:7753:2112_2:N:0:TCTGCAAG+GCTGCACT
ACACGAAGAGATCATCCGCAAACGACTCGAAACCGAGACGCCGTAGATCAAAGTCCCTTCTAACTTTA
GATTATGAATCCTGCCTTTACTCATTTTATAGCAGTTATTTATGTTGT
+
FFFFFFFFFFFFFFFFFFFFFFFFFFFFFFFFFFFFFFFFFFFFFFFFFFFFFFFFFFFFFFFFFFFF
FFFFFFFFFFFFFFFFFFFFFFFFFFFFFFFFFFFFFFFFFFFFFFFF
@A00721:190:HGJW7DSXY:4:2244:25726:9502_1:N:0:TCTGCAAG+GCTGCACT
TTTATAACCTTCTTATACTTTTGGTTATGTTTTGTGAAAAACATGAGATACCCAAGTCTATCGAAGAC
ATTATCTCTTATGTACTTATTGTAACGATCATTCTTTCGAGTTTTGTCATTCTTATTCTTTTATTCCT
TTA
+
FFFF:FFFFFFFFF:FFFFFFF:F,FFFFF:FF:FFFFFF:F:FFFFFFF:F:F:FFFFFF,:FFFF:
FF::FFF:FFFFFFFFFF:FFF,F:FFFFFFFFFFFFFFFFF,F:FFFFFFFFFFFFFFFFFFFFFFF:
FFF
@A00721:190:HGJW7DSXY:4:1430:2618:10692_2:N:0:TCTGCAAG+GCTGCACT
TTTATAATGCTTTTCGATAGTGATTGTTATCAATTTAAATACATTACTGTTTCAAATCATCTTTACTC
ACAGACTCCGAAAATGGTTTCAAATGAGGTCAAAGCTCTATTTATTAA
+
FFFFFFFFFFFFFFFFFFFFFFFFFFFFF:FFFFFFFFFFFFFFFFFFFFFFFFFFFFFFFFFFFFFF
FFFFFFFFFFFFFFFFFFFFFFFFFFFFFFFFFF:FFFFFFFFFFFFF
@A00721:190:HGJW7DSXY:4:2246:1904:1470_1:N:0:TCTGCAAG+GCTGCACT
CACTACCACTGATCTGCCGACTTTAGTCAAGGAACTTCAATTCCTTCGATGTTTATATTTTTCTATAA
GGGAAAATATCAATAATCAAATTTCGACAAATTTTAGAACTTTATTTCAGAACTCTCTCCGATCACCT
TGG
+
FFFFFFFFFFFFFFFFFFFFFFFFFFFFFFFFFFFFFF::F,FFFFFFFFFFFFFFFFFFFF::FFFFF
FFFFFFFFFFFFFFF:FFFFFFFFFFFFFFFFFFFFFFFFFFFFFFFFFFFFFFFFFFFFFFFFFFFF
FFF
@A00721:190:HGJW7DSXY:4:2478:8024:18051_1:N:0:TCTGCAAG+GCTGCACT
TCTTTTCAATAAGATGTATGATGATAATCTACTATTTTTAAATAGTGATATTCAGCGTATACTTGAAT
ACTTTATCAAAGATTTGGCTTGGTCTATTTACCGCAAAAAGTTGGAAGAGGTCTATGATATTTATTTT
ACT
+
FFFFFFFFFFFFFFFFFFFFFFFFFFFFFFFFFFFFFFFFFFFFFFFFFFFFFFFFFFFFFFFFFFFF
```

```
FFFFFFFFFFFFFFFFFFFFFFFFFFFFFFFFFFFFFFFFFFFFFFFFFFFFFFFFFFFFFFFFFFFFFF
FFF
@A00721:190:HGJW7DSXY:4:1158:24469:13808_2:N:0:TCTGCAAG+GCTGCACT
CCGATTATAACTTGACTGTTGATAATATTACAATAGCTCTTTCTCAGAAGATTAGAGAAGTAACCGGT
GAACACGTCGGTAGCATTGATTTACTTTTACCGTTAGAACTGAAAAAG
+
FFFFFFFFFFFFFFFFFFFFFFF:FFFFFFFFFFFFFFFFFFFFFFFFFF:FFFFFFFFFFFFFFFFF
FFFFFFFFFF::FFF::FFFFFF:F:FFFFFFFFFFFFF,FF:FFFFF
@A00721:190:HGJW7DSXY:4:1653:28393:30091_1:N:0:TCTGCAAG+GCTGCACT
ACCTATAAGTTACAAACGATTCTTCGACTATATTAAAGAAATAAAAATACTTCAAGTACCTGAACTTT
ACATTTTTAATGGTATGGTATAATGTGTCTTACAAATAGAACCTTCACTCTCGGTAATGAGAGTGTAT
TCC
+
FFFFFFFF:FFFFFFFFFF::FFFFFF:FFFFFFFFFFFFFFFFFFFFFFFFFFFFFFFFFFFFFF:FF
FFFFFFFFFFFFFFFFFFFFFFFFFFFFFFFFFFFFFFFFFFFFFFFFFFFFFFFFFFFFFFFFFFFFFF
FFF
@A00721:190:HGJW7DSXY:4:1547:24623:25911_1:N:0:TCTGCAAG+GCTGCACT
GGCATTACTAGGATTTATCACACTCCTAAATTTGATAGCAATTTATGTTTTAAATGCTAGGAAAATTT
TAGAAAAACTCCATTGTATAGTTCAAGGAGTACTTAATTCAATAGCTTTAGTATTATTTACTATCGCC
TTT
+
FFFFFFFFFFFFFF:FFFFFFFFFFFFFFFFFFFFFFFFFFFFFFFFFFFFFFFFFFFFFFFFFFF:FF
FFFFFFFFFFFFFFFFFFFFFFFFFFFFFFFFFFFFFFFFFFFFFFFFFFFFFFFFFFFFFFFFFFFFFF
FFF
@A00721:190:HGJW7DSXY:4:1361:28926:6089_2:N:0:TCTGCAAG+GCTGCACT
AGTTAATTGCTTACACAAAAGTGACGCGCGAAGGAATCTTAATTGATTGAAGCTAGGCGTTGGGGAGA
ATGAACTGCCGCTTCCCGGAAGAAACGATCTATTTACAGAGTATTTAG
+
FFFFFFFFFFFFFFFFFFF:FF::,FF,F:FFFFFFFFF:FFFFF:FF:FFFFFFF:,FFFF:,:FF,
FF,FFFFF:FFFFFF,FFFFFFFF:FFFFFFFFFFF:FFFFF:FFFFF
@A00721:190:HGJW7DSXY:4:1153:4155:14857_2:N:0:TCTGCAAG+GCTGCACT
TACACTCCCCAAACATAACATACCATAACCTACCATGACCAATCATCGCTCCAACTGGCACGCCAGAC
GTAGGGTCAAACCTACTACCGCCTGTCGGCAGATCGGCACCACTGGTC
+
FFFFFFFFFFFFFFFFFFFFFFFFFFFFFFFFFFFFFFFFFFFFFFFFFFFFFFFFFFFFFFFFFFFF:F
FFFFFF:FF:FFFFFFFFFFFFFFFFFFFFFFFFFFFFFFFFFFFFFF
@A00721:190:HGJW7DSXY:4:2249:24153:5869_2:N:0:TCTGCAAG+GCTGCACT
ACCCGCTGTGCAGAATAACCTTCCCAAATCTAGATCTGCACAGAAGAAGGCTGAAGGTCGTTACCGGA
CGACCACTCAAAGGAAACGGGCTGTTGCCCAGAGGAAAGCAGCGACCA
+
FFFFFFFFFFFFFFFFFFFFFFFFFFFFFFFFFFFFFFFFFFFFFFFFFFFFFFFFFFFFFFFFFFFFFF
FFFFFFFFFFFFFFFFFFFFFFFFF:FFFFFFFFFFFF:FFFFFFFFFF
@A00721:190:HGJW7DSXY:4:2172:27091:34538_2:N:0:TCTGCAAG+GCTGCACT
GTAACATTGACGGCAGTCCCGTCATCTCTGTGGGAACTGAGGCAGTACCTCAGTCAAAAGTCACATTC
ACCACTCCCATACCAATCAATGATGTGACTATACAGTTCGGCCATTTC
+
FFFFFFFFFFFFFFFFFFFFFFFFFFFFFFFFFFFFFFFFFFFFFFFFFFFFFFFFFFFFFFFFFFFFFF
FFFFFFFFFFFFFFFFFFFFFFFFFFFFFFF,FFFFFFFFFFFFF:FFF
@A00721:190:HGJW7DSXY:4:1504:6705:29011_1:N:0:TCTGCAAG+GCTGCACT
ATACGTCGTGGGAGGTACTGAAGCCTTCCCCTCCGCTGGCACGAAGAACACTCCCAAGCCAATCAATA
GTTCTTCTGACCAGAATGAAAAGGGGTTTACCAGGGTAGTCTCTAAGTCTACCAGGAAGAGGGGAAAT
GTC
+
FFFFFFFFFFFFFFFFFFFFFFFFFFFFFFFFFFFFFFFFFFFFFFFFFFFFFFFFF:FFFFFFFFFFF
```

FFFFFFFFFFFFFFFFFFFFFFFFFFFFFFFFFFFF:FFFFFFFFFFFFFFFFFFFFFFFFFFFFFFF
FFF
@A00721:190:HGJW7DSXY:4:2659:25156:19257_1:N:0:TCTGCAAG+GCTGCACT
AAAGGGAAAGGTCCATCAGGACCTAAGAAGGGGACATTACCCCTAATTCTGAAGGAAGATCTCCCACC
ATCTGGGGTTGGGAATAAGAAATCTGCACGGGCCAAAGAGGCCGCTGCTACGAAGTCGCTGAAGGCGG
CTT
+
FFFFFFFFFFFFFFFFFFFFFFFFFFFFFFFFFF,FFFFFFFFFFF:FFFFFFFFFFFFFFFFFFFFFF
FFFFFFFFFFFFFFFFFFFFF:,FFFFFFFFFFFFFFFFFFFFFFFFFFFFFFFFFFFFFFFFFFFFF
FFF
@A00721:190:HGJW7DSXY:4:1452:3323:34554_2:N:0:TCTGCAAG+GCTGCACT
TGTAAAGTTGCGCCTGCCGACCCCATCTTTGAGACCAAGACTCTCTACAGTAGGTTCGTGCCTACTAT
GAGCAAATCTGTTGAATTCGTCACTGAGGACGAAGGTGAGAGGACTTT
+
FFFFFFFFFFFFFFFFFFFFFFFFFFFFFFFFFFFFFFFFFFFFFFFFFFFFFFFFFFFFFFFFFFFF
FFFFFFFFFFFFFFFFFF:FFFFFFFFFFFFFFFFFFFFFFFFFFFFF
@A00721:190:HGJW7DSXY:4:1127:4173:6057_1:N:0:TCTGCAAG+GCTGCACT
CTGAGAGCAGTCGCTGGGGAAAGAGAATTGCGTGGGTTGAAGGCCGACGCTTCGAGCTTGAGTGTGAC
GAGGCCGGTGACGTTTTTGTCAACGGCGTTTGGAGGATTTTATCCGCGCTTAACTATGGGGCGAAGAT
CCC
+
FFF:F:FFFFFFFFFFFFFF:FFFFFF,:FFFFFFFF:FFFFFFFFFFFFFFFFFFFFFFFFFFFFFF
FFFFFFFFFFFFFFFFFFFFFFFFFFFFFFFFFFFFFFFFFFFFFFFFFFFFFFFFFFFFFFFFFFFF
FFF
@A00721:190:HGJW7DSXY:4:1259:26648:33802_2:N:0:TCTGCAAG+GCTGCACT
AGGTTGGCATATTTATGCCGACGATAATAAGCATGTCACCCTTAAGCACCGAGGTAAGCTCGGGACTT
ACAAGAGAGAGTGTGAGAGAGTCGCTCTCACTATTTTTGCCCAGAATG
+
FFFFFFFFFFFFFFFFFFFFFFFFFF:FFFFFFFFFFFFFFFFFFFFFFFFFFFFFFFFFFFFFFFFF
FFFFFFFFFFFFFF:FFFFFFFFFFFFFFFFFFFFFFFFFFFFFFFFF
@A00721:190:HGJW7DSXY:4:2544:31069:3724_1:N:0:TCTGCAAG+GCTGCACT
CTGATGAGGTTAAAACTCTTGCTCCTTCTGTGGGTGAGGGTTTTTGTTATATTCACTGGTTCTGGGAG
GCTGCTTTCCTCCATAATTACAACCTCTCTTTAGAGATCATTAGAAGGAAGTTGGGCAAATTCCCACA
TAT
+
FFFFFFFFF,FFF,FFFFFFFFFFF,FFFF:FFFFFFFFFFFFFFFFFFFFFFFFFFFFFFFFFFFFF
FFFFFFFFFFFFFFFFFFFFFFFFFFFFFFFFFFFFFFFFFFFFFFFFFFFFFFFFFFFFFFFFFFFF
:FF
@A00721:190:HGJW7DSXY:4:2570:4001:3662_1:N:0:TCTGCAAG+GCTGCACT
CACACTTTTCTTGAAAATTTTTCCTTTCCTCCGTCATTGGGGATTTCGATGTGAGAAGGTGGAAGGTG
TCAAGGGTCTTCTTCATGCTGATCTTTCAGGACCTAATCACATCATGATTGATAGTTACGGCTCGTAT
CGA
+
FFFFFFF:FFFFFFFFFFFFFFFF::FFFFFFFFFFFFFFFFFFFFFFFFFFFFFFFFFFFFFFFFFF
FFFFFFFFFFFFFFFFFFFFFFFFFFFFFFFFFFFFFFFFFF:FFFFFFFFFFFFFFF,FFFFFFFFFFFF
FFF
@A00721:190:HGJW7DSXY:4:1233:5240:16705_2:N:0:TCTGCAAG+GCTGCACT
CGAAATTAAAGACGGGGGGATTAAGAGTCCAGCTGTAACTGTCATTTCAGAGAGGGGAGCTCTGAAAA
GGAAACTTCGTCAGAAGATGAGGAAGTCTAAGAGGAGGAGTTTGAGGA
+
FFFFFFFFFFFFFFFFFFFF:FFFFF:FFF,FFFF:FFF,FFFFFFFFFFFFFF:FFFFF,FFFFFF
FFFFFFFFF:FFFFFFFFFFFFFFFFFF:F,FFFFFF:FFF:FFFFFF
@A00721:190:HGJW7DSXY:4:1318:2492:15013_2:N:0:TCTGCAAG+GCTGCACT
GAAGTTGATGAAGAAGGATTATTTGAAGCAGGGGTTACCACTTCCTAAGGTGGATGAGGGAGGTTGTT

TCTTTGGAGTTTTAGATAATCTTTTGAGAAGGGGTCATAGAGCCAGCA
+
FFFFFFFFFFF:FFFFFFFFFFFFFFFFFFFFFFFFFFFFFFFFFFFF,FFFFFFF:FFFFFFFFFFFFF
:FFFFFFFFFFFFFFFFFFFFFFFFFFFFF:FFFFFFFFFFFFFFFFFF
@A00721:190:HGJW7DSXY:4:2121:31222:35963_2:N:0:TCTGCAAG+GCTGCACT
CCTTGAGAAGACAAGCCACATTAAGATATAATTTAGGTCAACTTGGGGTTTCGAATGTCATTAGTGCC
TTACGATTGAAAGAGGTCCTTTTGAGTATCGACAGATCTTTCGATCTT
+
FFFFFFFFFFFFFFFFFFFFFFFFFFFFFFFFFFFFFFFFFFFFFFFFFFFFFFFFFFFFFFFFFFFF
FFFFFFFFFFFF::FFFFF:FFFFFFFFFFFFFFFFFFFFFF:FFFFF
@A00721:190:HGJW7DSXY:4:2308:18909:12383_2:N:0:TCTGCAAG+GCTGCACT
GTGGCAGAATTCGGTAACTTACCATATTGTTCCTTCCGGGAAGTTGTGGGATTTAACTTTGACTCAAC
TACTCCGATCAGATCCGGATGGTTTGATTGGTATGGACTACTCGACTT
+
FFFFFFFFFFFFFFFFFFFFFFFFFF:FFFFFFFFFFFFFF:FFFFFFFFFFFFFFFF:FFFFFFFFF
FFFFFFFFFFFFFFFFFFFF,FFFF:FFFFFFFFFFFFFFFFFFFFFFF
@A00721:190:HGJW7DSXY:4:2566:9869:25629_1:N:0:TCTGCAAG+GCTGCACT
TAGGGTCATGGCAGATACTACCGATAAGGTGAAAAATATGCAGGTCAAAGATTCTGTAGCTCTGAGGG
CGGTTGAGGGAGCCGTTTTGGCCGACTTTAACTATTCTAAGAGTCTTTATGATTCAAAACCTATCGTT
GTC
+
FFFFFFFFFFFFFFFFFFFFFFFFFFFF:FFFFFFFFFFFFFFFFFFFFFFFFFFFF:FFFFFFFFFF
FFFFFFFFFFFFFFFFFFFFFFFFFFFFFFFFFFFFFFFFFFFFFFFFFFFFFFFFFFFFFFFFFFFF
FFF
@A00721:190:HGJW7DSXY:4:1236:17381:13902_2:N:0:TCTGCAAG+GCTGCACT
GCGGCTTATTGTCGCGCAGTTTCCAGAATTGCGGTTGAAGTTCACCCATTCTATCTTGAATGCTCACG
CCGTCGCAGCCATATCGAGACTATGTTATAATCACTTGTTAAAGTACA
+
FFFFFFFFFFFFFFFFFFFFFFFFFFFFFFFFFFFFFFFFFFFFFFFFFFFFFFFFFFFFFFFFFFFF
FFFFFFFFFFFFFFFFFFFFFFFFFFFFFFFFFFFFFFFFFFFFFFFF
@A00721:190:HGJW7DSXY:4:1239:32425:6856_2:N:0:TCTGCAAG+GCTGCACT
TTAGATATTATTGATATTGGAGGCGATATTCGTTTTCATATCCAAAACTCCTTGAATGGAGTACATAT
TTGCAACCCTATTCTTGATGTGAAAGATGCATCGAGGTATACGTCTAG
+
FF,F:FFFFFFFF,FFF:FFFFFFFFFFFF::FFFFFFFFFFFFFFFFFFF:FFFFF,FFFFFFFFFF
FFFF,F,F::FFFFFFFFF:FF::FFFF,FFFF:FFFFFFFF,FFFFF
@A00721:190:HGJW7DSXY:4:2525:3405:32346_1:N:0:TCTGCAAG+GCTGCACT
GGGAACGTACCTACTCACGAGACGTCATGAAAGCTATCGGAGGGAAGCAAAATGTTTCGTTCTGTTAC
AAGAACGCTTTGGATTGTGACCATAAAGCCTCAGTTGCGATAGCTGTAGAGGTATACGATATCTCTCT
TGA
+
FFFFFFFFFFFFFFFFFFFFFFFFFFFFFFFFFFFFFFFFFFFFFFFFFFFFFFFFFFFFFFFFFFFF
FFFFFFFFFFFFFFFFFFFFFFFFFFFFFFFFFFFFFFFFFFFFFFFFFFFFFFFFFFFFFFFFFFFF
FFF
@A00721:190:HGJW7DSXY:4:1166:19669:28604_2:N:0:TCTGCAAG+GCTGCACT
AATGCTTAAGAGAGAGATTGATGTATGTCATTTCGTCTTAGTTTGTCCCGGCGAATTATTGGACACGA
TGAAAAATGACATACCCGTTCCTGACCTTGGTATCGTTATCCGTCATG
+
FFFFFFFFFFFFFFFFFFFFFFFFFFFFFFFFFFFFFFFFFFFFFFFFFFFFFFFFFFFFFFFFFFFF
FFFFFFFFFFFF:FFFFFFFFFFFFFFFFFFFFFFFFFFFFFFFFFFF
@A00721:190:HGJW7DSXY:4:2335:16197:30044_2:N:0:TCTGCAAG+GCTGCACT
GGTTATTCTCACAACAAAGATAAGATTTTATCTTTCTTCAAACACCCTGTTTTCCTTAAGGGCGGACA
ATTGTTCCATTGTGAACTCTTGGGGACTAGATTGAGCATTAATGAGTT
+

FFFFFFFFFFFFFFFFFFFFFFFFFFFFFFFF:FFFFFFFFFFFFFFFFFFFFFFFFFFFFFFFFFFF
FFFFFFFFFFFFFFFFFFFF:FFFFF,FFFFFFFFFFFFFFFFFFFFF
@A00721:190:HGJW7DSXY:4:1621:16477:16282_1:N:0:TCTGCAAG+GCTGCACT
TCTCTAGTGGACAATTCAGAATACCTATTTCCGTACCAAGAGCCACTGCCAATCTCATCGCTATACGC
ATACCTGATTGGAAGAAGCGAGGTTCGTTCCTCAATAAAAATTTTTCTCAAGAGATTATGGTTGATAA
AGT
+
FFFFFFFFFFFFFFFFFFFFFFFFFFFFFFFFFFFFFFFFFFFFFFFFFFFFFFFF:FFFFFFFFFFF
FFFFFFFFFFFFFFFFFFFFFFFFFFF:FFFFFFFFFFFFFFFFFFFFFF,FFFFFFFFFFFFFFFFF
FFF
@A00721:190:HGJW7DSXY:4:1458:2121:22294_1:N:0:TCTGCAAG+GCTGCACT
AGGGCTATTCAGTACGTCATGGGGATCACTTCTTCGGTCAATGACAAAACCTTCGAATATGTTACGAC
ATGGCTTCGTAACAACAGTGCGAGGATTGTCATCTCGGGGAAAGTGATTCACCAAGATGTTCATATAG
AT
+
FFF:FFFFF:FFFF,FF:FFFFF,FFFF::FFFF::FFFF:FFFF,FFF,,FF,FFFFFFFFFFFF,F
:F:FFFF,FFFFFFFFFFFFF:F:FFFFFFFFF:F,FFFFFFFFFF:FFFFF:FF:FFFFF:FFFF:F
FF
@A00721:190:HGJW7DSXY:4:1355:11342:24455_1:N:0:TCTGCAAG+GCTGCACT
GAAGGTCGCAGCGATCTTACTCGTTATCGGAGCTAAGAAGAAGACTTTGGGTACACGTTATGCTCGCA
AACTTAACGTTAGTACCGGAGGTGATCTCTCTGCTTCTGCGTTACTGAGAGAAAAGATTCAGGACGCC
TAC
+
FFFFFFFFFFFFFFFFFFFFFFFF:FFFFFFFFFFFFFFFFFFFFF:FFFFFFFFFFFFFFFFFFFFF
FFFFFFFFFFFFFFFFFFFFFFFFFFFFFFFFFFFFFFFFFFFFFFFFFFFFFFFFFFFFFFFFFFFF
FFF
@A00721:190:HGJW7DSXY:4:2558:14380:16125_2:N:0:TCTGCAAG+GCTGCACT
TTTAGGTTTAGTAGGTGATCTTGACGTCGATTCTTTGTATAGGGAGTGTGAGAAGACAACGAATCTTG
AAACTACGGTCCTTATCAAAGAAAATGGTGAACCGGTCTCTAAATCTT
+
FFFFFFFFFFFFFFFFFFFFFFFFFFFFFFFFFFFFFFFFFFFFFFFFFFFFFFFFFFFFFF:FFFFF
FFFFFFFFFFFFFFFFFFFFFFFFFFFFFFF:FFFFFFFFFFFFFFFF
@A00721:190:HGJW7DSXY:4:1617:2239:28855_1:N:0:TCTGCAAG+GCTGCACT
GATTGAAGAACACGTCAAGAAGTCGCTCGGCGTTGTTCTTGAAAACCAAGTTAAGAAGGAGGCTGATC
ATGGAGTCTTTGTACCTTCTGTCAAACTTCGAGGTTCAGCTACGAGAGGTGATCCCGGATGTGGATTG
AAG
+
FFFFFFFFFFFFFFFFFFFFFFFFFFFFFFFFFFFFFFFFFFFFFFFFFFFFFFFF:FF:FFFFFFFF
F:FFFFFFF:FF,FFFFFFFFFFFF:FFFFFFFFFFFFFFFFFFFFFFFFFFFFFFFFFFFFFFFFFF
FFF
@A00721:190:HGJW7DSXY:4:2436:15275:10535_2:N:0:TCTGCAAG+GCTGCACT
TCCTTCTTCTTGAATGTTGTAAAGAATTGGGTTGAGAAGTGTGCTTTACAAGGAGTTAGACTCTTCTT
CAAGTTTTTAGATCTTCTTGATAATCTTAAAGAAAAATTTTCGAATAA
+
FFFFFFFFFFFFFFFFFFFFFFFFFFFFFFFFFFFFFFFFFFFFFFFFFFFFFFFFFFFFFFFFFFFF
FFFFFFFFFFFFFFFF,FFFFFFFF:FFFFFF:FFFFFFFFFFFFFFF
@A00721:190:HGJW7DSXY:4:2647:19922:13072_2:N:0:TCTGCAAG+GCTGCACT
CTGTACTTACATTCTTTACGCCAGATTCTTCATGGGGCGACGCTGTTTCTCTTGGTATTTCTGAAATA
GTTTATCAGAGCGTCATTTTCCTTTGGGATCTTTCCTCTGGTAAGGGA
+
FFFFFFFFFFFFFFFFFFFFFFFFFFFFFFFFFFFFFFFFFFFFFFFF:FFFFFFFFFFFFFFFFFFF
FFFFFFFFFFFFFFFFFFFFF:,FFFFFFFFFFFF:FFFFFFFFFFFF
@A00721:190:HGJW7DSXY:4:1249:4896:13792_2:N:0:TCTGCAAG+GCTGCACT
CGAGAATTATTACTGTTTTCTCTACCCATAACTTCTTTTTCAAAAATGCGGATGGGTCTACTGATGTT

```
TTCCTACAGAGTGCAGTCGTTTCAACACTTATCACCACTTTTGAAAGG
+
FFFFFFFFFFFFFFFFFFFFFFFFFFFFFFFFFFFFFFFFFFFFF:FFFFFFFFFFFFFFFFFFFFFF
FFFFFFFFFFFFFFFFFFFFFFFFFFFFFFFFFFFFFFF:,FFF:FFFF
@A00721:190:HGJW7DSXY:4:1565:5159:36041_2:N:0:TCTGCAAG+GCTGCACT
AATACGTATGTCGGTGGGGTTCTCAAGGCTCTCATCGCCACTGGGATTCTAAGGGGTTCCATAAGATA
TTATCTGACATTGTGGTTCAATGATCAGGATTCTTCTTTTGGTCCTTA
+
FFFFFFFFFFFFFFFFFFFFFFFFFFFFF:F,:FFF:FFFF:FFFFF,FF:FFFFFFFFFFFFFFFFF
FFFFFF:FFFF:FFFFFFFFFFFFFF,FFFF:FFFFFFF:FFFFFFFF
@A00721:190:HGJW7DSXY:4:2574:2899:22545_1:N:0:TCTGCAAG+GCTGCACT
ATTTGGATGTTGTCGAAAACTATCAGGTTCTTGTCACGATTCTTAAAAGAAGTTTATTAAAACTTCAG
AGTCTCTTGGAAGTCCAGGTAGCTAATGTTCTTGCAATAGCCACAGACTTTCTGGGAAAAGAGACCTC
TGT
+
FFFFFFFFFFFFFFFFFFFFFFFFFFFFFF:FFFFFFFFFFFFFFFFFFFFFFFFFF:FF:FFFFFFFF
FFFFFFFFFFFFFFFFFFFFFFFFFFFFFF:FF:FFFFFFFFFFFFFFFFFFFFFFFFFFFFFFFFFFF
FFF
@A00721:190:HGJW7DSXY:4:1674:31747:5744_1:N:0:TCTGCAAG+GCTGCACT
GAGAATTACTCAGATTCAATCTGGCATCACTGAACGTCTACAACGAGTTAGGGAGTGGCTAGGTTTTA
AAAGATCCTTTGCACAAGCAATACCTTCCACTGAGTTCATCACCCAGAGTTTCGACGTCGCGGAAAAC
GAT
+
FFFFFFFFFFFFFFFFFFFFFFFFFFFFFFFFFFFFFFFFFFFFFFFFFFFFFFFFFFFFFFFF:FFF
FFFFFFFFFFFFFFFFFFFFFFFFFFFFFFFFFFFFFFFFFFFFFFFFFFFFFFFFFFFFFFFFFFFF
FFF
@A00721:190:HGJW7DSXY:4:2149:7780:3599_2:N:0:TCTGCAAG+GCTGCACT
ACTGTTCGAGAATCTTCTGATGCTGAATTTGAGGAAAATTTTTCGGAAGTTTTTGGTATAAATCCTGA
TGGCTCAGTACAGGGAGGAGGTAATAGTTATAAAACCTTCTTTTATCA
+
FFFFFFFFFFFFFFFFFFFF:FFFFFFFFFFFFFFFFFF:F:FFFFFFFFF:FFFFFFFFFFFFFFFF
FFFFFFFFFFFFFFFFFFFFFFFFFFFFFFFFFFFFFFFFFFFFFFFF
@A00721:190:HGJW7DSXY:4:1259:1741:12493_2:N:0:TCTGCAAG+GCTGCACT
GGACAAAATCTGGGATCATAGGTATGTGTCACGCTACACTCGAAATATGGGCCCTCATAAGGAAGTAT
AGTTGGCGTCTTGTCGTTGACGTCATTACAGTCTGGAAGTTAGATCAT
+
FFFFFFFFFFFFFFFFFFFFFF:FFFFF:FFFFFF:FFFFFFFFFF:FFFFFFFFFF,FFFFFFFFFF
:FFFFFFFFF:FFFFFFFFFFFFFFFF:FFFFFFFFFFFFFFFFFFFF
@A00721:190:HGJW7DSXY:4:2374:22327:15859_2:N:0:TCTGCAAG+GCTGCACT
CGAGAGTTGTGATCTAGTTCTAAATTTAGGTCGACGGACTTTAGCAACTTTAGATAAATTCTTTCCGG
GCTCAATGACCTTTGCTATGTACGTGGATCTAATCCTTGGTGATCTGA
+
FFFFFFFFFFFFFFFFFFFFFFFFFFFFFFFFFFFFFFFFFFFFFFFFFFFFFFFFFFFFFFFF:FFFF
FFFFFFFFFFFFFF:FFFFFFFF:FFFFFFFFFFFFFFFFFFFFF:FF
@A00721:190:HGJW7DSXY:4:2126:30653:22514_2:N:0:TCTGCAAG+GCTGCACT
TATTAGTTCATGAAGTTCTTACACTTCTCGCGACTAATGTCAGCTTCACTACAAAGGCGTTACGTAAA
GTAGTTTTCACGCTTTATCTTAAAGCATTACCACCAAATTTAGCACTT
+
FFFFFFFFFFFFFFFFFFFFFF:FFFFFFFFFFFFFFFFFFFFFFFFFFFFFFFF:FFFFFFFFFFFFF
FFFFFFFFFFF:FFFF:FFFFFF,FFFFFFFF:F:FFFFFFFF:F:FF
@A00721:190:HGJW7DSXY:4:2515:18123:5478_1:N:0:TCTGCAAG+GCTGCACT
CTCGCCATATCAGATTAGAGACATGCCAGTTAGAGTTCTTCTAACCAGGACTGATGGTGAGATTCTTT
TACCTTTTGGAGGTCCAGGAACCTTTACTCCTAGTGTCTTTTCTAGAGTCCAAGGACGTTTCCGTCGG
AGG
```

+
FFFFFFFFFFFFFFFFFFFFFFFFFFFFFFFFFFFFFFFFFFFFFFFFFFFFFFFFFFFFFFFFFFFFFFFFF
FFFFFFFFFFFFFFFFFFFFFFFFFFFFFFFFFFFFFFFFFFFFFFFFFFFFFFFFFFFFF:FFFFFFFFF
FFF
@A00721:190:HGJW7DSXY:4:2134:30942:19664_2:N:0:TCTGCAAG+GCTGCACT
GACCGCTCAATAACACTCTATTGAATCCCTTTTCCACGATGAAAGCTCTTTATGAGGTGCTCCATGCA
AATTTACAGTTCCTCTTTGGTGATATCGCCTCTGTCCTTTCTCCTTTA
+
FFFFFFFFFFFFFFFFFFFFFFFFFFFFFFFFFFFFFFFFFFFFFFFFFFFFFFFFFFFFFFF:FFFFF
FFFFFFFFFFFFFFFFFFFFFFFFFFFFFFFFFFFFFFFFFFFFFFFF
@A00721:190:HGJW7DSXY:4:1519:20030:13886_2:N:0:TCTGCAAG+GCTGCACT
ACTTGGGTTTATCGGTTGTATTTTGATAAATTTGTTGGAATCGATAATGATACTGACGAAGGTTCTAT
TACCACCCTTGTCATTGATACAAACATTCTAGGTGATGCTTTCTTAGA
+
FFFFF:FF::FFFFFFFF:FFFFF:FFFFFFF,FF:F:FFFF:F,FFFF:FF,F,FFFFFFFFFFFF:
FFF:FFFFFF,,FFFF,F:FFFFFFFF,FFF,,FFFFFFFFFFFFFFF
@A00721:190:HGJW7DSXY:4:2565:22842:23140_2:N:0:TCTGCAAG+GCTGCACT
TTACAATCTGTAGAGAGAGAGGTTAATCAGAATTTGAGTGATAACGATTTAGTACTGGGAAATGGTGG
TAGTCAGTTTACTACTTTTCTTTCCCTCCCCTTTTTCGATTGGATTCG
+
FFFFFFF,FFFFFFFFFFFFFFF,FFFFFFFFFFFFFFFFFFFF,F:,:FFFFFF::FFFFFFFFFFF
FF:FFFF:FFFFFFFFF:FFFFFF,FFFFF:FF:FFF:FFFFFFFFF,
@A00721:190:HGJW7DSXY:4:1568:10782:16282_2:N:0:TCTGCAAG+GCTGCACT
TTGATAAGGTTTTTCTTTTATGGGACTCTTGTTTGTACTTATGAAGGAGGTATTACTTCGGAAGTTCT
TAATGTAGGTCTCCTAGTACTTAAGGGTTTATGTTTCGGTTTCAGCTG
+
FFFFFFFFFFFFFFFFFFFFFFFFFFFFFFFFFFFFFFF:FFFFFFFFFFFFFFFFF,FFFFFFFFFFF
FFFFFFFFFFFFFFFFFFFFFFFFFFFFFFFFFFFFFFFFFFFFFFFF
@A00721:190:HGJW7DSXY:4:1636:11858:33395_2:N:0:TCTGCAAG+GCTGCACT
TGTTTCACATTGCGTGATCACTTCCCGTGGACTTGGGAAGAGATTAAACATAAAATCCAGCGATTCTA
TTATGTCGCTAAAGCCTGTCCAGTTGTCGATGATGTTCGGGCTGTGGG
+
FFFFFFFFFFFFFFFFFFFFFFFFFFFFFFFFFFFFFFFFFFFFFFFFFF:FFFFFFFF:FFFFFFFFF
FFFFFFFFFFFFFFFFFFFFFFFFFFFFFFFFF,FFFFFFFFFFFFFF
@A00721:190:HGJW7DSXY:4:2442:12906:19429_2:N:0:TCTGCAAG+GCTGCACT
AAACTTACGACGATGTTCTCGCTCGCATTTCACAAGGATTACTAAGTCTGAGATCTGCCACTTCTAAT
TTTAGGAAACGTCGCGAAAAGAAAGTTCATGAGGATGTCTTGGTCGAA
+
FFFFFFFFFFFFFFFFFFFFFFFFFFFFFFFFFFFFFFFFFFFFFFFFFFFFFFFFFFFFFFFFFFFFF
FFFFFFFFFFFFFFFFFFFFFFFFFFFFFFFFFFFFFFFFFFFFFFFF
@A00721:190:HGJW7DSXY:4:2536:22679:20071_2:N:0:TCTGCAAG+GCTGCACT
GAACTTCTAATAGAGGATCCTGATGACATTATTTCAACGGATACCGATCTCAGTTCTACCACAGAAAC
CGTCTCAGGAACTGTCTCCACTGATTCGGAATTAGAGAAAGACGACCT
+
FFFFFFFFFFFFFFFFFFFFFFFFFFF:FFFFFFFFFFFFFFFFFFFFFFFFFFFFFFFFFFFFFFFFF
FFFFFFFFFF:FFFFFFFFFFFFFFFFFFF,FFFFFFFFFFFFFFFFF
@A00721:190:HGJW7DSXY:4:1340:26738:9878_1:N:0:TCTGCAAG+GCTGCACT
AGACCTCTGATTTTTCAGGGTGCCCTGATGAGGACGTTGAAAGGGATAATTTCAACTCTGAGACTTCT
AAGAGTGTTTCAAAAGACCAGAAACGTAACAAGAAGAATAAAGGTAGGAAGGGGAGAAAACAGAACAA
GC
+
F,F,,F:F,F,FFFFFFF:FF:F,FFFFFFF:FFF::F,FFF:FFFFF:FFFFFF,FFF,F,F,FF:F
FF:FFFFFFF,FFFFFFF::FFFFFFFFF:FF:F::FFFFFF:,FFF:F:FFFFFFFFFFFFF,:FFF
FF

@A00721:190:HGJW7DSXY:4:2405:10845:5713_2:N:0:TCTGCAAG+GCTGCACT
ATGGTTGCGGCTGATCGTACCCCAATTCCTGCTAGAAAGATCACTCTCGGCGATACCACTGATCTTCC
AGAGTTACGTAGTGGTCGTTCTTTTGGTGAGATCGGAAGAGCGTCGTG
+
FFF:FF:FFFFFFFFFFFFFFFFFFFFFFF:F:FFF:FFFFF:FFFF:FFFFFFFFFFFFFFFF:F:
FFFFFF,FFFFF,FF:FFFF:FFFFFFFFFFFFFFFFFFFFF:F:,:F
@A00721:190:HGJW7DSXY:4:1421:25129:11381_1:N:0:TCTGCAAG+GCTGCACT
CAAGTTAAACTTCGAACTGACCACTTACAAGAGTGTTGAAGTACCGCAACATGGATCAGTTTTCCAAA
AAGCTATTAATAGCAGTTGCGGGGCTATAATACCGCCTCCAGATTACTCTAAACTGAATCCACTCTCT
TGG
+
FFFFFFFFFFFFFFFFFFFFFFFFFFFFFFFFFFFFFFFFFFFFFFFFFFFFFFFFFFFFFFFFFFFF
FFFFFFFFFFFFFFFFFFFFFFFFFFFFFFFFFFFFFFFFFFFFFF:FFFFFFFFFFFFFFFFFFFFF
FFF
@A00721:190:HGJW7DSXY:4:2419:10574:36777_1:N:0:TCTGCAAG+GCTGCACT
AGAGTTTTGGAACTGTGTAATGTCCTACGTACTGTTCAAGGTGTTTGGGCTTCTCTAAAGAACAGCAA
CTTTCAACATGAATTAGTTATTGAAAGTCTTGACCCTCACGTCTCACTTTATCACCTTGGAGATGGTA
AAG
+
FFFFFFFFFFFFFFFFFFFFFFFFFFFFFFFFFFFFFFFFFFFFFFFFFFFFFFFFFFFFFFFFFFFF
FFF:FFFFFFFFFFFFFFFFFFFFFFFFFFFFFFFFFFFFFFFFFFFFFFFFFFFFFFFFFFFFFFFF
FFF
@A00721:190:HGJW7DSXY:4:2535:16532:14747_2:N:0:TCTGCAAG+GCTGCACT
AGACCTTTCTTGGGGAGTAAAGAGGGTTTGAAAATTACGGAACCTTTCCTCGTTCTTCATGAAGATCT
TCGAGTGTTTTATAGTGAGTTATTCTTGCGAAGGTTCAAGTCGATTGA
+
FFFFFFFFF:FFFFFFFFFFFFFFFFFFFFFFFFFFFFFFFFFFFFFFFFFFFFFFFFFFFFFFFF:F
FFFFFFFFFFFFFFFFFFFFFFFFFFFF:FFFFFFFFFFFFFFF,FFF
@A00721:190:HGJW7DSXY:4:2220:15528:4836_2:N:0:TCTGCAAG+GCTGCACT
AATAAGAAGTTCTTCGCAGTTGAATCTCCACCAGGTGGAGGGAAGACCACTGAACTTATAGCGACTTT
TGTCGAATTTTTCGCAAAAGGTCTTGAAGTACGTATTGTCACTGCTAA
+
FFFFFFFFFFFFFFFFFFFFFFFFFFFFFFFFFFFFFFFFFFFFFFFFFFFFFFFF:,FFFFFFFFFF
FFFFFFFFFFFFFFFFFFFFFFFFFFFFFFFFFFFFFFFFFFFFFFFF
@A00721:190:HGJW7DSXY:4:2462:13874:4131_2:N:0:TCTGCAAG+GCTGCACT
TTGGCACTTCTAGTTCATCGCGGTATTTGTGAAATTAAGACAGACGTGAAAGTTATGATGAGTGATCG
TATTAGAACAGCTGATTCTTATTGCATGAACGTCGCTGAGGTCAAACC
+
FFFFFFFFFFFFFFFFFFFFFFFFFFFFFFFFFFFFFFFFFFFFFFFFFF:FFFFFFFFFFFFFFF:F
FFFFFFFF,FFFFFFFFFFFFFF:FFFFFFFFF,FFFFF:FFFFFFFF
@A00721:190:HGJW7DSXY:4:1347:15528:10880_2:N:0:TCTGCAAG+GCTGCACT
TTCATCGACGAATTATTCATGATTCACGCTGGTCAGACCCTTTTTCTTACACATCTACTTAAGGCGGC
TTATACCATAGGTTACGGTGATTCCAATCAAATCGGATTCATCAATCG
+
FFFFFFFFFFF:FFFFFFFFFFFFFFFFFFFF:FFFFFFFFFF:F:FFFFFFFFFFFFFFFFFFFFFF
FFFFFFFFFF:FFFFFFFFFFFFF:FFFFFFFFFFFFFFFFFFFFFF,F
@A00721:190:HGJW7DSXY:4:1235:32479:36323_2:N:0:TCTGCAAG+GCTGCACT
CAAGTCGCAAACTACTTCAACATCAACGACATCATTCTTGATGAGTGTCGTTCTTATCGAAGTCTCAG
TTATCGCTGCCCGGCCGACGTTTGTCGCGAACTCTCAGAGGTTTACGG
+
FFFFFFFFFF:FFFFFFFFFFFFF:FFFFF:FF:FFFFFFFFFFFFFF:FFFFFF:FFFFFFFFFF,F
FFFFFFFFFFFFFFFFFFFFFFFFFFFFFFFFFFFFF:FFFFFFFFFFF
@A00721:190:HGJW7DSXY:4:2324:1515:20181_2:N:0:TCTGCAAG+GCTGCACT
CTATGGAAATTAGACCTATCAGATGTCTTGACGACTTCGAATTTGAGGACGGAGTTAAATATATTACT

TTCACTCAAGGTGAAAAGTTTGAAATTAACAAAGTCCTTGGTAAGAGT
+
FFFFFFFFFFFFFFFFFFFFFFFFFFFFFFFFFFFFFFFFFFFFFFFFFFFFFFFFFFFFF:FFFFFFFFF
FFFFFFFFFFFFFFFFFFFFFFFFFFFFFFFFFFFFFFFFFFFFFFFF
@A00721:190:HGJW7DSXY:4:1515:7988:12007_2:N:0:TCTGCAAG+GCTGCACT
ACTTACCAGCACGTTCGGCTGGTTAGAATTAAACCTCAAGATGACCCTGTTTTTGCATCACAAACACA
TATTACCGTTGCTCTATCACGACACGTTGAATCTTTAAGATACTTTGT
+
FFFFFFFFFFFFF,FFFF:F,,FFFF:FFFFFFF,FFFF:FFFFFFFF,:FFFF,FFF,F,FFFFFF:
:FFFFF:FFF:,:FFFF:FF,F:FF:FF:FFFF:F:F,FFFF:FF,F:
@A00721:190:HGJW7DSXY:4:1669:13096:33755_1:N:0:TCTGCAAG+GCTGCACT
TTAAACGATGCTACTTGTGAGAGGATCAATCACAGTAAGTAACTGTCACGTTTAGCACGGGAGGTTGC
AGATTCAACCCCTTATATCGAGGCTATCGAGGACGATGAGTATCTTGAAGTTGTTGACGACAAGCCTC
AAG
+
FFFFFFF:FFFFFFFFFFFFFFFFFFFFFFFFFFFFFFFFFFFFFFFFFFFFFFFFFFFFFFFFFFFF
FFFFFFFFFFFFFF:FFFFFFFFFFFFFFFFFFFFFFFFFFFFFFFFFFFFFFFFFFFFFFFFFFFFF
FFF
@A00721:190:HGJW7DSXY:4:2512:10872:15624_2:N:0:TCTGCAAG+GCTGCACT
TCTTCTACTGTCTCTGCCTTCCAAGATTTCTTAGAAGAGGTTATTCCTGGAACCACCTTAACGTCACT
CGGTAATCTTTCAGCCGAATTAAGTACTAATGAGTTCAAGTCGGTTGT
+
FFFFFFFFFFFF:FFFFFFFFFFFFFFFFFFF:FFFFFFFFFFFFFFFFFFFFFFFFFFF:FFFFFFF
FFFFFFFFFFFFFFFFFFFFFFFFFFFFFFFFFF,FFFFFFFFFFF:FFF
@A00721:190:HGJW7DSXY:4:1507:17074:13150_1:N:0:TCTGCAAG+GCTGCACT
GATCAAGGCTAATACCTTTATAGGTAAGAAGCCCCTTGAGCCGCGTAGTCAGCGCGTTTAGCTGTATC
ATCAGTCAGAGTATACCCGATAGGGTACCGAGTCTCCAAGAGAATATTTATTCTTATGAGGCTCGAAA
CTT
+
:FFFFFFFFFFFFFFFFFFFFFFFFFFFFFFFFFFFFFFFFFFFFFFFFFFFFFFFFFFFFFFFFFFF
FFFFFFFFFFFFFFFFFFFFFFFFFFFFFFFFFFFF:FFFFFFFFFFFFFFFFFFFFFFFFFF:FFFFFF
FF:
@A00721:190:HGJW7DSXY:4:1546:18991:5948_1:N:0:TCTGCAAG+GCTGCACT
CAACGGAAAGACATATCGCACCCGAAATTTTTGGGCAGAATGCTCTTAACGCGGTTCTTGAACGGTGT
TTTGATCAGTCTAAGTTGGATAAAGTTATAGGAGACTCAGTTAACCTGAGAGCTCCTCAATTGATAGA
TTG
+
:FFFFFFFFFFFFFFFFFFFFFFFFFFFFFFFFFF:FFFFFFFFFFFFFFFFFFFFFFFFFFFFFFF:FF
:FFFFFFFFFFFFFFFFFFFFFFFFFFFFFFF:FFFFFFFFFFFFFFFFFFFFFFFFFFFFFFFFFFFF
FFF
@A00721:190:HGJW7DSXY:4:2120:19443:12367_2:N:0:TCTGCAAG+GCTGCACT
CTAGTATACTTCGTAAAGTCGACAGCGAAATGGTCAATATTGATAATTGGACCGACTATATGACCTAT
TTTAAGCTCATGGTGAAAAGGGAAGCTAAGGTTAAACTTGATGCTTCA
+
FFFFFFFFFFFFFFFFFFFFFFFFFFFFFFFFFFFFFFFFFFFFFFFFFFFFFFFFFFFFFFFFFFFF
FFFFFFFFFFFFFFFFFFFFFFFFFFFFFFFFFFFFFFFFFFFFFFFF
@A00721:190:HGJW7DSXY:4:1567:5647:4570_2:N:0:TCTGCAAG+GCTGCACT
CCTATCTTTAAGGTTCTTGTGAGGAGAGTCAAACAGTGTCTTTTACCCAACATTTTTATGTATACAGA
GATGACCAGTGACGATTTCTCCCGGAAGCTCTTCTCTCTCTTAGGAGG
+
FFF:FFFFFFFF:FFFFFF,FFFFFFF:FFFFFFFFFFFFFFFFFFFFFFFFFFFFFFFFFFFFFFFF
FFFFFFFFFFFFFFFFFFFFFFFF:FFF::FFFFFFFFFFF,FFFFFFF
@A00721:190:HGJW7DSXY:4:2212:2094:9471_2:N:0:TCTGCAAG+GCTGCACT
TTTCTTTTGTAAAGCCTATGAGATAAAGATGTATTCTTATTTAGGCTTCGATGCTGAATTTCTTGAAC

```
TTTGGAGTGCCAGTGAATATTTTTGTCACGCCAGGAGTATCCATAACG
+
:,FFFFFFFFFFFFFFFFFFFFFFFF:FFFFFF:FFFFFFFFFFFFFFFFFFFFFFFFFFFFFFFFFFF
:FFFFFFFFFFFF:FFFFFFFFFFFFFFFFFFFFFFFFFFFFFFFFFFF
@A00721:190:HGJW7DSXY:4:1630:1099:6339_2:N:0:TCTGCAAG+GCTGCACT
TACCACCTTTGCTCAACGACGAAGTGGAACGGCCAACACTTTCTTTGGTAATTGTATGATCACTTTGA
TGATTTTATCACTTTATTATGACCTTGGAAAGTTTTCGGCTGTGGTTG
+
FFFFFFFFFFFFFFFFFFFFFFFFFFFF:FFFFFFFFFFFFFFFFFFFFFFFFFFFFF,FFFFFFFFF
FFFFFFFFFFFFFFFFFF:FFFFFFFFFF:FFFF,F:FFFFFFFFFFF
@A00721:190:HGJW7DSXY:4:1234:23873:23703_2:N:0:TCTGCAAG+GCTGCACT
CTGTTTTCAAAGACTCCAATAACTGATCATTCTGATCATATGATTTTGGACAATGGTTTCGAAGAGAA
ATTTTTGAGGGATAAACCAGCGTACTTCTGCTCGCGATTTATTCTTTT
+
FF,FFFFFFFFF:FFF,FFFFFFFFF::,FFFFFFF:FFFFFFFFFFFFFFFFFF::F:::FF:,FFF
FFFFFF:FF,FFFFF:F:F,,:F:FF:::FF,,,,FFFFFFFF:FFFF
@A00721:190:HGJW7DSXY:4:2128:14100:13448_2:N:0:TCTGCAAG+GCTGCACT
CCTGATCCTTACAAGATCATCGTTAAGTTAGGTAAACCCCTAACCGATGTTACTCTTGTAGGACTTTG
GGAGAGATTCGTTAGTTTTAAAGACTATACTAAATACCTCCACTACGA
+
FFFFFFFFFFFFFFFFFFF,FFFFFFFFFFFFFFF,FFFFF,FFFFFFFFFFFFFFFFFFFFF:F:FF
FFFFFFF,FFFFF::FFFFFF:F:FFFFFFF,FFFFFFFFFFFFFFFF
@A00721:190:HGJW7DSXY:4:1505:17173:19366_2:N:0:TCTGCAAG+GCTGCACT
GCGACTAATATTCGATATTCGATTGAGGGTCGTTATACCTATTCGGCTATCTGTGCTTTGAATTGTAT
AAGATCAAATTTTAAACGATTCCGAGAGGTTTATCCTGATGTGGAGGG
+
FFFFFFFFFFFFFFFFFFFFFF:FFFFFFFFFFFFFFFFFFFFFFFFFFFFFFFFFFFFFFFFFFFFF
FFFFFFFFFFFFFFFFFFFFFFFFFFF,FFF:FFFFFFFFFFFFFFFFF
@A00721:190:HGJW7DSXY:4:1609:17888:34225_1:N:0:TCTGCAAG+GCTGCACT
ATCCCGACCTTTCCTTGTGAAAATTTTGATGCGGTTTCATAAGATTATCAGTAATCCTGATTATACTA
CGGATTCATGGACCTTCCATCCTGTACCTCTTCTGGAGAGACGTATCTCAAAGATCGACTTCGAGACA
GGT
+
F:F:F:F,:FF:,,FFFF,FFFFFFFFF,FF:FFFFF,FFFFF:FFF:FF,:FFFFFFF,FF,FFFFF
:FFFF:,FF:FFFFFF:FFFFF:FFFFFF:F,:FFFFF:F:FFF::FF,FF,FFFFFF:F:FFFFFF,
FF:
@A00721:190:HGJW7DSXY:4:1410:22200:8938_2:N:0:TCTGCAAG+GCTGCACT
GCGAGAGCGACCAAGATCTAAATTCGGTAACGGTGCGTGGCGCTTTATTTTAAAAAGCTCCCTCCTCT
CGCAAGTTTGATCTGATACTTATCACGAAAGGACTTGAGTCTTTTGAT
+
FFF:FFFFFFFFFFFFFFFFFFFFFFFFFFFFFFFFFFFFFFFFFFFFFFFFFFFFFFFFFFFFFFFF
FFFFFFFFFFFFFFFFFFFFFFFFFFFFFFFFFFFFFFFFFFFFFFFF
@A00721:190:HGJW7DSXY:4:1547:15402:14982_2:N:0:TCTGCAAG+GCTGCACT
TCTTTCATTTTAAATGACGATCATTAAGACTCTCTTTCTTCTTTGTTTCCTTCTTTGCACTGATTTTT
TCCTCATCAGCTTGTGTTTTACCTAATCTGTGTTTTTACGATAACAAC
+
FFFFFFFFFFFFFFFFFFFFFFFFFFFFF:FFFFFFF:FFFFF:FFFFFFFFFFFFFFFFFFFFFFFF
FFFFFFFFFFFFFFFFFFFFFFFFFFFFFFFFFFFFFFFFFFFFFFFF
@A00721:190:HGJW7DSXY:4:1155:29306:35305_2:N:0:TCTGCAAG+GCTGCACT
TCTTCCGAAAGGCGAACTTTGGGAATTTGAAGCTCAGGGTGAAGCACAACGTTTGACCGTGACGAACT
GTGGTGTTCTTGATCTACGTTACGGATATACTCTCCTTGAGTTCACTC
+
FFFFFFFFFFFFFFFFFFFFFFFFFFFFF:FFFFFFFFFFF:FFFFFFFFFFFFFFFFFFFFFFFFFF:
FFFFFFFFFFFFFFFFFFFFF:FFFFFFF,FFFFFFFFFFFFFFFFFF
```

```
@A00721:190:HGJW7DSXY:4:1202:14552:20118_1:N:0:TCTGCAAG+GCTGCACT
CACTTATTACGATATTTCTTTAGTCGATGGGTTTTCGACGCCTTTAAGCGTCTTCTGTTCATCGCAAC
CAGATGATATCTTCTATTCGAACTTATGTTTCGATCAGTGTTTCTTCTATCAGGAGGAAAACACTTGT
TCT
+
FF:FFFFFF,FFFFFF:FFFFFFF,FFFFFFFFFFFFFFFFFFFFFFFFFFFFFFFFFFFFFFFFFFF
FFFFFFFFFFFFFFFFFFFFFFFFFF:FFFFFFFFFFFFFFFFFFFFFFFFFFFFFFFFFFFFFFFFF
FFF
@A00721:190:HGJW7DSXY:4:1272:6578:19680_2:N:0:TCTGCAAG+GCTGCACT
TCTTCTAATGTGGCCTGAACTACGTTTCACTTTGTGTGTACTTGTCTCTATTTTAAGTTTTGTCTTCC
TTGTTTGTCTTATTCTATTAATCACTCATTTTAGTGAGATTATTAAAA
+
FFFFFFFFFFFFFFF:FFFFFFFFFFFFFFFFF,,FFF,FFFFFFFFFFFFFFFFFFFFFFFFFFFFF
FFFFFF:FFFFFFFFF,FFFF::FFFFFF:F:FFFFFFFFF:FFFFFF
@A00721:190:HGJW7DSXY:4:2270:3821:16783_1:N:0:TCTGCAAG+GCTGCACT
TCTTCGACAACAGCAGGGGTTTTCGAATAGTGATAGCCACCCGCCTTACAGAGGGGCAGCAGCATGAC
CATAATTGGTATTGACTTTGGTACCACCTTTTCTACTGTCTGTCTCGCCTCTTCGAATGAAGTTTTAA
TGC
+
FFFFFFFFFFFFFFFFFFFFFFFFFFFFFFFFFFFFFFFFFFFFFFFFFFFFFFFFFFFFFFFFFFFF
FFFFFFFFFFFFFFFFFFFFFFFFFFFFFFFFFFFFFFFFFFFFFFFFFF:FFFFFFFFFFFFFFFFF
FFF
@A00721:190:HGJW7DSXY:4:2318:19153:22294_2:N:0:TCTGCAAG+GCTGCACT
CTACAATCATTGGTTTTTCTAATAATGATGACACTATTGTTTATGGATATGATGCTATTACTCATGAT
GGTTACGGTCAGGGAGGATTCTCAGTTTACAAGGACCTTAAGCGATGG
+
FFFFFFFFFFFFFFFFFFFFFFFFFFFFFFFFFFFFFFFFFFF:FF:FFFFF:F:FFF,FFFFFFFFFF
FFFFFFFFFFFFFFFFFFFFFFFFFFFFFFFFFFFFFFFFF:F:FFFF:
@A00721:190:HGJW7DSXY:4:1553:13268:8249_2:N:0:TCTGCAAG+GCTGCACT
CCGAAAGACGTGCTAAATTAAAACCATTGTACGATGTCGTCTGTCTCGGCGATCACTTTTCACTAAGA
ATTGGTCCTACTTTTGGACAAGGCCAACTTCGTAATCTTCATGAGATC
+
FFFFFFFFFF:FFFFFFFFFFFFFFFFFFFFFFFFFFFFFFFFFFFFFFFFFFFFFFFFFFF:FFFFFF
FFFFFFFFFFF:FFFFFFFFFFFFFFFFFFFFFFFFFFFFFFFFFFFFF
@A00721:190:HGJW7DSXY:4:2604:14461:23657_1:N:0:TCTGCAAG+GCTGCACT
TGTTAGGGATTTTGAGAGAGTGAAAGGCTTCAATTGTAGTGGTTTGGTCGTTTCAGTACCCAGTCAGT
ATACCTCTACGCAAAGATATTTCATGAAAACTACAGAGGAACGTACCGGATTACCTGTCTTTCATATT
ATG
+
FFFFFFFFFFFFFFFFFFFFFFFFFFFFFFFFFFFFFFFFFFFFF:FFFFFFFFFFFFFFFFFFFFFFFF
FFFFFFFFFFFFFFFFFFFFFFFFFFFFFFFFFFFFFFFFFFFFFFFFFFFFFFFFFFFFFFFFFFFF
FFF
@A00721:190:HGJW7DSXY:4:1672:10176:10379_1:N:0:TCTGCAAG+GCTGCACT
CTCTTTGCTTCGATGTTGGATGTCAAGAAGGGTTCAGAATGGGATTCCTACGTAGTTTATGACTTTGG
TGGTGGTACTTTCGATACTTCTATCGTCTTACGTTACGGTAATTACTATAGTGTTATTTATTCTGGTG
GTG
+
FF:FFFFFFFFFFFFFFFFFFFFFFF:FFFF:FF:FFFFFFFFFFFFFFFF:FF:FFFFF:FFFFFFFF
FFFFFFFFFFFFFFFFFFFF:FFFFFFF:FFFFFFFFFFFFFFFFF,FFFFFFFFFFFFFFFFFFFFF
FFF
@A00721:190:HGJW7DSXY:4:2163:6750:9079_2:N:0:TCTGCAAG+GCTGCACT
AGGTCGTGACGTTGATGAAGCTATAAGAGCGTTCTTATTACGGCGCACTTCTTTACCCGCCACCACAC
AACTTTCAGTTTCACAACTGAAGGAGGAGGTTTCTGTTACTGGAAAAC
+
```

FFFFFFFFFFFFFFFFFFFFFFF,FFFFFFFFFFFFFFFFFFFFFFFFFFFFFFFF:::FFF:FFFFFFF
FFFF:FFFFFFFFFFFFFFFFF:FFFFFFFFFFF:FFFFFFF:FFFFF
@A00721:190:HGJW7DSXY:4:1445:21441:31360_1:N:0:TCTGCAAG+GCTGCACT
ACTTTTCAATAATATACGTCTTGAACTCACTTATGACGATCTAGTGAAATTAACGCGTCCTTTTCTGG
AACGTAGTTTTACTATTATGAACAAAGTTATCAAAGATAGTGGCATTCAAGGCAAACTTACCCTGATT
CCG
+
FFFFFFFFFFFFFFFFFFFFFFFFFFFFFFFF:FFFFFF:FFFFFFFFFFFFFFFFFFFFFFFFFFFFF
FFFFFFFFFFFFFFFFFFFFFFFFFFFFFFFFFFFFFFFFFFFFFFFFFFFFFFFFFFFFFFFFFFFF
FFF
@A00721:190:HGJW7DSXY:4:1212:30861:17613_1:N:0:TCTGCAAG+GCTGCACT
TTCTACCAGGGGCTATTACTCTTGCTGAAACAGCTATTAAAGCTGTTGAGCGGGTAATTACACCTAAA
CGAGCTTGATCTGCCATCGCTGAAGGATGTGCACTCTTTTCTGCTACTTTAACGGAGCGTAGTGTACT
CTT
+
FFFF,,FFFF:F,FFF:FFFFFFFFFFFFFFFFFFFFFFFFFFFFFFFFF:F,FFFFFFFF:FFFFF,
FFFFFFFFFF:FFFFFFFFFFFFFFFFFFFFFFFFFFFFFF:FFFFFFFFFFFFFFF:FFFFFFFFFF
FFF
@A00721:190:HGJW7DSXY:4:1456:27254:16438_1:N:0:TCTGCAAG+GCTGCACT
CAACAGTAACATTACTTCTAATAAAGGTCTCTATCAAGCTCGTATTATCGTCGCTGCTGGATCTGCGT
TACCGTTGAAGGCTAAACTCCAAGGTAATTATGAAGACTGTGATTTATCTTTCACTCTGGCCTTGTAT
GAG
+
FFFFFFFFFF:F:FF:FFFFFFFFFFFFFFFFFFFFFFFFFFFFFFFFFFFFFFFFF,FFFFFFFFFF
,FFFFFFFFFFFFFFFFFFFFFFFFFFFFFFFFFFFFFFFFFFFFFFFFFFFFFFFFFFFFFFFFFFF
FFF
@A00721:190:HGJW7DSXY:4:2220:32371:13182_2:N:0:TCTGCAAG+GCTGCACT
ATCGTAACATTAATAATTTCAATCTTCTTAAAAAGAAGATAGAGAGTCATACTTTGGGCTATCGTTCA
GGTGCATGGGGTTGGATAGCCAATATTGAAGTGGACACCTCTGGTAGT
+
FF:::FFFFFFFFFFFFFF,FFFFF,:F::FF,FFFFFFFFFF:FFFFFFFFFFFFFFFFFFFFFFFFF
FF:FF:FFFFFFFFFFFFFFFFFFFFFFFFFFFFFF:FFF:FFFFFFFF
@A00721:190:HGJW7DSXY:4:2104:5394:36385_1:N:0:TCTGCAAG+GCTGCACT
ATACTATGGTTGAATCAACTAAGCATACTACTTTTGCTTTGATTACTCTACCGCCTTACAAGTCTAAG
GTTAAAGAGTCGATTAAACAAGTTGTACTTATCAATGATTTTATCGCTGGTTGTTATCTCGAAAAGTC
TCT
+
FFFFFFF:FF,FFFFFFF:FFFF:FFFFFFFFFFFFFFFFFFFFFFFFFFFFFFF:FFFFFFFFFF:FF
FFFFF:FFFFF,FFFF,FFFFFFFFFFFFFFFFFFFF:FFFFFFFFFFFFFFFFFFFFFFFFFFFFFFF
FFF
@A00721:190:HGJW7DSXY:4:2149:26006:1376_1:N:0:TCTGCAAG+GCTGCACT
ACCCAAAGAGAATATCTAAAATATTTACAAAATCTTACCACTATCTACGAACCTAAAGTTCTGAAGAC
GTTGCTTGTCGCTTCCTCCTTTAATGGCACTACCTCGGTACAATCTTACTTGGAACGAAGGCGACCTG
TTC
+
FFF:FFFFF:FFFFFFFFFFFFFFFFFFFFFFFFFFFFFFFFFFFFFF:FFFFFFFFFFFFFFFFFFFFF
FFFFFFFFFFFFFFFFFFFFFFFFFFFFFFFFFFFFFFFFFFFFFFFFFFFFFFFFFFFFFFFFFFFFF
FFF
@A00721:190:HGJW7DSXY:4:1237:22064:13432_1:N:0:TCTGCAAG+GCTGCACT
TGTTCTGTCTATATTAGGTGAAGCGAAATACGATTTCGAACGTCAACGTTCACCGAGTTTCGTTTCGT
ACCTTGATGCTGACTTTCGTCAATATGATTTGATCTGGTTGCTCAATTCTAAGCGTGTTCCGCTTAGT
GCG
+
FFFFFFFFFFFFFFFFFFFFFFFF:FFFFFFFFFFFFFFFFFFFFFFFFFFFFFFFFFFFFFFFFFFF

```
FFFFFFFFFF:FFFFFFFFFFFFFFFFFFF:FFFFFFFFFFFFF:FFFFF:FFFFFFFFFFFFFFFFF
FFF
@A00721:190:HGJW7DSXY:4:1660:26829:4679_2:N:0:TCTGCAAG+GCTGCACT
GAGTATTGCTATTTATACGGTTACGAGAAGAAGGTTCGAAAGTTTGAGAACTTAGTTTACGAACTTGA
CTCTACTATACGTGACATACTAAATACTAGATATTTGGATGATCTTGA
+
FFFFFFFFFFFFFFFFFFFFFFFFFFFFFFFFFFFFFFFFFFFFFFFFFFFFFFFFFFFFFFFFFFFF
FFFFFFFFFFFFFFFFFFFFFFFFFFFFFFFFFFFFFFFFFFFFFFFF
@A00721:190:HGJW7DSXY:4:1646:16875:17785_2:N:0:TCTGCAAG+GCTGCACT
CATGAATTTTCAGAGGATGAAAAATTTTCTTCCTTGGATTTTAATATAAAGAATCTTTACCAGGACTC
CTACTACAGGAGGCAGTCTTCTGATCGTAAGAAGTTTATAAGGAACAT
+
FFF:FFFFF:FFFFFFFF:FF,FFFFFFFF:F:FFFFFFFF,FF,FFF,FF:FFFFF,FF,FF:FFFF
,FF,FFFFFFFF:FFFFFFF,FFFFF:FF,,FFF:,:FFFF,:FFFFF
@A00721:190:HGJW7DSXY:4:2344:31340:13495_1:N:0:TCTGCAAG+GCTGCACT
ACTCGAAACTCTAAGTGGTAATACTGTCTTTCCAAGCTTACCTGTATCTTCAATGAAATTGGACTCTT
CGATAGGGGACATTTTATCGTGGGCTTTCCGTAAAGGAAGATCTGTTATCGACGGTTTATCGTTTACG
AAG
+
FFFFFFFFFFFFFFFFFFFFFFFFFFFFF:FF:FFFFFFFFFFFFF:FF:FFFFFFFFFFFFFFFFFF
FFFFFFFFFFFFFFFFFFFFFFFFFFFFFFFFFFFFFFFFFFFFFFFFFFFFFFFFFFFFFFFFFFFF
FFF
@A00721:190:HGJW7DSXY:4:1107:7916:1924_1:N:0:TCTGCAAG+GCTGCACT
GTTTAAGAATACTATAAATAGAATAGCTATTACTACTAATGGCGTTGGCCTAGTGGATGTTATTAGTG
ACATCTGGTCAATTTCGTTTTTAGGTGGTCTTTATTCTATTATTGATTTTTATGAGATTGATACGTTA
ACA
+
FFFFFFFFFFFFFFFFFFFFFFFFFFFFFFFFFFFFFFFFFFFFFFFFFFFFFFFFF,FFFFFFFFFF
FFFFFFFFFFFF,FFFFFFFFFFFFFF:FFFFFFFF:FF:FFFFF:FFFFFFF:FFFFF,FFFFFFFF
FFF
@A00721:190:HGJW7DSXY:4:1202:13295:27899_1:N:0:TCTGCAAG+GCTGCACT
TAGAGTAGAATTCCTGAAGAGTCCTTCCGTAACCAACTTCTTGACAATTTTCTTCAATCCTTCGATAA
TAGTTAAATCTAGGATTATGTCTGTGTTGAAAGACTTTCTACCGATAAGGGTCACTAGTCTTACACAA
TTA
+
FFFFFFFFFFFFFFFFFFFFFFFFFFFFFFFFFFFF,FFFF:FFFFFFFFFFFFFFFFFFFFFFFFFF
FFFFFFFFFFFFFFFFFFFFFFFFFFFFFFFFFFFFFFFFFFFFF:FFFFFFFFFFFFFFFFFFFFFF
FFF
@A00721:190:HGJW7DSXY:4:1215:17906:14434_2:N:0:TCTGCAAG+GCTGCACT
ATTGACTGATTATCTAAAGATTGAAGGAAAATTTTATCCTCGTTTAACTTACTTAGACTTTCCTACGA
TACAAATGATAACTTCTAATTTACGTTTGCTACTCAGAGCTGGTCCTC
+
FFFFFFFFFFFFFFFFFFFFFFFFFFFFFFF:FFFFFFFFFFFFFFFFFFFFFFFFFFFFFFFFFFFF
FFFFF:FFFFFFFFFFFFFFFFFFFFFFFFFFFFFFFFFFFFFFFFFF
@A00721:190:HGJW7DSXY:4:1139:2446:19727_1:N:0:TCTGCAAG+GCTGCACT
GATGCATGGTGTTATCTCCTCAGGAGTCCCGGCATTTGATAATAATGACGAGACTTTCGTCATAATCG
CTGCATATTACGGATTACACGGTACCAGCGCATCTCGTGTCGAGCCTCGACCCGATTATATGGTATTA
ACT
+
FFFFFFFFFFFFFFFFFFFFFFFFFFFFFFFFFFFFFFFFFFFFFFFFFFFFFFFFFFFFFFFF:FFF
FFFFFFFFFFFFFFFFFFFFFFFFFFFFFFFFFFFFFFFFFFFFFFFFFFFFFFFFFFFFFFFFFFFF
FFF
@A00721:190:HGJW7DSXY:4:1157:26738:26224_1:N:0:TCTGCAAG+GCTGCACT
TGAAATTTATAGAAGATTATTTTGACAGAATCAATAAGAATCATCGATTCTCGATTCGTAGAGCTTAT
```

```
CTAGGTACTCTCATTTACTATGTTGATTTAGTCTACATTTATGCTGATCTAAAATTTCGATCTAGATG
GAT
+
FFFFFFFFFFFFF:FFFFF:FFFFFFFFFFFFFFFFFFFFFFFFFFFFFFFFFFFFFFFFFFFFFFFF
FFFFFFFFFFFFFFFFFFFFFFFFFFFFFFFFFFFFFFFFFFFFFFFFFFFFFFFFFFFFFFFFFFFF
FFF
@A00721:190:HGJW7DSXY:4:2568:29894:7012_2:N:0:TCTGCAAG+GCTGCACT
GATTTACAACGATATAGGAGTAATCTACGAGAAGATTCTAGAATTCTTAATTATAATATTCAACCTCA
AAAGTTACGTGTTTACAGTAACGAGATCACACATTTTGAGAACTAACT
+
FFFFFFFFFFF:FFFFFFFFFFFFFFF:FFFFFFFFFFFFFFFFFFFFFFFFFFFFFFFFFFFFFFFF
FFFFFFF:FFFFFFFFF:FFFFFFFFFFFFFFFFFFFFFFFFFFF:FFFF
@A00721:190:HGJW7DSXY:4:1238:15103:4445_1:N:0:TCTGCAAG+GCTGCACT
TCTCTTCTTATAATAAATAAATAAATAATATTACTAATGGCAAATAGTGGAAAGGCTCCAGTCAACAC
TAGTTCGAGTGCTGCTTTGGATTACGATGACGTGAGAATAGGTGAAGGGATAGTTTTTACCTCGACCA
ACA
+
FFFFFFFFFFFFFFFFFFFFFFFFFFF:FF,FF:FFFFFFFFFFFFFFF:FFFFFFFFFFFFFFFF
FFFFFFFFFFFFFFFFFFFFFFFFFFFFFFFFFFFFFFFFFFFFFFFFFFFFFFFFF:FFFFFFFFF
FFF
@A00721:190:HGJW7DSXY:4:1452:14940:7138_2:N:0:TCTGCAAG+GCTGCACT
GCGACTAATCTTAAAGCTTCACCCAAAATGAAGGACACTTCGAAACTGAATAATGATGCACTACAAAC
AAGTATCGCTAATCATCTGAAAAAGGCTCTAAAGGACACTATACAATA
+
FFFFFFFFFFFFFFFFFFFFFFFFFFFFFFFFFFFFFFFFFFFFFFFFFFFFFFFFFFFFFFFFFF:F
FFFFFFFFFFFFF:FFFFFFFFFFFFFFFFFFFFFFFFFFFFFFFFFF
@A00721:190:HGJW7DSXY:4:1223:12744:25473_2:N:0:TCTGCAAG+GCTGCACT
ATTCAGCACCACATTTTGGTCAAGTCAATCCGTTGAGAAAATTTCTTAGAGGATATTCGCCATTGTGG
TACGAATTGGTCCAACATTTACCACCAAATTCTTTTGATACAACTCTA
+
FFFFFFFFFFFFFFFFFFFFFFFFFFF:FFFFFFFFFFFFFFFFFFFFFFFFFFFFFFFFFFFFFFFFF
FFFFFFFFFFFFFFFFFFFFFFFFFFFFFFFFFFFFFFFFFFFFFFFF
@A00721:190:HGJW7DSXY:4:1644:24334:19586_2:N:0:TCTGCAAG+GCTGCACT
CGGAATACGCTTGCTATTCACCAGATTGGTTCGAACCCGTTGATGCTATGTCAGAAGAAGCTATTCAA
GCTTGTCGTTTAAAGACTGCAGAGGCTGTCAAACAAGCCCAGACTCAA
+
FFFFFFFFFF,FFFFFF:FFFF,,FFFFFF,F,FFF,FFF:FFFFFF,:FFFFFFFFFFF:FF:::FF
,FFFFFFF,:FFF:,F,:FFFFFF:,F,,FFF:FF,:FFFFF:FFFFF
@A00721:190:HGJW7DSXY:4:2445:32886:24095_1:N:0:TCTGCAAG+GCTGCACT
AATTTAGCTCAACTGGGGAGACGTGCTTAAATGGGTGATAAAAATTATTTAGAGATCTTTTCTACTTA
CAAAGGACAATTCAGTTTCATTAATTCTACGGAAAAGTCCGGAGTATATTGTATGTTAATAGATAGTG
GGA
+
FFFFFFFFFFF,FFFFFFFFFFFFFFF:FFFFFFFFFFFFFFFFFFFFFFFF:FFFFFFFFFFFFFFF
FFFF:FFFFFFFFFFFFFFFFFFFFFFFFFFFFFFFFFFFFFFFFFFFFFFFFFFFFFFFFFFFFFFF
FFF
@A00721:190:HGJW7DSXY:4:1324:24713:1423_2:N:0:TCTGCAAG+GCTGCACT
GAACGCGTTCGTATAACCAAGTACTTTCCGTCTGAAGAGTTTAGAGCTGCTATTGATTATGTAACCGA
TAGGAGTTGTATAGTTGAAGATGTGGCTTTAGACGATAATCCTTTAAG
+
FFFFFFFFFFFFF,FFFFFFFFFFFFFF,FFFF,F:::FFFFFFFFFFFFFFFFFFF:FFFFF:FFFF
,FFFFF::FFFFFFFFFFF:FF:FF,FFFFFF:FFFFFFFF,F:FF:F
@A00721:190:HGJW7DSXY:4:1362:28230:6699_1:N:0:TCTGCAAG+GCTGCACT
GAAGATTTTCCCACAGATTTGTCTACGTTGCAGATCGCCACTCCAAACACTATATACATTATAAAATA
```

TAAAAATAAGGGAGTCATAACTTTTGTTACCAAATTCTTGGCTTTCAACGAAGTCTCTGATTTTATTA
ATC
+
FFFFFFFFFFFFFFFFFFFFFFFFFFFFFFFFFFFFFFFFFFFFFFFFF,FFFFFFFFFFFFFFFFFFFF
FFFFFFFFFFFFFFFFFFFFFFFFFFFFF:FFFFFFFFFFFFFFFFFFFFFFFFFFFFFFFFFFFFFFFF
FFF
@A00721:190:HGJW7DSXY:4:1267:18376:19852_2:N:0:TCTGCAAG+GCTGCACT
GATCTCTCGGTCATTTACTCTGTACTTGATCTACGTCGTTTAACTGTCGGAACAACCTTTACTTAGAA
TGACTCTATCTTTTCAGACACTTCTTATATTATTTACAATAGTTAATA
+
FFFFFFFFFFFFFFFFFFFFFFFFFFFFFFFFFFFFFFFFFFFFFFFFFFFFFFFFFFFFFFF:FFFFF
FFFFFFFFFFFFFFFFFFFFFFFF:FFFFFFFFFFFFFFFFFFFFFFFFFF
@A00721:190:HGJW7DSXY:4:1147:31430:22670_1:N:0:TCTGCAAG+GCTGCACT
AATACTCACTATCGTTTTCTGCTTTTTCTTAGCTAATAGATTAATAAAGGATGAGTACGAAAACCAAC
TCTTCGTTACGAGACCTGATGGTAGAATAGATTTAGTCTCTACACCCATCTCAACGAGGATAAAGCGA
GTT
+
FFFFFFFFFFFFFFFFFFFFFF,FFFFFFFFFFFFFF:FFFFFFFFFFFFFFFFFF,FFFFFFFFFFFF
:FFFFFFFFFFFFFFFFFFFFFFFFFFFFFFFFFFFFFFFFFFFFFFFFFFFFFFFFFFFFFFFFFFFFFFF
FFF
@A00721:190:HGJW7DSXY:4:2569:30779:4570_2:N:0:TCTGCAAG+GCTGCACT
ACACGAAGAGATCATCCGCAAACGACTCGAAACCGAGACGCCGTAGATCAAAGTCCCTTCTAACTTTA
GATTATGAATCCTGCCTTTACTCATTTTATAGCAGTTATTTATGTTGT
+
FFFFFFFFFFFFFFFFFFFFFFFFFFFFFFFFFFFFFFFFFFFFFFFFFFFFFFFFFFFFFFF:FFFFF
FFFFFFFFFFFFFFF:FFFF,FFFFF:FFFFF:FFF:FFFFFFFFFFF
@A00721:190:HGJW7DSXY:4:2344:4535:4335_1:N:0:TCTGCAAG+GCTGCACT
TTTATAACCTTCTTATACTTTTGGGTATGTTTTGTGAAAAACATGAGATACCCAAGTCTATCGAAGAC
ATTATCTCTTATGTACTTATTGTAACGATCATTCTTTCGAGTTTTGTCATTCTTATTCTTTTATTCCT
TTA
+
FFFFFFFFFFFFFFFFFFFFFFFFFFFFFFFFFFFFFFFFFFFFFFFFFFFFFFFFFFFFFFFFFFFFF
FFFFFFFFFFFFFFFFFFFFFFFFFFFFFFFFFFFFFFFFFFFFFFFFFFFFFFFFFFFFFFFF:FFFF
FFF
@A00721:190:HGJW7DSXY:4:2127:16288:22811_2:N:0:TCTGCAAG+GCTGCACT
TTTATAATGCTTTTCGATAGTGATTGTTATCAATTTAAATACATTACTGTTTCAAATCATCTTTACTC
ACAGACTCCGAAAATGGTTTCAAATGAGGTCAAAGCTCTATTTATTAA
+
FFFFFFFFFFFFFFFFFFFFFFFFFFFFFFFFFFFFFFFFFFFFFFFFFFFFFFFFFFFFFFFFFFFFF
FFFFFFFFFFFFFFFFFFFFFFFFFFFFFFFFFFFFFFFFFFFFFFFF
@A00721:190:HGJW7DSXY:4:2278:19750:36886_1:N:0:TCTGCAAG+GCTGCACT
CACTACCACTGATCTGCCGACTTTAGTCAAGGAACTTCAATTCCTTCGATGTTTATATTTTTCTATAA
GGGAAAATATCAATAATCAAATTTCGACAAATTTTAGAACTTTATTTCAGAACTCTCTCCGATCACCT
TGG
+
FFFFFF:::F:FFF:F:FFFFFFF,FFFFFFFFFFFFF:FFF:FFFFFFFFFFFF:F,FFF:FFFFFF
FFFFFFFFFFFFFFFFFFFFFFF:FFFFFFF:FFFFFF:F:FFFFFFFFFFFFFFFFFFFFFFFFFFFFF
F:F
@A00721:190:HGJW7DSXY:4:2549:1796:4883_1:N:0:TCTGCAAG+GCTGCACT
TCTTTTCAATAAGATGTATGATGATAATCTACTATTTTTAAATAGTGATATTCAGCGTATACTTGAAT
ACTTTATCAAAGATTTGGCTTGGTCTATTTACCGCAAAAAGTTGGAAGAGGTCTATGATATTTATTTT
ACT
+
F:FFFFFFFFFFFFFFFFFFFFFFFFFFFFF:FFFFFFFFFFFFFFFFFFFFFFFFFFFFFFFFFFFFFF

```
FFFFFFFFFFFFFFFFFFFFFFFFFFFFFFFFFFFFFFFFFFFFFFFFFFFFFFFFFFFFFFFFFFFFFFF
FFF
@A00721:190:HGJW7DSXY:4:1302:29839:28432_2:N:0:TCTGCAAG+GCTGCACT
CCGATTATAACTTGACTGTTGATAATATTACAATAGCTCTTTCTCAGAAGATTAGAGAAGTAACCGGT
GAACACGTCGGTAGCATTGATTTACTTTTACCGTTAGAACTGAAAAAG
+
FFFFFFFFFFFFFFFFFFFFFFFFFFFFFFFFFFFFFFFFFFFFFFFFFFFFFF:FFFFFFFFFFFFFFFFF
FFFFFFFFFFFFFFFFFFFF:FFFFFFFFFFFFFFFFF:FFFFFFFFF:FF
@A00721:190:HGJW7DSXY:4:2262:28736:4476_1:N:0:TCTGCAAG+GCTGCACT
ACCTATAAGTTACAAACGATTCTTCGACTATATTAAAGAAATAAAAATACTTCAAGTACCTGAACTTT
ACATTTTTAATGGTATGGTATAATGTGTCTTACAAATAGAACCTTCACTCTCGGTAATGAGAGTGTAT
TCC
+
FFFFFFF,F:FFFF:FFFFFFFF:FFF:FFFF::FFF:FFFFFFFFFFFFFFF,FFFFFFFFFFFFFF
FFFFFFFFFFFFFFFFFFFFFFFFFFFFFFFFFFFFFFFFFFFFFFFFFFFFFFFFFFFFFFFFFFFFFFF
FFF
@A00721:190:HGJW7DSXY:4:1564:31268:31814_1:N:0:TCTGCAAG+GCTGCACT
GGCATTACTAGGATTTATCACACTCCTAAATTTGATAGCAATTTATGTTTTAAATGCTAGGAAAATTT
TAGAAAAACTCCATTGTATAGTTCAAGGAGTACTTAATTCAATAGCTTTAGTATTATTTACTATCGCC
TTT
+
FFFFFFF:FFFFFFFFFFFF:FFFFFFFFFFFFFFFF::FFFFFFFF::FFFFFFFFFFFFFFFFFFF
FFFFFFFFFFFFFFFFFFFFFFFFFFFFFFFFFFFFFFFFFFFFFFFFFFFFFFFFFFFFFFFFFFFFFFF
FFF
@A00721:190:HGJW7DSXY:4:1369:12427:25708_2:N:0:TCTGCAAG+GCTGCACT
AGTTAATTGCTTACACAAAAGTGACGCGCGAAGGAATCTTAATTGATTGAAGCTAGGCGTTGGGGAGA
ATGAACTGCCGCTTCCCGGACGAAACGATCTATTTACCGAGTATTTAG
+
FFFFFFFFFFFFFFFF:FFFFFF:FFFFFFFFFFF,FFFFFFFFFFFFFFFFFFFFFFFFF,FFFFFFF
FFFFFFFFFFFFFFFFFFFFF,FFFFFFFFFFFFFFFF:FFFFFFFFFF
@A00721:190:HGJW7DSXY:4:1258:1334:25254_2:N:0:TCTGCAAG+GCTGCACT
TACACTCCCCAAACATAACATACCATAACCTACCATGACCAATCATCGCTCCAACTGGCACGCCAGAC
GTAGGGTCAAACCTACTACCGCCTGTCGGCAGATCGGCACCACTGGTC
+
FFFFFFFFFFFFFFFFFFFFFFFFFFFFFFFFFFFFFFFFFFFFFFFFFFFFFFFFFFFFFFFFFFFFFFF
FFFFFFFFFFFFFFFFFFFFFFFFFF:FFFFFF:FFFFFFFFFFFFFFF
@A00721:190:HGJW7DSXY:4:2251:16034:30984_2:N:0:TCTGCAAG+GCTGCACT
ACCCGCTGTGCAGAATAACCTTCCCAAATCTAGATCTGCACAGAAGAAGGCTGAAGGTCGTTACCGGA
CGACCACTCAAAGGAAACGGGCTGTTGCCCAGAGGAAAGCAGCGACCA
+
FFFFFFFFFFFFFFFFFFFFFFFFFFFFFFFFFFFFFFFFFFFFFFFFFFFFFFFFF:FFFFFFFFFFFFF
FFFFFFFFFFFFFFFFFFFFFFFFFFFFFFFFFFFFFF:FFFFFFFFFFF
@A00721:190:HGJW7DSXY:4:2246:28836:8312_2:N:0:TCTGCAAG+GCTGCACT
GTAACATTGACGGCAGTCCCGTCATCTCTGTGGGAACTGAGGCAGTACCTCAGTCAAAAGTCACATTC
ACCACTCCCATGCCAATCAATGATGTGACTATACAGTTCGGCCATTTC
+
FFFFFFFFFFFFFFFFFFFFFFFFFFFFFFFFFF:FFFFFFF:F:FFFFFFFFFFFFFFFFFFFFFFFFFF
FFFFFFFFFFFFFF,,FFFFFFFFFFFFFF:FFFFFFFFFFFFFFFFF
@A00721:190:HGJW7DSXY:4:1536:30291:33943_1:N:0:TCTGCAAG+GCTGCACT
ATACGTCGTGGGAGGTACTGAAGCCTTCCCCTCCGCTGGCACGAAGAACACTCCCAAGCCAATCAATA
GTTCTTCTGACCAGAATGAAAAGGGGTTTACCAGGGTAGTCTCTAAGTCTACCAGGAAGAGGGGAAAT
GTC
+
FFFFFFFFFFFFFFFFFFFFFFFFFFFFFFFFFFFFFFFFFFFFFFFFFFFFFFFFFFFFFFFFFFFFFFF
```

FFFFFFFFFFFFFFFFFFFFFFFFFFFF:FFFFFFFFFFFFFFFFFFFFFFFFFFFFFFFFFFFFFFF
FFF
@A00721:190:HGJW7DSXY:4:1203:26820:26052_1:N:0:TCTGCAAG+GCTGCACT
GCATACGAGATCTTGCAGAGTGACTGGAGTTCAGACGTGTGCTCTTCCGATCTGGAAGATCTCCCACC
ATCTGGGGTTGGGAATAAGAAATCTGCACGGGCCAAAGAGGCCGCTGCTACGAAGTCGCTGAAGGCGG
CTT
+
:FF::F,,FFFFF:FFFFFFFFFFFFFFFFFFFFFFFFFFFFFFFFFFFFFFFFFFFFFFFFFFFFFF
FFFFFFFFF:FFFFFFFFFFFFFFFFFFFFFFFFFF,FFFFFFFFFFFFFFFFFFFFFFFFFFFFFFF
FFF
@A00721:190:HGJW7DSXY:4:2169:15365:7404_2:N:0:TCTGCAAG+GCTGCACT
TGTAAAGTTGCGCCTGCCGACCCCATCTTTGAGACCAAGACTCTCTACAGTAGGTTCGTGCCTACTAT
GAGCAAATCTGTTGAATTCGTCACTGAGGACGAAGGTGAGAGGACTTT
+
FFFFFFFFFFFFFFFFFFFFFFFFFFFFFFFFFFFFFFFFFFFFFFFFFFFFFFFFFFFFFFFFFFFF
FFFFFFFFFFFFFFFFFF:FFFFFFFFFFFFFFFFFFFFFFFFFFFFF
@A00721:190:HGJW7DSXY:4:1356:19587:4695_1:N:0:TCTGCAAG+GCTGCACT
CTGAGAGCAGTCGCTGGGGAAAGAGAATTGCGTGGGTTGAAGGCCGACGCTTCGAGCTTGAGTGTGAC
GAGGCCGGTGACGTTTTTGTCAACGGCGTTTGGAGGATTTTATCCGCGCTTAACTATGGGGCGAAGAT
CCC
+
FFF:FFFFFFFFFFFFFFFFFFFFFFF,:FFFFFFFFFFFFFFFFFFFFFFFFFFFFFFFFFFFFF,F
FFFFFFFFFFFFFFFFFFFFFFFFFFFFFFFFFFFFFFFFFFFFFFFFFFFFFFFFFFFFFFFFFFFF
FFF
@A00721:190:HGJW7DSXY:4:1641:8386:6402_2:N:0:TCTGCAAG+GCTGCACT
AGGTTGGCATATTTATGCCGACGATAATAAGCATGTCACCCTTAAGCACCGAGGTAAGCTCGGGACTT
ACAAGAGAGAGTGTGAGAGAGTCGCTCTCACTATTTTTGCCCAGAATG
+
FFFFFFFFFFFFFFFFFFFFFFFFFFFFFFFFFFFFFFFFFFFFFFFFFFFFFFFFFFFFFFFFFFFF
FFFFFFFFFFFFFFFFFFFFFFFFFFFFFFFFFFFFFFFFFFFF:FFF
@A00721:190:HGJW7DSXY:4:2502:2727:4961_2:N:0:TCTGCAAG+GCTGCACT
GATGAGGTTAAAACTCTTGCTCCTTCTGTGGGTGAGGGTTTTTGTTATATTCACTGGTTCTGGGAGGC
TGAGATCGGAAGAGCGTCGTGTAGGGAAAGAGTGAAGTGGAGGGGGGG
+
FFFFFFFFFFFFFFFFFFFFFFFFFF:FFFFFFFFFFFFFFFFFFFFFFFFF:FFFF,FFFFFFFFFF
FFFFF:FFFFFFFFF:FFFFF,:FFF:FFFFFF:,FF::F,F,F:FFF
@A00721:190:HGJW7DSXY:4:1207:27859:27164_1:N:0:TCTGCAAG+GCTGCACT
TCCCACATATGGTAGACGTTGTCACACTTTTCTTGAAAATTTTTCCTTTCCTCCGTCATTGGGGATTT
CGATGTGAGAAGGTGGAAGGTGTCAAGGGTCTTCTTCATGCTGATCTTTCAGGACCTAATCACATCAT
GAT
+
FFFFFFF,::FFFFF:FFFFFFFFFFFFFFFFFFFFFFFFFF:FFFFFFFFFFFFFFFFFFFFFFFFF
FFFFFFFFFFFFFF:FFFFFFFFFFFFFFFFFFFFFFFFFFFFFFFFFFFFFFFFFFFFFFFFFFFFF
FFF
@A00721:190:HGJW7DSXY:4:1351:2094:31391_1:N:0:TCTGCAAG+GCTGCACT
GCTCGTATCGAGTTGGAGGGAGGAAGTTCTCCGAAATTAAAGACGGGGGGATTAAGAGTCCAGCTGTA
ACTGTCATTTCAGAGAGGGGAGCTCTGAAAAGGAAACTTCGTCAGAAGATGAGGAAGTCTAAGAGGAG
GAG
+
FFFFFFFFFFFFFFFFFFFFFFFFFFFFFFFFFFFFFFFFFFFFFFFFFFFFFFFFFFFFFFFFFFFF
FFFFFFFFFFFFFFFFFFFFFFFFFFFFFFFFFFFFFFFFFFFFFFFFFFFFFFFFFFFFFFFFFFFF
FFF
@A00721:190:HGJW7DSXY:4:1504:18005:9846_1:N:0:TCTGCAAG+GCTGCACT
CTCTAGAGAAGATCTCACCACCTGGAGAAGGCAAGTGGAGAGTCCAGAAGTTGATGAAGAAGGATTAT

```
TTGAAGCAGGGGTTACCACTTCCTAAGGTGGATGAGGGAGGTTGTTTCTTTGGAGTTTTAGATAATCT
TTT
+
FFFFFFFFFFFFFFFF,FFFFFFFFFFFFFFFFFFFFFFFFFFFFFFFFFFFFFFFFFFFFFFFFFFF
FFFFFFFFFFFFFFFFFFFFFFFFFFFFFFFFFFFFFFFFFFFFFFFFFFFFFFFFFFFFFFFFFFFF
FFF
@A00721:190:HGJW7DSXY:4:2131:21893:6652_2:N:0:TCTGCAAG+GCTGCACT
CCTTGAGAAGACAAGCCACATTAAGATATAATTTAGGTCAACTTGGGGTTTCGAATGTCATTAGTGCC
TTACGATTGAAAGAGGTCCTTTTGAGTATCGACAGATCTTTCGATCTT
+
FFFFFFFFFFFFFFFFFFFFF:FFFFFFFFFFFFFFFFFFFFFFFFFFFFFFFFFFFFFFFFFFFFFF
FFFFFFFFFFFFFFFFFFFFFFFFFFFFFFFFFFFFFFFFFFFFFFFF
@A00721:190:HGJW7DSXY:4:2366:26142:32174_2:N:0:TCTGCAAG+GCTGCACT
GTGGCAGAATTCGGTAACTTACCATATTGTTCCTTCCGGGAAGTTGTGGGATTTAACTTTGACTCAAC
TACTCCGATCAGATCCGGATGGTTTGATTGGTATGGACTACTCGACTT
+
FFFFFFFFFFFFFFFFFFFFFFFFFFF:FFFFFFFFFFFFFFFFFFFFFFFFFFFFFFFFFFFFFFFF
FFFFFFFFFFFFFFFFFF::FFF:FF:FFFFFFFFFFFFFFFFFFFFF,
@A00721:190:HGJW7DSXY:4:1144:18819:33802_1:N:0:TCTGCAAG+GCTGCACT
AGGGTCATGGCAGATACTACCGATAAGGTGAAAAATATGCAGGTCAAAGATTCTGTAGCTCTGAGGGC
GGTTGAGGGAGCCGTTTTGGCCGACTTTAACTATTCTAAGAGTCTTTATGATTCAAAACCTATCGTTG
TC
+
FFFFFFFFFFFFFFFFFFFF:FFFFFFFFFFFFFFFFFFFFFFFFFFFF:FFFFFFFFFFFFFFFFFFF
FFFFFFFFFFFFFFFFFFFFFFFFFFFFFFFFFFFFFFFFFFFFFFFFFFFFFFFFFFFFFFFFFFFF
FF
@A00721:190:HGJW7DSXY:4:1406:11342:32847_2:N:0:TCTGCAAG+GCTGCACT
GCGGCTTATTGTCGCGCAGTTTCCAGAATTGCGGTTGAAGTTCACCCATTCTATCTTGAATGCTCACG
CCGTCGCAGCCATATCGAGACTATGTTATAATCACTTGTTAAAGTACA
+
FF:FFFFFF::FF,FF:F::FF::,FFFFFFFFFFF:FF,FFFFFFFF,,,:FFF::,FFFFF:FF::
FF:FFF:FFFFF,FF:FFFFFF,FFFFFF,FFFF,,:FFFFFFFFF,,
@A00721:190:HGJW7DSXY:4:1406:7663:32456_2:N:0:TCTGCAAG+GCTGCACT
TTAGATATTATTGATATTGGAGGCGATATTCGTTTTCATATCCAAAACTCCTTGAATGGAGTACATAT
TTGCAACCCTATTCTTGATGTGAAAGATGCATCGAGGTATACGTCTAG
+
FFFFFFFFFFFFFFFFFFFFFFFFFFFFFFFFFFFFFFFFFFFFFFFFFFFFFFFFFFFFFFF:FFFF
FF:FFF:FFFFFFFFFFFFFFFFFFFFFFFFFFFFFFFFFFFF,F:FFF
@A00721:190:HGJW7DSXY:4:2572:25554:18693_1:N:0:TCTGCAAG+GCTGCACT
AACGTACCTACTCACGAGACGTCATGAAAGCTATCGGAGGGAAGCAAAATGTTTCGTTCTGTTACAAG
AACGCTTTGGATTGTGACCATAAAGCCTCAGTTGCGATAGCTGTAGAGGTATACGATATCTCTCTTGA
TGA
+
::F,FFFF,F,FFFF::F:FFF,FFF::FFFF,F:FFFFFFFFF:FF:FF,FFFFFFF:FFFFFFF::
FFFF:FFFFFFFFFFFFFFFFFFFFFFFFF:FFFF,FF:FFFFFFFFFFFFFFF,FFFFFFFFF:FFFF
FF:
@A00721:190:HGJW7DSXY:4:1311:21748:9032_2:N:0:TCTGCAAG+GCTGCACT
AATGCTTAAGAGAGAGATTGATGTATGTCATTTCGTCTTAGTTTGTCCCGGCGAATTATTGGACACGA
TGAAAAATGACATACCCGTTCCTGACCTTGGTATCGTTATCCGTCATG
+
FFFFFFFFFFFFFFFFFFFFFFFFFFFFFFFFFFFFFFFFFFFFFFFFFFFFFFFF:FFFFFFFFFFF
FFFFFFFFFFFFFFFFFFFFFFFFFFFFFFFFFFFFFFFFFFFFFFFF
@A00721:190:HGJW7DSXY:4:2349:31937:15499_2:N:0:TCTGCAAG+GCTGCACT
GGTTATTCTCACAACAAAGATAAGATTTTATCTTTCTTCAAACACCCTGTTTTCCTTAAGGGCGGACA
```

ATTGTTCCATTGTGAACTCTTGGGGACTAGATTGAGCATTAATGAGTT
+
FFFFFFFFFFFFFFFFFFFFFFFFFFFFFFFFFFFFFF::FFFFFFFFFFFFFFFFFFFFFFFFFF
FFFFFFFFFFFFFF:FFFFFF:F:FFFFFFFFFF:FFFFFFFFFFFF
@A00721:190:HGJW7DSXY:4:2330:19271:5118_1:N:0:TCTGCAAG+GCTGCACT
TCTCTAGTGGACAATTCAGAATACCTATTTCCGTACCAAGAGCCACTGCCAATCTCATCGCTATACGC
ATACCTGATTGGAAGAAGCGAGGTTCGTTCCTCAATAAAAATTTTTCTCAAGAGATTATGGTTGATAA
AGT
+
FFFF:FFF,FF,,FFFFFF::F:F,FFF:F:FFFFF,FFFFFFFFFFF:FFFFFFFFFFF,F:F:FFF
FF,:FF:FFFFFFFFFF:,FFFFFFFFFFFFFFFFFFF:FFFFFF,FF,FF:FFFFFFFFFFFFFF:F
FFF
@A00721:190:HGJW7DSXY:4:2454:17644:5556_1:N:0:TCTGCAAG+GCTGCACT
AGGGCTATTCATTACGTCATGGGGATCACTTCTTCGGTCAATGACAAAACCTTCGAATATGTTACGAC
ATGGCTTCGTAACAACAGTGCGAGGATTGTCATCTCGGGGAAAGTGATTCACCAAGATGTTCATATAG
AT
+
FFFF:F:FFFF,:FF:F:,F,FFFFF,FFFFFFFF,FFF,,FF,FFFFFF:FF,FF,FFFFFFFFFFF
FFF,:FFFFFF::FFFFFF:FFF:FFFFFFF:F,FFFF:F:FFFFFFFF:F:FFFF:F,FF:FFFFFF
:F
@A00721:190:HGJW7DSXY:4:1430:2130:18396_1:N:0:TCTGCAAG+GCTGCACT
GAAGGTCGCAGCGATCTTACTCGTTATCGGAGCTAAGAAGAAGACTTTGGGTACACGTTATGCTCGCA
AACTTAACGTTAGTACCGGAGGTGATCTCTCTGCTTCTGCGTTACTGAGAGAAAAGATTCAGGACGCC
TAC
+
FFFFFFFFFFFFFFFFFFFFFFFFFFFFFFFFFFFFFFFFFFFFFFFFFFFFFFFFFFFFFFFFFFFF
FFFF:FFFFFFFFFFFFFFFFFFFFFFFFFFFFFFFFFFFFFFFFFF:F:FFFFFFFFFFFFFFFFFF
FFF
@A00721:190:HGJW7DSXY:4:2573:18602:8844_2:N:0:TCTGCAAG+GCTGCACT
TTTAGGTTTAGTAGGTGATCTTGACGTCGATTCTTTGTATAGGGAGTGTGAGAAGACAACGAATCTTG
AAACTACGGTCCTTATCAAAGAAAATGGTGAACCGGTCTCTAAATCTT
+
FFFFFFFFFFFFFFFFFFFFFFFFFFFFFFFFFFFFFFF:FFFFFFFFFFFFFFFFFFFF:FFFFFFF
FFFFFFFFFFFFFFFFFF:F,FFFFFFFFFFFFFFFFFFFFFFFFFFF
@A00721:190:HGJW7DSXY:4:2552:4942:29465_1:N:0:TCTGCAAG+GCTGCACT
GATTGAAGAACACGTCAAGAAGTCGCTCGGCGTTGTTCTTGAAAACCAAGTTAAGCAGGAGGCTGATC
ATGGAGTCTTTGTACCTTCTGTCAAACTTCGAGGTTCAGCTACGAGAGGTGATCCCGGATGTGGATTG
AAG
+
FFFFFFFFFFFFFFFFFFFFFFFFFFFF:FFFFFFFFFFFFFFFFFFFFFFFFFFF,F:FFFFFFFFFF
FFFFFFFFFFFFFFFFFFFFFFFFFFFFFFFFFFFFFFFFFFFFFFFFFFFFFFFFFFFFFFFFFFFF
FFF
@A00721:190:HGJW7DSXY:4:2456:28791:27618_2:N:0:TCTGCAAG+GCTGCACT
TCCTTCTTTTTGAATGTTGTAAAGAATTGGGTTGAGAAGTGTGCTTTACAAGGAGTTAGACTCTTCTT
CAAGTTTTTAGATCTTCTTGATAATCTTAAAGAAAAATTTTCGAATAA
+
FFFFFFFFFFFFFFFFFFFFFFFFF:FFFFFFFFFFFFFFFFFFFFFFFFFFFFFFFFFFFFFFFF:FF
FFFFFFFFFFFFF:FFFFFFFFFFFFFFF:F,FFFFFFFFFF,FFFFF
@A00721:190:HGJW7DSXY:4:1168:20799:2910_1:N:0:TCTGCAAG+GCTGCACT
GTTTCTCTTGGTATTTCTGAAATAGTTTATCAGAGCGTCATTTTCCTTTGGGATCTTTCCTCTGGTAA
GGGAATTATTAAATCATTCTTCTCACTCTTGACGAGAATTATTACTGTTTTCTCTACCCATAACTTCT
TTT
+
::FFFF:FFFFFFFFF,FFFFF:FF::FFFFFFFFFF:FFFFFFFFFFFF,FFFFF:F:FFFFFFFFF

FFFFFFFFFFFFFFFFFFFFFFFFFFFFFFFFFFFFFFFFFFFFFFFFFFFFFFFFFFFFFFFFFFFFFF
FFF
@A00721:190:HGJW7DSXY:4:1541:31195:3912_1:N:0:TCTGCAAG+GCTGCACT
ACTGGAGTTCAGACGTGTGCTCTTCCGATCTGTTTCAACACTTATCACCACTTTTGAAAGGAAAATAA
TTGGTGATGAAAATACGTATGTCGGTGGGGTTCTCAAGGCTCTCATCGCCACTGGGATTCTAAGGGGT
TCC
+
FFFFFFFFFFFFFFFFFFFFFFFFFFFFFFFFFFFFFFFFFFFFF:FFFFFFFFFFFFF:FFFFFFFFFF
FFFFFFFFFFFFFFFFFFFF:FFFFFFFFFFFFFFFFFFFFFFFFFFFFFFFFFFFFFFFFFFFFFFFFF
FFF
@A00721:190:HGJW7DSXY:4:2574:3323:22686_2:N:0:TCTGCAAG+GCTGCACT
TTCAATGATCAGGATTCTTCTTTTGGTCCTTACGTCTTAAGTAATTTGGATGTTGTCGAAAACTATCA
GGTTCTTGTCACGATTCTTAAAAGAAGTTTATTAAAACTTCAGAGTCT
+
FFFFFFFFFFFFFFFFFFFFFFFFFFFFFFFFFFFFFFFFFFFFFFFFFFFFFFFFFFFFFFFFFFFFFF
FFFFFFFFFFFFFFFFFFFFFFFFFFFFFFFFFFFFFFF:FFFFFFFFFF
@A00721:190:HGJW7DSXY:4:1348:16947:19727_1:N:0:TCTGCAAG+GCTGCACT
GGTAGATAATGTTCTTGCAATAGCCACAGACTTTCTGGGAAAAGAGACCTCTGTTGTAGAGGAGTTGA
GAGAATTACTCAGATTCAATCTGGCATCACTGAACGTCTACAACGAGTTAGGGAGTGGCTAGGTTTTA
AAA
+
FFFFF,FFFFFFFFFFFFFFFFFFFFFFFFFFFFFFFFFFFF:,FFFFFFFFFFFFFFFFFFFFFFF,FF
FFFFFFFFFFF:FFFFFF:FFFFFFFFFF:FFFFFFF:FFFFF:FFFFFFFFFFFFFF,FFFFFFFFFF
FFF
@A00721:190:HGJW7DSXY:4:1230:29794:2394_1:N:0:TCTGCAAG+GCTGCACT
CAATACCTTCCACTGAGTTCATCACCCAGAGTTTCGACGTCGCGGAAAACGATGAAGATTTCCACACT
GTTCGAGAATCTTCTGATGCTGAATTTGAGGAAAATTTTTCGGAAGTTTTTGGTATAAATCCTGATGG
CTC
+
FFFFFFFFFFFFFFFFFFFFFFFFFFFFFFFFFFFFFFFFFFFFFFFFFFFFFFFFFFFFF:FFFFFFFF
FFFFFFFFFFFFFFFFFFFFFFFFFFFFFFFFFFFFFFFFFFFFFFFFFFFFFFFFFFFFFFFFFFF:FF
FFF
@A00721:190:HGJW7DSXY:4:2514:20410:36652_1:N:0:TCTGCAAG+GCTGCACT
TAAAACCTTCTTTTATCATTGCAACAGGACTTTGAGAAGACTGTTGAAATGGACAAAATCTGGGATCA
TAGGTATGTGTCACGCTACACTCGAAATATGGGCCCTCATAAGGAAGTATAGTTGGCGTCTTGTCGTT
GAC
+
FFF:FFFFFFFFFFFFFF:FFFFFFFFFFFFFFFFFFFFFFFFFFFFFFFFFFFFFFFFFFFFFFFFFFF
FFFFFFFFFFFFFFFFFFFFFFFFFFFFFFFFFFFFFFFFFFFFFFFFFFFFFFFFFFFFFFFFFFFFFF
FFF
@A00721:190:HGJW7DSXY:4:2560:15826:3975_2:N:0:TCTGCAAG+GCTGCACT
AGTTAGATCATTTGTTCAGGAAGATCTCTACAGAGATTATCGAGAGTTGTGATCTAGTTCTAAATTTA
GGTCGACGGACTTTAGCAACTTTAGATAAATTCTTTCCGGGCTCAATG
+
FFFFF:F:FFFFFFFFFFFFFFFFFFFFFFFFFFFFFFFFFFFFFFFFFFFFFFFFFFFFFFFFFFFFFF
F:FFFFFFFFFFFFFFFFFFFFFFFFFFF:FFFFFFFFFFFFFFFFFFFF
@A00721:190:HGJW7DSXY:4:2468:20419:26710_1:N:0:TCTGCAAG+GCTGCACT
TCTGACCATCGCAAAGAAGACTTTATTAGTTCATGAAGTTCTTACACTTCTCGCGACTAATGTCAGCT
TCACTACAAAGGCGTTACGTAAAGTAGTTTTCACGCTTTATCTTAAAGCATTACCACCAAATTTAGCA
CTT
+
FFFFF:FFF,F:F,FFFF,FF,FFFFFFFFFFFFFFFFFFFF:FF:FFFF:FFFF,F:FFFF:,FFFFF
FF:F:FFFF::F:F:FF:FFFFF:FFFFFFF:F,FFFFF:FFFF:FFFFFFFFFF,FFFFFFFFFF:,
FFF

@A00721:190:HGJW7DSXY:4:1126:18475:24095_1:N:0:TCTGCAAG+GCTGCACT
CGTGTGCTCTTCCGATCTAGACATGCCAGTTAGAGTTCTTCTAACCAGGACTGATGGTGAGATTCTTT
TACCTTTTGGAGGTCCAGGAACCTTTACTCCTAGTGTCTTTTCTAGAGTCCAAGGACGTTTCCGTCGG
AGG
+
FFFFFFFFFFFFFFFFFFFFFFF:FFFFFFFFFFFFFFFFFFFFFFFFFFFFFFFFFFFFFFFFF,FF
F:FF:FFFFFFFFFFFFFFFFFF:FFFFFFFFFFFFFFFFFFFFFFFFFFFFFFFFFF:FFFFFFFFF
FFF
@A00721:190:HGJW7DSXY:4:2350:18231:24612_2:N:0:TCTGCAAG+GCTGCACT
GACCGCTCAATAACACTCTATTGAATCCCTTTTCCACGATGAAAGCTCTTTATGAGGTGCTCCATGCA
AATTTACAGTTCCTCTTTGGTGATATCGCCTCTGTCCTTTCTCCTTTA
+
FFFFFFFFFFFFFFFFFFFFFFFFFFFFFFFFFFFFFFFFFFFFFFFFFFFFFFFFFFFFFFFFFFFF
FFFFFFFFFFFFFFFFFFFFFFFFFFF:FFFFFFFFFFFFFFFFFFFF
@A00721:190:HGJW7DSXY:4:1627:29387:19633_2:N:0:TCTGCAAG+GCTGCACT
ACTTGGGTTTATCGGTTGTATTTTGATAAATTTGTTGGAATCGATAATGATACTGACGAAGGTTCTAT
TACCACCCTTGTCATTGATACAAACATTCTAGGTGATGCTTTCTTAGA
+
FFFFFFFFFFFFFFFFFFFFFFFFFFFFFFFFFFFFFFFFFFFFFFFFFFFFFFF:FFFFFFFFFFFF
FFFFFFFFFFFFFFFFFFFFFFFF:FFFFFFFFFFFFFF:FFFFFFFFF
@A00721:190:HGJW7DSXY:4:1103:4218:3192_2:N:0:TCTGCAAG+GCTGCACT
TACAATCTGTAGAGAGAGAGGTTAATCAGAATTTGAGTGATAACGATTTAGTACTGGGAAATGGTGGT
AGTCAGTTTACTACTTTTCTTTCCCTCCCCTTTTTCGATTGGATTCGA
+
FFFFFFFFFFFFFFFFFFFFFFFFFFFFFFFFFFFFFFFFFFFFF,FFFFF,FFFFFFF,FFFFFFF
FFFFFFFFFFFFFFFFFFFFFFFFFFFF:FFFF,FF,FFFFFF:FFF,
@A00721:190:HGJW7DSXY:4:2219:32181:26099_2:N:0:TCTGCAAG+GCTGCACT
TTGATAAGGTTTTTCTTTTATGGGACTCTTGTTTGTACTTATGAAGGAGGTATTACTTCGGAAGTTCT
TAATGTAGGTCTCCTAGTAATTAAGGGTTTATGTTTCGGTTTCAGCTG
+
F:FFFFF,FFFFFFFF:FFFFF:,F,F:FFFFFF,FF:FFFF:FF,FF,:FF:FFFFF::FFFF,F,F
FFF:F,F::FFF,FFF:FF,:FFF,:,FFFFFF:F:F:,FFF,F:,FF
@A00721:190:HGJW7DSXY:4:2147:20211:16986_2:N:0:TCTGCAAG+GCTGCACT
TGTTTCACATTGCGTGATCACTTCCCGTGGACTTGGGAAGAGATTAAACATAAAATCCAGCGATTCTA
TTATGTCGCTAAAGCCTGTCCAGTTGTCGATGATGTTCGGGCTGTGGG
+
FFFFFFFFFFFFFFFF:FFFFFFFFFFFFFFFFFFFFFFFFFFFFFFF:FFFFFFFFFFFFFFFFFFF
:FFFFFF:FFFFFFFFFFFFFFFFFFFFFFFFFFFFF:FFFFFFFFFFF
@A00721:190:HGJW7DSXY:4:2442:13440:19194_2:N:0:TCTGCAAG+GCTGCACT
AAACTTACGACGATGTTCTCGCTCGCATTTCACAAGGATTACTAAGTCTGAGATCTGCCACTTCTAAT
TTTAGGAAACGTCGCGAAAAGAAAGTTCATGAGGATGTCTTGGTCGAA
+
FFFFFFFFFFFFFFFFFFFFFFFFFFFFFFFFFFFFFFFFFFFFFFFFFFFFFFFFFFFFF:FFFFFF
FFFFFFFFFFFFFFFFFFFFFFFFFFFFFFFFFFFFFFF,F,FFFFFFFF
@A00721:190:HGJW7DSXY:4:2649:22227:19194_2:N:0:TCTGCAAG+GCTGCACT
GAACTTCTAATAGAGGATCCTGATGACATTATTTCAACGGATACCGATCTCAGTTCTACCACAGAAAC
CGTCTCAGGAACTGTCTCCACTGATTCGGAATTAGAGAAAGACGACCT
+
FFFFFFFFFFFFFFFFFFFFFFFFFFFFFFFFFFFFFFFFFFFFFFFFFFFFFFFFFFFFFFFFFFFF
FFFFFFFFFFFFFFFFFFFFFFFFFFFFFFF:FFFFFFFFFFFFFFFFF
@A00721:190:HGJW7DSXY:4:2517:24659:24408_1:N:0:TCTGCAAG+GCTGCACT
AGACCTCTTAATTTTCAGGGTGCCCTGATGAGGACGTTGAAAGGGATAATTTCAACTCTGAGACTTCT
AAGAGTGTTTCAAAAGACCAGAAACGTAACAAGAAGAATAAAGGTAGGAAGGGGAGAAAACAGAACAA
GC

+
FFF,FF,F,:,FFFFFF::FFFFF:FF,FFFFFF,FFF,:FF:FFFF,FFFFFFFFF:F,:FFFFFFF
FFFFFF,FFFFFFFF,FF,FFFFFFFFFFFFFFF,:F:FF,F:FF:FFF,:FF:FFFFF:FF:F:FFF
:F
@A00721:190:HGJW7DSXY:4:2628:1154:34679_2:N:0:TCTGCAAG+GCTGCACT
ATGGTTGCGGCTGATCGTACCCCAATTCCTGCTAGAAAGATCACTCTCGGCGATACCACTGATCTTCC
AGAGTTACGTAGTGGTCGTTCTTTTGGTGATTTTATCAAAGAGAAAGT
+
FFFFFFFFFFFFFFFFFFFFFFFFFFFFFFFFFFFFFFFFFFFFFFFFFFFFFFFFFFFFFFFFFFFF
FFFFFFFFFFFFFFFFFFFFFFFFFFFFFFFFFFFFFFFFFFFFFFFF
@A00721:190:HGJW7DSXY:4:1422:2672:1329_1:N:0:TCTGCAAG+GCTGCACT
CAAGTTAAACTTCGAACTGACCACTTACAAGAGTGTTGAAGTACCGCAACATGGATCAGTTTTCCAAA
AAGCTATTAATAGCAGTTGCGGGGCTATAATACCGCCTCCAGATTACTCTAAACTGAATCCACTCTCT
TGG
+
FFFFFFFFFFFFFFFFFFFFFFFFFFFFFFFFFFFFFFFFFFFFFFFFFFFFFFFFFFFFFFFFFFFF
FFFFFFFFFFFFFFFFFFFFFFFFF,FFFFFFFFFFFFFFF:FF,FFFFFFFFFFFFFFFFFFFFFFFF
FFF
@A00721:190:HGJW7DSXY:4:2420:11288:1063_1:N:0:TCTGCAAG+GCTGCACT
AGAGTTTTGGAACTGTGTAATGTCCTACGTACTGTTCAAGGTGTTTGGGCTTCTCTAAAGAACAGCAA
CTTTCAACATGAATTAGTTATTGAAAGTCTTGACCCTCACGTCTCACTTTATCACCTTGGAGATGGTA
AAG
+
FFFFFFFFFFFFFFFFFFFFFFFFFFFFFFFFFFFFFFFFFFFFFFFFFFFFFFFFFFFFFFFFFFFF
FFFFFFFFFFFFFFFFFFFFFFFFFFFFF:FFFFFFFFFFFFFFFFFFFFFFFFFFFFFFFFFFFFFF
FFF
@A00721:190:HGJW7DSXY:4:2628:1624:24345_2:N:0:TCTGCAAG+GCTGCACT
AGACCTTTCTTGGGGAGTAAAGAGGGTTTGAAAATTACGGAACCTTTCCTCGTTCTTCATGAAGATCT
TCGAGTGTTTTATAGTGAGTTATTCTTGCGAAGGTTCAAGTCGATTGA
+
FFFFFFFFFFFFFFFFFFFFFFFFFFFFFFFFFFFFFFFFFFFFFFFFFFFFFFFFFFFFFFFFFFFF
FFFFFFFFFFFFFFFFFFFFFFFFFFFFFFFFFFFFFFFFFFFFFFFF
@A00721:190:HGJW7DSXY:4:2524:32208:4257_2:N:0:TCTGCAAG+GCTGCACT
AATAAGAAGTTCTTCGCAGTTGAATCTCCACCAGGTGGAGGGAAGACCACTGAACTTATAGCGACTTT
TGTCGAATTTTTCGCAAAAGGTCTTGAAGTACGTATTGTCACTGCTAA
+
FFFFFFFFFFFFFFFFFFFFFFFFFFFFFFFFFFFFFFFFFFFFFFFFFFFFFFF:FFFFFFFFFFFFF
FFFFFFFFFFFFFFFFFF,FFFFFFFFFFFFFFFFFFF:FFFFFFFFF
@A00721:190:HGJW7DSXY:4:2468:3694:10958_2:N:0:TCTGCAAG+GCTGCACT
TTGGCACTTCTAGTTCATCGCGGTATTTGTGAAATTAAGACAGACGTGAAAGTTATGATGAGTGATCG
TATTAGAACAGCTGATTCTTATTGCATGAACGTCGCTGAGGTCAAACC
+
FFFFFFFFFFFFFFFFFFFFFFFFFFFFFFFFFFFFFFFFFFFFFFFF,FFFFFFFFFFFFFFFFFFFF
FFFFFFF:FFFFFFFFFFFFFFF:FFFFFFFFFFF:FFFFF:FFF:FF
@A00721:190:HGJW7DSXY:4:1401:7148:35415_2:N:0:TCTGCAAG+GCTGCACT
TTCATCGACGAATTATTCATGATTCACGCTGGTCAGACCCTTTTTCTTACACATCTACTTAAGGCGGC
TTATACCATAGGTTACGGTGATTCCAATCAAATCGGATTCATCAATCG
+
FFFFFFFFFFFFFFFFFFFF:FFFFF:FFFFFFFFFFFFFFFFFFFFFFFFFFFF:FFFFFFF:FFFFF
:FFFFFFF:FFFFFF:FFFFF,FFF:F::FFFFF:FFFFFFF:FFF:F
@A00721:190:HGJW7DSXY:4:1263:30282:23218_2:N:0:TCTGCAAG+GCTGCACT
CAAGTCGCAAACTACTTCAACATCAACGACATCATTCTTGATGAGTGTCGTTCTTATCGAAGTCTCAG
TTATCGCTGCCCGGCCGACGTTTGTCGCGAACTCTCAGAGGTTTACGG
+

FFFFFFFFFFFFFFFFFFFFFFFFFFFFFFFFFFFFFFFFFFFFFFFFFFFFFFFFFFFFFFFFFFFF
FFFFFFFFFFFFFFFFFFFFFFFFFFFFFFFFFFFFFFFFFFFFFFFF
@A00721:190:HGJW7DSXY:4:2535:3830:9815_2:N:0:TCTGCAAG+GCTGCACT
CTATGGAAATTAGACCTATCAGATGTCTTGACGACTTCGAATTTGAGGACGGAGTTAAATATATTACT
TTCACTCAAGGTGAAAAGTTTGAAATTAACAAAGTCCTTGGTAAGAGT
+
FFFFFFFFFFFFFFFFFFFFFFFFFFFFFFFFFFF,FFFFFFFFFFFFFFFFFFFFFFFFFFFFFFFF
FFFFFFFFFFFFFFFFFFFFFFFFFFFFFFFFF:FFFFFFFFFFFFFF
@A00721:190:HGJW7DSXY:4:1646:10899:18865_2:N:0:TCTGCAAG+GCTGCACT
ACTTACCAGCACGTTCGGCTGGTTAGAATTAAACCTCAAGATGACCCTGTTTTGCATCACAAACACA
TATTACCGTTGCTCTATCACGACACGTTGAATCTTTACGATACTTTGT
+
FFFFFFFFFFFFFFFFFFFFFF::FFFF:FFFFFFFFFFFFFFFFFFFF,:FFFFFFFFFFFFFF:FF
FFFFFFFFFFFFFFFFFFFFFFFFFFFFFFFFFFFFFFFFFFF:::FFF
@A00721:190:HGJW7DSXY:4:2159:17345:27586_2:N:0:TCTGCAAG+GCTGCACT
AAACGATGCTACTTGTGAGAGGATCAATCACAGTAAGGAACTGTCACGTTTAGCACGGGAGGTTGCAG
ATTCAACCCCTTATATCGAGGCTATCGAGGACGATGAGTATCTTGAAG
+
FFFFFFFFFFFFFFFFFFFFFFFFFFFFFFFFFFFFFFFFF:FFFFFFFFFFFFFFFFFFFFFFFFFF
FFFFFFFFFFFFFFFFFFFFFFFFFFFFFFFFFFFFFFFFFFFFFF:,F
@A00721:190:HGJW7DSXY:4:1630:4535:32080_1:N:0:TCTGCAAG+GCTGCACT
TAGAGGACCACCTGGATCTTCTACTGTCTCTGCCTTCCAAGATTTCTTAGAAGAGGTTATTCCTGGAA
CCACCTTAACGTCACTCGGTAATCTTTCAGCCGAATTAAGTACTAATGAGTTCAAGTCGGTTGTCGAT
CGA
+
FFFFFFFFFFFFFFFFFFFFFFFFFFFFFFFFFFFFFFFFFFFFFFFFFFFFFFFFFFFFFFFFFFFF
FFFFFFFFFFFFFFFFFFFFFFFFFFFFFFFFFFFFFFFFFFFFFFFFFFFFFFFFFFFFFFFFFFFF
FFF
@A00721:190:HGJW7DSXY:4:2440:15736:36855_2:N:0:TCTGCAAG+GCTGCACT
GGCTAATACCTTTATAGGTAAGAAGCCCCTTGAGCCGCGTAGTCAGCGCGTTTAGCTGTATCATCAGT
CAGAGTATACCCGATAGGGTACCGAGTCTCCAAGAGAATATTTATTCT
+
FFFFFFFFFF::FFFFFFF:FFFFFFFFFFFFFFFFF:F,FFFFFFFFFFFFFFFFFFFFFF:FFFFF
FFFFFFFFFFFFFFFFFFFFFFFFFFFFFFFFFFFFFFFFFF,FFFFFF
@A00721:190:HGJW7DSXY:4:1314:7997:1877_2:N:0:TCTGCAAG+GCTGCACT
GTTTTCACAACGGAAAGACATATCGCACCCGAAATTTTTGGGCAGAATGCTCTTAACGCGGTTCTTGA
ACGGTGTTTTGATCAGTCTAAGTTGGATAAAGTTATAGGAGACTCAGT
+
FFFFFFFFFFFFFF:FFFF:FFFFFFFFFFFF:FFFFFFFFFFFFFFFFFFFFFFFFFFFFFFFFFFF
FFFFFFF::FFFFFFFFF:FFFFFFFFFF,FFFFFFFFFFFFFFFFFF
@A00721:190:HGJW7DSXY:4:1170:31114:8469_2:N:0:TCTGCAAG+GCTGCACT
GCTCCTCAATTGATAGATTGGGTCAACAATAGGGACGCTAGTATACTTCGTAAAGTCGACAGCGAAAT
GGTCAATATTGATAATTGGACCGACTATATGACCTATTTTAAGCTCAT
+
FFF:FFFFFF:FFFFF:FFFFFFFF:FFFFFFFFFFFFFFFFFFFFFFFF:FFF:FFFFFFFFFFFFFF
,FF:FFFFF:::FFFFF::FF:FFFFFFFFFFF:FFFFFFFFFFFFFF
@A00721:190:HGJW7DSXY:4:1246:15754:6981_2:N:0:TCTGCAAG+GCTGCACT
CTAAGGTTAAACTTGATGCTTCAGCCTTTTCGAAGCATAACCCCGCACAGAATATCGTTTATCACGCA
AAATTTATTAATGCAATTTTCGGACCTATCTTTAAGGTTCTTGTGAGG
+
FFFFF:FFFFFF:,FFFF,FF:FFFFFFFFFFFFFFFFFFFFF,FFFFFFFFFFFFF:F,FFFFFFF,F
FF:FFFFFFFF:FF:F,FFFFF:F:FFFF,F:FFF::FFFFF:F,F:F
@A00721:190:HGJW7DSXY:4:1639:15393:27618_1:N:0:TCTGCAAG+GCTGCACT
TGTCTTTTACCCAACATTTTTATGTATACAGAGATGACCAGTGACGATTTCTCCCGGAAGCTCTTCTC

```
TCTATTAGGAGGTCATGATGACTACTATAAGGGTGAAATCGATTTTTCGAAGTTTGACAAATCTCAAG
ATT
+
FFFFFFFFFF,:FF:F:FFFFFF,FFF:F,FFFFFF:FFFFFF,,:FFFFFFFF:::FFF,F:FFFFF
:,F,FFFFFF,:F:FFFFFFF,FFFFFF,FFFFF,FFFFFFFFFFFFF:FFFFFFFFF,:FFF:FFFF
,FF
@A00721:190:HGJW7DSXY:4:2346:15148:29356_2:N:0:TCTGCAAG+GCTGCACT
AGCCTATGAGATAAAGATGTATTCTTATTTAGGCTTCGATGCTGAATTTCTTGAACTTTGGAGTGCCA
GTGAATATTTTTGTCACGCCAGGAGATCGGAAGAGCGTCGTGTAGGGA
+
FFFFFFFFFFFFFFFFFFFFFFFF:FFFFFFFFFFFFFFFFFFFFFFFFFFFFFFFFFFFFFFFFFFF
FFFFFFFFFFFFFFFFFFFFFFFFFFFFFFFFFFFFFFFF:FFFFFFF
@A00721:190:HGJW7DSXY:4:1122:8495:4241_1:N:0:TCTGCAAG+GCTGCACT
TAACACATTCTTTGGTAATTGTATGATCACTCTTATGATGCTCTCTCTTTATTACGACCTTCGGAAGT
TTAGAGCCGTCGCTGTCGCCGGAGACGATTCGATCCTCTTTTCGGACGTACCGGTCCAGGATTATTCA
GAT
+
FFFFFFFFFFFFFFFFFFFFFFFFFFFFFFFFFFFFFFFFFFFFFFFFFFFFFFFFFFF:FFFFFFFF
FFFFFFFFFFFFFFFFFFFFFFFFFFFFFFFFFFFFFFFFFFFFFFFFFFFFFFFFFFFFFFFFFFFF
FFF
@A00721:190:HGJW7DSXY:4:2261:3233:28917_2:N:0:TCTGCAAG+GCTGCACT
CAGCGTACTTCTGCTCGCGATTTATTCTTTTTGTCAATGATTACATCTACTTCGTTCCTGATCCTTAC
AAGATCATCGTTAAGTTAGGTAAACCCCTAACCGATGTTACTCTTGTA
+
FFFFFFFFFFFFFFFFFFFFFFFFFFFFFFFFFFFFFFFFFFFFFFFFFFFFFFFFFFFFFFFFFFFF
FFFFFFFFFFFFFFFFFFFFFFFFFFFFFFFFFFFFFFFFFFFFFFFF
@A00721:190:HGJW7DSXY:4:2153:22950:32597_1:N:0:TCTGCAAG+GCTGCACT
TTTAAAGACTATAATAAATATCTCCACTACGAGGTTGTTGTTTGGCTATTAGCTATTGCGACTAATAT
TCGATATTCGATTGAGGGTCGTTATACCTATTCGGCTATCTGTGCTTTGAATTGTATAAGATCAAATT
TTA
+
FF:FFF,:,F:FF,FFFFF:F,F,F:,FFF::F,FF:FFFFF,FF:,:FF,F,F,FFF::F,F::F,F
FFF:F,FF,,F,F:,FFFFF:FF,FFF:F,F:FFF,FFF,F,FF,FFF,F,FF,F,FF:FFF:F::FF
FFF
@A00721:190:HGJW7DSXY:4:2646:10520:7341_2:N:0:TCTGCAAG+GCTGCACT
CCTGATGTGGAGGGAGTTCTTGAAGCATCCCGACCTTTCCTTGTGAAAATTTTGATGCGGTTTCATAA
GATTATCAGTAATCCTGATTCTACTACGGATTCATGGACCTTCCATCC
+
FFFFFFFFFFFFFFFFFFFFFFFFFFFFFFFFFFFFFFFFFFFFFFFFFFFFFFFFFFFFFFFFFFFF
FFFFFF:FFFFFFFFFFFFFFFFFFFFFFFFFFFFFFFFFFF:FFF:F
@A00721:190:HGJW7DSXY:4:1669:12093:20556_2:N:0:TCTGCAAG+GCTGCACT
GACGTATCTCAAAGATCGACTTCGAGACAGGTGATATTGTTGAGCGAGAGCGACCAAGATCTAAATTC
GGTAACGGTGCGTGGCGCTTTATTTTAAAAAGCTCCCTCCTCTCCCAA
+
FFFFFFFFFFFFFFFFFFFFFFFFFFFFFFFFFFFFFFFFFFFFFFFFFFFFFFFFFFFFFFFFFFFF
FFFFFFFFFFFFFFFFFFFFFFFFFFFFFFFFFFFFFFFFFFFFFFFF
@A00721:190:HGJW7DSXY:4:1308:3649:31109_2:N:0:TCTGCAAG+GCTGCACT
ACTTGAGTCTTTTGATCAGTCTCTGACTTGACCATCTTTCATTTTAAATGACGATCATTAAGACTCTC
TTTCTTCTTTGTTTCCTTCTTTGCACTGATTTTTCCTCATCAGCTTGT
+
FFFFFFFFF:FFFFFFFFFFFFFFFFFFFFFFFFFFFFFFFFFFFF:FFFFFFFFFFFFFFFFFFFFFF
,FF:FF::,FFFFF::FFFFFFF:FFFFFFFFFFFF:FFFFFFF,FFF
@A00721:190:HGJW7DSXY:4:2567:6912:12399_1:N:0:TCTGCAAG+GCTGCACT
GTTTTTACGATAACAACTATCAGTTGATTAAGACTCTTCCGAAAGGCGAACTTTGGGAATTTGAAGCT
```

CAGGGTGAAGCACAACGTTTGACCGTGACGAACTGTGGTGTTCTTGATCTACGTTACGGATATACTCT
CCT
+
FFFFFFFFFFFFFFFFFFFFFFFFFFFFFFFFFFFFFFFFFFFFFFFFFFFFFFFFFFFFFFFFFFFFFF
FFFFFFFFFFFFFFFFFFFFFFFFFFFFFFFFFFFFFFFFFFFFFFFFFFFFFFFFFFFFFFFFFFFFFF
FFF
@A00721:190:HGJW7DSXY:4:1331:10122:15765_1:N:0:TCTGCAAG+GCTGCACT
CACTACCACTTATTACGATATTTCTTTAGTCGATGGGTTTTCGACGCCTTTAAGCGTCTTCTGTTCAT
CGCAACCAGATGATATCTTCTATTCGAACTTATGTTTCGATCAGTGTTTCTTCTATCAGGAGGAAAAC
ACT
+
FFFFFFFFFFFFFFFFFFFFFFFFFFFFFFFFFFFF:FFFFFFFFFFFFFFFFFFFFFFFFFFFFFFFFF
FFFFFFFFFFFFFFFFFFFFFFFFFFFFFF,FF:FFFFFFFFFFFFFFFFFFFFFFFFF:FFFFFFFFFF
FFF
@A00721:190:HGJW7DSXY:4:1305:14127:27680_2:N:0:TCTGCAAG+GCTGCACT
TCTTCTAATGTGGCCTGAACTACGTTTCACTTTGTGTGTACTTGTCTCTATTTTAAGTTTTGTCTTCC
TTGTTTGTCTTATTCTATTAATCACTCATTTTAGTGAGATTATTAAAA
+
FFFFFFFFFFFFFFFFFFFFF:FFFFFFFFFFFFFFFFF:FFFFFFFFFFFF:FFFFFFFFFFFFFFF
FFFFFFF:FFFFFFFFFFFFFFFFFFF:FFFFFFFFFFFFFFFFFFFFFF
@A00721:190:HGJW7DSXY:4:1134:31331:23500_2:N:0:TCTGCAAG+GCTGCACT
CGACAACAGCAGGGGTTTTCGAATAGTGATAGCCACCCGCCTTACAGAGGGGCAGCAGCATGACCATA
ATTGGTATTGACTTTGGTACCACCTTTTCTACTGTCTGTCTCGCCTCT
+
FFFFFFFFFFFFFFFFFFFFFFFFFFFFFFFFFFFFFFFFF:F:FFFFFFFFFFFFFFFFFFFFFF:FFF
:FFFFFFFFFFFFFFFFFFFFFFFFFFFFFFFFFFFFFFFFFFFFFFF
@A00721:190:HGJW7DSXY:4:1449:32398:30702_1:N:0:TCTGCAAG+GCTGCACT
CGTGTGCTCTTCCGATCTGTGAATTTATACCTACAATCATTGGTTTTTCTAATAATGATGACACTATT
GTTTATGGATATGATGCTATTACTCATGATGGTTACGGTCAGGGAGGATTCTCAGTTTACAAGGACCT
TAA
+
FFFFFFFF:FFFFFFFFFFFF:FFFFFFFFFFFFFFFFFFFFFFF:FFFFFFFFFFFFFFFFFFFFFFFF
FFFFFFFFFFFFFFFFFFFFFFFFFFFFFFFFFFFFFFFFFFFFFFFFFFFFFFFFFFFFFFFFFFFFF:
FFF
@A00721:190:HGJW7DSXY:4:1553:5005:17832_2:N:0:TCTGCAAG+GCTGCACT
CCGAAAGACGTGCTAAATTAAAACCATTGTACGATGTCGTCTGTCTCGGCGATCACTTTTCACTAAGA
ATTGGTCCTACTTTTGGACAAGGCCAACTTCGTAATCTTCATGAGATC
+
FFFFFFFFFFFFFFFFFFFF:FFFFFFFFFFFFF:FFFF:FFFFFFFFFFFFFFFFFFFFFFFFFFFFF
FFFFFFFFFFFFF:FFFFFFFFFFFFFFFFFFFFFFFFF:FFFFFFFFFF
@A00721:190:HGJW7DSXY:4:1121:17273:20666_1:N:0:TCTGCAAG+GCTGCACT
TTAGGGATTTTGAGAGAGTGAAAGGCTTCAATTGTAGTGGTTTGGTCGTTTCAGTACCCAGTCAGTAT
ACCTCTACGCAAAGATATTTCATGAAAACTACAGAGGAACGTACCGGATTACCTGTCTTTCATATTAT
GAA
+
FFFFFFFFFFFFFFFFFFFFFFFFFFFFFFFFFFFFFFFFFFFFFFFFFFFFFFFFFFFFFFFFFFFFFF
FFFFFFFFFFFFFFFFFFFFFF:FFFFFFFFFFFFFFFFFFFFFFFFFFFFFFFFFFFFFFFFFFFFFF
FFF
@A00721:190:HGJW7DSXY:4:2668:6840:2785_1:N:0:TCTGCAAG+GCTGCACT
CTCTTTGCTTCGATGTTGGATGTCAAGTTGGTTTCAGAATTGGATTCCTACGTAGTTTATGACTTTGG
TGGTGGTACTTTCGATACTTCTATCGTATTACGTTACGGTAATTACTATAGTGTTATTTATTCTGGTG
GTG
+
FF:,FF,,FF,:FFF,FFF,,:F,,,:,,:,,F,:,:FFF,,:,FF,,F,,,FFF:F:,:FF,FF:F,

F:FFFFF,,F,FFF,FF::FFF,FF:F,FFF:FFF:,:,F,F,FF,F,:,:F,FF,F:FFFFFFFFFF
FF,
@A00721:190:HGJW7DSXY:4:2310:9760:5838_2:N:0:TCTGCAAG+GCTGCACT
AGGTCGTGACGTTGATGAAGCTATAAGAGCGTTCTTATTACGGCGCACTTCTTTACCCGCCACCACAC
AACTTTCAGTTTCACAACTGAAGGAGGAGGTTTCTGTTACTGGAAAAC
+
FFFFFFFFFFFFFFFFFFFFFFF:FFFFFFFFFFFFFFFFFFFFFFFFFFFFFFFFFFFFFFFFFFFFF
FFFFFFFFFFFFFFFFFFFFFFFFFFFFFFFFFFFFFFFFFFFFF:FFF
@A00721:190:HGJW7DSXY:4:1629:16016:25567_1:N:0:TCTGCAAG+GCTGCACT
ACTTTTCAATAATATACGTCTTGAACTCACTTATGACGATCTAGTGAAATTAACGCGTCCTTTTCTGG
AACGTAGTTTTACTATTATGAACAAAGTTATCAAAGATAGTGGCATTCAAGGCAAACTTACCCTGATT
CCG
+
FFFFFFFFFFF:FF:FFFFFFFFFFFFFF:FFFFFFFFFFFFFFFFFFFFFFFFFFFFFFFFFFFFFFF
FFFFFFFFFFFFFFFFFFFFFFFFFFFFFFFFFFF:FFFFFFFFFFFFFFF:FFFFFFFFFFFFFFFFFFF
FFF
@A00721:190:HGJW7DSXY:4:1425:14995:4006_1:N:0:TCTGCAAG+GCTGCACT
TTCTACCAGGGGCTATTACTCTTGCTGAAACAGCTATTAAAGCTGTTGAGCGGGTAATTACACCTAAA
CGAGCTTGATCTGCCATCGCTGAAGGATGTGCACTCTTTTCTGCTACTTTAACGGAGCGTAGTGTACT
CTT
+
FFFFFFF:FFFFFFFFFFFFFFFFFFFFFFFFFFFFFFFFFFFFFFFFFFFFFFFF:FFFFFFFFFFFFF
FFFFFFFFFFFFFFFFFFFFFFFFFFFFFFFFFFFFFFFFFFFFFFFFF:FFFFFFFFFFFFFFFFFFFF
FFF
@A00721:190:HGJW7DSXY:4:1477:26458:18223_1:N:0:TCTGCAAG+GCTGCACT
CAACAGTAACATTACTTCTAATAAAGGTCTCTATCAAGCTCGTATTATCGTCGCTGCTGGATCTGCGT
TACCGTTGAAGGCTAAACTCCAAGGTAATTATGAAGACTGTGATTTATCTTTCACTCTGGCCTTGTAT
GAG
+
FFFFFFFFFFFFFFFFFFFFFFFFFFFFFFFFFFFFFFFFFFFFFFFFFFFFFFFFFFFFFFFFFFFFFF
FFFFFFFFFFFFFFFFFFFFFFFFFFFFFFFFFFFFFFFFFFFFFFFFFFFFFFFFFFFFFFFF:FFFFFF
FFF
@A00721:190:HGJW7DSXY:4:2502:10881:3615_2:N:0:TCTGCAAG+GCTGCACT
ATCGTAACATTAATAATTTCAATCTTCTTAAAAAGAAGATAGAGAGTCATACTTTGGGCTATCGTTCA
GGTGCATGGGGTTGGATAGCCAATATTGAAGTGGACACCTCTGGTAGT
+
FF:,F,F::FFFFFFFFFFFFFFF:FFFFF:FF:FFF:FFFFFFF:FFFFFFFFFFFFFFFFFFFFFFFF
FFF:F:FFFFFFFF::FFFFF:FFFFFFFFFFFF:::FFFFFFFFFFF
@A00721:190:HGJW7DSXY:4:2133:14100:25097_1:N:0:TCTGCAAG+GCTGCACT
ATACTATGGTTGAATCAACTAAGCATACTACTTTTGCTTTGATTACTCTACCGCCTTACAAGTCTAAG
GTTAAAGAGTCGATTAAACAAGTTGTACTTATCAATGATTTTATCGCTGGTTGTTATCTCGAAAAGTC
TCT
+
FFFFFFFFFFFFFFFFFFFFFFF:FFFFFFFFFFFFFFFFFFFFFFFFFFFFF,FFFFFFFFFFFFFFFFFF
FFFFFFFFFFFFFFFFFFFFFFFFFFFFFFFFFFFFFFFFFFFFFFFFFFFFFFFFFFFFFFFFFFFFFFFFF
FFF
@A00721:190:HGJW7DSXY:4:2250:9616:9032_1:N:0:TCTGCAAG+GCTGCACT
ACCCAAAGAGAATATCTAAAATATTTACAAAATCTTACCACTATCTACGAACCTAAAGTTCTGAAGAC
GTTGCTTGTCGCTTCCTCCTTTAATGGCACTACCTCGGTACAATCTTACTTGGAACGAAGGCGACCTG
TTC
+
FFFFFFFFFFFFF:FFFFFFFFFFFFFFFF:,FFFFFFFFF,:FFF,FFFFFFFFFFF,FFFFFFFFF
FFFFFFFFFFFFFFFFFFFF:FFFFFFFF:FF:FFFFFFFFFF:FFFFFFF:FFFFFFFFFFFFFFFFF
FFF

```
@A00721:190:HGJW7DSXY:4:1453:13720:29418_1:N:0:TCTGCAAG+GCTGCACT
TGTTCTGTCTATATTAGGTGAAGCGAAATACGATTTCGAACGTCAACGTTCACCGAGTTTCGTTTCGT
ACCTTGATGCTGACTTTCGTCAATATGATTTGATCTGGTTGCTCAATTCTAAGCGTGTTCCGCTTAGT
GCG
+
FFFFFFFFFFFFFFFFFFFFFFFFFFFFFFFFFFFFFFFFFFFFFFFFFFFFFFFFFFFFFFFFFFFF
FFFFFFFFFFFFFFFFFFFFFFFFFFFFFFFFFFFFFFFFFFFFFFFFF:FFFFFFFFFFFFFFFFFF
FFF
@A00721:190:HGJW7DSXY:4:2204:12463:27305_2:N:0:TCTGCAAG+GCTGCACT
GAGTATTGCTATTTATACGGTTACGAGAAGAAGGTTCGAAAGTTTGAGAACTTAGTTTACGAACTTGA
CTCTACTATACGTGACATACTAAATACTAGATATTTGGATGATCTTGA
+
FFFF::FFFFFFFFFFFFFFFFFFFFFFF:FFFFFFFFFFFFFFFFFFFFFFFFFFFFFFFFF:FFFF
FFFFFFFFFFFFFFFFFFFFFFFFFFFFF:FFFF:FFFFFFFFFFFFF
@A00721:190:HGJW7DSXY:4:2139:24279:33708_2:N:0:TCTGCAAG+GCTGCACT
CATGAATTTTCAGAGGATGAAAAATTTTCTTCCTTGGATTTTAATATAAAGAATCTTTACCAGGACTC
CTACTACAGGAGGCAGTCTTCTGATCGTAAGAAGTTTATAAGGAACAT
+
FFFFFFFFFFFFFFFFFFFFFFFFFFFFFFFFFFFFFFFFFFFFFFFFFFFFFFFFFFFFFFFFFFFF
FFFFFFFFFFFFFFFFFFFFFFFFF:FFFFFFFFFFFFFFF,F:FFFF
@A00721:190:HGJW7DSXY:4:2359:10077:17284_1:N:0:TCTGCAAG+GCTGCACT
ACTCGAAACTCTAAGTGGTAATACTGTCTTTCCAAGCTTACCTGTATCTTCAATGAAATTGGACTCTT
CGATAGGGGACATTTTATCGTGGGCTTTCCGTAAAGGAAGATCTGTTATCGACGGTTTATCGTTTACG
AAG
+
FFFFFFFFFFFF:FFFFFFFFFFFFFFFFFFFFFFFFFFFFFFFFFFFFFFFFFFFFFFFFFFFFFFF
FFFFFFFFFFFFFFFFFFFFFFFFFFFFFFFFFFFFFFFFFFFFFFFFFFFFFFFFFFFFFFFFFFFF
FFF
@A00721:190:HGJW7DSXY:4:1272:17490:30060_1:N:0:TCTGCAAG+GCTGCACT
GTTTAAGAATACTATAAATAGAATAGCTATTACTACTAATGGCGTTGGCCTAGTGGATGTTATTAGTG
ACATCTGGTCAATTTCGTTTTTAGGTGGTCTTTATTCTATTATTGATTTTTATGAGATTGATACGTTA
ACA
+
F,:FFFFFF:FFFFFFFFFFFF:FFFFFFFFFFFFFFFFF,:,FF:FFFFFFFFFFFFFF:FFFFF:F
FF:FFFFFFFFFFFFF,::FFFFF:FFFFFFFF,F:,F:FFFFFF:FFFFF:FFF,FFF,F,FF::FF
,FF
@A00721:190:HGJW7DSXY:4:1619:32859:17159_1:N:0:TCTGCAAG+GCTGCACT
TAGAGTAGAATTCCTGAAGAGTCCTTCCGTAACCAACTTCTTGACAATTTTCTTCAATCCTTCGATAA
TAGTTAAATCTAGGATTATGTCTGTGTTGAAAGACTTTCTACCGATAAGGGTCACTAGTCTTACACAA
TTA
+
FFFFFFFFFFFFFFFFFFFFFFFFFFFFFFFFFF:F:FFFFFFFFFFFFFFFFFFFFFFFFFFFFFFF
FFFFFFFFFFFFFFFFFFFFFFFFFFFFF:FFFFFFFFFFFFFFFFFFFFFFFFFFFFFFFFFFFFFF
FFF
@A00721:190:HGJW7DSXY:4:1525:12924:21997_2:N:0:TCTGCAAG+GCTGCACT
ATTGACTGATTATCTAAAGATTGAAGGAAAATTTTATCCTCGTTTAACTTACTTAGACTTTCCTACGA
TACAAATGATAACTTCTAATTTACGTTTGCTACTCAGAGCTGGTCCTC
+
,FFFFFFFFFFFFFFF:FFFFFFFFFFFFFFFFFFFFFFFFFFFFFFF:FFFFFFFFFFFFFFFFFFF
FFFFFFFFFFFF:FFFFFFFF:FFFF:FFFFFFFFFFFFFFFFFFFFF
@A00721:190:HGJW7DSXY:4:1154:31494:10066_1:N:0:TCTGCAAG+GCTGCACT
GATGCATGGTGTTATCTCCTCAGGAGTCCCGGCATTTGATAATAATGACGAGACTTTCGTCATAATCG
CTGCATATTACGGATTACACGGTACCAGCGCATCTCGTGTCGAGCCTCGACCCGATTATATGGTATTA
ACT
```

+
FFFFFFFFF:FFFFFFFFF:FFFFFFFFFFFFFFFFFFFFFFFFFFFFFFFFFFFFFFFFFFFFFFFFF
FFFFFFFFFFFFFFFFFFFFFFFFFFFFFFFFF:FFFFFFFFFFFFFFFFFFFFFFFFFFFFFFFFFFF
FFF
@A00721:190:HGJW7DSXY:4:1363:29839:2910_1:N:0:TCTGCAAG+GCTGCACT
TGAAATTTATAGAAGATTATTTGACAGAATCAATAAGAATCATCGATTCTCGATTCGTAGAGCTTAT
CTAGGTACTCTCATTTACTATGTTGATTTAGTCTACATTTATGCTGATCTAAAATTTCGATCTAGATG
GAT
+
FFFFFFFFFFFFFFFFFFFFFFFFFFFFF,FFFFFFFFFFFFFFFFFFFFFFFFFFFFFFFFFFF,FFF
FFFFFFFFFFFFFFFFFFFFFFFFFFFFFFFFFFFFFFFFFFFFFFFFFFFFFFFFFFFFFFFFFFFF
FFF
@A00721:190:HGJW7DSXY:4:2675:9254:19085_2:N:0:TCTGCAAG+GCTGCACT
GATTTACAACGATATAGGAGTAATCTACGAGAAGATTCTAGAATTCTTAATTATAATATTCAACCTCA
AAAGTTACGTGTTTACAGTAACGAGATCACACATTTTGAGAACTAACT
+
FFFFFFFFFFFFFFFFFFFFFFFFFFFFFFFFFFFFFFFFFFFFFFFFFFFFFFFFFFFFFFFFFFFF
FFFFFFFFFFFFFFFFFFFFFFFFFFFFFFFFFFFFFFFFFFFFFFFF
@A00721:190:HGJW7DSXY:4:1251:24713:6277_1:N:0:TCTGCAAG+GCTGCACT
TCTCTTCTTATAATAAATAAATAAATAATATTACTAATGGCAAATAGTGGAAAGGCTCCAGTCAACAC
TAGTTCGAGTGCTGCTTTGGATTACGATGACGTGAGAATAGGTGAAGGGATAGTTTTTACCTCGACCA
ACA
+
FFF,FF:FFFF:FFFFFFFFFFF:FF:FFFFFFFFFFFF:FFFFFFFFFFFFFFFFFF,:FFFF:FF,
FFFFFFFFFFFFFFFFF::FFFF::FFFFF:FF::F:FFFFFFFFFFFFFF:FFFFF,FFFFFFFFFF
FFF
@A00721:190:HGJW7DSXY:4:1560:27995:17315_2:N:0:TCTGCAAG+GCTGCACT
GCGACTAATCTTAAAGCTTCACCCAAAATGAAGGACACTTCGAAACTGAATAATGATGCACTACAAAC
AAGTATCGCTAATCATCTGAAAAAGGCTCTAAAGGACACTATACAATA
+
FFFFFFFFFFFFF:FFFFFFFFF:FFFFFFFFFFFFFF:FFF:FFFFFF:FFFFFFFFFFFFFFFFFF
FFFFFFFFFFFFFFFFFFFFFFFF:FFFFF:FFFFFFF,FFFFFFFF:F
@A00721:190:HGJW7DSXY:4:1512:16875:5415_2:N:0:TCTGCAAG+GCTGCACT
ATTCAGCACCACATTTTGGTCAAGTCAATCCGTTGAGAAAATTTCTTAGAGGATATTCGCCATTGTGG
TACGAATTGGTCCAACATTTACCACCAAATTCATTTGATACAACTCTA
+
FFFFFFFFFFFFFFFFFFFFFFFFFFFFFFFFFFFFFFFFFFFFFFFFFFFFFFFFFFFFFFFFFFFF
FFFFF:FFFFFFFFFFFFFFFFFFFFFFFFFFF,:FFFFFFFFFFFFFF
@A00721:190:HGJW7DSXY:4:2470:25120:4131_2:N:0:TCTGCAAG+GCTGCACT
CGGAATACGCTTGCTATTCACCAGATTGGTTCGAACCCGTTGATGCTATGTCAGAAGAAGCTATTCAA
GCTTGTCGTTTAAAGACTGCAGAGGCTGTCAAACAAGCCCAGACTCAA
+
FFFFFFFFFFFFFFFFFFFFFFFFFFFFFFFFFFFFFFFFFFFFFFFFFFFFFFFFFFFFFF,FFFFF
FFFFFFFFFFFFFFFFFFFFFFFFFFF:FFFFFFFFFFFFFFFFFFFF
@A00721:190:HGJW7DSXY:4:2326:31241:31798_2:N:0:TCTGCAAG+GCTGCACT
TGGGTGATAAAAATTATTTAGAGATCTTTTCTACTTACAAAGGACAATTCAGTTTCATTAATTCTACG
GAAAAGTCCGGAGTATATTGTATGTTAATAGATAGTGGGATAATAGTT
+
FFFFFFFFFFFFFFFFFFFFFFFFFFFFFFFFFFFFFFFFFFF:FFFFFFFFFFFFFFFFFFFF:FFFF
FFFFF:FFFFFFFFFFFFFFFFFF,FFFFFF:FFF:FFFFFFFFFFFFF
@A00721:190:HGJW7DSXY:4:1365:25979:20181_2:N:0:TCTGCAAG+GCTGCACT
GAACGCGTTCGTATAACCAAGTACTTTCCGTCTGAAGAGTTTAGAGCTGCTATTGATTATGTAACCGA
TAGGAGTTGTATAGTTGAAGATGTGGCTTTAGACGATAATCCTTTAAG
+

FFFFFFFFFFFFFFFFFFFFFFFFFFFFFFFFFFF:FFFFFFFFFFFFFFFFFFFFFFFFFFFFFFFFF
FFFFFFF:FFFFFFFFFFFFFFFFFFFFFFFFFFFFFFFFFFFFFFFFF
@A00721:190:HGJW7DSXY:4:1478:2003:19398_1:N:0:TCTGCAAG+GCTGCACT
GAAGATTTTCCCACAGATTTGTCTACGTTGCAGATCGCCACTCCAAACACTATATACATTATAAAATA
TAAAAATAAGGGAGTCATAACTTTTGTTACCAAATTCTTGGCTTTCAACGAAGTCTCTGATTTTATTA
ATC
+
FFFFFFFFFFFFFFFFFFFFFFFFFFFFFFFFFFFFFFFFFFFFFFFFFFFFFFFFFFFFFFFFFFFF
FFFFFFFFFFFFFFFFFFFFFFFFFFF:FFFFFFFFFFFFFFFFFFFFFFFFFFFFFFFFFFFFFFFF
FFF
@A00721:190:HGJW7DSXY:4:1277:29270:2268_2:N:0:TCTGCAAG+GCTGCACT
GATCTCTCGGTCATTTACTCTGTACTTGATCTACGTCGTTTAACTGTCGGAACAACCTTTACTTAGAA
TGACTCTATCTTTTCAGACACTTCTTATATTATTTACAATAGTTAATA
+
FFFFFFFF:FFFFFFFFFF:FFFFFFFFFFFFFFFFFFFFFFFFFFFFFFFFFFFFFFFFFFFFFFFF
FFFFFFFFFFFFFFFFFFFFFFFFFFFFFFFFFFFFFFFFFFFFFFFF
@A00721:190:HGJW7DSXY:4:1265:26241:27398_1:N:0:TCTGCAAG+GCTGCACT
AATACTCACTATCGTTTTCTGCTTTTTCTTAGCTAATAGATTAATAAAGGATGAGTACGAAAACCAAC
TCTTCGTTACGAGACCTGATGGTAGAATAGATTTAGTCTCTACACCCATCTCAACGAGGATAAAGCGA
GTT
+
FF:FF:F,FF:FFFFFFFFFF:FF:FF,FF,,FFF:FF:FFF,FF:FFFFFFFF::FFFF:F:FFFFF
FFFFFFFFFFFFFFFFFFFFF:F::FFFFF,FFFFFFFFFF::FF::FFFFFF,:FFFFFFFFFFFFF
FFF
@A00721:190:HGJW7DSXY:4:2318:23366:17096_1:N:0:TCTGCAAG+GCTGCACT
ACACGAAGATATCATCCGCAAACGACTCGAAACCGAGACGCCGTAGATCAAAGTCCCTTCTAACTTTA
GATTATGAATCCTGCCTTTACTCATTTTATAGCAGTTATTTATGTTGTACTTTTTATAATTTATAACC
TT
+
FF::,F,,,,,:,:,FF,,F,FFFFFFFFFFFFFFFFFFFFFFFFFFFFFFFFFFFFFFFFFFFFFFF
FFFFFFFFFFFFFFFFFFFFFFFFFFFFFFFFFFFFFFFFFFFFFFFFFFFFFFFFFFFFFFFFFFFF
FF
@A00721:190:HGJW7DSXY:4:1364:11234:17785_1:N:0:TCTGCAAG+GCTGCACT
TACTTATTGTAACGATCATTCTTTCGAGTTTTGTCATTCTTATTCTTTTATTCCTTTAACTGTGTATG
AGGAGGATTTATAATGCTTTTCGATAGTGATTGTTATCAATTTAAATACATTACTGTTTCAAATCATC
TTT
+
FFFFFFFFFFFFFFF:FFFFFFFFFFFFFFFFFFFFFFFFFFFFFFFFFFFFFF:FFFFFFFFFFFFF
FFFFFFFFFFFFFFF:FFFFFFFFFFFFFFFFFFFFFFFFFFFFFFFFFFFFFFFFFFFFFFFFFFFF
FF:
@A00721:190:HGJW7DSXY:4:1578:2157:25958_1:N:0:TCTGCAAG+GCTGCACT
GAAAATGGTTTCAAATGAGGTCAAAGCTCTATTTATTAAACGAACTATTATCCGATTCTTCGAGGGTA
TCACTACCACTGATCTGCCGACTTTAGTCAAGGAACTTCAATTCCTTCGATGTTTATATTTTTCTATA
AGG
+
FFFFFFFFFFFFFFFFFFFFFFFFFFFFFFFFFFFFFFFFFFFFFFFFFFFFFFFFFFFFFFFFFFFF
FFFFFFFFFFFFFFFFFFFFFFFFFFFFFFFFFFFFFFFFF,FFFFFFFFFFFFFFFFFFFFFFFFFF
FFF
@A00721:190:HGJW7DSXY:4:1233:19153:18255_2:N:0:TCTGCAAG+GCTGCACT
TAATCAAATTTCGACAAATTTTAGAACTTTATTTCAGAACTCTCTCCGATCACCTTGGTGTGATCTCC
CGGAGGATCTTTTCAATAAGATGTATGATGATAATCTACTATTTTTAA
+
FFFFFFFF:FFFFFFFFFFFFFFFFFFFFFFFFFFFFFFFFFFFFFFFFFFF:,FFFFFFFFFFFF
FFFFFFFFFFFFFFFFFFFFF:FFFFF:FFFFFFFFFFFFFFFFFFFF

@A00721:190:HGJW7DSXY:4:1635:29993:33896_1:N:0:TCTGCAAG+GCTGCACT
AGCGTATACTTGAATACTTTATCAAAGATTTGGCTTGGTCTATTTACCGCAAAAAGTTGGAAGAGGTC
TATGATATTTATTTTACTTATACTTTAAATAGCACCGATTATAACTTGACTGTTGATAATATTACAAT
AGC
+
FFFFFFFFFFFFFFFFFFFFFFFFFFFFFFFFFFFF,FFFFFF,FFFFFFFFFFFFFF:FFFFFFFFF
FFFFFFFFFFFFFFFFFFFFFFFFFFFFFFFFFFFFFFFFF:FFFFFFFFFFFFFFFFFFFFFFFFFF
FFF
@A00721:190:HGJW7DSXY:4:1210:18765:1924_2:N:0:TCTGCAAG+GCTGCACT
AAGATTAGAGAAGTAACTCGGTGAACACGTCGGTAGCATTGATTTACTTTTACCGTTAGAACTGAAAA
AGATTCTTTTCGAAAAGAAACCTATAAGTTACAAACGATTCTTCGACT
+
FFFFFFFFFFFF,FFFFFFFFFFFFFFFFFFFFFFFFFFFFFFFFFFFFFFFFFFFFFFFFFFFFFFF
FFFFFFFFFFFFFFFFFFFFFFFFFFFFFFFFFFFFFFFFFFFFFFFF
@A00721:190:HGJW7DSXY:4:1144:28492:19022_1:N:0:TCTGCAAG+GCTGCACT
AATAAAAATACTTCAAGTACCTGAACTTTACATTTTTAATGGTATGGTATAATGTGTCTTACAAATAG
AACCTTCACTCTCGGTAATGAGAGTGTATTCCTTAATATTCTAGTAACCGCTATGTGGGCATTACTAG
GAT
+
FFFFFFFFFFFFFFF:FFFFFFF:FFFFFFFFFFFFFFFFFFFFFFFFFFFFFFFFFFFFFFFFFFFF
FFFFFFFF:FFFFFFFFFFFFFFFFFFFFFFFFFF:FFFFFFFFFFFFFFFFFFFFFFFFFFFFFFFF
FFF
@A00721:190:HGJW7DSXY:4:1170:25111:18427_2:N:0:TCTGCAAG+GCTGCACT
TTTAAATGCTAGGAAAATTTTAGAAAAACTCCATTGTATAGTTCAAGGAGTACTTAATTCAATAGCTT
TAGTATTATTTACTATCGCCTTTATTGAAAATTGTATAAGAGTTAATT
+
FFFFFFFFFFFFFFFFFFFFFFFFFFFFFFFFFFFFFFFFFFFFFFFFFFFFFFFFFFFFFFFFFFFF
FFFFFFFFFFFFFFFFFF:FFFFFFFFFFFFFFFFFFFFFFFFFFFFF
@A00721:190:HGJW7DSXY:4:1361:28827:4194_1:N:0:TCTGCAAG+GCTGCACT
GGGAGAATGAACTGCCGCTTCCCGGAAGAAACGATCTATTTACAGAGTATTTAGAGTAGTCCAAAGTT
TGTATAAATTTATATGATTTTCTATATATATACCACATATATATATATATAAAATCCTATAAATTTGT
ACA
+
FFFFFFFFFFFFFFFFFFFFFFFFFFFFFFFFFFF:F:FFFFFFFFFFFFFFFFFFFFFFFFFFF:,F
:FFFFFFFFFFFFFFFF,F:FFFFFFFFFFFFFFFFFFFFFFFFFFFFFFFFFFFFFFFFFFFFFFFF
FFF
@A00721:190:HGJW7DSXY:4:1328:2980:23907_2:N:0:TCTGCAAG+GCTGCACT
TACACTCCCCAAACATAACATACCATAACCTACCATGACCAATCATCGCTCCAACTGGCACGCCAGAC
GTAGGGTCAAACCTACTACCGCCTGTCGGCAGATCGGCACCACTGGTC
+
FFFFFFFFFFFFFFFFFFFFFFFFFFFFFFFFFFFFFFFFFFFFFFFFFFFFFFFFFFFFFFFFFFFF
FFFFFFFFFFFFFFFFFFFFFFFFFFFFFFFFFFFFFFFFFFFFFFFF
@A00721:190:HGJW7DSXY:4:2378:11885:15562_2:N:0:TCTGCAAG+GCTGCACT
ACCCGCTGTGCAGAATAACCTTCCCAAATCTAGATCTGCACAGAAGAAGGCTGAAGGTCGTTACCGGA
CGACCACTCAAAGGAAACGGGCTGTTGCCCAGAGGAAAGCAGCGACCA
+
FFFFFFFFFFFFFFFFFFFFFFFFFFFFFFFFFFFFFFFFFFFFFFFFFFFFFFFFFFFFFFFFFFFF
FFFFFFFFFFFFFFF,FFFFFFFFFFFFFFFFFFFF:FFFFFFFFFFF
@A00721:190:HGJW7DSXY:4:2260:17969:30044_2:N:0:TCTGCAAG+GCTGCACT
GTAACATTGACGGCAGTCCCGTCATCTCTGTGGGAACTGAGGCAGTACCTCAGTCAAAAGTCACATTC
ACCACTCCCATGCCAATCAATGATGTGACTATACAGTTCGGCCATTTC
+
FFFFFFFFFFFFFFFFFFFFFFFFFFFFFFFFFFF:FFFFFFFFFFFFFFFFFFFFFFFFFFFFFFFF
FFFFFFFFFFFFFFFFFFFFFFFFFFFFFFFFFFFFFFFFFFFFFFFF

```
@A00721:190:HGJW7DSXY:4:1541:28031:6417_1:N:0:TCTGCAAG+GCTGCACT
ATACGTCGTGGGAGGTACTGAAGCCTTCCCCTCCGCTGGCACGAAGAACACTCCCAAGCCAATCAATA
GTTCTTCTGACCAGAATGAAAAGGGGTTTACCAGGGTAGTCTCTAAGTCTACCAGGAAGAGGGGAAAT
GTC
+
FFFFFFFFFFFFFFFFFFFFFFFFFF:FFFFFFFFFFFFFFFFFFFFFFFFFFFFFFFFFFFFFFFFF
FFFFFFFFFFFFFFFFFFFFFFFFFFFFFFFFFFFFFFFFFFFFFFFFFF:FFFFFFFFFFFFFFFFFF
FFF
@A00721:190:HGJW7DSXY:4:1369:14127:2942_1:N:0:TCTGCAAG+GCTGCACT
GCATACGAGATCTTGCAGAGTGACTGGAGTTCAGACGTGTGCTCTTCCGATCTGGAAGATCTCCCACC
ATCTGGGGTTGGGAATAAGAAATCTGCACGGGCCAAAGAGGCCGCTGCTACGAAGTCGCTGAAGGCGG
CTT
+
FFF:FFF,F,:FFFFFFFFFFFFF:FFFFFFFFFFFFFFFFFFFFFFFFFFFFFFFFFFFFFFFFF:FF
FFFFFFFFFFFFFFFFFFFFFFFFFFFFFFFFFFFFFFFF:FFFFFFFFFFFFFFFFFFFFFFFFFFFF
FFF
@A00721:190:HGJW7DSXY:4:2367:21432:6167_2:N:0:TCTGCAAG+GCTGCACT
TGTAAAGTTGCGCCTGCCGACCCCATCTTTGAGACCAAGACTCTCTACAGTAGGTTCGTGCCTACTAT
GAGCAAATCTGTTGAATTCGTCACTGAGGACGAAGGTGAGAGGACTTT
+
FFFFFFFFFFFFFFFFFFFFFFFFFF:FFFFFFFFFFFFFFFFFFFFFFFFFFFFFFFFFFFFFFFFF
FFFFFFFFFFFFFFFFFFFFFFFFFFFFFFFFFFFFFFFFFFF:FFFF
@A00721:190:HGJW7DSXY:4:1529:2013:10394_1:N:0:TCTGCAAG+GCTGCACT
CTGAGAGCAGTCGCTGGGGAAAGAGAATTGCGTGGGTTGAAGGCCGACGCTTCGAGCTTGAGTGTGAC
GAGGCCGGTGACGTTTTTGTCAACGGCGTTTGGAGGATTTTATCCGCGCTTAACTATGGGGCGAAGAT
CCC
+
:FFFFFFFFFFFFFFFFFFFFFFFFFFFF,FFFFFFFFFFFFFFFFFFFFFFFFFFFFFFF:F:FFFFF
FFFFFFFFFFFFFFFFFFFFFFFFFFFFF:FFFFFFFFFFFF:FFFFFFFFFFFFFFFFFFFFFFFFFF
FFF
@A00721:190:HGJW7DSXY:4:2128:3423:26146_2:N:0:TCTGCAAG+GCTGCACT
AGGTTGGCATATTTATGCCGACGATAATAAGCATGTCACCCTTAAGCACCGAGGTAAGCTCGGGACTT
ACAAGAGAGAGTGTGAGAGAGTCGCTCTCACTATTTTTGCCCAGAATG
+
FFFFFFFFFFFFFFFFFFFFFFFFFFFFFFFFFFFFFFFF:FFFFFFFFFFFFFFFFFFFFFFFFFFFF
FFFFFFFFFFFFFFFFFFFFFFFFFFFFF:FFFFFFFFFFFFFFFFFF
@A00721:190:HGJW7DSXY:4:2366:14281:17644_1:N:0:TCTGCAAG+GCTGCACT
ATGAGGTTAAAACTCTTGCTCCTTCTGTGGGTGAGGGTTTTTGTTATATTCACTGGTTCTGGGAGGCT
GCTTTCCTCCATAATTACAACCTCTCTTTAGAGAGCATTAGAAGGAAGTTGGGCAAATTCCCACATAT
GGT
+
FFFFFFFFFFFFF:FFFFFFFFFFFFFFFFFFFFFFFFFFFFFFFFFFFFFFFFFFFFFFFFFFFFFFF
FFFFFFFFFFFFFFFFFFFFFFFFF:FFFFFFFFFFFFFFFFFFFFFFFFFFFFFFFFF:FFFFFFFFF
FFF
@A00721:190:HGJW7DSXY:4:1126:2944:24408_2:N:0:TCTGCAAG+GCTGCACT
ACACTTTTCTTGAAAATTTTTCCTTTCCTCCGTCATTGGGGATTTCGATGTGAGAAGGTGGAAGGTGT
CAAGGGTCTTCTTCATGCTGATCTTTCAGGACCTAATCACATCATGAT
+
FFFFFFFFFFFFFFFFFFFFFFFFFFFFFFFFFFFFFFFFFFFFFFFFFFFFFFFFFFFFFFFFFFFFF
FFFFFFFFFFFFFFFFFFFFFFFFFFFFFFFFFFFFFFFFFFFFFFFF
@A00721:190:HGJW7DSXY:4:1558:21169:16423_1:N:0:TCTGCAAG+GCTGCACT
GCTCGTATCGAGTTGGAGGGAGGAAGTTCTCCGAAATTAAAGACGGGGGGATTAAGAGTCCAGCTGTA
ACTGTCATTTCAGAGAGGGGAGCTCTGAAAAGGAAACTTCGTCAGAAGATGAGGAAGTCTAAGAGGAG
GAG
```

+
F,F,:FFF,FFFF:::F,FFFF:FFFFF:F:,FFFFFF:FF,F:FFFFFFFFFFF::FFFFFF,FFF:
F,FFF,FFFF,FFF:FFFFF:FFFFFFFFF,FF,F,:FFFFFFFFFFFFF:FFFFFFFFFFFFFFF,F
FFF
@A00721:190:HGJW7DSXY:4:1504:19443:9768_1:N:0:TCTGCAAG+GCTGCACT
CTCTAGAGAAGATCTCACCACCTGGAGAAGGCAAGTGGAGAGTCCAGAAGTTGATGAAGAAGGATTAT
TTGAAGCAGGGGTTACCACTTCCTAAGGTGGATGAGGGAGGTTGTTTCTTTGGAGTTTTAGATAATCT
TTT
+
FFF:FFFFFFFFFFFF:FFFFFFFFFFFFFFF:FFFFFFFFFFFFFFFFFFFFFFFFFFFFFFFFFFF
FFFFFFFFFFFFFFFFFFFFFFFFFFFFFFFFFFFFFFFFFFFFFFFFFFFFFFFFFFFFFFFFFFFF
FFF
@A00721:190:HGJW7DSXY:4:2227:18159:8265_2:N:0:TCTGCAAG+GCTGCACT
CCTTGAGAAGACAAGCCACATTAAGATATAATTTAGGTCAACTTGGGGTTTCGAATGTCATTAGTGCC
TTACGATTGAAAGAGGTCCTTTTGAGTATCGACAGATCTTTCGATCTT
+
FFFFFFFFFFFFFFFFFFFFFFFFFFFFFFFFFFFFFFFFFFFFFFFFFFFFFFFFFFFFFFFFFFFF
FFFFFFFFFFFFFFFFFFF:FFFFFFFFFFFFFFFFFFFFFFFFFFFF
@A00721:190:HGJW7DSXY:4:2461:9670:14450_2:N:0:TCTGCAAG+GCTGCACT
GTGGCAGAATTCGGTAACTTACCATATTGTTCCTTCCGGGAAGTTGTGGGATTTAACTTTGACTCAAC
TACTCCGATCAGATCCGGATGGTTTGATTGGTATGGACTACTCGACTT
+
FFFFFFFFFFFFFFFFFFFFFFFF:FFFFFFFFFFFF,FFFFFFFFFFFFFFFFFFFFFFFFFFFFFF
:FFFFFFFFFFFFFFFFFFFFFFFFFFFFFFFFFFFFFFFFFFFFFFF
@A00721:190:HGJW7DSXY:4:1249:16767:17628_2:N:0:TCTGCAAG+GCTGCACT
TCATGGCAGATACTACCGATAAGGTGAAAAATATGCAGGTCAAAGATTCTGTAGCTCTGAGGGCGGTT
GAGGGAGCCGTTTTGGCCGACTTTAACTATTCTAAGAGTCTTTATGAT
+
FFFFFFFFFFFFFFFFFFFFFFF:FFFFFFFFFFFFFF:FFFF:FFFFFFFFFFFFFFFFFFFFFFFF
FFFFFFFFFFFFFFF:FFFFFFFFFFFFF:F:FFFFF,FFFFFFF:FF
@A00721:190:HGJW7DSXY:4:1122:12310:25535_2:N:0:TCTGCAAG+GCTGCACT
CGTTGTCGTACCTTACAAGGTTGAGGAGACTTTACAGCGGCTTATTGTCGCGCAGTTTCCAGAATTGC
GGTTGAAGTTCACCCATTCTATCTTGAATGCTCACGCCGTCGCAGCCA
+
FFFFFFFFFFFFFFFFFFFFFFFFFFFFFFFFFFFFFFFFFFFFF:FFFFFFFFFFFFFFFFFFFFFF
FFF:FFFFFFFFFFFFFFFFFFFFFFFFFFFFFFFFFFFF,FFFFFFFF
@A00721:190:HGJW7DSXY:4:2619:21124:27931_2:N:0:TCTGCAAG+GCTGCACT
TTATAATCACTTGTTAAAGTACAGATTTAGTGATTTAGATATTATTGATATTGGAGGCGATATTCGTT
TTCATATCCAAAACTCCTTGAATGGAGTACATATTTGCAACCCTATTC
+
FFFFFFFFFFFFFFFFFFFFFFFFFFF:FFFFFF:FFFFFFFFFFFFFFF,FFFFFFFFFFFFFFFFF
FFFFFFFFFFFFFFFFFFFFFFFF:FFFFFFFFFFFFFFFFFFFFFFFF
@A00721:190:HGJW7DSXY:4:2604:22978:30545_1:N:0:TCTGCAAG+GCTGCACT
GATGCATCGAGGTATACGTCTAGGAACATTGATTGGGAACGTACCTACTCACGAGACGTCATGAAAGC
TATCGGAGGGAAGCAAAATGTTTCGTTCTGTTACAAGAACGCTTTGGATTGTGACCATAAAGCCTCAG
TTG
+
FFFFFFFFFFFFFFFFFFFFFFFFFFFFFFFFFFFFFFFFFFFFF:FFFFFFFFFFFFFFFFFFFFFF
:FFFFFFFFFFFFFFFFFFFFFFFFFFFFFFFFFFFFFFFFF:FFFFFFFFFFFFFFFFFFFFFFFFF
FFF
@A00721:190:HGJW7DSXY:4:1656:1145:32597_1:N:0:TCTGCAAG+GCTGCACT
CTCTCTTGATGATGTAGCCACTATAATGCTTAAGAGAGAGATTGATGTATGTCATTTCGTCTTAGTTT
GTCCCGGCGAATTATTGGACACGATGAAAAATGACATACCCGTTCCTGACCTTGGTATCGTTATCCGT
CAT

```
+
FFFFFFFFFFFFFFFFFFFFFFFFFFFFFFFFFFFFFFFFFFFFFFFFFFFFFFFFFFFFFFFFFFFFFF
FFFFFFFFFF:FFFF:FFFFFFFFFFFFFFFFFFFFFFFFFFFFFFFFFFFFFFFFFFFFFFFFFFFFF
FFF
@A00721:190:HGJW7DSXY:4:2636:24153:18239_2:N:0:TCTGCAAG+GCTGCACT
GGTTATTCTCACAACAAAGATAAGATTTTATCTTTCTTCAAACACCCTGTTTTCCTTAAGGGCGGACA
ATTGTTCCATTGTGAACTCTTGGGGACTAGATTGAGCATTAATGAGTT
+
FFFFFFFFFFFFFFFFFFFFFFFFFFFFFFFFFFFFFFFFFFFFFFFFFFFFFFFFFFFFFFFFFFFFFF
FFFFFFFFF:FFFFFFFFFFFFFFFFFFFFFFFFFFFFFFF:FFFFFF
@A00721:190:HGJW7DSXY:4:2627:1642:19993_1:N:0:TCTGCAAG+GCTGCACT
TCTCTAGTGGACAATTCAGAATACCTATTTCCGTACCAAGAGCCACTGCCAATCTCATCGCTATACGC
ATACCTGATTGGAAGAAGCGAGGTTCGTTCCTCAATAAAAATTTTTCTCAAGAGATTATGGTTGATAA
AGT
+
:FFFFFFFF:FFFF:FFFFFF,FFFFF:FFFFFFFFFFFFFFFFFFFFFFFFFFFFFFFFFFFFFFFF
FFFFFFFFFFFFFFFFFFFFFFFFFFFFFFFFFFFFFFFFFFFFFFFFFFFFFFFFFFFFFFFFFFFFFF
FFF
@A00721:190:HGJW7DSXY:4:2617:12391:32972_1:N:0:TCTGCAAG+GCTGCACT
CGGCATACGAGATCTTGCAGAGTGACTGGAGTTCAGACGTGTGCTCTTCCGATCTGTTACGACATGGC
TTCGTAACAACAGTGCGAGGATTGTCATCTCGGGGAAAGTGATTCACCAAGATGTTCATATAGATACG
AGA
+
FFF,FFFFFFFFFFFFFFFFFFFFFFFFFFFFFFFFFFFFFFFFFFFFFFFFFFFFFFFFFFFFFFFFFF
FFFFFFFFFFFFFFFFFFFFFFFFFFFFFFFFFFFFFFFFFFFFFFFFFFFFFFFFFFFFFFFFFFFFFF
FFF
@A00721:190:HGJW7DSXY:4:2261:4237:6167_1:N:0:TCTGCAAG+GCTGCACT
AGGTCGCAGCGATCTTACTCGTTATCGGAGCTAAGAAGAAGACTTTGGGTACACGTTATGCTCGCAAA
CTTAACGTTAGTACCGGAGGTGATCTCTCTGCTTCTGCGTTACTGAGAGAAAAGATTCAGGACGCCTA
CG
+
FFFFFFFFFFFFFFFFFFFFFFFFFFFFFFFFFFFFFFFFFFFFFFFFFFFFFFFFFFFFFFFFFFFFFF
FFFFFFFFFFFFFFFFFFFFFFFFFFFFFFFFFFFFFFFFFFFFFFFFFFFFFFFFFFFFFFFFFFFFFF
FF
@A00721:190:HGJW7DSXY:4:2657:6470:7623_2:N:0:TCTGCAAG+GCTGCACT
TTTAGGTTTAGTAGGTGATCTTGACGTCGATTCTTTGTATAGGGAGTGTGAGAAGACAACGAATCTTG
AAACTACGGTCCTTATCAAAGAAAATGGTGAACCGGTCTCTAAATCTT
+
FFFFFF,:FFFFFFFFFFFF:FFFFFFFFFFFFFFFFFFFFF:FFFFFFFFFFFFFF::FFF,FFF:
FFFFFFFFFFFFFFFFFFF:FFFFFF:FFFFFFF:FFFFFF:FFFF:F
@A00721:190:HGJW7DSXY:4:2629:12816:30953_1:N:0:TCTGCAAG+GCTGCACT
GATTGAAGAACACGTCAAGAAGTCGCTCGGCGTTGTTCTTGAAAACCAAGTTAAGAAGGAGGCTGATC
ATGGAGTCTTTGTACCTTATGTCAAACTTCGAGGTTCAGCTACGAGAGGTGATCCCGGATGTGGATTG
AAG
+
FFF,:FFFFF,F,FF:F:,FFFFFFFF,:F:FFF,FF,FFFFFFF,FF:,,FF::F:FF:F,:F:F,:
FF::F,F,F:FFFF::FF,FFF,FFFFFFFFFFFFFFFFFFFFF:FF,FFFFFFFFFFF:F:FFFFF
FF,
@A00721:190:HGJW7DSXY:4:2130:7292:22294_2:N:0:TCTGCAAG+GCTGCACT
CTTTTTGAATGTTGTAAAGAATTGGGTTGAGAAGTGTGCTTTACAAGGAGTTAGACTCTTCTTCAAGT
TTTTAGATCTTCTTGATAATCTTAAAGAAAAATATTAGAATAAAATAA
+
FFFFF:FFFFF:FFFFFF:FFF,F:F,FFFFFF:,::FF,FFF:FF,:F:FFF:F,F::FFF,,FF,F
,:FFF,F:,::,::,F,FF::,,FFF,FFFFF,,,F,,FF:FFF,,FF
```

```
@A00721:190:HGJW7DSXY:4:1548:29894:11365_1:N:0:TCTGCAAG+GCTGCACT
GTTTCTCTTGGTATTTCTGAAATAGTTTATCAGAGCGTCATTTTCCTTTGGGATCTTTCCTCTGGTAA
GGGAATTATTAAATCATTCTTCTCACTCTTGACGAGAATTATTACTGTTTTCTCTACCCATAACTTCT
TTT
+
FFFFFFFFFF:FFFFFFFFFFFFFFFFFFFFFFFFFFFFFFFFFFFFFFFFFFFFFFFFFFFFFFFFFFF
FFFFFFFFFFFFFFFFFFFFFFFFFFFFFFFFFFFFFFFFFFFF:FFFFFFFFFFFFFFFFFFFFFFFFF
FFF
@A00721:190:HGJW7DSXY:4:1634:8712:2895_1:N:0:TCTGCAAG+GCTGCACT
ACTGGAGTTCAGACGTGTGCTCTTCCGATCTGTTTCAACACTTATCACCACTTTTGAAAGGAAAATAA
TTGGTGATGAAAATACGTATGTCGGTGGGGTTCTCAAGGCTCTCATCGCCACTGGGATTCTAAGGGGT
TCC
+
:FFFFFFFFFFFFFFFFFFFFFFFFFFFFFFFFFFFFFFFFFFFFFFFFFFFFFFFFFFFFFFFFFFFFF
FFFFFFFFFFFFFFFFFFFFFFFFFFFFFFFFFFFFFFFFFFFFFFFFFFFFFFFFFFFFFFFFFFFFFF
FFF
@A00721:190:HGJW7DSXY:4:2641:18240:14450_2:N:0:TCTGCAAG+GCTGCACT
TTCAATGATCAGGATTCTTCTTTTGGTCCTTACGTCTTAAGTAATTTGGATGTTGTCGAAAACTATCA
GGTTCTTGTCACGATTCTTAAAAGAAGTTTATTAAAACTTCAGAGTCT
+
FFFFFFF:FFFFFFFFFFFFFFFFFFFFFFFFFFFFFFFFFFFFFFFFFFFFFFFFFFFFFFFFFFFFFF
FFFFFFFFFFFFFFFFFFFFFFFF::FFFFFFFFFFFFFFFFFF,FFF
@A00721:190:HGJW7DSXY:4:1477:30165:10864_1:N:0:TCTGCAAG+GCTGCACT
GGTAGCTAATGTTCTTGCAATAGCCACAGACTTTCTGGGAAAAGAGACCTCTGTTGTAGAGGAGTTGA
GAGAATTACTCAGATTCAATCTGGCATCACTGAACGTCTACAACGAGTTAGGGAGTGGCTAGGTTTTA
AAA
+
FF:FFFFFFFFFF,FFFFFFFFFFFFFFFFFFFFFFFFFFFFFFFFFFFFFFFFFFFFFFFFFFFFFFFF
FFFFFFFFFFFFFFFFFFFFFFFFFFFFFFFFFFFFFFFFFFFFFFFFFFFFFFFFF:FFFFFFFFFFFF
FFF
@A00721:190:HGJW7DSXY:4:2102:31928:29888_1:N:0:TCTGCAAG+GCTGCACT
CAATACCTTCCACTGAGTTCATCACCCAGAGTTTCGACGTCGCGGAAAACGATGAAGATTTCCACACT
GTTCGAGAATCTTCTGATGCTGAATTTGAGGAAAATTTTTCGGAAGTTTTTGGTATAAATCCTGATGG
CTC
+
FFFFFFFF:FFFFFFFFFFF:FFFFFFFFFFFFFFFFFFFFFFFFFFFFFFFF:FFFFFFFFFFFFFFFF
FFFFFFFFFFFFFFFFFFFFFFFFFFFFFFFFFFFFFFFFFFFFFFFFFFFFFFFFFFFFFFFFFFFFFF
FFF
@A00721:190:HGJW7DSXY:4:2658:26169:31626_1:N:0:TCTGCAAG+GCTGCACT
TAAAACCTTCTTTTATCATTGCAACAGGACTTTGAGAAGACTGTTGAAATGGACAAAATCTGGGATCA
TAGGTATGTGTCACGCTACACTCGAAATATGGGCCCCTCATAAGGAAGTATAGTTGGCGTCTTGTCGTT
GAC
+
:F,FF:,FF:F:FF,F:,F:FF:,F:,FF,::FFFFFFF,F:FFFFFFFF,FF,:FFFF,FFF:::FF:,
F:FFF,FFFFF:F,:FF:,FFFF::,FFF:,,F:F:F,:F:,F:FFFFFFFFFF:F::F,FFF:,,FF
FFF
@A00721:190:HGJW7DSXY:4:1305:13973:25911_1:N:0:TCTGCAAG+GCTGCACT
GATCATTTGTTCAGGAAGATCTCTACAGAGATTATCGAGAGTTGTGATCTAGTTCTAAATTTAGGTCG
ACGGACTTTAGCAACTTTAGATAAATTCTTTCCGGGCTCAATGACCTTTGCTATGTACGTGGATCTAA
TGC
+
FFFFFFFFFFFFFFFFFFFFFFFF::FFFFFFFFFFFFFFFFFFFFFFFFFFFFFFFFFFFFFFFFFFFF
FFFFFFFF:FFFFFFFFFFFFFFFFFFFFFFFFFFFFFFFFFFFFFFFFFFFFFFFFFFFFFFFFFFFFF
FFF
```

```
@A00721:190:HGJW7DSXY:4:2476:12744:33144_1:N:0:TCTGCAAG+GCTGCACT
TCTGACCATCGCAAAGAAGACTTTATTAGTTCATGAAGTTCTTACACTTCTCGCGACTAATGTCAGCT
TCACTACAAAGGCGTTACGTAAAGTAGTTTTCACGCTTTATCTTAAAGCATTACCACCAAATTTAGCA
CTT
+
FFFFFFFFFFFFFFFFFFFFFFFFFFFFFFFFFFFFFFFFFFFFFFFFFFFFFFFFFFFFFFF:FFFF
FFFFFFFFFFFFFFFFFFFFFFFF:FFFFFFFFFFFFFFFFFFFFFFFFFFFFFFFFFFFFFFFFFFF
FFF
@A00721:190:HGJW7DSXY:4:1372:30454:7545_1:N:0:TCTGCAAG+GCTGCACT
CGTGTGCTCTTCCGATCTAGACATGCCAGTTAGAGTTCTTCTAACCAGGACTGATGGTGAGATTCTTT
TACCTTTTGGAGGTCCAGGAACCTTTACTCCTAGTGTCTTTTCTAGAGTCCAAGGACGTTTCCGTCGG
AGG
+
FFFFFFFF:FFFFF,FFFFFFFF:FFFFFFFFFFFFFFFFFFFFFF:FFFFFFFF:FFFFFFFFFFFF
FFFF:FFFFFFFFFFFFFFFFFFF:FFFFFFFFFFFFFFFFFFFFFFFFFFFFFFFFFFF:FFFFFFFF
FFF
@A00721:190:HGJW7DSXY:4:2671:28664:4946_2:N:0:TCTGCAAG+GCTGCACT
GACCGCTCAATAACACTCTATTGAATCCCTTTTCCACGATGAAAGCTCTTTATGAGGTGCTCCATGCA
AATTTACAGTTCCTCTTTGGTGATATCGCCTCTGTCCTTTCTCCTTTA
+
FFFFFFFFFFFFFFFFFFFFFFFFFFFFFFFFFFFFFFFFFFFFFFFFFFFFFFFFFFFFFFFFFFFF
FFFFFFFFFF:FFFFFFFFFFFFFFF:FFFFFF:FFFFFFFFF:FFFF
@A00721:190:HGJW7DSXY:4:1628:31873:29919_2:N:0:TCTGCAAG+GCTGCACT
ACTTGGGTTTATCGGTTGTATTTTGATAAATTTGTTGGAATCGATAATGATACTGACGAAGGTTCTAT
TACCACCCTTGTCATTGATACAAACATTCTAGGTGATGCTTTCTTAGA
+
FFFFFFFFFFFFFFFFFFFFFFFFF,FFFFFFFFFFFFF,FFF:F:FFFF:FFFFFFFFFFFFFFFFFF
FFFFFFFFFFFFFFFFFFFFFFFFFFFFFFFF:F:FFFFFFFFFFFFFF
@A00721:190:HGJW7DSXY:4:1103:4237:3192_2:N:0:TCTGCAAG+GCTGCACT
TACAATCTGTAGAGAGAGAGGTTAATCAGAATTTGAGTGATAACGATTTAGTACTGGGAAATGGTGGT
AGTCAGTTCACTACTTTTCTTTCCCTCCCCTTTTTCGATTGGATTCGA
+
FFFFFF:,,FFFFFF:FFFF,FF:F:FFFFFF,,,FFF:FFFF,FFFF,FFFF:F,FFFFFF:FF::F
F:FFFF:FFFF,F:FF,,F:FF,:FFF:FFFFF:FF:FFFFFFFFFFF
@A00721:190:HGJW7DSXY:4:2253:25301:24330_2:N:0:TCTGCAAG+GCTGCACT
TTGATAAGGTTTTTCTTTTATGGGACTCTTGTTTGTACTTATGAAGGAGGTATTACTTCGGAAGTTCT
TAATGTAGGTCTCCTAGTACTTAAGGGTTTATGTTTCGGTTTCAGCTG
+
FFFFFFFFFFFFFFFFFFFFF:FFFFFFFFFFFFFFFFFFFFFFFF:FFFFFFFFFFFFFFFFFFFFF
FFFFFFFFFFFFFFFFFFFFFFFF:FF:FFF:FFFFFF:FFFFFFFFF,
@A00721:190:HGJW7DSXY:4:2244:16966:8766_2:N:0:TCTGCAAG+GCTGCACT
TGTTTCACATTGCGTGATCACTTCCCGTGGACTTGGGAAGAGATTAAACATAAAATCCAGCGATTCTA
TTATGTCGCTAAAGCCTGTCCAGTTGTCGATGATGTTCGGGCTGTGGG
+
FFFFFFFFFF:FFFFFFFFFFFFFFFFFFFFFFFFF:FF,FF:FFF:F:FFFFFFFFFFFFFFF:FFFF
FFFFFFFFFFFFFFFFFFFFFFFFF:FFFFFFFFFFFFFFFFFFFFFFF
@A00721:190:HGJW7DSXY:4:2502:31593:35540_2:N:0:TCTGCAAG+GCTGCACT
AAACTTACGACGATGTTCTCGCTCGCATTTCACAAGGATTACTAAGTCTGAGATCTGCCACTTCTAAT
TTTAGGAAACGTCGCGAAAAGAAAGTTCATGAGGATGTCTTGGTCGAA
+
FFFFFFFFFFFFFFFFFFFFFFFFFFFFFFFFFFFFFFFFFFFF,FFFFFFFFFFFFFF:FFFFFFFFF
FFFFFFFFFFFFFFFF::FFFFFFFFFFFFFFFFFFFFFFFFFFFFFF
@A00721:190:HGJW7DSXY:4:2649:22462:19194_2:N:0:TCTGCAAG+GCTGCACT
GAACTTCTAATAGAGGATCCTGATGACATTATTTCAACGGATACCGATCTCAGTTCTACCACAGAAAC
```

CGTCTCAGGAACTGTCTCCACTGATTCGGAATTAGAGAAAGACGACCT
+
FFFFFFFFFFFFFF:FFFFFFF,FFFFFFFFFFFFFFFFFF,FFF:FFFFFFF:FFF,FFFFF:FFFF
FFFFFFFFFFFFFFFFFFFFFFFFFFFF:FFFFFFFFFFFF:FFFFFF
@A00721:190:HGJW7DSXY:4:2111:3803:35665_1:N:0:TCTGCAAG+GCTGCACT
GACCTCTGAATTTTCAGGGTGCCCTGATGAGGACGTTGAAAGGGATAATTTCAACTCTGAGACTTCTA
AGAGTGTTTCAAAAGACCAGAAACGTAACAAGAAGAATAAAGGTAGGAAGGGGAGAAAACAGAACAAG
C
+
FFF:F,F,FFFF:F::FFFFFF::F:FF:FFF:::FFFFF:FF,FFFFFFFFFFFFFFFFFFFFFFFF:
FFFFF:FFFFFFFFFFFFFFFFFFFFFFFFFFFFF:FFFFFFFFFFFFFFFFFFFFFFFFFFFFFFFF
F
@A00721:190:HGJW7DSXY:4:1310:26793:23563_2:N:0:TCTGCAAG+GCTGCACT
TGGTTGCGGCTGATCGTACCCCAATTCCTGCTAGAAAGATCACTCTCGGCGATACCACTGATCTTCCA
GAGTTACGTAGTGGTCGTTCTTTTGGTGATTTTATCAAAGAGAAAGTT
+
FFFFFFFFFFFFF:FFFFFFFFFFFFFFFFFFFFFFFFFFFFFFFFFFFFFFFFFFFFFFFFFFFFFF
F:FFFFFFFFFFFFFFFFF,FFFFFFFFFFFFFFFFFFFFFFFFFFFFF
@A00721:190:HGJW7DSXY:4:1520:25970:5008_1:N:0:TCTGCAAG+GCTGCACT
CAAGTTAAACTTCGAACTGACCACTTACAAGAGTGTTGAAGTACCGCAACATGGATCAGTTTTCCAAA
AAGCTATTAATAGCAGTTGCGGGGCTATAATACCGCCTCCAGATTACTCTAAACTGAATCCACTCTCT
TGG
+
FFF,F,FFFFFF::::FF::,F,FFFFF:F,FF:FF,,:FFFFF:F,,,FFFFFF:,F:FFFFFF:,
FFF:FFFF,,F,,,FFFFFF:F:,,,FFF,FFFFFF,F,F,,:F:F:F::FFF,,::,F,FF:F::,F
FFF
@A00721:190:HGJW7DSXY:4:1506:25120:6386_2:N:0:TCTGCAAG+GCTGCACT
TTTGGAACTGTGTAATGTCCTACGTACTGTTCAAGGTGTTTGGGCTTCTCTAAAGAACAGCAACTTTC
AACATGAATTAGTTATTGAAAGTCTTGACCCTCACGTCTCACTTTATC
+
FFFFFFFFFFFFFFFFFFFFFFFFFFFFFFFFFF,FFFFFFFFFFFFFFFFFFFFFFFFFFFFFFFFFF
FFFFFFFFFFFFFFFFFFFFFFFFFFFFFFFFFFFFFFFFFFFFFFFF
@A00721:190:HGJW7DSXY:4:2635:16613:13479_2:N:0:TCTGCAAG+GCTGCACT
AGACCTTTCTTGGGGAGTAAAGAGGGTTTGAAAATTACGGAACCTTTCCTCGTTCTTCATGAAGATCT
TCGAGTGTTTTATAGTGAGTTATTCTTGCGAAGGTTCAAGTCGATTGA
+
FFFFFFFFFFFFFFFFFFFFFFFFFFFFFFFFFFFFFFFFFFFFFFFFFFFFFFFFFF:FFFFFFFFF
FFFFFFFFFFFFFFFFFFFFFFFFFFFFFFFFFFFFFFFFFFFFFFFF
@A00721:190:HGJW7DSXY:4:2642:17734:8688_2:N:0:TCTGCAAG+GCTGCACT
AATAAGAAGTTCTTCGCAGTTGAATCTCCACCAGGTGGAGGGAAGACCACTGAACTTATAGCGACTTT
TGTCGAATTTTTCGCAAAAGGTCTTGAAGTACGTATTGTCACTGCTAA
+
FFFFFFFFFFFFF,:FFFFFFFFFFFFFFFFFFFFFFFFFFFFFFFFFFFFFFFFFFFFFFFFFFFFF
FFFFFFFFFFFFFFFFFFFFFFFFFFFFFFFFFFFFFFFFFFFFFFFF
@A00721:190:HGJW7DSXY:4:2631:2754:11115_2:N:0:TCTGCAAG+GCTGCACT
TTGGCACTTCTAGTTCATCGCGGTATTTGTGAAATTAAGACAGACGTGAAAGTTATGATGAGTGATCG
TATTAGAACAGCTGATTCTTATTGCATGAACGTCGCTGAGGTCAAACC
+
FFFFFFFFFFFFFFFFF:FFFFFF:FFFFFFFFFFFFFFFFFFFFFFFFFFFFFFFFFFFFFFFFFFF
FFFFFFF:FFFFFFFFFFFFFFF,FFFFFFFFFFFFFFFFFFFFFFFFF
@A00721:190:HGJW7DSXY:4:1514:9833:3020_2:N:0:TCTGCAAG+GCTGCACT
TTCATCGACGAATTATTCATGATTCACGCTGGTCAGACCCTTTTTCTTACACATCTACTTAAGGCGGC
TTATACCATAGGTTACGGTGATTCCAATCAAATCGGATTCATCAATCG
+

FFFFFFFFFF:FFFFFFFFFFFFFFFFFFFFFFFFF:FFFFFFFFFFFFFFFFFFFFFFFFFFFF:FFFF
FFFFFFFFFFFFFFFFFFFF:FFFFFFFFFFFFF:FFFFFFFF:FFF:
@A00721:190:HGJW7DSXY:4:2177:4336:6120_2:N:0:TCTGCAAG+GCTGCACT
CAAGTCGCAAACTACTTCAACATCAACGACATCATTCTTGATGAGTGTCGTTCTTATCGAAGTCTCAG
TTATCGCTGCCCGGCCGACGTTTGTCGCGAACTCTCAGAGGTTTACGG
+
FFF,FFFFFFFFFFFFFFFFFFFFFFFFFFFFFFFFFFF:FFFFFFFFFFF:FFFFFFFFFFFFFFFFFF
FFFFFFFFFFFFF,FFFFFFFFFFFFFFFFFFFFFFFFFFFFFFF:F:
@A00721:190:HGJW7DSXY:4:1502:23041:5791_1:N:0:TCTGCAAG+GCTGCACT
TGGAAATTAGACCTATCAGATGTCTTGACGACTTCGAATTTGAGGACGGAGTTAAATATATTACTTTC
ACTCAAGGTGAAAAGTTTGAAATTAACAAAGTCCTTGGTAAGAGTAAGGTTAAATGTGAAGATGCTCA
GAC
+
FFFFFFFFFFFFFFFFFFFFFFFFFFFFFFFFFFFFFFFFFFFFFFFFFFFFFFFFFFFFFFFFFFFFFF
FFFFFFFFFFFFFFFFFFFFFFFFFFFFFFFFFFFFFFFFFFFFFFFFFFFFFFFFFFFFFFFFFFFFFF
FFF
@A00721:190:HGJW7DSXY:4:2432:31376:3129_2:N:0:TCTGCAAG+GCTGCACT
ACTTACCAGCACGTTCGGCTGGTTAGAATTAAACCTCAAGATGACCCTGTTTTTGCATCACAAACACA
TATTACCGTTGCTCTATCACGACACGTTGAATCTTTACGATACTTTGT
+
FFFFFFFFFFFFFFFFFFFFFFFFFFFFFFFFFFFFFFFFFFFFFFFFFFFFFFFFFFFFFFFFF:FFFF
FFFFFFFFFFFFFFFFFFFFFFFFFFFFF:FFFFFFFFFFFF:FFFFF
@A00721:190:HGJW7DSXY:4:2159:17870:27774_2:N:0:TCTGCAAG+GCTGCACT
AAACGATGCTACTTGTGAGAGGATCAATCACAGTAAGGAACTGTCACGTTTAGCACGGGAGGTTGCAG
ATTCAACCCCTTATATCGAGGCTATCGAGGACGATGAGTATCTTGAAG
+
FFFFFFFFFFFFFFFFFFFFFFFFFFFFFFFFFFFFFFFFFFFFFFFFFFFFFFFFFFFFFFFFFFFFFF
FFFFFFFFFFFFFFFFFFFFFFFFFFFFFFFFFFFFFFFFFFFFFFFF
@A00721:190:HGJW7DSXY:4:2221:32660:4726_1:N:0:TCTGCAAG+GCTGCACT
TAGAGGACCACCTGGATCTTCTACTGTCTCTGCCTTCCAAGATTTCTTAGAAGAGGTTATTCCTGGAA
CCACCTTAACGTCACTCGGTAATCTTTCAGCCGAATTAAGTACTAATGAGTTCAAGTCGGTTGTCGAT
CGA
+
FFFFFFF:FFFFFFFFFFF:FFFFFFFFFFFFFFFFFFFFFFFFFFFFFFFFFFFFFFFFFFFFFFFFFF
FFFFFFFFFFFFFFFFFFFFFFFFFFF:FFFFFFFFFFFFFFFFFFFFFFFFFFFFFFFFFFFFFFFFFF
FFF
@A00721:190:HGJW7DSXY:4:2521:1796:21981_2:N:0:TCTGCAAG+GCTGCACT
GGCTAATACCTTTATAGGTAAGAAGCCCCTTGAGCCGCGTAGTCAGCGCGTTTAGCTGTATCATCAGT
CAGAGTATACCCGATAGGGTACCGAGTCTCCAAGAGAATATTTATTCT
+
::F:FF:F:FFFFFFFFF,,FF:FFFF:FFFFFFFFF,FFFFFFFF:FFF:F,:,FFFF,,FF:FFF:F,
FFFFFFFFF:FF:F:FFFFF:FFFFFFFFF,F:F:FFFFF:,FFFFFF
@A00721:190:HGJW7DSXY:4:2242:14651:16689_2:N:0:TCTGCAAG+GCTGCACT
GTTTTCACAACGGAAAGACATATCGCACCCGAAATTTTTGGGCAGAATGCTCTTAACGCGGTTCTTGA
ACGGTGTTTTGATCAGTCTAAGTTGGATAAAGTTATAGGAGACTCAGT
+
F,F,FFFFFFFFFFFFFF:FFFF:FFF:FFFFFFFFFFFF::,FFF:FF:FFFF::F:::F,:,FFFFFF
F:F:FFF,FFFFFFF:F:F,:F:,:FFFFFFF::FFFF,FFFFFFFFF
@A00721:190:HGJW7DSXY:4:1242:27778:7983_2:N:0:TCTGCAAG+GCTGCACT
GCTCCTCAATTGATAGATTGGGTCAACAATAGGGACGCTAGTATACTTCGTAAAGTCGACAGCGAAAT
GGTCAATATTGATAATTGGACCGACTATATGACCTATTTTAAGCTCAT
+
FFFFFFFFFFFFFFFFFFFFFFFFFFFFF:FFFFFFFFFFFFFFFFFFFFFFFFFFFFFFFFFFFFFFFF
FFFFFFFFFFFFFFF:FFFFFFFFFFFFFFFFFFFFFFFFFFFFFFF,

```
@A00721:190:HGJW7DSXY:4:1368:6596:22842_2:N:0:TCTGCAAG+GCTGCACT
CTAAGGTTAAACTTGATGCTTCAGCCTTTTCGAAGCATAACCCCGCACAGAATATCGTTTATCACGCA
AAATTTATTAATGCAATTTTCGGACCTATCTTTAAGGTTCTTGTGAGG
+
FFFFFFF:FFFFFFFFFFFFFFFFFFFFFFFFFFFFFFFFFFFFFFFFFFFFFFFFFFFFFFFFFFFF
FFFFF:FFFFFFFFFFFFFFFFFFFFFFFFFF:FFF:F:FFF:FFFFFF
@A00721:190:HGJW7DSXY:4:2302:15899:21230_1:N:0:TCTGCAAG+GCTGCACT
TGTCTTTTACCCAACATTTTTATGTATACAGAGATGACCAGTGACGATTTCTCCCGGAAGCTCTTCTC
TCTCTTAGGAGGTCATGATGACTACTATAAGGGTGAAATCGATTTTTCGAAGTTTGACAAATCTCAAG
ATT
+
FFFFFFFFFFFFFFFFFFFFFFFFFFFFFFFFFFFFFFFFFFFFFFFFFFFFFFFFFFFFFFFFFFFF
FFFFFFFFFFFFFFFFFFFFFFFFFFFFFFFFFFFFFFFFFFFFFFFFFFFFFFFFFFFFFFFFFFFF
FFF
@A00721:190:HGJW7DSXY:4:2417:19162:24408_2:N:0:TCTGCAAG+GCTGCACT
AGCCTATGAGATAAAGATGTATTCTTATTTAGGCTTCGATGCTGACTTTCTTGAACTTTGGAGTGCCA
GTGAATATTTTTGTCACGCCAGGAGATCGGAAGAGCGTCGTGTAGGGA
+
FFF::FFFFFFFFF:F:FFF:FFFFFFF:FFFF:FFF:FFFF:FF,FFF::FFFFFFFFFFF:FF::F
FFFFFFFFFFFFFFFFFF,FFFF:FFF:FFFF:::FF,FFFFFFFF,FF
@A00721:190:HGJW7DSXY:4:1376:25328:7592_1:N:0:TCTGCAAG+GCTGCACT
TAACACATTCTTTGGTAATTGTATGATCACTCTTATGATGCTCTCTCTTTATTACGACCTTCGGAAGT
TTAGAGCCGTCGCTGTCGCCGGAGACGATTCGATCCTCTTTTCGGACGTACCGGTCCAGGATTATTCA
GAT
+
FFFFFFFFFFFFFFFFFF:FFFFFFFFFFFFFFFFFFFFFFFFFFFFFFFFFFFFFFFFFFFFFFFFF
FFFFFFFFFFFFFFFFFFFFFFFFFFFFFFFFFFFFFFFFFFFFFFFFFFFFFFFFFFFFFFFFFFFF
FFF
@A00721:190:HGJW7DSXY:4:2353:3106:27383_2:N:0:TCTGCAAG+GCTGCACT
CAGCGTACTTCTGCTCGCGATTTATTCTTTTTGTCAATGATTACATCTACTTCGTTCCTGATCCTTAC
AAGATCATCGTTAAGTTAGGTAAACCCCTAACCGATGTTACTCTTGTA
+
FFFFF:FFFFFFFFFFFFFF:FFFFFFFFFFFFFFFFFFFFFFFFFFFFFFFFFFFFFFFFFFFFFFF
FFFFFFFFFFFFFFF:F,FFFF:FFFFFFFFFFFFFFFFFFFF:FFFF
@A00721:190:HGJW7DSXY:4:2315:13187:32252_1:N:0:TCTGCAAG+GCTGCACT
TTTAAAGACTATACTAAATATCTCCACTACGTGGTTGTTGTTTGGCGATTAGCTCTTGCGACTAATAT
TCGATATTCGATTGAGGGTCGTTATACCTATTCGGCTATCTGTGCTTTGAATTGTATAAGATCAAATT
TTA
+
FFFFF::F:FFFF,FFF,FF:,FF,F,F,F,,F,FFFFF,FFFFF:,,FF,F,::FFFF:F,FF:FFF
F,,FF,F::,:FF,,FF:F,FFF:FFF::,F:,F:,F,::F:FF,FFF:F,FF,FFFF,,F:,FF,FF
FFF
@A00721:190:HGJW7DSXY:4:1378:10845:5181_2:N:0:TCTGCAAG+GCTGCACT
TGATGTGGAGGGAGTTCTTGAAGCATCCCGACCTTTCCTTGTGAAAATTTTGATGCGGTTTCATAAGA
TTATCAGTAATCCTGATTCTACTACGGATTCATGGACCTTCCATCCTG
+
F:F,FF:FFFFFF:FFFFFFFF:F,FFFFFFFFFFFFFFFFFFFFFFFFFFFFFFFFF:FFFFFFFFFF
FFFFFFFFFFFFFFFF:FFFF:F,FFF:FF:FFFF::,FF::,FFFF:
@A00721:190:HGJW7DSXY:4:2105:8938:24330_2:N:0:TCTGCAAG+GCTGCACT
GACGTATCTCAAAGATCGACTTCGAGACAGGTGATATTGTTGAGCGAGAGCGACCAAGATCTAAATTC
GGTAACGGTGCGTGGCGCTTTATTTTAAAAAGCTCCCTCCTCTCCCAA
+
FFFFFFFFFFFFFFFFFFFFFFFFFFFFFFFFFFFFFFFFFFFFFFFFFFFFFFFFFFFFFFFFFFFF
FFFFFFFFFFFFFFFFFFFFFFFFFFFFFFFFFFFFFFFFFFFFFFFF
```

```
@A00721:190:HGJW7DSXY:4:1533:27697:20588_2:N:0:TCTGCAAG+GCTGCACT
ACTTGAGTCTTTTGATCAGTCTCTGACTTGACCATCTTTCATTTTAAATGACGATCATTAAGACTCTC
GTTCTTCTTTGTTTCCTTCTTTGCACTGATTTTTCCTCATCAGCTTGT
+
FFFFFFFFFFF:FFFFFFFFFFFFFFF:FFFFFFFFFFFFFFFFFFF:FFFFFF:FFFFF:FFFFFFF
,:FFFFF:,FFFFFFFF:FFFFFFFF:FFFFFF:FF:FF,FFFF,:FF
@A00721:190:HGJW7DSXY:4:2567:7111:12868_1:N:0:TCTGCAAG+GCTGCACT
GTTTTTACGATAACAACTATCAGTTGATTAAGACTCTTCCGAAAGGCGAACTTTGGGAATTTGAAGCT
CAGGGTGAAGCACAACGTTTGACCGTGACGAACTGTGGTGTTCTTGATCTACGTTACGGATATACTCT
CCT
+
FFFFFFFFFFFFFFFFFFFFFFFFFFFFFFFFFFFFFFFFFFFFFFFFFFFFFFFFFFFFFFFFFFF:
FFFFFFFFFFFFFFFFFFFFFFFFFFFFFFFFFFFFFFFFFFFFFFFFFFFFFFFFFFFFFFFFFFFF
FFF
@A00721:190:HGJW7DSXY:4:1465:30011:5744_1:N:0:TCTGCAAG+GCTGCACT
CACTACCACTTATTACGATATTTCTTTAGTCGATGGGTTTTCGACGCCTTTAAGCGTCTTCTGTTCAT
CGCAACCAGATGATATCTTCTATTCGAACTTATGTTTCGATCAGTGTTTCTTCTATCAGGAGGAAAAC
ACT
+
FFFFFFFFF:FFFFFFFFFFFFFFFFFFFFFFFFFFFFFFFFFFFFFFFFFFFFFF:FFFFFFFFFFF
FFFFFFFFF:FFFFFFFFFFFFFFFFFFFF:FFFFFFFFFFFFFFFFFFFFFFFFFFFFFFFFFFFFF
FFF
@A00721:190:HGJW7DSXY:4:1358:28348:18584_2:N:0:TCTGCAAG+GCTGCACT
TCTTCTAATGTGGCCTGAACTACGTTTCACTTTGTGTGTACTTGTCTCTATTTTAAGTTTTGTCTTCC
TTGTTTGTCTTATTCTATTAATCACTCATTTTAGTGAGATTATTAAAA
+
FFFFFFFFFFFFFFFFFFFFFF:FFFFFFFFFF:FFFFF:FFFFFFFFFFFFFFFFFFFFFFFFFFFFFF
FFFFFFFFFFFFFFFFFFFFFFFFFFFFF:FFFFFFFFFFFFFFFFFF
@A00721:190:HGJW7DSXY:4:1227:24985:10441_2:N:0:TCTGCAAG+GCTGCACT
CGACAACAGCAGGGGTTTTCGAATAGTGATAGCCACCCGCCTTACAGAGGGGCAGCAGCATGACCATA
ATTGGTATTGACTTTGGTACCACCTTTTCTACTGTCTGTCTCGCCTCT
+
FFFFFFFFFFFFFFFFFFFFFFFFFFFFFFFFFFFFFFFFFFFFFFFFFFFFFFFFFFFFFFFFFFFF
FFFFFFFFFFFFFFF:FFFFFFFFFFFFFFFFFFFFFFFFFFFFFFFF
@A00721:190:HGJW7DSXY:4:1606:4652:34068_1:N:0:TCTGCAAG+GCTGCACT
CGTGTGCTCTTCCGATCTGTGAATTTATACCTACAATCATTGGTTTTTCTAATAATGATGACACTATT
GTTTATGGATATGATGCTATTACTCATGATGGTTACGGTCAGGGAGGATTCTCAGTTTACAAGGACCT
TAA
+
FFFFFFFFFFFFFFFFFFFFFFFFFFFFFFFFFFFFFFFFFFFFFFFFFFFFFFFFFFFFFFFFFFFF
FFFFFFFFFFFFFFFFFFFFFFFFFFFFFFFFFFFFFFFFFFFFFFFFFFFFFFFFFFFFFFFFFFFF
FFF
@A00721:190:HGJW7DSXY:4:1566:26368:11303_2:N:0:TCTGCAAG+GCTGCACT
CCGAAAGACGTGCTAAATTAAAACCATTGTACGATGTCGTCTGTCTCGGCGATCACTTTTCACTAAGA
ATTGGTCCTACTTTTGGACAAGGCCAACTTCGTAATCTTCATGAGATC
+
FFFFFFFFFFFFFFFFFFFFFFFFFFFFFFFFFFFFFFFFFFFFFFFFFFFFFFFFFFFFFFFFFFFF
FFFFFFFFFFFFFFFFFFFFFFFFFFFFFFFFFFFFFFFFFFFFFFFF
@A00721:190:HGJW7DSXY:4:1162:13169:9987_1:N:0:TCTGCAAG+GCTGCACT
TTAGGGATTTTGAGAGAGTGAAAGGCTTCAATTGTAGTGGTTTGGTCGTTTCAGTACCCAGTCAGTAT
ACCTCTACGCAAAGATATTTCATGAAAACTACAGAGGAACGTACCGGATTACCTGTCTTTCATATTAT
GAA
+
FFFFFFFFFFFFFFFFFFFFFFFFFFFFFFFFFFFFFFFFF:FFFFFFFFFFFFF:F:FFFFFFFFFF
```

FFFFFFFF:FFFFFFFFFFFFFFFFFFFF,FFFFFFFFFFFF:FFFFFF:FFFFFFFFFFFFFFFFFF
FFF
@A00721:190:HGJW7DSXY:4:1666:3251:33395_2:N:0:TCTGCAAG+GCTGCACT
TCTTTGCTTCGATGTTGGATGTCAAGAAGGGTTCAGAATGGGATTCCTACGTAGTTTATGACTTTGGT
GGTGGTACTTTCGATACTTCTATCGTCTTACGTTACGGTAATTACTAT
+
FFFFFFFFFFFFFF:FFFFFFFFFFFFFFFFFFFFFFFFFF:FFFFFFFFFFFFFFFFF:FFF:FFFFF
FFFFFFFFFFFFFFFFFFFFFFFFFFFFFFFFFFFFFFFFFFFFFFFF
@A00721:190:HGJW7DSXY:4:1159:27308:4351_1:N:0:TCTGCAAG+GCTGCACT
ATAATCTTGGAGGTCGTGACGTTGATGAAGCTATAAGAGCGTTCTTATTACGGCGCACTTCTTTACCC
GCCACCACACAACTTTCAGTTTCACAACTGAAGGAGGAGGTTTCTGTTACTGGAAAACCCTCGTCAAT
ACT
+
FFFFFFFFFFFFFFFFFFFFFFFFFFFFFFFFFFFFFFFFFFFFF:FFFFFFFFFFFFFFFFFFFFFFFFF
FFFFFFFFFFFFFFFFFFFFFFFFFFFFFFFFFFFFFFFFFFFFFFFFFFFFFFFFFFFFFFFFFFFF
FFF
@A00721:190:HGJW7DSXY:4:2246:29116:2879_1:N:0:TCTGCAAG+GCTGCACT
TCTTATGACGATCTAGTGAAATTAACGCGTCCTTTTCTGGAACGTAGTTTTACTATTATGAACAAAGT
TATCAAAGATAGTGGCATTCAAGGCAAACTTACCCTGATTCCGGCCGGTGGTAGCTCATTTCTACCAG
GGG
+
FF:FFFFFFFFF:FFFFFFFFFFFFFFFFFFFFFFFFFFFFFFFFFFFFFFFFFFFFFFFFFFFFFFF
FFFFFFFFFFFFFFFFFFFFFFFFFFFFFF:FFFFFFFFFFFFFFFFF,FFFFFFFFFFFFFF:FFFF
FFF
@A00721:190:HGJW7DSXY:4:1136:12084:4319_2:N:0:TCTGCAAG+GCTGCACT
TGCTGAAACAGCTATTAAAGCTGTTGAGCGGGTAATTACACCTAAACGAGCTCGATCTGCCATCGCTG
AAGGATGTGCACTCTTTTCTGCTACTTTAACGGAGCGTAGTGTACTCT
+
FFFFFFFFFFFFFFFFFFFFFFFFFFFFFFFFFFFFFFFFFFFFFF:FFFFFFFFFFFFFFFFFFFFFF
FFFFFFFFFFFFFFF:FFFFFFFFFFFFFFFFFFFFFFFFFFFFFFFF
@A00721:190:HGJW7DSXY:4:1522:12029:28432_1:N:0:TCTGCAAG+GCTGCACT
CAACAGTAACATTACTTCTAATAAAGGTCTCTATCAAGCTCGTATTATCGTCGCTGCTGGATCTGCGT
TACCGTTGAAGGCTAAACTCCAAGGTAATTATGAAGACTGTGATTTATCTTTCACTCTGGCCTTGTAT
GAG
+
F,:FF:FFF,FFFFFFFFF:FFFFFFFF:F:FFFFF:FFF:FFFFFFFFFF:FFFFFFFFFFF:FFF
F,FFFFFFFFF,FFFFFFFF:FFFFFFFFFFFFFFFFFFFFFFFFFFFFFFFFFFFFFFFFFFFFF:FF
F:F
@A00721:190:HGJW7DSXY:4:1144:29731:37043_2:N:0:TCTGCAAG+GCTGCACT
CGTAACATTAATAATTTCAATCTTCTTAAAAAGAAGATAGAGAGTCATACTTTGGGCTATCGTTCAGG
TGCATGGGGTTGGATAGCCAATATTGAAGTGGACACCTCTGGTAGTAT
+
FFFFFFF:FFFFFFFFFFFFFFFFFFFFFFFFFFFFFFFFFFFFFFFFFFFFFFFFFF:FFFFFFFFF
FFFF:FFFFFFFFFFFFFFFF:FFFFFFFFFFFFFFFFFFFFFFFFFFF
@A00721:190:HGJW7DSXY:4:2247:29803:17315_1:N:0:TCTGCAAG+GCTGCACT
ATACTATGGTTGAATCAACTAAGCATACTACTTTTGCTTTGATTACTCTACCGCCTTACAAGTCTAAG
GTTAAAGAGTCGATTAAACAAGTTGTACTTATCAATGATTTTATCGCTGGTTGTTATCTCGAAAAGTC
TCT
+
FFFFFFFF:FFFFFFFFFFFFFFFFFFFFFFFFFFFFFFFFF:FFFFFFFFFFFFF:FFFFFFFFFFFFF
FFFFFFFFFFFFFFFFFFFFFFFFFFFFFFFFFFFFFFFFFFFFFFFFFFFFFFFFFFFFFFFFFFFF
FFF
@A00721:190:HGJW7DSXY:4:2372:12183:25316_1:N:0:TCTGCAAG+GCTGCACT
ACCCAAAGAGAATATCTAAAATATTTACAAAATCTTACCACTATCTACGAACCTAAAGTTCTGAAGAC

```
GTTGCTTGTCGCTTCCTCCTTTAATGGCACTACCTCGGTACAATCTTACTTGGAACGAAGGCGACCTG
TTC
+
FFFFFF:FFFFFFFFFFFFFFFFFFFFFFFFFFFFFFFFFFF:FFFFFFFFFFFFFFFFFFFFFFFFF
FFFFFFFFFFFF:FFFFFFFFFFFFFFFFF:FFFFFFFFFFF:FFFFFFFFFFFFFFFFFFFFFFFFF
FFF
@A00721:190:HGJW7DSXY:4:1622:32931:35321_1:N:0:TCTGCAAG+GCTGCACT
TGTTCTGTCTATATTAGGTGAAGCGAAATACGATTTCGAACGTCAACGTTCACCGAGTTTCGTTTCGT
ACCTTGATGCTGACTTTCGTCAATATGATTTGATCTGGTTGCTCAATTCTAAGCGTGTTCCGCTTAGT
GCG
+
FFFFFFFFFFFFFFFFFFFFFFFFFFFFFFFFFFFFFFFFFFFFFFFFFFFFFFFFFFFFFFFFFFFF
FFFFFFFFFFFFFFFFFFFFFFFFFFFFFFFFFFFFFFFFFFFFFFFFF:FFFFFF,FFFFFFFFFFF
FFF
@A00721:190:HGJW7DSXY:4:2224:31575:4507_2:N:0:TCTGCAAG+GCTGCACT
GAGTATTGCTATTTATACGGTTACGAGAAGAAGGTTCGAAAGTTTGAGAACTTAGTTTACGAACTTGA
CTCTACTATACGTGACATACTAAATACTAGATATTTGGATGATCTTGA
+
FFFFFFFFFFFFFFFFFFFFFFFFFFFFFFFFFFFFFFFFFFFFFFFFFFFFFFFFFFFFFFFFFFFF
FFFFFFFFFFFFFFFFFFFFFFFFFFFFFFFFFFFFFFFF:FFFFFFF
@A00721:190:HGJW7DSXY:4:2273:26530:32690_2:N:0:TCTGCAAG+GCTGCACT
CATGAATTTTCAGAGGATGAAAAATTTTCTTCCTTGGATTTTAATATAAAGAATCTTTACCAGGACTC
CTACTACAGGAGGCAGTCTTCTGATCGTAAGAAGTTTATAAGGAACAT
+
FFFFFFFFFFFFFFFFFFFFFFFFFFFFFFFFFFFFFFFFFFFF:FFFFFFFFFFFFFFFFFFFFFFF
FFFFFFFFFFFFFFFFFFFFFFFFFFFFFFFFFFFFFFFFFFFFFFFF
@A00721:190:HGJW7DSXY:4:2502:17499:7686_1:N:0:TCTGCAAG+GCTGCACT
ACTCGAAACTCTAAGTGGTAATACTGTCTTTCCAAGCTTACCTGTATCTTCAATGAAATTGGACTCTT
CGATAGGGGACATTTTATCGTGGGCTTTCCGTAAAGGAAGATCTGTTATCGACGGTTTATCGTTTACG
AAG
+
FFFFFFFFFFFFFFFFFFFFFFFFFFFFFFFFFFFFFFFFFFFFFFFFFFFFFFFF:FFFFFFFFFF:
FFFFFFFFFFFFFFFFFFFFFFFFFFFFFFFFFFFFFFFFFFFFFFFFFFFFFFFFFFFFFFFFFFFF
FFF
@A00721:190:HGJW7DSXY:4:1647:16640:15624_1:N:0:TCTGCAAG+GCTGCACT
TAAGAATACTATAAATAGAATAGCTATTACTACTAATGGCGTTGGCCTAGTGGATGTTATTAGTGACA
TCTGGTCAATTTCGTTTTTAGGTGGTCTTTATTCTATTATTGATTTTTATGAGATTGAGATCGGAAGA
GCA
+
FFFFFFFFFFFFFFFFFFFFFFFFFFFFFFFFFFFFFFFFFFFFFFFFFFFFFFFF:FFFFFFFFFFF
FFFFFFFFFFFFFFFFFFFFFFFFFFFFFFF,F:FFFFFFFFFFFFFFFFFFFFFF,:FFFFFFFFFFFF
FFF
@A00721:190:HGJW7DSXY:4:1149:26784:1908_2:N:0:TCTGCAAG+GCTGCACT
TTCCTGAAGAGTCCTTCCGTAACCAACTTCTTGACAATTTTCTTCAATCCTTCGATAATAGTTAAATC
TACGATTATGTCTGTGTTGAAAGACTTTCTACCGATAAGGGTCACTAG
+
FFFFFFFFFFF:FFFFFFFFF:FFFFFFFFFFFFFFFFFFFFFFFFFFFFFFFFFFFFFFFFFF,FF:
FFFFF::FFFFFFFFFFF:FFFFFFFFFFFF:FFFFFFFFFFFFFFFF
@A00721:190:HGJW7DSXY:4:2412:5394:26177_2:N:0:TCTGCAAG+GCTGCACT
TAAGAAATAATCCTACATTGACTGATTATCTAAAGATTGAAGGAAAATTTTATCCTCGTTTAACTTAC
TTAGACTATCCTACGATACAAATGATAACTTCTAATTTACGTTTGCTA
+
FFF,FF:FFFFF,,FFF:FFFFFFFFFFFFFFFF:F:F:FF:,,FF:,F,FFFF,F::FF:FFF,FFF
F:FFFFF,FFFFFFF:FF,,FF::FFFFF:FFFFFF,FFFFF,FF:::
```

```
@A00721:190:HGJW7DSXY:4:1221:3396:11663_2:N:0:TCTGCAAG+GCTGCACT
AAGGGATGCATGGTGTTATCTCCTCAGGAGTCCCGGCATTTGATAATAATGACGAGACTTTCGTCATA
ATCGCTGCATATTACGGATTACACGGTACCAGCGCATCTCGTGTCGAG
+
FFFFFFFFFFFFFFFFFFFFFFFFFFFFFFFFFFFFFFFFFFFFFFFFFFFFFFFFFFFFFFFFFFFF
FFFFFFFFFFFFFFFFFFFFFFFFFFFFFFFFFFFFFFFFFFFFFFFF
@A00721:190:HGJW7DSXY:4:2130:24668:4194_1:N:0:TCTGCAAG+GCTGCACT
TGAAATTTATAGAAGATTATTTGACAGAATCAATAAGAATCATCGATTCTCGATTCGTAGAGCTTAT
CTAGGTACTCTCATTTACTATGTTGATTTAGTCTACATTTATGCTGATCTAAAATTTCGATCTAGATG
GAT
+
:,FFF:FFFFFFFF:,FFFF,:FFFFF:F:FFFFFFFFF,FFFF:FFFFFFFFFFFFFFFFFFFFFFF
FFFFFFFFFFFFFFFFFFFFFFFFFFFFFFFFFFFFFFFFFFFFFFFFFFFFFFFFFFFFFFFFFFFF
FFF
@A00721:190:HGJW7DSXY:4:1319:17508:24455_2:N:0:TCTGCAAG+GCTGCACT
TTTACAACGATATAGGAGTAATCTACGAGAAGATTCTAGAATTCTTAATTATAATATTCAACCTCAAA
AGTTACGTGTTTACAGAAACGAGATCACACATTATGATAACTAACTAT
+
F,FFFFFFFFFFFFFFF:FFF:F,F,:FFFF,FFFF,F,FF,FF,:FFF,F,FFFF,FFFFFFFFFFF
F,,,F,,F:FFFFFF,,FFF,F:F,,F:FFFF,,,,F,FFF,FF,FF,
@A00721:190:HGJW7DSXY:4:1371:27299:28541_1:N:0:TCTGCAAG+GCTGCACT
TCTCTTCTTATAATAAATAAATAAATAATATTACTAATGGCAAATAGTGGAAAGGCTCCAGTCAACAC
TAGTTCGAGTGCTGCTTTGGATTACGATGACGTGAGAATAGGTGAAGGGATAGTTTTTACCTCGACCA
ACA
+
FFFFFFFFFFFFFFFFFFFFFFFF:FF,FFFF,FFFFFFFFFFFFFFFFFFFFFFFFFFFFFFFFFFF
FFFFFFFFFFFFFFFFFFFFFFFFFFFFFFFFFFFFFFFFFFFFFFFFFFFFFFFFFFFFFFFFFFFF
FFF
@A00721:190:HGJW7DSXY:4:1569:2917:16438_2:N:0:TCTGCAAG+GCTGCACT
GCGACTAATCTTAAAGCTTCACCCAAAATGAAGGACACTTCGAAACTGAATAATGATGCACTACAAAC
AAGTATCGCTAATCATCTGAAAAAGGCTCTAAAGGACACTATACAATA
+
FFFFFFFFFFFFFFFFFFFFFFFFFFFFFFFFFFFFFFFFFFFFFFFFFFFFFFFFFFFFFFFFFFFF
FFFFFFFFFFFFFFFFFFFFFFFFFFFFFFFFFFFFFFFFFFFFFFF:
@A00721:190:HGJW7DSXY:4:2430:32940:16235_2:N:0:TCTGCAAG+GCTGCACT
ATTCAGCACCACATTTTGGTCAAGTCAATCCGTTGAGAAAATTTCTTAGAGGATATTCGCCATTGTGG
TACGAATTGGTCCAACATTTACCACCAAATTCTTTTGATACAACTCTA
+
FFFFFFFFFFFFFFFFFFFFFFFFFFFFFFFFF,FFFFFFFFFFFFFFFFFFFFFFFFFFF:FFFFFF
FFFFFFFFFFFFFFFFFFFFFFFFFFFFFFFFFFFFFFFFF:FFFFFF
@A00721:190:HGJW7DSXY:4:1113:19714:16376_1:N:0:TCTGCAAG+GCTGCACT
ATACGCTTGCTATTCACCAGATTGGTTCGAACCCGTTGATGCTATGTCAGAAGAAGCTATTCAAGCTT
GTCGTTTAAAGACTGCAGAGGCTGTCAAACAAGCCCAGACTCAAGGAGTCCAATCAAAGGTTTTGAAT
TTA
+
FFFF:FFFF:FFFFFFFFFFFFFF:FFFFFFFFFFF:FFFFFFFFFFFFF:FFFFFFFFFFFFFFFFF
FFFF,FFFFFFFFFFFFFFFFFFFFFFFFFFFFFFFFFFFFFFFFFFFFFFFF:FFFFFFFFFFFFFF
FFF
@A00721:190:HGJW7DSXY:4:2424:2031:36010_2:N:0:TCTGCAAG+GCTGCACT
TGGGTGATAAAAAATTATTTAGAGATCTTTTCTACTTACAAAGGACAATTCAGTTTCATTAATTCTACG
GAAAAGTCCGGAGTATATTGTATGTTAATAGATAGTGGGATAATAGTT
+
FFFFFFFFFFFFF:FFFFFFFFFFFFFFFFFFFFFFFFF:FFF,FFFFFFFFFFFFFFFFFFFFFFFF
FF:FFFFFFFFFF:FFFFFFFF,FFFFFFFFFFFFFFFFFFFFFFFFF
```

@A00721:190:HGJW7DSXY:4:2242:25455:16172_2:N:0:TCTGCAAG+GCTGCACT
GAACGCGTTCGTATAACCAAGTACTTTCCGTCTGAAGAGTTTAGAGCTGCTATTGATTATGTAACCGA
TAGGAGTTGTATAGTTGAAGATGTGGCTTTAGACGATAATCCTTTAAG
+
FFFFFFFFFFFFFFFFFFFFFFFFFFFFFFFFFFFFFFFFFFFFFFFFFFF:FFFFFFFFFFFFFFFF
FFFFFFFFFFFFFFFFFFFFFFFFFFFFFFFFFFFFFFFFFFFF:FFFF
@A00721:190:HGJW7DSXY:4:1516:18882:22545_1:N:0:TCTGCAAG+GCTGCACT
GAAGATTTTCCCACAGATTTGTCTACGTTGCAGATCGCCACTCCAAACACTATATACATTATAAAATA
TAAAAATAAGGGAGTCATAACTTTTGTTACCAAATTCTTGGCTTTCAACGAAGTCTCTGATTTTATTA
ATC
+
FFFFFFFFFFFFF:FFFFFFFFFF:FFFFF:FFFF:FF:FFF:FFFF:FFF:FFFFF:FFFFFFFFFF
FFFFFFFFF::FFFFFFFFF:FFFFFFFFFFFFFFFFF,::FFFFFFFFFFFFFFFFFFFFFFFFFF:
FFF
@A00721:190:HGJW7DSXY:4:2211:29405:18349_2:N:0:TCTGCAAG+GCTGCACT
GATCTCTCGGTCATTTACTCTGTACTTGATCTACGTCGTTTAACTGTCGGAACAACCTTTACTTAGAA
TGACTCTATCTTTTCAGACACTTCTTATATTATTTACAATAGTTAATA
+
F::FFFFF::FFFFFFFFFFF:,FFF:FFFFFFFFFFFFFFFFF,FFFFFFFFFFFF::,FFFFFF::
F:F::FFFFF::FFFFFFFFFFFFFFFFFFFFFFFFF:FFF:FFFFFFFF
@A00721:190:HGJW7DSXY:4:1344:21052:24079_1:N:0:TCTGCAAG+GCTGCACT
AATACTCACTATCGTTTTCTGCTTTTTCTTAGCTAATAGATTAATAAAGGATGAGTACGAAAACCAAC
TCTTCGTTACGAGACCTGATGGTAGAATAGATTTAGTCTCTACACCCATCTCAACGAGGATAAAGCGA
GTT
+
FFFFFFFFFFFFFFFFFFFFFFFFFFFFFFFFFFFFFFFF:FFFFFFFFFFFFFFFF:FFFFFFFFFFFF
FFF:FFFFFFFFFFFFFFFFFFFFFFFFFFFFFFFFFFFFFFFFFFFFFFFFFFFFFFFFFFFFFFFFF
FFF
@A00721:190:HGJW7DSXY:4:2407:12102:7106_1:N:0:TCTGCAAG+GCTGCACT
ACACGAAGAGAACATCCGCAAACGACTCGAAACCGAGACGCCGTAGATCAAAGTCCCTTCTAACTTTA
GATTATGAATCCTGCCTTTACTCATTTTATAGCAGTTATTTATGTTGTACTTTTTATAATTTATAACC
TT
+
::::,,:,:,F,FF,FF,,:,FFFFF:FFFFFFFFFFFFFFFFFFFFFFFFFFFFFFFFFFFFFFFFF
FFFFFFFFFFFFFFFFFFFFFFFFFFFFFFFFFFFFFFFFFFFFFFFFFFFFFFFFFFFFFFFFFFFF
FF
@A00721:190:HGJW7DSXY:4:2136:11993:25989_1:N:0:TCTGCAAG+GCTGCACT
TACTTATTGTAACGATCATTCTTTCGAGTTTTGTCATTCTTATTCTTTTATTCCTTTAACTGTGTATG
AGGAGGATTTATAATGCTTTTCGATAGTGATTGTTATCAATTTAAATACATTACTGTTTCAAATCATC
TTT
+
FFFFFFFFFFFFFFFFF:FFFFFFFFFFFFFFFFFFFFFFFFFFFFFFFFFFFFFFFFFFFFFFFFFF
FFFFFFFFFFFFFFFFFF:FFFFFFFFFF:FFFFFFFFFFFF:FFFFFFFFFFFFFFFFFFFFFFFFFF
FFF
@A00721:190:HGJW7DSXY:4:2253:21947:11287_1:N:0:TCTGCAAG+GCTGCACT
GAAAATGGTTTCAAATGAGGTCAAAGCTCTATTTATTAAACGAACTATTATCCGATTCTTCGAGGGTA
TCACTACCACTGATCTGCCGACTTTAGTCAAGGAACTTCAATTCCTTCGATGTTTATATTTTTCTATA
AGG
+
FFFFFFFFFFFFFFFFFFFFFFFFFFFFFFFFFFFFFFFFFFFFFFFFFFFFFFFFFFFFFFFFFFFFFF
FFFFFFFFFFFFFFFFFFFFFFFFFFFFFFFFFFFFFFFFFFF,FF:FFFFFFFFFFFFFFFFFFFF,FF
FFF
@A00721:190:HGJW7DSXY:4:1249:16631:25222_2:N:0:TCTGCAAG+GCTGCACT
TAATCAAATTTCGACAAATTTTAGAACTTTATTTCAGAACTCTCTCCGATCACCTTGGTGTGATCTCC

CGGAGGATCTTTTCAATAAGATGTATGATGATAATCTACTATTTTTAA
+
FFFFFFFFFFFFFFF:FF:FFFFFFFFFFF:FFFFFFFFFFFFFFFFFFFFFFF,FFFFFFFFFFFF
FFFFFFFFFFFFFFFFFFFF:FFFFFFFFFFFFFFFFFFFFFFFFFFFF
@A00721:190:HGJW7DSXY:4:1635:30201:33223_1:N:0:TCTGCAAG+GCTGCACT
AGCGTATACTTGAATACTTTATCAAAGATTTGGCTTGGTCTATTTACCGCAAAAAGTTGGAAGAGGTC
TATGATATTTATTTTACTTATACTTTAAATAGCACCGATTATAACTTGACTGTTGATAATATTACAAT
AGC
+
FFF,FFFFFFFFFFFFFFF:FFFF:FFFFFFFFFFFFFFFFFFF,F:FFFFFFFFFFFFFFFFFFFFF
FFFFFFF:FFFFFFFFFFFFFFFFFFFFF:FFFFFFFFFFFFFFFFFFFFFFFFFF:FFFFFFFFFFFFFF
,FF
@A00721:190:HGJW7DSXY:4:1448:3812:10441_2:N:0:TCTGCAAG+GCTGCACT
AAGATTAGAGAAGTAACTCGGTGAACACGTCGGTAGCATTGATTTACTTTTACCGTTAGAACTGAAAA
AGATTCTTTTCGAAAAGAAACCTATAAGTTACAAACGATTCTTCGACT
+
FFFFFFFFFFFFFFFFFFFFFFFFFFFFFFFFFFF:FFFF:FFFFFFFFFFFFFFFFFFFFFFFFFFF
FFFFFFFFFFFFFFFFFFF,FFFFFF:FFFFFFFFFFFFFFFF:FFFF
@A00721:190:HGJW7DSXY:4:2111:4562:14246_1:N:0:TCTGCAAG+GCTGCACT
AATAAAAATACTTCAAGTACCTGAACTTTACATTTTTAATGGTATGGTATAATGTGTCTTACAAATAG
AACCTTCACTCTCGGTAATGAGAGTGTATTCCTTAATATTCTAGTAACCGCTATGTGGGCATTACTAG
GAT
+
FFFFFFFFFFFFFFFFFFFFFFFFFFFFFFFFFFFFFFFFFFFFFFFFFFFFFFFFFFFFFFFFFFFF
FFFFFFFFFFFFFFFFFFFFFFFFFFFFFFFFFFFFFFFFFFFFFFFFFFFFFFFFFFFFFFFFFFFF
FFF
@A00721:190:HGJW7DSXY:4:1311:23493:14810_2:N:0:TCTGCAAG+GCTGCACT
TTTAAATGCTAGGAAAATTTTAGAAAAACTCCATTGTATAGTTCAAGGAGTACTTAATTCAATAGCTT
TAGTATTATTTACTATCGCCTTTATTGAAAATTGTATAAGAGTTAATT
+
FFFFFFFFF,FFFFFFFFFFFFFFFFFFFF:FFF:FFFFFFFFFFFFFFFFFFFFFFFFFFFFFFFFF
FFFFFFFFFFFFFFFF:,FFFFFFFFF:FFFFFFFFFFF:FFFFFF:F
@A00721:190:HGJW7DSXY:4:2122:11252:34475_1:N:0:TCTGCAAG+GCTGCACT
GGGAGAATGAACTGCCGCTTCCCGGAAGAAACGATCTATTTACAGAGTATTTAGAGTAGTCCAAAGTT
TGTATAAATTTATATGATTTTATATATATATACCACATATATATATATATAAAATCCTATAAATTTGT
ACA
+
F:FF,FFF:F,F:,FFF,F,FFF:::F,FF,,:F,FF:F::F:F,,,FFFFFFFF,F:,,,FFF,FFF
F,FF,FFFFFFFF::,FFFF:,FFFFFFFF:F:FF,FFFFFFFFFFFFFFFFFFFFF:FFFFFFFFFF
FFF
@A00721:190:HGJW7DSXY:4:1362:22399:29042_2:N:0:TCTGCAAG+GCTGCACT
TACACTCCCCAAACATAACATACCATAACCTACCATGACCAATCATCGCTCCAACTGGCACGCCAGAC
GTAGGGTCAAACCTACTACCGCCTGTCGGCAGATCGGCACCACTGGTC
+
FFFFFFFFFFFFFFFFFFFFFFFFFFFFFFFFFFFFFFFFFFFFFFFFFFFFFFFFFFFFFFFFFF:F
FFFFFFFFFFFFFFFFFFFFFFFFFFFFFFFFFFFFFFFFFFFFFFFF
@A00721:190:HGJW7DSXY:4:2474:7527:14058_2:N:0:TCTGCAAG+GCTGCACT
ACCCGCTGTGCAGAATAACCTTCCCAAATCTAGATCTGCACAGAAGAAGGCTGAAGGTCGTTACCGGA
CGACCACTCAAAGGAAACGGGCTGTTGCCCAGAGGAAAGCAGCGACCA
+
FFFFFFFFFFFFFF,FFFFFFFFFFFF:FFFFFFFFFFFFFFFF:FFFFFFFFFFFFFFFFFFFFFFFF
FFFFFFFFFFFFFFFFFFFFFFFFFFFFFFFFFFFFFFFFFFFFFFFF
@A00721:190:HGJW7DSXY:4:2432:12210:22357_2:N:0:TCTGCAAG+GCTGCACT
GTAACATTGACGGCAGTCCCGTCATCTCTGTGGGAACTGAGGCAGTACCTCAGTCAAAAGTCACATTC

```
ACCACTCCCATACCAATCAATGATGTGACTATACAGTTCTGCCATTTC
+
,:,FFF,F:FFFFFFFFFF:FFF:FFFFFFF,F:FF:FFF:FFF:FFFFFFFFFFFFFFFFFFFF,FF
FFFF:FFFFFFF:FFFFFFFF:FF:FFFFFFFFF,,:F:,:F,FFFFF
@A00721:190:HGJW7DSXY:4:1549:21612:10958_1:N:0:TCTGCAAG+GCTGCACT
ATACGTCGTGGGAGGTACTGAAGCCTTCCCCTCCGCTGGCACGAAGAACACTCCCAAGCCAATCAATA
GTTCTTCTGACCAGAATGAAAAGGGGTTTACCAGGGTAGTCTCTAAGTCTACCAGGAAGAGGGGAAAT
GTC
+
FFFFFFFFFFFFFFFFFFFFFFFFFFFFFFFFFFFFFFFFFFFFFFFFFFFFFFFFFFFFFFFFFFFF
FFFFFFFFFFFFFFFFFFFFFFFFFFF:FFFFFFFFFFFFFFFFFFFFFFFFFFFFFFFFFFFFFFFF
FFF
@A00721:190:HGJW7DSXY:4:1147:2031:12493_1:N:0:TCTGCAAG+GCTGCACT
AAGGGAAAGGTCCATCAGGACCTAAGAAGGGGAAATTACCCCTAATTCTGAAGGAAGATCTCCCACCA
TCTGGGGTTGGGAATAAGAAATCTGCACGGGCCAAAGAGGCCGCTGCTACGAAGTCGCTGAAGGCGGC
TT
+
F:FFFFF,F:FFFFFFFFFFFFFFFFF:FFFFFFFFFFFFFFFFF:FFFFFFFFFFFFFFFFFFFFFF
FFFFFFFFFFFFFFFFFFFF:FFFFFFFFFFFFFFFFFFFFFFFFFFFFFFFFFFFFFFFFFFFFFFF
FF
@A00721:190:HGJW7DSXY:4:2376:15483:2848_2:N:0:TCTGCAAG+GCTGCACT
TGTAAAGTTGCGCCTGCCGACCCCATCTTTGAGACCAAGACTCTCTACAGTAGGTTCGTGCCTACTAT
GAGCAAATCTGTTGAATTCGTCACTGAGGACGAAGGTGAGAGGACTTT
+
FFFFFF:FFF:FFFFFFFFFFFF:FFFFFFFFFFFFFFFFFFF:::FFFFFFF:FF:F:FFFFFFF,F
FFFFFF:FFFFFF:FFFFFFFFFFFFFFFFFFFFF:FF:FFFFFFF,F
@A00721:190:HGJW7DSXY:4:1573:23583:18474_1:N:0:TCTGCAAG+GCTGCACT
CTGAGAGCAGTCGCTGGGGAAAGAGAATTGCGTGGGTTGAAGGCCGACGCTTCGAGCTTGAGTGTGAC
GAGGCCGGTGACGTTTTTGTCAACGGCGTTTGGAGGATTTTATCCGCGCTTAACTATGGGGCGAAGAT
CCC
+
FFFFFFFFFFFFFFFFFFF:FFFFFFFF:FFFFFFFF:F:FFFFFFFFFFFFFFFFFFFFFFFFFFFF
FFFFFFFFFFFFFFFFFFFFFFF,FFFFFFFFFFFFFFFFFFFFFFFFFFFFFFFFFFFFFFFFFFFF
FFF
@A00721:190:HGJW7DSXY:4:2240:18683:18035_2:N:0:TCTGCAAG+GCTGCACT
AGGTTGGCATATTTATGCCGACGATAATAAGCATGTCACCCTTAAGCACCGAGGTAAGCTCGGGACTT
ACAAGAGAGAGTGTGAGAGAGTCGCTCTCACTATTTTTGCCCAGAATG
+
FFFFFFFFFFFFFFFFFFFFFFFFFFFFFFFFFFFFFFFFFFFFFFFFFF:FFFFFFFFFFFFFFFFF
FFFFFFFFFFFFFFFFFFFFFFFFFFFFFFFF:FFFFFF:FFFFFFF:,
@A00721:190:HGJW7DSXY:4:2451:13883:4022_1:N:0:TCTGCAAG+GCTGCACT
ATGAGGTTAAAACTCTTGCTCCTTCTGTGGGTGAGGGTTTTTGTTATATTCACTGGTTCTGGGAGGCT
GCTTTCCTCCATAATTACAACCTCTCTTTAGAGAGCATTAGAAGGAAGTTGGGCAAATTCCCACATAT
GGT
+
FFFFFFFFF:FFFFF:FFFFFFFFFFFFFFFFFFFFFFFFFFFFFF:FFFFFFFFFFFFFFFFFFFFFFF
FFFFFFFFFFFFFFFFFFFFFFFFFFFFFFFFFFFFFFF:FFFFFFFFFFFFFFFFFFFFFFFFFFFFF
FFF
@A00721:190:HGJW7DSXY:4:1250:19479:11647_2:N:0:TCTGCAAG+GCTGCACT
ACACTTTTCTTGAAAATTTTTCCTTTCCTCCGTCATTGGGGATTTCGATGTGAGAAGGTGGAAGGTGT
CAAGGGTCTTCTTCATGCTGATCTTTCAGGACCTAATCACATCATGAT
+
FFFFFFFFFFFFFFFFFFFFFFFFFFFFFFFFFFFFFFFFFFFFFFFFFFFFFFFFFFFFFFFFFFFF
FFFFFFFFFFFFFFFFFFFFFFFFFFFFFFFFFFFFFFFFFFFFFFFF
```

```
@A00721:190:HGJW7DSXY:4:1616:19153:36980_1:N:0:TCTGCAAG+GCTGCACT
GCTCGTATCGAGTTGGAGGGAGGAAGTTCTCCGAAATTAAAGACGGGGGGATTAAGAGTCCAGCTGTA
ACTGTCATTTCAGAGAGGGGAGCTCTGAAAAGGAAACTTCGTCAGAAGATGAGGAAGTCTAAGAGGAG
GAG
+
FFFFFFFFFFFFF,FFFFFFF,FFFFFFFFF:FFFFFFFFFFFFFFFFFFFFFFFFFFFFFFFFFFFF
FFFFFFFFFFFFFFFFFFFFFFFFFFFFFFFFFFFFFFFFFFFFFFFFFFFFFFFFFFFFFFFFFFFF
FFF
@A00721:190:HGJW7DSXY:4:1619:7654:25426_1:N:0:TCTGCAAG+GCTGCACT
CTCTAGAGAAGATCTCCCCACCTGGAGAAGGCAAGTGGAGAGTCCAGAAGTTGATGAAGAAGGATTAT
TTGAAGCAGGGGTTACCACTTCCTAAGGTGGATGAGGGAGGTTGTTTCTTTGGAGTTTTAGATAATCT
TTT
+
FFFFFFFFFFFFFFFF,FFFFFFFFFFFFFFFFFFFFFFFFFFFFFFFFFF:FFFFFFFFFFFFFFF
FFFFFFFFFFFFFFFFFFFFFFFFFFFFFFFFFFFFFFFFFFFFFFFFFFFFFFFFFFFFFFFFFFFF
FFF
@A00721:190:HGJW7DSXY:4:2269:22191:24643_2:N:0:TCTGCAAG+GCTGCACT
CCTTGAGAAGACAAGCCACATTAAGATATAATTTAGGTCAACTTGGGGTTTCGAATGTCATTAGTGCC
TTACGATTGAAAGAGGTCCTTTTGAGTATCGACAGATCTTTCGATCTT
+
FFFFFFFFFFFFFFFFFFFFFFFFFFFFFFFFFFFFFFFFFFFFFFFFFFFFFFFFFFFFFFFFFFFF
FFFFFFFFFFFFFFFFFFFFFFFFFFFF:F:FFFFFFFFFFF:FFFFF
@A00721:190:HGJW7DSXY:4:2461:9923:14575_2:N:0:TCTGCAAG+GCTGCACT
GTGGCAGAATTCGGTAACTTACCATATTGTTCCTTCCGGGAAGTTGTGGGATTTAACTTTGACTCAAC
TACTCCGATCAGATCCGGATGGTTTGATTGGTATGGACTACTCGACTT
+
F,FFFFFF:,:F,FF:FFFFFFFF,,FFFF:F,::F:FFFFFFFFF:FFFFFFFFFFF,:,FFFFF:F
FFFFFFFF,FF:F,FF,,F,FF,:FFF,FFF,FF:FFF::,FFF:FFF
@A00721:190:HGJW7DSXY:4:1368:7048:23500_2:N:0:TCTGCAAG+GCTGCACT
TCATGGCAGATACTACCGATAAGGTGAAAAATATGCAGGTCAAAGATTCTGTAGCTCTGAGGGCGGTT
GAGGGAGCCGTTTTGGCCGACTTTAACTATTCTAAGAGTCTTTATGAT
+
FFFFFFFFFFFFFFFFFFFFFFFFFFFFFFFFFFFFFFFFFFFFFFFFFFFFFFFFFFFFFFFFFFFF
FFFFFFFFFFF:FFFFFFFFF:FFFFFFFFFFFFFFFFFFFFF:FFFF
@A00721:190:HGJW7DSXY:4:1122:8314:36871_2:N:0:TCTGCAAG+GCTGCACT
CGTTGTCGTACCTTACAAGGTTGAGGAGACTTTACAGCGGCTTATTGTCGCGCAGTTTCCAGAATTGC
GGTTGAAGTTCACCCATTCTATCTTGAATGCTCACGCCGTCGCAGCCA
+
FFFFFFFFFFFFFFFF:FFFFFFFFFFFFFFFFFFFFFFFFFFFFFFFFFFFFFFFFFFFFFF,FFFF
FFF:FFFFFFFFFFFFFFFFFFFFFFFFFFFFFFFFFFFFFFFFFFFF
@A00721:190:HGJW7DSXY:4:1178:17833:32503_1:N:0:TCTGCAAG+GCTGCACT
ATCACTTGTTAAAGTACAGATTTAGTGATTTAGATATTATTGATATTGGAGGCGATATTCGTTTTCAT
ATCCAAAACTCCTTGAATGGAGTACATATTTGCAACCCTATTCTTGATGTGAAAGATGCATCGAGGTA
TAC
+
FFFFFFFFFFFFFFFFFFFFFFFFFFFFFFFFFFFFFFFFFFFFFFFFFFFFFFFFFFFFFFFFFFFF
FFFFFF:FFFFFFFFFFFFFFFFFFFFFFFFFFFFFFFFFFFFFFFFFFFFFFFFFFFFFFFFFFFFF
FFF
@A00721:190:HGJW7DSXY:4:1436:7817:22357_1:N:0:TCTGCAAG+GCTGCACT
ATTGATTGGGAACGTACCTACTCACGAGACGTCATGAAAGCTATCGGAGGGAAGCAAAATGTTTCGTT
CTGTTACAAGAACGCTTTGGATTGTGACCATAAAGCCTCAGTTGCGATAGCTGTAGAGGTATACGATA
TCT
+
FFFFFFFFFFFFFFFFFFFFFFFFFFFFFFFFFFFFFFFFFFFFFFFFFFFFFFFFFFFFFFFFFFFF
```

FFFFFFFF,F:FFFF,FFFFFFFFFFFFFFFFFFFFFFFFFFFFFFFFFFFFFFFFFFFFFFFFFFF
FFF
@A00721:190:HGJW7DSXY:4:2103:19840:6042_2:N:0:TCTGCAAG+GCTGCACT
GCCACTATAATGCTTAAGAGAGAGATTGATGTATGTCATTTCGTCTTAGTTTGTCCCGGCGAATTATT
GGACACGATGAAAAATGACATACCCGTTCCTGACCTTGGTATCGTTAT
+
FFFFFFFFFFFFFFFFFFFFFFFFFFFFFFFFFFFFFFF:FFFFFFFFFFFFFFFFFFFFFFFF:FFFFF
FFFFFFFFFFFFFFFFFF:FFFFFFFFFFFFFFFFFFFFFFFF:FFFFFFF
@A00721:190:HGJW7DSXY:4:1364:24026:4022_1:N:0:TCTGCAAG+GCTGCACT
GGTTATTCTCACAACAAAGATAAGATTTTATCTTTCTTCAAACACCCTGTTTTCCTTAAGGGCGGACA
ATTGTTCCATTGTGAACTCTTGGGGACTAGATTGAGCATTAATGAGTTCCGAATAACTCGTAATAAAT
CTT
+
FFFFFFFFFFFFFF:FFFFFFFFFFFFFFFFFFFF:FFFFFFFFFFFFFFFFFF:FFFFFFFFFFFFFFFF
FFFFFFFFFFFFFFFFFFFFFFFFFFFFFFFFFFFFFFFFFFFFFFFFFFFFFFFFFFFFFFFFFFFFF
FFF
@A00721:190:HGJW7DSXY:4:1140:11433:12242_1:N:0:TCTGCAAG+GCTGCACT
ATTCAGAATACCTATTTCCGTACCAAGAGCCACTGCCAATCTCATCGCTATACGCATACCTGATTGGA
AGAAGCGAGGTTCGTTCCTCAATAAAAATTTTTCTCAAGAGATTATGGTTGATAAAGTTTTCTTCATA
AGG
+
FFFFFFFFFFFFFFFF:FFFFFFFFFFFFFFFFFFFFFFFFFFFFFFFFFFFFFFFFFFFFFFFFFFFF
FFFFFFFFFFFFFFFFFFFFFFFFFFFFFFFFFFFFFFFFFFFFFFFFFFFFFFFFFFFFFFFFFFFFF
:FF
@A00721:190:HGJW7DSXY:4:1178:17933:7404_2:N:0:TCTGCAAG+GCTGCACT
ACGTCATGGGGATCACTTCTTCGGTCAATGACAAAACCTTCGAATATGTTACGACATGGCTTCGTAAC
AACAGTGCGAGGATTGTCATCTCGGGGAAAGTGATTCACCAAGATGTT
+
FFFFFFFFFFFFFFFFFFFFFFFFFFFFFFFFFFFFFFFFFFFFFFFFFFFFFFFFFFFFFFFFFFFFF
FFFFFFFFFFFFFFFFFFFFFFFFFFFFFFFFFFFFFFF:FFFFFFFFFF
@A00721:190:HGJW7DSXY:4:2210:15203:2863_1:N:0:TCTGCAAG+GCTGCACT
GAGATACCTAGAGAAGGTCGCAGCGATCTTACTCGTTATCGGAGCTAAGAAGAAGACTTTGGGTACAC
GTTATGCTCGCAAACTTAACGTTAGTACCGGAGGTGATCTCTCTGCTTCTGCGTTACTGAGAGAAAAG
ATT
+
FF:FFFFFFFFFFFFFFFFFFFF::FFFFFFFFF:FFFFFFFFF:FFFFFFFFFFFFFFFFFFF:FFF
FFFFFFFFFFFFFFFFFFFFFFFFFFFFFFFFFFFFFFFFFFFFFFFFFFFFFFFFFFFFFFFFFFFFF
FFF
@A00721:190:HGJW7DSXY:4:2114:26323:36151_2:N:0:TCTGCAAG+GCTGCACT
ACGGTGACTTTATGGACAAGATGCGAGTTCTTGGGAAATACTTGTTTCCCTTTTTAGGTTTAGTAGGT
GATCTTGACGTCGATTCTTTGTATAGGGAGTGTGAGAAGACAACGAAT
+
:FFFFFFFFFFFFFFFFFFFFFFFFF,FFFFFFFFFFFFFFFFFFFFFFFFFFFFFFFFFF,F,FFF:FFF
FFFFFFFFFFFFFFFFFFFFFF:FFFFFFFFFFFFFFFFF:FFFFFFFF
@A00721:190:HGJW7DSXY:4:1159:10004:3975_1:N:0:TCTGCAAG+GCTGCACT
GAACCGGTCTCTAAATCTTTTGAGATTGAGAAGATTGAAGAACACGTCAAGAAGTCGCTCGGCGTTGT
TCTTGAAAACCAAGTTAAGAAGGAGGCTGATCATGGAGTCTTTGTACCTTCTGTCAAACTTCGAGGTT
CAG
+
FFFFFFFFFFFFFFFFFFFFFFFFFFFFFFFFFFFFFFFFFFFFFFFFFFFFFFFFFFFFFFFFFFFFF
FFFFFFFFFFFFFFFF:FFFFFFFFFFFFFFFFFFFFFFFFFFFFFFFFFFFFFFFFFFFFFFFFFFF
FFF
@A00721:190:HGJW7DSXY:4:1650:28248:30342_2:N:0:TCTGCAAG+GCTGCACT
GATCCCGGATGTGGATTGAAGGCTGGCTCTAAGGGAGACTCCTTCTTTTTGAATGTTGTAAAGAATTG

```
GGTTGAGAAGTGTGCTTTACAAGGAGAGATCGGAAGAGCGTCGTGTAG
+
FFFFFFFFFFFFFFFFFFFFFFFFFFFFFFFFFFFFFFFFFFFFFFFFFFFFFFFFFFFFFFFFFFFFF
:FFFFFFFFFFFFFFFFFFFFFF:FFFFFFFFFFFFFF:FFFFFFFFFF
@A00721:190:HGJW7DSXY:4:1548:29894:11365_2:N:0:TCTGCAAG+GCTGCACT
TAAAGAAAAATTTTCGAATAAACTAATGAGAGGTATAGGCTTAGTCAAGTCTGTACTTACATTCTTTA
CGCCAGATTCTTCATGGGGCGACGCTGTTTCTCTTGGTATTTCTGAAA
+
FFFFFFF:FFFFFFFFFFFFF:FFFFFFFFFF::FFFFFFFFFFFFFFFFFFFFFFFFFFFFFFFFFF
FFFFFFFFFFFFFFFFFFF:FFFFFFFFFF::FFFFFFFFFFFFFFFF
@A00721:190:HGJW7DSXY:4:1314:6488:2707_2:N:0:TCTGCAAG+GCTGCACT
CGTCATTTTCCTTTGGGATCTTTCCTCTGGTAAGGGAATTATTAAATCATTCTTCTCACTCTTGACGA
GAATTATTACTGTTTTCTCTACCCATAACTTCTTTTTCAAAAATGCGG
+
FFFFFFFFFFFFF:FFFFFFFFFFFFFFFFFFFFFFFFFFFFFFFFFF:FFFFFFFFFFFFFFFFFFF
FFFFFFFFF:FFFFFFFFFFFF:FFFFFFF:FFFFFFFFFFFFFF:FF
@A00721:190:HGJW7DSXY:4:1410:24731:28510_2:N:0:TCTGCAAG+GCTGCACT
TGATGTTTTCCTACAGAGTGCAGTCGTTTCAAAACTTATAACCACTTTTGAAAGGAAAATAATTGGTG
TTGTAAATAAGTATGTCGGTGGGGTTATCAAGGCTCTCATCGCCACAG
+
FFFFFFFFFFFFFFFFFFFFFFFFF:F,::FF,FF,FFF,F:FFF,,FF,FFF,FFFF:FFF:F:F:,
,,,,FF,FF,::F:FF,FF,F:FF,F,:F:F,F,FF,,,FF:,FFF,F
@A00721:190:HGJW7DSXY:4:1609:3369:8202_2:N:0:TCTGCAAG+GCTGCACT
GGGTTCCATAAGATATTATCTGACATTGTGGTTCAATGATCAGGATTCTTCTTTTGGTCCTTACGTCT
TAAGTAATTTGGATGTTGTCGAAAACTATCAGGTTCTTGTCACGATTC
+
FFFFFFFFFFFFFFFFFFFFFFFFFFFFFFFFFFFFFFFFFFFFFFFFFFFFFFFFFFFFFFFFFFFF
FFFFFFFFFFFFFFFFFFFFFFFFFFFFFFFFFFFFFFFFFFFFFFFF
@A00721:190:HGJW7DSXY:4:1111:27769:13166_2:N:0:TCTGCAAG+GCTGCACT
TTAAAACTTCAGAGTCTCTTGGAAGTCCAGGTAGCTAATGTTCTTGCAATAGCCACAGACTTTCTGGG
AAAAGAGACCTCTGTTGTAGAGGAGTTGAGAGAATTACTCAGATTCAA
+
FFFFFFFFFFFFFFFFFFFFFFFFFFFFF:FFFFFFF,F,FFFFFFFFFFFFFFFFFFFFFFFFFFFF
F:FFFFFFFFFFFFFFFFFFFFFFFFFFFFFFFFFFFFFFFFFFFFFF
@A00721:190:HGJW7DSXY:4:1259:12255:28667_2:N:0:TCTGCAAG+GCTGCACT
ACAACGAGTTAGGGAGTGGCTAGGTTTTAAAAGATCCTTTGCACAAGCAATACCTTCCACTGAGTTCA
TCACCCAGAGTTTCGACGTCGCGGAAAACGATGAAGATTTCCACACTG
+
FFF:FFFFF:FFFFFFFFFF:FFFFFFFFFFFFFF:FFFFFFFFFFFFFFFFFFFFFFFFFFFFFFFF
FFFFFFFFFFFFFFFFFFFFFFFFFFFFFFFFFFFFFFFFF:FFFFFF
@A00721:190:HGJW7DSXY:4:1243:31810:13088_2:N:0:TCTGCAAG+GCTGCACT
TCTGATGCTGAATTTGAGGAAAATTTTTCGGAAGTTTTTGGTATAAATCCTGATGGCTCAGTACAGGG
AGGAGGTAATAGTTATAAAACCTTCTTTTATCATTGCAACAGGACTTT
+
FFFFFFFFFFFFFFFFFFFFFFFFFFFFFFFFFFFF:FFFFFFFFFFFFFFFFFFFFFFFFFFFFFFF
FFF,FFFF:FFFFFFFFF:FFFFFFFFFFFFFFFFFFFFFFFFFFFF,F
@A00721:190:HGJW7DSXY:4:1619:5267:27806_2:N:0:TCTGCAAG+GCTGCACT
GGACAAAATCTGGGATCATAGGTATGTGTCACGCTACACTCGAAATATGGGCCCTCATAAGGAAGTAT
AGTTGGCGTCTTGTCGTTGACGTCATTACAGTCTGGAAGTTAGATCAT
+
F:FFFFFFFFFFF:FFFFFFFFFFFFFFFFFF:F:FFFFF::FFFFF,FFFFFFFFFFFF:FFF:FF,
FFFFF:F::FFFFFF:FF:FF:FFFFFFFFF,FFFFFF,,:F:F:FFF
@A00721:190:HGJW7DSXY:4:2575:23773:13260_2:N:0:TCTGCAAG+GCTGCACT
CGAGAGTTGTGATCTAGTTCTAAATTTAGGTCGACGGACTTTAGCAACTTTAGATAAATTCTTTCCGG
```

GCTCAATGACCTTTGCTATGTACGTGGATCTAATCCTTGGTGATCTGA
+
FFFFFFFFFFFFFFFFFFFFFF:FFFFF:FFFFFFFFFF:FFFFFFFFFFFF:FFFFFFFFFFFFFFF
FFFFFFFFFFFFFFF:FFFFFFFFFFFFFFFFFFFFFFFFFFFFFF:FF
@A00721:190:HGJW7DSXY:4:2150:7862:31516_2:N:0:TCTGCAAG+GCTGCACT
TATTAGTTCATGAAGTTCTTACACTTCTCGCGACTAATGTCAGCTTCACTACAAAGGCGTTACGTAAA
GTAGTTTTCACGCTTTATCTTAAAGCATTACCACCAAATTTAGCACTT
+
FFFFFFFFFFFFFFFFFFFFFFFFFFFFFFFFFFFFFFFFFFFFFFFFFFFFFFFFFFFFFFFFFFFF
FFFFFFFFFFFFFFFFFFFFFFFFFFFFFFFFFFFFFFFFFFFFFFFF
@A00721:190:HGJW7DSXY:4:2573:17761:25520_1:N:0:TCTGCAAG+GCTGCACT
CGTGTGCTCTTCCGATCTAGACATGCCAGTTAGAGTTCTTCTAACCAGGACTGATGGTGAGATTCTTT
TACCTTTTGGAGGTCCAGGAACCTTTACTCCTAGTGTCTTTTCTAGAGTCCAAGGACGTTTCCGTCGG
AGG
+
FFFFFFF:F:FFFFFFFFFFFFFFFFFFFFFFF:FFFFFFFFF:FFFFFFFFFFFFFFFFFFFFFF:F
:FFFFFFFFFFFFFFFFFFFFFF:FFFFFFF:FFFFFFFFFFFFFFFFFFFFFFFFFFFFFFFFFFFF
FFF
@A00721:190:HGJW7DSXY:4:2671:28980:4993_2:N:0:TCTGCAAG+GCTGCACT
GACCGCTCAATAACACTCTATTGAATCCCTTTTCCACGATGAAAGCTCTTTATGAGGTGCTCCATGCA
AATTTACAGTTCCTCTTTGGTGATATCGCCTCTGTCCTTTCTCCTTTA
+
FFFFFFFFFFFFFFFFFFFFF:FFFFFFFFFFFFFFFFFFFFFFFFFFFFFFFF:FFFFFFFFFFFFFF
FFFFFFFFFFFFFFF:FFF:FFFFFFFFFFFFFFFFFFFFFFFFFF:FF
@A00721:190:HGJW7DSXY:4:1649:10212:28917_2:N:0:TCTGCAAG+GCTGCACT
ACTTGGGTTTATCGGTTGTATTTTGATAAATTTGTTGGAATCGATAATGATACTGACGAAGGTTCTAT
TACCACCCTTGTCATTGATACAAACATTCTAGGTGATGCTTTCTTAGA
+
FFFFFFFFFFFFFFFFFFFFFFFFFF:FFFFFFFFFFFFFFFFFFFFFFFFFFFFFFFFFFFF:FFFFF
:FFFFFFFFF:FFFFFFFFFFFFFFFFFFFFFFFFFFFFFFFFFFFFF
@A00721:190:HGJW7DSXY:4:1309:18285:6668_2:N:0:TCTGCAAG+GCTGCACT
TACAATCTGTAGAGAGAGAGGTTAATCAGAATTTGAGTGATAACGATTTAGTACTGGGAAATGGTGGT
AGTCAGTTTACTACTTTTCTTTCCCTCCCCTTTTTCGATTGGATTCGA
+
FFFFFF:,FFFFFFFFFFFFFFFFFFFFFFFFFFFFFFFFFFFFFFFFFFFFF:FFFFFFFFFFFFFF
FFFFFFFFFFFFFFF:FFFFFFFFFFFFFFFFFFFFFFFFFF:FFFF,F
@A00721:190:HGJW7DSXY:4:2274:9498:34037_2:N:0:TCTGCAAG+GCTGCACT
TTGATAAGGTTTTTCTTTTATGGGACTCTTGTTTGTACTTATGAAGGAGGTATTACTTCGGAAGTTCT
TAATGTAGGTCTCCTAGTACTTAAGGGTTTATGTTTCGGTTTCAGCTG
+
FFFFFFFFFFFFFFFFFFFFFFFFFFFFFFFFFFFFFFFFFFFFFFFFFFFFFFFFFFF:FFFFFFFF
FFFFFFFFFFFFFFFFFFFFFFF,FFFFF:FFFFFFFFFFFFFFFFFFF
@A00721:190:HGJW7DSXY:4:1370:1380:21668_1:N:0:TCTGCAAG+GCTGCACT
TTCACATTGCGTGATCACTTCCCGTGGACTTGGGAAGAGATTAAACATAAAATCCAGCGATTCTATTA
TGTCGCTAAAGCCTGTCCAGTTGTCGATGATGTTCGGGCTGTGGGGATTAACTTCCCTCGTGAAACTT
ACG
+
F:FFFFFFFFFFFFFFFF:FFFFFFFFFFFFFFFFFFFFFFFFFFFFFFFFFFFFFFFFFFFF:FFFF:
FFFFFFFFFFFFFFFFFFFFFFFFFFFFFFFFFFFFFFFFFFFFFFFFFFFF:FFFFFFFFFFFFFFF
FFF
@A00721:190:HGJW7DSXY:4:1313:30789:3803_1:N:0:TCTGCAAG+GCTGCACT
CACAAGGATTACTAAGTCTGAGATCTGCCACTTCTAATTTTAGGAAACGTCGCGAAAAGAAAGTTCAT
GAGGATGTCTTGGTCGAAAAATATCGCCGGGAACTTCTAATAGAGGATCCTGATGACATTATTTCAAC
GGA

```
+
FFFFFF:FFFFF:FFFFFFFFFFFFFFFFFFFFFFFFFFFFFFFFFFFFFFFFFFFFFFFFFFFFFFFFF
FFFFFFFFFFFFFFFFFFFFFFFFFFFFFFFFFFF:FFFFFFFFFFFFFFFFFFFFFFFFFFFFFFFFFF
FFF
@A00721:190:HGJW7DSXY:4:2256:24243:22905_2:N:0:TCTGCAAG+GCTGCACT
AGTTCTACCACAGAAACCGTCTCAGGAACTGTCTCCACTGATTCGGAATTAGAGAAAGACGACCTTCT
TGTGGGGACTGAGACCTCTGAATTTTCAGGGTGCCCTGATGAGGACGT
+
FFFFFFFFFFFFFFFFFFFFFFFFFFFFFFFFFFFFFFFFFFFFFFFFFF,FFFFFFFFFFFFFFFFFF:
FFFFFFFFFFFFFFFFFFFFFFFFFFFFFFFFFFFFFFFFFFFFFFFF
@A00721:190:HGJW7DSXY:4:1443:26386:36323_1:N:0:TCTGCAAG+GCTGCACT
ACTCTGAGACTTCTAAGAGTGTTTCAAAAGACCAGAAACGTAACAAGAAGAATAAAGGTAGGAAGGGG
AGAAAACAGAACAAGCGAGCGGTTGAAGAGAAAACTGATATGGTTGCGGCTGATCGTACCCCAATTCC
TGC
+
:FFFFFFFFFFFFFFFFFFFFFF:FFFFFFFFFFFFFFFFFFFFFFFFFFFFFFF:FFFFFFFFFFFFF
F:FFFFFFFFFFFFFFFFFFFFFFFFFFFFFFFFFFFFFFFFFF::FFFFFFFFFFFFFFFFFFFFFFF
FFF
@A00721:190:HGJW7DSXY:4:2535:17020:27273_1:N:0:TCTGCAAG+GCTGCACT
TCTCGGCGATACCACTGATCTTCCAGAGTTACGTAGTGGTCGTTCTTTTGGTGATTTTATCAAAGAGA
AAGTTTCTGCTAGTCGCGACGATAGTATTTATCGGAGTGATATTAACAAGTTAAACTTCGAACTGACC
ACT
+
FFFFFFFFFFFFFFFFFFFFFFFFFFFFFFFFFFF:FFFFFFFFFFFFF:FFFFFFFFFFFFFFFFFFF
FFFFFFFFFFFFFFFFFFFFFFFFFFFF:FFFFF:FFFFFFFFFFFFFFFFFFFFFFFFFFFFFFFFFFF
FFF
@A00721:190:HGJW7DSXY:4:2472:24279:9533_2:N:0:TCTGCAAG+GCTGCACT
TTGAAGTACCGCAACATGGATCAGTTTTCCAAAAAGCTATTAATAGCAGTTGCGGGGCTATAATACCG
CCTCCAGATTACTCTAAACTGAATCCACTCTCTTGGGATATAAATGAG
+
FFFFFFFFFFFFFFFFFFFFFFFFFFFFFFFFFFFFFFFFFFFFFFFFFFFFFFFFFFFFFFFFFFFFFF
FFFFFFFFFFFFFFFFFFFFFFFFFFFFFFFFFFFFFFFFFFFFFF:F
@A00721:190:HGJW7DSXY:4:1569:10068:29794_2:N:0:TCTGCAAG+GCTGCACT
TTTGGAACTGTGTAATGTCCTACGTACTGTTCAAGGTGTTTGGGCTTCTCTAAAGAACAGCAACTTTC
AACATGAATTAGTTATTGAAAGTCTTGACCCTCACGTCTCACTTTATC
+
FFFFFFFFFFFFFFFFFFFFFFFFFFFFFFFFFFFFFFFFFFFFFFFFFFFFFFFFFFFFFFFFFFFFFF
FFFFFFFFFFFFFF:FFFFFFFFFFFFFFFFFFFFFFFFFFFFFFFFF
@A00721:190:HGJW7DSXY:4:2635:16721:13542_2:N:0:TCTGCAAG+GCTGCACT
AGACCTTTCTTGGGGAGTAAAGAGGGTTTGAAAATTACGGAACCTTTCCTCGTTCTTCATGAAGATCT
TCGAGTGTTTTATAGTGAGTTATTCTTGCGAAGGTTCAAGTCGATTGA
+
:FFFFFFFFFFFFFFFFFFF:F:FFFFFFFFFFFFFFFFFFFFFFFFFFFFFFFFF:FFFFFFFFFFFF
FFFFFFFFFFF:FFFFFFFFFFFFF:FFFFFFFFFF:FFFFFFFFFF
@A00721:190:HGJW7DSXY:4:1512:29324:16141_2:N:0:TCTGCAAG+GCTGCACT
AAGAAGTTCTTCGCAGTTGAATCTCCACCAGGTGGAGGGAAGACCACTGAACTTATAGCGACTTTTGT
CGAATTTTTCGCAAAAGGTCTTGAAGTACGTATTGTCACTGCTAATCG
+
FFFFFFFFFFFFFFFFFFFFFFFFFFFFFFFFFFFFFFFFFFFFFFFFFFFFFFFFFFFFFFFFFFFFFF
FFFFFFFFFFFFFFFFFFFF,FFFFFF:FFFFFFFFFFFFFFFFFFFF
@A00721:190:HGJW7DSXY:4:1146:19217:1673_2:N:0:TCTGCAAG+GCTGCACT
CACTTCTAGTTCATCGCGGTATTTGTGAAATTAAGACAGACGTGAAAGTTATGATGAGTGATCGTATT
AGAACAGCTGATTCTTATTGCATGAACGTCGCTGAGGTCAAACCTGTG
+
```

FFFFFFFFFFFFFFFFFFFFFFFFFFFFFFFFFFFFFFFFFFFFFF:FFFFFFFFFFFF:FFFFFFFFF
FFFFFFFFFFFFFFFFFFFFFFFFFFFFFFFFFFFFFFFFFFFFFFFF
@A00721:190:HGJW7DSXY:4:1155:30355:3176_1:N:0:TCTGCAAG+GCTGCACT
CATCGACGAATTATTCATGATTCACGCTGGTCAGACCCTTTTTCTTACACATCTACTTAAGGCGGCTT
ATACCATAGGTTACGGTGATTCCAATCAAATCGGATTCATCAATCGTATTGATCTCCAAGTCGCAAAC
TAC
+
FFFFFFFFFFFFFFFFFFFFFFFFFFFFFFFFFFFFFFFFFFFFFFFFFFFF:FFFFFFFFFFFFFF
FFFFFFFFFFFFFFFFFFFFFFFFFFFFFFFFFFFFFFFFFFFFFFFFFFFFFFFFFFFFFFFFFFFF
FFF
@A00721:190:HGJW7DSXY:4:2273:24542:7388_2:N:0:TCTGCAAG+GCTGCACT
TTGATGAGTGTCGTTCTTATCGAAGTCTCAGTTATCGCTGCCCGGCCGACGTTTGTCGCGAACTCTCA
GAGGTTTACGGCAGAAAAATTTCTACTGCTTGTAATTCGAGAGAGGAC
+
FF:FF,F:FFFF:FFF:FFFF:FF:F:FFF:FFF,::FF:FF:F:FFFFF::FF,F::F,FF:F::FF
,F,,:FFF,::FF,FFFFF,::,,,,:,,,,::FF:::,F:F,F,,F,
@A00721:190:HGJW7DSXY:4:1266:5882:7200_1:N:0:TCTGCAAG+GCTGCACT
TTAGACCTATCAGATGTCTTGACGACTTCGAATTTGAGGACGGAGTTAAATATATTACTTTCACTCAA
GGTGAAAAGTTTGAAATTAACAAAGTCCTTGGTAAGAGTAAGGTTAAATGTGAAGATGCTCAGACTAT
TCA
+
:FFFFF:FFFFFFFFFFFFFF:FFFFFFFFFFFFFFFFFFFF:FFFFFFFFFFFFFFFFFFFFFFFFFF
FFFFFFFFF:,FFFFFFFFFFFFFFFFFFFFFFF:FFFFFF,FFFFFFFFFFFF:FFFFFFFFFFFF:F
FFF
@A00721:190:HGJW7DSXY:4:1104:5014:13777_1:N:0:TCTGCAAG+GCTGCACT
TGACCCTGTTTTTGCATCACAAACACATATTACCGTTGCTCTATCACGACACGTTGAATCTTTACGAT
ACTTTGTTATCAATGTAAAGTTAAACGATGCTACTTGTGAGAGGATCAATCACAGTAAGGAACTGTCA
CGT
+
FFFFFFFFFFFFF:,F,FF:F,F,F:FFFFFFF:FFFFFF,FF,FFFFFF:F:F:FFFFF:FFFFFFF
FFFFF,FFFFFFFFFFFFFFFFFFFFFFFFFFFFFFFFFFFFFFFFFFFFFFFFFFFFFFF:FFFFFF
FFF
@A00721:190:HGJW7DSXY:4:2430:9136:15969_1:N:0:TCTGCAAG+GCTGCACT
TGCAGATTCAACCCCTTATATCGAGGCTATCGAGGACGATGAGTATCTTGAAGTTGTTGACGACAAGC
CTCAAGAGGGTAGAGGACCACCTGGATCTTCTACTGTCTCTGCCTTCCAAGATTTCTTAGAAGAGGTT
ATT
+
FFFFFFFFFFFFFFFFFFFFFFFFFFFFFFFFFFFFFFFFFFFFFFFFFFFFFFFFFFFFFFFFFFFF
FFFFFFFFFFFFFFFFFFFFFFFFFFFFFFFFFFFFFFFFFFFFFFFFFFFFFFFFFFFFFFFFFFFF
FFF
@A00721:190:HGJW7DSXY:4:2531:20066:3583_1:N:0:TCTGCAAG+GCTGCACT
CTTAACGTCACTCGGTAATCTTTCAGCCGAATTAAGTACTAATGAGTTCAAGTCGGTTGTCGATCGAA
TTAAGATCAAGGCTAATACCTTTATAGGTAAGAAGCCCCTTGAGCCGCGTAGTCAGCGCGTTTAGCTG
TAT
+
FFFFFFFFFFFFF:FFF:FFFFFFFFFFFFFFF:FFFFFFFFFFFFFFFFFFFFFFFFFFFFFFFFFF
FFFFFFFFFFFFFFFFFF:FFFFFFFFFFFFFFFFFFFFFFFFFFFFFFFFFFFFFFFFFFFFFFFFF
FFF
@A00721:190:HGJW7DSXY:4:2558:1958:33411_1:N:0:TCTGCAAG+GCTGCACT
TATACCCGATAGGGTACCGAGTCTCCAAGAGAATATTTATTCTTATGAGGCTCGAAACTTCAACGTTT
TCACAACGGAAAGACATATCGCACCCGAAATTTTTGGGCAGAATGCTCTTAACGCGGTTCTTGAACGG
TGT
+
FFFFFFFFFFFFFFFFFFFFFFFFFFFFFFFFFFFFFFFFFFFFFFFFFFFFFFFFFFFFFFFFFFFF

FFFFF:FFFFFFFFFFFFFFFFFFFFFFFFFFFFFFFFFFFFFFFFFFFFFFFFFFFFFFFFFFFFFF
FFF
@A00721:190:HGJW7DSXY:4:2263:10294:14685_2:N:0:TCTGCAAG+GCTGCACT
CTAAGTTGGATAAAGTTATAGGAGACTCAGTTAACCTGAGAGCTCCTCAATTGATAGATTGGGTCAAC
AATAGGGACGCTAGTATACTTCGTAAAGTCGACAGCGAAATGGTCAAT
+
FFFFFFFFFFFFFFFFFFFFFFFFFFFFFFFFFFFFFFFFFFFFFFFFFFFFFFFFFFF:FFFFFFFFF
FFFFFFFFFFFFFFFFFFFFFFFFFFFFFFFFFFF:FFFFFFFFFFFFF
@A00721:190:HGJW7DSXY:4:2604:25753:31125_1:N:0:TCTGCAAG+GCTGCACT
ATATGACCTATTTTAAGCTCATGGTGAAAAGGGAAGCTAAGGTTAAACTTGATGCTTCAGCCTTTTCG
AAGCATAACCCCGCACAGAATATCGTTTATCACGCAAAATTTATTAATGCAATTTTCGGACCTATCTT
TAA
+
FFFFFFFFFFFFFFFFFFFFFFFFFFFFFFFFFFFFFFFFFFFFFFFFFFFFFFFFFFFFFFF:FFFFF
FFFFFFFFFFFFFFFFFFFFFFFFFFFFFFFFFFF:FFFFFFFFFFFFFFFFFFFFFFFFFFFFFFFFFF
FFF
@A00721:190:HGJW7DSXY:4:2308:29423:25989_1:N:0:TCTGCAAG+GCTGCACT
TGTCTTTTACCCAACATTTTTATGTATACAGAGATGACCAGTGACGATTTCTCCCGGAAGCTCTTCTC
TCTCTTAGGAGGTCATGATGACTACTATAAGGGTGAAATCGATTTTTCGAAGTTTGACAAATCTCAAG
ATT
+
FFFFFFFFFFFFFFFFFFFFFFFFFFFFFFFFFFFFFFFF:FFFFFFFFFFFFFFFFFFFFFFFFFFFFF
FFFFF:FFFFFFFFFFFFFFFFFFFFFFFFFFFFFFFFFFFFFFFFFFFFFFFFFFFFFFFFFFFFFFFF
FFF
@A00721:190:HGJW7DSXY:4:1158:24560:13369_1:N:0:TCTGCAAG+GCTGCACT
GATAAAGATGTATTCTTATTTAGGCTTCGATGCTGAATTTCTTGAACTTTGGAGTGCCAGTGAATATT
TTTGTCACGCCAGGAGTATCCATAACGATTTGTCATTTACCACCTTTGCTCAACGACGAAGTGGAACG
GCC
+
FFFFFFFFFFFFFFFFFFFFFFFFFFFFFFFFFFFFFFFFFFFFFFFFFFFFFFFFFFFFFFFFFFFFF
FFFFFFFFFFFFFFFFFFFFFFFFFFFFFFFFFFFFFFFFFFFFFF:F:FFFFFFFFFFFFFFFFFFFFF
FFF
@A00721:190:HGJW7DSXY:4:2469:6334:28651_2:N:0:TCTGCAAG+GCTGCACT
TGGTAATTGTATGATCACTTTGATGATTTTATCACTTTATTATGACCTTGGAAAGTTTTCGGCTGTGG
TTGTCGCTGGGGACGATTCCATTCTGTTTTCAAAGACTCCAATAACTG
+
FFFFFFFFFFFFFFFFFFFFFFFFFFFFFFFFFFFFFFFFFFFFFFFFFFFFFFFFFFFFFFFFFFFFF
FFFFFFFFFFFFFFF:FFFFFFFFFFFFFFFF,FF:FFFFFFFFFFFF
@A00721:190:HGJW7DSXY:4:2369:3369:2566_2:N:0:TCTGCAAG+GCTGCACT
CAGCGTACTTCTGCTCGCGATTTATTCTTTTTGTCAATGATTACATCTACTTCGTTCCTGATCCTTAC
AAGATCATCGTTAAGTTAGGTAAACCCCTAACCGATGTTACTCTTGTA
+
FFFFFF:FFFFFFFFFFFFFFFFFFFFFFFFFFFFFFFFFFFFFFFFFFFFFFFFFFFFFFFFFFFFFFF
FFFFFFFFFFFFFFFFFFFFFFFFFFFFF:FFFFFFFFFF:FFFFFFFF
@A00721:190:HGJW7DSXY:4:2556:29234:18646_1:N:0:TCTGCAAG+GCTGCACT
TTTAAAGACTATACTAAATATCTCCACTACGAGGTTGTTGTTTGGCGATTAGCTCTTGCGACTAATAT
TCGATATTCGATTGAGGGTCGTTATACCTATTCGGCTATCTGTGCTTTGAATTGTATAAGATCAAATT
TTA
+
FFFFFF:F,FFFFFFFFFF:FFFF:F:FF:F,FFFFFFFFFFFFF:FFFF:F:F::FFF,F,FFFFFF
FFFFFFFFF:FFFFFFFFFF:FFFFFFFFFFFFFFFFFF::FFF:FFFFFFFFFFFFFF:F,,FFFFF
FFF
@A00721:190:HGJW7DSXY:4:1524:30101:16110_2:N:0:TCTGCAAG+GCTGCACT
TGATGTGGAGGGAGTTCTTGAAGCATCCCGACCTTTCCTTGTGAAAATTTTGATGCGGTTTCATAAGA

TTATCAGTAATCCTGATTCTACTACGGATTCATGGACCTTCCATCCTG
+
FFFFFFFFFFFFFFF:FFFFFFFFFFFFFFFFFF,FFFFFFFFFFFFFFFFF:FFFFFFFFFFFFFFF
FFFFFFFFFFFFFFFFFFFFFFFFFFFFFFFFFFFFFFFFFFFFFFFF
@A00721:190:HGJW7DSXY:4:2256:4345:8923_2:N:0:TCTGCAAG+GCTGCACT
GACGTATCTCAAAGATCGACTTCGAGACAGGTGATATTGTTGAGCGAGAGCGACCAAGATCTAAATTC
GGTAACGGTGCGTGGCGCTTTATTTTAAAAAGCTCCCTCCTCTCCCAA
+
FFFFFF,F:FFFFFFFFFFFF,FFFFFFFFF,FFFF:FF:FFF::FFFFF:FFFFFF:FFFFFFFFFF
,FFFFFFFFFF:FFFFFFFFFFFFFFFFFFFFFFF:FF,FFFFF,:FFF
@A00721:190:HGJW7DSXY:4:1635:6858:18912_2:N:0:TCTGCAAG+GCTGCACT
ACTTGAGTCTTTTGATCAGTCTCTGACTTGACCATCTTTCATTTTAAATGACGATCATTAAGACTCTC
TTTCTTCTTTGTTTCCTTCTTTGCACTGATTTTTCCTCATCAGCTTGT
+
FFFFFFFFFFFFFFFFFFFFFFFFFFFFFFFFFF::FFFFFFFFFFFFFFFFFFFFFFFFF:FFFF:::
F:FFFF:F:FFFFFFFFFFFFFFFFFFFFFFFFFF:FFFFFFFFFFFFFF
@A00721:190:HGJW7DSXY:4:1405:22363:11381_2:N:0:TCTGCAAG+GCTGCACT
TTTTACGATAACAACTATCAGTTGATTAAGACTCTTCCGAAAGGCGAACTTTGGGAATTTGAAGCTCA
GGGTGAAGCACAACGTTTGACCGTGACGAACTGTGGTGTTCTTGATCT
+
FFFFFFFFFFFFFFFFFFFFFFFFFFFFFFFFFFFFFFFFFFFFFF,FFFFFFFFFFFFFFFFFF,FFFF
:FFFFFFFFFFFFF:FFFFFFFFFFFF:FFFFFFFFFFFFFFFFFFFF:
@A00721:190:HGJW7DSXY:4:1336:15628:24424_1:N:0:TCTGCAAG+GCTGCACT
ATACTCTCCTTGAGTTCACTCGTAAGGACACTACCACTTATTACGATATTTCTTTAGTCGATGGGTTT
TCGACTCCTTTAAGCGTCTTCTGTTCATCGCAACCAGATGATATCTTCTATTCGAACTTATGTTTCGA
TCA
+
FFFFFFF,:FFF:FF:FFFFFFFFFFFFFFFFFFFFFFFFFFFF:F:FFF:FFFFFFFFFFFFFFFFFF
:FFFFFF:FFF:F:,FFFFF,FFFFFFF:FF:FFFFF,F:FFFFFFFFFFFFFFFF,FFFF,FFFF:F
FFF
@A00721:190:HGJW7DSXY:4:1376:26657:23547_2:N:0:TCTGCAAG+GCTGCACT
TCTTCTAATGTGGCCTGAACTACGTTTCACTTTGTGTGTACTTGTCTCTATTTTAAGTTTTGTCTTCC
TTGTTTGTCTTATTCTATTAATCACTCATTTTAGTGAGATTATTAAAA
+
:F,FFFFFFF:FFFFFFFFFFFFFF,FFFF,FFFFFFFF:F:F:FFFFFFFFFFFFFFF:FFFFF,FF
FFF:,FFFF:FF,FFFFF:FF:FFFFFF:F,FFFFFFFFF,FFFFFFF
@A00721:190:HGJW7DSXY:4:1242:30246:25441_2:N:0:TCTGCAAG+GCTGCACT
CGACAACAGCAGGGGTTTTCGAATAGTGATAGCCACCCGCCTTACAGAGGGGCAGCAGCATGACCATA
ATTGGTATTGACTTTGGTACCACCTTTTCTACTGTCTGTCTCGCCTCT
+
FFFFFFFFFFFFFFFFFFFFFFFFFFFFFFFFFFFFFFFFFFFF:F:FFFFFFF,FFFFFFFFFFFFFF
FFFFFFFFFFFFFFFFFFFFFFFFFFFFFFFFFFFFFFFFFFFFFFFF
@A00721:190:HGJW7DSXY:4:2361:31494:6183_1:N:0:TCTGCAAG+GCTGCACT
CGTGTGCTCTTCCGATCTGTGAATTTATACCTACAATCATTGGTTTTTCTAATAATGATGACACTATT
GTTTATGGATATGATGCTATTACTCATGATGGTTACGGTCAGGGAGGATTCTCAGTTTACAAGGACCT
TAA
+
FFFFFF:F:FFFFFFFFFFF:FFFFFFFFFFFFFFFFFFFFFFFFFFFFFFFFFFFFFFFFFFFFFFF
F:FFFFFFFFFFFFFFFFFFFFFFFFFFFFFFFFFFFFFFFFFFFFFFFFFFFFFFFFFFFFFFFFFF
FFF
@A00721:190:HGJW7DSXY:4:1566:26756:10598_2:N:0:TCTGCAAG+GCTGCACT
CCGAAAGACGTGCTAAATTAAAACCATTGTACGATGTCGTCTGTCTCGGCGATCACTTTTCACTAAGA
ATTGGTCCTACTTTTGGACAAGGCCAACTTCGTAATCTTCATGAGATC
+

FFFFFFFFFFFFFFFFFFFFFFF:FFFFFFFFFFFFFFFFFFFFFFFFFFFFFFFFFFFFFFFFFFFFF
FFFFF:FFFFFF:::FFFFFFFFFFFF:F:FFFFFF:FFFFFFFFFFF
@A00721:190:HGJW7DSXY:4:1224:7997:1532_1:N:0:TCTGCAAG+GCTGCACT
TTAGGGATTTTGAGAGAGTGAAAGGCTTCAATTGTAGTGGTTTGGTCGTTTCAGTACCCAGTCAGTAT
ACCTCTACGCAAAGATATTTCATGAAAACTACAGAGGAACGTACCGGATTACCTGTCTTTCATATTAT
GAA
+
F,FFFFFFFFFFFFFFFFFFFFFFFFFFFFFFFFFFFFFF,FFFFFFFFFFFFFFFFFFFFFFFFFFFF
FFFFFFFFFFFFFFFFFFFFFFFFFFFFFFFFFFFFFFFFFFFFFFFFFFFFFFFFFFFFFFFFFFFF
FFF
@A00721:190:HGJW7DSXY:4:2234:10375:16924_2:N:0:TCTGCAAG+GCTGCACT
TCTTTGCTTCGATGTTGGATGTCAAGAAGGGTTCAGAATGGGATTCCTACGTAGTTTATGACTTTGGT
GGTGGTACTTTCGATACTTCTATCGTCTTACGTTACGGTAATTACTAT
+
FFFFFFFFFFFFFFFFFFFFFFFFFFFFFFFFFFFF,FFFFFFFFFFFFFFFFFFF:FFFFF,FFF:
FFFFFFFFFFFFFFFFFFFFFFFFFFFFFFFFFFFFFFFFFFFF::F,F
@A00721:190:HGJW7DSXY:4:1464:8160:23547_1:N:0:TCTGCAAG+GCTGCACT
ATAATCTTGGAGGTCGTGACGTTGATGAAGCTATAAGAGCGTTCTTATTACGGCGCACTTCTTTACCC
GCCACCACACAACTTTCAGTTTCACAACTGAAGGAGGAGGTTTCTGTTACTGGAAAACCCTCGTCAAT
ACT
+
FFFFFFFFFFFFFFFFFFFFFFFFFFFFFFFFFFFFFFFFFFFFFFFFFFFFFFFFFFFFFFFFFFFF
FFFFFFFFFFFFFFFFFFFFFFFFFFF:FFFFFFFFFFFFFFFFFFFFFFFFFFFFFFFFFFFF:FFFF
FFF
@A00721:190:HGJW7DSXY:4:2277:11143:4633_1:N:0:TCTGCAAG+GCTGCACT
TCTTATGACGATCTAGTGAAATTAACGCGTCCTTTTCTGGAACGTAGTTTTACTATTATGAACAAAGT
TATCAAAGATAGTGGCATTCAAGGCAAACTTACCCTGATTCCGGCCGGTGGTAGCTCATTTCTACCAG
GGG
+
FFF:FFFFFFFFFFFFFFFFFFFFFFFFFFFFFF:FFFFFFFFFFFFFFFFFFFFFFFFFFFFFFFFF
FFFFFFFFFFFFFFFFFFFFFFFFFFFFFFFFFFFFFFFFFFFFFFFFFFFFFFFFFFFF:FFFFFFFF
FFF
@A00721:190:HGJW7DSXY:4:1515:16423:2848_2:N:0:TCTGCAAG+GCTGCACT
TGCTGAAACAGCTATTAAAGCTGTTGAGCGGGTAATTACACCTAAACGAGCTCGATCTGCCATCGCTG
AAGGATGTGCACTCTTTTCTGCTACTTTAACGGAGCGTAGTGTACTCT
+
FFFFFFFFFFFFFFF:FFFFFFFFFFFFFFFFFFF:FFFFFFFFFFFFFFFFFFFFFFFFFFFFFFFFF
FFFFFFFFFFFFFFFFFFFFFFFFFFFFFFFFFFFFFFFFFFFFFFFF
@A00721:190:HGJW7DSXY:4:1647:8296:27132_1:N:0:TCTGCAAG+GCTGCACT
CAACAGTAACATTACTTCTAATAAAGGTCTCTATCAAGCTCGTATTATCGTCGCTGCTGGATCTGCGT
TACCGTTGAAGGCTAAACTCCAAGGTAATTATGAAGACTGTGATTTATCTTTCACTCTGGCCTTGTAT
GAG
+
:FFFFFFFFFFFFFFFFFFFFFFFFFFFFFFFFFFFFFFFFFFFFFFFFFFFFFFFFFFFFFFFFFFF
FFFFFFFFFFFF,FFFFFFFFFFFFFFFFFFFFFFFFFFFFFFFFFFFFFFFFFFFFFFFFFFFFFFF
FFF
@A00721:190:HGJW7DSXY:4:1432:28637:2206_2:N:0:TCTGCAAG+GCTGCACT
CGTAACATTAATAATTTCAATCTTCTTAAAAAGAAGATAGAGAGTCATACTTTGGGCTATCGTTCAGG
TGCATGGGGTTGGATAGCCAATATTGAAGTGGACACCTCTGGTAGTAT
+
FFFFFFFFFFFFFFFFFFFFFFFFFFF:FFFF:FFFFFFFFFFFFFF,FFFFFFFFFFFFFFFFFFFFF
FFFFFFFFFFFFFF:FFFFFFFFFFFFFFFFFFFFFFFFFFFFFFFFFF
@A00721:190:HGJW7DSXY:4:2411:19976:10410_1:N:0:TCTGCAAG+GCTGCACT
ATACTATGGTTGAATCAACTAAGCATACTACTTTTGCTTTGATTACTCTACCGCCTTACAAGTCTAAG

GTTAAAGAGTCGATTAAACAAGTTGTACTTATCAATGATTTTATCGCTGGTTGTTATCTCGAAAAGTC
TCT
+
F,FFFFFFFFFFFFFFFFFFFFFFFFFFFFFFFFFFFFFFFFFFFFFFFFFFFFFFFFFFFFFFFFFFF
FFFFFFFFFFFFFFFFFFFFFFFFFFFFFFFFFFFFFFFFFFFFFFFFFFFFFFFFFFFFFFFFFFFFF
FFF
@A00721:190:HGJW7DSXY:4:1140:8305:3881_1:N:0:TCTGCAAG+GCTGCACT
CCCAAAGAGAATATCTAAAATATTTACAAAATCTTACCACTATCTACGAACCTAAAGTTCTGAAGACG
TTGCTTGTCGCTTCCTCCTTTAATGGCACTACCTCGGTACAATCTTACTTGGAACGAAGGCGACCTGT
TCC
+
FFFFFFFFFFFFFFFFFFFFFFFFFFFFFFFFFFFFFFFFFFFFFFFFFFFFFFFFFFFFFFFFFFFFF
FFFFFFFFFFFFFFFFFFFFFFFFFFFFFFFFFFFFFFFFFFFFFFFFFFFFFFFFFFFFFFFFFFFFF
FFF
@A00721:190:HGJW7DSXY:4:2333:16830:6903_1:N:0:TCTGCAAG+GCTGCACT
TGTTCTGTCTATATTAGGTGAAGCGAAATACGATTTCGAACGTCAACGTTCACCGAGTTTCGTTTCGT
ACCTTGATGCTGACTTTCGTCAATATGATTTGATCTGGTTGCTCAATTCTAAGCGTGTTCCGCTTAGT
GCG
+
FFFF,FFFFFFFFFFFF:FFFFF:F:FFFFFFFFFFFFFFFFFFFFFFFFFF:FFF:FFFFFFFFFFF
FFFFFFFFFFFFFFFFFFFFFFFFFFFFFFFFFFFFFFFFFFFFF,FF::FF,F:FFFFFFFFFFFFF
FFF
@A00721:190:HGJW7DSXY:4:2351:30951:2143_2:N:0:TCTGCAAG+GCTGCACT
GAGTATTGCTATTTATACGGTTACGAGAAGAAGGTTCGAAAGTTTGAGAACTTAGTTTACGAACTTGA
CTCTACTATACGTGACATACTAAATACTAGATATTTGGATGATCTTGA
+
FFFFFFFFFFFFFFFFFFFFFFFFFFFFFFFFFFFFFFFFFFFFFFFFFFF:FFFFFFFFFFFFFFFF
FFFFFFFFFFFFFFFFFFFFFFFFFFFFF:F:FFFFF:,FFFFFFFFFF
@A00721:190:HGJW7DSXY:4:2616:15492:32456_2:N:0:TCTGCAAG+GCTGCACT
CATGAATTTTCAGAGGATGAAAAATTTTCTTCCTTGGATTTTAATATAAAGAATCTTTACCAGGACTC
CTACTACAGGAGGCAGTCTTCTGATCGTAAGAAGTTTATAAGGAACAT
+
FFFFFFFFFFFFFFFFFFFFFFFFFFFFFFFFFFFFFFFFFFFFFFFFFFF:FFFFFFFFFFFFFFFF
FFFFFFFFFFFFFFFFFFFFFFFFFFFFFFFFFFFFFFFFFF,FFFFF:
@A00721:190:HGJW7DSXY:4:1142:20021:8484_2:N:0:TCTGCAAG+GCTGCACT
CGAAACTCTAAGTGGTAATACTGTCTTTCCAAGCTTACCTGTATCTTCAATGAAATTGGACTCTTCGA
TAGGGGACATTTTATCGTGGGCTTTCCGTAAAGGAAGATCTGTTATCG
+
FFFFFFFFFFF:FFFFFFFFFFFFFFFFFFFFFFF:FFFF:FFFFFFFFFFFFF,FFFFFFFFFFFF:F
FFFFFFFFFFFFFFFFFFFFFFFFFFFFFF,F,FFFFFF:FFFFFFFFF
@A00721:190:HGJW7DSXY:4:1142:20021:8484_1:N:0:TCTGCAAG+GCTGCACT
ACCGGTGAATGGTTTAAGAATACTATAAATAGAATAGCTATTACTACTAATGGCGTTGGCCTAGTGGA
TGTTATTAGTGACATCTGGTCAATTTCGTTTTTAGGTGGTCTTTATTCTATTATTGATTTTTATGAGA
TTG
+
FFFFFFFFFFFFFFFFFFFFFFFFFFFFFFFFFFFFFFFFFFFFFFFFFFFFFFFFFFFFFFFFFFFFF
FFFFFF:FFFFFFFFFFFFFFFFFFFFFFFFFFFFFFFFFFFFFFFFFFFFFFFFFFFFFFFFFFFFFF
FFF
@A00721:190:HGJW7DSXY:4:1152:24722:13651_2:N:0:TCTGCAAG+GCTGCACT
TGTTAGAGTAGAATTCCTGAAGAGTCCTTCCGTAACCAACTTCTTGACAATTTTCTTCAATCCTTCGA
TAATAGTTAAATCTAGGATTATGTCTGTGTTGAAAGACTTTCTACCGA
+
FFFFF:FFFFF:F:FFFFFFFFFFFFFFF,::FFFF,FF:FFFFFFF:,FFFFFFFFFFFF:FFFFFF
FFFFF:FF,::F:FFFF:FFFFFF:FFFFFFFFFFFFFFFFFF:FFFF

```
@A00721:190:HGJW7DSXY:4:1167:32127:20118_2:N:0:TCTGCAAG+GCTGCACT
TACACAATTAAGAAATAATCCTACATTGACTGATTATCTAAAGATTGAAGGAAAATTTTATCCTCGTT
TAACTTACTTAGACTTTCCTACGATACAAATGATAACTTCTAATTTAC
+
FFFFFFFFFFFFFFFFFFFFFFFFFFFFFFFFFFFFFFFFFFFFF:FFFFFFFFFFFFFFFFFFFFFFFF
FFFFFFFFFFFF:FFFFFFFFFFFF:FFFFFFFFFFFFFFFFFFFFFFF
@A00721:190:HGJW7DSXY:4:1559:21296:3803_2:N:0:TCTGCAAG+GCTGCACT
AAGGGATGCATGGTGTTATCTCCTCAGGAGTCCCGGCATTTGATAATAATGACGAGACTTTCGTCATA
ATCGCTGCATATTACGGATTACACGGTACCAGCGCATCTCGTGTCGAG
+
FFFFFFFF:F,F:FFFF,:F:F:FF:F:FFFFFFFF:FFF:FFFFFF::FFFFFFFFFFFFF:FFFFF
F,,F:FFFF:F:FFF:FFFFFFFFF:FFFFF:FFFFFF,,FFFFFFFF
@A00721:190:HGJW7DSXY:4:1131:23032:7748_1:N:0:TCTGCAAG+GCTGCACT
AAATTTATAGAAGATTATTTTGACAGAATCAATAAGAATCATCGATTCTCGATTCGTAGAGCTTATCT
AGGTACTCTCATTTACTATGTTGCTTTAGGCTTCAGTGCTGCTGATCGAAAATTTCGATCTAGCTGGA
TCC
+
FFFFFFFFFFFFFFFFFFFFFFFFFFFFF:FFFFFFFFFFFFFFFFFFFFFFFFFFFFFFFFFFFFFF
FFFFFFFFFFFFFFFFFFFFFFFFFFFF,F:FF,FF,,,FFFF,FFFFFFF,FFFFFFFFFFFFFFFFF
FFF
@A00721:190:HGJW7DSXY:4:1509:10330:27085_2:N:0:TCTGCAAG+GCTGCACT
TTTACAACGATATAGGAGTAATCTACGAGAAGATTCTAGAATTCTTAATTATAATATTCAACCTCAAA
AGTTACGTGTTTACAGTAACGAGATCACACATTTTGAGAACTAACTAT
+
:FFFFFFFFF:F,F:FFFFFFFFFFFFFFFFFFFFFFFFFFFFFFFFFFFFFFFFFFFFFFFFFFFFF
FFFFFFFFFFFFFFFFFFFFFFFFFFFFFFFFFFFFFFFFFFFFFFFF
@A00721:190:HGJW7DSXY:4:1440:27037:22138_1:N:0:TCTGCAAG+GCTGCACT
TCTCTTCTTATAATAAATAAATAAATAATATTACTAATGGCAAATAGTGGAAAGGCTCCAGTCAACAC
TAGTTCGAGTGCTGCTTTGGATTACGATGACGTGAGAATAGGTGAAGGGATAGTTTTTACCTCGACCA
ACA
+
FFF:FFFFFFFFFFFFFFFFFFFFFFFFFFFFFFFF:FFFFFFFFFFFFFFFFFFFFF:FFFFFFFFFF
FFFFFFFF:FFFFFFFFFFFFFFFFFFFFFFFFFFFF:FFFF:FFFFFFFFFFFF:FFFFFFFFFFFFFF
FFF
@A00721:190:HGJW7DSXY:4:2105:15890:12446_2:N:0:TCTGCAAG+GCTGCACT
GCGACTAATCTTAAAGCTTCACCCAAAATGAAGGACACTTCGAAACTGAATAATGATGCACTACAAAC
AAGTATCGCTAATCATCTGAAAAAGGCTCTAAAGGACACTATACAATA
+
F:FFFFFFFFFFFFFFFFFFFFFFFFFFFFFFFFFFFFFFFFFFFFFFFFFFFFFFFFFFFFFFFFFF
FFF:FFFFFFFFFFFFFFFFFFFFFFFFFFFFFF,FFFFFF,FFFF:F
@A00721:190:HGJW7DSXY:4:2505:10185:11835_2:N:0:TCTGCAAG+GCTGCACT
ATTCAGCACCACATTTTGGTCAAGTCAATCCGTTGAGAAAATTTCTTAGAGGATATTCGCCATTGTGG
TACGAATTGGTCCAACATTTACCACCAAATTCTTTTGATACAACTCTA
+
FFFFFFFFFFFFFFFFFFFFFFFFFFFFFFFFF:FFFFFFFFFFFFFFFFFFFFFFFFFFFFFFFFFF
FFFFFFFFFFFFFFFFFFFFFFFFFFFFFFFFFFFFFFFFFFFFFFFF
@A00721:190:HGJW7DSXY:4:1329:7780:20353_1:N:0:TCTGCAAG+GCTGCACT
ATACGCTTGCTATTCACCAGATTGGTTCGAACCCGTTGATGCTATGTCAGAAGAAGCTATTCAAGCTT
GTCGTTTAAAGACTGCAGAGGCTGTCAAACAAGCCCAGACTCAAGGAGTCCAATCAAAGGTTTTGAAT
TTA
+
FFFFFFFFFFFFFFFFFFFFFFFFFFFFFFFFFFFFF::FFFFF:FFFFFFFFFFFFFFFFFFFFFFFFF
FFFFFFFFFFFFFFFFFFFFFFFFFFFFFFFFFFFFFFFFFFFFFFFFFFFFFFFFFFFFF:FFFFFFF
FFF
```

```
@A00721:190:HGJW7DSXY:4:2557:2437:1736_2:N:0:TCTGCAAG+GCTGCACT
TGGGTGATAAAAATTATTTAGAGATCTTTTCTACTTACAAAGGACAATTCAGTTTCATTAATTCTACG
GAAAAGTCCGGAGTATATTGTATGTTAATAGATAGTGGGATAATAGTT
+
FFFFFFFFFFFF:FFFFFFFFFFFFFFFFFFFFFFFFFFFFFFFFF,F:FFFFF:FFFFFFFFFFFFFF
FFFFFFFFFFFFFFFFFFFFFFFFFFFFFFF:FFFFFFFFFFF:F:FFF
@A00721:190:HGJW7DSXY:4:2351:32678:13745_2:N:0:TCTGCAAG+GCTGCACT
GAACGCGTTCGTATAACCAAGTACTTTCCGTCTGAAGAGTTTAGAGCTGCTATTGATTATGTAACCGA
TAGGAGTTGTATAGTTGAAGATGTGGCTTTAGACGATAATCCTTTAAG
+
FFFFFFFFFFFFFFFFFFFFFFFFFFFFFFFFF:FFFFFFFFFFFFFFFFFFFFFFFFFFFFFFFFFF
FFFFFFFFFFFFFFFFFFFFFFFFFFFFFFFFFFFFFFFFFFFFFFFF
@A00721:190:HGJW7DSXY:4:1654:29785:28213_1:N:0:TCTGCAAG+GCTGCACT
GAAGATTTTCCCACAGATTTGTCTACGTTGCAGATCGCCACTCCAAACACTATATACATTATAAAATA
TAAAAATAAGGGAGTCATAACTTTTGTTACCAAATTCTTGGCTTTCAACGAAGTCTCTGATTTTATTA
ATC
+
FFFFFFFFFFFFFFFFFFFFFFFFFFFF,FFFFFFFFFFFFFFFFFFFFFFFFFFFFFF:FFFFFFFFF
FFFFFFFFF:FFFFFFFFFF:FFFFFFFFFFFFFFFFFFFFFFFFFFFFFFFFFFFFFFFFFFFFFFFF
FFF
@A00721:190:HGJW7DSXY:4:2170:19840:19069_1:N:0:TCTGCAAG+GCTGCACT
GATCTCTCGGTCATTTACTCTGTACTTGATCTACGTCGTTTAACTGTCGGAACAACCTTTACTTAGAA
TGACTCTATCTTTTCAGACACTTCTTATATTATTTACAATAGTTAATATCATATTAGTAATACTCACT
AT
+
FFFFFFFFFFFFFFFFFFFFFFFFFFFFFFFFFFFFFF,FFFFFFFFFFFFFFFFFFFFFFFFFFFFF:
FFFFFFFFFFFFFFFFFFFFFFFFFFFFFFFFFFFFFFFFFFFFFFFFFFFFFFFFFFFFFFFFFFFF
FF
@A00721:190:HGJW7DSXY:4:1409:5773:35446_2:N:0:TCTGCAAG+GCTGCACT
TTTTCTTAGCTAATAGATTAATAAAGGATGAGTACGAAAACCAACTCTTCGTTACGAGACCTGATGGT
AGAATAGATTTAGTCTCTACACCCATCTCAACGAGGATAAAGCGAGTT
+
FFFFFFFFFFFFFFFFFFFFFFFFFFFFFFFFFFFFFFFFFFFFFFFFFFFFFFFFFFFFFFFFFF:FF
FFFFFFFFFFFFFFFFFFFFFFF:FFFFFFFFFFFFFFF:FFFFF:FFF
@A00721:190:HGJW7DSXY:4:2523:25201:17425_1:N:0:TCTGCAAG+GCTGCACT
ACACTAATATATAATCCGCACACGACTCGAAACCGAGACGCCGTAGATCAAAGTCCCTTCTAACTTTA
GATTATGAATCCTGCCTTTACTCATTTTATAGCAGTTATTTATGTTGTACTTTTTATAATTTATAACC
TT
+
F:,,,F,:,,F:,,,FF:,F,FFFFFFFFFFFFFFFFF:FFFFFFFFFFFFFFFFFFFFFFFFFFFFF
FFFFFFFFFFFFFFFFFFFFFFFFFFFFFFFFFFFFFFFFFFFFFFFFFFFFFFFFFFFFFFFFFFFF
FF
@A00721:190:HGJW7DSXY:4:2136:13856:26083_1:N:0:TCTGCAAG+GCTGCACT
TACTTATTGTAACGATCATTCTTTCGAGTTTTGTCATTCTTATTCTTTTATTCCTTTAACTGTGTATG
AGGAGGATTTATAATGCTTTTCGATAGTGATTGTTATCAATTTAAATACATTACTGTTTCAAATCATC
TTT
+
FFFFFFFFFFFF,FFFFFFFFFFF,FFFFFFFFF:FFFFFFFFFFFFFFF:F:FFFFFFFFFFFFFFF
FFFF:F:FFFFFFFFFFFFFF:FFFFFFFFFFFFFFFFFFFFFFFFFFFFFFFFFFFFFFFFFFFFF
FFF
@A00721:190:HGJW7DSXY:4:2269:8124:18317_1:N:0:TCTGCAAG+GCTGCACT
GAAAATGGTTTCAAATGAGGTCAAAGCTCTATTTATTAAACGAACTATTATCCGATTCTTCGAGGGTA
TCACTACCACTGATCTGCCGACTTTAGTCAAGGAACTTCAATTCCTTCGATGTTTATATTTTTCTATA
AGG
```

+
FFFFFFFFFFFFFFFFFFFFFFFFFFFFFFFFFFF:FFFFFFFFFFFF:FFFFFFFFFFFFFFFFF
FFFFFFFFFFFFFFFFFFFFFFFFFFFFFFFFFFFFFFFF:FFF:FFFFFFFFFFFFFFFFFFFFF
:FF
@A00721:190:HGJW7DSXY:4:1423:28863:25426_2:N:0:TCTGCAAG+GCTGCACT
TAATCAAATTTCGACAAATTTTAGAACTTTATTTCAGAACTCTCTCCGATCACCTTGGTGTGATCTCC
CGGAGGATCTTTTCAATAAGATGTATGATGATAATCTACTATTTTTAA
+
FFFFFFFFFFFFFFFFFFFFFFFFFFFFFFFFFFFFFFFFFFFFFFFFFFFFF:FFFFFFFFFFFF
FFFFFFFFFFFFFFFFFF:FF:FFFFFFFFFFFFFFFFFFFFFFFFFFF
@A00721:190:HGJW7DSXY:4:1670:14850:6230_1:N:0:TCTGCAAG+GCTGCACT
AGCGTATACTTGAATACTTTATCAAAGATTTGGCTTGGTCTATTTACCGCAAAAAGTTGGAAGAGGTC
TATGATATTTATTTTACTTATACTTTAAATAGCACCGATTATAACTTGACTGTTGATAATATTACAAT
AGC
+
FFFFFFFFFFFFFFFFFFFFFFFFFFFFFFFFFFFFFFFFFFFFFFFFFFFFFFFFFFFFFFFFFFF,
FFFFFFFFFFFFFFFFFFFFFFFFFFFFFFFFFFFFFFFFFFFFFFFFFFFFFFFFFFFFFFFFFFFF
FFF
@A00721:190:HGJW7DSXY:4:2217:8106:28181_2:N:0:TCTGCAAG+GCTGCACT
AAGATTAGAGAAGTAGCTCGGTGAACACGTCGGTAGCATTGATTTACTTTTACCGTTAGAACTGAAAA
AGATTCTTTTCGAAAAGAAACCTATAAGTTACAAACGATTCTTCGACT
+
F:F:FFFFFFFFFFF,FFFFFFFFFFFFFFFFFFFFFFFFFFFFFFFF::FFFF:FFFFFFFFFFFF
:FFFFFFFFFFFFFFFFFFFFFFFFFFFFFFF:FFFFFFFFFFFFFFF
@A00721:190:HGJW7DSXY:4:1126:26323:20306_1:N:0:TCTGCAAG+GCTGCACT
AATAAAAATACTTCAAGTACCTGAACTTTACATTTTTAATGGTATGGTATAATGTGTCTTACAAATAG
AACCTTCACTCTCGGTAATGAGAGTGTATTCCTTAATATTCTAGTAACCGCTATGTGGGCATTACTAG
GAT
+
FFFFFFFFFFFFFFFFFFFFFFFFFFFFFFFFFFFFFFFFFFFFFFFFFFFFFFFFF:FFFFFFFFFF
FFFFFFFFFFFFFFFFFFFFFFFFFFFFFFFFF:FFFFFFFFFFFFFFFFFFFFFFFFFFFFFFFFFF
FFF
@A00721:190:HGJW7DSXY:4:1362:6750:2597_2:N:0:TCTGCAAG+GCTGCACT
TTTAAATGCTAGGAAAATTTTAGAAAAACTCCATTGTATAGTTCAAGGAGTACTTAATTCAATAGCTT
TAGTATTATTTACTATCGCCTTTATTGAAAATTGTATAAGAGTTAATT
+
:FFFFFFFF:FFFFFFFFFFFFFFFFFFFFFFFFFFFFFFFFFFFFFFFFFFFFFFFFFFF:FFFFFF
FFFFFFFFFFFFFFFFFFFFFFFFFFFFFFFFFFFFFFFFFFFFFFFF
@A00721:190:HGJW7DSXY:4:2175:14009:7592_1:N:0:TCTGCAAG+GCTGCACT
GGGAGAATGAACTGCCGCTTCCCGGAAGAAACGATCTATTTACAGAGTATTTAGAGTAGTCCAAAGTT
TGTATAAATTTATATGATTTTCTATATATATACCACATATATATATATATAAAATCCTATAAATTTGT
ACA
+
FFFFFFFFFFFFFFFFFFFFFFFFFFFFF:FFFFF,FFFFF:F:FFFFF:FFFFFFFFF:FFFF,::F
FFFFFFFFFFFFFFFFFFF:FFFFFFFFFFFFFFFFF:FFFFFFFFFFFFFFFFFFFFFFFFFFFFFF
FFF
@A00721:190:HGJW7DSXY:4:1403:8938:27242_2:N:0:TCTGCAAG+GCTGCACT
TACACTCCCCAAACATAACATACCATAACCTACCATGACCAATCATCGCTCCAACTGGCACGCCAGAC
GTAGGGTCAAACCTACTACCGCCTGTCGGCAGATCGGCACCACTGGTC
+
FFFFFFFFFFFFFFFFFFFFFFFFFFFFFFFFFFFFFFFFFFFFFFFFFFFFFFFFFFFFFFFFFF:F
FFFFFFFFFFFFFFFFF:FFFFFFFFFFFFFFF,FFFFFFFFFFFFFF
@A00721:190:HGJW7DSXY:4:2561:28185:18552_2:N:0:TCTGCAAG+GCTGCACT
ACCCGCTGTGCAGAATAACCTTCCCAAATCTAGATCTGCACAGAAGAAGGCTGAAGGTCGTTACCGGA

CGACCACTCAAAGGAAACGGGCTGTTGCCCAGAGGAAAGCAGCGACCA
+
FFFFFFFFFFFFFFFFFFFFFFFFFFFFFFFFFFFFFFFFFFFFFFFFFFFFFFFFFFFFFFFFFFFFF
FFFFFFFFFFFFFFFFFFFFFFFFFFFFFFFFFFFFFFFFFFFFFFFF
@A00721:190:HGJW7DSXY:4:1427:4227:29356_2:N:0:TCTGCAAG+GCTGCACT
ACGGCAGTCCCGTCATCTCTGTGGGAACTGAGGCAGTACCTCAGTCAAAAGTCACATTCACCACTCCC
ATACCAATCAATGATGTGACTATACAGTTCGGCCATTTCAAGAGTGGT
+
FFFFFFFFFFFFFFFFFF:F:FFFFFFFFFFFFFFFFFFFFFF:FFF:FFFFFFFFFFFF:FFFFFF
FFFFFFF::F:FFF,:FFFFFFFFFF:::FFFFFFFFFFFFFFFFFFF
@A00721:190:HGJW7DSXY:4:1245:3486:35368_2:N:0:TCTGCAAG+GCTGCACT
GGAGGTACTGAAGCCTTCCCCTCCGCTGGCACGAAGAACACTCCCAAGCCAATCAATAGTTCTTCTGA
CCAGAATGAAAAGGGGTTTACCAGGGTAGTCTCTAAGTCTACCAGGAA
+
FFFFFFFFFFFFFFFFFFFFFFFFFFFFFF:FFFFFF,FFFFFFFFFFFFFFFFFFFFFFFFFFFFFF
FFFFFFFFF:F:FFFFFFFFFFFFFFF:FFFFF,FFFFFFFFFFFFFF
@A00721:190:HGJW7DSXY:4:1163:20066:25347_2:N:0:TCTGCAAG+GCTGCACT
CAAAACTGATAAGAATAAAGGGAAAGGTCCATCAGGACCTAAGAAGGGGAAATTACCCCTAATTCTGA
AGGAAGATCTCCCACCATCTGGGGTTGGGAATAAGAAATCTGCAGATC
+
FFFFFFFFFFFFFFFFFFFFFFFFFFFFFFFFFFFFFFFFFFFFFFFFFFF,FFFFFFFFFFFFFFFF
FFFFFFFFFFFFFFFF,FFFFFFFFFFFFF:FFFFFFFFFFFFFFFFF
@A00721:190:HGJW7DSXY:4:1407:7003:22326_2:N:0:TCTGCAAG+GCTGCACT
CTACGAAGTCGCTGAAGGCGGCTTGGGTCATTAAGGGTAGCTGTAAAGTTGCGCCTGCCGACCCCATC
TTTGAGACCAAGACTCTCTACAGTAGGTTCGTGCCTACTATGAGCAAT
+
FFFFF,FFFFFFFF:FFFFFFFFFFFFF,FFFFFFFFFFFFFFFFF:FFFFFFFFFFFFFFF:FFFFF
FFFFFFFFF:FFFFFFFFFFFFFFFFFFFFFFFFF:FF:FFFF,FFFFF
@A00721:190:HGJW7DSXY:4:2160:17815:16031_2:N:0:TCTGCAAG+GCTGCACT
TCGTCACTGAGGACGAAGGTGAGAGGACTTTTGGCTACTGGCGACAAACTGAGAGCAGTCGCTGGGGA
AAGAGAATTGCGTGGGTTGAAGGCCGACGCTTCGAGCTTGAGTGTGAC
+
FFFFFFFFFFFFFFFFFFFFFFF:FFFFFFFFFF:FFFFFFFFFFFFFFFFFFFFFFFFFFFFFFFFF
F:FFFF:FFFFF,FFF:FFFFFFFFFFFFFFFFFFFFF,FFFFFFF:F
@A00721:190:HGJW7DSXY:4:2416:7817:1908_2:N:0:TCTGCAAG+GCTGCACT
GTTTTTGTCAACGGCGTTTGGAGGATTTTATCCGCGCTTAACTATGGGGCGAAGATCCCCTATGGAGT
CTTGAAGGACTTAGGTTGGCATATTTATGCCGACGATAATAAGCATGT
+
FFFFFFFFF:FFFFFFFFFFFFFFFFFFFFFFFFFFFFFFFFFFFFFFFFFFFFFFFFFFFFFFFFFF
FFFFFF:FFF,FFFFFFFFFFFFFFFFFFFFFFFFF:FFFFFFFFFFF
@A00721:190:HGJW7DSXY:4:1167:19741:16172_2:N:0:TCTGCAAG+GCTGCACT
CGGGACTTACAAGAGAGAGTGTGAGAGAGTCGCTCTCACTATTTTTGCCCAGAATGCGGGCTTTCTGA
CTGATGAGGTTAAAACTCTTGCTCCTTCTGTGGGTGAGGGTTTTTGTT
+
FFFFFFFFFFFFFFFFFFFFFFFFFFFFFFFFFFF:FFF,FFFFFFFFFFFFFFFFFFFFFFFFFFFF
FFFFFFFFFFFFFFFFFFFFFFFF:FFFF:FFFFFFF:FFFFFFFFFFF
@A00721:190:HGJW7DSXY:4:2110:29243:29340_2:N:0:TCTGCAAG+GCTGCACT
TCCTCCATAATTACAACCTCTCTTTAGAGAGCAGTAGAAGGAAGTTGGGCAAATTCCCACATATGGTA
GACGTTGTCACACTTTTCTTGAAAATTTTTCCTTTCCTCCGGCATTGG
+
FFFFFFFFFFFFFFFFFFFFFFFFFF:FFFFFFF,FFFFFFFFFFFFFFFFFFFFFFFFF:FFFFFFFFF
FFFFFFFFFFFFFFFFFFFFFFFF:FFFFFFFFFFFFFFFFF,FF,FF:
@A00721:190:HGJW7DSXY:4:1135:29695:26866_2:N:0:TCTGCAAG+GCTGCACT
AAGGTGGAAGGTGTCAAGGGTCTTCTTCATGCTGATCTTTCAGGACCTAATCACATCATGATTGATAG

```
TTACGGCTCGTATCGAGTTGGAGGGAGGAAGTTCTCCGAAATTAAAGA
+
FFFFFFFFFFFFFFFFFFFFFFFFFFFFFFFFFFFFFFFFFFFFFFFFFFFFFFFFFFFFFFFFFFFFFF
FFFFFFFFFF:FFFFFFFFFFFFFFFFFFFFFFFFFFFFFFFFFFFFF:F
@A00721:190:HGJW7DSXY:4:1507:27742:27680_2:N:0:TCTGCAAG+GCTGCACT
TCAGAGAGGGGAGCTCTGAAAAGGAAACTTCGTCAGAAGATGAGGAAGTCTAAGAGGAGGAGTTTGAG
GAAGGCTCTAGAGAAGATCTCACCACCTGGAGAAGGCAAGTGGAGAGT
+
FFFFFFFFFFFFFFFFFFFFFFFFFF:FFFFFFFFFFFFFF:FFFFFFFFFF:FFFFFFFFFFFF:FFF
FF:FFFFFFFFFFF,FFFFFFFFFFFFFFFFF:FFFFF,FFFFFFFF:
@A00721:190:HGJW7DSXY:4:1405:26196:22060_2:N:0:TCTGCAAG+GCTGCACT
AAGAAGGATTATTTGAAGCAGGGGTTACCACTTCCTAAGGTGGATGAGGGAGGTTGTTTCTTTGGAGT
TTTAGATAATCTTTTGAGAAGGGGTCATAGAGCCAGCAAGAGAAGCTT
+
FFFFFFFFFFFFFFFFFFFFFFFFFFFFFFFFFFFFFFFFFFFFFFFFFFFFFFFFFFFFFFFFFFFFFF
FFFF:FF:FFFFFFF:FFFFFFF:FFFFFFFFFFFFFFFFFFF:FFFF
@A00721:190:HGJW7DSXY:4:2323:9516:22858_2:N:0:TCTGCAAG+GCTGCACT
CCTTGAGAAGACAAGCCACATTAAGATATAATTTAGGTCAACTTGGGGTTTCGAATGTCATTAGTGCC
TTACGATTGAAAGAGGTCCTTTTGAGTATCGACAGATCTTTCGATCTT
+
FFFFFFFFFFFFFFFFFFFFFFFFFFFFFFFFFFFFFFFFFFFFFFFFFFFFFFFFFFFFFF:FFFFFF
FFFFFFFFFFFFFFFFFFFFFFFFFFFFFFFFFFFFFFFFFFFFFFFF
@A00721:190:HGJW7DSXY:4:2557:22788:26960_2:N:0:TCTGCAAG+GCTGCACT
GTGGCAGAATTCGGTAACTTACCATATTGTTCCTTCCGGGAAGTTGTGGGATTTAACTTTGACTCAAC
TACTCCGATCAGATCCGGATGGTTTGATTGGTATGGACTACTCGACTT
+
FFFFFFFFFFFFFFFFFFFFFFFFFF:FFFFFFFFFFFFFFFFFFFFFFFFFFFFFFFFFFFFFFFFFFF
FFFFFFFFFFFFFFFFFFFFFFFF:FFFFFFFFFFFFFFFFFFFFFFF
@A00721:190:HGJW7DSXY:4:1426:4616:28087_2:N:0:TCTGCAAG+GCTGCACT
TCATGGCAGATACTACCGATAAGGTGAAAAATATGCAGGTCAAAGATTCTGTAGCTCTGAGGGCGGTT
GAGGGAGCCGTTTTGGCCGACTTTAACTATTCTAAGAGTCTTTATGAT
+
FFFFFFFFFFFFFFFFFFFFFFFFFFFFFFFFFFFFFFFFFFFFFFFFFFFFFFFFFFFFFFFFFFFFFF
FFFFFFFFFFFFFFFFFFFFFFFFFFFFFFFFFFFFFFFF,FFF:FFFF
@A00721:190:HGJW7DSXY:4:1132:19768:11960_2:N:0:TCTGCAAG+GCTGCACT
CGTTGTCGTACCTTACAAGGTTGAGGAGACTTTACAGCGGCTTATTGTCGCGCAGTTTCCAGAATTGC
GGTTGAAGTTCACCCATTCTATCTTGAATGCTCACGCCGTCGCAGCCA
+
FFFFFFFFFFFFFFFFFFFFFFFFFFFFFFFFFFFFFFFFFFFFFFFF:FFFF:FFFFFFFFFFFFFFFF
FFFFFF,FFFFFFFF,FFFFFFFFFFFFFFFFFFFFFFFFFFFFFFFF
@A00721:190:HGJW7DSXY:4:1268:24840:23844_1:N:0:TCTGCAAG+GCTGCACT
ATCACTTGTTAAAGTACAGATTTAGTGATTTAGATATTATTGATATTGGAGGCGATATTCGTTTTCAT
ATCCAAAACTCCTTGAATGGAGTACATATTTGCAACCCTATTCTTGATGTGAAAGATGCATCGAGGTA
TAC
+
FFFFFFFFFFFFFFFFFFFFFFFFFFFFFFFFFFFFFFFFFFFFFFFFFFFFFFFFFFFFFFFFFFFFFF
FFFFFFFFFFFFFFFFFFFFFFFFFFFFFFFFFFFFFFFFFFFFFFFFFFFFFFFFFFFFFFFFFFFFFF
FFF
@A00721:190:HGJW7DSXY:4:2158:30002:11052_1:N:0:TCTGCAAG+GCTGCACT
ATTGATTGGGAACGTACCTACTCACGAGACGTCATGAAAGCTATCGGAGGGAAGCAAAATGTTTCGTT
CTGTTACAAGAACGCTTTGGATTGTGACCATAAAGCCTCAGTTGCGATAGCTGTAGAGGTATACGATA
TCT
+
:FFFFFFFFFFF,FFFFFF,FF:FFFFFFFFFFFFFFFFFFF:F,FFFFFFFFFFFFFFFFFFFFFFFF
```

FFFFFFFFFFFFFFFFFFFFFFFF:FFFFFFFFFFFF:FFFFFFFFFF:FFFFFFFFFFFF:FFFFFFFF
FFF
@A00721:190:HGJW7DSXY:4:2255:20663:5400_2:N:0:TCTGCAAG+GCTGCACT
GCCACTATAATGCTTAAGAGAGAGATTGATGTATGTCATTTCGTCTTAGTTTGTCCCGGCGAATTATT
GGACACGATGAAAAATGACATACCCGTTCCTGACCTTGGTATCGTTAT
+
FF:F::FFFF,::,:FF:F:F:F:FF:FFF,,F:,F:FF,FF,F,,,FFF,::,,FF,FFF:F:,FFF
::F:FF:FF,FFFFF,,F,F:F:,F:,:F,FFFF,F::FFFF::F:F:
@A00721:190:HGJW7DSXY:4:1506:15754:19914_1:N:0:TCTGCAAG+GCTGCACT
GGTTATTCTCACAACAAAGATAAGATTTTATCTTTCTTCAAACACCCTGTTTTCCTTAAGGGCGGACA
ATTGTTCCATTGTGAACTCTTGGGGACTAGATTGAGCATTAATGAGTTCCGAATAACTCGTAATAAAT
CTT
+
FFFFFFFFFFFFFFFF:FFFF:FFFFFFFFFFFFFFFFFFFFF:F:FFFFF,FFFFFFFFFFFFFFFFFF
FFFFFFFFFFFFFFFFFFFFFFFFFFFFFFFFFFFFFFFFFFFFFFFFFFFFFFFFFFFFFFFFFFFFFF
FFF
@A00721:190:HGJW7DSXY:4:2155:22254:17394_1:N:0:TCTGCAAG+GCTGCACT
ATTCAGAATACCTATTTCCGTACCAAGAGCCACTGCCAATCTCATCGCTATACGCATACCTGATTGGA
AGAAGCGAGGTTCGTTCCTCAATAAAAATTTTTCTCAAGAGATTATGGTTGATAAAGTTTTCTTCATA
AGG
+
FFFFFFFFFFFFFF:FFFFFFFFFFFFFFFFFFFFFFFFFFFFFF,FFFFFFFFFFFFFFFFFFFFFFFF
FFFFFFFFFFFFFFFFFFFFFFFFFFFFFFFFFFFFFFFFFFFFFFFFFFFFFFFFFFFFFFFFFFFFFF
FFF
@A00721:190:HGJW7DSXY:4:1258:25274:31266_2:N:0:TCTGCAAG+GCTGCACT
ACGTCATGGGGATCACTTCTTCGGTCAATGACAAAACCTTCGAATATGTTACGACATGGCTTCGTAAC
AACAGTGCGAGGATTGTCATCTCGGGGAAAGTGATTCACCAAGATGTT
+
FFFFFFFFFFFFFFFFFFFFFFFFFFFFFFFFFFFFFFFFFFFFFFFFFFFFFFFFFFFFFFFFFFFFFF
FFFFFFFFFFFFFFFFFFFFFFFFFFFFFFFFFFFFFFFFFFFFFFFF
@A00721:190:HGJW7DSXY:4:2368:30083:26694_1:N:0:TCTGCAAG+GCTGCACT
GAGATACCTAGAGAAGGTCGCAGCGATCTTACTCGTTATCGGAGCTAAGAAGAAGACTTTGGGTACAC
GTTATGCTCGCAAACTTAACGTTAGTACCGGAGGTGATCTCTCTGCTTCTGCGTTACTGAGAGAAAAG
ATT
+
FFFFFFFFFFFFFFFFFFFFFFFFFFFFFFFFFFFFFFFFFFFFFFFFFFFFFFFFFFFFFFFFFFFFFF
FFFFFFFFFFFFFFFFFFFFFFFFFFFFFFFFFFFFFFFFFFFFFFFFFFFFFFFFFFFFF:FFFFFFFF
FFF
@A00721:190:HGJW7DSXY:4:2620:31629:18881_2:N:0:TCTGCAAG+GCTGCACT
ACGGTGACTTTATGGACAAGATGCAAGTTCTTGGGAAATACTTGTTTCCCTTTTTAGGTTTAGTAGGT
GATCTTGACGTCGATTCTTTGTATAGGGAGTGTGAGAAGACAACGAAT
+
FFFFFFFFFFFFFFFFFFFFFFFFFFFFFFFFFFFFFFFFFFFFFFFFFFFFFFFFFFFF:FFFFFFFFF
FFFFFFF:FFFFFFFFFFFFFFFFFFFFFFFFFFFFFFF:FFFFFFFF
@A00721:190:HGJW7DSXY:4:1320:3369:12931_1:N:0:TCTGCAAG+GCTGCACT
GAACCGGTCTCTAAATCTTTTGAGATTGAGAAGATTGAAGAACACGTCAAGAAGTCGCTCGGCGTTGT
TCTTGAAAACCAAGTTAAGAAGGAGGCTGATCATGGAGTCTTTGTACCTTCTGTCAAACTTCGAGGTT
CAG
+
FFFFFFFFFFFFFF,FFFFFFFFFFFFFFFFFFFFFFFFFFFFFFFFFFFFFFFFFFFFFFFFFFFFFFF
FFFFFFFFFFFFFFFFFFF,FFFFFFFFFFFFFFFFFFFFFFFFFFFFFFFFFFFFFFFFFFFFFFFFFF
FFF
@A00721:190:HGJW7DSXY:4:2121:7862:18709_2:N:0:TCTGCAAG+GCTGCACT
GATCCCGGATGTGGATTGAAGGCTGGCTCTAAGGGAGACTCCTTCTTTTTGAATGTTGTAAAGAATTG

GGTTGAGAAGTGTGCTTTACAAGGAGAGATCGGAAGAGCGTCGTGTAG
+
FFFFFFFFFFFFFFFFFFFFFFFFFFFFFFFFFFFFFFFFFFFFFFFFFFFFFFFFFFFFFFFFFFFFFFF
FFFFFFFFFFFFFFFFFFFFFFFFFFFFFFFFFFFFFFFFFFFFFFFF
@A00721:190:HGJW7DSXY:4:1548:30445:11381_2:N:0:TCTGCAAG+GCTGCACT
TAAAGAAAAATTTTCGAATAAACTAATGAGAGGTATAGGCTTAGTCAAGTCTGTACTTACATTCTTTA
CGCCAGATTCTTCATGGGGCGACGCTGTTTCTCTTGGTATTTCTGAAA
+
FFFFFFFFFFFFFFFFFFFFFFFFFFFF:FFFFFFFFFFFFFFFFFFFF:FFFFFFFFFFFFFF:FFFF
FFFFFFFFFFFFFFFFF:FFFFFFFFFFFF:FFFFFFFFFFFF:FFFFF
@A00721:190:HGJW7DSXY:4:2522:8250:34632_2:N:0:TCTGCAAG+GCTGCACT
CGTCATTTTCCTTTGGGATCTTTCCTCTGGTAAGGGAATTATTAAATCATTCTTCTCACTCTTGACGA
GAATTATTACTGTTTTCTCTACCCATAACTTCTTTTTCAAAAATGCGG
+
FFFFFFFF:F:F,FFFFFFFFFFFFFFFFFFFFFFFFF:FFFFFFFFFFFFFFFFFFFFFFFFFFFFFFF
FFFF,FFFFFFFFFFFFFFFFFFFFF:FFFF:FFFFFFF,FFFFFFFF:
@A00721:190:HGJW7DSXY:4:1526:16224:17628_2:N:0:TCTGCAAG+GCTGCACT
TGATGTTTTCCTACAGAGTGCAGTCGTTTCAACACATATCACCACTTTTGAAAGGAAAATAATTGGTG
ATGAAAATACGTATGTCGGTGGGGTTCTCAAGGCTCTCATCGCCACGG
+
FFFFFFF:FFFFFFFFFFFFFFFFFFFFF:FF:FF,FFF,FFFF,:,:F,:FF:F,FFF,FF:F,FFF
FF:FFFF:FF::F:FF:F:,:F::,F,F,FFF,FF:,FF,:FFF,F,F
@A00721:190:HGJW7DSXY:4:2415:15474:35775_2:N:0:TCTGCAAG+GCTGCACT
GGGTTCCATAAGATATTATCTGACATTGTGGTTCAATGATCAGGATTCTTCTTTTGGTCCTTACGTCT
TAAGTAATTTGGATGTTGTCGAAAACTATCAGGTTCTTGTCACGATTC
+
FFF:FFFFFF,FFFFFFFFFFFFFFFFFFFFFFFFFF,FFFFFFFFFFFFFFFFFFFFFFFFFFFFFF:F
FF:FFF,,FFFFFFF:FFFFFF:FFFFFFFFFFFF,FFFFFFF:F,FF
@A00721:190:HGJW7DSXY:4:1158:11379:29716_2:N:0:TCTGCAAG+GCTGCACT
TTAAAACTTCAGAGTCTCTTGGAAGTCCAGGTAGCTAATGTTCTTGCAATAGCCACAGACTTTCTGGG
AAAAGAGACCTCTGTTGTAGAGGAGTTGAGAGAATTACTCAGATTCAA
+
FFFFFFFFFFFFFFFFFFFFFFFF:FFFFFFFFFFFFFF:F:FF,FFFFFFFFFFFFFFFFFFF:FFFF
FFF:FFFFFFFFFFFFFFFFFFFFFFFFFFFFFFFFFFFF:FFFFFFFF
@A00721:190:HGJW7DSXY:4:1465:7536:12477_2:N:0:TCTGCAAG+GCTGCACT
ACAACGAGTTAGGGAGTGGCTAGGTTTTAAAAGATCCTTTGCACAAGCAATACCTTCCACTGAGTTCA
TCACCCAGAGTTTCGACGTCGCGGAAAACGATGAAGATTTCCACACTG
+
FFFFFFFFF:FFFFFFFFFFFFFFFFFFFFFFFFFFFFFFFFFFFFFFFFFFFFFFFFFFFF:FFFFFFF
FFFFFFFFFFFFFFFFFFFFFFFFFFFFFFFFFFFFFFFFFFFFFFFFFF
@A00721:190:HGJW7DSXY:4:1402:24894:18333_2:N:0:TCTGCAAG+GCTGCACT
TCTGATGCTGAATTTGAGGAAAATTTTTCGGAAGTTTTTGGTATAAATCCTGATGGCTCAGTACAGGG
AGGAGGTAATAGTTATAAAACCTTCTTTTATCATTGCAACAGGACTTT
+
FFFFFFFFFFFFFFFFFFFFFFFFFFFFFFFFFFFF:FFFFFFFFFFFFFFFFFFFFFFFFFFFFFFFF
FFFFFFF:FFFFFFFFFFFFFFFFFFFFFFFFFFFFFFFFFF:FFFFFFF
@A00721:190:HGJW7DSXY:4:2116:4716:30765_2:N:0:TCTGCAAG+GCTGCACT
GGACAAAATCTGGGATCATAGGTATGTGTCACGCTACACTCGAAATATGGGCCCTCATAAGGAAGTAT
AGTTGGCGTCTTGTCGTTGACGTCATTACAGTCTGGAAGTTAGATCAT
+
FFFFFFFFFFFFFFFFFFFFFFFFFFFFFFFFFFFFFFFFFFFFFFFFFFFFFFFFFFFFFFFFFFFF
FFFFFFFFFFFFFFFFFFFFFFFFFFFFFFFFFFFFFFFFFFFFFFFF
@A00721:190:HGJW7DSXY:4:1218:19208:33035_1:N:0:TCTGCAAG+GCTGCACT
TTTAGATAAATTCTTTCCGGGCTCAATGACCTTTGCTATGTACGTGGATCTAATGCTTGGTGATCTGA

```
ATCTGACCATCGCAAAGAAGACTTTATTAGTTCATGAAGTTCTTACACTTCTCGCGACTAATGTCAGC
TTC
+
FFFFFFFFFFFFFFFFFFFFFFFFFFFFFFFF:FFFFFFFFFFFFFFFFFFFFFFFFFFFFFFFFFFF
FFFFFFFFFFFFFFFFFFFFFFFFFFFFFFFFFFFFFFFFFFFFFFFFFFFFFFFFFFFFFFFFFFFF
FFF
@A00721:190:HGJW7DSXY:4:1157:14678:13385_2:N:0:TCTGCAAG+GCTGCACT
CCAAATTTAGCACTTTTTGAATATGACAGAAGCTCGCCATATCAGATTAGAGACATGCCAGTTAGAGT
TCTTCTAACCAGGACTGATGGTGAGATTCTTTTACCTTTTGGAGGTCC
+
FFFFFFFFFFFFFFFFFFFFFFFFFFFFFFFFFFFFFFFFFFFFFFFFFFFFFFFFFFFFFFFFFFFF
FFFFFFFFFFFFFFF:FFFFFFFFFFFFFFFFFFFF:FFFFFFFFFFFF
@A00721:190:HGJW7DSXY:4:2261:18710:22216_2:N:0:TCTGCAAG+GCTGCACT
CCTAGTGTCTTTTCTAGAGTCCAAGGACGTTTCCGTCGGAGGATGACAGGTGGGAGACCGCTCAATAA
CACTCTATTGAATCCCTTTTCCACGATGAAAGCTCTTTATGAGGTGCT
+
FF:FFFFFFFFFFFFFFFFF:FFFFFFFFFFFFFFFFFFFFFFFFFFFFFFFFFFFFFFFFFFFF:FFF
FFFFFFF:FFFFFFFFFF,FFFFFFFFFFFFFFFFFFFFFFFFFFFFF:F
@A00721:190:HGJW7DSXY:4:2134:30942:19664_1:N:0:TCTGCAAG+GCTGCACT
GATATCGCCTCTGTCCTTTCTCCTTTAATCGTGAGGTTACGAGAGTTTCGTACTTGGGTTTATCGGTT
GTATTTTGATAAATTTGTTGGAATCGATAATGATACTGACGAAGGTTCTATTACCACCCTTGTCATTG
ATA
+
FFFFFFFFFFFFFF,FFFFFFF:FFF:FFFF:FFFFFFFFFFFFFFFFFFFFFFFFFFFFFFFFFFFF
FFFFFFF:FFFFFFFFFFFFFFFFFFFFFFFFFFFFFFFFFFFFFFFFFFFFFFFFFFFFFFFFFF:FF
FFF
@A00721:190:HGJW7DSXY:4:2660:26476:36699_2:N:0:TCTGCAAG+GCTGCACT
TGATGCTTTCTTAGAGGCTCGTCTAGCCGCTGAGATTCGTCTTACCTATTTACAATCTGTAGAGAGAG
AGGTTAATCAGAATTTGAGTGATAACGATTTAGTACTGGGACATGGTG
+
FFFFFFFFFFFFFFFFFFFFFFFFFFFFFFFFFFFFFFFFFFFFFFFFFFFFFFFFFFFFFFFFFFFF
FFFFFFFFFFFFFFFFFFFFFFFFFFFF:FFFFFFFFFFFFF,FFFFFF
@A00721:190:HGJW7DSXY:4:1428:27941:30029_1:N:0:TCTGCAAG+GCTGCACT
CCTTTTTCGATTGGATTCGACGGGGTCTTCGTCTCACGACTAATTTGATAAGGTTTTTCTTTTATGGG
ACTCTTGTTTGTACTTATGAAGGAGGTATTACTTCGGAAGTTCTTAATGTAGGTCTCCTAGTACTTAA
GGG
+
FFFFF:FFFFFFFFFFFFFFFFFFFFFFFFFFFFFFFFFFFFFFFFFFFFFFFFFFFFFFFFF:FFFFF
FFFFFFFFFFFFFFFFFFFFFFFFFFFFFFF:F:FFFFFFFFFFFFFFFFFFFFFFFFFFFFFFFFFF
FFF
@A00721:190:HGJW7DSXY:4:1418:23249:28134_2:N:0:TCTGCAAG+GCTGCACT
GTTTCAGCTGGATGGATCTTTTGATGGTATGTTTCACATTGCGTGATCACTTCCCGTGGACTTGGGAA
GAGATTAAACATAAAATCCAGCGATTCTATTATGTCGCTAAAGCCTGT
+
FFFFFFFFFFFFFFFFFFFFFFFFFFFFFFFFFFFFFFFFFFFFF:FFFFFFFFFFFFFFFFFFFFFFF
FFFFFFFF:FFFFFFFFFFFFFFFFFFFFFFFFFFFFFFFFFFFFFFF
@A00721:190:HGJW7DSXY:4:1404:30933:36871_2:N:0:TCTGCAAG+GCTGCACT
GTTCGGGCTGTGGGGATTAACTTCCCTCGTGAAACTTACGACGATGTTCTCGCTCGCATTTCACAAGG
ATTACTAAGTCTGAGATCTGCCACTTCTAATTTTAGGAAACGTCGCGA
+
FFFFFFFFFFFFFFFFFFFFFFFFFFFFFFFFFFFFFFFFFFF:FF:FFFFFFFFFFFFFFFFFFFFFFFF
FFFFFFFFFFFFFFFFFFFF:FFFFFFFFFFFFFFFFFFFFFF,F:FFFF
@A00721:190:HGJW7DSXY:4:2307:2519:24392_2:N:0:TCTGCAAG+GCTGCACT
ATGAGGATGTCTTGGTCGAAAAATATCGCCGGGAACTTCTAATAGAGGATCCTGATGACATTATTTCA
```

ACGGATACCGATCTCAGTTCTACCACAGAAACCGTCTCAGGAACTGTC
+
F:FFFFFFFFFFFFFFFFFFFFFFFFFFFFFFFFF,FFFFFF,FFFFFFFFFFFFFFFFFFFFFFFFFFF
FF:FFFFFFFFFFFFFFFFFFFFFFFFFFFFFFFFFFFFFFF,FFFFF
@A00721:190:HGJW7DSXY:4:1369:8015:10426_1:N:0:TCTGCAAG+GCTGCACT
GGAATTAGAGAAAGACGACCTTCTTGTGGGGACTGAGACCTCTGAATTTTCAGGGTGCCCTGATGAGG
ACGTTGAAACGGATAATTTCAACTCTGAGACTTCTAAGAGTGTTTCAAAAGACCAGAAACGTAACAAG
AAG
+
FFFFFFFFFFFFFFFFFFFFFFFFFFFFFFFFFFFFFFFFFFFFFFFFFFFFFFFFF,FFFFFFFFFFFF
FFFFFFFFFFFFFFFFFFFFFFFFFFFFFFFFFFFFFFFFFFFFFFFFFFFFFFFFF:FFFFFFFFFFFF
FFF
@A00721:190:HGJW7DSXY:4:1502:23312:5321_2:N:0:TCTGCAAG+GCTGCACT
GGAAGGGGAGAAAACAGAACAAGCGAGCGGTTGAAGAGAAAACTGATAAGGATGCGGCTGATAGTACC
CCAATTCCTGCAAGAAAGATCACTCTCGTCGATACCACTGATCTTCCA
+
:,FF,F,FF,FFF:FF,F,FFF,F,:,F,,,FFFF,F,::FF,:,FF:,,:,F,:,,::FFF,F,FFF
,F,F,:,,,,F,FFFF:FF,:,:,F,F,,F:FFFF,F:F,F,FF,FFF
@A00721:190:HGJW7DSXY:4:2362:8287:19820_1:N:0:TCTGCAAG+GCTGCACT
GGTCGTTCTTTTGGTGATTTTATCAAAGAGAAAGTTTCTGCTAGTCGCGACGATAGTATTTATCGGAG
TGATATTAACAAGTTAAACTTCGAACTGACCACTTACAAGAGTGTTGAAGTACCGCAACATGGATCAG
TTT
+
FFF:FFFFFFFFFFFFFFFFFFFFFFFFF:FFFFFFFFFFFFFFFFFFFFFFFFFFFF:FFFFFFFFFFF
FFFFFFFFFFFFFFFFFFFFFFFFFFFFFFFFFFFFFFFFFFFFFFFFFFFFFFFFFFFFFFFFFFFFFF
FFF
@A00721:190:HGJW7DSXY:4:2345:16089:13291_2:N:0:TCTGCAAG+GCTGCACT
AAACTGAATCCACTCTCTTGGGATATAAATGAGTACATCTATAATAGAGTTTTGGAACTGTGTAATGT
CCTACGTACTGTTCAAGGTGTTTGGGCTTCTCTAAAGAACAGCAACTT
+
FFFFFFFFFFFFFFFFFF,FFFFFFFFFFFFFF:F:FFFFFFFFFFFFFFFFFFFFFFFFFFFFFFFFFF
FFFFFFFFFFF:F:FFFFFFFFFFFFFFFFFF:FFFFFFFFFFF,FFF
@A00721:190:HGJW7DSXY:4:2635:18738:25238_2:N:0:TCTGCAAG+GCTGCACT
AGACCTTTCTTGGGGAGTAAAGAGGGTTTGAAAATTACGGAACCTTTCCTCGTTCTTCATGAAGATCT
TCGAGTGTTTTATAGTGAGTTATTCTTGCGAAGGTTCAAGTCGATTGA
+
FFFFFFFFFFFFFFFFFFFFFFFFFFF:FFFFFFFFFFFFFFFFFFFFFFFFFFFFFFFFFFFFFFFFFF
FFFFFFFFFFFFFFFFFFFFFFFFFF:FFFFFFF,FF:FFFF:FFFFF
@A00721:190:HGJW7DSXY:4:1520:7545:6230_2:N:0:TCTGCAAG+GCTGCACT
AAGAAGTTCTTCGCAGTTGAATCTCCACCAGGTGGAGGGAAGACCACTGAACTTATAGCGACTTTTGT
CGAATTTTTCGCAAAAGGTCTTGAAGTACGTATTGTCACTGCTAATCG
+
FFFFFFFFFF:FFFFF:FFF:FFFFFFFFFFFFFFFFFFFFFFFFFFFFFFFFFFFFFFFFFFFFFFFFF
FFFFFFFFFFFFFF:FFFFFFFFFFFFFFFFFFFFFFFFFFFFFFFFF
@A00721:190:HGJW7DSXY:4:1505:30680:31454_2:N:0:TCTGCAAG+GCTGCACT
CACTTCTAGTTCATCGCGGTATTTGTGAAATTAAGACAGACGTGAAAGTTATGATGAGTGATCGTATT
AGAACAGCTGATTCTTATTGCATGAACGTCGCTGAGGTCAAACCTGTG
+
FFFFFFFFFFFFFFFFFFFFFFFFFFFFFFFFFFFFFFFFFFFFFFFFFFFFFFFFFFFFFFFFFFFFFF
FFFFFFFFFFFFFFFFFFFFFFFFFFFFFFFFF:FFFFFFF:FFFFFF
@A00721:190:HGJW7DSXY:4:2321:9815:7279_1:N:0:TCTGCAAG+GCTGCACT
CATCGACGAATTATTCATGATTCACGCTGGTCAGACCCTTTTTCTTACACATCTACTTAAGGCGGCTT
ATACCATAGGTTACGGTGATTCCAATCAAATCGGATTCATCAATCGTATTGATCTCCAAGTCGCAAAC
TAC

+
:FF,FFFFFFFFFFF:FFFFFFFFFFFFFFFFFFFFFFFFFFFFFFFFFFFFFFFFFFFFFFFFFFFF
FFFFFFFF:FFFFFFFFFFFFFFFFFFFFFFFFFFFFFFFFFFFFFFFFFFFFFFFFFFFFFFFFFFF
FFF
@A00721:190:HGJW7DSXY:4:2308:3775:28980_2:N:0:TCTGCAAG+GCTGCACT
TTGATGAGTGTCGTTCTTATCGAAGTCTCAGTTATCGCTGCCCGGCCGACGTTTGTCGCGAACTCTCA
GAGGTTTACGGCAGAAAAATTTCTCCTGCTTGTAATTCGAGAGAGGAC
+
FFFFFFFFFFFFFFFFFFFFFFFFFFFFFFFFFFFFFFFFFFFFFFFFFFFFFFFFFFFFFFFFFFFF
FFFFFFFFFFFFFFFFFFFFFFFFFFFFFFFFFFFFFFFFFFFFFFFF
@A00721:190:HGJW7DSXY:4:1470:26955:22216_1:N:0:TCTGCAAG+GCTGCACT
TTAGACCTATCAGATGTCTTGACGACTTCGAATTTGAGGACGGAGTTAAATATATTACTTTCACTCAA
GGTGAAAAGTTTGAAATTAACAAAGTCCTTGGTAAGAGTAAGGTTAAATGTGAAGATGCTCAGACTAT
TCA
+
FFFFFFFFFFFFFFFFFFFFFFFFFFFFFFFFFFFFFFFFFFFFFFFFFFFFF:FFFFFFFFFFFFFF
FFFFFFFFF:FFFFFFFFFFFFFFFFFFFFFFFFFFFFFFFFFFFFFFFFFFFFFFFFFFFFFFFFFF
FFF
@A00721:190:HGJW7DSXY:4:1303:23294:29183_1:N:0:TCTGCAAG+GCTGCACT
TGACCCTGTTTTTGCATCACAAACACATATTACCGTTGCTCTATCACGACACGTTGAATCTTTACGAT
ACTTTGTTATCAATGTAAAGTTAAACGATGCTACTTGTGAGAGGATCAATCACAGTAAGGAACTGTCA
CGT
+
FFFF,FFFFFFFFF,FFFFFF:FF:F:FFFFFF,FFFFFF,FF:FFFFFFF:FFF:FFFFFFF:F:,F
:FFFFFFFFF,FFFFFF:FFFFFFFFFFFFFFF:FFF:FFFFFFFFF:FFFFFFFFFFFFFFFFFFFF
FFF
@A00721:190:HGJW7DSXY:4:1402:22589:16501_2:N:0:TCTGCAAG+GCTGCACT
AGATTCAACCCCTTATATCGAGGCTATCGAGGACGATGAGTATCTTGAAGTTGTTGACGACAAGCCTC
AAGAGGGTAGAGGACCACCTGGATCTTCTACTGTCTCTGCCTTCCAAG
+
FFFFFFFFFFFFFFFFFFFFFFFFFFFFFFFFFFFFFFFFFFFFFFFF,FFFFFFFFFFFFFFFFFFF
FFFF:FFFFFFFFFFFFFFFFFFFFFFFFFFFFFFFFFFFFFFFFFFF
@A00721:190:HGJW7DSXY:4:1137:16957:6277_2:N:0:TCTGCAAG+GCTGCACT
TTATTCCTGGAACCACCTTAACGTCACTCGGTAATCTTTCAGCCGAATTAAGTACTAATGAGTTCAAG
TCGGTTGTCGATCGAATTAAGATCAAGGCTAATACCTTTATAGGTAAG
+
FFFFFFFFFFFFFFFFFFFFFFFFFFFFFFFFFFFFFFFFFFFFFFF:FFFFFFFFFFFFFF:FFFFFF
FF:FFFFFFFFFFFFFFFFFFFFFF:FFFFFFFFFFFFFFFFFFFFFF
@A00721:190:HGJW7DSXY:4:2642:24532:14450_1:N:0:TCTGCAAG+GCTGCACT
TCATCAGTCAGAGTATACCCGATAGGGTACCGAGTCTCCAAGAGAATATTTATTCTTATGAGGCTCGA
AACTTCAACGTTTTCACAACGGAAAGACATATCGCACCCGAAATTTTTGGGCAGAATGCTCTTAACGC
GGT
+
FFFFFFFFFFFFFFFFFFFFFFFFFFFFFFFFFFFFFFFFFFFFFFFFFFFFFFFFFFFFFFFFFFFF
FFFFFFFFFFFFFFFFFFFFFFFFFFFFFFFFFFFFFFFFFFFFFFFFFFFFFFFFFFFFFFFFFFFF
FFF
@A00721:190:HGJW7DSXY:4:2375:15130:26725_2:N:0:TCTGCAAG+GCTGCACT
GATCAGTCTAAGTTGGATAAAGTTATAGGAGACTCAGTTAACCTGAGAGCTCCTCAATTGATAGATTG
GGTCAACAATAGGGACGCTAGTATACTTCGTAAAGTCGACAGCGAAAT
+
FFFFFFFFFFFFFFFFFFFFFFFFFFFFFFFFFFFFFFFFFFFFFFFFFFFF:FFFFFFFFFFFFFFF
FFFFFFFFFFFFFFFFFFFFFFFF:FFFFF:FFFFFFF:FFFFFFFFFF
@A00721:190:HGJW7DSXY:4:2431:13060:19413_2:N:0:TCTGCAAG+GCTGCACT
TAATTGGACCGACTATATGACCTATTTTAAGCTCATGGTGAAAAGGGAAGCTAAGGTTAAACTTGATG

CTTCAGCCTTTTCGAAGCATAACCCCGCACAGAATATCGTTTATCACG
+
FFFFFFFFFFFFFFFFFFFFFFFFFFFFFFFFFFFFFFFFFFFFFFFFFFFFFFFFF,FFFFFF:FFFF
FFFFFFFFFFFFFFFFFFFFFFFFFF,FFFFFFFFFFFFFF:FFFFFFF
@A00721:190:HGJW7DSXY:4:1567:6298:4633_2:N:0:TCTGCAAG+GCTGCACT
CCTATCTTTAAGGTTCTTGTGAGGAGAGTCAAACAGTGTCTTTTACCCAACATTTTTATGTATACAGA
GATGACCAGTGACGATTTCTCCCGGAAGCTCTTCTCTCTCTTAGGAGG
+
FFFFFFFFFFFFFFFFFFFFFFFFFFFFFFFFFFFFFFFFFFFFFFFFFFFFFFFFFFFFFFFFFFFF
FFFFFFFFFFFFFFFFFFFFFFFFFF:FFFFFFFFFFFFFF:FFFFFFF
@A00721:190:HGJW7DSXY:4:2263:3839:18161_2:N:0:TCTGCAAG+GCTGCACT
TTTCTTTTGTAAAGCCTATGAGATAAAGATGTATTCTTATTTAGGCTTCGATGCTGAATTTCTTGAAC
TTTGGAGTGCCAGTGAATATTTTTGTCACGCCAGGAGTATCCATAACG
+
FFFFFFFFFFFFFFFFFFFFFFFFFFFFFFFFFFFFFFFFFFFFFFFFFFFFFFFFFFFFFFFFFFFF
FFFFFFFFFFFFFFFFFFFFFFFFFFFFFFFFFFFFFFFFFFFFFFFF
@A00721:190:HGJW7DSXY:4:1671:31548:1110_2:N:0:TCTGCAAG+GCTGCACT
TACCACCTTTGCTCAACGACGAAGTGGAACGGCCAACACTTTCTTTGGTAATTGTATGATCACTTTGA
TGATTTTATCACTTTATTATGACCTTGGAAAGTTTTCGGCTGTGGTTG
+
FFFFFFFFFFFFFFFFFFFFFFFFFFFFFFFFFFFFFFFFF:FFFFFFFFFFFFFFFFFFFFFFFFFF
FFFF,FFFFF:FF:FFFFFFFFFFFFFFF:FFFFFFFFFFFFFFFFFF
@A00721:190:HGJW7DSXY:4:1420:27498:29888_2:N:0:TCTGCAAG+GCTGCACT
CTGTTTTCAAAGACTCCAATAACTGATCATTCTGATCATATGATTTTGGACAATGGTTTCGAAGCGAA
ATTTTTGAGGGATAAACCAGCGTACTTCTGCTCGCGATTTATTCTTTT
+
FFFFFFFFFFFFFFF:FFFFFFFFFFFFF,FFFFFFFFFFFFFFFFFFFFFFF,FFFFFFFFFFFFFF
FFFFF:FFFFFFFFFFFFFFFFFFFF,FFFFFFFFFF,FF:FFFFFFF
@A00721:190:HGJW7DSXY:4:2326:32226:5791_2:N:0:TCTGCAAG+GCTGCACT
CCTGATCCTTACAAGATCATCGTTAAGTTAGGTAAACCCCTAACCGATGTTACTCTTGTAGGACTTTG
GGAGAGATTCGTTAGTTTTAAAGACTATACTAAATATCTCCACTACGA
+
FFFFFFFFFFFFFFFFFFFF:FFFFFFFFFFFFFFFFFFFFFFF:FFF:,FFFFFFFFFF,FFFFFFFFF
FFFFFFFFFFFFFFFFFFFFFFFFFFFFFFFFFFFFFFFFFFFF:FFF
@A00721:190:HGJW7DSXY:4:1545:12617:35243_2:N:0:TCTGCAAG+GCTGCACT
GCGACTAATATTCGATATTCGATTGAGGGTCGTTATACCTATTCGGCTATCTGTGCTTTGAATTGTAT
AAGATCAAATTTTAAACGATTCCGAGAGGTTTATCCTGATGTGGAGGG
+
FFFFFF:FFFFFFFFFFFFFFFFFFFFFFFFFFFFFFFFFF,FFFFFFFFFFFFFFFFFFFFFFFFFF
FFFFFFFFF,FFFFFFFFFFFFFFFFFFF:FF:FFFFFF:FFFFFFFF
@A00721:190:HGJW7DSXY:4:2517:15456:1705_1:N:0:TCTGCAAG+GCTGCACT
ATCCCGACCTTTCCTTGTGAAAATTTTGATGCGGTTTCATAAGATTATCAGTAATCCTGATTCTACTA
CGGATTCATGGACCTTCCATCCTGTACCTCTTCTGGAGAGACGTATCTCAAAGATCGACTTCGAGACA
GGT
+
F:FFFFFFFF:FFFFFFFFFFFFFFFFFFFFFFFFFFFFFFFFFFFFFFFFFFFFFFFFFFFFFFFFFF
FFFFFFFFFFFFFFFFFFFFFFFFFFFFFFFFFF,FFFFFFFFFFFFFFFFFFFFFFFFFFFFFFFFFF
FFF
@A00721:190:HGJW7DSXY:4:2267:6017:22060_2:N:0:TCTGCAAG+GCTGCACT
GCGAGAGCGACCAAGATCTAAATTCGGTAACGGTGCGTGGCGCTTTATTTTAAAAAGCTCCCTCCTCT
CGCAAGTTTGATCTGATACTTATCACGAAAGGACTTGAGTCTTTTGAT
+
FFFFFFFFFFFFFFFFFFFFFFF:FFFFFFFFFFFFFFFFFFFFFFFFFFFFFFFFFF:FFFFFFFFFF
FFFFFFFFFF:FFFFFFFFFFFFFFFFFFF::FFFFFFFFFFFFFFFF

@A00721:190:HGJW7DSXY:4:2145:3577:10974_2:N:0:TCTGCAAG+GCTGCACT
TCTTTCATTTTAAATGACGATCATTAAGACTCTCTTTCTTCTTTGTTTCCTTCTTTGCACTGATTTTT
TCCTCATCAGCTTGTGTTTTACCTAATCTGTGTTTTTACGATAACAAC
+
FFFFFFFFFFFFFFFFF:FFFFFFFF:FF:FFFFFFF:FF::FFFFFFF:FF,:FFFFFFFFFFFFFF
F::FFFFFFFFFFFFFFFFFFFFFFFFF:FFFFFFFFFFFFFFFFFFF,
@A00721:190:HGJW7DSXY:4:1232:17065:15139_2:N:0:TCTGCAAG+GCTGCACT
TCTTCCGAAAGGCGAACTTTGGGAATTTGAAGCTCAGGGTGAAGCACAACGTTTGACCGTGACGAACT
GTGGTGTTCTTGATCTACGTTACGGATATACTCTCCTTGAGTTCACTC
+
FFFFFFFFFFFFFFFFF:FFFFFFFFFFFFFFFFFFFFFFFFFFFFFFFFFFFFFFFFFFFFFFFFFF
FFFF:F,FFFFFFFFFFFFFFFFFFFFFFFFFFFFFFFFFFFFFFFFF
@A00721:190:HGJW7DSXY:4:1473:15664:26741_1:N:0:TCTGCAAG+GCTGCACT
CACTTATTACGATATTTCTTTAGTCGATGGGTTTTCGACGCCTTTAAGCGTCTTCTGTTCATCGCAAC
CAGATGATATCTTCTATTCGAACTTATGTTTCGATCAGTGTTTCTTCTATCAGGAGGAAAACACTTGT
TCT
+
FFFFFFFFFFFFFFFFFFFFFFFFFFFFFFFFFFFFFFFFFFFFFFFFFFF,FFFFFFFFFFFFFFFF
FFFFFFFFFFFFFFFFFFFFFFFFFFFFFFFFFFFFFFFFFFFFFFFFFFFFFFFFFFFFFFFFFFFF
,FF
@A00721:190:HGJW7DSXY:4:1441:27055:31782_2:N:0:TCTGCAAG+GCTGCACT
TCTTCTAATGTGGCCTGAACTACGTTTCACTTTGTGTGTACTTGTCTCTATTTTAAGTTTTGTCTTCC
TTGTTTGTCTTATTCTATTAATCACTCATTTTAGTGAGATTATTAAAA
+
FFFFFFFFFFFFFFFFFFFFFF:FFFFFFFFFFFFFFFFFFFFFFFFFFFFF:FFFFFFFFFFFFFFFFFF
FFFFFFFFFFFFFFFFFFFFFFFFFFFFFFFFFFFFFFFFFF:FFFFFF
@A00721:190:HGJW7DSXY:4:1325:2636:22748_2:N:0:TCTGCAAG+GCTGCACT
CGACAACAGCAGGGGTTTTCGAATAGTGATAGCCACCCGCCTTACAGAGGGGCAGCAGCATGACCATA
ATTGGTATTGACTTTGGTACCACCTTTTCTACTGTCTGTCTCGCCTCT
+
FFFFFFFFFFFFFFFFFFFFFFFFFFFFFFFFFFFFFFFFFFFFFFFFFFFFFFFFFFFFFFFFFFFF
FFFFFFFFFFFFFFFFFFFFFFFFFFFFFFFFFFFFFFFFFFFFFFFF
@A00721:190:HGJW7DSXY:4:2573:32181:2832_1:N:0:TCTGCAAG+GCTGCACT
CGTGTGCTCTTCCGATCTGTGAATTTATACCTACAATCATTGGTTTTTCTAATAATGATGACACTATT
GTTTATGGATATGATGCTATTACTCATGATGGTTACGGTCAGGGAGGATTCTCAGTTTACAAGGACCT
TAA
+
FFFFFFFFFFFFFFFFFFFFFFFFFFFFFFFFFFFFFFFFFFFFF:FFFFFFFFFFFFFFFFFFFFFFFF
FFFFFFFFFFFFFFFFFFFFFFFFFFFFFFFFFFFFFFFFFFFFFFFFFFFFFFFFFFFFFFFFFFFF
FFF
@A00721:190:HGJW7DSXY:4:1668:16776:8093_2:N:0:TCTGCAAG+GCTGCACT
CCGAAAGACGTGCTAAATTAAAACCATTGTACGATGTCGTCTGTCTCGGCGATCACTTTTCACTAAGA
ATTGGTCCTACTTTTGGACAAGGCCAACTTCGTAATCTTCATGAGATC
+
FFFFFFF,FFFFFFFFFFFFFFFFFFFFFFFFFFFFFFFFFF:FFFFFFFFFFFFFFFFFFFFFFF:FFF
FFFFFFFFFFFFFFFFFFFFFFFFFFFFF:FFFFFFFFFFFFFFFFFF
@A00721:190:HGJW7DSXY:4:1577:16089:8750_1:N:0:TCTGCAAG+GCTGCACT
TTAGGGATTTTGAGAGAGTGATAGGCTTCAATTGTAGTGGTTTGGTCGTTTCAGTACCCAGTCAGTAT
ACCTCTACGCAAAGATTTTTCATGAAAACTACAGAGGAACGTACCGGATTACCTGTCTTTCATATTAT
GAA
+
FFF::FFF:FF,FF,FF:FFF,,FFFFFFFFF,FF:FFFFFFFFFF:FFFFFF:FF,FFFFFFFFFFF
FF,F,FFF,F,F::::,FFFFFFFFF,FFF,FFF,F:FF::F::::F,FF,:,F:FFFFFFFFFFF:F
F:F

```
@A00721:190:HGJW7DSXY:4:2276:14000:16939_2:N:0:TCTGCAAG+GCTGCACT
TCTTTGCTTCGATGTTGGATGTCAAGAAGGGTTCAGAATGGGATTCCTACGTAGTTTATGACTTTGGT
GGTGGTACTTTCGATACTTCTATCGTCTTACGTTACGGTAATTACTAT
+
FFFFFFFFFFFFFFFFFFFFFFFF:FFFFFFFFFFFFFFFFFFFFFFFFFFFFFFFFFFFFFF:FFFF:
FFFFFFFFFFFFFFFFFFFFFFFFFFFFFFFFFFFFFFFFFFFFFFFF
@A00721:190:HGJW7DSXY:4:1477:18909:16329_1:N:0:TCTGCAAG+GCTGCACT
ATAATCTTGGAGGTCGTGACGTTGATGAAGCTATAAGAGCGTTCTTATTACGGCGCACTTCTTTACCC
GCCACCACACAACTTTCAGTTTCACAACTGAAGGAGGAGGTTTCTGTTACTGGAAAACCCTCGTCAAT
ACT
+
:FFFFFFFFFFFFFFFFFFFFFFFFFFFFFFFFFF:FFFFFFFFFFFFFFFFFFFFFFFFFFFFFFF:FF
FFFFFFFFFF:FFFFFFFFFFFFFFFFFFFFFFFFFFFFFFFFFFFFFFFFFFFF:FFFFFFFFFFFFF
:FF
@A00721:190:HGJW7DSXY:4:1169:3974:36025_2:N:0:TCTGCAAG+GCTGCACT
GATCTAGTGAAATTAACGCGTCCTTTTCTGGAACGTAGTTTTACTATTATGAACAAAGTTATCAAAGA
TAGTGGCATTCAAGGCAAACTTACCCTGATTCCGGCCGGTGGTAGCTC
+
FFFFFFF:FFFFFFFFFFFFFFFFFFFFFFFFFFFFFFFFFFFFFFFFFFFFFFFFFFFFFFFFFFFF
FFFFF,FFFFFFFFF:FFFFFFFFFFFFFFFFF,FFFFFFFFFFFF:F
@A00721:190:HGJW7DSXY:4:1126:13810:12696_2:N:0:TCTGCAAG+GCTGCACT
GGCTATTACTCTTGCTGAAACAGCTATTAAAGCTGTTGAGCGGGTAATTACACCTAAACGAGCTCGAT
CTGCCATCGCTGAAGGATGTGCACTCTTTTCTGCTACTTTAACGGAGC
+
FFFFFFFFFFFFFFFFFFFFFFFFFFFFFFFFFFFFFFFFFFFFFFFFFFF:FFFFF:FFFFFFFFFF
FFFFFFFFFFFFFFFFFFFFFFFFFFF:FFFFFFFFFF:FF:FFFFFF
@A00721:190:HGJW7DSXY:4:1476:11252:8703_2:N:0:TCTGCAAG+GCTGCACT
TCGATTGTATCAACAGTAACATTACTTCTAATAAAGGTCTCTATCAAGCTCGTATTATCGTCGCTGCT
GGATCTGCGTTACCGTTGAAGGCTAAACTCCAAGGTAATTATGAAGAC
+
FFFFFFFFFFFFFFFFFFFFFFFFFFFFFFFFFFFFFFFFFFFFFFFFFFFFFFFFFFFFFFFFFFFF
FFFFFFFFFFFFFFFFFFFFFFFFFFFF:FFF:FFFFFFFFFFFFFFFF
@A00721:190:HGJW7DSXY:4:1424:1452:3223_1:N:0:TCTGCAAG+GCTGCACT
GTATGAGGGTGTTTCTAATCGTAACATTAATAATTTCAATCTTCTTAAAAAGAAGATAGAGAGTCATA
CTTTGGGCTATCGTTCAGGTGCATGGGGTTGGATAGCCAATATTGAAGTGGACACCTCTGGTAGTATC
GAA
+
FFFFF:F:FFFFFF:FFFF:FFFFFF,FFFFFFFFFFFFF:FFF:FFFFFFFFFFFFFFFFFFFFFFFF
FFFFFFFFFFFFFFFFFFFFFFFFFFFFFFFFFFFFFFFFFFFFFFFFFFFFFFFFFFFFFFFFFFFF
FFF
@A00721:190:HGJW7DSXY:4:1257:7193:4272_1:N:0:TCTGCAAG+GCTGCACT
CTAAGCATACTACTTTTGCTTTGATTACTCTACCGCCTTACAAGTCTAAGGTTAAAGAGTCGATTAAA
CAAGTTGTACTTATCAATGATTTTATCGCTGGTTGTTATCTCGAAAAGTCTCTTCTAAAAGGAACGTC
AGT
+
:FFFFFFFFFFFFFF:FFFFFFFFFFFFFFFFFFFFFFFFFFFFFFF,FFFFFFFFFFFFFFFFFFFFFFF
FFFFFFFFFFF:FFFFFFFFFFFFFFFFFFFFFFFFFFFFFFFFFFFFFFFFF:FFFFFFFFFFFFFF
FFF
@A00721:190:HGJW7DSXY:4:1147:4887:30185_2:N:0:TCTGCAAG+GCTGCACT
CTAAAATATTTACAAAATCTTACCACTATCTACGAACCTAAAGTTCTGAAGACGTTGCTTGTCGCTTC
CTCCTTTAATGGCACTACCTCGGTACAATCTTACTTGGAACGAAGGCG
+
FFFFFFFFFFFFFFFFFFFFFFFFFFFFFFFFFFFFFFFFFFFFFFFFFFFFFFFFFFFFFFFFFFFF
FFFFFFFFFFFFFFFF:FFFFFFFFFF:FFFFFFFFFFFFFFFFFFFF
```

```
@A00721:190:HGJW7DSXY:4:2438:11713:30608_1:N:0:TCTGCAAG+GCTGCACT
TGTTCTGTCTATATTAGGTGAAGCGAAATACGATTTCGAACGTCAACGTTCACCGAGTTTCGTTTCGT
ACCTTGATGCTGACTTTCGTCAATATGATTTGATCTGGTTGCTCAATTCTAAGCGTGTTCCGCTTAGT
GCG
+
,FFFFFFFFFFFFFFFFFFFFFFFFFFFFFFFFFFFFFFFFFF:FFFFFF:FFFFFFFFFFFFFFF:F
FFFFFFFF:FFFFFF:FFFFFFFFF:FFFFFFFFFFF:FFFF:FFFFFF:FF,,FFFFFFFFFFFFF,
F:F
@A00721:190:HGJW7DSXY:4:2415:4155:4805_2:N:0:TCTGCAAG+GCTGCACT
GAGTATTGCTATTTATACGGTTACGAGAAGAAGGTTCGAAAGTTTGAGAACTTAGTTTACGAACAAGA
CTCTACTATACGTGACATACTAAATACTAGATATTTGGATGATCTTGA
+
FFFFF:,FF,FFFFFFFFFFFFFFFFFFFFFFFF,FFFFFF:FFFF,FFFFFFFFFFFFFFFFFF,,FF
FFFF:F,FFFFFFFF:FFFF,,FFFF:FF:F,:F,F:,FFFFFFF,FF
@A00721:190:HGJW7DSXY:4:1521:2465:18129_2:N:0:TCTGCAAG+GCTGCACT
AAAATTTTCTTCCTTGGATTTTAATATAAAGAATCTTTACCAGGACTCCTACTACAGGAGGCAGTCTT
CTGATCGTAAGAAGTTTATAAGGAACATATCCATTTGTTGTAATAGGG
+
FFFFFFFFFFFFFFFFFFFFFFFFF,FFFFFFFFFFFFFFFFFFFFFFFFFFFFFF:FFFFFFFFFFF
FFF:FFFFFFFFFFFFFFFFFFFFFFFFFFFFFF,FFFFFFFFFFFFFF
@A00721:190:HGJW7DSXY:4:1336:22073:34240_2:N:0:TCTGCAAG+GCTGCACT
CGAAACTCTAAGTGGTAATACTGTCTTTCCAAGCTTACCTGTATCTTCAATGAAATTGGACTCTTCGA
TAGGGGACATTTTATCGTGGGCTTTCCGTAAAGGAAGATCTGTTATCG
+
FFFFFFFFFFFF:FFFFFFFFFFFFFFFFFFFFFFFFFFFFFF,FFFFFFFFF:F:FFFFFFFFFFFF
,FFFFFFF,FF:FFFFFFFFFFFFFFFFFFF:FFF:FFFF,FFFFFF:
@A00721:190:HGJW7DSXY:4:1336:22073:34240_1:N:0:TCTGCAAG+GCTGCACT
ACCGGTGAATGGTTTAAGAATACTATAAATAGAATAGCTATTACTACTAATGGCGTTGGCCTAGTGGA
TGTTATTAGTGACATCTGGTCAATTTCGTTTTTAGGTGGTCTTTATTCTATTATTGATTTTTATGAGA
TTG
+
FFFFFFFFFFFFFFFFFFFFFFFFFFFFFFFFFFFFFFFFFFFFFFFFFFFFFFFFFFFFFFFFFFFF
FFFFFFFFFFFFFFFFFFFFFFFFFFFFFFFFFFFFFFFFFFFFFFFFFFFFFFFFFFFFFFFFFFFF
,FF
@A00721:190:HGJW7DSXY:4:1171:21721:9330_2:N:0:TCTGCAAG+GCTGCACT
TGTTAGAGTAGAATTCCTGAAGAGTCCTTCCGTAACCAACTTCTTGACAATTTTCTTCAATCCTTCGA
TAATAGTTAAATCTAGGATTATGTCTGTGTTGAAAGACTTTCTACCGA
+
FFFFFFFFFFFFFFFFFFFFFFFFFFFFFFF:FFFFFFFFFFFFFFFFFFFFFFFFFFFFFFFFFFFF
FFFFFFFFFFFFFFFFFFFFFFFFFFFFFFFFFFFFFFFFFFFFFFFF
@A00721:190:HGJW7DSXY:4:1204:28492:4586_2:N:0:TCTGCAAG+GCTGCACT
TACACAATTAAGAAATAATCCTACATTGACTGATTATCTAAAGATTGAAGGAAAATTTTATCCTCGTT
TAACTTACTTAGACTTTCCTACGATACAAATGATAACTTCTAATTTAC
+
FFFFFFFFFFFFFFFF:FFFFFFFFFFFFFFFFFFFFFFFFFFFFFFFFFFFF:FF,FFFFFFFFFFFF
FFFFFFFFFFFFFFFFFFFFFFFFFFFFFF:FFFFFFFFFFFFFFFFF
@A00721:190:HGJW7DSXY:4:2106:32289:29919_2:N:0:TCTGCAAG+GCTGCACT
AAGGGATGCATGGTGTTATCTCCTCAGGAGTCCCGGCATTTGATAATAATGACGAGACTTTCGTCATA
ATCGCTGCATATTACGGATTACACGGTACCAGCGCATCTCGTGTCGAG
+
FFFFFFFFFFFFFFFFFFFFFFFFFFFFFFFFFFFFFFFFFFFFFFFFFFFFFFFFFFFFFFFFFFFF
FFFFFFFFFFFFFFFFFFFFFFFFFFFFFFFFFFFFFFFFFFFFFFFF
@A00721:190:HGJW7DSXY:4:1149:12933:7607_1:N:0:TCTGCAAG+GCTGCACT
AAATTTATAGAAGATTATTTTGACAGAATCAATAAGAATCATCGATTCTCGATTCGTAGAGCTTATCT
```

```
AGGTACTCTCATTTACTATGTTGATTTAGTCTACATTTATGCTGATCTAAAATTTCGATCTAGATGGA
TCC
+
:FFFFFFFFFFFFFFFFFFFFFFFFFFFFFFFFFFFFFFFFFFFFFFFFFFFFFFFFFFFFFFFFFFF
FFFFFFFFFFFFFFFFFFFFFFFFFFFFFFFFFFFFFFFFFFFFFFFFFFFFFFFFFFFFFFFFFFFF
FFF
@A00721:190:HGJW7DSXY:4:2443:16785:7044_2:N:0:TCTGCAAG+GCTGCACT
TTTACAACGATATAGGAGTAATCTACGAGAAGATTCTAGAATTCTTAATTATAATATTCAACCTCAAA
AGTTACGTGTTTACAGTAACGAGATCACACATTTTGAGAACTAACTAT
+
FFFFFFFFFFFFFFFFFFFFFFFFFFFFFFFFFFFFFFFFFFFFFFFFFFFFFFFFFFFFFFFFFFFF
FFFFFFFFFFFFFFFFFFFFFFFFFFFFFFFFFFFFFFFF:F:,FFF
@A00721:190:HGJW7DSXY:4:1531:14371:11725_1:N:0:TCTGCAAG+GCTGCACT
TCTCTTATTATAATAAATAAATAAATAATATTACTAATGGCAAATAGTGGAAAGGCTCCAGTCAACAC
TAGTTCGAGTGCTGCTTTGGGTTACGATGACGTGAGAATAGGTGAAGGGATAGTTTTTACCTCGACCA
ACA
+
FFFFFF,FFFFF:FFFFFF,FFFF:FFFF:FFFFF:FFFFFFFFFFFFFFFFFFFF:FFFFFFFFFF
FFFFF:FFFFFFFFFFFFFFFFFFFFFFFFFFFFFFFFFF:FFFFF:,FFFFFFFFFFFFFFFFF,F:
FFF
@A00721:190:HGJW7DSXY:4:2433:9155:25426_2:N:0:TCTGCAAG+GCTGCACT
GCGACTAATCTTAAAGCTTCACCCAAAATGAAGGACACTTCGAAACTGAATAATGATGCACTACAAAC
AAGTATCGCTAATCATCTGAAAAAGGCTCTAAAGGACACTATACAATA
+
FFFFFFFFFFFFFFFFFFFFFFFFFFFFFFFFFFFFFFFFFFFFFFFFFFFFFFFFFFFFFFFFFFFF
FFFFFFFFFFFFFFFFFFFFFFFF:FFFFFFFFF,FFFFFFFFFFF:F
@A00721:190:HGJW7DSXY:4:1169:23538:10316_2:N:0:TCTGCAAG+GCTGCACT
CAGCACCACATTTTGGTCAAGTCAATCCGTTGAGAAAATTTCTTAGAGGATATTCGCCATTGTGGTAC
GAATTGGTCCAACATTTACCACCAAATTCTTTTGATACAACTCTAGCC
+
FFFFFFFFFFFFFFFFFFFFFFFFFFFFFF:FFFF,FFFFFFFFFFFFFFFFFFFFFFFFFFFFFFFF
FFFFFFFFFFF:FFFFFFFFFFFFFFFFFF:FFFFFFFFFFFFFFFFF
@A00721:190:HGJW7DSXY:4:1421:19804:28745_1:N:0:TCTGCAAG+GCTGCACT
ATACGCTTGCTATTCACCAGATTGGTTCGAACCCGTTGATGCTATGTCAGAAGAAGCTATTCAAGCTT
GTCGTTTAAAGACTGCAGAGGCTGTCAAACAAGCCCAGACTCAAGGAGTCCAATCAAAGGTTTTGAAT
TTA
+
F,FFFFFFFFFFFFFFFFFFFFFFFFFFFFFFFFFFFFFFFFF:FFFFFFFFFFFFFFFFFFFFFFFF
FFFFFFFFFFFFFFFFFFFFFF,FFFFFFFFFFFFFFFFFFFFFFFFFFFFFFFFFFFFFF:FFFFFF
FFF
@A00721:190:HGJW7DSXY:4:1225:11143:15655_2:N:0:TCTGCAAG+GCTGCACT
GGTGATAAAAATTATTTAGAGATCTTTTCTACTTACAAAGGACAATTCAGTTTCATTAATTCTACGGA
AAAGTCCGGAGTATATTGTATGTTAATAGATAGTGGGATAATAGTTCT
+
FFFFFFFFFFF:FFFFFFFFFFFFFFFFFFFFFFFFFFFFFFFFFFFFFFFFFFFFFFFFFFFFF:FF
FFFFFFFFFFFFFFFFFFFFFFFFFFFFFFFFFFFFFFFFFFF:FFFF
@A00721:190:HGJW7DSXY:4:2662:19687:1360_2:N:0:TCTGCAAG+GCTGCACT
GAACGCGTTCGTATAACCAAGTACTTTCCGTCTGAAGAGTTTAGAGCTGCTATTGATTATGTAACCGA
TAGGAGTTGTATAGTTGAAGATGTGGCTTTAGACGATAATCCTTTAAG
+
FFFFFFFFFFFFFFFFFFFFFFFFFFFFFFFFFFFFFFFFFFFFFFFFFFFFFFFFFFFFFFFFFFFF
FFFFFFFFFFFFFFFFFFFFFFFFFFFFFFFFFFFFFFFFFFFFFFFF
@A00721:190:HGJW7DSXY:4:2114:25545:25692_1:N:0:TCTGCAAG+GCTGCACT
GAAGATTTTCCCACAGATTTGTCTACGTTGCAGATCGCCACTCCAAACACTATATACATTATAAAATA
```

```
TAAAAATAAGGGAGTCATAACTTTTGTTACCAAATTCTTGGCTTTCAACGAAGTCTCTGATTTTATTA
ATC
+
FFF:FFFFFFFFFFF:FFFFFFFFFFFFFFFFFFFFF:FFFFFFFFFFF:FFFFFFFFFFFFFFFFFF
FFFFFFFFFFFFFFFFFFFFFFFFFFFFFFFFFFFFFFFFFFFFFFFFFFFFFFFFFFFFFFFFFFFF
FFF
@A00721:190:HGJW7DSXY:4:1121:24831:11365_2:N:0:TCTGCAAG+GCTGCACT
ATCTCTCGGTCATTTACTCTGTACTTGATCTACGTCGTTTAACTGTCGGAACAACCTTTACTTAGAAT
GACTCTATCTTTTCAGACACTTCTTATATTATTTACAATAGTTAATAT
+
FFFFFFFFFFFFFFFFFFFFFFFFFFFFFFFF:FF:FFFFFFFFFFFFFFFFFFFF,FFFFFFFFFFF
F:FFFFFFFFFFFFFFFFFFFFFFFF:FFFFFFFFFFFFFFFFFFFFF
@A00721:190:HGJW7DSXY:4:2460:10212:10347_1:N:0:TCTGCAAG+GCTGCACT
ATACTCTCTATCGTTTTCTGCTTTTTCTTAGCTAATAGATTAATTAAGGATGAGTACGAAAACCAACT
CTTCGTTACGAGACCTGATGGTATTATAGATTTAGTCTCTACACCCATCTCAACGAGGATAAAGCGAG
TT
+
FFFFF:,,F,F,,FF:FFF,FFF:FFFFFFFFFFF:,,,FF:FF,:F,F,F::FF,FFF,FFFFF:FF
,:F:FFFF,FFF:,F,FFFF,:F,,FFFFFFFFFFFFFF:FFFFFFFFFF:FFFFFFFFFFFFFFFFF
:F
@A00721:190:HGJW7DSXY:4:2463:22092:24659_1:N:0:TCTGCAAG+GCTGCACT
CACGAAGATAACATCCGCAAACGACTCGAAACCGAGACGCCGTAGATCAAAGTCCCTTCTAACTTTAG
ATTATGAATCCTGCCTTTACTCATTTTATAGCAGTTATTTATGTTGTACTTTTTATAATTTATAACCT
T
+
FF:,F,,F,F,,F:FF:,FFFFFFFFFFFFFFFFFFFFFFFFFFFFFFFFFFFFF:FFFFFFFFFFFF
FFFFFFFFFFFFFFFFFFFFFFFFFFFFFFFFFFFFFFFFFFFFFFFFFFFFFFFFFFFFFFFFFFFF
F
@A00721:190:HGJW7DSXY:4:2247:8901:1125_1:N:0:TCTGCAAG+GCTGCACT
TACTTATTGTAACGATCATTCTTTCGAGTTTTGTCATTCTTATTCTTTTATTCCTTTAACTGTGTATG
AGGAGGATTTATAATGCTTTTCGATAGTGATTGTTATCAATTTAAATACATTACTGTTTCAAATCATC
TTT
+
FF:FFFFFFFFF,FFFFFFFFFFFFFFFFFFFFFFFFFFFFFFFFFFFFFFFFFF:FFFFFFFFFFFF
FFFFFFFFFFFFFFFFFFFFFFFFFFFFFFFFFFFFFFFFFFFFFFFFFFFFFFFFFFFFFFFFFFFF
FFF
@A00721:190:HGJW7DSXY:4:2456:10384:6292_1:N:0:TCTGCAAG+GCTGCACT
GAAAATGGTTTCAAATGAGGTCAAAGCTCTATTTATTAAACGAACTATTATCCGATTCTTCGAGGGTA
TCACTACCACTGATCTGCCGACTTTAGTCAAGGAACTTCAATTCCTTCGATGTTTATATTTTTCTATA
AGG
+
FFFFFFFFFF:FF:FFFFFFF:FFFFF:FFFFFFFFFFFFFFFFFFFFFFFFFFFFFFFFFFFFFFFF
FFFFFFFFFFFFFFFFFFFFFFFFFFFFFFFFFFFFFFFFFF,FFFFFFFFFFFFFFF:FFFFFFFFFF
FFF
@A00721:190:HGJW7DSXY:4:2143:31033:17284_2:N:0:TCTGCAAG+GCTGCACT
TAATCAAATTTCGACAAATTTTAGAACTTTATTTCAGAACTCTCTCCGATCACCTTGGTGTGATCTCC
CGGAGGATCTTTTCAATAAGATGTATGATGATAATCTACTATTTTTAA
+
:FFFFFFFFFF,FFFFFF,:FF:FFFFFFFFFFFFFFFFFF::FFFFFF::F:FFFFFFFFF:FFFF,
:FFFFFFFFFFFFF:FFFF:F:FFF::FFF:FFFF:FFFF:FFFFF:F
@A00721:190:HGJW7DSXY:4:2351:25753:32346_1:N:0:TCTGCAAG+GCTGCACT
AGCGTATACTTGAATACTTTATCAAAGATTTGGCTTGGTCTATTTACCGCAAAAAGTTGGAAGAGGTC
TATGATATTTATTTTACTTATACTTTAAATAGCACCGATTATAACTTGACTGTTGATAATATTACAAT
AGC
```

+
FF:FFFFFFFFFFFFFFFFFFFFFFFFFFFFFFFFFFFFFFFFFFFFFFF:FFFFFFFFFFFFFFFFFFF
FFFFFFFFFFF,FFFFFFFFFFFFFFFFFFFFFFFFFFFFFFFFFFFFFFFFFFFFFFFFFFFFFFFFFF
FFF
@A00721:190:HGJW7DSXY:4:2221:24424:16955_2:N:0:TCTGCAAG+GCTGCACT
AAGATTAGAGAAGTAACTCGGTGAACACGTCGGTAGCATTGATTTACTTTTACCGTTAGAACTGAAAA
AGATTCTTTTCGAAAAGAAACCTATAAGTTACAAACGATTCTTCGACT
+
FF,FFFF:F,FF:FF:FF:F,F:FF::,FF,,,FF,,:FF:FFFFF,FFFFF:,:FFF:FF:FFFFFF
F,FFF,FFFF::FFFFFFFFF:FFFFFFFFFFFFFFFFFFFFFFFFFFF
@A00721:190:HGJW7DSXY:4:1264:32913:11146_1:N:0:TCTGCAAG+GCTGCACT
AATAAAAATACTTCAAGTACCTGAACTTTACATTTTTAATGGTATGGTATAATGTGTCTTACATATAG
AACCTTCACTCTCGGTAATGAGAGTGTATTCCTTAATATTCTAGTAACCGCTATGTGGGCATTACTAG
GAT
+
FFFFFFFFF:F:,F:,FFF,:F:FF,FFFFFF:F:FF,FF,FFFFFF,FFFFFFFF::FFF,,,FFF:
F,:,FFF:,F,F:FF,FFF:FFF:F,FFFF,,FFFFFF::FFF:,FF::FFFF:FFFFF::FFFF,FF
F:F
@A00721:190:HGJW7DSXY:4:1509:13838:31375_2:N:0:TCTGCAAG+GCTGCACT
TTTAAATGCTAGGAAAATTTTAGAAAAACTCCATTGTATAGTTCAAGGAGTACTTAATTCAATAGCTT
TAGTATTATTTACTATCGCCTTTATTGAAAATTGTATAAGAGTTAATT
+
FFFFFFFFF:FFFFFFFFFFFFFFFFFFFFFFFFFFFFFFFFFFFFFFFFFFFFFFFFFFFFFFFFFFFF
FFFFFFFFFFFFFFFFFFFFFFFFFFFFFFFFFFFFFFFFFFFFFFFFFF
@A00721:190:HGJW7DSXY:4:2573:6994:6120_1:N:0:TCTGCAAG+GCTGCACT
GGGAGAATGAACTGCCGCTTCCCGGAAGAAACGATCTATTTACAGAGTATTTAGAGTAGTCCAAAGTT
TGTATAAATTTATATGATTTTCTATATATATACCACATATATATATATATAAAATCCTATAAATTTGT
ACA
+
FFFFFFFFFFFFFFFFFFFFFFFFFFFFFFFFFFFFFFFFFFFFFFFFFFFF,FFFFFFFFFFFF:FFFF
::FFFFFFFFFFFFFFFF:FFFFFFFFFFFFFFFFFFFFFFFFFFFFFFFFFFFFFFFFFFFFFFFFFF
FFF
@A00721:190:HGJW7DSXY:4:2231:15953:18004_2:N:0:TCTGCAAG+GCTGCACT
TACACTCCCCAAACATAACATACCATAACCTACCATGACCAATCATCGCTCCAACTGGCACGCCAGAC
GTAGGGTCAAACCTACTACCGCCTGTCGGCAGATCGGCACCACTGGTC
+
FFFFFFFFFFFFFFFFFFFFFFFFFFFFFFFFFFFFFFFFFFFFFFFFFFFFFFFFFFFFFFFFFFFFFF
FFFFFFFFFFFFFFFFFFFFFFFFFFFFFFFFFFFFFFFFFFFFFFFFFF
@A00721:190:HGJW7DSXY:4:2606:2410:6355_2:N:0:TCTGCAAG+GCTGCACT
ACCCGCTGTGCAGAATAACCTTCCCAAATCTAGATCTGCACAGAAGAAGGCTGAAGGTCGTTACCGGA
CGACCACTCAAAGGAAACGGGCTGTTGCCCAGAGGAAAGCAGCGACCA
+
FFFFFFFFFFFFFFFFFFFFF:FFFFFFFFFFFFFFFFFFFFFFFFFFFFFFFFFFFFFFFFFF:FFFFF
FFFFFFFFFFFFFFFFFFFFFFFFFFFFFFFFFFFFFF:FFFFFFFFFFF
@A00721:190:HGJW7DSXY:4:1658:4562:18192_2:N:0:TCTGCAAG+GCTGCACT
ACGGCAGTCCCGTCATCTCTGTGGGAACTGAGGCAGTACCTCAGTCAAAAGTCACATTCACCACTCCC
ATACCAATCAATGATGTGACTATACAGTTCGGCCATTTCAAGAGTGGT
+
FFFFFFFFFFFFFFFFFFFFFFFFFFFFFFFFFFFFFFFFFFFFFFFFFFFFFFFFFFFFFFFFFFFFFF
FFFFFFFFFFFFFFFFFFFFF:FFFFFFFFFFFFFFFFFFFFFFFFFFF:
@A00721:190:HGJW7DSXY:4:1272:17978:15749_2:N:0:TCTGCAAG+GCTGCACT
GGAGGTACTGAAGCCTTCCCCTCCGCTGGCACGAAGAACACTCCCAAGCCAATCAATAGTTCTTCTGA
CCAGAATGAAAAGGGGTTTACCAGGGTAGTCTCTAAGTCTACCAGGAA
+

FFFFFFFFFF:FFFFFFFFFFFFFFFFFFFFFFFFFFFFFFFFFFFFFFFFFFFFFFFFFFFFFFFFF
FFFFFFFFFFF,FFFFFFFFFFFFFFFFFFFFFFFFFFFFFFFFFFFF
@A00721:190:HGJW7DSXY:4:1619:13150:10488_2:N:0:TCTGCAAG+GCTGCACT
CAAAACTGATAAGAATAAAGGGAAAGGTCCATCAGGACCTAAGAAGGGGAAATTACCCCTAATTCTGA
AGGAAGATCTCCCACCATCTGGGGTTGGGAATAAGAAATCTGCAGATC
+
FFFFFFFFFFFFFFFFFFFFFFFFFFFFFFFFFFFFFFFFFFFFFFFFFFFFFFFFFFFFFFFFFFFF
FFFFFFFFFFFFFFFFFFFFFFFFFFFFFFFFFFFFFFFFFFFFFFFF
@A00721:190:HGJW7DSXY:4:1427:13774:4867_2:N:0:TCTGCAAG+GCTGCACT
CTACGAAGTCGCTGAAGGCGGCTTGGGTCATTAAGGGTAGCTGTAAAGTTGCGCCTGCCGACCCCATC
TTTGAGACCAAGACTCTCTACAGTAGGTTCGTGCCTACTATGAGCAAT
+
FFFFFFFFFFFFF:FFFFFFFFF:FF:FFFF:FFFFFFFFF:F:FF,F:FFF:FFFFFFFFFFFF:FF
FFFFFFFFFFF:FFFFFFFFFFFFF:FFFFF:FFFFFFFFFFFFFF:F
@A00721:190:HGJW7DSXY:4:1111:11180:29810_1:N:0:TCTGCAAG+GCTGCACT
TCGTCACTGAGGACGAAGGTGAGAGGACTTTTGGCTACTGGCGACAAACTGAGAGCAGTCGCTGGGGA
AAGAGAATTGCGTGGGTTGAAGGCCGACGCTTCGAGCTTGAGTGTGACGAGGCCGGTGACGTTTTTGT
CAA
+
FFFFFFFFFFFFFFFFFFFFFFFFFFFFFFFFFFFFFFFFFFFFFFFFFFFFFFFFFFFFFFFFFFFF
FFFFFFF,FFFFFFFFFFFFFFFFFFFFFFFFFFFFFFFFFFFFFFFFFFFFFFFFFFFFFFFF
FFF
@A00721:190:HGJW7DSXY:4:2239:12328:20243_1:N:0:TCTGCAAG+GCTGCACT
GATTTTATCCGCGCTTAACTATGGGGCGAAGATCCCCTATGGAGTCTTGAAGGACTTAGGTTGTCATA
TTTATGCCGACGATAATAAGCATGTCACCCTTAAGCACCGAGGTAAGCTCGGGACTTACAAGAGAGAG
TGT
+
FFFFFF:F:FFF,,FFFF,FFFFF,FF,,FFFFF:FFFFF,,FFFFFF:F:F:FFFFF,:FFF,,:FF
FFFFF::F,,F,FFF,FF:FF:F:FFF:::FFFFFFF:FF::,FFFF:FFF,F,:FFF,,,,FFFFFF
FFF
@A00721:190:HGJW7DSXY:4:2178:23303:24126_2:N:0:TCTGCAAG+GCTGCACT
CTCTCACTATTTTTGCCCAGAATTCGGGCTTTCTTACTGATGAGGTTAAAACTCTTGCTCCTTCTGGG
GGTGAGGGTTTTTGTTATATTAACTGGTTCTGGGAGGCTGCTTTCCTC
+
FF:FFF,FF:FFFF,:FFF:FFF,F:FFFFFF:F,FFF,F,FFFFF:FFFF:F,F:FFFFFFFF,F,F
::FFF,FFFFFFF:FFFFFFF,F,,:FFF:FFFF:F,:FFFFFF:FFF
@A00721:190:HGJW7DSXY:4:1174:5511:2394_2:N:0:TCTGCAAG+GCTGCACT
TTTAGAGAGCATTAGAAGGAAGTTGGGCAAATTCCCACATATGGTAGACGTTGTCACACTTTTCTTGA
AAATTTTTCCTTTCCTCCGTCATTGGGGATTTCGATGTGAGAAGGTGG
+
FFFFFFFFFFFFFFFFFFFF:FFFFFFFFFFFFFFFFFFFFFFFFFFFFFFFFFFFFFFFFFFFFFFF
FFFFFFFFFFFFFFFFFFFFFF,FFFFFFFFFFFFFFFFFFFFFFFFF
@A00721:190:HGJW7DSXY:4:1558:21169:16423_2:N:0:TCTGCAAG+GCTGCACT
GGGTCTTCTTCATGCTGATCTTTCAGGACCTAATCACATCATGATTGATAGTTACGGCTCGGATCGAG
TTGGAGGGAGGAAGTTCTCCGAAATTAAAGACGGGGGGATTAAGAGTC
+
F::F:FFFFFFFF:FFFF:FFFFFFFFFFFFFFFFFFF:FFFFFFFFFFFF:F,F:F:FFF,FFFFF,
FFFF,FFFF:FFFFFFFFF::F,FFFFFFFFFFF::F:F:FFFFFFFF
@A00721:190:HGJW7DSXY:4:1670:25220:3646_2:N:0:TCTGCAAG+GCTGCACT
TCAGAGAGGGGAGCTCTGAAAAGGAAACTTCGTCAGAAGATGAGGAAGTCTAAGAGGAGGAGTTTGAG
GAAGGCTCTAGAGAAGATCTCACCACCTGGAGAAGGCAAGTGGAGAGT
+
FFFFFFFFFFFFFFFFFFFFFFFFFFFFFFFFFFFFFFFFFFFFFFFFFFFFFFFFFFFFFFFFFFFF
FFFFFFFFFFFFFFFFFFFFFFFFFFFFFFFFFFFFFFFFFFFFFFFF

@A00721:190:HGJW7DSXY:4:1449:15365:23093_2:N:0:TCTGCAAG+GCTGCACT
AAGAAGGATTATTTGAAGCAGGGGTTACCACTTCCTAAGGTGGATGAGGGAGGTTGTTTCTTTGGAGT
TTTAGATAATCTTTTGAGAAGGGGTCATAGAGCCAGCAAGAGAAGCTT
+
FFFFFFFFFFFFFFFFFFFFFFFFFFFFFFFFFFFFFFFFFFFFFFFFFFFFFFFFFFFFFFFFFFFF
FFFFFFFFFFFFFFFFFFFFF,FFFFFFFFFFFFFFFFFFFFFFFFFF
@A00721:190:HGJW7DSXY:4:2341:16984:13839_2:N:0:TCTGCAAG+GCTGCACT
CCTTGAGAAGACAAGCCACATTAAGATATAATTTAGGTCAACTTGGGGTTTCGAATGTCATTAGTGCC
TTACGATTGAAAGAGGTCCTTTTGAGTATCGACAGATCTTTCGATCTT
+
FFFFFFFFFFFFFFFFFFFFFFFFFFFFFFFFFFFFFFFFFFFFFFFFFFFFFFFFFFF:FFFFFFFF
FFFFFFFFFFFFFFFFFFFF,FFFFFFFFFFFFFFFFFFFFFFFFFFF
@A00721:190:HGJW7DSXY:4:2612:18376:12054_2:N:0:TCTGCAAG+GCTGCACT
GTGGCAGAATTCGGTAACTTACCATATTGTTCCTTCCGGGAAGTTGTGGGATTTAACTTTGACTCAAC
TACTCCGATCAGATCCGGATGGTTTGATTGGTATGGACTACTCGACTT
+
FFFFFFFFFFFFFFFFFFFFFFFFFFFFFFFFFFFFFFFFFFFFFFFFFFFFFFFFFFFFFF:FFFFFF
FFFFFFFFFFFFFFFFFFFFFFFF,FFFFFFFFFFFFFFFFFFFFFFF:
@A00721:190:HGJW7DSXY:4:1439:19587:11898_2:N:0:TCTGCAAG+GCTGCACT
TCATGGCAGATACTACCGATAAGGTGAAAAATATGCAGGTCAAAGATTCTGTAGCTCTGAGGGCGGTT
GAGGGAGCCGTTTTGGCCGACTTTAACTATTCTAAGAGTCTTTATGAT
+
FFFFFFFFFFFFFFFFFFF:FFFFFFFFFFFFFFFFFFFFFFFFFFFFFFFFFFFFFFFFFFFFFFFF:
FFFFFFFFFFFFFFFFFFFFFFFFFFFFFFFFF:FFFFFFFFFFFFFF
@A00721:190:HGJW7DSXY:4:1224:16911:17221_2:N:0:TCTGCAAG+GCTGCACT
CGTTGTCGTACCTTACAAGGTTGAGGAGACTTTACAGCGGCTTATTGTCGCGCAGTTTCCAGAATTGC
GGTTGAAGTTCACCCATTCTATCTTGAATGCTCACGCCGTCGCAGCCA
+
FFFFFFFFFFFFFFFF:FFFFFFFFFFFFFFFFFFFFFFFFFF:F,FFFFFFFFFFFFFFFFFFFFFFF
FFF,FFFF:FFFFFFFFFFFFFFFFFFFFFFFFFFFFFFF,FFFFFFFF
@A00721:190:HGJW7DSXY:4:1268:24894:23156_1:N:0:TCTGCAAG+GCTGCACT
ATCACTTGTTAAAGTACAGATTTAGTGATTTAGATATTATTGATATTGGAGGCGATATTCGTTTTCAT
ATCCAAAACTCCTTGAATGGAGTACATATTTGCAACCCTATTCTTGATGTGAAAGATGCATCGAGGTA
TAC
+
FFFFFFFF:FF:FFFFFFFFFFFFFFFFFFFFFFFFFFFFFFFFFFFFFFFFFFFFFFFFFFFFFFFFF
FFFFFFFFFFFFFFFFFFFFFFFFFFFFFFFFFFFFFFFFFFFFFFFFFFFFFFFFFFFFFFFFFFFF
FFF
@A00721:190:HGJW7DSXY:4:2158:30472:11772_1:N:0:TCTGCAAG+GCTGCACT
ATTGATTGGGAACGTACCTACTCACGAGACGTCATGAAAGCTATCGGAGGGAAGCAAAATGTTTCGTT
CTGTTACAAGAACGCTTTGGATTGTGACCATAAAGCCTCAGTTGCGATAGCTGTAGAGGTATACGATA
TCT
+
FFFFFFFFFFFFFFFFFFFFFFFFFFFFFFFFFFFFFFFFFFFFFFFFFFFFFFFFFFFFFFFFFFFF
FFFFFFFFFF:FFFFFFFFFFFFFFFFFFFFFFFFFFFFFFFFFFFFFFFFFFFFFFFFFFFFFFFFF
FFF
@A00721:190:HGJW7DSXY:4:2347:30816:16595_2:N:0:TCTGCAAG+GCTGCACT
GCCACTATAATGCTTAAGAGAGAGATTGATGTATGTCATTTCGTCTTAGTTTGTCCCGGCGAATTATT
GGACACGATGAAAAATGACATACCCGTTCCTGACCTTGGTATCGTTAT
+
FFFFFFFFFFFFFFFF:FFFFFFFFFFFFFFFFFFFFFFF:FFFFFFFFFFFFFFFFFFFFFFF:FFFFF
FFFFFF:FFFFFFFFFFFFFFFFFFFFFFFFFFFF:FFFFFFFFFFF:F
@A00721:190:HGJW7DSXY:4:1524:18258:27070_1:N:0:TCTGCAAG+GCTGCACT
GGTTATTCTCACAACAAAGATAAGATTTTATCTTTCTTCAAACACCCTGTTTTCCTTAAGGGCGGACA

```
ATTGTTCCATTGTGAACTCTTGGGGACTAGATTGAGCATTAATGAGTTCCGAATAACTCGTAATAAAT
CTT
+
:FFFF:FFFFFFFFFFFFFFFFFFFFFFFFFFFFFFFFFFFFF,FFFFFFFFFFFFFFFFFFFFFFFF
FFFFFFFFFFFFFFFFFFFFFFFFFFFFFFFFFFFFFFFFFFFFFFF:FFFFFFFFFFFFFFFFFFFF
FFF
@A00721:190:HGJW7DSXY:4:2610:21965:5901_1:N:0:TCTGCAAG+GCTGCACT
ATTCAGAATACCTATTTCCGTACCAAGAGCCACTGCCAATCTCATCGCTATACGCATACCTGATTGGA
AGAAGCGAGGTTCGTTCCTCAATAAAAATTTTTCTCAAGAGATTATGGTTGATAAAGTTTTCTTCATA
AGG
+
FFFFFFFFFFFFFFFFFFFFFFFFFFFFFFFFFFFFFFFFFFFFFFFFFFFFFFFFFFFFFFFFFFFF
FFFFFFFFFFFFFFFFFFFFFFFFFFFFFFFFFFFFFFFFFFFFFFFFFFFFFFFFFFFFFFFFFFFF
FFF
@A00721:190:HGJW7DSXY:4:1262:17372:26256_2:N:0:TCTGCAAG+GCTGCACT
ACGTCATGGGGATCACTTCTTCGGTCAATGACAAAACCTTCGAATATGTTACGACATGGCTTCGTAAC
AACAGTGCGAGGATTGTCATCTCGGGGAAAGTGATTCACCAAGATGTT
+
FFFFFFFFFFFFFFFFFFFFFFFFFFFFFFFFFFFFFFFFFFFFFFFFFFFFFFFFFFFFFFFFFFFF
FFFFFFFFFFFFFFFFFFFFFFFFFFFFFFFFFFFFFFFFFFFFFFFF
@A00721:190:HGJW7DSXY:4:2377:30535:11350_1:N:0:TCTGCAAG+GCTGCACT
GAGATACCTAGAGAAGGTCGCAGCGATCTTACTCGTTATCGGAGCTAAGAAGAAGACTTTGGGTACAC
GTTATGCTCGCAAACTTAACGTTAGTACCGGAGGTGATCTCTCTGCTTCTGCGTTACTGAGAGAAAAG
ATT
+
FFFFFFFFFFFFFFFFFFFFFFFFFFFFFFFFFFFFFFFFFFFFFFFFFFFFFFFFFFFFFFFFFFFF
FFFFFFFFFFFFFFFFFFFFFFFFFFFFFFFFFFFFFFFFFFFFFFFFFFFFFFFFFFFFFFFFFFFF
FFF
@A00721:190:HGJW7DSXY:4:1317:15637:19648_1:N:0:TCTGCAAG+GCTGCACT
ACTTTATGGACAAGATGCAAGTTCTTGGGAAATACTTGTTTCCCTTTTTAGGTTTAGTAGGTGATCTT
GACGTCGATTCTTTGTATAGGGAGTGTGAGAAGACAACGAATCTTGAAACTACGGTCCTTATCAAAGA
AAA
+
:,FFFFFFFF:FFFFFFFF:FFFFFFF:FFFFFFFFFFFFFFFFFFFFFF:FFFFFFFFFFFFFFFF
F:FFFFFFF:FFFFFFFFFFFFFFFFFFFFFFFFFFF:FFFFFFFFFFFFFFFFFFF:FFFFFFFFFFF
FFF
@A00721:190:HGJW7DSXY:4:1128:14859:35430_1:N:0:TCTGCAAG+GCTGCACT
TCTAAATCTTTTGAGATTGAGAAGATTGAAGAACACGTCAAGAAGTCGCTCGGCGTTGTTCTTGAAAA
CCAAGTTAAGAAGGAGGCTGATCATGGAGTCTTTGTACCTTCTGTCAAACTTCGAGGTTCAGCTACGA
GAG
+
:FFFF:FFFFFFFF::FFFFFFFFFFFFFFFFF::FFFFFFFFFFFFFFFFFF:FFFFFFFFFFFFFF
FFFFFFFFFF:F:FF:FFFFFFFFFFFFFFFFFFFFFFFFFFFFFF:FFFFFFFFFFFFFF:F,FFFFFF
FFF
@A00721:190:HGJW7DSXY:4:2404:6551:10300_1:N:0:TCTGCAAG+GCTGCACT
TGGATTGAAGGCTGGCTCTAAGGGAGACTCCTTCTTTTTGAATGTTGTAAAGAATTGGGTTGAGAAGT
GTGCTTTACAATGAGTTAGACTCTTCTTCAAGTTTTTAGATCTTCTTGATAATCTTAAAGAAAAATTT
TCG
+
F,:FFF,FF:,:F,,FFFF::F,FF:F,F,,F,F,FFFFFF:F,F::FFF:F:FFF,FFFFFF:::FF
:F::FFFF:::,,F,FFFF::F:FF,FFFFFFFFFFFFFFF:FF,FF,FF:FFFFFFF:F:FF:FFFF
FF,
@A00721:190:HGJW7DSXY:4:1548:30445:11381_1:N:0:TCTGCAAG+GCTGCACT
GTTTCTCTTGGTATTTCTGAAATAGTTTATCAGAGCGTCATTTTCCTTTGGGATCTTTCCTCTGGTAA
```

GGGAATTATTAAATCATTCTTCTCACTCTTGACGAGAATTATTACTGTTTTCTCTACCCATAACTTCT
TTT
+
FF:FFFFFFFFFFFFFFFFFFF:FFFFFFFFFFFFFFFFFFFFFFFFFFFFFFFFFFFFFFFFFFFFFF
FFFFFFFFFFFFFFFFFFFFFFFFFFFFFFFFFFFFFFFFFFFFFFFFFFFFFFFFFFFFFFFFFFFFF
FFF
@A00721:190:HGJW7DSXY:4:1643:15600:15515_1:N:0:TCTGCAAG+GCTGCACT
ACTGGAGTTCAGACGTGTGCTCTTCCGATCTGTTTCAACACTTATCACCACTTTTGAAAGGAAAATAA
TTGGTGATGAAAATACGTATGTCGGTGGGGTTCTCAAGGCTCTCATCGCCACTGGGATTCTAAGGGGT
TCC
+
FFFFFFFFFFFFFFFFFFFFFFFFFFFFFFFFFFFFFFFFFFFFFFFFFFFFFFFFFFFFFFFFFFFFF
FFFFFFFFFFFFFFFFFFFFFFFFFFFFFFFFFFFFFFFFFFFFFFFFFFFFFFFFFFFFFFFFFFFFF
FFF
@A00721:190:HGJW7DSXY:4:2662:30327:29904_2:N:0:TCTGCAAG+GCTGCACT
TTCAATGATCAGGATTCTTCTTTTGGTCCTTACGTCTTAAGTAATTTGGATGTTGTCGAAAACTATCA
GGTTCTTGTCACGATTCTTAAAAGAAGTTTATTAAAACTTCAGAGTCT
+
FFFFFFFFFFFFFFFFFFFFFFFFFFFFFFF:FFFFFFFFFFF:FFFFFFFFFFFFFFFFF:FFFFFF
FFFFFFFFFFFFFFFFFFF:FFFFF:FFFFFFFFF:F,FFFFFFFFFFF
@A00721:190:HGJW7DSXY:4:2215:13087:4366_1:N:0:TCTGCAAG+GCTGCACT
GGTAGCTAATGTTCTTGCAATAGCCACAGACTTTCTGGGAAAAGAGACCTCTGTTGTAGAGGAGTTGA
GAGAATTACTCAGATTCAATCTGGCATCACTGAACGTCTACAACGAGTTAGGGAGTGGCTAGGTTTTA
AAA
+
FFFFF:FFFFFFFFFFFFFFFFFFFFFFFFFFFFFFFFFFFFFFFFF:FFFFFFFFFFFFFFFFFFFFFFFF
FFFFFFFFFFFFFFFFFFFFFFFFFFFFFFFFFFFFFFFFFFFFFFFFFFFFFFFFFFFFFFFFFFFFF
FFF
@A00721:190:HGJW7DSXY:4:2176:15854:15327_1:N:0:TCTGCAAG+GCTGCACT
CAATACCTTCCACTGAGTTCATCACCCAGAGTTTCGACGTCGCGGAAAACGATGAAGATTTCCACACT
GTTCGAGAATCTTCTGATGCTGAATTTGAGGAAAATTTTTCGGAAGTTTTTGGTATAAATCCTGATGG
CTC
+
FFFFFFFFFFFFFFFFFFFFFFFFFFFFFFFFFFFFFFFFFFFFFFFFFFFFFFFFFFF:FFFFFFFFF
FFFFFFFFFFFFFFFFFFFFFFFFFFFFFFFFFFFFFFFFFFFFFFFFFFFFFFFFFFFFFFFFFFFFF
FFF
@A00721:190:HGJW7DSXY:4:2265:16351:2440_2:N:0:TCTGCAAG+GCTGCACT
AAAACCTTCTTTTATCATTGCAACAGGACTTTGAGTAGACTGTTGAAATGGACAAAATCTGGGATCAT
AGGTATGTGTCACGCTACACTCGAAATATGGGCCCTCATAAGGAAGTA
+
FFFFFFFFFFFFFFF:FFFFFFFFFFFFFFFFFFFFFFFFFFFFFFFFFFFFFFFFFFFFFFFFFFFF:
FFFF:FFFFFFFFFFFFFFFFFF:FFFFF:F:FFFFFFFFFFFFFFFF
@A00721:190:HGJW7DSXY:4:2131:24044:9314_1:N:0:TCTGCAAG+GCTGCACT
CGTCATTACAGTCTGGAAGTTAGATCATTTGTTCAGGAAGATCTCTACAGAGATTATCGAGAGTTGTG
ATCTAGTTCTAAATTTAGGTCGACGGACTTTAGCAACTTTAGATAAATTCTTTCCGGGCTCAATGACC
TTT
+
:FFFFFFFFFFFFFFF:FFFFFFFFFFFFFFFFFFFFFFFFFFFFFFFFFFFFFFFFFFFFFFFFFFFF
FFFFFFFF:FFFFFFFFFFFFFFFFFFFFFFFFFFFFFFFFFFFFFFFFFFFFFFFFFFFFFFFFFFFF
FFF
@A00721:190:HGJW7DSXY:4:2537:24451:11741_1:N:0:TCTGCAAG+GCTGCACT
TCTGACCATCGCAAAGAAGACTTTATTAGTTCATGAAGTTCTTACACTTCTCGCGACTAATGTCAGCT
TCACTACAAAGGCGTTACGTAAAGTAGTTTTCACGCTTTATCTTAAAGCATTACCACCAAATTTAGCA
CTT

+
FFFFFFFFFFFFFFFFFFFFFFFFFFFFFFFFFFFFFFFFFFFFFFFFFFFFFFFFFFFFFFFFFFFF
FFFFFFFFFFFFFFFFFFFFFFFFFFFFFFFFFFFFFFFFFFFFFFFFFFFFFFFFFFFFFFFFFFFF
FFF
@A00721:190:HGJW7DSXY:4:2617:5068:20102_1:N:0:TCTGCAAG+GCTGCACT
CGTGTGCTCTTCCGATCTAGACATGCCAGTTAGAGTTCTTCTAACCAGGACTGATGGTGAGATTCTTT
TACCTTTTGGAGGTCCAGGAACCTTTACTCCTAGTGTCTTTTCTAGAGTCCAAGGACGTTTCCGTCGG
AGG
+
FFFFFFFFFFFFFFF:F,FFFF:FF:FFFFFFFFFFFFFFFFFFFFFFF,FFFFFFFFFFFF:FF:FF
,FFFFFFFFFFFFFFFFFFFFFFFFFFFFFFFFFFFFFFFF:FFFFFFFFFFFFFFFFFFFFFFFFFF
FFF
@A00721:190:HGJW7DSXY:4:2678:31919:10175_2:N:0:TCTGCAAG+GCTGCACT
GACCGCTCAATAACACTCTATTGAATCCCTTTTCCACGATGAAAGCTCTTTATGAGGTGCTCCATGCA
AATTTACAGTTCCTCTTTGGTGATATCGCCTCTGTCCTTTCTCCTTTA
+
,FF:FFFFFFFFFFFFFFFF:FFFFFF:FFF:FFFFFFFFFFFFFFFFFFFFFFF:FFFFFFFFFFFF
FFFFFFFFF:F,:F,F:F::FFFFFFF,:FFFFFF,:FFFFF,FF,,F
@A00721:190:HGJW7DSXY:4:2406:9109:36902_2:N:0:TCTGCAAG+GCTGCACT
ACTTGGGTTTATCGGTTGTATTTTGATAAATTTGTTGGAATCGATAATGATACTGACGAAGGTTCTAT
TACCACCCTTGTCATTGATACAAACATTCTAGGTGATGCTTTCTTAGA
+
FFF:::FF:FF:FFFF,FFFFFFFFFFFFFF:F,FF,FFF::,F,FFF:::FF::FF,FF,:FFF:FF
FFF,F::F:F,::FFF:FFF:FFFFFF::FF::F:F:,F,F:FFFF,F
@A00721:190:HGJW7DSXY:4:1309:18575:6605_2:N:0:TCTGCAAG+GCTGCACT
TACAATCTGTAGAGAGAGAGGTTAATCAGAATTTGAGTGATAACGATTTAGTACTGGGAAATGGTGGT
AGTCAGTTTACTACTTTTCTTTCCCTCCCCTTTTTCGATTGGATTCGA
+
FFF,FF,:F:FFFFFFFFF,,FFFFFFF:FFFFF:F,,FFFFFFFFFF:F:F:FFF,:FFFFF,F::F
FFF:FFFFFFFF:FFFFF:FFFFFF,F::F::F:FFFFFFF,FFFFFF
@A00721:190:HGJW7DSXY:4:2512:1118:35837_2:N:0:TCTGCAAG+GCTGCACT
TTGATAAGGTTTTTCTTTTATGGGACTCTTGTTTGTACTTATGAAGGAGGTATTACTTCGGAAGTTCT
TAATGTAGGTCTCCTAGTACTTAAGGGTTTATGTTTCGGTTTCAGCTG
+
FFFFFFFFFFFFFFFFFFFFFFFFFFFFFFFFFFF:FFFFFFFFFFFFFFF:FFFFFFFFFFFFFFFF
FFFFFFFFFF:F:FFFFFFFFFFFF:FFFFFFFFFFFFFFFFFFF,FF
@A00721:190:HGJW7DSXY:4:1674:22146:25755_1:N:0:TCTGCAAG+GCTGCACT
TTCACATTGCGTGATCACTTCCCGTGGACTTGGGAAGAGATTAAACATAAAATCCAGCGATTCTATTA
TGTCGCTAAAGCCTGTCCAGTTGTCGATGATGTTCGGGCTGTGGGGATTAACTTCCCTCGTGAAACTT
ACG
+
FFFFFFFFFFFFFFFFFFFFFFFFFFFFFFFFFFFFFFFFFFFFFFFFFFFFFFFFFFFFFFFFFFF,
FFFFFFFFFFFFFFFFFFFFFFFFFFFFFFFFFFFFFFFFFFFFFFFFFFFF,FFFFFFFFFFFFFFF
FFF
@A00721:190:HGJW7DSXY:4:1521:17996:12806_1:N:0:TCTGCAAG+GCTGCACT
CACAAGGATTACTAAGTCTGAGATCTGCCACTTCTAATTTTATGAAACGTCGCGAAAATAAAGTTCAT
GAGGATGTCTTGGTCGAAAAATATCGCCGGGAACTTCTAATAGAGGATCCTGATGACATTATTTCAAC
GGA
+
FFFFFF,,:FFF:,,::FFF,:FFFF:FF::FFF::,FFFF,,:FF:,:FF,,FFF:F,FFF,FF,FF
::FF,F,FF:FF:F::F,,FFFFFF,,F,F::,FFF,:FFFF,F:,FF,:FF:F:,::FFFFF::F,,
FF:
@A00721:190:HGJW7DSXY:4:2256:24605:23844_2:N:0:TCTGCAAG+GCTGCACT
AGTTCTACCACAGAAACCGTCTCAGGAACTGTCTCCACTGATTCGGAATTAGAGAAAGACGACCTTCT

TGTGGGGACTGAGACCTCTGAATTTTCAGGGTGCCCTGATGAGGACGT
+
FFFFFFFFFFFFFFFFFFFFFFFFFFFFFFFFFFFFFFFFFFFFFFFFFFFFFFFFFFFFFFFFFFFFFF
FFFFFFFFFFFFFFFFFF,FFFFFFFFFFFFFFFFFFFFFFFFFFFFFFF
@A00721:190:HGJW7DSXY:4:1457:26738:23907_1:N:0:TCTGCAAG+GCTGCACT
ACTCTGAGACTTCTAAGAGTGTTTCAAAAGACCAGAAACGTAACAAGAAGAATAAAGGTAGGAAGGGG
AGAAAACAGAACAAGCGAGCGGTTGAAGAGAAAACTGATATGGTTGCGGCTGATCGTACCCCAATTCC
TGC
+
FFFFFFFFFFFFFFF:F:FFFFFFFFF:FFFFFFFFFFFFFFFFFFFFFFFFFFFFFFFFFFFFFFFFFF
FFFFFFFFFFFFFFFFFFFFFFFFFFFFFFFFFFFFFFFFFFFFF,FFFFFFFFFFFFFFFFFFFFFFFF
FFF
@A00721:190:HGJW7DSXY:4:1513:10267:3959_2:N:0:TCTGCAAG+GCTGCACT
CTCGGCGATACCACTGATCTTCCAGAGTTACGTAGTGGTCGTTCTTTTGGTGATTTTATCAAAGAGAA
AGTTTCTGCGAGTCGCGACGATAGTATTTATCGGAGTGATATTAACAA
+
FFFFFFFFFFFFFFFFF:FFFFFFFFFFFFFFFFFFFFFFFFFFFFFF,FFFFFFFF::FFFFFFFFFF
FFFFFFFFF,FF:FFFFFFFFF::F:FFFFFFFFFFF:FFFFFFFFFFFF
@A00721:190:HGJW7DSXY:4:1167:26431:21590_2:N:0:TCTGCAAG+GCTGCACT
CACTTACAAGAGTGTTGAAGTACCGCAACATGGATCAGTTTTCTAAAAAGCTATTAATAGCAGTTGCG
GGGCTATAATACCGCCTCCAGATTACTCTAAACTGAATCCACTCTCTT
+
FFFFFFFFFF:FFFFFFFFFFFFFFFF:FFFFFFFFFFFFFFFFFFFFFFFFFFFFFFFFFFFFFFFFFF
FFFFFFFFFFFFFFFFFFFFFFFFFFFFFFFFFFFFFFFFFFFFFFFFFF
@A00721:190:HGJW7DSXY:4:2332:17345:14465_2:N:0:TCTGCAAG+GCTGCACT
TTTGGAACTGTGTAATGTCCTACGTACTGTTCAAGGTGTTTGGGCTTCTCTAAAGAACAGCAACTTTC
AACATGAATTAGTTATTGAAAGTCTTGACCCTCACGTCTCACTTTATC
+
FFFFFF,FFFFFFFFFFFFFFFFFFFFFFFFFFFFFFFFFFFFFFFFFFFFFFFFFFFFFFFFFFFFFFF
FFFFFFFFFFFFFFFFFFFFFFFFFFFFFFFFFFFFFFFFFFFFFFFFFF
@A00721:190:HGJW7DSXY:4:2635:18783:14231_2:N:0:TCTGCAAG+GCTGCACT
AGACCTTTCTTGGCGAGTAAAGAGGGTTTGAAAATTACGGAACCTTTCCTCGTTCTTCAAGAAGATCT
TCGAGTGTTTTATAGTGAGTTATTCTTGCGAAGGATCAATTCTATTGA
+
FFF:,FFF,,,F,,,FFFF::FF,FFFFF::FFF:,F,F,F,:FFFFFF,F,FFF,FFF,,FF,:F,,
F:FF,,:F,,:FFF,F,FFFF,:F,,F,FFF::,,FFFF,,F,FF,,,
@A00721:190:HGJW7DSXY:4:2346:12888:33364_2:N:0:TCTGCAAG+GCTGCACT
AAGAAGTTCTTCGCAGTTGAATCTCCACCAGGTGGAGGGAAGACCACTGAACTTATAGCGACTTTTGT
CGAATTTTTCGCAAAAGGTCTTGAAGTACGTATTGTCACTGCTAATCG
+
FFFFFFFFFF:FFFFFFFFFFFFFFFFFFFFFFFFFFFFFFFFFFFFFFFFFFFFFFFFFFFFFFFFFFF
FF:FFFFFFFFFFFFFFFFFFFFFFFFFFFFFFFFFFFFFFFFFFFFFFF
@A00721:190:HGJW7DSXY:4:1544:24008:35399_2:N:0:TCTGCAAG+GCTGCACT
CACTTCTAGTTCATCGCGGTATTTGTGAAATTAAGACAGACGTGAAAGTTATGATGAGTGATCGTATT
AGAACAGCTGATTCTTATTGCATGAACGTCGCTGAGGTCAAACCTGTG
+
FFFFFF,FFFFFFFFFFFFFFFFFFFFFFFFFFFFF:FFFFFFFFFFFFFFFFFFFFFFFFFFF:FFFFF
FFFFFFFFFFFFFFF:FFFF:FFFFF::FFFFF,F,F,:FF,FFF:::
@A00721:190:HGJW7DSXY:4:2442:23086:34741_1:N:0:TCTGCAAG+GCTGCACT
CATCGACGAATTATTCATGATTCACGCTGGTCAGACCCTTTTTCTTACACATCTACTTAAGGCGGCTT
ATACCATAGGTTACGGTGATTCCAATCAAATCGGATTCATCAATCGTATTGATCTCCAAGTCGCAAAC
TAC
+
FFFFFFFFFFFFFFFFFFFF,FFFFFF:FFFFFFFFFFFFFFFFFFFFFFFFFFFFFFFFFFFFFFFFFF

F:FFFFFFFFFFFFFFFFFFFFFFFFFFFFFF:FFFFFFFFFFFFFFFFFFFFFFFFFFFFFFFFFF
FFF
@A00721:190:HGJW7DSXY:4:2308:3775:29074_2:N:0:TCTGCAAG+GCTGCACT
TTGATGAGTGTCGTTCTTATCGAAGTCTCAGTTATCGCTGCCCGGCCGACGTTTGTCGCGAACTCTCA
GAGGTTTACGGCAGAAAAATTTCTCCTGCTTGTAATTCGAGAGAGGAC
+
FFFFFFFFFFFFFFF:FFFFFFFFFFFFFFFFFFFFFFFFFFFFFFFFFFFFFFFFFFFFFFFFFFFF
FFFFFFFFFFFFFFFFFFFFFFFFFFFFFF:FFFFFFFFFFFFFFFFFF
@A00721:190:HGJW7DSXY:4:1510:30391:18333_1:N:0:TCTGCAAG+GCTGCACT
TTAGACCTATCAGATGTCTTGACGACTTCGAATTTGAGGACGGAGTTAAATATATTACTTTCACTCAA
GGTGAAAAGTTTGAAATTAACAAAGTCCTTGGTAAGAGTAAGGTTAAATGTGAAGATGCTCAGACTAT
TCA
+
FFFFFFFFFFFFFFFFFFFFFFF::FFFFFFFFFFFFFFFFFFFFFFFFFFFFFFFFFFF:FFFFFFF
FFFFFFFFFFFFFFFFFFFFFFFFFFF::FFFFFFFFFFF:FF:FFFF:FFFFFFFFFFFFFFFFF:FFF
FFF
@A00721:190:HGJW7DSXY:4:1417:8323:33974_1:N:0:TCTGCAAG+GCTGCACT
TGACCCTGTTTTTGCATCACAAACACATATTACCGTTGCTCTATCACACACGTTGAATCTTTACGATA
CTTTGTTATCAATGTAAAGTTAAACGATGCTACTTGTGAGAGGATCAATCACAGTAAGGAACTGTCAC
GT
+
FFFFF,FFFFF:FFFFFFF:FFFFFFFFFFF:FFFFFFFFFFFFFFFFFFFFFF,FFFFFFFFFFFFF
FFFFFFFFFFFFFFFFFFFFFFFFFFFFFFFFF:FFFFFFFFFFFFFFFFFFFFFFFFFFFFFFFFFF
FF
@A00721:190:HGJW7DSXY:4:1530:19497:32315_2:N:0:TCTGCAAG+GCTGCACT
AGATTCAACCCCTTATATCGAGGCTATCGAGGACGATGAGTATCTTGAAGTTGTTGACGACAAGCCTC
AAGAGGGTAGAGGACCACCTGGATCTTCTACTGTCTCTGCCTTCAAAG
+
FFFF:FFFFFFFFFFFFFFFFFFFFFFFFFFFFFFFF::FFFFFFFFFFFF:FFFFFFFFFFF,FFFF
FFFFFFFFFFFFFFFFFFFFFFFFFFFFFFFFFFFFFFFF:FFFF,:FF
@A00721:190:HGJW7DSXY:4:1463:1154:23844_2:N:0:TCTGCAAG+GCTGCACT
TTATTCCTGGAACCACCTTAACGTCACTCGGTAATCTTTCAGCCGAATTAAGTACTAATGAGTTCAAG
TCGGTTGTCGATCGAATTAAGATCAAGGCTAATACCTTTATAGGTAAG
+
FFFFFFFFFFFFFFFFFFFFFFFFFFFFFFFFFFFFFFFFFFFFFFFFFFFFFFFFFFFFFFFFF:FF
FFFFFFFFFF:FFFFFFFFF,FFFFFF:FFFFFFFFFFFFFFFFFFF:
@A00721:190:HGJW7DSXY:4:2321:25961:10974_1:N:0:TCTGCAAG+GCTGCACT
CATCAGTCAGAGTATACCCGATAGGGTACCGAGTCTCCAAGAGAATATTTATTCTTATGAGGCTCGAA
ACTTCAACGTTTTCACAACGGAAAGACATATCGCACCCGAAATTTTTGGGCAGAATGCTCTTAACGCG
GT
+
F:FFFFFFFFFFFFFFFFFFFFFFFFFFFFFFFFFFFFFFFFFFFFFFFFFFFFFFFFFFFFFFFFFF
FFFFFFFFFF:FFFFFFFFFFFFFFFFFFFFFFFFFFFFFFFFFFFF:FFFFFFFFFFFFFFFFFFFFF
FF
@A00721:190:HGJW7DSXY:4:2413:2428:29309_2:N:0:TCTGCAAG+GCTGCACT
GATCAGTCTAAGTTGGATAAAGTTATAGGAGACTCAGTTAACCTGAGAGCTCCTCAATTGATAGATTG
GGTCAACAATAGGGACGCTAGTATACTTCGTAAAGTCGACAGCGAAAT
+
FFFFFFFFFFFFFFFFFFFFFFFFFFFFFFFFFFFFFFFFFFFFFFFFF:FFFFFFF:FF:FFFFFFF
FFFFFFFFFFFFFFFFFFFF:FFFFFFFFFFFF,FFFFFFFFFFFFFFF
@A00721:190:HGJW7DSXY:4:1612:10556:20682_2:N:0:TCTGCAAG+GCTGCACT
AATTGGACCGACTATATGACCTATTTTAAGCTCATGGTGAAAAGGGAAGCTAAGGTTAAACTTGATGC
TTCAGCCTTTTCGAAGCATAACCCCGCACAGAATATCGTTTATCACGC
+

FFFFFFFFFFFFFFFFFFFFFFFFFFFFFFFFFFFFFFFFFFFFFFFFFFFFFFFFFFFFFFFFFFFF
FFFFFFFFFFFFFFFFFFFFFFFFFFFFFFFFFFFFFFFFFFFFFFFFF
@A00721:190:HGJW7DSXY:4:1629:9914:25488_2:N:0:TCTGCAAG+GCTGCACT
CCTATCTTTAAGGTTCTTGTGAGGAGAGTCAAACAGTGTCTTTTACCCAACATTTTTATGTATACAGA
GATGACCAGTGACGATTTCTCCCGGAAGCTCTTCTCTCTCTTAGGAGG
+
FFFFFFFFFFFFFFFFFFFFFFFFFFFFFFFFFFFFFFFFFF:FFFFFF:FFFFFFFFFFFFFFF
FFFFFFFFFFFFFFFFFFFFFFFFFFF:FFFFFFFFFF,FFFFFFFFFF
@A00721:190:HGJW7DSXY:4:2443:9082:33880_2:N:0:TCTGCAAG+GCTGCACT
TTTCTTTTGTAAAGCCTATGAGATAAAGATGTATTCTTATTTAGGCTTCGATGCTGAATTTCTTGAAC
TTTGGAGTGCCAGTGAATATTTTTGTCACGCCAGGAGTATCCATAACG
+
FFFFFFFFFFFFFFFFFFFFFFFFFFFFFFFFFFFFFFFF:FFFFFFFFFFFFFFFFFFFFFFFFFFF
FFFFFFFFFFFFFFFFFFFFFFFFFFFFFF:FFFFFFFFFFFFFFFFFF
@A00721:190:HGJW7DSXY:4:1108:2736:3380_2:N:0:TCTGCAAG+GCTGCACT
CGATTGGTCAACGTCGGAGTGGTACTGCTAACACATTCTTTGGTAATTGTATGATCACTCTTATGATG
CTCTCTCTTTATTACGACCTTCGGAAGTTTAGAGCCGTCGCTGTCGCC
+
FFFFFFFFFFFFFFFFFFFFFFFFFFFFFFFFFFFFFFFFFFFFFFFFFFFFFFFFFFFFFFFFFFFF
FFFFFFFF:FFFFFFFFFFFFFFFFFFFFFFFFFFFFFFFFFFFFFFF
@A00721:190:HGJW7DSXY:4:1566:13856:23547_2:N:0:TCTGCAAG+GCTGCACT
CTGTTTTCAAAGACTCCAATAACTGATCATTCTGATCATATGATTTTGGACAATGGTTTCGAAGCGAA
ATTTTTGAGGGATAAACCAGCGTACTTCTGCTCGCGAGTTATTCTTTT
+
FFFFFFFFFFFFFFF:FFFFFFFFFFFFFFFFFFFFFFFFFFFFFFFFFFFFFFFFFFFFFFFFFFFF
FFFFFF:FFFFFFFFFFFFFFF,FFFFFFFFFFFFFF,F:FFFFFFFF
@A00721:190:HGJW7DSXY:4:2529:12020:25285_2:N:0:TCTGCAAG+GCTGCACT
CCTGATCCTTACAAGATCATCGTTAAGTTAGGTAAACCCCTAACCGATGTTACTCTTGTAGGACTTTG
GGAGAGATTCGTTAGTTTTAAAGACTATACTAAATATCTCCACTACGA
+
FFFFFFFFFFFFFFFFFFFFFFFFFFFFFFFFFFFFFFFFFFFFFFFFFFFFFFFFFFFFFFFFFFFF
FFFFFFFFFFFFFFFFFFFF:FFFFFFFFFFFFFFFFFFFFFFFFF:FF
@A00721:190:HGJW7DSXY:4:1546:30454:21919_2:N:0:TCTGCAAG+GCTGCACT
GCGACTAATATTCGATATTCGATTGAGGGTCGTTATACCTATTCGGCTATCTGTGCTTTGAATTGTAT
AAGATCAAATTTTAAACGATTCCGAGAGGTTTATCCTGATGTGGAGGG
+
FFFFFFFFFFFFFFFFFFFFFFFFFFFFFFFFFFFFFFFFFFFFFFFFFF:FFFFFFFFFFFFFFFFFF
FFFFFFFFFFFFFF:FFFFFFFFF:FFFFFF:FFFFFFFFFFFFFFFF
@A00721:190:HGJW7DSXY:4:1365:5638:32174_1:N:0:TCTGCAAG+GCTGCACT
CCCGACCTTTCCTTGTGAAAATTTTGATGCGGTTTCATAAGATTATCAGTAATCCTGATTCTACTACG
GATTCATGGACCTTCCATCCTGTACCTCTTCTGGAGAGACGTATCTCAAAGATCGACTTCGAGACAGG
TGA
+
FFFFFFFFFFFFFFFFFFFFFFFFFFFFFFFFFFFFFFFFFFFFFFFFFFFFFFFFFFFFFFFFFFFF
FFFFFFFFFFFFFFFFFFFFFFFFFFFFFFFFFFFFFFFFFFFFFFFFF:FFFFFFFFFFFFFFFFFF
FFF
@A00721:190:HGJW7DSXY:4:1230:30960:1219_1:N:0:TCTGCAAG+GCTGCACT
CGAGAGCGACCAAGATCTAAATTCGGTAACGGTGCGTGGCGCTTTATTTTAAAAAGCTCCCTCCTCTC
CCAAGTTTGATCTGATACTTATCACGAAAGGACTTGAGTCTTTTGATCAGTCTCTGACTTGACCATCT
TTC
+
FFFFFFFFFFFFFFFFFFFFFFFFFFFFFFFFFFFFFFFFF,FFF:FF:FFFFFF:FFFFFFFFFFFFF
FFFFFFFFFFFFFFFFFFFFFFFFFFFFFFFFFFFFFFFFFFFFFFFFFFFFFFFFFFFFFFFFFFFF
FFF

@A00721:190:HGJW7DSXY:4:1430:23466:35994_2:N:0:TCTGCAAG+GCTGCACT
TTTCTTCTTTGTTTCCTTCTTTGCACTGATTTTTCCTCATCAGCTTGTGTTTTACCTAATCTGTGTTT
TTACGATAACAACTATCAGTTGATTAAGACTCTTCCGAAAGGCGAACT
+
,FFFFFFF:FFFFFFFF:FFFFFFFFFFFFFFFFFF:FFFFFFFFFFF:FFFFFFFFFF:FFFFFFF
FFFFFFFFFFFFFFFFFFFFFFFFFFFFF:F:FFFFFFFFFFFFFFF:F
@A00721:190:HGJW7DSXY:4:1609:4698:15859_2:N:0:TCTGCAAG+GCTGCACT
GAAGCTCAGGGTGAAGCACAACGTTTGACCGTGACGAACTGTGGTGTTCTTGATCTACGTTACGGATA
TACTCTCCTTGAGTTCACTCGTAAGGACACTACCACTTATTACGATAT
+
FFFFFFFFFFFFFFF:FFFFFFFF:FFFFFFFFFFFFFFF,FFFFFFFFFFFFFFF:FFFFFFFF:
FFFFFFFFFFFFFFFFFFFFFFFFFF:FFFFFFFFFFFFFFFFFFFFFF
@A00721:190:HGJW7DSXY:4:1304:14642:16423_1:N:0:TCTGCAAG+GCTGCACT
AGCGTCTTCTGTTCATCGCAACCAGATGATATCTTCTATTCGAACTTATGTTTCGATCAGTGTTTCTT
CTATCAGGAGGAAAACACTTGTTCTAGTCCTTGTAAGATTAATCCTAACGTAGAAGATTGTTGTTTAC
CAC
+
FF,FF,FFFFFFFFFFF:FFF:FFFFFFFFFFFFFFFFFF:FFFFFFFFFFFF::FFFFFFFFFFFF
:FFF:FFFFFFFFF:FFFFFFFF:FFFFF:FFFFFFFFFFFFFF:FFFFFFFFFFFFFFFFFFFFFFF
FFF
@A00721:190:HGJW7DSXY:4:1449:31711:25786_2:N:0:TCTGCAAG+GCTGCACT
TCTTCTAATGTGGCCTGAACTACGTTTCACTTTGTGTGTACTTGTCTCTATTTTAAGTTTTGTCTTCC
TTGTTTGTCTTATTCTATTAATCACTCATTTTAGTGAGATTATTAAAA
+
FFFFFFFFFFFFFFFFFFFFFFFFFFFFFFFFFFFFFFFFFFFFFFFFFFFFFFF:FFFFFFFFFF,
FFFFFFFFFFFFFFFFFFFFFFFFFFFFFFFFFF:FFFFFFFFFFFFFF
@A00721:190:HGJW7DSXY:4:1336:11993:23578_2:N:0:TCTGCAAG+GCTGCACT
CGACAACAGCAGGGGTTTTCGAATAGTGATAGCCACCCGCCTTACAGAGGGGCAGCAGCATGACCATA
ATTGGTATTGACTTTGGTACCACCTTTTCTACTGTCTGTCTCGCCTCT
+
FFFFFFFFFFFFFFFFFFF:FFFFFFFFFFFFFFFFFFFFFFFFFFFFFFFFFFFFFFFFFFFF:FFF
F,FFFFFFFF:FFFFFFFFFFFFFFFFFFFFFFFFFFFFFFFFFFFFFF
@A00721:190:HGJW7DSXY:4:2644:10646:7404_1:N:0:TCTGCAAG+GCTGCACT
CGTGTGCTCTTCCGATCTGTGAATTTATACCTACAATCATTGGTTTTTCTAATAATGATGACACTATT
GTTTATGGATATGATGCTATTACTCATGATGGTTACGGTCAGGGAGGATTCTCAGTTTACAAGGACCT
TAA
+
FFFFFFFFFFFFF:FFFFFFFFFFFFFFFFFFFFFFFFFFFFFFFFFFFFFFFFFFFFFFFFFFFFFF
FFFFFFFFFFFFFFFFFFFFFFFFFFFFFFFFFFFFFFFFFFFFFFFFFFFFFFFFFFFFFFFFFFFF
FFF
@A00721:190:HGJW7DSXY:4:2152:1588:5588_2:N:0:TCTGCAAG+GCTGCACT
CCGAAAGACGTGCTAAATTAAAACCATTGTACGATGTCGTCTGTCTCGGCGATCACTTTTCACTAAGA
ATTGGTCCTACTTTTGGACAAGGCCAACTTCGTAATCTTCATGAGATC
+
FFFFFFFFFFFFFFFFFFFFFF::FFFFFFFFFFFFFFFFFFFFFFFFFFFFFFFFFFFFFFFFFFFF
FFFFFFFFFFFFFFFFFFFFFFFFFFFFFF:FFFFFFFFFFFFFFFFFF
@A00721:190:HGJW7DSXY:4:1577:17146:8265_1:N:0:TCTGCAAG+GCTGCACT
TTAGGGATTTTGAGAGAGTGAAAGGCTTCAATTGTAGTGGTTTGGTCGTTTCAGTACCCAGTCAGTAT
ACCTCTACGCAAAGATATTTCATGAAAACTACAGAGGAACGTACCGGATTACCTGTCTTTCATATTAT
GAA
+
FFFFFFFFFFFFFFFFFFFF:FFFF,FFFF,FFFFFFFFFFFFFFFFFFFFFFFFFF:FFFFFFFFFFF
FFFFFFFFFFFFFFFFFFFFFFFFFFFFFFFFFFF,FFFFFFFFFFFFFFFF:FFFF:FFF:FFFFFFF
FFF

```
@A00721:190:HGJW7DSXY:4:2276:15817:15765_2:N:0:TCTGCAAG+GCTGCACT
TCTTTGCTTCGATGTTGGATGTCAAGAAGGGTTCAGAATGGGATTCCTACGTAGTTTATGACTTTGGT
GGTGGTACTTTCGATACTTCTATCGTCTTACGTTACGGTAATTACTAT
+
FFFFFFFFFFFFFFFFFFFFFFFFFF:FFFFFFFFFFFFFFFFFFFFFFFFFFFFFFFFFFFF:FFF:
FFFFFFFFFFFFFFFFFFFFFFFFFFFFFFFFFFFFFFFFFFFFFFFF
@A00721:190:HGJW7DSXY:4:1531:18846:12994_1:N:0:TCTGCAAG+GCTGCACT
ATAATCTTGGAGGTCGTGACGTTGATGAAGCTATAAGAGCGTTCTTATTACGGCGCACTTCTTTACCC
GCCACCACACAACTTTCAGTTTCACAACTGAAGGAGGAGGTTTCTGTTACTGGAAAACCCTCGTCAAT
ACT
+
FFFFFFFFFFFFFFFFFFFFFFFFFFFFFFFFFFFFFFFFFFFFFFFFFFFFFFFFFFFFFFFFFFFF
FFFFFFFFFFFFFFFFFFFFFFFFFFFFFFFFFFFFFFFFFFFFFFFFFFFFFFFFFFFFFFFFFFFF
FFF
@A00721:190:HGJW7DSXY:4:1176:31575:2848_2:N:0:TCTGCAAG+GCTGCACT
GATCTAGTGAAATTAACGCGTCCTTTTCTGGAACGTAGTTTTACTATTATGAACAAAGTTATCAAAGA
TAGTGGCATTCAAGGCAAACTTACCCTGATTCCGGCCGGTGGTAGCTC
+
FFFFFFFFFFFFFFF:FFFFFFFFFFFFFFFFFFFFFFFFFFFFFFFFFFFFFFFFFFFFFFFFFFFF
FFFFFFFFFFFFFFFFFF:FFFFFFFFFFFFFFFFFFFFFFFFFFFFF
@A00721:190:HGJW7DSXY:4:1226:10013:27132_2:N:0:TCTGCAAG+GCTGCACT
GGCTATTACTCTTGCTGAAACAGCTATTAAAGCTGTTGAGCGGGTAATTACACCTAAACGAGCTCGAT
CTGCCATCGCTGAAGGATGTGCACTCTTTTCTGCTACTTTAACGGAGC
+
FFFFFFFFFFFFFFFFFFFFFFFFFFFFFFFFFFFFFFFFFFFFFFFFFFFFFFFFFFFFFFFFFFFF
FFFFFFFFFFFFFFFFFFFFFFFFFFFFFFFFFFFFFFFFFFFFFFFF
@A00721:190:HGJW7DSXY:4:1611:27706:12211_2:N:0:TCTGCAAG+GCTGCACT
TCGATTGTATCAACAGTAACATTACTTCTAATAAAGGTCTCTATCAAGCTCGTATTATCGTCGCTGCT
GGATCTGCGTTACCGTTGAAGGCTAAACTCCAAGGTAATCATGAAGAC
+
FFFFFFFFFFFFFFFFFFFFFFFFFFFFFFFFFFFFFFFFFFFFF:FFFFFF:FFFFFFFFFFFFFFF
FFFFFFFFFFFFFFFFFFFFFFFFFFFFFFFFF:FFFFFF:F:FFFFFF
@A00721:190:HGJW7DSXY:4:2126:11324:9799_1:N:0:TCTGCAAG+GCTGCACT
GTATGAGGGTGTTTCTAATCGTAACATTAATAATTTCAATCTTCTTAAAAAGAAGATAGAGAGTCATA
CTTTGGGCTATCGTTCAGGTGCATGGGGTTGGATAGCCAATATTGAAGTGGACACCTCTGGTAGTATC
GAA
+
FFFFFFFFFFFFFFFFFFFFFFFFFFFFFFFFFFFFFFF:FFFFFFFF,FFFFFFFFFFFFFFFFFFF
FFFFFFFFFFFFFFFFFFFFFFFFFFFFFFFFFFFFFFFFFFFFFFFFFFFFFFFFF:FFFFFFFFFF
FFF
@A00721:190:HGJW7DSXY:4:1270:3676:3443_1:N:0:TCTGCAAG+GCTGCACT
CTAAGCATACTACTTTTGCTTTGATTACTCTACCGCCTTACAAGTCTAAGGTTAAAGAGTCGATTAAA
CAAGTTGTACTTATCAATGATTTTATCGCTGGTTGTTATCTCGAAAAGTCTCTTCTAAAAGGAACGTC
AGT
+
FFFFFFFFFFFFFFFFFFFFFFFFFFFFFFFFFFFFFFFFFFFFFFFFFFFFFFFFFFFFFFFFFFFF
FFFFFFFFFFFFFFFFFFFFFFFFFFFFFFFFFFFFFFFFFFFFFFFFFFFFFFFFFFFFFFFFFFFF
FFF
@A00721:190:HGJW7DSXY:4:1177:4707:29778_2:N:0:TCTGCAAG+GCTGCACT
CTAAAATATTTACAAAATCTTACCACTATCTACGAACCTAAAGTTCTGAAGACGTTGCTTGTCGCTTC
CTCCTTTAATGGCACTACCTCGGTACAATCTTACTTGGAACGAAGGCG
+
F:FFFFFFFFFFFFF:FFFFFFFFFFFFFFFFFFFFFFFFFFFFFFFFFFFFFFFFFF:FFFFFF:FF
FFFFFF:F:FF:FFFFFFFFFFFFFFFF,FFFFFFFFFFFFFFFFFFF
```

```
@A00721:190:HGJW7DSXY:4:1151:15058:7185_1:N:0:TCTGCAAG+GCTGCACT
GTTCTGTCTATATTAGGTGAAGCGAAATACGATTTCGAACGTCAACGTTCACCGAGTTTCGTTTCGTA
CCTTGATGCTGACTTTCGTCAATATGATTTGATCTGGTTGCTCAATTCTAAGCGTGTTCCGCTTAGTG
CGA
+
FFFFFFF:FFFFFFFFFFFFFFFFFFFFFFFFFFFFFFFFFFFFFFFFFFFFFFFFFFFFFFFFFFFF
FFFFFFFFFFFFFFFFFFFFFFF:FFFFFFFFFFFFFFFFFFFFFFFFFFFFFFFFFFFFFFFFFFFF
FFF
@A00721:190:HGJW7DSXY:4:2636:23023:31501_2:N:0:TCTGCAAG+GCTGCACT
GAGTATTGCTATTTATACGGTTACGAGAAGAAGGTTCGAAAGTTTGAGAACTTAGTTTACGAACTTGA
CTCTACTATACGTGACATACTAAATACTAGATATTTGGATGATCTTGA
+
FFFFFFFFFFFFFFFFFFFFFFFFFFFFFFFFFFFFFFFFFFFFFFFFFFFFFFFFFFFFFFFFFFFF
FFFFFFFFFFFFFFFFFFFF:FFFFFFFFFFFFFFFFFFFFFFFFFFF
@A00721:190:HGJW7DSXY:4:1569:2718:12837_2:N:0:TCTGCAAG+GCTGCACT
AAAATTTTCTTCCTTGGATTTTAATATAAAGAATCTTTACCAGGACTCCTACTACAGGAGGCAGTCTT
CTGATCGTAAGAAGTTTATAAGGAACATATCCATTTGTTGTAATAGGG
+
FFFFFFFFFFFFFFFFFFFFFFFFFFFFFFFFFFFFFFFFFFFFFFFFFFFFFFFFFFFFFFFFFFFF
FFFFFFFFFFFFFFFFFFFFFFFFFFFFFFFFFFFFFFFFFFFFFFFF
@A00721:190:HGJW7DSXY:4:1455:19642:1939_2:N:0:TCTGCAAG+GCTGCACT
CGAAACTCTAAGTGGTAATACTGTCTTTCCAAGCTTACCTGTATCTTCAATGAAATTGGACTCTTCGA
TAGGGGACATTTTATCGTGGGCTTTCCGTAAAGGAAGATCTGTTATCG
+
FFFFFFFFFFFFFFFFFFFFFFFFFFFFFFFFFFFFFFFFFFFFFFFFFFFFFF:FFFFFFFFFFFFF
FFF:FFFFFFFFFFF:FFFFF:FFFFFFFFFFFFFFFFFFFF,FFFF:
@A00721:190:HGJW7DSXY:4:1455:19642:1939_1:N:0:TCTGCAAG+GCTGCACT
ACCGGTGAATGGTTTAAGAATACTATAAATAGAATAGCTATTACTACTAATGGCGTTGGCCTAGTGGA
TGTTATTAGTGACATCTGGTCAATTTCGTTTTTAGGTGGTCTTTATTCTATTATTGATTTTTATGAGA
TTG
+
FFF::F:FFFF:FFFFFF:FF:FFFFF:F:FFFF:FFFFFF:FFFF:FFFFFFFFFFFFFF:FFF,FF
FFFFFFFFFFFFFFFFFFFFFFFFFF:F:FFFFFFFFFFFF:FFFFFFFFFFFFFFFFFFFFFFFFFF:
FFF
@A00721:190:HGJW7DSXY:4:1171:21793:9424_2:N:0:TCTGCAAG+GCTGCACT
TGTTAGAGTAGAATTCCTGAAGAGTCCTTCCGTAACCAACTTCTTGACAATTTTCTTCAATCCTTCGA
TAATAGTTAAATCTAGGATTATGTCTGTGTTGAAAGACTTTCTACCGA
+
FFFFFFFFFFFFF:FFFFFFFFFFFFFF:FFFFFFFFFFFF,FFFFFFFFFFFFFFFFFFFFFFFFFF
FFFFFFFFFFFFFFFFFFFFFFFFFFFFFFFFFFFFFFFFFFFFFFFF
@A00721:190:HGJW7DSXY:4:1244:28646:7326_2:N:0:TCTGCAAG+GCTGCACT
TACACAATTAAGAAATAATCCTACATTGACTGATTATCTAAAGATTGAAGGAAAATTTTATCCTCGTT
TAACTTACTTAGACTTTCCTACGATACAAATGATAACTTCTAATTTAC
+
FFFFFFFFFFFFFFFFFFFFFFFFFFFFFFFFFFFFFFFFFFFFFFFFFFFFFFFFFFFFFFFFFFFF
FFFFFF:FFFFFFFFFFF:FFFFFFFFFFFFFFFFF:FFFFFFFFFFF
@A00721:190:HGJW7DSXY:4:2162:12509:7153_2:N:0:TCTGCAAG+GCTGCACT
AAGGGATGCATGGTGTTATCTCCTCAGGAGTCCCGGCATTTGATAATAATGACGAGACTTTCGTCATA
ATCGCTGCATATTACGGATTACACGGTACCAGCGCATCTCGTGTCGAG
+
FFFFFFFFFFFFFFFFFFFFFFFFFFFFFFFFFFFFFFFFFFFFFFFF:FFFFFFFFFFFFFFFFFFF
FFFFFFFFF:FFFFFFFFFFFFFFFFFFFFFFFFFFFFFFFFFFFFFF
@A00721:190:HGJW7DSXY:4:1341:21477:31454_1:N:0:TCTGCAAG+GCTGCACT
AAATTTATAGAAGATTATTTTGACAGAATCAATAAGAATCATCGATTCTCGATTCGTAGAGCTTATCT
```

```
AGGTACTCTCATTTACTATGTTGATTTAGTCTACATTTATGCTGATCTAAAATTTCGATCTAGATGGA
TCC
+
FFFFFFFF:F:FF,F:F:FFFFFF,FF,FFFFFFFFFFFFFFFFFFFFFFFFFFFFFFFFFFFFFFF:F
FFFFFFFF:FFFFFFFFFFFFFFFFFFFFF:FFFFFFFFFFFFFFFFFFFFFFFFFFFFFFFFFFFFFF
FFF
@A00721:190:HGJW7DSXY:4:2635:11948:29199_2:N:0:TCTGCAAG+GCTGCACT
ACGATATAGGAGTAATCTACGAGAAGATTCTAGAATTCTTAATTATAATATTCAACCTCAAAAGTTAC
GTGTTTACAGTAACGAGATCACACATTTTGAGAACTAACTATGTTATG
+
FF:FFFFFFFFFFFFFFFFFFFFFFFFFFFFFFFFFFF:FFFFFFFFFFFFFFFF:FFFFFFF:FFF
,FFFFFFFFFFFFFFFFFFFFFFFFFFFFFFFFFFFFFFF:FFFFFF:
@A00721:190:HGJW7DSXY:4:1550:3206:21230_1:N:0:TCTGCAAG+GCTGCACT
TCTCTTCTTATAATAAATAAATAAATAATATTACTAATGGCAAATAGTGGAAAGGCTCCAGTCAACAC
TAGTTCGAGTGCTGCTTTGGATTACGATGACGTGAGAATAGGTGAAGGGATAGTTTTTACCTCGACCA
ACA
+
FFFFFFFFFFFFFFFFFFFFFFFFFFFFFFFFFFFF,FFFFFFFFFFFFFFFFFFFFFFFFFFFFFFF
FFFFFFFFFFFFFFFFFFFFFFFFFFFFFFFFFFFFFFFFFFFFFFFFFFFFFFFFFFFFFFFFFFFF
FFF
@A00721:190:HGJW7DSXY:4:2440:26883:4147_2:N:0:TCTGCAAG+GCTGCACT
GCGACTAATCTTAAAGCTTCACCCAAAATGAAGGACACTTCGAAACTGAATAATGATGCACTACAAAC
AAGTATCGCTAATCATCTGAAAAAGGCTCTAAAGGACACTATACAATA
+
FFFFFFFFFFFFFFFFFFFFFFFFFFFFFFFFFFFFFFFFFFFFFFFFFFFFFFFFFFFFFFFFFFFF
FFFFFFFFFFFFFFFFFFFFFFFFFFFFFF:FFFFFFFFFFFFFFFFF
@A00721:190:HGJW7DSXY:4:1255:23176:18646_2:N:0:TCTGCAAG+GCTGCACT
CAGCACCACATTTTGGTCAAGTCAATCCGTTGAGAAAATTTCTTAGAGGATATTCGCCATTGTGGTAC
GAATTGGTCCAACATTTACCACCAAATTCTTTTGATACAACTCTAGCC
+
FFFFFFFFFFFFFFFFFFFFFFFFFFFFFF:FFFFFFFFFFFFFFFFFFFFFFFFFFFFFFFFFFFFF
FFFFFFFFFF:FFFF:FFFFFFFFFFFFFF:FFFFFFFFFF:FFFFFF
@A00721:190:HGJW7DSXY:4:1521:27127:32440_1:N:0:TCTGCAAG+GCTGCACT
ATACGCTTGCTATTCACCAGATTGGTTCGAACCCGTTGATGCTATGTCAGAAGAAGCTATTCAAGCTT
GTCGTTTAAAGACTGCAGAGGCTGTCAAACAAGCCCAGACTCAAGGAGTCCAATCAAAGGTTTTGAAT
TTA
+
FFFFFFFF:FFFF,:F,FFFFFF:FFF:FF:FFFFFFFFF,:F:FFFFFFFFFFFF,FFFF,FFFFFF
FFFFFFFFFFFFFFFFFFFF:FFFFF:F:FFFFFFFFFFFFFFFFFFFFFFFFF:::FFFFFFFFFFF
FF:
@A00721:190:HGJW7DSXY:4:1239:4381:14121_2:N:0:TCTGCAAG+GCTGCACT
GGTGATAAAAATTATTTAGAGATCTTTTCTACTTACAAAGGACAATTCAGTTTCATTAATTCTACGGA
AAAGTCCGGAGTATATTGTATGTTAATAGATAGTGGGATAATAGTTCT
+
FFFFFFFF:FF:FFFFFFFFFFFFFFFFFFFFFFFFFFFFFFFFFFFFFFFFF:FF:FFFFFFFFFF,FF
:FFFFFFFFFFFFFF:FFFFFFFFFFFF:FFFFFFFFFFFFFFFFFFF
@A00721:190:HGJW7DSXY:4:1110:5981:4398_1:N:0:TCTGCAAG+GCTGCACT
CGTCTGAAGAGTTTAGAGCTGCTATTGATTATGTAACCGATAGGAGTTGTATAGTTGAAGATGTGGCT
TTAGACGATAATCCTTTAAGGACTATGTTGACTGACGAAGATTTTCCCACAGATTTGTCTACGTTGCA
GAT
+
FFFFFFFFFFFFFFFFFFFFFF,FFFFFFFFFFFFFFFFFFFFFFFFFFFFFFFFFFFFFFFFF:FFFF
FFFFFFFFFFFFFF:FFFFFFFFFFFFFFFFFFFFFFFFFFFFFFFFFFFFFFFFFFFFFFFFFFFFF
FFF
```

```
@A00721:190:HGJW7DSXY:4:1422:4906:30311_2:N:0:TCTGCAAG+GCTGCACT
CACTATATACATTATAAAATATAAAAATAAGGGAGTCATAACTTTTGTTACCAAATTCTTGGCTTTCA
ACGAAGTCTCTGATTTTATTAATCATTACTTTAACGATCTCTCGGTCA
+
FFFF,FFFFFFFFFFFFFFFFFFFFFFFFFFFFFFFFFFFFFFFFFFFFFFFFFFFFFFFFFFFFFFFF
FFFFFFFFFFFFFFFFFFFFFFFFFFFFFFFFFFFFFFFFFFFFFFFF
@A00721:190:HGJW7DSXY:4:1464:14009:33395_2:N:0:TCTGCAAG+GCTGCACT
TTGATCTACGTCGTTTAACTGTCGGAACAACCTTTACTTAGAATGACTCTATCTTTTCAGACACTTCT
TATATTATTTACAATAGTTAATATCATATTAGTAATACTCACTATCGT
+
FFFFFFFFFFFFFFFFFFFFFFFFFFFF,FFFFFFFFFFFFFFFFFFFFFFFFFFFFFFF:FFFFFF:
FFFFFFFFFFFFF:FFFFFFFFFFFFFFFFF:FFFFFFFFFFFFFFFF
@A00721:190:HGJW7DSXY:4:2141:13006:12774_1:N:0:TCTGCAAG+GCTGCACT
GCTCTTCCGATCTGATTAATAAAGGATGAGTACGAAAACCAACTCTTCGTTACGAGACCTGATGGTAG
AATAGATTTAGTCTCTACACCCATCTCAACGAGGATAAAGCGAGTTTTCCGTCCTGAACGTAATACAC
GAA
+
FFF,FFF:FFF,F:FFFFFFFFFFFF:FFFFFFFFFFFF:FFFFFFF:FFF:F:FFFF:FFFFFFFFF
FFFFFFFFFFFFFFFFFFF:FF,FFFFFFFFF:FFFFFFFFFFF:FFFFFFFFFF:FFFFFFFFFFFF
FFF
@A00721:190:HGJW7DSXY:4:1637:31458:9408_2:N:0:TCTGCAAG+GCTGCACT
CAAACGACTCGAAACCGAGACGCCGTAGATCAAAGTCCCTTCTAACTTTAGATTATGAATCCTGCCTT
TACTCATTTTATAGCAGTTATTTATGTTGTACTTTTTATAATTTATAA
+
FFFFFFFFFFFFFFFFFFFFFFFFFFFFFFFFFFFFFFFFFFFFFFFFFFFFFFFFFFFFFFFFFFFF
FFFFFFFFFFFFFFFFFFF:FFFFFFFFFFFFFFFFFFFFFFFFFFFF
@A00721:190:HGJW7DSXY:4:1443:5538:24674_1:N:0:TCTGCAAG+GCTGCACT
TTTGGGTATGTTTTGTGAAAAACATGAGATACCCAAGTCTATCGAAGACATTATCTCTTATGTACTTA
TTGTAACGATCATTCTTTCGAGTTTTGTCATTCTTATTCTTTTATTCCTTTAACTGTGTATGAGGAGG
ATT
+
FFFFFFFFFFFFFFFFFFFF,FFFFFFFFFFFFFFFFFFFFFFFFFFFFFF:FFFFFFFFFFFFFFFF
FFFFFF:FFFFFFFFFFFFFFFFFFFFFF:FFF:FFFFF:FFFFFFFF,FFFFFFFFFFFFFF:FFFFF
FFF
@A00721:190:HGJW7DSXY:4:1239:9833:26725_1:N:0:TCTGCAAG+GCTGCACT
TCGATAGTGATTGTTATCAATTTAAATACATTACTGTTTCAAATCATCTTTACTCACAGACTCCGAAA
ATGGTTTCAAATGAGGTCAAAGCTCTATTTATTAAACGAACTATTATCCGATTCTTCGAGGGTATCAC
TAC
+
FFFFFFFFFFFFFFFFFFFFFFFFFFFFFFFFFFFFFFFFFFFFFFFFFFF:FFFFFFFFFFFFFFFF
FFFFFFFFFFFFFFFFFFFF:FFFFFFFFFFFFFFFFFFFFFFFFFFFFFFFFFFFFFFFFFFFFFFFF
FFF
@A00721:190:HGJW7DSXY:4:1377:1208:2362_1:N:0:TCTGCAAG+GCTGCACT
GACTTTAGTCAAGGAACTTCAATTCCTTCGATGTTTATATTTTTCTATAAGGGAAAATAGCAATAATC
AAATTTCGACAAATTTTAGAACTTTATTTCAGAACTCTCTCCGATCACCTTGGTGTGATCTCCCGGAG
GAT
+
FF,FFFFFFFFF:FFF:FFFFF:FFFFF:FFFFFFFFFFFFFFFFFFFFFFFFFFFFFFFFFFFFFFFF
FFFFFFFFFFFFFFFFFFFFFFFFFFFFFFFFFF:FFFFFFFFFFFFFFFFFFFFFFFFFFFFFFFFFF
FFF
@A00721:190:HGJW7DSXY:4:1377:22426:1438_2:N:0:TCTGCAAG+GCTGCACT
ATGTATGATGATAATCTACTATTTTTAAATAGTGATATTCAGCGTATACTTGAATACTTTATCAAAGA
TTTGGCTTGGTCTATTTACCGCAAAAAGTTGGAAGAGGTCTATGATAT
+
```

```
:FFFFFFFFFFFFFFFFFFFFFFFFFFFFFFFFFFFFFFFFFFFFFFFFFFFFFFFFFFFFFFFFFFFFF:
FFFFFFFFFFFFFFF:FFFF:FFFFFF:FFFFFFF:F,:FFFFFFFFF
@A00721:190:HGJW7DSXY:4:1646:15899:10457_2:N:0:TCTGCAAG+GCTGCACT
TATACTTTAAATAGCACCGATTATAACTTGACTGTTGATAATATTACAATAGCTCTTTCTCAGAAGAT
TAGAGAAGTAACCGGTGAACACGTCGGTAGCATTGATTTACTTTTACC
+
:FFFFF:FFFFFFFFFFFFFFFFFFFFFFFF:FFF,FFFFFFFFF:FFF:FFFFFFFFFFF,FFFF:FF
FFFFF,F,FFFFFFFFFFFFF:FFFFFFFFFFFFFFFFFFFFFFFFFFF
@A00721:190:HGJW7DSXY:4:1416:22580:1517_2:N:0:TCTGCAAG+GCTGCACT
TTTCGAAAAGAAACCTATAAGTTACAAACGATTCTTCGACTATATTAAAGAAATAAAAATACTTCAAG
TACCTGAACTTTACATTTTTAATGGTATGGTATAATGTGTCTTACAAA
+
FFFFFFFF:FFFFFFFF:FFFFFFF:F:FFFFFFFFFFFFFFFFFFFFFF:FFFFFFF:FFFFFFFFF
FF:FFFFFFFFFF:FFFFFFF,FFFFFF:FFFFFFFFF:FFFF:FFFF
@A00721:190:HGJW7DSXY:4:1202:21269:34319_2:N:0:TCTGCAAG+GCTGCACT
CTCTCGGTAATGAGAGTGTATTCCTTAATATTCTAGTAACCGCTATGTGGGCATTACTAGGATTTATC
ACACTCCTAAATTTGATAGCAATTTATGTTTTAAATGCTAGGAAAATT
+
FFFFFF:FFFFFFFFFFFFFFFFFFFF:FFFFFFFFFFFF:FFFFFFF:FFFFFFFFFFFFFFFFFFF
FFFFFF,FFFFFF:FFFF:FFFFFF:FFFFFFF:FFFFFFFFFFFFFF
@A00721:190:HGJW7DSXY:4:2452:9625:7232_2:N:0:TCTGCAAG+GCTGCACT
TTGTATAGTTCAAGGAGTACTTAATTCAATAGCTTTAGTATTATTTACTATCGCCTTTATTGAAAATT
GTATAAGAGTTAATTGCTTACACAAAAGTGACGCGCGAAGGAATCTTA
+
FFFFFFFF:FFFFFFFFFFFFFFFFFFFFFFFFFFFFFF:FFFFFFFFFFFFFFFFFFFFFFFFFFFFF
FFFFFFFFFFFFFFF:FFF:FFFFFFFFFFFFFFFFFFFFFFFFFFFF,
@A00721:190:HGJW7DSXY:4:2605:1551:7216_1:N:0:TCTGCAAG+GCTGCACT
GGGAGAATGAACTGCCGCTTCCCGGAAGAAACGATCTATTTACAGAGTATTTAGAGTAGTCCAAAGTT
TGTATAAATTTATATGATTTTCTATATATATACCACATATATATATATATAAAATCCTATAAATTTGT
ACA
+
F,FF,FFF:F,F:FFF:F:FFFF,,FF,FF::FFF,F,FF:FFFF,:F,FFFF,:,FF:,FFFF:,F:
F,FFFFF,FFF:FFF:,FFFF:FF:FFFFFF:FFFF:FFF:FFF:FFFFFFFFFFF:FFFFF:FFFFF
FFF
@A00721:190:HGJW7DSXY:4:2361:31331:7028_2:N:0:TCTGCAAG+GCTGCACT
TACACTCCCCAAACATAACATACCATAACCTACCATGACCAATCATCGCTCCAACTGGCACGCCAGAC
GTAGGGTCAAACCTACTACCGCCTGTCGGCAGATCGGCACCACTGGTC
+
FFFFF,FFFFFFFFFFFFFFFFFFFFFFFFFFFFFFFFFFFFFFFFFFFFFFFFFFFFFFFFFFFFF:F
FFFFFFFFFFFFFFFFFFFFFFFFFFFFFFFFFFFFFFFFFFFFFFFF
@A00721:190:HGJW7DSXY:4:2664:16251:28260_2:N:0:TCTGCAAG+GCTGCACT
ACCCGCTGTGCAGAATAACCTTCCCAAATCTAGATCTGCACAGAAGAAGGCTGAAGGTCGTTACCGGA
CGACCACTCAAAGGAAACGGGCTGTTGCCCAGAGGAAAGCAGCGACCA
+
FFFFFFFFFFFFFFFFFFFFFFFFFFFFFFFFFFFFFFFFFFFFFFFFFFFFFFFFFFFFFFFFFFFF
FFFFFFFFFFFFFFFFFFFFFFFFFFFFFFFFFFFFFFFFFFFFFFFF
@A00721:190:HGJW7DSXY:4:2102:14027:24439_2:N:0:TCTGCAAG+GCTGCACT
ACGGCAGTCCCGTCATCTCTGTGGGAACTGAGGCAGTACCTCAGTCAAAAGTCACATTCACCACTCCC
ATACCAATCAATGATGTGACTATACAGTTCGGCCATTTCAAGAGTGGT
+
FFFFFFFFFFFFFFFFFFFFFFFFFFF,FFFFFFFFFFFFFFFFFFFFFFFFFFFFFF,FFFFFFFFF:F
FFFFFFF:FFFFFFFFFFFFFFFFFFFFFFFFFFF:FFF:FF,FFFFFFF
@A00721:190:HGJW7DSXY:4:1413:14651:33348_2:N:0:TCTGCAAG+GCTGCACT
GGAGGTACTGAAGCCTTCCCCTCCGCTGGCACGAAGAACACTCCCAAGCCAATCAATAGTTCTTCTGA
```

CCAGAATGAAAAGGGGTTTACCAGGGTAGTCTCTAAGTCTACCAGGAA
+
FFFFFF:FFFFFFFFFFFFFFFFFFFFFFFFFFFFFFFFFFFFFFFFFFFFFFFFFFFFFFFFF:
FFFFFFFFF:F:FFFFFFFFFFFFFFFFFFFFFFF:,FFFFFFFFFFFF
@A00721:190:HGJW7DSXY:4:2443:22101:15405_2:N:0:TCTGCAAG+GCTGCACT
CAAAACTGATAAGAATAAAGGGAAAGGTCCATCAGGACCTAAGAAGGGGAAATTACCCCTAATTCTGA
AGGAAGATCTCCCACCATCTGGGGTTGGGAATAAGAAATCTGCAGATC
+
FF:FFF:FFFFFFF:FFFFFFFF,FFFFFFFFFFFFFFFFFFFF:FFFFF:FFFFFFFFFFFFFFFFF
FFFFFFFFFFFFFFFFFFFFFFFFF:FFFFFFFFFFFFFFFFFFFFFFFF
@A00721:190:HGJW7DSXY:4:1427:13937:5087_2:N:0:TCTGCAAG+GCTGCACT
CTACGAAGTCGCTGAAGGCGGCTTGGGTCATTAAGGGTAGCTGTAAAGTTGCGCCTGCCGACCCCATC
TTTGAGACCAAGACTCTCTACAGTAGGTTCGTGCCTACTATGAGCAAT
+
FFFFFFFFFFFFFFFFFFFFFFFFFFFFFFFFFFFFFFFFFFFFFFFFFFFFFFFFFFFFFFFFFFFF
F:FF,FFFFFFFFFFFFFFFFFFFFFFFFFFFFFFFFFFFFF,FFFFFFFF
@A00721:190:HGJW7DSXY:4:1127:13259:15749_1:N:0:TCTGCAAG+GCTGCACT
TCGTCACTGAGGACGAAGGTGAGAGGACTTTTGGCTACTGGCGACAAACTGAGAGCAGTCGCTGGGGA
AAGAGAATTGCGTGGGTTGAAGGCCGACGCTTCGAGCTTGAGTGTGACGAGGCCGGTGACGTTTTTGT
CAA
+
FFFFFFFFFFFFFFFFFFFFFFFFFFFFFFFFFFFFFFFFFFFFFFFF:,FFFFFFFFFFFFFFFFFFF
FFFFF:FFFFFFFFFFFFFFFFFFFFFFFFFFFFFFFFFFFFF,FFFFFFFFFFFFFFFFFFFFFFFFF
FFF
@A00721:190:HGJW7DSXY:4:1240:26223:3850_1:N:0:TCTGCAAG+GCTGCACT
TTTATCCGCGCTTAACTATGGGGCGAAGATCCCCTATTGAGTCTTGAAGGACTTAGGTTGGCATATTT
ATGCCGACGATAATAAGCATGTCACCCTTAAGCACCGAGGTAAGCTCGGGACTTACAAGAGAGAGTGT
GAG
+
FFFFFFFFFFFFFFFFFFFFFFFFFFFFFFFFFFF:FFFFFFFFFFFFFFFFFFFFFFFFFFFFFFF:FF
FFFFFFFFFFFFFFFFFFFFFFFFFFFFFFFFFFFFFFFFFFFFFFFFFFFFFFFFFFFFFFFFFFFFFFF
FFF
@A00721:190:HGJW7DSXY:4:1655:26106:32581_1:N:0:TCTGCAAG+GCTGCACT
CACTATTTTTGCCCAGAATGCGGGCTTTCTGACTGATGAGGTTAAAACTCTTGCTCCTTCTGTGGGTG
AGGGTTTTTGTTATATTCACTGGTTCTGGGAGGCTGCTTTCCTCCATAATTACAACCTCTCTTTAGAG
AGC
+
FFFFFFF:FFFFFFFFFFFFFFFFFFFFFFFFFFFFFFFFFF:FFFFFFFFFFFFFFFFFFFFFFFFF:F
FFFFFFFFFFFFFFFFFFFFFFFFFFFFFFFFFFFFFFFFFFFFFFFFFFFF:FFFFFFFFFFFFFFFFF
FFF
@A00721:190:HGJW7DSXY:4:1429:3531:23015_2:N:0:TCTGCAAG+GCTGCACT
AGTTGGGCAAATTCCCACATATGGTAGACGTTGTCACACTTTTCTTGAAAATTTTTCCTTTCCTCCGT
CATTGGGGATTTCGATGTGAGAAGGTGGAAGGTGTCAAGGGTCTTCTT
+
,F:FFFFFFFFFFFFFFFFFFFFFFFFFFFFFFFFFFFFFFFFFFFFFFFFFFFFFFFFFFFFFFFFFF
F:FFFFFFFFFFFFFFFFFFFFFFFFFFFFFFFFFFFFF,FFFFFFFFFF
@A00721:190:HGJW7DSXY:4:1602:32190:34194_2:N:0:TCTGCAAG+GCTGCACT
TTTCAGGACCTAATCACATCATGATTGATAGTTACGGCTCGTATCGAGTTGGAGGGAGGAAGTTCTCC
GAAATTAAAGACGGGGGGATTAAGAGTCCAGCTGTAACTGTCATTTCA
+
F,FFFFFFFFFFFFFFFFFFF:FFF:FF:FFFFFFFFF:FF::FFFFFF:FFFFFFF:FFFFFFFFFF
FFFF:FFFF::F:::,FF,:FF:FFF,FFF,F,:,FFF:FFFF,:FF,
@A00721:190:HGJW7DSXY:4:1374:18882:30248_2:N:0:TCTGCAAG+GCTGCACT
AAGGAAACTTCGTCAGAAGATGAGGAAGTCTAAGAGGAGGAGTTTGAGGAAGGCTCTAGAGAAGATCT

CACCACCTGGAGAAGGCAAGTGGAGAGTCCAGAAGTTGATGAAGAAGG
+
FFFFFFFFFFFFFFFFFFFFFF:FFFFFFF:FF:,FFFFFFFFFFFFFFF,FFFFFFFFFFFF:FFFF
FFFFFFFF:FFFFFFFFFFFFFFF:FFFFFFFFFFFFF::FFFFFFFF
@A00721:190:HGJW7DSXY:4:1558:30996:11052_2:N:0:TCTGCAAG+GCTGCACT
CAGGGGTTACCACTTCCTAAGGTGGATGAGGGAGGTTGTTTCTTTGGAGTTTTAGATAATCTTTTGAG
AAGGGGTCATAGAGCCAGCAAGAGAAGCTTATATGCTGGTTTAAGTGA
+
FFFFFFFFFFFFFFFFF:FFFFFFFFFFFFFFFFFFFFFFFFFFFFFFFFFFFFFFFFFFFFF,FFFF
FFFFFFFFFFFFFFFFFFFF:FFFFFFFFF:FFFFFFFFFFFFFFFFF
@A00721:190:HGJW7DSXY:4:2441:10610:23594_2:N:0:TCTGCAAG+GCTGCACT
CCTTGAGAAGACAAGCCACATTAAGATATAATTTAGGTCAACTTGGGGTTTCGAATGTCATTAGTGCC
TTACGATTGAAAGAGGTCCTTTTGAGTATCGACAGATCTTTCGATCTT
+
FF,FFFFFFFFFFFFFFFFFFFFFFFFFFFFFFFF:FFFFFFFFFFFFFFFFFFFFFFFFFFFFFFFF
FFFFFFFFFFFFFFFFFFFFFFFFFFFFFFFFFFFFFFFFFFFFFFFF
@A00721:190:HGJW7DSXY:4:2636:7726:28494_2:N:0:TCTGCAAG+GCTGCACT
GTGGCAGAATTCGGTAACTTACCATATTGTTCCTTCCGGGAAGTTGTGGGATTTAACTTTGACTCAAC
TACTCCGATCAGATCCGGATGGTTTGATTGGTATGGACTACTCGACTT
+
FFFFFFFFFFFFFFFFFFFFFFFFFFFFFFFFFFFFFFFFFFFFFFFFFFFFFFFFFFFFFFFFFFFF
FFFFFFFFFFFFFFFFFFFFFFFFFFFFFFFFF:FFFFFFFFFFFFFF
@A00721:190:HGJW7DSXY:4:1439:19623:12242_2:N:0:TCTGCAAG+GCTGCACT
TCATGGCAGATACTACCGATAAGGTGAAAAATATGCAGGTCAAAGATTCTGTAGCTCTGAGGGCGGTT
GAGGGAGCCGTTTTGGCCGACTTTAACTATTCTAAGAGTCTTTATGAT
+
FFFFFFFFFFFFFFFFFFFFFFFFFFFFFFFFFFFFFFFFFFFFFFFFFFFFFFFFFFFFFFFFFF:F
FFFFFFFFFF:,FFFFFFFFFFFFFFFFFFFFFFFFFFFFFFFFFFFF
@A00721:190:HGJW7DSXY:4:1251:9046:14497_2:N:0:TCTGCAAG+GCTGCACT
CGTTGTCGTACCTTACAAGGTTGAGGAGACTTTACAGCGGCTTATTGTCGCGCAGTTTCCAGAATTGC
GGTTGAAGTTCACCCATTCTATCTTGAATGCTCACGCCGTCGCAGCCA
+
FFFFFFFFFFFFFFFFFFFFFFFFFFFFFFFFFFFFFFFFFFFFFF:FFFFFFFFFFFFFFFFFFFFF
FFFFFFFFFFFFFFFFFFF,FFFFFFFFFFFFFFFFFFFFFFFF:FFF:
@A00721:190:HGJW7DSXY:4:1373:8341:10676_1:N:0:TCTGCAAG+GCTGCACT
ATCACTTGTTAAAGTACAGATTTAGTGATTTAGATATTATTGATATTGGAGGCGATATTCGTTTTCAT
ATCCAAAACTCCTTGAATGGAGTACATATTTGCAACCCTATTCTTGATGTGAAAGATGCATCGAGGTA
TAC
+
FFFFFFFFFFFFFFFFFFFFFFFFFFFFFFFFFFFFFFFFFFFFF:FFFFFFFFFFFFFFFFFFFFFF
FFFFFFFFFFFFFFFFFFFFFFFFFFFFFFFFFFFFFFFFFFFFFFFFFFFFFFFFFFFFFFFFFFFF
FFF
@A00721:190:HGJW7DSXY:4:1272:12924:37059_2:N:0:TCTGCAAG+GCTGCACT
TTGATTGGGAACGTACCTACTCACGAGACGTCATGAAAGCTATCGGAGGGAAGCAAAATGTTTCGTTC
TGTTACAAGAACGCTTTGGATTGTGACCATAAAGCCTCAGTTGCGATA
+
FFFFFFFFFFFFFFFFFFFFFFFFFFFFFFFFFFFFFFFFFFFFFFFFFFFF,FFFFFFFFFFFFFFF
FFFFFFFF:FFFFFFFFFFFF:FFFFFFFFFFF:FFFFFFFFFFFFFF
@A00721:190:HGJW7DSXY:4:2204:11351:5525_1:N:0:TCTGCAAG+GCTGCACT
CTCTCTTGATGATGTAGCCACTATAATGCTTAAGAGAGAGATTGATGTATGTCATTTCGTCTTAGTTT
GTCCCGGCGAATTATTGGACACGATGAAAAATGACATACCCGTTCCTGACCTTGGTATCGTTATCCGT
CAT
+
FFFFFFFFFFFFFFFFFFFF:FFFFFF::FF:FFFFFF:FFFFFFFFFFFFFFFFFFFFFFFFFFFFF

```
FFFFFFFFFFF:FFFF:FFFFFFFFFFFFFFFFFFFFFFFFF:FFFFFFFFF:FFFFFFFF,FFFFFF
FFF
@A00721:190:HGJW7DSXY:4:1527:18756:15311_1:N:0:TCTGCAAG+GCTGCACT
GGTTATTCTCACAACAAAGATAAGATTTTATCTTTCTTCAAACACCCTGTTTTCCTTAAGGGCGGACA
ATTGTTCCATTGTGAACTCTTGGGGACTAGATTGAGCATTAATGAGTTCCGAATAACTCGTAATAAAT
CTT
+
FFFFFFFFFFFFFFFF:FFFFFFFFFFFFFFFFFFFFFFFFFFFFFFFFFFFFFFFFFFFFFFFFFF
FFFF:FFFFFFFFFFFFFFFFFFFFFFFFFFFFFFFFFFFFFFFFFFFFFFFFFFFFFFFFFFFFFF
FFF
@A00721:190:HGJW7DSXY:4:1604:31015:23202_1:N:0:TCTGCAAG+GCTGCACT
ACCTATTTCCGTACCAAGAGCCACTGCCAATCTCATCGCTATACGCATACCTGATTGGAAGAAGCGAG
GTTCGTTCCTCAATAAAAATTTTTCTCAAGAGATTATGGTTGATAAAGTTTTCTTCATAAGGGCTATT
CAG
+
FFFFFF:FFFFFFFFFFFFFFFFFFFFFFFFFF:F,FFFFFFFFFFFFFFFFFFFFFFFFFFFFFFFF
FFFFFFFFFFFFFFFFFFFFFFFFFFFFFFFFFFFFFFFFFFFFFFFFFFFFFFFFFFFFFFFFFFFF
FFF
@A00721:190:HGJW7DSXY:4:1350:10176:6965_1:N:0:TCTGCAAG+GCTGCACT
CTTCGGTCAATGACAAAACCTTCGAATATGTTACGACATGGCTTCGTAACAACAGTGCGAGGATTGTC
ATCTCGGGGAAAGTGATTCACCAAGATGTTCATATAGATACGAGATACCTAGAGAAGGTCGCAGCGAT
CTT
+
FFFFFFFFFFFFFFFFFFFFFFFFFF:FFFFFFFFFFFFFFFFFFFFFFFFFFFFFFFFFFFFFFFFF
FFFFFFFFFFFFFFFFFFFFFFFFFFFFFFFFFFFFFFFFFFFFFF:FFFFFFFFFFFFFFFFFFFFF
FFF
@A00721:190:HGJW7DSXY:4:1357:31358:27806_2:N:0:TCTGCAAG+GCTGCACT
AGCTAAGAAGAAGACTTTGGGTACACGTTATGCTCGCAAACTTAACGTTAGTACCGGAGGTGATCTCT
CTGCTTCTGCGTTACTGAGAGAAAAGATTCAGGACGCCTACGGTGACT
+
FFFFFFFFFFFFFFFFFFFFFFFFFFFFFFFFFFFFFFFFFFFFFFFFFFFFFFFFFFFFFFFFFFFF
FFFFFFFFFFFFFFFFFFFFFFFFFFFFFFFFFFFFFFFFFFFFFF:F
@A00721:190:HGJW7DSXY:4:1567:18656:16736_2:N:0:TCTGCAAG+GCTGCACT
TCTTTGTATAGGGAGTGTGAGAAGACAACGAATCTTGAAACTACGGTAATTAACAAAGAGATTGGTGT
AGCGGACTCTTATACTTTTGAGATTGAGAAGATTGATGAACACGTCAA
+
FFFFFFFFFFFFFFFFFFFF:FFFFFFFFFFFFFFFFFFFFFFFFFFFF,,F,,,:,FFFF,F,F:FFF,
F,,F,:,F,,,,,,,F,,,F,F,,,,,:,F,FF:,F,::,::FFF,FF
@A00721:190:HGJW7DSXY:4:2201:23294:27649_2:N:0:TCTGCAAG+GCTGCACT
TTGTTCTTGAAAACCAAGTTAAGAAGGAGGCTGATCATGGAGTCTTTGTACCTTCTGTCAAACTTCGA
GGTTCAGCTACGAGAGGTGATCCCGGATGTGGATTGAAGGCTGGCTCT
+
FFFFFFFFFFFFFFFFFFFFFF:FFFFFFFFFFFFFFFFFFFFFFFFFFFFFFFFFFFFFFFF:FFFF
FFFFFFFFFFFFFFFFFFFFFFFFFFFFFFFFFFFF:FFFFFFFFFFF
@A00721:190:HGJW7DSXY:4:2370:25726:10285_2:N:0:TCTGCAAG+GCTGCACT
CTTTTTGAATGTTGTAAAGAATTGGGTTGAGAAGTGTGCTTTACAAGGAGTTAGACTCTTCTTCAAGT
TTTTAGATCTTCTTGATAATCTTAAAGAAAAATTTTCGAATAAACTAA
+
FFFF:FFFFFFFFFFFFFFFFFFFFFFFFFFFFFFFFFFFFFFFFFFFFFFFFFFFFFFFFFFFFFFF
FFFFFFFFFFFFFFFFFFFFFFFFFFFFFFF,FF:FFFFFFFFFFFFFF
@A00721:190:HGJW7DSXY:4:1654:11487:33442_1:N:0:TCTGCAAG+GCTGCACT
GTTTCTCTTGGTATTTCTGAAATAGTTTATCAGAGCGTCATTTTCCTTTGGGATCTTTCCTCTGGTAA
GGGAATTATTAAATCATTCTTCTCACTCTTGACGAGAATTATTACTGTTTTCTCTACCCATAACTTCT
TTT
```

+
FFFFFFFFFFFFFFFFFFFFFFFFFFFFF:FFFFFF:FFFFFFFFFFFFFFFFFF:FFFFFFFFFFFFF
FFFFFFFFFFFFFFFFFFFFFFFFFFFFFFFFFFFFFFFFFFFFFFFFFFFFFFFFFFFFFFF:FFFFF
FFF
@A00721:190:HGJW7DSXY:4:2477:24388:22623_1:N:0:TCTGCAAG+GCTGCACT
ACTGGAGTTCAGACGTGTGCTCTTCCGATCTGTTTCAACACTTATCACCACTTTTGAAAGGAAAATAA
TTGGTGATGAAAATACGTATGTCGGTGGGGTTCTCAAGGCTCTCATCGCCACTGGGATTCTAAGGGGT
TCC
+
F:FFF,FFFFFFFFFFFFFFFFFF,FFFFFFFFFFFF,FF,FFFF,F,FFFF:FFFFFFFFFFFFFF,
FFFFFFFFFFFFFFFFFF,:FFFFFFFFFFFFFFFF:FFFFFFFFFFFFFFFFFFFFFFFFF:FFFFFF
F:F
@A00721:190:HGJW7DSXY:4:1118:24026:12915_2:N:0:TCTGCAAG+GCTGCACT
AGGATTCTTCTTTTGGTCCTTACGTCTTAAGTAATTTGGATGTTGTCGAAAACTATCAGGTTCTTGTC
ACGATTCTTAAAAGAAGTTTATTAAAACTTCAGAGTCTCTTGGAAGTC
+
FFFFFFFFFFFFFFFFFFFFFFFFFFFFFFFFFFFFFFFFFFFFFFFFFFFFFFFFFFFFFFFFFFFF
FFFFFFFFFFFFFFFFFFFFFFFFFFFFFFFFF:FFFFFFFF:FFFFFF
@A00721:190:HGJW7DSXY:4:1147:30147:2566_2:N:0:TCTGCAAG+GCTGCACT
TGTTCTTGCAATAGCCACAGACTTTCTGGGAAAAGAGACCTCTGTTGGTAGAGGAGTTGAGAGAATTA
CTCAGATTCAATCTGGCATCACTGAACGTCTACAACGAGTTAGGGAGT
+
FFFFFFFFFF:FFFFFFFFFFFF,FF,FFFF:F:FFFFFFFFFFFFFFFFFFFFFFFFFFFFFFFFFF:
FFFFFF,FFFFFFFFFFFFFFFFFFFFFFFFFFFFF:FFFFFF:FFFFF
@A00721:190:HGJW7DSXY:4:1241:2437:6151_1:N:0:TCTGCAAG+GCTGCACT
AAAAGATCCTTTGCACAAGCAATACCTTCCACTGAGTTCATCACCCAGAGTTTCGACGTCGCGGAAAA
CGATGAAGATTTCCACACTGTTCGAGAATCTTCTGATGCTGAATTTGAGGAAAATTTTTCGGAAGTTT
TTG
+
FFFFFFFFFFFFFFFFFFFFF:FFFFFFFFFFFFFFFFFFFFFFFFFFFFFFFFFFFFFFFFFFFFFF
FFFFFFFFFFFFFFFFFFFFFFFFFFFFFFFFFFFFFFFFFFFFFFFFFFFFFFFFFFFFFFFFFFFF
FFF
@A00721:190:HGJW7DSXY:4:1119:10981:14497_1:N:0:TCTGCAAG+GCTGCACT
TGATGGCTCAGTACAGGGAGGAGGTAATAGTTATAAAACCTTCTTTTATCATTGCAACAGGACTTTGA
GAAGACTGTTGAAATGGACAAAATCTGGGATCATAGGTATGTGTCACGCTACACTCGAAATATGGGCC
CTC
+
FFFFFF:FFFFFFFFFFFFFFFFFFFFFFFFFFFFFFFFFFFFFFFFFFFFFFFFFFFFFFFFFFFFF
FFFFFFFFFFFFFFFFFFFFFFFFFFFFFFFFFFFFFFFFFFFFFFFFFFFFFFFFFFFFFFFFFFFF
FFF
@A00721:190:HGJW7DSXY:4:1501:1425:10441_2:N:0:TCTGCAAG+GCTGCACT
GTTGGCGTCTTGTCGTTGACGTCATTACAGTCTGGAAGTTAGATCATTTGTTCAGGAAGATCTCTACA
GAGATTATCGAGAGTTGTGATCTAGTTCTAAATTTAGGTCGACTGACT
+
FFF,FFFFFFFFFF,FFFF:FFFFFFFFFFFFFFFFFFFFFFFFFFFFF:FFFFFFFF:F:FFFFFFF
FFFFFFFFFFFFF:FF:FFFFFFFFFFFFFFFFFFFF,FFFFF,FFFF
@A00721:190:HGJW7DSXY:4:2537:24451:11741_2:N:0:TCTGCAAG+GCTGCACT
ATGTACGTGGATCTAATGCTTGGTGATCTGAATCTGACCATCGCAAAGAAGACTTTATTAGTTCATGA
AGTTCTTACACTTCTCGCGACTAATGTCAGCTTCACTACAAAGGCGTT
+
FFFFFFFFFFFFFFFFFFFFFFFFFFFFFFFFF,FFFFFFFFFFFFFFFF:FFFFF:FFFFFFFFFFFFF
FFFFFFFFFFFFFFFFFFFFFFFFFFFFFFFFFFFFFFFFFFFFFF:F
@A00721:190:HGJW7DSXY:4:1169:22987:5290_2:N:0:TCTGCAAG+GCTGCACT
CCAAATTTAGCACTTTTTGAATATGACAGAAGCTCGCCATATCAGATTAGAGACATGCCAGTTAGAGT

```
TCTTCTAACCAGGACTGATGGTGAGATTCTTTTACCTTTTGGAGGTCC
+
FFFFFFFFFFFFFFFFFFFFFFFFFFFFFFFFFFFFFFFFFFFFFFFFFFFFFFFFFFFFFFFFFFFF
FFFFFFFFFFFFFFFFFFFFFFFFFFFFFFFFFFFFFFFFFFFFFFFF
@A00721:190:HGJW7DSXY:4:2337:14036:13025_2:N:0:TCTGCAAG+GCTGCACT
CCTAGTGTCTTTTCTAGAGTCCAAGGACGTTTCCGTCGGAGGATGACAGGTGGGAGACCGCTCAATAA
CACTCTATTGAATCCCTTTTCCACGATGAAAGCTCTTTATGAGGTGCT
+
FFFFFFFFFFFFFFFFFFFFFFFFFFFFFFFFFFFFFFFFFFFFFFFFFFFFFFFFFFFFFFFFFFFF
FFFFFFF:FFFFFFFFFFFFF:FFFFFFFFFFFFFFFFFFFFFFFFFF
@A00721:190:HGJW7DSXY:4:2350:18231:24612_1:N:0:TCTGCAAG+GCTGCACT
GATATCGCCTCTGTCCTTTCTCCTTTAATCGTGAGGTTACGAGAGTTTCGTACTTGGGTTTATCGGTT
GTATTTTGATAAATTTGTTGGAATCGATAATGATACTGACGAAGGTTCTATTACCACCCTTGTCATTG
ATA
+
FFFFFFFFFFFFFFFFFFFFFFFFFFFFFFFFFFFFFFFFFFFFFFFFFFFFFFFFFFFFFFFFFFFF
FFFFFFFFFFFFFFFFFFFFFFFFFFFFFFFFFFFFFFFFFFFFFFFFFFFFFFFFFFFFFFFFFFFF
FFF
@A00721:190:HGJW7DSXY:4:2666:32199:31767_2:N:0:TCTGCAAG+GCTGCACT
TGATGCTTTCTTAGAGGCTCGTCTAGCCGCTGAGATTCGTCTTACCTATTTACAATCTGTAGAGAGAG
AGGTTAATCAGAATTTGAGTGATAACGATTTAGTACTGGGAAATGGTG
+
FFFFFFFFFFFFF:FFFFFFFFFFFF:FFFFFFFFF:F:FFFFFFFFFFFFFFFFFFFFFFFFFFFFF
FF:FFFFFFFFFFFFFFFFFFFFFFFFF:FFFFFFFFFFFFF:F:F:,
@A00721:190:HGJW7DSXY:4:2265:14922:11522_1:N:0:TCTGCAAG+GCTGCACT
CCTTTTTCGATTGGATTCGACGGGGTCTTCGTCTCACGACTAATTTGATAAGGTTTTTCTTTTATGGG
ACTCTTGTTTGTACTTATGAAGGAGGTATTACTTCGGAAGTTCTTAATGTAGGTCTCCTAGTACTTAA
GGG
+
FFF:FFF:FFFFFFFFFFFFFFFFFFFFFFFF:FFFFFFFFFFFFFFFFFFF:FFFFFFFFFFFFFFF
FFFFFFFFFFFFFFFFFFFFFFFFFFFFFFFFFFFFFFFFFFFFFFFFFFFFFFFFFFFFFFFFFFFF
FFF
@A00721:190:HGJW7DSXY:4:1641:14959:25833_2:N:0:TCTGCAAG+GCTGCACT
GTTTCAGCTGGATGGATCTTTTGATGGTATGTTTCACATTGCGTGATCACTTCCCGTGGACTTGGGAA
GAGATTAAACATAAAATCCAGCGATTCTATTATGTCGCTAAAGCCTGT
+
FFFFFFFFFFFFFFFFFFFF:FFFFFFFFFFFFFFFFFFFFFFFFFFFFFFFFFFFFFFFFFFFFFFF
FFFFFFFFFFFFFF:FF:FFFFFF,FFFFFFFFFFFFFF:FF,FFFFF
@A00721:190:HGJW7DSXY:4:1405:32009:1470_2:N:0:TCTGCAAG+GCTGCACT
GTTCGGGCTGTGGGGATTAACTTCCCTCGTGAAACTTACGACGATGTTCTCGCTCGCATTTCACAAGG
ATTACTAAGTCTGAGATCTGCCACTTCTAATTTTAGGAAACGTCGCGA
+
FFFFFFFFFFFFFFFFFFFFFFFFFFFFFFFFFFFFFFFFFFFFFFFFFFFFFFFFFFFFFFFFFFFF
FFFFFFFFFFFFFFFFFFFFFFFFFFF::FFFFFF::FFFFFFFFFFF
@A00721:190:HGJW7DSXY:4:1349:14696:4586_1:N:0:TCTGCAAG+GCTGCACT
TCTTGGTCGAAAAATATCGCCGGGAACTTCTAATAGAGGATCCTGATGACATTATTTCAACGGATACC
GATCTCAGTTCTACCACAGAAACCGTCTCAGGAACTGTCTCCACTGATTCGGAATTAGAGAAAGACGA
CCT
+
FFFFFFFFFFFFFFFFFFFFFFFFFFFFFFFFFFFFFFFFFFFFFFFFFFFFFFFFFFFFFFFFFFFF
FFFFFF:FFFFFFFFFFFFFFFFFFFFFFFFFFFFFFFFFFFFFFFFFFFFFFFFFFFFFF,FFFFFF
FFF
@A00721:190:HGJW7DSXY:4:2311:32452:27665_1:N:0:TCTGCAAG+GCTGCACT
GACCTCTTAATTTTCAGGGTGCCCTGATGAGGACGTTGAAAGGGATAATTTCAACTCTGAGAATTCTA
```

AGAGTGTTTCAAAAGACCATAAACGTAACAAGAAGAATAAAGGTAGGAAGGGGAGAAAACAGAACAAG
C
+
::,F:,::,,FF:F:,FF::FF,,F,:,F,F,,,F:F,::FFF:FF:FFF,:,FFFFFFFFF,FF,:F
:FFFF,FFF,FFF,FF::F,F,F:FFFFFFF:FF:FFFF,,FFFFF,FFFFF:FFFFFFFFFFFFFFF
,
@A00721:190:HGJW7DSXY:4:1316:29288:22654_2:N:0:TCTGCAAG+GCTGCACT
TGGTTGCGGCTGATCGTACCCCAATTCCTGCTAGAAAGATCACTCTCGGCGATACCACTGATCTTCCA
GAGTTACGTAGTGGTCGTTCTTTTGGTGATTTTATCAAAGAGAAAGTT
+
F:FFFFFFFFFFFFFFFFFFFFFFFFFFFFFFFFFFFFFFFFFFFFFFFFFFFFFFFFFFFFFFFFFF
FFFFFFFFF:FFFFFF:FF:FFFFFFFFFFFFFFFFFFFFFFFFFFF,
@A00721:190:HGJW7DSXY:4:2260:8196:30937_1:N:0:TCTGCAAG+GCTGCACT
CAAGTTAAACTTCGAACTGACCACTTACAAGAGTGTTGAAGTACCGCAACATGGATCAGTTTTCCAAA
AAGCTATTAATAGCAGTTGCGGGGCTATAATACCGCCTCCAGATTACTCTAAACTGAATCCACTCTCT
TGG
+
FFFFFFFFFFFFFFFFFFFFFFFFFFFFFFFFFFFFFFFFFFFFFFFFFFFFFFFFFFFFFFFF:FFF
FFFFFFFFFFFFFFFFFFFFFFFFF:FFFFFFFFFFFFFFFFFFFFFFFFFFFFFFFFFFFFFFFFFF
FFF
@A00721:190:HGJW7DSXY:4:2525:30156:5243_2:N:0:TCTGCAAG+GCTGCACT
TTTGGAACTGTGTAATGTCCTACGTACTGTTCAAGGTGTTTGGGCTTCTCTAAAGAACAGCAACTTTC
AACATGAATTAGTTATAGAAAGTCTTTACCCTCACGTCTCACTTTATC
+
FFFFF:F,:FFFF:FFF:FFFFFFFFFFFFFFF,F:FFFFFF,FFFFFF:FFFFFFFFFF:FFFF:F:
FFFFFFFFFF:FFFFF,F:FFFFFFF,:,,:FFFFFFF:FFFFFFFF:
@A00721:190:HGJW7DSXY:4:1262:20302:20932_2:N:0:TCTGCAAG+GCTGCACT
AAAGAGGGTTTGAAAATTACGGAACCTTTCCTCGTTCTTCATGAAGATCTTCGAGTGTTTTATAGTGA
GTTATTCTTGCGAAGGTTCAAGTCGATTGATTTTGTTGACTACGATCT
+
FFFFFFFFFFFFFFFFFFFFFFFFFFFFFFFFFFFFFFFFFFFFFFFFFFFFFFFFFFFFFFFFFFFF
FFFFFFFFFFFFFFFFFFFFFFFFFFFFFFFFFFFF:FFFFF:FFFFF
@A00721:190:HGJW7DSXY:4:2432:15709:27070_2:N:0:TCTGCAAG+GCTGCACT
AAGAAGTTCTTCGCAGTTGAATCTCCACCAGGTGGAGGGAAGACCACTGAACTTATAGCGACTTTTGT
CGAATTTTTCGCAAAAGGTCTTGAAGTACGTATTGTCACTGCTAATCG
+
FFFFFFFFFFFFFFFFFFFFFFFFFFFFFFFFFFFFFFFFFFFFFFFFFFFFFFFFFFFFFFFFFFFF
FFFFFFFFFFFFFFFFFFFFFFFFFFFFFFFFFFFFFFFFFFFFFFFF
@A00721:190:HGJW7DSXY:4:1666:10122:2300_2:N:0:TCTGCAAG+GCTGCACT
CACTTCTAGTTCATCGCGGTATTTGTGAAATTAAGACAGACGTGAAAGTTATGATGAGTGATCGTATT
AGAACAGCTGATTCTTATTGCATGAACGTCGCTGAGGTCAAACCTGTG
+
F:F:FFFFFFFFFFFFFFFFFFFFFFFFFFFFFF:FF,FFFFFFFF:F:FFFFFFFFFFFFFFFFFFF
FFF:FFFFFFFFFFFFFFFFFFFFFFFFFFFFFFFF:FFFFFF:FFFFFF
@A00721:190:HGJW7DSXY:4:1331:24216:13025_1:N:0:TCTGCAAG+GCTGCACT
TCGTCGAATTATTCATGATTCACGCTGGTCAGACCCTTTTTCTTACACATCTAATTAAGGCGGCTTAT
ACCATAGGTTACGGTGATTACAATCAAATCGGATTCATCAATCGTATTGATCTCCAAGTCGCAAACTA
C
+
F,F,:,F,F,:FF,FFFFFF,FF,:F,FF,,:::,,FF,FF,FFF,F::FFFF,:FF,F::,,,FF::
F,:FF,,FFF,,,FF,FFF,FFFF,,FFFF,FFFF,,F,FF:FFFFFFFFFFF,,FFFF,F:F,,F,
:
@A00721:190:HGJW7DSXY:4:2556:10710:9925_2:N:0:TCTGCAAG+GCTGCACT
TTGATGAGTGTCGTTCTTATCGAAGTCTCAGTTATCGCTGCCCGGCCGACGTTTGTCGCGAACTCTCA

```
GAGGTTTACGGCAGAAAAATTTCTCCTGCTTGTAATTCGAGAGAGGAC
+
FFFFFFFFFFFFFFFF:FFFFF,,FFFFFFFFFFFFFF:FFFFFFFFFFFFFFFFFFFFFFFFFFFFF
FFFF:FFFFFFFFFFFFFFFFFFFFFFFFFFFFFF:FFFFFFFF,FFFF
@A00721:190:HGJW7DSXY:4:1363:12328:10128_1:N:0:TCTGCAAG+GCTGCACT
CTTGACGACTTCGAATTTGAGGACGGAGTTAAATATATTACTTTCACTCAAGGTGAAAAGTTTGAAAT
TAACAAAGTCCTTGGTAAGAGTAAGGTTAAATGTGAAGATGCTCAGACTATTCATGAAGTTCAAGGTA
ATA
+
:FFFFFFF:F:FFFFFFFFFFFFF:FFFFFFFFFFFFFFFFFFFFFFFFFFFFFFFFFFF:FFFFFFF
FFFFFFFFFFFFFFFFFFFFFFFFF,:FFFFFFFFFFFFFFFFFFFFFFFFFFFFFFFFFFFFFFFFF
FFF
@A00721:190:HGJW7DSXY:4:1629:27407:22341_1:N:0:TCTGCAAG+GCTGCACT
TGACCCTGTTTTTGCATCACAAACACATATTACCGTTGCTCTATCACGACACGTTGAATCTTTACGAT
ACTTTGTTATCAATGTAAAGTTAAACGATGCTACTTGTGAGAGGATCAATCACAGTAAGGAACTGTCA
CGT
+
FFFFFFFFFFFFFFFFFFFFF:FFFFFFFFFFFFFFFFFFFFFFFFFFFFFFFFFFFFFFFFFFFFFF
FFFFFFFFFFFFFFFFFFFFFFFFFFFFFFFFFFFFFFFFFFFFFFFFFFFFFFFFFFFFFFFFFFFF
FFF
@A00721:190:HGJW7DSXY:4:2305:3215:24846_2:N:0:TCTGCAAG+GCTGCACT
AGATTCAACCCCTTATATCGAGGCTATCGAGGACGATGAGTATCTTGAAGTTGTTGACGACAAGCCTC
AAGAGGGTAGAGGACCACCTGGATCTTCTACTGTCTCTGCCTTCCAAG
+
FFFFFFFFFFFFFFFFFFFFFFFFFFFFFFFFFFFFFFFFFFFFFFFFFFFFFFFFFFFFFFFFFFFF
FFFFFFFFFFFFFFFFFFFFFFFFFFFFFFFFFFFFFFFFFFFFFFFF
@A00721:190:HGJW7DSXY:4:1674:15103:2033_2:N:0:TCTGCAAG+GCTGCACT
TTATTCCTGGAACCACCTTAACGTCACTCGGTAATCTTTCAGCCGAATTAAGTACTAATGAGTTCAAG
TCGGTTGTCGATCGAATTAAGATCAAGGCTAATACCTTTATAGGTAAG
+
FFFFFFFFFFFFFFFFFFFFFFFFFFFFFFFFFFFFF:FFFFFFFFFFFFFFFFFFFFFFFFFFFFFF
FFFFFFFFFFFFFFFFFFFFFFFFFFFFFFFFFFFFFFFFFFFFFFFF
@A00721:190:HGJW7DSXY:4:1364:32371:28494_1:N:0:TCTGCAAG+GCTGCACT
CCCGATAGGGTACCGAGTCTCCAAGAGAATATTTATTCTTATGAGGCTCGAAACTTCAACGTTTTCAC
AACGGAAAGACATATCGCACCCGAAATTTTTGGGCAGAATGCTCTTAACGCGGTTCTTGAACGGTGTT
TTG
+
FF:FFFFFFFFFFFFFFFFFFF:FFFFFFFFFFFFFFFFFFFFFFFFFFFFFFFFFFFFFFFFFFFFF
FFFFFFFFFFFFFFFFFFFFFFFFFFFFFFFFFFFFFFFFFFFFFFFFFFFFFFFFFFFFFFFFFFFF
FFF
@A00721:190:HGJW7DSXY:4:1110:27100:14575_1:N:0:TCTGCAAG+GCTGCACT
GATAAAGTTATAGGAGACTCAGTTAACCTGAGAGCTCCTCAATTGATAGATTGGGTCAACAATAGGGA
CGCTAGTATACTTCGTAAAGTCGACAGCGAAATGGTCAATATTGATAATTGGACCGACTATATGACCT
ATT
+
:FFFFFFF:FFFFF:,FFFFFFFFFFFFFFFFFFFFFFFFFF:FFFF:,FF,FFFFFFFFFF:FFFFFF
FFFFFFFFFFFFFFFFFFFFFFFFFFFFFFFFFFFFFFFFFFFFFFFFFFFFFFFFFFFFFFFFFFFF
FFF
@A00721:190:HGJW7DSXY:4:1612:10556:20682_1:N:0:TCTGCAAG+GCTGCACT
AAGGGAAGCTAAGGTTAAACTTGATGCTTCAGCCTTTTCGAAGCATAACCCCGCACAGAATATCGTTT
ATCACGCAAAATTTATTAATGCAATTTTCGGACCTATCTTTAAGGTTCTTGTGAGGAGAGTCAAACAG
TGT
+
FFFFFFFFFFFFFFFFFFFFFFFFFFFFFFFFFFFFFFFFFFFFFFFFFFFFFFFFFFFFFFFFFFFF
```

FFFFFFFFFFFFFFFFFFFFFFFFFFFFFFFFFFFFFFFFFFFFFFFFFFFFFFFFFFFFFFFFFFFFF
FFF
@A00721:190:HGJW7DSXY:4:1164:10728:5885_1:N:0:TCTGCAAG+GCTGCACT
ACATTTTTATGTATACAGAGATGACCAGTGACGATTTCTCCCGGAAGCTCTTCTCTCTCTTAGGAGGT
CATGATGACTACTATAAGGGTGAAATCGATTTTTCGAAGTTTGACAAATCTCAAGATTTCTTTTGTAA
AGC
+
FFFFFFFFFFFFFFFFFFFFFFFFFFFFFFFFFFFFFFFFFFFFFFFFFFFFFFFFFFFFFFFFFFFFF
FFFFFFFFFFFFFFFFFFFFFFFFFFFFFFFFFFFFFFFFFFFFFFFFFFFFFFFFFFFFFFFFFFFFF
FFF
@A00721:190:HGJW7DSXY:4:1225:20112:9737_1:N:0:TCTGCAAG+GCTGCACT
ATTTAGGCTTCGATGCTGAATTTCTTGAACTTTGGAGTGCCAGTGAATATTTTTGTCACGCCAGGAGT
ATCCATAACGATTTGTCATTTACCACCTTTGCTCAACGACGAAGTGGAACGGCCAACACTTTCTTTGG
TAA
+
FF:FFFFFFFFFFFFFFFFFFFFFFFFFFFFFFFFFFFFFFFFFFFFFFFFFFFFFFFFFFFFFFFFFF
FFFFFFFFFFFFFFFFFFFFFFFFFFFFFFFFFFFFFFFFFFFFFFFFFFFFFFFFFFFFFFFFFFFFF
FFF
@A00721:190:HGJW7DSXY:4:1376:7545:28902_1:N:0:TCTGCAAG+GCTGCACT
TCTGATGATTTTATCACTTTATTATGACCTTGGAAAGTTTTCGGCTGTGGTTGTCGCTGGGGACGATT
CCATTCTGTTTTCAAAGACTCCAATAACTGATCATTCTGATCATATGATTTTGGACAATGGTTTCGAA
GCG
+
FFFFFFFFFFFFF:FFFFFFFFFFFFFFFFFFFFFFFFFFFFFFFFFFFFFFFFFFFFFFFFFFFFFFF
FFFFF:FFFFFFFFFFFFFFFFFFFFFFFFFFFFFFFFFFFFFFFFFFFFFFFFFFFFFFFFFFFFFFF
FFF
@A00721:190:HGJW7DSXY:4:2577:2166:33865_2:N:0:TCTGCAAG+GCTGCACT
CAGCGTACTTCTGCTCGCGATTTATTCTTTTTGTCAATGATTACATCTACTTCGTTCCTGATCCTTAC
AAGATCATCGTTAAGTTAGGTAAACCCCTAACCGATGTTACTCTTGTA
+
:F:FFF:F:FFFF:FFFF,F,F,FF,FFFFFFFFFFFF:FFF:FFFFFFFFFFFFF,F:FFFF,FFFF
FF:FFFFFFF::FFFFFFFFFFFFFFFFFF:FFFFF,FFFFFFF,FFF
@A00721:190:HGJW7DSXY:4:1107:24985:1830_2:N:0:TCTGCAAG+GCTGCACT
TTAAAGACTATACTAAATATCTCCACTACGAGGTTGTTGTTTGGCGATTAGCTCTTGCGACTAATATT
CGATATTCGATTGAGGGTCGTTATACCTATTCGGCTATCTGTGCTTTG
+
FFFFFFFFFFFFFFFFFFFFFFFFFFFFFFFFFFFF:FFFFFFFFFFFFFFFFFFFFFFFFFFFFFFF
FFFFFFFFFFFFFFFFFFFFFFFFFFFFFFFFFFFF,FF:FF:FFFFF
@A00721:190:HGJW7DSXY:4:1121:23095:23390_2:N:0:TCTGCAAG+GCTGCACT
TCAAATTTTAAACGATTCCGAGAGGTTTATCCTGATGTGGAGGGAGTTCTTGAAGCATCCCGACCTTT
CCTTGTGAAAATTTTGATGCGGTTTCATAAGATTATCAGTAATCCTGA
+
FFFFFFFFFFFFFFFFFFFFFFFFFFFFFFFFFFFFFFFFFFFFFFFFFFFFFFFFFFFFFFFFFFFF
FFF::FFFFFFFFFFFFFFFFFFFFFFFFFFFFFFFFFFFFFFFFFFF
@A00721:190:HGJW7DSXY:4:1220:1208:34898_1:N:0:TCTGCAAG+GCTGCACT
ATTCATGGACCTTCCATCCTGTACCTCTTCTGGAGAGACGTATCTCAAAGATCGACTTCGAGACAGGT
GATATTGTTGAGCGAGAGCGACCAAGATCTAAATTCGGTAACGGTGCGTGGCGCTTTATTTTAAAAAG
CTC
+
FFFFFFFFFFFFFFFFFFFFFFFFFFFFFFFFFFFFFFFFFFFFFFFF:FFFFFFFFFFFFFFFFFFFF
FFFFFFFFFFFFFFFFFFFFFFFFFFFFFFFFFF:FFFFFFFFFFFFFFFFFFFFFFFFFFFFFFFFFF
FFF
@A00721:190:HGJW7DSXY:4:1546:5710:34898_2:N:0:TCTGCAAG+GCTGCACT
CTGATACTTATCACGAAAGGACTTGAGTCTTTTGATCAGTCTCTGACTTGACCATCTTTCATTTTAAA

TGACGATCATTAAGACTCTCTTTCTTCTTTGTTTCCTTCTTTGCACTG
+
FFFFFFFFFFFFFFFFFFFFFFFFFFFFFFFFFFFFFFFFFFFFFFFFFFFFFFFFFFFFFFFFFFFF
FFFFFFFFFFFFFFFFFFFFFFFFFFFFFFFFFFFFFFFFFF:FFFFF
@A00721:190:HGJW7DSXY:4:1531:1244:22842_1:N:0:TCTGCAAG+GCTGCACT
CTCTTCCGATCTCTAATCTGTGTTTTTACGATAACAACTATCAGTTGATTAAGACTCTTCCGAAAGGC
GAACTTTGGGAATTTGAAGCTCAGGGTGAAGCACAACGTTTGACCGTGACGAACTGTGGTGTTCTTGA
TCT
+
FFFFFFF:FFFF:,FFFFFFFFFFFFFFFFFFFFFFFFFFFFFFFFFFFFFFFFFFFFFFFFFFFFFF
FFFFFFFFFFFFFFFFFFFFFFFFFFFFFFFFFFFFFF:FFFFFFFFFFFFFFFFFFFFFFFFFFFFF
FFF
@A00721:190:HGJW7DSXY:4:1372:4544:21919_1:N:0:TCTGCAAG+GCTGCACT
ATACTCTCCTTGAGTTCACTCGTAAGGACACTACCACTTATTACGATATTTCTTTAGTCGATGGGTTT
TCGACTCCTTTAAGCGTCTTCTGTTCATCGCAACCAGATGATATCTTCTATTCGAACTTATGTTTCGA
TCA
+
FFFFFFFFFFFFFFFFFFFFFFFFFFFFF:FFFFFFFFF:FFFFFFFFFFFFFFFFFFFFFFFFF,FF
FFFFFFFFFFFFFFFFFFFFFFFFFFFFFFFF:FF:FFFFFFFFFFFFFFFFFFFFFFFFFFFFFFFF
FFF
@A00721:190:HGJW7DSXY:4:1529:20220:8985_2:N:0:TCTGCAAG+GCTGCACT
TCTTCTAATGTGGCCTGAACTACGTTTCACTTTGTGTGTACTTGTCTCTATTTTAAGTTTTGTCTTCC
TTGTTTGTCTTATTCTATTAATCACTCATTTTAGTGAGATTATTAAAA
+
FFFFF:FFFFFFFFFF:FFFFFFFFFFFFFFFFFFFFFFF,FFFFFFFFFF:FF,FFFFFFFFFF:FF
,FFFFFFFFFFFFFFFFFFFFFFFFFFFFFFFFFFFFFFFFFFFFFFF
@A00721:190:HGJW7DSXY:4:1363:15112:36464_2:N:0:TCTGCAAG+GCTGCACT
CGACAACAGCAGGGGTTTTCGAATAGTGATAGCCACCCGCCTTACAGAGGGGCAGCAGCATGACCATA
ATTGGTATTGACTTTGGTACCACCTTTTCTACTGTCTGTCTCGCCTCT
+
FFFFFFFFFFFFFFFFFFFFFFFFFFFFFFFFFFFFFFFFFFFFFFFFFFFFFFFFFFFFFFFFFFF:
FFFFFFFFFFFFFFFFFFFFFFFFFFFFFFFFFFFFFFFFFFFFFFFF
@A00721:190:HGJW7DSXY:4:1164:12653:35430_1:N:0:TCTGCAAG+GCTGCACT
CTTAACAACAATGATAGTGAATTTATACCTACAATCATTGGTTTTTCTAATAATGATGACACTATTGT
TTATGGATATGATGCTATTACTCATGATGGTTACGGTCAGGGAGGATTCTCAGTTTACAAGGACCTTA
AGC
+
F:FFFFFFFFFFFFF:F,FFF,,FFFFFFFFF:FFFFFFFFFFFFFF:FFFFFFFFFFFFFFFFFFFF
FFFFFFFFFFFFFFFFFFFFFFFFFFFFFFFFFFFFFFFFFFFFFFFFFFFFFFFFFFFFFFFFFFFF
FFF
@A00721:190:HGJW7DSXY:4:2259:19696:21449_2:N:0:TCTGCAAG+GCTGCACT
CCGAAAGACGTGCTAAATTAAAACCATTGTACGATGTCGTCTGTCTCGGCGATCACTTTTCACTAAGA
ATTGGTCCTACTTTTGGACAAGGCCAACTTCGTAATCTTCATGAGATC
+
FFFFFFFFFFFFFF:FFFFFFFFFFF,FFFFFFFFFFFFFFFFFF:FFFFFFFFFFFFFFF:FFFFFF
FFFFFFFFFFFF:FFF,FFFFFFFFFFFFFFFFFFFFFFFF:FFFFFF
@A00721:190:HGJW7DSXY:4:2229:6840:4789_1:N:0:TCTGCAAG+GCTGCACT
TTAGGGATTTTGAGAGAGTGAAAGGCTTCAATTGTAGTGGTTTGGTCGTTTCAGTACCCAGTCAGTAT
ACCTCTACGCAAAGATATTTCATGAAAACTACAGAGGAACGTACCGGATTACCTGTCTTTCATATTAT
GAA
+
FFFFFFFFFFFFFFFFFFFFFFFFFFFFFFFF,FFFFFFF,FFFFFFFFFFFFFFFFFFFFFFFFFFF
FFFFFFFFFFFFFFFFFFFFFFFFFFFFFFFFFFFFFFFFFFFFFFFFFFFFFFFFFFFFFFFFFFFF
FFF

@A00721:190:HGJW7DSXY:4:2313:18493:35556_2:N:0:TCTGCAAG+GCTGCACT
TCTTTGCTTCGATGTTGGATGTCAAGAAGGGTTCAGAATGGGATTCCTACGTAGTTTATGACTTTGGT
GGTGGTACTTTCGATACTTCTATCGTCTTACGTTACGGTAATTACTAT
+
FF::FFFFF:FFFF::FFFFFF:FFF:FF:F,FFFFFFFF,FFFFFFFFF,FFFFFFFFFFFFFFFF
F,F,,FFFFFFFFFFFFFFFFFFFFFFFFF,FFFFF:FFFFFFFFFFF
@A00721:190:HGJW7DSXY:4:1570:20103:34585_1:N:0:TCTGCAAG+GCTGCACT
ATAATCTTGGAGGTCGTGACGTTGATGAAGCTATAAGAGCGTTCTTATTACGGCGCACTTCTTTACCC
GCCACCACACAACTTTCAGTTTCACAACTGAAGGAGGAGGTTTCTGTTACTGGAAAACCCTCGTCAAT
ACT
+
FFFFFFFFFFFFFFFFFFFFFFFFFFFFFFFFFFFFFFFFFFFFFFFFFFFFFFFFFFFFF,FFFFFF
FFF:FFFFFFFFFFFFFFFFFFFFFFFFFFFFFFFFFFFFFFFFFFFFFFFFFFFFFFFFFFFFFFF:
:FF
@A00721:190:HGJW7DSXY:4:1351:16613:4586_2:N:0:TCTGCAAG+GCTGCACT
GATCTAGTGAAATTAACGCGTCCTTTTCTGGAACGTAGTTTTACTATTATGAACAAAGTTATCAAAGA
TAGTGGCATTCAAGGCAAACTTACCCTGATTCCGGCCGGTGGTAGCTC
+
FFFFFFFFFFFFFFFFFFFFFFFFFFFFFFFFFFFFFFFFFFFFFFFFFFFFFFFFFFFFFFFFF:FF
FFFFFFFFFFFFFFFFFFFFFFFFFFFFFFFFFFFFFFFFFFFFFFFF
@A00721:190:HGJW7DSXY:4:1459:18765:12226_2:N:0:TCTGCAAG+GCTGCACT
GGCTATTACTCTTGCTGAAACAGCTATTAAAGCTGTTGAGCGGGTAATTACACCTAAACGAGCTCGAT
CTGCCATCGCTGAAGGATGTGCACTCTTTTCTGCTACTTTAACGGAGC
+
FFFFFFFFFFFFFFFFFFFFFFFFFFFFFFFFFFFFFFFFFFFFFFFFFFFFFFFFFFFFFFFFFFFF
FFFFFFFFFF:FFFFFFFFFFFFFFFFFFFFFFFFFFFFFFFFFFFFF
@A00721:190:HGJW7DSXY:4:1629:20889:15843_2:N:0:TCTGCAAG+GCTGCACT
TCGATTGTATCAACAGTAACATTACTTCTAATAAAGGTCTCTATCAAGCTCGTATTATCGTCGCTGCT
GGATCTGCGTTACCGTTGAAGGCTAAACTCCAAGGTAATTATGAAGAC
+
FFFFFFFFFFFFFFFFFFFFFFFFFFFFFFFFFFFFFFFFFFFFFFFFFFFFF:FFFFFFFFFFFFFF
FFFFFFFFFFFFFFFFFFFFFFFFFF,FFFFFF,FFFFFFFFFFFFF:F
@A00721:190:HGJW7DSXY:4:2126:11731:12446_1:N:0:TCTGCAAG+GCTGCACT
GTATGAGGGTGTTTCTAATCGTAACATTAATAATTTCAATCTTCTTAAAAAGAAGATAGAGAGTCATA
CTTTGGGCTATCGTTCAGGTGCATGGGGTTGGATAGCCAATATTGAAGTGGACACCTCTGGTAGTATC
GAA
+
:FFFFFFFFFFFFF:FFFFFFF:FFFFFFFF:FFFFFFFFFFFFFFFFFFFFFFFFFFFFFFFFFFFF
FFFFFFFFFFFFFFFFFFFFFFFFFFFFFFFFFFFFFFFFFFFFFFFFFFFFFFFFFFFFFF:FFFFF
FFF
@A00721:190:HGJW7DSXY:4:1354:30770:25441_1:N:0:TCTGCAAG+GCTGCACT
CTAAGCATACTACTTTTGCTTTGATTACTCTACCGCCTTACAAGTCTAAGGTTAAAGAGTCGATTAAA
CAAGTTGTACTTATCAATGATTTTATCGCTGGTTGTTATCTCGAAAAGTCTCTTCTAAAAGGAACGTC
AGT
+
FFFFFFFFFF:FFFFFFFFFFFFFFFFFFFFFFFFFFFFFFFFFFFFFFFFFFFFF:FFFFFFFFFF:FF
FFFFFFFFFFFFFFFFFFFFFFFFFFFFFFFFFFFFFFFFFFFFFFFFFFF:FFFFFFFFFFFFFFFF
FFF
@A00721:190:HGJW7DSXY:4:1202:4001:34632_2:N:0:TCTGCAAG+GCTGCACT
CTAAAATATTTACAAAATCTTACCACTATCTACGAACCTAAAGTTCTGAAGACGTTGCTTGTCGCTTC
CTCCTTTAATGGCACTACCTCGGTACAATCTTACTTGGAACGAAGGCG
+
FFFFFFFFFFFFFFFFFFFFFFFFFFFFFFFFFFFFFFFFFFFFFFFFFFFFFFFFFFFFFFFFFFFF
FFFFFFFFFFFFFFFFFFFFFFFFFFFFFFFFFFFFFFFFFFFFFFFF

```
@A00721:190:HGJW7DSXY:4:1156:30318:31172_1:N:0:TCTGCAAG+GCTGCACT
GTTCTGTCTATATTAGGTGAAGCGAAATACGATTTCGAACGTCAACGTTCACCGAGTTTCGTTTCGTA
CCTTGATGCTGACTTTCGTCAATATGATTTGATCTGGTTGCTCAATTCTAAGCGTGTTCCGCTTAGTG
CGA
+
FFFFFFFFFFFFFFFFFFFFFFFFFFFFFFFFFFF:FFFFFFFFFFFFFFFFFFFFFFFFFFFFFFFFF
FFFFFFFFFFFFFFFFFFFFFFFFFFFFFFFFFFFFFFFFFFFFFFFFFFFFFFFFFFFFFFFFFFFFF
FFF
@A00721:190:HGJW7DSXY:4:2460:13639:15029_2:N:0:TCTGCAAG+GCTGCACT
AGTTTACGAACTTGACTCTACTATACGTGACATACTAAATACTAGATATTTGGATGATCTTGATAAAG
AACTTCATGAATTTTCAGAGGATGAAAAATTTTCTTCCTTGGATTTTA
+
FFFFFFFFFFFFFFFFFFFFFFFFFFFFFFFFFFFFF:FFFFFFFFFFFFFFFFFFFFFFFFFFFFFFFF
FFFFFFFFFFFFFFFFFFFFFFFFFFFFFFFF::FFFFF:FFFFF:F:FF
@A00721:190:HGJW7DSXY:4:2135:31033:5948_1:N:0:TCTGCAAG+GCTGCACT
TACAGGAGGCATTCTTCTGATCGTAAGAAGTTTATAAGGAACATATCCATTTGTTGTAATAGGGTCAT
CAACTTAGAACTCGAAACTCTAAGTGGTAATACTGTCTTTCCAAGCTTACCTGTATCTTCAATGAAAT
TGG
+
FFFF,::FF,F,F,FFFFFFF:,FFFFFF,FFFFFFF,FFF:FFFF,FFFFF,FF,FFFFF,:FFFFF
F::::FF:,FFFFFFFF,F,FFF,F,FFFFFF,FFF:F:FFFFFF:FFF:FFFFFF:FF:FFFFFFFF
FFF
@A00721:190:HGJW7DSXY:4:2109:4010:14011_2:N:0:TCTGCAAG+GCTGCACT
TAAAGGAAGATCTGTTATCGACGGTTTATCGTTTACGAAGTATCACTCCACCGGTGAATGGTTTAAGA
ATACTATAAATAGAATAGCTATTACTACTAATGGCGTTGGCCTAGTGG
+
FFFFFFFFFFFFFFFFFFFFFFFFFFFFFFFFFFFFFFFFFFFFFFFFFFFFFFFFFFF:FFFFFFFFF
FFFFFFFFFFFFFFFFFFFFFFFFFFFFFFFFFFFFFFFFFFF::FFFFF
@A00721:190:HGJW7DSXY:4:1557:16450:22748_1:N:0:TCTGCAAG+GCTGCACT
TTTAGGTGGTCTTTATTCTATTATTGATTTTTATGAGATTGATACGTTAACAGAAGATGATGTTAGAG
TAGAATTCCTGAAGAGTCCTTCCGTAACCAACTTCTTGACAATTTTCTTCAATCCTTCGATAATAGTT
AAA
+
,FFFFFFFFFFFFFFFFFFFFFFFFFFFFFFFFFFFFFFFFFFFFFFFFFFFFFFFFFFFFFFFFFFF
FFFFFFFFFFFFFFFFFFFFFFF:FFFFFFFFFFFFFF:FFF:F:F:FFFFFFFFFFFFFFFFFFFFFF
FFF
@A00721:190:HGJW7DSXY:4:2226:4010:1517_1:N:0:TCTGCAAG+GCTGCACT
CCGATAAGGGTCACTAGTCTTACACAATTAAGAAATAATCCTACATTGACTGATTATCTAAAGATTGA
AGGAAAATTTTATCCTCGTTTAACTTACTTAGACTTTCCTACGATACAAATGATAACTTCTAATTTAC
GTT
+
FFFFFFFFFFFFFFFFFFFFFFFFFFFFFFFFFFFFFFFFFFFFFFFFFFFFFFFFFFFFFFFFFFFF
FFFFFFFFFFFFFFFFFFFFFFFFFFFFFF:FFFFFFFFFFFFFFFFFFFFFFFFFFFFFFFFFFFFFF
FFF
@A00721:190:HGJW7DSXY:4:2321:31873:6746_2:N:0:TCTGCAAG+GCTGCACT
AAGGGATGCATGGTGTTATCTCCTCAGGAGTCCCGGCATTTGATAATAATGACGAGACTTTCGTCATA
ATCGCTGCATATTACGGATTACACGGTACCAGCGCATCTCGTGTCGAG
+
F:FFFFFFFFFFFFFFFFFFFFFFFF:FFFFFFFFFFFFFFFFFFFFFFFFFFFFFFFFFFFFFFFFFF
FFFFFFFFFFFFFFFFFFFFFFF:F:FFFFFFFF:FFFFFFFFFFFFFF
@A00721:190:HGJW7DSXY:4:1442:26259:16658_1:N:0:TCTGCAAG+GCTGCACT
AAATTTATAGAAGATTATTTTGACAGAATCAATAAGAATCATCGATTCTCGATTCGTAGAGCTTATCT
AGGTACTCTCATTTACTATGTTGATTTAGTCTACATTTATGCTGATCTAAAATTTCGATCTAGATGGA
TCC
```

+
F:FFFFFF:FF::F,:,:FFFFF,,:F:F,,:F:,,FFF:FF,FFFFFFF:FFF:,FFFFFFFFFFFF
FF:FFFFFFFFFFFFFFF,FFFFFFFF,FFFF:FF:FFFFFFFFFFFFFFFFFFFF,FFFFFFFFFFF
FFF
@A00721:190:HGJW7DSXY:4:1222:28320:25739_2:N:0:TCTGCAAG+GCTGCACT
TATAGGAGTAATCTACGAGAAGATTCTAGAATTCTTAATTATAATATTCAACCTCAAAAGTTACGTGT
TTACAGTAACGAGATCACACATTTTGAGAACTAACTATGTTATGTTCT
+
FFFF:FFF,FFFFFFFFFFFFFFFFFFFFFF,FFFFF:FFFFFFFFFFFFFFFFF,FFFFFFFFFFFF
F:FFFFFFFFFFFFFFFFFFFFFF,FFFFF:FFFFFF:FFFFFFFFFFF
@A00721:190:HGJW7DSXY:4:1550:3405:21136_1:N:0:TCTGCAAG+GCTGCACT
TCTCTTCTTATAATAAATAAATAAATAATATTACTAATGGCAAATAGTGGAAAGGCTCCAGTCAACAC
TAGTTCGAGTGCTGCTTTGGATTACGATGACGTGAGAATAGGTGAAGGGATTGTTTTTACCTCGACCA
ACA
+
FFFFFF:FFFFF:FFFFFFFFF,FFFFFFF:F:FFFF:FFFF:FF::FFFFFF:FFFFFFFFFFF,FF
FFFFFFFFFFFFFFFFFFFFFFFFFFFFFFFFFFFFFFFFFFFFFFFFF:FFFFF:FFFFFF:FFF
FFF
@A00721:190:HGJW7DSXY:4:2475:3974:6934_2:N:0:TCTGCAAG+GCTGCACT
GCGACTAATCTTAAAGCTTCACCCAAAATGAAGGACACTTCGAAACTGAATAATGATGCACTACAAAC
AAGTATCGCTAATCATCTGAAAAAGGCTCTAAAGGACACTATACAATA
+
FFFFFFFFFFFFFFFFFFFFFFFFFFFFFFFFFFF:FFFFFFFFFFFFFFFFFFFFFFFFFFFFFFFF
FFFFFFFFFFFFFFFFFFFFFFFFFFFFFFFFFFFFFFFFFFFFFFFF
@A00721:190:HGJW7DSXY:4:1255:24849:19163_2:N:0:TCTGCAAG+GCTGCACT
CAGCACCACATTTTGGTCAAGTCAATCCGTTGAGAAAATTTCTTAGAGGATATTCGCCATTGTGGTAC
GAATTGGTCCAACATTTACCACCAAATTCTTTTGATACAACTCTAGCC
+
FFFFFFFFFFF:FFFFFF:FFFFFFFFFFFFFFFF:FFFFFFFFFFFFFFFFFFFFFFFFFFFFFFFF
FFFFFFFFFFFFFFFFFFFFFFFFFFFFFFFFFFFFFFFFFFFFFFFF
@A00721:190:HGJW7DSXY:4:2515:18945:3677_1:N:0:TCTGCAAG+GCTGCACT
ATACGCTTGCTATTCACCAGATTGGTTCGAACCCGTTGATGCTATGTCAGAAGAAGCTATTCAAGCTT
GTCGTTTAAAGACTGCAGAGGCTGTCAAACAAGCCCAGACTCAAGGAGTCCAATCAAAGGTTTTGAAT
TTA
+
FFFFFFFFFFFF:FFFFFFFFFFFFFFFFFFFFFFFFFF:FFFFF:FFFFFFFFFFFFFF:FFFFFFFF
FFFFFF:FFFFFFFFFFFFFFFFFF:FFFFFFFFFFFFFFFFFFFFFFFFFFFFF:FFFFF:FFFFFFFFF
FFF
@A00721:190:HGJW7DSXY:4:1308:13349:14841_2:N:0:TCTGCAAG+GCTGCACT
GGTGATAAAAATTATTTAGAGATCTTTTCTACTTACAAAGGACAATTCAGTTTCATTAATTCTACGGA
AAAGTCCGGAGTATATTGTATGTTAATAGATAGTGGGATAATAGTTCT
+
FFFFFFFFFFF:FFFFFFFFFFFFFFFFFFFFFFFFFFF,FFFFFFFFFFFFFFFFFFFFFFFFFFFFF
FFFFFFFFFFFFFF:FFFFFFFFFFFFFFFFFFFFFFF:FFFFFFFF:F
@A00721:190:HGJW7DSXY:4:1311:28999:29763_1:N:0:TCTGCAAG+GCTGCACT
CGTCTGAAGAGTTTAGAGCTGCTATTGATTATGTAACCGATAGGAGTTGTATAGTTGAAGATGTGGCT
TTAGACGATAATCCTTTAAGGACTATGTTGACTGACGAAGATTTTCCCACAGATTTGTCTACGTTGCA
GAT
+
FFFFFFFFFFF,FFFFFFFFFFFFFFFFFFFFFFFFFFFFFFFFFFFFFFFFFFFFFFFFFFFFFFFF
FFFFFFFFFFFFFFFFFFFFFFFFFFFFFFFFFFFFFFFFFFFFFFFFFFFFFFFFFFFFFFFFFFFF
FFF
@A00721:190:HGJW7DSXY:4:2559:22263:29590_2:N:0:TCTGCAAG+GCTGCACT
CACTATATACATTATAAAATATAAAAATAAGGGAGTCATAACTTTTGTTACCAAATTCTTGGCTTTCA

```
ACGAAGTCTCTGATTTTATTAATCATTACTTTAACGATCTCTCGGTCA
+
FFFFFFFFFFFFFFFFFFFFFFFFF:FFFFFFFFFFFFFFFFFFFFF:FFFFF:FFFFFFFFFFFFF:
FFFFFFFFFFFFFFFFFFFF:FFFFFF:FFFFFFFFFFFFFFFFFFFF
@A00721:190:HGJW7DSXY:4:1107:17833:10864_1:N:0:TCTGCAAG+GCTGCACT
TTGATCTACGTCGTTTAACTGTCGGAACAACCTTTACTTAGAATGACTCTATCTTTTCAGACACTTCT
TATATTATTTACAATAGTTAATATCATATTAGTAATACTCACTATCGTTTTCTGCTTTTTCTTAGCTA
ATA
+
FFFFFFFFFFFFFFFFFFFFFFFFFFFFFFFFFFFFFFFFFFFFFFFFFFFFFFFFFFFFFFFFFFFF
FFFFFFFFFFFFFFFFFFFFFFFFFFFFFFFFFFFFFFFFFFFFFFFFFFFFFFFFFFFFFFFFFFFF
FFF
@A00721:190:HGJW7DSXY:4:1677:26738:23876_1:N:0:TCTGCAAG+GCTGCACT
GGATGAGTACGAAAACCAACTCTTCGTTACGAGACCTGTTGGTAGAATAGATTTAGTCTCTACACCCA
TCTCAACGAGGATAAAGCGAGTTTTCCGTCCTGAACGTAATACACGAAGAGATCATCCGCAAACGACT
CGA
+
F::F:,,FF,,FFFFFF,F,:,F::,FFF::,,FF,FF,FFFF,,FFF,F:FF:,F:::,,F,FFF:F
:,::FFFF,::,FF:,F:,,:FFF:FFFFF,F,FF,,FFF:F,,,,F,F,F:F,,,FF::FFFFFFFF
FFF
@A00721:190:HGJW7DSXY:4:2247:8901:1125_2:N:0:TCTGCAAG+GCTGCACT
AAAGTCCCTTCTAACTTTAGATTATGAATCCTGCCTTTACTCATTTTATAGCAGTTATTTATGTTGTA
CTTTTTATAATTTATAACCTTCTTATACTTTTGGGTATGTTTTGTGAA
+
FFFFFFFFFFFFFFFFFFFFFFFF:FFFFFFFFFFFFFFFFFFFF,FFFFF:FFFFFFFFFFFFFFFF
FFFFFFFFFFFFFFF:FFFFFFFFFFFFFFFFFFFFFFFFFFFFFFFF
@A00721:190:HGJW7DSXY:4:2373:21549:4867_1:N:0:TCTGCAAG+GCTGCACT
TACTTATTGTAACGATCATTCTTTCGAGTTTTGTCATTCTTATTCTTTTATTCCTTTAACTGTGTATG
AGGAGGATTTATAATGCTTTTCGATAGTGATTGTTATCAATTTAAATACATTACTGTTTCAAATCATC
TTT
+
FFFFFFFFFFFFFFFFFFFF:FFFFFFFFFFFFFFFFFFF:FFFFFFFFFFFFFFFFFFFFFFFFFFFF
FFFFFFFFFFFFFFFFFFFFFF:F:FFFFFFFFFFFFFFFFFFFFFFFFFFFFFFFFFFFFFFFFFFFF
FFF
@A00721:190:HGJW7DSXY:4:2650:1588:14011_1:N:0:TCTGCAAG+GCTGCACT
GAAAATGGTTTCAAATGAGGTCAAAGCTCTATTTATTAAACGAACTATTATCCGATTCTTCGAGGGTA
TCACTACCACTGATCTGCCGACTTTAGTCAAGGAACTTCAATTCCTTCGATGTTTATATTTTTCTATA
AGG
+
FFFF,FF,:FFFFFFFFFFFFFFFFFFFFFF,FFFFFF:FFFFFF,FFFFFFF,:FFFF:FFFFFFFF:
FFF:FF::::FFFFFF:FF:FFFFFFFF,FFF:FF,FFFFFFFF:FF,,FFFFFFFFFFFF:F,:FFF
FFF
@A00721:190:HGJW7DSXY:4:2357:17237:14653_2:N:0:TCTGCAAG+GCTGCACT
TAATCAAATTTCGACAAATTTTAGAACTTTATTTCAGAACTCTCTCCGATCACCTTGGTGTGATCTCC
CGGAGGATCTTTTCAATAAGATGTATGATGATAATCTACTATTTTTAA
+
FFFFFFFFFFFFF:F:FFFFF,FF:F:FFF:FFFFFFFFFFFFFFFFFFFFFF:FF::FFFFFFFF:F:
FFFFFFFFFF,,::F,FF:F:FFFFFFFF:FF:FFFFF:FFF,FFFF,
@A00721:190:HGJW7DSXY:4:2412:2691:25692_1:N:0:TCTGCAAG+GCTGCACT
AGCGTATACTTGAATACTTTATCAAAGATTTGGCTTGGTCTATTTACCGCAAAAAGTTGGAAGAGGTC
TATGATATTTATTTTACTTATACTTTAAATAGCACCGATTATAACTTGACTGTTGATAATATTACAAT
AGC
+
F::FFFFFFFFFFFFFFFFFFFFFFFFF:FFFFFFFFFFFFFFFFF:FFFFFFFFFFFFFFFFFFFFFF:
```

```
FFFFFFFFFFFFFFFFFFFFFFFFFFFFFFFFFFFFFFFFFFFF:FFFFFFFFFF:FFFFFFFFFFFFFF
FFF
@A00721:190:HGJW7DSXY:4:2266:17255:8735_2:N:0:TCTGCAAG+GCTGCACT
AAGATTAGAGAAGTAACTCGGTGAACACGTCGGTAGCATTGATTTACTTTTACCGTTAGAACTGAAAA
AGATTCTTTTCGAAAAGAAACCTATAAGTTACAAACGATTCTTCGACT
+
:FFFFFFFFFFFFFFFFFFFFFFFFFFFFFFFFFFFFFFFFFFFFFF:FFF:FFFFFFFFFFFFFFFFF
FFFFFFFFFFFFFF:FFFFFFFFFFFFFFFFF:FFFFFFFFFFFFFFFF
@A00721:190:HGJW7DSXY:4:1340:29243:10300_1:N:0:TCTGCAAG+GCTGCACT
AATAAAAATACTTCAAGTACCTGAACTTTACATTTTTAATGGTATGGTATAATGTGTCTTACAAATAG
AACCTTCACTCTCGGTAATGAGAGTGTATTCCTTAATATTCTAGTAACCGCTATGTGGGCATTACTAG
GAT
+
FFFFFFFFFFFFFFFFFFFF:FFFFFFFFFFFFFFFFFFFFFFFFFFFFFFFFFFFFFFFFFFFFFFFF
F:FFFFFFFFFFFFFFFFFFFFFFFFFFFFFFFFFFFFFFFFFFFFFFFFFFFFFFFFFFFFFFFFFFF
FFF
@A00721:190:HGJW7DSXY:4:1509:15248:26396_2:N:0:TCTGCAAG+GCTGCACT
TTTAAATGCTAGGAAAATTTTAGAAAAACTCCATTGTATAGTTCAAGGAGTACTTAATTCAATAGCTT
TAGTATTATTTACTATCGCCTTTATTGAAAATTGTATAAGAGTTAATT
+
FFFFFFFFFFFFFFFFFFFFFFFFFFFFFFFFFFFFFFFFFFFFFFFFFFF:FFFFFFFFFFFFFFFFF
FFFFFFFFFFFFFFFFFFFFFFFFFFFFFFFFFFFFFFFFFFFFFFFF
@A00721:190:HGJW7DSXY:4:1309:17788:1235_1:N:0:TCTGCAAG+GCTGCACT
GTGAGAATGAACTGCCGCTTCCCGGAAGAAACGATCTATTTACAGAGTATTTAGAGTAGTCCAAAGTT
TGTATAAATTTATATGATTTTCTATATATATACCACATATATATATATATAAAATCCTATAAATTTGT
ACA
+
F,FFFFFFF:F:FFFFF:F,FFFF:FF:FFFF:FFFFFF,FFFF:F,FFF,FFFF:FFFFFFFFF:F:
,:F:FFFFFFFFF:FF,FFFF::FFFFFFFFFFFFF:F,F:FFFFFFFFFFFFFFF:FFFFF:FFFFF
F:F
@A00721:190:HGJW7DSXY:4:2371:5113:26005_2:N:0:TCTGCAAG+GCTGCACT
TACACTCCCCAAACATAACATACCATAACCTACCATGACCAATCATCGCTCCAACTGGCACGCCAGAC
GTAGGGTCAAACCTACTACCGCCTGTCGGCAGATCGGCACCACTGGTC
+
FFFFFFFFFFFFFFFFFFFFFFFFFFFFFFFFFFFFFFFFFFFFFFFFFFFFFFFFFFFFFFFFFFFF
FFFFFFFF,FFFFFFFFFFFFFFFFFFFFFFFFFFFFFFFFFFFFFFF
@A00721:190:HGJW7DSXY:4:1101:8513:32643_1:N:0:TCTGCAAG+GCTGCACT
CAGACGTGTGCTCTTCCGATCTCCCAAATCTAGATCTGCACAGAAGAAGGCTGAAGGTCGTTACCGGA
CGACCACTCAAAGGAAACGGGCTGTTGCCCAGAGGAAAGCAGCGACCACCGAGGTCGCTCGTAACATT
GAC
+
FFFF:FFFFFFFFFFFFFFFFFFFFFFFFF:FFFFFF:FFFFFFFFFFFFFFFFF:FFF:FFFFFFFFF
FFFFFFFFFFFFFFFFFFFFFFFFFFFFFFFFFFFFFFFFFFFFFFFFFFFFFFFFFFFFFFFFFFFF
FFF
@A00721:190:HGJW7DSXY:4:1403:11433:12399_1:N:0:TCTGCAAG+GCTGCACT
CATCTCTGTGGGAACTGAGGCAGTACCTCAGTCAAAAGTCACATTCACCACTCCCATGCCAATCAATG
ATGTGACTATACAGTTCGGCCATTTCAAGAGTGGTCAATACGTCGTGGGAGGTACTGAAGCCTTCCCC
TCC
+
FFFFF:FFFFFFFFFFFFFFFFFFFFFFFFFFFFFFFFFFFFFFFFF:FFFFFFFFFFFFFFFFFFFFFFFFFF
FFFFFFFFFFFFFFFFFFFFFFFFFFFFFFFFFFFFFFFFFFFFFFFFFFFFFFFFFFFFFFFFFFFF
FFF
@A00721:190:HGJW7DSXY:4:1130:8549:4210_2:N:0:TCTGCAAG+GCTGCACT
CTCCCAAGCCAATCAATAGTTCTTCTGACCAGAATGAAAAGGGGTTTACCAGGGTAGTCTCTAAGTCT
```

```
ACCAGGAAGAGGGGAAATGTCCCCAAAACTGAGAAGAATAAAGGGAAA
+
FFFFFFFFFFFFFFFFFFFFFFFFFFFFFFFFFFFFFFFFFFFFFFFFFFFFFFFFFFFFFFFFFFFFFF
FFFFFFFFFFFFFFF:FFFFFFFFFFFFFFF:FFFFFFFFFF:FFFFF
@A00721:190:HGJW7DSXY:4:1472:11822:31892_2:N:0:TCTGCAAG+GCTGCACT
CCTAAGAAGGGGAAATTACCCCTAATTCTGAAGGAAGATCTCCCACCATCTGGGGTTGGGAATAAGAA
ATCTGCACGGGCCAAAGAGGCCGCTGCTACGAAGTCGCTGAAGGCGGC
+
FFFFFFFFFFFFFFFFFF:FFFFFFFFFFF:FFFFF:FFFFFFFFFFFF:FFFFFFFFFFFFFFFFFF
F:FFFFFFFFFFFFFFF,FFFFFFFFFFFF:FFFFFFFFFFFFFFF:F
@A00721:190:HGJW7DSXY:4:1309:15582:9251_2:N:0:TCTGCAAG+GCTGCACT
AAGGGTAGCTGTAAAGTTGCGCCTGCCGACCCCATCTTTGAGACCAAGACTCTCTACAGTAGGTTCGT
GCCTACTATGAGCAAATCTGTTGAGATCGGAGGAGCGTCGTGTAGGGA
+
FFFFFFFFFFFFFFFFFFFFFFF,FFFFFFFFFFFFFFFFFFFFFFFFFFFFFFFFFFFFFFFFFFFF
FFFFFFFFFFFFFFFFFFFFFFFFFFFFFFFFF,FFFFFFFFFF:FFFF:
@A00721:190:HGJW7DSXY:4:1429:7654:5729_2:N:0:TCTGCAAG+GCTGCACT
GGCTACTGGCGACAAACTGAGAGCAGTCGCTGGGGAAAGAGAATTGCGTGGGTTGAAGGCCGACGCTT
CGAGCTTGAGTGTGACGAGGCCGGTGACGTTTTTGTCAACGGCGTTTG
+
FFFFFFFFFFFFFFFFFFFFFFFFFFFFFFFFFFFFFFFFFFFFFFFFF:FFFFFFFFFFFFFFFFFF
FFFFF,FFFFFFFF:FFFFFFFFFFF,FFFFFF:FFFFFFFFFF:FFF
@A00721:190:HGJW7DSXY:4:1167:8929:34147_2:N:0:TCTGCAAG+GCTGCACT
CTTAACTATGGGGCGAAGATCCCCTATGGAGTCTTGAAGGACTTAGGTTGGCATATTTATGCCGACGA
TAATAAGCATGTCACCCTTAAGCACCGAGGTAAGCTCGGGACTTACAA
+
FFFFFFFFFFFFFFFFFFFFFFFFFFFFFFFFF:FFFFFFFFF:FFFFFFFFFFFFFFFFFFFFFFFFF
FFFFFFFFFFFFFFFFFFFFFFFFFFFFFFFFFFFFFFFFFFFFFFFF
@A00721:190:HGJW7DSXY:4:1178:28076:20149_1:N:0:TCTGCAAG+GCTGCACT
GATCTTGCAGAGTGACTGGAGTTCAGACGTGTGCTCTTCCGATCTGTTAAAACTCTTGCTCCTTCTGT
GGGTGAGGGTTTTTGTTATATTCACTGGTTCTGGGAGGCTGCTTTCCTCCATAATTACAACCTCTCTT
TAG
+
F:FFFFFFFFFFFFFFFFFFFFFFFFFFFFFFFFFFFFFFFFFFFFFFFFFFFF:FFFFFF:FFFFF
FFFFFFFFF:FFFFFFFFF:FFFFFFFFFFFFFFFFFFFFFF:FFFFFFFFFFFFF:FFFFFFFFFFF
FFF
@A00721:190:HGJW7DSXY:4:1526:19316:1470_2:N:0:TCTGCAAG+GCTGCACT
AGTTGGGCCAATTCCCACAGATGGTAGACGTTGTCACACTTTTCTTGAAAATTTTTCCTTTCCTCCGT
CATTGGGGATTTCGATGTGAGAAGGTGGAAGGTGTCAAGGGTCTTCTT
+
:FFFFFFF,FFFFFFFFFF,:FFFFFFFFFFFFFF,FFF:FFFFF:FFFFFFFFFFFFFFFFFFF,FF
F:::,FFFFFFF:FF:FFFFFFFFFF,FFFF:::FFFFFF:FFFFFFF
@A00721:190:HGJW7DSXY:4:1650:14678:20369_2:N:0:TCTGCAAG+GCTGCACT
TTTCAGGACCTAATCACATCATGATTGATAGTTACGGCTCGTATCGAGTTGGAGGGAGGAAGTTCTCC
GAAATTAAAGACGGGGGGATTAAGAGTCCAGCTGTAACTGTCATTTCA
+
::FFFFFFFFFFFFFFFFFFF:FFFFFFFFFFFFFFFF:FFFFFFFFFFFFFFFFFFF:FFFFFFFFF
FFFFFFFFFFFFFFFFFFF:FFFFFFFFFFFFFFFFFFFFFFFFFFFF
@A00721:190:HGJW7DSXY:4:1471:17110:25676_2:N:0:TCTGCAAG+GCTGCACT
AAGGAAACTTCGTCAGAAGATGAGGAAGTCTAAGAGGAGGAGTTTGAGGAAGGCTCTAGAGAAGATCT
CACCACCTGGAGAAGGCAAGTGGAGAGTCCAGAAGTTGATGAAGAAGG
+
FFFFFFFFFFFFFFFFFFFFFFFFFFFFFFFFFFFFFFFFFFFFFFFFFFFFFFFFFFFFFF,FFFFFF
FFFFFFFFFFFFFFFFFFFFFFFFFFFFFFFFFFFFFFFFFFFFFF:FF
```

```
@A00721:190:HGJW7DSXY:4:2369:18394:23547_2:N:0:TCTGCAAG+GCTGCACT
CAGGGGTTACCACTTCCTAAGGTGGATGAGGGAGGTTGTTTCTTTGGAGTTTTAGATAATCTTTTGAG
AAGGGGTCATAGAGCCAGCAAGAGAAGCTTATATGCTGGTTTAAGTGA
+
FFFFFFFFFFFFF:F:FFF:FFFFFFFF:FFFFFFFFFFFFFFFFFFFFFFFFFFFFFFF::FFF,FF
FFFFF:FF:FFFFFFFFFFFFFFFFFFFFFFFFF:FFFFFF:FFFFFF
@A00721:190:HGJW7DSXY:4:2472:1108:25269_2:N:0:TCTGCAAG+GCTGCACT
CCTTGAGAAGACAAGCCACATTAAGATATAATTTAGGTCAACTTGGGGTTTCGAATGTCATTAGTGCC
TTACGATTGAAAGAGGTCCTTTTGAGTATCGACAGATCTTTCGATCTT
+
FFFFFFFFFFFFFFFFFFFFFFFFFFFFFFFFFFFFFFFFFFFFFFFFFFFFFFFFFFFFFFFFFFFF
FFFFFFFFFFFFFFFFFF:FFFFFFFFFFFFFFFFFFFFFFFFFFFFF
@A00721:190:HGJW7DSXY:4:2664:4598:12524_2:N:0:TCTGCAAG+GCTGCACT
GTGGCAGAATTCGGTAACTTACCATATTGTTCCTTCCGGGAAGTTGTGGGATTTAACTTTGACTCAAC
TACTCCGATCAGATCCGGATGGTTTGATTGGTATGGACTACTCGACTT
+
FFFFFFFFFFFFFFFFFFFFFFFFFFFFFFFFFFFFFFFFFFFFFFFFFFFFFFFFFFFFFFFFFFFF
FFFFFFFFFF:FFFFFFFFFFF,FFFF,FFFFFFFFFFFFFFFFFFFF
@A00721:190:HGJW7DSXY:4:1463:30120:4679_2:N:0:TCTGCAAG+GCTGCACT
TCATGGCAGATACTACCGATAAGGTGAAAAATATGCAGGTCAAAGATTCTGTAGCTCTGAGGGCGGTT
GAGGGAGCCGTTTTGGCCGACTTTAACTATTCTAAGAGTCTTTATGAT
+
FFFFFFFFFFFFFFFFFFFFFFFFFFFFFFFFFFFFFFFFFFFFFFFFFFFFFFFFF:FFFFFFFFFF
F:FFFFFFFFFFFFFFFFFFFFFFFFFFFFFFFFFFFFFFFFFFFFFF
@A00721:190:HGJW7DSXY:4:1322:11198:22013_2:N:0:TCTGCAAG+GCTGCACT
CGTTGTCGTACCTTACCAGGTTGAGGAGACTTTACAGCGGCTTATTGTCGCGCAGTTTACAGAATTGC
GGTTGAAGTTCACCCATTCTATCTTGAATGCTCACGCCGTCGCAGCCA
+
F:,FF,FFFFF:,FFF,F,:,FFFF:FFF:,F:FFFFFF,FF:F:FFFF::FFFFFFF,:FFF:,FFF
FFF,::FFFFFFFF,F,FFFFFFFFFFFF:FFFFFFFF:FFFFFF:FF
@A00721:190:HGJW7DSXY:4:1564:8043:35556_1:N:0:TCTGCAAG+GCTGCACT
ATCACTTGTTAAAGTACAGATTTAGTGATTTAGATATTATTGATATTGGAGGCGATATTCGTTTTCAT
ATCCAAAACTCCTTGAATGGAGTACATATTTGCAACCCTATTCTTGATGTGAAAGATGCATCGAGGTA
TAC
+
FFFFFFFFFFFFFFFFFFFFFFFFFFFFFFFFFFFFFFFFFFFFFFFFFFFFFFFFFFFFFFFFFFFF
FFFFF::FFFFFFFFFFFFFFFFFFFFFFFFFFFFFFFFFFFFFFFFFFFFFFFFFFFFFFFFFFFFF
FFF
@A00721:190:HGJW7DSXY:4:1344:25681:29465_2:N:0:TCTGCAAG+GCTGCACT
TTGATTGGGAACGTACCTACTCACGAGACGTCATGAAAGCTATCGGAGGGAAGCAAAATGTTTCGTTC
TGTTACAAGAACGCTTTGGATTGTGACCATAAAGCCTCAGTTGCGATA
+
FFFFFFFFFF,FFFFFFFFFFFFFFFFFFFFFFFFFFFFFF,FFFFFFFFFFFFFFFFFFFFFFFFFF
FFFFFFFFFFFFFFFFFFFFFFFFFFFFFFFFFFFFFFFFFFFF:FFF
@A00721:190:HGJW7DSXY:4:2270:17653:5666_1:N:0:TCTGCAAG+GCTGCACT
CTCTCTTGATGATGTAGCCACTATAATGCTTAAGAGAGAGATTGATGTATGTCATTTCGTCTTAGTTT
GTCCCGGCGAATTATTGGACACGATGAAAAATGACATACCCGTTCCTGACCTTGGTATCGTTATCCGT
CAT
+
FFFFFFFFFFFFFF:FFFFFFFFFF:FFFFFFFFFFFFFFFFFFFFFFFFFFFFFFFFFFFFFFFFFF
FFFFFFF:FFFFFFFFFFFFFFFFFFFFFFFFFFFFFFFFFFFFFFFFFFFFFFFFFFFFFFFFFFFF
F:F
@A00721:190:HGJW7DSXY:4:1541:7048:5337_1:N:0:TCTGCAAG+GCTGCACT
GGTTATTCTCACAACAAAGATAAGATTTTATCTTTCTTCAAACACCCTGTTTTCCTTAAGGGCGGACA
```

```
ATTGTTCCATTGTGAACTCTTGGGGACTAGATTGAGCATTAATGAGTTCCGAATAACTCGTAATAAAT
CTT
+
FFFFFFFFFFFFF:FFFFFFFFFFFFFFFFFFFFFFFFFFFFFFFFFFF:FFFFFFFFFFFFFFFFFF
FFFFFFFFFFFFFFFFFFFFFFFFFFFFFFFFFFFFFFFFFF:FFFFFFFFFFFFFFFFFFFFFFFFFF
FFF
@A00721:190:HGJW7DSXY:4:1649:28565:28729_1:N:0:TCTGCAAG+GCTGCACT
ACCTATTTCCGTACCAAGAGCCACTGCCAATCTCATCGCTATACGCATACCTGATTGGAAGAAGCGAG
GTTCGTTCCTCAATAAAAATTTTTCTCAAGAGATTATGGTTGATAAAGTTTTCTTCATAAGGGCTATT
CAG
+
FFFFFFFFFFF:FFFFFFFFFFFFFFFFFFFFFFFFFFF:FFFFFFFFFFFFFFFFFFFFFFFFFFFFF
FFFFFFFFFFFFFFFFFFFFFFFFFFFFFFFFFFFFFFFFFFFFFFFFFFFFFFFFFFFFFFFFFFFFF
FFF
@A00721:190:HGJW7DSXY:4:1572:25780:15295_1:N:0:TCTGCAAG+GCTGCACT
CTTCGGTCAATGACAAAACCTTCGAATATGTTACGACATGGCTTCGTAACAACAGTGCGAGGATTGTC
ATCTCGGGGAAAGTGATTCACCAAGATGTTCATATAGATACGAGATACCTAGAGAAGGTCGCAGCGAT
CTT
+
FFFFFFFFFFFFFFFFFFFFFFFFFFFFFFFFFFFFFFFFFFFFFFFFFFFFFFFFFFFFFFFFFFFFF
FFFFFFFFFFFFFFFFFFFFFFFFFFFFFFFFFFFFFFFFFFFFFFFFFFFFFFFFFFFFFFFFFFFFF
FFF
@A00721:190:HGJW7DSXY:4:1456:17897:15577_2:N:0:TCTGCAAG+GCTGCACT
AGCTAAGAAGAAGACTTTGGGTACACGTTATGCTCGCAAACTTAACGTTAGTACCGGAGGTGATCTCT
CTGCTTCTGCGTTACTGAGAGAAAAGATTCAGGACGCCTACGGTGACT
+
FFFFFFFFFFFFFFFFFFFFFFFFFFFFFFFFFFFFFFFFFFFFFFFFFFFFFFFFFFFFFFFFFFFFF
FFFFFFFFFFFFFFFFFFF:FFFFFFFFFFFFFFFFFFFFFFFFFFFF
@A00721:190:HGJW7DSXY:4:1344:10031:18865_1:N:0:TCTGCAAG+GCTGCACT
CGTGTGCTCTTCCGATCTAACGAATCTTGAAACTACGGTCCTTATCAAAGAAAATGGTGAACCGGTCT
CTAAATCTTTTGAGATTGAGAAGATTGAAGAACACGTCAAGAAGTCGCTCGGCGTTGTTCTTGAAAAC
CAA
+
FFFFFFFFFFFF,FFFFFFFFFFFFFFFFFFFFFFFFFFFF:FFFFFFFFFFFFFFFFFFFFFFFFFFF
FFFFFFFFFFFFFFFFFFFFFFFFFFFFFFFFFFFFFFFFFFFFFFFFFFFFFFFFFFFFFFFFFFFFF
FFF
@A00721:190:HGJW7DSXY:4:1638:32994:8469_1:N:0:TCTGCAAG+GCTGCACT
GCTGATCATGGAGTCTTTGTACCTTCTGTCAAACTTCGAGGTTCAGCTACGAGAGGTGATCCNNGATG
TGGATTGAAGGCTGGCTCTAAGGGAGACTCCTTCTTTTT
+
FFFFF:FF,FFFF,FFFFF:FFFFFFFF,FFFFFFFFFFFFFFFFFFFFFFFFFFFFFFFFF##FFFF
FFFFFFFFFFFFFFFFFFFFFFFFFFFFFFFF:FFFFFF
@A00721:190:HGJW7DSXY:4:2665:27001:20509_1:N:0:TCTGCAAG+GCTGCACT
AGAATTGGGTTGAGAAGTGTTCTTTACAAGGATTTAGACTCTTCTTCAAGTTTTTAGATCTTCTTGAT
ATTCTTAAAGAAAAATTTTCGAATAAACTAATGAGAGGTATAGGCTTATTCAAGTCTGTACTTTCAT
+
FF,F:FFF,F:FF:FFFFFF,,F:F:,,FFFF,:FFFF:FFFF:FF,,F,FFFFFFFFF,FF:FFFFF
F,F:FFFF:FFF,F,FF:FFFFFFFF,:FF:FFFFFFFFFFFFF:FFF,FFF:FF,::FFFFF,FFF
@A00721:190:HGJW7DSXY:4:2319:8757:12305_1:N:0:TCTGCAAG+GCTGCACT
GTTTCTCTTGGTATTTCTGAAATAGTTTATCAGAGCGTCATTTTCCTTTGGGATCTTTCCTCTGGTAA
GGGAATTATTAAATCATTCTTCTCACTCTTGACGAGAATTATTACTGTTTTCTCTACCCATAACTTCT
TTT
+
FFFFFFFFF:FFFFFFFFFFFFFFFFFFFFFFFFFFFFFFFFFFFFFFFFFFFFFFFFFFFFFFFFFFF
```

FFFFFFFFFFFFFFFFFFFFFFFFFF:FFFFFFFFFFFFFFFFFFFFFFFFFFFFFFFFFFFFFFFFFF
FFF
@A00721:190:HGJW7DSXY:4:2574:14669:9173_1:N:0:TCTGCAAG+GCTGCACT
ACTGGAGTTCAGACGTGTGCTCTTCCGATCTGTTTCAACACTTATCACCACTTTTGAAAGGAAAATAA
TTGGTGATGAAAATACGTATGTCGGTGGGGTTCTCAAGGCTCTCATCGCCACTGGGATTCTAAGGGGT
TCC
+
FFFFFFFFFFFFFFFFFFFFF:FFFFFFFFFFFFFFFFFFFFFFFFFFFFFFFFFFFFFFFFFFFFFF
FFFFFFFFFFFFFFFFFFF:FFFFFFFFFFFFFFFFFFFFFFFFFFFFFFFFFFFFFFFFFFFFFFFF
FFF
@A00721:190:HGJW7DSXY:4:1262:20916:34867_2:N:0:TCTGCAAG+GCTGCACT
AGGATTCTTCTTTTGGTCCTTACGTCTTAAGTAATTTGGATGTTGTCGAAAACTATCAGGTTCTTGTC
ACGATTCTTAAAAGAAGTTTATTAAAACTTCAGAGTCTCTTGGAAGTC
+
FFFFFFFFFFFFFFFFFFFFFFFFFFFFFFFFFFF,FFFFFFFFFFFFFFFFFFFFFFFFFFF:FFFFF
FFFFFFFFFFFFFFFFFFFFFFFFFFFFFFFFFFFFFFFFFFFFFFFF
@A00721:190:HGJW7DSXY:4:1454:32913:29465_2:N:0:TCTGCAAG+GCTGCACT
TGTTCTTGCAATAGCCACAGACTTTCTGGGAAAAGAGACCTCTGTTGGTAGAGGAGTTGAGAGAATTA
CTCAGATTCAATCTGGCATCACTGAACGTCTACAACGAGTTAGGGAGT
+
FFFFFFFFFFFF:FFFFFF:FFFFFFF:FFFFFFFFFFFFFFFFFFFFFFFFFFFF:FF,FFFFFFFFF
FFFFFFF:FFFFFFFF:FF,::FF:FFF:FFFFFF:FFFFFFFFFFFF
@A00721:190:HGJW7DSXY:4:1305:18927:6402_1:N:0:TCTGCAAG+GCTGCACT
AAAAGATCCTTTGCACAAGCAATACCTTCCACTGAGTTCATCACCCAGAGTTTCGACGTCGCGGAAAA
CGATGAAGATTTCCACACTGTTCGAGAATCTTCTGATGCTGAATTTGAGGAAAATTTTTCGGAAGTTT
TTG
+
FFFFFFFFFF:FFFFFFFFFFFFFFFFFFFFFFFFFF:FFFFFFFFFFFFFFFFFFFFFFFFFFFFFFF
FFFFFFFFFF:FFFFFFFFFFFFFFFFFFFFFFFFFFFFFFFFFFFFFFFFFFFFFFFFFFFFFFFFFF
FFF
@A00721:190:HGJW7DSXY:4:1121:27073:7952_1:N:0:TCTGCAAG+GCTGCACT
TGATGGCTCAGTACAGGGAGGAGGTAATAGTTATAAAACCTTCTTTTATCATTGCAACAGGACTTTGA
GAAGACTGTTGAAATGGACAAAATCTGGGATCATAGGTATGTGTCACGCTACACTCGAAATATGGGCC
CTC
+
FFFFFFFFFFF:FFFFFFFFFFFFFFFFFFFFFFFFFFFFFFFFFFFFFFFFFFFFFFFFFFFFFFFFF
FFFFFFFFFFFFFFFFFFFFFFFFFFFFFFFFFFFFFFFFFFFFFFFFFFFFFFFFFFFFFFFFFFFFF
FFF
@A00721:190:HGJW7DSXY:4:1617:6451:11130_2:N:0:TCTGCAAG+GCTGCACT
GTTGGCGTCTTGTCGTTGACGTCATTACAGTCTGGAAGTTAGATCATTTGTTCAGGAAGATCTCTACA
GAGATTATCGAGAGTTGTGATCTAGTTCTAAATTTAGGTCGACGGACT
+
FFFFFFFFFFFFFFFFFFFFFFFFFFFFFFFFFFFFFFFFFFFFFF:F:FFFFFFFFFFFFFFFFFFFF
FFFFFFFFFFF:FFFFFFFFFFFFFFFFFFFFFFFFFF:FFFFFFFFFF
@A00721:190:HGJW7DSXY:4:2616:2211:3067_1:N:0:TCTGCAAG+GCTGCACT
TCTGACCATCGCAAAGAAGACTTTATTAGTTCATGAAGTTCTTACACTTCTCGCGACTAATGTCAGCT
TCACTACAAAGGCGTTACGTAAAGTAGTTTTCACGCTTTATCTTAAAGCATTACCACCAAATTTAGCA
CTT
+
FFFFF:FFFF,,,FFFFFF,:FFFFFF:FFF,FFF,FFFFF:FFF,FFF:FF,:FFFF:FFFF:F,FF
FFFFFFFFF::F,,FFFFFFFFF:FF:FFFFF:::FFFFFFFFFFF:,:FFFF,,FF,FFFFFFF::F
:FF
@A00721:190:HGJW7DSXY:4:1365:13747:1157_2:N:0:TCTGCAAG+GCTGCACT
TCGCCATATCAGATTAGAGACATGCCAGTTAGAGTTCTTCTAACCAGGACTGATGGTGAGATTCTTTT

```
ACCTTTTGGAGGTCCAGGAACCTTTACTCCTAGTGTCTTTTCTAGAGT
+
FFFFFFFFFFFFFFFFFFFFFFFFFFFFFFFFFFFFFFFFFFFFFFFFFFFFFFFFFFFFFFFFFFFF
FFFFFFFFFFFFFFFFFFFFFFFFFFFFFFFFFFFFFF:FFFFFFFFF
@A00721:190:HGJW7DSXY:4:1616:20745:30780_2:N:0:TCTGCAAG+GCTGCACT
GTCGGAGGATGACAGGTGGGAGACCGCTCAATAACACTCTATTGAATCCCTTTTCCACGATGAAAGCT
CTTTATGAGGTGCTCCATGCAAATTTACAGTTCATCTTTGGTGATATC
+
FFFFFFFFFFFFFFFFFFFFFFFFFFFFFFFFFFFFFFFFFFFFFFFFFFFFFFFFFFFFFFFFFFFF
FFFF:F::FFFFFFFFFFF:FFFFFFFFFFF,:,FFFFF:FF:FF::F
@A00721:190:HGJW7DSXY:4:1339:12906:6057_2:N:0:TCTGCAAG+GCTGCACT
TTCTCCTTTAATCGTGAGGTTACGAGAGTTTCGTACTTGGGTTTATCGGTTGTATTTTGATAAATTTG
TTGGAATCGATAATGATACTGACGAAGGTTCTATTACCACCCTTGTCA
+
FFFFFFFFFFFFFFFFFFFFFFFFFFFFFFFFFFFFFFFFFFFFFFFFFFFFFFFFFFFFFFFFFFFF
FFFFFFFFFFFFFFFFFFFF:FFFFFFFFFFFFFFFFFFFFFFFFFFF
@A00721:190:HGJW7DSXY:4:1159:17680:14418_1:N:0:TCTGCAAG+GCTGCACT
GTCTAGCCGCTGAGATTCGTCTTACCTATTTACAATCTGTAGAGAGAGAGGTTAATCAGAATTTGAGT
GATAACGATTTAGTACTGGGAAATGGTGGTAGTCAGTTTACTACTTTTCTTTCCCTCCCCTTTTTCGA
TTG
+
F:F:FFFFFFF:FFFFFFFFFFFFFFFFFFFFFF:F::FFFFFF:FFFFFFFFFFFFFFFFFFFFFFF
FFFFFFF:FFF:FFFFFFFFFFFFFFFFFFFF,FFFFFFFFFFFFFFFFF:FF:FF:FF:FFF:FFFF
FFF
@A00721:190:HGJW7DSXY:4:1365:16062:3317_2:N:0:TCTGCAAG+GCTGCACT
AGGTTTTTCTTTTATGGGACTCTTGTTTGTACTTATGAAGGAGGTATTACTTCGGAAGTTCTTAATGT
AGGTCTCCTAGTACTTAAGGGTTTATGTTTCGGTTTCAGCTGGATGGA
+
FFFFFFFFFFFFFFFF:FFFFFFF:FFFFFFFFFFFFFF,FF,FFFFFF:FFFFFFFFFFFFFFFFFF
FF,FFFFFFFFFFFFFFFFFF,FFFFFFFFFFFFFFFFFFFFFFFFFF
@A00721:190:HGJW7DSXY:4:2369:30147:35634_1:N:0:TCTGCAAG+GCTGCACT
TTCACATTGCGTGATCACTTCCCGTGGACTTGGGAAGAGATTAAACATAAAATCCAGCGATTCTATTA
TGTCGCTAAAGCCTGTCCAGTTGTCGATGATGTTCGGGCTGTGGGGATTAACTTCCCTCGTGAAACTT
ACG
+
:FFFFFFFFFFFFFFFFFFFFFFFF::::FF:,FFF,F,FFFFFF::FFFFFFF:FF:FFFF,F:FF,
FFFFFFFF,FFFFFFF:FFFFFFF:FF,:FFFFFFFFFFFFFFFFFFFF,FFFF:FFFFFF,:,:F:F
F,F
@A00721:190:HGJW7DSXY:4:1522:11406:32737_1:N:0:TCTGCAAG+GCTGCACT
CACAAGGATTACTAAGTCTGAGATCTGCCACTTCTAATTTTAGGAAACGTCGCGAAAAGAAAGTTCAT
GAGGATGTCTTGGTCGAAAAATATCGCCGGGAACTTCTAATAGAGGATCCTGATGACATTATTTCAAC
GGA
+
::FFF:FFFFFFFFFFFFFFFFFFFFFFFFFFFFFFFFFFFFFFFFFFFFFFFFFFFF:FFFFFFFFF
FFFFFFFFFFFFFFFFFFFFFFFFFFFFFFFFFFFFFFFFFFFFFFFFFFFFFFFFFFFFFFFFFFFF
FFF
@A00721:190:HGJW7DSXY:4:2448:13223:31563_2:N:0:TCTGCAAG+GCTGCACT
AGTTCTACCACAGAAACCGTCTCAGGAACTGTCTCCACTGATTCGGAATTAGAGAAAGACGACCTTCT
TGTGGGGACTGAGACCTCTGAATTTTCAGGGTGCCCTGATGAGGACGT
+
FFFFFFFFFFFFFFFFFFFFFFFFFFFFFFFFFFFFFFFFFFFFFFFFFFFFFFFFFFFFFFFFFFFF
FFFFFFFFFFFFFFFF:F:FFFFFFFFFFFFFFFFFFFFFFFFFFFFF
@A00721:190:HGJW7DSXY:4:1627:13648:5619_1:N:0:TCTGCAAG+GCTGCACT
ACTCTGAGACTTCTAAGAGTGTTTCAAAAGACCAGAAACGTAACAAGAAGAATAAAGGTAGGAAGGGG
```

AGAAAACAGAACAAGCGAGCGGTTGAAGAGAAAACTGATATGGTTGCGGCTGATCGTACCCCAATTCC
TGC
+
FFFFFFFFFFFFFFFFFFFFFFFF,FFFFFFFFFFFFFFFFFFFFFFFFFFFFFFFFFFFFFFF:FFFF
FFFFFFFFFFFFFFFFFFFFFFFFFFFFFFFFFFFFFFF:FFFF:FFFFFFFFFFFFFFFFFFFFFFFF
FFF
@A00721:190:HGJW7DSXY:4:1553:28393:18693_2:N:0:TCTGCAAG+GCTGCACT
CTCGGCGATACCACTGATCTTCCAGAGTTACGTAGTGGTCGTTCTTTTGGTGATTTTATCAAAGAGAA
AGTTTCTGCTAGTCGCGACGATAGTATTTATCGGAGTGATATTAACAA
+
FFFFFFFFFFFFFFFFFFFFFFFFFFFFFFFFFFFFFFFFFFFF,FFFFFFFFFFFFFFFF:FFFF,
FF,FFFFFFFFF:FFFFFFFFFFFFFFFFFFFFFFFFFFFFFFFFFFF
@A00721:190:HGJW7DSXY:4:1355:8061:9815_2:N:0:TCTGCAAG+GCTGCACT
CACTTACAAGAGTGTTGAAGTACCGCAACATGGATCAGTTTTCTAAAAAGCTATTAATAGCAGTTGCG
GGGCTATAATACCGCCTCCAGATTACTCTAAACTGAATCCACTCTCTT
+
FFFFFFFFFFFFFFFFFFFFFFFFFFFFFFFFFFFFFFFFFFFFFFFFFFFFFFFFFFFFFFFFFFFF
FFFFFFFFFFFFFFFFFFFFFFFFFFFFFFF:FFFFFFFFFFFFFFFF
@A00721:190:HGJW7DSXY:4:2602:27850:32189_2:N:0:TCTGCAAG+GCTGCACT
TTTGGAACTGTGTAATGTCCTACGTACTGTTCAAGGTGTTTGGGCTTCTCTAAAGAACAGCAACTTTC
AACATGAATTAGTTATTGAAAGTCTTGACCCTCACGTCTCACTTTATC
+
FFFFFFFFFFFFFFFFFFFFFFFFFFFFFFFFFFFFFFFFFFFFFFFFFFFFFFFFFFFFFFFFFFFF
FFFFFFFFFFFFFFFFFFFFFFFFFFFFFFFFFFFFFFFFFFFFFFFF
@A00721:190:HGJW7DSXY:4:1433:28519:26929_2:N:0:TCTGCAAG+GCTGCACT
AAAGAGGGTTTGAAAATTACGGAACCTTTCCTCGTTCTTCATGAAGATCTTCGAGTGTTTTATAGTGA
GTTATTCTTGCGAAGGTTCAAGTCGATTGATTTTGTTGACTACGATCT
+
FFFFFFFFFFFFFFFFFFFFFFFFFFFFFFFFFFFFFFFFFFFFFFFFFFFFFFFFFFFFFFFFFFFF
FFFFFFFFFFFFFFFFFFFFFF:FFFF:FFFFFF:FFFFFFF:FFFFF
@A00721:190:HGJW7DSXY:4:1244:15745:8375_1:N:0:TCTGCAAG+GCTGCACT
AGAAGTTCTTCGCAGTTGAATCTCCACCAGGTGGAGGGAAGACCACTGAACTTATAGCGACTTTTGTC
GAATTTTTCGCAAAAGGTCTTGAAGTACGTATTGTCACTGCTAATCGTAATTCGCGAGAAGAGATAGT
GAC
+
FFFFFFFFFFFFFFFFFFFFFFFFFFFFFFFFFFFFFFFFFFFFF:FFFFFFFFFFFFFFFFFFFFFF
FFFFFFFFFFFFFFFFFFFFFFFFFFFFFFFFFFFFFFFFFFFFFFFFFFFFFFFFFFFFFFFFFFFF
FFF
@A00721:190:HGJW7DSXY:4:2104:10872:28056_2:N:0:TCTGCAAG+GCTGCACT
CACTTCTAGTTCATCGCGGTATTTGTGAAATTAAGACAGACGTGAAAGTTATGATGAGTGATCGTATT
AGAACAGCTGATTCTTATTGCATGAACGTCGCTGAGGTCAAACCTGTG
+
FFFFFFFFFFFFFFFFFFFFFFFF:FFFFFFFFFFFFFFFFFFFFFFFFFFFFFFFFFFF:FFFFFFFF
FFFF:FFFFFFFFFFFFFFFFFF:FFFFFFFFFFFFFFFFFFFFFFFFF
@A00721:190:HGJW7DSXY:4:1146:19217:1673_1:N:0:TCTGCAAG+GCTGCACT
CGACGAATTATTCATGATTCACGCTGGTCAGACCCTTTTTCTTACACATCTACTTAAGGCGGCTTATA
CCATAGGTTACGGTGATTCCAATCAAATCGGATTCATCAATCGTATTGATCTCCAAGTCGCAAACTAC
TTC
+
FFFFFFFFFFFFFFFFFFFFFFFFFFFFFFFFFFFFFFFFFFFFFFFFFFFFFFFFFFFFFFFFFFFF
FFFFFFFFFFFFFFFFFFFFFFFFFFFFFFFFFFFFFFFFFFFFFFFFFFFFFFFFFFFFFFFFFFFF
FFF
@A00721:190:HGJW7DSXY:4:1127:16396:22874_2:N:0:TCTGCAAG+GCTGCACT
GATGAGTGTCGTTCTTATCGAAGTCTCAGTTATCGCTGCCCGGCCGACGTTTGTCGCGAACTCTCAGA

```
GGTTTACGGCAGAAAAATTTCTCCTGCTTGTAATTCGAGAGAGGACAC
+
FFF:FF,:F:F:,F,FFFFFFFFFF:,FF,:F,,::,:FFFFFFF,FFF,F,FFF,FFFFF,FFFF:F
F:FFFFF:,,:FFFFFF:,F:F:,F:F,F,FF:FF,FF,FFF:,FFFF
@A00721:190:HGJW7DSXY:4:1509:28655:9784_1:N:0:TCTGCAAG+GCTGCACT
CTTGACGACTTCGAATTTGAGGACGGAGTTAAATATATTACTTTCACTCAAGGTGAAAAGTTTGAAAT
TAACAAAGTCCTTGGTAAGAGTAAGGTTAAATGTGAAGATGCTCAGACTATTCATGAAGTTCAAGGTA
ATA
+
:FFFFFFFFFF:FFFFFFFFFFFFF:FFFFFF:FFFFFFFFFFFFFFFFFFFFFFFFFF:FFFFFFFFFF
FFFF:FFFF:FFFFFFFFFFFFFFFFFFFFFFFFFFFFFFFFFFFFFFFFFFFFFFFFFFFFFFFFFFFF
FFF
@A00721:190:HGJW7DSXY:4:2320:3215:30326_1:N:0:TCTGCAAG+GCTGCACT
TGACCCTGTTTTTGCATCACAAACACATATTACCGTTGCTCTATCACGACACGTTGAATCTTTACGAT
ACTTTGTTATCAATGTAAAGTTAAACGATGCTACTTGTGAGAGGATCAATCACAGTAAGGAACTGTCA
CGT
+
FFFFFFFFFFFFFFFFFFFFF,FFF:FFFFFFFFFFFFFFFFFFFFFFFFFFFFFFFFFFFFFFFFFFFF
FFFFFFFFFFFFFFFFF:FFFFFFFFFFFFFFF:FFFFFFFFFFFFFFFFFFFFFFFFFFFFFFFFFFFF
FFF
@A00721:190:HGJW7DSXY:4:1105:8250:4476_2:N:0:TCTGCAAG+GCTGCACT
GATTCAACCCCTTATATCGAGGCTATCGAGGACGATGAGTATCTTGAAGTTGTTGACGACAAGCCTCA
AGAGGGTAGAGGACCACCTGGATCTTCTACTGTCTCTGCCTTCCAAGA
+
FFFFFFFFFFFFFFFFFFFFFFFFFFFFFFFFFF::FFFFFFFFFFFFFFFFFFFFFFFFFFFFFFFFFF
:FFFFFF:FFFFFFFFFFFFFFFFFFFFF:FFFFFFFFFFFFFFFFFF
@A00721:190:HGJW7DSXY:4:2158:17987:9784_2:N:0:TCTGCAAG+GCTGCACT
TTATTCCTGGAACCACCTTAACGTCACTCGGTAATCTTTCAGCCGAATTAAGTACTAATGAGTTCAAG
TCGGTTGTCGATCGAATTAAGATCAAGGCTAATACCTTTATAGGTAAG
+
FFFFFFFFFFFFFFFFFFFFFFFFFFFFFFFFFFFFFFFFFFFFFFFFFFFFFFFFFFFFFFFFFFFFFF
FFFFFFFFFFFFFFFFFFFFFFFFFFFFFFFF:FFFFFFFFFFFFFFF
@A00721:190:HGJW7DSXY:4:1527:23511:11647_1:N:0:TCTGCAAG+GCTGCACT
CCCGATAGGGTACCGAGTCTCCAAGAGAATATTTATTCTTATGAGGCTCGAAACTTCAACGTTTTCAC
AACGGAAAGACATATCGCACCCGAAATTTTTGGGCAGAATGCTCTTAACGCGGTTCTTGAACGGTGTT
TTG
+
FFFFFFFFFFFFFFFFFFFFFFFFFFFFFFFFFFFFFFFFFFFFFFFFFFFFFFF:FFFFFFFFFFFFFF
FFFFFFFFFFFFFFFFFFFFFFFFFFFFFFFFFFFFFFFFFFFFFFFFFFFFFFFFFFFFFFFFFFFFFF
FFF
@A00721:190:HGJW7DSXY:4:1268:18728:36182_1:N:0:TCTGCAAG+GCTGCACT
GATAAAGTTATAGGAGACTCAGTTAACCTGAGAGCTCCTCAATTGATAGATTGGGTCAACAATAGGGA
CGCTAGTATACTTCGTAAAGTCGACAGCGAAATGGTCAATATTGATAATTGGACCGACTATATGACCT
ATT
+
FFFFFFFFFFFFFFFF,FFFFFFFFFFFFFFFFFFFFFFFFFFFFFFFFFFFFFFFFFFFFFFFFFFFFF
FFFFFFFF:FFFFFFFFFFFFFFFFFFFFFFFFFFFFFFFFFFFFFFFFFFFFFFFFFFFFFFFFFFFFF
F:F
@A00721:190:HGJW7DSXY:4:2164:28592:31000_1:N:0:TCTGCAAG+GCTGCACT
AAGGGAAGCTAAGGTTAAACTTGATGCTTCAGCCTTTTCGAAGCATAACCCCGCACAGAATATCGTTT
ATCACGCAAAATTTATTAATGCAATTTTCGGACCTATCTTTAAGGTTCTTGTGAGGAGAGTCAAACAG
TGT
+
FFFFFFFFFFFFFF:FFFFFFFFFFFFF:FFFFFFFFFFFFFFFFFFFFFFFFFFFFFFFFFFFFFFFFF
```

FFFFFFFFFFFFFFFFFFFFFFFFFFFFFFFFFFFFFFFFFFFFFFFFFFFFFFFFFFFFFFFFFFFF
FFF
@A00721:190:HGJW7DSXY:4:2160:7039:30279_1:N:0:TCTGCAAG+GCTGCACT
ACATTTTTATGTATACAGAGATGACCAGTGACGATTTCTCCCGGAAGCTCTTCTCTCTCTTAGGAGGT
CATGATGACTACTATAAGGGTGAAATCGATTTTTCGAAGTTTGACAAATCTCAAGATTTCTTTTGTAA
AGC
+
FFFFFFFFFFFFFFFFFFFFFFFFFFFFFFFFFFFFFFFFFFFFFFFFFFFFFFFFFFFFFFFFFFFF
FFFFFFFFFFFFFFFFF:FFFFFFFFFFFFFFFFFFFFFFFFFFFFFFFFFFFFFFFFFFFFFF:FFF
F,F
@A00721:190:HGJW7DSXY:4:1307:21386:17801_1:N:0:TCTGCAAG+GCTGCACT
ATTTAGGCTTCGATGCTGAATTTCTTGAACTTTGGAGTGCCAGTGAATATTTTTGTCACGCCAGGAGT
ATCCATAACGATTTGTCATTTACCACCTTTGCTCAACGACGAAGTGGAACGGCCAACACTTTCTTTGG
TAA
+
FFFFFFFFFFFFFFF:FF:FFFFFFFFFFFFFFFFFFFFFFFFFFFFFFFFFFFFFFFFFFFFFFFFF
FFFFFFFFFFFFFFFFFFFFFFFFFFFFFFFFFFFFFFFFFFFFFFFFFFFFFFFFFFFFFFFFFFFF
FFF
@A00721:190:HGJW7DSXY:4:1376:9182:29356_1:N:0:TCTGCAAG+GCTGCACT
TCTGATGATTTTATCACTTTATTATGACCTTGGAAAGTTTTCGGCTGTGGTTGTCGCTGGGGACGATT
CCATTCTGTTTTCAAAGACTCCAATAACTGATCATTCTGATCATATGATTTTGGACAATGGTTTCGAA
GCG
+
FFFFFFFFFFFFFFFFFFFF:FFFFFFFFFFFFFFFFFFFFFFFFFFFFFFFFFFFFFFFFFFFFFFF
FFFFFFFFFFFFFFFFFFFFFFFFFFFFFFFFFFFFFFFFFFFFFFFFFFFFFFFFFFFFFFFFFFFF
FFF
@A00721:190:HGJW7DSXY:4:2615:16495:9987_2:N:0:TCTGCAAG+GCTGCACT
CAGCGTACTTCTGCTCGCGATTTATTCTTTTTGTCAATGATTACATCTACTTCGTTCCTGATCCTTAC
AAGATCATCGTTAAGTTAGGTAAACCCCTAACCGATGTTACTCTTGTA
+
FFFFFFFF:FFFFFFFFFFFFFFFFFFFFFFFFFFFFFFFFFFFFFFFFFFFFFFFFF:FFFFFFFFFF
FFFFFFFFFFFFFFFFFFFFFFFFFFFFFFFFFFFF:FFFFFFFFFFF
@A00721:190:HGJW7DSXY:4:1118:18611:21637_2:N:0:TCTGCAAG+GCTGCACT
TTAAAGACTATACTAAATATCTCCACTACGAGGTTGTTGTTTGGCGATTAGCTCTTGCGACTAATATT
CGATATTCGATTGAGGGTCGTTATACCTATTCGGCTATCTGTGCTTTG
+
F:FFFFFFFFFFFFFFFFFFFFFFFFFFFFFFFFFFF:FF,FFFFFF:FF,FFFFFFFFFFFFFFFFF
F:FFFFFFFFFFFFFFFFFFFFFFFFFFFFFFFFFF:FFFFFFFFFFF
@A00721:190:HGJW7DSXY:4:1544:1425:16799_2:N:0:TCTGCAAG+GCTGCACT
TCAAATTTTAAACGATTCCGAGAGGTTTATCCTGATGTGGAGGGAGTTCTTGAAGCATCCCGACCTTT
CCTTGTGAAAATTTTGATGCGGTTTCATAAGATTATCAGTAATCCTGA
+
FFFFFFFFFFF:FFFFFFFFFFFFFFFFFFFFFFF:FFFFFFFFFFFFFFFFFFFFFFFFFFFFFFFFF
FF:FFFFFFFFFFFFFFFFFFF,:FFF,FFFFFFFFFFFFFFF:FFFF
@A00721:190:HGJW7DSXY:4:1505:17173:19366_1:N:0:TCTGCAAG+GCTGCACT
ATTCATGGACCTTCCATCCTGTACCTCTTCTGGAGAGACGTATCTCAAAGATCGACTTCGAGACAGGT
GATATTGTTGAGCGAGAGCGACCAAGATCTAAATTCGGTAACGGTGCGTGGCGCTTTATTTTAAAAAG
CTC
+
FFFFFFFFFFF:FFFFFFFFFFFFFF:FFFFFFFFFFFFFFFFFFFFF:FFFFFFFFFFFFFFFFFFFF
FFFFFFFFFFFFFFFFFFFFFFFFFFFFFFFFFFFFFFFFFFFFFFFFFFFFFFFFFFFFFFFFFFFF
FFF
@A00721:190:HGJW7DSXY:4:2143:18593:4507_2:N:0:TCTGCAAG+GCTGCACT
CTGATACTTATCACGAAAGGACTTGAGTCTTTTGATCAGTCTCTGACTTGACCATCTTTCATTTTAAA

TGACGATCATTAAGACTCTCTGTCTTCTTTGTTTCCTTCTTTGCACTG
+
FFFFFFFFFFFF:FFFFFFFFFFFFFF:FFFFFFFFFFFFFFFFFFFFFFFFFFFFFFFFFFFFFFFF
FFFFFFFFFFFFFFF,FFFF:,:FFFFFFFFFFFFFF:FFF:FF:FFF
@A00721:190:HGJW7DSXY:4:1640:20464:10379_1:N:0:TCTGCAAG+GCTGCACT
CTCTTCCGATCTCTTATATGTGTTTTTACTATAACAACTATCAGTTGATTAAGACTCTTCCGAAAGGC
GAACTTTGGGAATTTGAAGCTCAGGGTGAAGCACAACGTTTGACCGTGACGAACTGTGGTGTTCTTGA
TCT
+
:F:FF,FFF::FFF,FF,F,F:FFFFFFF,FF:F:FF,FF:,FFFFFF:FFFF:FF:FF,,,FFFF,F
:,FFFFFF:,FF:FFFFFFFF:,F:,:FFFF:FFF:FFFFFFF,FFFFF,FFF,FFFFFF,FF,FFF,
F::
@A00721:190:HGJW7DSXY:4:1533:19922:5901_1:N:0:TCTGCAAG+GCTGCACT
ATACTCTCCTTGAGTTCACTCGTAAGGACACTACCACTTATTACGATATTTCTTTAGTCGATGGGTTT
TCGACTCCTTTAAGCGTCTTCTGTTCATCGCAACCAGATGATATCTTCTATTCGAACTTATGTTTCGA
TCA
+
FFFFFFFFFFFFFFFFFFFFFFFFFFFFFFFFFFFFFFFFF:FFFFFFFFFFFFFFFFFFFFFFFFFF
FFFFFFFFFFF,FFFFFFFFFFFFFFFFFFFF:FFFFFFFFFFFFFFFFFFFFFFFFFFF:FFFFFFF
FFF
@A00721:190:HGJW7DSXY:4:1610:27154:26788_2:N:0:TCTGCAAG+GCTGCACT
TCTTCTAATGTGGCCTGAACTACGTTTCACTTTGTGTGTACTTGTCTCTATTTTAAGTTTTGTCTTCC
TTGTTTGTCTTATTCTATTAATCACTCATTTTAGTGAGATTATTAAAA
+
FFFFFFFFFFFFFFFFFFFFFFFFFFFFFFF,FFFFFFFFFFFFFFFFFFFFFFFFFFFFFFFFFFFF
FFFFFFFFFFFFFFFFFFFFFFFFFFFFFFFFFFFFFFFFFFFFFFFF
@A00721:190:HGJW7DSXY:4:1415:23095:21512_2:N:0:TCTGCAAG+GCTGCACT
CGACAACAGCAGGGGTTTTCGAATAGTGATAGCCACCCGCCTTACAGAGGGGCAGCAGCATGACCATA
ATTGGTATTGACTTTGGTACCACCTTTTCTACTGTCTGTCTCGCCTCT
+
FFFFFFFFFFFFFFFFF:FFFFFFFFFFFFFFFFFFFFFFFFFFFFFFFFFFFFFFFFFFFFFFFFFF
FFFFFFFFFFFFFFFFFFFFFFFFFFFFFFFFFFFFFFFFFFFFFFFF
@A00721:190:HGJW7DSXY:4:1175:14036:34319_1:N:0:TCTGCAAG+GCTGCACT
CTTAACAACAATGATAGTGAATTTATACCTACAATCATTGGTTTTTCTAATAATGATGACACTATTGT
TTATGGATATGATGCTATTACTCATGATGGTTACGGTCAGGGAGGATTCTCAGTTTACAAGGACCTTA
AGC
+
FFFFFFFFFFFFFFFFFFFFF:FFFFFFFFFFFFFFFFFFFFFFFFFFFFFF:FFFFFFFFFFFFFFF
FFFFFFFFFFFFFFFFFFFFF:F,FFFFFFFFFFFFFFFFFFFFFFFFFFFFFFFFFFFFFFFFFFFF
FFF
@A00721:190:HGJW7DSXY:4:2347:21585:25598_2:N:0:TCTGCAAG+GCTGCACT
CCGAAAGACGTGCTAAATTAAAACCATTGTACGATGTCGTCTGTCTCGGCGATCACTTTTCACTAAGA
ATTGGTCCTACTTTTGGACAAGGCCAACTTCGTAATCTTCATGAGATC
+
FFFFFFFFFFFFFFFFFFFFFFFFFFFFFFFFFFFFFFFFFFFFFFFFFFFFFFFFFFFFFFFFFFF:
FFFFFFFFFFFFFF:FFFFFFFFFFFFFF,FFFFFFFFFFFFFFFFFF
@A00721:190:HGJW7DSXY:4:2359:13006:13432_1:N:0:TCTGCAAG+GCTGCACT
TTAGGGATTTTGAGAGAGTGAAAGGCTTCAATTGTAGTGGTTTGGTCGTTTCAGTACCCAGTCAGTAT
ACCTCTACGCAAAGATATTTCATGAAAACTACAGAGGAACGTACCGGATTACCTGTCTTTCATATTAT
GAA
+
FFFF:FFFFFFFFFFFFFFFFFFFFFFFFFFFFFFFFFFFFFFFFFFFFFFFFFFFFFFFFFFFFFFF
FFFFFFFFFFFFFFFFFFFFFFFFFFFFFFFFFFFFFFFFFFFFFFFFFFFFFFFFFFFFFFFFFFFF
FFF

@A00721:190:HGJW7DSXY:4:2447:5936:34726_2:N:0:TCTGCAAG+GCTGCACT
TCTTTGCTTCGATGTTGGATGTCAAGAAGGGTTCAGAATGGGATTCCTACGTAGTTTATGACTTTGGT
GGTGGTACTTTCGATACTTCTATCGTCTTACGTTACGGTAATTACTAT
+
F,,FFFFFFFFF:,FFFFFFFFFFFFFF,FFFFFFFFFFF:FFFFFFFFFFFFFFF:FFF:F:FFFFF
FFFFFFFF:F:FF:FF:F,,FFFF,FF:FFF,FF::F:FFFFFFFFF:
@A00721:190:HGJW7DSXY:4:1604:26476:36229_1:N:0:TCTGCAAG+GCTGCACT
ATAATCTTGGAGGTCGTGACGTTGATGAAGCTATAAGAGCGTTCTTATTACGGCGCACTTCTTTACCC
GCCACCACACAACTTTCAGTTTCACAACTGAAGGAGGAGGTTTCTGTTACTGGAAAACCCTCGTCAAT
ACT
+
FFFFFFFFFFFFFFFFFFFFFFFFFFFFFFFFFFFFFFFFFFFFFFFFFFFFFFFFFFFFFFFFFFFF
FFFFFFFFFFFFFFFFFFFFFFFFFFFFFFFFFFFFFF:FFFFFFFFFFFFFFFFFFFFFFFFFFFFF
FFF
@A00721:190:HGJW7DSXY:4:1375:23484:3051_2:N:0:TCTGCAAG+GCTGCACT
GATCTAGTGAAATTAACGCGTCCTTTTCTGGAACGTAGTTTTACTATTATGAACAAAGTTATCAAAGA
TAGTGGCATTCAAGGCAAACTTACCCTGATTCCGGCCGGTGGTAGCTC
+
FFFFFFFFFFFFFFFFFFFFFFFFFFFFFFFFFFFFFFFFFFFFFFFFFFFFFFFFFFFFFFFFFFFF
FFFFFFFFFFFFFFFFFFFFFFFFFFFFF:FFFFFFFFFFFFFFFFFF
@A00721:190:HGJW7DSXY:4:2152:2175:14372_2:N:0:TCTGCAAG+GCTGCACT
GGCTATTACTCTTGCTGAAACAGCTATTAAAGCTGTTGAGCGGGTAATTACACCTAAACGAGCTCGAT
CTGCCATCGCTGAAGGATGTGCACTCTTTTCTGCTACTTTAACGGAGC
+
FFFF:FFFFFFFFFFFFFFFFFFFF:FFFFFFFFFFFFFFFFFFF:FFFFFFFFFFFFFFFFFFFFFF
FFFFFFFFFFFFFFFFFFFFFFFFFFF,F:FFFFFFFFFFFFFFFFFF
@A00721:190:HGJW7DSXY:4:1638:7410:31923_2:N:0:TCTGCAAG+GCTGCACT
TCGATTGTATCAACAGTAACATTACTTCTAATAAAGGTCTCTATCAAGCTCGTATTATCGTCGCTGCT
GGATCTGCGTTACCGTTGAAGGCTAAACTCCAAGGTAATTATGAAGAC
+
FFFFFFFFFF:FFFFFFFFFFFFFFFFFFFFFFF:FFFFFFFFFFFFFFFFFFFFFFFFFFFFFFFFF
FFFFFF:FFFFF,FFFFFFFF:FFFF:FFFFFF:FFFFFFFFFFFFFF
@A00721:190:HGJW7DSXY:4:2126:11975:12430_1:N:0:TCTGCAAG+GCTGCACT
GTATGAGGGTGTTTCTAATCGTAACATTAATAATTTCAATCTTCTTAAAAAGAAGATAGAGAGTCATA
CTTTGGGCTATCGTTCAGGTGCATGGGGTTGGATAGCCAATATTGAAGTGGACACCTCTGGTAGTATC
GAA
+
FFFFFFFFFFFFFFFFFFFFFFFFFFFFFFFFFFFFFFFFFFFFFFFFFFFFFFFFFFFFFFFFFFFF
FFFFFFFFFFFFFFFFFFFFFFFFFFFFFFFFFFFFFFFFFFFFFFFFFFFFFFFFFFFFFFFFFFFF
FFF
@A00721:190:HGJW7DSXY:4:1410:16432:26663_1:N:0:TCTGCAAG+GCTGCACT
CTAAGCATACTACTTTTGCTTTGATTACTCTACCGCCTTACAAGTCTAAGGTTAAAGAGTCGATTAAA
CAAGTTGTACTTATCAATGATTTTATCGCTGGTTGTTATCTCGAAAAGTCTCTTCTAAAAGGAACGTC
AGT
+
FFFFFFFFFFFFFFFFF:FFFF,FFFFFFFFFFFFFFFFFFFFFFFFFFFFFFFFFFFFFFFFFFFFF
FFFFFFFFFFFFFFFFFF:FFFFFFFFFFF:FFFFFFFFFFFFFFFFFF,FFFFFFFFFFFFFFFFFF
FFF
@A00721:190:HGJW7DSXY:4:1202:4056:34726_2:N:0:TCTGCAAG+GCTGCACT
CTAAAATATTTACAAAATCTTACCACTATCTACGAACCTAAAGTTCTGAAGACGTTGCTTGTCGCTTC
CTCCTTTAATGGCACTACCTCGGTACAATCTTACTTGGAACGAAGGCG
+
FF,FFFFFFFFFFFFFFFFFFFFFFFFFFFFFFFFFFFFFFFFFFFFFFFFFFFFFF:FFFFFFF,F
FFFFFFFFFFFFFFFFFFFFFFFFFFFFFFFFFF:FFFFFFFFFFFFF

@A00721:190:HGJW7DSXY:4:1315:24650:9204_1:N:0:TCTGCAAG+GCTGCACT
GTTCTGTCTATATTAGGTGAAGCGAAATACGATTTCGAACGTCAACGTTCACCGAGTTTCGTTTCGTA
CCTTGATGCTGACTTTCGTCAATATGATTTGATCTGGTTGCTCAATTCTAAGCGTGTTCCGCTTAGTG
CGA
+
FFFFFFFFFFFFFFFFFFFFFFFFFFF:FFFFFFFFFFFF:FFFFFFFFFFFFFFFFFFFFFFFFFFF:F
FFFFFFFFFFFFFFFFFFF:FFFFFFFFFFFFFFFFFFFFFFFFFFFFFFFFFFFFFFFFFFFFFFFFFF
FFF
@A00721:190:HGJW7DSXY:4:1130:6705:23124_1:N:0:TCTGCAAG+GCTGCACT
TACGAACTTGACTCTACTATACGTGACATACTAAATACTAGATATTTGGATGATCTTGATAAAGAACT
TCATGAATTTTCAGAGGATGAAAAATTTTCTTCCTTGGATTTTAATATAAAGAATCTTTACCAGGACT
CCT
+
FFFFFFFFFFFFFFFFFFFFFFFFFFFFFFFFFFFFFFFFFFFFFFFFF:FFFFFFFFFFFFFFFFFFFF
FFFFFFFFFFFFFFFFFFFFFFFFFFFFFFF,FFFFFFFFFFFFFFFFFFFFFFFFFFFFFFFFFFFFFF
FFF
@A00721:190:HGJW7DSXY:4:1335:25843:15060_1:N:0:TCTGCAAG+GCTGCACT
TGATCGTAAGAAGTTTATAAGGAACATATCCATTTGTTGTAATAGGGTCATCAACTTAGAACTCGAAA
CTCTAAGTGGTAATACTGTCTTTCCAAGCTTACCTGTATCTTCAATGAAATTGGACTCTTCGATAGGG
GAC
+
FFFFFFFFFFFFFFFFFFFFF,FFFFFFFFFFFFFF:FF,FFFFFFFFFFFFFFFFFFFFFFFFFFFFFF
FFFFFFFFFFFFFFFFFFFFFFFFFFFFFFFFFFFFFFFFFFFFFFFFFF:FFFFFFFFFFFFFFFFFFF
FFF
@A00721:190:HGJW7DSXY:4:2218:13648:9659_2:N:0:TCTGCAAG+GCTGCACT
TAAAGGAAGATCTGTTATCGACGGTTTATCGTTTACGAAGTATCACTCCACCGGTGAATGGTTTAAGA
ATACTATAAATAGAATAGCTATTACTACTAATGGCGTTGGCCTAGTGG
+
FFFFFFFFFFFF:FFFFFFFFFFF:FFFFFFFFFFFFFFFFFFFFFFFFF:FFFFFFFFFFFFFFFFFFF
FFFFFFFFFFFFFFFFFFFFF:FFFFFFFFFFFFF:FFFFF::,FFFFF
@A00721:190:HGJW7DSXY:4:2364:28058:11224_1:N:0:TCTGCAAG+GCTGCACT
TTTAGGTGGTCTTTATTCTATTATTGATTTTTATGAGATTGATACGTTAACAGAAGATGATGTTAGAG
TAGAATTCCTGAAGAGTCCTTCCGTAACCAACTTCTTGACAATTTTCTTCAATCCTTCGATAATAGTT
AAA
+
FFFFFFFFFFFFFFFFF:F:FFFFFFFFFFFFFFFFFFFFFFFFFFFFFFFFFFFFFFFFFFFFFFF:FF
FFFFFFFFFFFF::FFFFFFFF:FFFFFFFFFFFFFFFFFFFFFF:FFFFFFFFF:FFFFFFFFFFFFFF
FFF
@A00721:190:HGJW7DSXY:4:2458:16324:25222_1:N:0:TCTGCAAG+GCTGCACT
CCGATAAGGGTCACTAGTCTTACACAATTAAGAAATAATCCTACATTGACTGATTATCTAAAGATTGA
AGGAAAATTTTATCCTCGTTTAACTTACTTAGACTTTCCTACGATACAAATGATAACTTCTAATTTAC
GTT
+
FFFFFFFFFFFFFF,FFFFFFFFFFFFF,FFFFFFFFFFFFFFFFFFFFFFFFFFFFFFFFFFFFFFFFF
FFFFFFFFFFFFFFFFFFFFFFFFFFFFFFFFFFFFFFFFFFFFFFFFFFFFFFFFFFFFFFFFFFFFFF
FFF
@A00721:190:HGJW7DSXY:4:2427:16288:33332_2:N:0:TCTGCAAG+GCTGCACT
AAGGGATGCATGGTGTTATCTCCTCAGGAGTCCCGGCATTTGATAATAATGACGAGACTTTCGTCATA
ATCGCTGCATATTACGGATTACACGGTACCAGCGCATCTCGTGTCGAG
+
FFFFFFFFFFFFFFFFFFFFFFFFFFFFFFFFFFFFFFFFFFFFFFFFFFFFFFFFFFFFFFFFFFFFFF
FFFFFFFFFFFFFFFFFFFFFFFFFFFFFFFFFFFFFFFFFFFFFFFFF
@A00721:190:HGJW7DSXY:4:1614:15962:22654_1:N:0:TCTGCAAG+GCTGCACT
AAATTTATAGAAGATTATTTTGACAGAATCAATAAGAATCATCGATTCTCGATTCGTAGAGCTTATCT

AGGTACTCTCATTTACTATGTTGATTTAGTCTACATTTATGCTGATCTAAAATTTCGATCTAGATGGA
TCC
+
FFFFFFFFFFF:FF,F,:FFFFFF:FFFFFFFFFFFFFFFFFFFFFFFFFFFFFFFFFFFFFFFFFFFF
FFFFFFFFFFFFFFFFFFFFFFFF:FFFFFFFFFFFFFFFFFFFFFFFFFFFFFFFFFFFFFFFFFFF:
FFF
@A00721:190:HGJW7DSXY:4:2261:30942:7921_2:N:0:TCTGCAAG+GCTGCACT
TATAGGAGTAATCTACGAGAAGATTCTAGAATTCTTAATTATAATATTCAACCTCAAAAGTTACGTGT
TTACAGTAACGAGATCACACATTTTGAGAACTAACTATGTTATGTTCT
+
FFFFFFFFFFFFFFFFFFFFFFFFFFFFFFFFFFFFFFFFFFFFFFFFFFFFFFFFFFFF:FFFFFFFF
FFFFFFFFFFFFFFFFF:FFFF:FFFFFFFFFFFFFFFFFFFFFFFFFFF
@A00721:190:HGJW7DSXY:4:1602:11605:27508_1:N:0:TCTGCAAG+GCTGCACT
TCTCTTCTTATAATAAATAAATAAATAATATTACTAATGGCAAATAGTGGAAAGGCTCCAGTCAACAC
TAGTTCGAGTGCTGCTTTGGATTACGATGACGTGAGAATAGGTGAAGGGATAGTTTTTACCTCGACCA
ACA
+
FFF:F:FFFFFFFFFFFFFFFFFFFFFFFFFFFFFFFFFFFFFFFFFFFFFFFFFFFFFFFFFFFFFFF
FFFFFFFFFFFFFFFFFFFFFFFFFFFFFFFFFFFFFFFFFFFFFFFFFFFFFFFFFFFFFFFFFFFFF
FFF
@A00721:190:HGJW7DSXY:4:2521:20654:19539_2:N:0:TCTGCAAG+GCTGCACT
GCGACTAATCTTAAAGCTTCACCCAAAATGAAGGACACTTCGAAACTGAATAATGATGCACTACAAAC
AAGTATCGCTAATCATCTGAAAAAGGCTCTAAAGGACACTATACAATA
+
FFFFFFFFFFFFFFFFFFFFFFFFFFFFFFFFFFFFFFFFFFFFFFFFF:FFFFFFFFFFFFFFFFF:F
FFFFFFFFFFFFFFFFFFFFFFFFFFFFFFFFFFFFFFFFFFFFF:FFFF
@A00721:190:HGJW7DSXY:4:1662:7591:10911_2:N:0:TCTGCAAG+GCTGCACT
CAGCACCACATTTTGGTCAAGTCAATCCGTTGAGAAAATTTCTTAGAGGATATTCGCCATTGTGGTAC
GAATTGGTCCAACATTTACCACCAAATTCTTTTGATACAACTCTAGCC
+
FFFFFFFFFFFFFFFFFFFFFFFFFFFFFFFFFFFFFFFFFFFFFFFFFFFFFFFFFFFFFFFFFFFFF
FFFFFFFFFFFFFF:FFFFFFFF:FFFFFFFFFFFFFFFFFFFFFFFFF
@A00721:190:HGJW7DSXY:4:2648:22037:5650_1:N:0:TCTGCAAG+GCTGCACT
ATACGCTTGCTATTCACCAGATTGGTTCGAACCCGTTGATGCTATGTCAGAAGAAGCTATTCAAGCTT
GTCGTTTAAAGACTGCAGAGGCTGTCAAACAAGCCCAGACTCAAGGAGTCCAATCAAAGGTTTTGAAT
TTA
+
FFFFFFFFFFFF:FFFFFFFF,FFFFFFFFFFFFF:,FFFFFFFFFFFFFFFFFFFFFFFFFFFFFFFF
FFFFFFFFFFFFFFFFFF:FFFFFFFFFFFFFFFFFFFFFFFFFFFFFFFFFFFFFFFFFFFFFFFFFF
FFF
@A00721:190:HGJW7DSXY:4:1308:13693:14967_2:N:0:TCTGCAAG+GCTGCACT
GGTGATAAAAATTATTTAGAGATCTTTTCTACTTACAAAGGACAATTCAGTTTCATTAATTCTACGGA
AAAGTCCGGAGTATATTGTATGTTAATAGATAGTGGGATAATAGTTCT
+
FFFFFFFFFFFFFFFFFFFFFFFFFFFFFFFFFFFFFFFFFFFFFFFFFFFFFFFFFFFFFFFFFFFFF
FFFFFFFFFFFF:FFFFFFFFFFFFFFF:FFFFFF,FFFFF:FFFF,F
@A00721:190:HGJW7DSXY:4:1324:24713:1423_1:N:0:TCTGCAAG+GCTGCACT
CGTCTGAAGAGTTTAGAGCTGCTATTGATTATGTAACCGATAGGAGTTGTATAGTTGAAGATGTGGCT
TTAGACGATAATCCTTTAAGGACTATGTTGACTGACGAAGATTTTCCCACAGATTTGTCTACGTTGCA
GAT
+
:FFFF::FFFFFFFFFF:FFFFF:FFFFFFFFFFFFFFF,FFF:F,FFFFFFFFFF:,F:FFFFFF,F
FFFFFFFFFFFFFFFFFFFFFFFFFFFFFFFFFFFFFFF,FFFFFFFF:FFFFFFFFFF:FFFFFFFFF
FFF

@A00721:190:HGJW7DSXY:4:2676:28890:21652_2:N:0:TCTGCAAG+GCTGCACT
CACTATATACATTATAAAATATAAAAATAAGGGAGTCATAACTTTTGTTACCAAATTCTTGGCTTTCA
ACGAAGTCTCTGATTTTATTAATCATTACTTTAACGATCTCTCGGTCA
+
FFFFFFFFFFFFFFFFFFFFFFFFF:FFFFFFFFFFF:FFFFFFFFFFFFFFFFFFFFFFFFFFFFFF
FFFFFFFFFFFFFFFFFFFFF,FFFFFFFFFFFFF::FF:FFFFFFFF
@A00721:190:HGJW7DSXY:4:1140:14660:26631_1:N:0:TCTGCAAG+GCTGCACT
TTGATCTACGTCGTTTAACTGTCGGAACAACCTTTACTTAGAATGACTCTATCTTTTCAGACACTTCT
TATATTATTTACAATAGTTAATATCATATTAGTAATACTCACTATCGTTTTCTGCTTTTTCTTAGCTA
ATA
+
FFFFFFF:,FFFF:FF:FFFFFFFFFFFFFFFFFFFFFFFFFFFFF:FFFFFF,FFFFFFFFFFFFFF
FFFFFFF:FFFFFFFFFFFFFFFFFFFFFFFFFFFFFFFFFFFFFFFFFFFFFFFFFFFFFFFFFFFF
FFF
@A00721:190:HGJW7DSXY:4:2461:20012:35869_1:N:0:TCTGCAAG+GCTGCACT
GTATGAGTACTAAAACAAACTCTTCTTTACTAGACCTGTTGGTAGAATATTTTTAGTCTCTACACCCT
TCTCACCGAGGATAACGCTATTTTTCCGTCCTTAACTTAATACACGAAGATATCAGCCGCAAACGACT
CGA
+
::F::,,:F,,FFFFF,F,,,,F:,,FFF,,,,::,F,,FFF:F,F,F,,,FFF,:,,F:,:,,FF,,
:,F,F,:,,:,FFF:,F,,,,FFF:F,:,F,F,F,,,:FF,:F:,:F,::,FF:F,F::,:,FFFFFF
FFF
@A00721:190:HGJW7DSXY:4:2373:21549:4867_2:N:0:TCTGCAAG+GCTGCACT
AAAGTCCCTTCTAACTTTAGATTATGAATCCTGCCTTTACTCATTTTATAGCAGTTATTTATGTTGTA
CTTTTTATAATTTATAACCTTCTTATACTTTTGGGTATGTTTTGTGAA
+
FFFFFFFF:FFFFFFFFFFFFFFFFFFFFFFFFFFFFFFFFFFFFFFFFFFF:FFFFFFFFF:FFFFF
FFFFFFFFFFFFFFFFF:FFFFFFFFFFFFF:FFFF:FFFFFFFFFFF
@A00721:190:HGJW7DSXY:4:1251:16450:7185_2:N:0:TCTGCAAG+GCTGCACT
CTTATTGTAACGATCATTCTTTCGAGTTTTGTCATTCTTATTCTTTTATTCCTTTAACTGTGTATGAG
GAGGATTTATAATGCTTTTCGATAGTGATTGTTATCAATTTAAATACA
+
FFFFFFFFFFFFFFF:FFFFFFFFFFFFF:FFFFFF,F::FFFFFFFFFFFFFFFFFFFFFFF:FFFF
FFFFFFFFFFFFFFFFFFFF,FFFFFFFFFF:FFFFFFFFFF,FFFFF
@A00721:190:HGJW7DSXY:4:1443:5538:24674_2:N:0:TCTGCAAG+GCTGCACT
CTTTACTCACAGACTCCGAAAATGGTTTCAAATGAGGTCAAAGCTCTATTTATTAAACGAACTATTAT
CCGATTCTTCGAGGGTATTACTACCACTGATCTGCCGACTTTAGTCAA
+
FFFFFFFFFFFFFFFFFFFFFFFFFFFFFFFFFFFF:FFFFFFF:FFFFFFF,FFFFFFFFFFFFFFF
FFFFFFFFFFFFFFFFFFFFFFFFFFFFFFFFFFFFFFFFFFF:FF:F
@A00721:190:HGJW7DSXY:4:1461:25156:4758_2:N:0:TCTGCAAG+GCTGCACT
TTCCTTCGATGTTTATATTTTTCTATAAGGGAAAATAGCAATAATCAAATTTCGACAAATTTTAGAAC
TTTATTTCAGAACTCTCTCCGATCACCTTGGTGTGATCTCCCGGAGGA
+
FFFFFFFFFFFFFFFFFFFFFFFFFFFFFFFFFFFFFFFFFFFFFFFFFFFFFFFFFFFFFFFFFFFF
FFF,FFFFFFFFFFFFFFFFFFFFFF:FFFFFFFFFFFFFFFFFFFFF
@A00721:190:HGJW7DSXY:4:1418:2130:9784_2:N:0:TCTGCAAG+GCTGCACT
ATGTATGATGATAATCTACTATTTTTAAATAGTGATATTCAGCGTATACTTGAATACTTTATCAAAGA
TTTGGCTTGGTCTATTTACCGCAAAAAGTTGGAAGAGGTCTATGATAT
+
FFFFFFFFFFFFFFFFFFFFFFFFFFFFFFFFFFFFFFFFFFFFFFFFFFFFFFFFFFFFFFFFFFFF
FFFFFFFFFFFFFFFFFFFFFFFFFFFFFF:FFFFFFFFFFFFFFFFF
@A00721:190:HGJW7DSXY:4:2207:3233:31172_2:N:0:TCTGCAAG+GCTGCACT
TATACTTTAAATAGCACCGATTATAACTTGACTGTTGATAATATTACAATAGCTCTTTCTCAGAAGAT

TAGAGAAGTAACCGGTGAACACGTCGGTAGCATTGATTTACTTTTACC
+
FFFFFFFFFFFFFFFFFFFFFFFFFFFFFFFFFFFFFFFFFFFFFFFFFFFFFFFFFFFFFFFFFFFFFF
FFFFFFFFFFFFFFFFFFFFFFFFFFFFFFFFFFFFFFFFFFFFFFFFFF
@A00721:190:HGJW7DSXY:4:1436:29152:9111_2:N:0:TCTGCAAG+GCTGCACT
TTTCGAAAAGAAACCTATAAGTTACAAACGATTCTTCGACTATATTAAAGAAATAAAAATACTTCAAG
TACCTGAACTTTACATTTTTATTGGTATGGTATAATGTGTCTTACAAA
+
FFFFFFFFFFFFFFFFFFFFFFFFFFFFFFFFFFFFFFFFFFFFFFFFFFFFFFFFFFFFFFFFFFFFFF
FFFFFFFF:FFFFFFFFFFFF,FFFFFFFFFFFFFFFFFFFFFFF:FF
@A00721:190:HGJW7DSXY:4:1424:6840:34194_2:N:0:TCTGCAAG+GCTGCACT
CTCTCGGTAATGAGAGTGTATTCCTTAATATTCTAGTAACCGCTATGTGGGCATTACTAGGATTTATC
ACACTCCTAAATTTGATAGCAATTTATGTTTTAAATGCTAGGAAAATT
+
FFFFFFFFFFFFFFFFFFFFFFFFFFFFFFFFFFFFFFFFFFFFFFFFFFFFFFFFFFFFFFFFFFFFFF
FFFFFFFFFFFFFFFFFFFFFFF:FFFFFFFFFFFFFFFFFFFFFFFFF
@A00721:190:HGJW7DSXY:4:1317:5466:12649_1:N:0:TCTGCAAG+GCTGCACT
TGTATAGTTCAAGGAGTACTTAATTCAATAGCTTTAGTATTATTTACTATCGCCTTTATTGAAAATTG
TATAAGAGTTAATTGCTTACACAAAAGTGACGCGCGAAGGAATCTTAATTGATTGAAGCTAGGCGTTG
GGG
+
FFFFFFF:FFFFFFFFFFFFFFFFFFFF,FFFFFFFFFFFFFFFFFFFFFFFFFFFFFFFFFFFFFFFFF
FFFFFFFFFFFFFFFFFFFFFFFFFFFFFFFFFFFFFF:FFFF,F:FFFFFFFFFFFFFFFFFFFFFFF
FFF
@A00721:190:HGJW7DSXY:4:1513:5864:34225_1:N:0:TCTGCAAG+GCTGCACT
ACTCCCCAAACATAACATACCATAACCTACCATGACCAATCATCGCTCCAACTGGCACGCCAGACGTA
GGGTCAAACCTACTACCGCCGGTCGGCAGATCGGCACCACTGGTCTCTACGTTCAGGGACCCGCTGTG
CAG
+
FFFFFFFF:,F,F:,F:,F:F:F::FF::FF,,,FFFFF,F,FFFFFFFF:FFFFFF,FF:FF:FFF:
FFFF:FF,:F:,:FF:FFF:FF,:FF,F,,F::::FFFFFFFFFFFFFF:FFFF,FFFF:FFFFFFFF
F:F
@A00721:190:HGJW7DSXY:4:1133:5095:17394_1:N:0:TCTGCAAG+GCTGCACT
CCAAATCTAGATCTGCACAGAAGAAGGCTGAAGGTCGTTACCGGACGACCACTCAAAGGAAACGGGCT
GTTGCCCAGAGGAAAGCAGCGACCACCGAGGTCGCTCGTAACATTGACGGCAGTCCCGTCATCTCTGT
GGG
+
FFFFFFFF,F,FF,FFFFFFFFFFFFFFFFFFFFFFFFF,FFFFFFFF:FFFFFFF:FFFF,FFFFFFF
FFFFFFFFFFFFF:FFFFFFFFFFFFFFFFFFFFFFFFFFFFFFFFFFFFFFFFFFFFFFFFFFFFFFF
FFF
@A00721:190:HGJW7DSXY:4:1452:1940:13589_1:N:0:TCTGCAAG+GCTGCACT
GCTCTTCCGATCTATTCACCACTCCCATACCAATCAATGATGTGACTATACAGTTCGGCCATTTCAAG
AGTGGTCAATACGTCGTGGGAGGTACTGAAGCCTTCCCCTCCGCTGGCACGAAGAACACTCCCAAGCC
AAT
+
FFFFFFFFFFFFFFFFFFFFFFFFFFF:F:FFFFFFFFFFFFFFFFFFFFFFFFFFFFFFFFFFFFFFFF
FF,FFFFFFFFFFFFFFFFFFFFFFFF:FFFFFFFFFFFFFFFFFFFFFFFFFFFFFFFFFFFFFFFFF
FFF
@A00721:190:HGJW7DSXY:4:1259:22761:19774_2:N:0:TCTGCAAG+GCTGCACT
TGACCAGAATGAAAAGGGGTTTACCAGGGTAGTCTCTAAGTCTACCAGGAAGAGGGGAAATGTCCCCA
AAACTGAGAAGAATAAAGGGAAAGGTCCATCAGGACCTAAGAAGGGGA
+
FFFFFFFFFFFFFFFFFFFFFFFFFFFFFFFFFFFFFFFFFFFFFFFFFFFFFFFFFFFFFFFFFFFFFF
FFFFFFFFFFFFFFF:FFFFFFFFFFFFFFFFFFFFFFFFFFFFFFFFFF

```
@A00721:190:HGJW7DSXY:4:1231:26151:27743_2:N:0:TCTGCAAG+GCTGCACT
AATTCTGAAGGAAGATCTCCCACCATCTGGGGTTGGGAATAAGAAATCTGCACGGGCCAAAGAGGCCG
CTGCTACGAAGTCGCTGAAGGCGGCTTGGGTCATTAAGGGTAGCTGTA
+
FF:FFFFFFFFF:FFFFFFFFFFFFFF:FFFFFFFFFFFFFFFF:FFFFFFFFFFFFFFFFFFFFFFFFF
FFFFFFFFFFFFFFFFF:FFFFFFFF:FFFFFFFFFFFFFFFFFFFFF
@A00721:190:HGJW7DSXY:4:1130:19316:4539_1:N:0:TCTGCAAG+GCTGCACT
CCGACCCCATCTTTGAGACCAAGACTCTCTACAGTAGGTTCGTGCCTACTATGAGCAAATCTGTTGAA
TTCGTCACTGAGGACGAAGGTGAGAGGACTTTTGGCTACTGGCGACAAACTGAGAGCAGTCGCTGGGG
AAA
+
FFFFFFFFFFFFFFFFFFFFFFFFFFFFFFFFFFFFFFFFFFFFFFFFFFFFFFFFFFFFFFFFFFFFFF
FFFFFFFFFFFFFFFFFFFFFFFFFFFFFFFFFFFFFFFFFFFFFFFFF:FFFFFFFFFFFFFFFFFFFF
FFF
@A00721:190:HGJW7DSXY:4:1154:15935:29058_2:N:0:TCTGCAAG+GCTGCACT
ACGCTTCGAGCTTGAGTGTGACGAGGCCGGTGACGTTTTTGTCAACGGCGTTTGGAGGATTTTATCCG
CGCTTAACTATGGGGCGAAGATCCCCTATGGAGTCTTGAAGGACTTAG
+
FFFFFFFFFFFFFFFFFFFFFFFFFFFFFFFFFFFFFFFF:FFFFFFFFFFFF:FFFFFFFFFFFFFFFF
FFFFFFFFFFFFFFFFFFFFFFFFFFFFFFFFFFFFFFF:FFFF:FFF
@A00721:190:HGJW7DSXY:4:2424:29324:18145_1:N:0:TCTGCAAG+GCTGCACT
GCATGTCACCCTTAAGCACCGAGGTAAGCTCGGGACTTACAAGAGAGAGTGTGAGAGAGTCGCTCTCA
CGATTTTTGCCCAGAATGCGGGCTTTATGACTGATGAGGTTAAAAATCATGCTCCGTCTGTGGGTGCG
GG
+
FFFFFFFF:FFFFFFFFFFFFFFF,FFFFFFF:FFFFFF:FFFFFFFFFFFFFFFFFF:FFFFFFF,,FF
F,:,,FFF:,F,:,FFF,:FFFFFFF,,F::,,::,F,FF::FFF,:,,,:,F,,,:,,F,FFF,F,,
FF
@A00721:190:HGJW7DSXY:4:1105:3459:27712_2:N:0:TCTGCAAG+GCTGCACT
TTCACTGGTTCTGGGAGGCTGCTTTCCTCCATAATTACAACCTCTCTTTAGAGAGCATTAGAAGGAAG
TTGGGCAAATTCCCACATATGGTAGACGTTGTCACACAGATCGGAAGA
+
FFFFFFFFFFFFFFFFFFFFFFFFFFFFFFFFFFFFFFFFFFFFFFFFFFFFFFFFFFFFFFFFFFFFFF
FFFFFFFFFFFFFFFFFFFFFFFFFFFFFFFFFFFFFFFFFFFFFFFF
@A00721:190:HGJW7DSXY:4:2477:31349:14231_1:N:0:TCTGCAAG+GCTGCACT
TCCTCCGTCATTGGGGATTTCGATGTGAGAAGGTGGAAGGTGTCAAGGGTCTTCTTCATGCTGATCTT
TCAGGACCTAATCACATCATGATTGATAGTTACGGCTCGTATCGAGTTGGAGGGAGGAAGTTCTCCGA
A
+
FFFFFFFFFFFFFFFFFFFFFFFFFFFFFFFFFFFFFFFFFFFF:FFFFFFFFF:FFFFFFFFFFFFFFF
FFFFFFFFFFFFFFFFFFFFFFFFFFFFFFFF:FFFFFFFFFFFFFFFFFFFFFFFFFFFFFFFFFFFF:
F
@A00721:190:HGJW7DSXY:4:2115:11053:10551_2:N:0:TCTGCAAG+GCTGCACT
TCAGAGCGGGGAGCTCTGAAAAGGAAACTTCGTCAGAAGATGTGGAAGTCTAAGAGGAGGAGGTTGAG
GAAGGCTCTAGAGAAGATCTCACCACCTGGAGAAGGCAAGTGGAGAGT
+
FFF:F:,,FFFFFFFFFF:::,:FFFFFFF::,F,:F,FFFF,F:FFFFF::F:F:F::,FF,F:FF:
FFF,FFFFFFFFFFFFFFFFFFFFFF::FFFFF,FFFF,FFFF:FFFF
@A00721:190:HGJW7DSXY:4:1453:15655:34773_2:N:0:TCTGCAAG+GCTGCACT
AAGAAGGATTATTTGAAGCAGGGGTTACCACTTCCTAAGGTGGATGAGGGAGGTTGTTTCTTTGGAGT
TTTAGATAATCTTTTGAGAAGGGGTCATAGAGCCAGCAAGAGAAGCTT
+
FFFFFFFF:FFFFFFFFFFFFFFFFFFFFFFFFFFF:FFFFFFFF:FFF:FFFFFFFFFFFFFFFFFFFF
FFFFFFFFFFFFFFF:FFFFFFF:FFF:F:FFFFFFFF:FFFF:FFFF
```

@A00721:190:HGJW7DSXY:4:2477:2908:9314_2:N:0:TCTGCAAG+GCTGCACT
CCTTGAGAAGACAAGCCACATTAAGATATAATTTAGGTCAACTTGGGGTTTCGAATGTCATTAGTGCC
TTACGATTGAAAGAGGTCCTTTTGAGTATCGACAGATCTTTCGATCTT
+
FFF:FFFFFFFF:FFFFFFFFFFFFFFFFFFFFFFFFFFFFFFFFFFFFFFFFFFFFFFFFFFFFFF
FFFFFFFFFFFFFFFFFFFF,FF:FFFFFFFFFFFFFFFFFFFFFFFF
@A00721:190:HGJW7DSXY:4:2664:5728:12070_2:N:0:TCTGCAAG+GCTGCACT
GTGGCAGAATTCGGTAACTTACCATATTGTTCCTTCCGGGAAGTTGTGGGATTTAACTTTGACTCAAC
TACTCCGATCAGATCCGGATGGTTTGATTGGTATGGACTACTCGACTT
+
FFFFFFFFFFFFFFFFFFFFFFFFFF:FFFFFFFFFFFFFFFFFFFFFFFFFFFFFFFFFFFFFFFFF
FFFFFFFFFFFFFFFFFFFFFFFFFFFFFFFFFFFFFFFFFFFFFFFF
@A00721:190:HGJW7DSXY:4:2437:3947:35884_2:N:0:TCTGCAAG+GCTGCACT
TCATGGCAGATACTACCGATAAGGTGAAAAATATGCAGGTCAAAGATTCTGTAGCTCTGAGGGCGGTT
GAGGGAGCCGTTTTGGCCGACTTTAACTATTCTAAGAGTCTTTATGAT
+
FFFFFFFFFFFFFFFFFFFFFFFFFFFFFFFFFFFFFFFFFFFFFFFFFFFFFFFFFFFFFFFFFFFF
FFFFFFFFFFFF:FFFFFFFFFFFFFFFFFFF,FFFF:FFFFFFFFFF
@A00721:190:HGJW7DSXY:4:1608:5113:36683_2:N:0:TCTGCAAG+GCTGCACT
CGTTGTCGTACCTTACAAGGTTGAGGAGACTTTACAGCGGCTTATTGTCGCGCAGTTTCCAGAATTGC
GGTTGAAGTTCACCCATTCTATCTTGAATGCTCACGCCGTCGCAGCCA
+
FFFFFFFFFFFFFFFFFFFFFFFFFFFFFFFFFFFFFFFFFFFFFFFFFFFFFFFFFFFFFFFFFFFF
FFFFFFFFFFFFFFFFFFFFFFFFFFFFFFFFFFFFFFFFFFFFFFF:
@A00721:190:HGJW7DSXY:4:1574:32560:29543_1:N:0:TCTGCAAG+GCTGCACT
ATCACTTGTTAAAGTACAGATTTAGTGATTTAGATATTATTGATATTGGAGGCGATATTCGTTTTCAT
ATCCAAAACTCCTTGAATGGAGTACATATTTGCAACCCTATTCTTGATGTGAAAGATGCATCGAGGTA
TAC
+
FF,FFF:,FFFF::F,,FFFFFFFFFFFFFFFFFFFFFFFFFFFFFFFFFFFFFFF:FFFF:FFFFFF
FFFFFFFFFFFFFFFFFFFFFFFFFFFFFFFFFFFF:FFFFFFFFFFFFFFFFFFFFFFFFFFFFF:F
FFF
@A00721:190:HGJW7DSXY:4:1344:25861:29434_2:N:0:TCTGCAAG+GCTGCACT
TTGATTGGGAACGTACCTACTCACGAGACGTCATGAAAGCTATCGGAGGGAAGCAAAATGTTTCGTTC
TGTTACAAGAACGCTTTGGATTGTGACCATAAAGCCTCAGTTGCGATA
+
:FFFFFFFFFFFFFFFFFFFFFFFFFFFFFFFFFFFFFFFFFFFFFFFFFF:FFFFFFFFFFFFFFFF
FFFFFFFFFFFFFFFFFFF:FFFFFFFFFFFFFFFFFFFFFFFFFFFF
@A00721:190:HGJW7DSXY:4:2357:30373:26224_1:N:0:TCTGCAAG+GCTGCACT
CTCTCTTGATGATGTAGCCACTATAATGCTTAAGAGAGAGATTGATGTATGTCATTTCGTCTTAGTTT
GTCCCGGCGAATTATTGGACACGATGAAAAATGACATACCCGTTCCTGACCTTGGTATCGTTATCCGT
CAT
+
FF:FFFFFFFFFFFFF:FFFFFFFFFFFFFFFFFFFFFFFFFFFFFFFFFFFFFFFFFF:FFFFFFF:
FFFFFFFFFFFF:FF:FFFFFFFFF:FFFFFFFFF::FFF,F:FFFFFFFFFFFF:FFFFFFFFFFFF
FFF
@A00721:190:HGJW7DSXY:4:1546:20039:27899_1:N:0:TCTGCAAG+GCTGCACT
GGTTATTCTCACAACAAAGATAAGATTTTATCTTTCTTCAAACACCCTGTTTTCCTTAAGGGCGGACA
ATTGTTCCATTGTGAACTCTTGGGGACTAGATTGAGCATTAATGAGTTCCGAATAACTCGTAATAAAT
CTT
+
FFFFFFFF:FFFF:FFFFFFFFFFFFFFFFFFFFFFFFFFF:FFFFFFFFFF:FFFFFFFFFFFFFFF
FFFFFFFFFFFFFFFFFFFFFFFFFFFFFFFFFFFFFFFFFFFFFFFFFFFFFFFFFFFFFFFFFFFF
FFF

```
@A00721:190:HGJW7DSXY:4:1125:13702:10692_1:N:0:TCTGCAAG+GCTGCACT
CCTATTTCCGTACCAAGAGCCACTGCCAATCTCATCGCTATACGCATACCTGATTGGAATAAGCGAGG
TTCGTTCCTCAATAAAAATTTTTCTCAAGAGATTATGGTTGATAAAGTTTTCTTCATAAGGGCTATTC
AGT
+
FFFFFFFFFFFFFFFFFFFFFFFFFFFFFFFFFFFFFFFFFFFFFFFFFFFFFFFFFFFFFFFFFFFF
FFFFFFFFFFFFFFFFFFFFFFFFFF,FFFFFFFFFFFFFFFFFFFFFFFFFFFFFFFFFFFFFFFFF
FFF
@A00721:190:HGJW7DSXY:4:1622:32832:1141_1:N:0:TCTGCAAG+GCTGCACT
CTTCGGTCAATGACAAAACCTTCGAATATGTTACGACATGGCTTCGTAACAACAGTGCGAGGATTGTC
ATCTCGGGGAAAGTGATTCACCAAGATGTTCATATAGATACGAGATACCTAGAGAAGGTCGCAGCGAT
CTT
+
FFFFFF:FFFFFFFFFFFFFFFFFFFFFFFFFFFFFFFFFFFFFFFFFFFFFFFFFFFFFFFFFFFFF
FFFFFFFFFFFFFFFFFFFFFFFFFFFFFFFFFFFFFFFFFFFFFFFFFFFFFFFFFFFFFFFFFFFF
FFF
@A00721:190:HGJW7DSXY:4:1576:8693:36526_2:N:0:TCTGCAAG+GCTGCACT
AGCTAAGAAGAAGACTTTGGGTACACGTTATGCTCGCAAACTTAACGTTAGTACCGGAGGTGATCTCT
CTGCTTCTGCGTTACTGAGAGAAAAGATTCAGGACGCCTACGGTGACT
+
FFFFFFFFFFFFFFFFFFFFFFFFFFFFFFFFFFFFFFFFFFFFFFFFFFFFFFFFFFFFFFFFFFFF
FFFFFFFFFFFFFFF:FFFFFFFFFFFFFFFFFFFFFFFFFFFFFFFF
@A00721:190:HGJW7DSXY:4:1350:10619:6198_1:N:0:TCTGCAAG+GCTGCACT
CGTGTGCTCTTCCGATCTAACGAATCTTGAAACTACGGTCCTTATCAAAGAAAATGGTGAACCGGTCT
CTAAATCTTTTGAGATTGAGAAGATTGAAGAACACGTCAAGAAGTCGCTCGGCGTTGTTCTTTAAAAC
CAA
+
FFFFFFFFFFFFFFFFFFFFFFFFFFFFFFFFFFFFFFFFFFFFFFFFFFFFFFFFFFFFFFFFFFFF
FFFFFFFFFFFFFFFFFFFFFFFFFFFFFFFFFFFFFFFFFFFFFFFFFFFFFFFFFFFFFFF:FFFFF
FFF
@A00721:190:HGJW7DSXY:4:1232:14714:22091_1:N:0:TCTGCAAG+GCTGCACT
GCTGATCATGGAGTCTTTGTACCTTCTGTCAAACTTCGAGGTTCAGCTACGAGAGGTGATCCCGGATG
TGGATTGAAGGCTGGCTCTAAGGGAGACTCCTTCTTTTTGAATGTTGTAAAGAATTGGGTTGAGAAGT
GTG
+
:F,F:,FFFFFFF:FFFFFFFF:FFFFF,FFFFFFFFFFFFFFFFF,FFFFFFFFFFFFFFFFFFFFF
FFF:FFFFFFFFFFFFFFFFFFFFFFFFFFFFF:FFFFFFFFFFFFFFFFF:FFFFFFFFFFFFFFFFF
FFF
@A00721:190:HGJW7DSXY:4:2304:12237:25160_1:N:0:TCTGCAAG+GCTGCACT
AGTTTTTAGATCTTCTTGATAATCTTAAAGAAAAATTTTCGAATAAACTAATGAGAGGTATAGGCTTA
GTCAAGTCTGTACTTACATTCTTTACGCCAGATTCTTCATGGGGCGACGCTGTTTCTCTTGGTATTTC
TGA
+
FFFFFF:FFFFFFFFFFFFFFFFFFFFFFFFFFFFFFFFFFFFFFFFFFFFFFFFFFFFFFFFFFFFF
FFFFFFFFFFFFFFFFFFFFFFFFFFFFFFFFFFFFFFFFFFFFFFFFFFFFFFFFFFFFFFFFFFFF
FFF
@A00721:190:HGJW7DSXY:4:1522:30843:36151_2:N:0:TCTGCAAG+GCTGCACT
CAGAGCGTCATTTTCCTTTGGGATCTTTCCTCTGGTAAGGGAATTATTAAATCATTCTTCTCACTCTT
GACGAGAATTATTACTGTTTTCTCTACCCATAACTTCTTTTTCAAAAA
+
F:FFFFFFFFFFFFF:,FFF:FFFFFFFFFFFFFFFFFFFFFFFFFFFFFFFFFFFFFFFFFFFFFFF
FFFFFFF,:FFFFFF:FFFF,FFFFFFFFFFFF,FFFFFFFF::FFFF
@A00721:190:HGJW7DSXY:4:1249:4896:13792_1:N:0:TCTGCAAG+GCTGCACT
CTGATGTTTTCCTACAGAGTGCAGTCGTTTCAACACTTATCACCACTTTTGAAAGGAAAATAATTGGT
```

GATGAAAATACGTATGTCGGTGGGGTTCTCAAGGCTCTCATCGCCACTGGGATTCTAAGGGGTTCCAT
AAG
+
FFFFFFFFFFFF:FFFFFFFFFFFFFFFFFFFFFFFFF:FFFFFFFFFFFFFFFFFFFFFFFFFFFFFF
FFFFFFFFFFFFFFFFFFFFFFFFFFFFFFFFFFFFFFFFFFFFFFFFFFFFFFFFFFFFFFFFFFFFF
FFF
@A00721:190:HGJW7DSXY:4:1278:12590:10175_2:N:0:TCTGCAAG+GCTGCACT
AGGATTCTTCTTTTGGTCCTTACGTCTTAAGTAATTTGGATGTTGTCGAAAACTATCAGGTTCTTGTC
ACGATTCTTAAAAGAAGTTTATTAAAACTTCAGAGTCTCTTGGAAGTC
+
FFFFFFFFFFF,FFFFFFFFFFFFFFFFFFFFFFFFFFFFFFFFFFFFFFFFFFFFFFFFFFFFFFFFF
FFFFFFFFFFFFFFFFFFFFFFFFFFFFFFFFFFFF:FFFF:FFFFFFF
@A00721:190:HGJW7DSXY:4:1609:24777:19288_2:N:0:TCTGCAAG+GCTGCACT
TGTTCTTGCAATAGCCACAGACTTTCTGGGAAAAGAGACCTCTGTTGGTAGAGGAGTTGAGAGAATTA
CTCAGATTCAATCTGGCATCACTGAACGTCTACAACGAGTTAGGGAGT
+
F:,FFFFFFFFFFF:FFFFFFFFF:FF,F,FFFFFFFFFFFFFFFFFFFFFFFFFFFFFFFFFFFFFFF
:FFFFFFFFFFFF:F::FFFFFFFF:F,FF:F:FF,:FFFFFFFFFFF
@A00721:190:HGJW7DSXY:4:1358:24099:2331_1:N:0:TCTGCAAG+GCTGCACT
AAAAGATCCTTTGCACAAGCAATACCTTCCACTGAGTTCATCACCCAGAGTTTCGACGTCGCGGAAAA
CGATGAAGATTTCCACACTGTTCGAGAATCTTCTGATGCTGAATTTGAGGAAAATTTTTCGGAAGTTT
TTG
+
FF:FFFFFFFFFFFFFFFFFFFFFFF:FFFFFFFFFFFFFFFFFFFFFFFFFFFFFFFFFFFFFFFFFF
FFFFFFFFFFFFFFFFFFFFFFFFFFFFFFFFFFFFFFFFFFFFFFFFFFFFFFFFFFFFFFFFFFFFF
FFF
@A00721:190:HGJW7DSXY:4:2172:23954:15170_1:N:0:TCTGCAAG+GCTGCACT
TGATGGCTCAGTACAGGGAGGAGGTAATAGTTATAAAACCTTCTTTTATCATTGCAACAGGACTTTGA
GAAGACTGTTGAAATGGACAAAATCTGGGATCATAGGTATGTGTCACGCTACACTCGAAATATGGGCC
CTC
+
FFFFFFFFFFFFFFFFFFFFFFFFFFFFFFFFFFFFFFFFFFFFF:FFFFFFFFFFFFFFFFFFFFFFF
FFFFFFFFFFFFFFFFFFFFFFFFFFFFFFFFFFFFFFFFFFFFFFFFFFFFFFFFFFFFFFFFFFFFF
FFF
@A00721:190:HGJW7DSXY:4:1634:7979:12085_2:N:0:TCTGCAAG+GCTGCACT
GTTGGCGTCTTGTCGTTGACGTCATTACAGTCTGGAAGTTAGATCATTTGTTCAGGAAGATCTCTACA
GAGATTATCGAGAGTTGTGATCTAGTTCTAAATTTAGGTCGACGGACT
+
FFFFFFFFFFFFFFFFFFFFFFFFFFFFFFFFFFFFFFFFFFFF:::FFFFFFFFFFFFFFFFFFFFFF
FFFF:FFFFFFFFFFFFFFFFFFFFFFFFF,FF:F:FFFFFFFFFFFF
@A00721:190:HGJW7DSXY:4:2327:29351:25238_1:N:0:TCTGCAAG+GCTGCACT
CTGACCATCGCAAAGAAGACTTTATTAGTTCATGAAGTTCTTACACTTCTCGCGACTAATGTCAGCTT
CACTACAAAGGCGTTACGTAAAGTAGTTTTCACGCTTTATCTTAAAGCATTACCACCAAATTTAGCAC
TT
+
FFFFFFFF,FFFFFFFFFFFFF:FF:FFFFFFFFFFFFFFFFFFFFFFFFFFFFFFFFFFFFFFFFFFF
FFFFFFFFFFFFFFFFFFFFFFFFFFFFFFFFFFFFFFFFFFFFFFFFFFFFFFFF:FFFFFFFFFFFF
FF
@A00721:190:HGJW7DSXY:4:1616:10691:4319_2:N:0:TCTGCAAG+GCTGCACT
TCGCCATATCAGATTAGAGACATGCCAGTTAGAGTTCTTCTAACCAGGACTGATGGTGAGATTCTTTT
ACCTTTTGGAGGTCCAGGAACCTTTACTCCTAGTGTCTTTTCTAGAGT
+
FFFFFFFFFFFFFFFFFFFFFFFFFFFFFFFFFFFFFFFFFFFF:FFFFFFFF:FFFFFFFFFFFFFF
FFFFFFFFFFFFFFFFFFFFFFFF:FFFFFFFFFFFFFFFFFFFFFFFFFF

@A00721:190:HGJW7DSXY:4:2242:21368:9502_2:N:0:TCTGCAAG+GCTGCACT
GTCGGAGGATGACAGGTGGGAGACCGCTCAATAACACTCTATTGAATCCCTTTTCCACGATGAAAGCT
CTTTATGAGGTGCTCCATGCAAATTTACAGTTCCTCTTTGGTGATATC
+
FFFFFF:FFFFFFFFF,FFFFF,FFFFFF:FFFF:FFFFFFFFFFF,FFFFFFF:FF:FFFFFFFFFF
FF:FF:FFFFFFFFFFFFFFFFF:FFFF,FFFF,F:FFFFFFFFFFFF
@A00721:190:HGJW7DSXY:4:1649:30490:24267_2:N:0:TCTGCAAG+GCTGCACT
TTCTCCTTTAATCGTGAGGTTACGAGAGTTTCGTACTTGGGTTTATCGGTTGTATTTTGATAAATTTG
TTGGAATCGATAATGATACTGACGAAGGTTCTATTACCACCCTTGTCA
+
FFFFFFFFFFFFFFFFFFFFFFFFFFFFF:FFF,F:FFFFFFFFFFFF:FFFFFFFFFFFFFFFFFF
F::FFFFFFFFFFF,FFFFFFFFFFFFFFFFFFF:FFFFFFFFF:FFF
@A00721:190:HGJW7DSXY:4:1562:27814:14810_1:N:0:TCTGCAAG+GCTGCACT
GTCTAGCCGCTGAGATTCGTCTTACCTATTTACAATCTGTAGAGAGAGAGGTTAATCAGAATTTGAGT
GATAACGATTTAGTACTGGGAAATGGTGGTAGTCAGTTTACTACTTTTCTTTCCCTCCCCTTTTTCGA
TTG
+
FFFFFFFFFFFFFFFFFFFFFFFFFFFFFFFFFFFFFFFFFFFFFFFFFFFFFFFFFFFFFFFFFFFF
FFFFFFFFFFFFFFFFFFFFFFFFFFFFFFFFF:FFFFFFFFFFFFF:FFF:FFFFFFFFFFFFFFFF
F:F
@A00721:190:HGJW7DSXY:4:1443:25509:34240_2:N:0:TCTGCAAG+GCTGCACT
AGGTTTTTCTTTTATGGGACTCTTGTTTGTACTTATGAAGGAGGTATTACTTCGGAAGTTCTTAATGT
AGGTCTCCTAGTACTTAAGGGTTTATGTTTCGGTTTCAGCTGGATGGA
+
FFFFFFFFFFFFFFFFFF:FFFF:FFFFFFFFF,FFFFFFFFFFFFFF:FFFFFFFFFFFFFFFFFF
FFFFFFFFFF:FFFFFFFFFFFFFFFFFFFFFFFFFFFFFFF:FFFFFF
@A00721:190:HGJW7DSXY:4:1366:20211:24158_1:N:0:TCTGCAAG+GCTGCACT
ATACGAGATCTTGCAGAGTGACTGGAGTTCAGACGTGTGCTCTTCCGATCTTATTATGTCGCTAAAGC
CTGTCCAGTTGTCGATGATGTTCGGGCTGTGGGGATTAACTTCCCTCGTGAAACTTACGACGATGTTC
TCG
+
F,FF::FFFFF:FFFFFFFFFFFFFFFFFFFFFFFFFFFFFFFFFFFFFFFFFF:FF:FFFFFFFF:FFF
FFFFFFFFFFFFFFF:F:FFFFFFFFFFFFFFFFFFFFFFFFFFFFFFFFFFFFF:FFFFFFFFFFFFF
FFF
@A00721:190:HGJW7DSXY:4:1123:3152:14935_1:N:0:TCTGCAAG+GCTGCACT
ATTACTAAGTCTGAGATCTGCCACTTCTAATTTTAGGAAACGTCGCGAAAAGAAAGTTCATGAGGATG
TCTTGGTCGAAAAATATCGCCGGGAACTTCTAATAGAGGATCCTGATGACATTATTTCAACGGATACC
GAT
+
FFFFFFFFFFFFFFFFFFFFFFFFFFFFFFFFFFFFFFFFFFFFFFFFFFFFFFFFFFFFFFFFFFFF
FFFFFFFFFFFFFFFFFFFFFFFFFFFFFFFFFFFFFFFFFFFFFFFFFFFFFFFFFFFFFFFFFFFF
FFF
@A00721:190:HGJW7DSXY:4:1114:30156:34460_2:N:0:TCTGCAAG+GCTGCACT
CACAGAAACCGTCTCAGGAACTGTCTCCACTGATTCGGAATTAGAGAAAGACGACCTTCTTGTGGGGA
CTGAGACCTCTGAATTTTCAGGGTGCCCTGATGAGGACGTTGAAAGGG
+
FFFFFFFFFFFFFFFFFF:FFFFFFFFFFFFFFFFFFFFFFFFFFFFFFFFFFFFFFFFFFFFFFFFF
FFFFFFFFFF,FFFFFFFFFFFFFFFFFFFFFFFFFFFFFFFFFFFFF
@A00721:190:HGJW7DSXY:4:1625:11234:6511_2:N:0:TCTGCAAG+GCTGCACT
AGACTTCTAAGAGTGTTTCAAAAGACCAGAAACGTAACAAGAAGAATAAAGGTAGGAAGGGGAGAAAA
CAGAACAAGCGAGCGGTTGAAGAGAAAACTGATATGGTTGCGGCTGAT
+
FFFFFFFFFFFFFFFFF:FFFFFFFFFFFFFFFFFFFFFFFFFFFFFFFFFFFFFFFFF:FFFFFFFFFF
FFFFFFFFFFFFFFFFFFFFFFFFFFFFFFFFFFFFFFFFFFFFFFFF

```
@A00721:190:HGJW7DSXY:4:1160:8495:30545_2:N:0:TCTGCAAG+GCTGCACT
TAGAAAGATCACTCTCGGCGATACCACTGATCTTCCAGAGTTACGTAGTGGTCGTTCTTTTGGTGATT
TTATCAAAGAGAAAGTTTCTGCTAGTCGCGACGATAGTATTTATCGGA
+
FFFFFFFFFFFFFFFFFFFFFFFFFFFFFFFFFFFFFFFFFFFFFFFFFFFFFFFFFFFF:FFFFFFFF
FFFFFFF:FFFFFFFFFFFFFFFFFFFFFFFFFFFFFFFFFFFFFFFF
@A00721:190:HGJW7DSXY:4:1108:24569:2049_1:N:0:TCTGCAAG+GCTGCACT
AAGTTAAACTTCGAACTGACCACTTACAAGAGTGTTGAAGTACCGCAACATGGATCAGTTTTCCAAAA
AGCTATTAATAGCAGTTGCGGGGCTATAATACCGCCTCCAGATTACTCTAAACTGAATCCACTCTCTT
GG
+
FFF:FFFFFFFFFFFFFFFFFFFFFFFFFFFFFFF:FFFFFFFFFFFFFFFFFFFFFFFFFFFFFFFFF
FFFFFFFFFFFFFFFFFFFFFFF:FFFFFFFFFFFFFFFFFFFFFFFFFFFFFFFFFFFFFFFFFFFFF
FF
@A00721:190:HGJW7DSXY:4:2602:28339:32064_2:N:0:TCTGCAAG+GCTGCACT
TTTGGAACTGTGTAATGTCCTACGTACTGTTCAAGGTGTTTGGGCTTCTCTAAAGAACAGCAACTTTC
AACATGAATTAGTTATTGAAAGTCTTGACCCTCACGTCTCACTTTATC
+
FFFFFFFFFFFFFFFFFFFFFFFFFFFFFFFFFFFFFFFFFFFFFFFFFFFFFFFFFFFFFFFFFFFFF
FF:FFFFFFFFFFFFFFFFFFFFFFFFFFFFFFFFFFFFFF:FFFFFFF
@A00721:190:HGJW7DSXY:4:1441:26693:28025_2:N:0:TCTGCAAG+GCTGCACT
AAAGAGGGTTTGAAAATTACGGAACCTTTCCTCGTTCTTCATGAAGATCTTCGAGTGTTTTATAGTGA
GTTATTCTTGCGAAGGTTCAAGTCGATTGATTTTGTTGACTACGATCT
+
FF:FFFFFFFFFFFFFF::FFFFFFFFFFFFFFFFFFFFFFFFFFFFF:FFFFFFFFFFFFFFFFFFFFF
FFFFFFFFFFFFFFFFFFFFFFFFFFFFFFFFFF,FF,FFFF:FFFF:
@A00721:190:HGJW7DSXY:4:1633:12581:32894_1:N:0:TCTGCAAG+GCTGCACT
AGAAGTTCTTCGCAGTTGAATCTCCACCAGGTGGAGGGAAGACCACTGAACTTATAGCGACTTTTGTC
GAATTTTTCGCAAAAGGTCTTGAAGTACGTATTGTCACTGCTAATCGTAATTCGCGAGAAGAGATAGT
GAC
+
FFFFF:FF:FFFFFFFFFFFFFFFFFFFFFFFFFFFFFFFFFFFFFFFFFFFFFFFFFFFFFFFFFFFF
FFFFFFFFFFFFFFFFFFFFFFFFFFFFFFFFFFFFFFFFFFFFFFFFFFFFFFFFFFFFFFFFFFFFF
FFF
@A00721:190:HGJW7DSXY:4:2165:16423:20854_2:N:0:TCTGCAAG+GCTGCACT
CACTTCTAGTTCATCGCGGTATTTGTGAAATTAAGACAGACGTGAAAGTTATGATGAGTGATCGTATT
AGAACAGCTGATTCTTATTGCATGAACGTCGCTGAGGTCAAACCTGTG
+
FFFFFFFFFFFFFFFFFFFFFFFFFFFFFFFFFFFFFFFFFFFFFFFFFFFFFFFFFFFFFFFFFFFFF
FFFFFFFFFFFFFFFFFFFFFFFFFFFFFFFFFFFFFFFFFFFFFFFF
@A00721:190:HGJW7DSXY:4:1307:14181:12305_1:N:0:TCTGCAAG+GCTGCACT
CGACGAATTATTCATGATTCACGCTGGTCAGACCCTTTTTCTTACACATCTACTTAAGGCGGCTTATA
CCATAGGTTACGGTGATTCCAATCAAATCGGATTCATCAATCGTATTGATCTCCAAGTCGCAAACTAC
TTC
+
FFFFF:FFFFFFFFF,FFFF:F:FFFFFF:FFF:,FFFFFFFFFFF:FFFFF:FFFFFFFFF,FFFFF
F,FFFFFFFFFFFFFFFFF:FFFF:FFFFFFFFFFFFFFFFF:FFFFFFFFFFFFFFFFFFFFFFFFFF
FFF
@A00721:190:HGJW7DSXY:4:1170:23538:17613_2:N:0:TCTGCAAG+GCTGCACT
GATGAGTGTCGTTCTTATCGAAGTCTCAGTTATCGCTGCCCGGCCGACGTTTGTCGCGAACTCTCAGA
GGTTTACGGCAGAAAAATTTCTCCTGCTTGTAATTCGAGAGAGGACAC
+
FFFFFFFFFFFFFFFFFFFFFFFFFFFFFFFFFFFFFFFFFFFFFFFFFFFFFFFFFFFFFFFFFFFFF
FFFFFFFFFF:FFFFF:FFFFFFFFFF:FFFFFFFFFFFFFFFFFFFF
```

```
@A00721:190:HGJW7DSXY:4:2513:26169:20134_1:N:0:TCTGCAAG+GCTGCACT
CTTGACGACTTCGAATTTGAGGACGGAGTTAAATATATTACTTTCACTCAAGGTGAAAAGTTTGAAAT
TAACAAAGTCCTTGGTAAGAGTAAGGTTAAATGTGAAGATGCTCAGACTATTCATGAAGTTCAAGGTA
ATA
+
FFFFFFFFFFFFFFFFFFFFFFFFFFFFFFFFFFFFFFFFFFFFFFFFFFFFFFFFFFFFFFFFFFFFFF
FFFFFFFFFFFFFFFFFFFFFFFFFFFFFFFFFFFFFFFFFFFFFFFFFFFFFFFFFFFFFFFFFFFFFF
FFF
@A00721:190:HGJW7DSXY:4:2456:15356:15718_1:N:0:TCTGCAAG+GCTGCACT
TGACCCTGTTTTTGCATCACAAACACATATTACCGTTGCTCTATCACGACACGTTGAATCTTTACGAT
ACTTTGTTATCAATGTAAAGTTAAACGATGCTACTTGTGAGAGGATCAATCACAGTAAGGAACTGTCA
CGT
+
FFFFFFFF:FFFFFFF,FFF,FFFFFFFFFFFF:FFFFFFFFFFFFFFFFFFFFFFFFFFFFFFFFFFFF
FFFFFFFFFFFFFFFFFFFFFFFFFFFF:FFFFFFFFFFFFFFFFFFFFFFFFFFFFFFFFFFFFFFFFFFF
FFF
@A00721:190:HGJW7DSXY:4:1354:30472:1313_2:N:0:TCTGCAAG+GCTGCACT
GATTCAACCCCTTATATCGAGGCTATCGAGGACGATGAGTATCTTGAAGTTGTTGACGACAAGCCTCA
AGAGGGTAGAGGACCACCTGGATCTTCTACTGTCTCTGCCTTCCAAGA
+
FFF:FFFFFFFFFFFFFFFFFFFFFFF:FFFFFFFFFFFFF:FFFFFF:FFFFFFFFFFFFFFFFFFFFF
FF:FFFFFFFFFFFFFFFFFFFFFFFFFFF:FFFFFFFFFFFFFFFFF
@A00721:190:HGJW7DSXY:4:2421:10538:29011_2:N:0:TCTGCAAG+GCTGCACT
TTATTCCTGGAACCACCTTAACGTCACTCGGTAATCTTTCAGCCGAATTAAGTACTAATGAGTTCAAG
TCGGTTGTCGATCGAATTAAGATCAAGGCTAATACCTTTATAGGTAAG
+
FFFFFFFFFFFFFFFFFFFFFFFFFFFFFFFFFFFFFFFFFFFFFFFFFFFFFFFFFFFFFFFFFFFFFF
FFFFFFFFFFFFFFFFFFFFFFFFFFFFFFFFFFFFFF,FFFFFFFFF
@A00721:190:HGJW7DSXY:4:2104:3685:35430_1:N:0:TCTGCAAG+GCTGCACT
CCCGATAGGGTACCGAGTCTCCAAGAGAATATTTATTCTTATGAGGCTCGAAACTTCAACGTTTTCAC
AACGGAAAGACATATCGCACCCGAAATTTTTGGGCAGAATGCTCTTAACGCGGTTCTTGAACGGTGTT
TTG
+
FFF:FFFFFFFFFFFFFFFFFFFFFFFFFF:FFFFFFFFFFFFFFFFFFFFFFFFFFFFFFF:FFFFFFF
FFFFFFFFFFFFF:FFFFFFFFFFFFFFFFFFFFFFFFFFFFFFFFFFFFFFFFFFFFFFFFFFFFFFFF
FFF
@A00721:190:HGJW7DSXY:4:1277:30436:7827_1:N:0:TCTGCAAG+GCTGCACT
GATAAAGTTATAGGAGACTCAGTTAACCTGAGAGCTCCTCAATTGATAGATTGGGTCAACAATAGGGA
CGCTAGTATACTTCGTAAAGTCGACAGCGAAATGGTCAATATTGATAATTGGACCGACTATATGACCT
ATT
+
FFFFFFFFFFFFFFFFFFFFFFFFFFFFFFFFFFFFFFFFFFFFFFFFFF,FFFFFFFFFFFFFFFFFFF
FFFFFFFFFFFFFFFFFFFFFFFFFFFFFFFFFFFFFFFFFFFFFFFFFFFFFFFFFFFFFFFFFFFFFF
FFF
@A00721:190:HGJW7DSXY:4:2237:18801:4460_1:N:0:TCTGCAAG+GCTGCACT
AAGGGAAGCTAAGGTTAAACTTGATGCTTCAGCCTTTTCGAAGCATAACCCCGCACAGAATATCGTTT
ATCACGCAAAATTTATTAATGCAATTTTCGGACCTATCTTTAAGGTTCTTGTGAGGAGAGTCAAACAG
TGT
+
FFFFFFFFFFFFFF:FFFFFFFFFFFFFFFFFFFFFFFFFFFFFFFFFFFFFFFFFFFFFFFFFFFFFFFF
FFFFFFFFFFFFFFFFFFFFFFFFFFFFFFFFFFFFFFFFFFFFFFFFFFFFFFFFFFFFFFFFFFFFFF
FFF
@A00721:190:HGJW7DSXY:4:2441:31403:24784_1:N:0:TCTGCAAG+GCTGCACT
ACATTTTTATGTATACAGAGATGACCAGTGACGATTTCTCCCGGAAGCTCTTCTCTCTCTTAGGAGGT
```

CATGATGACTACTATAAGGGTGAAATCGATTTTTCGAAGTTTGACAAATCTCAAGATTTCTTTTGTAA
AGC
+
FFFFFFFFFFFFFFFFFFFFFFFFFFFFFFFFFFFFFFFFFFFFFFFFFFFFFFFFFFFFFFFFFFFFFF
FFFFFFF::FFFFFFFFFFFFF:F,F,FFFFFFF:::F:FFFF:FFFFF:FFFFFFFFF,FFFFFFFF
F,F
@A00721:190:HGJW7DSXY:4:1225:20112:9737_2:N:0:TCTGCAAG+GCTGCACT
TTCGATGCTGAATTTCTTGAACTTTGGAGTGCCAGTGAATATTTTTGTCACGCCAGGAGTATCCATAA
CGATTTGTCATTTACCACCTTTGCTCAACGACGAAGTGGAACGGCCAA
+
FFFFF:FFFFFFFFFFFFFFFFFFFFFFFFFFFFFFFFFFFFFFFFFFFFFFFFFFFFFFFFFFFFFF
FFFFFFFFFFFFFFFFFFFFFFFFFFFFFFFFFFFFFFFF:FFFFFFF
@A00721:190:HGJW7DSXY:4:2534:18873:16172_2:N:0:TCTGCAAG+GCTGCACT
TAATTGTATGATCACTTTGATGATTTTATCACTTTATTATGACCTTGGAAAGTTTTCGGCTGTGGTTG
TCGCTGGGGACGATTCCATTCTGTTTTCAAAGACTCCAATAACTGATC
+
FFFFFFFFFFFFFFFFFFFFFFFFFFFFFFFFFFFFFFFFFFFFFFFFFF:FFFFFF:FFFFFFFF
FFFFFFF:FFFF:FFFFFFFF:FFFFFFFFFF:FF:FFFFFFF,FFFF
@A00721:190:HGJW7DSXY:4:1231:10484:29982_2:N:0:TCTGCAAG+GCTGCACT
TGCTCGCGATTTATTCTTTTTGTCAATGATTACATCTACTTCGTTCCTGATCCTTACAAGATCATCGT
TAAGTTAGGTAAACCCCTAACCGATGTTACTCTTGTAGGACTTTGGGA
+
FFFFFFFFFFFF:FFFFFF:FFFFFFFFFFFFFFFFFFFFFFFFFFFFFFFFFFFFFFFFFFFFFFFFF
FFFFFF:FF:FFFFF:FFFFF:FFFFFFFFFFFFFFFFFFFFFFFF:FF
@A00721:190:HGJW7DSXY:4:1177:28302:11898_2:N:0:TCTGCAAG+GCTGCACT
TTAAAGACTATACTAAATATCTCCACTACGAGGTTGTTGTTTGGCGATTAGCTCTTGCGACTAATATT
CGATATTCGATTGAGGGTCGTTATACCTATTCGGCTATCTGTGCTTTG
+
FFFFFFFFFFFFFFFFFFFFFFFFFFFFFFFFFFFFFFFFFFFFFFFFFFFFFFFFFFFFFFFFFFFFFF
FFFFFFFFFFFFFFFFFFFFFFFFFFFFFFFFFFFFFFFFFFFF:FFF
@A00721:190:HGJW7DSXY:4:1562:20582:7200_2:N:0:TCTGCAAG+GCTGCACT
TCAAATTTTAAACGATTCCGAGAGGTTTATCCTGATGTGGAGGGAGTTCTTGAAGCATCCCGACCTTT
CCTTGTGAAAATTTTGATGCGGTTTCATAAGATTATCAGTAATCCTGA
+
FFFF:FFFFFFFFFFFFFFFFFFFFFFFFFFFFFFFFFFFFFFFFFFFFFFFFFFFFFFFFFFFFFFFF
FFFFFFFFFFFFFFFFFF:FFFFFFFFFFFFFFFFFFFFFFFFFFFFF
@A00721:190:HGJW7DSXY:4:1545:12617:35243_1:N:0:TCTGCAAG+GCTGCACT
ATTCATGGACCTTCCATCCTGTACCTCTTCTGGAGAGACGTATCTCAAAGATCGACTTCGAGACAGGT
GATATTGTTGAGCGAGAGCGACCAAGATCTAAATTCGGTAACGGTGCGTGGCGCTTTATTTTAAAAAG
CTC
+
FFFFFFFFFFFFFFFFFFFFFFFFFFFFFFFFFFFFFFFFFFFFFFFFFFFFFFFFFFFFF:FFF:FFF
FFFFFFFFFFFFFFFFFFFFFFFFFFFFFFFFFFFFFFFFFFFFFFFFFFFFFFFFFFFFFFFFFFFFFF
FFF
@A00721:190:HGJW7DSXY:4:2369:12246:3411_2:N:0:TCTGCAAG+GCTGCACT
CTGATACTTATCACGAAAGGACTTGAGTCTTTAGATCAGTCTCTGACTTGACCATCTTTCATTTTAAA
TGACGATCATTAAGACTCTCTATCTTCTTTGTTTCCTTCTTTGCACTG
+
F,FFFF,FFFFFFF:FFF:,:F:F,FF:F,FF,FFFF,,F::FFFFFFFFFF,:FF,FFFFFF,,,FF
::FFFF,FF,:FFFFF,FFFF,,FF,F,FF,FFFFFF:F,FFF:F,F:
@A00721:190:HGJW7DSXY:4:1642:1090:5666_1:N:0:TCTGCAAG+GCTGCACT
CTCTTCCGATCTCTAATCTGTGTTTTTACGATAACAACTATCAGTTGATTAAGACTCTTCCGAAAGGC
GAACTTTGGGAATTTGAAGCTCAGGGTGAAGCACAACGTTTGACCGTGACGAACTGTGGTGTTCTTGA
TCT

```
+
F:FFFFF:::FFF:::FF:FFF::FFFFFFFFFFFFFFFF,:FFFFFFFFFFFFF:FFFFFFF:FFFF
FFFFFFFFFFFFFFFFFFFFFFFFFF:FFFFFFFFFFFFFFFFFFFFFFFFFFFFFFFFFFFFFFFFF
FFF
@A00721:190:HGJW7DSXY:4:1609:4698:15859_1:N:0:TCTGCAAG+GCTGCACT
ATACTCTCCTTGAGTTCACTCGTAAGGACACTACCACTTATTACGATATTTCTTTAGTCGATGGGTTT
TCGACTCCTTTAAGCGTCTTCTGTTCATCGCAACCAGATGATATCTTCTATTCGAACTTATGTTTCGA
TCA
+
FFFFFFFFFFFFFFF:FFFF:FFFFFFFFFFFFFFFFFFFFFFFFFFFFFFFFFFFFFFFFFFFFFFF
FFFFFFFFFF:FFFFFFFFFFFFFFFFFFFFFFFFFFFFFFFFFFFFFFFFFFFFFFFFFFFFFFFFF
FF:
@A00721:190:HGJW7DSXY:4:1636:21730:35931_2:N:0:TCTGCAAG+GCTGCACT
TCTTCTAATGTGGCCTGAACTACGTTTCACTTTGTGTGTACTTGTCTCTATTTTAAGTTTTGTCTTCC
TTGTTTGTCTTATTCTATTAATCACTCATTTTAGTGAGATTATTAAAA
+
FFFFFFFFFFFFFFFFFFFFFFFFFFFFFFFFFFFFFFFF:FFFFFFFFFFFFFFFFFFFFFFFFFFFF
FFFFFFFFFFFFFFFFFFFFFFFFFFFFFFFFFFFFFFFFFFF:FFFFF
@A00721:190:HGJW7DSXY:4:1429:29134:34100_2:N:0:TCTGCAAG+GCTGCACT
CGACAACAGCAGGGGTTTTCGAATAGTGATAGCCACCCGCCTTACAGAGGGGCAGCAGCATGACCATA
ATTGGTATTGACTTTGGTCCCACCTTTTCTACTGTCTGTCTCGCCTCT
+
FFFFFFFFFFFFFFFFFFFFFFFFFFFFFFFFFFF:FFFFFFFFFFFFFFFFFFFFFFFFFFFFFFFF
F:FFFFFFFF:FFFFFFF,FFFFFFFFFFFFFFFFFFFFFFFFFFFFF
@A00721:190:HGJW7DSXY:4:1178:21169:31015_1:N:0:TCTGCAAG+GCTGCACT
CTTAACAACAATGATAGTGAATTTATACCTACAATCATTGGTTTTTCTAATAATGATGACACTATTGT
TTATGGATATGATGCTATTACTCATGATGGTTACGGTCAGGGAGGATTCTCAGTTTACAAGGACCTTA
AGC
+
FFFFFFFFFFFFFFFFFFFFFFFFFFFFFFFFFFFFFFFFFFFFFFFFFFFFFFFFFFFFFFFFFFFF
FFFFFFFFFFFFFFFFFFFFFFFFFFFFFFFFFFFFFFFFFFFFFFFFFFFFFFFFFFFFFFFFFFFF
FFF
@A00721:190:HGJW7DSXY:4:2366:9019:29606_2:N:0:TCTGCAAG+GCTGCACT
CCGAAAGACGTGCTAAATTAAAACCATTGTACGATGTCGTCTGTCTCGGCGATCACTTTTCACTAAGA
ATTGGTCCTACTTTTGGACAAGGCCAACTTCGTAATCTTCATGAGATC
+
FFFFFFFFFFFFFFFFFFFFFFFFFFFFFFFFFFFFFFFFFFFFFFFFFFFFFFFFFFFFFFFFFFFF
FFFFFFFFFFFFFFFFFFFFFFFFFFFFFFFFFFFFFFFFFFFFFFFF
@A00721:190:HGJW7DSXY:4:2452:18954:6668_1:N:0:TCTGCAAG+GCTGCACT
TTAGGGATTTTGAGAGAGTGAAAGGCTTCAATTGTAGTGGTTTGGTCGTTTCAGTACCCAGTCAGTAT
ACCTCTACGCAAAGATATTTCATGAAAACTACAGAGGAACGTACCGGATTACCTGTCTTTCATATTAT
GAA
+
FFFFFFFFFFFFFFFFFFFFFFFFFFFFFFFFFFFFFFFFFFFFFFFFFFFFFFFFFFFFFFFFFFFF
FFFFFFFFFFFFFFFFFFFFFFFFFFFFFFFFFFFFFFFFFFFFFFFFFFFFFFFFFFFFFFFFFFFF
FFF
@A00721:190:HGJW7DSXY:4:2541:29930:25050_2:N:0:TCTGCAAG+GCTGCACT
TCTTTGCTTCGATGTTGGATGTCAAGAAGGGTTCAGAATGGGATTCCTACGTAGTTTATGACTTTGGT
GGTGGTACTTTCGATACTTCTATCGTCTTACGTTACGGTAATTACTAT
+
FFFFFFFFFFFFFFF:FFFFFFFFFFFFFFFFFFFFFFFFFFFFFFFFFFFFFFFFFFFFFFF,FFFF
FFFFFFFFFFFFFFFFFFFFFFFFF:FFFFFFFFFFFFFFFFFFFFFF:
@A00721:190:HGJW7DSXY:4:1633:26350:12555_1:N:0:TCTGCAAG+GCTGCACT
ATAATCTTGGAGGTCGTGACGTTGATGAAGCTATAAGAGCGTTCTTATTACGGCGCACTTCTTTACCC
```

```
GCCACCACACAACTTTCAGTTTCACAACTGAAGGAGGAGGTTTCTGTTACTGGAAAACCCTCGTCAAT
ACT
+
FFFFFFFFFFFFFFFFFFFFFFFFFFFFFFFFFFFFFFFFFFFFFFFFFFFFFFFFFFFF:FFFFFFFF
FFFFFFFFFFFFFFFFFFFFFFFFFFFFFFFFFFFFFFFFFFFFFFFFFFFFFFFFFFFFFFFFFFFFF
FFF
@A00721:190:HGJW7DSXY:4:1624:14371:31297_2:N:0:TCTGCAAG+GCTGCACT
GATCTAGTGAAATTAACGCGTCCTTTTCTGGAACGTAGTTTTACTATTATGAACAAAGTTATCAAAGA
TAGTGGCATTCAAGGCAAACTTACCCTGATTCCGGCCGGTGGTAGCTC
+
FFFFFFFFFFFFFFFFFFFFFFFFFFFFFFFFFFFFFFFFFFFFFFFFFFFFFFFFFFFFFFFFFFFFF
FFFFFFFFFFFFFFFFFFFFFFFFFFFFFFFFFFFFFFFFFFFFFFFF
@A00721:190:HGJW7DSXY:4:1519:2926:28698_2:N:0:TCTGCAAG+GCTGCACT
TGCTGAAACAGCTATTAAAGCTGTTGAGCGGGTAATTACACCTAAACGAGCTCGATCTGCCATCGCTG
AAGGATGTGCACTCTTTTCTGCTACTTTAACGGAGCGTAGTGTACTCT
+
FFFFFFFFFFFFFF:FFFFFF:::FFFFFFFFFFFF:FFFFFFFFFFFFFFFFFFFFFFFFFFFFFFFF
FFFFFFFFFFFFFFFFFFFFFFFFFF:FFF:FFFFFFF:FFFFFFFFFF
@A00721:190:HGJW7DSXY:4:1663:29749:28338_1:N:0:TCTGCAAG+GCTGCACT
CAACAGTAACATTACTTCTAATAAAGGTCTCTATCAAGCTCGTATTATCGTCGCTGCTGGATCTGCGT
TACCGTTGAAGGCTAAACTCCAAGGTAATTATGAAGACTGTGATTTATCTTTCACTCTGGCCTTGTAT
GAG
+
FFFFFFFFFFFFFFFFFF:FFFFFFFFFFFFFFFFFFFFFFFFFFFFFFFFFFFFFFFFFFFFFFFFFF
FFFFFFFFFFFFFFFFFFFFFFFFFFFFFFFFFFFFFFFFFFFFFFFFFFFFFFFFFFFFFFFFFFFFF
FFF
@A00721:190:HGJW7DSXY:4:1569:18674:8907_2:N:0:TCTGCAAG+GCTGCACT
CGTAACATTAATAATTTCAATCTTCTTAAAAATAATATAGAGAGTCATAATTTGGGCTATCGTTCAGG
TGCATGGGTTTGGATAGCCAATATTGAAGTGGACACCTCTGGTAGTAT
+
F,FFFFFFFFF,FFF::::FFF:,:,FFFFFF,FF,F:F,F,:FFFFFF,FF::F,,,F:,,,:F:FF
::,FF:,:,FF,,FFF:,FFFF:FF:FFFF,FFFFFFF,,,,FFF,F:
@A00721:190:HGJW7DSXY:4:1460:3631:7153_1:N:0:TCTGCAAG+GCTGCACT
CTAAGCATACTACTTTTGCTTTGATTACTCTACCGCCTTACAAGTCTAAGGTTAAAGAGTCGATTAAA
CAAGTTGTACTTATCAATGATTTTATCGCTGGTTGTTATCTCGAAAAGTCTCTTCTAAAAGGAACGTC
AGT
+
FF:FFFFFFFFFFFFFFFFFFFFFFFFFFFFFFFFFFFFFFFFFFFFFFFFFFFFFFFFFFFFFFFFFF
FFFFFFFFFFFFFFFFFFFFFFFFFFFFFFFFFFFFFFFFFFFFFFFFFFFFFFFFFFFFFFFFFFFFF
FFF
@A00721:190:HGJW7DSXY:4:1344:13494:9236_2:N:0:TCTGCAAG+GCTGCACT
CTAAAATATTTACAAAATCTTACCACTATCTACGAACCTAAAGTTCTGAAGACGTTGCTTGTCGCTTC
CTCCTTTAATGGCACTACCTCGGTACAATCTTACTTGGAACGAAGGCG
+
FFFFFFFFFFFFFFFFFFFFFFFFFFFFFFFFFFFFFFFF:FFFFFFFFFFFFFFFFFFFFFFFFFF:FF
FFFFFFFFFFF:FFFFFFFFFFFFF:FFFFFFFFFFFFFFFFFFFFFF
@A00721:190:HGJW7DSXY:4:1326:31358:35415_1:N:0:TCTGCAAG+GCTGCACT
GTTCTGTCTATATTAGGTGAAGCGAAATACGATTTCGAACGTCAACGTTCACCGAGTTTCGTTTCGTA
CCTTGATGCTGACTTTCGTCAATATGATTTGATCTGGTTGCTCAATTCTAAGCGTGTTCCGCTTAGTG
CGA
+
:FF,:F,FFFF,FFFF:FFFFFFFFFFFFFFFFFFFFFFFFFFF:FFFFFFFFFFF:FFF:FF:FFFFF
F:FFFFFFFFFFFFFFFFFFFFF:FFFFFFF:FFFFFFFFFFFFFFFF:FFFFFFFFFFFFFFF:FFF
FFF
```

```
@A00721:190:HGJW7DSXY:4:1344:32398:13041_1:N:0:TCTGCAAG+GCTGCACT
TACTAACTTGACTCTACTATACGTGACATACTAAATACTAGATATTTGGATGATCTTGATAAAGAACT
TCATGAATTTTCAGAGGATGAAAAATTTTCTTCCTTGGATTTTAATATAAAGAATCTTTACCAGGACT
CCT
+
FFF,:F:FFFF,FFFFFFFFF,FFFFFFFFFFFFF::F:FFF:FFF:FFFFFFF:FFFFFFF::FFF
FFFFFF:FFFFFFFFFFFFFFF,FFFFFFFFFFFFF,,FFFFFFFFFFF:FFFFFFFFFFFFFFFFFF
FFF
@A00721:190:HGJW7DSXY:4:1461:4571:4492_1:N:0:TCTGCAAG+GCTGCACT
TGATCGTAAGAAGTTTATAAGGAACATATCCATTTGTTGTAATAGGGTCATCAACTTAGAACTCGAAA
CTCTAAGTGGTAATACTGTCTTTCCAAGCTTACCTGTATCTTCAATGAAATTGGACTCTTCGATAGGG
GAC
+
FF:FFFFFFFFFF,FF:FFFFFFFFFFFF:FFFFFFFFFFFFFFFFFFFFFFFF,FFFFFFFFFFFFF
FFFFFFFFFFFFFFFFFFFFFFFFFFFFFF:FFFF:FFFFFFF:FF:FFFFFFFFFFFFFFFFFFFFF
FFF
@A00721:190:HGJW7DSXY:4:2325:27543:28181_2:N:0:TCTGCAAG+GCTGCACT
TAAAGGAAGATCTGTTATCGGCGGTTTATCGTTTACGAAGTATCACTCCCCCGGTGAATGGTTTAAGA
ATACTATAAATAGAATAGCTATTACTACTAATGGCGTTGGCCTAGTGG
+
FFFFF:FFFFFF::FFFFFF,F:FFF,FFF,F,F:FFFF,FFFFF,FFF,:FF,FFF,F,,,F,:F:F
:,,FF:FFFF,FFFFFF:FF,F:F::F,:FFFFFF,FF:F,F:FFFFF
@A00721:190:HGJW7DSXY:4:2612:32832:27696_1:N:0:TCTGCAAG+GCTGCACT
TTTAGGTGGTCTTTATTCTATTATTGATTTTTATGAGATTGATACGTTAACAGAAGATGATGTTAGAG
TAGAATTCCTGAAGAGTCCTTCCGTAACCAACTTCTTGACAATTTTCTTCAATCCTTCGATAATAGTT
AAA
+
FFFFFFFFFFFFFFFFFFFFFFFFFFFFFFFFFFFFFFFFFFFFFFFFFFFFFFFF:FFFFFFFFFF:F
FFFFFFFFFFFFFFF:FFFFFFFFFFF,FFFFFFFFFFFFFF:FFF:F::FF:,FFFF,FFFFFFFFF
FFF
@A00721:190:HGJW7DSXY:4:2517:10004:25708_1:N:0:TCTGCAAG+GCTGCACT
CCGATAAGGGTCACTAGTCTTACACAATTAAGAAATAATCCTACATTGACTGATTATCTAAAGATTGA
AGGAAAATTTTATCCTCGTTTAACTTACTTAGACTTTCCTACGATACAAATGATAACTTCTAATTTAC
GTT
+
FF,FFFF,F:FFFFFFF:FFFFFF:FFFFFFFFFFFFFFFFF:FFFFFFFF:FFFF:FFFFFFFFFFF
FFFFFF:FFFFFFFFFF:FFFFFFFFFFFFFFFFFFFFFFFFFFFFFF,FFFFFFFFFFFFFFFFFFFF
FFF
@A00721:190:HGJW7DSXY:4:2521:5339:21825_2:N:0:TCTGCAAG+GCTGCACT
AAGGGATGCATGGTGTTATCTCCTCAGGAGTCCCGGCATTTGATAATAATGACGAGACTTTCGTCATA
ATCGCTGCATATTACGGATTACACGGTACCAGCGCATCTCGTGTCGAG
+
FFFFFFFFFFFFFFFFFFFFFFFFFFFFFFFFFFFFFFFFFFFFFFFFFFFFFFFFFFFFFFFFFFF:
FFFFFFFF:,FFF:FFFFFFFFFFFFF:FFFFFFFFFFFFFFFF:FF
@A00721:190:HGJW7DSXY:4:2113:11017:23390_1:N:0:TCTGCAAG+GCTGCACT
AAATTTATAGAAGATTATTTTGACAGAATCAATAAGAATCATCGATTCTCGATTCGTAGAGCTTATCT
AGGTACTCTCATTTACTATGTTGATTTAGTCTACATTTATGCTGATCTAAAATTTCGATCTAGATGGA
TCC
+
FFFFF,FF:FFFF:FFFFFF,FFFF:,F:FFFFFFFFFFFFFFFFFFFFFFFFFFFFFFFFFFFFFFF
FFFFFFFFFFFFFFFFFFFFFFFFFFFFFFFFFFFFFFFFFFFFFFFFFFFFFFFFFFFFFFFFFFFF
FFF
@A00721:190:HGJW7DSXY:4:1319:17508:24455_1:N:0:TCTGCAAG+GCTGCACT
TTATAGAATTATTAATTATAATATTCAACCTCAAAAGTTACGTGTTTACAGTAACGAGATCACACATT
```

CTGAGAACTAACTATGTTATGTTCTCTGATCATTTTGACAATCTCTTCTTATAATAAATAAATAAATA
ATA
+
FF,F,FFFFF,FFFFFFFFFFFFFFFFF,FF:::FFFFFF::F:FFF:,FFFFF,,F:FFFFFF,FFF
,FFFF:,FFFFFF:FFFFFFFFF,FFFF:FFFFFFFFF:F:FFFFFF,FF,FFFFFF:FFFFFFF:F:
,FF
@A00721:190:HGJW7DSXY:4:1362:31403:9815_2:N:0:TCTGCAAG+GCTGCACT
CAAATAGTGGAAAGGCTCCAGTCAACACTAGTTCGAGTGCTGCTTTGGATTACGATGACGTGAGAATA
GGTGAAGGGATAGTTTTTACCTCGACCAACATTAAGGCGGCGGCGACT
+
FFFFFFFFFFFFFFFFFFFFFFFFFFFFFFFFFFFFFFFFFFFFFFFFFFFFFFFFF:FFFFFFFFFFF
FFFFFFFFFFFFFFFFFFFFFFFFFFFFFFFFFFFFFFFFFFFFFFFF
@A00721:190:HGJW7DSXY:4:1323:2266:7169_2:N:0:TCTGCAAG+GCTGCACT
CTTCACCCAAAATGAAGGACACTTCGAAACTGAATAATGATGCACTACAAACAAGTATCGCTAATCAT
CTGAAAAAGGCTCTAAAGGACACTATACAATATCCAGACGACGACGAA
+
FFFFFFFFFFFFFFFFFFFFFFFFFFFFFFFFFFFFFFFFFFFFFFFFFFFFFFFFFFFF:FFFFFFFF
FFFFFFFFFFFFFFFFFFFFFFFFFF:FFFFFFFFFFFFFFFFFFFFFF
@A00721:190:HGJW7DSXY:4:2117:5692:30420_2:N:0:TCTGCAAG+GCTGCACT
CAGCACCACATTTTGGTCAAGTCAATCCGTTGAGAAAATTTCTTAGAGGATATTCGCCATTGTGGTAC
GAATTGGTCCAACATTTACCACCAAATTCTTTTGATACAACTCTAGCC
+
FFFFFFFFFFFFFFFFFFFFFFFFFFFFFFFFFFFFFFFFFFFFFFFFFFFFF:FFFFFFFFFFFFFFF
FFFFFFFFFFFFFF:FFFFF:FF:FFFFFFFFFFFFFFF:FFFFFFFF
@A00721:190:HGJW7DSXY:4:2648:22227:4914_1:N:0:TCTGCAAG+GCTGCACT
ATACGCTTGCTATTCACCAGATTGGTTCGATCACGTTGTTGCTATGTCAGAAGTAGCTATTCAAGCTT
GTCGTTTTAAGTCTGCAGAGGCTGTCATTCAAGCCAAGACTCTAGGTGTCCAATCAAAGGTTTTGAAT
TTA
+
FF,FF,FFF::,FF:,:FF,,:F,FFF,F,,,,,FF,,,FF,F,FF,,::,FF,F:FFF::FF:F:FF
FF,:FFF,,FF,:F::,::FF:,,FFF,,,FF,,,,::FFF:,,FF,F:,,,::,F,FF:FFF:F,,F
,FF
@A00721:190:HGJW7DSXY:4:1408:24985:21214_2:N:0:TCTGCAAG+GCTGCACT
GGTGATAAAAATTATTTAGAGATCTTTTCTACTTACAAAGGACAATTCAGTTTCATTAATTCTACGGA
AAAGTCCGGAGTATATTGTATGTTAATAGATAGTGGGATAATAGTTAT
+
F:FFFFFFFFFFFFFFFFFF:FFFFFFFFFFF:FFFFFFF:F:FF:F:FFFFF:FFFFFFFFFF,::F
FFF,F,:F,FF:FFFF:,FFF,FFFFFF,FFF::::,FFFFFF,FF,F
@A00721:190:HGJW7DSXY:4:1365:25979:20181_1:N:0:TCTGCAAG+GCTGCACT
CGTCTGAAGAGTTTAGAGCTGCTATTGATTATGTAACCGATAGGAGTTGTATAGTTGAAGATGTGGCT
TTAGACGATAATCCTTTAAGGACTATGTTGACTGACGAAGATTTTCCCACAGATTTGTCTACGTTGCA
GAT
+
FF:FFFFFFFFFFFFFFFFFFFFFFFFFFFFFFFF:FFFFFFFFFFFFFFFFFFFFFFFFFFFFFFFFF
FFFFFFFFFFFFFFFFFF:FFFFFFFFFFFFFFFFFFFFFFFFFF:FFFFFFFFFFFFFFFFFFFFFFFFF
FFF
@A00721:190:HGJW7DSXY:4:1102:5249:19820_2:N:0:TCTGCAAG+GCTGCACT
CTATATACATTATAAAATATAAAAATAAGGGAGTCATAACTTTTGTTACCAAATTCTTGGCTTTCAAC
GAAGTCTCTGATTTTATTAATCATTACTTTAACGATCTCTCGGTCATT
+
FFFFFFFFFFFFFFFFFFFFFFFFFFFFFFFFFFFFFFFFFFFFFFFFFFFFFFFFFFFFFFFFFFFF
FFFFFFFFFFFFFFFFFFFFFFFFFFFFFFFFFFFFFFFFFFFFFF:F
@A00721:190:HGJW7DSXY:4:1176:14127:32158_1:N:0:TCTGCAAG+GCTGCACT
TTGATCTACGTCGTTTAACTGTCGGAACAACCTTTACTTAGAATGACTCTATCTTTTCAGACACTTCT

TATATTATTTACAATAGTTAATATCATATTAGTAATACTCACTATCGTTTTCTGCTTTTTCTTAGCTA
ATA
+
FFFFFFFFFFFFFFFFFFFFFFFFFFFFFFFFFFFFFFFFFFFFFFFFFFFFFFFFFFFFFFFF:FFFF
FFFFFFFFFFFFFFFFFFFFFFFFFFFFFFFFFFFFFFFFFFFFFFFFFFFFFFFF:FFFFFFFFFFF
FFF
@A00721:190:HGJW7DSXY:4:2475:19633:29262_1:N:0:TCTGCAAG+GCTGCACT
TTCCGATCTCCAAAACCAACTCTTCGTTACGAGACCTGATGGTAGAATAGATTTAGTCTCTACACCCA
TCTCAACGAGTATAAAGCGTGTTTTCCGTCCTGAACGTAATACACGAAGAGATCAACCGCAAACGACT
CGA
+
::F,,::F,:,FFFF::F:::,F::,F,F,F:,::,F::,F,F,,FFFF,,FFF,:::,,,F:,FF:F
F,F:F:FF,F,,FFF,F:,,,FFF,F:,,::F,F:,,FFF,:F::,F,,:,F::F,FFFFF,FFFFFF
FFF
@A00721:190:HGJW7DSXY:4:1642:1949:8030_2:N:0:TCTGCAAG+GCTGCACT
TTTAGATTATGAATCCTGCCTTTACTCATTTTATAGCAGTTAGTTATGTTGTACTTTTTATAATTTAT
AACCTTCTTATACTTTTGGGTATGTTTGTGAAAAACATGAGATACCC
+
FFFFFFFFFFFFFFFFFFFFFFFFFFFFFF:FFFFFFFF,FF,FFFFFFFFFFFFFFFFFFFFFFFFF
FFFFFFFFFFFF::FFFFFFFFFFFFFFF:FFFFF,FFFFFFFFFFFF
@A00721:190:HGJW7DSXY:4:1336:24722:18474_2:N:0:TCTGCAAG+GCTGCACT
CTTATTGTAACGATCATTCTTTCGAGTTTTGTCATTCTTATTCTTTTATTCCTTTAACTGTGTATGAG
GAGGATTTATAATGCTTTTCGATAGTGATTGTTATCAATTTAAATAAA
+
F::F,FFFFFFFF:F::F,FF,F,FF:,::,FF:FF:FF,F:FFFF::FFF,FFFFF,,,F:FFFFF,
:FFFFFFFF:FFFF,F:FF,FFFFFFF,,FF:,FFF,F:FFFF,FF,F
@A00721:190:HGJW7DSXY:4:2112:1154:11099_2:N:0:TCTGCAAG+GCTGCACT
CTTTACTCACAGACTCCGAAAATGGTTTCAAATGAGGTCAAAGCTCTATTTATTAAACGAACTATTAT
CCGATTCTTCGAGGGTATTACTACCACTGATCTGCCGACTTTAGTCAA
+
FFFFFFFFFFFFFFFFFFFFFFFFFFFFFFFFFFFFFFFFFFFFFFFFFFFF:FFFFFFFFFFFFFFF
FFFFFFFFFFFF:FFFFFFFFFFFFFFFFFFFFFFFFFFFFFFFFFFF
@A00721:190:HGJW7DSXY:4:1621:32271:1391_2:N:0:TCTGCAAG+GCTGCACT
TTCCTTCGATGTTTATATTTTTCTATAAGGGAAAATAGCAATAATCAAATTTCGACAAATTTTAGAAC
TTTATTTCAGAACTCTCTCCGATCACCTTGGTGTGATCTCCCGGAGGA
+
FFFFFFFFFFFFFFFFFFFFFFFFFFFFFFFFFFFFFFFFF:FF:FFFFFFFFFFFFFFFFFFFFFFF
FFFFF:FFFFFFFFFFFFFFFFFFFFFFF:F:FFFFFFFFF:FFFFFFF
@A00721:190:HGJW7DSXY:4:1503:13946:29277_2:N:0:TCTGCAAG+GCTGCACT
ATGTATGATGATAATCTACTATTTTTAAATAGTGATATTCAGCGTATACTTGAATACTTTATCAAAGA
TTTGGCTTGGTCTATTTACCGCAAAAAGTTGGAAGAGGTCTATGATAT
+
FFFFFFFFFFFFFFFFFFFFFFFFFFFFFFFFFFFFFFFFF::FFFFFFF:FFFFFFFFFFFFFF,F
FFFF:,FFFFFFFFFFFF:FFFFFF:F::FFFFFF:FFFFFF::,F:F
@A00721:190:HGJW7DSXY:4:2334:28592:14372_2:N:0:TCTGCAAG+GCTGCACT
TATACTTTAAATAGCACCGATTATAACTTGACTGTTGATAATATTACAATAGCTCTTTCTCAGAAGAT
TAGAGAAGTAACCGGTGAACACGTCGGTAGCATTGATTTACTTTTACC
+
FFFFFFFFFFFFFFFFFFFFFFFFFFFFFFFFFFFFFFFFFFFFFFFFFFFFFF:FFFFFFFFFFFFF:
FFFFFFFFFFFFFFFFFFFFFFFFFFFFFFFFFFFFFFFFFFFFFFFF
@A00721:190:HGJW7DSXY:4:1436:31557:6605_2:N:0:TCTGCAAG+GCTGCACT
TTTCGAAAAGAAACCTATAAGTTACAAACGATTCTTCGACTATATTAAAGAAATAAAAATACTTCAAG
TACCTGAACTTTACATTTTTAATGGTATGGTATAATGTGTCTTACAAA
+

```
FFFFFFFFFFFFFFFFFFFFFFFFFFFF,FFFFFF:FFFFFFFFFFFFFFFFFF:FFFFFFFFFFFFFF
FFF:FFFFFFFFFF:FFF,FFFF:FFFFFFFFFFFF:FFFFFFFFFFF
@A00721:190:HGJW7DSXY:4:1505:1850:18756_2:N:0:TCTGCAAG+GCTGCACT
CTCTCGGTAATGAGAGTGTATTCCTTAATATTCTAGTAACCGCTATGTGGGCATTACTAGGATTTATC
ACACTCCTAAATTTGATAGCAATTTATGTTTTAAATGCTAGGAAAATT
+
FFFFFFFFF:FFFFFFFFFF:FFFFFF,FFFFFFFFFFFFFFFF:FFFFFFFFFFF:FFFFFFFFFFF
FFFFFFFFFFFFF,FFFFFFFFF:FFFFFFFFFF,FFFFFFFFFFFFF
@A00721:190:HGJW7DSXY:4:1478:22453:6558_1:N:0:TCTGCAAG+GCTGCACT
TGTATAGTTCAAGGAGTACTTAATTCAATAGCTTTAGTATTATTTACTATCGCCTTTATTGAAAATTG
TATAAGAGTTAATTGCTTACACAAAAGTGACGCGCGAAGGAATCTTAATTGATTGAAGCTAGGCGTTG
GGG
+
FFFFFFFFFFFFFFF:FFFFFFFFFFFFFFFFF:FFFFFFFFFFFFFFFFFFFFFFFFFFFFFFFFFF
FFFF:FFFFFFFFFFFFFFFFFFFFFFFFFFFFFFFF,FFFF,,FFFFFFFFFFFFFFFFFFFFFFFF
FFF
@A00721:190:HGJW7DSXY:4:2464:21007:9596_1:N:0:TCTGCAAG+GCTGCACT
ACTCCCCAAACATAACATACCATAACCTACCATGACCAATCATCGCTCCAACTGGCACGCCAGACGTA
GGGTCAAACCTACTACCGCCGGTCGGCAGATCGGCACCACTGGTCTCTACGTTCAGGGACCCGCTGTG
CAG
+
FFFFFFFF:FFFFF:FFFFF:FFFFFFFFFF,F,FFFF:FF:FFFFFFFFFFFFFFFFFFFFFFFFFF
FFFFFFFFFFFFFFFFFFFFFFFFFFFFFFFFFFFFFFFFFFFFF:FFFFFFFFFFFFFFFFFFFFFF
FFF
@A00721:190:HGJW7DSXY:4:1675:18385:5212_1:N:0:TCTGCAAG+GCTGCACT
CCAAATCTAGATCTGCACAGAAGAAGGCTGAAGGTCGTTACCGGACGACCACTCAAAGGAAACGGGCT
GTTGCCCAGAGGAAAGCAGCGACCACCGAGGTCGCTCGTAACATTGACGGCAGTCCCGTCATCTCTGT
GGG
+
FFFFFFFFFFFFF:FFFFFFFFFFFFFFFFFFFFFFFFFFFFFFFFFFFFFFFFFFFFFFFFFFFFFF
FFFFFFFFFFFFFFFFFFFFFFFFFFFFFFFFFFFFFFFFFFFFFFFFFFFFFFFFFFFFFFFFFFFF
FFF
@A00721:190:HGJW7DSXY:4:1554:10646:27132_1:N:0:TCTGCAAG+GCTGCACT
GCTCTTCCGATCTATTCACCACTCCCATACCAATCAATGATGTGACTATACAGTTCGGCCATTTCAAG
AGTGGTCAATACGTCGTGGGAGGTACTGAAGCCTTCCCCTCCGCTGGCACGAAGAACACTCCCAAGCC
AAT
+
FFFFFFFFFFFFFF,FFFFFFFFFFFFFFFFFFFFFF,FFFFFFFFFF:FFFFFFFFFFFFFFFFFFF
FFFFFFFFFFFFFFFFFFFFFFFFFFFFFFFFFFFFFFFFFFFFFFFFFFFFFFFFFFFFFFFFFFFF
FFF
@A00721:190:HGJW7DSXY:4:1449:13512:1407_2:N:0:TCTGCAAG+GCTGCACT
TGACCAGAATGAAAAGGGGTTTACCAGGGTAGTCTCTAAGTCTACCAGGAAGAGGGGAAATGTCCCCA
AAACTGAGAAGAATAAAGGGAAAGGTCCATCAGGACCTAAGAAGGGGA
+
FFFFFFF,FFFFFFFFFFFFFFFFFFFFFFFFFFFFFFFFFFFFFFFFFFFFFFFFFFFFFFFFFFFF
FFFFFFFFFFFFF,FFFFFFFFFFFFFFFFFFFFFFFFFFFFFFFFFF
@A00721:190:HGJW7DSXY:4:1267:19623:31093_2:N:0:TCTGCAAG+GCTGCACT
AATTCTGAAGGAAGATCTCCCACCATCTGGGGTTGGGAATAAGAAATCTGCACGGGCCAAAGAGGCCG
CTGCTACGAAGTCGCTGAAGGCGGCTTGGGTCATTAAGGGTAGCTGTA
+
FFFFFFFFFFFFFFFFFFFFFFFFFFFFFFFFFFFFFFFFFFFFFFFFFFFFFFFFFFFFFFF:FFFFF
FFFFFFFFFFFFFFFFFFFFFFFFFFFFFFFFFFFFFFFFFF:FFFFFF
@A00721:190:HGJW7DSXY:4:1259:28212:14810_1:N:0:TCTGCAAG+GCTGCACT
CCGACCCCATCTTTGAGACCAAGACTCTCTACAGTAGGTTCGTGCCTACTATGAGCAAATCTGTTGAA
```

TTCGTCACTGAGGACGAAGGTGAGAGGACTTTTGGCTACTGGCGACAAACTGAGAGCAGTCGCTGGGG
AAA
+
FFFFFFFFFFFFFFFFFFFFFFFFFFFFFFFFFF:FFFFFFFFFFFFFFF,FFFFFFF:FFFFFFFFF
FFFFFFFFFFFFFFFFFFFFFFFFFFFFFFFFFFFFFFFFFFFFFFFFFFFFFFFFFFFFFFFFFFFF
FFF
@A00721:190:HGJW7DSXY:4:1229:12599:6433_2:N:0:TCTGCAAG+GCTGCACT
ACGCTTCGAGCTTGAGTGTGACGAGGCCGGTGACGTTTTTGTCAACGGCGTTTGGAGGATTTTATCCG
CGCTTAACTATGGGGCGAAGATCCCCTATGGAGTCTTGAAGGACTTAG
+
FFFFFFFFFFFFFFFFFFFFFFFF:FF:FFFFFFF,,F,FFFFFFF:FFFFFFFFFFFFF:FFFF:F:
FF,FFFFFFFFFFFFFFFFFFFF,FF,:FFFF:FFFF:FFFFFFFFFF
@A00721:190:HGJW7DSXY:4:1623:1081:12571_1:N:0:TCTGCAAG+GCTGCACT
GCATGTCACCCTTAAGCACCGAGGTAAGCTCGGGACTTACAAGAGAGAGTGTGAGAGAGTCGCTCTCA
CTATTTTTGCCCAGAATGCGGGCTTTCTGACTGATGAGGTTAAAACTCTTGCTCCTTCTGTGGGTGAG
GGT
+
FFFFFFFFFFFFFFF:FFFFFFFFFFFFFFFFFFFFFFFFFFFFFFFFFFFFFFFFFFFFFFF,F,,,F
F,FFFFFF,,:FFFFF,,FFFF:,FF,,:,,::,F,,FFFF,FFF,FFFFF,F,,,F,,FFFFF,F,F
FF:
@A00721:190:HGJW7DSXY:4:1247:28330:24314_2:N:0:TCTGCAAG+GCTGCACT
TTCACTGGTTCTGGGAGGCTGCTTTCCTCCATAATTACAACCTCTCTTTAGAGAGCATTAGAAGGAAG
TTGGGCAAATTCCCACATATGGTAGACGTTGTCACACAGATCGGACGA
+
FFFFFFF,FFFFFFFFFFFFFFFFFFFFFFFF,FFFFFFFFFFF,:,FF:FFFFF:FFFFFFFFFFF:F
FFFFFFFFFFF,F,F:FFF::FFFFFFFFFFFFFFFFF,FFFFFF,FF
@A00721:190:HGJW7DSXY:4:1138:24695:8876_2:N:0:TCTGCAAG+GCTGCACT
CGTCATTGGGGATTTCGATGTGAGAAGGTGGAAGGTGTCAAGGGTCTTCTTCATGCTGATCTTTCAGG
ACCTAATCACATCATGATTGATAGTTACGGCTCGTATCGAGTTGGAGG
+
FF:FFFFFFFFFFFFFFFFFFFFFFFFFFFFFFFFFFFFFFFFFFFFFFFFFFFFFFFFFFFFFFFFF
FFFFFFFFFFFFFFFFFFFFFFFFFFFFFFFFFFFFFFFFFFFFF:FF
@A00721:190:HGJW7DSXY:4:1233:5258:16579_2:N:0:TCTGCAAG+GCTGCACT
CGAAATTAAAGACGGGGGGATTAAGAGTCCAGCTGTAACTGTCATTTCAGAGAGGGGAGCTCTGAAAA
GGAAACTTCGTCAGAAGATGAGGAAGTCTAAGAGGAGGAGTTTGAGGA
+
F,FFFFFFFFFFFFFFFFFF:FF::FFFFFFFFFFF:FF:FFFFFFFFFFF:FF:FFFFFFF,FFFFF
:FFFFF:FFFFFFFFFFFFFFFF:FFFF:FFFF:FFFFFFFFFFFFFF
@A00721:190:HGJW7DSXY:4:1623:18539:23015_2:N:0:TCTGCAAG+GCTGCACT
GAAGTTGATGAAGAAGGATTATTTGAAGCAGGGGTTACCACTTCCTAAGGTGGATGAGGGAGGTTGTT
TCTTTGGAGTTTTAGATAATCTTTTGAGAAGGGGTCATAGAGCCAGCA
+
FFFFFFFFFFFFFFFFFFFFFFFF:FFFFFFFFFFFFFFFFFFFFFFFFFFFFFFFFFFFFFFFFFFF
FFFFFFFFFFFFFFF:FFFFFFFFFFFFFFFFFFFFFFFFFFFFFFFF
@A00721:190:HGJW7DSXY:4:2515:31675:22592_2:N:0:TCTGCAAG+GCTGCACT
CCTTGAGAAGACAAGCCACATTAAGATATAATTTAGGTCAACTTGGGGTTTCGAATGTCATTAGTGCC
TTACGATTGAAAGAGGTCCTTTTGAGTATCGACAGATCTTTCGATCTT
+
FFFFFFFFFFFFFFFFFFFFFFFFFFFFFFFFFFFFFFFFFFFFFFFFFFFFFFFFFFFFFFFFFFFF
FFFFFFFFFFFFFFF:FF:FFFFFFFFFFFFFFFFFFFFFFFFFF:FFF
@A00721:190:HGJW7DSXY:4:1339:31991:21136_2:N:0:TCTGCAAG+GCTGCACT
TGGCAGAATTCGGTAACTTACCATATTGTTCCTTCCGGGAAGTTGTGGGATTTAACTTTGACTCAACT
ACTCCGATCAGATCCGGATGGTTTGATTGGTATGGACTACTCGACTTT
+

FFFFFFFFFFFFFFFFFFFFFFFFFFFFFFFFFFFFFFFFFFFFFFFFFFFFFFFFFFFFFFFFFFFFFF
FFFFFFFFF:FFFFFFFFFFFFFFFFFFFFFFFF:FFFFFFFFFFFFF
@A00721:190:HGJW7DSXY:4:2510:19560:14920_2:N:0:TCTGCAAG+GCTGCACT
TCATGGCAGATACTACCGATAAGGTGAAAAATATGCAGGTCAAAGATTCTGTAGCTCTGAGGGCGGTT
GAGGGAGCCGTTTTGGCCGACTTTAACTATTCTAAGAGTCTTTATGAT
+
FFFFFFFFFFFFFFFFFFFFFFFFFFFFFFFFFFFFFFFFFFFFFFFFFFFFFFFFFFFFFFFFFFFFFF
FFFFFFFFFFFFFFFFFFFFFFFFFFFFFFFFFFFFFFFFFFFFFFFF
@A00721:190:HGJW7DSXY:4:2101:15646:17534_2:N:0:TCTGCAAG+GCTGCACT
CGTTGTCGTACCTTACAAGGTTGAGGAGACTTTACAGCGGCTTATTGTCGCGCAGTTTCCAGAATTGC
GGTTGAAGTTCACCCATTCTATCTTGAATGCTCACGCCGTCGCAGCCA
+
FFFFFFFFFFFFFFFFFFFFFFFFFFF:FFFFFFFFFFFFFFFFFFFFFFFFFFFFFFFFFFFFFFFFFF
FFFFFFFFFFFFFFFFFFFFFFFFFFFFFFFFFFFFFFFFFFFFFFFF
@A00721:190:HGJW7DSXY:4:2137:1479:26694_1:N:0:TCTGCAAG+GCTGCACT
ATCACTTGTTAAAGTACAGATTTAGTGATTTAGATATTATTGATATTGGAGGCGATATTCGTTTTCAT
ATCCAAAACTCCTTGAATGGAGTACATATTTGCAACCCTATTCTTGATGTGAAAGATGCATCGAGGTA
TAC
+
FFFFFFFFFFFFFFFFFFF:FFFFFFFFFFFFFFFFFFFFFFFFFFFFFFFFFFFFFFFFFFFFFFFFFF
FFFFFFFFFFFFFFFFFFFFFFFFFFFFFFFFFFFFFFFFFFFFFFFFFFFFFFFFFFFFFFFFFFFFFF
FFF
@A00721:190:HGJW7DSXY:4:1439:9787:17284_2:N:0:TCTGCAAG+GCTGCACT
TTGATTGGGAACGTACCTACTCACGAGACGTCATGAAAGCTATCGGAGGGAAGCAAAATGTTTCGTTC
TGTTACAAGAACGCTTTGGATTGTGACCATAAAGCCTCAGTTGCGATA
+
:FFFFFFFFFFF:,FFFFFFFFFF,FFFFFFFFFFFFFFFFFFFFFFFFFFFF:FFFFFF,F,FFF:FFF
FFFFFFFFFFFFF:FFF:FFF:FFFFFFFFFFFFFFFFFFFF:FFFF,
@A00721:190:HGJW7DSXY:4:2525:15691:14231_1:N:0:TCTGCAAG+GCTGCACT
CTCTCTTGATGATGTAGCCACTATAATGCTTAAGAGAGAGATTGATGTATGTCATTTCGTCTTAGTTT
GTCCCGGCGAATTATTGGACACGATGAAAAATGACATACCCGTTCCTGACCTTGGTATCGTTATCCGT
CAT
+
FFFFFFFFFFFFFFFFFFFFFFFFFF:FFFFFFFFFFFFFFFFFFFFFFFFFFFFFFFFFFFFFFFFFFF
FFFFFFFFFFFFFFFFFFFFFFFFFFFFFFFFFFFFFFFFFFF:FFFFFFFFFFFFFFFFF:FFFFFFFF
FFF
@A00721:190:HGJW7DSXY:4:1671:31557:21073_1:N:0:TCTGCAAG+GCTGCACT
GGTTATTCTCACAACAAAGATAAGATTTTATCTTTCTTCAAACACCCTGTTTTCCTTAAGGGCGGACA
ATTGTTCCATTGTGAACTCTTGGGGACTAGATTGAGCATTAATGAGTTCCGAATAACTCGTAATAAAT
CTT
+
FFFFFFFFFFFFFFFFFFFFFFFFFFFFFFFFFFFFFFFFF:FF:FFFFFFFFFFFF,FFFFFFFFFFFF
FFFF:FFFFFFFFFFFFFFFFFFFFFFFFFFFFFFFFFFFFFFFFFFF:FFFFFFFFFFFFFFFFFFFFF
FFF
@A00721:190:HGJW7DSXY:4:2101:2709:4836_1:N:0:TCTGCAAG+GCTGCACT
CCTATTTCCGTACCAAGAGCCACTGCCAATCTCATCGCTATACGCATACCTGATTGGAATAAGCGAGG
TTCGTTCCTCAATAAAAATTTTTCTCAAGAGATTATGGTTGATAAAGTTTTCTTCATAAGGGCTATTC
AGT
+
FFFFF,FFFFFFFFFFFFFFFFFFFFFFFFFFFFFFF:FFFFFFFFFFFFFFFFFFFFFFFFFFFFFFFF
FFFFFFFFFFFFFFFFFFFFFFFFFFFFFFFFFFFFFFFFFFFFFFFFFFFFFFFFFFFFFFFFFFFFFF
FFF
@A00721:190:HGJW7DSXY:4:2136:9815:34334_1:N:0:TCTGCAAG+GCTGCACT
CTTCGGTCAATGACAAAACCTTCGAATATGTTACGACATGGCTTCGTAACAACAGTGCGAGGATTGTC

ATCTCGGGGAAAGTGATTCACCAAGATGTTCATATAGATACGAGATACCTAGAGAAGGTCGCAGCGAT
CTT
+
FFFFFFFF:FFFF,FFFFFFFFF,FFFFFFFFFF:FF:FFF:F:FFFFF:FFFFFFFFFFFF:FFFF:
FFFFF,FFF,FFFFFFFFF,FFFFF,FFFFFFFFFFF:FFFF,FFFFFFF:FFF,FFFFFF:FFFFFF
FFF
@A00721:190:HGJW7DSXY:4:2261:16288:7529_2:N:0:TCTGCAAG+GCTGCACT
AGCTAAGAAGAAGACTTTGGGTACACGTTATGCTCGCAAACTTAACGTTAGTACCGGAGGTGATCTCT
CTGCTTCTGCGTTACTGAGAGAAAAGATTCAGGACGCCTACGGTGACT
+
FFFFFFFFFFF:FFFFFFFFFFFFF:F::FFFFFFFFF:FFFFFFFFFF:FFFFFFFFFFFFFFFFFF
FFFFFFFFFFFFFFFFFFFFFFF,FFFFFFFFFFFFFFFFFFFFFFFFFF
@A00721:190:HGJW7DSXY:4:1350:11740:7263_1:N:0:TCTGCAAG+GCTGCACT
CGTGTGCTCTTCCGATCTAACGAATCTTGAAACTACGGTCCTTATCAAAGAAAATGGTGAACCGGTCT
CTAAATCTTTTGAGATTGAGAAGATTGAAGAACACGTCAAGAAGTCGCTCGGCGTTGTTCTTGAAAAC
CAA
+
:FFFFFFFFFFFFFFFFFFFFFFFFFFFFFFFFFFFFFFFFFFFFFFFFFFFFFFFFFFFFFFFFFFF
FF:FFFFFFFFFFFFFFFFFFFFFFFFFFFFFFFFFFFFFFFFFFFFFFFFFFFFFFFFFFFFFFFFF
FFF
@A00721:190:HGJW7DSXY:4:1236:27769:35430_1:N:0:TCTGCAAG+GCTGCACT
GCTGATCATGGAGTCTTTGTACCTTCTGTCAAACTTCGAGGTTCAGCTACGAGAGGTGATCCCGGATG
TGGATTGAAGGCTGGCTCTAAGGGAGACTCCTTCTTTTTGAATGTTGTAAAGAATTGGGTTGAGAAGT
GTG
+
FFFFFFF:,FFFFFFFFFFFFFFFFFFF:FFFFFFFFFFFFFFFFFFFFFFFFFFFFFFFFFFFFFFF
FFFFFFFFFFFFFFFFFFFFFFFFFFFFFFF:F:FFFFFFFFFFFFFFFFF:FFFFFFFFFFFFFFFFF
FFF
@A00721:190:HGJW7DSXY:4:2456:7654:30906_1:N:0:TCTGCAAG+GCTGCACT
AGTTTTTAGATCTTCTTGATAATCTTAAAGAAAAATTTTCGAATAAACTAATGAGAGGTATAGGCTTA
GTCAAGTCTGTACTTACATTCTTTACGCCAGATTCTTCATGGGGCGACGCTGTTTCTCTTGGTATTTC
TGA
+
FFFFFF::FFF,FF,FFFFF:FFFFF,FFFFFFFFFFFFFFFFFFFFFFFFFFFFFFFFFFFF::FF:
FF,FFFFFFFFFFFF:FFFFFFFFFFFFFFFFFFFFFFFFFFFF,FFFFF:FFFFF:FFFFFFFFFFFF
FFF
@A00721:190:HGJW7DSXY:4:1623:15157:32002_2:N:0:TCTGCAAG+GCTGCACT
CAGAGCGTCATTTTCCTTTGGGATCTTTCCTCTGGTAAGGGACTTATTAAATCATTCTTCTCACTCTT
GACGAGAATTATTACTGTTTTCTCTACCCATAACTTCTTTTTCAAAAA
+
FFFF,FFFFFFFFFF:FFFF:FFFFFF:FFFF:FFFF:FFFF,FFF:F,FFFFFF:FFFF:FFFFFFF
FFFFFFFFFF,FF,:F:FFFFFFFFFFFFFFFFFFFFFFFF,FFFFFF:
@A00721:190:HGJW7DSXY:4:1249:5421:27508_1:N:0:TCTGCAAG+GCTGCACT
CTGATGTTTTCCTACAGAGTGCAGTCGTTTCAACACTTATCACCACTTTTGAAAGGAAAATAATTGGT
GATGAAAATACGTATGTCGGTGGGGTTCTCAAGGCTCTCATCGCCACTGGGATTCTAAGGGGTTCCAT
AAG
+
FFFFFF:FFFFFFFFFFFFFFFFFFFFFFFFFFFFFFFFFFF:FFFFFFFFFFFFFFFFFFFFFFFFFF
FFFFFFFFFFFFFFFFFFFFFFFFFFFFFFFFFFFFFFFFFFFFFFFFFFFFFFFFFFFFFFFFFFFF
FFF
@A00721:190:HGJW7DSXY:4:1473:26006:9518_2:N:0:TCTGCAAG+GCTGCACT
AGGATTCTTCTTTTGGTCCTTACGTCTTAAGTAATTTGGATGTTGTCGAAAACTATCAGGTTCTTGTC
ACGATTCTTAAAAGAAGTTTATTAAAACTTCAGAGTCTCTTGGAAGTC
+

```
FFFFFFFFF:FFFFFFFFFFFFFFFFFFFFFFFFFFFFFFFFFFFFFFFFFFF:FFFFFFFFFFF:FFFF
FFFFFFF:FFFFFFFFFFFFFFFFFFFFFFFFFFFFFFFFFFFFFFFFF
@A00721:190:HGJW7DSXY:4:2365:22815:22404_2:N:0:TCTGCAAG+GCTGCACT
TGTTCTTGCAATAGCCACAGACTTTCTGGGAAAAGAGACCTCTGTTGGTAGAGGAGTTGAGAGAATTA
CTCAGATTCAATCTGGCATCACTGAACGTCTACAACGAGTTAGGGAGT
+
FF:FFFF,FFF:F:::FFF:FFFFFF:F:FFFFFFFFFF::F:FFFF:FF,FF:FFFFFFFF:FF,:F
,:FFFF::FFFFF:F::FFFFF::F::,FF:FFFF::FFF,FF,:FF,
@A00721:190:HGJW7DSXY:4:1440:12816:33176_1:N:0:TCTGCAAG+GCTGCACT
AAAAGATCCTTTGCACAAGCAATACCTTCCACTGAGTTCATCACCCAGAGTTTCGACGTCGCGGAAAA
CGATGAAGATTTCCACACTGTTCGAGAATCTTCTGATGCTGAATTTGAGGAAAATTTTTCGGAAGTTT
TTG
+
FFFFFFF:F:FFFFF:F:FF,F:FFFFF,FFFFF::FF,FFFFFF,FFF:FFFFFF:FFF,:F,F:FF
::FFFFFFFFFFF:FFFFFFFF:FFFF:FF:F:FFFFF:FFFFFFFFF:FFFFFFFFFF:FFFFFFFF
FF:
@A00721:190:HGJW7DSXY:4:2408:9372:17628_1:N:0:TCTGCAAG+GCTGCACT
TGATGGCTCAGTACAGGGAGGAGGTAATAGTTATAAAACCTTCTTTTATCATTGCAACAGGACTTTGA
GAAGACTGTTGAAATGGACAAAATCTGGGATCATAGGTATGTGTCACGCTACACTCGAAATATGGGCC
CTC
+
FFFFF:FF:FFFF:FFFFFFFFFFFFFFF:FFFFF:FFFFFFFFFFFFF:FFFFFFFFFFFFFFFFFF
FFFF:FFFFFFFFFFFF:FFFFFFFFFFFFFFFFFFFFFFFFFF:FFFFFFFFFFFFFFFFFFFFFFFF
FFF
@A00721:190:HGJW7DSXY:4:1635:5421:36307_2:N:0:TCTGCAAG+GCTGCACT
GTTGGCGTCTTGTCGTTGACGTCATTACAGTCTGGAAGTTAGATCATTTGTTCAGGAAGATCTCTACA
GAGATTATCGAGAGTTGTGATCTAGTTCTAAATTTAGGTCGACGGACT
+
FFFFFFFFFFFFFFFFFFFFFFFFFFFFFFFFFFFFFFFF:FFFFFFFFFFFF:FFFFFFFFFFFF:F
FFFFFFFFFFFFFFFF:F:FFFFFFFFFFFFFF:FFFFFFFFFFFFFF
@A00721:190:HGJW7DSXY:4:1227:10556:16736_2:N:0:TCTGCAAG+GCTGCACT
CATCGCAAAGAAGACTTTATTAGTTCATGAAGTTCTTACACTTCTCGCGACTAATGTCAGCTTCACTA
CAAAGGCGTTACGTAAAGTAGTTTTCACGCTTTATCTTAAAGCATTAC
+
FFFFFFFFFFFFFFFFFFFFFFFFFFFFFFFFF:FFFFFF:FFFFFFFFFFFFFFFFFFFFFF:FFFFF
FFFFFFFFFFFFFF:FFF:FFF:FFFFFFFF:FFFFFFFFFFFFFFF
@A00721:190:HGJW7DSXY:4:1114:9715:26647_2:N:0:TCTGCAAG+GCTGCACT
ACTTTTTGAATATGACAGAAGCTCGCCATATCAGATTAGAGACATGCCAGTTAGAGTTCTTCTAACCA
GGACTGATGGTGAGATTCTTTTACCTTTTGGAGGTCCAGGAACCTTTA
+
FFF:FFF:FFFFFF:FFF::FFFFFFFFFFF:FFFFFFFF:FF,:FFFFFFFFFFFFFFFFFFFFFFF
F:FFFFFFFFFFF:FF:FF:FFFFFFFFFFFFFFFFFFFFFFFFFFFFF
@A00721:190:HGJW7DSXY:4:1173:2537:34006_2:N:0:TCTGCAAG+GCTGCACT
CCAAGGACGTTTCCGTCGGAGGATGACAGGTGGGAGACCGCTCAATAACACTCTATTGAATCCCTTTT
CCACGATGAAAGCTCTTTATGAGGTGCTCCATGCAAATTTACAGTTCC
+
FFFFFFFFFFFFFFFFFFFFFFFFFFF:FFFFFFFFFFFFFFFFF,FFFFFFFFFFFFFFFFFF:FFF
FFFFFFFFFFF,FFFFFFFFFFFFFFFFFFFFFFFFFFFFFFFFFFFF
@A00721:190:HGJW7DSXY:4:2465:9109:34867_2:N:0:TCTGCAAG+GCTGCACT
GGTTTATCGGTTGTATTTTGATAAATTTGTTGGAATCGATAATGATACTGACGAAGGTTCTATTACCA
CCCTTGTCATTGATACAAACATTCTAGGTGATGCTTTCTTAGAGGCTC
+
FFFFFFFFFFFFFFFFFFFFFFFFFFFFFFFFFFFFFFFFFFFFFFFFFFFFFFFFFFFFFFFFFFFF
FFFFFFFFFFFFFFFFFFFFFFFFFFFFFFFFF:FFFFFFFFFFFFFFF
```

```
@A00721:190:HGJW7DSXY:4:1378:23321:13542_2:N:0:TCTGCAAG+GCTGCACT
TACAATCTGTAGAGAGAGAGGTTAATCAGAATTTGAGTGATAACGATTTAGTACTGGGAAATGGTGGT
AGTCAGTTTACTACTTTTCTTTCCCTCCCCTTTTTCGATTGGATTCGA
+
FFFFFFFFF:FFFFFFFFFFFFF:FFFFFFFFFFFFFFFFFFFF:FF:FFFFFFFFFFFFFFFFFFFF
FFFFFFFFFFFFFFFFFFFFFFFFFFFFFFFFFFFFFFFFFFFFFFFF
@A00721:190:HGJW7DSXY:4:1527:22399:6057_2:N:0:TCTGCAAG+GCTGCACT
AGGTTTTTCTTTTATGGGACTCTTGTTTGTACTTATGAAGGAGGTATTACTTCGGAAGTTCTTAATGT
AGGTCTCCTAGTACTTAAGGGTTTATGTTTCGGTTTCAGCTGGATGGA
+
FFFFFFF:FFFFFFFFFFFFFFFFFFFFFFFFFFFFFFFFFFFFF:FFFF:FFFFFFFFFFFFFFFFF
FFFFFFFFF:FF,FFFFFFFFFFFFFFFFFFF:FFFFFFFFFFFFFFFF
@A00721:190:HGJW7DSXY:4:1539:29613:20901_1:N:0:TCTGCAAG+GCTGCACT
ATACGAGATCTTGCAGAGTGACTGGAGTTCAGACGTGTGCTCTTCCGATCTTATTATGTCGCTAAAGC
CTGTCCAGTTGTCGATGATGTTCGGGCTGTGGGGATTAACTTCCCTCGTGAAACTTACGACGATGTTC
TCG
+
FFFF:,FFFFFFFFFFFFFFFFF:FFFFFFFFFFFFFFFFFFFFFFFFFFFFFFFFF:FFFFFFF:FFF
FF::FFFFF,FF::FFFFFFFF:FFFFFFFFFFFFFFFFF:FFFFFFFFF:FFF:F,FFFFFFFFFFF
FFF
@A00721:190:HGJW7DSXY:4:1477:24623:14638_1:N:0:TCTGCAAG+GCTGCACT
ATTACTAAGTCTGAGATCTGCCACTTCTAATTTTAGGAAACGTCGCGAAAAGAAAGTTCATGAGGATG
TCTTGGTCGAAATATATCGCCGGGATCTTCTAATAGAGGATCCTGATGACATTATTTCAACGGATACC
GAT
+
:FFFFF:FFFFFFFFFFFFFF,FFFF:FFFFFFF:F:FFFFFFFFFF:F:,F,FFFFFF:FFF,F:FF
F:FFFFF:FFFF,FFFFFF:FFFFF,F:F,FF:FFFFFFFFF:FFFF:F::FFFFFFFFF:FFFFFFF
F,F
@A00721:190:HGJW7DSXY:4:1143:28348:1548_2:N:0:TCTGCAAG+GCTGCACT
CACAGAAACCGTCTCAGGAACTGTCTCCACTGATTCGGAATTAGAGAAAGACGACCTTCTTGTGGGGA
CTGAGACCTCTGAATTTTCAGGGTGCCCTGATGAGGACGTTGAAAGGG
+
FFFFFFFFFFFFFFFFFFFFFFFFFFFFFFFFFFFFFFFFFFFFFFFFFFFFFFFFF:FFFFF:FFF
FFF,FFFFFFFFFFFFFFFFFFFFFFFFFFFFF,FFFFFFFFFFFFFF
@A00721:190:HGJW7DSXY:4:1657:17291:26365_2:N:0:TCTGCAAG+GCTGCACT
AGACTTCTAAGAGTGTTTCAAAAGACCAGAAACGTAACAAGAAGAATAAAGGTAGGAAGGGGAGAAAA
CAGAACAAGCGAGCGGTTGAAGAGAAAACTGATATGGTTGCGGCTGAT
+
:FFFF:F,FF,:FFFFF:F:FFFFFFF,FF,FFFFFFFF:FF:FFF,FF::FFF:F::FFFFFFFFFF
FFFFFFFFFFFFFFFFF,FFFFF:FFFFFFFFFFF:FFFFFFFFFFF
@A00721:190:HGJW7DSXY:4:1544:28103:6386_2:N:0:TCTGCAAG+GCTGCACT
TAGAAAGATCACTCTCGGCGATACCACTGATCTTCCAGAGTTACGTAGTGGTCGTTCTTTTGGTGATT
TTATCAAAGAGAAAGTTTCTGCTAGTCGCGACGATAGTATTTATCGGA
+
FFFFFFFFFFFFFFFFFFFFFFFFFFFFFFFFFFFFFFFFFFFFFFF,FFFFFFFFFFFFFFFFFFFF
FFFFFFFFFFFFFFFFFFFFFFFF,F:FFFFFFFFFFFFFFFFFF:FFF
@A00721:190:HGJW7DSXY:4:1628:7102:20588_2:N:0:TCTGCAAG+GCTGCACT
CACTTACAAGAGTGTTTAAGTACCGCAACATGGATCAGTTTTCTAAAAAGCTATTAATAGCAGTTGCG
GGGCTATAATACCGCCTCCAGATTACTCTAAACTGAATCCACTCTCTT
+
FFFFFFFFFFFFFF,:,FFFFFFFFFFFFFFFF:FFF:FFF,FFF:FFFFFFFFFFFFFFFFF,FFFF
,FFFFF:FFF::FFFFFFFF:FFFF:,FFFF:FF:FFFFFF:FFFFFF
@A00721:190:HGJW7DSXY:4:2613:10312:17534_2:N:0:TCTGCAAG+GCTGCACT
TTTGGAACTGTGTAATGTCCTACGTACTGTTCAAGGTGTTTGGGCTTCTCTAAAGAACAGCAACTTTC
```

AACATGAATTAGTTATTGAAAGTCTTGACCCTCACGTCTCACTTTATC
+
FFFF:FF:FFFFFFFFFFFFFFFFFFFFFFFFFFFFFFFFFFFFFFFFFFFFFFFFFFFFFFFFFF
FFFFFFFF:FFFF::FF:FFFFFFFFF:F,FFFFFFFFFF:FFFFFFF
@A00721:190:HGJW7DSXY:4:2252:16803:20322_2:N:0:TCTGCAAG+GCTGCACT
AAAGAGGGTTTGAAAATTACGGAACCTTTCCTCGTTCTTCATGAAGATCTTCGAGTGTTTTATAGTGA
GTTATTCTTGCGAAGGTTCAAGTCGATTGATTTTGTTGACTACGATCT
+
FFFFFFFFFFFFFFFFFFFFFFF:FFFFFFFFFFFFFFFFFFFFFFFFFFFFFFFFFFFFFFFFFFFF
FFFFFFFF:FFFFFFFFFFFFFFFFFFFFFFFFFF:FFF:FFFFFFF:F
@A00721:190:HGJW7DSXY:4:2234:31168:8343_1:N:0:TCTGCAAG+GCTGCACT
AGAAGTTCTTCGCAGTTGAATCTCCACCAGGTGGAGGGAAGACCACTGAACTTATAGCGACTTTTGTC
GAATTTTTCGCAAAAGGTCTTGAAGTACGTATTGTCACTGCTAATCGTAATTCGCGAGAAGAGATAGT
GAC
+
FFFFFF,FFFFFFFFFFFFF:FFFFFFFF,FFFFFFFFFFFFFFFFFFFFFFFFFFFFFFFFFFFF:F
FFFF:FFFFFFFFFFFFFFFFFFFFFFFFFFFFFFFFFFFFFFFFFFFFFFFFFFFFFFFFFFFFFFF
FFF
@A00721:190:HGJW7DSXY:4:2227:7581:28839_2:N:0:TCTGCAAG+GCTGCACT
CACTTCTAGTTCATCGCGGTATTTGTGAAATTAAGACAGACGTGAAAGTTATGATGAGTGATCGTATT
AGAACAGCTGATTCTTATTGCATGAACGTCGCTGAGGTCAAACCTGTG
+
FFFFFFFFFFFFFFFFFFFF:FFFFFFFFFFFFFFF:FFFFFFFFFFFFFFFFFFFFFFFFFFFFFFF
FFFFFFFFFFFFFFFFFFFFFFFFFFFFFFFFFFFFFFFFFFFFFFFF
@A00721:190:HGJW7DSXY:4:1361:4553:13761_1:N:0:TCTGCAAG+GCTGCACT
CGACGAATTATTCATGATTCACGCTGGTCAGACCCTTTTTCTTACACATCTACTTAAGGCGGCTTATA
CCATAGGTTACGGTGATTCCAATCAAATCGGATTCATCAATCGTATTGATCTCCAAGTCGCAAACTAC
TTC
+
FFFFFFFFFFFFFFFFFFFFFFFFFFFFFFFFFFFFFFFFFFFFFFFFFFFFFFFFFFFFFFFFFFFF
FFFFFFFFFFFFFFFFFFF:FFFFFFFFFFFFFFFFFFFFFFFFFFFFFFFFFFFFFFFFFFFFFFFF
FFF
@A00721:190:HGJW7DSXY:4:1473:19768:1282_2:N:0:TCTGCAAG+GCTGCACT
GATGAGTGTCGTTCTTATCGAAGTCTCAGTTATCGCTGCCCGGCCGACGTTTGTCGCGAACTCTCAGA
GGTTTACGGCAGAAAAATTTCTCCTGCTTGTAATTCGAGAGAGGACAC
+
FFFFFFFFFFFFFFFFFFFFFFF:FFFFFFFFFFFFFFFFFFFFFFFFFFFFFFFFFFFFFFFFFFFF
FFFFFFFFFFFFFFFFFFFFFFFFFFFFFFFFFFFF:FFFFFFFFFFF
@A00721:190:HGJW7DSXY:4:2578:1099:9565_1:N:0:TCTGCAAG+GCTGCACT
CTTGACGACTTCGAATTTGAGGACGGAGTTAAATATATTACTTTCACTCAAGGTGAAAAGTTTGAAAT
TAACAAAGTCCTTGGTAAGAGTAAGGTTAAATGTGAAGATGCTCAGACTATTCATGAAGTTCAAGGTA
ATA
+
FFFFFFFFFFFFFFFFFFFFFFFFFFFFFFFFFFFFFFFFFFFFFFFFFFFFFFFFFFFFFFFFFFFF
FFFFFFFFFFFFFFFFFFFFFFFFFFFFFFFFFFFFFFFFFFFFFFFFF:F:FFFFFFFFFFFFFFFF
FFF
@A00721:190:HGJW7DSXY:4:2605:1994:32659_1:N:0:TCTGCAAG+GCTGCACT
TGACCCTGTTTTTGCATCACAAACACATATTACCGTTGCTCTATCACGACACGTTGAATCTTTACGAT
ACTTTGTTATCAATGTAAAGTTAAACGATGCTACTTGTGAGAGGATCAATCACAGTAAGGAACTGTCA
CGT
+
FFFFFFFF:FFFFF:FFFFFFFFF:,FFFFFFFF:FFFFFFFFFFFFFFFFFFFFFFFFFFFF:FFFF
FFFFFFFFFFFFFFFFFFFFFFFFFFFFFFFFFFFFFFFFFFFFFFFFFFFFFFFFFFFFFFFFFFFF
FFF

@A00721:190:HGJW7DSXY:4:1660:29758:15922_2:N:0:TCTGCAAG+GCTGCACT
GATTCAACCCCTTATATCGAGGCTATCGAGGACGATGAGTATCTTGAAGTTGTTGACGACAAGCCTCA
AGAGGGTAGAGGACCACCTGGATCTTCTACTGTCTCTGCCTTCCAAGA
+
FFFFFFFFFFFFFFFFFFFFFFFFFFFFFFFFFFFFFFFFF,FF:FFFFFFFF:FFFFFFFFFFF:
FF:FFFFFFFFFFFFFFFFFFFFFF:FFFFFFFFFFFFFFFFFFFFFF:
@A00721:190:HGJW7DSXY:4:2672:31973:2002_2:N:0:TCTGCAAG+GCTGCACT
TTATTCCTGGAACCACCTTAACGTCACTCGGTAATCTTTCAGCCGAATTAAGTACTAATGAGTTCAAG
TCGGTTGTCGATCGAATTAAGATCAAGGCTAATACCTTTATAGGTAAG
+
FFFFFFFFFFFFFFFFFFFFFFFFFFFFFFFFFFFFFFFFFFFFFFFFFFFFFFFFFFFFFFFFFFF:
FFFFFFFFFFFFFFFFFFFFFFFFFF:FFFFFFFFFFFFFFFFFFFFF
@A00721:190:HGJW7DSXY:4:2107:12147:31391_1:N:0:TCTGCAAG+GCTGCACT
CCCGATAGGGTACCGAGTCTCCAAGAGAATATTTATTCTTATGAGGCTCGAAACTTCAACGTTTTCAC
AACGGAAAGACATATCGCACCCGAAATTTTTGGGCAGAATGCTCTTAACGCGGTTCTTGAACGGTGTT
TTG
+
FFFFFFFFFFFFFFFFFFFF:FFFFFFFFFFFFF,FFFFFFFFFFFFFFFF,FFFFFFFFFFFFFFFF
FFFFFFFFFFFFFFFFFFFFFFFFFFFFFFFFFFFFFFFFFFFFFFFFFFFFFFFFFFFFFFFFFFFF
FFF
@A00721:190:HGJW7DSXY:4:1569:18041:16830_1:N:0:TCTGCAAG+GCTGCACT
GATAAAGTTTTAGGAGACTCAGTTAACCTGAGAGCTCCTCAATTGATAGATTGGGTCAACAATAGGGA
CGCTAGTATACTTCGTAAAGTCGACAGCGAAATGGTCAATATTGATAATTGGACCGACTATATGACCT
ATT
+
:FFFFFFFF,FFFFFFFFFFFFFFFFFFFFFFFFFFFFFFFFFFFFF:FFFFFFFF:FFFF:FFFF:F
FFFFFFFFFFFFFFFFFFFFFFFFFFFFFFFFFFFFFFFFFFFFFFFFFFFFFFFF:FF:FFFFFFFFFF
FFF
@A00721:190:HGJW7DSXY:4:2304:30246:12540_1:N:0:TCTGCAAG+GCTGCACT
AAGGGAATCTAAGGTTAAACTTGATGCTTCAGCCTTTTCGAAGCATAACCCCGCACAGAATATATTTT
ATCACGCTAAATTTATTAATGCAATTTTCGGACCTATCTTTAAGGTTCTTGTGAGGAGAGTCAAACAG
TGT
+
FFFF::F,,FFF,,FFF:,,FF::FFFFFF:,FF:FFFFF,F,FFFFFF,F,,,F,,,::FFF,,FFF
F:F,FF,,,,FFFF:F::FF:,::FFFFFFF,:FFFF:F:F,F,,FF,FFFFF:F:::FFFFFFF:FF
F,F
@A00721:190:HGJW7DSXY:4:2664:16396:14763_1:N:0:TCTGCAAG+GCTGCACT
ACATTTTTATGTATACAGAGATGACCAGTGACGATTTCTCCCGGAAGCTCTTCTCTCTCTTAGGAGGT
CATGATGACTACTATAAGGGTGAAATCGATTTTTCGAAGTTTGACAAATCTCAAGATTTCTTTTGTAA
ATC
+
FFFF,FFFFFFFFFFFFFFFFFFFFFFFFFFFFFF:FFFFFFFFFFFFFFFFFFFFFFFFFFFFFFF:F
:FFFFF,FFF,:FF:FF:FFFFFFFF,FFFFFFFFFFF::FF,FFFFFF::FFFFFF,FFFF:F:FFF
F,:
@A00721:190:HGJW7DSXY:4:1267:16785:10958_2:N:0:TCTGCAAG+GCTGCACT
TTCGATGCTGAATTTCTTGAACTTTGGAGTGCCAGTGAATATTTTTGTCACGCCAGGAGTATCCATAA
CGATTTGTCATTTACCACCTTTGCTCAACGACGAAGTGGAACGGCCAA
+
FFFFFFFFFFFFFFFFFFFFFFFFFFFFFFFFFFFFFFFFFFFFFFFFFFFFFFFFFFFFFFFFFFFF
FFFFFFFFFFFFFFFFFFFFFFFFFFFFFFFFFFFFFFFFFFFFFFFF
@A00721:190:HGJW7DSXY:4:2558:7627:10942_2:N:0:TCTGCAAG+GCTGCACT
TAATTGTATGATCACTTTGATGATTTTATCACTTTATTATGACCTTGGAAAGTTTTCGGCTGTGGTTG
TCGCTGGGGACGATTCCATTCTGTTTTCAAAGACTCCAATAACTGATC
+

```
:FFFFFFFFFFFFFFFFFFFF:FFFFFFFFFFFFFFFFFFFFFFFFFFFFFFFFFFFFFF:FFFF,FF
:FFFFFFFFFFFFFFFFFFFFFFFFFFFFFFFFFFFFFFFF:FFFFFF
@A00721:190:HGJW7DSXY:4:1261:15483:19633_2:N:0:TCTGCAAG+GCTGCACT
TGCTCGCGATTTATTCTTTTTGTCAATGATTACATCTACTTCGTTCCTGATCCTTACAAGATCATCGT
TAAGTTAGGTAAACCCCTAACCGATGTTACTCTTGTAGGACTTTGGGA
+
FFFFFFFFF,FFFF:FF:FFFFFFFFFFFFFFFFFFFFFFFFF:FFF:FF,:::F:FFFF:FFFFFFF
,FFFFFFFFF:F:F:FFFFFFFFFFFFFFFFFF,FFFFF:FFFFFF:F
@A00721:190:HGJW7DSXY:4:1177:30047:13917_2:N:0:TCTGCAAG+GCTGCACT
TTAAAGACTATACTAAATATCTCCACTACGAGGTTGTTGTTTGGCGATTAGCTCTTGCGACTAATATT
CGATATTCGATTGAGGGTCGTTATACCTATTCGGCTATCTGTGCTTTG
+
FFFFFFFFFFFFFFFFFFFFFFFFFFFFFFFFFFFFFFFFFFFFFFFFFFFFFFFFFFFFFFFFFFFF
F:FFFFFFFFFFFFFFFFFFFFFFFFFFFFF,FFFFFFFFFFFFFFFF
@A00721:190:HGJW7DSXY:4:2159:20708:4006_2:N:0:TCTGCAAG+GCTGCACT
TCAAATTTTAAACGATTCCGAGAGGTTTATCCTGATGTGGAGGGAGTTCTTGAAGCATCCCGACCTTT
CCTTGTGAAAATTTTGATGCGGTTTCATAAGATTATCAGTAATCCTGA
+
FFFFFFFFFFFFFFFFFFFFFFFFFFFFFFFFFFFFFFFFFFFFFFFFFFFFFFFFFFFFFFFFFFFF
FFFFFFFFFFFFFFFFFFFFFFFFFFFFFFFFFFFFFFFFFFFFFFFF
@A00721:190:HGJW7DSXY:4:1546:30454:21919_1:N:0:TCTGCAAG+GCTGCACT
ATTCATGGACCTTCCATCCTGTACCTCTTCTGGAGAGACGTATCTCAAAGATCGACTTCGAGACAGGT
GATATTGTTGAGCGAGAGCGACCAAGATCTAAATTCGGTAACGGTGCGTGGCGCTTTATTTTAAAAAG
CTC
+
FFFFFFFFFFFFFFFFFFFFFFFFFFFFFFFFFFFFFFFFFFFFFFFFFFFFFFFFFFFFFFFFFFFF
FFFFFFFFFFFFFFFFFFFFFFFFFFFFFFFFFFFFFFFFFFFFFFFFFFF:FFFFFFFFFFFFFFFF
FFF
@A00721:190:HGJW7DSXY:4:2567:6912:12399_2:N:0:TCTGCAAG+GCTGCACT
CTGATACTTATCACGAAAGGACTTGAGTCTTTTGATCAGTCTCTGACTTGACCATCTTTCATTTTAAA
TGACGATCATTAAGACTCTCTTTCTTCTTTGTTTCCTTCTTTGCACTG
+
FFFFFFFFFFFFFFFFFFFFFFFFFFFFFFFFFFFFFFFFFFFFFFF:FFFFFFFFFFFFFFFFFFFF
FFF:FF,FFFFFFFFFFFFFFFFFFFFF,FFFFF:FFF:FFFFFFFFF
@A00721:190:HGJW7DSXY:4:2139:32570:13777_1:N:0:TCTGCAAG+GCTGCACT
CTCTTCCGATCTCTAATATGTGTTTTTACGATAACAACTATCAGTTGATTAAGACTCTTCCGAAAGGC
GAACTTTGGGAATTTGAAGCTCAGGGTGAAGCACAACGTTTGACCGTGACGAACTGTGGTGTTCTTGA
TCT
+
FFFFFF,FFFFFFF:FF,FFFFFFFFFFFFFFFFFFF:FFFFFFFFFFFFFFFFFFFFF:FFFFFFFF
FF:FFFFFFFFFFFF:FFFFFFFFFFFFFFFFFFFFFFFFFFF:FFFFFFFFFFF:FFFFFFFFFFFF
FFF
@A00721:190:HGJW7DSXY:4:2336:18240:25254_1:N:0:TCTGCAAG+GCTGCACT
ATACTCTCCTTGAGTTCACTCGTAAGGACACTACCACTTATTACGATATTTCTTTAGTCGATGGGTTT
TCGACTCCTTTAAGCGTCTTCTGTTCATCGCAACCAGATGATATCTTCTATTCGAACTTATGTTTCGA
TCA
+
FFFFFFFFFFFFFFFFFFFFFFFFFFFFFFF:FFFFFFFFFFFFFFFFFFFFFFFFFFFFFFFFFFFF
FFFFFFFFFFFFFFFFFFFFFFFFFF:FFFFFFFFFFFFFFFFFFFFFFFFFFFFFFFFFFFFFFFFF
FFF
@A00721:190:HGJW7DSXY:4:1642:10764:2534_2:N:0:TCTGCAAG+GCTGCACT
TCTTCTAATGTGGCCTGAACTACGTTTCACTTTGTGTGTACTTGTCTCTATTTTAAGTTTTGTCTTCC
TTGTTTGTCTTATTCTATTAATCACTCATTTTAGTGAGATTATTAAAA
+
```

FFFFFFFFFFFFFFF:FFFFFFFFFFFFFFFFFFFFFFFF:FFFFFFFFFFFFFFFFFFFFFFFFF:F
FFFFFFFFFFFFFFFFFFFFFFFFFFFFFFF:FFFFFFFFFFFFFFFF
@A00721:190:HGJW7DSXY:4:1445:17454:3537_2:N:0:TCTGCAAG+GCTGCACT
CGACAACAGCAGGGGTTTTCGAATAGTGATAGCCACCCGCCTTACAGAGGGGCAGCAGCATGACCATA
ATTGGTATTGACTTTGGTACCACCTTTTCTACTGTCTGTCTCGCCTCT
+
FFFFFFFFFFFFFFFFFFFFFFFFFFFFFFFFFFFFFFFFFFFFFFFFFFFFF:FFFFFFFFFFFFFF
FFFFFFFFFFFFFFFF:FFFFFFFFFFFFFFFFFF,FFFFFFFFF:FF
@A00721:190:HGJW7DSXY:4:1241:18177:2002_1:N:0:TCTGCAAG+GCTGCACT
CTTAACAACAATGATAGTGAATTTATACCTACAATCATTGGTTTTTCTAATAATGATGACACTATTGT
TTATGGATATGATGCTATTACTCATGATGGTTACGGTCAGGGAGGATTCTCAGTTTACAAGGACCTTA
AGC
+
FFFFFFFFFFFFFFFFFFFFF::FFFFFFFFFFFFFFFFFFFFFFFFFFFFFFFFFFFFFFFFFFFF:
FFFFFFFFFFF:FFFFFFFFFFFFFFFFFFFFFFFFFFFFFFFFFFFFFFFFFFFFFFFF
FFF
@A00721:190:HGJW7DSXY:4:2368:12997:21527_2:N:0:TCTGCAAG+GCTGCACT
CCGAAAGACGTGCTAAATTAAAACCATTGTACGATGTCGTCTGTCTCGGCGATCACTTTTCACTAAGA
ATTGGTCCTACTTTTGGACAAGGCCAACTTCGTAATCTTCATGAGATC
+
FFFFFFF:FFFFFFFFFFFFFF:FFF:FFFFFFFFFFFFFFFFFFFFFFFFFFFFFFFFFFFFFFFFF
FFFFFFFFFFFFFF:FFFFFFFFFFFFFFFFFFFF:FFFFFFFFFF:F
@A00721:190:HGJW7DSXY:4:1127:13358:2581_1:N:0:TCTGCAAG+GCTGCACT
TAGGGATTTTGAGAGAGTGAAAGGCTTCAATTGTAGTGGTTTGGTCGTTTCAGTACCCAGTCAGTATA
CCTCTACGCAAAGATATTTCATGAAAACTACAGAGGAACGTACCGGATTACCTGTCTTTCATATTATG
AAT
+
FFFFFFFFFFFFFFFFFFFFFFFFFFFFFFFFFFFFFFF:FFFFFFF:FFFFFFFFFFFFFFFFFFF:
FFFFFFFF:FFFFFFFFFFFFFFFFFFFFFFFFFFFFFFFFFFFFFFFFFFFFFFFFFFFFFF
FFF
@A00721:190:HGJW7DSXY:4:2552:1868:1846_2:N:0:TCTGCAAG+GCTGCACT
TCTTTGCTTCGATGTTGGATGTCAAGAAGGGTTCAGAATGGGATTCCTACGTAGTTTATGACTTTGGT
GGTGGTACTTTCGATACTTCTATCGTCTTACGTTACGGTAATTACTAT
+
F,FFFFFFFFFFF:F,FFFF:FFFFFFFFFFFFFFFFF:F::FFFF,FFFFFFFFFFF:FF:F:FFFF
FFFFFFFFFFFFFFFFFFFFFFFF:FFF:FF:FFFFFFFFFF:FF:FF
@A00721:190:HGJW7DSXY:4:1674:31919:6699_1:N:0:TCTGCAAG+GCTGCACT
ATAATCTTGGAGGTCGTGACGTTGATGAAGCTATAAGAGCGTTCTTATTACGGCGCACTTCTTTACCC
GCCACCACACAACTTTCAGTTTCACAACTGAAGGAGGAGGTTTCTGTTACTGGAAAACCCTCGTCAAT
ACT
+
FFFFFFFFFFFFFFFFFFFFFFFFFFFFFF:FFFFFFFFFFFFFFFFFFFFFFFFFFFFFFFFFFFFF
FFFFFFFFFFFFFFF:FFFFFFFFFFFFFFFFFFFFFFFFFFFFFFF:FFFFFFFFFFFFFFFFFFFF
FFF
@A00721:190:HGJW7DSXY:4:2102:20148:25801_2:N:0:TCTGCAAG+GCTGCACT
GATCTAGTGAAATTAACGCGTCCTTTTCTGGAACGTAGTTTTACTATTATGAACAAAGTTATCAAAGA
TAGTGGCATTCAAGGCAAACTTACCCTGATTCCGGCCGGTGGTAGCTC
+
FFFFFFFFFFFFFFFFFFFFFFFFFFFFFFFFFFFFFFFFFFFFFFFFFFFFFFFFFFFFFFFFFFFF
FFFFFFFFFFFFFFFFFFFFFFFFFFFFFFFFFFFFFFFFFFFFFFFF
@A00721:190:HGJW7DSXY:4:1537:29125:3709_2:N:0:TCTGCAAG+GCTGCACT
TGCTGAAACAGCTATTAAAGCTGTTGAGCGGGTAATTACACCTAAACGAGCTCGATCTGCCATCGCTG
AAGGATGTGCACTCTTTTCTGCTACTTTAACGGAGCGTAGTGTACTCT
+

FFFFFFFFFFFFFFFFFFFFFFFFFFFFFFFFFFFFFFFFFFFFFFFFFFFFFFFFFFFFFFFFFFFFFF
FFFFFFFFFFFFFFFFFFFFFFFFFFFFFFFFFFFFFFFFFFFFFFF
@A00721:190:HGJW7DSXY:4:2358:24108:3098_1:N:0:TCTGCAAG+GCTGCACT
CAACAGTAACATTACTTCTAATAAAGGTCTCTATCAAGCTCGTATTATCGTCGCTGCTGGATCTGCGT
TACCGTTGAAGGCTAAACTCCAAGGTAATTATGAAGACTGTGATTTATCTTTCACTCTGGCCTTGTAT
GAG
+
FFFFFFFFFFF,FFFFFFFFFFFFFFFFFFFFFFFFFFFFFFFFFFFFFFFFFFFFFFFFFFFFFFFFFF
FFFFFFFFFFFFFFFFFFFFFFFFFFFFFFFFFFFFFFFFFFFFFFFFFFFFFFFFFFFFFFFFFFFFFF
FFF
@A00721:190:HGJW7DSXY:4:2110:8712:19022_2:N:0:TCTGCAAG+GCTGCACT
CGTAACATTAATAATTTCAATCTTCTTAAAAAGAAGATAGAGAGTCATACTTTGGGCTATCGTTCAGG
TGCAGGGGGTTGGATAGCCAATATTGAAGTGGACACCTCTGGTAGTAT
+
:FFFFF::FF:F,FF,FF,FFFFFFF:FFFF,FFFFFF,FFFFFFFFFFF,:FF,:F:,,F:::FFFF
:F,F,FFFFFFFFFF:FFF,F,,,F,FFF,FF:FFFFFF,,,,FFFF
@A00721:190:HGJW7DSXY:4:2242:10113:3255_1:N:0:TCTGCAAG+GCTGCACT
CTAAGCATACTACTTTTGCTTTGATTACTCTACCGCCTTACAAGTCTAAGGTTAAAGAGTCGATTAAA
CAAGTTGTACTTATCAATGATTTTATCGCTGGTTGTTATCTCGAAAAGTCTCTTCTAAAAGGAACGTC
AGT
+
FFFFFFFFF,FFFFFFFFF:FFFFFF:F,:FFFFFF,F:,:F:F::FFFFFFFFFFFFFFFFFFFFFFFFF
F:FF:FFFFFFFFFFFFFF:FFFFFFFFFFFFFFFFFFFFFFFFFFFFFFFFF:FFFFFFFF:FFFFFFF
:FF
@A00721:190:HGJW7DSXY:4:1344:13747:10426_2:N:0:TCTGCAAG+GCTGCACT
CTAAAATATTTACAAAATCTTACCACTATCTACGAACCTAAAGTTCTGAAGACGTTGCTTGTCGCTTC
CTCCTTTAATGGCACTACCTCGGTACAATCTTACTTGGAACGAAGGCG
+
FFFFFFFFFFFFFFFFFFFFFFFFFFFFFFFFFFFFFFFFFFFFFFFFFFFFFFFFFFFFFFFFFFFFFF
FFFFF:FFFFFFFFFFFFFFFFFFFFFFFFFFFFFFFFFFFFFFFFF
@A00721:190:HGJW7DSXY:4:1340:31864:20071_1:N:0:TCTGCAAG+GCTGCACT
GTTCTGTCTATATTAGGTGAAGCGAAATACGATTTCGAACGTCAACGTTCACCGAGTTTCGTTTCGTA
CCTTGATGCTGACTTTCGTCAATATGATTTGATCTGGTTGCTCAATTCTAAGCGTGTTCCGCTTAGTG
CGA
+
FFFFFFFFFFFFFFFFFFFFFFFFFFFFFFFFFFFFFFFFFFFFFFFFFFFFFFFFFFFFFFFFFFFFFF
FFFFFFFFFFFFFFFFFFFFFFFFFFFFFFFFFFFFFFFFFFFFFFFFFFFFFFFFFFFFFFFFFFFFFF
FFF
@A00721:190:HGJW7DSXY:4:1546:21088:22294_1:N:0:TCTGCAAG+GCTGCACT
TACGAACTTGACTCTACTATACGTGACATACTAAATACTAGATATTTGGATGATCTTGATAAAGAACT
TCATGAATTTTCAGAGGATGAAAAATTTTCTTCCTTGGATTTTAATATAAAGAATCTTTACCAGGACT
CCT
+
F:FFFFFFFFFFFFFFFFFFFFFFFFFFFFFFFFFFFFFFF:FFFFFF:FFFFFFFFFFFFFFFFFFFFF
FFFFFFFFFFFFFFFFFFFFFFFFFFFFFFFFFFFFFFFFFFFFFFFFFFFFFFFFFFFFFFFFFFFFFF
FFF
@A00721:190:HGJW7DSXY:4:1509:21088:16282_1:N:0:TCTGCAAG+GCTGCACT
TGATCGTAAGAAGTTTATAAGGAACATATCCATTTGTTGTAATAGGGTCATCAACTTAGAACTCGAAA
CTCTAAGTGGTAATACTGTCTTTCCAAGCTTACCTGTATCTTCAATGAAATTGGACTCTTCGATAGGG
GAC
+
FFFFFFFFFFFFFFFFFFFFFFFFFFFFFFFFFFFFFFFFFFFFFFFFFFFFFFFFFFFFFFFFFFFFFF
FFFFFFFFFFFFFFFFFFFFFFFFFFFFFFFFFFFFF:FFFFFFFFFFFFFFFFFFFFFFFFFFFFFFFF
FFF

@A00721:190:HGJW7DSXY:4:2676:24478:27164_2:N:0:TCTGCAAG+GCTGCACT
TAAAGGAAGATCTGTTATCGACGGTTTATCGTTTACGAAGTATCACTCCACCGGTGAATGGTTTAAGA
ATACTATAAATAGAATAGCTATTACTACTAATGGCGTTGGCCTAGTGG
+
FFFFFFF:FFFFFFFFFFFFFFFFFFFFFFFFFFFFFFFFFFFFFFFFFFFFFFFFFFFFFFFFFFFF
FFFFFFFFFFFFF:FFFFFFFFFFFFFFFFFFFFF:FFF:F:FFFFFF
@A00721:190:HGJW7DSXY:4:2643:29731:35164_1:N:0:TCTGCAAG+GCTGCACT
TTTAGGTGGTCTTTATTCTATTATTGATTTTTATGAGATTGATACGCTAACAGAAGATGATGTTAGAG
TAGAATTCCTGAAGAGTCCTTCCGTAACCAACTTCTTGACAATTTTCTTCAATCCTTCGATAATAGTT
AAA
+
FFFFFFFFFFFFFFFFFFFFFFFFFFFFFFFFFFFFFFFFFFFFFFFFFFFFFFFFFFFFFFFF:FFF
FFFFFFFFFFFFFFFFFFFFFFF:FFFFF:FFFFFFF:FFFFF:FFFFFFFFF,F:FFFF:FFFF:FF
F:F
@A00721:190:HGJW7DSXY:4:2134:22643:9298_1:N:0:TCTGCAAG+GCTGCACT
CGATAAGGGTCACTAGTCTTACACAATTAAGAAATAATCCTACATTGACTGATTATCTAAAGATTGAA
GGAAAATTTTATCCTCGTTTAACTTACTTAGACTTTCCTACGATACAAATGATAACTTCTAATTTACG
TT
+
FFFFFFFFFFFFFFFFFFFFFFFFFFFFFFFFFFFFFFFFFFFFFFFFFFFFFFFFFFFFFFFFFFFF
FFFFFFFFFFFFFFFFFFFFFFFFFFFFFFFFFF,FFFFFFFFFFFFFFFFFFFFFFFFFFFFFFFFF
FF
@A00721:190:HGJW7DSXY:4:2576:31575:5040_2:N:0:TCTGCAAG+GCTGCACT
AAGGGATGCATGGTGTTATCTCCTCAGGAGTCCCGGCATTTGATAATAATGACGAGACTTTCGTCATA
ATCGCTGCATATTACGGATTACACGGTACCAGCGCATCTCGTGTCGAG
+
FFFFFFFFFFFFFFFFFFFFFFFFFFFFFFFFFFFFFF:FFF,FFFFFFFFFFFF:FFFFFFFFFFFF
FFFF:FFFFFFFFFF:FFFFFF:F,FFFFFF:F:FFF,FFFFFFF,FF
@A00721:190:HGJW7DSXY:4:2274:8603:8249_1:N:0:TCTGCAAG+GCTGCACT
AAATTTATAGAAGATTATTTTGACAGAATCAATAAGAATCATCGATTCTCGATTCGTAGAGCTTATCT
AGGTACTCTCATTTACTATGTTGATTTAGTCTACATTTATGCTGATCTAAAATTTCGATCTAGATGGA
TCC
+
FFFFFFFFFF,FF:FFFF:FFFFFFFF,FFFFFFFFFFFFFFFFFFFFFFFFFFFFFFFFFFFFFFFF
FFFFFFFFFFFFFFFFFFFFFFFFFFFFFFFFFFFFFFFFFFFFFFFFFFFFFFFFFFFFFFFFFFFF
FFF
@A00721:190:HGJW7DSXY:4:1509:10330:27085_1:N:0:TCTGCAAG+GCTGCACT
TTCTAGAATTCTTAATTATAATATTCAACCTCAAAAGTTACGTGTTTACAGTAACGAGATCACACATT
TTGAGAACTAACTATGTTATGTTCTCTGATCATTTTGACAATCTCTTCTTATAATAAATAAATAAATA
ATA
+
FF:FF:FFFF,FF:FF:FFFFFFFFFFFFFFFFFFFFFFFFFFF:FFFFFFFFFFFFFFFFFFFFFFF
FFFFFFFFFFFFF,FFFFFFFFFF:FFFFFFFFFFFFFFFFFFFFFFFFFFFFFFFFFFFFF:FFFFF
FFF
@A00721:190:HGJW7DSXY:4:1364:4779:29121_2:N:0:TCTGCAAG+GCTGCACT
CAAATAGTGGAAAGGCTCCAGTCAACACTAGTTCGAGTGCTGCTTTGGATTACGATGACGTGAGAATA
GGTGAAGGGATAGTTTTTACCTCGACCAACATTAAGGCGGCGGCGACT
+
FFFFFFFFF:FFFFFFFFFFFFFFFFFFFFFFFFFFFFFFFFFFFFFFFFF:FFFFFFFFFFFFF:FFF
FF:FFFFFFFFFFFFFFFFFFFFFFFFFFFFFFFFFFFFFFFFFFFFF
@A00721:190:HGJW7DSXY:4:1630:29279:24111_2:N:0:TCTGCAAG+GCTGCACT
CTTCACCCAAAATGAAGGACACTTCGAAACTGAATAATGATGCACTACAAACAAGTATCGCTAATCAT
CTGAAAAAGGCTCTAAAGGACACTATACAATATCCAGACGACGACGAA
+

FFFFFFFFFFFFFFFFFFFFFFFFFFFFFFFFFFFFFFFFFFFFFFFFFFFFFFFFFFFFFFFFFFFFFF
FFFFFFFFFFFFFFFFFF:FFFFFFFFFFFFFFFFFFFFFFFFFFFFFFF
@A00721:190:HGJW7DSXY:4:2212:13693:13933_2:N:0:TCTGCAAG+GCTGCACT
CAGCACCACATTTTGGTCAAGTCAATCCGTTGAGAAAATTTCTTAGAGGATATTCGCCATTGTGGTAC
GAATTGGTCCAACATTTACCACCAAATTCTTTTGATACAACTCTAGCC
+
FFFFFFFFFFFFFFFFFFFFFFFFFFFFFFFFFFFFFFFFFFFFFFFFFFFFFFFFFFFFFFFFFFFFFF
FFFFFFFFFFFFFFFFFFFFFFFFFFFFFFFFFFFFFFFFFFFFFFFFFF
@A00721:190:HGJW7DSXY:4:1161:9344:7780_2:N:0:TCTGCAAG+GCTGCACT
CGCTTGCTATTCACCAGATTGGTTCGAACCCGTTGATGCTATGTCAGAAGAAGCTATTCAAGCTTGTC
GTTTAAAGACTGCAGAGGCTGTCAAACAAGCCCAGACTCAAGGAGTCC
+
FFFFFFFFFFFFFFFFFFFFFFFFFFFFFFFFFFFFFFFFF,FFFFFFFFFFFFFFFF:FFFFF:FFFF
FFFFFFFFFFFFFFFFFFFFFFFFFFFFFFFFFF,FFFFFFFFFFFFFF
@A00721:190:HGJW7DSXY:4:1511:17472:33849_2:N:0:TCTGCAAG+GCTGCACT
GGTGATAAAAATTATTTAGAGATCTTTTCTACTTACAAAGGACAATTCAGTTTCATTAATTCTACGGA
AAAGTCCGGAGTATATTGTATGTTAATAGATAGTGGGATAATAGTTCT
+
FFFFFFFFFFFFFFFFFFFFFFFFFFFFFFFFFFFFFFFFFFFFFFFFFFFFFFFFFFFFFFFFFFFFFF
FFFFFFFFFFFFFFFFFFFFFFFFFFFFFFFFFFFFFFFFFFFFFFFFFF
@A00721:190:HGJW7DSXY:4:2242:25455:16172_1:N:0:TCTGCAAG+GCTGCACT
CGTCTGAAGAGTTTAGAGCTGCTATTGATTATGTAACCGATAGGAGTTGTATAGTTGAAGATGTGGCT
TTAGACGATAATCCTTTAAGGACTATGTTGACTGACGAAGATTTTCCCACAGATTTGTCTACGTTGCA
GAT
+
FFFFFFFFFFFFFFFFFFFFFFFFFFFFFFFFFFFF:FFFFFFFFFFFFFFFFFFFFFFFFFFFFFFFFF
FFFFFFFFFFFFFFFFFFFFFFFFFFFFFFFFFFFFFFFFFFFFFFFFFFFFF:FFFFFFFFFFF:FFFF
FFF
@A00721:190:HGJW7DSXY:4:1370:17788:8155_2:N:0:TCTGCAAG+GCTGCACT
CTATATACATTATAAAATATAAAAATAAGGGAGTCATAACTTTTGTTACCAAATTCTTGGCTTTCAAC
GAAGTCTCTGATTTTATTAATCATTACTTTAACGATCTCTCGGTCATT
+
FF:FFFFFFFFFFFFFFFFFFFFFFFFFFFFFFFFFFFFFFFFFFFFFFFFFFFFFFFFFFFFFFFFFFF
FFFFFFFFFFFFFFFFFFFFFFFFFFFFFFFFFFFFFFFFFFFFFFFFFF
@A00721:190:HGJW7DSXY:4:1223:13295:2660_1:N:0:TCTGCAAG+GCTGCACT
TTGATCTACGTCGTTTAACTGTCGGAACAACCTTTACTTAGAATGACTCTATCTTTTCAGACACTTCT
TATATTATTTACAATAGTTAATATCATATTAGTAATACTCACTATCGTTTTCTGCTTTTTCTTAGCTA
ATA
+
FF:FF:FFFFF,F:FFFFFFFFFF:FFFFFFFFFFFFF:FFFFFFFFFFFFFFFFFFFFFFFFF:FFF:F
FFFFFFFFFFFFF:FFFFFFFFFFFFFFFFFFFFFFFFFFFFFFFFFFFFFFFFFFFFFFFFFFFFFFFF
F,F
@A00721:190:HGJW7DSXY:4:2672:25220:16673_1:N:0:TCTGCAAG+GCTGCACT
TATGATTACTAAAACCAACTCTTCTTTACTTGACCTGTTGGTAGAATAGATTTAGTCTCTACACCCAT
CTCAACGAGGATAAAGCGAGTTTTCCGTCCTGAACGTAATACACGAATAGATCATCCGCAAACGACTC
GA
+
:,FF,,:FF,FFF:F,F:,:,F:,,F,F,,,,::,F,,,,,F:,F:F,,:FFF,,,,F,,,F:FF,F,
,:,F:,:,,,:,F:,:,,,:FFF,FFF,F,F,F,,::FF:::FF,F:,,,F,,F:FF,,FFFFFFFFF
FF
@A00721:190:HGJW7DSXY:4:2205:10013:28103_2:N:0:TCTGCAAG+GCTGCACT
TTTAGATTATGAATCCTGCCTTTACTCATTTTATAGCAGTTATTTATGTTGTACTTTTTATAATTTAT
AACCTTCTTATACTTTTGGGTATGTTTTGTGAAAAACATGAGATACCC
+

```
,FFFFFFFFFFFFFFFFFFFFFFFFFFF:,FFFFFFF:FF,FFFFFFFFF:FFFFFFFFFFFFFFFFF
FF:FFFFFFFFFFFFFFF:FFFFFFFFFFFFF:FFFFF:FFFFFFFFF
@A00721:190:HGJW7DSXY:4:1107:30761:20729_2:N:0:TCTGCAAG+GCTGCACT
TTGTAACGATCATTCTTTCGAGTTTTGTCATTCTTATTCTTTTATTCCTTTAACTGTGTATGAGGAGG
ATTTATAATGCTTTTCGATAGTGATTGTTATCAATTTAAATACATTAC
+
FFFFFFFFFFFFFFFFFFFFFFFFFFFF:FFFFFFFFF:FFFFFFFFFFFFFFFFFFFFFFF:FFFFF
FFFFFFFFFFFFF:FFFFFFFFFFFFFFFFFFFFFFFFFFFFF:FFF:
@A00721:190:HGJW7DSXY:4:2137:24126:18223_2:N:0:TCTGCAAG+GCTGCACT
CTTTACTCACAGACTCCGAAAATGGTTTCAAATGAGGTCAAAGCTCTATTTATTAAACGAACTATTAT
CCGATTCTTCGAGGGTATTACTACCACTGATCTGCCGACTTTAGTCAA
+
FFFFFFFFFFFFFFFFFFFFFFFFFFFFFF:FFFFFFFFFFFFFFFFFFFFFFFFFFFFFFFFF:FFF
FFFFFFF:FFFFFFFFFFFFFFFFFFFFFFFFFFFFFFFFFFFFFFFF
@A00721:190:HGJW7DSXY:4:1660:26404:16063_2:N:0:TCTGCAAG+GCTGCACT
TTCCTTCGATGTTTATATTTTTCTATAAGGGAAAATAGCAATAATCAAATTTCGACAAATTTTAGAAC
TTTATTTCAGAACTCTCTCCGATCACCTTGGTGTGATCTCCCGGAGGA
+
FFFFFFFFFFFFFFFFFFFFFFFFF:FFFFFFFFFFFFFFFFFFFFFFFFFFFFFFFFFF:FFFFFFF
FFFFFFFFFFFFFFFFFFFFFFFFFFFFFFFFFFFF:FFFFFF:FFFF
@A00721:190:HGJW7DSXY:4:1548:26422:5509_2:N:0:TCTGCAAG+GCTGCACT
ATGTATGATGATAATCTACTATTTTTAAATAGTGATATTCAGCGTATACTTGAATACTTTATCAAAGA
TTTGGCTTGGTCTATTTACCGCAAAAAGTTGGAAGAGGTCTATGATAT
+
FFFFFFF,FFFFFFFFFFFFFFFFFFFFFFFFFFFFFFFFFFFFF:FFFFFFFFFFFFFFFFFFFFFF
FFF,FFFFFFFFFFFFFFFFFFFF::,FFFFFFFFFFFFFFFFFFFFF
@A00721:190:HGJW7DSXY:4:2439:14362:5916_2:N:0:TCTGCAAG+GCTGCACT
TATACTTTAAATAGCACCGATTATAACTTGACTGTTGATAATATTACAATAGCTCTTTCTCAGAAGAT
TAGAGAAGTAACCGGTGAACACGTCGGTAGCATTGATTTACTTTTACC
+
:FFFFF:FFFFFFFFFFFFFFFFFFFFFFFFFFFFFFFFFFFFFFFFFFFFFFFFFFFFFFFFFFFFF
FF:FFFFFFFFFFFFFFFFFFFFFFFF:FFFFFFFFFFFFFFFFFFF:
@A00721:190:HGJW7DSXY:4:1523:18195:7232_2:N:0:TCTGCAAG+GCTGCACT
TTTCGAAAAGAAACCTATAAGTTACAAACGATTCTTCGACTATATTAAAGAAATAAAAATACTTCAAG
TACCTGAACTTTACATTTTTAATGGTATGGTATAATGTGTCTTACAAA
+
FFFFFFFFFFFFFFFFFFFFFFFFFFFFFFFFFFFFFFFFFFFFFFFFFFFFFFFFFFFFFFF:FFFF
FFFFFFFFFFFFFFFFFFFFFFF:FFFFFFFFFFFF:FFFFFFFFFFF
@A00721:190:HGJW7DSXY:4:2205:5177:29246_2:N:0:TCTGCAAG+GCTGCACT
CTCTCGGTAATGAGAGTGTATTCCTTAATATTCTAGTAACCGCTATGTGGGCATTACTAGGATTTATC
ACACTCCTAAATTTGATAGCAATTTATGTTTTAAATGCTAGGAAAATT
+
FFFFFFFF:FFFFFFFFFFFFFFFFFFFFFFFFFFFFFFFFFFFFFFFFFFFFFFFFFFFFFFFFFF
FFFFFFFFFF:FFFFFFFFFFFFFFFFFFFFFFFFFFFFFFFFFFFFF
@A00721:190:HGJW7DSXY:4:1502:32533:25864_1:N:0:TCTGCAAG+GCTGCACT
TGTATAGTTCAAGGAGTACTTAATTCAATAGCTTTAGTATTATTTACTATCGCCTTTATTGAAAATTG
TATAAGAGTTAATTGCTTACACAAAAGTGACGCGCGAAGGAATCTTAATTGATTGAAGCTAGGCGTTG
GGG
+
FFFFFFFFFFFFFFFFFFFFFFFFFFFFFFFFFFFFFFFFFFFFFFFFFFFFFFFFFFFFFFFFFFFF
FFFFFFFFFFFF:FFF:FFFFFFFFFFFFFFFFFFF,FFFFF:FFFFFFFFFFFFFFFFFFFFFFFFF
FFF
@A00721:190:HGJW7DSXY:4:1153:4155:14857_1:N:0:TCTGCAAG+GCTGCACT
CTCCCCAAACATAACATACCATAACCTACCATGACCAATCATCGCTCCAACTGGCACGCCAGACGTAG
```

GGTCAAACCTACTACCGCCTGTCGGCAGATCGGCACCACTGGTCTCTACGTTCAGGGACCCGCTGTGC
AGA
+
FFFFFF:FFF:FFFFFFFFFFF:FFFFFFFFFFFFFFFFFFFFFFFFFFF,FFFFFFFFFFFFFFFFFF
FFFFFF:FFFFFFFFFFFFFFFFFFFFFFFFFFFFFFFFFFFFFFFFFFFFFFFFFFFFFFFFFFFFFF
F:F
@A00721:190:HGJW7DSXY:4:1119:29830:9533_2:N:0:TCTGCAAG+GCTGCACT
AAATCTAGATCTGCACAGAAGAAGGCTGAAGGTCGTTACCGGACGACCACTCAAAGGAAACGGGCTGT
TGCCCAGAGGAAAGCAGCGACCACCGAGGTCGCTCGTAACATTGACGG
+
FFFFFFFFFFFFFFFFFFFFFFFFFFFFFFFFFFFFFFFFFFFFFFFFFFFFFFF,FFFFFFFFFFFFF
FFFFFFFFFFFFFFFFFF:FFFFFFFFFFFFFF:FFFFFFFFFFFFFF
@A00721:190:HGJW7DSXY:4:1175:32940:31516_2:N:0:TCTGCAAG+GCTGCACT
TCTGTGGGAACTGAGGCAGTACCTCAGTCAAAAGTCACATTCACCACTCCCATACCAATCAATAGATC
GGAAGAGCGTCGTGTAGGGAAAGAGTGTAGTGCAGCGGGTGGTGGGGG
+
FFFFFFFFFFFFFFFFFFFFFFFFFFFF:FFFFFFFFFFFFFFFFFFFFFFFFFFFFF:FFFFFFFFFF
FFFFFF:FF:FF,FF:FFF,FFFFF:FF,FFF:,F,,::,,F,,,,,F
@A00721:190:HGJW7DSXY:4:1518:10456:34538_2:N:0:TCTGCAAG+GCTGCACT
CCCTCCGCTGGCACGAAGAACACTCCCAAGCCAATCAATAGTTCTTCTGACCAGAATGAAAAGGGGTT
TACCAGGGTAGTCTCTAAGTCTACCAGGAAGAGGGGAAATGTCCCCAA
+
FFFFFFFFFFFFFFFFFFFFFFFFFFFFFFFFFFFFFFFFFFFFFFFFFFFFFFFFFFFFFFFFFFFF
FFFFFFFFFFFFFFFFFFFFFFFFFFFFFFFFFFFFFFFFFFFFFFFF
@A00721:190:HGJW7DSXY:4:2103:6027:13025_1:N:0:TCTGCAAG+GCTGCACT
CATCCGAGATCTTGCAGAGTGACTGGAGTTCAGACGTGTGCTCTTCCGATCTGGAAGATCTCCCACCA
TCTGGGGTTGGGAATAAGAAATCTGCACGGGCCAAAGAGGCCGCTGCTACGAAGTCGCTGAAGGCGGC
TT
+
FF,,,FFF,F,FF,FFF:FFFFFFFFFFF,:FF,FFFF:FFFFFFFFF,FF,FFF:FFF,FF:,,:
FF:FFFFFFFFFFFFF::F:,,,FF:,F,F::,F:,FFFF:FF,FFFFF:FFFFFF,FFF:FFFFFFF
FF
@A00721:190:HGJW7DSXY:4:2376:15492:3082_2:N:0:TCTGCAAG+GCTGCACT
TGTAAAGTTGCGCCTGCCGACCCCATCTTTGAGACCAAGACTCTCTACAGTAGGTTCGTGCCTACTAT
GAGCAAATCTGTTGAATTCGTCACTGAGGACGAAGGTGAGAGGACTTT
+
FFFFFFFFFFFFFFFFFFFFFFFFFFFFFFFFFFFFFFFFFFFFFFFFFFFFFFFFFFFFFFFFFFFF
FFFFFFFFFFFFFFFFFFFFFFFFFFFFFFFFFFFFFFFFFFFFFFFF
@A00721:190:HGJW7DSXY:4:2374:5113:16642_1:N:0:TCTGCAAG+GCTGCACT
CTGAGAGCAGTCGCTGGGGAAAGAGAATTGCGTGGGTTGAAGGCCGACGCTTCGAGCTTGAGTGTGAC
GAGGCCGGTGACGTTTTTGTCAACGGCGTTTGGAGGATTTTATCCGCGCTTAACTATGGGGCGAAGAT
CCC
+
FFFFFFFFFFFFFFFFFFFFFFFFFFFFFFFFFFFFFFFFFFFFFFFFFFFFFFFFFFFFFFFFFFFF
FFFFFFFFFFFFFFFFFFFFFFFFFFFFFFFFFFFFFFFFFFFFFFFFFFFFFFFFFFFFFFFFFFFF
FFF
@A00721:190:HGJW7DSXY:4:2470:23863:22310_2:N:0:TCTGCAAG+GCTGCACT
AGGTTGGCATATTTATGCCGACGATAATAAGCATGTCACCCTTAAGCACCGAGGTAAGCTCGGGACTT
ACAAGAGAGAGTGTGAGAGAGTCGCTCTCACTATTTTTGCCCAGAATG
+
FFFFFFFFFFFFFFFFFFFFFFFFFFFFFFFFFFFFFFFFFFFFFFFFFFFFFF:FFFFFFFF:FFF
:FF:FFFFF:FFFFFFFFFFFFFFFFFFFFF:,FFFFF:FFFFFFFFF
@A00721:190:HGJW7DSXY:4:2178:23303:24126_1:N:0:TCTGCAAG+GCTGCACT
TGAGGTTAAAACTCTTGCTCCTTCTGTGGGTGAGGGTTTTTGTTTTAGTCACTGGTTCTGGGAGGCTG

CTTTCCTCCATAATTACAACCTCTCTTTAGAGAGCATTAGAAGGAAGTTGGGCAAATTCCCACATATG
GT
+
FFFFFFFFF,,FFF,FFFF,F:F,FFF::FFFFF,FF:FFFFFF,FF,FF,FFF:FF,FFF,,,FFFF
,FFFFFF,::FF,FF,FF,,:FFF:FFFFF,FF:,,FF:FFFFFFF,FF,FFFF:FFF:::FFFFFFF
FF
@A00721:190:HGJW7DSXY:4:1309:21884:4413_2:N:0:TCTGCAAG+GCTGCACT
ACACTTTTCTTGAAAATTTTTCCTTTCCTCCGTCATTGGGGATTTCGATGTGAGAAGGTGGAAGGTGT
CAAGGGTCTTCTTCATGCTGATCTTTCAGGACCTAATCACATCATGAT
+
FFFFFFFFFFFFFFFFFFFFFFFFFFFFFFFFFFFF:FFFFFFFFFFFFFFFFFFFFFFFFFFFF::FF
FFFFFFFFFFFFFFFFFFFFFFFFFFFFFFFFFFFFFFFFFFFFFFFF
@A00721:190:HGJW7DSXY:4:2306:5267:19006_1:N:0:TCTGCAAG+GCTGCACT
GCTCGTATCGAGTTGGAGGGAGGAAGTTCTCCGAAATTAAAGACGGGGGGATTAAGAGTCCAGCTGTA
ACTGTCATTTCAGAGAGGGGAGCTCTGAAAAGGAAACTTCGTCAGAAGATGAGGAAGTCTAAGAGGAG
GAG
+
F:FF,FFFFFFF:F,:FF,FFFFFF,FFFF,F,FFFFFFFFF:F:F,FF,FFFFFFFFFFFFFFFFFF
FFF,FFFFFFFFFFFFF,FFFFFFFFFFFFF,FFFFFFFFFFFFFFF:FFFFFFFF,FFFF:FFFFFF
FFF
@A00721:190:HGJW7DSXY:4:2158:6072:4617_1:N:0:TCTGCAAG+GCTGCACT
CTCTAGAGAAGATCTCACCACCTGGAGAAGGCAAGTGGAGAGTCCAGAAGTTGATGAAGAAGGATTAT
TTGAAGCAGGGGTTACCACTTCCTAAGGTGGATGAGGGAGGTTGTTTCTTTGGAGTTTTAGATAATCT
TTT
+
FFFFFFFFFFFFFFFF,FFFFFFFFFFFFFFFFFFFFFFFFFFFFFFFFFFFFFFFFFFFFFFFF,FF
FFFF:FFFFFFFFFFFFFFFFFFFFFFFFFFFFFFFFFFFFFFFFFFFFFFFFF:FFFFFF:FFFFF
FFF
@A00721:190:HGJW7DSXY:4:2541:20211:1611_2:N:0:TCTGCAAG+GCTGCACT
CCTTGAGAAGACAAGCCACATTAAGATATAATTTAGGTCAACTTGGGGTTTCGAATGTCATTAGTGCC
TTACGATTGAAAGAGGTCCTTTTGAGTATCGACAGATCTTTCGATCTT
+
FF:FFFFFFFFFF:FFFFFFFFFFFFFFFFFFFFFFFFFFFFFFFFFFFFFFFFFFFFFFFFFFFFFF
FFFFFF,FFFFF:FFFFFFFFFFF:FFFFFFFFFFFFFFFFFFFFFFF
@A00721:190:HGJW7DSXY:4:1446:22752:31313_2:N:0:TCTGCAAG+GCTGCACT
TGGCAGAATTCGGTAACTTACCATATTGTTCCTTCCGGGAAGTTGTGGGATTTAACTTTGACTCAACT
ACTCCGATCAGATCCGGATGGTTTGATTGGTATGGACTACTCGACTTT
+
FFFFFFFFFFFFFFFFFFFFFFFFFFFFFFFFFFFFFFF:FFFFFFFFFFFFFF:FFFFFFFFFF:F
FFFFFFFFFFFFFFFFFFFF::FFFFFFFFFFFFFFFFFFFFFFFFFF
@A00721:190:HGJW7DSXY:4:1338:19859:1564_1:N:0:TCTGCAAG+GCTGCACT
CATGGCAGATACTACCGATAAGGTGAAAAATATGCAGGTCAAAGATTCTGTAGCTCTGAGGGCGGTTG
AGGGAGCCGTTTTGGCCGACTTTAACTATTCTAAGAGTCTTTATGATTCAAAACCTATCGTTGTCGTA
CCT
+
FFFFFFFFFFFFFFFFFFFFFFFFFFFFFFFFFFFFFFFFFFFFFFFFFFFFFFFFFFFFFFFFFFFF
FFFFFFFFFFFFFFFFFFFFFFFFFFFFFFFFFFFFFFFFFFFFFFFFFFFFFFFFFFFFFFFFFFFF
FFF
@A00721:190:HGJW7DSXY:4:1466:32488:13385_2:N:0:TCTGCAAG+GCTGCACT
GCGGCTTATTGTCGCGCAGTTTCCAGAATTGCGGTTGAAGTTCACCCATTCTATCTTGAATGCTAACG
CCGTCGCAGCCATATCGAGACTATGTTATAATCACTTGTTAAAGTACA
+
FFFFFFFFFFFFFFFFFFFFFFFFFFFFFFFFFFFFFFFFFFFFFFFFFFFFFFFFFFFFFFFF,FFF
FFFFFFFFFFF,FFFFFFFFFFFFF:FFFFFFFFFFFFF:FFFFFF

```
@A00721:190:HGJW7DSXY:4:1406:7717:31673_2:N:0:TCTGCAAG+GCTGCACT
TTAGATATTATTGATATTGGAGGCGATATTCGTTTTCATATCCAAAACTCCTTGAATGGAGTACATAT
TTGCAACCCTATTCTTGATGTGAAAGATGCATCGAGGTATACGTCTAG
+
FFFFFFFFFFFFFFFFFFFFFFFFFFFFFFFFFFFFFFFFFFFFFFFFFFFFFFFFFFFFFFFFFFFF
FF:FFFF:FFFFFFFFFFFFFFFFFFFFFFFFFFFFFFFFFFFFFFFF
@A00721:190:HGJW7DSXY:4:2677:29993:9471_1:N:0:TCTGCAAG+GCTGCACT
AACGTACCTACTCACGAGACGTCATGAAAGCTATCGGAGGGAAGCAAAATGTTTCGTTCTGTTACAAG
AACGCTTTGGATTGTGACCATAAAGCCTCAGTTGCGATAGCTGTAGAGGTATACGATATCTCTCTTGA
TGA
+
FF:FFFFF:FFFFFFFFFFFFFFFFFFFFFFFFFFFFFFFFFFFFFFFFFFFFFFFFFFFFFFFFFFF
FFFFFFFFFFFFFFFFFFFFFFFFFFFFFFFFFFFFFFFFFFFFFFFFFFFFFFFFFFFFFFFFFFFF
FFF
@A00721:190:HGJW7DSXY:4:2338:11171:12258_2:N:0:TCTGCAAG+GCTGCACT
AATGCTTAAGAGAGAGATTGATGTATGTCATTTCGTCTTAGTTTGTCCCGGCGAATTATTGGACACGA
TGAAAAATGACATACCCGTTCCTGACCTTGGTATCGTTATCCGTCATG
+
FFFFFF:FFFFFFFFFFFFFFFFFFFFFFFFFFFFFFFFFFFFFFFFFFFFFFFF:FFFFFFFFFF
FFFFFFFFFFFFFFFFFFFFFFFFFFFFFFFFFFFFFFF,FFF:FFFFF
@A00721:190:HGJW7DSXY:4:1233:17002:32565_2:N:0:TCTGCAAG+GCTGCACT
GTTATTCTCACAACAAAGATAAGATTTTATCTTTCTTCAAACACCCTGTTTTCCTTAAGGGCGGACAA
TTGTTCCATTGTGAACTCTTGGGGACTAGATTGAGCATTAATGAGTTC
+
F:FFFFFFFFFFFFFFFFFFFFFFFF,FFFFFFFFFFFFFFFFFFFFF:FF:FFFFFFFFF:FFFFF:F
FFFFFFFFFFFFFFFFFF::FF:FFFFFFFFFFFFFFFFFFFFFFFFFF
@A00721:190:HGJW7DSXY:4:2631:21242:31861_1:N:0:TCTGCAAG+GCTGCACT
TCTCTAGTGGACAATTCAGAATACCTATTTCCGTACCAAGAGCCACTGCCAATCTCATCGCTATACGC
ATACCTGATTGGAAGAAGCGAGGTTCGTTCCTCAATAAAAATTTTTCTCAAGAGATTATGGTTGATAA
AGT
+
FFFFF:FFFFFFFFFFFFFFFFFFFFFFFFFF:FFFFF,FFFF,FFFFFFFFFFFFF:F,FFFFFFFFF
FFFFFFF:FFFF,FFF:FFFFFFFFFFFFFFFF:FFFF,FFFFFFFFFFFFFFFFFFFFFFFFFFFFFF
FFF
@A00721:190:HGJW7DSXY:4:1105:16848:32831_1:N:0:TCTGCAAG+GCTGCACT
TTCAGTACGTCATGGGGATCACTTCTTCGGTCAATGACAAAACCTTCGAATATGTTACGACATGGCTT
CGTAACAACAGTGCGAGGATTGTCATCTCGGGGAAAGTGATTCACCAAGATGTTCATATAGATACGAG
ATA
+
FFFFFFFFFFFFFFFFFFFFFFFFFFFFFFFFFFFFFFFFFFFFFFFFFFFFFFFFFFFFFFFFFFFF
FFFFFFFFFFFFFFFFFFFFFFFFFFFFFFFFFFFFFFFFFFFFFFFFFFFFFFFFFFFFFFFFFFFF
FFF
@A00721:190:HGJW7DSXY:4:1103:24985:34178_2:N:0:TCTGCAAG+GCTGCACT
CAGCGATCTTACTCGTTATCGGAGCTAAGAAGAAGACTTTGGGTACACGTTATGCTCGCAAACTTAAC
GTTAGTACCGGAGGTGATCTCTCTGCTTCTGCGTTACTGAGAGAAAAG
+
FFFFFFFFFFFFFFFFFFFFFFFFFFFFFFFFFFFFFFFFFFFFFFFFFFFFFFFFFFFFFFFFFFFF
FFFFFFFFFFFFFFFFFFFFFFFFFFFFFFFFFFFFFFFFFFFFF:FFF
@A00721:190:HGJW7DSXY:4:1126:26332:24267_1:N:0:TCTGCAAG+GCTGCACT
CCTACGGTGACTTTATGGACAAGATGCAAGTTCTTGGGAAATACTTGTTTCCCTTTTTAGGTTTAGTA
GGTGATCTTGACGTCGATTCTTTGTATAGGGAGTGTGAGAAGACAACGAATCTTGAAACTACGGTCCT
TAT
+
FFFFFFFFFF:FFFFFFFFFF,FFFFFF:FFFFFFFFFFFFFFFFFFFFFFFFFFFFFFFFFFFFFFFF
```

```
FFFFFFFFFFFFFFFFFFFFFFFFFFFFFFFFFFFFFFFF:FFFFFFFFFFFFFFFFFFFFFFFFFFF
FF,
@A00721:190:HGJW7DSXY:4:1608:32244:10895_1:N:0:TCTGCAAG+GCTGCACT
GAACCGGTCTCTAAATCTTTTGAGATTGAGAAGATTGAAGAACACGTCAAGAAGTCGCTCGGCGTTGT
TCTTGAAAACCAAGTTAAGAAGGAGGCTGATCATGGAGTCTTTGTACCTTCTGTCAAACTTCGAGGTT
CAG
+
:FFFFFF,F,FFFFFFFFFFFFFFFFFFF:FF:FFFFFFFFFFFF::FFFFFFFFFFFFFFFFFFFFF
FFFFFFFFFFFFFFFFFFFFFFFFFFFFFFFFFFFFFFFFFFFFFFFFF:FFFFFFFFFFFFFFFFFF
FFF
@A00721:190:HGJW7DSXY:4:2649:7256:16720_2:N:0:TCTGCAAG+GCTGCACT
GATCCCGGATGTGGATTGAAGGCTGGCTCTAAGGGAGACTCCTTCTTTTTGAATGTTGTAAAGAATTG
GGTTGAGAAGTGTGCTTTACAAGGAGAGATCGGAAGAGCGTCGTGTAG
+
FFFFFFFFFFFFFFFFFFFFFFFFFFFFFFFFFFFFFFFFFFFFFFFFFFFFFFFFFFFFF:FFFFFF
FFFFFFFFFFFFFFFFFFFFFFFFFFFFFFFFFFFFFFFFFFFFF,FFFF
@A00721:190:HGJW7DSXY:4:1654:11487:33442_2:N:0:TCTGCAAG+GCTGCACT
TAAAGAAAAATTTTCGAATAAACTAATGAGAGGTATAGGCTTAGTCAAGTCTGTACTTACATTCTTTA
CGCCAGATTCTTCATGGGGCGACGCTGTTTCTCTTGGTATTTCTGAAA
+
FFFFFFFFFFFFFFFFFFFFFFFFFFFF:FFF,FFFFFFFFFFF:FFFFF:FFFFFFFFFFFFFFFF:F
FFFFFFFFFFFFFFFFFFFFFFFF:FFFFF:FFFFFFFF:FFFFFFFFF
@A00721:190:HGJW7DSXY:4:1111:12536:32221_1:N:0:TCTGCAAG+GCTGCACT
TTTCCTCTGGTAAGGGAATTATTAAATCATTCTTCTCACTCTTGACGAGAATTATTACTGTTTTCTCT
ACCCATAACTTCTTTTTCAAAAATGCGGATGGGTCTACTGATGTTTTCCTACAGAGTGCAGTCGTTTC
AAC
+
FFFFFFFFF:FFFFFFFFFFFFFF:F:FFFFFFFFFFFFFFFFFFFFFFFFFFFFFFFFFFFFFFFFF
FFFFFFFFFFFFFFFFFFFFFFFFFFFFFFFFFFFFFFFFFFFFFFFFFFFFFFFFFFFFFFFFFFFF
FFF
@A00721:190:HGJW7DSXY:4:1456:25771:32158_1:N:0:TCTGCAAG+GCTGCACT
AATAATTGGTGATGAAAATACGTATGTCGGTGGGGTTCTCAAGGCTCTCATCGCCACTGGGATTCTAA
GGGGTTCCATAAGATATTATCTGACATTGTGGTTCAATGATCAGGATTCTTCTTTTGGTCCTTACGTC
TTA
+
FFFFFFFFFFFFFFFFFFFFFFFFFFFFFFFFFFFFFFFFFFFFFFFFFFFFFFFFFFFFFFFFFFFF
FFFFFFFFFFFFFFFFFFFFFFFFFFFFFFFFFFFFFFFFFFFFFFF:FFFFFFFFFFFFFFFFFFFF
FFF
@A00721:190:HGJW7DSXY:4:1646:2844:3630_1:N:0:TCTGCAAG+GCTGCACT
TCTTGTCACGATTCTTAAAAGAAGTTTATTAAAACTTCAGAGTCTCTTGGAAGTCCAGGTAGCTAATG
TTCTTGCAATAGCCACAGACTTTCTGGGAAAAGAGACCTCTGTTGGTAGAGGAGTTGAGAGAATTACT
CAG
+
FF:FFF:F:FFFFFFFFFFFF:FFFFFFFFF:FFFFFFFFFFFFFF:FF:FFFF:FFFFFFFFFFFF
FFF,::FFF:F,FFFFFFFFFFFFFFFF,FFF:FFFFFFFFFFFFFFFFFFFFFFFFFFFFFFFFFFF
FFF
@A00721:190:HGJW7DSXY:4:1465:7835:13307_2:N:0:TCTGCAAG+GCTGCACT
ACAACGAGTTAGGGAGTGGCTAGGTTTTAAAAGATCCTTTGCACAAGCAATACCTTCCACTGAGTTCA
TCACCCAGAGTTTCGACGTCGCGGAAAACGATGAAGATTTCCACACTG
+
FFFFFFFFF,FFFFFFFFFFFFFFFFFFFFFFFFFFFFFFFFFFFFFFFFFFFFFFFFFFFFFFFFFF
FFFFFFFFFFFFFFFFFFFFFFFFFF:FFFFFFFFFFFFFFFFFFFFFF
@A00721:190:HGJW7DSXY:4:1461:15510:4867_2:N:0:TCTGCAAG+GCTGCACT
TCTGATGCTGAATTTGAGGAAAATTTTTCGGAAGTTTTTGGTATAAATCCTGATGGCTCAGTACAGGG
```

```
AGGAGGTAATAGTTATAAAACCTTCTTTTATCATTGCAACAGGACTTT
+
FFFFFFFFFFFFFFFFFFFFFFFFFFFFFFFFFFFFFFFFFFFFFFFFFFFFFFFFFFFFFFFFFFFFFF
FFF:FFFFFFFFFFFFFFF:FFFFFFFFFFFFFFFFFFFFFFFFFFFF
@A00721:190:HGJW7DSXY:4:1306:22309:26569_2:N:0:TCTGCAAG+GCTGCACT
ATCTGGGATCATAGGTATGTGTCACGCTACACTCGAAATATGGGCCCTCATAAGGAAGTATAGTTGGC
GTCTTGTCGTTGACGTCATTACAGTCTGGAAGTTAGATCATTTGTTCA
+
FFFFFFFFFFFFFFFFFFFFFFFFFFFFFFFFFFFFFFFFFFFFFFFFFFFFFFFFFFFFFFFFFFFFFF
FFFFFFFFFFFFFFFFFFFFFFFFFFFFFFFFFFFFFFFFFFFFFFFF
@A00721:190:HGJW7DSXY:4:1258:15239:27383_1:N:0:TCTGCAAG+GCTGCACT
TTTAGATAAATTCTTTCCGGGCTCAATGACCTTTGCTATGTACGTGGATCTAATGCTTGGTGATCTGA
ATCTGACCATCGCAAAGAAGACTTTATTAGTTCATGAAGTTCTTACACTTCTCGCGACTAATGTCAGC
TTC
+
F:FFFFFFFFFFFF:FFFFFFFFFFFFFFFFFFFFFFFFFFFFFFFFFFFFFFFFFFFFFFFFFFFFFFFF
FFFFFFFFFFFFFFFFFFFFFFFFFFFFFFFFFFFFFFFFFFFFFFFFFFFFFFFFFFFFFFFFFFFFFF
FFF
@A00721:190:HGJW7DSXY:4:1217:7392:2926_2:N:0:TCTGCAAG+GCTGCACT
CCAAATTTAGCACTTTTTGAATATGACAGAAGCTCGCCATATCAGATTAGAGACATGCCAGTTAGAGT
TCTTCTAACCAGGACTGATGGTGAGATTCTTTTACCTTTTGGAGGTCC
+
FFFFFFFFFFFFFFFFFFFFFFFFFFFFFFFFFFFFFFF:FFFFFFFFFFFFFFFFFFFFFFFFFFFFFF
FFFFFFFFFFFFFFFFFFFFFFFFFFFFFFFFFFFFFFFFFFFFF,FFF
@A00721:190:HGJW7DSXY:4:2418:19352:34287_2:N:0:TCTGCAAG+GCTGCACT
CCTAGTGTCTTTTCTAGAGTCCAAGGACGTTTCCGTCGGAGGATGACAGGTGGGAGACCGCTCAATAA
CACTCTATTGAATCCCTTTTCCACGATGAAAGCTCTTTATGAGGTGCT
+
FFF,FFFFFFFFFFFFFFFFFFF:FFFFFFFFFFFFFFFFFFFFFFF,FFFFFFFFFFFFFFFFFFFFFF
FFFFFFF:FFFFFFFFF:FFFFFF:F:FFFFFFFFFFFFFFFFFFFFF
@A00721:190:HGJW7DSXY:4:2671:28664:4946_1:N:0:TCTGCAAG+GCTGCACT
GATATCGCCTCTGTCCTTTCTCCTTTAATCGTGAGGTTACGAGAGTTTCGTACTTGGGTTTATCGGTT
GTATTTTGATAAATTTGTTGGAATCGATAATGATACTGACGAAGGTTCTATTACCACCCTTGTCATTG
ATA
+
FFFFFFFFFFF:FFFFFFF:FFFFFF:FFFFFFFFFFFFFFFFFFFFFFFFFFFFFFFFFFFFFFFFFFF
FFFFFFFFFFFFFFFFFFFFFFFFFFFFFFFFFFFFFFFFFFFFFFFFFFFFFFFFFFFFFFFFFFFFFF
FFF
@A00721:190:HGJW7DSXY:4:2136:27525:17127_1:N:0:TCTGCAAG+GCTGCACT
GTCTAGCCGCTGAGATTCGTCTTACCTATTTACAATCTGTAGAGAGAGAGGTTAATCAGAATTTGAGT
GATAACGATTTAGTACTGGGAAATGGTGGTAGTCAGTTTACTACTTTTCTTTCCCTCCCCTTTTTCGA
TTG
+
FFF:FFFF:FFFFFF:FFFFFFFFFFFFFFFFFFFFFFFFFFFFFFFFFFFFFFFFFFFFFFFFFFFFFF
FFFFFFFFFFFFF:FFFFFFFFFFFFFFFFFF,FFFFFFFFFFFFFFFFFFFFFFFFFFFFFFF:FFFF
:FF
@A00721:190:HGJW7DSXY:4:2233:18331:18020_2:N:0:TCTGCAAG+GCTGCACT
AGGTTTTTCTTTTATGGGACTCTTGTTTGTACTTATGAAGGAGGTATTACTTCGGAAGTTCTTAATGT
AGGTCTCCTAGTACTTAAGGGTTTATGTTTCGGTTTCAGCTGGATGGA
+
FFFFFFFFFFFFFFFFFFFFFFFFFFFFFFFFFFFFFFFFFFFFFFFFFFFFFFFFFFFFFFFFFFFFFF
FFFFFFF:FFFFFFFFFFFFFFFFFFFFFFFF:FFFFFFFFFFFFFFF
@A00721:190:HGJW7DSXY:4:2508:12563:6370_1:N:0:TCTGCAAG+GCTGCACT
ATACGAGATCTTGCAGAGTGACTGGAGTTCAGACGTGTGCTCTTCCGATCTTATTATGTCGCTAAAGC
```

```
CTGTCCAGTTGTCGATGATGTTCGGGCTGTGGGGATTAACTTCCCTCGTGAAACTTACGACGATGTTC
TCG
+
FF:FF,FFFFFFFFFFFFFFFFFFFFFFFF:FFFFFFFFFFFFFFFF:FFFFFFF:FFFFFFFFFFFF
FFFFFFFFFFFFFFFFFFFFFFFFFFFFFFFFFFFFFFFF:FFFFFF,FFF:FFFFFFFFFFFFFFFF
FFF
@A00721:190:HGJW7DSXY:4:2173:5963:27258_1:N:0:TCTGCAAG+GCTGCACT
ATTACTAAGTCTGAGATCTGCCACTTCTAATTTTAGGAAACGTCGCGAAAAGAAAGTTCATGAGGATG
TCTTGGTCGAAAAATATCGCCGGGAACTTCTAATAGAGGATCCTGATGACATTATTTCAACGGATACC
GAT
+
FFFFFFFFFFFFFFFFFFFFFFFFFFFFFFFFFFFFFFFFFFFFFFFFFFFFFFFFFFFFFFFFFFFF
FFFFFFFFFFFFFFFFFFFFFFFFFFFFFFFFFFFFFFFFFFFFFFFFFFFFFFFFFFFFFFFFFFFF
FFF
@A00721:190:HGJW7DSXY:4:1361:13024:2253_2:N:0:TCTGCAAG+GCTGCACT
CACAGAAACCGTCTCAGGAACTGTCTCCACTGATTCGGAATTAGAGAAAGACGACCTTCTTGTGGGGA
CTGAGACCTCTGAATTTTCAGGGTGCCCTGATGAGGACGTTGAAAGGG
+
FFFFFFFFFFFFFFFFFFFFFFFFFFFFFFFFFFFFFFF:FFFFFFFFFFFFFFFFFFFFFFFFFFFF
FFFFFFFFFFFFF:FFFFFFFFFFFFFFFFF,FFFFFFFFFFFFFFFF
@A00721:190:HGJW7DSXY:4:2414:9507:21120_2:N:0:TCTGCAAG+GCTGCACT
AGACTTCTAAGAGTGTTTCAAAAGACCAGAAACGTAACAAGAAGAATAAAGGTAGGAAGGGGAGAAAA
CAGAACAAGCGAGCGGTTGAAGAGAAAACTGATATGGTTGCGGCTGAT
+
FFFFFFFFFFFFFFFFFFFFFFFFFFFFFFFFFFFFFFFFFFFFFFFFFFFFFFFFFFFFFFFFFF:F
FFFFFFFFFFFFFFFFFFFFFFFFFFFFFFFFFFFFFFFFFFFFFFFF
@A00721:190:HGJW7DSXY:4:2319:12897:11522_2:N:0:TCTGCAAG+GCTGCACT
TAGAAAGAACACACTCGGCGATACCACTGATCTTCCAGAGTTACGTAGTGCTCGTTATTTTGGTGATT
TTATCAAAGAGAAAGTTTCTGCTAGTCGCGACTATAGAATTTATAGTA
+
,F,FFFFF,FFF,,FF,FF,F,FF,F,,,FFFFF:,F:FFFFFFFFF,F,,F:,F,,FF,,,,F,FF:
::F::FFF,F:F,F,F,:,F,FFF,FF,F:FF,FFF,,,,,:FF,,,F
@A00721:190:HGJW7DSXY:4:2516:1515:34522_2:N:0:TCTGCAAG+GCTGCACT
CACTTACAAGAGTGTTGAAGTACCGCAACATGGATCAGTTTTCTAAAAAGCTATTAATAGCAGTTGCG
GGGCTATAATACCGCCTCCAGATTACTCTAAACTGAATCCACTCTCTT
+
FFFFFFFFFFFFFFFFFFFFFFFFFFFFFFFFFF:FFF:FFFFFFFFFFFFFFFFFFFFFFFFFFF:F
F,FFFFFFFFFFFFFFFFFFFFF:FFFFFFFFFFF:FFFFFFFFFF:F
@A00721:190:HGJW7DSXY:4:2639:21594:28244_2:N:0:TCTGCAAG+GCTGCACT
TTTGGAACTGTGTAATGTCCTACGTACTGTTCAAGGTGTTTGGGCTTCTCTAAAGAACAGCAACTTTC
AACATGAATTAGTTATTGAAAGTCTTGACCCTCACGTCTCACTTTATC
+
FFFFFFFF:FFFFFFFFFFFFFFFFFFFFFFFFFFFFFFFFFFFFFFFFFFFFFFFFFFF:FFFFFF:
FFFFFFFFFFFFFFFFFFFFFFFFFFFFFFFFFFFFFF:F:FFFFFFF
@A00721:190:HGJW7DSXY:4:2263:3757:10598_2:N:0:TCTGCAAG+GCTGCACT
AAAGAGGGTTTGAAAATTACGGAACCTTTCCTCGTTCTTCATGAAGATCTTCGAGTGTTTTATAGTGA
GTTATTCTTGCGAAGGTTCAAGTCGATTGATTTTGTTGACTACGATCT
+
FFFFFFFFFFFFFFFFFFFFFFF:FFFFFFFFFFFFFFFFFFFFFFFFFFFFFFFFFFFFFFFFFFFF
FFFFFFFFFFFFFFFFFFFFFFFFFFFFFFFFFFFFF:FFFF:FFFFF
@A00721:190:HGJW7DSXY:4:2354:17897:36432_1:N:0:TCTGCAAG+GCTGCACT
AGAAGTTCTTCGCAGTTGAATCTCCACCAGGTGGAGGGAAGACCACTGAACTTATAGCGACTTTTGTC
GAATTTTTCGCAAAAGGTCTTGAAGTACGTATTGTCACTGCTAATCGTAATTCGCGAGAAGAGATAGT
GAC
```

+
FFFFFFFFFFFFFFF:FFFFFFFFFFFFFFFFFFFFFFFFFFFFFFFFFFFFFFFFFFFFFFFFFFFFFF
FFFFFFFFFFFFFFFFFFFFFFFFFFFFFFFFFFFFFFFFFFFFFFFFFFFFFFFFFFFFFFFFFFFFFF
FFF
@A00721:190:HGJW7DSXY:4:2355:9525:21527_2:N:0:TCTGCAAG+GCTGCACT
CACTTCTAGTTCATCGCGGTATTTGTGAAATTAAGACAGACGTGAAAGTTATGATGAGTGATCGTATT
AGAACAGCTGATTCTTATTGCATGAACGTCGCTGAGGTCAAACCTGTG
+
F:FFFFFFFFFFFFFFFFFFFFFFFFFFFFFFFFFFFFFFFFFFFFFFFFFFFFFFFFFFFFFFF:FFFF
FFFFFFFFFFFFFFFFFFFFFFFFFFFFFFFFFFFFFFFFFFFFFFFF
@A00721:190:HGJW7DSXY:4:1402:23023:17190_1:N:0:TCTGCAAG+GCTGCACT
CGACGAATTATTCATGATTCACGCTGGTCAGACCCTTTTTCTTACACATCTACTTAAGGCGGCTTATA
CCATAGGTTACGGTGATTCCAATCAAATCGGATTCATCAATCGTATTGATCTCCAAGTCGCAAACTAC
TTC
+
FF:F,FFFFFFFFFFF:FFFFFFFFFFFFF:FF::FFFFFFFFFFFFFF:FFFFFFFFFFFFFFFFFF
F:,FFF,FFFFFFFFF:FFFF:FFFFFFF,F:FF:FFFFFFFFFFFFFFFFFFFFFFFFFFFFFFFFFF
FFF
@A00721:190:HGJW7DSXY:4:2410:10655:4257_2:N:0:TCTGCAAG+GCTGCACT
GATGAGTGTCGTTCTTATCGAAGTCTCAGTTATCGCTGCCCGGCCGACGTTTGTCGCGAACTCTCAGA
GGTTTACGGCAGAAAAATTTCTCCTGCTTGTAATTCGAGAGAGGACAC
+
FFFFFFFFFFFFFFFFFFFFFFFFFFFFFFFFFFFFFFFFFFFFFFFFFFFFFFFFFFFFFFFFFFFFFF
FFFFFFFFFFFFFFF,FFFFFFFFFFFFFFFFFFFFFFFFFFFFFFFF
@A00721:190:HGJW7DSXY:4:2674:1425:3333_1:N:0:TCTGCAAG+GCTGCACT
CTTGACGACTTCGAATTTGAGGACGGAGTTAAATATATTACTTTCACTCAAGGTGAAAAGTTTGAAAT
TAACAAAGTCCTTGGTAAGAGTAAGGTTAAATGTGAAGATGCTCAGACTATTCATGAAGTTCAAGGTA
ATA
+
FFFFFFFFFFF:FFFFFFFF:FFFFFFFFFFFFFFFFFFFF:FFFFFFFFFFFFFFFFFF,FFFFFFF
FFFFFFFFFFFFFFFFFFFFFFFFFFFFFFFFFFFFFFFFFFFFFFFFFFFFFFFFFFFFFFFFFFFFFF
FFF
@A00721:190:HGJW7DSXY:4:2670:16089:25755_1:N:0:TCTGCAAG+GCTGCACT
TGACCCTGTTTTTGCATCACAAACACATATTACCGTTGCTCTATCACGACACGTTGAATCTTTACGAT
ACTTTGTTATCAATGTAAAGTTAAACGATGCTACTTGTGAGAGGATCAATCACAGTAAGGAACTGTCA
CGT
+
FFFFFFFFFF:FFFFFFFFFFFFFFFFFFFFFFFFFFFFFFFFFFFFFFFFFFFFFFFFFFFFFFFFFFF
FFFFFFFFFFFFFFFFF:FFFFFFFFFFFFFFFFFFFFFFFFFFFFFFFFFFFFFFFFFFFFFFFFFF
FFF
@A00721:190:HGJW7DSXY:4:2203:25789:19038_2:N:0:TCTGCAAG+GCTGCACT
GATTCAACCCCTTATATCGAGGCTATCGAGGACGATGAGTATCTTGAAGTTGTTGACGACAAGCCTCA
AGAGGGTAGAGGACCACCTGGATCTTCTACTGTCTCTGCCTTCCAAGA
+
FFFFFFFFFFFFF,FFFFFFFFFFFFFFFFFFFFFFFFFFFFFFFFFFFFFFFFFFFFFFFFFFFFFFF
FFFFFFFFFFFFFFFFFFFFFFFFFFFFFFFFFFFFFFFFFFFFFFFF
@A00721:190:HGJW7DSXY:4:1329:32678:14184_1:N:0:TCTGCAAG+GCTGCACT
CCGATCTCTTAACGTCACTCGGTAATCTTTCAGCCGAATTAAGTACTAATGAGTTCAAGTCGGTTGTC
GATCGAATTAAGATCAAGGCTAATACCTTTATAGGTAAGAAGCCCCTTGAGCCGCGTAGTCAGCGCGT
TTA
+
FFFFFFFF,FFFFFFFFFFFFFFFFFFFFFFFFFFFFFFFFFFFFFFFFFFFFFFFFFFFFFFFFFFFFF
FFFFFFFFFFFFFFFFFFFFFFFFFFFFFFFFFFFFFFFFFFFFFFFFFFFFFFFFFFFFFFFFFFFFFF
FFF

```
@A00721:190:HGJW7DSXY:4:2277:9914:27492_1:N:0:TCTGCAAG+GCTGCACT
CCCGATAGGGTACCGAGTCTCCAAGAGAATATTTATTCTTATGAGGCTCGAAACTTCAACGTTTTCAC
AACGGAAAGACATATCGCACCCGAAATTTTTGGGCAGAATGCTCTTAACGCGGTTCTTGAACGGTGTT
TTG
+
FFFFFFFFFFFFFFFFFFFFFFFFFFFFFF:FFFFFFFFFFFFFFFFFFFFFFFFFFFFFF,FFFFFF
FFFFFFFFFFFFFFFFFFFFFFFFFFFFFFFFFFFFFFFFFFFFFFFFFFFFFFFFFFFFFFFFFFFF
FFF
@A00721:190:HGJW7DSXY:4:1569:29695:14278_1:N:0:TCTGCAAG+GCTGCACT
GATAAAGTTATAGGAGACTCAGTTAACCTGAGAGCTCCTCAATTGATAGATTGGGTCAACAATAGGGA
CGCTAGTATACTTCGTAAAGTCGACAGCGAAATGGTCAATATTGATAATTGGACCGACTATATGACCT
ATT
+
FFFFFFFFFFFFFFFFFFFF:FFFFFFFFFFFFFFFFFFFFFFFFFFFF,FFFFFFFFFFFFFFFFFF
FFFFFFFFFFFFF:FFFFFFFFFFFFFFFFFFFFFFFFFFFFFFFFFFFFFFFFFFFFFFFFFFFFFF
FFF
@A00721:190:HGJW7DSXY:4:1670:26268:18740_2:N:0:TCTGCAAG+GCTGCACT
AGGGAAGCTAAGGTTAAACTTGATGCTTCAGCCTTTTCGAAGCATAACCCCGCACAGAATATCGTTTA
TCACGCAAAATTTATTAATGCAATTTTCGGACCTATCTTTAAGGTTCT
+
FFFFF:FFFFFFFFFFFFFFFFFFFFFFFFFFFFFFFFFFFFFFFFFFFFFFFFFFFFFFFFFFFFFF
FFFFFFFFFFFF:FFFFFFFF:FFFFFFFFFFFFFFFFFFFFFFFFFF
@A00721:190:HGJW7DSXY:4:1378:24080:36464_2:N:0:TCTGCAAG+GCTGCACT
CTTTTACCCAACATTTTTATGTATACAGAGATGACCAGTGACGATTTCTCCCGGAAGCTCTTCTCTCT
CTTAGGAGGTCATGATGACTACTATAAGGGTGAAATCGATTTTTCGAA
+
FFFFFFFFFFFFFFFFFFFFFFFFFFFF:FFFFFFFFFFFFFFFFFFFFFFFFFFFFFFFFFFFFFFF
FFFFFFFFFFFFFF:FFFFFFFFFFFFFFFFFFFFFFFFFFFFFFFFF
@A00721:190:HGJW7DSXY:4:2207:27263:35336_1:N:0:TCTGCAAG+GCTGCACT
TTTTGTAAAGCCTATGAGATAAAGATGTATTCTTATTTAGGCTTCGATGCTGAATTTCTTGAACTTTG
GAGTGCCAGTGAATATTTTTGTCACGCCAGGAGTATCCATAACGATTTGTCATTTACCACCTTTGCTC
AAC
+
FF:FFFFFFFFFFFFFFFF:FFFFFFFF::FFF:FFFFFFFFFFFFFFFFFFFFFFFFFFFFFFFFFF
FFFFFFFFFFFFFFFFFFFFFFFFFFFFFFFFFFFFFFFFFFFFFFFFFFFFFF:FFFFFFFFFFFFF
FFF
@A00721:190:HGJW7DSXY:4:1552:26946:10520_1:N:0:TCTGCAAG+GCTGCACT
TAACACATTCTTTGGTAATTGTATGATCACTCTTATGATGCTCTCTCTTTATTACGACCTTCGGAAGT
TTAGAGCCGTCGCTGTCGCCGGAGACGATTCGATCCTCTTTTCGGACGTACCGGTCCAGGATTATTCA
GAT
+
FFFFFFFFFFFFFFFFFFFFFFFFFFFFFFFFFFFFFFFFFFFFFFFFFFFFFFFFFFFFFFFFFFFF
FFFFFFFFFFFFFFFFFFFFFFFFFFFFFFFFFFFFFFFFFFFFFFFFFFFFFFFFFFFFFFFFFFFF
FFF
@A00721:190:HGJW7DSXY:4:1311:2392:31814_2:N:0:TCTGCAAG+GCTGCACT
TGCTCGCGATTTATTCTTTTTGTCAATGATTACATCTACTTCGTTCCTGATCCTTACAAGATCATCGT
TAAGTTAGGTAAACCCCTAACCGATGTTACTCTTGTAGGACTTTGGGA
+
FFFFFFFFF,FFFFFFFFFFFFFFFFFFFFFFFFFFFFFF:FFFFFFFF:FFFFFFFFFFFFF:FFF:F
FFFFFFFFFFFFFFF:FFFFFFFFFFFFFFFFFFFFFFFFFFFFFFFF
@A00721:190:HGJW7DSXY:4:1303:3803:34757_2:N:0:TCTGCAAG+GCTGCACT
TTAAAGACTATACTAAATATCTCCACTACGAGGTTGTTGTTTGGCGATTAGCTCTTGCGACTAATATT
CGATATTCGATTGAGGGTCGTTATACCTATTCGGCTATCTGTGCTTTG
+
```

FFFFFFFFFFFFFFFFFFFFFFFFFFFFFFFFFFFFFFFFF:FFFFFFFFFF:FFFFFFFFFFFFFF:FF
FFF::FFFFFFFFFFFFFFFFFFFFFFFFFFFFFFFFFFFFFFFFFFFF
@A00721:190:HGJW7DSXY:4:2415:26332:13745_2:N:0:TCTGCAAG+GCTGCACT
TCAAATTTTAAACGATTCCGAGAGGTTTATCCTGATGTGGAGGGAGTTCTTGAAGCATCCCGACCTTT
CCTTGTGAAAATTTTGATGCGGTTTCATAAGATTATCAGTAATCCTGA
+
FFFFFFFFFFFFFFFFFFFFFFFFFFFFFFFFFFFFFFFFFFFFFFFFFFFFFFFFFFFFFFFFFFFFFF
FFFFFFFFFFFFFFFFFFFFFFFFFFFFFFFFFFFFFFFFFFFFFFFFF
@A00721:190:HGJW7DSXY:4:1652:15004:27414_1:N:0:TCTGCAAG+GCTGCACT
ATTCATGGACCTTCCATCCTGTACCTCTTCTGGAGAGACGTATCTCAAAGATCGACTTCGAGACAGGT
GATATTGTTGAGCGAGAGCGACCAAGATCTAAATTCGGTAACGGTGCGTGGCGCTTTATTTTAAAAAG
CTC
+
FFFFFFFFFFFFFFFFFFFFFFFFFFFFFFFFFFFFFFFFFFFFFFFFFFFFFFFFFFFFFFFFFFFFFF
FFFFFFFFFFFFFFFFFFFFFFFFFFFFFFFFFFFFFFFFFFFFFFFFFFFFFFFFFFFFFFFFFFFFFF
FFF
@A00721:190:HGJW7DSXY:4:2567:7111:12868_2:N:0:TCTGCAAG+GCTGCACT
CTGATACTTATCACGAAAGGACTTGAGTCTTTTGATCAGTCTCTGACTTGACCATCTTTCATTTTAAA
TGACGATCATTAAGACTCTCTTTCTTCTTTGTTTCCTTCTTTGCACTG
+
FFFFFFFFFFFFFFFFFFFFFFFFFFFFFFFFFFFFFFFFFFFFFFFFFFFFFFFFFFFFFFFFFFFFFF
FFFFFFFFFFFFFFFFFFFFFFFFF:F:FFF:FFFFFFF:FFFFFFFFF
@A00721:190:HGJW7DSXY:4:2368:13286:8563_1:N:0:TCTGCAAG+GCTGCACT
CTCTTCCGATCTCTAATCTGTGTTTTTACGATAACAACTATCAGTTGATTAAGACTCTTCCGAAAGGC
GAACTTTGGGAATTTGAAGCTCAGGGTGAAGCACAACGTTTGACCGTGACGAACTGTGGTGTTCTTGA
TCT
+
FFFFFFFFFFFFF:F:FFFFFFFFFFFFFFFFFFFFFFFFFFFFFFFFFFFFFFFFFFFFFFFFFFFFFF
FFFFFFFFFFFFFFFFFFFFFFFFFFFFFFFFFFFFFFFFFFFFFFFFFFFFFFFFFFFFFFFFFFFFFF
FFF
@A00721:190:HGJW7DSXY:4:2551:23873:20885_1:N:0:TCTGCAAG+GCTGCACT
ATACTCTCCTTGAGTTCACTCGTAAGGACACTACCACTTATTACGATATTTCTTTAGTCGATGGGTTT
TCGACTCCTTTAAGCGTCTTCTGTTCATCGCAACCAGATGATATCTTCTATTCGAACTTATGTTTCGA
TCA
+
FFFFFFFFFFFFFFFFFFFF:FFFFFFFFFFFFFFFFFFFFFFFFFFFFFFFFFFF:FFFFFFFFFFFF
FFFFFFFF:FF:FFFFFFFFFFF:FFFFFFFFFFFFFFFFFFFFFFFFFFFFFFFFFFFFFFFFFFFFFF
FFF
@A00721:190:HGJW7DSXY:4:1673:28999:19304_2:N:0:TCTGCAAG+GCTGCACT
TCTTCTAATGTGGCCTGAACTACGTTTCACTTTGTGTGTACTTGTCTCTATTTTAAGTTTTGTCTTCC
TTGTTTGTCTTATTCTATTAATCACTCATTTTAGTGAGATTATTAAAA
+
FFFFFFFFFFFFFFFFF:FFF:FFFFFFFFFFFFFFFFFFFFFFFFFFFFFFFFFFFF:FFFFFFFFF:
FFFFFFFFFFFFFFFFFFFFFFFFFFFFFF:FFFFFFFFFFFFFFFFFF
@A00721:190:HGJW7DSXY:4:2141:15637:5713_2:N:0:TCTGCAAG+GCTGCACT
CGACAACAGCAGGGGTTTTCGAATAGTGATAGCCACCCGCCTTACAGAGGGGCAGCAGCATGACCATA
ATTGGTATTGACTTTGGTACCACCTTTTCTACTGTCTGTCTCGCCTCT
+
FFFFFFFFFFFFFFFF:FFFFFFFFFFFFFFFFFFFFFFFFFFFFFFFFFFFFFFFFFFFFFFFFFFF,
FFFFFFFFFFFFFFFFFFFFFFFFFFFFFFFFFFFFFFFFFFFFFFFFF
@A00721:190:HGJW7DSXY:4:2206:20708:27868_1:N:0:TCTGCAAG+GCTGCACT
CTTAACAACAATGATAGTGAATTTATACCTACAATCATTGGTTTTTCTAATAATGATGACACTATTGT
TTATGGATATGATGATATTACTCATGATGGTTACGGTCAGGGAGGATTCTCAGTTTACAAGGACCTTA
AGC

+
::FFFFFFF,FFFFF:FFF:FFFFFFF:FF::FFF,,FF,FFFFF:FFFFF,FFFFF,FF,FFFFF,,
FFFFF,FFFFFFFF,F:FF:,F:FFF:F::FFFFF,F:F:F,:FF,FFFF:FFFFF,FFFF:,:FFFF
FF:
@A00721:190:HGJW7DSXY:4:2468:31394:15749_2:N:0:TCTGCAAG+GCTGCACT
CCGAAAGACGTGCTAAATTAAAACCATTGTACGATGTCGTCTGTCTCGGCGATCACTTTTCACTAAGA
ATTGGTCCTACTTTTGGACAAGGCCAACTTCGTAATCTTCATGAGATC
+
FFFFFFFFFFFFFFFFFFFFFFFFFFFFFFFFFFF:FFFFFF:FFFFFFFFFFFFFFFFFFFFFFFFF
FFF::FFF:FFFFFF::FFFFFFFFFFFFF,FFFFFFFFFFFFFFFFF
@A00721:190:HGJW7DSXY:4:1235:6460:27868_1:N:0:TCTGCAAG+GCTGCACT
TAGGGATTTTGAGAGAGTGAAAGGCTTCAATTGTAGTGGTTTGGTCGTTTCAGTACCCAGTCAGTATA
CCTCTACGCAAAGATATTTCATGAAAACTACAGAGGAACGTACCGGATTACCTGTCTTTCATATTATG
AAT
+
FFFFFFFFFFFFFFFFFFFF:FFFF:FFF:,FFF:F,FFFFFF:FFFFFFFFF,FFFFFFFFFFFF:F
FFFFFFFFF:FFFFFFFFFFFFFFFFFFFFFFFF:FFFFFFFFFFFF:FFFFFFFFFFFFFFFFFFFF
FFF
@A00721:190:HGJW7DSXY:4:1232:6280:29027_1:N:0:TCTGCAAG+GCTGCACT
TTTGCTTCGATGTTGGATGTCAAGAAGGGTTCAGAATGGGATTCCTACGTAGTTTATGACTTTGGTGG
TGGTACTTTCGATACTTCTATCGTCTTACGTTACGGTAATTACTATAGTGTTATTTATTCTGGTGGTG
ATG
+
F,FFFFFFFFFFFFFFFFFFFF:FFFFFFFFFFFFFFFFFFFFFFFFFFFFFFFFFFFFFFFFFFFFF
FFFFFFFFFFFFFFFFFFFFFFFFFFFFFFFFFFFFFFFFFFFFFFFFFFFFFFFFFFFFFFFFFFFF
FFF
@A00721:190:HGJW7DSXY:4:1566:10737:18239_2:N:0:TCTGCAAG+GCTGCACT
GGTCGTGACGTTGATGAAGCTATAAGAGCGTTCTTATTACGGCGCACTTCTTTACCCGCCACCACACA
ACTTTCAGTTTCACAACTGAAGGAGGAGGTTTCTGTTACTGGAAAACC
+
FFFFFFFFFFFFFFFFFFFFF:FFFFFFFFFFFFFFFFFFFFFFFFFFFFFFFFFFFFFFFFFFFFFF
FFFFFFFFFFFFFFFFFFFFFFFFFFFFFFFFFFFFFFFFFFFFFFFF
@A00721:190:HGJW7DSXY:4:1554:20627:10723_2:N:0:TCTGCAAG+GCTGCACT
TCAATAATATACGTCTTGAACTCACTTATGACGATCTAGTGAAATTAACGCGTCCTTTTCTGGAACGT
AGTTTTACTATTATGAACAAAGTTATCAAAGATAGTGGCATTCAAGGC
+
FFF:FFFFFFFFFFFFFFFFFFFFFFFFFFFFFFFFFFFFFFFFFFFFFFFFF,FFFFFFFFF:F:FF
FFFFFFFFFF:FFFFFFFFFFFFFFFFF:FFFFFFFFFFFFFFFFFFF
@A00721:190:HGJW7DSXY:4:1155:11523:32127_1:N:0:TCTGCAAG+GCTGCACT
TGATTCCGGCCGGTGGTAGCTCATTTCTACCAGGGGCTATTACTCTTGCTGAAACAGCTATTAAAGCT
GTTGAGCGGGTAATTACACCTAAACGAGCTCGATCTGCCATCGCTGAAGGATGTGCACTCTTTTCTGC
TAC
+
FFFFFFFFFFFFFF:FF:FFFFFFFFFFFFFFFFFFFFFFFFFFFFFFFFFFFFFFFFFFFFFFFFFF
FFFFFFFFFFFFFFFFFFFFFFFFFFFFFFFFFFFFFFFFFFFFFFFFFFFFFFFFFFFFFFFFFFFF
FFF
@A00721:190:HGJW7DSXY:4:2110:31774:10081_2:N:0:TCTGCAAG+GCTGCACT
TCGATTGTATCAACAGTAACATTACTTCTAATAAAGGTCTCTATCAAGCTCGTATTATCGTCGCTGCT
GGATCTGCGTTACCGTTGAAGGCTAAACTCCAAGGTAATTATGAAGAC
+
FFFFFFFFFFFFFFFFFFFFFFFFFFFFFFFFFFFFFFFFFFFFFFFFFFFFFFFFFFFFFFFFFFFF
FFFFFFF:FFFFFFFFFFFFFFFFFF:FFF:FFFFFFFFFFFF,F:FF
@A00721:190:HGJW7DSXY:4:2161:21992:31000_1:N:0:TCTGCAAG+GCTGCACT
GTATGAGGGTGTTTCTAATCGTAACATTAATAATTTCAATCTTCTTAAAAAGAAGATAGAGAGTCATA

CTTTGGGCTATCGTTCAGGTGCATGGGGTTGGATAGCCAATATTGAAGTGGACACCTCTGGTAGTATC
GAA
+
FFFFFFFFFFFFFFFFFFFFFFFFF:FFFFFFFFF:FFFFFFFFFFFFFFFFFFFFFFFFFFFFFFFFF
FFFFFFFFFFFFFFFFFFFFFFFFFFFFFFFFFFFFFFFFFFFFFF:FFFFFFFFFFFFFFFFFFFFFF
FFF
@A00721:190:HGJW7DSXY:4:2615:27534:28729_1:N:0:TCTGCAAG+GCTGCACT
CTAAGCATACTACTTTTGCTTTGATTACTCTACCGCCTTACAAGTCTAAGGTTAAAGAGTCGATTAAA
CAAGTTGTACTTATCAATGATTTTATCGCTGGTTGTTATCTCGAAAAGTCTCTTCTAAAAGGAACGTC
AGT
+
FFFFFFFFF:F::FFFFFFFFFFFFFF:FFFFFFF,FFFFFFF:FFFFFFFFFFFFFFFFFFFFFFFFF
FFFFFFFFFFFFFFFFFFFFFFFFFFFFFFFFFFFFFFFFFFFFFFFFFFFFFFFFFFFFFFFFFFFFF
FFF
@A00721:190:HGJW7DSXY:4:1478:12102:25520_2:N:0:TCTGCAAG+GCTGCACT
CTAAAATATTTACAAAATCTTACCACTATCTACGAACCTAAAGTTCTGAAGACGTTGCTTGTCGCTTC
CTCCTTTAATGGCACTACCTCGGTACAATCTTACTTGGAACGAAGGCG
+
FFFFFFFFFFFFFFFFFFFFFFFFFFFFFFFFFFFFFFFFFFFFF:FFFFFFFFFFFFFFFFFFFFFFF
FFFFFFFFFF:FFFFFFFFFFFFFFFFFFFFFFFFFFFFFFFFFFFFF
@A00721:190:HGJW7DSXY:4:1410:5186:15890_1:N:0:TCTGCAAG+GCTGCACT
GTTCTGTCTATATTAGGTGAAGCGAAATACGATTTCGAACGTCAACGTTCACCGAGTTTCGTTTCGTA
CCTTGATGCTGACTTTCGTCAATATGATTTGATCTGGTTGCTCAATTCTAAGCGTGTTCCGCTTAGTG
CGA
+
FFFFFFFFFFFFF,:FFFFFFFFFFFFFFFFF::FFFFFFFFFFFFFFFFFFFFFFFFFFFFFFFFFFF
FFFFFFFFFFFFFFFFFFFFFFFFFFFFFFFFFFFFFFFFFFFFFFFFFFFFFFFFFFFFFFFFFFFFF
FFF
@A00721:190:HGJW7DSXY:4:2140:32750:3787_1:N:0:TCTGCAAG+GCTGCACT
TACGAACTTGACTCTACTATACGTGACATACTAAATACTAGATATTTGGATGATCTTGATAAAGAACT
TCATGAATTTTCAGAGGATGAAAAATTTTCTTCCTTGGATTTTAATATAAAGAATCTTTACCAGGACT
CCT
+
:FFFFFFFF,FFFF:FFFFFFF::FFFFFF:FFFFFF:FFFFFFFFF:FFFFFFFFF,FFFFFFFFFF
FFFFFFFFFFFFFFF:FFF:FFFFFFFF:FFFFFFFFFFFFFFFFFFFFFFFFFFFFFFFFFFFFFFFF
FFF
@A00721:190:HGJW7DSXY:4:1610:14290:20478_1:N:0:TCTGCAAG+GCTGCACT
TGATCGTAAGAAGTTTATAAGGAACATATCCATTTGTTGTAATAGGGTCATCAACTTAGAACTCGAAA
CTCTAAGTGGTAATACTGTCTTTCCAAGCTTACCTGTATCTTCAATGAAATTGGACTCTTCGATAGGG
GAC
+
FFFFFFFFFFFFFFFF:FF:FFF:FFFFFFFFFFFFFFFFFFFFFFFF:FFFFFFFFFFFFFFFFFFFF
FFFFFFFFFFFFFFFFFFFFFFFFFFFFFFFFFFFFFFFFFFFFFFFFFFFFFFFFFFFFFFFFFFFFF
FFF
@A00721:190:HGJW7DSXY:4:1445:10601:9862_1:N:0:TCTGCAAG+GCTGCACT
GGAAGATCTGTTATCGACGGTTTATCGTTTACGAAGTATCACTCCACCGGTGAATGGTTTAAGAATAC
TATAAATAGAATAGCTATTACTACTAATGGCGTTGGCCTAGTGGATGTTATTAGTGACATCTGGTCAA
TTT
+
FFFFFFFFFFFFFFFFFFFFFFFFFFFFFFFF:FFFFFFFFFFFFFFFFFFFFFFFFFFFFFFFFFFFF
FFFFFFFFFFF,FFFFFF:FFFFFFFFFFFFFFFFFFFFFFFFFFFFFFFFFFFFFFFFFFFFFFFFFF
FFF
@A00721:190:HGJW7DSXY:4:1176:20410:6339_1:N:0:TCTGCAAG+GCTGCACT
TGGTCTTTATTCTATTATTGATTTTTATGAGATTGATACGTTAACAGAAGATGATGTTAGAGTAGAAT

```
TCCTGAAGAGTCCTTCCGTAACCAACTTCTTGACAATTTTCTTCAATCCTTCGATAATAGTTAAATCT
AGG
+
FFFFFFFFFFF:FFFFFFF:FFFFFFFFFFFFFFFFFF:F:FFFFFFFFFFFFFFF:FFFFFFFFFFF
FFFFFFFFFFFFFFFFFFFFFFFFFFFFFFFFFFFFFFFFF:FFFFFFFFFFFFFFFFFFFFFFFFFF
FFF
@A00721:190:HGJW7DSXY:4:1342:4218:12555_2:N:0:TCTGCAAG+GCTGCACT
TACACAATTAAGAAATAATCCTACATTGACTGATTATCTAAAGATTGAAGGAAAATTTTATCCTCGTT
TAACTTACTTAGACTTTCCTACGATACAAATGATAACTTCTAATTTAC
+
FFFFFFFFFFFFFFFFFFFFFFFFFFFFFFFFFFFFFFFFFFFF,FFFFFFF:FFFFFFFFFFFFFFF
FFFFFFFFFFFFFFFFFFFFFFFFFFFFFFFFFFFFFFFFFFFFF:FFF
@A00721:190:HGJW7DSXY:4:1175:28357:36636_1:N:0:TCTGCAAG+GCTGCACT
GGGATGCATGGTGTTATCTCCTCAGGAGTCCCGGCATTTGATAATAATGACGAGACTTTCGTCATAAT
CGCTGCATATTACGGATTACACGGTACCAGCGCATCTCGTGTCGAGCCTCGACCCGATTATATGGTAT
TAA
+
FFFFFFFFFFFFFFFFFFFFFFFFFFFFFFFFFFFFFFFFFFFFFFFFFFFFFFFFFFFFFFFFFFFF
FFFFFFFFFFFFFFFFFFFFFFFFFFFFFFFFFFFF:FFFFFFFFFFFFFFFFFFFFFFFFFFFFFFF
FFF
@A00721:190:HGJW7DSXY:4:2459:2121:10895_1:N:0:TCTGCAAG+GCTGCACT
AAATTTATAGAAGATTATTTTGACAGAATCAATAAGAATCATCGATTCTCGATTCGTAGAGCTTATCT
AGGTACTCTCATTTACTATGTTGATTTAGTCTACATTTATGCTGATCTAAAATTTCGATCTAGATGGA
TCC
+
FFFFFFFFFFF,F,:FFFFFFFFFFF::FFFF,FFFFFFFFFFFFFFFFFFFFFFFFFFFFFFFFFFF
FFFFFFFFFFFFF::FFFFFF:FFFFFFFFFFFFFFFFFFFFFFFFFF:FFFFFFFFF:FFFFFFFFFF
FFF
@A00721:190:HGJW7DSXY:4:1638:24261:30138_1:N:0:TCTGCAAG+GCTGCACT
TTCTAGAATTCTTAATTATAATATTCAACCTCAAAAGTTACGTGTTTACAGTAACGAGATCACACATT
TTGAGAACTAACTATGTTATGTTCTCTGATCATTTTGACAATCTCTTCTTATAATAAATAAATAAATA
ATA
+
FF,FFFFFFFFFFFFFFFFFFFFFFFFFFFFFFFFFFFFFFFFFFFFFFFFFFFFFFFFFFFFFFFFF
FFFFFFFFFFFFFFFFFFFFFFFFFFFFFFFFFFFFFFFFFFFFFFFFFFFFFFFFFFFFFFFFFFFF
FFF
@A00721:190:HGJW7DSXY:4:1409:6677:31469_2:N:0:TCTGCAAG+GCTGCACT
CAAATAGTGGAAAGGCTCCAGTCAACACTAGTTCGAGTGCTGCTTTGGATTACGATGACGTGAGAATA
GGTGAAGGGATAGTTTTTACCTCGACCAACATTAAGGCGGCGGCGACT
+
FFFFFFFFFFFFFFFFFFFFFFFFFFFFFFFFFFFFFFFFFFFFFFFFFFFFF:FFFFF:FFFFFFFFFF
FFFFFFFFFFFFFFFFFFFFFF:FFFFFFFFFFFFFFFFFFFFFFFFFFF
@A00721:190:HGJW7DSXY:4:1636:16441:11553_2:N:0:TCTGCAAG+GCTGCACT
CTTCACCCAAAATGAAGGACACTTCGAAACTGAATAATGATGCACTACAAACAAGTATCGCTAATCAT
CTGAAAAAGGCTCTAAAGGACACTATACAATATCCAGACGACGACGAA
+
FFFFFF:FF:FFFFFFFFFF:FFFFFFFFF:FFFFFFFFFFFFF:FFFFFFFFFFFFF:FF:FFFFFF
FFFFFFFFFF:FFFFFFFFFFFFFFFFFFFFFFFF:FFFFFFFFFFFF:
@A00721:190:HGJW7DSXY:4:2431:32759:5682_2:N:0:TCTGCAAG+GCTGCACT
CAGCACCACATTTTGGTCAAGTCAATCCGTTGAGAAAATTTCTTAGAGGATATTCGCCATTGTGGTAC
GAATTGGTCCAACATTTACCACCAAATTCTTTTGATACAACTCTAGCC
+
FFFFFFFFFFFFFFFFFFFFFFFFFFFFFFFFFFFFFFFFFFFFF:FFFFFFFFFFFFFFFFFFFFFFF
FFFFFFFFFF,FFFFFFFFFFFF:FFFFFFFFFFFFFFFFFFFFFFFFF
```

```
@A00721:190:HGJW7DSXY:4:1469:1777:18411_2:N:0:TCTGCAAG+GCTGCACT
CGCTTGCTATTCACCAGATTGGTTCGAACCCGTTGATGCTATGTCAGAAGAAGCTATTCAAGCTTGTC
GTTTAAAGACTGCAGAGGCTGTCAAACAAGCCCAGACTCAAGGAGTCC
+
FFFFFFFFFFFFFFFFFFFFFFFFFFFFFFFFF:FFFFF,:,FFFFFFFFFFFFFFFF:FFFFFFFF
FFFFF:FF:FFFFF,FFFFFFFFFFFFFFFFFFFFFFFFFFFFFFFFF
@A00721:190:HGJW7DSXY:4:1627:2799:5682_2:N:0:TCTGCAAG+GCTGCACT
GGTGATAAAAATTATTTAGAGATCTTTTCTACTTACAAAGGACAATTCAGTTTCATTAATTCTACGGA
AAAGTCCGGAGTATATTGTATGTTAATAGATAGTGGGATAATAGTTCT
+
FFFFFFFFFFFFFFFFFFFFFFFFFFFFFFFFFFFFFFFFFFFFFFFFFFFFFFFFFFFFFFFFFFFF
FFFFFFFFFFFFF:FFFFFFFFFFFFFFFFFFFFFFFFFFFFFFFFFF
@A00721:190:HGJW7DSXY:4:2351:32678:13745_1:N:0:TCTGCAAG+GCTGCACT
CGTCTGAAGAGTTTAGAGCTGCTATTGATTATGTAACCGATAGGAGTTGTATAGTTGAAGATGTGGCT
TTAGACGATAATCCTTTAAGGACTATGTTGACTGACGAAGATTTTCCCACAGATTTGTCTACGTTGCA
GAT
+
FFFFFFFFFFFFF:FFFF:FFFFFFFFFFFFFFFFFFFFFFFFFFFFFFFFFFFFFFFFFFFFFFFFF
FFFFFFFFFFFFFFFFFFFFFFFFFFFFFFFFFFFFFFFFFFFFFFFFFFFFFFFFFFFFFFFFFFFF
FFF
@A00721:190:HGJW7DSXY:4:1617:4164:19852_2:N:0:TCTGCAAG+GCTGCACT
CTATATACATTATAAAATATAAAAATAAGGGAGTCATAACTTTTGTTACCAAATTCTTGGCTTTCAAC
GAAGTCTCTGATTTTATTAATCATTACTTTAACGATCTCTCGGTCATT
+
FFFFFFFFFFFFFFFFFFFFFFFFFFFFFFFFFFFFFFFFFFF:FFFFFFFFFFFFFFFFFFFFFFFFF
FFFFFFFFFFFFFFFFFFFFFFFFFFFFFFFFFFFF:FFFFFFFFFFF
@A00721:190:HGJW7DSXY:4:1248:20817:35102_1:N:0:TCTGCAAG+GCTGCACT
TTGATCTACGTCGTTTAACTGTCGGAACAACCTTTACTTAGAATGACTCTATCTTTTCAGACACTTCT
TATATTATTTACAATAGTTAATATCATATTAGTAATACTCACTATCGTTTTCTGCTTTTTCTTAGCTA
ATA
+
FFFFFFFFFFFFFFFFFFFFFFFFFFFFFFFFFFFFFFFFFFFFFFFFFFFFF:FFFFFFFFFFFFFF
FFFFFFFFFFFFFFFFFFFFFFFFFFFFFFFFFFFFFFFFFFFFFFFFFFFFFFFFFFFFFFFFFFFF
FFF
@A00721:190:HGJW7DSXY:4:1143:30400:23672_2:N:0:TCTGCAAG+GCTGCACT
ATGAGTACGAAAACCAACTCTTCGTTACGAGACCTGATGGTAGAATAGATTTAGTCTCTACACCCATC
TCAACGAGGATAAAGCGAGTTTTCCGTCCTGAACGTAATACACGAAGA
+
F:FFFFFFFFFFFFFFFFFFFFFFFFFFFF,FFFFFFFF,FF::FFFFFFFFFFFFFFFFFFFFFFFF
FFFF:FF,:FFFFF,FFFF:FFF:,FFF,F,FFFF:FF:FFFFFFF::
@A00721:190:HGJW7DSXY:4:2521:15447:22608_1:N:0:TCTGCAAG+GCTGCACT
ACACGACTCGAAACCGAGACGCCGTAGATCAAAGTCCCTTCTAACTTTAGATTATGAATCCTGCCTTT
ACTCATTTTATA
+
F,:F,F:FFFFFFFFFFFFF::F:F:FFFFFF,FFFFFF:FFFFF:FFF:FFFFFFFFFFFFFFFFFF
FFFFFFFFFFFF
@A00721:190:HGJW7DSXY:4:1507:21847:5196_2:N:0:TCTGCAAG+GCTGCACT
TTTTTATAATTTATAACCTTCTTATACTTTTGGGTATGTTTTGTGAAAAACATGAGATACCCAAGTCT
ATCGAAGACATTATCTCTTATGTACTTATTGTAACGATCATTCTTTCG
+
FFFFFFFFFFFFFFFFFFFFFFFFFFFFFFFFFFFF:FFFFFFFFFFFFF,FF:FFFFFFFFFFFFFF
FFF,FFFFFFFFFFFFFFFFFFFF:FFFFFFFFFF:FFFFFFFFFFF,
@A00721:190:HGJW7DSXY:4:2677:25301:30029_1:N:0:TCTGCAAG+GCTGCACT
TTCTTATTCTTTTATTCCTTTAACTGTGTATGAGGAGGATTTATAATGCTTTTCGATAGTGATTGTTA
```

TCAATTTAAATACATTACTGTTTCAAATCATCTTTACTCACAGACTCCGAAAATGGTTTCAAATGAGG
TCA
+
FFF:FFFFFFFFFFFFFFFFFFFFFFFFFFFFFFFF:FFFFFFFFF:F:FFFFFFFFFFFFFFFFFFF
FFFFFFFFFFFFFFF:F:,FFFFFFFFFFFFFFFFFFFFFFFFFFFF:FFFFFF,FFFFFFFFFFFFF
FFF
@A00721:190:HGJW7DSXY:4:2677:25301:30029_2:N:0:TCTGCAAG+GCTGCACT
TATTAAACGAACTATTATCCGATTCTTCGAGGGTATCACTACCACTGATCTGCCGACTTTAGTCAAGG
AACTTCAATTCCTTCGATGTTTATATTTTTCTATAAGGGAAAATAGCA
+
F:FFF:FFF:F,FFFFFF::FFFFFFFFF:FFFFFFFFFFFFFFFFFFFFFFFFFF:FFFFFFFFFFF
FFFFFFFFFFFFFFFFFFFFFFFFFFFFFFFFFFFFFFFF:FFFFFFF
@A00721:190:HGJW7DSXY:4:2252:21694:25097_2:N:0:TCTGCAAG+GCTGCACT
TTCGACAAATTTTAGAACTTTATTTCAGAACTCTCTCCGATCACCTTGGTGTGATCTCCCGGAGGATC
TTTTCAATAAGATGTATGATGATAATCTACTATTTTTAAATAGTGATA
+
FFFFFFFFFFFFFFFFFFFF:FFFFFFFF:FFFFFFFFFFFFFFFFFFFFFFFFFFFFFFFFFFFFFF
FFFFFFFFFFFFFFFFFFFFFFFFFFFFFFFFFFFFFFFFFFFFFFFF
@A00721:190:HGJW7DSXY:4:2473:21368:19961_1:N:0:TCTGCAAG+GCTGCACT
CTTTATCAAAGATTTGGCTTGGTCTATTTACCGCAAAAAGTTGGAAGAGGTCTATGATATTTATTTTA
CTTATACTTTAAATAGCACCGATTATAACTTGACTGTTGATAATATTACAATAGCTCTTTCTCAGAAG
ATT
+
FFFF,FFFFFF:FFFFFFFFFFFFFFFFF:FFFFFFFFFFFFFFFFFFFFFFFFFFFFFFFFFFFFFF
FFFFFFFFFFFFF,FFFFFFFFFFFFFFFFFFFFFFFFFFFFFFFFFFFFFFFFFFFFFFFFFFFFFF
FFF
@A00721:190:HGJW7DSXY:4:1616:17318:31109_2:N:0:TCTGCAAG+GCTGCACT
GGTGAACACGTCGGTAGCATTGATTTACTTTTACCGTTAGAACTGAAAAAGATTCTTTTCGAAAAGAA
ACCTATAAGTTACAAACGATTCTTCGACTATATTAAAGAAATAAAAAT
+
FFFFF:FFFFFFFFFFF:FFFFFFFFFFFFFFFF:FFFFFFFFFFFFFFFFFFFFFFFFFF:FFFFFFF
FF:FFFFF:FFFFFFFFFFFFFFFFFFFFFFFFFFFFFFFFFFFFFFF
@A00721:190:HGJW7DSXY:4:1460:7374:6464_2:N:0:TCTGCAAG+GCTGCACT
CTTTACATTTTTAATGGTATGGTATAATGTGTCTTACAAATAGAACCTTCACTCTCGGTAATGAGAGT
GTATTCCTTAATATTCTAGTAACCGCTATGTGGGCATTACTAGGATTT
+
F:FFFFFFFFFFFFFFFFFFFFFFFFF:FFFFFFFFFFFFFFFFFFFFFFFFFFFFFFFFFFFFFFFFF
FFFFFF,FFFFFFFFFFFFFFFFFFFFF:FFFFFFFFFF:FFFFFFFFF
@A00721:190:HGJW7DSXY:4:1510:10972:29105_2:N:0:TCTGCAAG+GCTGCACT
TTTAAATGCTAGGAAAATTTTAGAAAAACTCCATTGTATAGTTCAAGGAGTACTTAATTCAATAGCTT
TAGTATTATTTACTATCGCCTTTATTGAAAATTGTATAAGAGTTAATT
+
FFFFFFFFFFFFFFFFFFFFFFFFFFFFFFFFFFFFFFFFFFFFFFFFFFFFFFFFFFFFFFFFFFFF
FFFFFFFFFFFFFFFFFFFFFFFFFFFFFFFFFFFFFFFFFFFFFFFF
@A00721:190:HGJW7DSXY:4:1361:28926:6089_1:N:0:TCTGCAAG+GCTGCACT
AGAATGAACTGCCGCTTCCCGGAAGAAACGATCTAATTACAGATTATTTAGAGTAGTCAAAAGTTTGT
ATAAATTTATATGATTTTCTATATATATACCACATATATATATATATAAAATCCTATAAATTTGTACA
+
F,FFF:F,FF,FF,,F,:F:,,FF,FFF,:F,,,F,F,F:F,F,,FFFFFFF:FF:FF,FFF:,,F,F
F:F::FFFFFF:FF,F,FFFFF:FFFFFFF:FF::F:FFFFF:FFFFFFFFF,FFF,F,FF:FFFFFF
@A00721:190:HGJW7DSXY:4:1258:1334:25254_1:N:0:TCTGCAAG+GCTGCACT
CTCCCCAAACATAACATACCATAACCTACCATGACCAATCATCGCTCCAACTGGCACGCCAGACGTAG
GGTCAAACCTACTACCGCCTGTCGGCAGATCGGCACCACTGGTCTCTACGTTCAGGGACCCGCTGTGC
AGA

+
FFFFFFFFFFFFF:FFFFFFFFFFFFFFFFFFFFFFFFFFFFFFFFFFF:FFFFFFFFFFFFFFFFFF
FFFFFFFFFFFFFFFFFFFFFFFFFFFFFFFFFFFFFFFFFFFFFFFFFFFFFFFFFFFFFFFFFFFF
FFF
@A00721:190:HGJW7DSXY:4:1633:31819:1110_2:N:0:TCTGCAAG+GCTGCACT
AAATCTAGATCTGCACAGAAGAAGGCTGAAGGTCGTTACCGGACGACCACTCAAAGGAAACGGGCTGT
TGCCCAGAGGAAAGCAGCGACCACCGAGGTCGCTCGTAACATTGACGG
+
FFF,FFF,FF:FFFFFFFFF,FF:FFFFFFFFFFFFF,:F:F,FFF:FFFFFFFFFFFFFF:FFFFFF
F,:FFF,FFFF,FFFF:FFFFFFF,FFFFFF,FFF:FFFFFFF,FFF:
@A00721:190:HGJW7DSXY:4:2108:28854:2456_2:N:0:TCTGCAAG+GCTGCACT
TCTGTGGGAACTGAGGCAGTACCTCAGTCAAAAGTCACATTCACCACTCCCATACCAATCAATAGATC
GGAAGAGCGTCGTGTAGGGAAAGAGTGTAGTGGGGCGGGGGGGGGGGG
+
FFFFFFFFFFFFFFFFFFFFFFFFFFFFFFF:FFFFFFFFFFFFFFFFFFFFFFFFFFFFFFFFFFFF
FFFFFFFFFFFFFFF:FFF:F:FFFFF:FFFF,,:,FF:,FF,,:,,:
@A00721:190:HGJW7DSXY:4:1629:16251:32049_2:N:0:TCTGCAAG+GCTGCACT
CCCTCCGCTGGCACGAAGAACACTCCCAAGCCAATCAATAGTTCTTCTGACCAGAATGAAAAGGGGTT
TACCAGGGTAGTCTCTAAGTCTACCAGGAAGAGGGGAAATGTCCCCAA
+
FFFFFFFFFFFFFFFFFFFFFFFFFFFFFFFFFFFFF:FFFFFFFFFFFFFFFFFF,FFFF:FFFFFF
FFFF:FFF::FFFFFF:FFFFFFFFFFFFFFFFFFFF:,FFFFFFFFF
@A00721:190:HGJW7DSXY:4:2316:12156:17910_2:N:0:TCTGCAAG+GCTGCACT
AGGTCCATCAGGACCTAAGAAGGGGAAATTACCCCTAATTCTGAAGGAAGATCTCCCACCATCTGGGG
TTGGGAATAAGAAATCTGCACGGGCCAAAGAGGCCGCTGCTACGAAGT
+
FFFFFFFFFFFFFFFFFFFFFFFFFF:FFFFFFFFFFFFFFFFFFFFFFFFFFFFFFFFFFFF:FFFF
FFFFF,FFFFFF:FFFFFFFFFFFFF:FFFFF:FFFFFFFFFFFFFFF
@A00721:190:HGJW7DSXY:4:2375:7346:7639_1:N:0:TCTGCAAG+GCTGCACT
GCTTGGGTCATTAAGGGTAGCTGTAAAGTTGCGCCTGCCGACCCCATCTTTGAGACCAAGACTCTCTA
CAGTAGGTTCGTGCCTACTATGAGCAAATCTGTTGAATTCGTCACTGAGGACGAAGGTGAGAGGACTT
TTG
+
F:FFFFFFFF:FFFFFFFFFFFFFFFFFFFFFFFFFFFFFFFFFFFFFFFFFFFFFFFFFFFFFFFF
FFFFFFFFFFFFFFFFFFFFFFFFFFFFFFFFFFFFFFFFFFFFFFFFFFFFFFFFFFFFFFFFFFFF
FFF
@A00721:190:HGJW7DSXY:4:2375:30572:36965_1:N:0:TCTGCAAG+GCTGCACT
CTGAGAGCAGTCGCTGGGGAAAGAGAATTGCGTGGGTTGAAGGCCGACGCTTCGAGCTTGAGTGTGAC
GAGGCCGGTGACGTTTTTGTCAACGGCGTTTGGAGGATTTTATCCGCGCTTAACTATGGGGCGAAGAT
CCC
+
FFFFFFFFFFFFFFFFFFFFFFFFFFFFFFFFF:FFFFFFFFFFFF:FFFFFFFFFFFFFFF:FFFFF
FFFFFFFFFFFFFFF,FFFFFFFFFFFF,FFFFFFFFFFFFFFFFFFFFFFFFFFFFFFFFFFFFFFF
FFF
@A00721:190:HGJW7DSXY:4:2512:1208:14638_2:N:0:TCTGCAAG+GCTGCACT
AGGTTGGCATATTTATGCCGACGATAATAAGCATGTCACCCTTAAGCACCGAGGTAAGCTCGGGACTT
ACAAGAGAGAGTGTGAGAGAGTCGCTCTCACTATTTTTGCCCAGAATG
+
FFFFFFFFFFFFFFFFFFFFFFFFFFFFFFFFFFFFFFFFFFFFFFFFFFFFFFFFFFFFFFFFFFFF
FFFFFFFFFFFFFFFFFFFFFFFFFFFFFFFFFFFFFF,FFFFFFFFF
@A00721:190:HGJW7DSXY:4:1105:3459:27712_1:N:0:TCTGCAAG+GCTGCACT
GTGACTGGAGTTCAGACGTGTGCTCTTCCGATCTGTTTTTGTTATATTCACTGGTTCTGGGAGGCTGC
TTTCCTCCATAATTACAACCTCTCTTTAGAGAGCATTAGAAGGAAGTTGGGCAAATTCCCACATATGG
TAG

```
+
FFFFFFFFFFFFFFFFFFFFFFFFFFFFFFFFFFFFFFFFFFFFFFFFFFFFFFFFFFFFFFFFFFFFFFF
FFFFFFFFFFFFFFFFFF:FFFFFFFFFFFFFFFFFFFFFFFFFFFFFFFFFFFFFFFFFFFFFFFFFFF
FFF
@A00721:190:HGJW7DSXY:4:1257:1949:21496_2:N:0:TCTGCAAG+GCTGCACT
ACTTTTCTTGAAAATTTTTCCTTTCCTCCGTCATTGGGGATTTCGATGTGAGAAGGTGGAAGGTGTCA
AGGGTCTTCTTCATGCTGATCTTTCAGGACCTAATCACATCATGATTG
+
FFFFFFFFFFFFFFF,FFFFFFFFFFFFF:FFF:FFFFFF,FFFFFF:F:FFFFFFFFFFFF:,FFFF
FFFFFFFFFFFFF:FFFFFFFFF:FFFFFFFFFFFFFFFFFFF,FFFFF
@A00721:190:HGJW7DSXY:4:1144:8323:12618_2:N:0:TCTGCAAG+GCTGCACT
ATCGAGTTGGAGGGAGGAAGTTCTCCGAAATTAAAGACGGGGGGATTAAGAGTCCAGCTGTAACTGTC
ATTTCAGAGAGGGGAGCTCTGAAAAGGAAACTTCGTCAGAAGATGAGG
+
FFFFFFFFFFFFFFFFFFFFFFFFFFFFFFFFFFFFFFFFFFFFFFFFFFFFFFFFFFFFFFFFFFF:F
FFFFFFFFFFFFFFFFFFFFFFFFFFFFFFFFFFFFFFFFFFFFFFFF
@A00721:190:HGJW7DSXY:4:1370:25102:24549_2:N:0:TCTGCAAG+GCTGCACT
GGAGGAGTTTGAGGAAGGCTCTAGAGAAGATCTCACCACCTGGAGAAGGCAAGTGGAGAGTCCAGAAG
TTGATGAAGAAGGATTATTTGAAGCAGGGGTTACCACTTCCTAAGGTG
+
FFFFFFFFFFFFFFFFFFFFFFFFFFFFFFFFFFFFFFFFFFFFFFFFFFFFFFFFFFFFFFFFFFFFFFF
FFFFFFFFFFFFFFFFFFFFFF,FFFFFFFFFFFFFFFFFFFFFFFFFF
@A00721:190:HGJW7DSXY:4:1426:4001:1219_2:N:0:TCTGCAAG+GCTGCACT
TTCTTTGGAGTTTTAGATAATCTTTTGAGAAGGGGTCATAGAGCCAGCAAGAGAAGCTTATATGCTGG
TTTAAGTGATGATTATCACTCCTTGAGAAGACAAGCCACATTAAGATA
+
FFFFF:FFFFFFFFFFFFFFFFFFFFFFFFFFFFFFFFFFFFFFFFFFFFFFFFFFFFFFFFFFFFFFFF
FFFFFFFFFFFFFFFFFFFFFFFFFFFFFF:FFFFFFFFFFFFFFFFF
@A00721:190:HGJW7DSXY:4:1376:25373:23265_2:N:0:TCTGCAAG+GCTGCACT
CTTGGGGTTTCGAATGTCATTAGTGCCTTACGATTGAAAGAGGTCCTTTTGAGTATCGACAGATCTTT
CGATCTTGAGGACGTACATTTAACGTGGCAGAATTCGGTAACTTACCA
+
FFFFFFFFFFFFFFFFFFFFFFFFFFFFFFFFFFFFFFFFFFFFFFFFFFFFFFFFFFFFFFFFFFFFFFF
FFFFFFFFFFFFFFFFFFFFFFFFFFFFFF:FFFFFFFFFFFFF::FFF
@A00721:190:HGJW7DSXY:4:1150:30346:4100_2:N:0:TCTGCAAG+GCTGCACT
GGGAAGTTGTGGGATTTAACTTTGACTCAACTACTCCGATCAGATCCGGATGGTTTGATTGGTATGGA
CTACTCGACTTTTGATCTGGAGTTGTCTAGGGTCATGGCAGATACTAC
+
FFFFFF,:F:FFFFFFFFFFFFFFFFFFFFFFFFFFFFFFFFFFFFFFFFFFFFFFFFFFFFFFFFFFF
FFFFFF:FFFFFFFFFFFFFFFFFFFFFFFF:FFF:FFFFFFFFFFFFF
@A00721:190:HGJW7DSXY:4:2259:24966:22811_2:N:0:TCTGCAAG+GCTGCACT
AAAATATGCAGGTCAAAGATTCTGTAGCTCTGAGGGCGGTTGAGGGAGCCGTTTTGGCCGACTTTAAC
TATTCTAAGAGTCTTTATGATTCAAAACCTATCGTTGTCGTACCTTAC
+
FFFFFFFFFFFFFFFFFFFFFFFFF:FFFFFFFFFFFFF:FFFFFFFFFFFFFFFFFFFFFFFFFFF:F
FFFFFFFFFFFFFFFF:FFFFFFFFF:FFFFFFFFFFFFFFFFFFFFF:
@A00721:190:HGJW7DSXY:4:1471:6316:36699_2:N:0:TCTGCAAG+GCTGCACT
GCGGCTTATTGTCGCGCAGTTTCCAGAATTGCGGTTGAAGTTCACCCATTCTATCTTGAATGCTCACG
CCGTCGCAGCCATATCGAGACTATGTTATAATCACTTGTTAAAGTACA
+
FF:FF:FFFFFFFFFFFFFFFFFFFF:FFFFFFFFFFFFFFFFFF:FF:FFFFFFFFFFFFFFFFFFFF
FFFFFFFFF:FF:FFFFFFFFFFFFFFFFFFFFFFFFFFFFF:FFFFFF
@A00721:190:HGJW7DSXY:4:1437:32642:22294_2:N:0:TCTGCAAG+GCTGCACT
TTAGATATTATTGATATTGGAGGCGATATTCGTTTTCATATCCAAAACTCCTTGAATGGAGTACATAT
```

TTGCAACCCTATTCTTGATGTGAAAGATGCATCGAGGTATACGTCTAG
+
FFFFFFFFF:F:FFFFFFFFFFFFFFFFFFFFFFFFFFFFFFFFFFF:FFFFFFFFFFFFFFF:F:FFF
FFFFFFFFFFF:,FF:FF,FFFF,:F:FFFFFFFFFFF,FFFFFFFFF
@A00721:190:HGJW7DSXY:4:2572:25093:15828_1:N:0:TCTGCAAG+GCTGCACT
ACGTACCTACTAACGAGAAGTCATGAAAGCTATCGGAGTGAAGCAAAATGTTTCGTTCTGTTACAAGA
ACGCTTTGGATTGTGACCATAAAGCCTCAGTTGCGATAGCTGTAGAGGTATACGATATCTCTCTTGAT
GA
+
FF:F,,FF,FF,FFF,::,F,,F,FF,,F:FFF,FF,F,:,F,FFF:FFFFFF,FF::F,FFF,,FFF
FFFFFFF:F,FFFFFFF:,F::FFF,F:,F:FFFFFF:F,:FFFF::FFF:F,:FF,FF:FF,FFFFF
,,
@A00721:190:HGJW7DSXY:4:2438:22182:21778_2:N:0:TCTGCAAG+GCTGCACT
AATGCTTAAGAGAGAGATTGATGTATGTCATTTCGTCTTAGTTTGTCCCGGCGAATTATTGGACACGA
TGAAAAATGACATACCCGTTCCTGACCTTGGTATCGTTATCCGTCATG
+
FF,FFF:FFFFFFFFFFFFFFFFFFFFFFFFFFFFFFFFFFFFFFFFFFFFFFFFFFFFFFFFFFFFF
:FFFFFFFF:FFFFFFFFFFFFFFFFFF:FFFFFFFFFFFF:FFFFFF
@A00721:190:HGJW7DSXY:4:1528:13069:29794_2:N:0:TCTGCAAG+GCTGCACT
GTTATTCTCACAACAAAGATAAGATTTTATCTTTCTTCAAACACCCTGTTTTCCTTAAGGGCGGACAA
TTGTTCCATTGTGAACTCTTGGGGACTAGATTGAGCATTAATGAGTTC
+
FFFFFFFFFFFFFFFFFFFFFFFFFFFFFFFFFFFFFFFFFFFFFFF:FFFFFFFFFFFFFFFFFFFF
FFF:FFFFFFFFFFFF:F:FFFFFFFF,FFFFFFFFFFFFFFFFFFFF
@A00721:190:HGJW7DSXY:4:1149:8793:7106_1:N:0:TCTGCAAG+GCTGCACT
TCTAGTGGACAATTCAGAATACCTATTTCCGTACCAAGAGCCACTGCCAATCTCATCGCTATACGCAT
ACCTGATTGGAAGAAGCGAGGTTCGTTCCTCAATAAAAATTTTTCTCAAGAGATTATGGTTGATAAAG
TTT
+
FFFFFFFFFFFFFFFFFFFFFFFF:FFFFFFFFFFFFFFFFFFFFF:FFFFF:FFFFFF:FFFFFFFF
FFFFFFFFFFFFFFFF:FFFFFFFFFFFFFFFFFFFFFFFFFFFFFFF,FFFFF:FFFFFFFFFFFFFF
FFF
@A00721:190:HGJW7DSXY:4:1418:27462:13917_1:N:0:TCTGCAAG+GCTGCACT
TTCAGTACGTCATGGGGATCACTTCTTCGGTCAATGACAAAACCTTCGAATATGTTACGACATGGCTT
CGTAACAACAGTGCGAGGATTGTCATCTCGGGGAAAGTGATTCACCAAGATGTTCATATAGATACGAG
ATA
+
FFFFFFFFFFFFFFFFFFFFFFFFFFFFFFFFFF:FFFFFFFFFFFFFFF:FFFFF,FFFFFFFFFFF
FFFFFFFFFFFFFFFFFFFFFFFFFFFFFFFFFFFFFFFFFFFFFFFFFFFFFFFFFFFF:FFFFFFFF
F:F
@A00721:190:HGJW7DSXY:4:1160:6234:25817_2:N:0:TCTGCAAG+GCTGCACT
CAGCGATCTTACTCGTTATCGGAGCTAAGAAGAAGACTTTGGGTACACGTTATGCTCGCAAACTTAAC
GTTAGTACCGGAGGTGATCTCTCTGCTTCTGCGTTACTGAGAGAAAAG
+
FFFFFFFFFFFFFFFFFFFFFFFFFFFFFFFFFFFFFFFFFFFFFFFFFFFFFFFFFFFFFFFFFFFF
FF:FFFFFFFFFFFFFFFFFFFFFFFF:FFFFFFFFFFFFFFFFFFFFF
@A00721:190:HGJW7DSXY:4:1204:8458:21684_1:N:0:TCTGCAAG+GCTGCACT
CCTACGGTGACTTTATGGACAAGATGCAAGTTCTTGGGAAATACTTGTTTCCCTTTTTAGGTTTAGTA
GGTGATCTTGACGTCGATTCTTTGTATAGGGAGTGTGAGAAGACAACGAATCTTGAAACTACGGTCCT
TAT
+
FFFFFFFFFFFFFFFFFFFFFFFF,FFFFFFF:FFF:FFFFFF:FFFFFFFFFFFFFFFFFFF:FFFF
FFFFFFFFFFFFFFFFFFFFFFFFFFFFFFFFFFFFFFFFFFFFFFF:FFF:FFFFFFFFFFFFFFFFFF
FFF

@A00721:190:HGJW7DSXY:4:2140:18982:11725_1:N:0:TCTGCAAG+GCTGCACT
GAACCGGTCTCTAAATCTTTTGAGATTGAGAAGATTGAAGAACACGTCAAGAAGTCGCTCGGCGTTGT
TCTTGAAAACCAAGTTAAGAAGGAGGCTGATCATGGAGTCTTTGTACCTTCTGTCAAACTTCGAGGTT
CAG
+
FFFFFFFFFFFFFFFFFFFFFFFFFFFFFFFFFFFFFFF:FFFFFFFFFFFFFFFFFFFFFFFFFFFFF
FFFFFFFFFFFFFFFFFFFFFFFFFFFFFFFFFFFFFFFFFFFFFFFFFFFFFFFFFFFFFFFFFFFFF
FFF
@A00721:190:HGJW7DSXY:4:2649:7645:17832_2:N:0:TCTGCAAG+GCTGCACT
GATCCCGGATGTGGATTGAAGGCTGGCTCTAAGGGAGACTCCTTCTTTTTTGAATGTTGTAAAGAATTG
GGTTGAGAAGTGTGCTTTACAAGGAGAGATCGGAAGAGCGTCGTGTAG
+
FFFF:FFFFFFFF:F,FF:F:::,F:FFFFF:,::F,F:F,FFF,FFF,:F:FFFF:,FFF::FFF:F,
:F,,,F:FF:,FFFFF:FFFFFF,F:FFFF,FFF:FF,,FFF:FFFF,
@A00721:190:HGJW7DSXY:4:2319:8757:12305_2:N:0:TCTGCAAG+GCTGCACT
TAAAGAAAAATTTTCGAATAAACTAATGAGAGGTATAGGCTTAGTCAAGTCTGTACTTACATTCTTTA
CGCCAGATTCTTCATGGGGCGACGCTGTTTCTCTTGGTATTTCTGAAA
+
FFF:FFFFFFFFFFFFFFFFFFFFFFFFFFFFFFFFFFFFFFFFFFFFFFFFFFFFFFFFFFFFFFFFF
:FFFFFFFFFFFFF:FFFFF:FFFFFFFFFFFFFFFFF,FFFFFFFFF
@A00721:190:HGJW7DSXY:4:1578:24279:19366_1:N:0:TCTGCAAG+GCTGCACT
TTTCCTCTGGTAAGGGAATTATTAAATCATTCTTCTCACTCTTGACGAGAATTATTACTGTTTTCTCT
ACCCATAACTTCTTTTTCAAAAATGCGGATGGGTCTACTGATGTTTTCCTACAGAGTGCAGTCGTTTC
AAC
+
FFFFFFFFFFFFFFFFFFFFFFFF:FFFFFFFFFFFFFFFFFFFFFFFFFFFFFFFFFFFF:FFFFFFF
FFFFFFFFFFFFFFFFFF:FFFFFFFFFFFFFFFFFFFFFFFFFFFFFFFFFF:FFFFFFFFFFFFFFF
FFF
@A00721:190:HGJW7DSXY:4:1522:30843:36151_1:N:0:TCTGCAAG+GCTGCACT
AATAATTGGTGATGAAAATACGTATGTCGGTGGGGTTCTCAAGGCTCTCATCGCCACTGGGATTCTAA
GGGGTTCCATAAGATATTATCTGACATTGTGGTTCAATGATCAGGATTCTTCTTTTGGTCCTTACGTC
TTA
+
FFFFFFFFFFFFFFFFFFFFFFFFFFFFFFFFFFFF:FFFFFFFFFFFFFFFFFFFFFFFFFFFFFFFF
FFFFFFFFFFFFFFFFFFFFFFFFFFFFFFFFFFFFFFFFFFFFFFFFFFFFFFFFFFFFFFFFFFFFF
FFF
@A00721:190:HGJW7DSXY:4:2136:17598:12023_1:N:0:TCTGCAAG+GCTGCACT
TCTTGTCACGATTCTTAAAAGAAGTTTATTAAAACTTCAGAGTCTCTTGGAAGTCCAGGTAGCTAATG
TTCTTGCAATAGCCACAGACTTTCTGGGAAAAGAGACCTCTGTTGGTAGAGGAGTTGAGAGAATTACT
CAG
+
FFFF:FFFFFFFFFFFFFFFFFFFFFFFFFF,FFFFFFFFFFFFFFFFFFFFFFFFFFFFFFFFFFFFF
FFFFFFFFFFFFFFFFFFFFFFFFFFFFFFFFFFFFFFFFFFFFFFFFFFFFFFFFFFFFFFFFFFFFF
FFF
@A00721:190:HGJW7DSXY:4:2173:24632:24549_2:N:0:TCTGCAAG+GCTGCACT
ACAACGAGTTAGGGAGTGGCTAGGTTTTAAAAGATCCTTTGCACAAGCAATACCTTCCACTGAGTTCA
TCACCCAGAGTTTCGACGTCGCGGAAAACGATGAAGATTTCCACACTG
+
FFFFFFFFFFFFF:FFFFFFFFFFFFFFF:FFFFFFFFFFFFFFFF:FFFFFFF:FFFFFFFFFFFFFF
FFFFFFFFFFFFFFFFFFFFFFFFFFFFFF:FFFFFFFFFFFFFFFFF
@A00721:190:HGJW7DSXY:4:2216:23692:25520_2:N:0:TCTGCAAG+GCTGCACT
TCTGATGCTGAATTTGAGGAAAATTTTTCGGAAGTTTTTGGTATAAATCCTGATGGCTCAGTACAGGG
AGGAGGTAATAGTTATAAAACCTTCTTTTATCATTGCAACAGGACTTT
+

```
FFFFF:FFFFFF,FFFFFFFFF:FFF:FFFFFFFFFF,FFFFF:FFFFFFFFFFFFFFFFFF,::FFF
FFFFFFFFFFFFFFFFFFFF:FFFFFFFF,FFFFFFFF:FFFFFFFFF
@A00721:190:HGJW7DSXY:4:1317:30698:28260_2:N:0:TCTGCAAG+GCTGCACT
ATCTGGGATCATAGGTATGTGTCACGCTACACTCGAAATATGGGCCCTCATAAGGAAGTATAGTTGGC
GTCTTGTCGTTGACGTCATTACAGTCTGGAAGTTAGATCATTTGTTCA
+
FFFFFFFFFFFFFFFFFFFFFFFFFFFFFFFFFFFFFFFFFFFFFFFFFFFFFFFFFFFFFFFFFFFF
FFFFFFFFFFFFFFFFFFFFFFFFFFFFFFFFFFF,FF:FFFFFFF,F
@A00721:190:HGJW7DSXY:4:1317:11243:23782_1:N:0:TCTGCAAG+GCTGCACT
TTTAGATAAATTCTTTCCGGGCTCAATGACCTTTGCTATGTACGTGGATCTAATGCTTGGTGATCTGA
ATCTGACCATCGCAAAGAAGACTTTATTAGTTCATGAAGTTCTTACACTTCTCGCGACTAATGTCAGC
TTC
+
FFFFFFFFFFF:FFFFFFFFF:FFFFFFFF:FFFFFFFFFFF:FFFFFFFFFFFFFFF:FFFFFF:FF
FFFFFFFFFFFFFFFFFFFFFFFFFFFFFFFFFFFFFFFFFFFFFFFFFFFFFFFFFFFFFFFFFFFF
FFF
@A00721:190:HGJW7DSXY:4:1678:14669:32503_2:N:0:TCTGCAAG+GCTGCACT
CCAAATTTAGCACTTTTTGAATATGACAGAAGCTCGCCATATCAGATTAGAGACATGCCAGTTAGAGT
TCTTCTAACCAGGACTGATGGTGAGATTCTTTTACCTTTTGGAGGTCC
+
FFF:FFFFFFFFFFFFFFFFFFFFFFFFFFFFFFFFFFFFFFFFFFFFFFFFFFFFFFFFFFFFFFFF
FFFFFFFFFFFFFFFFFFFFFFFFFFFFFFFFFFFFFFFFFFFFFFFF
@A00721:190:HGJW7DSXY:4:2665:25825:33880_2:N:0:TCTGCAAG+GCTGCACT
CCTAGTGTCTTTTCTAGAGTCCAAGGACGTTTCCGTCGGAGGATGACAGGTGGGAGACCGCTCAATAA
CACTCTATTGAATCCCTTTTCCACGATGAAAGCTCTTTATGAGGTGCT
+
FFFFFFFFFFFFFFFFFFFFFFFFFFFFFFFFFFFFFFFFFFFFFFFFFFFFFFFFFFFFFFFFFFFF
FFFFFFFFFFFFFFFFFFFFFFFFFFFFFFFFFFFFFFFFFFFFFFFF
@A00721:190:HGJW7DSXY:4:2671:28980:4993_1:N:0:TCTGCAAG+GCTGCACT
GATATCGCCTCTGTCCTTTCTCCTTTAATCGTGAGGTTACGAGAGTTTCGTACTTGGGTTTATCGGTT
GTATTTTGATAAATTTGTTGGAATCGATAATGATACTGACGAAGGTTCTATTACCACCCTTGTCATTG
ATA
+
FFFFFFFFFFFFFFF,F:FFFFFF:FFFFFFFFFFFFF:FFFF:FFFFFFF:FFFFFFFFFFFFFFF
FFFFFFFFFFFFFFF:FFFFFFFFFFFFFFFFFFFFFFFFFFFFFFFFFFFFFFFFFFFFFFFFFFFF
FFF
@A00721:190:HGJW7DSXY:4:2443:15501:5102_1:N:0:TCTGCAAG+GCTGCACT
GTCTAGCCGCTGAGATTCGTCTTACCTATTTACAATCTGTAGAGAGAGAGGTTAATCAGAATTTGAGT
GATAACGATTTAGTACTGGGAAATGGTGGTAGTCAGTTTACTACTTTTCTTTCCCTCCCCTTTTTCGA
TTG
+
FFFFFFFFFFFFFFFFFFFFFFFFFFFFFFFFFFFFFFFFFFFFFFFFFFFFFFFFFFFF:FFFFFFF
FFFFFFFFFFFFFF:FFFFFFFFFFFFFFFFF,FF:FFFFFFFFFFFFFFFFFFFFFFFFF::FFFFF
FFF
@A00721:190:HGJW7DSXY:4:2268:27019:19820_2:N:0:TCTGCAAG+GCTGCACT
AGGTTTTTCTTTTATGGGACTCTTGTTTGTACTTATGAAGGAGGTATTACTTCGGAAGTTCTTAATGT
AGGTCTCCTAGTACTTAAGGGTTTATGTTTCGGTTTCAGCTGGATGGA
+
FFFFFFFFF:FFFFFFFFFFFFFFFFFFFFFFFFFFFFFFFFFFFFFFFFF:FFFFFFFFFFFFFFFF
:FFFFFFFFFFFFFFFFFFFFFF:FFFFFFFFFFFFFFFFFFFFFFFFFF
@A00721:190:HGJW7DSXY:4:2425:22435:29136_2:N:0:TCTGCAAG+GCTGCACT
TGGACTTGGGAAGAGATTAAACATAAAATCCAGCGATTCTATTATGTCGCTAAAGCCTGTCCAGTTGT
CGATGATGTTCGGGCTGTGGGGATTAACTTCCCTCGTGAAACTTACGA
+
```

FFFFFFFFFFFFFFFFFFFFFFFFFFFFFFFFF:F::FFF:FFFFFFFFFFFFFFFFFFFFFFFFFFF
FFFFFFFFFFFFFFFFFFFFFFFFFFFFFFFFFFFFFFFFFFFFFFFF
@A00721:190:HGJW7DSXY:4:1533:27923:3161_1:N:0:TCTGCAAG+GCTGCACT
CACAAGGATTACTAAGTCTGAGATCTGCCACTTCTAATTTTAGGAAACGTCGCGAAAAGAAAGTTCAT
GAGGATGTCTTGGTCGAAAAATATCGCCGGGAACTTCTAATAGAGGATCCTGATGACATTATTTCAAC
GGA
+
FFFFFFFFFFFFFFFFFFFFFFFFFFFFFFFFFFFFFFFFFFFFFFFFFFFFFFF:FFFFFFFFFFFF
FFFFF:FFFFFFFFFFFFFFFFFFFFFFFFFFFFFFFFFFFFFFFFFFF:FFFFFFFF:FFFFF
FFF
@A00721:190:HGJW7DSXY:4:1101:4020:8891_2:N:0:TCTGCAAG+GCTGCACT
CTACCACAGAAACCGTCTCAGGAACTGTCTCCACTGATTCGGAATTAGAGAAAGACGACCTTCTTGTG
GGGACTGAGACCTCTGAATTTTCAGGGTGCCCTGATGAGGACGTTGAA
+
FFFFFFFFFFFFFFFFFFFFFFFFFFFFFFFFFFFFF::FFFFFFFFFFFFFFFFFFFFF,FF,,F,F
FFFFFFFFFFFFFFFFFFFFFFFFFFFFFFFFFFFFFFFFFFFF:FFF
@A00721:190:HGJW7DSXY:4:2201:28519:23641_1:N:0:TCTGCAAG+GCTGCACT
ACTCTGAGACTTCTAAGAGTGTTTCAAAAGACCAGAAACGTAACAAGAAGAATAAAGGTAGGAAGGGG
AGAAAACAGAACAAGCGAGCGGTTGAAGAGAAAACTGATATGGTTGCGGCTGATCGTACCCCAATTCC
TGC
+
FFFFFFFFF:FF,FFFFFFF,FF:FFFFF,FF:FFFFF:FFFFFFFFFFFFFFFFFFFFFFFF:FFFF
FFFFFFFFFFFFFFFFFFFFFFFFFFFFFFFFFFFFFFFFFFFFFFFFFFFFFFFFFFFFFFFFFFFF
FFF
@A00721:190:HGJW7DSXY:4:1642:18448:32440_2:N:0:TCTGCAAG+GCTGCACT
CTCGGCGATACCACTGATCTTCCAGAGTTACGTAGTGGTCGTTCTTTTGGTGATTTTATCAAAGAGAA
AGTTTCTGCTAGTCGCGACGATAGTATTTATCGGAGTGATATTAACAA
+
FFFFFFFFFFFFFFFFFFFFFFFFFFFFFFFFFFFFFFFFFFFFFFFFFFFFFFFFFFFFFFFFFFFF
FFFFFFFFFFFFFFFFFFFFFFFFFFFFFFFFFFFFFFFFFFFFFFFF
@A00721:190:HGJW7DSXY:4:2628:10673:9330_2:N:0:TCTGCAAG+GCTGCACT
CACTTACAAGAGTGTTGAAGTACCGCAACATGGATCAGTTTTCTAAAAAGCTATTAATAGCAGTTGCG
GGGCTATAATACCGCCTCCAGATTACTCTAAACTGAATCCACTCTCTT
+
FFFFFFFFFFFFFFFFFFFFFFFFFFFFFFFFFFFFFFF:FFFFFFFFFFFFFFF:FFFFF:F,FFFF
FFFFFFFFFFFFFFFFFFFFFFFFFFFFF,FFFFFFFFFFFFFFFFFF
@A00721:190:HGJW7DSXY:4:2439:23818:10895_2:N:0:TCTGCAAG+GCTGCACT
TATGGAACTGTGTAATGTCCTACCAACTGAACAATTTGTTAGGGCTTCTCTAAAGAACAGCAACTATC
AACAAGAATTAGTTATTGAAAGACTTGACCCTCACGTCTCACATTATC
+
F,FFFFFFFF:F,FFF,,::FF:,,FFFF,,FFF,,FFFF,,FF,FFFFFF:FFFFF,F:FFFF,,FF
:F,F,,,F:FF,FFF,F:FFF,,FFF,FFFFFFFFFFFFFFF,F:FF,
@A00721:190:HGJW7DSXY:4:2542:25907:1924_2:N:0:TCTGCAAG+GCTGCACT
AAAGAGGGTTTGAAAATTACGGAACCTTTCCTCGTTCTTCATGAAGATCTTCGAGTGTTTTATAGTGA
GTTATTCTTGCGAAGGTTCAAGTCGATTGATTTTGTTGACTACGATCT
+
FFF::FFFFFFFFFFFFFFFFFFFFFFFFFFFFFFF:FFFFFFFFFFF:FFFFFFFFFFFFFFFFFFF
FFFFFFFFF:FFFF,FFFFFF:FFFFFFFFFFFF:FF,FFFF::FFFF
@A00721:190:HGJW7DSXY:4:2354:26087:13823_1:N:0:TCTGCAAG+GCTGCACT
AGAAGTTCTTCGCAGTTGAATCTCCACCAGGTGGAGGGAAGACCACTGAACTTATAGCGACTTTTGTC
GAATTTTTCGCAAAAGGTCTTGAAGTACGTATTGTCACTGCTAATCGTAATTCGCGAGAAGAGATAGT
GAC
+
FFFFFFFFFF:FFFFFFFFFFFFFFFFFFFFFFFFFFFFFFFFFFFFFFFFFFFFFFFFFFFFFFFFF

FFFFFFFFFFFFFFFFFFFFFFFFFFFFFFFFFFFFFFFFFFFFFFFFFFFFFFFFFFFFFFFFFFFFFF
FFF
@A00721:190:HGJW7DSXY:4:2557:21097:5024_2:N:0:TCTGCAAG+GCTGCACT
CACTTCTAGTTCATCGCGGTATTTGTGAAATTAAGACAGACGTGAAAGTTATGATGAGTGATCGTATT
AGAACAGCTGATTCTTATTGCATGAACGTCGCTGAGGTCAAACCTGTG
+
FFFFFF:FFFFFFFFFFFFFFFFFFFFFFFFFFFFFFFFFFFFFFFFFFFFFFFFFFFFFFFFFFFFFFF
FFFFFFFFFFF,FFFFFFFFFFFFFFFF:FFFFFFFFFFFFFFFFFFFF
@A00721:190:HGJW7DSXY:4:1505:30680:31454_1:N:0:TCTGCAAG+GCTGCACT
CGACGAATTATTCATGATTCACGCTGGTCAGACCCTTTTTCTTACACATCTACTTAAGGCGGCTTATA
CCATAGGTTACGGTGATTCCAATCAAATCGGATTCATCAATCGTATTGATCTCCAAGTCGCAAACTAC
TTC
+
FFFF,FFFFFFFFFFFFFFFFFFFFFFFFFFFF:FFFFFFFFFFFFFFFFFFFFFFFFFFFFFFFFFFFF
FFFFFFFFFFFFFFFFFFFFFFFFFFFFFFFFFFFFFFFFFFFFFFFFFFFFFFFFFFFFFFFFFFFFFF
FFF
@A00721:190:HGJW7DSXY:4:2635:16007:16125_2:N:0:TCTGCAAG+GCTGCACT
GATGAGTGTCGTTCTTATCGAAGTCTCAGTTATCGCTGCCCGGCCGACGTTTGTCGCGAACTCTCAGA
GGTTTACGGCAGAAAAATTTCTCCTGCTTGTAATTCGAGAGAGGACAC
+
FFFFFFFFFFFFFFFFFFFFFFFFFFFFFFFFFFFFFFFFFFFFFFFFFFFFFFFFFFFFFFFFFFFFFF
FFFFFFFFFFFFFFFFFFFFFFFF:F:FFFFFFFFF:FF:FFF:FFFFF
@A00721:190:HGJW7DSXY:4:1378:13783:19601_1:N:0:TCTGCAAG+GCTGCACT
TTGACGACTTCGAATTTGAGGACGGAGTTAAATATATTACTTTCACTCAAGGTGAAAAGTTTGAAATT
AACAAAGTCCTTGGTAAGAGTAAGGTTAAATGTGAAGATGCTCAGACTATTCATGAAGTTCAAGGTAA
TA
+
FFFFFFF:FFFFFFFFFFFFFFFFF:FFFFFFFFF:F:FFFFFFFFFFFFFFFFFFFFFFFFFFFFFFFF
FFFFFFFFFFFFFFFFFFFF:FFFFFFFFFFFFFFFFFFFFFFFFFFFFFFFFFFFFFFFFFFFFFFFFF
FF
@A00721:190:HGJW7DSXY:4:1117:31919:13839_2:N:0:TCTGCAAG+GCTGCACT
TTTTGCATCACAAACACATATTACCGTTGCTCTATCACGACACGTTGAATCTTTACGATACTTTGTTA
TCAATGTAAAGTTAAACGATGCTACTTGTGAGAGGATCAATCACAGTA
+
FFFFF,FFFFF:FFFFFFFFFFF:FFFFFFFFFFFFFFFFFFFFFFFFFFFFFFFFFFFFFFFFFFFFFF
F:FFFFFF:FFFF:F:F:FFF:FFFFFFFFFFFFFFFFF,FF,FFFFF
@A00721:190:HGJW7DSXY:4:1375:19524:14982_2:N:0:TCTGCAAG+GCTGCACT
TTAGCACGGGAGGTTGCAGATTCAACCCCTTATATCGAGGCTATCGAGGACGATGAGTATCTTGAAGT
TGTTGACGACAAGCCTCAAGAGGGTAGAGGACCACCTGGATCTTCTAC
+
FFFFFFFFFFFFFFFFFFFFFFFFFFFFFFFFFFFFFFFFFFFFFFFFFFFFFFFFFFFFFFFFFFFFFF
FFFFFFFFFFFFFFFFF:FFFFFFF,FFFFFFFFFFFFFFFFFFFFFF
@A00721:190:HGJW7DSXY:4:1340:16486:2581_1:N:0:TCTGCAAG+GCTGCACT
CCGATCTCTTAACGTCACTCGGTAATCTTTCAGCCGAATTAAGTACTAATGAGTTCAAGTCGGTTGTC
GATCGAATTAAGATCAAGGCTAATACCTTTATAGGTAAGAAGCCCCTTGAGCCGCGTAGTCAGCGCGT
TTA
+
FFFFFFFFF:F:FFFFFFFFFFFFFFFFFFFFFFFFFFF:FFFFFFFF:F:FFFFFFFFFFFFFFFFF:
FFFFFFFFFF:FFFFFFFFFFFF,FFFFFFFFFFFFFFFFFFFFFFFFFFFFFFFFFFFFFFFFFFFFFF
FFF
@A00721:190:HGJW7DSXY:4:2440:15736:36855_1:N:0:TCTGCAAG+GCTGCACT
CCCGATAGGGTACCGAGTCTCCAAGAGAATATTTATTCTTATGAGGCTCGAAACTTCAACGTTTTCAC
AACGGAAAGACATATCGCACCCGAAATTTTTGGGCAGAATGCTCTTAACGCGGTTCTTGAACGGTGTT
TTG

```
+
FFFFFFFFF:FFFFFFFFFFFF:FFFFFFFFFFFFFFFFFFFFFFFFFFFFFFFFFFFFFFFFFFFFF
FFFFFFFFFFFFFFFFFFFFFFFFFFFFFFFFFFFFFFFFFFFFFFFF:FFFFFFFF:FFF:FFFFFFFF
FFF
@A00721:190:HGJW7DSXY:4:1620:26341:23249_1:N:0:TCTGCAAG+GCTGCACT
GATAAAGTTATAGGAGACTCAGTTAACCTGAGAGCTCCTCAATTGATAGATTGGGTCAACAATAGGGA
CGCTAGTATACTTCGTAAAGTCGACAGCGAAATGGTCAATATTGATAATTGGACCGACTATATGACCT
ATT
+
FFFFFF:FFFFFFFF,:FFFFFFFFFFFFFFFFFFFFFFFFFFFFFFFFFFFFFFFFFFFFFFFFFFF
FFFFFFFFFFFFFFFFFFFFFFFFFFFFFFFFFFFFFFFFFFFFFFFFFFFFFFFFFFFFFFFFFFFF
FFF
@A00721:190:HGJW7DSXY:4:2144:6343:34115_2:N:0:TCTGCAAG+GCTGCACT
AGGGAAGCTAAGGTTAAACTTGATGCTTCAGCCTTTTCGAAGCATAACCCCGCACAGAATATCGTTTA
TCACGCAAAATTTATTAATGCAATTTTCGGACCTATCTTTAAGGTTCT
+
FFFFF:FFFFFFFFFFFFFFFFFFFFFFFFFFFFFFFFFFFFFFFFFFFFFFF:FFFFFFFFF:FFF
FFFFFFFFFFFFFFFFFF:F:FFF:FFFFFFFFFFFFFFFFFFFFFFFF
@A00721:190:HGJW7DSXY:4:1534:32696:18787_2:N:0:TCTGCAAG+GCTGCACT
CTTTTACCCAACATTTTTATGTATACAGAGATGACCAGTGACGATTTCTCCCGGAAGCTCTTCTCTCT
CTTAGGAGGTCATGATGACTACTATAAGGGTGAAATCGATTTTTCGAA
+
FFFFFFF:FFFFFFFFFFFFFFFFF,FF:FFFFFFFFFFFFFF:F:FFFFFFF:FFFFFFFFFFFFFF
:FFFF:FFFFFFFFF,FFFFF:FFFFFFF:FFFFFFF:FFFFFF,F:F
@A00721:190:HGJW7DSXY:4:1263:16839:19194_1:N:0:TCTGCAAG+GCTGCACT
TAAAGCCTATGAGATAAAGATGTATTCTTATTTAGGCTTCGATGCTGAATTTCTTGAACTTTGGAGTG
CCAGTGAATATTTTTGTCACGCCAGGAGTATCCATAACGATTTGTCATTTACCACCTTTGCTCAACGA
CGA
+
FFFFFFFFFFFFFFFFFFFFFFFFFFFFFFFFFFFFFFFFFFFFFFFFFFFFFFFFFFFFFFFFFFFF
FFFFFFFFFFFFFFFFFFFFFFFFFFFFFFFFFFFFFFFFFFFFFFFFFFFFFFFFFFFFFFFF:FFF
FFF
@A00721:190:HGJW7DSXY:4:1566:5665:34475_1:N:0:TCTGCAAG+GCTGCACT
TAACACATTCTTTGGTAATTGTATGATCACTCTTATGATGCTCTCTCTTTATTACGACCTTCGGAAGT
TTAGAGCCGTCGCTGTCGCCGGAGACGATTCGATCCTCTTTTCGGACGTACCGGTCCAGGATTATTCA
GAT
+
FFFF:FFFFFFFFFFFFFFFFFFFFFFFFFFFFFFFFFFFFFFFFFFFFFFFFFFF:FFFFFFFF
FFFFFFFFFFFFFFFFFFFFFFFFFFFFFFFFFFFFFFFFFFFFFFFFFFFFFFFFFFFFFFFFFFFF
FFF
@A00721:190:HGJW7DSXY:4:1311:4282:27946_2:N:0:TCTGCAAG+GCTGCACT
TGCTCGCGATTTATTCTTTTTGTCAATGATTACATCTACTTCGTTCCTGATCCTTACAAGATCATCGT
TAAGTTAGGTAAACCCCTAACCGATGTTACTCTTGTAGGACTTTGGGA
+
FFFFFFFFFFFFFFFFFFFFFFFFF:FFFFFFFFFFFFFFFFFFFFFFFFFFFFF:FFFFFFFFFFFFF
FFFFFFFFFFFFFFFFFFFFFFFFFFFFFFFFFFFFFFFFFF:FFFFFF
@A00721:190:HGJW7DSXY:4:1469:3974:8155_2:N:0:TCTGCAAG+GCTGCACT
TTAAAGACTATACTAAATATCTCCACTACGAGGTTGTTGTTTGGCGATTAGCTCTTGCGACTAATATT
CGATATTCGATTGAGGGTCGTTATACCTATTCGGCTATCTGTGCTTTG
+
FFFFFFFFFFFFFFFFFFFFFFFFFFFFFFFFFFF:FFFFFFF::FFFFFFF:FFFFFFFFFFFFFFFF
FFFFFFFF:FFFFFFFFFFFFFFFFFFFFFFF:FFFFFFFFFFFFFFF
@A00721:190:HGJW7DSXY:4:2504:23014:18490_2:N:0:TCTGCAAG+GCTGCACT
TCAAATTTTAAACGATTCCGAGAGGTTTATCCTGATGTGGAGGGAGTTCTTGAAGCATCCCGACCTTT
```

```
CCTTGTGAAAATTTTGATGCGGTTTCATAAGATTATCAGTAATCCTGA
+
FFFFFFFFFFFFFFFFFFFFFFFFFFFFFFFFFFFFFFFFFFFFFFFFFFFFFFFFFFFFFFFFFFFF
FF:FFFFFFFFFFFFFFFFFFFFFFFFFFFFFFFFFFFFFFFFFFFFF
@A00721:190:HGJW7DSXY:4:2214:32325:27226_1:N:0:TCTGCAAG+GCTGCACT
ATTCATGGACCTTCCATCCTGTACCTCTTCTGGAGAGACGTATCTCAAAGATCGACTTCGAGACAGGT
GATATTGTTGAGCGAGAGCGACCAAGATCTAAATTCGGTAACGGTGCGTGGCGCTTTATTTTAAAAAG
CTC
+
FFFFFFFFFFFFFFFFFFFFFFFFFFFFFFF:FFFFFFFFFFFFFFFFFFFFFFFFFFFFFFFFFFFF
FFFFFFFFFFFFFFFFFFFFFFFFFFFFFFFFFFFFFFFFFFFFFFFFFFFFFFFFFFFFFFFFFFFF
FFF
@A00721:190:HGJW7DSXY:4:1336:28691:6433_1:N:0:TCTGCAAG+GCTGCACT
GATACTTATCACGAAAGGACTTGAGTCTTTTGATCAGTCTCTGACTTGACCATCTTTCATTTTAAATG
ACGATCATTAAGACTCTCTTTCTTCTTTGTTTCCTTCTTTGCACTGATTTTTCCTCATCAGCTTGTGT
TTT
+
FFFFFFFFFFFFFFFFFFFFFFFFFFFFFFFFFFFFFFFFFFFFFFFFFFFFFFFFFFFFFFFFFFFF
FFFFFFFFFFFFFFFFFFFFFFFFFFFFFFFFFFFFFFFFFFFFFFFFFFFFFF:FFFFFFFFFFFFF
FFF
@A00721:190:HGJW7DSXY:4:1467:4255:29684_2:N:0:TCTGCAAG+GCTGCACT
TTTTACGATAACAACTATCAGTTGATTAAGACTCTTCCGAAAGGCGAACTTTGGGAATTTGAAGCTCA
GGGTGAAGCACAACGTTTGACCGTGACGAACTGTGGTGTTCTTGATCT
+
FFFFFFFFFFFFFFFFFFFFFFFFFFFFFFFFFFFFFFFF:FFFFFFFFFFFFFFFFFFFFFFFFFFF
FFF:FFFFFFFFFFFFFFFFFFFFFFFFFFFFFFFFFFFFFFFFFFFF
@A00721:190:HGJW7DSXY:4:2618:31259:35055_1:N:0:TCTGCAAG+GCTGCACT
ATACTCTCCTTGAGTTCACTCGTAAGGACACTACCACTTATTACGATATTTCTTTAGTCGATGGGTTT
TCGACTCCTTTAAGCGTCTTCTGTTCATCGCAACCAGATGATATCTTCTATTCGAACTTATGTTTCGA
TCA
+
FFFFFFFFFFFFFFFFFFFFFFFFFFFFFFFFFFFFFFFFFFFFFFFFFFFFFFFFFFFFFFFFFFFF
FFFFFFFFFFFFFFFFFFFFFFFFFFFFFFFFFFFFFFFFFFFFFFFFFFFFFF:FFFFFFFFFFFFF
FFF
@A00721:190:HGJW7DSXY:4:2161:6470:15890_2:N:0:TCTGCAAG+GCTGCACT
TCTTCTAATGTGGCCTGAACTACGTTTCACTTTGTGTGTACTTGTCTCTATTTTAAGTTTTGTCTTCC
TTGTTTGTCTTATTCTATTAATCACTCATTTTAGTGAGATTATTAAAA
+
FFFFFFFFFFFFFFFFFFFFFFFFFFFFFFFF:FFFFFFFFFFFFFFFFFFFFFFFFFFFFFFFFFFF
FFFFFFFFFFFFFFFFFFFFFF:F:FFFFFFFFFFFFFFFFFFFFFFF
@A00721:190:HGJW7DSXY:4:2226:28429:10927_2:N:0:TCTGCAAG+GCTGCACT
CGACAACAGCAGGGGTTTTCGAATAGTGATAGCCACCCGCCTTACAGAGGGGCAGCAGCATGACCATA
ATTGGTATTGACTTTGGTACCACCTTTTCTACTGTCTGTCTCGCCTCT
+
FFFFFFFFFF:FFFF:FFFFFFFFFFFFFFFFFFFFFFFFFFFFFFFFFFFFFFFFFFFFFFFFFFFF
FFFFFFFFFFFFFFFFFFFFFFFFFFFFFFFFFFFFFFFFFFFFFFFF
@A00721:190:HGJW7DSXY:4:2210:29089:3051_1:N:0:TCTGCAAG+GCTGCACT
CTTAACAACAATGATAGTGAATTTATACCTACAATCATTGGTTTTTCTAATAATGATGACACTATTGT
TTATGGATATGATGCTATTACTCATGATGGTTACGGTCAGGGAGGATTCTCAGTTTACAAGGACCTTA
AGC
+
:FFFFFFF:FFFFFFFFFFF:FFFFFFFFFF:FFFFFFFFFFFFFFFFFFFF::FFFFFFFFFFFFFF
FFFFFFFFFFFFFFFFFFFFFFFFFFFFFFFFFFFFFFFFFFFFFFFFFFFFFFFFFFFFFFFFFFFF
FFF
```

@A00721:190:HGJW7DSXY:4:2539:31222:34741_2:N:0:TCTGCAAG+GCTGCACT
CCGAAAGACGTGCTAAATTAAAACCATTGTACGATGTCGTCTGTCTCGGCGATCACTTTTCACTAAGA
ATTGGTCCTACTTTTGGACAAGGCCAACTTCGTAATCTTCATGAGATC
+
FFFFFFFFFFFFFFFFFFFFFFF:FFFFFFFFFFFFFFFFFFFFFFFFFFFFFFFFFFFFFFFFFFFFF
FFFF:FFFFFFFFFFFFFFFFFFFFFFFFFFFFFFFFFFFFFFFFFFF
@A00721:190:HGJW7DSXY:4:1538:6650:27602_1:N:0:TCTGCAAG+GCTGCACT
TAGGGATTTTGAGAGAGTGAAAGGCTTCAATTGTAGTGGTTTGGTCGTTTCAGTACCCAGTCAGTATA
CCTCTACGCAAAGATATTTCATGAAAACTACAGAGGAACGTACCGGATTACCTGTCTTTCATATTATG
AAT
+
FFFFFFF:FFFFFFFFFFFFFFFFFFFFFFFFFFFFFFFFFFFFFFFFFFFFFFFFFFFFFFFFFFFF
FFFFFFFFFFFFFFFFFFFFFFFFFFFFFFFF:FFFFFFFFFFFFFFFFFFFFFFFFF:FFFFFFFFFFF
FFF
@A00721:190:HGJW7DSXY:4:1403:13205:18129_1:N:0:TCTGCAAG+GCTGCACT
TTTGCTTCGATGTTGGATGTCAAGAAGGGTTCAGAATGGGATTCCTACGTAGTTTATGACTTTGGTGG
TGGTACTTTCGATACTTCTATCGTCTTACGTTACGGTAATTACTATAGTGTTATTTATTCTGGTGGTG
ATG
+
FFFFFFFFFFFFFFFFFFFFFFFFFFFFFFFFFFFFFFFFFFFFFFFFFFFFFFFFFFFFFFFFFFFF
FFFFFFFFFFFFFFFFFFFFFFFFFFFFFFFFFFFFFFFFFFFFFFFFFFFFFFFFFFFFFFFFFFFF
FFF
@A00721:190:HGJW7DSXY:4:2316:29234:26162_2:N:0:TCTGCAAG+GCTGCACT
GGTCGTGACGTTGATGAAGCTATAAGAGCGTTCTTATTACGGCGCACTTCTTTACCCGCCACCACACA
ACTTTCAGTTTCACAACTGAAGGAGGAGGTTTCTGTTACTGGAAAACC
+
FFFFFFFFFFFFFFFFFFFFFFFFFFFFFFFFFFFFF:FFFFFFFFFFFF:FFFFFFFFFFFFFFFFF
FFFFFFFFFFFFFFFFFFFFFFFFFFFFFFFFFFFFFF:FFFFFF:FFF
@A00721:190:HGJW7DSXY:4:1555:22218:5118_2:N:0:TCTGCAAG+GCTGCACT
TCAATAATATACGTCTTGAACTCACTTATGACGATCTAGTGAAATTAACGCGTCCTTTTCTGGAACGT
AGTTTTACTATTATGAACAAAGTTATCAAAGATAGTGGCATTCAAGGC
+
FFFFFFFFFFFFFFFFFFFFFFFFFFFFFFFFFFFFFFFFFFFFFFFFFFFFFFFFFFFFFFFFFFFF
FFFFF:FFFFFFFFFFFFFFFFFFFFFFF:FFFFFFFFFFFFFFFFFFF
@A00721:190:HGJW7DSXY:4:2216:6424:23954_1:N:0:TCTGCAAG+GCTGCACT
TGATTCCGGCCGGTGGTAGCTCATTTCTACCAGGGGCTATTACTCTTGCTGAAACAGCTATTAAAGCT
GTTGAGCGGGTAATTACACCTAAACGAGCTCGATCTGCCATCGCTGAAGGATGTGCACTCTTTTCTGC
TAC
+
FFF:FFFFFFFFFFFFFFFFFFFFFFFFFFFFFFFFFFFFFFFFFFFFFFFFFFFFFFFFFFFFFFFF
FFFFFFFFFFFFFF:FFFFFFFFFFFFFFFFFFF:FFFFFFFFFFFFFFFFFFF:FFFFFFFFFFFFFFF
FFF
@A00721:190:HGJW7DSXY:4:2111:6686:35524_2:N:0:TCTGCAAG+GCTGCACT
TCGATTGTATCAACAGTAACATTACTTCTAATAAAGGTCTCTATCAAGCTCGTATTATCGTCGCTGCT
GGATCTGCGTTACCGTTGAAGGCTAAACTCCAAGGTAATTATGAAGAC
+
FFFF,FFFFFFFFFFFFFFFFFFFFFFFFFFFFFFFFFFFFFFFFFFFFFFFFFFFFFFFFFFFFF:F
FFFFF:FFFFFFFFFFFFFFFFFFFFFFFFFFF:FFFFF:FFFF:FFFFF
@A00721:190:HGJW7DSXY:4:2161:22327:31078_1:N:0:TCTGCAAG+GCTGCACT
GTATGAGGGTTTTTCTAATCGTAACATTAATAATTTCAATCTTCTTAAAAAGAAGATAGAGAGTCATA
CTTTGGGCTATCGTTCAGGTGCATGGGGTTGGATAGCCAATATTGTAGTGGACACCTCTGGTAGTATC
GAA
+
FFFFFFF,FF,FFF,F,,FF,:FFFFFFFFFFFFFF,FFFFFF,FF,:FFF,,FF::FF,F,:F,,F:

FFFFFFFFFF:FFFFFFFFFFFFFFFF,FFF::FFFF:FFFFFF,,,FFFF:FFFFFFFFFFFFFF,F
F:F
@A00721:190:HGJW7DSXY:4:2674:15176:15342_1:N:0:TCTGCAAG+GCTGCACT
CTAAGCATACTACTTTTGCTTTGATTACTCTACCGCCTTACAAGTCTAAGGTTAAAGAGTCGATTAAA
CAAGTTGTACTTATCAATGATTTTATCGCTGGTTGTTATCTCGAAAAGTCTCTTCTAAAAGGAACGTC
AGT
+
FFFFFFFFFFFFFFFFFFFFFFFFFFFFFFFFFFFFFFFFFFFFFFFFFFFFFFFFFFFFFFFFFFFF
FFFFFFFFFFFFF:FFFFFFFFFFFFFFFFFFFFFFFFFFFFFFFFFFFFFFFFFFFFFFFFFFFFFF
FFF
@A00721:190:HGJW7DSXY:4:1526:15320:6511_2:N:0:TCTGCAAG+GCTGCACT
CTAAAATATTTACAAAATCTTACCACTATCTACGAACCTAAAGTTCTGAAGACGTTGCTTGTCGCTTC
CTCCTTTAATGGCACTACCTCGGTACAATCTTACTTGGAACGAAGGCG
+
FFFFFFFFFFFFFFFFFFFFFFFFFFFFFFFFFFFFFFFFFFFFFFFFFFF::FFFFFFFFFFFFFFF
FFFFFFFFFFFFFFFFFFFFFFFFFFFFFFFFFFFFFFFFFFFFFFFF
@A00721:190:HGJW7DSXY:4:1608:30608:35744_1:N:0:TCTGCAAG+GCTGCACT
GTTCTGTCTATATTAGGTGAAGCGAAATACGATTTCGAACGTCAACGTTCACCGAGTTTCGTTTCGTA
CCTTGATGCTGACTTTCGTCAATATGATTTGATCTGGTTGCTCAATTCTAAGCGTGTTCCGCTTAGTG
CGA
+
FFFFFFFFFFFFFFFFFFFF:FFFFFFFFFFFFFFFFFFFFFFFFFFFFFFFFFFFFFFFFFFFFFFF
FFFFFFFFFFFFFFFFFFFFFFFFFFF:FFFFFFFFFFFFFFFFFFFFFFFFFFFFFFFFFFFFFFFFF
FFF
@A00721:190:HGJW7DSXY:4:2227:22227:8547_1:N:0:TCTGCAAG+GCTGCACT
TACGAACTTGACTCTACTATACGTGACATACTAAATACTAGATATTTGGATGATCTTGATAAAGAACT
TCATGAATTTTCAGAGGATGAAAAATTTTCTTCCTTGGATTTTAATATAAAGAATCTTTACCAGGACT
CCT
+
FFF:FF:FFF:FF,FFFFFFF,FFFF,FFF:FFFFFFFFFFFFFFFFFFFFFFFF:FFFFFFFFFFFF
FFFFFFFFFFFFFFFFFFFFFFF:FFFFFFFFFFFFFFFFFFFFFFFFFFFFFFFFFFFFFFFFFFFF
FFF
@A00721:190:HGJW7DSXY:4:2313:13765:7388_1:N:0:TCTGCAAG+GCTGCACT
TGATCGTAAGAAGTTTATAAGGAACATATCCATTTGTTGTAATAGGGTCATCAACTTAGAACTCGAAA
CTCTAAGTGGTAATACTGTCTTTCCAAGCTTACCTGTATCTTCAATGAAATTGGACTCTTCGATAGGG
GAC
+
FF:FFFFFF:FF,F,FFFFFFFF:,FF:F:,F:FFFFFFFFFF,:,FF:FFFFFFFF:FF,FFFFFFF
FFFFFF,FFFFFFF::FFF::FFFFFFFFFFFFFFFFFF,FFFFFFFFFFFFFFFF:FFFFFFFFFFF
FFF
@A00721:190:HGJW7DSXY:4:1608:31982:32017_1:N:0:TCTGCAAG+GCTGCACT
GGAAGATCTGTTATCGACGGTTTATCGTTTACGAAGTATCACTCCACCGGTGAATGGTTTAAGAATAC
TATAAATAGAATAGCTATTACTACTAATGGCGTTGGCCTAGTGGATGTTATTAGTGACATCTGGTCAA
TTT
+
FFFFFFFFFFFFFFFFFFFFFFFFFFFFFFFFFFFFFFFFFFFFFFFFFFFFFFFFFFFFFFFFFFFF
FFFFFFFFFFFFFFFFFFF,FFFFFFFFFFFFF:FFFFFFFFFFFFFFFFFFFFFFFFFFFFFFFFFFF
FFF
@A00721:190:HGJW7DSXY:4:1217:9579:16861_1:N:0:TCTGCAAG+GCTGCACT
TGGTCTTTATTCTATTATTGATTTTTATGAGATTGATACGTTAACAGAAGATGATGTTAGAGTAGAAT
TCCTGAAGAGTCCTTCCGTAACCAACTTCTTGACAATTTTCTTCAATCCTTCGATAATAGTTAAATCT
AGG
+
FFFFFFFFFFFFFFFFFFFFFFFFFF:FFFFFFFFFFFF:FFFF:FFFFFFFFFFFFFFFFFFFFFFFF

FFFFFFFFFFFFF:FFFFFFFFFFFFFFFFFFFFFFFFFFFFFFFFFFFFFFFFFFFFFFFFFFFFFFF
FFF
@A00721:190:HGJW7DSXY:4:1355:29785:31407_2:N:0:TCTGCAAG+GCTGCACT
TACACAATTAAGAAATAATCCTACATTGACTGATTATCTAAAGATTGAAGGAAAATTTTATCCTCGTT
TAACTTACTTAGACTTTCCTACGATACAAATGATAACTTCTAATTTAC
+
FFFFFFFFFFFFFF:FFFFFFFFFFFFFFFFFFFFFFFFFFFFF,FFFFFFFFFFFFFFFFFFFFFFF
FF,FFFF:,FFFFFFFFFFFFFFFF:FFFFF:FFFFFFFFFFFFFFFF
@A00721:190:HGJW7DSXY:4:1602:23348:24486_1:N:0:TCTGCAAG+GCTGCACT
GGGATGCATGGTGTTATCTCCTCAGGAGTCCCGGCATTTGATAATAATGACGAGACTTTCGTCATAAT
CGCTGCATATTACGGATTACACGGTACCAGCGCATCTCGTGTCGAGCCTCGACCCGATTATATGGTAT
TAA
+
FFFFFFFFFFFFFFFFFFFFFFFFFFFFFFFFFFFFFFFFFFFFFFFFFFFFFFFFFFFFFFFFFFFF
FFFFFFFFFFFFFFFFFFFFFFFFFFFFFFFFFFFFFFFFFFFFFFFFFFFFFFFFFFFFFFFFFFFF
FFF
@A00721:190:HGJW7DSXY:4:1349:13114:26396_1:N:0:TCTGCAAG+GCTGCACT
AATTTATAGAAGATTATTTTGACAGAATCAATAAGAATCATCGATTCTCGATTCGTAGAGCTTATCTA
GGTACTCTCATTTACTATGTTGATTTAGTCTACATTTATGCTGATCTAAAATTTCGATCTAGATGGAT
CC
+
FFFFFFFFFF:,FFF,FF,FF:,F:,FFFFF:FFFFFFFFFFFFFFFF:FF:FFFFFF:FFF:FFFFF
F,F,FFFF,FFFFF,FFFFFFFFFFF,FF:F::FFFFFFFFF,FF:FFFFFFFFFFFF:F,FFFFFFF
FF
@A00721:190:HGJW7DSXY:4:1678:28709:35055_1:N:0:TCTGCAAG+GCTGCACT
TTCTAGAATTCTTAATTATAATATTCAACCTCAAAAGTTACGTGTTTACAGTAACGAGATCACACATT
TTGAGAACTAACTATGTTATGTTCTCTGATCATTTTGACAATCTCTTCTTATAATAAATAAATAAATA
ATA
+
F:FFFFFFFF:FFFFFFFFFFFFFFFFFFFFFFFFFFFFFFFFFFFFFFFFFFFF,FFFFFFFFF:F
FFFFFFFFFFFFFFFFFFFFFFFFFFFF:FFFFFFFFFFFFFFFFFFFFFFFFFFFFFFFFFFFFFFF
FFF
@A00721:190:HGJW7DSXY:4:1660:6352:20478_2:N:0:TCTGCAAG+GCTGCACT
CAAATAGTGGAAAGGCTCCAGTCAACACTAGTTCGAGTGCTGCTTTGGATTACGATGACGTGAGAATA
GGTGAAGGGATAGTTTTTACCTCGACCAACATTAAGGCGGCGGCGACT
+
FFFFFFFFFFFFFFFFFFFFFFFFFFFFFFFFFFFFFFFFFFFFFFFFFFFFFFFFFFFFFFFFFFFF
FFFFFFFFFFFFFFFFFFFFFFFFFFFFFFFFFFFFFFFFFFFFFFFF
@A00721:190:HGJW7DSXY:4:2316:14651:19006_2:N:0:TCTGCAAG+GCTGCACT
CTTCACCCAAAATGAAGGACACTTCGAAACTGAATAATGATGCACTACAAACAAGTATCGCTAATCAT
CTGAAAAAGGCTCTAAAGGACACTATACAATATCCAGACGACGACGAA
+
FFFFFFFFFFFFFF:FFFFFFFFFFFFFFFFFFFFFFFFFFFFFFFFFFFFFFFFFFFFFFFFFFFFF
FFFFFFFFFFFFFFFFFFFFFFFFFFFFFFFFF:FFFFFFFFFFFFFF:
@A00721:190:HGJW7DSXY:4:2646:15655:13823_2:N:0:TCTGCAAG+GCTGCACT
CAGCACCACATTTTGGTCAAGTCAATCCGTTGAGAAAATTTCTTAGAGGATATTCGCCATTGTGGTAC
GAATTGGTCCAACATTTACCACCAAATTCTTTTGATACAACTCTAGCC
+
FFFFFFFFFFFFFFFFFFFFFFFFFFFFFFFFFFFFFFFFFFFFFFFFFFFFFFFFFFFFFFFFFFFF
FF:FFFFFFFFFFFFFFFFFFFFFFFFFFFF:FFFFFFFFFFFFFFFFF
@A00721:190:HGJW7DSXY:4:1413:13458:12399_2:N:0:TCTGCAAG+GCTGCACT
GCTTGCTATTCACCAGATTGGTTCGAACCCGTTGATGCTATGTCAGAAGAAGCTATTCAAGCTTGTCG
TTTAAAGACTGCAGAGGCTGTCAAACAAGCCCAGACTCAAGGAGTCCA
+

FFFFFFFFFFFFFFFFF:FFFFFFFFFFFFFFFFFFFFFFFFFFFFFFFFFFFFFFFFFFFFFFFFFF
FFFFFFFFFFFFFFFFFFFFFFFFFFFFF,FFFFFFFFFFFFFFFFFFF
@A00721:190:HGJW7DSXY:4:2122:29026:25739_2:N:0:TCTGCAAG+GCTGCACT
GGTGATAAAAATTATTTAGAGATCTTTTCTACTTACAAAGGACAATTCAGTTTCATTAATTCTACGGA
AAAGTCCGGAGTATATTGTATGTTAATAGATAGTGGGATAATAGTTCT
+
FFFFFFFFFFFFFFFFFFFFFFFFFFFFFFFFFFFFFFFFFFFFFFFFFFFFFFFFFFFFFFFFFFFF
FFFFFFFFFFFFFFFFFFFFFFFFFFFFFFFFFFFFFFFFFFFF:FFFF
@A00721:190:HGJW7DSXY:4:2662:19687:1360_1:N:0:TCTGCAAG+GCTGCACT
CGTCTGAAGAGTTTAGAGCTGCTATTGATTATGTAACCGATAGGAGTTGTATAGTTGAAGATGTGGCT
TTAGACGATAATCCTTTAAGGACTATGTTGACTGACGAAGATTTTCCCACAGATTTGTCTACGTTGCA
GAT
+
FFFFFFFFFFFF:FFFFFFFFFFFFFFFFFFFFFFFFFFFFFFFFFFFFFFFFFFFFFFFFFFFFFF
FFFFFFFFFF:FFFFFFFFFFFFFFFFFFFFFFFFFFFFFFFFFFFFFFFFFFFFFFFFFFFFFFFF
FFF
@A00721:190:HGJW7DSXY:4:2137:11686:11741_2:N:0:TCTGCAAG+GCTGCACT
CTATATACATTATAAAATATAAAAATAAGGGAGTCATAACTTTTGTTACCAAATTCTTGGCTTTCAAC
GAAGTCTCTGATTTTATTAATCATTACTTTAACGATCTCTCGGTCATT
+
FFFFFFFFFFFFF:FFFFFFF:FFFFFFFFFFFFFFFFFFFFFFFFFFFFFFFFFFFFFFFF:FFFFF
FF:FFFFFFFFFFFFFFFFFFFFFFFFFFFFFF:FFFFFFFFFFFFFF
@A00721:190:HGJW7DSXY:4:1248:25771:28902_1:N:0:TCTGCAAG+GCTGCACT
TTGATCTACGTCGTTTAACTGTCGGAACAACCTTTACTTAGAATGACTCTATCTTTTCAGACACTTCT
TATATTATTTACAATAGTTAATATCATATTAGTAATACTCACTATCGTTTTCTGCTTTTTCTTAGCTA
ATA
+
FFFFF,FFFFFFF:FFFFFFFFFFFFFFFFFFFFF:FFFFFF:FFFFFF:FFFFFFFFFFFFFFFFFF
FFFFFFFFFFFFFFFFFFFFF:FFFFFFFFFFFFFFFFFFFFFFFFFFFFFFFFFFFFFFFFFFFFFF
FFF
@A00721:190:HGJW7DSXY:4:1333:32036:20337_2:N:0:TCTGCAAG+GCTGCACT
ATGAGTACGAAAACCAACTCTTCGTTACGAGACCTGATGGTAGAATAGATTTAGTCTCTACACCCATC
TCAACGAGGATAAAGCGAGTTTTCCGTCCTGAACGTAATACACGAAGA
+
FFFFFFFFFFFFFFFFFFFFFFFFFFFFFFFFFFFF:FFFFFF:FFFFFFFFFFFFFFFFFFFFFFFF
FFFFFFFFFFFFFFFFFFFFFFFFFFFFFFFFFFFFFFFFFFFFFFFF
@A00721:190:HGJW7DSXY:4:2623:15691:17769_1:N:0:TCTGCAAG+GCTGCACT
AAACGACTCGAAACCGAGACGCCGTAGATCAAAGTCCCTTCTAACTTTAGATTATGAATCCTGCCTTT
ACTCATTTTATAGC
+
F,FFFF:FFFFFFFFFFFFFFFFFFFFFFFFFFFFFFFFFFFFFFFFFFFFFFFFFFFFFFFFFFFFF
FFFFFFFFFFFFFF
@A00721:190:HGJW7DSXY:4:1537:28366:30921_2:N:0:TCTGCAAG+GCTGCACT
TTTTTATAATTTATAACCTTCTTATACTTTTGGGTATGTTTTGTGAAAAACATGAGATACCCAAGTCT
ATCGAAGACATTATCTCTTATGTACTTATTGTAACGATCATTCTTTCG
+
FFFFFFFFFFFFFFFFFFFFFFFFFFFFFFFFFFF:FFFFFFFFFFFFF,FFFFFFFFFFFFFFFFFF
FF:FFFFFFFFFFFFFFFFFFFFF:FFFFFFFFFF:F:FFFFFFFFF,
@A00721:190:HGJW7DSXY:4:2264:2157:21324_1:N:0:TCTGCAAG+GCTGCACT
TTCTTTTATTCCTTTAACTGTGTATGAGGAGGATTTATAATGCTTTTCGATAGTGATTGTTATCAATT
TAAATACATTACTGTTTCAAATCATCTTTACTCACAGACTCCGAAAATGGTTTCAAATGAGGTCAAAG
CTC
+
FFFFFFFFFFFFFFFFFFFFFFFF:FFFFFFFFFFFFFFFFFFFFFFFF:FFFFFFFFFFFFFFF::FFF

FFFFFFFFFFFFFFFFFFFFFFFFFF:FFFFFFFFFFFFFFFFFFFFFFFF:FFF:FFFF:FF:FFFFFF
FFF
@A00721:190:HGJW7DSXY:4:1261:18484:16689_2:N:0:TCTGCAAG+GCTGCACT
AACTATTATCCGATTCTTCGAGGGTATCACTACCACTGATCTGCCGACTTTAGTCAAGGAACTTCAAT
TCCTTCGATGTTTATATTTTTCTATAAGGGAAAATAGCAATAATCAAA
+
FFFFFFFFFFFFFFFFFFFFFFFFFFFFFFFFFFFFFFFFFFFFFFFFFF:FFFFFFFFFFF:FFFFFFF
FFFFFFFFFFFFFFFFFFFFFFFFFFFFFFFFFFFFFFFFFFFFFFFFF
@A00721:190:HGJW7DSXY:4:1156:18584:3677_2:N:0:TCTGCAAG+GCTGCACT
AACTTTATTTCAGAACTCTCTCCGATCACCTTGGTGTGATCTCCCGGAGGATCTTTTCAATAAGATGT
ATGATGATAATCTACTATTTTTAAATAGTGATATTCAGCGTATACTTG
+
FFFFFFFFFFFFFFFFFFFFFFFFFFFFFFFFFFFFFFFFFFFFFFFFFFFFFFFFFFFFFFFFFFFFFF
FFFFFFFFFFFFFFFFFFFFFFFFFFFFFFFFFFFFFFFFFFFFFFFFF
@A00721:190:HGJW7DSXY:4:1417:14000:29152_1:N:0:TCTGCAAG+GCTGCACT
AAAGATTTGGCTTGGTCTATTTACCGCAAAAAGTTGGAAGAGGTCTATGATATTTATTTTACTTATAC
TTTAAATAGCACCGATTATAACTTGACTGTTGATAATATTACAATAGCTCTTTCTCAGAAGATTAGAG
AAG
+
FFFFFFFFFFFFF:FFFFFFFFFFFFFFFFFFFFF:FFFFFFFFFFFFFFFFFFFFFFFFFFFFFFFFFF
FFFFFFFFFFFFFFFFFFFFFFFFFFFFFFFFFFFFFFFFFFFFFFFFFFFFFFFFFFFFFFFFFFFFFF
FFF
@A00721:190:HGJW7DSXY:4:1202:6343:18991_2:N:0:TCTGCAAG+GCTGCACT
CGTCGGTAGCATTGATTTACTTTTACCGTTAGAACTGAAAAAGATTCTTTTCGAAAAGAAACCTATAA
GTTACAAACGATTCTTCGACTATATTAAAGAAATAAAAATACTTCAAG
+
FFFFFFFFFFFFFFFFFFFFFFFFFFFFFFFFFFFFFFF,FFFFFFFFFFFFFFF:FFFFFFFFFFFFFF
FFFFFFFFFFFFFFFFFFFFFFFFFFFFF::FFFFFFFFFFFFFF:FFF
@A00721:190:HGJW7DSXY:4:2110:24361:13338_2:N:0:TCTGCAAG+GCTGCACT
TTACATTTTTAATGGTATGGTATAATGTGTCTTACAAATAGAACCTTCACTCTCGGTAATGAGAGTGT
ATTCCTTAATATTCTAGTAACCGCTATGTGGGCATTACTAGGATTTAT
+
FFFFFFFFFFFFFFFFFFF:FFFFFFFFFFFFFFFFFFFFFFFFFFFFFFFFFFFFFFFFFFFFFFFFFF
FFFFFFFF:FFFFF:FFFFFFFFFFFFFFFFFFFFFFFFFFFFFFFFFF
@A00721:190:HGJW7DSXY:4:1526:10890:25676_2:N:0:TCTGCAAG+GCTGCACT
TTTAAATGCTAGGAAAATTTTAGAAAAACTCCATTGTATAGTTCAAGGAGTACTTAATTCAATAGCTT
TAGTATTATTTACTATCGCCTTTATTGAAAATTGTATAAGAGTTAATT
+
FFFFFFFFFF,FFFFFFFFFFFFFFFFFFFFFFFFFFFFFFFFFFFFFFFFFFFFFFFFFFFFFFFFFFF
FFFFFFFFFFFFFFFFFFFFFFFFFFFFFFFFFFFFFFFFFFFFFFFFF
@A00721:190:HGJW7DSXY:4:1328:2980:23907_1:N:0:TCTGCAAG+GCTGCACT
CTCCCCAAACATAACATACCATAACCTACCATGACCAATCATCGCTCCAACTGGCACGCCAGACGTAG
GGTCAAACCTACTACCGCCTGTCGGCAGATCGGCACCACTGGTCTCTACGTTCAGGGACCCGCTGTGC
AGA
+
FFFFF:FFFF:FFFFFFFFFFFFFFFFFFFFFFFFFF:FFF,FFFFFFFFFFF:FFFFFFFFFFFFFFFF
FFFFFFFFFFFFFFFFFFFFFFFFFFFFFFFFFFFFFFFFFFFFFFFFFFFFFFFFFFFFFFFFFFFFFF
FFF
@A00721:190:HGJW7DSXY:4:2517:30192:14356_2:N:0:TCTGCAAG+GCTGCACT
AAATCTAGATCTGCACAGAAGAAGGCTGAAGGTCGTTACCGGACGACCACTCAAAGGAAACGGGCTGT
TGCCCAGAGGAAAGCAGCGACCACCGAGGTCGCTCGTAACATTGACGG
+
FFFFFFFFFFFFFFFFFFFFFFFFFFFFFFFFFFF:FFFFFFFFFFFFFFFFFFFFFFFFFFFFFFFFFF
FFFFFFFFFFFFFFFFFFFFFFFFFFFFFFFFFFFFFFFFFFF:FFFFF

```
@A00721:190:HGJW7DSXY:4:1258:11568:9940_1:N:0:TCTGCAAG+GCTGCACT
CAGACGTGTGCTCTTCCGATCTCTCAGTCAAAAGTCACATTCACCACTCCCATGCCAATCAATGATGT
GACTATACAGTTCGGCCATTTCAAGAGTGGTCAATACGTCGTGGGAGGTACTGAAGCCTTCCCCTCCG
CTG
+
FFFFFFFFFFFFFFFFFFFFF:FFF:FFFFFFFFFFFFFFFFFFFFFFFFFFFFFFFFFFFFFFFFFF
FFFFFFFFFFFFFFFFFFFFFFFFFFFFFFFFFFFFFFFFFFFFFFFFFFFFFFFFFFFFFFFFFFFF
FFF
@A00721:190:HGJW7DSXY:4:2605:27615:16313_2:N:0:TCTGCAAG+GCTGCACT
CTCCCAAGCCAATCAATAGTTCTTCTGACCAGAATGAAAAGGGGTTTACCAGGGTAGTCTCTAAGTCT
ACCAGGAAGAGGGGAAATGTCCCCAAAACTGAGAAGAATAAAGGGAAA
+
FFFFFFFFFFFFFFFFFFFFFFFFFFFFFFFFFFFFFFFFFFFFFFFFFFFFFFF:FFFFFFFFFFFF
FFFFFFF,FFFFFFFFFFFFFFFFFFFFFFFFFFFFFF:FFFFFFFF,F
@A00721:190:HGJW7DSXY:4:1659:27091:10363_2:N:0:TCTGCAAG+GCTGCACT
GAAGGGGAAATTACCCCTAATTCTGAAGGAAGATCTCCCACCATCTGGGGTTGGGAATAAGAAATCTG
CACGGGCCAAAGAGGCCGCTGCTACGAAGTCGCTGAAGGCGGCTTGGG
+
FFFFFFFFFFFFFFFFFFFFFFFFFFFFFFF,FFFFFFFFFFFFFFFFFFFFFFFFFFFFFFFFFFFF
FFFFFFFFFFFFFFFFFFFFFFFFFFFFFFFFFFFFFFFFFFFFFFFF
@A00721:190:HGJW7DSXY:4:2521:20292:36793_2:N:0:TCTGCAAG+GCTGCACT
TGTAAAGTTGCGCCTGCCGACCCCATCTTTGAGACCAAGACTCTCTACAGTAGGTTCGTGCCTACTAT
GAGCAAATCTGTTGAATTCGTCACTGAGGACGAAGGTGAGAGGACTTT
+
FFFFFFFFFFFFFFFFFFFFFFFFFFFFFFFFFFFFFFFFFFFFFFFFFFFFFFFFFFFFFFFFFFFF
FFFFFFFFFFFFFFFFFFFFFFFFFFFFFFFFFFFFFFFFFFFFFFFF
@A00721:190:HGJW7DSXY:4:2425:19732:34381_1:N:0:TCTGCAAG+GCTGCACT
CTGAGAGCAGTCGCTGGGGAAAGAGAATTGCGTGGGTTGAAGGCCGACGCTTCGAGCTTGAGTGTGAC
GAGGCCGGTGACGTTTTTGTCAACGGCGTTTGGAGGATTTTATCCGCGCTTAACTATGGGGCGAAGAT
CCC
+
FFFFFFFFFFFFFF:FFFFFFFFFFFF,FFFFFFFFFFFFFFFFFFFFFFFFFFFFFFFFFFFFFFFF
F:FFFFFFFFFFFFF:FFFFFFFFFFFFFFFFFFFFFFFFFFFFFFFFFFFFFFFFFFFFFFFFFFFF
FFF
@A00721:190:HGJW7DSXY:4:2577:21404:9251_2:N:0:TCTGCAAG+GCTGCACT
AGGTTGGCATATTTATGCCGACGATAATAAGCATGTCACCCTTAAGCACCGAGGTAAGCTCGGGACTT
ACAAGAGAGAGTGTGAGAGAGTCGCTCTCACTATTTTTGCCCAGAATG
+
FFFFFFFFFFFFFFFFFFFFFFFFFFFFFFFFFFFFFFFFFFFFFFFFFFFFFFFFFFFFFFFFFFFF
FFFFFFFFFFFFFFFFFFFFFFFFFFFFFFFFFFFFFFFFFFFFFFFF
@A00721:190:HGJW7DSXY:4:1247:28330:24314_1:N:0:TCTGCAAG+GCTGCACT
GTGACTGGAGTTCAGACGTGTGCTCTTCCGATCTGTTTTTGTTATATTCACTGGTTCTGGGAGGCTGC
TTTCCTACATAATTACAACCTCTCTTTAGAGAGCATTAGAAGGAAGTTGGGCAAATTCCCACATATGG
TAG
+
:F:::F,F:FFF,FFFF:FFFF,F::FF,F,F::FFFFFF:FFFF:FF:FFFFFFF,FFFFFFF:FFF
FFF,:F,FFFF:FF,F,FF,F,FFFFF::FFFFFFFFFF:FFFFFFFFFF,:F::FFF:FFF,FFFFF
F:,
@A00721:190:HGJW7DSXY:4:1320:9182:25723_2:N:0:TCTGCAAG+GCTGCACT
ACTTTTCTTGAAAATTTTTCCTTTCCTCCGTCATTGGGGATTTCGATGTGAGAAGGTGGAAGGTGTCA
AGGGTCTTCTTCATGCTGATCTTTCAGGACCTAATCACATCATGATTG
+
FFFFFFFFFFFFFFFFFFFFFFFFFFFFFFFFF:FFFFFFFFFFFFFFFFFFFFFFFFFFFFFFFFFF
FFFFFFFFFFFFFFFFFFFFFFFFFFFFFFFFFFFFFFFFFFFFFFFF
```

```
@A00721:190:HGJW7DSXY:4:1244:14461:29324_2:N:0:TCTGCAAG+GCTGCACT
ATCGAGTTGGAGGGAGGAAGTTCTCCGAAATTAAAGACGGGGGGATTAAGAGTCCAGCTGTAACTGTC
ATTTCAGAGAGGGGAGCTCTGAAAAGGAAACTTCGTCAGAAGATGAGG
+
:FFFFFFFFFFFFFFFFFFFFFFFFFFFFFFFFFFFFFFFFFFF:FFFFFFFFFFFFFFFFFFF:F
FFFFFFFFFFFFFFF:FFFFFFFFFFFFFFFFFFFFFFFFFFFFFFFF
@A00721:190:HGJW7DSXY:4:1375:2546:36025_2:N:0:TCTGCAAG+GCTGCACT
GGAGGAGTTTGAGGAAGGCTCTAGAGAAGATCTCACCACCTGGAGAAGGCAAGTGGAGAGTCCAGAAG
TTGATGAAGAAGGATTATTTGAAGCAGGGGTTACCACTTCCTAAGGTG
+
FFFFFFFFFFFFFFFFFFFFFFFFFFF,FFFFFFFFFFFF:FFFFFFFFFFFFFFFFFFFFFF:FFFF
FFFFFFFFFFFFFFFFFFFFFFFFFFFFFFFFFFFFFFFFFFFFFFFF
@A00721:190:HGJW7DSXY:4:1477:17065:1955_2:N:0:TCTGCAAG+GCTGCACT
TTCTTTGGAGTTTTAGATAATCTTTTGAGAAGGGGTCATAGAGCCAGCAAGAGAAGCTTATATGCTGG
TTTAAGTGATGATTATCACTCCTTGAGAAGACAAGCCACATTAAGATA
+
FFFFFFFFFFF::FFFFFFFFFFFFFFFFFFFFFFFFFFFFFFFFFFFFFFFFFFFFFFFFFFFFFFF
FFFFFFFFFFFF:FF:FFFFFFFFFFFFF:FFFFFFFFFF:FFFFFFF
@A00721:190:HGJW7DSXY:4:1376:25608:4539_2:N:0:TCTGCAAG+GCTGCACT
CTTGGGGTTTCGAATGTCATTAGTGCCTTACGATTGAAAGAGGTCCTTTTGAGTATCGACAGATCTTT
CGATCTTGAGGACGTACATTTAACGTGGCAGAATTCGGTAACTTACCA
+
FFFFFFFFFFFFFFFFFFFFFFFFFFFFFFFFFFFFFFFFFFFFFFFFFFFFFFFFFFFFFFFFFFFF
FFFFFFFFFFFFFFFFFFFFFFFFFFFFFFFFFFFFFFFFFFFFFFFF
@A00721:190:HGJW7DSXY:4:1607:19316:7263_2:N:0:TCTGCAAG+GCTGCACT
GGGAAGTTGTGGGATTTAACTTTGACTCAACTACTCCGATCAGATCCGGATGGTTTGATTGGTATGGA
CTACTCGACTTTTGATCTGGAGTTGTCTAGGGTCATGGCAGATACTAC
+
FFFFFFFFFFFFFFFFFFFFFFFFFFFFFFFFFFFFFFFFFFFFFFFFFF:,FFFF::FFFFFFFF
FFFFFFFFFFFFFFFFFFFFFFFFFF:FFFFFFFFFFFFFFFFFFF:F
@A00721:190:HGJW7DSXY:4:2675:10719:26631_2:N:0:TCTGCAAG+GCTGCACT
AAAATATGCAGGTCAAAGATTCTGTAGCTCTGAGGGCGGTTGAGGGAGCCGTTTTGGCCGACTTTAAC
TATTCTAAGAGTCTTTATGATTCAAAACCTATCGTTGTCGTACCTTAC
+
FFFFFFFFFFFFFFFFFF,FFFFFFFFFFFFFFFFFFFFFFFFFFFFFFFFF,FFFFFFFFFFFF,F
FF:FFFFFF:FFFFFFFFFFFFFFFFFFFF:FFFFFFFFFF:FFFFFF
@A00721:190:HGJW7DSXY:4:2201:24361:25958_2:N:0:TCTGCAAG+GCTGCACT
GCGGCTTATTGTCGCGCAGTTTCCAGAATTGCGGTTGAAGTTCACCCATTCTATCTTGAATGCTCACG
CCGTCGCAGCCATATCGAGACTATGTTATAATCACTTGTTAAAGTACA
+
FFFFFFFFFFFFFFFFFFFFFFFFFFFFF:FFFFF:FFFFFFFFFFFF::FFFFFFFFFFFFFFFFFF
FFFFFFFFFFFFFFFFFFFFFFFFFFFFFFFFFFFFFFFF:FFFFFFF
@A00721:190:HGJW7DSXY:4:1644:14579:13745_2:N:0:TCTGCAAG+GCTGCACT
TTAGATATTATTGATATTGGAGGCGATATTCGTTTTCATATCCAAAACTCCTTGAATGGAGTACATAT
TTGCAACCCTATTCTTGATGTGAAAGATGCATCGAGGTATACGTCTAG
+
FFFFFFFFFFFFFFFFFFFFFFFFFFFFFFFFFFFFFFFFFFF:FFFFFFF:FFFFFFFFFFFFFFFF
FFFFFFFFFFFFFFFF:FFFFFFFFFFF:FFFFFFFFFFFFFFFFFFF
@A00721:190:HGJW7DSXY:4:1130:27615:9267_2:N:0:TCTGCAAG+GCTGCACT
TCACGAGACGTCATGAAAGCTATCGGAGGGAAGCAAAATGTTTCGTTCTGTTACAAGAACGCTTTGGA
TTGTGACCATAAAGCCTCAGTTGCGATAGCTGTAGAGGTATACGATAT
+
,FFFF:FFFFFF:,F:FFFFF,:FFFFFF,,FFFFFFFFF::F::FFFF,FFF,FF:FFFF:FFFFFF
F,::FFFF:FFFFFFF:FFFFF:F:F:FFFFFFFFFFFFFFFF,FFFF
```

@A00721:190:HGJW7DSXY:4:1224:8413:15436_2:N:0:TCTGCAAG+GCTGCACT
CTTAAGAGAGAGATTGATGTATGTCATTTCGTCTTAGTTTGTCCCGGCGAATTATTGGACACGATGAA
AAATGACATACCCGTTCCTGACCTTGGTATCGTTATCCGTCATGGAGA
+
FFFFFFFFFFFFFFFFFFFF,:FFFFFFFFFFF,FFFFFFFFFFFFFFF::FFFFFFFFFFFFFFF
FFFFFFFFFFFFFFFFFFFFFFFFFFFFFFFFFF:FFFFFFFFFFFFF
@A00721:190:HGJW7DSXY:4:1604:15709:36526_2:N:0:TCTGCAAG+GCTGCACT
GTTATTCTCACAACAAAGATAAGATTTTATCTTTCTTCAAACACCCTGTTTTCCTTAAGGGCGGACAA
TTGTTCCATTGTGAACTCTTGGGGACTAGATTGAGCATTAATGAGTTC
+
FFFFFFFFFFFFFFFFFF:FF:,FFFFFFFFFF:FFFFF,FFF,FF:,FFFFF:FFF:F:F:FF:FFF
FFFFFF:FFFFFFFFFFFFFFF:FFFF:FFF,:::,F,FFFFFF,FF,
@A00721:190:HGJW7DSXY:4:2201:7202:10488_1:N:0:TCTGCAAG+GCTGCACT
TCTAGTGGACAATTCAGAATACCTATTTCCGTACCAAGAGCCACTGCCAATCTCATCGCTATACGCAT
ACCTGATTGGAAGAAGCGAGGTTCGTTCCTCAATAAAAATTTTTCTCAAGAGATTATGGTTGATAAAG
TTT
+
F:FFFF:F::FFF:FFF:F:F:FFFF,FFFFFFF:FFFFF:FF:FF,FFFFFFFFFFF:FFFFFFFFF
FFFFFFFF:FFFFFFFFFFFFFFFFFFFFFFFFFFFFFFFFFFFFFFF:FFFFFFFFFFFFFFFFFFFF
FFF
@A00721:190:HGJW7DSXY:4:1507:14290:18004_1:N:0:TCTGCAAG+GCTGCACT
TTCAGTACGTCATGGGGATCACTTCTTCGGTCAATGACAAAACCTTCGAATATGTTACGACATGGCTT
CGTAACAACAGTGCGAGGATTGTCATCTCGGGGAAAGTGATTCACCAAGATGTTCATATAGATACGAG
ATA
+
FFFFFFFFFFFFFFFFFFFFFFFFFFFFFFFFFFFFFFFFFFFFF:FFFFFFFFFFFFFFFFFFFFFFF
FFFFFFF:FFFFFFFFFFFFFFFFFFFFFFFFFFFFFFFFFFFFFFFFFFFFFFFFFFFFFFFFFFFF
FFF
@A00721:190:HGJW7DSXY:4:1425:10601:33129_2:N:0:TCTGCAAG+GCTGCACT
CAGCGATCTTACTCGTTATCGGAGCTAAGAAGAAGACTTTGGGTACACGTTATGCTCGCAAACTTAAC
GTTAGTACCGGAGGTGATCTCTCTGCTTCTGCGTTACTGAGAGAAAAG
+
FFFFFFFFFFFFFFFFFFFFFFFFFFFFF:FFFFFFFFFFFFFFFFFFFFFFFFFFFFFFFFFFFFFF
FFFFFFFFFFFFFFFFFFFFFFFFFFFFFFFFFFFFFFFFFFFFFFFF
@A00721:190:HGJW7DSXY:4:2115:9643:21699_1:N:0:TCTGCAAG+GCTGCACT
CCTACGGTGACTTTATGGACAAGATGCAAGTTCTTGGGAAATACTTGTTTCCCTTTTTAGGTTTAGTA
GGTGATCTTGACGTCGATTCTTTGTATAGGGAGTGTGAGAAGACAACGAATCTTGAAACTACGGTCCT
TAT
+
FFF:F,FFFFFFFFFFFFFFFFFFFFFFFFFFFFFFFFFFFFFFFFFFFFFFFFFFFFFFFFFFFFFF
FFFFFFFFFFFFFFFFFFFFFFFFFFFFFFFFFFFFFFFFFFFFF:FFFFFFFFFFFFFFFFFFFFFFF
FFF
@A00721:190:HGJW7DSXY:4:2157:25500:19319_1:N:0:TCTGCAAG+GCTGCACT
GAACCGGTCTCTAAATCTTTTGAGATTGAGAAGATTGAAGAACACGTCAAGAAGTCGCTCGGCGTTGT
TCTTGAAAACCAAGTTAAGAAGGAGGCTGATCATGGAGTCTTTGTACCTTCTGTCAAACTTCGAGGTT
CAG
+
FFFFF:FFFF,FFFFFFFFFFFFF:FFFFFFFFFFFFFFFFFFFFFFFFFFFFFFFFFFFFFFFFFFF
FFFFFFFFFFFFFFFFFFFFFFFFFFFFFFFFFFFFFFFFFFFFFFFFFFFFFFFFFFFFFFFFFFFFF
FFF
@A00721:190:HGJW7DSXY:4:1212:13241:10332_1:N:0:TCTGCAAG+GCTGCACT
TCCCGGATGTGGATTGAAGGCTGGCTCTAAGGGAGACTCCTTCTTTTTGAATGTTGTAAAGAATTGGG
TTGAGAAGTGTGCTTTACAAGGAGTTAGACTCTTCTTCAAGTTTTTAGATCTTCTTGATAATCTTAAA
GAA

+
FFFFFFFFFFFFFFFFFFFFFFFFFFFFFFFFFFFFFFFFFFFFFFFFFFFFFFFFFFFFFFFFFFFFFF
FFFFFFFFFFFFFFFFFFFFFFFFFFFFFFFFFFFFFFFFFFFFFFFFFFFFFFFFFFFFFFFFFFFFFF
FFF
@A00721:190:HGJW7DSXY:4:2412:26386:30561_1:N:0:TCTGCAAG+GCTGCACT
GTTTCTCTTGGTATTTCTGAAATAGTTTATCAGAGCGTCATTTTCCTTTGGGATCTTTCCTCTGGTAA
GGGAATTATTAAATCATTCTTCTCACTCTTGACGAGAATTATTACTGTTTTCTCTACCCATAACTTCT
TTT
+
FFFFFFFFFFFFFFFFFFFFFFFFFFFFFFFFFFFFFFFFFFFFFFFFFFFFFFFFFFFFFFFFFFFFFF
FFFFFFFFFFFFFFFFF:FFFFFFFFFFFFFFFFFFFFFFFFFFFFFFFFFFFFFFFFFFFFFFFFFFFF
:FF
@A00721:190:HGJW7DSXY:4:2574:16351:8735_1:N:0:TCTGCAAG+GCTGCACT
ACTGGAGTTCAGACGTGTGCTCTTCCGATCTGTTTCAACACTTATCACCACTTTTGAAAGGAAAATAA
TTGGTGATGAAAATACGTATGTCGGTGGGGTTCTCAAGGCTCTCATCGCCACTGGGATTCTAAGGGGT
TCC
+
FFFFFFFFFFFFFFFFFFFFFFFFFFFFFFFFFFFFFFFFFFFFFFFFFFFFFFFFFFFFFFFFFFFFFF
FFFFFFFFFFFFFFFFFFFFFFFFFFFFFFFFFFFFFFFFFFFFFFFFFFFFFFFFFFFFFFFFFFFFFF
FFF
@A00721:190:HGJW7DSXY:4:2238:2022:33238_2:N:0:TCTGCAAG+GCTGCACT
AGGATTCTTCTTTTGGTCCTTACGTCTTAAGTAATTTGGATGTTGTCGAAAACTATCAGGTTCTTGTC
ACGATTCTTAAAAGAAGTTTATTAAAACTTCAGAGTCTCTTGGAAGTC
+
FFFFFFFFFFFFFFFFFFFFFFFFFFFFFFFFFFFFFFFFFFFFFFFFFFFFFFFFFFFFFFFF:FFFF
FFFF:FFF,FFFFFFFFF:FFFFFFFFFFFFFFFFFFFFFFFFFFFFFF
@A00721:190:HGJW7DSXY:4:1113:9607:24737_1:N:0:TCTGCAAG+GCTGCACT
CAATAGCCACAGACTTTCTGGGAAAAGAGACCTCTGTTGGTAGAGGAGTTGAGAGAATTACTCAGATT
CAATCTGGCATCACTGAACGTCTACAACGAGTTAGGGAGTGGCTAGGTTTTAAAAGATCCTTTGCACA
AGC
+
FFFFFFF:FFFFFFFFFFFF,FFFFFFFFFFFFFFFFFFFFFFF:FF:FFFFFFF:FFFFFFFFFFFF
FFFFFFFFFFFFFFFFFFFFFFFFF,FFFFFFFFFFFFFFFFFFFFFFFF:FFFFFFFFF:FFFFFFFFF
FFF
@A00721:190:HGJW7DSXY:4:1235:19162:29387_2:N:0:TCTGCAAG+GCTGCACT
TTTCGACGTCGCGGAAAACGATGAAGATTTCCACACTGTTCGAGAATCTTCTGATGCTGAATTTGAGG
AAAATTTTTCGGAAGTTTTTGGTATAAATCCTGATGGCTCAGTACAGG
+
FFFFFFFFFFFFFFFFFFFFFFFFFFFFFFFFFFFFFFFFFFFFFFFFFFFFFFFFFFFFFFFFFFFFFF
FFFFFFFFFFFFFFFFFFFFFFFFF:FFFFFFFFFFFFFFFFFFFFFF
@A00721:190:HGJW7DSXY:4:1305:13973:25911_2:N:0:TCTGCAAG+GCTGCACT
AAACCTTCTTTTATCATTGCAACAGGACTTTGAGAAGACTGTTGAAATGGACAAAATCTGGGATCATA
GGTATGTGTCACGCTACACTCGAAATATGGGCCCTCATAAGGAAGTAT
+
FFFFFFFFFFFFF:FFFFFFFFFFFFFFFFFFFFFFFFFFFFFFFFFFFFFFFFFFFFFFF:FFF:FFF
FFFFFFFFFFFFFFFF:FFFFFFFFFFFFFFFFFFFFFFFFFFFF:FFFF
@A00721:190:HGJW7DSXY:4:2140:21314:29105_1:N:0:TCTGCAAG+GCTGCACT
CGTCATTACAGTCTGGAAGTTAGATCATTTGTTCAGGAAGATCTCTACAGAGATTATCGAGAGTTGTG
ATCTAGTTCTAAATTTAGGTCGACGGACTTTAGCAACTTTAGATAAATTCTTTCCGGGCTCAATGACC
TTT
+
FFFFF:FFFFFFFFFFFFFFFFFFFFFFFFFFFF,FFFFFFFFFFFFFFFFFFFFFFFFFFFFFF:FFFF
FFFFFFFFFFFFFFFFFFFFFFF:FFFFFFFFFFFFFFFFFFFFFFFFFFFFFFFFFFFFFFFFFFFFFF
FFF

```
@A00721:190:HGJW7DSXY:4:1227:12156:15186_2:N:0:TCTGCAAG+GCTGCACT
CATCGCAAAGAAGACTTTATTAGTTCATGAAGTTCTTACACTTCTCGCGACTAATGTCAGCTTCACTA
CAAAGGCGTTACGTAAAGTAGTTTTCACGCTTTATCTTAAAGCATTAC
+
FFFFFFFFFFFFFFFFFFFFFFFFFFFFFFFFFFF:FFFFFFFFFFFFFFFFFFFFFFFFFFFFFFFF
FFFFFFFFFFFFFFFFFFFFFFFFFFFFFFFFFFFFFFFFFFFFFFFF
@A00721:190:HGJW7DSXY:4:1138:10682:36652_2:N:0:TCTGCAAG+GCTGCACT
ACTTTTTGAATATGACAGAAGCTCGCCATATCAGATTAGAGACATGCCAGTTAGAGTTCTTCTAACCA
GGACTGATGGTGAGATTCTTTTACCTTTTGGAGGTCCAGGAACCTTTA
+
FFFF::::FFFFFFFFF,FF:FF:FFFFFFFFFFFFF:FFFFFFF,FFF:,F:FF:FFFFFFFFFFFF
F:FF:FFF:F:FF:FF:FFF,:FFF:FFF:FF,F,FFF,:FFF,::,F
@A00721:190:HGJW7DSXY:4:1362:16152:7200_2:N:0:TCTGCAAG+GCTGCACT
CCAAGGACGTTTCCGTCGGAGGATGACAGGTGGGAGACCGCTCAATAACACTCTATTGAATCCCTTTT
CCACGATGAAAGCTCTTTATGAGGTGCTCCATGCAAATTTACAGTTCC
+
FFFFFFFFFFFFFFFFFFFFFFFFFFFFFFFFFFFFFFFFFFFFFFFFFFFF:FFFFFFFFFFFFFF
FFFFFFFF:FFFFFFF:FFFFFFFFFFFFFFFFFF,FFFFFFFFFFFF
@A00721:190:HGJW7DSXY:4:1478:24252:28025_1:N:0:TCTGCAAG+GCTGCACT
GTATTTTGATAAATTTGTTGGAATCGATAATGATACTGACGAAGGTTCTATTACCACCCTTGTCATTG
ATACAAACATTCTAGGTGATGCTTTCTTAGAGGCTCGTCTAGCCGCTGAGATTCGTCTTACCTATTTA
CAA
+
FFFFFFFFFFFFFFFFFFFFFFFFFFFFFFFFFFFFFFFFF:FFFFFFFFFFFFFFFFFFFFFFFFFF
FFFFFFFFFFFFFFFFFFFFFFFFFFFFFFFFFFFFFFFFFFFFFFFFFFFFFFFFFFFFFFFFFFFF
FFF
@A00721:190:HGJW7DSXY:4:2161:5891:1360_2:N:0:TCTGCAAG+GCTGCACT
TTTGAGTGATAACGATTTAGTACTGGGAAATGGTGGTAGTCAGTTTACTACTTTTCTTTCCCTCCCCT
TTTTCGATTGGATTCGACGGGGTCTTCGTCTCACGACTAATTTGATAA
+
FFFF:FFFFFFFFFFF,F:FFFFFFFFFFFFFFFFFFFFFFFFFF:FFFFFFFFFFFFFFFFFFFF:FF
FFFFFFFFFF,FFFFFF:FFFFFFFFF:FF:F,F::FF:FFFFFFFFF
@A00721:190:HGJW7DSXY:4:1459:2908:21997_2:N:0:TCTGCAAG+GCTGCACT
TACTTATGAAGGAGGTATTACTTCGGAAGTTCTTAATGTAGGTCTCCTAGTACTTAAGGGTTTATGTT
TCGGTTTCAGCTGGATGGATCTTTTGATGGTATGTTTCACATTGCGTG
+
FFFFFFFFFFFFFFFFFFFFFFFFFFFFFFFFFFFFFFFFFFFFFFFFF:FFFFFFFFFFFFFFFFFFF
FFFFFFFFFFFFFF:FFFFF:FFFFFFFFFFFFFFFFFFFFFFFFFFFF
@A00721:190:HGJW7DSXY:4:1374:3721:4805_1:N:0:TCTGCAAG+GCTGCACT
TTGGGAAGAGATTAAACATAAAATCCAGCGATTCTATTATGTCGCTAAAGCCTGTCCAGTTGTCGATG
ATGTTCGGGCTGTGGGGATTAACTTCCCTCGTGAAACTTACGACGATGTTCTCGCTCGCATTTCACAA
GGA
+
FFFFFFFFFFFFFFFFF:FFFFFFFFFFFFFFFFFFFFFFFFFFFFFFFFFFFFFFFFF:FFFFFFFFF
FFFFFFFFF:FFFFFFFFFFFFFFF,FFFFFFFFFFFFFFFFFFFFFFFFFFF:FFFFF:FFFFFFFFF
FFF
@A00721:190:HGJW7DSXY:4:1253:19099:23171_1:N:0:TCTGCAAG+GCTGCACT
GAGATCTGCCACTTCTAATTTTAGGAAAAGTCGCGAAAAGAAAGTTCATGAGGATGTCTTGGTCGAAA
AATATCGCCGGGAACTTCTAATAGAGGATCCTGATGACATTATTTCAACGGATACCGATCTCAGTTCT
ACC
+
F:,:F,F:,F:,FF,::FFFFFF,:F:F,:F:F,,FF:FF::FFFF,,FFF:FFF,F,FFF:F,,::F
,:F:FF,F,FFF::FFF:FFFFF:FF,FFFFFFFFF::FFFFFFFFF:FFF:FFFFFFFFFFFFFFFF
FFF
```

@A00721:190:HGJW7DSXY:4:1369:16432:21465_2:N:0:TCTGCAAG+GCTGCACT
CAGGAACTGTCTCCACTGATTCGGAATTAGAGAAAGACGACCTTCTTGTGGGGACTGAGACCTCTGAA
TTTTCAGGGTGCCCTGATGAGGACGTTGAAAGGGATAATTTCAACTCT
+
FFFFFFFFFFFFFFFFFF:FFFFFFFFFFFFFFFFFFFFFFFFF:FFFFFFFFFFFFFFFFFFFFF:F
,FFFFFFFFFFFFF:FF,F:FFFFFFFFF:FFFFFFFFFFFFFFFFFF
@A00721:190:HGJW7DSXY:4:1237:27579:24330_1:N:0:TCTGCAAG+GCTGCACT
TGTTTCAAAAGACCAGAAACGTAACAAGAAGAATAAAGGTAGGAAGGGGAGAAAACAGAACAAGCGAG
CGGTTGAAGAGAAAACTGATATGGTTGCGGCTGATCGTACCCCAATTCCTGCTAGAAAGATCACTCTC
GGC
+
FFFFFFFFFFFFFFFFF,FFFFFFFFFFFFFFFFFFFFFFFFFFFFFFFFFFFFFFFFF:FF:FFFFF
FFF:FFFFFFFFFFFFFFF:FFFFFFFFFFFFFFFFFFFFFFFFF:FFFFFFFFFFFFFFFFFFFFFF
FFF
@A00721:190:HGJW7DSXY:4:1307:30020:22733_2:N:0:TCTGCAAG+GCTGCACT
AGAGTTACGTAGTGGTCGTTCTTTTGGTGATTTTATCAAAGAGAAAGTTTCTGCTAGTCGCGACGATA
GTATTTATCGGAGTGATATTAACAAGTTAAACTTCGAACTGACCACTT
+
FFFFFFFFFFFFFFFFFFFFFF:FFFFFFFFFFFFFFFFFFFF:FFFFFFFFFF:,FFFFFFFFFFF,
FFFF:FFFFFFFFF:FFFFFF:FFFFFFFFFFFFFFFFFFFFFFFFFF
@A00721:190:HGJW7DSXY:4:1240:8404:22435_2:N:0:TCTGCAAG+GCTGCACT
TCAGTTTTCCAAAAAGCTATTAATAGCAGTTGCGGGGCTATAATACCGCCTCCAGATTACTCTAAACT
GAATCCACTCTCTTGGGATATAAATGAGTACATCTATAATAGAGTTTT
+
FFFFFFFFFFFFFFFFFFFFFFFFF:FFFFFFFFFFFFFFFFFFFFFFFFFFFFFFFFFFFFFFFFFF
FFFFFFFFFFFFFFFFFFFFFFFFFF,FFFFFFFFFFFFFF:FFFFFF
@A00721:190:HGJW7DSXY:4:1564:9109:26788_1:N:0:TCTGCAAG+GCTGCACT
GGCTTCTCTAAAGAACAGCAACTTTCAACATGAATTAGTTATTGAAAGTCTTGACCCTCACGTCTCAC
TTTATCACCTTGGAGATGGTAAAGTCATTAACAAAGGAAATGTTAAGGCTGATGACTTAAATTTCGCA
TTC
+
FFFFF:FFFFFFFFFFFFFFFFFFFFFFFFFFFFFF:FFFFFFFFFFFFFFFFFFFF:FFFFFFFF
FFFFFFFFFFFFFFFFFFFFFFFFFFFF:FFFFFFFFFFFFFFFFFFFFFFFFFFFFFFFFFFFFFFF
FFF
@A00721:190:HGJW7DSXY:4:2649:10556:7216_2:N:0:TCTGCAAG+GCTGCACT
AAAGAGGGTTTGAAAATTACGGAACCTTTCCTCGTTCTTCATGAAGATCTTCGAGTGTTTTATAGTGA
GTTATTCTTGCGAAGGTTCAAGTCGATTGATTTTGTTGACTACGATCT
+
FFFFFFFFFFFFFFFFFFFF:FFFFFFFFFFFFFFFFFFFFFFFFFFFFFFFFFFFFFFFFFFFFFFF
FFFFFFFFFF,FFFFFFFFFFFFFFFFFFFFFFFFFF,FFFFFFFFFF
@A00721:190:HGJW7DSXY:4:2359:14633:15468_1:N:0:TCTGCAAG+GCTGCACT
AGAAGTTCTTCGCAGTTGAATCTCCACCAGGTGGAGGGAAGACCACTGAACTTATAGCGACTTTTGTC
GAATTTTTCGCAAAAGGTCTTGAAGTACGTATTGTCACTGCTAATCGTAATTCGCGAGAAGAGATAGT
GAC
+
FFFFFFFFFFFFFFFFFFFFFFFFFFFFFFFFFFFFFFFFFFFFFFFFFFFFFFFFFFFFFFFFFFFF
FFF:FFFFFFFFFFFFFFFFFFFFFFFFFFFFFFFFFFFFFFFFFFFFFFFFFFFFFFFFFFFFFFFF
FFF
@A00721:190:HGJW7DSXY:4:2664:8901:7200_2:N:0:TCTGCAAG+GCTGCACT
CACTTCTAGTTCATCGCGGTATTTGTGAAATTAAGACAGACGTGAAAGTTATGATGAGTGATCGTATT
AGAACAGCTGATTCTTATTGCATGAACGTCGCTGAGGTCAAACCTGTG
+
FFFFFFFFFFFFF,:FFFFF:FFFFFFFF:FFFFFFFFFFFFFFFFFFF:FFF,FFFFFFFFFFFFFF
FFFFFFFFFFFFF:FFFFFFFFFFF:FFFFFFFFFFFFFFF::FFFFF

```
@A00721:190:HGJW7DSXY:4:1527:17969:2206_1:N:0:TCTGCAAG+GCTGCACT
CGACGAATTATTCATGATTCACGCTGGTCAGACCCTTTTTCTTACACATCTACTTAAGGCGGCTTATA
CCATAGGTTACGGTGATTCCAATCAAATCGGATTCATCAATCGTATTGATCTCCAAGTCGCAAACTAC
TTC
+
FFFFFFFFFFFFFFF:FFFF:F:FFFFFFFFFFF:FFFFFFFFFFFFFFFFFFFFFFFFFFFFFFFFF
:FFFFFFFFFFFFFFFFFFFFFFFFFFFFFFFFFFFFFFFFFFFFFFFFFFFFFFFFFFFFFFFFFFF
FFF
@A00721:190:HGJW7DSXY:4:2646:32741:31297_2:N:0:TCTGCAAG+GCTGCACT
GATGAGTGTCGTTCTTATCGAAGTCTCAGTTATCGCTGCCCGGCCGACGTTTGTCGCGAACTCTCAGA
GGTTTACGGCAGAAAAATTTCTCCTGCTTGTAATTCGAGAGAGGACAC
+
F:FFFFFFFFFFFF::FFFFFFFFFFFFF,FFFFFFFFFFFFFFFFFFF:F:FFFFFF:FFFFFFFF:
FFFFFFFFFFFFFFFFFFFFFFFFFFFFFFFFF,FFFF:FFFFFFFFFFF
@A00721:190:HGJW7DSXY:4:1216:30418:20572_2:N:0:TCTGCAAG+GCTGCACT
TATTACTTTCACTCAAGGTGAAAAGTTTGAAATTAACAAAGTCCTTGGTAAGAGTAAGGTTAAATGTG
AAGATGCTCAGACTATTCATGAAGTTCAAGGTAATACTTACCAGCACG
+
FFFFFFFFFFFFFF:FFFFFFFF:FFFFFFFFFFFFFFFFFFFFFFFFFFFFFFFFFFFFFFFFFFFF
FFFFFFFFFFFFFFF:FFFFFFFFFFFFFFFFFFFFFFFFFFFFFFFF
@A00721:190:HGJW7DSXY:4:1263:6370:9893_2:N:0:TCTGCAAG+GCTGCACT
TTTTGCATCACAAACACATATTACCGTTGCTCTATCACGACACGTTGAATCTTTACGATACTTTGTTA
TCAATGTAAAGTTAAACGATGCTACTTGTGAGAGGATCAATCACAGTA
+
F,FFFFFFFFFFF:FFFFFFFFFFFFFFFFFFFFF:FFFFFFFFFFFFFFFFFFFFF,FFFFF,FFFFF
FFFFF:FFFFFFFFF:F:FFFFFFF:FFFFFFFF:F,FF:FFFFFF:F
@A00721:190:HGJW7DSXY:4:1475:22354:26835_2:N:0:TCTGCAAG+GCTGCACT
TTAGCACGGGAGGTTGCAGATTCAACCCCTTATATCGAGGCTATCGAGGACGATGAGTATCTTGAAGT
TGTTGACGACAAGCCTCAAGAGGGTAGAGGACCACCTGGATCTTCTAC
+
FFFFFFFFFFFFFFFFFFFFFFFFFFFFFFFFFFFFFFFFFFFFFFFFFFFFFFFFFFFFFF:FFFFFF
FFFFFFFFFFFFFFFFFFFFFFFFFFF:FFFFFFFFFFFFFFFFF:FF
@A00721:190:HGJW7DSXY:4:1568:16568:10488_1:N:0:TCTGCAAG+GCTGCACT
CCGATCTCTTAACGTCACTCGGTAATCTTTCAGCCGAATTAAGTACTAATGAGTTCAAGTCGGTTGTC
GATCGAATTAAGATCAAGGCTAATACCTTTATAGGTAAGAAGCCCCTTGAGCCGCGTAGTCAGCGCGT
TTA
+
F:FFFFFF::FFFFFFFFFFFFFFFFF:FFFFFFFFFFFFFFFFFFFFFFFFFFFFFFFFFFFFFFFFF
FFFFFFF:FFFFFFFFFFFF:FFFFFF:FFFFFFFFFFFFFFFFFFFFFFFFFFFFFFFFFFFFFFFFF
FFF
@A00721:190:HGJW7DSXY:4:2521:1796:21981_1:N:0:TCTGCAAG+GCTGCACT
CCCGATAGGGTACCGAGTCTCCAAGAGAATATTTATTCTTATGAGGCTCGAAACTTCAACGTTTTCAC
AACGGAAAGACATATCGCACCCGAAATTTTTGGGCAGAATGCTCTTAACGCGGTTCTTGAACGGTGTT
TTG
+
F,FFFFFFFFFF,FF:F:FFFF::FFFFFFFF:FFFFFFF:FFFFFFFFFF,FFFF:,:FFFFFFFFF
F,F:FFF,FFFFFFFFFF::FFF:F,:FFF:FFFFF:FFFFFF:FFF:FFFFFFF:FFFF::FFFFFF
FFF
@A00721:190:HGJW7DSXY:4:2242:22589:29904_1:N:0:TCTGCAAG+GCTGCACT
GATAAAGTTATAGGAGACTCAGTTAACCTGAGAGCTCCTCAATTGATAGATTGGGTCAACAATAGGGA
CGCTAGTATACTTCGTAAAGTCGACAGCGAAATGGTCAATATTGATAATTGGACCGACTATATGACCT
ATT
+
FFFFFFFFFFFFFFFFFFFFFFFFFFFFFFFFFFFFFFFFFFFFFFFFFFFFFFFFFFFFFFFFFFFF
```

FFFFFFFFFFFFFFFFFFFFFFFFFF:FFFFFFFFFFFFFFFFFFFFFFFFFFFFFFFFFFFFFFFFF
FFF
@A00721:190:HGJW7DSXY:4:2268:1832:14559_2:N:0:TCTGCAAG+GCTGCACT
AGGGAAGCTAAGGTTAAACTTGATGCTTCAGCCTTTTCGAAGCATAACCCCGCACAGAATATCGTTTA
TCACGCAAAATTTATTAATGCAATTTTCGGACCTATCTTTAAGGTTCT
+
FFFFF:FFFFFFFFFFFFFFFFFFFFFFFFFFFFFFFFFFFFFFFFFFFFFFFFFFFFFFFFFFFFFFFF
FFFFFFFFFFFFFFFF:FFFFF:FFFFFFFFFFFFFFFFFFFFFFFFF
@A00721:190:HGJW7DSXY:4:2402:31602:12759_2:N:0:TCTGCAAG+GCTGCACT
CTTTTACCCAACATTTTTATGTATACAGAGATGACCAGTGACGATTTCTCCCGGAAGCTCTTCTCTCT
CTTAGGAGGTCATGATGACTACTATAAGGGTGAAATCGATTTTTCGAA
+
FFFFFFF::FFFFFFFFFFFFFFFFFFFF:FFFFFFFFFFFFFFF,FFFFF:FFFFFFFFF:FFFF:
F:FFFF,FFF,FFF:F::FFF,FFFFF,,FFFFFF,:F:::FFFF,,F
@A00721:190:HGJW7DSXY:4:1355:32859:4038_1:N:0:TCTGCAAG+GCTGCACT
TAAAGCCTATGAGATAAAGATGTATTCTTATTTAGGCTTCGATGCTGAATTTCTTGAACTTTGGAGTG
CCAGTGAATATTTTTGTCACGCCAGGAGTATCCATAACGATTTGTCATTTACCACCTTTGCTCAACGA
CGA
+
FFFF:FFFFFFFFFFFFFFFFFFFFFFFFFF:FFFFF:FFFFFFFFFFFFFFFFFFFFFFFFFFFFFFF
FFFFFFFFFFFFFFFFFFFFFFFFFFFFFFFFFFFFFFFFFFFFFFFFFFFFFFFFFFFFFFFFFFFFF
FFF
@A00721:190:HGJW7DSXY:4:1631:26928:28056_1:N:0:TCTGCAAG+GCTGCACT
TAACACATTCTTTGGTAATTGTATGATCACTCTTATGATGCTCTCTCTTTATTACGACCTTCGGAAGT
TTAGAGCCGTCGCTGTCGCCGGAGACGATTCGATCCTCTTTTCGGACGTACCGGTCCAGGATTATTCA
GAT
+
FFFFFFFFFF:FFFFFFFFFFFFFFFFFFFFFFFFFFFFFFFFFFFFFFFFFFFFFFFFFFFFFFFFFF
FFFFFFFFFFFFFFFFFFFFFFFFFFFFFFFFFFFFFFFFFFFFFFFFFFFFFFFFFFFFFFFFFFFFF
FFF
@A00721:190:HGJW7DSXY:4:1353:24316:12571_2:N:0:TCTGCAAG+GCTGCACT
TGCTCGCGATTTATTCTTTTTGTCAATGATTACATCTACTTCGTTCCTGATCCTTACAAGATCATCGT
TAAGTTAGGTAAACCCCTAACCGATGTTACTCTTGTAGGACTTTGGGA
+
FFFFFFFFFFFFFF:FFFFFFF:FFFFFFFFFFFFFFFFFFFFFFFFFFFFFFFFFFFFFFFFFFFFFF
FFFFFFFFF:FF:FFFFFF:FFFFFFF:FFFFFFFFFF:FFFFFFFFF
@A00721:190:HGJW7DSXY:4:1641:32136:5697_2:N:0:TCTGCAAG+GCTGCACT
TTAAAGACTATACTAAATATCTCCACTACGAGGTTGTTGTTTGGCGATTAGCTCTTGCGACTAATATT
CGATATTCGATTGAGGGTCGTTATACCTATTCGGCTATCTGTGCTTTG
+
FFFFF:FFFFFFFFFFFFFFFFFFF:FFFF,FFFF::FFFFFFFFFFFFFFFFFFFFFFFFFFF:FFFF
:FFFFFFF,FFFFFFFFFFFFFFF:FFFFF:FFFFFFFFFFFFFFF
@A00721:190:HGJW7DSXY:4:2517:30807:36213_2:N:0:TCTGCAAG+GCTGCACT
TCAAATTTTAAACGATTCCGAGAGGTTTATCCTGATGTGGAGGGAGTTCTTGAAGCATCCCGACCTTT
CCTTGTGAAAATTTTGATGCGGTTTCATAAGATTATCAGTAATCCTGA
+
FFFFFFFFFFFFFFFFFFFFFFFFFFFFFFFFFFFFFFFFFFFFFFFFFFFFFFFFFFFFFFFFFFFFF
FFFFFFFFFFFFFFFFFFFFFFFFFFFFFFFFFFFFFFFFFFFFFFFF
@A00721:190:HGJW7DSXY:4:2222:26585:21887_1:N:0:TCTGCAAG+GCTGCACT
ATTCATGGACCTTCCATCCTGTACCTCTTCTGGAGAGACGTATCTCAAAGATCGACTTCGAGACAGGT
GATATTGTTGAGCGAGAGCGACCAAGATCTAAATTCGGTAACGGTGCGTGGCGCTTTATTTTAAAAAG
CTC
+
FFFFFFFFFFF:FFFFFFFFFFFFFFF:FFFFFFFFFFFFFFFFFFFFFFFFFFFFFFFFFFFFFFFFF

```
FFFF:FFFFFFFFFFFFFFFFFFFFFFFFFFFFFFFFFFFFFFFFFFFFFFFFFFFFFFFFFFFFFF
FFF
@A00721:190:HGJW7DSXY:4:1678:21621:15796_1:N:0:TCTGCAAG+GCTGCACT
GATACTTATCACGAAAGGACTTGAGTCTTTTGATCAGTCTCTGACTTGACCATCTTTCATTTTAAATG
ACGATCATTAAGACTCTCTTTCTTCTTTGTTTCCTTCTTTGCACTGATTTTTCCTCATCAGCTTGTGT
TTT
+
FFFFFFFFFFFFFFFFFFFFFFFFFFFFFFFFFFFFFFFFFFFFFFFFFFFFFFF:FFFFFFFFFFFF
FFFFFFFFFFFFFFFFFFFFFFFFFFFFFFFFFFF:FFFFFFFFFFFFFFFFFFFFF:FFFFFFFFFFF
FFF
@A00721:190:HGJW7DSXY:4:1608:8205:17300_2:N:0:TCTGCAAG+GCTGCACT
TTTTACGATAACAACTATCAGTTGATTAAGACTCTTCCGAAAGGCGAACTTTGGGAATTTGAAGCTCA
GGGTGAAGCACAACGTTTGACCGTGACGAACTGTGGTGTTCTTGATCT
+
FFFFFFFFFFFFFFFFFFFFFFFFFFFFFFFFFFFFFFFFFFFFFFFFFFFFFFFFFFFFFFFFFFFF
FFFFFFFFFFFFFFFFFFFFFFF:FFFFFFFFFFFF:FFFFFFFFFFF
@A00721:190:HGJW7DSXY:4:2107:20283:7874_1:N:0:TCTGCAAG+GCTGCACT
CACTACCACTTATTACGATATTTCTTTAGTCGATGGGTTTTCGACGCCTTTAAGCGTCTTCTGTTCAT
CGCAACCAGATGATATCTTCTATTCGAACTTATGTTTCGATCAGTGTTTCTTCTATCAGGAGGAAAAC
ACT
+
FFFFFFFFFFFFFFF:FFFFFFFFFFFFFFFFFFFFFFFFFFFFFFFFFF:FFFF:F:FFFFFFFFFFF
FFFFFFFFFFFFFFF:FFFFFFFFFFFFFFFFFFFFFFFFFFFFFFFFFFFFFFFFFFFFFFFFFFFF
FFF
@A00721:190:HGJW7DSXY:4:2203:24641:22999_2:N:0:TCTGCAAG+GCTGCACT
TCTTCTAATGTGGCCTGAACTACGTTTCACTTTGTGTGTACTTGTCTCTATTTTAAGTTTTGTCTTCC
TTGTTTGTCTTATTCTATTAATCACTCATTTTAGTGAGATTATTAAAA
+
FFFFFFFFFFFFFFFFFFFFFF:FFFFFFFFFFFFFFFFFFFFFFFFFFFFFFFFFFFFFFFFFFFFF:
FFFFFFFFFFFFFFFFFFFFFF,FFFFFFFFFFFFFFFFFFFFFF:FF
@A00721:190:HGJW7DSXY:4:2313:9941:18740_2:N:0:TCTGCAAG+GCTGCACT
CGACAACAGCAGGGGTTTTCGAATAGTGATAGCCACCCGCCTTACAGAGGGGCAGCAGCATGACCATA
ATTGGTATTGACTTTGGTACCACCTTTTCTACTGTCTGTCTCGCCTCT
+
FFFFFF:FF:FFFFFFFFFFFFFFFFFFFFFFFFFFFFFFFFFFF:FFFFFFFFFFFFFFFFFFFF:F
:FFFFF,FFFFFFFFFFF,FFFFFFFFFFFFFFFFFFFFFFFFFFFFF
@A00721:190:HGJW7DSXY:4:2210:29306:3114_1:N:0:TCTGCAAG+GCTGCACT
CTTAACAACAATGATAGTGAATTTATACCTACAATCATTGGTTTTTCTAATAATGATGACACTATTGT
TTATGGATATGATGCTATTACTCATGATGGTTACGGTCAGGGAGGATTCTCAGTTTACAAGGACCTTA
AGC
+
FFFFFFFFFFFFFFF:F:FFFFFFF:FF:FFFFFFFFFFFFFFFFFFFF:FFFFFF:FFF:FFFFFFF
FFFFFFFFFFFFFFFFFFFFFF:FFFFFFFFFFFFFFFFFFFFFFFFFFFFFFFFFFFFFFFFFFFFFF
FFF
@A00721:190:HGJW7DSXY:4:2539:32000:33270_2:N:0:TCTGCAAG+GCTGCACT
CCGAAAGACGTGCTAAATTAAAACCATTGTACGATGTCGTCTGTCTCGGCGATCACTTTTCACTAAGA
ATTGGTCCTACTTTTGGACAAGGCCAACTTCGTAATCTTCATGAGATC
+
FFFFFFF:FFFFFFFFFFFFFFFF:F:FFFFFFFFFFFFFFFFFFFFFFFFFFFFFFFFFFF:FFFFFF
FFF:FF:FFFFFFF:FFFFF:,FFFFFFFFFFFFFFFFFF:FF,F:FF
@A00721:190:HGJW7DSXY:4:2241:6587:5259_1:N:0:TCTGCAAG+GCTGCACT
TAGGGATTTTGAGAGAGTGAAAGGCTTCAATTGTAGTGGTTTGGTCGTTTCAGTACCCAGTCAGTATA
CCTCTACGCAAAGATATTTCATGAAAACTACAGAGGAACGTACCGGATTACCTGTCTTTCATATTATG
AAT
```

+
F:F,FF:FFF::FFFFFFFF,FFFFFFFFFFFFFFFFFFF:FFF,FFFFFFFFF:F,FFFFFFFFFFF
FFFFF,FFFFF:F:FFFFFF:FFFFFF::,FFFFFFFFFFFFFFFFFFFFFFFFFFFFFFFFFFFFFF
FFF
@A00721:190:HGJW7DSXY:4:1431:9977:25034_1:N:0:TCTGCAAG+GCTGCACT
TTTGCTTCGATGTTGGATGTCAAGAAGGGTTCAGAATGGGATTCCTACGTAGTTTATGACTTTGGTGG
TGGTACTTTCGATACTTCTATCGTCTTACGTTACGGTAATTACTATAGTGTTATTTATTCTGGTGGTG
ATG
+
F:FFFFFFFFFFFFFFF:FFFFFFFFFFFFFFFFFFFFFFFFFFFFFFFFFFFFFFFFFFFFFFFFFF
FFFFFFFFFFFFFFFFFFFFFFFFFFFFFFFFFFFFFFFFFFFFFFFFFFFFFFFF:FFFFFFFFFF
FFF
@A00721:190:HGJW7DSXY:4:2361:28122:33849_2:N:0:TCTGCAAG+GCTGCACT
GGTCGTGACGTTGATGAAGCTATAAGAGCGTTCTTATTACGGCGCACTTCTTTACCCGCCACCACACA
ACTTTCAGTTTCACAACTGAAGGAGGAGGTTTCTGTTACTGGAAAACC
+
FFFFFFFFFFFFFFFFFFFFF:FFFFFFFFFFFFFFFFFFFFFFF:FFFFFFFFFFFFFFFFFFFFFF
FFFFFFFFFFFFFFFFFFFFFFFFF:FFFFFFFFFFFFFFFFFFFFFFF
@A00721:190:HGJW7DSXY:4:1562:7410:32706_2:N:0:TCTGCAAG+GCTGCACT
TCAATAATATACGTCTTGAACTCACTTATGACGATCTAGTGAAATTAACGCGTCCTTTTCTGGAACGT
AGTTTTACTATTATGAACAAAGTTATCAAATATAGTGGCATACAAGGC
+
FFFF,FF:FFFFF,FFF,FFF,,F:F:FFFF:,FF::F:FFFFFF:FF,FF:,:F::FF:FF,FF,,:
F:FFFFF::FF,FF,FF,FFF,FFFF,FFF,F,F:F,,FFF,:FF,F:
@A00721:190:HGJW7DSXY:4:2224:30101:9220_1:N:0:TCTGCAAG+GCTGCACT
TGATTCCGGCAGGTGGTAGCTCATTTCTACCAGGGGCTATTACTCTTGCTGAAACAGCTATTAAAGCT
GTTGAGCGGGTAATTACACCTAAACGAGCTCGATCTGCCATCGCTGAAGGATGTGCACTCTTTTCTGC
TAC
+
:FFFF,F,FF,FFFFFFF,FF:,F:FF::F,FFF,F:FF:,FFF,FFF,F:,,F,:,:FFFFFFFF,F
,FFFFF:FF:FFFFFF:F:FF:FF,FFFFFFFFF,FF,F:FFFFF,,FFF,FFF:F,:FFFFFFFFF:
F,,
@A00721:190:HGJW7DSXY:4:2137:26549:17785_2:N:0:TCTGCAAG+GCTGCACT
TCGATTGTATCAACAGTAACATTACTTCTAATAAAGGTCTCTATCAAGCTCGTATTATCGTCGCTGCT
GGATCTGCGTTACCGTTGAAGGCTAAACTCCAAGGTAATTATGAAGAC
+
FFFFFFFFFFFFFFFFFFFFFFFFFFFFFFFFFFFFFFFFFFFFFFFFFFFFFFFFFFFFFFFFFFFF
F:FF,FFFFFFF:FFFFFFFFFFF:FFFFFFFFFFFFFFFF:FFFF,
@A00721:190:HGJW7DSXY:4:2206:12292:2331_1:N:0:TCTGCAAG+GCTGCACT
GTATGAGGGTGTTTCTAATCGTAACATTAATAATTTCAATCTTCTTAAAAAGAAGATAGAGAGTCATA
CTTTGGGCTATCGTTCAGGTGCATGGGGTTGGATAGCCAATATTGAAGTGGACACCTCTGGTAGTATC
GAA
+
FF,FF:FFFFFFFFFF::F:FFFFFFFFFFFFFF:FFFFFF:FF,FFF:FFFFFFFFFFFFFFFFFFF
FFFFFFFFFFFFFFFFFFFFFFFFFFFFFFFFFF,FFFFFFFFF:FFFFFFF::FFFFFFFFFFFFFF
FFF
@A00721:190:HGJW7DSXY:4:1346:3613:1955_1:N:0:TCTGCAAG+GCTGCACT
TAAGCATACTACTTTTGCTTTGATTACTCTACCGCCTTACAAGTCTAAGGTTAAAGAGTCGATTAAAC
AAGTTGTACTTATCAATGATTTTATCGCTGGTTGTTATCTCGAAAAGTCTCTTCTAAAAGGAACGTCA
GT
+
FFFFFFFFFFFFFFFFFFFFFFFFFFFFFFFFFFFFFFFFFFFFFFFFFFFFFFFFFFF:FFFFFFFF
FFFFFFFFFFFFFF:FFFFFFFFFFFFFFFFFFFFFFFFFFFFFFFFFFFFFFFFFF:FFFFFFFF:F
FF

```
@A00721:190:HGJW7DSXY:4:1531:8115:11819_2:N:0:TCTGCAAG+GCTGCACT
CTAAAATATTTACAAAATCTTACCACTATCTACGAACCTAAAGTTCTGAAGACGTTGCTTGTCGCTTC
CTCCTTTAATGGCACTACCTCGGTACAATCTTACTTGGAACGAAGGCG
+
FFFFFFFFFFFFFFFFFFFFFFFFFFFFFFFFFFFFFFFFFFFFFFFFFFFFFFFF:FFFFFFFFFFF
FFFFFFFFFFFFFFFFFFFFFFFFFFFFFFFFFFF:FFFFFFFFFFFFF
@A00721:190:HGJW7DSXY:4:2120:18078:25504_1:N:0:TCTGCAAG+GCTGCACT
GTTCTGTCTATATTAGGTGAAGCGAAATACGATTTCGAACGTCAACGTTCACCGAGTTTCGTTTCGTA
CCTTGATGCTGACTTTCGTCAATATGATTTGATCTGGTTGCTCAATTCTAAGCGTGTTCCGCTTAGTG
CGA
+
FFFFFFFFFFFFFFFFFFFFFFFFFF:FFFFFFFFFFFFFFFFFFFFFFFFFFFFFFFFFFFFFFFFF
FFFFFFFFFFFFFFFFFFFFFFFFFFFFFFFFFFFFFFF:FFFFFFFFFFFFFFFFFFFFFFFFFFFF
FFF
@A00721:190:HGJW7DSXY:4:2264:31919:27524_1:N:0:TCTGCAAG+GCTGCACT
TACGAACTTGACTCTACTATACGTGACATACTAAATACTAGATATTTGGATGATCTTGATAAAGAACT
TCATGAATTTTCAGAGGATGAAAAATTTTCTTCCTTGGATTTTAATATAAAGAATCTTTACCAGGACT
CCT
+
FFF,FFFFFFFFFFFFFFFFFF:FFF:FFF:FFFFFFFFFFFFFFFFFFFFFFFFFFFFFFFFFFFFF
F:FFFFFFFFFFFFFFFFFFFFFFFFFFFFFFFFFFFFFFFFFFFFFFFFFFFFFFFFFFFFFFFFFF
FFF
@A00721:190:HGJW7DSXY:4:2326:15374:22106_1:N:0:TCTGCAAG+GCTGCACT
TGATCGTAAGAAGTTTATAAGGAACATATCCATTTGTTGTAATAGGGTCATCAACTTAGAACTCGAAA
CTCTAAGTGGTAATACTGTCTTTCCAAGCTTACCTGTATCTTCAATGAAATTGGACTCTTCGATAGGG
GAC
+
FFFFFFFFFFFFFFFFFFFFFFFFFFFFFFFFFFFFFFF:FFFFFFFFFFFFFFFFFFFFFFFFFFFF
FFFFFFFFFFFFFFFFFFFFFFFFFFFFFFFFFFFFFFFFF:FFFFFFFFFFFFFFF:FFFFFFFFFF
FFF
@A00721:190:HGJW7DSXY:4:2156:10538:8343_1:N:0:TCTGCAAG+GCTGCACT
GGAAGATCTGTTATCGACGGTTTATCGTTTACGAAGTATCACTCCACCGGTGAATGGTTTAAGAATAC
TATAAATAGAATAGCTATTACTACTAATGGCGTTGGCCTAGTGGATGTTATTAGTGACATCTGGTCAA
TTT
+
FFFFFFFF:FFFFFFFF,F:FFFFFFFFFFFFF:F,:FFF:FFFFFFF,FFFF:,:FFFFFFFFFFFF
F:F:FFF:FFF,F,FFFF:FFFFFFF,:FFFFFFF:FF:,FFFFFFFFF:FFF,FFFFFFFFFFFFF,
FFF
@A00721:190:HGJW7DSXY:4:1335:21133:15577_1:N:0:TCTGCAAG+GCTGCACT
TGGTCTTTATTCTATTATTGATTTTTATGAGATTGATACGTTAACAGAAGATGATGTTAGAGTAGAAT
TCCTGAAGAGTCCTTCCGTAACCAACTTCTTGACAATTTTCTTCAATCCTTCGATAATAGTTAAATCT
AGG
+
FFFFFFFFFFFFFFFFFFFFFFFFFFFFFFFFFFFFFFFFFFF,FFFFFFFFFFFFFFFFFFFFFFFF
FFFFFFFFFFFFFFFFFFFFFFFFFFFFFFFFFFFFFFFFFFFFFFFFFFFFFFFFFFFFFFFFFFFF
FFF
@A00721:190:HGJW7DSXY:4:1376:21025:13917_2:N:0:TCTGCAAG+GCTGCACT
TACACAATTAAGAAATAATCCTACATTGACTGATTATCTAAAGATTGAAGGAAAATTTTATCCTCGTT
TAACTTACTTAGACTTTCCTACGATACAAATGATAACTTCTAATTTAC
+
FFFFFFFFFFFFFFFFFFFFFFFFFFFFFFFFFFFFFFFFFFFFFFFFFFFFFFF:F,FFFFFFFFFFF
FFF:FFFFFFFFFFFFFFFFFFFFFFFFFFFFFFFFF,F:FFFFFFFFF
@A00721:190:HGJW7DSXY:4:1602:24560:28557_1:N:0:TCTGCAAG+GCTGCACT
GGGATGCATGGTGTTATCTCCTCAGGAGTCCCGGCATTTGATAATAATGACGAGACTTTCGTCATAAT
```

CGCTGCATATTACGGATTACACGGTACCAGCGCATCTCGTGTCGAGCCTCGACCCGATTATATGGTAT
TAA
+
FFFFFFFFFFFFFFFFFFFFFFFFFFFFFFFFFFFFFFFFFFFFFFFFFFFFFFFFFFFFFFFFFFFFFF
FFFFFFFFFFFFFFFFFFFFFFFFF:FFFFFFFFFFFFFFFFFFFFFFFFFFFFFFFFFFFFFFFFFFFF
FFF
@A00721:190:HGJW7DSXY:4:2206:26268:5995_1:N:0:TCTGCAAG+GCTGCACT
AATTTATAGAAGATTATTTTGACAGAATCAATAAGAATCATCGATTCTCGATTCGTAGAGCTTATCTA
GGTACTCTCATTTACTATGTTGATTTAGTCTACATTTATGCTGATCTAAAATTTCGATCTAGATGGAT
CC
+
FFFFFF:FFFF,FF:FFF,FFFFFFFF:FFFFFFFFFFFFFFF:FFFFFF:FFFFFFFFFF:FFFFF
FFFFFFFFFFFFF,FF,F:FFF:FFFFFFFF,FFFFFFFFFFFFFFFFFFFFFFFFFFFFFFFFFFFFFF
FF
@A00721:190:HGJW7DSXY:4:2355:24659:22686_1:N:0:TCTGCAAG+GCTGCACT
TTCTAGAATTCTTAATTATAATATTCAACCTCAAAAGTTACGTGTTTACAGTAACGAGATCACACATT
TTGAGAACTAACTATGTTATGTTCTCTGATCATTTTGACAATCTCTTCTTATAATAAATAAATAAATA
ATA
+
FFFFFF:FFF,FFFFFFFFFFFFFFFFFFFFFFFFFFFFFFFFFFFFFFFF:FFFFF:FFFFFFF:FFF
FFFFFFFFFFFFFFFFFFFFFFFFF:FFFFFFFFFFFFFFFFFFFFFFFFFFFFFFFFFFFFFFFFFFFF
FFF
@A00721:190:HGJW7DSXY:4:2357:10601:24956_2:N:0:TCTGCAAG+GCTGCACT
CAAATAGTGGAAAGGCTCCAGTCAACACTAGTTCGAGTGCTGCTTTGGATTACGATGACGTGAGAATA
GGTGAAGGGATAGTTTTTACCTCGACCAACATTAAGGCGGCGGCGACT
+
FFFF:FFFFFFFFFFFFFFFFFFFFFFFFFFFFFFFFFFFFFFFFFFFFFFFFFFFFFFFFFFFFFFFFF
FFFFFFFFF:FFFFFFFFFFFFFFFFFFFFFFFF,FFFFFFFFFFFF
@A00721:190:HGJW7DSXY:4:2433:18873:10692_2:N:0:TCTGCAAG+GCTGCACT
CTTCACCCAAAATGAAGGACACTTCGAAACTGAATAATGATGCACTACAAACAAGTATCGCTAATCAT
CTGAAAAAGGCTCTAAAGGACACTATACAATATCCAGACGACGACGAA
+
FFFFFFFFFFFFFFFFFFFFFFFFFFFFFFFFFFFFFFFFFFFFFFFFFFFFFFFFFFFFFFF:FFFFFF
FFFFFFFFFFFFFFFFFFFFFFFFFFFFFFFFFFFFFFFFFFFFFFFF
@A00721:190:HGJW7DSXY:4:2329:31141:25989_2:N:0:TCTGCAAG+GCTGCACT
CACCACATTTTGGTCAAGTCAATCCGTTGAGAAAATTTCTTAGAGGATATTCGCCATTGTGGTACGAA
TTGGTCCAACATTTACCACCAAATTCTTTTGATACAACTCTAGCCTTC
+
FFFFFFFFFFFFFFF:FFFFF,FFFFFFFFFF:::FFFFFFFFFFFFFFFFFFFFFFFFFFFFFFFFF,
FFFFFFFFF:FFFFFFFFF:FFFFFFFFFFFFF:FFFFFF:FFFFF:F
@A00721:190:HGJW7DSXY:4:2378:29713:2597_2:N:0:TCTGCAAG+GCTGCACT
GCTTGCTATTCACCAGATTGGTTCGAACCCGTTGATGCTATGTCAGAAGAAGCTATTCAAGCTTGTCG
TTTAAAGACTGCAGAGGCTGTCAAACAAGCCCAGACTCAAGGAGTCCA
+
FFFFFFFFFFFFFFFFFF:FFFFFFFFFFFFFF:FFFFFFFFFFFFFFFFFFFFFFFFFFFFFFFFFFFF
FFFFFFFFFFFFFFFFFFFFFFFFFFFFFFFFFFFFFFFFFFFFFFFF
@A00721:190:HGJW7DSXY:4:2169:17418:12994_2:N:0:TCTGCAAG+GCTGCACT
GGTGATAAAAATTATTTAGAGATCTTTTCTACTTACAAAGGACAATTCAGTTTCATTAATTCTACGGA
AAAGTCCGGAGTATATTGTATGTTAATAGATAGTGGGATAATAGTTCT
+
FFFFFFFFFFFFFF:FFFFFFFF:FFFFFFFFFFFFFFFFFF:FFFFFFFFFFFFFFFFFFFFFFF,FF
FFFFFFFFFFFFFFF:FFFFFFFFFFFFFFFF:FFF:FFFFFF:FFFF
@A00721:190:HGJW7DSXY:4:1106:13612:29262_2:N:0:TCTGCAAG+GCTGCACT
TGCTATTGATTATGTAACCGATAGGAGTTGTATAGTTGAAGATGTGGCTTTAGACGATAATCCTTTAA

GGACTATGTTGACTGACGAAGATTTTCCCACAGATTTGTCTACGTTGC
+
FFFF:FFFFFFFFFFFFFFFF:FFFFFFFFFFFFFFFFFFFFFFFFFFFFFFFFFFFFFFFFFFFFFF:
FFFFFFFFFFFFFFFFFFFFFFFFFFFFFFFFFFFFFF,FFFFFFFFFF
@A00721:190:HGJW7DSXY:4:2137:11939:11741_2:N:0:TCTGCAAG+GCTGCACT
CTATATACATTATAAAATATAAAAATAAGGGAGTCATAACTTTTGTTACCAAATTCTTGGCTTTCAAC
GAAGTCTCTGATTTTATTAATCATTACTTTAACGATCTCTCGGTCATT
+
FFFFFFFFFFFFFFFFFFFFFFFFFFFFFFFFFFFFFFFFFFFFFFFFFFFFFFFFFFFFFFFFFFFF
FFFFFFFFFFFFFFFFFFFFFFFFFFF:FFFFFFFF:FFFFFFFFFFFF
@A00721:190:HGJW7DSXY:4:1432:26422:8108_1:N:0:TCTGCAAG+GCTGCACT
TTGATCTACGTCGTTTAACTGTCGGAACAACCTTTACTTAGAATGACTCTATCTTTTCAGACACTTCT
TATATTATTTACAATAGTTAATATCATATTAGTAATACTCACTATCGTTTTCTGCTTTTTCTTAGCTA
ATA
+
FFFFFFFFFFFFF:FFFFFFFFF:FFFFFFFFFFFFFFFFFFFFFFFFFFFFFFFFFFFFFFFFFFFF
FFFFFFFFFFFFFFFFFFFFFFFFFFFFFFFFFFFF:FFFFFFFFFFFFFFFFFFFFFFFFFFFFFFF
F:F
@A00721:190:HGJW7DSXY:4:2141:13006:12774_2:N:0:TCTGCAAG+GCTGCACT
ATGAGTACGAAAACCAACTCTTCGTTACGAGACCTGATGGTAGAATAGATTTAGTCTCTACACCCATC
TCAACGAGGATAAAGCGAGTTTTCCGTCCTGAACGTAATACACGAAGA
+
FFFFFFFFFFFFFFFFFFFFFFFFFFFFFFFFFFFFFFFFFFFFF:FFFFFFFFFFFFF:FFFFFFFF
FFFFFFFFFFFFFFFFFFFFFFFFFFFFFFFFFFFFF:FFFFFFFFFFFF
@A00721:190:HGJW7DSXY:4:1509:16260:11052_1:N:0:TCTGCAAG+GCTGCACT
AAACGACTCGAAACCGAGACGCCGTAGATCAAAGTCCCTTCTAACTTTAGATTATGAATCCTGCCTTT
ACTCATTTTATAGCAGTTATTTATGTTGTACTTTTTATAATTTATAACCTTCTT
+
F,FFFFFFFFF:FFFFFFFFFFFFFFFFFFFFFFFFFFFFFFFFFFFFFFFFFFFFFFFFFFFFF:FF
FFFFFFFFFFFFFFFFFFFFFFFFFFFFFFFFFFFFFFFFFFFFFFFFFFFFFF
@A00721:190:HGJW7DSXY:4:1110:3694:15186_2:N:0:TCTGCAAG+GCTGCACT
TTGTAACGATCATTCTTTCGAGTTTTGTCATTCTTATTCTTTTATTCCTTTAACTGTGTATGAGGAGG
ATTTATAATGCTTTTCGATAGTGATTGTTATCAATTTAAATACATTAC
+
FFFFFFFFFFFFFFFFFFFFFFFFFFFFFFFFFF:FFFFFFFFFFFFFFFFFFFFFFFFFFFFFFFFF
FFFFFFFFFFFFFFFFFFFFFFFFF,FFFFFFFFFFFFFFFFFFFFFF
@A00721:190:HGJW7DSXY:4:2340:10375:9690_2:N:0:TCTGCAAG+GCTGCACT
CTTTACTCACAGACTCCGAAAATGGTTTCAAATGAGGTCAAAGCTCTATTTATTAAACGAACTATTAT
CCGATTCTTCGAGGGTATTACTACCACTGATCTGCCGACTTTAGTCAA
+
FFF:FFFFFFFFFFFFFFFF:FFFFFFFFFFFFFF:FFFFFFFFFFFFFFFFFFFFFFFFFFFFFFFFF
FFFFFFFFFFFFFF:FFFFFFF:FFFFFFFFFFFFFFFFFFFFFFFFFF
@A00721:190:HGJW7DSXY:4:2254:32000:11913_2:N:0:TCTGCAAG+GCTGCACT
TTCCTTCGATGTTTATATTTTTCTATAAGGGAAAATAGCAATAATCAAATTTCGACAAATTTTAGAAC
TTTATTTCAGAACTCTCTCCGATCACCTTGGTGTGATCTCCCGGAGGA
+
FFFFFFFFFFFFFFFFFFFFFFFFFFFFFFFFFFFFFFFFFFFFFFFFFFFFFF:FFFFFFFFFFFFF:
FFFFFFFFFFFFFFFFFFFF:FF:FFFF:FFFFFF:FFFFFF:FFFFF
@A00721:190:HGJW7DSXY:4:1565:6054:28322_2:N:0:TCTGCAAG+GCTGCACT
ATGTATGATGATAATCTACTATTTTTAAATAGTGATATTCAGCGTATACTTGAATACTTTATCAAAGA
TTTGGCTTGGTCTATTTACCGCAAAAAGTTGGAAGAGGTCTATGATAT
+
FFFFFFFFFF:FFFFFFFFFFF:FFFFFFFFFFFFFFFFFFFFFFFFFFFFFFFFFFFFFFFFFFFFF:
FFFFFFFFFFFFFFFFFFFFFFFFFF:FFFFFF,:FFFFFFF,FFF:F

```
@A00721:190:HGJW7DSXY:4:2565:12970:26772_2:N:0:TCTGCAAG+GCTGCACT
TATACTTTAAATAGCACCGATTATAACTTGACTGTTGATAATATTACAATAGCTCTTTCTCAGAAGAT
TAGAGAAGTAACCGGTGAACACGTCGGTAGCATTGATTTACTTTTACC
+
FFFFFFFFF:FFFFFFFFFFFFFFFFFFFFFFFFFFFFFFFFFFFFFFFFF:FFFFFFFFFFFFFFFF
FFFFFFFFFFFFFFFFFFFFFFFFFFFFFFFFFFFFFFFFFFFFFFFF
@A00721:190:HGJW7DSXY:4:1539:30002:30561_2:N:0:TCTGCAAG+GCTGCACT
TTTCGAAAAGAAACCTATAAGTTACAAACGATTCTTCGACTATATTAAAGAAATAAAAATACTTCAAG
TACCTGAACTTTACATTTTTAATGGTATGGTATAATGTGTCTTACAAA
+
FFFFFFFFFFFFFFFFF:FFFFFFFFFFFFFFFFF,FFFFFFFFFFFFFFFFFFFFFF:FFFFFFFFF
FFFF,,FFF:FFFFFFFFFFFFFFFFFFFFFFFFFFF,:FFFFF:FFFF
@A00721:190:HGJW7DSXY:4:2215:27516:2550_2:N:0:TCTGCAAG+GCTGCACT
CTCTCGGTAATGAGAGTGTATTCCTTAATATTCTAGTAACCGCTATGTGGGCATTACTAGGATTTATC
ACACTCCTAAATTTGATAGCAATTTATGTTTTAAATGCTAGGAAAATT
+
FFFFFFFFFFFFFFFFFFFF:FFFFFFFFFFFFFFFFFFFFFFFFFFFFFFFFFFFFFFFFFFFFFFF
:FFFFFFFFF:FFFFF:FFFFFFFFFFFFFFFFF:FFFFFFFFFFFFF
@A00721:190:HGJW7DSXY:4:1610:7717:1673_1:N:0:TCTGCAAG+GCTGCACT
TGTATAGTTCAAGGAGTACTTAATTCAATAGCTTTAGTATTATTTACTATCGCCTTTATTGAAAATTG
TATAAGAGTTAATTGCTTACACAAAAGTGACGCGCGAAGGAATCTTAATTGATTGAAGCTAGGCGTTG
GGG
+
FFFFFFFFFFFF:FFFFFFFFFFFFFFF:FFFFFFFFFFFFFFFFFFFFFFFFFFFF:FFFFFFFFFF
FFFFFFFFFFFFFFFFFFFFFFFFFFFFFFFFFFFFFFFFFFFF,FFFFFFFFFFFFF,FFFFFFFFFF
FFF
@A00721:190:HGJW7DSXY:4:1362:22399:29042_1:N:0:TCTGCAAG+GCTGCACT
CTCCCCAAACATAACATACCATAACCTACCATGACCAATCATCGCTCCAACTGGCACGCCAGACGTAG
GGTCAAACCTACTACCGCCTGTCGGCAGATCGGCACCACTGGTCTCTACGTTCAGGGACCCGCTGTGC
AGA
+
FFFFFFFFFFFFFFFFFFFFFFFFFFFFFFF,FFFFFF,FFFFFFFFFFFFFFFFFFFFFFFFFFFFFF
FFFFFFFFFFFFFFFFFFFFFFFFFFFFFFFFFFFFFFFFFFFFFFFFFFFFFFFFFFFFFFFFFFFFF
FFF
@A00721:190:HGJW7DSXY:4:1602:27832:31782_2:N:0:TCTGCAAG+GCTGCACT
TAGATCTGCACAGAAGAAGGCTGAAGGTCGTTACCGGACGACCACTCAAAGGAAACGGGCTGTTGCCC
AGAGGAAAGCAGCGACCACCGAGGTCGCTCGTAACATTGACGGCAGTC
+
,FFFFFFFFFFF,FFFFFFFFFFFFFFFFFFFFFFFFFFF:FFFFFFFFFFF,::FFFF:FFFFFFFFF
FFFFFFF,FFFFF:FFFFFFFFFF:FFF:FFFFFFF,F:FF::FFFFF
@A00721:190:HGJW7DSXY:4:1427:4227:29356_1:N:0:TCTGCAAG+GCTGCACT
TGTGGGAACTGAGGCAGTACCTCAGTCAAAAGTCACATTCACCACTCCCATACCAATCAATGATGTGA
CTATACAGTTCGGCCATTTCAAGAGTGGTCAATACGTCGTGGGAGGTACTGAAGCCTTCCCCTCCGCT
GGC
+
FFFFFFFFFFFFFFFFFFFFFFFFFFFFF,FFFFFFFFFFFFFFFFFFFF:FFFFFFFF,,FFFFFFF
FFFFFFFFFFFFFFFF,FFFFFFFF:FFFFFFFFFFFFFFFFFFFFFFFFFFFFFF:FFFFFFFFFFF
FFF
@A00721:190:HGJW7DSXY:4:1147:2031:12493_2:N:0:TCTGCAAG+GCTGCACT
CCAAGCCAATCAATAGTTCTTCTGACCAGAATGAAAAGGGGTTTACCAGGGTAGTCTCTAAGTCTACC
AGGAAGAGGGGAAATGTCCCCAAAACTGAGAAGAATAAAGGGAAAGGT
+
FFFFFFFFFFFFFFFFFFFFFFFFFFFFFFFFFFFFFFFFFFFFFFFFFFFFFFFFFFFFFFFFFFFF
FFFFFFFFFFFFFFFFFFFFFFFFFFFFFFFFFFFF,:FFF:FF:FFFF
```

```
@A00721:190:HGJW7DSXY:4:2350:26756:21778_2:N:0:TCTGCAAG+GCTGCACT
GAAGGGGAAATTACCCCTAATTCTGAAGGAAGATCTCCCACCATCTGGGGTTGGGAATAAGAAATCTG
CACGGGCCAAAGAGGCCGCTGCTACGAAGTCGCTGAAGGCGGCTTGGG
+
FFFFFFFFFFFFFFFFFFFFFFFFFFF:F:FFFF:FFFFFFFFFFFFFFFFFFF:FFFFFFFFFFF
FFFFFFFFFFFFFF:FFFFFFFFFFFFFFFFFFFFFFFF:FFFFFFFFF
@A00721:190:HGJW7DSXY:4:2521:21567:36338_2:N:0:TCTGCAAG+GCTGCACT
TGTAAAGTTGCGCCTGCCGACCCCATCTTTGAGACCAAGACTCTCTACAGTAGGTTCGTGCCTACTAT
GAGCAAATCTGTTGAATTCGTCACTGAGGACGAAGGTGAGAGGACTTT
+
FFFFFFFFFFFFFFFFFFFFFFFFFFFFFFFFFFFFFFFFFFFFFFFFFFFFFFFFFFFFFFFFFFFF
FFFFFFFFFFFFFFFFFFFFFFFFFFFFFFFFFFFFFFFFFFFFFFFFF
@A00721:190:HGJW7DSXY:4:2443:2474:28260_1:N:0:TCTGCAAG+GCTGCACT
CTGAGAGCAGTCGCTGGGGAAAGAGAATTGCGTGGGTTGAAGGCCGACGCTTCGAGCTTGAGTGTGAC
GAGGCCGGTGACGTTTTTGTCAACGGCGTTTGGAGGATTTTATCCGCGCTTAACTATGGGGCGAAGAT
CCC
+
FFFFFFFFFFFFFFFFFFFFFFFFFFFFFFFFFFFFFFFFFFFFFFFFFFFFFFFFFFFFFFFFFF:F
FFFFFFFFFFFFFFFFFFFFFFFFFFFFF:FFFFFFFFFFFFF:FFFFFFFFFF:FFFFFFFFFFFFFFF
FFF
@A00721:190:HGJW7DSXY:4:1619:16170:7639_2:N:0:TCTGCAAG+GCTGCACT
GTTGGCATATTTATGCCGACGATAATAAGCCTGTCACCCTTAAGCACCGAGGTAAGCTCGGGACTTAC
AAGAGAGAGTGTGAGAGAGTCGCTCTCACTATTTTTGCCCAGAATGCG
+
FFFFFFFFFFFFFFFFFFFFFFF:FFFF:F,F:FFFFFFFFFFFFFFFFFFFFF:FFFFF:FFFFFFF
FF:,FFFFFFFFFFFFFFFFFFFFFFFFFFFFFFFFFFFF:FFFFFFF
@A00721:190:HGJW7DSXY:4:1668:16098:19789_1:N:0:TCTGCAAG+GCTGCACT
GTGACTGGAGTTCAGACGTGTGCTCTTCCGATCTGTTTTTGTTATATTCACTGGTTCTGGGAGGCTGC
TTTCCTCCATAATTACAACCTCTCTTTAGAGAGCATTAGAAGGAAGTTGGGCAAATTCCCACATATGG
TAG
+
FFFFFFFFFFFFFFFFFFFFFFF:FFFFFFFFFFFFFFFFFFFFFFFFFFFFFFFFFFFFFFFFFFFF
FFFFFFFFFFFFFFFFFFFFFFFFFFFFFFFFFFFFFFFFFFFFFFFFFFFFFFF:FFFFFFFFFFFF
FFF
@A00721:190:HGJW7DSXY:4:1360:31675:18208_2:N:0:TCTGCAAG+GCTGCACT
ACTTTTCTTGAAAATTTTTCCTTTCCTCCGTCATTGGGGATTTCGATGTGAGAAGGTGGAAGGTGTCA
AGGGTCTTCTTCATGCTGATCTTTCAGGACCTAATCACATCATGATTG
+
FFFFFFFFFFFFFFFFFFFFFFFFFFFFFF::,FFFFFFF:FFFFFFFFFFFFFFFFFFFF,FFFFFF
FFFFFFFFFFFFFFFFFFFFFFFFFFFFFFFFFFFFFFFFFFFFFFFFF
@A00721:190:HGJW7DSXY:4:1447:29541:17394_2:N:0:TCTGCAAG+GCTGCACT
ATCGAGTTGGAGGGAGGAAGTTCTCCGAAATTAAAGACGGGGGGATTAAGAGTCCAGCTGTAACTGTC
ATTTCAGAGAGGGGAGCTCTGAAAAGGAAACTTCGTCAGAAGATGAGG
+
:FFFFFFFFFFFFFFFFFFFFFFFFFFFFFFFFFFFFFFFFFFFFF,FFFFFFFFFFFF:FFF:FFF:F
FFFFFFFFFFFFFFFFFFFFFFFFFFFFF:FFFFFFFFFFFFFFFFFFF
@A00721:190:HGJW7DSXY:4:1228:19388:27054_2:N:0:TCTGCAAG+GCTGCACT
GAGTTTGAGGAAGGCTCTAGAGAAGATCTCACCACCTGGAGAAGGCAAGTGGAGAGTCCAGAAGTTGA
TGAAGAAGGATTATTTGAAGCAGGGGTTACCACTTCCTAAGGTGGATG
+
FFFFFFFFFFFFFFFFFFFFFFFFFFFFFFFFFFFFFFFFFFFFFFFF:FFFFFFFFFFFF:FFFFFFF
FFFFF:FFFFFFFFFFFFFFFFFFFFFFFFFFFFFFFFFFFFFFFFF:FF
@A00721:190:HGJW7DSXY:4:2230:8549:1141_2:N:0:TCTGCAAG+GCTGCACT
TTCTTTGGAGTTTTAGATAATCTTTTGAGAAGGGGTCATAGAGCCAGCAAGAGAAGCTTATATGCTGG
```

TTTAAGTGATGATTATCACTCCTTGAGAAGACAAGCCACATTAAGATA
+
FFFFFFFFFF:FFFFFFFFFFFFFFFFFFFFFFFFFFFFFFFFFFFFFFFFFFFFFFFFFFFFFF
FFFFFFFFFFFFFFFFFFFF:FFFFF:FFFFFFF:FFFFFFFFFFFFF
@A00721:190:HGJW7DSXY:4:2208:16116:13683_2:N:0:TCTGCAAG+GCTGCACT
CTTGGGGTTTCGAATGTCATTAGTGCCTTACGATTGAAAGAGGTCCTTTTGAGTATCGACAGATCTTT
CGATCTTGAGGACGTACATTTAACGTGGCAGAATTCGGTAACTTACCA
+
FFFFFFFFFFFFFFFFFFFFFFFFFFFFFFFFFFFFFFFFFFFFFFFFFFFFFFFFFFFFFFFFFFFF
FFFFFFFFFFFFFFFFFFFFFFFFFFFFFFFFFFFFFFFFFFF:FFFF
@A00721:190:HGJW7DSXY:4:1677:24189:26349_2:N:0:TCTGCAAG+GCTGCACT
GGGAAGTTGTGGGATTTAACTTTGACTCAACTACTCCGATCAGATCCGGATGGTTTGATTGGTATGGA
CTACTCGACTTTTGATCTGGAGTTGTCTAGGGTCATGGCAGATACTAC
+
FFFFFFFFFFFFFFFFFFFFFFFFFFFFFFFFFF:FFFFFFFFFFFFFFFFFFFFFFFFFFFFFFFFF
F:FFFFFFFFFFFFFFFFFFFFFFFFFFFFFFFFFFFFFFFFFFFFFF:
@A00721:190:HGJW7DSXY:4:1161:7817:15123_2:N:0:TCTGCAAG+GCTGCACT
AATATGCAGGTCAAAGATTCTGTAGCTCTGAGGGCGGTTGAGGGAGCCGTTTTGGCCGACTTTAACTA
TTCTAAGAGTCTTTATGATTCAAAACCTATCGTTGTCGTACCTTACAA
+
FFFFFFFFFFFFFFFFFFFFFFFFFFFFFFFFFFFFFFFFFFFFFFFFFFFFFFFFFFFFFFFFFFFF
FFFFFF:FFFF:FFFFFFFF,FFFF,::FF::F:,FF:FF,F,FF,FF
@A00721:190:HGJW7DSXY:4:2275:9579:25723_2:N:0:TCTGCAAG+GCTGCACT
GCGGCTTATTGTCGCGCAGTTTCCAGAATTGCGGTTGAAGTTCACCCATTCTATCTTGAATGCTCACG
CCGTCGCAGCCATATCGAGACTATGTTATAATCACTTGTTAAAGTACA
+
FFFFFFFFFFFFF:FFFFFFFFFFFFFFFFFFFFFFFFFFFFFFFFFFF:FFFFFFFFFFFFFFF:FF
:FFFFFFFFFFFF:FFFFF::FFFFFFFFFFFFFF,FFFF:FFFFFFF
@A00721:190:HGJW7DSXY:4:2168:12988:2472_2:N:0:TCTGCAAG+GCTGCACT
TTAGATATTATTGATATTGGAGGCGATATTCGTTTTCATATCCAAAACTCCTTGAATGGAGTACATAT
TTGCAACCCTATTCTTGATGTGAAAGATGCATCGAGGTATACGTCTAG
+
FFFFFFFFFFFFFFFFFFFFFFFFFFFFFFFFFFFFFF:FFFFFFFFFFFFFFFF:FFFFFFFFFFFF
FFFFFFFFFF:FFFFFFFF:,FFFFF:FFFFF,FF::FFFF:FFFFFF
@A00721:190:HGJW7DSXY:4:1173:32325:24314_2:N:0:TCTGCAAG+GCTGCACT
TCACGAGACGTCATGAAAGCTATCGGAGGGAAGCAAAATGTTTCGTTCTGTTACAAGAACGCTTTGGA
TTGTGACCATAAAGCCTCAGTTGCGATAGCTGTAGAGGTATACGATAT
+
FFFFF:FFFFFFFFFFFFFFFFFFFFFFFFFF:FFF:FFFFFFFFFFFFFFFFFFFFFFFFFFFFFFF
,FFFFFFFFFFFFFF:FFFFFFFFFF,FFFFFFFFFFFFFFFFF:FFF
@A00721:190:HGJW7DSXY:4:1405:2058:16924_2:N:0:TCTGCAAG+GCTGCACT
CTTAAGAGAGAGATTGATGTATGTCATTTCGTCTTAGTTTGTCCCGGCGAATTATTGGACACGATGAA
AAATGACATACCCGTTCCTGACCTTGGTATCGTTATCCGTCATGGAGA
+
FFFFFFFFFFFFFFF:FF:FFFFFFFFF:FFFFFFFFFFFFFF::F:F:FFFFFFFF:FFFFFFF:FF
FF:FFFFF:FFFFFFFFFFFF:FFFFFFFFFFFFFFFF::FFF:FFFF
@A00721:190:HGJW7DSXY:4:1620:30346:24706_2:N:0:TCTGCAAG+GCTGCACT
GTTATTCTCACAACAAAGATAAGATTTTATCTTTCTTCAAACACCCTGTTTTCCTTAAGGGCGGACAA
TTGTTCCATTGTGAACTCTTGGGGACTAGATTGAGCATTAATGAGTTC
+
FFFFFFFFFFFFFFFFFFFFFFFFFFFFFFFFFFFFFFFF:FFFFFFFFFFFFF:FF:FFFFFFFFFFF
FFFFFFFFFFFFFFFFFFFF:FFFFFFFFFFFFFFFFFFF:FFFFFFF
@A00721:190:HGJW7DSXY:4:1149:10664:20275_2:N:0:TCTGCAAG+GCTGCACT
TGGACAATTCAGAATACCTATTTCCGTACCAAGAGCCACTGCCAATCTCATCGCTATACGCATACCTG

```
ATTGGAAGAAGCGAGGTTCGTTCCTCAATAAAAATTTTTCTCAAGAGA
+
FFFFFFFFFFFFFFFFFFFFFFFFFFFFFFFFFFFFFFFFFFFFFFFFFFFFF:FFFFFFFFFFFFFFF
FFFFFFFFFFFFFFFFFFFFFFFFFFFFFFFFFFFFFFFFFFFFFFFF
@A00721:190:HGJW7DSXY:4:1223:28628:34194_2:N:0:TCTGCAAG+GCTGCACT
TTTCTTCATAAGGGCTATTCAGTACGTCATGGGGATCACTTCTTCGGTCAATGACAAAACCTTCGAAT
ATGTTACGACATGGCTTCGTAACAACAGTGCGAGGATTGTCATCTCGG
+
FFFFFFFFF:FFFFFFFFFFFFFFFFFFFFFFFFFFFFFFFFFFFFFFFFFFFFFFFFFFFFFFFFFFF
FFFFFFFFFFFFFFFFFFFFFFFFFFFFFFFFFFFFFFFFFFFFFFFF
@A00721:190:HGJW7DSXY:4:1366:1678:22780_1:N:0:TCTGCAAG+GCTGCACT
TCACCAAGATGTTCATATAGATACGAGATACCTAGAGAAGGTCGCAGCGATCTTACTCGTTATCGGAG
CTAAGAAGAAGACTTTGGGTACACGTTATGCTCGCAAACTTAACGTTAGTACCGGAGGTGATCTCTCT
GCT
+
FFFFFFFFFFFFFFFFFFFF:F:FFFFFFFFFFFFFF:FFFFFFFFFFFF:FFFFFFFFFF:FFFFFFF
FFFFFFFFFFFFFFFFFFFFFFFFFFFFFFFFFFFFFFFFFFFFFFFFFFFFFFFFFFFFFFFFFFFFF
FFF
@A00721:190:HGJW7DSXY:4:1406:6659:4883_1:N:0:TCTGCAAG+GCTGCACT
GAGAGAAAAGATTCAGGACGCCTACGGTGACTTTATGGACAAGATGCAAGTTCTTGGGAAATACTTGT
TTCCCTTTTTAGGTTTAGTAGGTGATCTTGACGTCGATTCTTTGTATAGGGAGTGTGAGAAGACAACG
AAT
+
FFFFFF:FFFFFFFFFFFFFFFFFFFFFFFFFFFFFFFFFFFFFFFFFFFFFFFFFFF:FFFFF:FFFF
FFFFFFFFFFFFFFFFFFFFFFFFFFFFFFFFFFFFFFFFFFFFFFFFFFFFFFFFFFFFF:FFFFFFF
FFF
@A00721:190:HGJW7DSXY:4:1328:27507:6230_1:N:0:TCTGCAAG+GCTGCACT
TCTCCGGTCTCTAAATCTTTTGAGATTGAGAAGATTGAAGAACACGTCAAGAAGTCGCTCGGCGTTGT
TCTTGAAAACCAAGTTAAGAAGGAGGCTGATCATGGAGTCTTTGTACCTTCTGTCAAACTTCGAGGTT
CAG
+
FFFFFFFFFFFFFFFFFFFFFFFFFFFFFFFFFFFFFFFFFFFFFFFFFFFFFFFFFFFFFFFFFFFFF
FFFFFFFFFFFFFFFFFFFFFFFFFFFFFFFFFFFFFFFFFFFFFFFFFFFFFFFFFF:FFFFFFFFFF
FFF
@A00721:190:HGJW7DSXY:4:1361:5132:19523_1:N:0:TCTGCAAG+GCTGCACT
TCCCGGATGTGGATTGAAGGCTGGCTCTAAGGGAGACTCCTTCTTTTTGAATGTTGTAAAGAATTGGG
TTGAGAAGTGTGCTTTACAAGGAGTTAGACTCTTCTTCAAGTTTTTAGATCTTCTTGATAATCTTAAA
GAA
+
FFFFF:FFFFF:FFFFFF:FFFFFFFFFFFFFFFFFF:FF,:FFFFF:FFFFFFFFF:FFF::FFFFFF
FFFFFFFFFFFFFFFFFFF:FFF:FF:FFFFFFFFFFFFFFF:FFFFFFFF:FFFFFFFFFFFFFFFFF
FFF
@A00721:190:HGJW7DSXY:4:2527:32226:26334_1:N:0:TCTGCAAG+GCTGCACT
GTTTCTCTTGGTATTTCTGAAATAGTTTATCAGAGCGTCATTTTCCTTTGGGATCTTTCCTCTGGTAA
GGGAATTATTAAATCATTCTTCTCACTCTTGACGAGAATTATTACTGTTTTCTCTACCCATAACTTCT
TTT
+
FF:FFFFFF,FFFFFF:FFFFFFFFFFFFFFFFFFF:FFFFFFFFFFFFFFFFFFFFFFFFF:FFFFFF
FFFFFFFFFFFFFFFFFFFFFFFFFFFFFFFFFFFFFFFFFFFFFFFFFF:FFFFFFFFFFFFFFFFF:
FFF
@A00721:190:HGJW7DSXY:4:2574:16631:7404_1:N:0:TCTGCAAG+GCTGCACT
ACTGGAGTTCAGACGTGTGCTCTTCCGATCTGTTTCAACACTTATCACCACTTTTGAAAGGAAAATAA
TTGGTGATGAAAATACGTATGTCGGTGGGGTTCTCAAGGCTCTCATCGCCACTGGGATTCTAAGGGGT
TCC
```

+
FFFFFFFFFFFFFFFFFFFFFFFFFFFFFFFFFFFFFFFFFFFFFFFFFFFFFFFFFFFFFFFFFFFFFF
FFFFFFFFFFFFFFFFFFFFFFFFFFFFFFFFFFFFFFFFFFFFFFFFFFFFFFFFFFFFFFFFFFFFFF
FFF
@A00721:190:HGJW7DSXY:4:2238:7726:25708_2:N:0:TCTGCAAG+GCTGCACT
AGGATTCTTCTTTTGGTCCTTACGTCTTAAGTAATTTGGATGTTGTCGAAAACTATCAGGTTCTTGTC
ACGATTCTTAAAAGAAGTTTATTAAAACTTCAGAGTCTCTTGGAAGTC
+
FFFFFFFFF:FF,FFFFFFFFFFFFFFFFFFFFF,FFFFFFFFFFFFFFFFFFF:FFFFF,FFFFFFF
:FFF,F:F:FFFFFFFFF:FF:FFFFFFFFFFFFFFFFF,FFFF,FFF
@A00721:190:HGJW7DSXY:4:1139:19108:22467_1:N:0:TCTGCAAG+GCTGCACT
CAATAGCCACAGACTTTCTGGGAAAAGAGACCTCTGTTGGTAGAGGAGTTGAGAGAATTACTCAGATT
CAATCTGGCATCACTGAACGTCTACAACGAGTTAGGGAGTGGCTAGGTTTTAAAAGATCCTTTGCACA
AGC
+
FFFFFFFFFFFFFFFFFFFFFFFFFFFFFFFFFFFFFFFFFFFFFFFFFFFFFFFFFFFFFFFFFFFFFF
FFFFFFFFFFFFFFFFFFFFFFFFFFFFFFFFFFFFFFFFFFFFFFFFFFFFFFFFFFFFFFFFFFFFFF
FFF
@A00721:190:HGJW7DSXY:4:1552:15176:33129_2:N:0:TCTGCAAG+GCTGCACT
TTTCGACGTCGCGGAAAACGATGAAGATTTCCACACTGTTCGAGAATCTTCTGATGCTGAATTTGAGG
AAAATTTTTCGGAAGTTTTTGGTATAAATCCTGATGGCTCAGTACAGG
+
FFFFFFFFFFFFFFFFF:FFFFFF:FFFFFFFFFFFFFFFFFFFFFFFFFFFFFFFFFFFFFFFFFFFFF
FFFFFFFFFFFFFFFFF,FFFFFFFFFFFFFFFF:FFFFFFFFFFFF:
@A00721:190:HGJW7DSXY:4:1404:3052:31485_2:N:0:TCTGCAAG+GCTGCACT
AAACCTTCTTTTATCATTGCAACAGGACTTTGAGAAGACTGTTGAAATGGACAAAATCTGGGATCATA
GGTATGTGTCACGCTACACTCGAAATATGGGCCCTCATAAGGAAGTAT
+
FFFFFFFFFFFFFFFF:FFFFFFFF,F:F:FF:FFF:FF:FFFFFFFFFFFFFFFFFFFF:FFFFFF:
FFFFF:FFFFFFFFFFF::FF::FF,FFFFF:FFFF:FFFFFFFFFFF
@A00721:190:HGJW7DSXY:4:2364:5086:4539_1:N:0:TCTGCAAG+GCTGCACT
CGTCATTACAGTCTGGAAGTTAGATCATTTGTTCAGGAAGATCTCTACAGAGATTATCGAGAGTTGTG
ATCTAGTTCTAAATTTAGGTCGACGGACTTTAGCAACTTTAGATAAATTCTTTCCGGGCTCAATGACC
TTT
+
FFFFFFFFFFFFFFFFFFFFFFFFFFFFFFFFFFFFFFFFFFFFFFFFFFFFFFFFFFFFFFFFFFFFFF
FFFFFFFFFFFFFFF:FFFFFFFFFFFFFFFFFFFFFFFFFFFFFFFFFFFFFFFFFFFFFFFFFFFFFF
FFF
@A00721:190:HGJW7DSXY:4:1311:23981:15969_2:N:0:TCTGCAAG+GCTGCACT
CATCGCAAAGAAGACTTTATTAGTTCATGAAGTTCTTACACTTCTCGCGACTAATGTCAGCTTCACTA
CAAAGGCGTTACGTAAAGTAGTTTTCACGCTTTATCTTAAAGCATTAC
+
FFFFFFFFFFFFFFFFFFFF:FFF:FFFFF:FFFFFFFFFFFFFFFFFFFFFFFFFFFFFFFFFFFFFFF
FFF:FFFFFFFFFFFFFFFFFFFFFFFFFFFFFFFFFFF:FFFFFFFFF
@A00721:190:HGJW7DSXY:4:1266:5403:12602_2:N:0:TCTGCAAG+GCTGCACT
ACTTTTTGAATATGACAGAAGCTCGCCATATCAGATTAGAGACATGCCAGTTAGAGTTCTTCTAACCA
GGACTGATGGTGAGATTCTTTTACCTTTTGGAGGTCCAGGAACCTTTA
+
FFFFFFFFFFFFFFFF:FFFFFF:FFF,FFFFFFFFFFFFFFFFFFFF:FFFFFFFFFFFFFFFFFFF
FFFFFFFFFFFFFFFFFFFFFFFFFFFFFFFFFFFFFFFFFF:FFFFFF
@A00721:190:HGJW7DSXY:4:1477:17517:8531_2:N:0:TCTGCAAG+GCTGCACT
CCAAGGACGTTTCCGTCGGAGGATGACAGGTGGGAGACCGCTCAATAACACTCTATTGAATCCCTTTT
CCACGATGAAAGCTCTTTATGAGGTGCTCCATGCAAATTTACAGTTCC
+

FFFFFFFFFFFFFFFFFFFFFFFFFFFFFF:FFFFFFFFFFFFFFFFFFFFFF:FFFFFFFFFFFFFFFF
FFFFFFFFFFFFFFFFF:FFFFFFFFFFFFFFFFFFFFFFFFFFFFFFFFFF
@A00721:190:HGJW7DSXY:4:1519:20030:13886_1:N:0:TCTGCAAG+GCTGCACT
GTATTTTGATAAATTTGTTGGAATCGATAATGATACTGACGAAGGTTCTATTACCACCCTTGTCATTG
ATACAAACATTCTAGGTGATGCTTTCTTAGAGGCTCGTCTAGCCGCTGAGATTCGTCTTACCTATTTA
CAA
+
FF:FFFFFFF:FFFFFFFFF,FFF,:FFFFFFFFFFFFFFFFFFFFFFFFFFF:FFFFFF:FFF,FFFF
FFFFFFFFFFFFFFFFFFFFFFFFFFFFFFFFFFFFFFFFFFFFF:FFFFFFFF:FFFFF:,FFFFFF
:FF
@A00721:190:HGJW7DSXY:4:2174:28456:2957_2:N:0:TCTGCAAG+GCTGCACT
TTTGAGTGATAACGATTTAGTACTGGGAAATGGTGGTAGTCAGTTTACTACTTTTCTTTCCCTCCCCT
TTTTCGATTGGATTCGACGGGGTCTTCGTCTCACGACTAATTTGATAA
+
FFFFFFFFFFFFFF:FFFFFFFFFFFFFFFFFFFFFFFFFFFFFFF:FFFF:FFFFFFFFFFFFFFFFFF
FFFFFFFFFFFFFFFFFFFFFFFFFF:F:FFFFFFFFFFFF,FFFFFFF
@A00721:190:HGJW7DSXY:4:2124:3260:2284_2:N:0:TCTGCAAG+GCTGCACT
TACTTATGAAGGAGGTATTACTTCGGAAGTTCTTAATGTAGGTCTCCTAGTACTTAAGGGTTTATGTT
TCGGTTTCAGCTGGATGGATCTTTTGATGGTATGTTTCACATTGCGTG
+
FFFFFFFFFFFFFFFFFFFFFFFFFFFFFFFFFFFFFFFFFFFFFFFFFFFFFFFFF:FFFFFFFFFF
FFFFFFFFFFFFFFFFFF:FFFFFFFFFFFFF:FFFFFFFFFFFFFFFF
@A00721:190:HGJW7DSXY:4:1418:23249:28134_1:N:0:TCTGCAAG+GCTGCACT
TTGGGAAGAGATTAAACATAAAATCCAGCGATTCTATTATGTCGCTAAAGCCTGTCCAGTTGTCGATG
ATGTTCGGGCTGTGGGGATTAACTTCCCTCGTGAAACTTACGACGATGTTCTCGCTCGCATTTCACAA
GGA
+
FFFFFFFFFFFFFFFFFFFFFFFFFFFFFFFFFFFFFFFFFFFFFFFFFFFFFFFFFFFFFFFFFFFF
FFFFFFFFFFFFFFFFFFFFFFFF:FFFFFFFFFFFFFFFFFFFFFFFFFFFFFFFFFFFFFFFFFFF
FFF
@A00721:190:HGJW7DSXY:4:1266:26847:17456_1:N:0:TCTGCAAG+GCTGCACT
GAGATCTGCCACTTCTAATTTTAGGAAACGTCGCGAAAAGAAAGTTCATGAGGATGTCTTGGTCGAAA
AATATCGCCGGGAACTTCTAATAGAGGATCCTGATGACATTATTTCAACGGATACCGATCTCAGTTCT
ACC
+
FFFFF:FFFFF:FF,FFFFFFFFFFFFFFFFFFFFFFFFFFFFFFF:FFFFFFFFFFFFFFFFFFFFFF
FFFFFFFFFFFFF:FFFFFFFFFFFFFFFFFFFFFFFFFFFFFFFFFFFFFFFFFFFFFFFFFFFFFF
FFF
@A00721:190:HGJW7DSXY:4:1445:28031:23516_2:N:0:TCTGCAAG+GCTGCACT
CAGGAACTGTCTCCACTGATTCGGAATTAGAGAAAGACGACCTTCTTGTGGGGACTGAGACCTCTGAA
TTTTCAGGGTGCCCTGATGAGGACGTTGAAAGGGATAATTTCAACTCT
+
FFFFF:FFFFFFFFFFFFF:FFFFFFFFFFFF:FFFFFFFFFFF,FFFFFFFFFFFFFFFFFFFFFFF
FFFFFFFFFFFFFFFFFFFFFFFFFFFFFF,FFFFFF:,FFFFFFFFFF
@A00721:190:HGJW7DSXY:4:1337:19777:11287_1:N:0:TCTGCAAG+GCTGCACT
TGTTTCAAAAGACCAGAAACGTAACAAGAAGAATAAAGGTAGGAAGGGGAGAAAACAGAACAAGCGAG
CGGTTGAAGAGAAAACTGATATGGTTGCGGCTGATCGTACCCCAATTCCTGCTAGAAAGATCACTCTC
GGC
+
FFF,FFFFFFFFFF:FF,F,FFFFFFF:F:FFFFF:FFFF::FFFFF,FF:FFFFFFFFFFF,FFFFF
FFFFFFFFFFFFFF,FFF:F,FFFFFFFFFFFFFFFFFFFFFFFFFFFFFFF:F:FF::FFFFFFFFFF
FFF
@A00721:190:HGJW7DSXY:4:1448:21766:25911_2:N:0:TCTGCAAG+GCTGCACT
AGAGTTACGTAGTGGTCGTTCTTTTGGTGATTTTATCAAAGAGAAAGTTTCTGCTAGTCGCGACGATA

GTATTTATCGGAGTGATATTAACAAGTTAAACTTCGAACTGACCACTT
+
FFFFFFFFFFFFFFFFFFFFFFFFFFFFFFFFFFFFFFFFFFFFFFFFFFFFFFFFFFFF:FFFFFF
FFFFFFFFF,FFF:FFFFFFFFFFFFFFFF:FFF:FFFFFFFF,FFFF
@A00721:190:HGJW7DSXY:4:1240:9787:18098_2:N:0:TCTGCAAG+GCTGCACT
TCAGTTTTCCAAAAAGCTATTAATAGCAGTTGCGGGGCTATAATACCGCCTCCAGATTACTCTAAACT
GAATCCACTCTCTTGGGATATAAATGAGTACATCTATAATAGAGTTTT
+
FFFFFFFF:FFFFF:FFFFFFFFFFFFFFFFFFFFFFFFFFFFFFFFFFFFFFFFFFFF:FFFFFFF
FFF:FFFFFFFFFFFFFFFFFFFFFFFFFF:FFFFF:F:FFFFFFFF:F
@A00721:190:HGJW7DSXY:4:1630:4996:25488_1:N:0:TCTGCAAG+GCTGCACT
GGCTTCTCTAAAGAACAGCAACTTTCAACATGAATTAGTTATTGAAAGTCTTGACCCTCACGTCTCAC
TTTATCACCTTGGAGATGGTAAAGTCATTAACAAAGGAAATGTTAAGGCTGATGACTTAAATTTCGCA
TTC
+
FF:FFFFFFFFFFFFFFFFFFFFFFFFFFFFFFFFFFFFF:FFFFFFFFF:FFFFFFFFFFFFFFFFF
FFFFFFFFFFFFFFFFFFFFFFFFFFFFFFFFFFFFFFFFFFFFFFFFFFFFFFFFFF:FFFFFFFFF
FFF
@A00721:190:HGJW7DSXY:4:2677:32172:24893_2:N:0:TCTGCAAG+GCTGCACT
AAAGAGGGTTTGAAAATTACGGAACCTTTCCTCGTTCTTCATGAAGATCTTCGAGTGTTTTATAGTGA
GTTATTCTTGCGAAGGTTCAAGTCGATTGATTTTGTTGACTACGATCT
+
FFFFFFFFFFFFFFFFFFFFFFFFFFFFF:FFFFFFFFFFFFFFFF:FFFFFFFFFFFFFFFFFFFFFF
FFFFFFFFFFF:FFFFFFFFFFFF:FFF,F:FFF:FF,FFFF:FFFFF
@A00721:190:HGJW7DSXY:4:2425:29577:23406_1:N:0:TCTGCAAG+GCTGCACT
AGAAGTTCTTCGCAGTTGAATCTCCACCAGGTGGAGGGAAGACCACTGAACTTATAGCGACTTTTGTC
GAATTTTTCGCAAAAGGTCTTGAAGTACGTATTGTCACTGCTAATCGTAATTCGCGAGAAGAGATAGT
GAC
+
FFFFFFFF,FFFFFFFFFFFFFFFFFFFFFFFFFFFFFFFFFFFFFFFFFFFFFFFFFFFFFFFFFFF
FFFFFFFFFFFFFFFFFFFFFFFFFFFFFFFFFFFFFFFFFFFFFFFFFFFFFFFFFFFFFFFF:FFF
FFF
@A00721:190:HGJW7DSXY:4:1125:14968:22028_2:N:0:TCTGCAAG+GCTGCACT
CTAGTTCATCGCGGTATTTGTGAAATTAAGACAGACGTGAAAGTTATGATGAGTGATCGTATTAGAAC
AGCTGATTCTTATTGCATGAACGTCGCTGAGGTCAAACCTGTGGACGT
+
FFFFFFFFFFFFFFFFFFFFFFFFFFF:FFFFFFFFFFFFFFFFFFFFFFFFFFFFFFFFFFFFFFFF
FFFFFFFFFFFFFFF:FFFFF:FFFFFFFFFFFFFFFFFFFFFFFFFFF
@A00721:190:HGJW7DSXY:4:1138:24306:25363_1:N:0:TCTGCAAG+GCTGCACT
ACGAATTATTCATGATTCACGCTGGTCAGACCCTTTTTCTTACACATCTACTTAAGGCGGCTTATACC
ATAGGTTACGGTGATTCCAATCAAATCGGATTCATCAATCGTATTGATCTCCAAGTCGCAAACTACTT
CAA
+
FFFFFFFFFF:FF:FFFFFFFFFFFFFFFFF,F:FFFFFFFFFFFFFFFFFFFFFFFFFFFFFFFFFF
FFFFFFFFFFFFFFFFFFFFFFFFFFFFFFFFFFFFFFFFFFFFFFFFFFFFFFFFFF:FFFFFFFFF
FFF
@A00721:190:HGJW7DSXY:4:1519:3513:32440_2:N:0:TCTGCAAG+GCTGCACT
TGAGTGTCGTTCTTATCGAAGTCTCAGTTATCGCTGCCCGGCCGACGTTTGTCGCGAACTCTCAGAGG
TTTACGGCAGAAAAATTTCTCCTGCTTGTAATTCGAGAGAGGACACTA
+
FFFFFFFFFFFF,FFFFFFFFFFFF:FFFFFFFFFFFFFFFFFFFFFFFFFFFFFFFFFFFFFFFFFFF
FFFFFFFFFFFFFFFFFFFFFFFFFF:FFFF,FF:FFF:FFFFFFFFFF
@A00721:190:HGJW7DSXY:4:1264:5728:17049_2:N:0:TCTGCAAG+GCTGCACT
TATTACTTTCACTCAAGGTGAAAAGTTTGAAATTAACAAAGTCCTTGGTAAGAGTAAGGTTAAATGTG

AAGATGCTCAGACTATTCATGAAGTTCAAGGTAATACTTACCAGCACG
+
FFFFFFFFFFFFFFF:FFFFFFF::FFFFFFFFFFFFFFFFFFFFFFFFFFFFFFFF:FFFFFFFFFF
FFFFF:FFFFFFFF:FFFFFFFFFFFFFFFFFFFFFFFFFFFF:FFFFF
@A00721:190:HGJW7DSXY:4:1463:27073:29465_2:N:0:TCTGCAAG+GCTGCACT
TTTTGCATCACAAACACATATTACCGTTGCTCTATCACGACACGTTGAATCTTTACGATACTTTGTTA
TCAATGTAAAGTTAAACGATGCTACTTGTGAGAGGATCAATCACAGTA
+
FFFFFFFFFFFFFFFFFFFFFFFFFFFFFFFFFFFFFFFFFFF:FFFFFFFFFFFFFFF:FFFFF
FFFF,FFFFFFFFFFFF::FFFFFFFFFFFFFFFFFFFFFFFFFFFF:
@A00721:190:HGJW7DSXY:4:2239:27037:20917_2:N:0:TCTGCAAG+GCTGCACT
TTAGCACGGGAGGTTGCAGATTCAACCCCTTATATCGAGGCTATCGATGACGATGAGTATCTTGAAGT
GGTTGACGACAAGCCTCAAGAGGGTAGAGGACCACCTGGATCTTCTAC
+
FFF:FF:FFF,:FFF,FFFFFFFFF,FF::FF,F:FFF:,FFFFFFF,FFFFFFFF::FFF,FFFFFF
,:FFFFFF:F:FFFFFFF,:FFFFF,FFFFFFFFF:::FFFF:FF:FF
@A00721:190:HGJW7DSXY:4:1205:13277:14528_1:N:0:TCTGCAAG+GCTGCACT
AACCACCTTAACGTCACTCGGTAATCTTTCAGCCGAATTAAGTACTAATGAGTTCAAGTCGGTTGTCG
ATCGAATTAAGATCAAGGCTAATACCTTTATAGGTAAGAAGCCCCTTGAGCCGCGTAGTCAGCGCGTT
TAG
+
FFFFFFFFFFFFFFFFFFFFFFFFFFFFFFFFFFFFFFFFFFFFFFFFFFFFFFFFFFFFFFFFFFFF
FFFFFFFFFFFFFFFFFFFFFFFFFFFFFFFFFFFFFFFFFFFFFFFFFFFFFFFFFFFFFFFFFFFF
FFF
@A00721:190:HGJW7DSXY:4:2521:1904:22326_1:N:0:TCTGCAAG+GCTGCACT
CCCGATAGGGTACCGAGTCTCCAAGAGAATATTTATTCTTATGAGGCTCGAAACTTCAACGTTTTCAC
AACGGAAAGACATATCGCACCCGAAATTTTTGGGCAGAATGCTCTTAACGCGGTTCTTGAACGGTGTT
TTG
+
FF,FFFFF,FFF,:FFFFFFF::FFFFFFFFFFFFF:FFFFFFF:FFFFFFFF:FFF,F:,FFFFFF
:FFF,F:F:FFFFFF,F:FF,F:F,,FF:FFF:F,FFFFFFFF:FFFFFFFF,:F:FF,:,:F,:FFF
FFF
@A00721:190:HGJW7DSXY:4:2276:10004:12461_1:N:0:TCTGCAAG+GCTGCACT
GATAAAGTTATAGGAGACTCAGTTAACCTGAGAGCTCCTCAATTGATAGATTGGGTCAACAATAGGGA
CGCTAGTATACTTCGTAAAGTCGACAGCGAAATGGTCAATATTGATAATTGGACCGACTATATGACCT
ATT
+
FFFFFFFFFFFFFFF::FFFFFFFFFFFFFFFFFFFFFFFFFFFFFFF:F:FFFFFFFFFFFFFFFFF
FFFFFFFFFFFFFFFFFFFFFFFFFFFFFFFFFFFFFFFFFFFFFFFFFFFFFFFFFFFFFFFFFFFF
FFF
@A00721:190:HGJW7DSXY:4:2564:18159:36855_2:N:0:TCTGCAAG+GCTGCACT
AGGGAAGCTAAGGTTAAACTTGATGCTTCAGCCTTTTCGAAGCATAACCCCGCACAGAATATCGTTTA
TCACGCAAAATTTATTAATGCAATTTTCGGACCTATCTTTAAGGTTCT
+
FFFFF,FFFFFFFFFFFFFFFFFFFFFFFFFFFFFFFFFFFFFFFFFFFFFFFFFFFFFFFFFFFFFF
FFFFFFFFFFFF:FFFF,FFFF,:FFFFFFFFFFFFFFFFFFFFFFFFF
@A00721:190:HGJW7DSXY:4:2428:10176:18490_2:N:0:TCTGCAAG+GCTGCACT
CTTTTACCCAACATTTTTATGTATACAGAGATGACCAGTGACGATTTCTCCCGGAAGCTCTTCTCTCT
CTTAGGAGGTCATGATGACTACTATAAGGGTGAAATCGATTTTTCGAA
+
FF::FFFFFFFFFFFFFFFFFFFFFFFFFFFFFFFFFFFFFFFFF:FFFFFFFFFFFFFFFFFFFFFFFFF
FFFF::FFFFFFFFFFFFFFFFFF:FFFFFFFFFFFFFFFFFFFFFFF
@A00721:190:HGJW7DSXY:4:1456:30563:6887_1:N:0:TCTGCAAG+GCTGCACT
TAAAGCCTATGAGATAAAGATGTATTCTTATTTAGGCTTCGATGCTGAATTTCTTGAACTTTGGAGTG

CCAGTGAATATTTTTGTCACGCCAGGAGTATCCATAACGATTTGTCATTTACCACCTTTGCTCAACGA
CGA
+
FFFFFFFFFFFFFFFFFFFFFFFFFFFFFFFFFFFFFFFFFFFFFFFFFFFFFFFFFFFFFFFFFFFF
FFFFFFFFFFFFFF:FFFFFFFFFFFFFFFFFFFFFFFFFFFFFFFFFFFFFFFFFFFFFFFFFFFFF
FFF
@A00721:190:HGJW7DSXY:4:2107:32271:25629_1:N:0:TCTGCAAG+GCTGCACT
TAACACATTCTTTGGTAATTGTATGATCACTCTTATGATGCTCTCTCTTTATTACGACCTTCGGAAGT
TTAGAGCCGTCGCTGTCGCCGGAGACGATTCGATCCTCTTTTCGGACGTACCGGTCCAGGATTATTCA
GAT
+
FFFFFFFFFFFFFFFFFFFFFFFFFFFFFFFFFFFFFFFFFFFFFFFFFFFFFFFFFFF:FFFFFFFF
FFFFFFFFFFFFFFFFFFFFFFFFFFFFFFFFFFFFFFFFFFFFFFFFFFFFFFFFFFFFFFFFFFFF
FFF
@A00721:190:HGJW7DSXY:4:1377:23809:26256_2:N:0:TCTGCAAG+GCTGCACT
TGCTCGCGATTTATTCTTTTTGTCAATGATTACATCTACTTCGTTCCTGATCCTTACAAGATCATCGT
TAAGTTAGGTAAACCCCTAACCGATGTTACTCTTGTAGGACTTTGGGA
+
FFF,FFFFFFFFFFFF:FFFFFFFFFFFFFFFFFFF:FFFFFFF:FFFFFFFFF:FFFFF:FFFFF:F
FFFF::FFF:FFF::FF:FFFFFFFFFFFF:FF::FF,:FFFF:FFFF
@A00721:190:HGJW7DSXY:4:1664:30020:10081_2:N:0:TCTGCAAG+GCTGCACT
TTAAAGACTATACTAAATATCTCCACTACGAGGTTGTTGTTTGGCGATTAGCTCTTGCGACTAATATT
CGATATTCGATTGAGGGTCGTTATACCTATTCGGCTATCTGTGCTTTG
+
FFFFFFFFFFFFFFFFFFFFFFFFFFFF,FFFFF:FFFF:F:FFFFFFF:F:FFFFF,FFF:FFFFFF:
F,FFFFFF:FFFFFFFFFF::FFFFF,FFFF::F:FFFFFF::FF:FF
@A00721:190:HGJW7DSXY:4:1215:30807:8343_2:N:0:TCTGCAAG+GCTGCACT
TTTTAAACGATTCCGAGAGGTTTATCCTGATGTGGAGGGAGTTCTTGAAGCATCCCTACCTTTCCTTG
TGAAAATTTTGATGCGGTTTCATAAGATTATCAGTAATCCTGATTCTA
+
FFFFFFFFFFFFFFFFFFFFFFFFFFFFFFFFFFFFFFFFFFFFFFFFFFFFFFFFFFFFFFFFFFFF
FFFFFFFFFFFFFFFFFFFFFFFFFFFFFFFFFFFFFFFFFFFFFFFF
@A00721:190:HGJW7DSXY:4:2268:5005:27571_1:N:0:TCTGCAAG+GCTGCACT
GACTGGAGTTCAGACGTGTGCTCTTCCGATCTCAAAGATCGACTTCGAGACAGGTGATATTGTTGAGC
GAGAGCGACCAAGATCTAAATTCGGTAACGGTGCGTGGCGCTTTATTTTAAAAAGCTCCCTCCTCTCG
CAA
+
FFFFFFFFFFFFFFFFFFFFFFFFFFFFFFFFFF,FFFFFFFFFFFFFFFFFFFFFFFFFFFFFFFFF
FFFFFFFFFFFFFFFFFFFFFFFFFFFFFFFFFFFFFFFFFFFFFFFFFFFFFFFFFFFFFFFFFFFF
FFF
@A00721:190:HGJW7DSXY:4:2204:7238:29183_2:N:0:TCTGCAAG+GCTGCACT
ACTTGAGTCTTTTGATCAGTCTCTGACTTGACCATCTTTCATTTTAAATGACGATCATTAAGACTCTC
TTTCTTCTTTGTTTCCTTCTTTGCACTGATTTTTCCTCATCAGCTTGT
+
FFFFFFFFFFFFFFFFFFFFFFFFFFFFFFFFFFFFFFFFFFFFFFFFFFFFFFFFFFFFFFFFFFFF
FFFFFFFFFFFFFFFFFFFFFFFFFFFFFFFFFFFFFFFFFFFFFFFF
@A00721:190:HGJW7DSXY:4:2318:20139:31078_2:N:0:TCTGCAAG+GCTGCACT
TTTTACGATAACAACTATCAGTTGATTAAGACTCTTCCGAAAGGCGAACTTTGGGAATTTGAAGCTCA
GGGTGAAGCACAACGTTTGACCGTGACGAACTGTGGTGTTCTTGATCT
+
FFFFFFFFFFFFFFFFFFFFFFFFFFFFFFFFFFFFFFFFFFFFFFFF:FFFFFFFFFFFFFFFFFFF
FFFFFFFFFFFFFFFFFFFFFFFFFFFFFFFFFFFFFFFFFFFFFFFF
@A00721:190:HGJW7DSXY:4:2171:26992:30107_1:N:0:TCTGCAAG+GCTGCACT
CACTACCACTTATTCCGATATTTCTTTAGTCGATGGGTTTTCGACGCCTTTAAGCGTCTTCTGTTCAT

CGCAACCAGATGATATCTTCTATTCGAACTTATGTTTCGATCAGTGTTTCTTCTATCAGGAGGAAAAC
ACT
+
FFFFFFF:FFFFFF,:FFFFFFFFFFFFFFFFFFFFFFFFFFFFFFFFFFFFFFFFFFFFFFFFFFFFF
FFFF:FFFFFFFFFFFFFF:FFFFFFF:FF::FFFFFFFFFFFFFFFFFFFFFFFFFFFFFFFFFFFFF
FFF
@A00721:190:HGJW7DSXY:4:2203:24804:23782_2:N:0:TCTGCAAG+GCTGCACT
TCTTCTAATGTGGCCTGAACTACGTTTCACTTTGTGTGTACTTGTCTCTATTTTAAGTTTTGTCTTCC
TTGTTTGTCTTATTCTATTAATCACTCATTTTAGTGAGATTATTAAAA
+
FFF:FFFFFFFFFFFFFFFFF:FFFFFFFFFFFFFFFFFFFFFFFFFFFFFFFFFFFFFFFFFFFFFFF
,FFFFFFFFFFFFFFFFFFFFFFFFFFFFFFFFF:FFFFFFFFFFFFFF
@A00721:190:HGJW7DSXY:4:1652:4074:28557_1:N:0:TCTGCAAG+GCTGCACT
CGACAACAGCAGGGGTTTTCGAATAGTGATAGCCACCCGCCTTACAGAGGGGCAGCAGCATGACCATA
ATTGGTATTGACTTTGGTACCACCTTTTCTACTGTCTGTCTCGCCTCTTCGAATGAAGTTTTAATGCT
TAA
+
FFFFFFFFFFFFFFFFFFF:FFF,FFFFFFFFFFFFFFFFFFFFFFFFFFFFFFFFFFFFFFFFFFFFF
FFFFFFFFFFFFFFFFFFFFFFFFFFFFFFFFFFFFFFFFFFFFFFFFFFFFFFFFFFFFFFFFFFFFF
FFF
@A00721:190:HGJW7DSXY:4:2321:25870:25786_2:N:0:TCTGCAAG+GCTGCACT
CTACAATCATTGGTTTTTCTAATAATGATGACACTATTGTTTATGGATATGATGCTATTACTCATGAT
GGTTACGGTCAGGGAGGATTCTCAGTTTACAAGGACCTTAAGCGATGG
+
FFFFFF:FFFFFFFFFFFFFFFFFFFFFFFFFFFFF:FFFFFFFFFFFFFFFFFFFFFFFFFFFFFFFFF
FFFFFFFFFFF:FFFFFFFFFFFFFFFFFFFFFFFFFFFFFFF:FFF::
@A00721:190:HGJW7DSXY:4:2554:21504:6198_2:N:0:TCTGCAAG+GCTGCACT
CCGAAAGACGTGCTAAATTAAAACCATTGTACGATGTCGTCTGTCTCGGCGATCACTTTTCACTAAGA
ATTGGTCCTACTTTTGGACAAGGCCAACTTCGTAATCTTCATGAGATC
+
FFFFFFFFFFFFFFFFFF:FFF:FFFFFFFFFFFFFFFFFFFFFFFFFFFFFFFFFFFFFFFFFFFFFF
FFFFFFFFFFFFFFFFFFFFFFFFFFFFFFFFFFFFFFFFFFFFFFFF
@A00721:190:HGJW7DSXY:4:2307:10565:26584_1:N:0:TCTGCAAG+GCTGCACT
TAGGGATTTTGAGAGAGTGAAAGGCTTCAATTGTAGTGGTTTGGTCGTTTCAGTACCCAGTCAGTATA
CCTCTACGCAAAGATATTTCATGAAAACTACAGAGGAACGTACCGGATTACCTGTCTTTCATATTATG
AAT
+
::FFFFFFFFFFFFFFFF,:FFFF:::FFFFFFFFFFF:FFF:FFFFFFFFFFFFFFFFF:FFFFFFF
FFFFFFFF:FFFFFFFFFFFFFFFFFFFF,FFFFFFF,FFFFFFFFFFFFFFFFFFFFFFFFFFFFFFF
FFF
@A00721:190:HGJW7DSXY:4:1533:26702:26663_1:N:0:TCTGCAAG+GCTGCACT
TTTGCTTCGATGTTGGATGTCAAGAAGGGTTCAGAATGGGATTCCTACGTAGTTTATGACTTTGGTGG
TGGTACTTTCGATACTTCTATCGTCTTACGTTACGGTAATTACTATAGTGTTATTTATTCTGGTGGTG
ATG
+
FFFFFFFFFFFFFFFFFFFFFFFFFFFFFFFFFFFFFFFFFFFFFFFFFFFFFFFFFFFFFFF:FFFFF
FFFFFFFFFFFFFFFFFFFFFFFFFFFFFFFFFFFFFFFFFFFFFFFFFFFFFFFFFFFFFFFFFFFFF
FFF
@A00721:190:HGJW7DSXY:4:2442:29740:20650_2:N:0:TCTGCAAG+GCTGCACT
GGTCGTGACGTTGATGAAGCTATAAGAGCGTTCTTATTACGGCGCACTTCTTTACCCGCCACCACACA
ACTTTCAGTTTCACAACTGAAGGAGGAGGTTTCTGTTACTGGAAAACC
+
FFFFFFFFFFFFFFFFF:FFFFFFFFFFFFFFFFFFFFFFFFFFFFFFFFFFFFFFFFFFFFFFFFFFF
FFFFFFFFFFFFFFFFFFFFFFFFFFFFFFFFFFFFFFFFFFFFFFF:

```
@A00721:190:HGJW7DSXY:4:1376:3070:25097_1:N:0:TCTGCAAG+GCTGCACT
CTGGAGTTCAGACGTGTGCTCTTCCGATCTGTGAAATTAACGCGTCCTTTTCTGGAACGTAGTTTTAC
TATTATGAACAAAGTTATCAAAGATAGTGGCATTCAAGGCAAACTTACCCTGATTCCGGCCGGTGGTA
GCT
+
FFFFFFFFFFFFFFFFFFFFFFFFFFFFFFFFFFFFFFFFFFFFF:FFFFFFFFFFFFFFFFFFFFFF
FFFFFFFFFFF:FFFFFFFFFFFFFFFFFFF:FFFFFFFFFFFFFFFFFFFFFFFFFFFFFFFFFFFF
FFF
@A00721:190:HGJW7DSXY:4:2333:26512:35634_2:N:0:TCTGCAAG+GCTGCACT
AGGGGCTATTACTCTTGCTGAAACAGCTATTAAAGCTGTTGAGCGGGTAATTACACCTAAACGAGCTC
GATCTGCCATCGCTGAAGGATGTGCACTCTTTTCTGCTACTTTAACGG
+
FFFFFFFFFFFFFFFFFFFFFFFFFFFFFFFFFFFFFFFFFFFFFFFFFFFFFFFFFFFFFFF:FFFF
FFFFFFFFFFFFFF,FFFFFFFFFFFFFFFFFFFFFFFFFFFFFFFFFF
@A00721:190:HGJW7DSXY:4:2303:6858:35102_2:N:0:TCTGCAAG+GCTGCACT
TCGATTGTATCAACAGTAACATTACTTCTAATAAAGGTCTCTATCAAGCTCGTATTATCGTCGCTGCT
GGATAAGCGTTACCGTTGAAGGCTAAACTACAAGGTAATTATGAAGAC
+
,,FFF:FFFF:F:FFFF,F,,FFFF,F,FF:,F,F:FF,F,:F:FFF,FFFFF,FF:FF,F,FF,,,,
,F,:,,F:::,FF,F,F,:F,,::F:,,,,,FF,F:FF,:F,,FFFFF
@A00721:190:HGJW7DSXY:4:2665:6280:28119_1:N:0:TCTGCAAG+GCTGCACT
GTATGAGGGTGTTTCTAATCGTAACATTAATAATTTCAATCTTCTTAAAAAGAAGATAGAGAGTCATA
CTTTGGGCTATCGTTCAGGTGCATGGGGTTGGATAGCCAATATTGAAGTGGACACCTCTGGTAGTATC
GAA
+
FFFFFFFFFFFFFFFFFFFFFFFFFFFFFFFFFFFFFFFFFFFFFFFFFFFFFFFFFFFFFFFFFFFF
FFFFFFFFFFFFFFFFFFFFFFFFFFFFFFFFFFFFFFFFFFFFFFFFFFFFFFFFFFFFFFFFFFFF
FFF
@A00721:190:HGJW7DSXY:4:1221:2266:25520_2:N:0:TCTGCAAG+GCTGCACT
AAGCATACTACTTTTGCTTTGATTACTCTACCGCCTTACAAGTCTAAGGTTAAAGAGTCGATTAAACA
AGTTGTACTTATCAATGATTTTATCGCTGGTTGTTATCTCGAAAAGTC
+
FFFFFFFFFFFFFFFFFFFFFFFFFFFFFFFFFFFFFFFFFFFFFFFFFFFFFFFFFFFFF:FFFF,F
FFFFFFFF:FFFFFF:FFFFFF:F,FF,FFFFFFFFFFF:FFFFFFF,
@A00721:190:HGJW7DSXY:4:1163:6180:3928_1:N:0:TCTGCAAG+GCTGCACT
CCCAAAGAGAATATCTAAAATATTTACAAAATCTTACCACTATCTACGAACCTAAAGTTCTGAAGACG
TTGCTTGTCGCTTCCTCCTTTAATGGCACTACCTCGGTACAATCTTACTTGGAACGAAGGCGACCTGT
TCC
+
FFFFFFFFFFFFFFFFFFFFFFFFFFFFFFFFFFFFFFFFFFFFF:FFFFFFFFFFFFFFFFFFFFFF
FFFFFFFFFFFFFFFFFFFFFFFFF:FFFFFFFFFFFFFFFFFFFFFFFFFFFFFFFFFFFFFFFFFF
FFF
@A00721:190:HGJW7DSXY:4:2120:18304:25614_1:N:0:TCTGCAAG+GCTGCACT
GTTCTGTCTATATTAGGTGAAGCGAAATACGATTTCGAACGTCAACGTTCACCGAGTTTCGTTTCGTA
CCTTGATGCTGACTTTCGTCAATATGATTTGATCTGGTTGCTCAATTCTAAGCGTGTTCCGCTTAGTG
CGA
+
FFFFFFFFFFFFFFFFF:F:FFFFFFFFFFFFFFFFFFFFFFFFFFFFFFFFFFFFFFFFFFFFFFFF
FFFFFFFFFFFFFFFFFFFFFFFFFFFFFFFFFFFFFFFFFFFFFFFFFFFFFFFFFFFFFFFFFFFF
FFF
@A00721:190:HGJW7DSXY:4:2370:4101:2738_1:N:0:TCTGCAAG+GCTGCACT
TACGAACTTGACTCTACTATACGTGACATACTAAATACTAGATATTTGGATGATCTTGATAAAGAACT
TCATGAATTTTCAGAGGATGAAAAATTTTCTTCCTTGGATTTTAATATAAAGAATCTTTACCAGGACT
CCT
```

+
FFF,FFFFFFFFF:FFFFFFFFFFFFFFFFFFFFFFFFFF:FFFFFFFFFFFFFFFFFFFFFFFFFFF
FFFFFFFFFFFFFFFFFFFFFFFFFFFFFFFFFFFFFFFFFFFFFFFFFF:FFFFFFFFFFFFFFFFF
FFF
@A00721:190:HGJW7DSXY:4:2360:7789:7341_1:N:0:TCTGCAAG+GCTGCACT
TGATCGTAAGAAGTTTATAAGGAACATATCCATTTGTTGTAATAGGGTCATCAACTTAGAACTCGAAA
CTCTAAGTGGTAATACTGTCTTTCCAAGCTTACCTGTATCTTCAATGAAATTGGACTCTTCGATAGGG
GAC
+
FF:FFFFFFFFFFFFFFFFFFFF:FFFFFFFFFFFFFFFFFFFFFFFFFFFFFFFFFFFFFFFFFFFF
FFFFFFFFFFFFFFFFFFFFFFFFFFFFFFFFFFFFFFFFFFFFFFFFFFFFFFFFFFFFFFFFFFFF
FFF
@A00721:190:HGJW7DSXY:4:2608:6343:32894_1:N:0:TCTGCAAG+GCTGCACT
GGAAGATCTGTTATCGACGGTTTATCGTTTACGAAGTATCACTCCACCGGTGAATGGTTTAAGAATAC
TATAAATAGAATAGCTATTACTACTAATGGCGTTGGCCTAGTGGATGTTATTAGTGACATCTGGTCAA
TTT
+
FFFFFFFFFFFFFFFFFFFFFFFFFFFFFFFFFFFFFFFFFFFFFFFFFFFFFFFFF:FFFFFFFFFF
FFF:F:FFFFF,FFFFF,FFFFFFFFFFFFFFFFFFFFFFFFFFFFFFF:FFFFFFFFFFFFFFFFFF
FFF
@A00721:190:HGJW7DSXY:4:1645:17490:9048_1:N:0:TCTGCAAG+GCTGCACT
TGGTCTTTATTCTATTATTGATTTTTATGAGATTGATACGTTAACAGAAGATGATGTTAGAGTAGAAT
TCCTGAAGAGTCCTTCCGTAACCAACTTCTTGACAATTTTCTTCAATCCTTCGATAATAGTTAAATCT
AGG
+
F:F:FFFFFFFFFFFFFFFFFFFFFFFFFFFFFFFFFFFF:FFFFFFFFFFFFFFFFFFFFFFFFFFF
FFFFFFFFFFFFFFFFFFFFFFFFFFFFFFFFFFFFFFFFFFFFFFFFFFFFFFFFFFFFFFFFFFFF
FFF
@A00721:190:HGJW7DSXY:4:1376:21712:13855_2:N:0:TCTGCAAG+GCTGCACT
TACACAATTAAGAAATAATCCTACATTGACTGATTATCTAAAGATTGAAGGAAAATTTTATCCTCGTT
TAACTTACTTAGACTTTCCTACGATACAAATGATAACTTCTAATTTAC
+
FFFFFFFFFF:FFFFFFFFFFFFFFFFFFFFFFFFFFFFFFFFFFFFFFFFFFF:FFFFFFFFFFF,F
:FFFFFFFFFF:FFFFFF:FFFFFFFFFFF::FFFFFFFFFFFFFFFF,
@A00721:190:HGJW7DSXY:4:2102:9299:12962_1:N:0:TCTGCAAG+GCTGCACT
GGGATGCATGGTGTTATCTCCTCAGGAGTCCCGGCATTTGATAATAATGACGAGACTTTCGTCATAAT
CGCTGCATATTACGGATTACACGGTACCAGCGCATCTCGTGTCGAGCCTCGACCCGATTATATGGTAT
TAA
+
FFFFFFFFFFFFFFFFFFFFFFFFFFFFFFFFFFFFFFFFFFFFFFFFFF:FFFFFFFFFFFFFFFFF
FFFFFFFFFFFFFFFF:FFF,FFFFFFFFFFFFFFFFFFFFFFFFFFFFFFFFFFFFFFFFFFFFFFF
FFF
@A00721:190:HGJW7DSXY:4:1166:10022:16219_2:N:0:TCTGCAAG+GCTGCACT
GATTCTCGATTCGTAGAGCTTATCTAGGTACTCTCATTTACTATGTTGATTTAGTCTACATTTATGCT
GATCTAAAATTTCGATCTAGATGGATCCACTCCTCGAGTTATCATGAT
+
FFFFFFFFFFFFFFFFFFFFFFFFFFFFFFFFFFFFFFFFFFFFFFFFFFFFFFFFFFFFFFFF:FFF
FFFFFFFFFFFFF:FFFFFFFFFFFFFFFFFFFFFFFFFFFFFFFFFF
@A00721:190:HGJW7DSXY:4:2443:16785:7044_1:N:0:TCTGCAAG+GCTGCACT
TTCTAGAATTCTTAATTATAATATTCAACCTCAAAAGTTACGTGTTTACAGTAACGAGATCACACATT
TTGAGAACTAACTATGTTATGTTCTCTGATCATTTTGACAATCTCTTCTTATAATAAATAAATAAATA
ATA
+
FF,FFF:FFF:FFFFFFFFFF:FF:FFF,FF:FFFFFFFF:FFFFFFFFFFFFFF:F,FFFFFFFFFF

FFFFFFFFFFFFFFFFFFFFFFFFFFFFFFFFFFFFFFFFF:FFFFFFFFFFFFFFFFFFFFFFFFFFF:
FFF
@A00721:190:HGJW7DSXY:4:2511:7762:33473_2:N:0:TCTGCAAG+GCTGCACT
CAAATAGTGGAAAGGCTCCAGTCAACACTAGTTCGAGTGCTGCTTTGGATTACGATGACGTGAGAATA
GGTGAAGGGATAGTTTTTACCTCGACCAACATTAAGGCGGCGGCGACT
+
FFFFFFFFFFFFFFFFFFFFFFFFFFFFFFFFFFFFFFFFFFFFFFFF:FF:FFFFFFFFFFFFFFF
FFFFFFFFFFFFFFFFFFFFFFFFFFFFFFFFFFFFFFFFFFFFFFFF
@A00721:190:HGJW7DSXY:4:1323:2266:7169_1:N:0:TCTGCAAG+GCTGCACT
AGGACACTTCGAAACTGAATAATGATGCACTACAAACAAGTATCGCTAATCATCTGAAAAAGGCTCTA
AAGGACACTATACAATATCCAGACGACGACGAAGCTTATACTACTTTAATTTTGGGTATTATGTGGAG
ATA
+
FFFFFFFFFFFFFFFFFFFFF:FFFFFFFFFFFFFFFF:FFFFF::FFFFFFFFFFF:FFFFFFFFFF
:FFFFFFFFFF:FFFFFFFFFFFFFFFFFFFFFFFFFFFFFFFFFFFFFFFFFFFFFFFF:FFFFFFF
FFF
@A00721:190:HGJW7DSXY:4:2357:32307:25034_2:N:0:TCTGCAAG+GCTGCACT
CACCACATTTTGGTCAAGTCAATCCGTTGAGAAAATTTCTTAGAGGATATTCGCCATTGTGGTACGAA
TTGGTCCAACATTTACCACCAAATTCTTTTGATACAACTCTAGCCTTC
+
FFFFFFFFFFFFFFFFFFFFFFFFFFFFF:FFFFFFFFFFFFFFFFFFFFFFFFFFFFFFFFFFFFFF
FFFFFF:FFFFFFFFFFFFFFFFFFFFFFFFF:FFFF:FFFFFFF:FFF
@A00721:190:HGJW7DSXY:4:2510:10981:21605_2:N:0:TCTGCAAG+GCTGCACT
GCTTGCTATTCACCAGATTGGTTCGAACCCGTTGATGCTATGTCAGAAGAAGCTATTCAAGCTTGTCG
TTTAAAGACTGCAGAGGCTGTCAAACAAGCCCAGACTCAAGGAGTCCA
+
FFFFFFFFFF:F::FF:FFFFFFFFF:FFF:F,F:FFF:,FFFFFFFFFF:FFF,F:FFF,FFFFF:F
F:FFFF:FFFF::FF,F:FFF:FFFFFF:FFFFFF:FFFF,,F:F,FF
@A00721:190:HGJW7DSXY:4:2219:26042:7921_2:N:0:TCTGCAAG+GCTGCACT
GGTGATAAAAATTATTTAGAGATCTTTTCTACTTACAAAGGACAATTCAGTTTCATTAATTCTACGGA
AAAGTCCGGAGTATATTGTATGTTAATAGATAGTGGGATAATAGTTCT
+
FFFFFFFFFFFFFFFFFFFFFFFFFFFFFFFFFFFFFFFFFFFFFFFFFFFFFFFFFFFFFFFFFFFF
FFF:FFFFFFFFFFFFFFFFFFFFFFFFF:FFF:FFFFFFFFF:FFFFF
@A00721:190:HGJW7DSXY:4:1171:14335:26757_2:N:0:TCTGCAAG+GCTGCACT
TGCTATTGATTATGTAACCGATAGGAGTTGTATAGTTGAAGATGTGGCTTTAGACGATAATCCTTTAA
GGACTATGTTGACTGACGAAGATTTTCCCACAGATTTGTCTACGTTGC
+
FFFFFFFFFFFFFFFFFFFFFFFFFFF:FFF:FFFFFFFFFFFFFFFFFFFFFFFFFFFFFFFFFFFF
FFFFFFFFFFFFFFFFFFFFFFFFFFFFFFFFFFFFFFFFFFFFFFFF
@A00721:190:HGJW7DSXY:4:2544:2718:36824_2:N:0:TCTGCAAG+GCTGCACT
CTATATACATTATAAAATATAAAAATAAGGGAGTCATAACTTTTGTTACCAAATTCTTGGCTTTCAAC
GAAGTCTCTGATTTTATTAATCATTACTTTAACGATCTCTCGGTCATT
+
FFFFFFFFFFFFFFFFFFFFFFFFFFFFFFFFFFFFFFFFFFFFFFFFFFFFFFFFFFFFFFFFFFFF
FFFFFFFFFFFFFFFFFFFFFFFFFFFFFFFFFFFFFFFF:FFFFFFFF
@A00721:190:HGJW7DSXY:4:2361:2962:22592_1:N:0:TCTGCAAG+GCTGCACT
TTGATCTACGTCGTTTAACTGTCGGAACAACCTTTACTTAGAATGACTCTATCTTTTCAGACACTTCT
TATATTATTTACAATAGTTAATATCATATTAGTAATACTCACTATCGTTTTCTGCTTTTTCTTAGCTA
ATA
+
FFFFFFFFFFFFFFFFFFFFFFFFFFFFFFFFFFFFFFFFFFFFFFFFFF:FFFFFFFFFFFFFFFFF
FFFFFFFFFFFFFFFFFFFFFFFFFFFFF:FFFFFFFFFFFFFFFFFFFFFFFFFFFFF:FFFFFFFFFF
FFF

```
@A00721:190:HGJW7DSXY:4:2451:18466:24142_2:N:0:TCTGCAAG+GCTGCACT
ATGAGTACGAAAACCAACTCTTCGTTACGAGACCTGATGGTAGAATAGATTTAGTCTCTACACCCATC
TCAACGAGGATAAAGCGAGTTTTCCGTCCTGAACGTAATACACGAAGA
+
FFFFFFFFFFFFFFFFFFFFFFFFFFFFFFFFFFFFFFFFFFFFFFFFFFFFFFFFFFFFFFF:FFFF
FFFFFFFFFFFFFFFFFFFFFFFFFFFFFF:FFFFFFFFFFFFFFFFFF
@A00721:190:HGJW7DSXY:4:2404:16658:6480_1:N:0:TCTGCAAG+GCTGCACT
CACGACTCGAAACCGAGACGCCGTA
+
:FFFFFFFFFFFFFFFFFFFFFFFF
@A00721:190:HGJW7DSXY:4:2269:4209:20556_2:N:0:TCTGCAAG+GCTGCACT
TTTAGATTATGAATCCTGCCTTTACTCATTTTATAGCAGTTATTTATGTTGTACTTTTTATAATTTAT
AACCTTCTTATACTTTTGGGTATGTTTTGTGAAAAACATGAGATACCC
+
FFFFFFFFFFFFFFFFFFFFFFFFFFFF,FFFFFF:FFFF:FFFFFFFFFF:FFFFFFFFFFFFFFFF
FFF:FFFFFFFFFFFFFFFFFFFFFFFFFFFFFFFFFFFFFFFFFF:F
@A00721:190:HGJW7DSXY:4:1261:2220:20400_2:N:0:TCTGCAAG+GCTGCACT
TTGTAACGATCATTCTTTCGAGTTTTGTCATTCTTATTCTTTTATTCCTTTAACTGTGTATGAGGAGG
ATTTATAATGCTTTTCGATAGTGATTGTTATCAATTTAAATACATTAC
+
FFFFFFFFFFFFFFFFFFFFFFFFFFFFFFFFFFFFFFFFFFFFFFFF:FFFFFFFFFFF::FFFFFFF
FFFFFFFFFFFFFFFF:FFFFFFFFFFF:,FFFFFFFFFFFFFFFFFF
@A00721:190:HGJW7DSXY:4:2464:4896:15922_2:N:0:TCTGCAAG+GCTGCACT
CTTTACTCACAGACTCCGAAAATGGTTTCAAATGAGGTCAAAGCTCTATTTATTAAACGAACTATTAT
CCGATTCTTCGAGGGTATTACTACCACTGATCTGCCGACTTTAGTCAA
+
FFFFFFFFFFFFFFFFFFFFFFFFFFFFFFFFFFFF:FFFFFFFFFFFFFFFFFFFFFFFFFFFFFFF
FFFFFFFFFFFFFFFFFFFFFFFFFFFFFFFFFFFFFFFFFFFFFFFF
@A00721:190:HGJW7DSXY:4:2419:8947:5901_2:N:0:TCTGCAAG+GCTGCACT
TTCCTTCGATGTTTATATTTTTCTATAAGGGAAAATAGCAATAATCAAATTTCGACAAATTTTAGAAC
TTTATTTCAGAACTCTCTCCGATCACCTTGGTGTGATCTCCCGGAGGA
+
FFFFFFFFFFFFFFFFFFFFFFFFFFFFFFFFFFFFFFFFFFFFFFFFFFFFFF::FFFFFF:FFFFFF
FFF:FFFFFFFFFFFFFFF,FFFFF:FFFFFFFFFFFFFFFFFFFFFFF
@A00721:190:HGJW7DSXY:4:1567:12635:21465_2:N:0:TCTGCAAG+GCTGCACT
ATGTATGATGATAATCTACTATTTTTAAATAGTGATATTCAGCGTATACTTGAATACTTTATCAAAGA
TTTGGCTTGGTCTATTTACCGCAAAAAGTTGGAAGAGGTCTATGATAT
+
FFFFFFFFFFFFFFFFFFFFFFFFFFFFFFFFFFF:FFFF,FFFFFFFFFFFFFFFFFFF:FFFFFFF:
FFFFFFFFFFFFFFFFFFFFFFFFFF::F,F:FFF,FFFFFF:FFFF:F
@A00721:190:HGJW7DSXY:4:1112:5629:6511_2:N:0:TCTGCAAG+GCTGCACT
TTTAAATAGCACCGATTATAACTTGACTGTTGATAATATTACAATAGCTCTTTCTCAGAAGATTAGAG
AAGTAACCGGTGAACACGTCGGTAGCATTGATTTAATTTTACCGTTAG
+
FF:FF::FFFFF:,FFFFFFFFFFFF:FFFFFFFFFF:FFF,FF:FF:FFFFFFFFFFFFFFFFFF,,
FFFFFFFFF:FFFFFFFFFF:FFFFFF::,FFF:F,,FFFFF::FFF:
@A00721:190:HGJW7DSXY:4:2164:9064:13714_2:N:0:TCTGCAAG+GCTGCACT
TTTCGAAAAGAAACCTATAAGTTACAAACGATTCTTCGACTATATTAAAGAAATAAAAATACTTCAAG
TACCTGAACTTTACATTTTTAATGGTATGGTATAATGTGTCTTACAAA
+
FFFFFFFFFFFFFFFFFFFFFFFFFFFFFFFFFFFFFFFFFFFFFFF:FFFFFFFFFFFFFFFFFFFF:
FFFFFFFFFFFFFFFFFFFFFF,FFFFFFFFF:FFFFFFFFFFFFFFFF
@A00721:190:HGJW7DSXY:4:1128:16387:28620_2:N:0:TCTGCAAG+GCTGCACT
TCTCGGTAATGAGAGTGTATTCCTTAATATTCTAGTAACCGCTATGTGGGCATTACTAGGATTTATCA
```

CACTCCTAAATTTGATAGCAATTTATGTTTTAAATGCTAGGAAAATTT
+
FFFFFFFFFFFFFFFFFFFFFFFFFF:FFFFFFFFFFFFFFFFFFFFFFFFFFFFFFFFFFFFFFFFFF
FFFFFFFFFFFFFFFFFFFFFFFFFFFFFFFFFFFFFFFFFFFFFFFFF
@A00721:190:HGJW7DSXY:4:2272:13467:8938_1:N:0:TCTGCAAG+GCTGCACT
TGTATAGTTCAAGGAGTACTTAATTCAATAGCTTTAGTATTATTTACTATCGCCTTTATTGAAAATTG
TATAAGAGTTAATTGCTTACACAAAAGTGACGCGCGAAGGAATCTTAATTGATTGAAGCTAGGCGTTG
GGG
+
FFFFFFFFFFF:FFFFFF:F::FFF:F:FFFFF:FFFFFFFFFFFFF:FFFFFF:FFFFFFFFFFFFF
FFFFFF,F,FFF:FFF:FFF,FFFF:F:FFFFFFFFF:FFFF,FF:FFFFFFFFFFFFFFFFFFFFFF
FFF
@A00721:190:HGJW7DSXY:4:1403:8938:27242_1:N:0:TCTGCAAG+GCTGCACT
CTCCCCAAACATAACATACCATAACCTACCATGACCAATCATCGCTCCAACTGGCACGCCAGACGTAG
GGTCAAACCTACTACCGCCTGTCGGCAGATCGGCACCACTGGTCTCTACGTTCAGGGACCCGCTGTGC
AGA
+
FFFFFFF::F,F:FFFFFFF:F,FFFFFFFFFFFFFFFFFFFFFFFFFFFFFFFFFFFFFFFFFFFFF
FFFFFFFFFFFFFFFFFFFFFFFFFFFFFFFFFFFFFFFFFFFFFFFFFFFFFFFFFFFFFFFFFFFF
FFF
@A00721:190:HGJW7DSXY:4:1101:8513:32643_2:N:0:TCTGCAAG+GCTGCACT
TCTGCACAGAAGAAGGCTGAAGGTCGTTACCGGACGACCACTCAAAGGAAACGGGCTGTTGCCCAGAG
GAAAGCAGCGACCACCGAGGTCGCTCGTAACATTGACGGCAGTCCCGT
+
FFFFFFFFFFFFFFFFFFFFFF:FFFFFFFFFFFFFFFFFFFFFFFFFFFFFFFFFFFFFFFFFFFFF
FFFFFFFFFFFFFFFFFFFFFFFF,FFFFFFFFFF:FFFFFFFFFFF
@A00721:190:HGJW7DSXY:4:1431:14009:8093_1:N:0:TCTGCAAG+GCTGCACT
AACTGAGGCAGTACCTCAGTCAAAAGTCACATTCACCACTCCCATACCAATCAATGATGTGACTATAC
AGTTCGGCCATTTCAAGAGTGGTCAATACGTCGTGGGAGGTACTGAAGCCTTCCCCTCCGCTGGCACG
AAG
+
FFFFFFFFFFFFFFFFFFFFFFFFFFFFFFFFFFFFFFFFFFF,FFFF:FF::FFFFFFFFFFFFFF
FFFFFFFFFFFFFFFFFFFFFFFFFFFFFFFFFFFFFFFFFFFFFFFFFFFFFFFFFFFFFFFFFFFF
FFF
@A00721:190:HGJW7DSXY:4:1223:7609:6151_2:N:0:TCTGCAAG+GCTGCACT
GCCAATCAATAGTTCTTCTGACCAGAATGAAAAGGGGTTTACCAGGGTAGTCTCTAAGTCTACCAGGA
AGAGGGGAAATGTCCCCAAAACTGAGAAGAATAAAGGGAAAGGTCCAT
+
FFFFFFFFFFFFFFFFFFFFFFFFFFFFFFFF:FFFFFFFFFFFFFFFFFFFFFFFFFFFFFFFFFFF
FFFFFFFF:FFFFFFFF:FFFFFFFFFFFFFFFFFFFFFFFFFFFFFF
@A00721:190:HGJW7DSXY:4:2508:22073:17989_2:N:0:TCTGCAAG+GCTGCACT
GAAGGGGAAATTACCCCTAATTCTGAAGGAAGATCTCCCACCATCTGGGGTTGGGAATAAGAAATCTG
CACGGGCCAAAGAGGCCGCTGCTACGAAGTCGCTGAAGGCGGCTTGGG
+
FFFFFFFFFFFFFFFFFFFFFFFFFFFFFFFFFFFFFFFFFFFFFFFFFFFFFFFFFFFFFFFFFFFF
FFFFFFFFFFFFFFFFFFFFFFFFFFFFFFFFFFFFFFFFFFFFFFFF
@A00721:190:HGJW7DSXY:4:1123:12264:4006_2:N:0:TCTGCAAG+GCTGCACT
GTAAAGTTGCGCCTGCCGACCCCATCTTTGAGACCAAGACTCTCTACAGTAGGTTCGTGCCTACTATG
AGCAAATCTGTTGAATTCGTCACTGAGGACGAAGGTGAGAGGACTTTT
+
FFFFFFFFFFFFFF,FF:FFFFFFFFF:,FF,FFFFFFFF:F,,FF:FFFFF:FFFFFFFF:FFFFF:
FFFFFFF:F:FF:FFFFF:FFF:FFFF,FFFFFF,,FFF:F,FFFFFF
@A00721:190:HGJW7DSXY:4:2458:30300:31140_1:N:0:TCTGCAAG+GCTGCACT
CTGAGAGCAGTCGCTGGGGAAAGAGAATTGCGTGGGTTGAAGGCCGACGCTTCGAGCTTGAGTGTGAC

GAGGCCGGTGACGTTTTTGTCAACGGCGTTTGGAGGATTTTATCCGCGCTTAACTATGGGGCGAAGAT
CCC
+
FFFFFFFFFFFFFFFFFFFFFFFFFFF,FFFFFFFFFFFFFFFFFFFFFFFFFFFFFFFFFFFFFFFFF
FFFFFFFFFFFFF,FFFFFFFFFFFFFFFFFFFFFFFFFFFFFFFFFFFFFFFFFFFFFFFFFFFFFFF
FFF
@A00721:190:HGJW7DSXY:4:2544:31069:3724_2:N:0:TCTGCAAG+GCTGCACT
GTTGGCATATTTATGCCGACGATAATAAGCATGTCACCCTTAAGCACCGAGGTAAGCTCGGGACTTAC
AAGAGAGAGTGTGAGAGAGTCGCTCTCACTATTTTTGCCCAGAATGCG
+
FFFFFFFFF:FFFFFFFFFFFFFFFFFFFFFFFFFFFFFFFFFFFFFFFFFFFFFFFFF:FFFFFFFF
FFFFFFFFFFFFFFFFFFFFFFFFFFFFFFFF:FFFFFFFFFFF,FF:F
@A00721:190:HGJW7DSXY:4:1668:16098:19789_2:N:0:TCTGCAAG+GCTGCACT
TTCACTGGTTCTGGGAGGCTGCTTTCCTCCATAATTACAACCTCTCTTTAGAGAGCATTAGAAGGAAG
TTGGGCAAATTCCCACATATGGTAGACGTTGTCACACAGATCGGAAGA
+
FFFFF:FFFFFFFFFFFFFFFFFFFFFFFFFFFFFFFFFFFFFFFFFFFFFFFFFFFFFFFFFFFFFF
FFFFFFFFFFFFFFFFFFFFFFFFFFFFFFFFFFFFFFFFFFFFFFFF
@A00721:190:HGJW7DSXY:4:1169:20139:22623_2:N:0:TCTGCAAG+GCTGCACT
CGTCATTGGGGATTTCGATGTGAGAAGGTGGAAGGTGTCAAGGGTCTTCTTCATGCTGATCTTTCAGG
ACCTAATCACATCATGATTGATAGTTACGGCTCGTATCGAGTTGGAGG
+
FFFFFFFFFFFFFFFFFFFFFFFFFFFFFFFFFFFFFFFFFFFFFFFFFFFFFFFFFFFFFFFFFFFF
FFFFFFFFFFFFFFFFFFFFFFFFFFFFFFFFFFFFFFFFFFFFFFFF
@A00721:190:HGJW7DSXY:4:1356:27489:32878_2:N:0:TCTGCAAG+GCTGCACT
CGAAATTAAAGACGGGGGGATTAAGAGTCCAGCTGTAACTGTCATTTCAGAGAGGGGAGCTCTGAAAA
GGAAACTTCGTCAGAAGATGAGGAAGTCTAAGAGGAGGAGTTTGAGGA
+
FFFFFFFFFFFFFFFFFFFFFFFFFFFFFFFFFFFFFFFFFFFFFFFFFFF:FFFFFFFFF:FFF:F
FFF:FFFFFFFFFFFFFFFFFFFFFFFFFFFFFFFFFFFFF:FFFFFFF
@A00721:190:HGJW7DSXY:4:1642:7762:33035_2:N:0:TCTGCAAG+GCTGCACT
GAAGTTGATGAAGAAGGATTATTTGAAGCAGGGGTTACCACTTCCTAAGGTGGATGAGGGAGGTTGTT
TCTTTGGAGTTTTAGATAATCTTTTGAGAAGGGGTCATAGAGCCAGCA
+
FF,FFFFFFFFFFFFFFFFFFFFFFFFFFFFFFFFFFFFFFFFFFFFFFFFFFFFFFFFFFFFFFFFF
FFFFFF,FFFFFFFFFFFFFFFFFFFFFFFFFFFFFFFFFFFFFFFFF
@A00721:190:HGJW7DSXY:4:2602:21649:15123_2:N:0:TCTGCAAG+GCTGCACT
CCTTGAGAAGACAAGCCACATTAAGATATAATTTAGGTCAACTTGGGGTTTCGAATGTCATTAGTGCC
TTACGATTGAAAGAGGTCCTTTTGAGTATCGACAGATCTTTCGATCTT
+
FFFFFFFFFFFFFFFFFFFFF:FFFFFFFFFFFFFFFFFFFFFFFFFFFFFFFFFFFFFFFFFFFFFF
FFFFFFFFFFFFFFFFFFFFFFFFFFFFFFFFFFFFFFFFFFFFFFFF
@A00721:190:HGJW7DSXY:4:1451:21567:29700_2:N:0:TCTGCAAG+GCTGCACT
TGGCAGAATTCGGTAACTTACCATATTGTTCCTTCCGGGAAGTTGTGGGATTTAACTTTGACTCAACT
ACTCCGATCAGATCCGGATGGTTTGATTGGTATGGACTACTCGACTTT
+
FFFFFFFF:FFFFFFFFFFFFFF:FFFFFFFFFFFFFFFFFF:FFFFFFFFFFFFFFFFFFFFFFFFF
FFFFFFFFF,FFFFFFFFFFFFFFFFF:FFF,FFFFFFFFFFFFFFFF
@A00721:190:HGJW7DSXY:4:2146:6271:32299_1:N:0:TCTGCAAG+GCTGCACT
CATGGCAGATACTACCGATAAGGTGAAAAATATGCAGGTCAAAGATTCTGTAGCTCTGAGGGCGGTTG
AGGGAGCCGTTTTGGCCGACTTTAACTATTCTAAGAGTCTTTATGATTCAAAACCTATCGTTGTCGTA
CCT
+
FFFFFFFFFFFFFFFFFFFFFFFF:FFFFFFFFFFFFF:FFFFFFFFFFFFFFFFFFFFFFFFFFFFFF

FFFFFFFFF:FFFFFFFFFF:FFFFFFFFFFFFFFFFFFFFFFFFFFFFFFFFFFFFFFFFFFFFFFF
FFF
@A00721:190:HGJW7DSXY:4:1145:7374:34491_2:N:0:TCTGCAAG+GCTGCACT
GGCTTATTGTCGCGCAGTTTCCAGAATTGCGGTTGAAGTTCACCCATTCTATCTTGAATGCTCACGCC
GTCGCAGCCATATCGAGACTATGTTATAATCACTTGTTAAAGTACAGA
+
FFF:FFFFFFFFFFFFFFFFFFFFFFFFFFFFFFFFFFFFFFFFFFFFFFFFFFFFF:FFFFFFFF
FFFFFFFFFFFFFFFFFFFFF:FFFFFFFFFFFFFF:FFFFFFFFFFFF
@A00721:190:HGJW7DSXY:4:1477:13503:16297_2:N:0:TCTGCAAG+GCTGCACT
AGATATTATTGATATTGGAGGCGATATTCGTTTTCATATCCAAAACTCCTTGAATGGAGTACATATTT
GCAACCCTATTCTTGATGTGAAAGATGCATCGAGGTATACGTCTAGGA
+
FF:FFFFFFFFFFFFFFFFFFFFFFFFFFFFFFFFFFFFFFFFFFFFFFFFFFFFFFFFFFFFFFFF
FFFFFFFFFFFFFFFFFFFFFFFFFFFFFFFFFFFFFFFFFFFFFFFF
@A00721:190:HGJW7DSXY:4:1525:2953:28025_2:N:0:TCTGCAAG+GCTGCACT
TCACGAGACGTCATGAAAGCTATCGGAGGGAAGCAAAATGTTTCGTTCTGTTACAAGAACGCTTTGGA
TTGTGACCATAAAGCCTCAGTTGCGATAGCTGTAGAGGTATACGATAT
+
FFFFFFFFFFFFFFFFFFFFFFFFFFFFFFF:FFFFFFFFFF,FFFFFFFFFFFFFFFFFFFFFFFFF
FFFFFFFFFFFFFFFFFFFFFFFFFFF:FFFF:FFFFFF,FFFFFFFF
@A00721:190:HGJW7DSXY:4:2202:4209:27320_2:N:0:TCTGCAAG+GCTGCACT
CTTAAGAGAGAGATTGATGTATGTCATTTCGTCTTAGTTTGTCCCGGCGAATTATTGGACACGATGAA
AAATGACATACCCGTTCCTGACCTTGGTATCGTTATCCGTCATGGAGA
+
FFFFFFFFFFFFFFFFFFFFFFFFFFFF:FFFFFFFFFFFFFFFFFFFFFFFFFFFFFFFFFFFFFFF
FF:FFFFFFFF:F::FFFFFF,FFFFFFFFFFFFFFFFFFFF,:FF:F
@A00721:190:HGJW7DSXY:4:1659:12029:9862_2:N:0:TCTGCAAG+GCTGCACT
GTTATTCTCACAACAAAGATAAGATTTTATCTTTCTTCAAACACCCTGTTTTCCTTAAGGGCGGACAA
TTGTTCCATTGTGAACTCTTGGGGACTAGATTGAGCATTAATGAGTTC
+
FFFFFFFFFFFFFFFFFFFFFFFFFFFFFFFFFFFFFFFFFFFFFFFFFFFFFFFFFFFFFFFFFFFF
FFFFFFFFFFFFFFFFFFFFFFFFFFFFFFFFFFFFFFFFFFFFFFFF
@A00721:190:HGJW7DSXY:4:1247:10691:15969_2:N:0:TCTGCAAG+GCTGCACT
CTATTTCCGTACCAAGAGCCACTGCCAATCTCATCGCTATACGCATACCTGATTGGAAGAAGCGAGGT
TCGTTCCTCAATAAAAATTTTTCTCAAGAGATTATGGTTGATAAAGTT
+
FFFFFFFFFFFFFFFFFFFFFFFFFFFFFFFFFFFFFFFFFFFFFFFFFFFFFFFFF:F:FFFFFFFF
FFFFFFFFFFFFFFFFFFFFFFFFFFFFFFFFFF,:FFFFFFFFFFFFF
@A00721:190:HGJW7DSXY:4:1547:21721:8766_1:N:0:TCTGCAAG+GCTGCACT
TTCAGTACGTCATGGGGATCACTTCTTCGGTCAATGACAAAACCTTCGAATATGTTACGACATGGCTT
CGTAACAACAGTGCGAGGATTGTCATCTCGGGGAAAGTGATTCACCAAGATGTTCATATAGATACGAG
ATA
+
FFFFFFFFFFF,:FFFFFFFFFFFFFFFFFFFFFFFFFFFFFFFFFFFFFFFFFFFFFFFFFFFFFFF
:FFFFFFFFFFFFFFFFFFFFFFFFFFFFFFFFFFFFFFFFFFFFFFFFFFFFFFFFFFFFF:FFFFF
FFF
@A00721:190:HGJW7DSXY:4:1452:15881:15280_2:N:0:TCTGCAAG+GCTGCACT
CAGCGATCTTACTCGTTATCGGAGCTAAGAAGAAGACTTTGGGTACACGTTATGCTCGCAAACTTAAC
GTTAGTACCGGAGGTGATCTCTCTGCTTCTGCGTTACTGAGAGAAAAG
+
FFFFFFFFFFFFFFFFFFFFFFFFFFFFFF:,F,FFFF:FFFFFFFFFFFF:FFFFFFFFF:FFFFFFF
FFFFFFFFFFF:FFFFFFFFFFFFFFFFFFFFFF:FFFFFFFFF:FFFF
@A00721:190:HGJW7DSXY:4:2234:16459:1251_1:N:0:TCTGCAAG+GCTGCACT
CCTACGGTGACTTTATGGACAAGATGCAAGTTCTTGGGAAATACTTGTTTCCCTTTTTAGGTTTAGTA

GGTGATCTTGACGTCGATTCTTTGTATAGGGAGTGTGAGAAGACAACGAATCTTGAAACTACGGTCCT
TAT
+
FFFFFFFFFFFFFFFFFFFFFFFF,FFFFFFFFFFFFFFFFFFFFFFFFFFFFFFFFFFFFFFFFFFFF
FFFFFFFFFFFFFFFFFFFFFFFFFFFFFFFFFFFFFFFFFF::FFFFFFFFFFFFFFFFFFFFFFFFFF
FFF
@A00721:190:HGJW7DSXY:4:1238:28628:28275_2:N:0:TCTGCAAG+GCTGCACT
ACCGGTCTCTAAATCTTTTGAGATTGAGAAGATTGAAGAACACGTCAAGAAGTCGCTCGGCGTTGTTC
TTGAAAACCAAGTTAAGAAGGAGGCTGATCATGGAGTCTTTGTACCTT
+
FFFFFFFFFFFF:F:FFFFF:FFFFFFFFFFFFFFFFFFFFFFFFFFFFFFFFFFFFFFFFFFFFFFFF
FFFFFFFFFFFFFF,FFFFFFFFFFFFF,FFFFFFFFFFF::FFFFFF
@A00721:190:HGJW7DSXY:4:1308:30924:5509_2:N:0:TCTGCAAG+GCTGCACT
GGTTCAGCTACGAGAGGTGATCCCGGATGTGGATTGAAGGCTGGCTCTAAGGGAGACTCCTTCTTTTT
GAATGTTGTAAAGAATTGGGTTGAGAAGTGTGCTTTACAAGGAGTTAG
+
FFFFFFFFFFFFFFFFFFFFFF,FFFFFFFFFFFFFFFFFFFFFFFFF:FFFFFFFFFF:FFFFFFFF
FFFFFFFFFFFFFFFFFFFFFFFFFFFFFFFFF:FFFFFFFFFFFFFF
@A00721:190:HGJW7DSXY:4:2505:17535:23312_1:N:0:TCTGCAAG+GCTGCACT
AGTTTTTAGATCTTCTTGATAATCTTAAAGAAAAATTTTCGAATAAACTAATGAGAGGTATAGGCTTA
GTCAAGTCTGTACTTACATTCTTTACGCCAGATTCTTCATGGGGCGACGCTGTTTCTCTTGGTATTTC
TGA
+
FFFFFFFFFFFFFF:FF,FFFFFF::FFFFFFFFF:FFFFFFFFFF,FFFF:F:FFFFFF:FFFFFFF
FFFFFFFFFFFFFF:FFFFFFFFFFFF:FFFFFFFF:FFFFFFFFFFFFFFFFFFFFFFFFFFFFFFFF
FFF
@A00721:190:HGJW7DSXY:4:1623:15619:32550_2:N:0:TCTGCAAG+GCTGCACT
CAGAGCGTCATTTTCCTTTGGGATCTTTCCTCTGGTAAGGGAATTATTAAATCATTCTTCTCACTCTT
GACGAGAATTATTACTGTTTTCTCTACCCATAACTTCTTTTTCAAAAA
+
FFFFFFFFFFFFFFFFFF:FFFFFFFFFFFFFFFFFFFFFFFFFFFFFFFFFFFFFFFFFFFFFFFFFF
FFFFFFF:FFFFFFFFFFFFFFFFFFFFFFFFFFFFFFFFFFFFFFFF
@A00721:190:HGJW7DSXY:4:1254:9896:5509_1:N:0:TCTGCAAG+GCTGCACT
CTGATGTTTTCCTACAGAGTGCAGTCGTTTCAACACTTATCACCACTTTTGAAAGGAAAATAATTGGT
GATGAAAATACGTATGTCGGTGGGGTTCTCAAGGCTCTCATCGCCACTGGGATTCTAAGGGGTTCCAT
AAG
+
FFFFFFFFFFFFFFFFFFFFFFFFFFFFFFFFFFFFFFFFFFFFFFFFFFFFFFFFFFFFFFFFFFFF
FFFFFFFFFFFFFFFFFFFFFFFFFFFFFFFFFFFFFFFFFFFFFFFFFFFFFFFFFFFFFFFFFFFF
FFF
@A00721:190:HGJW7DSXY:4:2430:31638:5212_2:N:0:TCTGCAAG+GCTGCACT
AGGATTCTTCTTTTGGTCCTTACGTCTTAAGTAATTTGGATGTTGTCGAAAACTATCAGGTTCTTGTC
ACGATTCTTAAAAGAAGTTTATTAAAACTTCAGAGTCTCTTGGAAGTC
+
FFFFFFFFFFFFFFFFFFFFFFFFFFFFF:FFFFFFFFFFFFFFFFFFFFF:FFFF:FFFFFFFFFFF
FF:FFFFFFFF::FFFFFFFFFF:F:FFFF:FFFFFFF,FFF,FFFF:
@A00721:190:HGJW7DSXY:4:1265:3161:3145_1:N:0:TCTGCAAG+GCTGCACT
CAATAGCCACAGACTTTCTGGGAAAAGAGACCTCTGTTGGTAGAGGAGTTGAGAGAATTACTCAGATT
CAATCTGGCATCACTGAACGTCTACAACGAGTTAGGGAGTGGCTAGGTTTTAAAAGATCCTTTGCACA
AGC
+
:F:F,F,FFFFF:FFFF:FF:FFFF:F,FFFFFFFFFFFFFFFFFFFFFF:FFFFF:FF:FFF:FFFF
FFFFF:FFFFF:FFFFFFFFFFF::FFFFFFFFFFFFFFFFF,FFFFFFFF:FFFFFFFFFFF:FFF,
,FF

@A00721:190:HGJW7DSXY:4:1635:4725:2002_2:N:0:TCTGCAAG+GCTGCACT
TTTCGACGTCGCGGAAAACGATGAAGATTTCCACACTGTTCGAGAATCTTCTGATGCTGAATTTGAGG
AAAATTTTTCGGAAGTTTTTGGTATAAATCCTGATGGCTCAGTACAGG
+
FFFFFFFFFFFFFFFFFFFFFFFFFFFFFFFFFFFFFFFFFFFFFFFFFFFFFFFFFFFFFFF,::FFFFF
FFFFFFFFFFFFFFFFFFFFFFFFFFFFFFFFFFFFFFFFFFFFFFFF
@A00721:190:HGJW7DSXY:4:1528:2293:27195_2:N:0:TCTGCAAG+GCTGCACT
AAACCTTCTTTTATCATTGCAACAGGACTTTGAGAAGACTGTTGAAATGGACAAAATCTGGGATCATA
GGTATGTGTCACGCTACACTCGAAATATGGGCCCTCATAAGGAAGTAT
+
FFFFFFFFFFFFFFFFFFFFF:FFFF:FFFFFF:FFFFF:FFFFFFFFFFFFFFFFFFFFFFFFFFF:F
F:F:FFFFFFFFFFF:FFFFFFFFFFFFFFFFFFFFFFFFFFFFFFFF
@A00721:190:HGJW7DSXY:4:1404:3052:31485_1:N:0:TCTGCAAG+GCTGCACT
GATCATTTGTTCAGGAAGATCTCTACAGAGATTATCGAGAGTTGTGATCTAGTTCTAAATTTAGGTCG
ACGGACTTTAGCAACTTTAGATAAATTCTTTCCGGGCTCAATGACCTTTGCTATGTACGTGGATCTAA
TGC
+
FFF::FFFFFFFFFFFFFFFFFF:,:FFFFFFFFFFFFFFFFFFFFFFFFFFFFFFFFFFF:F,FFFFF
FF:FFFFFFF,,FFFFFFFFFFF:::FFFFFFF:F:FFFFFFFFFFF:FFFFFFF,:FFFFFFFFFFF
FFF
@A00721:190:HGJW7DSXY:4:1508:22182:21966_2:N:0:TCTGCAAG+GCTGCACT
CATCGCAAAGAAGACTTTATTAGTTCATGAAGTTCTTACACTTCTCGCGACTAATGTCAGCTTCACTA
CAAAGGCGTTACGTAAAGTAGTTTTCACGCTTTATCTTAAAGCATTAC
+
FFFFFFFFFFFFFFFFFFFFFFFFFFFFFFFFFFFFF:FFFF:FFFFF:FFFFFFFFFFFFFFFFFF
FFFFFFFFFFFFFFF,FFFFFFFFFFFFFF,FFFFFFFFFFFFFFFFF
@A00721:190:HGJW7DSXY:4:2246:7554:19586_2:N:0:TCTGCAAG+GCTGCACT
ACTTTTTGAATATGACAGAAGCTCGCCATATCAGATTAGAGACATGCCAGTTAGAGTTCTTCTAACCA
GGACTGATGGTGAGATTCTTTTACCTTTTGGAGGTCCAGGAACCTTTA
+
FFFFFFFFFFFFFFFFFFFFFFFFFFFF:FFFFFFFFFFFFFFFFFFFF:F:F:FFFFFFFFFFFFFF
FFFFFFFFFFFFFFFFFFFFFFFFFFFFFFFFFFFFFFFFFFFF:FFF
@A00721:190:HGJW7DSXY:4:1621:22290:29543_2:N:0:TCTGCAAG+GCTGCACT
CCAAGGACGTTTCCGTCGGAGGATGACAGGTGGGAGACCGCTCAATAACACTCTATTGAATCCCTTTT
CCACGATGAAAGCTCTTTATGAGGTGCTCCATGCAAATTTACAGTTCC
+
FFFFFFFFFFFFFFFFFFFFFFFFFFFFFFFFFFFFFFFFFFFFFFFFFFFFFFFFFFFFFFFFFFFFFFFFFFFFFFFFFFFFFFFFFFFFFFFFFFFFFFFFFFFFFFFFFFFFFFFFFFFFFFFFFFFFFF
FFF,FFFFFFFFFFFFFFFFFFFFFFFFFFFFFFFFFFFFFFFFFFFF
@A00721:190:HGJW7DSXY:4:1627:29387:19633_1:N:0:TCTGCAAG+GCTGCACT
GTATTTTGATAAATTTGTTGGAATCGATAATGATACTGACGAAGGTTCTATTACCACCCTTGTCATTG
ATACAAACATTCTAGGTGATGCTTTCTTAGAGGCTCGTCTAGCCGCTGAGATTCGTCTTACCTATTTA
CAA
+
FFFFFFFFFFFFFFFFFFFFF::FFFFFFFFFFFFFFFFFFFFFFFFFFFFFFFFFFFFFFFFFFFFF
FFFFFFFFFFFFFFFFFFFFFFFFFFFFFFFFFFFFFFFFFFFFFFFFFFFFFFFFFFFFFFFFFFFF
FFF
@A00721:190:HGJW7DSXY:4:2357:28845:31814_2:N:0:TCTGCAAG+GCTGCACT
TTTGAGTGATAACGATTTAGTACTGGGAAATGGTGGTAGTCAGTTTACTACTTTTCTTTCCCTCCCCT
TTTTCGATTGGATTCGACGGGGTCTTCGTCTCACGACTAATTTGATAA
+
FFFFFFFFFFFFFFFFFFFFFFFFFFFFFF,FFFFFFFFFFFFFFFFFFFFFFFFFFFFFFFFFFFFF
FFFFFFFFFFFFFFFFF:FFFFFFFFFFFFFFFFFF:F,,FFFFFFFF
@A00721:190:HGJW7DSXY:4:1256:21739:9173_2:N:0:TCTGCAAG+GCTGCACT
TATTACTTCGGAAGTTCTTAATGTAGGTCTCCTAGTACTTAAGGGTTTATGTTTCGGTTTCAGCTGGA

TGGATCTTTTGATGGTATGTTTCACATTGCGTGATCACTTCCCGTGGA
+
FFFFFFFFFFFFFFFFFFFFFFFFFFFFFFFFFFFFFFFFFFFFFFFFFFFFFFFFFFFFFFFFFFFFFF
F:FFFFFFFFFFFFFFFFFFFFFFFFFFFFFFFFFFFFFFFFFFFFFF
@A00721:190:HGJW7DSXY:4:1473:3323:34491_1:N:0:TCTGCAAG+GCTGCACT
GATTAAACATAAAATCCAGCGATTCTATTATGTCGCTAAAGCCTGTCCAGTTGTCGATGATGTTCGGG
CTGTGGGGATTAACTTCCCTCGTGAAACTTACGACGATGTTCTCGCTCGCATTTCACAAGGATTACTA
AGT
+
FFFFFFFFFFF:FFFFFFFFFFFFFFFFFFFFFFFFFFFFFFFFFFFFFFFFFFFFFFFFFFFFFFFFF
FFFFFFFFFFFFFFFFFFFFFFFFFFFFFFFFFFFFFFFFFFFFFFFFFFFFFFFFFFFFFFFFFFFFF
FFF
@A00721:190:HGJW7DSXY:4:1266:26847:17456_2:N:0:TCTGCAAG+GCTGCACT
CCACTTCTAATTTTAGGAAACGTCGCGAAAAGAAAGTTCATGAGGATGTCTTGGTCGAAAAATATCGC
CGGGACCTTCTAATAGAGGATCCTGATGACATTATTTCAACGGATACC
+
FFFFFFFFFFFFFFFFFFFFFFFFFFFFFFFFFFFFFFFFFFFFFFF:FFFFFFFFFFFFFFFFFFFFF
FFFFF,FFFFF:FFFFFFFFFFFFF:FFFFFFFFFFFFFFFFFFFFFF
@A00721:190:HGJW7DSXY:4:1119:23366:25801_2:N:0:TCTGCAAG+GCTGCACT
CTACCACAGAAACCGTCTCAGGAACTGTCTCCACTGATTCGGAATTAGAGAAAGACGACCTTCTTGTG
GGGACTGAGACCTCTGAATTTTCAGGGTGCCCTGATGAGGACGTTGAA
+
FFFFFFFFFFFFFFF:F:FFFFFFFFFFFFFFFFFFFFFFFFF:FFFFFFFFFFFFFFFFFFFFFFFFF
FFFFFFFFFFFFFFFFFFFFFFFFFFFFFFFFFFFFFFFFFFFFFFFF
@A00721:190:HGJW7DSXY:4:2443:27715:30639_1:N:0:TCTGCAAG+GCTGCACT
ACTCTGAGACTTCTAAGAGTGTTTCAAAAGACCAGAAACGTAACAAGAAGAATAAAGGTAGGAAGGGG
AGAAAACAGAACAAGCGAGCGGTTGAAGAGAAAACTGATATGGTTGCGGCTGATCGTACCCCAATTCC
TGC
+
FFFFFFFFFFFFFFFFFFFFFFFFFFFFFFFFFFFFFFFFFFFFFFFFFFFFFFFFFFFFFFFFFFFFF
FFF:FFFFFF,FF,FFFFFFFFF,FFFFFFFFFFFFFFFFFFFFFFFFFFFFFFFFFFFFFFFFF:FFF
FFF
@A00721:190:HGJW7DSXY:4:2126:6144:2769_2:N:0:TCTGCAAG+GCTGCACT
CTCGGCGATACCACTGATCTTCCAGAGTTACGTAGTGGTCGTTCTTTTGGTGATTTTATCAAAGAGAA
AGTTTCTGCTAGTCGCGACGATAGTATTTATCGGAGTGATATTAACAA
+
FFFFFFFFFFFFFFFFFFFFFFFFFFFFFFFFFFFFFFFFFFFFFFFFFFFFFFFFFFFFFFF:F:FFF
FFFFFFFFF::FFFFFFFFFFFFFFFFFFFFFFFFFFFF:FFFFFFFFF
@A00721:190:HGJW7DSXY:4:1354:20166:19163_2:N:0:TCTGCAAG+GCTGCACT
TGTTGAAGTACCGCAACATGGATCAGTTTTCCAAAAAGCTATTAATAGCAGTTGCGGGGCTATAATAC
CGCCTCCAGATTACTCTAAACTGAATCCACTCTCTTGGGATATAAATG
+
FFFFFFFFFFFFFFFFFFFFFFFFFFFFFFFFFFFFFFFFFFFFFFFFFFFFFFFFFFFFFFFFFFFFF
FFFFFFFFFFFFFFFFFFFFFFFFFFFFFFFFFFF:FFFFFFFF:FFF:
@A00721:190:HGJW7DSXY:4:2226:4770:9126_1:N:0:TCTGCAAG+GCTGCACT
GGCTTCTCTAAAGAACAGCAACTTTCAACATGAATTAGTTATTGAAAGTCTTGACCCTCACGTCTCAC
TTTATCACCTTGGAGATGGTAAAGTCATTAACAAAGGAAATGTTAAGGCTGATGACTTAAATTTCGCA
TTC
+
F:FFFFFFFF:FFFFFF:FFFFFFFFFFFFFFFFFFFFFFFFFFFFFFFFFFFFFFFFFFFFFFFFFFF
FFFFFFFFFFFFFFFFFFFFFFFFFFFFFFFFFFFFFFFFFFFFFFFFFFFFFFFFFFFFFFFFFFFFF
FFF
@A00721:190:HGJW7DSXY:4:1305:23312:20478_2:N:0:TCTGCAAG+GCTGCACT
TTTGAAAATTACGGAACCTTTCCTCGTTCTTCATGAAGATCTTCGAGTGTTTTATAGTGAGTTATTCT

```
TGCGAAGGTTCAAGTCGATTGATTTTGTTGACTACGATCTCTCTTGTA
+
FFFFFFFFFFFFFFFFFFFFFFFFFFFFFFFFFFFFFFFFFFFFFFFFFFFFFFFFFFFFFFFFFFFFFF
FFFFFFFFFFFFFFFFFF,FFFFFFFFFF:FFFF:FFFFFFFFF,FFF
@A00721:190:HGJW7DSXY:4:1512:29324:16141_1:N:0:TCTGCAAG+GCTGCACT
AGACCACTGAACTTATAGCGACTTTTGTCGAATTTTTCGCAAAAGGTCTTGAAGTACGTATTGTCACT
GCTAATCGTAATTCGCGAGAAGAGATAGTGACTAAAGTTTTGGCACTTCTAGTTCATCGCGGTATTTG
TGA
+
FFFFFFFFFFFFFFFFFFFFFFFFFFFFFFFFFFFFFFFFFFFFFFFFFFFFFFFFFFFFFFFFFFFFFF
FFFFFFFFFFFFFFFFFFFFFFFFFFFFFFFFFFFFFFFFFFFFFFFFFFFFFFFFFFFFFFFFFFFFFF
FFF
@A00721:190:HGJW7DSXY:4:1109:14073:21605_2:N:0:TCTGCAAG+GCTGCACT
CGTGAAAGTTATGATGAGTGATCGTATTAGAACAGCTGATTCTTATTGCATGAACGTCGCTGAGGTCA
AACCTGTGGACGTTCTTTTCATCGACGAATTATTCATGATTCACGCTG
+
FFFFFFFFFFFFFFFFFFFFFFFFFFFFFFFF:FFFFFFFFFFFFFFFFFFFFFFFFFFFFFFFFFFFFF
FFFFFFFFFFFFFFFFFFFFFFFFFFFFFFFFFFFFFFFFFFF:FFFF
@A00721:190:HGJW7DSXY:4:1224:14145:27398_2:N:0:TCTGCAAG+GCTGCACT
CATCTACTTAAGGCGGCTTATACCATAGGTTACGGTGATTCCAATCAAATCGGATTCATCAATCGTAT
TGATCTCCAAGTCGCAAACTACTTCAACATCAACGACATCATTCTTGA
+
FFFFFFFFFFFFFFFFFFFFFFFFFFFFFFFFFFFFFFFFFFFFFFFFFFFFFFFFFFFFFFFFFFFFFF
FFFFFFFFFF:FFFFFF:FFFFFFF,FFFFFFFFFFFFFFFFFFFFFF
@A00721:190:HGJW7DSXY:4:1201:24876:14920_2:N:0:TCTGCAAG+GCTGCACT
TTATCGAAGTCTCAGTTATCGCTGCCCGGCCGACGTTTGTCGCGAACTCTCAGAGGTTTACGGCAGAA
AAATTTCTCCTGCTTGTAATTCGAGAGAGGACACTATGGAAATTAGAC
+
FFFF:FFFFFFFFFFFFFFFFFFFFFFFFFFFFFFFFFFFFF:FFFFFFFFF:FFFFFFFFFFFFFFFFF
FF:FFFFF:FFFFFFFFFFFF:FFFFFFFFFFFFFFFFFFFFFFFFFFF
@A00721:190:HGJW7DSXY:4:1351:30318:14951_2:N:0:TCTGCAAG+GCTGCACT
TATTACTTTCACTCAAGGTGAAAAGTTTGAAATTAACAAAGTCCTTGGTAAGAGTAAGGTTAAATGTG
AAGATGCTCAGACTATTCATGAAGTTCAAGGTAATACTTACCAGCACG
+
FFFFFFFFFFFFFFFFFFFFFFFFFFFFFFFFFFFFFFFFFFFFFFFFFFFFFFFFFFFFFFFFFFFFFF
FFFFFFFFFFFFFFFFFFFFF:FFFFFFFFFFFFFFFFFFFFFFFFFFF
@A00721:190:HGJW7DSXY:4:1540:6442:9204_2:N:0:TCTGCAAG+GCTGCACT
TTTTGCATCACAAACACATATTACCGTTGCTCTATCACGACACGTTGAATCTTTACGATACTTTGTGA
TCAATGTAAAGTTAAACGATGCTACTTGTGAGAGGATCAATCACAGTA
+
F,F:FFFFFFFFFFFFFF:FF,FFF:F:FFFF:FFFFFFFFFFF:FFF::FFF,FFFFF:FFFFF:,F
:FF:FF:FFFF,FF:FFFFFFFFFFF,:,FFFFFFF:FFFFF,FF:F:
@A00721:190:HGJW7DSXY:4:2517:3396:16485_2:N:0:TCTGCAAG+GCTGCACT
TTAGCACGGGAGGTTGCAGATTCAACCCCTTATATCGAGGCTATCGAGGACGATGAGTATCTTGAAGT
TGTTGACGACAAGCCTCAAGAGGGTAGAGGACCACCTGGATCTTCTAC
+
FFFFFFFFFFFFFFFFFFFFFFFFFFFFFFFFF,FFFFFFFFFFFFFFF:FFFFFFFFFFFFFFFFF:
FFFFFFFFFFFFFFFFFFFFFFFFFFFFFFFFFFFFFFFFFFFFFF:F
@A00721:190:HGJW7DSXY:4:1322:26937:8061_1:N:0:TCTGCAAG+GCTGCACT
AACCACCTTAACGTCACTCGGTAATCTTTCAGCCGAATTAAGTACTAATGAGTTCAAGTCGGTTGTCG
ATCGAATTAAGATCAAGGCTAATACCTTTATAGGTAAGAAGCCCCTTGAGCCGCGTAGTCAGCGCGTT
TAG
+
FFFFFFFFFFFFFFFFFFFFFFFFFFFF:FFFFFFFFFFFFFFFFFFFFFFFFFFFFFFFFFFFFFFFFF
```

FFFFFFFFFFFFFFFFFFFFFFFFFFFFFFFFFFFFFFFFFFFFFFFFFFFFFFFFFFFFFFFFFFFFFF
FFF
@A00721:190:HGJW7DSXY:4:1132:25003:28166_2:N:0:TCTGCAAG+GCTGCACT
CCGATAGGGTACCGAGTCTCCAAGAGAATATTTATTCTTATGAGGCTCGAAACTTCAACGTTTTCACA
ACGGAAAGACATATCGCACCCGAAATTTTTGGGCAGAATGCTCTTAAC
+
FFFFFFFFFFFFFFFFFFFFFF:F:FFFFFFFFFFFFF:FFFFFFFFFFFFFFFFFF,FFFFFFFFFF
FFFF:FFFFFFFFFFFFFF:FFFFFFFFFFFFFFFFFF:FFFFFFFF:
@A00721:190:HGJW7DSXY:4:2540:1118:14575_2:N:0:TCTGCAAG+GCTGCACT
GATCAGTCTAAGTTGGATAAAGTTATAGGAGACTCAGTTAACCTGAGAGCTCCTCAATTGATAGATTG
GGTCAACAATAGGGACGCTAGTATACTTCGTAAAGTCGACAGCGAAAT
+
:FFFFFFFFFFFFF:FFFFFFFFFFFFFFFFFFFFFFFFF:FFFFFFFFFFFFFFFFFFFFFFFFFFFF
FFFFFFFFFFF:FFFFFFFFFFFFFFFFF:FFFFFFFFFFF:F:FFFF
@A00721:190:HGJW7DSXY:4:2164:28592:31000_2:N:0:TCTGCAAG+GCTGCACT
AATTGGACCGACTATATGACCTATTTTAAGCTCATGGTGAAAAGGGAAGCTAAGGTTAAACTTGATGC
TTCAGCCTTTTCGAAGCATAACCCCGCACAGAATATCGTTTATCACGC
+
FFFFFFFFFFFFFFFFFFFFFFFFFFFFFFFFFFFFFFFFFFFFFF:FFFFFFFFFFF:FFFFFFFF
FFFFFFFFFFFFFFFFFFFFFFFFFFFFFFFFFFFFFFFFFFFFFFFF
@A00721:190:HGJW7DSXY:4:1629:9968:25488_2:N:0:TCTGCAAG+GCTGCACT
CCTATCTTTAAGGTTCTTGTGAGGAGAGTCAAACAGTGTCTTTTACCCAACATTTTTATGTATACAGA
GATGACCAGTGACGATTTCTCCCGGAAGCTCTTCTCTCTCTTAGGAGG
+
FFFFFFFFFFFFFFFFFFFFFFFFFFFFFFFFFFFFFFFFFFFFFFFFFFFFFFFFFFFFFFFFFFFFFF
FFFFFFFFFFFFFFFFFFFFFFFFFFF:FFFFFFFFFFFFFFFFFFFF
@A00721:190:HGJW7DSXY:4:1511:29071:18928_1:N:0:TCTGCAAG+GCTGCACT
TAAAGCCTATGAGATAAAGATGTATTCTTATTTAGGCTTCGATGCTGAATTTCTTGAACTTTGGAGTG
CCAGTGAATATTTTTGTCACGCCAGGAGTATCCATAACGATTTGTCATTTACCACCTTTGCTCAACGA
CGA
+
FFFFFFFF,F:F,FFFFFFFFFF,F::FF,:FFFFFFFFFF:FFFFFFFFFFFFFFFFFFFFFF:FFF
FFFFFFFFFFFFFFFFFFFFFF:FFF,FFFFFFFFFFFFFFFFFFFFFFF:F:FF:FFFFFF:FFFFF
FFF
@A00721:190:HGJW7DSXY:4:2469:27959:17253_1:N:0:TCTGCAAG+GCTGCACT
TAACACATTCTTTGGTAATTGTATGATCACTCTTATGATGCTCTCTCTTTATTACGACCTTCGGAAGT
TTAGAGCCGTCGCTGTCGCCGGAGACGATTCGATCCTCTTTTCGGACGTACCGGTCCAGGATTATTCA
GAT
+
FFFFFFFFFFFFFFFFFFFFFFFFFFFFFFFFFFFFFFFFFFFFFFFFFFFFFFFFFFFFFFFFFFFF
FFFFFFFFFFFFFFFFFFFFFFFFFFFFFFFFFFFFFFFFFFFFFFFFFFFFFFFFFFFFFFFFFFFF
FFF
@A00721:190:HGJW7DSXY:4:1459:19849:10410_2:N:0:TCTGCAAG+GCTGCACT
TGCTCGCGATTTATTCTTTTTGTCAATGATTACATCTACTTCGTTCCTGATCCTTACAAGATCATCGT
TAAGTTAGGTAAACCCCTAACCGATGTTACTCTTGTAGGACTTTGGGA
+
FFFFFFFFF:FFFFFF:FFFFFFFFFFFFFFF:FFFF:FFFFFFFFFFFFFF:F:FFFFF:FFFFF:F
F:FFFFFFFF,FFF:FFFF:FFFFFFF:FFFFFFFFFFFFFFFFFFFF
@A00721:190:HGJW7DSXY:4:2213:15619:18020_2:N:0:TCTGCAAG+GCTGCACT
TTAAAGACTATACTAAATATCTCCACTACGAGGTTGTTGTTTGGCGATTAGCTCTTGCGACTAATATT
CGATATTCGATTGAGGGTCGTTATACCTATTCGGCTATCTGTGCTTTG
+
FFFFFFFFFFFFFFFFFFFFFFFFFFFFFFFFFFFFFFFFFFFFFFFFFFFFFFFFFFFFFFFFFFFF
FFFFFFFFFFFFFFFFFFFFFFFFFFFFFFFFFFFFFFFFFFFFFFFF

```
@A00721:190:HGJW7DSXY:4:1226:32714:2284_2:N:0:TCTGCAAG+GCTGCACT
TTTTAAACGATTCCGAGAGGTTTATCCTGATGTGGAGGGAGTTCTTGAAGCATCCCTACCTTTCCTTG
TGAAAATTTTGATGCGGTTTCATAAGATTATCAGTAATCCTGATTCTA
+
FFFFFFFFFFFFFFFFFFFFFFFFFFFFFFFFFFFFFFFFFF:FFFFFFFFFFFFFFFFFFFFFFFFF:F
FFFFFFFFFFF:FFFFFFFFFFFFFFFFFFFFFFFFFFFFFFFFFFFF
@A00721:190:HGJW7DSXY:4:2435:31665:8735_1:N:0:TCTGCAAG+GCTGCACT
GACTGGAGTTCAGACGTGTGCTCTTCCGATCTCAAAGATCGACTTCGAGACAGGTGATATTGTTGAGC
GAGAGCGACCAAGATCTAAATTCGGTAACGGTGCGTGGCGCTTTATTTTAAAAAGCTCCCTCCTCTCG
CAA
+
FFFFFFF:FFFFFF:FFFF:FF:FFFFFFFF:F,F:FFFFFFFFFFFFFFFFFFFFFFFFFFFFFFFF
FFFFFFFFFFFFFFFFFFFFFFFFFFFFFFFFFFFFFFFFFFFFFFFFFFFFFFFFFFFFFFFFFFFF
FFF
@A00721:190:HGJW7DSXY:4:2215:16071:3270_2:N:0:TCTGCAAG+GCTGCACT
ACTTGAGTCTTTTGATCAGTCTCTGACTTGACCATCTTTCATTTTAAATGACGATCATTAAGACTCTC
TTTCTTCTTTGTTTCCTTCTTTGCACTGATTTTTCCTCATCAGCTTGT
+
FFFFFFFFFFFFFFFFFFFFFFFFFFFFFFFFFFFFFFFFFFFFFFFFFFFFFFFFFFFFFFFFFFF:
FFFFFFF:FFFFFFFFFFFFFFFF:FFFFFFFFFFFFFFFFFF:FFFF
@A00721:190:HGJW7DSXY:4:2336:31910:7435_2:N:0:TCTGCAAG+GCTGCACT
TTTTACGATAACAACTATCAGTTGATTAAGACTCTTCCGAAAGGCGAACTTTGGGAATTTGAAGCTCA
GGGTGAAGCACAACGTTTGACCGTGACGAACTGTGGTGTTCTTGATCT
+
FFFFFFFFFFFFFFFFFFFFFFFFFFFFFF:FFFFFFFFFFFFFFFFFFFFFFFFFFFFFFFFFFFFF
FFFFFFFFFFFFFFFFFFFFFFFFFFFFFFFFFFFFFFFFFFF:FFFF
@A00721:190:HGJW7DSXY:4:2211:8034:11209_1:N:0:TCTGCAAG+GCTGCACT
CACTACCACTTATTACGATATTTCTTTAGTCGATGGGTTTTCGACGCCTTTAAGCGTCTTCTGTTCAT
CGCAACCAGATGATATCTTCTATTCGAACTTATGTTTCGATCAGTGTTTCTTCTATCAGGAGGAAAAC
ACT
+
FFFFFFFFFFFFFFFFFFFFFFFFFFFFFFFFFFFFFFFFFFFFFFFFFFFFFFFFFFFFFFFFFFFF
FFFFFFFFFFFFFFFFFFFFFFFFFFFFFFFFFFFFFFFFFFFFFFFFFFFFFFFFFFFFFFFFFFFF
FFF
@A00721:190:HGJW7DSXY:4:2203:25229:23829_2:N:0:TCTGCAAG+GCTGCACT
TCTTCTAATGTGGCCTGAACTACGTTTCACTTTGTGTGTACTTGTCTCTATTTTAAGTTTTGTCTTCC
TTGTTTGTCTTATTCTATTAATCACTCATTTTAGTGAGATTATTAAAA
+
FFFFFFFFFFFFFFFFFFFFFFFFFFFFFFFFFF:FFFFFFFFFFFFFFFFFFFFFFFF:FFFFFFFFFFFF
FFFFFFFFFFFFFF:FFFFFFFFFFF:FFFFFFFFF,FF:FFFFF::F
@A00721:190:HGJW7DSXY:4:1368:16315:25927_2:N:0:TCTGCAAG+GCTGCACT
GACAACAGCAGGGGTTTTCGAATAGTGATAGCCACCCGCCTTACAGAGGGGCAGCAGCATGACCATAA
TTGGTATTGACTTTGGTACCACCTTTTCTACTGTCTGTCTCGCCTCTT
+
FFF:FFFFF:FFFFFFFFFFFFF:FFFFFFFFFFFFFFFFFFFFFFFFFFFFFFFFFFFFFFFFFFFF
,FFFFFF:FFFFFFFFFFFFFFFFFFFFFFFF:FFFFFFFFFFFFFFF:F
@A00721:190:HGJW7DSXY:4:2438:15754:15812_1:N:0:TCTGCAAG+GCTGCACT
CTTAACAACAATGATAGTGAATTTATACCTACAATCATTGGTTTTTCTAATAATGATGACACTATTGT
TTATGGATATGATGCTATTACTCATGATGGTTACGGTCAGGGAGGATTCTCAGTTTACAAGGACCTTA
AGC
+
FFFFF:FF,FFF,FFFFFFFFFFFFFFF:FF:FFFFFFFF:FFFFF,FFFFFFF::FFFFF:F::FF,
FFFF,FFFFF:FFFFFFFFFF:FFF,FF:FFF,F:FFFFF:::F,FFFFFFFFFFFFFFFFFFFFFFF
FF:
```

@A00721:190:HGJW7DSXY:4:2603:21549:28792_2:N:0:TCTGCAAG+GCTGCACT
CCGAAAGACGTGCTAAATTAAAACCATTGTACGATGTCGTCTGTCTCGGCGATCACTTTTCACTAAGA
ATTGGTCCTACTTTTGGACAAGGCCAACTTCGTAATCTTCATGAGATC
+
FFFFFFFFFFFFFFFFFFFFFFFFFFFFFFFFFFFFFFFFFFFFFFFFFFFFFFFFFFFFFFFFFFFFFF
:FFFFFFFFFFFFFFFFFFFFFFFFFFFFFFFFFFFFFFFFFFFFFFF
@A00721:190:HGJW7DSXY:4:2536:2790:6417_1:N:0:TCTGCAAG+GCTGCACT
TAGGGATTTTGAGAGAGTGAAAGGCTTCAATTGTAGTGGTTTGGTCGTTTCAGTACCCAGTCAGTATA
CCTCTACGCAAAGATATTTCATGAAAACTACAGAGGAACGTACCGGATTACCTGTCTTTCATATTATG
AAT
+
FF::F:FFFFFF:F:FFFFFF,,FFFFF::FFFFFF:FFFFFF:FFFFF:FFF:FFFF::F:FFFF::,F
::FFFFF:FF,:F:F,:FF:FFFF::FF:F:FFFFFFF:FFFFFF,F:F:FFF:FFFFFFFF:FFFF:FF
FFF
@A00721:190:HGJW7DSXY:4:1571:26124:18897_1:N:0:TCTGCAAG+GCTGCACT
TTTGCTTCGATGTTGGATGTCAAGAAGGGTTCAGAATGGGATTCCTACGTAGTTTATGACTTTGGTGG
TGGTACTTTCGATACTTCTATCGTCTTACGTTACGGTAATTACTATAGTGTTATTTATTCTGGTGGTG
ATG
+
F:FFFFFFFFFFFFFFFFFFFFFFFFFFFFFFFFFFFFFFFFFFFFFFFFFFFFFFFFFFFFFFFFFFFF
FFFFFFFFFFFFFFFFFFFFFFFFFFFFFFFFFFFFFFFFFFFFFFFFFFFFFFFF:FFFFFFFFFFFFF
FFF
@A00721:190:HGJW7DSXY:4:1202:30101:32487_1:N:0:TCTGCAAG+GCTGCACT
GGTCGTGACGTTGATGAAGCTATAAGAGCGTTCTTATTACGGCGCACTTCTTTACCCGCCACCACACA
ACTTTCAGTTTCACAACTGAAGGAGGAGGTTTCTGTTACTGGAAAACCCTCGTCAATACTTTTCAATA
ATA
+
FFFFFFFFFFFFFFFFFFFFFFFFFFFFFFFFFFFFFFFFFFFFFFFFFFFFFFFFFFFFFFFFFFFFFF
FFFFFFFFFFFFFFFFFFFFFFFFFFFFFFFFFFFFFFFFFFFFFFFFFFFFFFFFFFFFFFFFFFFFFF
FFF
@A00721:190:HGJW7DSXY:4:2134:14398:29747_2:N:0:TCTGCAAG+GCTGCACT
GATCTAGTGAAATTAACGCGTCCTTTTCTGGAACGTAGTTTTACTATTATGAACAAAGTTATCAAAGA
TAGTGGCATTCAAGGCAAACTTACCCTGATTCCGGCCGGTGGTAGCTC
+
FFFFFFFFFFFFFFFF:FFFFF:FFFFFFFFFFFFFFFFFFFFF:FFFFFFFFFFFFFFFFFFFFF:FFF
FFFFF:F:FFFFFFFFFF:FFFFFFFFFFFFFFFFFFFFFFFFF:FFF
@A00721:190:HGJW7DSXY:4:1558:31991:8234_2:N:0:TCTGCAAG+GCTGCACT
TGCTGAAACAGCTATTAAAGCTGTTGAGCGGGTAATTACACCTAAACGAGCTCGATCTGCCATCGCTG
AAGGATGTGCACTCTTTTCTGCTACTTTAACGGAGCGTAGTGTACTCT
+
FFFFFFFFFFFFFFFFFFFFFFFFFFFFFF:FFFFFFFFFFFFFFFFFF::FFFFFFFFFFFFFFFFFFF
FFFFFFFFFFFFFFFFFFFFFFFFFF:FFFFF:FFFFFFFFFFFFFFF
@A00721:190:HGJW7DSXY:4:2521:21377:14685_1:N:0:TCTGCAAG+GCTGCACT
CAACAGTAACATTACTTCTAATAAAGGTCTCTATCAAGCTCGTATTATCGTCGCTGCTGGATCTGCGT
TACCGTTGAAGGCTAAACTCCAAGGTAATTATGAAGACTGTGATTTATCTTTCACTCTGGCCTTGTAT
GAG
+
FFFFFF::FFFFFFF:FFFF,FFFFFFFFFFFFFFFFFFFFFFFFF:FFFFFFFFFFFFFFFFFFFFFFF
,FFFFFFFFFFFFFFFFFFFFFFFFFFFFFFFFFFFFFF:F:FFFFFFFFFFFFFFFFFFFFFFFFFFFF
FFF
@A00721:190:HGJW7DSXY:4:2608:11677:29763_2:N:0:TCTGCAAG+GCTGCACT
CGTAACATTAATAATTTCAATCTTCTTAAAAAGAAGATAGAGAGTCATACTTTGGGCTATCGTTCAGG
TGCATGGGGTTGGATAGCCAATATTGAAGTGGACACCTCTGGTAGTAT
+

FFFF:FFFFFFFFFFFFFFFFFFFFFFFFFFFFFFFFFFFFFFFFFFFFFFFFFFFFFFFFFFFFFFF
FFFFFFFFFFFFF,,FFFFFFFFFFF:FFFFFFFFFFFFFFFFFFFFF
@A00721:190:HGJW7DSXY:4:1242:1280:28948_2:N:0:TCTGCAAG+GCTGCACT
AAGCATACTACTTTTGCTTTGATTACTCTACCGCCTTACAAGTCTAAGGTTAAAGAGTCGATTAAACA
AGTTGTACTTATCAATGATTTTATCGCTGGTTGTTATCTCGAAAAGTC
+
FFFFFFFFFFFFFFFFFF,:FFFFFFFFFFFFFFFFFFFFFFFFFFFFF:F:FFFFFFFFF,FFFF,F
FF,FFFFFFFFF,FFF:FFFFFFF,FF,:FF,FFF,:,F:F:FFF:FF
@A00721:190:HGJW7DSXY:4:1172:18900:12837_1:N:0:TCTGCAAG+GCTGCACT
CCCAAAGAGAATATCTAAAATATTTACAAAATCTTACCACTATCTACGAACCTAAAGTTCTGAAGACG
TTGCTTGTCGCTTCCTCCTTTAATGGCACTACCTCGGTACAATCTTACTTGGAACGAAGGCGACCTGT
TCC
+
FFFF:FFFFFFFFFFFFFFFFFF:FFFFFFFFFFFFFFFFF:FFFFFFFFFFFFF:FFFFFFFFFFFF
FFFFFFFFFFFFFFFFFFFF:FFFFFFFFFFFFFFFFFFFFFFFFFFF:FFFFFFFFFFFFFFFFFFFF
FFF
@A00721:190:HGJW7DSXY:4:2131:19723:28573_1:N:0:TCTGCAAG+GCTGCACT
GTTCTGTCTATATTAGGTGAAGCGAAATACGATTTCGAACGTCAACGTTCACCGAGTTTCGTTTCGTA
CCTTGATGCTGACTTTCGTCAATATGATTTGATCTGGTTGCTCAATTCTAAGCGTGTTCCGCTTAGTG
CGA
+
FFFFFFFFFFFFFFFFFFFFFFFFFFFFFFFFFFFFFFFFFFFFFFFFFFFFFFFFFFFFFFFFFFFF
FFFFFFFFFFFFFFFFFFFFFFFFFFFFFFFFFFFFFFFFFFFFFFFFFFFFFFFFFFFFFFFFFFFF
FFF
@A00721:190:HGJW7DSXY:4:2370:4381:3129_1:N:0:TCTGCAAG+GCTGCACT
TACGAACTTGACTCTACTATACGTGACATACTAAATACTAGATATTTGGATGATCTTGATAAAGAACT
TCATGAATTTTCAGAGGATGAAAAATTTTCTTCCTTGGATTTTAATATAAAGAATCTTTACCAGGACT
CCT
+
FFF::FFFFFFFF:FFFFFFFFF:FFFFFFFFFFFF:FFFFFFFFFFF:FFFFFFFFFFFFF:FFFFF
FFFFFFFFFFFFFFFFFFFFFFFFFFFFFFF:FFFFFFFFFFFFFFFFFFFFFFFFFFFFFFFFFFFF
FFF
@A00721:190:HGJW7DSXY:4:1220:3070:36370_1:N:0:TCTGCAAG+GCTGCACT
GATCGTAAGAAGTTTATAAGGAACATATCCATTTGTTGTAATAGGGTCATCAACTTAGAACTCGAAAC
TCTAAGTGGTAATACTGTCTTTCCAAGCTTACCTGTATCTTCAATGAAATTGGACTCTTCGATAGGGG
ACA
+
FFFFFFFFFFFFFFFFFFFFFFFFFFF:FFFFFFFFFFFFFFFFFFFFFFFFFFFFFFFFFFFFFFFF
FFFF:FFFFFFF:FFFFFFFFFFFFFFFFFFFFFFFFFFFFFFFFFFFFFFFFFFFFFFFFFFFFFFF
FFF
@A00721:190:HGJW7DSXY:4:2629:11080:24502_1:N:0:TCTGCAAG+GCTGCACT
GGAAGATCTGTTATCGACGGTTTATCGTTTACGAAGTATCACTCCACCGGTGAATGGTTTAAGAATAC
TATAAATAGAATAGCTATTACTACTAATGGCGTTGGCCTAGTGGATGTTATTAGTGACATCTGGTCAA
TTT
+
:FFFFFFFFFFFFFFF,FFFFFFFFFFFFFFFFFFFFFFFFFFFFFFFFFFFFFFFFF,FFFFFFFFFF
FFFFFF:FFFFFFFFFFFFFFFFFFFFFFFFFFFFFFFFF:FFFFFFFFFFFFFFFFFFFFFFFFFFFF
FFF
@A00721:190:HGJW7DSXY:4:1650:11749:12226_1:N:0:TCTGCAAG+GCTGCACT
TGGTCTTTATTCTATTATTGATTTTTATGAGATTGATACGTTAACAGAAGATGATGTTAGAGTAGAAT
TCCTGAAGAGTCCTTCCGTAACCAACTTCTTGACAATTTTCTTCAATCCTTCGATAATAGTTAAATCT
AGG
+
FFFFFFFFFFFFFFFFFFFFFFFFFFFFFFFFFFFFFFFFFFFFFFFFFFFFFFFFFFFFFFFFFFFF

FFFFFFFFFFFFFFFFFFFFFFFFFFFFFFFFFFFFFFFFFFFFFFFFFFFFFFFFFFFFFFFFFFFF
FFF
@A00721:190:HGJW7DSXY:4:1412:3929:19820_2:N:0:TCTGCAAG+GCTGCACT
TACACAATTAAGAAATAATCCTACATTGACTGATTATCTAAAGATTGAAGGAAAATTTTATCCTCGTT
TAACTTACTTAGACTTTCCTACGATACAAATGATAACTTCTAATTTAC
+
FFFFFFF:FFFFFFFFFF:FFFFFFFFFFFFFFFFFFFFFFFFFFFFFFFFFFFFFFFFFFFFFFFFF:
FFFFFFFF:FFFFFFFFFFFFFFF,FFFFFFFFFFFFFFFF,F,FF,F
@A00721:190:HGJW7DSXY:4:2129:31123:31720_1:N:0:TCTGCAAG+GCTGCACT
GGGATGCATGGTGTTATCTCCTCAGGAGTCCCGGCATTTGATAATAATGACGAGACTTTCGTCATAAT
CGCTGCATATTACGGATTACACGGTACCAGCGCATCTCGTGTCGAGCCTCGACCCGATTATATGGTAT
TAA
+
FFFFFFFFFFFFFFFFFFFFFFF:FFFFFFFF:F:FFFFFFFFFFFFFFFF:FFF:FFFFFFF:FFFF
,FFFFF:FFFFFFFFFFFFFF:FFFFFFFFFFFFFFFFF:FFF:FFFFFFFFFFFFFFFFFFFFFFFFFF
F:F
@A00721:190:HGJW7DSXY:4:1178:13910:35509_2:N:0:TCTGCAAG+GCTGCACT
GATTCTCGATTCGTAGAGCTTATCTAGGTACTCTCATTTACTATGTTGATTTAGTCTACATTTATGCT
GATCTAAAATTTCGATCTAGATGGATCCACTCCTCGAGTTATCATGAT
+
FFFFFFFFFFFFFFFFFF:FFFFFFFFFFFFFFFFFFFFFFFFFFFFFFFFFFFFFFFFFFFFFFFFFF
FFFFFFFFFFFFFFFFFFFFFFFFFFFFFFFFFFFF:FFFFFFFFFFF
@A00721:190:HGJW7DSXY:4:2456:29677:33849_1:N:0:TCTGCAAG+GCTGCACT
TTCTAGAATTCTTAATTATAATATTCAACCTCAAAAGTTACGTGTTTACAGTAACGAGATCACACATT
TTGAGAACTAACTATGTTATGTTCTCTGATCATTTTGACAATCTCTTCTTATAATAAATAAATAAATA
ATA
+
FF,FF:FFFF:FF:FFFFFFFFFFF,FFFFFFFFFFFFFFFFFFFFFFFFFFFFFFFFFFFFFFFFFF
FFFFFFFFFFFFFFFFFFFFFFFFFFFFFFFFFFFFFFFFFFFFFFFFFFFFFFFFFFFFFFFFFFFF
FFF
@A00721:190:HGJW7DSXY:4:2516:3694:24706_2:N:0:TCTGCAAG+GCTGCACT
CAAATAGTGGAAAGGCTCCAGTCAACACTAGTTCGAGTGCTGCTTTGGATTACGATGACGTGAGAATA
GGTGAAGGGATAGTTTTTACCTCGACCAACATTAAGGCGGCGGCGACT
+
FFFFFFFFFFFFFFFFFFFFFFFFFFFFFFFFFFFFFFFF:FFFFFFFFFFFFFFFFFFFFFFFFFFFF
FFFFFFFFFFFFFFFFFFFFFFFFFFFFFFFFFFFFFF:FFFFFFFFFF
@A00721:190:HGJW7DSXY:4:1452:14940:7138_1:N:0:TCTGCAAG+GCTGCACT
AGGACACTTCGAAACTGAATAATGATGCACTACAAACAAGTATCGCTAATCATCTGAAAAAGGCTCTA
AAGGACACTATACAATATCCAGACGACGACGAAGCTTATACTACTTTAATTTTGGGTATTATGTGGAG
ATA
+
F:FFFFFFFFFFFFFF:FFFFFFFFFFFFFFFFFFFFFFFFFFFFFFFFFFFFFFFFFFFFFFFFFFF
FFFFFFFFFFFFFFFFFFFFFF:FFFFFFFFFFFFFFFFFFFFFFFFF:FFFFFFFFFFFFFFFFFFFFF
FFF
@A00721:190:HGJW7DSXY:4:1210:23909:14215_2:N:0:TCTGCAAG+GCTGCACT
ACCACATTTTGGTCAAGTCAATCCGTTGAGAAAATTTCTTAGAGGATATTCGCCATTGTGGTACGAAT
TGGTCCAACATTTACCACCAAATTCTTTTGATACAACTCTAGCCTTCA
+
FFFFFFFFFFFFFFFFFFFFFFFFFFFFFFFFFFFFFFFFFFFFFFFFFFFFFFFFFFFFFFFFFFFF
FFFFFFFFFFFFFFFFFFFFFFFFFFFFFFFFFFFFFFFFFFFFFFFF
@A00721:190:HGJW7DSXY:4:1127:23068:6433_2:N:0:TCTGCAAG+GCTGCACT
CTTGCTATTCACCAGATTGGTTCGAACCCGTTGATGCTATGTCAGAAGAAGCTATTCAAGCTTGTCGT
TTAAAGACTGCAGAGGCTGTCAAACAAGCCCAGACTCAAGGAGTCCAA
+

```
FFFFFFFFFFFFFFF:FFFFFFFFFFFFFFFFFFFFFFF:FFFFFFFFFFFFFFFFFFFFFFFFFFFFF
FFFFF,FFFFFFFFFFFFFFFFFFFFFFFFFFFFFFFFFFFFF:FFFF
@A00721:190:HGJW7DSXY:4:2271:32750:35383_2:N:0:TCTGCAAG+GCTGCACT
GGTGATAAAAATTATTTAGAGATCTTTTCTACTTACAAAGGACAATTCAGTTTCATTAATTCTACGGA
AAAGTCCGGAGTATATTGTATGTTAATAGATAGTGGGATAATAGTTCT
+
FFFFFFFFFFFFFFFFFFFFFFFFFFFFFFFFFFFFFFFFFFFFFFFFFFFFFFFFFFFFFFFFFFFF
FFFFFFFFFFF:FFFFFFFF:FFFFFFFFFFFFFFFFFFFFFFF:FFF
@A00721:190:HGJW7DSXY:4:1305:22290:17143_2:N:0:TCTGCAAG+GCTGCACT
TGCTATTGATTATGTAACCGATAGGAGTAGTATAGTTGAAGATGTGTCTTTAGACGATAATCCTTTAA
GGACTATGTTGACTGACGAAGATTTTACCACAGATTTGTCTACGTTGC
+
::,FF:F,FFFFF:FFF:F,FFFFFF,F,,:FFFF,:,FF,F:,,F,FF,FF,FF,:,FFF,:::,:F
,:FFFFFF:,FF,F::F,FFF:,FFF,:FF,F,FFF,,,,FF,FFF:F
@A00721:190:HGJW7DSXY:4:2620:9245:30279_2:N:0:TCTGCAAG+GCTGCACT
CTATATACATTATAAAATATAAAAATAAGGGAGTCATAACTTTTGTTACCAAATTCTTGGCTTTCAAC
GAAGTCTCTGATTTTATTAATCATTACTTTAACGATCTCTCGGTCATT
+
FFFFFFFFFFFFFFFFFFFFFF:FFFFFFFFFFFFFFFFFFFFFFFFFFFFFFFFFFFFFFFFFFFFF
FFFFFFFFFFFFFFFFFFFFFFFFFFFFFFFFFFFFFFFFFFFFFF,FF
@A00721:190:HGJW7DSXY:4:2411:5122:24893_1:N:0:TCTGCAAG+GCTGCACT
TTGATCTACGTCGTTTAACTGTCGGAACAACCTTTACTTAGAATGACTCTATCTTTTCAGACACTTCT
TATATTATTTACAATAGTTAATATCATATTAGTAATACTCACTATCGTTTTCTGCTTTTTCTTAGCTA
ATA
+
FFFFFFF:FFF:FFFFFFFFFFFFFFFFFFFF:FFFFFFF:FFFFFFFFFFFFFFFFFFFFFFFFFFFF
FFFFFFFFFFFFFFFFFFFFFFFFFFFFFFFFFFFFFFFF:FFFFFFFFFFFFFFFFFFFFFFFFFFFF
FFF
@A00721:190:HGJW7DSXY:4:2516:15157:3912_2:N:0:TCTGCAAG+GCTGCACT
ATGAGTACGAAAACCAACTCTTCGTTACGAGACCTGATGGTAGAATAGATTTAGTCTCTACACCCATC
TCAACGAGGATAAAGCGAGTTTTCCGTCCTGAACGTAATACACGAAGA
+
FFFFFFFFFFFFFFFFFFFFFFFFFFFFFFFFFFFFFFFFFFFFFFFFFFFFFFFFFFFFFFFFFFFF
FFFFFFFFFFFFFFFFFFFFFFFFFFFFFFFFFFFFFFFFFFFFFFFF
@A00721:190:HGJW7DSXY:4:1118:6903:7310_1:N:0:TCTGCAAG+GCTGCACT
CACGACTCGAAACCGAGACGCCGTAGATCAAAGTCCCTTCTAACTTTAGATTATGAATCCTGCCTTTA
CTCATTTTATAGCAGTTATTTA
+
:FF:FFFF,FFFFFFFFFFFFFFF:FF,FFF:,FFFFFF:FFFFFF:,FFFFFFFFFFFFF,FFFFFF
FF::FFFFFFFFFFFFFF,F::
@A00721:190:HGJW7DSXY:4:2378:4951:36933_1:N:0:TCTGCAAG+GCTGCACT
TTTATAACCTTCTTATACTTTTGGGTATGTTTTGTGAAAAACATGAGATACCCAAGTCTATCGAAGAC
ATTATCTCTTATGTACTTATTGTAACGATCATTCTTTCGAGTTTTGTCATTCTTATTCTTTTATTCCT
TTA
+
FFFFFFFFFFFFFFFFFFFFFFFFFFFFFFFFFFFFFFFFFFFFFFFFFFFFFFFFFFFFFFFFFFFF
FFFFFFFFFFFFFFFFFFFFFFFFFFFFFFFFFFFFFFFFFFFFFFFFFFFFFFFFFFFFFFFFFFFF
FFF
@A00721:190:HGJW7DSXY:4:2334:15040:25598_2:N:0:TCTGCAAG+GCTGCACT
TTTATAATGCTTTTCGATAGTGATTGTTATCAATTTAAATACATTACTGTTTCAAATCATCTTTACTC
ACAGACTCCGAAAATGGTTTCAAATGAGGTCAAAGCTCTATTTATTAA
+
FFFFFFFFFFFFFFFFFFFFFFFFFFFFFFFFFFFFFFFF:FFFFFFFFFFFFFFFFFFFFFFFFFFF
FFFFFFFFFFFFFFFFFFFFFFFFFFFFFFFFFFFFFFFFF,FFFFFFF
```

```
@A00721:190:HGJW7DSXY:4:2367:19913:35697_1:N:0:TCTGCAAG+GCTGCACT
CACTACCACTGATCTGCCGACTTTAGTCAAGGAACTTCAATTCCTTCGATGTTTATATTTTTCTATAA
GGGAAAATATCAATAATCAAATTTCGACAAATTTTAGAACTTTATTTCAGAACTCTCTCCGATCACCT
TGG
+
:F:FFF:FFFFFFF:F:FFFFFFFF,F:FF,FF:FFFFFFFF,F:F,FF:F,FFFFF,:FFF:FFF:F
FFFFF:FFFFFFFFFFFFFFFF:,,FF::::FFFFF:FFFFFFFFFFF:F:F::FFFF::FFFFF,:F
FF:
@A00721:190:HGJW7DSXY:4:2668:22978:31610_1:N:0:TCTGCAAG+GCTGCACT
TCTTTTCAATAAGATGTATGATGATAATCTACTATTTTAAATAGTGATATTCAGCGTATACTTGAAT
ACTTTATCAAAGATTTGGCTTGGTCTATTTACCGCAAAAAGTTGGAAGAGGTCTATGATATTTATTTT
ACT
+
:FFFFFFFF:FFFFFFFFFFFFFFFFFFFFFFFFFFFFFFFFFFFFFFFFFFFFFFFFFFFFFFFFFF
FFFFFFFFFFFFFFFFFFFFFFFFFFFFFFFFFFFFFFFFFFFFFFFFFFFFFFFFFFFFFFFFFFFF
FFF
@A00721:190:HGJW7DSXY:4:1552:5846:9831_2:N:0:TCTGCAAG+GCTGCACT
CCGATTATAACTTGACTGTTGATAATATTACAATAGCTCTTTCTCAGAAGATTAGAGAAGTAACCGGT
GAACACGTCGGTAGCATTGATTTACTTTTACCGTTAGAACTGAAAAAG
+
FFFFFFFFFFFFFFFFFFFFF:FFFFFFFFFFFFFFFFFFFFFFFFFFFFFFFFFFFFFFFFFFFFFF
FFFFFFFFFFFFFFFFFFFFFFFFFFFFFFFFFFFFFFFFFFFFFFFF
@A00721:190:HGJW7DSXY:4:2402:14597:14497_1:N:0:TCTGCAAG+GCTGCACT
ACCTATAAGTTACAAACGATTCTTCGACTATATTAAAGAAATAAAAATACTTCAAGTACCTGAACTTT
ACATTTTTAATGGTATGGTATAATGTGTCTTACAAATAGAACCTTCACTCTCGGTAATGAGAGTGTAT
TCC
+
FF:FFF:F,FFFFFFFFFFFF:FFFFFFFFFFFFFFFFFFFFFFFFFFFFFFFFFFFFFFFFFFFFFF
FFFFFFFFFFFFFFFFFFFFFFFFFFFFFFFFFFFFFFFFFFFFFFFFFFFFFFFFFFFFFFFFFFFF
FFF
@A00721:190:HGJW7DSXY:4:1575:11243:29450_1:N:0:TCTGCAAG+GCTGCACT
GGCATTACTAGGATTTATCACACTCCTAAATTTGATAGCAATTTATGTTTTAAATGCTAGGAAAATTT
TAGAAAAACTCCATTGTATAGTTCAAGGAGTACTTAATTCAATAGCTTTAGTATTATTTACTATCGCC
TTT
+
FFFFFFFFFFFFFFFFFFFFFFFFFFFFFFFFFFFFFFFFFFFFFFFFFFFFFFFFFFFFFFFFFFFF
FFFFFFFFFFFFF:FFFFFFFFFFFFFFFFFFFFFFFFFFF::FFFFFFFFFFFFFFFFFFFFFFFFF
FFF
@A00721:190:HGJW7DSXY:4:1453:6596:31015_2:N:0:TCTGCAAG+GCTGCACT
AGTTAATTGCTTACACACAAGTGACGCGCGAAGGAATCTTAATTGATTGAAGCTAGGCGTTGGGGAGA
ATGAACTGCCGCTTCCCGGAAGAAACGATCTATTTACAGAGTATTTAG
+
FF:FFFFFFFFFFFFFF,FFFFFFFFFFFFFFFFFFFFFFFFFFFFFFFFFFFFFFFFF:FFFFFFFF
FFFFFFFFFFFFFFFFFFFFFFFFFFFFFFFFFFFFFFFFFFFFFFFF
@A00721:190:HGJW7DSXY:4:2231:15953:18004_1:N:0:TCTGCAAG+GCTGCACT
CTCCCCAAACATAACATACCATAACCTACCATGACCAATCATCGCTCCAACTGGCACGCCAGACGTAG
GGTCAAACCTACTACCGCCTGTCGGCAGATCGGCACCACTGGTCTCTACGTTCAGGGACCCGCTGTGC
AGA
+
FFFFFFFFFFFF,FFFFFFFFFFFFFF:FFFFFFFFFFFFFFFFFFFFFFFFFFFFFFFFFFFFFFFF
FFFFFFFFFFFFFFFFFFFFFFFFFFFFFFFFFFFFFFFFFFFFFFFFFFFFFFFFFFFFFFFFFFFF
FFF
@A00721:190:HGJW7DSXY:4:1176:32253:28948_2:N:0:TCTGCAAG+GCTGCACT
TCTGCACAGAAGAAGGCTGAAGGTCGTTACCGGACGACCACTCAAAGGAAACGGGCTGTTGCCCAGAG
```

```
GAAAGCAGCGACCACCGAGGTCGCTCGTAACATTGACGGCAGTCCCGT
+
FFFFFFFFFFFFFFFFFFFFFFFFFFFFFFFFFFFFFFFFFFFF:FFFFFFFFFFFFFFFFFFFFFFFF
FFFFFFFFFFFFFFFFFFFFFFFFF:FFFFFFFFFFFFFFFFFFFFFFF
@A00721:190:HGJW7DSXY:4:1538:2329:16892_1:N:0:TCTGCAAG+GCTGCACT
AACTGAGGCAGTACCTCAGTCAAAAGTCACATTCACCACTCCCATACCAATCAATGATGTGACTATAC
AGTTCGGCCATTTCAAGAGTGGTCAATACGTCGTGGGAGGTACTGAAGCCTTCCCCTCCGCTGGCACG
AAG
+
FFFFFFFFFFF:FFFFFFFFFFF:FFFFFFFFFFFFFFFFFFFFFFFFFF:FFF,FFFFFFFFFFFFFF
FFFFFFFFFFFFFFFFFFFFFFFFFFFFFFFFFFFFFFFFFFFFFFFFFFFFFFFFFFFFFFFFFFFFF
FFF
@A00721:190:HGJW7DSXY:4:1228:3676:32784_2:N:0:TCTGCAAG+GCTGCACT
GCCAATCAATAGTTCTTCTGACCAGAATGAAAAGGGGTTTACCAGGGTAGTCTCTAAGTCTACCAGGA
AGAGGGGAAATGTCCCCAAAACTGAGAAGAATAAAGGGAAAGGTCCAT
+
FFFFFFFFFFFFFFFFFFFFFFFFFFFFFFFFFFFFFFFFFFFFFFFFFFFFFFFFFFFFFFFFFFFFF
FFFFFFFFFFFFFFFFFFFFF:FFFFFFFFFF,:FFFFFFFFFFFFFFF
@A00721:190:HGJW7DSXY:4:1103:30743:28557_2:N:0:TCTGCAAG+GCTGCACT
AGGGGAAATTACCCCTAATTCTGAAGGAAGATCTCCCACCATCTGGGGTTGGGAATAAGAAATCTGCA
CGGGCCAAAGAGGCCGCTGCTACGAAGTCGCTGAAGGCGGCTTGGGTC
+
FFFFFFFFFFFFFFFFFFFFFFFFFFFFF,FFFFFFFFF:FFFFFFFF:FFFFFFFFFF:FFFFFFFF
FFFFFFFFFFFFFFFFFFFFFFFFFFFFFFFFFFFFFFFFFFFFFFFF
@A00721:190:HGJW7DSXY:4:2174:29414:29951_2:N:0:TCTGCAAG+GCTGCACT
GTAAAGTTGCGCCTGCCGACCCCATCTTTGAGACCAAGACTCTCTACAGTAGGTTCGTGCCTACTATG
AGCAAATCTGTTGAATTCGTCACTGAGGACGAAGGTGAGAGGACTTTT
+
FFFFFFFFF,FFFFFFF:FFFFFF,:F::,FFFFFFF:FFFFF:FFFFFF:FFFF:F:F:FFFFFF::
F:FFFF::FFFFFFFFF:FFFFFF:FF:FFFF:FF:FFFFFFFFFFF
@A00721:190:HGJW7DSXY:4:2239:12328:20243_2:N:0:TCTGCAAG+GCTGCACT
CTGGGGAAAGAGAATTGCGTGGGTTGAAGGCCGACGCTTCGAGCTTGAGTGTGACGAGGCCGGTGACG
TTTTTGTCAACGGCGTTTGGAGGATTTTATCCGCGCTTAACTATGGGG
+
F::FFFFF:FFF:FFF:FF:,FF:,F::FFFFFFFFFF:FFFFF:FFFFFFFFFF:FFFFFFFFFFF
F,FF:FFFFFFFFFFF,FF,FFFFF:FF::FFFF:F:FFFFF:FFFFF
@A00721:190:HGJW7DSXY:4:1414:32533:21355_2:N:0:TCTGCAAG+GCTGCACT
CTATGGAGTCTTGAAGGACTTAGGTTGGCATATTTATGCCGACGATAATAAGCATGTCACCCTTAAGC
ACCGAGGTAAGCTCGGGACTTACAAGAGAGAGTGTGAGAGAGTCGCTC
+
FFFFFFFFFFFFFFFFFFFFFFFFFFFFFFFFFFFFFFFFFFFF:::FFFFFFFFFFFFFFFFFFFFFF
FFFFFFFFFFFFFFFFFFFFFFF:F:FFFFFFFFFFFFFFFFFFFFFFF
@A00721:190:HGJW7DSXY:4:1174:32235:16642_2:N:0:TCTGCAAG+GCTGCACT
TTCTGACTGATGAGGTTAAAACTCTTGCTCCTTCTGTGGGTGAGGGTTTTTGTTATATTCACTGGTTC
TGGGAGGCTGCTTTCCTCCATAATTACAACCTCTCTTTAGAGAGCATT
+
FFFFF:FFFFFF:FFFFF,:FFFFFFFFFFFFFFFFF:FFFF:FFFFFFFF:FF,FFFFFF::FF,FF
F:FFFFFFFFFFFFFFFFFFFFFFFFFFFFFFFFF:FFFFFFFFFFFFF
@A00721:190:HGJW7DSXY:4:1353:1416:12618_2:N:0:TCTGCAAG+GCTGCACT
AAATTCCCACATATGGTAGACGTTGTCACACTTTTCTTGAAAATTTTTCCTTTCCTCCGTCATTGGGG
ATTTCGATGTGAGAAGGTGGAAGGTGTCAAGGGTCTTCTTCATGCTGA
+
FFFFFFFFFFF,FFFFFFFFFFFFFFFFFFFFFFFFF,FFFFFFFFFFFF:FFFFFFFFF:::FFFFFF
FFFFFFF:F:FFFFFFFFFFF:FFFFFFF:FFFFFFFFFFFFFFFFFF
```

```
@A00721:190:HGJW7DSXY:4:1154:23547:8265_1:N:0:TCTGCAAG+GCTGCACT
TTCAGACGTGTGCTCTTCCGATCTCGGCTCGTATCGAGTTGGAGGGAGGAAGTTCTCCGAAATTAAAG
ACGGGGGGATTAAGAGTCCAGCTGTAACTGTCATTTCAGAGAGGGGAGCTCTGAAAAGGAAACTTCGT
CAG
+
FFFFFFFFFFFFFFFFF:FFFFFFFFF:FFFFFFFFFFFFFFFF:FFFFFFFFFFFFFFFFFFFFFF
FFF:FFF:FFFFFFFFFFFFFFFFFFFFFFFFFFFFFFFFFFFFFFFFFFFFFFFFFFFFFFFFFFFF
FFF
@A00721:190:HGJW7DSXY:4:2141:19388:26741_2:N:0:TCTGCAAG+GCTGCACT
AGAGGGGAGGAGTTTGAGGAAGGCTCTAGAGAAGATCTCACCACCTGGAGAAGGCAAGTGGAGAGTCC
AGAAGTTGATGAAGAAGGATTATTTGAAGCAGGGGTTACCACTTCCTA
+
FFFFFFFFFFFFFFFFFFFF:FFFFFFFFFFF:FFFFFFFFFFFFFFFFFFFFFFFFFFFFFFFFF:FF
FFFFFF:FFFFFF:FFF:FF,FFF:FFFFFFFFFFFFFFFFF:FFFFF
@A00721:190:HGJW7DSXY:4:2649:4463:29105_2:N:0:TCTGCAAG+GCTGCACT
TTCTTTGGAGTTTTAGATAATCTTTTGAGAAGGGGTCATAGAGCCAGCAAGAGAAGCTTATATGCTGG
TTTAAGTGATGATTATCACTCCTTGAGAAGACAAGCCACATTAAGATA
+
FFFFFFFFFFFF:FFFFFFFFFFFFFFFFFFFFFFFFFFFFFFFFFFFFFFFFFFFFFFFFFFFFFFF
F,FFFFFFFFFFFFFFFFFFFF,FFFFFFFFFF:FFFFFFFFFFFFF
@A00721:190:HGJW7DSXY:4:2516:20302:16078_2:N:0:TCTGCAAG+GCTGCACT
CTTGGGGTTTCGAATGTCATTAGTGCCTTACGATTGAAAGAGGTCCTTTTGAGTATCGACAGATCTTT
CGATCTTGAGGACGTACATTTAACGTGGCAGAATTCGGTAACTTACCA
+
FFFFFF:FFFFFFFFFFFFFFFFFFFFFFFFFFFFFFFFF:FFFFFFFFFFF:FFFFFFFFFFFFFFFF
F:FFFFFFFFFFFFFFFFFFFFFFF:FFFFFFFFFFFF:FFFFFFFFF:
@A00721:190:HGJW7DSXY:4:2328:3296:8641_2:N:0:TCTGCAAG+GCTGCACT
GGGAAGTTGTGGGATTTAACTTTGACTCAACTACTCCGATCAGATCCGGATGGTTTGATTGGTATGGA
CTACTCGACTTTTGATCTGGAGTTGTCTAGGGTCATGGCAGATACTAC
+
:F,F:FFFF,FFFFF:FFFFFF,FFFFFFFFFFFFFFFFFF:FF::F,:FFF:FFFFFFFF:FFFFFF
F:FFFFFFFF,F,FFFFF:FFF,,FFF:FF,:FFFFFFFF:FFFF,FF
@A00721:190:HGJW7DSXY:4:1339:21838:28009_2:N:0:TCTGCAAG+GCTGCACT
AATATGCAGGTCAAAGATTCTGTAGCTCTGAGGGCGGTTGAGGGAGCCGTTTTGGCCGACTTTAACTA
TTCTAAGAGTCTTTATGATTCAAAACCTATCGTTGTCGTACCTTACAA
+
FFFFFFFFFFFFFFFFFFFFFFFFFFFFFFFFFFFF:FFFF:FFFFFFFFFFFFFFFFFFFFFFFFFF
FFFFFFFFFFFFF:FFFFFFFFFFFFFFFFF,FFFFF:FFFFFFFFFF
@A00721:190:HGJW7DSXY:4:1241:27064:10504_2:N:0:TCTGCAAG+GCTGCACT
GGCTTATTGTCGCGCAGTTTCCAGAATTGCGGTTGAAGTTCACCCATTCTATCTTGAATGCTCACGCC
GTCGCAGCCATATCGAGACTATGTTATAATCACTTGTTAAAGTACAGA
+
FFFFF:FFFFFFFFFFFFFFFFFFFFFFFFFFFFFF,FFFFFFFFFF:FFFFFFFFFFFFFFFFFFFFF
FFFFFFFFFFFFFFFFFFFFFFFFFFFFFFFFFFFFFFFFFFFFFFFF
@A00721:190:HGJW7DSXY:4:2428:1208:11663_2:N:0:TCTGCAAG+GCTGCACT
AGATATTATTGATATTGGAGGCGATATTCGTTTTCATATCCAAAACTCCTTGAATGGAGTACATATTT
GCAACCCTATTCTTGATGTGAAAGATGCATCGAGGTATACGTCTAGGA
+
FFFFFFFFFFFFFFFFFFFFFFFFFFFFFFFFFFFFFFFFFFFFFFFFFFFFFFFFFFFFFFFFFFFF
FFFFFFFFFFFFFFFFFFFFFFFFFFFFFFFFFFFFFFFFFFFFFFFF
@A00721:190:HGJW7DSXY:4:1268:24840:13698_1:N:0:TCTGCAAG+GCTGCACT
CACGAGACGTCATGAAAGCTATCGGAGGGAAGCAAAATGTTTCGTTCTGTTACAAGAACGCTTTGGAT
TGTGACCATAAAGCCTCAGTTGCGATAGCTGTAGAGGTATACGATATCTCTCTTGATGATGTAGCCAC
TAT
```

+
FFFFFFFFFFFFFFFFFFFFFFFFFFFFFFFFFFFFFFFFFFFFFFFFFFFFFFFFFFFFFFFFFFFFF
FFFFFFFFFFFFFFFFFFFFFFFFFFFFFFFFFFFFFFFFFFFFFFFFFFFFF:FFFFFFFFFFFFFFF
FFF
@A00721:190:HGJW7DSXY:4:1167:7943:33129_2:N:0:TCTGCAAG+GCTGCACT
GATTGATGTATGTCATTTCGTCTTAGTTTGTCCCGGCGAATTATTGGACACGATGAAAAATGACATAC
CCGTTCCTGACCTTGGTATCGTTATCCGTCATGGAGATGATGGCGCAT
+
FFFFFFFFFFFFFFFFFFFFFFFFFFFFFFFFFFFFFFFFFFFFFFFFFFFFFF:FFFFFFFFFFFFFF
FFFFFFFFFFFFFFFFFFFFFFFFFFFFFFFFFFFFFFFFFFFFFFFFF
@A00721:190:HGJW7DSXY:4:2309:31819:20118_2:N:0:TCTGCAAG+GCTGCACT
GTTATTCTCACAACAAAGATAAGATTTTATCTTTCTTCAAACACCCTGTTTTCCTTAAGGGCGGACAA
TTGTTCCATTGTGAACTCTTGGGGACTAGATTGAGCATTAATGAGTTC
+
FFFFFFFFFFFFFFFFFFFFFFFFFFFFFFFFFFFFFFFFFFFFFFFF:FFFFFFFFFFFFF:FFFFFF
FFFFFFFFFFFFFFF:FFFF:::FFFFFFFFFFFFF:FFFFFF,FFFFF
@A00721:190:HGJW7DSXY:4:2146:14751:36025_2:N:0:TCTGCAAG+GCTGCACT
CTATTTCCGTACCAAGAGCCACTGCCAATCTCATCGCTATACGCATACCTGATTGGAAGAAGCGAGGT
TCGTTCCTCAATAAAAATTTTTCTCAAGAGATTATGGTTGATAAAGTT
+
FFFFFFFFFFFFF:FFFFFFFFFFFFFFFFFFFFFFFFFFFFFFFFFFFFFFFFFFFFFFFFFFFFFFF
FFFFFFFFFFFFFFFFFFFFFFFFFFFFFFFFFFFFFF::FFFFFFFFF
@A00721:190:HGJW7DSXY:4:1626:3323:6308_1:N:0:TCTGCAAG+GCTGCACT
TTCAGTACGTCATGGGGATCACTTCTTCGGTCAATGACAAAACCTTCGAATATGTTACGACATGGCTT
CGTAACAACAGTGCGAGGATTGTCATCTCGGGGAAAGTGATTCACCAAGATGTTCATATAGATACGAG
ATA
+
FFFFFFFFFFFFFFFFFFFFFFFFFFFFFFFFFFFFFFFFFFFFFFFFFFFFFFFFFFFFFFFFFFFFF
FFFFFFFFFFFFFFFFFFFFFFFFFFFFFFFFFFFFFFFFFFFFFFFFFFFFFFFFFFFFFFFFFFFFF
FFF
@A00721:190:HGJW7DSXY:4:1457:15628:2597_2:N:0:TCTGCAAG+GCTGCACT
CAGCGATCTTACTCGTTATCGGAGCTAAGAAGAAGACTTTGGGTACACGTTATGCTCGCAAACTTAAC
GTTAGTACCGGAGGTGATCTCTCTGCTTCTGCGTTACTGAGAGAAAAG
+
FFFFFFFFFFFFFFFFFFFFFFFFFFFFFFFFFFFFFFFFFFFFFFFFFFFF:FFFFFFFF,FFFFFFF
FFFFFFFFFFFFFFFFFFFFFFFFFFFFFFFFFFFFFFFFFFFFFFFFF
@A00721:190:HGJW7DSXY:4:2465:20591:35524_1:N:0:TCTGCAAG+GCTGCACT
CCTACGGTGACTTTATGGACAAGATGCAAGTTCTTGGGAAATACTTGTTTCCCTTTTTAGGTTTAGTA
GGTGATCTTGACGTCGATTCTTTGTATAGGGAGTGTGAGAAGACAACGAATCTTGAAACTACGGTCCT
TAT
+
FFFFFFFFFFFFFFFFFFFFF,FF:FFFFFFFFFFFFFFFFFFFFFFFFFFFFFFFFFFFFF,FFFFFF
F:FFFFFFFFFFFFFFFFFFFFFFFFFFFFFFFFFFFFFFFFFFFFFFFFFFFFFFFFFFFFFFFFFFF
FFF
@A00721:190:HGJW7DSXY:4:1238:28800:30514_2:N:0:TCTGCAAG+GCTGCACT
ACCGGTCTCTAAATCTTTTGAGATTGAGAAGATTGAAGAACACGTCAAGAAGTCGCTCGGCGTTGTTC
TTGAAAACCAAGTTAAGAATGAGGCTGATCATGGAGTCTTTGTACCTT
+
:F,F:FFFFFFFFF,FFFF:::FFFFF,::FF,F:FF,,FFF:FF,FF:FF,FFF:FFFFFF,F:FFF
FFF:FFFF,F:F:FFFFFF,:FFFFF,,FF:F:,FFFFFFF,FFF,:F
@A00721:190:HGJW7DSXY:4:1314:21206:30733_2:N:0:TCTGCAAG+GCTGCACT
GGTTCAGCTACGAGAGGTGATCCCGGATGTGGATTGAAGGCTGGCTCTAAGGGAGACTCCTTCTTTTT
GAATGTTGTAAAGAATTGGGTTGAGAAGCGTGCAGATCGGAAGAGCGT
+

```
FFFFFFFFFFFFFFFFFFFFFFFFFFF:F:FFFFFFFFFFFFFFFFFFFFFFFFFFFFFFFFFFFFF:F
FFF,:FFF:FF:FFFFFFFFFFFFFFF:FFFFF:FFFFFFFFFFFFFF
@A00721:190:HGJW7DSXY:4:2603:22164:15671_1:N:0:TCTGCAAG+GCTGCACT
AGTTTTTAGATCTTCTTGATAATCTTAAAGAAAAATTTTCGAATAAACTAATGAGAGGTATAGGCTTA
GTCAAGTCTGTACTTACATTCTTTACGCCAGATTCTTCATGGGGCGACGCTGTTTCTCTTGGTATTTC
TGA
+
F:FFFFFFFFFFFFFFFFFFFFF:FFFFFFFFFFFFFFFFFFFFFFFFFFFFFFFFFFFFFFFFFFFF
FFFFFFFFFFFFFFFFFFFFFFFFFFFFFFFFFFFFFFFFFFFFFFFFFFFFFFFFFFFFFFFFFFFF
FFF
@A00721:190:HGJW7DSXY:4:1671:3170:36824_2:N:0:TCTGCAAG+GCTGCACT
CAGAGCGTCATTTTCCTTTGGGATCTTTCCTCTGGTAAGGGAATTATTAAATCATTCTTCTCACTCTT
GACGAGAATTATTACTGTTTTCTCTACCCATAACTTCTTTTTCAAAAA
+
FFFFFFFFFFFFFFFFFFFFFFFFFFFFFFFFFFFFFFFFFFF,FFFFFF:FFFFFFFFFFFFFFFFF
FFFFFFF::FFFFFFFFFFFFFFFFFFFFFFFFFFFFFFFFFFFFFFF
@A00721:190:HGJW7DSXY:4:1539:5240:19523_1:N:0:TCTGCAAG+GCTGCACT
CTGATGTTTTCCTACAGAGTGCAGTCGTTTCAACACTTATCACCACTTTTGAAAGGAAAATAATTGGT
GATGAAAATACGTATGTCGGTGGGGTTCTCAAGGCTCTCATCGCCACTGGGATTCTAAGGGGTTCCAT
AAG
+
FFFFFFFFFFFFFFFFFFFFFFFFFFF:FFF:FFFF,FFFFFFFFFFFFFFFFFFFFFFFFFFFFFFF
FFFFFFFFFFFFFFFFFFFFFFFFFFF:F:FFFF,FFFFFFFFFFFFFFFFFFFFFFFFFFFFFFFFF
FFF
@A00721:190:HGJW7DSXY:4:2659:19741:16454_2:N:0:TCTGCAAG+GCTGCACT
AGGATTCTTCTTTTGGTCCTTACGTCTTAAGTAATTTGGATGTTGTCGAAAACTATCAGGTTCTTGTC
ACGATTCTTAAAAGAAGTTTATTAAAACTTCAGAGTCTCTTGGAAGTC
+
FFFFFFFFFFF,FFFFFFFFFFFFFFFFFFFFFFFFFFF:FFFFFFFFFFFF:FFFFFFFFFFFFFFF
FFF:FFFFFFF:FFFFF:FFFFFFFFFFFFFFFFF:FFFFFFFFFFFF
@A00721:190:HGJW7DSXY:4:1411:20003:8516_1:N:0:TCTGCAAG+GCTGCACT
CAATAGCCACAGACTTTCTGGGAAAAGAGACCTCTGTTGGTAGAGGAGTTGAGAGAATTACTCAGATT
CAATCTGGCATCACTGAACGTCTACAACGAGTTAGGGAGTGGCTAGGTTTTAAAAGATCCTTTGCACA
AGC
+
:FFFFFFF:,FFFFFFFFFFFF:FFFFFFF:FFFFFFFFFFFFFFFFFFFFFFFFFFFFFFFFFFFFF
FFFFF:FF:FFF:FFFFFFFFFFFFFFFFFFFFFFFFFFFFFFFFFFFFFFFFFF:FFFFFFFFFFF
,FF
@A00721:190:HGJW7DSXY:4:2140:13612:35055_2:N:0:TCTGCAAG+GCTGCACT
TTTCGACGTCGCGGAAAACGATGAAGATTTCCACACTGTTCGAGAATCTTCTGATGCTGAATTTGAGG
AAAATTTTTCGGAAGTTTTTGGTATAAATCCTGATGGCTCAGTACAGG
+
FFFFFFFFFFFFFFFFFFFFFFFFFFFFFFFFFFFFFFFFFFFFFFFFFFFFFFFFFFFF:FFFFFFF
FFFFFFFFFFFFF:FFFFFFFFFFFFFFFFFFFFFFFFFFFFFFFFFF
@A00721:190:HGJW7DSXY:4:2270:19180:34178_2:N:0:TCTGCAAG+GCTGCACT
AAACCTTCTTTTATCATTGCAACAGGACTTTGAGAAGACTGTTGAAATGGACAAAATCTGGGATCATA
GGTATGTGTCACGCTACACTCGAAATATGGGCCCTCATAAGGAAGTAT
+
FFFFFFFFFFFFFFFFFF::FFFFFFFFFFFFFF:FFFFFFFFFFFFFFFFFFFFFF:FFFFFFFFFF
FFFFFFFFFFFFFFFFFFFFFF:FFF:FFFFFFFFFFFFFFFFFFFFFF
@A00721:190:HGJW7DSXY:4:1528:2293:27195_1:N:0:TCTGCAAG+GCTGCACT
GATCATTTGTTCAGGAAGATCTCTACAGAGATTATCGAGAGTTGTGATCTAGTTCTAAATTTAGGTCG
ACGGACTTTAGCAACTTTAGATAAATTCTTTCCGGGCTCAATGACCTTTGCTATGTACGTGGATCTAA
TGC
```

+
FFFFFFFFFFFFFFFFFFFFFFF,,FFFFFFFFFFFFFFFFFFFFFFFFFFFFFFFFFFF:FFFFFFF
FFFFFFFFFFFFFFFFFFFFFFFFFFFFF:FFFFFFFFFFFFFFFFFFFFFFFFFFFFFFFFFFFFF
FFF
@A00721:190:HGJW7DSXY:4:1670:32362:1861_2:N:0:TCTGCAAG+GCTGCACT
CATCGCAAAGAAGACTTTATTAGTTCATGAAGTTCTTACACTTCTCGCGACTAATGTCAGCTTCACTA
CAAAGGCGTTACGTAAAGTAGTTTTCACGCTTTATCTTAAAGCATTAC
+
FFFFFFF:FFFFFFFFFFFFFFFFFFFFFFFFFFFFFFFFFFFFFFFFFFFFFFFFFFFFFFFFFFF
FFFFFFFFFFFFFFFFFFFFFF,FFFFFFFFFFFFFFF:FFFFFFFF
@A00721:190:HGJW7DSXY:4:2264:1371:30890_2:N:0:TCTGCAAG+GCTGCACT
ACTTTTTGAATATGACAGAAGCTCGCCATATCAGATTAGAGACATGCCAGTTAGAGTTCTTCTAACCA
GGACTGATGGTGAGATTCTTTTACCTTTTGGAGGTCCAGGAACCTTTA
+
FFFFFFFFFFFFFF:FFFFFFFFFFFFFFFFFFFFFFFFFFFFFFFFFFFFFFFFFFFFFFFFFFFF
FFFFFFFFFFFFFFFFFFFFF:FFFFFFFFFFFFFFFFFFFFFFFFFF
@A00721:190:HGJW7DSXY:4:1644:14461:27633_2:N:0:TCTGCAAG+GCTGCACT
CCAAGGACGTTTCCGTCGGAGGATGACAGGTGGGAGACCGCTCAATAACACTCTATTGAATCCCTTTT
CCACGATGAAAGCTCTTTATGAGGTGCTCCATGCAAATTTACAGTTCC
+
FFFFFFFFFFFFFFFFFFFFFFFFFFFFFFFFFFFFFFFFFFFFFFFFFFFFFFF:FFFFFFFFFFFF
:FFFFFFFFFFFFFFFFFFFFFFFFFFFFFFFFFFF:FFFFFFFFFF:F
@A00721:190:HGJW7DSXY:4:1628:31873:29919_1:N:0:TCTGCAAG+GCTGCACT
GTATTTTGATAAATTTGTTGGAATCGATAATGATACTGACGAAGGTTCTATTACCACCCTTGTCATTG
ATACAAACATTCTAGGTGATGCTTTCTTAGAGGCTCGTCTAGCCGCTGAGATTCGTCTTACCTATTTA
CAA
+
FFFFFF:FFFFFFFFFFFFFFFFFFFFFFFFFFFFFFFFFFFFFFFFFFFFFFFFFFFFFFFFFFFFF
FFFFFF:FFFFFFFFFFFFFFFFFFFFFFFFFFFFFFFFFFFFFFFFFFFFFFFFFFFFFFFFFFFFF
FFF
@A00721:190:HGJW7DSXY:4:2662:31656:32017_2:N:0:TCTGCAAG+GCTGCACT
TTTGAGTGATAACGATTTAGTACTGGGAAATGGTGGTAGTCAGTTTACTACTTTTCTTTCCCTCCCCT
TTTTCGATTGGATTCGACGGGGTCTTCGTCTCACGACTAATTTGATAA
+
FFFFFFFFFFFFFFFFFFFFFFFFFFFFFFFFFFFFFFFFFFFFFFFFFFFFFFFFFFFFFFFF:FFF
FFFFFFFFFFFFFFFFFF:FFFFFFFFFFFFFFFFFFFFFFFFFFF,FF
@A00721:190:HGJW7DSXY:4:2171:14055:27524_2:N:0:TCTGCAAG+GCTGCACT
TATTACTTCGGAAGTTCTTAATGTAGGTCTCCTAGTACTTAAGGGTTTATGTTTCGGTTTCAGCTGGA
TGGATCTTTTGATGGTATGTTTCACATTGCGTGATCACTTCCCGTGGA
+
FFFFFFFFFFFFFFFFFFFFFFFFFFFFFFFFFFFFFFFFFFFFFFFFFFFFFFFFFFFFFFFFFFFF
FFFFFFFFFFFFFFFFFFFFFFFFFFFFFFFFFFFFFFFFFFFFFFFF
@A00721:190:HGJW7DSXY:4:1606:1118:4617_1:N:0:TCTGCAAG+GCTGCACT
GATTAAACATAAAATCCAGCGATTCTATTATGTCGCTAAAGCCTGTCCAGTTGTCGATGATGTTCGGG
CTGTGGGGATTAACTTCCCTCGTGAAACTTACGACGATGTTCTCGCTCGCATTTCACAAGGATTACTA
AGT
+
:FFFFFFFFFFFFFFF::FFFFFFFFFFFFFFFFFFFFFFFFFFFFFFFFFFFFFFFFFFFFFFFFFF
FFFFFFF:FFFFFFFFFFFFFFFFFFFFFFFFFFFFFFFFFFFFFFFFFFFFFFFFFFFFFFFFFFFF
FFF
@A00721:190:HGJW7DSXY:4:1338:8241:19899_2:N:0:TCTGCAAG+GCTGCACT
CCACTTCTAATTTTAGGAAACGTCGCGAAAAGAAAGTTCATGAGGATGTCTTGGTCGAAAAATATCGC
CGGGAACTTCTAATAGAGGATCCTGATGACATTATTTCAACGGATACC
+

FFFFFFFFFFFFFFFFFFFFFFFFFFFFFFFF:FFFFFFFFFFFFFFFFFFFFFFFFFFFFFFFFFF
F:FFFFFFFFFFFFFFFFFFFFFFFFFFFFFFFFFFFFFFFF:FFFFFF
@A00721:190:HGJW7DSXY:4:1162:15854:30013_2:N:0:TCTGCAAG+GCTGCACT
CTACCACAGAAACCGTCTCAGGAACTGTCTCCACTGATTCGGAATTAGAGAAAGACGACCTTCTTGTG
GGGACTGAGACCTCTGAATTTTCAGGGTGCCCTGATGAGGACGTTGAA
+
FFFFFFFFFFFFFFFFFFFFFFFFFFFFFFFFFFFF,FFFFFFFFFFFF:FF:FFFFF:FFFFF,F
FFF:FFFFFFFFFFFFFFFFFFFFFFFFFFFFFFFF:FFFFFF:FFF
@A00721:190:HGJW7DSXY:4:1669:28953:9173_1:N:0:TCTGCAAG+GCTGCACT
ATCTTCTAAGAGTGTTTCAAAAGACCAGAAACGTAACAAGAAGAATAAAGGTAGGAAGGGGAGAAAAC
AGAACAAGCGAGCGGTTGAAGAGAAAACTGATATGGTTGCGGCTGATCGTACCCCAATTCCTGCTAGA
AAG
+
FFFFFFFFFFFFFFFF:FFFFFFFFFFFFFFFFFFFFFFFFFFFFFFFFFFFFFFFFFFFFFFFFFFF
FFFFFFFFFFFFFFF:FFFFFFFF:FFFFFFFFFFFFFFFFFFFFFFFFFFFFFFFFFFFFFFFFFFF
FFF
@A00721:190:HGJW7DSXY:4:2103:22245:14372_1:N:0:TCTGCAAG+GCTGCACT
ATACCACTGATCTTCCAGAGTTACGTAGTGGTCGTTCTTTTGGTGATTTTATCAAAGAGAAAGTTTCT
GCTAGTCGCGACGATAGTATTTATCGGAGTGATATTAACAAGTTAAACTTCGAACTGACCACTTACAA
GAG
+
FFFFFFFFFFFFFFFFFFFFFFFFFFFFFFFFFFFFFFFFFFF:FFFFFFFFFFFFFFFFFFFFFFFFF
FFFFFFFFFFFFFFFFFFFFFFFFFFFFFFFFFFFFFFFFFFFFFFFFFFFFFFFFFFFFFFFFFFFF
FFF
@A00721:190:HGJW7DSXY:4:1263:31611:7420_2:N:0:TCTGCAAG+GCTGCACT
TCAGTTTTCCAAAAAGCTATTAATAGCAGTTGCGGGGCTATAATACCGCCTCCAGATTACTCTAAACT
GAATCCACTCTCTTGGGATATAAATGAGTACATCTATAATAGAGTTTT
+
FFFFFFFFFFFFFFFFFFFFFFFFFFFFFFFFFFFFFFFFFFFFFFFFF:FFFFFFFFFFFFFFFFFFF
FFFFFFFFFFFFFFFFFFFFFFFF:FF:FFFFFFFFFFFFFFFFFFFFF
@A00721:190:HGJW7DSXY:4:2367:3097:35196_1:N:0:TCTGCAAG+GCTGCACT
GGCTTCTCTAAAGAACAGCAACTTTCAACATGAATTAGTTATTGAAAGTCTTGACCCTCACGTCTCAC
TTTATCACCTTGGAGATGGTAAAGTCATTAACAAAGGAAATGTTAAGGCTGATGACTTAAATTTCGCA
TTC
+
FFFFFFFFFFFFFFFFFFFFFFFFFFFFFFFFFFFFF:FFF:FFFFFFFFFFFFFFFFFFFFFFFFFF
FFFFFFFFFFFFFFFFFFFFFFFFFFFFFFFFFFFFFFFFFFFFFFFFFFFFFFFFFFFFFFFFFFFF
FFF
@A00721:190:HGJW7DSXY:4:1324:7392:25160_2:N:0:TCTGCAAG+GCTGCACT
TTTGAAAATTACGGAACCTTTCCTCGTTCTTCATGAAGATCTTCGAGTGTTTTATAGTGAGTTATTCT
TGCGAAGGTTCAAGTCGATTGATTTTGTTGACTACGATCTCTCTTGTA
+
FFFFFFFFFFFFFFFFFFFFFFFFFFFFFFFFFFFFFFFFFFFFFFFFFFFFFFFFFFFFFFFFFFFF
FFFFFFFFFFFFFFFFFF:FFFFFFFFFFFFFFF:FFFFFFFFFFFFF
@A00721:190:HGJW7DSXY:4:1520:7545:6230_1:N:0:TCTGCAAG+GCTGCACT
AGACCACTGAACTTATAGCGACTTTTGTCGAATTTTTCGCAAAAGGTCTTGAAGTACGTATTGTCACT
GCTAATCGTAATTCGCGAGAAGAGATAGTGACTAAAGTTTTGGCACTTCTAGTTCATCGCGGTATTTG
TGA
+
FFFFF:FFFFF:FFFFFFFFFF,FFFFFFFFFFFFFFFFFFFFFF:FFFFFFFFFFFFFFFFFFFFFFFF
FFFFFFFFFFFFF::FFFFFFFFFFFFFFFFFFFFFFF:FFFFFFFFFFFFFFFFFFFFFFFFFFFFFF
FFF
@A00721:190:HGJW7DSXY:4:1239:1579:5196_2:N:0:TCTGCAAG+GCTGCACT
CGTGAAAGTTATGATGAGTGATCGTATTAGAACAGCTGATTCTTATTGCATGAACGTCGCTGAGGTCA

AACCTGTGGACGTTCTTTTCATCGACGAATTATTCATGATTCACGCTG
+
FF,FFFFFFFFFFFFFFFFFFFFFFFFFFFFFFFFFFFF:FFFFFFFFFFFFFFFFFF:FFFFFFFFF
FFFFFFFFFFFFFFFFFFFFFFFFFFFFFFFFF:FFFFFFFFFFFFFFF
@A00721:190:HGJW7DSXY:4:1248:5249:6198_2:N:0:TCTGCAAG+GCTGCACT
CATCTACTTAAGGCGGCTTATACCATAGGTTACGGTGATTCCAATCAAATCGGATTCATCAATCGTAT
TGATCTCCAAGTCGCAAACTACTTCAACATCAACGACATCATTCTTGA
+
FFFFFFFFFFFFFFFFFFFFFFFFFFFFFFFFFFFFFFFFFFFFFFFFFFFFFFFFFFFFFFFF:FFF
F:FFFFFFFFFFFF:FFFFFFFFFFFFFFFFFFFFFFFFFFFFFFFFFF
@A00721:190:HGJW7DSXY:4:1527:19226:9173_2:N:0:TCTGCAAG+GCTGCACT
TTATCGAAGTCTCAGTTATCGCTGCCCGGCCGACGTTTGTCGCGAACTCTCAGAGGTTTACGGCAGAA
AAATTTCTCCTGCTTGTAATTCGAGAGAGGACACTATGGAAATTAGAC
+
FFFFFFFFFFFFFFFFFFFFFFFFFFFFFFFFFFFFFFFFFFFFFFFFFFFFFFFFFFFFFFFFFFFF
FFFFFFFFFFFFFFFFFFFFFFFFFFFFFFFFF,FFFFFFFFFFFFFFF
@A00721:190:HGJW7DSXY:4:1372:16161:30796_2:N:0:TCTGCAAG+GCTGCACT
TATTACTTTCACTCAAGGTGAAAAGTTTGAAATTAACAAAGTCCTTGGTAAGAGTAAGGTTAAATGTG
AAGATGCTCAGACTATTCATGAAGTTCAAGGTAATACTTACCAGCACG
+
FFFFFFFFFFFFFFFFFFFFFFFFFFFFFFFFFFFFFFFFFFFFFFFFFFFFFFFFFFFFFFFFFFFF
FFFFFFFFFFFFFFFFFFFFFFFFFFFFFFFFFF:FFFFFFFFFFFFFF
@A00721:190:HGJW7DSXY:4:2101:12400:19116_2:N:0:TCTGCAAG+GCTGCACT
TTTTGCATCACAAACACATATTACCGTTGCTCTATCACGACACGTTGAATCTTTACGATACTTTGTTA
TCAATGTAAAGTTAAACGATGCTACTTGTGAGAGGATCAATCACAGTA
+
FFFFFFFFFFFFFFFFFFFFFFFFFFFFFFFFFFFFFFFFFFFFFFFFFFFFFFFFFFFFFFFFFFFF
FFFFFFFFFFFFFFFFFFFFFFFFFFFFFFFFFFFFFFFFFFFFFFFFF
@A00721:190:HGJW7DSXY:4:2533:32805:29590_2:N:0:TCTGCAAG+GCTGCACT
TTAGCACGGGAGGTTGCAGATTCAACCCCTTATATCGAGGCTATCGAGGACGATGAGTATCTTGAAGT
TGTTGACGACAAGCCTCAAGAGGGTAGAGGACCACCTGGATCTTCTAC
+
FFFFFFFFFFFFFFFFFFFFFFFFFFFFFFFFFFFFFFFFFFFFFFFFFFFFFF:FFFFFFFFFFFF,FF
FFFFF:FFFFFFFF:FFF:F:FFFFFF:FFFFFFFFFFFFFFFFFFFF
@A00721:190:HGJW7DSXY:4:1504:12138:18411_1:N:0:TCTGCAAG+GCTGCACT
AACCACCTTAACGTCACTCGGTAATCTTTCAGCCGAATTAAGTACTAATGAGTTCAAGTCGGTTGTCG
ATCGAATTAAGATCAAGGCTAATACCTTTATAGGTAAGAAGCCCCTTGAGCCGCGTAGTCAGCGCGTT
TAG
+
FFFFFFFFFFFFFFFFFFFFFFFFFF:FFFFFFFFFFFFFFFFFFFFFFFFFFFFFFFFFFFFFFFFF
FFFFFFFFFFFFFFFFFFFFFFFFFFFFFFFFFFFFFFFFFFFFFFFFFFFFFFFFFFFFFFFFFFFF
FFF
@A00721:190:HGJW7DSXY:4:1570:21251:27524_2:N:0:TCTGCAAG+GCTGCACT
CCGATAGGGTACCGAGTCTCCAAGAGAATATTTATTCTTATGAGGCTCGAAACTTCAACGTTTTCACA
ACGGAAAGACATATCGCACCCGAAATTTTTGGGCAGAATGCTCTTAAC
+
FFFFFFFFFFFFFFFFFF:FFFFF:FFFFF:FFFFFFFFFFFFFFFFFFFFFFFFFFFFFFFFFFFFF
FFFFFFFFFFFFFFFFFFFFFFFFFFF,FFFFFFFFF,FFFFFFFFFFF
@A00721:190:HGJW7DSXY:4:2540:1172:14512_2:N:0:TCTGCAAG+GCTGCACT
GATCAGTCTAAGTTGGATAAAGTTATAGGAGACTCAGTTAACCTGAGAGCTCCTCAATTGATAGATTG
GGTCAACAATAGGGACGCTAGTATACTTCGTAAAGTCGACAGCGAAAT
+
FFFFFFFFFFFFFFFFFFFFFFFFFFFFFFFFFFFFFFFFFFFFFFFFFFFFFFFFFFFFFFFFFFFF
FFFFFFFFFFF:FFFFFFFFFFFFFFFFFFFFF::FFFFFF,FFFFFF

```
@A00721:190:HGJW7DSXY:4:2237:18801:4460_2:N:0:TCTGCAAG+GCTGCACT
AATTGGACCGACTATATGACCTATTTTAAGCTCATGGTGAAAAGGGAAGCTAAGGTTAAACTTGATGC
TTCAGCCTTTTCGAAGCATAACCCCGCACAGAATATCGTTTATCACGC
+
FFFFFFFFFFFFFFFFFFFFFFFFFFFFFFFFFFFFFFFFF:FFFF,FFFFFFFFFFFFFFFFFFFFF
FFFFFFFFFFFFFFFFFFFFFFFFFFFFFFFFFFFFFF:FFF:FFF:F
@A00721:190:HGJW7DSXY:4:2125:17137:6089_2:N:0:TCTGCAAG+GCTGCACT
CCTATCTTTAAGGTTCTTGTGAGGAGAGTCAAACAGTGTCTTTTACCCAACATTTTTATGTATACAGA
GATGACCAGTGACGATTTCTCCCGGAAGCTCTTCTCTCTCTTAGGAGG
+
FFFFFFFFFFFFFFFFFFFFFFFFFFFFFFFFFFFFFFFFFFFFFFFFFFFFFFFFFFFFFFFFFFFF
FFFFFFFFFFFFFFFFFFFFFFFFFF:FFFFFFFFFFFFFFFFFFFFF
@A00721:190:HGJW7DSXY:4:1566:4526:18662_1:N:0:TCTGCAAG+GCTGCACT
TAAAGCCTATGAGATAAAGATGTATTCTTATTTAGGCTTCGATGCTGAATTTCTTGAACTTTGGAGTG
CCAGTGAATATTTTTGTCACGCCAGGAGTATCCATAACGATTTGTCATTTACCACCTTTGCTCAACGA
CGA
+
FFFFFFFFFFFFFFFFFFFFFFFFFFFFFFFFFFFFFFFFFFFFFFFFFFFFFFFFFFFFFFFFFFFF
FFFFFFFFFF:FFFFFFFFFFFFFFFFFFFFFFFFFFFFFFF:FFFFFFFFFFFFFFFFFFFFFFFFF
FFF
@A00721:190:HGJW7DSXY:4:2673:10294:9267_1:N:0:TCTGCAAG+GCTGCACT
TAACACATTCTTTGGTAATTGTATGATCACTCTTATGATGCTCTCTCTTTATTACGACCTTCGGAAGT
TTAGAGCCGTCGCTGTCGCCGGAGACGATTCGATCCTCTTTTCGGACGTACCGGTCCAGGATTATTCA
GAT
+
FFFFFFFFFFFFFFFFFFFFFFFFFFFFFFFFFFFFFFFFFFFFFFFFFFFFF:FFFFFFFFFFFFFF
FFFFFFFFFFFFFFFFFFFFFFFFFFFFFFFFFFFF:FFFFFFFFFFFFFFFFFFFFFFFFFFFFFFF
FFF
@A00721:190:HGJW7DSXY:4:1459:20618:10144_2:N:0:TCTGCAAG+GCTGCACT
TGCTCGCGATTTATTCTTTTTGTCAATGATTACATCTACTTCGTTCCTGATCCTTACAAGATCATCGT
TAAGTTAGGTAAACCCCTAACCGATGTTACTCTTGTAGGACTTTGGGA
+
FFFFFFF:FF,FFF::FFFF:FFFFFF,FFFFFFFFFFFFFF,,FF,F:FFFFFFFFFFFFFFFFF,F
FFFF:FFFFFFFFF:::FFF,F::F,FFFFFFF:FFF,FF:FFF:FFF
@A00721:190:HGJW7DSXY:4:2224:32895:37012_2:N:0:TCTGCAAG+GCTGCACT
TTAAAGACTATACTAAATATCTCCACTACGAGGTTGTTGTTTGGCGATTAGCTCTTGCGACTAATATT
CGATATTCGATTGAGGGTCGTTATACCTATTCGGCTATCTGTGCTTTG
+
FFFF:FFFFFFFFFFFFFFFFFFFFFFFFFFFFFFFFFFFFFFFFFFFFFFFFFFF:FFFFFFFFFF
FFFFFFFFFFFFFFFFFFFFFFFFFFFFFFFFFFFFFFFFFFFFFFFF
@A00721:190:HGJW7DSXY:4:1377:29875:19413_2:N:0:TCTGCAAG+GCTGCACT
TTTTAAACGATTCCGAGAGGTTTATCCTGATGTGGAGGGAGTTCTTGAAGCATCCCGACCTTTCCTTG
TGAAAATTTTGATGCGGTTTCATAAGATTATCAGTAATCCTGATTCTA
+
FFFFFFFFFFFFFFFFFFFFFFFFFFFFFFFFFFFFFFFF:FFFFFFFFFFFFFFFFFFFFFFFFFF:F
FFFFFFFFFFFFFFFFFFFFFFFFFFFFFFFFFFFFFFFFFFFFFFFF
@A00721:190:HGJW7DSXY:4:2565:29270:32174_1:N:0:TCTGCAAG+GCTGCACT
GACTGGAGTTCAGACGTGTGCTCTTCCGATCTCAAAGATCGACTTCGAGACAGGTGATATTGTTGAGC
GAGAGCGACCAAGATCTAAATTCGGTAACGGTGCGTGGCGCTTTATTTTAAAAAGCTCCCTCCTCTCG
CAA
+
FFFFFFFFFFFFFF,FFFFFFFFFFFFFFFFFF,,FFFFFF,,FFFFFFFFFFFFFFFFFFFFFFFFFF
FFFFFFFFFFFFFFFFFFFFFFFFFFFFFFFFFFFFFFFFFFF:FFFFF:FFF:FFFFFFFFFFFFF
FFF
```

```
@A00721:190:HGJW7DSXY:4:2255:15447:27962_2:N:0:TCTGCAAG+GCTGCACT
ACTTGAGTCTTTTGATCAGTCTCTGACTTGACCATCTTTCATTTTAAATGACGATCATTAAGACTCTC
TTTCTTCTTTGTTTCCTTCTTTGCACTGATTTTTCCTCATCAGCTTGT
+
FFFFFFFFFFFFFFFFFFFFFFFFFFFFFFFFFFFFFFFFFFFFFFFF:FF:FFFFFFFFF:FF::
FFFFFFFFFFFFFFFFFFFFFFFF,F:FFFFFFFFF:FFFFFFFFFFFF
@A00721:190:HGJW7DSXY:4:2436:24126:2534_2:N:0:TCTGCAAG+GCTGCACT
TTTTACGATAACAACTATCAGTTGATTAAGACTCTTCCGAAAGGCGAACTTTGGGAATTTGAAGCTCA
GGGTGAAGCACAACGTTTGACCGTGACGAACTGTGGTGTTCTTGATCT
+
FFFFFFFFFFFFFFFFFFFFFFFFFFFFFFFFFFFFFFFFFFFFF:FFFFFFFFFFFFF:FFFFFFFFFFF
FFFFFFFFFFFFFFFFFFFFFFFFFFFFFFFFF::FFFFFFFFFFFFFF
@A00721:190:HGJW7DSXY:4:2237:20021:7169_1:N:0:TCTGCAAG+GCTGCACT
CACTACCACTTATTACGATATTTCTTTAGTCGATGGGTTTTCGACGCCTTTAAGCGTCTTCTGTTCAT
CGCAACCAGATGATATCTTCTATTCGAACTTATGTTTCGATCAGTGTTTCTTCTATCAGGAGGAAAAC
ACT
+
FFFFFFF:FFFFFF:FFFFFFFFFFFFFFFFFFFFFFFFFFFFFFFFFFFFFFFFF:FFFFFFFFFFFFFF
FFF,FFF:FF:FFFFFFFFFF:FFFFFFF,FFFFFFFFFFFFFFFFFFFFFFFFF:FFFFFFFFFFFFFF
FFF
@A00721:190:HGJW7DSXY:4:2248:14931:26068_2:N:0:TCTGCAAG+GCTGCACT
TCTTCTAATGTGGCCTGAACTACGTTTCACTTTGTGTGTACTTGTCTCTATTTTAAGTTTTGTCTTCC
TTGTTTGTCTTATTCTATTAATCACTCATTTTAGTGAGATTATTAAAA
+
FFFFFFFFFFFFFFFFFFFFFF:FFFFFFFFFFFFFFFFFFFFFFFFFFFFF:FFFFFFFFFFFF:FFFFF
FFFFFFFFFFFFFFFFFFFFFFFFFF::FFFFFFFFFFFFFFFFFFFFF
@A00721:190:HGJW7DSXY:4:1607:5502:26303_2:N:0:TCTGCAAG+GCTGCACT
GACAACAGCAGGGGTTTTCGAATAGTGATAGCCACCCGCCTTACAGAGGGGCAGCAGCATGACCATAA
TTGGTATTGACTTTGGTACCACCTTTTCTACTGTCTGTCTCGCCTCTT
+
FFFFFFFFFFFFFFFFFFFFFFFFFFFFFFFFFFFFFFFFFFFFFFFFFFFFFFFFFFFFFFFFFFFFF
FFFFFFFFFFFFFFFFFFFFFFFFFFFFFFFFFFFFFFFFFFFFFFFFF
@A00721:190:HGJW7DSXY:4:2450:7762:16532_1:N:0:TCTGCAAG+GCTGCACT
CTTAACAACAATGATAGTGAATTTATACCTAAAATCATTGGTTTTTCTAATAATGTTGACACTATTGT
TTATGGATATGATGCTATTACTCATGATGGTTACGGTCAGGGAGGATTCTCAGTTTACAAGGACCTTA
AGC
+
F:F,FFF,FFFFF:FFFF,FF::FFFF,:F:,F:FFFFFFFFFFFF:FF,FFFF,,,F,FFFFFFFFF
FF,F:F,FFFF:FF,FFFFFFFFFFFFFFFFFF,FFFF:FF,FFFFFF:FFF,FFFF:FFF,FFFFFF
,:F
@A00721:190:HGJW7DSXY:4:2655:26901:5963_2:N:0:TCTGCAAG+GCTGCACT
CCGAAAGACGTGCTAAATTAAAACCATTGTACGATGTCGTCTGTCTCGGCGATCACTTTTCACTAAGA
ATTGGTCCTACTTTTGGACAAGGCCAACTTCGTAATCTTCATGAGATC
+
FFFFFFFFFFFFFFFFFFFFFFFFFFFFFFFFFFFFFFFFFFFFFFFFFFFFFFFFFFFFFFFFFFFFF
FFFFFFFFFFFFFFFFFFFFFFFFFFFFFFFFFFFFFFFFFFFFFFFFF
@A00721:190:HGJW7DSXY:4:2536:2889:6151_1:N:0:TCTGCAAG+GCTGCACT
AGGGATTTTGAGAGAGTGAAAGGCTTCAATTGTAGTGGTTTGGTCGTTTCAGTACCCAGTCAGTATAC
CTCTACGCAAAGATATTTCATGAAAACTACAGAGGAACGTACCGGATTACCTGTCTTTCATATTATGA
AT
+
FFFFFFFF:FFFFFFFFFFFFFFF,FFFF:FFFFFFFFF,FFFFFFFFFFFFFFFFFFFFFFFFFFFFF
:FFFFFFFFFFFFFFFFFFFFFFFFFF:FFFFFFFFFFFFFFFFFFFFFFFFFFFFFFFFFFFFFFFFF
FF
```

@A00721:190:HGJW7DSXY:4:2160:4092:31344_1:N:0:TCTGCAAG+GCTGCACT
TTTGCTTCGATGTTGGATGTCAAGAAGGGTTCAGAATGGGATTCCTACGTAGTTTATGACTTTGGTGG
TGGTACTTTCGATACTTCTATCGTCTTACGTTACGGTAATTACTATAGTGTTATTTATTCTGGTGGTG
ATG
+
FFF:FFFFFFFFFFFFFFFF:FFFFFFFFF:FFFFFFFFFFFFFFFFFFFFFFFFFFFFFFFFFFFFFF
FFFFFFFFFFFFFFFFFFFFFFFFFFFFFFFFFFFFFF:FFF:FFFFFFFFFFFFFFFFFFFFFFFFF
FFF
@A00721:190:HGJW7DSXY:4:1260:15167:21715_1:N:0:TCTGCAAG+GCTGCACT
GGTCGTGACGTTGATGAAGCTATAAGAGCGTTCTTATTACGGCGCACTTCTTTACCCGCCACCACACA
ACTTTCAGTTTCACAACTGAAGGAGGAGGTTTCTGTTACTGGAAAACCCTCGTCAATACTTTTCAATA
ATA
+
FFFFFFFFFFFFFFFFFFFFFFFFFFFFFFFFFFFFFFFFFFFFFFFFFFFFFFFFFFFFFFFFFFFF
FFFFFFFFFFFFFFFFFFFFFFFFFFFFFFFFFFFFFFFFFFFFFFFFFFFFFFFFFFFFFF:FFFFF
FFF
@A00721:190:HGJW7DSXY:4:2135:5312:23124_2:N:0:TCTGCAAG+GCTGCACT
GATCTAGTGAAATTAACGCGTCCTTTTCTGGAACGTAGTTTTACTATTATGAACAAAGTTATCAAAGA
TAGTGGCATTCAAGGCAAACTTACCCTGATTCCGGCCGGTGGTAGCTC
+
FFFFFFFFFFFFFFFFFFFFFFFFFFFFFFFFFFFFFFFFFFFFFFFFFFFFFFFFFFFFFFFFFFFF
FFFFFFFFFFFFFFFFFF:FFFFFFFFFFFFFFFFFFFFFFFFFF:FFFF
@A00721:190:HGJW7DSXY:4:1627:21811:3380_2:N:0:TCTGCAAG+GCTGCACT
TGCTGAAACAGCTATTAAAGCTGTTGAGCGGGTAATTACACCTAAACGAGCTCGATCTGCCATCGCTG
AAGGATGTGCACTCTTTTCTGCTACTTTAACGGAGCGTAGTGTACTCT
+
FFFFFFFFFFFFFFFFFFFF:FFFFF:FFFFFFFFFFFFFFFFFF:FFFFFFFFFFFFFFFFFFFF:F
FFFFFFFFFFFFFFFFFFFFFFFFFFFFFFFFFFFFFFFFFFFFFFFF
@A00721:190:HGJW7DSXY:4:1101:5439:2080_2:N:0:TCTGCAAG+GCTGCACT
AAAGGTCTCTATCAAGCTCGTATTATCGTCGCTGCTGGATCTGCGTTACCGTTGAAGGCTAAACTCCA
AGGTAATTGTGAAGACTGTGATTTATCTTTCACTCTGGCCTTGTATGA
+
FFFFFFFFFFFFFFFFFF:FFFFFFFFFFFFFFFFFFFFFFFFFFFFFFFFFFFFF:FFFFFFFFFFFFF
FFFFFFFFFFFFF,FFF:FFFFFFFFFFFFFFFFFFFF:FFFFFFFFF
@A00721:190:HGJW7DSXY:4:2625:30951:8719_2:N:0:TCTGCAAG+GCTGCACT
CGTAACATTAATAATTTCAATCTTCTTAAAAAGAAGATAGAGAGTCATACTTTGGGCTATCGTTCAGG
TGCATGGGGTTGGATAGCCAATATTGAAGTGGACACCTCTGGTAGTAT
+
FFFFFFFFFFFFFFFFFFFFFFFFFFFFF:FFFFFFFFFFFFFFFFFFFFFFFFFFFFFFFFFFFFFF
FFFFFFFFFFFFFFFFFFFFFFFFFFF,FFFFFFFFFFF:FFFFFFF:F
@A00721:190:HGJW7DSXY:4:1401:20654:28150_2:N:0:TCTGCAAG+GCTGCACT
AAGCATACTACTTTTGCTTTGATTACTCTACCGCCTTACAAGTCTAAGGTTAAAGAGTCGATTAAAAA
AGTTGTACTTATCAATGATTTTATCGCTGGTTGTTATCTCGAAAAGTC
+
FFFFFFFFFFFFFFFFFFFFFFFFFFFFFFFFFFFFFFFFFFFFFFFFFFFFFFFFFFFF,FFF,,:
F:FFFF,:FFFF:FF,,FFFFFF:,::::F,:FFF:F,FFF,FFF,F,
@A00721:190:HGJW7DSXY:4:1671:7871:25081_1:N:0:TCTGCAAG+GCTGCACT
CCCAAAGAGAATATCTAAAATATTTACAAAATCTTACCACTATCTACGAACCTAAAGTTCTGAAGACG
TTGCTTGTCGCTTCCTCCTTTAATGGCACTACCTCGGTACAATCTTACTTGGAACGAAGGCGACCTGT
TCC
+
:FFFFFFFFFFFFFFFFFFFFFFFFFFFFFFFFFFFFFFFFFFFFFFFFFFFFFFFFFFFFFFFFFFF
FFFFFFFFFFFFFFFFFFFFFFFFFFFFFFFFFFFFFFFFFFFFFFFFFFFFFFFFFFFFFFFFFFFF
FFF

```
@A00721:190:HGJW7DSXY:4:2325:26503:7592_1:N:0:TCTGCAAG+GCTGCACT
GTTCTGTCTATATTAGGTGAAGCGAAATACGATTTCGAACGTCAACGTTCACCGAGTTTCGTTTCGTA
CCTTGATGCTGACTTTCGTCAATATGATTTGATCTGGTTGCTCAATTCTAAGCGTGTTCCGCTTAGTG
CGA
+
F:FFFFFFFFFFFF,:FFFFFFFFF:FFFFFFFFFFFFFFFFFFFFFFFFFFFFFFFFFFFFFFFFFF
FFFFFFFFFFFFFFF:FFFFFFFFFFFFFFFFFFFFFFFFFFFFFFFFFFFFFFFFFFFFFFFFFFFF
FFF
@A00721:190:HGJW7DSXY:4:2403:8223:20838_1:N:0:TCTGCAAG+GCTGCACT
TACGAACTTGACTCTACTATACGTGACATACTAAATACTAGATATTTGGATGATCTTGATAAAGAACT
TCATGAATTTTCAGAGGATGAAAAATTTTCTTCCTTGGATTTTAATATAAAGAATCTTTACCAGGACT
CCT
+
FFF:FFFFF:FFFFFFFFFF,:FF,FFFFFFFFFFFFFFFFFFFFFFFFFF:FFFFFFFFFFFFFFFF
:FFFFFFFFFFFFFFFFFFFFFFFFFFFFFFFFFFFFFFFFFFFFFFFFFFFFFFFFFFFFFFFFFFF
FFF
@A00721:190:HGJW7DSXY:4:1310:4137:16892_1:N:0:TCTGCAAG+GCTGCACT
GATCGTAAGAAGTTTATAAGGAACATATCCATTTGTTGTAATAGGGTCATCAACTTAGAACTCGAAAC
TCTAAGTGGTAATACTGTCTTTCCAAGCTTACCTGTATCTTCAATGAAATTGGACTCTTCGATAGGGG
ACA
+
FFF:FFFFFFFFFFFFF:FF:,FFFFFFFFFF:FFFFFFF,FFFFFFFFFFFFFFFFFFFFFFFFF:F
FFFFFFFFFF:FF:FFFFFFFFFFFFFFFFFFFFFFF,FF:FFF,FFFFFFFFFF::FFFFFF:FFFFF
FFF
@A00721:190:HGJW7DSXY:4:1627:1958:13902_1:N:0:TCTGCAAG+GCTGCACT
ACCGGTGGATGGTTTAAGAATACTATAAATAGAATAGCTATTACTACTAATGGCGTTGGCCTAGTGGA
TGTTATTAGTGACATCTGGTCAATTTCGTTTTTAGGTGGTCTTTATTCTATTATTGATTTTTATGAGA
TTG
+
FFFFF:FFFFF:FFFFFFFF,FFFFFF:F:FFFF,FFFFFFFFFFFFFFFFFFFFFFFF,F,FFFFFF
FFFFFFFFFFFFFFFFFFFFFFFFFFFFFFFFFFFFFFFFFFFFFFFFFFFFFF:FFFF:FFFFFFFF
F,F
@A00721:190:HGJW7DSXY:4:1224:16758:2112_2:N:0:TCTGCAAG+GCTGCACT
TGTTAGAGTAGAATTCCTGAAGAGTCCTTCCGTAACCAACTTCTTGACAATTTTCTTCAATCCTTCGA
TAATAGTTAAATCTAGGATTATGTCTGTGTTGAAAGACTTTCTACCGA
+
FFFFFFFFFFFFFFFFFFFFFFFFFFFFFFFFFFFFFFFFFFFFFFFFFFFFFFFFFFFFFFFFFFFF
FFFF:FFFFFFFFFFFFFFFFFF:FFFFF:FFFFFFFFF:FFFFFFFF
@A00721:190:HGJW7DSXY:4:1424:23511:21919_2:N:0:TCTGCAAG+GCTGCACT
TACACAATTAAGAAATAATCCTACATTGACTGATTATCTAAAGATTGAAGGAAAATTTTATCCTCGTT
TAACTTACTTAGACTTTCCTACGATACAAATGATAACTTCTAATTTAC
+
FFFFFFF:FFFFFFFFFFFFFF:FFFFF,FFFFFFFFFF:FFFFFFFFFFFFFFFFFFFFFF:FFFF:
FFFF,FFF,FFFFFF:FFFFFFFFFFFFFFFFFFFFFFFFFFFFFFFFF
@A00721:190:HGJW7DSXY:4:2270:6677:13808_1:N:0:TCTGCAAG+GCTGCACT
GGGATGCATGGTGTTATCTCCTCAGGAGTCCCGGCATTTGATAATAATGACGAGACTTTCGTCATAAT
CGCTGCATATTACGGATTACACGGTACCAGCGCATCTCGTGTCGAGCCTCGACCCGATTATATGGTAT
TAA
+
FFFFFFFFFFFFFFFFFFFFFFFFFFFFFFFFFFFFFFFFFFFFFFFFFFFFFFFFFFFFFFFFFFFF
FFFFFFFFFFFFFFFFFFFFFFFFFFFFFFFFFFFFFFFFFFFFFFFFFFFFFFFFFFFFFFFFFFFF
FFF
@A00721:190:HGJW7DSXY:4:1538:11243:24721_2:N:0:TCTGCAAG+GCTGCACT
GATTCTCGATTCGTAGAGCTTATCTAGGTACTCTCATTTACTATGTTGATTTAGTCTACATTTATGCT
```

```
GATCTAAAATTTCGATCTAGATGGATCCACTCCTCGAGTTATCATGAT
+
FF,FFFFFFFFFFFF:FFFFFFFFFFFFFFFFFFFFFFFFF:FFFFFFFFFFFFFFFFF:FFFFFF:FF
,FFFFFFFFFF:F,FF:FF:FFFFFFF:FFFFFFFFFFFFFF,FFFFF
@A00721:190:HGJW7DSXY:4:2529:22399:2707_1:N:0:TCTGCAAG+GCTGCACT
TTCTAGAATTCTTAATTATAATATTCAACCTCAAAAGTTACGTGTTTACAGTAACGAGATCACACATT
TTGAGAACTAACTATGTTATGTTCTCTGATCATTTTGACAATCTCTTCTTATAATAAATAAATAAATA
ATA
+
FFFFFF:FFFFFFFFFFFFFFFFFFFFFFFFFFFF,FFFFFFFFFFFFFFFFFFFFFFFFFFFFFFFFF
FFFFFFFFFFFFFFFFFFFFFFFFF:FFFFFF:FFFFFFFFFFFFFFFFFFFFF:FFFFFFFF:FFFFF
FFF
@A00721:190:HGJW7DSXY:4:1160:19081:23985_2:N:0:TCTGCAAG+GCTGCACT
AGTGGAAAGGCTCCAGTCAACACTAGTTCGAGTGCTGCTTTGGATTACGATGACGTGAGAATAGGTGA
AGGGATAGTTTTTACCTCGACCAACATTAAGGCGGCGGCGACTAATCT
+
FFFFFFFFFFFFFFFFFFFFFFFFFFFFFFFFFFFFFFFFFFFFFFFFFFFFFFFFFFFFFFFFFFFF
FFFFFFFFFFFFFFFFFFFFFFFFFFFFFFFFFFFFFFFFFFFFFFFF
@A00721:190:HGJW7DSXY:4:1569:2917:16438_1:N:0:TCTGCAAG+GCTGCACT
AGGACACTTCGAAACTGAATAATGATGCACTACAAACAAGTATCGCTAATCATCTGAAAAAGGCTCTA
AAGGACACTATACAATATCCAGACGACGACGAAGCTTATACTACTTTAATTTTGGGTATTATGTGGAG
ATA
+
FFFFFFFFFFFFF::FFF:FFFFFFFFFFFFFF:FFFF,FFFFFF,FFFFFFF:FFFFFFFFFFFFFF
FFFFFFFFFFFFFFFFFFFFFFFFFFFFFFFFFFFFFFFFFFFFFFFFFFFFFFFFF:FFFFFFFFFF
FFF
@A00721:190:HGJW7DSXY:4:1268:12020:36902_2:N:0:TCTGCAAG+GCTGCACT
ACCACATTTTGGTCAAGTCAATCCGTTGAGAAAATTTCTTAGAGGATATTCGCCATTGTGGTACGAAT
TGGTCCAACATTTACCACCAAATTCTTTTGATACAACTCTAGCCTTCA
+
FFFFFFFFFFFFFFFFFFFFFFFFFFFFFFFFFFFFFFFFFFFFFFFFFFFFFFFFFFFFFFFFFFFF
FFFFFFFFFFFFFFFFFFFFFFFFFFFFFFFFFFFFFFFFFFFFFFFF
@A00721:190:HGJW7DSXY:4:2257:13928:3161_2:N:0:TCTGCAAG+GCTGCACT
CTTGCTATTCACCAGATTGGTTCGAACCCGTTGATGCTATGTCAGAAGAAGCTATTCAAGCTTGTCGT
TTAAAGACTGCAGAGGCTGTCAAACAAGCCCAGACTCAAGGAGTCCAA
+
F:FFFFFFFFFFF:FFF,FFFFFFFFFFFFFFFFFFFFFFFFFFFFFFFFFFFFF:FFFFFFFFFFFF
:FFFF:FFFFFFFFFFFFFFFFFFFFFFFFFFF:FFFFFFFFFFFFFF
@A00721:190:HGJW7DSXY:4:2320:5466:20165_2:N:0:TCTGCAAG+GCTGCACT
GGTGATAAAAATTATTTAGAGATCTTTTCTACTTACAAAGGACAATTCAGTTTCATTAATTCTACGGA
AAAGTCCGGAGTATATTGTATGTTAATAGATAGTGGGATAATAGTTCT
+
:FFFFFFFFFFFFFFFFFFFFFFFFFFFFFFFFFFFFFFFFFFFFFFFFFFFFFFFFFFFFFFFFF:FF
FFFFFFF:FFFFFFFFFFFFFFFFFFFF:FFFFFFFFFFFF::FFFFF
@A00721:190:HGJW7DSXY:4:1605:19497:34444_2:N:0:TCTGCAAG+GCTGCACT
TGCTATTGATTATGTAACCGATAGGAGTTGTATAGTTGAAGATGTGGCTTTAGACGATAATCCTTTAA
GGACTATGTTGACTGACGAAGATTTTCCCACAGATTTGTCTACGTTGC
+
FFFFFFFFF:FFFFFFFFFFFFFFFFFFFFFFFFF:FFFFFFFFFFFFFFFF::FFFFFFFFFFFFFF
::FFFFFFFFFFFFFFFFFFFFFFFFFFF,FF:FFFFF:FFFFFFFF,F
@A00721:190:HGJW7DSXY:4:1118:14895:10598_1:N:0:TCTGCAAG+GCTGCACT
CTATATACATTATAAAATATAAAAATAAGGGAGTCATAACTTTTGTTACCAAATTCTTGGCTTTCAAC
GAAGTCTCTGATTTTATTAATCATTACTTTAACGATCTCTCGGTCATTTACTCTGTACTTGATCTACG
TCG
```

+
FF:FFF:FFFFFFFFFFFFFFFFFFFFF:FFFFFF:FFFFFFFFFFFFFFFFFFFFFFFFF:FF:FFFFF
FFFFF:FFFFFFFFFFFFF:FFFFFFFFFFFFF,FFFFFFFFFFFFFFFFFFFFFFF:FFFFFFFFFF
FFF
@A00721:190:HGJW7DSXY:4:2460:10212:10347_2:N:0:TCTGCAAG+GCTGCACT
CTTAGAATGACTCTATCTTTTCAGACACTTCTTATATTATTTACAATAGTTAATATCATATTAGTAAT
ACTCACTATCGTTTTCTGCTTTTTCTTAGCTAATAGATTAATAAAGGA
+
FFFFFF:FFFF,FFFFF:,:FF:F::FF:F::FFFFFFFF:FFFF::FFF:FF:FFFFFF:,FF,,FF
F,FFFF,FFF:,FF:FF,FFFFFFFF:F:FFFFF:FFFFFFFFFFF:F
@A00721:190:HGJW7DSXY:4:1337:23411:10911_1:N:0:TCTGCAAG+GCTGCACT
CCAACTCTTCGTTACGAGACCTTTTGTTATAATATTTTTATTCTCTACACCCATCTCACCGAGTATAA
AGCTAGTTTTCCGTCCTGAACTTAATACACGAAGATATCATCCGCAAACGACTCGAAACCGAGACGCC
GTA
+
FFF:,:,FF,,FFF:,,,F:,F,,:F,F,,F:F,,,FFF,,F,,F:F,FFFFF,:FFF,F,,:,,F:F
,:,,,:FFF:F,::F,F,F:,,::,F,:,:,F,,,,F,,,,F:,F::FFFFFFFFFFFFFFFFFFFFF
FFF
@A00721:190:HGJW7DSXY:4:2339:32768:8484_2:N:0:TCTGCAAG+GCTGCACT
TTTAGATTATGAATCCTGCCTTTACTCATTTTATAGCAGTTATTTATGTTGTACTTTTTATAATTTAT
AACCTTCTTATACTTTTGGGTATGTTTTGTGAAAAACATGAGATACCC
+
FFFFFF:FFFFFFFFFFFFFFFFFFFFFFFFFFFFFFFFFFFFFFFFFFFF:F,FFFFFFFFFFFFFF
FFF:FFFFFFFFF:FFFFFFFF:FFFFFFFF,F:FFFFFF:,FFF::F
@A00721:190:HGJW7DSXY:4:2101:22327:9627_2:N:0:TCTGCAAG+GCTGCACT
TTGTAACGATCATTCTTTCGAGTTTTGTCATTCTTATTCTTTTATTCCTTTAACTGTGTATGAGGAGG
ATTTATAATGCTTTTCGATAGTGATTGTTATCAATTTAAATACATTAC
+
FFFFFFFFFFFFFFFFFFFFFFFFFFFFFFFFFFFFFFFFFFFFFFFFFFFFFFFFFFFFFFFFFFFF
FFFFFFFFFFFFFFFFFFFFFFFFFFFFFFFFFFFFFFFFFFFFFFFF
@A00721:190:HGJW7DSXY:4:2630:20175:1110_1:N:0:TCTGCAAG+GCTGCACT
TCTTCCGATCTCCGAAAATTGTTTCAAATGAGGTCAAAGCTCTATTTATTAAACGAACTATTATCCGA
TTCTTCGAGGGTATCACTACCACTGATCTGCCGACTTTAGTCAAGGAACTTCAATTCCTTCGATGTTT
ATA
+
F,FFF,,FF,F,,FF,:FF,FFFF,FF:F,:FFFFFFF,,F,FFFFF:FFFFFFFFFFFFFFFF:FFF
FFFFF:::F:FFF:F,:FFFF,FFFFF,FFFFFF,:FFF:FFFF,FFFFFFFFFFFF:FFFFFFFFFF
FFF
@A00721:190:HGJW7DSXY:4:1324:19343:35775_2:N:0:TCTGCAAG+GCTGCACT
GGAAAATAGCAATAATCAAATTTCGACAAATTTTAGAACTTTATTTCAGAACTCTCTCCGATCACCTT
GGTGTGATCTCCCGGAGGATCTTTTCAATAAGATGTATGATGATAATC
+
FFFFFFFFFFFFFFFFFFFFFFFFFFFFFFFFFFFFFFFFFFFFFFFFFFFFFFFFFFFFFFFFFFFF
FFFFFFFFFFFFFFFFFFFFFFFFFFFFFFFFFFFFFFFFFFFFFFFF
@A00721:190:HGJW7DSXY:4:1211:8874:19241_2:N:0:TCTGCAAG+GCTGCACT
AGTGATATTCAGCGTATACTTGAATACTTTATCAAAGATTTGGCTTGGTCTATTTACCGCAAAAAGTT
GGAAGAGGTCTATGATATTTATTTTACTTATACTTTAAATAGCACCGA
+
FFFFFFFFFFFFFFFFFFFF:FFFFFFFFFFFFFFFFFFFFFFFFFFFF:FFFFFFFF:FFFFFFFFF:
FFFFFFFFFFFFFFFFFFFFFFFFFFFFFF:FFFFFFFFFFFF:FF:FF
@A00721:190:HGJW7DSXY:4:1137:3721:25128_2:N:0:TCTGCAAG+GCTGCACT
TGTTGATAATATTACAATAGCTCTTTCTCAGAAGATTAGAGAAGTAACCGGTGAACACGTCGGTAGCA
TTGATTTACTTTTACCGTTAGAACTGAAAAAGATTCTTTTCGAAAAGA
+

:FFFFFFFFFFFFFFFFFFFFFFFFFFFFFFFFFFFFFFFFFFFFFFFFFF:FFFFFFFFFFFFFFFF
FFFFFFFFFFFFFFFFFFFFFFFFFFFFFFFFFFFFFFFFFFFFFFFF
@A00721:190:HGJW7DSXY:4:1128:10999:14278_1:N:0:TCTGCAAG+GCTGCACT
AAACGATTCTTCGACTATATTAAAGAAATAAAAATACTTCAAGTACCTGAACTTTACATTTTTAATGG
TATGGTATAATGTGTCTTACAAATAGAACCTTCACTCTCGGTAATGAGAGTGTATTCCTTAATATTCT
AGT
+
FFFFFFFFFFFFFFFFFF:FFFFFFFFFFFFFF:FFFFFFFFFFFFFFFFFF:FFFFF:FFFFFFFFF
FFFFFFFFFFFFFFFFFFFFFFFFFFFFFFFFFFFFFFFFFFFFFFFFFFFFFFFFFFFFFFFFFFFF
FFF
@A00721:190:HGJW7DSXY:4:2136:25030:36918_1:N:0:TCTGCAAG+GCTGCACT
GGCATTACTAGGATTTATCACACTCCTAAATTTGATAGCAATTTATGTTTTAAATGCTAGGAAAATTT
TAGAAAAACTCCATTGTATAGTTCAAGGAGTACTTAATTCAATAGCTTTAGTATTATTTACTATCGCC
TTT
+
F:FFFFFFFF,,FFFFFFF::FF:,F:FFFF:F,FFFF:::FFF,FFF:FFFFFFF:FF:FFFF:FFF
FFFF,FFF:F,,:FFFFF:FFFF,,FFFFFFFFFFFFFF:FF,:FF:FFF:F:FFFFFFFFFFFFFF:
FFF
@A00721:190:HGJW7DSXY:4:2122:11252:34475_2:N:0:TCTGCAAG+GCTGCACT
AGTTAATTGCTTACACAAAAGTGACGCGCGAAGGAATCTTAATTGATTGAAGCTAGGCGTTGGGGAGA
ATGAACTGCCGCTTCCCGGAAGAAACGATCTATTTACAGAGTATTTAG
+
FFFFFFFFFF:FFFFFFFFFFFFFF:FFFFFFFFFFFFFFFFFFFFFFFFFFFFFFFFFF,FFFFFFFF
FFFFFFFFFFFFFFFFFFFFFFF,F:FFFFFF:FFF::FFFFF,FFFFF
@A00721:190:HGJW7DSXY:4:2361:31331:7028_1:N:0:TCTGCAAG+GCTGCACT
CTCCCCAAACATAACATACCATAACCTACCATGACCAATCATCGCTCCAACTGGCACGCCAGACGTAG
GGTCAAACCTACTACCGCCTGTCGGCAGATCGGCACCACTGGTCTCTACGTTCAGGGACCCGCTGTGC
AGA
+
FFFFFFFFFFFFFFFF:FFFFFFFFFFFFFFFFFFFFFFFFFFFFFFFFFFFFFFFFFFFFFFFFFFF
FFFFFFFFFFFFFFFFFFFFFFFFFFFFFFFFFFFFFFFFFFFFFFFFFFFFFFFFFFFFFFFFFFFF
FFF
@A00721:190:HGJW7DSXY:4:1359:26431:33959_2:N:0:TCTGCAAG+GCTGCACT
TCTGCACAGAAGAAGGCTGAAGGTCGTTACCGGACGACCACTCAAAGGAAACGGGCTGTTGCCCAGAG
GAAAGCAGCGACCACCGAGGTCGCTCGTAACATTGACGGCAGTCCCGT
+
FFFFFFFFFFFFFFFFFFFFFFFFFFFFFFFFFFFFFFFFFFFFFFFFFFFFFFFFFFFFFFFFFF:F
FFFFFFFFFFFFFFFFFFFFFFFFFFFF:FFFFFFFFFFFFFFFFFFFF
@A00721:190:HGJW7DSXY:4:1650:29152:36949_1:N:0:TCTGCAAG+GCTGCACT
AACTGAGGCAGTACCTCAGTCAAAAGTCACATTCACCACTCCCATACCAATCAATGATGTGACTATAC
AGTTCGGCCATTTCAAGAGTGGTCAATACGTCGTGGGAGGTACTGAAGCCTTCCCCTCCGCTGGCACG
AAG
+
FFF:FFFF:FFFFFFFFFFFFFFFFFFFFFFFFFFFFFFFFFFFFFFFFFFF:FFFFFFFFFFFFFF
FFFFFFFFFFFFFFFFFFFF:FFFFFFFFFFFFFFFFFFFFFFFFFFFFFFFFFFFFFFFFFFFFFFF
FFF
@A00721:190:HGJW7DSXY:4:1228:4300:34209_2:N:0:TCTGCAAG+GCTGCACT
GCCAATCAATAGTTCTTCTGACCAGAATGAAAAGGGGTTTACCAGGGTAGTCTCTAAGTCTACCAGGA
AGAGGGGAAATGTCCCCAAAACTGAGAAGAATAAAGGGAAAGGTCCAT
+
FFFFFFFFFFFFFFFFFFFFFFFFFFFFFFFFFFFFFFFFFFFFFFFFFFFFFFFFFFFFFFFFFFFF
FFFFFFFFFFFFFFFFFFFFFFFFFFFFFFFFFFFFFFFFFFFFFFFF
@A00721:190:HGJW7DSXY:4:1332:7283:19272_2:N:0:TCTGCAAG+GCTGCACT
AGGGGAAATTACCCCTAATTCTGAAGGAAGATCTCCCACCATCTGGGGTTGGGAATAAGAAATCTGCA

CGGGCCAAAGAGGCCGCTGCTACGAAGTCGCTGAAGGCGGCTTGGGTC
+
FFFFFFFFFFFFFFFFFFFFFFFFFFFFFFFFFFFFFFFFFFFF:FFFF:FFFFF:FFFFFFFFFFFF
FFFFFFFFFFFFFFFFFFFFFFFFFFFFFFFFFFFFFFFFFFFFFFFF
@A00721:190:HGJW7DSXY:4:1612:20338:13510_2:N:0:TCTGCAAG+GCTGCACT
GTTGCGCCTGCCGACCCCATCTTTGAGACCAAGACTCTCTACAGTAGGTTCGTGCCTACTATGAGCAA
ATCTGTTGAATTCGTCACTGAGGACGAAGGTGAGAGGACTTTTGGCTA
+
FFFFFFFFFFFFFFFFFFFFFFFFFFFFFFFFFFFFFFFFFFFFFFFFFFFFFFFFFFFFFFFFFFF:
FFFFFFFFFFFFFFFFFFFFFFFFF:FFFFFFFFFFFFFFFFFFFFFF
@A00721:190:HGJW7DSXY:4:1438:5141:34194_1:N:0:TCTGCAAG+GCTGCACT
CTGAAAGAGAATTGCGTGGGTTGAAGGCCGACGCTTCGAGCTTGAGTGTGACGAGGCCGGTGACGTTT
TTGTCAACGGCGTTTGGAGGATTTTATCCGCGCTTAACTATGGGGCGAAGATCCCCTATGGAGTCTTG
AAG
+
FFFFFFFFFFFF,FFFFFFFFFFFFFFFFF:FFFFFFFFFFFFFFFFF:FFFFFFFFFFFFFFFFFFF
FFFFFFFFFFFFFFFFFFFFFFFFFFFFFFFFFFFFFFFFFFFFFFFFFFFFFFFFFFFFFFFFFFFF
FFF
@A00721:190:HGJW7DSXY:4:1354:29017:10097_2:N:0:TCTGCAAG+GCTGCACT
GGCATATTTATGCCGACGATAATAAGCATGTCACCCTTAAGCACCGAGGTAAGCTCGGGACTTACAAG
AGAGAGTGTGAGAGAGTCGCTCTCACTATTTTTGCCCAGAATGCGGGC
+
FFFFFFFFFFFFFFFFFFFFFFFFFFFFFFFFFFFFFFFFFFFFFFFFFFFFFFFFFFFFFFFFFFFF
FFFFFFFFFFFFFFFFFFFFFFFFF:F,FFFFFFFFFF:FFFFFFFFFF
@A00721:190:HGJW7DSXY:4:2246:22037:35336_2:N:0:TCTGCAAG+GCTGCACT
TTCACTGGTTCTGGGAGGCTGCTTTCCTCCATAATTACAACCTCTCTTTAGAGAGCATTAGAAGGAAG
TTGGGCAAATTCCCACATATGGTAGACGTTGTCACACTTTTCTTGAAA
+
FFFFFFFFFFFFFFFFFFFFFFFFFFFFFFFFFFFFFFFFFFFFFFFFFFFFFFFFF,FFFFFFFFF:
:FFFFFFF:FFFFFFFF:FFFFFFFFFFFFFFFFFFFFFFFFFFFFF:
@A00721:190:HGJW7DSXY:4:1473:4544:36542_2:N:0:TCTGCAAG+GCTGCACT
CGTCATTGGGGATTTCGATGTGAGAAGGTGGAAGGTGTCAAGGGTCTTCTTCATGCTGATCTTTCAGG
ACCTAATCACATCATGATTGATAGTTACGGCTCGTATCGAGTTGGAGG
+
FFF:FFFFFFFFFFFFFFFFFFFFFFFFFFFFFFFFFFFFFFFFFFFFFFFFFFFFFFFFFFFFFFFF
FFFFFFFFFFFFFFFFFFFFFFFFFFFFFFFFFFFFFFFFFFFFFFFF
@A00721:190:HGJW7DSXY:4:1416:1280:8594_2:N:0:TCTGCAAG+GCTGCACT
CGAAATTAAAGACGGGGGGATTAAGAGTCCAGCTGTAACTGTCATTTCAGAGAGGGGAGCTCTGAAAA
GGAAACTTCGTCAGAAGATGAGGAAGTCTAAGAGATCGGAAGAGCGTC
+
FFFFFFFFFFFFFFFFFFFF:FFFFFFFFFFFFFFFFFFFFFFFFFFFFFF:FFFFFFFFFFFFFFFF
FFFFFFFFFFFFFFFFFFFFFFFFF:FFFFF:FFFFFFFFFFFFFFFF:
@A00721:190:HGJW7DSXY:4:2141:30011:7310_2:N:0:TCTGCAAG+GCTGCACT
GAAGTTGATGAAGAAGGATTATTTGAAGCAGGGGTTACCACTTCCTAAGGTGGATGAGGGAGGTTGTT
TCTTTGGAGTTTTAGATAATCTTTTGAGAAGGGGTCATAGAGCCAGCA
+
FFFFFFFFFFFFFFFFFFFFFFFFFFFFFFFFFFFFFFFFFFFFFFFFFFFFFFFFFFFFFFFFFFFF
FFFFFFFFF:FFFFFFFFFFFFFFFFFFFFFFFFFFFFFFFFFFFFF:
@A00721:190:HGJW7DSXY:4:2626:8865:28369_2:N:0:TCTGCAAG+GCTGCACT
CCTTGAGAAGACAATCCACATTAAGATATAATTTAGGTCAACTTGGTGTTTCGAATGTCATTAGTGAC
TTACGATTGAAAGAGGTCCTTTTGAGTATCGACAGATCTTTCGATCTT
+
,FFF:F,FFFF,FF,FFF:FF:FF,:FFFFFFFFFF,,FFF,,F,F,FF,,,,FFFFF,F::FFFF,,
:FFF:FFF,F:FF:,,::FFFFFFFF:FF,FF,F:,:,FFF::FF::F

```
@A00721:190:HGJW7DSXY:4:1540:27037:15248_2:N:0:TCTGCAAG+GCTGCACT
TGGCAGAATTCGGTAACTTACCATATTGTTCCTTCCGGGAAGTTGTGGGATTTAACTTTGACTCAACT
ACTCCGATCAGATCCGGATGGTTTGATTGGTATGGACTACTCGACTTT
+
FFFFFFFFFFFFFFFFFFFFFFFF:FFFFFFFFFFFFFFFFFFFFFFFFFFFFFFFFFFFFFFFFFFF
FFFFFFFFFFFFFFFFFFFFFFFFFFFFFFFFFFFFF:FFFFFFFFFF
@A00721:190:HGJW7DSXY:4:2663:6343:19774_1:N:0:TCTGCAAG+GCTGCACT
CATGGCAGATACTACCGATAAGGTGAAAAATATGCAGGTCAAAGATTCTGTAGCTCTGAGGGCGGTTG
AGGGAGCCGTTTTGGCCGACTTTAACTATTCTAAGAGTCTTTATGATTCAAAACCTATCGTTGTCGTA
CCT
+
FFFFFFFFFFFFFFFFFFFFFFFFFFFFFFFFFFFFFFFFFFFFFFFFFFFFFFFFFFFFFFFFFFFF
FFFFFFFFF:FFFFFFFFFFFFFFFFFFFFFFFFFFFFFFFFFFFFFFFFFFFFFFFFFFFFFFFFFF
FFF
@A00721:190:HGJW7DSXY:4:1312:27163:2065_2:N:0:TCTGCAAG+GCTGCACT
GGCTTATTGTCGCGCAGTTTCCAGAATTGCGGTTGAAGTTCACCCATTCTATCTTGAATGCTCACGCC
GTCGCAGCCATATCGAGACTATGTTATAATCACTTGTTAAAGTACAGA
+
FFFFFFFFFFFFFFFFFFFFFFFFF:FFFFFF:FFFFFFFFFFFFFFFFFFFFFFFFFFFFFFFFFFF
FFFFFFFFFFFFFFFFFFFFFFFFFFFFFFFFFFF:FFFFFFF:FFFF
@A00721:190:HGJW7DSXY:4:1275:31385:24408_2:N:0:TCTGCAAG+GCTGCACT
ATTATTGATATTGGAGGCGATATTCGTTTTCATATCCAAAACTCCTTGAATGGAGTACATATTTGCAA
CCCTATTCTTGATGTGAAAGATGCATCGAGGTATACGTCTAGGAACAT
+
FFFFFFFFFFFFFFFFFFFFFFFFFFFFFFFFFFFFFFFFFFFFFFFFFFFFF:FFFFFFFFFFFFFF
F:FFFFFFFFFFFFFF,F::FFFFFFFFFFFFFFFFFFFFFFFFFFFF
@A00721:190:HGJW7DSXY:4:2365:6578:10316_1:N:0:TCTGCAAG+GCTGCACT
CACGAGACGTCATGAAAGCTATCGGAGGGAAGCAAAATGTTTCGTTCTGTTACAAGAACGCTTTGGAT
TGTGACCATAAAGCCTCAGTTGCGATAGCTGTAGAGGTATACGATATCTCTCTTGATGATGTAGCCAC
TAT
+
FFFFFFFFFFFFFFFFFFFFFFFFFFFFFFFFFFFFFFFFFFFFFFFFFFFFFFFFFFFF:FFFFF:F
FFFFFFFFFFFFFFFFFFFFFFFFFFFFFFFFFFFFFFFFFFFFFFFFFFFFFFFFFFFFFFFFFFFF
FFF
@A00721:190:HGJW7DSXY:4:1327:12057:8782_2:N:0:TCTGCAAG+GCTGCACT
GATTGATGTATGTCATTTCGTCTTAGTTTGTCCCGGCGAATTATTGGACACGATGAAAAATGACATAC
CCGTTCCTGACCTTGGTATCGTTATCCGTCATGGAGATGATGGCGCAT
+
FFFFFFFFFFFFFFFFFFFFFFFFFFFFFFFFFFFFFFFFFFFFFFFFFFFFFFFFFFFFFFFFFFFF
FFFFFFFFFFFFFFFFFFFFFFFFFFFFFFFFFFFFFFFFFFFFFFFF
@A00721:190:HGJW7DSXY:4:2620:31313:17801_2:N:0:TCTGCAAG+GCTGCACT
GTTATTCTCACAACAAAGATAAGATTTTATCTTTCTTCAAACACCCTGTTTTCCTTAAGGGCGGACAA
TTGTTCCATTGTGAACTCTTGGGGACTAGATTGAGCATTAATGAGTTC
+
FFFFFFFFFFFFFFFFFF,FFFFFFFFFFFFFFFFFFFF:FFFFFFFFFFFFFFFFFF:FFFFFFFFF
FFFFFFF:FFFFF:FFFFFF:FFFF:FFFFFFFFFFFFFFFFFFFFFFF
@A00721:190:HGJW7DSXY:4:1147:6451:31485_2:N:0:TCTGCAAG+GCTGCACT
TATTTCCGTACCAAGAGCCACTGCCAATCTCATCGCTATACGCATACCTGATTGGAAGAAGCGAGGTT
CGTTCCTCAATAAAAATTTTTCTCAAGAGATTATGGAGATCGGAAGAG
+
FFFFFFFFFFFFFFFFFFFFFFFFFFFFFFFFFFFFF:FFFFFFFFFFFFFFFFFFFFFFFFFFFFFF
FFFFFFFFF:FFFFFFFFFFFFFFFFF:FF,F,FFFFFFFFFFFFFFF
@A00721:190:HGJW7DSXY:4:2374:14027:18239_1:N:0:TCTGCAAG+GCTGCACT
TTCAGTACGTCATGGGGATCACTTCTTCGGTCAATGACAAAACCTTCGAATATGTTACGACATGGCTT
```

CGTAACAACAGTGCGAGGATTGTCATCTCGGGGAAAGTGATTCACCAAGATGTTCATATAGATACGAG
ATA
+
FFFFFFFFFFFFFFFFFFFF:FFFFFFFFFFFFFFFFFFFFFFFFFFFFFF:FFFFFFFFFFFFFFFF
FFFFFFFFFFFFFFFFFFFFFFFFFFFFFFFFFFFFFFFFFFFFFFFFFFFFFFFFFFFFFFFFFFFF
FFF
@A00721:190:HGJW7DSXY:4:1549:25554:34663_2:N:0:TCTGCAAG+GCTGCACT
CAGCGATCTTACTCGTTATCGGAGCTAAGAAGAAGACTTTGGGTACACGTTATGCTCGCAAACTTAAC
GTTAGTACCGGAGGTGATCTCTCTGCTTCTGCGTTACTGAGAGAAAAG
+
FFFFFFFFF,FF:FFFF:FFF:FF,FFFFFFFFFFFF:FF:F:FFFFFF:FFFF::FFFFF,F,FFFF
FF:FFFF::F:FFFFFFFFFFFFFFFFF:FFFFFFFFFFFFF:FFFFF
@A00721:190:HGJW7DSXY:4:1354:9724:12571_1:N:0:TCTGCAAG+GCTGCACT
ACTTTATGGACAAGATGCAAGTTCTTGGGAAATACTTGTTTCCCTTTTTAGGTTTAGTAGGTGATCTT
GACGTCGATTCTTTGTATAGGGAGTGTGAGAAGACAACGAATCTTGAAACTACGGTCCTTATCAAAGA
AAA
+
FFFFFFFFFFFF:FFF:FFFFFFFFFFFFFFFFFFFFFFFFFFFFFFF:FFFFFFFFFFFFFFFFFF
FFFFFFFFFFFFFFFFFFFFFFFFFFFFFFFFFFFFFFFFFFFFFFFFFFFFFFFFFFFFFFFFFFFF
FFF
@A00721:190:HGJW7DSXY:4:1407:17671:27179_1:N:0:TCTGCAAG+GCTGCACT
TCTAAATCTTTTGAGATTGAGAAGATTGAAGAACACGTCAAGAAGTCGCTCGGCGTTGTTCTTGAAAA
CCAAGTTAAGAAGGAGGCTGATCATGGAGTCTTTGTACCTTCTGTCAAACTTCGAGGTTCAGCTACGA
GAG
+
FFFFF:FFFFFFFFFFFFFFFFFFFFFFFFFFFFFFFFFFFFFFFFFFFFFFFFFFFFFFFFFFFFFF
FFFFFFF:FFF::FFFFF:FFFFF:FFFFFFFFFFFFFFFFFFF:FFFFFFFFFFFFFFFFFFFFFFF
FFF
@A00721:190:HGJW7DSXY:4:2560:1090:17221_1:N:0:TCTGCAAG+GCTGCACT
TGGATTGAAGGCTGGCTCTAAGGGAGACTCCTTCTTTTTGAATGTTGTAAAGAATTGGGTTGAGAAGT
GTGCTTTACAAGGAGTTAGACTCTTCTTCAAGTTTTTAGATCTTCTTGATAATCTTAAAGAAAAATTT
TCG
+
FFFFFFFFFFFFFFFFFFFFFFFFFFFFFFFFFFFFF:FFFFFFFFFFFFFFFFFFFFFFFFFFFFFF
FFFFFFFFFFFFFFFFFFFFFFFFFFFFFFFFFFFFFFFFFFFFFFFFFFFFFFFFFFFFFFFFFFFF
FFF
@A00721:190:HGJW7DSXY:4:2630:15736:9111_1:N:0:TCTGCAAG+GCTGCACT
GTTTCTCTTGGTATTTCTGAAATAGTTTATCAGAGCGTCATTTTCCTTTGGGATCTTTCCTCTGGTAA
GGGAATTATTAAATCATTCTTCTCACTCTTGACGAGAATTATTACTGTTTTCTCTACCCATAACTTCT
TTT
+
FFFFFFFFFF:FFFFFFFFFFFFFFFFFFFFFFFFFFFFFFFFFFFFFFFFFFFFFFFFFFFFFFFFF
FFFFFFFFFFFFFFFFFF:FFFFFFFFFFFFFFFFFFFFFFFFFFFFFFFFFFFFFFFFFFFFFFFFF
FFF
@A00721:190:HGJW7DSXY:4:2635:20799:33849_1:N:0:TCTGCAAG+GCTGCACT
ACTGGAGTTCAGACGTGTGCTCTTCCGATCTGTTTCAACACTTATCACCACTTTTGAAAGGAAAATAA
TTGGTGATGAAAATACGTATGTCGGTGGGGTTCTCAAGGCTCTCATCGCCACTGGGATTCTAAGGGGT
TCC
+
FFFFFFFFFFFFFFFFFFFFFFFFFFFFFFFFFFFFFFFFFFFFFFFFFFFFFFFFFFFFFFFFFFFF
FFFFFFFFFFFFFFFFFFFFFFFFFFFFFFF,FFFFFFFFFFFFFFFFFFFFFFFFFFFFFFFFFFFF
FFF
@A00721:190:HGJW7DSXY:4:1162:23963:11397_2:N:0:TCTGCAAG+GCTGCACT
TTTGGTCCTTACGTCTTAAGTAATTTGGATGTTGTCGAAAACTATCAGGTTCTTGTCACGATTCTTAA

AAGAAGTTTATTAAAACTTCAGAGTCTCTTGGAAGTCCAGGTAGCTAA
+
F:FFFFFFFFFFFFFFFFFFFFFFFFFFFFFFFFFFFFFFFFFFFFFFFFFFFFFFFFFFFFFFFFFFF
F::FFFFFFFFFFFFFFFFFFFFFFFFFFFFFFFFFFFFFFFFFFFFF
@A00721:190:HGJW7DSXY:4:1614:25066:25739_2:N:0:TCTGCAAG+GCTGCACT
GCCACAGACTTTCTGGGAAAAGAGACCTCTGTTGGTAGAGGAGTTGAGAGAATTACTCAGATTCAATC
TGGCATCACTGAACGTCTACAACGAGTTAGGGAGTGGCTAGAGATCGG
+
FFFFFFFFFFFFFFFFFFFFFFFFFFFFFFFFFFFFFFFFFFF:FFFFFFFFFFFFFFFFF:FFFFFF
FFFFFFFFFFFFF:FFFFFFFFFFFFFFFFFFFFFFFFFFFFFFFFFFF
@A00721:190:HGJW7DSXY:4:2534:4399:8547_1:N:0:TCTGCAAG+GCTGCACT
CAATACCTTCCACTGAGTTCATCACCCAGAGTTTCGACGTCGCGGAAAACGATGAAGATTTCCACACT
GTTCGAGAATCTTCTGATGCTGAATTTGAGGAAAATTTTTCGGAAGTTTTTGGTATAAATCCTGATGG
CTC
+
FFFFFFFF:FFFFFFFFFFFFFFFFFFFFFFFFFFFFFFFFFFFFFFFFFFFFFFFFF,FFFFFFFFF
FFFFFFFFFFFFFFFFFFFFFFFFFFFFFFFFFFFFFFFFFFFFFFFFFFFFFFFFFFFFFFFFFFFF
FFF
@A00721:190:HGJW7DSXY:4:2437:1895:8437_2:N:0:TCTGCAAG+GCTGCACT
AAACCTTCTTTTATCATTGCAACAGGACTTTGAGAAGACTGTTGAAATGGACAAAATCTGGGATCATA
GGTATGTGTCACGCTACACTCGAAATATGGGCCCTCATAAGGAAGTAT
+
FFFFFFFFFFFFFFFFFFFFFFFFFFFFF::FFFFFFFFFFFFFFFFFFFFFFFFFFFFFFFFFFFFF
FFFFFFFFFFFFF:FF:FFFFF,FFFFFFFFFFFF:FFFFFFFF,:FF:
@A00721:190:HGJW7DSXY:4:2270:19180:34178_1:N:0:TCTGCAAG+GCTGCACT
GATCATTTGTTCAGGAAGATCTCTACAGAGATTATCGAGAGTTGTGATCTAGTTCTAAATTTAGGTCG
ACGGACTTTAGCAACTTTAGATAAATTCTTTCCGGGCTCAATGACCTTTGCTATGTACGTGGATCTAA
TGC
+
FFFFFFFFFFFFFFFFFFFFFFF:FFFFFFFFFFFFFFFFFFFFFFFFFFFFFFFFFFFFFFFFFFFF
FFFFFFFFFFFFFFFFFFFFFFFFFFFFFFFFFFFFFFFFFFFFFFFFFFFFFFFFFFFFFFFFFFFF
FFF
@A00721:190:HGJW7DSXY:4:2174:11116:14888_2:N:0:TCTGCAAG+GCTGCACT
CATCGCAAAGAAGACTTTATTAGTTCATGAAGTTCTTACACTTCTCGCGACTAATGTCAGCTTCACTA
CAAAGGCGTTACGTAAAGTAGTTTTCACGCTTTATCTTAAAGCATTAC
+
FFFFFFFFFFFFFFFFFFFFFFFFFFFFFFFFFFFFFFFFFFFFFFFFFFFFFFF,FFFFFFFFFFFFF
FFF:FFFFFFFFFFFFFFFFFFFFFFF:FFFFFFFFFFFF:FFFFFFFF
@A00721:190:HGJW7DSXY:4:2264:1524:30405_2:N:0:TCTGCAAG+GCTGCACT
ACTTTTTGAATATGACAGAAGCTCGCCATATCAGATTAGAGACATGCCAGTTAGAGTTCTTCTAACCA
GGACTGATGGTGAGATTCTTTTACCTTTTGGAGGTCCAGGAACCTTTA
+
FFFFFFFFFFFFFFFFFFFFFFFFFFFFFFFFFFFFFFFFFFFFFFFFFFFFFFFFFFFFFFFFFFFF
FFFFFFFFFFFFFFFFFFFFFFFFFFFFFFFFFFFFFFFFFFFFFFFF
@A00721:190:HGJW7DSXY:4:2576:4887:3067_2:N:0:TCTGCAAG+GCTGCACT
CCAAGGACGTTTCCGTCGGAGGATGACAGGTGGGAGACCGCTCAATAACACTCTATTGAATCCCTTTT
CCACGATGAAAGCTCTTTATGAGGTGCTCCATGCAAATTTACAGTTCC
+
FFFFFFFFFFFFFFFFFFFFFFFFFFFFFFFFFFFFFFFFFFFFFFFFFFFFFFFFFFFFFFFFFFFF
FFFF,FFFFFFFFFFFFFFFFFFF:FFFFFFFFFFFFFFFFFFFFFFFF
@A00721:190:HGJW7DSXY:4:1649:10212:28917_1:N:0:TCTGCAAG+GCTGCACT
GTATTTTGATAAATTTGTTGGAATCGATAATGATACTGACGAAGGTTCTATTACCACCCTTGTCATTG
ATACAAACATTCTAGGTGATGCTTTCTTAGAGGCTCGTCTAGCCGCTGAGATTCGTCTTACCTATTTA
CAA

```
+
FFFFFFFFFFFFFFFFFFFFFFFFFFFFFFFF:F:FFFFF:FFFFFFFFFFFFFFFFFFFFFFFFFFFF
FFFFFFFFFFFFFFFFFFFFFFFFFFFFFFFFFF,FFFFFFFFFFF,FFFF:FFFFF:FFFFFFFFFF:
FFF
@A00721:190:HGJW7DSXY:4:1103:4218:3192_1:N:0:TCTGCAAG+GCTGCACT
GATAACGATTTAGTACTGGGAAATGGTGGTAGTCAGTTTACTACTTTTCTTTCCCTCCCCTTTTTCGA
TTGGATTCGACGGGGTCTTCGTCTCACGACTAATTTGATAAGGTTTTTCTTTTATGGGACTCTTGTTT
GTA
+
FFFFFFFFFFFFFFFFFFFFFFFFFFFFFFFFFFFFFFFFFFFFFFFFFFFFF:FFFFF:FFFFFF:
FFFF:FFFFFFFFFFFFFFFFFFFFFFFFFFFFFFFFFFF:FFFFFFFFFFFFFFFFFFFFFFFFFFFF
FFF
@A00721:190:HGJW7DSXY:4:2436:20356:24377_2:N:0:TCTGCAAG+GCTGCACT
TATTACTTCGGAAGTTCTTAATGTAGGTCTCCTAGTACTTAAGGGTTTATGTTTCGGTTTCAGCTGGA
TGGATCTTTTGATGGTATGTTTCACATTGCGTGATCACTTCCCGTGGA
+
FFFFFFFFFFFFFFFFFFFFFFFFFFFFFFFFFFFFFFFFFFFF:FFFFFFFFF:FFFFFFFFFFFF
FFFFFFF,FFFFFFFFFFFFFFFFFFFFFFFF:FFFFFFFFFFFFFFFF
@A00721:190:HGJW7DSXY:4:2121:27362:14841_1:N:0:TCTGCAAG+GCTGCACT
GATTAAACATAAAATCCAGCGATTCTATTATGTCGCTAAAGCCTGTCCAGTTGTCGATGATGTTCGGG
CTGTGGGGATTAACTTCCCTCGTGAAACTTACGACGATGTTCTCGCTCGCATTTCACAAGGATTACTA
AGT
+
FFFFFF:FFFFFFFFFFFFFFFFFF:FFFFFFFFFF:FFFFFFFFFFFFFFFFFFFFFFFFFF:FFFFFFF
F:FFFFFFFFFFFFFFFFFFFFFFFFFFFFFFFFFFFFFF:FFFFFFFFFFFFFFFFFFFFFFFFFFFF:
FFF
@A00721:190:HGJW7DSXY:4:1455:6840:2973_2:N:0:TCTGCAAG+GCTGCACT
CCACTTCTAATTTTAGGAAACGTCGCGAAAAGAAAGTTCATGAGGATGTCTTGGTCGAAAAATATCGC
CGGGAACTTCTAATAGAGGATCCTGATGACATTATTTCAACGGATACC
+
FFFFFFFFFFFFFFFFFFFFFFFFFFFFFFFFFFFFFFFFFFFFFFFFFFFFFFFFFFFFFFFFFFFF
FFFF:,FFFFFF:FFFFFFFFFFFFFFFFFFFFFFFFFFFFFFFFFFF
@A00721:190:HGJW7DSXY:4:1223:26087:20400_2:N:0:TCTGCAAG+GCTGCACT
CTACCACAGAAACCGTCTCAGGAACTGTCTCCACTGATTCGGAATTAGAGAAAGACGACCTTCTTGTG
GGGACTGAGACCTCTGAATTTTCAGGGTGCCCTGATGAGGACGTTGAA
+
FFFFFFFFFFFFFFFFFFFFFFFFFFFFFFFFFFFFFFFFFFFFFFFFFFFF:FFFFFFFFFFFFFFF
FFFFFFFFFFFFFFFFFFFFFFFFFFFFFFFFFFFFFFFFFFFFFFFF
@A00721:190:HGJW7DSXY:4:2347:8585:4836_1:N:0:TCTGCAAG+GCTGCACT
ATCTTCTAAGAGTGTTTCAAAAGACCAGAAACGTAACAAGAAGAATAAAGGTAGGAAGGGGAGAAAAC
AGAACAAGCTAGCGGTTGAAGAGAAAACTGATATGGTTGCGGCTGATCGTACCCCAATTCCTGCTAGA
AAG
+
F:FFFFFFFFFFFFFFFFFFFFFFFFFFFFFF:FFFFFFFFFFFFFFFFFFFFFFFFFFFFFFFFFFFF
FFFFFFFFFFFFFFFFFFFFFFFFFFFFFFFFFFFFFFFFFFFFFFFFFFFFFFFFFFFFFFFFFFFF
FFF
@A00721:190:HGJW7DSXY:4:2202:1479:29481_1:N:0:TCTGCAAG+GCTGCACT
ATACCACTGATCTTCCAGAGTTACGTAGTGGTCGTTCTTTTGGTGATTTTATCAAAGAGAAAGTTTCT
GCTAGTCGCGACGATAGTATTTATCGGAGTGATATTAACAAGTTAAACTTCGAACTGACCACTTACAA
GAG
+
FFFF:FFFFFFFFFFFFFFFFFFFFFFFFFFFFFFFFFFFFFFFFFFFFFFFFFFFFFFFFF:FFFFFF
FFFFFFFFFFFFFFFFFFFFFFFFFFFFFFFFFFFFFFFF:FFFFFFFFFFFFFFFFFFFFFFFFFFFF
FFF
```

```
@A00721:190:HGJW7DSXY:4:1530:30092:3724_2:N:0:TCTGCAAG+GCTGCACT
TCAGTTTTCCAAAAAGCTATTAATAGCAGTTGCGGGGCTATAATACCGCCTCCAGATTACTCTAAACT
GAATCCACTCTCTTGGGATATAAATGAGTACATCTATAATAGAGTTTT
+
FFFFFFFFFFFFFFFFFF:FFFFFFFFFFFFFFFFFFFFFFFFFFFFFFFFFFFFFFFFFFFFFFFFF
FFFFFFFFFFFFFFFFFFFFFF:FFFFFFFFFF:FF:FFFFFFFFFFF
@A00721:190:HGJW7DSXY:4:2367:3622:35352_1:N:0:TCTGCAAG+GCTGCACT
GGATTCTCTAAAGAACAGCAACTTTCAACATGAATTAGTTATTGAAAGTCTTGACCCTCACGTCTCAC
TTTATCACCTTGGAGATGGTAAAGTCATTAACAAAGGTAATGTTAAGGCTGATGACTTAAATTTCGCA
TTC
+
FF,FF,F:FFF:,FFFFF,:,FFFF,FFFFFFF,FFF,FFF:FFFFFFF:FFFFF,FFFFF,FFFFF:
:FF:F:FFFF:FF,F,F:,FFFF,F::FFF,FF,::F,:::,FFFF:,:F,:FFF,FFF::FFF:FFF
FFF
@A00721:190:HGJW7DSXY:4:1522:17689:4633_2:N:0:TCTGCAAG+GCTGCACT
TTTGAAAATTACGGAACCTTTCCTCGTTCTTCATGAAGATCTTCGAGTGTTTTATAGTGAGTTATTCT
TGCGAAGGTTCAAGTCGATTGATTTTGTTGACTACGATCTCTCTTGTA
+
FFFFFFFFFFFFFFFFFFFFFFFFFFF:FFFFFFFFFFFFFFFFFFFFFFFFFFFFFFFFFFFFFFFF
FFFFFFFFFFFFFF:FF:,F:FFFFFFFF:FF:::FFFFFFF:F:F:F
@A00721:190:HGJW7DSXY:4:2346:12888:33364_1:N:0:TCTGCAAG+GCTGCACT
AGACCACTGAACTTATAGCGACTTTTGTCGAATTTTTCGCAAAAGGTCTTGAAGTACGTATTGTCACT
GCTAATCGTAATTCGCGAGAAGAGATAGTGACTAAAGTTTTGGCACTTCTAGTTCATCGCGGTATTTG
TGA
+
FFFFFFFFFFFFFFFFFFFFFFFFFFFFFFFFFFFFFFFFFFFFFFF:FFFFFFFFFFFFFFFFFFFF
FFFFFFFFFFFFFFFFFFFFFFFFFFFFFFFFFFFFFFFFFFFFFFFFFFFFFFFFFFFFFFFFFFFF
FFF
@A00721:190:HGJW7DSXY:4:2172:32344:15201_2:N:0:TCTGCAAG+GCTGCACT
CGTGAAAGTTATGATGAGTGATCGTATTAGAACAGCTGATTCTTATTGCATGAACGTCGCTGAGGTCA
AACCTGTGGACGTTCTTTTCATCGACGAATTATTCATGATTCACGCTG
+
FFFFFFFFFFFFFFFFFFFFFFFFFFFFFFFFFFFFFFFFFFFFFFFFFFFFFFFFFFFFFFFF:FFF
FFFFFFFFFFFFFFFFFFFFFFFFFFFFFFFFFFFFFFFFFF,FFFFF
@A00721:190:HGJW7DSXY:4:1363:6234:25128_2:N:0:TCTGCAAG+GCTGCACT
CATCTACTTAAGGCGGCTTATACCATAGGTTACGGTGATTCCAATCAAATCGGATTCATCAATCGTAT
TGATCTCCAAGTCGCAAACTACTTCAACATCAACGACATCATTCTTGA
+
FFFFFFFFFFFFFFFFFFFFFFFFFFFFF:FFFFFFFFFFFFFFFFFFFFF:FFFFFFFFFFFFFFFF
F,FFFFF:FF,FFFFFFFFF:,FFFFFFFFFFFFFFFFFFFFFFFFFF
@A00721:190:HGJW7DSXY:4:2115:13874:1063_2:N:0:TCTGCAAG+GCTGCACT
TTATCGAAGTCTCAGTTATCGCTGCCCGGCCGACGTTTGTCGCGAACTCTCAGAGGTTTACGGCAGAA
AAATTTCTCCTGCTTGTAATTCGAGAGAGGACACTATGGAAATTAGAC
+
FFFFFFFFFFFFFFFFFFFFFFFFFFFFFFFFFFFFFFFFFFFFFFFFFFFFFFFFFFFFFFFFFFFF
FFFFFFFFFFFFF:FFFFFFFFFFFFFFFFFFFFFFFFFFFFF:FFFF
@A00721:190:HGJW7DSXY:4:1515:2175:15374_2:N:0:TCTGCAAG+GCTGCACT
TATTACTTTCACTCAAGGTGAAAAGTTTGAAATTAACAAAGTCCTTGGTAAGAGTAAGGTTAAATGTG
AAGATGCTCAGACTATTCATGAAGTTCAAGGTAATACTTACCAGCACG
+
FFFFFFFFFFFFFFFFFFFFFFFFFFFFFFFFFFFFFFFFFFFFFF,FFFFFFFFFFFFFFFFFFFFF
FFFFFFFFFFFFFFFFFFFFFFFFFFFFFFFFFFFFFFFFFFFFFFFF
@A00721:190:HGJW7DSXY:4:2138:29107:1673_2:N:0:TCTGCAAG+GCTGCACT
TTTTGCATCACAAACACATATTACCGTTGCTCTATCACGACACGTTGAATCTTTACGATACTTTGTTA
```

TCAATGTAAAGTTAAACGATGCTACTTGTGAGAGGATCAATCACAGTA
+
FFFFFFFFFFFFF,FFFFFFFFFFFFFFFFFFFFFFFFFFFFFFFFFF:FFFFFFFFFFFF,FFF:FF
FFFFFFF,FFFFFFFFFFFFFFFFFFFFFFFFFFFFFFFFFFFFFFFF
@A00721:190:HGJW7DSXY:4:2650:18096:4648_2:N:0:TCTGCAAG+GCTGCACT
TTAGCACGGGAGGTTGCAGATTCAACCCCTTATATCGAGGCTATCGAGGACGATGAGTATCTTGAAGT
TGTTGACGACAAGCCTCAAGAGGGTAGAGGACCACCTGGATCTTCTAC
+
FFFFFFFFFFFFFFFFFFFFFFFFFFFF:FFFFFFFFFFFF:FFFFFFFFFFFFFFFFFFFF:FFF
FFFFFFFF:FFFFFFFFFFFFFFF:FFFFFFFFFFFFFFFFFFFFFFFF
@A00721:190:HGJW7DSXY:4:1525:2365:1767_1:N:0:TCTGCAAG+GCTGCACT
AACCACCTTAACGTCACTCGGTAATCTTTCAGCCGAATTAAGTACTAATGAGTTCAAGTCGGTTGTCG
ATCGAATTAAGATCAAGGCTAATACCTTTATAGGTAAGAAGCCCCTTGAGCCGCGTAGTCAGCGCGTT
TAG
+
F,:FFFFFF:,FFFFF:FFF:FF:F,F,F,FF,:FF:FFF::FF,FFF::,FFF:,:FFFFFFFFF:,
FFFFFFF:FFFFFF:FFFFFFFF,,FFFF:F:FFFFFF,,F,:FFFFF,FFFF,FFFF::FFFFFFFF
F,F
@A00721:190:HGJW7DSXY:4:1603:2510:28541_2:N:0:TCTGCAAG+GCTGCACT
CCGATAGGGTACCGAGTCTCCAAGAGAATATTTATTCTTATGAGGCTCGAAACTTCAACGTTTTCACA
ACGGAAAGACATATCGCACCCGAAATTTTTGGGCAGAATGCTCTTAAC
+
FFFFFFFFFFFFFFFFFFFFFFFFFFFFFFFFFFFFFFFFFF:F:FFFFFFFFFFFFFFF:FFFFFF
FFFFFFFFFFF:FFF:FFF:FFFFFF:FFFFFFFFFFFFFFFFFFFF:
@A00721:190:HGJW7DSXY:4:2623:3197:29982_2:N:0:TCTGCAAG+GCTGCACT
GATCAGTCTAAGTTGGATAAAGTTATAGGAGACTCAGTTAACCTGAGAGCTCCTCAATTGATAGATTG
GGTCAACAATAGGGACGCTAGTATACTTCGTAAAGTCGACAGCGAAAT
+
FFFFFFFFFFFFFFFFFFFFFFFFFFFFFFFFFFFFFFFFFFFFFFFFF:FFFFFFFFFFFFFFFFFF
FFFFFFFFFFFF:FFFFFFFFFF:FFFFFFFFFFFFFFFFFFFFF:FF
@A00721:190:HGJW7DSXY:4:2304:30246:12540_2:N:0:TCTGCAAG+GCTGCACT
AATTGGACCGACTATATGACCTATTTTAAGCTCATGGTGAAAAGGGAAGCTAAGGTTAAACTTTATGC
TTCAGCCTTTTCGAAGCATAACCCCGCACAGAATATCGTTTATCACGC
+
FFFFF:FF::FFF::FFFFFFFFFFFFFFFF:,FF::FFFFFFF,:F:,FFFFF,:,FFF:F,,FFF:
FFFFF,FFFFFFFFFFFFFFFFF,FF,F,F,F:FFF:FF:FFF,,:,F
@A00721:190:HGJW7DSXY:4:2271:9055:33959_2:N:0:TCTGCAAG+GCTGCACT
CCTATCTTTAAGGTTCTTGTGAGGAGAGTCAAACAGTGTCTTTTACCCAACATTTTTATGTATACAGA
GATGACCAGTGACGATTTCTCCCGGAAGCTCTTCTCTCTCTTAGGAGG
+
FF:FFFFFFFFFFFFFFFFFFFFFFFFFFFFFFFFFFFFFFFFFFFFFFFFFFFFFFFFFFFFFFFFF
FFFFFFFFFFFFFFFFF:FF:FFFFFFFFFFFFFFF:FFFFFFFFFFF
@A00721:190:HGJW7DSXY:4:2675:26494:20760_1:N:0:TCTGCAAG+GCTGCACT
TAAAGCCTATGAGATAAAGATGTATTCTTATTTAGGCTTCGATGCTGAATTTCTTGAACTTTGGAGTG
CCAGTGAATATTTTTGTCACGCCAGGAGTATCCATAACGATTTGTCATTTACCACCTTTGCTCAACGA
CGA
+
FFFF:FFFFF:FFFFFFF:FFFFFFFFFFFFFFFF:FFFFFFFF:FFFFFFFFFFFFFFFFFFFFFF,
FFFFFFFFFF:FFFFFFFFFFFFFF:FFFFFFFFFFFFFFFFFFFFFFFFFFFFFFFFFFFF:FFFFFF
FFF
@A00721:190:HGJW7DSXY:4:2347:15944:19272_1:N:0:TCTGCAAG+GCTGCACT
GTTCAGACGTGTGCTATTCCGATCTATCACTTTGATGATTTTATCACTTTATTATGACCTTGGAAAGT
TTTCGGCTGTGGTTGTCGCTGGGGACGATTCCATTCTGTTTTCAAAGACTCCAATAACTGATCATTCT
GAT

+
:FF,,FFF:F,FFFF,:FFF,FF,FFF,:F::FFFFFFFFFFFF::FFFFFF:FFFF:F,FF:FFFF
FFFFFFFFFFF:FFFFF::FFFFFFFF,,F,FF:FFFFFF,FFFF:F,:FFFFFF,FFFFFFFF:FF:
,FF
@A00721:190:HGJW7DSXY:4:1509:27661:4429_2:N:0:TCTGCAAG+GCTGCACT
TGCTCGCGATTTATTCTTTTTGTCAATGATTACATCTACTTCGTTCCTGATCCTTACAAGATCATCGT
TAAGTTAGGTAAACCCCTAACCGATGTTACTCTTGTAGGACTTTGGGA
+
FFFFFFFFFFFFFF:FFFFFFFFFFFFFFFFFFFFFFFFFFFFFFFFFFFFF:FF:FF:FFFF::FFFF
FFFFFF:FFF:FFF:F:FFFFFFFFFFFFFFFFFFFFFFFFFFFFFFF:
@A00721:190:HGJW7DSXY:4:2304:4969:4398_2:N:0:TCTGCAAG+GCTGCACT
TTAAAGACTATACTAAATATCTCCACTACGAGGTTGTTGTTTGGCGATTAGCTCTTGCGACTAATATT
CGATATTCGATTGAGGGTCGTTATACCTATTCGGCTATCTGTGCTTTG
+
FFFFFFFFFFFFFFFFFFFFFFFFFFFFFFFFFFFFFFFFFFFFFFFFFFFFFFFFFFFFFFFFFFFF
FFFFFFFFFFFFFFFFFFFFFFFFFFFFFFFFFFFFFFFFFFFFFFFF
@A00721:190:HGJW7DSXY:4:1521:8115:11350_2:N:0:TCTGCAAG+GCTGCACT
TTTTAAACGATTCCGAGAGGTTTATCCTGATGTGGAGGGAGTTCTTGAAGCATCCCTACCTTTCCTTG
TGAAAATTTTGATGCGGTTTCATAAGATTATCAGTAATCCTGATTCTA
+
FFFFFFFFFFFFFFFFFFFFFFFFFFFFFFFFFFFFFFFFFFFFFFFFFFFFFFFFFFFFFFFFFFFF
FFFFFFFFFFFFFFFFFFFFFFFFFFFFFFFFFFFFFFFFFFFFFFFF
@A00721:190:HGJW7DSXY:4:2617:31006:25911_1:N:0:TCTGCAAG+GCTGCACT
GACTGGAGTTCAGACGTGTGCTCTTCCGATCTCAAAGATCGACTTCGAGACAGGTGATATTGTTGAGC
GAGAGCGACCAAGATCTAAATTCGGTAACGGTGCGTGGCGCTTTATTTTAAAAAGCTCCCTCCTCTCG
CAA
+
FFFFFFFFFFFFFFFFFFF:FFFFFFFFFFFFF:,F:FF:FFFFFFFFFFFFF:FFFFFF:FFFFFF
FFFFFFFFFFFFFFFFFFFFFFFFFFFFFFFFFFFFFFFFFFFFFFFFFFFFFFFFFFFFFFFFFFFF
FFF
@A00721:190:HGJW7DSXY:4:2405:4444:22310_2:N:0:TCTGCAAG+GCTGCACT
ACTTGAGTCTTTTGATCAGTCTCTGACTTGACCATCTTTCATTTTAAATGACGATAATTAAGACTCTC
TTTCTTCTTTGTTTCCTTCTTTGCACTGATTTTTCCTCATCAGCTTGT
+
FFFFFFFFF,,:F,F:,FFFF:,:FFFF:FFFFF,FFFFFFF:FFFF:FF:FFFF,FFFFF:,:FF,,
F,,F:F,F,F:F:FFF::FF,FFFFFF,F:FF:FF,F:FFFFFFFFF:
@A00721:190:HGJW7DSXY:4:1531:1244:22842_2:N:0:TCTGCAAG+GCTGCACT
TTTACGATAACAACTATCAGTTGATTAAGACTCTTCCGAAAGGCGAACTTTGGGAATTTGAAGCTCAG
GGTGAAGCACAACGTTTGACCGTGACGAACTGTGGTGTTCTTGATCTA
+
FFFFFFFFFFFFFFFFFFFFF:FFFFFFFFFFFFFFFFFFFFFFFFFFFFFFFFFFFFFFFFFFFFFF
FFFFFFFFFFFFFFFFFFFFFFFFFFFFFFF:FFFFFFFFFFFFFFFF
@A00721:190:HGJW7DSXY:4:2331:7102:15796_1:N:0:TCTGCAAG+GCTGCACT
CACTACCACTTATTACGATATTTCTTTAGTCGATGGGTTTTCGACGCCTTTAAGCGTCTTCTGTTCAT
CGCAACCAGATGATATCTTCTATTCGAACTTATGTTTCGATCAGTGTTTCTTCTATCAGGAGGAAAAC
ACT
+
FFFFFFFFFFFFF:FFFFFFFFFFFFFFFFFFFFFFFFFFFFFFFFFFFFFF:FFFFFFFFFFFFFFFF
FFFFFFFFFFFFFFFFFFFFFFFFFFFFFFFFFFFFFFFFFFFFFFFFFFFFFFFFFFFFFFFFFFFF
FFF
@A00721:190:HGJW7DSXY:4:2624:19750:32409_2:N:0:TCTGCAAG+GCTGCACT
TCTTCTAATGTGGCCTGAACTACGTTTCACTTTGTGTGTACTTGTCTCTATTTTAAGTTTTGTCTTCC
TTGTTTGTCTTATTCTATTAATCACTCATTTTAGTGAGATTATTAAAA
+

:FFFFFFFFFFFFFFFFFFFFFFFFF:FFFFFFFFFFFFFFFF::FFFFF:F:FFFFFFFFFF:FFFFF
FF:FFFFFFFFFFFFFFFFFFFFFFFFF:FFFF:FFFFFFFFFFFFFFFF
@A00721:190:HGJW7DSXY:4:1607:5511:26287_2:N:0:TCTGCAAG+GCTGCACT
GACAACAGCAGGGGTTTTCGAATAGTGATAGCCACCCGCCTTACAGAGGGGCAGCAGCATGACCATAA
TTGGTATTGACTTTGGTACCACCTTTTCTACTGTCTGTCTCGCCTCTT
+
FFFFFFFFFFFFFFFFFFFFFFFFFFFFFFFFFFFFFFFFFFFFFFFFFFFFFFFFFFFFFFFFFFFF
FFFFFFFFFFFFFFFFFFFFFFFFFFFFFFFFFFFFFFFFFFFFFFFF
@A00721:190:HGJW7DSXY:4:2540:28411:34350_1:N:0:TCTGCAAG+GCTGCACT
CTTAACAACAATGATAGTGAATTTATACCTACAATCATTGGTTTTTCTAATAATGATGACACTATTGT
TTATGGATATGATGCTATTACTCATGATGGTTACGGTCAGGGAGGATTCTCAGTTTACAAGGACCTTA
AGC
+
FFFFFFFF:FFF:FFF:FFFFFFFF,FF:FFFFFF,FFFF:FFFFFFFFFFFFFFFFFFFFFFFFFFF
FFFFFFFFFFFFFFFFFFFFFFFFFFFFFFFFFFFFFFFFFFFFFFFFFFFFFFFFFFFFFFFFFFFF
FFF
@A00721:190:HGJW7DSXY:4:1176:17137:6057_1:N:0:TCTGCAAG+GCTGCACT
AGACGTGCTAAATTAAAACCATTGTACGATGTCGTCTGTCTCGGCGATCACTTTTCACTAAGAATTGG
TCCTACTTTTGGACAAGGCCAACTTCGTAATCTTCATGAGATCGTTTCTTTCTTTATCTCTTTGGTTG
TTA
+
F:FF:FF,FFFFFF,FFFF,:FFFF,::FFFF::FFFFF,F,F:FFFFFFF,FFF,FF,FFFFFFFF:
F:FF:FF,FFFFF,:F:F:FFF,FF,,FFFFFFFF::FFFFF,FFFF,FFFFFFFFFFF:FFFFFFFF
FFF
@A00721:190:HGJW7DSXY:4:1110:17282:8563_2:N:0:TCTGCAAG+GCTGCACT
GAGTGAAAGGCTTCAATTGTAGTGGTTTGGTCGTTTCAGTACCCAGTCAGTATACCTCTACGCAAAGA
TATTTCATGAAAACTACAGAGGAACGTACCGGATTACCTGTCTTTCAT
+
FFFFFFFFFFFFFFFFFFFFFFFFFFFFFFFFFFFFFFFFFFFFFFFFFFFFFFFFFFFFFFFFFFFF
FFFFFFFFFFFFFFFFFFFFFFFFFFFFFFFFFFFFFFFFFFFFFFFF
@A00721:190:HGJW7DSXY:4:1663:29514:3161_1:N:0:TCTGCAAG+GCTGCACT
CTTCGGCCGCTCTCTTTGCTTCGATGTTGGATGTCAAGAAGGGTTCAGAATGGGATTCCTACGTAGTT
TATGACTTTGGTGGTGGTACTTTCGATACTTCTATCGTCTTACGTTACGGTAATTACTATAGTGTTAT
TTA
+
FFFFFFFFFFFFFFFFFFFFFFFFFFFFFFFFFFFFFFFFFFFFFFFFFFFFFFFFFFFFFFFFFFFF
FFFFFFFFFFFFFFFFFFFFFFFFFFFFFFFFFFFFFFFFFFFFFFFFFFFFFFFFFFFF:F,FFFFF
FFF
@A00721:190:HGJW7DSXY:4:2165:5945:36871_1:N:0:TCTGCAAG+GCTGCACT
ATAATCTTGGAGGTCGTGACGTTGATGAAGCTATAAGAGCGTTCTTATTACGGCGCACTTCTTTACCC
GCCACCACACAACTTTCAGTTTCACAACTGAAGGAGGAGGTTTCTGTTACTGGAAAACCCTCGTCAAT
ACT
+
FFFFFFFFFFFF:FFFFFFFFFF:FFF:FFFFFF:F:FFFFFF:FFFFFFFFFFFFFFFF,FFFFF,F
FFFFFF:FFF:FFFFFF:FFFFFFFFFFFFFFFFFFFFFFFFFFFFFFFFFFFFFFFFFFFFFFF:FF
FFF
@A00721:190:HGJW7DSXY:4:2223:15962:9565_2:N:0:TCTGCAAG+GCTGCACT
GATCTAGTGAAATTAACGCGTCCTTTTCTGGAACGTAGTTTTACTATTATGAACAAAGTTATCAAAGA
TAGTGGCATTCAAGGCAAACTTACCCTGATTCCGGCCGGTGGTAGCTC
+
FFFFFFFFFFFFFFFFFFFFFFFFF:FFFFFFFFFF:FFFFFFFFFFFFFFFFFFFFFFFFFFFFFFFF
FFFFFFFFFFFFFFFFFFFFFFFFFFFFFFFFFFFFFFFFFFFFFFFF
@A00721:190:HGJW7DSXY:4:2112:21920:25801_2:N:0:TCTGCAAG+GCTGCACT
TGCTGAAACAGCTATTAAAGCTGTTGAGCGGGTAATTACACCTAAACGAGCTCGATCTGCCATCGCTG

AAGGATGTGCACTCTTTTCTGCTACTTTAACGGAGCGTAGTGTACTCT
+
FFFFFFFFFFFFFFFFFFFFFFFFFFFFFFFFFFFFFFFFFFFFFFFFFFFFFFFFFFFFFFFFFF:FFFF
FFFFFFFFFFFFFFFFFFFFFFFFFFFFFFFFFFFFFFFFFFFFFFFF
@A00721:190:HGJW7DSXY:4:1101:6587:4758_2:N:0:TCTGCAAG+GCTGCACT
AAAGGTCTCTATCAAGCTCGTATTATCGTCGCTGCTGGATCTGCGTTACCGTTGAAGGCTAAACTCCA
AGGTAATTATGAAGACTGTGATTTATCTTTCACTCTGGCCTTGTATGA
+
FFFFFFFFFFFFFF:FFFFF:FFFFFFFFFFFFFFFFFFFFFFFFFFFFFFFFFFFFFFF,FFFFFFFF
FFFFFFFFFFFFFFFF:FFFFFFFFFFF:FFFFFFFFFFFFFFFFFFF
@A00721:190:HGJW7DSXY:4:1476:5032:32565_2:N:0:TCTGCAAG+GCTGCACT
CGTAACATTAATAATTTCAATCTTCTTAAAAAGAAGATAGAGAGTCATACTTTGGGCTATCGTTCAGG
TGCATGGGGTTGGATAGCCAATATTGAAGTGGACACCTCTGGAGATCG
+
FFFFFFFFFFFFFFFFFFFFFFFFFFFFFFFFFFFFFFFFFFFFFFFFFFFFFFFFFFFFFFFFFFFF
FFFFFFFFFFFFFFFFFFFFFFFFFFFFFFFFFFFFFFFFFFFFFFFF
@A00721:190:HGJW7DSXY:4:1460:10022:34131_2:N:0:TCTGCAAG+GCTGCACT
AAGCATACTACTTTTGCTTTGATTACTCTACCGCCTTACAAGTCTAAGGTTAAAGAGTCGATTAAACA
AGTTGTACTTATCAATGATTTTATCGCTGGTTGTTATCTCGAAAAGTC
+
FFFFFFFFFFFFFFFFFFFFFFFFFFFFFFFFFFFFFFFFFFFFFFFFFFFFFFFFFFFFFFFFFF,F
F,FF:FF,,FF:,FFF,F:FFFFF,FF,:FFFFFFF::::FFFFF,F,
@A00721:190:HGJW7DSXY:4:2248:27154:3082_1:N:0:TCTGCAAG+GCTGCACT
CCCAAAGAGAATATCTAAAATATTTACAAAATCTTACCACTATCTACGAACCTAAAGTTCTGAAGACG
TTGCTTGTCGCTTCCTCCTTTAATGGCACTACCTCGGTACAATCTTACTTGGAACGAAGGCGACCTGT
TCC
+
FFFF,FFFFFFFFFFFFF:FFF:FFFFFF:F:FFFFFF:F,FFFFFFFFFFFFFFFFFFFFFFFFFFF
F:FFFFFFFFFFFFFFFFFFFFFFFFFFFFFFFFFFFF:FFF:FFFFFFFFFFFFFFFFFFFFFFFFFF
FFF
@A00721:190:HGJW7DSXY:4:2416:12509:36620_1:N:0:TCTGCAAG+GCTGCACT
GTTCTGTCTATATTAGGTGAAGCGAAATACGATTTCGAACGTCAACGTTCACCGAGTTTCGTTTCGTA
CCTTGATGCTGACTTTCGTCAATATGATTTGATCTGGTTGCTCAATTCTAAGCGTGTTCCGCTTAGTG
CGA
+
FFFFFFFFFFFFFFF:FF:FFF:FFFFFFFFFFFFFFFFF:FFF:FFF:F:F,,FFFFF:F,:FFFF
F,FFFFFFFF:F,FFFF:FF:FFFFFFFFFFF:FF:FFFF:FFF:,,FFFF::FFFFF:FF:FFFFF
FFF
@A00721:190:HGJW7DSXY:4:2409:9959:19680_1:N:0:TCTGCAAG+GCTGCACT
TACGAACTTGACTCTACTATACGTGACATACTAAATACTAGATATTTGGATGATCTTGATAAAGAACT
TCATGAATTTTCAGAGGATGAAAAATTTTCTTCCTTGGATTTTAATATAAAGAATCTTTACCAGGACT
CCT
+
FFFFFFFFFFFF:FFFFFFFFFFFFFFFFFFFFFFFFFFFFFFFFFFF:FFFFFFFFFFFFFFFFFFF
FFFFFFFFFFFFFFFFFFFFFFFFFFFFFFFFFFFFFF:FFFF:FFFFFFFFFFFFFFFFFFFFFFFF
FFF
@A00721:190:HGJW7DSXY:4:1338:18367:6214_1:N:0:TCTGCAAG+GCTGCACT
GATCGTAAGAAGTTTATAAGGAACATATCCATTTGTTGTAATAGGGTCATCAACTTAGAACTCGAAAC
TCTAAGTGGTAATACTGTCTTTCCAAGCTTACCTGTATCTTCAATGAAATTGGACTCTTCGATAGGGG
ACA
+
FFFFFFFFFFFFFFF:FFFFFFFFFFFFFFFFFFFFFFFFFFFFFFFF:FFFFFFFFFFFFFFFFFFFF
FFFFFFFFFFFFFFFFFFFFFFFFFFFFFFFFFFFFFFFFFFFFFFFFFFFFFFFFFFFFFFFFFFFF
FFF

@A00721:190:HGJW7DSXY:4:2263:1597:24643_1:N:0:TCTGCAAG+GCTGCACT
ACCGGTGAATGGTTTAAGAATACTATAAATAGAATAGCTATTACTACTAATGGCGTTGGCCTAGTGGA
TGTTATTAGTGACATCTGGTCAATTTCGTTTTTAGGTGGTCTTTATTCTATTATTGATTTTTATGAGA
TTG
+
FFFFFFFFFFFFFFFFFFFFFFFFFFFFFFFFFF,FFFFFFFFFFFFFFFFFFFFFFFFFFFFFFFFF
FFFFFFFFFFFFFFFFFFFFFFFFFFFFFFFFFFFFFFFFFFFFFFFFFFFFFFFFFFFFFFFFFFFF
:FF
@A00721:190:HGJW7DSXY:4:1542:19614:24940_2:N:0:TCTGCAAG+GCTGCACT
TGTTAGAGTAGAATTCCTGAAGAGTCCTTCCGTAACCAACTTCTTGACAATTTTCTTCAATCCTTCGA
TAATAGTTAAATCTAGGATTATGTCTGTGTTGAAAGACTTTCTACCGA
+
FFFFFFFFFFFFFFFFFFFFFFFFFFFFFFFFFFFFFFFFFFFFFFFFFFFFFFFFFFFFFFFFFFFF
FFFFFFFFFFFFFFFFFFFFFFFFFFFFFFFFFFFFFFFFFFFFFFFF
@A00721:190:HGJW7DSXY:4:1458:16957:8500_2:N:0:TCTGCAAG+GCTGCACT
TACACAATTAAGAAATAATCCTACATTGACTGATTATCTAAAGATTGAAGGAAAATTTTATCCTCGTT
TAACTTACTTAGACTTTCCTACGATACAAATGATAACTTCTAATTTAC
+
FFFFFFFFFFF:FFFFFFFFFFFFFFFFFFFFFFFFFFFFFFFFFFFFF:FFFF,FFFFFFFFFFFFFF
FFFFFFFFFFFFFFFFFFFFFFFFFFFFFFFFFFFFFFFFFFFFFFFF
@A00721:190:HGJW7DSXY:4:2305:15438:24439_1:N:0:TCTGCAAG+GCTGCACT
GGGATGCATGGTGTTATCTCCTCAGGAGTCCCGGCATTTGATAATAATGACGAGACTTTCGTCATAAT
CGCTGCATATTACGGATTACACGGTACCAGCGCATCTCGTGTCGAGCCTCGACCCGATTATATGGTAT
TAA
+
F:,FF,FFF:FF:FFFFFF:FFF::FFFFF:F:F,:FFF:FFFFFFFFFFFFFFF:FFFFFFFFFF:F
:FFFFFFFFFFFFFFFFFFFF:FFFFFFFFFFFFFFFFFFFFFFFF,FFFFFFFFFFFFFFFFFFFFFF
FFF
@A00721:190:HGJW7DSXY:4:1620:3396:20932_2:N:0:TCTGCAAG+GCTGCACT
GATTCTCGATTCGTAGAGCTTATCTAGGTACTCTCATTTACTATGTTGATTTAGTCTACATTTATGCT
GATCTAAAATTTCGATCTAGATGGATCCACTCCTCGAGTTATCATGAT
+
FFFFFFFFFFFFFFFFFFFFFFFFFFFFFFFFFFFFFFFFFFFFFFFFFFFFFFFFFFFFFFFFFFFF
FFFFFFFFFFFFFFFFFFFFFFFFFFFFFFFFFFFFFF:FFFFFFFFF
@A00721:190:HGJW7DSXY:4:2532:2456:7623_1:N:0:TCTGCAAG+GCTGCACT
TTCTAGAATTCTTAATTATAATATTCAACCTCAAAAGTTACGTGTTTACAGTAACGAGATCACACATT
TTGAGAACTAACTATGTTATGTTCTCTGATCATTTTGACAATCTCTTCTTATAATAAATAAATAAATA
ATA
+
FFFFFFFF,FFFFFFFFFFFFFFFFFFFFFFFFFFFFFFFFFFFFFFFFFFFFFFFFFFFFFFFFFFF
FFFFFFFFFFFFFFFF:FFFF:FFFFFFFFFFFFFFFFFFFFFFFFFFFFFFFFFFFFFFFFFFFFFF
FFF
@A00721:190:HGJW7DSXY:4:1443:27905:4257_2:N:0:TCTGCAAG+GCTGCACT
AGTGGAAAGGCTCCAGTCAACACTAGTTCGAGTGCTGCTTTGGATTACGATGACGTGAGAATAGGTGA
AGGGATAGTTTTTACCTCGACCAACATTAAGGCGGCGGCGACTAATCT
+
FFFFFFFFFFFFFFFFFFFFFFFFFFFFFFFFFFFFFFFFFFFFFFFFFFFFFFFFFFFF:FFFFFFF
:FFFFFFFFFFFFFFFFFFFFFFFFFFFFFFFFFFFFFFFFFFFFFFF
@A00721:190:HGJW7DSXY:4:1630:29279:24111_1:N:0:TCTGCAAG+GCTGCACT
AGGACACTTCGAAACTGAATAATGATGCACTACAAACAAGTATCGCTAATCATCTGAAAAAGGCTCTA
AAGGACACTATACAATATCCAGACGACGACGAAGCTTATACTACTTTAATTTTGGGTATTATGTGGAG
ATA
+
FFFFFFFFFFFFFFFFFFFFFFFFFFFFFFFFFFFFFFFFF:FFFFFFFFFFFFFFFFFFFFFFFFFF

FFFFFFFFFFFFFFFFFFFFFFFFFFFFFFFFFFFFFFFFFFFFFFFFFFFFFFFFFFFFFFFFFFFFFF
FFF
@A00721:190:HGJW7DSXY:4:1320:25925:29857_2:N:0:TCTGCAAG+GCTGCACT
ACCACATTTTGGTCAAGTCAATCCGTTGAGAAAATTTCTTAGAGGATATTCGCCATTGTGGTACGAAT
TGGTCCAACATTTACCACCAAATTCTTTTGATACAACTCTAGCCTTCA
+
FFFFFFFFFFFFFFFFFFFFFFFFFFFFFFFFFFFFFFFFFFFFFFFFFFFFFFFFFFFFF::FFFFF
FFFFFFFFFF,FFFFFFFFFFFFFFFF:FFFFFFFFFFFFFFFFFFFF
@A00721:190:HGJW7DSXY:4:2445:32886:24095_2:N:0:TCTGCAAG+GCTGCACT
CTTGCTATTCACCAGATTGGTTCGAACCCGTTGATGCTATGTCAGAAGAAGCTATTCAAGCTTGTCGT
TTAAAGACTGCAGAGGCTGTCAAACAAGCCCAGACTCAAGGAGTCCAA
+
FFFFFFFFFFFFFFFFFFFFFFFFFFFFFFFFFFFFFFFFFFFFF:FFFFFFFFFFFFF:FFFFFFFF
FFFFFFFFF:FFFFFFFFFFFFF::FF:F:FFFFFF:FFFFFFFFFFF
@A00721:190:HGJW7DSXY:4:2651:27181:24173_2:N:0:TCTGCAAG+GCTGCACT
GGTGATAAAAATTATTTAGAGATCTTTTCTACTTACAAAGGACAATTCAGTTTCATTAATTCTACGGA
AAAGTCCGGAGTATATTGTATGTTAATAGATAGTGGGATAATAGTTCT
+
FFFFFFFFFFFFFFFFFFFFFFFFFFFFFFFFFFFFFFFFFFFFF:FFFFFFFFFFFFFFFFFFFFFF
FFFFFFFFFFFF:FFFFFFFFFFFFFFFFFFFFFFFFFFFFFFFFFFF
@A00721:190:HGJW7DSXY:4:2256:30861:31829_2:N:0:TCTGCAAG+GCTGCACT
TGCTATTGATTATGTAACCGATAGGAGTTGTATAGTTGAAGATGTGGCTTTAGACGATAATCCTTTAA
GGACTATGTTGACTGACGAAGATTTTCCCACAGATTTGTCTACGTTGC
+
FFFFFFFFFFFFFFFFFFFFFFFFFFFFFFFFFFFFFFFFFFFFFFFFFFFF:FFFFFFFFFFFFFFF
FFFFFFFFFFFFFFFF:FFF,FFFFFF,FFFFFFFFFFFFFFF:FFFF
@A00721:190:HGJW7DSXY:4:1251:25889:34867_1:N:0:TCTGCAAG+GCTGCACT
CTATATACATTATAAAATATAAAAATAAGGGAGTCATAACTTTTGTTACCAAATTCTTGGCTTTCAAC
GAAGTCTCTGATTTTATTAATCATTACTTTAACGATCTCTCGGTCATTTACTCTGTACTTGATCTACG
TCG
+
:FFFFFFFFFFFFFFFFFFFFFF:FFFFFFFFFFFFFFFFFFFFFFFFFFFF,FFFFFFFFFFFFFFF
FFFFFFFFFFFFFFFFFFFFFFFFFFFFFFFF::FFFFFFFFFFFFFFFFFFF:FFFFFFFFFFFFFFFF
FFF
@A00721:190:HGJW7DSXY:4:1428:8124:16438_2:N:0:TCTGCAAG+GCTGCACT
GAATGACTCTATCTTTTCAGACACTTCTTATATTATTTACAATAGTTAATATCATATTAGTAATACTC
ACTATCGTTTTCTGCTTTTTCTTAGCTAATAGATTAATAAAGGATGAG
+
FFFFFFFFFFFFFFFFFFFFFFFFFF:FFFFFFFFFFFFFFFFFF,FFFFFFFFFFFFFFFFFFFFFF
FFFFFFFF,FFFFF:F:FFFFFFFFFFFFFFFFFFFFFFFFF,FFF:F:
@A00721:190:HGJW7DSXY:4:1623:22498:19038_1:N:0:TCTGCAAG+GCTGCACT
AACTCTTGGTTACGAGACCTTTTGGTATAATTTATTTAGTCTCTACACCCATCACACCGTGCATAACG
CGTTTTTTCCGTCCTTAACTTAATACACTAATATATCATCCGCAAACGACTCGAAACCGAGACGCCGT
A
+
F:::,F:,,FF,,,,,,:,F,,,F,F,:F:F,,:FFF,,,F:,,F:,FF,:,,,:F,::,,,F,F:,,
,,:,FFF,F,F,F,,,FF,:,F:,F:::,F,,,,F,,,,FF,,:,FFFFF:FFFFFFFFFFFFFFFFF
F
@A00721:190:HGJW7DSXY:4:2461:3812:8938_2:N:0:TCTGCAAG+GCTGCACT
TTTAGATTATGAATCCTGCCTTTACTCATTTTATAGCAGTTATTTATGTTGTACTTTTTATAATTTAT
AACCTTCTTATACTTTTGGGTATGTTTTGTGAAAAACATGAGATACCC
+
FFFFFFFFFFFFFFFFFFFFFFFFFFFFFFFFFFFFFFFFFFFFFFFFFFFFFFFFFFFFFFFFFFFF
FFFFFFFFFFFFFFFFFFFFFFFFFFFFFFFFFFFFFFFFFFFFFFFF

```
@A00721:190:HGJW7DSXY:4:2135:25563:17644_2:N:0:TCTGCAAG+GCTGCACT
TTGTAACGATCATTCTTTCGAGTTTTGTCATTCTTATTCTTTTATTCCTTTAACTGTGTATGAGGAGG
ATTTATAATGCTTTTCGATAGTGATTGTTATCAATTTAAATACATTAC
+
FFFFFFFFFFFFFFFFFFFFFFFF:FFFFF:F:FFFFF:FFFFFFFFFFFFF,FFFFF:FFFFFFF:
FFFFFFFFFFFFFFFFFFFFFF:FFF:FFFFFFFFFFFFFFF,FFFFF
@A00721:190:HGJW7DSXY:4:2630:20546:1125_1:N:0:TCTGCAAG+GCTGCACT
TCTTCCGATCTCCGAAAATGGTTTCAAATGAGGTCAAAGCTCTATTTATTAAACGAACTATTATCCGA
TTCTTCGAGGGTATCACTACCACTGATCTGCCGACTTTAGTCAAGGAACTTCAATTCCTTCGATGTTT
ATA
+
FFFFFFFFFFFFFFFFFFFFFFFFFFFFFFFFFFFFFFFFFFFFFFFFFFFFFFFFFFFFFFFFFFFF
FFFFFFFFFFFFFFFFFFFFFFFFFFFFFFFFFFFFFFFFFFFFFFFFFFFFFFFFFFFFFFFFFFFF
FFF
@A00721:190:HGJW7DSXY:4:1412:18050:30405_2:N:0:TCTGCAAG+GCTGCACT
GGAAAATAGCAATAATCAAATTTCGACAAATTTTAGAACTTTATTTCAGAACTCTCTCCGATCACCTT
GGTGTGATCTCCCGGAGGATCTTTTCAATAAGATGTATGATGATAATC
+
FFFFFFFFFFFFFFFFFFFFFFFFFFFFFFFFFFFFFFFFFFFFFFFFFFFFFFFFFFFFFFFFFFFF
FFFFFFFFFFFFFFFFFFFFFFFFFFFFFFFFFFFFFFFFFFFFFFFF
@A00721:190:HGJW7DSXY:4:1227:24921:26303_2:N:0:TCTGCAAG+GCTGCACT
AGTGATATTCAGCGTATACTTGAATACTTTATCAAAGATTTGGCTTGGTCTATTTACCGCAAAAAGTT
GGAAGAGGTCTATGATATTTATTTTACTTATACTTTAAATAGCACCGA
+
FFFFFFFFFFFFFFFFFFFFFFFFFFFFFFFFFFFFFFFFFFFFF:FFFFFFFFFFFFFFFFFFFFF:F
:FFFFFFFFFFFFFFFFFFFFFFFFFFFFFFFFFFFFFFFFFFFF:FF
@A00721:190:HGJW7DSXY:4:2415:23285:14732_2:N:0:TCTGCAAG+GCTGCACT
TGTTGATAATATTACAATAGCTCTTTCTCAGAAGATTAGAGAAGTAACCGGTGAACACGTCGGTAGCA
TTGATTTACTTTTACCGTTAGAACTGAAAAAGATTCTTTTCGAAAAGA
+
FFFFF:FFFFF:F:FFF,FFFFFFFFFFFFF:FF:FF,FFFFFFFFFFFFFFFFFFFFFFFFFF:FFF
F:FFF:FFFFF,FFFFFFFF:FFFFFFFFFFFFFFFFFFFFFFFFFFF,
@A00721:190:HGJW7DSXY:4:1131:17879:19116_1:N:0:TCTGCAAG+GCTGCACT
AAACGATTCTTCGACTATATTAAAGAAATAAAAATACTTCAAGTACCTGAACTTTACATTTTTAATGG
TATGGTATAATGTGTCTTACAAATAGAACCTTCACTGTCGGTAATGAGAGTGTATTCCTTAATATTCT
AGT
+
FFF:F:FFFFFF:FF:FFFFFFFFFFFFFFFFFFFFFFFFFF:FFFFFFFFFFFFFF:FFFFFF:FFFF
FFF:FFFF:FFFFFFFFFFFFFFFFFF,FFFF:FF,,FFFFFFFFFFFFF:FF:FFFFFF:F,FFFF
FFF
@A00721:190:HGJW7DSXY:4:2216:14968:29951_1:N:0:TCTGCAAG+GCTGCACT
GGCATTACTAGGATTTATCACACTCCTAAATTTGATAGCAATTTATGTTTTAAATGCTAGGAAAATTT
TAGAAAAACTCCATTGTATAGTTCAAGGAGTACTTAATTCAATAGCTTTAGTATTATTTACTATCGCC
TTT
+
FFFFFFFFFF:FFFFFFFFFFFFFFFFFFFFFFFFFFFFFFFFFFFFFFFFFFFFFFFFFFFFFFFFF
FFFFFFFFFFFF:FFFFFFFFFFFFFFFFFFFFFFFFF:FFF:FFFFFF,FFFFFFFFFFFFFFFFFF
FFF
@A00721:190:HGJW7DSXY:4:2175:14009:7592_2:N:0:TCTGCAAG+GCTGCACT
AGTTAATTGCTTACACAAAAGTGACGCGCGAAGGAATCTTAATTGATTGAAGCTAGGCGTTGGGGAGA
ATGAACTGCCGCTTCCCGGAAGAAACGATCTATTTACAGAGTATTTAG
+
FFFFFFFFFFFFFFFFFFFFFFFFFFFFFFFFFFFFFFFFFFFFFFFFFFFFFFFFFFFFFFFFFFFF
FFFFFFFFFFFFFFFFFFFFFFFFFFFFFFFFFFFFFFFFFF,FFFFF
```

```
@A00721:190:HGJW7DSXY:4:2371:5113:26005_1:N:0:TCTGCAAG+GCTGCACT
CTCCCCAAACATAACATACCATAACCTACCATGACCAATCATCGCTCCAACTGGCACGCCAGACGTAG
GGTCAAACCTACTACCGCCTGTCGGCAGATCGGCACCACTGGTCTCTACGTTCAGGGACCCGCTGTGC
AGA
+
FFFFFFFF,FFF,FFFFFFFFFFFFFF,FFFFFFFFFFFFFFFFFFFFFFFFFFFFFFFFFFFFFFFF
FFFFFFFFFFFFFFFFFFFFFFFFFFFFFFFFFFFFFFFFFFFFFFFFFFFFFFFFFFFFFFFFFFFF
FFF
@A00721:190:HGJW7DSXY:4:1561:11822:29293_2:N:0:TCTGCAAG+GCTGCACT
TCTGCACAGAAGAAGGCTGAAGGTCGTTACCGGACGACCACTCAAAGGAAACGGGCTGTTGCCCAGAG
GAAAGCAGCGACCACCGAGGTCGCTCGTAACATTGACGGCAGTCCCGT
+
FFFFFFFFFFFFFFFFFFFFFFFFFFFFFFFFFFFFFFFFFFFFFFFFFFFFFFFFFFFFFFFFFFFF
FFFFFFFFFFFFFFFFFFFFFFFFFFFFFFFFFFFFFFFFFFFFFFFF
@A00721:190:HGJW7DSXY:4:1653:1163:33473_1:N:0:TCTGCAAG+GCTGCACT
AACTGAGGCAGTACCTCTGTCAAAAGTCACATTCACCACTCCCATACCAATCAATGATGTGAATATAC
AGTTCGGCCATTTCAAGAGTGGTCAATACGTCGTGGGAGGTTCTGAAGCCTTCCCCTCCGCTGGCACG
AAG
+
:FFFFFF::FFF,FFFF,,FFF,,F,F,::F,:FF,FFF,:FFFFFF:F,FFF:FFF:FFF:,F,F:,
,FFFF,F:F::FFFFFF:FFFFF:F,FFF:F,FF,FFFFFF,:FFFFF,FF::,F:F,FFFFFF,F::
FFF
@A00721:190:HGJW7DSXY:4:1318:22851:36683_2:N:0:TCTGCAAG+GCTGCACT
GCCAATCAATAGTTCTTCTGACCAGAATGAAAAGGGGTTTACCAGGGTAGTCTCTAAGTCTACCAGGA
AGAGGGGAAATGTCCCCAAAACTGAGAAGAATAAAGGGAAAGGTCCAT
+
FFFFFFFFFFFFFFFFFFFFFFFFFFFFFFFFF:FFFFFFFFFFFFFFFFFFFFF:FFFFFFFFFFFFF
FFFFFFFFFFFFFFFFFFFFFFFFFFFFFFFFFFFFFFFFFFFFFFFF
@A00721:190:HGJW7DSXY:4:1529:21115:4617_2:N:0:TCTGCAAG+GCTGCACT
AGGGGAAATTACCCCTAATTCTGAAGGAAGATCTCCCACCATCTGGGGTTGGGAATAAGAAATCTGCA
CGGGCCAAAGAGGCCGCTGCTACGAAGTCGCTGAAGGCGGCTTGGGTC
+
FFFFFF:FFFFFFFFFFFFFFFF:FFFFFFFFFFFFFFFFF:FFFFFFF:FFFF:FFFFFFFFFFFFF
FFFFFFFFFF:FFFFFFFFFF,FFFFFFFFFFFFFF:FFFFFFFFFFF
@A00721:190:HGJW7DSXY:4:2263:32732:2973_2:N:0:TCTGCAAG+GCTGCACT
GTTGCGACTGCCGACCCCATCTTTGAGACCAATACTCTCTACAGTAGGTTCGTGACTACTATGATCAA
ATCTGTTGAATTCGTCACAGAGGACGAAGGTGAGAGGAATATTGGCTA
+
FFF:F,,F,:F:F:F:FFFFF,F,,,FF,FFF,F,FF::,FFF,FF,,FF:,F,:FFFFFFF:F,FFF
F,,,F,F,:FFFFFF,FF,:F,:F,:FFF,,:FF:,:F,,,:F,F,FF
@A00721:190:HGJW7DSXY:4:1470:4264:20337_1:N:0:TCTGCAAG+GCTGCACT
CTGAAAGAGAATTGCGTGGGTTGAAGGCCGACGCTTCGAGCTTGAGTGTGACGAGGCCGGTGACGTTT
TTGTCAACGGCGTTTGGAGGATTTTATCCGCGCTTAACTATGGGGCGAAGATCCCCTATGGAGTCTTG
AAG
+
FFFFFFFFFFFFFFFFFFFFFFFFFFFFFFFFFFFFFFFFFFFFFFFFFFFFFFFFFFFFFFFFFFFF
FFFFFFFFFFFFFFFFFFFFFFFF:FF:FFFFFFFFF:FFFFFFFFFFFFFFFFFFFFFFFFFFFFFF
FFF
@A00721:190:HGJW7DSXY:4:1546:18114:14137_2:N:0:TCTGCAAG+GCTGCACT
GGCATATTTATGCCGACGATAATAAGCATGTCACCCTTAAGCACCGAGGTAAGCTCGGGACTTACAAG
AGAGAGTGTGAGAGAGTCGCTCTCACTATTTTTGCCCAGAATGCGGGC
+
FFFFFFFFFFFFFFFFFFFFFFFF:FFFFFFFFFFFFFFFFFFFFFFFFFFFFFFFFFFFFFFFFFFF
FFFFFFFFFFFFFFFFFFFFFFFFFFFFFFFFFFFFFFFFFFFFFFFF
```

```
@A00721:190:HGJW7DSXY:4:2553:22318:14935_2:N:0:TCTGCAAG+GCTGCACT
TTCACTGGTTCTGGGAGGCTGCTTTCCTCCATAATTACAACCTCTCTTTAGAGAGCATTAGAAGGAAG
TTGGGCAAATTCCCACATATGGTAGACGTTGTCACACTTTTCTTGAAA
+
FFFFFFFFFFFFFFFFFFFFFFFFFFFFFFFFFFF:,FFFFFFFFFFF:FFFFFFF:FFFFFFF
:FFFFFFFFFFFFFFFF:F,FFFFFFFF:FFFFFFFFFFFFFFFFFFF
@A00721:190:HGJW7DSXY:4:2124:8006:32612_2:N:0:TCTGCAAG+GCTGCACT
CGTCATTGGGGATTTCGATGTGAGAAGGTGGAAGGTGTCAAGGGTCTTCTTCATGCTGATCTTTCAGG
ACCTAATCACATCATGATTGATAGTTACGGCTCGTATCGAGTTGGAGG
+
FFFFFFFFFFFFFFFFFFFFFFFFFFFFFFFFFFFFFFFFFFFFFFFFFFFFFFFFFFFFFFFFFFFF
FFFFFFFFFFFFFFFFFFFFFFFFFFFF,FFFFFFFFFFFFFFFFFF:
@A00721:190:HGJW7DSXY:4:1427:20980:20228_2:N:0:TCTGCAAG+GCTGCACT
CGAAATTAAAGACGGGGGGATTAAGAGTCCAGCTGTAACTGTCATTTCAGAGAGGGGAGCTCTGAAAA
GGAAACTTCGTCAGAAGATGAGGAAGTCTAAGAGGAGGAGTTTGAGGA
+
FFFFFFFFFFFFFFFFFFFFFFFFFFFFFFFFFFFFFFFFFFF:FFFFFFFFFFFFFFFFFFFFFFFFF
FFFFFFFFFFFFFFFFFFFFFFFFFFFFFFFFFFFFFFFFFFFFFFFF
@A00721:190:HGJW7DSXY:4:2352:10221:10802_2:N:0:TCTGCAAG+GCTGCACT
GAAGTTGATGAGGAAGGATTATTTGAAGCAGGGGTTACCACTTCCTAAGGTGGATGAGGGAGGTTGTT
TCTTTGGAGTTTTAGATAATCTTTTGAGAAGGGGTCATAGAGCCAGCA
+
FFFFFFFFFFF,FFFFFFFFFFFFFFFFFFFFFFFFFFFFFFFFFFFFFFFFFFFFFFFFFFFFFFFF
FFFFFFFFFFFFFFFFFFFFFFFFFFFFFFFF:FFFFFFFFF,FFFFFF
@A00721:190:HGJW7DSXY:4:1158:26630:25003_2:N:0:TCTGCAAG+GCTGCACT
TATAATTTAGGTCAACTGGGGTTTTCGAATGTCATTATTGCCTTACGCTTGAAAGAGGTCCTTTTGAG
TATCGACAGATCTTTCGATATTGAGGACGTACATTTAACGTGGCAGAA
+
FF,FF,FFF,,,,FFFF,,F:,,FF:,FF,,:FFFFF,:FFFF:F,FFF::FFFFF,FFFFF,,,FFF
FFFFFFF:FFF:F:F:FFF,F:FF,,FFFFFFFFFFFFF:FFF,FFFF
@A00721:190:HGJW7DSXY:4:1134:30951:26443_2:N:0:TCTGCAAG+GCTGCACT
ATATTGTTCCTTCCGGGAAGTTGTGGGATTTAACTTTGACTCAACTACTCCGATCAGATCCGGATGGT
TTGATTGGTATGGACTACTCGACTTTTGATCTGGAGTTGTCTAGGGTC
+
FFF:FFFFFFFFFFFFFFFFFFFFFFFFFFFFFFFFFFFFFFFFFFFFFFFFFFFFFFFFFFFFFFFF
,FFFFFFFFFFFFFFFFFFFFFFFFFFFFFFFFFFFFFFFFFFFFFFF
@A00721:190:HGJW7DSXY:4:2217:7907:11271_2:N:0:TCTGCAAG+GCTGCACT
CGATAAGGTGAAAAATATGCAGGTCAAAGATTCTGTAGCTCTGAGGGCGGTTGAGGGAGCCGTGTTGG
CCGACTTTAACTATTCTAAGAGTCTTTATGATTCAAAACCTATCGTTG
+
FFFFFF:FFF:,:FFFFFFF:FF:FFFFFFF:FFFFFF:FFFF,:F:FFF,FFFF:FF:F,FF,F,:F
F:FFFF,:F::FF:F::FFFFFFFFF,F:,FFFFFFFFFFF,:FFFF
@A00721:190:HGJW7DSXY:4:1606:20835:27586_2:N:0:TCTGCAAG+GCTGCACT
GGCTTATTGTCGCGCAGTTTCCAGAATTGCGGTTGAAGTTCACCCATTCTATCTTGAATGCTCACGCC
GTCGCAGCCATATCGAGACTATGTTATAATCACTTGTTAAAGTACAGA
+
FFFFFFFFFFFFFFFFFFFFFFFFFFFFFFFFFFFFFFFFFFFFFFFFFFFFFF:F:FFFFFFFFFF
FFFFFFFFFFFFFFFFFFFFFFFFFFFFFFF,FFFFFFFFFFFFFFF:F
@A00721:190:HGJW7DSXY:4:1320:13829:24220_2:N:0:TCTGCAAG+GCTGCACT
ATTATTGATATTGGAGGCGATATTCGTTTTCATATCCAAAACTCCTTGAATGGAGTACATATTTGCAA
CCCTATTCTTGATGTGAAAGATGCATCGAGGTATACGTCTAGGAACAT
+
FFFFFFFFFFFFFFFFFFFFFFFFFFFFFFFFFFFFFFFFFFFFFFFFFFFFFFFFFFFFFFFFFFFF
FFFFFFFFFFFFFFFFFFFFFFFFFFFFFFFFFFFFFFFFFFFF,FF:
```

@A00721:190:HGJW7DSXY:4:2365:7509:10770_1:N:0:TCTGCAAG+GCTGCACT
CACGAGACGTCATGAAAGCTATCGGAGGGAAGCAAAATGTTTCGTTCTGTTACAAGAACGCTTTGGAT
TGTGACCATAAAGCCTCAGTTGCGATAGCTGTAGAGGTATACGATATCTCTCTTGATGATGTAGCCAC
TAT
+
FFFFFFFFFFFFFFFFFFFFFFFFFFFFFFFFFFFFFFFFFFFFFFFFFFFFFFFFFFFFFFFFFFFFFF
FFFFFFFFFFFFFFFFFFFFFFFFFFFFFFFFFFFFFFFFFFFFFFFFFFFFFFFFFF:FFFFFFFFF
FFF
@A00721:190:HGJW7DSXY:4:2571:12744:21715_2:N:0:TCTGCAAG+GCTGCACT
GATTGATGTATGTCATTTCGTCTTAGTTTGTCCCGGCGAATTATTGGACACGATGAAAAATGACATAC
CCGTTCCTGACCTTGGTATCGTTATCCGTCATGGAGATGATGGCGCAT
+
FFFFFFFFFFFFFFFF:FFFFFFFFFFFFFFFFFFFFFFFFFFFFFFFFFFFFFFFFFFFFFFFFFFF
FFFFFFFFFFFFFFFFFFFFFFFFFFFFFFFFFFFFFFFFFFFFFFFF
@A00721:190:HGJW7DSXY:4:2647:2130:23688_1:N:0:TCTGCAAG+GCTGCACT
TTATTCTCACAACAAAGATAAGATTTTATCTTTCTTCAAACACCCTGTTTTCCTTAAGGGCGGACAAT
TGTTCCATTGTGAACTCTTGGGGACTAGATTGAGCATTAATGAGTTCCGAATAACTCGTAATAAATCT
T
+
:FFFF:F,,,F,,,,,F,F::F:FFFF:FFFFFFFF:FF,,,F,,,F:FFF,,FFF,F,FFFFF,:FFF
FFFFF:,FFFFFFFF:FFFF:FF:FFFFFF:FF:FFFFF,,FFFFFFF,:,:FFFFFFFFFFFFFFFF:F
F
@A00721:190:HGJW7DSXY:4:1331:13973:4586_2:N:0:TCTGCAAG+GCTGCACT
TATTTCCGTACCAAGAGCCACTGCCAATCTCATCGCTATACGCATACCTGATTGGAAGAAGCGAGGTT
CGTTCCTCAATAAAAATTTTTCTCAAGAGATTATGGAGATCGGAAGAG
+
FFFFFFFFFFFFFFFFFFFFFFFFFFFFFFFFFFFFFFFFFFFFFFFFFFF:FFFFFFFFFFFFFFF
FFFFFFFF,FF:FFFFFFFFFFFFF:FFFF,FFFFFFFFFFF:FF:FF
@A00721:190:HGJW7DSXY:4:2529:10095:35978_1:N:0:TCTGCAAG+GCTGCACT
TTCAGTACGTCATGGGGATCACTTCTTCGGTCAATGACAAAACCTTCGAATATGTTACGACATGGCTT
CGTAACAACAGTGCGAGGATTGTCATCTCGGGGAAAGTGATTCACCAAGATGTTCATATAGATACGAG
ATA
+
FFF:FFFFFFFFFFFFFFFFFFF:FFFF:FFFFFFFFFFFFFFFFFFFFFFFFFFFFFFFFFFFFFFF
FFFFFFFFFFFF,FFFFFFFFFFFFFFFFFFFFFFFFFFFFFFFFFFFFFFFFFFFFFFFFFFFFFF
FFF
@A00721:190:HGJW7DSXY:4:2160:4833:29152_2:N:0:TCTGCAAG+GCTGCACT
CAGCGATCTTACTCGTTATCGGAGCTAAGAAGAAGACTTTGGGTACACGTTATGCTCGCAAACTTAAC
GTTAGTACCGGAGGTGATCTCTCTGCTTCTGCGTTACTGAGAGAAAAG
+
FFFFFFFFFFFFFFFFFFFFFFFFFFFFFFFFFFFFFFFFFFFFFFFFFFFFFFFFFFFFFFFFFFFFFF
FFFFFFFFFFFFFFFFFFFFFFFFFFFFFFFFFFFFFFFFFFFFFFFF
@A00721:190:HGJW7DSXY:4:2410:23538:6277_1:N:0:TCTGCAAG+GCTGCACT
ACTTTATGGACAAGATGCAAGTTCTTGGGAAATACTTGTTTCCCTTTTTAGGTTTAGTAGGTGATCTT
GACGTCGATTCTTTGTATAGGGAGTGTGAGAAGACAACGAATCTTGAAACTACGGTCCTTATCAAAGA
AAA
+
FFFFFFFFFFF:FFFFF:FFFFFFFFFFFFFFFFFFFFFF,FFFFFFFFFFFFFFFFFF:FFFFFFFFF
FFFFFFFFFFFFFFFFFF:FFFFFFFFFFFFFFFFFFFFFFFFFFFFFFFFFFFFFFFFFFFFFFFFF
FFF
@A00721:190:HGJW7DSXY:4:1573:17924:5822_1:N:0:TCTGCAAG+GCTGCACT
TCTAAATCTTTTGAGATTGAGAAGATTGAAGAACACGTCAAGAAGTCGCTCGGCGTTGTTCTTGAAAA
CCAAGTTAAGAAGGAGGCAGATCATGGAGTCTTTGTACCTTCTGTCAAACTTCGAGGTTCAGCTACGA
GAG

```
+
F,:FFF:FF:F,FFFFFF,F:FFFFFFFFFFFFFFFFF,FFFFFFFFF,FFFFF,,FFFF,:FFF,FF
:FFFFFF:FF:,:FF:,F,,FFF:F,:F,F,:FFF:F:FFF:FFFFFF:,FFF:F,FFFFFFFFF,FF
F:F
@A00721:190:HGJW7DSXY:4:2642:14760:36448_1:N:0:TCTGCAAG+GCTGCACT
TGGATTGAAGGCTGGCTCTAAGGGAGACTCCTTCTTTTTGAATGTTGTAAAGAATTGGGTTGAGAAGT
GTGCTTTACAAGGAGTTAGACTCTTCTTCAAGTTTTTAGATCTTCTTGATAATCTTAAAGAAAAATTT
TCG
+
FFFFFFFFFFFFFFFFFFF:FFFFFFFFFFFFFFFFFFFFFFFFFFFFFFFFFFFFFFFFF,FFFFFFF
FFFFFFFFFFFFFFFFFFFFFFFFFFFFFFFFFFFFFFFFFFFFFFFFFFFFFFFFFFFFFFFFFFFF
FFF
@A00721:190:HGJW7DSXY:4:2471:8703:11741_1:N:0:TCTGCAAG+GCTGCACT
TTTCTCTTGGTATTTCTGAAATAGTTTATCAGAGCGTCATTTTCCTTTGGGATCTTTCCTCTGGTAAG
GGAATTATTAAATCATTCTTCTCACTCTTGACGAGAATTATTACTGTTTTCTCTACCCATAACTTCTT
TTT
+
FFFFFFFF:FFFFFFFFFFFFFFFFFFFFFFFFFFFFFFF:FFFFFFFFFFFFFFFFFFFFFFFFFFFF
FFFFFFFFFFFFFFFFFFFFFFFFFFFFFFFFFFFFFFFFFFFFFFFFFFFFFFFFFFFFFFFFFFFF
FFF
@A00721:190:HGJW7DSXY:4:1544:29044:7576_1:N:0:TCTGCAAG+GCTGCACT
CTGATGTTTTCCTACAGAGTGCAGTCGTTTCAACACTTATCACCACTTTTGAAAGGAAAATAATTGGT
GATGAAAATACGTATGTCGGTGGGGTTCTCAAGGCTCTCATCGCCACTGGGATTCTAAGGGGTTCCAT
AAG
+
FFFFFFFFFFFFFFFFFFFFFFFFFFFFFFFFFFF:FFFFFFFFFFFFFFFFFFFFFFFFFFFF:FFF
FFFFFFFFFFFFFFFFFFFFFFFFFFFFFFFFFFFFFFFFFFFFFFFFFFFFFFFFFFFFFFFFFFFF
FFF
@A00721:190:HGJW7DSXY:4:1405:10872:2722_2:N:0:TCTGCAAG+GCTGCACT
TTTGGTCCTTACGTCTTAAGTAATTTGGATGTTGTCGAAAACTATCAGGTTCTTGTCACGATTCTTAA
AAGAAGTTTATTAAAACTTCAGAGTCTCTTGGAAGTCCAGGTAGCTAA
+
FFFFFFFFFFFFFFFFFFFFFFFFFFFFFFFFFFFFFFFFFFFFFFFFFFFFF:FFFFFFFFFFFFFF
FFFFFFFFFFFFFFFFFFFFFFFFFFFFFFFFFFFFFFFFFFFFFFFF
@A00721:190:HGJW7DSXY:4:1616:30834:21637_2:N:0:TCTGCAAG+GCTGCACT
GCCACAGACTTTCTGGGAAAAGAGACCTCTGTTGGTAGAGGAGTTGAGAGAATTACTCAGATTCAATC
TGGCATCACTGAACGTCTACAACGAGTTAGGGAGTGGCTAGAGATCGG
+
FFFFFFFFFFFFFFFFFFFFFFFFFFFFFFFFFFFFFFFFFFFFFFFFFFFFFFFFFFFFFFFFFFFF
FFFFFFFFFFFFFFFFFFFFFFFFFFFFFFFFFFFFFFFFFFFFFFFF
@A00721:190:HGJW7DSXY:4:2534:4472:8578_1:N:0:TCTGCAAG+GCTGCACT
CAATACCTTCCACTGAGTTCATCACCCAGAGTTTCGACGTCGCGGAAAACGATGAAGATTTCCACACT
GTTCGAGAATCTTCTGATGCTGAATTTGAGGAAAATTTTTCGGAAGTTTTTGGTATAAATCCTGATGG
CTC
+
FFFFF:FFFFFFFFFFFFFFFFFFFFFFFFFFFFFFFFFFFFFFFFFFFFFFFFFFF:F:FFFFFFFFF
FFFFFFFFFFFFFFFFFFFFFFFFFFFFFFFFFFFFFFFFFFFFFFFFFFFFFFFFF:FFFFFFFFFF
FFF
@A00721:190:HGJW7DSXY:4:1334:5186:36996_1:N:0:TCTGCAAG+GCTGCACT
AACCTTCTTTTATCATTGCAACAGGACTTTGAGAAGACTGTTGAAATGGACAAAATCTGGGATCATAG
GTATGTGTCACGCTACACTCGAAATATGGGCCCTCATAAGGAAGTATNGTTGGCGTCTTGTCGTTGAC
GTC
+
FFFFFFFFFFFFFFFFFFFFFFFFFFFFFFFFFFF:FFFFFFFFFFFFFFFFFFFFFFFFFFFFFFFFF
```

```
FFFFFFFFFFFFFFFFFFFFFFFFFFFFFFFFFFFFFFFFFFFFFFF#FFFFFFFFFFFFFFFFFFF
FFF
@A00721:190:HGJW7DSXY:4:2437:1895:8437_1:N:0:TCTGCAAG+GCTGCACT
GATCATTTGTTCAGGAAGATCTCTACAGAGATTATCGAGAGTTGTGATCTAGTTCTAAATTTAGGTCG
ACGGACTTTAGCAACTTTAGATAAATTCTTTCCGGGCTCAATGACCTTTGCTATGTACGTGGATCTAA
TGC
+
FFFFFFFFFFFFFFFFFFFFFFFFFFFFFFFFF:FFFFFFFFFFFFFFFFFFFFFFFFFFFFFFFFFFF
FFFFFFFFFFFFFFFFFFFFFFFFFFFFFFFFFFFFFFFFFFFFFFFFFFFFFFFFFFFFFFFFFFFF
F:F
@A00721:190:HGJW7DSXY:4:2323:19343:30295_2:N:0:TCTGCAAG+GCTGCACT
CATCGCAAAGAAGACTTTATTAGTTCATGAAGTTCTTACACTTCTCGCGACTAATGTCAGCTTCACTA
CAAAGGCGTTACGTAAAGTAGTTTTCACGCTTTATCTTAAAGCATTAC
+
FFFFFFFFFFFFFFFFFFFFFFFFFFFFFFFFFFFFFFFFFFFFFFFFFFFFFFFFFFFFFFFFFFFF
FFFFFFFFFFFFFFFFF:FFFFFFFFFFFFFFFFFFFFFFFFFFFFFF
@A00721:190:HGJW7DSXY:4:1118:12789:29904_1:N:0:TCTGCAAG+GCTGCACT
ACTTTTTGAATATGACAGAAGCTCGCCATATCAGATTAGAGACATGCCAGTTAGAGTTCTTCTAACCA
GGACTGATGGTGAGATTCTTTTACCTTTTGGAGGTCCAGGAACCTTTACTCCTAGTGTCTTTTCTAGA
GTC
+
FFFFFFFFFFFFFFFFFFF,FFFFFFFFFFFFFFFFFFFFFFFFFFFFFFFFFFFFFFFFFFFFFFFF
FFFFFFFFFFFFFFFFFFFFFFFFFFFFFFFFFFFFFFFFFFFFFFFFFFFFFFFFFFFFFFFFFFFF
FFF
@A00721:190:HGJW7DSXY:4:2555:19868:26036_2:N:0:TCTGCAAG+GCTGCACT
GTCGGAGGATGACAGGTGGGAGACCGCTCAATAACACTCTATTGAATCCCTTTTCCACGATGAAAGCT
CTTTATGAGGTGCTCCATGCAAATTTACAGTTCCTCTTTGGTGATATC
+
FFFFFFFFFFFFFFFFFFFFFFFFFFFFFFFFFFFFFFFFFFFFFFFFFFFFFFFFFFFFFFFFFFFF
FFFFFFFFFFFFFFFFFFFFFFFFFFFFFFFFFFFFFFFFFFFFFFFF
@A00721:190:HGJW7DSXY:4:2406:9109:36902_1:N:0:TCTGCAAG+GCTGCACT
GTATTTTGATAAATTTGTTGGAATAGATAATGATACTGACGAAGGTTCTATTACCACCCTTGTCATTG
ATACAAACATTCTAGGTGATGCTTTCTTAGAGGCTCGTCTAGCCGCTGAGATTCGTCTTACCTATTTA
CAA
+
:FFFFFFFFFF:FFFF:FFFFFFF,FFFFFF:FFFFF:F,FFFFFFF:FF,FFF:FFFFFF:FFFFFF
FFF:FFFFFFFF:FFFFFFFFFFFFFFFFFFFFFFF,:F:FF,,F:FF:FFFFFFFFFFFFFFFFFFF
:FF
@A00721:190:HGJW7DSXY:4:1103:4237:3192_1:N:0:TCTGCAAG+GCTGCACT
GATAACGATTTAGTACTGGGTAATGGTGGTAGTCAGTTCACTAATTTTCTTTCCCTACCCTTTTTCGA
TTTGATTCGACGGGGTCTTCGTCTCACGAATATTTTGATAAGGTTTTTCTTTTATGGGACTCTTGTTT
GTA
+
FFF,F,F,FFFFFF:FF:FF,FFFF:F,FFFFF:FFFFFF:FF,:FFFF,FF:::F,:,,FFFFF:,F
F:,:FFFFFFF:F:FF,FF:FF:FFFFFF,FF,FFFFFF,FFFFFFFFFF:FFFFF:FF:F:FF:FFF
FFF
@A00721:190:HGJW7DSXY:4:2474:32009:16125_2:N:0:TCTGCAAG+GCTGCACT
TATTACTTCGGAAGTTCTTAATGTAGGTCTCCTAGTACTTAAGGGTTTATGTTTCGGTTTCAGCTGGA
TGGATCTTTTGATGGTATGTTTCACATTGCGTGATCACTTCCCGTGGA
+
FFFFFFFFFFFFFFFFFFFFFFFFFF:FFFFF:FFFFFFFFF:FFFFFFFFFFFFFF,FFFFFFFFFFF
FFFFFFFFFFFFFFFFFFFFFFFFFF:FFFFFFFFFFFFF:FF,FFFFF
@A00721:190:HGJW7DSXY:4:2425:22435:29136_1:N:0:TCTGCAAG+GCTGCACT
GATTAAACATAAAATCCAGCGATTCTATTATGTCGCTAAAGCCTGTCCAGTTGTCGATGATGTTCGGG
```

```
CTGTGGGGATTAACTTCCCTCGTGAAACTTACGACGATGTTCTCGCTCGCATTTCACAAGGATTACTA
AGT
+
FFFFFFFFFFFFFFFFFFFFFFFFFFFFFFFFFFFFFFFFFFFFFFFFFFFFFFFFFFFFFFFFFFFF
FFFFFFFFFFFFFFFFFFFFFFFFFFFFFFFFFFFFFFFFFFFFFFFFFFFFFFFFFFFFFFFFFFFF
FFF
@A00721:190:HGJW7DSXY:4:2146:20537:17707_2:N:0:TCTGCAAG+GCTGCACT
CCACTTCTAATTTTAGGAAACGTCGCGAAAAGAAAGTTCATGAGGATGTCTTGGTCGAAAAATATCGC
CGGGAACTTCTAATAGAGGATCCTGATGACATTATTTCAACGGATACC
+
FFFFFFFFFFFFFFFFFFFFFFFFFFFFFFFFFFFF:FFFFFFFFFFFFFFFFFFFFFFFFFFFFFFF
FFFF,:FFFFFF:FFFFFFFFFFFFFFFFFFFFFFFFFFFFFFFFFFF
@A00721:190:HGJW7DSXY:4:1352:13422:28213_2:N:0:TCTGCAAG+GCTGCACT
CTACCACAGAAACCGTCTCAGGAACTGTCTCCACTGATTCGGAATTAGAGAAAGACGACCTTCTTGTG
GGGACTGAGACCTCTGAATTTTCAGGGTGCCCTGATGAGGACGTTGAA
+
FFFFFFFFFFFFFFFFFFF:FFFFFFFFFFFFFFFFFFFFFFFFFFFFFFFFFFFFFFFFFFFF:FFFF
FFFFFFFFFFFFFFFFFFFFFFFFFFFFFFFFFFFFFFFFFFFFFFFF
@A00721:190:HGJW7DSXY:4:1217:30011:12289_2:N:0:TCTGCAAG+GCTGCACT
CTTCTAAGAGTGTTTCAAAAGACCAGAAACGTAACAAGAAGAATAAAGGTAGGAAGGGGAGAAAACAG
AACAAGCGAGCGGTTGAAGAGAAAACTGATATGGTTGCGGCTGATCGT
+
FFFFFFFFFFFFFFFFFFFFFFFFFFFFFFFFFFFFFFFFFFFFFFFFFFFFFFFFFFFFFFFFFFF:
FFFFFFFFFFFFFFFFF:FFFFFFFFFFFFFFFFFFF:FFFFFFFFFF
@A00721:190:HGJW7DSXY:4:2535:17020:27273_2:N:0:TCTGCAAG+GCTGCACT
TAGAAAGATCACTCTCGGCGATACCACTGATCTTCCAGAGTTACGTAGTGGTCGTTCTTTTGGTGATT
TTATCAAAGAGAAAGTTTCTGCTAGTCGCGACGATAGTATTTATCGGA
+
FFFFFFFFFFFFFFFFFFFFFFFFFFFFFFFFFFFFFFFFFFFFFFFFFFFFFFFFFFFFFFFFFFFF
FFFFFFFFFFFFFFFFFFFFFFFFFFFF:FFFFFFFFFFFFFFFFFFF
@A00721:190:HGJW7DSXY:4:1455:24306:35571_2:N:0:TCTGCAAG+GCTGCACT
TGTTGAAGTACCGCAACATGGATCAGTTTTCCAAAAAGCTATTAATAGCAGTTGCGGGGCTATAATAC
CGCCTCCAGATTACTCTAAACTGAATCCACTCTCTTGGGATATAAATG
+
FFFFFFFFFFFFFFFFFFFFFFFFFFFFFFFFFFFFFFFFFFFFFFFFFFFFFFFFFFFFFFFFFFFF
FFFFFFFFFFFFFFFFFFFFFFFFFFFFFFFFFFFFFFFFFFFFFFFF
@A00721:190:HGJW7DSXY:4:2464:17942:35446_1:N:0:TCTGCAAG+GCTGCACT
GGCTTCTCTAAAGAACAGCAACTTTCAACATGAATTAGTTATTGAAAGTCTTGACCCTCACGTCTCAC
TTTATCACCTTGGAGATGGTAAAGTCATTAACAAAGGAAATGTTAAGGCTGATGACTTAAATTTCGCA
TTC
+
FFFFFFFFFF:FFFFFFFFFFFFFFFFFFFFFFFFFFFFFFFFFFFFFFFFFFFFFFFFFFFFFF:FFF
FFFFFFFFFFFFFFFFFFFFFFFFFFFFFFFFFF:FFFFFFFFFFFFFFFFFFFFFFFFFFFFFFFFF
FFF
@A00721:190:HGJW7DSXY:4:2272:28085:16501_2:N:0:TCTGCAAG+GCTGCACT
TTTGAAAATTACGGAACCTTTCCTCGTTCTTCATGAAGATCTTCGAGTGTTTTATAGTGAGTTATTCT
TGCGAAGGTTCAAGTCGATTGATTTTGTTGACTACGATCTCTCTTGTA
+
FFFFFFFFFFFFFFFFFFFFFFFFFFFFFFFFFFFFFFFFFFFFFFFFFF:FFFFFFFFFFFFFFFFF
FFFFFFFFFFFFFFFFFF,FFFFFFFFFF:F:FFFFFFFFFFFFFFFF
@A00721:190:HGJW7DSXY:4:2432:15709:27070_1:N:0:TCTGCAAG+GCTGCACT
AGACCACTGAACTTATAGCGACTTTTGTCGAATTTTTCGCAAAAGGTCTTGAAGTACGTATTGTCACT
GCTAATCGTAATTCGCGAGAAGAGATAGTGACTAAAGTTTTGGCACTTCTAGTTCATCGCGGTATTTG
TGA
```

+
FFFFFFFFFFF:FFFFFFFFFFFFFFFFF:FFFFFFFFFFFFFFFFFFFFFFFFFFFFFFFFFFFFFF
FFFFFFFFFFFFFFFFFFFFFFFFFFFFFFFFFFFFFFFFFFFFFFFFFFFFFFFFFFFFFFFFFFFF
FFF
@A00721:190:HGJW7DSXY:4:2215:5900:15718_2:N:0:TCTGCAAG+GCTGCACT
CGTGAAAGTTATGATGAGTGATCGTATTAGAACAGCTGATTCTTATTGCATGAACGTCGCTGAGGTCA
AACCTGTGGACGTTCTTTTCATCGACGAATTATTCATGATTCACGCTG
+
FFFFFFFFFFFFFFFFFFFF::FFFFFFFFFF:FFFFFFFFF:FFFFFFFFFFFFFFFFFFFFFFFFF
,,FFFFFFFFFFFFFFFFFFFFFFFFFFFFFFFFFFFFFFFF:F:FFF
@A00721:190:HGJW7DSXY:4:1419:27489:36730_2:N:0:TCTGCAAG+GCTGCACT
CATCTACTTAAGGCGGCTTATACCATAGGTTACGGTGATTCCAATCAAATCGGATTCATCAATCGTAT
TGATCTCCAAGTCGCAAACTACTTCAACATCAACGACATCATTCTTGA
+
FFFFFFFFFFFFFFFFF:FFFFFF:FFFFFFFFFFFFFFFFFFFFFFFFFFFFF:FFFFFF::FFFFF
FFFFFFFFFFFFFF:FFFFF:FFFFF:FFFFFFFFFFFFFFFFFFFFF
@A00721:190:HGJW7DSXY:4:2412:9408:25739_2:N:0:TCTGCAAG+GCTGCACT
TTATCGAAGTCTCAGTTATCGCTGCCCGGCCGACGTTTGTCGCGAACTCTCAGAGGTTTACGGCAGAA
AAATTTCTCCTGCTTGTAATTCGAGAGAGGACACTATGGAAATTAGAC
+
FFFFFFFFFFFFFFFFFFFFFFFFFFFFFFFFFFFFFFFFFFFFFFFFFFFFFFFFFFFFFFFFFFFF
FFFFFFFFFFFFFFFFFFFFFFFFFFFFFFFFFFFFFFFFFFFFFFFF
@A00721:190:HGJW7DSXY:4:1631:4761:14184_2:N:0:TCTGCAAG+GCTGCACT
TATTACTTTCACTCAAGGTGAAAAGTTTGAAATTAACAAAGTCCTTGGTAAGAGTAAGGTTAAATGTG
AAGATGCTCAGACTATTCATGAAGTTCAAGGTAATACTTACCAGCACG
+
FFFFFFFFFFFFFFFFFFFFFFF:FFFFFFFFFFFFFFFFFFFFFFFFFFFFFFFFFFFFFFFFFFFF
FFFFFFFFFFFFFFFFFFFFFFFFFFFFFFFFFFFFFFFFFFFFFFFF
@A00721:190:HGJW7DSXY:4:2208:17598:15843_2:N:0:TCTGCAAG+GCTGCACT
TTTTGCATCACAAACACATATTACCGTTGCTCTATCACGACACGTTGAATCTTTACGATACTTTGTTA
TCAATGTAAAGTTAAACGATGCTACTTGTGAGAGGATCAATCACAGTA
+
FFFFFFFFFFF:F,FF:FFF,FFFF:F::FFFF:FFFFFFFF,FFFFFFFFFFFFFFFFFFFFF,FFF
FFFFF,FFFF,FF:F::FFFFFFFFFFFFFF:FFFFFFF,FFF:FF::
@A00721:190:HGJW7DSXY:4:1511:21902:14779_2:N:0:TCTGCAAG+GCTGCACT
ACGGGAGGTTGCAGATTCAACCCCTTATATCGAGGCTATCGAGGACGATGAGTATCTTGAAGTTGTTG
ACGACAAGCCTCAAGAGGGTAGAGGACCACCTGGATCTTCTACTGTCT
+
FFFFFFFFFFFFFFFFFFFFFFFFFFFFFFFFFFFFFFFFFFFFFFFFFFFFFFFFFFFFFFFFFFFF
FFFFFFFFFFFFFFFFFFFFFFFFFFFFFFFFFFFFFFFFFFFFFFFF
@A00721:190:HGJW7DSXY:4:1540:11840:20995_1:N:0:TCTGCAAG+GCTGCACT
AACCACCTTAACGTCACTCGGTAATCTTTCAGCCGAATTAAGTACTAATGAGTTCAAGTCGGTTGTCG
ATCGAATTAAGATCAAGGCTAATACCTTTATAGGTAAGAAGCCCCTTGAGCCGCGTAGTCAGCGCGTT
TAG
+
FFFFFFFFFFFFFFFFFFFFFFFFFFFFFFFFFFFFFFFFFFFFFFFFFFFFF:FFFFFFFFFFFFFF
FFFFFFFFFFFFFFFFFFFFFFFFFFFFFFFFFFFFFFFFFFFFFFFFFFFFFFFFFFFFFFFFFFFF
FFF
@A00721:190:HGJW7DSXY:4:1674:31819:28416_2:N:0:TCTGCAAG+GCTGCACT
CCGATAGGGTACCGAGTCTCCAAGAGAATATTTATTCTTATGAGGCTCGAAACTTCAACGTTTTCACA
ACGGAAAGACATATCGCACCCGAAATTTTTGGGCAGAATGCTCTTAAC
+
FFFFFFFFFFFF:FF:FFFFFFFFFFFFFFFFFFFFFFFFFFFFFFFFFFFFFFFFFFF:FFFFFFFF
FFF:FFFFFFFFFFFFFFF:FFFFFFFFFFFFFFFFFFF:FFFFFFF:

```
@A00721:190:HGJW7DSXY:4:2270:13322:29857_2:N:0:TCTGCAAG+GCTGCACT
CTAAGTTGGATAAAGTTATAGGAGACTCAGTTAACCTGAGAGCTCCTCAATTGATAGATTGGGTCAAC
AATAGGGACGCTAGTATACTTCGTAAAGTCGACAGCGAAATGGTCAAT
+
FFFFFFFFFFFFFFFFFFFFFFFFFFFFFFFFFFFFFFFFFFFFFF:FFFFFFFFFFFFFFFFFFFFF
FFFFFFFFFFFFFFFFFFFFFFFFFFFFFFFFFFFFFFFFFFFFFFFF
@A00721:190:HGJW7DSXY:4:1262:25545:27164_1:N:0:TCTGCAAG+GCTGCACT
TGACCTATTTTAAGCTCATGGTGAAAAGGGAAGCTAAGGTTAAACTTGATGCTTCAGCCTTTTCGAAG
CATAACCCCGCACAGAATATCGTTTATCACGCAAAATTTATTAATGCAATTTTCGGACCTATCTTTAA
GGT
+
FFFFFFFFFFFFFFFFFFFFFFFFFFFFFFFFFFFFFFFF:FFFFFFFFFFFFF:FFFFFFFFFFFFFF
FFFFFFFFFFFFFFFFFFFFFFFFFFFFFFFFFFFFFFFFFFFFFFFFFFFFFFFFFFFFFFFFFFFF
FF:
@A00721:190:HGJW7DSXY:4:2555:26928:10050_2:N:0:TCTGCAAG+GCTGCACT
CTTTTACCCAACATTTTTATGTATACAGAGATGACCAGTGACGATTTCTCCCGGAAGCTCTTCTCTCT
CTTAGGAGGTCATGATGACTACTATAAGGGTGAAATCGATTTTTCGAA
+
FF,,:FF:FFF:FF:,::FF:FF:FFFFF:F::F,:FF::FFFFFFFFFF::,:FFF:FFFFFFF:FF
F,,FF:F,F:FFF:FF:FFFFFFF,FFF::,,FFF:FFFF:FFF:FFF
@A00721:190:HGJW7DSXY:4:1511:2419:16736_1:N:0:TCTGCAAG+GCTGCACT
GATAAAGATGTATTCTTATTTAGGCTTCGATGCTGAATTTCTTGAACTTTGGAGTGCCAGTGAATATT
TTTGTCACGCCAGGAGTATCCATAACGATTTGTCATTTACCACCTTTGCTCAACGACGAAGTGGAACG
GCC
+
FFFFFFFF:FFFFFFFFFFFFFFFFFFFFF:FFFFFFFFFFFFFFFFFFFFFFFFFFFFFFFFFFFFF
FFFFFFFFFFFFFFFFFFFFFFFFFFFFFFFFFFFFFFFFFFFFFF::FFFFFFFFFFFFFFFFFFFFF
FFF
@A00721:190:HGJW7DSXY:4:2652:4824:26318_2:N:0:TCTGCAAG+GCTGCACT
TAATTGTATGATCACTTTGATGATTTTATCACTTTATTATGACCTTGGAAAGTTTTCGGCTGTGGTTG
TCGCTGGGGACGATTCCATTCTGTTTTCAAAGACTCCAATAACTGATC
+
FFFFFFFFFFFFFFFFFFFFFFFFFFFFFFFFFFFFFFFFFFFFFFFFFFFFFFFFFF,FFFFFFFFFF
FFFFFFFFFFFFF:FFFF::FFFFFFFFFFFFFFF:,F:FFFFFFFFF
@A00721:190:HGJW7DSXY:4:2112:29396:3583_2:N:0:TCTGCAAG+GCTGCACT
TGCTCGCGATTTATTCTTTTTGTCAATGATTACATCTACTTCGTTCCTGATCCTTACAAGATCATCGT
TAAGTTAGGTAAACCCCTAACCGATGTTACTCTTGTAGGACTTTGGGA
+
FFFFFFFFF:FFFF:FFFFFFFFFFFFFFFFFFFFFFFFFFFFFFFFFFFFFFFFFFFFFFFFFFFFF
FFFFFFFFFFFFFF:FFFFF:FFFFFFFFFFFFF:FFFFFFFFFFFFF
@A00721:190:HGJW7DSXY:4:2345:14244:11162_2:N:0:TCTGCAAG+GCTGCACT
TTAAAGACTATACTAAATATCTCCACTACGAGGTTGTTGTTTGGCGATTAGCTCTTGCGACTAATATT
CGATATTCGATTGAGGGTCGTTATACCTATTCGGCTATCTGTGCTTTG
+
FFFFFFFFFFFFFFF:F:FFFFFFFFFFFFFFF,FFFFFFFFFFFFFFFF:FFFFFFFFFFFFFFFFF
FFFFFFFFFFFFFFFFFFFFFFFFFFFFFFFFFFF:FF:FFFFFFFFF,
@A00721:190:HGJW7DSXY:4:2363:3152:24831_2:N:0:TCTGCAAG+GCTGCACT
TTTTAAACGATTCCGAGAGGTTTATCCTGATGTGGAGGGAGTTCTTGAAGCATCCCGACCTTTCCTTG
TGAAAATTTTGATGCGGTTTCATAAGATTATCAGTAATCCTGATTCTA
+
FFFFFFFFFFFFFFFFFFFFFFFFFFFFFFFFFFFFFFFFFFFFFFFFFFFFFFFFFFFFFFFFFFFF
FFFFFFFFFFFFFFFFFFFFFFFFFFFF:FFFFFFFFFFFFFFFFFFFF
@A00721:190:HGJW7DSXY:4:1619:24189:28917_1:N:0:TCTGCAAG+GCTGCACT
ACCTCTTCTGGAGAGACGTATCTCAAAGATCGACTTCGAGACAGGTGATATTGTTGAGCGAGAGCGAC
```

```
CAAGATCTAAATTCGGTAACGGTGCGTGGCGCTTTATTTTAAAAAGCTCCCTCCTCTCGCAAGTTTGA
TCT
+
FFFFFFFFFFFFFFFFF:FFFFFFFFF:FFFFFFFFFFFFFFFFFF:FFFFFFFFFFFFFFFFFFF
FFFFFFFFFFFFFFF:FFFFFFFFFFFFFFFFFFFFFFFFFFFFFFFFFFFFFFFFF:FFFFFFFF
FFF
@A00721:190:HGJW7DSXY:4:2405:4806:22498_2:N:0:TCTGCAAG+GCTGCACT
ACTTGAGTCTTTTGATCAGTCTCTGACTTGACCATCTTTCATTTTAAATGACGATCATTAAGACTCTC
TTTCTTCTTTGTTTCCTTCTTTGCACTGATTTTTCCTCATCAGCTTGT
+
FFFFFFFFFF:FFFFFFFFFFFFFF,FFFFFFFFFF:FFFFFFFFF:FFFFFFFFFFFFFFF:FFFF
FFFF:FFFFFFFFFFFFFFFFFFFFFFFFFFFFFF:F:FFFFFF:,,FF
@A00721:190:HGJW7DSXY:4:1640:20464:10379_2:N:0:TCTGCAAG+GCTGCACT
TTTACGATAACAACTATCAGTTGATTAAGACTCTTCCGAAAGGCGAACTTTGGGAATTTGAAGCTCAG
GGTGAAGCACAACGTTTGACCGTGACGAACTGTGGTGTTCTTGATCTA
+
FF:FFFF:FFFFFFF,F,FFFFFFF,FFFFFF::FFF:FFF:FFFFFF:,FF,:F:FFFFFF::FFFF
,F,:FFF:FFF:FF,FF,F::F:::F:FF,:FFF::F,F,F,,FFFFF
@A00721:190:HGJW7DSXY:4:2446:19235:23531_1:N:0:TCTGCAAG+GCTGCACT
CACTACCACTTATTACGATATTTCTTTAGTCGATGGGTTTTCGACGCCTTTAAGCGTCTTCTGTTCAT
CGCAACCAGATGATATCTTCTATTCGAACTTATGTTTCGATCAGTGTTTCTTCTATCAGGAGGAAAAC
ACT
+
FFFFFFFFF,FFFFFFFFFFFFFFFFFFFFFFFFFFFFFFFFFFFFFFFFFFFFFFFFFFFFFFFFFF
FFFFFFFFFFFFFFFFFFFFFFFFFFFFF:FFFFFFFFFFFFFFFFFFFFFFFFFFFFFFFFFFFFFF
FFF
@A00721:190:HGJW7DSXY:4:1652:4074:28557_2:N:0:TCTGCAAG+GCTGCACT
TAATGTGGCCTGAACTACGTTTCACTTTGTGTGTACTTGTCTCTATTTTAAGTTTTGTCTTCCTTGTT
TGTCTTATTCTATTAATCACTCATTTTAGTGAGATTATTAAAAACGCA
+
FFFFFFFFFFFFFFFF:FFFFFFFFFFFFFFFFFFFFF:FFFF:FFFFFFFFFFFFFFFFFFFFFFFF
FFFFFFFFFFFFFFFFFFFFFFFFFFFFFFFFFFFFFFFFFF::FF:F
@A00721:190:HGJW7DSXY:4:1607:6045:30436_2:N:0:TCTGCAAG+GCTGCACT
GACAACAGCAGGGGTTTTCGAATAGTGATAGCCACCCGCCTTACAGAGGGGCAGCAGCATGACCATAA
TTGGTATTGACTTTGGTACCACCTTTTCTACTGTCTGTCTCGCCTCTT
+
FFFFFFFFFFFFFFFFFFFFFFFFFFFFFFFFFFFFFFFFFFFFFFFFFFFFFFFFFFFFFFFFFFFF
FFFFFFFFFFFFFFFFFFFFFFFFFFFFFFFFFFFFFFFFFFFFFF:F
@A00721:190:HGJW7DSXY:4:2659:21911:14074_1:N:0:TCTGCAAG+GCTGCACT
CTTAACAACAATGATAGTGAATTTATACCTACAATCATTGGTTTTTCTAATAATGATGACACTATTGT
TTATGGATATGATGCTATTACTCATGATGGTTACGGTCAGGGAGGATTCTCAGTTTACAAGGACCTTA
AGC
+
FFFFFFFFFFFFFF:FFFFFFFFFF,FFFFFFFFFFFFFFFFFFFFFFFFFFFFFFFFFFF:FFFFFFF
FFFFFFFFFFFFFFFFFFFFFFFFFFFFFFFFFFFFFFFFFFFFFFFFFFFFFFFFFFFFFFFFFFFF
FFF
@A00721:190:HGJW7DSXY:4:1406:28908:15013_1:N:0:TCTGCAAG+GCTGCACT
AGACGTGCTAAATTAAAACCATTGTACGATGTCGTCTGTCTCGGCGATCACTTTTCACTAAGAATTGG
TCCTACTTTTGGACAAGGCCAACTTCGTAATCTTCATGAGATCGTTTCTTTCTTTATCTCTTTGGTTG
TTA
+
FFFFFF:FFFFFFFFFFFFFFFFFFFFFFFFFFFFFFFFFFFFFFFFFFFFFFFFFFFFFFFFFFFFF
FFFFFFFFFFFFFFFFFFFFFFFFFFFFFFFFFFFFFFFFFFFFFFFFFFFFFFFFFFFFFFFFFFFF
FFF
```

@A00721:190:HGJW7DSXY:4:1155:5927:31360_2:N:0:TCTGCAAG+GCTGCACT
GAGTGAAAGGCTTCAATTGTAGTGGTTTGGTCGTTTCAGTACCCAGTCAGTATACCTCTACGCAAAGA
TATTTCATGAAAACTACAGAGGAACGTACCGGATTACCTGTCTTTCAT
+
FFFFFFFFFFFFFFFFFFF:,FFFFFFFFFFFFFFFFFFFFFFFFFFFFFFFFFFFFFFFFFFFFFFF
FFFFFFFFFFFFFFFFFFFFFFFF,FFFFFFFFFFFFFFFFFFFFFF:F
@A00721:190:HGJW7DSXY:4:2106:9263:20165_1:N:0:TCTGCAAG+GCTGCACT
CTTCGGCCGCTCTCTTTGCTTCGATGTTGGATGTCAAGAAGGGTTCAGAATGGGATTCCTACGTAGTT
TATGACTTTGGTGGTGGTACTTTCGATACTTCTATCGTCTTACGTTACGGTAATTACTATAGTGTTAT
TTA
+
FFFFFFFFFFFFFFFFFFFFFFFFFFFFFFFF:FFFF:FFFFFFFFFFFFFFFFFFFFFFFFFFFFFF
FFFFFFFFFFFFFFFFFFFFFFFFFFFFFFFFFFFFFFFFFFFFFFFFFFFFFFFFFFFFF:FFFFFFF
FFF
@A00721:190:HGJW7DSXY:4:2214:16622:30686_1:N:0:TCTGCAAG+GCTGCACT
ATAATCTTGGAGGTCGTGACGTTGATGAAGCTATAAGAGCGTTCTTATTACGGCGCACTTCTTTACCC
GCCACCACACAACTTTCAGTTTCACAACTGAAGGAGGAGGTTTCTGTTACTGGAAAACCCTCGTCAAT
ACT
+
FFFFFFFFFFFFFFFFFFFFFFFFFFFFFFFFFFFFFFFFFFFFFFFFFFFFFFFFFFFFFFFFFFF:
FFFFFFFFFFFFFFFFFFFFFFFFFFFFFFFFFFFFFFFFFFFFFFFFFFFFFFFFFFFFFFFFFFFF
FFF
@A00721:190:HGJW7DSXY:4:2328:1732:13823_2:N:0:TCTGCAAG+GCTGCACT
GATCTAGTGAAATTAACGCGTCCTTTTCTGGAACGTAGTTTTACTATTATGAACAAAGTTATCAAAGA
TAGTGGCATTCAAGGCAAACTTACCCTGATTCCGGCCGGTGGTAGCTC
+
FFFFFFFFFFFF:FFFFFFF:FFF:FFFFFFFFFFFFFFF:FFFFFFFFFF:FFFFFFFFFFFFFF
FFF:FFFFFFFFFFF:FFFFFFFFFFFFFFFFFFFFF:FFFFFFFFF
@A00721:190:HGJW7DSXY:4:2231:13973:14512_2:N:0:TCTGCAAG+GCTGCACT
TGCTGAAACAGCTATTAAAGCTGTTGAGCGGGTAATTACACCTAAACGAGCTCGATCTGCCATCGCTG
AAGGATGTGCACTCTTTTCTGCTACTTTAACGGAGCGTAGTGTACTCT
+
FFFFFFFFFFFFFFFFFFFFFFFFFFFFFFFFFFF:FFFFFFFFFFFFFFFFFFFFFFFFFFFFFFF:F
FFFFFFFFFFFFFFFFFFFFFFFFFFFFFF:FFFFFFFFFFFFFFFFFF
@A00721:190:HGJW7DSXY:4:1113:7979:1720_2:N:0:TCTGCAAG+GCTGCACT
AAAGGTCTCTATCAAGCTCGTATTATCGTCGCTGCTGGATCTGCGTTACCGTTGAAGGCTAAACTCCA
AGGTAATTATGAAGACTGTGATTTATCTTTCACTCTGGCCTTGTATGA
+
FFFFFFFFFFFFFFFFFFFFFFFFFFFFFFFFFFFFFFFFFFFFFFFFFFFFFF:FFFFFFFFFFFFF
:FF:FFFFFFFFFFF::F:FFFFFFFFFFFFFFFFFFFFFFFFFFFFF
@A00721:190:HGJW7DSXY:4:2452:12780:22717_2:N:0:TCTGCAAG+GCTGCACT
CGTAACATTAATAATTTCAATCTTCTTAAAAAGAAGATAGAGAGTCATACTTTGGGCTATCGTTCAGG
TGCATGGGGTTGGATAGCCAATATTGAAGTGGACACCTCTGGAGATCG
+
F::FF:FFFFF:FF,:FFFFFFFF:F:FFFFF::FF:FFFF:FF,:FFF,F,:F,FFF,FF:FFF:FF
FFF:,FF:FFFF,FFFFF,FF:FFF::FFFF,FFFF:FF:,F:FFFFF
@A00721:190:HGJW7DSXY:4:1558:10655:18662_2:N:0:TCTGCAAG+GCTGCACT
AAGCATACTACTTTTGCTTTGATTACTCTACCGCCTTACAAGTCTAAGGTTAAAGAGTCGATTAAACA
AGTTGTACTTATCAATGATTTTATCGCTGGTTGTTATCTCGAAAAGTC
+
FFFFFFFFFFFFFFFFFFFFFFFFFFFFFFFFFFFFFFFFFFFFFFFFFFFFFFFFFFFFFFFFFF,F
F,FFFFF:FFFFFFFF,FFFFFF:,FFF:F:FFFFFF:F::FFFFFFF
@A00721:190:HGJW7DSXY:4:2276:13404:1689_1:N:0:TCTGCAAG+GCTGCACT
CCCAAAGAGAATATCTAAAATATTTACAAAATCTTACCACTATCTACGAACCTAAAGTTCTGAAGACG

TTGCTTGTCGCTTCCTCCTTTAATGGCACTACCTCGGTACAATCTTACTTGGAACGAAGGCGACCTGT
TCC
+
FFFFF,FFFFFFFFFFFFFFFFFFFFFFFFFFFFFFFFFFFFFFFFFFFFFFFFFFFFFFFFFFFFF
FFFFFFFFFFFFFFFFFFF:FFFFFFFFFFFFFFFFFFFFFFFFFFFFFFFFFFFFFFFFFFFFFFF
FFF
@A00721:190:HGJW7DSXY:4:2467:20898:17581_1:N:0:TCTGCAAG+GCTGCACT
GTTCTGTCTATATTAGGTGAAGCGAAATACGATTTCGAACGTCAACGTTCACCGAGTTTCGTTTCGTA
CCTTGATGCTGACTTTCGTCAATATGATTTGATCTGGTTGCTCAATTCTAAGCGTGTTCCGCTTAGTG
CGA
+
FFFFFFFFFFFFFFFFFFFFFFFFFFFFFFFFFFFFFFFFFFFFFFFFFFFFFFFFFFFFFFFFFFFF
FFFFFFFFFFFFFFFFFFFFFFFFFFFFFFFFFFFFFFFFFFFFFFFFFFFFFFFFFFFFFFFFFFFF
FFF
@A00721:190:HGJW7DSXY:4:2646:12201:4053_1:N:0:TCTGCAAG+GCTGCACT
TACGAACTTGACTCTACTATACGTGACATACTAAATACTAGATATTTGGATGATCTTGATAAAGAACT
TCATGAATTTTCAGAGGATGAAAAATTTTCTTCCTTGGATTTTAATATAAAGAATCTTTACCAGGACT
CCT
+
FF,:FFFFFFFFFFFFFFFFFFFFFFFFFFFFFFFFFFFFFFFFFFFFFFFFF:FF:FFFFFF:FFF
FFFFFFFFFFFFFFFFFFFFFFFFFFFFFFFFFFFFFFFFFFFFFFFFFFFFFFFFFFFFFFFFFFFF
FFF
@A00721:190:HGJW7DSXY:4:1455:22941:35556_1:N:0:TCTGCAAG+GCTGCACT
GATCGTAAGAAGTTTATAAGGAACATATCCATTTGTTGTAATAGGGTCATCAACTTAGAACTCGAAAC
TCTAAGTGGTAATACTGTCTTTCCAAGCTTACCTGTATCTTCAATGAAATTGGACTCTTCGATAGGGG
ACA
+
FFFFFFFFFFFFFFFFFFFFF,FFFFFFFFFFFFFFFFFFFFFF:FFFF:FFFFFFFFFFFFFFFFF
FFFFFFFFFFFFFFFFFFFFFFFFFFFFFFFFFFFFFFFFFFFFFFFFFF:FFFFFFFFFFFFFFFFF
FFF
@A00721:190:HGJW7DSXY:4:2519:12201:8281_1:N:0:TCTGCAAG+GCTGCACT
ACCGGTGAATGGTTTAAGAATACTATAAATAGAATAGCTATTACTACTAATGGCGTTGGCCTAGTGGA
TGTTATTAGTGACATCTGGTCAATTTCGTTTTTAGGTGGTCTTTATTCTATTATTGATTTTTATGAGA
TTG
+
FFFFFFFFFFFFFFFFFFFFFFFFFFFFFFFFFFFF,FFFFFF,FFFFFFFF,FFFFFFFFFFFFFFFF
FFFFFFFFFFFFFFFFFFFFFFFFFFFFFFFFFFFFFFFFFFFFFFFFFFFFFFFFFFFFFFFFFFFF
FFF
@A00721:190:HGJW7DSXY:4:2152:25319:22075_2:N:0:TCTGCAAG+GCTGCACT
TGTTAGAGTAGAATTCCTGAAGAGTCCTTCCGTAACCAACTTCTTGACAATTTTCTTCAATCCTTCGA
TAATAGTTAAATCTAGGATTATGTCTGTGTTGAAAGACTTTCTACCGA
+
F:FFF,:,:F:FFFFFFFF,FFFFF:FF:,FFFF:F:FF,FFFF,FF,,F,FFFFFFFF,F,,,FFFF
FFFFF:FF:FFF:F:FFFFFFFFF,:F::F:FF,FFFFFF:F:FFFF:
@A00721:190:HGJW7DSXY:4:1526:22218:13949_2:N:0:TCTGCAAG+GCTGCACT
TACACAATTAAGAAATAATCCTACATTGACTGATTATCTAAAGATTGAAGGAAAATTTTATCCTCGTT
TAACTTACTTAGACTTTCCTACGATACAAATGATAACTTCTAATTTAC
+
FFFFFF:FFFFFFFFFF:FFFF:FFFFF:FFFFFFFFFFFFFFFFFFFFFFFFF,FFFFFFFFFFFFF:
FFFFFFFFFFF:FFFFFFFFFFFFFFFFFFFFFFFFFFFFFFFFFF:FFFF
@A00721:190:HGJW7DSXY:4:2347:16269:24283_1:N:0:TCTGCAAG+GCTGCACT
GGGATGCATGGTGTTATCTCCTCAGGAGTCCCGGCATTTGATAATAATGACGAGACTTTCGTCATAAT
CGCTGCATATTACGGATTACACGGTACCAGCGCATCTCGTGTCGAGCCTCGACCCGATTATATGGTAT
TAA

```
+
FFFFFFFFFFFFFFFFFFFFFFFFFFFFFFFFFFFFFFFFFFFFFFFFFFFFFFFFFFFFFF:FFFFFFF
FFFFFFFFFFF:FFFFFFF::FFFFFFFFF:FFFFFFFFFFFFFFFFFFFFFFFFFFFFFFFFF:FFFFF
FFF
@A00721:190:HGJW7DSXY:4:2609:8901:32127_2:N:0:TCTGCAAG+GCTGCACT
GATTCTCGATTCGTAGAGCTTATCTAGGTACTCTCATTTACTATGTTGATTTAGTCTACATTTATGCT
GATCTAAAATTTCGATCTAGATGGATCCACTCCTCGAGTTATCATGAT
+
FFFFFFFFFFFFFFFFFFFFFFFFFFFFFFFFFFFFFFFFFFFFFFFFFFFFFFFFFFFFFFFFFFFFFF
FFFFFFFFFFFFFFFFFFFFFFFFFFFFFFFFFFFFFFFFFFFFFFFF
@A00721:190:HGJW7DSXY:4:2559:2248:20760_1:N:0:TCTGCAAG+GCTGCACT
TTCTAGAATTCTTAATTATAATATTCAACCTCAAAAGTTACGTGTTTACAGTAACGAGATCACACATT
TTGAGAACTAACTATGTTATGTTCTCTGATCATTTTGACAATCTCTTCTTATAATAAATAAATAAATA
ATA
+
FFFFFFFF:FFFFFFFFFFFFFFFFFFFFFFFFFFFFFFFFFFFFFFFFFFFFFF:FFFFFFFFFFFFF
FFFFFFFFFFFFFFFFF:FFFFFFFFFFFFFFFFFFFFFFFFFFFFFFFFFFFFFFFFFFFFFFFFFFFF
FFF
@A00721:190:HGJW7DSXY:4:1659:2962:11036_2:N:0:TCTGCAAG+GCTGCACT
AGTGGAAAGGCTCCAGTCAACACTAGTTCGAGTGCTGCTTTGGATTACGATGACGTGAGAATAGGTGA
AGGGATAGTTTTTACCTCGACCAACATTAAGGCGGCGGCGACTAATCT
+
FFFFFFFFFFFFFFFFFFFFFFFFFFFFFFFFFFFFFFFFFFFFFFFFFFFFFFFFFFFFFFFFFFFFFF
FFFFFFFFFFFFFFFFFFFFFFFFFFFFFFFFFFFFFFFFFFFFFFFF
@A00721:190:HGJW7DSXY:4:1636:16441:11553_1:N:0:TCTGCAAG+GCTGCACT
AGGACACTTCGAAACTGAATAATGATGCACTACAAACAAGTATCGCTAATCATCTGAAAAAGGCTCTA
AAGGACACTATACAATATCCAGACGACGACGAAGCTTATACTACTTTAATTTTGGGTATTATGTGGAG
ATA
+
F:,F,:,FFFFFF:FF:FFFFFFF,F,,FF:FF:FFFF::F,:FFFFFF:FFF:FFFFF:FFF:F,FF
,F:FFFFFFFFFFFF,:FFFF:FFFF:::FFFFFF:F:FFFF,F:FFFF:FFFF,:FFFFF:FFFFFF
FFF
@A00721:190:HGJW7DSXY:4:1323:10917:30232_2:N:0:TCTGCAAG+GCTGCACT
ACCACATTTTGGTCAAGTCAATCCGTTGAGAAAATTTCTTAGAGGATATTCGCCATTGTGGTACGAAT
TGGTCCAACATTTACCACCAAATTCTTTTGATACAACTCTAGCCTTCA
+
FFFFFFFFFFFFFFFFFFFFFFFFFFFFFFFFFFFFFFFFFFFFFFFFFFFFFFFFFFFFFFFFFFFFFF
FFFFFFFFFFFFFFFFFFFFFFFFFFFFFFFFFFFF:FFFFFFFFFFF
@A00721:190:HGJW7DSXY:4:1224:26621:27211_2:N:0:TCTGCAAG+GCTGCACT
TCACCAGATTGGTTCGAACCCGTTGATGCTATGTCAGAAGAAGCTATTCAAGCTTGTCGTTTAAAGAC
TGCAGAGGCTGTCAAACAAGCCCAGACTCAAGGAGTCCAATCAAAGGT
+
FFFFFFFFFFFFFFFFFFFFFFF:FFFFFF:FFFFFFFFFFFFFFF,FFFFFFFFFFFFFFFFFFF,FF
FFFFFFFFFFFFFFFFFFFFFFFFFFFFFFFFFFFFFFFFFFFFFFFF
@A00721:190:HGJW7DSXY:4:1344:7699:20838_1:N:0:TCTGCAAG+GCTGCACT
ATTATTTAGAGATCTTTTCTACTTACAAAGGACAATTCAGTTTCATTAATTCTACGGAAAAGTCCGGA
GTATATTGTATGTTAATAGATAGTGGGATAATAGTTCTACCGATACGTAGTGAACGCGTTCGTAGAAC
CAA
+
FFF:FFFFFFFFFFFFFFF,FFFFFFFFFFFFFFFFFFFFF:FFFFFFFFFFFFFFFFFFFFFFFFFFFF
FFFFFFFFFFFFFFFFFFFFFFFFFFFFFFFFFFFFFFFFFFFFFFFF:FFFFFFFFFFFFFFFFFFFF
FFF
@A00721:190:HGJW7DSXY:4:1370:29803:18067_2:N:0:TCTGCAAG+GCTGCACT
TATTGATTATGTAACCGATAGGAGTTGTATAGTTGAAGATGTGGCTTTAGACGATAATCCTTTAAGGA
```

CTATGTTGACTGACGAAGATTTTCCCACAGATTTGTCTACGTTGCAGA
+
FF:FFFFFFFFFFFF:FFFFFFFFFFFFFFFFFFFF:FFFFFF:FFFFFFFFF:FF::FFFFFFFFFF
FFFFFFFFFFFF,FFFFFFFFF:FFFFFFFFFFFFFFFFFFFFFFF:FF
@A00721:190:HGJW7DSXY:4:1646:10339:19241_1:N:0:TCTGCAAG+GCTGCACT
CTATATACATTATAAAATATAAAAATAAGGGAGTCATAACTTTTGTTACCAAATTCTTGGCTTTCAAC
GAAGTCTCTGATTTTATTAATCATTACTTTAACGATCTCTCGGTCATTTACTCTGTACTTGATCTACG
TCG
+
FFFFFFFFFFFFFFFFFFFFFFFFFFFFFFFFFFFFFFFFFFFFFFFFFFFFFFFFFFFFFFFFFFFF
FFFFFFFFFFFFFFFFFFFFFFFFFFFFFFFFFFFFFFFFFFFFFFFFFFFFFFFFFFFFFFFFF:FF
FFF
@A00721:190:HGJW7DSXY:4:1666:25771:32659_2:N:0:TCTGCAAG+GCTGCACT
GAATGACTCTATCTTTTCAGACACTTCTTATATTATTTACAATAGTTAATATCATATTAGTAATACTC
ACTATCGTTTTCTGCTTTTTCTTAGCTAATAGATTAATAAAGGATGAG
+
FFFFFFFFFFFF:FFFFFFFFFF:FFFFFFFFFFFFFFFFFFFFFFFFF:FF:FFFFFFFFFFFF:F:
FF:FFFFFFFFFFF:FFFFF:F:FFFFFF:FFFFFFF:FFF:,F::FF
@A00721:190:HGJW7DSXY:4:1173:28330:12352_2:N:0:TCTGCAAG+GCTGCACT
ACTCTTCGTTACGAGACCTGATGGTAGAATAGATTTAGTCTCTACACCCATCTCAACGAGGATAAAGC
GAGTTTTCCGTCCTGAACGTAATACACGAAGAGATCATCCGCAAACGA
+
FFFFFFFFFFFFFFFFFFFFFFFFFFFFFFFFFFFFFFFFFFFFFFFFFF:FFFFF:FFFFFFFFFFF
FFFFFFFFFFFFFFFFFFFFFFFFFFFFFFFFFFFFFFFFFFFFF:FFF
@A00721:190:HGJW7DSXY:4:2504:4851:15718_2:N:0:TCTGCAAG+GCTGCACT
TTTAGATTATGAATCCTGCCTTTACTCATTTTATAGCAGTTATTTATGTTGTACTTTTTATAATTTAT
AACCTTCTTATACTTTTGGGTATGTTTTGTGAAAAACATGAGATACCC
+
FFFFFFFFFFFFFFFFFFFFFFFFFFFFFFFFFFFFFFFFFFF:FFFFFFFF:FFFFFFFFFFFFFFF
FFF:FFFFFFFFFFFFF:FFFFFFFFFF:FF:F:F::FFF:FFFFFFF
@A00721:190:HGJW7DSXY:4:2178:16260:24549_2:N:0:TCTGCAAG+GCTGCACT
TTGTAACGATCATTCTTTCGAGTTTTGTCATTCTTATTCTTTTATTCCTTTAACTGTGTATGAGGAGG
ATTTATAATGCTTTTCGATAGTGATTGTTATCAATTTAAATACATTAC
+
FFFFFFFFFFFFFFFFFFFFFFFFFFFFFFFF:FFFFFFFFFFFFFFFFFFFFFFFFF::FFFFFFFFF
FFFFFFFFFFF:FFFF:FFF:FFFFFFFFFFFFFF:F::FFFFFFFFF
@A00721:190:HGJW7DSXY:4:1609:1524:6292_2:N:0:TCTGCAAG+GCTGCACT
TCACAGACTCCGAAAATGGTTTCAAATGAGGTCAAAGCTCTATTTATTAAACGAACTATTATCCGATT
CTTCGAGGGTATCACTACCACTGATCTGCCGACTTTAGTCAAGGAACT
+
FFFFFFFFFFFFFFFFFFFFFFFFFF:FFFFFFFFF,FF,FFFFFFFFFFFFFFFFFFFFFFFFFFFFF
FFFFFFFFFFFFFFFFFFFFFFFFFFFFFFFFFFF:FFFFFFFFFFFFF
@A00721:190:HGJW7DSXY:4:1410:15619:25692_2:N:0:TCTGCAAG+GCTGCACT
CGATGTTTATATTTTTCTATAAGGGAAAATAGCAATAATCAAATTTCGACAAATTTTAGAACTTTATT
TCAGAACTCTCTCCGATCACCTTGGTGTGATCTCCCGGAGGATCTTTT
+
FFFFFF:FFFFFFFFFFFF:FFFFFFFFF,FFFFFFFFFFFFFFFFFFFFFFFFF:F,FFFFFFFFFF:
:FF:FFFFFFF,FFFFFFFFFF,FF:FFFFFFF,FFFFFFFFFFFFFF
@A00721:190:HGJW7DSXY:4:2123:9959:4492_2:N:0:TCTGCAAG+GCTGCACT
ATTTTTAAATAGTGATATTCAGCGTATACTTGAATACTTTATCAAAGATTTGGCTTGGTCTATTTACC
GCAAAAAGTTGGAAGAGGTCTATGATATTTATTTTACTTATACTTTAA
+
FFFFFFFFFFFFFFFFFFFFFFFFFFFFFFFFFFFFFFFFFFFFFFFFFFFFFFFFFFFFFFFFFFFF
FFF:FFFFFFFFF,FFFFFFFF:FFFFFFFFFFFFFFFFFFFFFFFF,

```
@A00721:190:HGJW7DSXY:4:1639:28546:22247_1:N:0:TCTGCAAG+GCTGCACT
AACTTGACTGTTGATAATATTACAATAGCTCTTTCTCAGAAGATTAGAGAAGTAACCGGTGAACACGT
CGGTAGCATTGATTTACTTTTACCGTTAGAACTGAAAAAGATTCTTTTCGAAAAGAAACCTATAAGTT
ACA
+
FF:FFFFFFFFFFFFF:FFFFFFFFFFFFF,FFFFFFFFFFFFFFFFFFF:FFFFF:FFFFFF:FFFF
F:FFFFFFFFFFFFFFFFFFFFFFFFFF:FFFFFFFFFFF::FFFFFFFFFFFFFFFFFFFFFFFFFFF
FFF
@A00721:190:HGJW7DSXY:4:1505:1850:18756_1:N:0:TCTGCAAG+GCTGCACT
TATATTAAAGAAATAAAAATACTTCAAGTACCTGAACTTTACATTTTTAATGGTATGGTATAATGTGT
CTTACAAATAGAACCTTCACTCTCGGTAATGAGAGTGTATTCCTTAATATTCTAGTAACCGCTATGTG
GGC
+
FFFFF:FFFFFFFFFFFFFFFFFFFFFFFFFFFFFFFFFFFFFFFFFFFFFFFFFFFFFFFFFFFFFFF
FF:FFFFFFFFFFFFFFFFFFFFFFFFFFFFFFFFFFFFFFFFFFFFFFFFFFF:FFFFFFF,:FFFF
FFF
@A00721:190:HGJW7DSXY:4:1638:30472:3787_2:N:0:TCTGCAAG+GCTGCACT
TTTAAATGCTAGGAAAATTTTAGAAAAACTCCATTGTATAGTTCAAGGAGTACTTAATTCAATAGCTT
TAGTATTATTTACTATCGCCTTTATTGAAAATTGTATAAGAGTTAATT
+
FFFFFF:FFFFFFFFFFFFFFFFFFFFFFFFFFFFFFFFFFFFFFFFFFFFFFFFFFFFFFFFFFFFF
FFFFFFFFF:FFFFFFFFFFFFF:FFFFFFFFFFFFFFFFFFFFFFFFF
@A00721:190:HGJW7DSXY:4:1363:21730:7404_1:N:0:TCTGCAAG+GCTGCACT
ACATACCATAACCTACCATGACCAATCATCGCTCCAACTGGCACGCCAGACGTAGGGTCAAACCTACT
ACCGCCGGTCGGCAGATCGGCACCACTGGTCTCTACGTTCAGGGACCCGCTGTGCAGAATAACCTTCC
CAA
+
FFFFFFFFFFFFFFFFFFFFFFFFFFFFFFFFFFFFFFFFFFFFFFFFFFFFFFFFFFFFFFFFFFFF
FFFFFFFFFFFFF:FFFFFFFFFFFFFFFFFFFFFFFFFFFFFF:FFFFFFFFFFFFFFFFFFFFFFFF
FFF
@A00721:190:HGJW7DSXY:4:1304:28330:21245_2:N:0:TCTGCAAG+GCTGCACT
CACAGAAGAAGGCTGAAGGTCGTTACCGGACGACCACTCAAAGGAAACGGGCTGTTGCCCAGAGGAAA
GCAGCGACCACCGAGGTCGCTCGTAACATTGACGGCAGTCCCGTCATC
+
FFFFFFFFFFFFFFF:FFFFFFFFFFFFFFFFFFFFFFFFFF,FF:FFFFFFFFFFFFFFFFFFFFFFF
FFFFFFFFFFFFFFFFFFFFFFFFFFFFFFFFFFFFFFFFFFFFFFFFF
@A00721:190:HGJW7DSXY:4:1117:15691:3991_1:N:0:TCTGCAAG+GCTGCACT
CTGAGGCAGTACCTCAGTCAAAAGTCACATTCACCACTCCCATACCAATCAATGATGTGACTATACAG
TTCGGCCATTTCAAGAGTGGTCAATACGTCGTGGGAGGTACTGAAGCCTTCCCCTCCGCTGGCACGAA
GAA
+
FFFFFFFFFFFFFFFFFFFFFFFFFFFFFFFFFFFFFFFFFFFFFFFFFFFF,FFFFFFFFFFFFFFF
FFFFFFFFFFFFFFFFF:FFFFFFFFFFFFFFFFFFFFFFFFFFFFFFFFFFFFFFFFFFFFFFFFFF
FFF
@A00721:190:HGJW7DSXY:4:1142:3125:7999_1:N:0:TCTGCAAG+GCTGCACT
CCAATCAATAGTTCTTCTGACCAGAATGAAAAGGGGTTTACCAGGGTAGTCTCTAAGTCTACCAGGAA
GAGGGGAAATGTCCCCAAAACTGAGAAGAATAAAGGGAAAGGTCCATCAGGACCTAAGAAGGGGAAAT
TAC
+
F,FFFFFFFF:FF:FFFFFFFFFFFFFFF:FFFFF,FFFFFFFFFFFFFFFFFFFFFFFFFFFFFFFFF
FFFF:FFFFFFFFFFFFFFFFFFFFFFFFFFFFFFFFFFF:FFFFFFFFFFFFFFFFFFFF:FFFFFFF
FFF
@A00721:190:HGJW7DSXY:4:1114:4689:25895_2:N:0:TCTGCAAG+GCTGCACT
GGAAGATCTCCCACCATCTGGGGTTGGGAATAAGAAATCTGCACGGGCCAAAGAGGCCGCTGCTACGA
```

AGTCGCTGAAGGCGGCTTGGGTCATTAAGGGTAGCTGTAAAGTTGCGC
+
FFFFFFFFFFFFFFFFFFFFFFFFFFFF,:FFFFFFFFFFFFFFFFFFFFFFFFFFFFFFFFFFFFF
FFFFFFFFFFFFFFFF,FFFFFFFFFF:FFFFFFF:FFF:FFFFFFFF
@A00721:190:HGJW7DSXY:4:1217:13114:1939_1:N:0:TCTGCAAG+GCTGCACT
ATCTTTGAGACCAAGACTCTCTACAGTAGGTTCGTGCCTACTATGAGCAAATCTGTTGAATTCGTCAC
TGAGGACGAAGGTGAGAGGACTTTTGGCTACTGGCGACAAACTGAGAGCAGTCGCTGGGGAAAGAGAA
TTG
+
FFFFFFFFFFFFFFFFFFFFFFFFFFFFFFFFFFFFFFFFFFFFFFFFFFFFFFFFFFFFFFFFFFFF
FFFFFFFFFFFFFFFFFFFFFFFFFFFFFFFFFFFFFFFFFFFFFFFFFFFFFFFFFFFFFFFFFFFF
FFF
@A00721:190:HGJW7DSXY:4:2307:11270:28087_2:N:0:TCTGCAAG+GCTGCACT
ACGCTTCGAGCTTGAGTGTGACGAGGCCGGTGACGTTTTTGTCAACGGCGTTTGGAGGATTTTATCCG
CGCTTAACTATGGGGCGAAGATCCCCTATGGAGTCTTGAAGGACTTAG
+
FFFFFFFFFFFFFFFFFFFFFFFFFFFFFFFFFFFFFFFFFFFFFFFFFFFFFFFFFFFFFFFFFFFF
FFFFFFFFFFFFFFFFFFFFFFFFFFFFFFFFFFFFFFFFFFFFFFFF
@A00721:190:HGJW7DSXY:4:2174:32416:6151_1:N:0:TCTGCAAG+GCTGCACT
GCATGTCACCCTTAAGCACCGAGGTAAGCTCGGGACTTACAAGAGAGAGTGTGAGAGAGTCGCTCTCA
CTATTTTTGCCCAGAATGCGGGCTTTCTGACTGATGAGGTTAAAACTCTTGCTCCTTCTGTGGGTGAG
GGT
+
FFF:FFFFFFFFFFFFFFFFFFFFFFFFFFFFFFFFFFFFFFFFFFFFFFFFFFFFFFFFFFFFFFFF
F:FFFFFF,FFFFFFFFFFFFFFFFFFFFFFFFFFFFFFFFFFFFFF,FFFFF:FFF:FFFFFFFFFF:F
FFF
@A00721:190:HGJW7DSXY:4:1249:30761:20040_2:N:0:TCTGCAAG+GCTGCACT
GGTTCTGGGAGGCTGCTTTCCTCCATAATTACAACCTCTCTTTAGAGAGCATTAGAAGGAAGTTGGGC
AAATTCCCACATATGGTAGACGTTGTCACACTTTTCTTGAAAATTTTT
+
FFFFFFFFFFFFFFFFFFFFF:FFFFFFFFFFFFFFFFFFFFFFFFFFFFFFFFFFFFFFFFFFFFFF
FFFFFFFFFFFFFFFFFFFFFFFFFFFFFFFFFFFFFFFFFF:FFFFF
@A00721:190:HGJW7DSXY:4:2112:13123:9439_1:N:0:TCTGCAAG+GCTGCACT
TTGGGGATTTCGATGTGAGAAGGTGGAAGGTGTCAAGGGTCTTCTTCATGCTGATCTTTCAGGACCTA
ATCACATCATGATTGATAGTTACGGCTCGTATCGAGTTGGAGGGAGGAAGTTCTCCGAAATTAAAGAC
GGG
+
F:FFF::FFFFF,F,F:F,:FFFFFF::FFFF:FF::FFF,F:,FF,:FF:F:FF:FFFFFFFF::FF
FFFF,FF,,F,:FFFFFFFF::FFF:FF:F:FF:F:FFFFFF,F:FFFFFFFF:FF,FFFFFFF,FFF
FFF
@A00721:190:HGJW7DSXY:4:2261:23231:7247_2:N:0:TCTGCAAG+GCTGCACT
TCAGAGAGGGGAGCTCTGAAAAGGAAACTTCGTCAGAAGATGAGGAAGTCTAAGAGGAGGAGTTTGAG
GAAGGCTCTAGAGAAGATCTCACCACCTGGAGAAGGCAAGTGGAGAGT
+
FFFFFF:FFFFFFFFFFFFFFFFFFFFFFFFFFFFFFFFFFFFFFFFFFFFFFFFFFFFFFFFFFFFF
FFFFFFFFF:FFFFFFFFFFFFFFFFFFFFFFFFFFFFFFFFFFFFFF
@A00721:190:HGJW7DSXY:4:1501:8296:4523_2:N:0:TCTGCAAG+GCTGCACT
AAGAAGGATTATTTGAAGCAGGGGTTACCACTTCCTAAGGTGGATGAGGGAGGTTGTTTCTTTGGAGT
TTTAGATAATCTTTTGAGAAGGGGTCATAGAGCCAGCAAGAGAAGCTT
+
FFFFFFFFFFFFFFFFFFFFFFFFFFFFFFFF:FFFFFFFFFFFFFFFFFFFFFFFFFFFFFFFFFFF
FFFFFFFF,:FFFFF:FF:FFFFF:FFFFFFFFFFFFFFFFFF:FFFF
@A00721:190:HGJW7DSXY:4:1540:14913:12978_2:N:0:TCTGCAAG+GCTGCACT
TATAATTTAGGTCAACTTGGGGTTTCGAATGTCATTAGTGCCTTACGCTTGAAAGAGGTCCTTTTGAG

TATCGACAGATCTTTCGATCTTGAGGACGTACATTTAACGTGGCAGAA
+
FFFFFFFFFFFFFFFFFFFFFFFFFFFFFFFFFFFFFFFFFFFFFFFFFFFFFFFFF:FFFFFFFFFFF
FFFFFFFFFFF:FFFFFFFFFF::FFFFFFFFFFFFFFFF,FFF:FFF
@A00721:190:HGJW7DSXY:4:1436:7817:7232_2:N:0:TCTGCAAG+GCTGCACT
ATATTGTTCCTTCCGGGAAGTTGTGGGATTTAACTTTGACTCAACTACTCCGATCAGATCCGGATGGT
TTGATTGGTATGGACTACTCGACTTTTGATCTGGAGTTGTCTAGGGTC
+
FFFFFFFFFFFFFFFFFF,FFFF:FFFFFFFFFF:FFFFFFFFFFFFFFFFFFFF:FFFFFFFF,FF:
FFFF:FFFFFFFFFFFFFFFFFFFFFFFFFFFFFFFFFFFFFFFFFF
@A00721:190:HGJW7DSXY:4:2533:12608:14935_2:N:0:TCTGCAAG+GCTGCACT
CGATAAGGTGAAAAATATGCAGGTCAAAGATTCTGTAGCTCTGAGGGCGGTTGAGGGAGCCGTTTTGG
CCGACTTTAACTATTCTAAGAGTCTTTATGATTCAAAACCTATCGTTG
+
FF:FFFFFFFFFFFFFFFFFFFFFFFFFFFFFFFFFFFFFFFFFFFFFFFFFFFFFFFFFFFFFFFFF
FFFFFF:FFF,FFFFFFFFFFFFFFFFFFFFFFFFFF:FFFFFFFFFF
@A00721:190:HGJW7DSXY:4:2107:27778:22576_2:N:0:TCTGCAAG+GCTGCACT
GGCTTATTGTCGCGCAGTTTCCAGAATTGCGGTTGAAGTTCACCCATTCTATCTTGAATGCTCACGCC
GTCGCAGCCATATCGAGACTATGTTATAATCACTTGTTAAAGTACAGA
+
FFFFFFFFFFFFFFFFFFFFFFFFFFFFFFFFFF:FFFFFFFFFFFFFFFFFFFFFFFFFFFFFFFFFF
FFFFFFFFFFFFFFFFFFFFFFFFFFFFFFFFFFFFFFFFFFFFFFFF
@A00721:190:HGJW7DSXY:4:1320:15628:24298_2:N:0:TCTGCAAG+GCTGCACT
ATTATTGATATTGGAGGCGATATTCGTTTTCATATCCAAAACTCCTTGAATGGAGTACATATTTGCAA
CCCTATTCTTGATGTGAAAGATGCATCGAGGTATACGTCTAGGAACAT
+
FFFFFFFFF,FFFFFFFFFFFFFFFFFFFFFFFFFFFFFFFFFFFFFFFFFFFFFFFFFFFFFFFFFF
FFFFFFFFFFFFFF:F,FFF,FFFFF:FFFFFFFFFFFFFFFFFFFFF
@A00721:190:HGJW7DSXY:4:2519:11080:15890_1:N:0:TCTGCAAG+GCTGCACT
CACGAGACGTCATGAAAGCTATCGGAGGGAAGCAAAATGTTTCGTTCTGTTACAAGAACGCTTTGGAT
TGTGACCATAAAGCCTCAGTTGCGATAGCTGTAGAGGTATACGATATCTCTCTTGATGATGTAGCCAC
TAT
+
FFFFFFFFFFFF:FFFFFFFFFFFFFFFFFFFFFFFFFFFFFFFFFFFFFFFFFFFFFFFFFFFFFFF
FFFFFFFFFFFFFFFFFFFFFFFFFFFFFFFFFFFFFFFFFFFFFFFFFFFFFFFFFFFFFFFFFFFF
FFF
@A00721:190:HGJW7DSXY:4:2636:4146:9580_2:N:0:TCTGCAAG+GCTGCACT
GATTGATGTATGTCATTTCGTCTTAGTTTGTCCCGGCGAATTATTGGACACGATGAAAAATGACATAC
CCGTTCCTGACCTTGGTATCGTTATCCGTCATGGAGATGATGGCGCAT
+
FFFFFFFFFFFFFFFFFFFFFFFFFFFFFFFFFFFFFFFF:FFFFFFFFFFFFFFFFFFFFFF:FFFFF
FFFFFFFFFFF:FFFFFFFFFFFFFFFFFFFFF:FFFFFFFFFFFF:FF
@A00721:190:HGJW7DSXY:4:1530:8142:35697_2:N:0:TCTGCAAG+GCTGCACT
AACAAAGATAAGATTTTATCTTTCTTCAAACACCCTGTTTTCCTTAAGGGCGGACAATTGTTCCATTG
TGAACTCTTGGGGACTAGATTGAGCATTAATGAGTTCCGAATAACTCG
+
FFFFFFFFFFFFFFFFFFFFFFFF:FFFFFFFFFFFFFFFFFFFFFFFFFFFFFFFFFFFFFFFFFFF
FFFFFFF:FFFFFFFFFFF:FFFFFFFFF,FFFFFFFFFFFFFFFFFF
@A00721:190:HGJW7DSXY:4:1331:14299:3740_2:N:0:TCTGCAAG+GCTGCACT
TATTTCCGTACCAAGAGCCACTGCCAATCTCATCGCTATACGCATACCTGATTGGAAGAAGCGAGGTT
CGTTCCTCAATAAAAATTTTTCTCAAGAGATTATGGAGATCGGAAGAG
+
FFFFFFFFFFFFFFFFFFFFFFFFFFFFFFFFFFFFFFFFFFFFFF:FFFF,FFFF:FFF,FFFFFFFF
FFF:FFFFFF,FF,FFFFFFFF:FFFFFF:F:::FFFFFFFFFFFFF

@A00721:190:HGJW7DSXY:4:2538:32805:14685_1:N:0:TCTGCAAG+GCTGCACT
TTCAGTACGTCATGGGGATCACTTCTTCGGTCAATGACAAAACCTTCGAATATGTTACGACATGGCTT
CGTAACAACAGTGCGAGGATTGTCATCTCGGGGAAAGTGATTCACCAAGATGTTCATATAGATACGAG
ATA
+
FFFFFF,FFFFFFFFFFFFFFFFFFFFFFFFFFFFFFFFFFFFFFFFFFFFFFFFFFFFFFFFFFFFF
FFFFFFFFFFFFFFFFFFFFFFFFFFFFFFFFFFFFFFFFFFFFFFFFFFFFFFFFFFFFFFFFFFFF
FFF
@A00721:190:HGJW7DSXY:4:2218:31819:13761_2:N:0:TCTGCAAG+GCTGCACT
CAGCGATCTTACTCGTTATCGGAGCTAAGAAGAAGACTTTGGGTACACGTTATGCTCGCAAACTTAAC
GTTAGTACCGGAGGTGATCTCTCTGCTTCTGCGTTACTGAGAGAAAAG
+
FFFFFFFFFFFFFFFFFFFFFFFFFFFFFFFFFFFFFFFFFFFFFFFFFFFFFFFFFFFFFFFFFFFF
FFFFFFFFFFFFFFFFFFFFFFFF:FFFFFFFFFFFFFFFFFFFFFFF
@A00721:190:HGJW7DSXY:4:1111:27344:29684_1:N:0:TCTGCAAG+GCTGCACT
CTTTATGGACAAGATGCAAGTTCTTGGGAAATACTTGTTTCCCTTTTTAGGTTTAGTAGGTGATCTTG
ACGTCGATTCTTTGTATAGGGAGTGTGAGAAGACAACGAATCTTGAAACTACGGTCCTTATCAAAGAA
AAT
+
FFFFFFFFFFFFF,FFFFFFFFFFFFFFFFFFFFFFFFF:FFFFFFFFFFFFFFFFFFFFFFF,FFFFF
FFFFFFFFFFFFFFFFFFFFFFFFFFFFFFFFFFFFFFFFFFFFFFFFFFF:FFFFFFFFFFFFFFFF
FFF
@A00721:190:HGJW7DSXY:4:2569:5041:9533_1:N:0:TCTGCAAG+GCTGCACT
TCTAAATCTTTTGAGATTGAGAAGATTGAAGAACACGTCAAGAAGTCGCTCGGCGTTGTTCTTGAAAA
CCAAGTTAAGAAGGAGGCTGATCATGGAGTCTTTGTACCTTCTGTCAAACTTCGAGGTTCAGCTACGA
GAG
+
FFFFFFFF:FFFFFFFFFFFFFFFFFFF:FFFFFFFFFFFFFFFFFFFFFFFFFFFFFFFFFFFFFFF
FFFFFFF:FFF:FFFFFF:FFFFF:FFFFFFFFFFFFFFFFFFFFFFFFFFFFFFFFFFFFFFFFFFF
FFF
@A00721:190:HGJW7DSXY:4:1202:11189:37027_2:N:0:TCTGCAAG+GCTGCACT
TTGAAGGCTGGCTCTAAGGGAGACTCCTTCTTTTTGAATGTTGTAAAGAATTGGGTTGAGAAGTGTGC
TTTACAAGGAGTTAGACTCTTCTTCAAGTTTTTAGATCTTCTTGATAA
+
FFFFFF:FFFF:FFFF:F,FF:FFFFFF:F,FFFFF,FFFFFFFFFFFFFFFFF:FFFFFFFFFFFFF
FFFFFFF,:F:FFF,F:FFF::F:FFF:FFFFFF:FF,FFFFFFFFFF
@A00721:190:HGJW7DSXY:4:1224:1949:24659_1:N:0:TCTGCAAG+GCTGCACT
CTTGGTATTTCTGAAATAGTTTATCAGAGCGTCATTTTCCTTTGGGATCTTTCCTCTGGTAAGGGAAT
TATTAAATCATTCTTCTCACTCTTGACGAGAATTATTACTGTTTTCTCTACCCATAACTTCTTTTTCA
AAA
+
FFFFFFFFFFFFFFFFFFFFFFFFFFFFFF:FFFFFFFFFFFFFFFFFFFFFFFFFFFFFFFFFFFFF
FFFFFF,FFFFFFFFFFFFFFFFFFFFFFFFFFFFFFFFFFF:FFFFFFFFFFFFFFFFFFFFFFFFF
FFF
@A00721:190:HGJW7DSXY:4:1544:29794:7091_1:N:0:TCTGCAAG+GCTGCACT
CTGATGTTTTCCTACAGAGTGCAGTCGTTTCAACACTTATCACCACTTTTGAAAGGAAAATAATTGGT
GATGAAAATACGTATGTCGGTGGGGTTCTCAAGGCTCTCATCGCCACTGGGATTCTAAGGGGTTCCAT
AAG
+
FFFFFFFFFFFFFFFFFFFFFFFFFFFFFFFFFFFFFFFFFFFFFFFFFFFFFFFFFFFFFFFFFFFF
FFFFFFFFF:FFFFFFFFFFFFFFFFFFFFFFFFFFFFFFFFFFFFFFFFFFFFFFFFFFFFFFFFFF
FFF
@A00721:190:HGJW7DSXY:4:1439:23366:31876_2:N:0:TCTGCAAG+GCTGCACT
TTTGGTCCTTACGTCTTAAGTAATTTGGATGTTGTCGAAAACTATCAGGTTCTTGTCACGATTCTTAA

AAGAAGTTTATTAAAACTTCAGAGTCTCTTGGAAGTCCAGGTAGCTAA
+
:FFF:FFFFFFFFFFFFFFFFFFFFFFFFFFFFFFFFFFFFFFFFFFFFFFFFFFFFFFFFFFFFFFF
FFF:FFFFFFFFFFFFFFFFFFFFFFFFFFFFFFFFFFFFFFFFFFFF
@A00721:190:HGJW7DSXY:4:1646:1452:16971_2:N:0:TCTGCAAG+GCTGCACT
GGGAAAAGAGACCTCTGTTGGTAGAGGAGTTGAGAGAATTACTCAGATTCAATCTGGCATCACTGAAC
GTCTACAACGAGTTAGGGAGTGGCTAGGTTTTAAAAGATCCTTTGCAC
+
FFFFFF:F:FFFFFFFFFFFFF:FFFFFFFFFFFFFFFFFFFFFFFFFFFFFFFFFFFFFFFFFFFFF
FFFFFFFFFFFFFFFFFFFFFFFFFFFFFFFFFFFFFFFFFFFFFFFF
@A00721:190:HGJW7DSXY:4:2332:30544:6918_2:N:0:TCTGCAAG+GCTGCACT
TTTCGACGTCGCGGAAAACGATGAAGATTTCCACACTGTTCGAGAATCTTCTGATGCTGAATTTGAGG
AAAATTTTTCGGAAGTTTTTGGTATAAATCCTGATGGCTCAGTACAGG
+
FFFFFFFFFFFFFFFFFFFFFFFFFF:FFFFFFFFFFFFFFFFFFFFFFFFFF:FFFF:,FFF:FFFF
FFFFFFFFFFFFFFFFFFFFFFFFFFFFFFFFFFFFFFF:FF:FFF:
@A00721:190:HGJW7DSXY:4:1455:13693:6198_1:N:0:TCTGCAAG+GCTGCACT
AACCTTCTTTTATCATTGCAACAGGACTTTGAGAAGACTGTTGAAATGGACAAAATCTGGGATCATAG
GTATGTGTCACGCTACACTCGAAATATGGGCCCTCATAAGGAAGTATAGTTGGCGTCTTGTCGTTGAC
GTC
+
:FFFFF:FFFFFFFFFFFFFFFFFFFF:FFFFFFFFFFFFFFFFFFFFFFFFFFFFFFFFFFFFFFFF
FFFFFFFFFFFFFFFFFFFFFFFFFFFFFFFFFFFFFFFFFFFFFFFFFFF,FFFFFFFFFFFFFFFF
FFF
@A00721:190:HGJW7DSXY:4:1211:21151:10316_1:N:0:TCTGCAAG+GCTGCACT
TGTTCAGGAAGATCTCTACAGAGATTATCGAGAGTTGTGATCTAGTTCTAAATTTAGGTCGACGGACT
TTAGCAACTTTAGATAAATTCTTTCCGGGCTCAATGACCTTTGCTATGTACGTGGATCTAATGCTTGG
TGA
+
FFFF:F:FFFFFFFFFF:FFFFF:F:FFFFFFF:FFFFFFFF:F:FFFFFFFFFFFFFF:FFFFFFF:
FFFFFFFFFFFFFFFFFFFFFFFFFFFFFFFFFFFFFFFFFFFFFFFFFFFFFFFFFFFFFFFFF:FF
FFF
@A00721:190:HGJW7DSXY:4:2447:5719:2691_2:N:0:TCTGCAAG+GCTGCACT
CATCGCAAAGAAGACTTTATTAGTTCATGAAGTTCTTACACTTCTCGCGACTAATGTCAGCTTCACTA
CAAAGGCGTTACGTAAAGTAGTTTTCACGCTTTATCTTAAAGCATTAC
+
FFFFFFFFFFFFFFFFFFFFFFFFFFFFFFFFFFFFFFFFFFFFFFFFFFFFFFFFFFFFFFFFFFFF
FFFFFFFFFFFFFFFFFF:FFFFFFFFFFFFFFFFFFFFFFFFFFFFF
@A00721:190:HGJW7DSXY:4:1248:26946:21324_1:N:0:TCTGCAAG+GCTGCACT
ACTTTTTGAATATGACAGAAGCTCGCCATATCAGATTAGAGACATGCCAGTTAGAGTTCTTCTAACCA
GGACTGATGGGGAGATTCTTTTACCTTTTGGAGGTCCAGGAACCTTTACTCCTAGTGTCTTTTCTAGA
GTC
+
FFFFFFFFFFFFFFFFFFFFFFFFFFFFFFFFFFFFFFFFFFFFFFFFFFFFFFFFFFFFFFFFFFFF
FFFFFFFFFF,FFFFFFFFFFFFFFFFFF:FFFFFFFFFFFFFFFFFFFFFFFFF:FFFFFFFFFFFF
FFF
@A00721:190:HGJW7DSXY:4:1239:19036:32800_2:N:0:TCTGCAAG+GCTGCACT
CGGAGGATGACAGGTGGGAGACCGCTCAATAACACTCTATTGAATCCCTTTTCCACGATGAAAGCTCT
TTATGAGGTGCTCCATGCAAATTTACAGTTCCTCTTTGGTGATATCGC
+
FFFFFFFFFFF,FFFFFFFFFFFFFFF:FFFFFFFFFFFFFFFFFFFF:FFFFFFFFFFFFFFFFFFF
FFFFFFFFFFFFFFFFFFF,FFFFFFFFFFFFFFFFFFFFFFFFFFFF:
@A00721:190:HGJW7DSXY:4:1239:19036:32800_1:N:0:TCTGCAAG+GCTGCACT
TTTGATAAATTTGTTGGAATCGATAATGATACTGACGAAGGTTCTATTACCACCCTTGTCATTGATA

```
CAAACATTCTAGGTGATGCTTTCTTAGAGGCTCGTCTAGCCGCTGAGATTCGTCTTACCTATTTACAA
TCT
+
FFFFFFFFFFFFFFFF,FFFF,FFFFFFFFFFFFFFFFFFFFFFFFFFFFFFFFFFFFFFFFFFFFFF
FFFFFFFFFFFFFFFFFFFFFFFFFFFFFFFFFFFFFFFFFFFFFFFFFFFFFFFFFFFFFFFFFFFF
FFF
@A00721:190:HGJW7DSXY:4:1120:22200:33458_1:N:0:TCTGCAAG+GCTGCACT
GATAACGATTTAGTACTGGGAAATGGTGGTAGTCAGTTTACTACTTTTCTTTCCCTCCCCTTTTTCGA
TTGGATTCGACGGGGTCTTCGTCTCACGACTAATTTGATAAGGTTTTTCTTTTATGGGACTCTTGTTT
GTA
+
FFF:,,FFFFF,:F:FFF,:FFFFF:FFFFFFFFFFFFFFFF:FFFFFFFFFF:FFFFFF,FFFFFFF
FF,FFFF:::F:F,FFFFFFFF::F:,FFFFFFFFFFFFFFF:FFFFFFFFFFFF:FFFFFFFFFF
:FF
@A00721:190:HGJW7DSXY:4:2541:12644:29935_2:N:0:TCTGCAAG+GCTGCACT
TATTACTTCGGAAGTTCTTAATGTAGGTCTCCTAGTACTTAAGGGTTTATGTTTCGGTTTCAGCTGGA
TGGATCTTTTGATGGTATGTTTCACATTGCGTGATCACTTCCCGTGGA
+
FFFFFFFFFFFFFFFFFFFFFFFFFFFFFFFFFFFFFFFFFFFFFFFFFFFFFFFFFFFFFFFFFFFF
FFFFFFFFFFFFFFFFFFFFFFFFFFFFFFFFFFFFFFFFFFFFFFFF
@A00721:190:HGJW7DSXY:4:1129:32235:28698_1:N:0:TCTGCAAG+GCTGCACT
ATTAAACATAAAATCCAGCGATTCTATTATGTCGCTAAAGCCTGTCCAGTTGTCGATGATGTTCGGGC
TGTGGGGATTAACTTCCCTCGTGAAACTTACGACGATGTTCTCGCTCGCATTTCACAAGGATTACTAA
GTC
+
FFFFFFFFFFFFFFFFFFFFFFFFFFFFFFFFFFFFFFFFFFFFFFFFFFFFFFFFFFFFFFFFFFFF
FFFFFFFFFFFFF:FFFFFFFFFFFFFFFFFFFFFFFFFFFFFFFFFFFFFFFFFFFF:FFFFFFFFF
FFF
@A00721:190:HGJW7DSXY:4:1105:3088:22561_2:N:0:TCTGCAAG+GCTGCACT
ACTTCTAATTTTAGGAAACGTCGCGAAAAGAAAGTTCATGAGGATGTCTTGGTCGAAAAATATCGCCG
GGAACTTCTAATAGAGGATCCTGATGACATTATTTCAACGGATACCGA
+
FFFFFFFFFFFFFFFFFFFFFFFFFFFFFFFFFFFFFFFFFFFFFFFFFFFFFFFFFFFFFFFFFFFF
FFFFFFFFFFFFFFFFFFFFFFFFFFFFFFFFFFFFFFFFFFFFFFFF
@A00721:190:HGJW7DSXY:4:2665:10348:28682_2:N:0:TCTGCAAG+GCTGCACT
CACAGAAACCGTCTCAGGAACTGTCTCCACTGATTCGGAATTAGAGAAAGACGACCTTCTTGTGGGGA
CTGAGACCTCTGAATTTTCAGGGTGCCCTGATGAGGACGTTGAAAGGG
+
FFFFFFFFFFFFFFFFFFF:FFFFFFFFFFFFFFFFFFFFFFFFFFFFFFFFFFFFFFFFFFFFFFFF
FFFFFFFFFFFFFFFFFFFFFFFFFFFFFFFFFFFFFFFFFFFFFFFF
@A00721:190:HGJW7DSXY:4:1511:3414:1266_2:N:0:TCTGCAAG+GCTGCACT
CTTCTAAGAGTGTTTCAAAAGACCAGAAACGTAACAAGAAGAATAAAGGTAGGAAGGGGAGAAAACAG
AACAAGCGAGCGGTTGAAGAGAAAACTGATATGGTTGCGGCTGATCGT
+
FFFFFFFFFFFFFFFFFFFFFFFFFFFFFFFFFFFFFFFFFFFFFFFFFFFFFFFFFFFFFFFFFFF,
FFF:FFFFFFFFFFFFFFFFFFFFFFFFFFFFFFFFFFFFFFFFFFFF
@A00721:190:HGJW7DSXY:4:2126:6334:3850_2:N:0:TCTGCAAG+GCTGCACT
CTCGGCGATACCACTGATCTTCCAGAGTTACGTAGTGGTCGTTCTTTTGGTGATTTTATCAAAGAGAA
AGTTTCTGCTAGTCGCGACGATAGTATTTATCGGAGTGATATTAACAA
+
FFFFFFFFFFFFFFFFFFFFFFFFFFFFFFFFFFFFFFFFFFFFF:FFFFFFFFFFFFFFFFFFFFFF
FFFFFFFFFF:FFFFFFFFFFFFFFFFFFFF:FFFFFFFFFFFFFFFF
@A00721:190:HGJW7DSXY:4:1578:15736:9392_2:N:0:TCTGCAAG+GCTGCACT
TGTTGAAGTACCGCAACATGGATCAGTTTTCCAAAAAGCTATTAATAGCAGTTGCGGGGCTATAATAC
```

CGCCTCCAGATTACTCTAAACTGAATCCACTCTCTTGGGATATAAATG
+
FFFFFFFFFFFFFFFFFF:FFFFFFFFFFFFFFFFFFFFFFFFFFFFFFFFFFFFFFFFFFFFFFFFFFFF
FFFFFFFFFFFFFFFFFFFF:FFFFFFFFFFFFFFFFFFFFFFFFFFFFF
@A00721:190:HGJW7DSXY:4:1506:25120:6386_1:N:0:TCTGCAAG+GCTGCACT
AACATGAATTAGTTATTGAAAGTCTTGACCCTCACGTCTCACTTTATCACCTTGGAGATGGTAAAGTC
ATTAACAAAGGAAATGTTAAGGCTGATGACTTAAATTTCGCATTCTGCTTCGACGGTAATTCCATAAG
ACC
+
FFFFFFFFF:FFFFFFFFFFFFFFFFFFFFFFFFFFFFFFFFFFFFFFFFFFFFFFFFFFFFFFFFFFFFFFFFFF
FFFFFFFFFFFFFFFFFFFFFFFFFFFFFFFFFFFFFFFFFFFFFFFFFFFFFFFFFFFFFFFFFFFFFFFF
FFF
@A00721:190:HGJW7DSXY:4:1262:20302:20932_1:N:0:TCTGCAAG+GCTGCACT
AACCTTTCCTCGTTCTTCATGAAGATCTTCGAGTGTTTTATAGTGAGTTATTCTTGCGAAGGTTCAAG
TCGATTGATTTTGTTGACTACGATCTCTCTTGTAATAAGAAGTTCTTCGCAGTTGAATCTCCACCAGG
TGG
+
FFFF,:FFFFFFFFFFFFFFFFFFFFFFFFFFFFFFFFFF:FFFFFFFFFFF:FFFFFFFFFFFFFF
FFFFFFFFFFFFFFFFFFFFFFFFFFFFFFFFFFFFFFFFFFFFFF:FFFFFFFFFFFFFFFFFFFFFFF
FFF
@A00721:190:HGJW7DSXY:4:1140:19822:14121_2:N:0:TCTGCAAG+GCTGCACT
GCGACTTTTGTCGAATTTTTCGCAAAAGGTCTTGAAGTACGTATTGTCACTGCTAATCGTAATTCGCG
AGAAGAGATAGTGACTAAAGTTTTGGCACTTCTAGTTCATCGCGGTAT
+
FFFFFFFFFFFFFFFFFFFFFFFFFFFFFFFFFFFFFFFFFFFFFFFFFFFFFFFFFFFFFFFFFFFFFFFF
FFFFFFFFFFFFFFFF,FFFFFFFFFFFFFFFFFFFFFFFFFFFFFFF
@A00721:190:HGJW7DSXY:4:1413:12147:7028_2:N:0:TCTGCAAG+GCTGCACT
CAGACGTGAAAGTTATGATGAGTGATCGTATTAGAACAGCTGATTCTTATTGCATGAACGTCGCTGAG
GTCAAACCTGTGGACGTTCTTTTCATCGACGAATTATTCATGATTCAC
+
FFFFFFFFFFFFFFFFFFFFFFFFFFFFFFFFFFFFFFFFFFFFFFFFFFFFFFFFFFFFFFFFFFFFFFFF
FFFFFFFFFFFFFFFFFFFFFFFFFFFF:FFFFFFFFFFFFFFFFFFF
@A00721:190:HGJW7DSXY:4:1448:21603:23500_2:N:0:TCTGCAAG+GCTGCACT
CATCTACTTAAGGCGGCTTATACCATAGGTTACGGTGATTCCAATCAAATCGGATTCATCAATCGTAT
TGATCTCCAAGTCGCAAACTACTTCAACATCAACGACATCATTCTTGA
+
:FFFFFFFFFFFFFFFFFFFFFFFFFFFFFFFFFFFFFFFFFFFFFFFFFF:FFFFFFFFFFFFFFF
FFFFFFFFFFFFFFFFFFFFFFFFF:FFFFFFFFFFFFFFFFFFFFFF:
@A00721:190:HGJW7DSXY:4:2443:14452:13714_2:N:0:TCTGCAAG+GCTGCACT
TTATCGAAGTCTCAGTTATCGCTGCCCGGCCGACGTTTGTCGCGAACTCTCAGAGGTTTACGGCAGAA
AAATTTCTCCTGCTTGTAATTCGAGAGAGGACACTATGGAAATTAGAC
+
FFFFFFFFFFFFFFFFFFFFFFFFFFFFFFFFFFFFFFFFFFFFFFFFFFFFFFFFFFFFFFFFFFFFFFFF
FF:FFFFFFFFFFFFFFFFFFFFFFFFFFFFFFFFF:FFFFFFFFFFFF
@A00721:190:HGJW7DSXY:4:2215:8006:19805_2:N:0:TCTGCAAG+GCTGCACT
TATTACTTTCACTCAAGGTGAAAAGTTTGAAATTAACAAAGTCCTTGGTAAGAGTAAGGTTAAATGTG
AAGATGCTCAGACTATTCATGAAGTTCAAGGTAATACTTACCAGCACG
+
FFFFFFFFFFFFFFFFFFFFFFFFFFFFFFFFFFFFFFFF,FFF::FFFFFFFFFFFFFFFFFFFFFFFF
F:FFFFFFFFFFFFFFFFFFFFFFFFFFFFFFFFFFFFFFFFFFFF:F
@A00721:190:HGJW7DSXY:4:2208:18810:17691_2:N:0:TCTGCAAG+GCTGCACT
TTTTGCATCACAAACACATATTACCGTTGCTCTATCACGACACGTTGAATCTTTACGATACTTTGTTA
TCAATGTAAAGTTAAACGATGCTACTTGTGAGAGGATCAATCACAGTA
+

```
FFFFFFFFFFFFFFFFFFFFFFFFFFFFFFFFFFFFFFF:FFFFFF:FFFFFFFFFFFFF,FFFFFFFF
FFFFFFFFFFFFFFFFFFFFFFFFFFFFFFFFFF::FFFFFFFFFFFF
@A00721:190:HGJW7DSXY:4:1563:3513:31563_2:N:0:TCTGCAAG+GCTGCACT
ACGGGAGGTTGCAGATTCAACCCCTTATATCGAGGCTATCGAGGACGATGAGTATCTTGAAGTTGTTG
ACGACAAGCCTCAAGAGGGTAGAGGACCACCTGGATCTTCTACTGTCT
+
FFFFFFFFFFFFFFFFFFFFFFFFFFFFFFFFFFFFFFFFFFFFFFFFFFFFFFFFFFFFFFFFFFFF
FFFFFFFFFFFFFFFFFFFFFFFFFFFFFFFFFFFFFFFFFFFFFFFF
@A00721:190:HGJW7DSXY:4:1565:2410:22294_1:N:0:TCTGCAAG+GCTGCACT
AACCACCTTAACGTCACTCGGTAATCTTTCAGCCGAATTAAGTACTAATGAGTTCAAGTCGGTTGTCG
ATCGAATTAAGATCAAGGCTAATACCTTTATAGGTAAGAAGCCCCTTGAGCCGCGTAGTCAGCGCGTT
TAG
+
FFFFFFFFFFFFFFFFFFFFFFFFFFFFF:,FFFFFFFFFFFFFFFFFFFFFFFFFFFFFFFFFFFFF
FFFFFFFFFFFFFFFFFFFFFFFFFFFFFFFFFFFFFFFFFFFFFFFFFFFFFFFFFFFFFFFFFFFF
FFF
@A00721:190:HGJW7DSXY:4:2171:9914:5979_2:N:0:TCTGCAAG+GCTGCACT
CCGATAGGGTACCGAGTCTCCAAGAGAATATTTATTCTTATGAGGCTCGAAACTTCAACGTTTTCACA
ACGGAAAGACATATCGCACCCGAAATTTTTGGGCAGAATGCTCTTAAC
+
FFFFFFFFFFFFFFFFFFFFFFFFFFFF:FFFFFFFFFFFFFFFF::FFFFFFFFFFFFFFFF:FFFFF
FFFFFFFF:FFFFFFFFFF,FFFFFFFFFFFFFF:FFFFFFFFFFFFF
@A00721:190:HGJW7DSXY:4:2603:28230:18380_2:N:0:TCTGCAAG+GCTGCACT
CTAAGTTGGATAAAGTTATAGGAGACTCAGTTAACCTGAGAGCTCCTCAATTGATAGATTGGGTCAAC
AATAGGGACGCTAGTATACTTCGTAAAGTCGACAGCGAAATGGTCAAT
+
F,FF,:FFFFFFFFF,:FFFF:FFFFFFFFFFFFFFFFFFF,FFFFFFFF:F:FFF:FFFFFFFFFF,
FFFFF:FFFF,FF:FFFFF,FFF:FFFF,F:FFFFFFFFFF,F:FFFF
@A00721:190:HGJW7DSXY:4:1338:7789:11537_1:N:0:TCTGCAAG+GCTGCACT
TGACCTATTTTAAGCTCATGGTGAAAAGGGAAGCTAAGGTTAAACTTGATGCTTCAGCCTTTTCGAAG
CATAACCCCGCACAGAATATCGTTTATCACGCAAAATTTATTAATGCAATTTTCGGACCTATCTTTAA
GGT
+
FFFFFFFFFFFFFFFFFFFFFFFFF:FFFFFFFFFF:FFF,FFFFFFFFFFFFFFFFFFF:FFFFFFFF
FFFFFFFFFFFFFFFFFFFFFFFFFFFFFFFFFFFFFFFFFFFFFFFFFFFFFFFFFFFFFFF:FFFF
FFF
@A00721:190:HGJW7DSXY:4:2441:31403:24784_2:N:0:TCTGCAAG+GCTGCACT
CCAGTGACGATTTCTCCCGGAAGCTCTTCTCTCTCTTAGGAGGTCATGATGACTACTATAAGGGTGAA
ATCGATTTTTCGAAGTTTGACAAATCTCAAGATTTCTTTTGTAAAGCC
+
FFFFFFFFFFFFFFFFFFFFFFFFFFFFFFFFFFFFFFFFFFFFFFFFFFFFFFFFFFFFFFFFFFFF
FFFFFFFFFFFFFFFFFFFFFFFFFFFFFFFFFFFFFFFFFFFFFFFF
@A00721:190:HGJW7DSXY:4:1267:17363:10802_2:N:0:TCTGCAAG+GCTGCACT
TTCGATGCTGAATTTCTTGAACTTTGGAGTGCCAGTGAATATTTTTGTCACGCCAGGAGTATCCATAA
CGATTTGTCATTTACCACCTTTGCTCAACGACGAAGTGGAACGGCCAA
+
FFFFFFFFFFFFFFFFFFFFFFFFFFFFFFFFFFFFFFFFFFFFFFFFFFFFFFFFFFFFFFFFFFFF
FFFFFFFFFFFFFFFFFFFFFFFFFFFFFFFFFFFFFFFFFFFFFFFF
@A00721:190:HGJW7DSXY:4:2670:24162:33880_2:N:0:TCTGCAAG+GCTGCACT
TAATTGTATGATCACTTTGATGATTTTATCACTTTATTATGACCTTGGAAAGTTTTCGGCTGTGGTTG
TCGCTGGGGACGATTCCATTCTGTTTTCAAAGACTCCAATAACTGATC
+
FFFFFFFFFFFFFFFFFFFFFFFFFFFFFFFFFFFFFFFFFFFFFFFFFFFFFFFFFFFFFFFFFFFF
FFFFFFFFFFFFFFFFFFFFFFFFFFFFFFFFFFFFFFFFFFFFFFFF
```

```
@A00721:190:HGJW7DSXY:4:2146:20347:8202_2:N:0:TCTGCAAG+GCTGCACT
TGCTCGCGATTTATTCTTTTTGTCAATGATTACATCTACTTCGTTCCTGATCCTTACAAGATCATCGT
TAAGTTAGGTAAACCCCTAACAGATGTTACTCTTGTAGGACTTTGGGA
+
FFFFFFFFFFFFFFFFFFFFFFFFFFFFFFFFFFFFFFFFFFFFFFFFFFFFFFFFFFFFFFFFFFFF
FFFFFFFFFFFF:FFFFF,FF,FFFFFFFFFFFFFFFFFFFFFFFFFF
@A00721:190:HGJW7DSXY:4:1301:28646:7921_2:N:0:TCTGCAAG+GCTGCACT
AGGTTGTTGTTTGGCGATTAGCTCTTGCGACTAATATTCGATATTCGATTGAGGGTCGTTATACCTAT
TCGGCTATCTGTGCTTTGAATTGTATAAGATCAAATTTTAAACGATTC
+
FFFFFFFFFFFFFFFFFFFFFFFFFFFFFFFFFFFFFFFFFFF:FFFFF:FFFFFFFFFFFFFF:FFF
FFFFFFFFFFFFFFFFFFFFFFFFFFFFFFFFFFFFFFFFFFF:FFFF
@A00721:190:HGJW7DSXY:4:1106:31530:2644_1:N:0:TCTGCAAG+GCTGCACT
TGATGTGGAGGGAGTTCTTGAAGCATCCCGACCTTTCCTTGTGAAAATTTTGATGCGGTTTCATAAGA
TTATCAGTAATCCTGATTCTACTACGGATTCATGGACCTTCCATCCTGTACCTCTTCTGGAGAGACGT
ATC
+
FFFFFFFFFFFFFFFFFFFFFFFFFFFF::FFFFFFFFFFFFFFFFFFFFFFFFFFFFFFFFFFFFFF
FFFFFFFFFFFFFFFFFFFFFFFFFFFFFFFFFFFFFFFFFFFFFFFFFFFFFFFFFFFFFFFFFFFF
FFF
@A00721:190:HGJW7DSXY:4:1105:13358:32424_2:N:0:TCTGCAAG+GCTGCACT
CTTCGAGACAGGTGATATTGTTGAGCGAGAGCGACCAAGATCTAAATTCGGTAACGGTGCGTGGCGCT
TTATTTTAAAAAGCTCCCTCCTCTCGCAAGTTTGATCTGATACTTATC
+
FFFFFFFFFFFFFF:FFFFFFFFFFFFFFFFFFFFFFFFFFFFFFFFFFFFFFFFFFFFFFFFF::F:
FFFFFFFFFFFFFFF::FFFFFFFFFFFFFFFFFFF:FFFFFFFFFF,
@A00721:190:HGJW7DSXY:4:2177:1506:34726_2:N:0:TCTGCAAG+GCTGCACT
TGATCAGTCTCTGACTTGACCATCTTTCATTTTAAATGACGATCATTAAGACTCTCTTTCTTCTTTGT
TTCCTTCTTTGCACTGATTTTTCCTCATCAGCTTGTGTTTTACCTAAT
+
FFFFFFFFFFFFFFFFFFFFFFFFFF:FFFFFFFFFFFFFFFFFFFFFFFFFF:FF,FF,FFFFFF:FFF
FFFFFFFFFFFFFFFFFFFFFFFFFFFFFFF:FFFFFFFFFFFFFFFFF
@A00721:190:HGJW7DSXY:4:1331:10122:15765_2:N:0:TCTGCAAG+GCTGCACT
GATAACAACTATCAGTTGATTAAGACTCTTCCGAAAGGCGAACTTTGGGAATTTGAAGCTCAGGGTGA
AGCACAACGTTTGACCGTGACGAACTGTGGTGTTCTTGATCTACGTTA
+
FFFFFFFFFFFFFFF:FFFFFFFFFFFFFFFFFFFFFFFFFFFFFFFFFFFFF:FFFFFFFFFFFFFFFF
FFFFFFFFFFFFFFFFFFFFFFFFFFFF,FFFFFFFFFFFFFFF:F:FF
@A00721:190:HGJW7DSXY:4:2450:6198:19022_1:N:0:TCTGCAAG+GCTGCACT
CACTACCACTTATTACGATATTTCTTTAGTCGATGGGTTTTCGACGCCTTTAAGCGTCTTCTGTTCAT
CGCAACCAGATGATATCTTCTATTCGAACTTATGTTTCGATCAGTGTTTCTTCTATCAGGAGGAAAAC
ACT
+
FFFFFFFFFFFFFFFFFFFFFFFFFFFFFFFFFFFFFFFFFFFFFFFFFFFFFFFFFFFFFFFFFFFF
FFFFFFFFFFFFFFFFFFFFFFFFFFFF:FFFFFFFFFFFFFFFFFFFFFFFFFFFFFFFFFFFFFFF
FFF
@A00721:190:HGJW7DSXY:4:1477:22860:6668_2:N:0:TCTGCAAG+GCTGCACT
TCACTTTGTGTGTACTTGTCTCTATTTTAAGTTTTGTCTTCCTTGTTTGTCTTATTCTATTAATCACT
CATTTTAGTGAGATTATTAAAAACGCACTAAAACGTCTTCGACAACAG
+
FFFFFFFFFFFFFFFF:FFFFFFFFFFFFFF:FFFFFFFFFFFFFFFFFFFFFFFFFFFFFFFFFFFFF
FFFFF:FFFFFFFFFFF:FFFF,F:FFFFFFFFFFFFFFF:FFFFFF,
@A00721:190:HGJW7DSXY:4:1459:20021:23140_1:N:0:TCTGCAAG+GCTGCACT
GCCTTACAGAGGGGCAGCAGCATGACCATAATTGGTATTGACTTTGGTACCACCTTTTCTACTGTCTG
```

```
TCTCGCCTCTTCGAATGAAGTTTTAATGCTTAACAACAATGATAGTGAATTTATACCTACAATCATTG
GTT
+
FFFFFFFFFFFFFFFFFFFFFFFFFFFFFFFFFFFFFFFFFFFFFFFFFFFFF:FFFFFFFFFFFFFF
FFFFFFFFFFFFFFFFFFFFFFFFFFFFFFFFFFFFFFFFFFFF:FFF:FFFFFFFFFFFFFFFFFFF
FFF
@A00721:190:HGJW7DSXY:4:1205:31946:21026_2:N:0:TCTGCAAG+GCTGCACT
TGTTTATGGATATGATGCTATTACTCATGATGGTTACGGTCAGGGAGGATTCTCAGTTTACAAGGACC
TTAAGCGATGGATCGGGGTTAATTCTAAAACATTACCCGAAAGACGTG
+
FFFFFFFFFFFFFFFFFFFFFFFFFFFFFFFFFFFFFFFFFFFFFFFFFFFFFFFFFFF:FFF:FFFF
FFFFFFFF:FFFFFFFFFFFFFFFFFFFF:F:FFFFFFFFFFFFFFFF
@A00721:190:HGJW7DSXY:4:1162:13169:9987_2:N:0:TCTGCAAG+GCTGCACT
GTACGATGTCGTCTGTCTCGGCGATCACTTTTCACTAAGAATTGGTCCTACTTTTGGACAAGGCCAAC
TTCGTAATCTTCATGAGATCGTTTCTTTCTTTATCTCTTTGGTTGTTA
+
FFFFFFFFFFFF:FFFFFFFFFFFFFFFFFFFFFFFFFFFFFFF:FFFFFFFFFF:FFFFFFFFFFFFFF
FFFFFFFFFFFFFFF,FFFF:FFFFFFFFFFFFFFFFFF:F:F:FFFF
@A00721:190:HGJW7DSXY:4:1155:7166:29309_2:N:0:TCTGCAAG+GCTGCACT
GAGTGAAAGGCTTCAATTGTAGTGGTTTGGTCGTTTCAGTACCCAGTCAGTATACCTCTACGCAAAGA
TATTTCATGAAAACTACAGAGGAACGTACCGGATTACCTGTCTTTCAT
+
FFFFFFFFFFF:FFFFFFFFFFFFFFFFFFFFFFFFFFFFFFFFFFF:FFFFFFFFFFF:FFFFFFFFF
FFFFFFFFFFFFFFFFFFFFFFFF:FFFFFFFFF:FFFFFFFFFFFFFF
@A00721:190:HGJW7DSXY:4:2153:26892:30592_1:N:0:TCTGCAAG+GCTGCACT
CTTCGGCCGCTCTCTTTGCTTCGATGTTGGATGTCAAGAAGGGTTCAGAATGGGATTCCTACGTAGTT
TATGACTTTGGTGGTGGTACTTTCGATACTTCTATCGTCTTACGTTACGGTAATTACTATAGTGTTAT
TTA
+
FFFFFFFFFFFFFFFFFFFFFFFFFFFFFFFFFFFFFFFFFFFFFFFFFFFFFFFFFFFFFFFFFFFF
FFFFFFFFFFFFFFFFFFFFFFFFFFFFFFFFFFFFFFFFFFFFFFFFFFFFFFFFFFFFFFFFFFFF
FFF
@A00721:190:HGJW7DSXY:4:2252:26494:26741_1:N:0:TCTGCAAG+GCTGCACT
ATAATCTTGGAGGTCGTGACGTTGATGAAGCTATAAGAGCGTTCTTATTACGGCGCACTTCTTTACCC
GCCACCACACAACTTTCAGTTTCACAACTGAAGGAGGAGGTTTCTGTTACTGGAAAACCCTCGTCAAT
ACT
+
FFFFFFFF:FFFFFFFFFF:FFFFFFFFFFFFFFFFFFFFFFFFFFFFFFFFFFF:FF:FFF:FFFFFFF
FFFFFFFFFFFFFFFFFFFFFFFFFFFFFFFFFFFFFFFFFFFFFFFFFFFF:FFFFFFFFFFFFFF
FFF
@A00721:190:HGJW7DSXY:4:2421:24623:27759_2:N:0:TCTGCAAG+GCTGCACT
GATCTAGTGAAATTAACGCGTCCTTTTCTGGAACGTAGTTTTACTATTATGAACAAAGTTATCAAAGA
TAGTGGCATTCAAGGCAAACTTACCCTGATTCCGGCCGGTGGTAGCTC
+
FFFFFFFF:FFF,FFF:,FFFFF,:FFFF,FFF,,FFF::FFF,FF:FFF:FF,,FF,FFFFFFFFFF
FF,FF:,FFF:FF,::FFF:FFF,FFFFF:FF,,:FFF:,F:FFF,FF
@A00721:190:HGJW7DSXY:4:1523:14787:6934_2:N:0:TCTGCAAG+GCTGCACT
TGAAACAGCTATTAAAGCTGTTGAGCGGGTAATTACACCTAAACGAGCTCGATCTGCCATCGCTGAAG
GATGTGCACTCTTTTCTGCTACTTTAACGGAGCGTAGTGTACTCTTCG
+
FFFFFFFFFFFFFFFFFFFFFFFFFFFFFFFFFFFFFFFFFFFFFFFFFFFFFFFFFFFFFFFFFFFF
FFFFFFFFFFFFFFFFFFFFFFFFFFFFFFFFFFFFFFFFFFFFFFFF
@A00721:190:HGJW7DSXY:4:1147:10610:32675_2:N:0:TCTGCAAG+GCTGCACT
AAAGGTCTCTATCAAGCTCGTATTATCGTCGCTGCTGGATCTGCGTTACCGTTGAAGGCTAAACTCCA
```

AGGTAATTATGAAGACTGTGATTTATCTTTCACTCTGGCCTTGTATGA
+
FFFFFFFFFFFFFFFFFFFFFF,FFFFFFFFFFFFFFFFFFFFFFFFFFFFFFFFF:FFFFFFFFFF,FF
FFFFF,FFFFFFFFF::FFFFFFFFFFF,FF:FFFFF:FF,FFFFFFF
@A00721:190:HGJW7DSXY:4:2111:18539:1877_1:N:0:TCTGCAAG+GCTGCACT
CATTAATAATTTCAATCTTCTTAAAAAGAAGATAGAGAGTCATACTTTGGGCTATCGTTCAGGTGCAT
GGGGTTGGATAGCCAATATTGAAGTGGACACCTCTGGTAGTATCGAAGTAAAGAGATCGGAAGAGCAC
ACG
+
FFFFFFFFFFFFFFFFFFFFFFFFFFFFFFFFFFFFFFFFFFFFFFFFFFFFFFFFFFFFFFFFFFFFF:
FFFFFFFFFFFFFFFFFFFFFFFFFFFFFFFFFFFFFFFFFFFFFFFFFFFFF,:FFFFFFFFFFFFFFF
FFF
@A00721:190:HGJW7DSXY:4:2113:1334:22780_2:N:0:TCTGCAAG+GCTGCACT
AAGCATACTACTTTTGCTTTGATTACTCTACCGCCTTACAAGTCTAAGGTTAAAGAGTCGATTAAACA
AGTTGTACTTATCAATGATTTTATCGCTGGTTGTTAACACGAAAAGTC
+
FFFFFFFFFFF::FFFFF,FFFFFFFFFFFFFFFFF:FFFFFFF:FFFF:FFFFFFFFFFFFF,FFFFFF
F,::FFF::FFF:FF,:FFFFFFF,,,,,F:FFFF,,:,F:F,FF,F,
@A00721:190:HGJW7DSXY:4:2311:6623:12806_1:N:0:TCTGCAAG+GCTGCACT
CCCAAAGAGAATATCTAAAATATTTACAAAATCTTACCACTATCTACGAACCTAAAGTTCTGAAGACG
TTGCTTGTCGCTTCCTCCTTTAATGGCACTACCTCGGTACAATCTTACTTGGAACGAAGGCGACCTGT
TCC
+
:FFF:FFF,FFFFFFFFFFFFFFFFFFFFF,FFFFFFFFFF:FFFFFFFFFFFF:FFF:FFFFFFFFFFF
:FFFFFFFFFF:FFFFFFFFFFF,FFFFFFFFFFFFFFFFFF:FFFFFFFFFFFFFFFFFFFFFFFFFFF
FFF
@A00721:190:HGJW7DSXY:4:2515:24406:7623_1:N:0:TCTGCAAG+GCTGCACT
GTTCTGTCTATATTAGGTGAAGCGAAATACGATTTCGAACGTCAACGTTCACCGAGTTTCGTTTCGTA
CCTTGATGCTGACTTTCGTCAATATGATTTGATCTGGTTGCTCAATTCTAAGCGTGTTCCGCTTAGTG
CGA
+
FFFFFFFFFFFFFFFFFFFFFFFFFFFFFFFFFFFFFFFFFFFFFFFFFFFFFFFFFFFFFFFFFFFFFF
FFFFFFFFFFFFFFFFFFFFFFFFFFFFFFFFFFFFFFFFFFFFFF:FFFFFFFFFFFFFFFFFFFFFFF
FFF
@A00721:190:HGJW7DSXY:4:2477:20690:31031_1:N:0:TCTGCAAG+GCTGCACT
TTCGATCTTGACTATACTATACGTGACATACTAAATACTAGATATTTGGATGATCTTGATAAAGAACT
TCATGAATTTTCAGAGGATGAAAAATTTTCTTCCTTGGATTTTAATATAAAGAATCTTTACCAGGACT
CCT
+
F,FFF,FFFFF,F,FFFFFF,FFFFF:FFFFFFF:FFFFF:FFFFFFF:FFF:FFFFFFF:FFFF:FF:F
:FFFFFFFFFFF:FF,FFFF:,,FFF:FFFFF:,FFFFFFFFFFFFF,FFFFFFFF:FFF:FFFFFFFFF
F,F
@A00721:190:HGJW7DSXY:4:2232:4571:7028_1:N:0:TCTGCAAG+GCTGCACT
GATCGTAAGAAGTTTATAAGGAACATATCCATTTGTTGTAATAGGGTCATCAACTTAGAACTCGAAAC
TCTAAGTGGTAATACTGTCTTTCCAAGCTTACCTGTATCTTCAATGAAATTGGACTCTTCGATAGGGG
ACA
+
FFFFFFFFFFFFFFFFFFFFFFFFFFFFFFFFFFFFFFFFFFFFFFFFFFFFFFFFFFFFFFFFFF:FFF
FFFFFFFFFFFFFFFFFFFFFFFFFFFFFFFFFFFFFFFFFFFFFFFFFFFFFFFFFFFFFFFFFFFFFF
FFF
@A00721:190:HGJW7DSXY:4:2252:8269:12900_1:N:0:TCTGCAAG+GCTGCACT
TAAGAATACTATAAATAGAATAGCTATTACTACTAATGGCGTTGGCCTAGTGGATGTTATTAGTGACA
TCTGGTCAATTTCGTTTTTAGGTGGTCTTTATTCTATTATTGATTTTTATGAGATTGAGATCGGAAGA
GCA

+
FFFFFFFFFFFFFFFFFFFFF:FFFFFFFFFFFFFFFFFFFF:FFFFFFFFFFFFFFFFFFFFF:FFFF
FFFF:FFFFFFFFFF:F,FFFFFFFFFFFFFFFFFF,FFFFFFFF:FFFFFFFFFFFFFFF:FFFFFF
FFF
@A00721:190:HGJW7DSXY:4:1163:2166:20807_2:N:0:TCTGCAAG+GCTGCACT
TTCCTGAAGAGTCCTTCCGTAACCAACTTCTTGACAATTTTCTTCAATCCTTCGATAATAGTTAAATC
TACGATTATGTCTGTGTTGAAAGACTTTCTACCGATAAGGGTCACTAG
+
FFFFFFFFFFFFFFFFFFFFFFFFFFFFFFFFFFFFFFFF:FFFFFFFFFFFFFFFFFFFFFFFFFFF
FFF:FFFFFFFFFFFFFFFFFFFFFFFFFFFFFFFFFFFFFFFFFFFF
@A00721:190:HGJW7DSXY:4:2546:14931:20932_2:N:0:TCTGCAAG+GCTGCACT
TAAGAAATAATCCTACATTGACTGATTATCTAAAGATTGAAGGAAAATTTTATCCTCGTTTAACTTAC
TTAGACTTTCCTACGATACAAATGATAACTTCTAATTTACGTTTGCTA
+
FFFFFFFFFFFFFFFFFFFFFFFFFFFFFFFFFFFFFFFFFFFFFFFFFFFFFFFFFFFFFFFFFFFF
FFFFFFFFFFFFFFFFFFFFFFFFFFFFFFFFFFFFFFFFFFFFFFFF
@A00721:190:HGJW7DSXY:4:1343:20609:31454_1:N:0:TCTGCAAG+GCTGCACT
GATGCATGGTGTTATCTCCTCAGGAGTCCCGGCATTTGATAATAATGACGAGACTTTCGTCATAATCG
CTGCATATTACGGATTACACGGTACCAGCGCATCTCGTGTCGAGCCTCGACCCGATTATATGGTATTA
ACT
+
FFFFFFFFF:FFFFFFFFF:F:FFFFFFFFFFFFFFFFFFFFFFFFFFFFFFFFFFFFFFFFFFFFFF
FFFFFFFFFFFFFF,FFFFFFFFFFFFFFFFFFFFFFFFFFFFFFFFFFFFFFFFFFFFFFFFFFFFF
FFF
@A00721:190:HGJW7DSXY:4:2630:5249:35008_2:N:0:TCTGCAAG+GCTGCACT
GATTCTCGATTCGTAGAGCTTATCTAGGTACTCTCATTTACTATGTTGATTTAGTCTACATTTATGCT
GATCTAAAATTTCGATCTAGATGGATCCACTCCTCGAGTTATCATGAT
+
FFFFFFFFFFFFFFFFF:FFFFFFFFFFFFFFFF::FFFFFFFFFFFF,FF:FFFFFFFF:FFFFF:F
FFFFFFFFFFFF,FFFFFFFFF:FFFFFFFFFFFFFFF,FFFFFFFFF
@A00721:190:HGJW7DSXY:4:2568:29894:7012_1:N:0:TCTGCAAG+GCTGCACT
TTCTAGAATTCTTAATTATAATATTCAACCTCAAAAGTTACGTGTTTACAGTAACGAGATCACACATT
TTGAGAACTAACTATGTTATGTTCTCTGATCATTTTGACAATCTCTTCTTATAATAAATAAATAAATA
ATA
+
FF:FFFFFFFFFFFFFFFFFFFFFFFFFFFFFFFFF:FFFFFFFFFFFFFFFFFFFFFFFFFFFFFFF
FFFFFFFFFFFFFFFFFFFFFFFFFFFF:FFFFFFFFFFFFFFFFFFFFFFFFFFFFFFFFFFFFFFFF
FFF
@A00721:190:HGJW7DSXY:4:2251:12888:24565_2:N:0:TCTGCAAG+GCTGCACT
AGTGGAAAGGCTCCAGTCAACACTAGTTCGAGTGCTGCTTTGGATTACGATGACGTGAGAATAGGTGA
AGGGATAGTTTTTACCTCGACCAACATTAAGGCGGCGGCGACTAATCT
+
FFFFFFFFFFFFFF:FFFFFFFFFFFFFFFFFFFFFFFFFFFF:FFFFFFFFFFFFFFFFFFFFFFFFF
FFFFFFFFFFFFFFFFFFFFFFFFFFFFFFFFFFFFFFFFFFFFFFFF
@A00721:190:HGJW7DSXY:4:2105:15890:12446_1:N:0:TCTGCAAG+GCTGCACT
AGGACACTTCGAAACTGAATAATGATGCACTACAAACAAGTATCGCTAATCATCTGAAAAAGGCTCTA
AAGGACACTATACAATATCCAGACGACGACGAAGCTTATACTACTTTAATTTTGGGTATTATGTGGAG
ATA
+
:F,F:FFFF,FFF:FF,F:FFFFFFF,FFFFF,FFFF::,FFF:F,FF,FFF:FFFFFFFFFFFFFFF
FFFFFFF:FFFFFFFFFFFFF:FFFF:FFFFFFF:FFFFFFFFFFFFFFFFFFFFFFFFFF:FFFFFFF
FFF
@A00721:190:HGJW7DSXY:4:1432:5565:31735_2:N:0:TCTGCAAG+GCTGCACT
ACCACATTTTGGTCAAGTCAATCCGTTGTGAAAATTTCTTAGAGGATATTCGCCATTGTGGTACGAAT

```
TGGTCCAACATTTACCACCAAATTCTTTTGATACAACTCTAGCCTTCA
+
FF:FFFFF:FFF:FFFFFFFFFFF:F:F,F:FFFFF::FFFF::FFFFFFFFFFFFFFFFFFFFFFFF
FFFFFFF,FFFFFFFFFFFFFFFF:FFFFFFFFFFFFFF:FFF:F::F
@A00721:190:HGJW7DSXY:4:1224:26666:26976_2:N:0:TCTGCAAG+GCTGCACT
TCACCAGATTGGTTCGAACCCGTTGATGCTATGTCAGAAGAAGCTATTCAAGCTTGTCGTTTAAAGAC
TGCAGAGGCTGTCAAACAAGCCCAGACTCAAGGAGTCCAATCAAAGGT
+
FFFFF:FFFFFFFFFFFFFFFFFFFFFFFFFFFFFFFFFFFFFFFFFFFFFFFFFFFFFFFFFFF:FF
FFFF:F:FFFFFF:FFFFFFFFFFF:FFF:FFFF:FFFFFFFFFFFFF
@A00721:190:HGJW7DSXY:4:1556:15935:33661_1:N:0:TCTGCAAG+GCTGCACT
ATTATTTAGAGATCTTTTCTACTTACAAAGGACAATTCAGTTTCATTAATTCTACGGAAAAGTCCGGA
GTATATTGTATGTTAATAGATAGTGGGATAATAGTTCTACCGATACGTAGTGAACGCGTTCGTAGAAC
CAA
+
FFFFFFFFFFF:FFFFFFFFFFFFFFFFFFFFFFFFFFFFFFFF:FFFFFFFFFFFFFFFFFFFFFFF
FFFFFFFFFFFFFFFFF:FFFFFFFFFFFFFFFFFFFFFFFFFFFFFFFFFFFFFFFFFFFFFFFFFF
FFF
@A00721:190:HGJW7DSXY:4:2337:17454:10457_2:N:0:TCTGCAAG+GCTGCACT
TATTGATTATGTAACCGATAGGAGTTGTATAGTTGAAGATGTGGCTTTAGACGATAATCCTTTAAGGA
CTATGTTGACTGACGAAGATTTTCCCACAGATTTGTCTACGTTGCAGA
+
FFFFFFFFFFFFFFFFFFFFFFFFFFFFFFFFFFFFFFFFFFFFFFFFFFFFFFFFFFFFFFFFFFFF
FF:FFFFFFFFFFFFFFFFFFFFFFFFFFFFFFFFFFFFFFFFFFFFF
@A00721:190:HGJW7DSXY:4:2115:25256:18239_1:N:0:TCTGCAAG+GCTGCACT
CTATATACATTATAAAATATAAAAATAAGGGAGTCATAACTTTTGTTACCAAATTCTTGGCTTTCAAC
GAAGTCTCTGATTTTATTAATCATTACTTTAACGATCTCTCGGTCATTTACTCTGTACTTGATCTACG
TCG
+
:FFFFFFFFFFFFFFFFFFFFFFFF:FFFFFFFFFFFFFFFFFFFFFFFFFFFFFFFFFFFFFFFFFF
FFFFFFFFFFFFFFFFFFFFFFFFFFFFFFFFFFFFFFFFFFFFFFFFFFFFFFFFFFFFFFFFFFFF
FFF
@A00721:190:HGJW7DSXY:4:2231:13847:5838_2:N:0:TCTGCAAG+GCTGCACT
GAATGACTCTATCTTTTCAGACACTTCTTATATTATTTACAATAGTTAATATCATATTAGTAATACTC
ACTATCGTTTTCTGCTTTTTCTTAGCTAATAGATTAATAAAGGATGAG
+
FFFFFFFFFFFFFFFFFFFFFFFFFFFFFFFFFFFFFFFFFFFFFFFFFFFFFFFFFFFFFFFFFFFF
FFFFFFFFFFFFFFFFFFFFFFFFFFFFFFF:FFFFFFFFFF,FFFFF
@A00721:190:HGJW7DSXY:4:1271:21314:35963_2:N:0:TCTGCAAG+GCTGCACT
ACTCTTCGTTACGAGACCTGATGGTAGAATAGATTTAGTCTCTACACCCATCTCAACGAGGATAAAGC
GAGTTTTCCGTCCTGAACGTAATACACGAAGAGATCATCCGCAAACGA
+
FFFFFFFFFFFFFFFFFFFFFFFFF:FFFFFFFFFFFFFFFFFFFFFFFFFFFFFFFFFFFFFFFFFF
FFFFFFFFFFFFFFFFFFFFFFFFFFFFFFFFFFFFFFFFFFFFFFF:
@A00721:190:HGJW7DSXY:4:2535:31150:9627_2:N:0:TCTGCAAG+GCTGCACT
TTTAGATTATGAATCCTGCCTTTACTCATTTTATAGCAGTTATTTATGTTGTACTTTTTATAATTTAT
AACCTTCTTATACTTTTGGGTATGTTTTGTGAAAAACATGAGATACCC
+
FFFFFFFFFFFFFFFFFFFFFFFFFFFFFFFFFFFFFFFFFF:FFFFF:FF:FFFFFFFFFFFFFFFF
FFFFFFFFFFFF:FFFF,FFFFFFFFFFFFFFFFFFFFFFFFFFF:F,F
@A00721:190:HGJW7DSXY:4:2303:16369:14497_2:N:0:TCTGCAAG+GCTGCACT
TTGTAACGATCATTCTTTCGAGTTTTGTCATTCTGATTCTTTTATTCCTTTAACTGTGTATGAGGAGG
ATTTATAATGCTTTTCGATAGTGATTGTTATCAATTTAAATACATTAC
+
```

F:FFFFFFFFF:FFFFFFFFFFFFFFFFFFFF::,F:,F,FFFFFFFFF:::FFFFFFFFFFF:FFF
FFFFF:FFFFFFF:FF:FFFFFFFFF:FF:FF:F:FFF:FFFFF:FFF
@A00721:190:HGJW7DSXY:4:2246:31647:13369_2:N:0:TCTGCAAG+GCTGCACT
TCACAGACTCCGAAAATGGTTTCAAATGAGGTCAAAGCTCTATTTATTAAACGAACTATTATCCGATT
CTTCGAGGGTATCACTACCACTGATCTGCCGACTTTAGTCAAGGAACT
+
FFFF:FFFFFFFFFF,FFFFFFFFFFFFFFFFFFFFFFFFFFFFFFF:FFFFFFFFFFFFFFFFFFFF
FFFF:FFFFF:FFFFFFFFFF:FFFFFFF::FFF,FF:FFF:F:FFFF
@A00721:190:HGJW7DSXY:4:1668:13946:37043_2:N:0:TCTGCAAG+GCTGCACT
CGATGTTTATATTTTTCTATAAGGGAAAATAGCAATAATCAAATTTCGACAAATTTTAGAACTTTATT
TCAGAACTCTCTCCGATCACCTTGGTGTGATCTCCCGGAGGATCTTTT
+
FFFFFFFFFFFFFFFFFFFFFFFFFF:FFFFFFFFFFFFFFFFF:FFFFFFFFFFFFFFFFFFFFFFFF
FFFFFFFFFF:FFFFFFFFFFFFFFFFFFFFFFFFFFFFFFFFFFFFF
@A00721:190:HGJW7DSXY:4:2174:20546:7670_2:N:0:TCTGCAAG+GCTGCACT
ATTTTTAAATAGTGATATTCAGCGTATACTTGAATACTTTATCAAAGATTTGGCTTGGTCTATTTACC
GCAAAAAGTTGGAAGAGGTCTATGATATTTATTTTACTTATACTTTAA
+
FFFFFFFFFFFFFFFFFFFFFFFFFFFFFF:FFFFFFFFFFFFFFFFFFFFFFFF:FFFFFFFFFFFFF
FFFF:F:FFFFFFFFF:FFFFFFFFFF:FFFFFFFFFFFFFFFFFFFF:
@A00721:190:HGJW7DSXY:4:2207:3233:31172_1:N:0:TCTGCAAG+GCTGCACT
AACTTGACTGTTGATAATATTACAATAGCTCTTTCTCAGAAGATTAGAGAAGTAACCGGTGAACACGT
CGGTAGCATTGATTTACTTTTACCGTTAGAACTGAAAAAGATTCTTTTCGAAAAGAAACCTATAAGTT
ACA
+
FF,FFFF:FFFF:FFF:FFFFFFFFFFFFFFFFF,F:F:FFF,FFFFFFFFFFFFF:F,F,FF,FF,F
:F,FFF,:FF:FFFFF:FFFFFFF:FFF:FF:F:FFFFFF:FF:FFFFFFFF:FFFFFFFFFFFFFFF
FFF
@A00721:190:HGJW7DSXY:4:2205:5177:29246_1:N:0:TCTGCAAG+GCTGCACT
TATATTAAAGAAATAAAAATACTTCAAGTACCTGAACTTTACATTTTTAATGGTATGGTATAATGTGT
CTTACAAATAGAACCTTCACTCTCGGTAATGAGAGTGTATTCCTTAATATTCTAGTAACCGCTATGTG
GGC
+
FFFFFFFFFFFFFFFFFFFFFFFFFFFFFFFFFFFFFFFFFFFFFFFFFFFFFFFFFFFFFFFFFFFF
FFFFFFFFFFFFFFFFFFFFFFFFFFFFFFFFFFFFFFFF:FFFFFFFFFFFFFFFFFFFFFFFFFFF
FFF
@A00721:190:HGJW7DSXY:4:2210:3115:28432_2:N:0:TCTGCAAG+GCTGCACT
TTTAAATGCTAGGAAAATTTTAGAAAAACTCCATTGTATAGTTCAAGGAGTACTTAATTCAATAGCTT
TAGTATTATTTACTATCGCCTTTATTGAAAATTGTATAAGAGTTAATT
+
FFFFFFFFFFFFFFFFFFFFFFFFFFFFFFFFF::FFFFFFFFFFFFFFFFFFFFFFFFFFFFFFFFF
FFFF,FFFFFFFFFFFFFFFFF:FFFFFFFF:FFFFFF:FFFFF:FFF
@A00721:190:HGJW7DSXY:4:2403:32452:2206_1:N:0:TCTGCAAG+GCTGCACT
ACATACCATAACCTACCATGACCAATCATCGCTCCAACTGGCACGCCAGACGTAGGGTCAAACCTACT
ACCGCCGGTCGGCAGATCGGCACCACTGGTCTCTACGTTCAGGGACCCGCTGTGCAGAATAACCTTCC
CAA
+
:FFFFF:FFFFFFF,FFFF,FF:FFF,,FFF:FF:FFFFFFFFFFF,:FF:FF,FF:F,FFFF:FF:F
FFFFF:FFFFFFF,FFF:FFFFFFFFFFFFFFFFFFFFFFFFFFFFFFFFFFFFFFFFFFFFFFFFFF
FFF
@A00721:190:HGJW7DSXY:4:1307:22110:29637_2:N:0:TCTGCAAG+GCTGCACT
CACAGAAGAAGGCTGAAGGTCGTTACCGGACGACCACTCAAAGGAAACGGGCTGTTGCCCAGAGGAAA
GCAGCGACCACCGAGGTCGCTCGTAACATTGACGGCAGTCCCGTCATC
+

FFFFFFFFFFFFFFFFFFFFFFFFFFFFFFFFFFFFFFFFFF:FFFFFFFFFFFFFFFFFFFFFFFFFF
FFFFFFFFFFFFFFFFFFFFFFFFFFFFFFFFFF:FFFFFF:FFFFFFF
@A00721:190:HGJW7DSXY:4:1208:30255:29810_1:N:0:TCTGCAAG+GCTGCACT
CTGAGGCAGTACCTCAGTCAAAAGTCACATTCACCACTCCCATACCAATCAATGATGTGACTATACAG
TTCGGCCATTTCAAGAGTGGTCAATACGTCGTGGGAGGTACTGAAGCCTTCCCCTCCGCTGGCACGAA
GAA
+
FFFFFFFFFFFFFFFFFFFFFFFFFFFFFFFFFFFFFFFFFFFFFFF:FFF,FFFFFFFFFFFFFFFF
FFFFFFFFFFFFF,FFFFFFFFFFFFFFFFFFFFFFFFFFFFFFFFFFFFFFFFFFFFFFFFFFFFFF
FFF
@A00721:190:HGJW7DSXY:4:1261:8458:8500_1:N:0:TCTGCAAG+GCTGCACT
CCAATCAATAGTTCTTCTGACCAGAATGAAAAGGGGTTTACCAGGGTAGTCTCTAAGTCTACCAGGAA
GAGGGGAAATGTCCCCAAAACTGAGAAGAATAAAGGGAAAGGTCCATCAGGACCTAAGAAGGGGAAAT
TAC
+
FFFFFFFFFFF:FFFFFFFF:FFFF:FFF:FFFFFF:FFFFFFFFFFFFFFFFFFFFFFFFFFFFFFF
FFFFFFFFFFFFFFFFFFFF:FFFFFFFFFFFFFFFFFFFFFFFFFF:FFFFFFFFFFFFFFFFFFFF
FFF
@A00721:190:HGJW7DSXY:4:1265:3812:15076_2:N:0:TCTGCAAG+GCTGCACT
GGAAGATCTCCCACCATCTGGGGTTGGGAATAAGAAATCTGCACGGGCCAAAGAGGCCGCTGCTACGA
AGTCGCTGAAGGCGGCTTGGGTCATTAAGGGTAGCTGTAAAGTTGCGC
+
FFFFFFFFFFFFFFFF:F,FFFFFFFFFF:FFFF:FFFFFFFFFFFFFFFFFFFFFFFFFFFFFFFFF
FFFFFFFFFFFFFFFFF:FFFFFF:F:FFFFFFFF:FFF,FFFFFFFF
@A00721:190:HGJW7DSXY:4:1263:10411:22999_1:N:0:TCTGCAAG+GCTGCACT
ATCTTTGAGACCAAGACTCTCTACAGTAGGTTCGTGCCTACTATGAGCAAATCTGTTGAATTCGTCAC
TGAGGACGAAGGTGAGAGGACTTTTGGCTACTGGCGACAAACTGAGAGCAGTCGCTGGGGAAAGAGAA
TTG
+
FFFFFFFFFFFFFFFFFFFFFFFFFFFFFFFFFFFFFFFFFFFFFFFFFFFFFFFFFFFFFFFFFFFF
FFFFFFFFFFFFFFFFFFFFFFFFFFFFFFFFFFFFFFFFF:FFFFFFFFFFFFFFFFFFFFFFFFFF
FFF
@A00721:190:HGJW7DSXY:4:2363:13702:33614_2:N:0:TCTGCAAG+GCTGCACT
ACGCTTCGAGCTTGAGTGTGACGAGGCCGGTGACGTTTTTGTCAACGGCGTGTGGAGGATTTTATCCG
CGCTTAACTATGGGGCGAAGATCCCCTATGGAGTCTTGAAGGACTTAG
+
FF::F,FFFF:FFFFFFFF:FFFF,FFF::F:FF:,F,:F,FFFF:,,:FF,FF,FFFFFFFFF,F:F
,F:F,FFFF:,FF,,,FFFFFFF:FF,F,::F,:FF,F,:::F:FFF:
@A00721:190:HGJW7DSXY:4:2206:22761:30514_1:N:0:TCTGCAAG+GCTGCACT
GCATGTCACCCTTAAGCACCGAGGTAAGCTCGGGACTTACAAGAGAGAGTGTGAGAGAGTCGCTCTCA
CAATTTTTGCCCAGAAAGAGGGCGTTCTGAAAGATGAGGTTAAAACTAATGCTCAATCTGTTGGTGAG
GGT
+
FFFFFFFFFFFFFFFFFFF:FFFFFFFFF:FFFFFFFFFFFFFFFFFFFFFFFFFFFFFFFFFFF,,,,
FFF,,FF:,:,F,,,F,,,,FFF,FF,,::,,,F:,:,FFF:FFF,,,,,F,F:,,,,,F:,::,:,:
F:F
@A00721:190:HGJW7DSXY:4:1273:25409:17942_2:N:0:TCTGCAAG+GCTGCACT
GGTTCTGGGAGGCTGCTTTCCTCCATAATTACAACCTCTCTTTAGAGAGCATTAGAAGGAAGTTGGGC
AAATTCCCACATATGGTAGACGTTGTCACACTTTTCTTGAAAATTTTT
+
FFFFFFFFFFFFFFFFFFFFFFFFFFFFFFFFFFFFFFFF,FFFFFFFFFFFFFFFFFFFFFFFFFFF
FFFFFFFFFFFFFFFFFFFFFFFFFFFFFFFFFFFFFFFF:FF:FFF:
@A00721:190:HGJW7DSXY:4:2466:5620:33082_1:N:0:TCTGCAAG+GCTGCACT
TTGGGGATTTCGATGTGAGAAGGTGGAAGGTGTCAAGGGTCTTCTTCATGCTGATCTTTCAGGACCTA

```
ATCACATCATGATTGATAGTTACGGCTCGTATCGAGTTGGAGGGAGGAAGTTCTCCGAAATTAAAGAC
GGG
+
FFFF:FFFFFFF,FFFFF,F:,FFFF:FFFF:F,FF:,,F:F:FFF,FFFFFFFF,FFF:FFFF:,FF
:F:F:FFFFFFFFFFFFF,FF,,:F,F:FFFFFFFFFF::FFFFF,,FFFFFFF:::F::FFF:F,FF
F:F
@A00721:190:HGJW7DSXY:4:2440:8169:4116_2:N:0:TCTGCAAG+GCTGCACT
TCAGAGAGGGGAGCTCTGAAAAGGAAACTTCGTCAGAAGATGAGGAAGTCTAAGAGGAGGAGTTTGAG
GAAGGCTCTAGAGAAGATCTCACCACCTGGAGAAGGCAAGTGGAGAGT
+
FFFFFFFFFFFFFFFFFFFFFFFFFFFFFFFFFFFFFFFFFFFFFFFFFFFFFFFFFFFFFFFFFFFF
FFFFFFFFFFFFFFFFFFFFFFFFFFFFFFFFFFFFFFFFFFFFFFFF
@A00721:190:HGJW7DSXY:4:1535:18349:34741_2:N:0:TCTGCAAG+GCTGCACT
AAGAAGGATTATTTGAAGCAGGGGTTACCACTTCCTAAGGTGGATGAGGGAGGTTGTTTCTTTGGAGT
TTTAGATAATCTTTTGAGAAGGGGTCATAGAGCCAGCAAGAGAAGCTT
+
FFFFFFF:FFFFFFFFFFFFFFFFFFFF:FFFFFFFFFFFFFFFFFFFFFFFFFFFFFFFFFFFFFFF
FFFF,FFFFFFFFFFF:FFFFF:FFFFFF:FFFFFFFFFFFFFF:FF:
@A00721:190:HGJW7DSXY:4:1677:20410:7686_2:N:0:TCTGCAAG+GCTGCACT
TATAATTTAGGTCAACTTGGGGTTTCGAATGTCATTAGTGCCTTACGCTTGAAAGAGGTCCTTTTGAG
TATCGACAGATCTTTCGATCTTGAGGACGTACATTTAACGTGGCAGAA
+
FFFFFFFFFFFFFFFFFFFFFFFFFFFFFFFFFFFFFFFFFFFFFFFFFFFFFFFFFFFFFFFFF:FF
FFFFFFFFFFFFFFFFFFFFFFFFF:FFFFFFFFFFFFFFFF:FFFFF
@A00721:190:HGJW7DSXY:4:1469:16043:25708_2:N:0:TCTGCAAG+GCTGCACT
ATATTGTTCCTTCCGGGAAGTTGTGGGATTTAACTTTGACTCAACTACTCCGATCAGATCCGGATGGT
TTGATTGGTATGGACTACTCGACTTTTGATCTGGAGTTGTCTAGGGTC
+
FFFFFFFFFFFFFF:FFFFF,FF,FFFF,FFFFFFFFFFFFFFF:FFFFFFFFFFFFFFFFFF::FF,
::FFFFFFFFFFFFFFF:FF:FF:FFFFFFFFFFFF:FFFFFFFFFFF
@A00721:190:HGJW7DSXY:4:2620:26313:32346_2:N:0:TCTGCAAG+GCTGCACT
CGATAAGGTGAAAAATATGCAGGTCAAAGATTCTGTAGCTCTGAGGGCGGTTGAGGGAGCCGTTTTGG
CCGACTTTAACTATTCTAAGAGTCTTTATGATTCAAAACCTATCGTTG
+
FFFFFFFFFFFFFFFFFFFFFFFFFFFFFFFFFFFFFFFFFFFF:FFFFFFFFFFFFFFFFFFFFFFF
FFFFFFFFFFFFFFFFFFFFFFFFFFFFFFFFFFFFFFFFFFFFFFFF
@A00721:190:HGJW7DSXY:4:2226:31982:28917_2:N:0:TCTGCAAG+GCTGCACT
GGCTTAATGTCGCGCAGTTTCCAGAATTGCGGTTGAAGTTCACCCATTCTATCTTGTATGATCACGCC
GTCGCAGCCATATCGAGACTATGTTATAATCACTTGTTAAAGTACAGA
+
:FFFFF,:F:FFFFFF:FFF,FFFFFFFFF:FFF,,FFFF,F,,FFFF::F:F:F,,,:F,FFFFFFF
FFF:FF:FFF,FF,FF,::FFF:F::,,FFFF,F::,FF:FFFFFFF:
@A00721:190:HGJW7DSXY:4:1368:10402:27806_2:N:0:TCTGCAAG+GCTGCACT
ATTATTGATATTGGAGGCGATATTCGTTTTCATATCCAAAACTCCTTGAATGGAGTACATATTTGCAA
CCCTATTCTTGATGTGAAAGATGCATCGAGGTATACGTCTAGGAACAT
+
FFFFFFFFFFFFFFFFFFFFFFFFFFFFFFFFFFFFFFFFF:FFFFFFFFFFFFFFFFFFFFFFFFFFF
FFFFFFFFFFFFF:FFFF:FFFFFFFFFFFFFFFFFFFFFFFFFFFFFF
@A00721:190:HGJW7DSXY:4:1335:9417:18145_2:N:0:TCTGCAAG+GCTGCACT
GCTATCGGAGGGAAGCAAAATGTTTCGTTCTGTTACAAGAACGCTTTGGATTGTGACCATAAAGCCTC
AGTTGCGATAGCTGTAGAGGTATACGATATCTCTCTTGATGATGTAGC
+
FFFFFFFFFFFFFFFFFFF:FFF:FFFFFFFFFFFFFFFFFFFFFFFFFFFFFFFFFFFFFFFFFFFFF
FFFFFFFFFFFFFFFFFFFFFFFFFFFFFFFFFFFFFFFFFFFFFFFF
```

```
@A00721:190:HGJW7DSXY:4:1333:27905:27367_1:N:0:TCTGCAAG+GCTGCACT
TGATGTATGTCATTTCGTCTTAGTTTGTCCCGGCGAATTATTGGACACGATGAAAAATGACATACCCG
TTCCTGACCTTGGTATCGTTATCCGTCATGGAGATGATGGCGCATCACTTGATTACTATATGCCCGAA
GGA
+
FFFFFFFFFFFFFFFFFFFFFFFFFFFFFFFFFFFFFFFFFFFFFFFFFFFFFFFFFFFFFF:FFFFF
FFFFFFFFFFFFFFFFFFFFFFFFFFFFFFFFFFFFFFFFFFFFFFFFFFFFFFFFFFF::FFFFFFF
FFF
@A00721:190:HGJW7DSXY:4:1554:23204:5321_2:N:0:TCTGCAAG+GCTGCACT
AACAAAGATAAGATTTTATCTTTCTTCAAACACCCTGTTTTCCTTAAGGGCGGACAATTGTTCCATTG
TGAACTCTTGGGGACTAGATTGAGCATTAATGAGTTCCGAATAACTCG
+
FFFFFFF:FFFFFFFFFFFFFFFFFFFFFFFFFFFFFFFFFFFFFFFFFFFFFFFFFFFFFFFFFFFF
FFFFFFFFFFFFFFFFFFFFFFFFFFFFFFFFFFFFFFFFFFFFFFFF
@A00721:190:HGJW7DSXY:4:1331:17553:6151_2:N:0:TCTGCAAG+GCTGCACT
TATTTCCGTACCAAGAGCCACTGCCAATCTCATCGCTATACGCATACCTGATTGGAAGAAGCGAGGTT
CGTTCCTCAATAAAAATTTTTCTCAAGAGATTATGGAGATCGGAAGAG
+
FFFFFFFFFFFFFFFFFFFFFFFFFFFFFFFFFFFFFFFFFFFFFFFFFFFFFFFF:FFFFFFFFFFF
FFFFFF:FF:FFFFFFFFFFFFFF:FFFFF::FFFFFFFFFFFFFFFF
@A00721:190:HGJW7DSXY:4:2616:13006:13213_1:N:0:TCTGCAAG+GCTGCACT
TTCAGTACGTCATGGGGATCACTTCTTCGGTCAATGACAAAACCTTCGAATATGTTACGACATGGCTT
CGTAACAACAGTGCGAGGATTGTCATCTCGGGGAAAGTGATTCACCAAGATGTTCATATAGATACGAG
ATA
+
FFFFFFFFFFFFFFFFFFFFFFFFFFFFFFFFFFFFFFFFFFFFFFFFFFFFFFFFFFFFFFFFFFFF
FFFFFFFFFFFFFFFFFFFFFFFFFFFFFFFFFFFFFFFFFFFFFFFFFFFFFFFFFFFFFFFFFFFF
FFF
@A00721:190:HGJW7DSXY:4:2259:22200:23563_2:N:0:TCTGCAAG+GCTGCACT
CAGCGATCTTACTCGTTATCGGAGCTAAGAAGAAGACTTTGGGTACACGTTATGCTCGCAAACTTAAC
GTTAGTACCGGAGGTGATCTCTCTGCTTCTGCGTTACTGAGAGAAAAG
+
FFFFFFFFFFFFFFFFFFFFFFFFFFFFFFFFF:F:FFFFFFFFFFFFFFF:FFFFFFFFFFF:FFFF
F:FFFFFFFFFFFFFFFFFFFFFFFFFFFFFFFFFFFFFFFFFFFFFF
@A00721:190:HGJW7DSXY:4:1231:12111:25535_1:N:0:TCTGCAAG+GCTGCACT
CTTTATGGACAAGATGCAAGTTCTTGGGAAATACTTGTTTCCCTTTTTAGGTTTAGTAGGTGATCTTG
ACGTCGATTCTTTGTATAGGGAGTGTGAGAAGACAACGAATCTTGAAACTACGGTCCTTATCAAAGAA
AAT
+
FFFFFFFFFFF:FFFFFFFFFFFFFFFFFFFFFFFF:FFFFFFFFFFFFFFFFFFFFFFFFFFFFFFF
FFFFFF:FFFFFFFFFFFFFFFFFFFFFFFFFFFFFFFFFFFFFFFFFFFFFFFFFFFFFFFFFFFFF
FFF
@A00721:190:HGJW7DSXY:4:1174:13494:1939_2:N:0:TCTGCAAG+GCTGCACT
AAATCTTTTGAGATTGAGAAGATTGAAGAACACGTCAAGAAGTCGCTCGGCGTTGTTCTTGAAAACCA
AGTTAAGAAGGAGGCTGATCATGGAGTCTTTGTACCTTCTGTCAAACT
+
FFFFF:FFFFFF,,FFFFFFFFFFFFFFFFFFFFFFFFFFFFFFFF:FFFFF,FFFFFFFFFFFFFFF
FFFFFFFFFFFFFFF:FFFFFFFFFFFFFFFFF:FFFFFFFFFFFFFF
@A00721:190:HGJW7DSXY:4:1529:18973:17253_2:N:0:TCTGCAAG+GCTGCACT
GCTACGAGAGGTGATCCCGGATGTGGATTGAAGGCTGGCTCTAAGGGAGACTCCTTCTTTTTGAATGT
TGTAAAGAATTGGGTTGAGAAGTGTGCTTTACAAGGAGTTAGACTCTT
+
FFFFFFFFFFFFFFFFFFFFFFFFFFFFFFFFFFFFFFFFFFFFFFFFFFFFFFFFFFFFFFFFFFFF
FFFFFFFFFFFFFFFFFFFFFFFFFFFFFFFFFFFFFFFFFFFFFFFF
```

```
@A00721:190:HGJW7DSXY:4:2471:8703:11741_2:N:0:TCTGCAAG+GCTGCACT
TCTTGATAATCTTAAAGAAAAATTTTCGAATAAACTAATGAGAGGTATAGGCTTAGTCAAGTCTGTAC
TTACATTCTTTACGCCAGATTCTTCATGGGGCGACGCTGTTTCTCTTG
+
FFFFFFFFFFFFFFFFFFFFFFFFFFFFFFFFFFFFFFFFFFFFFFFFF:FFFFFFFFFFFFFFFFFF
FFFFFFFFFFFFFFFFFFFFFFFFFFFFFFFFFFFFFFFFFFFFF:FF
@A00721:190:HGJW7DSXY:4:1623:14434:32283_2:N:0:TCTGCAAG+GCTGCACT
AACAGCGTCATTTTCAGATGCTAACTTTCCTCTTGTAAGTTAATTATTAAATAATTCTTCTCACTCTT
GACGAGAATTAATACTGTTTACTCTACCCATAACTTATTTTTCAAAAA
+
,:,FF:,FFFF:FFF,,:FF,,F,,FFF:FFF,,F:FF,,,FF:F:FF:FFF,F:FFFFFFFF,:,:F
,:,:F,FF:,F,,FFF,F:F,,,,FFF:,FFFF,:,F:FF:FFFF:FF
@A00721:190:HGJW7DSXY:4:2448:14597:4507_1:N:0:TCTGCAAG+GCTGCACT
CTGATGTTTTCCTACAGAGTGCAGTCGTTTCAACACTTATCACCACTTTTGAAAGGAAAATAATTGGT
GATGAAAATACGTATGTCGGTGGGGTTCTCAAGGCTCTCATCGCCACTGGGATTCTAAGGGGTTCCAT
AAG
+
FFFFFFFF:FFFFFFFFFFFFFFFFFFFFFFFFFFFFFF,FFFFFFF:FFFFFFFFFFFFFFFFFFFF
FFFFFFFFFFFFFFFFFFFFFFFFFFFFFFFFFFFFFFFFFFFFFFFFFFFFFFFFFFFFFFFFFFFF
FFF
@A00721:190:HGJW7DSXY:4:1502:24071:7044_2:N:0:TCTGCAAG+GCTGCACT
TTTGGTCCTTACGTCTTAAGTAATTTGGATGTTGTCGAAAACTATCAGGTTCTTGTCACGATTCTTAA
AAGAAGTTTATTAAAACTTCAGAGTCTCTTGGAAGTCCAGGTAGCTAA
+
FFFFFFFFFFFFFFFFFFFFFFFFFFFFFFFFFFFFFFFFFFFFFFFFFFFFFFFFFFFFFFFFFFFF
:FFFFFFFFFFFFFFFFFFFFFFFFFFFFFFFFFFFFFFFFFFFFFFF
@A00721:190:HGJW7DSXY:4:1661:1154:19742_2:N:0:TCTGCAAG+GCTGCACT
GGGAAAAGAGACCTCTGTTGGTAGAGGAGTTGAGAGAATTACTCAGATTCAATCTGGCATCACTGAAC
GTCTACAACGAGTTAGGGAGTGGCTAGGTTTTAAAAGATCCTTTGCAC
+
FFF:FFFFFFFFFFFFFFFFFFFFFFFFF:FFFFFFFFFFFFFFFFFFFFFFFFFFFFFFFFFFFFFF
FFFFFFFFFFFFFFFFF:FFFFFFFFFFFFFFFFFFFFFFFFFFFFFF
@A00721:190:HGJW7DSXY:4:1345:12527:17143_2:N:0:TCTGCAAG+GCTGCACT
ACGTCGCGGAAAACGATGAAGATTTCCACACTGTTCGAGAATCTTCTGATGCTGAATTTGAGGAAAAT
TTTTCGGAAGTTTTTGGTATAAATCCTGATGGCTCAGTACAGGGAGGA
+
:FFFFFFFFFFFFFFFFFFFFFFFFFFFFFFFFFFFFFFFFFFFFFFFFFFFFFFF,FF,FF:FFFF
FFFF:FFFFFFFFFFF:FFFFFFFFFFFFFFFFFFFFFFFF:FFFFFFF
@A00721:190:HGJW7DSXY:4:1455:14163:5791_1:N:0:TCTGCAAG+GCTGCACT
AACCTTCTTTTATCATTGCAACAGGACTTTGAGAAGACTGTTGAAATGGACAAAATCTGGGATCATAG
GTATGTGTCACGCTACACTCGAAATATGGGCCCTCATAAGGAAGTATAGTTGGCGTCTTGTCGTTGAC
GTC
+
FFFFFFFFFFFFFFFFFFFFFFFFFFFFFFFFFFFFFFFFFFFFFFFFFFFFFFFFFFFFFFFFFFFF
FFFFFFFFFFFFFFFFFFFFFFFFFFFFFFFFFFFFFFFFFFFFFFFFFFFFFFFFFFFFFFFFFFFF
FFF
@A00721:190:HGJW7DSXY:4:1350:28094:27289_1:N:0:TCTGCAAG+GCTGCACT
TGTTCAGGAAGATCTCTACAGAGATTATCGAGAGTTGTGATCTAGTTCTAAATTTAGGTCGACGGACT
TTAGCAACTTTAGATAAATTCTTTCCGGGCTCAATGACCTTTGCTATGTACGTGGATCTAATGCTTGG
TGA
+
FFFFFFFFFFFFFFFF,FFFFFFFFFFFFFFFFF:FFFFFFFFFFFFFFFFFFFFFFFFFFFFFFFFFF
FFFFFFFFFFFFFFFFFFFFFFFFFFFFFFFFFFFFFFFFFFFFFFFFFFFFFFFFFFFFFFFFFFFF
FFF
```

@A00721:190:HGJW7DSXY:4:2459:11632:18349_2:N:0:TCTGCAAG+GCTGCACT
CATCGCAAAGAAGACTTTATTAGTTCATGAAGTTCTTACACTTCTCGCGACTAATGTCAGCTTCACTA
CAAAGGCGTTACGTAAAGTAGTTTTCACGCTTTATCTTAAAGCATTAC
+
FF:FFFFFFFFFFF:FFFFFFFFFFFFFFF:FFFF:FFFFFFFFFFF::FF,FFFFFFFFFF,FFFFF
FFF:F:FFFFFF:F,:,:FFF:FFFFFFFFFFFFF:F:FFFFF:FFFF
@A00721:190:HGJW7DSXY:4:1372:5837:7874_1:N:0:TCTGCAAG+GCTGCACT
ACTTTTTGAATATGACAGAAGCTCGCCATATCAGATTAGAGACATGCCAGTTAGAGTTCTTCTAACCA
GGACTGATGGTGAGATTCTTTTACCTTTTGGAGGTCCAGGAACCTTTACTCCTAGTGTCTTTTCTAGA
GTC
+
FFFFFFFFFFFFFFFFFFFFFFFFFFFFFFFFFFFFFFFFFFFFFFFFFFFFFFFFFFFFFFFFFFFF
FFFFFFFFFF,FFFFFFFFFFFFFFFFFFFFFFFFFFFFFF:FFFFFFFFFFFFFFFFFFFFFFFFFF
FFF
@A00721:190:HGJW7DSXY:4:1239:29098:33254_2:N:0:TCTGCAAG+GCTGCACT
CGGAGGATGACAGGTGGGAGACCGCTCAATAACACTCTATTGAATCCCTTTTCCACGATGAAAGCTCT
TTATGAGGTGCTCCATGCAAATTTACAGTTCCTCTTTGGTGATATCGC
+
FFFFFFFFF:FFFFFFFFFFFFFFFFFFFFFFFFFFFFFFFFFFFFFFFFFFFFFFFFFFFFFFFFFF
:FFFFFFFFFFFFFFFFFFFFFFFFFFFFFFFFFFF,FFFFFFFFFFFF
@A00721:190:HGJW7DSXY:4:1239:29098:33254_1:N:0:TCTGCAAG+GCTGCACT
TTTTGATAAATTTGTTGGAATCGATAATGATACTGACGAAGGTTCTATTACCACCCTTGTCATTGATA
CAAACATTCTAGGTGATGCTTTCTTAGAGGCTCGTCTAGCCGCTGAGATTCGTCTTACCTATTTACAA
TCT
+
FFFFFFFFFFFFFFFFFF:FFFFFFFFFFFFFFFFFFFFFFFFFFFFFFFFFFFFFFFFFFFFFFFFF
FFFFFFFFFFFFFFFFFFFFFFFFFFFFFFFFFFFFFFFFFFFFFFFFFFFFFFFFFFFFFFFFFFFF
FFF
@A00721:190:HGJW7DSXY:4:1214:4698:13260_1:N:0:TCTGCAAG+GCTGCACT
GATAACGATTTAGTACTGGGAAATGGTGGTAGTCAGTTTACTACTTTTCTTTCCCTCCCCTTTTTCGA
TTGGATTCGACGGGGTCTTCGTCTCACGACTAATTTGATAAGGTTTTTCTTTTATGGGACTCTTGTTT
GTA
+
FFFFFFFFFFFFFFFFFFFFFFFFFFFFFFFFFFFFFFFF:FFFFFFFFF:FFFFFFFFFFFFFFFFF
FFFFFFFFFFFFFFFFFFFFFFFFFFFFFFFFFFFFFFFFFFFFFFFFFFFFFFFFFFFFFFFFFFFF
FFF
@A00721:190:HGJW7DSXY:4:2618:7229:3959_2:N:0:TCTGCAAG+GCTGCACT
TATTACTTCGGAAGTTCTTAATGTAGGTCTCCTAGTACTTAAGGGTTTATGTTTCGGTTTCAGCTGGA
TGGATCTTTTGATGGTATGTTTCACATTGCGTGATCACTTCCCGTGGA
+
FFFFFFFFFFFFFFFFFFFFFFFFFFFFFFFFFFFFFFF:FFFFFFFFFFFFFFFFFFFFFFFFFFFF
FFFFFFFFFFFFFFFFFFFFFFFFFFFFFFF,FFFFFFFFFFFFFFFF
@A00721:190:HGJW7DSXY:4:1129:32606:26584_1:N:0:TCTGCAAG+GCTGCACT
ATTAAACATAAAATCCAGCGATTCTATTATGTCGCTAAAGCCTGTCCAGTTGTCGATGATGTTCGGGC
TGTGGGGATTAACTTCCCTCGTGAAACTTACGACGATGTTCTCGCTCGCATTTCACAAGGATTACTAA
GTC
+
FFFFFFFFFFFFFFFFFFFFFF:FFFFFFFFFFFFFFFFFFFFFFFFFFF:FF:FFFFFFFFFFFFFFF
FFFFFFFFFFFFFFFFFFFFFFFFFFFFFFFFFFFFFFFFFFFFFFFFFFFFFFFFFFFFFFFFFFFF
FFF
@A00721:190:HGJW7DSXY:4:1468:29649:35524_2:N:0:TCTGCAAG+GCTGCACT
ACTTCTAATTTTAGGAAACGTCGCGAAAAGAAAGTACATGATGATGTCTTTGTCGAAAATTATCGACG
GGAAGTTCTAATATAGGATGCTGATGACATTATGTCAACGGATACCCA
+

```
,:FFF,:FF:::F,,FF,:F,:F,:::F,,,F,FF,::,FF,F:::FF,,,FFFFFFFF,FFF,,,FF
,,FF,F,F,F:,:,::,,:,:,,FF,,,F:,::,,F:,,F,F,,:F,F
@A00721:190:HGJW7DSXY:4:1335:10719:17143_1:N:0:TCTGCAAG+GCTGCACT
AGAAACCGTCTCAGGAACTGTCTCCACTGATTCGGAATTAGAGAAAGACGACCTTCTTGTGGGGACTG
AGACCTCTGAATTTTCAGGGTGCCCTGATGAGGACGTTGAAAGGGATAATTTCAACTCTGAGACTTCT
AAG
+
FFFFFFFFFFFFFFFFFFFFFFFFFFFFFFFFFFFFFFFFFFFFF:FFFFFFFFFFFFFFFFFFFFFFFF
FFFFFFFFFFFFFFFFFFFFFFFFFFFFFFFFFFFFFFFFFFFFFFFFFFFFFFFFFFFFFFFFFFFFFF
FFF
@A00721:190:HGJW7DSXY:4:1125:9073:17519_1:N:0:TCTGCAAG+GCTGCACT
AGACCAGAAACGTAACAAGAAGAATAAAGGTAGGAAGGGGAGAAAACAGAACAAGCGAGCGGTTGAAG
AGAAAACTGATATGGTTGCGGCTGATCGTACCCCAATTCCTGCTAGAAAGATCACTCTCGGCGATACC
ACT
+
FFFFFFFFFFFFFFFFFFFFFFFFFFFFFFFFFFFFFFFFFFFFFFFFFFFFFFFFFFFFFFF:FFFFF
FFF:FFFFFFFFFFFFFFFFFFFFFFFFFFFFFFFFFFFFFFFFFFFFFFFFFFFFFFFFFFFFFFFFFF
FFF
@A00721:190:HGJW7DSXY:4:1154:3821:36730_2:N:0:TCTGCAAG+GCTGCACT
TAGTGGTCGTTCTTTTGGTGATTTTATCAAAGAGAAAGTTTCTGCTAGTCGCGACGATAGTATTTATC
GGAGTGATATTAACAAGTTAAACTTCGAACTGACCACTTACAAGAGTG
+
FFFFFFFFFFFFF:FFFFFFFFFFF:FF:F,FFFFFFFFFFFFFF:FF,FFFFFFF:FFFFFF:FFFF
FFFFFFFFFFFFFFFFFF:FFFFFFFFFFFFFFFFFFFFFFFFFFFFFF
@A00721:190:HGJW7DSXY:4:1564:9109:26788_2:N:0:TCTGCAAG+GCTGCACT
TCAGTTTTCCAAAAAGCTATTAATAGCAGTTGCGGGGCTATAATACCGCCTCCAGATTACTCTAAACT
GAATCCACTCTCTTGGGATATAAATGAGTACATCTATAATAGAGTTTT
+
FFFFFFFFFFFFFFFFFFFFFFFFFFFFFFFFFFFFFFFF:FFFFFFFFFFFFFFFFFFFF:FFFFFFF
FFFFFFFFFF:FF,FFFFFFFFFFFFFFFFFFFFFFFFFF,FFFF,FFF
@A00721:190:HGJW7DSXY:4:1569:10068:29794_1:N:0:TCTGCAAG+GCTGCACT
AACATGAATTAGTTATTGAAAGTCTTGACCCTCACGTCTCACTTTATCACCTTGGAGATGGTAAAGTC
ATTAACAAAGGAAATGTTAAGGCTGATGACTTAAATTTCGCATTCTGCTTCGACGGTAATTCCATAAG
ACC
+
FFFFFFFFFFFFFFFFFFFFFFFFFFFFFFFFFF:FFFFFFFFFFFFFFFFFFFFFFFFFFFFFFFFFFF
FFFFFFFFFFFFFFFFFFFFFFFFFFFFFFFFFFFFFFFFFFFFFFFFFFFFFFFFFFFFFFFFFFFFFF
FFF
@A00721:190:HGJW7DSXY:4:1433:28519:26929_1:N:0:TCTGCAAG+GCTGCACT
AACCTTTCCTCGTTCTTCATGAAGATCTTCGAGTGTTTTATAGTGAGTTATTCTTGCGAAGGTTCAAG
TCGATTGATTTTGTTGACTACGATCTCTCTTGTAATAAGAAGTTCTTCGCAGTTGAATCTCCACCAGG
TGG
+
FFFF::FFFFFFFFFFFFFFFFFFFFFFFFFFFFFF,FFF:FFFFFFFFFFFFFFFFFFFFFFFFFFFF
FFFFFFFFFFFFFFFFFFFFFFFFFFFFFFFFFFFFFFFFFFFFFFFFFFFFFFFFFFFFFFFFFFFFFF
FFF
@A00721:190:HGJW7DSXY:4:1242:13214:3490_2:N:0:TCTGCAAG+GCTGCACT
GCGACTTTTGTCGAATTTTTCGCAAAAGGTCTTGAAGTACGTATTGTCACTGCTAATCGTAATTCGCG
AGAAGAGATAGTGACTAAAGTTTTGGCACTTCTAGTTCATCGCGGTAT
+
FFFFFFFFFFFFFFFFFFFFFFFFFFFFFFFFFFFFFFFFFFFFFFFFFFFFFFFFFFFFFFFFFFFFFF
FFFFFFFFFFFFFFFFFFFFFFFFFFFFFFFFFFFFFFFFFFFFFF,F
@A00721:190:HGJW7DSXY:4:1438:4164:10394_2:N:0:TCTGCAAG+GCTGCACT
CAGACGTGAAAGTTATGATGAGTGATCGTATTAGAACAGCTGATTCTTATTGCATGAACGTCGCTGAG
```

GTCAAACCTGTGGACGTTCTTTTCATCGACGAATTATTCATGATTCAC
+
FFFFFFFFFFFFFFFFFFFFFFFFFFFFFFFFFFFFFFFFFFFFFFFFFFFFFFFFFFFFFFFFFFFFFFFF
FFFFFFFFFFFFFFFFFFFFFFFFFFFFFFFFFFFFFFFFFFFFFFFF
@A00721:190:HGJW7DSXY:4:1476:28971:4758_2:N:0:TCTGCAAG+GCTGCACT
CATCTACTTAAGGCGGCTTATACCATAGGTTACGGTGATTCCAATCAAATCGGATTCATCAATCGTAT
TGATCTCCAAGTCGCAAACTACTTCAACATCAACGACATCATTCTTGA
+
:F:,FF,,FFFFF,FFF,FFFFFFF,:,,,FFF:FFFFF:FFFFF:FFFFFFFF,F,FF:FF:F:FFF
F,FFFFFFFFFFF,FFFFFFFF,F,F:FFFFFFFFFFFFFFF:,,FFF
@A00721:190:HGJW7DSXY:4:2514:13313:20635_2:N:0:TCTGCAAG+GCTGCACT
TTATCGAAGTCTCAGTTATCGCTGCCCGGCCGACGTTTGTCGCGAACTCTCAGAGGTTTACGGCAGAA
AAATTTCTCCTGCTTGTAATTCGAGAGAGGACACTATGGAAATTAGAC
+
FFFFFFFFFFFFFFFFFFFFFFFFFFFFFFFFFFFFFFFFFFFFFFFFFFFFFFFFFFFFFFFFFFFFFFFF
FFFFFFFFFFF:FFFFFF:FF,FFF:FFFFFFFFFFFFFFFFFFFFFF
@A00721:190:HGJW7DSXY:4:2333:9787:21104_2:N:0:TCTGCAAG+GCTGCACT
TATTACTTTCACTCAAGGTGAAAAGTTTGAAATTAACAAAGTCCTTGGTAAGAGTAAGGTTAAATGTG
AAGATGCTCAGACTATTCATGAAGTTCAAGGTAATACTTACCAGCACG
+
FFFFFFFFFFFFFFFFFFFFFFFFFF:FFFFFFFFFFFFFFFFFFFFFFFFFFFFFFFFFFFFFFFFFFFFF
FFFFFFFFFFFFFFFFFFFFFFFFFFFFFFFFFFFFFFFFFFFFFFF:
@A00721:190:HGJW7DSXY:4:2228:2727:24752_2:N:0:TCTGCAAG+GCTGCACT
TTTTGCATCACAAACACATATTACCGTTGCTCTATCACGACACGTTGAATCTTTACGATACTTTGTTA
TCAATGTAAAGTTAAACGATGCTACTTGTGAGAGGATCAATCACAGTA
+
FFF:FFFFFFFFFFFFFFFFFFFFFFFFFFFFFFFFFFFFFFFF:F:FFFFFFFFFFFFF:FFFFFFFFF
FFFFFF:FFFFFFFFFFFFFFFFFFFFFFFFFFFFFFFFFFFFFFFF:
@A00721:190:HGJW7DSXY:4:1563:3866:31391_2:N:0:TCTGCAAG+GCTGCACT
ACGGGAGGTTGCAGATTCAACCCCTTATATCGAGGCTATCGAGGACGATGAGTATCTTGAAGTTGTTG
ACGACAAGCCTCAAGAGGGTAGAGGACCACCTGGATCTTCTACTGTCT
+
FFFFFFFFFFFFFFFFFFFFFFFFFFFFFFFFFFFFF,FFFFFFFFFFFFFFFFFFFFFFFFFFFFFFFF
FFFFFFFFFFFFF:FFFFFFFFFFFFFFFFFFFFFFFFFFFFFFFFFF
@A00721:190:HGJW7DSXY:4:1606:7075:27398_1:N:0:TCTGCAAG+GCTGCACT
AACCACCTTAACGTCACTCGGTAATCTTTCAGCCGAATTAAGTACTAATGAGTTCAAGTCGGTTGTCG
ATCGAATTAAGATCAAGGCTAATACCTTTATAGGTAAGAAGCCCCTTGAGCCGCGTAGTCAGCGCGTT
TAG
+
FFFFFFFFFFFFFFFFFFFFFFFFFFFFFFFFFFFFFFFFFFFFFFFFFFFFFFFFFFFFFFFFFFFFFFFF
FFFFFFFFFFFFFFFFFFFFFFFFFFFFFFFFFFFFFFFFFFFFFFFFFFFFFFFFFFFFFFFFFFFFFFFF
FFF
@A00721:190:HGJW7DSXY:4:1512:24578:3787_1:N:0:TCTGCAAG+GCTGCACT
TACCGAGTCTCCAAGAGAATATTTATTCTTATGAGGCTCGAAACTTCAACGTTTTCACAACGGAAAGA
CATATCGCACCCGAAATTTTTGGGCAGAATGCTCTTAACGCGGTTCTTGAACGGTGTTTTGATCAGTC
TAA
+
FFFFFFFFFFFFFFFFFFFFFFFFFF:FFFFFFFFFFFFFFFFFFFFFFFFFFFFFFFFFFFFFFFFFFFFF
FFFFFFFFFFFFFFFFFFFFFFFFFFFFFFFFFFFFFFFFFFFFFFFFFFFFFFFFFFFFFFFFFFFFFFFF
FFF
@A00721:190:HGJW7DSXY:4:1267:11677:15765_2:N:0:TCTGCAAG+GCTGCACT
TATAGGAGACTCAGTTAACCTGAGAGCTCCTCAATTGATAGATTGGGTCAACAATAGGGACGCTAGTA
TACTTCGTAAAGTCGACAGCGAAATGGTCAATATTGATAATTGGACCG
+

FFFFFFFFFFFFFFFFFFFFFFFFFFFFFFFFFFFFFFFFFFFFFFFFFFFFFFFFFFFFFFFFFFFFFF
FFFFFFFFFFFFFFFFFF:FFFFFF:FFFFFFFFFFFFFFFFFF,FFFF
@A00721:190:HGJW7DSXY:4:1128:21992:10958_1:N:0:TCTGCAAG+GCTGCACT
AGCTCATGGTGAAAAGGGAAGCTAAGGTTAAACTTGATGCTTCAGCCTTTTCGAAGCATAACCCCGCA
CAGAATATCGTTTATCACGCAAAATTTATTAATGCAATTTTCGGACCTATCTTTAAGGTTCTTGTGAG
GAG
+
FFFFFFFFFFFFFFFFFFFFFFFF:FFFFFFFFFFFFFFFFFFFFFFFFFFFF:FFFFFFFF:FF:FFFF
FFFFFFFFFFFFFFFFFFFFFF:FFFFFFFFFFFF:FFFFFFFFFFFFFFFFFFF,FFFFFFFFFFFFF
FFF
@A00721:190:HGJW7DSXY:4:2664:16396:14763_2:N:0:TCTGCAAG+GCTGCACT
CCAGTGACGATTTCTCCCGGAAGCTCTTCTCTCTCTTAGGAGGTCATGATGACTACTATAAGGGTGAA
ATCGATTTTTCGAAGTTTGACAAATCTCAAGATTTCTTTTGTAAAGCC
+
FFFFFF:FFFFFFFFFFFFFFFFFFFFFFFFFFF,FFFFFFFFFFFFFFFFFFFFFFFFFFFFFFFFFFF
FFFFFFFFFFF:FFF,FFFFFFFFFFFFFFFFFFFFFFFFFFFFFFFF
@A00721:190:HGJW7DSXY:4:1302:21658:20650_2:N:0:TCTGCAAG+GCTGCACT
TTCGATGCTGAATTTCTTGAACTTTGGAGTGCCAGTGAATATTTTTGTCACGCCAGGAGTATCCATAA
CGATTTGTCATTTACCACCTTTGCTCAACGACGAAGTGGAACGGCCAA
+
FFFFFFFFFFFFFFFFFFFFFFFFFFFFFFFFFFFFFFFFFFFFFFFFFFFFFFFFFFFFFFFFFFFFFF
FFFFFFFFFFFFFFFFFFFFFFFFFFFFFFFFFFFFFFFFFFFFFFFF
@A00721:190:HGJW7DSXY:4:2469:6334:28651_1:N:0:TCTGCAAG+GCTGCACT
TAATTGTATGATCACTTTGATGATTTTATCACTTTATTATGACCTTGGAAAGTTTTCGGCTGTGGTTG
TCGCTGGGGACGATTCCATTCTGTTTTCAAAGACTCCAATAACTGATCATTCTGATCATATGATTTTG
GAC
+
FFFFFFFFFFFFFFFFFFFFFFFFFFFFFFFFFFFFFFFFFFFFFFFFFFFFFFFFFFFFFFFFFFFFFF
FFFFFFFFFFFFFFFFFFFFFFFFFFFFFFFFFFFFFFFFFFFFFFFFFFFFFFFFFFFFFFFFFFFFFF
FFF
@A00721:190:HGJW7DSXY:4:2313:5538:2096_2:N:0:TCTGCAAG+GCTGCACT
TGCTCGCGATTTATTCTTTTTGTCAATGATTACATCTACTTCGTTCCTGATCCTTACAAGATCATCGT
TAAGTTAGGTAAACCCCTAACCGATGTTACTCTTGTAGGACTTTGGGA
+
FFFFFFFFF:FFFFFFFFFF,FF:FFFFFFF:FFFF:FFFFFFFFFFFFFFFFFFFFFFFFFFFFFFFF
FFFFFF:F,F:FFF,F:FFFF:FFFFFFFFFFFFFFFFFFFFFFFFFF
@A00721:190:HGJW7DSXY:4:1328:22218:1986_2:N:0:TCTGCAAG+GCTGCACT
AGGTTGTTGTTTGGCGATTAGCTCTTGCGACTAATATTCGATATTCGATTGAGGGTCGCTATACCTAT
TCGGCTATCTGTGCTTTGAATTGTATAAGATCAAATTTTAAACGATTC
+
FFFFFFFFFFFFFFFFFFFFFFFFFFFFFFFFFFFFFFFFFFFFFFFFFFFFFFFFFFFFFFFFFFFFFF
FFFFFF:FFFFFFFFFFFFFFFFFFFFFFFFFFFFFFFFFFFFFFFFF
@A00721:190:HGJW7DSXY:4:1322:4869:36886_1:N:0:TCTGCAAG+GCTGCACT
TGATGTGGAGGGAGTTCTTGAAGCATCCCGACCTTTCCTTGTGAAAATTTTGATGCGGTTTCATAAGA
TTATCAGTAATCCTGATTCTACTACGGATTCATGGACCTTCCATCCTGTACCTCTTCTGGAGAGACGT
ATC
+
FFFFFFFFFFFFFFFFFFFFFFFFFFFFFFFFFFFFFFFFFFFFFFFFFFFFFFFFFFFFFFF:FFFFFF
FFFFFFFFFFFFFFFFFFFFFFFFFFFFFFFFFFFFFFFFFFFFFFFFFFFFFFFFFFFFFFFFFFFFFF
FFF
@A00721:190:HGJW7DSXY:4:1571:19352:27931_2:N:0:TCTGCAAG+GCTGCACT
CTTCGAGACAGGTGATATTGTTGAGCGAGAGCGACCAAGATCTAAATTCGGTAACGGTGCGTGGCGCT
TTATTTTAAAAAGCTCCCTCCTCTCGCAAGTTTGATCTGATACTTATC
+

FFFFFFFFFFFFFFFFFFFFFFFFFFFFFFFFFFFFFFFFFFFFFFFFFFFFFFFFFFFFFFFFFFFF
FFFFFFFFFFF:FFFFFFFFFFFFFFFFFFFFFFFFFFFFFFFFFFFF
@A00721:190:HGJW7DSXY:4:2402:20066:3302_2:N:0:TCTGCAAG+GCTGCACT
TGATCAGTCTCTGACTTGACCATCTTTCATTTTAAATGACGATCATTAAGACTCTCTTTCTTCTTTGT
TTCCTTCTTTGCACTGATTTTTCCTCATCAGCTTGTGTTTTACCTAAT
+
FFFFFFFFFFFFFFFFFFFFFFFFFFFFFFFFFFFFFFFFFFFFFFFFF:FFFFFFFFFFFF:FFFFF
FFFFFFFFFF:FFFFFFFFFFFFFFFFFFFFFFFFFFFF:FFF:FF:F
@A00721:190:HGJW7DSXY:4:1465:30011:5744_2:N:0:TCTGCAAG+GCTGCACT
GATAACAACTATCAGTTGATTAAGACTCTTCCGAAAGGCGAACTTTGGGAATTTGAAGCTCAGGGTGA
AGCACAACGTTTGACCGTGACGAACTGTGGTGTTCTTGATCTACGTTA
+
FFFFFFFFFFFFF:FFFFFFFFFFFFFFFFFFFFF:FFFFF:FFFFFFFFFFFFFFFFFFFFFFFFFF
FFFFFFFFFFFFFFFFFFF:FFFFFFFFFFFFFFFFFFFFFFFFFFFF
@A00721:190:HGJW7DSXY:4:2464:30689:33505_1:N:0:TCTGCAAG+GCTGCACT
CACTACCACTTATTACGATATTTCTTTAGTCGATGGGTTTTCGACGCCTTTAAGCGTCTTCTGTTCAT
CGCAACCAGATGATATCTTCTATTCGAACTTATGTTTCGATCAGTGTTTCTTCTATCAGGAGGAAAAC
ACT
+
FFFFFFFF:FFFFFFFFFFFFFFFFFFFFFFFFFFFFFFFFFFFFFFFFFFFF:FFFFFFFFFF
FFFFFFFFFFFFFFFFFFFFFFFFFFFFFFFFFFFFFFFFFFFFFFFFFFFFFFFFFFFFFFFFFFFF
FFF
@A00721:190:HGJW7DSXY:4:1508:31937:30686_2:N:0:TCTGCAAG+GCTGCACT
TCACTTTGTGTGTACTTGTCTCTATTTTAAGTTTTGTCTTCCTTGTTTGTCTTATTCTATTAATCACT
CATTTTAGTGAGATTATTAAAAACGCACTAAAACGTCTTCGACAACAG
+
FFFFFFFFFFFFFF:FFFFFFFFFFFFF:FFFF:FFFFFFFFFFFFFFFFFFFFFFFFFFFFFFFFFF
FFFFFFFFFFFFFFFFFFFFFF,FFFFFFFFFFFFFFFFFFFFFFFFF
@A00721:190:HGJW7DSXY:4:1611:5077:6778_1:N:0:TCTGCAAG+GCTGCACT
GCCTTACAGAGGGGCAGCAGCATGACCATAATTGGTATTGACTTTGGTACCACCTTTTCTACTGTCTG
TCTCGCCTCTTCGAATGAAGTTTTAATGCTTAACAACAATGATAGTGAATTTATACCTACAATCATTG
GTT
+
FFFFFFFFFFFFFFFFFFFFFFFFFFFFFFFFF:FFFFFFFFFFFFFFFFFFFFFFFFFFFFFFFFFF
FFFFFFFFFFFFFF:FFFFFFFFFFFFFFFFFFFFFFFFFFFFFFFFFFFFFFFFFFFFFFFFFFFFF
FFF
@A00721:190:HGJW7DSXY:4:1252:3378:28134_2:N:0:TCTGCAAG+GCTGCACT
TGTTTATGGATATGATGCTATTACTCATGATGGTTACGGTCAGGGAGGATTCTCAGTTTACAAGGACC
TTAAGCGATGGATCGGGGTTAATTCTAAAACATTACCCGAAAGACGTG
+
FFFFFFFFFFFFFFFFFFFFFFFFFFFFFFFFFFFFFFFFFFFFFFFFFFFFFFFFFFFFFFFFFFFF
FFFFFFFFFFFFFFFFFFFFFFFFFFFFFFFFFFFFFFFFFFFFFFFF
@A00721:190:HGJW7DSXY:4:1224:7997:1532_2:N:0:TCTGCAAG+GCTGCACT
GTACGATGTCGTCTGTCTCGGCGATCACTTTTCACTAAGAATTGGTCCTACTTTTGGACAAGGCCAAC
TTCGTAATCTTCATGAGATCGTTTCTTTCTTTATCTCTTTGGTTGTTA
+
FFFFFFFFFFFFFFFFFFF:FFFFFFFFFFFFFFFFFFFFFFFFFFFFFFFFFFFFFFFFFFF:FFFF
F,FFFFFFF:FFFFFFFFF:FFFFFFFFFFFFFFFFFFFFFF:FFFFF
@A00721:190:HGJW7DSXY:4:1165:25382:13009_2:N:0:TCTGCAAG+GCTGCACT
GAGTGAAAGGCTTCAATTGTAGTGGTTTGGTCGTTTCAGTACCCAGTCAGTATACCTCTACGCAAAGA
TATTTCATGAAAACTACAGAGGAACGTACCGGATTACCTGTCTTTCAT
+
FF:F:FFF:FFFFFFFFFFFFFFFFFFFFFFFFFFFFF:FFF,FFFF:F:FFFFFFFFFFFFFF,F:F
FFF:FF:FFFFF,FFFFF:FFFF:FF:FFFF:FF,FFFF::F:FFF:F

```
@A00721:190:HGJW7DSXY:4:2172:20681:3959_1:N:0:TCTGCAAG+GCTGCACT
CTTCGGCCGCTCTCTTTGCTTCGATGTTGGATGTCAAGAAGGGTTCAGAATGGGATTCCTACGTAGTT
TATGACTTTGGTGGTGGTACTTTCGATACTTCTATCGTCTTACGTTACGGTAATTACTATAGTGTTAT
TTA
+
FFFFFFFFFFFFFFF:FF:FFFFFFFFFFF:FFFFFFFFFFFFFFFFFFFFFFFFFFFFFFFFFFFFF
FFFFFFFFFFFFFFFFFFFFFFFFFFFFFFFFFFFFFFFFFFFFFFFFFFFFFFFFF:F:FFFFFFFF
FFF
@A00721:190:HGJW7DSXY:4:2412:21748:1297_1:N:0:TCTGCAAG+GCTGCACT
ATAATCTTGGAGGTCGTGACGTTGATGAAGCTATAAGAGCGTTCTTATTACGGCGCACTTCTTTACCC
GCCACCACACAACTTTCAGTTTCACAACTGAAGGAGGAGGTTTCTGTTACTGGAAAACCCTCGTCAAT
ACT
+
FFFFFFFFFFF,FFFFFFFFFFF:FFFFFFFFFFFFFFFFFF:FFFFFFFFFFFFFFFF:FFFFFFF:
FFFFFFFFFFFFFFFFFF:FFFFFFFFFFFFFFFFFFFFFFFFFFFFFFFFFFFFFFFFFFFFFFFFF
FFF
@A00721:190:HGJW7DSXY:4:2550:4426:7968_2:N:0:TCTGCAAG+GCTGCACT
GATCTAGTGAAATTAACGCGTCCTTTTCTGGAACGTAGTTTTACTATTATGAACAAAGTTATCAAAGA
TAGTGGCATTCAAGGCAAACTTACCCTGATTCCGGCCGGTGGTAGCTC
+
FFFFFFFFFFFFFFFFFFFFFFFF:FFFFFFFF:FFFFFFFFFFFF,FFFFFFFFFFFFFF,FFFFFFF
FFFFFFFFFFFFFFFFFFFFFFFFFFFFFFFFFFFFFFFFFFFFFFFF
@A00721:190:HGJW7DSXY:4:1604:32181:23093_2:N:0:TCTGCAAG+GCTGCACT
TGAAACAGCTATTAAAGCTGTTGAGCGGGTAATTACACCTAAACGAGCTCGATCTGCCATCGCTGAAG
GATGTGCACTCTTTTCTGCTACTTTAACGGAGCGTAGTGTACTCTTCG
+
FFFFFFFFFFFFFFFFFFFFFFFFFFFFFFFFFFFFFFFFFFFFFFFFFFFFFFFFFFFFFFFFFFFF
FFFFFFFFFFFFF:FFFFFFFFFFFFFFFFFFFFFFFFFFFFFFFFFF
@A00721:190:HGJW7DSXY:4:1241:11849:31093_2:N:0:TCTGCAAG+GCTGCACT
AAAGGTCTCTATCAAGCTCGTATTATCGTCGCTGCTGGATCTGCGTTACCGTTGAAGGCTAAACTCCA
AGGTAATTATGAAGACTGTGATTTATCTTTCACTCTGGCCTTGTATGA
+
FFFFFFFFFFFFFFFFFFFFFFFFFFFFFFFFFFFFFFFFFFFFFFFFFFFFFFFFFFFFFFFFFFFF
:FFFFFFFFFFFFFFFFFF:FFFFFFFFFFFFFF:FF:FFFFFFFFFF
@A00721:190:HGJW7DSXY:4:2145:15510:12665_1:N:0:TCTGCAAG+GCTGCACT
CATTAATAATTTCAATCTTCTTAAAAAGAAGATAGAGAGTCATACTTTGGGCTATCGTTCAGGTGCAT
GGGGTTGGATAGCCAATATTGAAGTGGACACCTCTGGTAGTATCGAAGTAAAGAGATCGGAAGAGCAC
ACG
+
FFFFFFFFFFFFFFFFFFFFFFFFFFFFFFFFFFFFFFFFFFFFFFFFFFFFFFFFFFFFFFF:FFFF:
FFFFFFFFFFFFFFFFFFFFFFFFFFFFFFFFFFFFFFFFFFFFFFFFFFF,FFFFFFFFFFFFFFFFF
FF:
@A00721:190:HGJW7DSXY:4:2610:30228:5932_2:N:0:TCTGCAAG+GCTGCACT
AAGCATACTACTTTTGCTTTGATTACTCTACCGCCTTACAAGTCTAAGGTTAAAGAGTCGATTAAACA
AGTTGTACTTATAAATGATTTTATCGCTGGTTGTTATATCGAAAAGTC
+
FFFFFFFFFFFFFFFFFFFFFFFFFFFFFFFFFFFFFFFFFFFFFFFFFFFFFFFFFFFFFFFFFF,F
F,FF,F:,,FFF,FF:,FFFFF,F,F,F:F,F,FF,F,F:F,FFF,F,
@A00721:190:HGJW7DSXY:4:2311:8124:15499_1:N:0:TCTGCAAG+GCTGCACT
CCCAAAGAGAATATCTAAAATATTTACAAAATCTTACCACTATCTACGAACCTAAAGTTCTGAAGACG
TTGCTTGTCGCTTCCTCCTTTAATGGCACTACCTCGGTACAATCTTACTTGGAACGAAGGCGACCTGT
TCC
+
:FFFFF:FFFFFFFFFF:FFFFFFFF:FFF,:FFFFFFFF,FF:FFF:FFF,F,,F::FFFFFFFFFF
```

```
FFFFFFFFFFFFFFFFF:FFF:FFF,FFF:F:FFFFFFF:F:F:FFF,FF:,FF,FFF::FFFF:FFF
FFF
@A00721:190:HGJW7DSXY:4:2618:30291:9455_1:N:0:TCTGCAAG+GCTGCACT
GTTCTGTCTATATTAGGTGAAGCGAAATACGATTTCGAACGTCAACGTTCACCGAGTTTCGTTTCGTA
CCTTGATGCTGACTTTCGTCAATATGATTTGATCTGGTTGCTCAATTCTAAGCGTGTTCCGCTTAGTG
CGA
+
FFFFFFFFFFFFFFFFFFFFFFFFFFFFFFFFFFFFFFFFFFFFFFFFFFFFFFFFFFFFFFFFFFFF
FFFFFFFFFFFFFFFFFFFFFFFFFFFFFFFFFFFFFFFFFFFFFFFFFFFFFFFFFFFFFFFFFFFF
FFF
@A00721:190:HGJW7DSXY:4:2110:25482:14372_1:N:0:TCTGCAAG+GCTGCACT
CCGAACTTGCCACGACTCTCCGTGACATACTACATACTAGATATTTGGATGATCTTGATAAAGAACTT
CATGAATTTTCAGAGGATGAAAAATTTTCTTCCTTGGATTTTAATATAAAGAATCTTTACCAGGACTC
CT
+
:F,F,F:,:,F,F,,F,,,,F:,,:F:,:F,,:,,,F,F:F,F,,:FFF,:F,F:F:F,F:F,FFF:,
FFFFFF,F:FFFFFFFFFFFFFFFFFFFFFFFFFFFFFFFFF:F:FFF:FFFFFFFFFFFFFFFFFFF
FF
@A00721:190:HGJW7DSXY:4:2401:26313:10802_1:N:0:TCTGCAAG+GCTGCACT
GATCGTAAGAAGTTTATAAGGAACATATCCATTTGTTGTAATAGGGTCATCAACTTAGAACTCGAAAC
TCTAAGTGGTAATACTGTCTTTCCAAGCTTACCTGTATCTTCAATGAAATTGGACTCTTCGATAGGGG
ACA
+
FFFFFFFFFFFFFFFFFFFF:FFFFFFFFFF:FFFFFFFFFFFFFFFFFFFFFFFFFFFFFFFFFFFF
FFFFFFFFFFFFFFFFFFFFFFFFFFFFFFFFFFFFFFFF:FFFFFFFFFFFFFFFFFFFFFFFFFFF
FFF
@A00721:190:HGJW7DSXY:4:2319:7889:26303_1:N:0:TCTGCAAG+GCTGCACT
TAAGAATACTATAAATAGAATAGCTATTACTACTAATGGCGTTGGCCTAGTGGATGTTATTAGTGACA
TCTGGTCAATTTCGTTTTTAGGTGGTCTTTATTCTATTATTGATTTTTATGAGATTGAGATCGGAAGA
GCA
+
FFFFFFFFFFFFFFFFFFFFFFFFFFFFFFFFFFFFFFFFFFFFFFFFFFFFFFFF,FFFFFFFFFFF
:FFFFFFFFFFFFFFFFFFFFFFFFFFF,FFFFFFFFFFFFFFFFFFFFFFFFF:,FFFFFFFF:FFF
:FF
@A00721:190:HGJW7DSXY:4:1516:14244:3834_2:N:0:TCTGCAAG+GCTGCACT
TTCCTGAAGAGTCCTTCCGTAACCAACTTCTTGACAATTTTCTTCAATCCTTCGATAATAGTTAAATC
TACGATTATGTCTGTGTTGAAAGACTTTCTACCGATAAGGGTCACTAG
+
FFFFFFFFFFFFFFFFFFFFFFFFFFFFFFFFFFFFFFFFFFFFFFFFFFFFFFFF:FFFFFFFFFFF
FFFFFFFFFFFFFFFFFFFFFFFFFFFFFFFFFFFFFFFFFFFFFFFF
@A00721:190:HGJW7DSXY:4:2674:27597:26271_2:N:0:TCTGCAAG+GCTGCACT
TAAGAAATAATCCTACATTGACTGATTATCTAAAGATTGAAGGAAAATTTTATCCTCGTTTAACTTAC
TTAGACTTTCCTACGATACAAATGATAACTTCTAATTTACGTTTGCTA
+
FFFFFFFFFFFFFFFFFFFFFFFFFFFFFFFF:FFFFFFFFFFFFF:FFFFFFFFFFFFFFFFFFFFF
FFFFFFFFFFFFFFFF:FFFFFFFFFF:FFFFFFFFFFFFFFFFFFFF
@A00721:190:HGJW7DSXY:4:1573:25889:27571_1:N:0:TCTGCAAG+GCTGCACT
GATGCATGGTGTTATCTCCTCAGGAGTCCCGGCATTTGATAATAATGACGAGACTTTCGTCATAATCG
CTGCATATTACGGATTACACGGTACCAGCGCATCTCGTGTCGAGCCTCGACCCGATTATATGGTATTA
ACT
+
FFF:FFFFFFFFFFFFFFFFFFFF:FFFFFFF,FFFFFFF:FFFFFF:FFFFFFFFFFFFFFFFFFFF
FFFFFFFFFF:FFFFFFFFFFFFFFFFFFFFFF:FFFFFFFFFFFFFF,FFFFFFFFFFFFFFFFFF:
FFF
```

@A00721:190:HGJW7DSXY:4:2675:9254:19085_1:N:0:TCTGCAAG+GCTGCACT
TTCTAGAATTCTTAATTATAATATTCAACCTCAAAAGTTACGTGTTTACAGTAACGAGATCACACATT
TTGAGAACTAACTATGTTATGTTCTCTGATCATTTTGACAATCTCTTCTTATAATAAATAAATAAATA
ATA
+
FFFFFFFFFFFFFFFFFFFFFFFFFFFFFFFFFFFFFFFFFFFFFFFFFFFFFFFFFFFFFFFFFFFFFF
FFFFFFFFFFFFFFFFFFFFFFFFFFFFFFFFFFFFFFFFFFFFFFFFFFFFFFFFFFFFFFFFFFFFFF
FFF
@A00721:190:HGJW7DSXY:4:2434:15537:31093_2:N:0:TCTGCAAG+GCTGCACT
AGTGGAAAGGCTCCAGTCAACACTAGTTCGAGTGCTGCTTTGGATTACGATGACGTGAGAATAGGTGA
AGGGATAGTTTTTACCTCGACCAACATTAAGGCGGCGGCGACTAATCT
+
FFFFFFFFFFFFFFFFFFFFFFFFFFFFFFFFFFFFFFFFFFFFFFFFFFFFFFFFFFFFFFFFFFFFFF
:FFFFFFFF:FFFFFFF:FFFFFFFFFFFFFFFFFFFFFFFFFFFFFFF
@A00721:190:HGJW7DSXY:4:2316:14651:19006_1:N:0:TCTGCAAG+GCTGCACT
AGGACACTTCGAAACTGAATAATGATGCACTACAAACAAGTATCGCTAATCATCTGAAAAAGGCTCTA
AAGGACACTATACAATATCCAGACGACGACGAAGCTTATACTACTTTAATTTTGGGTATTATGTGGAG
ATA
+
FFFFFFFF:F:FFFFFFFFFFFFFFFFFFFFFFFFFFF:F:FFFFFFFFFF:FFFFFFFFFFFFFFFFFF
:FFFFFFFFF:FFFFFFFFFFFFFFFFFFFFFFFFFFFFFFFFFFFFFFFFFFFFFFFFFFFFFFFFFFF
FFF
@A00721:190:HGJW7DSXY:4:1513:18448:28056_2:N:0:TCTGCAAG+GCTGCACT
ACCACATTTTGGTCAAGTCAATCCGTTGAGAAAATTTCTTAGAGGATATTCGCCATTGTGGTACGAAT
TGGTCCAACATTTACCACCAAATTCTTTTGATACAACTCTAGCCTTCA
+
FFFFFFFFFFFFFFFFFFFFFFFFFFFFFFFFFFFFFFFFFFFFFFFFFFFFFFFFFFFFFFFFFFFFFF
FFFFFFFFFFFFFFF:FFFFFFFFFFFFFFFFFFFFFFFFFFFFFFFF:
@A00721:190:HGJW7DSXY:4:2370:26142:19899_2:N:0:TCTGCAAG+GCTGCACT
TCACCAGATTGGTTCGAACCCGTTGATGCTATGTCAGAAGAAGCTATTCAAGCTTGTCGTTTAAAGAC
TGCAGAGGCTGTCAAACAAGCCCAGACTCAAGGAGTCCAATCAAAGGT
+
FFFFFFFFFFFFFFFFFFFFFFFFFFFFFFFFFFFFFFFFFFFFFFFFFFFFFFFFFFFF:FFFFFFFFF
FFFFFFFFFFFFFFF:FFFFFFFFFFFFFFFFFFFFFFFFFFFFFFFFF
@A00721:190:HGJW7DSXY:4:1636:25373:7858_1:N:0:TCTGCAAG+GCTGCACT
ATTATTTAGAGATCTTTTCTACTTACAAAGGACAATTCAGTTTCATTAATTCTACGGAAAAGTCCGGA
GTATATTGTATGTTAATAGATAGTGGGATAATAGTTCTACCGATACGTAGTGAACGCGTTCGTAGAAC
CAA
+
FFFFFFFFFFFFFFFFFFFFFFFFFFFFFFFFFFFFFFFFFFFFFFFFFFFFFFFFFFFFFFFFFFFFFF
FFFFFFFFFFFFFFFFFFFFFFFFFFFFFFFFFFFFFFFFFFFFFFFFFFFFFFFFFFFFFFFFFFFFFF
FFF
@A00721:190:HGJW7DSXY:4:2424:2031:36010_1:N:0:TCTGCAAG+GCTGCACT
TAACCGATAGGAGTTGTATAGTTGAAGATGTGGCTTTAGACGATAATCCTTTAAGGACTATGTTGACT
GACGAAGATTTTCCCACAGATTTGTCTACGTTGCAGATCGCCACTCCAAACACTATATACATTAAAAT
ATA
+
FFFFFFFFFFFFFFF:FFFFFFFFFFFFFFFFFFFFFFFFFFFFFFFFFF:FFFFFFFFFFFFFFFF:FF
FFFFFFFFFFFFFFFFFFFFFFFFFFFFFFF:FFFFFFFFFFFFFFFFFFFFFFFFFFFFFFFFFFFFFF
FFF
@A00721:190:HGJW7DSXY:4:1617:4164:19852_1:N:0:TCTGCAAG+GCTGCACT
CATAACTTTTGTTACCAAATTCTTGGCTTTCAACGAAGTCTCTGATTTTATTAATCATTACTTTAACG
ATCTCTCGGTCATTTACTCTGTACTTGATCTACGTCGTTTAACTGTCGGAACAACCTTTACTTAGAAT
GAC

+
FFFFFFFFFFFFFF:FFFFFFFFFFFFFFFFFFFFFFFFFFFFFFFFFFFFFFFFFFFFF:FFFFFFFFF
FFFFFFFFFFFFFFFFFFFFFFFFFFFFFFFFFFFFFFFFFFFFFFFFFFFFFFFFFFFFFFFFFFFFFF
FFF
@A00721:190:HGJW7DSXY:4:1618:17246:12383_2:N:0:TCTGCAAG+GCTGCACT
TTCTTATATTATTTACAATAGTTAATATCATATTAGTAATACTCACTATCGTTTTCTGCTTTTTCTTA
GCTAATAGATTAATAAAGGATGAGTACGAAAACCAACTCTTCGTTACG
+
FFFFFFFFFFFFFFFFFFFFFFFFFFFFFFFFFFFFFFFFFFFFFFFFFFFFFFFFFFFFFFFFFFFFFF
FFFFFFFFFFFFFFFFFFFFFFFFFFFFFFFFFFFFFFFFFFFFFFFFF
@A00721:190:HGJW7DSXY:4:2675:2537:22232_1:N:0:TCTGCAAG+GCTGCACT
GTAGAATAGATTTAGTCTCTACACCCATCTCAACGAGGATAACGCGAGTTTTCCGTCCTGAACGTAAT
ACACGAAGAGATCATCCGCAAACGACTCGAAACCGAGACGCCGTAGATCAAAGTCCCTTCTAACTTTA
GAT
+
FF,,F:F:,,FFF,F::F,,FF,FF:,,:F::,:F,F,::F:,FF,,FFFF,::F:FFF,F,,,,FF:
:F,F:F,,,,F,F,,F::,F:FFFFFFFFF:FFFFFFFFFFFFFFFFFFFFFFFFFFFFFFFFFFFFFFF
FFF
@A00721:190:HGJW7DSXY:4:2550:12789:16313_1:N:0:TCTGCAAG+GCTGCACT
CTTTACTCATTTTATAGCAGTTATTTATGTTGTACTTTTTATAATTTATAACCTTCTTATACTTTTGG
GTATGTTTTGTGAAAAACATGAGATACCCAAGTCTATCGAAGACATTATCTCTTATGTACTTATTGTA
ACG
+
FFFF:FFFFFFFFFFFFFFFFFF,:FFFFFFFFFFFFF:FFFFFFFFFFFFFFFFFFFFFFFFFFFFF:F
FFFFFF::FFFFFFFFFFFFFFFFFFFFFFFFFFFFFFFFFFFFFFFFFFFFFFFFFFFFFFFFFFFFFF
:FF
@A00721:190:HGJW7DSXY:4:1320:28438:36839_1:N:0:TCTGCAAG+GCTGCACT
TTCTTTTATTCCTTTAACTGTGTATGAGGAGGATTTATAATGCTTTTCGATAGTGATTGTTATCAATT
TAAATACATTACTGTTTCAAATCATCTTTACTCACAGACTCCGAAAATGGTTTCAAATGAGGTCAAAG
CTC
+
FFFFFFFFFFFF:FFFFFFFFFFFFFFFFFFFFFFFFFFFFFFFFFFFFFFFFFFFFFFFFFFFFFFFF
FFFFFFFFFFFFFFFFFFFFFFFFFFFFFFFFFFFFFFFFFFFFFFFFFFFFFFFFFFFFFFFFFFFFFF
FFF
@A00721:190:HGJW7DSXY:4:1621:29975:30890_2:N:0:TCTGCAAG+GCTGCACT
AACTATTATCCGATTCTTCGAGGGTATCACTACCACTGATCTGCCGACTTTAGTCAAGGAACTTCAAT
TCCTTCGATGTTTATATTTTTCTATAAGGGAAAATAGCAATAATCAAA
+
FFFF:FFFFFFFFFFFFFFFFFFFFF:FFFFFFFFFFFFFFFFFFFFFFFFFFFFFFFFFFFFFFFFFFF
FF:FFFFFFFFFFFFFFFFFFFFFFFFFFFFFFFFFFFFFFFFF:FFFF
@A00721:190:HGJW7DSXY:4:1235:31910:15389_2:N:0:TCTGCAAG+GCTGCACT
AACTTTATTTCAGAACTCTCTCCGATCACCTTGGTGTGATCTCCCGGAGGATCTTTTCAATAAGATGT
ATGATGATAATCTACTATTTTTAAATAGTGATATTCAGCGTATACTTG
+
FFFFFFFFFFFFFFFFFFFFFFFFFFFFFFFFFFFFFFFFFFFFFFFFFFFFFFFFFFFFFFFFFFFFFF
FFFFFFFFFFFFFFFFFFFFF:FFFFFFFFF:FFFF:FFFFFFFFFFFF
@A00721:190:HGJW7DSXY:4:1513:21730:23500_1:N:0:TCTGCAAG+GCTGCACT
AAAGATTTGGCTTGGTCTATTTACCGCAAAAAGTTGGAAGAGGTCTATGATATTTATTTTACTTATAC
TTTAAATAGCACCGATTATAACTTGACTGTTGATAATATTACAATAGCTCTTTCTCAGAAGATTAGAG
AAG
+
FFFFFFFFFF:FFFF:FFFFF:FFFFFFFFFFFFFFFFFFFF:FFFFFFFFFFFFFFFFFFFFFFFFFFF
FFFFFFFFFFFFFFFFFFFFFFFFFFFFFFF:FFFFFFFFFFFFFFFFFFFFFFFFFFFFFFFFFFFFFF
FFF

```
@A00721:190:HGJW7DSXY:4:1507:4218:15186_2:N:0:TCTGCAAG+GCTGCACT
CGTCGGTAGCATTGATTTACTTTTACCGTTAGAACTGAAAAAGATTCTTTTCGAAAAGAAACCTATAA
GTTACAAACGATTCTTCGACTATATTAAAGAAATAAAAATACTTCAAG
+
FFFFFFFFFFFFFFFFFFFFF::FFFFFFFFFFFFFFFFFFFFFFFFFFFFFFFF:FFFFFFFF:F,FF
FFFFFFFFFFFFFFF:FFFFFF:FFFFFFFFFFFFFFFFFFFFFFFFF:FF
@A00721:190:HGJW7DSXY:4:2126:22652:10755_2:N:0:TCTGCAAG+GCTGCACT
TTACATTTTTAATGGTATGGTATAATGTGTCTTACAAATAGAACCTTCACTCTCGGTAATGAGAGTGT
ATTCCTTAATATTCTAGTAACCGCTATGTGGGCATTACTAGGATTTAT
+
FF::F:F:FF:FFFFFFFFFFFFFFFFFFFFFFFFFFFFFFFFFF:,FFFFFFF:FFFFFFFFFFFFFF
FFFF:FFFFFFFFFFFFFFFFFFFFFFFFFFFFFFFFFFFFFFFFFFF
@A00721:190:HGJW7DSXY:4:2510:12780:33238_2:N:0:TCTGCAAG+GCTGCACT
TTTAAATGCTAGGAAAATTTTAGAAAAACTCCATTGTATAGTTCAAGGAGTACTTAATTCAATAGCTT
TAGTATTATTTACTATCGCCTTTATTGAAAATTGTATAAGAGTTAATT
+
FFFFFFFFFFFFFFFFFFFFFFFFFFFFFFFFFFF,FFFFFFFFFFFFFFFFFFFFFFFFFFFFFFFFF
FFFFFFFFFFF:FFFFF:FFFFFFFFFFFFFFFFFFFFFFFFFFFFFF
@A00721:190:HGJW7DSXY:4:2522:23511:11866_1:N:0:TCTGCAAG+GCTGCACT
ACATACCATAACCTACCATGACCAATCATCGCTCCAACTGGCACGCCAGACGTAGGGTCAAACCTACT
ACCGCCGGTCGGCAGATCGGCACCACTGGTCTCTACGTTCAGGGACCCGCTGTGCAGAATAACCTTCC
CAA
+
FFFFFFFF:FFFFFFFFFFFF:FFFFFFFFFFFFFFFF:FFFFFFFFFFFFFFFFF:FFFFFFFFFFF
FFFFFFFFFFFFFFFFFFFFFFFFFFFFFFFFFFFFFFFFFFFFFFFFFFFFFFFFFFFFFFFFFFFFF
FFF
@A00721:190:HGJW7DSXY:4:1307:22191:29747_2:N:0:TCTGCAAG+GCTGCACT
CACAGAAGAAGGCTGAAGGTCGTTACCGGACGACCACTCAAAGGAAACGGGCTGTTGCCCAGAGGAAA
GCAGCGACCACCGAGGTCGCTCGTAACATTGACGGCAGTCCCGTCATC
+
FFFFFFFFFFFFFFFFFFFFFFFFFFFFFFFFFFFFFFFFFFFFFF:FFFFFFFFFFFFFFFFFFFF,F
FFFFFFFFFFFFFFFFFFFFFFFFFFFFFFFFFFFFFFFFFFFFFFFF
@A00721:190:HGJW7DSXY:4:1304:28330:21245_1:N:0:TCTGCAAG+GCTGCACT
CTGAGGCAGTACCTCAGTCAAAAGTCACATTCACCACTCCCATACCAATCAATGATGTGACTATACAG
TTCGGCCATTTCAAGAGTGGTCAATACGTCGTGGGAGGTACTGAAGCCTTCCCCTCCGCTGGCACGAA
GAA
+
FFFFFFFFFFFFFFFFFFFFFFFFFFFFFFFFFFFFFFFFFFF:FFFFFFF,FFFFFFFFFFFFFFFF
FFFFFFFFFFFFFFFFFFFFFFFFFFFFFFFFFFFFFFFFFFFFFFFFFFFFFFFFFFFFFFFFFFFFF
FFF
@A00721:190:HGJW7DSXY:4:1512:9390:16532_1:N:0:TCTGCAAG+GCTGCACT
CCAATCAATAGTTCTTCTGACCAGAATGAAAAGGGGTTTACCAGGGTAGTCTCTAAGTCTACCAGGAA
GAGGGGAAATGTCCCCAAAACTGAGAAGAATAAAGGGAAAGGTCCATCAGGACCTAAGAAGGGGAAAT
TAC
+
F:FFFFFF:FFF,FF:FFFFFFFFFFFFF::FFFFFF:FFFFFFFFFFFFFFFFFFFFFFF:FFFFFFF
FFFFFFFFFFFFFFFFFFFFFFF,FFFFFFFFFFFFFFFFFFFFFFFFFFFFFFFFFFFFFFFFFFFFF
FFF
@A00721:190:HGJW7DSXY:4:1555:25518:4570_2:N:0:TCTGCAAG+GCTGCACT
GGAAGATCTCCCACCATCTGGGGTTGGGAATAAGAAATCTGCACGGGCCAAAGAGGCCGCTGCTACGA
AGTCGCTGAAGGCGGCTTGGGTCATTAAGGGTAGCTGTAAAGTTGCGC
+
FFFFFFFFFFFFFFFFFFFFFFFFFFFFFFFFFFFFFFFFFFFFFFFFFFFFFFFFFFFFFFFFFFFFF
FFFFFFFFFFFFFFFFFFFFFFFFFFFFFFFFFFFFFFFFFFFFFFFF
```

@A00721:190:HGJW7DSXY:4:1268:19407:36730_1:N:0:TCTGCAAG+GCTGCACT
ATCTTTGAGACCAAGACTCTCTACAGTAGGTTCGTGCCTACTATGAGCAAATCTGTTGAATTCGTCAC
TGAGGACGAAGGTGAGAGGACTTTTGGCTACTGGCGACAAACTGAGAGCAGTCGCTGGGGAAAGAGAA
TTG
+
FFFFFFFFFFFFFFFFFFFFFFFF,FFFFFFFFFFFFFFFFFFFFFFFFFFFFFFFFFFFFFFFFFFF
FFFFFFFFFFFFFFFFFFFFFFFFFFFFFFFFFFFFFFFFFFFFFFFFFFFFFFFFFFFFFFFFFFFF
:FF
@A00721:190:HGJW7DSXY:4:2406:13711:12587_2:N:0:TCTGCAAG+GCTGCACT
ACGCTTCGAGCTTGAGTGTGACGAGGCCGGTGACGTTTTTGTCAACGGCGTTTGGAGGATTTTATCCG
CGCTTAACTATGGGGCGAAGATCCCCTATGGAGTCTTGAAGGACTTAG
+
FFFFFFFFFFFFFFFFFFFFFFFF:FFFFF:F:FFFFFFFFFFFFFF:FFF,FFFFFFF,FFFFFFF
FFFF:FFFFFFFFFFFFFFFFFFFFFFF:FFFFFFFFFF,FFFF,FFFF
@A00721:190:HGJW7DSXY:4:2452:16929:12336_1:N:0:TCTGCAAG+GCTGCACT
GCATGTCACCCTTAAGCACCGAGGTAAGCTCGGGACTTACAAGAGAGAGTGTGAGAGAGTCGCTCTCA
CTATTTTTGCCCAGAATGCGGGCTTTCTGACTGATGAGGTTAAAACTCTTGCTCCTTCTGTGGGTGAG
GGT
+
FFFFFFFFFFFFFFFFFFFFFFFFFFFFFFFFFFFFFFFFFFFFF,FFFFFFFFFFFFFFFFFFFF,:FF
FFF:FFFFF:FF,:FFF,FFFFFFFF:FF,:::,F:,FF:FF,FF:FFFF::FFF,F:FF::FF::,F
FF:
@A00721:190:HGJW7DSXY:4:1433:4372:3959_2:N:0:TCTGCAAG+GCTGCACT
GGTTCTGGGAGGCTGCTTTCCTCCATAATTACAACCTCTCTTTAGAGAGCATTAGAAGGAAGTTGGGC
AAATTCCCACATATGGTAGACGTTGTCACACTTTTCTTGAAAATTTTT
+
FFFFFFFFFFFFFFFFFFFFFFFFFFFFFFFFFFFFFFFFFFFFFFFFFFFFFFFFFFFFFFFFFFFF
FFFFFFFFFFFFFFFFFFFFFFFFFFFFFFFFFFFFFFFFFFFFFFFF
@A00721:190:HGJW7DSXY:4:2652:32877:17942_1:N:0:TCTGCAAG+GCTGCACT
TTGGGGATTTCGATGTGAGAAGGTGGAAGGTGTCAAGGGTCTTCTTCATGCTGATCTTTCAGGACCTA
ATCACATCATGATTGATAGTTACGGCTCGTATCGAGTTGGAGGGAGGAAGTTCTCCGAAATTAAAGAC
GGG
+
FFF,FFFFFFF,,F,F:FF:FF:FFFFF,:F,FFFF:FFFFFFFFFFFFFF:FFFFFFFFFFFF:F:
FFFFFFFF:FFFFFFFF,:FF:FFFFFFFFFFFFFFFFFFFFFF:FFFFFFFFFFF:FFFFFFF:FF:
FFF
@A00721:190:HGJW7DSXY:4:2538:21920:17159_2:N:0:TCTGCAAG+GCTGCACT
TCAGAGAGGGGAGCTCTGAAAAGGAAACTTCGTCAGAAGATGAGGAAGTCTAAGAGGAGGAGTTTGAG
GAAGGCTCTAGAGAAGATCTCACCACCTGGAGAAGGCAAGTGGAGAGT
+
FFFFFFFFFFFFFFFFFF:FFFFFFFFFFFFFFFFFFFFFF:FFFFF,FFFF,FFFFFFFFFFFFFFFF
FFFFFFFF:FFFFFFFF:FFFFFFFFFFFFFFF:FFFFFFFFFFFFFF
@A00721:190:HGJW7DSXY:4:1537:20401:26929_2:N:0:TCTGCAAG+GCTGCACT
AAGAAGGATTATTTGAAGCAGGGGTTACCACTTCCTAAGGTGGATGAGGGAGGTTGTTTCTTTGGAGT
TTTAGATAATCTTTTGAGAAGGGGTCATAGAGCCAGCAAGAGAAGCTT
+
FFFFFFFFFFFFFFFFFFFFFFFFFFFFFFFFFFFFFFFFFFFFFFFFFF:FFFFFFFFFFFFFFFFFF
FFFF::FFFFFFFFF:FFFFFFFFFFFFFFFFFFF:FFFFFFF:FFFF
@A00721:190:HGJW7DSXY:4:2159:5972:8108_2:N:0:TCTGCAAG+GCTGCACT
TATAATTTAGGTCAACTTGGGGTTTCGAATGTCATTAGTGCCTTACGCTTGAAAGAGGTCCTTTTGAG
TATCGACAGATCTTTCGATCTTGAGGACGTACATTTAACGTGGCAGAA
+
FFFFFFFFFFFFFFFFFFFFFFFFFFFFFFFFFFFFFFFFFFFFFF:FFFFFFFF,FFFFFFFFFFFF
FFFFFFFFFFFFFFFF:FFFFFFFFFFFFFFFFFFFFFFFFFFFFFFF

```
@A00721:190:HGJW7DSXY:4:1547:2437:3208_2:N:0:TCTGCAAG+GCTGCACT
ATATTGTTCCTTCCGGGAAGTTGTGGGATTTAACTTTGACTCAACTACTCCGATCAGATCCGGATGGT
TTGATTGGTATGGACTACTCGACTTTTGATCTGGAGTTGTCTAGGGTC
+
F,FFFFFFFFFFFFFFFFFFFFFFFFFFFFFFFFFFFFFFFFFFFFFFFFFFFFFFF,FFFFFF:FFF
FFFF::FFFFFFFFFFFFFFFFFFFFFFFFFFFFFFFFFFF::FFFFF
@A00721:190:HGJW7DSXY:4:2653:32452:9565_2:N:0:TCTGCAAG+GCTGCACT
CGATAAGGTGAAAAATATGCAGGTCAAAGATTCTGTAGCTCTGAGGGCGGTTGAGGGAGCCGTTTTGG
CCGACTTTAACTATTCTAAGAGTCTTTATGATTCAAAACCTATCGTTG
+
FFFFFFFFFFFFFFFFFFFFFFFFFFFFFFFFFFFFFFFFFFFFFFFFFFFFFFFFFFFFFFFFFFFF
FFFFFFFFFFFFFFFFFFFFFFFFFFFFF:FFFFFFFF,FFFFFFFFF
@A00721:190:HGJW7DSXY:4:2237:1380:21793_2:N:0:TCTGCAAG+GCTGCACT
GGCTTATTGTCGCGCAGTTTCCAGAATTGCGGTTGAAGTTCACCCATTCTATCTTGAATGCTCACGCC
GTCGCAGCCATATCGAGACTATGTTATAATCACTTGTTAAAGTACAGA
+
FFF,FFFFFFFFFFFFFFFFFFFFFFFFFFFFFFFFFFFF:FFFFFFFFFFFFFFFFFFF:FFFFFFF
FFFFFFFFF,FFFFFFFFFFFFFFFFFFFFFFFFFFFFFFFFFFFFFF
@A00721:190:HGJW7DSXY:4:1368:11731:30389_2:N:0:TCTGCAAG+GCTGCACT
ATTATTGATATTGGAGGCGATATTCGTTTTCATATCCAAAACTCCTTGAATGGAGTACATATTTGCAA
CCCTATTCTTGATGTGAAAGATGCATCGAGGTATACGTCTAGGAACAT
+
FFFFFFFFFFFFFFFFFFFFFFFFFFFFFFFFFFFFFFFFFFFFFFFFFFFFFFFFF:FFFFFFFFFF
FFFFFFFFFFFFFFFFFFFFFFFFFFFFFFFFFFFFFFFFFFFFFFFF
@A00721:190:HGJW7DSXY:4:1472:12698:26428_2:N:0:TCTGCAAG+GCTGCACT
GCTATCGGAGGGAAGCAAAATGTTTCGTTCTGTTACAAGAACGCTTTGGATTGTGACCATAAAGCCTC
AGTTGCGATAGCTGTAGAGGTATACGATATCTCTCTTGATGATGTAGC
+
FFFFFFFFFFFFFFFFFFFFFFFFFFFFFFFFFFFFFFFFFFFFFFFFFFFFFFFFFFFFFFFFFFFF
FF:FFFFFFFFFFFFFFFFFFFFFFFFFFFFFFFFFFFFFFFFFFFFF
@A00721:190:HGJW7DSXY:4:2103:19840:6042_1:N:0:TCTGCAAG+GCTGCACT
TGATGTATGTCATTTCGTCTTAGTTTGTCCCGGCGAATTATTGGACACGATGAAAAATGACATACCCG
TTCCTGACCTTGGTATCGTTATCCGTCATGGAGATGATGGCGCATCACTTGATTACTATATGCCCGAA
GGA
+
FFFFFFFFFFFF:FFFFFFFFFFFFFFFFFFFFFFFFFFFFFFFFFFFFFFFFFFFFFFFFFFFFFFF
FFFFFFFFFFFFFFFFFFFFFFFFFFFFFFFFFFFFFFFFFFFFFFFFFFFFFFFFFFFFFFFFFFFF
FFF
@A00721:190:HGJW7DSXY:4:1641:5267:26240_2:N:0:TCTGCAAG+GCTGCACT
AACAAAGATAAGATTTTATCTTTCTTCAAACACCCTGTTTTCCTTAAGGGCGGACAATTGTTCCATTG
TGAACTCTTGGGGACTAGATTGAGCATTAATGAGTTCCGAATAACTCG
+
FFFFFFFFFFFFFFFFFFFFFFFFFFFFF:FFFFFFFFFFFFFFFFFFFFFFFFFFFFFFFFFFFFFF
FFFFFFFFFFFFFFFFFFFFFFFFFFFFFFFFFFFFFFFFFFFFFFFF
@A00721:190:HGJW7DSXY:4:1339:7798:9956_2:N:0:TCTGCAAG+GCTGCACT
TATTTCCGTACCAAGAGCCACTGCCAATCTCATCGCTATACGCATACCTGATTGGAAGAAGCGAGGTT
CGTTCCTCAATAAAAATTTTTCTCAAGAGATTATGGTTGATAAAGTTT
+
FFFFFFFFFFFFFFFFFFF:FFFFFFFFFFFFFFFFFFFFFFFFFFFFFFFFFFFFFFFFFFFFFFFF
FFFFFFFFFFFFFFFFFFFFFFFFFFFFFFFFFFFFFFFFFFFFFFFF
@A00721:190:HGJW7DSXY:4:1131:4878:7748_1:N:0:TCTGCAAG+GCTGCACT
TACGTCATGGGGATCACTTCTTCGGTCAATGACAAAACCTTCGAATATGTTACGACATGGCTTCGTAA
CAACAGTGCGAGGATTGTCATCTAGGGTAAAGAGAGAAACAAAGATGTTCCTATAGATACGAGATACC
TAG
```

```
+
FFFFFFFFFFFFFFFFF,FFFFFFFFFF:FFFFFFFFFFFFFFF,FFFFFFFFFFFFFFFFFFFFFF
FFFFFFFFFFFFFFF:FFFFFFFFFFF,F:FF:FF:FFFFFFFFFFFFF:FFFFFFFFFFFFFFFFF
FFF
@A00721:190:HGJW7DSXY:4:1211:5999:11036_2:N:0:TCTGCAAG+GCTGCACT
AGCGATCTTACTCGTTATCGGAGCTAAGAAGAAGACTTTGGGTACACGGTATGCTCGCAAACTTAACG
TTAGTACCGGAGGTGATCTCTCTGCTTCTGCGTTACTGAGAGAAAAGA
+
FFF:F:FFFF:FFF:FFFFFFFFF:FFFFF:FFFFF:FFFFF,FFFF:,F,:F,,FFFFFFFFFFFF
FFFF:F:FFF,FFFFFFFFF,:FFFFFFF:FF,FFFFF:FFFFFFFFF
@A00721:190:HGJW7DSXY:4:1258:2474:25191_1:N:0:TCTGCAAG+GCTGCACT
CTTTATGGACAAGATGCAAGTTCTTGGGAAATACTTGTTTCCCTTTTTAGGTTTAGTAGGTGATCTTG
ACGTCGATTCTTTGTATAGGGAGTGTGAGAAGACAACGAATCTTGAAACTACGGTCCTTATCAAAGAA
AAT
+
FFFFFFFFFFF:FFFFFFFFFFFFFFFFFFFFFFFFFFFFFFFFFFFFFFFFFFFFFFFFFFFFFFFF
FFFFFFF:FFFFFFFFFFFFFFFFFFFFFFFFFFFF:FFFFFFFFFFFFFFFFFFFFFFFFFFFFFFF
FFF
@A00721:190:HGJW7DSXY:4:1178:15275:4554_2:N:0:TCTGCAAG+GCTGCACT
AAATCTTTTGAGATTGAGAAGATTGAAGAACACGTCAAGAAGTCGCTCGGCGTTGTTCTTGAAAACCA
AGTTAAGAAGGAGGCTGATCATGGAGTCTTTGTACCTTCTGTCAAACT
+
FFFFFFFFFFFFFFFFFFFFFFFFFFFFFFFFFFFFFFFFFFFFFFFFFFFFFFFFFFFFFFFF:FFF
FFFFFFFFFFF:FFFFFFFFFFFF:FFFFF:FFFFFFFF:FFFFFFFF
@A00721:190:HGJW7DSXY:4:2120:26096:15906_2:N:0:TCTGCAAG+GCTGCACT
GCTACGAGAGGTGATCCCGGATGTGGATTGAAGGCTGGCTCTAAGGGAGACTCCTTCTTTTTGAATGT
TGTAAAGAATTGGGTTGAGAAGTGTGCTTTACAAGGAGTTAGACTCTT
+
FFFFFFFFF:F:FFFFF:FFF:FFFFFFFF:FFFFFFFFFFF,FFFFFFFFFFFFFFFFFFFFFFF:F
FFF,FFFFFFFFFFFFFFFFFFFFFFFFFFFFFFFFFFFFFF:FFFFFF
@A00721:190:HGJW7DSXY:4:2412:26386:30561_2:N:0:TCTGCAAG+GCTGCACT
TAAAGAAAAATTTTCGAATAAACTAATGAGAGGTATAGGCTTAGTCAAGTCTGTACTTACATTCTTTA
CGCCAGATTCTTCATGGGGCGACGCTGTTTCTCTTGGTATTTCTGAAA
+
FFFFFFFFFFFFFFFFFFFFFFFFFFFFFFFFFFFFFFFFFF:FF:FFFFFFFFFFFFFFFFFFFFFFF
FFFFFFFFFFFFFFFFFFFFFFFFFFFFFFFFFFFFFFFFFF:FFFFF
@A00721:190:HGJW7DSXY:4:2270:10447:6590_1:N:0:TCTGCAAG+GCTGCACT
TTTCCTCTGGTAAGGGAATTATTAAATCATTCTTCTCACTCTTGACGAGAATTATTACTGTTTTCTCT
ACCCATAACTTCTTTTTCAAAAATGCGGATGGGTCTACTGATGTTTTCCTACAGAGTGCAGTCGTTTC
AAC
+
FFFFFFFFFFFFFFFFFFFFFFFFFFFFFFFFFFFFFFFFFFFFFFFFFFFFFFFFFFFFFFFFFFF:
FFFFFFFFFFFFFFFFFFFFFFFFFFFFFFFFFFFFFFFFFFFFFFFFFFFFFFFFFFFFFFFFFFFF
FFF
@A00721:190:HGJW7DSXY:4:1623:14434:32283_1:N:0:TCTGCAAG+GCTGCACT
AATAATTGGTGATGTAAATACGTATGTCGGTGGGGTTCTCAAGGCTCTCATCGCCACTGGGATTCTAA
GGGGTTCCATAAGATATTATCTGACATTGTGGTTCAATGATCAGGATTCTTCTTTTGGTCCTTACGTC
TTA
+
:FF,,FFF:F,,FF,F:FF::,F,FFFF:FF:FF:FF:FF,,FF,F,FFFFF:,F:,:,FF,:F:F,,
FF,,FFF,FFF,FFF:FFFF,FF:F:FF:F:FFF,FFFFF::FF:FFF::F:FFFFFFF:FFFF,F:F
FFF
@A00721:190:HGJW7DSXY:4:2177:23999:33505_1:N:0:TCTGCAAG+GCTGCACT
TCTTGTCACGATTCTTAAAAGAAGTTTATTAAAACTTCAGAGTCTCTTGGAAGTCCAGGTAGCTAATG
```

TTCTTGCAATAGCCACAGACTTTCTGGGAAAAGAGACCTCTGTTGGTAGAGGAGTTGAGAGAATTACT
CAG
+
FFFFF:,,FFFF:FFF,F,FFFF:FFFFFFF:FFFFFFF,FFFF:::,FFFFFFFF:FFFFFFFF:::
FFFF:F:FFFFFFFFFFFFF:FFFFFFFFFFFFFFFFF,FFFFFFFFFF,FFFFFFFFFFFFFFFFFF
FFF
@A00721:190:HGJW7DSXY:4:1173:7880:33238_1:N:0:TCTGCAAG+GCTGCACT
AGTTAGGGAGTGGCTAGGTTTTAAAAGATCCTTTGCACAAGCAATACCTTCCACTGAGTTCATCACCC
AGAGTTTCGACGTCGCGGAAAACGATGAAGATTTCCACACTGTTCGAGAATCTTCTGATGCTGAATTT
GAG
+
FFFFFFFFFFFFFFFFFF:FFFFF,:FFFFFF,FF:FFFFFFFFFFFFFFFFFFFFFFFFFFFF:FFF
FFF,FFFFFFFFFF:F:FFFFFFFFFFFFF:FFFFFFFFFFFFFFF:FFF:,FFFFFFFFFFFFF
FFF
@A00721:190:HGJW7DSXY:4:2305:9652:7968_1:N:0:TCTGCAAG+GCTGCACT
TGTGCTCTTCCGATCTCCTGATGGCTCAGTACAGGGAGGAGGTAATAGTTATAAAACCTTCTTTTATC
ATTGCAACAGGACTTTGAGAAGACTGTTGAAATGGACAAAATCTGGGATCATAGGTATGTGTCACGCT
ACA
+
:FFFFFFFFFFFFFFFFFFFFFFFFFFFFFFFFFFFFFFFFFFFFFFFFFF:FFFFFFFFFFFFFFFF
FFFFFFFFFFFFFFFFFFFFFFFFFFFFFFFFFFFFFFFFFFFFFFFFFFFFFFFFFFFFFFFFFFFF
FFF
@A00721:190:HGJW7DSXY:4:1677:17598:1501_2:N:0:TCTGCAAG+GCTGCACT
GTTGGCGTCTTGTCGTTGACGTCATTACAGTCTGGAAGTTAGATCATTTGTTCAGGAAGATCTCTACA
GAGATTATCGAGAGTTGTGATCTAGTTCTAAATTTAGGTCGACGGACT
+
FFFFFFFFFFFFFFFFFFFFFFFFFFFFFFFFFFFFFFFFFFFFFFFFFFFFFFFFFFFFFFFFFFFF
FFFFFFFFFFFFFFFFFFFFFFFFFFFFFFFFFFFFFFFFFFFFFFFF
@A00721:190:HGJW7DSXY:4:2532:28510:30076_2:N:0:TCTGCAAG+GCTGCACT
CATCGCAAAGAAGACTTTATTAGTTCATGAAGTTCTTACACTTCTCGCGACTAATGTCAGCTTCACTA
CAAAGGCGTTACGTAAAGTAGTTTTCACGCTTTATCTTAAAGCATTAC
+
FFFFFFFF:FFFFFFFFFFF:FFFFFFFFFFFFFFFFFFFFFFFFFFFFFFFFFFFFFFFFFFFFFFF
FFFFFFFFFFFFFFF,FFFFFFFFFFFFFFFFFFFFFFFFF::F:FFFF
@A00721:190:HGJW7DSXY:4:1421:1768:8281_1:N:0:TCTGCAAG+GCTGCACT
ACTTTTTGAATATGACAGAAGCTCGCCATATCAGATTAGAGACATGCCAGTTAGAGTTCTTCTAACCA
GGACTGATGGGGAGATTCTTTTACCTTTTGGAGGTCCAGGAACCTTTACTCCTAGTGTCTTTTCTAGA
GTC
+
FFFFFFFFFFFFFFFFFFFFFFFFFFFFFFFFFFFFFFFFFFFFFFFFFFFFFFFFFFFFFFF:FFFFF
FFFFFFFFFF,FFFFFFFFFFFFFFFFFFFFFFFFFFFFFFFFFFFFFFFF:F:FFFFFFFFFFFFFF
:FF
@A00721:190:HGJW7DSXY:4:1241:25066:5822_2:N:0:TCTGCAAG+GCTGCACT
CGGAGGATGACAGGTGGGAGACCGCTCAATAACACTCTATTGAATCCCTTTTCCACGATGAAAGCTCT
TTATGAGGTGCTCCATGCAAATTTACAGTTCCTCTTTGGTGATATCGC
+
FFFFFFFFFFFFFFFFFFFFFFFFFFFFFFFFFFFFFFFFFFFFFFFFFFFFFFFFFFFFF:FFFFFFF
FFFFFFFFFFFFFFFFFFFFF:FFFFFFFFFFFFFF:FFFFFFFFFFFF
@A00721:190:HGJW7DSXY:4:1241:25066:5822_1:N:0:TCTGCAAG+GCTGCACT
TTTTGATAAATTTGTTGGAATCGATAATGATACTGACGAAGGTTCTATTACCACCCTTGTCATTGATA
CAAACATTCTAGGTGATGCTTTCTTAGAGGCTCGTCTAGCCGCTGAGATTCGTCTTACCTATTCACAA
TCT
+
FFFFFFFFFFFFFFFFFFFFFFFFFFFFFFFFFFFFFFFFFFFFFFFFFFFFFFFFFFFFFFFFFFFF

FFFFFFFFFFFFFFFFFFFFFFFFFFFFFFFFFFFFFFFFFFFFFFFFFFFFFFFFFFFFFFF,FFFF
FFF
@A00721:190:HGJW7DSXY:4:1309:18285:6668_1:N:0:TCTGCAAG+GCTGCACT
GATAACGATTTAGTACTGGGAAATGGTGGTAGTCAGTTTACTACTTTTCTTTCCCTCCCCTTTTTCGA
TTGGATTCGACGGGGTCTTCGTCTCACGACTAATTTGATAAGGTTTTTCTTTTATGGGACTCTTGTTT
GTA
+
FFFFFFFFFFFFFFFFFFFFFFFFFFFFFFFFFFFFFFF:FFFFFFFFFFFFFFFFFFFFFF:FFFFF
FFFFFFFFFFFFFFFFFFFFFFFFFFFFFFFFFFFFFFFFFFFFFFFFFFFFFFFFFFFFFFF:FFFF
FFF
@A00721:190:HGJW7DSXY:4:1146:8269:34820_2:N:0:TCTGCAAG+GCTGCACT
AAGTTCTTAATGTAGGTCTCCTAGTACTTAAGGGTTTATGTTTCGGTTTCAGCTGGATGGATCTTTTG
ATGGTATGTTTCACATTGCGTGATCACTTCCCGTGGACTTGGGAAGAG
+
FFFFFFFFF:FFFFFFFFFFFFFFFFFFF:FFFFFFFFFFFFFFFFF:FFFFFFFFFFFFFFFF:FFF
:,FFFFFFFFFFFFFFFFFFFFFFFFFFFFFFFFFFFFFFFFFF,F:F
@A00721:190:HGJW7DSXY:4:1106:25635:2707_1:N:0:TCTGCAAG+GCTGCACT
CGATTCTATTATGTCGCTAAAGCCTGTCCAGTTGTCGATGATGTTCGGGCTGTGGGGATTAACTTCCC
TCGTGAAACTTACGACGATGTTCTCGCTCGCATTTCACAAGGATTACTAAGTCTGAGATCTGCCACTT
CTA
+
FFFFFFFFFFFFFFFFFFFFFFFFFFFFFFFFFFFFFFFFFFFFFFFFFFFFFFFFFFFFFFFFFFFF
FFFFFFFFFFFFFFFFFFFFFFFFFFFFFFFFFFFFFFFFFFFFFFFFF:FFFFFFFFFFFFFFFFFF
FFF
@A00721:190:HGJW7DSXY:4:2462:29170:25426_2:N:0:TCTGCAAG+GCTGCACT
AGAAAGTTCATGAGGATGTCTTGGTCGAAAAATATCGCCGGGAACTTCTAATAGAGGATCCTGATGAC
ATTATTTCAACGGATACCGATCTCAGTTCTACCACAGAAACCGTCTCA
+
FFFFFFFFFFFFFFFFFF:FFFFFFFFFFFFFFFFFFFFFFFF,FFFFFFFF:FFFFFFFFFF:FFFF
FFFFFFFFFFFFFFFFFFFFFFFFFFFFFFFFF,FFFFFFFFFFFFFF
@A00721:190:HGJW7DSXY:4:2104:21305:36385_2:N:0:TCTGCAAG+GCTGCACT
ACTGATTCGGAATTAGAGAAAGACGACCTTCTTGTGGGGACTGAGACCTCTGAATTTTCAGGGTGCCC
TGATGAGGACGTTGAAAGGGATAATTTCAACTCTGAGACTTCTAAGAG
+
FFFFFFFFFFFFFFFFFFFFFFFFFFFFFFFFFFFFFFFFFFFFFFFFFFFFFFFFFFFFFFFFFFFF
FFFFFFFFFFFFFFFFFFFFFFFFFFFFFFFFFFFFFFFFFFFFFFFF
@A00721:190:HGJW7DSXY:4:2127:9851:32518_2:N:0:TCTGCAAG+GCTGCACT
ACCAGAAACGTAACAAGAAGAATAAAGGTAGGAAGGGGAGAAAACAGAACAAGCGAGCGGTTGAAGAG
AAAACTGATATGGTTGCGGCTGATCGTACCCCAATTCCTGCTAGAAAG
+
:FFFFFFFFFFFFFFFFFFFFFFFFFFFFFFFFFFFFFFFFFFFFFFFFFFFFFFFFFFF:FFFFFFF
FFFFFFFFFFFFFFFFFFFFFFFFFFFFFFFFFFFFFFFFFFFFFFFF
@A00721:190:HGJW7DSXY:4:2270:11424:16391_1:N:0:TCTGCAAG+GCTGCACT
ATACCACTGATCTTCCAGAGTTACGTAGTGGTCGTTCTTTTGGTGATTTTATCAAAGAGAAAGTTTCT
GCTAGTCGCGACGATAGTATTTATCGGAGTGATATTAACAAGTTAAACTTCGAACTGACCACTTACAA
GAG
+
FFFFFFFFFFFFFFFFFFFFFFFFFFFFFFFFFFFFFFFFFFFFFFFFFFFFFFFFFFFFFFFFFFFF
FFFFFFFFFFFFFFFFFFFFFF:FFFFFFFFFFFFFFFFFFF:FFFFFFFFFFFFFFFFFFFFFFFFF
FFF
@A00721:190:HGJW7DSXY:4:1630:4996:25488_2:N:0:TCTGCAAG+GCTGCACT
TCAGTTTTCCAAAAAGCTATTAATAGCAGTTGCGGGGCTATAATACCGCCTCCAGATTACTCTAAACT
GAATCCACTCTCTTGGGATATAAATGAGTACATCTATAATAGAGTTTT
+

```
FFFFFFFFFFFFFFFFFF:FFFFFFFFFFFFFFFFFFFFFFFFFFFFFFFFFFFFFFFFFFFFFFFFF
FFFFFFFFFFFFF:FF:FFFFFFF:FFFFFFFFFFFFFF:FFFFFFFF
@A00721:190:HGJW7DSXY:4:2332:17345:14465_1:N:0:TCTGCAAG+GCTGCACT
AACATGAATTAGTTATTGAAAGTCTTGACCCTCACGTCTCACTTTATCACCTTGGAGATGGTAAAGTC
ATTAACAAAGGAAATGTTAAGGCTGATGACTTAAATTTCGCATTCTGCTTCGACGGTAATTCCATAAG
ACC
+
FFFFFFFFFFFFFFFFFFFFFFFFFFFFFFFFFFFFFFFFFFFFFFFFFFFFFFFFFFFFFFFFFFFF
FFFF:FFFFFFFFFFFFFFFFFFFFFFFFFFFFFFFFFFFFFFFFFFFFFFFFFFFFFFFFFFFFFFF
FFF
@A00721:190:HGJW7DSXY:4:1441:26693:28025_1:N:0:TCTGCAAG+GCTGCACT
AACCTTTCCTCGTTCTTCATGAAGATCTTCGAGTGTTTTATAGTGAGTTATTCTTGCGAAGGTTCAAG
TCGATTGATTTTGTTGACTACGATCTCTCTTGTAATAAGAAGTTCTTCGCAGTTGAATCTCCACCAGG
TGG
+
FFFFFFFFFFFFFF:FFFFFFFFFFFFFFFFFFFFFFFFFFFFFFFFFFFFFFFFFFFFFFFFFFFFF
FFFFFFFFFFFFFFFFFFFFFFFFFFFFFFFFFFFFFFFFFFFFFFFFFFFFFFFFFFFFFFFFFFFF
FFF
@A00721:190:HGJW7DSXY:4:1308:21414:35164_2:N:0:TCTGCAAG+GCTGCACT
GCGACTTTTGTCGAATTTTTCGCAAAAGGTCTTGAAGTACGTATTGTCACTGCTAATCGTAATTCGCG
AGAAGAGATAGTGACTAAAGTTTTGGCACTTCTAGTTCATCGCGGTAT
+
FFFFFFFFFFFFFFFFFFFFFFFFFFFFFFFFFFFFFFFFFFFFFFFFFFFFFFFFFFFFFF,FFFFF
FFFFFFFFFFFFFFF:FFFFFFFF:FFFFFFFFFFFFFFFFFFFFFF,
@A00721:190:HGJW7DSXY:4:2272:5493:17832_2:N:0:TCTGCAAG+GCTGCACT
CAGACGTGAAAGTTATGATGAGTGATCGTATTAGAACAGCTGATTCTTATTGCATGAACGTCGCTGAG
GTCAAACCTGTGGACGTTCTTTTCATCGACGAATTATTCATGATTCAC
+
FFFFFF:FFFFFFFFFFFFFFFFFFFFFFF:FFFFFFFFF:FFFFF:FFFFFFFFFFFFFFFFF:FFF
FFFFFFFFFFFFFFFFFFFFFFFFFFFFFFFFFFFFFFFFFFFFFFFF
@A00721:190:HGJW7DSXY:4:1631:2926:20055_2:N:0:TCTGCAAG+GCTGCACT
CATCTACTTAAGGCGGCTTATACCATAGGTTACGGTGATTCCAATCAAATCGGATTCATCAATCGTAT
TGATCTCCAAGTCGCAAACTACTTCAACATCAACGACATCATTCTTGA
+
FFFFFFFFFFFFFFFFFFFFFFFFFFFFFFFFFFFFFFFFF:F:FFFFFFFFFFFFFFFFFFFFF:FF
FFFFFFF:F::FFFFF,FFFFFFFFFFFFF:FFFFFFFFFFF:FFFFF
@A00721:190:HGJW7DSXY:4:2623:14235:31000_2:N:0:TCTGCAAG+GCTGCACT
TTATCGAAGTCTCAGTTATCGCTGCCCGGCCGACGTTTGTCGCGAACTCTCAGAGGTTTACGGCAGAA
AAATTTCTCCTGCTTGTAATTCGAGAGAGGACACTATGGAAATTAGAC
+
FFFFFFFFF:FFFFFFFFFFFFFFFFFFFFFFFFFFFFFFFFFFFFFFFFFFFFFFFFFFFFFFFFFF
FFFFFFFFFFFFFFFFFFFFFFFFFFFFFFFFFFF:FFFFFFFFFFFF
@A00721:190:HGJW7DSXY:4:2348:15320:26240_2:N:0:TCTGCAAG+GCTGCACT
TATTACTTTCACTCAAGGTGAAAAGTTTGAAATTAACAAAGTCCTTGGTAAGAGTAAGGTTAAATGTG
AAGATGCTCAGACTATTCATGAAGTTCAAGGTAATACTTACCAGCACG
+
FFFFFFFFFFFFFFFFFFFFFFFFFFFFFFFF:FFF:FFFFFFFF,:FFFFFFF:FF:FFFFFF:FFF
FFFFFF:FF:FFF:FFFFFFFFFFFFFFFF,FFFFFFFFFFFF::FFF
@A00721:190:HGJW7DSXY:4:2243:16387:18380_2:N:0:TCTGCAAG+GCTGCACT
TTTTGCATCACAAACACATATTACCGTTGCTCTATCACGACACGTTGAATCTTTACGATACTTTGTTA
TCAATGTAAAGTTAAACGATGCTACTTGTGAGAGGATCAATCACAGTA
+
FFFFFFFFFFFFFFFFFFFFFFFFFFFFFFFFFFFFFFFFFFFFFFFFFFFF:FF:FFFFFFFFFFFF
FFFFFFFF:FFFFFFFF,FF,FFFFFFFFFFFFFFFFFFFFFFFFFFF
```

@A00721:190:HGJW7DSXY:4:1646:31123:34256_2:N:0:TCTGCAAG+GCTGCACT
ACGGGAGGTTGCAGATTCAACCCCTTATATCGAGGCTATCGAGGACGATGAGTATCTTGAAGTTGTTG
ACGACAAGCCTCAAGAGGGTAGAGGACCACCTGGATCTTCTACTGTCT
+
FFFFFFFFFFFFFFFFFFFFFFFFFFFFFFFFFFFFFFFFFFFFFFFFFFFFFFFFFFFFFFFFFFFF
FFFFFFFFFFFFFFFFFFFFFFFFFFFFFFFFFFFFFFFFFFFFFFFF
@A00721:190:HGJW7DSXY:4:2358:3450:24126_1:N:0:TCTGCAAG+GCTGCACT
AACCACCTTAACGTCACTCGGTAATCTTTCAGCCGAATTAAGTACTAATGAGTTCAAGTCGGTTGTCG
ATCGAATTAAGATCAAGGCTAATACCTTTATAGGTAAGAAGCCCCTTGAGCCGCGTAGTCAGCGCGTT
TAG
+
FFFFFFFFFFFFFFFFFFFFFFFFFFFFFF:FFFFFFFFFFFFFFFFFFFFFFFFFFFFFFFFFFFFF
FFFFFFFFFFFFFFFFFFFFFFFFFFFFFFFFFFFFFFFFFFFFFFFFFFFFFFFFFFFFFFFFFFFF
FFF
@A00721:190:HGJW7DSXY:4:1664:31521:28526_1:N:0:TCTGCAAG+GCTGCACT
TACCGAGTCTCCAAGAGAATATTTATTCTTATGAGGCTCGAAACTTCAACGTTTTCACAACGGAAAGA
CATATCGCACCCGAAATTTTTGGGCAGAATGCTCTTAACGCGGTTCCTGAACGGTGTTTTGATCAGTC
TAA
+
FFFFFFFFFFFFFFFFFFFFFFFFFFFFFFFFFFFFFFFFFFFFFFFFFFFFFFFFFFFFFFFFFFFF
FFFFFFFFFFFFFFFFFFFFF,FFFFFFFFFFFFFFFFFFFFFFFFFF,FFFFFFFFFFFFFFFFFFFF
FFF
@A00721:190:HGJW7DSXY:4:2315:24252:4038_2:N:0:TCTGCAAG+GCTGCACT
TATAGGAGACTCAGTTAACCTGAGAGCTCCTCAATTGATAGATTGGGTCAACAATAGGGACGCTAGTA
TACTTCGTAAAGTCGACAGCGAAATGGTCAATATTGATAATTGGACCG
+
FFFFFFFFFFFFFFFFFFFFFFFFFFFFFFFFFFFFFFFFFFFFFFFFFFFFF:FFFFFFFFFFFFFF
FFFFFFFFFFFFFFFFFFFFFFFFFFFFFFFFFFFFFFFFFFFFFFFF
@A00721:190:HGJW7DSXY:4:1140:19072:28855_1:N:0:TCTGCAAG+GCTGCACT
AGCTCATGGTGAAAAGGGAAGCTAAGGTTAAACTTGATGCTTCAGCCTTTTCGAAGCATAACCCCGCA
CAGAATATCGTTTATCACGCAAAATTTATTAATGCAATTTTCGGACCTATCTTTAAGGTTCTTGTGAG
GAG
+
FFFFFFFFFFFFFFFFFFFF:FFFFF,FFFFF:FFFFFFFFFFFFFFFFFFFFFFFFFFFFFFFFFFF
FFFFFFF:FFFFFFFFFFFFFFFFFFFFFFFFFFFFFFFFFFFFFFFFFFFFFFFFFFFFFFFFFFFF
FFF
@A00721:190:HGJW7DSXY:4:1378:24080:36464_1:N:0:TCTGCAAG+GCTGCACT
CAGTGACGATTTCTCCCGGAAGCTCTTCTCTCTCTTAGGAGGTCATGATGACTACTATAAGGGTGAAA
TCGATTTTTCGAAGTTTGACAAATCTCAAGATTTCTTTTGTAAAGCCTATGAGATAAAGATGTATTCT
TAT
+
FF:FFF:FFFFFFFFFFFFFFFFFFF:FFFFFFFFFFFFFFFFFFFFFFFFFFFFFFFFFFFFFFFFF
FFFFFFFFFFFFFF:FFFFFFFFFFFFFFFFFFFFFFFFFFFFFFFFFFFFFFFFFFFFFFFFFFFFF
FFF
@A00721:190:HGJW7DSXY:4:1348:31675:6621_2:N:0:TCTGCAAG+GCTGCACT
AACTTTGGAGTGCCAGTGAATATTTTTGTCACGCCAGGAGTATCCATAACGATTTGTCATTTACCACC
TTTGCTCAACGACGAAGTGGAACGGCCAACACTTTCTTTGGTAATTGT
+
FFFFFFFFFF:FF:FFFFFFFFFFFFFFFFFFFFFFFFFFFFFFFFFFFFFFFFFFFFFFFFFFFFFF
FFFFFFFFFFFFFF,FFFFFFFFFFFFFFFFFFFFFFFFFFFFFFFFF
@A00721:190:HGJW7DSXY:4:1142:30816:19539_1:N:0:TCTGCAAG+GCTGCACT
GATGATTTTATCACTTTATTATGACCTTGGAAAGTTTTCGGCTGTGGTTGTCGCTGGGGACGATTCCA
TTCTGTTTTCAAAGACTCCAATAACTGATCATTCTGATCATATGATTTTGGACAATGGTTTCGAAGCG
AAA

```
+
FFFFFFFFFFFFFFFFFFFFFFFFF:FFFFFFFFFFFFFFFFFFFFFFFFFFFFFFFFFFFFFFFFFFFFF
FFFFF:FFFFFFFFFFFFFFFFFFFFFFFFFFFFFFFFFFFFFFFFFFFFFFFFFFFFFFFFFFFFFFFFF
FFF
@A00721:190:HGJW7DSXY:4:2622:12454:19335_2:N:0:TCTGCAAG+GCTGCACT
TGCTCGCGATTTATTCTTTTTGTCAATGATTACATCTACTTCGTTCCTGATCCTTACAAGATCATCGT
TAAGTTAGGTAAACCCCTAACCGATGTTACTCTTGTAGGACTTTGGGA
+
FFFFFFFFFFFFF:FFFFF:FFFFFFFFFFFFFFFFFFFFFFFFFFFFFFFFFFFF:FFFFFFFFFF:
FFF:FFFFF:FF:F:FF:F,FFFFFFFFFFFFFFFFF:FFFFFFFFFF
@A00721:190:HGJW7DSXY:4:1442:15510:25535_2:N:0:TCTGCAAG+GCTGCACT
AGGTTGTTGTTTGGCGATTAGCTCTTGCGACTAATATTCGATATTCGATTGAGGGTCGTTATACCTAT
TCGGCTATCTGTGCTTTGAATTGTATAAGATCAAATTTTAAACGATTC
+
FFFFFFFFFFFFFFFFFFFFFFFFFFFFFFFFFFFFFFFFFFFFFFFFFFFFFFF:FFFFFFFFFFFFF
FFFFFFFFFFFFFFFFFFFFFFFFFFFFFFFFFFF:FFFFFFFFFFFFF:
@A00721:190:HGJW7DSXY:4:1633:22833:30733_1:N:0:TCTGCAAG+GCTGCACT
TGATGTGGAGGGAGTTCTTGAAGCATCCCGACCTTTCCTTGTGAAAATTTTGATGCGGTTTCATAAGA
TTATCAGTAATCCTGATTCTACTACGGATTCATGGACCTTCCATCCTGTACCTCTTCTGGAGAGACGT
ATC
+
FFFFFFFFFFFFFFFFFF:FFFFFFFFFFFFFFF:FFFFFFFFFFFFF:FFFFFFFFFFFFFFFF:FFFFFF
FFFFFFFFFFFFFFFFFFFFFFFFFFFFFFFFFFFFFFFFFFFFFFFFFFFFFFFFFFFFFFFFFFFFFFF
FFF
@A00721:190:HGJW7DSXY:4:2431:26368:7044_2:N:0:TCTGCAAG+GCTGCACT
CTTCGAGACAGGTGATATTGTTGAGCGAGAGCGACCAAGATCTAAATTCGGTAACGGTGCGTGGCGCT
TTATTTTAAAAAGCTCCCTCCTCTCGCAAGTTTGATCTGATACTTATC
+
:FFFFFFFFF::FFFFFFFFFFFF,:FFFF:F:FFFFFFF:FFFFFFF,::FFFF:FFF:FF:F:F:F
FFFFFFFFFFF:FFF,:FFF:FFFFFFFFFFFFFFF:F,FFF,FFFF:
@A00721:190:HGJW7DSXY:4:2619:30020:23484_2:N:0:TCTGCAAG+GCTGCACT
TGATCAGTCTCTGACTTGACCATCTTTCATTTTAAATGACGATCATTAAGACTCTCTTTCTTCTTTGT
TTCCTTCTTTGCACTGATTTTTCCTCATCAGCTTGTGTTTTACCTAAT
+
FFFFFFFFFFFFFFFFFFFFFFFFFFFFFFFFFFFFFFFF:FFFFFFFF::F::F:FFFFF:FFFFFFFF
FFFFFFFFFFFFFFFF,FFFFFFF:FFFFFFFFFFFFFFFFF,:FFFF
@A00721:190:HGJW7DSXY:4:2107:20283:7874_2:N:0:TCTGCAAG+GCTGCACT
GATAACAACTATCAGTTGATTAAGACTCTTCCGAAAGGCGAACTTTGGGAATTTGAAGCTCAGGGTGA
AGCACAACGTTTGACCGTGACGAACTGTGGTGTTCTTGATCTACGTTA
+
FFFFFFFFFFFFFFFFFF,FFFFFF:FF:FFFFFFFFFFFFFFFFFFFFFFFFFFFFF,FFFFFFFFFFF:
FFFFFFFFFFF:FFFFFFFFFFFFFF,FFFFFFFFFF:FFFF:FFFFF
@A00721:190:HGJW7DSXY:4:2554:20799:25958_1:N:0:TCTGCAAG+GCTGCACT
CACTACCACTTATTACGATATTTCTTTAGTCGATGGGTTTTCGACGCCTTTAAGCGTCTTCTGTTCAT
CGCAACCAGATGATATCTTCTATTCGAACTTATGTTTCGATCAGTGTTTCTTCTATCAGGAGGAAAAC
ACT
+
FFFFFFFFFFFF:FFFFFFFFFFFFFFFFFFFFFFFFFFFFFFFFFFFFFFFFFFFFFFFFFFFFFFFFF
FFFFFFF:F,FFFFFFFFFFFFFFFFFFFFFFFFFFFFFFFFFFFFFFFFFFFFFFFFFFFFFFFFFFFF
FFF
@A00721:190:HGJW7DSXY:4:2255:18511:17049_2:N:0:TCTGCAAG+GCTGCACT
TCACTTTGTGTGTACTTGTCTCTATTTTAAGTTTTGTCTTCCTTGTTTGTCTTATTCTATTAATCACT
CATTTTAGTGAGATTATTAAAAACGCACTAAAACGTCTTCGACAACAG
+
```

FFFFF:FF:F:FFFFFFFFFFFFFFF:FFFF:FFFFFFFFFF:FFFFFFFFFFF:FF:FFFFFFFFF
FFFFFFFFFFFFFFFFFFFFFFFFFFFFFFFF:FFFFFFFFFFFF,FFF
@A00721:190:HGJW7DSXY:4:2473:6054:10003_1:N:0:TCTGCAAG+GCTGCACT
GCCTTACAGAGGGGCAGCAGCATGACCATAATTGGTATTGACTTTGGTACCACCTTTTCTACTGTCTG
TCTCGCCTCTTCGAATGAAGTTTAATGCTTAACAACAATGATAGTGAATTTATACCTACAATCATTG
GTT
+
F:FFF:FFFFF,F::FFFFFFFFFFFFFFFFFFF,FFFFFFFFFFF:FFFFFFFFFFFFFFFFFF:FF
F:F:FF::F:FFFFFFFFFFFFFFF:FFF:FFFFFFFFFFFFFFFFFFFF:FFFFFFFFFFFFFFFFF
FFF
@A00721:190:HGJW7DSXY:4:1419:6614:29919_2:N:0:TCTGCAAG+GCTGCACT
TGTTTATGGATATGATGCTATTACTCATGATGGTTACGGTCAGGGAGGATTCTCAGTTTACAAGGACC
TTAAGCGATGGATCGGGGTTAATTCTAAAACATTACCCGAAAGACGTG
+
:FFFFFFFFFFFFFFFFFFFFFFFFFFFFFFFFFFFFFFFFFFFFFFFFFF,FFFFFFFFFFFFFF:FF
FFFFFFFFFFFFFFFFFFFFFFFFFFFFFFFFFFFFFFFFFFF:FFFFF
@A00721:190:HGJW7DSXY:4:1577:16089:8750_2:N:0:TCTGCAAG+GCTGCACT
GTACGATGTCGTCTGTCTCGGCGATCACTTTTCACTAAGAATTGGTCCTACTTTTGGACAAGGCCAAC
TTCGTAATCTTCATGAGATCGTTTCTTTCTTTATCTCTTTGGTTGATA
+
FFFFFFFFF:::FFFFFFFFFFFFFFF:FFFFFFFFFFFFFFF,F:FFFFF,FF,FFFF,FFFFFFFF
,:FFFFFF:FFFF,FFFFFF,FFFFFFFFFFFFFFF::FFF,:F,,:F
@A00721:190:HGJW7DSXY:4:1239:9064:18098_2:N:0:TCTGCAAG+GCTGCACT
GAGTGAAAGGCTTCAATTGTAGTGGTTTGGTCGTTTCAGTACCCAGTCAGTATACCTCTACGCAAAGA
TATTTCATGAAAACTACAGAGGAACGTACCGGATTACCTGTCTTTCAT
+
FFFFFFFFFFFFFFFFFFFFFFFFFFFFFFFF:FFFFFFFFF:FFFFFFFFFFFFFFFFFFFFF:F:FF
FFFFFFFFFFFFFFFFFFFFFFFF,FFFFFFFFFFFFF:FFFFFFFF:F
@A00721:190:HGJW7DSXY:4:2254:11134:13479_1:N:0:TCTGCAAG+GCTGCACT
CTTCGGCCGCTCTCTTTGCTTCGATGTTGGATGTCAAGAAGGGTTCAGAATGGGATTCCTACGTAGTT
TATGACTTTGGTGGTGGTACTTTCGATACTTCTATCGTCTTACGTTACGGTAATTACTATAGTGTTAT
TTA
+
FFFFFFFFFFFFFFFFFFFFFFFFFFFFFFFFFFFFFFFFFFFFFFFFFFFFFFFFFFFFFFFFFFFF
FFFFFFFFFFFFFFFFFFFFFFFFFFFFFFFFFFFFFFFFFFFFFFFFFFFFFFFFFFF:FFFFFFFF
FFF
@A00721:190:HGJW7DSXY:4:2424:8350:29794_1:N:0:TCTGCAAG+GCTGCACT
ATAATCTTGGAGGTCGTGACGTTGATGAAGCTATAAGAGCGTTCTTATTACGGCGCACTTCTTTACCC
GCCACCACACAACTTTCAGTTTCACAACTGAAGGAGGAGGTTTCTGTTACTGGAAAACCCTCGTCAAT
ACT
+
FFFFFFFFFFFFFFFFFFFFFFFFFFFFFF:FFFFFFFF:FFFFFFFFFFFFFFFFFFFFFFFFFFFFF
FFFFFFFFFFFFFFFFFFFFFFFFFFFFFFFFFFFFFFFFFFFFFFFFFFFFFFFFFFFFFFFFFFFF
FFF
@A00721:190:HGJW7DSXY:4:2570:14895:24189_2:N:0:TCTGCAAG+GCTGCACT
GATCTAGTGAAATTAACGCGTCCTTTTCTGGAACGTAGTTTTACTATTATGAACAAAGTTATCAAAGA
TAGTGGCATTCAAGGCAAACTTACCCTGATTCCGGCCGGTGGTAGCTC
+
FFFFFFFFFFFFFFFFFFFFFFFFFFFFFFFFFFFFFFFFFFFFFFFFFFFFFFFFFFFFFFFFFFFF
FFFFFFFFFFFFFFFFFFFFFFFFFFFFFFFFFFFFFFFFFFFFFFFF
@A00721:190:HGJW7DSXY:4:2523:32877:3443_2:N:0:TCTGCAAG+GCTGCACT
TGAAACAGCTATTAAAGCTGTTGAGCGGGTAATTACACCTAAACGAGCTCGATCTGCCATCGCTGAAG
GATGTGCACTCTTTTCTGCTACTTTAACGGAGCGTAGTGTACTCTTCG
+

```
FFFFFFFFFFFFFFF:FFFFFFFFFFFFFFFFFFFFFFFFFFFFFFFFFFFFFFFFFFFFFFFFFFFFF
FFFFFFFFFFFFFFFFFFFFFFFFFFF,FFFFFFFFFFFFF:FFFFFFF
@A00721:190:HGJW7DSXY:4:1547:29550:9142_2:N:0:TCTGCAAG+GCTGCACT
AAAGGTCTCTATCAAGCTCGTATTATCGTCGCTGCTGGATCTGCGTTACCGTTGAAGGCTAAACTCCA
AGGTAATTATGAAGACGGTGATTTATCTTTCACTCTGGCCTTGTATGA
+
FF:FFFFFFFFFFFFFFFFFFFFFFFFF:FFFFFFFFFFFFFFFFFFFFFFFFFFFFFFFFFFFFFFFF
FFFFFFFFFFFFFFFF,FFFFFFFFFFFFFFFFFFFFFFFFFFFF:FFF
@A00721:190:HGJW7DSXY:4:2220:32371:13182_1:N:0:TCTGCAAG+GCTGCACT
CATTAATAATTTCAATCTTCTTAAAAAGAAGATAGAGAGTCATACTTTGGGCTATCGTTCAGGTGCAT
GGGGTTGGATAGCCAATATTGAAGTGGACACCTCTGGTAGTATCGAAGTAAAGAGATCGGAAGAGCAC
ACG
+
FFFFFFFFFFFFFFFFFFFFFFFFFFFFFFFFFFFFFFFFFFFFFFFFFFFFFFFFFFFFFFFFFFFF
FFFFFFFFFFFFFFFFFFFFFFFFFFFFFFFFFFFFFFFFFFFFFFFFFFFFFFFFFFFFFFFFFFFF
FFF
@A00721:190:HGJW7DSXY:4:2619:12915:33066_2:N:0:TCTGCAAG+GCTGCACT
AAGCATACTACTTTTGCTTTGATTACTCTACCGCCTTACAAGTCTAAGGTTAAAGAGTCGATTAAACA
AGTTGTACTTATCAATGATTTTATCGCTGGTTGTTATCTCGAAAAGTC
+
FFFFFFFFFFFFF,FFFFFFFFFFFFFFFFFFFFFFFFFFFFFFFFFFFF:FFFFFFFFFFFF,FF,F
F,,FF:,,FFFFFFFF,FFFFFFF:FFFFFFFFFF,F,F,FFF:F:FF
@A00721:190:HGJW7DSXY:4:2505:10194:35336_1:N:0:TCTGCAAG+GCTGCACT
CCCAAAGAGAATATCTAAAATATTTACAAAATCTTACCACTATCTACGAACCTAAAGTTCTGAAGACG
TTGCTTGTCGCTTCCTCCTTTAATGGCACTACCTCGGTACAATCTTACTTGGAACGAAGGCGACCTGT
TCC
+
FF:FFFFFF:FFFF:FFFFFFFFFFFFFFFFFFF:FFFFFF,FFF:FFFFFFFFF:FFFFFFFFFFFF
FFFFFFFFFF:FFFFFFFFFFFFFFFFFFFFFFFFFFFFFFFFFFFFFFFFFFFFFFFFFFFFFFFFF
FFF
@A00721:190:HGJW7DSXY:4:2659:11315:25661_1:N:0:TCTGCAAG+GCTGCACT
GTTCTGTCTATATTAGGTGAAGCGAAATACGATTTCGAACGTCAACGTTCACCGAGTTTCGTTTCGTA
CCTTGATGCTGACTTTCGTCAATATGATTTGATCTGGTTGCTCAATTCTAAGCGTGTTCCGCTTAGTG
CGA
+
FFFFFFFFFFFFFFFFFFFFFFFFFFFFFFFFFFFFFFFFFFFFFFFFFFFFFFFFFFFFFFFFFFFF
FFFFFFFFFFFFFFFFFFFFFFFFFFFFFFFFFFFFFFFFFFFFFFFFFFFFFFFFFFFFFFFFFFFF
FFF
@A00721:190:HGJW7DSXY:4:1255:12346:11350_2:N:0:TCTGCAAG+GCTGCACT
AAATACTAGATATTTGGATGATCTTGATAAAGAACTTCATGAATTTTCAGAGGATGAAAAATTTTCTT
CCTTGGATTTTAATATAAAGAATCTTTACCAGGACTCCTACTACAGGA
+
FFFFFFFFFFFFFFFFFFFFFFFFFFFFFFFFFFFFFFFFFFFFFFFFFF:FFFFFFFFFF:FFFFFF
FFFFFFFFFFFFFFFFFFFFFFFFFFFFFFFFFFFFFFFF:FFFFFFF
@A00721:190:HGJW7DSXY:4:2460:13639:15029_1:N:0:TCTGCAAG+GCTGCACT
GATCGTAAGAAGTTTATAAGGAACATATCCATTTGTTGTAATAGGGTCATCAACTTAGAACTCGAAAC
TCTAAGTGGTAATACTGTCTTTCCAAGCTTACCTGTATCTTCAATGAAATTGGACTCTTCGATAGGGG
ACA
+
FFFFFFFFFFFFFFFFFFFFFFFFFFFFFFFFFFFFFFFFFFFFFFFFFFFFFFFFFFFFFFFFFFFF
FFFFFFFFFFFFFFFFFFFFFFFFFFFFFFFFFFFFF:FFFFFFFFFF,FFFFFFFFFFFFFFFFFFFF
FFF
@A00721:190:HGJW7DSXY:4:2414:2600:4178_1:N:0:TCTGCAAG+GCTGCACT
TAAGAATACTATAAATAGAATAGCTATTACTACTAATGGCGTTGGCCTAGTGGATGTTATTAGTGACA
```

```
TCTGGTCAATTTCGTTTTTAGGTGGTCTTTATTCTATTATTGATTTTTATGAGATTGAGATCGGAAGA
GCA
+
FFFFFFFFFFFFF:FFFFF:FFFFFFFFFFFFFFFFFFFFFFFFFFFFFFFFFFFF,FFF:FFFFFFFFF
FF:FFFFFFFF:FFF:,::FFF,FFFF:F:F:FF,F::FFFFFFF:FFFF:F:F:FFFFFFFFFFFFF
FFF
@A00721:190:HGJW7DSXY:4:1634:21757:24330_2:N:0:TCTGCAAG+GCTGCACT
TTCCTGAAGAGTCCTTCCGTAACCAACTTCTTGACAATTTTCTTCAATCCTTCGATAATAGTTAAATC
TACGATTATGTCTGTGTTGAAAGACTTTCTACCGATAAGGGTCACTAG
+
FFFFFFFFFFFFFFFFFFFFFFFFFFFFFFFFFFFFFFFFFFFFFFFFFFFFFFFFFFFFFFFFFFFF
FFFFFFFFFFFFFFFFFFFFFFFFFFFFFFFFFFFFFFFFFFFFFFFF
@A00721:190:HGJW7DSXY:4:2674:8892:2988_2:N:0:TCTGCAAG+GCTGCACT
TAAGAAATAATCCTACATTGACTGATTATCTAAAGATTGAAGGAAAATTTTATCCTCGTTTAACTTAC
TTAGACTTTCCTACGATACAAATGATAACTTCTAATTTACGTTTGCTA
+
FFFFFFFFFFFFFFFFFFFFFFFFFFFFFFFFFFFFFFFFFFFFFFFFFFFFFFF:FFFFFFFFFFFFF
FFFFFFFFFFFFFFF:FFFFFFFFFF::FFFFFFFFFFFFFFFFFFFF
@A00721:190:HGJW7DSXY:4:2103:25861:10394_1:N:0:TCTGCAAG+GCTGCACT
GATGCATGGTGTTATCTCCTCAGGAGTCCCGGCATTTGATAATAATGACGAGACTTTCGTCATAATCG
CTGCATATTACGGATTACACGGTACCAGCGCATCTCGTGTCGAGCCTCGACCCGATTATATGGTATTA
ACT
+
FFFFFFFFFFFFFFFFFFFFFFFFFFFFFFFFFFFFFFFFFFFFFFFFFFFFFFFFFFFFFFFFFFFF
FFFFFFFFFFFFFFFFFFFFFFFFFFFFFFFFFFFFFFFFFFFFFFFFFFFFFFFFFFFFFFFFFFFF
FFF
@A00721:190:HGJW7DSXY:4:1141:22164:24815_2:N:0:TCTGCAAG+GCTGCACT
TCTAGAATTCTTAATTATAATATTCAACCTCAAAAGTTACGTGTTTACAGTAACGAGATCACACATTT
TGAGAACTAACTATGTTATGTTCTCTGATCATTTTGACAATCTCTTCT
+
FFFFFFFFFFFFFFFFFFFFFFFFFFFFFFFFFFFFFFFFFFFFFFFFFFFFFFFFFFFFFFFFFFFF
FFFFFFF:FFFFFFFFFFFFFFFFFFFFFFFFFFFFFFFF:FFFFFFF
@A00721:190:HGJW7DSXY:4:1429:27841:12164_2:N:0:TCTGCAAG+GCTGCACT
AAATAAATAATATTACTAATGGCAAATAGTGGAAAGGCTCCAGTCAACACTAGTTCGAGTGCTGCTTT
GGATTACGATGACGTGAGAATAGGTGAAGGGATAGTTTTTACCTCGAC
+
FFFFFFFFFFFFFFFFFFFFFFFFFFFFFFFFFFFFFFFFFFFFFFFFFFFFFFFFFFFFFFFFFFFF
FFFFFFFFF:FFFFFFFFFFFF:FFFFFF:FFFFFFFFFFFFFFFFFF
@A00721:190:HGJW7DSXY:4:2538:9670:18333_2:N:0:TCTGCAAG+GCTGCACT
GCGACTAATCTTAAAGCTTCACCCAAAATGAAGGACACTTCGAAACTGAATAATGATGCACTACAAAC
AAGTATCGCTAATCATCTGAAAAAGGCTCTAAAGGACACTATACAATA
+
FFFFFFFFFFFFFFFFFFFFFFFFFFFFFFFFFFFFFFFFFFFFFFFFFFFFFFFFFFFFFFFFFFFF
FFFFFFFFFFFFFFFFFFFFFFFFFFFFFFFFFFFFFFFFFFFFFFFF
@A00721:190:HGJW7DSXY:4:1518:23764:35571_2:N:0:TCTGCAAG+GCTGCACT
ACCACATTTTGGTCAAGTCAATCCGTTGAGAAAATTTCTTAGAGGATATTCGCCATTGTGGTACGAAT
TGGTCCAACATTTACCACCAAATTCTTTTGATACAACTCTAGCCTTCA
+
FFFFFFFFFFFFFFFFFFFF:FFFFFFF:FFFFFFFFFFFFFFFFFFFFFFFFFFFFFFFFFFFFFFF
FFFFFFFFFFFFFFFFFFFFFFFFFFFFFFFFFFFFFFFFF:FF:F,
@A00721:190:HGJW7DSXY:4:1423:15582:35931_2:N:0:TCTGCAAG+GCTGCACT
ACCAGATTGGTTCGAACCCGTTGATGCTATGTCAGAAGAAGCTATTCAAGCTTGTCGTTTAAAGACTG
CAGAGGCTGTCAAACAAGCCCAGACTCAAGGAGTCCAATCAAAGGTTT
+
```

FFFFFF:FFFFFFFFFFFFFFFFFFFFF,FFFFFFFFFFFFFFFFFFFFFFFFFFFFFFFFF:,FFFF
FFFFF:FFFFFFFFFFF:FFFF,FF:FFF::FFFFFF:F:FFFFFFFF
@A00721:190:HGJW7DSXY:4:1471:15393:31062_1:N:0:TCTGCAAG+GCTGCACT
TTATTTAGAGATCTTTTCTACTTACAAAGGACAATTCAGTTTCATTAATTCTACGGAAAAGTCCGGAG
TATATTGTATGTTAATAGATAGTGGGATAATAGTTCTACCGATACGTAGTGAACGCGTTCGTAGAACC
AAG
+
FFFFFFFF:,::FFFFFFFFFFFFFFFFFFFFFFFFFFFFFFFFFFFFFFFFFFFFFFFFFFFFFFFF
FFFFFFFFFFFFFFFFFFFFFFFFFFFFFFFFFFFFFFFFFFFFFFFF:FFFFFFFFFFFFFFFFFFF
FFF
@A00721:190:HGJW7DSXY:4:2557:2437:1736_1:N:0:TCTGCAAG+GCTGCACT
TAACCGATAGGAGTTGTATAGTTGAAGATGTGGCTTTAGACGATAATCCTTTAAGGACTATGTTGACT
GACGAAGATTTTCCCACAGATTTGTCTACGTTGCAGATCGCCACTCCAAACACTATATACATTAAAAT
ATA
+
FFFFFFFFFFFFFFFFFFFFFFFFFFFFFFFFFFFFFFFFFFFFFFFFFFFFFFFFFFFFFFFFFFFF
FFFFFFFFFFFFFFFFFFFFFFFFFFFFFFFFFFFFFFFFFFFFFFFFFFFFFFFFFFFFFFFFFFFF
FFF
@A00721:190:HGJW7DSXY:4:2137:11686:11741_1:N:0:TCTGCAAG+GCTGCACT
CATAACTTTTGTTACCAAATTCTTGGCTTTCAACGAAGTCTCTGATTTTATTAATCATTACTTTAACG
ATCTCTCGGTCATTTACTCTGTACTTGATCTACGTCGTTTAACTGTCGGAACAACCTTTACTTAGAAT
GAC
+
FFFFF:FFFFFFFFF:FFFFFFFFFFFFFFFFFFFFFFFFFFFFFFFFFFFFFFFFFFFFFFFFFFFF
FFFFF,FFFFFFFFFFFFFFFFFFFFFFFFFFFFFFFFFFFFFFFFFFFFFFFFFFFFFFFFFFFFFF
FFF
@A00721:190:HGJW7DSXY:4:1618:17987:10347_2:N:0:TCTGCAAG+GCTGCACT
TTCTTATATTATTTACAATAGTTAATATCATATTAGTAATACTCACTATCGTTTTCTGCTTTTTCTTA
GCTAATAGATTAATAAAGGATGAGTACGAAAACCAACTCTTCGTTACG
+
:FFFFFFFFFFFFFFFFFFFFFFFFFFFFFFFFF:FFFFFFFFFFFFFFFFFFFFFFFFFFF,FFFFF
FFFF:FFFF:FFFFFFF:FFFFFFFF,FFFFFFFFF:FFFFFFFFFFF
@A00721:190:HGJW7DSXY:4:2675:2582:22373_1:N:0:TCTGCAAG+GCTGCACT
GTAGAATAGATTTAGTCTCTACACCCATCTCAACGAGGATAACGCGAGTTTTCCGTCCTGAACGTAAT
ACACGAAGAGATCATCCGCAAACGACTCGAAACCGAGACGCCGTAGATCAAAGTCCCTTCTAACTTTA
GAT
+
:,:,F:F:,:FFF::,,:::FFFFF:F:F::F,:F,F,FFFF,:F,,,FFF,F:FFF,F,F,:,FFF,
FF,:,F,,,F:,:,FF::F:FFFFFFFFFFFFFFFFFFFFFFFFFFFFFFFFFFFFFFFFFFFFFFFF
FFF
@A00721:190:HGJW7DSXY:4:1240:1732:22091_1:N:0:TCTGCAAG+GCTGCACT
ACTCATTTTATAGCAGTTATTTATGTTGTACTTTTTATAATTTATAACCTTCTTATACTTTTGGGTAT
GTTTTGTGAAAAACATGAGATACCCAAGTCTATCGAAGACATTATCTCTTATGTACTTATTGTAACGA
TCA
+
FFFFFFFFFFFFFFFFFFFFFFFFFFFFFFFFFFFFFFFFFFFFFFFFFFFFFFFFFF:FFFFFFFFF
FF:FFFFFFFFFFFFFFFFFFFFFFFFFFFFFFFFFFFFFFFFFFFFFFFFFFFFFFFFFFFFF:FFF
FFF
@A00721:190:HGJW7DSXY:4:1467:31259:27915_1:N:0:TCTGCAAG+GCTGCACT
TTCTTTTATTCCTTTAACTGTGTATGAGGAGGATTTATAATGCTTTTCGATAGTGATTGTTATCAATT
TAAATACATTACTGTTTCAAATCATCTTTACTCACAGACTCCGAAAATGGTTTCAAATGAGGTCAAAG
CTC
+
FFFFFFFFFFFFFFFFFFFFFFFFFFFFFFFFFFFFFFFFFFFFFFFFFFFFFFFFFFFFFFFFFFFF

FFFFFFFFFFFFFFFFFFFFFFFFFFFFFFFFFFFFFFFFFFFFFFFFFFFFFFFFFFFFFFFFFFFFFFFF
FFF
@A00721:190:HGJW7DSXY:4:1631:14235:10770_2:N:0:TCTGCAAG+GCTGCACT
AACTATTATCCGATTCTTCGAGGGTATCACTACCACTGATCTGCCGACTTTAGTCAAGGAACTTCAAT
TCCTTCGATGTTTATATTTTTCTATAAGGGAAAATAGCAATAATCAAA
+
FFF:FFF:F,FFFFFFFFFFFFFFFFFFFFFFFFFFFFFFF:F:FFFFFF,:FFFFFFFFFFF,FF:FF
,FFFFFFFFFFFFFFFFFFFFFFFFFFFFFFF,:FFFF:FFFFFFFFFFF
@A00721:190:HGJW7DSXY:4:1402:4725:34976_2:N:0:TCTGCAAG+GCTGCACT
AACTTTATTTCAGAACTCTCTCCGATCACCTTGGTGTGATCTCCCGGAGGATCTTTTCAATAAGATGT
ATGATGATAATCTACTATTTTTAAATAGTGATATTCAGCGTATACTTG
+
FFFFFFFFFFFFFFFFFFFFFFF:FFFFFFFFFFFFFFFFFFFFFFFFFFFFFFFFFFFFFFFFFFFF:F
FFFFFFFFFFFFFFFFFFFFFFFFFFFFFFFFFFFFF:FFFFFFFFFFF
@A00721:190:HGJW7DSXY:4:1434:26576:23531_2:N:0:TCTGCAAG+GCTGCACT
TTTGGCTTGGTCTATTTACCGCAAAAAGTTGGAAGAGGTCTATGATATTTATTTTACTTATACTTTAA
ATAGCACCGATTATAACTTGACTGTTGATAATATTACAATAGCTCTTT
+
FFFFFFFFFFFFFFFFFFFFFFFFFFFFFFFFFFFFFFFFFFFFFFFFFFFFFFFFFFFFFFFFFFFFFFFF
FFFFFFFFFFFFFFFFFFFFFFFFFFFFFFFFFFFFFFFFFFFFFFFFF
@A00721:190:HGJW7DSXY:4:1232:15908:27101_2:N:0:TCTGCAAG+GCTGCACT
AGTAACCGGTGAACACGTCGGTAGCATTGATTTACTTTTACCGTTAGAACTGAAAAAGATTCTTTTCG
AAAAGAAACCTATAAGTTACAAACGATTCTTCGACTATATTAAAGAAA
+
FFFFFFFFFFFFFFFFFFFFFFFFFFFFFFFFFFFFFFFFFFFFFFFFFFFFFFFFFFFFFFFFFFFFFFFF
FFFFFFFFFFFFFFFFFFFFFFFFFFFFFFFFFFFFF:FFFFFFFFFFF
@A00721:190:HGJW7DSXY:4:1342:32913:27336_2:N:0:TCTGCAAG+GCTGCACT
TCAAGTACCTGAACTTTACATTTTTAATGGTATGGTATAATGTGTCTTACAAATAGAACCTTCACTCT
CGGTAATGAGAGTGTATTCCTTAATATTCTAGTAACCGCTATGTGGGC
+
FFFFFFFFFFFFFFFFFFFFFFFFFFFFFFFFFFFFFFFFFFFFFFFFFFFFFFFFFFFFFFFFFFFFFFFF
FFFFFFFFFFFFFFFFFF::FFF:FFFFFFFFFFFFFFFFFFFFFFFFF
@A00721:190:HGJW7DSXY:4:1304:15438:29136_2:N:0:TCTGCAAG+GCTGCACT
TGCTAGGAAAATTTTAGAAAAACTCCATTGTATAGTTCAAGGAGTACTTAATTCAATAGCTTTAGTAT
TATTTACTATCGCCTTTATTGAAAATTGTATAAGAGTTAATTGCTTAC
+
FFFFFFFFFFFFFFFFFFFFFFFFFFFFFFFFFFFFFFFFFFFFFFFFFFFFFFFFFFFFFFFFFFFFFFFF
FFFFFFFFFFF:FFFFFFFFFFFFFFFFFFFFFFFFFFFFFFFFFFFFF
@A00721:190:HGJW7DSXY:4:2660:7048:31923_1:N:0:TCTGCAAG+GCTGCACT
ACATACCATAACCTACCATGACCAATCATCGCTCCAACTGGCACGCCAGACGTAGGGTCAAACCTACT
ACCGCCGGTCGGCAGATCGGCACCACTGGTCTCTACGTTCAGGGACCCGCTGTGCAGAATAACCTTCC
CAA
+
FFFFFFFFFFFFFFFFF:FFFFFF:FFFFFFFFFFFFFFFFFFFFFFFFFFFFFFFFFFFFFFFFFFFFF
FFFFFFFFFFFFF:FFFFFFFFFFFFFFFFFFFFFFFFFFFFFFFFFFFFFFFFFFFFFFFFFFFFFFFF
FFF
@A00721:190:HGJW7DSXY:4:1344:6008:2347_2:N:0:TCTGCAAG+GCTGCACT
CACAGAAGAAGGCTGAAGGTCGTTACCGGACGACCACTCAAAGGAAACGGGCTGTTGCCCAGAGGAAA
GCAGCGACCACCGAGGTCGCTCGTAACATTGACGGCAGTCCCGTCATC
+
FFFF:FFFFFFFFFFFFFFFFFFFFFFFFFFFFFFFF:FFFFFFFFFFF:FFFFFFFFF::FFF:FFF
:FFFF:FFFFFFFFFFFFFFFFFFFFFFFFFFFFF:FFFFFF:FFFFFFF
@A00721:190:HGJW7DSXY:4:1307:22110:29637_1:N:0:TCTGCAAG+GCTGCACT
CTGAGGCAGTACCTCAGTCAAAAGTCACATTCACCACTCCCATACCAATCAATGATGTGACTATACAG

TTCGGCCATTTCAAGAGTGGTCAATACGTCGTGGGAGGTACTGAAGCCTTCCCCTCCGCTGGCACGAA
GAA
+
FFFFFFFFFFFFFFFFFFFFFFFFFFFFFFFFFFFFFFFFFFFFFFFFFFFFFFFFFFFFFFFFFFFFF
FFFFFFFFFFFFFFFFFFFFF::FFFFFFFFFFFFFFFFFFFFFFFFFFFFFFFFFFFFFFFFFFFFFF
FFF
@A00721:190:HGJW7DSXY:4:1606:8838:3991_1:N:0:TCTGCAAG+GCTGCACT
CCAATCAATAGTTCTTCTGACCAGAATGAAAAGGGGTTTACCAGGGTAGTCTCTAAGTCTACCAGGAA
GAGGGGAAATGTCCCCAAAACTGAGAAGAATAAAGGGAAAGGTCCATCAGGACCTAAGAAGGGGAAAT
TAC
+
FFFF:FFFFFF,FFFFFFFF:FFFFFFFFFFFFFFF:FFFFFFFFFFFFFF:FFFFFFFFFFFFFFFF
FFFFFF:FFFFFFFFFFFFFFFFFFFFFFFFFF:FFFFFFFFFFFFFFFFFFFFFFFFFFFFFFFFFF
:FF
@A00721:190:HGJW7DSXY:4:2236:25003:2581_2:N:0:TCTGCAAG+GCTGCACT
GGAAGATCTCCCACCATCTGGGGTTGGGAATAAGAAATCTGCACGGGCCAAAGAGGCCGCTGCTACGA
AGTCGCTGAAGGCGGCTTGGGTCATTAAGGGTAGCTGTAAAGTTGCGC
+
FFFFFFFFFFFFFFFFFFFFFFFFFFFF:FFFFFFFFFFFFFFFFFFFFFFFFFFFFFFFFFFFFFFF
FFFFFFFFFFFFFFFFFFFFFFFFFFF:FFFFFFFFFFFFFFFFFFFF
@A00721:190:HGJW7DSXY:4:1517:30680:16736_1:N:0:TCTGCAAG+GCTGCACT
ATCTTTGAGACCAAGACTCTCTACAGTAGGTTCGTGCCTACTATGAGCAAATCTGTTGAATTCGTCAC
TGAGGACGAAGGTGAGAGGACTTTTGGCTACTGGCGACAAACTGAGAGCAGTCGCTGGGGAAAGAGAA
TTG
+
FFFFFFFFFFFFFFFFFFFFFFFFFFFFFFFFFFFFFFFFFFFFFFFFFFFFFFFFFFFFFFFFFFFF
FFFFFFFFFFFFFFFFFFFFFFFFFFFFFFFFFFFFFFFFFFFFFFFFFFFFFFFFFFFFFFFFFFFF
FFF
@A00721:190:HGJW7DSXY:4:1238:2392:26584_1:N:0:TCTGCAAG+GCTGCACT
CTTGAGTGTGACGAGGCCGGTGACGTTTTTGTCAACGGCGTTTGGAGGATTTTATCCGCGCTTAACTA
TGGGGCGAAGATCCCCTATGGAGTCTTGAAGGACTTAGGTTGGCATATTTATGCCGACGATAATAAGC
ATG
+
:FFFFFFFFFFFFFFFFFFFFFFFFFFFFFFFFFFFFF,F:FF:FFFFFFFFF,FFF:FFFFFFFFF
FFFFFFFFFFFFFFFFFFF::FFFFFFFFFFFFFFFFFFFFFFFFFFFFFFFFFF:FFFFFFFF,FFF
FFF
@A00721:190:HGJW7DSXY:4:1216:28266:17409_2:N:0:TCTGCAAG+GCTGCACT
CGGGACTTACAAGAGAGAGTGTGAGAGAGTCGCTCTCACTATTTTTGCCCAGAATGCGGGCTTTCTGA
CTGATGAGGTTAAAACTCTTGCTCCTTCTGTGGGTGAGGGTTTTTGTT
+
FFFFFFFFFFFFFFFFFFFFFFFFFFFFFFFFFFF:FFFFFFFFFFF:FFFFF:F,F:FFF,:FF,F,:
:FFFFF::FFFFFFFFF,FF,:::F:F,:F:FFF,::FFFFFF,FFFF
@A00721:190:HGJW7DSXY:4:2213:23836:24643_2:N:0:TCTGCAAG+GCTGCACT
TCCTCCATAATTACAACCTCTCTTTAGAGAGCATTAGAAGGAAGTTGGGCAAATTCCCACATATGGTA
GACGTTGTCACACTTTTCTTGAAAATTTTTCCTTTCCTCCGTCATTGG
+
FFFFFFFFFFFFFFFFFFFFFFFFFFFFFFFFFFFFFFFFFFFFFFFFFFFFFFFFFFFFFFFFFFFF
FFFFFFFFFFFFFFFFFFFFFFFFFFFFFFFFFFFFFFFFFFFFFFFF
@A00721:190:HGJW7DSXY:4:1314:29875:24580_2:N:0:TCTGCAAG+GCTGCACT
AAGGTGGAAGGTGTCAAGGGTCTTCTTCATGCTGATCTTTCAGGACCTAATCACATCATGATTGATAG
TTACGGCTCGTATCGAGTTGGAGGGAGGAAGTTCTCCGAAATTAAAGA
+
FFFFFFFFFFFFFFFFFFFFFFFFFFFFFFFFFFFFFFFFFFFFFFFFFFFFFFFFFFFFFFFFFFFF
FFFFFFFFFFFFFFFFFFFFFFFFFFFFFFFFFFFFFFFF:FFFFFFF

```
@A00721:190:HGJW7DSXY:4:2553:19407:36135_2:N:0:TCTGCAAG+GCTGCACT
TCAGAGAGGGGAGCTCTGAAAAGGAAACTTCGTCAGAAGATGAGGAAGTCTAAGAGGAGGAGTTTGAG
GAAGGCTCTAGAGAAGATCTCACCACCTGGAGAAGGCAAGTGGAGAGT
+
FFFFFFFFFFFFFFFFFFFFFFFFFFFFFFFFFFFFF:FFFFFFFFF:FFFFFFFFFFFFFFFF
FF:FFFFFFFFFFFFFFFFFFFFFFFFFFFFFFFFFFFFFFFFFFFFF
@A00721:190:HGJW7DSXY:4:2104:14136:33771_2:N:0:TCTGCAAG+GCTGCACT
AAGAAGGATTATTTGAAGCAGGGGTTACCACTTCCTAAGGTGGATGAGGGAGGTTGTTTCTTTGGAGT
TTTAGATAATCTTTTGAGAAGGGGTCATAGAGCCAGCAAGAGAAGCTT
+
FFFFFFFFFFFFFFFFFFFFFFFFFFFFFFFFFFFFFFFFFFFFFFFFFFFFFFFFFFFFFFFFFFFF
FFFFFFFFFFFFFFFFFFFFFFFFFFFFFFFFFFFFFFFFFFFFFFFF
@A00721:190:HGJW7DSXY:4:2170:20618:27430_2:N:0:TCTGCAAG+GCTGCACT
TATAATTTAGGTCAACTTGGGGTTTCGAATGTCATTAGTGCCTTACGCTTGAAAGAGGTCCTTTTGAG
TATCGACAGATCTTTCGATCTTGAGGACGTACATTTAACGTGGCAGAA
+
FFFFFFFFFFFFFFFFFFFFFFFFFFFF:FFFFFFFFFFFFFFFFFFFFFFFFFFFFFFFFFFFFFFF
FFFFFFFFF:FFFFFFFFF:FFFF:FFFFFFFFFFFF:FFFFFFFFFF
@A00721:190:HGJW7DSXY:4:2223:11315:33019_2:N:0:TCTGCAAG+GCTGCACT
ATATTGTTCCTTCCGGGAAGTTGTGGGATTTAACTTTGACTCAACTACTCCGATCAGATCCGGATGGT
TTGATTGGTATGGACTACTCGACTTTTGATCTGGAGTTGTCTAGGGTC
+
FFFFFFFFFFFFFFFFFFFFFFFFFFFFFFFFFFFFFFFFFFFFFFFFFFFFFFFFFFFFFFFF,FFF
FFFFFFFFFFFFFFFFFFFFFFFFFFFFFFFFFFFFFFFFFFFFFFFF
@A00721:190:HGJW7DSXY:4:2653:32579:9439_2:N:0:TCTGCAAG+GCTGCACT
CGATAAGGTGAAAAATATGCAGGTCAAAGATTCTGTAGCTCTGAGGGCGGTTGAGGGAGCCGTTTTGG
CCGACTTTAACTATTCTAAGAGTCTTTATGATTCAAAACCTATCGTTG
+
FF:FFFFFFFFFFFFFFFFFFFFFFFFFFFFFFFFFFFFFFFFFFFFFFFFFFFFFFFFFFF:FFFFF
FFFFFF,FF:FFFFFFFF:FFFFFFFFFFFFFFFFFFFFFFFFFFFFF,
@A00721:190:HGJW7DSXY:4:2262:12011:30937_2:N:0:TCTGCAAG+GCTGCACT
GGCTTATTGTCGCGCAGTTTCCAGAATTGCGGTTGAAGTTCACCCATTCTATCTTGAATGCTCACGCC
GTCGCAGCCATATCGAGACTATGTTATAATCACTTGTTAAAGTACAGA
+
FFFFFFFFFFFFFFFFFFFFFFFFFFFFFFFFFFFFFFFFFFFFFFFFFFFFFFFFFFFFFFFFFFFF
FFFFFFFFFFFFFFFFFFFFFFFFFFFFFFFFFFFFFFFFFFFFFFFF
@A00721:190:HGJW7DSXY:4:1415:19000:36965_2:N:0:TCTGCAAG+GCTGCACT
ATTATTGATATTGGAGGCGATATTCGTTTTCATATCCAAAACTCCTTGAATGGAGTACATATTTGCAA
CCCTATTCTTGATGTGAAAGATGCATCGAGGTATACGTCTAGGAACAT
+
FFFFFFFFFFFFFFFFFFFFFFFFFFFFFFFFFFFFFFFFFFFFFFFFFFFFFFFFFFFFFFFFFFFF
FFFFFFFFFFFFFFFFFFFFFFFFFFFFFFFFFFF:FFFFFFFF,FFF
@A00721:190:HGJW7DSXY:4:1578:29505:25160_2:N:0:TCTGCAAG+GCTGCACT
GCTATCGGAGGGAAGCAAAATGTTTCGTTCTGTTACAAGAACGCTTTGGATTGTGACCATAAAGCCTC
AGTTGCGATAGCTGTAGAGGTATACGATATCTCACTTGATGATGTAGC
+
,FFF:::,F:::F,,:FF:,FFF:FF:F,F:F,,F,:F,FF,,FFFF:FFFFFFFF,:FFFFFF,,FF
FFF:FFFF:FFFFF,FF:FFFFFFF:FFF,FF:,,,F,FF:F:,:FFF
@A00721:190:HGJW7DSXY:4:2255:20663:5400_1:N:0:TCTGCAAG+GCTGCACT
TGATGTATGTCATTTAGTCTTAGTTTGTCACGGCGAATTATTGGACACGATGAAAAATGACATACCCG
TTCCTGACCTTGGTATCGTTATCCGTCATGGAGATGATGGCGCATCACTTGATTACTCTATGCCCGAA
GGA
+
FFFF,FFF::,F:FF,FFFFF:FFFFFF,,F:F:FF:FF,F:,F:FF,,,F,F,:,FFF,:FFFF,:,
```

FF:FFFF:,:,F:F::F,,FFF:F,FFFFFFF:FFFF,:FFF,F:,,FFFF:FFF:F,FFF,F:FF:,
:F:
@A00721:190:HGJW7DSXY:4:1676:26937:34616_2:N:0:TCTGCAAG+GCTGCACT
AACAAAGATAAGATTTTATCTTTCTTCAAACACCCTGTTTTCCTTAAGGGCGGACAATTGTTCCATTG
TGAACTCTTGGGGACTAGATTGAGCATTAATGAGTTCCGAATAACTCG
+
FFFFFFFFFFFFFFFFFFFFFFFFFFFFFFFFFFFFFFFFFFFFFFFFFFFFFFFFFFFFFFFFF:FFF
FFFFFFFFFFFFFFFFFFFFFFFFFFFFFFFFFFFFFFFFFFFF:FFFF
@A00721:190:HGJW7DSXY:4:1404:14805:11913_2:N:0:TCTGCAAG+GCTGCACT
TATTTCCGTACCAAGAGCCACTGCCAATCTCATCGCTATACGCATACCTGATTGGAAGAAGCGAGGTT
CGTTCCTCAATAAAAATTTTTCTCAAGAGATTATGGTTGATAAAGTTT
+
FFFFFFFFFFFFFF,:FFFFFFFFFFF:FFFFFFF:FF:FFFFFFFFFFFF:FFFFFFFFFFFFF:FF
FFFFFFFFFFFFFFFFFFFFFFFFFFF:FFFFFFFFFFFFFFFFFFFFF
@A00721:190:HGJW7DSXY:4:1247:3260:33880_1:N:0:TCTGCAAG+GCTGCACT
TACGTCATGGGGATCACTTCTTCGGTCAATGACAAAACCTTCGAATATGTTACGACATGGCTTCGTAA
CAACAGTGCGAGGATTGTCATCTCGGGGAAAGTGATTCACCAAGATGTTCATATAGATACGAGATACC
TAG
+
FFFFFFFFFFFFFFFFFF:FFFFFFFFFFFFFFFFFFFFFFFFFFFFFFFFFFFFFFFFFFFFFFFFFF
FFFFFFFFFFFFFFFFFFFFFFFFFFFFFFFFFFFFFFFFFFFFFFFFFFFFFFFFFFFFFFFFFFFFF
FFF
@A00721:190:HGJW7DSXY:4:1458:14733:12414_2:N:0:TCTGCAAG+GCTGCACT
AGCGATCTTACTCGTTATCGGAGCTAAGAAGAAGACTTTGGGTACACGTTATGCTCGCAAACTTAACG
TTAGTACCGGAGGTGATCTCTCTGCTTCTGCGTTACTGAGAGAAAAGA
+
FFFFFFFFFFFFFFFFFFFFF:FFFFFFFFFFFFFFFFFFFFFFFFFFFFFF,FFFFFFFFFFFFFFFF
FFFFFFFFFFFFFFFFFFFFFFFFFFFFFFFFFFFFFFFFFFFFFFFF
@A00721:190:HGJW7DSXY:4:1325:11107:24768_1:N:0:TCTGCAAG+GCTGCACT
CTTTATGGACAAGATGCAAGTTCTTGGGAAATACTTGTTTCCCTTTTTAGGTTTAGTAGGTGATCTTG
ACGTCGATTCTTTGTATAGGGAGTGTGAGAAGACAACGAATCTTGAAACTACGGTCCTTATCAAAGAA
AAT
+
FFFFFFFFFFFF:F:FFFFFFFFFFF:FFFFFFFFFFF:FFFFFFFFFFFFFFFFFFFFFFFFFFFFFF
FFFFFFFFFFFFFFFFFFFFF:FFFFFFFFFFFFFFFFFFFFFFFFFFFFFFFFFFFFFFFFFFFFFFF
FFF
@A00721:190:HGJW7DSXY:4:1218:26386:24236_2:N:0:TCTGCAAG+GCTGCACT
AAATCTTTTGAGATTGAGAAGATTGAAGAACACGTCAAGAAGTCGCTCGGCGTTGTTCTTGAAAACCA
AGTTAAGAAGGAGGCTGATCATGGAGTCTTTGTACCTTCTGTCAAACT
+
FFFFFFFFFFFFFFFFFFFFFFFFFFFFFFFFFFFFFFFFFFFFFFFFFFFFFFFFFFFFFFFFFFFFF
FFFFFFFFFFFFFFFFFFFFFFFFFFFFFFFFFFFFFFFFFFFF:FFFF
@A00721:190:HGJW7DSXY:4:1630:3685:10786_2:N:0:TCTGCAAG+GCTGCACT
TACGAGAGGTGATCCCGGATGTGGATTGAAGGCTGGCTCTAAGGGAGACTCCTTCTTTTTGAATGTTG
TAAAGAATTGGGTTGAGAAGTGTGCTTTACAAGGAGTTAGACTCTTCT
+
FFFFFFFFFFF:FFFFFFFFF:FF:FFFFFF:FFFFFFFF:FFFFFFFFFFFFFFFFFFFFFFF,FFFF
,FFFFFFFFFFFFFFFF:FFFFFFFFFFFFFFFFFFFFFFFFFFFF:FFF
@A00721:190:HGJW7DSXY:4:2527:32226:26334_2:N:0:TCTGCAAG+GCTGCACT
TAAAGAAAAATTTTCGAATAAACTAATGAGAGGTATAGGCTTAGTCAAGTCTGTACTTACATTCTTTA
CGCCAGATTCTTCATGGGGCGACGCTGTTTCTCTTGGTATTTCTGAAA
+
FFFFFFFFFFFFFFFFFFFFF:FFFFFFFFFFFFFFFFFFFFFFFFFFFFFFFFFFFFFFFFF:FFFFFF
FFFFFFFFFFFFFFF:FFFFFFFFFFFFFFFFFFFFFF:FFFF,FFFFF

```
@A00721:190:HGJW7DSXY:4:2364:4164:25614_1:N:0:TCTGCAAG+GCTGCACT
TTTCCTCTGGTAAGGGAATTATTAAATCATTCTTCTCACTCTTGACGAGAATTATTACTGTTTTCTCT
ACCCATAACTTCTTTTTCAAAAATGCGGATGGGTCTACTGATGTTTTCCTACAGAGTGCAGTCGTTTC
AAC
+
F,FFFFFFFFFFFFFFFFFFFF:FFFFFFFFFFFFFFFFFFFFFFFFFFFFFFFFFFFFFFFFFFFFF
FFFFFFFFFFFFFFFFFFFFFFFFFFFFFFFFFFFFFFFFFFFFFFFFFF:FFFFFFFFFFFFFFFFF
FFF
@A00721:190:HGJW7DSXY:4:1623:15157:32002_1:N:0:TCTGCAAG+GCTGCACT
AATAATTGGTGATGAAAATACGTATGTCGGTGGGGTTCTCAAGGCTCTCATCGCCACTGGGATTCTAA
GGGGTTCCATAAGATATTATCTGACATTGTGGTTCAATGATCAGGATTCTTCTTTTGGTCCTTACGTC
TTA
+
FFFFF::FFFFFFFF:FFFFFFFFFFFFFFFFFFFFFFFFFFFFFFFFFFFFFFFFFFFFFFFFFFFF
FFFFFFFFFFFFFFFFFFFFFFFFFFF:FFFF::FFFFFFFFFFFFFFFFFFFFFFFFFFFFFFFFFF
FFF
@A00721:190:HGJW7DSXY:4:2427:4598:11240_1:N:0:TCTGCAAG+GCTGCACT
TCTTGTCACGATTCTTAAAAGAAGTTTATTAAAACTTCAGAGTCTCTTGGAAGTCCAGGTAGCTAATG
TTCTTGCAATAGCCACAGACTTTCTGGGAAAAGAGACCTCTGTTGGTAGAGGAGTTGAGAGAATTACT
CAG
+
F:FFFFFFFFFFFFFFFFFFFFFFFFFFFFFFF:FFFFFFFF:FFFFFFFFFFFFFFFFFFFFFFFFF
FFFFFFFFFFFFFFFFFFFFFFFFFFFFFFFFFFFFFFFFFFFFFFFFFFFFFFFFFFFFFFFFFFFF
FFF
@A00721:190:HGJW7DSXY:4:1214:27651:17848_1:N:0:TCTGCAAG+GCTGCACT
AGTTAGGGAGTGGCTAGGTTTTAAAAGATCCTTTGCACAAGCAATACCTTCCACTGAGTTCATCACCC
AGAGTTTCGACGTCGCGGAAAACGATGAAGATTTCCACACTGTTCGAGAATCTTCTGATGCTGAATTT
GAG
+
FFFFFFFFFFFFFFFFFFFFFFFFFFFFFFFFFFFFFFFFFFFFFFFFFFFFFFFFFFFFFFFFFFFF
FFFFFFFFFFFFFFFFFFFFFFFFFFFFFFFFFFFFFFFFFFFFFFFFFFFFFFFFFFFFFFFFFFFF
FFF
@A00721:190:HGJW7DSXY:4:2660:11134:27696_1:N:0:TCTGCAAG+GCTGCACT
TGTGCTCTTCCGATCTCCTGATGGCTCAGTACAGGGAGGAGGTAATAGTTATAAAACCTTCTTTTATC
ATTGCAACAGGACTTTGAGAAGACTGTTGAAATGGACAAAATCTGGGATCATAGGTATGTGTCACGCT
ACA
+
FFF:FF:FF::FFF:F::FF,FF:FFFFFFFFFFFFFFF:,FFFFFF:FFFFFFFFFFFF:FFFFFFF
FFFFFFFFFFF::FFFFFFFFFFFFFFFFFFFFFFFFF,:,FF:FFFFFFFFFFFFFFFFFFFFFF,,FF
FFF
@A00721:190:HGJW7DSXY:4:1468:8323:31657_1:N:0:TCTGCAAG+GCTGCACT
TGTTCAGGAAGATCTCTACAGAGATTATCGAGAGTTGTGATCTAGTTCTAAATTTAGGTCGACGGACT
TTAGCAACTTTAGACAAATTCTTTCCGGGCTCAATGACCTTTGCTATGTACGTGGATCTAATGCTTGG
TGA
+
FFFFFFFFFFFFF:FFFFFFFFFFFFFF:FFFFFFFFFFFFFFFFFFFFFFFFFFFFFFFFFFFFFFF:
FFFFFFFFFFFFFF,FFFFFFFFFFFFFFFFFFFFFFFFFFFFFFFFFFFFFFFFFFF:FFFFFFFFFF
FFF
@A00721:190:HGJW7DSXY:4:2338:22200:12571_2:N:0:TCTGCAAG+GCTGCACT
TATTAGTTCATGAAGTTCTTACACTTCTCGCGACTAATGTCAGCTTCACTACAAAGGCGTTACGTAAA
GTAGTTTTCACGCTTTATCTTAAAGCATTACCACCAAATTTAGCACTT
+
FFFFFF:FFFFFF:FFF:FFFFF,FFFFF:FFFFFFF:FFFFFFFF:FFFFFFF:FFFFFFFFFFFFF
FFF:FFFFFFFFFFFFFFF:FFFFF:F:FF,FFFFFFFFFFF,F,FF
```

```
@A00721:190:HGJW7DSXY:4:2304:21296:7498_2:N:0:TCTGCAAG+GCTGCACT
TCGCCATATCAGATTAGAGACATGCCAGTTAGAGTTCTTCTAACCAGGACTGATGGTGAGATTCTTTT
ACCTTTTGGAGGTCCAGGAACCTTTACTCCTAGTGTCTTTTCTAGAGT
+
FFFFFFFFFFFFFFFFFFFFFFFFFFFFFFFFFFFFFFFFFFFFFFFFFFFFFFFFFFFFFFFFFFFF
FFF:FFFFFFFFFFFFFFFFFFFFFFFFFFFFFFFFFFF:FFFFFFFF
@A00721:190:HGJW7DSXY:4:1327:3016:16016_2:N:0:TCTGCAAG+GCTGCACT
CGGAGGATGACAGGTGGGAGACCGCTCAATAACACTCTATTGAATCCCTTTTCCACGATGAAAGCTCT
TTATGAGGTGCTCCATGCAAATTTACAGTTCCTCTTTGGTGATATCGC
+
FFFFFFFFFFF,FFFFFFFFFFFFFFF,FFFFFFFFF:FF:FFFFFFFFFF,FFFFFFFFFFFFFFFF
,FFFFFFFFFFFFFFFFFFFFFFFFF:FF:FF,FFFF::FFFF:FFFF,
@A00721:190:HGJW7DSXY:4:1327:3016:16016_1:N:0:TCTGCAAG+GCTGCACT
TTTTGATAAATTTGTTGGAATCGATAATGATACTGACGAAGGTTCTATTACCACCCTTGTCATTGATA
CAAACATTCTAGGTGATGCTTTCTTAGAGGCTCGTCTAGCCGCTGAGATTCGTCTTACCTATTTACAA
TCT
+
:FFFFFF:FFFFFFFFFFFFFF:FFFFFFFFFFFFF:FF:FFFF:F:F:FFFFF,:FFFF:FFF::FF
FFFFFFFF:FFFFFFFFFFFFF:FF:FFFFFFFFFF:FFFFFFFFFF:FFFFFFFFFFFFF:F:FFFF
FF:
@A00721:190:HGJW7DSXY:4:1309:18575:6605_1:N:0:TCTGCAAG+GCTGCACT
GATATCGATTTAGTACTGGGAAATGGTGGTAGTCAGTTTACTACTTTTCTTTCCCTCCCCTTTTTCGA
TTGGATTCGACGGGGTCTTCGTCTCACTACTAATTTGATAAGGTTTTTCTTTTATGGGACTCTTGTTT
GTA
+
F:F,:FFF:FFFFF::FF:FF,:FFFFFF:,F,,FFF,F,FFF,FFFF:FFF:FFF:F:FF,F:F:FF
FFFFFFFFFF,F,F:F:FF:FFFFFF:,,FFF:FFFF,F,,F,FFFF,,F,FFFFF,F,FFFFFFFFF
FFF
@A00721:190:HGJW7DSXY:4:2555:32615:24064_2:N:0:TCTGCAAG+GCTGCACT
AAGTTCTTAATGTAGGTCTCCTAGTACTTAAGGGTTTATGTTTCGGTTTCAGCTGGATGGATCTTTTG
ATGGTATGTTTCACATTGCGTGATCACTTCCCGTGGACTTGGGAAGAG
+
FFFFFFFFFFFFFFFFFFFFFFFFFFFFFFFFFFFFFFFFFFFFFFFFFFFFFFFFFFFFFFFFFFFF
FFFFFFFFFFFFFFFFFFFFFFFF:FFFFFFFFFFFFFFFFFFFFFFF
@A00721:190:HGJW7DSXY:4:1162:21495:33959_1:N:0:TCTGCAAG+GCTGCACT
CGATTCTATTATGTCGCTAAAGCCTGTCCAGTTGTCGATGATGTTCGGGCTGTGGGGATTAACTTCCC
TCGTGAAACTTACGACGATGTTCTCGCTCGCATTTCACAAGGATTACTAAGTCTGAGATCTGCCACTT
CTA
+
FFFFFFFFFFFFFFFFFFFFFFFFFFFFFFFFFFFFFFFFFFFFFFFFFFFFFFFFFFF:FFFFFFFF
FFFFFFFFFFFFFFFFFFFFFFFFFFFFFFFFFFFFFFFFFFFFFFFFFFFFFFFFFFFFFFFFFFFF
FFF
@A00721:190:HGJW7DSXY:4:1117:16016:11350_1:N:0:TCTGCAAG+GCTGCACT
TCTGTTCATGAGGATGTCTTGGTCGAAAAATATCGCCGGGAACTTCTAATAGAGGATCCTGATGACAT
TATTTCAACGGATACCGATCTCAGTTCTACCACAGAAACCGTCTCAGGAACTGTCTCCACTGATTCGG
AAT
+
FFFFFFFFFFFFFFFFFFFFFFFFFFFFFFFFFFFFFFFFFFFFFFFFFFFFFFFFFFFFFFFFFFFF
FFFFFFFFFFFFFFFFFFFFFFFFFFFFFFFFFFFFFFFFFFFFFFFFFFFFFFFFFFFFFFFFFFFF
FFF
@A00721:190:HGJW7DSXY:4:1275:11388:36057_2:N:0:TCTGCAAG+GCTGCACT
CGACCTTCTTGTGGGGACTGAGACCTCTGAATTTTCAGGGTGCCCTGATGAGGACGTTGAAAGGGATA
ATTTCAACTCTGAGACTTCTAAGAGTGTTTCAAAAGACCAGAAACGTA
+
```

FFFFFFFFFFFFFFFFFFFFFFFFFFFFFFFFFFFFFFFFFFFFFFFFFFFFFFFFFFFFFFFFFFFFFF
FFFFFFFFFFFFFFFFFFFFFFFFFFFFFFFFFFFFFFFF:FFFFFFFFFFF
@A00721:190:HGJW7DSXY:4:2225:14136:17425_1:N:0:TCTGCAAG+GCTGCACT
TAAAGGTAGGAAGGGGAGAAAACAGAACAAGCGAGCGGTTGAAGAGAAAACTGATATGGTTGCGGCTG
ATCGTACCCCAATTCCTGCTAGAAAGATCACTCTCGGCGATACCACTGATCTTCCAGAGTTACGTAGT
GGT
+
FFF,:,F,F:,,FF,FF:,FFFF:FFFFF,FF:FFFF:FFFFFFFFFFFF:FFFFFF,FFFFFFFFFF
FF::F,FFF,FFFF:FFF:FFFFFFFFF:::FFFFF:FFFFFFFFFFFFFFFFFFFFFFFFFFFF:FF
FFF
@A00721:190:HGJW7DSXY:4:1508:16278:35540_1:N:0:TCTGCAAG+GCTGCACT
TTTTATCAAAGAGAAAGTTTCTGCTAGTCGCGACGATAGTATTTATCGGAGTGATATTAACAAGTTAA
ACTTCGAACTGACCACTTACAAGAGTGTTGAAGTACCGCAACATGGATCAGTTTTCCAAAAAGCTATT
AAT
+
FFFFFFFFFFFFFFFFFFFFFFFFFFFFFFFFFFFFFFFFFF:FFFFFFFFFFFFFFFFFFFFFFFFFF
FFFFFFFFFFFFFFFFFFFFFFFFFFFFFFFFFF:FFFF:FFFFFFFFFFFFFFFFFFFFFFFFFFFFF
FFF
@A00721:190:HGJW7DSXY:4:2419:10574:36777_2:N:0:TCTGCAAG+GCTGCACT
AAACTGAATCCACTCTCTTGGGATATAAATGAGTACATCTATAATAGAGTTTTGGAACTGTGTAATGT
CCTACGTACTGTTCAAGGTGTTTGGGCTTCTCTAAAGAACAGCAACTT
+
FFFFFFFFFFFFFFFFFFFFFFFFFFFFFFFFFFF:F:FFFFF:F:FFFFFFFFFFFFFFF:FFF:FFF
FFFFFFFFFFFFFF,FFFFF:FFFFFFF:FFFFFFFFFFFFFFFFFFF
@A00721:190:HGJW7DSXY:4:2252:16803:20322_1:N:0:TCTGCAAG+GCTGCACT
AACCTTTCCTCGTTCTTCATGAAGATCTTCGAGTGTTTTATAGTGAGTTATTCTTGCGAAGGTTCAAG
TCGATTGATTTTGTTGACTACGATCTCTCTTGTAATAAGAAGTTCTTCGCAGTTGAATCTCCACCAGG
TGG
+
FFFFF:FFFFFFFFFFFFFFFFFFFFFFFFFFFFFFFFFFFFFFFFFFFFFFFFFFFFFFFFFFFFFFF
FFFFFFF::FFFFFFFFFFFFFFFFFFFFFFFFFFFFFFFFFFFFFFFFFFFFFFFFFFFFFFFFFFFF
FFF
@A00721:190:HGJW7DSXY:4:1372:9028:33599_2:N:0:TCTGCAAG+GCTGCACT
GCGACTTTTGTCGAATTTTTCGCAAAAGGTCTTGAAGTACGTATTGTCACTGCTAATCGTAATTCGCG
AGAAGAGATAGTGACTAAAGTTTTGGCACTTCTAGTTCATCGCGGTAT
+
FFFFFFFFFFFFFFFFFFFFFFFFFFF,FFFFFFFFFFFFFFFFFFFFFFFFFFFFFFFFFFFFFFFFF
FFFFFFFFFFFFFFFFFFFFFFFFFFFFFFFFFFFFFFFF:FFFFFFFF
@A00721:190:HGJW7DSXY:4:2449:24840:15515_2:N:0:TCTGCAAG+GCTGCACT
CAGACGTGAAAGTTATGATGAGTGATCGTATTAGAACAGCTGATTCTTATTGCATGAACGTCGCTGAG
GTCAAACCTGTGGACGTTCTTTTCATCGACGAATTATTCATGATTCAC
+
FFFFFF:::FFFFFFFFFFFFFFFFFF:FFFFF,FFFF:FFFFF,FFFF:F:FF:::FFFFFFFFFF:
FF:FFFFFFF:FFFFF:F:FFFFFFF:FFFFFFFFFFFFFFFF:::FF
@A00721:190:HGJW7DSXY:4:1665:6759:7999_2:N:0:TCTGCAAG+GCTGCACT
CATCTACTTAAGGCGGCTTATACCATAGGTTACGGTGATTCCAATCAAATCGGATTCATCAATCGTAT
TGATCTCCAAGTCGCAAACTACTTCAACATCAACGACATCATTCTTGA
+
FFFFFFFFFFFFFFFFFFFFFFFFFFFFFFFFFFFFFFFFFFFFFFFFFFFFFFFFFFFFFFFFFFFFF
:FFFFFFFFFFFFFFFFFFF:FFFFFFFFFFFFFFFFFFFFFFFFFFFF
@A00721:190:HGJW7DSXY:4:2623:15980:31610_2:N:0:TCTGCAAG+GCTGCACT
TTATCGAAGTCTCAGTTATCGCTGCCCGGCCGACGTTTGTCGCGAACTCTCAGAGGTTTACGGCAGAA
AAATTTCTCCTGCTTGTAATTCGAGAGAGGACACTATGGAAATTAGAC
+

FFFFFFFFF:FFFFFFFFFFFFF:FFFFFFFF:FFFFFFFFFFFFFFFFFF:FFF:FFFFFF:F:FF
FF:FFFF:FFFFFFFFFFFFFFFFFFFFFFFFFFFFFFFFF,FFFFFFF
@A00721:190:HGJW7DSXY:4:2445:8024:34929_2:N:0:TCTGCAAG+GCTGCACT
TATTACTTTCACTCAAGGTGAAAAGTTTGAAATTAACAAAGTCCTTGGTAAGAGTAAGGTTAAATGTG
AAGATGCTCAGACTATTCATGAAGTTCAAGGTAATACTTACCAGCACG
+
FFFFFFFFFFFFFFF:FFFFFFFFFFFFFFFFFFFFF,FFFFFFFFFFFFFFFFFFFFFFFFFFFFFF
FFFFFFFFFFFFFFFFFFFFFFFFFFFFFFFFFFFFFFFFFFFFFFFF
@A00721:190:HGJW7DSXY:4:1214:29912:33661_1:N:0:TCTGCAAG+GCTGCACT
ACAAACACATATTACCGTTGCTCTATCACGACACGTTGAATCTTTACGATACTTTGTTATCAATGTAA
AGTTAAACGATGCTACTTGTGAGAGGATCAATCACAGTAAGGAACTGTCACGTTTAGCACGGGAGGTT
GCA
+
FFF,FFFFFFF,FFFFFFFFFFFFFFFFFF:F:FFFFFFFFFFFFFFFFFFFFFFFFFFFFFF:FFFF
FFFFFFFFFFFFFFFFFFFFFFFFFFFFFFFFFFFFFFFFFFFFFFFFFFFFFFFFFFFFFFFFFFFF
FFF
@A00721:190:HGJW7DSXY:4:1148:15501:13088_2:N:0:TCTGCAAG+GCTGCACT
TATATCGAGGCTATCGAGGACGATGAGTATCTTGAAGTTGTTGACGACAAGCCTCAAGAGGGTAGAGG
ACCACCTGGATCTTCTACTGTCTCTGCCTTCCAAGATTTCTTAGAAGA
+
FFFFFFFFFFFFFFFFFFFFFFFF:FFFFFFF:FFFFFFFFFFFFFFFFFFFFFFFFFFFFFFFFFFF
FFFFFFFFFFFFFFFFFFFFFFFFFFFFFFFFFFFFFFFFFFFFFFFF
@A00721:190:HGJW7DSXY:4:2374:1090:10019_1:N:0:TCTGCAAG+GCTGCACT
AACCACCTTAACGTCACTCGGTAATCTTTCAGCCGAATTAAGTACTAATGAGTTCAAGTCGGTTGTCG
ATCGAATTAAGATCAAGGCTAATACCTTTATAGGTAAGAAGCCCCTTGAGCCGCGTAGTCAGCGCGTT
TAG
+
FFFFFFFFFFFFFFF,FFFF:FFFFFFFFFFFFFFFFFFFFFFFFFFFFFFFFFFFFFFFFFFFFFFF
FFFFFFFFFFFFFFFFFFFFFFFFFFFFFFFFFFFFFFFFFFFFFFFFFFFFFFFFFFFFFFFFFFFF
FFF
@A00721:190:HGJW7DSXY:4:2306:7446:25379_1:N:0:TCTGCAAG+GCTGCACT
TACCGAGTCTCCAAGAGAATATTTATTCTTATGAGGCTCGAAACTTCAACGTTTTCACAACGGAAAGA
CATATCGCACCCGAAATTTTTGGGCAGAATGCTCTTAACGCGGTTCTTGAACGGTGTTTTGATCAGTC
TAA
+
FFFFFFFFFFFF:F:FFFFFFF,F:FFFFFFFFFFFFFFFFFFF::FFFFF:FFFFFFFFFFFFFFFF
FF:FFFFFFFFFFFFFFFFFFFFFFFFFFFFFFFFFFFFFFFFFFFFFFFFFFFFFFFFFFFFFFFFF
FFF
@A00721:190:HGJW7DSXY:4:1213:1280:2174_1:N:0:TCTGCAAG+GCTGCACT
ATAGGAGACTCAGTTAACCTGAGAGCTCCTCAATTGATAGATTGGGTCAACAATAGGGACGCTAGTAT
ACTTCGTAAAGTCGACAGCGAAATGGTCAATATTGATAATTGGACCGACTATATGACCTATTTTAAGC
TCA
+
FFF:FF:FFFFFFFFFFFFFFFFFFFFFFFFFFFFFF:FFFFFFFFFFFFFFFFFFFFFFFFFFFFFF
FFFFFFFFFFFFFFFFFFFFFFFFFFF,FF:FFF:FF:FFF:FFFFFFFFFFFFFFFFFFFFFFFFFF
FFF
@A00721:190:HGJW7DSXY:4:1373:3079:1720_2:N:0:TCTGCAAG+GCTGCACT
CTAAGGTTAAACTTGATGCTTCAGCCTTTTCGAAGCATAACCCCGCACAGAATATCGTTTATCACGCA
AAATTTATTAATGCAATTTTCGGACCTATCTTTAAGGTTCTTGTGAGG
+
FFFFFFFFFFFFFFFFFFFFFFFFFFFFFFFFFFFFFFFFFFFFFFFFFFFF:FFFFFFFFFFFFFFF
FFFF:FFFF:FFFFF:FFFFFFFF:F,FF::FFF,FFFFFFFFFFFFF
@A00721:190:HGJW7DSXY:4:1534:32696:18787_1:N:0:TCTGCAAG+GCTGCACT
CAGTGACGATTTCTCCCGGAAGCTCTTCTCTCTCTTAGGAGGTCATGATGACTACTATAAGGGTGAAA

TCGATTTTTCGAAGTTTGACAAATCTCAAGATTTCTTTTGTAAAGCCTATGAGATAAAGATGTATTCT
TAT
+
FFFFFFFFFFFFFF:F:FFFF:FFFF:FFFFF,F,FFFFFF,,FFFFFF,F:FF:FFFFFFFFFFFF
FFFFFF:FFFFFFF:FFFFFFFFFFFFFFFF:FF:F:FFFFF:FFFFF::FFFFFFFFFFFFF:FFF
FF:
@A00721:190:HGJW7DSXY:4:2228:10185:24706_2:N:0:TCTGCAAG+GCTGCACT
AACTTTGGAGTGCCAGTGAATATTTTTGTCACGCCAGGAGTATCCATAACGATTTGTCATTTACCACC
TTTGCTCAACGACGAAGTGGAACGGCCAACACTTTCTTTGGTAATTGT
+
FFFFFFFFFFFFFF:FFFFFFFFFFFFFFFFFFFFFFFFFFFFFFFFFFFFFFFFFFF:FFFFFFFF
FFFFFFFFFFFFFFFFFFFFFFFFFFFFFFFFFF:FFF:FFFFFFFFFF
@A00721:190:HGJW7DSXY:4:1251:20338:35399_1:N:0:TCTGCAAG+GCTGCACT
GATGATTTTATCACTTTATTATGACCTTGGAAAGTTTTCGGCTGTGGTTGTCGCTGGGGACGATTCCA
TTCTGTTTTCAAAGACTCCAATAACTGATCATTCTGATCATATGATTTTGGACAATGGTTTCGAAGCG
AAA
+
FFFFFFFFFFFFFFFFFFFFFFFFFFFFFFF:FFFFFFFF:FFFFFFFFFFFFFFFFFFFFFFFFFFF
FFFFFFFFFFFFFFFFFFFFFFFFFFFFFFFFFFFFFFFFFFFFFFFFFFFFFFFFFFFFFFFFFFFF
FFF
@A00721:190:HGJW7DSXY:4:2638:7518:9377_2:N:0:TCTGCAAG+GCTGCACT
TGCTCGCGATTTATTCTTTTTGTCAATGATTACATCTACTTCGTTCCTGATCCTTACAAGATCATCGT
TAAGTTAGGTAAACCCCTAACCGATGTTACTCTTGTAGGACTTTGGGA
+
FFFFFFFFFFFFFFFFFFFFFFFFFFFFFFFFFFFFFFFFFFFFFFFFFFFFFFFFFFFFFFFFFFFF
FFFFFFFFFF:FFF:FFFFFF:FFFFFFFFFFFFFFFFFFFFFFFFFFF
@A00721:190:HGJW7DSXY:4:1532:12346:36307_2:N:0:TCTGCAAG+GCTGCACT
AGGTTGTTGTTTGGCGATTAGCTCTTGCGACTAATATTCGATATTCGATTGAGGGTCGTTATACCTAT
TCGGCTATCTGTGCTTTGAATTGTATAAGATCAAATTTTAAACGATTC
+
FFF:FFFFFFFFFFFFFFFFFFFFFFFFFFFFFFFFFFFFFFFFFFFFFFFFFFFFFFFFFFF:FFFF
FF:FFFFFFFFFFFFFFFFFFFFFFFFFFFFFFFF,FFFFFFFFFFFF
@A00721:190:HGJW7DSXY:4:1645:7129:14873_1:N:0:TCTGCAAG+GCTGCACT
TGATGTGGAGGGAGTTCTTGAAGCATCCCGACCTTTCCTTGTGAAAATTTTGATGCGGTTTCATAAGA
TTATCAGTAATCCTGATTCTACTACGGATTCATGGACCTTCCATCCTGTACCTCTTCTGGAGAGACGT
ATC
+
F:FFFFFFFFFFFFFFFFFFFFFFFFFFFFFFF,FFFF:FFFFFFFFFFFFFFFFFFFFFFFFFFFFF
FFFFFFFFFFFFFFFFFFFFFFFFFFFFFFFFFFFFFFFFFFFFFFFFFFFFFFFFFFFFFFFFFFFF
FFF
@A00721:190:HGJW7DSXY:4:1116:27651:27398_2:N:0:TCTGCAAG+GCTGCACT
TCGAGACAGGTGATATTGTTGAGCGAGAGCGACCAAGATCTAAATTCGGTAACGGTGCGTGGCGCTTT
ATTTTAAAAAGCTCCCTCCTCTCGCAAGTTTGATCTGATAGATCGGAA
+
:FFFFFFFFFFFFF:,FF:FFFFFFFFFFFFFFFFFFF,FF:F::FF:FFFFFFFFFFFFFFFFF:FF
FFFF,FFFFFFFFFFFFFFFFFFFFFFFFF:FFFFFFF,FFFFFFFF:
@A00721:190:HGJW7DSXY:4:1168:20112:30655_2:N:0:TCTGCAAG+GCTGCACT
GATCAGTCTCTGACTTGACCATCTTTCATTTTAAATGACGATCATTAAGACTCTCTTTCTTCTTTGTT
TCCTTCTTTGCACTGATTTTTCCTCATCAGCTTGTGTTTTACCTAATC
+
FFFFFFFFFFFFFFFFFFFFFFFFFFFFFFFFFFFFFFFFFFFFFFFFFFFFFFFFFFFFFFFFFFFF
FF,FFFFFFFFFFFFFFFFFFFFFFFFFFFFFFFFFFFFFFFFFFFFF
@A00721:190:HGJW7DSXY:4:2171:26992:30107_2:N:0:TCTGCAAG+GCTGCACT
GATAACAACTATCAGTTGATTAAGACTCTTCCGAAAGGCGAACTTTGGGAATTTGAAGCTCAGGGTGA

AGCACAACGTTTGACCGTGACGAACTGTGGTGTTCTTGATCTACGTTA
+
FFFF:FFFFFFFFFFFFFFFFFFFFFFFFFFFFFF,FFFFFFFFF:FFFFF:FFFFFFFFFFFF:FFFF
FFFFFFFFFFFFFFFFFFFFFFFF,F:FFFFFFFFFFFFFFFFFFFFFF:
@A00721:190:HGJW7DSXY:4:2606:28962:27477_1:N:0:TCTGCAAG+GCTGCACT
CACTACCACTTATTACGATATTTCTTTAGTCGATGGGTTTTCGACGCCTTTAAGCGTCTTCTTTTCAT
CGCAACCAGATGATATCTTCTATTCGAACTTATGTTTCGATCAGTGTTTCTTCTATCAGGAGGAAAAC
ACT
+
:,::FFF::,F:FF,F:FFFFFFFF:F:FFFF:FFFFF:FF:F:FFFF:FFFFFFFFFFF,F,,F:FF
F:FFFF:FF,FFF::F,FF,FF,FF::,,,F:FFFFFFFFF,:FFFF,,:FFFFFFF,FFFF:FFFFF
,,F
@A00721:190:HGJW7DSXY:4:2270:3821:16783_2:N:0:TCTGCAAG+GCTGCACT
TCACTTTGTGTGTACTTGTCTCTATTTTAAGTTTTGTCTTCCTTGTTTGTCTTATTCTATTAATCACT
CATTTTAGTGAGATTATTAAAAACGCACTAAAACGTCTTCGACAACAG
+
FFFFF:FFFFFFFFF:FFFFFFF:FFFFFFFFFFFFFFFFFFFFFFFFFFFFFFFFFFFFFFFFFFFF
FFFFFFFFFFFFFFFFFFF:FFFFFFFFFFFFFF:FFFFFFFF,FFFF
@A00721:190:HGJW7DSXY:4:2475:17137:29450_1:N:0:TCTGCAAG+GCTGCACT
GCCTTACAGAGGGGCAGCAGCATGACCATAATTGGTATTGACTTTGGTACCACCTTTTCTACTGTCTG
TCTCGCCTCTTCGAATGAAGTTTTAATGCTTAACAACAATGATAGTGAATTTATACCTACAATCATTG
GTT
+
FFFFFFFFFFFFFFFFFFFFFFFFFFFFFFF,FFFFFFFFFFFFFFFFFFFFFFFFFFFFFFFFFFFF
FFFFFFFFFFFFFFFFFFFFFFFFFFFFFFFFFFFFFFFFFFFFFFFFF:,:FFFFFFFFFFFFFFFF
FFF
@A00721:190:HGJW7DSXY:4:2138:23556:9408_2:N:0:TCTGCAAG+GCTGCACT
TGTTTATGGATATGATGCTATTACTCATGATGGTTACGGTCAGGGAGGATTCTCAGTTTACAAGGACC
TTAAGCGATGGATCGGGGTTAATTCTAAAACATTACCCGAAAGACGTG
+
FFFFFFFFFFFFFFFFF:FFFFFFFFFFFFFFFFFFFFFFFFFFFFFFFFFFFFFFFFFFFFFFFFFF
FFFFFFFFFFFFFFFFFFFFFFF:FFFFFFFFFFFF,F:FFFFFFFF:F
@A00721:190:HGJW7DSXY:4:1577:17146:8265_2:N:0:TCTGCAAG+GCTGCACT
GTACGATGTCGTCTGTCTCGGCGATCACTTTTCACTAAGAATTGGTCCTACTTTTGGACAAGGCCAAC
TTCGTAATCTTCATGAGATCGTTTCTTTCTTTATCTCTTTGGTTGTTA
+
FFFFFFFFFFFFFFFFFFFFFFFFFFFFFFFFFFFFFFF,FFFFF,FFFFF:F:F,FFFFFF,FFFFFF
:FFFFFFFFFFFFFFFFFFFFFFFFFFF,FFFFFFFF,:FFFFFF,FFF
@A00721:190:HGJW7DSXY:4:1263:11360:12900_2:N:0:TCTGCAAG+GCTGCACT
GAGTGAAAGGCTTCAATTGTAGTGGTTTGGTCGTTTCAGTACCCAGTCAGTATACCTCTACGCAAAGA
TATTTCATGAAAACTACAGAGGAACGTACCGGATTACCTGTCTTTCAT
+
FFFFFFFFFFFFFFFFFFFFFFFFFFFFFFFFFFFFFFFFFFFFFFFFFFFFFFFFFFFFFFFFFFFF
FFFFFFFFFFFFFF,FFFFFFFF:FFFFFFFFFFFFFFFFFFFFFFFFF
@A00721:190:HGJW7DSXY:4:2345:16957:31140_1:N:0:TCTGCAAG+GCTGCACT
CTTCGGCCGCTCTCTTTGCTTCGATGTTGGATGTCAAGAAGGGTTCAGAATGGGATTCCTACGTAGTT
TATGACTTTGGTGGTGGTACTTTCGATACTTCTATCGTCTTACGTTACGGTAATTACTATAGTGTTAT
TTA
+
FFFFFFFF:FFFFFFFFFFF:FFFFFFFFFFFFFFFFFFFFFFFFFFFFFFFFFFFFFFFFFFFFFFF
FFFFFFFFFFFFFFFFFFFFFFFFFFFFFFFFFFFFFFFFFFFFFFFFFFFFFFFFFFFFFFFFFFFF
FFF
@A00721:190:HGJW7DSXY:4:2526:3857:30154_1:N:0:TCTGCAAG+GCTGCACT
ATAATCTTGGAGGTCGTGACGTTGATGAAGCTATAAGAGCGTTCTTATTACGGCGCACTTCTTTACCC

```
GCCACCACACAACTTTCAGTTTCACAACTGAAGGAGGAGGTTTCTGTTACTGGAAAACCCTCGTCAAT
ACT
+
FFFFFFFFFFFFFFFFFFFFFFFF:FFFFFFFFFFFFFFFFFFFFFFFFFFFFFFFFFFFFFFFFFFF
FFFFFFFFFFFFFFFFFFFFFFFFFFFFFFFFFFFFFFFFFFFFFFFFFFFFFFFFFFFFFFFFFFFF
FFF
@A00721:190:HGJW7DSXY:4:1248:1561:14403_2:N:0:TCTGCAAG+GCTGCACT
TCTAGTGAAATTAACGCGTCCTTTTCTGGAACGTAGTTTTACTATTATGAACAAAGTTATCAAAGATA
GTGGCATTCAAGGCAAACTTACCCTGATTCCGGCCGGTGGTAGCTCAT
+
FFFFFFFFFFFFFFFFFFFFFF::FFFFFFFFF:FFFFFFFFFFFFFFFFFFFFFFFFFFFFFFFFFF
FFFFFF:FFFFFFFFF:FFFFFFFFFF,FFFFFFFFFFFFFFFFFFFF
@A00721:190:HGJW7DSXY:4:2636:32353:6324_2:N:0:TCTGCAAG+GCTGCACT
TGAAACAGCTATTAAAGCTGTTGAGCGGGTAATTACACCTAAACGAGCTCGATCTGCCATCGCTGAAG
GATGTGCACTCTTTTCTGCTAGTTTAACGGAGCGTAGTGTACTCTTCG
+
FFFFFFFFF::F,FFFFFFF,FFFFFFFFFF:FFFFFFFFFF:FFFFFFFFFFFFFFFFFFFFFFF,F
FFFFFFFFFFF:FFFFFFFFF,FFFF,FFFFF:FF:FFFFF,FFFFFF
@A00721:190:HGJW7DSXY:4:1570:8811:35947_2:N:0:TCTGCAAG+GCTGCACT
AAAGGTCTCTATCAAGCTCGTATTATCGTCGCTGCTGGATCTGCGTTACCGTTGAAGGCTAAACTCCA
AGGTAATTATGAAGACTGTGATTTATCTTTCACTCTGGCCTTGTATGA
+
FFFFFFFFFFFFFFFFFFFFFFFFFFFFFFFFFFFFFFFFFFFFFFFFFFFFFFFFFFFFFFFFFFFF
FFF:FFFFFFFFFFFFFFFFFFFFFFFFFFFFFFFFFFFFFFFF:FFF
@A00721:190:HGJW7DSXY:4:2502:10881:3615_1:N:0:TCTGCAAG+GCTGCACT
CATTAATAATTTCAATCTTCTTAAAAAGAAGATAGAGAGTCATACTTTGGGCTATCGTTCAGGTGCAT
GGGGTTGGATAGCCAATATTGAAGTGGACACCTCTGGTAGTATCGAAGTAAAGAGATCGGAAGAGCAC
ACG
+
:F,FFFFFF,F,FFFFFFF:F:FFFFFFFF:FFFFFF:F:FFFFFFFF:FFFF:F,FFFFFF:F,FFF
FFFFFFFFFFFF::FF,FFFFFFFF:F:FFFFFFF:FFF:F:FFFF:,:,:FFF:FFF,FFF:FF,FF
FFF
@A00721:190:HGJW7DSXY:4:1221:2266:25520_1:N:0:TCTGCAAG+GCTGCACT
CTCTACCGCCTTACAAGTCTAAGGTTAAAGAGTCGATTAAACAAGTTGTACTTATCAATGATTTTATC
GCTGGTTGTTATCTCGAAAAGTCTCTTCTAAAAGGAACGTCAGTTCTAACCCAAAGAGAATATCTAAA
ATA
+
FFFFFFFFFFFFFFFFFFFFFFFFFFFFFFFFFFFFFFFFFFFFFFFFFFFFFFFFFFFFFFFFFFFF
FFFFFFFFFFFFFFFFFFFFFFFFFF:FFFFFFFFFFFFFFFFFFFFFFFFFFFFFFFFFF:FFFFFFF
FFF
@A00721:190:HGJW7DSXY:4:2307:28782:21402_1:N:0:TCTGCAAG+GCTGCACT
TACCACTATCTACGAACCTAAAGTTCTGAAGACGTTGCTTGTCGCTTCCTCCTTTAATGGCACTACCT
CGGTACAATCTTACTTGGAACGAAGGCGACCTGTTCCGGAGTTTTTACGGGATCCGGAATGTTCTGTC
TAT
+
FFFFFFFFFFFFFFFFF:FFFFFFFFFFFFFFFFFFFFFFFFFFFFFFFFFFFFFFFFFFFFFFFFFF
FFFFFFFFFFFFFFFFFFFFFFFFFFFFFFFFFFFFFFFFFFFFFFFFFFFFFFFFFFFFFFFFFFFF
FFF
@A00721:190:HGJW7DSXY:4:2102:14190:23563_1:N:0:TCTGCAAG+GCTGCACT
AATACGATTTCGAACGTCAACGTTCACCGAGTTTCGTTTCGTACCTTGATGCTGACTTTCGTCAATAT
GATTTGATCTGGTTGCTCAATTCTAAGCGTGTTCCGCTTAGTGCGAGGCCTCTCTTCTACGAGTATTG
CTA
+
FFFFFFFFFFFFFFFFF:FFFFFFFFFFFFFFFFFFFFFFFFFFFFFFFFFFFFFFFFFFFFFFFFFF
```

FFF:FFFFFFFFFFFFFFFFFFFFFFFFFFFFFFFFFFFFFFFFFFFFFFFFFFFFFFFFFFFFFFFFFF
FFF
@A00721:190:HGJW7DSXY:4:1444:19135:36323_2:N:0:TCTGCAAG+GCTGCACT
AAATACTAGATATTTGGATGATCTTGATAAAGAACTTCATGAATTTTCAGAGGATGAAAAATTTTCTT
CCTTGGATTTTAATATAAAGAATCTTTACCAGGACTCCTACTACAGGA
+
FFFFFFFFFFFFFFFFFFFFFFFFFFFFFFFFFFFFFFFFFFFFFFFFFFFFFFFFFFFFFFFFFFFF
FFFFFFFFFFFFFFFFFFFFFFFFFFFFFFFFFFFFFFFFFFFFFFFF
@A00721:190:HGJW7DSXY:4:2523:20898:28103_1:N:0:TCTGCAAG+GCTGCACT
GATCGTAAGAAGTTTATAAGGAACATATCCATTTGTTGTAATAGGGTCATCAACTTAGAACTCGAAAC
TCTAAGTGGTAATACTGTCTTTCCAAGCTTACCTGTATCTTCAATGAAATTGGACTCTTCGATAGGGG
ACA
+
FFFFFFFFFFFFFF:FFFFFFFFFFFFFF:FFFFFFFFFFFFFFFFFFFFFFFFFFFFFFFFFFFFFF
FFFFFFFFFFFFFFFFFFFFFFFFFFFF::FFFFFFFFFFFFFFFFFFFFFFFFFFFFFFFFFFFFFF
FFF
@A00721:190:HGJW7DSXY:4:2452:3314:11115_1:N:0:TCTGCAAG+GCTGCACT
TAAGAATACTATAAATAGAATAGCTATTACTACTAATGGCGTTGGCCTAGTGGATGTTATTAGTGACA
TCTGGTCAATTTCGTTTTTAGGTGGTCTTTATTCTATTATTGATTTTTATGAGATTGAGATCGGAAGA
GCA
+
FFFFFFFFFFFFFFFFFFFFFFFFFFFFFFFFFFFFFFFFFFFFFFFF:FFFFFFFFFFFFFFFFFFF
FFFFFFFFFFFFFFF:FFFFFFFFFFFFFFFFF:FFFFFFFFFFFFFFFFFFFF:,FFFFFFFFFFFF
FFF
@A00721:190:HGJW7DSXY:4:2533:17173:24596_2:N:0:TCTGCAAG+GCTGCACT
TTCCTGAAGAGTCCTTCCGTAACCAACTTCTTGACAATTTTCTTCAATCCTTCGATAATAGTTAAATC
TACGATTATGTCTGTGTTGAAAGACTTTCTACCGATAAGGGTCACTAG
+
FFFFFFFFFFFFFFFFFFFFFFFFFFFFFFFFFFFFFFFFFFFFFFFFFFFFFFFFFFFFFFFFFFFF
FFFFFFFFFFFFFFFFFFFFFF:FFFFFFFFFFFFFFFFFFFFFFFFF
@A00721:190:HGJW7DSXY:4:1146:26476:17409_2:N:0:TCTGCAAG+GCTGCACT
ATCCTACATTGACTGATTATCTAAAGATTGAAGGAAAATTTTATCCTCGTTTAACTTACTTAGACTTT
CCTACGATACAAATGATAACTTCTAATTTACGTTTGCTACTCAGAGCT
+
FFFFFFFFFFF:FFFFFFFFFFFFFFFFFFFFFFFFFF:FFFFFFFFFFFFFFFFFFFFFFFFFFFFF
FFFFFFFFFFFF:FFFFFFFFFFFFFFFFFFFFFFFFFFFFFFFFF:F
@A00721:190:HGJW7DSXY:4:2146:6036:26130_1:N:0:TCTGCAAG+GCTGCACT
GATGCATGGTGTTATCTCCTCAGGAGTCCCGGCATTTGATAATAATGACGAGACTTTCGTCATAATCG
CTGCATATTACGGATTACACGGTACCAGCGCATCTCGTGTCGAGCCTCGACCCGATTATATGGTATTA
ACT
+
FFFFF:FFFFFFFFFFFFFFFFFFFFFFFFFF:FFFFFFFFFFFFFFFFFFFFFFFFFFFFFFFFFFF
FFFFFFFFFFFFFFFFFFFFFFFFFFFFFFFFFFFFFFFFFFFFFFFFFFFFFFFFFFFFFFFFFFFF
FFF
@A00721:190:HGJW7DSXY:4:1152:22318:28307_2:N:0:TCTGCAAG+GCTGCACT
TCTAGAATTCTTAATTATAATATTCAACCTCAAAAGTTACGTGTTTACAGTAACGAGATCACACATTT
TGAGAACTAACTATGTTATGTTCTCTGATCATTTTGACAATCTCTTCT
+
FFFFFFFFFFFFFFFFFFFFFFFFFFFFFFFFFFFFFFFFFFFFFFFFFFFFFFFFFFFFFFFFFFFF
FFFFFFFFFFFF:FF,FFFFFFFFFFFFFFFFFFFFFFFFFFFFFFFF
@A00721:190:HGJW7DSXY:4:1475:32696:17347_2:N:0:TCTGCAAG+GCTGCACT
AAATAAATAATATTACTAATGGCAAATAGTGGAAAGGCTCCAGTCAACACTAGTTCGAGTGCTGCTTT
GGATTACGATGACGTGAGAATAGGTGAAGGGATAGTTTTTACCTCGAC
+

```
FFFFFFFFFFFFFFFFFF:FFFFFF::FFFFFFFFFFFFFFFFFF:FFFFFFFFFFFFFFFFFF,F
FFF:FFFFFFFFFFFFFFFFFFFFFFFFFFFFFFFFFFFFFFFFFFF
@A00721:190:HGJW7DSXY:4:1115:14344:31219_1:N:0:TCTGCAAG+GCTGCACT
TCTTAAAGCTTCACCAAAAATGAAGGACACTTCGAAACTGAATAATGATGCAATACAAAAAGTATCG
CTAATCTTCTGAAAAAGGCTCTAAAGGACACTATACAATATCCAGACGACGACGAAGCTTATACAATA
TCC
+
F,FFFF:,,:FFF::,FFFFF,F:F:F,,,FFF,FF:,FFFFFFFFFFFFFF,FFF,F,,F,FFFF:F
,F::FF,FFFFFFF:FFFFF,F:FF:FF,F,FFFF:F:FFFFFFFF,,F,,:F,FF,,FF,FFF,:FF
FF:
@A00721:190:HGJW7DSXY:4:1525:2456:21684_2:N:0:TCTGCAAG+GCTGCACT
ACCACATTTTGGTCAAGTCAATCCGTTGAGAAAATTTCTTAGAGGATATTCGCCATTGTGGTACGAAT
TGGTCCAACATTTACCACCAAATTCTTTTGATACAACTCTAGCCTTCA
+
FFFFFFFFFFFFFFFFFFFFFFFFFFFFFFFFFFFFFFFFFFFFFFFFFFFFFFFFFFFFFFFFFFFF
FFFFFFFFFFFFFFFFFFFFFFFFFFFFFFFFFFFFFFFFFFFFFFFF
@A00721:190:HGJW7DSXY:4:1424:13810:15107_2:N:0:TCTGCAAG+GCTGCACT
ACCAGATTGGTTCGAACCCGTTGATGCTATGTCAGAAGAAGCTATTCAAGCTTGTCGTTTAAAGACTG
CAGAGGCTGTCAAACAAGCCCAGACTCAAGGAGTCCAATCAAAGGTTT
+
FFFFFF,FFFFFFFFFFFFFFFFFFFFF:FFFFFFFFFFFFFFFFFFFFFFFFFFFFFFFFFFFFFFF
FFFFFF:FFFFFFFFFFFFFFFFFFFFFFF,FFFFFFFFFFFFF,FFFF
@A00721:190:HGJW7DSXY:4:1522:25211:31876_1:N:0:TCTGCAAG+GCTGCACT
TTATTTAGAGATCTTTTCTACTTACAAAGGACAATTCAGTTTCATTAATTCTACGGAAAAGTCCGGAG
TATATTGTATGTTAATAGATAGTGGGATAATAGTTCTACCGATACGTAGTGAACGCGTTCGTAGAACC
AAG
+
FFFFFFFFFF:FFFFFFFFFFFFFFFFFFFFFFFFFFFFFFFFFFFFFFFFFFFFFFFFFFFFFFFFF
FFFFFFFFFFFFFFFFFFFFFFFFFFFFFFFFFFFFFFFFFFFFFFFFFFFFFFFFFFFFFFFFFFFF
FFF
@A00721:190:HGJW7DSXY:4:1145:10203:5071_1:N:0:TCTGCAAG+GCTGCACT
ACCGATAGGAGTTGTATAGTTGAAGATGTGGCTTTAGACGATAATCCTTTAAGGACTATGTTGACTGA
CGAAGATTTTCCAACAGATTTGTCTACGTTGCAGATCGCCACTCCAAACACTATATACATTATAAAAT
ATA
+
FFFFFFFFFFFFFFFFFFFFFFFFFFFFFFF:FFFFF:FFFFFFFFFFFFFFFFFFFFFFFFFFFFFF
FFFFFFFFFFFFFFFFF:FFFFFFFFFFFFFFFFFFFFFFFFFFFFFFFFFFFFFFFFFFFFFFFFFF
F:F
@A00721:190:HGJW7DSXY:4:2137:11939:11741_1:N:0:TCTGCAAG+GCTGCACT
CATAACTTTTGTTACCAAATTCTTGGCTTTCAACGAAGTCTCTGATTTTATTAATCATTACTTTAACG
ATCTCTCGGTCATTTACTCTGTACTTGATCTACGTCGTTTAACTGTCGGAACAACCTTTACTTAGAAT
GAC
+
FFFFFFFFFFFFFFFFFFFFFFFFFFFFFFFFFFFFFFFFFFFFFFFFFFFFFFFFFFFFFFFFFFFF
FFFFFFFFFFFFFFF:FFFFFFFFFFFFFFFFFFFFFFFFFFFFF:FFFFFFFFFFFFFFFFFF:FFFFFF
FFF
@A00721:190:HGJW7DSXY:4:2204:8395:24330_2:N:0:TCTGCAAG+GCTGCACT
TTCTTATATTATTTACAATAGTTAATATCATATTAGTAATACTCACTATCGTTTTCTGCTTTTTCTTA
GCTAATAGATTAATAAAGGATGAGTACGAAAACCAACTCTTCGTTACG
+
F,,:FFFF:FFFFFFF:FFFFFFFFFFFFFFFFFF:FFFFFFFFFFFFFFFFFFFFF:FF:FFFFF,:
,FFFFFFFF,FFFFFF::FFFFFFFFFFF:FFF:FF,FFF:FFFFFFF
@A00721:190:HGJW7DSXY:4:1166:13711:3724_1:N:0:TCTGCAAG+GCTGCACT
TAGAATAGATTTAGTCTCTACACCCATCTCAACGAGTATAACGCGTGTTTTCCGTCCTGAACGTAATA
```

```
CACGAAGAGATCATCCGCAAACGACTCGAAACCGAGACGCCGTAGATCAAAGTCCCTTCTAACTTTAG
AT
+
:F,FFF::::F:,F,,:FFFF,FF::,,,FF,:,,:,F:F:,FF,,,FFF:F:::F:F,F::,,F,F,
:,:,:,,:,:,::,FF,,F:FFFFFFFFFFFFFFFFFFFFFFFFFFFFFFFFFFFFFFFFFFFFFFFFFF
FF
@A00721:190:HGJW7DSXY:4:1544:19027:24518_1:N:0:TCTGCAAG+GCTGCACT
CATTTTATAGCAGTTATTTATGTTGTACTTTTTATAATTTATAACCTTCTTATACTTTTGGGTATGTT
TTGTGAAAAACATGAGATACCCAAGTCTATCGAAGACATTATCTCTTATGTACTTATTGTAACGATCA
TTC
+
FFFFFFFFFFFFFFFFFFFFFFFFFFFFFFFFFFFFFFFF:FFFFFFFFFFFFFFFFFFFFFFFFFFFFF
FFFFFFFFFFFFFFFFFFFFFFFFFFFFFFFFFFFFFFFFFFFFFFFFFFFFFFFFFFFFFFFFFFFFFF
FFF
@A00721:190:HGJW7DSXY:4:1556:2320:35884_1:N:0:TCTGCAAG+GCTGCACT
TTCTTTTATTCCTTTAACTGTGTATGAGGAGGATTTATAATGCTTTTCGATAGTGATTGTTATCAATT
TAAATACATTACTGTTTCAAATCATCTTTACTCACAGACTCCGAAAATGGTTTCAAATGAGGTCAAAG
CTC
+
FFF:FFFF:FFFFFFFFFFFFF,FFFFFFFFFFFFFFFFFFFFFFFFF:FFFFFFFFFFFFFFFFFFFFF
FFFFFFFFF:FFFFFFFFFFFFFFFFFFFFFFFFFFFFFFFFFFFFFFFFFFFFFFFFFFFFFFFFFFFF
FFF
@A00721:190:HGJW7DSXY:4:1631:14425:10786_2:N:0:TCTGCAAG+GCTGCACT
AACTATTATCCGATTCTTCGAGGGTATCACTACCACTGATCTGCCGACTTTAGTCAAGGAACTTCAAT
TCCTTCGATGTTTATATTTTTCTATAAGGGAAAATAGCAATAATCAAA
+
FFFFFFFFFFFFFFFFFFFFFFFFFFFFFFFFFFFFFFFFFFFFFFFFFFFFFFFFFFFFFFFFFFFFFF
FFFFFFFFFFFFFFFFFF,FFFFFFFFFFFFFFFFFFFFFFFFFFFFFFF
@A00721:190:HGJW7DSXY:4:1435:12057:18458_2:N:0:TCTGCAAG+GCTGCACT
AACTTTATTTCAGAACTCTCTCCGATCACCTTGGTGTGATCTCCCGGAGGATCTTTTCAATAAGATGT
ATGATGATAATCTACTATTTTTAAATAGTGATATTCAGCGTATACTTG
+
FFFFFFFFFFFFFFFFFFFFFFFFFFFFFFFFFFFFFFFFFFFFFFFFFFFFFFFFFFFFFFFFFFFFFF
FFFFFFFFFFFFFFFFFFFFFFFFFFFFFFFFFFFFFFFFFFFFFFFFFF
@A00721:190:HGJW7DSXY:4:1468:19913:12336_2:N:0:TCTGCAAG+GCTGCACT
TTTGGCTTGGTCTATTTACCGCAAAAAGTTGGAAGAGGTCTATGATATTTATTTTACTTATACTTTAA
ATAGCACCGATTATAACTTGACTGTTGATAATATTACAATAGCTCTTT
+
FFFFFFFFFFFFFFFFFFFFFFFFFFFFFFFFFF:FFFFFFFF:FFFFFFFFFFFFFFFFFFFFFFFFFF
FFFFFFFFFFFFFFFFFFFFFFFFFFFFFFFFFFFFFFFFFFFFFFFFFF
@A00721:190:HGJW7DSXY:4:1677:11324:17910_2:N:0:TCTGCAAG+GCTGCACT
AGTAACCGGTGAACACGTCGGTAGCATTGATTTACTTTTACCGTTAGAACTGAAAAAGATTCTTTTCG
AAAAGAAACCTATAAGTTACAAACGATTCTTCGACTATATTAAAGAAA
+
FFFFFFFFFFFF:FFFFFFFFFFFFFFFFFFFFFFFFFFFFFFFFFFFFFFFFFFFFFFFFFFFFFFFFF
FFFFFFFFFFFFFFFFFFFFFFFFFFFFFFFFFFFFFFFFFFFFF:F:FF
@A00721:190:HGJW7DSXY:4:1403:22607:31375_2:N:0:TCTGCAAG+GCTGCACT
TCAAGTACCTGAACTTTACATTTTTAATGGTATGGTATAATGTGTCTTACAAATAGAACCTTCACTCT
CGGTAATGAGAGTGTATTCCTTAATATTCTAGTAACCGCTATGTGGGC
+
:FFFFFFFFFFFFFFFFFFFFFFFFFFFFFFFFFFFFFFFFFF:FFFFFFFFF:FFFFFFF:FFFFFF:F
F:FFFFFFFFFFFFFFFFF:FFFFFFFFFFFFFFFFFFFFFFFFFFFFFF
@A00721:190:HGJW7DSXY:4:1317:5466:12649_2:N:0:TCTGCAAG+GCTGCACT
TGCTAGGAAAATTTAGAAAAACTCCATTGTATAGTTCAAGGAGTACTTAATTCAATAGCTTTAGTAT
```

TATTTACTATCGCCTTTATTGAAAATTGTATAAGAGTTAATTGCTTAC
+
FFF,FFFFFFFFFFFFFFFFFFFFFFFF:FFFFFF:FFFFFFFFFFFFFFF:FFFFFFFF,FFFFFFFF
FFFFF,FFFFF:FFFFFFFF:FFFFFFFFFFFF:,FFFFFFFFFFFFF
@A00721:190:HGJW7DSXY:4:1435:29215:7310_2:N:0:TCTGCAAG+GCTGCACT
CATACCATAACCTACCATGACCAATCATCGCTCCAACTGGCACGCCAGACGTAGGGTCAAACCTACTA
CCGCCGGTCGGCAGATCGGCACCACTGGTCTCTACGTTCAGGGACCCG
+
FFFFFFFFFFFFFFFFFFFFFFF:FFFFFFFFFFFFFF:FFFFFFFFFFFFFFFFFFFFFFFFFFF:FF:
FFFFFFFFFFFFFFFFFFFFFFFFFFFFFFFFFFFFFFFFFFFFFFFF
@A00721:190:HGJW7DSXY:4:2242:19705:34867_2:N:0:TCTGCAAG+GCTGCACT
TAACCTTCCCAAATCTAGATCTGCACAGAAGAAGGCTGAAGGTCGTTACCGGACGACCACTCAAAGGA
AACGGGCTGTTGCCCAGAGGAAAGCAGCGACCACCGAGGTCGCTCGTA
+
FFFFFFFFFFFFFFFFFFFFFFFFFFFFFFFFFFFFFFFFFFFFFFFFFFFFFFFFFFFFFFFFFFFF
FFFFFFFFFFFFFFFFFFFFFFFFFFFFFFFFFFFFFFFFFFFFFFFF
@A00721:190:HGJW7DSXY:4:2575:20817:8923_1:N:0:TCTGCAAG+GCTGCACT
AGTCCCGTCATCTCTGTGGGAACTGAGGCAGTACCTCAGTCAAAAGTCACATTCACCACTCCCATGCC
AATCAATGATGTGACTATACAGTTCGGCCATTTCAAGAGTGGTCAATACGTCGTGGGAGGTACTGAAG
CCT
+
FFFFFFFFFFFFFFFFFFFFFFFFFFFFFFFFFFFFFFFFFFFFFFFFFFFF:FFFFFFFFFFFFFFF
FFFFF:FFFFFFFFFFFFFFFFFFFFFFFFFFFFFFFFFFFFFFFFFFFFFFFFFFFFFFFFFFFFFF
FFF
@A00721:190:HGJW7DSXY:4:2127:21459:17895_1:N:0:TCTGCAAG+GCTGCACT
ATCTAAGAACACTCCCAAGCCAATCAATAGTTCTTCTGACCAGAATGAAAAGGGGTTTACCAGGGTAG
TCTCTAAGTCTACCAGGAAGAGGGGAAATGTCCCCAAAACTGAGAAGAATAAAGGGAAAGGTCCATCA
GGA
+
FFFFFF:FFFFFFFFFF,FF,FFFF:F,FFFF:::FFFFFFFFFF:,,:F:FFFF::F:FFFFFFFFF
FFF::FFFFFFFFFFFFFF:FFFFFF:FFFFFFFFFFFFFFFFFFFF:F:FFFFFFFF:F:::FFFFF
FFF
@A00721:190:HGJW7DSXY:4:2320:29071:3897_2:N:0:TCTGCAAG+GCTGCACT
ATTACCCCTAATTCTGAAGGAAGATCTCCCACCATCTGGGGTTGGGAATAAGAAATCTGCACGGGCCA
AAGAGGCCGCTGCTACGAAGTCGCTGAAGGCGGCTTGGGTCATTAAGG
+
FFFFFFFFFFFFFFFFFFFFFFFFFFFFFFFFFFFFFFF:FFFFF,FFFFFFFFFFFFFFFFFFFFFF
F,FFFFFFFFFFFFFFFFFFFFFFFFFF:FFFFFFFFFFFFFF,F,FFF
@A00721:190:HGJW7DSXY:4:2331:8766:34397_2:N:0:TCTGCAAG+GCTGCACT
GTTGCGCCTGCCGACCCCGTCTTTGAGACCAAGACTCTCTACAGTAGGTTCGTGCCTACTATGAGCAA
ATCTGTTGAATTCGTCACTGAGGACGAAGGTGAGAGGACTTTTGGCTA
+
FFFFFFFFFFFFFFFFFF,FFFFFFFFFFFFFFFFFFFFFFFFFFFFFFFFFFFFFFFFF:FFFFFFFF
FFFFFFFFFFFFF:FFFFFFFFFFFFFFFFFFFFFFFFFFFFFFFFFFF
@A00721:190:HGJW7DSXY:4:2213:1796:26021_1:N:0:TCTGCAAG+GCTGCACT
CTGAAAGAGAATTGCGTGGGTTGATGGCAGACGCTTCGAGCTTGTGTGTGACGAGGCCGGTGAAGTTT
TTGTCAACGGCGATTGGAGGATTTTATCCGCGCTTAACTTTGGGGCGAAGATCCCCTATGGAGTCTTG
AAG
+
FFFFFFFF,F::F,,F:,F:FF:,,,F:,,,,,F:FFFF,FFF,,,F,F,:,,,,F,,,FF,F,F:FF
:F,F,:F:,:F,,F:,:,,,:F,F::F:,,,,FFFFF,F,F,:,,,:F:F,FF,,,F,F::F,F,F::
:F,
@A00721:190:HGJW7DSXY:4:1678:6623:4382_2:N:0:TCTGCAAG+GCTGCACT
GGCATATTTATGCCGACGATAATAAGCATGTCACCCTTAAGCACCGAGGTAAGCTCGGGACTTACAAG

AGAGAGTGTGAGAGAGTCGCTCTCACTATTTTTGCCCAGAATGCGGGC
+
FFFFFFFFFFFFF,FFFFFFFFFFFFFFFFFFFFFFFFFFFFFFFFFFFFFFFFFFFFFFFFFFFF:F
FFFFFFFFFFFFFFFFFFFFFFFFFFF:FFFFF,FFFFFFFFFFFFFF
@A00721:190:HGJW7DSXY:4:2553:28727:7153_2:N:0:TCTGCAAG+GCTGCACT
GGTTCTGGGAGGCTGCTTTCCTCCATAATTACAACCTCTCTTTAGAGAGCATTAGAAGGAAGTTGGGC
AAATTCCCACATATGGTAGACGTTGTCACACTTTTCTTGAAAATTTTT
+
FFFFFFFFFFFFFFFFFFFFFFFFFFFFFFFFFFFFFFFFFFFFFFFFFFFFFFFFFFFFFFFFFFFF
FFFFFFFFFFFFFFFFFFFFFFFFFFFFFFFFFFFFFFFFFFFFFFFF
@A00721:190:HGJW7DSXY:4:2466:5954:33473_1:N:0:TCTGCAAG+GCTGCACT
TGGGGATTTCGTTGTGAGAATGTGTAAGGTGTCAAGTGTCTTCTTCATGCTGATATTTCAGGACCTAA
TCACATCTTGATTGTTAGTTACTGGTCGTATCGAGTTGGAGGGAGGATGTTCTCCGAAATTAAAGACG
GG
+
F,:F,,FFF,F,:,F:,,FF,,F,,,F,FF,F:,FF,,FFFFFFF,FF,FFFFF,F,,,FFFFF,F,F
F:,:,F,,FFFFF,,F,::,,,,,,::,F:F:F:,F:,F:FFFFF,F,,FF,F:FFF,FFFF:FF,::
:,
@A00721:190:HGJW7DSXY:4:2558:14362:29622_2:N:0:TCTGCAAG+GCTGCACT
TCAGAGAGGGGAGCTCTGAAAAGGAAACTTCGTCAGAAGATGAGGAAGTCTAAGAGGAGGAGTTTGAG
GAAGGCTCTAGAGAAGATCTCACCACCTGGAGAAGGCAAGTGGAGAGT
+
FFFFFFFFFFFFFFF:FFFFFFFFFFFFFFFFFFFFFFFFFFFFFF:FFFFFFF:FFFFFFFFFFFFF
FFFFFFFFFFFFFFFFFFFFFFFFFFFFFFFFFFFFFFFFFFFFFFFF
@A00721:190:HGJW7DSXY:4:2147:6497:36385_2:N:0:TCTGCAAG+GCTGCACT
AAGAAGGATTATTTGAAGCAGGGGTTACCACTTCCTAAGGTGGATGAGGGAGGTTGTTTCTTTGGAGT
TTTAGATAATCTTTTGAGAAGGGGTCATAGAGCCAGCAAGAGAAGCTT
+
FFFFFFFFFFFFFFFFFFFFFFFFFFFFFFFFFFFFFFFFFFFFFFFFFFFFFFFFFFFFFFFFFFFF
FFFFFFFFFFFFFFFFFFFFFFFFFFFFFFFFFFFFFF,FFFF::F:F
@A00721:190:HGJW7DSXY:4:2201:8350:28855_2:N:0:TCTGCAAG+GCTGCACT
TATAATTTAGGTCAACTTGGGGTTTCGAATGTCATTAGTGCCTTACGCTTGAAAGAGGTCCTTTTGAG
TATCGACAGATCTTTCGATCTTGAGGACGTACATTTAACGTGGCAGAA
+
FFFFF:FFFFFFFFFFFFFFFFFFFFFFFFFFFFFFFFFFFFF:FFFFFFFFFFFFFFFFFFFFFFFF
FFFFFFFFFFFFFFFFFFFFFFFFFFFFFFFFFF:FFFFF:FFFFFFF
@A00721:190:HGJW7DSXY:4:2228:2663:3035_2:N:0:TCTGCAAG+GCTGCACT
ATATTGTTCCTTCCGGGAAGTTGTGGGATTTAACTTTGACTCAACTACTCCGATCAGATCCGGATGGT
TTGATTGGTATGGACTACTCGACTTTTGATCTGGAGTTGTCTAGGGTC
+
FFFFFFFFFFFFFFFFFF:FFFFFFFFFFFFFFFFFFFFFFFFFFFFFFFFFFFFFFFFFFFFFFFFF
FFFFFFFFFFFFFFFFFFFFFFFFFFFFFFFFFFFFFFFFFFFFFFFF
@A00721:190:HGJW7DSXY:4:2216:5602:17707_2:N:0:TCTGCAAG+GCTGCACT
AATATGCAGGTCAAAGATTCTGTAGCTCTGAGGGCGGTTGAGGGAGCCGTTTTGGCCGACTTTAACTA
TTCTAAGAGTCTTTATGATTCAAAACCTATCGTTGTCGTACCTTACAA
+
FFFFFFFFFFFFFFFFFFFFFFFFFFFFFFFFFFFFFFFFF:FFFFFFFFFFFFFFFFFF:FFF:F:F
FFFFFFFFFFFFFFF:F::FFFFFFFFFFFFFFFFFFFFFFFFFFFFF
@A00721:190:HGJW7DSXY:4:2456:22318:7138_2:N:0:TCTGCAAG+GCTGCACT
GGCTTATTGTCGCGCAGTTTCCAGAATTGCGGTTGAAGTTCACCCATTCTATCTTGAATGCTCACGCC
GTCGCAGCCATATCGAGACTATGTTATAATCACTTGTTAAAGTACAGA
+
FFFFFFFFFFFFFFFFFFFFFFFFFFFFFFFFFFFFFFFFFFFFFFFFFFFFFFFFFFFFFFFFFFFF
FFFFFFFFFFFFFFFFFFFFFFFFFFFFFFFFFFFFFFFFFFFFFFFF

```
@A00721:190:HGJW7DSXY:4:1515:19732:22733_2:N:0:TCTGCAAG+GCTGCACT
ATTATTGATATTGGAGGCGATATTCGTTTTCATATCCAAAACTCCTTGAATGGAGTACATATTTGCAA
CCCTATTCTTGATGTGAAAGATGCATCGAGGTATACGTCTAGGAACAT
+
FFFFFFFFFFFFFFFFFFFFFFFFFFFF:FFFFFFFFFFFFFFFFFFFFFFFFFFFFFFFFFFFFFFF
FFFFFFF:FF,FFFF:FFFFFFFF:FF:FFFFFFFF:FFFFFFFFFFF
@A00721:190:HGJW7DSXY:4:1578:29839:24831_2:N:0:TCTGCAAG+GCTGCACT
GCTATCGGAGGGAAGCAAAATGTTTCGTTCTGTTACAAGAACGCTTTGGATTGTGACCATAAAGCCTC
AGTTGCGATAGCTGTAGAGGTATACGATATCTCTCTTGATGATGTAGC
+
FFFFFFFFFFFFFFFFFFFFFFFFFFFFFFFFFFFFFFFFFFFFFFFFFFFFFFFFFFFFFFFFFFFF
FFFFFFFFFFFFFFFFFFFFF:FFFFFFFFFFFFFFFFFFFFFFFFFF
@A00721:190:HGJW7DSXY:4:2347:30816:16595_1:N:0:TCTGCAAG+GCTGCACT
TGATGTATGTCATTTCGTCTTAGTTTGTCCCGGCGAATTATTGGACACGATGAAAAATGACATACCCG
TTCCTGACCTTGGTATCGTTATCCGTCATGGAGATGATGGCGCATCACTTGATTACTATATGCCCGAA
GGA
+
FFFFFFFFFFFF,FF:F:FFFFFFFFFFFFFFFFFFFFFFFFFFFFFFFFFFFFFFFFFFFFFFFFFF
FFFFFFFFFFFFFFFFFFFFFFFFFFFFFFFFFFFFFFFFFFFFFFFFFFFFFFFFFFFFFFFFFFFF
FFF
@A00721:190:HGJW7DSXY:4:2516:8703:28087_2:N:0:TCTGCAAG+GCTGCACT
AACAAAGATAAGATTTTATCTTTCTTCAAACACCCTGTTTTCCTTAAGGGCGGACAATTGTTCCATTG
TGAACTCTTGGGGACTAGATTGAGCATTAATGAGTTCCGAATAACTCG
+
FFFFFFFFFFFFFFFFFFFFFFFFFFFFFFFFFFFFFFFFFFFFFF:FFFFFFFFF::FFFFFFFFFF
FFFFFFFFFFFFFFFFFFF:FFFFFFFFFFFFFFFFFFFFFFFFFFFF
@A00721:190:HGJW7DSXY:4:1443:19741:17895_2:N:0:TCTGCAAG+GCTGCACT
TATTTCCGTACCAAGAGCCACTGCCAATCTCATCGCTATACGCATACCTGATTGGAAGAAGCGAGGTT
CGTTCCTCAATAAAAATTTTTCTCAAGAGATTATGGTTGATAAAGTTT
+
FFFFFFFFFFFFFFF:FFFFFFFFFFFFFFFFFFFFFFFFFFFFFFFFFFFFFFFFFFFFFFFFFFFF
FFFFFFFFFFFFFFF:FFFFFFFFFFFFFFFFFFFFFFFFFFFFFFFF
@A00721:190:HGJW7DSXY:4:1445:19578:16266_1:N:0:TCTGCAAG+GCTGCACT
TACGTCATGGGGATCACTTCTTCGGTCAATGACAAAACCTTCGAATATGTTACGACATGGCTTCGTAA
CAACAGTGCGAGGATTGTCATCTCGGGGAAAGTGATTCACCAAGATGTTCATATAGATACGAGATACC
TAG
+
FFFFFFFFFFFFFFFFFFFFFFFFF:FFFFFFFFFFFFFFFFFFFFFFFF:FFFFFFFFFFFFFFFFFF
FFFF:FFFFFFFFFFFFFFFFFFFFFFFF:FFFFFFFFFFFFFFFFF:FFFFFFFFFF:FFFFFFFFF
FFF
@A00721:190:HGJW7DSXY:4:1459:8992:30843_2:N:0:TCTGCAAG+GCTGCACT
AGCGATCTTACTCGTTATCGGAGCTAAGAAGAAGACTTTGGGTACACGTTATGCTCGCAAACTTAACG
TTAGTACCGGAGGTGATCTCTCTGCTTCTGCGTTACTGAGAGAAAAGA
+
FFFFFFFFFFFFFFFFFFFFFFF:FFFFFFFF:FFFFFFFFFFFFFFFFFFFFFFFFFFFFFFFFFFF:
FFFFFFFFFFFFFFFFFFFFFFFFFFFFFFFFFFFFFFFFFFFFFFF:
@A00721:190:HGJW7DSXY:4:1406:24957:3443_1:N:0:TCTGCAAG+GCTGCACT
CTTTATGGACAAGATGCAAGTTCTTGGGAAATACTTGTTTCCCTTTTTAGGTTTAGTAGGTGATCTTG
ACGTCGATTCTTTGTATAGGGAGTGTGAGAAGACAACGAATCTTGAAACTACGGTCCTTATCAAAGAA
AAT
+
FFFFFFFFFFFFFFFFFFFFFFFFFFFFFFFFFFFFFFFFFFFFFFFFFFFFFFFFFFFFFFFFFFFF
FFFFFFFFFFFFFFFFFFFFFFFFFFFFFFFFFFFFFFFFFFFFFFFFFFFFFFFFFFFFFFFFFFFF
FFF
```

```
@A00721:190:HGJW7DSXY:4:1218:26982:24455_2:N:0:TCTGCAAG+GCTGCACT
AAATCTTTTGAGATTGAGAAGATTGAAGAACACGTCAAGAAGTCGCTCGGCGTTGTTCTTGAAAACCA
AGTTAAGAAGGAGGCTGATCATGGAGTCTTTGTACCTTCTGTCAAACT
+
FFFF:FFFFFFFFFFFFFFFF:FFFFFFFFFFFFFFFFFFFFFFFFFFFFFFFFFFFFFFFFFFFFFF
FFFFFFFFFFFFFFFFFFFFFFFFFFFFFFFFFFFFFFFFFFFFFFFF
@A00721:190:HGJW7DSXY:4:2319:5023:31454_2:N:0:TCTGCAAG+GCTGCACT
TACGAGAGGTGATCCCGGATGTGGATTGAAGGCTGGCTCTAAGGGAGACTCCTTCTTTTTGAATGTTG
TAAAGAATTGGGTTGAGAAGTGTGCTTTACAAGGAGTTAGACTCTTCT
+
FFFFFFFFFFFFFFFFFFF:FFFFFFFFFFFFFFFFFFFFFFFFFFFFFFFFFFFFFFFFFFFFFFFF
FFFFFFFFFFFFFFFFFFFFFFFFFFFFFFFFFFF:FFFFFFFFFFFF
@A00721:190:HGJW7DSXY:4:2630:15736:9111_2:N:0:TCTGCAAG+GCTGCACT
TAAAGAAAAATTTTCGAATAAACTAATGAGAGGTATAGGCTTAGTCAAGTCTGTACTTACATTCTTTA
CGCCAGATTCTTCATGGGGCGACGCTGTTTCTCTTGGTATTTCTGAAA
+
FFFFFFFFFFFF:FFFFFFFFFFFFFFFFFFFFFFFFF:FFFFFFFFFFFFFFFF:FF:FFFFFFFF
,FFFFFFFFFFFFFFF,FFF:FFFFFFFFFFFF,FFFFFFFFFFFF:F
@A00721:190:HGJW7DSXY:4:2634:28809:13244_1:N:0:TCTGCAAG+GCTGCACT
TTTCCTCTGGTAAGGGAATTATTAAATCATTCTTCTCACTCTTGACGAGAATTATTACTGTTTTCTCT
ACCCATAACTTCTTTTTCAAAAATGCGGATGGGTCTACTGATGTTTTCCTACAGAGTGCAGTCGTTTC
AAC
+
F:F:FFFFFFFFFF:F:FFFFFFFF,,FFFFFFFFF:FFFFFFFFFFFFFFFFFFFFFFFFFFF,FFFF
FFFFFFFFFFFFF,FFFFFFFFFFFFFFFFFFFFFFFFFFFFFFFFFFFFFFFFFFFFFFFFFFFFFFFF
FFF
@A00721:190:HGJW7DSXY:4:1623:15619:32550_1:N:0:TCTGCAAG+GCTGCACT
AATAATTGGTGATGAAAATACGTATGTCGGTGGGGTTCTCAAGGCTCTCATCGCCACTGGGATTCTAA
GGGGTTCCATAAGATATTATCTGACATTGTGGTTCAATGATCAGGATTCTTCTTTTGGTCCTTACGTC
TTA
+
FFFFFFFFFFFFFFFFFFFFFFFFFFFFFFFFFFFFFFFFFFFFFFFFFFFFFFFFFFFFFFFFFFFF
FFFFFFFFFFFFFFFFFFFFFFFFFFFFFFFFFFFFFFFFFFFFFFFFFFFFFFFFFFFFFFFFFFFF
FFF
@A00721:190:HGJW7DSXY:4:2470:4408:32769_1:N:0:TCTGCAAG+GCTGCACT
TCTTGTCACGATTCTTAAAAGAAGTTTATTAAAACTTCAGAGTCTCTTGGAAGTCCAGGTAGCTAATG
TTCTTGCAATAGCCACAGACTTTCTGGGAAAAGAGACCTCTGTTGGTAGAGGAGTTGAGAGAATTACT
CAG
+
FFFFFFFFFFFFFFFFFFFFFFFFFFFFFFFFF:FFFFFFFFFFFFFFFFFFFFFFFFFFFFFFFFFF
FFFFFFFFFFFFFFFFFFFFFFFFFFFFFFFFFFFFFFFFFFFFFFFFFFFFFFFFFFFFFFFFFFFF
FFF
@A00721:190:HGJW7DSXY:4:1669:30535:35243_1:N:0:TCTGCAAG+GCTGCACT
AGTTAGGGAGTGGCTAGGTTTTAAAAGATCCTTTGCACAAGCAATACCTTCCACTGAGTTCATCACCC
AGAGTTTCGACGTCGCGGAAAACGATGAAGATTTCCACACTGTTCGAGAATCTTCTGATGCTGAATTT
GAG
+
FFFFFFFFFFFFFFFFFF:FFFFFFFFFFFF:FFFFFFFFFFFFFFFF:FFFFFFFFFFFFFFFFFFF
FFFFFFFFFFFFFFFFFFFFFFFFFFFFFFFFFFFFFFFFFFFFFFFFFFFFFFFFFFFFFFFFFF:FF
FFF
@A00721:190:HGJW7DSXY:4:1122:3007:30185_1:N:0:TCTGCAAG+GCTGCACT
GTTTTTGGTATAAATCCTGATGGCTCAGTACAGGGAGGAGGTAATAGTTATAAAACCTTCTTTTATCA
TTGCAACAGGACTTTGAGAAGACTGTTGAAATGGACAAAATCTGGGATCATAGGTATGTGTCACGCTA
CAC
```

```
+
FFFFFFFFFFFFFFFFFFFFFFFFFFFFFFFFFFFFFFFFFFFFFFFFF:FFFFFFFFFFFFFFFFFF
FFFFFFF,FFFFFFFFFFFFFFFFFFFFFFFFFFFFFFFFFFFFFFFFF:FFFFFFFFFFFFFFFFFF
FFF
@A00721:190:HGJW7DSXY:4:1632:14796:2284_1:N:0:TCTGCAAG+GCTGCACT
TGTTCAGGAAGATCTCTACAGAGATTATCGAGAGTTGTGATCTAGTTCTAAATTTAGGTCGACGGACT
TTAGCAACTTTAGATAAATTCTTTCCGGGCTCAATGACCTTTGCTATGTACGTGGATCTAATGCTTGG
TGA
+
FFFFFFFFFFFFFFFFFFFFFFFFFFFFFFFFFFFFFFFFFFF:FFFFFFFFFFFFFFFFFFFFFFFF
FFFFFFFFFFFFFFFFFFFFFFFFFFFFFFFFFFFFFFFFFFFFFFFFFFFFFFFFFFFFFFFFFFFF
FFF
@A00721:190:HGJW7DSXY:4:2342:23484:23030_2:N:0:TCTGCAAG+GCTGCACT
TATTAGTTCATGAAGTTCTTACACTTCTCGCGACTAATGTCAGCTTCACTACAAAGGCGTTACGTAAA
GTAGTTTTCACGCTTTATCTTAAAGCATTACCACCAAATTTAGCACTT
+
FFFFFFFFFFFFFFFFFFFFFFFFFFFFFFFFFFFFFFFFFFFFFFFFFFFFFFFF::FFFFFFFFFF
FFFFFFFFFFF,FFFFFF,FFFFF:FFFFF,FFFFFFF,FFFFFF::F
@A00721:190:HGJW7DSXY:4:2565:29776:1266_2:N:0:TCTGCAAG+GCTGCACT
TCGCCATATCAGATTAGAGACATGCCAGTTAGAGTTCTTCTAACCAGGACTGATGGTGAGATTCTTTT
ACCTTTTGGAGGTCCAGGAACCTTTACTCCTAGTGTCTTTTCTAGAGT
+
FFFFF:FFFFFFFF:FFFFFFFFFFFFFFFFFFFFFFFFFFFFFFFFFFFFFFFFFFFFFFFFF:FFF
FFF,FFFFFFFFFFFFFFFFFFFFFFFFFFFFF:FFFFF:FFF,F:FF
@A00721:190:HGJW7DSXY:4:1331:12057:18020_2:N:0:TCTGCAAG+GCTGCACT
CGGAGGATGACAGGTGGGAGACCGCTCAATAACACTCTATTGAATCCCTTTTCCACGATGAAAGCTCT
TTATGAGGTGCTCCATGCAAATTTACAGTTCCTCTTTGGTGATATCGC
+
FFFFFFFFFFFFFFF:FFFFFFFFFFFFFFFFFFFFFFFFFFFFFFFFFFFFFFFFFFFFFFFFFFF:
FFFFFFFFFFFFFFFFFFF:F:FFFFFFFFFFFF:FFFFFFFFFFFF
@A00721:190:HGJW7DSXY:4:1331:12057:18020_1:N:0:TCTGCAAG+GCTGCACT
TTTTGATAAATTTGTTGGAATCGATAATGATACTGACGAAGGTTCTATTACCACCCTTGTCATTGATA
CAAACATTCTAGGTGATGCTTTCTTAGAGGCTCGTCTAGCCGCTGAGATTCGTCTTACCTATTTACAA
TCT
+
FFFFFFFF:FFFFFFFFFFFFFFFFFFFFFFFFFFFFFFFFFFFFFFFFF:FFFFFFFFFF:FFFFFFF
FFFFFFFFFFFFFFFFFFFFFFFFF:FFFFFFFFFFFFFFFFFFFFFFFFFFFFFFFFFFFFFFFFFF:
FFF
@A00721:190:HGJW7DSXY:4:1378:23321:13542_1:N:0:TCTGCAAG+GCTGCACT
GATAACGATTTAGTACTGGGAAATGGTGGTAGTCAGTTTACTACTTTTCTTTCCCTCCCCTTTTTCGA
TTGGATTCGACGGGGTCTTCGTCTCACGACTAATTTGATAAGGTTTTTCTTTTATGGGACTCTTGTTT
GTA
+
FFFFFFFFFFFFFFFFFFFFFFFFFFFFFFFFFFFFFFFFFFFFFFFFFFFFFFFFFFFFFFFFFFFF
FFFFFFFFFFFFFFFFFFFFFFFFFFFFFFFFFFFFFFFFFFFFFFFFFFFFFFFFFFFFFFFFFFFF
FFF
@A00721:190:HGJW7DSXY:4:2619:27868:7576_2:N:0:TCTGCAAG+GCTGCACT
AAGTTCTTAATGTAGGTCTCCTAGTACTTAAGGGTTTATGTTTCGGTTTCAGCTGGATGGATCTTTTG
ATGGTATGTTTCACATTGCGTGATCACTTCCCGTGGACTTGGGAAGAG
+
FFFFFFFFFFFFFFFFFFFFFFFFFFFFFFFFFFF,FFFFFFFFFFFFFFFFFFFFFFFFFFFFFFFF
FFFFFFFFFFFFFFFFFFFFFFFFFFFFFFFFFFFFFFFFFFFFFFFF
@A00721:190:HGJW7DSXY:4:1327:20980:24893_1:N:0:TCTGCAAG+GCTGCACT
CGATTCTATTATGTCGCTAAAGCCTGTCCAGTTGTCGATGATGTTCGGGCTGTGGTGATTAACTTCCC
```

TCGTGAAACTTACGACGATGTTCTCGCTCGCATTTCACAAGGATTACTAAGTCTGAGATCTGCCACTT
CTA
+
F:,FFFF:FFF:F:,F:FFFF,,,FF:F,FFFF:F,FFFF:FFFF:,F:FFF:FF,FFFFF,,,F:F,
FFFF,::FFFF:::F:,F:::F,FFF,FFF,:FFFF,:FF:,:,FFFFFFFF,FFF::F,::F,FFFF
,FF
@A00721:190:HGJW7DSXY:4:1362:19940:12790_1:N:0:TCTGCAAG+GCTGCACT
TCTGTTCATGAGGATGTCTTGGTCGAAAAATATCGCCGGGAACTTCTAATAGAGGATCCTGATGACAT
TATTTCAACGGATACCGATCTCAGTTCTACCACAGAAACCGTCTCAGGAACTGTCTCCACTGATTCGG
AAT
+
FFFFFFFFFFFFFFFFFF:FFFFFFFFFFFFFFFFFFFFFFFFFFFFFFFFFFFFFFFFFFFFFFFFF
FFFFFFFFFFFFFFFFFFFFFFFFFFFFFFFFFFFFFFFFFFFFFFFFFFFFFFFFFFFFFFFFFFFF
FFF
@A00721:190:HGJW7DSXY:4:1533:16721:21183_2:N:0:TCTGCAAG+GCTGCACT
CGACCTTCTTGTGGGGACTGAGACCTCTGAATTTTCAGGGTGCCCTGATGAGGACGTTGAAAGGGATA
ATTTCAACTCTGAGACTTCTAAGAGTGTTTCAAAAGACCAGAAACGTA
+
FFFFFFFFFFFFFFFFFFFFFFFFFFFFFFFF,FFFFFFFFFFFFFFFFFFFFFFFFFFFFFF:FFF:
FFFFFFF:FFFFFFFFFFFFF:FFF:FFFFFFFFF:FFFFFFFFFFFF
@A00721:190:HGJW7DSXY:4:1627:6876:7639_1:N:0:TCTGCAAG+GCTGCACT
AAAGGTAGGAAGGGGAGAAAACAGAACAAGCGAGCGGTTGAAGAGAAAACTGATATGGTTGCGGCTGA
TCGTACCCCAATTCCTGCTAGAAAGATCACTCTCGGCGATACCACTGATCTTCCAGAGTTACGTAGTG
GTC
+
FFFFFFF:FF:FFFFFFFFFFFFFFFF,F:FFFFF:FFFFFFFFFFFFFFFFFFFFFFFFFFFFFFFF
FFFFFFFFFFF:FFFFFFFFFFFFFFFFFFFFFFFFFFFFFFFFFFFFFFFFFFFFFFFFFFFFFFFF
FFF
@A00721:190:HGJW7DSXY:4:1508:16306:35462_1:N:0:TCTGCAAG+GCTGCACT
TTTTATCAAAGAGAAAGTTTCTGCTAGTCGCGACGATAGTATTTATCGGAGTGATATTAACAAGTTAA
ACTTCGAACTGACCACTTACAAGAGTGTTGAAGTACCGCAACATGGATCAGTTTTCCAAAAAGCTATT
AAT
+
FFFFFFFFFFFFFFFF:FFF,F,FFFFFF:,FFFFFFFFFF:FFFF:FFFFFFFFFFFF:FFFFFFFF
F,FFFFFF:FFFFF:FFFF:FFFFFFFFFFFFFFFF:FFFFF:FFFFFFFFFFFFF:FFFFFFFFFFF
FF:
@A00721:190:HGJW7DSXY:4:2420:11288:1063_2:N:0:TCTGCAAG+GCTGCACT
AAACTGAATCCACTCTCTTGGGATATAAATGAGTACATCTATAATAGAGTTTTGGAACTGTGTAATGT
CCTACGTACTGTTCAAGGTGTTTGGGCTTCTCTAAAGAACAGCAACTT
+
FFFFFFFFFFFFFFFFFFFFFFFFFFFFFFFFFFFFFFFFFFFFFFFFFFFFFFFFFFFFFFFFFFFF
FFFFFFFFFFFFFFFFFF,FFFFFFFFFFFFFFFFFFFFFFFFFFFFF
@A00721:190:HGJW7DSXY:4:2263:3757:10598_1:N:0:TCTGCAAG+GCTGCACT
AACCTTTCCTCGTTCTTCATGAAGATCTTCGAGTGTTTTATAGTGAGTTATTCTTGCGAAGGTTCAAG
TCGATTGATTTTGTTGACTACGATCTCTCTTGTAATAAGAAGTTCTTCGCAGTTGAATCTCCACCAGG
TGG
+
:FFFFFFFFFFFFFFFFFFFFFFFFFFFFFFFFFFFFFFFFFFFFFFFFFFFFFFFFFFFFFFFFFFF
FFFFFFFFFFFFFFFFFFFFFFFFFFFFFFFFFFFFFFFFFFFFFFFFFFFFFFFFFFFFFFFFFFFF
FFF
@A00721:190:HGJW7DSXY:4:1613:13503:3145_2:N:0:TCTGCAAG+GCTGCACT
GCGACTTTTGTCGAATTTTTCGCAAAAGGTCTTGAAGTACGTATTGTCACTGCTAATCGTAATTCGCG
AGAAGAGATAGTGACTAAAGTTTTGGCACTTCTAGTTCATCGCGGTAT
+

FFFFFFFFFFFFFFFFFFFFFFFFFFFFFFFFFFFFFFFFFFFFFFFFFFFFFFFFFFFFFFFFFFFF
FFFFFFFFFFFFFFFFFFFFFFFFFFFFFFFFFFFFFFFFFFFFFFFF
@A00721:190:HGJW7DSXY:4:2452:26078:13056_2:N:0:TCTGCAAG+GCTGCACT
CGTGAAAGTTATGATGAGTGATCGTATTAGAACAGCTGATTCTTATTGCATGAACGTCGCTGAGGTCA
AACCTGTGGACGTTCTTTTCATCGACGAATTATTCATGATTCACGCTG
+
FFFFFFFFFFFFFFFFFFFFFFFFFFFFFFFFFFFFFFFFFFFFFFFFFFFFFFFFFFFFFFFFFFFF
FFFFFFFFFFFFFFFFFFFFFFF:FFFFFFFFFFFFFFFFFFFFFFFF
@A00721:190:HGJW7DSXY:4:2159:10321:2801_2:N:0:TCTGCAAG+GCTGCACT
CATCTACTTAAGGCGGCTTATACCATAGGTTACGGTGATTCCAATCAAATCGGATTCATCAATCGTAT
TGATCTCCAAGTCGCAAACTACTTCAACATCAACGACATCATTCTTGA
+
FFFFFFFFFFFFFFFFFFFFFFFFFFFFFFFFFFFFFFFFFFFFFFFFFFFFFFFFFFFFFFFFFFFF
FFFFFFFFFFFFFFFFFFF:FFFFFFFFFF:FFFFFFFFFFFFFFFFF
@A00721:190:HGJW7DSXY:4:2667:22824:17879_2:N:0:TCTGCAAG+GCTGCACT
TTATCGAAGTCTCAGTTATCGCTGCCCGGCCGACGTTTGTCGCGAACTCTCAGAGGTTTACGGCAGAA
AAATTTCTCCTGCTTGTAATTCGAGAGAGGACACTATGGAAATTAGAC
+
FFFFFFFFFFFFFFFFFFFFFFFFFFFFFFFFFFFFFFFFFFFFFFFFFFFFFFFFFFFFFFFFFFFF
FFFFFFFFFFFFFFFFFFFFFFFFFFFFFFFFFFFFFFFFFFFFFFFF
@A00721:190:HGJW7DSXY:4:2455:13449:31735_2:N:0:TCTGCAAG+GCTGCACT
TATTACTTTCACTCAAGGTGAAAAGTTTGAAATTAACAAAGTCCTTGGTAAGAGTAAGGTTAAATGTG
AAGATGCTCAGACTATTCATGAAGTTCAAGGTAATACTTACCAGCACG
+
FFFFFFFFFFFFFFFFFFFFFFFFFFFFFFFFFFFFFFFFFFFFFFFFFFFFFFFFFFFFFFFFFFFF
FFFFF:FFFFFFFFFFFFFFFFFFFFFFFFFFFFFFFFFFFFFFFFFF
@A00721:190:HGJW7DSXY:4:1442:20347:22357_1:N:0:TCTGCAAG+GCTGCACT
ACAAACACATATTACCGTTGCTCTATCACGACACGTTGAATCTTTACGATACTTTGTTATCAATGTAA
AGTTAAACGATGCTACTTGTGAGAGGATCAATCACAGTAAGGAACTGTCACGTTTAGCACGGGAGGTT
GCA
+
:FFFFF:FFFFFFFFFF,FFFFFFFFF:FFFFFFFFFFFFF:FFFFFFFFFFFFFFFFFFFFFF:FFF
FFFFFFFFFFFFFFFFFFFFFFFFFFFFFFFFFFFFFFFFFFFFFFFFFFFFFFFFFFFFFFFFFFFF
FFF
@A00721:190:HGJW7DSXY:4:1273:28113:21339_2:N:0:TCTGCAAG+GCTGCACT
TATATCGAGGCTATCGAGGACGATGAGTATCTTGAAGTTGTTGACGACAAGCCTCAAGAGGGTAGAGG
ACCACCTGGATCTTCTACTGTCTCTGCCTTCCAAGATTTCTTAGAAGA
+
FFFFFFFFFFFF:FFFFFFFFFFFFFFFFFFFFFFFFFFFFFFFFFFFFFFFFFFFFFFFFFFFFFF
FFF:FFFFFFFFFFFFFFFFFFFFFFFFFFFFFFFFFFFF:F:FFFFF
@A00721:190:HGJW7DSXY:4:2442:28818:1799_1:N:0:TCTGCAAG+GCTGCACT
AACCACCTTAACGTCACTCGGTAATCTTTCAGCCGAATTAAGTACTAATGAGTTCAAGTCGGTTGTCG
ATCGAATTAAGATCAAGGCTAATACCTTTATAGGTAAGAAGCCCCTTGAGCCGCGTAGTCAGCGCGTT
TAG
+
FFFFFFFFFFFFFFFFFFFFFFFFFFFFFFFFF:FFFFFFFFFFFFFFFFFFFFFFFFFFFFFFFFFF
FFFFFFFFFFFFFFFFFFFFFF:FFFFFFFFFFFFFFFFFFFFFFFFFFFFFFFFFFFFFFFFFFFFF
FFF
@A00721:190:HGJW7DSXY:4:2306:7726:26303_1:N:0:TCTGCAAG+GCTGCACT
TACCGAGTCTCCAAGAGAATATTTATTCTTATGAGGCTCGAAACTTCAACGTTTTCACAACGGAAAGA
CATATCGCACCCGAAATTTTTGGGCAGAATGCTCTTAACGCGGTTCTTGAACGGTGTTTTGATCAGTC
TAA
+
:FFFFFFFFFFFFF:FFFFFFFFFF,FFFFFFFFFFFFFFFFFFFFFFFFF:FFFFFFF:FFFFFFFF

```
FFFF:FFFFFFFFFFF:FFF,FFFFFFFFFFFFFFFFFFFFFFFFFFFFFFFFFFFFFFFFFFFFFFF
FFF
@A00721:190:HGJW7DSXY:4:1347:17363:7169_1:N:0:TCTGCAAG+GCTGCACT
ATAGGAGACTCAGTTAACCTGAGAGCTCCTCAATTGATAGATTGGGTCAACAATAGGGACGCTAGTAT
ACTTCGTAAAGTCGACAGCGAAATGGTCAATATTGATAATTGGACCGACTATATGACCTATTTTAAGC
TCA
+
FFFFFFFFFFFFFFFFFFFFFFFFFFFFFFFFFFFF:FFFFFFFFF:FFFFFFFFFFFFFFFFFFFFFF
FFFFFFFFFFFFFFFFFFFFFFFFFFF:FFFFFFFFF:FFFFFFFFFFFFFFFFFFFFFFFFFFFFFFF
FFF
@A00721:190:HGJW7DSXY:4:1544:3540:15013_2:N:0:TCTGCAAG+GCTGCACT
CTAAGGTTAAACTTGATGCTTCAGCCTTTTCGAAGCATAACCCCGCACAGAATATCGTTTATCACGCA
AAATTTATTAATGCAATTTTCGGACCTATCTTTAAGGTTCTTGTGAGG
+
FFFFFFFFF:FFFFFFFF:FFFFFFFFFFFFFFFFFFFFFFFF:FFFFFFFFFFFFFFFFFFFFFFFFF
FFFFFFFFF,FFFFFFFFFFFFFFFFFFF:F:FFFFFFFFFFFFFFFF
@A00721:190:HGJW7DSXY:4:2402:31602:12759_1:N:0:TCTGCAAG+GCTGCACT
CAGTGACGATTTCTCCCGGAAGCTCTTCTCTCTCTTAGGAGGTCATGATGACTACTATAAGGGTGAAA
TCGATTTTTCGAAGTTTGACAAATCTCAAGATTTCTTTTGTAAAGCCTATGAGATAAAGATGTATTCT
TAT
+
FFFFFF:FFFFFFFFFF,FFFFFFFFFF:F,FFFFFFFFF:FFFFF:FF:FFFFFFFF::F,FFFFFF
FF:FFFFFFFFFFFFFFFFFFFFFFFFFFFFFFFFFFFFFFFFFF:F:FFFFF:FFFFFFFFFFFFFFF
FFF
@A00721:190:HGJW7DSXY:4:2316:10050:12633_2:N:0:TCTGCAAG+GCTGCACT
AACTTTGGAGTGCCAGTGAATATTTTTGTCACGCCAGGAGTATCCATAACGATTTGTCATTTACCACC
TTTGCTCAACGACGAAGTGGAACGGCCAACACTTTCTTTGGTAATTGT
+
FFFFFFFFFFFFFFFFFFFFFFFFF:FFFFFFFFFFFFFFFFFFFFFFFFFFFFFFFFFFFFFFFFFFF
FFFFFFFFFFFFFFF:FFFFFFFFFFFFFFFFFFFFFFFF,FFFFFFFFF
@A00721:190:HGJW7DSXY:4:1309:21061:14638_1:N:0:TCTGCAAG+GCTGCACT
GATGATTTTATCACTTTATTATGACCTTGGAAAGTTTTCGGCTGTGGTTGTCGCTGGGGACGATTCCA
TTCTGTTTTCAAAGACTCCAATAACTGATCATTCTGATCATATGATTTTGGACAATGGTTTCGAAGCG
AAA
+
FFFFFFFFFFFFFFFFFFFFFFFFFFFFFFFFFFFFFFFFFFFFFFFFFFFFFFFFFFFFFFFFFFFFF
FFFFFFFFFFFFFFFFFFFFFFFFFFFFFFFFFFFFFFFFFFFFFFFFFFFFFFFFFFFFFFFFFFFFF
FFF
@A00721:190:HGJW7DSXY:4:2643:4734:21433_2:N:0:TCTGCAAG+GCTGCACT
TGCTCGCGATTTATTCTTTTTGTCAATGATTACATCTACTTCGTTCCTGATCCTTACAAGATCATCGT
TAAGTTAGGTAAACCCCTAACCGATGTTACTCTTGTAGGACTTTGGGA
+
FFFFFFFFFFFF:FFFFFFFFFF:FFFFFFFFFFFFFFFFFFFFFFFFFFFFFFFFFFFFFFF:FFFFF
FFFFFFFFFFFFFFF:FFFFFFFFFFFFFFFFFFFFFFFFFFFFFF:FFF
@A00721:190:HGJW7DSXY:4:1643:15944:27759_2:N:0:TCTGCAAG+GCTGCACT
AGGTTGTTGTTTGGCGATTAGCTCTTGCGACTAATATTCGATATTCGATTGAGGGTCGTTATACCTAT
TCGGCTATCTGTGCTTTGAATTGTATAAGATCAAATTTTAAACGATTC
+
FFF,FFFFFFFFFFFFFFFFFFFFFFFFFFFFFFFFFFFFFFFFFFFFFFFFFFFFFFFFFFFFFFFFF
FFFFFFFFFFF:FFFFFFFFFFFFFFFFFFFFFFFFFFFFFFF:FF:FF:
@A00721:190:HGJW7DSXY:4:2128:20175:7999_1:N:0:TCTGCAAG+GCTGCACT
TGATGTGGAGGGAGTTCTTGAAGCATCCCGACCTTTCCTTGTGAAAATTTTGATGCGGTTTCATAAGA
TTATCAGTAATCCTGATTCTACTACGGATTCATGGACCTTCCATCCTGTACCTCTTCTGGAGAGACGT
ATC
```

```
+
FFFFFFF:F:FFFFFFFFF:FFFFFFFFFFFFFFFFF,:FFF,FFFFFFFFFFFFF,:FFFFFFFFFF
F:FFFFFF,FFFFFFFFFFFFFFFFFFFFFFFFFFFFFFFFFFFF:FFFFFFFFFFFFFFFFFFF:
FFF
@A00721:190:HGJW7DSXY:4:1139:10791:15608_2:N:0:TCTGCAAG+GCTGCACT
TCGAGACAGGTGATATTGTTGAGCGAGAGCGACCAAGATCTAAATTCGGTAACGGTGCGTGGCGCTTT
ATTTTAAAAAGCTCCCTCCTCTCGCAAGTTTGATCTGATACTTATCAC
+
FFFFFFF:FFFFFFFFFFFFFFFF,FFFFFFFF:FFFFFFFFF::FF:FFFFFFFFFFFFFFF:FFFF
FFFFFFFFFFFFFFFFFFFFFFFFFFFFFFFFFFFFFFFFFFF:FFFFF
@A00721:190:HGJW7DSXY:4:1259:11740:29590_2:N:0:TCTGCAAG+GCTGCACT
GATCAGTCTCTGACTTGACCATCTTTCATTTTAAATGACGATCATTAAGACTCTCTTTCTTCTTTGTT
TCCTTCTTTGCACTGATTTTTCCTCATCAGCTTGTGTTTTACCTAATC
+
FFFFFFFFFFFFFFFFFFFFF:FFFFFFFFFFFFFFFFFFFFFFFFFFFFFFFFFFFFFFFFFFFFFF,
FFFFFFFFFFFFFFFFFFFFFFF:FFFFFF:FFFFFFFFFFFFFFFFFF
@A00721:190:HGJW7DSXY:4:2211:8034:11209_2:N:0:TCTGCAAG+GCTGCACT
GATAACAACTATCAGTTGATTAAGACTCTTCCGAAAGGCGAACTTTGGGAATTTGAAGCTCAGGGTGA
AGCACAACGTTTGACCGTGACGAACTGTGGTGTTCTTGATCTACGTTA
+
FFFFFFFFFFFFFFFFFFF:F,:FFFFFFFFFFFFFF:FFFF,FFFFFFF,FFFFFFFFFFFFFFFFF
F:FFFFFFFFFFFFFFF,FFFFFFFF:FFFFFFFFFFFFFFFFFF,FF
@A00721:190:HGJW7DSXY:4:2344:24108:15123_1:N:0:TCTGCAAG+GCTGCACT
CACTTATTACGATATTTCTTTAGTCGATGGGTTTTCGACGCCTTTAAGCGTCTTCTGTTCATCGCAAC
CAGATGATATCTTCTATTCGAACTTATGTTTCGATCAGTGTTTCTTCTATCAGGAGGAAAACACTTGT
TCT
+
FF,FFFFFFF:FFFFFFFFFFFFFFFFFFFFFFFFF:FF:::F:FFFFFFFFFFF:FFFFFFFF:FFFF
FF:FFFFFFF,FF:FFF:FFFFFF,FFFFFFFFFFFFFFFFFFFFFFFFFFFFFFFFFFF:FFFFFFF
FFF
@A00721:190:HGJW7DSXY:4:1105:19325:24846_1:N:0:TCTGCAAG+GCTGCACT
TCTCTATTTTAAGTTTTGTCTTCCTTGTTTGTCTTATTCTATTAATCACTCATTTTAGTGAGATTATT
AAAAACGCACTAAAACGTCTTCGACAACAGCAGGGGTTTTCGAATAGTGATAGCCACCCGCCTTACAG
AGG
+
FFF:FFFFFFFFFFFFFF:FFFFFFFFFFFFFFFFF:FFF:FFFFFFFFFFFF:F:FFFFFFFFFFFF
F,FFFFFFFFFFFFFFFFFFFFFFFFFFFFFFFFFFFFFFFFFFFFFFFFFFFFFFFFFFFFFFFFFF
F:F
@A00721:190:HGJW7DSXY:4:1209:14742:4413_2:N:0:TCTGCAAG+GCTGCACT
GACTTTGGTACCACCTTTTCTACTGTCTGTCTCGCCTCTTCGAATGAAGTTTTAATGCTTAACAACAA
TGATAGTGAATTTATACCTACAATCATTGGTTTTTCTAATAATGATGA
+
FFFFFFFFFFFFFFFFFFFFFFFFFFFFFFFFFFFFFFFFFFFFFFFFFFFFFFFFFFFFFFFFFFFF
FFFFFFFFFFFFFFFFFFFFFFFFFFFFFFFFFFFFFFFFFFFFFFFF
@A00721:190:HGJW7DSXY:4:2478:26350:22482_1:N:0:TCTGCAAG+GCTGCACT
CTTGGATATGATGCTATTACTCATGATGGTTACGGTCAGGGAGGATTCTCAGTTTACAAGGACCTTAA
GCGATGGATCGGGGTTAATTCTAAAACATTACCCGAAAGACGTGCTAAATTAAAACCATTGTACGATG
TCG
+
FFFFFFFFFFFFFFFFFFFFFFFFFFFFFFFFFFFFFFFFFFFFFFFFFFFFFFFFFFFFFFFFFFFF
FFFFFFFFFFFFFFFFFFFFFFFFFFFFFFFFFFFFFFFFFFFFFFFFFFFFFFFFFFFFFFFFFFFF
FFF
@A00721:190:HGJW7DSXY:4:1260:1199:35978_1:N:0:TCTGCAAG+GCTGCACT
TCACTTTTCACTAAGAATTGGTCCTACTTTTGGACAAGGCCAACTTCGTAATCTTCATGAGATCGTTT
```

CTTTCTTTATCTCTTTGGTTGTTAGGGATTTTGAGAGAGTGAAAGGCTTCAATTGTAGTGGTTTGGTC
GTT
+
FFFFFFFFFFFFFFFFFFFFFFFFFFFFFFFFFFFFFFFFFFFFFFFFFFFFFFFFFFFFFFFFFFFF
FFF:FFFFFFFFFFFFFFFFFFFFFFFFFFFFFFFFFFFFFFFFFFFFFFFFFFFFFFFFFFFFFFFF
FFF
@A00721:190:HGJW7DSXY:4:1207:2031:36636_2:N:0:TCTGCAAG+GCTGCACT
ACCTCTACGCAAAGATATTTCATGAAAACTACAGAGGAACGTACCGGATTACCTGTCTTTCATATTAT
GAATGAACCTTCGGCCGCTCTCTTTGCTTCGATGTTGGATGTCAAGAA
+
FFFFFFFFFFFFFFFFFFFFFFFFFFFFFFFFFFFFFFFFFFFFFFFFFFFFFFFFFFFFFF:FFFFFF
FFFFFFFF:FFFFFFFFFFFFFFFFF,FFFFFFFFFFFFFFFF:FFFF
@A00721:190:HGJW7DSXY:4:1666:3251:33395_1:N:0:TCTGCAAG+GCTGCACT
ATTCCTACGTAGTTTATGACTTTGGTGGTGGTACTTTCGATACTTCTATCGTCTTACGTTACGGTAAT
TACTATAGTGTTATTTATTCTGGTGGTGATGATAATCTTGGAGGTCGTGACGTTGATGAAGCTATAAG
AGC
+
FFFFFFFFFFFFFFFFFFFFFFFFFFFFFFF:FFFFFFFFFFFFFFFFFFFFFFFFFFFFFFFFFFFF
FFFFFFFFFFFFFFFFFFFFFFFFFFFFF:FFFFFFFFFFFFFFFFFFFFFFFFFFFFFFFFFFFFFF
FFF
@A00721:190:HGJW7DSXY:4:1247:2094:7373_2:N:0:TCTGCAAG+GCTGCACT
CACAACTTTCAGTTTCACAACTGAAGGAGGAGGTTTCTGTTACTGGAAAACCCTCGTCAATACTTTTC
AATAATATACGTCTTGAACTCACTTATGACGATCTAGTGAAATTAACG
+
FFFFFFFFFFFFFFFFFFFFFFFF:FFFFFFFFFFFFFFFFFFFFFFFFFFFFFFFFFFFFFFFFFFF
FFFFFFFFFFFFFFFFFFFFFFFFFFFFFFFFFFFFFFFFFFFFFFFF
@A00721:190:HGJW7DSXY:4:2341:19081:11960_1:N:0:TCTGCAAG+GCTGCACT
CTTGCAGAGTGACTGGAGTTCAGACGTGTGCTCTTCCGATCTGGCAAACTTACCCTGATTCCGGCCGG
TGGTAGCTCATTTCTACCAGGGGCTATTACTCTTGCTGAAACAGCTATTAAAGCTGTTGAGCGGGTAA
TTA
+
FFFFFFFFFFFFFFFFFFFFFFFFFFFFFFFFFFFFFFFFFFFFFFFFFFFFFFFFFFFFFFFFFFFF
:FFFFFFFFFFFFFFFFFFFFFFFFFFFFFFFFFFFFFFFFFFFFFFFFFFFFFFFFFFFFFFFFFFF
FFF
@A00721:190:HGJW7DSXY:4:1110:24804:35931_2:N:0:TCTGCAAG+GCTGCACT
ATCGCTGAAGGTTGTGCACTCTTTTCTGCTACTTTAACGGCGCGTAGTGTACTCTTCGTCGATGGTAG
CAACCGTACCATTACTTCTAATAAAGGTCTCTGTCACGCTCGGAGTAT
+
FFFFFFF::FF,,FFFF,FFF:::F:,F:FF::F::::FF,FFF,FF,F,:FF,FFFFFFF,:::FF,
F:,:,FF,,,F,,F,:F,F,,,:,,FF:F,FF,:FF,FF:FF,,,FF:
@A00721:190:HGJW7DSXY:4:1101:5439:2080_1:N:0:TCTGCAAG+GCTGCACT
TTACCGTTGAAGGCTAAACTCCAAGGTAATTGTGAAGACTGTGATTTATCTTTCACTCTGGCCTTGTA
TGAGGGTGTTTCTAATCGTAACATTAATAATTTCAATCTTCTTAAAAAGAAGATAGAGAGTCATACTT
TGG
+
FFFFF:FFF:FFFFFFFFFF:FFFFFF:FFFFFFFFF,FFFFFFFFFFFFFFFF:FFFFFFFFF::FF
FFFFFFFFFFFFFFFFFFFFFFFFFFFFF:FFFFFFFFFFFFFFF:FFFFFFFFFFFFFFFFFFFFFFF
FFF
@A00721:190:HGJW7DSXY:4:2136:22959:19460_2:N:0:TCTGCAAG+GCTGCACT
GATAGCCAATATTGAAGTGGACACCTCTGGTAGTATCGAAGTAAAGATAACCAATAAGGAAGGAGCTT
ATACTATGGTTGAATCAACTAAGCATACTACTTTTGCTTTGATTACTC
+
FFFFFFFFFFFFFFFFFFFFFFFFFFFFFFFFFFFFFFFFFFFFFFFFFFFFFFFFFFFFFFFFFF:FF
FFFFFFFFFFFFFFFFFFFFFFFFFFFFFFFFFFFFFFFFFFFFFFFF

@A00721:190:HGJW7DSXY:4:1117:14425:33520_1:N:0:TCTGCAAG+GCTGCACT
GTCTAAGGTTAAAGAGTCGATTAAACAAGTTGTACTTATCAATGATTTTATCGCTGGTTGTTATCTCG
AAAAGTCTCTTCTAAAAGGAACGTCAGTTCTAACCCAAAGAGAATATCTAAAATATTTACAAAATCTT
ACC
+
FFFFFF,FFFFFFFFFF::FFFFFFFFFFFF:FFFFFFFFFFFFFFFFFFFFFFFFFFFFFFFFFFFF
FFFFFFFFFFFFFFFFFFFFFFFFFFFFFFFFFFFFFFFFFFFFFFFFFFFFFFFFFFFFFFFFFFFF
FFF
@A00721:190:HGJW7DSXY:4:1257:7355:33865_2:N:0:TCTGCAAG+GCTGCACT
AGACGTTGCTTGTCGCTTCCTCCTTTAATGGCACTACCTCGGTACAATCTTACTTGGAACGAAGGCGA
CCTGTTCCGGAGTTTTTACGGGATCCGGAATGTTCTGTCTATATTAGG
+
FFFFFFFFFFFFFFFFFFFFFFFFFFFFFF:FFFFFFFFFFFFFFFFF::FFFFFFFFFFFF:FFFF
FF:FFFFFFFFFFFFFFFFFFFFFFFFFFF:FF,FFFF,:FFFFFFFFF
@A00721:190:HGJW7DSXY:4:1318:28330:6527_2:N:0:TCTGCAAG+GCTGCACT
TCGAACGTCAACGTTCACCGAGTTTCGTTTCGTACCTTGATGCTGACTTTCGTCAATATGATTTGATC
TGGTTGCTCAATTCTAAGCGTGTTCCGCTTAGTGCGAGGCCTCTCTTC
+
FFFFFFFFFFFFFFFFFFFFFFFFFFFFFFFFFFFFFFFFFFFFFFFF:FFFFFFFFFFFFFFFFFFF
FFFFFFFFFFFFFFFFFF,FFFFFFFFFFF,FFFFFFFFFFFFFFFFF
@A00721:190:HGJW7DSXY:4:1445:26865:23531_2:N:0:TCTGCAAG+GCTGCACT
AAATACTAGATATTTGGATGATCTTGATAAAGAACTTCATGAATTTTCAGAGGATGAAAAATTTTCTT
CCTTGGATTTTAATATAAAGAATCTTTACCAGGACTCCTACTACAGGA
+
FFFFFFFFFFFFFFFFFFFFFFFFFFFFFF:FFFFFFFFFFFFFFFFFFFFFFFFFFFFFFFFFFFFF
FFFFFFFFFFFFFFFFF::FFFFFFFFF:,FFFFFFFFFFFFFFFFFF
@A00721:190:HGJW7DSXY:4:2647:2003:7686_1:N:0:TCTGCAAG+GCTGCACT
GATCGTAAGAAGTTTATAAGGAACATATCCATTTGTTGTAATAGGGTCATCAACTTAGAACTCGAAAC
TCTAAGTGGTAATACTGTCTTTCCAAGCTTACCTGTATCTTCAATGAAATTGGACTCTTCGATAGGGG
ACA
+
FFF:FFFFFFFFFFFFFFFFFFFFFFFFFFFFFFFFFFFFFFFFFFFFFFFFFFFFFFFFFFFFFFFF
FFFFFFFFFFFFFFFFFFFFFFFFFFFFFFFFFFFFFFFFFFFFFFFFFFFFFFFFFFFFFFFFFFFF
FFF
@A00721:190:HGJW7DSXY:4:2678:26838:6887_1:N:0:TCTGCAAG+GCTGCACT
TAAGAATACTATAAATAGAATAGCTATTACTACTAATGGCGTTGGCCTAGTGGATGTTATTAGTGACA
TCTGGTCAATTTCGTTTTTAGGTGGTCTTTATTCTATTATTGATTTTTATGAGATTGAGATCGGAAGA
GCA
+
FFFFFFFFFFFFFFFFFFFFFFFFFFFFFF:FFF:FFFFFFFFFFFFFFFFFFFFFFFFFFFFFFFFF
FFFFFFFFF:FFFFFFFFFFFFFFFFFFFFFFFFFFFFFFFFFFFFFFFFF:F:FFFFFFFFFFFFFFF
FFF
@A00721:190:HGJW7DSXY:4:2552:21169:12320_2:N:0:TCTGCAAG+GCTGCACT
TTCCTGAAGAGTCCTTCCGTAACCAACTTCTTGACAATTTTCTTCAATCCTTCGATAATAGTTAAATC
TACGATTATGTCTGTGTTGAAAGACTTTCTACCGATAAGGGTCACTAG
+
FFFFFFFFFFFFFFFFFFFFFFFFFFFFFFFFFF:FFFFFFFFFFFFFFF:FFFFFFFFFF:FFFFFFF
FFFFFFFFFFFFFFFFFFFFFFFFFFFFFFFFFFFFFFFFFFFFFFFF
@A00721:190:HGJW7DSXY:4:1414:3676:18630_2:N:0:TCTGCAAG+GCTGCACT
ATCCTACATTGACTGATTATCTAAAGATTGAAGGAAAATTTTATCCTCGTTTAACTTACTTAGACTTT
CCTACGATACAAATGATAACTTCTAATTTACGTTTGCTACTCAGAGCT
+
FFFFFFFFFFFFFFFFFFFFFFFFFFFFFFFFFFFFFF:F:FFFFFFFFFF:FFFFFFFFFFFFFFFFF
FFFFFFFFFFFFFFFFFFFFFFFFFFFFFFFFFFFFFFFFFFFFFFFF

```
@A00721:190:HGJW7DSXY:4:2217:7545:34507_1:N:0:TCTGCAAG+GCTGCACT
GATGCATGGTGTTATCTCCTCAGGAGTCCCGGCATTTGATAATAATGACGAGACTTTCGTCATAATCG
CTGCATATTACGGATTACACGGTACCAGCGCATCTCGTGTCGAGCCTCGACCCGATTATATGGTATTA
ACT
+
FFFFF:FFFFFFFFFFFFFFFFFFFFFFFFFFFF:FFFFFFFFFFFFFFFFFFFFFFFFFFFFFFFFF
FFFFFFFF:FFFFF:FFF:FFFFFFFFFFFFFFFFFFFFFFFFFFFFFFFFFFFFFFFFFFFFFFFFF
FFF
@A00721:190:HGJW7DSXY:4:1152:23520:29230_2:N:0:TCTGCAAG+GCTGCACT
TCTAGAATTCTTAATTATAATATTCAACCTCAAAAGTTACGTGTTTACAGTAACGAGATCACACATTT
TGAGAACTAACTATGTTATGTTCTCTGATCATTTTGACAATCTCTTCT
+
FFFFFFFFFFFFFFFFFFFFFFFFFFFFFFFFFFFFFFFFFFFFFFFFFFFF:FFFFFFFFFFFFFFF
FFFFFFFFFFFFFFFFFFFFFFFFFFFFFFFFFFFFFFFFFFFFFFFF
@A00721:190:HGJW7DSXY:4:1516:18728:9377_2:N:0:TCTGCAAG+GCTGCACT
AAATAAATAATATTACTAATGGCAAATAGTGGAAAGGCTCCAGTCAACACTAGTTCGAGTGCTGCTTT
GGATTACGATGACGTGAGAATAGGTGAAGGGATAGTTTTTACCTCGAC
+
FFFFFFFFFFFFFFFFFFFFFFFFFFFFFFFFFFFFFFFFFFFFFFFFFFFFFFFFFFFFFFFFFFFF
FFFFFFFFFFFFFFF:FFFFFFFFFFFFFFFFFF:FFFFFFFFFFFFFFF
@A00721:190:HGJW7DSXY:4:1328:6171:12430_1:N:0:TCTGCAAG+GCTGCACT
TCTTAAAGCTTCACCCAAAATGAAGGACACTTCGAAACTGAATAATGATGCACTACAAACAAGTATCG
CTAATCATCTGAAAAAGGCTCTAAAGGACACTATACAATATCCAGACGACGACGAAGCTTATACAATA
TCC
+
:,FFFFFFFFF:FF:FF:FFFFFFFFFF:FFF:FFFFFF,FFFFFFFFFFFFFFFFFFFFFFFFF:F:F
FFFFFF:FFFFFFFFFFFFFFFFF:FFFFFFFFFFFFF:FFFFFFFFFFFFFFFFFFFFFFFFFFFFFFF
FFF
@A00721:190:HGJW7DSXY:4:1603:3929:34413_2:N:0:TCTGCAAG+GCTGCACT
ACCACATTTTGGTCAAGTCAATCCGTTGAGAAAATTTCTTAGAGGATATTCGCCATTGTGGTACGAAT
TGGTCCAACATTTACCACCAAATTCTTTTGATACAACTCTAGCCTTCA
+
FFFFFFFFFFFFFFFFFFFFFFFFFFFFFFFFFFFFFFFF:FFFFFFFFFFFFFFFFFFFFFFFFFFF:
FFFFFFFFFFFFFFFFFFFFFFFFFFFFFFFFFFFFFFFFFFFFFFFF
@A00721:190:HGJW7DSXY:4:1554:28158:23923_2:N:0:TCTGCAAG+GCTGCACT
ACCAGATTGGTTCGAACCCGTTGATGCTATGTCAGAAGAAGCTATTCAAGCTTGTCGTTTAAAGACTG
CAGAGGCTGTCAAACAAGCCCAGACTCAAGGAGTCCAATCAAAGGTTT
+
FFF:FFFFFFFFFFFFFFFFFFFFFFFFF:FFFFFFFFFFFFFFFFFFFFFFFFFFFFFFFFFFFFFF
FFFFFFFFFFFFFFFFFFFFFFFFFFFFFFFFFFFFFFFFFFFFFFFF
@A00721:190:HGJW7DSXY:4:1553:24388:26381_1:N:0:TCTGCAAG+GCTGCACT
TTATTTAGAGATCTTTTCTACTTACAAAGGACAATTCAGTTTCATTAATTCTACGGAAAAGTCCGGAG
TATATTGTATGTTAATAGATAGTGGGATAATAGTTCTACCGATACGTAGTGAACGCGTTCGTAGAACC
AAG
+
FFFF:FFFF:FFFFFFFFFFF:FFFFFFFFFFFFFFFFFFFFFFFFFFFFFFFFFFFFFFFFFFFFFF
FFFFFFFFFFFFFFFFFFFFFFFFFFFFFFFFFFFFFFFFFFFFFFFFFFFFFFFFFFFFFFFFFFFF
FFF
@A00721:190:HGJW7DSXY:4:1146:18756:11584_1:N:0:TCTGCAAG+GCTGCACT
ACCGATAGGAGTTGTATAGTTGAAGATGTGGCTTTAGACGATAATCCTTTAAGGACTATGTTGACTGA
CGAAGATTTTCCCACAGATTTGTCTACGTTGCAGATCGCCACTCCAAACACTATATACATTATAAAAT
ATA
+
FFFFFFFFFFFFF:FFFFFFFFFFFFFFFFFFFFFFFFFFFFF:FFFF:FFFFFFFFFFFFFFFFFFFF
```

```
FFFFFFFFFFFFFFFFFFFFFFFFFFFFFFFFFFFFFFFFFFFFFFFFFFFFFFFFFFFFFFFFFFFFFF
FFF
@A00721:190:HGJW7DSXY:4:2315:30445:2425_1:N:0:TCTGCAAG+GCTGCACT
CATAACTTTTGTTACCAAATTCTTGGCTTTCAACGAAGTCTCTGATTTTATTAATCATTACTTTAACG
ATCTCTCGGTCATTTACTCTGTACTTGATCTACGTCGTTTAACTGTCGGAACAACCTTTACTTAGAAT
GAC
+
FFFFFFFFFFFFFFF,FFFFFFF:F:FFFFFFFFFFFFF:FFFFFFFFFFFFFFF:FF:FFFFFFFFF
FFFFFFFFFFFFFFFFFFFFFFFFFFFFFFFFF:FFFFFFFFFFFFFFFFFFFFFFFFFFFFFFFFFF
FFF
@A00721:190:HGJW7DSXY:4:2557:29658:8891_2:N:0:TCTGCAAG+GCTGCACT
TTCTTATATTATTTACAATAGTTAATATCATATTAGTAATACTCACTATCGTTTTCTGCTTTTTCTTA
GCTAATAGATTAATAAAGGATGAGTACGAAAACCAACTCTTCGTTACG
+
FFFFFFFFF:FFFFFFFFFFFFFFFFFFFFFFFFFFFFFFFFFFFFFF:FFFFFFFFFFFFFFFFFFF
FFFFFFF:FFFFFFFFFFFFFFFFFFF:FFFFFFFFFFFFFFFFFFFF
@A00721:190:HGJW7DSXY:4:1453:15845:5259_1:N:0:TCTGCAAG+GCTGCACT
TAGAATATATTTAGTCTCTACACCAATCTAACCGCGTATAACGCGATTTTTCCGACCTGACCGTAATA
CACGAAGATATCATCCGCACACGACTCGAAACCGAGACGCCGTAGATCAAAGTCCCTTCTAACTTTAG
AT
+
F:,F,F,,:FFF,,,,::::,FFF,,,:F,F,F:,F,,:FF,,,,,:FFF:F,F,F,F,F,:,,FF::
,F:,:,,,,:,,,:FF:FF,FFFFFFFFFFFFFFFFFFFFFFFFFFFFFFFFFFFFFFFFFFFFFFFF
FF
@A00721:190:HGJW7DSXY:4:1642:1949:8030_1:N:0:TCTGCAAG+GCTGCACT
CATTTTATAGCAGTTATTTATGTTGTACTTTTTATAATTTATAACCTTCTTATACTTTTGGGTATGTT
TTGTGAAAAACATGAGATACCCAAGTCTATCGAAGACATTATCTCTTATGTACTTATTGTAACGATCA
TTC
+
FFFFFFFFFFFFFFFFFF,FFFFFFFFFF:FFFFFFFFFFFFFFFFFFFFFFFFFFFFFFFFFFFFFFF
FFFFFF:FFFFFFFFFFFFFFFFFFFFFFFFFFFFFFFFFFFFFFFFFFFFFFFFFFFFFFFFFFFFF
FFF
@A00721:190:HGJW7DSXY:4:2210:3170:17503_1:N:0:TCTGCAAG+GCTGCACT
TTCTTTTATTCCTTTAACTGTGTATGAGGAGGATTTATAATGCTTTTCGATAGTGATTGTTATCAATT
TAAATACATTACTGTTTCAAATCATCTTTACTCACAGACTCCGAAAATGGTTTCAAATGAGGTCAAAG
CTC
+
FFFFFFFFFFFF:FF,FFF:FFFFFFFFFFFFFFFFFFFFFFFFFFFFFFFFFFFFFFFFFFFFFFFF
FFFFFFFFFFFFFFFFFFFFFFFFFFFFFFFFFFFFFFFFFFFFFFFF:FFFFFFFFFFFFFFFFFFF
FFF
@A00721:190:HGJW7DSXY:4:2218:27914:4961_2:N:0:TCTGCAAG+GCTGCACT
AACTATTATCCGATTCTTCGAGGGTATCACTACCACTGATCTGCCGACTTTAGTCAAGGAACTTCAAT
TCCTTCGATGTTTATATTTTTCTATAAGGGAAAATAGCAATAATCAAA
+
FFFFFFFFFFFFFFFFFFFFFFFFFFFFFFFFFFFFFFFFFFFFFFFFFFFFFFFFFFFFFFFFFFFF
FFFFFFFFFFFFFFFFFFFFFFFFF:FFFFFFFFFFFFFFFFFFFFFF
@A00721:190:HGJW7DSXY:4:1540:27859:34178_2:N:0:TCTGCAAG+GCTGCACT
AACTTTATTTCAGAACTCTCTCCGATCACCTTGGTGTGATCTCCCGGAGGATCTTTTCAATAAGATGT
ATGATGATAATCTACTATTTTTAAATAGTGATATTCAGCGTATACTTG
+
FFFFFFFFFFFFFFFFFFFFFFFFFFFFFFFFFFFFFFFFFFFFFFFFFFFFFFFFFFFFFFFFFFFF
FFFFFFFFFFFFFFFFFFFFFFFFFFFFFFFFFFFFFFFFFFFFFFFF
@A00721:190:HGJW7DSXY:4:1610:2266:14810_1:N:0:TCTGCAAG+GCTGCACT
GGCTTGGTCTATTTACCGCAAAAAGTTGGAAGAGGTCTATGATATTTATTTTACTTATACTTTAAATA
```

GCACCGATTATAACTTGACTGTTGATAATATTACAATAGCTCTTTCTCAGAAGATTAGAGAAGTAACC
GGT
+
FFFFFFFFFFFFFFFFFF:FFFFFFFFFFFFFFFFFFFFF:FFFFFFFFFFF:FFFFFFFFFFFFF
FFFFFFFFFF,FFFFFFFFFFFFFFFFFFFFFFFF:FFFFFFFFFFF:FFFFFFFFFFFFFFFFFF
FFF
@A00721:190:HGJW7DSXY:4:1158:24469:13808_1:N:0:TCTGCAAG+GCTGCACT
GTAGCATTGATTTACTTTTACCGTTAGAACTGAAAAAGATTCTTTTCGAAAAGAAACCTATAAGTTAC
AAACGATTCTTCGACTATATTAAAGAAATAAAAATACTTCAAGTACCTGAACTTTACATTTTTAATGG
TAT
+
FFF:F,FF::FFFF::FF:::,::FF,FF:F:FFFFFFFFFF:FF:FFFFFFFF,F::FFFF,FFFFF
FFFFFF,FFFFFFF:FFFFFFFFFF:FFFFFFFFFF:FFF,FFFF:FFF::FFFF,:FFFFFF:FFFF
FFF
@A00721:190:HGJW7DSXY:4:1202:6343:18991_1:N:0:TCTGCAAG+GCTGCACT
TTACAAATAGAACCTTCACTCTCGGTAATGAGAGTGTATTCCTTAATATTCTAGTAACCGCTATGTGG
GCATTACTAGGATTTATCACACTCCTAAATTTGATAGCAATTTATGTTTTAAATGCTAGGAAAATTTT
AGA
+
FFFFFFFFFFFFFF,FFFFFFFFFFFFFFFFFFFFFFFFFFFFFFFFFFFFFFFFFFFFFFFFFFFFF
FFFFFFFFFFFFFFFFFFFFFFFFFFFFFFFFFFFFFFFFFFFFFFFFFFFFFFFFFFFFFFFFFFFF
FFF
@A00721:190:HGJW7DSXY:4:2364:16197:14700_1:N:0:TCTGCAAG+GCTGCACT
TGTATAGTTCAAGGAGTACTTAATTCAATAGCTTTAGTATTATTTACTATCGCCTTTATTGAAAATTG
TATAAGAGTTAATTGCTTACACAAAAGTGACGCGCGAAGGAATCTTAATTGATTGAAGCTAGGCGTTG
GGG
+
FFFFFFFFFFFFFFFFFFFFFFFFFFFFFFFFFFFFFFFFFFFFFFFFFFFFFFFFFFFFFFFFFFFF
FFFFFFFFFFFFFFFFFFFFFFFFFFFFFFFFFFFFF,FFF:,FFFFFFFFFFFFFFFFFFFFFFFFF
FFF
@A00721:190:HGJW7DSXY:4:2274:26485:27571_2:N:0:TCTGCAAG+GCTGCACT
CATACCATAACCTACCATGACCAATCATCGCTCCAACTGGCACGCCAGACGTAGGGTCAAACCTACTA
CCGCCGGTCGGCAGATCGGCACCACTGGTCTCTACGTTCAGGGACCCG
+
FFFFFFFFFFFFFFFFFFFFFFFFFFFFFFFFFFFFFFFFFFFFFFFFFFFFFFFFFFFF:FFFFFFF
FFFFFFFFF:FFFF:FFFFFFFFFFFFFFFFFFFFFFFFFF:FFFFFFFF
@A00721:190:HGJW7DSXY:4:2565:20835:33254_2:N:0:TCTGCAAG+GCTGCACT
TAACCTTCCCAAATCTAGATCTGCACAGAAGAAGGCTGAAGGTCGTTACCGGACGACCACTCAAAGGA
AACGGGCTGTTGCCCAGAGGAAAGCAGCGACCACCGAGGTCGCTCGTA
+
FFFFFFFFFFFFFFFFFFFFFFFFFFFFF:FFFFFFFFFFFFFFFFFFFFFFFFFFFFFFFFF:FFFF
FFFFFFFFFFFFFFFFFFFFFF,FFFFFFFFFFFFFFFFFFFFFFFFFF
@A00721:190:HGJW7DSXY:4:2569:14244:3051_2:N:0:TCTGCAAG+GCTGCACT
TCCCGTCATCTCTGTGGGAACTGAGGCAGTACCTCAGTCAAAAGTCACATTCACCACTCCCATGCCAA
TCAATGATGTGACTATACAGTTCGGCCATTTCAAGAGTGGTCAATACG
+
FFFFFFFFFFFFFFFFFFFF:FFFFFFFFFFFFFFFFFFFFFFFFFFFFFFFFFFFFFFFFFFFFFFF
FFFFFFFFFFFFFFFFFFFFFFFFFFFFFFFFFF:FFFFFFFFFFFFFF
@A00721:190:HGJW7DSXY:4:1361:11089:12680_2:N:0:TCTGCAAG+GCTGCACT
ACTGAAGCCTTCCCCTCCGCTGGCACGAAGAACACTCCCAAGCCAATCAATAGTTCTTCTGACCAGAA
TGAAAAGGGGTTTACCAGGGTAGTCTCTAAGTCTACCAGGAAGAGGGG
+
FFFFFFFFFFFFFFFFFFFFFFFFFFFFFFFFFFFFFFFF:FFFFFFFFFFFFFFFFFFFFFFFFFFF
FFF:F:FFFFFFFFFFFFFFFFFFFFFFFFFFFFFFFFFFFFFFFFFFF

```
@A00721:190:HGJW7DSXY:4:1621:26106:34522_1:N:0:TCTGCAAG+GCTGCACT
AAAACTGAGAAGAATAAAGGGAAAGGTCCATCAGGACCTAAGAAGGGGAAATTACCCCTAATTCTGAA
GGAAGATCTCCCACCATCTGGGGTTGGGAATAAGAAATCTGCACGGGCCAAAGAGGCCGCTGCTACGA
AGT
+
FFFFFFFFFFFFFFFFFFFFFFFFFFFFFFFFFFFFFFFFFFFFF:FFFFFFFFFFFFFFFFFFFFFF
FFFFFFFFFFFFFFFFFFFFFFFFF:FFFFFFFFFFFFFFFFFFFFFFFFFFFFFF:FFFFFFFFFFFF
FFF
@A00721:190:HGJW7DSXY:4:1324:17291:26271_1:N:0:TCTGCAAG+GCTGCACT
GATTGGGTCATTAAGGGTAGCTGTATAGTTGCGCCTGCCGACCCCATCTTTGAGACCAAGACTCTCTT
CAGTAGGTTCGTGCCTACTATGAGCAATTCTGTTGAATTCGTCACTGTGGACGAAGGTGAGAGGACTT
TTG
+
F,FFF:FF,,FF::F,FFFFFFFF,,,FF:FFF,:FFFFF:FF,,FF:FFF:F,:F,FFF:,F,F,F,
F,FF::FFF:FFF,:F,FFFFF:::FF,FFFFFF:,FFF::FF,:FF,FF:FFF,,FFFFF,F:FFFF
FF:
@A00721:190:HGJW7DSXY:4:2522:26838:22701_1:N:0:TCTGCAAG+GCTGCACT
CTGAAAGAGAATTGCGTGGGTTGAAGGCCGACGCTTCGAGCTTGAGTGTGACGAGGCCGGTGACGTTT
TTGTCAACGGCGTTTGGAGGATTTTATCCGCGCTTAACTATGGGGCGAAGATCCCCTATGGAGTCTTG
AAG
+
FFFFFFFFFFF,FFFFFFFFFFFFFFFFFFFFFFFFFFFFFFFFFFFFFFFFFFFFFFFFFFFFFFFF
FFFFFFFFFFFFFFFFFFFFFFFFFFFFFFFFFFFFFFFFFFFFFFFFFFFFFFFFFFFFFFFFFFFF
FFF
@A00721:190:HGJW7DSXY:4:2240:12726:33207_2:N:0:TCTGCAAG+GCTGCACT
GGCATATTTATGCCGACGATAATAAGCATGTCACCCTTAAGCACCGAGGTAAGCTCGGGACTTACAAG
AGAGAGTGTGAGAGAGTCGCTCTCACTATTTTTGCCCAGAATGCGGGC
+
FFFFFFFFFFFFFFFFFFFFFFFFFFFFFFFFFFFFFFFFFFFFFFFFFFF:FFFFFFFFFFFFFFFF
FFFFFFFFFFFFFFFFFFFFFFFFFFFFF:FFFFFFFFFFFFFFFFFF
@A00721:190:HGJW7DSXY:4:2220:10348:35289_2:N:0:TCTGCAAG+GCTGCACT
TCCTCCATAAGTACAACCTCTCTTTAGAGAGCATTAGAAGGAAGTTGGGCAAATTCCCTCATATGGTA
GACGTTGTCACACTTTTCTTGAAAATTTTTCATTTCCTCCGTCATTGG
+
FFFFFFFF,F,FFFFFFFFFFF,:F,,,F,:FFFFFFFF:,F:F,FFFFFFFF:F:FF,FFFFFFF,:
FFFF,FFFFFFFF:F:FFFFFFFF::,F,FF,FFFF,FFFFF::,,F,
@A00721:190:HGJW7DSXY:4:1406:13241:31344_2:N:0:TCTGCAAG+GCTGCACT
AAGGTGGAAGGTGTCAAGGGTCTTCTTCATGCTGATCTTTCAGGACCTAATCACATCATGATTGATAG
TTACGGCTCGTATCGAGTTGGAGGGAGGAAGTTCTCCGAAATTAAAGA
+
FFFFFFFFFFFFFFFF:FFFFFFFFFFFFFFFFFFFFFFFFFF:FFFFFFFFFFFFFFFFFFFFFFFFF
FFFFFFFFFFFFFFFFFFFFFFFFFFFFFFFF:FFFFFFF:FFFFFFFF
@A00721:190:HGJW7DSXY:4:1103:31421:11318_1:N:0:TCTGCAAG+GCTGCACT
TCAGAGAGGGGAGCTCTGAAAAGTAAACTTCGTCAGAAGATGGGGAAGTCTAAGAGGAGGAGTTTGAG
GAAGGCTCTAGAGAAGATCTCACCACCTGGAGAAGGCAAGTGGAGAGTCCAGAAGTTGATGAAGAAGG
ATT
+
FFFF,FFFF,F:FFFFFFFFFFF,:FFFFF,FF,FFFFFFFFFFFFFFF,FFFF,:FFF,FFFFFFFF
FFFFFFFFFFFFF:FFFFFFF,::FF:F:FFFFFFFF:FFFF:FFFFFFFFFFFFFFFFFFFFFFFF:F
F:F
@A00721:190:HGJW7DSXY:4:1120:27778:11741_2:N:0:TCTGCAAG+GCTGCACT
GTTACCACTTCCTAAGGTGGATGAGGGAGGTTGTTTCTTTGGAGTTTTAGATAATCTTTTGAGAAGGG
GTCATAGAGCCAGCAAGAGAAGCTTATATGCTGGTTTAAGTGATGATT
+
```

FFFFFFFFFFFFF,:FFFFF:FFFFFFFFFFFFFFFF,FFFFFFFFFFF:F:FFFFFF:FFFFF:FFF
FFF,FFFFFFFFFFFFFFFF:FFFFFF,FFF,FFF:FF:FFFF:FF,,
@A00721:190:HGJW7DSXY:4:2675:17219:33880_2:N:0:TCTGCAAG+GCTGCACT
TATAATTTAGGTCAACTTGGGGTTTCGAATGTCATTAGTGCCTTACGCTTGAAAGAGGTCCTTTTGAG
TATCGACAGATCTTTCGATCTTGAGGACGTACATTTAACGTGGCAGAA
+
FFFFFFFFF:FFFF:FFFFFFFF,FFFFFFFFFFFFF:FF:FFFFFFFFFFFFFFFFFFFFF:FFFF:
FFFF,FFF:FFFFFFF:FFFFFFFFFFFFFFFFFFFFF::FFFFFFFF
@A00721:190:HGJW7DSXY:4:2539:16676:16658_2:N:0:TCTGCAAG+GCTGCACT
ATATTGTTCCTTCCGGGAAGTTGTGGGATTTAACTTTGACTCAACTACTCCGATCAGATCCGGATGGT
TTGATTGGTATGGACTACTCGACTTTTGATCTGGAGTTGTCTAGGGTC
+
FFFFFFFFFFFFFFFFFFFFFFFFFFFF,FFFFFFFFFFFFFFFFFFFFFFF:FFFFFFFFFFFFFFF
,FFFFFFFFFFFFFFFFFFFFFFFFFFFFFFFFFFFFFFFF:FFFFFF
@A00721:190:HGJW7DSXY:4:2522:2130:28322_2:N:0:TCTGCAAG+GCTGCACT
AATATGCAGGTCAAAGATTCTGTAGCTCTGAGGGCGGTTGAGGGAGCCGTTTTGGCCGACTTTAACTA
TTCTAAGAGTCTTTATGATTCAAAACCTATCGTTGTCGTACCTTACAA
+
FFFFFFFFFFFFFFFFFFFFFFFFFFFFFFFFFFFFFFFFFFFFFFFFF:,FFFFFFFFFFFFFFF:F
FFFF:FFFFFFFFFFFFFFFFFFFFFFFFFFFFFFFFFFFFFFFFFFF
@A00721:190:HGJW7DSXY:4:1165:8341:12994_1:N:0:TCTGCAAG+GCTGCACT
CGCAGTTTCCAGAATTGCGGTTGAAGTTCACCCATTCTATCTTGAATGCTCACGCCGTCGCAGCCATA
TCGAGACTATGTTATAATCACTTGTTAAAGTACAGATTTAGTGATTTAGATATTATTGATATTGGAGG
CGA
+
FFFFFFFFFFFFFFFFFFFFFFFFFF,FFFFFFFFFFFFFFFFFFFFFFFFFFFFFFFFFFFFFFFFF
FFFFFFFFFFFFFFFFFFFFFFFF:FFFFFFFFFFFFFFFFFFFFFFFFFFFFFFFFFFFFFFFFFF
FFF
@A00721:190:HGJW7DSXY:4:2206:31801:6057_2:N:0:TCTGCAAG+GCTGCACT
ATCCAAAACTCCTTGAATGGAGTACATATTTGCAACCCTATTCTTGATGTGAAAGATGCATCGAGGTA
TACGTCTAGGAACATTGATTGGGAACGTACCTACTCACGAGACGTCAT
+
FFFFFFFFFFF:FFFF:FFFFFFFFFFFFFFFFFFFFFFFFFFFFF,,F:FFF:FF:FFFFFFFFFFF
FFFFFFFFFFFFFFFFFFFFFFFFFFFFFFFFFFFFFF:FFFFFFFFF
@A00721:190:HGJW7DSXY:4:2670:20907:11835_1:N:0:TCTGCAAG+GCTGCACT
ATGTTTCGTTCTGTTACAAGAACGCTTTGGATTGTGACCATAAAGCCTCAGTTGCGATAGCTGTAGAG
GTATACGATATCTCTCTTGATGATGTAGCCACTATAATGCTTAAGAGAGAGATTGATGTATGTCATTT
CGT
+
FFFFFFFFFFFFFFFFFFFFFFFFFFFFFFFFFFFFFFFFFFFFFFFFFFFFFFFFFFFFFFFFFFFF
FFFFFFFF:FFFFFFFFFFFFFFFFFFFFFFFFFFFFFFFFFFFFFFFFFFFFFFFFFFFFFFFFFFF
FFF
@A00721:190:HGJW7DSXY:4:2367:15456:6277_1:N:0:TCTGCAAG+GCTGCACT
GAATTATTGGACACGATGAAAAATGACATACCCGTTCCTGACCTTGGTATCGTTATCCGTCATGGAGA
TGATGGCGCATCACTTGATTACTATATGCCCGAAGGATTGGGTTATTCTCACAACAAAGATAAGATTT
TAT
+
FFFFFFFFFFFFFFFFFFFFFFFFFFFF:FFFFFFFFFFFFFFFFFFFFFFFFFFFFFFFFFFFFFFF
FFFFFFFFFFFFFFFFFFFFFFFFFFFFFFFFFFFFFFFFFFFFFFFFFFFF:FFFFFFFF:FFFFFF
FFF
@A00721:190:HGJW7DSXY:4:2349:31937:15499_1:N:0:TCTGCAAG+GCTGCACT
TAAGGGCGGACAATTGTTCCATTGTGAACTCTTGGGGACTAGATTGAGCATTAATGAGTTCCGAATAA
CTCGTAATAAATCTTTCTCTAGTGGACAATTCAGAATACCTATTTCCGTACCAAGAGCCACTGCCAAT
CTC

```
+
FFFFFFFFFFFFFFFFFFFFFFFFFFFFFFFFFFFFFFFFFFFFFFFFFFFFFFFFFFFFFFFFFFFF
FFFFFFFFFFFFFFFFFFFFFFFFFFFFFFFFFFFFFFFFFFFFFFFFFFFFFFFFFFFFFFFFFFFF
FFF
@A00721:190:HGJW7DSXY:4:1445:15989:22764_2:N:0:TCTGCAAG+GCTGCACT
AAATTTTTCTCAAGAGATTATGGTTGATAAAGTTTTCTTCATAAGGGCTATTCAGTACGTCATGGGGA
TCACTTCTTCGGTCAATGACAAAACCTTCGAATATGTTACGACATGGC
+
FFFFFFFFFFFFFFFFFFF,:FFFFFFFFFFFFF:FFFFFFF,FFFFFFF:FFFFFFFFFFFFFFFFF
FFFFFFFFFFFFFFFFFFFFFFFFFFF:FFFFFFFFFFFFFFFFFFFF
@A00721:190:HGJW7DSXY:4:2459:31150:5588_2:N:0:TCTGCAAG+GCTGCACT
AGGATTGTCATCTCGGGGAAAGTGATTCACCAAGATGTTCATATAGATACGAGATACCTAGAGAAGGT
CGCAGCGATCTTACTCGTTATCGGAGCTAAGAAGAAGACTTTGGGTAC
+
FFFFFFFFFFFFFFFFFFF:FFFFFFFFFFFFFF,FFFFFFFFFFFFFFFFFFFFFFFFF:FFFFFFF
FFFFFFFFFFFFF:FFFFFFFFFFFFFFFFF,,FFF::::FFFFFFFF
@A00721:190:HGJW7DSXY:4:2220:6659:1595_2:N:0:TCTGCAAG+GCTGCACT
TAACGTTAGTACCGGAGGTGATCTCTCTGCTTCTGCGTTACTGAGAGAAAAGATTCAGGACGCCTACG
GTGACTTTATGGACAAGATGCAAGTTCTTGGGAAATACTTGTTTCCCT
+
FFFFFFFFFFFFFFFFFFFFFFFFFFFFFFFFFFFFFFFFFFF:FFFFFFFFFFFF:FFFFFFFFFFF
FFFFFF,FFFFFFFFFFFFFFF,FFFFFFFFFFFFFFFFFFFFFFFFF
@A00721:190:HGJW7DSXY:4:1354:5665:33880_1:N:0:TCTGCAAG+GCTGCACT
CGTGTGCTCTTCCGATCTAACGAATCTTGAAACTACGGTCCTTATCAAAGAAAATGGTGAACCGGTCT
CTAAATCTTTTGAGATTGAGAAGATTGAAGAACACGTCAAGAAGTCGCTCGGCGTTGTTCTTGAAAAC
CAA
+
FFFFFFFFFFFFFFFFFFFFFFFFFFFFFFFFFFFFFFFF:FFFFFFFFFFFFFFFFFFFFFFFF:FF
FFFFFFFFFFFFFFFFFFFFFFFFFFFFFFFFFFFFFFFFFFFFFFFFFFFFFFFFFFFFFFFFFFFF
FFF
@A00721:190:HGJW7DSXY:4:1363:29351:10332_1:N:0:TCTGCAAG+GCTGCACT
GCTGATCATGGAGTCTTTGTACCTTCTGTCAAACTTCGAGGTTCAGCTACGAGAGGTGATCCCGGATG
TGGATTGAAGGCTGGCTCTAAGGGAGACTCCTTCTTTTTGAATGTTGTAAAGAATTGGGTTGAGAAGT
GTG
+
F:,F:FF,FF,,FFFF:FF,F:FF,FFFF:,F:FFFFFFFFFFFFFFFFFFFFFFFF:FFFFFFFFFF
F:F,FFFFFFFFFFFFF:FFFFFFFFFFFFFFF:FFFFFFF,F::FFF:F:FFFFF,FFFFFFFF:FF
FFF
@A00721:190:HGJW7DSXY:4:1260:3061:9204_1:N:0:TCTGCAAG+GCTGCACT
CTTGGTATTTCTGAAATAGTTTATCAGAGCGTCATTTTCCTTTGGGATCTTTCCTCTGGTAAGGGAAT
TATTAAATCATTCTTCTCACTCTTGACGAGAATTATTACTGTTTTCTCTACCCATAACTTCTTTTTCA
AAA
+
FFFFFFF,F:FFF:FF:FFFFF:FFFFFFFFFFFFFFFFFFFFFFFFFFFFFFFFFFFFFFFFFFFFF
FFFFFFFFFFFFFFFFFF:FFFFFFFFFFFFFFFFFFFFFFFFFFFFFFFFF:FFFFFFFF:FFFFFF
F:F
@A00721:190:HGJW7DSXY:4:2553:4978:11960_1:N:0:TCTGCAAG+GCTGCACT
CTGATGTTTTCCTACAGAGTGCAGTCGTTTCAACACTTATCACCACTTTTGAAAGGAAAATAATTGGT
GATGAAAATACGTATGTCGGTGGGGTTCTCAAGGCTCTCATCGCCACTGGGATTCTAAGGGGTTCCAT
AAG
+
FFF:FFFFFFFFFFFFFFFF:FFFFF,FFFFFFFFFFFFFFFFFFFFFFFFFFFF:FFFFFFFFF:FF
FFFFFFFFFFFFFFFFFFFFFFF::FFF:FFF:FFFFFFFFFFFFFFFFFFFFFFFFFFFFFFFFFFF
FFF
```

```
@A00721:190:HGJW7DSXY:4:1502:24542:6073_2:N:0:TCTGCAAG+GCTGCACT
TTTGGTCCTTACGTCTTAAGTAATTTGGATGTTGTCGAAAACTATCAGGTTCTTGTCACGATTCTTAA
AAGAAGTTTATTAAAACTTCAGAGTCTCTTGGAAGTCCAGGTAGCTAA
+
:FFFFFFFFFFFF:FFF,FFFF:FFFFFFFFFFFFFFFFFFFFFFFFFFFFFFFFFFFFFFFFFFFFF
FFF,FFFFFFFFFFF,FFFFFFFFFFFFFFFFFFFFFFFFF:FFFFFF
@A00721:190:HGJW7DSXY:4:2307:24668:8735_2:N:0:TCTGCAAG+GCTGCACT
GGGAAAAGAGACCTCTGTTGGTAGAGGAGTTGAGAGAATTACTCAGATTCAATCTGGCATCACTGAAC
GTCTACAACGAGTTAGGGAGTGGCTAGGTTTTAAAAGATCCTTTGCAC
+
FFFFFFFFFFFFFFFFFFFFFFFFFFFFFFFFFFFF:FFFFFFFFFFFFFFFFFFFFFFFFFFFFFFF
FFFF:FFFFFF:FFFFFFFFFFFFFFFFFFFFFFFFFFFFFFFFFFFF
@A00721:190:HGJW7DSXY:4:2144:23095:19006_2:N:0:TCTGCAAG+GCTGCACT
ACGTCGCGGAAAACGATGAAGATTTCCACACTGTTCGAGAATCTTCTGATGCTGAATTTGAGGAAAAT
TTTTCGGAAGTTTTTGGTATAAATCCTGATGGCTCAGTACAGGGAGGA
+
FFFFFFFFFFFFFFFFFFFFFFFFFFFFFFFFFFFFFFFFFFFFFFFFFFFFFFFFF:FFFFFF,FFF
FFFFFFFFFFFFFFFFFFFFFFFFFFFFFFFFFFFFFFFFFFFFFFFF
@A00721:190:HGJW7DSXY:4:2445:1217:11021_1:N:0:TCTGCAAG+GCTGCACT
AACCTTCTTTTATCATTGCAACAGGACTTTGAGAAGACTGTTGAAATGGACAAAATCTGGGATCATAG
GTATGTGTCACGCTACACTCGAAATATGGGCCCTCATAAGGAAGTATAGTTGGCGTCTTGTCGTTGAC
GTC
+
FFFFFFFFFFFFFFFFFFFFFFFFFFFFFFFFFFFFFFFFFFFFFFFFFFFFFFFFFFFFFFFFFFFF
FFFFFFFFFFFFFFFFFFFFFFFFFFFFFFFFFFFFFFFFFFFFFFFFFFFFFFFFFFFFFFFFFFFF
FFF
@A00721:190:HGJW7DSXY:4:2120:12744:36839_1:N:0:TCTGCAAG+GCTGCACT
TGTTCAGGAAGATCTCTACAGAGATTATCGAGAGTTGTGATCTAGTTCTAAATTTAGGTCGACGGACT
TTAGCAACTTTAGATAAATTCTTTCCGGGCTCAATGACCTTTGCTATGTACGTGGATCTAATGCTTGG
TGA
+
FFFFFFFFFFFFFFFF:FFFFFFFFFFFFFFFFFFFFFFFFFFFFFFFFFFFFFFFFFFFFFFFFFFF
FFFFFFFFFFFFFFFFFFFFFFFFFFFFFFFFFFFFFFFFFFFFFFFFFFFFFFFFFFFFFFFFFFFF
FFF
@A00721:190:HGJW7DSXY:4:2514:13838:30060_2:N:0:TCTGCAAG+GCTGCACT
TATTAGTTCATGAAGTTCTTACACTTCTCGCGACTAATGTCAGCTTCACTACAAAGGCGTTACGTAAA
GTAGTTTTCACGCTTTATCTTAAAGCATTACCACCAAATTTAGCACTT
+
FFFFFFFFFFFFFFFFFFFFFFFFFFFFFFFFFFF:F:FFFFFFFFFFFFFFFFFFFFFFFFFFF:FF
FFFFFFFFFFF:F,FFFFFFFFFFFFF:FFFFFFFFF,FFFF:FFFFF
@A00721:190:HGJW7DSXY:4:2608:14687:3129_2:N:0:TCTGCAAG+GCTGCACT
TCGCCATATCAGATTAGAGACATGCCAGTTAGAGTTCTTCTAACCAGGACTGATGGTGAGATTCTTTT
ACCTTTTGGAGGTCCAGGAACCTTTACTCCTAGTGTCTTTTCTAGAGT
+
FFFFFFFFFFFFFFFFFFFFFFFFFFFFFFFFFFFFFFFFFFFFFFFFFFFFFFFFFFFFFFFFFFFF
FFFFFFFFFFFFFFFFFFFFFFFFFFFFFFFFFFFFFFFFFFFFFFFF
@A00721:190:HGJW7DSXY:4:1605:9507:9345_2:N:0:TCTGCAAG+GCTGCACT
CGGAGGATGACAGGTGGGAGACCGCTCAATAACACTCTATTGAATCCCTTTTCCACGATGAAAGCTCT
TTATGAGGTGCTCCATGCAAATTTACAGTTCCTCTTTGGTGATATCGC
+
FFFFFFFFF:FFFFFFFF:FFFFFFFFFFFFFFFF:FFFFFFFFFFFFFFFFFFFFFFFFFFFFFFFF
F:FFFFFFFFFFFFFFFFFFFFFFFFFFFFFFFFF,FFFFF:FFFFFF
@A00721:190:HGJW7DSXY:4:1605:9507:9345_1:N:0:TCTGCAAG+GCTGCACT
TTTTGATAAATTTGTTGGAATCGATAATGATACTGACGAAGGTTCTATTACCACCCTTGTCATTGATA
```

CAAACATTCTAGGTGATGCTTTCTTAGAGGCTCGTCTAGCCGCTGAGATTCGTCTTACCTATTTACAA
TCT
+
FFFFFFFFFFFFFFFFFFFFFFFFFFFFFFFFFFFFFFFFFFFFFFFFFFFFFFFFFFFFFFFFFFFF
FFFFFFFFFF,FFFFFFFFFFFFFFFFFFFFFFFFFFFFFFFFFFFFFFFFFFFFFFFFFFFFFFFF
FFF
@A00721:190:HGJW7DSXY:4:1510:10737:5337_1:N:0:TCTGCAAG+GCTGCACT
GATAACGATTTAGTACTGGGAAATGGTGGTAGTCAGTTTACTACTTTTCTTTCCCTCCCCTTTTTCGA
TTGGATTCGACGGGGTCTTCGTCTCACGACTAATTTGATAAGGTTTTTCTTTTATGGGACTCTTGTTT
GTA
+
FFFFFFFFFFFFFFFFFFFFFFFFFFFF:FFFFFFFFFFFFFFFFFFFFFFFFFFFFFFFFFFFFFFF
FFFFFFFFFFFFFFFFFFFFFFFFFFFFFFFFFFFFFFFFFFFFFFFFFFFFFFFFFFFFFFFFFFFF
FFF
@A00721:190:HGJW7DSXY:4:2666:22453:6308_2:N:0:TCTGCAAG+GCTGCACT
AAGTTCTTAATGTAGGTCTCCTAGTACTTAAGGGTTTATGTTTCGGTTTCAGCTGGATGGATCTTTTG
ATGGTATGTTTCACATTGCGTGATCACTTCCCGTGGACTTGGGAAGAG
+
FFFFFFFFFFFFFFFFFFFFFFFFFFFFFFFFFFFFFFFFFFFFFFFFFFFFFFFFFFFFFFFFFFFF
FFFFFFFFFFFFFFFFFFFFFFFFFFFFFFFFFFFFFFFFFFFFFFFF
@A00721:190:HGJW7DSXY:4:1330:7184:4100_1:N:0:TCTGCAAG+GCTGCACT
CGATTCTATTATGTCGCTAAAGCCTGTCCAGTTGTCGATGATGTTCGGGCTGTGGGGATTAACTTCCC
TCGTGAAACTTACGACGATGTTCTCGCTCGCATTTCACAAGGATTACTAAGTCTGAGATCTGCCACTT
CTA
+
FF,FFFFFFF:FF:,F:FF,F,,:FFFF:F:FF:FFF,F,:FFFFF,FF,FFF,F,,FFFFF:FFFFF
FF,F:F,F,FFF:F:F:FFFFFFF,FFF:FFFFFFFF:FF:F,F,FFFFFFFFF:F:FFFFFFFFFFF
FF:
@A00721:190:HGJW7DSXY:4:2109:28718:36135_1:N:0:TCTGCAAG+GCTGCACT
TCTGTTCATGAGGATGTCTTGGTCGAAAAATATCGCCGGGAACTTCTAATAGAGGATCCTGATGACAT
TATTTCAACGGATACCGATCTCAGTTCTACCACAGAAACCGTCTCAGGAACTGTCTCCACTGATTCGG
AAT
+
FFFFFFFFFFFF,FFFFFFF:FFF:FFFFFFFFFFFFFFFFFFFFFFFFFFFFFFFFFFFFFFFFFFF
FFFFFFFFFFFFFFFFFFFFFFFFFFFFFFFFFFFFFFFFFFFFFFFFFFFFFFFFFFFFFFFFFFFF
FFF
@A00721:190:HGJW7DSXY:4:1545:25337:12524_2:N:0:TCTGCAAG+GCTGCACT
CGACCTTCTTGTGGGGACTGAGACCTCTGAATTTTCAGGGTGCCCTGATGAGGACGTTGAAAGGGATA
ATTTCAACTCTGAGACTTCTAAGAGTGTTTCAAAAGACCAGAAACGTA
+
FFFFFFFFFFFFFFFFFFFFFFFFFF:FF:FFFFFFFFFFFFFFFFFFFFFFFFFFFFF,FFFFFFFF
FFFFFFFFFFFFFFF:FFFFFFF:FFFFFFFFFFFFFFFFFFFFFFFFF
@A00721:190:HGJW7DSXY:4:1676:1398:20666_1:N:0:TCTGCAAG+GCTGCACT
AAAGGTAGGAAGGGGAGAAAACAGAACAAGCGAGCGGTTGAAGAGAAAACTGATATGGTTGCGGCTGA
TCGTACCCCAATTCCTGCTAGAAAGATCACTCTCGGCGATACCACTGATCTTCCAGAGTTACGTAGTG
GTC
+
F,FFF:FFFFFFFFF:FF:F:FFF:FF:FFFF:FFFFFFFFFFFFF,FFFFFFFFFFFFFFFFFFFFF
FFFFFFFFFF:FFFFFFFFFFF:FFFFFFFFFFFFFFFFFFFFFFFFFF,FFFFFFFFFFFFFFFFFFF
FFF
@A00721:190:HGJW7DSXY:4:1561:30373:22905_1:N:0:TCTGCAAG+GCTGCACT
TTTTATCAAAGAGAAAGTTTCTGCTAGTCGCGACGATAGTATTTATCGGAGTGATATTAACAAGTTAA
ACTTCGAACTGACCACTTACAAGAGTGTTGAAGTACCGCAACATGGATCAGTTTTCCAAAAAGCTATT
AAT

+
FFFFFFFFFFFFFFFFFF:FFFFFFFFFFFFFFFFF,FFFFFFFFFFFFFFFFFFFFFFFFFFFFFFF
FFFFFFFFFFFFFFFFFFFFFFFFFFFFFFFFFFFFFFFFFFFFFFFFFFFFFFFFFFFFFFFFFFFF
FFF
@A00721:190:HGJW7DSXY:4:1654:18936:23516_1:N:0:TCTGCAAG+GCTGCACT
ACTGAATCCACTCTCTTGGGATATAAATGAGTACATCTATAATAGAGTTTTGGAACTGTGTAATGTCC
TACGTACTGTTCAAGGTGTTTGGGCTTCTCTAAAGAACAGCAACTTTCAACATGAATTAGTTATTGAA
AGT
+
FFFFFFFFFF,FFFFFFF:FFFFFFFFFFFFF:FFFFFFFF:FFFFFFFFFFFFFFFFFFFFF:FF:F
FFFFFFFFFFFFFFFFFFFFFFFFFFFFFFFFFFFFFFFFFFFFFFFFFFFFFFFFFFFFFFFFFFFF
FFF
@A00721:190:HGJW7DSXY:4:2542:25907:1924_1:N:0:TCTGCAAG+GCTGCACT
AACCTTTCCTCGTTCTTCATGAAGATCTTCGAGTGTTTATAGTGAGTTATTCTTGCGAAGGTTCAAG
TCGATTGATTTTGTTGACTACGATCTCTCTTGTAATAAGAAGTTCTTCGCAGTTGAATCTCCACCAGG
TGG
+
FF:FF,FFFFFFF:FFFFFFFFFFFFFFFFFFFFFFFFFFFFFFFFF,FFFFFFFFFFFFFFFFFFFF
F:F:FFFFFFFFFFFFFFFFFFFFFFFFFFFFFFFFFFFFFF:FFFFFFFFFFFFFFFFFFFFFFFFFF
FFF
@A00721:190:HGJW7DSXY:4:2226:27181:14121_2:N:0:TCTGCAAG+GCTGCACT
GCGACTTTTGTCGAATTTTTCGCAAAAGGTCTTGAAGTACGTATTGTCACTGCTAATCGTAATTCGCG
AGAAGAGATAGTGACTAAAGTTTTGGCACTTCTAGTTCATCGCGGTAT
+
FFFFFFFFFFFFFFFFFFFFFFFFFF:FFFFFFFFFFFFFFFF:FFFFFFFFFFFFFFFFFFFFFFFFF
FFFFFFFFFFFFFFFFFFFFFFFFFF:FFFFFFFFFFFFFFFFFFFFF:
@A00721:190:HGJW7DSXY:4:2452:26946:12336_2:N:0:TCTGCAAG+GCTGCACT
CGTGAAAGTTATGATGAGTGATCGTATTAGAACAGCTGATTCTTATTGCATGAACGTCGCTGAGGTCA
AACCTGTGGACGTTCTTTTCATCGACGAATTATTCATGATTCACGCTG
+
FFFFFFFFFFFFFFFFFFFFFFFFFFFFFFFF:FFFFFFFFFFFFFFFFFFFFFFFFFFFFFFFFFFF
F:F:FFFFFFFFFFFFFFFFFFFFFFFFFFFFFFFFFFFFFFFFFFFF
@A00721:190:HGJW7DSXY:4:2206:22761:10723_2:N:0:TCTGCAAG+GCTGCACT
CATCTACTTAAGGCGGCTTATACCATAGGTTACGGTGATTCCAATCAAATCGGATTCATCAATCGTAT
TGATCTCCAAGTCGCAAACTACTTCAACATCAACGACATCATTCTTGA
+
FFFFFFFFFFFFFFFFFFFFFFFFFFFFFFFFFFFFFFFFFFFFFFFFFFFF:FFFFFFFFFFFFFFF
FFFFFFF:FFFFFFFFFFFFF:FFFFFFFF:FF::FFFF:F:FFFFFF
@A00721:190:HGJW7DSXY:4:1112:30644:9283_1:N:0:TCTGCAAG+GCTGCACT
TTATCGAAGTCTCAGTTATCGCTGCCCGGCCGACGTTTGTCGCGAACTCTCAGAGGTTTACGGCAGAA
AAATTTCTCCTGCTTGTAATTCGAGAGAGGACACTATGGAAATTAGACCTATCAGATGTCTTGACGAC
TTC
+
FFFFFFFFFFFFFFFFFFFFFFFFFFFFFFFFFFFFFFFFFFFFFFFFFFFFFF:FFFFFFFFFFFFF
FFFFFFFFFFFFFFFFFFFFFFFFFFFFFFFFFFFFFFFFFFFFFFFFFFFFFFFFFFFFFFFFFFFF
FFF
@A00721:190:HGJW7DSXY:4:2515:4833:17409_2:N:0:TCTGCAAG+GCTGCACT
TATTACTTTCACTCAAGGTGAAAAGTTTGAAATTAACAAAGTCCTTGGTAAGAGTAAGGTTAAATGTG
AAGATGCTCAGACTATTCATGAAGTTCAAGGTAATACTTACCAGCACG
+
FFFFFFFFFFFFFFFFFF:FFFFFFFFFFFFFFFFFFFFFFFFFFFFFFFFFFFFFFFFFFFFFFFFF
FFFFFFFFFFFFFFFFFFFFFFFFFFFFFFFFFFFFFFFFFFFFFFFF
@A00721:190:HGJW7DSXY:4:1646:10899:18865_1:N:0:TCTGCAAG+GCTGCACT
ACAAACACATATTACCGTTGCTCTATCACGACACGTTGAATCTTTACGATACTTTGTTATCAATGTAA

```
AGTTAAACGATGCTACTTGTGAGAGGATCAATCACAGTAAGGAACTGTCACGTTTAGCACGGGAGGTT
GCA
+
FFFF:FFFFFF:FFFFFFFFFFFFFFFFFFFFFFFFFFFFFFFFFFFFFFFFFFFFFFFFFFF:FFFF
FFFFFFFFFF:FFFFFFFFFFFFFFFFFFFFFFFFFFFFFFFFFFFFFFFFFFFFFFFFFFFFFFFFF
FFF
@A00721:190:HGJW7DSXY:4:1358:4354:19993_2:N:0:TCTGCAAG+GCTGCACT
TATATCGAGGCTATCGAGGACGATGAGTATCTTGAAGTTGTTGACGACAAGCCTCAAGAGGGTAGAGG
ACCACCTGGATCTTCTACTGTCTCTGCCTTCCAAGATTTCTTAGAAGA
+
FFFFFFFFFFFFFFFF::FFFFFFFFFFF:FF:FFFF,FFFFFFFFFFFF,FFFFFFFFF,FFF:FFF
FFFFFFFFFFF,:FFFFF,F::FFFFF,FFF,FFFF,FFFF,:FFF:F
@A00721:190:HGJW7DSXY:4:2472:2745:1485_1:N:0:TCTGCAAG+GCTGCACT
AACCACCTTAACGTCACTCGGTAATCTTTCAGCCGAATTAAGTACTAATGAGTTCAAGTCGGTTGTCG
ATCGAATTAAGATCAAGGCTAATACCTTTATAGGTAAGAAGCCCCTTGAGCCGCGTAGTCAGCGCGTT
TAG
+
FFFFFFFF:F,FFFFFFFFFFFFFFFFFFFFFFFFFFFFFFFFFFFFFFFFF,FFFFFFFFFFFFF:F
FFFFFFFFFFFFFFFFFFFFFFFF,FFFFFFFFFFFFFFFFFFFFFFFFFFFFFFFFFFFFFFFFFFF
FFF
@A00721:190:HGJW7DSXY:4:2639:16432:3051_1:N:0:TCTGCAAG+GCTGCACT
TACCGAGTCTCCAAGAGAATATTTATTCTTATGAGGCTCGAAACTTCAACGTTTTCACAACGGAAAGA
CATATCGCACCCGAAATTTTTGGGCAGAATGCTCTTAACGCGGTTCTTGAACGGTGTTTTGATCAGTC
TAA
+
:FFFFFFFFFFFFFFFFFFFFFFFFFFFFFFF:FFFFFFFFFFFFFFFF,FFFFFFFFFFFFFFFFFFF
FFF,FF:FFFFFFFFFFFFFFFFFFFFFF:FFFFFFFFFFFFFFFFFFFFFFFFFFFFFFFFFFFFFF
FFF
@A00721:190:HGJW7DSXY:4:1623:2049:36542_1:N:0:TCTGCAAG+GCTGCACT
ATAGGAGACTCAGTTAACCTGAGAGCTCCTCAATTGATAGATTGGGTCAACAATAGGGACGCTAGTAT
ACTTCGTAAAGTCGACAGCGAAATGGTCAATATTGATAATTGGACCGACTATATGACCTATTTTAAGC
TCA
+
FFFFF:FFFFFFFFFFFFFFFFFFFFFFFFFFFFFFFFFFFFFFFFFFFFFFFFFFFFFFFFFFFFFF
FFFFFFFFFFFFFFFFFFFFFFF:FFFFF:FFFFFFF:F:FFFFFFFFFFFFFFFFFFFFFFFFFFFFF
FFF
@A00721:190:HGJW7DSXY:4:1577:10719:10066_2:N:0:TCTGCAAG+GCTGCACT
CTAAGGTTAAACTTGATGCTTCAGCCTTTTCGAAGCATAACCCCGCACAGAATATCGTTTATCACGCA
AAATTTATTAATGCAATTTTCGGACCTATCTTTAAGGTTCTTGTGAGG
+
FFFFFFFFFFFFFFFFFFFFFFFFFFFFFFFFFFFFFFFFFFFFFFFFFFFFFFFFFF:FFFFFFFFF
FFFFFFFFFFFFFFFF,FFFFFFFFFFFFFF:FFFFFFFF:FFFFFFF
@A00721:190:HGJW7DSXY:4:2428:10176:18490_1:N:0:TCTGCAAG+GCTGCACT
CAGTGACGATTTCTCCCGGAAGCTCTTCTCTCTCTTAGGAGGTCATGATGACTACTATAAGGGTGAAA
TCGATTTTTCGAAGTTTGACAAATCTCAAGATTTCTTTTGTAAAGCCTATGAGATAAAGATGTATTCT
TAT
+
FFFFFFFFF:FFFFFFFFFFFFFFFFFFFFFFFFFFFFFFFFFFFFFFFFFFFFFFFFFFFFFFFFFF
FFFFF:FFF:FFFFFFFFFFFFFFFFFFFFFFFFFFFFFFFFFFFFFFFFFFFFFFFFFFFFFFFFFF
FFF
@A00721:190:HGJW7DSXY:4:2505:19895:8202_2:N:0:TCTGCAAG+GCTGCACT
AACTTAGGAGTCCAAGTGAATATTTTTGACACGCCATGAGTATCCATAACGATTAGTCAATTACCACC
ATTGATCAACGACGAAGTGGAACGGCCAACACTTTATTTGGTAATTGT
+
```

```
FF,F,,:F,F:,F,,:F,FFFF:F,F,,,FFF,FF,,,:,,F,F:FFFFF,:FF,,,FF,,FFF,FFF
,F,,,FF,F,FFF,FFFFF,FF,FFF,FF,FFFFF,:FFFFFFFFF,,
@A00721:190:HGJW7DSXY:4:1441:10556:33677_1:N:0:TCTGCAAG+GCTGCACT
GATGATTTTATCACTTTATTATGACCTTGGAAAGTTTTCGGCTGTGGTTGTCGCTGGGGACGATTCCA
TTCTGTTTTCAAAGACTCCAATAACTGATCATTCTGATCATATGATTTTGGACAATGGTTTCGAAGCG
AAA
+
FFFFFFFFFFFFFFFFFFFFFFFFFFFFFFFFFFFFFFFFFFFFFFFFFFFFFFFFFFFFFFFFFFFF
:FFFFFFFFFFFFFFFFFFFFFFFFFFFFFFFFFFFFFFFFFFFFFFFFFFFFFFFFFFFFFFFFFFF
FFF
@A00721:190:HGJW7DSXY:4:1369:24144:32315_1:N:0:TCTGCAAG+GCTGCACT
CTTTACAAGATCATCGTTAAGTTAGGTAAACCCCTAACCGATGTTACTCTTGTAGGACTTTGGGAGAG
ATTCGTTAGTTTTAAAGACTATACTAAATATCTCCACTACGAGGTTGTTGTTTGGCGATTAGCTCTTG
CGA
+
FFFFF::FFFFFFFFFFF::FFFFFFFFFFFFF:FFFFFF,FFFFFFFFFFFFFFFFFFFFFFFFFFF
F:FFFFFFFFFFFFFFFFFFFFFFFFFFFFFFFFFFFF:FF:FFFFFFFFFFFFFFFFFFFFFFFFFF
:FF
@A00721:190:HGJW7DSXY:4:2638:10610:7811_2:N:0:TCTGCAAG+GCTGCACT
ATTGAGGGTCGTTATACCTATTCGGCTATCTGTGCTTTGAATTGTATAAGATCAAATTTTAAACGATT
CCGAGAGGTTTATCCTGATGTGGAGGGAGTTCTTGAAGCATCCCGACC
+
FFFFFFFFFFFFFFFFFFFFFFFFFFFFFFFFFFFFFFFFFFFFFFFFFFFFFFFF:FFFFF:F:FFF
FFFFFFFFFFFFFFFFFFFFFFFFFFFFFFFFFFFFFFFFFF:FFFFF
@A00721:190:HGJW7DSXY:4:2260:25500:16877_2:N:0:TCTGCAAG+GCTGCACT
GGTTTCATAAGATTATCAGTAATCCTGATTCTACTACGGATTCATGGACCTTCCATCCTGTACCTCTT
CTGGAGAGACGTATCTCAAAGATCGACTTCGAGACAGGTGATATTGTT
+
FFFFFFFFFFFFFFFFFFFFFFFFFFFFFFFFFFFFFFFFFFFFFFFFFFFFFFFFFFFFFFFFFFFF
FFFFFFFFFFFFFFFFFFFFFFFFFFFFFFFFFFFFFFFFFFFFFFFF
@A00721:190:HGJW7DSXY:4:1449:10004:30373_1:N:0:TCTGCAAG+GCTGCACT
ACCAAGATCTAAATTCGGTAACGGTGCGTGGCGCTTTATTTTAAAAAGCTCCCTCCTCTCGCAAGTTT
GATCTGATACTTATCACGAAAGGACTTGAGTCTTTTGATCAGTCTCTGACTTGACCATCTTTCATTTT
AAA
+
F,F,FFFFFFFFFFFFFFFFFFFFF:FFFFF::FFFFFFFFFFFFF:FFFFF:FF,FFFFFF:FFFFF
FFFFFF,FFFFFFFFF:FFF:FFF:FFFFFFFFFFFFFFFFFFFFFFFFFFFFFFF:F:FFFFFFFFF
FF:
@A00721:190:HGJW7DSXY:4:1430:23710:36699_2:N:0:TCTGCAAG+GCTGCACT
TTTCTTCTTTGTTTCCTTCTTTGCACTGATTTTTCCTCATCAGCTTGTGTTTTACCTAATCTGTGTTT
TTACGATAACAACTATCAGTTGATTAAGACTCTTCCGAAAGGCGAACT
+
,FFFFFFFFFFFFFFFFFFFFFFFFFFFFFFFFFFFFFFFFFFFFFFFFFFFFFFFFFFFFFFFFFFF
FFFFFFFFFFFFFFFFFFFFFFFFFFFFFFFFFFF:FFFF:FFFFFFF
@A00721:190:HGJW7DSXY:4:2336:18240:25254_2:N:0:TCTGCAAG+GCTGCACT
GAAGCTCAGGGTGAAGCACAACGTTTGACCGTGACGAACTGTGGTGTTCTTGATCTACGTTACGGATA
TACTCTCCTTGAGTTCACTCGTAAGGACACTACCACTTATTACGATAT
+
FFFFFFFFFFFFFFFFFFFFFFFFFFFFFFFFFFFFFFFFFFFFFFFFFFFFFFFFFFFFFFFFFFFF
FFFFFFFFFFFFFFFFFFFFFFFFFFFFFFFFFFFFFFFFFFFFFFFF
@A00721:190:HGJW7DSXY:4:2224:12002:28166_1:N:0:TCTGCAAG+GCTGCACT
AGCGTCTTCTGTTCATCGCAACCAGATGATATCTTCTATTCGAACTTATGTTTCGATCAGTGTTTCTT
CTATCAGGAGGAAAACACTTGTTCTAGTCCTTGTAAGATTAATCCTAACGTAGAAGATTGTTGTTTAC
CAC
```

```
+
FFFF:FFFFFFFFFFFF:FFFF:FFFF:FFFFFFFFFFFFFFFFFFFFFFFFFFFFFFFFFFFFFFFFF
FFFFFFFFFFFFFFFFFFFFFFFFFFFFFFFFFFFFFFFFFFFFFFFFFFFFFFFFFFFFFFFF:,FFF
FFF
@A00721:190:HGJW7DSXY:4:1203:24044:32111_1:N:0:TCTGCAAG+GCTGCACT
TCTCTATTTTAAGTTTTGTCTTCCTTGTTTGTCTTATTCTATTAATCACTCATTTTAGTGAGATTATT
AAAAACGCACTAAAACGTCTTCGACAACAGCAGGGGTTTTCGAATAGTGATAGCCACCCGCCTTACAG
AGG
+
FFFFFFFFFFFFFFFFFFFFFFFFFFFFFFFFFFFFFFFFFFFFFFFFFF:FFFFFFFFFFFFFFFFFF
FFFFFFFFFFFFFFFFFFFFFFFFFFFFFFFFFFFFFFFFFFFFFFFFFFFFFFFFFFFFFFFFFFFFF
FFF
@A00721:190:HGJW7DSXY:4:1314:26241:26365_2:N:0:TCTGCAAG+GCTGCACT
GACTTTGGTACCACCTTTTCTACTGTCTGTCTCGCCTCTTCGAATGAAGTTTTAATGCTTAACAACAA
TGATAGTGAATTTATACCTACAATCATTGGTTTTTCTAATAATGATGA
+
FFFFFFFFFFFFFFFFFFFFFFFFFFFFFFFFFFFFFFFFFFFFFFFFFFFFFFFFFFFFFF:FFFFFF
FFFFFFFFFFFFFFFFFFFFFFFFFFFFFFFFFFFFFFFFFFFFFFFF
@A00721:190:HGJW7DSXY:4:2565:14669:23578_1:N:0:TCTGCAAG+GCTGCACT
CTTGGATATGATGCTATTACTCATGATGGTTACGGTCAGGGAGGATTCTCAGTTTACAAGGACCTTAA
GCGATGGATCGGGGTTAATTCTAAAACATTACCCGAAAGACGTGCTAAATTAAAACCATTGTACGATG
TCG
+
FFFFFFFFFFFFFFFFFFFFFFFFFFFFFFFFFFFFFFFFFFFFFFFFFFFFFFFFFFFFFFFFFFFFF
FFFFFFFFFFFFFFFFFFFFFFFFFFFFFFFFFFFFFFFFFFFFFFFFFFFFFFFFFFFFFFFFFFFFF
FFF
@A00721:190:HGJW7DSXY:4:1260:1416:35008_1:N:0:TCTGCAAG+GCTGCACT
TCACTTTTCACTAAGAATTGGTCCTACTTTTGGACAAGGCCAACTTCGTAATCTTCATGAGATCGTTT
CTTTCTTTATCTCTTTGGTTGTTAGGGATTTTGAGAGAGTGAAAGGCTTCAATTGTAGTGGTTTGGTC
GTT
+
FFFFFF,FFFFFFFFFFFFFFFFFFFFFFFFFFFFFFFFFFFFFFFFFFFFFFFF:FFFFFFFFF:F
FFFFFFFFFFFFFFFFFFFFFFFFFFFFFFFFFFFFFFFFFFFFFFFFFFFFFFFFFFFFFFFFFFFFF
FFF
@A00721:190:HGJW7DSXY:4:1361:10059:3662_2:N:0:TCTGCAAG+GCTGCACT
ACCTCTACGCAAAGATATTTCATGAAAACTACAGAGGAACGTACCGGATTACCTGTCTTTCATATTAT
GAATGAACCTTCGGCCGCTCTCTTTGCTTCGATGTTGGATGTCAAGAA
+
FFFFFFFFFFFFFFFFFFFFFFFFFFFFFFFFFFFFFFFFFFFFFFFFFFFFFFFFFFFFFF:FFFFFF
FFFFFFFFFFFFFFFFFFFFFFFFFFF,FFFFFFF:FFFF:FFFFFF:
@A00721:190:HGJW7DSXY:4:2234:10375:16924_1:N:0:TCTGCAAG+GCTGCACT
ATTCCTACGTAGTTTATGACTTTGGTGGTGGTACTTTCGATACTTCTATCGTCTTACGTTACGGTAAT
TACTATAGTGTTATTTATTCTGGTGGTGATGATAATCTTGGAGGTCGTGACGTTGATGAAGCTATAAG
AGC
+
F,FFFFFFFFFFFFFFFFFFFFFFFFFFFF:FFFFF:FFFFFFFFFFFFFFFFFFFFFFFFFFFFFFFF
FFFFFFFFFFFFFFFFFFFFFFFFFFFFFFFFFFFFFFFFFFFFFFFFFFFFFFFFFFFFFFFFFFFFF
FFF
@A00721:190:HGJW7DSXY:4:2113:26395:31986_2:N:0:TCTGCAAG+GCTGCACT
CACAACTTTCAGTTTCACAACTGAAGGAGGAGGTTTCTGTTACTGGAAAACCCTCGTCAATACTTTTC
AATAATATACGTCTTGAACTCACTTATGACGATCTAGTGAAATTAACG
+
FFFFFFFFFFFFFFFFFFFFFFFFFFFFFFFFFFFFFFFFFFFFFFFFFFFFFFFFFFFFFFFFFFFFF
FFFFFFFFFFFFFFFFFFFFFFFFFFFFFFFFFFFFFFFFFFFFFFFF
```

@A00721:190:HGJW7DSXY:4:2470:24569:22905_1:N:0:TCTGCAAG+GCTGCACT
CTTGCAGAGTGACTGGAGTTCAGACGTGTGCTCTTCCGATCTGGCAAACTTACCCTGATTCCGGCCGG
TGGTAGCTCATTTCTACCAGGGGCTATTACTCTTGCTGAAACAGCTATTAAAGCTGTTGAGCGGGTAA
TTA
+
FFFFFFFFFFFFFFFFFFFFFFFFFFFFFFFFFFFFFFFFFFFFFFFFFFFFFFFFFFFFFFFFFFFF
FFF:FFFFFFFFFF:FFFFFFFFFFFFFFFFFFFFFFFFFFFFFFFFFFFFFFFFFFFFFFFFFFFFF
FFF
@A00721:190:HGJW7DSXY:4:1139:20726:29403_2:N:0:TCTGCAAG+GCTGCACT
ATCGCTGAAGGATGTGCACTCTTTTCTGCTACTTTAACGGAGCGTAGTGTACTCTTCGTCGATTGTAT
CAACAGTAACATTACTTCTAATAAAGGTCTCTATCAAGCTCGTATTAT
+
FFFFF:F:FFFFFFFFFFFFFFFFFFFFFFFFFFFFFFFFFFFFFFFFFFFFFFFFFFFFFFFFFFFF
FFFFFFFFFFFFFFFFFFFFFFFFFFFFFFFFFFFFFFFFFFFFFFFF
@A00721:190:HGJW7DSXY:4:1101:6587:4758_1:N:0:TCTGCAAG+GCTGCACT
TTACCGTTGAAGGCTAAACTCCAAGGTAATTATGAAGACTGTGATTTATCTTTCACTCTGGCCTTGTA
TGAGGGTGTTTCTAATCGTAACATTAATAATTTCAATCTTCTTAAAAAGAAGATAGAGAGTCATACTT
TGG
+
F,FFFFFFFFFFFFFFFFFFFFFFFFFFFFFFFFFFFFFFFFF:FFFFFFFFFFFFFFFFFFFFFFFF
FFFFFFFFFFFFFFFFFFFFFFFFFFFFFFFF:FFF:FFFFFFFFFFFFFFFFFFFFFFFFFFFFFFF
FFF
@A00721:190:HGJW7DSXY:4:2247:29803:17315_2:N:0:TCTGCAAG+GCTGCACT
GATAGCCAATATTGAAGTGGACACCTCTGGTAGTATCGAAGTAAAGATAACCAATAAGGAAGGAGCTT
ATACTATGGTTGAATCAACTAAGCATACTACTTTTGCTTTGATTACTC
+
:FFFFFFFFFFFFFFFFFFFFFFFFFFFFFFFFFFFFFFFFFFFFFFFFFFFFFFFFFFFFFFFFFFF
FFFFFFFFFFFFFFFFFFFFFFFFFFFFFFFFFFF:FFFFFFFFFFFF
@A00721:190:HGJW7DSXY:4:1527:5068:13369_1:N:0:TCTGCAAG+GCTGCACT
GTCTAAGGTTAAAGAGTCGATTAAACAAGTTGTACTTATCAATGATTTTATCGCTGGTTGTTATCTCG
AAAAGTCTCTTCTAAAAGGAACGTCAGTTCTAACCCAAAGAGAATATCTAAAATATTTACAAAATCTT
ACC
+
FF,FFFFFFFFFFFFFFFFFFFFFFFFFFFFFFFFFFFFFFFFFFFFFFFFF:FFFFFFFFFFFFF:F
FFFFFFFFFFFFFFFFFFFFFFFFFFFFFFFFFFFFF:FFFFFFFFFFFFFFFFFFFFFFFFFFFFFF
FFF
@A00721:190:HGJW7DSXY:4:1261:2510:3834_2:N:0:TCTGCAAG+GCTGCACT
AGACGTTGCTTGTCGCTTCCTCCTTTAATGGCACTACCTCGGTACAATCTTACTTGGAACGAAGGCGA
CCTGTTCCGGAGTTTTTACGGGATCCGGAATGTTCTGTCTATATTAGG
+
FFFFFFFFFFFFFFFFFFFFFFFFFFFFFFFFFFFFFFFFFFFFFFFFFFFFFFFFFFFFFFFFFFFF
FFFFFFFFFFFFFFFFFFFFFFFFFFFFFFFFFFFFFFFFF::FFF:F
@A00721:190:HGJW7DSXY:4:1323:7898:20525_2:N:0:TCTGCAAG+GCTGCACT
TCGAACGTCAACGTTCACCGAGTTTCGTTTCGTACCTTGATGCTGACTTTCGTCAATATGATTTGATC
TGGTTGCTCAATTCTAAGCGTGTTCCGCTTAGTGCGAGGCCTCTCTTC
+
FFFFFFFFFFFFFFFFFFFFFFFFFFFFFFFF:FFFFFFFFFFFFFFFFFFFFF:FFF:FFFFFFFFF
:F:FFFFFFF:FFFFFFFFFFFFFFFFF,FFFFFFFFFFFFFFFFFFF
@A00721:190:HGJW7DSXY:4:1523:16604:32189_2:N:0:TCTGCAAG+GCTGCACT
AAATACTAGATATTTGGATGATCTTGATAAAGAACTTCATGAATTTTCAGAGGATGAAAAATTTTCTT
CCTTGGATTTTAATATAAAGAATCTTTACCAGGACTCCTACTACAGGA
+
:FF:F,:FFFFFFFFFFFFFF:F:FF::FFFFFFFF,FF,:FFFF,F:FFFF,FFF:F:FFFFFF,FF
,FF:F:FFFFFFFFFFFF:FFFFFFFFFFFF::F:F,:FFF,F:FF,F

```
@A00721:190:HGJW7DSXY:4:1421:6542:28228_1:N:0:TCTGCAAG+GCTGCACT
ATCGTAAGAAGTTTATAAGGAACATATCCATTTGTTGTAATAGGGTCATCAACTTAGAACTCGAAACT
CTAAGTGGTAATACTGTCTTTCCAAGCTTACCTGTATCTTCAATGAAATTGGACTCTTCGATAGGGGA
CAT
+
FFFFFFFFFFFFF:FFFFFFFFFFFFFFFFFFFFFFFFFFF:FFFFFFFFFFFFFFFFFFFFFFFFFFF
FFFFFFFFFFFFFFFFFFFFFFFFFFFFFFFFFFFFFFFFFFFFFFFFFFFFFFFFFFFFFFFFFFFF
FFF
@A00721:190:HGJW7DSXY:4:1155:4038:20071_1:N:0:TCTGCAAG+GCTGCACT
CTACTAATGGCGTTGGCCTAGTGGATGTTATTAGTGACATCTGGTCAATTTCGTTTTTAGGTGGTCTT
TATTCTATTATTGATTTTTATGAGATTGATACGTTAACAGAAGATGATGTTAGAGTAGAATTCCTGAA
GAG
+
FFFFFF:FFFFFFFFFFFFFFFFFFFFFFFFFFFFFFFFFFFFFFFFFFFFFFFFFFFFFFFFFFFFF
FFFFFFFFFFFFFFFFFFFFFFFFFFFFFFFFFFFFFFFFFFFFFFFFFFFFFFFFFFFFFFFFFFFF
FFF
@A00721:190:HGJW7DSXY:4:1101:15212:28526_2:N:0:TCTGCAAG+GCTGCACT
CCAACTTCTTGACAATTTTCTTCAATCCTTCGATAATAGTTAAATCTAGGATTATGTCTGTGTTGAAA
GACTTTCTACCGATAAGGGTCACTAGTCTTACACAATTAAGAAATAAT
+
FFFFFFFFFFFFFFFFFF:FFFFFFF:FFFFFFFFFFFFFFFFFFFFFFFFFFFFF:FFF:FFFFFFF
FFFFFFFFFFFFFFFFFFFFFFFFFFFFFFFFFFFFFFFFFFFFFFFF
@A00721:190:HGJW7DSXY:4:1169:1588:9909_2:N:0:TCTGCAAG+GCTGCACT
GATTATCTAAAGATTGAAGGAAAATTTTATCCTCGTTTAACTTACTTAGACTTTCCTACGATACAAAT
GATAACTTCTAATTTACGTTTGCTACTCAGAGCTGGTCCTCTCAGCAA
+
FFFFFFFFFFFFFFFFFFFFFFFFFFFFFFFFFFFFFFFFFFFFFFFFF,FFFFFFFFFFFFFFFFFF
FFFFFFFFFFFFF:FFF:FFFFFFFFFFFFFFFFFFFFFFFFFFFFFF
@A00721:190:HGJW7DSXY:4:1371:18502:10520_2:N:0:TCTGCAAG+GCTGCACT
TCCTCAGGAGTCCCGGCATTTGATAATAATGACGACTTTCGTCATAATCGCTGCATATTACGGATTAC
ACGGTACCAGCGCATCTCGTGTCGAGCCTCGACCCGATTATATGGTAT
+
FFFFFFFFFFFFFFFFFFFFFFFFFFFFFFFFFFFFF:FFFFFFFFFFFFFFFFFFFFFFFFFFFFFF
FFFFFFFF:FFFFFFFFFFFFFFFFFFFFFFFFFFFFFFFFFFFFFFF
@A00721:190:HGJW7DSXY:4:1176:12961:16172_2:N:0:TCTGCAAG+GCTGCACT
TCTAGAATTCTTAATTATAATATTCAACCTCAAAAGTTACGTGTTTACAGTAACGAGATCACACATTT
TGAGAACTAACTATGTTATGTTCTCTGATCATTTTGACAATCTCTTCT
+
FFFFFFFFFFFFFFFFFFFFFFFFFFFFFFFFFFFFFFFFFFFFFFFFFFFFFFFFFFFFFFFFFFFF
FFFFFFFFFFFFFFFFFFFFFFFFFFFFFFFFFFFFFFFFFFFFFFFF
@A00721:190:HGJW7DSXY:4:1552:17951:25034_2:N:0:TCTGCAAG+GCTGCACT
AAATAAATAATATTACTAATGGCAAATAGTGGAAAGGCTCCAGTCAACACTAGTTCGAGTGCTGCTTT
GGATTACGATGACGTGAGAATAGGTGAAGGGATAGTTTTTACCTCGAC
+
FFFFFFFFFFFFFFFFFFFFFFFFFF:FFFFFFFFFFFFFFFFFFFFFFFFFF:FFFFFFFFFFFFFF,F
FFFFFFFF,FFF::FFFFFFFFFFFFFFFFFFFFFFFFFFFFFFFF,FF
@A00721:190:HGJW7DSXY:4:1364:16857:3912_1:N:0:TCTGCAAG+GCTGCACT
TCTTAAAGCTTCACCCAAAATGAAGGACACTTCGAAACTGAATAATGATGCACTACAAACAAGTATCG
CTAATCATCTGAAAAAGGCTCTAAAGGACACTATACAATATCCAGACGACGACGAAGCTTATACAATA
TCC
+
FFFFFFFFFFF:FFFFFFFFFF:FFFFFFFFFFFFFFFFFFFFFFFFFFFFFFFFFFFFFFFFF:FFFFF
FFFFFFFFFFFFFFFFFFFFFFFFFFFFFFFFFFFFFFFFFFFFFFFFFFFFFFFFFFFFFFFFFFFF
FFF
```

@A00721:190:HGJW7DSXY:4:2124:27001:31125_2:N:0:TCTGCAAG+GCTGCACT
ACCACATTTTGGTCAAGTCAATCCGTTGAGAAAATTTCTTAGAGGATATTCGCCATTGTGGTACGAAT
TGGTCCAACATTTACCACCAAATTCTTTTGATACAACTCTAGCCTTCA
+
FFFFFF,:FFFFFFFFFFFF:F:F,FFFFFFFFFFFF:FFF,F,:F,FFFF:FFF:F,F,FFFF,:FF
FFFFF:FF::F:FFF:F,F:FFFFF,FFFF:FFFFFFF,,F:,FF,,F
@A00721:190:HGJW7DSXY:4:1557:11586:11851_2:N:0:TCTGCAAG+GCTGCACT
ACCAGATTGGTTCGAACCCGTTGATGCTATGTCAGAAGAAGCTATTCAAGCTTGTCGTTTAAAGACTG
CAGAGGCTGTCAAACAAGCCCAGACTCAAGGAGTCCAATCAAAGGTTT
+
FFFFFFFFFFFFFFFFFFFFFFFFFFFFFF:FFFFFFFFFFFFFFFFFFFFFFFFFFFFFFFFFFFFF
FFFFFFFFFFFFFFFFFFFFFFFFFFFFFFFFFFFFFFFFFFFFFFFF
@A00721:190:HGJW7DSXY:4:2415:3812:11005_1:N:0:TCTGCAAG+GCTGCACT
TTATTTAGAGATCTTTTCTACTTACAAAGGACAATTCAGTTTCATTAATTCTACGGAAAAGTCCGGAG
TATATTGTATGTTAATAGATAGTGGGATAATAGTTCTACCGATACGTAGTGAACGCGTTCGTAGAACC
AAG
+
FF:FFFFFFFFFFFFFFFF,FFFFFFF:,F:FFFFFFFFFFFFFFFFFFFFFFFFFFFFFFFFFFFFF
FFF:FFFFFFFFFFF:FFFFFFFFFFFFFFFFFFFF:FFFF,FFFFFFFFF::FFFFFFFFFFFFFFF
FFF
@A00721:190:HGJW7DSXY:4:1232:16432:30921_1:N:0:TCTGCAAG+GCTGCACT
ACCGATAGGAGTTGTATAGTTGAAGATGTGGCTTTAGACGATAATCCTTTAAGGACTATGTTGACTGA
CGAAGATTTTCCAACAGATTTGTCTACGTTGCAGATCGCCACTCCAAACACTATATACATTATAAAAT
ATA
+
FFFFFFFFFFFFFFFFFFFFFFFFFFFFFFFF,FFFFFFFFFFFFFFFFFFFFFFFFFFFFFFFFFFF
FFFFFFFFFFFFFFFFFFFFF:FFFFFFFFFFFFFFFFFFFFFFFFFFFFFFFFFFFFFFFFFFFFFF
FFF
@A00721:190:HGJW7DSXY:4:2544:2718:36824_1:N:0:TCTGCAAG+GCTGCACT
CATAACTTTTGTTACCAAATTCTTGGCTTTCAACGAAGTCTCTGATTTTATTAATCATTACTTTAACG
ATCTCTCGGTCATTTACTCTGTACTTGATCTACGTCGTTTAACTGTCGGAACAACCTTTACTTAGAAT
GAC
+
FF:FFFFFFFFFFFFFFFFFFFFFFFFFFFFFFF:FFFFFFFFFFFFFFFFFFFFFFFFFFFFFFFFFF
FFFFF,FFFFFFFFFFFFFFFFFFFFFFFFFFFFFFFFFFFFFFFFFFFFFFFFFFFFFFFFFFFFFF
FFF
@A00721:190:HGJW7DSXY:4:2614:32551:10300_2:N:0:TCTGCAAG+GCTGCACT
TTCTTATATTATTTACAATAGTTAATATCATATTAGTAATACTCACTATCGTTTTCTGCTTTTTCTTA
GCTAATAGATTAATAAAGGATGAGTACGAAAACCAACTCTTCGTTACG
+
FFFFFFFFFFFFFFFFFF:FFFFFFFFFFFFFFFF,FFFFFFFFFFFFFFFFFFFFFFFFFFFFFFFF
FFFF:FFFFFFFFFFFFF:FFF:FFFFFFFFFFFFF:FFFFFFFFFFF
@A00721:190:HGJW7DSXY:4:2153:9679:31720_1:N:0:TCTGCAAG+GCTGCACT
TAGAATAGATTTAGTCTATACACCCAAATCAACGTGTATAACGCTATTTTTCCGTCCTCAACTTAATA
CACTAATAGATCATCCGAAAACGACTCGAAACCGAGACGCCGTAGATCAAAGTCCCTTCTAACTTTAG
AT
+
FF,F,FF,:FFF,,,,:,,F::FF,:,,,,:,:F,:,F:FF,:,,,,FFF,F,,:F:F,F:,,,FF:F
:::,F,,,,F,::,FFF,F:FFFFFFFFFFFFFFFFFFFFFFFFFFFFFFFFFFFFFFFFFFFFFFFF
FF
@A00721:190:HGJW7DSXY:4:2205:10013:28103_1:N:0:TCTGCAAG+GCTGCACT
CATTTTATAGCAGTTATTTATGTTGTACTTTTTATAATTTATAACCTTCTTATACTTTTGGGTATGTT
TTGTGAAAAACATGAGATACCCAAGTCTATCGAAGACATTATCTCTTATGTACTTATTGTAACGATCA
TTC

```
+
FFFFFFF:F:FFFFFFFFFFFFFFFFFFFFFFFFFFFFFFFFFFFFFFFFFFFFFFFFFFFFFFFFFFF
FFFFFFFFFFFFFFFFFFFFFFFFFFFFFFFFFFFFFFFFFFFFFFFFFFFFFFFFFFFFFFFFFFFFF
FFF
@A00721:190:HGJW7DSXY:4:2319:8097:9721_1:N:0:TCTGCAAG+GCTGCACT
TTCTTTTATTCCTTTAACTGTGTATGAGGAGGATTTATAATGCTTTTCGATAGTGATTGTTATCAATT
TAAATACATTACTGTTTCAAATCATCTTTACTCACAGACTCCGAAAATGGTTTCAAATGAGGTCAAAG
CTC
+
FFFFFFFFFFFFFFFFFFFFFFFFFFFFFFFFFFFFFFFFFFFFF:FFFFFFFFFFFFFFFFFFFFFFF
FFFFFFFFFFFFFFFFFFFFFFFFFFFFFFFFFFFFFFFFFFFFFFFFFFFFFFFFFFFFFFFFFFFFF
FFF
@A00721:190:HGJW7DSXY:4:2369:9652:12352_2:N:0:TCTGCAAG+GCTGCACT
AACTATTATCCGATTCTTCGAGGGTATCACTACCACTGATCTGCCGACTTTAGTCAAGGAACTTCAAT
TCCTTCGATGTTTATATTTTTCTATAAGGGAAAATAGCAATAATCAAA
+
FFFFFFFFFFFFFFFFFFFFFFFFFFFFFFFFFFFFFFFFFFFFFFFFFFFFFFFFFFFFFFFFFFFFF
FFFFFFFFFFFFFFFFFFFFFFFFFFFFFFFFFFFFFFFFFFFFFFFFF
@A00721:190:HGJW7DSXY:4:1564:11261:4085_2:N:0:TCTGCAAG+GCTGCACT
AACTTTATTTCAGAACTCTCTCCGATCACCTTGGTGTGATCTCCCGGAGGATCTTTTCAATAAGATGT
ATGATGATAATCTACTATTTTTAAATAGTGATATTCAGCGTATACTTG
+
FFFFFFFFFFFFFFFFFFFFFFFFFFFFFFFFFFFFFFFFFFFFFFFFFFFFFFFFFFFFFFFFFFFFF
FFFFFFFFFFFFFFFFFFFFFFFFF:FFFF:FFFFFFFFFFFFFFFFFF
@A00721:190:HGJW7DSXY:4:2321:26259:23328_1:N:0:TCTGCAAG+GCTGCACT
GGCTTGGTCTATTTACCGCAAAAAGTTGGAAGAGGTCTATGATATTTATTTTACTTATACTTTAAATA
GCACCGATTATAACTTGACTGTTGATAATATTACAATAGCTCTTTCTCAGAAGATTAGAGAAGTAACC
GGT
+
FFFFFFFFFF:FFFFFFFFFFF:FFFFFFFFFFFFF:FFFFFFFFFFFFFFFFFFFFFFFFFFFFFF,F
FFFFFFFFFFFFFFFFFFFFFFFFFFFFFFFFFFFFFFFFFFFFFFFFFFFFFFFFFFFFFFFFFFFFF
FFF
@A00721:190:HGJW7DSXY:4:1302:29839:28432_1:N:0:TCTGCAAG+GCTGCACT
GTAGCATTGATTTACTTTTACCGTTAGAACTGAAAAAGATTCTTTTCGAAAAGAAACCTATAAGTTAC
AAACGATTCTTCGACTATATTAAAGAAATAAAAATACTTCAAGTACCTGAACTTTACATTTTTAATGG
TAT
+
FFFFFFFF:FFFFFFFFF::FFFFFF:FFF::FFFFFFFFFF:F,FFFF:FFFFFFFFFFFFF:FFFF
FFFFFFFFFFFFFFFFF:FFFF,FFFFFFFFFFFFFFFFFFFFFFFFFFFFFFFFFFFFFFFFFFFFFF
FFF
@A00721:190:HGJW7DSXY:4:1507:4218:15186_1:N:0:TCTGCAAG+GCTGCACT
TTACAAATAGAACCTTCACTCTCGGTAATGAGAGTGTATTCCTTAATATTCTAGTAACCGCTATGTGG
GCATTACTAGGATTTATCACACTCCTAAATTTGATAGCAATTTATGTTTTAAATGCTAGGAAAATTTT
AGA
+
FFFFFFF,FF:FFFFFFFFFFFFFFFFFFFFFFFFFFFFFFFFFFF:FFFFFFFFFFFFFFFFFFFFFFF
FFFFFFFFFFFFFFFFFFFFFFFFFFFFFFFFFFFFFFFFFFFFFFFFFFFFFFFFFFFFFFFFFFFFF
FFF
@A00721:190:HGJW7DSXY:4:2365:7907:24173_1:N:0:TCTGCAAG+GCTGCACT
TGTATAGTTCAAGGAGTACTTAATTCAATAGCTTTAGTATTATTTACTATCGCCTTTATTGAAAATTG
TATAAGAGTTAATTGCTTACACAAAAGTGACGCGCGAAGGAATCTTAATTGATTGAAGCTAGGCGTTG
GGG
+
FFFF:FFFFFFFFFFFFFFFFFFFFFFFFFFFFFFFFFFFFFFFFFFFFFFFFFFFFFFFFFFFFFFFF
```

FFFFFFFFFFFFFFFFFFFFFFFFFFFFFFFFFFFFFFFFFF:FFFFFFFFFFFFFFFFFFFFFFFFFF
FFF
@A00721:190:HGJW7DSXY:4:2542:29152:33254_2:N:0:TCTGCAAG+GCTGCACT
CATACCATAACCTACCATGACCAATCATCGCTCCAACTGGCACGCCAGACGTAGGGTCAAACCTACTA
CCGCCGGTCGGCAGATCGGCACCACTGGTCTCTACGTTCAGGGACCCG
+
FFFFFFFFFFFFFFFFFFFFFFFFFFFFFFFFFFFFFFFFFFFFFFFFFFFFFFFFFFFFFFF,FFFFFF,
FFFFFFFFFFFFFFFFFFFFFFFFFFFFFFFFF:,FFFFFFFFF:,FFF
@A00721:190:HGJW7DSXY:4:2613:32434:2769_2:N:0:TCTGCAAG+GCTGCACT
TAACCTTCCCAAATCTAGATCTGCACAGAAGAAGGCTGAAGGTCGTTACCGGACGACCACTCAAAGGA
AACGGGCTGTTGCCCAGAGGAAAGCAGCGACCACCGAGGTCGCTCGTA
+
FFFFFFFFFFFFFFFFFFFFFFFFF:FFFFFFFFFFFFFFFFFFFFFFFFFFFFFFFFFFFFFFFFFFFFFFFFFFF
FFFFFFFFFFFFFFFFFFFFFFFFFFFFFFFFFFFFFFFFFFFFFFFF
@A00721:190:HGJW7DSXY:4:1117:15691:3991_2:N:0:TCTGCAAG+GCTGCACT
CCCGTCATCTCTGTGGGAACTGAGGCAGTACCTCAGTCAAAAGTCACATTCACCACTCCCATACCAAT
CAATGATGTGACTATACAGTTCGGCCATTTCAAGAGTGGTCAATACGT
+
FFFFFFFFFFFFFFFFFFFFFFFFFFFFFFFFFFFFFFFFFFFFFFFFFFFFFFFFFFFFFFFFFFFF
FFFFFFFFFFFFFFFFFFFFFFFFFFFFFFFFFFFFFFFFFFFFFFFF
@A00721:190:HGJW7DSXY:4:1148:15962:14700_2:N:0:TCTGCAAG+GCTGCACT
CCTTCCCCTCCGCTGGCACGAATAACACTCCCAAGCCAATCAATAGTTCTTCTGACCAGAATGAAAAG
GTGTTTACCAGGGTAGTCTCTAAGTCTACCAGGAAGAGGGGAAATGTC
+
,FFF,FFF,,F::,F,,FF,FF,FFFFF::FFFF,,FFFFFFF:FF:F,F:,F,FF,F,FFF:F:FFF
F,FFFFF:,:,F,,,,,:FFFFF,:,FFF,F,,F:FFF,FFFFFF,,,
@A00721:190:HGJW7DSXY:4:2420:23149:6981_2:N:0:TCTGCAAG+GCTGCACT
AGGTCCATCAGGACCTAAGAAGGGGAAATTACCCCTAATTCTGAAGGAAGATCTCCCACCATCTGGGG
TTGGGAATAAGAAATCTGCACGGGCCAAAGAGGCCGCTGCTACGAAGT
+
FFFFFFFFFFFFFFFFFFFFFFFFFFFFFFFFFFFFFFFFFFFFFFFFFFFFFFFFFFFFFFFFFFFF
FFFFFFFFFFFFFFFFFFFFFFFFFFFFFFFFFFFFFFFFFFFFFFFF
@A00721:190:HGJW7DSXY:4:1316:22137:7138_1:N:0:TCTGCAAG+GCTGCACT
TCTTGCAGAGTGACTGGATTTCAGACGTGTGCTCTTCCGATCTCTTTGAGACCAAGACTCTCTACAGT
AGGTTCGTGCCTACTATGAGCAAATCTGTTGAATTCGTCACTGAGGACGAAGGTGAGAGGACTTTTGG
CTA
+
F::F,,,FFFFFFFFFFF,FFFFFF:FF:FF,F:FFF,FFFFFF,FFFFFFF::FFF:F:FF,:FFFF
FFFFFFFF:,FFFFFFFFF::,,FFFFFFFFFFFFFFFFFFFFFFFFF,FFFFFFFFF:,FFFFFFFFF
FFF
@A00721:190:HGJW7DSXY:4:2667:6596:31892_1:N:0:TCTGCAAG+GCTGCACT
CTGAAAGAGAATTGCGTGGGTTGAAGGCCGACGCTTCGAGCTTGAGTGTGACGAGGCCGGTGACGTTT
TTGTCAACGGCGTTTGGAGGATTTTATCCGCGCTTAACTATGGGGCGAAGATCCCCTATGGAGTCTTG
AAG
+
FFFFFFFFF::FFFFFFFFFFFFFFFFFFFFFFFFFFFFFFFFFFFFFFFFFFFFFFFFFFFFFFF:FF
FFFFFFFFFFFFFFFFFFFFFFFFFFFFFFFFFFFFFFFFFFFFFFFFFFFFFFFFFFFFFFFFFFFF
FFF
@A00721:190:HGJW7DSXY:4:2318:18927:23249_2:N:0:TCTGCAAG+GCTGCACT
GGCATATTTATGCCGACGATAATAAGCATGTCACCCTTAAGCACCGAGGTAAGCTCGGGACTTACAAG
AGAGAGTGTGAGAGAGTCGCTCTCACTATTTTTGCCCAGAATGCGGGC
+
FFFFFFFFFFFFFFFFFFFFFFFFFFFFFFFFFFFFFFFFFFFFF:FFF::FFFFFFFFFFFFFFFF
FFFFFFFFFFFFFFFFFFFFFFFFFFFFFFFFFFFFFFFFFF:F:FFFF

```
@A00721:190:HGJW7DSXY:4:2265:19696:6198_2:N:0:TCTGCAAG+GCTGCACT
TCCTCCATAATTACAACCTCTCTTTAGAGAGCATTAGAAGGAAGTTGGGCAAATTCCCACATATGGTA
GACGTTGTCACACTTTTCTTGAAAATTTTTCCTTTCCTCCGTCATTGG
+
FFFFFFFFFFFFFFFFFFFFF:F::FFFFFFFFFFFFFFFFFFFFFFFFFFFFFFFFFFFFFFFFFFF
F:FF:FFFFFFFFFFFFFFF:FFFFFFFFFFFFFFF:F::FFFF,FFF
@A00721:190:HGJW7DSXY:4:1470:13033:13197_2:N:0:TCTGCAAG+GCTGCACT
AAGGTGGAAGGTGTCAAGGGTCTTCTTCATGCTGATCTTTCAGGACCTAATCACATCATGATTGATAG
TTACGGCTCGTATCGAGTTGGAGGGAGGAAGTTCTCCGAAATTAAAGA
+
FFFFFFFFFFF:FFFFFFFF:FFFFFFFFFFFFFFFFF:FFFFFFFFFFFFFFFFFFF:FFFFFFFFF
FFFF:FF::FFF:FFFFFFFFFFFFFFFF:FFFFFFFFFFFFFFFFFF
@A00721:190:HGJW7DSXY:4:1233:5240:16705_1:N:0:TCTGCAAG+GCTGCACT
TCAGAGAGGGGAGCTCTGAAAAGGAAACTTCGTCAGAAGATGAGGAAGTCTAAGAGGAGGAGTTTGAG
GAAGGCTCTAGAGAAGATCTCACCACCTGGAGAAGGCAAGTGGAGAGTCCAGAAGTTGATGAAGAAGG
ATT
+
F,FFFF,FF:FFFF:FF,:F,F:FF:FFFFFFFFF:FF,FFFFFFFFFFFFFFFFFFFFFF:FFFFFF
:F::FFFFFFFFFFFFFFFFF,FFFFFFFFFF,F::FFFFF,FFF::FFFFFFFFFF:FFFFFFFF:F
FF,
@A00721:190:HGJW7DSXY:4:1258:4824:35368_2:N:0:TCTGCAAG+GCTGCACT
GTTACCACTTCCTAAGGTGGATGAGGGAGGTTGTTTCTTTGGAGTTTTAGATAATCTTTTGAGAAGGG
GTCATAGAGCCAGCAAGAGAAGCTTATATGCTGGTTTAAGTGATGATT
+
FFFFFFFFFFFFFFFFFFFFF:FFFFFFFF::FFFFFFFFFFFFFFFFFFFFFFFF::FFFFFFFFFF
FFFFFFFFFFFFFFF::FFFFFFFFF,FFFFFFFFFFFFFFFFFFFF:
@A00721:190:HGJW7DSXY:4:1165:26178:25880_1:N:0:TCTGCAAG+GCTGCACT
ATTTAGGTCAACTTGGGGTTTCGAATGTCATTAGTGCCTTACGATTGAAAGAGGTCCTTTTGAGTATC
GACAGATCTTTCGATCTTGAGGACGTACATTTAACGTGGCAGAATTCGGTAACTTACCATATTGTTCC
TTC
+
FFFFFFFFFFFFFFFFF,FFF,FFFFFFF,FFFFFFFFFFFF:FFFF,FFFF,FFFFF:FF:FFFFFF
FFFFFFFFFFFFFFFFFF:FFFFFFFFFFFFFFFFFFFFFFFFFFFFFFFFFFFFFF:FF,FFFFFFFFF
FFF
@A00721:190:HGJW7DSXY:4:2373:11704:19194_1:N:0:TCTGCAAG+GCTGCACT
TCTTGCAGAGTGACTGGAGTTCAGACGTGTGCTCTTCCGATCTTTTGATTGGTATGGACTACTCGACT
TTTGATCTGGAGTTGTCTAGGGTCATGGCAGATACTACCGATAAGGTGAAAAATATGCAGGTCAAAGA
TTC
+
FFFFFFFFFFFFFFFFFFFFFFFFFFFFFFFFFFFFFFFFFFFFFFFFFFFFFFFFFFFFFFFFFFFF
FFFFFFFFFFFFFFFFFFFFFFFFFFFFFFFFFFFFFFFFFFFFFFFFFFFFFFFFFFFFFFFFFFFF
FFF
@A00721:190:HGJW7DSXY:4:1227:15383:34648_2:N:0:TCTGCAAG+GCTGCACT
GGGCGGTTGAGGGAGCCGTTTTGGCCGACTTTAACTATTCTAAGAGTCTTTATGATTCAAAACCTATC
GTTGTCGTACCTTACAAGGTTGAGGAGACTTTACAGCGGCTTATTGTC
+
FFFFFFFFFFFFFFFFFFFFFFFFFFFFFFFFFFFFFFFFFFFFFF:FFFFFFFFFFFFFFFFFFF:FF
FFFFFFFF:FFFFFFFF:FFFFFFFFFFFFFFFFFFFFFFF,FFFF,F,
@A00721:190:HGJW7DSXY:4:1212:2889:14826_1:N:0:TCTGCAAG+GCTGCACT
AGAATTGCGGTTGAAGTTCACCCATTCTATCTTGAATGCTCACGCCGTCGCAGCCATATCGAGACTAT
GTTATAATCACTTGTTAAAGTACAGATTTAGTGATTTAGATATTATTGATATTGGAGGCGATATTCGT
TTT
+
FFFFFFFFFFFFFFFFFFFFFFFFFFFFFFFFFFFFFFFFFFFFFFFFFFFFFFFFFFFFFFFFFFFF
```

```
FFFFFFFFFFFFFFFFFFFFFFFFFFFFFFFFFFFFFFFFFFFFF:FFFFFFFFFFFFFFFFFFFFFFFFF
FFF
@A00721:190:HGJW7DSXY:4:1510:22761:2863_2:N:0:TCTGCAAG+GCTGCACT
ACATATTTGCAACCCTATTCTTGATGTGAAAGATGCATCGAGGTATACGTCTAGGAACATTGATTGGG
AACGTACCTACTCACGAGACGTCATGAAAGCTATCGGAGGGAAGCAAA
+
FFFFFFFFFFFFFFFFF:FFFFFFFFFFFFFFFFFFFFFFFFFFF:FFFFFFFFFFF:FFFFFFFFFFF
:FFFFFFFFFFFFFFFFFFFFFFFFFFFFFFFFFFFFFFFFF::FFFF,
@A00721:190:HGJW7DSXY:4:1228:9019:11819_1:N:0:TCTGCAAG+GCTGCACT
TTACAAGAACGCTTTGGATTGTGACCATAAAGCCTCAGTTGCGATAGCTGTAGAGGTATACGATATCT
CTCTTGATGATGTAGCCACTATAATGCTTAAGAGAGAGATTGATGTATGTCATTTCGTCTTAGTTTGT
CCC
+
FFFFFFFFFFFFFFFFFFFF:FFFFFFFFFFFFFFFFFFFFFFFFFFFFFFFFFFFFFFFFFFFFFFFF
FFFFFFFFFFFFFFFFFFFFFFFFFFFFFFFFFFFFFFFFFFFFFFFFFFFFFFFFFFFFFFFFFFFF
FFF
@A00721:190:HGJW7DSXY:4:2252:12500:26365_2:N:0:TCTGCAAG+GCTGCACT
GACACGATGAAAAATGACATACCCGTTCCTGACCTTGGTATCGTTATCCGTCATGGAGATGATGGCGC
ATCACTTGATTACTATATGCCCGAAGGATTGGGTTATTCTCACAACAA
+
FFFF:FFFFFFFFF:FFFFFFF:FF:FFF,FFFFFFF:FFFFFFF:F:FFFFFF,FFFF:FFFFFFFF
FFFFFFFFF,FFFFFFFFFFFFF,FFF:FFFF:FFF:FF:FFFFFFF
@A00721:190:HGJW7DSXY:4:2636:24153:18239_1:N:0:TCTGCAAG+GCTGCACT
TAAGGGCGGACAATTGTTCCATTGTGAACTCTTGGGGACTAGATTGAGCATTAATGAGTTCCGAATAA
CTCGTAATAAATCTTTCTCTAGTGGACAATTCAGAATACCTATTTCCGTACCAAGAGCCACTGCCAAT
CTC
+
FFFFFFFFFFFFFFFFFFFFFFFFFFFFFFFFFFFFFFFFFFFFF:FFFFFFFFFFFFFFFFFFFFFFFFF
FFFFFFFFFFFFFFFFFFFFFFFFFFFFFFFFFFFFFFFFFFFFFFFFFFFFFFFFFFFFFFFFFFFF
FFF
@A00721:190:HGJW7DSXY:4:1458:2121:22294_2:N:0:TCTGCAAG+GCTGCACT
AAATTTTTCTCAAGAGATTATGGTTGATAAAGTTTTCTTCATAAGGGCTATTCAGTACGTCATGGGGA
TCACTTCTTCGGTCAATGACAAAACCTTCGAATATGTTACGACATGGC
+
FFFFFFFFFFF:FFFFFF:FF:FFFFFFFF:FF:FFFFFFF,FFFFFF:FF:FF:FFFF:FFFFFFFF
FFFFFFFFFFFFFFFFFFFFFFFFFFFFFFFFFFF::,FFF:FFFFFFF
@A00721:190:HGJW7DSXY:4:2459:32190:5447_2:N:0:TCTGCAAG+GCTGCACT
AGGATTGTCATCTCGGGGAAAGTGATTCACCAAGATGTTCATATAGATACGAGATACCTAGAGAAGGT
CGCAGCGATCTTACTCGTTATCGGAGCTAAGAAGAAGACTTTGGGTAC
+
FFFFFFFFFFFFFFFFFFFFFFFFFFFFFFFF,FFFFFFFFFFFFF:FFFFFFFFFFFFFFFFFFFFFF
F:FFFFFFFFFFFFFFFFFFFFFFFFFFF:FFFFFFF:FFFFFFFFFF
@A00721:190:HGJW7DSXY:4:2448:5936:19820_2:N:0:TCTGCAAG+GCTGCACT
TAACGTTAGTACCGGAGGTGATCTCTCTGCTTCTGCGTTACTGAGAGAAAAGATTCAGGACGCCTACG
GTGACTTTATGGACAAGATGCAAGTTCTTGGGAAATACTTGTTTCCCT
+
FFFFFFFFFFFFFFFFFFFFFFFFFFFFFFFFFFFFFFFFFFFFFFFFFFFFFFFFFFFFFFFFFFFF
FFFFFFFFFFFFFF:FFFFFFFFFFFFFFFFFFF,FF:FFFFFFFFFF
@A00721:190:HGJW7DSXY:4:2345:32805:2942_1:N:0:TCTGCAAG+GCTGCACT
CGTGTGCTCTTCCGATCTAACGAATCTTGAAACTACGGTCCTTATCAAAGAAAATGGTGAACCGGTCT
CTAAATCTTTTGAGATTGAGAAGATTGAAGAACACGTCAAGAAGTCGCTCGGCGTTGTTCTTGAAAAC
CAA
+
FFFFFF,FFFFF,:F:FFFFFFFF,FFF,FF:FFFF:FFF,FFFFFFFFFFFFFFFFFFFFFFFF,FF
```

:FFFF,F:FFFFFF:FF::FFFFFFFFFFFFFFFFFFFFFFFFFFFFFFFFFFFFFFFF:FFFFFFFFF
FFF
@A00721:190:HGJW7DSXY:4:1476:14109:31532_1:N:0:TCTGCAAG+GCTGCACT
GCTGATCATGGAGTCTTTGTACCTTCTGTCAAACTTCGAGGTTCAGCTACGAGAGGTGATCCCGGATG
TGGATTGAAGGCTGGCTCTAAGGGAGACTCCTTCTTTTTGAATGTTGTAAAGAATTGGGTTGAGAAGT
GTG
+
FFFFFFF:FFFFFFF:FFFFFFFFFFFF,FFFFFFFFFFFF:FFFFFFFFFFFFFFFFFFFFFFFFFFF
FFFFFFFFFFFFFFFFFFFFFFFFFFFFFFFFF:FFFFFFFFFFFFFFFFFFFFFFFFFFFFFFFFFFF
FFF
@A00721:190:HGJW7DSXY:4:1446:3640:32847_1:N:0:TCTGCAAG+GCTGCACT
CTTGGTATTTCTGAAATAGTTTATCAGAGCGTCATTTTCCTTTGGGATCTTTCCTCTGGTAAGGGAAT
TATTAAATCATTCTTCTCACTCTTGACGAGAATTATTACTGTTTTCTCTACCCATAACTTCTTTTTCA
AAA
+
FFFFFFFFFFFF,FFFFFFFFFFFFFFFFFFFFFFFFFFFFFFFFFFFFFFFFFFFFFFFFFFFFFFF
FFFFFFFFFFFFFFFFFFFFFFFFFFFFFFFFFFFFF:FFFFFFFFFFFFFFF:FFFFFFF:FFFFF
FFF
@A00721:190:HGJW7DSXY:4:2615:26241:5290_1:N:0:TCTGCAAG+GCTGCACT
CTGATGTTTTCCTACAGAGTGCAGTCGTTTCAACACTTATCACCACTTTTGAAAGGAAAATAATTGGT
GATGAAAATACGTATGTCGGTGGGGTTCTCAAGGCTCTCATCGCCACTGGGATTCTAAGGGGTTCCAT
AAG
+
FFFFFFFFFFFFFFFFFFFFFFFFFF,FFFFFFFFFFFFFFFFFFFFFFFFFFFFFFFFFFFFFFFFF
FFFFFFFFFFFFFFFFFFFFFFFFFFFFFFFFFFFFFFFFFFFFFFFFFFFFFFFFFFFFFFFFFFFF
FFF
@A00721:190:HGJW7DSXY:4:1504:29098:17566_2:N:0:TCTGCAAG+GCTGCACT
TTTGGTCCTTACGTCTTAAGTAATTTGGATGTTGTCGAAAACTATCAGGTTCTTGTCACGATTCTTAA
AAGAAGTTTATTAAAACTTCAGAGTCTCTTGGAAGTCCAGGTAGCTAA
+
FFFFFFFFFFFFFFFFFFFFFFFF:FFFFFFFFFFFFFFFFFFFFFFFFFFFFFFFFFFFFFFFFFFF
FFFFFFFFFFFFFFFFFFFFFFFFFFFFFFFFFFFFFFFFFFFFFFFF
@A00721:190:HGJW7DSXY:4:2339:14714:20369_2:N:0:TCTGCAAG+GCTGCACT
GGGAAAAGAGACCTCTGTTGGTAGAGGAGTTGAGAGAATTACTCAGATTCAATCTGGCATCACTGAAC
GTCTACAACGAGTTAGGGAGTGGCTAGGTTTTAAAAGATCCTTTGCAC
+
FFFFFF,FFFFFFFFFFFFFFFFFFFFFFFFFFFFFFFFFFFFFFFFFFFFFFFFFFFFFFFFFFFFF
FFFFFFFFFFFFFFFFFFFFFFFFFFFFFFFFFFFFFFFFFFFFFFFF
@A00721:190:HGJW7DSXY:4:1220:3043:7294_2:N:0:TCTGCAAG+GCTGCACT
CGTCGCGGAAAACGATGAAGATTTCCACACTGTTCGAGAATCTTCTGATGCTGAATTTGAGGAAAATT
TTTCGGAAGTTTTTGGTATAAATCCTGATGGCTCAGTACAGGGAGGAG
+
FFFFFFFFF:FFFFFFFFFFFFFFFFFFFFFFFFFF:FFFFFFFFFFFFFFF:FFFFFFFFFFFFFFF
FFFF:FFFFF,FFFFFFFFFFFFFFFFFFFFFFFFFFFFFFFFFFFF
@A00721:190:HGJW7DSXY:4:2532:7862:13009_1:N:0:TCTGCAAG+GCTGCACT
AACCTTCTTTTATCATTGCAACAGGACTTTGAGAAGACTGTTGAAATGGACAAAATCTGGGATCATAG
GTATGTGTCACGCTACACTCGAAATATGGGCCCTCATAAGGAAGTATAGTTGGCGTCTTGTCGTTGAC
GTC
+
FF:FFFFFFFFFFFFFFFFFFFFFFFFFFFFFFFFFFFFFFFFFFFFFFFFFFFFFFFFFFFFFFFFF
FFFFFFFFFFFFFFFFFFFFFFF:FF:FFFFFFFFFFFFFFFFFFFFFFFFFFFFFFFFFFFFFFFFF
FFF
@A00721:190:HGJW7DSXY:4:2121:13268:1297_1:N:0:TCTGCAAG+GCTGCACT
TGTTCAGGAAGATCTCTACAGAGATTATCGAGAGTTGTGATCTAGTTCTAAATTTAGGTCGACGGACT

```
TTAGCAACTTTAGATAAATTCTTTCCGGGCTCAATGACCTTTGCTATGTACGTGGATCTAATGCTTGG
TGA
+
FFFFFFFFFFFF,FFFFFFFFFFF,FFFFFFFFFFFFFFFFFFFFFFFFFFFFFFFFFFFFFFFFFFF
FFFFFFFFFFFFFFFFFF:FF:FFFFFFFFFFFFFFFFF:FFFFFFFFFFFFFFFFFFFFFFFFFFFF
FFF
@A00721:190:HGJW7DSXY:4:2634:9625:14841_2:N:0:TCTGCAAG+GCTGCACT
TATTAGTTCATGAAGTTCTTACACTTCTCGCGACTAATGTCAGCTTCACTACAAAGGCGTTACGTAAA
GTAGTTTTCACGCTTTATCTTAAAGCATTACCACCAAATTTAGCACTT
+
FFFFFF:FFFFFFFFFFFFFFFFF:FFFFFFFFFFFFFFFFFFFFFFFFFFFFFF:FFFFFFFFFFFF
F:FFFFFFFFFFFFFFFFFFFFFFFFFFF:FFFFFFF:FF,FFFFFFF,
@A00721:190:HGJW7DSXY:4:2654:21459:14450_2:N:0:TCTGCAAG+GCTGCACT
TCGCCATATCAGATTAGAGACATGCCAGTTAGAGTTCTTCTAACCAGGACTGATGGTGAGATTCTTTT
ACCTTTTGGAGGTCCAGGAACCTTTACTCCTAGTGTCTTTTCTAGAGT
+
FFFFFFFFFFFFFFFFFFFFFFFFFFFFFFFFFFFFFFFFFFFFFFFFFFFFFFFFFFFFFFFFFFFF
FFFFFFFFFFFFFFFFFFFFFFFFFFFFFFFFFFFFFFF:FFFFFFFF
@A00721:190:HGJW7DSXY:4:1663:24062:5963_2:N:0:TCTGCAAG+GCTGCACT
CGGAGGATGACAGGTGGGAGACCGCTCAATAACACTCTATTGAATCCCTTTTCCACGATGAAAGCTCT
TTATGAGGTGCTCCATGCAAATTTACAGTTCCTCTTTGGTGATATCGC
+
FFFFFFFFFFFFFFFFFFFFFFFFFFFFFFFFFFFFFFFFFFFFFFFFFFFFFFFFFFFFFFFFFFFF
FFFFFFFFFFFFF:FFFFF,F:FFFFFFFFF,FFFFFFFFFFFFFF:F
@A00721:190:HGJW7DSXY:4:1663:24062:5963_1:N:0:TCTGCAAG+GCTGCACT
TTTTGATAAATTTGTTGGAATCGATAATGATACTGACGAAGGTTCTATTACCACCCTTGTCATTGATA
CAAACATTCTAGGTGATGCTTTCTTAGAGGCTCGTCTAGCCGCTGAGATTCGTCTTACCTATTTACAA
TCT
+
FFFFFFFFFFFFFFFFFFFFFFFFFFFFFFFFFFFFFFFFFFFFFFFFFFFFFFFFFFFFFFFFFFFF
FFFFFFFFFFFFFFFFFFFFFFFFFFFFFFFFFFFFFFFFFFFFFFFFFFFFFFFFFFFFFFFFFFF,
FFF
@A00721:190:HGJW7DSXY:4:2170:10936:2581_1:N:0:TCTGCAAG+GCTGCACT
GATAACGATTTAGTACTGGGAAATGGTGGTAGTCAGTTTACTACTTTTCTTTCCCTCCCCTTTTTCGA
TTGGATTCGACGGGGTCTTCGTCTCACGACTAATTTGATAAGGTTTTTCTTTTATGGGACTCTTGTTT
GTA
+
FFFFFFFFFFFFFFFFFF:FFFFFFFFFFFFFFFFFFFFFFFFFFFFFFFFFFFFFFFFFFFFFFFFF
FFFFFFFFFFFFFFFFFFFFFFFFFFFFFFFFFFFFFFFFFFFFFFFFFFFFFFFFFFFFFFFFFFFF
FFF
@A00721:190:HGJW7DSXY:4:1532:15953:6136_2:N:0:TCTGCAAG+GCTGCACT
AGTTCTTAATGTAGGTCTCCTAGTACTTAAGGGTTTATGTTTCGGTTTCAGCTGGATGGATCTTTTGA
TGGTATGTTTCACATTGCGTGATCACTTCCCGTGGACTTGGGAAGAGA
+
FFFFFFFFFFFFFFFFFFFFFFFFFFFFFFFFFFFFFFF:FFFFFFFFFFFFFFFFFFFFFFFFFFFF
FFFFFFFFFFFF:FFFFFFFFFFFFFFFFFFF:FFFFFFFFFFF:FFF
@A00721:190:HGJW7DSXY:4:1464:2609:29747_1:N:0:TCTGCAAG+GCTGCACT
CGATTCTATTATGTCGCTAAAGCCTGTCCAGTTGTCGATGATGTTCGGGCTGTGGGGATTAACTTCCC
TCGTGAAACTTACGACGATGTTCTCGCTCGCATTTCACAAGGATTACTAAGTCTGAGATCTGCCACTT
CTA
+
FFFFFFFFFFFFFFFFFFFF:FFFFFFF:FFFFFFFFFFFFFFFFFFFFFFFFFFFFFFFFFFF:FFFF
FFFFFFFFF:FFFFFFFFFFFFFFFFFFF:FFFFFFFFFFFFFFFFFFFFFFFFFFFFFFFFFFFFFFF
FF:
```

```
@A00721:190:HGJW7DSXY:4:1102:6479:36166_2:N:0:TCTGCAAG+GCTGCACT
AAGTTCATGAGGATGTCTTGGTCGAAAAATATCGCCGGGAACTTCTAATAGAGGATCCTGATGACATT
ATTTCAACGGATACCGATCTCAGTTCTACCACAGAAACCGTCTCAGGA
+
FFFFFFFFFFFFFFFFFFFFFFFFFFFFFFFFFFFFFFFFFF,FFFFFFFFFFFFF:FF:FFFFFFFFF
FFFFFFFFFFFFFFFFFFFFFFFFFFFFFFFFFFFFFFFFFFFFFFFF
@A00721:190:HGJW7DSXY:4:1134:14091:13714_2:N:0:TCTGCAAG+GCTGCACT
CTGATTCGGAATTAGAGAAAGACGACCTTCTTGTGGGGACTGAGACCTCTGAATTTTCAGGGTGCCCT
GATGAGGACGTTGAAAGGGATAATTTCAACTCTGAGACTTCTAAGAGT
+
FFFFFFFFFFFFFFFFFFFFF:FFFFFFFFFFFFFFFFFFFFFFFFFFFFFFFFFFFFFFFFFFFFFF
FF:FFFFFFFFFFFFFF:FFFFFFFFFFFFFFFFFFFFFFFFFFFFFFF
@A00721:190:HGJW7DSXY:4:2207:1118:25817_2:N:0:TCTGCAAG+GCTGCACT
ACCAGAAACGTAACAAGAAGAATAAAGGTAGGAAGGGGAGAAAACAGAACAAGCGAGCGGTTGAAGAG
AAAACTGATATGGTTGCGGCTGATCGTACCCCAATTCCTGCTAGAAAG
+
:FF:FF:FFFFFFFFFFFFFFFFFF:F:FFFFFFFFFFFFFFFFFFFFFFFFFFFFFFFFFFFFFFFFF
FFFFFFF:FFFFFFFFFFFFFF:FFFF:FFF:F:FFFFFFFF:FFFFF
@A00721:190:HGJW7DSXY:4:2421:4517:34084_1:N:0:TCTGCAAG+GCTGCACT
ATACCACTGATCTTCCAGAGTTACGTAGTGGTCGTTCTTTTGGTGATTTTATCAAAGAGAAAGTTTCT
GCTAGTCGCGACGATAGTATTTATCGGAGTGATATTAACAAGTTAAACTTCGAACTGACCACTTACAA
GAG
+
F:FFFFFFFFFFFFFFFFFF:FFFFF,FFFFFFFFFFFFFFFFFFFFFFFFFFFFFFFFFFFFFFFFFF
FFFFFFFFFFFFFFFFFFFFFFFF:FFFFFFFFFFFFFFFFFFFFFFFFFFFFFFFFFFFFFFFFFFF
FFF
@A00721:190:HGJW7DSXY:4:2226:4770:9126_2:N:0:TCTGCAAG+GCTGCACT
TCAGTTTTCCAAAAAGCTATTAATAGCAGTTGCGGGGCTATAATACCGCCTCCAGATTACTCTAAACT
GAATCCACTCTCTTGGGATATAAATGAGTACATCTATAATAGAGTTTT
+
FFFFFFFFFFFFFFFFFFFFFFFFFFFFFFFFFFFFFFFFFFFFFFFFFFFFFFFFFFFFFFFFFFFF
FFFFFFFFFFFFFFFFFFFFFFFFFFFFFFFFFF:FFFFFFFFFFFFF
@A00721:190:HGJW7DSXY:4:2525:30156:5243_1:N:0:TCTGCAAG+GCTGCACT
AACATGAATTAGTTATTGAAAGTCTTGACCCTCACGTCTCACTTTATCACCTTGGAGATGGTAAAGTC
ATTAACAAAGGAAATGTTAAGGCTGATGACTTAAATTTCGCATTCTGCTTCGACGGTAATTCCATAAG
ACC
+
FFFFFFF:FFF,F,FF,FFFFFFFFFFFFFFFFF:F:FFFF:FFFFFFFF,FFF,FFFFF:FFFFFF,
FFFFFFFFF,F,F:FFFFFF:FFF:FFFF:FF:F:FFFFF:FFF,FF:FFFFF:FFFFFFFFFFFFFF
FFF
@A00721:190:HGJW7DSXY:4:2649:10556:7216_1:N:0:TCTGCAAG+GCTGCACT
AACCTTTCCTCGTTCTTCATGAAGATCTTCGAGTGTTTTATAGTGAGTTATTCTTGCGAAGGTTCAAG
TCGATTGATTTTGTTGACTACGATCTCTCTTGTAATAAGAAGTTCTTCGCAGTTGAATCTCCACCAGG
TGG
+
FFFFF,FFFFFFFFFFFFF:FFFFFFFFFFFFFFFF:FFFFFFFFFFFFFFFFFFFFFFFFFFFFFFF
FFFFFFFFFFFFFFFFFFFFFFFFFFFFFFFFFFFFFFFFFFFFFFFFFFFFFFFFFFFFFFFFFFFF
FFF
@A00721:190:HGJW7DSXY:4:2646:2844:23829_2:N:0:TCTGCAAG+GCTGCACT
GCGACTTTTGTCGAATTTTTCGCAAAAGGTCTTGAAGTACGTATTGTCACTGCTAATCGTAATTCGCG
AGAAGAGATAGTGACTAAAGTTTTGGCACTTCTAGTTCATCGCGGTAT
+
FFFFFFFFFFFFFFFFFFFFFFFFFFFFFFFFFFFFFFFFFFFFFFFFFFFFFFFFFFFFFFFFFFFF
FFFFFFFFFFFFFFFFFFFFFFFFFFFFFFFFFFFFFFFFFFFFFFFF
```

```
@A00721:190:HGJW7DSXY:4:1109:24912:13197_1:N:0:TCTGCAAG+GCTGCACT
CGTGAAAGTTATGATGAGTGATCGTATTAGAACAGCTGATTCTTATTGCATGAACGTCGCTGAGGTCA
AACCTGTGGACGTTCTTTTCATCGACGAATTATTCATGATTCACGCTGGTCAGACCCTTTTTCTTACA
CAT
+
FFFFFFFFFFFFFFFFFFFFFFFFFFFFFFFFFFFFFFFFFFFFFFFFFFFFFFFFFFFFFFFFFFFF
FFFFFFFFFFFFFFFFFFFFFFFFFFFFFFFFFFFFFFFFFFFFFFFFFFFFFFFFFFFFFFF:FFFF
FFF
@A00721:190:HGJW7DSXY:4:1574:7636:23140_2:N:0:TCTGCAAG+GCTGCACT
GTTACGGTGATTCCAATCAAATCGGATTCATCAATCGTATTGATCTCCAAGTCGCAAACTACTTCAAC
ATCAACGACATCATTCTTGATGAGTGTCGTTCTTATCGAAGTCTCAGT
+
FFFFFFFFFFFFFFFFFFFFFFFFFFFFFFFFFFFFFFFFFFFFFFFFFFFFFFFFFFFFFFFFFFFF
FFFFFFFFFFFFFFFFFFFFFFFFFFFFFFFFFFFFFFFFFFFFF:FF
@A00721:190:HGJW7DSXY:4:1502:23041:5791_2:N:0:TCTGCAAG+GCTGCACT
CGGCCGACGTTTGTCGCGAACTCTCAGAGGTTTACGGCAGAAAAATTTCTCCTGCTTGTAATTCGAGA
GAGGACACTATGGAAATTAGACCTATCAGATGTCTTGACGACTTCGAA
+
FFFFFFFFFFFFFFFFFFFFFFFFFFFFFF:FFFFFFFFFFFFFF:FFFFFFFFF:FFFFFFFFFFFF
FFFFFFFFFFFFFFFFFFFFFFFFFFFFFFFFFFFFFFFFFFFFFFFF
@A00721:190:HGJW7DSXY:4:2532:22299:8359_2:N:0:TCTGCAAG+GCTGCACT
TATTACTTTCACTCAAGGTGAAAAGTTTGAAATTAACAAAGTCCTTGGTAAGAGTAAGGTTAAATGTG
AAGATGCTCAGACTATTCATGAAGTTCAAGGTAATACTTACCAGCACG
+
FFFFFFFFFFFFFFFFFFFFFFFFFFFFFFFFFFF:FFFFFFFFFFFFFFFFFFFFFFFFFFFFFFFF
FFFFFFFFFFFFFFFFFFFFFFFFFFFFFFFFFFFFFF:FFFFFF:F:
@A00721:190:HGJW7DSXY:4:2432:31376:3129_1:N:0:TCTGCAAG+GCTGCACT
CAAACACATATTACCGTTGCTCTATCACGACACGTTGAATCTTTACGATACTTTGTTATCAATGTAAA
GTTAAACGATGCTACTTGTGAGAGGATCAATCACAGTAAGGAACTGTCACGTTTAGCACGGGAGGTTG
CA
+
FF:FFFFFFFFFFFFFFFF:FFFFFFFF:FFFFFFF:FF:FFFFFFFFFFFFFFFFFFFFFFFFFFFF
FFFFFFFFFFFFFFFFFFFFFFFFFFFFFFFFFFFFFFFFFFFFFFFFFFFFFFFFFFFFFFFFFFFF
FF
@A00721:190:HGJW7DSXY:4:2218:5086:7513_2:N:0:TCTGCAAG+GCTGCACT
TATATCGAGGCTATCGAGGACGATGAGTATCTTGAAGTTGTTGACGACAAGCCTCAAGAGGGTAGAGG
ACCACCTGGATCTTCTACTGTCTCTGCCTTCCAAGATTTCTTAGAAGA
+
FF:FFFFFFFFFFFFFFFF:FF,FFFFFFFFFFFFFFFFFFFFFFFFFFFFFFFFFFFFFFFFFFFFF
FFFFFFFFFFFFFFFFFFFFFFFFFFFFFFFFFFFFFFFFFFFFFFFF
@A00721:190:HGJW7DSXY:4:1120:14760:1501_2:N:0:TCTGCAAG+GCTGCACT
ACCACCTTAACGTCACTCGGTAATCTTTCAGCCGAATTAAGTACTAATGAGTTCAAGTCGGTTGTCGA
TCGAATTAAGATCAAGGCTAATACCTTTATAGGTAAGAAGCCCCTTGA
+
FFFFFFFFFFFFFFFFFFFFFFFFFFFFFFFFFFFFFFFFFFFFFFFFFFFFFFFFFFFFFFFFFFFF
FFFFF:FFFFFFFFFFFFFFFFFFFFF:FFFFFFFFFFFFFFFFFFFF
@A00721:190:HGJW7DSXY:4:2655:16161:11162_1:N:0:TCTGCAAG+GCTGCACT
ACCGAGTCTCCAAGAGAATATTTATTCTTATGAGGCTCGAAACTTCAACGTTTTCACAACGGAAAGAC
ATATCGCACCCGAAATTTTTGGGCAGAATGCTCTTAACGCGGTTCTTGAACGGTGTTTTGATCAGTCT
AA
+
FFFF:FFFFF,FFFFFFFFFFFFFFF:FFFFFF:,FF:FF,FFFFF:::FFFFFFFFF:FFFFFF,FF
FFFFFFFFF:FFFFFFFFFFF:FFFFFFFFFF:FFFFFFFFFFFFFFFFFFFFFFFFFFFFFF::FFF
FF
```

@A00721:190:HGJW7DSXY:4:2375:15130:26725_1:N:0:TCTGCAAG+GCTGCACT
ATAGGAGACTCAGTTAACCTGAGAGCTCCTCAATTGATAGATTGGGTCAACAATAGGGACGCTAGTAT
ACTTCGTAAAGTCGACAGCGAAATGGTCAATATTGATAATTGGACCGACTATATGACCTATTTTAAGC
TCA
+
F::::F,FFFFF:FFFF,FFFFFFFFFFFFFF:FFF:FFFFFFF,FFFFF,FFF:F,FFFF,F,FFFF
FFFFF:FFF:FFFFFF:FFFFFFFFF:FFFF,FFFF,F,FFFF:FFFFFFFFFFFF::F:,,FFFFFF
F:F
@A00721:190:HGJW7DSXY:4:2254:17309:4476_2:N:0:TCTGCAAG+GCTGCACT
CTAAGGTTAAACTTGATGCTTCAGCCTTTTCGAAGCATAACCCCGCACAGAATATCGTTTATCACGCA
AAATTTATTAATGCAATTTTCGGACCTATCTTTAAGGTTCTTGTGAGG
+
FFFFFFFFFF:F,FFFFFF:FF:FFFFFFFFFFFFFFFFFFFFFFFFFFFF,FF:FFFFFFFFFFFFF
FFF:FFFFF::FFF:FF:FFFFF:F:FFFFFF:FFFFFFFFFFFFFFF
@A00721:190:HGJW7DSXY:4:2555:26928:10050_1:N:0:TCTGCAAG+GCTGCACT
CAGTGACGATTTCTCCCGGAAGCTCTTCTCTCTCTTAGGAGGTCATGATGACTACTATAAGGTTGAAA
TCGATTTTTCGAAGTTTGACTAATCTCAAGATTTCTTTTGTAAAGCCTATGAGATAAAGATGTATTCT
TAT
+
FF:F:FF,,FFFFFF:F,,F,::F:FFFF,FF:,,FFFFFF:FFFF,F,F::F:,FFFFF:,,F:,FF
F,FFFFFFF:,FF,FFFFFF,:FFFF,,,F:FFF,FFFFFFFF:FF,:,F:F:,FFF,FFF:FF:FFF
FFF
@A00721:190:HGJW7DSXY:4:1119:22544:9032_2:N:0:TCTGCAAG+GCTGCACT
TTGGAGTGCCAGTGAATATTTTTGTCACGCCAGGAGTATCCATAACGATTTGTCATTTACCACCTTTG
CTCAACGACGAAGTGGAACGGCCAACACTTTCTTTGGTAATTGTATGA
+
FFFFFFFFFFFFFFFFFFFFFFFFFFFFFFFFFFFFFFFFFFFFFFFFFFFFFFFFFFFFFFFFF:FF
FFF:FFFFFFFFFFFFFFFFFFFFFFFFFFFFF,FFFFF,FFFFFFFF
@A00721:190:HGJW7DSXY:4:1135:22200:8187_2:N:0:TCTGCAAG+GCTGCACT
ATTTTATCACTTTATTATGACCTTGGAAAGTTTTCGGCTGTGGTTGTCGCTGGGGACGATTCCATTCT
GTTTTCAAAGACTCCAATAACTGATCATTCTGATCATATGATTTTGGA
+
FFFFFFFFFFFFFFFFFFFFFFFFFFFFFFFFFFFFFFFFFFFFFFFFFFFFFFFFFFF,FFFFFFFF
FFFFFFFFFFFFF:FFFFFFFFFFFFFFFFFFFFFFFFFFFFFFFF:F
@A00721:190:HGJW7DSXY:4:2120:10059:32002_1:N:0:TCTGCAAG+GCTGCACT
CTTTACAAGATCATCGTTAAGTTAGGTAAACCCCTAACCGATGTTACTCTTGTAGGACTTTGGGAGAG
ATTCGTTAGTTCTAAAGACTATACTAAATATCTCCACTACGAGGTTGTTGTTTGGCGATTAGCTCTTG
CGA
+
FFFFFFFFFFFFFFFFFFFFFFFF:FFFFFFFFFFFFFF:FFFFFFFFFFFFFFFFFFFFFFFFFFFF
FFFFFFFFFFF,FFFFFFFFFFFFFFFFFFFFFFFFFFFFFFFFFFFFFFFFFFFFFFFFFFFFFFFF
FFF
@A00721:190:HGJW7DSXY:4:2673:12418:29763_2:N:0:TCTGCAAG+GCTGCACT
ATTGAGGGTCGTTATACCTATTCGGCTATCTGTGCTTTGAATTGTATAAGATCAAATTTTAAACGATT
CCGATAGGTTTATCCTGATGTGGAGGGAGTTCTTGAAGCATCCCGACC
+
FFF,FF,FF:,FFFFFFFFF,:F,F:,FF:F:F::FFF,FFFF,FFFFF,FF:FFFFFFFFFFFFFFF
FF,F,FF,FFFFF::FFFFF:FFFFFFF,FF:FFF:F,,FF:::FFFF
@A00721:190:HGJW7DSXY:4:2421:28230:27712_2:N:0:TCTGCAAG+GCTGCACT
GGTTTCATAAGATTATCAGTAATCCTGATTCTACTACGGATTCATGGACCTTCCATCCTGTACCTCTT
CTGGAGAGACGTATCTCAAAGATCGACTTCGAGACAGGTGATATTGTT
+
FFFFFFFFFFFFFFFFFFFFFFFFFFFFFFFFFFFFFFFFFFFFFFFF:FFFFFFFFFFFFFFFFFFF
FFFFFFFFFFFFFFFFFFFFFF,FFFFFFFFFFFFFFFFFFFF,FFFF

```
@A00721:190:HGJW7DSXY:4:1528:32859:30718_1:N:0:TCTGCAAG+GCTGCACT
ACCAAGATCTAAATTCGGTAACGGTGCGTGGCGCTTTATTTTAAAAAGCTCCCTCCTCTCGCAAGTTT
GATCTGATACTTATCACGAAAGGACTTGAGTCTTTTGATCAGTCTCTGACTTGACCATCTTTCATTTT
AAA
+
FFFF:FF:FFF:FFFFFFFFFFFFFFFFFFFFFFFFFFFFFFFFFFFFFFFFFFFFFFFFFFFFFFFF
FFFFFFFFFFFFFFFFFFFFFFFFFFFFFF:FFFFFFFFFFFFFFFFFFFFFFFFFFFFFFFFFFFFF
FFF
@A00721:190:HGJW7DSXY:4:1539:31665:14027_2:N:0:TCTGCAAG+GCTGCACT
TTTCTTCTTTGTTTCCTTCTTTGCACTGATTTTTCCTCATCAGCTTGTGTTTTACCTAATCTGTGTTT
TTACGATAACAACTATCAGTTGATTAAGACTCTTCCGAAAGGCGAACT
+
FFFFFFFFFF,FFFFFFFFFFFFFFF:FFFFFFFFFFFFFFFFFFFFFFFFF:FFF:FFFFFFFFFFFF
FFFF:FFFFFFF,FFFFFFFFFFFFFFFFFFFFFFFFFFFFFFFFFF,FF
@A00721:190:HGJW7DSXY:4:2551:23873:20885_2:N:0:TCTGCAAG+GCTGCACT
GAAGCTCAGGGTGAAGCACAACGTTTGACCGTGACGAACTGTGGTGTTCTTGATCTACGTTACGGATA
TACTCTCCTTGAGTTCACTCGTAAGGACACTACCACTTATTACGATAT
+
FFFFFFFFFFFFFFFFFFFFFFFFFFFFFFFFFFFFFFFFFFFFFFFFFFFFFFFFFFFFFFFFFFFF
FFFFFFFFFFFFFFFFFFFFFFFFFFFFFFFFFFFFFFFFFFFFFFFF
@A00721:190:HGJW7DSXY:4:2371:4300:20462_1:N:0:TCTGCAAG+GCTGCACT
AGCGTCTTCTGTTCATCGCAACCAGATGATATCTTCTATTCGAACTTATGTTTCGATCAGTGTTTCTT
CTATCAGGAGGAAAACACTTGTTCTAGTCCTTGTAAGATTAATCCTAACGTAGAAGATTGTTGTTTAC
CAC
+
FFFFFFFFFFFFFFFFFFFFFFFFFFFFFFFFFFFFFFFF:FFFFFFFFFFFFFFFFFFFFFFFFFFF
FFFFFFFFFFFFFFFFFFFFFFFFFFFFFFFFFFFFFFFFFFFFFFFFFFFFFFFFFFFFFFFFFFFF
FFF
@A00721:190:HGJW7DSXY:4:1210:12500:16532_1:N:0:TCTGCAAG+GCTGCACT
TCTCTATTTTAAGTTTTGTCTTCCTTGTTTGTCTTATTCTATTAATCACTCATTTTAGTGAGATTATT
AAAAACGCACTAAAACGTCTTCGACAACAGCAGGGGTTTTCGAATAGTGATAGCCACCCGCCTTACAG
AGG
+
FFFFFFFFFFFFFFFFFFFFFFFFFFFFFFFFFFFFFFFFFFFFFFFFFFFFFFFFFFFFFFFFFFFF
FFFFFFFFFFFFFFFFFFFFFF:FFFFFFFFFFFFFFFFFFFFFFFFFFFFFFFFFFFFFFFFFFFFF
FFF
@A00721:190:HGJW7DSXY:4:2339:26268:20556_2:N:0:TCTGCAAG+GCTGCACT
GACTTTGGTACCACCTTTTCTACTGTCTGTCTCGCCTCTTCTAATGAAGTTTTAATGCTTAACAACAA
TGATAGTGAATTTATACCTACAATAATTGTTTTTTCTAATAATGATTA
+
FF,F:,F,FFFFFFF::,F,FF,F:::::FF:F,FFFF,FF,FF,,FF,F,F:FFFFF,:FF,FFFFF
F,FFF,,FFF,:,F:F,,:F,FFF,F,:F,FFFFF:FFFFFF,,FF,F
@A00721:190:HGJW7DSXY:4:1166:11080:3145_1:N:0:TCTGCAAG+GCTGCACT
GTGACTGGAGTTCAGACGTGTGCTCTTCCGATCTGTCAGGGAGGATTCTCAGTTTACAAGGACCTTAA
GCGATGGATCGGGGTTAATTCTAAAACATTACCCGAAAGACGTGCTAAATTAAAACCATTGTACGATG
TCG
+
FFFFFFFFFFFFFFFFFFFFFFFFFFFFFFFFFFFFFFFFFFFFFFFFFFFFF:FFFFFFFFFFFFFF
FFFFFFFFFFFFFFFFFFFFFFFFFFFFFFFFFFFFFFFFFFFFFFFFFFFFFFFFFFFFFFFFFFFF
FFF
@A00721:190:HGJW7DSXY:4:1271:7780:17002_1:N:0:TCTGCAAG+GCTGCACT
TCACTTTTCACTAAGAATTGTTCCTACTTTTGGACAAGTCCAACTTCGTAATCTTCATGAGATCGTTT
CTTTCTTTATCTCTTTGGTTGTTAGGGATTTTGAGAGAGTGAAAGGCTTCAATTGTAGTGGTTTGGTC
GTT
```

```
+
:FF,FFFF:::FFF,FFFFF:F:::,,FFFFF,F:FFF,,,F:FFFFFFFFF,FFFFFFFFFF:FFFF
FFF:FFFF:FFFFFFF:FFFFFFFFFFFFFFFFF:FFFFFFFFFFFFFF:FFFFFFFFF:FFFFFFFF
FFF
@A00721:190:HGJW7DSXY:4:1571:26124:18897_2:N:0:TCTGCAAG+GCTGCACT
ACCTCTACGCAAAGATATTTCATGAAAACTACAGAGGAACGTACCGGATTACCTGTCTTTCATATTAT
GAATGAACCTTCGGCCGCTCTCTTTGCTTCGATGTTGGATGTCAAGAA
+
FFFFFFFFFFFFFFFFFFFFFFFFFFFFFFFFFFFFFFF,FFFFFFFFFFFFFFFFFFFFFFFFFFFF
FFFFFFFFFFFFFFFFFFFFFF:FFFFFFFFFFFFFFFFFFFFFFFFFF
@A00721:190:HGJW7DSXY:4:2276:14000:16939_1:N:0:TCTGCAAG+GCTGCACT
ATTCCTACGTAGTTTATGACTTTGGTGGTGGTACTTTCGATACTTCTATCGTCTTACGTTACGGTAAT
TACTATAGTGTTATTTATTCTGGTGGTGATGATAATCTTGGAGGTCGTGACGTTGATGAAGCTATAAG
AGC
+
FFFFFFFF:FFFFFFF,F:FFFFF:FFFFFFFFFFFFFFFFFFFFFFFFFFFFFFFFFFFFFFFFFFF
FFFFFFFFFFFFFFFFFFFFF:FFFFFFFFFFFFFFFFFFFFFFFFFFFFFFFFFFFFFFFFFFFF:FF
FFF
@A00721:190:HGJW7DSXY:4:2146:22408:32941_2:N:0:TCTGCAAG+GCTGCACT
CACAACTTTCAGTTTCACAACTGAAGGAGGAGGTTTCTGTTACTGGAAAACCCTCGTCAATACTTTTC
AATAATATACGTCTTGAACTCACTTATGACGATCTAGAGAAATTAACG
+
FFFFFF:F::F,FF,,FF:FF:,F::,FF:F::F,F:F,FFFF::,F:FF,:,,F:FFFF,FFFFF,F
FFFFFFF,F,,F,,FFFFF,,F,F:FF:FF,FFFFFF,,,FFFFF,FF
@A00721:190:HGJW7DSXY:4:2527:7310:8328_1:N:0:TCTGCAAG+GCTGCACT
CTTGCAGAGTGACTGGAGTTCAGACGTGTGCTCTTCCGATCTGGCAAACTTACCCTGATTCCGGCCGG
TGGTAGCTCATTTCTACCAGGGGCTATTACTCTTGCTGAAACAGCTATTAAAGCTGTTGAGCGGGTAA
TTA
+
FFFFFFFFFFFFFFFFFFFFFFFFFFFFFFFFFFFFFFFFFFFFFFFFFFFFFFFFFF,FFFFFFFFF
,FF:FFFFFFFFFFF:FFFFFFFFFFFFFF:FFFFFFFFFFFFFFFFFFFFF,FFFFFFFFFFFFFFFF
FFF
@A00721:190:HGJW7DSXY:4:1139:20808:27853_2:N:0:TCTGCAAG+GCTGCACT
ATCGCTGAAGGATGTGCACTCTTTTCTGCTACTTTAACGGAGCGTAGTGTACTCTTCGTCGATTGTAT
CAACAGTAACATTACTTCTAATAAAGGTCTCTATCAAGCTCGTATTAT
+
FFFFFFFFFFFFFFFFFFFFFFFFFFFFFFFFFFFFFFFFFFFFFFFFFFFFFFFFFFFFFFFFFFFF
FFFFFFFFFFFFFFFFFFFFFFFFFFFFFFFFFFFFFFFFFFFFFFFF
@A00721:190:HGJW7DSXY:4:1110:24804:35931_1:N:0:TCTGCAAG+GCTGCACT
TTACCGGTGAAGGCTAAACTCCAAGGTAATTATGAAGACTGTGAGTTATCTTTCACTCTGGCCTTGTA
TGAGGGTGTTTCTAATCGTAACATTAATAATTTCAATCTTCTTAAAAAGAAGATAGAGAGTCATACTT
TGG
+
F::FFF,,F,FF,F,F,FF,FF,,F:,F:F,FFFFF,FF,,,FFFFF,,:F,FF,FFFFFFFFFFFFF
FFFFFF:F:F,F:F:FFFFFFFFFFFFFFF,F,FF,FFF:FF,FFF:FFFFFF:FFFFFFFFFFFFFF
FF:
@A00721:190:HGJW7DSXY:4:2359:31024:23156_2:N:0:TCTGCAAG+GCTGCACT
GATAGCCAATATTGAAGTGGACACCTCTGGTAGTATCGAAGTAAAGATAACCAATAAGGAAGGAGCTT
ATACTATGGTTGAATCAACTAAGCATACTACTTTTGCTTTGATTACTC
+
FFFFFFFFFFFFFFFFFFFFFFFFFFFFFFFFFFFFFFFFFFFFFFFFFFFFFFFFFFFFFFFFFFFF
FFFFFFFFFFFFFFF:FFFFFFFFFFFFFFFFFFFF,FFFFFFFFFFF
@A00721:190:HGJW7DSXY:4:1677:9977:17143_1:N:0:TCTGCAAG+GCTGCACT
GTCTAAGGTTAAAGAGTCGATTAAACAAGTTGTACTTATCAATGATTTTATCGCTGGTTGTTATCTCG
```

AAAAGTCTCTTCTAAAAGGAACGTCAGTTCTAACCCAAAGAGAATATCTAAAATATTTACAAAATCTT
ACC
+
FFFFFFFFFFFFFFFFFFFFFFFFFFFFFFFFFFFFFFFFFFFFFFFFFFFFFFFFFFFFFFFFFFFF:F
FFFFFFFFFFFFFFFFFFFFFFFFFFFF:FFFFF:FFFFFFFFFFFFFFFFFFFFFFFFFFFFFFFFF
FFF
@A00721:190:HGJW7DSXY:4:1261:2564:3646_2:N:0:TCTGCAAG+GCTGCACT
AGACGTTGCTTGTCGCTTCCTCCTTTAATGGCACTACCTCGGTACAATCTTACTTGGAACGAAGGCGA
CCTGTTCCGGAGTTTTTACGGGATCCGGACTGTTCGGTCTATATTAGG
+
FFFFFFFFF,FFFFFFFFFFFFFFFFFFFFFFFFFFFFFFFFFFFFFF:FFFFFFFF:FFF:FFFFF
FFFFFFFFFFFF:FFFFFFFFFF:FFFFF,FF,FF,FFFFFFFFFFFF
@A00721:190:HGJW7DSXY:4:1328:21893:21339_2:N:0:TCTGCAAG+GCTGCACT
TCGAACGTCAACGTTCACCGAGTTTCGTTTCGTACCTTGATGCTGACTTTCGTCAATATGATTTGATC
TGGTTGCTCAATTCTAAGCGTGTTCCGCTTAGTGCGAGGCCTCTCTTC
+
FFFFFFFFFFFFFFFFFFFFFFFFFFFF:FFFFFFFFFFFFFFFFF:FFFFFFFFFFFFFFFFFFFFF
FFFFFFFFFFFFFF:FF:FFFFFFFFFFFFFFFFFFFFFFFFFFFFFFF
@A00721:190:HGJW7DSXY:4:2110:30987:26036_2:N:0:TCTGCAAG+GCTGCACT
AAATACTAGATATTTGGATGATCTTGATAAAGAACTTCATGAATTTTCAGAGGATGAAAAATTTTCTT
CCTTGGATTTTAATATAAAGAATCTTTACCAGGACTCCTACTACAGGA
+
FFFFFFFFFFFFFFFFFFFFFFFFFFFFFFF:FFFFFFFFFFFFFFFFFFFFFFFFFF:FF::FFFFFFF
FFFFFFFFFFFF:F:FFFFFFFFFFFFFFFFFFFFFFFFFFFFFFF:FFF
@A00721:190:HGJW7DSXY:4:2221:32750:26083_1:N:0:TCTGCAAG+GCTGCACT
ATCGTAAGAAGTTTATAAGGAACATATCCATTTGTTGTAATAGGGTCATCAACTTAGAACTCGAAACT
CTAAGTGGTAATACTGTCTTTCCAAGCTTACCTGTATCTTCAATGAAATTGGACTCTTCGATAGGGGA
CAT
+
FF,FFFF:FF,FFFFFFFFFFF,FFFFFFFFFFFFFFFFFFFFFFFFFFFFFFFFFFFFFFFFFFFFFF
FF,FFFFFFFFFFFFFFFFF:FFFFFFFFFFFFFFFFFFFFFFF:FFFFFFFFFFFFFFFFFFFFFFFFFF
FFF
@A00721:190:HGJW7DSXY:4:1470:28320:35446_1:N:0:TCTGCAAG+GCTGCACT
CTACTAATGGCGTTGGCCTAGTGGATGTTATTAGTGACATCTGGTCAATTTCGTTTTTAGGTGGTCTT
TATTCTATTATTGATTTTTATGAGATTGATACGTTAACAGAAGATGATGTTAGAGTAGAATTCCTGAA
GAG
+
FFFFFFFFFFFFFFFFFFFFFFFFFFFFFFFFFFFFFFFFFFFFFFFFFFFFF:FFFFFFFFFFFFFF
FFFFFFFFFFFFFF:FFFFFFFFFF,FFFFFFFFFFFFFFFFFFFFFFFFFFFFFFFFFFFFFFFFFFF
FFF
@A00721:190:HGJW7DSXY:4:1244:26214:2237_2:N:0:TCTGCAAG+GCTGCACT
CCAACTTCTTGACAATTTTCTTCAATCCTTCGATAATAGTTAAATCTAGGATTATGTCTGTGTTGAAA
GACTTTCTACCGATAAGGGTCACTAGTCTTACACAATTAAGAAATAAT
+
FFFFFFFFFFFFFFFFFFFFFFFFFFFF:FFFFFFFFFFFFFFFFFFFFFFFFFFFFFFFFFFFFFFF:F
FFFFFFFF:FFFFFFFFFFFFFFFFFFFFFFFFFFFFFFFFFFFFFFFF
@A00721:190:HGJW7DSXY:4:1440:7455:36323_2:N:0:TCTGCAAG+GCTGCACT
GATTATCTAAAGATTGAAGGAAAATTTTATCCTCGTTTAACTTACTTAGACTTTCCTACGATACAAAT
GATAACTTCTAATTTACGTTTGCTACTCAGAGCTGGTCCTCTCAGCAA
+
FFFFFFFFFFFFFFFFFFFFFFFF:FFF:FFFFFFFFFFFFFFFFFFFFFFFFFFFFFFF:FFFFFFFFFF
FFFFFFFFFFFFFFFFFFFFFFFFFFFFFFFFFFFFFFFFFFFFFFFF
@A00721:190:HGJW7DSXY:4:1568:21612:21042_2:N:0:TCTGCAAG+GCTGCACT
TCCTCAGGAGTCCCGGCATTTGATAATAATGACGACTTTCGTCATAATCGCTGCATATTACGGATTAC

```
ACGGTACCAGCGCATCTCGTGTCGAGCCTCGACCCGATTATATGGTAT
+
FFFFFFFFFFFFFFFFFFFFFFFFFFFFFFFFFFFFFFFFFFFFFFFFFFFFFFFFFFFFFFFFFFFFFF
FFFFFFFFFFFFFFFFFFFFFFFFFFFFFFFFFFFFFFFFFFFFFFFF
@A00721:190:HGJW7DSXY:4:1348:10592:6872_2:N:0:TCTGCAAG+GCTGCACT
TCTAGAATTCTTAATTATAATATTCAACCTCAAAAGTTACGTGTTTACAGTAACGAGATCACACATTT
TGAGAACTAACTATGTTATGTTCTCTGATCATTTTGACAATCTCTTCT
+
FFFFFFFFFFFFFFFFFFFFFFFFFFFFFFFFFFFFFFFFFFFFFFFFFFFFFFFFFFFFFFFFFFFFFF
FFFF:FF:FFFFFFFFFFFFFF,FFFFFFFFFFFFFFFFFFFFFFFFF
@A00721:190:HGJW7DSXY:4:1602:2645:9298_2:N:0:TCTGCAAG+GCTGCACT
AAATAAATAATATTACTAATGGCAAATAGTGGAAAGGCTCCAGTCAACACTAGTTCGAGTGCTGCTTT
GGATTACGATGACGTGAGAATAGGTGAAGGGATAGTTTTTACCTCGAC
+
FFFFFF:FFFFFFFFFFFFFFFFFFFFFFFFFFFFFFFFFFFFFFFFFFF:FFFFFFFFFFFFFF:F
FFFFFFFFFFFFFFFFFFFFFFFFFFFFFFFFFFFFFFFF:FFFFFFF
@A00721:190:HGJW7DSXY:4:1445:15456:16454_1:N:0:TCTGCAAG+GCTGCACT
TCTTAAAGCTTCACCCAAAATGAAGGACACTTCGAAACTGAATAATGATGCACTACAAACAAGTATCG
CTAATCATCTGAAAAAGGCTCTAAAGGACACTATACAATATCCAGACGACGACGAAGCTTATACAATA
TCC
+
FFFFFFFFFFFFFFFFFFFFFFFFFFF:FFFFFFFFF:FFFFFFFFFFFFFFFFFFF:FFFFFFFFF
FFFFFFFFFFFFFF:FFFFFFFFFFFFFFFFFF,FFF,FFFFFFFFFFFFFFFFFFFFFFFFFFFFFF
FFF
@A00721:190:HGJW7DSXY:4:2124:27136:30921_2:N:0:TCTGCAAG+GCTGCACT
ACCACATTTTGGTCAAGTCAATCCGTTGAGAAAATTTCTTAGAGGATATTCGCCATTGTGGTACGAAT
TGGTCCAACATTTACCACCAAATTCTTTTGATACAACTCTAGCCTTCA
+
FFFFFFFFFFFFFFFFFFFFFFFFFFFFFFFFFFFFFFFFFFFFFFFFFFFFFFFFFFFFFFFFFFFFFF
FFFFFFFFFFFFFFFFFFFFFFFFFF,FFFFFFFFFFFFFFFFFFFFF
@A00721:190:HGJW7DSXY:4:1557:9200:14387_2:N:0:TCTGCAAG+GCTGCACT
ACCAGATTGGTTCGAACCCGTTGATGCTATGTCAGAAGAAGCTATTCAAGCTTGTCGTTTAAAGACTG
CAGAGGCTGTCAAACAAGCCCAGACTCAAGGAGTCCAATCAAAGGTTT
+
FF:FFFFFFFFFFFFFFFFFFFFFFFF:FFFFFFFFFFFFFFFFFFFFFFFFFFFFFFFFFFFFFFFFFF
FFFFFFFFFFFFF:FFFFFFFFFFFFFFFFFFFFFFFFFFFFFFFFFF
@A00721:190:HGJW7DSXY:4:2470:5990:10614_1:N:0:TCTGCAAG+GCTGCACT
TTATTTAGAGATCTTTTCTACTTACAAAGGACAATTCAGTTTCATTAATTCTACGGAAAAGTCCGGAG
TATATTGTATGTTAATAGATAGTGGGATAATAGTTCTACCGATACGTAGTGAACGCGTTCGTAGAACC
AAG
+
FFFF,F:FFFF:FFFFFFFFFFFFFFFFFFFFFFFFFFFFFFFFFFFFFFFFFFFFFFFFFFFFFFFFFF
FFFFFFFFFFFFFFFFFFFFFFFFFFFFFFFFFFFFFFFFFFFFFFFFFFFFFFFFFFFFFFFFFFFFFF
FFF
@A00721:190:HGJW7DSXY:4:1238:23421:16564_1:N:0:TCTGCAAG+GCTGCACT
ACCGATAGGAGTTGTATAGTTGAAGATGTGGCTTTAGACGATAATCCTTTAAGGACTATGTTGACTGA
CGAAGATTTTCCCACAGATTTGTCTACGTTGCAGATCGCCACTCCAAACACTATATACATTATAAAAT
ATA
+
FFFFFFFFFFFFFFFFFFFFFFFFFFFFFFFFFFFFFFFFFFFFFFFFFFFFFFFFFFFFFFFFFFFFFF
FFFFFFFFFFFFFFFFFFFFFFFFFFFFFFFFFFFFFFFFFFFFFFFFFFFFFFFFFFFFFFFFFFFFFF
FFF
@A00721:190:HGJW7DSXY:4:2620:9245:30279_1:N:0:TCTGCAAG+GCTGCACT
CATAACTTTTGTTACCAAATTCTTGGCTTTCAACGAAGTCTCTGATTTTATTAATCATTACTTTAACG
```

ATCTCTCGGTCATTTACTCTGTACTTGATCTACGTCGTTTAACTGTCGGAACAACCTTTACTTAGAAT
GAC
+
FFFFFFFFFFFFFFFFFFFFFFFFFFFFFFFFFFFFFFFFFFFFFFFFFFFFFFFFFFFFFFFFFFFF
FFFFF,FFFFFFFFFFFFFFFFFFFFFFFFFFFFFFFFFFFFFFFFFFFFFFFFFFFFFFFF:F
FFF
@A00721:190:HGJW7DSXY:4:1316:31602:32988_2:N:0:TCTGCAAG+GCTGCACT
TCTTATATTATTTACAATAGTTAATATCATATTAGTAATACTCACTATCGTTTTCTGCTTTTTCTTAG
CTAATAGATTAATAAAGGATGAGTACGAAAACCAACTCTTCGTTACGA
+
FFFFFFFFFFFFFFFFFFFFFFFFFFFFFFFFFFFFFFFFFFFFFFFFFFFFFF:FFFFFFFF:FFFF
:FFFFFFFFFFFFFFFFFFFFFFFFFFFFFFF:FF,FFFFF:FFFFFF
@A00721:190:HGJW7DSXY:4:2274:31430:32064_1:N:0:TCTGCAAG+GCTGCACT
TAGAATATATTTAGTCTCTACACCCATATCAACGAGTATAAAGCGAGTTTTCCGTCCTGAACTTAATA
CACGAAGATATCATCCGCAAACGACTCGAAACCGAGACGCCGTAGATCAAAGTCCCTTCTAACTTTAG
AT
+
F:,FFF,,,FFF:,FFF:,::FF:,F,,,,F:,,,,,F:F,,,:,,,FFF,F:,,F,:,FF,,FFF,:
,::,F,,,,F,FF,F:FFF,FFFFFFFFFFFFFFFFFFFFFFFFFFFFFFFFFFFFFFFFFFFFFFFF
FF
@A00721:190:HGJW7DSXY:4:2269:4209:20556_1:N:0:TCTGCAAG+GCTGCACT
CATTTTATAGCAGTTATTTATGTTGTACTTTTTATAATTTATAACCTTCTTATACTTTTGGGTATGTT
TTGTGAAAAACATGAGATACCCAAGTCTATCGAAGACATTATCTCTTATGTACTTATTGTAACGATCA
TTC
+
FFFFFFFFF:FFFFFFFFFFFFFFFFFFFFFFFFFFFFFFFFFF:FFFFFFFFFFF:FFFFFFFFFFF
FFFFFFFFFFFFFFFFFFFFFFFFFFFFFFFFFFFFFFF:FFFFFF:FFFFFFFFFFFFFFFFFFFFF
FFF
@A00721:190:HGJW7DSXY:4:2547:4661:8375_1:N:0:TCTGCAAG+GCTGCACT
TTCTTTTATTCCTTTAACTGTGTATGAGGAGGATTTATAATGCTTTTCGATAGTGATTGTTATCAATT
TAAATACATTACTGTTTCAAATCATCTTTACTCACAGACTCCGAAAATGGTTTCAAATGAGGTCAAAG
CTC
+
FFFFFFFF:FFFFFFFFFFFFFFFFFFFFFFFFFFFFFFFFFFF:FFFFFFFFF:FFFFFFFFF:FFF
FFFFFFFFFFFFFFFFFFFF,FFFFFFFFFFFFFFFF:FFFFFFFFFFFFFFFFFF:FFFFFFFFFFF
FFF
@A00721:190:HGJW7DSXY:4:1109:3920:1861_2:N:0:TCTGCAAG+GCTGCACT
TATTATCCGATTCTTCGAGGGTATCACTACCACTGATCTGCCGACTTTAGTCAAGGAACTTCAATTCC
TTCGATGTTTATATTTTTCTATAAGGGAAAATAGCAATAATCAAATTT
+
FFFFFFFFFFFFFFFFFFFFFFFFFFFFFF:FFFFFFFFFFFFFFFFFFFFFFFFFFFFFFFFFFFFF
FFFFFFFFFFFFFFFFFFFFFF:FFFFFFFFFFFFFFFFFFFFFFFFF
@A00721:190:HGJW7DSXY:4:1642:32099:16031_2:N:0:TCTGCAAG+GCTGCACT
AACTTTATTTCAGAACTCTCTCCGATCACCTTGGTGTGATCTCCCGGAGGATCTTTTCAATAAGATGT
ATGATGATAATCTACTATTTTTAAATAGTGATATTCAGCGTATACTTG
+
FFFFFFFFFFFFFFFFFFFFFFFFFFFFFFFFFFFFFF:FFFFFFFFFFFFF:FFFFFFFFFFFFFFF
FFFFFFFFFFFFFFFFFFFFFF::FFFFFFFFFFFFFFFFFFFFFFFF
@A00721:190:HGJW7DSXY:4:2548:17345:23296_1:N:0:TCTGCAAG+GCTGCACT
GGCTTGGTCTATTTACCGCAAAAAGTTGGAAGAGGTCTATGATATTTATTTTACTTATACTTTAAATA
GCACCGATTATAACTTGACTGTTGATAATATTACAATAGCTCTTTCTCAGAAGATTAGAGAAGTAACC
GGT
+
FFFFFFFFFFFFFFFFFFFFFFFFFFFFFFFFFFFFFFFFFFFFFFFFFFFFFFFFFFFFFFFFFFFF

FFFFFFFFFFFFFFFFFFFFFFFFFFFFFFFFFFFFFFFFFFFFFFFFFFFFFFFFFFFFFFFFFFFFFFFFF
FFF
@A00721:190:HGJW7DSXY:4:1552:5846:9831_1:N:0:TCTGCAAG+GCTGCACT
GTAGCATTGATTTACTTTTACCGTTAGAACTGAAAAAGATTCTTTTCGAAAAGAAACCTATAAGTTAC
AAACGATTCTTCGACTATATTAAAGAAATAAAAATACTTCAAGTACCTGAACTTTACATTTTTAATGG
TAT
+
FFFF:FFFFF:FFF,::FFFFFF:FFFFFFFFFFFFFFFFFFFFFFFFFFFFFFFFFFFFFFFF::FFF
FFFFFFFFFFFFFFFFFFFFFFFF,FFFFFFFFFFFFFFFFFFFFFFFFFFFFFFFFF:FFFFFFFFF
FFF
@A00721:190:HGJW7DSXY:4:1677:21875:22561_1:N:0:TCTGCAAG+GCTGCACT
TTACAAATAGAACCTTCACTCTCGGTAATGAGAGTGTATTCCTTAATATTCTAGTAACCGCTATGTGG
GCATTACTAGGATTTATCACACTCCTAAATTTGATAGCAATTTATGTTTTAAATGCTAGGAAAATTTT
AGA
+
FFFFFFFFFFFFFFFFFFFFFFFFFFFFFFFFFFFFFFFFFFFFFFFFFFFFFFFFFFFFFFFFFFFF
FFFFFFFFFFFFFFFFFFFFFFFFFFFFFFFFFFFFFFFFFFFFFFFFFFFFFFFFFFFFFFFFFFFF
FF:
@A00721:190:HGJW7DSXY:4:2517:4010:32831_1:N:0:TCTGCAAG+GCTGCACT
TGTATAGTTCAAGGAGTACTTAATTCAATAGCTTTAGTATTATTTACTATCGCCTTTATTGAAAATTG
TATAAGAGTTAATTGCTTACACAAAAGTGACGCGCGAAGGAATCTTAATTGATTGAAGCTAGGCGTTG
GGG
+
FFFFFFFFFFFFFFFFFFFFFFFFFF:FFFFFFFFFFFFFFFFFFFFFFFFFFFFFFFFFFFFFFFFF
FFFFFFFFFFFFFFFFFFFFFFFFFFFFFFFFFFFFF,FFF:FFFFFFFFFFFFFF:FFFFFFFFFFF
FFF
@A00721:190:HGJW7DSXY:4:2565:4562:36041_2:N:0:TCTGCAAG+GCTGCACT
CATACCATAACCTACCATGACCAATCATCGCTCCAACTGGCACGCCAGACGTAGGGTCAAACCTACTA
CCGCCGGTCGGCAGATCGGCACCACTGGTCTCTACGTTCAGGGACCCG
+
FFFFFFFFFFFFFFFFFFFFFFFFFFFFFFFFFFFFFFFFFFFFFFFFFFFFFFFFFFFFFFFFFFFF
FFFFFFFFFFFFFFFFFFFFFFFFFFFFFFFFFFFFFFFFFFFFFFFF
@A00721:190:HGJW7DSXY:4:1318:24316:19053_1:N:0:TCTGCAAG+GCTGCACT
CCTTCCCAAATCTAGATCTGCACAGAAGAAGGCTGAAGGTCGTTACCGGACGACCACTCAAAGGAAAC
GGGCTGTTGCCCAGAGGAAAGCAGCGACCACCGAGGTCGCTCGTAACATTGACGGCAGTCCCGTCATC
TCT
+
FFF:FFFFFFFF:FFFFFFFFFFFFFFFFFFFFFFFFFFFFFFFFFFFFFFFFFFFFFFFFFFFFFFF
FFFFFFF:FFFFFFFFFFFFFFFFFFFFFFFFFFFFFFFFFFFFFFFFFFFFFFFFFFFFFFFFFFFF
FFF
@A00721:190:HGJW7DSXY:4:1329:23366:30154_2:N:0:TCTGCAAG+GCTGCACT
GCAGTACCTCAGTCAAAAGTCACATTCACCACTCCCATACCAATCAATGATGTGACTATACAGTTCGG
CCATTTCAAGAGTGGTCAATACGTCGTGGGAGGTACTGAAGCCTTCCC
+
FFFFF:FF:FFFFF,FFFF:FFFF,FFFFFFFFFFFFFFFFFFFFFFFF:FFFFFFFFFFFFF:FFFFF
FFF::FFF:F,F:FFFFFF:FFFFFFFFFFFFF:FF:FFFFF:FFFFF
@A00721:190:HGJW7DSXY:4:1245:24162:7513_2:N:0:TCTGCAAG+GCTGCACT
CCAAGCCAATCAATAGTTCTTCTGACCAGAATGAAAAGGGGTTTACCAGGGTAGTCTCTAAGTCTACC
AGGAAGAGGGGAAATGTCCCCAAAACTGAGAAGAATAAAGGGAAAGGT
+
FFFFFFFFFFFFFFFFFFFFFFFFFFFFFFFFFFFFF:FFFFFFFFFFFFFFFFFFFFFFFFFFFFFFF
FFFFFFFFFFFFFFFFFF:FFFFFFFFFFFFFFFFFFFFFFFFF,FFFF
@A00721:190:HGJW7DSXY:4:1608:31268:35383_2:N:0:TCTGCAAG+GCTGCACT
AGGGGAAATTACCCCTAATTCTGAAGGAAGATCTCCCACCATCTGGGGTTGGGAATAAGAAATCTGCA

CGGGCCAAAGAGGCCGCTGCTACGAAGTCGCTGAAGGCGGCTTGGGTC
+
FFFFFFFFFFFFFFFFFFFFFFFFFFFFFFFFFFFFFFFFFFFFFFFFFFFFFFFFFFFFFFFFFFFF
FFFFFFFFFFFFFFFFFFFFFFFFFFFFFFFFFFFFFFFFF:FFFFFF
@A00721:190:HGJW7DSXY:4:2333:2672:8343_2:N:0:TCTGCAAG+GCTGCACT
GTTGCGCCTGCCGACCCCATCTTTGAGACCAAGACTCTCTACAGTAGGTTCGTGCCTACTATGAGCAA
ATCTGTTGAATTCGTCACTGAGGACGAAGGTGAGAGGACTTTTGGCTA
+
FFFFFFFFFFFFFFFFFFFFFFFFFFFFFFFFFFFFFFFFFFFFFFFFFFFFFFFFFFFFFFFFFFFF
FFFFFFFFFFFFFFFFFFFFFFFFFFFFFFFFFFFFFFFFFFFFFFFF
@A00721:190:HGJW7DSXY:4:1474:26856:31657_2:N:0:TCTGCAAG+GCTGCACT
GGAAAGAGAATTGCGTGGGTTGAAGGCCGACGCTTCGAGCTTGAGTGTGACGAGGCCGGTGACGTTTT
TGTCAACGGCGTTTGGAGGATTTTATCCGCGCAAGATCGGAAGAGCGT
+
FFFF:FFFFFFFFFFFFFF:FFFFFFFFFFFFFFFFFFFFFFFFFFFFFFFFFF,FFFFFFFFFFFFF
FFFFFF:FFFFFFFF:FFFFFFFFFFFFFFFFFFFFFFFFFFFFFFFF
@A00721:190:HGJW7DSXY:4:1127:10800:22013_2:N:0:TCTGCAAG+GCTGCACT
GAGTCTTGAAGGACTTAGGTTGGCATATTTATGCCGACGATAATAAGCATGTCACCCTTAAGCACCGA
GGTAAGCTCGGGACTTACAAGAGAGAGTGTGAGAGAGTCGCTCTCACT
+
F:FFFFFFFFFFFFFFFFFFFFFFFFFFFFFFFFFFFFFFFFFFFFFFFFFFFFF:FFFFFFFFFFFF
FFFFFFFFFFFFFFFFFFFFFFFFFFFFFFFFFFFFFFF:FFFFFFFF:
@A00721:190:HGJW7DSXY:4:1501:22905:12007_2:N:0:TCTGCAAG+GCTGCACT
TTCTGACTGATGAGGTTAAAACTCTTGCTCCTTCTGTGGGTGAGGGTTTTTGTTATATTCACTGGTTC
TGGGAGGCTGCTTTCCTCCATAATTACAACCTCTCTTTAGAGAGCATT
+
FFF,FFFFFFFFFFFFFFFFFFFFFFFFFFFF:FF:F:FFF,FFFFFFFF:FFFFFFFFFF,FFFFFFF
FFFFF:FFFFFF:FFFFFFFFFFFFFFFFFFFFFFF:::FF,FFFFFFF
@A00721:190:HGJW7DSXY:4:2262:18846:32409_2:N:0:TCTGCAAG+GCTGCACT
AAATTCCCACATATGGTAGACGTTGTCACACTTTTCTTGAAAATTTTTCCTTTCCTCCGTCATTGGGG
ATTTCGATGTGAGAAGGTGGAAGGTGTCAAGGGTCTTCTTCATGCTGA
+
FFFFFFFFFFFFFFFFFFFFFFFFFFFFFFFFFFFFFFFFFFFFFFFFFFFFFFFFFFFFFFFFFFFF
FFFFFFFFFFFFFFFFFFFFFFFFFFFFFFFFFFFFFFFF:FFFFFFF
@A00721:190:HGJW7DSXY:4:1168:28031:34068_1:N:0:TCTGCAAG+GCTGCACT
TTCAGACGTGTGCTCTTCCGATCTCGGCTCGTATCGAGTTGGAGGGAGGAAGTTCTCCGAAATTAAAG
ACGGGGGGATTAAGAGTCCAGCTGTAACTGTCATTTCAGAGAGGGGAGCTCTGAAAAGGAAACTTCGT
CAG
+
FFFFFFFFFFFFFFFFFFFFFFFFFFFFFFFFFFFFFFFFFFFFFFFFFFFFFFFFFFFFFFFFFFFF
FFFFFFFFFFFFFFFFFFFFFFFFFFFFFFFFFFFFFFFFFFFFFFFFFFFFFFFFFFFFFFFFFFFF
FFF
@A00721:190:HGJW7DSXY:4:2141:27850:29778_2:N:0:TCTGCAAG+GCTGCACT
AGAGGGGAGGAGTTTGAGGAAGGCTCTAGAGAAGATCTCACCACCTGGAGAAGGCAAGTGGAGAGTCC
AGAAGTTGATGAAGAAGGATTCTTTGAAGCAGGGGTTACCACTTCCTA
+
F,FFFFFFFFFFFFFFFFFFFFFFFFF,FFFFFF:FFFFFFFFFFFFFFFFFFFFFFFFFF,FFFFFF
FFF,F,FF:FF,,:FFFFFF:,,FFFFFFF,FFFFF:FFFFFFFFF::
@A00721:190:HGJW7DSXY:4:1135:28583:34867_2:N:0:TCTGCAAG+GCTGCACT
TCTTTGGAGTTTTAGATAATCTTTTGAGAAGGGGTCATAGAGCCAGCAAGAGAAGCTTATATGCTGGT
TTAAGTGATGATTATCACTCCTTGAGAAGACAAGCCACATTAAGATAT
+
FFFFFFFFF:FFFFFFFFFFFFFF:FFFFFFFFFFFFFFF:FFFFFFFFFFFFFFFFFFFFFFFFFFF
FFFFFFFF,FFFFFFFFFFFFF:FFFFFFFFFFFFFF,F::F:FFF

@A00721:190:HGJW7DSXY:4:2666:26964:3035_2:N:0:TCTGCAAG+GCTGCACT
CTTGGGGTTTCGAATGTCATTAGTGCCTTACGATTGAAAGAGGTCCTTTTGAGTATCGACAGATCTTT
CGATCTTGAGGACGTACATTTAACGTGGCAGAATTCGGTAACTTACCA
+
FFFFFFFFFFFFFFFFFFF:FFFFFFFF:FFF:FFFFFFFFFFFFFF:FFFFFFF:FFFF:FFFFFFF
FFF:F:FF:FFFFFFFFFFFFFFFFFFFFFFFF:FFFFFFFFFFFFFFF
@A00721:190:HGJW7DSXY:4:2622:3947:13056_1:N:0:TCTGCAAG+GCTGCACT
TCTTGCAGAGTGACTGGAGTTCAGACGTGTGCTCTTCCGATCTTTTGATTGGTATGGACTACTCGACT
TTTGATCTGGAGTTGTCTAGGGTCATGGCAGATACTACCGATAAGGTGAAAAATATGCAGGTCAAAGA
TTC
+
FFFFFFFFFFFFFFFFFFFFFFFFFFFFFFFFFFFFFFFFFFFFFFFFFFFFFFFFFFFFFFFFFFFF
FFFFFFFFFFFFFFFFFFFFFFFFFFFFFFFFFFFFFFFFFFFFFFFFFFFFFFFFFFFFFFFFFFFF
FFF
@A00721:190:HGJW7DSXY:4:1376:17716:36464_2:N:0:TCTGCAAG+GCTGCACT
GGGCGGTTGAGGGAGCCGTTTTGGCCGACTTTAACTATTCTAAGAGTCTTTATGATTCAAAACCTATC
GTTGTCGTACCTTACAAGGTTGAGGAGACTTTACAGCGGCTTATTGTC
+
FFFFFFFFFFFFFFFFFFF:FFFFFFFFFFFFFFFFFFFFFFFFFFFFFFFFFFFFFFFFFFFFFFFF
FFFFFFFFFFFFFFFFFFFFFFFFFFFFFFFFFFFFFFFFFFFFFFFF
@A00721:190:HGJW7DSXY:4:1601:18349:21840_1:N:0:TCTGCAAG+GCTGCACT
AGAATTGCGGTTGAAGTTCACCCATTCTATCTTGAATGCTCACGCCGTCGCAGCCATATCGAGACTAT
GTTATAATCACTTGTTAAAGTACAGATTTAGTGATTTAGATATTATTGATATTGGAGGCGATATTCGT
TTT
+
FFFFFFFFFFFFFFFFFFFFFFFFFFFFFFFFFFFFFFFFFFFFFFFFFFFFFFFFFFFFFFFFFFFF
FFFFFFFFFFFFFF:FFFFFFFFFFFFFFFFFFFFFFFFFFFFFFFFFFFFFFFFFFFFFFFFFFFFF
FFF
@A00721:190:HGJW7DSXY:4:2326:32479:33129_2:N:0:TCTGCAAG+GCTGCACT
ACATATTTGCAACCCTATTCTTGATGTGAAAGATGCATCGAGGTATACGTCTAGGAACATTGATTGGG
AACGTACCTACTCACGAGACGTCATGAAAGCTATCGGAGGGAAGCAAA
+
FFFFFFFFFFFFFFFFFFFFFFFFFFFFFFFFFFFFFFFFFFFFFFFFFFFFFFFFF:FFFFFFFFFF
FFFFFFFFFFFFFFFFFFFFFFFFFFFFFFFFF,FFFFFFFFFFFFF,:
@A00721:190:HGJW7DSXY:4:1324:28556:3255_1:N:0:TCTGCAAG+GCTGCACT
TTACAAGAACGCTTTGGATTGTGACCATAAAGCCTCAGTTGCGATAGCTGTAGAGGTATACGATATCT
CTCTTGATGATGTAGCCACTATAATGCTTAAGAGAGAGATTGATGTATGTCATTTCGTCTTAGTTTGT
CCC
+
::FFFFF:FFFF,FFFFFFFFFFFFFFF:FFFFFFFF:FFFFFFFFFFFFFFFFFFFFFFFFFFFFFF
FFFFFFFFFFFFFFFFFFFFFFFFFFFFFFFFFFFFFFFFFFFFFFFFFFFFFFFFFFFFFFFFFFFF
FFF
@A00721:190:HGJW7DSXY:4:2268:13376:20149_2:N:0:TCTGCAAG+GCTGCACT
GACACGATGAAAAATGACATACCCGTTCCTGACCTTGGTATCGTTATCCGTCATGGAGATGATGGCGC
ATCACTTGATTACTATATGCCCGAAGGATTGGGTTATTCTCACAACAA
+
FFFFFFFFFFFFFFFFFFFFFFFFFFFFFFFFFFF:FFFFFFFF:FFFFFFFFFFFFFFFFFFFFFFFF
FFFFFFFFFFFFFFFFFFFFFFFFFFFFFFFFFFFFFFFFFFFFFFFF
@A00721:190:HGJW7DSXY:4:1159:9254:23030_2:N:0:TCTGCAAG+GCTGCACT
AAGGGCGGACAATTGTTCCATTGTGAACTCTTGGGGACTAGATTGAGCATTAATGAGTTCCGAATAAC
TCGTAATAAATCTTTCTCTAGTGGACAATTCAGAATACCTATTTCCGT
+
FFFFFFFFFFFFFFFFFFFFFFFFFFFFFFFFFFFFFFFFFFFFFFFFFFFFFFFFFFFFFFFFFFFF
FFFFFFFFFFFFFFFFFFFFFFFFFFFFF:FFFFFFFFFFFFFFFFFFF

```
@A00721:190:HGJW7DSXY:4:1339:7798:9956_1:N:0:TCTGCAAG+GCTGCACT
CTGCCAATCTCATCGCTATACGCATACCTGATTGGAAGAAGCGAGGTTCGTTCCTCAATAAAAATTTT
TCTCAAGAGATTATGGTTGATAAAGTTTTCTTCATAAGGGCTATTCAGTACGTCATGGGGATCACTTC
TTC
+
FFFFFFFFFFFFFFFFFFFFFFFFFFFFFFFFF:FFFFFFFFFFFFFFFFFFFFFFFFFFFFFFFFFF:
FF,FF:FFFFFFFFFFFFFFFFFFFFFFFFFFFFF:FFFFFFFFFFFFFFFFFFFFFFFFFFFFFFFF
FFF
@A00721:190:HGJW7DSXY:4:1104:9236:1485_1:N:0:TCTGCAAG+GCTGCACT
ACCTTCGAATATGTTACGACATGGCTTCGTAACAACAGTGCGAGGATTGTCATCTCGGGGAAAGTGAT
TCACCAAGATGTTCATATAGATACGAGATACCTAGAGAAGGTCGCAGCGATCTTACTCGTTATCGGAG
CTA
+
FFF:FFFF:,,F::FFFFFFFFFFFFFFFFFFFFFFFFFFFFFFFFFFFFFFFFFFFFFFFFFFFFFF
FFFFF:FFFFFFFFFFFF,FFFFFFFFFFFFFFFFFFFFFFFFFFFFFFFFFFFFFFFFFFFFFFFFF
FFF
@A00721:190:HGJW7DSXY:4:2549:12436:16203_2:N:0:TCTGCAAG+GCTGCACT
GTTATGCTCGCAAACTTAACGTTAGTACCGGAGGTGATCTCTCTGCTTCTGCGTTACTGAGAGAAAAG
ATTCAGGACGCCTACGGTGACTTTATGGACAAGATGCAAGAGATCGGA
+
FFF,FFFFFFFFFFFFFFFFFFFFFFFFFFFFFFFFFFFFFFFFFFFFFFFFFFFFFFFFFFFFF,FF
FFFFFFFFFFFFFFFFFFFFFFF:FFFFFFFFFFFFFFFFFFFFFFFF
@A00721:190:HGJW7DSXY:4:2622:27371:21778_1:N:0:TCTGCAAG+GCTGCACT
CGTGTGCTCTTCCGATCTAACGAATCTTGAAACTACGGTCCTTATCAAAGAAAATGGTGAACCGGTCT
CTAAATCTTTTGAGATTGAGAAGATTGAAGAACACGTCAAGAAGTCGCTCGGCGTTGTTCTTGAAAAC
CAA
+
FFFFFFFFFFFFFFFF,FFFFFFFFFFFFFFFFFFFFFFFFFFFFFFFFFFFFFFFFFFFFFF:FFFF
:FFFFFFFFFFFFFF,FFFFFF:FFFFFFFFFFFFFFFFFFFFFFFFFFFFF:FFF:FFFFFFFFFFFF
FFF
@A00721:190:HGJW7DSXY:4:1538:32949:33285_1:N:0:TCTGCAAG+GCTGCACT
GCTGATCATGGAGTCTTTGTACCTTCTGTCAAACTTCGAGGTTCAGCTACGAGAGGTGATCCCGGATG
TGGATTGAAGGCTGGCTCTAAGGGAGACTCCTTCTTTTTGAATGTTGTAAAGAATTGGGTTGAGAAGT
GTG
+
FFFFFFFFFFFFFFFFFFFFFFFFFFFFFFFFFFFFFFFFFFFFFFFFFFFFFFFFFF:FFFFFFFFF
FFFFFFFFFFFFFFFFFFFFFFFFFFFFFFFFFFFFFFFFFFFFFFFF:FFFFFFFFFFFFFFFFFFF
FFF
@A00721:190:HGJW7DSXY:4:1543:29803:31501_1:N:0:TCTGCAAG+GCTGCACT
CTTGGTATTTCTGAAATAGTTTATCAGAGCGTCATTTTCCTTTGGGATCTTTCCTCTGGTAAGGGAAT
TATTAAATCATTCTTCTCACTCTTGACGAGAATTATTACTGTTTTCTCTACCCATAACTTCTTTTTCA
AAA
+
FFFFFFF:FFFFFFFFFFFFFFFFFFFFFFFFFFF::FFFFFFFFFFFFFFFFFFFFFFFFFFFFFFF
FFFFFFFFFFFFFFFFFFFFFFFFFFFFFFFFFFFFFFFFFFFFFFFFFFFFFFFFFFFFFFFFFFFF
FFF
@A00721:190:HGJW7DSXY:4:1534:4788:18521_2:N:0:TCTGCAAG+GCTGCACT
TGATGTTTTCCTACAGAGTGCAGTCGTTTCAACACTTATCACCACTTTTGAAAGGAAAATAATTGGTG
ATGAAAATACGTATGTCGGTGGGGTTCTCAAGGCTCTCATCGCCACTG
+
FF:FFFFFFFFFFFFFFFFFFFFFFFFFFFFFFFFF,FF:,FFFFF:FFF:FFFFFFFFFFFFFFFFFF
FFF,FFFFFF,FFF,F,FFFFFFFFFFFFFFFFF:FFFF:FFFFFFF:
@A00721:190:HGJW7DSXY:4:1527:24704:12085_2:N:0:TCTGCAAG+GCTGCACT
TTTGGTCCTTACGTCTTAAGTAATTTGGATGTTGTCGAAAACTATCAGGTTCTTGTCACGATTCTTAA
```

```
AAGAAGTTTATTAAAACTTCAGAGTCTCTTGGAAGTCCAGGTAGCTAA
+
FFFFFFFFFFFFFFFFFF:FFFFF:FFFFFFFFFFFFFFFFFFFFFFFFFFFFFFFFFFFFFFFFFFF
FFF:FFFFFFFFFFFFFFFFFFFFFFFFFFFF:FFFFF:FFFFFFFFFF
@A00721:190:HGJW7DSXY:4:2458:17761:36824_2:N:0:TCTGCAAG+GCTGCACT
GGGAAAAGAGACCTCTGTTGGTAGAGGAGTTGAGAGAATTACTCAGATTCAATCTGGCATCACTGAAC
GTCTACAACGAGTTAGGGAGTGGCTAGGTTTTAAAAGATCCTTTGCAC
+
FFFF,FFFFFFFFFFFFFFFF:FFFFFFFFFFFFFFFFFFFFFFFFFFFFFFFFFFFFFFFFFFFFFF
FFFFFFFFFFFFF:FFFFFFFFFFFFFFFFFFFFFFFFFFFFFFFFFFF
@A00721:190:HGJW7DSXY:4:2128:3549:10833_2:N:0:TCTGCAAG+GCTGCACT
CGTCGCGGAAAACGATGAAGATTTCCACACTGTTCGAGAATCTTCTGATGCTGAATTTGAGGAAAATT
TTTCGGAAGTTTTTGGTATAAATCCTGATGGCTCAGTACAGGGAGGAG
+
FFFFFFFFFFFFFF::FFFFFFFFFFFFFFFFFFFFFF:F:FFFFFFFFFFFFF:FFFFFFFF:FFFF
FFFFFFFFFF,FFFFFF,,FFFFFFFFFFFFFFFFF:FFFFFFFFFFF
@A00721:190:HGJW7DSXY:4:1150:2510:36151_2:N:0:TCTGCAAG+GCTGCACT
ACCTTCTTTTATCATTGCAACAGGACTTTGAGAAGACTGTTGAAATGGACAAAATCTGGGATCATAGG
TATGTGTCACGCTACACTCGAAATATGGGCCCTCATAAGGAAGTATAG
+
FFFFFFFFFFFFFFFFFFFFFFFFFFFFFFFFFF:FFFFFFFFFFFFFFFFFFFFFFFFFF:FFF,FFF
::FFFFFFFFFFFFFFFFFFFFFF,FFFFFFFFFF,FFFFFFFF,FFF
@A00721:190:HGJW7DSXY:4:2526:24695:9533_1:N:0:TCTGCAAG+GCTGCACT
TGTTCAGGAAGATCTCTACAGAGATTATCGAGAGTTGTGATCTAGTTCTAAATTTAGGTCGACGGACT
TTAGCAACTTTAGATAAATTCTTTCCGGGCTCAATGACCTTTGCTATGTACGTGGATCTAATGCTTGG
TGA
+
FFFFFFFFFFFFFFFF,FFFFFFFFFFFFFFFFFFFFFFFFF:FFFFFFFFFFFFFFFFFFFFFFFFFF
FFFFFFFFFFFFFFFFFFFFFFFFFFFFFFFFFFFFFFFFFFFFFFFFFFFFFFFFFFFFFFFFFFFF
FFF
@A00721:190:HGJW7DSXY:4:1227:10556:16736_1:N:0:TCTGCAAG+GCTGCACT
TTAGTTCATGAAGTTCTTACACTTCTCGCGACTAATGTCAGCTTCACTACAAAGGCGTTACGTAAAGT
AGTTTTCACGCTTTATCTTAAAGCATTACCACCAAATTTAGCACTTTTTGAATATGACAGAAGCTCGC
CAT
+
FFFFFFFFFFFFFFFFFFFFFFF:FFFFFFFFFFFF:FFFFFFFFFFFFFFF:FFFFFFFFFFFFFFFF
FFFFFFFFFFFFFFFFFFFFFFFFFFFFFFFFFFFFFFFFFFFFF:FFFFFFFFFFFFFFFFFFFFFFF
FFF
@A00721:190:HGJW7DSXY:4:1252:14027:28416_2:N:0:TCTGCAAG+GCTGCACT
CCAGTTAGAGTTCTTCTAACCAGGACTGATGGTGAGATTCTTTTACCTTTTGGAGGTCCAGGAACCTT
TACTCCTAGTGTCTTTTCTAGAGTCCAAGGACGTTTCCGTCGGAGGAT
+
FFFFFFF:FFFF:FF:FFFF:FFFFFFF,,FFFFFFFFFFFFFFF,FFFFFF:FFFF:FFFFFF,FF,
FFFF,FFFFF:F:FFFFFFFFFFFFFFFF:F:F,FF::FF,F:FFFFF
@A00721:190:HGJW7DSXY:4:1123:6885:18521_1:N:0:TCTGCAAG+GCTGCACT
GCTCAATAACACTCTATTGAATCCCTTTTCCACGATGAAAGCTCTTTATGAGGTGCTCCATGCAAATT
TACAGTTCCTCTTTGGTGATATCGCCTCTGTCCTTTCTCCTTTAATCGTGAGGTTACGAGAGTTTCGT
ACT
+
FFFFFFFFFFFF:FFFFFFFFFFFFFFFFFFFFFFFFFFFFFFF:FFFFFFFFFFFFFFFFFFFFFFFF
FFFFFFFFFFFFFFFFFFFFFFFFFFFFFFFFFFFFFFFFFFFFFFFFFFFFFFFFFFFFFFFFFFFF
FFF
@A00721:190:HGJW7DSXY:4:2121:5900:28494_1:N:0:TCTGCAAG+GCTGCACT
TTTTGATAAATTTGTTGGAATCGATAATGATACTGACGAAGGTTCTATTACCACCCTTGTCATTGATA
```

CAAACATTCTAGGTGATGCTTTCTTAGAGGCTCGTCTAGCCGCTGAGATTCGTCTTACCTATTTACAA
TCT
+
FFFFFFFFF:FFFFFFFFFFFFFFFFFFFFFFFFFFFFFFFFF:FFFFFFFFFFFFFFFFFFF:FFFFF
FFFFFFFFFFFFFFFFFFFFFFFFFFFFFFFFFFFFFFFFFFFFFFFFFFFFFFFFFFFFFF:FFFFFF
FFF
@A00721:190:HGJW7DSXY:4:2212:13819:32189_1:N:0:TCTGCAAG+GCTGCACT
GATAACGATTTAGTACTGGGAAATGGTGGTAGTCAGTTTACTACTTTTCTTTCCCTCCCCTTTTTCGA
TTGGATTCGACGGGGTCTTCGTCTCACGACTAATTTGATAAGGTTTTTCTTTTATGGGACTCTTGTTT
GTA
+
:FFFFFFF,FFFFFFFFFFFFFFFFFFFFFF:FFFFFFFF:FFFFFFFFFFFFFFFFFFF::F:FFFF
FFFFFFFFFFFFFFFFFFFFFFFFFFFFFFFFFFFFFFFFFFF,FFFFFFFFFFFFFFFFFFFFFFFF
F:F
@A00721:190:HGJW7DSXY:4:2430:13114:14090_2:N:0:TCTGCAAG+GCTGCACT
AGTTCTTAATGTAGGTCTCCTAGTACTTAAGGGTTTATGTTTCGGTTTCAGCTGGATGGATCTTTTGA
TGGTATGTTTCACATTGCGTGATCACTTCCCGTGGACTTGGGAAGAGA
+
FFFFFFFFFFFFFFFFFFFFFFFFFFFFFFFFFFFFFFFFFFFFFFFFFFFFFFFFFFFFFFFFFFFF
FFFFFFFFFFFFFFFFFFFFFFFFFFFFFFFFFFFFFFFFFFFFFFFF
@A00721:190:HGJW7DSXY:4:1558:13675:2566_1:N:0:TCTGCAAG+GCTGCACT
CGATTCTATTATGTCGCTAAAGCCTGTCCAGTTGTCGATGATGTTCGGGCTGTGGGGATTAACTTCCC
TCGTGAAACTTACGACGATGTTCTCGCTCGCATTTCACAAGGATTACTAAGTCTGAGATCTGCCACTT
CTA
+
FFFFFFFFFFFFFFFFFFFFFFFFFFFFFFFFFFFFFFFFFFFFFFFFFFFFFFFFFFFFFFFFFFFF
FFFFFFFFFFFFFFFFFFFFFFFFFFFFFFFFFFFFFFFFFFFFFFFFFFFFFFFFFFFFFFFFFFFF
FFF
@A00721:190:HGJW7DSXY:4:1239:2474:34240_2:N:0:TCTGCAAG+GCTGCACT
AAGTTCATGAGGATGTCTTGGTCGAAAAATATCGCCGGGAACTTCTAATAGAGGATCCTGATGACATT
ATTTCAACGGATACCGATCTCAGTTCTACCACAGAAACCGTCTCAGGA
+
FFFFFFFF:FFFFFFFFFFFFFFFFFFFFFFF:FFFFFFFF:FFFFF,,FFFFFFFFFFFFFFFFFFF
:FFFFFFFF:FFF:FFFFFFFFFFFFF:FFFFFFFFFFF,FFFF:FFF
@A00721:190:HGJW7DSXY:4:1262:23637:13495_2:N:0:TCTGCAAG+GCTGCACT
CTGATTCGGAATTAGAGAAAGACGACCTTCTTGTGGGGACTGAGACCTCTGAATTTTCAGGGTGCCCT
GATGAGGACGTTGAAAGGGATAATTTCAACTCTGAGACTTCTAAGAGT
+
FFFF:FFFFFFFFFFFFFFFFFFFFFFFFFF,FFFFFF:FF:FFFFFFFFFFF:FFFFFFFFFFFFF,
FFFFFFFFFFFFFFFFFFFFFFFFFFFFFFFFFFFFFFFFFFFFFFF,
@A00721:190:HGJW7DSXY:4:2224:5529:3145_2:N:0:TCTGCAAG+GCTGCACT
ACCAGAAACGTAACAAGAAGAATAAAGGTAGGAAGGGGAGAAAACAGAACAAGCGAGCGGTTGAAGAG
AAAACTGATATGGTTGCGGCTGATCGTACCCCAATTCCTGCTAGAAAG
+
FFFFFFF:FFFFFFFF:FFFFFFFFF:FF:FFFFF::F:FFFFFFFFFFFFFFF:FFFFFF:FF:FFF
FFFFF:FFFFFFFFFF:FFF:FFFF:::FFFFFFFFFFFFFFFFFFFF
@A00721:190:HGJW7DSXY:4:2431:13720:28447_1:N:0:TCTGCAAG+GCTGCACT
ATACCACTGATCTTCCAGAGTTACGTAGTGGTCGTTCTTTTGGTGATTTTATCAAAGAGAAAGTTTCT
GCTAGTCGCGACGATAGTATTTATCGGAGTGATATTAACAAGTTAAACTTCGAACTGACCACTTACAA
GAG
+
FFFF,F:F,:F,FF,FFF,FFFF,,F,,FF,F,FFF,F:F:F:F,F:FFFFF:FFF,F,,FF:,FFFF
FFFFF:FFF:FFF:F:FF,FFF:F:F,F:F:FFFFFFFFFFFFFFF,FFFFFFFFF:FFF:FFFF:F:
FFF

```
@A00721:190:HGJW7DSXY:4:2367:3097:35196_2:N:0:TCTGCAAG+GCTGCACT
TCAGTTTTCCAAAAAGCTATTAATAGCAGTTGCGGGGCTATAATACCGCCTCCAGATTACTCTAAACT
GAATCCACTCTCTTGGGATATAAATGAGTACATCTATAATAGAGTTTT
+
FFFFFFFFFFFFFFFFFFFFFFFFFFFFFFFFFFFFFFFFFFFFFFFFFFFFFFFFFFFFFFFFFFFF
FFFFFFFFFFFFFFFFFFFFFFFFFFF:FFFFFFFFFFFFFFFFFFFF
@A00721:190:HGJW7DSXY:4:2602:27850:32189_1:N:0:TCTGCAAG+GCTGCACT
AACATGAATTAGTTATTGAAAGTCTTGACCCTCACGTCTCACTTTATCACCTTGGAGATGGTAAAGTC
ATTAACAAAGGAAATGTTAAGGCTGATGACTTAAATTTCGCATTCTGCTTCGACGGTAATTCCATAAG
ACC
+
FFFFFFFFFFFFFFFFF:FFFFFFFFFFFFFFFFFFFFFFFFFFFFFFFFFFFFFFFFFFFFFFFFFF
FFFFFFFFFFFFFFFFFFFFFFFFFFFFFFFFFFFFFFFFFFFFFFFFFFFFFFFFFFFFFFFFFFFF
FFF
@A00721:190:HGJW7DSXY:4:2677:32172:24893_1:N:0:TCTGCAAG+GCTGCACT
AACCTTTCCTCGTTCTTCATGAAGATCTTCGAGTGTTTTATAGTGAGTTATTCTTGCGAAGGTTCAAG
TCGATTGATTTTGTTGACTACGATCTCTCTTGTAATAAGAAGTTCTTCGCAGTTGAATCTCCACCAGG
TGG
+
FFFFF:F:FFFFFFFFF:FFFFFFFFFFF:FFFFFFFFFFFFFFFFFFFFFFFFFFFFFFFFFFF:FFF
FFFFFFFF:FFFFFFFFFFFFFFFFFFF:FFFFFFFFFFFFFFFFFFFFFFFFF:FFFFFFFFFFFFFF
FFF
@A00721:190:HGJW7DSXY:4:1140:19822:14121_1:N:0:TCTGCAAG+GCTGCACT
GACTTTTGTCGAATTTTTCGCAAAAGGTCTTGAAGTACGTATTGTCACTGCTAATCGTAATTCGCGAG
AAGAGATAGTGACTAAAGTTTTGGCACTTCTAGTTCATCGCGGTATTTGTGAAATTAAGACAGACGTG
AAA
+
FFFFFFFFFFFFFFFFFFFFFFFFFFFFFFFFFFFFFFFFFFFFFFFFFFFFFFFFFFFF:FFFFFFF
FFFFFFFFFFFFFFFFFFFFFFFFFFFFFFFFFFFFFFFFFFFFFFFFFFFFFFFFFFFFFFFFFFFF
FFF
@A00721:190:HGJW7DSXY:4:1226:29668:25661_1:N:0:TCTGCAAG+GCTGCACT
TCGTATTAGAACAGCTGATTCTTATTGCATGAACGTCGCTGAGGTCAAACCTGTGGACGTTATTTTCA
TCGACGAATTATTCATGATTCACGCTGGTCAGACCCTTTTTCTTACACATCTACTTAAGGCGGCTTAT
ACC
+
F:FF:FFFFFF::FFF:FFFFFF,FFFFFFFFFFFFFFFFF:FFFFFFF:FFFFFF:FFFF,FFFFFF
FFFFFFF:FFFFFFFFFFFFFF:FFFFFFF:FFFFFFFFFFFFFFFFFFFFFFFFFFF:FFFFFFFFFF
FFF
@A00721:190:HGJW7DSXY:4:1135:30951:29575_1:N:0:TCTGCAAG+GCTGCACT
GTGATTCCAATCAAATCGGATTCATCAATCGTATTGATCTCCAAGTCGCAAACTACTTCAACATCAAC
GACATCATTCTTGATGAGTGTCGTTCTTATCGAAGTCTCAGTTATCGCTGCCCGGCCGACGTTTGTCG
CGA
+
FFFFFFFFFFFFFFFFFFFFFFFFFFFFFFFFFFFFFFFFFFFFFFFFFFFFFFFFFFFF:FFFFFFF
FFFFFFFF:FFFFFFFFFFFF:FFFFFFFFFFFFFFFFFFFFFFFFFFFFFFFFFFFFFFFFFFFF::F
FFF
@A00721:190:HGJW7DSXY:4:1510:25870:31548_2:N:0:TCTGCAAG+GCTGCACT
TACGGCAGAAAAATTTCTCCTGCTTGTAATTCGAGAGAGGACACTATGGAAATTAGACCTATCAGATG
TCTTGACGACTTCGAATTTGAGGACGGAGTTAAATATATTACTTTCAC
+
FFFFFFFFFFFFFFFFFFFFFFFFF:FFF:FFFFFFFFFFFFFFF,:FFFFFFFFFFFFFF,FFFFFFF
FFFFFFF:FF::FFFF,FFFFFFFFFFFFFFFFFFFFFFFFFFFFFFF
@A00721:190:HGJW7DSXY:4:2665:31864:18662_2:N:0:TCTGCAAG+GCTGCACT
AATTAACAAAGTCCTTGGTAAGAGTAAGGTTAAATGTGAAGATGCTCAGACTATTCATGAAGTTCAAG
```

```
GTAATACTTACCAGCACGTTCGGCTGGTTAGAATTAAACCTCAAGATG
+
FFFFF:FFFFFFFFF:FFFFFF:FFFFFFFFFFFFF:FFFFFFFFFFFFFFFFFFFFFFFFFFFFF:F
FFFFFFFFFFFFFFFFFFFFFFFFFFF:FFFFFFFF:FFFFFFFFFF:
@A00721:190:HGJW7DSXY:4:1515:7988:12007_1:N:0:TCTGCAAG+GCTGCACT
AACACATATTACCGTTGCTCTATCACGACACGTTGAATCTTTACGATACTTTGTTATCAATGTAAAGT
TAAACGATGCTACTTGTGAGAGGATCAATCACAGTAAGGAACTGTCACGTTTAGCACGGGAGGTTGCA
+
FF:F:FF:FF,F:FFFF,F:F:FFFFFF,FF,FFFFFFF:FF,,:FF,FFFF:FFFF,FFFFFF:FF,
FFFFF:FFFFF,F:FFFFFFFFFFF:F:FF::FFFFFF:FF:FFF:FFFFFFFFF:F:FFFFFFFFFF
@A00721:190:HGJW7DSXY:4:2243:17336:30389_2:N:0:TCTGCAAG+GCTGCACT
TATATCGAGGCTATCGAGGACGATGAGTATCTTGAAGTTGTTGACGACAAGCCTCAAGAGGGTAGAGG
ACCACCTGGATCTTCTACTGTCTCTGCCTTCCAAGATTTCTTAGAAGA
+
FFFFFFFFFFFFFFFFFFFF:FFFFFFFFFFF:FFFFFFFFFFFFFFFFFFFFFFFFFFFFFFFFFFF
FFFFFFFFFFFFFFFFFFFFFFFFFFFFFFFF::FFFFFFFFFFFFFFF
@A00721:190:HGJW7DSXY:4:1253:13792:29418_2:N:0:TCTGCAAG+GCTGCACT
ACCACCTTAACGTCACTCGGTAATCTTTCAGCCGAATTAAGTACTAATGAGTTCAAGTCGGTTGTCGA
TCGAATTAAGATCAAGGCTAATACCTTTATAGGTAAGAAGCCCCTTGA
+
FFFFFFFFFFFFFFFFFFFFFFFFFFFFFFFFFFFFFFFFFFFFFFFFFFFFFFFFFFFFFFFFFFFF
FFFFFFFFFFFFFFFFFFFFFFFFFFFFFFFFFFFFFFFFFFFFFFFF
@A00721:190:HGJW7DSXY:4:1343:27597:7670_2:N:0:TCTGCAAG+GCTGCACT
GAGTCTCCAAGAGAATATTTATTCTTATGAGGCTCGAAACTTCAACGTTTTCACAACGGAAAGACATA
TCGCACCCGAAATTTTGGGGCAGAATGCTCTTAACGCGGTTCTTGAAC
+
FFFFF,FFFFFFFF,FFFFFFFFFFFFFFF:FFFFFF,FFFFFFFFFFFFFF:FFFFFFFFFFFFFF
:FFF:FFFF::FF,,F,FFFFFFF:FFFFFFFFFFFFF,FFFFFFFF
@A00721:190:HGJW7DSXY:4:2629:26377:26819_2:N:0:TCTGCAAG+GCTGCACT
CTAAGTTGGATAAAGTTATAGGAGACTCAGTTAACCTGAGAGCTCCTCAATTGATAGATTGGGTCAAC
AATAGGGACGCTAGTATACTTCGTAAAGTCGACAGCGAAATGGTCAAT
+
FFFFFFFFFFFFFFFFFFFFFFFFFFFFFFFFFFFFFFFFFFFFFFFFFFFFFFFFFFFFFFFFFFFF
FFFFFFFFFFFFFFFFFFFFFFFFFFFFFFFFFFFFFFFFFFFFFFFF
@A00721:190:HGJW7DSXY:4:1468:30653:19351_1:N:0:TCTGCAAG+GCTGCACT
TGACCTATTTTAAGCTCATGGTGAAAAGGGAAGCTAAGGTTAAACTTGATGCTTCAGCCTTTTCGAAG
CATAACCCCGCACAGAATATCGTTTATCACGCAAAATTTATTAATGCAATTTTCGGACCTATCTTTAA
GGT
+
FFFFFFFFFFFFFFFFFFFFFFFFFFFFFFFFFFFFFFFFFFFFFFFFFFFFFFFFFFFFFF:FFFFF
FFFFFFFFFFFFFFFFFFFFFFFFFFFFFFFFFFFFFFFFFFFFFFFFFFFFFFFFFFFFFFFFFFFF
FFF
@A00721:190:HGJW7DSXY:4:1143:31684:10989_1:N:0:TCTGCAAG+GCTGCACT
GACGATTTCTCCCGGAAGCTCTTCTCTCTCTTAGGAGGTCATGATGACTACTATAAGGGTGAAATCGA
TTTTTCGAAGTTTGACAAATCTCAAGATTTCTTTTGTAAAGCCTATGAGATAAAGATGTATTCTTATT
TAG
+
FF,FFFF:F:FFFFF:FFFF,F:FFFF:F:FF:FFFFFFFFFFFFFFFFF:FFF,:F,FFFFFFF:FF
::FFFFFFF::F,FF,FFFFFFFFF::F:FFFFFFFFF,FFFFFFFFFFFFFFFFFFFFFFFFFFFFF
FFF
@A00721:190:HGJW7DSXY:4:1161:12599:19742_2:N:0:TCTGCAAG+GCTGCACT
TTGGAGTGCCAGTGAATATTTTTGTCACGCCAGGAGTATCCATAACTATTTGTCATTTACCACCTTTG
CTCAACGACGAAGTGGAACGGCCAACACTTTCTTTGGTAATTGTATGA
+
```

F:FFF,FFF,:FFFFFFF,FFFFFFFFF:F,FFFFFFFF,FFFFFF,:FFFF:FF:::F,FFFFF:F:
FFFFFFFFF:FFFF,FFFF,,FFFFFF,FFFFF:::FFFF,F:,FFF:
@A00721:190:HGJW7DSXY:4:1545:19877:13056_2:N:0:TCTGCAAG+GCTGCACT
ATTTTATCACTTTATTATGACCTTGGAAAGTTTTCGGCTGTGGTTGTCGCTGGGGACGATTCCATTCT
GTTTTCAAAGACTCCAATAACTGATCATTCTGATCATATGATTTTGGA
+
FFFFFFF:FFFFFFFFFFFF:FFF,FFFFFFFFFFFFFFFFFFFFFFFFFFFFF:FFFFFFFFFFF,F
FFFF:FFFFFFFFFFFFFFFFFFFFFFFFFFFFF:FFFFFFFFFFFF:
@A00721:190:HGJW7DSXY:4:2206:5104:15468_1:N:0:TCTGCAAG+GCTGCACT
CTTTACAAGATCATCGTTAAGTTAGGTAAACCCCTAACCGATGTTACTCTTGTAGGACTTTGGGAGAG
ATTCGTTAGTTTTAAAGACTATACTAAATATCTCCACTACGAGGTTGTTGTTTGGCGATTAGCTCTTG
CGA
+
FFFFFFF:FFFFFFFFFFFFFFFFFFFF:FFFFFFFFFFFFFFFFFFFFFFFFFFFFFFFFFFFFFFF
FFFFFFFFFFFFFFFFFFFFFFFFFFFFFFFFFFFFFFFFFFFFFFFFFFFFFFFFFFFFFFFFFFFF
FFF
@A00721:190:HGJW7DSXY:4:1375:15573:36573_2:N:0:TCTGCAAG+GCTGCACT
GAGGGTCGTTATACCTATTCGGCTATCTGTGCTTTGAATTGTATAAGATCAAATTTTAAACGATTCCG
AGAGGTTTATCCTGATGTGGAGGGAGTTCTTGAAGCATCCCGACCTTT
+
FFFFFFFFFFFFFFFFFFFFFFFFFFFFFFFFFFFFFFFFFFFFFFFFFFFFFFFFFFFFFFFFFFFF
F:FF:FFFFFFFFFF,FFFFFFFFFFFFFFFFFFFFFFFFFFFFFFFF
@A00721:190:HGJW7DSXY:4:2421:28257:26850_2:N:0:TCTGCAAG+GCTGCACT
GGTTTCATAAGATTATCAGTAATCCTGATTCTACTACGGATTCATGGACCTTCCATCCTGTACCTCTT
CTGGAGAGACGTATCTCAAAGATCGACTTCGAGACAGGTGATATTGTT
+
FFFFFFFFFFFFFFFFFFFFFFFFFFFFFFFFFFFFFFFFFFFFFFFFFFFFFFFFFFFFFFF:FFFF
FFFFFFFFFFFFFFFFFFFFFFFFFFFFFFFFFFFFFFFFFFFFFFFF
@A00721:190:HGJW7DSXY:4:1538:29080:27774_1:N:0:TCTGCAAG+GCTGCACT
ACCAAGATCTAAATTCGGTAACGGTGCGTGGCGCTTTATTTTAAAAAGCTCCCTCCTCTCGCAAGTTT
GATCTGATACTTATCACGAAAGGACTTGAGTCTTTTGATCAGTCTCTGACTTGACCATCTTTCATTTT
AAA
+
FFFFFFFFFFFFFFFFFFFFFFFFFFFFFFF:F,FFFFFFFFFFFFFFFFFFFFFFFFFFFFFFFFFF
FFFFFFFFFFFFFFFFFFFFFFFFFFFFFFFFFFFFFFFFFFFFFFFFFFFFFFFFFFFFFFFFFFFF
FFF
@A00721:190:HGJW7DSXY:4:1574:30798:16532_2:N:0:TCTGCAAG+GCTGCACT
TTTCTTCTTTGTTTCCTTCTTTGCACTGATTTTTCCTCATCAGCTTGTGTTTTACCTAATCTGTGTTT
TTACGATAACAACTATCAGTTGATTAAGACTCTTCCGAAAGGCGAACT
+
FFFFFFFFFFFFFFFFFFFFFFFFFFFFFFFFFFFFFFFFF:FFFFFFFFFFFFFFFFFFFFFFFFFF
FF:FFFFFF:FFFFFFFFFFF:FFFFFFFFFFFFF:FFFFF:FFFFFF
@A00721:190:HGJW7DSXY:4:2618:31259:35055_2:N:0:TCTGCAAG+GCTGCACT
GAAGCTCAGGGTGAAGCACAACGTTTAACCGTGACGAACTGTGGTGTTCTTGATCTACGTTACGGATA
TACTCTCCTTGAGTTCACTCGTAAGGACACTACCACTTATTACGATAT
+
FFFFFFFFFFFFFFFFFFFFFFFFFFFFFFFFFFFFFFFFFFFFFFFFFFFFFFFFFFFFFFFFFFFF
FFFFFFFFFFFFFFFFFFFFF:FFFFFFFFFFFFFFFFFFFFFFFFFF
@A00721:190:HGJW7DSXY:4:2572:10691:23923_1:N:0:TCTGCAAG+GCTGCACT
AGCGTCTTCTGTTCATCGCAACCAGATGATATCTTCTATTCGAACTTATGTTTCGATCAGTGTTTCTT
CTATCAGGAGGAAAACACTTGTTCTAGTCCTTGTAAGATTAATCCTAACGTAGAAGATTGTTGTTTAC
CAC
+
FFFFFFFFFFFFFFFFFFFFFFFFFFFFFFFFFFFFFFFFFFFFFFFFFFFFFFFFFFFFFFFFFFFF

FFFFFFFFFFFFFFFFFFFFFFFFFFFFFFFFFFFFFFFFFFFFFFFFFFFFFFFFFFFFFFFFFFFF
FFF
@A00721:190:HGJW7DSXY:4:1228:30436:3756_1:N:0:TCTGCAAG+GCTGCACT
TCTCTATTTTAAGTTTTGTCTTCCTTGTTTGTCTTATTCTATTAATCACTCATTTTAGTGAGATTATT
AAAAACGCACTAAAACGTCTTCGACAACAGCAGGGGTTTTCGAATAGTGATAGCCACCCGCCTTACAG
AGG
+
FFFFFFFFFFFFFFFFFFFFFF:FFFF:FFFFFFFFFFFFFFFFFFFFFFFFFFFFFFFFFFFFFFFF
FFFFFFFFFFFFFFFFFFFFFFFFFFFFFFFFFFFFFFFFFFFFFFFFFFFFFFFFFFFFFFFFFFFF
FFF
@A00721:190:HGJW7DSXY:4:2411:28763:4554_2:N:0:TCTGCAAG+GCTGCACT
GACTTTGGTACCACCTTTTCTACTGTCTGTCTCGCCTCTTCGAATGAAGTTTTAATGCTTAACAACAA
TGATAGTGAATTTATACCTACAATCATTGGTTTTTCTAATAATGATGA
+
FFFFFFFFFFFFFFFFFFFFFFFFFFFFFFFFFFFFFFFFFFFFFFFFFFFFFFFFFFFFFFFFFFFF
FFFFFFFFFFFFFFFFFFFFFFFFFFF:FFFFFFFFFFFF:FF:FFFFF
@A00721:190:HGJW7DSXY:4:1204:7229:9565_1:N:0:TCTGCAAG+GCTGCACT
GTGACTGGAGTTCAGACGTGTGCTCTTCCGATCTGTCAGGGAGGATTCTCAGTTTACAAGGACCTTAA
GCGATGGATCGGGGTTAATTCTAAAACATTACCCGAAAGACGTGCTAAATTAAAACCATTGTACGATG
TCG
+
F:::FFFFF:FFF:F:F:,FFFFF:FF:F,,FFFFF:FF,F,FFFFFFFFF:F:F,F:,:F:F,:F,:
F:FFFF::FFFFFFFFFF:FFFF:FFFFFFFFFFFFFFFF:FF,FFFFFFFF,FF:FFFFFFFFFFFF
FFF
@A00721:190:HGJW7DSXY:4:1363:6135:11616_1:N:0:TCTGCAAG+GCTGCACT
TCACTTTTCACTAAGAATTGGTCCTACTTTTGGACAAGGCCAACTTCGTAATCTTCATGAGATCGTTT
CTTTCTTTATCTCTTTGGTTGTTAGGGATTTTGAGAGAGTGAAAGGCTTCAATTGTAGTGGTTTGGTC
GTT
+
FFFFFFFFFFFFFFFFFFFFFFFFFFFFFFFFFFFFFFFF:FFFFFFFFFFFFFFFFFFFFFFFFFFF
FFFFFFFFFFFFFFFFFFFFFFFFFFFFFFFFFFFFFFFFFFFFFFFFFFFFFFFFFFFFFFFFFFFF
FFF
@A00721:190:HGJW7DSXY:4:2160:4092:31344_2:N:0:TCTGCAAG+GCTGCACT
ACCTCTACGCAAAGATATTTCATGAAAACTACAGAGGAACGTACCGGATTACCTGTCTTTCATATTAT
GAATGAACCTTCGGCCGCTCTCTTTGCTTCGATGTTGGATGTCAAGAA
+
FFFFFFFFFFFF:FFFFFFFFFFF:FFFFF,FFFFFFFFFFFFFFFFFFFFFFFFFFFFFFFFFFFF:F
FFFFF:FFFFFFFFFFFFFFFFFFFF:FFFFF:FFFFFFFFFFFFFFF
@A00721:190:HGJW7DSXY:4:2276:15817:15765_1:N:0:TCTGCAAG+GCTGCACT
ATTCCTACGTAGTTTATGACTTTGGTGGTGGTACTTTCGATACTTCTATCGTCTTACGTTACGGTAAT
TACTATAGTGTTATTTATTCTGGTGGTGATGATAATCTTGGAGGTCGTGACGTTGATGAAGCTATAAG
AGC
+
FFFFFFFFFFFFFFFFFFFFFFFFFFFFFFFFFFFFFFFFFFFFFFFFFFFFFFFFFFFFFFFFFFFF
FFFFFFFFFFFFFFFFFFFFFFFFFFFFFFFFFFFFFFFFFFFFFFFFFFFFFFFFFFFFFFFFFFFF
FFF
@A00721:190:HGJW7DSXY:4:2249:16785:34569_2:N:0:TCTGCAAG+GCTGCACT
CACAACTTTCAGTTTCACAACTGAAGGAGGAGGTTTCTGTTACTGGAAAACCCTCGTCAATACTTTTC
AATAATATACGTCTTGAACTCACTTATGACGATCTAGTGAAATTAACG
+
FFFFFFFFFFFFFFFFFFFFFFFF:,FFFFFFFFFFFFFFFFFFFFFFFFF,FFFFFF:FF:FFFF:FFF
FFFFFFFFFFFFFFFF:FFFFFFF,FFFFFFF:F::FFFF,F::FFFF
@A00721:190:HGJW7DSXY:4:1175:2302:29590_1:N:0:TCTGCAAG+GCTGCACT
TACTATTATGAACAAAGTTATCAAAGATAGTGGCATTCAAGGCAAACTTACCCTGATTCCGGCCGGTG

```
GTAGCTCATTTCTACCAGGGGCTATTACTCTTGCTGAAACAGCTATTAAAGCTGTTGAGCGGGTAATT
ACA
+
:FFFFFFFFFFFFFFFFFFFFFFFFFFFFFFFFFFFFFFFFFFFFFFFFFFFFFFFFFFFFFFFFFFF
FFFFFFFFFFFFFFFFFFFFFFFFFFFFFFFFFFFFFFFFFFFFFFFFFFFFFFFFFFFFFFFFFFFF
FFF
@A00721:190:HGJW7DSXY:4:1255:8793:36949_2:N:0:TCTGCAAG+GCTGCACT
ATCGCTGAAGGATGTGCACTCTTTTCTGCTACTTTAACGGAGCGTAGTGTACTCTTCGTCGATTGTAT
CAACAGTAACATTACTTCTAATAAAGGTCTCTATCAAGCTCGTATTAT
+
FFFFFFFFFFFFFFFFFFFFFFFFFFFFFFFFFFFFFFFFF:FFFFFFFFFFFFFFFFFFFFFFFFFF
FFFFFFFFFFFFFFFFFFFFFFFFFFFFFFFFFFFFFFFFFFFFFFFF
@A00721:190:HGJW7DSXY:4:1113:7979:1720_1:N:0:TCTGCAAG+GCTGCACT
TTACCGTTGAAGGCTAAACTCCAAGGTAATTATGAAGACTGTGATTTATCTTTCACTCTGGCCTTGTA
TGAGGGTGTTTCTAATCGTAACATTAATAATTTCAATCTTCTTAAAAAGAAGATAGAGAGTCATACTT
TGG
+
FFFFFFFFFFFFF:FFFFFFFFFF:FFFFFFFFFFFFFFFFFFFFFFFFFFFFFFFFFFFFFFFFF:FF
FFFFFFFFFFFFF::FFFFFFFFFFFFFFFFFFF::FFFFFFF:FF:F:FFFFFFFFFFFFFFF,FFF
FFF
@A00721:190:HGJW7DSXY:4:2411:19976:10410_2:N:0:TCTGCAAG+GCTGCACT
GATAGCCAATATTGAAGTGGACACCTCTGGTAGTATCGAAGTAAAGATAACCAATAAGGAAGGAGCTT
ATACTATGGTTGAATCAACTAAGCATACTACTTTTGCTTTGATTACTC
+
FFFFFFFFFFFFFFFFFFFFFFFFFFFFFFFFFFFFFFFFFFFFFFFFFFFFFFFFFFFFFFFFFFFF
FFFFFFFFFFFFFFFFFFFFFFFFFFFFFFFFFFFFFFFFFFFFFFFF
@A00721:190:HGJW7DSXY:4:2164:29487:5055_1:N:0:TCTGCAAG+GCTGCACT
GTCTAAGGTTAAAGAGTCGATTAAACAAGTTGTACTTATCAATGATTTTATCGCTGGTTGTTATCTCG
AAAAGTCTCTTCTAAAAGGAACGTCAGTTCTAACCCAAAGAGAATATCTAAAATATTTACAAAATCTT
ACC
+
FFFFFFFFFFFFFFFFFFFFFFFFFFFFFFFFFFFFFFFFFFFFFFFFFFFFFF:FF:FFFFFFFFFFFF
FFFFFFFF:FFFFFFFFFFFFFFFFFFFFFFFFFFFFFFFFFFFFFFFFFFFFFFFFFFFFFF:FFFF
FFF
@A00721:190:HGJW7DSXY:4:1261:2582:3646_2:N:0:TCTGCAAG+GCTGCACT
AGACGTTGCTTGTCGCTTCCTCCTTTAATGGCACTACCTCGGTACAATCTTACTTGGAACGAAGGCGA
CCTGTTCCGGAGTTTTTACGGGATCCGGAATGTTCTGTCTATATTAGG
+
FFFFFFFFFFFFFFFFFFFFFFFFFFFFFFFFFFFFFFFFFFFFFFFFFF:FFFFFFFFFFFFFFFFF
FFFFFFFFFFFFFF,FFFFFFF:FFFFFF,FFF:F,FFF::F:FFFFF
@A00721:190:HGJW7DSXY:4:1343:27227:6809_2:N:0:TCTGCAAG+GCTGCACT
TCGAACGTCAACGTTCACCGAGTTTCGTTTCGTACCTTGATGCTGACTTTCGTCAATATGATTTGATC
TGGTTGCTCAATTCTAAGCGTGTTCCGCTTAGTGCGAGGCCTCTCTTC
+
FFFFFFFFFFFFFFFFFFFFFFFFFFFFFFFFFFFFFFFFFFFFFF:F:FFFFFFFFFFFFFFFFFFFF
FFF,F:FFFFFFFFFFFF:FFFFFFFFFFFFFFFFFFFFFFFFFFFFF
@A00721:190:HGJW7DSXY:4:2259:31919:8328_2:N:0:TCTGCAAG+GCTGCACT
AAATACTAGATATTTGGATGATCTTGATAAAGAACTTCATGAATTTTCAGAGGATGAAAAATTTTCTT
CCTTGGATTTTAATATAAAGAATCTTTACCAGTACTCCTACTACAGGA
+
F:FFFFFFFFFFFFFFFFFFFFFFFFFFFFFFFFFFFFFFFFFFFFF:FFFFFFFFFFFFFFFFFFFF
F:FFFFFFFFFF:FFFFFF::FFFFFFFFFFF,FFFF:FFFFF:FFFF
@A00721:190:HGJW7DSXY:4:2362:31964:34428_1:N:0:TCTGCAAG+GCTGCACT
ATCGTAAGAAGTTTATAAGGAACATATCCATTTGTTGTAATAGGGTCATCAACTTAGAACTCGAAACT
```

CTAAGTGGTAATACTGTCTTTCCAAGCTTACCTGTATCTTCAATGAAATTGGACTCTTCGATAGGGGA
CAT
+
FFF:F:FFFF:FFFFFFF:FFFFFF:FFF:FFFFFFFF:FFFFFFFFFFFFFFFFFFFFFFFFFFFFFF
FFF:FFF:FFFFFFFF:FFFFFFFFFFFFFFFFFFF:FFFFFFFFFFFFFFFFF:FFFFFFFFFFF
FFF
@A00721:190:HGJW7DSXY:4:2139:31168:10285_1:N:0:TCTGCAAG+GCTGCACT
CTACTAATGGCGTTGGCCTAGTGGATGTTATTAGTGACATCTGGTCAATTTCGTTTTTAGGTGGTCTT
TATTCTATTATTGATTTTTATGAGATTGATACGTTAACAGAAGATGATGTTAGAGTAGAATTCCTGAA
GAG
+
FFFFFFF:FF,FFFFFFFFFFFFFFFFFFFFFFFFFFFFFFFFFFFFFFFFFFFFF:FFFFF::FF
FFFFFFFFFFFFFFFFFFFFFFFFFFFFFFFFFFFFFFFFFFFFFFFFFFFFFFFFFFFFFFFFFFFF
FFF
@A00721:190:HGJW7DSXY:4:1638:32606:6261_2:N:0:TCTGCAAG+GCTGCACT
CCAACTTCTTGACAATTTTCTTCAATCCTTCGATAATAGTTAAATCTAGGATTATGTCTGTGTTGAAA
GACTTTCTACCGATAAGGGTCACTAGTCTTACACAATTAAGAAATAAT
+
FFFFFFFFFFFFFFFFFFFFFFFFFFFFFFFFFFFFFFFF::FFFFFFFFFF:FFFFFFFFFFF
FFFFFFFFFFFFFFFFFFFFFFFFFFFFFFFFFFFFFFFF:FFFFFFF
@A00721:190:HGJW7DSXY:4:1660:10384:24048_2:N:0:TCTGCAAG+GCTGCACT
GATTATCTAAAGATTGAAGGAAAATTTTATCCTCGTTTAACTTACTTAGACTTTCCTACGATACAAAT
GATAACTTCTAATTTACGTTTGCTACTCAGAGCTGGTCCTCTCAGCAA
+
FFFFFFFFFFFFFFFFFFFFF:FFFFFFFFFFFFFFFFFFFFFFFFFFFFFFFFFFF:FFFFFFFFFFF
FFFFFFFFFFFFFFFFFFFFFFFFFFFFFFFFF:FFFFFFFFFFFFFF
@A00721:190:HGJW7DSXY:4:1620:23827:21120_2:N:0:TCTGCAAG+GCTGCACT
TCCTCAGGAGTCCCGGCATTTGATAATAATGACGACTTTCGTCATAATCGCTGCATATTACGGATTAC
ACGGTACCAGCGCATCTCGTGTCGAGCCTCGACCCGATTATATGGTAT
+
FFFFFFFFFFFFFFFFFFFFFFFFFFFFFFFFFFFFFFFFFFFFFFFFFFFFFFFFFFFFFFFFFFFF
FFFFFFFFFFFFFFFFFFFFFFF:FFFFFFFFFFFFFFFFFFFFFFFF
@A00721:190:HGJW7DSXY:4:1360:8974:22576_2:N:0:TCTGCAAG+GCTGCACT
TCTAGAATTCTTAATTATAATATTCAACCTCAAAAGTTACGTGTTTACAGTAACGAGATCACACATTT
TGAGAACTAACTATGTTATGTTCTCTGATCATTTTGACAATCTCTTCT
+
FFFFFFFFFFFFFFFFFFFFFFFFFFFFFFFFFFFFFFFFFFFFFFFFFFFFFFFFFFFFFFFFFFFF
FFFFFFFFFFFFFFFFFFFFFFFFFFFFFFFFFFFFFFFFFFFFFFFF
@A00721:190:HGJW7DSXY:4:1663:11460:32831_2:N:0:TCTGCAAG+GCTGCACT
AAATAAATAATATTACTAATGGCAAATAGTGGAAAGGCTCCAGTCAACACTAGTTCGAGTGCTGCTTT
GGATTACGATGACGTGAGAATAGGTGAAGGGATAGTTTTTACCTCGAC
+
FFFFFFFFFFFFFFFFFFFFFFFFFFFFFFFFFFFFFFFFFFFFFFFFFFFF:FFFFFFFFFFFFFFF
FFFFFFFFFFFFFFFFFFFFFFFFFFFFFFFFFFFFFFFFFFFFFFFF
@A00721:190:HGJW7DSXY:4:1461:16495:28714_1:N:0:TCTGCAAG+GCTGCACT
TCTTAAAGCTTCACCCAAAATGAAGGACACTTCGAAACTGAATAATGATGCACTACAAACAAGTATCG
CTAATCATCTGAAAAAGGCTCTAAAGGACACTATACAATATCCAGACGACGACGAAGCTTATACAATA
TCC
+
FFFFFFFFF:FFFFFFFFFFFFFFFFFFFFFFFFFFFFFFFFFFFFFFFFFFFFFFFFFFFFFFFFFF
FFFFFFFF,FFFFFFFFFFFFFFFF:FFFFF:FFFFFFFFFFFFFFFFFFFFFFFFFFFFFFFFFFFF
FFF
@A00721:190:HGJW7DSXY:4:2130:19180:16736_2:N:0:TCTGCAAG+GCTGCACT
ACCACATTTTGGTCAAGTCAATCCGTTGAGAAAATTTCTTAGAGGATATTCGCCATTGTGGTACGAAT

```
TGGTCCAACATTTACCACCAAATTCTTTTGATACAACTCTAGCCTTCA
+
FFFFF:FFFFFFFFFFFFFFFFFFFFFFFFFF:FFFFFF:FFFFFFFFFFFFFFFFFFFFFFFFFFFF
:FFFFFFFFFFFFFFFFFFF:FFFFFFFFFFFFFFFFFFFFFFFFFFFF
@A00721:190:HGJW7DSXY:4:2269:31675:3082_2:N:0:TCTGCAAG+GCTGCACT
ACCAGATTGGTTCGAACCCGTTGATGCTATGTCAGAAGAAGCTATTCAAGCTTGTCGTTTAAAGACTG
CAGAGGCTGTCAAACAAGCCCAGACTCAAGGAGTCCAATCAAAGGTTT
+
FFFFFFFFFFFFFFFFFFFFFFFFFFF:FFFFFF:FFFFFFFFFFFFFFFFFFFFFFFFFFFF:FFFF
FFFFFFFFFFFFFF,FFFFFFFFFFFFFF,:::FFF:FFFFFF,FFFFF
@A00721:190:HGJW7DSXY:4:2657:13268:28291_1:N:0:TCTGCAAG+GCTGCACT
TTATTTAGAGATCTTTTCTACTTACAAAGGACAATTCAGTTTCATTAATTCTACGGAAAAGTCCGGAG
TATATTGTATGTTAATAGATAGTGGGATAATAGTTCTACCGATACGTAGTGAACGCGTTCGTAGAACC
AAG
+
FFFFFFFFFFFFFF,FFFFFFFFFFF:FFFFFFFFFFFFFFFFFFFFFFFFFFFFFFFFFFFFFFFFF
FFFFFFFFFFFFFFFFFFFFFFFFFFFFFFFFFFFF:FFFFFFFFFFFFFFFFFFFFFFFFFFFFFFF
FFF
@A00721:190:HGJW7DSXY:4:1303:9001:16203_1:N:0:TCTGCAAG+GCTGCACT
ACCGATAGGAGTTGTATAGTTGAAGATGTGGCTTTAGACGATAATCCTTTAAGGACTATGTTGACTGA
CGAAGATTTTCCCACAGATTTGTCTACGTTGCAGATCGCCACTCCAAACACTATATACATTATAAAAT
ATA
+
FFFFFF:FFFFFFFFFFFFFFFFFFFFFF:FFFFFFFFFFFFFFF:FFFFFFFFFFFFFFFFFFFFFFF
FFFFFFFFFFF:FFFFFFFFFFFFFFFFFFFFFFFFFFFFFFFFFFFFFFFFFFFFFFFFFFFFFFFF
FFF
@A00721:190:HGJW7DSXY:4:2503:7193:4648_1:N:0:TCTGCAAG+GCTGCACT
ATAACTTTTGTTACCAAATTCTTGGCTTTCAACGAAGTCTCTGATTTTATTAATCATTACTTTAACGA
TCTCTCGGTCATTTACTCTGTACTTGATCTACGTCGTTTAACTGTCGGAACAACCTTTACTTAGAATG
AC
+
FF,F,F:FF,FFFF::FFFF,FF:F:FFF,,:,,FFFF:FFF,,FFFFFFFFFFF,FFFFFFFFF:FF
FFF:FFFFFFF:FFFFFFFFFFFFFFFFFFFFF,FF:FFFFF:FFF:F,FF,FFFFFFFFFFFFFFFFF
::
@A00721:190:HGJW7DSXY:4:1316:31882:32315_2:N:0:TCTGCAAG+GCTGCACT
TCTTATATTATTTACAATAGTTAATATCATATTAGTAATACTCACTATCGTTTTCTGCTTTTTCTTAG
CTAATAGATTAATAAAGGATGAGTACGAAAACCAACTCTTCGTTACGA
+
FFFFFFFFFFFFFFFFFFFFFFFFFFFFFFFFFFFFFFFFFFFFFFFFFFFFFFFFFFFFFFFFFFFF
FFFFFFFFFFFFFFFFFFFFF:FFFFFFFF:FFFFFFFFFFFFFFFFF
@A00721:190:HGJW7DSXY:4:2676:18792:8860_1:N:0:TCTGCAAG+GCTGCACT
TATAATAGTTTTAGTCTCTACACCCATCTCAACGAGGATAAAGCGTGTTTTCCGTCCTGAACGTAATA
CACGAAGAGATCATCCGCAAACGACTCGAAACCGAGACGCCGTAGATCAAAGTCCCTTCTAACTTTAG
AT
+
FF,F:F,,,FFF,FF,F,:FFFFF,FFFF,:F,,,F,:FF:,F,,,,FFFFFF,,F,F:F:,,FF,:,
F::FF,,,,F,,FFF:FFF:FFFFFFFFFFFFFFFFFFFFFFFFFFFFFFFFFFFFFFFFFFFFFFFF
FF
@A00721:190:HGJW7DSXY:4:2339:32768:8484_1:N:0:TCTGCAAG+GCTGCACT
CATTTTATAGCAGTTATTTATGTTGTACTTTTTATAATTTATAACCTTCTTATACTTTTGGGTATGTT
TTGTGAAAAACATGAGATACCCAAGTCTATCGAAGACATTATCTCTTATGTACTTATTGTAACGATCA
TTC
+
F:FFFFFFFF:FFFFFFFFFFFFFFFFF:FFFFFFFFFFFFFFFFFFFFFFFFFFFFFFFFFFFFFFF
```

FFFFFFFFFFFFFFFFFFFFFFFFFFFFFFFFFFFFFFFFFFFFFFFFFFFFFFFFFFFFFFFFFFFFFFF
FFF
@A00721:190:HGJW7DSXY:4:2547:5882:6856_1:N:0:TCTGCAAG+GCTGCACT
TTCTTTTATTCCTTTAACTGTGTATGAGGAGGATTTATAATGCTTTTCGATAGTGATTGTTATCAATT
TAAATACATTACTGTTTCAAATCATCTTTACTCACAGACTCCGAAAATGGTTTCAAATGAGGTCAAAG
CTC
+
FFF::FFF,FFFF:F:FFFFF:FFFFFFFF:FFFFFFFFFFFF:FFFFFFFFFFFFFFFFFFFFFFF,F
FFFFFFFFFFFFFFFFFFFFFFFFF:F:FFFFFFFFFFFFFFFFFFFFFFFFFFFFFFFFFFFFFFFFF
F:F
@A00721:190:HGJW7DSXY:4:1551:16179:30796_2:N:0:TCTGCAAG+GCTGCACT
TATTATCCGATTCTTCGAGGGTATCACTACCACTGATCTGCCGACTTTAGTCAAGGAACTTCAATTCC
TTCGATGTTTATATTTTTCTATAAGGGAAAATAGCAATAATCAAATTT
+
FFFFFFFFFFFFF:FFFFFFFF,FFFFFFFFFFFFFFFFFFFFFFF:FFFFFFFFFF:FFFFFFFFFF
,FFFFFFFFFFFFFFFFFFFFFFFFFFFFFF:FFFFF:FFFFFFFFFFF
@A00721:190:HGJW7DSXY:4:2134:21079:30608_2:N:0:TCTGCAAG+GCTGCACT
AACTTTATTTCAGAACTCTCTCCGATCACCTTGGTGTGATCTCCCGGAGGATCTTTTCAATAAGATGT
ATGATGATAATCTACTATTTTTAAATAGTGATATTCAGCGTATACTTG
+
FFFFFFFFFFFFFFFFFFFFFFFFFFFFFFF:FFFFFFF,FFFFFFFFFFFFFFFFFFFFFFFFFFFFF
FFFFF:FF:FF:FFFFFFFFFFFFFFFF:FFFFF:FFF::FFFFFFFF
@A00721:190:HGJW7DSXY:4:2572:17300:19210_1:N:0:TCTGCAAG+GCTGCACT
GGCTTGGTCTATTTACCGCAAAAAGTTGGAAGAGGTCTATGATATTTATTTTACTTATACTTTAAATA
GCACCGATTATAACTTGACTGTTGATAATATTACAATAGCTCTTTCTCAGAAGATTAGAGAAGTAACC
GGT
+
FFFFFFFFFFFFFFFFFFFFFFFFFFFFFFFFFFFFFFFFFFFFFFFFFFFFFFFFFFFFFFFFFFFFF
FFFFFFFFFFFFFFFFFFFFFFFFFFFFFFFFFFFFFFFFFFFFFFFFFFFFFFFFFFFFFFFFFFFFF
FFF
@A00721:190:HGJW7DSXY:4:1660:7780:35947_1:N:0:TCTGCAAG+GCTGCACT
GTAGCATTGATTTACTTTTACCGTTAGAACTGAAAAAGATTCTTTTCGAAAAGAAACCTATAAGTTAC
AAACGATTCTTCGACTATATTAAAGAAATAAAAATACTTCAAGTACCTGAACTTTACATTTTTAATGG
TAT
+
FFF:FFFFFFFFFFFFFFFFF,FFFFFFFFFFFFFFFFFFF,,FFFFFFFFFFFFFFFFFF:FFFFFF
FFFFFFFFFFFFFFFFFFFFFFFFFFFFFFFF:FF,FFFFFFFFFFFFFFFFF:FFFFFFFFFFFFFFF
FFF
@A00721:190:HGJW7DSXY:4:2534:17978:2284_1:N:0:TCTGCAAG+GCTGCACT
TTACAAATAGAACCTTCACTCTCGGTAATGAGAGTGTATTCCTTAATATTCTAGTAACCGCTATGTGG
GCATTACTAGGATTTATCACACTCCTAAATTTGATAGCAATTTATGTTTTAAATGCTAGGAAAATTTT
AGA
+
FFFFF:FFFFFFFFFFFFFFFFFFFFFFFFFFFFFFFFF:FFFFFFFFFFFFFFFF,FFFFFFFFFFF
FFFFFFFFFFFFFFFFFFFFFFFFFFFFFF::FFFFFFFFFFFFFFFFFFFFFFFFFFFFFFFFFFFFF
FFF
@A00721:190:HGJW7DSXY:4:1304:15438:29136_1:N:0:TCTGCAAG+GCTGCACT
GTATAGTTCAAGGAGTACTTAATTCAATAGCTTTAGTATTATTTACTATCGCCTTTATTGAAAATTGT
ATAAGAGTTAATTGCTTACACAAAAGTGACGCGCGAAGGAATCTTAATTGATTGAAGCTAGGCGTTGG
GG
+
FFFFFFFFFFFFFFFFFFFFFFFFFFFFFFFFFFFFFFFFFFFFFFFFFFFFFFFFFFFFFFFFFFFFF
FFFFFFFFFFFFFFFFFFFFFFFFFFFFFFFFFFFFF:FFFFFFF:FFFFFFFFFFFFFFFFFFFFFFFFF
FF

```
@A00721:190:HGJW7DSXY:4:1363:21730:7404_2:N:0:TCTGCAAG+GCTGCACT
ATACCATAACCTACCATGACCAATCATCGCTCCAACTGGCACGCCAGACGTAGGGTCAAACCTACTAC
CGCCGGTCGGCAGATCGGCACCACTGGTCTCTACGTTCAGGGACCCGC
+
FFFFFFFFFFFFFFFFFFFFFFFFFFFFFFFFFFFFFFFFFFFFFFFF:FFFFFFFFFFFFFFFF:FFFF
FFFFFFFFFFFFF:FFFFFFFFFFFFFFFFFFFFFFFFFFFFFFFFFF
@A00721:190:HGJW7DSXY:4:1414:4110:13182_1:N:0:TCTGCAAG+GCTGCACT
CCTTCCCAAATCTAGATCTGCACAGAAGAAGGCTGAAGGTCGTTACCGGACGACCACTCAAAGGAAAC
GGGCTGTTGCCCAGAGGAAAGCAGCGACCACCGAGGTCGCTCGTAACATTGACGGCAGTCCCGTCATC
TCT
+
FFF,FFFFFFFFF,::FFFFFFFFFFF:FFFFFFFFFFFFFFFFFFFFFFF:FFFFFFFFFFF:FFFFFF
FFFFFFFFFFFFFFFFFFFFFFFFFFFFFFFFFFFFFFFFFFFFFFFFFFFFFFFFFFFFFFFFFFFFFF
FFF
@A00721:190:HGJW7DSXY:4:1343:30798:27273_2:N:0:TCTGCAAG+GCTGCACT
GCAGTACCTCAGTCAAAAGTCACATTCACCACTCCCATACCAATCAATGATGTGACTATACAGTTCGG
CCATTTCAAGAGTGGTCAATACGTCGTGGGAGGTACTGAAGCCTTCCC
+
FFFFFFFF:FFFFFFFFFFFFFFFFFFFFFFFFFFFFFFFFFFFFFFFFFFFFFFFFFFFFFFFFFFFFF
FFFFFFFFFFFFFFFFFFFFFFFFFFFFFFFFFFFFFFFFFFFFFFFF
@A00721:190:HGJW7DSXY:4:1371:25183:29763_2:N:0:TCTGCAAG+GCTGCACT
CCAAGCCAATCAATAGTTCTTCTGACCAGAATGAAAAGGGGTTTACCAGGGTAGTCTCTAAGTCTACC
AGGAAGAGGGGAAATGTCCCCAAAACTGAGAAGAATAAAGGGAAAGGT
+
FFFFFFFFFFFFFFFFFFFFFFFFFFFFFFFFFFFFFFFFFFFFFFFFFFFFFF:FFFFFFFFFFFFFFF
FFFFFFFFFFFFFFFFFFFFFFFFFFFFFFFFFFFFFFFFFFFFFFFF
@A00721:190:HGJW7DSXY:4:1622:20645:36589_2:N:0:TCTGCAAG+GCTGCACT
AGGGGAAATTACCCCTAATTCTGAAGGAAGATCTCCCACCATCTGGGGTTGGGAATAAGAAATCTGCA
CGGGCCAAAGAGGCCGCTGCTACGAAGTCGCTGAAGGCGGCTTGGGTC
+
FFFFFF:FFFFFFFFFFFFFFFFFFFFFFFFFFFFFFFFFFFFF:FFFFFFFFFFFFFFFFFFFFFFFFF
FFFFFFFFFFFFFFFFFFFFFFFFFFFFFFFFFFFFFFFFFFFFFFFF
@A00721:190:HGJW7DSXY:4:2350:14045:19116_2:N:0:TCTGCAAG+GCTGCACT
GTTGCGCCTGCCGACCCCATCTTTGAGACCAAGACTCTCTACAGTAGGTTCGTGCCTACTATGAGCAA
ATCTGTTGAATTCGTCACTGAGGACGAAGGTGAGAGGACTTTTGGCTA
+
FFFFFFFFFFFFFFFFFFFFFFFFFFFFFFFFFFFFFFFFFFFFFFFFFFFF:FFFFFFF:FF,:FFF:,
FFFFF:,FFFFFFF:FFFFF,FFFFFF:FF:FFFFF:FFF,FFF:FFF
@A00721:190:HGJW7DSXY:4:2123:16179:35493_2:N:0:TCTGCAAG+GCTGCACT
GGAAAGAGAATTGCGTGGGTTGAAGGCCGACGCTTCGAGCTTGAGTGTGACGAGGCCGGTGACGTTTT
TGTCAACGGCGTTTGGAGGATTTTATCCGCGCAAGATCGGAAGAGCGT
+
FFFFFFFFFFFFFFFFFFFFFFFFFFFFFFFFFFFFFFFFFFFFFFFFFFFFFFFFFFFFFFFFFFFFFF
FFFFFFFFFFFFFFFFFFFFFFFFFFFFFFFFFFFFFFFFFFFFFFFF
@A00721:190:HGJW7DSXY:4:1165:3152:32784_2:N:0:TCTGCAAG+GCTGCACT
GAGTCTTGAAGGACTTAGGTTGGCATATTTATGCCGACGATAATAAGCATGTCACCCTTAAGCACCGA
GGTAAGCTCGGGACTTACAAGAGAGAGTGTGAGAGAGTCGCTCTCACT
+
FFFFFFFFFFFFFFFFFFFFFFFFFFFFFFFFFFFFFFFFFFFFFFFFFFFFFFFFFFFFFFFFFFFFFF
FFFFFFFFFFFFFFFFFFFFF:FFFFFFFFFFFFFFFFFFFFFF::FF
@A00721:190:HGJW7DSXY:4:1569:3197:2550_2:N:0:TCTGCAAG+GCTGCACT
TTCTGACTGATGAGGTTAAAACTCTTGCTCCTTCTGTGGGTGAGGGTTTTTGTTATATTCACTGGTTC
TGGGAGGCTGCTTTCCTCCATAATTACAACCTCTCTTTAGAGAGCATT
+
```

```
FFFFFFFFFFFFFFFFFFFFF:FFFFFFFFFFFF:FFFFFFFFFFFFFFF:FFFFFFFFFFFFFFFFFF
FFFFFFFFFFFFFFFFFFFFFFFFFFFFFFFFFFFFFFFFFFFFFFFF
@A00721:190:HGJW7DSXY:4:2262:21206:31328_2:N:0:TCTGCAAG+GCTGCACT
AAATTCCCACATATGGTAGACGTTGTCACACTTTTCTTGAAAATTTTTCCTTTCCTCCGTCATTGGGG
ATTTCGATGTGAGAAGGTGGAAGGTGTCAAGGGTCTTCTTCATGCTGA
+
FFFFFFFFFFFFFFFFFFFFFFFFFFFFFFFFFFFFFFFFFFFFFFFFFFFFFFFFFFFFFFF,:FFFF
FFFFFFFFFFF,FFFFFFFFF:FFFFFFFFFFFFFFFFFFFFFFFFFF
@A00721:190:HGJW7DSXY:4:1561:10529:6480_1:N:0:TCTGCAAG+GCTGCACT
TTCAGACGTGTGCTCTTCCGATCTCGGCTCGTATCGAGTTGGAGGGAGGAAGTTCTCCGAAATTAAAG
ACGGGGGGATTAAGAGTCCAGCTGTAACTGTCATTTCAGAGAGGGGAGCTCTGAAAAGGAAACTTCGT
CAG
+
FFFFFFFFFFFFFFF,FFFFFFFFFFFFFFFFFFFFFFFFFFFFFFFFFFFFF:FFFFFFFFFFFFFFF
FFFFFFFFFFFFFFFFFFFFFFFFFFFFFFFFFFFFFFFFFFFFFFFFFFFFFFFFFFFFFFFFFFFFF
FFF
@A00721:190:HGJW7DSXY:4:2313:19425:17378_2:N:0:TCTGCAAG+GCTGCACT
AGAGGGGAGGAGTTTGAGGAAGGCTCTAGAGAAGATCTCACCACCTGGAGAAGGCAAGTGGAGAGTCC
AGAAGTTGATGAAGAAGGATTATTTGAAGCAGGGGTTACCACTTCCTA
+
F:F:FF:FFFFFFFFFFFFFFF,FFFF:FFFFFFFFFFF:FF:FFFFFFFFFFFFFFFFF::FFFF:FF
FFFFFFFFFFFFFFFFFFFFFFF::FFFFFF:,::FFF::FF::F:FF
@A00721:190:HGJW7DSXY:4:2161:27353:19147_2:N:0:TCTGCAAG+GCTGCACT
TCTTTGGAGTTTTAGATAATCTTTTGAGAAGGGGTCATAGAGCCAGCAAGAGAAGCTTATATGCTGGT
TTAAGTGATGATTATCACTCCTTGAGAAGACAAGCCACATTAAGATAT
+
FFFFFFFFFF,FFFFFFFFFFFFFFFFFFFFFFFFFFFFFFFFFFFFFFFFFFFFFFFFFFFFFFFFFF
FFFFFFFFFFFFFFFFFFFFFFFFFFFFFFFFFFFFFFFFFFFFFFFF
@A00721:190:HGJW7DSXY:4:1319:18304:15436_2:N:0:TCTGCAAG+GCTGCACT
AATGTCATTAGTGCCTTACGATTGAAAGAGGTCCTTTTGAGTATCGACAGATCTTTCGATCTTGAGGA
CGTACATTTAACGTGGCAGAATTCGGTAACTTACCATATTGTTCCTTC
+
FFFFFFFFFFFFFFFFFFF:FFFFFFFFFFFFFFFFFFFF:FFFFFFFFFFFFFFFF::FFFFFFFFFF
FFFFFFFFFFF:FFFFFFFFFFFFFFFFFFFFF:FFFFFFF:FFFFFF
@A00721:190:HGJW7DSXY:4:1165:1850:5259_1:N:0:TCTGCAAG+GCTGCACT
ATTTAACTTTGACTCAACTACTCCGATCAGATCCGGATGGTTTGATTGGTATGGACTACTCGACTTTT
GATCTGGAGTTGTCTAGGGTCATGGCAGATACTACCGATAAGGTGAAAAATATGCAGGTCAAAGATTC
TGT
+
FFFFF:FFFFFFFFFFFFFFFFFFFFFFFFFFFFFFFFFFFFFFFFFFFFFFFFFFFFFFFFFFFFFFF
FFFFFFFFFFFFFFFFFFFFFFFFFFFFFFFFFFFFFFFFFFFFFFFFFFFFFFFFFFFFFFFFFFFFF
FFF
@A00721:190:HGJW7DSXY:4:1577:17635:7138_2:N:0:TCTGCAAG+GCTGCACT
GAGGGAGCCGTTTTGGCCGACTTTAACTATTCTAAGAGTCTTTATGATTCAAAACCTATCGTTGTCGT
ACCTTACAAGGTTGAGGAGACTTTACAGCGGCTTATTGTCGCGCAGTT
+
FFFFFFFFFFFFFFFFFFFFFFFFF:FFFFFFFFFFFFFFFFFFFFFFFFFFF:FFFFFFFFFFFFFFF
FFFFFFFFFFFFFFFFFFFFFF:FFFFFFFFFFFF:FFFFFFFFFFFF
@A00721:190:HGJW7DSXY:4:1122:12310:25535_1:N:0:TCTGCAAG+GCTGCACT
TATCTTGAATGCTCACGCCGTCGCAGCCATATCGAGACTATGTTATAATCACTTGTTAAAGTACAGAT
TTAGTGATTTAGATATTATTGATATTGGAGGCGATATTCGTTTTCATATCCAAAACTCCTTGAATGGA
GTA
+
FFFFFFFFFFFFFFFFFFFFFFFFFFFFFFFFFFFFFFFFFFFFFFFFFFFFFFFFFFFFFFFFFFFFF
```

FFFFFFFFFFFFFFFFFFFFFFFFFFFFFFFFFFFFFFFFFFFFFFFFFFFFF:FFFFFFFFFFFFFF
FFF
@A00721:190:HGJW7DSXY:4:1175:17074:13933_1:N:0:TCTGCAAG+GCTGCACT
ACCCTATTCTTGATGTGAAAGATGCATCGAGGTATACGTCTAGGAACATTGATTGGGAACGTACCTAC
TCACGAGACGTCATGAAAGCTATCGGAGGGAAGCAAAATGTTTCGTTCTGTTACAAGAACGCTTTGGA
TTG
+
FFFFFFFFFFFFFFFFFF,FFFFFF:FFFFFFFFF,FFF,FFFFF:FFFFFFFFFFF:FFF:FFFF:F
FFFFFFF:FFFFFFFFF:FFFFFFFFFFFFF,FFF:FFFFFFFFFFFFFFFF,FFFFFFF:FFFFFFF
FFF
@A00721:190:HGJW7DSXY:4:1269:20148:13307_2:N:0:TCTGCAAG+GCTGCACT
CTCAGTTGCGATAGCTGTAGAGGTATACGATATCTCTCTTGATGATGTAGCCACTATAATGCTTAAGA
GAGAGATTGATGTATGTCATTTCGTCTTAGTTTGTCCCGGCGAATTAT
+
FFFFFFFFFFFFFFFFFFFF,FFFFFFFFFFFFFFFFFFFFFFFFFFFFFFFFFFFFFFFFFFFFFFF
FFFFFFFFFFFFFFFFFFFFFFFFFFFFFFFFFFFFFFFFFFFFFFFF
@A00721:190:HGJW7DSXY:4:1209:19877:26772_2:N:0:TCTGCAAG+GCTGCACT
AAAAATGACATACCCGTTCCTGACCTTGGTATCGTTATCCGTCATGGAGATGATGGCGCATCACTTGA
TTACTATATGCCCGAAGGATTGGGTTATTCTCACAACAAAGATAAGAT
+
FFFFFFFFFFFFFFFFFFFFFFFFFFFFFFFFFFFFFFFFFFFFFFFFFFFFFFFFFFFFFFFFFFFF
,FFFFFFFFFFFFFFFFFFFFFFFFFFFFFFFFFFFFFFFFFFF:FFF
@A00721:190:HGJW7DSXY:4:1177:20428:27445_2:N:0:TCTGCAAG+GCTGCACT
AAGGGCGGACAATTGTTCCATTGTGAACTCTTGGGGACTAGATTGAGCATTAATGAGTTCCGAATAAC
TCGTAATAAATCTTTCTCTAGTGGACAATTCAGAATACCTATTTCCGT
+
FFFFFFFFFFFFFFFFFFFFFFFFFFFFFFFFFFFFFFFFFFFFFFFFFFFFFFFFFFFFFFFFFFFF
FFFFFFFFFFFFFFFFFFFFFFFFFFFFFFFFFFFFFFFFFFFFFFFF
@A00721:190:HGJW7DSXY:4:1404:14805:11913_1:N:0:TCTGCAAG+GCTGCACT
CTGCCAATCTCATCGCTATACGCATACCTGATTGGAAGAAGCGAGGTTCGTTCCTCAATAAAAATTTT
TCTCAAGAGATTATGGTTGATAAAGTTTTCTTCATAAGGGCTATTCAGTACGTCATGGGGATCACTTC
TTC
+
FFFFF:FFFFF:FFFFFFF,F,FF:FFFFFFFFFFFFFFFFFF,:FFFFFFFFFFFFFF:FF:,,FFF
F:FFFFFFFFFFFFF:FFF:FF,:FFFFFFFFF:F:FFFFFFFFFFFFFFFFF:FFFFFFFFFFFFFF
F:F
@A00721:190:HGJW7DSXY:4:1509:6813:32863_1:N:0:TCTGCAAG+GCTGCACT
ACCTTCGAATATGTTACGACATGGCTTCGTAACAACAGTGCGAGGATTGTCATCTCGGGGAAAGTGAT
TCACCAAGATGTTCATATAGATACGAGATACCTAGAGAAGGTCGCAGCGATCTTACTCGTTATCGGAG
CTA
+
F:FFF,FF,F,FF,F,FF:F,FFFFFF,FFFFFF::FFFFFFFFFFFFFFFFFFFFF:FFFFFFFFFF
F:F,F,F,FFFFFFFFFF:FFFFFFFFFFFFFF:FFF,FFFFFFFFFFFFFFFFFFFFFFFFFFF:FFF
:FF
@A00721:190:HGJW7DSXY:4:2621:5927:18928_2:N:0:TCTGCAAG+GCTGCACT
GTTATGCTCGCAAACTTAACGTTAGTACCGGAGGTGATCTCTCTGCTTCTGCGTTACTGAGAGAAAAG
ATTCAGGACGCCTACGGTGACTTTATGGACAAGATGCAAGAGATCGGA
+
FFFFFFFFFFFFFFFFFFFFFFFFFFFFFFFFFFFFFFFFFFFFFFFFFFFFFFFFFFFFFFFFFFFF
FFFFFFFFFFFFFFFFFFFFFFFFFFFFFFFFFFFFFFFFFFFFFFFF
@A00721:190:HGJW7DSXY:4:1109:31421:6214_1:N:0:TCTGCAAG+GCTGCACT
GCTCTTCCGATATGACAACGAATATTGAAACTACGGTCCTTATCAAAGAAAATGGTGAACCGGTCTCT
AAATCTTTTGAGATTGAGAAGATTGAAGAACACGTCAAGAAGTCGCTCGGCGTTGTTCTTGAAAACCA
AGT

+
FFFFFF,FF:F,FFFFFF::FF:,FFFFFFFFF:FFF:FFFFF,FFFFF,FF:FFFF:F:F:F:FF:F
FFFF:FFFFFFFFFFFFF,FF:FFF:FFFFF,FFFFFFFFFFFFFFF:FF:FFFFFFFFF:FFFF:FF
F:F
@A00721:190:HGJW7DSXY:4:1570:3477:12117_1:N:0:TCTGCAAG+GCTGCACT
GCTGATCATGGAGTCTTTGTACCTTCTGTCAAACTTCGAGGTTCAGCTACGAGAGGTGATCCCGGATG
TGGATTGAAGGCTGGCTCTAAGGGAGACTCCTTCTTTTTGAATGTTGTAAAGAATTGGGTTGAGAAGT
GTG
+
FFFFFFFFFFFFFFFFFFF:FFFFFFFFFFFFFFFFFFFFFFFFFFFFFFFFFFFFFFFFFFFFFFFF
FFFFFFFFFFFFFFFFFFFFFFFFFFFFFFFFFFFFFFFFFFFFFFFFF,FFFFFFFFFFFFFFFFFF
FFF
@A00721:190:HGJW7DSXY:4:2101:18927:13886_1:N:0:TCTGCAAG+GCTGCACT
CTTGGTATTTCTGAAATAGTTTATCAGAGCGTCATTTTCCTTTGGGATCTTTCCTCTGGTAAGGGAAT
TATTAAATCATTCTTCTCACTCTTGACGAGAATTATTACTGTTTTCTCTACCCATAACTTCTTTTTCA
AAA
+
FFFFFFFFFFFFFFFFFFFFFFFFFFFFFFFFFFFFFFFFFFFFFFFFFFFFFFFFFFFFFFFFFFFF
FFFFFFFFFFFFFFFFFFFFFFFFFFFFFFFFFFFFFFFFFFFFFFFFFFFFFFFFFFFFFFFFFFFF
FFF
@A00721:190:HGJW7DSXY:4:1308:28384:1924_2:N:0:TCTGCAAG+GCTGCACT
TTTCCTACAGAGTGCAGTCGTTTCAACACTTATCACCACTTTTGAAAGGAAAATAATTGGTGATGAAA
ATACGTATGTCGGTGGGGTTCTCAAGGCTCTCATCGCCACTGGGATTC
+
FFFFFFFFFFFFFFFFFFFFF,F,F:FFF:FFF,F:FFFFFFF:,FFFF:FFFF:FF:,FF,,F:,FF
F::,FF:F:F,FF:FFFFFF:F,FFFF:F:F:FF,,FFF:,FFFFFF,
@A00721:190:HGJW7DSXY:4:1533:29613:29356_2:N:0:TCTGCAAG+GCTGCACT
TTTGGTCCTTACGTCTTAAGTAATTTGGATGTTGTCGAAAACTATCAGGTTCTTGTCACGATTCTTAA
AAGAAGTTTATTAAAACTTCAGAGTCTCTTGGAAGTCCAGGTAGCTAA
+
:FFFFFFFF:FFFFFFFFFFFFFFFFFFFFFFFFFFFFFFFFFFFFFFFFFFFFFFFFFFFFFFFFFF
FFFFFFFFFFFFFFFFFFFFFFFFFFFFFFFFFFFFFFFFFFFFFFFF
@A00721:190:HGJW7DSXY:4:2570:14588:29700_2:N:0:TCTGCAAG+GCTGCACT
GGGAAAAGAGACCTCTGTTGGTAGAGGAGTTGAGAGAATTACTCAGATTCAATCTGGCATCACTGAAC
GTCTACAACGAGTTAGGGGGTGGCTAGGTTTTAAAAGATCCTTTGCAC
+
FFFF,:FFF:FFFFFFFFFFF:FFFFFFFFFFFFFFFFFFFFFFFFF:FFFFFFFFFFFFFFFFFFFF
FFFFFFFFFFFF:FFFFFFFFFFF::FFFFF:FFFFFFFFFF:FFFFF
@A00721:190:HGJW7DSXY:4:2246:9579:2268_2:N:0:TCTGCAAG+GCTGCACT
CGTCGCGGAAAACGATGAAGATTTCCACACTGTTCGAGAATCTTCTGATGCTGAATTTGAGGAAAATT
TTTCGGAAGTTTTTGGTATAAATCCTGATGGCTCAGTACAGGGAGGAG
+
FFFFFFFFFFFFFFFFFFFFFFF:FFFFFF:FF:,FFFFF:FFFFFFFF,FFFFF:::FFFFFFFFF,
F:FF:FFFFF,:FFFF::FFFFFF,,,,FF,FF,FFFFF:F,FFFF:F
@A00721:190:HGJW7DSXY:4:1150:2519:36198_2:N:0:TCTGCAAG+GCTGCACT
ACCTTCTTTTATCATTGCAACAGGACTTTGAGAAGACTGTTGAAATGGACAAAATCTGGGATCATAGG
TATGTGTCACGCTACACTCGAAATATGGGCCCTCATAAGGAAGTATAG
+
FFFFFFFFFFFFFFFFFFFFFFFFFFFFFFFFFFFFFFFFFFFFFFFFFFFFFFFFFFFFFFFFFFFF
FFFFFFFFFFFFF:FFFFFFFFFFFFFFFF:FFFF:FFFFFFFFFFFF
@A00721:190:HGJW7DSXY:4:1211:21151:10316_2:N:0:TCTGCAAG+GCTGCACT
GGAAGATCTCTACAGAGATTATCGAGAGTTGTGATCTAGTTCTAAATTTAGGTCGACGGACTTTAGCA
ACTTTAGATAAATTCTTTCCGGGCTCAATGACCTTTGCTATGTACGTG
+

FFFFFFFFFF:FFFFFFFFFFFFFFFFFFFFFFFFFFFFF:FFF:FFFFFFFFFF:F:F:FF,FFFF:F
F:FFFFFFFFFFFFFFFFFFFFFFFF:FFF:FFFFFFFFFFF:FFFFFF
@A00721:190:HGJW7DSXY:4:1227:12156:15186_1:N:0:TCTGCAAG+GCTGCACT
TTAGTTCATGAAGTTCTTACACTTCTCGCGACTAATGTCAGCTTCACTACAAAGGCGTTACGTAAAGT
AGTTTTCACGCTTTATCTTAAAGCATTACCACCAAATTTAGCACTTTTTGAATATGACAGAAGCTCGC
CAT
+
FFFFFFFFFFFFFFFFFFFFFFFFFFFFF:FFFFFFFFFFFFFFFFFFFFFFFFFFFFFFFFFFFFFFF
FFFFFFFFFFFFFFFFFFFFFFFFFFFFFFFFFFFFFFFFFFFFFFFFFFFFFFFFFFFFFFFFFFFF
FFF
@A00721:190:HGJW7DSXY:4:1516:23240:23390_2:N:0:TCTGCAAG+GCTGCACT
CCAGTTAGAGTTCTTCTAACCAGGACTGATGGTGAGATTCTTTTACCTTTTGGAGGTCCAGGAACCTT
TACTCCTAGTGTCTTTTCTAGAGTCCAAGGACGTTTCCGTCGGAGGAT
+
FFFFFFFFFFFFFFFFFFFFFFFFFFFFFFFFFFFFFFFFFFFF:FFFFFFFFFFFFFFF:FFFF
FFFFFF:,FFFFF:FFFFFFFFFFFFFFFFFFFFFFFFFFFFFFFFFFF
@A00721:190:HGJW7DSXY:4:2461:21278:8750_1:N:0:TCTGCAAG+GCTGCACT
GCTCAATAACACTCTATTGAATCCCTTTTCCACGATGAAAGCTCTTTATGAGGTGCTCCATGCAAATT
TACAGTTCCTCTTTGGTGATATCGCCTCTGTCCTTTCTCCTTTAATCGTGAGGTTACGAGAGTTTCGT
ACT
+
FFFFFFFFFFFFFFFFFFFFFFFFFFFFFFFFFFFFFFFFFFFFFFFFFFFFFFFFFFFFFFFFFFFF
FFFFFFFFFFFFFFFFFFFFFFFFFFFFFFFFFFFFFFFFFFFFFFFFFFFFFFFFFFFFFFFFFFFF
FFF
@A00721:190:HGJW7DSXY:4:2257:23637:32002_1:N:0:TCTGCAAG+GCTGCACT
TTTTGATAAATTTGTTGGAATCGATAATGATACTGACGAAGGTTCTATTACCACCCTTGTCATTGATA
CAAACATTCTAGGTGATGCTTTCTTAGAGGCTCGTCTAGCCGCTGAGATTCGTCTTACCTATTTACAA
TCT
+
FFFFFFFFFFFFFFF::FFFF:FFFFFFFFF:FFFFFFFFFFFFFFFFFFFFFFFFFFFFFFFFFFFF
FFFFFFFFFFFFFFFFFFFFFFFFFFFFFFFFFFFFFFFFFFFFFFFFFFFFFFFFFFFFF:FFFFFF
FFF
@A00721:190:HGJW7DSXY:4:2236:31105:9236_1:N:0:TCTGCAAG+GCTGCACT
GATAACGATTTAGTACTGGGAAATGGTGGTAGTCAGTTTACTACTTTTCTTTCCCTCCCCTTTTTCGA
TTGGATTCGACGGGGTCTTCGTCTCACGACTAATTTGATAAGGTTTTTCTTTTATGGGACTCTTGTTT
GTA
+
FFFFFF:FFFFFFFFFFFFFFFFFFFFF:FFFFFFFFFFFFFFFFFFFFFFFFFFFFFFFFFFFFFFF
FF:FFFFFFFFFFFFFFFFFFFFFF:FFFFFFFFFFFFFFFFFFF:FFFFFFFFFFFFFFFFFFFFFFF
FFF
@A00721:190:HGJW7DSXY:4:1270:18177:15374_2:N:0:TCTGCAAG+GCTGCACT
GTTCTTAATGTAGGTCTCCTAGTACTTAAGGGTTTATGTTTCGGTTTCAGCTGGATGGATCTTTTGAT
GGTATGTTTCACATTGCGTGATCACTTCCCGTGGACTTGGGAAGAGAT
+
FFFFFFFFFFFFFFFFFFFFFFFFFFFFFFFFFFFFF,FFFFFFFFFFFFFFFFFFFFFFFFF:FFFFF
FFFFFFFFFFFFFFFFFFFFFFFFFFFFFFFFFFFFF:F,FFFFFFFFF:
@A00721:190:HGJW7DSXY:4:2511:22182:25942_1:N:0:TCTGCAAG+GCTGCACT
CGATTCTATTATGTCGCTAAAGCCTGTCCAGTTGTCGATGATGTTCGGGCTGTGGGGATTAACTTCCC
TCGTGAAACTTACGACGATGTTCTCGCTCGCATTTCACAAGGATTACTAAGTCTGAGATCTGCCACTT
CTA
+
F:FFFFFFF:::FFFFFFFFFFFFFFFFFFFFFFFFFFFFFFFFFFFFFFFFFF:F:FFFFFFFFFF:F
FFF,FFFF:FFFFFFFFFFFFFFFFFFFFFFFFFFF:FF:FFFFFFFFFFFF,FFFFFFFFFFFFFFFFF
FFF

```
@A00721:190:HGJW7DSXY:4:1264:27760:4225_2:N:0:TCTGCAAG+GCTGCACT
AAGTTCATGAGGATGTCTTGGTCGAAAAATATCGCCGGGAACTTCTAATAGAGGATCCTGATGACATT
ATTTCAACGGATACCGATCTCAGTTCTACCACAGAAACCGTCTCAGGA
+
FFFFFFF:FFFFFFFFFFFFFFFFFFFFFFFFFFFFFFF::F:FF:FFFFFFFFFFFFFFF:FFF:FF
:FFFFFFFFFFFFFFFFFFFFFFFFFFFFFFFFFFFFFFFFFFFFFFF
@A00721:190:HGJW7DSXY:4:1355:27082:23782_2:N:0:TCTGCAAG+GCTGCACT
CTGATTCGGAATTAGAGAAAGACGACCTTCTTGTGGGGACTGAGACCTCTGAATTTTCAGGGTGCCCT
GATGAGGACGTTGAAAGGGATAATTTCAACTCTGAGACTTCTAAGAGT
+
F,:::F:FFFFFF:,FFFFFF::,F,FFFF,:FF:::FFF:FF,FFFF:,,F,:,:FFFF,:FF:FFF
FFFFFF,F:FF,FF,F:FFF:FFF:FFFFF:FFFF,F,,FF,,F::FF
@A00721:190:HGJW7DSXY:4:2237:26847:29387_2:N:0:TCTGCAAG+GCTGCACT
ACCAGAAACGTAACAAGAAGAATAAAGGTAGGAAGGGGAGAAAACAGAACAAGCGAGCGGTTGAAGAG
AAAACTGATATGGTTGCGGCTGATCGTACCCCAATTCCTGCTAGAAAG
+
FFFFFFFFFFFFFFFFFFFFFFFFFF:FFFFFFFFFFFFFFFFFF:FFFFFFFFFFFFFFFFFFFFFFF
FFFFFFFFFFFFFFFFFFFFFFFFFFFFFFFFF:FFFFFFF:FFFFFF
@A00721:190:HGJW7DSXY:4:2618:3604:5384_1:N:0:TCTGCAAG+GCTGCACT
ATACCACTGATCTTCCAGAGTTACGTAGTGGTCGTTCTTTTGGTGATTTTATCAAAGAGAAAGTTTCT
GCTAGTCGCGACGATAGTATTTATCGGAGTGATATTAACAAGTTAAACTTCGAACTGACCACTTACAA
GAG
+
FFFFFFFFFFFFFFFFFFFFFFFFFFFFFFFFFFFF:FFFFFFFFFFFFFFFFFFFFFFFFFFFFFFF
FFFFFFFFFF:FFFFFFFFF:FF:FFFFFFFFFFFFFFFFFFFFFFFFFFFFFFFFFFFFFFFFFFFF
FFF
@A00721:190:HGJW7DSXY:4:2367:3622:35352_2:N:0:TCTGCAAG+GCTGCACT
TCAGTTTTCCAAAAAGCTATTAATAGCAGTTGCGGGGCTATAATACCGCCTCCAGATTACTCAAAACT
GAATCAACTCTCTTGGGATATAAATGAGTACATCTATAATAGAGTTTT
+
F:,:,FFFF:FFFFFFFFFF,FF:F:FFFFFFFF,:F,FFFFF,F:F,FFF,FF:F,FFF,F,FFFFF
FFF,:,F,:FFFF:,F,FFF:FFF,,FFFFFFFFFFFFF::FF:F,:F
@A00721:190:HGJW7DSXY:4:2602:28339:32064_1:N:0:TCTGCAAG+GCTGCACT
AACATGAATTAGTTATTGAAAGTCTTGACCCTCACGTCTCACTTTATCACCTTGGAGATGGTAAAGTC
ATTAACAAAGGAAATGTTAAGGCTGATGACTTAAATTTCGCATTCTGCTTCGACGGTAATTCCATAAG
ACC
+
FF:FFFFFFFFFFFFFFFFFFFF:FFFFF:FFFF:FF:FFFFFFFFFFFFFFFFFFFFFFFFFFFFFF
FFFFFFFFFFFFFFFFFFFFFFFFFFFFFFFFFF:FFFFFFFFFFFFFFFFFFFFFFFFFFFFFFFFF
FFF
@A00721:190:HGJW7DSXY:4:1324:29803:28839_1:N:0:TCTGCAAG+GCTGCACT
AGAGTGACTGGAGTTCAGACGTGTGCTCTTCCGATCTGTGAGTTATTCTTGCGAAGGTTCAAGTCGAT
TGATTTTGTTGACTACGATCTCTCTTGTAATAAGAAGTTCTTCGCAGTTGAATCTCCACCAGGTGGAG
GGA
+
FFFFFFFFF:FFFFFFF:F:FFFFFFF:FFFFFFFFFFFFFFFFFFFFFFFFFFFFFFFFFFFFFFFF
FFFFFFFFFFFFFFFFF:FFF:FFFFFFFFFF:F,:F:FFFFFFFF,FFFFFFFFFFFFF:FFFFFFF
FFF
@A00721:190:HGJW7DSXY:4:1242:13214:3490_1:N:0:TCTGCAAG+GCTGCACT
GACTTTTGTCGAATTTTTCGCAAAAGGTCTTGAAGTACGTATTGTCACTGCTAATCGTAATTCGCGAG
AAGAGATAGTGACTAAAGTTTTGGCACTTCTAGTTCATCGCGGTATTTGTGAAATTAAGACAGACGTG
AAA
+
FFFFFFFFFFFFFFFFFFFFFFFFFFFFFFFFFFFFFFFFFFFFFFFFFFFFFFFFFFFFFFFFFFFF
```

FFFFFFFFFFFFFFFFFF:FFFFFFFFFFFFFFFFFFFFFFFFFFFFFFFFFFFFFFFFFFFFFFFFF
FFF
@A00721:190:HGJW7DSXY:4:1305:9019:33395_1:N:0:TCTGCAAG+GCTGCACT
TCGTATTAGAACAGCTGATTCTTATTGCATGAACGTCGCTGAGGTCAAACCTGTGGACGTTCTTTTCA
TCGACGAATTATTCATGATTCACGCTGGTCAGACCCTTTTTCTTACACATCTACTTAAGGCGGCTTAT
ACC
+
FFFFFFFFFFFFFFFFFFFFFFFFFFFFFFFFFFFFFFFFFFFFFFFFFFFFFFFFFFFFFFFFFFFF
FFFFFFFFFFFFFFFFFFFFFFFFFFFFFFFFFFFF:FFFFFFFFFFFFFFFFFFFFFFFFFFFFFFF
FFF
@A00721:190:HGJW7DSXY:4:1470:27263:3552_1:N:0:TCTGCAAG+GCTGCACT
GTGATTCCAATCAAATCGGATTCATCAATCGTATTGATCTCCAAGTCGCAAACTACTTCAACATCAAC
GACATCATTCTTGATGAGTGTCGTTCTTATCGAAGTCTCAGTTATCGCTGCCCGGCCGACGTTTGTCG
CGA
+
FFFFFFFFFFFFFFFFFFFFFFFFFFFFFFFFFFFFFFFFFFFFFFFFFFFFFFFFFFFFFFFFFFFF
FFFFFFFFFFFFFFFFFFFFFFFFFFFFFFFFFFFFFFFFFFFFFFFFF:FFFFFFFFFFFFFFFFFF
FFF
@A00721:190:HGJW7DSXY:4:1620:9091:11162_2:N:0:TCTGCAAG+GCTGCACT
TACGGCAGAAAAATTTCTCCTGCTTGTAATTCGAGAGAGGACACTATGGAAATTAGACCTATCAGATG
TCTTGACGACTTCGAATTTGAGGACGGAGTTAAATATATTACTTTCAC
+
FFFFFFFFFFFFFFFFFFFFFFFFFFF:FFFFFFFFFFFFFFFFFFFFFFFFFFFFFFFFFFFFFFF:F
FFFFFFFFFFFFFFFFFFFFFFFFFFFFFFFFFFFFFFFFFFFFFFFF
@A00721:190:HGJW7DSXY:4:1220:18511:19648_2:N:0:TCTGCAAG+GCTGCACT
CAAAGTCCTTGGTAAGAGTAAGGTTAAATGTGAAGATGCTCAGACTATTCATGAAGTTCAAGGTAATA
CTTACCAGCACGTTCGGCTGGTTAGAATTAAACCTCAAGATGACCCTG
+
FFFFFFFFFFFFFFFFFFFFFFFFFFFFFFFFFFFFFFFFFFFFFFFFFFFFFFFFFFFFFFF:FFFF
FFFFFFFFFFFFFFF:FFFFFFFFFFFFFFFFFFFF:FFFFFFFFFFF
@A00721:190:HGJW7DSXY:4:1270:21278:4053_1:N:0:TCTGCAAG+GCTGCACT
CTTCCGATCTCTCTATCACGACACGTTGAATCTTTACGATACTTTGTTATCAATGTAAAGTTAAACGA
TGCTACTTGTGAGAGGATCAATCACAGTAAGGAACTGTCACGTTTAGCACGGGAGGTTGCAGATTCAA
CCC
+
FFFFFFFFFFFFF,FFFFFFFFFFFFFFFFFFFFFFFFFFFFFFFFFFFFFFFFFFFFFFFFFFFFFF
FFFFFFFFFFFFFFFFFFFFFFFFFFFFFFFFFFFFFFFFFFFFFFFFFFFFFFFFFFFFFFFFFFFF
FFF
@A00721:190:HGJW7DSXY:4:1344:1434:30373_1:N:0:TCTGCAAG+GCTGCACT
AGACGTGTGCTCTTCCGATCTCTTGAAGTTGTTGACGACAAGCCTCAAGAGGGTAGAGGACCACCTGG
ATCTTCTACTGTCTCTGCCTTCCAAGATTTCTTAGAAGAGGTTATTCCTGGAACCACCTTAACGTCAC
TCG
+
FFFFFFFFFFFFFFFFFFFFFFFFFFFFFFFFFFFFFFFFFFFFFFFFFFFFFFFFFFFFFFFFFFFF
FFFFFFFFFFFFFFFFFFFFFFFFFFFFFFFFF:FFFFFFFFFFFFFFFFFFFFFFFFFFFFFFFFFF
FFF
@A00721:190:HGJW7DSXY:4:2163:8974:22514_2:N:0:TCTGCAAG+GCTGCACT
TGAGTTCAAGTCGGTTGTCGATCGAATTAAGATCAAGGCTAATACCTTTATAGGTAAGAAGCCCCTTG
AGCCGCGTAGTCAGCGCGTTTAGCTGTATCATCAGTGAGATCGGAAGA
+
FFFFFFFFFFFFFFFFFFFFFFFFFFFFFFFFFFFFFFFFFFFFFFF:FFFFFFFFFFFFFFFFFFFF
FFFFFFFFFFFFFFFFFFFFFFFFFFFFFFFFFFFFFFFFFFFFFFFF
@A00721:190:HGJW7DSXY:4:2319:1298:23563_2:N:0:TCTGCAAG+GCTGCACT
GAGTCTCCAAGAGAATATTTATTCTTATGAGGCTCGAAACTTCAACGTTTTCACAACGGAAAGACATA

TCGCACCCGAAATTTTTGGGCAGAATGCTCTTAACGCGGTTCTTGAAC
+
FFFFFFFFFFFFFFFFFFFFFFFFFFFFFFFFFFFFFFFFFFFFFFFFFFFFFFFFFFFFFFFFFFFFFFF
FFFFFFFFFFFFFFFFFFFFFFFFFFFFFFFFFFFFFFFFFFFFFFFF
@A00721:190:HGJW7DSXY:4:2653:31060:12978_2:N:0:TCTGCAAG+GCTGCACT
CTAAGTTGGATAAAGTTATAGGAGACTCAGTTAACCTGAGAGCTCCTCAATTGATAGATTGGGTCAAC
AATAGGGACGCTAGTATACTTCGTAAAGTCGACAGCGAAATGGTCAAT
+
FFFFFFF:FFFFFFFFFFFFFFFFFFFFFFFFFFFFFFFFFFFFFFFFFFFFFFFFFFFFFFF:FFFFF
FFFF:FFFFFFFFFFFFFFFFFFFFFFFFFFFFFFF:FFF,FFFFFFFF
@A00721:190:HGJW7DSXY:4:1570:15420:30326_1:N:0:TCTGCAAG+GCTGCACT
TGACCTATTTTAAGCTCATGGTGAAAAGGGAAGCTAAGGTTAAACTTGATGCTTCAGCCTTTTCGAAG
CATAACCCCGCACAGAATATCGTTTATCACGCAAAATTTATTAATGCAATTTTCGGACCTATCTTTAA
GGT
+
FFFFFFFFFFFFFFFFFFFFFFFFFFFFFFFFFFFFFFF,FFFFFFFFFFFFFFFFFFF:FFFFFFFF
FFFFFFFFFFFFFFFFFFFFFFFFFFFFFFFFFFFFFFFFFFFFFFFFFFFFFFFFFFFFFFFFFFFF
FFF
@A00721:190:HGJW7DSXY:4:1143:32117:13338_1:N:0:TCTGCAAG+GCTGCACT
GACGATTTCTCCCGGAAGCTCTTCTCTCTCTTAGGAGGTCATGATGACTACTATAAGGGTGAAATCGA
TTTTTCGAAGTTTGACAAATCTCAAGATTTCTTTTGTAAAGCCTATGAGATAAAGATGTATTCTTATT
TAG
+
FFFFFFFFFFFFF:FFFFFFFFFFFFFF:FFFFFFFFFFFFFFFFFFFFFFFFFFFFFFFFFFFFFFFF
:FFFFFFFF::FFFFFFFFFFFFFFFFFFFFFF:FFFFFFFFFFFFFFFFFFFFFFFFFFFFFFFFFFF
FFF
@A00721:190:HGJW7DSXY:4:1223:11586:12665_2:N:0:TCTGCAAG+GCTGCACT
TTGGAGTGCCAGTGAATATTTTTGTCACGCCAGGAGTATCCATAACGATTTGTCATTTACCACCTTTG
CTCAACGACGAAGTGGAACGGCCAACACTTTCTTTGGTAATTGTATGA
+
:FFFFFFFFF:FFFFFFFFFFFFFFFFF:FFFFFFFFFFFFFFFFFFF:FFFFFFFFFFFFFFFFFFF
:FFFFFFFFFFFF:FFFFFFFFFFFFFFFFFFFFFFFFF:FFFFFFF:F
@A00721:190:HGJW7DSXY:4:1549:9173:23171_2:N:0:TCTGCAAG+GCTGCACT
ATTTTATCACTTTATTATGACCTTGGAAAGTTTTCGGCTGTGGTTGTCGCTGGGGACGATTCCATTCT
GTTTTCATAGACTCCAATAACTGATCATTCTGATCATATGATTTTGGA
+
FFFFFFFFFFFFFFFFFFFFFFFFFFFFFFFFFFFFFFFFFFFFFFFFFFFFFFFFFFFFFFFFFFFF
FF:FFFF,:,FFFFFF:FFFFFFFFFFFF:FFFFFFFFF:FFFFFFFF
@A00721:190:HGJW7DSXY:4:1261:15483:19633_1:N:0:TCTGCAAG+GCTGCACT
CTTACAAGATCATCGTTAAGTTAGGTAAACCCCTAACCGATGTTACTCTTGTAGGACTTTGGGAGAGA
TTCGTTAGTTTTAAAGACTATACTAAATATCTCCACTACGAGGTTGTTGTTTGGCGATTAGCTCTTGC
GA
+
FFFFFFFFFF::F,FFFFFF:FF:FFFFFF:FFFFFF:FFFFFFFFFFFFFFFF:FFFFF:,FFFFFF
,FFFFFFFFFFF:FFFF:FFF:FFFFFFFFFFFF:FFF:FFFFFFFFFFFFFFFFFFFFF::FFFFF:
FF
@A00721:190:HGJW7DSXY:4:1462:26042:11992_2:N:0:TCTGCAAG+GCTGCACT
GAGGGTCGTTATACCTATTCGGCTATCTGTGCTTTGAATTGTATAAGATCAAATTTTAAACGATTCCG
AGAGGTTTATCCTGATGTGGAGGGAGTTCTTGAAGCATCCCGACCTTT
+
FFFFFFFFFFFFFFFFFFFFFFFFFFFFFFFFFFFFFFFFFFFFFFFFFFFFFFFFFFFFFFFFFFFF
FFFFFFFFFFFFFFFFFFFFFFFFFFFFFFFFFFFFFFFFFFFFFFFF
@A00721:190:HGJW7DSXY:4:2508:19153:35289_2:N:0:TCTGCAAG+GCTGCACT
GGTTTCATAAGATTATCAGTAATCCTGATTCTACTACGGATTCATGGACCTTCCATCCTGTACCTCTT

```
CTGGAGAGACGTATCTCAAAGATCGACTTCGAGACAGGTGATATTGTT
+
FFFFFF::FFFFFFFFFFFFFFFFFFFFFFFFFFFFFFFFFFFFFFFFFFFFFFFFFFFFFFFFFFFF
FFFFFFFFFFFFFFFFFFF:FFFFFFF:FFFFFFFFFFFFFFFFFFFF
@A00721:190:HGJW7DSXY:4:1559:27914:9815_1:N:0:TCTGCAAG+GCTGCACT
ACCAAGATCTAAATTCGGTAACGGTGCGTGGCGCTTTATTTTAAAAAGCTCCCTCCTCTCGCAAGTTT
GATCTGATACTTATCACGAAAGGACTTGAGTCTTTTGATCAGTCTCTGACTTGACCATCTTTCATTTT
AAA
+
FFFFFFFFFFFFF,FFFFFFFFFFFFFFFF:FFFFFFFFFFFFFFFFFFFFFFFFFFFFFFFFFFFFF
FFFFFFFFFFFFFFFFFFFFFFFFFFFFFFFFFFFFFFFFFFFFFFFFFFFFFFFFFFFFFFFFFFFF
FFF
@A00721:190:HGJW7DSXY:4:1611:26883:33144_2:N:0:TCTGCAAG+GCTGCACT
TTTCTTCTTTGTTTCCTTCTTTGCACTGATTTTTCCTCATCAGCTTGTGTTTTACCTAATCTGTGTTT
TTACGATAACAACTATCAGTTGATTAAGACTCTTCCGAAAGGCGAACT
+
FFFFFFFFFFFFFFFFFFFFFFFFFFFFFFFFFFFFFFFFFFFFFFFFFFFFFF:FFFFFFFFFFFF
FFFFFFFFFFFFFFFF,F:FFFFF:FF:FFFF:F,::FFF:FF:FFFF
@A00721:190:HGJW7DSXY:4:1233:7925:7764_1:N:0:TCTGCAAG+GCTGCACT
AGCTCAGGGTGAAGCACAACGTTTGACCGTGACGAACTGTGGTGTTCTTGATCTACGTTACGGATATA
CTCTCCTTGAGTTCACTCGTAAGGACACTACCACTTATTACGATATTTCTTTAGTCGATGGGTTTTCG
ACT
+
FFFFFFFFFFFFFFFFFFFFFFFFFFFFFFFFFFFFFFFFFFFFFFFFFFFFFFFFFFFFFFFFFFFF
FFFFFFFFFFFFFFFFFFFFFFFFFFFFFFFFFFFFFFFFFFFFFFFFFFFFFFFFFFFFFFFFFFFF
FFF
@A00721:190:HGJW7DSXY:4:1441:27055:31782_1:N:0:TCTGCAAG+GCTGCACT
TCTCTATTTTAAGTTTTGTCTTCCTTGTTTGTCTTATTCTATTAATCACTCATTTTAGTGAGATTATT
AAAAACGCACTAAAACGTCTTCGACAACAGCAGGGGTTTTCGAATAGTGATAGCCACCCGCCTTACAG
AGG
+
FFF:FFFFFFFFFFFFFFFFFFFFFFFFFFFFFFFFF:FFFFFFFFFFFFFF:FFFFFFFFFFFFFF:F
FFFFFFFFFFFFFFFFFFFFFFFFFFFFFFFFFFFFFFFFFFFFFFFFFFFFFFFFFFFFFFFFFFFF
FFF
@A00721:190:HGJW7DSXY:4:2603:2510:15421_2:N:0:TCTGCAAG+GCTGCACT
GACTTTGGTACCACCTTTTCTACTGTCTGTCTCGCCTCTTCGAATGAAGTTTTAATGCTTAACAACAA
TGATAGTGAATTTATACCTACAATCATTGGTTTTTCTAATAATGATGA
+
FFF:FFFFFFFFFFFFFFFFFFFFFFFFFFFFFFFFFFFFFFFFFFFFFF:FFFFFFFFFFFFFFFFF
FFFFFFFFFFFFFFFFFF,FFFFFFFFF:FFFF:FFFFFFFFFFFFFF
@A00721:190:HGJW7DSXY:4:1270:26811:6590_1:N:0:TCTGCAAG+GCTGCACT
GTGACTGGAGTTCAGACGTGTGCTCTTCCGATCTGTCAGGGAGGATTCTCAGTTTACAAGGACCTTAA
GCGATGGATCGGGGTTAATTCTAAAACATTACCCGAAAGACGTGCTAAATTAAAACCATTGTACGATG
TCG
+
FFFFFFFFFFFFFFFFFFFFFFFFFFFFFFFFFFFFFFFFFFFFFFFFFFFFFFFFFFFF:FFFFFFF
FFFFFFFFFFFFFFFFFFFFFFFFFFFFFF:FFFFFFFFFFFFFFFFFFFFFFFF:FFFFFFFFFFFFF
FFF
@A00721:190:HGJW7DSXY:4:1553:5005:17832_1:N:0:TCTGCAAG+GCTGCACT
TCACTTTTCACTAAGAATTGGTCCTACTTTTGGACAAGGCCAACTTCGTAATCTTCATGAGATCGTTT
CTTTCTTTATCTCTTTGGTTGTTAGGGATTTTGAGAGAGTGAAAGGCTTCAATTGTAGTGGTTTGGTC
GTT
+
F:F:FFFFFFFFFFFFFFFFFFFFFFFFFFFFFFFFFFFFFFFFFFFFFF:FFFFFFFFFFFFFFFFF
```

```
FFF:FFFFFFFFFFFFFFFFFFFFFFFFFFFFFFFFFFFFFFFFFFFFFFFFFFFFFFFFFFFFF:FFFF
FFF
@A00721:190:HGJW7DSXY:4:2615:25545:8625_2:N:0:TCTGCAAG+GCTGCACT
ACCTCTACGCAAAGATATTTCATGAAAACTACAGAGGAACGTACCGGATTACCTGTCTTTCATATTAT
GAATGAACCTTCGGCCGCTCTCTTTGCTTCGATGTTGGATGTCAAGAA
+
FFFFFFFFFFFFFFFFFFFFFFFFFFFFFFFFFFFFFFFFFFFFFFFFFFFFFFFFFFFFFFFFFFFF
FFFFFFFFFFFFFFFFFFFFFFFFFFFFFFFFFFFFFFFFFFFFFFFF
@A00721:190:HGJW7DSXY:4:2313:18493:35556_1:N:0:TCTGCAAG+GCTGCACT
ATTCCTACGTAGTTTATGACTTTGGTGGTGGTACTTTCGATACTTCTATCGTCTTACGTTACGGTAAT
TACTATAGTGTTATTTATTCTGGTGGTGATGATAATCTTGGAGGTCGTGACGTTGATGAAGCTATAAG
AGC
+
F,FFFF::FFFF:FFFF:FFFFFFFFF:FFFFFFFFF:FF:FFF,FFF,FFFFFFFFFFFFFFF::F
FFFFFF,F:FFFFFFF:FF:FFFFFFFFFFFFFFFFFFFFFFF:FFFFFFF:FFFF:FFFFFFFFFFFF
FFF
@A00721:190:HGJW7DSXY:4:2609:3287:11757_2:N:0:TCTGCAAG+GCTGCACT
CACAACTTTCAGTTTCACAACTGAAGGAGGAGGTTTCTGTTACTGGAAAACCCTCGTCAATACTTTTC
AATAATATACGTCTTGAACTCACTTATGACGATCTAGTGAAATTAACG
+
FFFFFFFFFFFFFFFFFFFFFFFFFFFFFFFFFF:FFFFFFFFFFFFFFF:FFF:FF,FFFFFFFFFFF
FFFFFFFFFFFFFFFFFFFFFFFFFFFFFFFFFFFF,FFFF:FFFFFFF
@A00721:190:HGJW7DSXY:4:1227:16785:16877_1:N:0:TCTGCAAG+GCTGCACT
TACTATTATGAACAAAGTTATCAAAGATAGTGGCATTCAAGGCAAACTTACCCTGATTCCGGCCGGTG
GTAGCTCATTTCTACCAGGGGCTATTACTCTTGCTGAAACAGCTATTAAAGCTGTTGAGCGGGTAATT
ACA
+
FFFFFFFFFFFFFFFFFFFFFFFFFFFFFFFFFFFFFFFFFFFFFFFFFFFFFFFFFFFFFFFFFFF,F
FFFFFFFFFFFFFFFFFFFFFFFFFFFFFFFFFFFFFFFFFFFFFFFFF:FFFFFFFFFFFFFFFFFFF
FFF
@A00721:190:HGJW7DSXY:4:1348:17616:33379_2:N:0:TCTGCAAG+GCTGCACT
ATCGCTGAAGGATGTGCACTCTTTTCTGCTACTTTAACGGAGCGTAGTGTACTCTTCGTCGATTGTAT
CAACAGTAACATTACTTCTAATAAAGGTCTCTATCAAGCTCGTATTAT
+
FFFFFFFF:FFFFFFFFFFFFFFFFFFFFFFFFFFFFFFFFFFFFFFFFFFFFFFFFFFFFFF:FFFFF
FF,FFFFFFFFFFFFFFFFFFFFFFFFFFFFFFFFFFFFFFFFFFFFF
@A00721:190:HGJW7DSXY:4:1147:10610:32675_1:N:0:TCTGCAAG+GCTGCACT
TTACCGTTGAAGGCTAAACTCCAAGGTAATTATGAAGACTGTGATTTATCTTTCACTCTGGCCTTGTA
TGAGGGTGTTTCTAATCGTAACATTAATAATTTCAATCTTCTTAAAAAGAAGATAGAGAGTCATACTT
TGG
+
FFFFFFFFFFF,FFF:FFFF::F,FFFFFFFFFFFFFFFFFFFFFFFFF:FFFF:FFFFF:FFFFFFF
FFFFFFFFFFFFFF,F:FFF:FFFFFFFFFFFFFFFF:FFFFFFFFFFFFFFFFFFF:FFFFFFFFFF
FFF
@A00721:190:HGJW7DSXY:4:2423:29288:19774_2:N:0:TCTGCAAG+GCTGCACT
GATAGCCAATATTGAAGTGGACACCTCTGGTAGTATCGAAGTAAAGATAACCAATAAGGAAGGAGCTT
ATACTATGGTTGAATCAACTAAGCATACTACTTTTGCTTTGATTACTC
+
,FFFFFFFFFFFF:FFFFF:FFFFFF:FF:FFFFFF,:FFFFFFFFF:FFF,F::FFF,FFFFF,FFF
FFFFFFF:FFFFFFF:FFFFFFFFFFFF,F:,,FFFFF:FFFFFF:FF
@A00721:190:HGJW7DSXY:4:2453:4110:5321_1:N:0:TCTGCAAG+GCTGCACT
GTCTAAGGTTAAAGAGTCGATTAAACAAGTTGTACTTATCAATGATTTTATCGCTGGTTGTTATCTCG
AAAAGTCTCTTCTAAAAGGAACGTCAGTTCTAACCCAAAGAGAATATCTAAAATATTTACAAAATCTT
ACC
```

+
FF:FFF,F:F,FF:FFF::FFFFFF,FF,FFFFF,FFFFFFFFFFFFFFFFFFFFFFFFFFFFFFFFF
FFFFFFFFFFF:FFFFF,FFFFFFFFFFFFFFFFFFFFFFFFFFFFFFFFFFFFFFFFFFFFFFFFFF
FFF
@A00721:190:HGJW7DSXY:4:1350:5810:36229_2:N:0:TCTGCAAG+GCTGCACT
AGACGTTGCTTGTCGCTTCCTCCTTTAATGGCACTACCTCGGTACAATCTTACTTGGAACGAAGGCGA
CCTGTTCCGGAGTTTTTACGGGATCCGGAATGTTCTGTCTATATTAGG
+
FFF:FFFFFFFFFFFFFFF:FFFF,FF:F:FFFFFFFFFFFF,FFFFFFFFF,FFF:FFFFFFF::FF
F,FFFFFFFFFFFFFF:FFFF::F:FF:FF:FF::F:F:FFFFFFFF:
@A00721:190:HGJW7DSXY:4:1372:3323:26788_2:N:0:TCTGCAAG+GCTGCACT
TCGAACGTCAACGTTCACCGAGTTTCGTTTCGTACCTTGATGCTGACTTTCGTCAATATGATTTGATC
TGGTTGCTCAATTCTAAGCGTGTTCCGCTTAGTGCGAGGCCTCTCTTC
+
FFFFFFFFFFFFFFFFFFFFFFFFFFFFFFFFFFFFFFFFFF:FFFF:FFFFFFFFFFFFFFFFFFF:
FFFFFFFFFFFFFFFFF:FFFFFFFFFFFFFFFFFFFFFFFFFFFFFF
@A00721:190:HGJW7DSXY:4:1147:21088:21856_2:N:0:TCTGCAAG+GCTGCACT
ATATTTGGATGATCTTGATAAAGAACTTCATGAATTTTCAGAGGATGAAAAATTTTCTTCCTTGGATT
TTAATATAAAGAATCTTTACCAGGACTCCTACTACAGGAGGCAGTCTT
+
FFFFFFFFFFFFFFFFFFFFFFFFFFFFFFFFFFFFFFFFFFFFFFFFFFFFFFFFFFFFFFFFFFFF
FFFFFFFFFFFFFFFFFFFFFFFFFFFFFFFFFFFFFFFFFFFFFFFF
@A00721:190:HGJW7DSXY:4:1243:26060:18474_1:N:0:TCTGCAAG+GCTGCACT
ATTTGTTGTAATAGGGTCATCAACTTAGAACTCGAAACTCTAAGTGGTAATACTGTCTTTCCAAGCTT
ACCTGTATCTTCAATGAAATTGGACTCTTCGATAGGGGACATTTTATCGTGGGCTTTCCGTAAAGGAA
GAT
+
FFFFFFFFFFFFFFFFFFFFFFFFFFFFFFFFFFFF,FFFFFFFFFFFFFFFFFFFFFFFFF:FFFF:F
FFFFFFFFFFF:F:FFFFFFFFFFF:FFFFFFFFFFFFFFFFFFFFFFFFFFFFFFFFFFFFFFFFFF
FFF
@A00721:190:HGJW7DSXY:4:2233:14588:19961_1:N:0:TCTGCAAG+GCTGCACT
CTACTAATGGCGTTGGCCTAGTGGATGTTATTAGTGACATCTGGTCAATTTCGTTTTTAGGTGGTCTT
TATTCTATTATTGATTTTTATGAGATTGATACGTTAACAGAAGATGATGTTAGAGTAGAATTCCTGAA
GAG
+
FFFFFF:FFFFFFFFFFFFFFFFFFFFFFFFFFFFFFFFFFFFFFFFFFFFFFFFFFFFFFFFFFFFF
FFFFFFFFFFFFFFFFFFFFFFFFFFFFFFFFFFFFFFFFFFFFFFFFFFFFFFFFFFFFFFFFFFFF
FFF
@A00721:190:HGJW7DSXY:4:1658:18530:33144_2:N:0:TCTGCAAG+GCTGCACT
CCAACTTCTTGACAATTTTCTTCAATCCTTCGATAATAGTTAAATCTAGGATTATGTCTGTGTTGAAA
GACTTTCTACCGATAAGGGTCACTAGTCTTACACAATTAAGAAATAAT
+
FF:FFFF:FFFFFFFFFFFFFFFFFF:FFFFFFFFFFFFFFFFFF,FFF:FFFFFF,:FFFFFFFFFF
FF:FFF,FFFF,FFFF:FFFFFFFFFFFFFFFFFFFFFFFFFFFFFFF
@A00721:190:HGJW7DSXY:4:2348:27100:6151_2:N:0:TCTGCAAG+GCTGCACT
GATTATCTAAAGATTGAAGGAAAATTTTATCCTCGTTTAACTTACTTAGACTTTCCTACGATACAAAT
GATAACTTCTAATTTACGTTTGCTACTCAGAGCTGGTCCTCCCAGCAA
+
FFFFFFFFFFFFFFFFFFFFFFFFF::F:FFFFFFFFFFFFFFFFFFF:FFFFFFF:FFFFFFFF,FF
F:FFF:FFFFFFFFFFFFFFFFFFFFFFFFFFFFFFFFFFF:FFFFFF
@A00721:190:HGJW7DSXY:4:1651:20654:36479_2:N:0:TCTGCAAG+GCTGCACT
TCCTCAGGAGTCCCGGCATTTGATAATAATGACGACTTTCGTCATAATCGCTGCATATTACGGATTAC
ACGGTACCAGCGCATCTCGTGTCGAGCCTCGACCCGATTATATGGTAT
+

```
FFFFF,FFFFFFFFFFFFF:FFFFFFFFFFFFFFFFFFFFFFFFFFFFFFFF:FFF:FFFFFFFFFFFF
FFFFFFFFFFFFFFFFFFFFFFFFFFFFFFFFFFFFFFFFFFFFFFFF
@A00721:190:HGJW7DSXY:4:2302:18123:34695_2:N:0:TCTGCAAG+GCTGCACT
TCTAGAATTCTTAATTATAATATTCAACCTCAAAAGTTACGTGTTTACAGTAACGAGATCACACATTT
TGAGAACTAACTATGTTATGTTCTCTGATCATTTTGACAATCTCTTCT
+
FFFFFFFFFFFFFFFFFFFFFFFFFFFFFFFFFFFFFFFFFFFFF:FFFFFFF:FFFFF:FFFFFF:F
FFFFFFFFFF,FFF:FFF:FFFFFFFFFFFFF:FF:FFFFFFF:FF:F
@A00721:190:HGJW7DSXY:4:1663:12029:33066_2:N:0:TCTGCAAG+GCTGCACT
AAATAAATAATATTACTAATGGCAAATAGTGGAAAGGCTCCAGTCAACACTAGTTCGAGTGCTGCTTT
GGATTACGATGACGTGAGAATAGGTGAAGGGATAGTTTTTACCTCGAC
+
FFFFFFFFFFFFFFFFFFFFFFFFFFFFFFFFFFFFFFFFFFFFFFFFFFFFFFFFFFFFFFFFFFFF
FFFFFFFFFFFFFFFFFFFFFFFFFFFFFF:FFFFFFFFFFFFFFFFF
@A00721:190:HGJW7DSXY:4:2152:6460:4695_1:N:0:TCTGCAAG+GCTGCACT
TCTTAAAGCTTCACCCAAAATGAAGGACACTTCGAAACTGAATAATGATGCACTACAAACAAGTATCG
CTAATCATCTGAAAAAGGCTCTAAAGGACACTATACAATATCCAGACGACGACGAAGCTTATACAATA
TCC
+
FFF:FFFFFF:FFF:FFFFFF:FF:F::FFFFFFFFFFFFFFFFFFFFFFFFFFFF:FFFFFFFFFFFF
FFFFFFFFFFFFFFFFFF,FFFFFFFFFFFFFFFFFFFFFFFFFFFFFFFFFFFFFFFFFFFFFFFFF
FF:
@A00721:190:HGJW7DSXY:4:2207:4399:18787_2:N:0:TCTGCAAG+GCTGCACT
ACCACATTTTGGTCAAGTCAATCCGTTGAGAAAATTTCTTAGAGGATATTCGCCATTGTGGTACGAAT
TGGTCCAACATTTACCACCAAATTCTTTTGATACAACTCTAGCCTTCA
+
FFFFFFFFFFFFFFFFFFFFFFFFFFFFFFFFFFFFFFFFFFFFFFFFFFFFFFFFFFFFFFFFFFFF
FFFFFFFFFFF:FFFFFFFFFFFFFFFFFFFFFFFFFFFFFFFFFFFF
@A00721:190:HGJW7DSXY:4:2302:25816:18709_2:N:0:TCTGCAAG+GCTGCACT
ACCAGATTGGTTCGAACCCGTTGATGCTATGTCAGAAGAAGCTATTCAAGCTTGTCGTTTAAAGACTG
CAGAGGCTGTCAAACAAGCCCAGACTCAAGGAGTCCAATCAAAGGTTT
+
FFFFF:FFFFFFFFFFFFFFFFFFFFFFFFFFFFFFFFFFFFFFFFFFFFFFFFFFF:FFFFFFFFF
FFFFFFFFFFFFFFFFFFFFFFFFFFFFFFFFFFFFFFF:FFFFFFFFF
@A00721:190:HGJW7DSXY:4:1143:20971:2707_1:N:0:TCTGCAAG+GCTGCACT
CTTGCAGAGTGACTGGAGTTCAGACGTGTGCTCTTCCGATCTGAAAAGTCCGGAGTATATTGTATGTT
AATAGATAGTGGGATAATAGTTCTACCGATACGTAGTGAACGCGTTCGTAGAACCAAGTACTTTCCGT
CTG
+
FFFFFFFFFFFFFFFFFFFFFFFFFFFFFFFFFFFFFFFFFFFFFFFFFFFFFFFFFFFFFFFFFFFF
FFFFFFFFFFFFFFFFFFFFFFFFFFFFFFFFFFFFFFFFFFFFFFFFFFFFFFFFFFFFFFFFFFFF
FFF
@A00721:190:HGJW7DSXY:4:1374:16884:8469_1:N:0:TCTGCAAG+GCTGCACT
ACCGATAGGAGTTGTATAGTTGAAGATGTGGCTTTAGACGATAATCCTTTAAGGACTATGTTGACTGA
CGAAGATTTTCCAACAGATTTGTCTACGTTGCAGATCGCCACTCCAAACACTATATACATTATAAAAT
ATA
+
FFFFFFFFF:FF:FFFFFFFFFFFFFFFFFFFFFFFFFFFFFFFFFFF,FFFFFFFFFFFFFFFFFFFF
:FFFFFFFFF:FFFFFFFFFFFFFFFFFFFFFFFFFFFFFFFFFFFFFFFFFFFFFFFFFFFFFFFFFF
FFF
@A00721:190:HGJW7DSXY:4:1202:20076:31156_1:N:0:TCTGCAAG+GCTGCACT
ATAACTTTTGTTACCAAATTCTTGGCTTTCAACGAAGTCTCTGATTTTATTAATCATTACTTTAACGA
TCTCTCGGTCATTTACTCTGTACTTGATCTACGTCGTTTAACTGTCGGAACAACCTTTACTTAGAATG
ACT
```

```
+
FFF:F:FFFFFF:FFFFFFFFFFFFFFF:FFFFFFFF::FFFFFF:F:FFFFFFFFFFFFFFFFFFFF
FFFFFFFFFFFFFFFFFFFFFFFFFFFFFFFFFFFF:FFFFFFFFFFFFFFFFFFFFFFFFFFFFFFF
FFF
@A00721:190:HGJW7DSXY:4:1568:8531:8375_2:N:0:TCTGCAAG+GCTGCACT
TCTTATATTATTTACAATAGTTAATATCATATTAGTAATACTCACTATCGTTTTCTGCTTTTTCTTAG
CTAATAGATTAATAAAGGATGAGTACGAAAACCAACTCTTCGTTACGA
+
FFFFFFFFFFFFFFFFFFFFFFFFFFFFFFFFFFFFF:FFFFFFFFFFFFFFFFFFFFFFFFFFFFFF
FFFFFFFFFFFFFFFFFFFFFFFFFFFFFF:FFFFFFFFFFFFFFFFF
@A00721:190:HGJW7DSXY:4:1269:13214:25598_1:N:0:TCTGCAAG+GCTGCACT
TTCCGATCTTTTAGTCTCTACACCCATCTCAACGAGGATAACGCGAGTTTTCCGTCCTGAACGTAATA
CACGAAGAGATCATCCGCAAACGACTCGAAACCGAGACGCCGTAGATCAAAGTCCCTTCTAACTTTAG
AT
+
F,FF:,F,FF:F,FFFF,FFFFFF,,:F,:,:F,,F,FFF:,F,,,:FFF,F::,F,F,F:F,FF,:,
F::FF,:,,F:FF,F:F,F,FFFFFFFFFFFFFFFFFFFFFFFFFFFFFFFFFFFFFFFFFFFFFFFF
FF
@A00721:190:HGJW7DSXY:4:2461:3812:8938_1:N:0:TCTGCAAG+GCTGCACT
CATTTTATAGCAGTTATTTATGTTGTACTTTTTATAATTTATAACCTTCTTATACTTTTGGGTATGTT
TTGTGAAAAACATGAGATACCCAAGTCTATCGAAGACATTATCTCTTATGTACTTATTGTAACGATCA
TTC
+
FFFFFFFFFFFFFFFFFFFFFFFFFFFFFFF:FFFFFFFFFFFFFFFFFFFFFFFFFFFFFFFFFFFF
FFFFFFFFFFFFFFFFFFFFFFFFFFFFFFFFFFFFFFFFFFFFFFFFFFFFFFFFFFFFFFFFFFFF
FFF
@A00721:190:HGJW7DSXY:4:2548:8449:26553_1:N:0:TCTGCAAG+GCTGCACT
TTATTCCTTTAACTGTGTATGAGGAGGATTTATAATGCTTTTCGATAGTGATTGTTATCAATTTAAAT
ACATTACTGTTTCAAATCATCTTTACTCACAGACTCCGAAAATGGTTTCAAATGAGGTCAAAGCTCTA
TTT
+
FFFFFFFFFFFFFFFFFFFFFFFFFFFFFFFFFFFFFFFFFFFFFFFFFFFFFFFFFFFFFFFFFFFF
FFFFFFFFFFFFFFFFFFFFFFFFFFFFFFFFFFFFFFFFFFFFFFFFFFFFFFFFFFFFFFFFFFFF
FFF
@A00721:190:HGJW7DSXY:4:2236:10041:22294_2:N:0:TCTGCAAG+GCTGCACT
TATTATCCGATTCTTCGAGGGTATCACTACCACTGATCTGCCGACTTTAGTCAAGGAACTTCAATTCC
TTCGATGTTTATATTTTTCTATAAGGGAAAATAGCAATAATCAAATTT
+
FFFFFF:FFFFFFF:,FF:FFFFF:FFFFFFFFFFFFF:F,FFF::FFFFF:FFFFFFFFFF:FFFFF
:FFF,FFFFFFFFFFFFF:,F:FFFF:FFFFFFFFFF::FFFF::F::
@A00721:190:HGJW7DSXY:4:2454:17318:23406_2:N:0:TCTGCAAG+GCTGCACT
AACTTTATTTCAGAACTCTCTCCGATCACCTTGGTGTGATCTCCCGGAGGATCTTTTCAATAAGATGT
ATGATGATAATCTACTATTTTTAAATAGTGATATTCAGCGTATACTTG
+
FFFFF,FFFFFFFFFFFFFFFFFFFF:FFFF::FFFFFFFFFFFFFFFF:FFFFFFF,FFFFFFFFFF
FFFFFFFFFFFF:FFFF:FFFFFFFFFFFFFFFFFFFFF,FFFFFFFFF
@A00721:190:HGJW7DSXY:4:1419:23339:36307_1:N:0:TCTGCAAG+GCTGCACT
CTTGGTCTATTTACCGCAAAAAGTTGGAAGAGGTCTATGATATTTATTTTACTTATACTTTAAATAGC
ACCGATTATAACTTGACTGTTGATAATATTACAATAGCTCTTTCTCAGAAGATTAGAGAAGTAACCGG
TGA
+
FFFFFFFFFFF:FFFFFFFFFFFFFFFFFFFFFFFFFFFFFFFFFFFFFFFFFFFFFFF:FFFFFFFF
FFFFFFFFFFFFFFFFFFFFFFFFFFFFFFFFFFFFFFFFFFFFFFFFFFFFFFFFFFFFFFFFFFFF
FFF
```

```
@A00721:190:HGJW7DSXY:4:1222:1108:8390_2:N:0:TCTGCAAG+GCTGCACT
CATTGATTTACTTTTACCGTTAGAACTGAAAAAGATTCTTTTCGAAAAGAAACCTATAAGTTACAAAC
GATTCTTCGACTATATTAAAGAAATAAAAATACTTCAAGTACCTGAAC
+
FFFFFFFFFFFF:FFFFFF,FF::FFFFFFFFFFFF:FFFFFFFFFFFFFFFFFFFFF:F:FFF:FF
FFFFFFFFFF:FFFFFFFFFFFFFFFFFFFFFFFFFFFFFFFFFFFFF
@A00721:190:HGJW7DSXY:4:1427:9634:31673_1:N:0:TCTGCAAG+GCTGCACT
ATGGTATAATGTGTCTTACAAATAGAACCTTCACTCTCGGTAATGAGAGTGTATTCCTTAATATTCTA
GTAACCGCTATGTGGGCATTACTAGGATTTATCACACTCCTAAATTTGATAGCAATTTATGTTTTAAA
TGC
+
FFFFFFFFF:FFFFFFFFFFFFFFFFFFFFFFFFFFFFFFFFFFFFFFFFFFFFFFFFFFFFFFFFFF
FFFFFFFFFFFFFFFFFFFFFFFFFFFFFFFFFFF:FFFFFFFFFFFFFFFFFFFFFFFFFFFFFFFF
FFF
@A00721:190:HGJW7DSXY:4:1614:20943:27398_2:N:0:TCTGCAAG+GCTGCACT
AAAAACTCCATTGTATAGTTCAAGGAGTACTTAATTCAATAGCTTTAGTATTATTTACTATCGCCTTT
ATTGAAAATTGTATAAGAGTTAATTGCTTACACAAAAGTGACGCGCGA
+
FFFFFFFFFF:FFFFFFFFFFFFFFFFFFFFFFFFFFFFFFFFF:FFFFFFFFFFFFFFFFFFFFFFF
FFFFFFFFFFFFFFFFFFFFFFFFFF:FFFFFFFF,FFFFFFFFFFFFF
@A00721:190:HGJW7DSXY:4:2403:32452:2206_2:N:0:TCTGCAAG+GCTGCACT
ATACCATAACCTACCATGACCAATCATCGCTCCAACTGGCACGCCAGACGTAGGGTCAAACCTACTAC
CGCCGGTCGGCAGATCGGCACCACTGGTCTCTACGTTCAGGGACCCGC
+
FFFFFFFFFFFFFFFFFFFFFFFFFFFFFFFFFFFFFFFFFFFFFFFFFFFFFFFFFFFFFFFFFFFF
FFFFF:FFFFFFFFFFFFFFFFFFFFFFFFFFFFFFFFFFFFFFFFFF
@A00721:190:HGJW7DSXY:4:2132:10104:25034_1:N:0:TCTGCAAG+GCTGCACT
CCTTCCCAAATCTAGATCTGCACAGAAGAAGGCTGAAGGTCGTTACCGGACGACCACTCAAAGGAAAC
GGGCTGTTGCCCAGAGGAAAGCAGCGACCACCGAGGTCGCTCGTAACATTGACGGCAGTCCCGTCATC
TCT
+
FFF,FFFF,,FFFFFF,F,FFFFFF:FFF,FFFFF:FFFFFF,,FFFFFFFFFFFFFFFF:FFFF,:F
FFFFFFF:FFFFFFFFFFF,FFFFFFFFFFFFFFFFFFFFFFFF:FFFFFFFFFFFFFFFFFFF:FFFF
FFF
@A00721:190:HGJW7DSXY:4:1440:2175:21543_2:N:0:TCTGCAAG+GCTGCACT
GCAGTACCTCAGTCAAAAGTCACATTCACCACTCCCATACCAATCAATGATGTGACTATACAGTTCGG
CCATTTCAAGAGTGGTCAATACGTCGTGGGAGGTACTGAAGCCTTCCC
+
FFFFFFFFFFFF:FFFFFFFFFFFFFFFFFFFFFFFFFFFFFFFFF,FFFFFFFFFFFFFFFFFFFFF
FFFFFFFFFFFFFFFFFFFFF:FFFFFFFFFFFFFFFFFFFFFFFFFF
@A00721:190:HGJW7DSXY:4:2505:25220:3114_2:N:0:TCTGCAAG+GCTGCACT
CCAAGCCAATCAATAGTTCTTCTGACCAGAATGAAAAGGGGTTTACCAGGGTAGTCTCTAAGTCTACC
AGGAAGAGGGGAAATGTCCCCAAAACTGAGAAGAATAAAGGGAAAGGT
+
FFFFFFFFFFFFFFFFFFFFFFFFFFFFFFFFFFF:FFFFFFFFFFFFFFFFFFFFFFFFFFFFFFFF
FFFFFFFFFFFFFFFFFFFFFFFF,FFFFFFFFFFFFFFFFFFFF:FFF
@A00721:190:HGJW7DSXY:4:2258:32334:22232_2:N:0:TCTGCAAG+GCTGCACT
AGGGGAAATTACCCCTAATTCTGAAGGAAGATCTCCCACCATCTGGGGTTGGGAATAAGAAATCTGCA
CGGGCCAAAGAGGCCGCTGCTACGAAGTCGCTGAAGGCGGCTTGGGTC
+
FFFFFFFFFFFFFFFFFFFFFFFFFFFFFFFFFFFFFFFFFFFFFFFFFFFFFFFFFFFF:FFFFFFF
FFFFFFFFFFFFFFFFFFFFFFFFFFFFFFFFFF:FFFFFFFFFFFFFF
@A00721:190:HGJW7DSXY:4:2515:3278:2566_1:N:0:TCTGCAAG+GCTGCACT
GTTGCGCCTGCCGACCCCATCTTTGAGACCAAGACTCTCTACAGTAGGTTCGTGCCTACTATGAGCAA
```

```
ATCTGTTGAATTCGTCACTGAGGACGAAGGTGAGAGGACTTTTGGCTACTGGCGACAAACTGAGAGCA
GT
+
FFFFFFFFFFFFFFFFFFFFFFF:FFFFFFFFFFFFFFFFFFFFFFFFFFFFFFFFFFFFFFFFFFFF
FFFFFFFFFFFFFFFFFFFFFFFFFFFFFFFFFFFFFFFFFFFFFFFFFFFFFFFFFFFFFFFFFFFF
FF
@A00721:190:HGJW7DSXY:4:1165:29017:29105_2:N:0:TCTGCAAG+GCTGCACT
CGTGGGTTGAAGGCCGACGCTTCGAGCTTGAGTGTGACGAGGCCGGTGACGTTTTTGTCAACGGCGTT
TGGAGGATTTTATCCGCGCTTAACTATGGGGCGAAGATCCCCTATGGA
+
FFFFFFFFFFFFFFFFFFFFFFFFFFFFFFFFFFFFFFFFFFFFFFFFFFFFFFFFFFFFFFFFFFF:
FFFFFFFFFFF:FFFFFFFFFFFFFFFFFFFFFFFFFFFFFFFFFFFF:
@A00721:190:HGJW7DSXY:4:1276:31801:32550_2:N:0:TCTGCAAG+GCTGCACT
TTGGCATATTTATGCCGACGATAATAAGCATGTCACCCTTAAGCACCGAGGTAAGCTCGGGACTTACA
AGAGAGAGTGTGAGAGAGTCGCTCTCACTATTTTTGCCCAGAATGCGG
+
FFFFFFFFFFFFFFFFFFFFFFFFFFFFFFFFFFFFFFFFFFFFFFFFFFFFFFFFFFFFFFFFFFFF
FFFFFFFFFFFFFFFFFFFFFFFFFFFFF:FFFFFFFFFFFFFFFFFFF
@A00721:190:HGJW7DSXY:4:2306:22697:28714_2:N:0:TCTGCAAG+GCTGCACT
TCCTCCATAATTACAACCTCTATTTAGAGAGCATTAGAAGGAAGTTGGGCAAATTCCCACATATGGTA
GACGTTGTCACACTTTTCTTGAAAATGTTTCATTTCCTCCGTCATTGG
+
FF,FFFFFF:F,F:FF,FF:F,,,,,::FFFFF:FFFFFFFF:,,,:F,FF:F:::F:FFF,F:F,,:
FFF,,FF:FFFFF:,F:F,,,FFF:,,,FFF,FFF::,FFF,:F:,:,
@A00721:190:HGJW7DSXY:4:2316:12735:34131_2:N:0:TCTGCAAG+GCTGCACT
AAGGTGGAAGGTGTCAAGGGTCTTCTTCATGCTGATCTTTCAGGACCTAATCACATCATGATTGATAG
TTACGGCTCGTATCGAGTTGGAGGGAGGAAGTTCTCCGAAATTAAAGA
+
::FFFFFFFFFFFFFFFFFFFFFFFF,FFFFFFFFFFF:FFFF:FFFFF:FFFFFFFFF,FFFFFFFFF
FFFFFFFFFFFFFFFFF:FFFFFFFFFFFFFFFFFFFFFFFFFFF:FFF
@A00721:190:HGJW7DSXY:4:1345:6126:18051_1:N:0:TCTGCAAG+GCTGCACT
TCAGAGAGGGGAGCTCTGAAAAGGAAACTTCGTCAGAAGATGAGGAAGTCTAAGAGGAGGAGTTTGAG
GAAGGCTCTAGAGAAGATCTCACCACCTGGAGAAGGCAAGTGGAGAGTCCAGAAGTTGATGAAGAAGG
ATT
+
FFFF::,FFFFF:FFFF,:FFFFF:FFFF,FFFFFFFFFF:FFFFFFF::FF,FFFFFFFFFFF:FFF
FFFFFFFFFFFFFFFFFFFFFF,FFFFFFFFFFFFFFFFFFFFFFFFFFFFFFFFFFFFFFFFFFFFF
FFF
@A00721:190:HGJW7DSXY:4:1377:24505:23202_2:N:0:TCTGCAAG+GCTGCACT
GTTACCACTTCCTAAGGTGGATGAGGGAGGTTGTTTCTTTGGAGTTTTAGATAATCTTTTGAGAAGGG
GTCATAGAGCCAGCAAGAGAAGCTTATATGCTGGTTTAAGTGATGATT
+
FFFFFFFFFFFF:FFFF:FFF,FFFFFFFF,FFFFFFFFFFFFFFFFFFFFFFFFFFFFFFFFFFFFF
FFFFFFF:FFFFFFFFFFFFFFFFFFF:FFFFFFF:FFFFFFFF:FFFF
@A00721:190:HGJW7DSXY:4:1212:4490:27461_1:N:0:TCTGCAAG+GCTGCACT
ATTTAGGTCAACTTGGGGTTTCGAATGTCATTAGTGCCTTACGATTGAAAGAGGTCCTTTTGAGTATC
GACAGATCTTTCGATCTTGAGGACGTACATTTAACGTGGCAGAATTCGGTAACTTACCATATTGTTCC
TTC
+
F:FFFFFFFFFFFFFFFFFFFFFFFFFFFFFF:FFFFFFFFFFFFFFFFFFFFFFFFFFFFF:FFFFFFF
FFFFFFFFFFFFF:FFFFFFFFFFFFFFFFFFFFFFFFFFFFFFFFFFFFFFFFFFFFFFFFFFFFFFFF
FFF
@A00721:190:HGJW7DSXY:4:1510:8766:9408_1:N:0:TCTGCAAG+GCTGCACT
ATTTAACTTTGACTCAACTACTCCGATCAGATCCGGATGGTTTGATTGGTATGGACTACTCGACTTTT
```

```
GATCTGGAGTTGTCTAGGGTCATGGCAGATACTACCGATAAGGTGAAAAATATGCAGGTCAAAGATTC
TGT
+
FFFFFFF:FFFFFFFFFFFFFFFFFFFFFFFFFFFFFFFFFFFFFFFFFFFFFFFFFF,FFFFFFFFFFF
FFFFFFFFFFFFFFFFFFFFFFFFFFFFFFFFFFFFFFFFFFFFFFFFFFFFFFFFFFFFFFFFFFFFFF
FFF
@A00721:190:HGJW7DSXY:4:1612:18367:36933_2:N:0:TCTGCAAG+GCTGCACT
GAGGGAGCCGTTTTGGCCGACTTTAACTATTCTAAGAGTCTTTATGATTCAAAACCTATCGTTGTCGT
ACCTTACAAGGTTGAGGAGACTTTACAGCGGCTTATTGTCGCGCAGTT
+
FFFFFFFFFFFFFFFFFFFFFFFFFFFFFFFF,FFFFF,F:FFFFFFFFFF:FFFFFFFF::F,FF,FFFF
FF:FFF,FF,F,F:FFFFFFFFFFF:FFFFFFFFFFF,F:F:FFFFFFFF
@A00721:190:HGJW7DSXY:4:1122:8314:36871_1:N:0:TCTGCAAG+GCTGCACT
TATCTTGAATGCTCACGCCGTCGCAGCCATATCGAGACTATGTTATAATCACTTGTTAAAGTACAGAT
TTAGTGATTTAGATATTATTGATATTGGAGGCGATATTCGTTTTCATATCCAAAACTCCTTGAATGGA
GTA
+
FFFFFFFFFFFFFFFFFFFFFFFFFFFFFFFFFFFFFFFFFFFFFFFFFFFFFFFFFFFFFFFFFFFFFF
FFFFFFFFFFFFFFFFFFFFFFFFFFFFFFFFFFFFFFFFFFFFFFFFFFFFFFFFFFFFFFFFFFFFFF
FFF
@A00721:190:HGJW7DSXY:4:2202:32009:20572_1:N:0:TCTGCAAG+GCTGCACT
ACCCTATTCTTGATGTGAAAGATGCATCGAGGTATACGTCTAGGAACATTGATTGGGAACGTACCTAC
TCACGAGACGTCATGAAAGCTATCGGAGGGAAGCAAAATGTTTCGTTCTGTTACAAGAACGCTTTGGA
TTG
+
FF,FFFFFFFFFFFFFFFFFFFFFFFFFFFFFFFFF:FFFFFFFFFFFFFFFFFFFFFFFFFFFFFFFFF
FFFFFFFFFFFFFFFFFFFFFFFFFFFFFFFFFFFFFFFFFFFFFFFFFFFFFFFFFFFFFFFFFFFFFF
FFF
@A00721:190:HGJW7DSXY:4:2466:11614:11898_2:N:0:TCTGCAAG+GCTGCACT
CTCAGTTGCGATAGCTGTAGAGGTATACGATATCTCTCTTGATGATGTAGCCACTATAATGCTTAAGA
GAGAGATTGATGTATGTCATTTCGTCTTAGTTTGTCCCGGCGAATTAT
+
FFFFFFFFFFFFFFFFFFFFFFFFFFFFFFFFFFFFFFFFFFFFFFFFFFFFFFFFFFFFFFFFFFFFFF
FFFFFFF:FFFFFFFFFFFFFFFFFFFFFFFFFFFFFFFFFFFFFFFFFF
@A00721:190:HGJW7DSXY:4:2214:12915:29966_2:N:0:TCTGCAAG+GCTGCACT
AAAAATGACATACCCGTTCCTGACCTTGGTATCGTTATCCGTCATGGAGATGATGGCGCATCACTTGA
TTACTATATGCCCGAAGGATTGGGTTATTCTCACAACAAAGATAAGAT
+
FFFFFFFFFFFFFFFFFFFFFFF:FFFFFFFFFFFFFFFFFFFFFFFFFFFFFFFFFFFFFF:F:FFF
:FFF,FFFFFFFFFFFFFFF:FFFFF,FFFFFF:FFFFFFFFFFFFFFF:
@A00721:190:HGJW7DSXY:4:1201:18295:20212_2:N:0:TCTGCAAG+GCTGCACT
AAGGGCGGACAATTGTTCCATTGTGAACTCTTGGGGACTAGATTGAGCATTAATGAGTTCCGAATAAC
TCGTAATAAATCTTTCTCTAGTGGACAATTCAGAATACCTATTTCCGT
+
FFFFFFF:FFFFFFFFFFFFFFFFFFFFFFFFFFFFFFFFFFFFFFFFF:FFFFFFFFFFFFFFFFFFFF
FFFFFFFFFFFFFFFFFFFFFFFFFFFFFFFFFFFFFFFFFFFFF:FFFF
@A00721:190:HGJW7DSXY:4:1443:19741:17895_1:N:0:TCTGCAAG+GCTGCACT
CTGCCAATCTCATCGCTATACGCATACCTGATTGGAAGAAGCGAGGTTCGTTCCTCAATAAAAATTTT
TCTCAAGAGATTATGGTTGATAAAGTTTTCTTCATAAGGGCTATTCAGTACGTCATGGGGATCACTTC
TTC
+
:FFFFFFFFFFFFFFFFFFFFFFFFFFFF:FFFFFFFFFFFFFFFFFFFFFFFFFFFFFFFFFFFFFFFFF
FFFFFFFFFFFFFFFFFFFFFFFFFFFFFFFFFFFFFFFFFFFFFFFFFFFFFFFFFFFFFFFFFFFFFF
FFF
```

```
@A00721:190:HGJW7DSXY:4:2135:27688:27336_1:N:0:TCTGCAAG+GCTGCACT
ACCTTCGAATATGTTACGACATGGCTTCGTAACAACAGTGCGAGGATTGTCATCTCGGGGAAAGTGAT
TCACCAAGATGTTCATATAGATACGAGATACCTAGAGAAGGTCGCAGCGATCTTACTCGTTATCGGAG
CTA
+
FFFFFFFFFFFFFFFFFFFFFFFFFFFFFFFFFFFFFFFFFFFFFFFFFFFFFFFFFFFFFFFFFFFF
FFFFFF,FFFFFFFFFFFFF:FFFFFFFFFFFFFFFFFFFFFFFFFFFFFFFFFFFFFFFFFFFFFFF
FFF
@A00721:190:HGJW7DSXY:4:1103:24985:34178_1:N:0:TCTGCAAG+GCTGCACT
TCGCAAACTTAACGTTAGTACCGGAGGTGATCTCTCTGCTTCTGCGTTACTGAGAGAAAAGATTCAGG
ACGCCTACGGTGACTTTATGGACAAGATGCAAGTTCTTGGGAAATACTTGTTTCCCTTTTTAGGTTTA
GTA
+
FFFFFFFFFFFFFFFFFFFFFFFFFFFFFFFFFFFFFFFFFFFFFFFFFFFFFFFFFFFFFFFFFFFF
FFFFFFFFFFFFFFFFFFFFFFFFFFFFFFFFFFFFFFFFFFFFFFFFFFFFFFFFFFFFFFFFFFFF
FFF
@A00721:190:HGJW7DSXY:4:1109:31882:6511_1:N:0:TCTGCAAG+GCTGCACT
GCTCTTCCGATCTGACAACGAATCTTGAAACTACGGTCCTTATCAAAGAAAATGGTGAACCGGTCTCT
AAATCTTTTGAGATTGAGAAGATTGAAGAACACGTCAAGAAGTCGCTCGGCGTTGTTCTTGAAAACCA
AGT
+
FFFFFFF:FFFFFFFFFFFFFFFFFFFFF:FFFFFFFFFFFFFFFFFFFFFFFFFFFFFFFFF:FFFF
FFFFFFFFFFFF:FFFFFFFFFFFFFFFFFFFFFFFFFFFFFFFFFFFFFFFFFFFFFFFFFFFFFFF
FFF
@A00721:190:HGJW7DSXY:4:1615:8043:4241_1:N:0:TCTGCAAG+GCTGCACT
GCTGATCATGGAGTCTTTGTACCTTCTGTCAAACTTCGAGGTTCAGCTACGAGAGGTGATCCCGGATG
TGGATTGAAGGCTGGCTCTAAGGGAGACTCCTTCTTTTTGAATGTTGTAAAGAATTGGGTTGAGAAGT
GTG
+
FF:FFFFFFFFFFFFFFFFFFFFFFFFFF:FFFFFFFFFFFFFFFFFFFFFFFFFFFFFFFFFFFFFF
FFFFFFFFFFFFFFFFFFFFFFFFFFFFFFFFF:FFFFFFFFFFFFF:FFFFFFFFFFFFFFFFFFFFF
FFF
@A00721:190:HGJW7DSXY:4:2265:26006:24424_1:N:0:TCTGCAAG+GCTGCACT
CTTGGTATTTCTGAAATAGTTTATCAGAGCGTCATTTTCCTTTGGGATCTTTCCTCTGGTAAGGGAAT
TATTAAATCATTCTTCTCACTCTTGACGAGAATTATTACTGTTTTCTCTACCCATAACTTCTTTTTCA
AAA
+
FFFFFFFFF:FFFFFFFFFFFFFFFFFFFFFFFFFFFFFFFFFFFFFFFFFFFFFFFFFFFFFFFFFF
FFFFFFFFFFFFFFFFFFFFFFFFFFFFFFFFFFFFFFFFFFFFFFFFFFFFFFFFFFFFF:FFFFFF
FFF
@A00721:190:HGJW7DSXY:4:1443:25355:9549_2:N:0:TCTGCAAG+GCTGCACT
TTTCCTACAGAGTGCAGTCGTTTCAACACATATCACCACAATTGAAAGGGAAATAATTGGTGTTGAAA
ATACGTATGTAGGTGGGGTTATCAAGGCTCTCAACGCCACAGGGATTC
+
:F,FFFFFFFF,FFFFFF:F,:FFFF,FF,FF,,,FF:F,,,,,:FFF,,,:FFFF:,F,FF,:::F,
FF,,::F,F,,FF,:::FFF,,F,F,::F:F,:,,,,::,,:,FF,F,
@A00721:190:HGJW7DSXY:4:1674:31747:5744_2:N:0:TCTGCAAG+GCTGCACT
TTTGGTCCTTACGTCTTAAGTAATTTGGATGTTGTCGAAAACTATCAGGTTCTTGTCACGATTCTTAA
AAGAAGTTTATTAAAACTTCAGAGTCTCTTGGAAGTCCAGGTAGCTAA
+
FFFFFFFFFFFFFFFFFFFFFFFFFFFFFFFFFFFFFFFFF,FFFFFFFFFFFFFFFFFFFFFFFFFF
F:FFFFF,FFFFFFFFFFFFFFFFFFFFFFFFFFFFFFFFFFFFFF:F
@A00721:190:HGJW7DSXY:4:2570:16043:28808_2:N:0:TCTGCAAG+GCTGCACT
GGGAAAAGAGACCTCTGTTGGTAGAGGAGTTGAGAGAATTACTCAGATTCAATCTGGCATCACTGAAC
```

```
GTCTACAACGAGTTAGGGAGTGGCTAGGTTTTAAAAGATCCTTTGCAC
+
FFFFFFFFFFFFFFFFFFFFFFFFFFFFFFFFFFFFFFFFFFFFFFFFFFFFFFFFFFFFFFFFFFFFFF
FFFFFFFFFFFF:FFFFFFFFFFFFFFFFFFFFFFFFFF:FFFFFF:FFF
@A00721:190:HGJW7DSXY:4:2475:5945:36714_2:N:0:TCTGCAAG+GCTGCACT
CGTCGCGGAAAACGATGAAGATTTCCACACTGTTCGAGAATCTTCTGATGCTGAATTTGAGGAAAATT
TTTCGGAAGTTTTTGGTATAAATCCTGATGGCTCAGTACAGGGAGGAG
+
FFFFFFFFFFFFFFFFFFFFFFFFFFFFFFFFFFFFFFFFFFFFFFFFFFFFFFFF,FFFFFFFFFF:
FFFFFFFFFFFFFFFFFFFFFFFFFFFFFFFFFFFFFFFFF:FFFFFFFF
@A00721:190:HGJW7DSXY:4:1150:3549:36354_2:N:0:TCTGCAAG+GCTGCACT
ACCTTCTTTTATCATTGCAACAGGACTTTGAGAAGACTGTTGAAATGGACAAAATCTGGGATCATAGG
TATGTGTCACGCTACACTCGAAATATGGGCCCTCATAAGGAAGTATAG
+
FFFFFFFFFFFFFFFFFFFFFFFFFFFFFFFFFFFFFFFFFFFFFFFFFFFFFFFFFFFFFFFFFFFF:
FFFFFFFFFFFFFFFFFFFFFFF:FFFFFFFFFFFFFFFFFFFFFFFFFF
@A00721:190:HGJW7DSXY:4:1350:28094:27289_2:N:0:TCTGCAAG+GCTGCACT
GGAAGATCTCTACAGAGATTATCGAGAGTTGTGATCTAGTTCTAAATTTAGGTCGACGGACTTTAGCA
ACTTTAGATAAATTCTTTCCGGGCTCAATGACCTTTGCTATGTACGTG
+
FFFFFFFFFFFFFFFFFFFFF:FFFFFFFF:FFFFFFFFFFFFFFFFFFFFFFFFFFFFFFFFFFFFFF
FFFFFFFFFFFFFFFFFFFFFFFFFF:FFFFFFFF:FFFF:FFFFFFFF
@A00721:190:HGJW7DSXY:4:1311:23981:15969_1:N:0:TCTGCAAG+GCTGCACT
TTAGTTCATGAAGTTCTTACACTTCTCGCGACTAATGTCAGCTTCACTACAAAGGCGTTACGTAAAGT
AGTTTTCACGCTTTATCTTAAAGCATTACCACCAAATTTAGCACTTTTTGAATATGACAGAAGCTCGC
CAT
+
F,FFFFFFFFFFFFFFFFFFFFFFFFFFFFFFFFFFFFFFFFFFFFFFFFFFFFFFFFFFFFFFFFFF
FFFFFFFFFFFFFFF:F:FFFFFFFFFFFFFFFFFFFFFFFFFFFFFFFFFFFFFFFFFFFFFFFFFF
FFF
@A00721:190:HGJW7DSXY:4:1636:22336:3035_2:N:0:TCTGCAAG+GCTGCACT
CCAGTTAGAGTTCTTCTAACCAGGACTGATGGTGAGATTCTTTTACCTTTTGGAGGTCCAGGAACCTT
TACTCCTAGTGTCTTTTCTAGAGTCCAAGGACGTTTCCGTCGGAGGAT
+
F,FFFFFFFFF:FFFFFFF:FFF:FF,FFF,:FFF:FFFFFF:FF,:F,:FF:F,FF,FFF:FFFF:F
FF:FF:FFFFFF:FF:FFF:FFFFFFFF:FFFFF:FFFFFFFFFFFFF
@A00721:190:HGJW7DSXY:4:2657:15872:19680_1:N:0:TCTGCAAG+GCTGCACT
GCTCAATAACACTCTATTGAATCCCTTTTCCACGATGAAAGCTCTTTATGAGGTGCTCCATGCAAATT
TACAGTTCCTCTTTGGTGATATCGCCTCTGTCCTTTCTCCTTTAATCGTGAGGTTACGAGAGTTTCGT
ACT
+
FFFFFFFFFFFFFFFFFFFFFFFFFF:FFFFF:FFFFFFFFFFFFFFFFFFFFFFFFFFFFFFFFFFF
FFFF:FFFFFFFFFFFFFFFFFFFFFFFFFFFFFFFFFFFFFFFFFFFFFFFFFFFFFFFFFFFFFFF
FFF
@A00721:190:HGJW7DSXY:4:2438:3323:32863_1:N:0:TCTGCAAG+GCTGCACT
TTTTGATAAATTTGTTGGAATCGATAATGATACTGACGAAGGTTCTATTACCACCCTTGTCATTGATA
CAAACATTCTAGGTGATGCTTTCTTAGAGGCTCGTCTAGCCGCTGAGATTCGTCTTACCTATTTACAA
TCT
+
FFFFFFFFFFFFFFFFFFFFFFFFFFFFFFFFFFFFFFFFFFFFFFFFFFFFFFFFFFFFFFFF:FFF
FFFFFFFFFFFFFFFFFFFFFFFFFFFFFFFFFFFFFFFFFFFFFFFFFFFFFFFFFFFFFFFFFFFF
FFF
@A00721:190:HGJW7DSXY:4:2533:13810:29825_1:N:0:TCTGCAAG+GCTGCACT
GATAACGATTTAGTACTGGGAAATGGTGGTAGTCAGTTTACTACTTTTCTTTCCCTCCCCTTTTTCGA
```

TTGGATTCGACGGGGTCTTCGTCTCACGACTAATTTGATAAGGTTTTTCTTTTATGGGACTCTTGTTT
GTA
+
FFFFFFFFFFFFFFFFFFFFFFFFFFFFFFFFFFFFFFFFFFFFFFFFFFFFFFFFFFFFFFFFFFFFFF
FFFFFFFFFFFFFFFFFFFFFFFFFFFFFFFFFFFFFFFFFFFFF:FFFFFFFFFFFFFFFFFFFFFFFF
FFF
@A00721:190:HGJW7DSXY:4:1405:5276:27195_2:N:0:TCTGCAAG+GCTGCACT
GTTCTTAATGTAGGTCTCCTAGTACTTAAGGGTTTATGTTTCGGTTTCAGCTGGATGGATCTTTTGAT
GGTATGTTTCACATTGCGTGATCACTTCCCGTGGACTTGGGAAGAGAT
+
FFFFFFFFFFFFFFFFFF:FFFFFFF,FFFFFFFFF:F,FFFFFFFFFFFFFFFFFFFFFFFFFFFFFF:
FFFFFF:FFFFFFFFFFFFFFFFFFFFFFFFFFFF:FFFFF:FFFFFFF
@A00721:190:HGJW7DSXY:4:2641:4255:29089_1:N:0:TCTGCAAG+GCTGCACT
CGATTCTATTATGTCGCTAAAGCCTGTCCAGTTGTCGATGATGTTCGGGCTGTGGGGATTAACTTCCC
TCGTGAAACTTACGACGATGTTCTCGCTCGCATTTCACAAGGATTACTAAGTCTGAGTTCTGCCACTT
CTA
+
FFFFFFFFFFFFFFFF:FFFFFFFFFFFFFFFFFFFFFFFFFFFFFFFFFFFFFFFFFFFFFFFF,FFFF
FFFFFFFFFFFFFFFFFFFFFFFFFFFFFFFFFFFFFFFFFFFFFFFFFFFFFFFFFFFF,FFFFFFFFF
FFF
@A00721:190:HGJW7DSXY:4:1309:11442:21057_2:N:0:TCTGCAAG+GCTGCACT
AAGTTCATGAGGATGTCTTGGTCGAAAAATATCGCCGGGAACTTCTAATAGAGGATCCTGATGACATT
ATTTCAACGGATACCGATCTCAGTTCTACCACAGAAACCGTCTCAGGA
+
FFFFFFFFFFFFFFFFFFFFFFFFFFFFFFFFFFFFFFFFFFFFFFFFF:FFFFFFFFFFFFFFFFFFFF
FFFFFFFFFFFFFFFFFFFFFFFFFFFFFFFFFFFFFFFFFFFFFFFF
@A00721:190:HGJW7DSXY:4:1443:26386:36323_2:N:0:TCTGCAAG+GCTGCACT
CTGATTCGGAATTAGAGAAAGACGACCTTCTTGTGGGGACTGAGACCTCTGAATTTTCAGGGTGCCCT
GATGAGGACGTTGAAAGGGATAATTTCAACTCTGAGACTTCTAAGAGT
+
FFFF:FFFFFFFFFFFFFFFF:FFFFFFFFFFFFFFFFFFFFFFFFFFFFFFF:FFFFFFFFFFFFFFFF
FFFFFFFFFFFFFFFFFFFFFFFFFFFFFFFFFFF,FFFFFFFFFFFF
@A00721:190:HGJW7DSXY:4:2508:2808:6668_2:N:0:TCTGCAAG+GCTGCACT
ACCAGAAACGTAACAAGAAGAATAAAGGTAGGAAGGGGAGAAAACAGAACAAGCGAGCGGTTGAAGAG
AAAACTGATATGGTTGCGGCTGATCGTACCCCAATTCCTGCTAGAAAG
+
FFFFFF:FFF:FFFFFFFFFFFFFFFFFFFFFFFFFFFFF:FFFFFFFFF:FFFF:FFFFFFFFFFFFFF
FFFFFFFFFFFFFFF::FFFFFFFFFFFFFFF:FFFFFFFFFFFFFFF
@A00721:190:HGJW7DSXY:4:1308:21242:22905_2:N:0:TCTGCAAG+GCTGCACT
TACCACTGATCTTCCAGAGTTACGTAGTGGTCGTTCTTTTGGTGATTTTATCAAAGAGAAAGTTTCTG
CTAGTCGCGACGATAGTATTTATCGGAGTGATATTAACAAGTTAAACT
+
FFFFFFFFFFFFFFFFFFFFFFFFFFFFFFFFFFFFFFFFFFFFFFFFFFFFFFFFFFFFFFFFFFFFFF
FFFFFFFFFFFFFFFFFFFFFFFFFFFFFFFFFFFFFFFFFFFFFFFF
@A00721:190:HGJW7DSXY:4:2174:30237:16313_2:N:0:TCTGCAAG+GCTGCACT
TGTTGAAGTACCGCAACATGGATCAGTTTTCCAAAAAGCTATTAATAGCAGTTGCGGGGCTATAATAC
CGCCTCCAGATTACTCTAAACTGAATCCACTCTCTTGGGATATAAATG
+
FFFFFFFFFFFFFFFFFFFFFFFFFFFFFFFFFFFFFFFFFFFFFFFFFFFFFFFFFFFFFFFFFFFFFF
FFFFFFFFFFFFFFFFFFFFFFFFFFFFFFFFFFFFFFFFFFFFFFFF
@A00721:190:HGJW7DSXY:4:2613:10312:17534_1:N:0:TCTGCAAG+GCTGCACT
AACATGAATTAGTTATTGAAAGTCTTGACCCTCACGTCTCACTTTATCACCTTGGAGATGGTAAAGTC
ATTAACAAAGGAAATGTTAAGGCTGATGACTTAAATTTCGCATTCTGCTTCGACGGTAATTCCATAAG
ACC

+
FFFFFFFFFFFFFFFFFFFFFFFFFFFFFFFFFFFFFFFFFFFFFFFFFFFFFFFFFFFFFFFFFFFFFFFFFFFF
FFFFFFFFFFFFFFFFFFFFFFFFFFFFFFFFFFFFFFFFFFFFFFFFFFFFFFFFFFFFFFFFFFFFFFFFFFFF
FFF
@A00721:190:HGJW7DSXY:4:1459:20094:26553_1:N:0:TCTGCAAG+GCTGCACT
TTCTTCATGAAGATCTTCGAGTGTTTTATAGTGAGTTATTCTTGCGAAGGTTCAAGTCGATTGATTTT
GTTGACTACGATCTCTCTTGTAATAAGAAGTTCTTCGCAGTTGAATCTCCACCAGGTGGAGGGAAGAC
CAC
+
F:FFFFFFF:FFFFFFF:F,FF:F,FFFF:FFFF:FFFFFFFFFF:FFFFF:FFFFFFFF,FFFFFF:
:FFFFFFFFFFFF:F:F,FFFFFFFF:FFFF:FFFF:FFFFFFFFFFFFFFF:FFFFFFFFFFFFFF
FFF
@A00721:190:HGJW7DSXY:4:1308:21414:35164_1:N:0:TCTGCAAG+GCTGCACT
GACTTTTGTCGAATTTTTCGCAAAAGGTCTTGAAGTACGTATTGTCACTGCTAATCGTAATTCGCGAG
AAGAGATAGTGACTAAAGTTTTGGCACTTCTAGTTCATCGCGGTATTTGTGAAATTAAGACAGACGTG
AAA
+
FFFFFFFFFFFFFFFFFFFFFFFFFFFFFFFFFFFFFF:FFFFFFFFFFFFFFFFFFFFF:FFFFFFFF
FFFFFFFFFFFFFFFFFFFFFFFFFFFF:FFFFFFFFFFFFFFFFFFFFFFFFFFFFFFFFFFFFFFFF
FFF
@A00721:190:HGJW7DSXY:4:1357:3341:20713_1:N:0:TCTGCAAG+GCTGCACT
TCGTATTAGAACAGCTGATTCTTATTGCATGAACGTCGCTGAGGTCAAACCTGTGGACGTTCTTTTCA
TCGACGAATTATTCATGATTCACGCTGGTCAGACCCTTTTTCTTACACATCTACTTAAGGCGGCTTAT
ACC
+
FFFFFFFFFFFFFFFFFFFFF:FFFFFFFFFFFFFFFFFFFFFFF:FFFFFF:FFFFFFFFFFFFFFFFF
FFFFFFFFFFFFFFFFFFFFFFFFFFF:FFFFFFFFF:FFFFFFFFFF:FFFFFFFFFF:FFFFFFFFFF
FFF
@A00721:190:HGJW7DSXY:4:2136:6307:30389_1:N:0:TCTGCAAG+GCTGCACT
GTGATTCCAATCAAATCGGATTCATCAATCGTATTGATCTCCAAGTCGCAAACTACTTCAACATCAAC
GACATCATTCTTGATGAGTGTCGTTCTTATCGAAGTCTCAGTTATCGCTGCCCGGCCGACGTTTGTCG
CGA
+
FFFFFFFFFFFFFFFFFFFFFFFFFFFFFFFFFFFFFFFFFFFFFFFFFFFFFFFFFFFFFFFFFFFFFFFFFFFF
FFFFFFFFFFFFFFFFFFFFFFFFFFFFFFFFFFFFFFFFFFFFFFFFFFFFFFFFFFFFFFFFFFFFFFFFFFFF
FFF
@A00721:190:HGJW7DSXY:4:2322:30065:31986_2:N:0:TCTGCAAG+GCTGCACT
TACGGCAGAAAAATTTCTCCTGCTTGTAATTCGAGAGAGGACACTATGGAAATTAGACCTATCAGATG
TCTTGACGACTTCGAATTTGAGGACGGAGTTAAATATATTACTTTCAC
+
FFFFFFFFFFFFFFFFFFFFFFFF::FFF,FFFFFFFFFFFFF:FF:FFFFFFFFFFFFFF,FFFFFFF
FFFFFFFFFF:FFFFFFFFFFFFFFFFFFFFFFFFFFFFFFFFFFFFF
@A00721:190:HGJW7DSXY:4:1241:31620:15640_2:N:0:TCTGCAAG+GCTGCACT
CAAAGTCCTTGGTAAGAGTAAGGTTAAATGTGAAGATGCTCAGACTATTCATGAAGTTCAAGGTAATA
CTTACCAGCACGTTCGGCTGGTTAGAATTAAACCTCAAGATGACCCTG
+
FFFFFFFFFFFFFFFFFFFFFFFFFFFFFFFFFFFFFFFF:FFFFFFFFFF:FF:FFFFFFFF:FFFFFF
FFFF:FF:FFFFFFFF:FFFFFFFFFFFFFFF,FFFFFFFFFFF:FFF
@A00721:190:HGJW7DSXY:4:1375:24804:12759_1:N:0:TCTGCAAG+GCTGCACT
CTTCCGATCTCTCTATCACGACACGTTGAATCTTTACGATACTTTGTTATCAATGTAAAGTTAAACGA
TGCTACTTGTGAGAGGATCAATCACAGTAAGGAACTGTCACGTTTAGCACGGGAGGTTGCAGATTCAA
CCC
+
FFFFFFFFFFF:FFFFFFFFFFFFFFFFFFF:FFFFFFF:FFFFFFFFFFFFFFFFFFFFFFFFFFFFF

```
FFFFFFFFFFFFFFFFFFFFFFFFFFFFFFFFF,FFFFFFFFFFFFFFFFFFFFFFFFFFFFFFFFF
FFF
@A00721:190:HGJW7DSXY:4:1539:8513:26850_1:N:0:TCTGCAAG+GCTGCACT
AGACGTGTGCTCTTCCGATCTCTTGAAGTTGTTGACGACAAGCCTCAAGAGGGTAGAGGACCACCTGG
ATCTTCTACTGTCTCTGCCTTCCAAGATTTCTTAGAAGAGGTTATTCCTGGAACCACCTTAACGTCAC
TCG
+
FFFFFFFFFFFFFFFFFFFFFFFFF:FFFFFFFFFFFFFFFFFFF:FFFFFFFFFFFFFFFFFFFFFF
FFFFFFFFFFFFFFFFFFFFFFFFFFFFFFF:,FFFFFFFFFFFFFFFFFFFFFFFFFFFFFFFFFFF
FFF
@A00721:190:HGJW7DSXY:4:2222:5457:28354_2:N:0:TCTGCAAG+GCTGCACT
TGAGTTCAAGTCGGTTGTCGATCGAATTAAGATAAAGGCTAATACCTTTATAGGTAAGAAGCCCCTTG
ATCAGCGTAGTCAGCGCGTTTAGCTGTATCATCAGTGAGATCGGAAGA
+
F,FFFF:FF:F,,FFFFF,,FF,::F,,FF,FF,FF,,,FFF,FFFFFFF,F,::FFFFF,,FF:FF,
:,F,,,F,FF,,FFF,FFF,FF,,,,:FF,FF,FFF:F,FF,,FFF,F
@A00721:190:HGJW7DSXY:4:1108:26530:14967_1:N:0:TCTGCAAG+GCTGCACT
TCCAAGAGAATATTTATTCTTATGAGGCTCGAAACTTCAACGTTTTCACAACGGAAAGACATATCGCA
CCCGAAATTTTTGGGCAGAATGCTCTTAACGCGGTTCTTGAACGGTGTTTTGATCAGTCTAAGTTGGA
TAA
+
FFFFFFFFFFFFFFFFFFFFFFFFFFFFFFFFFFFFFFFFFFF,FFFFFF:FFFFFFFFFFFFFFFFF
FFFFFFFFFFF:FFFFFFFFFFFFFFFFFFFFFFFFFFFFFFFFFFFFFFFFFFFFFFFFFFFFFFFF
FFF
@A00721:190:HGJW7DSXY:4:1140:19072:28855_2:N:0:TCTGCAAG+GCTGCACT
ACTCAGTTAACCTGAGAGCTCCTCAATTGATAGATTGGGTCAACAATAGGGACGCTAGTATACTTCGT
AAAGTCGACAGCGAAATGGTCAATATTGATAATTGGACCGACTATATG
+
FFF:FFFFFFFFFFFFFFFFFFFFFFF:FFFFFFFFFFFFFFFFFFFFFFFF:FFFFFFFF:FFFFFFFF
FFFFFFFFFFFFFF:FFFFFFFFFFFFFFFFFFFFFFFFFFFFFFFFF
@A00721:190:HGJW7DSXY:4:1245:27588:22404_1:N:0:TCTGCAAG+GCTGCACT
AGCTCATGGTGAAAAGGGAAGCTAAGGTTAAACTTGATGCTTCAGCCTTTTCGAAGCATAACCCCGCA
CAGAATATCGTTTATCACGCAAAATTTATTAATGCAATTTTCGGACCTATCTTTAAGGTTCTTGTGAG
GAG
+
FFFFFFFFFFFFFFFFFFFFFFFF,FFFFFFFFFFFFFFFFFFFFFFFFFFFFF:FFFFF:FFFFFFF
FFFFFFFFFFF:FFFFFFFFFFFFFFFFFFFFFFFFFFFFFFFFFFFFFFFFFF:FFFFFFFFFFFFF
FFF
@A00721:190:HGJW7DSXY:4:1640:6741:7654_1:N:0:TCTGCAAG+GCTGCACT
GACGATTTCTCCCGGAAGCTCTTCTCTCTCTTAGGAGGTCATGATGACTACTATAAGGGTGAAATCGA
TTTTTCGAAGTTTGACAAATCTCAAGATTTCTTTTGTAAAGCCTATGAGATAAAGATGTATTCTTATT
TAG
+
FFFFFFFFFFFFFFFFFFFFFFFFFFFFFFFFFFFFFFFFFFFFFFFFFFFFFFFFFFFFFFFFFFFF
FFFFFFFF:FFFFFFFFFFFFFFFF:FFFFFFFFFFFFFFFFFFF:FFFFFFFFFFFFFFFFFFFFFF
F:F
@A00721:190:HGJW7DSXY:4:2544:3269:3114_2:N:0:TCTGCAAG+GCTGCACT
TTGGAGTGCCAGTGAATATTTTTGTCACGCCAGGAGTATCCATAACGATTTGTCATTTACCACCTTTG
CTCAACGACGAAGTGGAACGGCCAACACTTTCTTTGGTAATTGTATGA
+
FFFFFFFFFFF,FFFFF:FFFFFFFFFFF:F,F:FFFFF:FFFFFF:FFF,:FFFFFFFFFFF:FF::
FFFFFFFFF:FFFFFFFFFFF,FFFFFFFFFFFFFF,FFFF:FFFFFF
@A00721:190:HGJW7DSXY:4:2347:15944:19272_2:N:0:TCTGCAAG+GCTGCACT
ATTTTATCACTTTATTATGACCTTGGAAAGTTTTCGGCTGTGGTTGTCGCTGGGGACGATTCCATTCT
```

GTTTTCAAAGACTCCAATAACTGATCATTCTGATCATATGATTTTGAG
+
FFFFFF:FFF,FFFF,FFFF,:FFFFFFFFFFFF:FF:FFFFFFFFFFF:,F:FFFFFFFFFFFFFFF
FFF:FFFFFFFFFFFFF:FFFFF:F,FFFF::F:FFFF:FFFF:F:F:
@A00721:190:HGJW7DSXY:4:2324:31684:2691_1:N:0:TCTGCAAG+GCTGCACT
TTTACAAGATCATCGTTAAGTTAGGTAAACCCCTAACCGATGTTACTCTTGTAGGACTTTGGGAGAGA
TTCGTTAGTTTTAAAGACTATACTAAATATCTCCACTACGAGGTTGTTGTTTGGCGATTAGCTCTTGC
GA
+
FFFFFFFFFFF:FF,FFFF,FFF::FFFFF:F:FFFFFFFFFF:FFFFFFFFFFFFFFFFFFFFFFF:F
FF,FFFFFFFFFFFFFFFFFFFFFFFFFFFFFFFFFFFFFFFFFFFFFFFF:FFFFFFFFFFFFFF
F:
@A00721:190:HGJW7DSXY:4:1570:31602:8750_2:N:0:TCTGCAAG+GCTGCACT
GAGGGTCGTTATACCTATTCGGCTATCTGTGCTTTGAATTGTATAAGATCAAATTTTAAACGATTCCG
AGAGGTTTATCCTGATGTGGAGGGAGTTCTTGAAGCATCCCGACCTTT
+
FFFFFFFFFFFFFFFFFFFFFFFFFFFFFFFFFFFFFFFFFFFFFFFFFFFFFFFFFF:FFFFFFFF
FFF:FFFFFFFFFFFFFFFFFFFFFFFFFFFFFFFFF,FFFFFFFFFFF
@A00721:190:HGJW7DSXY:4:2565:3152:32534_2:N:0:TCTGCAAG+GCTGCACT
GGTTTCATAAGATTATCAGTAATCCTGATTCTACTACGGATTCATGGACCTTCCATCCTGTACCTCTT
CTGGAGAGACGTATCTCAAAGATCGACTTCGAGACAGGTGATATTGTT
+
FFFFFFFFFFFFFFFFFFFFFFFFFFFFFFFFFFFFFFFFFFFFFFFFFFFFFFFFFFFFFFFFFFFF
FFFFFFFFFFFFFFFFFFFFFFFFFFFFFFFFFFFFFFFFFFFFFFFF
@A00721:190:HGJW7DSXY:4:1336:28691:6433_2:N:0:TCTGCAAG+GCTGCACT
CTAAATTCGGTAACGGTGCGTGGCGCTTTATTTTAAAAAGCTCCCTCCTCTCGCAAGTTTGATCTGAT
ACTTATCACGAAAGGACTTGAGTCTTTTGATCAGTCTCTGACTTGACC
+
FFF:FFFFFFFFFFFFFFFFFFFFFFFFFFFFFFFFFFFFFFFFFFFFFFFFFFFFFFFFFFFFFFFF
F:FFFFFFFFFFFFFFFFFFFFFFFFFFFFFFFFFFFFFFFFFFFFFF
@A00721:190:HGJW7DSXY:4:1465:10520:4335_2:N:0:TCTGCAAG+GCTGCACT
TAAATGACGATCATTAAGACTCTCTTTCTTCTTTGTTTCCTTCTTTGCACTGATTTTTCCTCATCAGC
TTGTGTTTTACCTAATCTGTGTTTTTACGATAACAACTATCAGTTGAT
+
FFFFFFFFFFFFFFFFFFFFFFFF:FFFFFFF:FFFFFFFFFFFFFFFFFFFFFFFFFFFFFFFFFFF
FFF:FFFFFFFFFFFFF:FFFFFFFFFFFFFFFFFFFFFFFF:F,FFFF
@A00721:190:HGJW7DSXY:4:1112:30897:9533_1:N:0:TCTGCAAG+GCTGCACT
CCGAAAGGCGAACTTTGGGAATTTGAAGCTCAGGGTGAAGCACAACGTTTGACCGTGACGAACTGTGG
TGTTCTTGATCTACGTTACGGATATACTCTCCTTGAGTTCACTCGTAAGGACACTACCACTTATTACG
ATA
+
FFFF,FFF,,FFFFFFFFFFFFF,FFF:FFFFFF:F:FFFFFFF:F:FFFFF,FFF:FFFFFFFFF:F
FFFFFFFFFF,FFF:FFFFF,:FFFFFFFFFFFFFFFFFFF:FFFFFFFFFFFFFFFFF:FFFFFFF:
FFF
@A00721:190:HGJW7DSXY:4:2609:30318:21621_1:N:0:TCTGCAAG+GCTGCACT
AGCGTCTTCTGTTCATCGCAACCAGATGATATCTTCTATTCGAACTTATGTTTCGATCAGTGTTTCTT
CTATCAGGAGGAAAACACTTGTTCTAGTCCTTGTAAGATTAATCCTAACGTAGAAGATTGTTGTTTAC
CAC
+
FFFFFFFFFFFFFFFF:FFFFFFFFFFFFFFF:FFFFFFFFFFFFFFFFFFFFFFFF:FFFFFFFFFFF
FFFFFFFFFFFFFFFFFFFFFFFFFFFFFFFFFFFFFFFFFFFFFFFFFFFFFFFFFFFFFFF:FFFF
FFF
@A00721:190:HGJW7DSXY:4:1529:20220:8985_1:N:0:TCTGCAAG+GCTGCACT
TCTCTATTTTAAGTTTTGTCTTCCTTGTTTGTCTTATTCTATTAATCACTCATTTTAGTGAGATTATT

AAAAACGCACTAAAACGTCTTCGACAACAGCAGGGGTTTTCGAATAGTGATAGCCACCCGCCTTACAG
AGG
+
FFF:FFFFFF,FFFFFFFF:FFFFFFFFFFFFFFFFFFFFFFFFFFFFFFF:FFFFFFFFFFFFFFF
FFFFFFFFFFFFFFFFFFFFFFFFFFFFFF:FFFFFFFFFFFFFFFFFFFFFFFFFFFFFFFFFF:F
FFF
@A00721:190:HGJW7DSXY:4:2666:25183:13823_2:N:0:TCTGCAAG+GCTGCACT
GACTTTGGTACCACCTTTTCTACTGTCTGTCTCGCCTCTTCGAATGAAGTTTTAATGCTTAACAACAA
TGATAGTGAATTTATACCTACAATCATTGGTTTTTCTAATAATGATGA
+
FFFFFFFFFFFFFFFFFFFFFFFFFFFFFFFFFFFFFFFFFFFFFFFFF:FFFFFFFFFFFFFFFFFF
FFFFFFFFFFFFFFFFFFFF:FFFFFFFFFFFFFFFFFFFFFFFFFFFFF
@A00721:190:HGJW7DSXY:4:1469:6488:32049_1:N:0:TCTGCAAG+GCTGCACT
GTGACTGGAGTTCAGACGTGTGCTCTTCCGATCTGTCAGGGAGGATTCTCAGTTTACAAGGACCTTAA
GCGATGGATCGGGGTTAATTCTAAAACATTACCCGAAAGACGTGCTAAATTAAAACCATTGTACGATG
TCG
+
FFFFFFFFFFFFFFFFFFFFFFFFFFFFFFFFFFFFFFFFFFFFFFFFFFFFFF,FFFFFFFFFFFFF
FFFFFFFFFFFFFFFFFFFFFFFFFFFFFFFFFFFFFFFFFFFFFFFFFFFFFFFFFFFFFFFFFFFF
FFF
@A00721:190:HGJW7DSXY:4:2347:21585:25598_1:N:0:TCTGCAAG+GCTGCACT
TCACTTTTCACTAAGAATTGGTCCTACTTTTGGACAAGGCCAACTTCGTAATCTTCATGAGATCGTTT
CTTTCTTTATCTCTTTGGTTGTTAGGGATTTTGAGAGAGTGAAAGGCTTCAATTGTAGTGGTTTGGTC
GTT
+
FFF:FFFFFFFFFFFFFFFFFFFFFFFFFFFFFFFFFFFFFFFFFFFFFFFFFFFFFFFFFFFFFFFF
FFFFFFFFFFFFFFFFFFFFFFFFFFFFFFFFFFFFFFFFFFFFFFFFFFFFFFFFFFFFFFFFFFFF
FFF
@A00721:190:HGJW7DSXY:4:2620:26467:23563_2:N:0:TCTGCAAG+GCTGCACT
ACCTCTACGCAAAGATATTTCATGAAAACTACAGAGGAACGTACCGGATTACCTGTCTTTCATATTAT
GAATGAACCTTCGGCCGCTCTCTTTGCTTCGATGTTGGATGTCAAGAA
+
FF:FFFFFFFFF:F:FFFFFF:FF:FFFFFFFF,F,FFF:FFFFFF:FFFF:FFFFFFFFFFFFFFFF
,FFFFFFF,F,:FF,FFFFFFFFF:F:,F::FF,FF::FFFF:FFFFF
@A00721:190:HGJW7DSXY:4:2447:5936:34726_1:N:0:TCTGCAAG+GCTGCACT
ATTCCTACGTAGTTTATGACTTTGGTGTTGGTACTTTCGATACTTCTATCGTCTTACGTTACGGTAAT
TACTATAGTGTTATTTATTCTGGTGGTGATGATAATCTTGGAGGTCGTGACGTTGATGAAGCTATAAG
AGC
+
:FF:FFFFFF,FFFF,FFF,:FFFFFF,F,:FFFF:FFFFF:FFF:FFFFFF:FFF,FFF,FFFF:F:
FF:FFF,FFF:FF:FFFFFFFFFF:FFFFFFFFF,FFFFFF:F:F:FFFF:FFFFFFFFF:FFFF:,F
FFF
@A00721:190:HGJW7DSXY:4:2648:1787:4085_2:N:0:TCTGCAAG+GCTGCACT
CACAACTTTCAGTTTCACAACTGAAGGAGGAGGTTTCTGTTACTGGAAAACCCTCGTCAATACTTTTC
AATAATATACGTCTTGAACTCACTTATGACGATCTAGTGAAATTAACG
+
FFFFFFFFFFFFFFFFFFFFFFFFFFFFFFFFFFFFFFFFFFFFFFFFFFFFFFFFFFFFFFFFFFFF
FFFFFFFFFFFFFFFFFFFFFFFFFFFFFFFFFFFFFFFFFFFFFFFF
@A00721:190:HGJW7DSXY:4:1244:4354:1642_1:N:0:TCTGCAAG+GCTGCACT
TACTATTATGAACAAAGTTATCAAAGATAGTGGCATTCAAGGCAAACTTACCCTGATTCCGGCCGGTG
GTAGCTCATTTCTACCAGGGGCTATTACTCTTGCTGAAACAGCTATTAAAGCTGTTGAGCGGGTAATT
ACA
+
FFFF:FFFFFFFFFFFFFFFFFFFFFFFFFFFFFFFFFFFFFFFFFFFFFFFFFFFFFFFFFFFFFFF

```
FFFF,FFF:FFFFFFFFFFFFFFFFFFFFFFFFFFFFFFFFFFFFFFFFFFFFFFFFFFFFFFFFF:F
FFF
@A00721:190:HGJW7DSXY:4:1409:27109:36385_2:N:0:TCTGCAAG+GCTGCACT
ATCGCTGAAGGATGTGCACTCTTTTCTGCTACTTTAACGGAGCGTAGTGTACTCTTCGTCGATTGTAT
CAACAGTAACATTACTTCTAATAAAGGTCTCTATCAAGCTCGTATTAT
+
FFFFF:FFFFFFFFFFFFFFFFFFFFFFFFFFFFFF,FFFFFFFFFFFFFFFFFFFFFFFFFFFFF,F
FFFFFFFFFFFFFFFFFFFFFFFFFFFFFFFFFFFFFFFFFF,FFFFF
@A00721:190:HGJW7DSXY:4:1241:11849:31093_1:N:0:TCTGCAAG+GCTGCACT
TTACCGTTGAAGGCTAAACTCCAAGGTAATTATGAAGACTGTGATTTATCTTTCACTCTGGCCTTGTA
TGAGGGTGTTTCTAATCGTAACATTAATAATTTCAATCTTCTTAAAAAGAAGATAGAGAGTCATACTT
TGG
+
F:FFFFF:FFFFFFFFFFFFFFFFFFFFFFFFFFFFFFFFFFFFFFFFFFFFFFFFFFFFFFFFFFFF
FFFFFFFFFFFFFFFFFFFFFFFFFFFFFFFFFFFFFFFFFFFFFFF:FFFFFFFFFFFFFFFFFFFF
FFF
@A00721:190:HGJW7DSXY:4:1247:7446:16016_2:N:0:TCTGCAAG+GCTGCACT
CAATATTGAAGTGGACACCTCTGGTAGTATCGAAGTAAAGATAACCAATAAGGAAGGAGCTTATACTA
TGGTTGAATCAACTAAGCATACTACTTTTGCTTTGATTACTCTACCGC
+
FFFFFFFFFFFFFFFFFFFFFFFFFFFFFFFFFFFFFFFFFFFFF:FFFFFFFFFFFFFFFF:FFFFFFF
FFFFFFFFFF:FFFFFF,:FFFFFFFFFFFF:FFFFFFFFFFFFFFF
@A00721:190:HGJW7DSXY:4:2307:28782:21402_2:N:0:TCTGCAAG+GCTGCACT
TAAAGAGTCGATTAAACAAGTTGTACTTATCAATTATTTTATCGCTGGTTGTTATATCGAAAAGTCTC
TTCTAAAAGGAACGTCAGTTCTAAACCAAAGAGAATATCTAAAATATT
+
FFFFFFFFFFF,FFFF:FF,FFFF:,,FF,,,F,,FFFFFF:,FF:,F:FFFFFF,,,F,F,F,:F:F
FF,,FFFF,F,F:,F,:::F,,FF:FFFFF,F:::,F,,,FFFF,F,F
@A00721:190:HGJW7DSXY:4:2402:14687:8766_1:N:0:TCTGCAAG+GCTGCACT
TACCACTATCTACGAACCTAAAGTTCTGAAGACGTTGCTTGTCGCTTCCTCCTTTAATGGCACTACCT
CGGTACAATCTTACTTGGAACGAAGGCGACCTGTTCCGGAGTTTTTACGGGATCCGGAATGTTCTGTC
TAT
+
FFFFFFFF:FFFFFFFFFFFFFFFFFFFFFFFFFFFFFFFFFFF:FFFFFFFFFFFFFFFFFFFFFFFF
FFFFFFFFFFFFFF:FFFFFFFFFFFFFFFFFFFFFFFFFFFFFFFFFFFFFFFFFFFFFFFFFFFFF
FFF
@A00721:190:HGJW7DSXY:4:2305:8006:4679_1:N:0:TCTGCAAG+GCTGCACT
AATACGATTTCGAACGTCAACGTTCACCGAGTTTCGTTTCGTACCTTGATGCTGACTTTCGTCAATAT
GATTTGATCTGGTTGCTCAATTCTAAGCGTGTTCCGCTTAGTGCGAGGCCTCTCTTCTACGAGTATTG
CTA
+
FFFFFFFFFFFFFFFF:FFFFFFFFFFFFFFFFFFFFFFFFFFFFFFFFFFFFFFFFFFFFFFFFFFF
FFFFFFFFFFFF:FFFFFFFFFFFFFFFFFFFFFFFFFFFFFFFFFFFFFFFFFFFFFFFFFFFFFFF
FFF
@A00721:190:HGJW7DSXY:4:1148:15953:36636_2:N:0:TCTGCAAG+GCTGCACT
ATATTTGGATGATCTTGATAAAGAACTTCATGAATTTTCAGAGGATGAAAAATTTTCTTCCTTGGATT
TTAATATAAAGAATCTTTACCAGGACTCCTACTACAGGAGGCAGTCTT
+
FFFFFFFFFFFFFFFFFFFFFFFFFFFFFFFFFFFFFFFFFFFFFFFFFFFFFFFFFFFFFFFFFFFF
FFFFFFFFFFFFFFFFFFFFFFFFFFFFFFFFFFFFFFFFFFFFFFFF
@A00721:190:HGJW7DSXY:4:1533:10149:15843_1:N:0:TCTGCAAG+GCTGCACT
ATTTGTTGTAATAGGGTCATCAACTTAGAACTCGAAACTCTAAGTGGTAATACTGTCTTTCCAAGCTT
ACCTGTATCTTCAATGAAATTGGACTCTTCGATAGGGGACATTTTATCGTGGGCTTTCCGTAAAGGAA
GAT
```

+
FFFFFFFFFFF:FFFFFFFFFFFFFFFFFFFFFFFFFFFFFFFFFFFFFFFFFFFFFFFF
FFFFFFFFFFFFFFFFFFFFFFFFFFFFFFFFFFFFFFFFFFFFFFFFFFFFFFFFFFFFFFFFFFFF
FFF
@A00721:190:HGJW7DSXY:4:2269:24288:2503_1:N:0:TCTGCAAG+GCTGCACT
CTACTAATGGCGTTGGCCTAGTGGATGTTATTAGTGACATCTGGTCAATTTCGTTTTTAGGTGGTCTT
TATTCTATTATTGATTTTTATGAGATTGATACGTTAACAGAAGATGATGTTAGAGTAGAATTCCTGAA
GAG
+
FFFFF:FFFFFFFFFF:FFF:FFFFFFFFFFFFFFFFF:F,FFFFF:FFFFFFFFFFFFFFFFFF,FF
FFFFFFFFFFFF:FFFFFFFFFFFFFFFFFFFFFFFFFFFFFFFFFFFFFFFFFFFFFFFFFFFFFFF
FFF
@A00721:190:HGJW7DSXY:4:2169:24867:10989_2:N:0:TCTGCAAG+GCTGCACT
CCAACTTCTTGACAATTTTCTTCAATCCTTCGATAATAGTTAAATCTAGGATTATGTCTGTGTTGAAA
GACTTTCTACCGATAAGGGTCACTAGTCTTACACAATTAAGAAATAAT
+
FFFFFFFFFFFFFFFFFFF:FFFFFFFFF:FFFFFFF:FFFFFFFFFFFFFFFFFFFFFFFFFFFFFF
FFFFFFFF:FFFFFFFFFFFFFFFFFFFFFFFFFFFFFFFFFFFFFFF
@A00721:190:HGJW7DSXY:4:1124:6506:22373_2:N:0:TCTGCAAG+GCTGCACT
ACTATCTAAAGATTGAAGGAAAATTTTATCCTCGTTTAACTTACTTAGACTTTCCTACGATACAAATG
ATAACTTCTAATTTACGTTTGCTACTCAGAGCTGGTCCTCTCAGCAAA
+
FFFFFFFFF,FFFFFFFFFFFFFFFFFFFFFFFFFFFFFF:FFFFFFFFFFFFFFFFFFFFFFFFFFF
FFFFFFFFFFFFFFF:FFFFFFFFFFFFFFFFFFFFFFFFFFFFFF,F
@A00721:190:HGJW7DSXY:4:2143:9742:15953_2:N:0:TCTGCAAG+GCTGCACT
TCCTCAGGAGTCCCGGCATTTGATAATAATGACGACTTTCGTCATAATCGCTGCATATTACGGATTAC
ACGGTACCAGCGCATCTCGTGTCGAGCCTCGACCCGATTATATGGTAT
+
FFFFFFFFFFFFFFFFFFFFFFFFFFFFFFFFFF,F,:F:FFF:FFFFFFFFFFFFFFFFFFFFFFF:
FFFFF:FFFFFFFFF:FFFFFFFFFFFFFF:FFFFFFFFFFFFF:FFF
@A00721:190:HGJW7DSXY:4:2345:6017:29293_2:N:0:TCTGCAAG+GCTGCACT
TCTAGAATTCTTAATTATAATATTCAACCTCAAAAGTTACGTGTTTACAGTAACGAGATCACACATTT
TGAGAACTAACTATGTTATGTTCTCTGATCATTTTGACAATCTCTTCT
+
FFFFFFFFFFFFFFFFFFFFFFFFFFFFFFFFFFFFFFFFFFFFFFFFFFFFFFFFFFFFFFFFFFFF
FFFFFFFFFFFFFFFFFFFFFFFFFFFFFFFFFFFFFFFFFFFFFFFF
@A00721:190:HGJW7DSXY:4:2206:4562:10770_2:N:0:TCTGCAAG+GCTGCACT
AAATAAATAATATTACTAATGGCAAATAGTGGAAAGGCTCCAGTCAACACTAGTTCGAGTGCTGCTTT
GGATTACGATGACGTGAGAATAGGTGAAGGGATAGTTTTTACCTCGAC
+
FFFFFFFFFFFFFFFFFFFFFFFFFFFFFFFFFFFFFFFFFFFFFFFFFFFFFFFFFFFFFFFFFFFF
FFFFFFFFFFFFFFFFFFFFFFFFFFFFFFFFFFFFFFFFFFFFFFFF
@A00721:190:HGJW7DSXY:4:2473:20735:36307_1:N:0:TCTGCAAG+GCTGCACT
TCTTAAAGCTTCACCCAAAATGAAGGACACTTCGAAACTGAATAATGATGCACTACAAACAAGTATCG
CTAATCATCTGAAAAAGGCTCTAAAGGACACTATACAATATCCAGACGACGACGAAGCTTATACAATA
TCC
+
:F:FFFFF:F:FF,F:FFFFFFFF,FF:F,FFF::FFFF:FFFF:FF:FFF:FFFFFFFFFFFF:FF:
FFFFFFFFFFFFFFFFFFFFFFFFFFFFFFFFFFFFFFFFFFFFFFFFFFFFFFFFFFFF:FFFFFFF
FFF
@A00721:190:HGJW7DSXY:4:2233:14922:12179_2:N:0:TCTGCAAG+GCTGCACT
ACCACATTTGGGTCAAGTCAATCCGTTGAGAAAATTTCTTAGAGGATATTCGCCATTGTGGTACGAAT
TGGTCCAACATTTACCACCAAATTCTTTTGATACAACTCTAGCCTTCA
+

FFFFFFFFF,FFFFFFFFFFFFFFFFFFFFFFFFFFFFFFFFFFFFFFFFFFFF:FFFFFFFFFFFF
FFFFFFFFFFFFFFFFFFFFFFFFFFFFFFFFFFFFFFFFFFFFFFFF
@A00721:190:HGJW7DSXY:4:2423:1344:36229_2:N:0:TCTGCAAG+GCTGCACT
ACCAGATTGGTTCGAACCCGTTGATGCTATGTCAGAAGAAGCTATTCAAGCTTGTCGTTTAAAGACTG
CAGAGGCTGTCAAACAAGCCCAGACTCAAGGAGTCCAATCAAAGGTTT
+
FFFFFFFFFFFFFFFFFFFFFFFFFFFFFFFFFFFFFFFFFFFFFFFFFFFFFFFFFFFFFFFFFFFF
FFFFFFFFFFFFFFFFFFFFFFFFFFFFFFFFFFFFFFFFFFFFFFFF
@A00721:190:HGJW7DSXY:4:1436:8630:18098_1:N:0:TCTGCAAG+GCTGCACT
CTTGCAGAGTGACTGGAGTTCAGACGTGTGCTCTTCCGATCTGAAAAGTCCGGAGTATATTGTATGTT
AATAGATAGTGGGATAATAGTTCTACCGATACGTAGTGAACGCGTTCGTAGAACCAAGTACTTTCCGT
CTG
+
F:FF:FFFF::F:FFF,FFFFFFF,FFFFFF::FFFFFFFFFFFFF:FFFF,FFFFFF::FFF:FFFF
FFFFFFFFFFFFFFFFFF:FFFFFFFFFFFFFFFFFFFFFFFF:FFFFFFFFFFFF:FFFFFFFF:FFF
FFF
@A00721:190:HGJW7DSXY:4:1506:18475:24095_1:N:0:TCTGCAAG+GCTGCACT
ACCGATAGGAGTTGTATAGTTGAAGATGTGGCTTTAGACGATAATCCTTTAAGGACTATGTTGACTGA
CGAAGATTTTCCAACAGATTTGTCTACGTTGCAGATCGCCACTCCAAACACTATATACATTATAAAAT
ATA
+
FFFFFFFFFFFFFFFFFFFFFFFF:FFFFFFFFFFFFFFFFFFFFFFFFFFFFFFFFFFFFFFFFFFF
FFFFFFFFFFFFFFFFFFFFFFFFFFFFFFFFFFFFFFFFFFFFFFFFFFFFFFFFFFFFFFFFFFFF
FFF
@A00721:190:HGJW7DSXY:4:1270:9146:16016_1:N:0:TCTGCAAG+GCTGCACT
ATAACTTTTGTTACCAAATTCTTGGCTTTCAACGAAGTCTCTGATTTTATTAATCATTACTTTAACGA
TCTCTCGGTCATTTACTCTGTACTTGATCTACGTCGTTTAACTGTCGGAACAACCTTTACTTAGAATG
ACT
+
FFFFFFFFFFFFFFFFFFFFFFFFFFFFFFFFFFFFFFFFFFFFFFFFFFFFFFFFFFFFFFFFFFFF
FFFFFFFFFFFFFFFFFFFFFFFFFFFFFFFFFFFFFFFFFFFFFFFFFFFFFFFFFFFFFFFFF:FF
FFF
@A00721:190:HGJW7DSXY:4:1617:28655:24533_2:N:0:TCTGCAAG+GCTGCACT
TCTTATATTATTTACAATAGTTAATATCATATTAGTAATACTCACTATCGTTTTCTGCTTTTTCTTAG
CTAATAGATTAATAAAGGATGAGTACGAAAACCAACTCTTCGTTACGA
+
FFFFFFFFFFFFFFFFFFFFFFFFFFFFFFFFFFFFFFFFFFFFFFFFFFFF:FFFFFFFFFFFFFFF
FFFFFFFFF:FFFFFFFFFFFFFFFFFFFFFFFFFFFF:FFFFFFFFFFF
@A00721:190:HGJW7DSXY:4:1505:31747:32330_1:N:0:TCTGCAAG+GCTGCACT
TTTAATATATTTTTTCTAAAAACCAATATAAACTATTATAATGCTTGTTTTCCGTCGTTAATTTAAAA
CACTATGATAACATCCCAAAACGACTCGAAACCGAGACGCCGTAGATCAAAGTCCCTTCTAACTTTAG
AT
+
F,::,F,FFFFF,,F,F,,:,FFF:::,F,F,:,,,,F,F,,,,,,:FFF,,,,,,,F,:,,,:,:,F
,,,,F,,F,:,,:,,F,,F,FFFFFFFFFFFFFFFF:FFFFFFFFFFFFFFF:FFFF:FFFFFFFFFFF
FF
@A00721:190:HGJW7DSXY:4:2504:4851:15718_1:N:0:TCTGCAAG+GCTGCACT
CATTTTATAGCAGTTATTTATGTTGTACTTTTTATAATTTATAACCTTCTTATACTTTTGGGTATGTT
TTGTGAAAAACATGAGATACCCAAGTCTATCGAAGACATTATCTCTTATGTACTTATTGTAACGATCA
TTC
+
FFFFFFFFFFFFFFFFFFFFFFFFFFFFFFFFFFFFFFFFFFFFFFFFFFFFFFFFFFFFFFFFFFFF
FFFFFFFFFFFFFFFFFFFFFFFFFFFFFFFFFFFFFFFFFFFFFFFF:FFFFFFFFFFFFFFFFFFF
FFF

@A00721:190:HGJW7DSXY:4:1206:17508:2534_2:N:0:TCTGCAAG+GCTGCACT
TATTCCTTTAACTGTGTATGAGGAGGATTTATAATGCTTTTCGATAGTGATTGTTATCAATTTAAATA
CATTACTGTTTCAAATCATCTTTACTCACAGACTCCGAAAATGGTTTC
+
FFFFFFFFFFFFFFFFFFFFF:FFFFFFF:FFFFFFFFFFFFFFFFFFFFFFFFFFFFFFFFFFFFFF
FFFFFFFFFFFFFFFF:FFFFFFFFFFFFFFFFFFFFFF:FFFFFFFF
@A00721:190:HGJW7DSXY:4:1267:10330:34601_1:N:0:TCTGCAAG+GCTGCACT
TATTTATTAAACGAACTATTATCCGATTCTTCGAGGGTATCACTACCACTGATCTGCCGACTTTAGTC
AAGGAACTTCAATTCCTTCGATGTTTATATTTTTCTATAAGGGAAAATAGCAATAATCAAATTTCGAC
AAA
+
FFFFFFFFFFFFFFFFFFFFFFFFFFFFFFFFFFFFFFFFFFFFFFFFFFFFFFFFFFFF,FFFFFFF
FFFFFFFFFFFF,FFFFFFFFFFFFFFFFFFFFFFFFFFFF:FFFFFFFFFFFFFFFFFFFFFFFFFF
FFF
@A00721:190:HGJW7DSXY:4:1252:8784:33990_2:N:0:TCTGCAAG+GCTGCACT
ATTTCAGAACTCTCTCCGATCACCTTGGTGTGATCTCCCGGAGGATCTTTTCAATAAGATGTATGATG
ATAATCTACTATTTTTAAATAGTGATATTCAGCGTATACTTGAATACT
+
FFFFFFFFFFFFFFFFFFFFFFFF:FFFFFFFFF:FFFFFFFFFFFFFFFFFFFFFFFFFFFFFFFFFF
FFFFFFFFFFFFFFFFFFFFFFFFFFFFFFFFFFFFFFFFF:FF:FFF
@A00721:190:HGJW7DSXY:4:1512:8169:3145_1:N:0:TCTGCAAG+GCTGCACT
CTTGGTCTATTTACCGCAAAAAGTTGGAAGAGGTCTATGATATTTATTTTACTTATACTTTAAATAGC
ACCGATTATAACTTGACTGTTGATAATATTACAATAGCTCTTTCTCAGAAGATTAGAGAAGTAACCGG
TGA
+
FFFFFF,FFFF:FFFFFFFFFFFFFFFFFFFFFFFFFFFFFFFFFFFFFFFFFFFFFF:FFFFFFFFF
FFFFFFFFFFFFFFFFFFFFFFFFFFFFFFFFFFFFFFFFFFFFFFFFFFFFFFFFFFFF,FFFFFFFF
FFF
@A00721:190:HGJW7DSXY:4:1318:30490:19194_2:N:0:TCTGCAAG+GCTGCACT
CATTGATTTACTTTTACCGTTAGAACTGAAAAAGATTCTTTTCGAAAAGAAACCTATAAGTTACAAAC
GATTCTTCGACTATATTAAAGAAATAAAAATACTTCAAGTACCTGAAC
+
FFFFFFFFFFFFFFFFFFFFFFFFFFFFFFFFFFFFFFFFFFFFFFFFFFFFFFFFFFFFFFFFFFFF
FFFFFFFFFFFFFFFFFFFFFFFFFFFFFFFFFFFFFFFFFFFFFFFF
@A00721:190:HGJW7DSXY:4:1672:17155:23938_1:N:0:TCTGCAAG+GCTGCACT
ATGGTATAATGTGTCTTACAAATAGAACCTTCACTCTCGGTAATGAGAGTGTATTCCTTAATATTCTA
GTAACCGCTATGTGGGCATTACTAGGATTTATCACACTCCTAAATTTGATAGCAATTTATGTTTTAAA
TGC
+
FFFFFF,F:FFFF:FFFFFFF:FFFF,F:FFFFFFFFFFFFFFFFFFF:FFFFFFFFFF:FFFFFFFF
FFFFFFFFFFFFFFFFFFFFFFFFFFFFFFFFF:FF:F:FFF,:::FFF:FFF:F,:FFFFFFFFFF,
FFF
@A00721:190:HGJW7DSXY:4:1614:21766:27633_2:N:0:TCTGCAAG+GCTGCACT
AAAAACTCCATTGTATAGTTCAAGGAGTACTTAATTCAATAGCTTTAGTATTATTTACTATCGCCTTT
ATTGAAAATTGTATAAGAGTTAATTGCTTACACAAAAGTGACGCGCGA
+
FFFFFFFFFFFFFFFFFFFFFFFFFFFFFFFFFFFFFFFFFFFFFFFFFF,FFFFFFFFFFFFFFFF:F
FFFFFFFFFFFFFFFFFFF:FF:FFFFFFFFFFFFFFFFFFFFFFFFFF
@A00721:190:HGJW7DSXY:4:2522:23511:11866_2:N:0:TCTGCAAG+GCTGCACT
ATACCATAACCTACCATGACCAATCATCGCTCCAACTGGCACGCCAGACGTAGGGTCAAACCTACTAC
CGCCGGTCGGCAGATCGGCACCACTGGTCTCTACGTTCAGGGACCCGC
+
:FFFFFFFFFFFFFFFFFFFFFFFFFFFFFFFFFFFFFFFFFFFFFFFFFFFFFFFFFFFFFFFFFFF
FFFFFFFFFFFFFFFFFFFFFFFFFFFFFFFFFFFFFFFFFFFFFFFF

```
@A00721:190:HGJW7DSXY:4:2241:23502:35806_1:N:0:TCTGCAAG+GCTGCACT
CCTTCCCAAATCTAGATCTGCACAGAAGAAGGCTGAAGGTCGTTACCGGACGACCACTCAAAGGAAAC
GGGCTGTTGCCCAGAGGAAAGCAGCGACCACCGAGGTCGCTCGTAACATTGACGGCAGTCCCGTCATC
TCT
+
FFFFFFFFFFFFFFFFFFFFFFFFFFFFFFFFFFFFFFFFFFFFFFFFFFFFFFFFFFFFFFFFFFFF
FFFFFFFFFFFFFFFFFFFFFFFFFFFFFFFFFFFFFFFFFFFFFFFFFFFFFFFFFFFFFFFFFFFF
FFF
@A00721:190:HGJW7DSXY:4:1549:2329:35837_2:N:0:TCTGCAAG+GCTGCACT
GCAGTACCTCAGTCAAAAGTCACATTCACCACTCCCATACCAATCAATGATGTGACTATACAGTTCGG
CCATTTCAAGAGTGGTCAATACGTCGTGGGAGGTACTGAAGCCTTCCC
+
FFFFFFFFFFFFFFFFFFFFFFFFFFFFFFFFFFFFFFFFFFFFFFFFFFFFFFFFFFFFFFFFFFFF
FFFFFFFFFFFFFFFFFFFFFFFFFFF:FFFFFFFFFFFFFFFF:FFF
@A00721:190:HGJW7DSXY:4:2659:25156:19257_2:N:0:TCTGCAAG+GCTGCACT
CCAAGCCAATCAATAGTTCTTCTGACCAGAATGAAAAGGGGTTTACCAGGGTAGTCTCTAAGTCTACC
AGGAAGAGGGGAAATGTCCCCAAAACTGAGAAGAATAAAGGGAAAGGT
+
FFFFFFFFFFFFFFFFFFFFFFFFFFFFFFFFFFF:F:FFFFFFFFFFFFFFFFFFFFFFF:FFFFFFF
FFFFFFFFFFFFFFFFFFFFF:::FFFFFFF:FFFFF,FFFFF:FFFF
@A00721:190:HGJW7DSXY:4:2332:21820:27070_2:N:0:TCTGCAAG+GCTGCACT
AGGGGAAATTACCCCTAATTCTGAAGGAAGATCTCCCACCATCTGGGGTTGGGAATAAGAAATCTGCA
CGGGCCAAAGAGGCCGCTGCTACGAAGTCGCTGAAGGCGGCTTGGGTC
+
FFFFFFFFFFFFFFFFFFFFFFFFFFFFFF,FFFFFFFFFFFFFFFFFF,FFFF,FFFFFFFFFFFFF
FFFFFFFFFF:FFFFFFFFF:FFFFFFFFFFFFFFFFFFFFF:FFFFF
@A00721:190:HGJW7DSXY:4:2136:26323:5744_2:N:0:TCTGCAAG+GCTGCACT
TTGCGCCTGCCGACCCCATCTTTGAGACCAAGACTCTCTACAGTAGGTTCGTGCCTACTATGAGCAAA
TCTGTTGAATTCGTCACTGAGGACGAAGGTGAGAGGACTTTTGGCTAC
+
FFFFFFFFFFFFFFFFFFFFFFFFFFFFFFFFFFFFFFFFFFFFFFFFFFFFFFFFFFFFFFFFFFFF
FF:FFFFFFFFFFFFFFFFFFFFFFFF:FFFFFFFFFFFFFFFFFFF:F
@A00721:190:HGJW7DSXY:4:1357:23854:17378_2:N:0:TCTGCAAG+GCTGCACT
CGTGGGTTGAAGGCCGACGCTTCGAGCCTGAGTGTGACGAGGCCGGTGACGTTTTTGTCAACGGCGTT
TGGAGGATTTTATCCGCGCTTAACTATGGGGCGAAGATCCCCTATGGA
+
FF:FFFFFFFFFFFFFFFFFFFFFFFFFFFFFFFFFFFFFFFFFFFFFFFFFFFFFFFFFFFFFFFFF
FFFFFFFFFFFFFFFFFFF:FFFFFFFFFFFF:FFFFFFFFFFFFFFF
@A00721:190:HGJW7DSXY:4:1446:16161:17801_2:N:0:TCTGCAAG+GCTGCACT
TTGGCATATTTATGCCGACGATAATAAGCATGTCACCCTTAAGCACCGAGGTAAGCTCGGGACTTACA
AGAGAGAGTGTGAGAGAGTCGCTCTCACTATTTTTGCCCAGAATGCGG
+
FFFF:FF:FFFFFFFFFFF:FFFFFFFFFFFFFFFFFFFFFFFFFFFFFFFFFF,FFFFFFFFFF:FF:
FFFFFFFFFFFFFFFFFFFFFFFFFFFFF:F:FFFF:FFFFFFF:F::F
@A00721:190:HGJW7DSXY:4:2601:12481:18756_2:N:0:TCTGCAAG+GCTGCACT
TCCTCCATAATTACAACCTCTCTGTAGAGAGCATTAGAAGGAAGTTGGGCAAATTCCCACATATGGTA
GACGTTGTCACACTTTTCTTGAAAATTTTTCCTTTCCTCCGTCATTGG
+
FFFFFFFFFFFFFFFFFFFFFFFF,FFFFFFFFFFFFFFFFFFFFFFFFF,FFFFFFFFFFFFFF,FFFF
FFFFFFFFFFFFFFFFFFFFFFFFFFFFFFFFFFFFFFFFFFFF:,FFF
@A00721:190:HGJW7DSXY:4:2327:25590:13056_2:N:0:TCTGCAAG+GCTGCACT
AAGGTGGAAGGTGTCAAGGGTCTTCTTCATGCTGATCTTTCAGGACCTAATCACATCATGATTGATAG
TTACGGCTCGTATCGAGTTGGAGGGAGGAAGTTCTCCGAAATTAAAGA
+
```

FFFFFFFFFFFFFFFFFFFFFFFFFFFFFFFFFFFFFFFFF,FFFFFFFFFFFFFFFFFFFFFFFFFF
FFFFFFFFFF:FFFFFFFFF:FFFFFFFFFFFFFFFFFFFFFFFFF,FF
@A00721:190:HGJW7DSXY:4:1427:20980:20228_1:N:0:TCTGCAAG+GCTGCACT
TCAGAGAGGGGAGCTCTGAAAAGGAAACTTCGTCAGAAGATGAGGAAGTCTAAGAGGAGGAGTTTGAG
GAAGGCTCTAGAGAAGATCTCACCACCTGGAGAAGGCAAGTGGAGAGTCCAGAAGTTGATGAAGAAGG
ATT
+
FF:FFFFFFFFFFFFFFFFFFFFFFFFFFFFFFFFFFFFFFFFFFFFFFFFFFFFFFFFFFFFFFFF
FFFFFFFFFFFFFFFFFFFFF,FFFFFFFFFFFFFFFFFFFFFFFFFFFFFFFFFFFFFFFFFFFFFF
FFF
@A00721:190:HGJW7DSXY:4:1532:13584:17221_2:N:0:TCTGCAAG+GCTGCACT
GTTACCACTTCCTAAGGTGGATGAGGGAGGTTGTTTCTTTGGAGTTTTAGATAATCTTTTGAGAAGGG
GTCATAGAGCCAGCAAGAGAAGCTTATATGCTGGTTTAAGTGATGATT
+
FFFFFFFFFFFFFFFFFFFF,FFFFFFFFFFFFFFFFFFFFF:FFFFFFFFFFFFFFFFFFFFFFFFFF
FFFFFFFFFFFFFFFFFFFFFFFFF,FFFFFF:FFFFFFFFFFFFFFFF
@A00721:190:HGJW7DSXY:4:1319:29568:7576_1:N:0:TCTGCAAG+GCTGCACT
ATTTAGGTCAACTTGGGGTTTCGAATGTCATTAGTGCCTTACGATTGAAAGAGGTCCTTTTGAGTATC
GACAGATCTTTCGATCTTGAGGACGTACATTTAACGTGGCAGAATTCGGTAACTTACCATATTGTTCC
TTC
+
FFFFFF:FFFFFFFFFFFFFFFFFF,FFFFFFFFFFFFFFFFFFFFFFFFFFFFFFFFFFFFFFF:FFF
FFFFFFFFFFFFF:FFFFFFFFFFFFFFFFFFFFFFFFFFFFFFFFFFFFFFFFFFFFFFFFFFFFFF
FFF
@A00721:190:HGJW7DSXY:4:2647:2003:4679_1:N:0:TCTGCAAG+GCTGCACT
ATTTAACTTTGACTCAACTACTCCGATCAGATCCGGATGGTTTGATTGGTATGGACTACTCGACTTTT
GATCTGGAGTTGTCTAGGGTCATGGCAGATACTACCGATAAGGTGAAAAATATGCAGGTCAAAGATTC
TGT
+
:FFFFFFFFFFFFFFFFFFFFFFFFFFFFFFFFFFFFFFFFFFFFFFFFFFFFFFFFFFFFFFFFFF
FFFFFFFFFFFFFFFFFFFFFFFFFFFFFFFFFFFFFFFFFFFFFFFFFFFFFFFFFFFFFFFFFFFF
FFF
@A00721:190:HGJW7DSXY:4:1612:19678:22764_2:N:0:TCTGCAAG+GCTGCACT
GAGGGAGCCGTTTTGGCCGACTTTAACTATTCTAAGAGTCTTTATGATTCAAAACCTATCGTTGTCGT
ACCTTACAAGGTTGAGGAGACTTTACAGCGGCTTATTGTCGCGCAGTT
+
FFFFFFFFFFFFFFFFFFFFFFFFFFFFFFFFFFFFFFFFFFFFFFFFFFFFFFFFFFFFFFFFFFFF
FF:FFFF:FF:FFFFFFFFFFFFFFFF:FFF:FFFFFFFFFFFFFFFF
@A00721:190:HGJW7DSXY:4:1132:19768:11960_1:N:0:TCTGCAAG+GCTGCACT
TATCTTGAATGCTCACGCCGTCGCAGCCATATCGAGAATATGTTATAATCACTTGTTAAAGTACAGAT
TTAGTGATTTAGATATTATTGATATTGGAGGCGATATTCGTTTTCATATCCAAAACTCCTTGAATGGA
GTA
+
FFFFFFF,FFFFFF,:F:FFFFFFFFF,FFFFFFFFF,FFF:FFFFFFFFFFFF:F:FFFFFFFFFF
FFFFFFFFFFFFFFFFFFFFFFFFFFFF,FFF,FFFFFFFFFFFFFFFFFFFF:FFFFFFFFFFF:FF
FFF
@A00721:190:HGJW7DSXY:4:2601:12554:3756_1:N:0:TCTGCAAG+GCTGCACT
ACCCTATTCTTGATGTGATAGATGCTGCGAGGTATACGTCTAGGAACATTGATTGGGAACGTACCTAC
TCACGAGACGTATTGAAAGCTATCGGAGGGAAGCAAAATGTTTCGTTCTGTTACAAGAACGCTTTGGA
TTG
+
FF,F,F:F,FFFFF,FFF,FFF,,,,,,F,FF,,FF,:F,:F,FF,,FFFF:FFF:,,F,FF,F,FF:
FF:FFFF:,FF,,FFFFFFFFF:F,F,F:F,:FFF,,FFF:FFF,FFFFFFF:,FFFFFFF,,:F:FF
FF,

```
@A00721:190:HGJW7DSXY:4:1155:3161:6997_1:N:0:TCTGCAAG+GCTGCACT
CTCAGTTGCGATAGCTGTAGAGGTATACGATATCTCTCTTGATGATGTAGCCACTATAATGCTTAAGA
GAGAGATTGATGTATGTCATTTCGTCTTAGTTTGTCCCGGCGAATTATTGGACACGATGAAAAATGAC
ATA
+
FFFFFFFFFFFFFFFFFFFFFFFFFFFFFFFFFFFFFFFFFFFFFFFFFFFFFFFFFFFFFFFFFFFFF
FFFFFFFFFFFFFFFFFFFFFFFFFFFFFFFFFFFFFFFFFFFFFFFFFFFFFFFFFFFFFFFFFFFFF
FFF
@A00721:190:HGJW7DSXY:4:1506:15754:19914_2:N:0:TCTGCAAG+GCTGCACT
TCATGGAGATGATGGCGCATCACTTGATTACTATATGCCCGAAGGATTGGGTTATTCTCACAACAAAG
ATAAGATTTTATCTTTCTTCAAACACCCTGTTTTCCTTAAGGGCGGAC
+
FFFFFFFFFFFFFFFFFFFFFFFFFFFFFFFFFFFFFFFFFFFFFFFFFFFFFFFFFFFFFFFFFFFF:
FFF:FFFFFFFFFF:FFFFFFFFFFFFFFFFFFFFFFFFFFFFFFFFF
@A00721:190:HGJW7DSXY:4:1649:28565:28729_2:N:0:TCTGCAAG+GCTGCACT
TGTGAACTCTTGGGGACTAGATTGAGCATTAATGAGTTCCGAATAACTCGTAATAAATCTTTCTCTAG
TGGACAATTCAGAATACCTATTTCCGTACCAAGAGCCACTGCCAATCT
+
FFFFFFFFFF:FFFFFFFFFFFFF:FFFFFFFFFFFFFFFFFFFFFFFFFFFFFFFFFFFFFFFFFFFF
FFFFFFFFFFFFF:FFFFFFFFFFFFFFFFFFFFFFFFFFFFFFFFFF
@A00721:190:HGJW7DSXY:4:1573:5882:34538_2:N:0:TCTGCAAG+GCTGCACT
AAATTTTTCTCAAGAGATTATGGTTGATAAAGTTTTCTTCATAAGGGCTATTCAGTACGTCATGGGGA
TCACTTCTTCGGTCAATGACAAAACCTTCGAATATGTTACGACATGGC
+
FFFFFFFFFFFFFFFFFFFFFFFFFF,FFF,FFFFFFFFFFF:FFFFFFFFFFFFFFFFFFFFFFFFFF
FFFFFFFFFFFFFFFFFFFFFFFFF:FFFFFFFFFFFFFFFFFFFFFF
@A00721:190:HGJW7DSXY:4:1149:19958:36839_1:N:0:TCTGCAAG+GCTGCACT
GTATTGTCATCTCGGGGAAAGTGATTCACCAAGATGTTCATATAGATACGAGATACCTAGAGAAGGTC
GCAGCGATCTTACTCGTTATCGGAGCTAAGAAGAAGACTTTGGTTACACGTTATGCTCGCAAACTTAA
CGT
+
FFFFFFFFFFFFFFFFFFFFFFFFFFFFFFFFFFFFFFFFFF,FFF:FFFFFFF:FFF,FFFFFFFFFF
FFFFFFFFFFFFFFFFFFFFFFFFFFFFFFFFFFFFFFFFFFFFFFFFFFFFFFFFFFFFFFFFFFFFF
FFF
@A00721:190:HGJW7DSXY:4:1111:30861:14074_2:N:0:TCTGCAAG+GCTGCACT
CTCTGCTTCTGCGTTACTGAGAGAAAAGATTCAGGACGCCTACGGTGACTTTATGGACAAGATGCAAG
TTCTTGGGAAATACTTGTTTCCCTTTTTAGGTTTAGTAGGTGATCTTG
+
FFFFFFFFFFFFFF,FFFFFFFFF,FFF::FFFFF:FFF:FFFFFFFFFFF,FFFFFF:FFFFFFF::F
,:F,FFFFFFFFFF:FFFFFFF:F,FF::FF,::FF:,FFFFFFFF:F
@A00721:190:HGJW7DSXY:4:2103:12201:28072_1:N:0:TCTGCAAG+GCTGCACT
GCTCTTCCGATCTGACAACGAATCTTGAAACTACGGTCCTTATCAAAGAAAATGGTGAACCGGTCTCT
AAATCTTTTGAGATTGAGAAGATTGAAGAACACGTCAAGAAGTCGCTCGGCGTTGTTCTTGAAAACCA
AGT
+
:FF:FFFFFFFFFFF:FFF,FFFFFFFFFFFFFFFFFFFFFFFFFFFFFFFFF:FFFFFFFFFFF:FFF
FFFFFFFFFF:FFFFFFFFFFFFFFFFFFFFFFFFFFFFFFFFFFFFFFFFFFFFFFFFFFFFFFFFFF
FFF
@A00721:190:HGJW7DSXY:4:1626:22761:28416_1:N:0:TCTGCAAG+GCTGCACT
GCTGATCATGGAGTCTTTGTACCTTCTGTCAAACTTCGAGGTTCAGCTACGAGAGGTGATCCCGGATG
TGGATTGAAGGCTGGCTCTAAGGGAGACTCCTTCTTTTTGAATGTTGTAAAGAATTGGGTTGAGAAGT
GTG
+
F:FFFFFFFFFFFFFFFFFFFFFFFFFFFFFFFFFFFFFFFFFFFFFFFFFFFFFFFFFFFFFFFFFFF
```

FFFFFFFFFFFFFFFFFFFF:FFFFFFFFFFF:FFFFFFFFFFFFFFFFFFFFFFFFFFFFFFFFFFF
FFF
@A00721:190:HGJW7DSXY:4:2337:25491:16297_1:N:0:TCTGCAAG+GCTGCACT
CTTGGTATTTCTGAAATAGTTTATCAGAGCGTCATTTTCCTTTGGGATCTTTCCTCTGGTAAGGGAAT
TATTAAATCATTCTTCTCACTCTTGACGAGAATTATTACTGTTTTCTCTACCCATAACTTCTTTTTCA
AAA
+
FFF,FFFFFF,F:FFFF,FFFFFFF,FFFFFF,,FFFFF:,FFFF:FF,FFFF:F:FF,FFFFFF::F
FFFFFFFFFFFFFFF,FFFFFFFFFFFFFFF:FFFFFF:FFFF,FFF::F:FFF::,,FFFFFFFF::
FF:
@A00721:190:HGJW7DSXY:4:1169:6424:6668_1:N:0:TCTGCAAG+GCTGCACT
GTGCTCTTCCGATCTGTCGTTTCAACACTTATCACCACTTTTGAAAGGAAAATAATTGGTGATGAAAA
TACGTATGTCGGTGGGGTTCTCAAGGCTCTCATCGCCACTGGGATTCTAAGGGGTTCCATAAGATATT
ATC
+
FFFFFFFFFFFFFFFFFFFFFF:FFFFFFFFFFFFFFFFFFFFFFFFFFFFFFFFFFFFFFFFFFFFF
FFFFFFFFFFFFFFFFFFFFFFFFFFFFFFFFFFFFFFFFFFFFFFFFFFFFFFFFFFFFFFFFFFFF
FFF
@A00721:190:HGJW7DSXY:4:2233:29586:24768_2:N:0:TCTGCAAG+GCTGCACT
TTTGGTCCTTACGTCTTAAGTAATTTGGATGTTGTCGAAAACTATCAGGTTCTTGTCACGATTCTTAA
AAGAAGTTTATTAAAACTTCAGAGTCTCTTGGAAGTCCAGGTAGCTAA
+
FFFFFFFFFFFFFFFFFFFFFFFFFFFFFFFFFF:FFFFFFFFFFFFFFFFFFFFFFFFFFFFFFFFF
FFFFFFFFFFFFFF:FFFFFFFFFFFFFFFFFFFFFFFFFFFFFFFFFF
@A00721:190:HGJW7DSXY:4:2570:16260:28651_2:N:0:TCTGCAAG+GCTGCACT
GGGAAAAGAGACCTCTGTTGGTAGAGGAGTTGAGAGAATTACTCAGATTCAATCTGGCATCACTGAAC
GTCTACAACGAGTTAGGGAGTGGCTAGGTTTTAAAAGATCCTTTGCAC
+
FFFFFFFFFFFFFFFFFFFFFFFFFFFFFFFFFFFFFFFFFFFFFFFFFFFFFFFFFFFFFFFFFFFF
FFFFFFFFFFFFFFFFFFFFFFFFFFFFFFFFFFFFFFFFFFFFFFFF
@A00721:190:HGJW7DSXY:4:2569:25617:11130_2:N:0:TCTGCAAG+GCTGCACT
CGTCGCGGAAAACGATGAAGATTTCCACACTGTTCGAGAATCTTCTGATGCTGAATTTGAGGAAAATT
TTTCGGAAGTTTTTGGTATAAATCCTGATGGCTCAGTACAGGGAGGAG
+
FFFFFFFF,FFFFFFFFFFFFFFFFFFFFFFFFFFFFFFFFFFFF:FFFFFFFF,:FFFFFFFFF:FF
FFFFFFF:FF:FFFFFFFFFFFFFFFFFFFFFFFFFFFFFFFFFFFFF
@A00721:190:HGJW7DSXY:4:1276:5231:1658_2:N:0:TCTGCAAG+GCTGCACT
ACCTTCTTTTATCATTGCAACAGGACTTTGAGAAGACTGTTGAAATGGACAAAATCTGGGATCATAGG
TATGTGTCACGCTACACTCGAAATATGGGCCCTCATAAGGAAGTATAG
+
FFFFFFFFFFFFFFFFFFFFFFFFFFFFFFFFFFFFFFFFFF:FFFFFFFFFFFFFFFFFFFFFFF:FF
FFFFFFFFFFFFFFFFFFFFFFFFFFFFFFFFFFFFFFFFFFFFF:FF
@A00721:190:HGJW7DSXY:4:1468:8323:31657_2:N:0:TCTGCAAG+GCTGCACT
GGAAGATCTCTACAGAGATTATCGAGAGTTGTGATCTAGTTCTAAATTTAGGTCGACGGACTTTAGCA
ACTTTAGAAAAATTCTTTCCGGGCTCAATGACCTTTGCTATGTACGTG
+
FFFFFFFFFF:FFFFFFFFFFFFFFFFFFFFFFFFFFFFFFFFFFFFFFFFFFFFFFFFFFFF:FFFFF
FFFFFFFFFFFFFFFF,FFFFFFFFFFFFFFFFFF,FFFFFFFFFFFF
@A00721:190:HGJW7DSXY:4:1508:22182:21966_1:N:0:TCTGCAAG+GCTGCACT
TTAGTTCATGAAGTTCTTACACTTCTCGCGACTAATGTCAGCTTCACTACAAAGGCGTTACGTAAAGT
AGTTTTCACGCTTTATCTTAAAGCATTACCACCAAATTTAGCACTTTTTGAATATGACAGAAGCTCGC
CAT
+
FFFFFFFFFFFFFFFFFFFFFFFFFFFFFFFFFFFFFFFFFFFFFFFFFFFFFFF:F:FFFFFFFFFFF

FFFFFFFFFFFFFFFFFFFFFFFFFFFFFFFFFFFFFFFFFFFFFFFFFFFFFFFFFFFFFFFFFFFF
FFF
@A00721:190:HGJW7DSXY:4:2112:25030:6073_2:N:0:TCTGCAAG+GCTGCACT
CCAGTTAGAGTTCTTCTAACCAGGACTGATGGTGAGATTCTTTTACCTTTTGGAGGTCCAGGAACCTT
TACTCCTAGTGTCTTTTCTAGAGTCCAAGGACGTTTCCGTCGGAGGAT
+
FFFFFFFFFFF:FFFF:FFFFFFFFFFFFFFFFFFFF,FFFFFFFFFFFFFFFFFFFFFFFFF:FFFF
FFFFFFFFFFFF:FF:FFFFFFFFFFFFFFFFFFFFFFFFFFFFFFFF
@A00721:190:HGJW7DSXY:4:1261:22281:28369_1:N:0:TCTGCAAG+GCTGCACT
TCAATAACACTCTATTGAATCCCTTTTCCACGATGAAAGCTCTTTATGAGGTGCTCCATGCAAATTTA
CAGTTCCTCTTTGGTGATATCGCCTCTGTCCTTTCTCCTTTAATCGTGAGGTTACGAGAGTTTCGTAC
TTG
+
FFFFFFFFFFFFFFFFFFFFFFFFFFFFFFFFFFFFFFFFFFFF:FFFFFFFFFFFFFFFFFFFFFFF
FFFFFFFFFFFFFFFFFFFFFFFFFFFFFFFFFFFFFFFFFFFFFFFFFFFFFFFFFFFFFFFFFFFF
FFF
@A00721:190:HGJW7DSXY:4:2530:26313:22482_1:N:0:TCTGCAAG+GCTGCACT
TTTTGATAAATTTGTTGGAATCGATAATGATACTGACGAAGGTTCTATTACCACCCTTGTCATTGATA
CAAACATTCTAGGTGATGCTTTCTTAGAGGCTCGTCTAGCCGCTGAGATTCGTCTTACCTATTTACAA
TCT
+
FFFFFFFFFFFFFFFFFFFFFFFFFFFFFF:FFFFFFFFFFFFFFFFFFFFFFFFFFFFFFFFFFFFF
FFFFFFFFFFFFFFFFFFFFFFFFFFFFFFFFFFFFFFFFFFFFFFFFFFFFFFFFFFFFFFFFFFFF
FFF
@A00721:190:HGJW7DSXY:4:2542:19515:33223_1:N:0:TCTGCAAG+GCTGCACT
GATAACGATTTAGTACTGGGAAATGGTGGTAGTCAGTTTACTACTTTTCTTTCCCTCCCCTTTTTCGA
TTGGATTCGACGGGGTCTTCGTCTCACGACTAATTTGATAAGGTTTTTCTTTTATGGGACTCTTGTTT
GTA
+
FFFFFFFFFFFFFFFFFFFFFFFFFFFFFFFFFFFFFFFFFFFFFFF:FFFFFFFFFFFFFFFFFFFF
FFFFFFFFFFFFFFFFFFFFFFFFFFFFFFFFFFFFFFFFFFFFFFFFFFFFFFFFFFFFFFFFFFFF
FFF
@A00721:190:HGJW7DSXY:4:1440:3106:29731_2:N:0:TCTGCAAG+GCTGCACT
GTTCTTAATGTAGGTCTCCTAGTACTTAAGGGTTTATGTTTCGGTTTCAGCTGGATGGATCTTTTGAT
GGTATGTTTCACATTGCGTGATCACTTCCCGTGGACTTGGGAAGAGAT
+
FFFFFFFFFFFFFFFFFFFFFFFFFFFFFFFFFFFFFFFFFFFFFFFFFFFFFFFFFFFFFFFFFFFF
FFFFFFFFFFFFFFFFFFFFFFFFFFFFF:FFFFFFFFFFFFFFFFFF
@A00721:190:HGJW7DSXY:4:1632:32786:20134_1:N:0:TCTGCAAG+GCTGCACT
TTCTATTATGTCGCTAAAGCCTGTCCAGTTGTCGATGATGTTCGGGCTGTGGGGATTAACTTCCCTCG
TGAAACTTACGACGATGTTCTCGCTCGCATTTCACAAGGATTACTAAGTCTGAGATCTGCCACTTCTA
ATT
+
FFFFFFFFFFFFFFFFFFFFFFFFFFFFFFFFFFFFFFFFFFFFFFFFFFFFFFFFFFFFFFFFFFFF
FFFFFFFFFFFFFFFFFFFFFFFFFFFFFFFFFFFFFFFFFFFFFFFFFFFFFFFFFFFFFFFFFFFF
FFF
@A00721:190:HGJW7DSXY:4:1374:29993:21245_2:N:0:TCTGCAAG+GCTGCACT
AAGTTCATGAGGATGTCTTGGTCGAAAAATATCGCCGGGACCTTCTAATAGAGGATCCTGATGACATT
ATTTCAACGGATACCGATCTCAGTTCTACCACAGAAACCGTCTCAGGA
+
FFFFFFFFFFFFFFFFFFFFFFFFFFFFFFFFFFFFFFFF,FFFFFFFFFFFFFFFFFFFFFFFFFFF
FFFFFFFFFFFFFFFFFFFFFFFFFFFFFFFFFFFFFFFFFFFFFFFF
@A00721:190:HGJW7DSXY:4:1457:26738:23907_2:N:0:TCTGCAAG+GCTGCACT
CTGATTCGGAATTAGAGAAAGACGACCTTCTTGTGGGGACTGAGACCTCTGAATTTTCAGGGTGCCCT

```
GATGAGGACGTTGAAAGGGATAATTTCAACTCTGAGACTTCTAAGAGT
+
FFFF:FFFFFFFFFFFFFFFFFFFFFFFFFF:FFFFFFFFFFFFFFFFFFFFFFFFFFFFFFFFFFFFF
FFFFFFFFFFFFFF:FFFFFFFFF:FFFFFFFFFFFFFFFFFFFFFFFF
@A00721:190:HGJW7DSXY:4:1121:26323:32299_2:N:0:TCTGCAAG+GCTGCACT
CAGAAACGTAACAAGAAGAATAAAGGTAGGAAGGGGAGAAAACAGAACAAGCGAGCGGTTGAAGAGAA
AACTGATATGGTTGCGGCTGATCGTACCCCAATTCCTGCTAGAAAGAT
+
FFFFFFFFFFFFFFFFFFFFFFFFFFFFFFFFFFFFFFFFFFFFFFFFFFFFFFFFFFFFFFFFFFFF
FFFFFFFFFFFFFFFFFFFFFFFFFFFFFFFFFFFFFFFFFFFFFFFF
@A00721:190:HGJW7DSXY:4:1659:31991:17159_2:N:0:TCTGCAAG+GCTGCACT
TACCACTGATCTTCCAGAGTTACGTAGTGGTCGTTCTTTTGGTGATTTTATCAAAGAGAAAGTTTCTG
CTAGTCGCGACGATAGTATTTATCGGAGTGATATTAACAAGTTAAACT
+
FFFFFFFF:FFFFFFFFFFFFFFFFFFFFFFFFFFF:FFFFFFFF:FFFFFFFFFFFFFFFFFFFFFFF
FF,FFFFFFFFFFF:FFFFFFFFFF:FFFFFFFFFF:FFFFFFFFFFF
@A00721:190:HGJW7DSXY:4:2177:29631:24972_2:N:0:TCTGCAAG+GCTGCACT
TGTTGAAGTACCGCAACATGGATCAGTTTTCCAAAAAGCTATTAATAGCAGTTGCGGGGCTATAATAC
CGCCTCCAGATTACTCTAAACTGAATCCACTCTCTTGGGATATAAATG
+
FFFFFFFFFFFFFFFFFFFFFFFFFFFFFFFFFFFFFFFFFFFFFFFFFFFFFFFFFFFFFFFFFFFF
FFFFFFFFFFFFFFFFFFFFFFFFFFFFFFFFFFFFFFFFFFFFFFFF
@A00721:190:HGJW7DSXY:4:2639:21594:28244_1:N:0:TCTGCAAG+GCTGCACT
AACATGAATTAGTTATTGAAAGTCTTGACCCTCACGTCTCACTTTATCACCTTGGAGATGGTAAAGTC
ATTAACAAAGGAAATGTTAAGGCTGATGACTTAAATTTCGCATTCTGCTTCGACGGTAATTCCATAAG
ACC
+
FFFFFFFFFFFFFFFFFFFFFFFFFFFFFFFFFFFFFFFFFFFFFFFFFFFFFFFFFFFFFFFFFFFF
FFFFFFFFFFFFFFFFFFFFFFF:FFFFFFFFFFFFFFFFFFFFFFFFFFFFFFFFFFFFFFFFFFFF
FFF
@A00721:190:HGJW7DSXY:4:2376:11812:22733_1:N:0:TCTGCAAG+GCTGCACT
TTCTTCATGAAGATCTTCGAGTGTTTTATAGTGAGTTATTCTTGCGAAGGTTCAAGTCGATTGATTTT
GTTGACTACGATCTCTCTTGTAATAAGAAGTTCTTCGCAGTTGAATCTCCACCAGGTGGAGGGAAGAC
CAC
+
FFFFFFFFFFFFFFFFFFFFFFFFFFFFFFFFFFFFFFFFFFFFFFFFFFFFFFFFFFFFFFFFFFFF
FFFFFFFFFFFFFFFFFFFFFFFFFFFFFFFFFFFFFFFFFFFFFFFFFFFFFFFFFFFFFFFFFFFF
FFF
@A00721:190:HGJW7DSXY:4:1372:9028:33599_1:N:0:TCTGCAAG+GCTGCACT
GACTTTTGTCGAATTTTTCGCAAAAGGTCTTGAAGTACGTATTGTCACTGCTAATCGTAATTCGCGAG
AAGAGATAGTGACTAAAGTTTTGGCACTTCTAGTTCATCGCGGTATTTGTGAAATTAAGACAGACGTG
AAA
+
FFFFFFFFFFFFFFFFFFFFFFFFFFFFFFFFFFFFFFFFFFFFFFFFFFFFFFFFFFFFFFFFFFFF
FFFFFFFFFFFFFFFFFFFFFFFFFFFFFFFFFFFFFFFFFFFFFFFFFFFFFFFFFFFFFFFFFFFF
FFF
@A00721:190:HGJW7DSXY:4:1417:17806:33927_1:N:0:TCTGCAAG+GCTGCACT
TCGTATTAGAACAGCTGATTCTTATTGCATGAACGTCGCTGAGGTCAAACCTGTGGACGTTCTTTTCA
TCGACGAATTATTCATGATTCACGCTGGTCAGACCCTTTTTCTTACACATCTACTTAAGGCGGCTTAT
ACC
+
FFFFFFFFFFFFFFFFFFFFFFFFF:FFFFFFFFFFFFFFFFFFFFFFFFFFFFFFFFFFFFF:FFFFF:
FFFFFFFFFFFFFFFFFFFFFFFFFFFFFFFFFFFFFFFFFFFFFFFFFFFFFFFFFFFFFFFFFFFF
FFF
```

@A00721:190:HGJW7DSXY:4:2150:8639:4210_1:N:0:TCTGCAAG+GCTGCACT
GTGATTCCAATCAAATCGGATTCATCAATCGTATTGATCTCCAAGTCGCAAACTACTTCAACATCAAC
GACATCATTCTTGATGAGTGTCGTTCTTATCGAAGTCTCAGTTATCGCTGCCCGGCCGACGTTTGTCG
CGA
+
FFFFFFFFFFFFFFFFFF:FFFFFFFFFFFFFFFFFFFFFFFFFFFFFFFFFFFFFFFFFFFFFFFFF
FFFFFFFFFFFFFFFFFFFFFFFFFFFFFFFFFFFFFFFFFFFFFFFFFFFFFFFFFFFFFFFFFFFF
FFF
@A00721:190:HGJW7DSXY:4:2636:25337:3662_2:N:0:TCTGCAAG+GCTGCACT
TACGGCAGAAAAATTTCTCCTGCTTGTAATTCGAGAGAGGACACTATGGAAATTAGACCTATCAGATG
TCTTGACGACTTCGAATTTGAGGACGGAGTTAAATATATTACTTTCAC
+
FFFFFFFFFFFFFFFFFFFFFFF:FFF:FFFFFFFFF:FFFFFFFFFFFFFFFF:FFFFFFFFF:FF
FFFFFFFFFFFFFFFFF:FFFFFFFFFFFFFFFF:FFFFFFFFFFFFF
@A00721:190:HGJW7DSXY:4:1438:4399:27117_2:N:0:TCTGCAAG+GCTGCACT
CAAAGTCCTTGGTAAGAGTAAGGTTAAATGTGAAGATGCTCAGACTATTCATGAAGTTCAAGGTAATA
CTTACCAGCACGTTCGGCTGGTTAGAATTAAACCTCAAGATGACCCTG
+
FFFFFFFFFFFFFFFFFFFFFFFFFFFFFFFFFFFFFFFFFFFFFFFFFFFFFFFFFFFFFFFFFFFF
FFFFFFFFFFFFFFFFFFFFFFF:FFFFFFFFFFFFFFFFFFFFFFFF
@A00721:190:HGJW7DSXY:4:2220:7826:34616_1:N:0:TCTGCAAG+GCTGCACT
CTTCCGATCTCTCTATCACGACACGTTGAATCTTTACGATACTTTGTTATCAATGTAAAGTTAAACGA
TGCTACTTGTGAGAGGATCAATCACAGTAAGGAACTGTCACGTTTAGCACGGGAGGTTGCAGATTCAA
CCC
+
FFFFFFFFFFFFFFFFFF:FFFFFFFFFFFFFFFFFFFFFFFFFFFFFFFFFFFFFFFFFFFFFFFFF
FFFFFFFFFFFFFFFFFFFFFFFFFFFFFFFFFFFFFFFFFFFFFFFFFFFFFFFFFFFFFFFFFFFF
FFF
@A00721:190:HGJW7DSXY:4:2148:28556:2566_1:N:0:TCTGCAAG+GCTGCACT
AGACGTGTGCTCTTCCGATCTCTTGAAGTTGTTGACGACAAGCCTCAAGAGGGTAGAGGACCACCTGG
ATCTTCTACTGTCTCTGCCTTCCAAGATTTCTTAGAAGAGGTTATTCCTGGAACCACCTTAACGTCAC
TCG
+
FFFFFFFFFFFFFFFFFFFFFFFFFFFFFFFFFFFFFFFFFFFFFFFFFFFFFFFFFFFFFFFFFFFF
FFFFFFFFFFFFFFFFFFFFFFFFFFFFFFF,,FFFFFFFFFFFFFFFFFFFFFFFFFFFFFFFFFFF
FFF
@A00721:190:HGJW7DSXY:4:2236:18096:19554_2:N:0:TCTGCAAG+GCTGCACT
TGAGTTCAAGTCGGTTGTCGATCGAATTAAGATCAAGGCTAATACCTTTATAGGTAAGAAGCCCCTTG
AGCCGCGTAGTCAGCGCGTTTAGCTGTATCATCAGTGAGATCGGAAGA
+
FFFFFFFFFFFFFF:FFFFFFFFFFFFFFFF:FFFFFFFFFFFFFF:FFFFFFFFFFFFFFFFFFFFF
FFFFFFFFFFFFFFFFFFFFFFFFFFFFFFFFFFFFFFFFFFFFFFFF
@A00721:190:HGJW7DSXY:4:1430:19623:7420_1:N:0:TCTGCAAG+GCTGCACT
TCCAAGAGAATATTTATTCTTATGAGGCTCGAAACTTCAACGTTTTCACAACGGAAAGACATATCGCA
CCCGAAATTTTTGGGCAGAATGCTCTTAACGCGGTTCTTGAACGGTGTTTTGATCAGTCTAAGTTGGA
TAA
+
F,FFFFFFFFFFFFFFFFFFFFFFFFFFFFFFFFFFFFFFFFFFFFFFFFFFFFFFFFFFFFFFFFFF
FFFFFFFFFFFFFFFFFFFFFFFFFFFFFFFFFFFFFFFFFFFFFFFFFFFFFFFFFFFFFFFFFFFF
FFF
@A00721:190:HGJW7DSXY:4:1245:27588:22404_2:N:0:TCTGCAAG+GCTGCACT
ACTCAGTTAACCTGAGAGCTCCTCAATTGATAGATTGGGTCAACAATAGGGACGCTAGTATACTTCGT
AAAGTCGACAGCGAAATGGTCAATATTGATAATTGGACCGACTATATG
+

```
FFFFFFFF::FFFFFFFFFFFFFFFFFFFFFFFFF:FFFFFFFFFF,FFFFFFFF:FFF:FFFFFFFF
:FFFFFFFFFFFF:FFFFFFFFFF:FFFF:FFF:FFFFFFFFFFFFFF
@A00721:190:HGJW7DSXY:4:1306:4001:28087_1:N:0:TCTGCAAG+GCTGCACT
AGCTCATGGTGAAAAGGGAAGCTAAGGTTAAACTTGATGCTTCAGCCTTTTCGAAGCATAACCCCGCA
CAGAATATCGTTTATCACGCAAAATTTATTAATGCAATTTTCGGACCTATCTTTAAGGTTCTTGTGAG
GAG
+
FFFFFFFFFFFFFFFFFFF:FFFFFFF,FFFFF,FFFFFFF:FFFFFFFFFFFFFFFFFFFFFFFFFF
FFFFFFFFFFFFFFFFFFFFFFFFFFFFF:FFFFFFFFFFFFFFFFFFFFFFFFFFFFFFFFFFFFFF
FFF
@A00721:190:HGJW7DSXY:4:2431:13060:19413_1:N:0:TCTGCAAG+GCTGCACT
GACGATTTCTCCCGGAAGCTCTTCTCTCTCTTAGGAGGTCATGATGACTACTATAAGGGTGAAATCGA
TTTTTCGAAGTTTGACAAATCTCAAGATTTCTTTTGTAAAGCCTATGAGATAAAGATGTATTCTTATT
TAG
+
FFFFFFFFFFFFFFFFFFFFFFFFFFFFFFFF:FFFFFFFFFFFFFFFFFFFFFFFFFFFFFFFFFFF
FFFFFFFFFFFFFFFFFFFFFFFFFFFFFFFFFFFFFFFFFFFFFFFFFFFFFFFFFFFFFFFFFFFF
FFF
@A00721:190:HGJW7DSXY:4:2551:14615:29089_2:N:0:TCTGCAAG+GCTGCACT
TTGGAGTGCCAGTGAATATTTTTGTCACGCCAGGAGTATCCATAACGATTTGTCATTTACCACCTTTG
CTCAACGACGAAGTGGAACGGCCAACACTTTCTTTGGTAATTGTATGA
+
FFFFFFFFFFFFFFFFFFFFFFFFFFFFFFFFFFFFFFFFFFFFFFFFFFFFFFFFFFFFFFFFFFFF
FFFFFFFFFFFFFFFFF:F:FFFFFFFFFFF:FFFFFFFFFFFFFFFF
@A00721:190:HGJW7DSXY:4:2406:19714:7326_2:N:0:TCTGCAAG+GCTGCACT
ATTTTATCACTTTATTATGACCTTGGAAAGTTTTCGGCTGTGGTTGTCGCTGGGGACGATTCCATTCT
GTTTTCAAAGACTCCAATAACTGATCATTCTGATCATATGATTTTGGA
+
FFFFFFFFFFFFFFFFFFFFFFFFFFFFFFFFFFFFFFFFFFFFFFFF:FFFFFFFFFF:FFFFFFFF
FFFFFFFF:FFFF:FFFFFFFFFFFFFFFFFFFFFFFFFFFFFFFFFFF
@A00721:190:HGJW7DSXY:4:1459:20618:10144_1:N:0:TCTGCAAG+GCTGCACT
TTACAAGATCATCGTTATGTTAGTTAAACCCCTAACCGATGTTACTCTTGTAGGACTTTGGGAGAGTT
TCGTTAGTTTTATAGACTATACTAAATATCTCCACTACGAGGTTGTTGTTTGGCGATTAGCTCTTGCG
A
+
FFF,,F:,:,FFF:FF,,FFF,F,FFFFF,F,F,FFFFFFFFFF,F,FF,F,F,,:FFF,,FFF,:,F
F,FFFFF:FF:F,,:,,FFFF,FF::F,FFF,FFF:,,:FFFFFFFFF:FFFFF:FFF:FFFFFFF,F
,
@A00721:190:HGJW7DSXY:4:1641:12626:22639_2:N:0:TCTGCAAG+GCTGCACT
GAGGGTCGTTATACCTATTCGGCTATCTGTGCTTTGAATTGTATAAGATCAAATTTTAAACGATTCCG
AGAGGTTTATCCTGATGTGGAGGGAGTTCTTGAAGCATCCCGACCTTT
+
FFFFFFFFFFFFFFFFFFFFFFFFFFFFFFFFFFFFFFFFFFFFFFFFFFFFFFFFFFFFFFFFFFFF
FFFFFFFFFFFFFFFFFFFFFFFFFFFFFFFFFFFFFFFFFFFFFFFF
@A00721:190:HGJW7DSXY:4:2326:15673:2801_1:N:0:TCTGCAAG+GCTGCACT
TTTCATAAGATTATCAGTAATCCTGATTCTACTACGGATTCATGGACCTTCCATCCTGTACCTCTTCT
GGAGAGACGTATCTCAAAGATCGACTTCGAGACAGGTGATATTGTTGAGCGAGAGCGACCAAGATCTA
AAT
+
FFFFFFFFFFFFFFFFFFF:FFFFFFFFFFFFFFFFFFFFFFFFFFFFFFFFFFFFFFFFFFFFFFF:
FFFFFFFFFFFFFFFFFFFFFFFFFFFFFFFFFFFFFFFFFFFFFFFFFFFFFFFFFFFFFFFFFFFF
FFF
@A00721:190:HGJW7DSXY:4:2414:15013:34726_1:N:0:TCTGCAAG+GCTGCACT
CGTGGCGCTTTATTTTAAAAAGCTCCCTCCTCTCGCAAGTTTGATCTGATACTTATCACGAAAGGACT
```

TGAGTCTTTTGATCAGTCTCTGACTTGACCATCTTTCATTTTAAATGACGATCATTAAGACTCTCTTT
CTT
+
FFFFFFFFFFFFFFFFFFFFFFFFFFFFF:FFFFFFFFFFFFFFFFFFFFFFFFFFFFFFFFFFFFFFFF
FFFFFFFFFFFFFFFFFFFFFFFFFFFFFFFFFFFFFFFFFFFFFFFFFFFFFFFFFFFFFFFFFFFFFF
FFF
@A00721:190:HGJW7DSXY:4:2177:5348:25692_2:N:0:TCTGCAAG+GCTGCACT
CTTTGCACTGATTTTTCCTCATCAGCTTGTGTTTTACCTAATCTGTGTTTTTACGATAACAACTATCA
GTTGAATAAGACTCATCCGAAAGGCGAACTTTGGGAATTTGAAGCTCA
+
FFFFFFFFF:F:F:FFFFFFFFFFF::,:FF:,FFFFF,FF,F::FFF:FFFF:FF,FFFFFF,FFFF
,,FFF,FFF,FF,,,:F:FFFF:F,FFF,,:FFFFFFF,F:FF:FFFF
@A00721:190:HGJW7DSXY:4:2166:10755:8187_2:N:0:TCTGCAAG+GCTGCACT
ACCGTGACGAACTGTGGTGTTCTTGATCTACGTTACGGATATACTCTCCTTGAGTTCACTCGTAAGGA
CACTACCACTTATTACGATATTTCTTTAGTCGATGGGTTTTCGACGCC
+
FFFFFFFFFFFFFFFFFFFFFFFFFFFFFFFFFFFFFFFFFFFFFFFFFFFFFFFFFFFFFFFFFFFFFF
FFFFFFFFF,FFFFFFFF:FFFFFFFFFFF:FFFFFFFFFFFFFFFFF
@A00721:190:HGJW7DSXY:4:1636:21730:35931_1:N:0:TCTGCAAG+GCTGCACT
TCTCTATTTTAAGTTTTGTCTTCCTTGTTTGTCTTATTCTATTAATCACTCATTTTAGTGAGATTATT
AAAAACGCACTAAAACGTCTTCGACAACAGCAGGGGTTTTCGAATAGTGATAGCCACCCGCCTTACAG
AGG
+
FFFFFFFFFFFFFFFFFFFFFFFFFFFFFFFFFFFFFFFFFFFFFFFFFFFFFFFFFFFFFFFFFFFFFF
FFFFFFFFFFFFFFFFFFFFFFFFFFFFFFFFFFFFFFFFFFFFFFFFFFFFFFFFFFFFFFFFFFFFFF
FFF
@A00721:190:HGJW7DSXY:4:1209:14742:4413_1:N:0:TCTGCAAG+GCTGCACT
GACTTTGGTACCACCTTTTCTACTGTCTGTCTCGCCTCTTCGAATGAAGTTTTAATGCTTAACAACAA
TGATAGTGAATTTATACCTACAATCATTGGTTTTTCTAATAATGATGACACTATTGTTTATGGATATG
ATG
+
FFFFFFFFFF:FFFFFFFF:FFFFFFFFFFFFFFFFFFFFFFFFFFFFFFFFFFFFFFFFFFFFFFFFFF
FFFFFFFFFF:FFFFFFFFFFFFFFFFFFFFFFFFFFFFFFFFFFFFFFFFFFFFFFFFFFFFFFFFFFF
FFF
@A00721:190:HGJW7DSXY:4:2520:24297:13542_1:N:0:TCTGCAAG+GCTGCACT
TGGTTACGGTCAGGGAGGATTCTCAGTTTACAAGGACCTTAAGCGATGGATCGGGGTTAATTCTAAAA
CATTACCCGAAAGACGTGCTAAATTAAAACCATTGTACGATGTCGTCTGTCTCGGCGATCACTTTTCA
CTA
+
FFFFFFFFFFFFFFFFFFFFFFFFFFFFFFFFFFFFFFFFFFFFFFFFFFFFFFFFFFFFFFFFFFFFFF
FFFFFFFFFFFFFFFFFFFFFFFFFFFFFFFFFFFFFFFFFFFFFFFFFFFFFFFFFFFFFFFFFFFFFF
FFF
@A00721:190:HGJW7DSXY:4:1202:31259:20431_1:N:0:TCTGCAAG+GCTGCACT
AGACGTGTGCTCTTCCGATCTATCGTTTCTTTCTTTATCTCTTTGGTTGTTAGGGATTTTGAGAGAGT
GAAAGGCTTCAATTGTAGTGGTTTGGTCGTTTCAGTACCCAGTCAGTATACCTCTACGCAAAGATATT
TCA
+
FFFFFFFFFFFFFFFFFFFFFFFFFFFFFFFFFFFFFFFFFFFFFFFFFFFFFFFFFFFFFFFFFFFFFF
FFFFFFFFFFFFFFFFFFFFFFFFFFFFFFFFFFFFFFFFFFFFFFFFFFFFFFFFFFFFFFFFFFFFFF
FFF
@A00721:190:HGJW7DSXY:4:1571:25816:32800_2:N:0:TCTGCAAG+GCTGCACT
GAGGAACGTACCGGATTACCTGTCTTTCATATTATGAATGAACCTTCGGCCGCTCTCTTTGCTTCGAT
GTTGGATGTCAAGAAGGGTTCAGAATGGGATTCCTACGTAGTTTATGA
+

```
FFFFFFFFFFFFFFFFFFFFFFFFFFFFF:FFFFFFFFFFFFF:FFF:FFFFFFFFFFFFFFFFFFFFF
FFF:FFFFFFFFFFFFFFFFFFFFFFF:FFFFFFFFFFFFFFFFFFFFF
@A00721:190:HGJW7DSXY:4:1453:25672:36652_1:N:0:TCTGCAAG+GCTGCACT
TACTTTCGATACTTCTATCGTCTTACGTTACGGTAATTACTATAGTGTTATTTATTCTGGTGGTGATG
ATAATCTTGGAGGTCGTGACGTTGATGAAGCTATAAGAGCGTTCTTATTACGGCGCACTTCTTTACCC
GCC
+
FFFFFFFFFFFFFFFFFFFFFFFFFFFFFFFFFFFFFFFFFF,FFFFF:FFFFFFFFFFF:FFFFFFF
FFFFFFFFFFFFFFFFFFFFFFFFFFFFFFFFFFFFFFFFFFFFFFFFFFFFFFFFFFFFFFFFFFFF
FFF
@A00721:190:HGJW7DSXY:4:1573:2193:7639_1:N:0:TCTGCAAG+GCTGCACT
TTTCAGTTTCACAACTGAAGGAGGAGGTTTCTGTTACTGGAAAACCCTCGTCAATACTTTTCAATAAT
ATACGTCTTGAACTCACTTATGACGATCTAGTGAAATTAACGCGTCCTTTTCTGGAACGTAGTTTTAC
TA
+
FFFFFFFFFFFFFF:FFFFFFFFFFFFFFFFFFFFFFFFFFFFFFFFFFFFFFFFFFFFFFFF,F:FF
FFFFFFFFFFFFFFFFFFFFFFFFFFFFFFFFFFFFFFFFFFFFFFFFFFFFFFFFFFFFFFFFFFFF
FF
@A00721:190:HGJW7DSXY:4:1155:11523:32127_2:N:0:TCTGCAAG+GCTGCACT
AAAGATAGTGGCATTCAAGGCAAACTTACCCTGATTCCGGCCGGTGGTAGCTCATTTCTACCAGGGGC
TATTACTCTTGCTGAAACAGCTATTAAAGCTGTTGAGCGGGTAATTAC
+
FFFFFFFFFFFFFFFFFFFFFFFFFFFFFFFFFFFFFFFFFFFFFFFFFFFFFFFFFFFFFFFFFFFF
FFFFFFFFFFFFFFFFFFFFFFFFFFFFFFFFFFFFFFFFFFFFFFFF
@A00721:190:HGJW7DSXY:4:1530:10059:14559_2:N:0:TCTGCAAG+GCTGCACT
ATCGCTGAAGGATGTGCACTCTTTTCTGCTACTTTAACGGAGCGTAGTGTACTCTTCGTCGATTGTAT
CAACAGTAACATTACTTCTAATAAAGGTCTCTATCAAGCTCGTATTAT
+
FFFFFFFFFFFFFFFFFFFFFFFFFFFFFFFF:FFFFFFFFFFFFFFFFFFFFFFFFFF:FFFFFFFFFF
FF:FFFFFFFFFFFF:FFFFFFFFFFFFFFFFFFFFFFFFFFF:FFFFF
@A00721:190:HGJW7DSXY:4:1409:27109:36385_1:N:0:TCTGCAAG+GCTGCACT
TTACCGTTGAAGGCTAAACTCCAAGGTAATTATGAAGACTGTGATTTATCTTTCACTCTGGCCTTGTA
TGAGGGTGTTTCTAATCGTAACATTAATAATTTCAATCTTCTTAAAAAGAAGATAGAGAGTCATACTT
TGG
+
FF:FFFFFFFFFFFFF:FFFFFFFF:FFFFFFFFFF:FFFFFFFFFFFFFFFFFFFFFFFFFFFFFFF
FF:FFFFFFFFFFFFFFFFFFFFFFFFFFFFFFFFFFFFFFFFF,FFFFFFFFFFFFFFFFFFFFFFFF
FFF
@A00721:190:HGJW7DSXY:4:2262:19777:36338_2:N:0:TCTGCAAG+GCTGCACT
CAATATTGAAGTGGACACCTCTGGTAGTATCGAAGTAAAGATAACCAATAAGGAAGGAGCTTATACTA
TGGTTGAATCAACTAAGCATACTACTTTTGCTTTGATTACTCTACCGC
+
FFFFFFFFFFFFFFFFFFFFFFFFFFFFFFFFFFFFFFFFFFFFFFFFFFFFFFFFFFFFFFFFFFFF
FFFFFFFFFFFFFFFFFFFFFFFFFFFFFFFFFFFFFFFFFFFFFFFF
@A00721:190:HGJW7DSXY:4:2402:14687:8766_2:N:0:TCTGCAAG+GCTGCACT
TAAAGAGTCGATTAAACAAGTTGTAATTATCAATGATTTTATCGCTGGGTGTTATCTCGAAAAGTCTA
TTATAAAAGGAACGGCAGTTCGAAACCAAAGAGAATATATAAAATGTT
+
FF:FF,FFFFF,F,FF,:F,FFFFF,,F,F:FF,:,:FFF:F,F,:FF,:,,F,F,F,,,:FF,F:,,
:F,FFFFF,:,FF,,,,,,F:,F,,FF,,F,,FFFF,:,,F:FF,,,F
@A00721:190:HGJW7DSXY:4:2510:25346:36683_1:N:0:TCTGCAAG+GCTGCACT
TACCACTATCTACGAACCTAAAGTTCTGAAGACGTTGCTTGTCGCTTCCTCCTTTAATGGCACTACCT
CGGTACAATCTTACTTGGAACGAAGGCGACCTGTTCCGGAGTTTTTACGGGATCCGGAATGTTCTGTC
TAT
```

```
+
FF:FFFFFFFF:FFFFFFFFF:FFFFFFFFFFFFFFFFFFFFFFF:FFFFFFFFFFFFFFFF:FFFF
FFFFFFFFFFFFFFFFFFFFFFFFFFFFFFFFFFFFFFFFFFFFFFFFFFFFFFFFFFFFFFFFFFF
FFF
@A00721:190:HGJW7DSXY:4:2528:27245:29951_1:N:0:TCTGCAAG+GCTGCACT
AATACGATTTCGAACGTCAACGTTCACCGAGTTTCGTTTCGTACCTTGATGCTGACTTTCGTCAATAT
GATTTGATCTGGTTGCTCAATTCTAAGCGTGTTCCGCTTAGTGCGAGGCCTCTCTTCTACGAGTATTG
CTA
+
FF,FFFFFFFF:F:FFFFFFFFFFFFFFFFFFFFFFF:FFFFFFFFF:FFFFFFFFFFFFFFFFFFF
FFFFFFFFFFFFFFFFFFFFFFFFFFFFFFFFFFFFFFFFFFFFFFFFFFFFFFFFFFFFFFFFFFF
FFF
@A00721:190:HGJW7DSXY:4:1419:24605:28009_2:N:0:TCTGCAAG+GCTGCACT
ATATTTGGATGATCTTGATAAAGAACTTCATGAATTTTCAGAGGATGAAAAATTTTCTTCCTTGGATT
TTAATATAAAGAATCTTTAACAGGACTCCTACTACAGGAGGCAGTCTT
+
F:FF:FFFFFFFFFFFFFFFFFFFFFFFFFFFFFFFFFFFFFFFF:FFFFFFFFFF:F,FFFFF:FF
FFFFFFFFF:FFFFFFFFF,FF::FFFFFFFFFFFF:FFFF:FFF:FF
@A00721:190:HGJW7DSXY:4:2139:24279:33708_1:N:0:TCTGCAAG+GCTGCACT
ATTTGTTGTAATAGGGTCATCAACTTAGAACTCGAAACTCTAAGTGGTAATACTGTCTTTCCAAGCTT
ACCTGTATCTTCAATGAAATTGGACTCTTCGATAGGGGACATTTTATCGTGGGCTTTCCGTAAAGGAA
GAT
+
FFFFFFFFFFFFFFFFFFFFFFFFFFFFFFFFFFFFFFFFFFFFFFFFFFFFFFFFFFFFFFFFFFF
FFFFFFFFFFFFFFFFFFFFFFFFFFFFFFFFFFFFFFFFFFFFFFFFFFFFFFFFFFFFFFFFFFF
FFF
@A00721:190:HGJW7DSXY:4:2303:30436:31876_1:N:0:TCTGCAAG+GCTGCACT
CTACTAATGGCGTTGGCCTAGTGGATGTTATTAGTGACATCTGGTCAATTTCGTTTTTAGGTGGTCTT
TATTCTATTATTGATTTTTATGAGATTGATACGTTAACAGAAGATGATGTTAGAGTAGAATTCCTGAA
GAG
+
:FFFFFF:FFFFFFFFFFFFFFFFFFFFFFFFFFFFFFFFFFFFF:FFFFFFFF:FFFFFFFFFFFFF
FFFFFFFFFFFFFFFFFFFFFFFFFFFFFFFFFFFFFFFFFFFFFFFFFFFFFFFFFFFFFFFFFFF
FFF
@A00721:190:HGJW7DSXY:4:2206:9733:36699_2:N:0:TCTGCAAG+GCTGCACT
CCAACTTCTTGACAATTTTCTTCAATCCTTCGATAATAGTTAAATCTAGGATTATGTCTGTGTTGAAA
GACTTTCTACCGATAAGGGTCACTAGTCTTACACAATTAAGAAATAAT
+
FFFFFFFFFFFFFFFFFFFFF:FFFFFFFF:FFFFFFFFFFFFFFFFFFFFFFFFFF:FFFFFFFFF
FFFFFFFFFFFFFFFFF,FF:FFFFFFFFFFFFFFFFFFFFFFFFFFFF
@A00721:190:HGJW7DSXY:4:1168:15817:31360_2:N:0:TCTGCAAG+GCTGCACT
ACTATCTAAAGATTGAAGGAAAATTTTATCCTCGTTTAACTTACTTAGACTTTCCTACGATACAAATG
ATAACTTCTAATTTACGTTTGCTACTCAGAGCTGGTCCTCTCAGCAAA
+
FFFFFFF:FFFFF:FFFF:FFFFF,FFFF:FF:FFFFFFFFFFFF,FFF:FFFFFFFFFFFFFFFFF
FFFFFFF,F:FFFFFFFFFFFFFFFFFF:FFFFFFFFFFFFFFF:FFF
@A00721:190:HGJW7DSXY:4:2215:29984:29152_2:N:0:TCTGCAAG+GCTGCACT
TCCTCAGGAGTCCCGGCATTTGATAATAATGACGACTTTCGTCATAATCGCTGCATATTACGGATTAC
ACGGTACCAGCGCATCTCGTGTCGAGCCTCGACCCGATTATATGGTAT
+
FFFFFFFFFFFFFFFFFFFFFFFFFFFFFFFFFFFFFFFFFFFFFFFFFFFFFFFFFFFFFFFFFFF
FFFFFFFFFFFFFFFFFFFFFFFFFFFFFFFFFFFFFFFFFFFFFFFF
@A00721:190:HGJW7DSXY:4:1266:25518:2816_2:N:0:TCTGCAAG+GCTGCACT
AGAATTCTTAATTATAATATTCAACCTCAAAAGTTACGTGTTTACAGTAACGAGATCACACATTTTGA
```

```
GAACTAACTATGTTATGTTCTCTGATCATTTTGACAATCTCTTCTTAT
+
F:FFFFFFFFFFFFFFFFFFFFFFFFFFFFFFFFFFFFFFFFFFFFFFFFF:FFFFFFFFFFFFFFFFF
FFFFFFFFFFFFFFFFFFFFFFFFFFFFFFFFFFFFFFFFFFFFFFFF
@A00721:190:HGJW7DSXY:4:1145:21522:18161_1:N:0:TCTGCAAG+GCTGCACT
TAAATAATATTACTAATGGCAAATAGTGGAAAGGCTCCAGTCAACACTAGTTCGAGTGCTGCTTTGGA
TTACGATGACGTGAGAATAGGTGAAGGGATAGTTTTTACCTCGACCAACATTAAGGCGGCGGCGACTA
ATC
+
F:FFFFF:FFFFFFFFFFFFFFFFFFFFFFFFFFFFFFFFFFFFFFFFFFFFFFFFFFFFFFFFFFFFF
FFFFFFFFFFFFFFFFFFFFFFFFFFFFFFFFFFFFFFFFFFFFFFFFFFFFFFFFFFFFFFFFFFFFF
FFF
@A00721:190:HGJW7DSXY:4:2433:18873:10692_1:N:0:TCTGCAAG+GCTGCACT
AGGACACTTCGAAACTGAATAATGATGCACTACAAACAAGTATCGCTAATCATCTGAAAAAGGCTCTA
AAGGACACTATACAATATCCAGACGACGACGAAGCTTATACTACTTTAATTTTGGGTATTATGTGGAG
ATA
+
FFFFFFFFFF:FFFFFFFFFFFFFFFFFFFFFFFFFFFFF,FFFFFFFFFFFFFFFFFFFFFFFFFFFF
FFFFFFFFFF:FFFFFFFFFFFFFFFFFFFFFFFFFFFFFFFFFFFFFFFF:FFFFFFFFFFFFFFFFF
FFF
@A00721:190:HGJW7DSXY:4:2252:26657:4288_2:N:0:TCTGCAAG+GCTGCACT
ACCACATTTTGGTCAAGTCAATCCGTTGAGAAAATTTCTTAGAGGATATTCGCCATTGTGGTACGAAT
TGGTCCAACATTTACCACCAAATTCTTTTGATACAACTCTAGCCTTCA
+
FFFFFFFFFFFFFFFFFFFFFFFFFFFFFFFFFFFFFFFFFFFFFFFFFFFFFFFFFFFFFF,FFFFF,
FFFFFFFFFFFFFFFFFFFFFFFFFFFFFFFFFFFFFFFFFFFFFFFF
@A00721:190:HGJW7DSXY:4:1232:31693:29606_1:N:0:TCTGCAAG+GCTGCACT
ACCAGATTGGTTCGAACCCGTTGATGCTATGTCAGAAGAAGCTATTCAAGCTTGTCGTTTAAAGACTG
CAGAGGCTGTCAAACAAGCCCAGACTCAAGGAGTCCAATCAAAGGTTTTGAATTTAGCTCAACTGGGG
AGA
+
FFFFFFFFFFFFFFFFFFFF::FFFFFFFFFFFFFFFFFFFFFFFF:FFFFFFFF:FF:F,FF:FFFFF
FFFFFFFFFFFFFFFFFFFFFFFFFFFFFFFFFFFFFF:FFFF:F::FFFFFFFFFFFFFFFFFFFFFF
FFF
@A00721:190:HGJW7DSXY:4:1514:21947:9972_1:N:0:TCTGCAAG+GCTGCACT
CTTGCAGAGTGACTGGAGTTCAGACGTGTGCTCTTCCGATCTGAAAAGTCCGGAGTATATTGTATGTT
AATAGATAGTGGGATAATAGTTCTACCGATACGTAGTGAACGCGTTCGTAGAACCAAGTACTTTCCGT
CTG
+
FFFFFFFFFFFFFFFFFFFFFFFFFFFFFFFFFFFFFFFFFFFFFFFFFFFFFFFFFFFFFFFFFFFFF
FFFFFFFFFFFFFFFFFFFFFFFF:FFFFFFFFFFFFFFF:FFFFFFFFFFFFFFFFFFFFFFFFFFFF
FFF
@A00721:190:HGJW7DSXY:4:1560:30056:13182_1:N:0:TCTGCAAG+GCTGCACT
ACCGATAGGAGTTGTATAGTTGAAGATGTGGCTTTAGACGATAATCCTTTAAGGACTATGTTGACTGA
CGAAGATTTTCCAACAGATTTGTCTACGTTGCAGATCGCCACTCCAAACACTATATACATTATAAAAT
ATA
+
FFFFFFFFFFFFFFFFFFFFFFFFFFFFFFFF:FFFFFFFFFFFFFFFFFFFFFFFFFFFFFFFFFFFF
FFFFFFFFFFFFFFFFFFFFFFFFFFFFFFFFFFFFFFFFFFFFFFFFFFFFFFFFFFFFFFFFFFFFF
FFF
@A00721:190:HGJW7DSXY:4:1512:14000:8077_1:N:0:TCTGCAAG+GCTGCACT
ATAACTTTTGTTACCAAATTCTTGGCTTTCAACGAAGTCTCTGATTTTATTAATCATTACTTTAACGA
TCTCTCGGTCATTTACTCTGTACTTGATCTACGTCGTTTAACTGTCGGAACAACCTTTACTTAGAATG
ACT
```

```
+
FFFFFFFFFFFFFFFFFFFFFFFFFFFFFFFFF:FFFFFFFFFFFFFFFFFFFFFFFFFFFFFFFFFF
FFFF::FFF:FFFFFFFFFFFFFF:FFFFFFFFFFFFFFFFFFFFFFFFFFFFFF:FFFFFFFFFFFF
FFF
@A00721:190:HGJW7DSXY:4:1617:28700:24455_2:N:0:TCTGCAAG+GCTGCACT
TCTTATATTATTTACAATAGTTAATATCATATTAGTAATACTCACTATCGTTTTCTGCTTTTTCTTAG
CTAATAGATTAATAAAGGATGAGTACGAAAACCAACTCTTCGTTACGA
+
FFFFFFFFFFFFFFFFFFFFFFFFFFFFFFFFFFFFFFFFFFFFFFF:FFFFFFFFFFFFFFFFFFFF
FFFFFFFFFFFFFFFFFFFFFFFFFFFFFFFFFFFFFFFFFFFFFFFF
@A00721:190:HGJW7DSXY:4:1215:3803:35352_1:N:0:TCTGCAAG+GCTGCACT
TATAATATTTTTAGTCTCTACACCAATCTCACCTAGTATAATGCTAGTTTTCCGTCCTTACCTTAATA
CACTAATAGATCATCCGCAAACGACTCGAAACCGAGACGCCGTAGATCAAAGTCCCTTCTAACTTTAG
ATT
+
FF,F,F,,,FFF,,::F,,FFFF:,:F,,,F,F,,,,F,F:,::,,,FFF,F::,:,F,F,,,:F,,F
:,:,,:,,,:,,:,FF::F,FFFFFFFFFFFFFFFFFFFFFFFFFFFFFFFFF:FFFFFFFFFFFFFF
FFF
@A00721:190:HGJW7DSXY:4:2535:31150:9627_1:N:0:TCTGCAAG+GCTGCACT
CATTTTATAGCAGTTATTTATGTTGTACTTTTTATAATTTATAACCTTCTTATACTTTTGGGTATGTT
TTGTGAAAAACATGAGATACCCAAGTCTATCGAAGACATTATCTCTTATGTACTTATTGTAACGATCA
TTC
+
FFFFFFFFFFFF:FFFFFFFFFFFF:FFFF:FFF:FFFFFFFFFFFFFFFFFFFFFFFFFF:FFFFFFF
FFFFFFFFFFFFFFFFFFFFFFFFFFFFFFFFFFFFFFFFFFFFFFFFFFFFFFFFFFFFFFFFFFFF
FFF
@A00721:190:HGJW7DSXY:4:1472:2410:12242_2:N:0:TCTGCAAG+GCTGCACT
TATTCCTTTAACTGTGTATGAGGAGGATTTATAATGCTTTTCGATAGTGATTGTTATCAATTTAAATA
CATTACTGTTTCAAATCATCTTTACTCACAGACTCCGAAAATGGTTTC
+
FFF:FFFFFFFFFFF::FFFFF:FFFFFFFFFFFFFFFFFFF:FFFFFFF:F:F:FFFFFFFFFFFFF
FFF:FFFFFFFFFFF::FFFFFFFFFFFFF:FFFF,:FFFFFFFFFFF
@A00721:190:HGJW7DSXY:4:1267:9362:35117_1:N:0:TCTGCAAG+GCTGCACT
TATTTATTAAACGAACTATTATCCGATTCTTCGAGGGTATCACTACCACTGATCTGCCGACTTTAGTC
AAGGAACTTCAATTCCTTCGATGTTTATATTTTTCTATAAGGGAAAATAGCAATAATCAAATTTCGAC
AAA
+
FFFFFFFFFFFFFFFFFFFFFFFFFFFFFFFFFFFFFFFFFFFFFFFFFFFFFFFFFFFFFFFFFFFF
FFFFFFFFFFFF:FFFFFFFFFF:FFFFFFFFFFFFFFFFFFFFFFFFFFFFFFFFFFFFFFFFFFFF
F:F
@A00721:190:HGJW7DSXY:4:1254:17987:33865_2:N:0:TCTGCAAG+GCTGCACT
ATTTCAGAACTCTCTCCGATCACCTTGGTGTGATCTCCCGGAGGATCTTTTCAATAAGATGTATGATG
ATAATCTACTATTTTTAAATAGTGATATTCAGCGTATACTTGAATACT
+
FFFFFFFFFFFFFFFFFFFFFFFFFFFFFFFFFFFFFFFFFFFFFFFFFF:FFFFFFFFFFFFFFFFF
FFFFFFFFFFFFFFFFFFFFFFFFFFFFFFFFFFFFFFFFFFFFFFFF
@A00721:190:HGJW7DSXY:4:1518:12310:32299_1:N:0:TCTGCAAG+GCTGCACT
CTTGGTCTATTTACCGCAAAAAGTTGGAAGAGGTCTATGATATTTATTTTACTTATACTTTAAATAGC
ACCGATTATAACTTGACTGTTGATAATATTACAATAGCTCTTTCTCAGAAGATTAGAGAAGTAACCGG
TGA
+
FFFFFFFFFFFFFFFFFFFFFFFFFFFFFFFFFFFFFFFFFFFFFFFFFFFFFFFFFFFFFFFFFFFF
FFFFFFFFFFFF:FFFFFFFFFFFFFFFFFFFFFFFFFFFFFFFFFFFFFFFFFFFFFFFFFFFFFFF
FFF
```

```
@A00721:190:HGJW7DSXY:4:2147:17580:33129_2:N:0:TCTGCAAG+GCTGCACT
CATTGATTTACTTTTACCGTTAGAACTGAAAAAGATTCTTTTCGAAAAGAAACCTATAAGTTACAAAC
GATTCTTCGACTATATTAAAGAAATAAAAATACTTCAAGTACCTGAAC
+
FFFFFFFFFFFFFFF:FFFFFFFFFFFFFFFFFFFFFFFFFFF:FFFFFFFFFFFFFFFFFFFFFFFFF
FFFFFFFFFFFFF:F:F:FFFFFFFFFFFFFFFFFFF:FFFFFFFFFFF
@A00721:190:HGJW7DSXY:4:2415:10547:32534_1:N:0:TCTGCAAG+GCTGCACT
ATGGTATAATGTGTCTTACAAATAGAACCTTCACTCTCGGTAATGAGAGTGTATTCCTTAATATTCTA
GTAACCGCTATGTGGGCATTACTAGGATTTATCACACTCCTAAATTTGATAGCAATTTATGTTTTAAA
TGC
+
:FF:FFFFF:FFF:FFFFFFFFFFFFFFF:FFFFFFFFFFFFFFFFFFFFFFFFFFFFFFFFFFFFFF
FFFFFFFFFFFFFFFFFFFFFFFFFFFFFFFFFFF:FFFFFFFFFFFFFFFFFFFFFFFFFF:FFF:F
FFF
@A00721:190:HGJW7DSXY:4:2105:3387:27242_2:N:0:TCTGCAAG+GCTGCACT
AAAAACTCCATTGTATAGTTCAAGGAGTACTTAATTCAATAGCTTTAGTATTATTTACTATCGCCTTT
ATTGAAAATTGTATAAGAGTTAATTGCTTACACAAAAGTGACGCGCGA
+
FFFFFFFFFFFFFFFFFFFFFFFFFFFFFFFFFFFFFFFFFFFFFFFFFFFFFFFFFFFFFFFFFFFF
FFFFFFFFFFFFFFFFFFFFFFFFFFFFFFFFFFF:FFFFFFFFFFFFF
@A00721:190:HGJW7DSXY:4:2660:7048:31923_2:N:0:TCTGCAAG+GCTGCACT
ATACCATAACCTACCATGACCAATCATCGCTCCAACTGGCACGCCAGACGTAGGGTCAAACCTACTAC
CGCCGGTCGGCAGATCGGCACCACTGGTCTCTACGTTCAGGGACCCGC
+
FFFFFFFFFFFFFFFFFFFFFFFFFFFFFFFFFFFFFFFFFFFFFFFF:FFFFFFFFFFFFFFFFFFFF
FFFFFFFFFFFFFFFFFFFFFFFFFFFFFFFFFFFFFFFFFFFFFFFF
@A00721:190:HGJW7DSXY:4:2253:15872:2143_1:N:0:TCTGCAAG+GCTGCACT
CCTTCCCAAATCTAGATCTGCACAGAAGAAGGCTGAAGGTCGTTACCGGACGACCACTCAAAGGAAAC
GGGCTGTTGCCCAGAGGAAAGCAGCGACCACCGAGGTCGCTCGTAACATTGACGGCAGTCCCGTCATC
TCT
+
F:FFFFFFF:FFFFFF:F:FFFFFFFFFFFFFFFFFFFFFFFFFFFFFFFFFFFFFFFFFFFFFFFFF
FFFFFFFFFFFFFFFFFFFFFFFFFFFFFFFFFFFFFFFFFFFFFFFFFFFFFFFFFFFFFFFFFFFF
FFF
@A00721:190:HGJW7DSXY:4:2324:2428:17378_2:N:0:TCTGCAAG+GCTGCACT
GCAGTACCTCAGTCAAAAGTCACATTCACCACTCCCATACCAATCAATGATGTGACTATACAGTTCGG
CCATTTCAAGAGTGGTCAATACGTCGTGGGAGGTACTGAAGCCTTCCC
+
FFFFFFFFFFFFFFFFFFFFFFFFFFFFFFFFFFFFFFFFFFFFFFFFFFFFF:FFFFFFFFFFFFFF
FFFFFFFF:FFFFFFFFFFFFFFFFFFFFF:FFF,FFFFFFFFFFFF
@A00721:190:HGJW7DSXY:4:1558:27670:14278_2:N:0:TCTGCAAG+GCTGCACT
AAGCCAATCAATAGTTCTTCTGACCAGAATGAAAAGGGGTTTACCAGGGTAGTCTCTAAGTCTACCAG
GAAGAGGGGAAATGTCCCCAAAACTGAGAAGAATAAAGGGAAAGGTCC
+
FFF:FFFFFFFFFFFFFFFF:FFFF:FFFFFFFF:FFFFFFFFF::FFFFFFFFFFF:FFFF,FF:,F
FFFFFFFFF:FFFFFFFFFFFFFFFFFFFFFFFFF:FFFFFF:FFFFFF
@A00721:190:HGJW7DSXY:4:2571:5330:16141_2:N:0:TCTGCAAG+GCTGCACT
AGGGGAAATTACCCCTAATTCTGAAGGAAGATCTCCCACCATCTGGGGTTGGGAATAAGAAATCTGCA
CGGGCCAAAGAGGCCGCTGCTACGAAGTCGCTGAAGGCGGCTTGGGTC
+
:FFFFF:FFFFFFFFFFFFFFFFFFFFFFFFFFFFFF:FF:FFFFFFFFFFFFF:FFFFFFFFFFFFF
FFFFFF:FFFFFFFFFFFFFFFFFFFFFFFFFFFFFFFFFFFFF,FFFFF
@A00721:190:HGJW7DSXY:4:2316:10972:18208_2:N:0:TCTGCAAG+GCTGCACT
TTGCGCCTGCCGACCCCATCTTTGAGACCAAGACTCTCTACAGTAGGTTCGTGCCTACTATGAGCAAA
```

```
TCTGTTGAATTCGACACTGAGGACGAAGGTGAGAGGACTTTTGGCTAC
+
::FFF:F:FFFFFFFFFFFF,FF:FFFFFFFFFFFFFFFFFFF:FFFFFFF,FFF:FFFFFFF,FFFF
,FFF:,FFFFFFF,F::,F:F:FFFFFFFFFFFFFFFFF,FFF:FF,F
@A00721:190:HGJW7DSXY:4:1438:5141:34194_2:N:0:TCTGCAAG+GCTGCACT
CGTGGGTTGAAGGCCGACGCTTCGAGCTTGAGTGTGACGAGGCCGGTGACGTTTTTGTCAACGGCGTT
TGGAGGATTTTATCCGCGCTTAACTATGGGGCGAAGATCCCCTATGGA
+
FFFFFFFFFFFFFFFFFFFFFFFFFFFFFFFFFFFFFFFFFFFFFFFFFFFFFFFFFFFFFFFFFF:F
FFFFFFFF,FFF:FFFFFFFFFFFFF:FFFFFFFFFFFFFFFF::FF:
@A00721:190:HGJW7DSXY:4:2126:14606:4335_2:N:0:TCTGCAAG+GCTGCACT
TTGGCATATTTATGCCGACGATAATAAGCATGTCACCCTTAAGCACCGAGGTAAGCTCGGGACTTACA
AGAGAGAGTGTGAGAGAGTCGCTCTCACTATTTTTGCCCAGAATGCGG
+
FFFFFFFFFFFFFFFFFFFFFFFFFFFFFFFFFFFFFFFFFFFFFFFFFFFFFFFFFFFFFFFFFFFF
FFFFFFFFFFFFFFFFFFFFFFFFFFFF:FFFFFF,FFFFFFFFFFFF
@A00721:190:HGJW7DSXY:4:2640:16514:32910_2:N:0:TCTGCAAG+GCTGCACT
TCCTCCATAATTACAACCTCTCTTTAGAGAGCATTAGAAGGAAGTTGGGCAAATTCCCACATATGGTA
GACGTTGTCACACTTTTCTTGAAAATTTTTCCTTTCCTCCGTCATTGG
+
FFFFFFFFFFFFFFFFFFFFFFFF:FFFFFFFFFFFFFFFFFFFFFFFFFFFFFFFFFFFFFFFFFFF
FFFFFFFFFFFFFFF:FF:FFF:FFFFFFFF:F:FF:FF,FFFF,:FF
@A00721:190:HGJW7DSXY:4:2327:25988:13369_2:N:0:TCTGCAAG+GCTGCACT
AAGGTGGAAGGTGTCAAGGGTCTTCTTCATGCTGATCTTTCAGGACCTAATCACATCATGATTGATAG
TTACGGCTCGTATCGAGTTGGAGGGAGGAAGTTCTCCGAAATTAAAGA
+
FFFFFFFFFFFFFFFFFFFFFFFFFFFFFFFFFFFFFFFFFFFFFFFFFFFFFFFFFFFFFFFFFFFF
FFFFFFFFFFFFFF:FFFFFFFFFFFFFF:FFFFFFFFFFFFFFFFFF
@A00721:190:HGJW7DSXY:4:1467:16206:8359_1:N:0:TCTGCAAG+GCTGCACT
TCAGAGAGGGGAGCTCTGAAAAGGAAACTTCGTCAGAAGATGAGGAAGTCTAAGAGGAGGAGTTTGAG
GAAGGCTCTAGAGAAGATCTCCCCACCTGGAGAAGGCAAGTGGAGAGTCCAGAAGTTGATGAAGAAGG
ATT
+
FFFFFFFFFFFFFFFFFFFFFFFFFFFFFF:FFFFFFFFFFFFFFFFFFFFFFFF:FFFFFFFFFFFF
FFFFFFFFFFFFFFFFFFFFF:FFFFFFFFFFFFFFFFFFFFFFFFFFFFFFFFFFFFFFFFFFFFFF
FFF
@A00721:190:HGJW7DSXY:4:2131:32570:16000_2:N:0:TCTGCAAG+GCTGCACT
GTTACCACTTCCTAAGGTGGATGAGGGAGGTTGTTTCTTTGGAGTTTTAGATAATCTTTTGAGAAGGG
GTCATAGAGCCAGCAAGAGAAGCTTATATGCTGGTTTAAGTGATGATT
+
FFFFFFFFFFFFFFFFFFFFFFFFFFFFFFFFFFFFF:FFFFFFFFFFFFFFFFFFFF:FFFFFFFFF
FFFFFFFFFFFFFFFFFFFFFFFFFFFFFFFFFFFFFFFFFFFFFFFF
@A00721:190:HGJW7DSXY:4:1323:32805:3098_1:N:0:TCTGCAAG+GCTGCACT
ATTTAGGTCAACTTGGGGTTTCGAATGTCATTAGTGCCTTACGATTGAAAGAGGTCCTTTTGAGTATC
GACAGATCTTTCGATCTTGAGGACGTACATTTAACGTGGCAGAATTCGGTAACTTACCATATTGTTCC
TTC
+
FFFFFFFF:FFFFFFFF:FFFFFFFFFFFFFFFFF:FFFFFFFFFFFFFFFF:FFFFFFFFFFFFFFF
:FFFFFFFFFFFFFFFFFFFFFFFFFFFFFFFFFFFFFFFFFFFFFFFFFFFFFFFFFFFFFFFFFFF
FFF
@A00721:190:HGJW7DSXY:4:1106:7771:5024_1:N:0:TCTGCAAG+GCTGCACT
TTTAACTTTGACTCAACTACTCCGATCAGATCCGGATGGTTTGATTGGTATGGACTACTCGACTTTTG
ATCTGGAGTTGTCTAGGGTCATGGCAGATACTACCGATAAGGTGAAAAATATGCAGGTCAAAGATTCT
GTA
```

+
FFFFF:FFFFFF,FFFFFFFFFFFFF::FFFFFFFFFFFFFFFFFFFFFFFFFFFFFFFFFFFFF:FFF
FFFFFFFFFFFFFFFFFFFFFFFFFFFFFFFFFFFFFFFF:FFFFFFFFFFFFFFFFFFFFFFFFFFFF
FFF
@A00721:190:HGJW7DSXY:4:2523:22670:34741_2:N:0:TCTGCAAG+GCTGCACT
GAGGGAGCCGTTTTGGCCGACTTTAACTATTCTAAGAGTCTTTATGATTCAAAACCTATCGTTGTCGT
ACCTTACAAGGTTGAGGAGACTTTACAGCGGCTTATTGTCGCGCAGTT
+
FFF:FFFFFFFFFFFFFFFFFFFFFFFFFFFF::FFFFFFFFFFF,FFFFFFFF:,F:FF:F:FFF::F
FFFFFFFFFFFF:FF:FFFFFFFFFFF::F::,FFFFFFFFFFFFFFF
@A00721:190:HGJW7DSXY:4:1224:16911:17221_1:N:0:TCTGCAAG+GCTGCACT
TATCTTGAATGCTCACGCCGTCGCAGCCATATCGAGACTATGTTATAATCACTTGTTAAAGTACAGAT
TTAGTGATTTAGATATTATTGATATTGGAGGCGATATTCGTTTTCATATCCAAAACTCCTTGAATGGA
GTA
+
FFFFFFFFFFFFFFFFFFFFFFFFFFFFFFFFFFFFFFFFFFFFFFFFFFF:FFFFFFFFFFFFFFFFF
FFFFFFFFFFFFFFFFFFFFFFFFFFFFFFFFFFFFFFFFFFFFFFFFFFFFFFFFFFFFF:FFFFFFF
FFF
@A00721:190:HGJW7DSXY:4:1175:17074:13933_2:N:0:TCTGCAAG+GCTGCACT
CCTATTCTTGATGTGAAAGATGCATCGAGGTATACGTCTAGGAACATTGATTGGGAACGTACCTACTC
ACGAGACGTCATGAAAGCTATCGGAGGGAAGCAAAATGTTTCGTTCTG
+
FFFFFFFFFFFFFFFFF,,FF,FFFFFFFFFFFFFFFFFFFFFFFF:FFFFF:FFFFFFFFFFFFFFFF
FF::FFFFFFFFFFFF:FFFFFFFFFFFFFFFFF:FFFFFFFFFFFFF
@A00721:190:HGJW7DSXY:4:1544:20220:6981_2:N:0:TCTGCAAG+GCTGCACT
AAGCCTCAGTTGCGATAGCTGTAGAGGTATACGATATCTCTCTTGATGATGTAGCCACTATAATGCTT
AAGAGAGAGATTGATGTATGTCATTTCGTCTTAGTTTGTCCCGGCGAA
+
FFFFFFFFFFFFFFFFFFFFFFFFFFFFFFFFFFFFFFFFFFFFFFFFFFFFFFFFFFFFFFFFFFFFF
:FFFFFFFFFFFFFFFFFFFFFFFFFFFFFFFFFFFFFFFFFFFFFFF
@A00721:190:HGJW7DSXY:4:1149:8793:7106_2:N:0:TCTGCAAG+GCTGCACT
CCGTTCCTGACCTTGGTATCGTTATCCGTCATGGAGATGATGGCGCATCACTTGATTACTATATGCCC
GAAGGATTGGGTTATTCTCACAACAAAGATAAGATTTTATCTTTCTTC
+
FFFFFFFFFFFFFFFFFFFFFFFFFF:FFFFFFFFFFFFFFFFFFFFFFFF:FF:FFFFFFFFFFFFFF
F::FFFFFFFFFFFFFFFFFFFFFFFFFFFFFFFFFFFFFFF:FFFFFFF
@A00721:190:HGJW7DSXY:4:1219:23583:29747_2:N:0:TCTGCAAG+GCTGCACT
AAGGGCGGACAATTGTTCCATTGTGAACTCTTGGGGACTAGATTGAGCATTAATGAGTTCCGAATAAC
TCGTAATAAATCTTTCTCTAGTGGACAATTCAGAATACCTATTTCCGT
+
FFFFFFFFFFFFFFFFFFFFFFFFFFFFFFFFFFFFFFFFFFFFFFFFFFFFFFFFFFFFFFFFFFFFF
FFFFF,FFFFFFFFFFFFFFFFFFFFFFFFFFFFFFFFFFFFFFFFFF
@A00721:190:HGJW7DSXY:4:1502:12545:2707_1:N:0:TCTGCAAG+GCTGCACT
CTGCCAATCTCATCGCTATACGCATACCTGATTGGAAGAAGCGAGGTTCGTTCCTCAATAAAAATTTT
TCTCAAGAGATTATGGTTGATAAAGTTTTCTTCATAAGGGCTATTCAGTACGTCATGGGGATCACTTC
TTC
+
FFFFFFFFFFFFFFF:FFFFFFFFFFFFFFFFFFFFFFFFFFFFFFFFFFFFFFFFFFFFFFFFFFFFF
FFFFFFFFFFFFFFFFFFFFFFFFFFFFFFFFFFFF:FFFFFFFFFFFFFFFFFFFFFFFFFFFFFFFF
FFF
@A00721:190:HGJW7DSXY:4:2437:15284:19789_1:N:0:TCTGCAAG+GCTGCACT
ACCTTCGAATATGTTACGACATGGCTTCGTAACAACAGTGCGAGGATTGTCATCTCGGGGAAAGTGAT
TCACCAAGATGTTCATATAGATACGAGATACCTAGAGAAGGTCGCAGCGATCTTACTCGTTATCGGAG
CTA

```
+
FFFFFFFFF:FFFFF:FFFFFFFFFFFFFFFFFFFFFFFFFFFFFFFFFFFFFFFFFFFFFFFFFFFFFFF
FFFFFFFFFFFFFFFFFFFFFFFFFFFFFFFFFFFFFFFFFFFFFFFFFFFFFFFFFFFFFFFFFFFFFFF
FFF
@A00721:190:HGJW7DSXY:4:1160:6234:25817_1:N:0:TCTGCAAG+GCTGCACT
TCGCAAACTTAACGTTAGTACCGGAGGTGATCTCTCTGCTTCTGCGTTACTGAGAGAAAAGATTCAGG
ACGCCTACGGTGACTTTATGGACAAGATGCAAGTTCTTGGGAAATACTTGTTTCCCTTTTTAGGTTTA
GTA
+
FFFFFFFFFFFFFFFFFFFFFFFFFFFFFFFFFFFFFFFFFFFFFFFFFFFFFFFFFFFFFFFFFFFFFFF
FFFFFFFFFFFFFFFFFFFFFFFFFFFFFFFFFFFFFFFFFFFFFFFFFFFFFFFFFFFFFFFFFFFFFFF
FFF
@A00721:190:HGJW7DSXY:4:1403:31204:32737_1:N:0:TCTGCAAG+GCTGCACT
CTCTTCCGATCTGACAACGAATCTTGAAACTACGGTCCTTATCAAAGAAAATGGTGAACCGGTCTCTA
AATCTTTTGAGATTGAGAAGATTGAAGAACACGTCAAGAAGTCGCTCGGCGTTGTTCTTGAAAACCAA
GT
+
FFFFF:F::F:FFFFFF:FFF::FFFFFFFFF,FFFF:FFFFFFFF::FF,FFFF:FFFF,FF:F:FF
FFFFFFFF:F,:FFFF,FFFFFFFFFFF:FFFFFFFFFFFFFFFFFFFFF,FFFFFFFFFFF::FFFF
FF
@A00721:190:HGJW7DSXY:4:1677:8693:19053_1:N:0:TCTGCAAG+GCTGCACT
GCTGATCATGGAGTCTTTGTACCTTCTGTCAAACTTCGAGGTTCAGCTACGAGAGGTGATCCCGGATG
TGGATTGAAGGCTGGCTCTAAGGGAGACTCCTTCTTTTTGAATGTTGTAAAGAATTGGGTTGAGAAGT
GTG
+
FFFFFFFFFFFFFFFFFFFF:FFFFFFFF:FFFFFFFFFFFFFFFFFFFFFFFFFFFFFFFFF:FFFFFF
FFFFFFFFFFFFFFFFFFFFFFFFFFFFFFFFF:FF:FFFFFFFFFFFFFFFFFFFFFFFFFFFFFFFFF
FFF
@A00721:190:HGJW7DSXY:4:2614:32497:19695_1:N:0:TCTGCAAG+GCTGCACT
CTTGGTATTTCTGAAATAGTTTATCAGAGCGTCATTTTCCTTTGGGATCTTTCCTCTGGTAAGGGAAT
TATTAAATCATTCTTCTCACTCTTGACGAGAATTATTACTGTTTTCTCTACCCATAACTTCTTTTTCA
AAA
+
FFFF:FFFFFFFFFFFFFFFFFFFFFFFFFFF::FFFFFFFFFFFFFFFFFFFFFFFFFFFFFFFFFFFF
FFFFFFFFFFFFFFFFFFFFFFFFFFFFFFFFFFFFFFFFFFFFFFFFFFFFFFFFFFFFFFFFFFFFFFF
FFF
@A00721:190:HGJW7DSXY:4:1174:10637:18443_2:N:0:TCTGCAAG+GCTGCACT
TACAGAGTGCAGTCGTTTCAACACTTATCACCACTTTTGAAATGAAAATAATTGGTGATGAAAATACG
TATGTCGGTGGGGTTCTCAAGGCTCTCATCGCCCCTGGGATTCTAAGG
+
,FF,FFFFFFFFFFFF,FFF:FFF::FF,FFF::,,:FFF,F,FFF:FFFF::F,F,,,F::FF,:F:
FF,FFF,F:FFF:,FF,FFF:::::,FF,:FF,,F,F:::FFF:F,,,
@A00721:190:HGJW7DSXY:4:2233:29993:25003_2:N:0:TCTGCAAG+GCTGCACT
TTTGGTCCTTACGTCTTAAGTAATTTGGATGTTGTCGAAAACTATCAGGTTCTTGTCACGATTCTTAA
AAGAAGTTTATTAAAACTTCAGAGTCTCTTGGAAGTCCAGGTAGCTAA
+
FFFFFFFFFFFFFFFFFFFFFFFFFFFFFFFFFFFFFFFFFFFFFFFFFFFFFFFFFFFFFFFFFFFFFFF
FFF:F:FFFFFFFFFFFFFFFFFFFFFFFFFFFFFFFFFFFFFF,FFFF
@A00721:190:HGJW7DSXY:4:1135:6497:26271_2:N:0:TCTGCAAG+GCTGCACT
ACCTCTGTTGGTAGAGGAGTTGAGAGAATTACTCAGATTCAATCTGGCATCACTGAACGTCTACAACG
AGTTAGGGAGTGGCTAGGTTTTAAAAGATCCTTTGCACAAGCAATACC
+
:FFFFFFFFFFFFFFFFFFFFFFFFFFFFFFFFFFFFFFFFFFFFFFFFFFFFFFFFFFFFFFFFFFFFFF
FFFFFFFFFFFF:FFFFFFFFFFFFFFFFFFFFFFFFFFFF,FFFFFFF
```

```
@A00721:190:HGJW7DSXY:4:2669:31286:1219_2:N:0:TCTGCAAG+GCTGCACT
CGTCGCGGAAAACGATGAAGATTTCCACACTGTTCGAGAATCTTCTGATGCTGAATTTGAGGAAAATT
TTTCGGAAGTTTTTGGTATAAATCCTGATGGCTCAGTACAGGGAGGAG
+
FFFFFFFFFFFFFF:FFFFFFFFFFFFFFFFFFFFF:FFFFFFFFFF:FF:FFFFFFFF:FFFFF,FF
FFFFFF:FFFFFFFFFFFFFFFFFFFFF:FFFFFFFFFF:FFFFFFFFF
@A00721:190:HGJW7DSXY:4:1357:8522:26209_2:N:0:TCTGCAAG+GCTGCACT
ACCTTCTTTTATCATTGCAACAGGACTTTGAGAAGACTGTTGAAATGGACAAAATCTGGGATCATAGG
TATGTGTCACGCTACACTCGAAATATGGGCCCTCATAAGGAAGTATAG
+
FFFFFFFFFFFFFFFFFFFFFFFFFFFFFFFFFFFFFFFFFFFFFFFFFFFF:FFFFFFFFFFFFFFF
F,FFFFFFFFFFFFFFFFFFFFFFF,FFFFFFFFF:FF,FFF:FFFFFF
@A00721:190:HGJW7DSXY:4:1632:14796:2284_2:N:0:TCTGCAAG+GCTGCACT
GGAAGATCTCTACAGAGATTATCGAGAGTTGTGATCTAGTTCTAAATTTAGGTCGACGGACTTTAGCA
ACTTTAGATAAATTCTTTCCGGGCTCAATGACCTTTGCTATGTACGTG
+
FFFFFFFFFFFFFFFFFFFFFFFFFFFFFFFFFFFFFFFFFFFFFFFFFF:FFFFFFFFFFFFFFFFFF
FFFFFFF,FFFFFFFF:FF:FFFFFFFFFFFFFFFFFFFFFFFFFFFFF
@A00721:190:HGJW7DSXY:4:1670:32362:1861_1:N:0:TCTGCAAG+GCTGCACT
TTAGTTCATGAAGTTCTTACACTTCTCGCGACTAATGTCAGCTTCACTACAAAGGCGTTACGTAAAGT
AGTTTTCACGCTTTATCTTAAAGCATTACCACCAAATTTAGCACTTTTTGAATATGACAGAAGCTCGC
CAT
+
FFFFFFFFFFFFFF:FFFFFFFFFFFFFFFFFFFFFFFFFFFFFFFFFFFFFF,:FFFFFFFFFFFFFFF
FFFFFFFFFFFFFFFFFFFFFFFFFFFFFFFFFFFF:FFFFFFFF:FFFFFFFFFFFFFFFFFFFFFFF
FFF
@A00721:190:HGJW7DSXY:4:2301:5204:26381_2:N:0:TCTGCAAG+GCTGCACT
CCAGTTAGAGTTCTTCTAACCAGGACTGATGGTGAGATTCTTTTACCTTTTGGAGGTCCAGGAACCTT
TACTCCTAGTGTCTTTTCTAGAGTCCAAGGACGTTTCCGTCGGAGGAT
+
FF:FFFF:FF:F::FFFFFFFF,,FF,,FF,FFFFF:F:F,FFFF:FFFFF:,F:FFFFFFFFF:FFF
FFF:FFFF,F:FFFF:F:FF,FFFFFF::FFF:,FFFF:F,FFFF:FF
@A00721:190:HGJW7DSXY:4:1403:31937:20791_1:N:0:TCTGCAAG+GCTGCACT
TCAATAACACTCTATTGAATCCCTTTTCCACGATGAAAGCTCTTTATGAGGTGCTCCATGCAAATTTA
CAGTTCCTCTTTGGTGATATCGCCTCTGTCCTTTCTCCTTTAATCGTGAGGTTACGAGAGTTTCGTAC
TTG
+
FFFFFFFFFFFFFFFFFFFFFFFF:FFFFFF:FFFFFFFFFFFFFFFFFFFFFFFFFFFFFFFFFFFFF
FFFFFF:FFFFF:FFFFFF:FFFFFFFFFFFFFFFFFFFFFFFFFFF:FFFF:FFFF:FFFFFFFFFFF
:FF
@A00721:190:HGJW7DSXY:4:2531:29821:29684_1:N:0:TCTGCAAG+GCTGCACT
TTTTGATAAATTTGTTGGAATCGATAATGATACTGACGAAGGTTCTATTACCACCCTTGTCATTGATA
CAAACATTCTAGGTGATGCTTTCTTAGAGGCTCGTCTAGCCGCTGAGATTCGTCTTACCTATTTACAA
TCT
+
FFFFFFFFFFFFFFFFFFFFFFFFFFFFFFFFFFFFFFFFFFF:FFFFFFFFFFFFFFFFFFFFFFFFF
FFFFFFFFFFFFFFFFFFFFFFFFFFFFFFF:FFFFFFFFFFFFFFFFFFFFFFFFFFFFFFFFFFFF:
FFF
@A00721:190:HGJW7DSXY:4:2565:22842:23140_1:N:0:TCTGCAAG+GCTGCACT
GATAACGATTTAGTACTGGGAAATGGTGGTAGTCAGTTTACTACTTTTCTTTCCCTCCCCTTTTTCGA
TTGGATTCGACGGGGTCTTCGTCTCACGACTAATTTGATAAGGTTTTTCTTTTATGGGACTCTTGTTT
GTA
+
FFF:F:FFFFFFFFFFFFFFFF,,FFFFFFFFF,F,FFFFFF::FFFFFFFFFFF,:F,FFFFFFFF
```

FFFF:FF:F,F,FFFFFFFF:F:FF:::,FF,FFFFFFF:FFFFFFFF:FFFFFFFFF:FF:FFFFFF
FF:
@A00721:190:HGJW7DSXY:4:2354:4634:32346_2:N:0:TCTGCAAG+GCTGCACT
GTTCTTAATGTAGGTCTCCTAGTACTTAAGGGTTTATGTTTCGGTTTCAGCTGGATGGATCTTTTGAT
GGTATGTTTCACATTGCGTGATCACTTCCCGTGGACTTGGGAAGAGAT
+
FFFFFFFF:FFFFFFFFFFFFFFFFF:FFFFF,FFFFFFFFFFFFFFFFFFFFFFFF:FFFFFFF:FF
FFFFFFFFFFFFFFFFFFFFFFFFFFFFFFFFFFF:FF:FF:FFFFF
@A00721:190:HGJW7DSXY:4:2159:3305:22247_1:N:0:TCTGCAAG+GCTGCACT
TTCTATTATGTCGCTAAAGCCTGTCCAGTTGTCGATGATGTTCGGGCTGTGGGGATTAACTTCCCTCG
TGAAACTTACGACGATGTTCTCGCTCGCATTTCACAAGGATTACTAAGTCTGAGATCTGCCACTTCTA
ATT
+
FFFFFFFFFFFFFFFFFFFFFFFFFFFFF:FFFFFFFFFFFFFFFFFFFFFFFFFFFFFFF:FFFFFF
FFFFFFFFFFFFFFFFFFFFFFFFFFFFFFFFFFFFFFFFFFFFFFFFFFFFFFFFFFFFFFFFFFFF
FFF
@A00721:190:HGJW7DSXY:4:1462:5122:35697_2:N:0:TCTGCAAG+GCTGCACT
AAGTTCATGAGGATGTCTTGGTCGAAAAATATCGCCGGGAACTTCTAATAGAGGATCCTGATGACATT
ATTTCAACGGATACCGATCTCAGTTCTACCACAGAAACCGTCTCAGGA
+
FFFFFFFFFFFFFFFFFFFFFFFFFFFFFFFFFFFFFFFF,FFFFFFFFFFFFFFFFFFFFFFFFFFF
:FFFFFFFFFFFFFFFFFFFFFFFFFFFFFFFFFFFFFFFFFFFFFF
@A00721:190:HGJW7DSXY:4:1627:13648:5619_2:N:0:TCTGCAAG+GCTGCACT
CTGATTCGGAATTAGAGAAAGACGACCTTCTTGTGGGGACTGAGACCTCTGAATTTTCAGGGTGCCCT
GATGAGGACGTTGAAAGGGATAATTTCAACTCTGAGACTTCTAAGAGT
+
F:FF,FFFFFFFFFFFFFFFFFFFFFFFFFFFFFFFFFFFFFFFFFFFFFFFFFFFFFFFFFFFFFFF
FFFFFFFFFFFFFFFFFFFFFFFFFFFFFFFFFFFFFFFFFFFFFFF
@A00721:190:HGJW7DSXY:4:1314:26530:35008_2:N:0:TCTGCAAG+GCTGCACT
CAGAAACGTAACAAGAAGAATAAAGGTAGGAAGGGGAGAAAACAGAACAAGCGAGCGGTTGAAGAGAA
AACTGATATGGTTGCGGCTGATCGTACCCCAATTCCTGCTAGAAAGAT
+
FFFFFFFFFFFFFFFFFFFFFFFFFFFFFFFFFFFFFFFFFFFFFFFFFFFFFFFFFFFFFFFFFFFF
F:FFFFFFFFFFFFFFFFFFFFFFFFFFFFFFFFFF:FFFFFFFFFFFF
@A00721:190:HGJW7DSXY:4:2362:8287:19820_2:N:0:TCTGCAAG+GCTGCACT
TACCACTGATCTTCCAGAGTTACGTAGTGGTCGTTCTTTTGGTGATTTTATCAAAGAGAAAGTTTCTG
CTAGTCGCGACGATAGTATTTATCGGAGTGATATTAACAAGTTAAACT
+
FFFFFFFFFFFFFFFFFFFFFFFFFFFFFFFFFFFFFF:FFFFFFFFFFFFFFFFFFFFFFFF:FFFFF
F,,F:FFFFFFFFFFFFFFFFFFFFFFFFFFFFFFFFF:FFFFFFFF:FF
@A00721:190:HGJW7DSXY:4:2246:30138:12414_2:N:0:TCTGCAAG+GCTGCACT
TGTTGAAGTACCGCAACATGGATCAGTTTTCCAAAAAGCTATTAATAGCAGTTGCGGGGCTATAATAC
CGCCTCCAGATTACTCTAAACTGAATCCACTCTCTTGGGATATAAATG
+
FFFFFFFFFFFFFFFFFFFFFFFFFFFFFFFFFFFFF:FFFFFFFFFFFFFFFFFFFFFFFFFFFFFF
FFFFFFFFFFFFFFFFFFFFFFFFF:FFFFFFFFFFFF:FFFFFFFFFF
@A00721:190:HGJW7DSXY:4:2439:23818:10895_1:N:0:TCTGCAAG+GCTGCACT
TGAATTAGTTATTGAAAGTCTTGACCCTCAATTCTGACTTTATCACCTTGGAGTTGGTAAAGTCATTA
ACAATGGTAATGTTAAGGCTGATGTCTTAAATTTCGCTTTCTGCTTCGTCGGTAATTCCATAAGACC
+
FF:FFFFFFFFFFF,FFFFFF:F:,,FF,,:,F,F,FF:FFF:F:,FFFFFFF,FF:FFF,FF:,FF,
,:,,,FF,FFF,FF,:FF,FFFFF,FFFFFFF:F,,F,FFFFF,,F,F,,:FF,F,FFFF,:,FFFF
@A00721:190:HGJW7DSXY:4:2417:18584:4773_1:N:0:TCTGCAAG+GCTGCACT
TTCTTCATGAAGATCTTCGAGTGTTTTATAGTGAGTTATTCTTGCGAAGGTTCAAGTCGATTGATTTT

GTTGACTACGATCTCTCTTGTAATAAGAAGTTCTTCGCAGTTGAATCTCCACCAGGTGGAGGGAAGAC
CAC
+
FFFFFFFFFFFFFFFFFFFFFFFFFFFFFFFFFFFFFFF,FFFFFFFFFFFFFFFFFFFFFFFFF:FFFF
FFFFFFFFFFFFFFFFFFFFFFFFFFFFFFFFFFFFFFFFFFFFFFFFFFFFFFFFFFFFFFFFFFFFFF
FFF
@A00721:190:HGJW7DSXY:4:1613:13503:3145_1:N:0:TCTGCAAG+GCTGCACT
GACTTTTGTCGAATTTTTCGCAAAAGGTCTTGAAGTACGTATTGTCACTGCTAATCGTAATTCGCGAG
AAGAGATAGTGACTAAAGTTTTGGCACTTCTAGTTCATCGCGGTATTTGTGAAATTAAGACAGACGTG
AAA
+
FFFFFFFFFFFFFFFFFFFFFFFFFFFFFFFFFFFFFFFFFFFFFFFFFFF:FFFFFFFFFFFFFFFFFF
FFFFFFFFFFFFFFFFFFFFFFFFFFFFFFFFFFFFFFFFFFFFFFFFFFFFFFFFFFFFFFFFFFFFFF
FFF
@A00721:190:HGJW7DSXY:4:1606:1741:4006_1:N:0:TCTGCAAG+GCTGCACT
TCGTATTAGAACAGCTGATTCTTATTGCATGAACGTCGCTGAGGTCAAACCTGTGGACGTTCTTTTCA
TCGACGAATTATTCATGATTCACGCTGGTCAGACCCTTTTTCTTACACATCTACTTAAGGCGGCTTAT
ACC
+
FFFFFFFFFFFFFFFFFFFFFFFF:FFFFFFFFFFFFFFFFFFFFFFFFFFFFFFFFFFFFFFFFFFFFF
FFF:FFFFFFFFFFFFFFF:FFFFFFFFFFFF:FFFFFFFFFFFFFFFFFFFFFFFFFFFFFFFFFFFFF
FFF
@A00721:190:HGJW7DSXY:4:2318:16550:30874_1:N:0:TCTGCAAG+GCTGCACT
GTGATTCCAATCAAATCGGATTCATCAATCGTATTGATCTCCAAGTCGCAAACTACTTCAACATCAAC
GACATCATTCTTGATGAGTGTCGTTCTTATCGAAGTCTCAGTTATCGCTGCCCGGCCGACGTTTGTCG
CGA
+
FFFFFFFFFFFFFFFFFFFFFFFFFFFFFFFFFFFFFFFFFFFFFFFFFFFFFFFFFFFFFFFFFFFF:F
F:FFFFFFFFFFFFFFFFFFFFFFFFFFFFFFFFFFFFFFFFFFFFFFFFFFFFFFFFFF:FFFFFFFFF
FFF
@A00721:190:HGJW7DSXY:4:1510:25870:31548_1:N:0:TCTGCAAG+GCTGCACT
ACGGCAGAAAAATTTCTCCTGCTTGTAATTCGAGAGAGGACACTATGGAAATTAGACCTATCAGATGT
CTTGACGACTTCGAATTTGAGGACGGAGTTAAATATATTACTTTCACTCAAGGTGAAAAGTTTGAAAT
TAA
+
FFFFFFFFFFFFFFFFFFFFFFFFFFFFFFFFFFFFFFFFFFFFF,FFFFFFFFFFFFFFFFFFFFFFFF
FFFFFFFFFFFFFFFFFFFFFFFFFFFFFFFFFFFFFFFFFFFFFFFFFFFFFFFFFFFFFFFFFFFFFF
FFF
@A00721:190:HGJW7DSXY:4:1216:30418:20572_1:N:0:TCTGCAAG+GCTGCACT
TAAGAGTAAGGTTAAATGTGAAGATGCTCAGACTATTCATGAAGTTCAAGGTAATACTTACCAGCACG
TTCGGCTGGTTAGAATTAAACCTCAAGATGACCCTGTTTTTGCATCACAAACACATATTACCGTTGCT
CTA
+
FFFFFFFFFFFFFFFFFFFFFFFFFFFFFFFFFFFFFFFFFFFFFFFFFFFFF:FFFFFFFFFFFFFFFF
F:FFFFFFFFFFFFFFFFFFFFFFF:FFFFFFFFFFFF,FFFFFFFFFFFFFFFFFFFFFF:FFFFFFFFF
FFF
@A00721:190:HGJW7DSXY:4:1133:9191:26490_1:N:0:TCTGCAAG+GCTGCACT
CTTTACGATACTTTGTTATCAATGTAAAGTTAAACGATGCTACTTGTGAGAGGATCAATCACAGTAAG
GAACTGTCACGTTTAGCACGGGAGGTTGCAGATTCAACCCCTTATATCGAGGCTATCGAGGACGATGA
GTA
+
FFFFFFFFFFFFFFFFFFFFFFFFFFFFFFFFFFFFFFFFFFFFFFFFFFFFFFFFFFFFFFFFFFFFFF
FFFFFFFFFFFFFFFFFFFFFFFFFFFFFFFFFFFFFFFFFFFFFFFFFFFFFFFFFFFFFFFFFFFFFF
FFF

```
@A00721:190:HGJW7DSXY:4:1112:27263:2581_2:N:0:TCTGCAAG+GCTGCACT
GACGACAAGCCTCAAGAGGGTAGAGGACCACCTGGATCTTCTACTGTCTCTGCCTTCCAAGATTTCTT
AGAAGAGGTTATTCCTGGAACCACCTTAACGTCACTCGGTAATCTTTC
+
FFFFFFFFFFFFFFF:FFFF:FFFFFFFFFFFFFFFFFFFFFFFFFFFFFFFFFFFFFFFFFFFFFFF
FFFFFFFFFFFFFFFFFFFFFFFFFFFFFFFFFFFFFFFFFFFFFFFF
@A00721:190:HGJW7DSXY:4:2471:5945:23688_2:N:0:TCTGCAAG+GCTGCACT
TGAGTTCAAGTCGGTTGTCGATCGAATTAAGATCAAGGCTAATACCTTTATAGGTAAGAAGCCCCTTG
AGCCGCGTAGTCAGCGCGTTTAGCTGTATCATCAGTGAGATCGGAAGA
+
FFFFFFFFFFFFFFF:FFFFFFFFFFFFFFFFFFFFFFFFFFFFFFFF:F:FFFFFFFFFFFFFFFFF
FFFFFFFFFFFFFFFFFFFFFFFFFFFFFFFFFFFFFFFFFFFFFFFF
@A00721:190:HGJW7DSXY:4:1529:17002:33724_1:N:0:TCTGCAAG+GCTGCACT
TCCAAGAGAATATTTATTCTTATGAGGCTCGAAACTTCAACGTTTTCACAACGGAAAGACATATCGCA
CCCGAAATTTTTGGGCAGAATGCTCTTAACGCGGTTCTTGAACGGTGTTTTGATCAGTCTAAGTTGGA
TAA
+
FFFFFFFFFFFFFFFFFFFFFFFFFFFFFFFFFFFFFFFFFFFFFFFFFFF,FFFFFFFFFFFFFFFF
F:FFFFF:FF:FFFFFFFFFFFFFFFFFFFFFFFFFFFF:FFFFFFFFFFFFFFFFFFFFFFFFFFFF
FFF
@A00721:190:HGJW7DSXY:4:1306:4001:28087_2:N:0:TCTGCAAG+GCTGCACT
ACTCAGTTAACCTGAGAGCTCCTCAATTGATAGATTGGGTCAACAATAGGGACGCTAGTATACTTCGT
AAAGTCGACAGCGAAATGGTCAATATTGATAATTGGACCGACTATATG
+
FFFFFFFFFFFFFFFFFFFFFFFFFFF:FFFFFFFFFFFFFFFFFFFFFFFFFFFF:FFFFFFFFFFF
FFFFFFFFFFFFFFFFFFFFFFFFFFFFFFFFFFFFFFFFFFFFFFFF
@A00721:190:HGJW7DSXY:4:1369:1389:4867_1:N:0:TCTGCAAG+GCTGCACT
AGCTCATGGTGAAAAGGGAAGCTAAGGTTAAACTTGATGCTTCAGCCTTTTCGAAGCATAACCCCGCA
CAGAATATCGTTTATCACGCAAAATTTATTAATGCAATTTTCGGACCTATCTTTAAGGTTCTTGTGAG
GAG
+
F,FFFFFFFFFF:FFFFFFFFFFFF,FFFFF,FFFFFFF:FFFFFFF,FFFFFFFFFFFFF:FFFFFF
FFFFFFFFFFFFFFFFFFFFFFFF,FFFFFFFFFFFFFFFFFFFFFFFFFFFF:FFF:F:FFF:FFFFFFF
F,F
@A00721:190:HGJW7DSXY:4:1130:19425:12179_1:N:0:TCTGCAAG+GCTGCACT
AGCTCTTCTCTCTCTTAGGAGGTCATGATGACTACTATAAGGGTGAAATCGATTTTTCGAAGTTTGAC
AAATCTCAAGATTTCTTTTGTAAAGCCTATGAGATAAAGATGTATTCTTATTTAGGCTTCGATGCTGA
ATT
+
FFFFFFFFFFFFFFFF:FFFFFFFFFFFFFFFFFFFFFFF:FFFFFFFFFFFF:FFFFFFFFFFF:FFF
FFFFFFFFFFFFFFFFFFFFFFFFFFFFFFFFFFFFFFFFFFFFFFFFFFFFFFFFFFFFFFFFFFFF
FFF
@A00721:190:HGJW7DSXY:4:1104:30599:1031_2:N:0:TCTGCAAG+GCTGCACT
AGTGCCAGTGAATATTTTTGTCACGCCAGGAGTATCCATAACGATTTGTCATTTACCACCTTTGCTCA
ACGACGAAGTGGAACGGCCAACACTTTCTTTGGTAATTGTATGATCAC
+
FFFFFFFFFFF,FFFFFFFFFFFFFFFFFFFFFFFFFFFFFFFFFFFFFFFFFFFFFFFFFFFFFFFF
FFFFFFFFFFFFFFFFFFFFFFFFFFFF:FFFFFFFF:FFFFFFFFFFF
@A00721:190:HGJW7DSXY:4:1136:17137:35556_2:N:0:TCTGCAAG+GCTGCACT
TATCACTTTATTATGACCTTGGAAAGTTTTCGGCTGTGGTTGTCGCTGGGGACGATTCCATTCTGTTT
TCAAAGACTCCAATAACTGATCATTCTGATCATATGATTTTGGACAAT
+
FFFFFFFFFFFFFFFFFFFFFFFFFFFFFFFFFFFFFFFFFFFFF:FFFFFFFFFFFFFFFFFFFFFFF
FFFFF:FFF::FFFFFFFFFFFFFFFFFFFFFFFFF:F:FFFFFFFFFF
```

```
@A00721:190:HGJW7DSXY:4:1229:31792:36793_2:N:0:TCTGCAAG+GCTGCACT
ATCGTTAAGTTAGGTAAACCCCTAACCGATGTTACTCTTGTAGGACTTTGGGAGAGATTCGTTAGTTT
TAAAGACTATACTAAATATCTCCACTACGAGGTTGTTGTTTGGCGATT
+
FFFFFFFFFFFFFFFFFFFFFFFFFFFFFFFFFFFFFFFF:FFFFFFFFFFFFFFFFFFFFF:FFFFF
FFF:FFFFFFFFF:FFFFFFFFFFFFFFFFFFFFFFFFFFFF:FFFFF
@A00721:190:HGJW7DSXY:4:1256:31033:12962_2:N:0:TCTGCAAG+GCTGCACT
ATATTCGATATTCGATTGAGGGTCGTTATACCTATTCGGCTATCTGTGCTTTGAATTGTATAAGATCA
AATTTTAAACGATTCCGAGAGGTTTATCCTGATGTGGAGGGAGTTCTT
+
FFFFFFFFFFFFFFFFFFFFFFFFFFFFFFFFFFFFFFFFFFFFFFFFFFFFFFFFFFFFFFFFFF:F
FFFFFFFFFFFFFFFFFFFFFFFFFFFFFFFFFFFFFFF:FFFFFFFF
@A00721:190:HGJW7DSXY:4:1166:18159:5415_1:N:0:TCTGCAAG+GCTGCACT
ACCTTTCCTTGTGAAAATTTTGATGCGGTTTCATAAGATTATCAGTAATCCTGATTCTACTACGGATT
CATGGACCTTCCATCCTGTACCTCTTCTGGAGAGACGTATCTCAAAGATCGACTTCGAGACAGGTGAT
ATT
+
FFFFFFFFFFFFFFFFFFFFFFFFFFFFFFFFFFFFFFFFFFFFFFFFFFFFFFFFFFFFFFFFFFFF
FFFFFFFFFFFFFFFFFFFFFFFFFFFFFFFFFFFFFFFFFFFFFFFFFFFFFFFFFFFFFFFFFFFF
FFF
@A00721:190:HGJW7DSXY:4:1678:21621:15796_2:N:0:TCTGCAAG+GCTGCACT
CTAAATTCGGTAACGGTGCGTGGCGCTTTATTTTAAAAAGCTCCCTCCTCTCGCAAGTTTGATCTGAT
ACTTATCACGAAAGGACTTGAGTCTTTTGATCAGTCTCTGACTTGACC
+
FFFFFFFFFFFFFFFFFFFFFFFFFFFFFFFFFFFFFFFFFFFFFFFFFFFFFFFFFFFFFFFFFFFF
FFFFFFFFFFFFFFFFFFFFFFFFFFFFFFFFFFFFFFFFFFFFFFFF
@A00721:190:HGJW7DSXY:4:1543:15664:11115_2:N:0:TCTGCAAG+GCTGCACT
TAAATGACGATCATTAAGACTCTCTTTCTTCTTTGTTTCCTTCTTTGCACTGATTTTTCCTCATCAGC
TTGTGTTTTACCTAATCTGTGTTTTTACGATAACAACTATCAGTTGAT
+
FFFFFFFFFFFFFFFFFFFFFFFF:FFFFF:FFFFFFFFFFFFFFFFFFFFFFFFFFFFFFFFFFFFF
FFFFFFFFFFFFFFFFFFFFFFFFFFFFFFFFFFFFFFFFFFFFFFFF
@A00721:190:HGJW7DSXY:4:1112:32316:10582_1:N:0:TCTGCAAG+GCTGCACT
CCGAAAGGCGAACTTTGGGAATTTGAAGCTCAGGGTGAAGCACAACGTTTGACCGTGACGAACTGTGG
TGTTCTTGATCTACGTTACGGATATACTCTCCTTGAGTTCACTCGTAAGGACACTACCACTTATTACG
ATA
+
FFFFFFFFFFFFFFFFFFFFFFFFFFFFFFFFFFFFFFFFFFFFFFFFFFFFFFFFFFFFFFFFFFFF
FFFFFFFFFFFFFFFFFFFFFFFFFFFFFFFFFFFFFFFFFFFFFFFFFFFFFFFFFFFFFFFFFFFF
FFF
@A00721:190:HGJW7DSXY:4:2161:6470:15890_1:N:0:TCTGCAAG+GCTGCACT
TCTCTATTTTAAGTTTTGTCTTCCTTGTTTGTCTTATTCTATTAATCACTCATTTTAGTGAGATTATT
AAAAACGCACTAAAACGTCTTCGACAACAGCAGGGGTTTTCGAATAGTGATAGCCACCCGCCTTACAG
AGG
+
FFFFFFFFFF:FF:FFFFFFFFFFFFFFFFFFF:FFFFFFFFFFFFFFFFFFFFF,FFFFFFFFFFFF
FFFFFFFFFFFFFFFFFFFFFFFFFFFFFFFFFFFFFFFFFFFFFFFFFFFFFFFFFFFFFFFFFFFF
FFF
@A00721:190:HGJW7DSXY:4:1314:26241:26365_1:N:0:TCTGCAAG+GCTGCACT
GACTTTGGTACCACCTTTTCTACTGTCTGTCTCGCCTCTTCGAATGAAGTTTTAATGCTTAACAACAA
TGATAGTGAATTTATACCTACAATCATTGGTTTTTCTAATAATGATGACACTATTGTTTATGGATATG
ATG
+
FFFFFFFFFFFFFFFFFFFFFFF:FFFFFFFFFFFFFFFFFFFFFFFFFFFFFFFFFFFFFFFFFFFF
```

FFFFFFFFFFFFFFFFFFFFFFFFFFFFFFFFFFFFFFFFFFFFFFFFFFFFFFFFFFFFFFFFFFFFFF
FFF
@A00721:190:HGJW7DSXY:4:2552:7274:33912_1:N:0:TCTGCAAG+GCTGCACT
TGGTTACGGTCAGGGAGGATTCTCAGTTTACAAGGACCTTAAGCGATGGATCGGGGTTAATTCTAAAA
CATTACCCGAAAGACGTGCTAAATTAAAACCATTGTACGATGTCGTCTGTCTCGGCGATCACTTTTCA
CTA
+
FFFFFFFFFFFFFF:FFFFFFFFFFFFFFFFFFFFFFFFFFFFFFFFFFFFFFFFFFFFFFFFFFFFFF
FFFFFFFFFFFFFFFFFFFFFFFFFFFFFFFFFFFFFFFFFFFFFFFFFFFFFFFFFFFFFFFFFFFFFF
FFF
@A00721:190:HGJW7DSXY:4:1325:1325:27305_1:N:0:TCTGCAAG+GCTGCACT
AGACGTGTGCTCTTCCGATCTATCGTTTCTTTCTTTATCTCTTTGGTTGTTAGGGATTTTGAGAGAGT
GAAAGGCTTCAATTGTAGTGGTTTGGTCGTTTCAGTACCCAGTCAGTATACCTCTACGCAAAGATATT
TCA
+
FFFFFFFFF,:FFFFFFFFFFFFF:FFFFFFFFFFFFFFFFFFFFFFFFFFFFFFF:FFFFFFFFFFFF
FFFFFFFFFFFFFFFFFFFFFFFFFFFFFFFFF:FFFFFFFFFFFFFFFFFFFFFFFFFFFFFFFFFFFF
FF:
@A00721:190:HGJW7DSXY:4:1669:3522:8844_2:N:0:TCTGCAAG+GCTGCACT
GAGGAACGTACCGGATTACCTGTCTTTCATATTATGAATGAACCTTCGGCCGCTCTCTTTGCTTCGAT
GTTGGATGTCAAGAAGGGTTCAGAATGGGATTCCTACGTAGTTTATGA
+
FFFFF:FFFFFFFFFFFFFFFFFFFFFFF:FFFFFFFFFFFFFFFFFFFFFFFFFFFFFFFFFFFFFFFF
FFFFFFFFFFFFFFFFFFFFFFFFFFFFFFFFFFFF:FFFFFFFFF:FFF
@A00721:190:HGJW7DSXY:4:2106:9182:33270_1:N:0:TCTGCAAG+GCTGCACT
TACTTTCGATACTTCTATCGTCTTACGTTACGGTAATTACTATAGTGTTATTTATTCTGGTGGTGATG
ATAATCTTGGAGGTCGTGACGTTGATGAAGCTATAAGAGCGTTCTTATTACGGCGCACTTCTTTACCC
GCC
+
FFFFFFFFFFFFFFFFFF:FFFFFFFFFFFFFFFFFFFFFFFFFFFFFFFFFFFFFFFFFFFFFFFFFFF
FFFFFFFFFFFFFFFFFFFFFFFFFFFFFFFFFFFFFFFFFFFFFFFFFFFFFFFFFFFFFFFFFFFFFF
FFF
@A00721:190:HGJW7DSXY:4:1109:30138:9815_2:N:0:TCTGCAAG+GCTGCACT
GTTTCACAACTGAAGGAGGAGGTTTCTGTTACTGGAAAACCCTCGTCAATACTTTTCAATAATATACG
TCTTGAACTCACTTATGACGATCTAGTGAAATTAACGCGTCCTTTTCT
+
FFFFFFFFFFFFFFFFFFFFFFFFFFFFFFFFFFFFFFFFFFFFFFFFFFFFFFFFFFFFFFFFFFFFFF
FFFFFFFFFFFFFFFFFFFFFFFFFFFFFFFFFFFFFFFFFFFFFFFFFF
@A00721:190:HGJW7DSXY:4:1338:14344:35289_1:N:0:TCTGCAAG+GCTGCACT
TACTATTATGAACAAAGTTATCAAAGATAGTGGCATTCAAGGCAAACTTACCCTGATTCCGGCCGGTG
GTAGCTCATTTCTACCAGGGGCTATTACTCTTGCTGAAACAGCTATTAAAGCTGTTGAGCGGGTAATT
ACA
+
FFFFFFFFFFFFFFFFFFFFFFFFFFFFFFFFFFFFFFFFFF:FFFFFFFFFFFFF:FFFFFFFFF,F
F:FFFFFFFFFFFFFFFFFFFFFFFFFFFFF:FFFFFFFFFFFFFFFFF,FFFFFF:FFFFFFFFFFFF
FFF
@A00721:190:HGJW7DSXY:4:1562:18502:15875_2:N:0:TCTGCAAG+GCTGCACT
ATCGCTGAAGGATGTGCACTCTTTTCTGCTACTTTAACGGAGCGTAGTGTACTCTTCGTCGATTGTAT
CAACAGTAACATTACTTCTAATAAAGGTCTCTATCAAGCTCGTATTAT
+
FFFFF:F:FFFFFFFFFFFFFFFFFFFFFFFFFF:FFFFFFFFFFFFFFFFFFFFFFFFFFFFFFFFFFF
FFFFFFFFFFFFFFFFFFFFFFFFFFFFFFFFFF,FFF:FFFFFFFFFFFF
@A00721:190:HGJW7DSXY:4:1530:10059:14559_1:N:0:TCTGCAAG+GCTGCACT
TTACCGTTGAAGGCTAAACTCCAAGGTAATTATGAAGACTGTGATTTATCTTTCACTCTGGCCTTGTA

TGAGGGTGTTTCTAATCGTAACATTAATAATTTCAATCTTCTTAAAAAGAAGATAGAGAGTCATACTT
TGG
+
FFFFFFFFFFFFFFFFFFFFFFFFFFFFFFFFFFFFFFFFFFFF:FFF:FFFFFFFFFFFFFF:FFFF
FFFFFFFFFFFFFFFFFFFFFFFFFFFFFFFFFFFFFFFFFFFFFFFFFFFFFFFFFFFFFFFFFFFF
FFF
@A00721:190:HGJW7DSXY:4:1361:32877:25426_2:N:0:TCTGCAAG+GCTGCACT
TGAAGTGGACACCTCTGGTAGTATCGAAGTAAAGATAACCAATAAGGAAGGAGCTTATACTATGGTTG
AATCAACTAAGCATACTACTTTTGCTTGGATTACTCTACCGCCTTACA
+
FFFFFFFFFFFFFFFFFFF,FFFFFFFFFFFF:FFFFFFFFFFFFFFFFFFFFFFFFFFFF:FFFFF,
FFFFFFFFFFFFF,FFFFFFFF:FFFF,FFF:F,,FFFF:FFFF:FFF
@A00721:190:HGJW7DSXY:4:2510:25346:36683_2:N:0:TCTGCAAG+GCTGCACT
TAAAGAGTCGATTAAACAAGTTGTACTTATCAATGATTTTATCGCTGGTTGTTATCTCGAAAAGTCTC
TTCTAAAAGGAACGTCAGTTCTAACCCAAAGAGAATATCTAAAATATT
+
FFFFFFFFFFFFFFFF,FF,FF,F:,:FFF,FF::FFFFFFF,F,::FFF:FF,F,F,FFFFF,F,:,
:F,FFFFF,F,FF,F,F,,F,:FF:FFFFF,F,FF,::,:FFFF,F:F
@A00721:190:HGJW7DSXY:4:2655:27670:15718_1:N:0:TCTGCAAG+GCTGCACT
TACCACTATCTACGAACCTAAAGTTCTGAAGACGTTGCTTGTCGCTTCCTCCTTTAATGGCACTACCT
CGGTACAATCTTACTTGGAACGAAGGCGACCTGTTCCGGAGTTTTTACGGGATCCGGAATGTTCTGTC
TAT
+
FFFF::FFFFF,FFFFFFFFFFFFFFFFFFFFFFFFFFFFFFFFF,FFFFFFFFFFFFFFFFF:FFFF
FFFFFFFFFFFFFFFFFFFFFFFFFFFFFFFFF:FFFFFFFFFFFFFFFFFFFFFFFFF:FFFFFFFF
FFF
@A00721:190:HGJW7DSXY:4:2528:28655:31109_1:N:0:TCTGCAAG+GCTGCACT
AATACGATTTCGAACGTCAACGTTCACCGAGTTTCGTTTCGTACCTTGATGCTGACTTTCGTCAATAT
GATTTGATCTGGTTGCTCAATTCTAAGCGTGTTCCGCTTAGTGCGAGGCCTCTCTTCTACGAGTATTG
CTA
+
FFFFFFFFFFFFFFFF:FFFFFFFFFFFFFFFFFFFFFFFFFFFFFFFFFF:FFFFFFFFFFFFFFFF
FFFFFFFFFFFFFFFFFFFFFFFFFF:FFFF:FFFFFFFFFFFFFFFFFFFFFFFFFFFFFFFFFFFF
FFF
@A00721:190:HGJW7DSXY:4:1440:29017:23062_2:N:0:TCTGCAAG+GCTGCACT
ATATTTGGATGATCTTGATAAAGAACTTCATGAATTTTCAGAGGATGAAAAATTTTCTTCCTTGGATT
TTAATATAAAGAATCTTTACCAGGACTCCTACTACAGGAGGCAGTCTT
+
F,FF,,:FFF,FFF:,FFFFFFFFFFF,:FFFFFFF,,FF:F::F:,FFFFFF,,F,F,FF,FF:FFF
FFFFFF,FFF,FF,F,,FFF,F,,F,:,,,FF,FFF,FF::,F,FF,F
@A00721:190:HGJW7DSXY:4:2273:26530:32690_1:N:0:TCTGCAAG+GCTGCACT
ATTTGTTGTAATAGGGTCATCAACTTAGAACTCGAAACTCTAAGTGGTAATACTGTCTTTCCAAGCTT
ACCTGTATCTTCAATGAAATTGGACTCTTCGATAGGGGACATTTTATCGTGGGCTTTCCGTAAAGGAA
GAT
+
FFFFFFFFFFFFFFFF:FFFFFFFFFFFFFFFFFFFFFFFFFFFFFFFFFFFFF:FFFFFFFFFFFF
FFFFFFFFFFFFFFFFFFFFFFFFFFFFFFFFFFFFFFFFFFFFFFFFFFFFFFFFFFFFFFFFFFFF
FFF
@A00721:190:HGJW7DSXY:4:2303:6958:29262_1:N:0:TCTGCAAG+GCTGCACT
CTACTAATGGCGTTGGCCTAGTGGATGTTATTAGTGACATCTGGTCAATTTCGTTTTTAGGTGGTCTT
TATTCTATTATTGATTTTTATGAGATTGATACGTTAACAGAAGATGATGTTAGAGTAGAATTCCTGAA
GAG
+
:FFFFFFFFFFFFFFFFF:FFFFFFFFFFFFFFFFFFFFFFFFFFFFFFFFFFFFFFFFFFFFF:FFFFF

```
FFFFFFFFFFFFFFFFFFFFFFFFFFFFF:FFFFFFFFFFFFFFFFFFFFFFFFFFFFFF:FFFFFFF
FFF
@A00721:190:HGJW7DSXY:4:2246:8350:30671_2:N:0:TCTGCAAG+GCTGCACT
CCAACTTCTTGACAATTTTCTTCAATCCTTCGATAATAGTTAAATCTAGGATTATGTCTGTGTTGAAA
GACTTTCTACCGATAAGGGTCACTAGTCTTACACAATTAAGAAATAAT
+
FFFFFFFFFFFF,FFFFFFFFFFFFFFFFFFFFFFFFFFFFFFFFFFFFFFFFFFFFFFFFFFFFFFF
FFFFFFFFFFFFFFFFFFFFFFFFFFFFFFFFFFFFFFFFFFFFFFFF
@A00721:190:HGJW7DSXY:4:1243:28420:14700_2:N:0:TCTGCAAG+GCTGCACT
ACTATCTAAAGATTGAAGGAAAATTTTATCCTCGTTTAACTTACTTAGACTTTCCTACGATACAAATG
ATAACTTCTAATTTACGTTTGCTACTCAGAGCTGGTCCTCTCAGCAAA
+
FFFFFFFFFFFFFFFFFFFFFFFFFFFFFFFFFFFFFFFFFFFFFFFFFFFFFFFFFFFFFFFFFFFF
FFFF:FFFFFFFFFFFFFFFFFFFFFFFFFFFFFFFFF:FFFFFFFFFF
@A00721:190:HGJW7DSXY:4:2422:11026:17174_2:N:0:TCTGCAAG+GCTGCACT
TCCTCAGGAGTCCCGGCATTTGATAATAATGACGACTTTCGTCATAATCGCTGCATATTACGGATTAC
ACGGTACCAGCGCATCTCGTGTCGAGCCTCGACCCGATTATATGGTAT
+
FFFFFFFFFF:FFFFFFFFFFFFFFFFFFFFFFFFFFFFFFFFFFFFFFF:FFFFFFFFFFFFFFFFF
FFFFF:FFFFFFFFFFFF:FFFF:FFFFFFFFFFFFFFFFFFFFFFFFF
@A00721:190:HGJW7DSXY:4:2654:9525:30076_2:N:0:TCTGCAAG+GCTGCACT
AGAATTCTTAATTATAATATTCAACCTCAAAAGTTACGTGTTTACAGTAACGAGATCACACATTTTGA
GAACTAACTATGTTATGTTCTCTGATCATTTTGACAATCTCTTCTTAT
+
FFFFFFFFFF:FFFFFFFFFFFFFFFFFFF:F:FFFFFFFFFFFFFFFFFFFFFFFFFFFFF:FFFFF
FFFFFFFFFFFFFFFFFFFFFFFFFFFFFFFFFF:FFFFFFFFFFFFF
@A00721:190:HGJW7DSXY:4:1156:16206:7294_1:N:0:TCTGCAAG+GCTGCACT
TAAATAATATTACTAATGGCAAATAGTGGAAAGGCTCCAGTCAACACTAGTTCGAGTGCTGCTTTGGA
TTACGATGACGTGAGAATAGGTGAAGGGATAGTTTTTACCTCGACCAACATTAAGGCGGCGGCGACTA
ATC
+
FFF:FFFF:FFFFFFFFFFFFFFF:FFFFFF:FFFFFFFFFFFFFFFFFFFFFFFFFFFFFFFFFFFF
:FFFFF:FFFFFFFFFFFFFFFFFFFFFFFFFFFFFFFFFFFFFFFFFFFFFFFFFFFFFFFFFFFFF
FFF
@A00721:190:HGJW7DSXY:4:2433:9155:25426_1:N:0:TCTGCAAG+GCTGCACT
AGGACACTTCGAAACTGAATAATGATGCACTACAAACAAGTATCGCTAATCATCTGAAAAAGGCTCTA
AAGGACACTATACAATATCCAGACGACGACGAAGCTTATACTACTTTAATTTTGGGTATTATGTGGAG
ATA
+
FFFFFF:FFFFFFFFF:FFFFFFFFFFFF,FFFFFFFF::FFFFF,FFFFFFFFF:FFFFFFFFFFFF
FF:FFFFFFFFFFFFFFFFFFFFFFFFFFFFFFFFFFFFFFFFFFFFFFFFFFFFFFFFFFFFFFFFF
FFF
@A00721:190:HGJW7DSXY:4:2507:17146:36198_2:N:0:TCTGCAAG+GCTGCACT
ACCACATTTTGGTCAAGTCAATCCGTTGAGAAAATTTCTTAGAGGATATTCGCCATTGTGGTACGAAT
TGGTCCAACATTTACCACCAAATTCTTTTGATACAACTCTAGCCTTCA
+
FFFFFFFFFFFFFFFFFFFFFFFFFFFFFFFFFFFFFFFFFFFFFFFFFFFFFFFFFFFFFFFFFFFF
FFFFFFFFFFFFFFFFFFF,FFFFFFFFFFFFFFFFFFFFFFFFFFFF
@A00721:190:HGJW7DSXY:4:1603:1976:34194_1:N:0:TCTGCAAG+GCTGCACT
ACCAGATTGGTTCGAACCCGTTGATGCTATGTCAGAAGAAGCTATTCAAGCTTGTCGTTTAAAGACTG
CAGAGGCTGTCAAACAAGCCCAGACTCAAGGAGTCCAATCACAGGTTTTGAATTTAGCTCAACTGGGG
AGA
+
FFFFF:FFFFF:FFF,FFFF::F,FFFFFFFF:FFFFFFFF:FFFFFFFFFFFFFFF:FFF:F:FFFF
```

FFFFFFFFFFFF:,:FF:FFFFFFFFF:FFFFFFFF:FF:F,FF:FFFFFF:FFFFFFFF:,FFFFFF
FFF
@A00721:190:HGJW7DSXY:4:1608:3405:34882_1:N:0:TCTGCAAG+GCTGCACT
CTTGCAGAGTGACTGGAGTTCAGACGTGTGCTCTTCCGATCTGAAAAGTCCGGAGTATATTGTATGTT
AATAGATAGTGGGATAATAGTTCTACCGATACGTAGTGAACGCGTTCGTAGAACCAAGTACTTTCCGT
CTG
+
:F:FFFFFFFFFFFFFFFFFFFFFFFFFFFF:FFFFFFFFFFFFFFFFFFFFFFFF,FFFFFFFFFF
FFFFFFFFFFFFFFFFFF:FFFFFFFFFFFFFFFFFFFFFFFFFF:FFFFFFFFFFFFFFFFFFFFFFF
FFF
@A00721:190:HGJW7DSXY:4:1668:18656:29105_1:N:0:TCTGCAAG+GCTGCACT
ACCGATAGGAGTTGTATAGTTGAAGATGTGGCTTTAGACGATAATCCTTTAAGGACTATGTTGACTGA
CGAAGATTTTCCCACAGATTTGTCTACGTTGCAGATCGCCACTCCAAACACTATATACATTATAAAAT
ATA
+
FFFFFFFFFFFFFFFFFFFFFFFFFFFFFFFFFFFFFFFFFFFFFFFFFFFFFFFFFFFFFFFFFFFF
FFFFFFFFFFF:FFFFFFFFFFFFFFFFFFFFFFFFFFFFFFFFFFFFFFFFFFFFFFFFFFFFFFFF
FFF
@A00721:190:HGJW7DSXY:4:1515:25473:14481_1:N:0:TCTGCAAG+GCTGCACT
ATAACTTTTGTTACCAAATTCTTGGCTTTCAACGAAGTCTCTGATTTTATTAATCATTACTTTAACGA
TCTCTCGGTCATTTACTCTGTACTTGATCTACGTCGTTTAACTGTCGGAACAACCTTTACTTAGAATG
ACT
+
FFFFFFFFFFFFFFFFFFFFFFFFFFFFFFFFFFFFFFFFFFFFFFFFFFFFFFFFFFFFFFF:FFFF
FFFFFFFFFFFFFFFFFFFFFFFFFFFFFFFFFFFFF:FFFFFFFFFFFFFFFFFFFFFFFFFFFFFFF
FFF
@A00721:190:HGJW7DSXY:4:1629:15004:27602_2:N:0:TCTGCAAG+GCTGCACT
TCTTATATTATTTACAATAGTTAATATCATATTAGTAATACTCACTATCGTTTTCTGCTTTTTCTTAG
CTAATAGATTAATAAAGGATGAGTACGAAAACCAACTCTTCGTTACGA
+
FFFFFFFFFFFFFFFFFFFFFFFFFFFFFFFFFFFFFFFFFFFFFFFFFFFFFFFFFFFFFFFFFFFF
FFFF,FFFFFFFFFFFF:FFFFFFFFFFFFFFFFFFFFFFFFFFFFFF
@A00721:190:HGJW7DSXY:4:1371:31656:1892_1:N:0:TCTGCAAG+GCTGCACT
TATAATAGATTTAGTCTCTACACCCATCTCACCGAGTATAAAGCGTGTTTTCCGTCCTGAACGTAATA
CACGAAGATATCATCCGCAAACGACTCGAAACCGAGACGCCGTAGATCAAAGTCCCTTCTAACTTTAG
ATT
+
FF,F:F,,:FFF,,FFF,:FFFFF,F,F:,F,F:,F,FFFFF::,,::F,:FFF,FFF:F:,,,FF,,
:,F:FF:F,F:FF,FF,FFFFFFFFFFFFFFFFFFFFFFFFFFFFFFFFFFFFFFFFFFFFFFFFFFFF
FFF
@A00721:190:HGJW7DSXY:4:1208:26702:21621_2:N:0:TCTGCAAG+GCTGCACT
CAGTTATTTATGTTGTACTTTTTATAATTTATAACCTTCTTATACTTTTGGGTATGTTTTGTGAAAAA
CATGAGATACCCAAGTCTATCGAAGACATTATCTCTTATGTACTTATT
+
FFFFFFFFFFFFFFFFFFFFFFFFFFFFFFFFFFFFFFFFFFFFFFFFFFFF,FFFFFFFFFFFFFFF:
FFFFFFFFF:FFFFFFFFFFFFFFFFFFFFFFFFFFFFFFFFFFF,FFFFF
@A00721:190:HGJW7DSXY:4:1513:9878:14528_2:N:0:TCTGCAAG+GCTGCACT
TATTCCTTTAACTGTGTATGAGGAGGATTTATAATGCTTTTCGATAGTGATTGTTATCAATTTAAATA
CATTACTGTTTCAAATCATCTTTACTCACAGACTCCGAAAATGGTTTC
+
FFFFFFFFFFFFFFFFFFFFFFFFFFFFFFFFFFFFFFFFFFF:F:FFFFF:FFFFFFFFFFFFFFFFF
FFF:FFFFFFFFFFFFFFFFFFFFFFFFFFFFFFFFFFFFFFF:FFFFFF
@A00721:190:HGJW7DSXY:4:1270:25138:10676_1:N:0:TCTGCAAG+GCTGCACT
TATTTATTAAACGAACTATTATCCGATTCTTCGAGGGTATCACTACCACTGATCTGCCGACTTTAGTC

```
AAGGAACTTCAATTCCTTCGATGTTTATATTTTTCTATAAGGGAAAATAGCAATAATCAAATTTCGAC
AAA
+
FFFFFFFFFFFFFFFFFFFFFFFFFFFFFFFFFFFFFFFFFFFFFFFFFFFFFFFFFFFF:FFFFFFF
FFFFFFFFFFFFFFFFFFFFFFFFFFFFFF:FFFFFFFFFFFFFFFFFFFFFFFFFFFFFFFFFFFFF
FFF
@A00721:190:HGJW7DSXY:4:1427:31114:34773_2:N:0:TCTGCAAG+GCTGCACT
ATTTCAGAACTCTCTCCGATCACCTTGGTGTGATCTCCCGGAGGATCTTTTCAATAAGATGTATGATG
ATAATCTACTATTTTTAAATAGTGATATTCAGCGTATACTTGAATACT
+
FFFFFFFFFFFFFFFFFFFFFFFFFFFFFFFFFFFFFFFFFFFFFFFFFFFFFFFFFFFFFFFFFFFF
FFFFFFFFFFFFFFFFFFFFFFFFFFFF:FFFFFFFFFFFF:FFFFFFF
@A00721:190:HGJW7DSXY:4:2305:6677:1752_1:N:0:TCTGCAAG+GCTGCACT
CTTGGTCTATTTACCGCAAAAAGTTGGAAGAGGTCTATGATATTTATTTTACTTATACTTTAAATAGC
ACCGATTATAACTTGACTGTTGATAATATTACAATAGCTCTTTCTCAGAAGATTAGAGAAGTAACCGG
TGA
+
FFFFFFFFFFFFFFFF,FFFFFFFFFFFFFFFFFFFFFFFFFFFFFFFFFFFFFFFFFFFFFFFFFFF
FFFFFFFFFFFFFFFFFFFFFF:FFFFFFFFFFFFFFFFFFFFFFFFFFFFFFFFFFFFFFFFFFFFF
FFF
@A00721:190:HGJW7DSXY:4:2158:15167:9596_2:N:0:TCTGCAAG+GCTGCACT
CATTGATTTACATTTACCGTTAGAACTGAAAAAGATTCTTTTCGAAAAGAAACCTATAAGTTACAAAC
GATTCTTCGACTATATTAAAGAAATAAAAATACTTCAAGTACCTGAAC
+
FF,FF:FFFFF,FFFFFFF,FF,FFFFFFFFFFFFFFFFFFFFFFFFFF:FFFFFFFFFFF,FFFFFFF
FFFFFFFF,F:FFFFF:FFFFFFFFFFFFFFFFFFFF::FFFFFFFFF
@A00721:190:HGJW7DSXY:4:2440:10529:28338_1:N:0:TCTGCAAG+GCTGCACT
ATGGTATAATGTGTCTTACAAATAGAACCTTCACTCTCGGTAATGAGAGTGTATTCCTTAATATTCTA
GTAACCGCTATGTGGGCATTACTAGGATTTATCACACTCCTAAATTTGATAGCAATTTATGTTTTAAA
TGC
+
FFF,FFFF,FFFFFFFFFFF:FFF:F:,FFFFFFFFFF:FFFFFFFFFFFFFFFFFFFFFFFFFFFFF
FFFFFFFFFFFFFFFFFFFFFFFFFF:FFFFFFFFFFFFFFFFFF,F:FFFFFFFF,:FFFFFFFFFF
FFF
@A00721:190:HGJW7DSXY:4:2171:17562:30655_2:N:0:TCTGCAAG+GCTGCACT
AAAAACTCCATTGTATAGTTCAAGGAGTACTTAATTCAATAGCTTTAGTATTATTTACTATCGCCTTT
ATTGAAAATTGTATAAGAGTTAATTGCTTACACAAAAGTGACGCGCGA
+
FFFFFFFFF:FFFFFFFFFFFFFFFFFFFFFFFFFFFFFFFFFFFFFFFF:FFFFFFFF:FFFF:FFF
FFFFFFFF:FFFFFFFFFFFFFFFFFFFFFFFFFF,FFFFF:FFFFFFF
@A00721:190:HGJW7DSXY:4:1173:15302:31250_2:N:0:TCTGCAAG+GCTGCACT
ACCATAACCTACCATGACCAATCATCGCTCCAACTGGCACGCCAGACGTAGGGTCAAACCTACTACCG
CCGGTCGGCAGATCGGCACCACTGGTCTCTACGTTCAGGGACCCGCTG
+
FFFFFFFFFFFFFFFFFFFFFFFFFFFFF:FFFFFFFFFFFFFFFFFFFFFFFFFFFFFFFFFFFFFF
FFFF,FFFFFF,FFFFFFFFFFFFFFFFFFFFFFFFFFFFFFFFFFFF
@A00721:190:HGJW7DSXY:4:2318:26494:22764_1:N:0:TCTGCAAG+GCTGCACT
CCTTCCCAAATCTAGATCTGCACAGAAGAAGGCTGAAGGTCGTTACCGGACGACCACTCAAAGGAAAC
GGGCTGTTGCCCAGAGGAAAGCAGCGACCACCGAGGTCGCTCGTAACATTGACGGCAGTCCCGTCATC
TCT
+
FFFFFFFFF,FF:FFFFFFFFFFFFFFFFFFFFFFFFFFFFFFFFFFFFFFFFFFFFFFFFFFFFFFF
FFFFFFFFFFFFFFFFFFFFFFFFFFFFFFFFFFFFFFFFFFFFFFFFFF:FFFFFFFFFFFFFFFFF
FFF
```

```
@A00721:190:HGJW7DSXY:4:1536:30291:33943_2:N:0:TCTGCAAG+GCTGCACT
CAGTACCTCAGTCAAAAGTCACATTCACCACTCCCATACCAATCAATGATGTGACTATACAGTTCGGC
CATTTCAAGAGTGGTCAATACGTCGTGGGAGGTACTGAAGCCTTCCCC
+
FFFFFFFFFFFFFFFFFFFFFFFFFFFFFFFFFFFFFFFFFFFFFFFFFFFFFFFFFFFFFFFFFFFF
FFFFFFFFFFFFFFFFFFFFFFFFFFFFFFFFFFFFF:FFFFFFFFFF
@A00721:190:HGJW7DSXY:4:1657:24523:33098_2:N:0:TCTGCAAG+GCTGCACT
AAGCCAATCAATAGTTCTTCTGACCAGAATGAAAAGGGGTTTACCAGGGTAGTCTCTAAGTCTACCAG
GAAGAGGGGAAATGTCCCCAAAACTGAGAAGAATAAAGGGAAAGGTCC
+
FFFFF:FFFFFFFFFFFFFFFFFFFFFFFFFFFFF:FFFFFFFFFFFFFFFFFFFFFFFFFFFFFFFF
FFFFFFFFFFFFFFFFFFFFFFFFFFFF:FFFFFFFFFFFFFFFFFFF
@A00721:190:HGJW7DSXY:4:1332:10312:14873_2:N:0:TCTGCAAG+GCTGCACT
GGGAAATTACCCCTAATTCTGAAGGAAGATCTCCCACCATCTGGGGTTGGGAATAAGAAATCTGCACG
GGCCAAAGAGGCCGCTGCTACGAAGTCGCTGAAGGCGGCTTGGGTCAT
+
FFFFFFFFFFFFFFFFFFFFFFFFFFFFFFFFFFFFFFF:FFFFFFFFFFFF,FFFFFFFFFFFFFFF
FFFF:FFFFFFFFFFFFFFFFFFFFFFFFFFFFFFFFFFFFFFFFF:F
@A00721:190:HGJW7DSXY:4:2520:32796:27884_2:N:0:TCTGCAAG+GCTGCACT
TTGCGCCTGCCGACCCCATCTTTGAGACCAAGACTCTCTACAGTAGGTTCGTGCCTACTATGAGCAAA
TCTGTTGAATTCGTCACTGAGGACGAAGGTGAGAGGACTTTTGGCTAC
+
FFFFFFFFFFFFFFFFFFFFFFFFFFFFFFFFFFFFFFFFFFFFFFFFFFFFFFFFFFFFFFFFFF,F
FFFFF:FFFFFFFFFFFF:FF:FFFFF:FFFFFFFFFFFFFFFFFFFF
@A00721:190:HGJW7DSXY:4:1470:4264:20337_2:N:0:TCTGCAAG+GCTGCACT
CGTGGGTTGAAGGCCGACGCTTCGAGCTTGAGTGTGACGAGGCCGGTGACGTTTTTGTCAACGGCGTT
TGGAGGATTTTATCCGCGCTTAACTATGGGGCGAAGATCCCCTATGGA
+
FFFFFFFFFFFFFFFFFFFFFFFFFFFFFFFFFFFFFFFFFFFF:FFFFFFFFFFFFFFFFFFFFFFF
FFFFFFFFFFFFFFFFFFFFFFFFFFFFFFFFFFFFFFFFFF,FFFFF
@A00721:190:HGJW7DSXY:4:2168:26241:20478_2:N:0:TCTGCAAG+GCTGCACT
TTGGCATATTTATGCCGACGATAATAAGCATGTCACCCTTAAGCACCGAGGTAAGCTCGGGACTTACA
AGAGAGAGTGTGAGAGAGTCGCTCTCACTATTTTTGCCCAGAATGCGG
+
FFFFFFFFFFFFFFFFFFFFFFFFFFFFFFFFFFFFFFFFFFFFFFFFFFFFFFFFFFFFFFFFFFFF
FFFFFFFFFFFFFFFFFFFFFFFFFFFFFFFFFFFFFFFFFFFFFFFF
@A00721:190:HGJW7DSXY:4:1122:6912:7545_2:N:0:TCTGCAAG+GCTGCACT
ATTACAACCTCTCTTTAGAGAGCATTAGAAGGAAGTTGGGCAAATTCCCACATATGGTAGACGTTGTC
ACACTTTTCTTGAAAATTTTTCCTTTCCTCCGTCATTGGGGATTTCGA
+
FFFFFFFFFFFFF:FFFFFFFFFF:FFFFFFFF,FFFFFFFFFFFFFFFFF:FFFFFF:,FFFFFFFF
FFFFFFFFFFFFFFFFFFFFFFFF:FFFF,:::FF:F:,FF:F:FFFF
@A00721:190:HGJW7DSXY:4:1138:24695:8876_1:N:0:TCTGCAAG+GCTGCACT
GGAAGGTGTCAAGGGTCTTCTTCATGCTGATCTTTCAGGACCTAATCACATCATGATTGATAGTTACG
GCTCGTATCGAGTTGGAGGGAGGAAGTTCTCCGAAATTAAAGACGGGGGGATTAAGAGTCCAGCTGTA
ACT
+
FFFFFFFFFFFFFFFFFFFFFFFFFFFFFF:FFFFFFFFFFFFF,FFFF:FFFFFFFFFFFFFFFFFF
FFFFFFFFFFFFFFFFFFFFFFFFFFFFFFFFFFFFFFFFFFFFFFFFFFFFFFFFFFFFFFFFFFFF
FFF
@A00721:190:HGJW7DSXY:4:1158:12201:12477_2:N:0:TCTGCAAG+GCTGCACT
AGAGGGGAGCTCTGAAAAGGAAACTTCGTCAGAAGATGAGGAAGTCTAAGAGGAGGAGTTTGAGGAAG
GCTCTAGAGAAGATCTCACCACCTGGAGAAGGCAAGTGGAGAGTCCAG
+
```

FFFFFFFFFFFFFFFFFFFFFFFFFFFFFFFFFFFFFFFFFFF,FFFFFFFFFFFFFFFFFFFFFFFF,F
FFFFFFFFFFFFFFFFFFFFFFFFFFF:FF:FFFF:FFFFFFFFFFFFFF
@A00721:190:HGJW7DSXY:4:1258:4824:35368_1:N:0:TCTGCAAG+GCTGCACT
CTTCCGATCTTTTGAAGCAGGGGTTACCACTTCCTAAGGTGGATGAGGGAGGTTGTTTCTTTGGAGTT
TTAGATAATCTTTTGAGAAGGGGTCATAGAGCCAGCAAGAGAAGCTTATATGCTGGTTTAAGTGATGA
TTA
+
FFFFFFFFFFFFFFFFFFFFFFFFFFFFFFFFFFFFFFFFFFFFFFFFFFFFFFFFFFFFFFFFFFFF
FFFFFFF:FFFFFFFFFFFFFFFFFFFFFFFFFFFFFFFFFFFFFFFFFFFFFFFFFFFFFFFFFFFF
FFF
@A00721:190:HGJW7DSXY:4:1414:14986:35180_1:N:0:TCTGCAAG+GCTGCACT
ATTTAGGTCAACTTGGGGTTTCGAATGTCATTAGTGCCTTACGATTGAAAGAGGTCCTTTTGAGTATC
GACAGATCTTTCGATCTTGAGGACGTACATTTAACGTGGCAGAATTCGGTAACTTACCATATTGTTCC
TTC
+
FFFFFFFFFFFFFFFFFFFFFFFFFFFFFFFFFFFFFFFFFFFFFFFFFFFFFFFFFFFFFFFFFFFF
FFFFFFFFFFFFFFFFFFFFFFFFFFFFF:FFFFFFFFFFFFFFFFFFFFFFFFFFFFFFFFFFFFFF
FFF
@A00721:190:HGJW7DSXY:4:1146:2835:5776_1:N:0:TCTGCAAG+GCTGCACT
TTTAACTTTGACTCAACTACTCCGATCAGATCCGGATGGTTTGATTGGTATGGACTACTCGACTTTTG
ATCTGGAGTTGTCTAGGGTCATGGCAGATACTACCGATAAGGTGAAAAATATGCAGGTCAAAGATTCT
GTA
+
FFFFFFFFFFFFFFFFFFFFFFFFFFFFFFFFFFFFFFFFFFFFFFFFFFFFFFFFFFFFFFFFFFFF
FFFFFFFFFFFFFFFFFFFFFFFFFFFFFFFFFFFFFFFFFFFFFFFFFFFFFFFFFFFFFFFFFFFF
FFF
@A00721:190:HGJW7DSXY:4:1111:32045:22451_2:N:0:TCTGCAAG+GCTGCACT
GTTTTGGCCGACTTTAACTATTCTAAGAGTCTTTATGATTCAAAACCTATCGTTGTCGTACCTTACAA
GGTTGAGGAGACTTTACAGCGGCTTATTGTCGCGCAGTTTCCAGAATT
+
FFFFFFFFFFFFFFFFFFFFFFFFFFFFFFFFFFFFFFFFFFFFF:FFFFFFFFFFF:FFFFFFFF:FF
,FFFFFFFFFFFFFFFFFFFFFFFFFFFF:FFFFFFF:FFFFF:F,FFF
@A00721:190:HGJW7DSXY:4:1251:9046:14497_1:N:0:TCTGCAAG+GCTGCACT
TATCTTGAATGCTCACGCCGTCGCAGCCATATCGAGACTATGTTATAATCACTTGTTAAAGTACAGAT
TTAGTGATTTAGATATTATTGATATTGGAGGCGATATTCGTTTTCATATCCAAAACTCCTTGAATGGA
GTA
+
FFFFFFFFFFFFFFFFFFFFFFFFFF:FFFFFFFFFFFFFFFFFFFFFFFFFFFFFFFFFFFFFFFFF
FFFFFFFFFFFFFFFFFFFFFFFFFFFFFFFFFFFFFFFFF:FFFFFFFFFFFFF,FFFFFFFFFFFF
FFF
@A00721:190:HGJW7DSXY:4:2202:32009:20572_2:N:0:TCTGCAAG+GCTGCACT
CCTATTCTTGATGTGAAAGATGCATCGAGGTATACGTCTAGGAACATTGATTGGGAACGTACCTACTC
ACGAGACGTCATGAAAGCTATCGGAGGGAAGCAAAATGTTTCGTTCTG
+
FFFFFF:FFFFFFFFFFFFFFFFFFFFFFFFFFFFFFFFFFF:FFFFFFFFFFFFFFFFFFFFFFFFF
FFF:FFFFFF:FFFFFFFFFFFFFFFF:F:FFF,,FFFFFFFFFFFF
@A00721:190:HGJW7DSXY:4:1560:18674:8907_2:N:0:TCTGCAAG+GCTGCACT
AAGCCTCAGTTGCGATAGCTGTAGAGGTATACGATATCTCTCTTGATGATGTAGCCACTATAATGCTT
AAGAGAGAGATTGATGTATGTCATTTCGTCTTAGTTTGTCCCGGCGAA
+
FFFFFFFFFFFFFFFFFFF:FFFFFFFFFFFFFFFFFFFFFFF:FF:FFF,FFF:FFFFFF::FFFFFF
FFFFFFF,F:FFFFFFFFFFFFFFFFFFF:FF:FFF,FFF:FFFFFFF
@A00721:190:HGJW7DSXY:4:2201:7202:10488_2:N:0:TCTGCAAG+GCTGCACT
CCGTTCCTGACCTTGGTATCGTTATCCGTCATGGAGATGATGGCGCATCACTTGATTACTATATGCCC

GAAGGATTGGGTTATTCTCACAACAAAGATAAGATTTTATCTTTCTTC
+
FFFFFFFFFFFFF:FFFFFFFF,FFFFFFF,FFFFFFFFFFFFFFF:FFF:F,FFFFFFFFFFFFFFF
FFFFF:FFFFFFFF,FFFFFFFFFFFFFFFFFFF,F,FF:FFFFF:FF
@A00721:190:HGJW7DSXY:4:1565:2320:10269_2:N:0:TCTGCAAG+GCTGCACT
AAGGGCGGACAATTGTTCCATTGTGAACTCTTGGGGACTAGATTGAGCATTAATGAGTTCCGAATAAC
TCGTAATAAATCTTTCTCTAGTGGACAATTCAGAATACCTATTTCCGT
+
FFFFFFFFFFFFFFFFFFFF:FFFFFFFFFF:FFFFFFFFFFFFFFFFFFFFFFFFFFFFFFFFFFFF
FFFFFFFFFFFFFFFFFFFFFFFFFFFFFFFFFFFFFFFFFFFFFFFF
@A00721:190:HGJW7DSXY:4:1507:14163:25645_1:N:0:TCTGCAAG+GCTGCACT
CTGCCAATCTCATCGCTATACGCATACCTGATTGGAAGAAGCGAGGTTCGTTCCTCAATAAAAATTTT
TCTCAAGAGATTATGGTTGATAAAGTTTTCTTCATAAGGGCTATTCAGTACGTCATGGGGATCACTTC
TTC
+
FFF:FFFFFFFFFFF:FFFFFFFFFFFFFFFFFFFFFFFFFFFFFFFFFFFFFFFFFFFFF:FFFFFF
FFFF,FFFFFFFFFFFFFFFFFFFFFFFFFFFFFFFFFFFFFFFFFF:FFFFFFFFFFFFFFFFFFF
FFF
@A00721:190:HGJW7DSXY:4:1239:5791:14058_1:N:0:TCTGCAAG+GCTGCACT
TCGAATATGTTACGACATGGCTTCGTAACAACAGTGCGAGGATTGTCATCTCGGGGAAAGTGATTCAC
CAAGATGTTCATATAGATACGAGATACCTAGAGAAGGTCGCAGCGATCTTACTCGTTATCGGAGCTAA
GAA
+
F:FF::FFFFFFFFFF:FFFFFFFFFFFFFFFFFFFFFFFFFFFFFFFFFFFFFFFFFFFFFFFFFFF
FFFFFFFFFFFFFFFFFFFFFFFFFFFFFFFFFFFFFFFFFFFFFFFFFFFFFFFFFFFFFFFFFFFF
FFF
@A00721:190:HGJW7DSXY:4:1425:10601:33129_1:N:0:TCTGCAAG+GCTGCACT
TCGCAAACTTAACGTTAGTACCGGAGGTGATCTCTCTGCTTCTGCGTTACTGAGAGAAAAGATTCAGG
ACGCCTACGGTGACTTTATGGACAAGATGCAAGTTCTTGGGAAATACTTGTTTCCCTTTTTAGGTTTA
GTA
+
FFFFFFF:FFFF:FFF:FFF:FFF:FF:FFFFFFFFFFFF:FFFFFFFF:FFFFFFFFFFFFFFFFFF
FFFF:FFFFFFFFFFFFFFFFFFF:FFFFFFFFFFFFFFFFFFFFFFFFFFFF,FFFFF:FFFFFFFFFFF
FFF
@A00721:190:HGJW7DSXY:4:1623:5746:33082_1:N:0:TCTGCAAG+GCTGCACT
CTCTTCCGATCTGACAACGAATCTTGAAACTACGGTCCTTATCAAAGAAAATGGTGAACCGGTCTCTA
AATCTTTTGAGATTGAGAAGATTGAAGAACACGTCAAGAAGTCGCTCGGCGTTGTTCTTGAAAACCAA
GT
+
FF,FF:F:FFFFFFFFFFFFFFFFFFFFFFFFFFF:FF,FFFFFFFFFFFFFFFFFFFF:FFFFFFFFF
F,FFFFFFFFFFFFFFFFFFFFFF:FFFFFFFFFFFFFFFFFFFFFFFFFFFFFFFFFFFFFFF,,:FF
FF
@A00721:190:HGJW7DSXY:4:2121:11975:19914_1:N:0:TCTGCAAG+GCTGCACT
GCTGATCATGGAGTCTTTGTACCTTCTGTCAAACTTCGAGGTTCAGCTACGAGAGGTGATCCCGGATG
TGGATTGAAGGCTGGCTCTAAGGGAGACTCCTTCTTTTTGAATGTTGTAAAGAATTGGGTTGAGAAGT
GTG
+
FFFFFFFFFFFFFFFFFFFFFFFFFFFFFFFFFFFFFFFFFFFFFFFFFFFFFFFFFFFFFFFFFFFF
FFFFFFFFFFFFFFFFFFFFFFFFFFFFFFFFFFF:FFFFFFFFFFFFFFFFFFFFFFFFFFFFFFFF
FFF
@A00721:190:HGJW7DSXY:4:2636:4065:17519_1:N:0:TCTGCAAG+GCTGCACT
CTTGGTATTTCTGAAATAGTTTATCAGAGCGTCATTTTCCTTTGGGATCTTTCCTCTGGTAAGGGAAT
TATTAAATCATTCTTCTCACTCTTGACGAGAATTATTACTGTTTTCTCTACCCATAACTTCTTTTTCA
AAA

```
+
:FFFFF:FFFFFFFFFFFFFFF:F,FFFF:FFFFFFFF::::FFFF:F,FFFFFFFF:FF:F::FF,F
FFFFFF:FFFF:FFFFF:FFFFFFFFFFFFFFFFFFFFFF:FFFFFFFFFFF:FFFFFFFFFFFFFF
FFF
@A00721:190:HGJW7DSXY:4:1310:11876:24001_2:N:0:TCTGCAAG+GCTGCACT
TACAGAGTGCAGTCGTTTAAACACTTATCACCACTTTTGAAAGGAAAATAATTTGTGATGAAAATACG
TATGTAGGTGGGGTTCTCAAGGCTCTCATCGCCACTGGGATTCTAAGG
+
FFFFFFFFFFFFFFFFFF,FF,:,,FFFFFFFFF:FFFF,FF:F:FFF,:F,F,F,:FFFFFFFFF,F
F:F,F,FF:FFFF,F:F:FF,F,FFF:F,,,F::F,FFFFFF,FFF,F
@A00721:190:HGJW7DSXY:4:2249:23909:13088_2:N:0:TCTGCAAG+GCTGCACT
TTTGGTCCTTACGTCTTAAGTAATTTGGATGTTGTCGAAAACTATCAGGTTCTTGTCACGATTCTTAA
AAGAAGTTTATTAAAACTTCAGAGTCTCTTGGAAGTCCAGGTAGCTAA
+
FFFFFFFFFFFFFFFFFFFFFFFFFFFFFFFFFFFFFFFFFFFFFFFFFFFFFFFFFFFFFFFFFFFF
FFF,FFFFFFFFFFFFFFFFFFFFFFFFFFFFFFFFFFFFFFFFFFFF
@A00721:190:HGJW7DSXY:4:1228:10936:36338_2:N:0:TCTGCAAG+GCTGCACT
ACCTCTGTTGGTAGAGGAGTTGAGAGAATTACTCAGATTCAATCTGGCATCACTGAACGTCTACAACG
AGTTAGGGAGTGGCTAGGTTTTAAAAGATCCTTTGCACAAGCAATACC
+
FFFFFFFFFFF:FFFFFFFFFFFFFFF,FFF:FFFFFFFFFFFFF:FFFFFFFFFFFFF:FFFFFF::
FFFFFFFFFFFFFFFFFFFFFFFFFFFFFFFFFFF:FFFFFFFFFFFF:
@A00721:190:HGJW7DSXY:4:1609:14850:14810_2:N:0:TCTGCAAG+GCTGCACT
GTCGCGGAAAACGATGAAGATTTCCACACTGTTCGAGAATCTTCTGATGCTGAATTTGAGGAAAATTT
TTCGGAAGTTTTTGGTATAAATCCTGATGGCTCAGTACAGGGAGGAGG
+
FFFFFFFFFFFFFFFFFFFFFFFFFFFFFFFFFFFFFFF:FFFFFFFF,FFFFFF:FFFFFF:FFFFFF
FFFFFF:FFFFFFFFFFFFFFFFFFFFFFFFFFFFFFFF:FF:FFFF:FF
@A00721:190:HGJW7DSXY:4:2106:21450:5353_2:N:0:TCTGCAAG+GCTGCACT
ACCTTCTTTTATCATTGCAACAGGACTTTGAGAAGACTGTTGAAATGGACAAAATCTGGGATCATAGG
TATGTGTCACGCTACACTCGAAATATGGGCCCTCAGAAGGAAGTATAG
+
FFFFFFFFFFFFFFFFFFFFFFFFFFFFFFFFFFFFFFFFFFFFFFFFFFFFFFFFFFFFFFFFFFFF
FFFFFFFFFFFFFFF:FFFFFFFFFFFFFFFFFFFF,FFFFF,FF,FFF
@A00721:190:HGJW7DSXY:4:2120:12744:36839_2:N:0:TCTGCAAG+GCTGCACT
GGAAGATCTCTACAGAGATTATCGAGAGTTGTGATCTAGTTCTAAATTTAGGTCGACGGACTTTAGCA
ACTTTAGATAAATTCTTTCCGGGCTCAATGACCTTTGCTATGTACGTG
+
FFFFFFFFFFFFFFFFFFFFFFFFFFFFFFFFFFFFFFFFFFFFFFFFFFFFFFFFFFFFFFFFFFFF
FFFFFFFFFFFF:FFFFFFFFFFFFFFFFFFFFFFFFFFFFFFFFF,FF
@A00721:190:HGJW7DSXY:4:2174:11116:14888_1:N:0:TCTGCAAG+GCTGCACT
TTAGTTCATGAAGTTCTTACACTTCTCGCGACTAATGTCAGCTTCACTACAAAGGCGTTACGTAAAGT
AGTTTTCACGCTTTATCTTAAAGCATTACCACCAAATTTAGCACTTTTTGAATATGACAGAAGCTCGC
CAT
+
FFFFFFFFFFFFFFFFFFFFFFFFFFFFFFFFFFFFFFFFFFFFFFFFFFF:FFFFFFFFFFFFFFFF
FFFFFFFFFFFFFFFFFFFFFFFFFFFFFFFFFFFFFFFFFFFFFFFFFFFFFFFFFFFFFFFFFFFF
FFF
@A00721:190:HGJW7DSXY:4:2363:24153:28948_2:N:0:TCTGCAAG+GCTGCACT
CCAGTTAGAGTTCTTCTAACCAGGACTGATGGTGAGATTCTTTTACCTTTTGGAGGTCCAGGAACCTT
TACTCCTAGTGTCTCTTCTAGAGTCCAAGGACGTTTCCGTCGGAGGAT
+
FFFFFFFFFFFFFFFFFFFFFFFFFFFFFFFFFFFFFFFFFFFFFFFFFFFFFFFFFFFFFFFFFFFF
FFFFFFFFFFFFFF:FFFFFFFFFFFFFFFFFFFFFFFFFFFFFFFFFF
```

```
@A00721:190:HGJW7DSXY:4:1623:18376:32878_1:N:0:TCTGCAAG+GCTGCACT
TCAATAACACTCTATTGAATCCCTTTTCCACGATGAAAGCTCTTTATGAGGTGCTCCATGCAAATTTA
CAGTTCCTCTTTGGTGATATCGCCTCTGTCCTTTCTCCTTTAATCGTGAGGTTACGAGAGTTTCGTAC
TTG
+
FFFFFFFFFFFFFFFFFFFFFFFFFFFFFFFFFFFFFFFFFFFF:FFFFFFFFFFFFFFFFFFFFFFF
FFF:FFF:FFFFFFFFFFFFFFFFFF,FFFFFFFFFFFFFFF:FFFFFFFFFFFFFFFFFFFFFFFFFF
FFF
@A00721:190:HGJW7DSXY:4:2567:29080:2347_1:N:0:TCTGCAAG+GCTGCACT
TTTTGATAAATTTGTTGGAATCGATAATGATACTGACGAAGGTTCTATTACCACCCTTGTCATTGATA
CAAACATTCTAGGTGATGCTTTCTTAGAGGCTCGTCTAGCCGCTGAGATTCGTCTTACCTATTTACAA
TCT
+
FFFFFFFFFFF:FFFFFFFFFFFFFFFFFFFFFFFFFFFFFFFF:FFFFFFFFFFFFFFFFFFFFFFF
FFFFFFFFF:FFFFFFFFFFFFFFFFFFFFFFFFFFFFFFFFFFFFFFFFFFFFFFFFFFFFFFFFFF
FFF
@A00721:190:HGJW7DSXY:4:2575:4490:1532_1:N:0:TCTGCAAG+GCTGCACT
GATAACGATTTAGTACTGGGAAATGGTGGTAGTCAGTTTACTACTTTTCTTTCCCTCCCCTTTTTCGA
TTGGATTCGACGGGGTCTTCGTCTCACGACTAATTTGATAAGGTTTTTCTTTTATGGGACTCTTGTTT
GTA
+
FFFFFFFFFFFFFFFFFFFFFFFFFFFFFFFFFFFFFFFFFFFFFFFFFFFFFFFFFFFF:FFFFFFF
FFFFFFFFFFFFFFFF:FFFFFFFFFFFFFFFFFFFFFFFFFFFFFFFFFFFFFFFFFFFFFFFFFFF
FFF
@A00721:190:HGJW7DSXY:4:2376:26286:6558_2:N:0:TCTGCAAG+GCTGCACT
GTTCTTAATGTAGGTCTCCTAGTACTTAAGGGTTTATGTTTCGGTTTCAGCTGGATGGATCTTTTGAT
GGTATGTTTCACATTGCGTGATCACTTCCCGTGGACTTGGGAAGAGAT
+
FFFFFFFFFFFF:FFFF:FFFFFFFFFFFFFFF,FFFFFFFFFFFFFFFFFFFFFFFFFFFFFFFFFF
FFFFFFFFFFFFFFFFFFFFFFFFFFFFFF:FFFFF:F:FFFFFFFFFF
@A00721:190:HGJW7DSXY:4:1313:28149:1830_2:N:0:TCTGCAAG+GCTGCACT
GCTAAAGCCTGTCCAGTTGTCGATGATGTTCGGGCTGTGGGGATTAACTTCCCTCGTGAAACTTACGA
CGATGTTCTCGCTCGCATTTCACAAGGATTACTAAGTCTGAGATCTGC
+
FFFFFFFFFFFFFFFF::FFFFFFFFFFFFFFFFFFFFFFFFFF:FFFFFFF:FFFFFFF,FFFFFF,F
FFFFFFFFFF:FFF:FFFFFFFFFFFFFFFFF,FF:FFFFFFFF:FFF
@A00721:190:HGJW7DSXY:4:1514:31684:3944_2:N:0:TCTGCAAG+GCTGCACT
AAGTTCATGAGGATGTCTTGGTCGAAAAATATCGCCGGGAACTTCTAATAGAGGATCCTGATGACATT
ATTTCAACGGATACCGATCTCAGTTCTACCACAGAAACCGTCTCAGGA
+
FFFFFFFFFFFFFFFFFFFFFFFFFFFFFFFFFFFFFFFFFFFF:FFFFF,FFFFFFFFFFFFFFFFFF
FFFFFFFFFFFFFFFFFFFFFFFFFFFFFFFFFFFFFFFF:FFFFFFFF
@A00721:190:HGJW7DSXY:4:2201:28519:23641_2:N:0:TCTGCAAG+GCTGCACT
CTGATTCGGAATTAGAGAAAGACGACCTTCTTGTGGGGACTGAGACCTCTGAATTTTCAGGGTGCCCT
GATGAGGACGTTGAAAGGGATAATTTCAACTCTGAGACTTCTAAGAGT
+
FFFF:FFFFFFFFFFFFFFFFFFFFFFFFF:FFFFFFFFFFFFFFFFFFFFFFFFFFFFFFFFFFFFFF
FFFFFFFFFFFFFF,:FFFFFFFFFFFFFFFFFFFFFFFFFFFFFFFFF
@A00721:190:HGJW7DSXY:4:1662:21974:20071_2:N:0:TCTGCAAG+GCTGCACT
CAGAAACGTAACAAGAAGAATAAAGGTAGGAAGGGGAGAAAACAGAACAAGCGAGCGGTTGAAGAGAA
AACTGATATGGTTGCGGCTGATCGTACCCCAATTCCTGCTAGAAAGAT
+
:F:FF,FFF:FFFFFFFFF:FFFFF:FFFFFFFF,FFFF:FFFFFFF:F,,FFFFFFFFFFFFFFFFF
FFFFF:FFFFFFFFF,FFFFFFFFFFFFFFFFF::FFFFFFFFFFFFF
```

```
@A00721:190:HGJW7DSXY:4:2571:20202:8328_2:N:0:TCTGCAAG+GCTGCACT
TACCACTGATCTTCCAGAGTTACGTAGTGGTCGTTCTTTTGGTGATTTTATCAAAGAGAAAGTTTCTG
CTAGTCGCGACGATAGTATTTATCGGAGTGATATTAACAAGTTAAACT
+
FFFFFFFFFFFFFFFFFFFFFFFFFFFFFFFFFFFFFFFFFFFFFFFFFFFFFFFFFFFFFFFFFFFF
FF:FFFFFFFFFFFFFFFFFFFFFFFFFFFFFFFFFFFFFFFFFFFFF
@A00721:190:HGJW7DSXY:4:2418:1714:26318_2:N:0:TCTGCAAG+GCTGCACT
TGTTGAAGTACCGCAACATGGATCAGTTTTCCAAAAAGCTATTAATAGCAGTTGCGGGGCTATAATAC
CGCCTCCAGATTACTCTAAACTGAATCCACTCTCTTGGGATATAAATG
+
FFFFFFFFFFFFFFFFFFFFFFFFFFFFFFFFFFFFFFFFFFFFFFFFFFFFFFFFFFFFFFFFFFFF
FFFFFFFFFFFFFFFFFFFFFFFFFFFFFFFFFFFFFFFFFFFFFFFF
@A00721:190:HGJW7DSXY:4:2628:1624:24345_1:N:0:TCTGCAAG+GCTGCACT
TTCTTCATGAAGATCTTCGAGTGTTTTATAGTGAGTTATTCTTGCGAAGGTTCAAGTCGATTGATTTT
GTTGACTACGATCTCTCTTGTAATAAGAAGTTCTTCGCAGTTGAATCTCCACCAGGTGGAGGGAAGAC
CAC
+
FFFFFFFFFFFFFFFFFFFFFFFFFFFFFFFFFFFFFFFFFFFFFFFFFFFFFFFFFFFFFFFFFFFF
FFFFFFFFFFFFFFFFFFFFFFFFFFFFFFFFFFFFFFFFFFFFFFFFFFFFFFFFFFFFFFFFFFFF
FFF
@A00721:190:HGJW7DSXY:4:2226:27181:14121_1:N:0:TCTGCAAG+GCTGCACT
GACTTTTGTCGAATTTTTCGCAAAAGGTCTTGAAGTACGTATTGTCACTGCTAATCGTAATTCGCGAG
AAGAGATAGTGACTAAAGTTTTGGCACTTCTAGTTCATCGCGGTATTTGTGAAATTAAGACAGACGTG
AAA
+
FFFFFFFFFFFFFFFFFFFFFFFF:FFFFFFFFFFFFFFFFFFFFFFFFFFFFFFFFF:FF:FFFFFFF
FFFFFFFFFFFFFFFFFFFFFFFFFFFFFFFFFFFFFFFFFFFFFFFFFFFFFFFFFFFFFFFFFFFF
FFF
@A00721:190:HGJW7DSXY:4:2174:16821:32503_1:N:0:TCTGCAAG+GCTGCACT
TCGTATTAGAACAGCTGATTCTTATTGCATGAACGTCGCTGAGGTCAAACCTGTGGACGTTCTTTTCA
TCGACGAATTATTCATGATTCACGCTGGTCAGACCCTTTTTCTTACACATCTACTTAAGGCGGCTTAT
ACC
+
:FFFFFFFFFFFFFFFFFFFFFFFFFFFFFFFFFFFFFFFFFFFFFFFFFFFFFFFFFFFFFFFFFFF
FFFFFFFFFFFFFFFFFFFFFFFFFFFFFFFFFFFFFFFFFFFFFFFFFFFFFFFFFFFFFFFFFFFF
FFF
@A00721:190:HGJW7DSXY:4:2373:10149:21605_1:N:0:TCTGCAAG+GCTGCACT
GTGATTCCAATCAAATCGGATTCATCAATCGTATTGATCTCCAAGTCGCAAACTACTTCAACATCAAC
GACATCATTCTTGATGAGTGTCGTTCTTATCGAAGTCTCAGTTATCGCTGCCCGGCCGACGTTTGTCG
CGA
+
FFFFFFFFFFFFFFFFFFFFFFFFFFFFFFFFFFFFFFFF:FFFFFFF:FFFFFFFFFFF:FFFFFFF
FFFFFFFFFFFFFFFFFFFFFFFFFFFFFFFFFFFFFFFFFFFFFFFFFFFFFFFFFFFFF:FFFFFF
FFF
@A00721:190:HGJW7DSXY:4:1620:9091:11162_1:N:0:TCTGCAAG+GCTGCACT
ACGGCAGAAAAATTTCTCCTGCTTGTAATTCGAGAGAGGACACTATGGAAATTAGACCTATCAGATGT
CTTGACGACTTCGAATTTGAGGACGGAGTTAAATATATTACTTTCACTCAAGGTGAAAAGTTTGAAAT
TAA
+
FFFFFFFFFFFFFFFFFFFFFF:FFFFFFFFFFFFFFFFFFFFFFFFFFFFFFFFFFFFFFFFFFFFF
FFFFFFFFFFFFFFFFFFFFFFFFFFFFFFFFFFFFFFFFFFFFFFFFFFFFFFFFFFFF:FFFFFFF
FFF
@A00721:190:HGJW7DSXY:4:1351:30318:14951_1:N:0:TCTGCAAG+GCTGCACT
TAAGAGTAAGGTTAAATGTGAAGATGCTCAGACTATTCATGAAGTTCAAGGTAATACTTACCAGCACG
```

```
TTCGGCTGGTTAGAATTAAACCTCAAGATGACCCTGTTTTTGCATCACAAACACATATTACCGTTGCT
CTA
+
FFFFFFFFFFFFFFF:FFFFFFFFFFFFFFFFFFFFFFFFFFFFFFFFFFFFFFFFFFFFFFFFFFFF
FFFFFFFFFFFFFFFFFFFFFFFFFFFFFFFFFFFF,FFFFFFFFFFFFFFFFFFFFFFFFFFFFFFF
FFF
@A00721:190:HGJW7DSXY:4:1258:25536:7764_1:N:0:TCTGCAAG+GCTGCACT
CTTTACGATACTTTGTTATCAATGTAAAGTTAAACGATGCTACTTGTGAGAGGATCAATCACAGTAAG
GAACTGTCACGTTTAGCACGGGAGGTTGCAGATTCAACCCCTTATATCGAGGCTATCGAGGACGATGA
GTA
+
:FFFFFFFFFFFFFFFFFFFFFFFFFFFFFFFFFFFFFFFFFFFFFFFFFFFFFFFFFFFFFFFFFFF
FFFFFFFFFFFFFFFFFFFFFFFFFFFFFFFFFFFFFFFFFFFFFFFFFFFFFFFFFFFFFFFFFFFF
FFF
@A00721:190:HGJW7DSXY:4:1128:27914:26162_2:N:0:TCTGCAAG+GCTGCACT
GACGACAAGCCTCAAGAGGGTAGAGGACCACCTGGATCTTCTACTGTCTCTGCCTTCCAAGATTTCTT
AGAAGAGGTTATTCCTGGAACCACCTTAACGTCACTCGGTAATCTTTC
+
FFFFFFFFFFFFFFFFFFFFFFFFF:FFFFFFFFFFFFFFF:FFFFFFFFFFFFFF,FFF:FFFFF
:FFFFFFFFF:FFFFFFFFFFFFFFFFFFFFFFFFFFFFFF,FFFF:FF
@A00721:190:HGJW7DSXY:4:1512:24578:3787_2:N:0:TCTGCAAG+GCTGCACT
TGTCGATCGAATTAAGATCAAGGCTAATACCTTTATAGGTAAGAAGCCCCTTGAGCCGCGTAGTCAGC
GCGTTTAGCTGTATCATCAGTCAGAGTATACCCGATAGGGTACCGAGT
+
FFFFFFFFFFFFFFFFFFFFFFFFFFF:FFFFFFF:FFFFF:FFFFFFFFFFFFFFFFFFFFFFFFFF
FFFFFFFFFFFFFFFFF:FF:FFFFFFF:FFFFFF:FFFFF,FFFFFF
@A00721:190:HGJW7DSXY:4:1529:2844:9768_2:N:0:TCTGCAAG+GCTGCACT
ATATTTATTCTTATGAGGCTCGAAACTTCAACGTTTTCACAACGGAAAGACATATCGCACCCGAAATT
TTTGGGCAGAATGCTCTTAACGCGGTTCTTGAACGGTGTTTTGATCAG
+
FFFFFFFFFFFFFFFFFFFFFFFFFFFFFFFFFFFFFFFFF:FFFFF,,FFFFFFFFFFFFFFFFFFF
FFFFFFFFFFFFFFFFFFFFFFFFF:FFFFFFFFFFFFFFFFFFFFFF
@A00721:190:HGJW7DSXY:4:1456:3513:30906_2:N:0:TCTGCAAG+GCTGCACT
TAAAGTTATAGGAGACTCAGTTAACCTGAGAGCTCCTCAATTGATAGATTGGGTCAACAATAGGGACG
CTAGTATACTTCGTAAAGTCGACAGCGAAATGGTCAATATTGATAATT
+
FFFFFFFFFF:FFFFFFFFFFFFFFFFFFFFFFFFFFFFFFFFFFFFFFFFFFFFFFFFFFFFFFFFF
FFFFFFFFFFFFFFFFFFFFFFFFFFFFFFFFFFFFFFFFFFFFFFFF
@A00721:190:HGJW7DSXY:4:2124:16504:9909_1:N:0:TCTGCAAG+GCTGCACT
TGACCTATTTTAAGCTCATGGTGAAAAGGGAAGCTAAGGTTAAACTTGATGCTTCAGCCTTTTCGAAG
CATAACCCCGCACAGAATATCGTTTATCACGCAAAATTTATTAATGCAATTTTCGGACCTATCTTTAA
GGT
+
FFFFFFFFFFFFFFFFFFFFFFFFFFFFFFFFF,FFFFFFF:FFFFFFFFFFFFFFFFFFF:FFFFFF:F
FFFFFFFFFFFFFFFFFFFFFFFFFFFFFFFFFFFFFFFFFFFFFFFFFFFFFFFFFFFFFFF,FFFF
F:F
@A00721:190:HGJW7DSXY:4:1136:6352:32753_1:N:0:TCTGCAAG+GCTGCACT
AGCTCTTCTCTCTCTTAGGAGGTCATGATGACTACTATAAGGGTGAAATCGATTTTTCGAAGTTTGAC
AAATCTCAAGATTTCTTTTGTAAAGCCTATGAGATAAAGATGTATTCTTATTTAGGCTTCGATGCTGA
ATT
+
FFFFFFFFFFFFFFFFFFFFFFFFFFFFFFFFFFFFFFFFFFFFFFFFFFFFFFFFFFFFFFFFFFFF
FFFFFFFFFFFFFFFFFFFFFFFFFFFFFFFFFFFFFFFFFFFFFFFFFFFFFFFFFFFFFFFFFFFF
FFF
```

@A00721:190:HGJW7DSXY:4:1503:24370:26036_2:N:0:TCTGCAAG+GCTGCACT
AGTGCCAGTGAATATTTTTGTCACGCCAGGAGTATCCATAACGATTTGTCATTTACCACCTTTGCTCA
ACGACGAAGTGGAACGGCCAACACTTTCTTTGGTAATTGTATGATCAC
+
FFFFFFFFFFFFFFFFFFFFFFFFFFFFFFFFFFFFFFFFFFF,,FFFFFF:FFFFFFFFFFFFFFF:
FFFFFFFFFFFFFFFFFFFFFFFFFFFFFFFFF::FFFFFFFFFFFF:
@A00721:190:HGJW7DSXY:4:1170:18991:30217_2:N:0:TCTGCAAG+GCTGCACT
TATCACTTTATTATGACCTTGGAAAGTTTTCGGCTGTGGTTGTCGCTGGGGACGATTCCATTCTGTTT
TCAAAGACTCCAATAACTGATCATTCTGATCATATGATTTTGGACAAT
+
FFFFFFFFFFFFFFFFFFFFFFFFFFFFFFFFFFFFFFFFFFFFFFFFFFFFFFFFFFFFFFFFFF:F
FFFF:FFFFFFFFFFFFFFFFFFFFFFFFFFFFFFFFFFFFFFFFFF:F
@A00721:190:HGJW7DSXY:4:1265:15167:7435_2:N:0:TCTGCAAG+GCTGCACT
ATCGTTAAGTTAGGTAAACCCCTAACCGATGTTACTCTTGTAGGACTTTGGGAGAGATTCGTTAGTTT
TAAAGACTATACTAAATATCTCCACTACGAGGTTGTTGTTTGGCGATT
+
FFFFFFFFFFFFFFFFFFFFFFFFFFFFFFFFFFFFFFFFFFFFF::FFFFFFFFFFFFFFFFFFFFF
FFFFFFFFFFFFFFFFFFFFFFFFFFFFFFFFF,FFF:FFFFFFFFFFF
@A00721:190:HGJW7DSXY:4:1263:3902:25254_2:N:0:TCTGCAAG+GCTGCACT
ATATTCGATATTCGATTGAGGGTCGTTATACCTATTCGGCTATCTGTGCTTTGAATTGTATAAGATCA
AATTTTAAACGATTCCGAGAGGTTTATCCTGATGTGGAGGGAGTTCTT
+
FFFFFFFFFFFFFFFFFFFFFFFFFFFFFFFFFFFFFFFFFFFFFFFFFFFFFFF,FFFFFFFFFFFFF
FFFFFFFFFFFFFFFFFFFFFFFFFFFFF:FFF:FFFFFFFFFFFFFFF
@A00721:190:HGJW7DSXY:4:1514:25581:14982_1:N:0:TCTGCAAG+GCTGCACT
ACCTTTCCTTGTGAAAATTTTGATGCGGTTTCATAAGATTATCAGTAATCCTGATTCTACTACGGATT
CATGGACCTTCCATCCTGTACCTCTTCTGGAGAGACGTATCTCAAAGATCGACTTCGAGACAGGTGAT
ATT
+
FFFFFFFFFFFFFFFFFFFFFFFFFFFFFFFFFFFFFFFFFFFFFFFFFFFFFFFFFFFFFFFFFFFF
FFFFFFFFFFFFFFFFFF:FFFFFFFFFFFFFFFFFFFFFFFFFFFFFFFFFFFFFFFFFFFFFFFFF
FFF
@A00721:190:HGJW7DSXY:4:2177:21938:22983_2:N:0:TCTGCAAG+GCTGCACT
CTAAATTCGGTAACGGTGCGTGGCGCTTTATTTTAAAAAGCTCCCTCCTCTCGCAAGTTTGATCTGAT
ACTTATCACGAAAGGACTTGAGTCTTTTGATCAGTCTCTGACTTGACC
+
FFFFFFFFFFFFFFFFFFFFFFFFFFFFFFFFFFFFFFFFFFFFFFFFFFFFFFFFFFFFFFFFFFFF
FFFFFFFFFFFFFF:FFFFFFFFFFFFFF:FFFFFFFFFFFFFFFFFF
@A00721:190:HGJW7DSXY:4:1618:8802:15796_2:N:0:TCTGCAAG+GCTGCACT
TAAATGACGATCATTAAGACTCTCTTTCTTCTTTGTTTCCTTCTTTGCACTGATTTTTCCTCATCAGC
TTGTGTTTTACCTAATCTGTGTTTTTACGATAACAACTATCAGTTGAT
+
FFFFFFFFFFFFFFFFFFFFFFFFFFF:FFFFFFFFFFFFFFFFFFFFFFFFFFFFFFFFFFFFFFFF
FFFFFFFFFF:::FFFFFFFFFFFFFFFFFFFFFFFFFFFFFFFFFFF
@A00721:190:HGJW7DSXY:4:1242:26919:33708_1:N:0:TCTGCAAG+GCTGCACT
CCGAAAGGCGAACTTTGGGAATTTGAAGCTCAGGGTGAAGCACAACGTTTGACCGTGACGAACTGTGG
TGTTCTTGATCTACGTTACGGATATACTCTCCTTGAGTTCACTCGTAAGGACACTACCACTTATTACG
ATA
+
::FFFFFFFFFFFFFFFFFFFFFFFFFFFFFFFFFFF:FFFFFFFFFF:FFFFFF,FFFFFFFFFFFFFF
FFFFFFFFFFFFFFFFFFFFFFFFFFFFFFFFFFFFFFFFFFFFFFFFFFFFFFFFFFFFFFFFFFFF
FFF
@A00721:190:HGJW7DSXY:4:2203:24641:22999_1:N:0:TCTGCAAG+GCTGCACT
TCTCTATTTTAAGTTTTGTCTTCCTTGTTTGTCTTATTCTATTAATCACTCATTTTAGTGAGATTATT

AAAAACGCACTAAAACGTCTTCGACAACAGCAGGGGTTTTCGAATAGTGATAGCCACCCGCCTTACAG
AGG
+
FFFFFFFFFF:FFFFFFFFF:FFFFFFFFFFFFFFFFFFFFFFFFFFFFFFFFFFFFFFFFFFFFFFF
FFFFFFFFFFFFFFFFFFFFFFFFFFFFFFFFFFFFFFFFFFFFFFFFFFFFFFFFFFFFFFFFFFFFF
FFF
@A00721:190:HGJW7DSXY:4:2339:26268:20556_1:N:0:TCTGCAAG+GCTGCACT
GACTTTGGTACCACCTTTTCTACTGTCTGTCTCGCCTCTTCGAATGAAGTTTTAATGCTTAACAACAA
TGATAGTGAATTTATACCTACAATCATTGGTTTTTCTAATAATGATGACACTATTGTTTATGGATATG
ATG
+
F,FFFF,,FF,F,,:FFF::F,,FFF,FFF:FFFFFF:FF,FF:FF:F:FFFFFFF,FFF:FFFF,,:
FFFFFFF,,FFFFFFF::F:FFFFF:FFFFFFFFFFFFFFFFFFFF:FFFFF,:F,FFFFF::FF,F:
:FF
@A00721:190:HGJW7DSXY:4:1336:6894:19664_1:N:0:TCTGCAAG+GCTGCACT
TCTGGATTCTCAGTTTACAAGGACCTTAAGCGATGGATCGGGGTTAATTCTAAAACATTACCCGAAAG
ACGTGCTAAATTAAAACCATTGTACGATGTCGTCTGTCTCGGCGATCACTTTTCACTAAGAATTGGTC
CTA
+
FFFFFFFFFFFFFFFF:FFFFFFFFFFFFFFFFFFFFFFFFFFFFFFFFFFFFFFFFFFFFFFFFFFF
FFFFFFFFFFFFFFFFFFFFFFFFFFFFFFFFFFFFFFFFFFFFFFFFFFFFFFFFFF:FFFFFFFFF
FFF
@A00721:190:HGJW7DSXY:4:1467:23204:23766_1:N:0:TCTGCAAG+GCTGCACT
AGACGTGTGCTCTTCCGATCTATCGTTTCTTTCTTTATCTCTTTGGTTGTTAGGGATTTTGAGAGAGT
GAAAGGCTTCAATTGTAGTGGTTTGGTCGTTTCAGTACCCAGTCAGTATACCTCTACGCAAAGATATT
TCA
+
FFFFFFFFFFFFFFFFFFFFFFFFFFFFFFFFFFFFFFFFFFFFFFFFFFFFFFFFFFFFFFFFFFFFF
FFFFFFFFFFFFFFFFFFFFFFFFFFFFFFFFFFFFFFFFFFFFFFFFFFFFFFFFFFFFFFFFFFFFF
FFF
@A00721:190:HGJW7DSXY:4:2325:31891:6872_2:N:0:TCTGCAAG+GCTGCACT
GAGGAACGTACCGGATTACCTGTCTTTCATATTATGAATGAACCTTCGGCCGCTCTCTTTGCTTCGAT
GTTGGATGTCAAGAAGGGTTCAGAATGGGATTCCAACGTAGTTTATGA
+
FFFFFFFFFFFFFFFFFF:FFFFFFFFF:FF:FFFF,FFFFFFFFFFFFFFFFFFF:FFFFFFF:F::
:FFFFFFFF:,FFFF:FFFFFFFFFFFFFFFF::,F:FFFFFFF:FFF
@A00721:190:HGJW7DSXY:4:2224:12084:6230_1:N:0:TCTGCAAG+GCTGCACT
TACTTTCGATACTTCTATCGTCTTACGTTACGGTAATTACTATAGTGTTATTTATTCTGGTGGTGATG
ATAATCTTGGAGGTCGTGACGTTGATGAAGCTATAAGAGCGTTCTTATTACGGCGCACTTCTTTACCC
GCC
+
FFFFFFFFFFFFFFFFFFFFFFFFFFFFFFFFFFFF:FFFFF:FFFFFFFFFFFFFFFFFFFFFFFFFF
FFFFFFFFFFFFFFFFFFFFFFFFFFFFFFFFFFFFFFFFFFFFFFFFFFFFFFFFFFFFFFFFFFFFF
FFF
@A00721:190:HGJW7DSXY:4:1176:18991:1783_2:N:0:TCTGCAAG+GCTGCACT
GTTTCACAACTGAAGGAGGAGGTTTCTGTTACTGGAAAACCCTCGTCAATACTTTTCAATAATATACG
TCTTGAACTCACTTATGACGATCTAGTGAAATTAACGCGTCCTTTTCT
+
FFFFFFFFFFFF:FFFFFFFFFFFFFFFFFFFFFFFFFFFFFFFFFFFFFFFFFFFFFFFFFFFFFFFF
FFFFFFFFFFFFFFFFFFFF:FFFFFFFFFFFFFFFFF:FFFFFFFFF
@A00721:190:HGJW7DSXY:4:1414:6723:15264_1:N:0:TCTGCAAG+GCTGCACT
TACTATTATGAACAAAGTTATCAAAGAGAGTGGCATTCAAGGCAAACTTACCCTGATTCCGGCCGGTG
GTAGCTCATTTCTACCAGGGGCTATTACTCTTGCTGAAACAGCTATTAAAGCTGTTGAGCGGGTAATT
ACA

```
+
FFFFFFF,FFF,F,FFFFFFF:FFFFF,FFFFF:FFFFFFFFFFFFFFFF:FFF,FFFFFFF:FFF:F
FFF,FFFFFFFFFFFF:FFF:FF::FFFFFFF:FFFFFFFFF:FFFF:FFFFFFFFFFFFF,FFFFFF
FFF
@A00721:190:HGJW7DSXY:4:2122:11722:8923_2:N:0:TCTGCAAG+GCTGCACT
ATCGCTGAAGGATGTGCACTCTTTTCTGCTACTTTAACGGAGCGTAGTGTACTCTTCGTCGATTGTAT
CAACAGTAACATTACTTCTAATAAAGGTCTCTATCAAGCTCGTATTAT
+
FFFFFFFFFFFFFFFFFFFFFFFFFFFFFFFFFFFFFFFF:FFFF:FFFFFFFFFFFFFFFF:FFF:F
FFFFFFFFFFFFFFFFFFFFFFFFF::FFFFFFFFFFFFFFFFFFFFFF
@A00721:190:HGJW7DSXY:4:1547:29550:9142_1:N:0:TCTGCAAG+GCTGCACT
TTACCGTTGAAGGCTAAACTCCAAGGTAATTATGAAGACTGTGATTTATCTTTCACTCTGGCCTTGTA
TGAGGGTGTTTCTAATCGTAACATTAATAATTTCAATCTTCTTAAAAAGAAGATAGAGAGTCATACTT
TGG
+
FFFFFFFFFFFFFFFFFF:FFFFFFFFFFFFFFFFFFFFFFFFFFFFFF:FFFFFFFFFFFFFFFFFFFFF
FFFFFFFFFFFFFFFFFFFFFFFFFFFFFFFFFFFF,:FFFF:FFFFFFFFFFFFFFFFFFFFFFFFFFF
FFF
@A00721:190:HGJW7DSXY:4:1446:19849:12352_2:N:0:TCTGCAAG+GCTGCACT
TGAAGTGGACACCTCTGGTAGTATCGAAGTAAAGATAACCAATAAGGAAGGAGCTTATACTATGGTTG
AATCAACTAAGCATACTACTTTTGCTTTGATTACTCTACCGCCTTACA
+
FFFFFFFFFFFFFFFFFFFFFFFFFFFFFFFFFFFFFFFF,FFFFFFFFFFFFFFFFFFFFFFFFFFF
FFFFFFFFFFFFFFFFFFFFFFFFFFFFFFFFFFFFFFFFFFFFFFFF
@A00721:190:HGJW7DSXY:4:2655:27670:15718_2:N:0:TCTGCAAG+GCTGCACT
TAAAGAGTCGATTAAACAAGTTGTACTTATCAATGATTTTATCGCTGGTTGTTATCTCGAAAAGTCTC
TTCTAAAAGGAACGTCAGTTCTAACCCAAAGAGAATATCTAAAATATT
+
FFFFFFFFFFFF:FFFFFF:,FFFF:,FFF,FF::,:FFFFF,FF,:FFF,F:FF:F,:FF,F,FF,:
FF,FFFFFFF:FF:,,F,:FF,FFF,FF,F,FF:::FFFF,:FF,:,F
@A00721:190:HGJW7DSXY:4:1403:14579:23923_2:N:0:TCTGCAAG+GCTGCACT
AGACGTTGCTTGTCGCTTCCTCCTTTAATGGCACTACCTCGGTACAATCTTACTTGGAACGAAGGCGA
CCTGTTCCGGAGTTTTTACGGGATCCGGAATGTTCTGTCTATATTAGG
+
FFFFFFFFFFFFFFFFFFFFFFFFFFFFFFFFFFFFFFFFFFFFFFFFFFFFFFFFFFFFFFFFFFFF
FF:FFFFFFFFFFFFFFFFFFF:FFFFFFFFFFFFFFFFFFFFFFFFF
@A00721:190:HGJW7DSXY:4:1639:26838:12085_2:N:0:TCTGCAAG+GCTGCACT
TCGAACGTCAACGTTCACCGAGTTTCGTTTCGTACCTTGATGCTGACTTTCGTCAATATGATTTGATC
TGGTTGCTCAATTCTAAGCGTGTTCCGCTTAGTGCGAGGCCTCTCTTC
+
FFFFFFFFFFFFFFFFFFFFFFFFFFFFFFFFFFFFFFFFF:FFFF:FFFFFFFFFF:FFFFFFFFFF
FFFFFFFFFFFFFFFFFFFFFFFFFFFFFFFFFFFFFFFFFFFFFFFF
@A00721:190:HGJW7DSXY:4:1506:21404:19523_2:N:0:TCTGCAAG+GCTGCACT
ATATTTGGATGATCTTGATAAAGAACTTCATGAATTTTCAGAGGATGAAAAATTTTCTTCCTTGGATT
TTAATATAAAGAATCTTTACCAGGACTCCTACTACAGGAGGCAGTCTT
+
FFFFFFFFFFFFFFFFFFFFFFFFFFFFFFFFFFF:FFFFFFFFFFFFFFFF,FF:FFFFFFFFFFFFFF
F:FFFFFFFFFFFFFFFFFFFFFFFFFFFFFFFFFFF:FF:FFFFFFFF
@A00721:190:HGJW7DSXY:4:2616:15492:32456_1:N:0:TCTGCAAG+GCTGCACT
ATTTGTTGTAATAGGGTCATCAACTTAGAACTCGAAACTCTAAGTGGTAATACTGTCTTTCCAAGCTT
ACCTGTATCTTCAATGAAATTGGACTCTTCGATAGGGGACATTTTATCGTGGGCTTTCCGTAAAGGAA
GAT
+
FFFFFFFFFFFFFF,:FFFF:FFFFFFFFFFFFFFFFFFFFFFFFFFFFFFFFFFFFFFFFFFFFFFF
```

```
FFFFFF:FFFFFFFFFFFFFFFFFFFFFFFFFFFFFFFFFFFFFFFFFFFFFFFFFFFFFFFFFFFFF
FFF
@A00721:190:HGJW7DSXY:4:2362:24379:12618_1:N:0:TCTGCAAG+GCTGCACT
CTACTAATGGCGTTGGCCTAGTGGATGTTATTAGTGACATCTGGTCAATTTCGTTTTTAGGTGGTCTT
TATTCTATTATTGATTTTTATGAGATTGATACGTTAACAGAAGATGATGTTAGAGTAGAATTCCTGAA
GAG
+
FFFFFF:FFFFFFFFFFFFFFFFFFFFFFFFFFFFFFFFFFFFFFFF:FFFF:FFFFFFFFFFFFFF
FFFFFFFFFFFFFFFFFFFFFFFFFFFFFFFFFFFFFFFFFFFFFFFFFFFFFFFFFFFFFFFFFFF:
FFF
@A00721:190:HGJW7DSXY:4:2335:9299:18662_2:N:0:TCTGCAAG+GCTGCACT
CCAACTTCTTGACAATTTTCTTCAATCCTTCGATAATAGTTAAATCTAGGATTATGTCTGTGTTGAAA
GACTTTCTACCGATAAGGGTCACTAGTCTTACACAATTAAGAAATAAT
+
F:FFFFFFF,:FFFFFFF:FFFFFFFFFFFFFFFFFFFFFFFFFFFFFFFFFFFFFFFFFFFFFFFFF
FFFFFFFFFFFFFFFFFFFFFFFFFFFFFFFFFFFFFFFFFFFFFFFF
@A00721:190:HGJW7DSXY:4:1243:28519:12399_2:N:0:TCTGCAAG+GCTGCACT
ACTATCTAAAGATTGAAGGAAAATTTTATCCTCGTTTAACTTACTTAGACTTTCCTACGATACAAATG
ATAACTTCTAATTTACGTTTGCTACTCAGAGCTGGTCCTCTCAGCAAA
+
FFFFFFFFFFFFFFFFFFFFFFF:FFFFFFFFFFFFFFFFFFFFFFFFFFFFFFFFFFFFFFFFF:FF
FFFFFFFFFF:FFFFFFFFFF:FFFFFFFFFFFFFFFFFFFFFFFFFFF
@A00721:190:HGJW7DSXY:4:2622:16902:2206_2:N:0:TCTGCAAG+GCTGCACT
TCCTCAGGAGTCCCGGCATTTGATAATAATGACGACTTTCGTCATAATCGCTGCATATTACGGATTAC
ACGGTACCAGCGCATCTCGTGTCGAGCCTCGACCCGATTATATGGTAT
+
FFFFFFFFFFFFFFFFFFFFFFFFFFFFFFFF:FFFFF,FFFFFFFFFFFFFFFFFFFFFFFF:FFFFF
FFFFFFFFFFFFFFFFFFFFFFFFFFFFFFFFFFFFFFFFFFFFFFFF
@A00721:190:HGJW7DSXY:4:2658:31711:24627_2:N:0:TCTGCAAG+GCTGCACT
AGAATTCTTAATTATAATATTCAACCTCAAAAGTTACGTGTTTACAGTAACGAGATCACACATTTTGA
GAACTAACTATGTTATGTTCTCTGATCATTTTGACAATCTCTTCTTAT
+
:FFFFFFFFFFFFFFFFFFFFFFFFFFFFFFFFFFFFFFFFFFFFFFFFFFFFFFFFFFFFFFFFFF:
FFFFFFFFFFFF:FFF:FFFFFFFFFFFFF:FFFFFFFFFFF::FFFF
@A00721:190:HGJW7DSXY:4:1233:17888:1251_1:N:0:TCTGCAAG+GCTGCACT
TAAATAATATTACTAATGGCAAATAGTGGAAAGGCTCCAGTCAACACTAGTTCGAGTGCTGCTTTGGA
TTACGATGACGTGAGAATAGGTGAAGGGATAGTTTTTACCTCGACCAACATTAAGGCGGCGGCGACTA
ATC
+
FFFFFFFFF:FFFFFFFFFFFFFFFFFFFFFFFFFFFFFFFFFFFFFFFFFFFFFFFFFFFFFFFFFF
FFFFFF:FFFFFFFFFFFFFFFFFFFFFFF:FFFFFFFFFFFFFFFFFFFFFFFFFFFFFFFFFFFFF
FFF
@A00721:190:HGJW7DSXY:4:2440:26883:4147_1:N:0:TCTGCAAG+GCTGCACT
AGGACACTTCGAAACTGAATAATGATGCACTACAAACAAGTATCGCTAATCATCTGAAAAAGGCTCTA
AAGGACACTATACAATATCCAGACGACGACGAAGCTTATACTACTTTAATTTTGGGTATTATGTGGAG
ATA
+
FFFFFF:FFF:FFFFFFFFFFFFFF,FFFFFFFFFFFF:FFFFFF,FFFFFFFFF:FFF:FFFFFFFF
FFFFFFFFFFFFFFFFFFFFFFFFFFFFFFFFFFFFFFFFFFFFFFFFFFFFFFFFFFFFFFFFFFFF
FFF
@A00721:190:HGJW7DSXY:4:1136:4237:11397_2:N:0:TCTGCAAG+GCTGCACT
CCACATTTTGGTCAAGTCAATCCGTTGAGAAAATTTCTTAGAGGATATTCGCCATTGTGGTACGAATT
GGTCCAACATTTACCACCAAATTCTTTTGATACAACTCTAGCCTTCAA
+
```

FFFFFF:FFFFFFFFF:FF,FFFF,:FFFFF:FFFFF,:F:F,FFF:FFFFF:FFFFFFFF:F,FFF:
F:FF:FF:F:FFFFFFF:FFF,FFFF,F:FF,:FF,:FF:FFF:FFF:
@A00721:190:HGJW7DSXY:4:1674:24153:29262_1:N:0:TCTGCAAG+GCTGCACT
ACCAGATTGGTTCGAACCCGTTGATGCTATGTCAGAAGAAGCTATTCAAGCTTGTCGTTTAAAGACTG
CAGAGGCTGTCAAACAAGCCCAGACTCAAGGAGTCCAATCACAGGTTTTGAATTTAGCTCAACTGGGG
AGA
+
F,:F:FFFFFFFFFFFFFFF:FF,:FFF:FFFFFF:F,:FFFF:FFF,FFFFFFFFFFFFFFFFFFFF
FFFFFFFFFFFFF:FFFFFFF:FFFF,FFFFFFFFFF::F,,FFF:FFFFFFFFF:F,:FFFFFFFFF
FFF
@A00721:190:HGJW7DSXY:4:2610:3260:6198_1:N:0:TCTGCAAG+GCTGCACT
CTTGCAGAGTGACTGGAGTTCAGACGTGTGCTCTTCCGATCTGAAAAGTCCGGAGTATATTGTATGTT
AATAGATAGTGGGATAATAGTTCTACCGATACGTAGTGAACGCGTTCGTAGAACCAAGTACTTTCCGT
CTG
+
F:FFFFFFFFFFFFFFFFFFFFFFFFFFFFFFFFFFFF:FFFFFFFFFFFFFFFF:FFFFFFFF:F:F
F:FFFFFFFFFFFFFFFFFFFFF:FFFFFFFFFFFFFFFFFFFFF:FFFFFFFFFFFFF:FFFFFFFF
FFF
@A00721:190:HGJW7DSXY:4:2125:32325:18709_1:N:0:TCTGCAAG+GCTGCACT
ACCGATAGGAGTTGTATAGTTGAAGATGTGGCTTTAGACGATAATCCTTTAAGGACTATGTTGACTGA
CGAAGATTTTCCAACAGATTTGTCTACGTTGCAGATCGCCACTCCAAACACTATATACATTATAAAAT
ATA
+
F:FFFF:FFFFFFFFFFFFFFFFFFFFFFFFF:FFFFFFFFFFFFFFFFFFFFFFFFFFFFFFFFFFF
FFFFFFFFFFFFFFFFFFFFFFFFFFFFFFFFFFFFFFFFFFFFFFFFFFFFFFFFFFFFFFFFFFFF
FFF
@A00721:190:HGJW7DSXY:4:2321:10764:21731_1:N:0:TCTGCAAG+GCTGCACT
ATAACTTTTGTTACCAAATTCTTGGCTTTCAACGAAGTCTCTGATTTTATTAATCATTACTTTAACGA
TCTCTCGGTCATTTACTCTGTACTTGATCTACGTCGTTTAACTGTCGGAACAACCTTTACTTAGAATG
ACT
+
FFFF:F:FFFFFF:,FFFFFFFFFFF:FFFFFFFFFFFFFFFF:FFFFFFFFFFFFFFFFFFFFFFFF
FFFFFFFFFFFFFFFFFFFFFFFFFFFFFFFFFFFFFFFFFFFFFFFFFFFFFFF:FFFFFFFFFF:F
FFF
@A00721:190:HGJW7DSXY:4:2212:6234:18020_2:N:0:TCTGCAAG+GCTGCACT
TCTTATATTATTTACAATAGTTAATATCATATTAGTAATACTCACTATCGTTTTCTGCTTTTTCTTAG
CTAATAGATTAATAAAGGATGAGTACGAAAACCAACTCTTCGTTACGA
+
FFFFFFFFFFFFFFFFFFFFFFFFFFFFFFFFFFFFFFFFFFFFFFFFFFFF:FFFFFFFFFFFFFFF
:F:FFFFFFFFFFFFFFFFFF:FFFFFFFFFFFFFFFFFF:FFFFFFF
@A00721:190:HGJW7DSXY:4:2247:25274:1360_1:N:0:TCTGCAAG+GCTGCACT
TATAATATTTTTATTCTATACACCCATCTCAACGAGGAAAAAGCGATTTTTCGTTCATTAACTTAATA
CACTAAGAGAAAAGCCGAAAACGACTCGAAACCGAGACGCCGTAGATCAAAGTCCCTTCTAACTTTAG
ATT
+
FF,F::,:,FF,,,,:F,FF,,F,:F,,,,F,,,,,,F,FF,,,,,,FFF,F,,,:,F:F,,F::,,,
FFF,F,,,,,,,:,,,,,FFFFFF:FFFFFFFF:FFFFFF,FFFFFFFFFFFF,F,:FFFFFFFFFFF
FFF
@A00721:190:HGJW7DSXY:4:1674:27651:34945_2:N:0:TCTGCAAG+GCTGCACT
CAGTTATTTATGTTGTACTTTTTATAATTTATAACCTTCTTATACTTTTGGGTATGTTTTGTGAAAAA
CATGAGATACCCAAGTCTATCGAAGACATTATCTCTTATGTACTTATT
+
FFFFFFFFFFFFFFFFFFFF:FFF:FFFFFFFFFFFFFFFFFFFFFFFFF:FFFFFFFFFFFFFFFFFF
FFFFF:FFFF:FFFFFFFFFFFFFFFFFFFFFFFFF,FFFFFFF:FF:FF

```
@A00721:190:HGJW7DSXY:4:2545:28863:27273_2:N:0:TCTGCAAG+GCTGCACT
TATTCCTTTAACTGTGTATGAGGAGGATTTATAATGCTTTTCGATAGTGATTGTTATCAATTTAAATA
CATTACTGTTTCAAATCATCTTTACTCACAGACTCCGAAAATGGTTTC
+
FFFFFFFFFFFFFFFFFFFFFFFFFFFFFFFFFF:FF:FF:FFFFFFFFFFFFFFFFFFFFFFFFFFF
FFF,FFFFFFFFFFFFFFF:FFFF:FFFFF:FFFF,FFFFFF,,FFF,
@A00721:190:HGJW7DSXY:4:1270:25165:11287_1:N:0:TCTGCAAG+GCTGCACT
TATTTATTAAACGAACTATTATCCGATTCTTCGAGGGTATCACTACCACTGATCTGCCGACTTTAGTC
AAGGAACTTCAATTCCTTCGATGTTTATATTTTTCTATAAGGGAAAATAGCAATAATCAAATTTCGAC
AAA
+
FFFFFFFFFFFFFFFFFFFFFFFFFFFFFFFFFFFFFFFFFFFFFFFFFFFFFFFFFFFFFFFFFFFF
FFFFFFFFFFFFFFFFFFFFFFFFFFFFFFF:FFFFFFFFFFFFFFFFF:FFFFFF,FFFFFFFFFFF
FF:
@A00721:190:HGJW7DSXY:4:1540:25789:27586_2:N:0:TCTGCAAG+GCTGCACT
ATTTCAGAACTCTCTCCGATCACCTTGGTGTGATCTCCCGGAGGATCTTTTCAATAAGATGTATGATG
ATAATCTACTATTTTTAAATAGTGATATTCAGCGTATACTTGAATACT
+
F:FFFFFFFFFFFFFFFFFFFFFFFFFFFFFFFFFFFFFFFFFFFFFFFFFFFFFFFFFFFFFFFFFF
FFFFFFFFFFFFFFFFFFFFFFFFFFFFFFF:FFFFFFFF:FFFFFFF
@A00721:190:HGJW7DSXY:4:2322:1244:10848_1:N:0:TCTGCAAG+GCTGCACT
CTTGGTCTATTTACCGCAAAAAGTGGGAAGAGGTCTATGATATTTATTTACTTATACTTTAAATAGC
ACCGATTATAACTTGACTGTTGATAATATTACAATAGCTCTTTCTCAGAAGATTAGAGAAGTAACCGG
TGA
+
FFFFFFFFFFFF:FFFFFFFFFFF:FFFFFFFFFFFFFFFFFFFFFFFFFFFFFFFFFFFFFFFFFFF
FFFFFFFFFFFFFFFFFFFFFFFFFFFFFFFFFFFFFFFFFFFFFFFFFFFFFFFFFFFFFFFFFFFF
FFF
@A00721:190:HGJW7DSXY:4:2158:15646:9956_2:N:0:TCTGCAAG+GCTGCACT
CATTGATTTACTTTTACCGTTAGAACTGAAAAAGATTCTTTTCGAAAAGAAACCTATAAGTTACAAAC
GATTCTTCGACTATATTAAAGAAATAAAAATACTTCAAGTACCTGAAC
+
FFFFFFFFFFFFFF:FFFFFFFFFF:FFFFFFFFFFFFFFFFFFFFFFFFFFFFFFFFFFFFFFFFFF
FFFFFFFFFFFFFF:FFFFFFFFFFFFFFFFFFFFFFFFFFFFFFFFF
@A00721:190:HGJW7DSXY:4:1511:27995:27336_2:N:0:TCTGCAAG+GCTGCACT
TGGTATAATGTGTCTTACAAATAGAACCTTCACTCTCGGTAATGAGAGTGTATTCCTTAATATTCTAG
TAACCGCTATGTGGGCATTACTAGGATTTATCACACTCCTAAATTTGA
+
,FFFFFFFFFFFFFFFFFF:FFFFFFFFFFFFFFFFFFFFFFFFFFFFFFFFFFFFFFFFFFFFF:FF
FFFFFFFFF,FFFFFFFFFFFFFFFFFFFFFFFFF,FFFF:FFFF:FFF
@A00721:190:HGJW7DSXY:4:1478:22453:6558_2:N:0:TCTGCAAG+GCTGCACT
TGCTAGGAAAATTTTAGAAAAACTCCATTGTATAGTTCAAGGAGTACTTAATTCAATAGCTTTAGTAT
TATTTACTATCGCCTTTATTGAAAATTGTATAAGAGTTAATTGCTTAC
+
FFFFFFFFFFFFFFFFFFFFFFFFFFFFFFFFFFFFFFFFFFFFFFFFFFFFFFFFFFFFFFFFFFFF
FFFFFFFFFFFFFFFFFFFFF:FFFFFFFFFFFFFFFFFFFF:FFFFFF
@A00721:190:HGJW7DSXY:4:1278:25952:27868_2:N:0:TCTGCAAG+GCTGCACT
ACCATAACCTACCATGACCAATCATCGCTCCAACTGGCACGCCAGACGTAGGGTCAAACCTACTACCG
CCGGTCGGCAGATCGGCACCACTGGTCTCTACGTTCAGGGACCCGCTG
+
FFFFFFFFFFFFFFFFFFFFFFFFFFFFFFFFFFFFFFFFFFFFFF:FFFFFFFFFFFFFFFFFFFFFF
FFFFFFFFFFFF:FFFFFFFFFFFFFFFFF:FFFFFFFFFFFFFFFFF
@A00721:190:HGJW7DSXY:4:2559:31638:24095_1:N:0:TCTGCAAG+GCTGCACT
CCTTCCCAACTCTAGATCTGCACAGAAGAAGGCTGAAGGTCGTTACCGGACGACCACTCAAAGGAAAC
```

GGGCTGTTGCCCAGAGGAAAGCAGCGACCACCGAGGTCGCTCGTAACATTGACGGCAGTCCCGTCATC
TCT
+
F,:FFFFFF,:FFFFFFFF:FF:FFFFFFFF,FFFFFFFFFFFFFFFFFFFFFF:F:::F,FF:FFF,
FFFFFFFFFF::F:FF:FFFFFFFFFFFFFFFFFF,FFFFFFFFFFFFFFFFFFFFFF,:FFFFFFFF
FFF
@A00721:190:HGJW7DSXY:4:1571:30382:36041_2:N:0:TCTGCAAG+GCTGCACT
CAGTACCTCAGTCAAAAGTCACATTCACCACTCCCATACCAATCAATGATGTGACTATACAGTTCGGC
CATTTCAAGAGTGGTCAATACGTCGTGGGAGGTACTGAAGCCTTCCCC
+
FFFFFFFFFFFFFFFFFFFFFFFFFFFFFFFFFFFFFFFFFFFFFFFFFFFFFFFFFFFFFFFFFFFF
FFFFFFFFFFFFFFFFFFFFFFFFFFFFFFFFFFFFFF:FFFFFFFFF
@A00721:190:HGJW7DSXY:4:1675:4327:10144_2:N:0:TCTGCAAG+GCTGCACT
AAGCCAATCAATAGTTCTTCTGACCAGAATGAAAAGGGGTTTACCAGGGTAGTCTCTAAGTCTACCAG
GAAGAGGGGAAATGTCCCCAAAACTGAGAAGAATAAAGGGAAAGGTCC
+
FFFFFFFFFFFFFFFFFFFFFFFFFFFFFFFFFF:FFFFFFFFFFFFFFFFFFFFFFFFFFFFFFFFF
FFFF:FFFFFFFFFFFFFFF:FFFFFFFFFFFFFFFFFFFFFFFFFFFF
@A00721:190:HGJW7DSXY:4:1422:28375:29340_2:N:0:TCTGCAAG+GCTGCACT
GGGAAATTACCCCTAATTCTGAAGGAAGATCTCCCACCATCTGGGGTTGGGAATAAGAAATCTGCACG
GGCCAAAGAGGCCGCTGCTACGAAGTCGCTGAAGGCGGCTTGGGTCAT
+
FFFFFFFFFFFFFFFFFFFFFFFFFFFFFFFFFFFFFFFFFFFFFFFFFFFFFFFFFFFFFFFFFFFF
FFFFFFFFFFFFFFFFFFFFFFFFFFFFFFFFFFFFFFFFFFFFFFF:
@A00721:190:HGJW7DSXY:4:2538:20130:36542_2:N:0:TCTGCAAG+GCTGCACT
TTGCGCCTGCCGACCCCATCTTTGAGACCAAGACTCTCTACAGTAGGTTCGTGCCTACTATGAGCAAA
TCTGTTGAATTCGTCACTGAGGACGAAGGTGAGAGGACTTTTGGCTAC
+
FFFFFFFFFFFFFFFFFFFFFFFFF:FFFFFFFFFFFFFFFFFFFFFFFFFFFFFFFFFFFFFFFFFF
FFFFFFFFF:FFFFFFFFFFFFFFFFFFFFFFFFFFFFFFFFFFF:FFF
@A00721:190:HGJW7DSXY:4:1664:24279:6840_2:N:0:TCTGCAAG+GCTGCACT
CGTGGGTTGAAGGCCGACGCTTCGAGCTTGAGTGTGACGAGGCCGGTGACGTTTTTGTCAACGGCGTT
TGGAGGATTTTATCCGCGCTTAACTATGGGGCGAAGATCCCCTATGGA
+
FF:FFFFFFFFFFFFFFFFFFFFFFFFFFFFFFFF:FFFFFFFFFFFFFFFFFFFFFFFFFFFFFFFF
FFFFFFFFFFFFFFFFFFFFFFFFFFFFFFFFFFFFFFFFFFFFFFFF
@A00721:190:HGJW7DSXY:4:2503:25518:3443_2:N:0:TCTGCAAG+GCTGCACT
TTGGCATATTTATGCCGACGATAATAAGCATGTCACCCTTAAGCACCGAGGTAAGCTCGGGACTTACA
AGAGAGAGTGTGAGAGAGTCGCTCTCACTATTTTTGCCCAGAATGCGG
+
FFFFFFFFFFFFFFFFFFFFFFFFFFFFFFFFFFFFFFFFFFFFFFFFFFF:FFFFFFFF,FFFFFF
FFFFFFFFFFFFFFFFFFFFFF,::FFFFFF:FFFF:FF:FFF,,FFF
@A00721:190:HGJW7DSXY:4:1428:19687:29857_2:N:0:TCTGCAAG+GCTGCACT
ATTACAACCTCTCTTTAGAGAGCATTAGAAGGAAGTTGGGCAAATTCCCACATATGGTAGACGTTGTC
ACACTTTTCTTGAAAATTTTTCCTTTCCTCCGTCATTGGGGATTTCGA
+
FFFFFFFFFFFFF:,FFFFFFFFFFFFFFFFFFFFFFFFFFFFFFFFFFFFFF:FFFFFFFFFFFFFF
FFFFFFFFFFFFFFFFFFFFFFFFFFFFFFFFFFFFFFFFFFFFFFFF
@A00721:190:HGJW7DSXY:4:1169:20139:22623_1:N:0:TCTGCAAG+GCTGCACT
GGAAGGTGTCAAGGGTCTTCTTCATGCTGATCTTTCAGGACCTAATCACATCATGATTGATAGTTACG
GCTCGTATCGAGTTGGAGGGAGGAAGTTCTCCGAAATTAAAGACGGGGGGATTAAGAGTCCAGCTGTA
ACT
+
FFFFFFFFFFFFFFFFFFFFFFFFFFFFFFFFFFFFFFFFFFFF,FF,FFFFFFFFFFF:FFFFFFF

FFFFFFFFFFFFFFFFFFFFFFFFFFFFFFFFFFFFFFFFFFFFFFFFFFFFFFFFFFFFFFFFFFFFFF
FFF
@A00721:190:HGJW7DSXY:4:1219:10456:9768_2:N:0:TCTGCAAG+GCTGCACT
AGAGGGGAGCTCTGAAAAGGAAACTTCGTCAGAAGATGAGGAAGTCTAAGAGGAGGAGTTTGAGGAAG
GCTCTAGAGAAGATCTCACCACCTGGAGAAGGCAAGTGGAGAGTCCAG
+
FFF:FFFFF,FFFFFFFFFFFFFFF:FFF:FFFFF:FFF:::,:FFFFFFFFFFFFFF,FF:FFFFF:
FFFFFFFF:F:FF::FFFFFFFFFFFFFFFF:FFFFFFF:FFFFFFFF
@A00721:190:HGJW7DSXY:4:1377:24505:23202_1:N:0:TCTGCAAG+GCTGCACT
CTTCCGATCTTTTGAAGCAGGGGTTACCACTTCCTAAGGTGGATGAGGGAGGTTGTTTCTTTGGAGTT
TTAGATAATCTTTTGAGAAGGGGTCATAGAGCCAGCAAGAGAAGCTTATATGCTGGTTTAAGTGATGA
TTA
+
FFFFFFFFFFFFFFFFFFFFFFFFFFFFFF:FFFF:FFFFFFFFFFFFFFFFFFFFFFFFFFFFFFFF
FFFFFFFFFFFFFFFFFFFFFFFFFFFFFFFFFFFFFFFFFFFFFFFFF:FFFFFFFFFFFFFFFFFF
FFF
@A00721:190:HGJW7DSXY:4:1441:12897:1470_1:N:0:TCTGCAAG+GCTGCACT
ATTTAGGTCAACTTGGGGTTTCGAATGTCATTAGTGCCTTACGATTGAAAGAGGTCCTTTTGAGTATC
GACAGATCTTTCGATCTTGAGGACGTACATTTAACGTGGCAGAATTCGGTAACTTACCATATTGTTCC
TTC
+
FFFFFFFFF:FFFFFFFFFFFFFFFFFFFFFFFFFFFFFFFFFFFFFFFFFFFFFFFFFFFFFFFFFF
FFFFFFFFFFFFFFFFFFFFFFFFFFFFFFFFFFFFFFFFFFFFFFFFFFFFFFFFFFFFFFFFFFFF
FFF
@A00721:190:HGJW7DSXY:4:1169:12933:10582_1:N:0:TCTGCAAG+GCTGCACT
TTTAACTTTGACTCAACTACTCCGATCAGATCCGGATGGTTTGATTGGTATGGACTACTCGACTTTTG
ATCTGGAGTTGTCTAGGGTCATGGCAGATACTACCGATAAGGTGAAAAATATGCAGGTCAAAGATTCT
GTA
+
FFFFFFFFFFFFFFFFFFFFFFFFFFFFFFFFFFFF,FFFFFFFFFFFFFFFFFFFFFFFFFFFFFFF
FFFFFFFFFFFFFFFFFFFFFFFFFFFFFFFFFFFFFFFFFFFFFFFFFFFFFFFFF:FFFFFFFFFF
FFF
@A00721:190:HGJW7DSXY:4:1234:1325:10050_2:N:0:TCTGCAAG+GCTGCACT
GTTTTGGCCGACTTTAACTATTCTAAGAGTCTTTATGATTCAAAACCTATCGTTGTCGTACCTTACAA
GGTTGAGGAGACTTTACAGCGGCTTATTGTCGCGCAGTTTCCAGAATT
+
FFFFFFFFFFFFFFFFFFFFFFFFFFFFFFFFFF:FFFFFFFFFFFFFFFFFFFFFFFFFFFFFFFFF
FFFFFFFFFFFFFFFFFFFF:F::FFF:FFFFFFFFFFFFFFFFFFFF
@A00721:190:HGJW7DSXY:4:1322:11198:22013_1:N:0:TCTGCAAG+GCTGCACT
TATCTTGAATGCTCACGCAGTCGCAGCCATATCGAGACTATGTTATAATCACTTGTTAAAGTACAGAT
TTAGTGATTTAGATATTATTGATATTGGAGGCGATATTCGTTTTCATATCCAACACTCCTTGAATGGA
GTA
+
F,FFF,,F:FFF,FF:FF,FFFFFFFFF,:FF,FFFFFFFFFFFFFFFFFFFFFF:F,FFFF,F:FFF
FF:FFFFF:F,F::FFFFFFFFF:FFF:FF,F,,FFFF:FF:FF:FF,FFFF:,FFF,FFFFF:F::F
FF,
@A00721:190:HGJW7DSXY:4:2601:12554:3756_2:N:0:TCTGCAAG+GCTGCACT
CCTATTCTTGATGTGAAAGATGCATCGAGGTATACGTCTAGGAACAGTGAGTTGGAACGTACCTACTC
ACGAGACGTCATGAAAGCTATCGGCGGGAAGCAAAATGTGTCGTTCTG
+
F:,FFFFF,FFFF,:,F,,F:,F,,FFF,,FF::FF,F,,:FFFF,,:,F,,,FFF:FF:FFF,FF,F
FF,FFF:F:FFF,FF::F::FFF:,F::F,FFFF:F:::,F:FFFFFF
@A00721:190:HGJW7DSXY:4:2559:30400:2221_2:N:0:TCTGCAAG+GCTGCACT
AAGCCTCAGTTGCGATAGCTGTAGAGGTATACGATATCTCTCTTGATGATGTAGCCACTATAATGCTT

```
AAGAGAGAGATTGATGTATGTCATTTCGTCTTAGTTTGTCCCGGCGAA
+
FFFFFFFFFFFFFFFFFFFFFFFFFFFFFFFFFFFFFFFFFFFFFFFFFFFFFFFFFFFFFFFFFFFFFF
FFFFFFFFFFFFFFFFFFFFFFFFFFFFFFFFFFFFFFFF:FFFFFFF
@A00721:190:HGJW7DSXY:4:1445:4616:8547_2:N:0:TCTGCAAG+GCTGCACT
TGACCTTGGTATCGTTATCCGTCATGGAGATGATGGCGCATCACTTGATTACTATATGCCCGAAGGAT
TGGGTTATTCTCACAACAAAGATAAGATTTTATCTTTCTTCAAACACC
+
FFFF:FFFFFFFFFFFFFFFFF::FFFFFFFFFFFFFFFFFFFFFFFFFFF:FFFFFFFFFFFFF:F
FFFFFFFFFFFFFFFFFFFFFFFFFFFFFFFFFFF:F:FFFF:FFFFFF
@A00721:190:HGJW7DSXY:4:2212:18701:12555_2:N:0:TCTGCAAG+GCTGCACT
AAGGGCGGACAATTGTTCCATTGTGAACTCTTGGGGACTAGATTGAGCATTAATGAGTTCCGAATAAC
TCGTAATAAATCTTTCTCTAGTGGACAATTCAGAATACCTATTTCCGT
+
FFFFFFFFFFFFFFFFFFFFFFFFFFFFFFFFFFFFFFFFFFFFFFFFFFFFFFFFFFFFFFFFFFFFFF
FFFFFFFFFFFFFFFFFFFFFFFFFFF:FFFFFFFFFFFFFFFFFFFFF
@A00721:190:HGJW7DSXY:4:1509:1588:24815_1:N:0:TCTGCAAG+GCTGCACT
CTGCCAATCTCATCTCTATACGCATACCTGATTGGAAGAAGCGAGGTTCGTTACTCAATAAAAATTTT
TCTCAAGAGATTATGGTTGATAAAGTTTTCTTCATAAGGGCTATTCAGTACGTCATGGGGATCACTTC
TTC
+
:FF:F:FFFFFFFF,FF:F,F:,FF:FFF:FFFFF:,F,,,FFF:FFFFF:F,,F,:,F:FFF:FFFF
FFF:F,F,,FFFFFFFFFF:F,FFFFFFF:FF,,FF:,FF,F,:F:FFF,:FFF:::FFFFFFFFF:F
FF:
@A00721:190:HGJW7DSXY:4:1466:32633:22968_1:N:0:TCTGCAAG+GCTGCACT
TCGAATATGTTACGACATGGCTTCGTAACAACAGTGCGAGGATTGTCATCTCGGGGAAAGTGATTCAC
CAAGATGTTCATATAGATACGAGATACCTAGAGAAGGTCGCAGCGATCTTACTCGTTATCGGAGCTAA
GAA
+
FFFFF:FFFFFFFFFFFFFFFF:FFFFFFFFFFFFFFFFFFFFFFFFFFFFFFFFFFFFFFFFFF:FFF
FFFFFFFFFFFFFFFFFFFFFFFF,:FFFFFFFFFFFFFFFFFFFFFFFFFFFFFFFFFFFFFFFFFFF
FFF
@A00721:190:HGJW7DSXY:4:1452:15881:15280_1:N:0:TCTGCAAG+GCTGCACT
TCGCAAACTTAACGTTAGTACCGGAGGTGATCTCTCTGCTTCTGCGTTACTGAGAGAAAAGATTCAGG
ACGCCTACGGTGACTTTATGGACAAGATGCAAGTTCTTGGGAAATACTTGTTTCCCTTTTTAGGTTTA
GTA
+
FFFFFFFFFFFFFFF:FFFFFFFFFFFFFFFFFFFFFFFFFFFFFFFFFFFFFFFFF:FFFFFFFFFFF
FFFFFFFFFFFFFFFFFFFFFFFFFFFFFFFFFFFFFFFFFFFFFFFFFFFFFFFFFFFFFFFFFFFFFF
FFF
@A00721:190:HGJW7DSXY:4:2232:18340:7545_1:N:0:TCTGCAAG+GCTGCACT
CTCTTCCGATCTGACAACGAATCTTGAAACTACGGTCCTTATCAAAGAAAATGGTGAACCGGTCTCTA
AATCTTTTGAGATTGAGAAGATTGAAGAACACGTCAAGAAGTCGCTCGGCGTTGTTCTTGAAAACCAA
GT
+
FFFFFF,,FFFFFFFFFFFFFFFFF,FF::FFF,FFF,FFFF:FF:FFFFFFFFFFFF:FFFFFFFFF
:FFFFFFFFFFFFFFFFFFFFFFFFFFFFFFFFFFFFFFFFFFFFFFFFFFFFFFFFFFFFFFFFFFFFF
FF
@A00721:190:HGJW7DSXY:4:2121:12084:20134_1:N:0:TCTGCAAG+GCTGCACT
GCTGATCATGGAGTCTTTGTACCTTCTGTCAAACTTCGAGGTTCAGCTACGAGAGGTGATCCCGGATG
TGGATTGAAGGCTGGCTCTAAGGGAGACTCCTTCTTTTTGAATGTTGTAAAGAATTGGGTTGAGAAGT
GTG
+
FFF:FFFFFFFFFFFFFFFFFFFFFFFFFFFFFFFFFFFFFFFFFFFFFFFFFFFFFFFFFFFFFFFFF
```

```
FFFFFFFFFFFFFFFFFFFFFFFFFFFFFFFF:FFFFFFFFFFFFFFFFFFFFFFFFFFFFFFFFFF
FFF
@A00721:190:HGJW7DSXY:4:2647:19922:13072_1:N:0:TCTGCAAG+GCTGCACT
CTTGGTATTTCTGAAATAGTTTATCAGAGCGTCATTTTCCTTTGGGATCTTTCCTCTGGTAAGGGAAT
TATTAAATCATTCTTCTCACTCTTGACGAGAATTATTACTGTTTTCTCTACCCATAACTTCTTTTTCA
AAA
+
FFFF:FFFFFFFFFFFFFFFFFFFFFFFF:FFFFFFFFFFFFFFFFFFFFFFFFFFFFFFFFFF:FFFF
FFFFFFFFFFFFFFFFFFFFFFFFFFFFFFFFFFFFFFFFFF:FFFFFFFFFFFFFFFFFFFFFFFFF
FFF
@A00721:190:HGJW7DSXY:4:2445:21802:30013_2:N:0:TCTGCAAG+GCTGCACT
TACAGAGTGCAGTCGTTTCAACACTTATCACCACTTTTGAAAGGAAAATAATTGGTGATGAAAATACG
TATGTCGGTGGGGTTCTCAAGGCTCTCATCGCCACTGGGATTCTAAGG
+
FFFFFFFFFFFFFFFFFF:FFFF,:FFFFFFF::,:FF,,FFFF:FFF:F:FF:FFF:FF:FFFFF::
:,F:FFFF:,FFF,:,,:FFFF,,,:FFF:F,F:F,FFF:FF::FFFF
@A00721:190:HGJW7DSXY:4:2323:26946:5008_2:N:0:TCTGCAAG+GCTGCACT
TTTGGTCCTTACGTCTTAAGTAATTTGGATGTTGTCGAAAACTATCAGGTTCTTGTCACGATTCTTAA
AAGAAGTTTATTAAAACTTCAGAGTCTCTTGGAAGTCCAGGTAGCTAA
+
FFFFFFFFFFFFFFFFFFFFFFFFFFFFFFFFFFFFFFFFFFFFFFFFFFFFFFFFFFFFFF:FFFFFF
FFFFFFFFFFFFFFFF,FF:FFFFFFFFFFFFFFFFFFFFFFF,FFF:F
@A00721:190:HGJW7DSXY:4:2377:25084:25457_2:N:0:TCTGCAAG+GCTGCACT
ACCTCTGTTGGTAGAGGAGTTGAGAGAATTACTCAGATTCAATCTGGCATCACTGAACGTCTACAACG
AGTTAGGGAGTGGCTAGGTTTTAAAAGATCCTTTGCACAAGCAATACC
+
FFFFFFFFFFFFFFFFFFFFFFFFFFFFFFFFFFFFFFFFFFFFFFFFFFFFFFFFFFFFFFFFFFFFF
FFFFFFFFFFFFFFFFFFFFFFFFFFFFFFFFFFFFFFFFFFFFFFFF
@A00721:190:HGJW7DSXY:4:1645:2121:28338_2:N:0:TCTGCAAG+GCTGCACT
GTCGCGGAAAACGATGAAGATTTCCACACTGTTCGAGAATCTTCTGATGCTGAATTTGAGGAAAATTT
TTCGGAAGTTTTTGGTATAAATCCTGATGGCTCAGTACAGGGAGGAGG
+
FFFFFFFFFFFFFFFFFFFFFFFFFFFFFFF:FFFFFFFFFFFFFFFFFFFFFF,F,:F:FFFFFFFFF
FFFFFFFFF,FF:FFFFFFFFFFFFFFFFFFFFFFFFFFFFFF:FFF:FF
@A00721:190:HGJW7DSXY:4:2514:20410:36652_2:N:0:TCTGCAAG+GCTGCACT
ACCTTCTTTTATCATTGCAACAGGACTTTGAGAAGACTGTTGAAATGGACAAAATCTGGGATCATAGG
TATGTGTCACGCTACACTCGAAATATGGGCCCTCATAAGGAAGTATAG
+
FFFFFFFFFFFFFFFFFFFFFFFFFFFFFFFFFFFFFFFFFFFFFFFFFFFFFFFFFFFFFFFFFFFFF
FFFFFFFFFFFFFFFFFFFFFFFFFFFFFFFFFFFF:FFFFFFFFFFF
@A00721:190:HGJW7DSXY:4:2121:13268:1297_2:N:0:TCTGCAAG+GCTGCACT
GGAAGATCTCTACAGAGATTATCGAGAGTTGTGATCTAGTTCTAAATTTAGGTCGACGGACTTTAGCA
ACTTTAGATAAATTCTTTCCGGGCTCAATGACCTTTGCTATGTACGTG
+
FFFFFFFFFFFFFFFFFFFFFFFFFFFFFFFFFFFFFFFFFFFFFFFFFFFFFFFFFFFFFFFFFFFFF
FFFFFFFFFFFFFFFFFFFFFFFFFFFFFFFFFFFFFFFFFFFFFFFF
@A00721:190:HGJW7DSXY:4:2323:19343:30295_1:N:0:TCTGCAAG+GCTGCACT
TTAGTTCATGAAGTTCTTACACTTCTCGCGACTAATGTCAGCTTCACTACAAAGGCGTTACGTAAAGT
AGTTTTCACGCTTTATCTTAAAGCATTACCACCAAATTTAGCACTTTTTGAATATGACAGAAGCTCGC
CAT
+
FFFFF:FFFFFFFFFFFFFFFFFFFFFFFFFFFFFFFFFFFFFFFFF:FFFFFFFFFFFFFFFFFFFFF
FFFFFFFFFFFFFFFFFFFFFFFFFFFFFFFFFFFFFFFFFFFFFFFFFFFFFFFFFFFFFFFFFFFFF
FFF
```

```
@A00721:190:HGJW7DSXY:4:2466:7139:23844_2:N:0:TCTGCAAG+GCTGCACT
CCAGTTAGAGTTCTTCTAACCAGGACTGATGGTGAGATTCTTTTACCTTTTGGAGGTCCAGGAACCTT
TACTCCTAGTGTCTTTTCTAGAGTCCAAGGACGTTTCCGTCGGAGGAT
+
FFFFFFFFFFFFFFFFFFFFFFFFFFFFFFFFFFFFFFFFFFFFFFFFFFFFFFFFFFFFFFFFFFFF
FFFFFFFFFFFFFF::FFFFFFFFFFFFFFFFFFFFFFFFFFFFFFFF
@A00721:190:HGJW7DSXY:4:1623:7780:36292_1:N:0:TCTGCAAG+GCTGCACT
TCAATAACACTCTATTGAATCCCTTTTCCACGATGAAAGCTCTTTATGAGGTGCTCCATGCAAATTTA
CAGTTCCTCTTTGGTGATATCGCCTCTGTCCTTTCTCCTTTAATCGTGAGGTTACGAGAGTTTCGTAC
TTG
+
FFFFFFFFFFFFFFFFFFFFFFFFFFFFFFFFFFFFFFFFFFF::,FFFFFFFFFFFFFFFFFFFFFF
FFFFFFFFFFFFFFFFFFFFFFFFFFF:FFFFFFFFFFF:FFFFFF:FFFFFFFFFFFFFFFFFFFFFF
FFF
@A00721:190:HGJW7DSXY:4:2606:20735:9846_1:N:0:TCTGCAAG+GCTGCACT
TTTTGATAAATTTGTTGGAATCGATAATGATACTGACGAAGGTTCTATTACCACCCTTGTCATTGATA
CAAACATTCTAGGTGATGCTTTCTTAGAGGCTCGTCTAGCCGCTGAGATTCGTCTTACCTATTTACAA
TCT
+
FFFF:FFFFFFFF:FFFFFFFFFFFFFFFF:FFFFFFFFFFFFFFFFFFFFFFFFFFFFFFFFFF:FFF
:FFF,FFFFFFFFFFFFFFFFFFFFFFFFFFFFFFFFFFFFFFFFFFFFFFFFFFFFFFFFFFFFFFFF
FFF
@A00721:190:HGJW7DSXY:4:1141:10429:20149_2:N:0:TCTGCAAG+GCTGCACT
GGGAAATGGTGGTAGTCAGTTTACTACTTTTCTTTCCCTCCCCTTTTTCGATTGGATTCGACGGGGTC
TTCGTCTCACGACTAATTTGATAAGGTTTTTCTTTTATGGGACTCTTG
+
FFFF:FFFFFFFFFFFFFFFFFFFFFFFFFFFFFFFFFFFFFFFFFFFFFFFFFFFFFFFFFFFFFFFF
FFFFFFFFFFFFFFFFFFFFFFFFFFFFFFFFFFFFFFFFFFFFFFFF
@A00721:190:HGJW7DSXY:4:1270:18177:15374_1:N:0:TCTGCAAG+GCTGCACT
ATGTAGGTCTCCTAGTACTTAAGGGTTTATGTTTCGGTTTCAGCTGGATGGATCTTTTGATGGTATGT
TTCACATTGCGTGATCACTTCCCGTGGACTTGGGAAGAGATTAAACATAAAATCCAGCGATTCTATTA
TGT
+
FFFFFFFFFFFFFFFFFFFFFFFFFFFFFFFFFFFFFFFFFFFFFFFFFFFFFFFFFFFFF:FFFFFFF
FFFFFFFFFFFFFFFFFF:FFFFFFFFFFFFFFFFFFFFFFFFFFFFFFFFFFFFFFFFFFFFFFFFFF
FFF
@A00721:190:HGJW7DSXY:4:2633:25373:24111_2:N:0:TCTGCAAG+GCTGCACT
TCGATGATGTTCGGGCTGTGGGGATTAACTTCCCTCGTGAAACTTACGACGATGTTCTCGCTCGCATT
TCACAAGGATTACTAAGTCTGAGATCTGCCACTTCTAATTTTAGGAAA
+
FFFFFFFFFFFFFFFFFFFFFFFFFFFFFFFFFFFFFFFFFFFFFFFFFFFFFFFFFFFFFFFFFFFF
FFFFFFFFFFFFFFFFFFFFFFFFFFFFFFF:FFFFFFFFFFFFFFFF
@A00721:190:HGJW7DSXY:4:1526:5448:36573_2:N:0:TCTGCAAG+GCTGCACT
AAGTTCATGAGGATGTCTTGGTCGAAAAATATCGCCGGGAACTTCTAATAGAGGATCCTGATGACATT
ATTTCAACGGATACCGATCTCAGTTCTACCACAGAAACCGTCTCAGGA
+
FFFFFFFFFFFFFFFFFFFFFFFFFFFFFFFFFFFFFFFF,,FFFFFFFFFF:FF:F:FFFFFFFFFF
FFFFFF:FFFFFFFFFFFFFFFFFFFFFFFFFFFFFFFFFFFFFFFFF
@A00721:190:HGJW7DSXY:4:2443:27715:30639_2:N:0:TCTGCAAG+GCTGCACT
CTGATTCGGAATTAGAGAAAGACGACCTTCTTGTGGGGACTGAGACCTCTGAATTTTCAGGGTGCCCT
GATGAGGACGTTGAAAGGGATAATTTCAACTCTGAGACTTCTAAGAGT
+
F:FF:FFFFF:FFFFFFFFFFFFFFFFFFFF,FFFFFFFFFFFFFFFFF,FFFFFFFFFFFFF:FFFF
FFFF:FFFFFFFFFFFFFFFFFFFFFFFFFFFFFFFFFFFFFF,FFFFF
```

@A00721:190:HGJW7DSXY:4:2225:14136:17425_2:N:0:TCTGCAAG+GCTGCACT
CAGAAACGTAACAAGAAGAATAAAGGTAGGAAGGGGAGAAAACAGAACAAGCGAGCGGTTGAAGAGAA
AACTGATATGGTTGCGGCTGATCGTACCCCAATTCCTGCTAGAAAGAT
+
FFFFFFFFFF,,,:FFFFF:FFF,FFFF:FF,F:FF,,FFF:FFFFFFFFF,FFF,FFFFFFFF:FFFFF
F:FFFFF:F,:FFFFFFFF,F:FFF:FF,FFFFFFFFFF:FFF,F:FF
@A00721:190:HGJW7DSXY:4:2628:4472:29403_2:N:0:TCTGCAAG+GCTGCACT
TACCACTGATCTTCCAGAGTTACGTAGTGGTCGTTCTTTTGGTGATTTTATCAAAGAGAAAGTTTCTG
CTAGTCGCGACGATAGTATTTATCGGAGTGATATTAACAAGTTAAACT
+
FFFFFFFFFFFFFFFFFFFFFFFFFFFFFFFFFFFFFFFFFFFFFFFFFFFFFFFFFFFFFFFFFFFFFF
FFFFFFFFFFFFFFFFFFFFFFFFFFFFFFFFFFFFFFFFFFFFFFFF
@A00721:190:HGJW7DSXY:4:2660:26811:12101_2:N:0:TCTGCAAG+GCTGCACT
TGTTGAAGTACCGCAACATGGATCAGTTTTCCAAAAAGCTATTAATAGCAGTTGCGGGGCTATAATAC
CGCCTCCAGATTACTCTAAACTGAATCCACTCTCTTGGGATATAAATG
+
FFFFFFFFF::FFFFFFFFFFFFFFFF::FFFFFFFFFFFFFFFFFFFF::FF:FF:F::FFFFF:FFFF
F:FFFFFFFFFFFFFFFFFFF,:FF:F:FFFFFF::,,:FFFFFFFF:
@A00721:190:HGJW7DSXY:4:2635:16613:13479_1:N:0:TCTGCAAG+GCTGCACT
TTCTTCATGAAGATCTTCGAGTGTTTTATAGTGAGTTATTCTTGCGAAGGTTCAAGTCGATTGATTTT
GTTGACTACGATCTCTCTTGTAATAAGAAGTTCTTCGCAGTTGAATCTCCACCAGGTGGAGGGAAGAC
CAC
+
FFFFFFFFFFFFFFFFFFFFFFFFFFFFFFFFFFFFFFFFFFFFFFFFFFFFFFFFFFFFFFFFFFFFFF
FFFFFFFFFFFFFFFFFFFFFFFFFFFFFFFFFFFFFFFFFFFFFFFFFFFFFFFFFFFFFFFFFFFFFF
FFF
@A00721:190:HGJW7DSXY:4:2646:2844:23829_1:N:0:TCTGCAAG+GCTGCACT
GACTTTTGTCGAATTTTTCGCAAAAGGTCTTGAAGTACGTATTGTCACTGCTAATCGTAATTCGCGAG
AAGAGATAGTGACTAAAGTTTTGGCACTTCTAGTTCATCGCGGTATTTGTGAAATTAAGACAGACGTG
AAA
+
FFFFFFFFFFFFFFFFFFFFFFFFFFFFF:FFFFFFFFFFFFFFFFFFFFFFFFFFFFFFFFFFFFFFFF
FFFFFFFFFFFFFFFFFFFFFFFFFFFFFFFFFFFFFFFFFFFFFFFFFFFFFFFFFFFFFFFFFFFFFF
FFF
@A00721:190:HGJW7DSXY:4:2174:16839:32503_1:N:0:TCTGCAAG+GCTGCACT
TCGTATTAGAACAGCTGATTCTTATTGCATGAACGTCGCTGAGGTCAAACCTGTGGACGTTCTTTTCA
TCGACGAATTATTCATGATTCACGCTGGTCAGACCCTTTTTCTTACACATCTACTTAAGGCGGCTTAT
ACC
+
FFFFFFFFFFFFFFFFFFFFFFFFF:FFFFFFFFFFFFFFFFFFFFFFFFFFFFFFFFFFFFFFFFFFFF
FFFFFFFFFFFFFFFFFFFFFFFFFFFFFFFFFFFFFFFFFFFFFFFFFFFFFFFFFFFFFFFFFFFFF:
FFF
@A00721:190:HGJW7DSXY:4:2638:5141:7921_1:N:0:TCTGCAAG+GCTGCACT
GTGATTCCAATCAAATCGGATTCATCAATCGTATTGATCTCCAAGTCGCAAACTACTTCAACATCAAC
GACATCATTCTTGATGAGTGTCGTTCTTATCGAAGTCTCAGTTATCGCTGCCCGGCCGACGTTTGTCG
CGA
+
FFFFFFFF:FFFFFFFFFFFFFFFFFFFFFFFFFFFFFFFFF:FFFFFFFFFFFFFFFFFFFFFFFFF:F
FFFFFFFFFFFFFFFFFFFFFFFFFFFFFFFFFFFFFFFFFFFFFFFFFFFFFFFFFFFFFFFFFFFFFF
FFF
@A00721:190:HGJW7DSXY:4:2322:30065:31986_1:N:0:TCTGCAAG+GCTGCACT
ACGGCAGAAAAATTTCTCCTGCTTGTAATTCGAGAGAGGACACTATGGAAATTAGACCTATCAGATGT
CTTGACGACTTCGAATTTGAGGACGGAGTTAAATATATTACTTTCACTCAAGGTGAAAAGTTTGAAAT
TAA

+
FFFFFFFFFFFFFFFFFFFFFFFFFFFFFFFFFFFFFFFFFFFFFFFFFFFFFFFFFFFFFFFFFFFFFF
FFFFFFFFFFFFFFFFFFFFFFFFFFFFFFFFFFFFFFFFFFFFFFFFFFFFFFFFFFFFFFFFFFFFFF
FFF
@A00721:190:HGJW7DSXY:4:1372:16161:30796_1:N:0:TCTGCAAG+GCTGCACT
TAAGAGTAAGGTTAAATGTGAAGATGCTCAGACTATTCATGAAGTTCAAGGTAATACTTACCAGCACG
TTCGGCTGGTTAGAATTAAACCTCAAGATGACCCTGTTTTTGCATCACAAACACATATTACCGTTGCT
CTA
+
FFFFFFFFFFFFFFFFFFFFFFFFFFFFFFFFFFFFFFFFFFFFFFFFFFFFFFFFFFFFFFFFFFFFFF
FFFFFFFFFFFFFFFFFFFFFFFFFFFFFFFFFFFFFFFFFFFFFFFFFFFFFFFFFFFFFFFFFFFFFF
F:F
@A00721:190:HGJW7DSXY:4:1501:24813:5415_1:N:0:TCTGCAAG+GCTGCACT
CTTTACTATACTTTGTTATCAATGTAAAGTTAAACGATGCTACTTGTGAGAGGATCAATCACAGTAAG
GAACTGTCACGTTTAGCACGGGAGGTTGCAGATTCAACCCCTTATATCGAGGCTATCGAGGACGATGA
GTA
+
:FFFFF,:FFFFFFFFFFF,FFF,FFF,,F:,FF:FFFFFFFFFFFFFFFFF:FF:FFFFFFFFFFFF
FFFFFFFFFF:FFFFFFFFFFFFFFFFFFFFF:FFFFFFFFFFFFF:FFFFFF:FFF:FFFFF::FFFF
FFF
@A00721:190:HGJW7DSXY:4:1340:19425:31469_2:N:0:TCTGCAAG+GCTGCACT
GACGACAAGCCTCAAGAGGGTAGAGGACCACCTGGATCTTCTACTGTCTCTGCCTTCCAAGATTTCTT
AGAAGAGGTTATTCCTGGAACCACCTTAACGTCACTCGGTAATCTTTC
+
FFFFFFFFFFFFFFFFFFFFFFFFFFFFFFFFFFFFFFFFFFFFFFFFFFFFFFFFF:FFFFFFFFFFF
FFFFFFFFFFFFFFFFFFF:FFFFFFFFFFFFFFFFFFFFFFFFFFFF
@A00721:190:HGJW7DSXY:4:1664:31521:28526_2:N:0:TCTGCAAG+GCTGCACT
TGTCGATCGAATTAAGATCAAGGCTAATACCTTTATAGGTAAGAAGCCCCTTGAGCCGCGTAGTCAGC
GCGTTTAGCTGTATCATCAGTCAGAGTATACCCGATAGGGTACCGAGT
+
FFFFFFF:FFFFFFFFFFFFFFFFFFFF:FFFFFFFFFFFFFFFFFFFFFFFFFFFFFFFFFFFFFFFF
FFFFFFFFFFFFFFFFFFFFFFFFFFFFFFFFFFFFFFFFFFFFFFFF
@A00721:190:HGJW7DSXY:4:1636:8883:32471_2:N:0:TCTGCAAG+GCTGCACT
ATATTTATTCTTATGAGGCTCGAAACTTCAACGTTTTCACAACGGAAAGACATATCGCACCCGAAATT
TTTGGGCAGAATGCTCTTAACGCGGTTCTTGAACGGTGTTTTGATCAG
+
FFFFFFFFFFFFFFFFFFFFFFFFFFFFFFFFFFFFFFFFFFFFFFFFFFFFFFFFFFFFFFFFFFFFFF
FFFFFFFFFFFFFFFFFFFFFFFFFFFFFFFFFFFFFFFFFFFFFFFF
@A00721:190:HGJW7DSXY:4:2150:1181:33254_2:N:0:TCTGCAAG+GCTGCACT
TAAAGTTATAGGAGACTCAGTTAACCTGAGAGCTCCTCAATTGATAGATTGGGTCAACAATAGGGACG
CTAGTATACTTCGTAAAGTCGACAGCGAAATGGTCAATATTGATAATT
+
FFFFFFFFFFFFF:FFFFFFFFFFFFFFFFFFFFFFFFFFFFFFF:FFFFFFFFFFFF:FFFFFFFFFFF
FFFFFFFFFFFFFFFFFFFFFFFFF,FFFFF,FFFFFFFFFFF:FFFFF
@A00721:190:HGJW7DSXY:4:2647:8712:4492_1:N:0:TCTGCAAG+GCTGCACT
TGACCTATTTTAAGCTCATGGTGAAAAGGGAAGCTAAGGTTAAACTTGATGCTTCAGCCTTTTCGAAG
CATAACCCCGCACAGAATATCGTTTATCACGCAAAATTTATTAATGCAATTTTCGGACCTATCTTTAA
GGT
+
FFFFFFFFFFFFFFFFFFFFFFFFFFFFFFFFFFFFFFFF:FFFFFFFFFFFFFFFFFFFFFFFFFFFFF
FFFFFFFFFFFFFFFFFFFFFFFFFFFFFFFFFFFFFFFFFFFFFFFFFFFFFFFFFFFFFFFFFFFFFF
FFF
@A00721:190:HGJW7DSXY:4:1324:32615:4523_1:N:0:TCTGCAAG+GCTGCACT
AGCTCTTCTCTCTCTTAGGAGGTCATGATGACTACTATAAGGGTGAAATCGATTTTTCGAAGTTTGAC

AAATCTCAAGATTTCTTTTGTAAAGCCTATGAGATAAAGATGTATTCTTATTTAGGCTTCGATGCTGA
ATT
+
FFF,F::FFFFFFFF,FFFFFFFFF:FF:FFFFFFFFFFFFFF:FFFFFFFF,FFFFFFFFFFFFFFF
FFFFFFFFFFFFFFFFFFFFFFFFFFFFFFFFFFFFFFFFFFFFFFFFFFFFFFFFFFFFFFFFFFFF
FFF
@A00721:190:HGJW7DSXY:4:2152:3495:9236_2:N:0:TCTGCAAG+GCTGCACT
AGTGCCAGTGAATATTTTTGTCACGCCAGGAGTATCCATAACGATTTGTCATTTACCACCTTTGCTCA
ACGACGAAGTGGAACGGCCAACACTTTCTTTGGTAATTGTATGATCAC
+
FFFFFFFFFFFFFFFFFFFFFFFFFFFFFFFFFFFFFFFFFFFFFFFFFFFFFFFFFFFFFFFFFFFF
FFFFFFFFFFFFFFFFFFFFFFFFFFFFFFFFFFFFFFFFFFFFFFFF
@A00721:190:HGJW7DSXY:4:1173:7084:5995_2:N:0:TCTGCAAG+GCTGCACT
TATCACTTTATTATGACCTTGGAAAGTTTTCGGCTGTGGTTGTCGCTGGGGACGATTCCATTCTGTTT
TCAAAGACTCCAATAACTGATCATTCTGATCATATGATTTTGGACAAT
+
FFFFFFFFFFFFFFFFFFFFFFF:FFFFFFFFFFFFFFFFFFFFFFFFFFFFFFFFFFFFFFFFFFFF
FFFFFFFFFFFFFFFFF:FFFFF:FFFFFFFFFFFFFFFFFFFFFF:F
@A00721:190:HGJW7DSXY:4:1274:8097:19085_2:N:0:TCTGCAAG+GCTGCACT
ATCGTTAAGTTAGGTAAACCCCTAACCGATGTTACTCTTGTAGGACTTTGGGAGAGATTCGTTAGTTT
TAAAGACTATACTAAATATCTCCACTACGAGGTTGTTGTTTGGCGATT
+
FFFFFFFFFFFFFFFFFFFFFFFFFFFFFFFFFFFFFFFFFFFFF:FFFFFFFFFF:FFFFFFFF:F
FFFFFFFFFFFFFFFFFFFFFFFFF:FFFFFF:,FFFFFFFFFFFFFF
@A00721:190:HGJW7DSXY:4:1358:27082:27477_2:N:0:TCTGCAAG+GCTGCACT
ATATTCGATATTCGATTGAGGGTCGTTATACCTATTCGGCTATCTGTGCTTTGAATTGTATAAGATCA
AATTTTAAACGATTCCGAGAGGTTTATCCTGATGTGGAGGGAGTTCTT
+
FFFFFFFFFFFFFFFFFFFFFFFFFFFFFFFFFFFFFFFFFFFFFFFFFFFFFFFFFFFFFFFFFFFF
FFFFFFFF:FFFFFFFFFFFFFFFFFFFFFFFFFFFFFFFFFFFFFFF
@A00721:190:HGJW7DSXY:4:1645:1461:28354_1:N:0:TCTGCAAG+GCTGCACT
ACCTTTCCTTGTGAAAATTTTGATGCGGTTTCATAAGATTATCAGTAATCCTGATTCTACTACGGATT
CATGGACCTTCCATCCTGTACCTCTTCTGGAGAGACGTATCTCAAAGATCGACTTCGAGACAGGTGAT
ATT
+
FFFFFFFFFFFFFFFFFF:FFFFFFFFFFF:FFFFFFFFFFFFFFFF:FFFFFF:FFFFF,FFFFFF:
FFFFFFFFFFFFFFFFFFFFFFFFFFFFFFFFFFFFFFFFFFFFF:FFFFFFFFFFFFFFFFFFFFFF
FFF
@A00721:190:HGJW7DSXY:4:2202:22616:23938_2:N:0:TCTGCAAG+GCTGCACT
CTAAATTCGGTAACGTTGCGTGGCGCTTTATTTTAAAAAGCTCCCTCCTCTCGCAAGTTTGATCTGAT
ACTTATAACGAAAGGACTTGAGTCTTTTTATCAGTCTCTGACTTGACC
+
FF:FFFFF:FFF:,F,::FFFFFFFFFFFFF,:FFFFFFFF:F,FFFFFFFFF::F,F:,,FFF,FFF
:FFF,F,F,:FFFFFF:FF,F,F:FF,:,FFFFFFFFF,F,:,:FFFF
@A00721:190:HGJW7DSXY:4:2225:23086:5494_2:N:0:TCTGCAAG+GCTGCACT
TAAATGACGATCATTAAGACTCTCTTTCTTCTTTGTTTCCTTCTTTGCACTGATTTTTCCTCATCAGC
TTGTGTTTTACCTAATCTGTGTTTTTACGATAACAACTATCAGTTGAT
+
FFFFFFFFFFFFFFFFFF,FFFFF::FFFFFF:FFFFFFFFFFFFFFFF:FFFFFFFFFFFFFFFFF
FFFFFFFFFF:FFFFFFFFFFFFFFFFF:FFFFFFF:FFF,FFFFFFF
@A00721:190:HGJW7DSXY:4:1274:28483:17315_1:N:0:TCTGCAAG+GCTGCACT
CCGAAAGGCGAACTTTGGGAATTTGAAGCTCAGGGTGAAGCACAACGTTTGACCGTGACGAACTGTGG
TGTTCTTGATCTACGTTACGGATATACTCTCCTTGAGTTCACTCGTAAGGACACTACCACTTATTACG
ATA

```
+
FFFFF:FFFFFFFFFFFFFF:FFFFF:FFFF:FFFFFFFFFFFFFFFFFFF:FFFFFFFFFFFFFFFF
FFF:FFFFFFFFFFFFFFFFFFFFFFFFFFFFFFFFFFFFFFFFFFFFFFFFFFFFFFFFFFFFFFFF
FFF
@A00721:190:HGJW7DSXY:4:2203:24804:23782_1:N:0:TCTGCAAG+GCTGCACT
TCTCTATTTTAAGTTTTGTCTTCCTTGTTTGTCTTATTCTATTAATCACTCATTTTAGTGAGATTATT
AAAAACGCACTAAAACGTCTTCGACAACAGCAGGGGTTTTCGAATAGTGATAGCCACCCGCCTTACAG
AGG
+
FF:FFFFFFFFFFFFFFFFFFFFFFFFFFFFFFFFFFFFFFFFFFFFFFFFFFFFFFFFFFFFFFFFF
FFFFFFFFFFFFFFFFFFFFFFFFFFFFFFFFFFFFFFFFFFFFFFFFFFFFFFFFFFFFFFFFFFFF
FFF
@A00721:190:HGJW7DSXY:4:2411:28763:4554_1:N:0:TCTGCAAG+GCTGCACT
GACTTTGGTACCACCTTTTCTACTGTCTGTCTCGCCTCTTCGAATGAAGTTTTAATGCTTAACAACAA
TGATAGTGAATTTATACCTACAATCATTGGTTTTTCTAATAATGATGACACTATTGTTTATGGATATG
ATG
+
FFFFFFFFFFFFF:FFFFFFFFFFFFFFFFFFFF:FFFFFFFFFFFFFFFFFFFFFFFFFFFFFFFFF
FFFFFFFFFFFFFFFFFFFFFFFFFFFFFFFFFFFFFFFFF:FFFFFFFFFFFFFFFFFFFFFFFFFF
FFF
@A00721:190:HGJW7DSXY:4:1465:7907:33974_1:N:0:TCTGCAAG+GCTGCACT
TCTGGATTCTCAGTTTACAAGGACCTTAAGCGATGGATCGGGGTTAATTCTAAAACATTACCCGAAAG
ACGTGCTAAATTAAAACCATTGTACGATGTCGTCTGTCTCGGCGATCACTTTTCACTAAGAATTGGTC
CTA
+
FF:FFFFFFFFFF:FFFFFFFFFFFFFFFFFFFFFFFFFFFFFFFFFFFFFFFFFFFFFFFFFFFFFF
FFFFFFFFFFFFFFFFFFFFFFFFFFFFFFFFFFFFFFFFFFFFFFFFFFFFFFFFFFFFFFFFFFFF
FFF
@A00721:190:HGJW7DSXY:4:1575:31485:1971_1:N:0:TCTGCAAG+GCTGCACT
AGACGTGTGCTCTTCCGATCTATCGTTTCTTTCTTTATCTCTTTGGTTGTTAGGGATTTTGAGAGAGT
GAAAGGCTTCAATTGTAGTGGTTTGGTCGTTTCAGTACCCAGTCAGTATACCTCTACGCAAAGATATT
TCA
+
F:F:FFFFFFFFFFF,FFFFFFFFFFFFFFFFFFFFFFFFFFFFFF:FFFFFFFFFFFFFFFFFFFFFF
FFFFF:FFFFFFFFFFFFFFF,FFFFFFFFFFFFFFFF:FFFFFFFFFFFFFFFFFFFFF:FFFFFFF
FFF
@A00721:190:HGJW7DSXY:4:2358:2257:9283_2:N:0:TCTGCAAG+GCTGCACT
GAGGAACGTACCGGATTACCTGTCTTTCATATTATGAATGAACCTTCGGCCGCTCTCTTTGCTTCGAT
GTTGGATGTCAAGAAGGGTTCAGAATGGGATTCCTACGTAGTTTATGA
+
FFFFFFFFF:FFFFFFFFFFFFFFFFFF:FFFFF:FFFFFFFFFFFFFFFFFFFFFFFFFFF:FFFFF
F,FFFFFFFFFFFFFFFFFFFFFFF,FFFFFFFFF:FFFFFFFFFFFF
@A00721:190:HGJW7DSXY:4:2364:21793:21574_1:N:0:TCTGCAAG+GCTGCACT
TACTTTCGATACTTCTATCGTCTTACGTTACGGTAATTACTATAGTGTTATTTATTCTGGTGGTGATG
ATAATCTTGGAGGTCGTGACGTTGATGAAGCTATAAGAGCGTTCTTATTACGGCGCACTTCTTTACCC
GCC
+
FFFFFFFFFFFFFFFFFFFFFFFFFFFFFFFFFFFFFFFFFFF,FFFFFFFFFFFFFFFFFFFFFFFF
FFFFFFFFFFFFFFFFFFFFFFFFFFFFFFFFFFFFFFFFFFFFFFFFFFFFFFFFFFFFFFFFFFFF
FFF
@A00721:190:HGJW7DSXY:4:1408:17553:15107_2:N:0:TCTGCAAG+GCTGCACT
GTTTCACAACTGAAGGAGGAGGTTTCTGTTACTGGAAAACCCTCGTCAATACTTTTCAATAATATACG
TCTTGAACTCACTTATGACGATCTAGTGAAATTAACGCGTCCTTTTCT
+
```

```
FFFFFFFFFFFFFFFFFFFFFFFFFFFFFFFFFFFFFFF:FFFF:FF:FFFFFFFFFFFFFFFFFFFF
FFFFFFFFFFFFFFFFFFFFFFFFFFFFFFFFFFFF:FFFFFFFFFFFF
@A00721:190:HGJW7DSXY:4:1451:16134:16313_1:N:0:TCTGCAAG+GCTGCACT
TACTATTATGAACAAAGTTATCAAAGATAGTGGCATTCAAGGCAAACTTACCCTGATTCCGGCCGGTG
GTAGCTCATTTCTACCAGGGGCTATTACTCTTGCTGAAACAGCTATTAAAGCTGTTGAGCGGGTAATT
ACA
+
FFFFFFFFFFFFFFFFFFFFFFFFFFFFFFFFFFFFFFFFFFFFFFFFFFFFFFFFFFFFFFFFFF,F
FFFFFFFFFFFF:FFFFFFFFFFFF:FFFFFFFFFFFFFFFFFFFFFFFFFFFFFFFFFFFFFFFFFF
FFF
@A00721:190:HGJW7DSXY:4:2134:29252:16110_2:N:0:TCTGCAAG+GCTGCACT
ATCGCTGAAGGATGTGCACTCTTTTCTGCTACTTTAACGGAGCGTAGTGTACTCTTCGTCGATTGTAT
CAACAGTAACATTACTTCTAATAAAGGTCTCTATCAAGCTCGTATTAT
+
FFFFFFFFFFFFFFFFFFFFFFFFFFFFFFFFFFFFFFFFFFFFFFFFFFFFFFFFFFFFFFFFFFFF
FFFFFFFFFFFFFFFFFFFFFFFFFF:FFFFFFFFFFFFFFFFFFFFFF
@A00721:190:HGJW7DSXY:4:1562:18502:15875_1:N:0:TCTGCAAG+GCTGCACT
TTACCGTTGAAGGCTAAACTCCAAGGTAATTATGAAGACTGTGATTTATCTTTCACTCTGGCCTTGTA
TGAGGGTGTTTCTAATCGTAACATTAATAATTTCAATCTTCTTAAAAAGAAGATAGAGAGTCATACTT
TGG
+
FFFFFFFFF:FFF:FF:FFFFFF:FFFFFFFFFFFFFFFFFFFFFFFFFFFFFFFFFFFFFFFFFFFF
FFFFFFFFFFFFFFFFFFFFFFFF:FFFFFFFFFFFFFFFFFFFFF,FFFFFFFFFFFFFFFFFFFFFFF
FFF
@A00721:190:HGJW7DSXY:4:2602:27959:29496_2:N:0:TCTGCAAG+GCTGCACT
TGAAGTGGACACCTCTGGTAGTATCGAAGTAAAGATAACCAATAAGGAAGGAGCTTATACTATGGTTG
AATCAACTAAGCATACTACTTTTGCTTTGATTACTCTACCGCCTTACA
+
FFFFFFFFFFFFFFFFFFFFFFFFFFFFFFFFFFFFFFFFFFFFFFFFFFFFFFFFFFFFFFFFFFFF
FFFFFFFFFFFFFFFFFFFFFFFFFFFFFFFFFFFFFFFFF:FFFFFFF
@A00721:190:HGJW7DSXY:4:1156:10719:22874_2:N:0:TCTGCAAG+GCTGCACT
ATTAAACAAGTTGTACTTATCAATGATTTTATCGCTGGTTGTTATCTCGAAAAGTCTCTTCTAAAAGG
AACGTCAGTTCTAACCCAAAGAGAATATCTAAAATATTTACAAAATCT
+
F:FFFF,FF:FFF:F:FFFF:FF:,FFFFFFF,F:,FFFFFFF:F:F:F:FFFFFFFF:F,FFFFF:F
FFFFF:F:FFFFFF,FF:FF,F:FF,F:F,FFFF:F:FFFFFFFFF,,
@A00721:190:HGJW7DSXY:4:1412:30002:5102_2:N:0:TCTGCAAG+GCTGCACT
AGACGTTGCTTGTCGCTTCCTCCTTTAATGGCACTACCTCGGTACAATCTTACTTGGAACGAAGGCGA
CCTGTTCCGGAGTTTTTACGGGATCCGGAATGTTCTGTCTATATTAGG
+
FFFFFFFFFFFFFFFFFFFFFFFFFFFFFFFFFFFF:FFFFFFFFFFFFFFFFFFFFFFFFFFFFFFF
FFFFFFFFFF:FFFFFFFFFFF:FFFFFFFFFFFFFFFFFFFFFFFFFF
@A00721:190:HGJW7DSXY:4:2258:8133:8061_2:N:0:TCTGCAAG+GCTGCACT
TCGAACGTCAACGTTCACCGAGTTTCGTTTCGTACCTTGATGCTGACTTTCGTCAATATGATTTGATC
TGGTTGCTCAATTCTAAGCGTGTTCCGCTTAGTGCGAGGCCTCTCTTC
+
FFFFFFFFFFFFFFFFFFFFFFFFFFFFFFFFFFFFFFFFFFFFFFFFFFFFFFFF:FFFFFFFFFFF
FF:FFFFFFFFFFFFFFF:FFFFFFFFFF:FFFF:FFFFFFFFFFFFF
@A00721:190:HGJW7DSXY:4:1617:13051:18646_2:N:0:TCTGCAAG+GCTGCACT
ATATTTGGATGATCTTGATAAAGAACTTCATGAATTTTCAGAGGATGAAAAATTTTCTTCCTTGGATT
TTAATATAAAGAATCTTTACCAGGACTCCTACTACAGGAGGCAGTCTT
+
FFF:FFFFFFFFFFFFFFFFFFFFFFFFFFFFFFFFFFFFFFFFFFFFFFFFFFFFFFFFFFF:FFFFFF
FFFFFFFFFFFFFFFFFFFFF:FFFFFFFFFFFFFFFFFF:FFFFFFFF
```

```
@A00721:190:HGJW7DSXY:4:1119:21016:27273_2:N:0:TCTGCAAG+GCTGCACT
TTTGTTGTAATAGGGTCATCAACTTAGAACTCGAAACTCTAAGTGGTAATACTGTCTTTCCAAGCTTA
CCTGTATCTTCAATGAAATTGGACTCTTCGATAGGGGACATTTTATCG
+
FFFFFFFFFF:FFFFFFFFF:FFFFFFFFFFFFFFFFFFF,FFFFF:FFFFFF:FFF:FFFFFFFFF
F,FFFFFFFFFFFFFFFFFFFFFFFFFFF,:FFFFFFFFF:FFFFFFF
@A00721:190:HGJW7DSXY:4:2545:7419:7513_1:N:0:TCTGCAAG+GCTGCACT
CTACTAATGGCGTTGGCCTAGTGGATGTTATTAGTGACATCTGGTCAATTTCGTTTTTAGGTGGTCTT
TATTCTATTATTGATTTTTATGAGATTGATACGTTAACAGAAGATGATGTTAGAGTAGAATTCCTGAA
GAG
+
FFFFFFFFFFFFFFFFFF:FFFFFFFFFFFFFFFFFFFFFFFFFFFFFFFFFFF:FFFFFFFFFFFFFF
FFFFFFFFFFFFFFFFFFFFFFFFFFFFFFFFFFFFFFFFFFFFFFFFFFFFFFFFFFFFFFFFFFFF
FFF
@A00721:190:HGJW7DSXY:4:2353:14091:17597_2:N:0:TCTGCAAG+GCTGCACT
CCAACTTCTTGACAATTTTCTTCAATCCTTCGATAATAGTTAAATCTAGGATTATGTCTGTGTTGAAA
GACTTTCTACCGATAAGGGTCACTAGTCTTACACAATTAAGAAATAAT
+
F:F:FFFFFFFF:FFFFF:FFFFFFFFFFFFFFFFFFFF:FFFFFFFF:FFFFFFFF:FFFFFFFF:F
FFFFFFFFFFFFFFFFFFFFFFFFFFFFFFFFFFFFFFF:FFFFF,FFF
@A00721:190:HGJW7DSXY:4:1243:29261:13808_2:N:0:TCTGCAAG+GCTGCACT
ACTATCTAAAGATTGAAGGAAAATTTTATCCTCGTTTAACTTACTTAGACTTTCCTACGATACAAATG
ATAACTTCTAATTTACGTTTGCTACTCAGAGCTGGTCCTCTCAGCAAA
+
FF:FFFFFFFFFFFFFFFFFFFFFF,FFFFFFFFFFFFFFFFFFFFFFFFFFFFFFFFFFFFFFF:FFFF
FFFFFFFFFFFFFFFF:FFFF:FFFFFFFFFFFFFFFFFFFFFFFFFFF
@A00721:190:HGJW7DSXY:4:2622:18159:2534_2:N:0:TCTGCAAG+GCTGCACT
TCCTCAGGAGTCCCGGCATTTGATAATAATGACGACTTTCGTCATAATCGCTGCATATTACGGATTAC
ACGGTACCAGCGCATCTCGTGTCGAGCCTCGACCCGATTATATGGTAT
+
FFFFFFFFFFFFFFFFFFFFFFFFFFFFFFFFFFFF::FFFFFFFFFFFFFFFFFFFFFFFFFFFFFFF
FFFFFFFF:FFFFFFFFFF:FFFFFFFFFFFFFFFFFFFFFFFFFFFF
@A00721:190:HGJW7DSXY:4:1141:22164:24815_1:N:0:TCTGCAAG+GCTGCACT
TTCTTAATTATAATATTCAACCTCAAAAGTTACGTGTTTACAGTAACGAGATCACACATTTTGAGAAC
TAACTATGTTATGTTCTCTGATCATTTTGACAATCTCTTCTTATAATAAATAAATAAATAATATTACT
AAT
+
FFFFFFFFFFFFFFFFFFFFFFFFFF,FFFFFFFFF:FFFFFFFFFFFFFFFFFFFFFFFFFFFFFFFF
FFF:FFFFFFFFFFF:FFFFFFFFFFFFFFFFFFFFFFFFFFFFFFFFFFFFFFFFFFFFFFFF:FFFF
FFF
@A00721:190:HGJW7DSXY:4:1233:19334:6731_1:N:0:TCTGCAAG+GCTGCACT
AAAGGCTCCAGTCAACACTAGTTCGAGTGCTGCTTTGGATTACGATGACGTGAGAATAGGTGAAGGGA
TAGTTTTTACCTCGACCAACATTAAGGCGGCGGCGACTAATCTTAAAGCTTCACCCAAAATGAAGGAC
ACT
+
FFFFFFFFFFFFF:FFFFFFFFFFFFFFFFFFFFF:FFFFFFFFFFFFFFFFFFFFFFFFFFFFFFFFFF
FFFFFFFFFFFFFFFFFFFFFFFFFFFFFFFFFFFFFFFFFFFFFFFFFFFFFFFFFFFFFFF:FFFFF
FFF
@A00721:190:HGJW7DSXY:4:1172:31358:17033_1:N:0:TCTGCAAG+GCTGCACT
GCTCTAAAGGACACTATACAATATCCAGACGACGACGAAGCTTATACTACTTTAATTTTGGGTATTAT
GTGGAGATATCGTGATATCAGTACGTCCAGATCCTCTGCTTTTACCGATAACGATAAATTCGTCATCA
AA
+
FFFFFF:::FFFFFFF:FFFFFFFFFF:FFFFFFFFFFFF:FFFFFFFFF,FFFFFFFFFF:FFFFFF
```

FFFFFFFFFFFFFFFFFFFFFFFFFFFFFFFFFFFFFFFFF:FFFFFFFFFFFFFFFFFFFFFFFFFFFFF
FF
@A00721:190:HGJW7DSXY:4:1156:18891:36213_2:N:0:TCTGCAAG+GCTGCACT
CCACATTTTGGTCAAGTCAATCCGTTGAGAAAATTTCTTAGAGGATATTCGCCATTGTGGTACGAATT
GGTCCAACATTTACCACCAAATTCTTTTGATACAACTCTAGCCTTCAA
+
FFFFFFFFFFFFF:FFFFFFFFFFFFFFFFFFF:FFFFFFFFFFFFFFFFFFFFF,FFFFFFFFFFFFF
FFFFFFFFFFFFFFFFFFFFFFFFFF,FFFFFFFFFFFFFFFFFFFFFFF
@A00721:190:HGJW7DSXY:4:2340:24831:17002_1:N:0:TCTGCAAG+GCTGCACT
ACCAGATTGGTTCGAACCCGTTGATGCTATGTCAGAAGAAGCTATTCAAGCTTGTCGTTTAAAGACTG
CAGAGGCTGTCAAACAAGCCCAGACTCAAGGAGTCCAATCAAAGGTTTTGAATTTAGCTCAACTGGGG
AGA
+
FFFFFFFFFFFFFFFFFFFFFFFFFFFFFFFFFFFF:FFFFFFFFFFFFFFFFFFFFFFFFFF:FFFFFF
FFFFFFFFFFFFFFFFFFFFFFFFFFFFFFFFFFFFFFFFFFFFFFFFFFFFFFFFFFFFFFFFFFFFF
FFF
@A00721:190:HGJW7DSXY:4:1230:1362:27806_2:N:0:TCTGCAAG+GCTGCACT
TTCTACTTACAAAGGACAATTCAGTTTCATTAATTCTACGGAAAAGTCCGGAGTATATTGTATGTTAA
TAGATAGTGGGATAATAGTTCTACCGATACGTAGTGAACGCGTTCGTA
+
FFFFFFFFFFFF,FFFFFFFFFFFFFFFFFFFFFFFFFFFFFF:F:FFFFFF:FFFFFFFFFFFFFFFF
FFFFFFFFFFFFFFFFFFFFFFFFFFFFFFFFFFFFFFFF:FFFFFFFF
@A00721:190:HGJW7DSXY:4:2171:3504:33270_1:N:0:TCTGCAAG+GCTGCACT
ACCGATAGGAGTTGTATAGTTGAAGATGTGGCTTTAGACGATAATCCTTTAAGGACTATGTTGACTGA
CGAAGATTTTCCCACAGATTTGTCTACGTTGCAGATCGCCACTCCAAACACTATATACATTATAAAAT
ATA
+
FFFFFFFFFFFFFFFFFFFFFFFFFFFFFFFFFFFFFFFFFFFFFFFFFFFFFFFFFFFFFFFFFFFFF
FFFFFFFFFFFFFFFFFFFFFFFFFFFFFFFFFFFFFFFFFFFFFFFFFFFFFFFFFFF,FFFFFFFFF
FFF
@A00721:190:HGJW7DSXY:4:2353:22914:25081_1:N:0:TCTGCAAG+GCTGCACT
ATAACTTTTGTTACCAAATTCTTGGCTTTCAACGAAGTCTCTGATTTTATTAATCATTACTTTAACGA
TCTCTCGGTCATTTACTCTGTACTTGATCTACGTCGTTTAACTGTCGGAACAACCTTTACTTAGAATG
ACT
+
FFF:FFFFFFFFFFFFFFFFFFFFFFF:FFFFFFFFFFF:FFFFFFFFFFFFFFFFFFFFFFFFFFFFF
FFFFFFFFFFFF:FFFFFFFFFFFFFFFFFF:FFFFFFFFFFFFFFFFFFFFFFFFFFFFFFFFFFFF:F
FFF
@A00721:190:HGJW7DSXY:4:2502:31367:31140_2:N:0:TCTGCAAG+GCTGCACT
TCTTATATTATTTACAATAGTTAATATCATATTAGTAATACTCACTATCGTTTTCTGCTTTTTCTTAG
CTAATAGATTAATAAAGGATGAGTACGAAAACCAACTCTTCGTTACGA
+
FFFFFFFFFFFFFFFFFFFFFFFFFFFFFFFFFFFFFFFFFFFFFFFFF:FFFFFFFFFFFFFFF:FF
FFFFFFFFFFFFFFFFFFFF:FFFFF:FFFFF:FFFFFFFFFFFFFFF
@A00721:190:HGJW7DSXY:4:2424:29821:22200_1:N:0:TCTGCAAG+GCTGCACT
TATAATATTTTTATACTCAACACCCAACACACCGAGTATAACGCTATTTTTCCGACCTTAACTAAATC
CCCCAAGAGAACACCCCCAAACGACTCGAAACCGAGACGCCGTAGATCAAAGTCCCTTCTAACTTTAG
ACT
+
::,F,F:,,FF,,,,:,,,:,:FF,,,,,:F::,,F,:,F:,:,,,,FF:,F,,,F,:,F,,,,F:,,
F,:,::,F,F,,:,FF,,,,FFF,FFFFFFFFFF:FFFFFFFFFFFFFFFFFFFFFFFFFFFFFFFFFF
FFF
@A00721:190:HGJW7DSXY:4:2534:3115:25488_2:N:0:TCTGCAAG+GCTGCACT
CAGTTATTTATGTTGTACTTTTTATAATTTATAACCTTCTTATACTTTTGGGTATGTTTTGTGAAAAA

CATGAGATACCCAAGTCTATCGAAGACATTATCTCTTATGTACTTATT
+
FFFFFFFFFFFFFFF:FFFFFFFFFFFFFFFFFFFFFFFFFFFFFFFF:FFFFFFFFFFFFFFFFFFF
FFFFF:FFFFF,FFFFFFFF:FFF:FFF:FFFFFFFFFF:FFFFFFFF
@A00721:190:HGJW7DSXY:4:1147:21160:3568_2:N:0:TCTGCAAG+GCTGCACT
CTGTGTATGAGGAGGATTTATAATGCTTTTCGATAGTGATTGTTATCAATTTAAATACATTACTGTTT
CAAATCATCTTTACTCACAGACTCCGAAAATGGTTTCAAATGAGGTCA
+
FFFFFFFFFFFFFFFFFFFFFFFFFFFFFFFFFFFFFFFF:FFFFFFFFFFFFFFFFFFFFFFFFFFF
FFFFFFFFFFF:FFFFFFFFFFFFFFFFFFFFFFFFF:FFFFFFFFFFF
@A00721:190:HGJW7DSXY:4:1237:18448:23109_2:N:0:TCTGCAAG+GCTGCACT
TTAAACGAACTATTATCCGATTCTTCGAGGGTATCACTACCACTGATCTGCCGACTTTAGTCAAGGAA
CTTCAATTCCTTCGATGTTTATATTTTTCTATAAGGGAAAATAGCAAT
+
FFFFFFFFFFFFFFFFFFFFFFFFFFFFFFFFFFFFFFFFFFFFFFFFFFFFF:FFFFFFFFFFFFFF
FFFF:FF::FFFFFFFFFFFFFFFFFFFFFFFFFFF::FFFFFF:FFFF
@A00721:190:HGJW7DSXY:4:1377:8513:19961_2:N:0:TCTGCAAG+GCTGCACT
CGACAAATTTTAGAACTTTATTTCAGAACTCTCTCCGATCACCTTGGTGTGATCTCCCGGAGGATCTT
TTCAATAAGATGTATGATGATAATCTACTATTTTTAAATAGTGATATT
+
FFFFF:FFFFFFF:FFFFFFFFFFFFFFFFFFFFFFFFFFFFFF:FFFFFFFFFFFFFFFFFFFFFFF
FFFFFFFF,FFFFFF:FFF:FFFFFFFFFFFFFFFFFFFFFFFFFFFF
@A00721:190:HGJW7DSXY:4:2537:32777:31266_1:N:0:TCTGCAAG+GCTGCACT
CTTTATCAAAGATTTGGCTTGGTCTATTTACCGCAAAAAGTTGGGAGAGGTCTATGATATTTATTTTA
CTTATACTTTAAATAGCACCGATTATAACTTGACTGTTGATAATATTACAATAGCTCTTTCTCAGAAG
ATT
+
FFFFFFFFFFFFFFFFFFFF:FF:FFFFFFFFF:FFFFFFFFFFFFFFFFFFFFFFFFFFFFF:FFFF
FFFFFFFFFFFFFFFFFFFFFFFFFFFFFFFFFFFFFFFFFFFFFFFFFFFFFFFFFFFFFFFFFFFF
FFF
@A00721:190:HGJW7DSXY:4:2502:14488:34601_2:N:0:TCTGCAAG+GCTGCACT
GGTGAACACGTCGGTAGCATTGATTTACTTTTACCGTTAGAACTGAAAAAGATTCTTTTCGAAAAGAA
ACCTATAAGTTACAAACGATTCTTCGACTATATTAAAGAAATAAAAAT
+
FFFFFFFFFFFFFFFFFFFFFFFFFFFFFFFFFFFF:FFFFFFFFFFFFFFFFFFFFFFFFFFFFFFF
FFFFFFFFFFFFFFFFFFFFFFFFFFFFFFFFFFFFFFFFFFFFFFFF
@A00721:190:HGJW7DSXY:4:1532:28176:7889_2:N:0:TCTGCAAG+GCTGCACT
CTTTACATTTTTAATGGTATGGTATAATGTGTCTTACAAATAGAACCTTCACTCTCGGTAATGAGAGT
GTATTCCTTAATATTCTAGTAACCGCTATGTGGGCATTACTAGGATTT
+
F:FFFFFFFFFFFFFFFFFFFFFFFFFFFFFFFFFFFFFFFFFFFFFFFFFFFFFFFFFFFFFFFFFF
FFFFFFFFFFFFFFFFFFFFFFFFFFFFFFFFF,FFFFFFFFFFFFFF
@A00721:190:HGJW7DSXY:4:1502:32533:25864_2:N:0:TCTGCAAG+GCTGCACT
TGCTAGGAAAATTTTAGAAAAACTCCATTGTATAGTTCAAGGAGTACTTAATTCAATAGCTTTAGTAT
TATTTACTATCGCCTTTATTGAAAATTGTATAAGAGTTAATTGCTTAC
+
FFFFFFFFF:FFFFFFFFFFFFFFFFFFF:FFFFFFFFFFFFFFFFFFFFFFFFFFFFFFFFFFFFF:F
FFFFFF:FFFFF,FFFFFF,FFFFFFFFFFFFFFFFFFFFFFFFFFFF
@A00721:190:HGJW7DSXY:4:1351:8495:4867_2:N:0:TCTGCAAG+GCTGCACT
ACCATAACCTACCATGACCAATCATCGCTCCAACTGGCACGCCAGACGTAGGGTCAAACCTACTACCG
CCGGTCGGCAGATCGGCACCACTGGTCTCTACGTTCAGGGACCCGCTG
+
FFFFFFFFFFFFFFFFFFFFFFFFFFFFFFFFFFFFFFFFFFFFFFFFFFFFFFFFFFFFFFFFFFFF
FFFFFFFFFFFFFFFFFFFFFFFFFFFFFFFFFFFFFFFFFFFFFFFF

```
@A00721:190:HGJW7DSXY:4:2608:24053:35196_1:N:0:TCTGCAAG+GCTGCACT
CCTTCCCAAATCTAGATCTGCACAGAAGAAGGCTGAAGGTCGTTACCGGACGACCACTCAAAGGAAAC
GGGCTGTTGCCCAGAGGAAAGCAGCGACCACCGAGGTCGCTCGTAACATTGACGGCAGTCCCGTCATC
TCT
+
FFF,FFFFF,FFF::F,FF:FFFFFF,FF:FFFFFF:FFFFFFFFFFFFFFFFFFF:FFFF:FFFFFF
FFFFFFF:FFFFFFFF,FFFFFFFFFFFFFFFFFFFFFFFFFFFFFFFFFFFFFFFFFFFFFFFFFFF
FFF
@A00721:190:HGJW7DSXY:4:1574:28655:35587_2:N:0:TCTGCAAG+GCTGCACT
CAGTACCTCAGTCAAAAGTCACATTCACCACTCCCATACCAATCAATGATGTGACTATACAGTTCGGC
CATTTCAAGAGTGGTCAATACGTCGTGGGAGGTACTGAAGCCTTCCCC
+
FFFFFFFFFFFFFFFFFFFFFFFFFFFFFFFFFFFFFFFFFFFFFFFFFFFFFFFFFFFFFFFFFFFF
FFFFFFFFFFFFFFFFFFFFFFFFFFFFFFFFFFFFFFFFFFFFFFFF
@A00721:190:HGJW7DSXY:4:2127:21459:17895_2:N:0:TCTGCAAG+GCTGCACT
AAGCCAATCAATAGTTCTTCTGACCAGAATGAAAAGGGGTTTACCAGGGTAGTCTCTAAGTCTACCAG
GAAGAGGGGAAATGTCCCCAAAACTGAGAAGAATAAAGGGAAAGGTCC
+
FFFFF:FFFFFFFFFFFFFFFFFFFFFFFFFFF:FFFFFFFFFFFFFFFFFFFFFFFFFFFFFFFFFF
FFFFFFFFFFFFFFFFFFFFFFFFFF,FFFFF,F,F,FFFFFFFFFFF
@A00721:190:HGJW7DSXY:4:2355:1985:2644_2:N:0:TCTGCAAG+GCTGCACT
ATTACCCCTAATTCTGAAGGAAGATCTCCCACCATCTGGGGTTGGGAATAAGAAATCTGCACGGGCCA
AAGAGGCCGCTGCTACGAAGTCGCTGAAGGCGGCTTGGGTCATTAAGG
+
FFFFFFFFFFFFFFFFFFFFFFFFFFFFFFFFFFFFFFFFFFF:FFFFFFFFFFFFFFFFFFFFFFFF
FFFFFFFFFFFFFFFFFFFFFFFFFFFFFFFFFFFFFFFFFFFFFFFF
@A00721:190:HGJW7DSXY:4:1243:28700:5196_2:N:0:TCTGCAAG+GCTGCACT
GCCTGCCGACCCCATCTTTGAGACCAAGACTCTCTACAGTAGGTTCGTGCCTACTATGAGCAAATCTG
TTGAATTCGTCACTGAGGACGAAGGTGAGAGGACTTTTGGCTACTGGC
+
FFFFFFFFFFFFFFFFFFFFFFFFFFFFFFFFFFFFFFFF:FFFFFFFFFFFFFFFFFF:FFFFFFFFF
FFFFFFFFFFFFFFFFFFFF,F:FFFFFFFFFFFFFFFFF:FFFFFFF
@A00721:190:HGJW7DSXY:4:1668:26702:31266_2:N:0:TCTGCAAG+GCTGCACT
CGTGGGTTGAAGGCCGACGCTTCGAGCTTGAGTGTGACGAGGCCGGTGACGTTTTTGTCAACGGCGTT
TGGAGGATTTTATCCGCGCTTAACTATGGGGCGAAGATCCCCTATGGA
+
FFFFFFFFFFFFFFFFFFFFFFFFFFFFFFFFFFFFFFFFFFFFFFFFFFFFFFFFFFFFFFFFFF:F
FFFFFFFFFFFF:FFFFFFFFFFFFFFFFFFFFFFFFFFFFFFFFFFF
@A00721:190:HGJW7DSXY:4:2508:11107:34663_2:N:0:TCTGCAAG+GCTGCACT
TTGGCATATTTATGCCGACGATAATAAGCATGTCACCCTTAAGCACCGAGGTAAGCTCGGGACTTACA
AGAGAGAGTGTGAGAGAGTCGCTCTCACTATTTTTGCCCAGAATGCGG
+
FFFFFFFFFFFFFFFFFFFFFFFFFFFFFFFFFFFFFFFFFFFFFFFFFFFFFFFFFFFFFFFFFFF:
FFFFFFFFFFFFFF,FFFFFFFFFFF:FFFFFFFF:FFFFFFFFFFFF
@A00721:190:HGJW7DSXY:4:1577:28890:14638_2:N:0:TCTGCAAG+GCTGCACT
ATTACAACCTCTCTTTAGAGAGCATTAGAAGGAAGTTGGGCAAATTCCCACATATGGTAGACGTTGTC
ACACTTTTCTTGAAAATTTTTCCTTTCCTCCGTCATTGGGGATTTCGA
+
FFFFFFFF:FFF:F:FFFFFFFFFFFF:FFFFFFFFFFFFFFFFFFFFFFF:::FFFFFFFFFFF,FFF
FFFFFFFFFFFFFF:FFFFFFFFF:FFFFFFFFFF:FFFFFFFF::F,
@A00721:190:HGJW7DSXY:4:1473:4544:36542_1:N:0:TCTGCAAG+GCTGCACT
GGAAGGTGTCAAGGGTCTTCTTCATGCTGATCTTTCAGGACCTAATCACATCATGATTGATAGTTACG
GCTCGTATCGAGTTGGAGGGAGGAAGTTCTCCGAAATTAAAGACGGGGGGATTAAGAGTCCAGCTGTA
ACT
```

```
+
FFFFFFFFFFFFFFFFFFF:FFFFFFFFFFFFF:FFFFFFFFFFF,FF:FFFFFFFF:FFFFFFFFFFF
FFFFFFFFFFFFFFFFFFFFFFFFFFFFFFFFFFFFFFFFFFFFFFFFFFFFFFFFFFFFFFFFFFFFF
FFF
@A00721:190:HGJW7DSXY:4:1532:25292:6308_2:N:0:TCTGCAAG+GCTGCACT
AGAGGGGAGCTCTGAAAAGGAAACTTCGTCAGAAGATGAGGAAGTCTAAGAGGAGGAGTTTGAGGAAG
GCTCTAGAGAAGATCTCACCACCTGGAGAAGGCAAGTGGAGAGTCCAG
+
FFFFFFFFFFFFFFFFFFFFFFFFFFFFFFFFFFFFFFFFFFF:FFFFFFFFFFFFFFFFFFFFFFFFF
FFFFFFFFFFFFFFFFFFFFFFFFFFFFFFFFFFFFFFFFFFFFFFFFF
@A00721:190:HGJW7DSXY:4:2414:9616:28354_1:N:0:TCTGCAAG+GCTGCACT
CTTCCGATCTTTTGAAGCAGGGGTTACCACTTCCTAAGGTGGATGAGGGAGGTTGTTTCTTTGGAGTT
TTAGATAATCTTTTGAGAAGGGGTCATAGAGCCAGCAAGAGAAGCTTATATGCTGGTTTAAGTGATGA
TTA
+
FF:FFFFFFF:FFFFFFFFFFFFFFFFFFFFFFFFFFFFFFFFFFFFFFFFFFFFFFFFFFFFFFFFFF
FFFFFFFFFFFFFFFFFFFFFFFFFFFFFFFFFFFFFFFFFFFFFFFFFFFFFFFFFFFFFFFFFFFFF
FFF
@A00721:190:HGJW7DSXY:4:1522:26060:8390_1:N:0:TCTGCAAG+GCTGCACT
ATTTAGGTCAACTTGGGGTTTCGAATGTCATTAGTGCCTTACGATTGAAAGAGGTCCTTTTGAGTATC
GACAGATCTTTCGATCTTGAGGACGTACATTTAACGTGGCAGAATTCGGTAACTTACCATATTGTTCC
TTC
+
FFFFF:FF:FFFFFFFFFFFFFFFFFFFFFFFFFFFFFFFFFFFFFFFFFFFFFFFFFFFFFF:,FFF
FFFFFFFFFFFFFFFFFFFFFFFFFFFFFFFFFFFFFFFFFFFFFFFFFFFFFFFFFFFFFFFFFFFFF
FFF
@A00721:190:HGJW7DSXY:4:1206:25274:33865_1:N:0:TCTGCAAG+GCTGCACT
TTTAACTTTGACTCAACTACTCCGATCAGATCCGGATGGTTTGATTGGTATGGACTACTCGACTTTTG
ATCTGGAGTTGTCTAGGGTCATGGCAGATACTACCGATAAGGTGAAAAATATGCAGGTCAAAGATTCT
GTA
+
FF:FFFFFFFFFFFFFFFFFFFFFFFFFFFFFFFFFFFFFFFFFFFFFFFFFFFFFFFFFFFFFF:FFF
FFFFFFFFFFFFFFFFFFFFFFFFFFFFFFFFFFFFFFFFFFFFFFFFFFFFFFFFFFFFFFFFF:FFF
FFF
@A00721:190:HGJW7DSXY:4:1246:13919:35023_2:N:0:TCTGCAAG+GCTGCACT
GTTTTGGCCGACTTTAACTATTCTAAGAGTCTTTATGATTCAAAACCTATCGTTGTCGTACCTTACAA
GGTTGAGGAGACTTTACAGCGGCTTATTGTCGCGCAGTTTCCAGAATT
+
FFFFF,FFFFFFFFFFFFFFFFFFF:FFFFFFFFFFF:FFFFFFFFFFFFFFFFFFFFFFFFF:FFFFF
FFFF:FFFFFFF:FFFFFFF:FFFFFFFFFF:FFFFFFFFF:FFFFFFF
@A00721:190:HGJW7DSXY:4:1608:5113:36683_1:N:0:TCTGCAAG+GCTGCACT
TATCTTGAATGCTCACGCCGTCGCAGCCATATCGAGACTATGTTATAATCACTTGTTAAAGTACAGAT
TTAGTGATTTAGATATTATTGATATTGGAGGCGATATTCGTTTTCATATCCAAAACTCCTTGAATGGA
GTA
+
FFFFFFFFFFFFFFFFFFFFFFFFFFFFFFFFFFFFFFFF:FFFFFFF,:FFF:FFFFFFFFFFFFFFF
FFFFFFFFFFFFFFFFFFFFFFFFFFFFFFFFFFFFFFFFFFFFFFFFFFFFFFF:FFFFF,FFFFFFF
FFF
@A00721:190:HGJW7DSXY:4:1108:17445:12727_1:N:0:TCTGCAAG+GCTGCACT
CTATTCTTGATGTGAAAGATGCATCGAGGTATACGTCTAGGAACATTGATTGGGAACGTACCTACTCA
CGAGACGTCATGAAAGCTATCGGAGGGAAGCAAAATGTTTCGTTCTGTTACAAGAACGCTTTGGATTG
TGA
+
FFFFFFFFFFFFFFFFFFFFFFFFFFFFFFFFFF,FFFFF,FF:FFFFFFFFFFFFFFFFFFFFFFFFF
```

```
:FFFF:,F:FFFFFFFFFFFFFFFF:F:F,FFFFFFFFFFFFFFFFFFFFFFFFFFFFFFFFFFFFFF
FF:
@A00721:190:HGJW7DSXY:4:1218:7518:10222_1:N:0:TCTGCAAG+GCTGCACT
GTTGCGATAGCTGTAGAGGTATACGATATCTCTCTTGATGATGTAGCCACTATAATGCTTAAGAGAGA
GATTGATGTATGTCATTTCGTCTTAGTTTGTCCCGGCGAATTATTGGACACGATGAAAAATGACATAC
CCG
+
FFFFFFFFFFFFFFFFFFFFFFFFFFFFFFFFFFFFFFFFFFFFFFFFFFFFFFFFFFFFFFFFFFFF
FFFFFFFFFFFFFFFFFFFFFFFFFFFFFFFFFFFFFFFFFFFFFFFFFFFFFFFFFFFFFFFFFFFF
FFF
@A00721:190:HGJW7DSXY:4:1524:18258:27070_2:N:0:TCTGCAAG+GCTGCACT
TCATGGAGATGATGGCGCATCACTTGATTACTATATGCCCGAAGGATTGGGTTATTCTCACAACAAAG
ATAAGATTTTATCTTTCTTCAAACACCCTGTTTTCCTTAAGGGCGGAC
+
FFFFFFFFFFFFFFFFFFFFFFFFFFFFFFFFFFFFFFFFFFFFFFFFFFFFFFFFFFFFFFFFFFFF
FFFFFFFFFFFFFFFFFFFFFF:FF:FFFFFFFFFFFFFFFFFFFFFFFF
@A00721:190:HGJW7DSXY:4:1527:11397:1658_1:N:0:TCTGCAAG+GCTGCACT
ACTGGAGTTCAGACGTGTGCTCTTCCGATCTATGAGTTCCGAATAACTCGTAATAAATCTTTCTCTAG
TGGACAATTCAGAATACCTATTTCCGTACCAAGAGCCACTGCCAATCTCATCGCTATACGCATACCTG
ATT
+
:FFFFFFFFFFF,,FF:FFFFFFFFFFFF:FFFFF,FFF:,FFFFFFFFFFFFFFFFFFFF:FFFFFF
FFFFFFFFFFFF,FFFFFFFFFFF:FFFFFFFFFFFFFFFFFFFFFFFFFFF:FFFFFF:FFFFFFFFF
FFF
@A00721:190:HGJW7DSXY:4:1608:1561:30405_2:N:0:TCTGCAAG+GCTGCACT
AAATTTTTCTCAAGAGATTATGGTTGATAAAGTTTTCTTCATAAGGGCTATTCAGTACGTCATGGGGA
TCACTTCTTCGGTCAATGACAAAACCTTCGAATATGTTACGACATGGC
+
FFFFFFFFFFFF:FFFFFFFFFFFFFFFFFFFFFFFFFFFFFF,:FFFFFFF,FFFFFFFFFFFFFFFF
FFFFFFFFFFFFFFFFFFFFFFFF,FFFFFFFFFFFFFFFFFFF:FF:F
@A00721:190:HGJW7DSXY:4:1367:1877:11036_1:N:0:TCTGCAAG+GCTGCACT
GTATTGTCATCTCGGGGAAAGTGATTCACCAAGATGTTCATATAGATACGAGATACCTAGAGAAGGTC
GCAGCGATCTTACTCGTTATCGGAGCTAAGAAGAAGACTTTGGTTACACGTTATGCTCGCAAACTTAA
CGT
+
FFFFFFFFFFFFFFFFFFFFFFFFFFFFFFFFFFFFFFFFFFFFFFFFFFFFFFFFFFFFFFFFFFFF
FFFFFFFFFFFFFFFFFFFFFFFFFFFFFFFFFFFFFFFFFFFFFFFFFFFFFFFFFFFFFFFFFFFF
FFF
@A00721:190:HGJW7DSXY:4:1159:10004:3975_2:N:0:TCTGCAAG+GCTGCACT
CTCTGCTTCTGCGTTACTGAGAGAAAAGATTCAGGACGCCTACGGTGACTTTATGGACAAGATGCCAG
TTCTTGGGAAATACTTGTTTCCCTTTTTAGGTTTAGTAGGTGATCTTG
+
FFFFFFFFFFFFFFFFF:FFFFFFF,FFFFFFFFFFFFFFFFFFFFF:FFFFFFFFFFFFFFFFF,FF
FFFFFFFFF:FFFFF:FFFFF:F,:F:FFFFF,FFFFFFFFFFFFFF
@A00721:190:HGJW7DSXY:4:1130:3025:37012_1:N:0:TCTGCAAG+GCTGCACT
CCGATCTAGAAGACAACGAATCTTGAAACTACGGTCCTTATCAAAGAAAATGGTGAACCGGTCTCTAA
ATCTTTTGAGATTGAGAAGATTGAAGAACACGTCAAGAAGTCGCTCGNCGTTGTTCTTGAAAACCAAG
TTA
+
:FFFFFFFFFFFFFFFFFFFFFFFFFFFFFFFFFFFFFF:FFFFFFFFFFFFFFFFFFFFFFFFFFFFF
FFFFFFFFFFFFFFFFFFF:FF:FFFFFFFFFFFFFFFFFFFFFFFFF#FFFFFFFFFFFFFFFFFFF
FFF
@A00721:190:HGJW7DSXY:4:1276:32425:26428_2:N:0:TCTGCAAG+GCTGCACT
AGTCTTTGTACCTTCTGTCAAACTTCGAGGTTCAGCTACGAGAGGTGATCCCGGATGTGGATTGAAGG
```

```
CTGGCTCTAAGGGAGACTCCTTCTTTTTGAATGTTGTAAAGAATTGGG
+
FFFFFFFFFFFFFFFFFFFFFFFFFFFFFFFFFFFFFFFFFFFFFFFFF:FFFFFFFFF:F:FF:FFF,FFF
FFFFFFFFFFFFFFFFFFFFFFFFFFFFFFFFFFFFFFFFFFFFFFFF
@A00721:190:HGJW7DSXY:4:1103:21911:35462_1:N:0:TCTGCAAG+GCTGCACT
ACAAGGAGTTAGACTCTTCTTCAAGTTTTTAGATCTTCTTGATAATCTTAAAGAAAAATTTTCGAATA
AACTAATGAGAGGTATAGGCTTAGTCAAGTCTGTACTTACATTCTTTACGCCAGATTCTTCATGGGGC
GAC
+
FFFFFFFFFFFFFFFFFFFFFFFFFFFFFFFFFFFFFFFFFFFFFFFFFFFFFFFFFFFFFFFFFFFF
FFFFFFFFFFFFFFFFFFFFFFFFFFFFFFFFFFFFFFFFFFFFFFFFFFFFFFFFFFFFFFFFFFFF
FFF
@A00721:190:HGJW7DSXY:4:1242:3341:26036_2:N:0:TCTGCAAG+GCTGCACT
AATTATTAAATCATTCTTCTCACTCTTGACGAGAATTATTACTGTTTTCTCTACCCATAACTTCTTTT
TCAAAAATGCGGATGGGTCTACTGATGTTTTCCTACAGAGTGCAGTCG
+
FFFFFFFFFFFFFFFFFFFFFFFFFFFFFFFFFFF:FFFFFFFFFFFFFFFFFFFFFFFFFFFFF:FFF
FFFFFFFFFFFFFFFF,FFFFFFFFFFFFFFFF:FFFFFFFFFFF:FF
@A00721:190:HGJW7DSXY:4:1360:14678:31234_2:N:0:TCTGCAAG+GCTGCACT
ATCACCACTTTTGAAAGGAAAATAATTGGTGATGAAAATACGTATGTCGGTGGGGTTCTCAAGGCTCT
CATCGCCACTGGGATTCTAAGGGGTTCCATAAGATATTATAGATCGGA
+
FFFFFFFFFFF:FFF:FFFFF:F:FFFFFFFFFFFFFFFFFFFFFFFFFFFFFFFFFFFFFFFFFFFF
FFFFFFF::FFFFFFFFF:FFFFFFFFFFFFFFFFFFFFFFFFFFFFFF
@A00721:190:HGJW7DSXY:4:2475:10248:30577_2:N:0:TCTGCAAG+GCTGCACT
TTTGGTCCTTACGTCTTAAGTAATTTGGATGTTGTCGAAAACTATCAGGTTCTTGTCACGATTCTTAA
AAGAAGTTTATTAAAACTTCAGAGTCTCTTGGAAGTCCAGGTAGCTAA
+
FFFFFFFFFFFFFFFFFFFFFFFFFFFFFFFFFFFFFFFFFFFFFFFFFFFFFFF:FFFFFF:FFFFFF
FFFFFFFFFFFFFFFFFFFFFFFFFFFFFFFFFFFFFFFFFFFFFFFF
@A00721:190:HGJW7DSXY:4:2530:28691:13103_2:N:0:TCTGCAAG+GCTGCACT
ACCTCTGTTGGTAGAGGAGTTGAGAGAATTACTCAGATTCAATCTGGCATCACTGAACGTCTACAACG
AGTTAGGGAGTGGCTAGGTTTTAAAAGATCCTTTGCACAAGCAATACC
+
FFFFFFFFFFFFFFFFFFFFFFFFFFFFFFFFFFFFFFFFFFFFFFFFFFFFFFFFFFFFFFFFFFFF
FF:FFFFFFFFFFFFFFFFFFFFFFFFFFFFFFFFFFFFFFFFFFFFF
@A00721:190:HGJW7DSXY:4:2127:16523:30232_2:N:0:TCTGCAAG+GCTGCACT
GTCGCGGAAAACGATGAAGATTTCCACACTGTTCGAGAATCTTCTGATGCTGAATTTGAGGAAAATTT
TTCGGAAGTTTTTGGTATAAATCCTGATGGCTCAGTACAGGGAGGAGG
+
FFFFFFFF:FFFFFFFFFFFFFFFFFFFFFFFFFFFFFFF:FFFFFFFFFFFFFF:FFFFFFFF,FFFF
FFFFFFFFF:F:FFFFF:FFFFFFFFFF:FF:F:FFFFFFFFFF:,FF
@A00721:190:HGJW7DSXY:4:2658:26169:31626_2:N:0:TCTGCAAG+GCTGCACT
ACCTTCTTTTATCATTGCAACAGGACTTTGAGAAGACTGTTGAAATGGACAAAATCTGGGATCATAGG
TATGTGTCACTCTACAATCGAAATATGGGACCTCATCAGGAAGTATAG
+
F,:,,FFFFFFFFF,:FFFF,F:FFF,FFFFFFFFF,FFF:FFFFFF,FFFFF,F,FFF,,F::,F,,
FF:FF,FFFF,F:F,F,,F,FFF,FFF,F,F,,FF,,F,:,:FFFF,:
@A00721:190:HGJW7DSXY:4:2526:24695:9533_2:N:0:TCTGCAAG+GCTGCACT
GGAAGATCTCTACAGAGATTATCGAGAGTTGTGATCTAGTTCTAAATTTAGGTCGACGGACTTTAGCA
ACTTTAGATAAATTCTTTCCGGGCTCAATGACCTTTGCTATGTACGTG
+
FFFFFFFFFFFFFFFFFFFFFFFFFFFFFFFFFFFFFFFFFFFFFFFFFFFFFFFFFFFFFFFFFFFF
FFFFFFFFFFFFFFFFFFFFFFFFFFFFFFFFFFFFFFFFFFFFFFFF
```

```
@A00721:190:HGJW7DSXY:4:2447:5719:2691_1:N:0:TCTGCAAG+GCTGCACT
TTAGTTCATGAAGTTCTTACACTTCTCGCGACTAATGTCAGCTTCACTACAAAGGCGTTACGTAAAGT
AGTTTTCACGCTTTATCTTAAAGCATTACCACCAAATTTAGCACTTTTTGAATATGACAGAAGCTCGC
CAT
+
FFFFFFFFFF,FFFFFFFFFFFFFFFFFFFFFFFFFFFFFFFFFFFFFFFF,FFFFFFFFFFFFFFFF
FFFFFFFFFFFFFFFFFFFFFFFFFFFFFFFFFFFFFFFFFFFFFFFFFFFF:FFFFFFFFFFFFFF
FFF
@A00721:190:HGJW7DSXY:4:2641:11695:20682_2:N:0:TCTGCAAG+GCTGCACT
CCAGTTAGAGTTCTTCTAACCAGGACTGATGGTGAGATTCTTTTACCTTTTGGAGGTCCAGGAACCTT
TACTCCTAGTGTCTTTTCTAGAGTCCAAGGACGTTTCCGTCGGAGGAT
+
FFFFFFFFFFFFFFFFFFFFFFFFFFFFFFFFFFFFFFFFFFFFF:F:FFFFFFFFFFFFFFFFFFFF
FFFFFFF,FFFF:FFFFFFFFFFFFFFFFFFFFFFFFFFFFFFFFFFF
@A00721:190:HGJW7DSXY:4:2209:5376:20697_1:N:0:TCTGCAAG+GCTGCACT
TCAATAACACTCTATTGAATCCCTTTTCCACGATGAAAGCTCTTTATGAGGTGCTCCATGCAAATTTA
CAGTTCCTCTTTGGTGATATCGCCTCTGTCCTTTCTCCTTTAATCGTGAGGTTACGAGAGTTTCGTAC
TTG
+
FFFFFFFFFFFFFFFFFFFFFFFFFFFFFFFFFFFFFFFFFFFFF:,FFFFFFFFFFFFFFFFFFFFFF
F:F:FFFFFFFFFFFFFFFFFFFFFFFFFFFFFFFFFFFFFFFFFFFFFFFFFFFFFFFFFFFFFFFF
FFF
@A00721:190:HGJW7DSXY:4:1453:11885:36260_1:N:0:TCTGCAAG+GCTGCACT
ACTGGAGTTCAGACGTGTGCTCTTCCGATCTCTGACGAAGGTTCTATTACCACCCTTGTCATTGATAC
AAACATTCTAGGTGATGCTTTCTTAGAGGCTCGTCTAGCCGCTGAGATTCGTCTTACCTATTTACAAT
CTG
+
FFFFFFFFFFFFFFFFFFFFFFFFFFFFFFFFFFFFFFFFFFFFFFFFFFFFFFFFFFFFFFFFFFFF
FF,FFFFFFFFFFFFFFFFFFFFFFFFFFFFFFFFFFFFFFFFFFFFFFFFFFFFFFFFFFFFFFFFF
FFF
@A00721:190:HGJW7DSXY:4:1156:24008:22874_2:N:0:TCTGCAAG+GCTGCACT
GGGAAATGGTGGTAGTCAGTTTACTACTTTTCTTTCCCTCCCCTTTTTCGATTGGATTCGACGGGGTC
TTCGTCTCACGACTAATTTGATAAGGTTTTTCTTTTATGGGACTCTTG
+
FFFFFFFFFFFFFFFFFFFFFFFFFFFFFFFFFFF:F:FF:FFFFFFFFFFFFFFFFFFFFFFFFF,FF
FFFFFFFFFFFFFFFFFFFFFFFFFFFFFFFF:F:FFFFF,FFFFFFFF
@A00721:190:HGJW7DSXY:4:1405:5276:27195_1:N:0:TCTGCAAG+GCTGCACT
ATGTAGGTCTCCTAGTACTTAAGGGTTTATGTTTCGGTTTCAGCTGGATGGATCTTTTGATGGTATGT
TTCACATTGCGTGATCACTTCCCGTGGACTTGGGAAGAGATTAAACATAAAATCCAGCGATTCTATTA
TGT
+
FFFFFFFFF:FFFFFFFFFFF:FFFFFF:F:FF:FFFFFFFFFFFFF:FFFFFFFFFFF:FF:F:FFF
FFFF:FFFFFFFFFFFFFF:FFFFFFFF:FFFF:FFFFFFFFFFFFFFFFFFFFFFFFFFFFFFFFFFF
:FF
@A00721:190:HGJW7DSXY:4:2675:29541:3928_2:N:0:TCTGCAAG+GCTGCACT
TCGATGATGTTCGGGCTGTGGGGATTAACTTCCCTCGTGAAACTTACGACGATGTTCTCGCTCGCATT
TCACAAGGATTACTAAGTCTGAGATCTGCCACTTCTAATTTTAGGAAA
+
FFFFFFFFFFFFFFFFFF:FFFFFFFFFFFFFFFFFFFFFFFFFFFFFFFFFFFFFFFFFFFFFFFFFF
FFFFFFFFFFFFFFF,FFFFFFFFFFFFFFFFFFFFFFFFF,FFF:FFF
@A00721:190:HGJW7DSXY:4:1571:17906:32064_2:N:0:TCTGCAAG+GCTGCACT
AAGTTCATGAGGATGTCTTGGTCGAAAAATATCGCCGGGAACTTCTAATAGAGGATCCAGATGACATT
ATTTCAACGGATACCGATCTCAGTTCTACCACAGAAACCGTCTCAGGA
+
```

```
F:FFFFFFFFFFFFFFFFFFFFFFFFFFFFFFFFFFFFFFF,FFFFFFFF:FFFFFFFF,FFFFFFFFF
FFFFFFFFFFFFFFFFFFFFFFFFFFFFFFFFFFFFFFFFFFFFFFFFF
@A00721:190:HGJW7DSXY:4:1603:30671:20415_1:N:0:TCTGCAAG+GCTGCACT
CTGATTCGGAATTAGAGAAAGACGACCTTCTTGTGGGGACTGAGACCTCTGAATTTTCAGGGTGCCCT
GATGAGGACGTTGATAGGGATAATTTCAACTCTGAGACTTCTAAGAGTGTTTCAAAAGACCAGAAACG
TAA
+
FFFFFFFFFFFFFFFFFFFFFFFFFFFFFFFFFFFFFFFFFFFFFFFFFFFFFFFFFFFFFFFFFFFF
F:FFFFFFFFFFFFFFFFFFFFFFFFFFFFFFFFFFFFFFFFFFFFFFFFFFFFFFFFFFFFFFFFFF
FFF
@A00721:190:HGJW7DSXY:4:2104:21305:36385_1:N:0:TCTGCAAG+GCTGCACT
AAAGGTAGGAAGGGGAGAAAACAGAACAAGCGAGCGGTTGAAGAGAAAACTGATATGGTTGCGGCTGA
TCGTACCCCAATTCCTGCTAGAAAGATCACTCTCGGCGATACCACTGATCTTCCAGAGTTACGTAGTG
GTC
+
FFFFF:FFFFFFFFFFFF:FFFFFFFFFFFFFFFFFFFFFFFFFFFFFFFFFFFFFFFFFFFFFFFFF
FFFFFFFFFFFFFFFFFFFFFFFFFFFFFFFFFFFFFFFFFFFFFFFFFFFFFFFFFFFFFFFFFFFF
FFF
@A00721:190:HGJW7DSXY:4:1571:32678:28025_1:N:0:TCTGCAAG+GCTGCACT
TTTTATCAAAGAGAAAGTTTCTGCTAGTCGCGACGATAGTATTTATCGGAGTGATATTAACAAGTTAA
ACTTCGAACTGACCACTTACAAGAGTGTTGAAGTACCGCAACATGGATCAGTTTTCCAAAAAGCTATT
AAT
+
FFFF:FFFFFFFFFFFFFFFFFFFFFFFFFFFFFFFFFFFFFFFFFFFFFFFFFFFFFFFFFFFFFFF
FFFFFFFFFFFFFFFFFFFFFFFFFFFFFFFFFFFFFFFFFFFFFFFFFFFF:FFFFFFFFFFFFFFF
FFF
@A00721:190:HGJW7DSXY:4:1409:14705:34381_1:N:0:TCTGCAAG+GCTGCACT
CTGAATCCACTCTCTTGGGATATAAATGAGTACATCTATAATAGAGTTTTGGAACTGTGTAATGTCCT
ACGTACTGTTCAAGGTGTTTGGGCTTCTCTAAAGAACAGCAACTTTCAACATGAATTAGTTATTGAAA
GTC
+
FFFFFFFFFFFFFFFF::FFFFFFFFFFFF:FFFF:FFFF,FFFFFFFFFFFFFFFFFFFFFFFFFFF
FFFFFFFFFFFFFFFFFFFFFFFF::FFFFFFFFFFFFFFFFFFFFFFFFFFFFFFFFFFFFFFFFFF
FFF
@A00721:190:HGJW7DSXY:4:2635:16721:13542_1:N:0:TCTGCAAG+GCTGCACT
TTCTTCATGAAGATCTTCGAGTGTTTTATAGTGAGTTATTCTTGCGAAGGTTCAAGTCGATTGATTTT
GTTGACTACGATCTCTCTTGTAATAAGAAGTTCTTCGCAGTTGAATCTCCACCAGGTGGAGGGAAGAC
CAC
+
FFFFFFFFFFFFFFFFFFFFFFFFFFFFFFFFFFFFFFFFFF:FFFFFFFFFFFFFFFFFFFFFFFFF
FFFFFFFFFFFFFFFFFFFFFFFFFFFFFFFFFFFFFFFFFFFFFFFFFFFFFFFFFFFFFFFFFFFF
FFF
@A00721:190:HGJW7DSXY:4:2239:29396:6402_1:N:0:TCTGCAAG+GCTGCACT
TTCGCAAAAGGTCTTGAAGTACGTATTGTCACTGCTAATCGTAATTCGCGAGAAGAGATAGTGACTAA
AGTTTTGGCACTTCTAGTTCATCGCGGTATTTGTGAAATTAAGACAGACGTGAAAGTTATGATGAGTG
ATC
+
:FFFFFFFFFFFFFFFFFFFFFFFFFFFFFFFFFFFFFFFFFFFFFFFFFFFFFFFFFFFFFFFFFFF
FFFFFFFFFFF:FFFFFFFFFFFFFFFFFFFFFFFFFFFFFFFFFFFFFFFFFFFFFFFFFFFFFFFF
FFF
@A00721:190:HGJW7DSXY:4:1422:21187:34021_2:N:0:TCTGCAAG+GCTGCACT
AGCTGATTCTTATTGCATGAACGTCGCTGAGGTCAAACCTGTGGACGTTCTTTTCATCGACGAATTAT
TCATGATTCACGCTGGTCAGACCCTTTTTCTTACACATCTACTTAAGG
+
```

```
FFFFFF:FFFFFFFFFFFFFFFFFFFFFFFFFFFFFFFFFFFFFFFF,FFFFFFFFFFFFFFFFFFFF
FFFFFFFFFF:FFFFFFFFFFFFFF::FFFFFFFFFF,,FFFFFFFFF
@A00721:190:HGJW7DSXY:4:2158:23249:26005_2:N:0:TCTGCAAG+GCTGCACT
GTTACGGTGATTCCAATCAAATCGGATTCATCAATCGTATTGATCTCCAAGTCGCAAACTACTTCAAC
ATCAACGACATCATTCTTGATGAGTGTCGTTCTTATCGAAGTCTCAGT
+
FFFFFFFFFFFFFFFFFFFFFFFFFFFFFFFFFFFFFFFFFFFFFFFFFFFFFFFFFFFFFFFFFFFF
FFFFFF:FFFFFFFFFFFFFFFFFFFFFFFFFFFFFFFFFFFFFFFFF
@A00721:190:HGJW7DSXY:4:1510:30391:18333_2:N:0:TCTGCAAG+GCTGCACT
CGGCCGACGTTTGTCGCGAACTCTCAGAGGTTTACGGCAGAAAAATTTCTCCTGCTTGTAATTCGAGA
GAGGACACTATGGAAATTAGACCTATCAGATGTCTTGACGACTTCGAA
+
FFFFFFFFF:FFFFFFFFFFFFFFFFFFFFFFFFFFFFFFFFFFFFFFFFFFFFFFFFFFFFFFFFFF
FFFFFFFF:FFFFFFFFFFFFFFFFFFFFFFFFFFFFFFF:FF:FFFFF
@A00721:190:HGJW7DSXY:4:2561:20627:5337_2:N:0:TCTGCAAG+GCTGCACT
TATTACTTTCACTCAAGGTGAAAAGTTTGAAATTAACAAAGTCCTTGGTAAGAGTAAGGTTAAATGTG
AAGATGCTCAGACTATTCATGAAGTTCAAGGTAATACTTACCAGCACG
+
FFFFFFFFFFFFFFFFFFFFFFFFFFFFFFFFFFFFFFFFFFFFF:FFFFF:FFFFFFFFFFFFFFFF
FFFFFFFFFFFFFFFFFFFFFFFFFFFFFFFFFF:FFFFFFFFFFFFFFF
@A00721:190:HGJW7DSXY:4:2577:11017:24330_1:N:0:TCTGCAAG+GCTGCACT
CTTCCGATCTCTCTATCACGACACGTTGAATCTTTACGATACTTTGTTATCAATGTAAAGTTAAACGA
TGCTACTTGTGAGAGGATCAATCACAGTAAGGAACTGTCACGTTTAGCACGGGAGGTTGCAGATTCAA
CCC
+
FFFFFFFFFF:FFFFFFFFFFFFFFFFFFF:FFFFFFFFFFFFFFFFFFFFFFFFFFFFFFFFFFFFF
FFFFFFFFFFFFFFFFFFFFFFFFFFFFFFFFFFFFFFFFFFFFFFFFFFFFFFFFFFFFFFFFFFFF
FFF
@A00721:190:HGJW7DSXY:4:2232:7012:32268_1:N:0:TCTGCAAG+GCTGCACT
AGACGTGTGCTCTTCCGATCTCTTGAAGTTGTTGACGACAAGCCTCAAGAGGGTAGAGGACCACCTGG
ATCTTCTACTGTCTCTGCCTTCCAAGATTTCGTAGAAGAGGTTATTCCTGGAACCACCTTAACGTCAC
TCG
+
FFFFFFFFFFFFFFFFFFFFFFFF,FFFFFFFFFFFFFFFFFFFFFFFFFFFFFF:FFFFFFFFFFFFFF
FFFFFFFFFFFFFFFFFFFFFFFFFFFFFFFF,FFFF:FFFFFFFFFFFFFFFFFFFFFFFFFFFFFFF
FFF
@A00721:190:HGJW7DSXY:4:2306:7446:25379_2:N:0:TCTGCAAG+GCTGCACT
TGTCGATCGAATTAAGATCAAGGCTAATACCTTTATAGGTAAGAAGCCCCTTGAGCCGCGTAGTCAGC
GCGTTTAGCTGTATCATCAGTCAGAGTATACCCGATAGGGTACCGAGT
+
:FFFFFFFFFFFFFFF:FFFFF:FFFF,FFFF:FFFFFFFFF:FFFFFFFF,FFFFFFFFFFFFFF:F
FFFFFFF:FFFF::FFFFFFFFFFFFFF:FFFFFFFFFFFF,F:FFF,
@A00721:190:HGJW7DSXY:4:2535:1289:23359_2:N:0:TCTGCAAG+GCTGCACT
ATATTTATTCTTATGAGGCTCGAAACTTCAACGTTTTCACAACGGAAAGACATATCGCACCCGAAATT
TTTGGGCAGAATGCTCTTAACGCGGTTCTTGAACGGTGTTTTGATCAG
+
FFFFFFFFFFFFFFFFFFFFFFFFFFFFFFFFFFFFFFFFFFFFFFFFFFFFFFFFFFFFFFFFFFFF
FFFFFFFFFFFFFFFFFFFFFFFFFFFFFFFFFFFFFFFFFFFFFFFF
@A00721:190:HGJW7DSXY:4:2174:25943:3020_2:N:0:TCTGCAAG+GCTGCACT
TAAAGTTATAGGAGACTCAGTTAACCTGAGAGCTCCTCAATTGATAGATTGGGTCAACAATAGGGACG
CTAGTATACTTCGTAAAGTCGACAGCGAAATGGTCAATATTGATAATT
+
FFFFFFFFFFFFFFFFFFFFFFFFFFF:FFFFFFFFFFFF:FF:FFFFFFF:F:FFFFFFFFFFFFFFF
,FFFFFFFFFFFFFFFFFFFFFFF:FFFFFFFFFFFFF:FFFFFFFFFF
```

@A00721:190:HGJW7DSXY:4:1372:12915:12023_1:N:0:TCTGCAAG+GCTGCACT
AGCTCATGGTGAAAAGGGAAGCTAAGGTTAAACTTGATGCTTCAGCCTTTTCGAAGCATAACCCCGCA
CAGAATATCGTTTATCACGCAAAATTTATTAATGCAATTTTCGGACCTATCTTTAAGGTTCTTGTGAG
GAG
+
FFFFFFFFFFFFFFFFFFFFFFFFFFFFFFFFFFFFFFFFFFFFFFFFFFFFFFFFFFFFFFFFFFFFFF
FFFF:FFFFFFFFFFFFFFFFFFFFFFFFFFFFFFFFFFFFFFFFFFFFFFFFFFFFFFFFFFFFFFFFF
FFF
@A00721:190:HGJW7DSXY:4:1516:22625:23390_1:N:0:TCTGCAAG+GCTGCACT
AGCTCTTCTCTCTCTTAGGAGGTCATGATGACTACTATAAGGGTGAAATCGATTTTTCGAAGTTTGAC
AAATCTCAAGATTTCTTTTGTAAAGCCTATGAGATAAAGATGTATTCTTATTTAGGCTTCGATGCTGA
ATT
+
FFFFF:FFFFFFFFFFF:FFFFFFFFFFFFFFFFFFFFFFFFFF,FFFFFFFFFFFFFFFFFFFFFFFFF
FFFFFFFFFFFFFFF:FFFFFFFFFFFFFFFFFFFFFFFFFFFFFFFFFFFFFFFFFFFFFFFFFFFFFF
FFF
@A00721:190:HGJW7DSXY:4:2218:9399:30076_2:N:0:TCTGCAAG+GCTGCACT
AGTGCCAGTGAATATTTTTGTCACGCCAGGAGTATCCATAACGATTTGTCATTTACCACCTTTGCTCA
ACGACGAAGTGGAACGGCCAACACTTTCTTTGGTAATTGTATGATCAC
+
FFF:FFFF,FFFFF:,FFFFFFFFFFFFF::FFFFFFFFFFF,FF,FF:FFFF:FFFFFFFFF,F:FFFF
FFFFFFFF:FFFFFF,FFFF:FFFFFFFF:F:FFFFFFFFFFFFF:FF
@A00721:190:HGJW7DSXY:4:1523:25744:36933_2:N:0:TCTGCAAG+GCTGCACT
TATCACTTTATTATGACCTTGGAAAGTTTTCGGCTGTGGTTGTCGCTGGGGACGATTCCATTCTGTTT
TCAAAGACTCCAATAACTGATCATTCTGATCATATGATTTTGGACAAT
+
FFFFFFFFFFFFFFFFFFFFFFFFFFFFFFFFFFFFFFFFFFFFFFFFFF:FFFFFFFFFFFFFFFFFFF
FFFFFFFFFFFFF:FFFFFFFFFFFFFFFFFFF:FFFFFF:FFFFFF,
@A00721:190:HGJW7DSXY:4:1536:1380:26772_2:N:0:TCTGCAAG+GCTGCACT
ATCGTTAAGTTAGGTAAACCCCTAACCGATGTTACTCTTGTAGGACTTTGGGAGAGATTCGTTAGTTT
TAAAGACTATACTAAATATCTCCACTACGAGGTTGTTGTTTGGCGATT
+
FFFFFFFFFFFFFFFFFFFFFFFFFFFFFF:FFFFFFFFFFFFFFFFFFFFFF:FFFFFFFFFFFFFFFF
FFFFFFFFFFFFFFFFFFFFFFFFFFFFFFFFFFFFFFFFFFFF:FFF
@A00721:190:HGJW7DSXY:4:1427:20726:24768_2:N:0:TCTGCAAG+GCTGCACT
ATATTCGATATTCGATTGAGGGTCGTTATACCTATTCGGCTATCTGTGCTTTGAATTGTATAAGATCA
AATTTTAAACGATTCCGAGAGGTTTATCCTGATGTGGAGGGAGTTCTT
+
FFFFFFFFFFFFFFFFFFFFFFFFFFFFFFFFFFFFFFFFFFFFFFFFFFFFFFFFFFFFFFFFFFFFFF
F:FFFFFFFFFFFFFFFFFFFFFFFFFFFFFFFFFFFFFFF:FFFFFF
@A00721:190:HGJW7DSXY:4:2253:15049:35509_1:N:0:TCTGCAAG+GCTGCACT
ACCTTTCCTTGTGAAAATTTTGATGCGGTTTCATAAGATTATCAGTAATCCTGATTCTACTACGGATT
CATGGACCTTCCATCCTGTACCTCTTCTGGAGAGACGTATCTCAAAGATCGACTTCGAGACAGGTGAT
ATT
+
FFFFFFF:FFF:FFFFFFFFFFFFFFFF,FFFFFF:FFFFFFFFFFFFF:FF:FFFFFFFFFFFFFFFFF
FFFFFFFFFFFFF:FFFFFFFFFFF:FFFFFFFFFFFFFFFFFFFF:F:FFFFFFFFFFFFFFFFFFFFF
FFF
@A00721:190:HGJW7DSXY:4:2332:8260:36526_2:N:0:TCTGCAAG+GCTGCACT
CTAAATTCGGTAACGGTGCGTGGCGCTTTATTTTAAAAAGCTCCCTCCTCTCGCAAGTTTGATCTGAT
ACTTATCACGAAAGGACTTGAGTCTTTTGATCAGTCTCTGACTTGACC
+
FFFFFFFFFFFFFFFFFFFFFFFFFFFFFFFFFFFFFFFFFFFFFFFFFFFFFFFFFFFFFFFFFFFFFF
FFFFFFFFFFFFFFFFFFFFFF:F:FFFFF:FFFFFFFFFFFFFFFFF

```
@A00721:190:HGJW7DSXY:4:2230:2483:16908_2:N:0:TCTGCAAG+GCTGCACT
TAAATGACGATCATTAAGACTCTCTTTCTTCTTTGTTTCCTTCTTTGCACTGATTTTTCCTCATCAGC
TTGTGTTTTACCTAATCTGTGTTTTTACGATAACAACTATCAGTTGAT
+
FFFFFFFFFFFFFFFFFFFF:FFFF:FF:FFF,FFFFFFFFFFFFFF:FFFFFFFFFFFFFFFFFFFF
FFFFFFFFFFFFFFFFFFFFFFFFFFFFF,FFFFFFFFFFFFFFFFFFF
@A00721:190:HGJW7DSXY:4:1430:10185:20008_1:N:0:TCTGCAAG+GCTGCACT
CCGAAAGGCGAACTTTGGGAATTTGAAGCTCAGGGTGAAGCACAACGTTTGACCGTGACGAACTGTGG
TGTTCTTGATCTACGTTACGGATATACTCTCCTTGAGTTCACTCGTAAGGACACTACCACTTATTACG
ATA
+
F:FFFF:FFFFFFF:FFFFFFFFFFFFFFFFFFFFFFFFFFFFFFFFFFFFFFFFFFFFFFF:FFFF:
FFFF:FFFFFFFFFFFFFFFFFFFFFFFFFF:FFFFFFF:FFFFFFF:FFFFFFFFFFFFFFFFFFFF
FF:
@A00721:190:HGJW7DSXY:4:2203:25229:23829_1:N:0:TCTGCAAG+GCTGCACT
TCTCTATTTTAAGTTTTGTCTTCCTTGTTTGTCTTATTCTATTAATCACTCATTTTAGTGAGATTATT
AAAAACGCACTAAAACGTCTTCGACAACAGCAGGGGTTTTCGAATAGTGATAGCCACCCGCCTTACAG
AGG
+
FFF:FFFFFFFF:FFFFFFFFFFFFF::FFFFFFFFFFFFFFFFFFFFFFFFF,FFFFFFFFFFFFFF
FFFFFFFFFFFFFFFFFFFFFFFFFFFFFFFFFFFFFFFFFFFFFFFFFFFFFFFFFFFFFFFFFFFF
FFF
@A00721:190:HGJW7DSXY:4:2603:2510:15421_1:N:0:TCTGCAAG+GCTGCACT
GACTTTGGTACCACCTTTTCTACTGTCTGTCTCGCCTCTTCGAATGAAGTTTTAATGCTTAACAACAA
TGATAGTGAATTTATACCTACAATCATTGGTTTTTCTAATAATGATGACACTATTGTTTATGGATATG
ATG
+
FFFF:FFFFFFFFFFFFFFFFFFFFFFFFFFFFFFFF:FFFFFF:FFFFFFF:FF:FFFFFFFFFFFF
FFFFFFFFFFFFFFFFFFFFFFFFFFFFFFFFFFFFFFF:FFFFFF:FFFFFFFFFFFFFFF:FFFFFF
FFF
@A00721:190:HGJW7DSXY:4:1465:7934:34021_1:N:0:TCTGCAAG+GCTGCACT
TCTGGATTCTCAGTTTACAAGGACCTTAAGCGATGGATCGGGGTTAATTCTAAAACATTACCCGAAAG
ACGTGCTAAATTAAAACCATTGTACGATGTCGTCTGTCTCGGCGATCACTTTTCACTAAGAATTGGTC
CTA
+
FFFFFFFFFFFFFFFFFFFFFFFFFFFFFFFFFFFFFFFFFFFFFFFFFFFF:FFFFFFFFFFFFFFF
FFFFFFFFFFFFFFFFFFFFFFFFFFFFFFFFFFFFFFFFFFFFFFFFFF:FFFFFFFFFFFFFFFFFF
FFF
@A00721:190:HGJW7DSXY:4:1640:24035:16063_1:N:0:TCTGCAAG+GCTGCACT
AGACGTGTGCTCTTCCGATCTATCGTTTCTTTCTTTATCTCTTTGGTTGTTAGGGATTTTGAGAGAGT
GAAAGGCTTCAATTGTAGTGGTTTGGTCGTTTCAGTACCCAGTCAGTATACCTCTACGCAAAGATATT
TCA
+
FFFFFFFFFFFFFFFFFFFFFFFFFFFFFF,FFFFFFFFFFFFFFFFFFFFFFFFFFFFFFFFFFFFF
FFFFFF,FFFFFFFFFFFFFFFFFFFFFFFFFFFFFFFFFFFFFFFFFFFFFFFFFFFFFFFFFFFFF
FFF
@A00721:190:HGJW7DSXY:4:2517:32045:21010_2:N:0:TCTGCAAG+GCTGCACT
GAGGAACGTACCGGATTACCTGTCTTTCATATTATGAATGAACCTTCGGCCGCTCTCTTTGCTTCGAT
GTTGGATGTCAAGAAGGGTTCAGAATGGGATTCCTACGTAGTTTATGA
+
FFFFFFFFFFFFFFFFFFFFFFFFFFFFFFFFFFFFFFFFFFFFFFFFFFFFFFFFFFFFFFFFFFFF
FFFFFFFFFFFF,FF:FFFF:FFFFF::FFFFFFFFFFFFFFFFFF,F
@A00721:190:HGJW7DSXY:4:2608:31467:16564_1:N:0:TCTGCAAG+GCTGCACT
TACTTTCGATACTTCTATCGTCTTACGTTACGGTAATTACTATAGTGTTATTTATTCTGGTGGTGATG
```

```
ATAATCTTGGAGGTCGTGACGTTGATGAAGCTATAAGAGCGTTCTTATTACGGCGCACTTCTTTACCC
GCC
+
FFFFFF:FFFFFFFFFFFFFFFFFFFFFFFFFFFFFFFFFFFFFFFFFFFFFFFFFFFFFFFFFFFFF
FFFFFFFFFFFFFFFFFFFFFFFFFFFFFFFFFFFFFFFFFFFFFFFFFFFFFFFFFFFFFFFFFFFF
FFF
@A00721:190:HGJW7DSXY:4:1535:28257:1673_2:N:0:TCTGCAAG+GCTGCACT
GTTTCACAACTGAAGGAGGAGGTTTCTGTTACTGGAAAACCCTCGTCAATACTTTTCAATAATATACG
TCTTGAACTCACTTATGACGATCTAGTGAAATTAACGCGTCCTTTTCT
+
FFFFFFFFFFFFFFFFFFFFFFFFFFFFFFFFFFFFFF:FFFFFF:FFFFFFFFFFFFFFFFFFFFFF
FFFFFFFFFFFFFFFFFFFFFFFFFFFFFFFFFFFFFFFFF:FFFFFF
@A00721:190:HGJW7DSXY:4:1453:31394:20384_1:N:0:TCTGCAAG+GCTGCACT
TACTATTATGAACAAAGTTATCAAAGATAGTGGCATTCAAGGCAAACTTACCCTGATTCCGGCCGGTG
GTAGCTCATTTCTACCAGGGGCTATTACTCTTGCTGAAACAGCTATTAAAGCTGTTGAGCGGGTAATT
ACA
+
FFFFFFFFFFFFFFFFFFFFFFFFFFF,FFFFFFFFFFFFFFFFFFFFFFFFFFFFF,FFFFFFFFFF
FFFFFFFFFFFFFFFFFFFFFFFFFFFFFFFFFFFFFFFFFFFFFFFFFFFFFFFFFFFFFFFFFFFF
FFF
@A00721:190:HGJW7DSXY:4:2146:7166:18255_2:N:0:TCTGCAAG+GCTGCACT
ATCGCTGAAGGATGTGCACTCTTTTCTGCTACTTTAACGGAGCGTAGTGTACTCTTCGTCGATTGTAT
CAACAGTAACATTACTTCTAATAAAGGTCTCTATCAAGCTCGTATTAT
+
F:FFF:FFFFFF:FFFFFFFFFFFFFFFFFFFFFFFFFFFFFFFFFFFFFFFFFFFFFFFF,FFFFFF
FFFFFFFFFFF:FFFF::FF:F:FFFFFFFFFFFFFFFFFFF:FFFFF
@A00721:190:HGJW7DSXY:4:1570:8811:35947_1:N:0:TCTGCAAG+GCTGCACT
TTACCGTTGAAGGCTAAACTCCAAGGTAATTATGAAGACTGTGATTTATCTTTCACTCTGGCCTTGTA
TGAGGGTGTTTCTAATCGTAACATTAATAATTTCAATCTTCTTAAAAAGAAGATAGAGAGTCATACTT
TGG
+
FFFFFFFFFFFFFFFFFFFFFFFFFFFFFFFFFFFFFFFFFFFFFFFFFFFFFFFFFFFFFFFFFFFF
FFFFFFFFFFFFFFFFFFFFFFFFFFFFFFFFFFF,FFFFFFFFFFFFFFFFFFFFFFFFFFFFFFFF
FFF
@A00721:190:HGJW7DSXY:4:2659:22860:11334_2:N:0:TCTGCAAG+GCTGCACT
TGAAGTGGACACCTCTGGTAGTATCGAAGTAAAGATAACCAATAAGGAAGGAGCTTATACTATGGTTG
AATCAACTAAGCATACTACTTTTGCTTTGATTACTCTACCGCCTTACA
+
FFFFFFFFFFFFFFFFFFFFFFFFFFFFFFFFFF:FFFFFFFFFFFFFFFFFFFFFFFFF,FFFFFFF
FFFFFFFFFFFFFFFFFFFFFFFFFFFFFFFFFFFFFFFFFFFFFFFF
@A00721:190:HGJW7DSXY:4:1224:27190:10974_2:N:0:TCTGCAAG+GCTGCACT
ATTAAACAAGTTGTACTTATCAATGATTTTATCGCTGGTTGTTATCTCGAAAAGTCTCTTCTAAAAGG
AACGTCAGTTCTAACCCAAAGAGAATATCTAAAATATTTACAAAATCA
+
F:FFFF,FF,F:,,:F:FFF,FF::FFFFFF:F::,FFFF,FFFF:FFFFFFFFF:,,:F:FFFFF,F
FFF::FFF:FF,FF:FF:FFFFFF:,FF:,FFFF,FFFFF::FFF,,,
@A00721:190:HGJW7DSXY:4:1505:3134:1752_2:N:0:TCTGCAAG+GCTGCACT
AGACGTTGCTTGTCGCTTCCTCCTTTAATGGCACTACCTCGGTACAATCTTACTTGGAACGAAGGCGA
CCTGTTCCGGAGTTTTTACGGGATCCGGAATGTGCTGTCTATATTAGG
+
FFFFFFFFFFFFFFFFFFFFFFFFFFFFFFFFFFFFFFFFFFFFFFFFFFFFFFFFFFFFFF:FFFFFF
FFFFFFF,F:FFF::FF:FFFF,FFFFFFF,FF,FFFFF:FF,FFFFF
@A00721:190:HGJW7DSXY:4:2378:31150:36307_2:N:0:TCTGCAAG+GCTGCACT
TCGAACGTCAACGTTCACCGAGTTTCGTTTCGTACCTTGATGCTGACTTTCGTCAATATGATTTGATC
```

```
TGGTTGCTCAATTCTAAGCGTGTTCCGCTTAGTGCGAGGCCTCTCTTC
+
FFFFFFFFF:FFFF:FFFFF:,FFFFFFFFFFFFFFFFF:FFFFF:FFFFFFFFFFFFFFFFFFFF,F
FFFFFFFFFFFFFFFF:FFFFFFFF:FFFF:FFFFFFF,FFFFF:FF:
@A00721:190:HGJW7DSXY:4:1661:22227:18912_2:N:0:TCTGCAAG+GCTGCACT
ATATTTGGATGATCTTGATAAAGAACTTCATGAATTTTCAGAGGATGAAAAATTTTCTTCCTTGGATT
TTAATATAAAGAATCTTTACCAGGACTCCTACTACAGGAGGCAGTCTT
+
FFFFFFFFFFFFFFFFFFFFFFFFFFFFFFFFFFFFFFFFFFFFFFFFFFFFFFFFFFFFF,FFFFFF
FFFFFFFFFFF:FFFFFFFFFFFFFFFFFFFFFFFFFFFFFFFFFFFF
@A00721:190:HGJW7DSXY:4:1532:29035:35336_2:N:0:TCTGCAAG+GCTGCACT
TTTGTTGTAATAGGGTCATCAACTTAGAACTCGAAACTCTAAGTGGTAATACTGTCTTTCCAAGCTTA
CCTGTATCTTCAATGAAATTGGACTCTTCGATAGGGGACATTTTATCG
+
FFFFFFFFFFFFFFFFFFFFFFFFFFFFFFFFFFFFFFFFFFFFFFFFFFFFFFFFFFFFFFFFFFFF
FFFFFFFFFF:FFFFFFFFFFFFFFFFFFF,F,FFFFFFFFFFFFFFFF
@A00721:190:HGJW7DSXY:4:2240:29432:8750_1:N:0:TCTGCAAG+GCTGCACT
TACTAATGGCGTTGGCCTAGTGGATGTTATTAGTGACATCTGGTCAATTTCGTTTTTAGGTGGTCTTT
ATTCTATTATTGATTTTTATGAGATTGATACGTTAACAGAAGATGATGTTAGAGTAGAATTCCTGAAG
AG
+
FFFFF::F,FFFF:FFFFFFFF,:FFFF:FFFFF:FFFFFF::F:FFFFFF,FFFFFFFFFFFFFFFF
FFFFFFFF,FFFFFFFFFFFFFFFFFF:FFFFFFFFFFFFFFFFFFFFFFFFF,FFFFFFFFFFF,FFF
FF
@A00721:190:HGJW7DSXY:4:2353:14597:17503_2:N:0:TCTGCAAG+GCTGCACT
CCAACTTCTTGACAATTTTCTTCAATCCTTCGATAATAGTTAAATCTAGGATTATGTCTGTGTTGAAA
GACTTTCTACCGATAAGGGTCACTAGTCTTACACAATTAAGAAATAAT
+
FFFFFFFFFFFFFFFFF:F:FFFFFFF:FF,FFFFFF:FF:FFFFFFFFFFFFFFFFFFFFFFFFFFF
FFFFFFFFFFFFFFFFFFFFFFFFFFFFFFFFFFFFFFFFFFFFFFFF
@A00721:190:HGJW7DSXY:4:1255:1814:31626_2:N:0:TCTGCAAG+GCTGCACT
ACTATCTAAAGATTGAAGGAAAATTTTATCCTCGTTTAACTTACTTAGACTTTCCTACGATACAAATG
ATAACTTCTAATTTACGTTTGCTACTCAGAGCTGGTCCTCTCAGCAAA
+
FFF,FFFFFFFFFFFFFFFFFFFFFFFFFFFFFFFFFFFFFFFFFFFFFFFFFFFFFFFFFFFFFF:F
FFFFFFFFFFFFFFFFFFFFFFFFFFFFFFFFFFFFFFFFFFFFFFFF
@A00721:190:HGJW7DSXY:4:2630:30626:4304_2:N:0:TCTGCAAG+GCTGCACT
TCCTCAGGAGTCCCGGCATTTGATAATAATGACGACTTTCGTCATAATCGCTGCATATTACGGATTAC
ACGGTACCAGCGCATCTCGTGTCGAGCCTCGACCCGATTATATGGTAT
+
FFFFFFFFFFFFFFFFFFFFFFFFFFFFFFFFFFFF:,FFFFFF,FFFFFFFFFFFFFFFFFFFFFFF
FFFFFFFFFFFFFF::FFFFFFFFFF:F:FFFFFFFF:,FFFFFFFFF
@A00721:190:HGJW7DSXY:4:1152:22318:28307_1:N:0:TCTGCAAG+GCTGCACT
TTCTTAATTATAATATTCAACCTCAAAAGTTACGTGTTTACAGTAACGAGATCACACATTTTGAGAAC
TAACTATGTTATGTTCTCTGATCATTTTGACAATCTCTTCTTATAATAAATAAATAAATAATATTACT
AAT
+
FFFFFFFFFFFFFFFF:FFFFFFFFFF:FFFFFFFFFFFFFFFFFFFFFFFFFFFFFFFFFFFFFFFF
FFFFFFFFFFFFFFFFFFFFFFFFFFFFFFFFF:FFFFFFFFFFFFFFFFFFFFFFFFFFFFFFFFFF
:FF
@A00721:190:HGJW7DSXY:4:1262:26666:14606_1:N:0:TCTGCAAG+GCTGCACT
AAAGGCTCCAGTCAACACTAGTTCGAGTGCTGCTTTGGATTACGATGACGTGAGAATAGGTGAAGGGA
TAGTTTTTACCTCGACCAACATTAAGGCGGCGGCGACTAATCTTAAAGCTTCACCCAAAATGAAGGAC
ACT
```

```
+
FFFFFFFFFFFF:FFFFFFFFFFFFFFFFFFFFFFFFFFFFFFFFFFFFFFFFFFFFFFFFFFFFFFFFF
FFFFFFFFFFFFFFFFFFFFFFFFFFFFFFFFFFFFFFFFFFFFF:FFFFFFFFFFFFFFFFFFFFFFFFFF
FFF
@A00721:190:HGJW7DSXY:4:2123:3251:21057_1:N:0:TCTGCAAG+GCTGCACT
TCTAAAGGACACTATACAATATCCAGACGACGACGAAGCTTATACTACTTTAATTTTGGGTATTATGT
GGAGATATCGTGATATCAGTACGTCCAGATCCTCTGCTTTTACCGATAACGATAAATTCGTCATCAAA
TAT
+
F:FFFFF:FFFFFFFFFFF:FFFFFFFFFFFFFFFFFFFFFFFFFFFFFFFFFFFFFFFFFFFFFFFFF
FFFFFFFFFFFFFFFFFFFFFFFFFFFFFFFFFFFFFFFFFFFFFFFFFFFFFFFFFFFFFFFFFFFFFFFF
FFF
@A00721:190:HGJW7DSXY:4:1656:29451:32675_2:N:0:TCTGCAAG+GCTGCACT
CCACATTTTGGTCAAGTCAATCCGTTGAGAAAATTTCTTAGAGGATATTCGCCATTGTGGTACGAATT
GGTCCAACATTTACCACCAAATTCTTTTGATACAACTCTAGCCTTCAA
+
F,,FFF,FFFF:F:FFFFFFFFFF,,FFFFF:F::FFFFFF:,FFFF,FFFF,FFF,,F:FFF,FF,,
:FF,F::FF,FF:F:FFFF,FF:FF,,F:F,F,F:FF:FF:FFFF,F,
@A00721:190:HGJW7DSXY:4:2469:24641:3396_1:N:0:TCTGCAAG+GCTGCACT
ACCAGATTGGTTCGAACCCGTTGATGCTATGTCAGAAGAAGCTATTCAAGCTTGTCGTTTAAAGACTG
CAGAGGCTGTCAAACAAGCCCAGACTCAAGGAGTCCAATCAAAGGTTTTGAATTTAGCTCAACTGGGG
AGA
+
FFFFFFFFFFFFFFFFFFFFFFFFFFFFFFFFFFFFFFFFFFFFFFFFFFFFFFFFF,FFFFFFFFFF
FFFFFFFFFFFFFFFFFFFFFFFFFFFFFFFFFFFFFFFFFFFFFFFFFFFFFFFFFFFFFFFFFFFFFFFF
FFF
@A00721:190:HGJW7DSXY:4:1230:1814:28025_2:N:0:TCTGCAAG+GCTGCACT
TTCTACTTACAAAGGACAATTCAGTTTCATTAATTCTACGGAAAAGTCCGGAGTATATTGTATGTTAA
TAGATAGTGGGATAATAGTTCTACCGATACGTAGTGAACGCGTTCGTA
+
FFFFFFFF:FFFFFFFFFF:FFFFFFFFFFFFFFFFFFFFFFFFFFFFFFFFFFFFFFFFFFFFFFF:F
FFFF:FFFFFFFFFFFFFFFFFFFFFFFFFFFF:FFFFFFFFFFFFFFF
@A00721:190:HGJW7DSXY:4:2218:22607:27680_1:N:0:TCTGCAAG+GCTGCACT
ACCGATAGGAGTTGTATAGTTGAAGATGTGGCTTTAGACGATAATCCTTTAAGGACTATGTTGACTGA
CGAAGATTTTCCAACAGATTTGTCTACGTTGCAGATCGCCACTCCAAACACTATATACATTATAAAAT
ATA
+
F:FFFF:F::FFFFFF,FFFFFFFFFFFFFFFFFFFFFFFFFFFFFFFFFFFFFFFFFFFFFFFFFFF
FFF:FFFFFFFFFFFFFFFFFFFFFFFFFFFFFFFFFFFFFFFFFFFFFFFFFFFFFFFFFFFFFFFFFFFF
FFF
@A00721:190:HGJW7DSXY:4:2373:6343:30044_1:N:0:TCTGCAAG+GCTGCACT
ATAACTTTTGTTACCAAATTCTTGGCTTTCAACGAAGTCTCTGATTTTATTAATCATTACTTTAACGA
TCTCTCGGTCATTTACTCTGTACTTGATCTACGTCGTTTAACTGTCGGAACAACCTTTACTTAGAATG
ACT
+
FFFFFFFFFFFFFFFFFFFFFFFFFFFFFFFFFFFFFFFFFFFFFFFFFFFFFFFFFFFFFFFFFFFFFFFF
FFFFFFFFFFFFFFFFFFFFFFFFFFFFFFFFFFFFFFFFFFFFFFFFFFFFFFFFFFFFFFFFFFFFFFFF
FFF
@A00721:190:HGJW7DSXY:4:1311:25635:23907_2:N:0:TCTGCAAG+GCTGCACT
TATTTACAATAGTTAATATCATATTAGTAATACTCACTATCGTTTTCTGCTTTTTCTTAGCTAATAGA
TTAATAAAGGATGAGTACGAAAACCAACTCTTCGTTACGAGACCTGAT
+
FFFFFFFFFFFFFFFFFFFFFFFFFFFFFFFFFFFFFFFFFFFFFFFFFFFFFFFFFFFFFFFFFFFFFFFF
FFFFFFFFFFFFF,FFFFFFFFFF:FF:FFFFFFFFFFFF,FFFFFFF
```

@A00721:190:HGJW7DSXY:4:1433:16794:27320_1:N:0:TCTGCAAG+GCTGCACT
ATTTCGTCTCTACACCACTCTCAACGAGTATAAAGCGAGTTTTTCGTCCTTAACGTAATACACTAAGA
GATCTTCCGCAAACGACTCGAAACCGAGACGCCGTAGATCAAAGTCCCTTCTAACTTTAGAT
+
FF:F,,F,,,,F,FFF,,F:F::,,:,F,::F,F::,,,FFFF:::,:,,,F::,,FF,,,,F,::,,
,F,F,,FF,FF,F::FFFFFFFFFFFFFFFFFFFFFFFFFF:F:FFFFFFFFFFFFFFFFFF
@A00721:190:HGJW7DSXY:4:2548:8449:26553_2:N:0:TCTGCAAG+GCTGCACT
CAGTTATTTATGTTGTACTTTTTATAATTTATAACCTTCTTATACTTTTGGGTATGTTTTGTGAAAAA
CATGAGATACCCAAGTCTATCGAAGACATTATCTCTTATGTACTTATT
+
FFFFFFFFFFFFFFFFFFFFFFFFFFFFFFFFFFFFFFFFFFFFFFFFFFFFFFFFFFFFFFFFFFF:
FFFFFFFFFFFFFFFFFFFFFFFFFFFFFFFFFFFFFFFFFFFFFFFF
@A00721:190:HGJW7DSXY:4:1230:29261:16282_2:N:0:TCTGCAAG+GCTGCACT
CTGTGTATGAGGAGGATTTATAATGCTTTTCGATAGTGATTGTTATCAATTTAAATACATTACTGTTT
CAAATCATCTTTACTCACAGACTCCGAAAATGGTTTCAAATGAGGTCA
+
FFFFFFFFFFFFFFFFFFFFFFF:FFFFFFFFFFFFFFFFFFFFFFFF:FFFFFFFFFFFFFFFFFFF
FFF:FFFFFFFFFFFFFFFFFFFFFFFFFFFFFFFF:FF:FFFFFF:F
@A00721:190:HGJW7DSXY:4:2233:8865:3505_2:N:0:TCTGCAAG+GCTGCACT
TTAAACGAACTATTATCCGATTCTTCGAGGGTATCACTACCACTGATCTGCCGACTTTAGTCAAGGAA
CTTCAATTCCTTCGATGTTTATATTTTTCTATAAGGGAAAATAGCAAT
+
FFFFFFFFFFFFFFFFFFFFFFFFFFFFFFFFFFFFFFFFFFFFFFFFFFFFFFFFFFF:FFFFFFF:
FFFFF::FFFFFFFFFFFFFFFFFFFFFFFFFFF:FFFFFFFFFFFFF
@A00721:190:HGJW7DSXY:4:1650:15799:31109_2:N:0:TCTGCAAG+GCTGCACT
CGACAAATTTTAGAACTTTATTTCAGAACTCTCTCCGATCACCTTGGTGTGATCTCCCGGAGGATCTT
TTCAATAAGATGTATGATGATAATCTACTATTTTTAAATAGTGATATT
+
FFFFFFF:FFFFFFFFFFFFFFFFFFFFFFFFFFFFFFFFFFFFFFFFFFFFFFFFFFFFFFFFFFFF
FFFFFFFFFFFFFFFFFFFFFFFFFFFFFFFFFFFFFFFFFFFFFFFF
@A00721:190:HGJW7DSXY:4:2570:4562:20823_1:N:0:TCTGCAAG+GCTGCACT
CTTTATCAAAGATTTGGCTTGGTCTATTTACCGCAAAAAGTTGGAAGAGGTCTATGATATTTATTTTA
CTTATACTTTAAATAGCACCGATTATAACTTGACTGTTGATAATATTACAATAGCTCTTTCTCAGAAG
ATT
+
:FFF:FFFFFFFFFFFFFFFFFFFFFFFFFFFFFFFFFFFFFFFFFFFFFFFFFFFFFFFFFFFFFFF
FFFFFFFFFFFFFFFFFFFFFFFFFFFFFFFFFFFFFFFFFFFFFFF:FFFFFFFFFFFFFFFFFFFF
FFF
@A00721:190:HGJW7DSXY:4:2577:21206:14857_2:N:0:TCTGCAAG+GCTGCACT
GGTGAACACGTCGGTAGCATTGATTTACTTTTACCGTTAGAACTGAAAAAGATTCTTTTCGAAAAGAA
ACCTATAAGTTACAAACGATTCTTCGACTATATTAAAGAAATAAAAAT
+
FFFFFFFFFFFFFFFFFFFFFFFFFFFFFFFFFF:FFFFFFFFFFFFFFFFFFFFFFFFFFFFFFFFF
FFFFFFFFFFFFFFFFFFFF:FFFFFFFFFFFFFFFFFFFFFFFFFFF
@A00721:190:HGJW7DSXY:4:1606:17011:3114_2:N:0:TCTGCAAG+GCTGCACT
CTTTACATTTTTAATGGTATGGTATAATGTGTCTTACAAATAGAACCTTCACTCTCGGTAATGAGAGT
GTATTCCTTAATATTCTAGTAACCGCTATGTGGGCATTACTAGGATTT
+
FFFFFFFFFFFFFFFFFFFFFFF:FFFFFFFFFFFFFFFFFFFFFFFFFFFF:FFFFFFFFFFFFFFF
FFFFFFFFFFFF:FFFFFFFF:FFFFFFFFFFFFFFFFFFFFF:F:FF
@A00721:190:HGJW7DSXY:4:1610:7717:1673_2:N:0:TCTGCAAG+GCTGCACT
TGCTAGGAAAATTTTAGAAAAACTCCATTGTATAGTTCAAGGAGTACTTAATTCAATAGCTTTAGTAT
TATTTACTATCGCCTTTATTGAAAATTGTATAAGAGTTAATTGCTTAC
+

```
FFF:FFFF:FFFFFFFFFFFFFFFFFFFFFFFFFFFFFFFFFFFFFFFFFFFFFFFFFFFFFFFFFF:
FFFFFFFFFFFFFFFFFFFFFFFFFFFFFF:FFFFFF,FFFFF:FFFF
@A00721:190:HGJW7DSXY:4:1527:26612:14011_2:N:0:TCTGCAAG+GCTGCACT
ACCATAACCTACCATGACCAATCATCGCTCCAACTGGCACGCCAGACGTAGGGTCAAACCTACTACCG
CCGGTCGGCAGATCGGCACCACTGGTCTCTACGTTCAGGGACCCGCTG
+
FFFFFFFFFFFFFFFFFFFFFFFF:FFFFFFFFFFFFFFFFFFFFFFFFFFFFFFFFFFFFFFFFFFF
FFFFFFFFFFFFFFFFFFFFFFFFFFFFFFFFFFFFFFFFFFFFFFFF
@A00721:190:HGJW7DSXY:4:1354:1814:14278_1:N:0:TCTGCAAG+GCTGCACT
CTTCCCAAATCTAGATCTGCACAGAAGAAGGCTGAAGGTCGTTACCGGACGACCACTCAAAGGAAACG
GGCTGTTGCCCAGAGGAAAGCAGCGACCACCGAGGTCGCTCGTAACATTGACGGCAGTCCCGTCATCT
CT
+
::FFFFFFFFFFF:FFFFFFFF,FFF:FFFF:FFFFFFFFFFFFFFFFFFFFFFFFF::FFFFF:FFF
FFFFFFFF,FFFFFFFFFFFFFFFFFFFFFFFFFFFFFFFFFFFFFFFFFFFFFFFFFFFFFFFFFFF
FF
@A00721:190:HGJW7DSXY:4:1674:14895:1423_2:N:0:TCTGCAAG+GCTGCACT
CAGTACCTCAGTCAAAAGTCACATTCACCACTCCCATACCAATCAATGATGTGACTATACAGTTCGGC
CATTTCAAGAGTGGTCAATACGTCGTGGGAGGTACTGAAGCCTTCCCC
+
FFFFFFFFFFFFFFFFFFFFFFFFFFFFFFFFFFFFFFFFFFFFFFFFFFFFFFFFFFFFFFFFFFFF
FFF:FF,FFFFFFFFFFFFFFFFFFFFFFFFFF:FFFFFFFFFFFFFF
@A00721:190:HGJW7DSXY:4:2344:16749:7952_2:N:0:TCTGCAAG+GCTGCACT
AAGCCAATCAATAGTTCTTCTGACCAGAATGAAAAGGGGTTTACCAGGGTAGTCTCTAAGTCTACCAG
GAAGAGGGGAAATGTCCCCAAAACTGAGAAGAATAAAGGGAAAGGTCC
+
FFFFFFFFFFFFFFFFFFFFFFFFFFFFFFFFFFFFFFFFFFFFFFFFFFFFFFFFFFFFFFFFFFFF
FFFFFFFFFFFFFFFFFFFFFFFFFFFFFFFFFFFFFFFFFFFFFFFF
@A00721:190:HGJW7DSXY:4:2554:14859:8249_2:N:0:TCTGCAAG+GCTGCACT
ATTACCCCTAATTCTGAAGGAAGATCTCCCACCATCTGGGGTTGGGAATAAGAAATCTGCACGGGCCA
AAGAGGCCGCTGCTACGAAGTCGCTGAAGGCGGCTTGGGTCATTAAGG
+
FFFFFFFFFFFFFFFFFFFFFFFFFFFFFFFFFFFF:FFFFFFFFFFFFFFFFFFFFFFFFFFFFFFF
FFFFFFFFFFFFFFFFFFFFFFFFFFFFFFFFFFFFFFFFFFF:FFFF
@A00721:190:HGJW7DSXY:4:1643:27724:12774_2:N:0:TCTGCAAG+GCTGCACT
GCCTGCCGACCCCATCTTTGAGACCAAGACTCTCTACAGTAGGTTCGTGCCTACTATGAGCAAATCGG
GTGAATTCGTCACTGAGGACGAAGGTGAGAGGACTTTTGGCTACTGGC
+
FFF:FFF::FFF:FFFFFFFFFFFFFFFFFF:FFFFFFFF:FF:FFF::F:FFF:FF:FFFFFFFF,:
,FFFFF:F::FFF,FFFFFFFFF:F,FFFFF,FFFFFFF,FF:FFFFF
@A00721:190:HGJW7DSXY:4:1672:21025:32643_2:N:0:TCTGCAAG+GCTGCACT
CGTGGGTTGAAGGCCGACGCTTCGAGCTTGAGTGTGACGAGGCCGGTGACGTTTTTGTCAACGGCGTT
TGGAGGATTTTATCCGCGCTTAACTATGGGGCGAAGATCCCCTATGGA
+
FFFFFF:FFFFFFFFFFFFFFFF:FFFFFFFFFFFFFFFFFFFFFFFFFFFFFFFFFFFFFFFFFFFF
FFFFFF,FFFFFFFFFFFF:FFFFFFFFFFFFFFFFFFFFFFFFFFFFF
@A00721:190:HGJW7DSXY:4:2405:19280:8202_2:N:0:TCTGCAAG+GCTGCACT
GGCATATTTATGCCGACGATAATAAGCATGTCACCCTTAAGCACCGAGGTAAGCTCGGGACTTACAAG
AGAGAGTGTGAGAGAGTCGCTCTCACTATTTTTGCCCAGAATGCGGGC
+
FFFFFFFFFFFFFFFFFFFFFFFFFFFFFFFFFFFFFFFFFFFFFFFFF:FF:FFFFFFFFFFFFFFF
FFFFFFFF:FFFFFFFFFFFFF:FFFFF:FFFF,:FFF:FF,,F:FFF
@A00721:190:HGJW7DSXY:4:1601:2871:3552_2:N:0:TCTGCAAG+GCTGCACT
ATTACAACCTCTCTTTAGAGAGCATTAGAAGGAAGTTGGGCAAATTCCCACATATGGTAGACGTTGTC
```

ACACTTTTCTTGAAAATTTTTCCTTTCCTCCGTCATTGGGGATTTCGA
+
FFFFFFFFFFFFFFFFFFFFFFFFFFFFFFFFFFFFFFFFFFFFFFFFFFFFFFFFFFFFFFFFFFFFFFF
FFFFFFFFFFFFFFFFFFFFFFFFFFFFFFFFFFFFFFFFFFFFFFFF
@A00721:190:HGJW7DSXY:4:2124:8006:32612_1:N:0:TCTGCAAG+GCTGCACT
GGAAGGTGTCAAGGGTCTTCTTCATGCTGATCTTTCAGGACCTAATCACATCATGATTGATAGTTACG
GCTCGTATCGAGTTGGAGGGAGGAAGTTCTCCGAAATTAAAGACGGGGGGATTAAGAGTCCAGCTGTA
ACT
+
FFFFFFFFFFFFFFFFFFFFFFFFFFFFFF::F,FFFFFFFFFFFFFFFF:FFF:FFFFFFFFFFFF
FFFFFFFFFFFFFFFFFFFFFFFFFFFFFFFFFFFFFFFFFFFFFFFFFFFFFFFFFFFFFFFFFFFFFFF
FFF
@A00721:190:HGJW7DSXY:4:1561:12762:1799_2:N:0:TCTGCAAG+GCTGCACT
AGAGGGGAGCTCTGAAAAGGAAACTTCGTCAGAAGATGAGGAAGTCTAAGAGGAGGAGTTTGAGGAAG
GCTCTAGAGAAGATCTCACCACCTGGAGAAGGCAAGTGGAGAGTCCAG
+
FFFFFFFFFFF:FFFFFFFFFFFFFFF:FFFFFFF:FFFFFFF:FFFFF:FFFFF:FFFFFFFFFFFF
FFFFFFFFFFFFFFFFFFFFFFFFFFFFFFFF:FFFFFFF:FFFFFFFF
@A00721:190:HGJW7DSXY:4:2446:14172:17206_1:N:0:TCTGCAAG+GCTGCACT
CTTCCGATCTTTTGAAGCAGGGGTTACCACTTCCTAAGGTGGATGAGGGAGGTTGTTTCTTTGGAGTT
TTAGATAATCTTTTGAGAAGGGGTCATAGAGCCAGCAAGAGAAGCTTATATGCTGGTTTAAGTGATGA
TTA
+
FFFF,FFF:FFFFFFFFFFFFF:FFFFFFFFFFFF:::FFFFFFFFFFFFFFFFFFFFFFFFF:FFFFFF:
F:FF,F:,FFFFFFFFFFFFFFF::FFFFFFFFFFFFFFFFFFFFFFFFFFFFFFF:FFFFFFFFFFFFF
FFF
@A00721:190:HGJW7DSXY:4:1537:32054:36558_1:N:0:TCTGCAAG+GCTGCACT
ATTTAGGTCAACTTGGGGTTTCGAATGTCATTAGTGCCTTACGATTGAAAGAGGTCCTTTTGAGTATC
GACAGATCTTTCGATCTTGAGGACGTACATTTAACGTGGCAGAATTCGGTAACTTACCATATTGTTCC
TTC
+
:FFFFFFFFFF,:FFFFFFFF,FF:FFFFFFFFFFF:FFFFF:FF:FFFFFFFFF,F::FFFFFFFFF
FFFFFFFFFFFFFFFFFFFFFFFFFFFFFFFFFFFFFFFF:FFFFFFFFFFFF:FFFFFFFFFFFFFFFF
FFF
@A00721:190:HGJW7DSXY:4:1313:2410:18411_1:N:0:TCTGCAAG+GCTGCACT
TTTAACTTTGACTCAACTACTCCGATCAGATCCGGATGGTTTGATTGGTATGGACTACTCGACTTTTG
ATCTGGAGTTGTCTAGGGTCATGGCAGATACTACCGATAAGGTGAAAAATATGCAGGTCAAAGATTCT
GTA
+
FFFFFFFFFFFF:FFFFFFFFFFFFFFFFFFFFFFFFFFFFFFFFFFFFFFFFFFFFFFFFFFFFFFFFF
FFFFFFFFFFFFFFFFFFFFFFFFFFFFF:FFFFFFFFFFFFFFFFFFFFFFFFFFFFFFFFFFFFF:FFF
FFF
@A00721:190:HGJW7DSXY:4:1312:1262:2581_2:N:0:TCTGCAAG+GCTGCACT
GTTTTTGCCGACTTTAACAATTCTAAGAGTCTTTATGATTCAAAAACTATCGTTGTCGTACCTTACAA
GGTTGAGGAGACTTTACAGCGGCTTATTGTCGCGCAGTTTCCAGAATT
+
FFFFF,FF,FFF,FFFFF,F,:F:FF:FFFFF,FF,,FFFFFFF:,FFF:,F,F,F,,:F,F:FF,FF
,F:FFFF,F,F,FFFF,F,F::FFFFFF,:,FF,FFFFFF:,FFFF::
@A00721:190:HGJW7DSXY:4:2101:15646:17534_1:N:0:TCTGCAAG+GCTGCACT
TATCTTGAATGCTCACGCCGTCGCAGCCATATCGAGACTATGTTATAATCACTTGTTAAAGTACAGAT
TTAGTGATTTAGATATTATTGATATTGGAGGCGATATTCGTTTTCATATCCAAAACTCCTTGAATGGA
GTA
+
FFFFFFFF:FFF:FFFFFFFFFFFFFFFFFFF:FFFFFFFFFFFFFFFFFFFFFFFFFFFFFFFFFFFF

FFFFFFFFFFFFFFFFFFFFFFFFFFFFFFFFFFFFFFFFFFFFFFFFFFFFFFFFFFFFFFFFFFFFFF
FFF
@A00721:190:HGJW7DSXY:4:1130:1063:10410_1:N:0:TCTGCAAG+GCTGCACT
CTATTCTTGATGTGAAAGATGCATCGAGGTATACGTCTAGGAACATTGATTGGGAACGTACCTACTCA
CGAGACGTCATGAAAGCTATCGGAGGGAAGCAAAATGTTTCGTTCTGTTACAAGAACGCTTTGGATTG
TGA
+
FFF,FFFFFFFFFFFFFFFFFFFFFFFFFFFFFFF,FFFFFFFFFFFFFFFFFFFFFFFFFF:FFFFF
FFFFFFFFFFFFFFFFFFFFFFFFFFFFFFFFFFFFFFF:FFFFFFFFFFFFFFFFFFFFFFFFFFFF
FFF
@A00721:190:HGJW7DSXY:4:1558:21685:21919_1:N:0:TCTGCAAG+GCTGCACT
GTTGCGATAGCTGTAGAGGTATACGATATCTCTCTTGATGATGTAGCCACTATAATGCTTAAGAGAGA
GATTGATGTATGTCATTTCGTCTTAGTTTGTCCCGGCGAATTATTGGACACGATGAAAAATGACATAC
CCG
+
FFFFFFFFFFFFFFFFFFFFFFFFFFFFFFFFFFFFFFFFFFFFFFFFFFFFFFFFFFFFFFFFFFFF
FFFFFFFFFFFFFFFFFFFFFFFFFFFFFFFFFFFFFFFFFFFFFFFFFFFFFFFFF:FFFFFFFFFF
FFF
@A00721:190:HGJW7DSXY:4:1527:18756:15311_2:N:0:TCTGCAAG+GCTGCACT
TCATGGAGATGATGGCGCATCACTTGATTACTATATGCCCGAAGGATTGGGTTATTCTCACAACAAAG
ATAAGATTTTATCTTTCTTCAAACACCCTGTTTTCCTTAAGGGCGGAC
+
[truncated: 447,834 more chars]
